# Peritumoral radiomics features predict distant metastasis in locally advanced NSCLC

**DOI:** 10.1371/journal.pone.0206108

**Published:** 2018-11-02

**Authors:** Tai H. Dou, Thibaud P. Coroller, Joost J. M. van Griethuysen, Raymond H. Mak, Hugo J. W. L. Aerts

**Affiliations:** 1 Department of Radiation Oncology, Dana-Farber Cancer Institute, Brigham and Women’s Hospital, Harvard Medical School, Boston, MA, United States of America; 2 Netherlands Cancer Institute (NKI), Amsterdam, the Netherlands; Baylor College of Medicine, UNITED STATES

## Abstract

**Purpose:**

Radiomics provides quantitative tissue heterogeneity profiling and is an exciting approach to developing imaging biomarkers in the context of precision medicine. Normal-appearing parenchymal tissues surrounding primary tumors can harbor microscopic disease that leads to increased risk of distant metastasis (DM). This study assesses whether computed-tomography (CT) imaging features of such peritumoral tissues can predict DM in locally advanced non-small cell lung cancer (NSCLC).

**Material and methods:**

200 NSCLC patients of histological adenocarcinoma were included in this study. The investigated lung tissues were tumor rim, defined to be 3mm of tumor and parenchymal tissue on either side of the tumor border and the exterior region extended from 3 to 9mm outside of the tumor. Fifteen stable radiomic features were extracted and evaluated from each of these regions on pre-treatment CT images. For comparison, features from expert-delineated tumor contours were similarly prepared. The patient cohort was separated into training and validation datasets for prognostic power evaluation. Both univariable and multivariable analyses were performed for each region using concordance index (CI).

**Results:**

Univariable analysis reveals that six out of fifteen tumor rim features were significantly prognostic of DM (p-value < 0.05), as were ten features from the visible tumor, and only one of the exterior features was. Multivariablely, a rim radiomic signature achieved the highest prognostic performance in the independent validation sub-cohort (CI = 0.64, p-value = 2.4×10^−5^) significantly over a multivariable clinical model (CI = 0.53), a visible tumor radiomics model (CI = 0.59), or an exterior tissue model (CI = 0.55). Furthermore, patient stratification by the combined rim signature and clinical predictor led to a significant improvement on the clinical predictor alone and also outperformed stratification using the combined tumor signature and clinical predictor.

**Conclusions:**

We identified peritumoral rim radiomic features significantly associated with DM. This study demonstrated that peritumoral imaging characteristics may provide additional valuable information over the visible tumor features for patient risk stratification due to cancer metastasis.

## Introduction

Lung cancer remains the leading cause in cancer-related mortality worldwide [1]. Histologically, adenocarcinoma represents the most common type of non-small cell lung cancer (NSCLC). Locally advanced NSCLC patients represent about 30% of newly diagnosed lung cancer [2]. These patients typically receive a combination of surgery, chemotherapy, and radiation therapy [3]. Despite these treatment approaches, the survival rate of these patients is limited to ~25% at five years due to disease progression [4, 5]. The limitations of the current treatment approach necessitate novel prognosticators that allow for further stratification of different risk groups and more refined therapeutic strategies.

Quantitative imaging has been increasingly employed to assess treatment response to cancer therapy. Especially for lung cancers, CT imaging remains the modality of choice as it is noninvasive, renders anatomical details in high resolution and can quickly capture patient thoracic anatomy so that artifacts due to respiratory motion can be minimized. Planning CT images are routinely acquired in lung cancer patients prior to radiation therapy. Recently, numerous studies have shown imaging-based radiomic features can quantify tumor heterogeneity and hold potential for their application as clinical biomarker for patient stratification [6–23]. In particular, radiomic studies have shown CT-derived image features may be prognostic for distant metastasis (DM) and treatment responses in NSCLC [13, 14, 17, 24].

Previous studies have predominantly investigated the association between clinical outcomes and radiomic features within the primary tumor volume [24–27]. However, recent cancer research has shown evidences that extratumoral lung parenchymal tissues surrounding the primary tumor can become involved as cancer infiltrates and metastasizes. Pathological studies have demonstrated that lung tumor can spread through blood and lymphatic vasculature as well as airspaces in lung parenchyma [28–35] and that extratumoral cancerous presence may lead to worse clinical performance. In all aforementioned modes of cancer spreading, study results have consistently found significantly stronger association with distant or local recurrences for the extratumoral cancerous presence than their intratumoral counterparts [28, 31, 33]. Thus, we hypothesized that tumor metastatic progression may manifest itself in the imaged peritumoral tissue characteristics and the underlying relationship may be explored using radiomics based profiling of the normal-appearing tissue beyond the identified tumor region. Given the lack of biomarkers for DM in NSCLC, an understanding of the peritumoral tissue radiomics as an imaging biomarker may provide additional information to the existing approaches that only quantify characteristics within the visible tumor volume for identifying patients at higher risk and facilitating improved treatment design.

In this study, we present a radiomics investigation on the association of DM with peritumoral tissues in a cohort of 200 adenocarcinoma NSCLC patients. For clinical utilization, their prognostic performances were compared to the tumor-only radiomic features and clinical factors.

## Materials and methods

### Patient characteristics

This study was conducted under Dana-Farber/Harvard Cancer Center IRB protocol. As the study was retrospective and involved no more than minimal risks to the subjects, the need for patient consent was waived. Our study cohort included patients with pathologically-confirmed lung adenocarcinoma with locally advanced NSCLC (overall stage II-III). Patients treated with surgery or chemotherapy prior to the CT simulation date were excluded from our analysis. Patients receiving SBRT treatment were also excluded. For unbiased validation purposes, our 200 cohort was temporally split into two halves, the training Dataset A (n = 100) and the independent validation Dataset B (n = 100).

### Clinical endpoints

The clinical outcome evaluated for this study was DM. Follow-up CT scans were performed every three to six months after treatment for tumor progression assessment. DM was considered as the disease spread to sites outside of the lungs. Time to DM was defined as the time interval between the start date of radiation therapy and the first scan date of radiographically-evident DM and censored at the date of last negative scan in patients without recurrence. Time to OS was defined as the time between radiotherapy start date and date of death, and censored at the last follow-up date.

### CT image acquisition and segmentation

Planning CT images were acquired on GE LightSpeed RT16 CT scanners (GE Medical Systems, Milwaukee, WI, USA) following clinical imaging protocol which mostly uses 120 kVp and reconstructed using standard convolution kernel. The most common voxel spacing on the CT image was 0.93mm, 0.93mm, 2.5mm. Exceptions to this protocol were two cases scanned using 140 kVp and 6 cases reconstructed using 3.75mm or 5mm slice thickness. All patients received contrast injection unless there existed contraindication. The primary tumors were contoured using Eclipse software (Varian Medical Systems, Palo Alto, CA, USA) by an experienced CT imaging researcher. Tumors contours were reviewed by an expert radiation oncologist (R.H.M). To ensure that the imaged tumor regions are of good quality for our analysis, cases with motion artifact were excluded.

### Peritumoral contour preparation

Based on the proximity to the primary tumor, two peripheral regions of tissue were designed and termed here as tumor rim and tumor exterior. Tumor rim was defined to be the region that included the outer 3 mm of the tumor and 3 mm of tumor-adjacent lung tissue on either side of the tumor contour boundary; tumor exterior the region of lung tissues extending from 3mm to 9mm outside of the tumor contour (Fig 1.1). Rationale for tumor rim tissue inclusion was to designate a “real invasive front” and account both for the aggressive invading front of the tumor tissue and the adjacent normal lung layer, where cancerous islets can be frequently found [34]. The designation for tumor exterior followed from the spatial extent of microscopic tumor presence as found in pathology studies [28, 36, 37]. The generation of masks for delineating the tumor edge and the exterior tissue was accomplished through mathematical morphology operations of erosion and dilation, implemented using SimpleITK toolbox [38]. Since we were interested in the lung parenchymal tissue characteristics that may show association to cancer metastasis, care was taken to only include the tissue contours that are within the lung masks.

**Fig 1:**
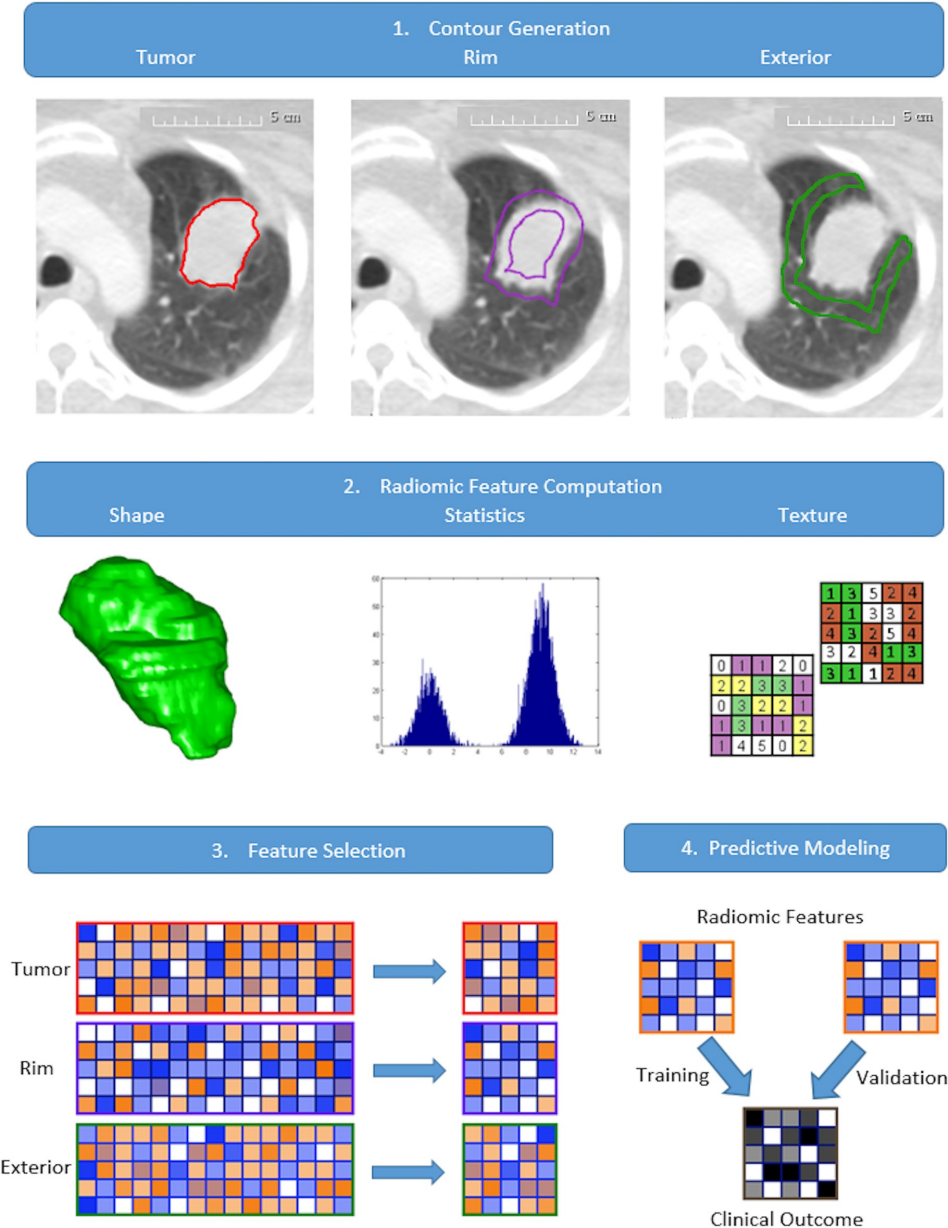
Schematic representation of the analysis workflow. 1. Generation of the rim and exterior contours based on segmented tumor contour. 2. Extraction of the radiomic features from each of the tissue contour region. 3. Dimension reduction of the radiomic features based on feature stability and relevance. 4. Prognostic power of the radiomic features is evaluated through predictive modeling and validation.

### Radiomic feature extraction and selection

A comprehensive radiomics computational toolbox, PyRadiomics, was employed for feature extraction [39] (Fig 1.2). Designed to facilitate data reproducibility, PyRadiomics platform provided open-source standardized algorithm for radiomic feature computation. Its implementation included built-in wavelet and Laplacian of Gaussian filters for image processing and computed a total of 2175 radiomic features, including first order statistics, shape, and texture classes. Texture classes included gray level co-occurrence matrix (GLCM), gray level run length matrix (GLRLM), gray level size zone matrix (GLSZM), neighboring gray tone difference matrix (NGTDM), gray level dependence matrix (GLDM), and gray level distance zone matrix (GLDZM). Feature computation was performed at resampled voxel dimensions of 3×3×3mm^3^ and an intensity bin width of 25 Hounsfield units. Radiomic feature extraction was performed for each of the three regions investigated in this study, i.e., tumor, tumor rim, and tumor exterior (data in S5–S10 Files). Feature selection followed a two-step procedure, feature stability and relevance, as shown in Fig 1.3. The selection of stable features was performed on Dataset A for each tissue region using the external test and retest RIDER dataset [40], subject to the intraclass correlation coefficient criterium (ICC > 0.85) [41] (description in S1 File). Subsequently, the set of stable features was processed using the minimal redundancy maximal relevance technique (mRMR) for dimension reduction, resulting in fifteen radiomic features for each region. Based on mutual information (MI), mRMR performed feature selection sequentially by determining the feature with maximum MI with the target variable and the minimum MI with the already selected features (Bioconductor “survcomp” package [42]).

The prognostic value of the peritumoral radiomic features was compared to the tumor ones as well as conventional and clinical parameters. The conventional variables considered for this study were the maximal 3D tumor diameter, and the volume of gross tumor volume (GTV). Clinical model included gender, age, overall stage, T-stage, N-stage, performance status, and tumor size.

### Univariable analysis

The prognostic value of the radiomic features was evaluated using concordance index (CI) from “survcomp” package [43]. Noether’s test was applied to assess the statistical significance of the computed CI from random chance (CI = 0.5) [42]. To account for multiple testing, a false-discovery-rate procedure by Benjamin and Hochberg was applied to adjust the p-values [44]. Univariable analysis was performed using the fifteen features selected using mRMR method for each of the tumor regions. All analyses were performed using R software (version 3.3.1) [45].

### Multivariable model construction and validation

Multivariable models were constructed using Cox regression method, where the model was trained using Dataset A and the model predictions were validated in Dataset B. The multivariable radiomics models were constructed with the mRMR selected features where the feature complementarity was explored for potential prediction enhancement. Based on the principle of parsimony, the fifteen features for each tumor region were included to the model incrementally according to their mRMR ranking and 1000 cross-validations were performed on Dataset A for each intermediate model in order to assess its predictive power. The cross validations were performed through random subset sampling with balanced event ratios of 70:30 using caret package [46]. For each tumor region, the optimal feature set was the combination that rendered the highest mean CI value before decreasing and was termed signature (Fig A in S2 File). To determine the improvement due to radiomic features, clinical factors were incorporated to the individual radiomics model to generate combined clinical-radiomics model. The statistical significance of the model performance between a pair of multivariable models was assessed using the cindex.comp function (“survcomp” package). To assess clinical efficacy, we evaluated the statistical significance of our best performing combined clinical and peritumoral radiomic signature to multivariable clinical model and combined clinical and tumor radiomics signature.

### Assessing patient stratification by peritumoral radiomics model

Finally, to demonstrate the clinical efficacy of our proposed peritumoral radiomics model, a Kaplan-Meier analysis was performed. We investigated the performance of three models, the clinical model, the combined clinical and tumor radiomics signature, and the combined clinical and rim radiomics signature, where the cox regression technique was used for combining separate models. For each model, the median prediction value from training Dataset A was used for stratifying the patients in Dataset B. A log rank test was performed to determine the statistical significance of risk to developing DM between the two groups.

## Results

### Clinical characteristics

A total of 200 NSCLC patients with adenocarcinoma were analyzed in this study. At the time of diagnosis, the median age was 64 years (range: 35–93 years). The median follow-up was 28.1 months (range: 1.8–142.1 months). The median time to DM was 13.5 months (range: 0.3–119.1 months), with 128 (64%) patients having developed DM versus 72 (36%) who did not. Patient characteristics and cancer progression information can be found in Table 1.

**Table 1:** Patient characteristics, treatment information, and outcomes.

	Total	Dataset A	Dataset B	p-value
Age	64 (35–93)	62 (40–85)	64 (35–93)	0.51
Gender (F/M)	127/73 (63.5/36.5)	66/34 (66/34)	61/39 (61/39)	0.56
Overall stage (IIA/IIB/IIIA/IIIB)	5/6/110/79 (2.5/3.0/55.0/39.5)	2/4/55/39 (2.0/4.0/55/39)	3/2/55/40 (3.0/2.0/55/40)	0.83
T stage (T1a, T1b, T2a, T2b, T3, T4, TX)	21/27/58/19/40/34/1 (10.5/13.5/29/9.5/20/17.0/0.5)	15/10/30/10/19/16/0 (15/10/30/10/19/16/0)	6/17/28/9/21/18/1 (6/17/28/9/21/18/1)	0.32
N stage (N0, N1, N2, N3)	14/16/108/62 (7/8/54/31)	5/10/54/31 (5/10/54/31)	9/6/54/31 (9/6/54/31)	0.54
Performance status (0/1/2/3)	95/95/8/2 (47.5/47.5/4.0/1.0)	39/56/5/0 (39/56/5/0)	56/39/3/2 (56/39/3/2)	0.04
Treatment modality (cCRT/Trimodality/other)	113/46/41 (56.5/ 23.0/20.5)	57/ 40/ 3 (57 40 3)	56 38 6 (56 38 6)	0.59
Follow-up [months]	27.7 (1.8–142.1)	28.6 (1.8–142.1)	25.2 (2.2–67.1)	0.07
Survival [months]	28.1 (1.8–142.1)	29.9 (1.8–142.1)	25.5 (2.4–67.1)	0.053
Time to DM [months]	13.5 (0.3–119.1)	13.8 (0.3–119.1)	12.8 (0.7–67.1)	0.51
DM [Yes/No]	128/72 (64/36)	65/35 (65/35)	63/37 (63/37)	0.88

Median (range) is reported for continuous and counts (percentage) for categorical variables. Statistical difference between Dataset A and Dataset B is determined using Wilcoxon- and Chi-Square tests for continuous and categorical variables, respectively.

### Radiomics analysis of tumor regions

**Fig 2:**
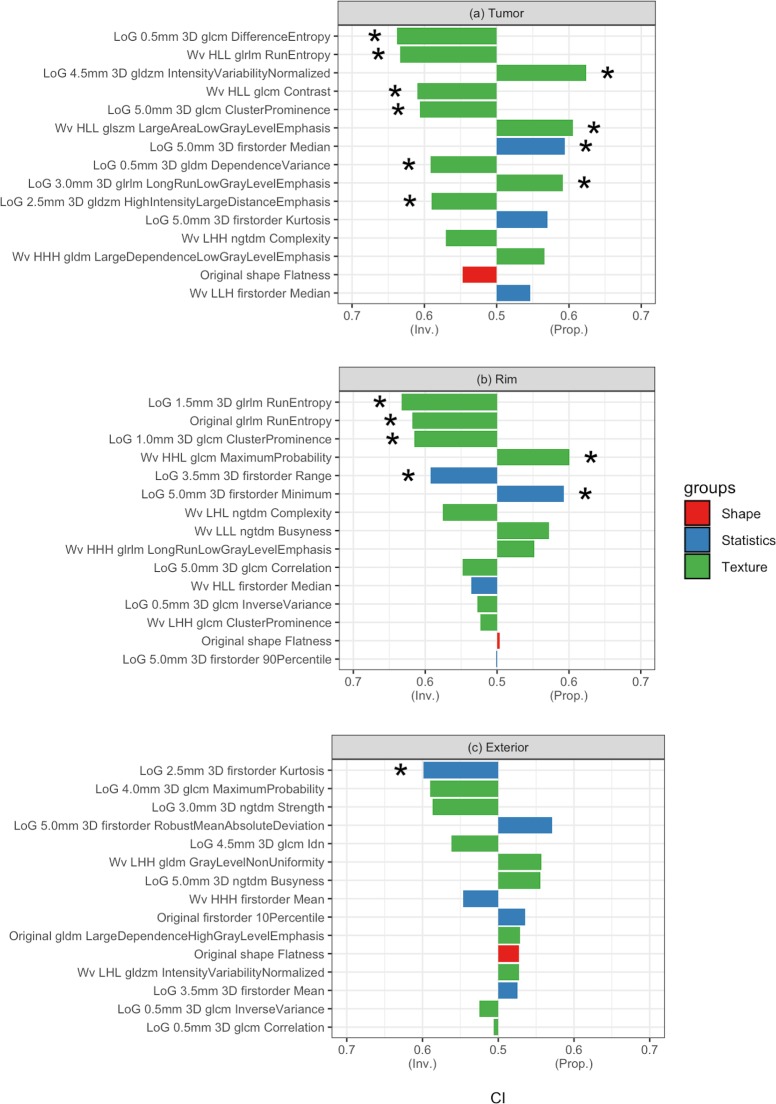
Univariable analysis of the selected radiomic features by region. a. tumor; b. tumor rim; c. tumor exterior. “Inv.” And “Prop” indicate inversely proportional and directly proportional, respectively. CI of 0.5 indicates equivalent to random guess. The types of radiomic features are shown by color: red (shape), blue (statistics), and green (texture). * indicates p-value < 0.05 (Noether’s test, p-value corrected by FDR).

Fig 2 displays the results of univariable analyses performed on Dataset A. In each tumor region, the full set of radiomic features was reduced to fifteen features based on relevance to DM events. In the tumor region (Fig 2A), ten features were significantly predictive of DM, had a CI range of 0.59–0.64, p-value < 0.05, and were all texture-based except for the 3D firstorder median, a statistics-type feature (Table B in S3 File). The top tumor feature was GLCM Difference Entropy, which measures the variability in neighborhood intensity differences. In the rim region (Fig 2B), six significant features were found with CI range: 0.59–0.63 (Table D in S3 File).

Four of these features were texture-based and two were statistics-based (first-order range and first-order minimum). The best performing feature in this region was GLRLM RunEntropy, providing a measure for the randomness in the distribution of run lengths and gray levels, with higher value indicating more heterogeneity in the texture pattern. In the exterior region, the only significantly predictive feature was first-order Kurtosis, which is a descriptor of the intensity distribution, with higher kurtosis value indicating the distribution is concentrated more towards the tails (Table F in S3 File). More detailed description of the selected features is provided (Tables A, C, E in S3 File). For comparison, the conventional features of tumor volume and maximum 2D axial and 3D diameters had CI values of 0.54, 0.55, and 0.54, respectively and were not statistically significant. In addition, the maximum 3D diameter and the contour volume of the rim and exterior regions were also not predictive. The feature expression trends between DM and non-DM cases were shown in Figs A-C in S4 File.

### Radiomic signature validation and inter-comparison

Multivariable models were generated based on cox proportional hazard method. The forward selected radiomic signature from each tumor region was validated using Dataset B. The tumor radiomic signature was log sigma 0.5mm 3D GLCM DifferenceEntropy, which quantifies the intensity variability in neighboring voxels, and wavelet HLL GLRLM RunEntropy, which measures the variation in the distribution of run lengths and gray levels. The tumor rim radiomic signature consisted of LoG 1.5mm 3D GLRLM RunEntropy and Wavelet LHL NGTDM complexity; these measure, respectively, the entropy of gray level runs and large intensity changes in neighboring pixels. The radiomic signature of the exterior region includes log sigma 2.5mm 3D firstorder Kurtosis and log sigma 3.0.mm 3D NGTDM Strength, which, respectively, measures the fourth moment of the intensity distribution and deviation from homogeneity. To account for the potential confounding effect of tumor size and volume, statistical significance was tested between our radiomic signatures and these factors. The prognostic performance of the tumor and rim radiomic signatures were determined to be significantly stronger than tumor dimension or volume.

**Fig 3:**
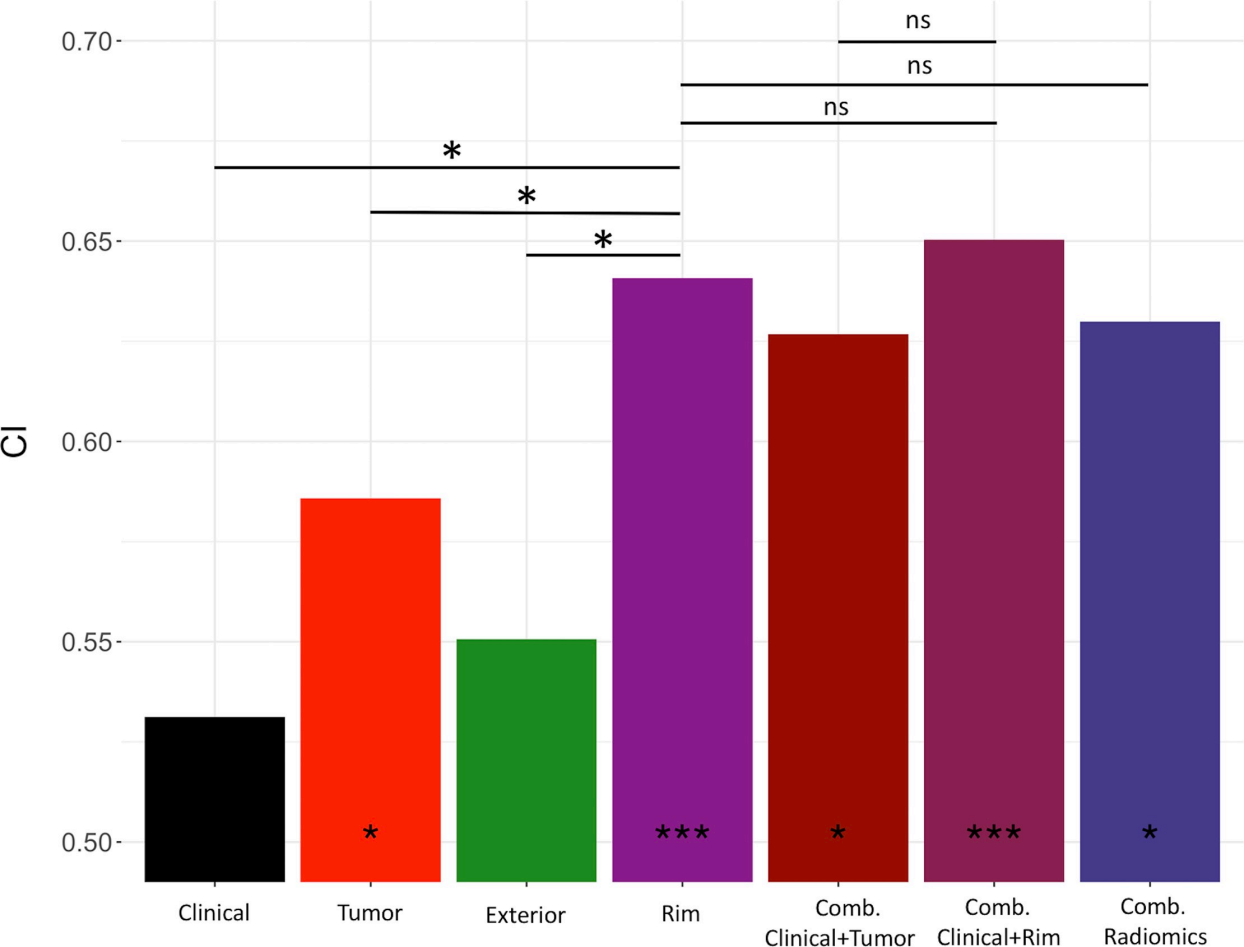
Comparison of prognostic performance across the different multivariable models in the validation cohort (n = 100). * indicates statistical significance (“*” indicates p-value <0.05, “***” indicates p-value <0.0001 from random prediction (Noether test)). From left to right, the compared multivariable models include clinical, visible tumor, exterior, tumor rim, combined clinical and tumor, combined clinical and rim, and the combined radiomics model. Crossbars indicate the comparison made between CI of two multivariable models, where * indicates significant difference and *ns* not statistically significant.

The performance of the radiomic signatures was compared to a clinical model constructed using Cox regression method. In Dataset B, this clinical model achieved a CI value of 0.53 (p-value < 0.44). In the radiomics models, the multivariable rim signature achieved a CI value of 0.64 (p-value < 2.37×10^−5^) in Dataset B, compared to the multivariable tumor signature CI value of 0.59 (p-value < 0.04) and the multivariable exterior model of CI value of 0.55 (p-value < 0.15). Incorporating the rim multivariable model to the clinical parameters yielded a CI value of 0.65 (p-value < 7.57×10^−6^). For comparison, this combined model was found to be significantly more predictive than the clinical model (p-value < 0.003). A composite radiomics model including the tumor, rim and exterior regions was also constructed (CI = 0.63), but was found to be less predictive than the tumor rim signature (p-value < 0.30). The prediction by combined clinical and tumor radiomics signature was found to be not statistically different from the combined clinical and rim predictor (p-value < 0.13), neither was the composite clinical, tumor and rim predictor (p-value < 0.38). The results of multivariable model validation and inter-model comparison were displayed in Fig 3.

**Fig 4:**
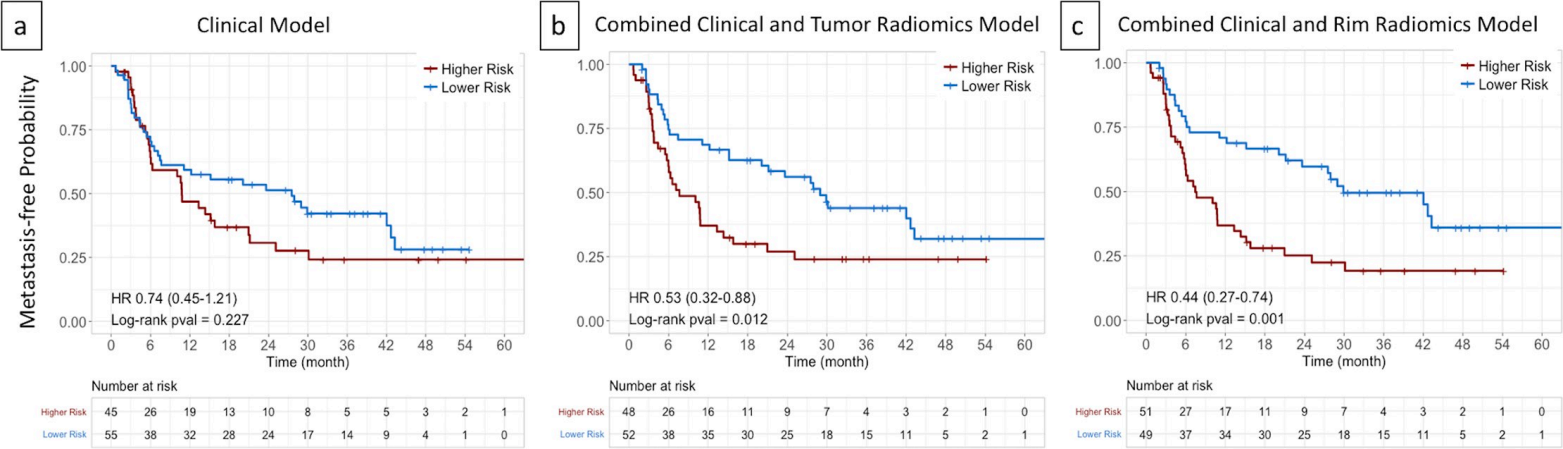
**Comparison of Kaplan-Meier curves for DM**: a) clinical model, b) the combined clinical and tumor radiomics model, and c) the combined clinical and tumor rim radiomics model for metastasis-free probability in validation dataset (n = 100). In a, the stratified patient groups did not show statistical significance (p-value < 0.227). In b, the incorporation of the tumor radiomics signature improved patient stratification (p-value < 0.012). In c, combining clinical and rim radiomics signature was shown to most significantly stratify patients (p-value < 0.001).

Fig 4 showed the patient stratification using the clinical parameters (Fig 4A). Neither the log rank test showed a significant p-value nor did the hazard ratio show significance in the validation cohort. Fig 4B showed risk stratification using the combined clinical and tumor radiomics model, which had a significant log rank p-value of 0.012. Fig 4C demonstrated the potential stratification that can be achieved using our proposed model combining clinical and rim radiomic signature. Using the patient risk scores derived from the training cohort, Kaplan-Meier analysis in validation Dataset B showed statistically significant difference (p-value < 1×10^−3^) for metastasis-free probability estimates. The lower risk group showed a hazard ratio of 0.44 compared to the higher risk group.

## Discussion

Advances in cancer biology research have provided insights on how the tumor proliferates through its interaction with the surrounding normal tissue [47–50]. Tumor invasion into the peripheral normal tissues on the cellular level may translate to tissue morphological changes, which, in turn, may inform us about the level of metastatic activity. Here, we leveraged radiomics analysis to quantitatively characterize the peritumoral imaging features on planning CT images and assessed their prognostic power on DM. To our knowledge, this is the first study that correlates radiomic features from the normal-appearing peritumoral tissues with cancer metastasis in NSCLC.

The use of radiomic features to predict DM for NSCLC has only been investigated in few studies [24, 25]. Fried et al [25] extracted 198 tumor features from averaged CT, 4DCT, and contrast-enhanced CT images; and showed qualitatively through Kaplan-Meier analysis that the combination of radiomic features with clinical parameters may enable patient stratification for DM in a 91-patient stage III cohort. Coroller et al [24] investigated intratumoral radiomic features for DM in a cohort of 182 NSCLC and demonstrated predictive power in a validation cohort (CI = 0.61). However, these were based on tumor-only radiomic features and did not account for the cancerous infiltration into the surrounding normal parenchymal tissues. This study explored the tumor rim and exterior radiomic features as potential DM prognosticator in a larger cohort of 200 patients with locally advanced NSCLC using a quantitative metric of CI. Importantly, we discovered a higher prognostic value for the tumor rim radiomic signature than the tumor-only one, where a comparison between the two showed statistical significance (p-value = 0.048).

Our data driven approach led us to the discovery of potentially important tumor rim radiomics signature for DM prediction and also the finding that the other tumor regions show relatively less prognostic power. In the exterior region, that the multivariable radiomics model was not significant was consistent with the overall weaker signal as evident in the univariable analysis. As for the visible tumor region, its significant features were similar to the rim region in terms of feature type and prognostic power. However, while the selected features from the tumor showed similar performance as the rim features univariablely, the rim radiomic signature showed the superior performance over the tumor one. This may be due to the complementary effect accomplished by combining top performing feature, LoG 1.5mm 3D GLRLM RunEntropy, and the moderately performing feature, Wavelet LHL NGTDM complexity. Interestingly, as larger value from either rim feature suggests reduced risk to developing DM, i.e. tumor rims with more heterogeneity in run lengths and gray levels as well as more rapid changes in gray level intensity would be less prone to tumor metastatic activities. Moreover, despite the fact that the CI of the combined clinical and rim radiomics model prediction was not shown to be statistically different from that of the combined clinical and tumor radiomics one, Kaplan-Meier analysis suggested that the former would allow for better risk stratification in terms of log rank p-value (Fig 4B vs. 4C). Furthermore, our identification of the tumor rim as the crucial imaging biomarker for DM was consistent with pathology and tumor biology findings and it was well known that the periphery of the tumor harbored many activities of cancer invasion and metastasis, e.g. epithelial-mesenchymal transition [51], tumor-associated macrophages [50, 52], tumor budding [53], and lymphovascular invasion [31, 54]. While a correlation with biological processes at the tumor periphery was beyond the scope of the present study, ample evidence from tumor biology literature supported our hypothesis that the tissue features on the tumor-normal interface may indicate tumor aggressiveness towards DM. Thus, our findings were hypothesis generating and may facilitate new discoveries in the DM prognostication using information originated from tumor rim.

Limitations of this study include the choice of 6mm shell of tissues around the tumor for our correlation analysis with DM. Given that there may exist individual variation in terms of the disease spread pattern and location of extratumoral cancer colonies, we attempted to address this by taking a relatively wide margin of 6mm and expand our region of interest radially outward from the tumor to mimic the disease spread pattern. Other limiting factor may be the variation in CT acquisition parameters as patient CT simulation dates spanned from 2001 to 2014. We sought to mitigate this by removing cases of motion artifacts and performed image resampling at 3×3×3 mm^3^ to reduce voxel noise. Lastly, our findings may be limited by our cohort size (n = 200) and patient cases collected in our institution. We had performed temporal split of our data to generate an independent validation cohort, of similar patient and treatment characteristics, for model testing. Future investigations would involve testing our hypothesis by expanding our study to include patients of other histology types and evaluating the generalizability of our findings using multi-institutional image data. In spite of these limitations, our investigation demonstrated differential predictiveness of imaging features between the tumor and its surrounding tissues for distant metastatic spread.

## Conclusion

In conclusion, we have demonstrated strong prognostic value of peritumoral radiomic features for DM in patients with locally advanced NSCLC. The presented rim radiomic signature was independently validated and was shown to have better predictive power compared to tumor radiomic signature. Such pretreatment imaging predictor may benefit patients susceptible to developing DM in precision medicine approach.

## Supporting information

S1 File **Fig A. Tumor regions generated for RIDER test/retest data**. Subfigures a., c., and e. represent the contours of the tumor, tumor rim, and the tumor exterior, respectively, from the test dataset. Subfigures, b., d., and f. represent the counterparts in in the re-test dataset.
(DOCX)

S2 File **Fig A. The results of cross-validation experiments for multivariable model construction**. The procedure was performed for each tumor region: a. tumor; b. tumor rim; c. tumor exterior.
(DOCX)

S3 File **Table A: Description of the selected radiomic features in the tumor region. Table B: Summary of the predictive power of the fifteen selected features for the tumor region**. Feature type, CI, and p-values (Noether test, multiple hypothesis testing correction) are reported. **Table C: Description of the selected radiomic features in the tumor rim region. Table D: Summary of the predictive power of the fifteen selected features for the tumor rim region**. Feature Type, CI, and p-values (Noether test, multiple hypothesis testing correction) are reported. **Table E: Description of the selected radiomic features in the tumor exterior region. Table F: Summary of the predictive power of the fifteen selected features for the tumor exterior region**. Feature Type, CI, and p-values (Noether test, multiple hypothesis testing correction) are reported.
(DOCX)

S4 File **Table A. Tumor radiomic feature expression statistics between DM and non-DM sub-cohort. Figure A. Feature trend between DM and non-DM in the tumor region**. Empirical Cumulative Distribution Function (eCDF) plotted against feature values for the top significant univariate tumor radiomic features between DM (red) and non-DM (blue) sub-cohorts. **Table B. Tumor rim radiomic feature expression statistics between DM and non-DM sub-cohort. Figure B. Feature trend between DM and non-DM in the tumor rim region**. Empirical Cumulative Distribution Function (eCDF) plotted against feature values for the top significant univariate tumor rim radiomic features between DM (red) and non-DM (blue) sub-cohorts. **Table C. Tumor exterior region radiomic feature expression statistics between DM and non-DM sub-cohort. Figure C. Feature trend between DM and non-DM in the tumor exterior region**. Empirical Cumulative Distribution Function (eCDF) plotted against feature values for the top significant univariate tumor exterior radiomic feature between DM (red) and non-DM (blue) sub-cohorts.
(DOCX)

S5 File Complete data of the tumor region of the training cohort. (PDF)

S6 File Complete data of the tumor region of the validation cohort. (PDF)

S7 File Complete data of the tumor rim region of the training cohort. (PDF)

S8 File Complete data of the tumor rim region of the validation cohort. (PDF)

S9 File Complete data of the tumor exterior region of the training cohort. (PDF)

S10 File Complete data of the tumor exterior region of the validation cohort. (PDF)
