# Supplementary material for: Peritumoral radiomics features predict distant metastasis in locally advanced NSCLC
Source: PLoS One. 2018 Nov 2;13(11):e0206108. doi: 10.1371/journal.pone.0206108 (PMC6214508; doi:10.1371/journal.pone.0206108)
Supplement: S1 File — Fig A. Tumor regions generated for RIDER test/retest data. Subfigures a., c., and e. represent the contours of the tumor, tumor rim, and the tumor exterior, respectively, from the test dataset. Subfigures, b., d., and f. represent the counterparts in in the re-test dataset. (DOCX) [file pone.0206108.s001.docx]

**S1: Selection of Stable Features**

| **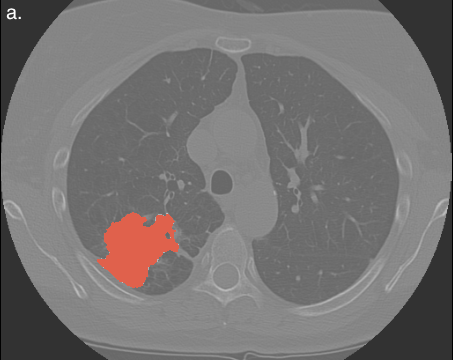** | **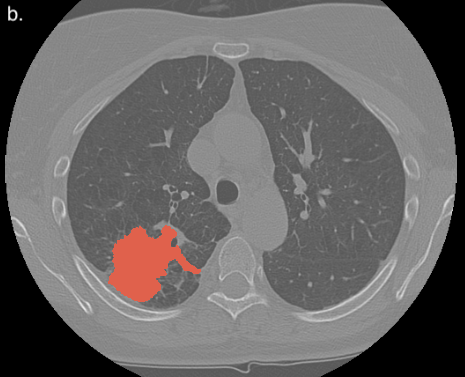** |
| --- | --- |
| **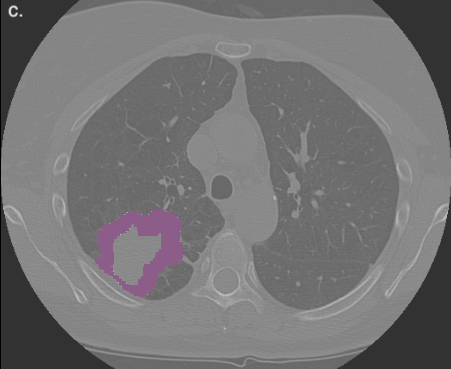** | **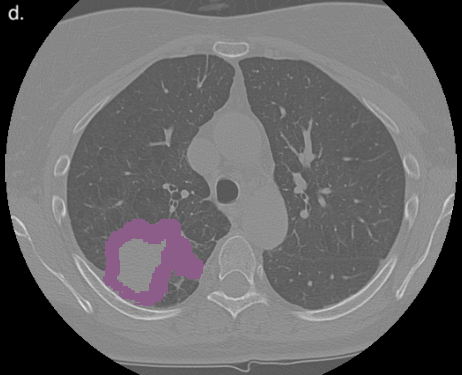** |
| **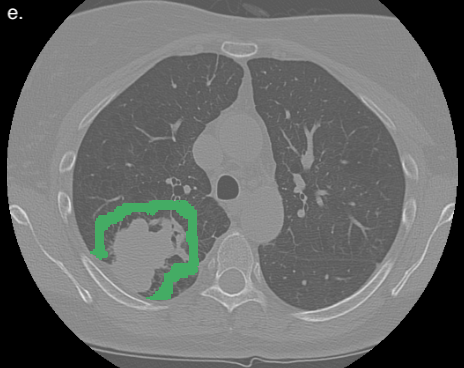** | **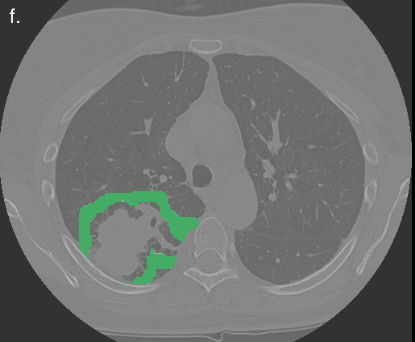** |

S1 Figure A. Tumor regions generated for RIDER test/retest data. Subfigures a., c., and e. represent the contours of the tumor, tumor rim, and the tumor exterior, respectively, from the test dataset. Subfigures, b., d., and f. represent the counterparts in in the re-test dataset.

The reproducibility of radiomic features in each of our tumor regions was determined before feature selection using the RIDER test/retest image dataset [1]. The procedure for selection of stable features of the tumors was previously described in previous publications of our group [2, 3]. Here we applied the same technique to each of our tumor regions. Peritumoral contours of the rim and exterior regions were generated based on the primary tumors contoured on both scans for twenty-three patients using the same image processing technique as done for the main dataset. A total of 2175 features were extracted and intraclass correlation coefficient (ICC) [4] was computed using R “irr” package [5]. The ICC measures consistency between the features obtained from the two datasets, with 0 representing non-reproducibility and 1 perfect reproducibility. A feature was considered as robust to variation due to image acquisition if ICC > 0.85 [6], which was set as threshold for this study, while non-robust features were excluded from the analysis workflow because of their sensitivity to image acquisition and hence for our predictive outcome analysis. Following the stability criteria, the number of stable features determined for a. the tumor region: 1113 stable features; b. tumor rim region: 721 stable features; c. tumor exterior region: 815 stable features.

**References**

1. Zhao, B., et al., *Evaluating variability in tumor measurements from same-day repeat CT scans of patients with non-small cell lung cancer.* Radiology, 2009. **252**(1): p. 263-72.

2. Aerts, H.J., et al., *Decoding tumour phenotype by noninvasive imaging using a quantitative radiomics approach.* Nat Commun, 2014. **5**: p. 4006.

3. Coroller, T.P., et al., *Radiomic phenotype features predict pathological response in non-small cell lung cancer.* Radiother Oncol, 2016. **119**(3): p. 480-6.

4. Barkto, J., *The Intraclass Correlation Coefficient as a Measure of Reliability.* Pschol Rep, 1966(19): p. 3-11.

5. Gamer M, L.J., Fellows I, Puspendra S, *Irr: Various Coefficients of Interrater Reliability and Agreement.* 2012.

6. Parmar, C., et al., *Robust Radiomics feature quantification using semiautomatic volumetric segmentation.* PLoS One, 2014. **9**(7): p. e102107.
