# Supplementary material for: Peritumoral radiomics features predict distant metastasis in locally advanced NSCLC
Source: PLoS One. 2018 Nov 2;13(11):e0206108. doi: 10.1371/journal.pone.0206108 (PMC6214508; doi:10.1371/journal.pone.0206108)
Supplement: S2 File — Fig A. The results of cross-validation experiments for multivariable model construction. The procedure was performed for each tumor region: a. tumor; b. tumor rim; c. tumor exterior. (DOCX) [file pone.0206108.s002.docx]

**S2. Cross-Validation Experiments for Multivariable Model Construction**

For the construction of multivariable model, we applied the same technique to construct for each of our defined tumor regions using Dataset A. Using the 15 ranked features generated by mRMR feature selection algorithm, forward selection method was applied to select the optimal feature combination. In forward selection process, the first combination consists of best-ranked mRMR feature, the second the top two ranked mRMR features, and so on. For each such combination, 1000 cross-validation experiments were performed. Based on the performance in terms of mean CI value across the 15 combinations, our radiomics signature is determined as the feature combination with the fewest features and highest mean CI values. In Fig. S2, cross-validation experiments revealed that final tumor radiomic signature consists of 2 features, final tumor rim signature consists of 2 features, and final tumor exterior signature consists of 2 features.

| 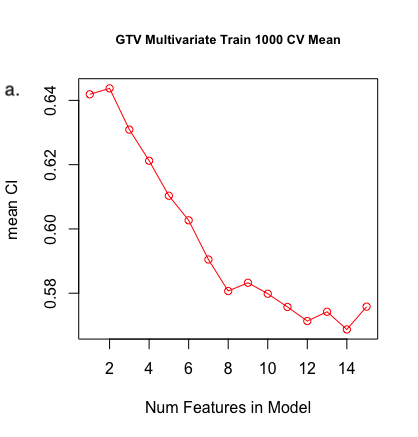 | 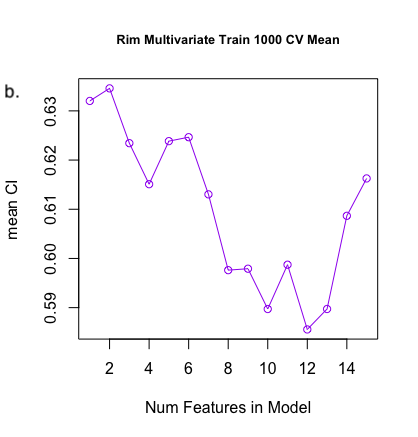 | 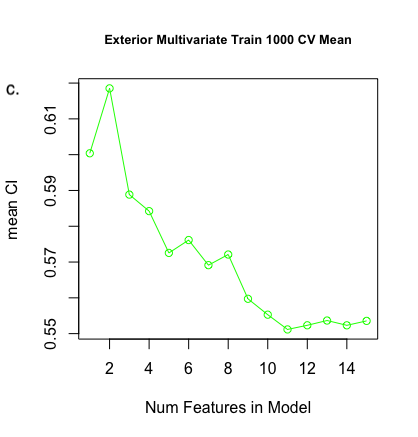 |
| --- | --- | --- |

S2 Figure A. The results of cross-validation experiments for multivariable model construction. The procedure was performed for each tumor region: a. tumor; b. tumor rim; c. tumor exterior.
