# Supplementary material for: Peritumoral radiomics features predict distant metastasis in locally advanced NSCLC
Source: PLoS One. 2018 Nov 2;13(11):e0206108. doi: 10.1371/journal.pone.0206108 (PMC6214508; doi:10.1371/journal.pone.0206108)
Supplement: S3 File — Table A: Description of the selected radiomic features in the tumor region. Table B: Summary of the predictive power of the fifteen selected features for the tumor region. Feature type, CI, and p-values (Noether test, multiple hypothesis testing correction) are reported. Table C: Description of the selected radiomic features in the tumor rim region. Table D: Summary of the predictive power of the fifteen selected features for the tumor rim region. Feature Type, CI, and p-values (Noether test, multiple hypothesis testing correction) are reported. Table E: Description of the selected radiomic features in the tumor exterior region. Table F: Summary of the predictive power of the fifteen selected features for the tumor exterior region. Feature Type, CI, and p-values (Noether test, multiple hypothesis testing correction) are reported. (DOCX) [file pone.0206108.s003.docx]

**S3: Univariate Analysis Results**

The top radiomic features for the tumor, tumor rim, and tumor exterior regions are described in Table A, C, and E, respectively. The results of univariable analysis are tabulated in Table B, D, and F, respectively.

**Table A: Description of the selected radiomic features in the tumor region.**

| Radiomic Feature | Feature Class | Associated Filter | Description |
| --- | --- | --- | --- |
| Median | Statistics | Wavelet LLH | Median voxel intensity value |
| Flatness | Shape | None | How flat the tumor is shaped |
| Large Dependence Low Gray Level Emphasis | GLDM | Wavelet HHH | Measures the joint distribution of large dependence with lower gray-level values |
| Complexity | NGTDM | Wavelet LHH | Higher complexity indicates heterogeneity and many rapid changes in gray level intensity. |
| Kurtosis | Statistics | 3D LoG 5mm | A measure of the “tailedness” of intensity distribution. |
| High Intensity Large Distance Emphasis | GLDZM | 3D LoG 2.5mm | Sensitive to large distance zones of higher gray-level values. |
| Long Run Low Gray Level Emphasis | GLRLM | 3D LoG 3mm | Sensitive to large consecutive voxels of low intensity levels. |
| Dependence Variance | GLDM | 3D LoG 0.5mm | Sensitive to the variance in the sizes of voxel groups of similar intensity |
| Median | Statistics | 3D LoG 5mm | Median voxel intensity value |
| Large Area Low Gray Level Emphasis | GLSZM | Wavelet HLL | Sensitive to larger size intensity zones of lower gray-level values. |
| ClusterProminence | GLCM | 3D LoG 5mm | Sensitive to flat zones (areas of connecting voxels with the same value). |
| Contrast | GLCM | Wavelet HLL | the spatial intensity change. |
| Intensity Variability Normalized | GLDZM | 3D LoG 4.5mm | Sensitive to variability of gray-level intensity values, where a lower value correlates with more homogeneity in intensity values. |
| Run Entropy | GLRLM | Wavelet HLL | A higher value indicates more heterogeneity in the texture patterns. |
| Difference Entropy | GLCM | 3D LoG 0.5mm | Sensitive to the variability in neighborhood intensity value differences. |

L, Low; H, High; LoG, Laplacian of Gaussian.

**Table B: Summary of the predictive power of the fifteen selected features for the tumor region. Feature Type, CI, and p-values (Noether test, multiple hypothesis testing correction) are reported.**

| Features | Feature Type | C-index | p-value |
| --- | --- | --- | --- |
| Wv.LLH_firstorder_Median | Statistics | 0.55 | 0.20 |
| original_shape_Flatness | Shape | 0.55 | 0.21 |
| Wv.HHH_gldm_LargeDependenceLowGrayLevelEmphasis | Texture | 0.57 | 0.11 |
| Wv.LHH_ngtdm_Complexity | Texture | 0.57 | 0.11 |
| LoG.5.0.mm.3D_firstorder_Kurtosis | Statistics | 0.57 | 0.10 |
| LoG.2.5.mm.3D_gldzm_HighIntensityLargeDistanceEmphasis | Texture | 0.59 | 0.02 |
| LoG.3.0.mm.3D_glrlm_LongRunLowGrayLevelEmphasis | Texture | 0.59 | 0.01 |
| LoG.0.5.mm.3D_gldm_DependenceVariance | Texture | 0.59 | 0.02 |
| LoG.5.0.mm.3D_firstorder_Median | Statistics | 0.59 | 0.02 |
| Wv.HLL_glszm_LargeAreaLowGrayLevelEmphasis | Texture | 0.61 | 0.02 |
| LoG.5.0.mm.3D_glcm_ClusterProminence | Texture | 0.61 | 0.003 |
| Wv.HLL_glcm_Contrast | Texture | 0.61 | 0.012 |
| LoG.4.5.mm.3D_gldzm_IntensityVariabilityNormalized | Texture | 0.62 | 0.00096 |
| Wv.HLL_glrlm_RunEntropy | Texture | 0.63 | 0.00096 |
| LoG.0.5.mm.3D_glcm_DifferenceEntropy | Texture | 0.64 | 0.00096 |

**Table C: Description of the selected radiomic features in the tumor rim region.**

| Radiomic Features | Feature Type | Associated Filter | Description |
| --- | --- | --- | --- |
| 90Percentile | Statistics | 3D LoG.5.0.mm. | The 90^th^ percentile of voxel intensity |
| Flatness | Shape | None | How flat the tumor region is shaped |
| ClusterProminence | GLCM | Wv.LHH | Sensitive to flat zones (area of similar intensity) |
| InverseVariance | GLCM | 3D LoG.0.5.mm. | measures the local homogeneity of an image through normalizing the difference between the neighboring intensity values by dividing over the total number of discrete intensity values. |
| Median | Statistics | Wv.HLL | Median voxel intensity value |
| Correlation | GLCM | 3D LoG.5.0.mm. | Measures gray level linear dependence between the pixels at the specified positions relative to each other |
| LongRunLowGrayLevelEmphasis | GLRLM | Wv.HHH | Sensitive to larger consecutive voxels of low gray levels. |
| Busyness | NGTDM | Wv.LLL | Higher “busyness” indicates more rapid changes of intensity between pixels and its neighbourhood. |
| Complexity | NGTDM | Wv.LHL_ | Higher complexity indicates more heterogeneity and many rapid changes in gray level intensity. |
| Minimum | Statistics | 3D LoG.5.0.mm. | The minimum gray value in the region of interest. |
| Range | Statistics | 3D LoG.3.5.mm. | The range of gray values in the region of interest |
| MaximumProbability | GLCM | Wv.HHL | Measures the occurrences of the most predominant pair of neighboring intensity values. |
| ClusterProminence | GLCM | 3D LoG.1.0.mm. | Sensitive to flat zones (area of similar intensity) |
| RunEntropy | GLRLM | None | A higher value indicates more heterogeneity in the texture patterns. |
| RunEntropy | GLRLM | 3D LoG.1.5.mm. | A higher value indicates more heterogeneity in the texture patterns. |

**Table D: Summary of the predictive power of the fifteen selected features for the tumor rim region.** Feature Type, CI, and p-values (Noether test, multiple hypothesis testing correction) are reported.

| Features | Feature Type | C-index | p-value |
| --- | --- | --- | --- |
| LoG.5.0.mm.3D_firstorder_90Percentile | Statistics | 0.5 | 0.98 |
| original_shape_Flatness | Shape | 0.5 | 0.98 |
| Wv.LHH_glcm_ClusterProminence | Texture | 0.52 | 0.62 |
| LoG.0.5.mm.3D_glcm_InverseVariance | Texture | 0.53 | 0.62 |
| Wv.HLL_firstorder_Median | Statistics | 0.54 | 0.43 |
| LoG.5.0.mm.3D_glcm_Correlation | Texture | 0.55 | 0.31 |
| Wv.HHH_glrlm_LongRunLowGrayLevelEmphasis | Texture | 0.55 | 0.28 |
| Wv.LLL_ngtdm_Busyness | Texture | 0.57 | 0.13 |
| Wv.LHL_ngtdm_Complexity | Texture | 0.58 | 0.1 |
| LoG.5.0.mm.3D_firstorder_Minimum | Statistics | 0.59 | 0.02 |
| LoG.3.5.mm.3D_firstorder_Range | Statistics | 0.59 | 0.03 |
| Wv.HHL_glcm_MaximumProbability | Texture | 0.6 | 0.02 |
| LoG.1.0.mm.3D_glcm_ClusterProminence | Texture | 0.62 | 0.0065 |
| original_glrlm_RunEntropy | Texture | 0.62 | 0.0081 |
| LoG.1.5.mm.3D_glrlm_RunEntropy | Texture | 0.63 | 0.0018 |

**Table E: Description of the selected radiomic features in the tumor exterior region.**

| Radiomic Features | Feature Type | Associated Filter | Feature Description |
| --- | --- | --- | --- |
| Correlation | GLCM | 3D LoG.0.5.mm. | Measures gray level linear dependence between the pixels at the specified positions relative to each other |
| Inverse Variance | GLCM | 3D LoG.0.5.mm. | measures the local homogeneity of an image through normalizing the difference between the neighboring intensity values by dividing over the total number of discrete intensity values. |
| Mean | Statistics | 3D LoG.3.5.mm. | Mean voxel intensity value |
| Intensity Variability Normalized | GLDZM | Wv.LHL_ | Sensitive to variability of gray-level intensity values, where a lower value correlates with more homogeneity in intensity values. |
| Flatness | Shape | None | Describes if the tumor region resembles a disk. |
| Large Dependence High Gray Level Emphasis | GLDM | None | Measures the joint distribution of large dependence with higher gray-level values. |
| 10Percentile | Statistics | None | The 10^th^ percentile of voxel intensity |
| Mean | Statistics | Wv.HHH | Mean voxel intensity value |
| Busyness | NGTDM | 3D LoG.5.0.mm. | Higher “busyness” indicates more rapid changes of intensity between pixels and its neighbourhood. |
| Gray Level NonUniformity | GLDM | Wv.LHH | Sensitive to the variability of gray-level intensity values in the image, with a lower value indicating more homogeneity in intensity values. |
| Idn | Texture | 3D LoG.4.5.mm. | measures local homogeneity of an image. IDN normalizes the difference between the neighboring intensity values by dividing over the total number of discrete intensity values. |
| Robust Mean Absolute Deviation | Statistics | 3D LoG.5.0.mm. | Computes the mean distance of all intensity values from the Mean Value calculated on the subset of image array with gray levels in between, or equal to the 10^th^ and 90^th^ percentile. |
| Strength | NGTDM | 3D LoG.3.0.mm. | Its value is high when the primitives are easily defined and visible, i.e. an image with slow change in intensity but more large coarse differences in gray level intensities. |
| Maximum Probability | Texture | .3D LoG.4.0.mm | Measures the occurrences of the most predominant pair of neighboring intensity values. |
| Kurtosis | Statistics | LoG.2.5.mm.3D | A measure of the “tailedness” of intensity distribution. |

**Table F: Summary of the predictive power of the fifteen selected features for the tumor exterior region.** Feature Type, CI, and p-values (Noether test, multiple hypothesis testing correction) are reported.

| Features | Feature Type | C-index | p-value |
| --- | --- | --- | --- |
| LoG.0.5.mm.3D_glcm_Correlation | Texture | 0.51 | 0.88 |
| LoG.0.5.mm.3D_glcm_InverseVariance | Texture | 0.53 | 0.55 |
| LoG.3.5.mm.3D_firstorder_Mean | Statistics | 0.53 | 0.55 |
| Wv.LHL_gldzm_IntensityVariabilityNormalized | Texture | 0.53 | 0.55 |
| original_shape_Flatness | Shape | 0.53 | 0.55 |
| original_gldm_LargeDependenceHighGrayLevelEmphasis | Texture | 0.53 | 0.55 |
| original_firstorder_10Percentile | Statistics | 0.54 | 0.54 |
| Wv.HHH_firstorder_Mean | Statistics | 0.55 | 0.34 |
| LoG.5.0.mm.3D_ngtdm_Busyness | Texture | 0.56 | 0.34 |
| Wv.LHH_gldm_GrayLevelNonUniformity | Texture | 0.56 | 0.34 |
| LoG.4.5.mm.3D_glcm_Idn | Texture | 0.56 | 0.20 |
| LoG.5.0.mm.3D_firstorder_RobustMeanAbsoluteDeviation | Statistics | 0.57 | 0.20 |
| LoG.3.0.mm.3D_ngtdm_Strength | Texture | 0.59 | 0.16 |
| LoG.4.0.mm.3D_glcm_MaximumProbability | Texture | 0.59 | 0.18 |
| LoG.2.5.mm.3D_firstorder_Kurtosis | Statistics | 0.6 | 0.01 |
