# Supplementary material for: Peritumoral radiomics features predict distant metastasis in locally advanced NSCLC
Source: PLoS One. 2018 Nov 2;13(11):e0206108. doi: 10.1371/journal.pone.0206108 (PMC6214508; doi:10.1371/journal.pone.0206108)
Supplement: S4 File — Table A. Tumor radiomic feature expression statistics between DM and non-DM sub-cohort. Figure A. Feature trend between DM and non-DM in the tumor region. Empirical Cumulative Distribution Function (eCDF) plotted against feature values for the top significant univariate tumor radiomic features between DM (red) and non-DM (blue) sub-cohorts. Table B. Tumor rim radiomic feature expression statistics between DM and non-DM sub-cohort. Figure B. Feature trend between DM and non-DM in the tumor rim region. Empirical Cumulative Distribution Function (eCDF) plotted against feature values for the top significant univariate tumor rim radiomic features between DM (red) and non-DM (blue) sub-cohorts. Table C. Tumor exterior region radiomic feature expression statistics between DM and non-DM sub-cohort. Figure C. Feature trend between DM and non-DM in the tumor exterior region. Empirical Cumulative Distribution Function (eCDF) plotted against feature values for the top significant univariate tumor exterior radiomic feature between DM (red) and non-DM (blue) sub-cohorts. (DOCX) [file pone.0206108.s004.docx]

**S4: Radiomic Feature Expression Trend**

Here we evaluated the radiomic feature expression trend between metastatic (DM) and non-metastatic (non-DM) sub-cohort based on entire dataset (N=200). Evaluated were the features significant for DM prediction as determined by univariable analysis. Two-sided t-test was performed and the statistics was presented. We also presented the feature expression trend between the two sub-cohorts using empirical cumulative distribution function (eCDF).

Table A. Tumor radiomic feature expression statistics between DM and non-DM sub-cohort.

| Radiomic feature | mean of sub-cohort with DM | mean of sub-cohort of non-DM | p-value | conf.low | conf.high |
| --- | --- | --- | --- | --- | --- |
| LoG.0.5.mm.3D_glcm_DifferenceEntropy | 1.20 | 1.27 | 0.01 | -0.13 | -0.01 |
| Wv.HLL_glrlm_RunEntropy | 4.50 | 4.61 | 0.03 | -0.22 | -0.01 |
| LoG.4.5.mm.3D_gldzm_IntensityVariabilityNormalized | 0.08 | 0.07 | 2.56E-3 | 0.00 | 0.01 |
| Wv.HLL_glcm_Contrast | 48.84 | 59.22 | 0.26 | -28.54 | 7.79 |
| LoG.5.0.mm.3D_glcm_ClusterProminence | 7399.03 | 12153.76 | 0.22 | -12363.05 | 2853.59 |
| Wv.HLL_glszm_LargeAreaLowGrayLevelEmphasis | 4.19 | 4.43 | 0.91 | -4.38 | 3.91 |
| LoG.5.0.mm.3D_firstorder_Median | -129.30 | -130.56 | 0.91 | -22.10 | 24.63 |
| LoG.0.5.mm.3D_gldm_DependenceVariance | 22.55 | 22.78 | 0.85 | -2.61 | 2.15 |
| LoG.3.0.mm.3D_glrlm_LongRunLowGrayLevelEmphasis | 0.04 | 0.03 | 0.26 | 0.00 | 0.01 |
| LoG.2.5.mm.3D_gldzm_HighIntensityLargeDistanceEmphasis | 158.85 | 204.00 | 0.06 | -92.56 | 2.27 |

conf.low = confidence interval lower bound; conf.high = confidence interval higher bound

In Figure A, we reported the feature expression trend for the ten features that were determined as significant using the training cohort. The figures show the eCDF plotted against the features values. Only three of these, LoG. 0.5mm 3D GLCM DifferenceEntropy, Wv.HLL GLRLM Run Entropy, LoG 4.5mm 3D GLDZM IntensityVariabilityNormalized showed statistically significant difference between the DM and non-DM sub-cohorts (N =200).

| **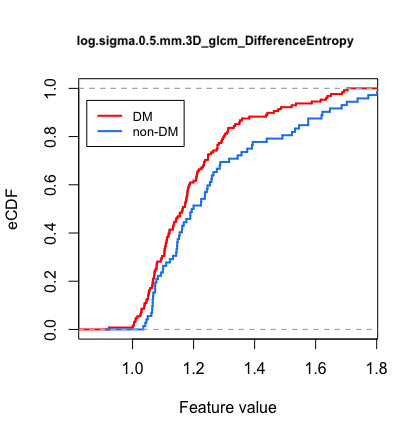**  p-value = 0.01 | **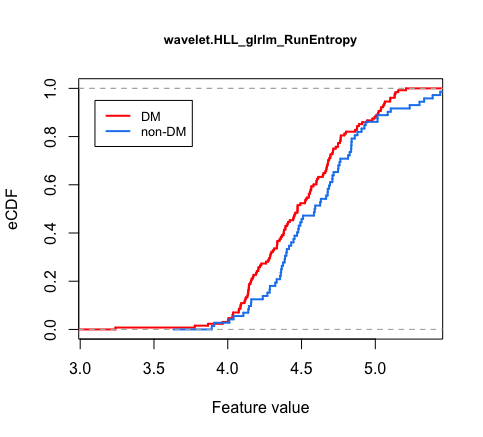**  p-value = 0.03 |
| --- | --- |
| **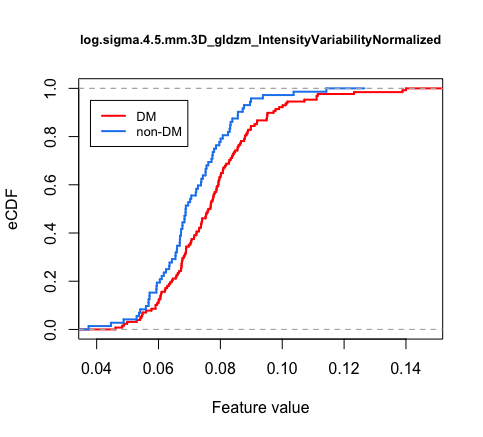**  p-value = 2.56E-3 | **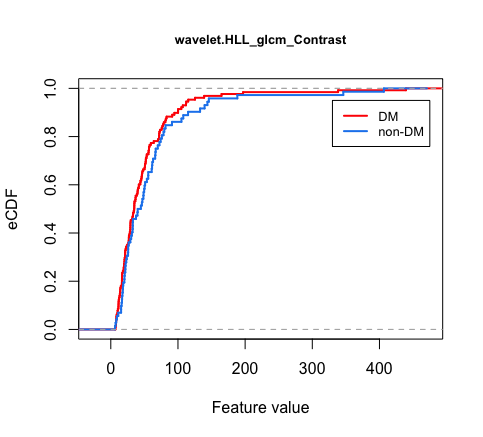**  p-value = 0.26 |
| **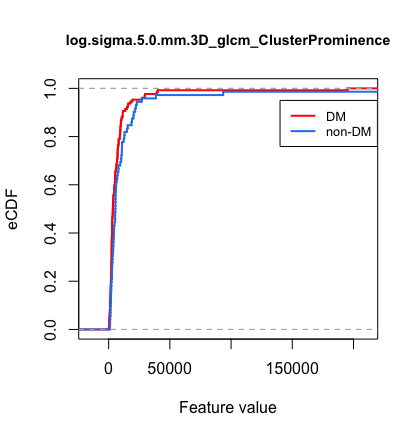**  p-value = 0.22 | **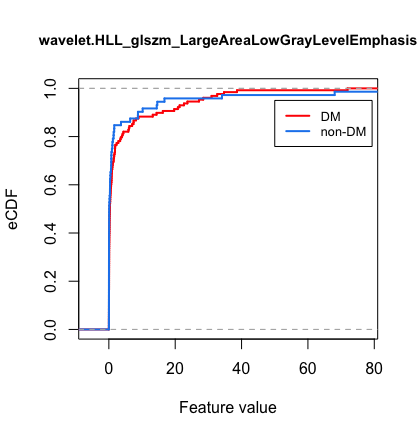**  p-value = 0.91 |
| **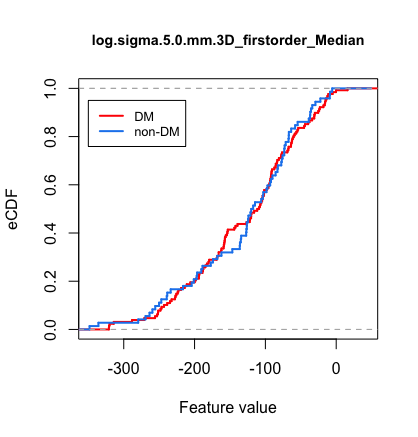**  p-value = 0.91 | **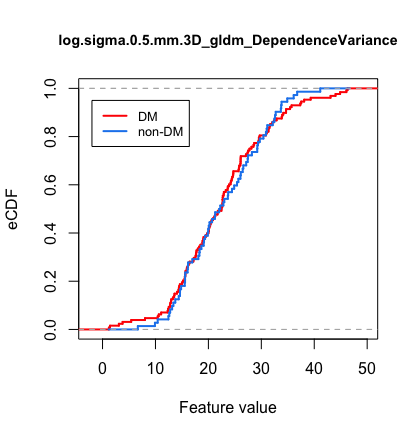**  p-value = 0.85 |
| **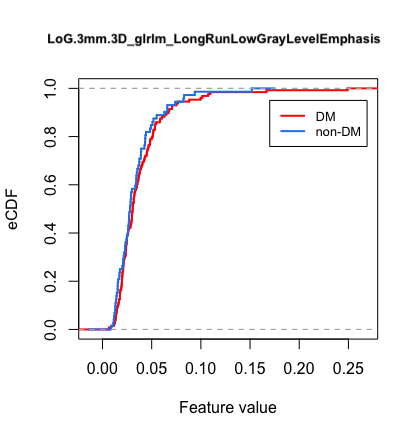**  p-value = 0.26 | **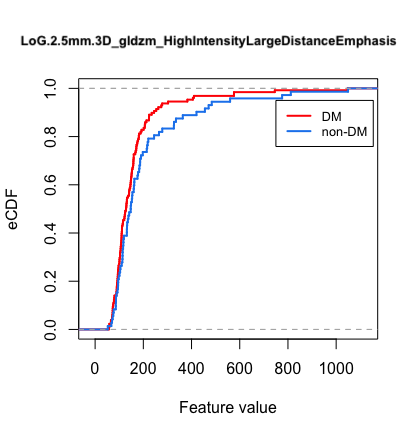**  p-value = 0.06 |

Figure A. Empirical Cumulative Distribution Function (eCDF) plotted against feature values for the top significant univariate tumor radiomic features between DM (red) and non-DM (blue) sub-cohorts.

Table B. Tumor rim radiomic feature expression statistics between DM and non-DM sub-cohort.

| feature | mean of sub-cohort with DM | mean of sub-cohort of non-DM | p-value | conf.low | conf.high |
| --- | --- | --- | --- | --- | --- |
| LoG.1.5.mm.3D_glrlm_RunEntropy | 4.57 | 4.67 | 2.67E-4 | -0.15 | -0.05 |
| original_glrlm_RunEntropy | 5.23 | 5.35 | 7.82E-5 | -0.17 | -0.06 |
| LoG.1.0.mm.3D_glcm_ClusterProminence | 1437.79 | 1887.49 | 8.65E-3 | -783.13 | -116.29 |
| Wv.HHL_glcm_MaximumProbability | 0.03 | 0.02 | 3.68E-3 | 0.00 | 0.01 |
| LoG.3.5.mm.3D_firstorder_Range | 727.47 | 758.91 | 0.06 | -64.38 | 1.50 |
| LoG.5.0.mm.3D_firstorder_Minimum | -356.10 | -372.74 | 0.18 | -7.57 | 40.83 |

In Table B, We report the t-test statistics between DM and non-DM sub-cohort for each of the top tumor rim features. Four featues, LoG.1.5.mm.3D_glrlm_RunEntropy, original glrlm RunEntropy, LoG.1.0.mm.3D glcm ClusterProminence, and Wv.HHL glcm MaximumProbability showed significant difference between DM and non-DM sub-groups using the entire 200 cases. LoG.3.5.mm.3D_firstorder_Range showed marginal significance with p-value of 0.06. In Fig B, we showed the expression trend for each of the top features.

| 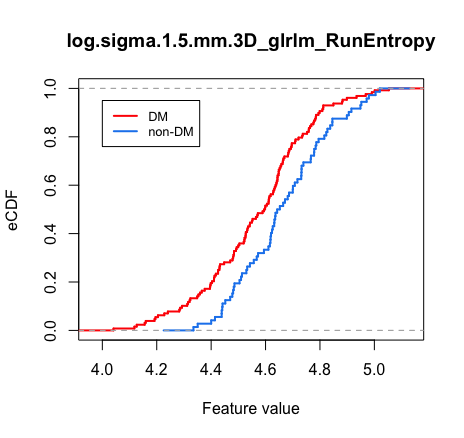  p-value = 2.67E-4 | 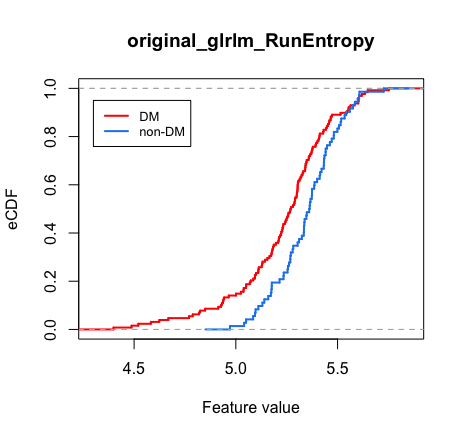  p-value = 7.82E-5 |
| --- | --- |
| 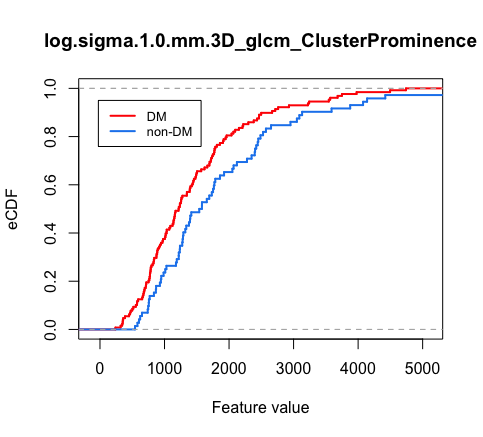  p-value = 8.65E-3 | 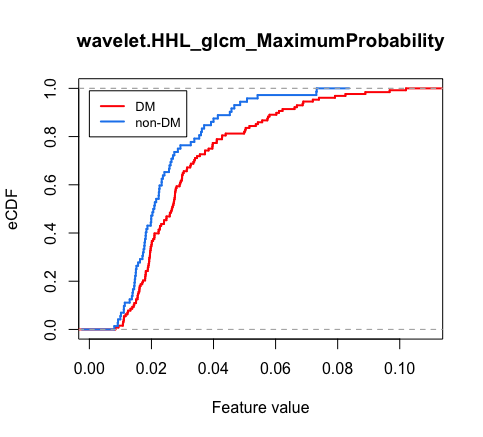  p-value = 3.68E-3 |
| 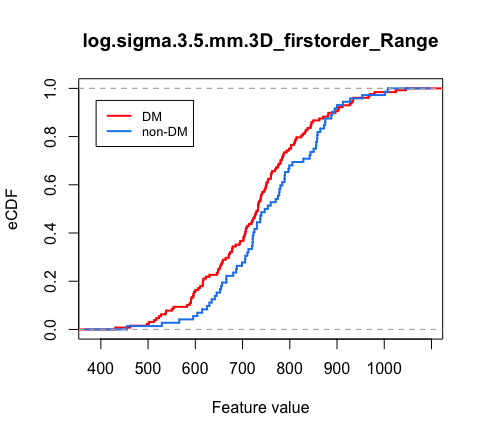  p-value = 0.06 | 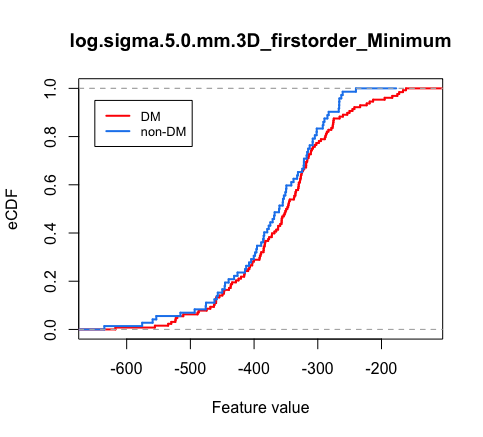  p-value = 0.18 |

Figure B. Empirical Cumulative Distribution Function (eCDF) plotted against feature values for the top significant univariate tumor rim radiomic features between DM (red) and non-DM (blue) sub-cohorts.

We analyzed the only feature for the tumor exterior region that was significant from univariable analysis. LoG.2.5.mm.3D firstorder Kurtosis was not found to be significant between the DM and non-DM sub-groups (N=200). We report the t-test statistics in Table C and feature expression trend plot in Fig. C.

Table C. Tumor exterior region radiomic feature expression statistics between DM and non-DM sub-cohort.

| Feature | mean of sub-cohort with DM | mean of sub-cohort of non-DM | p-value | conf.low | conf.high |
| --- | --- | --- | --- | --- | --- |
| LoG.2.5.mm.3D_firstorder_Kurtosis | 4.50 | 4.50 | 0.98 | -0.27 | 0.26 |


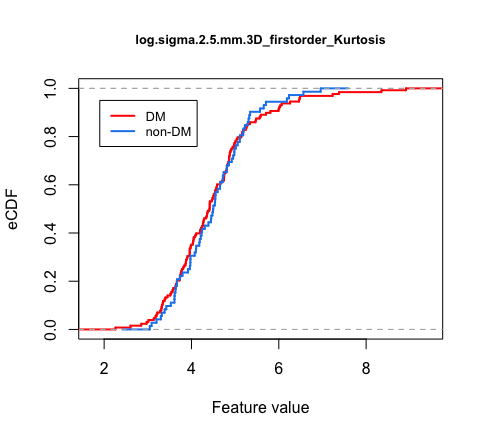


p-value = 0.98

Figure C. Empirical Cumulative Distribution Function (eCDF) plotted against feature values for the top significant univariate tumor exterior radiomic feature between DM (red) and non-DM (blue) sub-cohorts.
