# Supplementary material for: Peritumoral radiomics features predict distant metastasis in locally advanced NSCLC
Source: PLoS One. 2018 Nov 2;13(11):e0206108. doi: 10.1371/journal.pone.0206108 (PMC6214508; doi:10.1371/journal.pone.0206108)
Supplement: S5 File — (PDF) [file pone.0206108.s005.pdf]

X	general_info_BoundingBox
1	(6, 6, 5, 10, 10, 12)
2	(6, 6, 6, 17, 18, 15)
3	(6, 6, 5, 6, 10, 5)
4	(6, 6, 5, 16, 18, 19)
5	(6, 7, 6, 7, 6, 5)
6	(6, 6, 6, 9, 9, 7)
8	(5, 6, 6, 10, 8, 3)
9	(6, 6, 6, 13, 11, 10)
10	(6, 6, 6, 19, 16, 13)
11	(5, 6, 6, 24, 18, 22)
12	(6, 6, 5, 8, 11, 8)
13	(6, 6, 6, 12, 13, 17)
14	(6, 6, 6, 17, 15, 14)
15	(5, 5, 6, 12, 14, 10)
16	(5, 6, 6, 12, 10, 11)
17	(6, 6, 6, 33, 27, 34)
18	(6, 6, 6, 13, 7, 8)
19	(6, 6, 5, 18, 17, 19)
20	(6, 6, 6, 8, 9, 9)
21	(6, 5, 6, 19, 21, 22)
22	(6, 6, 6, 23, 22, 32)
23	(6, 6, 6, 13, 10, 19)
24	(5, 5, 6, 18, 26, 29)
25	(6, 6, 6, 8, 8, 7)
26	(6, 6, 5, 20, 20, 14)
27	(6, 6, 6, 13, 16, 8)
29	(5, 6, 5, 17, 25, 18)
30	(5, 5, 6, 25, 30, 28)
31	(5, 6, 5, 22, 22, 26)
32	(6, 6, 6, 17, 18, 18)
33	(6, 6, 6, 19, 26, 25)
34	(6, 5, 6, 34, 32, 30)
35	(6, 5, 6, 16, 15, 16)
36	(6, 5, 6, 3, 3, 3)
37	(6, 6, 5, 6, 7, 8)
38	(6, 6, 6, 15, 15, 13)
39	(5, 5, 6, 24, 30, 28)
40	(6, 6, 6, 20, 23, 16)
41	(6, 6, 5, 9, 12, 8)
42	(6, 6, 6, 12, 11, 11)
43	(6, 5, 6, 13, 17, 14)
44	(6, 6, 6, 14, 11, 10)
45	(5, 6, 6, 23, 12, 12)
46	(6, 5, 6, 11, 12, 13)
48	(5, 6, 5, 17, 15, 12)
49	(5, 6, 6, 30, 33, 24)
50	(6, 5, 2, 20, 19, 26)
51	(6, 5, 5, 8, 8, 7)
52	(6, 6, 5, 3, 3, 4)
53	(6, 5, 6, 6, 9, 8)
54	(6, 6, 5, 27, 23, 23)
55	(5, 5, 6, 10, 10, 9)
56	(6, 6, 6, 17, 21, 28)
57	(6, 6, 5, 9, 9, 9)
58	(5, 6, 6, 29, 43, 32)
59	(6, 6, 5, 10, 11, 9)
60	(5, 6, 6, 24, 18, 19)
61	(6, 6, 5, 16, 10, 13)
62	(6, 6, 5, 10, 11, 10)
63	(6, 6, 6, 14, 10, 7)
64	(6, 5, 5, 15, 17, 14)
65	(5, 6, 6, 25, 26, 31)
66	(6, 6, 6, 21, 19, 34)
67	(6, 7, 6, 21, 24, 17)
68	(6, 6, 6, 6, 5, 4)
69	(5, 5, 5, 21, 15, 14)
70	(6, 6, 6, 8, 6, 6)
71	(5, 6, 6, 23, 19, 21)
72	(6, 6, 6, 7, 7, 7)
73	(6, 6, 6, 17, 19, 18)
74	(6, 6, 5, 26, 23, 20)
75	(5, 6, 6, 12, 12, 10)
76	(6, 6, 6, 24, 29, 27)
77	(6, 5, 6, 34, 37, 35)
78	(6, 6, 5, 35, 38, 34)
79	(5, 6, 6, 13, 15, 10)
80	(6, 7, 6, 13, 14, 8)
81	(6, 5, 6, 8, 8, 9)
82	(6, 5, 5, 19, 17, 10)
83	(5, 5, 5, 19, 27, 20)
84	(6, 6, 6, 10, 9, 4)
85	(8, 6, 6, 15, 21, 9)
86	(5, 6, 6, 21, 22, 26)
87	(6, 5, 6, 4, 5, 3)
88	(5, 5, 6, 23, 25, 24)
89	(6, 6, 6, 31, 21, 21)
90	(6, 6, 6, 10, 9, 9)
91	(5, 6, 6, 14, 24, 11)
92	(7, 6, 6, 5, 7, 3)
93	(7, 6, 6, 4, 4, 1)
94	(6, 6, 5, 27, 31, 19)
95	(6, 6, 6, 9, 10, 12)
96	(6, 5, 6, 11, 13, 9)
107	(6, 6, 6, 22, 24, 21)
108	(6, 6, 5, 24, 27, 26)
112	(6, 6, 6, 5, 5, 5)
114	(6, 6, 6, 14, 12, 11)
126	(6, 6, 5, 3, 4, 4)
135	(6, 5, 5, 18, 11, 14)
139	(6, 6, 5, 15, 12, 10)

general_info_GeneralSettings

{'distances': [1], 'verbose': True, 'additionalInfo': True, 'enableCExtensions': True, 'force2D': False, 'interpolator': 3, 'resampledPixelSpacing': [3, 3, 3], 'label': 1, 'normalizeScale': 1, 'normalize': False, 'force2Ddimension': 0, 'removeOutliers': None, 'minimumROISi
{'distances': [1], 'verbose': True, 'additionalInfo': True, 'enableCExtensions': True, 'force2D': False, 'interpolator': 3, 'resampledPixelSpacing': [3, 3, 3], 'label': 1, 'normalizeScale': 1, 'normalize': False, 'force2Ddimension': 0, 'removeOutliers': None, 'minimumROISi
{'distances': [1], 'verbose': True, 'additionalInfo': True, 'enableCExtensions': True, 'force2D': False, 'interpolator': 3, 'resampledPixelSpacing': [3, 3, 3], 'label': 1, 'normalizeScale': 1, 'normalize': False, 'force2Ddimension': 0, 'removeOutliers': None, 'minimumROISi
{'distances': [1], 'verbose': True, 'additionalInfo': True, 'enableCExtensions': True, 'force2D': False, 'interpolator': 3, 'resampledPixelSpacing': [3, 3, 3], 'label': 1, 'normalizeScale': 1, 'normalize': False, 'force2Ddimension': 0, 'removeOutliers': None, 'minimumROISi
{'distances': [1], 'verbose': True, 'additionalInfo': True, 'enableCExtensions': True, 'force2D': False, 'interpolator': 3, 'resampledPixelSpacing': [3, 3, 3], 'label': 1, 'normalizeScale': 1, 'normalize': False, 'force2Ddimension': 0, 'removeOutliers': None, 'minimumROISi
{'distances': [1], 'verbose': True, 'additionalInfo': True, 'enableCExtensions': True, 'force2D': False, 'interpolator': 3, 'resampledPixelSpacing': [3, 3, 3], 'label': 1, 'normalizeScale': 1, 'normalize': False, 'force2Ddimension': 0, 'removeOutliers': None, 'minimumROISi
{'distances': [1], 'verbose': True, 'additionalInfo': True, 'enableCExtensions': True, 'force2D': False, 'interpolator': 3, 'resampledPixelSpacing': [3, 3, 3], 'label': 1, 'normalizeScale': 1, 'normalize': False, 'force2Ddimension': 0, 'removeOutliers': None, 'minimumROISi
{'distances': [1], 'verbose': True, 'additionalInfo': True, 'enableCExtensions': True, 'force2D': False, 'interpolator': 3, 'resampledPixelSpacing': [3, 3, 3], 'label': 1, 'normalizeScale': 1, 'normalize': False, 'force2Ddimension': 0, 'removeOutliers': None, 'minimumROISi
{'distances': [1], 'verbose': True, 'additionalInfo': True, 'enableCExtensions': True, 'force2D': False, 'interpolator': 3, 'resampledPixelSpacing': [3, 3, 3], 'label': 1, 'normalizeScale': 1, 'normalize': False, 'force2Ddimension': 0, 'removeOutliers': None, 'minimumROISi
{'distances': [1], 'verbose': True, 'additionalInfo': True, 'enableCExtensions': True, 'force2D': False, 'interpolator': 3, 'resampledPixelSpacing': [3, 3, 3], 'label': 1, 'normalizeScale': 1, 'normalize': False, 'force2Ddimension': 0, 'removeOutliers': None, 'minimumROISi
{'distances': [1], 'verbose': True, 'additionalInfo': True, 'enableCExtensions': True, 'force2D': False, 'interpolator': 3, 'resampledPixelSpacing': [3, 3, 3], 'label': 1, 'normalizeScale': 1, 'normalize': False, 'force2Ddimension': 0, 'removeOutliers': None, 'minimumROISi
{'distances': [1], 'verbose': True, 'additionalInfo': True, 'enableCExtensions': True, 'force2D': False, 'interpolator': 3, 'resampledPixelSpacing': [3, 3, 3], 'label': 1, 'normalizeScale': 1, 'normalize': False, 'force2Ddimension': 0, 'removeOutliers': None, 'minimumROISi
{'distances': [1], 'verbose': True, 'additionalInfo': True, 'enableCExtensions': True, 'force2D': False, 'interpolator': 3, 'resampledPixelSpacing': [3, 3, 3], 'label': 1, 'normalizeScale': 1, 'normalize': False, 'force2Ddimension': 0, 'removeOutliers': None, 'minimumROISi
{'distances': [1], 'verbose': True, 'additionalInfo': True, 'enableCExtensions': True, 'force2D': False, 'interpolator': 3, 'resampledPixelSpacing': [3, 3, 3], 'label': 1, 'normalizeScale': 1, 'normalize': False, 'force2Ddimension': 0, 'removeOutliers': None, 'minimumROISi
{'distances': [1], 'verbose': True, 'additionalInfo': True, 'enableCExtensions': True, 'force2D': False, 'interpolator': 3, 'resampledPixelSpacing': [3, 3, 3], 'label': 1, 'normalizeScale': 1, 'normalize': False, 'force2Ddimension': 0, 'removeOutliers': None, 'minimumROISi
{'distances': [1], 'verbose': True, 'additionalInfo': True, 'enableCExtensions': True, 'force2D': False, 'interpolator': 3, 'resampledPixelSpacing': [3, 3, 3], 'label': 1, 'normalizeScale': 1, 'normalize': False, 'force2Ddimension': 0, 'removeOutliers': None, 'minimumROISi
{'distances': [1], 'verbose': True, 'additionalInfo': True, 'enableCExtensions': True, 'force2D': False, 'interpolator': 3, 'resampledPixelSpacing': [3, 3, 3], 'label': 1, 'normalizeScale': 1, 'normalize': False, 'force2Ddimension': 0, 'removeOutliers': None, 'minimumROISi
{'distances': [1], 'verbose': True, 'additionalInfo': True, 'enableCExtensions': True, 'force2D': False, 'interpolator': 3, 'resampledPixelSpacing': [3, 3, 3], 'label': 1, 'normalizeScale': 1, 'normalize': False, 'force2Ddimension': 0, 'removeOutliers': None, 'minimumROISi
{'distances': [1], 'verbose': True, 'additionalInfo': True, 'enableCExtensions': True, 'force2D': False, 'interpolator': 3, 'resampledPixelSpacing': [3, 3, 3], 'label': 1, 'normalizeScale': 1, 'normalize': False, 'force2Ddimension': 0, 'removeOutliers': None, 'minimumROISi
{'distances': [1], 'verbose': True, 'additionalInfo': True, 'enableCExtensions': True, 'force2D': False, 'interpolator': 3, 'resampledPixelSpacing': [3, 3, 3], 'label': 1, 'normalizeScale': 1, 'normalize': False, 'force2Ddimension': 0, 'removeOutliers': None, 'minimumROISi
{'distances': [1], 'verbose': True, 'additionalInfo': True, 'enableCExtensions': True, 'force2D': False, 'interpolator': 3, 'resampledPixelSpacing': [3, 3, 3], 'label': 1, 'normalizeScale': 1, 'normalize': False, 'force2Ddimension': 0, 'removeOutliers': None, 'minimumROISi
{'distances': [1], 'verbose': True, 'additionalInfo': True, 'enableCExtensions': True, 'force2D': False, 'interpolator': 3, 'resampledPixelSpacing': [3, 3, 3], 'label': 1, 'normalizeScale': 1, 'normalize': False, 'force2Ddimension': 0, 'removeOutliers': None, 'minimumROISi
{'distances': [1], 'verbose': True, 'additionalInfo': True, 'enableCExtensions': True, 'force2D': False, 'interpolator': 3, 'resampledPixelSpacing': [3, 3, 3], 'label': 1, 'normalizeScale': 1, 'normalize': False, 'force2Ddimension': 0, 'removeOutliers': None, 'minimumROISi
{'distances': [1], 'verbose': True, 'additionalInfo': True, 'enableCExtensions': True, 'force2D': False, 'interpolator': 3, 'resampledPixelSpacing': [3, 3, 3], 'label': 1, 'normalizeScale': 1, 'normalize': False, 'force2Ddimension': 0, 'removeOutliers': None, 'minimumROISi
{'distances': [1], 'verbose': True, 'additionalInfo': True, 'enableCExtensions': True, 'force2D': False, 'interpolator': 3, 'resampledPixelSpacing': [3, 3, 3], 'label': 1, 'normalizeScale': 1, 'normalize': False, 'force2Ddimension': 0, 'removeOutliers': None, 'minimumROISi
{'distances': [1], 'verbose': True, 'additionalInfo': True, 'enableCExtensions': True, 'force2D': False, 'interpolator': 3, 'resampledPixelSpacing': [3, 3, 3], 'label': 1, 'normalizeScale': 1, 'normalize': False, 'force2Ddimension': 0, 'removeOutliers': None, 'minimumROISi
{'distances': [1], 'verbose': True, 'additionalInfo': True, 'enableCExtensions': True, 'force2D': False, 'interpolator': 3, 'resampledPixelSpacing': [3, 3, 3], 'label': 1, 'normalizeScale': 1, 'normalize': False, 'force2Ddimension': 0, 'removeOutliers': None, 'minimumROISi
{'distances': [1], 'verbose': True, 'additionalInfo': True, 'enableCExtensions': True, 'force2D': False, 'interpolator': 3, 'resampledPixelSpacing': [3, 3, 3], 'label': 1, 'normalizeScale': 1, 'normalize': False, 'force2Ddimension': 0, 'removeOutliers': None, 'minimumROISi
{'distances': [1], 'verbose': True, 'additionalInfo': True, 'enableCExtensions': True, 'force2D': False, 'interpolator': 3, 'resampledPixelSpacing': [3, 3, 3], 'label': 1, 'normalizeScale': 1, 'normalize': False, 'force2Ddimension': 0, 'removeOutliers': None, 'minimumROISi
{'distances': [1], 'verbose': True, 'additionalInfo': True, 'enableCExtensions': True, 'force2D': False, 'interpolator': 3, 'resampledPixelSpacing': [3, 3, 3], 'label': 1, 'normalizeScale': 1, 'normalize': False, 'force2Ddimension': 0, 'removeOutliers': None, 'minimumROISi
{'distances': [1], 'verbose': True, 'additionalInfo': True, 'enableCExtensions': True, 'force2D': False, 'interpolator': 3, 'resampledPixelSpacing': [3, 3, 3], 'label': 1, 'normalizeScale': 1, 'normalize': False, 'force2Ddimension': 0, 'removeOutliers': None, 'minimumROISi
{'distances': [1], 'verbose': True, 'additionalInfo': True, 'enableCExtensions': True, 'force2D': False, 'interpolator': 3, 'resampledPixelSpacing': [3, 3, 3], 'label': 1, 'normalizeScale': 1, 'normalize': False, 'force2Ddimension': 0, 'removeOutliers': None, 'minimumROISi
{'distances': [1], 'verbose': True, 'additionalInfo': True, 'enableCExtensions': True, 'force2D': False, 'interpolator': 3, 'resampledPixelSpacing': [3, 3, 3], 'label': 1, 'normalizeScale': 1, 'normalize': False, 'force2Ddimension': 0, 'removeOutliers': None, 'minimumROISi
{'distances': [1], 'verbose': True, 'additionalInfo': True, 'enableCExtensions': True, 'force2D': False, 'interpolator': 3, 'resampledPixelSpacing': [3, 3, 3], 'label': 1, 'normalizeScale': 1, 'normalize': False, 'force2Ddimension': 0, 'removeOutliers': None, 'minimumROISi
{'distances': [1], 'verbose': True, 'additionalInfo': True, 'enableCExtensions': True, 'force2D': False, 'interpolator': 3, 'resampledPixelSpacing': [3, 3, 3], 'label': 1, 'normalizeScale': 1, 'normalize': False, 'force2Ddimension': 0, 'removeOutliers': None, 'minimumROISi
{'distances': [1], 'verbose': True, 'additionalInfo': True, 'enableCExtensions': True, 'force2D': False, 'interpolator': 3, 'resampledPixelSpacing': [3, 3, 3], 'label': 1, 'normalizeScale': 1, 'normalize': False, 'force2Ddimension': 0, 'removeOutliers': None, 'minimumROISi
{'distances': [1], 'verbose': True, 'additionalInfo': True, 'enableCExtensions': True, 'force2D': False, 'interpolator': 3, 'resampledPixelSpacing': [3, 3, 3], 'label': 1, 'normalizeScale': 1, 'normalize': False, 'force2Ddimension': 0, 'removeOutliers': None, 'minimumROISi
{'distances': [1], 'verbose': True, 'additionalInfo': True, 'enableCExtensions': True, 'force2D': False, 'interpolator': 3, 'resampledPixelSpacing': [3, 3, 3], 'label': 1, 'normalizeScale': 1, 'normalize': False, 'force2Ddimension': 0, 'removeOutliers': None, 'minimumROISi
{'distances': [1], 'verbose': True, 'additionalInfo': True, 'enableCExtensions': True, 'force2D': False, 'interpolator': 3, 'resampledPixelSpacing': [3, 3, 3], 'label': 1, 'normalizeScale': 1, 'normalize': False, 'force2Ddimension': 0, 'removeOutliers': None, 'minimumROISi
{'distances': [1], 'verbose': True, 'additionalInfo': True, 'enableCExtensions': True, 'force2D': False, 'interpolator': 3, 'resampledPixelSpacing': [3, 3, 3], 'label': 1, 'normalizeScale': 1, 'normalize': False, 'force2Ddimension': 0, 'removeOutliers': None, 'minimumROISi
{'distances': [1], 'verbose': True, 'additionalInfo': True, 'enableCExtensions': True, 'force2D': False, 'interpolator': 3, 'resampledPixelSpacing': [3, 3, 3], 'label': 1, 'normalizeScale': 1, 'normalize': False, 'force2Ddimension': 0, 'removeOutliers': None, 'minimumROISi
{'distances': [1], 'verbose': True, 'additionalInfo': True, 'enableCExtensions': True, 'force2D': False, 'interpolator': 3, 'resampledPixelSpacing': [3, 3, 3], 'label': 1, 'normalizeScale': 1, 'normalize': False, 'force2Ddimension': 0, 'removeOutliers': None, 'minimumROISi
{'distances': [1], 'verbose': True, 'additionalInfo': True, 'enableCExtensions': True, 'force2D': False, 'interpolator': 3, 'resampledPixelSpacing': [3, 3, 3], 'label': 1, 'normalizeScale': 1, 'normalize': False, 'force2Ddimension': 0, 'removeOutliers': None, 'minimumROISi
{'distances': [1], 'verbose': True, 'additionalInfo': True, 'enableCExtensions': True, 'force2D': False, 'interpolator': 3, 'resampledPixelSpacing': [3, 3, 3], 'label': 1, 'normalizeScale': 1, 'normalize': False, 'force2Ddimension': 0, 'removeOutliers': None, 'minimumROISi
{'distances': [1], 'verbose': True, 'additionalInfo': True, 'enableCExtensions': True, 'force2D': False, 'interpolator': 3, 'resampledPixelSpacing': [3, 3, 3], 'label': 1, 'normalizeScale': 1, 'normalize': False, 'force2Ddimension': 0, 'removeOutliers': None, 'minimumROISi
{'distances': [1], 'verbose': True, 'additionalInfo': True, 'enableCExtensions': True, 'force2D': False, 'interpolator': 3, 'resampledPixelSpacing': [3, 3, 3], 'label': 1, 'normalizeScale': 1, 'normalize': False, 'force2Ddimension': 0, 'removeOutliers': None, 'minimumROISi
{'distances': [1], 'verbose': True, 'additionalInfo': True, 'enableCExtensions': True, 'force2D': False, 'interpolator': 3, 'resampledPixelSpacing': [3, 3, 3], 'label': 1, 'normalizeScale': 1, 'normalize': False, 'force2Ddimension': 0, 'removeOutliers': None, 'minimumROISi
{'distances': [1], 'verbose': True, 'additionalInfo': True, 'enableCExtensions': True, 'force2D': False, 'interpolator': 3, 'resampledPixelSpacing': [3, 3, 3], 'label': 1, 'normalizeScale': 1, 'normalize': False, 'force2Ddimension': 0, 'removeOutliers': None, 'minimumROISi
{'distances': [1], 'verbose': True, 'additionalInfo': True, 'enableCExtensions': True, 'force2D': False, 'interpolator': 3, 'resampledPixelSpacing': [3, 3, 3], 'label': 1, 'normalizeScale': 1, 'normalize': False, 'force2Ddimension': 0, 'removeOutliers': None, 'minimumROISi
{'distances': [1], 'verbose': True, 'additionalInfo': True, 'enableCExtensions': True, 'force2D': False, 'interpolator': 3, 'resampledPixelSpacing': [3, 3, 3], 'label': 1, 'normalizeScale': 1, 'normalize': False, 'force2Ddimension': 0, 'removeOutliers': None, 'minimumROISi
{'distances': [1], 'verbose': True, 'additionalInfo': True, 'enableCExtensions': True, 'force2D': False, 'interpolator': 3, 'resampledPixelSpacing': [3, 3, 3], 'label': 1, 'normalizeScale': 1, 'normalize': False, 'force2Ddimension': 0, 'removeOutliers': None, 'minimumROISi
{'distances': [1], 'verbose': True, 'additionalInfo': True, 'enableCExtensions': True, 'force2D': False, 'interpolator': 3, 'resampledPixelSpacing': [3, 3, 3], 'label': 1, 'normalizeScale': 1, 'normalize': False, 'force2Ddimension': 0, 'removeOutliers': None, 'minimumROISi
{'distances': [1], 'verbose': True, 'additionalInfo': True, 'enableCExtensions': True, 'force2D': False, 'interpolator': 3, 'resampledPixelSpacing': [3, 3, 3], 'label': 1, 'normalizeScale': 1, 'normalize': False, 'force2Ddimension': 0, 'removeOutliers': None, 'minimumROISi
{'distances': [1], 'verbose': True, 'additionalInfo': True, 'enableCExtensions': True, 'force2D': False, 'interpolator': 3, 'resampledPixelSpacing': [3, 3, 3], 'label': 1, 'normalizeScale': 1, 'normalize': False, 'force2Ddimension': 0, 'removeOutliers': None, 'minimumROISi
{'distances': [1], 'verbose': True, 'additionalInfo': True, 'enableCExtensions': True, 'force2D': False, 'interpolator': 3, 'resampledPixelSpacing': [3, 3, 3], 'label': 1, 'normalizeScale': 1, 'normalize': False, 'force2Ddimension': 0, 'removeOutliers': None, 'minimumROISi
{'distances': [1], 'verbose': True, 'additionalInfo': True, 'enableCExtensions': True, 'force2D': False, 'interpolator': 3, 'resampledPixelSpacing': [3, 3, 3], 'label': 1, 'normalizeScale': 1, 'normalize': False, 'force2Ddimension': 0, 'removeOutliers': None, 'minimumROISi
{'distances': [1], 'verbose': True, 'additionalInfo': True, 'enableCExtensions': True, 'force2D': False, 'interpolator': 3, 'resampledPixelSpacing': [3, 3, 3], 'label': 1, 'normalizeScale': 1, 'normalize': False, 'force2Ddimension': 0, 'removeOutliers': None, 'minimumROISi
{'distances': [1], 'verbose': True, 'additionalInfo': True, 'enableCExtensions': True, 'force2D': False, 'interpolator': 3, 'resampledPixelSpacing': [3, 3, 3], 'label': 1, 'normalizeScale': 1, 'normalize': False, 'force2Ddimension': 0, 'removeOutliers': None, 'minimumROISi
{'distances': [1], 'verbose': True, 'additionalInfo': True, 'enableCExtensions': True, 'force2D': False, 'interpolator': 3, 'resampledPixelSpacing': [3, 3, 3], 'label': 1, 'normalizeScale': 1, 'normalize': False, 'force2Ddimension': 0, 'removeOutliers': None, 'minimumROISi
{'distances': [1], 'verbose': True, 'additionalInfo': True, 'enableCExtensions': True, 'force2D': False, 'interpolator': 3, 'resampledPixelSpacing': [3, 3, 3], 'label': 1, 'normalizeScale': 1, 'normalize': False, 'force2Ddimension': 0, 'removeOutliers': None, 'minimumROISi
{'distances': [1], 'verbose': True, 'additionalInfo': True, 'enableCExtensions': True, 'force2D': False, 'interpolator': 3, 'resampledPixelSpacing': [3, 3, 3], 'label': 1, 'normalizeScale': 1, 'normalize': False, 'force2Ddimension': 0, 'removeOutliers': None, 'minimumROISi
{'distances': [1], 'verbose': True, 'additionalInfo': True, 'enableCExtensions': True, 'force2D': False, 'interpolator': 3, 'resampledPixelSpacing': [3, 3, 3], 'label': 1, 'normalizeScale': 1, 'normalize': False, 'force2Ddimension': 0, 'removeOutliers': None, 'minimumROISi
{'distances': [1], 'verbose': True, 'additionalInfo': True, 'enableCExtensions': True, 'force2D': False, 'interpolator': 3, 'resampledPixelSpacing': [3, 3, 3], 'label': 1, 'normalizeScale': 1, 'normalize': False, 'force2Ddimension': 0, 'removeOutliers': None, 'minimumROISi
{'distances': [1], 'verbose': True, 'additionalInfo': True, 'enableCExtensions': True, 'force2D': False, 'interpolator': 3, 'resampledPixelSpacing': [3, 3, 3], 'label': 1, 'normalizeScale': 1, 'normalize': False, 'force2Ddimension': 0, 'removeOutliers': None, 'minimumROISi
{'distances': [1], 'verbose': True, 'additionalInfo': True, 'enableCExtensions': True, 'force2D': False, 'interpolator': 3, 'resampledPixelSpacing': [3, 3, 3], 'label': 1, 'normalizeScale': 1, 'normalize': False, 'force2Ddimension': 0, 'removeOutliers': None, 'minimumROISi
{'distances': [1], 'verbose': True, 'additionalInfo': True, 'enableCExtensions': True, 'force2D': False, 'interpolator': 3, 'resampledPixelSpacing': [3, 3, 3], 'label': 1, 'normalizeScale': 1, 'normalize': False, 'force2Ddimension': 0, 'removeOutliers': None, 'minimumROISi
{'distances': [1], 'verbose': True, 'additionalInfo': True, 'enableCExtensions': True, 'force2D': False, 'interpolator': 3, 'resampledPixelSpacing': [3, 3, 3], 'label': 1, 'normalizeScale': 1, 'normalize': False, 'force2Ddimension': 0, 'removeOutliers': None, 'minimumROISi
{'distances': [1], 'verbose': True, 'additionalInfo': True, 'enableCExtensions': True, 'force2D': False, 'interpolator': 3, 'resampledPixelSpacing': [3, 3, 3], 'label': 1, 'normalizeScale': 1, 'normalize': False, 'force2Ddimension': 0, 'removeOutliers': None, 'minimumROISi
{'distances': [1], 'verbose': True, 'additionalInfo': True, 'enableCExtensions': True, 'force2D': False, 'interpolator': 3, 'resampledPixelSpacing': [3, 3, 3], 'label': 1, 'normalizeScale': 1, 'normalize': False, 'force2Ddimension': 0, 'removeOutliers': None, 'minimumROISi
{'distances': [1], 'verbose': True, 'additionalInfo': True, 'enableCExtensions': True, 'force2D': False, 'interpolator': 3, 'resampledPixelSpacing': [3, 3, 3], 'label': 1, 'normalizeScale': 1, 'normalize': False, 'force2Ddimension': 0, 'removeOutliers': None, 'minimumROISi
{'distances': [1], 'verbose': True, 'additionalInfo': True, 'enableCExtensions': True, 'force2D': False, 'interpolator': 3, 'resampledPixelSpacing': [3, 3, 3], 'label': 1, 'normalizeScale': 1, 'normalize': False, 'force2Ddimension': 0, 'removeOutliers': None, 'minimumROISi
{'distances': [1], 'verbose': True, 'additionalInfo': True, 'enableCExtensions': True, 'force2D': False, 'interpolator': 3, 'resampledPixelSpacing': [3, 3, 3], 'label': 1, 'normalizeScale': 1, 'normalize': False, 'force2Ddimension': 0, 'removeOutliers': None, 'minimumROISi
{'distances': [1], 'verbose': True, 'additionalInfo': True, 'enableCExtensions': True, 'force2D': False, 'interpolator': 3, 'resampledPixelSpacing': [3, 3, 3], 'label': 1, 'normalizeScale': 1, 'normalize': False, 'force2Ddimension': 0, 'removeOutliers': None, 'minimumROISi
{'distances': [1], 'verbose': True, 'additionalInfo': True, 'enableCExtensions': True, 'force2D': False, 'interpolator': 3, 'resampledPixelSpacing': [3, 3, 3], 'label': 1, 'normalizeScale': 1, 'normalize': False, 'force2Ddimension': 0, 'removeOutliers': None, 'minimumROISi
{'distances': [1], 'verbose': True, 'additionalInfo': True, 'enableCExtensions': True, 'force2D': False, 'interpolator': 3, 'resampledPixelSpacing': [3, 3, 3], 'label': 1, 'normalizeScale': 1, 'normalize': False, 'force2Ddimension': 0, 'removeOutliers': None, 'minimumROISi
{'distances': [1], 'verbose': True, 'additionalInfo': True, 'enableCExtensions': True, 'force2D': False, 'interpolator': 3, 'resampledPixelSpacing': [3, 3, 3], 'label': 1, 'normalizeScale': 1, 'normalize': False, 'force2Ddimension': 0, 'removeOutliers': None, 'minimumROISi
{'distances': [1], 'verbose': True, 'additionalInfo': True, 'enableCExtensions': True, 'force2D': False, 'interpolator': 3, 'resampledPixelSpacing': [3, 3, 3], 'label': 1, 'normalizeScale': 1, 'normalize': False, 'force2Ddimension': 0, 'removeOutliers': None, 'minimumROISi
{'distances': [1], 'verbose': True, 'additionalInfo': True, 'enableCExtensions': True, 'force2D': False, 'interpolator': 3, 'resampledPixelSpacing': [3, 3, 3], 'label': 1, 'normalizeScale': 1, 'normalize': False, 'force2Ddimension': 0, 'removeOutliers': None, 'minimumROISi
{'distances': [1], 'verbose': True, 'additionalInfo': True, 'enableCExtensions': True, 'force2D': False, 'interpolator': 3, 'resampledPixelSpacing': [3, 3, 3], 'label': 1, 'normalizeScale': 1, 'normalize': False, 'force2Ddimension': 0, 'removeOutliers': None, 'minimumROISi
{'distances': [1], 'verbose': True, 'additionalInfo': True, 'enableCExtensions': True, 'force2D': False, 'interpolator': 3, 'resampledPixelSpacing': [3, 3, 3], 'label': 1, 'normalizeScale': 1, 'normalize': False, 'force2Ddimension': 0, 'removeOutliers': None, 'minimumROISi
{'distances': [1], 'verbose': True, 'additionalInfo': True, 'enableCExtensions': True, 'force2D': False, 'interpolator': 3, 'resampledPixelSpacing': [3, 3, 3], 'label': 1, 'normalizeScale': 1, 'normalize': False, 'force2Ddimension': 0, 'removeOutliers': None, 'minimumROISi
{'distances': [1], 'verbose': True, 'additionalInfo': True, 'enableCExtensions': True, 'force2D': False, 'interpolator': 3, 'resampledPixelSpacing': [3, 3, 3], 'label': 1, 'normalizeScale': 1, 'normalize': False, 'force2Ddimension': 0, 'removeOutliers': None, 'minimumROISi
{'distances': [1], 'verbose': True, 'additionalInfo': True, 'enableCExtensions': True, 'force2D': False, 'interpolator': 3, 'resampledPixelSpacing': [3, 3, 3], 'label': 1, 'normalizeScale': 1, 'normalize': False, 'force2Ddimension': 0, 'removeOutliers': None, 'minimumROISi
{'distances': [1], 'verbose': True, 'additionalInfo': True, 'enableCExtensions': True, 'force2D': False, 'interpolator': 3, 'resampledPixelSpacing': [3, 3, 3], 'label': 1, 'normalizeScale': 1, 'normalize': False, 'force2Ddimension': 0, 'removeOutliers': None, 'minimumROISi
{'distances': [1], 'verbose': True, 'additionalInfo': True, 'enableCExtensions': True, 'force2D': False, 'interpolator': 3, 'resampledPixelSpacing': [3, 3, 3], 'label': 1, 'normalizeScale': 1, 'normalize': False, 'force2Ddimension': 0, 'removeOutliers': None, 'minimumROISi
{'distances': [1], 'verbose': True, 'additionalInfo': True, 'enableCExtensions': True, 'force2D': False, 'interpolator': 3, 'resampledPixelSpacing': [3, 3, 3], 'label': 1, 'normalizeScale': 1, 'normalize': False, 'force2Ddimension': 0, 'removeOutliers': None, 'minimumROISi
{'distances': [1], 'verbose': True, 'additionalInfo': True, 'enableCExtensions': True, 'force2D': False, 'interpolator': 3, 'resampledPixelSpacing': [3, 3, 3], 'label': 1, 'normalizeScale': 1, 'normalize': False, 'force2Ddimension': 0, 'removeOutliers': None, 'minimumROISi
{'distances': [1], 'verbose': True, 'additionalInfo': True, 'enableCExtensions': True, 'force2D': False, 'interpolator': 3, 'resampledPixelSpacing': [3, 3, 3], 'label': 1, 'normalizeScale': 1, 'normalize': False, 'force2Ddimension': 0, 'removeOutliers': None, 'minimumROISi
{'distances': [1], 'verbose': True, 'additionalInfo': True, 'enableCExtensions': True, 'force2D': False, 'interpolator': 3, 'resampledPixelSpacing': [3, 3, 3], 'label': 1, 'normalizeScale': 1, 'normalize': False, 'force2Ddimension': 0, 'removeOutliers': None, 'minimumROISi
{'distances': [1], 'verbose': True, 'additionalInfo': True, 'enableCExtensions': True, 'force2D': False, 'interpolator': 3, 'resampledPixelSpacing': [3, 3, 3], 'label': 1, 'normalizeScale': 1, 'normalize': False, 'force2Ddimension': 0, 'removeOutliers': None, 'minimumROISi
{'distances': [1], 'verbose': True, 'additionalInfo': True, 'enableCExtensions': True, 'force2D': False, 'interpolator': 3, 'resampledPixelSpacing': [3, 3, 3], 'label': 1, 'normalizeScale': 1, 'normalize': False, 'force2Ddimension': 0, 'removeOutliers': None, 'minimumROISi
{'distances': [1], 'verbose': True, 'additionalInfo': True, 'enableCExtensions': True, 'force2D': False, 'interpolator': 3, 'resampledPixelSpacing': [3, 3, 3], 'label': 1, 'normalizeScale': 1, 'normalize': False, 'force2Ddimension': 0, 'removeOutliers': None, 'minimumROISi
{'distances': [1], 'verbose': True, 'additionalInfo': True, 'enableCExtensions': True, 'force2D': False, 'interpolator': 3, 'resampledPixelSpacing': [3, 3, 3], 'label': 1, 'normalizeScale': 1, 'normalize': False, 'force2Ddimension': 0, 'removeOutliers': None, 'minimumROISi
{'distances': [1], 'verbose': True, 'additionalInfo': True, 'enableCExtensions': True, 'force2D': False, 'interpolator': 3, 'resampledPixelSpacing': [3, 3, 3], 'label': 1, 'normalizeScale': 1, 'normalize': False, 'force2Ddimension': 0, 'removeOutliers': None, 'minimumROISi

general_info_ImageHash	general_info_ImageSpacing	general_info_InputImages	general_info_MaskHash	general_info_Version	general_info_VolumeNum
7d71e808fe6671534a3019b7be487ba8a0f98390	(1.0, 1.0, 1.0)	{'LoG': {}, 'Original': {}, 'Wavelet': {}}	81ea194eace8c0a0e8b410c9961f6be4db1053bd	1.1.1-beta1	1
acd8228c36628cbfaefc90db997bb9ba7b57d5b7	(1.0, 1.0, 1.0)	{'LoG': {}, 'Original': {}, 'Wavelet': {}}	ed1acee93d3b6f972b33d5098b2bc669c92d61f9	1.1.1-beta1	1
a7529fcb4f8c9ad8d0d680fb4c32473100b7d25e	(1.0, 1.0, 1.0)	{'LoG': {}, 'Original': {}, 'Wavelet': {}}	7fcefdd8bc315f1b73cc51c9108f77e298e1b2f6	1.1.1-beta1	1
dbc259ac4a070f69d98a1a383291a01e3a986bc5	(1.0, 1.0, 1.0)	{'LoG': {}, 'Original': {}, 'Wavelet': {}}	08998bfb5a70086298af980fc48a523d8d6caf00	1.1.1-beta1	1
89de885ea7aa7e2148a38702fb9ea9a34295bb7a	(1.0, 1.0, 1.0)	{'LoG': {}, 'Original': {}, 'Wavelet': {}}	42c9c7dbe0d7d99a95a93e209a2e9070df3b9f45	1.1.1-beta1	1
60fced09f191524b644aded04cb0252ee2b6fc2b	(1.0, 1.0, 1.0)	{'LoG': {}, 'Original': {}, 'Wavelet': {}}	b3006a54f772b59ca23bfd4121c0465d6f90a20a	1.1.1-beta1	1
4995f6ecbf9ab9416d2b5b690525de6bf6f58dd9	(1.0, 1.0, 1.0)	{'LoG': {}, 'Original': {}, 'Wavelet': {}}	ba6d2282f9f79a34d6b917f1c4a83e72da83bd93	1.1.1-beta1	1
cd5f1a7045e07876b5e7fb747ce134ffc613ac6b	(1.0, 1.0, 1.0)	{'LoG': {}, 'Original': {}, 'Wavelet': {}}	a279de4189d295f9f4e97afc2bc0d4bf5271fa3d	1.1.1-beta1	1
de70f76ee5c3c953e9b5710409ec3282e7258cde	(1.0, 1.0, 1.0)	{'LoG': {}, 'Original': {}, 'Wavelet': {}}	978c3b0579f2c16106ff60ab768b79e68895fc13	1.1.1-beta1	1
be3af75292a035c2daeed04f83ce4a3e84e6f871	(1.0, 1.0, 1.0)	{'LoG': {}, 'Original': {}, 'Wavelet': {}}	63ae801cae4307e1a23126db2dcfbac20a9d1689	1.1.1-beta1	1
778f4b4a8ea6a18c8da5046951d7aed66d34a6b2	(1.0, 1.0, 1.0)	{'LoG': {}, 'Original': {}, 'Wavelet': {}}	fac203d585d218f43bce567c467a047f1d74a5f8	1.1.1-beta1	1
43820772bb86565ffd947ea76a7e037d208aa784	(1.0, 1.0, 1.0)	{'LoG': {}, 'Original': {}, 'Wavelet': {}}	7dbf67c04b05b93bbe70a69ad1d9d8009befa180	1.1.1-beta1	1
5ae037077e6ef4bfb56a0af8bd4e0f4ba3c34c57	(1.0, 1.0, 1.0)	{'LoG': {}, 'Original': {}, 'Wavelet': {}}	741faac1c9898c319c32037cb2462b7bfc8b7e38	1.1.1-beta1	1
4824fd3555f87f78f06c42bcaece5153075edba7	(1.0, 1.0, 1.0)	{'LoG': {}, 'Original': {}, 'Wavelet': {}}	25da6efa54c9e4a20db3bb8aeced4cce9a188060	1.1.1-beta1	1
069fd1be988755e457288f8d74b75c3c15fefbc4	(1.0, 1.0, 1.0)	{'LoG': {}, 'Original': {}, 'Wavelet': {}}	855e7b4e60b08595b7b211943364b4b933a21d09	1.1.1-beta1	1
cc30fa586f37ea761ac29cb87ac70d41ad928a62	(1.0, 1.0, 0.9999999999999999)	{'LoG': {}, 'Original': {}, 'Wavelet': {}}	4ad68e1625df8423f1569fae250e76ea3d61f483	1.1.1-beta1	1
24235a029862f96ddac62459ec91f1189d5dc2d9	(1.0, 1.0, 1.0)	{'LoG': {}, 'Original': {}, 'Wavelet': {}}	bf67230e5312c13babdf9b24d3528e7fde7d2cce	1.1.1-beta1	1
95880cb500e05e8a3cd7fd96ee5264dcb1e450d9	(1.0, 1.0, 0.9999999999999999)	{'LoG': {}, 'Original': {}, 'Wavelet': {}}	69fc83e0145a841c83613fdcb6e09a3721f623fa	1.1.1-beta1	1
d02f4eeadf32f7bc50c30c0d58b7691f5080827b	(1.0, 1.0, 1.0)	{'LoG': {}, 'Original': {}, 'Wavelet': {}}	22456cf613100b90b3dc77f854701ef6f0dc0cf9	1.1.1-beta1	1
1ba33fcb87a615e09a14f5560918e3602925af8c	(1.0, 1.0, 1.0)	{'LoG': {}, 'Original': {}, 'Wavelet': {}}	d97d840007d67e6276bdac5c2ccb43299db97e73	1.1.1-beta1	1
56955aa6d8ad23566e6500c3745a0238a7b67410	(1.0, 1.0, 1.0)	{'LoG': {}, 'Original': {}, 'Wavelet': {}}	87616e3ff5e52c3730211f0a0cca8e49396407d7	1.1.1-beta1	1
78c291ea9c332118a69a05e48f1c08b14d5e7c7f	(1.0, 1.0, 0.9999999999999999)	{'LoG': {}, 'Original': {}, 'Wavelet': {}}	21f66a609af7596531b66c6cee75fdf4b1780566	1.1.1-beta1	1
03fd1116f7ebbc9b0236bacd710d54e70f43222b	(1.0, 1.0, 1.0)	{'LoG': {}, 'Original': {}, 'Wavelet': {}}	c55745978b7739cd230364e2cfa348d6403bf9f0	1.1.1-beta1	1
e83cabcb67c6acdba6290c6523c55038a9d3bec9	(1.0, 1.0, 1.0)	{'LoG': {}, 'Original': {}, 'Wavelet': {}}	2914d29b44f83d60fdf4f5034bfc0227ab2da33a	1.1.1-beta1	1
bab90b53bb641677fc25b517e30253e88e6c1551	(1.0, 1.0, 0.9999999999999999)	{'LoG': {}, 'Original': {}, 'Wavelet': {}}	e33ee53b66b9f21500958b6c5e0b799f1af11359	1.1.1-beta1	1
331a674ae99f13aad88c4d4d11ba8a24a766d352	(1.0, 1.0, 1.0)	{'LoG': {}, 'Original': {}, 'Wavelet': {}}	01c41f61ea0e479bd4ce6145ce00ca03c99eb51a	1.1.1-beta1	2
df35a3918b83254f64f08cd4d9c7ea699dc12827	(1.0, 1.0, 1.0)	{'LoG': {}, 'Original': {}, 'Wavelet': {}}	3d56492f2ececb879c56742f732c1f817d20036e	1.1.1-beta1	1
90a2432541c8208da8e51d3eed094103df5926b0	(1.0, 1.0, 1.0)	{'LoG': {}, 'Original': {}, 'Wavelet': {}}	89a67efb35577e690f048f1606e8e054ee5fd632	1.1.1-beta1	1
1b368f9f9427ad60615923d9ece22534d031e675	(1.0, 1.0, 1.0)	{'LoG': {}, 'Original': {}, 'Wavelet': {}}	ea8b11de95f8e84fa9b49e3b6fa5e1fbbd2262d7	1.1.1-beta1	1
4b5e7d8d777c8fcef9804e75c9fc5e48ab1a0412	(1.0, 1.0, 1.0)	{'LoG': {}, 'Original': {}, 'Wavelet': {}}	f2a006e5fceecbed767c0800fd3710a4e35903c0	1.1.1-beta1	1
86c04aea820ab661dfd5c877cbf21e4cf8defd7d	(1.0, 1.0, 1.0)	{'LoG': {}, 'Original': {}, 'Wavelet': {}}	b295179057078e115779cad1c54140679c9a2fa2	1.1.1-beta1	1
3bc54655740cee2499184a8461d0ed43db11a9ac	(1.0, 1.0, 1.0)	{'LoG': {}, 'Original': {}, 'Wavelet': {}}	17344b36a7d76ffc60324546ec6f09d439f94cab	1.1.1-beta1	1
bd7e8746c1d829d9a88e7d61e8e554f2b32f7740	(1.0, 1.0, 1.0)	{'LoG': {}, 'Original': {}, 'Wavelet': {}}	140a6ef5052c66cdca2b421248431441fb6acae3	1.1.1-beta1	1
f3c7c875021eddbd98b64055ac1af6012a53427c	(1.0, 1.0, 1.0)	{'LoG': {}, 'Original': {}, 'Wavelet': {}}	1367d6a43288078e600046b075ba3593849accc9	1.1.1-beta1	1
b843096e4fc13a50b9dd6fd33ac8111282f33a52	(1.0, 1.0, 1.0)	{'LoG': {}, 'Original': {}, 'Wavelet': {}}	74e6d06a937cd03aaa8cadada8605ab9807e167c	1.1.1-beta1	1
62f24f52dfe1b55bef588eae84ed26a62bec8c90	(1.0, 1.0, 1.0)	{'LoG': {}, 'Original': {}, 'Wavelet': {}}	e9b8ea0df935c1d3d99a9a1e98fb68f17c90c063	1.1.1-beta1	1
001216bee1af2b3881edcf5e01759d697a0150ff	(1.0, 1.0, 1.0)	{'LoG': {}, 'Original': {}, 'Wavelet': {}}	989bea60b422dd3305e1108aba7f415fb4b9cb4a	1.1.1-beta1	1
425d9499496351afcb3b43d88e01590e34770cc2	(1.0, 1.0, 1.0)	{'LoG': {}, 'Original': {}, 'Wavelet': {}}	4030f7f5dee7b880a44f9efdf82cfc0f51847973	1.1.1-beta1	1
e5b1250a787ca1ed95ef710f82c70cfe8b39a0b0	(1.0, 1.0, 1.0)	{'LoG': {}, 'Original': {}, 'Wavelet': {}}	41256b0fbc7e3a3cfb951f6f9d4e34f8cd74be1a	1.1.1-beta1	1
92005832804dbdf44d0453ea2aaf4b3be7cf95ee	(1.0, 1.0, 1.0)	{'LoG': {}, 'Original': {}, 'Wavelet': {}}	a90b21f800a61a2aa0769c8c20bc1ec240fba74c	1.1.1-beta1	1
2c3510223208746f011c6cc80d234d225f19eb6c	(1.0, 1.0, 1.0)	{'LoG': {}, 'Original': {}, 'Wavelet': {}}	66cfc61bf4175890f9952acd70ed46127fae7b64	1.1.1-beta1	1
7b9e96024ec8928dc40f07539fda7467232b1db0	(1.0, 1.0, 1.0)	{'LoG': {}, 'Original': {}, 'Wavelet': {}}	7ab81729cf6d7d083da26be7496ff8e91b6e05a8	1.1.1-beta1	1
f1a89c80faf8e38162c556eca91776d271727649	(1.0, 1.0, 1.0)	{'LoG': {}, 'Original': {}, 'Wavelet': {}}	64f241a269b6a94831c1a3855fff7dc5d5e988f8	1.1.1-beta1	1
ecf3abdee7a7aa29590029351da7e80de30b5748	(1.0, 1.0, 1.0)	{'LoG': {}, 'Original': {}, 'Wavelet': {}}	1cbba30d0650e034de1eeaad127972e601f496bd	1.1.1-beta1	1
7e56a6fa7582b1efd41526d493b9dd57779f00b0	(1.0, 1.0, 1.0)	{'LoG': {}, 'Original': {}, 'Wavelet': {}}	70cf0af9a75113454883672d35748ee1e44e8e42	1.1.1-beta1	1
124810c6a78ec91138f06ece964dbe2c6aaa17fe	(1.0, 1.0, 1.0)	{'LoG': {}, 'Original': {}, 'Wavelet': {}}	1b00bd61166160a9a5684cbb7165f5eca123ca53	1.1.1-beta1	1
f60bb3e1c364756a8ccbe1d8f9fc0855f7ccd1f6	(1.0, 1.0, 1.0)	{'LoG': {}, 'Original': {}, 'Wavelet': {}}	b813e48077b7aee51ccd734abd6fa3d85de02ff6	1.1.1-beta1	1
6a36618ca2f75a85a5128fc656f10e6180d8cc57	(1.0, 1.0, 1.0)	{'LoG': {}, 'Original': {}, 'Wavelet': {}}	9dbe598a6eb611053690176af55f980538d9451c	1.1.1-beta1	1
08940100339b89405caae24ed3babf29aa4c22bb	(1.0, 1.0, 1.0)	{'LoG': {}, 'Original': {}, 'Wavelet': {}}	525ede987869f2ddb905dbab7ceb5b4349d58bf6	1.1.1-beta1	1
39ccd562aa9d39222569d219fb2eb8003df0d30c	(1.0, 1.0, 1.0)	{'LoG': {}, 'Original': {}, 'Wavelet': {}}	206aebef4e9c20d071cfcd94b697db5a72dd096b	1.1.1-beta1	1
2c2efec1dbcd2cd1429b1960dadedb922e5aa603	(1.0, 1.0, 1.0)	{'LoG': {}, 'Original': {}, 'Wavelet': {}}	db42c71c3723d5ea3462c3ffe286e2446e98169e	1.1.1-beta1	1
ff839a7865e4a66e9ed3f151a45992caa8c43eb4	(1.0, 1.0, 1.0)	{'LoG': {}, 'Original': {}, 'Wavelet': {}}	6da369e2fe4fc8f0d3a00e3185b7f39ff35aa07d	1.1.1-beta1	1
40b3697fef4a5dc9de3a9bc4bb29356337cf24da	(1.0, 1.0, 1.0)	{'LoG': {}, 'Original': {}, 'Wavelet': {}}	d8031befef2c2d0d207371c2508ee49d6f6c12bd	1.1.1-beta1	1
8b1df46b0cfc61193922dedcc4296bb21c2e773c	(1.0, 1.0, 1.0)	{'LoG': {}, 'Original': {}, 'Wavelet': {}}	53ee7351a53da57fe0197e0dd621a35bf81241a9	1.1.1-beta1	1
303aa75ad2b720c7d7329d77466a4c9967dcdd29	(1.0, 1.0, 1.0)	{'LoG': {}, 'Original': {}, 'Wavelet': {}}	f2ec84f2dbad4bf8d81ddb083d9dc217579096ec	1.1.1-beta1	1
dcd6cede20943bb052f20e3f05664f6ce47536a3	(1.0, 1.0, 1.0)	{'LoG': {}, 'Original': {}, 'Wavelet': {}}	caa1cf1d3b591dc3b572b3ecdf2ac8bb432cf202	1.1.1-beta1	1
882c441365cfbede7ef6456bba09d3eb7759da54	(1.0, 1.0, 1.0)	{'LoG': {}, 'Original': {}, 'Wavelet': {}}	9be3d0fc13e431a91a53df48ad5375f18faa6d92	1.1.1-beta1	1
082856f2a12425cee867d722afa3b93a8d1c8f30	(1.0, 1.0, 1.0)	{'LoG': {}, 'Original': {}, 'Wavelet': {}}	d447f60e84902c5080399fc39a5223fe86b2ccf4	1.1.1-beta1	1
0f850fec7bb5bc5714a52d63844862f8e428496d	(1.0, 1.0, 1.0)	{'LoG': {}, 'Original': {}, 'Wavelet': {}}	802ab05516e78f26017f7fc607d933e490b5fb08	1.1.1-beta1	1
425351daad40d6a5866008f88407c9c301ab9d74	(1.0, 1.0, 1.0)	{'LoG': {}, 'Original': {}, 'Wavelet': {}}	05ec6e730288f0eca8759f27e270975d0e9fb0aa	1.1.1-beta1	1
286477e11a09101dd019b73125ded81c1fb2f575	(1.0, 1.0, 1.0)	{'LoG': {}, 'Original': {}, 'Wavelet': {}}	112ff205e96906385e93affca1f6f8fe113f479e	1.1.1-beta1	1
05028e3b7ffbbe3f7d7095782ca680d0b92bd767	(1.0, 1.0, 1.0)	{'LoG': {}, 'Original': {}, 'Wavelet': {}}	a7e3e5ae2380087278a4b9d86c4525b6e18b3bac	1.1.1-beta1	1
a1ef95f7dc3da464757d168b8b06989cff9fa3d9	(1.0, 1.0, 1.0)	{'LoG': {}, 'Original': {}, 'Wavelet': {}}	3852c3b774256dde3506938263b65be8f38739b3	1.1.1-beta1	1
53e9cc2d3a433a1c0d3f6efa685675ffdc80dd24	(1.0, 1.0, 1.0)	{'LoG': {}, 'Original': {}, 'Wavelet': {}}	2739049555e6490b9e619b3626ed6a013fefd819	1.1.1-beta1	1
99adb35d859ae1c7f7c50bfcdd3d64a7cbf59bf0	(1.0, 1.0, 1.0)	{'LoG': {}, 'Original': {}, 'Wavelet': {}}	69a10f018197c58ed43596a7fb09bc63f1bf34f9	1.1.1-beta1	1
bec1de8d9329b8187163106b831361859cd7bf20	(1.0, 1.0, 1.0)	{'LoG': {}, 'Original': {}, 'Wavelet': {}}	81981d0473863de71308d9074097bcac3a387660	1.1.1-beta1	1
2ca04a708c5b413bac22cde7644c886624834bf3	(1.0, 1.0, 1.0)	{'LoG': {}, 'Original': {}, 'Wavelet': {}}	2fd0eec52f3ea20d2e3526d4361e5fb3d857104d	1.1.1-beta1	1
68faced8e8d9f36b5bf826127fc1ef66dc61dcfc	(1.0, 1.0, 1.0)	{'LoG': {}, 'Original': {}, 'Wavelet': {}}	3a606c00c8c58c79960d22f7fe32f38edd149308	1.1.1-beta1	1
5627858df0e21252bee88b75a293758b47022e9a	(1.0, 1.0, 1.0)	{'LoG': {}, 'Original': {}, 'Wavelet': {}}	2685accc10ae3b355bc380f897785e4f3fa8de6c	1.1.1-beta1	1
8d140ccfe8a2e02737dd8387970765181737d54f	(1.0, 1.0, 1.0)	{'LoG': {}, 'Original': {}, 'Wavelet': {}}	957340b48f815c97172340320b747938292b1efa	1.1.1-beta1	1
f8fcbac275778cd107756269b7306710466567cc	(1.0, 1.0, 1.0)	{'LoG': {}, 'Original': {}, 'Wavelet': {}}	245114e9e57cc170a4ef0ebefa964e0bab9b2d04	1.1.1-beta1	1
ceb6b308f0598622f4c495c05a54895ecfa308f4	(1.0, 1.0, 1.0)	{'LoG': {}, 'Original': {}, 'Wavelet': {}}	a17931260f38dfbd057b0f7260cc01ecef28b2f9	1.1.1-beta1	1
e567a92e50fcdc68a599ba598cdd80985307759c	(1.0, 1.0, 1.0)	{'LoG': {}, 'Original': {}, 'Wavelet': {}}	6b7e4daf2c0cdf141d93f885c0a4f5173bc653cf	1.1.1-beta1	1
bb83d60db485faac72a5af79b593e920ddc3806a	(1.0, 1.0, 1.0)	{'LoG': {}, 'Original': {}, 'Wavelet': {}}	1e308fcf35c51e93b15c6a22c9a5df295f14fcfd	1.1.1-beta1	1
a73d9a89b71cea5258e59ad0bfee7eb241a94258	(1.0, 1.0, 1.0)	{'LoG': {}, 'Original': {}, 'Wavelet': {}}	283a4503d14bd63c2d2b2c7f8f0c8e98e96e871d	1.1.1-beta1	1
6f7dbc63238a8f35b442abd4d0df881a5c90a25c	(1.0, 1.0, 1.0)	{'LoG': {}, 'Original': {}, 'Wavelet': {}}	58ba5a4f0d3db54f41eb81426c07c87d4f5fc7d9	1.1.1-beta1	1
ab6ef8e1c396418632c92d0f36915895773e5e7c	(1.0, 1.0, 1.0)	{'LoG': {}, 'Original': {}, 'Wavelet': {}}	046fa6ac08d210d76912301a1bb9a979a194123a	1.1.1-beta1	1
d9f46cc9c5c2019d299615d06d6993a53e2bd258	(1.0, 1.0, 1.0)	{'LoG': {}, 'Original': {}, 'Wavelet': {}}	0a4893fcb59445ac97df19e97b0c867e05cd050e	1.1.1-beta1	1
5b2de6388b14d2c1e149157e5ff79b32e4fcc5e4	(1.0, 1.0, 1.0)	{'LoG': {}, 'Original': {}, 'Wavelet': {}}	58c5bbb1b88fd497830b4a9ea325871e0d3d6bf1	1.1.1-beta1	1
341adba7f1fdbadb81a40490335321ac03811a01	(1.0, 1.0, 1.0)	{'LoG': {}, 'Original': {}, 'Wavelet': {}}	ae1d820d9ec42c0e57d98bdfec71c0e894402b53	1.1.1-beta1	1
7505a548e249926abe434b898d8fc2e5763f238d	(1.0, 1.0, 1.0)	{'LoG': {}, 'Original': {}, 'Wavelet': {}}	9353fc69bed149b7e45c80244e1d08c5e0949e88	1.1.1-beta1	1
eb16fbadd3bea123c4b0fae2635d350cb7791af5	(1.0, 1.0, 1.0)	{'LoG': {}, 'Original': {}, 'Wavelet': {}}	ef9d1632e1bd2282854b3c7729bc50650ad0b525	1.1.1-beta1	1
2f247bc2a882d7a7b134d0035e403c3a80a8d389	(1.0, 1.0, 1.0)	{'LoG': {}, 'Original': {}, 'Wavelet': {}}	9b01a68aca38e627bb88fa9b88c7681a52b0d685	1.1.1-beta1	1
88011fc8e4004e2da019b1e69eeb737f6b1b8a5c	(1.0, 1.0, 1.0)	{'LoG': {}, 'Original': {}, 'Wavelet': {}}	badf7bcd8b22b80872c195f2d37e3bfbd76d860c	1.1.1-beta1	1
27878584982c4556d58290555364c0bf9271a6b2	(1.0, 1.0, 1.0)	{'LoG': {}, 'Original': {}, 'Wavelet': {}}	cd81613dd8959efdb13c16adb7de5bd2d1dd5c21	1.1.1-beta1	1
bb975f93f3220af49485a0f8e3cbc6248da84fe9	(1.0, 1.0, 1.0)	{'LoG': {}, 'Original': {}, 'Wavelet': {}}	c60de3548033458f1155019aea5c490c6bc14628	1.1.1-beta1	1
c038c6ada1b41149a97d5d58676ae2c625c43d0e	(1.0, 1.0, 1.0)	{'LoG': {}, 'Original': {}, 'Wavelet': {}}	825a8c9055f156a787c44fb70811a646cd827cad	1.1.1-beta1	1
7b65b3b77f26f606f1ad35358001d7167203c041	(1.0, 1.0, 1.0)	{'LoG': {}, 'Original': {}, 'Wavelet': {}}	0e75dfe16ce5907d9237cdebed2916b2cef807c7	1.1.1-beta1	2
1e75d9bc15f8092472c67d8c924d8786f94c1e78	(1.0, 1.0, 1.0)	{'LoG': {}, 'Original': {}, 'Wavelet': {}}	1b8fa672c230819f9a322ba60b904dbe2f89d902	1.1.1-beta1	1
38e36f9da7fef61903791c86f3239e06c671c416	(1.0, 1.0, 1.0)	{'LoG': {}, 'Original': {}, 'Wavelet': {}}	6c337955ea98b61f885bd90ca3ad719cc70883d5	1.1.1-beta1	1
fd24cf5e6b7434ab0bfc65f6c8a8ca90b14ee927	(1.0, 1.0, 1.0)	{'LoG': {}, 'Original': {}, 'Wavelet': {}}	e7f583bc6c7ead8af8c1bf7e84678f5adb2f45a3	1.1.1-beta1	1
a2f8c625d6f5fe6dfeda8762d8a180e51e1e0860	(1.0, 1.0, 1.0)	{'LoG': {}, 'Original': {}, 'Wavelet': {}}	35566368c109cbd0ec25bf1f99023a979c4fb1b0	1.1.1-beta1	1
98c354e74e35b0436885e9fa927228ab2a4fab7d	(1.0, 1.0, 1.0)	{'LoG': {}, 'Original': {}, 'Wavelet': {}}	1a130461bfb1b7d33a7a40404ee26a33764712ed	1.1.1-beta1	1
c907b4bccf40f0ddf63e2ab0ea5b2631e2a3fa9e	(1.0, 1.0, 1.0)	{'LoG': {}, 'Original': {}, 'Wavelet': {}}	4731d5b23d25e6e2e21932c25fa76dd7ecf97bd2	1.1.1-beta1	1
bab82adc990efd6447998cba8f0e2c5158114246	(1.0, 1.0, 1.0)	{'LoG': {}, 'Original': {}, 'Wavelet': {}}	3de6e655fb7b9f62aa7cea0f4424524e093404bd	1.1.1-beta1	1
a82808907bd5f4f4342a69e2ffa3a4b2a00a0435	(1.0, 1.0, 1.0)	{'LoG': {}, 'Original': {}, 'Wavelet': {}}	0ff337f045e6af95e0ac4ba2ac3b4a9202610092	1.1.1-beta1	1
a3ef48a21870bef679038cdf1da0a554774524e5	(1.0, 1.0, 1.0)	{'LoG': {}, 'Original': {}, 'Wavelet': {}}	86bc40991c5657d3d30f445f1656b6ced844a0ba	1.1.1-beta1	1
060480befcbe7178b54f76651aef987231588545	(1.0, 1.0, 1.0)	{'LoG': {}, 'Original': {}, 'Wavelet': {}}	0b4d6e1d7e47b680172f4787ca0b8b09375a3d52	1.1.1-beta1	1
d271553e8e5eca7e73252b2e6e52b09a3019aca8	(1.0, 1.0, 1.0)	{'LoG': {}, 'Original': {}, 'Wavelet': {}}	fc38f0fccd5a6aad95d589a242de1c54370f0be1	1.1.1-beta1	2
1751d2ed951684b0961cc9a1157a921e2543d279	(1.0, 1.0, 1.0)	{'LoG': {}, 'Original': {}, 'Wavelet': {}}	24958630658f384b79d77d24cdda5db0c96e837f	1.1.1-beta1	1

general_info_VoxelNum	original_shape_Maximum3DDiameter	original_shape_Compactness2	original_shape_Maximum2DDiameterSlice	original_shape_Sphericity	original_shape_Compactness1	original_shape_Elongation
361	33.67491648	0.51122997	30	0.799598742	0.037932113	1.09688974
1403	56.68333088	0.370168664	55.07267925	0.718014504	0.032277412	1.109728469
177	28.61817604	0.467457488	27.16615541	0.77609349	0.036271869	1.524308363
1933	69.84268036	0.331811698	55.15432893	0.692304622	0.030559393	1.208858578
101	20.34698995	0.636533913	18.24828759	0.860215339	0.042326236	1.096013263
244	27.49545417	0.560355838	24.73863375	0.824431607	0.039712829	1.039235613
70	33	0.332420856	24.18677324	0.69272802	0.030587431	2.563770718
401	39.79949748	0.35737926	34.20526275	0.709648216	0.031714915	1.412741723
1476	56.60388679	0.42821041	53.16013544	0.753735679	0.034715825	1.210410642
2319	82.8673639	0.281809415	54.33231083	0.655619455	0.028162852	1.553324876
376	38.06573262	0.499394215	33.54101966	0.793379855	0.037490448	1.303889034
757	65.520989	0.326400762	36.24913792	0.688520784	0.030309199	1.706470251
1366	49.56813493	0.445935898	48.46648326	0.763995519	0.035427058	1.082003604
528	40.91454509	0.447131405	34.20526275	0.76467764	0.035474515	1.322133355
364	44.29446918	0.275834025	27.65863337	0.650952469	0.027862674	1.821897805
10828	121.2311841	0.250059051	96.18731725	0.63001012	0.026528956	1.323849293
217	40.02499219	0.288487272	36.49657518	0.660757679	0.028494576	1.635484725
1552	78.74642849	0.352928861	49.65883607	0.706690183	0.031516825	2.043457768
182	32.31098884	0.410420878	21.63330765	0.743149999	0.03398706	1.679612091
2774	78.05767099	0.393394622	52.39274759	0.732728031	0.033274619	1.475362
5075	98.49873096	0.239658785	64.89992296	0.621151852	0.025971412	1.703359114
909	61.91930232	0.263825696	37.94733192	0.641365652	0.02724943	1.461930169
2920	89.04493248	0.236188114	69.97142274	0.618138811	0.02578267	1.248948112
133	31.46426545	0.258862504	25.8069758	0.637318291	0.0269919	1.330698466
960	66.13622306	0.170769967	53.07541804	0.55480091	0.021923234	1.407649687
302	52.73518749	0.376400322	40.24922359	0.722021277	0.032547967	1.579700074
2289	74.45804188	0.278984721	69.26037828	0.653421579	0.028021352	1.026792046
3895	103.3585991	0.162751618	89.49860334	0.545977951	0.021402354	1.543579385
3716	86.37708029	0.223500616	61.84658438	0.606866142	0.025080618	1.323100778
1693	59.16924877	0.356828331	51.35172831	0.709283368	0.03169046	1.076214663
4201	96.04686356	0.314492221	64.13267498	0.680043409	0.029751155	1.757601844
8833	120.2247895	0.259987054	95.34149149	0.638239837	0.027050465	1.197414334
1389	57.31491952	0.437799742	48.46648326	0.759320574	0.035102385	1.24537177
15	9	0.958577381	6.708203932	0.9859973	0.051941258	1.120352613
113	27.82085549	0.435882189	15	0.758210349	0.035025427	1.893719217
1334	52.39274759	0.469807866	45.79301257	0.77739205	0.036362942	1.04882128
6691	94.58329662	0.301777911	81.88406438	0.670752782	0.02914356	1.136146565
3032	77.12976079	0.433900777	64.76109943	0.757059725	0.034945728	1.161898007
321	36.24913792	0.348783421	33.54101966	0.703912392	0.031331183	1.279809145
537	37.70941527	0.472534019	35.11409973	0.778892807	0.036468291	1.066321222
715	49.56813493	0.235257579	49.47726751	0.617325961	0.025731831	1.132999029
381	40.47221269	0.384585538	40.02499219	0.727217492	0.032899959	1.054755818
824	66.2721661	0.337462043	66.2721661	0.696212223	0.030818489	1.383119918
439	47.24404724	0.379676461	30.59411708	0.724110021	0.032689307	1.565310429
1115	56.68333088	0.404426695	45.69463864	0.739514349	0.033737957	1.108281313
6122	114.629839	0.232727165	84.53401682	0.615104673	0.025593072	1.410374521
2746	78.40280607	0.159278208	62.42595614	0.542065941	0.02117274	1.346112166
228	26.32489316	0.612776809	23.43074903	0.849377542	0.041528863	1.048126122
22	11.22497216	0.929771853	6.708203932	0.976020183	0.05115488	1.424457904
146	27	0.423798159	21.21320344	0.751137922	0.034536507	1.584030679
4080	83.03011502	0.363990652	75.53806987	0.713997586	0.032006928	1.190408773
403	31.46426545	0.668339159	27.65863337	0.874310383	0.04337079	1.182603243
3195	82.97590012	0.313574072	54.74486277	0.679380976	0.029707695	1.539945657
239	28.93095228	0.5153555	26.83281573	0.801743852	0.038084857	1.163046593
11306	151.3737097	0.2557245	124.7798061	0.634732564	0.026827799	1.882426395
335	32.86335345	0.39776925	29.54657341	0.735434053	0.033459117	1.112985964
872	97.85703858	0.099191859	53.16013544	0.462905147	0.016708478	2.157499729
458	60.29925373	0.164626671	43.26661531	0.548066679	0.021525288	1.865080299
354	33.67491648	0.346110953	28.46049894	0.702109929	0.031210918	1.137971474
466	40.47221269	0.564684729	40.02499219	0.826549145	0.03986593	1.372527415
1455	54.41507144	0.463251166	47.4341649	0.773758632	0.036108308	1.128568934
6776	99.94998749	0.31531787	70.22819946	0.680638004	0.029790183	1.408266434
4074	112.3699248	0.270733293	66.61080993	0.646915001	0.027603853	1.706231749
1782	71.62401832	0.224250859	66.61080993	0.607544423	0.025122678	1.264645033
53	15.58845727	0.795776111	15.29705854	0.926681087	0.047325404	1.203293211
1152	67.94850992	0.239349661	56.92099788	0.620884673	0.025954656	1.604431244
105	23.62202362	0.434783357	18.97366596	0.757572679	0.034981251	1.255922388
3467	72.18725649	0.490816637	63.5688603	0.788811243	0.037167086	1.436103954
143	21.21320344	0.708711424	20.1246118	0.891572117	0.04466153	1.12516168
1792	62.92853089	0.366646954	57.31491952	0.71573023	0.032123504	1.23127302
2599	83.30066026	0.191315163	74.09453421	0.576213104	0.023204573	1.062191719
567	41.78516483	0.506228599	38.41874542	0.796982696	0.037746111	1.547007106
7057	96.09370427	0.405221287	81.60882305	0.739998348	0.033771084	1.135954645
10834	119.1721444	0.273788202	109.2016483	0.649339133	0.027759155	1.151724195
14519	116.1507641	0.407222456	109.4897255	0.741214499	0.03385437	1.0228164
446	52.47856705	0.301381527	41.78516483	0.670458976	0.029124414	1.502843692
456	45.79301257	0.336103891	40.36087214	0.695276974	0.03075641	1.481933359
224	28.46049894	0.517625905	24.18677324	0.802919492	0.038168657	1.227144431
913	56.1248608	0.263743012	48.83646179	0.641298643	0.02724516	1.064431399
2704	82.97590012	0.311268982	72.99315036	0.677712166	0.029598302	1.024986278
149	36.24913792	0.338765715	36.12478374	0.697107599	0.03087796	1.599849715
865	63	0.231448553	56.04462508	0.613976134	0.02552267	1.499341448
4929	88.74119675	0.338786767	67.08203932	0.697122039	0.03087892	1.305460787
31	13.07669683	0.778493133	12.72792206	0.91992325	0.046808667	1.254444344
4962	80.61017306	0.347221061	72.62231062	0.702859771	0.031260931	1.113055665
3507	91.9782583	0.11772184	84.48076704	0.490101104	0.018202347	1.532236141
365	35.24202037	0.483554661	28.46049894	0.784901558	0.036891105	1.156199342
589	78.51751397	0.212639888	56.92099788	0.596872512	0.024463649	2.602193913
50	21	0.659164411	19.20937271	0.870291183	0.043072071	1.886083631
13	10.81665383	0.67300516	10.81665383	0.876340329	0.043521923	0
3429	119.4361754	0.257604169	86.37708029	0.636283941	0.026926216	1.531259238
304	39.57271787	0.440325147	21.84032967	0.760777797	0.035203482	1.779969341
416	40.36087214	0.328486325	35.11409973	0.689984124	0.030405876	1.209862722
3652	86.63717447	0.35471917	70.29224708	0.707883115	0.031596662	1.30638907
7222	85.53946458	0.468064941	78.23042886	0.776429517	0.036295429	1.033693612
63	18	0.697229087	12.72792206	0.886730879	0.044298256	1.311296104
680	43.05810028	0.508331064	37.94733192	0.798084513	0.037824413	1.464177173
24	13.07669683	0.801280772	6.708203932	0.928812904	0.047488805	1.668265066
163	62.13694553	0.27763441	21.63330765	0.652365667	0.027953457	4.481763927
280	51.35172831	0.227274645	40.02499219	0.610262939	0.025291487	1.586433773

original_shape_SurfaceVolumeRatio	original_shape_Volume	original_shape_SphericalDisproportion	original_shape_Flatness	original_shape_SurfaceArea	original_shape_Maximum2DDiameterColumn
0.283131616	9747	1.250627281	1.316029163	2759.683857	33.54101966
0.200544349	37881	1.392729526	1.229381183	7596.820486	50.28916384
0.369934135	4779	1.288504559	1.098151844	1767.915232	19.20937271
0.186919676	52191	1.444450851	1.363354377	9755.524836	57.31491952
0.402391282	2727	1.162499615	1.298150139	1097.321026	20.1246118
0.312904369	6588	1.212956892	1.146339074	2061.413984	24.73863375
0.564634532	1890	1.443567996	1.403433097	1067.159265	18.24828759
0.308038474	10827	1.409148896	1.158420142	3335.132561	39
0.187837231	39852	1.326725042	1.10373971	7485.689323	53.16013544
0.185756543	62613	1.525275054	1.061811633	11630.77445	79.20227269
0.281504766	10152	1.260430289	1.138930317	2857.836387	27.65863337
0.256889994	20439	1.452388981	1.446565394	5250.574588	44.59820624
0.190161163	36882	1.3089082	1.124555346	7013.52402	49.47726751
0.260819368	14256	1.307740605	1.035516137	3718.240905	35.11409973
0.346827184	9828	1.536210471	1.167762503	3408.617564	38.18376618
0.115655403	292356	1.587276089	1.257609593	33812.55114	111.3642672
0.405976355	5859	1.513414118	1.601230402	2378.615462	36.12478374
0.19701679	41904	1.41504725	1.131016353	8255.791574	49.65883607
0.382762391	4914	1.345623361	1.078266485	1880.894389	26.83281573
0.156573374	74898	1.364762855	1.260547697	11727.03259	67.41661516
0.151014878	137025	1.609912289	1.075387148	20692.81367	97.71898485
0.259459282	24543	1.559172989	1.515238608	6367.909148	54.74486277
0.182452257	78840	1.617759606	1.500785383	14384.53594	76.4852927
0.495514012	3591	1.56907469	1.68179276	1779.390818	23.43074903
0.294533904	25920	1.802448377	1.624196742	7634.318793	56.92099788
0.33276979	8154	1.385000737	1.410503295	2713.404871	21.21320344
0.187192078	61803	1.530405533	1.478945814	11569.03201	59.16924877
0.187651459	105165	1.831575795	1.458280132	19734.36565	94.15412896
0.17149234	100332	1.647809838	1.162127515	17206.16945	76.4852927
0.190688327	45711	1.409873748	1.083761913	8716.5541	54.08326913
0.146906836	113427	1.470494363	1.171540528	16663.20174	75.89466384
0.122182653	238491	1.566809125	1.207296429	29139.46316	99.72462083
0.190269986	37503	1.316966818	1.158514851	7135.695304	55.31726674
0.662913288	405	1.01420156	1.426439553	268.4798816	8.485281374
0.439757931	3051	1.318895214	1.231111351	1341.701448	25.8069758
0.188366715	36018	1.286352234	1.238726347	6784.592359	44.29446918
0.127537151	180657	1.490862247	1.249734896	23040.47905	85.38149682
0.147114787	81864	1.320899748	1.21616575	12043.40494	61.84658438
0.334458765	8667	1.420631332	1.110886988	2898.754113	25.8069758
0.254620723	14499	1.283873712	1.067355029	3691.74587	34.98571137
0.292020259	19305	1.619889755	1.342027434	5637.451094	41.78516483
0.30576673	10287	1.375104438	1.343975065	3145.422348	35.11409973
0.246970732	22248	1.436343641	1.627269121	5494.604841	66
0.292911758	11853	1.381005609	1.145115166	3471.883068	32.44996148
0.210212228	30105	1.352238806	1.300208205	6328.439132	56.60388679
0.143257071	165294	1.625739559	1.376146403	23679.53433	97.5807358
0.212362211	74142	1.844794009	1.107862739	15744.95905	77.88452991
0.31065891	6156	1.177332754	1.254232204	1912.416249	24.18677324
0.589426754	594	1.024568977	1.18371579	350.1194919	10.81665383
0.407560634	3942	1.331313425	1.06519591	1606.60402	21.84032967
0.141290427	110160	1.400564959	1.232998383	15564.55348	82.54089895
0.249610137	10881	1.143758578	1.082690946	2716.0079	29.69848481
0.161098885	86265	1.471928174	1.311167299	13897.19529	82.37718131
0.323987259	6453	1.247281157	1.017096309	2090.689779	24.18677324
0.113153788	305262	1.575466673	1.092557373	34541.55159	92.4175308
0.31560039	9045	1.359741224	1.155252013	2854.605532	28.3019434
0.364500978	23544	2.16026978	1.775444201	8581.811037	52.39274759
0.381570079	12366	1.824595507	1.416118485	4718.495591	42.63801121
0.324556268	9558	1.424278392	1.245157578	3102.108812	32.44996148
0.251554573	12582	1.209849415	1.451071414	3165.059635	39.11521443
0.183852567	39285	1.29239269	1.151871378	7222.648099	45.79301257
0.125157115	182952	1.469209762	1.186365688	22897.74445	90.44888059
0.156018215	109998	1.545798131	1.725769248	17161.69167	110.0227249
0.2188512	48114	1.645970175	1.299193934	10529.80661	57.07889277
0.463099111	1431	1.079119898	1.071326911	662.6948279	15.29705854
0.247666788	31104	1.610605067	1.537687161	7703.427761	60.74537019
0.4510335	2835	1.320005365	1.412889137	1278.679972	21.84032967
0.135022195	93609	1.26773041	1.014023763	12639.29266	64.76109943
0.34574909	3861	1.12161426	1.133452112	1334.937238	20.1246118
0.185424667	48384	1.397174464	1.262883982	8971.587081	49.47726751
0.203474964	70173	1.735469036	1.749525179	14278.44862	75.53806987
0.244372852	15309	1.254732386	1.186352804	3741.103995	32.44996148
0.113568724	190539	1.351354368	1.369035411	21639.27113	85.95929269
0.112191946	292518	1.540027314	1.114162343	32818.16379	105.3422992
0.089146999	392013	1.349137127	1.404406789	34946.78257	105.5130324
0.314687173	12042	1.491515567	1.452567907	3789.462935	36.12478374
0.301219726	12312	1.438275733	1.342977258	3708.617262	31.32091953
0.330578696	6048	1.245454881	1.033662881	1999.339956	28.3019434
0.259106887	24651	1.559335906	1.631204484	6387.243872	54.74486277
0.170732169	73008	1.475552676	1.214145008	12464.81422	60.74537019
0.43618189	4023	1.434498779	2.087924898	1754.759743	27.65863337
0.27555355	23355	1.628727804	1.229886121	6435.553165	41.78516483
0.135873402	133083	1.434469066	1.224266194	18082.44002	78
0.557815669	837	1.087047207	1.474171378	466.8917154	10.81665383
0.134464796	133974	1.422758908	1.076891112	18014.78654	74.09453421
0.216487055	94689	2.040395321	1.144392644	20498.94279	90.24965374
0.28737571	9855	1.27404512	1.049146131	2832.087621	27.65863337
0.322188123	15903	1.675399654	1.519940542	5123.757716	35.11409973
0.502776478	1350	1.149040711	1.268704721	678.7482451	13.41640786
0.782304126	351	1.141109187	0	274.5887483	9
0.168005208	92583	1.571625394	1.269822403	15554.4262	77.4209274
0.315123298	8208	1.314444249	1.182222685	2586.532026	33.54101966
0.312962759	11232	1.449308709	1.266199117	3515.197706	29.69848481
0.147873741	98604	1.412662598	1.335336472	14580.94237	74.09453421
0.107409247	194994	1.287946913	1.263857147	20944.15866	78.51751397
0.45686812	1701	1.127737878	1.373021188	777.1326723	16.97056275
0.229691196	18360	1.253000132	1.070318896	4217.130356	40.36087214
0.601678116	648	1.076643096	1.224053779	389.8874189	10.81665383
0.452351364	4401	1.53288263	1.445693868	1990.798355	60.74537019
0.40376301	7560	1.638637931	1.599261939	3052.448358	43.68065934

original_shape_Maximum2DDiameterRow	log.sigma.5.0.mm.3D_gldm_GrayLevelVariance	log.sigma.5.0.mm.3D_gldm_HighGrayLevelEmphasis	log.sigma.5.0.mm.3D_gldm_GrayLevelNonUniformityNormalized
33.13608305	23.37047751	138.2936288	0.057465796
51.26402247	10.71989307	112.6535994	0.087062699
27.65863337	10.81668741	76.27118644	0.089725175
66.61080993	18.9293639	193.4754268	0.065784484
17.49285568	41.81374375	198.6831683	0.049897069
25.63201124	12.83181604	76.6352459	0.078204784
12.36931688	7.179591837	30.77142857	0.132244898
33.9411255	29.01825237	131.9825436	0.055652639
46.57252409	78.81724347	454.4573171	0.035827072
58.24946352	9.825802969	117.9624838	0.090051417
32.44996148	15.83575713	74.2606383	0.073845632
50.28916384	14.92362084	103.7859974	0.072075861
46.95742753	14.03913919	56.35578331	0.091573435
40.02499219	20.86819545	129.967803	0.061380854
29.69848481	18.69704746	134.0824176	0.066100109
110.0227249	5.117323958	221.3115996	0.209316114
22.84731932	21.97825394	133.6359447	0.062243836
57.31491952	12.85525826	84.40592784	0.083623392
31.89043744	38.05808477	218.3846154	0.048605241
76.4852927	9.23246567	84.91744773	0.092801423
96.04686356	5.982374103	108.7440394	0.123298619
54.74486277	18.17037545	103.1386139	0.072322854
88.23264702	7.503809228	175.3393836	0.11193845
25.8069758	30.47521058	162.2932331	0.053536096
58.94064811	7.147638889	78.26875	0.110568576
37.58989226	12.94403754	102.5033113	0.081487654
63.28506933	6.086473172	105.9816514	0.125249426
87.20665112	8.88074115	138.7178434	0.136358947
77.88452991	12.79433132	197.6840689	0.104025765
55.15432893	13.52942818	136.7879504	0.077579792
90.69729875	7.550776526	113.0321352	0.115435217
94.86832981	7.136905875	159.4314502	0.145335971
44.59820624	17.73698622	120.3455724	0.066335773
8.485281374	14.37333333	57.93333333	0.12
24.18677324	18.14926776	130.7345133	0.069778369
51.6139516	13.30025017	112.2833583	0.081270834
94.15412896	8.166491856	135.0292931	0.143980293
66.2721661	12.25356259	103.5531003	0.081944744
34.20526275	29.60289593	213.834891	0.054357004
30.14962686	17.78716852	100.4040968	0.067333867
45.79301257	12.88557093	165.4685315	0.081711575
32.44996148	25.99319376	108.7506562	0.064569685
40.80441153	13.32913093	86.57281553	0.078565369
45	28.2813601	112.2186788	0.056304191
46.95742753	18.09469404	121.3130045	0.071563072
91.78235124	4.472402567	84.23930088	0.195101914
73.54590403	19.56903851	162.5047342	0.067504929
25.45584412	18.24576793	92.44736842	0.067751616
10.81665383	8.57231405	42.95454545	0.123966942
26.83281573	44.01731094	304.1986301	0.047288422
69.97142274	8.734139213	139.6066176	0.11310866
30	11.78346028	69.09925558	0.091097168
81.05553652	11.95528067	134.6065728	0.082331009
26.83281573	14.51644754	109.4769874	0.078237426
148.2194319	6.276556832	127.3797099	0.179130656
32.31098884	23.68666518	166.4	0.058445088
57.31491952	15.67994987	129.0584862	0.072016244
41.67733197	37.96177132	344.058952	0.047872848
31.89043744	16.8894395	104.3700565	0.071164097
28.46049894	13.8474691	80.65879828	0.075254656
51.26402247	10.25060639	69.76838488	0.095270958
93.1933474	15.40443368	252.1037485	0.086480045
90.7964757	6.633365941	74.55621011	0.119087746
70.03570518	6.055899121	65.56734007	0.113048429
12.36931688	10.55322179	72.75471698	0.094339623
43.26661531	13.7443388	130.4105903	0.078762478
19.20937271	25.55229025	152.7047619	0.060498866
59.54829972	16.54571742	124.8269397	0.073734479
20.1246118	20.22142892	129.958042	0.068609712
56.60388679	10.23346788	115.124442	0.086147386
79.88116173	10.86883803	112.3362832	0.084613151
36.24913792	16.30068836	103.0828924	0.075445816
87.36131867	8.3148764	128.4124982	0.120992086
104.3551628	6.909505289	76.84040982	0.136541172
111.3642672	7.139613316	161.4747572	0.177010209
39.11521443	23.34316495	147.970852	0.057310624
39.11521443	12.6875	80.75	0.079312865
25.8069758	18.55325255	86.32142857	0.069116709
48.09365863	9.328295558	53.8652793	0.095676543
75.95393341	9.32569763	213.5281065	0.097532474
24.73863375	21.40480159	162.2013423	0.065177244
60.07495318	16.73036319	149.5260116	0.071816633
77.88452991	10.67736548	187.6715358	0.102313184
12.36931688	5.723204995	39.06451613	0.146722164
77.82673063	6.636984086	105.0552197	0.120403661
65.7951366	7.199574861	83.60621614	0.105981934
31.89043744	17.6384012	83.09315068	0.07299681
59.77457654	28.28269837	275.1833616	0.054407199
13.41640786	6.4496	48.44	0.1336
9	6.284023669	29.76923077	0.136094675
82.37718131	14.77585747	236.3680373	0.08593838
39.11521443	19.67190746	69.36513158	0.076480263
35.11409973	8.574311206	47.45673077	0.095055936
77.4209274	10.45000174	106.2239869	0.08535961
81.9390017	8.681326217	111.6467737	0.119894778
16.97056275	27.46686823	231.0952381	0.062736206
36.61966685	23.02091479	169.0397059	0.060964533
12.72792206	4.331597222	16.875	0.15625
22.84731932	25.38311566	152.809816	0.062290639
30.59411708	17.48927296	117.4178571	0.070994898

log.sigma.5.0.mm.3D_gldm_DependenceEntropy	log.sigma.5.0.mm.3D_gldm_DependenceNonUniformity	log.sigma.5.0.mm.3D_gldm_GrayLevelNonUniformity	log.sigma.5.0.mm.3D_gldm_SmallDependenceEmphasis
6.367917341	63.40443213	20.74515235	0.252166769
6.948805526	137.044191	122.1489665	0.109839324
5.566069537	34.14124294	15.88135593	0.285568407
7.029018449	248.8592861	127.1614071	0.203061822
5.307680513	28.72277228	5.03960396	0.469130913
5.616096595	48.57377049	19.08196721	0.265805463
4.629372753	12.51428571	9.257142857	0.32226563
6.228846106	87.26932668	22.31670823	0.376705812
7.22358606	287.9945799	52.88075881	0.309554659
7.074340004	209.2828806	208.8292367	0.120423661
6.001848056	67.86702128	27.76595745	0.274897197
6.643410317	102.7463672	54.56142668	0.195987184
6.623679995	147.5051245	125.0893119	0.168759432
6.30848202	96.59090909	32.40909091	0.276592496
6.088750853	71.95054945	24.06043956	0.311833566
7.008545997	439.2007758	2266.47488	0.043079847
5.65334045	54.79723502	13.50691244	0.372433116
6.823246686	170.306701	129.7835052	0.191166872
5.915696429	48.43956044	8.846153846	0.415114469
7.073797774	224.850036	257.4311464	0.079261172
6.962937912	314.7462069	625.7404926	0.07056966
6.735415912	119.5786579	65.74147415	0.209005985
7.05492241	201.9130137	326.860274	0.088350852
5.635972992	33.85714286	7.120300752	0.41523183
6.805466922	84.95416667	106.1458333	0.125184498
5.826176104	54.90728477	24.60927152	0.305452403
7.031215065	158.2616863	286.6959371	0.092452021
7.024144087	213.0513479	531.1181001	0.072099415
7.155427978	260.3821313	386.5597417	0.113870484
7.013182301	158.9291199	131.3425871	0.11309343
7.090968579	267.8107593	484.9433468	0.072455987
7.183353732	418.4199026	1283.752632	0.077698212
6.858468681	183.1209503	92.14038877	0.199414745
3.189898095	5.4	1.8	0.522222222
5.260197272	30.7699115	7.884955752	0.382544248
6.558015452	178.2668666	108.4152924	0.210174507
7.060984063	320.0617247	963.3721417	0.068962781
7.201234586	251.3542216	248.4564644	0.10390512
6.195577991	70.42056075	17.44859813	0.303546832
6.443726862	79.27932961	36.15828678	0.227695813
6.645907508	86.35384615	58.42377622	0.179728982
6.130746387	71.37795276	24.60104987	0.345359758
6.243219642	124.0315534	64.73786408	0.2657233
6.249051062	85.72892938	24.71753986	0.341179504
6.714932336	150.2753363	79.79282511	0.201153322
6.820852979	257.0486769	1194.413917	0.03494332
6.934241097	387.7589221	185.3685361	0.196797644
5.797303724	41.5877193	15.44736842	0.321227755
3.55034071	11.18181818	2.727272727	0.482323232
5.814432395	46.28767123	6.904109589	0.494090563
7.13471418	255.345098	461.4833333	0.085567728
6.021383471	55.44168734	36.71215881	0.262064044
7.040265537	327.8826291	263.0475743	0.137270016
5.84157101	46.64016736	18.69874477	0.257219755
7.052472613	480.743499	2025.251194	0.057721282
6.000456897	76.68358209	19.57910448	0.375664425
6.239411237	167.766055	62.79816514	0.341801502
6.298810927	124.0524017	21.92576419	0.423403972
6.228074289	57.81920904	25.1920904	0.256979185
6.467383501	61.60085837	35.06866953	0.187810161
6.446303521	172.2659794	138.619244	0.174423206
7.442642093	468.6068477	585.9887839	0.127114984
7.193845468	252.0765832	485.1634757	0.079286166
6.808231269	144.4635241	201.4523008	0.097338162
4.56236503	14.81132075	5	0.453322851
6.64102401	162.3559028	90.734375	0.205117889
5.205885552	33.4952381	6.352380952	0.475579365
7.212451391	310.1987309	255.6374387	0.130923211
5.421748744	37.41958042	9.811188811	0.43958042
6.976602071	172.8325893	154.3761161	0.138399892
7.002165253	266.0496345	219.9095806	0.143443177
6.479596005	80.38977072	42.77777778	0.245185583
7.040110393	391.362477	853.8411506	0.066455171
7.131955443	509.0180912	1479.287059	0.050985048
7.125849972	615.6550038	2570.011227	0.063348386
6.118664841	98.78923767	25.56053812	0.334163263
5.979458817	75.43859649	36.16666667	0.296037684
5.669491137	46.33928571	15.48214286	0.307461677
6.442215434	109.6155531	87.35268346	0.196422826
6.979391097	208.5902367	263.7278107	0.114333644
5.325141068	41.57718121	9.711409396	0.415594705
6.689094871	121.8439306	62.12138728	0.256103059
7.335947873	338.1393792	504.3016839	0.109666913
4.115486633	7.258064516	4.548387097	0.343808244
7.133538619	307.6360339	597.4429665	0.084168112
6.846445504	318.1816367	371.6786427	0.133105183
6.11633033	58.33150685	26.64383562	0.279132656
6.315706413	136.9286927	32.04584041	0.392859532
4.67326969	8.68	6.68	0.322071882
3.238901257	7.461538462	1.769230769	0.769230769
7.304683051	278.3368329	294.6827063	0.134654394
5.969171928	51.71710526	23.25	0.258913726
6.131709947	56.88942308	39.54326923	0.165374657
7.229189992	294.8066813	311.7332968	0.094670455
7.181728247	388.8482415	865.8800886	0.066032193
5.040461669	21.76190476	3.952380952	0.504078483
6.429503679	118.0147059	41.45588235	0.268125292
3.772055209	7	3.75	0.309027778
5.641561824	41.14723926	10.15337423	0.361001941
6.305929561	42.55714286	19.87857143	0.269457303

log.sigma.5.0.mm.3D_gldm_DependenceNonUniformityNormalized	log.sigma.5.0.mm.3D_gldm_DependenceVariance	log.sigma.5.0.mm.3D_gldm_LargeDependenceEmphasis
0.175635546	3.621196891	14.57894737
0.097679395	12.01185932	45.30220955
0.192888378	2.440167257	11.30508475
0.128742517	7.888907474	25.90636317
0.284383884	1.472992844	6.089108911
0.19907283	3.008263908	12.55737705
0.17877551	3.08244898	12.42857143
0.217629244	2.355072419	9.224438903
0.195118279	2.943919331	11.54200542
0.090247038	11.90943257	46.44976283
0.180497397	9.82806134	23.02659574
0.135728358	5.443272739	21.9009247
0.107983254	8.826891952	32.35578331
0.182937328	3.731003214	13.80681818
0.197666345	2.335678058	10.59340659
0.040561579	60.20444871	255.6514592
0.25252182	1.795578585	7.783410138
0.109733699	9.211028005	31.4806701
0.266151431	1.216278227	6.241758242
0.081056249	18.64599804	67.65609229
0.062018957	36.84605806	117.9737931
0.131549679	6.407781372	23.18261826
0.069148292	22.85952149	80.74452055
0.254564984	1.356097009	6.684210526
0.088493924	14.36249566	50.3875
0.181812201	3.196701899	12.7615894
0.069140099	25.11710555	83.0139799
0.054698677	49.80601245	147.5848524
0.070070541	39.22309601	100.3455328
0.093874259	16.01338195	52.46131128
0.063749288	34.81872596	109.3546775
0.047370078	51.76545677	159.9296955
0.131836537	5.671897004	22.44708423
0.36	0.56	3.8
0.272300102	1.030934294	6.203539823
0.133633333	5.122857612	21.05097451
0.047834662	52.65480022	166.5420714
0.082900469	19.76977456	63.1853562
0.219378694	1.703923681	9.168224299
0.147633761	4.265791399	17.95716946
0.12077461	6.361238202	25.90629371
0.187343708	6.296953038	16.43307087
0.15052373	5.488806674	18.70873786
0.195282299	4.56939306	13.51480638
0.134776086	6.671900903	23.69955157
0.041987696	50.38235877	212.3975825
0.141208639	5.106069664	20.77567371
0.182402278	3.366497384	12.63157895
0.508264463	0.289256198	3.272727273
0.317038844	0.9581535	4.849315068
0.062584583	41.24917436	115.7328431
0.137572425	9.026519466	25.48635236
0.102623671	11.65872987	39.94647887
0.195147144	2.106265647	11.41004184
0.042521095	59.7262576	205.9338404
0.228906215	2.181599465	8.695522388
0.192392265	2.913559465	11.18577982
0.270856772	1.244903797	6.165938865
0.163331099	3.691053018	15.22033898
0.132190683	5.939066846	23.27038627
0.118395862	6.175512335	26.57113402
0.069156855	34.47065647	91.70336482
0.061874468	26.52301398	95.09474718
0.081068195	20.13356032	66.28507295
0.279458882	1.133499466	5.679245283
0.140933943	4.660659602	19.82465278
0.319002268	0.827755102	4.714285714
0.0894718	23.09713597	61.71473897
0.261675388	1.810064062	7.167832168
0.096446757	10.59476518	40.95535714
0.102366154	11.04167184	38.82377838
0.141780901	5.9405454	20.52028219
0.055457344	41.51648201	131.91597
0.046983394	56.48786451	186.7814288
0.042403403	64.92694733	214.0682554
0.221500533	1.957006978	9.004484305
0.165435519	5.335795629	16.74122807
0.206871811	4.232063138	13.28571429
0.120060847	7.383323996	26.83789704
0.07714136	30.72950822	81.20044379
0.279041485	0.967163641	5.724832215
0.140860035	5.428241505	19.39768786
0.068602025	29.93812095	86.90708054
0.234131113	1.602497399	8.096774194
0.061998395	33.26369631	104.2414349
0.090727584	11.80485532	44.28542914
0.159812348	4.257504222	15.85479452
0.232476558	2.293692224	8.650254669
0.1736	3.1744	13.16
0.573964497	0.213017751	1.923076923
0.08117143	30.89095573	77.19364246
0.170122057	3.687629848	14.90131579
0.136753421	4.571745562	22.47115385
0.080724721	19.06993992	65.72946331
0.053842182	49.44382227	150.3486569
0.34542706	0.630385488	4.079365079
0.173551038	3.588961938	14.17058824
0.291666667	0.909722222	6.75
0.252437051	1.647182807	7.699386503
0.151989796	5.129540816	17.93571429

log.sigma.5.0.mm.3D_gldm_LargeDependenceLowGrayLevelEmphasis	log.sigma.5.0.mm.3D_gldm_SmallDependenceHighGrayLevelEmphasis	log.sigma.5.0.mm.3D_gldm_LargeDependenceHighGrayLevelEmphasis
0.806965503	44.55363114	1807.157895
0.631055968	14.26788544	6221.697078
0.517004084	30.40727985	598.9830508
0.224125989	52.71796491	4651.930678
0.306851286	116.8318152	699.0594059
1.341392967	29.43793499	549.5368852
3.20168648	16.21962083	169.5714286
0.892247347	73.08696753	641.1246883
0.079731458	144.6997023	6445.466802
0.577296797	18.54142788	5455.464856
2.988643528	32.31433235	545.3297872
0.581705259	25.47540687	1924.792602
3.189650486	19.55093283	846.5153734
0.478883019	51.510384	1006.111742
0.305364802	56.48325055	975.0934066
1.122641547	9.614481542	60112.38733
0.579375383	67.07901562	637.1013825
1.518298965	26.38872991	1880.856959
0.27110192	107.6185333	1022.43956
1.212329919	7.051168574	7336.59589
0.996454596	8.15472541	16675.11606
1.195486405	32.940932	1383.825083
0.45005289	17.08264909	16763.58801
0.48038602	63.105967	1251.105263
1.023413353	11.75740598	4204.879167
0.45290265	40.74518843	895.615894
0.949431225	12.37205718	9169.371778
1.027148019	9.464456022	26592.68601
0.473968097	23.69918753	26111.23735
0.47635579	14.98749092	9957.625517
0.985908785	9.1520304	15691.36825
0.961425862	15.07256784	29206.63082
0.507099919	33.7123994	2542.764579
0.620305654	29.82222222	277.9333333
0.164001286	60.51488201	612.9115044
0.430641602	35.21019427	1691.958021
1.123988957	9.003421653	28396.51666
0.951253083	13.63473868	7712.121702
0.177040604	74.40390209	1830.700935
0.766603783	28.55578378	1364.646182
0.202948438	33.05061073	4680.106294
1.190638836	57.8532631	608.2939633
0.687373601	36.63020315	890.7524272
2.064469972	56.94424963	639.5353075
0.497723827	35.13695661	1976.412556
2.413477341	2.441286302	21811.88157
0.27952644	41.87906032	2788.876184
1.396803338	45.03169143	577.2631579
0.540389715	28.81818182	106.7727273
0.058262521	165.4581126	1174.253425
0.786112251	13.1332535	20376.9473
1.34250325	29.87898073	772.8014888
0.423865235	22.0548731	6307.213772
0.338747514	34.55072259	1081.669456
1.561166726	7.013220302	30383.16266
0.319913897	80.38216514	887.2119403
0.256946752	60.30615555	1011.569954
0.105655651	157.5726828	1873.347162
0.622472018	37.77184697	1088.646893
2.21914787	20.29830725	1465.796137
0.985007416	19.9344924	1563.779381
0.349177729	38.89782732	27877.00015
1.794499168	6.814345523	7941.175749
1.256396674	7.048833783	5480.804153
0.205507047	37.72775681	331.0188679
0.424049297	34.99871746	2454.056424
0.228611683	99.5151455	495.6380952
0.77649557	18.76830873	10725.48803
0.382527306	72.25533023	534.4195804
0.520800185	18.89666325	5319.94029
0.509231024	20.28273812	5008.054636
0.634780873	39.0483574	1372.299824
0.971601005	8.501053549	22176.74352
2.647601458	3.462307334	18797.34567
1.261619923	11.90503485	39107.97782
0.691225636	65.05551221	887.1636771
0.942147942	36.54510467	632.2763158
1.693118851	42.70378627	417.0089286
1.507262081	17.33675477	1162.719606
0.344925009	27.25392592	22026.59541
0.107045344	85.46821402	691.8389262
0.275383401	50.99790254	2290.716763
0.49884222	23.2138374	19501.47393
0.414219981	14.66585125	272.2258065
1.018810103	10.3396477	13366.16002
0.862210143	16.07857782	3139.561734
1.205746033	38.02869961	642.2876712
0.081023302	132.1494336	1657.886248
0.526353318	19.36694705	464.52
0.219378501	26.88461538	41.30769231
0.311327189	33.76738614	23469.04112
2.123050577	30.61483707	480.2664474
2.81656575	11.59349379	794.2788462
0.797435805	10.40622142	9071.148412
1.317194044	7.834748163	20944.27984
0.044462081	122.1090168	913.9206349
0.30580643	63.53728596	1808.430882
2.426440618	7.863715278	65.125
0.138536578	65.34552345	1237.521472
0.52412882	33.0378486	2177.046429

log.sigma.5.0.mm.3D_gldm_SmallDependenceLowGrayLevelEmphasis	log.sigma.5.0.mm.3D_gldm_LowGrayLevelEmphasis	log.sigma.5.0.mm.3D_gldzm_DistanceZoneVariabilityNormalized
0.003850472	0.037437358	0.961919125
0.00250943	0.02160204	0.985816327
0.007634593	0.041535074	1
0.001716989	0.010343485	0.927252088
0.008999322	0.036766719	0.967222222
0.005854067	0.051186826	0.974363299
0.015713489	0.149593294	1
0.006560564	0.046759536	0.967042227
0.002217945	0.006888375	0.503617106
0.001699639	0.014643237	0.992366524
0.006546089	0.069951481	0.95240928
0.004167474	0.026407272	0.987578125
0.00492674	0.076004617	0.913940115
0.00366633	0.023970256	0.976881776
0.006046031	0.021837705	0.930645412
0.000379987	0.005541191	0.962107708
0.006339302	0.034130756	0.980584391
0.003362503	0.039691874	0.961197195
0.006814522	0.029237868	0.918515165
0.001610488	0.025321731	0.957239691
0.001525005	0.013733215	0.973313381
0.003005335	0.035060485	0.955969483
0.001048176	0.008065832	0.991631947
0.011948901	0.049442439	1
0.003626997	0.025555767	1
0.005681974	0.029846432	0.963648834
0.00121302	0.016034737	0.979383681
0.001105864	0.013707265	0.992126107
0.001008888	0.008944161	0.962582021
0.00264014	0.017246463	0.933779592
0.001151077	0.013968529	0.984704142
0.000678	0.008673089	0.963092763
0.003288219	0.02385192	0.94506112
0.064159194	0.173919248	1
0.01425271	0.028804151	1
0.00321328	0.018350905	0.910066667
0.00104667	0.012195393	0.959821429
0.001756552	0.021146239	0.979616251
0.005892313	0.017206252	0.906476754
0.005538844	0.0329659	0.969704142
0.003125344	0.011554817	0.957766161
0.008732633	0.046296673	0.937173216
0.005927067	0.030214502	0.961255448
0.006897481	0.069709226	0.955578512
0.002970026	0.020355538	0.893872679
0.000924145	0.017492493	0.98726167
0.002007484	0.013032178	0.686379965
0.006451231	0.055318659	0.953514739
0.038662641	0.137885721	1
0.012146925	0.016738219	0.910311419
0.001065707	0.011225148	0.986949745
0.006436902	0.035917488	0.922688
0.001727972	0.014810805	0.894254609
0.00757126	0.027114043	0.972227733
0.00077809	0.011591607	0.974415832
0.006138578	0.02030226	0.935555556
0.004521283	0.017485281	0.966207174
0.003572269	0.014069235	0.872091952
0.005094441	0.031852207	0.962976148
0.004365091	0.055399467	0.942358592
0.003435767	0.034805433	0.894255647
0.000835111	0.005911621	0.906831938
0.002127486	0.028006685	0.993031444
0.003795137	0.028967411	1
0.03086305	0.04839837	0.933412604
0.002775843	0.020539972	0.950850929
0.009696775	0.040122373	0.967750605
0.002135458	0.022357483	0.881960629
0.012275939	0.031344408	0.948088889
0.002373358	0.015556934	0.958381212
0.002519653	0.017694933	0.958577286
0.00484287	0.024883925	0.961553249
0.000836657	0.012454738	0.973409086
0.001859421	0.029192373	0.986154252
0.000613236	0.008459146	0.948638996
0.0050931	0.030681096	0.956066153
0.007147479	0.031132182	0.949079414
0.009194593	0.047557922	0.976193932
0.008113032	0.064852409	1
0.001101675	0.006517577	0.973426748
0.010159631	0.019889195	0.975004006
0.004237949	0.014630526	0.951642647
0.000863949	0.008924475	0.902868128
0.045627399	0.081900554	1
0.001331395	0.014497477	0.978879018
0.002020598	0.018505321	0.961470986
0.005455769	0.046919392	0.983194484
0.003246546	0.010244968	0.964550561
0.028778531	0.053847205	1
0.119218244	0.139250296	0.702479339
0.001051933	0.007025537	0.928367117
0.009070756	0.086733893	1
0.00885588	0.079143557	0.973688889
0.002075392	0.019551634	0.949765779
0.001218505	0.014918599	0.96403275
0.020706547	0.024368626	1
0.003008948	0.018040046	0.858611296
0.040592335	0.258020479	1
0.010635033	0.02426558	1
0.014944125	0.042885234	1

log.sigma.5.0.mm.3D_gldzm_LowIntensityEmphasis	log.sigma.5.0.mm.3D_gldzm_LargeDistanceEmphasis	log.sigma.5.0.mm.3D_gldzm_HighIntensitySmallDistanceEmphasis
0.023270984	1.058252427	170.8276699
0.029763868	1.021428571	140.1517857
0.035166113	1	102.5967742
0.009935387	1.164319249	264.3604754
0.027337731	1.05	233.0833333
0.030314944	1.038961039	110.25
0.072148772	1	48.71428571
0.020863464	1.05027933	188.472067
0.007171058	2.397790055	253.3508287
0.016950285	1.011494253	166.7021073
0.033518086	1.073170732	117.2804878
0.026132235	1.01875	135.5453125
0.038089736	1.203252033	122.6686992
0.016832831	1.035087719	186.9210526
0.01910583	1.107913669	176.6151079
0.009538789	1.069879518	233.3239625
0.023661543	1.029411765	168.1102941
0.021351651	1.075657895	149.2055921
0.022757848	1.127659574	251.9308511
0.024630303	1.06557377	98.82240437
0.026922457	1.097643098	121.389263
0.01652614	1.067567568	164.5416667
0.013563123	1.012605042	197.8193277
0.036069043	1	155.4782609
0.029970714	1	98.60330579
0.023572601	1.055555556	132.6712963
0.018034003	1.03125	143.703125
0.020098881	1.011857708	131.5899209
0.011749021	1.080760095	210.0796384
0.03122071	1.102857143	132.2614286
0.018846529	1.069230769	136.975
0.009493735	1.064162754	210.3963224
0.021408135	1.084805654	173.6183746
0.150935164	1	56.1
0.035372328	1	150.6363636
0.016447448	1.23	168.850463
0.018285837	1.099489796	137.8003118
0.017844853	1.047945205	145.1938737
0.018681618	1.147540984	234.2868852
0.028832483	1.046153846	127.8384615
0.018261041	1.064748201	187.5881295
0.029550135	1.097402597	165.4902597
0.023569284	1.059288538	137.1116601
0.02675681	1.068181818	163.6306818
0.017137131	1.168674699	173.0742972
0.03139183	1.019230769	71.86698718
0.011819072	2.082332762	169.9534972
0.030114059	1.071428571	138.6577381
0.111921225	1	51.64285714
0.022091926	1.141176471	337.2205882
0.015321356	1.036065574	165.2237705
0.024104132	1.16	112.6148889
0.017105463	1.226506024	165.3005355
0.029673565	1.042253521	137.1161972
0.015626853	1.04805915	130.8754364
0.016888768	1.1	211.3716667
0.014109116	1.051575931	169.6769341
0.011355785	1.206008584	369.9881974
0.026137686	1.056603774	145.9764151
0.028396449	1.089108911	110.4975248
0.025911473	1.187250996	117.7849712
0.007105012	1.170035672	317.3947021
0.033012391	1.01048951	90.51048951
0.043094812	1	78.34375
0.062134267	1.103448276	82.35344828
0.015908678	1.075630252	177.3813025
0.030862061	1.049180328	190.9385246
0.020406644	1.27816092	151.0474457
0.028322635	1.08	161.27
0.017833703	1.084745763	144.9422081
0.019776237	1.07651715	148.5352536
0.020062394	1.058823529	163.2859477
0.014957496	1.040431267	142.3712938
0.04734807	1.032482599	72.13444187
0.00990373	1.090909091	205.7776449
0.01999156	1.06741573	192.491573
0.022636805	1.078431373	124.5179739
0.028090116	1.036144578	136.6174699
0.060085643	1	91.47089947
0.010486557	1.04040404	246.9646465
0.022728728	1.037974684	195.4208861
0.015689095	1.074380165	204.8904959
0.009906725	1.17254902	220.4344227
0.118280959	1	41.71428571
0.017498384	1.119680851	133.466117
0.015070144	1.058951965	129.720524
0.025410488	1.025423729	135.0021186
0.009893086	1.054151625	330.4891697
0.082300985	1	57.73684211
0.148785703	1.545454545	32.56818182
0.00939929	1.122270742	254.0440927
0.031256627	1	125.1264368
0.070514079	1.04	73.15
0.029145383	1.131410256	114.8264779
0.021145176	1.08616188	131.1515992
0.035058462	1	235.7179487
0.014292806	1.229665072	238.4150718
0.158275742	1	23.09090909
0.028771119	1	170.5066667
0.058480808	1	120.5978261

log.sigma.5.0.mm.3D_gldzm_LowIntensityLargeDistanceEmphasis	log.sigma.5.0.mm.3D_gldzm_HighIntensityEmphasis	log.sigma.5.0.mm.3D_gldzm_DistanceZoneVariability	log.sigma.5.0.mm.3D_gldzm_ZonePercentage
0.024439628	171.6796117	99.0776699	0.28531856
0.05119244	140.1571429	138.0142857	0.099786172
0.035166113	102.5967742	62	0.350282486
0.015061711	266.8497653	395.0093897	0.220382825
0.028118981	233.8833333	58.03333333	0.594059406
0.069275983	110.2597403	75.02597403	0.31557377
0.072148772	48.71428571	28	0.4
0.043675382	188.5307263	173.1005587	0.44638404
0.010803066	464.412523	273.4640884	0.367886179
0.016990058	167.532567	259.0076628	0.112548512
0.064981501	117.4634146	117.1463415	0.32712766
0.028215569	135.5875	158.0125	0.211360634
0.071342574	123.9796748	224.8292683	0.180087848
0.01899874	187.3157895	167.0467836	0.323863636
0.043088144	177.4892086	129.3597122	0.381868132
0.010494323	234.739759	399.2746988	0.038326561
0.053073308	168.1176471	100.0196078	0.470046083
0.023518369	150.1546053	292.2039474	0.195876289
0.057947958	253	86.34042553	0.516483516
0.026332122	99.53551913	175.1748634	0.065969719
0.028011365	122.5319865	289.0740741	0.058522167
0.018204075	165.6801802	212.2252252	0.244224422
0.013913263	197.9327731	236.0084034	0.081506849
0.036069043	155.4782609	69	0.518796992
0.029970714	98.60330579	121	0.126041667
0.051693314	133.2407407	104.0740741	0.357615894
0.019444593	143.90625	188.0416667	0.083879423
0.020196878	131.9486166	251.0079051	0.064955071
0.0127367	211.6365796	405.2470309	0.113293864
0.035768339	133.4228571	163.4114286	0.103366804
0.019376356	137.8230769	256.0230769	0.061890026
0.010427227	212.0109546	615.4162754	0.072342353
0.044318176	174.3816254	267.4522968	0.203743701
0.150935164	56.1	10	0.666666667
0.035372328	150.6363636	55	0.486725664
0.03291415	172.5466667	273.02	0.224887556
0.021432605	138.9336735	376.25	0.058586161
0.018739458	145.6883562	286.0479452	0.096306069
0.044716259	237.7295082	110.5901639	0.380062305
0.040370944	127.8846154	126.0615385	0.242085661
0.019657258	188.5971223	133.1294964	0.194405594
0.061476541	165.7532468	144.3246753	0.404199475
0.042838058	137.2332016	243.1976285	0.307038835
0.046391936	164.0909091	168.1818182	0.400911162
0.038515344	180.0923695	222.5742972	0.223318386
0.0506226	71.87179487	154.0128205	0.025481869
0.019659321	225.3207547	400.1595197	0.212308813
0.074756916	138.702381	80.0952381	0.368421053
0.111921225	51.64285714	14	0.636363636
0.07085143	337.7764706	77.37647059	0.582191781
0.025273997	165.8819672	301.0196721	0.074754902
0.053760595	113.52	115.336	0.310173697
0.022676249	168.5108434	371.1156627	0.129890454
0.071927086	137.1267606	69.02816901	0.29707113
0.017585717	131.3197782	527.1589649	0.047850699
0.044738768	211.7266667	140.3333333	0.447761194
0.017429918	170.3495702	337.2063037	0.400229358
0.034562029	372.193133	203.1974249	0.508733624
0.027847351	146.5	102.0754717	0.299435028
0.032266086	111.0693069	95.17821782	0.216738197
0.029226186	121.5657371	224.4581673	0.172508591
0.015353567	320.8739596	762.6456599	0.124114522
0.035634769	90.52097902	284.006993	0.070201276
0.043094812	78.34375	160	0.089786756
0.165582543	82.37931034	27.06896552	0.547169811
0.032400848	178.3235294	226.302521	0.206597222
0.080042389	190.9508197	59.03278689	0.580952381
0.031529891	155.0873563	383.6528736	0.125468705
0.078322635	161.32	71.10666667	0.524475524
0.034959409	145.4279661	226.1779661	0.131696429
0.032983019	149.4564644	363.3007916	0.145825317
0.040854711	163.6535948	147.1176471	0.26984127
0.024098768	142.7493261	361.1347709	0.052571914
0.054744958	72.44779582	425.0324826	0.039782167
0.016135502	207.7296651	793.062201	0.057579723
0.030635144	192.7696629	170.1797753	0.399103139
0.052354942	124.875817	145.2091503	0.335526316
0.064234694	136.626506	81.02409639	0.370535714
0.060085643	91.47089947	189	0.207009858
0.010858174	248.2424242	289.1077441	0.109837278
0.060703412	195.4303797	77.02531646	0.530201342
0.034942233	206.3099174	230.2975207	0.279768786
0.015350544	223.2019608	460.4627451	0.103469264
0.118280959	41.71428571	14	0.451612903
0.02096082	134.412234	368.0585106	0.075775897
0.025870671	130.1004367	440.3537118	0.130595951
0.02592934	135.3135593	116.0169492	0.323287671
0.014135577	331.7184116	267.1805054	0.470288625
0.082300985	57.73684211	19	0.38
0.489694794	32.90909091	7.727272727	0.846153846
0.012864174	257.4934498	425.1921397	0.133566638
0.031256627	125.1264368	87	0.286184211
0.110514079	73.16	73.02666667	0.180288462
0.042912393	116.4679487	296.3269231	0.08543264
0.032456662	131.9843342	369.2245431	0.053032401
0.035058462	235.7179487	39	0.619047619
0.040899631	240.4784689	179.4497608	0.307352941
0.158275742	23.09090909	11	0.458333333
0.028771119	170.5066667	75	0.460122699
0.058480808	120.5978261	92	0.328571429

log.sigma.5.0.mm.3D_gldzm_IntensityVariabilityNormalized	log.sigma.5.0.mm.3D_gldzm_LowIntensitySmallDistanceEmphasis	log.sigma.5.0.mm.3D_gldzm_IntensityVariability
0.061740032	0.022978823	6.359223301
0.086020408	0.024406725	12.04285714
0.090010406	0.035166113	5.580645161
0.056227821	0.008726964	23.95305164
0.051111111	0.027142419	3.066666667
0.085511891	0.020574684	6.584415584
0.130102041	0.072148772	3.642857143
0.054399051	0.015160485	9.737430168
0.037236009	0.006344628	20.21915285
0.071974868	0.016940342	18.78544061
0.079648357	0.025652233	9.796747967
0.06484375	0.025611402	10.375
0.070824245	0.029946617	17.42276423
0.071714374	0.016291354	12.26315789
0.063713058	0.013110251	8.856115108
0.08425606	0.009322032	34.96626506
0.065936178	0.016308602	6.725490196
0.077345914	0.020835353	23.51315789
0.049796288	0.01396032	4.680851064
0.099166891	0.024204849	18.14754098
0.087462731	0.026735956	25.97643098
0.070286503	0.016106656	15.6036036
0.078525528	0.013475588	18.68907563
0.059021214	0.036069043	4.072463768
0.085991394	0.029970714	10.40495868
0.085733882	0.016542423	9.259259259
0.081705729	0.017681355	15.6875
0.084597478	0.020074381	21.40316206
0.064934186	0.011536045	27.33729216
0.067755102	0.030083803	11.85714286
0.077011834	0.018769262	20.02307692
0.076618641	0.00926809	48.95931142
0.060470227	0.015680625	17.1130742
0.1	0.150935164	1
0.063801653	0.035372328	3.509090909
0.069888889	0.012439788	20.96666667
0.075372241	0.017606771	29.54591837
0.074333834	0.017659254	21.70547945
0.056839559	0.012172958	6.93442623
0.069112426	0.025947868	8.984615385
0.071787175	0.017911987	9.978417266
0.058610221	0.021568533	9.025974026
0.079566936	0.01875209	20.13043478
0.057592975	0.021848028	10.13636364
0.067498911	0.011792578	16.80722892
0.116206443	0.026584138	18.12820513
0.059828356	0.010162806	34.87993139
0.073979592	0.018953344	6.214285714
0.112244898	0.111921225	1.571428571
0.041107266	0.00990205	3.494117647
0.074076861	0.012849387	22.59344262
0.087872	0.016828906	10.984
0.06783568	0.015835187	28.15180723
0.078357469	0.019110185	5.563380282
0.088215497	0.015157675	47.7245841
0.060444444	0.009926268	9.066666667
0.070582343	0.013278915	24.63323782
0.049236494	0.005554225	11.472103
0.067817729	0.025710269	7.188679245
0.076365062	0.02742904	7.712871287
0.083982794	0.02510538	21.07968127
0.053485107	0.00506565	44.98097503
0.086141132	0.032356797	24.63636364
0.102890625	0.043094812	16.4625
0.093935791	0.036272198	2.724137931
0.06825083	0.011785635	16.24369748
0.064767536	0.018566979	3.950819672
0.064563351	0.017834594	28.08505747
0.073777778	0.015822635	5.533333333
0.081442114	0.013625841	19.22033898
0.074066597	0.016503858	28.07124011
0.068221624	0.014864315	10.4379085
0.08851287	0.012672178	32.83827493
0.101027665	0.04555146	43.54292343
0.068714086	0.008382962	57.44497608
0.061923999	0.017330664	11.02247191
0.087957623	0.01520727	13.45751634
0.071273044	0.019053971	5.915662651
0.078861174	0.060085643	14.9047619
0.07560453	0.010393652	22.45454545
0.06136837	0.013235057	4.848101266
0.063656854	0.010875811	15.40495868
0.06651288	0.008585562	33.92156863
0.12244898	0.118280959	1.714285714
0.082885355	0.016750254	31.16489362
0.092275128	0.012370013	42.26200873
0.07426027	0.025280775	8.762711864
0.056132623	0.008832463	15.54873646
0.113573407	0.082300985	2.157894737
0.123966942	0.06355843	1.363636364
0.063480864	0.008542545	29.07423581
0.067247985	0.031256627	5.850574713
0.086933333	0.060514079	6.52
0.077231262	0.025805267	24.09615385
0.074122804	0.018360757	28.38903394
0.053254438	0.035058462	2.076923077
0.058217532	0.0076411	12.16746411
0.140495868	0.158275742	1.545454545
0.060622222	0.028771119	4.546666667
0.058364839	0.058480808	5.369565217

log.sigma.5.0.mm.3D_gldzm_HighIntensityLargeDistanceEmphasis	log.sigma.5.0.mm.3D_gldzm_SmallDistanceEmphasis	log.sigma.5.0.mm.3D_glcm_SumVariance	log.sigma.5.0.mm.3D_glcm_Homogeneity1
175.0873786	0.985436893	299.3295045	0.389394478
140.1785714	0.994642857	265.0843107	0.514123154
102.5967742	1	134.911472	0.387675091
286.786385	0.970738785	468.6256856	0.425634382
237.0833333	0.9875	457.5963756	0.280473505
110.2987013	0.99025974	124.375461	0.37556889
48.71428571	1	37.16356238	0.449391298
188.7653631	0.987430168	234.997368	0.332212707
1615.362799	0.723258134	1498.232374	0.311004648
170.8544061	0.997126437	286.4341234	0.521362549
118.195122	0.981707317	110.4405795	0.390207038
135.75625	0.9953125	225.0127218	0.437840238
135.2601626	0.965136631	89.50447832	0.4652227
188.8947368	0.99122807	257.2161765	0.375434843
180.9856115	0.973021583	265.2150197	0.362772041
241.6987952	0.985207497	678.8020319	0.754577612
168.1470588	0.992647059	244.748312	0.323060051
156.0559211	0.984740497	162.9117633	0.457999949
257.2765957	0.968085106	439.5769829	0.278469215
102.3879781	0.983606557	197.2264401	0.559720509
136.023569	0.988800037	288.1770365	0.62864554
170.2342342	0.983108108	203.8408939	0.425880089
198.3865546	0.996848739	491.1791043	0.590305945
155.4782609	1	398.3650722	0.330114926
98.60330579	1	174.7180118	0.55977839
135.5185185	0.986111111	195.7215922	0.3885079
144.71875	0.9921875	260.9918231	0.588340661
133.3833992	0.997035573	387.4159832	0.65535649
221.1686461	0.985088414	551.7975088	0.565654877
138.0685714	0.974285714	354.2792922	0.526747654
149.6961538	0.993509615	293.727205	0.610008213
220.0344288	0.985698139	440.242528	0.648719738
177.434629	0.978798587	259.6492304	0.422859291
56.1	1	151.665493	0.332208532
150.6363636	1	260.569118	0.327412406
204.65	0.962986111	235.3845897	0.414334562
146.244898	0.983630952	373.801482	0.658918958
149.1883562	0.991818874	237.9712385	0.536629418
251.5	0.963114754	473.0644981	0.338996866
128.0692308	0.988461538	199.5893162	0.410468317
192.6330935	0.98381295	414.2226609	0.469552117
166.8051948	0.975649351	180.5388019	0.36436201
137.7193676	0.985177866	150.1344879	0.400267303
165.9318182	0.982954545	192.3860128	0.353177853
208.1646586	0.957831325	265.8574312	0.422504271
71.89102564	0.995192308	226.3633346	0.736374532
723.1372213	0.851867734	411.3138333	0.410929088
138.8809524	0.982142857	152.3037138	0.35481247
51.64285714	1	84.16735278	0.312200965
340	0.964705882	676.5643429	0.256658753
171.7934426	0.994626594	371.0879096	0.602459555
119.416	0.968888889	111.1768602	0.417327923
188.0240964	0.956760375	333.0366248	0.481619932
137.1690141	0.98943662	216.4580349	0.385588216
133.9186691	0.990039022	353.6637784	0.697171351
213.1466667	0.975	332.3319383	0.319057899
173.0401146	0.987106017	260.1695096	0.36483809
381.0128755	0.948497854	806.5350243	0.290098768
148.5943396	0.985849057	207.1734169	0.405834757
113.3564356	0.977722772	161.3025026	0.441585243
140.1593625	0.957613988	137.9856289	0.450531982
339.7110583	0.962775796	714.0674207	0.544532796
90.56293706	0.997377622	167.992428	0.602490807
78.34375	1	144.0456989	0.563111014
82.48275862	0.974137931	132.0112307	0.332765483
182.092437	0.981092437	292.1663403	0.426103692
191	0.987704918	271.166984	0.28421255
179.9724138	0.950893997	304.4000687	0.503500763
161.52	0.98	239.8704977	0.303898702
148.5762712	0.983521657	273.9895108	0.499482699
154.3139842	0.983802404	264.3982095	0.488429385
165.124183	0.985294118	196.0647181	0.410464399
144.2614555	0.989892183	346.5790844	0.624565012
74.20649652	0.994457334	189.4821594	0.68176524
216.3086124	0.979930888	458.7994167	0.68772152
193.8820225	0.983146067	293.141625	0.32954491
126.3071895	0.980392157	137.0829347	0.39345225
136.6626506	0.990963855	139.4642039	0.36391467
91.47089947	1	92.16521993	0.453520958
253.3535354	0.98989899	610.817273	0.553159975
195.4683544	0.990506329	327.3166865	0.298800426
211.9876033	0.981404959	335.2094296	0.416189235
236.1980392	0.961220044	513.0400726	0.557315067
41.71428571	1	72.79729904	0.410594157
153.1223404	0.990964096	264.0607727	0.597753925
131.6200873	0.985262009	181.4086382	0.515067436
136.559322	0.993644068	143.2432273	0.395493075
336.6353791	0.986462094	655.8115775	0.304647999
57.73684211	1	100.1099491	0.43074114
34.27272727	0.863636364	48.81453824	0.365740741
273.7751092	0.971858321	663.0712364	0.528877156
125.1264368	1	119.0262316	0.382298426
73.2	0.99	77.60707752	0.467778637
131.0128205	0.979723113	257.4475523	0.548498871
140.2793734	0.985802872	286.8836397	0.635853914
235.7179487	1	608.4780654	0.276513882
248.7320574	0.942583732	367.6533326	0.368366121
23.09090909	1	25.43737423	0.423015052
170.5066667	1	340.4890597	0.350524433
120.5978261	1	271.572411	0.43309675

log.sigma.5.0.mm.3D_glcm_Homogeneity2	log.sigma.5.0.mm.3D_glcm_ClusterShade	log.sigma.5.0.mm.3D_glcm_MaximumProbability	log.sigma.5.0.mm.3D_glcm_Idmn	log.sigma.5.0.mm.3D_glcm_SumVariance2
0.302538889	20.61776065	0.022949983	0.978794429	77.92436684
0.455864273	-79.70597285	0.04151828	0.98638312	37.06484126
0.298859599	44.42064947	0.036779429	0.963884049	29.77325983
0.349122524	52.84980178	0.021368054	0.986935962	57.71906416
0.18215348	623.1822541	0.028668138	0.960654089	130.2530581
0.284326158	35.09133646	0.025119771	0.957506083	34.80488644
0.375311786	70.85200889	0.094803731	0.954552652	15.46204011
0.241389569	463.7439672	0.017809604	0.974693568	77.87065228
0.221260995	2769.343761	0.008636907	0.98102251	316.3482346
0.464671021	-19.81091084	0.038495275	0.99029551	31.6856638
0.30570269	105.3299162	0.055813123	0.965804541	39.08512815
0.362135025	40.549069	0.026601408	0.984661636	45.91979057
0.396882317	253.9561429	0.043236833	0.984654778	35.50044499
0.289116573	203.2431196	0.022244725	0.975827867	60.82309875
0.272721584	106.7854073	0.023875405	0.975641867	53.86508478
0.738409148	-142.0159088	0.251288201	0.997547848	17.95883493
0.2247771	112.9264242	0.023098052	0.96383824	62.35947431
0.388086829	70.46914181	0.033822943	0.987271746	35.5257546
0.182034018	224.6361099	0.018030925	0.958527104	108.6020714
0.511279239	-39.73343502	0.060544787	0.989019375	34.21284338
0.594106841	-50.75367143	0.134434204	0.994641825	20.91653307
0.347979881	280.8082313	0.033142415	0.979947749	54.63798684
0.54991326	-96.1359541	0.081849177	0.994432428	25.9204272
0.234008763	2.429937091	0.028894633	0.966065638	104.9392956
0.514218823	-56.09622439	0.059203275	0.98847062	24.02543974
0.300660962	3.381581151	0.028800786	0.968649485	37.44644686
0.546591618	-40.51596803	0.104960548	0.992978101	19.12085904
0.621018908	-164.8014592	0.236209629	0.996610851	31.16136212
0.514078327	-187.9643769	0.160667893	0.993740869	44.56245595
0.470849092	-122.5388347	0.052854884	0.990342248	48.50354824
0.57078116	-78.02119796	0.132464985	0.993924117	26.49550351
0.615471616	-88.32868701	0.206513581	0.996041477	22.97723503
0.343385031	101.5075013	0.017963703	0.984113093	54.20433592
0.235772751	-2.791031072	0.163141026	0.902353902	30.28396902
0.229916894	66.87583144	0.029182036	0.963019952	51.92961994
0.333939643	78.29134922	0.026896763	0.98124649	34.41742015
0.626033503	-164.9293844	0.184878236	0.994695664	29.17320701
0.482769814	-22.83886851	0.059964624	0.990726944	41.08472148
0.245833763	65.95234091	0.016892242	0.973728272	100.024075
0.328381211	136.5331544	0.024655289	0.973722799	55.75318897
0.400189549	-69.50689439	0.037121319	0.988987919	44.43101187
0.278028875	525.6943966	0.045092022	0.972771902	65.81669311
0.318115164	89.86070591	0.030206978	0.977071162	31.46334646
0.265636868	479.1447989	0.025299842	0.975511672	77.71413005
0.343841858	425.0038702	0.033970818	0.984551532	60.20248675
0.718947546	-70.94256071	0.23310921	0.99435094	16.13911061
0.330565591	522.2922181	0.019179994	0.990903668	70.76022007
0.263790695	116.1988621	0.028724588	0.96208491	47.6610496
0.203229333	21.46887327	0.116519592	0.913020943	17.5454484
0.161573224	277.9182771	0.019150844	0.962599282	131.6269371
0.560755274	-91.13887472	0.158177446	0.993484524	30.41305423
0.33812873	103.5883992	0.051817535	0.970394476	25.95801173
0.415264652	-56.65767882	0.034583197	0.990193331	40.05804871
0.298289002	-1.259043838	0.02623323	0.971406022	44.10240442
0.670827258	-126.2721413	0.239929618	0.995362519	22.38692792
0.223691018	78.56989152	0.01777682	0.965326853	64.89362389
0.275193683	69.3748794	0.019801936	0.980677043	42.58701047
0.19454376	-402.7038803	0.015399457	0.972585984	121.1174414
0.32068148	134.0934904	0.029400793	0.980435393	52.13993105
0.361942298	30.02722915	0.027938487	0.980212838	46.04646953
0.376938649	102.3114821	0.035883368	0.982347974	29.42991686
0.491401285	-135.1918839	0.098308652	0.994598154	50.77816744
0.563676548	-57.07152198	0.093466777	0.993218265	22.98037867
0.514096196	-8.03836198	0.068425898	0.983401047	21.25853081
0.235045556	40.67554328	0.042306728	0.952492433	25.57196724
0.349171783	-54.58716873	0.021186778	0.984532051	43.57200412
0.185106151	89.90078496	0.025729443	0.952805468	62.83955445
0.441726502	-73.37099865	0.062142494	0.988933865	57.66730871
0.20736291	56.27025244	0.023415853	0.953111501	53.72957812
0.43751406	-24.24089955	0.02980538	0.986950544	34.99829868
0.424676923	-28.4130847	0.034327829	0.986894723	34.58244152
0.330452879	208.9913871	0.02514922	0.980404878	42.05247872
0.587099324	-117.3088171	0.128728256	0.993329798	30.35457503
0.653523637	-96.6460046	0.185398439	0.9936249	25.04990269
0.65955579	-117.6552851	0.250236078	0.996225834	24.22148559
0.23673665	113.3921781	0.014884598	0.966349774	65.26219701
0.308095377	87.01271707	0.030590631	0.97316945	31.99663436
0.276368424	199.5785771	0.032853834	0.96656591	47.68330872
0.382230753	48.05834223	0.030904997	0.97399387	23.64815937
0.50121079	-62.19688641	0.095174406	0.992919877	31.2679275
0.204307079	110.2826433	0.026950807	0.949970247	54.20364102
0.336686387	71.58685535	0.028769428	0.984871919	50.44221928
0.507698507	-145.8015684	0.087154748	0.993570767	36.19667187
0.324758333	7.759804701	0.094366211	0.938059299	13.26059583
0.557216298	-57.62678127	0.12139644	0.991914364	22.25587116
0.457339055	33.24908465	0.040749735	0.986440985	21.34548771
0.312408091	262.0555366	0.028297637	0.977962526	46.98256317
0.209872008	146.5762831	0.014493612	0.975665994	76.76975944
0.353063207	23.25017292	0.088720551	0.946239917	12.53372523
0.27791731	43.50509504	0.113095238	0.918049499	13.51713278
0.47134636	-206.6491722	0.078364913	0.993763903	53.38538543
0.296302643	425.5476551	0.037504053	0.974479697	54.26111129
0.397750441	40.01728388	0.034455294	0.974800602	24.69801253
0.498138886	-33.14772641	0.055318959	0.989306861	37.50004599
0.599081514	-89.60496365	0.181803856	0.993970106	30.05701126
0.175962628	-135.1486443	0.036772662	0.959779287	64.05854636
0.280688831	77.04510866	0.01858908	0.978777713	70.08954281
0.342300403	18.31240371	0.12801344	0.909037357	8.047915738
0.256870611	355.3360036	0.029326706	0.970792723	83.32453577
0.356337981	-60.5457787	0.03751428	0.980892034	53.70205214

log.sigma.5.0.mm.3D_glcm_Contrast	log.sigma.5.0.mm.3D_glcm_DifferenceEntropy	log.sigma.5.0.mm.3D_glcm_InverseVariance	log.sigma.5.0.mm.3D_glcm_Entropy	log.sigma.5.0.mm.3D_glcm_Dissimilarity
12.01561054	2.847991787	0.306022891	7.3435813	2.702180398
5.217044691	2.371595905	0.414691385	6.548788051	1.658436945
10.16307488	2.648879421	0.310654201	6.318074256	2.578198368
10.77857922	2.83588144	0.346545362	7.484635977	2.445886734
31.90527466	3.311196657	0.180555732	6.513649415	4.577233311
12.19473158	2.864190065	0.282413638	6.753831971	2.803141166
7.765974268	2.318239643	0.370002416	4.707985479	2.101207946
21.4830809	3.251932183	0.260215255	7.523807176	3.629341587
34.10323924	3.594527579	0.224636036	9.004199183	4.432681966
4.457658799	2.292881815	0.428875696	6.504288646	1.555372295
13.63639972	2.974814319	0.287463432	6.836560552	2.873663781
8.623552557	2.647931023	0.364540325	6.978518348	2.214065113
7.955175613	2.628951745	0.383812185	6.598775177	2.057826447
13.74340778	2.954853669	0.30435185	7.347118406	2.907605494
15.11536773	3.01721336	0.279539742	7.251615698	3.066151669
1.584063363	1.568572452	0.339638695	4.478708752	0.667178712
19.17471502	3.074215256	0.219517433	6.987158518	3.580660145
8.406714452	2.678845612	0.375940376	6.891438156	2.127033233
33.55673249	3.464578572	0.174223562	7.251363819	4.730237338
3.319999863	2.113523212	0.44152314	6.186405618	1.329353843
2.200016645	1.896479998	0.43787267	5.48132478	1.024863336
10.42749635	2.801266514	0.333226218	7.114893096	2.440118951
3.035621999	2.04979784	0.450594363	5.887030523	1.209707201
19.73020695	2.991329187	0.227918504	6.530806655	3.565187014
3.532306493	2.115334091	0.460792773	5.940035893	1.338611926
11.21260156	2.814482115	0.31077821	6.793456473	2.638475112
3.222620578	2.091339763	0.440053597	5.798492389	1.237918297
2.538498019	1.940666602	0.378430803	5.356956735	1.011550331
5.458460406	2.391811575	0.362923519	6.262208659	1.551878772
4.434530037	2.295660035	0.423990895	6.571913046	1.542564761
2.76932102	2.021861236	0.425659043	5.767296791	1.140142167
3.207486611	2.036837038	0.385829527	5.483402536	1.094140858
9.704548871	2.771996936	0.342867416	7.337524917	2.371014781
17.86987179	1.816830447	0.228999201	3.141025559	3.462179487
18.07298235	3.001601892	0.240788498	6.409439875	3.444755661
10.60855539	2.835944623	0.335733193	7.070856168	2.482083712
2.664610504	1.965715774	0.377655705	5.329070184	1.020912276
4.252797277	2.274002411	0.423961167	6.512379106	1.497518237
19.20743621	3.124094337	0.248146672	7.506869048	3.468079906
10.36607582	2.796185708	0.329857266	7.090187887	2.485499073
6.630067003	2.504418192	0.388503476	6.759668778	1.929441468
18.64904615	3.162348771	0.270999921	7.122964794	3.307925152
11.94082966	2.873532632	0.322011564	6.946705323	2.660384917
19.30902016	3.190099066	0.272035255	7.389925005	3.399901253
10.18215108	2.806230019	0.336977966	7.299162909	2.429621822
1.145389679	1.517844924	0.376471691	4.383482083	0.655242376
12.2761431	2.92085105	0.328260943	7.597735549	2.601716722
16.8304875	3.072133794	0.255379126	6.899287179	3.263252651
15.81909202	1.939862672	0.220833184	3.794511884	3.294272394
42.08512877	3.572164753	0.16325325	7.040254787	5.31075737
3.268376617	2.110660749	0.409336929	5.869030351	1.221571286
11.819475	2.893931492	0.317770567	6.49836808	2.591905052
6.374236101	2.508844721	0.390514237	6.829149885	1.876909732
11.18539599	2.804123697	0.313780887	6.792199628	2.662968568
2.121752789	1.818102995	0.363308183	4.95188772	0.868948755
21.97186498	3.244888267	0.228506268	7.499125944	3.76489288
14.95544452	3.00159924	0.283137015	7.324051996	3.040483464
30.2307963	3.417723923	0.20474883	7.995265784	4.418091835
10.09799156	2.737796326	0.319619418	6.943850676	2.479112141
6.789922339	2.483767948	0.351146061	6.743260307	2.062222736
7.568854789	2.622572811	0.373922471	6.687153202	2.080208279
6.450770805	2.494691427	0.376631754	6.71546184	1.690832662
2.809209508	1.994176631	0.4424484	5.751003428	1.159410791
3.493389781	2.14265988	0.426242794	5.86448174	1.344362689
15.46985343	2.779355387	0.25452194	5.412302331	3.241093672
9.424500651	2.72585517	0.359330916	7.136934817	2.33445604
28.07784495	3.291963892	0.190754951	6.453720944	4.351127123
6.719160312	2.555353631	0.37979317	6.894764534	1.843591116
23.09113868	3.231271143	0.2159267	6.785411534	3.956757986
5.512991453	2.415261787	0.408514797	6.647294827	1.730843973
6.757406762	2.52137473	0.397368704	6.780039565	1.872155945
12.16996611	2.904835408	0.327363856	7.051952882	2.614498533
2.772957519	2.022238656	0.409344838	5.611763186	1.109833459
1.91199646	1.81312075	0.384469686	5.198691122	0.879386374
2.853918954	1.932723767	0.354321139	5.140422573	0.967047967
21.3927661	3.202670669	0.245570451	7.578082025	3.655495216
11.62873135	2.843862832	0.309916157	6.719716116	2.671508997
16.32807081	3.062617294	0.281201004	6.718971429	3.158147708
8.276903855	2.667112872	0.37063355	6.542150012	2.146035957
4.588623962	2.315302535	0.400706072	6.207965871	1.490549052
27.67313552	3.295027458	0.213185769	6.699769041	4.23829156
10.80299814	2.842449985	0.337442638	7.293608803	2.489263722
5.239038734	2.351990405	0.400454898	6.332980016	1.534734291
8.935252354	2.307856156	0.318007951	4.277046532	2.379496145
3.398947531	2.113615688	0.426940485	5.807908792	1.235412642
5.188533932	2.374580355	0.417055944	6.298109086	1.652939224
12.60684952	2.915000393	0.318461791	6.897834414	2.717192383
26.60821969	3.404172477	0.211819391	7.884789475	4.14352176
9.029416951	2.384172094	0.33584564	4.71090639	2.334973082
10.3015873	2.020660448	0.369435019	3.782290755	2.603174603
6.186624466	2.49633473	0.373375427	6.712390119	1.724212341
14.81029598	2.991063006	0.288407704	6.681965705	2.948037081
6.199650103	2.451906283	0.391911448	6.291898051	1.900381115
4.076892857	2.222973095	0.434677725	6.442890231	1.434214466
2.760894673	2.015987503	0.39244656	5.668338212	1.084784322
27.57822781	3.167079576	0.185999724	5.823246303	4.379346749
15.32799806	3.045014067	0.28971049	7.60487357	3.05031692
7.579413962	2.013148077	0.363896988	3.778213004	2.184594252
15.26509185	2.9572682	0.262833829	6.693005461	3.151730329
9.929136917	2.733622427	0.340618385	6.715159511	2.361921156

log.sigma.5.0.mm.3D_glcm_DifferenceVariance	log.sigma.5.0.mm.3D_glcm_Idn	log.sigma.5.0.mm.3D_glcm_Idm	log.sigma.5.0.mm.3D_glcm_Correlation	log.sigma.5.0.mm.3D_glcm_Autocorrelation
4.46813374	0.900821408	0.302538889	0.732706522	115.3863047
2.349185454	0.924589014	0.455864273	0.753461132	104.5310519
3.08969135	0.869615477	0.298859599	0.495192815	57.63193675
4.584557526	0.924064883	0.349122524	0.68559777	170.5842708
9.728085659	0.863915996	0.18215348	0.603809831	156.0928336
4.122810763	0.860837478	0.284326158	0.48290349	53.9994001
2.770648017	0.863738613	0.375311786	0.36628811	17.9482893
7.9392174	0.892109234	0.241389569	0.566089442	90.60582877
13.79813922	0.908277225	0.221260995	0.807262132	478.0097174
1.978392958	0.93451377	0.464671021	0.75190095	111.5651774
5.033866936	0.877834334	0.30570269	0.481595988	48.25217222
3.411605578	0.917093346	0.362135025	0.682149212	90.87773319
3.590753467	0.919788582	0.396882317	0.630847999	41.39153254
4.971291728	0.894358397	0.289116573	0.630907039	100.9667058
5.368609715	0.893204621	0.272721584	0.564598733	103.1198666
1.118200326	0.975519454	0.738409148	0.836969102	218.4585659
5.456214263	0.86804081	0.2247771	0.538137073	93.98609463
3.740056557	0.926044859	0.388086829	0.616590594	69.55353306
10.00503825	0.859989469	0.182034018	0.531373447	153.8777046
1.49153387	0.931471116	0.511279239	0.82286709	82.0284706
1.123259251	0.953579834	0.594106841	0.809115259	108.8191958
4.213682967	0.906393673	0.347979881	0.675678205	83.29924402
1.531746257	0.952397087	0.54991326	0.789770561	172.660829
5.957147194	0.873928666	0.234008763	0.689229524	141.3720788
1.673124221	0.931364495	0.514218823	0.743213209	73.17730162
4.049052336	0.880226418	0.300660962	0.53954429	79.74187153
1.647190209	0.947294891	0.546591618	0.711225413	100.537634
1.474377032	0.965607627	0.621018908	0.848733613	138.7110533
2.935764844	0.9520501	0.514078327	0.781465288	191.7593509
1.997856892	0.935080809	0.470849092	0.83191876	134.0602363
1.431436688	0.951141185	0.57078116	0.810016652	111.6591698
1.967018685	0.964470664	0.615471616	0.753325574	154.2727299
3.916158903	0.915033873	0.343385031	0.696132052	103.162072
2.747964744	0.794640469	0.235772751	0.290502913	46.38653846
5.738112526	0.867481317	0.229916894	0.482309913	97.82146262
4.280183107	0.908310349	0.333939643	0.52987246	93.5826422
1.58517915	0.958321954	0.626033503	0.832133629	134.2672812
1.947985476	0.936890923	0.482769814	0.811262021	96.03033851
6.443318578	0.889159905	0.245833763	0.677605467	169.8508985
3.938651826	0.891727665	0.328381211	0.685331297	81.22048748
2.759754113	0.929304034	0.400189549	0.739918859	152.3127374
7.148407092	0.890991817	0.278028875	0.561599392	71.46599873
4.51727765	0.898783344	0.318115164	0.445809113	63.63025006
7.284234418	0.895102337	0.265636868	0.602192499	75.67961527
4.088191634	0.916271287	0.343841858	0.710688511	104.3440504
0.689612419	0.958236703	0.718947546	0.865780817	85.06268868
5.302342175	0.935782525	0.330565591	0.706153612	152.2749362
5.760524613	0.869043149	0.263790695	0.477038805	62.76164294
4.437063974	0.799907501	0.203229333	0.062221756	28.98909701
12.72372397	0.865857335	0.161573224	0.515953287	224.5223896
1.728427256	0.950274768	0.560755274	0.805546255	135.9782175
4.86611388	0.888825355	0.33812873	0.37728114	48.58594062
2.724101467	0.933594944	0.415264652	0.725591404	126.6335925
3.850563752	0.884319804	0.298289002	0.5967832	86.65472843
1.33808311	0.9627582	0.670827258	0.826242462	125.9171139
7.23971106	0.872670593	0.223691018	0.496575562	123.7358555
5.105044283	0.904137889	0.275193683	0.491171228	101.2770817
9.596289485	0.885431621	0.19454376	0.602346736	270.4635324
3.708794245	0.904258045	0.32068148	0.674985557	84.38245091
2.34875411	0.902592231	0.361942298	0.741710494	69.07098625
3.161879946	0.911308057	0.376938649	0.590275225	60.26257543
3.502589472	0.955092695	0.491401285	0.774231459	243.1596784
1.383834911	0.94807355	0.563676548	0.781469136	70.79898046
1.604236276	0.91837121	0.514096196	0.717933803	62.28889695
4.679888337	0.849614206	0.235045556	0.250983171	51.71125462
3.76595481	0.916201942	0.349171783	0.645141401	113.4206368
8.425975902	0.850763729	0.185106151	0.37877501	98.89339035
3.235616116	0.932885402	0.441726502	0.790884004	117.6572115
7.063518834	0.851224881	0.20736291	0.403081587	91.23025107
2.430327835	0.924870558	0.43751406	0.727909881	107.4169851
3.098177635	0.926331473	0.424676923	0.673077512	104.1009243
5.119485542	0.907972171	0.330452879	0.549233245	79.77306995
1.508391926	0.950438704	0.587099324	0.832403472	127.3234993
1.119390653	0.953936531	0.653523637	0.857526625	76.61218802
1.896825002	0.967552245	0.65955579	0.788751681	157.6897452
7.003483123	0.876350253	0.23673665	0.511066615	111.1503446
4.129847873	0.889413526	0.308095377	0.466488656	58.58563995
5.977431423	0.878102676	0.276368424	0.495343573	57.63320312
3.508209359	0.896021454	0.382230753	0.475926208	43.00439616
2.307198447	0.946672504	0.50121079	0.743601214	208.7260658
8.851880143	0.849672115	0.204307079	0.327894274	118.009569
4.447067029	0.917433736	0.336686387	0.647630127	127.3825287
2.770647268	0.950896049	0.507698507	0.747463166	180.5802763
3.005621728	0.836251145	0.324758333	0.198465787	29.58920012
1.830526118	0.945361159	0.557216298	0.734713341	101.653966
2.338016474	0.924825551	0.457339055	0.610379622	75.23370288
5.016433599	0.900851768	0.312408091	0.577556363	60.38719967
8.952834649	0.891465318	0.209872008	0.486081745	224.4734244
2.737445192	0.850856078	0.353063207	0.181214365	40.78358048
3.316830436	0.809652775	0.27791731	0.142463757	18.66666667
3.116501282	0.949946875	0.47134636	0.79188031	228.1242182
5.649119736	0.893757509	0.296302643	0.567399021	49.43506811
2.429348486	0.894955014	0.397750441	0.598929744	37.7272698
1.950681702	0.933906216	0.498138886	0.803552902	102.5965167
1.5546631	0.953714638	0.599081514	0.831120886	109.223219
7.744354866	0.858985507	0.175962628	0.39070371	198.3059694
5.733205719	0.90098979	0.280688831	0.641313914	137.6780389
2.535514803	0.805195318	0.342300403	0.029440845	11.04968237
5.028606127	0.881738869	0.256870611	0.688824955	125.3224675
4.058391892	0.909140167	0.356337981	0.690362451	105.8169631

log.sigma.5.0.mm.3D_glcm_SumEntropy	log.sigma.5.0.mm.3D_glcm_AverageIntensity	log.sigma.5.0.mm.3D_glcm_Energy	log.sigma.5.0.mm.3D_glcm_SumSquares	log.sigma.5.0.mm.3D_glcm_ClusterProminence
5.010845577	9.944730928	0.007496404	22.48499435	12776.39274
4.553757308	9.826795411	0.015117153	10.57047149	3324.93772
4.269068749	7.257398649	0.01518174	9.984083678	2479.392496
4.936123864	12.61112716	0.008042333	17.25926215	9958.625178
4.844921639	11.507645	0.012032715	41.37070671	38529.22243
4.442016054	6.951520809	0.010862913	11.7499045	2809.193313
3.352812285	3.987755127	0.046203174	5.807003594	1100.852238
4.958584869	8.749999211	0.006670674	24.96991678	19386.08304
5.992755588	20.37727068	0.002523069	88.69399058	236965.7087
4.509602626	10.23505446	0.01576827	9.03583065	2729.710474
4.497132472	6.467697241	0.012247377	13.18038197	3828.386565
4.678903958	9.030426188	0.010302638	13.63583578	4791.222699
4.399125693	5.873065058	0.015957959	10.86390515	5917.697014
4.874638432	9.443317709	0.007848876	18.64162663	9526.345574
4.793982875	9.664581447	0.008118384	17.24511313	8113.444704
3.575554522	14.64119609	0.113929943	4.885724573	2408.382837
4.736421484	9.11444777	0.009110928	20.38354733	9110.910537
4.559085944	7.922589799	0.012370429	10.98311726	4521.933114
5.055873335	11.64644755	0.007269512	36.02379155	27252.44848
4.472519575	8.619923205	0.019519942	9.38321081	2379.378692
4.061845737	10.20483386	0.039007589	5.779137428	1221.068903
4.78441255	8.497900886	0.010930273	16.2663708	9469.372258
4.271298009	12.9204635	0.027221701	7.239012299	2611.99982
4.787351798	10.95025402	0.012159218	31.16737564	20293.34766
4.223478531	8.24931603	0.023886095	6.889436559	1560.860941
4.528293844	8.553906688	0.011395676	12.16476211	3775.096268
4.10106433	9.826532457	0.032057842	5.585869904	1455.3332
4.065025741	11.46974675	0.069356457	8.426463258	3357.043645
4.458441912	13.49172837	0.036888067	12.52638309	5490.202093
4.698911244	11.09214501	0.015164423	13.23451957	5295.957045
4.217714568	10.28233744	0.035490294	7.316206133	1925.56284
4.013094073	12.22072451	0.060870319	6.557150741	2123.339504
4.853933055	9.593263547	0.007983077	15.9772212	7318.356132
2.198717867	6.538141026	0.127483974	12.0384602	1866.063486
4.461640024	9.447086891	0.013117682	17.50065057	6616.166148
4.545841113	9.360962398	0.010605773	11.25649388	3833.250495
4.031452787	11.2977486	0.063164709	7.959454379	2828.432913
4.603941652	9.317556969	0.016511875	11.33437969	3465.479063
5.153590619	12.22582768	0.006491605	29.83746339	22243.32394
4.724768785	8.358260419	0.009307698	16.5298162	6306.342705
4.675396624	11.95141781	0.012876051	12.76526972	5119.953123
4.738992175	7.722768824	0.010747413	21.11643482	16032.50086
4.434827021	7.662850462	0.010929425	10.85104403	2936.934744
4.922143599	7.813882574	0.00772325	24.30690532	17879.70418
4.825635136	9.582412658	0.00961929	17.59615946	13487.50733
3.535851447	9.017346615	0.100130951	4.321125072	856.5694525
5.01115198	11.7771351	0.007291477	21.34938717	27014.12516
4.609540462	7.416195758	0.010243451	16.12288427	5597.777914
2.530139924	5.337210012	0.078270158	8.341135104	825.5518924
5.091090203	14.26811155	0.008358605	43.83240091	40106.41321
4.27519167	11.36621493	0.039443432	8.420357712	2443.622685
4.191752691	6.711237623	0.016276601	9.444371683	2176.517239
4.628634172	10.87252458	0.012545708	11.6080712	3906.670719
4.583302473	8.855197796	0.010901486	13.8219501	4658.9157
3.78544093	10.99319734	0.088074665	6.127170178	1851.285126
4.90686235	10.62849825	0.006632789	21.71637222	10742.28367
4.677971455	9.714319663	0.00780787	14.43675888	5482.557537
5.299352021	15.8524455	0.004733271	38.60725114	39250.33389
4.738304458	8.592882854	0.01040888	15.55948065	7297.711702
4.65964	7.696977826	0.01175146	13.20909797	4413.923727
4.386152315	7.402450904	0.013763157	9.249692912	2754.489096
4.713813447	15.25652232	0.022549764	14.46369522	8142.009862
4.176007094	8.108987233	0.032231274	6.447397043	1397.345694
4.131018302	7.605468548	0.02393094	6.187980148	936.0261042
3.711890875	7.012071786	0.025353028	10.26045517	1791.042146
4.715509129	10.24062226	0.009665025	13.24912619	5419.757084
4.561593158	9.486268847	0.01241434	22.72934985	9907.049044
4.778443523	10.24295194	0.014823377	16.09661726	6708.400591
4.63996216	9.140674773	0.010230618	19.2051792	7259.036889
4.545333065	10.00202374	0.013415754	10.12782253	2809.200136
4.553093052	9.856058543	0.012800243	10.33496207	2976.5952
4.595668265	8.502207643	0.010387319	13.55561121	6784.583817
4.165318755	10.97392156	0.043722235	8.281883138	2343.5452
4.008890195	8.415798214	0.061883876	6.740474787	1614.109862
3.839323576	12.34356129	0.088706784	6.780248672	2692.056434
4.921849355	10.0071904	0.00625011	21.66374078	10380.44149
4.375369489	7.311385013	0.012249626	10.90634143	2713.195421
4.533012369	7.054772113	0.012004568	16.00284488	6186.576136
4.23811499	6.257433773	0.014474572	7.981265807	1760.468772
4.355534802	14.21460127	0.023327922	8.964137866	2839.182713
4.579406695	10.55073436	0.010994472	20.46919414	8051.821112
4.801838637	10.83851208	0.008990428	15.33896107	7826.43495
4.457104181	13.14851979	0.025382832	10.38464726	4453.725419
2.973767396	5.334575467	0.057494616	5.548962046	400.8575519
4.141608778	9.845758667	0.03321231	6.413704672	1346.940671
4.223938332	8.437535478	0.018183914	6.63350541	1321.469559
4.581974912	7.195218033	0.01099496	14.89735317	7433.201833
5.051851435	14.6845536	0.005324532	26.94391201	18674.02937
3.287312254	6.312015015	0.04518355	5.390785546	401.4802048
2.515289912	4.222222222	0.077790375	5.95468002	566.7314723
4.724297281	14.71940983	0.019907413	15.00542736	7999.934042
4.534489103	6.288915928	0.013130124	17.26785182	10943.75848
4.233030203	5.752676109	0.015953843	7.724415658	1465.2104
4.585002946	9.707720363	0.016693521	10.39423471	3160.839142
4.21273061	10.11918423	0.049362975	8.204476484	2154.347626
4.182910466	13.74867897	0.019050387	22.90919354	10711.66631
5.019822024	11.1373343	0.006730757	21.42628146	12997.92148
2.50921675	3.272530568	0.081660479	3.906832425	190.5496013
4.776927493	10.40428743	0.011100898	24.6474069	15605.12469
4.720731199	9.738516732	0.012503003	15.90779726	7016.18132

log.sigma.5.0.mm.3D_glcm_SumAverage	log.sigma.5.0.mm.3D_glcm_Imc2	log.sigma.5.0.mm.3D_glcm_Imc1	log.sigma.5.0.mm.3D_glcm_DifferenceAverage	log.sigma.5.0.mm.3D_glcm_Id
19.88946186	0.931807157	-0.248644809	2.702180398	0.389394478
19.65359082	0.883158018	-0.218987025	1.658436945	0.514123154
14.5147973	0.900410785	-0.239625243	2.578198368	0.387675091
25.20642509	0.839687056	-0.161183784	2.445886734	0.425634382
22.92398799	0.993593158	-0.503555212	4.577233311	0.280473505
13.90304162	0.846635415	-0.174600269	2.803141166	0.37556889
7.975510253	0.927133001	-0.351792235	2.101207946	0.449391298
17.48937781	0.904933591	-0.206768969	3.629341587	0.332212707
40.36783993	0.943080256	-0.223519587	4.432681966	0.311004648
20.47010892	0.866508056	-0.202556401	1.555372295	0.521362549
12.93539448	0.82703416	-0.160988849	2.873663781	0.390207038
18.06085238	0.868364356	-0.19308071	2.214065113	0.437840238
11.74613012	0.778037325	-0.142853989	2.057826447	0.4652227
18.88663542	0.874511	-0.187044133	2.907605494	0.375434843
19.32916289	0.890110961	-0.19868981	3.066151669	0.362772041
29.28239217	0.925143758	-0.366127874	0.667178712	0.754577612
18.22889554	0.949281052	-0.285338136	3.580660145	0.323060051
15.8451796	0.798563903	-0.146155564	2.127033233	0.457999949
23.23705389	0.980775686	-0.368561402	4.730237338	0.278469215
17.23984641	0.913445897	-0.262565602	1.329353843	0.559720509
20.40966772	0.901018415	-0.273391691	1.024863336	0.62864554
16.99580177	0.862398168	-0.187038256	2.440118951	0.425880089
25.84092701	0.898746428	-0.255163088	1.209707201	0.590305945
21.90050804	0.990347624	-0.470544551	3.565187014	0.330114926
16.49863206	0.877658713	-0.232315482	1.338611926	0.55977839
17.10781338	0.879204612	-0.198872642	2.638475112	0.3885079
19.65306491	0.84663856	-0.206546841	1.237918297	0.588340661
22.93926145	0.934622388	-0.334050828	1.011550331	0.65535649
26.98011282	0.910473309	-0.261743395	1.551878772	0.565654877
22.18429002	0.923871096	-0.263851964	1.542564761	0.526747654
20.56467487	0.904505381	-0.268107479	1.140142167	0.610008213
24.44010487	0.894538677	-0.267197101	1.094140858	0.648719738
19.18652709	0.842096428	-0.163770435	2.371014781	0.422859291
13.07628205	0.992083215	-0.82249007	3.462179487	0.332208532
18.89417378	0.975939897	-0.387244551	3.444755661	0.327412406
18.7219248	0.730011657	-0.109565826	2.482083712	0.414334562
22.5954972	0.921395337	-0.311782995	1.020912276	0.658918958
18.63511394	0.902042335	-0.239256638	1.497518237	0.536629418
24.44905226	0.95303726	-0.278671708	3.468079906	0.338996866
16.71652084	0.870317543	-0.188837965	2.485499073	0.410468317
23.90283561	0.90325945	-0.229009796	1.929441468	0.469552117
15.44553765	0.892818156	-0.205150909	3.307925152	0.36436201
15.32570092	0.718307578	-0.10963662	2.660384917	0.400267303
15.62431151	0.898431745	-0.203094726	3.399901253	0.353177853
19.16482532	0.851983273	-0.171041647	2.429621822	0.422504271
18.03469323	0.920795968	-0.369022641	0.655242376	0.736374532
23.46502174	0.846871887	-0.160986604	2.601716722	0.410929088
14.83239152	0.900450591	-0.218283283	3.263252651	0.35481247
10.67442002	0.989596182	-0.692285862	3.294272394	0.312200965
28.42469859	0.990964453	-0.44660693	5.31075737	0.256658753
22.73242985	0.908048167	-0.269168225	1.221571286	0.602459555
13.42247525	0.755860375	-0.12709624	2.591905052	0.417327923
21.74504916	0.846056715	-0.179921621	1.876909732	0.481619932
17.71039559	0.899142497	-0.22179047	2.662968568	0.385588216
21.98639469	0.909976198	-0.313098589	0.868948755	0.697171351
21.2569965	0.900227918	-0.20313707	3.76489288	0.319057899
19.42403975	0.787036618	-0.130328649	3.040483464	0.36483809
31.46481659	0.946955308	-0.250958846	4.418091835	0.290098768
17.18576571	0.91189058	-0.233336452	2.479112141	0.405834757
15.39395565	0.898554524	-0.230351321	2.062222736	0.441585243
14.80490181	0.747513287	-0.121907945	2.080208279	0.450531982
30.46814665	0.908792368	-0.23988367	1.690832662	0.544532796
16.21797447	0.882264465	-0.248503498	1.159410791	0.602490807
15.2109371	0.851309869	-0.209108812	1.344362689	0.563111014
14.02414357	0.979427373	-0.464403333	3.241093672	0.332765483
20.48124451	0.825827084	-0.159744389	2.33445604	0.426103692
18.97253769	0.984810793	-0.430405209	4.351127123	0.28421255
20.48590388	0.903881665	-0.226838479	1.843591116	0.503500763
18.28134955	0.951370288	-0.297837925	3.956757986	0.303898702
20.00404747	0.85847041	-0.191289798	1.730843973	0.499482699
19.71211709	0.822519032	-0.167540609	1.872155945	0.488429385
17.00441529	0.825663736	-0.155401688	2.614498533	0.410464399
21.94784313	0.917547045	-0.289941411	1.109833459	0.624565012
16.83159643	0.934060598	-0.338622393	0.879386374	0.68176524
24.68584029	0.90318069	-0.290427301	0.967047967	0.68772152
20.0143808	0.885678408	-0.188662378	3.655495216	0.32954491
14.62277003	0.805546581	-0.150891886	2.671508997	0.39345225
14.10954423	0.911540898	-0.235522697	3.158147708	0.36391467
12.51486755	0.716462002	-0.115182846	2.146035957	0.453520958
28.42920255	0.885943801	-0.228641115	1.490549052	0.553159975
21.10146872	0.969920638	-0.351231204	4.23829156	0.298800426
21.67462547	0.848482475	-0.166276748	2.489263722	0.416189235
26.29367861	0.880876788	-0.225002023	1.534734291	0.557315067
10.66915093	0.968542096	-0.509536966	2.379496145	0.410594157
19.69151733	0.866381559	-0.223409328	1.235412642	0.597753925
16.87507096	0.743480739	-0.136697541	1.652939224	0.515067436
14.39043607	0.863821419	-0.184867471	2.717192383	0.395493075
29.11346897	0.886668421	-0.179500574	4.14352176	0.304647999
12.62403003	0.92956642	-0.35895247	2.334973082	0.43074114
8.444444444	0.988692034	-0.706892635	2.603174603	0.365740741
29.41561508	0.91033631	-0.240702691	1.724212341	0.528877156
12.57783186	0.903142161	-0.22877838	2.948037081	0.382298426
11.50535222	0.812737162	-0.167805328	1.900381115	0.467778637
19.41544073	0.910232686	-0.251222558	1.434214466	0.548498871
20.23836845	0.924544364	-0.298652518	1.084784322	0.635853914
27.49735794	0.994509271	-0.563255945	4.379346749	0.276513882
22.26761118	0.874967218	-0.18013756	3.05031692	0.368366121
6.545061135	0.952421685	-0.523797837	2.184594252	0.423015052
20.80857485	0.978693153	-0.383887393	3.151730329	0.350524433
19.47703346	0.943468014	-0.288193908	2.361921156	0.43309675

log.sigma.5.0.mm.3D_glcm_ClusterTendency	log.sigma.5.0.mm.3D_firstorder_InterquartileRange	log.sigma.5.0.mm.3D_firstorder_Skewness	log.sigma.5.0.mm.3D_firstorder_Uniformity
77.92436684	176.4224701	-0.042719992	0.057465796
37.06484126	123.6477985	-0.362879372	0.087062699
29.77325983	100.7828522	0.248670411	0.089725175
57.71906416	147.2548141	0.161584729	0.065784484
130.2530581	227.697464	0.302219462	0.049897069
34.80488644	133.7296982	0.018065185	0.078204784
15.46204011	82.99454212	0.761321138	0.132244898
77.87065228	200.5261993	0.507521638	0.055652639
316.3482346	281.168438	0.633351803	0.035827072
31.6856638	109.8533325	0.020942896	0.090051417
39.08512815	164.7533455	0.308087498	0.073845632
45.91979057	146.9003563	0.23566738	0.072075861
35.50044499	110.3165531	1.134349563	0.091573435
60.82309875	178.6016846	0.24865868	0.061380854
53.86508478	153.7995558	0.231354305	0.066100109
17.95883493	36.77934003	-1.73813289	0.209316114
62.35947431	172.0007172	0.004602057	0.062243836
35.5257546	109.2122993	0.534227909	0.083623392
108.6020714	232.7642736	-0.027259567	0.048605241
34.21284338	122.956424	-0.212253686	0.092801423
20.91653307	90.41501617	-0.465654843	0.123298619
54.63798684	150.047657	0.644683514	0.072322854
25.9204272	93.81931591	-0.632381137	0.11193845
104.9392956	241.7350464	-0.06753344	0.053536096
24.02543974	89.67090988	-0.306589389	0.110568576
37.44644686	124.4592133	-0.026519398	0.081487654
19.12085904	75.06896782	-0.239337851	0.125249426
31.16136212	115.5367785	-0.786709707	0.136358947
44.56245595	137.5127101	-0.535141959	0.104025765
48.50354824	141.9265785	-0.342764027	0.077579792
26.49550351	103.2673759	-0.436325838	0.115435217
22.97723503	83.2706852	-0.471208891	0.145335971
54.20433592	153.3317642	0.285281766	0.066335773
30.28396902	170.0989723	-0.049714467	0.12
51.92961994	144.8092957	0.000687943	0.069778369
34.41742015	123.3908577	0.477993622	0.081270834
29.17320701	94.63299298	-0.949556564	0.143980293
41.08472148	144.1076212	-0.024843118	0.081944744
100.024075	195.0857391	-0.188926965	0.054357004
55.75318897	176.6806412	0.184113525	0.067333867
44.43101187	130.2024364	-0.220955894	0.081711575
65.81669311	183.0179749	0.694681493	0.064569685
31.46334646	134.0009117	0.442923164	0.078565369
77.71413005	200.2365417	0.469115598	0.056304191
60.20248675	138.3160934	0.676416486	0.071563072
16.13911061	71.06941263	-1.076396669	0.195101914
70.76022007	141.7395064	0.727954105	0.067504929
47.6610496	162.5002556	0.234689052	0.067751616
17.5454484	97.20629311	0.171732452	0.123966942
131.6269371	248.6741333	-0.05589925	0.047288422
30.41305423	116.2898359	-0.433332324	0.11310866
25.95801173	127.3490067	0.650967057	0.091097168
40.05804871	129.4459095	-0.127542676	0.082331009
44.10240442	134.4803562	-0.117738384	0.078237426
22.38692792	67.66759476	-1.140371121	0.179130656
64.89362389	186.2028809	0.045664602	0.058445088
42.58701047	136.6311569	0.267593316	0.072016244
121.1174414	214.2080402	-0.407391809	0.047872848
52.13993105	145.1681995	0.352314409	0.071164097
46.04646953	143.1791534	0.08837347	0.075254656
29.42991686	105.5966225	0.689911095	0.095270958
50.77816744	135.7444887	-0.105065852	0.086480045
22.98037867	88.1409359	-0.415358986	0.119087746
21.25853081	100.4184246	-0.128051086	0.113048429
25.57196724	117.117569	0.106811609	0.094339623
43.57200412	123.2399529	-0.025377702	0.078762478
62.83955445	192.59552	-0.002172638	0.060498866
57.66730871	169.3186789	-0.111244932	0.073734479
53.72957812	163.4323349	0.007945565	0.068609712
34.99829868	121.7034845	-0.095812258	0.086147386
34.58244152	119.2736568	-0.045391564	0.084613151
42.05247872	125.9049149	0.723214398	0.075445816
30.35457503	113.0789664	-0.666904083	0.120992086
25.04990269	97.73008162	-0.756750953	0.136541172
24.22148559	61.54883099	-0.676965273	0.177010209
65.26219701	185.8269119	0.124480124	0.057310624
31.99663436	138.0472374	0.407345843	0.079312865
47.68330872	171.8555374	0.482217168	0.069116709
23.64815937	100.0078583	0.509366698	0.095676543
31.2679275	113.3395176	-0.281272676	0.097532474
54.20364102	173.6422729	0.135569154	0.065177244
50.44221928	127.8276367	0.257895437	0.071816633
36.19667187	106.6550291	-0.471742524	0.102313184
13.26059583	78.67526054	0.04645435	0.146722164
22.25587116	90.98739505	-0.418652599	0.120403661
21.34548771	92.38001919	0.416587792	0.105981934
46.98256317	145.4094391	0.708161564	0.07299681
76.76975944	187.177063	0.157396424	0.054407199
12.53372523	94.59080029	0.26228779	0.1336
13.51713278	85.24024963	0.540616834	0.136094675
53.38538543	145.1027317	-0.457786345	0.08593838
54.26111129	155.400013	0.938721386	0.076480263
24.69801253	107.6607513	0.331822697	0.095055936
37.50004599	122.3367491	-0.140873742	0.08535961
30.05701126	113.4681613	-0.446599778	0.119894778
64.05854636	188.9994049	-0.312270874	0.062736206
70.08954281	168.2199078	0.119686689	0.060964533
8.047915738	69.80059052	0.653220785	0.15625
83.32453577	192.761444	0.244547202	0.062290639
53.70205214	157.4287744	-0.10722983	0.070994898

log.sigma.5.0.mm.3D_firstorder_MeanAbsoluteDeviation	log.sigma.5.0.mm.3D_firstorder_Energy	log.sigma.5.0.mm.3D_firstorder_RobustMeanAbsoluteDeviation	log.sigma.5.0.mm.3D_firstorder_Median
100.5430279	15679851.89	75.68470233	-162.0469971
67.19723426	26388973.29	49.56630615	-103.9174728
65.68580071	7257608.473	45.35845222	-193.8518066
86.70163054	45014803.68	60.64137269	-105.8097534
136.2300109	5725406.265	102.7208062	-204.0945282
74.68370646	18841107.82	55.88038184	-259.5412292
53.60526736	612456.8259	38.64629986	-83.65852737
109.5245749	29300520.06	78.35531964	-252.9199829
177.7575684	74000963.01	123.3472873	-72.82268906
63.00390978	38592044.63	44.50453016	-101.7311935
84.53561799	26587674.4	65.05999252	-251.3699341
79.91239409	19058957.15	60.10743674	-126.2535934
72.5487323	33313774.67	47.79604287	-146.6660843
95.80607528	18925997	72.63518758	-157.1279144
87.28362576	7404818.834	62.33003458	-95.7151947
38.08775884	38259673.94	18.87713255	-5.010635376
97.35113225	13971649.28	71.70866612	-211.0669403
69.01605563	47777512.52	45.9822012	-157.296669
129.3206698	9948970.529	95.8636399	-167.7818146
64.14951834	29404117.9	49.14039039	-61.29930115
50.1203259	54546487.57	36.96495332	-76.84919739
86.39587991	20636114.79	61.13612719	-124.4781265
53.84730738	36461957.45	37.97317305	-83.17770004
118.6797402	2828335.461	93.29240786	-54.4531517
53.29846035	14194251.92	37.30723425	-92.32361221
72.55013333	14757582.83	50.30391027	-196.4334488
47.46801602	17648208.58	31.83404727	-56.37356186
61.86089731	44804516.31	46.42532659	-60.05125809
73.78844915	64871711.09	55.33938805	-82.36061096
76.76516957	44954787.34	58.10873411	-126.7984161
56.16646226	47045874.39	41.27632624	-71.30812836
51.6790103	64696677.37	34.95813158	-37.71105194
86.49479659	40379291.45	63.38697194	-142.2446289
82.87807758	144249.2906	63.85249675	-24.03824615
86.25667455	4617828.774	61.09356152	-174.0276489
72.85892104	58535051.38	51.27489094	-199.8768539
57.72254434	54566235.35	41.2200661	-30.16950798
74.3044779	39469100.18	57.16076855	-66.97212219
114.0563153	10785510.25	85.88006412	-105.8437119
89.73533232	24300792.09	70.24039173	-187.4983368
73.59316208	13482426.49	53.78947027	-95.14821625
103.7287093	27229381.88	75.28841297	-255.7955475
75.11438336	48201681.8	55.15635712	-234.7351303
110.8595395	26765475.53	83.93550509	-219.0989532
84.45323949	32522139.17	57.90537769	-153.1283264
43.22964747	26109402.79	31.08206571	-16.09984016
85.16309679	44624408.55	58.29863552	-66.87311554
89.44336693	19371431.47	67.75684642	-279.5750427
60.29258799	411048.5647	39.5778189	-106.9541092
137.4337698	8965853.237	101.2629166	-180.285347
61.35783104	45955181.87	46.16284606	-62.73266792
71.4401633	38867518.23	53.07060384	-320.1044006
70.75499046	62790810.08	52.05860812	-103.6686707
79.31821634	5524970.059	58.53420482	-107.7216873
48.99845055	59340166.97	31.73901368	-12.42837477
100.9516603	24159885.6	73.6767208	-238.6360626
79.64478311	42182435.89	56.16615807	-201.1300278
123.5838121	29200698.62	86.38099994	-184.6876526
82.81115129	14974243.75	58.92536821	-188.7942429
78.17606332	12336884.13	58.72874882	-134.6964111
63.61976092	51084770.89	44.56191788	-182.5570374
78.13741689	105846333.4	55.44256033	-62.265522
51.97597351	20693863.33	37.06746113	-18.0474577
51.77530821	15288139.26	39.79375339	-67.26261902
66.01224518	2826108.088	46.40495334	-213.8679504
72.81074222	15089728.81	50.10233475	-63.61804008
104.5488766	7231427.367	75.29076382	-232.5020294
86.31013422	71988768.68	67.06744208	-90.69963837
92.61395335	16894078.89	67.17089468	-314.1331787
66.50170727	31758021.13	49.492008	-104.0662651
66.55906759	49251368.77	48.21200839	-108.8550949
79.16954583	35637907.83	53.57207749	-243.1071167
60.2658186	64501567.25	46.14950576	-44.15099335
55.20115702	77126077.18	41.25053908	-30.97235012
48.83922607	74355548.87	29.57927803	-5.687800407
100.8187337	30869032.95	75.83108829	-239.3007431
73.9438107	28013004.39	55.37441961	-238.566658
89.69736095	17409814.84	67.08859669	-271.026947
60.36656413	49678179.18	41.39991684	-225.7589874
62.40406887	45580000.71	46.62330046	-101.8123894
95.10922381	19258920.73	69.90834973	-348.2901306
79.74730702	28247636.47	54.07768059	-155.9938354
64.62345525	47665169.34	45.03317864	-38.7555542
48.60028345	258260.1704	35.93954946	-67.63511658
52.11558264	40758277.1	37.70897764	-54.77502251
53.79718981	32348732.74	38.24189941	-73.33790588
85.52836401	20052381.3	61.72784264	-227.6864319
107.8624862	53128709.64	77.1876918	-269.2327881
51.2397851	471620.2396	37.71156717	-87.38605881
55.0693321	313769.9449	31.60988758	-158.1386566
78.21258183	59543620.35	57.40651193	-75.68774414
88.60835029	20654043.51	61.73920327	-255.506279
59.71235055	9435170.655	43.83205751	-135.9260559
67.28453726	55375996.21	50.08889154	-93.42545319
62.13133924	63636320.93	46.83097646	-36.94255829
106.9413206	4408000.74	74.57560958	-216.2618866
96.8919316	22323060.86	69.14409149	-136.1845703
43.6661054	293106.9602	30.97092845	-120.0873146
105.0777555	4395609.955	78.72740629	-122.0995865
84.54262994	6592736.146	61.42908296	-103.015316

log.sigma.5.0.mm.3D_firstorder_TotalEnergy	log.sigma.5.0.mm.3D_firstorder_Maximum	log.sigma.5.0.mm.3D_firstorder_RootMeanSquared	log.sigma.5.0.mm.3D_firstorder_90Percentile	log.sigma.5.0.mm.3D_firstorder_Minimum
423356001.1	125.6305084	208.4094354	-16.9026432	-423.6070557
712502278.9	110.4570313	137.1457681	-12.76608238	-349.6224365
195955428.8	10.57273388	202.4930559	-71.42450562	-356.6855774
1215399699	268.9962158	152.6025368	27.55214005	-420.5271606
154585969.2	177.815567	238.0907196	63.31339264	-466.1383667
508709911.2	-67.83268738	277.8806489	-147.2664948	-432.8607178
16536334.3	104.7764435	93.53813783	32.72114258	-166.3795319
791114041.7	216.9400635	270.3120948	-42.09953308	-468.4841309
1998026001	511.5474548	223.9110399	342.7444611	-475.5308838
1041985205	162.904068	129.0026168	-4.185625267	-344.0487366
717867208.9	45.85198593	265.9170924	-110.8995705	-424.4159546
514591843.2	220.7097321	158.6724864	-0.209500003	-333.1751709
899471916	250.0553131	156.1660308	7.938313246	-273.8922729
511001919	157.1331329	189.3269429	3.144880891	-382.8885498
199930108.5	232.2554321	142.6285697	45.66846581	-329.3588257
1033011196	237.0324554	59.44242366	28.56527843	-351.9190063
377234530.4	56.29323196	253.7429418	-71.57694397	-465.6961365
1289992838	250.9879761	175.4550647	-39.53741112	-349.2285767
268622204.3	157.0686035	233.8047759	23.10036392	-487.5423889
793911183.3	141.8489227	102.9558075	21.80286961	-265.7252808
1472755164	168.8114471	103.6729298	-9.115769768	-311.4815063
557175099.4	219.0461884	150.6718171	39.46540375	-323.4521484
984472851.3	155.2089539	111.7451195	-8.057811069	-376.4219666
76365057.45	229.0911865	145.82757	129.2800949	-324.4385071
383244801.8	123.0152206	121.5963777	-23.19573612	-276.3450623
398454736.4	23.57508659	221.0569346	-92.8379364	-413.1448669
476501631.7	200.5670013	87.8066519	3.848697519	-294.759613
1209721940	306.595459	107.2524362	-2.61222806	-329.7709656
1751536200	293.9125061	132.1264675	-5.967617273	-413.7554321
1213779258	104.6431503	162.9519248	-20.03747482	-386.6417236
1270238609	182.7259216	105.824063	-6.722863674	-301.5285034
1746810289	327.8135376	85.58287532	2.260182524	-330.2314453
1090240869	206.1259308	170.5015079	2.214687443	-362.3525085
3894730.846	110.8400726	98.06436341	90.01460419	-172.4747772
124681376.9	91.68953705	202.1527674	-35.95634537	-414.283905
1580446387	129.8475494	209.4739813	-64.47775955	-401.677124
1473288354	218.1811981	90.30597397	3.539712191	-318.223999
1065665705	201.672287	114.0943174	32.94579964	-299.2250366
291208776.7	198.0095978	183.3022651	49.30227661	-429.7382507
656121386.3	66.88095093	212.7272236	-43.64580841	-383.4181213
364025515.2	184.3509064	137.3191194	-1.52205081	-382.4237061
735193310.7	150.451828	267.3353585	-47.87880325	-446.3042603
1301445409	102.0536728	241.8619165	-95.49287415	-421.0270691
722667839.3	228.1120148	246.919405	-12.30635319	-424.3555908
878097757.5	230.6707458	170.7859415	14.22932358	-356.0071106
704953875.4	75.93095398	65.3058079	7.974059153	-226.1424713
1204859031	541.81073	127.4782139	70.10780716	-338.1052856
523028649.6	5.376304626	291.4831362	-124.8148277	-466.6448669
11098311.25	43.32533264	136.6895229	-33.39332008	-233.6198578
242078037.4	220.977005	247.8103179	28.82651806	-554.1082764
1240789910	183.6621704	106.1297553	-0.346937823	-330.8377991
1049422992	-24.91174698	310.5566851	-175.0663239	-450.0609436
1695351872	246.4198303	140.188572	-11.56729202	-370.3661804
149174191.6	117.3640137	152.0428543	-9.03211422	-332.1437683
1602184508	208.2916565	72.44691412	17.66286182	-298.4160156
652316911.2	69.86597443	268.5499237	-75.23310699	-506.7993164
1138925769	213.4348602	219.9417038	-75.02063599	-446.1629028
788418862.6	170.5473022	252.5014532	-21.06905632	-619.9633789
404304581.3	143.8793945	205.6699385	-44.81474495	-382.5334167
333095871.5	101.8463287	162.7083289	-3.420511603	-321.7207336
1379288814	148.7819824	187.3761142	-56.57905731	-349.6957092
2857851003	398.8364868	124.9830765	13.66553926	-437.7503967
558734309.8	267.6526184	71.27057718	37.90251274	-220.1207123
412779760	87.2272644	92.6239857	9.861229992	-230.1676483
76304918.39	8.872081757	230.9172885	-118.7910263	-387.6362915
407422677.8	265.577179	114.4496523	42.75135612	-316.236969
195248538.9	56.9070282	262.4323472	-68.82391663	-498.6007996
1943696754	234.8069611	144.0971701	9.523664665	-345.2637329
456140129.9	-54.92046356	343.7155973	-186.4777588	-550.380127
857466570.5	146.3389893	133.1244155	-3.433187127	-325.6316223
1329786957	177.1464844	137.6594445	-7.502354336	-334.4685669
962223511.3	138.8155365	250.7059093	-90.74356842	-438.938385
1741542316	165.1593018	95.60378244	5.540915489	-320.97995
2082404084	155.0236664	84.37352734	10.18230829	-242.6341248
2007599819	338.1176147	71.56296719	23.72852936	-312.5830078
833463889.5	78.47826385	263.0837858	-73.80821228	-496.789978
756351118.6	72.99599457	247.854851	-112.8302765	-409.1134949
470065000.6	52.65500641	278.7873521	-106.2261116	-425.0961914
1341310838	28.82912636	233.2638536	-115.9885178	-372.5852661
1230660019	157.7985535	129.8326197	-13.56189747	-447.2629395
519990859.8	-78.77205658	359.5198208	-179.4882843	-601.6439209
762686184.6	205.4516602	180.7103404	-14.36930599	-403.5884094
1286959572	316.0688477	98.33795239	26.8116436	-374.9187012
6973024.602	53.73376846	91.27416524	12.03725147	-195.0429993
1100473482	189.5799408	90.63157533	3.885932875	-297.6104126
873415783.9	191.5812073	96.04190218	21.14807968	-252.4339905
541414295	171.4824066	234.3886323	-58.56790085	-390.9079285
1434475160	138.29599	300.3357195	-93.69451141	-643.5793457
12733746.47	53.74763489	97.12056833	4.811005557	-214.0175018
8471788.512	-4.102388859	155.3581334	-39.10442657	-245.1300812
1607677749	311.1950073	131.7752582	5.359745312	-432.0480042
557659174.9	164.4007416	260.6548151	-68.40704422	-399.6130676
254749607.7	80.53678131	150.6011245	-38.4646759	-273.1574097
1495151898	142.598465	123.1389346	9.917567444	-316.4776001
1718180665	202.5982819	93.86934547	13.1967494	-278.1679993
119016020	44.65192413	264.5151522	-69.84152832	-553.4043579
602722643.4	206.4309998	181.185073	20.14882259	-402.5069275
7913887.925	8.332927704	110.5114927	-25.28156643	-161.437561
118681468.8	157.5339355	164.2161144	66.05570068	-357.6609192
178003876	181.8856354	153.4453852	10.40903969	-345.6373291

log.sigma.5.0.mm.3D_firstorder_Entropy	log.sigma.5.0.mm.3D_firstorder_StandardDeviation	log.sigma.5.0.mm.3D_firstorder_Range	log.sigma.5.0.mm.3D_firstorder_Variance	log.sigma.5.0.mm.3D_firstorder_10Percentile
4.241723929	120.2733976	549.2375641	14465.69017	-339.1737061
3.695289598	81.63920385	460.0794678	6664.959605	-223.8264984
3.672785626	81.43625698	367.2583113	6631.863951	-284.0565674
4.146432493	108.3996153	689.5233765	11750.4766	-249.4848511
4.469864954	162.1049735	643.9539337	26278.02243	-374.4842224
3.786946935	88.61770378	365.0280304	7853.097423	-387.9372955
3.170343164	65.49171956	271.1559753	4289.165331	-145.735611
4.346866553	135.0035632	685.4241943	18225.96208	-399.7626038
4.996971583	222.132089	987.0783386	49342.66494	-288.4004059
3.68377071	78.21847415	506.9528046	6118.129699	-205.5670044
3.901930876	99.96158211	470.2679405	9992.317898	-370.4682159
3.930814312	95.99713689	553.884903	9215.450292	-250.7210999
3.734320843	93.47923038	523.9475861	8738.366512	-223.1098328
4.153286089	113.7709921	540.0216827	12943.83864	-292.2774353
4.108078669	107.5697181	561.6142578	11571.24425	-230.9137848
2.821534759	56.06431482	588.9514618	3143.207396	-96.17066498
4.163014359	117.3833381	521.9893684	13778.84806	-381.5665527
3.833253977	89.26616448	600.2165527	7968.44812	-260.7060455
4.529715841	153.5994565	644.6109924	23592.79304	-385.3053131
3.568928009	75.48673636	407.5742035	5698.247367	-177.3276337
3.228099623	61.22673501	480.2929535	3748.71308	-168.4162445
4.030421425	106.2588702	542.4983368	11290.9475	-226.0556885
3.412597328	68.28379883	531.6309204	4662.677183	-173.7777908
4.317292782	137.1183281	553.5296936	18801.43589	-230.1385651
3.405385941	66.75020955	399.3602829	4455.590475	-197.377211
3.818590116	89.92624717	436.7199535	8086.72993	-320.1518463
3.306741995	61.29684557	495.3266144	3757.303276	-140.0392242
3.306694	74.72503056	636.3664246	5583.830192	-177.2365509
3.649178498	89.37222854	707.6679382	7987.395234	-225.4814377
3.826662148	91.45926806	491.284874	8364.797714	-265.1302368
3.381748981	68.46669423	484.254425	4687.688219	-175.4976501
3.260171137	66.59475726	658.0449829	4434.861695	-147.8065002
4.0748768	104.9295093	568.4784393	11010.20191	-268.9449402
3.189898095	94.77473771	283.3148499	8982.250908	-151.8368134
4.019002087	105.517392	505.9734421	11133.92	-321.1887207
3.857496369	90.77814786	531.5246735	8240.67213	-290.9692078
3.230683073	71.01700495	536.4051971	5043.414992	-166.526123
3.771105177	87.27962798	500.8973236	7617.73346	-192.10858
4.370587992	136.1253207	627.7478485	18530.10292	-313.0488586
3.987724142	105.0114868	450.2990723	11027.41237	-323.5192566
3.830448504	89.76922116	566.7746124	8058.513067	-225.7885742
4.195844578	126.8583673	596.7560883	16093.04536	-380.5403748
3.84181392	90.90416352	523.0807419	8263.566945	-334.3262512
4.283118902	132.7561613	652.4676056	17624.19837	-372.1657959
4.04903375	106.049922	586.6778564	11246.58596	-256.0978882
2.753419085	52.26126901	302.0734253	2731.240239	-122.9193512
4.123730788	110.4915218	879.9160156	12208.3764	-200.5802383
4.007217812	106.5449273	472.0211716	11351.82153	-409.2766785
3.152391278	73.45106347	276.9451904	5395.058724	-217.6544525
4.589721361	165.9551021	775.0852814	27541.09593	-427.9499207
3.456831323	73.64699487	514.4999695	5423.879854	-180.3913773
3.668267816	85.37201549	425.1491966	7288.381028	-392.3765015
3.794651309	86.20702789	616.7860107	7431.651658	-227.4507416
3.873879278	95.07306396	449.507782	9038.887491	-255.9973633
2.998938004	62.19875551	506.7076721	3868.685187	-138.3549652
4.251195301	122.3811213	576.6652908	14977.13885	-388.8811829
3.991059052	98.67639626	659.5977631	9737.031179	-323.3359833
4.580987195	154.1272844	790.5106812	23755.2198	-427.1322906
4.008873303	102.0348074	526.4128113	10411.10192	-310.6600647
3.85718561	93.58429976	423.5670624	8758.021161	-260.3638
3.635039811	79.63846204	498.4776917	6342.284636	-260.4059875
3.886949388	97.81375863	836.5868835	9567.531378	-211.2685928
3.32572426	63.93279878	487.7733307	4087.402759	-125.374192
3.281160438	61.11820843	317.3949127	3735.435402	-150.7481506
3.564803418	81.39714709	396.5083733	6625.495555	-321.6175232
3.907350686	92.37363878	581.8141479	8532.889141	-187.8296631
4.209664658	126.9602551	555.5078278	16118.90636	-396.5560059
3.942273365	101.2706403	580.070694	10255.7426	-245.2606354
4.062539124	112.0554013	495.4596634	12556.41297	-473.0319336
3.691530678	80.06899223	471.9706116	6411.043516	-212.8621155
3.745137686	81.94761139	511.6150513	6715.411012	-218.727832
3.965960124	101.0730957	577.7539215	10215.77067	-346.8030151
3.337298977	71.22211725	486.1392517	5072.589986	-171.7465393
3.191153344	65.46067925	397.6577911	4285.100528	-155.5400909
3.097878868	66.08679764	650.7006226	4367.464822	-128.1232208
4.255887533	120.6275398	575.2682419	14551.00335	-389.591095
3.781599214	88.64548929	482.1094894	7858.022771	-340.1859741
3.991323995	108.0229584	477.7511978	11668.95954	-395.1799133
3.586782151	76.26544417	401.4143925	5816.417974	-314.2028137
3.562912616	75.97042339	605.0614929	5771.50523	-205.2124619
4.126249321	115.7959073	522.8718643	13408.69215	-481.5046875
4.046021878	101.6333569	609.0400696	10329.33923	-278.2008972
3.619100331	81.34962584	690.9875488	6617.761625	-171.7122162
2.9938447	59.36580913	248.7767677	3524.299294	-141.3892975
3.323102494	63.90907886	487.1903534	4084.370361	-155.5914063
3.439963285	66.78400307	444.0151978	4460.103065	-152.2469971
3.952068085	104.6282654	562.3903351	10947.07393	-332.2627808
4.414382055	132.9381819	781.8753357	17672.56021	-430.4568604
3.121624105	61.40920587	267.7651367	3771.090566	-140.2554626
3.026986833	67.96161497	241.0276923	4618.781109	-209.7465912
3.839493533	95.87995969	743.2430115	9192.96667	-220.7995667
3.927551841	110.9506305	564.0138092	12310.04241	-357.8734833
3.528597956	72.25240191	353.694191	5220.409582	-224.7658539
3.702986266	80.77455346	459.0760651	6524.528486	-203.1481354
3.417905322	73.44659018	480.7662811	5394.401609	-168.1391296
4.195711178	131.2315127	598.056282	17221.70993	-405.8102966
4.244764288	120.0489015	608.9379272	14411.73876	-296.882312
2.804585169	50.13601665	169.7704887	2513.620166	-154.3473434
4.186178686	125.4211443	515.1948547	15730.46343	-265.6064453
3.996674426	104.2113332	527.5229645	10860.00198	-260.8696747

log.sigma.5.0.mm.3D_firstorder_Kurtosis	log.sigma.5.0.mm.3D_firstorder_Mean	log.sigma.5.0.mm.3D_glrlm_ShortRunLowGrayLevelEmphasis	log.sigma.5.0.mm.3D_glrlm_GrayLevelVariance
2.221962328	-170.2022403	0.031698363	23.39476458
2.585761402	-110.1998281	0.02000974	10.7933607
2.633082279	-185.395722	0.038376993	10.92503276
2.982704305	-107.4106961	0.009544656	19.70686073
2.171662359	-174.3822477	0.034178988	41.59764272
2.141725798	-263.3715201	0.042650597	12.60890014
2.987673562	-66.78486278	0.123982905	7.269344541
2.802955404	-234.1851116	0.039747288	29.07748816
2.688134345	-28.16893426	0.006481854	76.5619217
2.890353731	-102.5843333	0.012997996	10.33187597
2.302501923	-246.4134374	0.055046655	15.58360331
2.581473136	-126.3388604	0.023805855	15.14072181
4.151963244	-125.0978124	0.061185364	14.82500816
2.232906607	-151.3302767	0.021058435	20.78141892
2.718452994	-93.65716541	0.019906594	18.85900041
7.463525957	-19.7533373	0.004826398	7.785274745
2.314303724	-224.9591796	0.029943594	21.68078251
3.714578448	-151.0497653	0.03274576	13.45655483
2.222345751	-176.2721765	0.027014849	37.92259532
2.170283041	-70.01179143	0.022708669	8.994521943
2.950007898	-83.66219749	0.012954234	5.93657045
3.013422539	-106.8225115	0.028561647	18.53257079
3.957049004	-88.45504253	0.00742111	7.882824986
1.90620378	-49.64115498	0.045208323	30.11616798
2.957944237	-101.6370435	0.022693931	7.499883074
2.636127848	-201.9391948	0.027080263	13.01048368
3.979341102	-62.87054032	0.01420848	6.887750168
3.394861141	-76.93669391	0.013706686	9.173346018
3.045979052	-97.31396704	0.008733338	13.1067029
2.315223376	-134.864866	0.016668389	13.23095846
2.97318303	-80.6910409	0.012655837	7.737251239
4.354690101	-53.75469146	0.007924951	8.681061879
2.674808638	-134.3895914	0.021603552	17.99282674
1.603335401	-25.18667234	0.168394149	14.27880167
2.473260075	-172.4291778	0.028026587	18.2409427
3.095974776	-188.7820879	0.016288756	13.69749988
3.44921223	-55.78309729	0.01221114	8.995953308
2.229173605	-73.48319395	0.01894942	12.55387734
2.26819946	-122.7583703	0.016215376	29.36970429
2.006324051	-185.0012413	0.029104877	17.58753721
2.881097772	-103.9135577	0.011149283	13.25685876
2.859857493	-235.3192481	0.039554671	26.09092217
2.67497183	-224.1285785	0.026737475	13.62533337
2.506619711	-208.1946065	0.056320302	27.97439096
3.19832304	-133.87028	0.018189912	18.53041191
3.231717143	-39.16131135	0.016057795	4.909767876
4.921581399	-63.57923116	0.011744956	20.1345295
2.217354944	-271.3127295	0.045209912	18.13713931
2.394085888	-115.2777817	0.131040118	8.59353951
2.287781995	-184.0349362	0.016703335	44.24012899
2.842728717	-76.41757071	0.010656032	9.19443776
2.717631504	-298.5918178	0.029729988	11.97640895
2.818302378	-110.5494643	0.013677207	12.07185041
2.432537504	-118.6513466	0.025068585	14.55147874
3.835108935	-37.14660387	0.011051776	7.665694952
2.370753126	-239.0437672	0.018101296	23.42093125
3.020878153	-196.5637858	0.015970818	15.78959727
2.821117105	-200.0044101	0.013146105	37.97116058
2.791494071	-178.5749749	0.028406027	17.05652359
2.181414685	-133.1013866	0.043121431	13.81018226
3.424539573	-169.6099158	0.030247732	10.44297568
3.71202374	-77.80255793	0.005564529	16.95152587
2.925414175	-31.4974985	0.024953136	7.20457911
2.181643947	-69.59717901	0.026914811	5.931425413
2.79373649	-216.0955774	0.047898588	10.64723163
3.203622559	-67.57095368	0.018368273	13.96416635
2.413105048	-229.6776665	0.037944709	25.65695193
2.259636655	-102.5097646	0.020653785	16.43802885
2.413495333	-324.9369152	0.028528653	19.9805174
2.425327638	-106.3534978	0.014095472	10.22390073
2.688727794	-110.6106308	0.01625809	11.05151317
3.388236377	-229.4290354	0.021680815	16.84169264
2.645118564	-63.77690201	0.011759883	8.344098215
2.595346754	-53.23336911	0.029726452	7.221176177
5.050494771	-27.45529915	0.007916946	9.487208682
2.292554208	-233.7992194	0.025370364	23.15498127
2.519409673	-231.4605893	0.026551752	12.62177398
2.613207151	-257.0086149	0.037861841	18.32739189
3.058240848	-220.444114	0.058138786	9.779933389
3.197729558	-105.2853452	0.006143787	9.327034302
2.36581632	-340.3612925	0.019346581	21.51794405
3.069331957	-149.4218455	0.013512709	17.2738085
3.809444052	-55.25026022	0.008188968	11.50146536
2.379095503	-69.33018063	0.081661471	5.859965369
3.007730643	-64.26283596	0.013123644	7.014706509
3.037908531	-69.02132937	0.015611946	7.540258283
3.156878813	-209.7402132	0.039584614	17.80313388
2.885719875	-269.3120572	0.009816591	28.49740184
2.360616455	-75.24170537	0.052833806	6.65732529
2.424584004	-139.7045758	0.138722819	6.452775723
3.192987467	-90.39774338	0.006776087	15.15470134
3.448863304	-235.8620152	0.074075023	19.96736689
2.57077163	-132.1373872	0.064097866	8.612275909
2.375612431	-92.9444389	0.018192273	10.29518487
2.491645899	-58.45555928	0.01429272	9.553005188
2.491369636	-229.6661834	0.0247992	27.90565174
2.623578772	-135.7066392	0.016222115	23.25843399
2.307737354	-98.48436344	0.237993623	4.345695314
2.154836378	-106.0022114	0.023727365	25.30886769
2.570844913	-112.6298551	0.041007056	17.98336762

log.sigma.5.0.mm.3D_glrlm_LowGrayLevelRunEmphasis	log.sigma.5.0.mm.3D_glrlm_GrayLevelNonUniformityNormalized	log.sigma.5.0.mm.3D_glrlm_RunVariance	log.sigma.5.0.mm.3D_glrlm_GrayLevelNonUniformity
0.035682803	0.05719184	0.104073389	18.81123836
0.022545327	0.085580577	0.35897463	98.05258884
0.041162218	0.089018767	0.09844215	14.56073
0.010438573	0.064080115	0.204071179	108.4159528
0.036042372	0.049757038	0.043739691	4.804153115
0.048041957	0.078566414	0.091554866	17.62661246
0.140702267	0.12658288	0.082342387	8.16516864
0.043970375	0.05523355	0.072493859	20.76630607
0.006877528	0.035955814	0.095586866	49.12808659
0.014915012	0.087378665	0.360712386	164.6334036
0.064017734	0.071901871	0.147187926	24.30004508
0.02625734	0.071313865	0.179017097	47.64080525
0.07217723	0.086545327	0.24744389	100.7553919
0.023158366	0.060942667	0.104739519	29.48481923
0.021407147	0.065637914	0.092180804	22.17140847
0.006465845	0.133701309	3.941302125	728.0975922
0.032529965	0.062556769	0.06147598	12.81712451
0.038094126	0.080933925	0.232247636	107.6781248
0.028599721	0.048494904	0.049380928	8.404962529
0.026457815	0.091418611	0.613868788	195.0845049
0.015319853	0.117887972	1.157859585	414.2363982
0.033009233	0.070103492	0.205082708	56.15350458
0.008655737	0.108059535	0.643792416	235.457269
0.048152024	0.053508461	0.055030228	6.757866315
0.026173072	0.106150508	0.363320799	82.35661236
0.029329764	0.081229234	0.107153342	22.55921818
0.016897334	0.113354561	0.681271659	193.7018388
0.016125466	0.09943315	1.573717876	255.1875074
0.009945475	0.083338038	1.002742097	228.9409388
0.018506774	0.076359087	0.420298657	104.1994502
0.015162762	0.10524241	1.021908947	312.4639117
0.00968454	0.106828525	1.940660716	603.7589002
0.02378471	0.065870534	0.190319489	80.59576562
0.173282313	0.118726464	0.030421818	1.726458157
0.029006395	0.069466721	0.050245491	7.465462915
0.018074519	0.0792963	0.169279009	93.6222689
0.014540578	0.105274272	1.895872262	443.5542515
0.021878357	0.078168488	0.533325246	186.0249566
0.017159476	0.054459932	0.080264345	16.31860539
0.03219059	0.0668319	0.142471427	32.15917909
0.012007983	0.079875332	0.224264021	49.62986723
0.043989379	0.061160969	0.115802583	21.34857257
0.02949711	0.076503545	0.146728974	56.65322367
0.063974066	0.055499499	0.10371476	22.49989363
0.020165024	0.069131812	0.180289457	67.83631286
0.020969428	0.142778299	2.617491411	481.2661636
0.012933603	0.066534851	0.164938428	161.9162669
0.051412283	0.067575024	0.095248223	14.19478843
0.135981943	0.123884095	0.026381841	2.650046032
0.016999033	0.046797094	0.037315794	6.576807019
0.012434942	0.094656292	1.17148556	273.2340605
0.034067998	0.086610115	0.166791536	30.80838978
0.015264788	0.081453974	0.323274098	217.0249491
0.026917846	0.077892531	0.098085871	17.15227766
0.013738916	0.116866046	2.980847841	760.6284844
0.019564295	0.058594108	0.070513212	18.45392299
0.017124961	0.071723795	0.097893632	58.03246991
0.01383535	0.047948293	0.054620201	20.93156042
0.031149437	0.070337173	0.121639102	22.6106574
0.051156975	0.074945697	0.202944368	30.69430957
0.034211111	0.092914973	0.203332805	116.9078838
0.006378753	0.074166643	0.902793143	376.3604856
0.030086873	0.10678403	0.896484594	314.2581012
0.030746304	0.112456879	0.564355093	155.7332219
0.049073281	0.093431831	0.043334431	4.735876173
0.020280124	0.078239203	0.176983045	80.09501836
0.039551531	0.060510834	0.037258492	6.116452367
0.023482775	0.069764918	0.512769108	193.4559883
0.030454042	0.068532063	0.051539652	9.304873796
0.015901528	0.086225488	0.335463569	127.7225045
0.018170242	0.083715929	0.312502419	181.8518626
0.024193347	0.07338095	0.145042211	37.09671026
0.014053634	0.101240935	1.409608104	481.449649
0.035902453	0.10454341	2.437083613	679.1743165
0.009853607	0.111210522	3.256686372	920.8534841
0.028506687	0.057539089	0.077305847	24.02323854
0.029519234	0.078656178	0.133613701	32.58213004
0.043354362	0.068610732	0.09340778	14.17962298
0.064851279	0.093029402	0.196918854	73.78221727
0.00697769	0.094938279	0.68089616	196.4254815
0.019982137	0.06475713	0.047405376	9.211536929
0.01465026	0.070029728	0.150051219	54.20885365
0.009553177	0.091124849	0.833837417	336.7510783
0.08405658	0.14416986	0.058815399	4.204696618
0.015616174	0.109252786	0.99763649	387.7366786
0.018230584	0.103281372	0.351437981	296.833469
0.044492873	0.071854951	0.120869458	23.80279265
0.010262653	0.054018941	0.069537542	29.95592642
0.055331659	0.128217088	0.096049295	5.882594524
0.141435493	0.13401956	0.038194444	1.676282051
0.007594654	0.077949808	0.640482398	207.7648586
0.082442195	0.074320696	0.13217685	20.55836237
0.07487021	0.094860586	0.185821429	34.5592533
0.020837383	0.085529091	0.571728391	242.2370382
0.017088466	0.09060433	1.726049747	427.1560472
0.024973992	0.062198547	0.035527863	3.789114924
0.017669376	0.059935843	0.117612281	37.22691894
0.251541238	0.155424795	0.069116771	3.527379143
0.024583834	0.061538352	0.058788751	9.469629075
0.04382214	0.069205393	0.138157521	17.46233859

log.sigma.5.0.mm.3D_glrlm_LongRunEmphasis	log.sigma.5.0.mm.3D_glrlm_ShortRunHighGrayLevelEmphasis	log.sigma.5.0.mm.3D_glrlm_RunLengthNonUniformity	log.sigma.5.0.mm.3D_glrlm_ShortRunEmphasis
1.310657262	131.5060218	274.4786761	0.930610036
1.874420663	94.60212616	823.6430326	0.86971825
1.278629435	74.80201795	141.4859922	0.942109342
1.51458234	181.0763608	1344.891183	0.91078187
1.143621636	198.1654016	88.36283118	0.964094591
1.275782151	75.7021815	190.5123175	0.937310811
1.278149171	31.15921262	55.14146681	0.93351521
1.212595465	132.2568482	332.579898	0.952525799
1.263931716	423.9856994	1184.679977	0.945236303
1.886116597	104.0985176	1333.374463	0.865166863
1.390785749	75.85272158	280.3493065	0.926729716
1.474998379	97.8471003	537.0455553	0.913309318
1.634920766	57.42510709	882.7692867	0.892096346
1.297854192	128.7694765	411.2305142	0.937239165
1.255709202	131.8557631	293.9986145	0.946575123
8.129059568	137.0071441	2321.021455	0.673270917
1.185316838	132.6013039	183.4724824	0.95709774
1.59946007	81.73936115	1017.796912	0.895933377
1.153548934	214.8135235	157.2009373	0.962615357
2.338629346	66.41547727	1438.921763	0.842993064
3.286069	77.93860603	2108.19707	0.799329013
1.511654768	101.9044925	647.1831465	0.914354425
2.47168865	136.7289132	1380.442282	0.820413784
1.166410345	154.4005372	114.3395041	0.961166266
1.917732519	67.05570934	543.1783764	0.859175929
1.294732218	100.477084	237.9288088	0.939972334
2.501678465	87.90791302	1092.986432	0.824743497
3.949475501	92.51373174	1496.570383	0.786993503
2.895860253	149.644541	1834.608324	0.838957441
1.971926671	109.8005567	976.3444512	0.867916098
3.063456211	83.73914998	1813.327026	0.806517898
4.455282271	116.810109	3207.360794	0.778688558
1.482421682	113.1303119	982.641446	0.91505151
1.101437025	55.73021274	13.70667794	0.974640744
1.158887198	128.4612224	97.27216689	0.961116143
1.450044509	108.5319107	948.1180396	0.915139701
4.507659104	86.23051013	2323.299616	0.767407955
2.176033661	86.54828826	1646.695273	0.854913139
1.232754626	205.1409217	263.0420416	0.94918573
1.392632642	96.0500361	394.7022887	0.92277501
1.562838271	148.4060281	488.1547024	0.904994489
1.309707084	111.2935792	298.2819114	0.93921083
1.390465513	86.55233586	612.785488	0.926096309
1.27875863	113.2210833	351.454877	0.944656851
1.477578291	117.0860108	782.4859606	0.911948956
6.120611219	46.45969509	1539.638244	0.691773494
1.441853774	154.2888765	1958.822908	0.915942691
1.278219102	92.8236853	180.3443336	0.940250605
1.101939237	42.8488962	20.41818574	0.974515191
1.117410031	299.3428852	130.1202174	0.970647492
3.214105848	104.4503549	1826.375732	0.819164745
1.456367618	70.17347663	284.2038472	0.912493015
1.774467994	116.6245604	1980.92243	0.883400974
1.279445903	105.3575744	188.9831497	0.940906828
6.111882977	81.21967372	3327.934008	0.740711499
1.204544115	164.9793263	280.2722187	0.95472607
1.263449074	126.8924342	706.6719794	0.947176085
1.156481734	335.0044542	398.0783383	0.964699578
1.341983627	101.6597693	269.3428633	0.930490494
1.54256925	76.77898503	328.8870479	0.908610178
1.545221749	65.84000143	970.1126404	0.899327693
2.72349893	201.3953669	3383.649954	0.841367347
2.923956621	57.82426507	1818.213314	0.805258267
2.24624114	52.368323	944.5779465	0.847883097
1.138920083	71.43706468	46.37004805	0.965269979
1.454263554	122.0693518	834.830122	0.919525297
1.117509564	152.1539445	93.61028488	0.970622609
2.091420481	101.3413522	1986.618024	0.869088705
1.161982242	129.5312031	122.2915927	0.960156883
1.815363708	99.61919009	1082.643043	0.876165955
1.759710843	98.4620047	1618.870811	0.883807256
1.40639383	101.4594425	409.4904885	0.918544478
3.677805072	86.85007317	2785.658053	0.790752306
5.266632027	46.23667059	3514.104647	0.76114577
6.409544001	110.3668641	4342.066838	0.75039919
1.221823339	145.684199	369.238356	0.951849857
1.353278546	81.25781632	350.3484349	0.933741204
1.271538399	87.70853606	177.8171778	0.941549322
1.532753936	51.66158114	619.4780746	0.902426043
2.406675443	171.9940335	1416.508646	0.850808734
1.148238774	160.0252006	129.8835762	0.964193978
1.403913851	143.5467353	633.8119195	0.922405703
2.66773187	148.5060307	2440.128245	0.836377849
1.195951264	37.66070991	25.81889022	0.951012184
2.995684177	80.05419267	2210.998056	0.814246988
1.856859931	76.96500212	2066.944459	0.870179088
1.33996745	83.58626811	277.4358612	0.930947774
1.199316145	270.7486848	494.6063617	0.956024304
1.29569744	46.97199957	38.94912465	0.934220265
1.125	30.09815705	11.58333333	0.96875
2.317332128	190.011957	1868.071439	0.85926421
1.350550514	69.95109561	234.6992879	0.934497602
1.503337811	45.29353599	287.6940032	0.906367828
2.257994761	83.74655177	1920.123557	0.846880478
4.125044833	77.34804311	2754.594842	0.789668211
1.106171695	226.0679333	57.08520591	0.974930698
1.320072071	164.894468	526.9834173	0.936287948
1.19562177	16.88564998	20.56071754	0.959641566
1.183995446	146.7572424	137.2868857	0.955668001
1.376859329	108.874747	208.5240689	0.925532929

log.sigma.5.0.mm.3D_glrlm_LongRunHighGrayLevelEmphasis	log.sigma.5.0.mm.3D_glrlm_RunPercentage	log.sigma.5.0.mm.3D_glrlm_LongRunLowGrayLevelEmphasis	log.sigma.5.0.mm.3D_glrlm_RunEntropy
177.9133559	0.911144257	0.052283277	4.639370443
222.5306516	0.81654696	0.036520207	4.49076537
93.96628183	0.92394611	0.053472528	3.998308427
285.3371765	0.875203948	0.015338321	4.745968097
219.6571886	0.955826352	0.043495909	4.594990921
92.82435801	0.919609079	0.07227017	4.141150306
36.41375034	0.920879121	0.20863961	3.481590575
151.309414	0.93765586	0.062631256	4.638738796
582.8252495	0.925682718	0.008691732	5.352954837
220.0036211	0.812419146	0.026368759	4.525701144
91.62871463	0.898731588	0.111439347	4.326902225
147.9915672	0.882430647	0.039033136	4.473524134
79.61817779	0.851897736	0.132357178	4.433559351
158.8713041	0.916375291	0.032776623	4.536799994
161.8700095	0.927937447	0.02822079	4.442597252
1882.965711	0.500760138	0.038149802	5.039851865
152.9437203	0.944345977	0.043914281	4.388006975
124.9978868	0.856958763	0.066457907	4.502183154
246.8193558	0.952240068	0.034960361	4.720578191
216.7668676	0.769203039	0.052858444	4.498098921
410.3652538	0.692034862	0.036152203	4.417524723
142.7090921	0.880934247	0.057282805	4.573808338
459.842523	0.745837724	0.017532479	4.487528638
191.551791	0.949681897	0.060068208	4.491357697
151.6655718	0.807612179	0.046159916	4.259901541
127.2588287	0.919510953	0.039356748	4.179873872
268.3483776	0.745807709	0.035381967	4.41720095
638.1743143	0.658141602	0.039033171	4.660863266
653.3413946	0.737766001	0.020091222	4.727704253
298.0360393	0.806261075	0.028997395	4.631269984
387.8379238	0.706406899	0.03553052	4.561200498
771.8234787	0.638453701	0.03131052	4.791157005
174.361263	0.880932602	0.035050292	4.6340751
64.64874613	0.969230769	0.19283497	3.232182619
148.549845	0.950987066	0.03293872	4.215849387
153.5054343	0.884961365	0.02708047	4.417171634
702.1969859	0.628007772	0.040190748	4.7448197
236.2233971	0.785036533	0.042112284	4.681324939
262.1818852	0.93338126	0.021163772	4.671437496
134.7372714	0.896146684	0.047642841	4.460710439
261.831398	0.868423884	0.01668439	4.433079299
128.5549938	0.916010499	0.065714159	4.575308554
110.7036858	0.898618372	0.043512088	4.324482223
132.486627	0.92342737	0.102641397	4.627433863
168.0827282	0.879751638	0.0302745	4.62390053
589.1312415	0.548903073	0.082095137	4.587315693
226.3370751	0.886212113	0.018900172	4.687524652
110.8860043	0.921390013	0.078629026	4.328604872
46.2029686	0.972027972	0.155749242	3.184210163
335.2594606	0.962592202	0.018181825	4.75365879
505.5950024	0.706523379	0.028786138	4.638342532
89.61291536	0.882420309	0.05672142	4.188178721
246.5278618	0.833899121	0.024446039	4.540318751
137.960543	0.9211458	0.034732236	4.221324453
858.9362794	0.57339874	0.053902385	4.798610312
192.7808033	0.940298507	0.025945841	4.514885205
156.0996847	0.927840508	0.023002912	4.348295695
393.372034	0.953140746	0.016667623	4.800801597
133.0575135	0.907866145	0.044123691	4.422025272
117.9345423	0.878342687	0.094605917	4.369422638
102.136497	0.864763415	0.054939964	4.274575976
745.8727216	0.747491145	0.013477256	4.960023975
228.7991046	0.719969034	0.069399709	4.515599742
157.9875483	0.777173444	0.057267644	4.180000738
81.47995626	0.956458636	0.053772055	3.727527973
186.4901032	0.888688568	0.030057435	4.438360673
166.1516033	0.962637363	0.04597882	4.341586192
293.0220081	0.799449757	0.03999759	4.779413309
145.6013641	0.94943518	0.038173722	4.268544194
214.1343619	0.826536745	0.026886535	4.449015129
202.1274142	0.835735638	0.029217427	4.486505265
135.5696464	0.891602225	0.036460285	4.472488557
547.0884233	0.672774441	0.035717022	4.632957496
485.8843525	0.599437668	0.101813418	4.729106438
1137.131274	0.56875606	0.042270586	4.957145109
174.2467848	0.936357365	0.041733805	4.546520806
100.1035303	0.908569501	0.04661681	4.181332248
101.0127557	0.922733516	0.070318651	4.31553914
78.30371236	0.868817929	0.097823282	4.205797378
563.0028218	0.765219618	0.013529729	4.48519954
181.7154336	0.954568921	0.022674208	4.312415579
201.4054404	0.894708759	0.020432297	4.559116486
538.5599609	0.748162367	0.020002809	4.695577963
46.13653845	0.94044665	0.093637019	3.205752828
343.7551585	0.714429665	0.036725247	4.498025162
147.6705513	0.819262574	0.034824948	4.261949035
102.243004	0.90748156	0.068324215	4.368898822
319.6223259	0.941491446	0.012218713	4.706104678
60.57615965	0.916923077	0.066574505	3.475544549
32.05128205	0.961538462	0.152286188	3.081914511
601.6208251	0.776745855	0.013636974	4.774964816
85.77602856	0.909665992	0.128106685	4.328568279
67.8323782	0.875739645	0.126509045	4.080922718
261.5982005	0.775739321	0.038005446	4.612889289
523.8420644	0.652110006	0.046630175	4.802244843
255.5066847	0.967032967	0.025686527	4.294672932
213.7917028	0.913348416	0.024486469	4.65288938
19.08722256	0.945512821	0.327099222	2.952348296
180.7942159	0.943841435	0.028030285	4.421843486
162.6767755	0.900824176	0.055762508	4.433433785

log.sigma.5.0.mm.3D_glrlm_HighGrayLevelRunEmphasis	log.sigma.5.0.mm.3D_glrlm_RunLengthNonUniformityNormalized	log.sigma.5.0.mm.3D_glszm_GrayLevelVariance
139.9170845	0.833660409	23.69120558
110.0348277	0.715582689	13.75530612
77.97317801	0.861897667	11.11680541
196.4470914	0.793656998	25.65282462
202.463759	0.912520617	39.28083333
78.90703553	0.848321821	11.2960027
32.17959513	0.84826656	6.464285714
135.8739345	0.883360244	28.41634156
450.9975242	0.866574418	62.49494487
119.1295261	0.705870661	16.459271
78.69041369	0.827725284	13.52290303
105.6960925	0.800377084	20.025
60.88448333	0.756547852	18.4583416
134.1651181	0.849019503	17.0972265
136.996727	0.869671918	20.24843435
210.9238042	0.422175036	14.91447815
136.3321862	0.894078079	19.85121107
88.21785711	0.763799304	15.39611106
220.989684	0.906318621	36.84744228
80.99464802	0.66893433	9.441189644
100.8747379	0.596510946	12.45868335
107.9235756	0.803524503	17.6524024
169.7093402	0.6298499	16.24955865
161.3283754	0.90385546	26.37597143
78.02516852	0.696433571	11.54361041
104.9372132	0.85530639	12.64780521
105.9972065	0.637006773	12.85145399
124.7527046	0.578761305	14.2304051
184.4164103	0.663754086	20.33022833
130.0588615	0.712758849	17.64525714
106.5480549	0.607474726	14.10023669
152.3730897	0.564779818	15.61639005
122.1193118	0.802178231	22.64797912
57.51391941	0.939156364	13.85
132.3073364	0.903590288	20.67636364
115.8607154	0.801985421	17.79306667
120.650851	0.548367944	14.99770929
101.7731188	0.688933758	14.61722415
214.9583771	0.876155063	27.00382962
102.475732	0.81853222	17.31130178
164.5363388	0.783015494	18.84809275
114.3658148	0.853877159	25.16141002
90.4802607	0.82585838	13.69832367
116.6518881	0.866084891	25.22611054
125.4181907	0.796102853	19.95300076
74.00372651	0.448386103	6.427843524
165.8125972	0.803752046	26.91202716
95.88856313	0.856502741	16.91539116
43.51971068	0.947236318	9.392857143
306.5262003	0.925368411	48.77647059
131.0371533	0.629408338	15.35722655
73.49717761	0.797493472	10.943616
132.8081976	0.740765238	18.43358397
110.7489936	0.857338744	15.19539774
116.3757743	0.509286728	11.01010315
169.9861152	0.888523402	22.20555556
131.8839134	0.871909255	16.70324546
345.9070763	0.911377146	37.44449152
107.1682407	0.835540571	19.40628337
82.90365122	0.794944284	13.82237036
71.74572285	0.769851628	13.68730655
243.0268045	0.66550117	29.6867214
72.68914115	0.608772208	11.2113673
63.12233006	0.677532597	8.307773438
73.445643	0.913331138	12.16646849
131.6972354	0.812689465	18.26913707
154.9534762	0.925483016	25.7651169
118.927868	0.714645776	19.9400502
132.6700738	0.900148049	17.63982222
114.077522	0.727462832	13.08049052
111.5237891	0.741955226	15.16566997
107.1567079	0.808932327	19.49668931
116.3933284	0.582823843	11.39672045
66.20985854	0.53857858	8.707392833
151.2546226	0.523114782	17.7779641
150.8471314	0.882441284	22.45249968
84.36484615	0.843068914	11.09803922
90.17797368	0.858877339	17.47481492
55.69194393	0.777854794	13.58327035
205.1001803	0.682361338	17.03862418
164.3257418	0.911372749	22.45793943
152.9633666	0.817520369	20.92256335
180.9054218	0.657261456	19.16034218
39.35587562	0.882369202	7.408163265
100.3472302	0.620028224	12.06274757
86.27146037	0.716066226	10.15489789
86.89389257	0.835957126	17.46229532
279.7507427	0.891204434	29.18450651
49.45085865	0.845542369	8.736842105
30.48878205	0.923611111	6.991735537
225.815449	0.698282946	22.02509487
72.51875434	0.845508849	20.94569956
48.85238067	0.785930447	11.0656
101.4880789	0.674449863	14.27658613
102.3933967	0.581706923	15.36689186
231.4357897	0.936353037	34.66009204
173.0169271	0.847277002	25.55445159
17.30887048	0.90330492	4.049586777
153.1325864	0.890934613	25.22382222
117.4347872	0.824573039	23.83211248

log.sigma.5.0.mm.3D_glszm_SmallAreaHighGrayLevelEmphasis	log.sigma.5.0.mm.3D_glszm_GrayLevelNonUniformityNormalized	log.sigma.5.0.mm.3D_glszm_SizeZoneNonUniformityNormalized
116.7450097	0.061740032	0.320765388
86.59357525	0.086020408	0.289795918
67.69990314	0.090010406	0.301768991
176.700201	0.056227821	0.344254888
174.589	0.051111111	0.431111111
72.00955828	0.085511891	0.307471749
35.72582957	0.130102041	0.380102041
144.2415977	0.054399051	0.415249212
291.9044183	0.037236009	0.344545852
106.5094955	0.071974868	0.345077142
80.07653907	0.079648357	0.333597726
83.28121429	0.06484375	0.32875
83.73643034	0.070824245	0.310562496
124.5943296	0.071714374	0.327382784
120.7852269	0.063713058	0.339992754
149.116578	0.08425606	0.343498331
121.0448397	0.065936178	0.356016917
106.018076	0.077345914	0.401553843
175.6699631	0.049796288	0.393390675
55.19682059	0.099166891	0.264295739
86.22837604	0.087462731	0.38026732
101.4778908	0.070286503	0.303709115
129.4389296	0.078525528	0.322258315
96.89212963	0.059021214	0.395505146
61.3815727	0.085991394	0.322040844
91.64037758	0.085733882	0.38494513
92.58928466	0.081705729	0.337727865
90.14671751	0.084597478	0.327407083
142.1185337	0.064934186	0.342127386
88.87889034	0.067755102	0.329763265
85.63643803	0.077011834	0.317840237
137.2099187	0.076618641	0.350070165
123.3099577	0.060470227	0.368777235
40.39444444	0.1	0.46
99.51573758	0.063801653	0.338842975
119.4207576	0.069888889	0.353977778
95.97333263	0.075372241	0.355997501
86.16192246	0.074333834	0.293676112
147.1495131	0.056839559	0.302338081
86.37100562	0.069112426	0.36852071
118.2332857	0.071787175	0.331608095
122.8575121	0.058610221	0.407066959
97.77499572	0.079566936	0.365682951
121.5440745	0.057592975	0.398824897
116.5388982	0.067498911	0.317107789
45.38997521	0.116206443	0.311143984
136.3872446	0.059828356	0.308756682
104.1167515	0.073979592	0.412698413
41.32142857	0.112244898	0.43877551
244.6236487	0.041107266	0.468788927
108.2266683	0.074076861	0.342219833
79.9932566	0.087872	0.360256
111.3280615	0.06783568	0.321875454
82.95920731	0.078357469	0.297361635
87.67735409	0.088215497	0.381961931
149.7317745	0.060444444	0.399111111
127.8591857	0.070582343	0.402270917
252.38472	0.049236494	0.403967655
99.52050046	0.067817729	0.339266643
64.43455255	0.076365062	0.277717871
89.78215767	0.083982794	0.380930461
230.8936935	0.053485107	0.400948138
58.9091962	0.086141132	0.325101472
47.58191488	0.102890625	0.29625
58.96222462	0.093935791	0.431629013
124.8469428	0.06825083	0.371478003
155.2754777	0.064767536	0.425423273
99.27080206	0.064563351	0.329634034
119.7203761	0.073777778	0.441066667
99.30965644	0.081442114	0.385305947
95.25804535	0.074066597	0.313204447
114.5724716	0.068221624	0.367251912
90.75583644	0.08851287	0.332524466
45.5352917	0.101027665	0.309532141
131.8396505	0.068714086	0.330266592
131.4035855	0.061923999	0.34648403
90.08304508	0.087957623	0.390747149
96.18360045	0.071273044	0.338655828
65.4054103	0.078861174	0.364071555
162.5031218	0.07560453	0.342629437
139.6904228	0.06136837	0.376061529
144.0419647	0.063656854	0.400109282
149.6333128	0.06651288	0.347643214
24.67478175	0.12244898	0.316326531
86.22249317	0.082885355	0.342222159
83.5548234	0.092275128	0.337827273
98.72349079	0.07426027	0.374317725
235.3966298	0.056132623	0.407916173
41.30342591	0.113573407	0.429362881
31.20454545	0.123966942	0.702479339
169.1364258	0.063480864	0.352043249
88.83717491	0.067247985	0.38697318
44.61923861	0.086933333	0.271822222
70.95321892	0.077231262	0.307897765
85.27718162	0.074122804	0.350844303
167.6641738	0.053254438	0.439842209
162.4629371	0.058217532	0.347267691
14.15659091	0.140495868	0.289256198
115.8455434	0.060622222	0.3376
74.50307999	0.058364839	0.340028355

log.sigma.5.0.mm.3D_glszm_SizeZoneNonUniformity	log.sigma.5.0.mm.3D_glszm_GrayLevelNonUniformity	log.sigma.5.0.mm.3D_glszm_LargeAreaEmphasis	log.sigma.5.0.mm.3D_glszm_ZoneVariance
33.03883495	6.359223301	35.36893204	23.08492789
40.57142857	12.04285714	814.5928571	714.1638265
18.70967742	5.580645161	18.17741935	10.0273153
146.6525822	23.95305164	239.5093897	218.9199508
25.86666667	3.066666667	4.05	1.216388889
23.67532468	6.584415584	25.79220779	15.75071682
10.64285714	3.642857143	16.57142857	10.32142857
74.32960894	9.737430168	13.72625698	8.707655816
187.0883978	20.21915285	23.18968692	15.80090148
90.0651341	18.78544061	1209.383142	1130.438895
41.03252033	9.796747967	38.74796748	29.40326525
52.6	10.375	123.19375	100.8090234
76.39837398	17.42276423	383.1788618	352.3447683
55.98245614	12.26315789	40.37426901	30.84025854
47.25899281	8.856115108	14.87769784	8.020081776
142.5518072	34.96626506	53423.78313	52743.0133
36.31372549	6.725490196	8.088235294	3.56218762
122.0723684	23.51315789	375.5789474	349.5152355
36.9787234	4.680851064	6.276595745	2.527840652
48.36612022	18.14754098	2899.540984	2669.761773
112.9393939	25.97643098	8724.393939	8432.409845
67.42342342	15.6036036	133.6081081	116.8424032
76.69747899	18.68907563	3035.008403	2884.482311
27.28985507	4.072463768	5.550724638	1.835328712
38.96694215	10.40495868	544.3140496	481.3675295
41.57407407	9.259259259	25.35185185	17.53257888
64.84375	15.6875	2647.255208	2505.124105
82.83399209	21.40316206	6771.395257	6534.381321
144.0356295	27.33729216	2443.809976	2365.90105
57.70857143	11.85714286	834.6342857	741.0424816
82.63846154	20.02307692	6376.296154	6115.225133
223.6948357	48.95931142	14248.27074	14057.19095
104.3639576	17.1130742	220.9151943	196.8254817
4.6	1	2.7	0.45
18.63636364	3.509090909	6.381818182	2.160661157
106.1933333	20.96666667	229.6866667	209.9138222
139.5510204	29.54591837	14030.08418	13738.7376
85.75342466	21.70547945	1768.123288	1660.304935
36.8852459	6.93442623	14.18852459	7.265587208
47.90769231	8.984615385	77.25384615	60.19059172
46.09352518	9.978417266	158.6690647	132.209513
62.68831169	9.025974026	27.81168831	21.69088379
92.51778656	20.13043478	78.97233202	68.3648237
70.19318182	10.13636364	21.52840909	15.3067859
78.95983936	16.80722892	149.875502	129.8238093
48.53846154	18.12820513	39856.85897	38316.79964
180.0051458	34.87993139	204.8027444	182.6175134
34.66666667	6.214285714	21.47619048	14.10884354
6.142857143	1.571428571	2.857142857	0.387755102
39.84705882	3.494117647	4.894117647	1.943806228
104.3770492	22.59344262	4878.045902	4699.100457
45.032	10.984	63.848	53.453824
133.5783133	28.15180723	1145.20241	1085.930962
21.11267606	5.563380282	30.23943662	18.90815314
206.6414048	47.7245841	38226.51756	37789.7771
59.86666667	9.066666667	12.58	7.592222222
140.3925501	24.63323782	21.32378223	15.08094351
94.12446352	11.472103	6.721030043	2.857190223
35.96226415	7.188679245	40.35849057	29.20541118
28.04950495	7.712871287	79.22772277	57.94000588
95.61354582	21.07968127	506.4741036	472.8710655
337.1973841	44.98097503	3156.444709	3091.528253
92.97902098	24.63636364	5127.412587	4924.499535
47.4	16.4625	1539.85	1415.806094
12.51724138	2.724137931	5.551724138	2.211652794
88.41176471	16.24369748	206.092437	182.6635831
25.95081967	3.950819672	4.31147541	1.348562214
143.3908046	28.08505747	1194.195402	1130.67267
33.08	5.533333333	7.373333333	3.737955556
90.93220339	19.22033898	678.720339	621.0633439
118.7044855	28.07124011	801.3852243	754.3596466
56.18954248	10.4379085	77.64052288	63.90695886
123.3665768	32.83827493	13686.57412	13324.75425
133.4083527	43.54292343	32032.83527	31400.97198
276.1028708	57.44497608	38716.18541	38414.56491
61.6741573	11.02247191	14.21348315	7.935361697
59.78431373	13.45751634	46.06535948	37.18262207
28.10843373	5.915662651	20.84337349	13.55987807
68.80952381	14.9047619	200.5661376	177.2305926
101.7609428	22.45454545	1539.93266	1457.042977
29.70886076	4.848101266	5.405063291	1.847780804
96.82644628	15.40495868	87.52479339	74.74859982
177.2980392	33.92156863	3172.739216	3079.332676
4.428571429	1.714285714	7.5	2.596938776
128.6755319	31.16489362	6108.707447	5934.551692
154.7248908	42.26200873	1568.386463	1509.753671
44.16949153	8.762711864	42.41525424	32.84724217
112.9927798	15.54873646	13.90252708	9.381146633
8.157894737	2.157894737	14.63157895	7.706371191
7.727272727	1.363636364	1.545454545	0.148760331
161.2358079	29.07423581	1251.783843	1195.730178
33.66666667	5.850574713	53.47126437	41.26146122
20.38666667	6.52	128.6666667	97.90115556
96.06410256	24.09615385	2519.801282	2382.791256
134.3733681	28.38903394	14352.14099	13996.57729
17.15384615	2.076923077	3.41025641	0.800788955
72.57894737	12.16746411	57.28229665	46.69645841
3.181818182	1.545454545	6.363636364	1.603305785
25.32	4.546666667	8.44	3.716622222
31.2826087	5.369565217	29.60869565	20.34593573

log.sigma.5.0.mm.3D_glszm_ZonePercentage	log.sigma.5.0.mm.3D_glszm_LargeAreaLowGrayLevelEmphasis	log.sigma.5.0.mm.3D_glszm_LargeAreaHighGrayLevelEmphasis	log.sigma.5.0.mm.3D_glszm_HighGrayLevelZoneEmphasis
0.28531856	1.182871626	4494.194175	171.6796117
0.099786172	9.488558005	99418.66429	140.1571429
0.350282486	0.751878648	923.6129032	102.5967742
0.220382825	1.929564569	39067.0939	266.8497653
0.594059406	0.181285251	604.7833333	233.8833333
0.31557377	1.718659392	1277	110.2597403
0.4	2.827235493	239.5714286	48.71428571
0.44638404	1.000825234	876.9329609	188.5307263
0.367886179	0.147451664	11304.97053	464.412523
0.112548512	13.13337007	131756.4483	167.532567
0.32712766	3.696048255	1113.138211	117.4634146
0.211360634	2.70501388	10225.4625	135.5875
0.180087848	24.93507605	8554.357724	123.9796748
0.323863636	1.061857857	2634.251462	187.3157895
0.381868132	0.376990824	1410.661871	177.4892086
0.038326561	230.9112052	12528599.47	234.739759
0.470046083	0.348212844	823.5196078	168.1176471
0.195876289	11.20149417	20723.36842	150.1546053
0.516483516	0.190487072	1090	253
0.065969719	50.76786488	266130.2678	99.53551913
0.058522167	81.63093697	1053374.673	122.5319865
0.244224422	4.215162228	6504.5	165.6801802
0.081506849	17.97489872	555513.7353	197.9327731
0.518796992	0.327881708	949.6376812	155.4782609
0.126041667	9.355225992	42645.30579	98.60330579
0.357615894	0.684885226	1832.259259	133.2407407
0.083879423	27.18631806	282791.0938	143.90625
0.064955071	42.45497694	1191921.439	131.9486166
0.113293864	11.22443337	605584.6318	211.6365796
0.103366804	6.990314444	137584.6629	133.4228571
0.061890026	56.70590771	823383.4769	137.8230769
0.072342353	86.24454345	2490759.102	212.0109546
0.203743701	3.841986773	20851.84452	174.3816254
0.666666667	0.482419183	172.7	56.1
0.486725664	0.155600629	693.8363636	150.6363636
0.224887556	3.722909486	16660.17333	172.5466667
0.058586161	93.9511609	2290159.324	138.9336735
0.096306069	28.76193355	174223.1473	145.6883562
0.380062305	0.226782801	2808.565574	237.7295082
0.242085661	2.912626241	5856.038462	127.8846154
0.194405594	1.248928121	27132.77698	188.5971223
0.404199475	1.759115135	997.5454545	165.7532468
0.307038835	2.712040541	3225.770751	137.2332016
0.400911162	2.168256156	995.2840909	164.0909091
0.223318386	2.886366572	10401.31727	180.0923695
0.025481869	433.0165662	3945209.853	71.87179487
0.212308813	2.028486105	28872.43225	225.3207547
0.368421053	1.345174819	1066.238095	138.702381
0.636363636	0.43015849	104.3571429	51.64285714
0.582191781	0.060175494	1172.741176	337.7764706
0.074754902	36.19294032	771034.3443	165.8819672
0.310173697	2.796198169	2006.888	113.52
0.129890454	10.21528475	168808.0337	168.5108434
0.29707113	0.723463078	2828.774648	137.1267606
0.047850699	280.3570115	5568546.272	131.3197782
0.447761194	0.300420114	1325.293333	211.7266667
0.400229358	0.434965048	1833.670487	170.3495702
0.508733624	0.08867848	2071.214592	372.193133
0.299435028	1.25425988	2786.330189	146.5
0.216738197	4.379184148	4768.168317	111.0693069
0.172508591	15.06205968	21324.08367	121.5657371
0.124114522	12.24122748	895769.2663	320.8739596
0.070201276	82.75748715	412456.2517	90.52097902
0.089786756	32.62372057	102832.5813	78.34375
0.547169811	0.197143741	335.862069	82.37931034
0.206597222	2.577260656	25118.09664	178.3235294
0.580952381	0.179207201	490.0983607	190.9508197
0.125468705	13.07148132	184662.4851	155.0873563
0.524475524	0.268336162	591.3066667	161.32
0.131696429	8.199678754	76381.72458	145.4279661
0.145825317	9.587295401	85950.49077	149.4564644
0.26984127	1.729256016	5084.352941	163.6535948
0.052571914	102.7747851	2116873.313	142.7493261
0.039782167	415.4384112	3019903.538	72.44779582
0.057579723	223.7322629	7005420.117	207.7296651
0.399103139	0.527238731	1327.398876	192.7696629
0.335526316	2.207947819	1614.104575	124.875817
0.370535714	1.667328344	773.1325301	136.626506
0.207009858	8.238908307	7739.645503	91.47089947
0.109837278	7.46139934	352384.1751	248.2424242
0.530201342	0.094940862	688.0253165	195.4303797
0.279768786	1.087586683	9798.272727	206.3099174
0.103469264	15.66632561	695151.398	223.2019608
0.451612903	0.431097025	262.3571429	41.71428571
0.075775897	60.40036799	702399.6968	134.412234
0.130595951	28.19197137	100600.6703	130.1004367
0.323287671	2.310261219	1553.09322	135.3135593
0.470288625	0.115493183	2563.801444	331.7184116
0.38	0.588720648	579.1578947	57.73684211
0.846153846	0.196134188	39.72727273	32.90909091
0.133566638	5.179821459	351137.0568	257.4934498
0.286184211	6.453078157	1420.517241	125.1264368
0.180288462	8.269644784	4443.546667	73.16
0.08543264	30.07947622	277699.6218	116.4679487
0.053032401	134.669105	1844255.621	131.9843342
0.619047619	0.050566021	783.1538462	235.7179487
0.307352941	0.842194074	7198.023923	240.4784689
0.458333333	2.210693671	70.81818182	23.09090909
0.460122699	0.15602846	1285.28	170.5066667
0.328571429	0.595808305	3748.119565	120.5978261

log.sigma.5.0.mm.3D_glszm_SmallAreaEmphasis	log.sigma.5.0.mm.3D_glszm_LowGrayLevelZoneEmphasis	log.sigma.5.0.mm.3D_glszm_ZoneEntropy	log.sigma.5.0.mm.3D_glszm_SmallAreaLowGrayLevelEmphasis
0.585799739	0.023270984	5.547663392	0.005571303
0.54658251	0.029763868	5.429991303	0.012657105
0.559921352	0.035166113	4.96574127	0.01084807
0.600546707	0.009935387	6.125607014	0.004225954
0.674597222	0.027337731	4.950489296	0.009172088
0.561876014	0.030314944	4.935236865	0.007942975
0.634473474	0.072148772	4.00862178	0.016891237
0.667112047	0.020863464	5.683279307	0.007484208
0.607747452	0.007171058	6.69482867	0.004593485
0.608823115	0.016950285	5.773963729	0.008084975
0.592738503	0.033518086	5.292448914	0.008339685
0.589584488	0.026132235	5.749101908	0.011753906
0.576726376	0.038089736	5.905404279	0.007599552
0.589997879	0.016832831	5.577936932	0.005530095
0.60262682	0.01910583	5.656740944	0.012172234
0.605352187	0.009538789	5.742536682	0.005959547
0.611214352	0.023661543	5.28137072	0.00716414
0.658204113	0.021351651	5.575852915	0.007228751
0.645550778	0.022757848	5.428541283	0.007842948
0.516657894	0.024630303	5.52992769	0.006614407
0.640855936	0.026922457	5.492640557	0.015927808
0.565539695	0.01652614	5.831609772	0.00499033
0.588104569	0.013563123	5.779770204	0.00817448
0.647999195	0.036069043	5.083465675	0.016314833
0.586497218	0.029970714	5.445146089	0.019047377
0.6383053	0.023572601	5.079821563	0.008688406
0.603505573	0.018034003	5.530607444	0.006085109
0.594027402	0.020098881	5.695112783	0.006682711
0.604611827	0.011749021	6.01833656	0.00430071
0.590341119	0.03122071	5.672000207	0.014929133
0.583631007	0.018846529	5.791864518	0.009727566
0.613335173	0.009493735	5.945544459	0.004406012
0.627998784	0.021408135	5.843978947	0.007661249
0.686111111	0.150935164	3.321928095	0.068982433
0.59699732	0.035372328	4.962822441	0.025618855
0.617506029	0.016447448	5.763571851	0.009312997
0.619759167	0.018285837	5.828589295	0.008210705
0.553054082	0.017844853	5.896363602	0.006196104
0.562696013	0.018681618	5.75268181	0.011951278
0.629266931	0.028832483	5.442565822	0.013350689
0.591553595	0.018261041	5.588265661	0.012086116
0.66252491	0.029550135	5.545341224	0.015149145
0.622209505	0.023569284	5.478084401	0.01299377
0.655536529	0.02675681	5.644516291	0.007602437
0.582611812	0.017137131	5.943828457	0.006946077
0.57873613	0.03139183	5.298015118	0.017320044
0.57327094	0.011819072	6.356940345	0.005172578
0.665914396	0.030114059	5.029871328	0.010179477
0.615079365	0.111921225	3.521640636	0.034566494
0.709334967	0.022091926	5.542935166	0.019707343
0.606280056	0.015321356	5.776068478	0.006515756
0.618746017	0.024104132	5.090449113	0.014514457
0.585561088	0.017105463	5.969378173	0.005592248
0.562840088	0.029673565	5.234342634	0.019591712
0.641592065	0.015626853	5.562892424	0.007641773
0.652570368	0.016888768	5.498838378	0.011085405
0.659369822	0.014109116	5.623902071	0.008169817
0.658905548	0.011355785	5.921529962	0.004662033
0.600670618	0.026137686	5.416264205	0.008835516
0.533309715	0.028396449	5.54677739	0.008092764
0.641185837	0.025911473	5.427957388	0.008111396
0.657807274	0.007105012	6.218271813	0.004221139
0.586870707	0.033012391	5.623808839	0.014639345
0.56107649	0.043094812	5.352349852	0.024125601
0.680136494	0.062134267	4.064203409	0.052192515
0.631588985	0.015908678	5.687547472	0.006070839
0.673349132	0.030862061	4.871242412	0.009271425
0.594469651	0.020406644	6.05680209	0.006911561
0.688086303	0.028322635	4.975616279	0.020313119
0.644802702	0.017833703	5.475006619	0.011559173
0.575164024	0.019776237	5.918975712	0.009363329
0.627837513	0.020062394	5.520021915	0.012411561
0.593121424	0.014957496	5.609583575	0.005763657
0.571941325	0.04734807	5.61516174	0.01866693
0.593065673	0.00990373	6.145084473	0.005274635
0.608915915	0.01999156	5.770454926	0.008264726
0.648261991	0.022636805	5.173196405	0.015861686
0.592261255	0.028090116	5.228551293	0.018178357
0.625871336	0.060085643	5.434823353	0.020292104
0.605474215	0.010486557	5.727177039	0.006948129
0.629396097	0.022728728	5.152695976	0.016984166
0.657270555	0.015689095	5.714985818	0.011938461
0.611655395	0.009906725	6.089580976	0.003816889
0.570813492	0.118280959	3.521640636	0.089878835
0.606085832	0.017498384	5.765744727	0.00965299
0.600263446	0.015070144	5.659793661	0.008694002
0.630684119	0.025410488	5.278841678	0.007928345
0.662325259	0.009893086	5.827184255	0.004808574
0.661121211	0.082300985	3.787143961	0.069223384
0.863636364	0.148785703	3.277613437	0.136948582
0.615764242	0.00939929	6.097030687	0.004325438
0.638929213	0.031256627	5.071411168	0.009914373
0.529578331	0.070514079	5.275141525	0.027400162
0.573867889	0.029145383	5.911864045	0.011941615
0.614343319	0.021145176	5.75592719	0.01168179
0.684116809	0.035058462	4.958353821	0.032501107
0.605535021	0.014292806	5.808113901	0.005230521
0.473964646	0.158275742	3.459431619	0.026621877
0.591621077	0.028771119	5.245503944	0.018892276
0.599540915	0.058480808	5.455748576	0.038430724

log.sigma.5.0.mm.3D_ngtdm_Coarseness	log.sigma.5.0.mm.3D_ngtdm_Complexity	log.sigma.5.0.mm.3D_ngtdm_Strength	log.sigma.5.0.mm.3D_ngtdm_Busyness	log.sigma.5.0.mm.3D_ngtdm_Contrast
0.033203215	0.838481127	5.566740487	0.148182429	0.000404195
0.00942528	0.098551038	1.117862861	0.515961873	4.41E-05
0.051766778	0.772970801	3.83531157	0.199532058	0.000773813
0.006567297	0.235628367	1.695643092	0.389460649	3.80E-05
0.085165159	5.848449386	18.7568686	0.061870355	0.003569003
0.032732694	0.655927586	2.530887334	0.326000208	0.000779935
0.099476316	1.067614426	5.513064375	0.233008306	0.002106394
0.023656747	1.459864273	5.70202394	0.219234422	0.000507813
0.010204196	0.862853407	6.185032092	0.174428257	0.000129526
0.00607104	0.071483015	0.909975149	0.687558778	1.88E-05
0.020848509	0.696983174	2.654866011	0.433280379	0.000481806
0.016112634	0.381785265	2.978541073	0.292925699	0.000104198
0.008551231	0.201005004	1.9930681	0.949879134	5.70E-05
0.020666425	0.567910495	3.119361649	0.296093065	0.000289455
0.028732594	1.040696794	5.257849685	0.147810468	0.000325356
0.001413029	0.011980811	0.610108809	1.193119873	7.16E-07
0.038221176	1.713980578	5.989246139	0.13423845	0.000936624
0.007080268	0.197786508	1.54937363	0.69681333	4.52E-05
0.045034677	3.88993828	10.56385527	0.077717889	0.001657404
0.005725504	0.031300777	0.54092357	1.147938311	1.85E-05
0.002990153	0.021720874	0.499829041	1.213498142	3.85E-06
0.013573942	0.272367479	2.166626248	0.484948549	0.000123012
0.005040603	0.057393474	1.132760608	0.495102568	7.40E-06
0.072664393	2.635863979	11.5464752	0.071794638	0.002038539
0.013000724	0.101287539	1.315454137	0.46880264	4.64E-05
0.028131746	0.65578263	2.798647633	0.235980019	0.00047564
0.006265816	0.061670943	1.068646338	0.57109823	9.51E-06
0.004217891	0.039634063	1.101003408	0.626628485	5.70E-06
0.003886965	0.087024821	1.271179625	0.476888188	8.63E-06
0.009760422	0.080830779	1.308787361	0.443679496	3.65E-05
0.003808689	0.030436867	0.663873532	0.909166325	5.77E-06
0.001704332	0.022065378	0.513055353	1.288575306	1.90E-06
0.00894456	0.232201637	1.734625643	0.503788944	6.57E-05
0.222783185	8.079406503	15.80191484	0.079964414	0.050727668
0.074131281	2.59180272	9.224514535	0.077337533	0.001699809
0.007693695	0.246322802	1.343028295	0.57749275	6.22E-05
0.002280829	0.020209082	0.499160308	1.202917722	3.37E-06
0.005381928	0.05158175	0.816796709	0.872879394	1.66E-05
0.032944544	1.433465438	7.575933742	0.094548943	0.000520943
0.019632132	0.377723565	2.220230042	0.430432368	0.000300145
0.01735344	0.372637663	3.614048398	0.165763618	7.38E-05
0.024177538	1.339098607	5.779341435	0.266369648	0.000481411
0.011013939	0.379028651	1.696095512	0.582883712	0.000135021
0.023098636	1.145839335	5.589585978	0.256858188	0.000432902
0.011075047	0.316908099	2.527132978	0.395810487	7.50E-05
0.00258832	0.005884688	0.250445492	2.214499646	3.34E-06
0.004777015	0.270430678	2.817057185	0.427887393	1.72E-05
0.035487859	1.178009062	4.070924302	0.242223361	0.000983129
0.208680172	4.223300909	8.524388777	0.131451978	0.01923266
0.051711296	7.282481476	15.99006985	0.047167758	0.002160923
0.003877565	0.036573963	0.726649596	0.773416389	6.74E-06
0.018645603	0.663039183	2.410918517	0.458383003	0.000318895
0.003847064	0.087450347	0.826065937	0.826911436	1.55E-05
0.038266564	0.870535262	4.545008846	0.152199566	0.000553953
0.001321801	0.009711721	0.285226905	2.170604258	1.48E-06
0.026730485	1.412233696	4.566092635	0.159984007	0.000578084
0.010077709	0.55903107	2.277613386	0.361501698	0.000114181
0.021050088	1.876253905	7.090631147	0.090885515	0.000412118
0.03227073	0.753427049	5.289861174	0.174216133	0.000299662
0.025328541	0.312791792	2.685604143	0.337322007	0.000237569
0.007048024	0.14946934	1.070591418	0.963659084	4.88E-05
0.002255408	0.074421099	1.174045475	0.606023309	4.02E-06
0.003593049	0.028400549	0.558561213	1.367633908	6.68E-06
0.006691485	0.036018056	0.417478517	1.392958508	3.14E-05
0.107072004	3.084484199	7.378913038	0.107048877	0.004733575
0.010324515	0.235542569	1.809465093	0.362948536	6.27E-05
0.062480593	4.64651201	10.01373039	0.076722143	0.002948119
0.004239493	0.076612789	0.862538448	0.831474123	1.91E-05
0.045774543	2.805786914	6.688469693	0.114735094	0.001690283
0.006985948	0.094627918	0.923268266	0.667399373	3.16E-05
0.004859852	0.077614272	0.700386422	0.911198971	2.27E-05
0.017664654	0.639227655	3.164855734	0.302457476	0.00020023
0.002172097	0.016497148	0.34921654	1.552651909	4.20E-06
0.001615438	0.005379579	0.1637645	3.573966585	2.80E-06
0.001090317	0.01353141	0.434147325	1.746405287	8.44E-07
0.01998111	1.044840407	3.498247943	0.231868801	0.000426153
0.019873197	0.455136476	2.055488814	0.470927622	0.000304219
0.03616876	1.572158378	5.680495358	0.217091624	0.000818629
0.01067366	0.173655057	1.005387206	0.94902664	0.000107803
0.005065365	0.092249022	1.279288629	0.419888711	1.05E-05
0.045566023	2.848545266	6.184033135	0.117287673	0.0018421
0.012940345	0.480449804	2.991047722	0.24776202	9.20E-05
0.002902446	0.062762411	0.970276838	0.654751558	5.19E-06
0.168971057	1.988328333	6.274616913	0.111731652	0.006324855
0.002772614	0.026537534	0.418255813	1.378848901	5.46E-06
0.003384432	0.043296128	0.414914599	1.637849985	1.22E-05
0.025216793	0.868974535	4.362274268	0.316506638	0.000412028
0.013804615	1.670109287	4.391499352	0.15520425	0.00027075
0.11040439	1.509516162	4.713183022	0.170000203	0.003859036
0.341880342	4.719003945	10.3432335	0.120332278	0.026639619
0.004055807	0.10933607	1.416181995	0.424023027	1.10E-05
0.029261106	1.090686537	5.683057491	0.368778135	0.000560292
0.026811125	0.233217548	1.918708955	0.506262959	0.000223951
0.004184656	0.033411868	0.489854389	1.285048153	1.44E-05
0.002376329	0.017346929	0.405786093	1.504411959	3.78E-06
0.102665234	8.163583217	18.03096229	0.035639733	0.005224878
0.016994492	0.640398246	3.588843078	0.201781987	0.000180399
0.186550478	1.671604712	4.132388366	0.41500486	0.01091882
0.055890389	2.009941957	9.567587528	0.094836846	0.001249187
0.036149745	0.825525878	4.897383321	0.151369151	0.000510102

log.sigma.4.5.mm.3D_gldm_GrayLevelVariance	log.sigma.4.5.mm.3D_gldm_HighGrayLevelEmphasis	log.sigma.4.5.mm.3D_gldm_GrayLevelNonUniformityNormalized	log.sigma.4.5.mm.3D_gldm_DependenceEntropy
19.61191212	140.2465374	0.063328243	6.352886589
10.06574243	118.3414113	0.091221387	6.899201827
11.24932171	69.50847458	0.09176801	5.57059499
17.96213129	227.7697879	0.068506826	7.028454928
40.55582786	213.960396	0.050877365	5.543070983
9.867844665	56.04098361	0.091306101	5.551648931
6.368163265	31.94285714	0.118367347	4.875731503
23.44349849	111.1296758	0.061697378	6.234956849
76.26381453	399.5325203	0.036798349	7.17355864
9.299543063	147.0961621	0.093919942	7.019165247
12.18568923	65.36702128	0.083875622	5.9363304
13.96395771	109.5746367	0.074686458	6.703444629
11.6729462	59.32942899	0.097343131	6.523307733
17.57162176	111.9337121	0.068669651	6.312172288
16.79376736	109.5137363	0.069556817	6.067392091
4.68127151	194.0003694	0.227138107	6.889232522
20.31349147	115.1290323	0.065599185	5.743205301
11.17370171	90.12242268	0.088168575	6.805053557
35.49088274	182.4065934	0.051141167	5.907044195
8.6355728	89.16258111	0.098984597	7.100300546
5.715130345	116.1659113	0.132516567	6.888233526
15.80992665	103.1265127	0.078894468	6.683344956
7.341840753	158.0174658	0.116299259	6.982584207
33.44632257	210.556391	0.053083837	5.502015988
6.586649306	66.19791667	0.114144965	6.712636403
11.19846717	87.09602649	0.083548967	5.994384607
5.890869413	109.6120577	0.133156256	6.943588134
8.145167251	122.8302953	0.152419563	6.927446839
12.03771055	206.4585576	0.120144785	7.001638534
12.59211743	149.1630242	0.083909318	6.976730215
7.146591807	118.6724589	0.12512754	6.984462023
6.573444921	138.5774935	0.15767233	7.079047102
16.22672847	127.6515479	0.069559705	6.855896887
18.35555556	72.13333333	0.093333333	3.506890596
21.24019109	154.2035398	0.065862636	5.234933972
12.10751895	104.3193403	0.08273412	6.497489336
7.528402887	139.3610821	0.158384659	6.945791223
10.85761889	108.0478232	0.088605882	7.170961469
27.23797323	186.5794393	0.056492076	6.282920728
16.08045941	90.29050279	0.070954229	6.504091242
12.86613526	171.3090909	0.08195413	6.685974187
20.38824478	129.4015748	0.072533256	6.149896848
11.24050187	95.12742718	0.085617165	6.304936752
22.79055215	112.284738	0.063578956	6.254750084
16.90518289	107.8466368	0.07486899	6.661518048
4.156843971	87.07628226	0.212095109	6.706062483
20.69325282	164.4311726	0.067706242	6.907141304
14.72245306	70.44736842	0.076523546	5.702594829
11.77066116	60.13636364	0.103305785	3.823067982
43.71945956	291.0273973	0.047100769	5.675901145
8.025878508	123.1882353	0.126232819	7.060492314
9.142485946	53.03722084	0.103534903	5.869661953
11.78958672	141.856651	0.084327086	6.962607933
13.93252919	92.31799163	0.07858756	5.954769687
5.879999478	130.2243941	0.195492992	6.973567053
21.52420584	150.9402985	0.060957897	6.00955236
15.89392728	152.1834862	0.071313968	6.161528152
34.34065426	280.8580786	0.049789287	6.267598258
15.32299148	94.50282486	0.074164512	6.14388269
12.51408204	86.12017167	0.080089889	6.411223569
9.697257236	77.34364261	0.095389048	6.450208784
14.19958121	228.9803719	0.093318674	7.343871128
6.151504723	75.82523319	0.126025547	7.154046713
6.201488951	69.13748597	0.113560471	6.805068806
12.50978996	85.88679245	0.091491634	4.508501766
13.59760802	133.3784722	0.078426408	6.715333114
23.81823129	167.1333333	0.061950113	5.280878913
15.61152234	130.9059706	0.078567887	7.173828501
16.21761455	83.5034965	0.07653186	5.37202512
9.876732651	101.9810268	0.088025151	6.995457124
10.72713378	118.2031551	0.085325534	6.98406543
12.94314891	92.39506173	0.08380069	6.296442931
7.791876746	156.6097492	0.131373713	6.992999396
6.398127903	80.30035075	0.151876324	7.04236832
6.607060473	139.8291205	0.186906426	7.096167229
20.10073096	151.5269058	0.063926482	6.135080974
10.55099646	72.11842105	0.090104648	6.074735713
14.01959104	57.37946429	0.086455676	5.549221817
9.631027545	97.45235487	0.092639002	6.468542574
8.968840814	195.8409763	0.103361829	6.921910972
20.04828611	149.6107383	0.065357416	5.409618796
16.74294497	158.3942197	0.070832971	6.649578795
10.44759735	220.6122946	0.109490616	7.257941316
9.798126951	66.29032258	0.100936524	4.438067278
6.463572851	109.0312374	0.126027702	7.070573143
7.052508857	69.23609923	0.107734265	6.87167517
13.88803903	70.40547945	0.082094202	6.046765527
25.94153712	281.0730051	0.05875401	6.176370802
5.2096	47.2	0.1312	4.57366069
9.017751479	39.69230769	0.112426036	3.238901257
13.94467905	243.0842811	0.091334687	7.207846694
18.1348576	62.06907895	0.080786877	6.061247981
8.502906574	52.87259615	0.095356416	6.115598615
9.948939665	134.1322563	0.089237814	7.188492447
7.941556945	115.2914705	0.133349352	7.113771684
27.1101033	224	0.066263542	4.953004923
21.51452422	155.0529412	0.061613322	6.455489959
6.138888889	23.5	0.135416667	3.386842188
27.43324928	169.2331288	0.058301028	5.688700931
15.07998724	138.2107143	0.075535714	6.248227543

log.sigma.4.5.mm.3D_gldm_DependenceNonUniformity	log.sigma.4.5.mm.3D_gldm_GrayLevelNonUniformity	log.sigma.4.5.mm.3D_gldm_SmallDependenceEmphasis
63.02216066	22.86149584	0.245478677
132.3114754	127.9836066	0.115236164
30.92090395	16.24293785	0.32640129
239.7656492	132.4236937	0.223878281
31.35643564	5.138613861	0.475368537
40.98360656	22.27868852	0.247247779
14.02857143	8.285714286	0.27922619
76.65586035	24.74064838	0.346597601
296.4403794	54.31436314	0.312089493
206.2100043	217.800345	0.121581039
61.69680851	31.53723404	0.267710067
100.664465	56.53764861	0.203656779
152.4699854	132.9707174	0.171016646
83.33333333	36.25757576	0.264124296
66.59340659	25.31868132	0.296046118
432.3952715	2459.451422	0.04449305
53.75576037	14.23502304	0.399923678
165.4368557	136.8376289	0.186434071
47.73626374	9.307692308	0.444752747
203.3496756	274.5832733	0.084417156
298.4947783	672.5215764	0.06955326
118.7293729	71.71507151	0.208221561
196.0767123	339.5938356	0.091870983
37.4962406	7.060150376	0.427032164
85.48958333	109.5791667	0.122754171
54.58278146	25.23178808	0.248454981
158.1716907	304.7946702	0.098626961
202.9620026	593.6741977	0.069384867
250.4542519	446.4580194	0.122431422
153.1039575	142.0584761	0.113683269
264.4744109	525.660795	0.074564623
408.7282916	1392.719688	0.079265644
181.0820734	96.61843053	0.201201687
7.8	1.4	0.7
35.77876106	7.442477876	0.389550147
177.2713643	110.3673163	0.204683629
316.878643	1059.751756	0.067008385
241.7255937	268.6530343	0.106523326
66.80685358	18.13395639	0.295784225
78.97392924	38.10242086	0.230360009
85.24335664	58.5972028	0.167598339
65.92913386	27.6351706	0.339158107
121.6286408	70.54854369	0.245289109
81.20501139	27.91116173	0.31258622
157.032287	83.47892377	0.216480486
255.299902	1298.446259	0.034205593
402.9344501	185.9213401	0.227316304
39.47368421	17.44736842	0.313041248
10.72727273	2.272727273	0.441919192
49.42465753	6.876712329	0.495089422
247.8764706	515.029902	0.083879124
52.6426799	41.72456576	0.247358193
327.4143975	269.4250391	0.144132318
43.08368201	18.78242678	0.203808416
469.0325491	2210.243764	0.054713154
77.93731343	20.42089552	0.355790139
174.5848624	62.18577982	0.339131236
123.9868996	22.80349345	0.402081192
57.20903955	26.25423729	0.259403485
62.02575107	37.32188841	0.174545017
172.1793814	138.7910653	0.176785171
455.1664699	632.3273318	0.126926693
243.742759	513.4280805	0.081064056
143.1919192	202.3647587	0.096925495
16.01886792	4.849056604	0.496331237
154.4357639	90.34722222	0.220192524
30.82857143	6.504761905	0.468007937
302.3642919	272.3948659	0.12584839
31.64335664	10.94405594	0.375529639
168.2332589	157.7410714	0.150920452
258.3035783	221.7610619	0.152359252
79.670194	47.51499118	0.224804047
372.3748052	927.1042936	0.069708582
486.9209895	1645.428097	0.052597447
593.717267	2713.6944	0.066800782
88.26008969	28.51121076	0.336571853
71.07894737	41.0877193	0.268730226
40.58035714	19.36607143	0.297683355
115.4315444	84.57940854	0.214136062
203.5525148	279.4903846	0.121028149
39.96644295	9.738255034	0.476020636
127.5156069	61.27052023	0.269308132
329.7660783	539.6792453	0.113483246
11.06451613	3.129032258	0.461191756
301.146715	625.3494559	0.087509142
319.540633	377.8240662	0.133199358
56.58356164	29.96438356	0.267374867
138.9286927	34.60611205	0.373999177
10.52	6.56	0.332497052
9.615384615	1.461538462	0.884615385
272.5707203	313.1866433	0.140674325
49.60526316	24.55921053	0.280213409
58.26442308	39.66826923	0.174574883
283.3893757	325.8964951	0.095487739
370.2171144	963.0490169	0.066384376
22.17460317	4.174603175	0.51675485
120.1205882	41.89705882	0.268306963
6.416666667	3.25	0.334490741
38.27607362	9.503067485	0.40810668
43.27142857	21.15	0.269081529

log.sigma.4.5.mm.3D_gldm_DependenceNonUniformityNormalized	log.sigma.4.5.mm.3D_gldm_DependenceVariance	log.sigma.4.5.mm.3D_gldm_LargeDependenceEmphasis
0.174576622	3.179195985	14.06371191
0.094306112	17.05722642	53.47754811
0.174694373	4.094608829	13.92655367
0.124038101	8.243813912	26.27884118
0.310459759	0.98009999	5.01980198
0.167965601	5.387664606	17.92622951
0.200408163	2.025306122	10.68571429
0.191161747	2.994322175	11.319202
0.200840365	2.718190231	11.00135501
0.088921951	13.62073138	48.98706339
0.164087257	6.210955183	18.7606383
0.132978157	6.076688032	23.14002642
0.111617852	7.380182598	29.49633968
0.157828283	5.382217057	18.08712121
0.182948919	2.915137061	12.21428571
0.039933069	60.86875289	256.2589583
0.247722398	1.900911041	7.709677419
0.106595912	8.700404865	31.99097938
0.262287163	1.512981524	6.538461538
0.073305579	22.69927938	77.5147801
0.058816705	40.79513116	126.800197
0.130615372	8.73893506	27.02420242
0.067149559	25.77421655	86.52876712
0.281926621	1.00514444	5.661654135
0.089051649	13.13766927	48.61458333
0.180737687	3.005876935	13.45033113
0.069100782	25.06169629	82.3088685
0.052108345	52.60172757	157.1766367
0.067398884	46.42039949	114.4251884
0.090433525	23.80224258	65.97932664
0.062955109	40.39563856	119.98786
0.046272874	53.72897847	166.063059
0.130368663	7.320465594	24.91936645
0.52	0.24	2.2
0.316626204	0.83577414	5.460176991
0.13288708	5.284951977	21.5862069
0.047358936	52.88297999	171.1043192
0.0797248	23.38527301	70.37467018
0.208121039	1.861433798	9.809968847
0.147065045	4.406520812	18.04283054
0.119221478	7.345446721	28.47972028
0.173042346	6.382086098	17.79265092
0.147607574	6.098430578	20.32524272
0.184977247	4.68836297	14.69931663
0.140836132	5.482635887	20.66816143
0.041702042	51.62454912	218.5161712
0.146735051	4.63254398	18.51128915
0.173130194	4.649738381	15.07017544
0.487603306	0.330578512	3.636363636
0.338525052	0.771626947	4.397260274
0.060754037	46.07325163	127.3735294
0.130626997	7.400205654	23.73945409
0.10247712	14.51413628	44.14929577
0.180266452	2.566411652	14.53974895
0.041485278	60.23958399	211.5430745
0.232648697	1.959313878	8.534328358
0.200211998	2.673343995	10.58027523
0.270713755	1.278389047	6.414847162
0.161607456	3.528360305	15.0960452
0.13310247	5.064027704	22.68240343
0.118336345	8.391025141	29.88109966
0.067173328	36.45477501	96.33441558
0.059828856	28.9077385	100.9950908
0.080354612	22.00078475	69.65881033
0.302242791	0.961908152	5.037735849
0.134058823	5.636959877	21.52604167
0.293605442	1.071746032	5.342857143
0.087212083	25.87596398	67.47418517
0.221282214	2.364125385	9.167832168
0.093880167	13.99414934	45.37165179
0.099385755	11.39156727	39.90034629
0.140511806	4.31091577	18.89065256
0.052766729	45.18533742	142.7712909
0.044943787	55.87175708	189.3324718
0.040892435	62.94692381	212.024313
0.197892578	2.769088459	10.85201794
0.155874885	6.938577255	20.35087719
0.181162309	4.239795918	14.57142857
0.126431045	6.134110074	23.35706462
0.075278297	36.86381287	91.25443787
0.268231161	1.579568488	6.395973154
0.147416887	4.628425941	17.39768786
0.066903242	35.47242279	96.80969771
0.356919875	0.869927159	4.741935484
0.060690592	38.00135719	112.3389762
0.091115094	12.43076713	45.06102082
0.155023457	4.348313004	16.4739726
0.235872144	2.66588647	9.387096774
0.2104	2.3024	9.92
0.73964497	0.130177515	1.461538462
0.079489857	36.87507298	87.22338874
0.163175208	4.492685249	16.15131579
0.140058709	5.164571006	22.10096154
0.077598405	21.77443259	71.69879518
0.051262409	50.47403542	155.9471061
0.351977828	0.58856135	3.920634921
0.176647924	2.955285467	13.17647059
0.267361111	0.993055556	6.833333333
0.234822538	2.089728631	8.202453988
0.154540816	4.556938776	16.90714286

log.sigma.4.5.mm.3D_gldm_LargeDependenceLowGrayLevelEmphasis	log.sigma.4.5.mm.3D_gldm_SmallDependenceHighGrayLevelEmphasis	log.sigma.4.5.mm.3D_gldm_LargeDependenceHighGrayLevelEmphasis
0.26471037	38.09445006	1913.792244
0.570977795	14.65772132	8184.777619
1.352557027	32.49984557	539.5536723
0.155715631	64.78032207	5973.466632
0.104255901	127.3629675	723.2871287
2.113826529	21.89565739	450.5901639
1.442003507	13.89432143	203.4571429
0.685035332	59.50565499	640.8004988
0.118763697	126.2356705	5345.852304
0.389957999	22.32828473	7660.421734
1.299107658	28.34592279	556.1276596
0.493967222	29.5092806	2198.140026
1.561314432	19.36212782	1090.036603
0.548427638	43.47830589	1134.4375
0.583342733	47.58738248	796.3653846
1.284796524	8.780078221	52697.61286
0.637340026	61.64448862	517.875576
0.842956019	26.28346181	2343.195876
0.721291201	107.2759646	815.6263736
1.069534148	7.573565009	8902.788392
0.961040907	8.262343619	18898.90049
0.769279047	30.3982936	1821.559956
0.527932893	15.34207609	16725.89144
0.299019507	87.0733208	1302.421053
1.245839335	10.46391706	3664.6125
0.479470874	28.36617038	978.7251656
0.826955675	13.09664932	9731.949323
1.202132765	7.778041483	24993.18151
0.479807265	25.49846213	30989.17707
0.43729512	15.9637484	13927.62847
0.942976206	9.855564782	18200.48393
1.165303139	13.10333349	26697.7952
0.417854006	33.69908685	3454.491001
0.167930618	51.93333333	152.9333333
0.13307522	74.01904867	671.3185841
0.446222446	31.65114353	1903.955022
1.102679567	8.489053705	29680.40009
0.825556829	14.42778099	9129.270778
0.207144466	69.46266788	1543.358255
0.848996375	24.97108663	1299.459963
0.213792716	30.89526909	5625.723077
0.40446482	65.73813164	1121.102362
0.449393388	34.72132798	1342.799757
0.630437451	51.84633317	778.9635535
0.522615264	30.43770234	1635.265471
2.319304464	2.342593019	22940.85348
0.246431417	51.47999251	2379.084487
1.48888601	36.40902724	407.9736842
0.475459606	38.17929293	174.2272727
0.07293421	150.9997698	1036.90411
0.962675159	10.64019957	19999.86814
1.406309986	23.34590897	680.3870968
0.361579545	22.61557744	7962.485759
0.750469028	24.58084406	1212.23431
1.545343779	6.631530591	31367.9127
0.230846614	67.98448782	901.9432836
0.161672285	69.85883946	1120.954128
0.151410969	129.9060837	1505.59607
0.851985883	33.64687905	1009.220339
0.82145327	19.37686803	1588.832618
0.680314966	19.66541665	2658.727835
0.398858974	34.29038927	26847.47772
1.613393991	6.930790963	8608.978891
1.179968746	7.009755691	6223.999439
0.1955943	50.99292453	372.3018868
0.370559675	36.15708413	2986.199653
0.185406873	95.85901587	692.447619
0.644328358	17.72465881	12362.42832
0.975508968	45.22231086	360.4125874
0.61573478	17.4169342	5774.787946
0.468048514	21.32952813	5668.221624
0.546407349	32.14202967	1252.86067
0.803073139	10.39005901	28441.05867
2.367598038	3.589322181	19316.1726
1.462254584	10.89677101	33342.99229
0.209377558	68.31456362	1088.042601
0.844958797	29.24552152	786.5657895
1.789802342	30.28505808	312.1473214
0.374069574	27.73217808	2220.998905
0.408447736	26.0551579	23136.99482
0.11396059	88.29003542	657.9328859
0.21333798	53.74226106	2289.564162
0.409712117	26.63249749	25418.39197
0.17446934	33.04771505	261.5806452
0.981361476	10.87997636	15177.74587
1.086712885	13.22328583	2779.577131
1.085173881	30.56734966	706.5808219
0.069181065	129.1585447	1850.278438
0.42447119	18.88358186	317.88
0.184853318	38.65384615	43.84615385
0.31798811	35.4196285	27562.02275
3.467480114	29.1347459	436.4835526
1.656633308	12.11048691	1131.829327
0.547351642	12.49095837	12393.24343
1.282301631	7.845148228	21988.23719
0.054543218	135.2751323	706.0952381
0.293256097	57.79043029	1532.847059
2.58803305	12.0306713	93.83333333
0.189213926	78.1314758	1230.263804
0.21783729	39.97145542	2306.403571

log.sigma.4.5.mm.3D_gldm_SmallDependenceLowGrayLevelEmphasis	log.sigma.4.5.mm.3D_gldm_LowGrayLevelEmphasis	log.sigma.4.5.mm.3D_gldzm_DistanceZoneVariabilityNormalized
0.004703481	0.023075918	0.9802
0.00200562	0.018576251	0.986302021
0.008976807	0.049470823	1
0.001762998	0.007277494	0.900706283
0.016842512	0.024826892	0.900277008
0.01090482	0.060854179	0.944489796
0.015252223	0.111960806	1
0.009803522	0.045821197	0.963203125
0.00283388	0.009299402	0.525943404
0.001518202	0.01015921	0.956385346
0.006651467	0.057280917	0.951369442
0.004766763	0.024760331	0.9753125
0.006327609	0.052281325	0.90506113
0.004671183	0.023327616	0.975613474
0.006296356	0.02764093	0.955578512
0.000374737	0.006555796	0.976680969
0.012767297	0.043926603	0.963315573
0.003134247	0.023830582	0.928437728
0.00788503	0.046304008	0.904091419
0.001994918	0.021116852	0.949273003
0.001212611	0.011833968	0.960684761
0.00458461	0.02365836	0.921528926
0.001033531	0.009663359	1
0.00822438	0.036222031	1
0.003504463	0.037466105	1
0.005244295	0.035538151	0.955578512
0.001436781	0.0142937	0.981396989
0.001546118	0.015288125	1
0.001285863	0.007560111	0.964620629
0.002131772	0.012586684	0.946277586
0.001101681	0.012847515	0.962243655
0.000846624	0.011056073	0.969976948
0.003526894	0.018979468	0.918567744
0.098698759	0.112545131	1
0.014679681	0.025586177	0.964923469
0.0033236	0.021784106	0.867988655
0.001105987	0.010953937	0.952417823
0.001632144	0.017424644	0.97288648
0.005886815	0.019393887	0.905
0.006998213	0.04062244	0.942906574
0.002946422	0.01128036	1
0.005907276	0.018632898	0.935967721
0.004570943	0.02127041	0.940504567
0.0068031	0.035715319	0.987879239
0.004222598	0.026224592	0.811805184
0.000982145	0.016353251	0.986487112
0.002139934	0.012633017	0.659090502
0.006473323	0.060512464	0.95240928
0.032001882	0.11937004	1
0.012374288	0.017783164	0.91322314
0.001541712	0.013828521	0.96287726
0.005593757	0.052649081	0.948884688
0.00209309	0.013488263	0.884936355
0.004564383	0.045198008	0.966676242
0.000764916	0.010858083	0.954755917
0.00649385	0.020225806	0.932966821
0.003953624	0.012482525	0.988506131
0.005281826	0.018880459	0.89066486
0.006930593	0.043857067	0.962278107
0.004073719	0.032334259	1
0.004306813	0.028031597	0.8858936
0.000878657	0.006794173	0.910929274
0.002018165	0.025907328	1
0.003125566	0.028241566	0.962976148
0.028405303	0.048817208	0.939453125
0.002950314	0.018623037	0.929679339
0.009318647	0.032855671	0.967222222
0.002080626	0.018645823	0.841814059
0.009490675	0.053850523	0.969704142
0.003068758	0.01914674	0.954502765
0.002393965	0.017241224	0.964923469
0.00476973	0.025133108	0.971836735
0.000820505	0.008738306	0.954798078
0.002088433	0.025872939	0.985982007
0.000824942	0.010730986	0.914998932
0.004645166	0.019211649	0.977014602
0.006266759	0.033287791	0.959476813
0.013443422	0.076287534	1
0.004066785	0.021195822	0.94155
0.001013268	0.007475584	0.957351182
0.015114306	0.022766805	0.954048443
0.004254227	0.013356888	0.977102309
0.000839215	0.006586698	0.884827603
0.04980365	0.063689922	1
0.001378566	0.013625611	0.96899563
0.003099528	0.026343606	0.978168599
0.008696038	0.05318624	0.965822306
0.004269399	0.008216268	0.927112029
0.029996592	0.051824188	1
0.120750754	0.133571267	0.722222222
0.001266047	0.006275623	0.943044181
0.012336924	0.110601898	0.9418
0.007125527	0.064253515	0.95125
0.001705566	0.012027491	0.974607376
0.001098238	0.013818631	0.958892623
0.019867656	0.024148305	1
0.003346287	0.019004928	0.9232
0.030102398	0.235988297	1
0.008936942	0.027292697	1
0.011042482	0.022344128	0.978497164

log.sigma.4.5.mm.3D_gldzm_LowIntensityEmphasis	log.sigma.4.5.mm.3D_gldzm_LargeDistanceEmphasis	log.sigma.4.5.mm.3D_gldzm_HighIntensitySmallDistanceEmphasis
0.024382356	1.03	157.7325
0.023582408	1.020689655	131.5051724
0.035528418	1	98.14925373
0.008032713	1.188720174	290.23349
0.031775981	1.157894737	258.2763158
0.047503886	1.085714286	89.55
0.076036317	1	49
0.034258475	1.05625	167.2109375
0.009272439	2.288848263	219.9435938
0.013880206	1.066914498	189.0250929
0.037263999	1.115702479	104.1528926
0.029738468	1.0375	145.6828125
0.0444245	1.190082645	119.0632461
0.018043539	1.037037037	165.6450617
0.021324704	1.068181818	154.4886364
0.010555627	1.058823529	207.4926797
0.03466348	1.056074766	147.6588785
0.016218525	1.111486486	146.2204392
0.02655134	1.151515152	222.3686869
0.026702192	1.078125	95.12369792
0.020554701	1.156146179	120.8273348
0.021135219	1.122727273	151.5
0.015197475	1	168.3904382
0.02833594	1	196.1285714
0.043187078	1	84.92307692
0.030596982	1.068181818	110.6647727
0.018636229	1.028169014	140.3908451
0.029845468	1	114.4541667
0.011459903	1.065168539	209.5117978
0.022211013	1.082872928	134.4654696
0.019193599	1.114942529	142.9521073
0.011867602	1.045731707	178.008003
0.019102137	1.161971831	169.0401017
0.12914277	1	73.33333333
0.033316062	1.053571429	180.9642857
0.02162009	1.321678322	153.0439005
0.018636295	1.086486486	132.0018769
0.017720715	1.041237113	147.7809278
0.01950791	1.15	222.6666667
0.03881309	1.088235294	103.8419118
0.018373808	1	187.3609023
0.017026517	1.099337748	191.1390728
0.020018123	1.113537118	140.8733624
0.028148872	1.018292683	164.3490854
0.024681617	1.332129964	133.3953069
0.035541883	1.020408163	67.42857143
0.010887384	2.21257485	168.5251996
0.028800239	1.073170732	116.0884146
0.10241516	1	72.92307692
0.022044249	1.136363636	307.8267045
0.022167412	1.072327044	132.0739867
0.028927049	1.12173913	92.35966184
0.019007658	1.264573991	155.2681396
0.035660373	1.050847458	118.3771186
0.016034825	1.132692308	127.4410048
0.018380204	1.104166667	186.0607639
0.011572047	1.01734104	200.3619942
0.014293599	1.174107143	316.3091518
0.038770589	1.057692308	130.6201923
0.028398679	1	117.3666667
0.031466176	1.201612903	109.5433468
0.007948089	1.168408827	275.9826107
0.032438028	1	89.93220339
0.03907099	1.056603774	73.00471698
0.055790465	1.09375	96.2890625
0.016824324	1.127272727	161.9072727
0.027248494	1.05	194.7875
0.021202279	1.304761905	144.5779762
0.03448904	1.046153846	115.2961538
0.022992421	1.108108108	118.242707
0.020454093	1.053571429	143.4502551
0.021275484	1.042857143	145.9946429
0.012568224	1.06940874	160.6793059
0.053862164	1.021176471	72.98470588
0.014659821	1.173480663	173.4004374
0.017984581	1.034883721	199.7936047
0.027556796	1.062068966	106.3827586
0.046442449	1	95.7654321
0.022117172	1.115	130.4751389
0.010990024	1.080745342	215.9760179
0.028571575	1.070588235	177.7205882
0.016021535	1.034749035	203.3957529
0.009222422	1.192949907	235.2805092
0.090900949	1	69.83333333
0.017296658	1.146214099	132.9128489
0.024844958	1.033112583	108.013245
0.039599087	1.052173913	111.9
0.010350243	1.113636364	340.7130682
0.076522246	1	56.52380952
0.135442946	1.5	41.9375
0.009292239	1.108559499	254.3604152
0.056359987	1.09	101.9575
0.056429506	1.075	72.04375
0.020055904	1.038585209	132.6672026
0.021122277	1.101827676	127.0695532
0.033546439	1	250.025641
0.014853018	1.12	219.69
0.142822788	1	30.83333333
0.026695529	1	182.0481928
0.037848636	1.032608696	147.9809783

log.sigma.4.5.mm.3D_gldzm_LowIntensityLargeDistanceEmphasis	log.sigma.4.5.mm.3D_gldzm_HighIntensityEmphasis	log.sigma.4.5.mm.3D_gldzm_DistanceZoneVariability	log.sigma.4.5.mm.3D_gldzm_ZonePercentage
0.02463029	158.64	98.02	0.27700831
0.044272063	131.5103448	143.0137931	0.103349964
0.035528418	98.14925373	67	0.378531073
0.01141487	294.0737527	415.2255965	0.238489395
0.091329627	259.0526316	51.31578947	0.564356436
0.133218172	89.57142857	66.11428571	0.286885246
0.076036317	49	25	0.357142857
0.073841808	167.2625	154.1125	0.399002494
0.014258121	393.3893967	287.691042	0.370596206
0.01425333	193.2713755	257.267658	0.115998275
0.06488567	104.5785124	115.1157025	0.321808511
0.032993676	145.8	156.05	0.211360634
0.10070905	120.5041322	219.0247934	0.17715959
0.020841893	165.8024691	158.0493827	0.306818182
0.046804502	155.1136364	126.1363636	0.362637363
0.011403528	208.32	415.0894118	0.039250092
0.090738246	147.6728972	103.0747664	0.493087558
0.028595892	148.1283784	274.8175676	0.190721649
0.046755465	223.1717172	89.50505051	0.543956044
0.03329036	95.82291667	182.2604167	0.069214131
0.021648309	123.1528239	289.166113	0.059310345
0.041615341	153.1227273	202.7363636	0.242024202
0.015197475	168.3904382	251	0.085958904
0.02833594	196.1285714	70	0.526315789
0.043187078	84.92307692	117	0.121875
0.03171359	111.7727273	84.09090909	0.291390728
0.020079891	140.5352113	209.0375587	0.093053735
0.029845468	114.4541667	240	0.061617458
0.0123015	211.0651685	429.2561798	0.119752422
0.024336678	135.6961326	171.2762431	0.106910809
0.021178728	144.9578544	251.1455939	0.062128065
0.012579779	179.1890244	636.304878	0.074266953
0.03455477	171.5105634	260.8732394	0.204463643
0.12914277	73.33333333	12	0.8
0.046708919	181.0178571	54.03571429	0.495575221
0.028096109	158.7797203	248.2447552	0.214392804
0.021910305	133.1378378	352.3945946	0.055298162
0.02122435	148.3298969	283.1099656	0.095976253
0.04634274	225.6666667	108.6	0.373831776
0.062711681	104.5367647	128.2352941	0.253258845
0.018373808	187.3609023	133	0.186013986
0.041932698	191.5165563	141.3311258	0.396325459
0.037979064	141.7467249	215.3755459	0.277912621
0.046441555	164.3536585	162.0121951	0.37357631
0.044920338	144.1227437	224.8700361	0.248430493
0.03586076	67.75510204	145.0136054	0.024011761
0.01686697	235.4251497	440.2724551	0.243262928
0.06767219	116.2439024	78.09756098	0.359649123
0.10241516	72.92307692	13	0.590909091
0.06886664	308.6363636	80.36363636	0.602739726
0.02436192	133.0628931	306.1949686	0.077941176
0.030352371	94.07826087	109.1217391	0.285359801
0.026004986	160.367713	394.6816143	0.139593114
0.03628812	119.4067797	57.03389831	0.246861925
0.017658985	128.975	496.4730769	0.045993278
0.046982458	186.5555556	134.3472222	0.429850746
0.012165259	200.7630058	342.0231214	0.396788991
0.042293064	318.375	199.5089286	0.489082969
0.039715412	131.5576923	100.0769231	0.293785311
0.028398679	117.3666667	90	0.193133047
0.033609742	114.8467742	219.7016129	0.170446735
0.014508885	279.9581882	784.3101045	0.127066116
0.032438028	89.93220339	295	0.072410407
0.039527217	74.98113208	153.1132075	0.089225589
0.149540465	96.3125	30.0625	0.603773585
0.031535595	163.1636364	255.6618182	0.238715278
0.077248494	194.8	58.03333333	0.571428571
0.036613942	149.5047619	353.5619048	0.121142198
0.080642887	115.3076923	63.03076923	0.454545455
0.036595906	119.2200772	247.2162162	0.14453125
0.024780547	144.3035714	378.25	0.150827241
0.042852865	146.7714286	136.0571429	0.24691358
0.023319849	161.4601542	371.4164524	0.055122573
0.061399419	73.09411765	419.0423529	0.039228355
0.023786287	177.078453	828.0740331	0.062332117
0.02062024	199.9418605	168.0465116	0.385650224
0.03272921	106.662069	139.1241379	0.317982456
0.046442449	95.7654321	81	0.361607143
0.025118345	132.37	188.31	0.21905805
0.011740217	218.5621118	308.2670807	0.11908284
0.072689222	177.7647059	81.09411765	0.570469799
0.017887687	203.7837838	253.0694981	0.299421965
0.01443397	238.9016698	476.9220779	0.10935281
0.090900949	69.83333333	18	0.580645161
0.018781103	134.3420366	371.1253264	0.077186618
0.034205599	108.2119205	443.1103753	0.129170231
0.06621843	112.226087	111.0695652	0.315068493
0.025870765	342.8636364	244.7575758	0.448217317
0.076522246	56.52380952	21	0.42
0.447942946	42.25	8.666666667	0.923076923
0.017144524	257.5448852	451.7181628	0.139690872
0.058542602	103.12	94.18	0.328947368
0.095429506	72.2875	76.1	0.192307692
0.021431693	133.0482315	303.1028939	0.085158817
0.032523513	128.3890339	367.2558747	0.053032401
0.033546439	250.025641	39	0.619047619
0.03953414	220.215	184.64	0.294117647
0.142822788	30.83333333	12	0.5
0.026695529	182.0481928	83	0.509202454
0.041471824	148.0543478	90.02173913	0.328571429

log.sigma.4.5.mm.3D_gldzm_IntensityVariabilityNormalized	log.sigma.4.5.mm.3D_gldzm_LowIntensitySmallDistanceEmphasis	log.sigma.4.5.mm.3D_gldzm_IntensityVariability
0.0696	0.024320373	6.96
0.080998811	0.018409994	11.74482759
0.086656271	0.035528418	5.805970149
0.055999172	0.007242275	25.81561822
0.044629117	0.016887569	2.543859649
0.103673469	0.026075315	7.257142857
0.1232	0.076036317	3.08
0.060078125	0.024362641	9.6125
0.037395266	0.008101693	20.45521024
0.0703003	0.013786925	18.91078067
0.08476197	0.030613657	10.25619835
0.06546875	0.028924666	10.475
0.072740933	0.030437221	17.60330579
0.073159579	0.017343951	11.85185185
0.068755739	0.014954755	9.075757576
0.081876817	0.010386864	34.79764706
0.067342126	0.020644788	7.205607477
0.078661432	0.013124184	23.28378378
0.048260382	0.021500309	4.777777778
0.094943576	0.02505515	18.22916667
0.083873246	0.020395478	25.24584718
0.066900826	0.016015189	14.71818182
0.076808305	0.015197475	19.27888446
0.055918367	0.02833594	3.914285714
0.08788078	0.043187078	10.28205128
0.080061983	0.03031783	7.045454545
0.079724041	0.018275314	16.98122066
0.0884375	0.029845468	21.225
0.06553213	0.011260601	29.16179775
0.067305638	0.021679597	12.18232044
0.073853878	0.018775939	19.27586207
0.078166828	0.011689558	51.27743902
0.060801428	0.015351157	17.26760563
0.083333333	0.12914277	1
0.059311224	0.029967848	3.321428571
0.073744437	0.020154702	21.09090909
0.078831264	0.01784261	29.16756757
0.07372374	0.016844806	21.45360825
0.060416667	0.012799202	7.25
0.077638408	0.032838443	10.55882353
0.068912884	0.018373808	9.165413534
0.062234113	0.010799972	9.397350993
0.075875746	0.015561582	17.37554585
0.06097561	0.023575701	10
0.071563555	0.019671458	19.82310469
0.111481327	0.035462163	16.3877551
0.056778658	0.009749372	37.92814371
0.085663296	0.019082251	7.024390244
0.088757396	0.10241516	1.153846154
0.048811983	0.010338651	4.295454545
0.07390926	0.021636612	23.50314465
0.098979206	0.028667337	11.3826087
0.068833075	0.017361987	30.69955157
0.077851192	0.035503437	4.593220339
0.089852071	0.01571814	46.72307692
0.062789352	0.011229641	9.041666667
0.068746032	0.011423744	23.78612717
0.049864477	0.007293733	11.16964286
0.068971893	0.038534384	7.173076923
0.077530864	0.028398679	6.977777778
0.087669095	0.030950197	21.74193548
0.054077788	0.006340642	46.56097561
0.091893134	0.032438028	27.10847458
0.100114711	0.038956934	15.91823899
0.091796875	0.032352965	2.9375
0.067557025	0.01325874	18.57818182
0.06	0.014748494	3.6
0.066655329	0.017437918	27.9952381
0.079289941	0.022950579	5.153846154
0.080782934	0.019687413	20.92277992
0.0740707	0.01937248	29.03571429
0.07877551	0.015881139	11.02857143
0.088956589	0.009880318	34.60411311
0.09506436	0.05197785	40.40235294
0.070226184	0.012477942	63.55469613
0.06726609	0.017325667	11.56976744
0.083186683	0.026263693	12.06206897
0.079408627	0.046442449	6.432098765
0.07145	0.021395224	14.29
0.070676286	0.010820081	22.75776398
0.062422145	0.017542163	5.305882353
0.060598381	0.015554997	15.69498069
0.061031733	0.007936572	32.8961039
0.086419753	0.090900949	1.555555556
0.082903285	0.017069889	31.75195822
0.087213524	0.022504798	39.50772627
0.076899811	0.032944251	8.843478261
0.057105142	0.006470113	15.07575758
0.111111111	0.076522246	2.333333333
0.111111111	0.057317946	1.333333333
0.063445505	0.00734903	30.39039666
0.0684	0.055814333	6.84
0.085625	0.046679506	6.85
0.075505836	0.019711957	23.48231511
0.075240816	0.018355735	28.81723238
0.067718606	0.033546439	2.641025641
0.05935	0.008682737	11.87
0.138888889	0.142822788	1.666666667
0.054434606	0.026695529	4.518072289
0.063563327	0.036942839	5.847826087

log.sigma.4.5.mm.3D_gldzm_HighIntensityLargeDistanceEmphasis	log.sigma.4.5.mm.3D_gldzm_SmallDistanceEmphasis	log.sigma.4.5.mm.3D_glcm_SumVariance	log.sigma.4.5.mm.3D_glcm_Homogeneity1
162.27	0.9925	315.0783758	0.40159292
131.5310345	0.994827586	290.1213467	0.522046828
98.14925373	1	119.6214952	0.39176594
313.4164859	0.960050615	584.739144	0.421267742
262.1578947	0.960526316	497.8928715	0.271429897
89.65714286	0.978571429	83.55032843	0.409676412
49	1	38.59154362	0.447653634
167.46875	0.9859375	191.5086259	0.350770802
1332.345521	0.742915905	1318.521426	0.303246035
210.2565056	0.983271375	384.8240988	0.523847038
107.6033058	0.980257117	96.81302151	0.399676727
146.26875	0.990625	245.9672645	0.442364045
130.3264463	0.961662075	99.79769011	0.460986994
166.4320988	0.990740741	215.9688348	0.397358768
157.6136364	0.982954545	202.8474863	0.370520932
214.16	0.990522876	590.3149371	0.754402453
147.728972	0.985981308	200.5851989	0.319980828
155.7601351	0.972128378	186.4794655	0.461578021
226.3838384	0.962121212	338.479534	0.282403814
98.61979167	0.98046875	213.4952218	0.571696487
150	0.983342562	316.9058035	0.63353867
159.6136364	0.969318182	208.7432716	0.434242652
168.3904382	1	440.2312838	0.594394556
196.1285714	1	548.7667345	0.317878648
84.92307692	1	144.9976309	0.557609389
116.2045455	0.982954545	165.7554992	0.399432914
141.1126761	0.992957746	276.8675323	0.586254623
114.4541667	1	341.9839098	0.665200106
219.5258427	0.986204744	592.1692199	0.576165328
140.6187845	0.979281768	402.5881975	0.539291475
161.7164751	0.98403576	317.5885405	0.615683138
183.9131098	0.988567073	378.6461699	0.653895619
184.0528169	0.967331768	287.7721061	0.424830559
73.33333333	1	184.9233046	0.287557775
181.2321429	0.986607143	317.8462938	0.310061708
200.9615385	0.944954351	222.7268989	0.416678371
139.1351351	0.981381381	394.6138805	0.664225394
150.5257732	0.989690722	255.180804	0.54263232
237.6666667	0.9625	398.614752	0.345286162
107.3161765	0.977941176	179.7709953	0.410629561
187.3609023	1	439.8935314	0.474912293
193.0264901	0.975165563	245.7569301	0.382948791
148.0349345	0.976467734	185.0803187	0.406323873
164.3719512	0.995426829	200.7940659	0.370904397
188.33213	0.92097874	237.0929985	0.406523897
69.06122449	0.994897959	240.1211108	0.740816451
834.3473054	0.835329341	426.2342647	0.393418119
116.8658537	0.981707317	106.5953781	0.381350205
72.92307692	1	122.1672661	0.28810391
311.875	0.965909091	634.3571099	0.247327605
139.7578616	0.985412299	325.1414081	0.611696445
104.8173913	0.979227053	81.67416315	0.421299397
196.4663677	0.952618959	363.1809873	0.482538975
123.5254237	0.987288136	176.1369208	0.402221141
145.3192308	0.981927885	367.6504773	0.702552159
188.5347222	0.973958333	300.4329047	0.324642089
202.367052	0.99566474	335.979037	0.357216195
326.6383929	0.956473214	633.8071252	0.29066272
135.3076923	0.985576923	185.7745819	0.405657021
117.3666667	1	180.0389043	0.452675302
140.0927419	0.954077061	168.0218312	0.45509966
301.0998839	0.964350239	645.2999932	0.549239378
89.93220339	1	174.3298284	0.608688524
82.88679245	0.985849057	156.8712496	0.564909401
96.40625	0.9765625	163.4429058	0.313938248
168.7709091	0.972222222	304.1458029	0.424654431
194.85	0.9875	313.5902168	0.283397539
174.3547619	0.934391534	328.7736249	0.510374858
115.3538462	0.988461538	133.9835352	0.334185282
126.2355212	0.981552982	240.1302516	0.501279082
147.7168367	0.986607143	288.7190462	0.490972799
149.8785714	0.989285714	180.4002348	0.416150115
164.5835476	0.982647815	445.435275	0.633612555
73.53176471	0.994705882	203.4025528	0.685095085
195.3867403	0.965876304	389.8086458	0.687265279
200.5348837	0.99127907	313.7653674	0.343833674
107.7793103	0.984482759	124.6466519	0.411509778
95.7654321	1	81.7040858	0.392623598
144.305	0.976805556	220.7655137	0.441072132
231.6118012	0.98326432	560.1401522	0.557860403
177.9411765	0.982352941	298.0500847	0.302182264
205.3359073	0.991312741	370.6097438	0.404933026
254.3840445	0.953823954	628.4355095	0.562455572
69.83333333	1	134.7545663	0.341470342
155.8720627	0.98685016	281.2018023	0.601862134
109.0066225	0.991721854	142.007953	0.513861717
113.5304348	0.986956522	120.4791996	0.406056165
351.4659091	0.971590909	692.6609663	0.308201419
56.52380952	1	98.16059556	0.423415174
43.5	0.875	66.6345695	0.314295163
274.782881	0.97749942	696.5757794	0.536909941
107.77	0.9775	107.9628568	0.391888916
73.2625	0.98125	93.09818528	0.459158611
134.5723473	0.990353698	349.1795769	0.554498183
137.4151436	0.983246301	303.5356209	0.641381353
250.025641	1	577.6979384	0.266684056
222.315	0.97	330.0678649	0.366487486
30.83333333	1	38.20612103	0.395399559
182.0481928	1	379.1213236	0.340449037
148.3478261	0.991847826	341.8149956	0.432568536

log.sigma.4.5.mm.3D_glcm_Homogeneity2	log.sigma.4.5.mm.3D_glcm_ClusterShade	log.sigma.4.5.mm.3D_glcm_MaximumProbability	log.sigma.4.5.mm.3D_glcm_Idmn	log.sigma.4.5.mm.3D_glcm_SumVariance2
0.318533895	12.37062177	0.025431328	0.978146152	64.85742575
0.464693529	-83.22193831	0.061627438	0.985061101	34.7935643
0.301679291	65.19120641	0.038665172	0.963467206	31.04414524
0.344194063	26.16519053	0.022190505	0.986710315	53.4603614
0.173515417	631.0793257	0.023297415	0.960409005	123.661973
0.327429547	49.45344805	0.036222876	0.959649579	25.1948
0.374037343	45.54336253	0.074158999	0.95342016	12.84822845
0.261927002	361.3366042	0.021854765	0.975457294	58.56199742
0.213485047	2387.407861	0.009770374	0.977285407	301.5064361
0.467904789	-29.71501943	0.048711876	0.991839276	29.61424241
0.317104253	87.73775898	0.039291795	0.967080041	27.38512121
0.366874784	18.00829917	0.024036186	0.985297296	41.91853581
0.391620925	159.8393857	0.0421661	0.983364535	28.69910853
0.315359502	206.5754554	0.026472906	0.977478359	48.31002192
0.282068863	109.8551029	0.021846103	0.97141881	46.20222358
0.738270767	-123.6650113	0.273470358	0.99728462	15.94409236
0.223382623	91.87733764	0.023569034	0.957806303	54.75911111
0.391688045	43.90979283	0.031313298	0.987588447	30.70460458
0.186916814	249.0022081	0.02121259	0.953065861	94.71368807
0.52539082	-48.22884885	0.075597278	0.989255715	31.6563849
0.598692003	-53.16668301	0.164339218	0.994927364	19.37671524
0.358013321	226.9243325	0.031786211	0.979847579	44.65565724
0.554051003	-103.9436774	0.100958521	0.993877014	24.93701381
0.220346497	-234.8841264	0.027233414	0.968825034	119.6552913
0.510127554	-55.20034947	0.054276537	0.987395796	21.84649325
0.314281592	-9.337741321	0.026697449	0.970223139	31.47446978
0.543945428	-43.06003204	0.092536611	0.993414305	18.46956171
0.632375921	-144.795369	0.262700491	0.996709769	27.95057529
0.525837014	-194.4069552	0.198589167	0.994032607	41.25902874
0.484583669	-135.6370895	0.087673476	0.990433786	44.26975825
0.576523926	-77.33149604	0.16593087	0.993682727	24.38182269
0.621076319	-88.96613252	0.225268309	0.995796819	20.94519807
0.345701996	44.96323201	0.02191576	0.985063821	49.50048176
0.189072389	57.35381508	0.1375	0.915366433	36.8552297
0.211146535	77.95634049	0.029416927	0.962791484	60.19048118
0.336018402	48.2758747	0.026277106	0.978340584	32.69128004
0.632095437	-153.6173158	0.193233811	0.994737406	26.42282065
0.48943612	-31.74902122	0.072306324	0.991330232	35.51375877
0.254187692	111.5286826	0.019746401	0.973785021	88.47679828
0.327468055	119.2825167	0.026230644	0.974751173	50.30720632
0.406009593	-100.0063951	0.032055594	0.988854626	44.46037219
0.299762901	381.528007	0.03627672	0.977552049	48.47024975
0.324025726	48.96046297	0.029924917	0.976005947	26.64000227
0.28601718	377.9684436	0.028154557	0.978012208	59.32140837
0.323589903	403.8537693	0.031795741	0.9802586	56.85402622
0.723515761	-68.51733374	0.238324799	0.994396912	14.52722681
0.310818893	737.0086353	0.016404839	0.990961718	75.00048062
0.297524984	126.8352844	0.036630177	0.960506362	35.5102603
0.17781399	35.07022194	0.096203796	0.909612344	23.80285996
0.151827006	221.1943894	0.020150007	0.956979473	126.704823
0.570537433	-93.26666357	0.186202342	0.992875049	27.43162973
0.342184139	58.2770496	0.056173102	0.966908471	18.74163779
0.415838943	-74.1362001	0.057106465	0.989798248	39.6558781
0.315824976	13.64481895	0.025867518	0.970318243	43.01159243
0.676971503	-120.2214298	0.261747329	0.994937097	20.60527532
0.230397132	91.28157492	0.018217633	0.963616207	57.00713213
0.266923058	105.1448221	0.017831635	0.980075622	42.01793209
0.19551263	-299.4802785	0.012590107	0.965951005	109.5871866
0.320242883	110.7321143	0.024864993	0.978403227	45.53308597
0.377395404	45.81505572	0.031282486	0.981993252	41.01815979
0.382291152	83.48617071	0.033784143	0.982895512	29.9850114
0.497185886	-146.068338	0.105990969	0.994133032	46.13289228
0.570924967	-58.58856655	0.098226613	0.993281976	20.91883813
0.51566784	-19.45080079	0.083596057	0.983155125	21.93429575
0.212750556	13.33269581	0.04767693	0.94552213	31.96786098
0.347000286	-64.20814504	0.024135853	0.984578894	43.0730535
0.185523333	8.989780327	0.028437726	0.946966213	58.65468413
0.450113268	-93.72236787	0.076773728	0.988929267	53.77171535
0.240043767	105.5476126	0.029483109	0.94992724	40.85061775
0.439443617	-29.99484533	0.035126623	0.98533823	33.5168073
0.427926944	-45.31094772	0.039332956	0.98564375	34.64030503
0.336392789	113.5545282	0.028239614	0.979128047	32.85604973
0.596968473	-114.5667299	0.147804429	0.994531932	27.88606688
0.657402511	-97.23047866	0.207993358	0.99277125	22.52736478
0.659225396	-108.7267948	0.24875721	0.995758528	21.82707381
0.254049156	116.0909977	0.022864253	0.970004279	52.4158353
0.330916247	75.19552009	0.04512017	0.974898699	25.23875296
0.310698934	159.6978618	0.047771096	0.967248657	32.2890117
0.366746826	9.527198882	0.028352658	0.97791399	24.86741156
0.505586728	-72.53403921	0.127540268	0.992601246	29.42204382
0.206986696	93.69303694	0.028596474	0.950590847	50.9843168
0.323761075	72.04050596	0.023017631	0.983171443	49.06227955
0.512953566	-157.6043186	0.112898107	0.993685311	34.99596862
0.246800943	9.482293399	0.0770051	0.922920929	23.04477169
0.561127493	-63.31782381	0.135379225	0.991096188	21.59191765
0.456318412	27.05768397	0.042601149	0.985571841	20.53421039
0.324085368	173.8389838	0.029765284	0.977089471	35.03240524
0.214435505	32.87802698	0.022480003	0.975419324	69.05866174
0.346317732	8.577896455	0.076835836	0.941794874	9.523734443
0.218291452	53.83005595	0.081349206	0.898610435	18.3568909
0.480642215	-203.4238755	0.089837703	0.993241064	49.74840114
0.306796107	374.9251654	0.03208005	0.972119534	48.98305453
0.387656373	25.960291	0.032626868	0.972147119	24.76548605
0.504731467	-54.38793955	0.065864116	0.990356429	35.31014148
0.605337256	-92.65455428	0.199681825	0.993385844	26.89443437
0.169308484	4.450968285	0.0343985	0.957869431	59.2016987
0.278319175	97.6464963	0.015423856	0.976687194	63.25878254
0.310148942	26.73406431	0.117316764	0.900020156	11.91188756
0.246388007	373.9447199	0.029695204	0.970984487	87.85573362
0.35569541	-95.52207859	0.038570333	0.982005996	45.59112664

log.sigma.4.5.mm.3D_glcm_Contrast	log.sigma.4.5.mm.3D_glcm_DifferenceEntropy	log.sigma.4.5.mm.3D_glcm_InverseVariance	log.sigma.4.5.mm.3D_glcm_Entropy	log.sigma.4.5.mm.3D_glcm_Dissimilarity
10.37120219	2.756784465	0.341185795	7.172299545	2.502588179
5.176174727	2.374726647	0.407074192	6.421508176	1.635042896
10.31091545	2.699395101	0.291809194	6.276875226	2.583377468
11.79739327	2.894760789	0.338096451	7.464922068	2.539131157
34.66433556	3.383819051	0.181524175	6.566796202	4.762681683
10.22019675	2.770639598	0.323399725	6.393133566	2.50159126
8.04046575	2.360421821	0.371962696	4.955906959	2.129960278
19.4201095	3.200694597	0.268855325	7.349100119	3.404184088
37.4332259	3.655686559	0.21852885	9.002978669	4.639261857
4.471930119	2.297974688	0.429574854	6.442432374	1.54959635
11.79470402	2.873499106	0.318119073	6.651203631	2.66182277
8.262519981	2.621892978	0.365385703	6.94114633	2.16336314
7.910442277	2.623900102	0.385845623	6.567391623	2.062325546
12.82753851	2.929901467	0.316631259	7.123031298	2.742314879
15.16541031	3.030632064	0.280062478	7.149041933	3.036562598
1.624564709	1.574495195	0.339581589	4.350351502	0.671318215
20.80437211	3.110571756	0.224942	6.976407392	3.6941755
8.201204859	2.667715172	0.372637473	6.805241494	2.099170219
33.14247143	3.465377793	0.184092001	7.191441214	4.668262553
3.25377736	2.110091506	0.435267844	6.059885227	1.2936439
2.298066951	1.922878998	0.42309115	5.382074763	1.028800357
10.56139291	2.820333662	0.334450458	6.997278459	2.409871342
3.064590629	2.059429302	0.441931543	5.801878237	1.205659011
21.19985718	3.049998068	0.221281713	6.58795452	3.718205373
3.436592422	2.111402607	0.453570676	5.871914497	1.33422896
10.63222932	2.796731617	0.322362522	6.752882136	2.545532373
3.312750385	2.105337171	0.438214427	5.739505897	1.252746453
2.466090462	1.92135753	0.37123002	5.169446404	0.982476427
5.565389727	2.401984942	0.349010593	6.070461421	1.540723683
4.396254143	2.301956382	0.40860855	6.401240189	1.511836663
2.890361346	2.044950532	0.411907943	5.647689231	1.143498045
3.177458255	2.02907191	0.379586295	5.348022537	1.079204944
9.879169948	2.791532802	0.339229191	7.274356853	2.380505355
23.70705128	1.719269229	0.234363195	3.214743508	4.010897436
21.86791443	3.135012581	0.220229347	6.471264154	3.781306847
10.32614488	2.821456511	0.334241731	7.052315468	2.45014778
2.646298155	1.956726801	0.37309792	5.165369063	1.007079876
4.358983291	2.294953259	0.414561952	6.402549565	1.496653395
19.29543089	3.134760134	0.258904186	7.446683674	3.427452032
9.917451194	2.772277131	0.327944491	7.031899468	2.447264577
6.740674213	2.516494511	0.381983756	6.743693898	1.920840792
16.48196544	3.089150741	0.2939102	6.935664	3.058159868
11.45640199	2.870952831	0.318667353	6.89999131	2.597203469
17.3084599	3.122571468	0.291816039	7.235948149	3.163757049
11.14738218	2.871827067	0.317104174	7.321218444	2.572939548
1.135882072	1.513540463	0.368489455	4.235028425	0.646132952
15.11481142	3.042341235	0.314777502	7.707025741	2.854115725
14.45360308	2.991189833	0.304920213	6.669280011	2.941501071
22.63296148	2.114175123	0.169457399	3.830833895	3.912887113
46.12147565	3.603426073	0.155243686	7.056671624	5.557949648
3.273619339	2.113203299	0.394890669	5.707430463	1.203136012
10.6922318	2.840395465	0.326283615	6.357234537	2.48567637
6.646018156	2.54184022	0.380665536	6.794268015	1.904528524
10.48566739	2.768977181	0.302330615	6.756550801	2.551813546
2.110241682	1.807402063	0.358382064	4.81726679	0.85608613
21.39877569	3.232115219	0.239481673	7.443765415	3.681992473
16.63932946	3.091560346	0.272267509	7.384049826	3.194288624
31.44859391	3.456525775	0.199123562	7.953198886	4.487674026
10.23591603	2.752893504	0.317436831	6.921931273	2.487591606
6.172739808	2.436523434	0.379017777	6.656184801	1.951014062
7.308851857	2.605977067	0.374154104	6.675512428	2.04740837
6.633102391	2.501883844	0.374127922	6.589342307	1.690643899
2.788099846	1.988608082	0.438876445	5.643163929	1.141339431
3.544956994	2.158055729	0.418922755	5.861342342	1.350009953
17.95869853	2.906724857	0.227243837	5.386154811	3.516253015
10.23015731	2.779258914	0.346071162	7.141179267	2.406669187
29.31640895	3.276242913	0.190370956	6.358777365	4.437399552
6.726724381	2.557116499	0.377445476	6.78139976	1.827212671
18.45237096	3.110048237	0.2446571	6.536680023	3.469006919
5.634844641	2.431655538	0.403762952	6.598598924	1.73928783
6.801457182	2.527004779	0.397747883	6.755917359	1.866907157
10.97003984	2.843369752	0.337602258	6.88769654	2.489380983
2.7338074	2.014994054	0.397895105	5.473440023	1.087520389
1.932518242	1.813555697	0.381198255	5.058577804	0.874950887
2.993497177	1.945362241	0.35407548	5.065027788	0.980194192
20.58198753	3.20152199	0.259228535	7.363515637	3.52493082
10.89363065	2.825790052	0.327347265	6.592654257	2.53845916
13.26413325	2.919079408	0.324403739	6.329666973	2.775423302
8.630997142	2.694316118	0.363815574	6.693943658	2.219634958
4.812673165	2.345004409	0.384769106	6.122377937	1.504840827
27.40969826	3.276366377	0.211570974	6.689570631	4.188714374
12.11604763	2.919364615	0.328656152	7.363717527	2.629268184
5.528720642	2.382377656	0.386632697	6.243143634	1.550692245
16.13208778	2.491956962	0.265474336	4.526319482	3.233437289
3.395617086	2.120006847	0.417069462	5.719515571	1.228396306
5.561624243	2.416728951	0.41445064	6.32914179	1.691105247
10.96368914	2.837083311	0.332821493	6.742440174	2.539709366
26.94215936	3.42677383	0.214074208	7.773081311	4.141749624
8.371249885	2.316839241	0.372102187	4.801115597	2.266159547
15.88888889	2.226424882	0.308137136	3.952925676	3.285714286
6.309208247	2.506189977	0.365480284	6.572042668	1.714965582
13.67965399	2.949086528	0.29919528	6.650324085	2.808049219
6.925913033	2.530469363	0.382730809	6.388100707	1.99940747
4.055747291	2.22755322	0.427381441	6.345865661	1.419713652
2.758490001	2.014183798	0.386567475	5.52515204	1.07334077
34.06596198	3.264822575	0.182527764	5.802919723	4.802862881
15.71542491	3.071305996	0.288509165	7.607063757	3.074959104
10.64711912	2.282315356	0.315436558	3.877961106	2.597277936
18.06467016	3.078763329	0.241913992	6.746137115	3.411982387
9.308455915	2.713045726	0.349874888	6.667355926	2.307307467

log.sigma.4.5.mm.3D_glcm_DifferenceVariance	log.sigma.4.5.mm.3D_glcm_Idn	log.sigma.4.5.mm.3D_glcm_Idm	log.sigma.4.5.mm.3D_glcm_Correlation	log.sigma.4.5.mm.3D_glcm_Autocorrelation
3.941222909	0.899675615	0.318533895	0.724714348	120.7187249
2.396064662	0.922145395	0.464693529	0.741003967	111.97486
3.290760012	0.869635508	0.301679291	0.502192319	52.1994779
5.134893785	0.924042423	0.344194063	0.638979712	205.3906263
10.87785294	0.863572569	0.173515417	0.55996542	168.0983591
3.802012844	0.867469707	0.327429547	0.423902429	38.78527896
3.075490383	0.862406677	0.374037343	0.258879775	19.04262083
7.5544995	0.894999249	0.261927002	0.500690524	76.11324794
15.22851281	0.90055145	0.213485047	0.78163743	423.5224305
2.017324438	0.939881719	0.467904789	0.735736196	142.0546877
4.457779732	0.880147123	0.317104253	0.395350069	43.63925572
3.299806209	0.918763753	0.366874784	0.668153229	98.07176208
3.534223293	0.916218399	0.391620925	0.565027908	45.80385999
5.09159727	0.900135586	0.315359502	0.579754803	86.75384188
5.62532277	0.886450697	0.282068863	0.509130698	81.54246423
1.155176861	0.974461905	0.738270767	0.813647704	191.5219281
6.081058629	0.859887953	0.223382623	0.464481782	78.63473707
3.666099642	0.926898705	0.391688045	0.577954961	77.42953973
10.11922775	0.853001006	0.186916814	0.486557399	121.4928364
1.525694153	0.933357463	0.52539082	0.813156436	87.0216821
1.214295261	0.955575666	0.598692003	0.787148228	116.8690045
4.538375722	0.907832011	0.358013321	0.612530327	84.71602318
1.568522762	0.950666274	0.554051003	0.780242869	156.6160793
6.469544095	0.877913887	0.220346497	0.702621316	188.1995182
1.601487832	0.927648233	0.510127554	0.727242209	62.68918135
3.963268734	0.884063791	0.314281592	0.49477584	69.19781167
1.702346955	0.948923918	0.543945428	0.695253427	105.0498429
1.463629653	0.966596255	0.632375921	0.836922507	123.5814078
3.087856949	0.953949873	0.525837014	0.761685701	202.0582175
2.059028208	0.936455957	0.484583669	0.818448538	148.1035271
1.546491909	0.951179467	0.576523926	0.786925594	118.1713231
1.974600839	0.963785753	0.621076319	0.734385325	134.6464599
4.056536173	0.917802547	0.345701996	0.667102807	112.2077509
4.277836538	0.804675088	0.189072389	0.244481937	54.50641026
7.045665242	0.86730129	0.211146535	0.467260924	115.6908227
4.167619703	0.901990737	0.336018402	0.521203641	89.31499987
1.597938697	0.958911254	0.632095437	0.817149811	139.322116
2.060955551	0.939647796	0.48943612	0.780010715	101.1485636
6.784291552	0.890667156	0.254187692	0.641610783	145.9200685
3.718956991	0.892900892	0.327468055	0.669411204	74.65132548
2.913576687	0.929763943	0.406009593	0.736362333	160.1611445
6.769771492	0.901683011	0.299762901	0.495714738	94.38378763
4.451114776	0.896927307	0.324025726	0.397491438	75.53625968
6.965633145	0.901741526	0.28601718	0.548567379	79.72305038
4.337972429	0.905201984	0.323589903	0.672385434	94.31465201
0.693854449	0.958843212	0.723515761	0.852517795	88.22682517
6.718048186	0.936635838	0.310818893	0.668193635	156.1460177
5.50944792	0.870095141	0.297524984	0.418346239	46.41575847
6.552147931	0.797983078	0.17781399	0.030867864	39.98716839
13.85027311	0.85719381	0.151827006	0.467029322	210.5658053
1.784575759	0.949023812	0.570537433	0.786128859	120.6132976
4.34113735	0.881973024	0.342184139	0.277161083	37.65327001
2.887347622	0.932821546	0.415838943	0.712962571	135.6988975
3.686634962	0.883608153	0.315824976	0.608502149	73.06138169
1.351474272	0.961693678	0.676971503	0.813147151	129.1551202
7.370971631	0.870885145	0.230397132	0.457246729	113.1884004
5.908636505	0.903163427	0.266923058	0.440973049	125.2230786
10.18596064	0.874249124	0.19551263	0.554603121	217.4257524
3.849909827	0.900180343	0.320242883	0.631022686	76.94072834
2.21455277	0.907272901	0.377395404	0.736830366	75.87619251
3.044133247	0.912493622	0.382291152	0.607943918	70.83789386
3.691128484	0.953983638	0.497185886	0.748129688	221.5746412
1.410178544	0.948914963	0.570924967	0.763683334	72.39024889
1.643753206	0.918168324	0.51566784	0.721174674	66.72966012
5.325643296	0.839202137	0.212750556	0.281311398	61.92752212
4.220558913	0.917154826	0.347000286	0.617030327	116.9512271
8.818812078	0.843118756	0.185523333	0.334706851	111.8472319
3.307945156	0.933564057	0.450113268	0.776986692	125.0878503
6.159920658	0.849583079	0.240043767	0.381481867	54.9994297
2.526173162	0.9212258	0.439443617	0.711988816	96.01043003
3.171248242	0.923584117	0.427926944	0.671126162	111.8564104
4.61809593	0.904842045	0.336392789	0.498442183	74.288464
1.521164437	0.955458762	0.596968473	0.820963324	156.323475
1.148607048	0.951703556	0.657402511	0.840906464	80.33150295
2.011151939	0.966066367	0.659225396	0.758120508	136.2488622
7.332794274	0.884600189	0.254049156	0.441923296	117.6039911
4.189669876	0.894613174	0.330916247	0.395640242	53.95070511
5.342024317	0.881863814	0.310698934	0.423078629	36.32073364
3.535103524	0.902178258	0.366746826	0.477405401	87.83599077
2.489223071	0.946365504	0.505586728	0.718196108	192.616008
8.966539978	0.851417998	0.206986696	0.308048365	108.3644375
5.04593937	0.913472358	0.323761075	0.603712486	138.3331993
3.015145227	0.952129339	0.512953566	0.727212846	214.0432342
5.290495033	0.816909066	0.246800943	0.170341426	49.15247829
1.847554132	0.943176024	0.561127493	0.727463171	106.4545452
2.591534752	0.923534619	0.456318412	0.574753983	61.67797931
4.393178057	0.898789177	0.324085368	0.522221305	52.69956639
9.364504966	0.891713521	0.214435505	0.439683371	234.9547345
2.782332788	0.84333813	0.346317732	0.066870381	39.64490571
4.976442429	0.788089186	0.218291452	0.075069685	22.94444444
3.278503197	0.948842816	0.480642215	0.774058178	236.5652747
5.416432993	0.89019997	0.306796107	0.558420068	45.7032829
2.778330708	0.890564202	0.387656373	0.563711166	43.57662149
1.976438899	0.937508804	0.504731467	0.79337839	131.4893384
1.579344769	0.952201272	0.605337256	0.813102584	113.4904582
9.911113992	0.857850129	0.169308484	0.270222774	187.8139138
6.007235764	0.897049091	0.278319175	0.602462822	125.1222471
3.436707243	0.797373432	0.310148942	0.070034912	15.06401739
6.060768983	0.882668533	0.246388007	0.657068333	136.7733214
3.820261327	0.910709462	0.35569541	0.661011871	128.1819581

log.sigma.4.5.mm.3D_glcm_SumEntropy	log.sigma.4.5.mm.3D_glcm_AverageIntensity	log.sigma.4.5.mm.3D_glcm_Energy	log.sigma.4.5.mm.3D_glcm_SumSquares	log.sigma.4.5.mm.3D_glcm_ClusterProminence
4.879255093	10.3484631	0.008522594	18.80715698	8606.413444
4.473198036	10.2258049	0.017754012	9.992434757	2928.986995
4.302091103	6.852908862	0.015938662	10.33876517	2659.10194
4.87716181	13.98011506	0.008363344	16.53814664	8756.710813
4.81107886	12.13368304	0.011419689	40.70732956	36856.57334
4.199754849	5.918299738	0.014736025	8.853749187	1590.425906
3.380257313	4.211019776	0.037347797	5.222173549	725.1412586
4.757700074	8.145604381	0.007801921	19.56483847	12339.39633
5.925639608	19.15615439	0.002554651	86.21185188	201372.9646
4.457229705	11.65190104	0.0169461	8.521543132	2562.618778
4.27450223	6.300913505	0.013451914	9.794956309	2281.023353
4.653176526	9.468582975	0.010452083	12.54526395	4368.443772
4.312330315	6.371653656	0.016042334	9.152387701	3783.767421
4.701837818	8.824387831	0.009703177	15.28439011	7040.486894
4.663287413	8.588371107	0.008529931	15.34190847	6153.88913
3.452371348	13.70918409	0.125644825	4.392164268	2018.733355
4.674618619	8.369952664	0.009281119	18.8908708	7117.6179
4.466284982	8.473583079	0.012535859	9.726452361	3184.930639
4.988611182	10.2956896	0.007778722	31.96403988	21279.84216
4.395106371	8.939686925	0.023081039	8.727540566	2045.412287
3.973693045	10.6111937	0.046956145	5.418695547	1082.30255
4.647531246	8.727517537	0.01187374	13.80426254	7154.687381
4.209874341	12.29413686	0.030411968	7.000401109	2477.917448
4.868002071	12.78313285	0.011619245	35.21378712	27997.33387
4.146156307	7.621254373	0.02417082	6.320771418	1300.086421
4.410439729	7.998641913	0.011327747	10.52667478	2488.688607
4.050939439	10.06271946	0.034120401	5.445578025	1375.449166
3.932010818	10.82634377	0.082658393	7.605739204	2592.183732
4.323194313	13.90031343	0.049762522	11.73510387	4861.757395
4.5773884	11.75268893	0.019910745	12.1665031	4518.596466
4.118225403	10.62057071	0.043931131	6.818046009	1705.730616
3.908548828	11.41087263	0.068738081	6.033893514	1824.201998
4.793079134	10.11420432	0.008487883	14.84491293	5994.330433
2.197869589	7.109294872	0.11940438	15.14057025	3030.785484
4.551912257	10.29546708	0.012562939	20.5145989	9039.918843
4.514902266	9.150003453	0.01037868	10.75435623	3119.103883
3.909627056	11.54887963	0.071977598	7.267279701	2435.055845
4.503311849	9.662066222	0.019106741	9.968185516	2727.337667
5.07376152	11.33576531	0.006802088	26.98284049	17732.33982
4.690662256	8.034043784	0.009612887	15.05616438	5310.347765
4.669504901	12.27636096	0.012997368	12.8002616	5286.355296
4.542411679	9.295296598	0.011716395	16.28541161	10415.11403
4.352146332	8.469090687	0.01134606	9.524101065	2176.560281
4.745038868	8.319849408	0.009114376	19.21434885	11988.21816
4.783123333	9.103782791	0.009281315	17.0003521	12679.1494
3.406318503	9.212869274	0.111513724	3.915777221	781.3704906
5.022343448	11.97152269	0.006867462	23.56359491	36041.35637
4.397589218	6.411942585	0.01256089	12.49096585	3538.413781
2.695457154	6.293095793	0.076362038	11.60895536	1527.450356
5.068456671	13.86694528	0.008222097	43.84883495	36657.77834
4.153635171	10.70384707	0.04898653	7.676312267	2116.841641
4.006785072	5.96970108	0.018061164	7.358467398	1257.638629
4.591890967	11.28913291	0.01384317	11.57547406	3695.613367
4.577862165	8.057442605	0.011041354	13.37431496	4277.070065
3.689599353	11.1593374	0.0996123	5.678879251	1707.273571
4.821823993	10.21081967	0.0069246	19.60147696	8120.477024
4.66184112	10.90868785	0.007526578	14.7965888	5575.885155
5.238310246	14.11265852	0.00474828	35.87954699	29724.12785
4.66378072	8.251790864	0.010311215	13.9422505	5770.462208
4.599919125	8.194914699	0.012548855	11.7977249	3659.816302
4.396892231	8.072612901	0.01335852	9.323465813	2555.776682
4.621950301	14.5702021	0.026069119	13.36814429	6905.856313
4.089321001	8.237540786	0.035867545	5.926734493	1224.648448
4.149206734	7.882003306	0.025333462	6.369813186	1015.455295
3.822316667	7.642871439	0.026118808	12.48163988	2369.488804
4.697840228	10.42733241	0.009677386	13.3258027	5018.409658
4.478153889	10.2133826	0.013437222	21.99277327	8300.555468
4.707954163	10.64535631	0.017507266	15.12460993	5993.56433
4.406885218	7.027489308	0.012588129	14.82574718	4500.766576
4.498361493	9.435797017	0.014058413	9.787912985	2523.624084
4.544644706	10.24163526	0.013162258	10.36044055	2973.454541
4.444321884	8.295118251	0.011362339	10.95652239	4104.078348
4.063858465	12.2488304	0.051090381	7.654968569	2061.666961
3.892780676	8.670667801	0.071789251	6.114970756	1455.452233
3.755381262	11.46926658	0.091942645	6.208372321	2352.2841
4.775633844	10.47016774	0.007744705	18.24945571	7350.345771
4.222074405	7.095717175	0.014251796	9.033095901	1959.383401
4.206533666	5.617140781	0.017007771	11.38828624	3342.450449
4.310665255	9.152693689	0.012914258	8.374602175	1805.355074
4.273161946	13.65509223	0.029469715	8.558679246	2534.009508
4.526273671	10.12015696	0.011106432	19.59850377	7329.972876
4.792268058	11.36255638	0.008450074	15.31695012	7352.589303
4.391941242	14.37978121	0.030761194	10.16989763	4229.158749
3.211262876	6.877738394	0.046570226	9.794214869	1116.645083
4.077398029	10.09476104	0.037550245	6.246883684	1288.585199
4.201982074	7.611305967	0.018382991	6.523958659	1272.237524
4.420840177	6.831658218	0.012224066	11.49902359	4554.520023
4.989201267	15.12634097	0.00606963	25.1840116	15289.59371
3.139664933	6.269591626	0.041174325	4.473746082	224.5048812
2.509298741	4.718253968	0.066043084	8.561444948	897.9637241
4.61443034	15.03066486	0.024489694	14.09405843	6998.511434
4.46727786	6.071031895	0.01318133	15.66567713	9041.427126
4.24243411	6.253677504	0.01497622	7.922849771	1429.285896
4.525702695	11.12089084	0.018899374	9.841472192	2884.947744
4.099768798	10.36604768	0.057600763	7.413231093	1888.578713
4.186487392	13.49445883	0.019338039	23.72803537	9800.001382
4.948048375	10.64028823	0.006448184	19.74355186	10646.95213
2.616088522	3.800701008	0.075614892	5.63975167	383.9853755
4.779804019	10.92092222	0.010877764	26.48010095	18201.32024
4.617411107	10.91244329	0.013107519	13.72489564	5507.355165

log.sigma.4.5.mm.3D_glcm_SumAverage	log.sigma.4.5.mm.3D_glcm_Imc2	log.sigma.4.5.mm.3D_glcm_Imc1	log.sigma.4.5.mm.3D_glcm_DifferenceAverage	log.sigma.4.5.mm.3D_glcm_Id
20.69692621	0.923815335	-0.239217886	2.502588179	0.40159292
20.4516098	0.881367762	-0.219938621	1.635042896	0.522046828
13.70581772	0.895526839	-0.233573933	2.583377468	0.39176594
27.92622271	0.820327169	-0.147838387	2.539131157	0.421267742
24.14247467	0.994922399	-0.521685017	4.762681683	0.271429897
11.83659948	0.805521925	-0.154780585	2.50159126	0.409676412
8.422039551	0.927344027	-0.338558794	2.129960278	0.447653634
16.28594848	0.870971542	-0.178596173	3.404184088	0.350770802
37.81209804	0.931612195	-0.206700407	4.639261857	0.303246035
23.30380209	0.857455184	-0.195708062	1.54959635	0.523847038
12.60182701	0.766528735	-0.130860847	2.66182277	0.399676727
18.93716595	0.85898562	-0.18696569	2.16336314	0.442364045
12.74330731	0.735522139	-0.12240774	2.062325546	0.460986994
17.64877566	0.849813594	-0.171147369	2.742314879	0.397358768
17.17674221	0.872974197	-0.18513812	3.036562598	0.370520932
27.41836819	0.90863722	-0.344488529	0.671318215	0.754402453
16.73990533	0.933193318	-0.259025857	3.6941755	0.319980828
16.94716616	0.770177124	-0.132643254	2.099170219	0.461578021
20.59137919	0.975077199	-0.347697856	4.668262553	0.282403814
17.87937385	0.909673242	-0.262002298	1.2936439	0.571696487
21.2223874	0.888747987	-0.261906134	1.028800357	0.63353867
17.45503507	0.826030445	-0.162554977	2.409871342	0.434242652
24.58827373	0.894226261	-0.253077816	1.205659011	0.594394556
25.56626571	0.990817114	-0.473632763	3.718205373	0.317878648
15.24250875	0.865291095	-0.219700624	1.33422896	0.557609389
15.99728383	0.851484269	-0.179267765	2.545532373	0.399432914
20.12543892	0.834140149	-0.197683954	1.252746453	0.586254623
21.6524297	0.923300688	-0.323410831	0.982476427	0.665200106
27.7944113	0.899371671	-0.25311122	1.540723683	0.576165328
23.50537785	0.915947526	-0.258777386	1.511836663	0.539291475
21.24114143	0.893621882	-0.258826089	1.143498045	0.615683138
22.82138237	0.882092732	-0.257074929	1.079204944	0.653895619
20.22840865	0.830289907	-0.156035527	2.380505355	0.424830559
14.21858974	0.995743155	-0.886354267	4.010897436	0.287557775
20.59093416	0.980130718	-0.407178378	3.781306847	0.310061708
18.30000691	0.720331285	-0.105230131	2.45014778	0.416678371
23.09775927	0.909199416	-0.300273655	1.007079876	0.664225394
19.32413244	0.886407756	-0.22460854	1.496653395	0.54263232
22.66813611	0.944437521	-0.266367566	3.427452032	0.345286162
16.06808757	0.859678474	-0.180288501	2.447264577	0.410629561
24.55272192	0.90272171	-0.228252704	1.920840792	0.474912293
18.58685424	0.866628394	-0.184995297	3.058159868	0.382948791
16.93818137	0.680542713	-0.095276785	2.597203469	0.406323873
16.63578725	0.876738208	-0.186385642	3.163757049	0.370904397
18.20756558	0.825486777	-0.151867235	2.572939548	0.406523897
18.42573855	0.907129153	-0.355064128	0.646132952	0.740816451
23.76305885	0.83134293	-0.148118107	2.854115725	0.393418119
12.82388517	0.862865963	-0.188998066	2.941501071	0.381350205
12.58619159	0.995020335	-0.775234806	3.912887113	0.28810391
27.58579888	0.990728973	-0.44191825	5.557949648	0.247327605
21.40769415	0.898674502	-0.262136668	1.203136012	0.611696445
11.93940216	0.699362412	-0.103978684	2.48567637	0.421299397
22.57826582	0.837153694	-0.174232493	1.904528524	0.482538975
16.11488521	0.904527585	-0.227375155	2.551813546	0.402221141
22.3186748	0.900906871	-0.306104262	0.85608613	0.702552159
20.42163934	0.888806223	-0.192377517	3.681992473	0.324642089
21.80198034	0.770573431	-0.119651487	3.194288624	0.357216195
28.12107628	0.938544317	-0.238418814	4.487674026	0.29066272
16.50358173	0.89271838	-0.21090245	2.487591606	0.405657021
16.3898294	0.891059571	-0.223911406	1.951014062	0.452675302
16.1452258	0.773425075	-0.133413138	2.04740837	0.45509966
29.09973361	0.899874749	-0.232276108	1.690643899	0.549239378
16.47508157	0.871749128	-0.241157996	1.141339431	0.608688524
15.76400661	0.859304711	-0.215587589	1.350009953	0.564909401
15.28574288	0.983643521	-0.48625432	3.516253015	0.313938248
20.85466482	0.822370106	-0.156290215	2.406669187	0.424654431
20.4267652	0.981769424	-0.418875241	4.437399552	0.283397539
21.29071262	0.89894322	-0.224292037	1.827212671	0.510374858
14.05497862	0.922862102	-0.258548469	3.469006919	0.334185282
18.87159403	0.846512609	-0.182753687	1.73928783	0.501279082
20.48327051	0.825463707	-0.168987551	1.866907157	0.490972799
16.5902365	0.796291607	-0.140460776	2.489380983	0.416150115
24.49766081	0.908682318	-0.283070765	1.087520389	0.633612555
17.3413356	0.921591993	-0.323250929	0.874950887	0.685095085
22.93820141	0.887076753	-0.272438328	0.980194192	0.687265279
20.94033548	0.868645904	-0.177205857	3.52493082	0.343833674
14.19143435	0.756025724	-0.126226611	2.53845916	0.411509778
11.23428156	0.861884627	-0.194591808	2.775423302	0.392623598
18.30538738	0.719040292	-0.11548114	2.219634958	0.441072132
27.31018446	0.877387697	-0.221970437	1.504840827	0.557860403
20.24031392	0.972590706	-0.360652995	4.188714374	0.302182264
22.72304584	0.831966689	-0.152912595	2.629268184	0.404933026
28.7523542	0.874175045	-0.219967567	1.550692245	0.562455572
13.75547679	0.99209768	-0.637121609	3.233437289	0.341470342
20.18952207	0.861349669	-0.221021938	1.228396306	0.601862134
15.22261193	0.726363861	-0.126543251	1.691105247	0.513861717
13.66331644	0.822589192	-0.158699101	2.539709366	0.406056165
29.96018104	0.876913137	-0.173636065	4.141749624	0.308201419
12.53918325	0.922898587	-0.342720993	2.266159547	0.423415174
9.436507937	0.993162646	-0.714877395	3.285714286	0.314295163
30.04660921	0.90474411	-0.238283176	1.714965582	0.536909941
12.14206379	0.887287544	-0.213104388	2.808049219	0.391888916
12.50735501	0.809451845	-0.161619931	1.99940747	0.459158611
22.24178169	0.90681584	-0.24961642	1.419713652	0.554498183
20.73209536	0.912078775	-0.285412206	1.07334077	0.641381353
26.93148152	0.990768558	-0.517467954	4.802862881	0.266684056
21.28057646	0.852389114	-0.161424053	3.074959104	0.366487486
7.601402017	0.971659668	-0.568985714	2.597277936	0.395399559
21.84184445	0.980666888	-0.390701912	3.411982387	0.340449037
21.82488657	0.931382071	-0.265227151	2.307307467	0.432568536

log.sigma.4.5.mm.3D_glcm_ClusterTendency	log.sigma.4.5.mm.3D_firstorder_InterquartileRange	log.sigma.4.5.mm.3D_firstorder_Skewness	log.sigma.4.5.mm.3D_firstorder_Uniformity
64.85742575	167.5886154	-0.036224522	0.063328243
34.7935643	121.6908951	-0.434414375	0.091221387
31.04414524	113.556282	0.335704661	0.09176801
53.4603614	141.4381447	0.169819619	0.068506826
123.661973	229.2808762	0.376972641	0.050877365
25.1948	123.5382576	0.223626016	0.091306101
12.84822845	81.88196087	0.629146931	0.118367347
58.56199742	166.6038208	0.622279981	0.061697378
301.5064361	286.4996109	0.610199754	0.036798349
29.61424241	103.1524906	-0.055465639	0.093919942
27.38512121	133.8453445	0.488676197	0.083875622
41.91853581	139.9526291	0.19185283	0.074686458
28.69910853	102.9396534	1.092481092	0.097343131
48.31002192	159.8644924	0.436558004	0.068669651
46.20222358	141.2202024	0.308341583	0.069556817
15.94409236	31.82187414	-1.799284073	0.227138107
54.75911111	163.6834869	0.04782663	0.065599185
30.70460458	103.3213615	0.498621632	0.088168575
94.71368807	225.0211105	0.096428485	0.051141167
31.6563849	119.3790646	-0.298430832	0.098984597
19.37671524	90.71999645	-0.540420997	0.132516567
44.65565724	131.0279427	0.754222527	0.078894468
24.93701381	93.86491585	-0.727139332	0.116299259
119.6552913	249.4936829	-0.2132996	0.053083837
21.84649325	84.81581211	-0.344374868	0.114144965
31.47446978	115.4396782	-0.018914265	0.083548967
18.46956171	73.05398941	-0.26468971	0.133156256
27.95057529	113.6090641	-0.804103944	0.152419563
41.25902874	133.607496	-0.650103608	0.120144785
44.26975825	143.354332	-0.436327555	0.083909318
24.38182269	100.6143856	-0.493474382	0.12512754
20.94519807	78.6987319	-0.606701306	0.15767233
49.50048176	151.0643806	0.201953847	0.069559705
36.8552297	199.7020483	0.155372247	0.093333333
60.19048118	155.025795	-0.018210492	0.065862636
32.69128004	123.0409641	0.33508315	0.08273412
26.42282065	90.05674645	-1.057135186	0.158384659
35.51375877	130.3880905	-0.060844591	0.088605882
88.47679828	192.9446373	-0.103268447	0.056492076
50.30720632	164.5652542	0.197711683	0.070954229
44.46037219	128.7851133	-0.31097974	0.08195413
48.47024975	146.6021271	0.831523177	0.072533256
26.64000227	115.1550903	0.383138556	0.085617165
59.32140837	177.2875214	0.606824383	0.063578956
56.85402622	127.8586006	0.699841561	0.07486899
14.52722681	62.68953784	-1.212174794	0.212095109
75.00048062	140.7126792	0.979708929	0.067706242
35.5102603	149.5695381	0.449143714	0.076523546
23.80285996	122.8705521	0.106048924	0.103305785
126.704823	243.6654758	-0.091608638	0.047100769
27.43162973	109.967288	-0.538218976	0.126232819
18.74163779	103.098114	0.721485208	0.103534903
39.6558781	132.4980335	-0.221000113	0.084327086
43.01159243	149.4699249	-0.078726317	0.07858756
20.60527532	58.38401687	-1.24132892	0.195492992
57.00713213	176.2755966	0.122534861	0.060957897
42.01793209	138.3226891	0.3800435	0.071313968
109.5871866	201.5000935	-0.383248076	0.049789287
45.53308597	135.967432	0.384382464	0.074164512
41.01815979	137.735961	0.178579712	0.080089889
29.9850114	107.0883331	0.543565535	0.095389048
46.13289228	129.2754052	-0.182580783	0.093318674
20.91883813	81.71492171	-0.511802454	0.126025547
21.93429575	100.6521497	-0.251282488	0.113560471
31.96786098	124.3428497	-0.054712795	0.091491634
43.0730535	124.0546167	-0.080571374	0.078426408
58.65468413	174.0705414	-0.135355182	0.061950113
53.77171535	163.5209961	-0.169971123	0.078567887
40.85061775	150.4872284	0.226422855	0.07653186
33.5168073	120.6965008	-0.15367957	0.088025151
34.64030503	123.0664387	-0.141704501	0.085325534
32.85604973	110.2649002	0.682078369	0.08380069
27.88606688	106.327482	-0.745516269	0.131373713
22.52736478	88.12020442	-0.886974665	0.151876324
21.82707381	55.02728605	-0.719579066	0.186906426
52.4158353	168.4220047	0.242981351	0.063926482
25.23875296	115.6445503	0.508585658	0.090104648
32.2890117	143.5385399	0.78918034	0.086455676
24.86741156	101.8728943	0.204451158	0.092639002
29.42204382	113.0670223	-0.32980568	0.103361829
50.9843168	171.8638763	0.133676859	0.065357416
49.06227955	129.4644165	0.284825826	0.070832971
34.99596862	103.8210485	-0.58922936	0.109490616
23.04477169	117.0859051	-0.101814525	0.100936524
21.59191765	90.99865508	-0.517268019	0.126027702
20.53421039	88.47938347	0.376197796	0.107734265
35.03240524	124.0888214	0.789664169	0.082094202
69.05866174	175.1474762	0.109624168	0.05875401
9.523734443	77.77416039	0.187376498	0.1312
18.3568909	87.14164734	0.265121998	0.112426036
49.74840114	140.2682939	-0.507297357	0.091334687
48.98305453	130.1351967	1.009875934	0.080786877
24.76548605	107.6879139	0.252879377	0.095356416
35.31014148	121.9100885	-0.261114212	0.089237814
26.89443437	103.5700204	-0.563497581	0.133349352
59.2016987	200.1481285	-0.092322447	0.066263542
63.25878254	159.8541851	0.177274907	0.061613322
11.91188756	100.7421741	0.489158472	0.135416667
87.85573362	190.4761591	0.213915018	0.058301028
45.59112664	133.3955412	-0.242351068	0.075535714

log.sigma.4.5.mm.3D_firstorder_MeanAbsoluteDeviation	log.sigma.4.5.mm.3D_firstorder_Energy	log.sigma.4.5.mm.3D_firstorder_RobustMeanAbsoluteDeviation	log.sigma.4.5.mm.3D_firstorder_Median
93.46502397	13914372.57	71.51764035	-156.1341553
65.47592033	23418771.39	48.87828875	-92.59667206
67.17196761	6490730.318	46.86908882	-180.9285583
84.14785333	41139659.54	58.56734303	-98.26805115
131.5269879	5928284.99	96.46109318	-204.894928
65.97899837	15880986.36	49.39025344	-245.4742279
49.88780692	794986.531	35.9879131	-89.40080261
97.18970822	26785233.94	68.466901	-244.3318481
176.1640762	72285875.8	124.8796376	-82.43948746
60.6355215	33706121.29	42.54586751	-91.28759003
72.25198034	22725878.27	53.50106819	-242.8038025
76.68126953	17029656.58	56.5386449	-117.8119736
65.73410359	27963957.81	43.36234839	-134.6249237
87.28387072	16805708.4	64.82367901	-156.9751587
82.81840916	7212724.567	59.08619213	-98.91452408
35.65971346	34733836.89	16.65556079	-4.408463955
93.36257359	13213266.11	68.88339922	-209.3232727
64.75398482	41286800.68	44.06810421	-144.6694565
125.3263634	10200735.74	93.1875024	-184.8371506
62.07303372	25888139.65	47.508158	-52.42295647
49.07923653	46110445.55	36.40049839	-65.84644318
78.43213238	18730388.13	53.0968386	-123.0521469
52.97290587	32077906.53	37.4171616	-73.3385582
124.1862731	3180546.192	96.02092404	-49.33525085
50.84988523	12412552.32	35.69888109	-87.57025528
67.29711641	13631751.25	47.63409718	-193.3098221
46.52259195	15911163.18	31.05815088	-49.23252869
59.8797001	38458382.43	45.53189434	-46.58825684
72.31111049	57073739.88	54.65345748	-68.55875778
74.44736672	37787066.87	56.8306524	-109.4861069
54.82333356	41091346.37	40.5365249	-61.34374237
49.37852748	57897247.09	33.25001914	-32.6328125
83.05461725	35338715.24	60.99659687	-130.1218872
94.51971049	268342.5804	71.25220244	-87.17256165
93.68056959	4941734.021	65.77737246	-178.825592
70.20632311	49620504.38	49.99489621	-180.5853729
55.56501186	48165650.66	39.40010773	-20.89940643
69.15178056	33598455.22	52.4207386	-57.77045441
110.3623857	10310419.29	83.80866433	-110.9095078
84.63540538	21251327.11	65.78247292	-175.9948578
72.99154809	12552992.89	53.05539091	-89.63835144
90.61568723	24243618.75	63.68611567	-258.5002747
67.7817501	41586326.95	48.09549891	-216.7525101
99.062296	23877136.02	73.80200164	-219.2613831
80.36047565	28966486.93	54.02878843	-142.0153198
40.88661319	22975679.57	28.22738731	-12.31879473
86.2232224	46102848.39	58.17097648	-71.14612579
79.92652808	16438817.29	60.06225239	-264.6673584
70.8501405	730145.187	46.17460024	-153.8917236
137.1034765	9498976.866	101.0225688	-189.2757797
58.86694577	39949893.41	44.04851024	-53.47550201
61.51369971	32105539.03	43.6648875	-288.9583435
70.84121282	56955825.59	52.91123101	-94.30195618
78.7903547	5328109.001	59.20749393	-105.4948044
46.58948047	53731188.84	28.74328771	-9.643909931
96.17282955	21890976.01	71.19757102	-234.3839569
80.00253398	42025862.07	56.72137499	-203.4522095
118.7373765	27413634.76	84.03814725	-177.6731339
78.83243761	13005467.98	55.50030491	-172.1595306
73.36364613	10617356.07	54.69094838	-129.818634
62.36692198	44601569.93	44.00830579	-168.6979218
74.80086483	93748000.32	52.89366521	-53.77398491
49.70292858	18924503.82	34.8033024	-14.03869295
52.35425974	14160694.35	40.16560186	-60.87981987
72.48457788	3063506.361	51.22496743	-236.4455872
73.41860752	14433799.26	51.40615888	-58.09539795
100.3705507	7556096.381	71.14123174	-229.5894012
83.73436891	64216420.74	65.09957288	-80.61185455
84.1856034	14988963.07	62.08792369	-298.4315796
65.22005072	28183731.48	48.66006263	-96.06662369
66.57953419	44922434.28	48.73024499	-101.6838379
69.4891698	30685778.41	47.0644536	-220.8276672
58.15735026	56978378.95	44.19366669	-34.97290039
52.43287078	67076431.31	38.18166351	-21.98457146
46.29717417	67448732.25	27.12713344	-3.787131548
93.47010342	28353230.97	69.94078604	-238.9144135
66.21042052	24327310.76	48.64653111	-223.3912354
75.69625987	15574369.95	55.41769703	-260.8605957
61.1284042	43012095.76	42.38675559	-202.5913086
61.28162603	39664046.95	45.75829707	-91.84764481
91.3698712	17937568.25	65.7943397	-333.4080505
80.52712699	25921145.4	55.08124309	-147.3059998
64.09960202	44456243.47	44.49823845	-29.06420898
64.39922765	500886.2032	49.0849704	-99.23252869
51.63034303	37125119.87	37.52447625	-47.60418129
52.73989526	30241511.75	37.16021267	-68.20336151
74.56329406	17641712.72	52.05870353	-213.9329834
102.4416313	50289820.17	72.3466574	-265.3667908
46.51253534	443575.5215	34.62928204	-83.66726303
60.18628255	464703.006	38.16550003	-194.5622864
76.1532401	53942363.36	56.26034735	-68.19133759
83.55947973	18333648.95	56.38879826	-244.2306442
59.93075569	8213018.536	44.11570707	-124.294014
65.58559736	48172353.17	49.04132689	-81.3900032
58.77789754	55895192.42	43.53237829	-29.17677784
105.8165329	4569405.665	75.87741752	-234.1299591
92.96504885	19402739.05	66.63238365	-122.2575378
50.8274004	535933.6086	34.57368625	-147.0458908
107.7165024	4905059.807	79.11398117	-127.8648834
76.97087457	5993612.372	54.05769062	-101.1074295

log.sigma.4.5.mm.3D_firstorder_TotalEnergy	log.sigma.4.5.mm.3D_firstorder_Maximum	log.sigma.4.5.mm.3D_firstorder_RootMeanSquared	log.sigma.4.5.mm.3D_firstorder_90Percentile	log.sigma.4.5.mm.3D_firstorder_Minimum
375688059.3	99.91651154	196.3261784	-22.24330139	-418.102478
632306827.6	93.08852386	129.1972346	-9.994631004	-341.4086609
175249718.6	37.36299133	191.4961952	-57.92788391	-346.3130188
1110770808	274.4463196	145.8862697	26.21725464	-427.1394653
160063694.7	191.9396362	242.2723488	41.4022789	-475.0978699
428786631.8	-48.11156082	255.1195988	-135.8961334	-382.828064
21464636.34	77.2417984	106.5689938	-1.793463826	-193.0717163
723201316.4	200.4273987	258.4494044	-55.48947525	-445.1486206
1951718647	482.0431213	221.3010858	334.7116699	-457.0112915
910065274.8	178.57341	120.5602154	-4.830167007	-356.5376587
613598713.4	33.71975327	245.8478506	-114.4983864	-389.6911316
459800727.5	224.4212189	149.9874789	2.247366047	-349.8734436
755026860.9	247.5883026	143.0783653	-5.632312775	-272.0367737
453754126.8	177.0597382	178.4068193	1.10151124	-354.7620239
194743563.3	212.7456665	140.7663929	35.23173828	-306.4697571
937813595.9	249.7086945	56.63726285	27.07653656	-345.3748779
356758184.9	65.44871521	246.7602627	-77.91945038	-443.7307739
1114743618	256.9295044	163.1021766	-38.40824509	-331.4057007
275419864.9	142.0209045	236.7445851	10.12887268	-461.0812683
698979770.6	132.3419037	96.60446351	22.66162987	-254.7019196
1244982030	184.6776428	95.31947377	-5.024501228	-304.581604
505720479.6	216.7310791	143.5461163	26.49460754	-300.7166138
866103476.2	162.9280396	104.8121387	-4.227607632	-368.8304138
85874747.19	229.4969788	154.6411366	127.6898102	-364.5334473
335138912.7	124.3029022	113.7090234	-19.33600292	-272.9029846
368057283.7	27.03040314	212.4576409	-90.54411087	-379.5536194
429601406	236.2323914	83.37350675	4.739061165	-289.8681335
1038376326	338.3512268	99.36690796	-0.947844279	-317.6472473
1540990977	304.6418457	123.9311025	-3.51513052	-402.1465149
1020250805	102.9696274	149.397425	-13.65284786	-381.1390686
1109466352	198.664505	98.90058144	-3.491645575	-309.7103577
1563225671	329.5792236	80.96080951	2.7088521	-318.7130737
954145311.5	227.1094666	159.5049831	5.63963995	-353.6054077
7245249.671	107.0528259	133.7516555	56.75726166	-246.1382141
133426818.6	116.7860413	209.1223553	-22.72684288	-445.5235596
1339753618	123.0218658	192.8646673	-53.80521698	-386.3916931
1300472568	201.0185547	84.84440352	3.74433732	-317.300293
907158291.1	233.4073181	105.2676812	31.11223278	-289.6351013
278381320.8	206.7236938	179.2196593	37.42058563	-401.036377
573785832.1	78.5793457	198.9325663	-34.43024979	-362.9569092
338930807.9	187.3838043	132.5014468	3.497568989	-396.9916077
654577706.3	152.7723999	252.2529391	-66.79589081	-458.4635315
1122830828	84.62391663	224.6527173	-91.84295654	-416.9357605
644682672.6	226.0832214	233.2162762	-27.78211594	-415.0516663
782095147	218.1689758	161.1797507	19.58030128	-345.415863
620343348.4	81.36328125	61.26148502	8.502187252	-228.5860596
1244776907	625.4428711	129.5727311	70.92420197	-334.1557007
443848066.7	3.455482721	268.5145728	-113.1963699	-417.504425
19713920.05	23.30469513	182.1768854	-62.89942894	-311.9936523
256472375.4	176.1748352	255.0715299	33.34464169	-558.473999
1078647122	190.5014343	98.95271872	0.796547174	-320.4871521
866849553.7	-9.628904343	282.2522807	-164.9243439	-419.6604004
1537807291	235.1953278	133.516102	-4.182876968	-368.8493652
143858943	106.2746658	149.3095546	-3.762834644	-313.0924377
1450742099	191.1283417	68.93800867	18.07967949	-293.1324463
591056352.2	70.01593018	255.6290223	-73.34444427	-483.5791016
1134698276	220.1517334	219.5331318	-64.15796738	-463.5080872
740168138.6	128.5483093	244.653015	-19.67542706	-572.9199219
351147635.3	131.795105	191.6731857	-34.37143669	-365.4883118
286668613.8	115.695343	150.9437839	1.932099819	-300.6408386
1204242388	143.1429443	175.0828374	-47.63240891	-339.6113586
2531196009	399.2024536	117.6235568	14.33585072	-420.98526
510961603.2	270.3418274	68.1556303	38.83964539	-223.826828
382338747.4	93.38349152	89.14324199	12.2978322	-239.6513062
82714671.75	-2.849956989	240.4204792	-113.3185852	-409.6981506
389712580	278.5249939	111.9345328	48.41797981	-303.585907
204014602.3	5.894629002	268.2588725	-69.17874985	-509.1761169
1733843360	246.3913879	136.09624	9.985800934	-335.7217102
404702002.9	-66.40657043	323.755963	-177.903714	-486.6274414
760960749.9	141.9700317	125.4094443	3.28580246	-300.8263855
1212905726	166.972229	131.4705565	-2.324021196	-349.2038879
828516016.9	124.5066376	232.6360648	-102.2402832	-401.8077698
1538416232	182.4583893	89.85556636	6.684012222	-327.723938
1811063645	141.1316223	78.68474808	9.664615726	-246.8595886
1821115771	326.1231995	68.15826755	24.5983593	-297.1706238
765537236.2	100.0666809	252.1354135	-67.62532806	-494.1489563
656837390.6	83.10254669	230.9748162	-106.9135208	-383.6422424
420507988.8	59.37262344	263.6824554	-122.3956032	-383.1247864
1161326586	49.28096008	217.0500615	-104.6387787	-406.4016418
1070929268	193.1588898	121.1142288	-7.346503019	-424.8642578
484314342.7	-70.75054932	346.9673819	-183.4272919	-589.0023193
699870925.7	222.564621	173.1087579	-2.756761885	-409.1950684
1200318574	304.0594482	94.97011678	27.94601784	-387.5629272
13523927.49	35.90781784	127.1126251	5.57159853	-267.8118591
1002378236	172.3142853	86.49789772	6.239788103	-282.8755493
816520817.4	203.8784332	92.86110897	21.51209869	-242.8916321
476326243.4	142.4671936	219.8487196	-68.29247742	-373.6638489
1357825145	144.2492981	292.2014683	-102.6215164	-625.3703613
11976539.08	41.02717972	94.18869587	11.88655558	-210.781723
12546981.16	-46.15087128	189.0671444	-76.40519867	-292.8490906
1456443811	288.6835327	125.424172	6.458793449	-426.0242004
495008521.6	131.9400024	245.5769854	-68.68007813	-368.2485046
221751500.5	90.70795441	140.5091919	-28.71838379	-270.5890503
1300653536	130.1331787	114.8506735	12.277878	-327.9598694
1509170195	191.4819641	87.97484001	11.54082708	-279.3304749
123373953	86.75429535	269.3144048	-77.46180573	-555.0065308
523873954.5	217.9293213	168.9184412	24.4949501	-394.5665894
14470207.43	-13.70308304	149.4341562	-40.48087349	-224.3265533
132436614.8	180.1984406	173.4715869	69.05305023	-396.3846741
161827534	174.4038544	146.3070691	2.134265208	-363.3719177

log.sigma.4.5.mm.3D_firstorder_Entropy	log.sigma.4.5.mm.3D_firstorder_StandardDeviation	log.sigma.4.5.mm.3D_firstorder_Range	log.sigma.4.5.mm.3D_firstorder_Variance	log.sigma.4.5.mm.3D_firstorder_10Percentile
4.107794988	110.4390893	518.0189896	12196.79244	-312.6051941
3.629323377	79.00246219	434.4971848	6241.389031	-213.8390198
3.650342929	83.64360214	383.6760101	6996.252179	-272.299585
4.10580132	105.6592295	701.5857849	11163.87278	-237.0642242
4.503786608	159.4765694	667.0375061	25432.77618	-385.9450989
3.578935745	78.49924958	334.7165031	6162.132185	-343.8731354
3.25366577	61.52710931	270.3135147	3785.585179	-159.6894165
4.18868611	120.8917738	645.5760193	14614.82097	-375.2689819
4.959884006	217.9797143	939.0544128	47515.15585	-291.0081177
3.641257664	75.6825414	535.1110687	5727.847072	-193.7854614
3.751190654	86.57957802	423.4108849	7496.02333	-331.2505951
3.895798853	93.13790435	574.2946625	8674.669227	-236.3055511
3.656547677	85.1073032	519.6250763	7243.253059	-207.7046814
4.023371785	104.9291626	531.8217621	11010.12917	-270.845166
4.022479514	101.9341879	519.2154236	10390.57867	-227.9901413
2.725135428	53.58607956	595.0835724	2871.467922	-88.16307831
4.103196737	112.3968992	509.1794891	12633.06295	-371.4249329
3.740422332	83.01003402	588.3352051	6890.665748	-247.8861313
4.442893245	149.0436832	603.1021729	22214.01951	-384.0826935
3.500377492	73.02211156	387.0438232	5332.228777	-167.9138977
3.166828139	59.87502209	489.2592468	3585.01827	-158.0764832
3.918714058	99.06874288	517.4476929	9814.615815	-209.7121216
3.37017742	67.18171491	531.7584534	4513.382818	-163.2854431
4.342856241	144.4870783	594.030426	20876.51579	-255.994043
3.339224187	63.65091808	397.2058868	4051.439373	-183.0501785
3.750170876	82.90759919	406.5840225	6873.670003	-308.75466
3.249697768	60.29357072	526.1005249	3635.31467	-134.1044495
3.186583299	71.62508804	655.9984741	5130.153236	-170.4320892
3.531159232	86.8779828	706.7883606	7547.783896	-214.718811
3.737918398	88.41415709	484.108696	7817.063173	-246.7962585
3.311668546	66.65662179	508.3748627	4443.105229	-164.5636749
3.167565336	63.72153598	648.2922974	4060.434148	-141.5595612
4.017348375	100.5613494	580.7148743	10112.585	-251.1880432
3.506890596	108.31808	353.19104	11732.80646	-201.816864
4.115959571	115.3770705	562.3096008	13311.86841	-335.5769653
3.809347742	86.87153996	509.413559	7546.664455	-272.0264374
3.126817458	68.15680862	518.3188477	4645.350561	-156.7827606
3.688329931	82.01099095	523.0424194	6725.802637	-178.922908
4.313580941	130.8577641	607.7600708	17123.75442	-306.2909241
3.924582273	99.58042251	441.5362549	9916.260547	-299.5544006
3.817919646	89.4900813	584.375412	8008.47465	-218.3142944
4.037123858	112.0018515	611.2359314	12544.41474	-345.5058594
3.746110574	83.59565202	501.5596771	6988.233036	-313.12146
4.163748692	119.52561	641.1348877	14286.37146	-337.5894531
3.994001043	102.549314	563.5848389	10516.3618	-246.2160675
2.655114379	50.36671158	309.9493408	2536.805635	-117.3661476
4.141634821	113.3274164	959.5985718	12843.10332	-195.6604004
3.852089263	95.5642419	420.9599078	9132.524329	-366.7377411
3.42511855	86.45479205	335.2983475	7474.431069	-259.6147156
4.568910306	165.0801822	734.6488342	27251.46655	-440.2695007
3.36088308	70.84926517	510.9885864	5019.618375	-168.8766861
3.512192936	75.51400029	410.031496	5702.364239	-353.5258484
3.7604071	85.43781276	604.044693	7299.61985	-220.2264587
3.848691536	93.55057953	419.3671036	8751.71093	-244.1753113
2.914951139	60.34284966	484.260788	3641.259505	-132.37854
4.191313611	115.677503	553.5950317	13381.2847	-371.802124
4.002684943	99.33211507	683.6598206	9866.869083	-319.594101
4.515723554	146.8839272	701.4682312	21574.88807	-405.063089
3.939961881	97.76346175	497.2834167	9557.694453	-293.1778992
3.794493349	88.05227782	416.3361816	7753.20363	-233.3286057
3.616488409	77.65528118	482.754303	6030.342694	-246.3921326
3.804125904	93.79971259	820.1877136	8798.386082	-202.1295242
3.260603928	61.75271004	494.1686554	3813.397198	-121.8748604
3.286122238	61.75050958	333.0347977	3813.125433	-147.2819489
3.626821465	88.3663348	406.8481936	7808.609127	-348.5412842
3.895750123	92.00491034	582.1109009	8464.903526	-186.4789337
4.154822514	122.302011	515.0707459	14957.7819	-423.8944275
3.881266126	98.22674992	582.1130981	9648.4944	-234.1078857
3.873031386	101.2204034	420.220871	10245.57006	-442.4209656
3.650117111	78.38301926	442.7964172	6143.897709	-205.6081863
3.730499646	81.50170685	516.1761169	6642.52822	-212.4667847
3.8172713	89.1738594	526.3144073	7951.977201	-322.0204529
3.255186438	68.89093633	510.1823273	4745.961109	-164.3688354
3.097942297	62.84858483	387.9912109	3949.944615	-146.8723465
3.028585181	63.6051985	623.2938232	4045.621276	-120.367511
4.138964056	111.9015658	594.2156372	12521.96042	-357.6109314
3.658564642	80.73517885	466.7447891	6518.169104	-313.084549
3.727086375	92.266022	442.4974098	8513.018816	-351.1931122
3.659930298	76.96509697	455.6826019	5923.626151	-303.2135193
3.514269551	74.93730395	618.0231476	5615.599523	-194.2685577
4.126505855	112.0973332	518.25177	12565.81212	-469.3117249
4.056011822	101.9118985	631.7596893	10386.03506	-265.4069092
3.569999551	80.45344953	691.6223755	6472.757541	-168.6758911
3.474380085	76.82300258	303.719677	5901.773726	-196.6130066
3.275271691	63.10070012	455.1898346	3981.698355	-150.1445572
3.434115395	65.90988579	446.7700653	4344.113044	-147.0038788
3.802533911	93.19581307	516.1310425	8685.459574	-303.8518799
4.33649217	127.2074717	769.6196594	16181.74085	-415.0408264
3.081218383	55.69555299	251.8089027	3101.994623	-141.0505905
3.238901257	70.70144968	246.6982193	4998.694987	-253.7620575
3.772441521	92.9157243	714.7077332	8633.331823	-212.3625244
3.855592116	105.9298651	500.1885071	11221.13632	-335.9537323
3.536614243	72.39999753	361.2970047	5241.759642	-211.8210754
3.651504533	78.50579431	458.0930481	6163.15974	-190.5394104
3.323115585	70.12227272	470.812439	4917.133132	-159.3242081
4.064936084	129.5243855	641.7608261	16776.56644	-390.4576721
4.208567164	115.0732999	612.4959106	13241.86436	-276.2154205
2.938721876	62.32010063	210.6234703	3883.794943	-209.5520477
4.278198267	130.4287058	576.5831146	17011.64729	-282.6748169
3.930366826	96.62483411	537.7757721	9336.358566	-248.8246048

log.sigma.4.5.mm.3D_firstorder_Kurtosis	log.sigma.4.5.mm.3D_firstorder_Mean	log.sigma.4.5.mm.3D_glrlm_ShortRunLowGrayLevelEmphasis	log.sigma.4.5.mm.3D_glrlm_GrayLevelVariance
2.155724381	-162.3181317	0.021848811	19.71685353
2.586870164	-102.2278651	0.017698182	10.01809263
2.674610865	-172.2629983	0.041672472	11.17489447
3.176842419	-100.592897	0.006776497	18.74373492
2.389291163	-182.3817831	0.024585502	40.81118389
2.22254837	-242.7424098	0.050360295	9.738206103
3.022715333	-87.0135924	0.101205657	6.473856445
3.079649155	-228.432208	0.041178192	23.78517437
2.516457018	-38.19705151	0.008644422	74.38117233
3.089661135	-93.84518351	0.00922985	9.870416938
2.660369474	-230.0981145	0.04990679	12.37872666
2.795659082	-117.565193	0.022761002	14.32092609
4.372230964	-115.0137624	0.045853005	12.38217241
2.513299973	-144.2874354	0.020372001	17.75420725
2.821700158	-97.08037246	0.023993272	16.90473411
7.970388196	-18.33880095	0.005920093	7.277177345
2.361224078	-219.6760439	0.039642451	20.20680245
3.788675323	-140.398199	0.020330639	11.63599374
2.209949921	-183.9401507	0.039664599	35.51254912
2.181407786	-63.24708366	0.019377948	8.52176079
3.028632804	-74.16726913	0.011086978	5.821165458
3.393581106	-103.8791205	0.020382732	16.42590701
3.980330481	-80.44999446	0.009073703	7.659112324
2.025268457	-55.11229763	0.033394548	32.97900285
3.009863155	-94.22474532	0.034133938	6.802507448
2.528658878	-195.613341	0.032532826	11.33833337
4.211553966	-57.58321767	0.012979738	6.655898455
3.339762133	-68.87400932	0.015668317	8.605044109
3.067523061	-88.38062154	0.00749459	12.44506403
2.346352554	-120.4264399	0.012228702	12.30491117
3.074042736	-73.06312189	0.012073071	7.433002263
4.493030038	-49.94215183	0.010401989	8.024983604
2.679666301	-123.8113671	0.017241347	16.44711268
1.70843714	-78.4646347	0.113068888	18.4187162
2.541139712	-174.4141367	0.025015611	21.3753764
2.908143962	-172.1920888	0.019838311	12.39857414
3.5493149	-50.52941962	0.010825011	8.399741576
2.38873292	-65.99607615	0.015851165	11.2823273
2.261913277	-122.4578778	0.018152844	27.17916572
2.037925649	-172.2147073	0.036255126	15.99962014
2.982436477	-97.71468035	0.010818999	13.08735339
3.319479622	-226.0246237	0.016729355	20.88191112
2.819662843	-208.5200478	0.019316409	11.66521308
2.852732891	-200.258483	0.032430678	23.02692643
3.4024663	-124.3485032	0.023844122	17.24200193
3.573424904	-34.87354172	0.015380646	4.757772843
5.900151284	-62.81711013	0.011460124	21.35532453
2.333276996	-250.9333607	0.051235889	14.72622678
2.432497654	-160.3558122	0.113362028	11.84794274
2.233402602	-194.4479849	0.017553583	43.56939674
2.964986701	-69.0798246	0.013588756	8.594369577
3.184118787	-271.9632065	0.045495462	9.544744375
2.686519436	-102.6008267	0.01283578	11.87185391
2.294297791	-116.3685188	0.039954323	13.96803235
4.138343931	-33.33450968	0.010424431	7.515516617
2.353201843	-227.9581373	0.018762503	21.47837536
3.078087162	-195.7751947	0.011579865	16.07783631
2.652476024	-195.6532895	0.017744352	34.38097653
2.855327038	-164.8663569	0.03864005	15.49750005
2.275134488	-122.6002539	0.028577198	12.5662126
3.116837512	-156.9192699	0.025635426	9.614333205
3.805206512	-70.97122676	0.006506368	15.74209472
3.053301065	-28.84081732	0.024117276	6.853558427
2.231087412	-64.29146257	0.02646848	6.109342533
2.550275721	-223.592034	0.04806655	12.51823826
3.052108573	-63.75293019	0.016871892	13.77720801
2.370278655	-238.7572842	0.031282109	23.82923646
2.315929403	-94.20027675	0.017868716	15.59588006
2.428043494	-307.5261834	0.046598709	16.04311174
2.414040671	-97.89602146	0.017568135	9.833103199
2.645622134	-103.159968	0.016033992	10.81555787
3.615494606	-214.8663804	0.022279012	13.38005436
2.750154657	-57.68935515	0.008210904	7.998547059
2.844603047	-47.34284492	0.027240775	7.056607875
5.413165836	-24.49343093	0.010477522	8.96919151
2.471102851	-225.9431485	0.018072668	20.24428318
2.837374863	-216.4051677	0.029541183	10.74109591
3.239809792	-247.0129925	0.065913795	14.18656423
3.013148927	-202.9460595	0.019919414	10.05870006
3.196669743	-95.14755326	0.007200501	9.045242799
2.458841912	-328.3603995	0.022543838	20.25268661
3.0901337	-139.9307222	0.012521906	17.19560346
3.779409383	-50.46350702	0.006279861	11.5227009
2.221380201	-101.2711496	0.064276485	9.90809534
3.027472121	-59.16238631	0.0126725	6.888026943
3.167934339	-65.41462003	0.022957925	7.476699295
3.523487216	-199.1180553	0.046537351	14.26912074
2.962154703	-263.0588475	0.007976632	26.27314874
2.474954788	-75.95732885	0.050734907	5.33442069
2.138758663	-175.3501927	0.132303076	9.060188609
3.198523054	-84.24898272	0.006106643	14.38993867
3.499759042	-221.5556802	0.089805653	18.44986629
2.567579871	-120.4204027	0.055309373	8.562215368
2.431962804	-83.8302897	0.011284911	9.898273447
2.668486392	-53.12663497	0.013389391	9.014389985
2.569620559	-236.1221765	0.024378871	27.26069686
2.66358995	-123.6591098	0.017317308	21.69829544
2.24414398	-135.8188944	0.208725285	6.132681025
2.29244402	-114.371081	0.026320729	27.41149538
2.80188307	-109.8608206	0.022271085	15.55646465

log.sigma.4.5.mm.3D_glrlm_LowGrayLevelRunEmphasis	log.sigma.4.5.mm.3D_glrlm_GrayLevelNonUniformityNormalized	log.sigma.4.5.mm.3D_glrlm_RunVariance	log.sigma.4.5.mm.3D_glrlm_GrayLevelNonUniformity
0.023283724	0.062581349	0.109970264	20.59398744
0.019724911	0.08887235	0.410777719	100.5788207
0.046670929	0.091007809	0.099036756	14.78564876
0.007385241	0.066631505	0.201581129	112.7393791
0.02512838	0.050308816	0.03863793	4.886056565
0.057163315	0.091231306	0.129193609	20.08087104
0.109684676	0.116987121	0.078677818	7.578028256
0.04464016	0.060918693	0.084902353	22.65890947
0.009210634	0.036805275	0.0888378	50.39942298
0.010484256	0.090537594	0.364211548	170.0063026
0.055455802	0.081402462	0.131339322	27.61452916
0.024989759	0.073850873	0.172800505	49.17749208
0.051794061	0.092519745	0.229117543	108.4187087
0.022611136	0.067324173	0.132420632	32.04279925
0.026479476	0.069395115	0.093014028	23.26927468
0.007813196	0.142330348	3.857588414	775.2932176
0.0424701	0.065633955	0.05702935	13.46837287
0.02327766	0.086378426	0.23501676	114.3516027
0.04351232	0.051030459	0.051204586	8.843390202
0.022523851	0.094612009	0.694373887	197.8855704
0.013183035	0.120085865	1.269803119	415.6664448
0.023035416	0.075740472	0.207035811	60.18769613
0.010536871	0.11108224	0.717100341	239.7601859
0.035335995	0.053206177	0.049311853	6.761454783
0.038954257	0.111618584	0.355117347	86.75772909
0.035201959	0.083222902	0.104355512	22.97340277
0.01520773	0.119180471	0.654847817	204.1509122
0.018345814	0.103053449	1.674140028	259.4661918
0.008506311	0.087099695	1.138616271	233.9611602
0.013629071	0.079922531	0.521911723	106.7183296
0.01426415	0.107784479	1.178712406	314.9998528
0.012652402	0.112163232	1.981969617	626.1057253
0.019016303	0.069069896	0.20043033	84.1427287
0.113639481	0.092987964	0.015306122	1.373626374
0.025771371	0.065375848	0.046996521	7.060559692
0.021790945	0.081095713	0.160057451	95.54050204
0.012997934	0.111404818	1.898937929	463.721943
0.018277946	0.08287079	0.56822082	194.6565418
0.019301771	0.056607044	0.089129607	16.90328358
0.039959787	0.070326979	0.144574555	33.85013439
0.011736608	0.080707268	0.220037941	49.74856826
0.018271317	0.06952526	0.120599115	24.07210224
0.021118739	0.08303128	0.154518881	61.13070437
0.035088454	0.061825464	0.111512198	24.88404145
0.02615419	0.072669361	0.158520716	72.00637698
0.01986945	0.151455278	2.693924566	503.8591371
0.012529294	0.066693382	0.148403379	163.941848
0.057198979	0.075166952	0.11178388	15.67222163
0.11799354	0.102784332	0.028440498	2.191566767
0.017931354	0.047059387	0.03464953	6.631573376
0.015772375	0.098928238	1.280196461	279.5979931
0.051163154	0.097250293	0.16093473	34.60857918
0.014172289	0.081871027	0.350410476	216.9312216
0.044205268	0.078270265	0.111480051	16.93251064
0.012929671	0.122421581	3.055822376	786.0141465
0.019878186	0.060920057	0.076487941	19.18067843
0.012329964	0.070808636	0.089267267	57.44223886
0.018574216	0.04977704	0.058324011	21.68728914
0.042672697	0.073762286	0.113975874	23.69664194
0.031890027	0.079326325	0.194276068	32.43157418
0.028399184	0.094663385	0.213159951	118.472427
0.007429248	0.077798442	0.947974356	391.3489486
0.028579574	0.110779993	0.955775239	322.4064133
0.030258501	0.111194789	0.589726367	153.1580949
0.049259442	0.091335737	0.042172253	4.649523369
0.018585825	0.077873464	0.171013254	79.40831836
0.032574858	0.061408989	0.042246834	6.183402297
0.020038944	0.072389183	0.582837037	198.4942433
0.051244499	0.07642259	0.061458925	10.25126363
0.019786512	0.087772034	0.354815624	129.4691065
0.017865509	0.084624272	0.32096061	183.2157788
0.024713091	0.082063205	0.138211308	41.48987181
0.009855785	0.104481087	1.524814756	486.3623397
0.03240598	0.108559455	2.384143837	699.4244449
0.012901714	0.117656651	3.010778973	974.6924988
0.019147877	0.063262979	0.09416312	26.21403957
0.032530466	0.087556609	0.157943448	35.8494107
0.072765082	0.083519051	0.116613243	17.12074759
0.021639515	0.090638493	0.17189162	72.71615742
0.008141433	0.096251737	0.795521168	196.4416811
0.023101792	0.064797863	0.050143351	9.212310834
0.013461448	0.069645669	0.136352477	54.29344869
0.007186952	0.09188718	0.935337719	334.6952653
0.065099586	0.099914293	0.037066122	2.982311645
0.014936214	0.110678932	1.066587866	388.6217131
0.026439681	0.104286195	0.372367039	299.5199469
0.051619083	0.080310526	0.122658599	26.51337296
0.008297555	0.057826905	0.076587946	31.97682772
0.052805354	0.12847667	0.070426686	5.990988306
0.134039187	0.112097304	0.019097222	1.429487179
0.006837508	0.079684579	0.735210866	209.3477247
0.102763768	0.078723777	0.130280128	21.7090139
0.062645627	0.095000357	0.168553006	34.78769117
0.012911239	0.087726981	0.605240914	245.6380386
0.015978005	0.094837569	1.747574403	441.3946858
0.024621796	0.066604424	0.03424191	4.062922222
0.018724408	0.061157946	0.106071113	38.07394653
0.226662294	0.135545283	0.053228953	3.076317885
0.027445084	0.057625824	0.058347068	8.862155192
0.023305559	0.073487937	0.127546051	18.59184067

log.sigma.4.5.mm.3D_glrlm_LongRunEmphasis	log.sigma.4.5.mm.3D_glrlm_ShortRunHighGrayLevelEmphasis	log.sigma.4.5.mm.3D_glrlm_RunLengthNonUniformity	log.sigma.4.5.mm.3D_glrlm_ShortRunEmphasis
1.316796242	131.9574332	276.4801025	0.932646615
1.962289882	96.79705376	804.0959347	0.86580616
1.291578264	69.03237788	138.1591016	0.936607842
1.513685005	210.9998222	1345.015109	0.910526149
1.123896922	212.4540935	89.72700811	0.96902577
1.361687812	56.27360616	182.4764811	0.926989482
1.255117445	31.67964163	55.66978335	0.93909541
1.248779478	111.6709788	322.3189529	0.944991601
1.251885174	372.0059739	1189.002876	0.945910239
1.898830352	127.3670065	1324.34354	0.863646262
1.366055904	66.02113541	281.4170947	0.926735457
1.476521193	102.7114507	528.6153066	0.908499064
1.595450882	58.45580931	894.8607626	0.895321777
1.36547907	111.1880752	393.6120658	0.926780384
1.274788724	108.5727685	286.8181548	0.939420343
8.022043092	120.7209252	2330.885075	0.67504451
1.178816227	114.4483506	183.8678418	0.956874071
1.616022065	85.19333601	1003.207091	0.892258329
1.156045924	180.9983282	157.5051766	0.963023912
2.49182889	67.79546174	1375.545525	0.833584648
3.460719122	81.97898539	2058.685387	0.796078762
1.529375135	100.5745943	630.2007766	0.90835715
2.586418279	120.5383561	1362.59074	0.81834106
1.146421371	202.2454073	116.4278982	0.966146778
1.901529488	56.23273693	545.9280117	0.860221999
1.305374568	83.5487825	232.9860967	0.933976299
2.468224585	89.51313025	1095.856563	0.82494042
4.141130733	79.02303555	1430.823823	0.778321292
3.106722899	151.9115463	1762.898676	0.833072326
2.139908909	116.6827088	937.4310499	0.861248291
3.285830055	86.42238785	1784.364763	0.806294267
4.549135329	98.83626016	3125.415331	0.774012238
1.505364673	117.5342485	974.1730445	0.912853734
1.049450549	71.38003663	14.34065934	0.987637363
1.143377876	151.6407353	98.8322159	0.965775853
1.444690667	98.88708643	937.1716094	0.91191294
4.574698934	87.50972264	2243.060995	0.758922286
2.254185223	88.73984085	1603.625917	0.849393975
1.252288694	180.7342693	261.2875309	0.947221856
1.395205555	85.95681607	395.1705676	0.923004772
1.580773549	151.1197108	475.4604382	0.897459961
1.335285161	130.5964812	290.2023553	0.931582063
1.412868217	92.80433546	601.5604386	0.921406504
1.302943281	112.7710962	343.3505735	0.938853568
1.430740597	103.3604921	800.2719197	0.916642788
6.278871503	47.32287228	1487.766287	0.686695817
1.399070617	157.8753107	2013.064202	0.92285198
1.312434901	71.82789887	176.9130097	0.93584033
1.120604396	59.74584062	20.30732601	0.969848901
1.109972096	286.1111631	131.3193353	0.972506976
3.405689732	88.74443015	1742.671964	0.809867552
1.446952239	53.19618802	283.9441256	0.912452596
1.818714959	119.8005159	1957.399324	0.880941557
1.336455991	87.37643234	178.8397858	0.925709332
6.274260301	82.5417417	3216.855582	0.733695673
1.210562373	148.4976366	280.6229791	0.955688492
1.247259691	149.6769739	710.0492083	0.948475948
1.165181512	274.5871483	396.6782297	0.963794138
1.334456507	91.72288763	268.4585894	0.929044808
1.524583892	81.07032104	325.1421392	0.90676777
1.569327553	69.96217386	958.3082958	0.896679371
2.802076148	180.1846503	3332.186659	0.838596071
3.032927935	57.95658696	1785.455158	0.801878024
2.291093044	54.07734433	935.7041701	0.845948465
1.128682834	84.46316918	47.16431484	0.969609918
1.458845129	122.6623235	821.7573405	0.914701868
1.13353738	164.8666611	92.83684738	0.967470356
2.198255197	104.1898459	1950.903802	0.866181442
1.199906831	83.91819319	117.8270154	0.950023292
1.845421861	86.20207369	1075.648047	0.875732341
1.77631427	101.73485	1603.592793	0.88169867
1.398982931	89.68083732	407.3777166	0.916892854
3.889500934	104.6164379	2661.023205	0.781780219
5.267669144	47.9381858	3388.203544	0.751195842
6.163080224	94.4887096	4240.709824	0.7419492
1.256989409	149.3626674	362.8175682	0.947972841
1.406086763	71.83840394	338.5218136	0.9258959
1.317382926	58.68385263	174.2801949	0.93665189
1.474088456	90.26249564	634.5025974	0.908327841
2.570495592	154.6622184	1391.165104	0.848751631
1.151453545	148.1107489	129.8683993	0.964597509
1.372009135	151.2463013	643.7410341	0.926053719
2.820476208	172.7863678	2399.401798	0.835235502
1.118093975	65.0030015	27.67137805	0.970476506
3.105159937	81.16737031	2175.476355	0.811866955
1.878801143	63.35527782	2075.210386	0.872128308
1.348230192	69.82270979	273.8833591	0.92769313
1.213105682	276.438328	491.5090942	0.954698584
1.224877436	46.25080525	40.58510072	0.945638686
1.0625	40.19110577	12.29166667	0.984375
2.459482741	191.7367444	1830.162994	0.856848246
1.353252475	62.92949607	231.2492127	0.930729822
1.466112333	49.07756549	290.3860397	0.909427216
2.330487457	103.8687877	1863.909352	0.839782801
4.209666276	79.0697103	2663.564416	0.782237123
1.101950271	221.7613868	57.3126806	0.975936934
1.301439661	150.7195731	527.9228949	0.936525457
1.185475394	23.55084209	20.42914477	0.953631151
1.184211552	163.6412195	136.8742612	0.955087169
1.359918742	128.8059245	209.540878	0.926238937

log.sigma.4.5.mm.3D_glrlm_LongRunHighGrayLevelEmphasis	log.sigma.4.5.mm.3D_glrlm_RunPercentage	log.sigma.4.5.mm.3D_glrlm_LongRunLowGrayLevelEmphasis	log.sigma.4.5.mm.3D_glrlm_RunEntropy
183.157302	0.911570424	0.029847197	4.512841612
250.6140753	0.806349032	0.032169399	4.445505785
84.35261093	0.917861799	0.070245756	3.994325697
341.6158218	0.875124358	0.010628965	4.709378083
234.6169092	0.961157654	0.027299892	4.648926116
69.95184088	0.902269861	0.091676098	3.982694689
38.08326834	0.925274725	0.145230058	3.542325157
130.1709089	0.92748897	0.059400548	4.536141523
507.7952294	0.927767355	0.011823398	5.315537344
281.5726315	0.809732312	0.018105581	4.502231539
81.20461901	0.902209493	0.081947212	4.193475985
157.4216833	0.879585408	0.035539548	4.457314071
85.67872191	0.857585314	0.08392614	4.339846392
142.0207797	0.901369464	0.0332977	4.475106077
132.4225362	0.921174979	0.037739481	4.381078574
1626.272012	0.500838283	0.043524584	4.957689241
130.363193	0.945763914	0.055358825	4.341718862
137.8406798	0.852844964	0.039721152	4.422231569
204.7938408	0.952240068	0.058976088	4.623047407
245.5706526	0.753701958	0.045423266	4.490573616
459.8244101	0.681773399	0.033155908	4.407064211
146.2576177	0.873995092	0.03724701	4.502852447
441.6905352	0.738672287	0.021190085	4.453370013
243.8185894	0.955465587	0.043131894	4.50048179
129.2273956	0.809375	0.065060757	4.180049133
111.0284056	0.914161997	0.046787285	4.149054036
277.3278108	0.74745438	0.031149998	4.370350837
593.0041232	0.645146638	0.044972515	4.616067468
738.0881161	0.721288399	0.018182572	4.684866554
361.4563941	0.788677359	0.022483588	4.592047816
442.54302	0.695328951	0.033831069	4.5300747
689.9109724	0.630816257	0.03891693	4.735575524
192.960104	0.87711137	0.027839331	4.587417371
75.54212454	0.984615385	0.11592185	3.510294451
173.5587443	0.955752212	0.028822131	4.287087561
144.8774656	0.883173798	0.031306091	4.377819336
729.381047	0.620270628	0.037691707	4.695902133
258.1013167	0.774812259	0.035029099	4.638261599
229.5595486	0.93002636	0.024251402	4.615341092
123.1944726	0.896433176	0.057632967	4.399853472
276.7643044	0.861646046	0.016473029	4.438040365
158.7190767	0.908540279	0.025628805	4.460512897
126.6333562	0.89339059	0.030290815	4.26570788
135.3260152	0.91676888	0.047763327	4.550589673
146.9560396	0.88858227	0.037358153	4.532601836
619.5495551	0.541590229	0.079001996	4.545141501
220.9125469	0.8951762	0.017807217	4.673380341
83.86499959	0.914304993	0.08745242	4.217491442
65.76318265	0.968531469	0.13651959	3.471315619
319.5065884	0.965226554	0.019442436	4.702037588
478.6332972	0.691666667	0.035692248	4.609837081
69.45200821	0.882992938	0.079376973	4.067469996
274.4061764	0.829083905	0.021894077	4.522048922
121.5187709	0.90537496	0.061856911	4.266804181
890.890645	0.565363524	0.052770185	4.783137545
176.4453782	0.939839265	0.025370213	4.46021471
182.4765099	0.930310515	0.01589809	4.357655992
322.2985945	0.951293248	0.022869351	4.749885267
120.4182909	0.907648848	0.060765233	4.363482659
126.3765132	0.87702212	0.05027456	4.341969967
121.283084	0.860163891	0.042729914	4.245221674
701.3772066	0.740838707	0.015566114	4.909824343
241.965101	0.71190665	0.064698065	4.490827473
172.7951253	0.772943106	0.055343404	4.197089758
95.83427087	0.960812772	0.05408047	3.754252853
194.6115484	0.885149573	0.026817356	4.441216239
185.9490054	0.958974359	0.037761048	4.310955404
327.2716508	0.790397373	0.033699779	4.752288852
94.95307128	0.938138784	0.069827659	4.128473747
198.8193819	0.823016827	0.032430262	4.4155587
218.1121399	0.833101489	0.028103263	4.476197585
122.5709027	0.891602225	0.035869049	4.335195652
698.6250804	0.658516912	0.02725251	4.620884282
495.8079028	0.594133852	0.091704956	4.710086959
940.1823491	0.568856724	0.049456876	4.920112646
182.6726389	0.929113487	0.024156242	4.469245188
93.02801892	0.897604588	0.048545184	4.123418689
68.08764904	0.914835165	0.110106109	4.110099345
141.687984	0.878844048	0.029898997	4.24389423
562.5397248	0.754807692	0.015919004	4.465443063
167.3295435	0.954052659	0.025534498	4.326017059
210.6421299	0.901022677	0.01798851	4.541958745
671.3571477	0.737238011	0.015521937	4.694431134
73.3533236	0.962779156	0.068391991	3.579462697
374.7444436	0.706848975	0.034933406	4.480236551
124.8609636	0.818758088	0.048293473	4.264385395
88.42996147	0.904531085	0.074641764	4.254710261
330.1393353	0.938748857	0.009767346	4.643825339
55.82581767	0.932307692	0.061551653	3.355185165
40.89423077	0.980769231	0.140983631	3.242083234
667.2713875	0.765551742	0.012746937	4.742192465
76.55793348	0.907135628	0.168237221	4.264596244
76.20557873	0.880177515	0.096094797	4.071711324
343.0413496	0.766703176	0.024149856	4.601256904
545.4116446	0.643461219	0.044377434	4.775595359
243.8440034	0.968253968	0.025607742	4.152023807
194.1250488	0.915497738	0.02532697	4.619999874
26.55523172	0.945512821	0.298410327	3.046434556
198.1767447	0.943369514	0.031973492	4.512843147
187.7572745	0.903296703	0.028005869	4.372577621

log.sigma.4.5.mm.3D_glrlm_HighGrayLevelRunEmphasis	log.sigma.4.5.mm.3D_glrlm_RunLengthNonUniformityNormalized	log.sigma.4.5.mm.3D_glszm_GrayLevelVariance
140.9309882	0.83894352	19.4
114.0290996	0.708152452	13.22596908
71.86615423	0.848280367	11.40922254
229.7559849	0.793429205	25.85320039
216.8866566	0.922724139	44.96952909
58.68100495	0.827417629	9.084897959
32.91529829	0.855666647	6.4896
115.0616559	0.865970495	25.94984375
396.0315474	0.867790003	60.24348198
147.0474285	0.70335077	17.53643537
68.69419927	0.827386664	12.03729253
111.4604554	0.790324225	21.4775
62.66228488	0.762356439	18.17669558
116.4539693	0.826318886	16.11781741
112.8571655	0.853975644	18.11725207
185.1049796	0.424323508	14.68458962
117.6262691	0.894010332	19.58127347
93.09882922	0.756470906	15.10021001
185.4531011	0.907760029	36.66993164
84.18861121	0.652696256	10.06759983
107.210639	0.5914633	13.28621097
107.4321059	0.789954949	19.08948347
151.1301872	0.626946498	15.45927842
209.7406069	0.915304238	28.98061224
65.5574462	0.698549885	10.99247571
88.37318812	0.842183198	13.13171488
108.6362226	0.637123758	13.36458815
109.1930327	0.564884447	13.36789931
190.2095367	0.653519344	20.19076379
140.0055532	0.700443872	17.8357193
110.6118486	0.607452375	15.46915048
130.9504658	0.557604959	15.06792832
128.0420992	0.798263585	22.48148929
72.21245421	0.969387755	19.55555556
155.7097089	0.914217198	25.21396684
106.6278443	0.794708392	16.53656658
124.5042643	0.535959244	13.97406136
105.4407818	0.679680991	15.32797204
188.8066502	0.872609392	24.97222222
91.77653666	0.819012553	15.26205666
169.5207577	0.768149442	17.96969868
135.4296504	0.837052038	22.51655629
98.27269927	0.815532958	14.61863809
116.7683939	0.852533529	23.84443783
110.6526993	0.805787781	18.71508817
76.49038228	0.440565263	6.97875885
168.1741657	0.818050505	30.00931371
74.03306467	0.846860171	11.80547293
60.94930902	0.943119004	13.75147929
292.7902481	0.930700941	43.09452479
113.883279	0.614011062	15.22435821
55.99315329	0.796773552	9.278185255
138.0045079	0.736157355	18.40529671
93.37706452	0.824438585	14.95145073
119.5499198	0.499385539	10.709375
153.5144235	0.890577891	21.14737654
155.3708437	0.874287422	18.12262354
283.1334861	0.909689073	34.51267538
96.90044538	0.832838243	18.46264793
87.99051725	0.78890106	13.55320988
77.60186475	0.764650312	13.71455515
219.2059306	0.661046742	28.32500361
73.5280649	0.60389533	10.08737719
65.82625551	0.674544197	8.338040426
86.53226131	0.924469596	13.046875
133.6664138	0.802664001	18.28958678
168.9292838	0.919937102	24.06222222
123.502699	0.709711634	19.87027211
86.12516881	0.87708215	14.38248521
99.58800053	0.726444362	13.10621487
116.2915321	0.737578466	15.14498516
95.3569457	0.804921602	15.83244898
142.1226728	0.568996508	11.60227596
69.89941121	0.523800395	9.432492734
131.2656932	0.510760958	17.34237172
154.9461254	0.873875511	20.81787453
75.26404417	0.825282706	12.10321046
60.39635987	0.848558988	15.20926688
98.44921951	0.78858902	15.9459
185.9010085	0.679177426	17.04557116
151.8580408	0.912126537	22.83543253
161.1471377	0.824752611	22.41880711
211.5524432	0.656031232	21.57171427
66.67306592	0.925867755	11.90432099
102.9042055	0.616560941	11.9595198
71.10779194	0.719688137	11.10460068
73.1328652	0.828349732	16.35644612
285.8816293	0.888241469	29.68089991
48.14202475	0.868525717	7.523809524
40.33173077	0.961805556	9.1875
229.7783435	0.694456686	22.09219799
65.17368887	0.836106329	19.9456
53.46013375	0.790913974	11.25234375
127.4776301	0.662236333	14.79134831
105.9845613	0.570159171	15.29719338
225.7220696	0.938952078	31.1374096
158.3569431	0.847196922	23.514375
24.15172002	0.893542094	5.833333333
170.3726554	0.888964347	28.64653796
138.6075738	0.826093278	20.5116966

log.sigma.4.5.mm.3D_glszm_SmallAreaHighGrayLevelEmphasis	log.sigma.4.5.mm.3D_glszm_GrayLevelNonUniformityNormalized	log.sigma.4.5.mm.3D_glszm_SizeZoneNonUniformityNormalized
94.3045012	0.0696	0.3242
87.30182822	0.080998811	0.318335315
72.71539079	0.086656271	0.414568946
208.6896095	0.055999172	0.400388667
198.858488	0.044629117	0.440443213
60.56923383	0.103673469	0.328979592
32.09873457	0.1232	0.2896
130.2343503	0.060078125	0.425078125
252.8545068	0.037395266	0.3409356
123.9694138	0.0703003	0.32115366
71.04385163	0.08476197	0.324226487
103.1941125	0.06546875	0.374453125
85.19458589	0.072740933	0.340516358
110.9104122	0.073159579	0.344459686
107.5233883	0.068755739	0.32805326
137.9452402	0.081876817	0.367806228
108.3249951	0.067342126	0.385972574
109.0061222	0.078661432	0.41268718
176.4720874	0.048260382	0.426793184
62.15859933	0.094943576	0.336425781
82.11932828	0.083873246	0.328373859
91.71770434	0.066900826	0.305950413
114.8872993	0.076808305	0.349311916
131.0648373	0.055918367	0.394693878
59.19892818	0.08788078	0.329096355
71.85789942	0.080061983	0.28822314
90.93595007	0.079724041	0.346690471
74.68280079	0.0884375	0.309340278
151.3497092	0.06553213	0.390884989
95.05591077	0.067305638	0.337199719
97.36183317	0.073853878	0.354560268
120.3967323	0.078166828	0.371012418
123.1626625	0.060801428	0.37306586
60.70833333	0.083333333	0.625
121.4811458	0.059311224	0.333545918
113.3577388	0.073744437	0.355909824
92.53956816	0.078831264	0.366588751
96.29767531	0.07372374	0.337938853
143.9755068	0.060416667	0.291111111
70.00822459	0.077638408	0.340073529
111.481932	0.068912884	0.30001696
143.4797881	0.062234113	0.425200649
98.27959806	0.075875746	0.34268988
115.4377798	0.06097561	0.362209994
88.96647234	0.071563555	0.31434008
45.66985967	0.111481327	0.354805868
160.7219447	0.056778658	0.361132525
86.95771613	0.085663296	0.404223676
57.39957265	0.088757396	0.384615385
209.8291709	0.048811983	0.43982438
82.90741929	0.07390926	0.30861121
69.78862108	0.098979206	0.384347826
108.5172492	0.068833075	0.348589354
68.97428118	0.077851192	0.240448147
82.51681389	0.089852071	0.340939349
127.9317283	0.062789352	0.359664352
147.3575876	0.068746032	0.391526613
216.9429252	0.049864477	0.37890625
90.09250875	0.068971893	0.365754438
65.5073925	0.077530864	0.265432099
87.95719345	0.087669095	0.410835068
196.2978656	0.054077788	0.372730572
59.94254604	0.091893134	0.346509624
47.85696523	0.100114711	0.301056129
76.82508681	0.091796875	0.474609375
116.2521567	0.067557025	0.370419835
149.7603218	0.06	0.448888889
94.60051505	0.066655329	0.316394558
85.34944584	0.079289941	0.388402367
86.13848355	0.080782934	0.413813151
101.9435062	0.0740707	0.383043524
103.427573	0.07877551	0.372244898
110.1731792	0.088956589	0.386377304
48.65964607	0.09506436	0.363598616
117.5576031	0.070226184	0.350781722
145.9856402	0.06726609	0.401297999
74.2403462	0.083186683	0.342306778
72.26299992	0.079408627	0.34613626
98.38535851	0.07145	0.4185
149.1265581	0.070676286	0.353670769
139.4518235	0.062422145	0.468512111
143.0962198	0.060598381	0.40796947
161.4316678	0.061031733	0.347764878
46.47839506	0.086419753	0.401234568
92.4468254	0.082903285	0.372986386
69.27502229	0.087213524	0.338196668
80.99645929	0.076899811	0.356975425
237.1881817	0.057105142	0.392705464
34.56045795	0.111111111	0.328798186
41.6875	0.111111111	0.847222222
173.9454095	0.063445505	0.364411766
74.14742653	0.0684	0.362
41.30948603	0.085625	0.265
85.63068005	0.075505836	0.333712431
85.88827228	0.075240816	0.365978362
191.5099715	0.067718606	0.47008547
154.8323644	0.05935	0.37115
20.77141204	0.138888889	0.319444444
128.8157999	0.054434606	0.393235593
92.96546887	0.063563327	0.34168242

log.sigma.4.5.mm.3D_glszm_SizeZoneNonUniformity	log.sigma.4.5.mm.3D_glszm_GrayLevelNonUniformity	log.sigma.4.5.mm.3D_glszm_LargeAreaEmphasis	log.sigma.4.5.mm.3D_glszm_ZoneVariance
32.42	6.96	41.89	28.8579
46.15862069	11.74482759	807.937931	714.3156243
27.7761194	5.805970149	22.19402985	15.21496993
184.5791757	25.81561822	200.527115	182.9453748
25.10526316	2.543859649	4.824561404	1.6848261
23.02857143	7.257142857	41.08571429	28.9355102
7.24	3.08	15.68	7.84
68.0125	9.6125	20.50625	14.22496094
186.4917733	20.45521024	22.31444241	15.03332453
86.39033457	18.91078067	1215.163569	1140.845068
39.23140496	10.25619835	43.17355372	33.51738269
59.9125	10.475	138.54375	116.1590234
82.40495868	17.60330579	413.1487603	381.286934
55.80246914	11.85185185	52.27160494	41.64883402
43.3030303	9.075757576	17.33333333	9.729109275
156.3176471	34.79764706	57131.70353	56482.59303
41.29906542	7.205607477	7.242990654	3.130055027
122.1554054	23.28378378	396.472973	368.9813733
42.25252525	4.777777778	5.535353535	2.155698398
64.59375	18.22916667	3049.895833	2841.153537
98.84053156	25.24584718	9267.325581	8983.050297
67.30909091	14.71818182	123.7954545	106.7235331
87.67729084	19.27888446	3129.378486	2994.040952
27.62857143	3.914285714	5.871428571	2.261428571
38.5042735	10.28205128	617.2820513	549.9579224
25.36363636	7.045454545	34.5	22.72262397
73.84507042	16.98122066	2500.530516	2385.043708
74.24166667	21.225	7972.879167	7709.493316
173.9438202	29.16179775	2731.761798	2662.029915
61.03314917	12.18232044	931.0552486	843.565581
92.54022989	19.27586207	7191.988506	6932.914197
243.3841463	51.27743902	15200.22409	15018.9195
105.9507042	17.26760563	205.4401408	181.5197753
7.5	1	1.75	0.1875
18.67857143	3.321428571	6.267857143	2.196109694
101.7902098	21.09090909	212.6923077	190.9362805
135.6378378	29.16756757	16883.43514	16556.4119
98.34020619	21.45360825	2057.642612	1949.081966
34.93333333	7.25	13.39166667	6.236041667
46.25	10.55882353	62.97794118	47.38705666
39.90225564	9.165413534	164.0827068	135.1819775
64.20529801	9.397350993	31.1986755	24.8322442
78.47598253	17.37554585	100.628821	87.68139433
59.40243902	10	29.5304878	22.36507287
87.07220217	19.82310469	140.8555957	124.652791
52.15646259	16.3877551	47439.14286	45704.73201
241.2365269	37.92814371	221.8772455	204.9787461
33.14634146	7.024390244	28.17073171	20.43961927
5	1.153846154	3.384615385	0.520710059
38.70454545	4.295454545	3.931818182	1.179235537
98.13836478	23.50314465	5225.918239	5061.304497
44.2	11.3826087	87.4173913	75.13693762
155.470852	30.69955157	1028.643498	977.3252277
14.18644068	4.593220339	41.81355932	25.4041942
177.2884615	46.72307692	43191.30385	42718.5759
51.79166667	9.041666667	12.42361111	7.011525849
135.4682081	23.78612717	22.26011561	15.90855024
84.875	11.16964286	7.464285714	3.283721301
38.03846154	7.173076923	42.78846154	31.2022929
23.88888889	6.977777778	103.3555556	76.54617284
101.8870968	21.74193548	476.1814516	441.7605196
320.9210221	46.56097561	3333.965157	3272.029536
102.220339	27.10847458	5633.925424	5443.204642
47.86792453	15.91823899	1630.012579	1504.403465
15.1875	2.9375	3.84375	1.100585938
101.8654545	18.57818182	118.3418182	100.7933355
26.93333333	3.6	4.783333333	1.720833333
132.8857143	27.9952381	1317.792857	1249.651763
25.24615385	5.153846154	10.29230769	5.452307692
107.1776062	20.92277992	660.3011583	612.4297193
150.1530612	29.03571429	831.9413265	787.9830735
52.11428571	11.02857143	102.9642857	86.56178571
150.3007712	34.60411311	14097.8329	13768.72294
154.5294118	40.40235294	36992.01412	36342.18405
317.4574586	63.55469613	37643.41105	37386.03019
69.02325581	11.56976744	24.1627907	17.43902109
49.63448276	12.06206897	49.07586207	39.18592152
28.03703704	6.432098765	27.38271605	19.73510136
83.7	14.29	177.265	156.425775
113.8819876	22.75776398	1623.15528	1552.637012
39.82352941	5.305882353	5.023529412	1.950726644
105.6640927	15.69498069	79.57915058	68.42509802
187.445269	32.8961039	3635.411874	3551.786108
7.222222222	1.555555556	4.055555556	1.089506173
142.8537859	31.75195822	6114.851175	5947.003252
153.2030905	39.50772627	1673.887417	1613.95316
41.05217391	8.843478261	45.05217391	34.97844991
103.6742424	15.07575758	18.125	13.14736857
6.904761905	2.333333333	10.28571429	4.616780045
10.16666667	1.333333333	1.25	0.076388889
174.5532359	30.39039666	1357.505219	1306.258751
36.2	6.84	36.62	27.3784
21.2	6.85	111.825	84.785
103.7845659	23.48231511	2819.62701	2681.734473
140.1697128	28.81723238	16345.5248	15989.9611
18.33333333	2.641025641	3.41025641	0.800788955
74.23	11.87	50.38	38.82
3.833333333	1.666666667	5.166666667	1.166666667
32.63855422	4.518072289	6.012048193	2.155320075
31.43478261	5.847826087	28.26086957	18.99810964

log.sigma.4.5.mm.3D_glszm_ZonePercentage	log.sigma.4.5.mm.3D_glszm_LargeAreaLowGrayLevelEmphasis	log.sigma.4.5.mm.3D_glszm_LargeAreaHighGrayLevelEmphasis	log.sigma.4.5.mm.3D_glszm_HighGrayLevelZoneEmphasis
0.27700831	0.767564864	5390.43	158.64
0.103349964	7.795320162	110665.6207	131.5103448
0.378531073	1.195659528	933.5970149	98.14925373
0.238489395	1.181862589	41657.60304	294.0737527
0.564356436	0.100765436	761.7192982	259.0526316
0.286885246	3.062258538	1377.514286	89.57142857
0.357142857	1.945053014	281.76	49
0.399002494	0.986409492	1155.44375	167.2625
0.370596206	0.174627885	11721.69653	393.3893967
0.115998275	9.650137209	170332.9368	193.2713755
0.321808511	2.53003701	1236.92562	104.5785124
0.211360634	2.168806569	13396.3875	145.8
0.17715959	16.50798797	13137.30579	120.5041322
0.306818182	1.389290771	3020.475309	165.8024691
0.362637363	0.589829212	1254.219697	155.1136364
0.039250092	282.9904907	11682584.18	208.32
0.493087558	0.478771417	550.7943925	147.6728972
0.190721649	8.215853655	26564.14865	148.1283784
0.543956044	0.361347821	795.4747475	223.1717172
0.069214131	43.35803503	305599.9323	95.82291667
0.059310345	76.83364219	1228951.043	123.1528239
0.242024202	2.616383216	7674.563636	153.1227273
0.085958904	19.91073031	535079.9084	168.3904382
0.526315789	0.197633052	1468.028571	196.1285714
0.121875	12.55477697	42274.03419	84.92307692
0.291390728	0.933700769	2490.852273	111.7727273
0.093053735	23.45444219	288094.6761	140.5352113
0.061617458	54.65796381	1261522.817	114.4541667
0.119752422	11.3441638	717380.5124	211.0651685
0.106910809	6.203624916	173409.3425	135.6961326
0.062128065	59.83930656	977102.9617	144.9578544
0.074266953	103.4384391	2350532.191	179.1890244
0.204463643	2.775323719	23200.61268	171.5105634
0.8	0.1637587	123.8333333	73.33333333
0.495575221	0.126720821	801.6964286	181.0178571
0.214392804	3.576054537	15399.05594	158.7797203
0.055298162	107.4277757	2833174.689	133.1378378
0.095976253	26.91188485	228665.8179	148.3298969
0.373831776	0.2654286	2057.316667	225.6666667
0.253258845	2.358494724	4781.514706	104.5367647
0.186013986	1.140597475	30639.42857	187.3609023
0.396325459	0.66028335	1941.907285	191.5165563
0.277912621	2.019797014	6097.50655	141.7467249
0.37357631	1.097464536	1443.945122	164.3536585
0.248430493	2.808509171	9187.115523	144.1227437
0.024011761	501.8592432	4776673.694	67.75510204
0.243262928	2.06798234	29822.33832	235.4251497
0.359649123	2.047395492	883.2195122	116.2439024
0.590909091	0.406241242	179.4615385	72.92307692
0.602739726	0.060002027	983.6818182	308.6363636
0.077941176	41.06334922	756849.283	133.0628931
0.285359801	4.623792462	2210.226087	94.07826087
0.139593114	7.766349129	172065.5067	160.367713
0.246861925	1.457860113	3476.372881	119.4067797
0.045993278	304.8073377	6370375.856	128.975
0.429850746	0.286451768	1276.034722	186.5555556
0.396788991	0.30087608	2302.988439	200.7630058
0.489082969	0.159757113	1716.120536	318.375
0.293785311	1.397148917	2704.115385	131.5576923
0.193133047	3.083204229	6894.711111	117.3666667
0.170446735	10.42500824	27251.06855	114.8467742
0.127066116	13.72084208	877258.7677	279.9581882
0.072410407	82.58549458	471711.4034	89.93220339
0.089225589	30.76214959	119083.2264	74.98113208
0.603773585	0.153852141	298.125	96.3125
0.238715278	1.370648065	16729.73091	163.1636364
0.571428571	0.158912631	644.6833333	194.8
0.121142198	11.54522088	223734.9548	149.5047619
0.454545455	0.791869802	472.5384615	115.3076923
0.14453125	8.435348114	69280.98842	119.2200772
0.150827241	8.727216519	100033.1224	144.3035714
0.24691358	2.174405356	6766.057143	146.7714286
0.055122573	82.91426963	2652845.139	161.4601542
0.039228355	434.5412936	3605601.854	73.09411765
0.062332117	250.7914179	5877289.986	177.078453
0.385650224	0.43364849	2157.319767	199.9418605
0.317982456	1.921670022	1787.544828	106.662069
0.361607143	3.162611446	597.3703704	95.7654321
0.21905805	2.415747468	16125	132.37
0.11908284	8.314867388	352631.8012	218.5621118
0.570469799	0.097182144	566.3529412	177.7647059
0.299421965	0.797103846	10081.57143	203.7837838
0.10935281	15.14457247	920606.564	238.9016698
0.580645161	0.176437083	242.6111111	69.83333333
0.077186618	55.39178609	751057.6736	134.3420366
0.129170231	37.91293444	87734.88962	108.2119205
0.315068493	2.378174663	1572.608696	112.226087
0.448217317	0.126270313	3374.44697	342.8636364
0.42	0.436537378	364.8571429	56.52380952
0.923076923	0.163220724	44.5	42.25
0.139690872	4.940382415	409053.8413	257.5448852
0.328947368	4.893535739	956.13	103.12
0.192307692	5.261468248	5074.9375	72.2875
0.085158817	23.32429821	408639.0193	133.0482315
0.053032401	141.606087	2170109.115	128.3890339
0.619047619	0.05564273	672.8205128	250.025641
0.294117647	0.975144924	5210.89	220.215
0.5	1.703200874	88.5	30.83333333
0.509202454	0.138120132	985.626506	182.0481928
0.328571429	0.34137464	3952.163043	148.0543478

log.sigma.4.5.mm.3D_glszm_SmallAreaEmphasis	log.sigma.4.5.mm.3D_glszm_LowGrayLevelZoneEmphasis	log.sigma.4.5.mm.3D_glszm_ZoneEntropy	log.sigma.4.5.mm.3D_glszm_SmallAreaLowGrayLevelEmphasis
0.583642553	0.024382356	5.441929057	0.009228346
0.585006956	0.023582408	5.474905423	0.006335462
0.666488666	0.035528418	4.825886445	0.01490462
0.657015523	0.008032713	6.060168798	0.00539036
0.68797987	0.031775981	5.280086944	0.027632358
0.583304426	0.047503886	4.771632125	0.024693241
0.542229938	0.076036317	4.13366069	0.016323817
0.677247978	0.034258475	5.570715108	0.01679646
0.604644857	0.009272439	6.733211604	0.005704162
0.585511663	0.013880206	5.881883629	0.008175982
0.581095643	0.037263999	5.202111663	0.009054207
0.633003703	0.029738468	5.632753428	0.015123315
0.604984498	0.0444245	5.771214705	0.01924352
0.6066082	0.018043539	5.507775483	0.010892611
0.589781612	0.021324704	5.541215025	0.012667841
0.629695648	0.010555627	5.684804231	0.004302788
0.64321824	0.03466348	5.264015562	0.018525674
0.666636826	0.016218525	5.489045578	0.010287243
0.677645662	0.02655134	5.501591565	0.009293448
0.596376822	0.026702192	5.33580979	0.014415994
0.592989588	0.020554701	5.729321524	0.011906447
0.569023063	0.021135219	5.944003165	0.013492241
0.612529616	0.015197475	5.600906651	0.005827809
0.64843254	0.02833594	5.206008867	0.010699024
0.593883937	0.043187078	5.203257242	0.012161357
0.545899792	0.030596982	5.141099022	0.00788523
0.60964032	0.018636229	5.535367163	0.008006591
0.574753918	0.029845468	5.712979606	0.013028997
0.649897309	0.011459903	5.911464328	0.007106074
0.602620371	0.022211013	5.738854642	0.012898976
0.617971442	0.019193599	5.680530412	0.008146604
0.63113135	0.011867602	5.794853269	0.005379735
0.634099542	0.019102137	5.875707972	0.010959638
0.8125	0.12914277	3.584962501	0.120488788
0.580671847	0.033316062	5.124057377	0.026566559
0.619575611	0.02162009	5.672154943	0.007907408
0.628677734	0.018636295	5.754095568	0.011469549
0.600845884	0.017720715	5.760039485	0.007450013
0.553025522	0.01950791	5.670892471	0.011517194
0.604044783	0.03881309	5.441569799	0.01704106
0.561568334	0.018373808	5.636462441	0.011989914
0.676407478	0.017026517	5.399736386	0.012192923
0.60177484	0.020018123	5.531173316	0.010909401
0.621354175	0.028148872	5.672751932	0.009623444
0.579203077	0.024681617	5.922680607	0.009454446
0.617386866	0.035541883	5.062078686	0.022095843
0.62326105	0.010887384	6.266286488	0.004718227
0.65827927	0.028800239	4.890714505	0.007536278
0.574786325	0.10241516	3.700439718	0.029258113
0.684379638	0.022044249	5.438132721	0.019333087
0.571466843	0.022167412	5.884092241	0.010601173
0.64264965	0.028927049	4.89562168	0.009115501
0.610092562	0.019007658	5.974442147	0.008272125
0.489837944	0.035660373	5.132394532	0.006917415
0.604398524	0.016034825	5.700888132	0.007565867
0.619289682	0.018380204	5.561932226	0.011765694
0.649224608	0.011572047	5.709348057	0.007710699
0.635112671	0.014293599	5.937199154	0.006859697
0.628010157	0.038770589	5.339238877	0.013720731
0.522101784	0.028398679	5.326652858	0.00910032
0.66392881	0.031466176	5.398145623	0.013896547
0.63317988	0.007948089	6.260381647	0.003686169
0.608502827	0.032438028	5.493039618	0.011745737
0.565622977	0.03907099	5.353188462	0.018096906
0.710069444	0.055790465	4.140319531	0.042631482
0.631642504	0.016824324	5.766722361	0.007115157
0.686108655	0.027248494	4.969146221	0.009988104
0.581339814	0.021202279	6.115524598	0.007480479
0.644237419	0.03448904	4.875762813	0.011780607
0.668673952	0.022992421	5.324502876	0.013738592
0.642960764	0.020454093	5.66990775	0.008455339
0.631829733	0.021275484	5.341718663	0.013384584
0.645611985	0.012568224	5.523676487	0.008317225
0.624962289	0.053862164	5.485436834	0.026220092
0.61313163	0.014659821	6.071613576	0.006398679
0.65841584	0.017984581	5.465530029	0.007355156
0.602682188	0.027556796	5.371371113	0.011911608
0.59986583	0.046442449	5.04761501	0.021786039
0.671903081	0.022117172	5.360277847	0.009670613
0.615881212	0.010990024	5.834715864	0.004520708
0.711414116	0.028571575	4.963580901	0.024824842
0.663510761	0.016021535	5.712614134	0.011377018
0.611899766	0.009222422	6.140866961	0.004008108
0.639567901	0.090900949	4.169925001	0.082110091
0.634132693	0.017296658	5.584188492	0.009928745
0.602466893	0.024844958	5.749872411	0.013583837
0.614745981	0.039599087	5.216853466	0.01796572
0.643790955	0.010350243	5.812931892	0.008449668
0.588451241	0.076522246	4.106603137	0.066023597
0.9375	0.135442946	3.251629167	0.128498502
0.627167368	0.009292239	6.082328479	0.006403201
0.618396305	0.056359987	5.328856631	0.017684064
0.519443191	0.056429506	5.320191026	0.017093442
0.596652486	0.020055904	5.768095671	0.012752941
0.626678765	0.021122277	5.673000492	0.009249738
0.69747151	0.033546439	4.695157347	0.031060599
0.633076045	0.014853018	5.740626648	0.00587508
0.519675926	0.142822788	3.084962501	0.024899852
0.646609772	0.026695529	5.361316956	0.012263634
0.60405438	0.037848636	5.481024967	0.029980693

log.sigma.4.5.mm.3D_ngtdm_Coarseness	log.sigma.4.5.mm.3D_ngtdm_Complexity	log.sigma.4.5.mm.3D_ngtdm_Strength	log.sigma.4.5.mm.3D_ngtdm_Busyness	log.sigma.4.5.mm.3D_ngtdm_Contrast
0.031183884	0.676560443	4.270611853	0.172074625	0.000396635
0.008981014	0.089901606	0.970068937	0.553805054	4.69E-05
0.049888934	0.800621943	3.925617888	0.222549304	0.000811056
0.005948836	0.256502819	1.559927681	0.380971601	3.89E-05
0.087029505	7.582412712	21.68271343	0.042256261	0.002922278
0.029645848	0.575004163	2.172938713	0.434556221	0.000653507
0.10550554	1.050749106	4.969565196	0.233288421	0.00193681
0.021714726	1.289669256	4.611860365	0.272491236	0.000481979
0.00895397	0.864998505	4.921169358	0.233133458	0.000155006
0.005840003	0.090450752	1.121406578	0.527096022	1.50E-05
0.02039616	0.60962305	2.224855787	0.477487645	0.000400859
0.015440962	0.354613706	2.639864007	0.308506861	0.000107709
0.007922316	0.180918925	1.517699678	0.927870534	5.37E-05
0.019152703	0.598157449	3.024281759	0.320624197	0.000246814
0.026349054	0.896287716	4.026253189	0.207232571	0.000363041
0.001291396	0.011386718	0.507166485	1.446181256	7.42E-07
0.033030343	1.699343965	4.862212343	0.180447066	0.001039369
0.006594444	0.204684476	1.436424526	0.675360539	3.96E-05
0.04337121	3.390882966	8.47328428	0.102110474	0.001860173
0.005400504	0.032093913	0.525380569	1.123697761	1.74E-05
0.002831268	0.024968555	0.553256899	1.123219579	3.42E-06
0.012092759	0.30137942	1.952228717	0.471574326	0.000113331
0.004729072	0.049764896	0.903361581	0.618664991	9.02E-06
0.071562072	3.112762349	13.16096112	0.057277071	0.002124552
0.012341569	0.091765541	1.095824836	0.575901734	4.95E-05
0.028646065	0.577687151	2.438659642	0.276298887	0.000457113
0.005668733	0.064244508	1.04958896	0.602771044	9.49E-06
0.004090702	0.033927862	0.964431109	0.751132236	6.19E-06
0.003514998	0.078297523	1.078484058	0.554321448	9.66E-06
0.009273211	0.093207882	1.409933692	0.378839708	3.10E-05
0.003589377	0.03112176	0.637161865	0.917694451	5.59E-06
0.001546138	0.020866983	0.443921364	1.568913129	1.95E-06
0.008244368	0.273241618	1.810283068	0.449770577	5.75E-05
0.282028031	11.95664179	23.31932844	0.053399802	0.048228393
0.07447482	3.172844868	10.07240504	0.067712038	0.001987794
0.007261539	0.201846283	1.004109506	0.730238741	6.87E-05
0.002072676	0.020803961	0.484787238	1.270873402	3.16E-06
0.004811346	0.061830398	0.856981697	0.826567921	1.41E-05
0.029136591	1.571771384	6.947383792	0.110153123	0.000512943
0.01812915	0.399871251	2.164568851	0.45038323	0.000274794
0.016532574	0.32323234	2.971629083	0.196128725	9.06E-05
0.023247034	1.24541408	4.881159025	0.217976108	0.000404515
0.01024716	0.344780195	1.316446402	0.572779571	0.00012829
0.022198837	1.140253375	5.323067565	0.248627242	0.000342325
0.009784252	0.284079555	1.817666663	0.539617725	9.11E-05
0.002386808	0.006045137	0.250975694	2.296857046	3.13E-06
0.004327822	0.400287022	3.80042404	0.402976403	1.62E-05
0.034690236	1.020025158	3.596606111	0.322242331	0.000848225
0.186789205	7.224198972	10.779026	0.095137177	0.022077114
0.045812423	7.060632497	12.82141215	0.060026153	0.002688036
0.003554339	0.034126826	0.61733416	0.924848783	6.99E-06
0.01805935	0.446322404	1.575980943	0.682175049	0.000345293
0.003682007	0.091382437	0.802709297	0.815465232	1.58E-05
0.037999387	0.755880058	4.044719242	0.184380134	0.000585545
0.001217838	0.008909573	0.245385291	2.444790118	1.56E-06
0.02519922	1.302055648	3.96609027	0.189449667	0.000579544
0.009609697	0.605245688	2.133277711	0.361722386	0.000125041
0.019933707	1.680514242	5.42435772	0.117230078	0.000451986
0.030938718	0.616368214	3.980885855	0.224282067	0.000338184
0.024307106	0.311187482	2.591704246	0.328501521	0.000210729
0.006858673	0.149228657	1.011679353	0.889141064	4.59E-05
0.002086264	0.07341369	1.041023376	0.696449282	4.10E-06
0.003354995	0.029327175	0.532791985	1.410216012	6.36E-06
0.006521643	0.036724735	0.414253646	1.358760612	3.28E-05
0.10681452	3.717006478	8.447499658	0.081023387	0.005042869
0.009559878	0.29162953	1.914632845	0.354660253	6.15E-05
0.059114123	4.481740067	8.473286406	0.087654228	0.003135191
0.003996824	0.07869425	0.816425878	0.823445417	1.84E-05
0.04416081	1.895227934	4.869095422	0.195517768	0.001717594
0.006535731	0.088303597	0.777830572	0.805696843	3.52E-05
0.004578678	0.079429439	0.657820958	0.919538476	2.31E-05
0.01585793	0.525460043	2.342552844	0.366296992	0.000189606
0.002032367	0.020858852	0.443695058	1.279962583	3.26E-06
0.001450426	0.004797219	0.133305497	4.129678077	3.08E-06
0.00097045	0.013378857	0.374410158	2.18469524	8.93E-07
0.018019572	1.231946166	3.642822905	0.217402723	0.00034425
0.018258992	0.470816006	1.992678802	0.468907181	0.00025572
0.033232733	1.118746587	4.413781047	0.375506545	0.000790519
0.010110973	0.227764047	1.102330707	0.554969251	9.32E-05
0.004753032	0.096312648	1.18439091	0.453331322	1.06E-05
0.045494586	3.189861149	6.477853566	0.110541209	0.001601469
0.01220054	0.468824093	2.447046213	0.279246686	0.000108266
0.002710148	0.070027598	1.013144718	0.605384352	4.89E-06
0.16543936	4.388877205	9.622230898	0.073887948	0.008499625
0.002606027	0.024276089	0.351594535	1.516613678	6.05E-06
0.003219607	0.046284157	0.420559748	1.857403855	1.26E-05
0.023719144	0.673228091	3.129798173	0.395385763	0.000389896
0.012536557	1.776430165	4.183456763	0.15670268	0.000257107
0.110316048	1.236594424	3.445203409	0.227531733	0.00388611
0.291370938	7.783925049	11.48338267	0.103279321	0.040572965
0.003840287	0.113979451	1.333536385	0.430305083	1.08E-05
0.028079543	0.881721749	4.242443644	0.514171114	0.000640552
0.025638709	0.241344714	1.708911047	0.474928755	0.000237918
0.003927183	0.039431377	0.541744633	1.043847954	1.26E-05
0.002115124	0.015909009	0.33633101	1.718842817	3.93E-06
0.083445251	8.67891495	14.86237685	0.052429587	0.007187197
0.015533977	0.628336394	3.010909966	0.248107025	0.000194709
0.178837364	2.074961324	4.546672259	0.372778177	0.01797133
0.05585015	2.694464552	10.65902404	0.081564024	0.001275308
0.034864139	0.834833077	4.648306794	0.136107767	0.000441384

log.sigma.4.0.mm.3D_gldm_GrayLevelVariance	log.sigma.4.0.mm.3D_gldm_HighGrayLevelEmphasis	log.sigma.4.0.mm.3D_gldm_GrayLevelNonUniformityNormalized	log.sigma.4.0.mm.3D_gldm_DependenceEntropy
16.23848804	148.0110803	0.070264961	6.312556214
9.379195076	125.1831789	0.099201436	6.795559244
11.16767212	63.05649718	0.094449232	5.433675979
17.14274818	206.7925504	0.071722679	6.989419241
36.52798745	183.6336634	0.051465543	5.501835369
7.521365224	52.91803279	0.105112873	5.637060457
5.48244898	34.02857143	0.130204082	4.614807753
18.25595612	114.2568579	0.070117723	6.191781199
71.62713396	383.6185637	0.038124904	7.079490994
8.676710624	155.9133247	0.099689629	6.952688341
9.216323846	74.28457447	0.100780896	5.843662382
12.74217039	117.2840159	0.078982775	6.630518755
9.818544489	63.81844802	0.101701219	6.504907928
14.63825399	80.62310606	0.078763487	6.329809387
14.89236656	88.97527473	0.075670209	5.882046062
4.230264255	168.563816	0.246262597	6.810650695
18.21814012	100.8018433	0.069336788	5.449362119
9.77049899	80.19201031	0.092213904	6.718164299
29.94831542	131.7142857	0.052107233	5.831168528
7.895028587	93.54758472	0.107593219	7.032768842
5.408781577	103.1166502	0.146351603	6.800384607
13.66911741	86.00330033	0.084398649	6.526064786
6.93925502	142.0719178	0.123482361	6.917231572
35.76561705	264.6165414	0.05217932	5.74123615
6.065815972	70.53333333	0.118194444	6.618260388
10.23250077	75.7781457	0.088526819	5.988597213
5.670220999	94.60113587	0.142787671	6.890544012
7.289692424	152.6051348	0.177271938	6.813604939
11.23658175	188.4359526	0.142787683	6.913462266
11.44777094	138.6190195	0.096162964	6.808486691
6.688876913	125.2763628	0.136083547	6.880938503
5.916383363	142.0706442	0.172194536	7.002906383
14.88971612	115.1202304	0.073009624	6.724648317
22	86	0.12	3.189898095
23.92278174	182.5044248	0.061633644	5.286918954
11.36506409	118.5667166	0.083660194	6.495647725
6.899564594	143.7731281	0.176809711	6.798865163
9.478084143	93.5280343	0.096826463	7.097237921
24.14017721	137.9844237	0.061887986	6.089447825
14.52745614	99.01675978	0.073693774	6.318816091
12.15937014	178.5874126	0.085119077	6.693201345
15.00507712	115.2677165	0.085153726	6.080220426
10.17760274	89.05097087	0.089157791	6.298731043
18.0628162	98.04100228	0.073437768	6.237748456
16.61339339	115.7542601	0.075234169	6.575900182
3.801156006	90.09196341	0.231912615	6.609043606
21.55904503	166.9257101	0.068772747	6.807536277
11.37140274	52.32017544	0.094028932	5.827888701
15.03512397	84.22727273	0.107438017	3.6412498
40.66766748	255.109589	0.048132858	5.574156261
7.38496678	129.1031863	0.140755839	6.96224874
8.096952755	54.18858561	0.109913859	5.795732312
11.43855584	149.484507	0.086854509	6.923024684
12.79774514	77.40167364	0.077362091	5.921703941
5.476428844	133.3828056	0.212236785	6.88057012
18.51583872	117.3253731	0.06511027	6.165092352
16.81129403	156.5768349	0.071292926	6.21335938
31.42236037	226.4454148	0.051114586	6.205186849
14.19876153	104.8248588	0.076494622	6.206140898
10.86640019	76.73390558	0.084354105	6.424050951
9.572237456	87.42405498	0.09411084	6.471497301
12.88220449	207.6033058	0.104105887	7.210385171
5.687589185	95.13721159	0.136801403	7.085501244
6.200205069	90.34118967	0.119480753	6.819743333
13.74866501	87.77358491	0.102883588	4.425664794
13.59230023	137.6284722	0.07810993	6.65081631
21.22412698	140.7619048	0.067029478	5.308865637
14.46512068	116.581771	0.086258846	7.120475951
12.25106362	63.93006993	0.086507898	5.49117527
9.632408921	108.6082589	0.090110935	6.879616167
10.49166393	147.8114659	0.087899407	6.945566128
10.49220347	84.5978836	0.090525648	6.317902476
7.135697701	138.4535922	0.145879976	6.930199297
5.776667955	83.68165036	0.172865942	6.89578437
6.0769503	166.3132447	0.197705095	7.035562736
17.61574232	137.9753363	0.070482013	6.262264709
9.301241728	82.18640351	0.096241151	6.062980248
9.224948182	37.02232143	0.107621173	5.574633107
10.45381486	134.7042716	0.089152788	6.518706539
8.665237206	180.029216	0.111570411	6.861390806
17.70109455	121.2214765	0.068960858	5.272855061
17.4057055	194.8034682	0.06976912	6.659752135
10.18994675	256.4996957	0.11818276	7.187238314
14.37044745	97.70967742	0.090530697	4.018081794
6.209815354	93.68621524	0.134019353	7.028931208
6.837791117	88.88822355	0.111124608	6.880431593
10.99535372	77.33972603	0.092272471	5.908203224
23.25796363	204.8896435	0.05956111	6.21004018
4.8084	34.62	0.1408	4.50597913
9.289940828	49.07692308	0.147928994	3.085055103
12.9607944	192.8366871	0.100044472	7.112983858
15.8742642	69.68421053	0.085028566	6.103470922
8.825813609	75.625	0.09522929	6.26078809
9.17691097	119.7606791	0.095856492	7.14807333
7.054830252	119.05054	0.148950662	6.992674118
26.96800202	195.5555556	0.073318216	4.913477542
19.45792388	142.2764706	0.065004325	6.462868381
9.901041667	41.54166667	0.149305556	3.522055209
28.02243216	216.7055215	0.059279612	5.579201106
13.25158163	140.1357143	0.083035714	6.161805601

log.sigma.4.0.mm.3D_gldm_DependenceNonUniformity	log.sigma.4.0.mm.3D_gldm_GrayLevelNonUniformity	log.sigma.4.0.mm.3D_gldm_SmallDependenceEmphasis
55.86426593	25.36565097	0.228102494
130.233072	139.1796151	0.12473516
36.06214689	16.71751412	0.256163196
239.3952406	138.6399379	0.226912625
32.92079208	5.198019802	0.545706821
33.81147541	25.64754098	0.248132982
17.48571429	9.114285714	0.319793651
75.33915212	28.11720698	0.300709657
301.3360434	56.27235772	0.327423377
206.2600259	231.1802501	0.120521901
58.55851064	37.89361702	0.266898516
100.014531	59.78996037	0.196526464
151.0395315	138.9238653	0.160457395
73.59469697	41.58712121	0.255044563
74.31868132	27.54395604	0.323784535
412.4977835	2666.5314	0.044735857
61.70967742	15.04608295	0.413156682
169.5412371	143.1159794	0.170515775
46.42857143	9.483516484	0.427776158
190.7887527	298.4635905	0.077406095
289.2175369	742.7343842	0.069472988
127.1452145	76.71837184	0.218187991
191.339726	360.5684932	0.090070324
37.06015038	6.939849624	0.480931495
85.86875	113.4666667	0.12967414
48.36423841	26.73509934	0.23974656
150.08519	326.8409786	0.096976348
191.9961489	690.4741977	0.071042108
240.9133477	530.5990312	0.119414466
149.8541051	162.8038984	0.108640798
256.7267317	571.6869793	0.076362369
398.7070078	1520.994339	0.080905285
187.6177106	101.4103672	0.191778279
5.4	1.8	0.522222222
33.44247788	6.96460177	0.4748353
178.8470765	111.6026987	0.206076677
315.9053953	1183.033777	0.069090223
228.4228232	293.5778364	0.109221016
69.87850467	19.86604361	0.289956167
86.0018622	39.5735568	0.21571206
83.77762238	60.86013986	0.167109024
62.95800525	32.44356955	0.298817489
124.1674757	73.46601942	0.25509318
71.77904328	32.23917995	0.297971022
173.993722	83.88609865	0.220530443
250.8703038	1419.76903	0.032932339
423.1427531	188.8499636	0.245057969
34.96491228	21.43859649	0.312070039
7.363636364	2.363636364	0.516414141
49.45205479	7.02739726	0.544425419
230.8813725	574.2838235	0.087703319
54.89578164	44.29528536	0.257995004
325.3974961	277.5001565	0.149026926
39.62761506	18.48953975	0.195396736
459.860605	2399.549089	0.055502509
69.16716418	21.8119403	0.356248242
185.1077982	62.16743119	0.368938356
121.7598253	23.41048035	0.418884606
57.27118644	27.07909605	0.255698701
59.01716738	39.30901288	0.146367812
170.9587629	136.9312715	0.168286853
449.3881346	705.4214876	0.130245277
229.5360825	557.3289151	0.083263097
135.4377104	212.9147026	0.100254308
15.86792453	5.452830189	0.398553459
151.5069444	89.98263889	0.209034653
27.43809524	7.038095238	0.449968254
281.2520911	299.0594174	0.133362073
28.21678322	12.37062937	0.420230611
173.8102679	161.4787946	0.149264134
248.3066564	228.4505579	0.153783634
77.42680776	51.32804233	0.230967062
347.2635681	1029.474989	0.069293497
484.8290567	1872.82961	0.051744567
584.9206557	2870.480267	0.068483994
88.23318386	31.43497758	0.304783527
65.92105263	43.88596491	0.259017904
34.13392857	24.10714286	0.298728824
119.4797371	81.39649507	0.234521858
196.5340237	301.6863905	0.121819862
40.31543624	10.27516779	0.421772931
132.8520231	60.35028902	0.279841123
319.7786569	582.5228241	0.117480341
12.5483871	2.806451613	0.634408602
289.5126965	665.0040306	0.085934462
311.9649273	389.7140006	0.146122364
57.69041096	33.67945205	0.269446758
135.4787776	35.08149406	0.391514494
10.16	7.04	0.358153997
5.615384615	1.923076923	0.585470085
268.8314377	343.0524934	0.143669254
47.17763158	25.84868421	0.28105961
54.375	39.61538462	0.200804621
270.5306681	350.067908	0.094197794
364.6410966	1075.721684	0.067374382
20.33333333	4.619047619	0.498844797
116.5382353	44.20294118	0.275802696
5.916666667	3.583333333	0.395115741
47.52760736	9.662576687	0.501271302
45.17857143	23.25	0.259435049

log.sigma.4.0.mm.3D_gldm_DependenceNonUniformityNormalized	log.sigma.4.0.mm.3D_gldm_DependenceVariance	log.sigma.4.0.mm.3D_gldm_LargeDependenceEmphasis
0.154748659	3.575509703	16.52354571
0.092824713	23.31760523	62.91874555
0.203740943	1.943502825	10.94350282
0.123846477	13.4222935	33.38696327
0.325948436	0.968336438	4.544554455
0.138571621	5.904797098	20.55737705
0.249795918	1.347755102	8.257142857
0.187878185	3.260253357	12.50124688
0.204157211	2.486883175	10.32384824
0.088943521	15.85147834	52.89909444
0.15574072	5.543090765	18.28191489
0.132119592	5.328162164	22.02245707
0.110570667	8.146290695	31.42020498
0.139383896	6.104281451	20.625
0.204172201	2.474006762	10.35714286
0.038095473	58.77951623	248.8719985
0.284376394	1.187411073	6.059907834
0.109240488	8.009816067	31.10180412
0.255102041	1.652578191	6.978021978
0.068777488	27.11616623	89.36337419
0.056988677	41.60047819	130.4872906
0.139873723	7.553137492	23.84488449
0.065527303	29.97074076	94.6630137
0.278647747	1.701509413	6.488721805
0.089446615	11.62860677	46.80833333
0.160146485	3.366738301	15.27152318
0.065568017	29.9120912	92.21100917
0.049292978	54.71681538	167.0985879
0.064831364	53.9285987	132.4198062
0.088513943	32.08623605	81.10277614
0.061110862	43.13283135	126.6196144
0.045138346	53.9468393	170.002151
0.135073946	11.77647991	30.96832253
0.36	0.56	3.8
0.295951132	0.998042133	5.17699115
0.134068273	6.092355621	22.57571214
0.04721348	52.16847477	172.5839187
0.075337343	27.56967196	79.07585752
0.217690046	2.070981454	9.984423676
0.160152444	3.552892301	16.27001862
0.1171715	7.167986699	28.22517483
0.165244108	5.450072678	17.11023622
0.150688684	5.248562541	18.3276699
0.163505793	4.440439807	15.45558087
0.156048181	4.003117698	17.08609865
0.040978488	50.18152483	218.096047
0.154094229	4.277585778	16.86380189
0.153354878	6.464373653	19.21052632
0.334710744	1.181818182	5.181818182
0.338712704	0.767498593	4.136986301
0.056588572	50.51288711	139.9544118
0.13621782	5.39252135	20.05210918
0.101845852	15.92258346	46.11236307
0.165805921	2.794944066	15.65271967
0.040674032	58.30236883	209.9324253
0.206469147	3.011218534	10.70149254
0.212279585	3.032799217	10.41972477
0.265851147	1.326690948	6.384279476
0.161783013	3.254811836	14.74576271
0.126646282	5.284330159	25.51502146
0.117497432	7.956538066	30.1532646
0.066320563	40.12041031	104.1818182
0.056341699	34.49632607	113.8610702
0.076003205	25.29282726	76.00448934
0.299394802	0.933428266	5.641509434
0.131516445	6.088249301	22.77604167
0.261315193	1.30521542	6.314285714
0.081122611	32.63369614	80.08739544
0.197320162	6.585554306	16.35664336
0.096992337	14.48396519	45.16517857
0.095539306	13.91317494	44.41977684
0.136555217	4.981949616	20.29805996
0.049208384	48.20004996	154.9387842
0.044750697	53.87095763	189.0732878
0.040286566	58.62635504	202.284386
0.197832251	6.152124515	16.66367713
0.144563712	5.950580948	19.94736842
0.15238361	4.714285714	16.96428571
0.130864991	5.432860387	21.19715225
0.072682701	39.30667037	97.41346154
0.270573398	1.421557587	6.476510067
0.153586154	4.982920913	17.19653179
0.064876985	39.37388935	104.8068574
0.404786681	0.616024974	3.322580645
0.058345969	39.98464509	118.0515921
0.088954927	13.93913497	46.928714
0.15805592	4.002807281	15.71232877
0.230014903	2.431337394	8.908319185
0.2032	2.88	10.72
0.431952663	0.390532544	3
0.078399369	39.2884728	92.41382327
0.155189578	4.492728532	16.55921053
0.130709135	7.232040496	25.00961538
0.074077401	27.15597629	82.31872946
0.050490321	50.95116279	159.7607311
0.322751323	0.965986395	4.777777778
0.171379758	3.240865052	13.59411765
0.246527778	1.493055556	7.333333333
0.291580413	1.314464225	5.588957055
0.161352041	3.79	15.35

log.sigma.4.0.mm.3D_gldm_LargeDependenceLowGrayLevelEmphasis	log.sigma.4.0.mm.3D_gldm_SmallDependenceHighGrayLevelEmphasis	log.sigma.4.0.mm.3D_gldm_LargeDependenceHighGrayLevelEmphasis
0.220799177	38.66043741	2205.34626
0.490032316	15.91616915	10607.13471
0.738744196	22.84768352	493.7344633
0.194601505	58.96020902	7655.318158
0.121319215	117.4676265	583.6930693
1.123726793	21.2464047	576.4508197
0.965688855	12.29297619	238.8857143
0.329489663	48.88019936	936.9650873
0.076171214	125.7043044	4780.759485
0.360673733	21.89286098	9209.620957
0.479877561	28.96119563	917.7898936
0.360300067	28.61567993	2479.054161
0.99019331	17.37732872	1716.383602
0.988477521	33.82267772	896.7215909
0.649929948	41.46533189	549.8873626
1.446035038	7.544772748	44282.58967
0.398769883	52.41875192	412.4423963
0.749727549	20.43884066	2387.532216
1.611971094	79.31060888	512.1868132
1.037001305	7.024799665	10759.77037
1.122643655	7.244429406	17105.59744
0.672204697	28.34910138	1519.664466
0.621455762	12.95284685	16859.60685
0.096901794	125.7778467	1770.721805
0.935958722	10.92669096	3766.6
0.550840922	24.01344344	1053.102649
1.021966508	11.30995986	9795.889035
0.953956166	9.592108233	31555.25263
0.594917353	21.1997388	32591.65231
0.524892438	13.10222601	16138.46663
0.896456109	10.26175524	20077.68484
1.120406433	12.83067659	27814.10076
0.459578357	29.17057732	4497.835853
0.217904538	44.25555556	267.2
0.07108845	103.6703663	788.380531
0.293285236	32.18354926	2798.088456
1.050898304	8.314675946	30420.61037
1.05245503	13.08132126	8955.564314
0.542364733	50.95968236	1000.632399
0.399632119	23.56222025	1502.882682
0.188775036	29.43262409	5668.721678
0.302062934	52.35977424	1258.627297
0.424237572	31.47943364	1290.580097
0.595782036	45.5440812	837.3667426
0.334782798	33.20514139	1567.133632
2.207278817	2.262755396	23260.80546
0.208160792	57.21684003	2143.442826
1.973542836	27.20209828	470.1622807
0.508917888	62.24368687	236.2272727
0.132053732	160.3615868	822.7260274
0.95829748	11.11673204	22632.34779
0.986408813	22.86072316	647.6476427
0.327772894	23.70814762	8838.584977
2.082264245	18.20069181	1007.334728
1.496970484	6.745509596	31294.31594
0.333343534	54.6667447	927.838806
0.134631854	74.60332704	1147.879587
0.242956585	111.7698987	1180.069869
0.327199741	36.37274001	1282.40113
0.873910333	15.3676155	1630.819742
0.502443345	17.62523417	3311.149141
0.463311241	31.1117251	26426.18418
1.253294121	8.653356669	11967.57413
0.852632106	8.480439945	8591.383838
0.29596356	42.9857652	459.6226415
0.294253068	34.41032687	3494.789931
0.333775957	83.23428042	634.2095238
0.851252434	15.84165906	13437.46149
1.215238389	40.71565118	390.4755245
0.481225217	17.52876105	6339.127232
0.341143967	24.87580372	8214.404771
0.519214885	28.61869702	1479.119929
0.986268113	8.980270871	27057.94686
2.174272474	3.560272354	19469.60024
1.153296129	12.68905515	37092.76713
0.283489563	55.14197305	1508.988789
0.465331846	30.27613793	1183.164474
2.512681988	21.17301373	282.5223214
0.223474629	36.69330403	2903.208105
0.460595574	23.57831264	22653.348
0.200331994	67.74653617	503.5973154
0.129793082	64.91191936	3174.810405
0.371395643	31.1038165	31494.1617
0.097369174	63.83870968	310.6129032
1.217795685	8.886956553	13805.9611
0.717326933	18.11748892	3961.923296
0.470497764	30.75223151	839.0273973
0.117699662	100.3914529	1295.473684
0.869210692	15.99352486	218.38
0.204555481	35.86752137	107.3846154
0.429783713	27.92966735	23705.37737
1.084082712	27.6958756	690.4539474
0.558602543	17.59205594	2038.596154
0.665556493	10.47859145	13076.7897
1.230685397	8.034871844	22875.64456
0.068937521	95.55891534	795.9365079
0.409573763	54.52321745	1428.132353
1.354571053	20.12907407	225.625
0.066128228	112.758819	1184.472393
0.162506088	39.44126036	2201.842857

log.sigma.4.0.mm.3D_gldm_SmallDependenceLowGrayLevelEmphasis	log.sigma.4.0.mm.3D_gldm_LowGrayLevelEmphasis	log.sigma.4.0.mm.3D_gldzm_DistanceZoneVariabilityNormalized
0.005441635	0.016103984	0.958781163
0.002774749	0.01542097	0.952186199
0.008373406	0.05688948	0.964297521
0.002171817	0.008371749	0.876994042
0.018499076	0.028256461	0.909297052
0.009110023	0.042821365	1
0.021583007	0.100035759	1
0.007135569	0.030247359	0.972608025
0.002845129	0.008254515	0.534429263
0.001552136	0.009436892	0.948203627
0.007644095	0.028247624	0.901096408
0.003943168	0.021617279	0.987097318
0.006763339	0.037663692	0.933961575
0.005039005	0.038900305	0.973943906
0.007900495	0.034935308	0.972227733
0.000479154	0.007770454	0.981144318
0.011862419	0.050021258	0.981983471
0.003560684	0.029470314	0.933412604
0.00819724	0.066816079	1
0.00178011	0.01854453	0.911791239
0.001584032	0.014354024	0.987159209
0.00607981	0.031636363	0.982457514
0.001802086	0.011063073	0.991902968
0.011772863	0.019681402	1
0.004068884	0.031797205	1
0.009933758	0.040772518	0.976193932
0.001767165	0.01789402	1
0.001186294	0.009279173	0.975907198
0.001538239	0.008555702	0.955756432
0.003733344	0.014756398	0.953573054
0.00138483	0.011793317	0.948470801
0.001078772	0.009713324	0.952268516
0.004214062	0.025340054	0.935567326
0.090566103	0.104922679	1
0.016214137	0.022258403	0.969704142
0.003325766	0.015493	0.885478132
0.001154567	0.010097268	0.88637006
0.002069391	0.021609217	0.930255308
0.00633013	0.039699471	0.933961575
0.008610302	0.027237831	0.908608
0.003302342	0.010353853	0.98449707
0.00612826	0.019086653	0.930645412
0.00471891	0.024485122	0.950265728
0.006889229	0.03892278	0.960622222
0.003750391	0.020937242	0.795117863
0.000868638	0.015150374	1
0.002466518	0.011933465	0.657166883
0.010737102	0.079844628	0.953514739
0.028172743	0.107326822	1
0.007074515	0.025079546	0.938207334
0.001759761	0.011903684	0.987302106
0.006741061	0.044651882	0.872890333
0.002182683	0.012157921	0.882045201
0.005329936	0.061018233	0.962976148
0.000818093	0.010265321	0.92986277
0.007056787	0.031629929	0.971836735
0.005186797	0.01274711	0.963267465
0.004993368	0.022991354	0.941719317
0.005923063	0.026264618	0.9802
0.003915354	0.034236339	1
0.004570165	0.021836778	0.834710744
0.001075054	0.007560685	0.909189137
0.001481652	0.015962081	0.993103531
0.002481116	0.017198538	0.963867665
0.017708842	0.065462072	1
0.003303308	0.017716529	0.876900427
0.010342477	0.041291019	0.965527855
0.002819757	0.025138848	0.817322335
0.014596866	0.050833241	0.971020761
0.003170386	0.017243242	0.929173875
0.001727607	0.011335739	0.9655375
0.004825499	0.026718867	0.931122449
0.000847722	0.010277707	0.942774414
0.001955007	0.022675869	0.963509516
0.000701356	0.007772068	0.922723607
0.006645093	0.021637386	0.988024387
0.006849003	0.024093018	0.985075471
0.017505454	0.123749268	0.972978045
0.003789516	0.01535914	0.940982987
0.001388622	0.007791144	0.969902588
0.009163672	0.032422857	1
0.003872335	0.009775789	0.978263495
0.000909018	0.005208764	0.883760566
0.048823591	0.05554585	1
0.00204334	0.018572859	0.948167744
0.002612353	0.017527329	0.988461927
0.007924881	0.029271074	0.945497423
0.004297668	0.014031015	0.964923469
0.04931962	0.085096633	1
0.0936714	0.111574712	0.654320988
0.001502122	0.00953202	0.885676972
0.01302195	0.051809371	0.978949751
0.00721964	0.02861255	0.959201389
0.002381496	0.015316468	0.969238281
0.001217342	0.012496538	0.956340065
0.020798995	0.025217724	1
0.003336374	0.022464863	0.91604997
0.032400277	0.152695479	1
0.010087371	0.015922552	0.979594006
0.007223788	0.017826033	1

log.sigma.4.0.mm.3D_gldzm_LowIntensityEmphasis	log.sigma.4.0.mm.3D_gldzm_LargeDistanceEmphasis	log.sigma.4.0.mm.3D_gldzm_HighIntensitySmallDistanceEmphasis
0.024047422	1.063157895	168.6210526
0.028847807	1.103658537	121.8140244
0.04488159	1.054545455	87.05909091
0.009832697	1.217758985	258.4258868
0.032173401	1.142857143	207.6111111
0.037643209	1	86.4057971
0.084432039	1	38.03333333
0.031264964	1.041666667	160.3489583
0.008627777	2.220103986	216.7463051
0.014547608	1.098484848	183.7961911
0.0290706	1.156521739	107.5608696
0.023983303	1.019480519	147.0454545
0.0453943	1.102564103	114.1848291
0.025366564	1.072368421	133.1578947
0.023020493	1.042253521	124.5950704
0.013192361	1.040380048	176.3563605
0.036078947	1.027272727	124.7022727
0.023766078	1.103448276	126.4894636
0.030561065	1	173.7526882
0.026296557	1.138728324	95.10260116
0.029816398	1.035483871	105.409767
0.028733347	1.026548673	133.2411504
0.023491273	1.012195122	143.4552846
0.023334628	1	262.1025641
0.045529828	1	83.71774194
0.047438759	1.036144578	95.87048193
0.021737367	1	123.5068493
0.019406267	1.036585366	131.0762195
0.014453647	1.101351351	173.6589089
0.03968721	1.100591716	115.6342867
0.022102078	1.116541353	143.1599833
0.014488872	1.122137405	168.678626
0.028229577	1.118081181	152.7291923
0.141638554	1	86.9
0.030389482	1.046153846	206.9692308
0.018092074	1.231543624	149.7323826
0.019399547	1.223057644	116.2406015
0.022400332	1.124590164	130.4052823
0.024632546	1.102564103	170.3782051
0.039881159	1.144	110
0.019888433	1.0234375	180.4160156
0.020797331	1.107913669	163.1438849
0.023078563	1.097457627	120.195033
0.031523655	1.093333333	152.6640741
0.020312058	1.415441176	137.8348652
0.03179298	1	68.18978102
0.010880762	2.16395664	166.9737748
0.046931856	1.071428571	84.07738095
0.083849846	1	106.5714286
0.01840797	1.095744681	285.8776596
0.022966482	1.019169329	131.3146965
0.031508914	1.285714286	83.13141923
0.017915996	1.252232143	154.7457372
0.04318698	1.056603774	94.54716981
0.016835372	1.127862595	122.5091709
0.026170121	1.042857143	148.9392857
0.013716444	1.056149733	195.3282086
0.014527551	1.090128755	257.6920601
0.030521222	1.03	138.87
0.033345054	1	108.5479452
0.031177197	1.272727273	100.3095238
0.009415038	1.148664344	244.7525487
0.020639616	1.010380623	109.516436
0.027377597	1.055214724	85.30368098
0.065154749	1	96.5
0.021646474	1.197674419	161.7180233
0.033090352	1.052631579	173.2149123
0.028667371	1.367483296	118.549802
0.034821033	1.044117647	93.93014706
0.025463912	1.110294118	113.5808824
0.013659997	1.065	162.0815278
0.026507107	1.107142857	119.5642857
0.015108007	1.117647059	132.8210784
0.050927288	1.094907407	69.80846515
0.010796651	1.16468039	196.2288582
0.023950466	1.018072289	181.9051205
0.026908658	1.022556391	114.8139098
0.066264553	1.04109589	69.45205479
0.018503314	1.091304348	152.35
0.012235266	1.06097561	195.9515583
0.028631554	1	154.2236842
0.013423981	1.032967033	232.4844322
0.008170249	1.19434629	263.968247
0.068490374	1	99.52173913
0.030006279	1.156084656	110.275463
0.019302826	1.017408124	128.5517408
0.031220263	1.08411215	112.5794393
0.01276065	1.053571429	248.4366071
0.12011423	1	43.36363636
0.141094028	1.666666667	56.61111111
0.0127992	1.202020202	190.2606622
0.050217792	1.031914894	98.04255319
0.037206685	1.0625	86.4609375
0.0325521	1.046875	111.8148438
0.020767536	1.079207921	126.8911579
0.036069721	1	192.6842105
0.01537789	1.131707317	202.7402439
0.116354386	1	47.38461538
0.019204892	1.030927835	222.1546392
0.02803164	1	145.6896552

log.sigma.4.0.mm.3D_gldzm_LowIntensityLargeDistanceEmphasis	log.sigma.4.0.mm.3D_gldzm_HighIntensityEmphasis	log.sigma.4.0.mm.3D_gldzm_DistanceZoneVariability	log.sigma.4.0.mm.3D_gldzm_ZonePercentage
0.026336895	169.5368421	91.08421053	0.263157895
0.047913539	123.7195122	156.1585366	0.116892373
0.050942196	87.18181818	53.03636364	0.310734463
0.014737309	262.6807611	414.8181818	0.244697362
0.086406205	208.1587302	57.28571429	0.623762376
0.037643209	86.4057971	69	0.282786885
0.084432039	38.03333333	30	0.428571429
0.053400381	160.4375	140.0555556	0.359102244
0.013512209	379.3795494	308.3656846	0.390921409
0.015110266	189.1780303	250.3257576	0.113842173
0.065669835	108.7478261	103.626087	0.305851064
0.024287686	147.3571429	152.012987	0.20343461
0.079262521	115.2136752	218.5470085	0.171303075
0.030950547	133.6513158	148.0394737	0.287878788
0.044321855	125.2394366	138.056338	0.39010989
0.014637788	176.9287411	413.0617577	0.03888068
0.063351674	124.7090909	108.0181818	0.506912442
0.028496172	128.394636	243.6206897	0.168170103
0.030561065	173.7526882	93	0.510989011
0.045910906	96.79768786	157.7398844	0.062364816
0.030026361	106.3032258	306.0193548	0.061083744
0.042278588	133.4070796	222.0353982	0.248624862
0.023830026	143.5650407	244.0081301	0.084246575
0.023334628	262.1025641	78	0.586466165
0.045529828	83.71774194	124	0.129166667
0.051454823	95.95180723	81.02409639	0.274834437
0.021737367	123.5068493	219	0.095674967
0.01966204	132.5	240.0731707	0.063157895
0.015297042	176.0022523	424.3558559	0.119483315
0.040815825	117.7751479	161.1538462	0.0998228
0.024228284	145.6428571	252.2932331	0.063318258
0.017558419	170.1725191	623.7358779	0.074153742
0.030961571	154.0848708	253.5387454	0.195104392
0.141638554	86.9	10	0.666666667
0.041927943	207.0153846	63.03076923	0.575221239
0.022181764	155.6342282	263.8724832	0.223388306
0.02273295	120.1177945	353.6616541	0.059632342
0.03144996	131.3442623	283.7278689	0.100593668
0.03278386	171.3846154	109.2735043	0.364485981
0.069990789	111.8	113.576	0.232774674
0.020366749	180.703125	126.015625	0.179020979
0.046035653	164.5251799	129.3597122	0.364829396
0.024377316	122.1059322	224.2627119	0.286407767
0.036501432	153.3466667	144.0933333	0.341685649
0.029969073	156.0110294	216.2720588	0.243946188
0.03179298	68.18978102	137	0.022378308
0.016374395	238.1775068	484.9891599	0.268754552
0.051628973	84.5952381	80.0952381	0.368421053
0.083849846	106.5714286	14	0.636363636
0.052098129	286.5957447	88.19148936	0.643835616
0.023545474	131.4920128	309.0255591	0.076715686
0.034434128	88.66386555	103.8739496	0.29528536
0.02095752	160.4821429	395.15625	0.140219092
0.046724715	94.77358491	51.03773585	0.221757322
0.019917431	124.870229	487.2480916	0.046347072
0.048036011	149.2071429	136.0571429	0.417910448
0.015093985	196.328877	360.2620321	0.428899083
0.034879131	258.4420601	219.4206009	0.508733624
0.032396222	138.99	98.02	0.282485876
0.033345054	108.5479452	73	0.156652361
0.035181981	108.1082251	192.8181818	0.158762887
0.015319265	248.116144	782.8118467	0.127066116
0.02070104	109.9550173	287.0069204	0.070937653
0.028640065	85.9202454	157.1104294	0.091470258
0.065154749	96.5	26	0.490566038
0.032540639	164.5116279	226.2403101	0.223958333
0.085721931	173.2280702	55.03508772	0.542857143
0.034246705	124.2249443	366.9777283	0.129506778
0.078938681	93.94117647	66.02941176	0.475524476
0.028503131	115.5220588	252.7352941	0.151785714
0.014225015	163.905	386.215	0.153905348
0.048565475	122.5428571	130.3571429	0.24691358
0.023823794	134.6740196	384.6519608	0.057814936
0.053905756	70.59259259	416.2361111	0.039874469
0.016922645	199.8569881	851.6738895	0.063571871
0.024673358	182.0180723	164.0120482	0.372197309
0.027187132	115.2706767	131.0150376	0.291666667
0.067406106	69.82191781	71.02739726	0.325892857
0.019835968	155.0956522	216.426087	0.251916758
0.01265421	198.2987805	318.1280488	0.121301775
0.028631554	154.2236842	76	0.510067114
0.014062717	233.8278388	267.0659341	0.315606936
0.01599685	269.4823322	500.2084806	0.114830594
0.068490374	99.52173913	23	0.741935484
0.040041272	111.9285714	358.4074074	0.07617896
0.020260273	128.6344294	511.0348162	0.147419447
0.038862397	114.0654206	101.1682243	0.293150685
0.016305479	250.0464286	270.1785714	0.475382003
0.12011423	43.36363636	22	0.44
0.511464398	57.44444444	5.888888889	0.692307692
0.014752869	197.0565657	438.410101	0.144356955
0.050716462	98.55319149	92.0212766	0.309210526
0.048491407	86.5625	92.08333333	0.230769231
0.033755001	112.346875	310.15625	0.08762322
0.029151679	128.1856436	386.3613861	0.055940183
0.036069721	192.6842105	38	0.603174603
0.038847403	203.8487805	187.7902439	0.301470588
0.116354386	47.38461538	13	0.541666667
0.020063998	222.4329897	95.02061856	0.595092025
0.02803164	145.6896552	87	0.310714286

log.sigma.4.0.mm.3D_gldzm_IntensityVariabilityNormalized	log.sigma.4.0.mm.3D_gldzm_LowIntensitySmallDistanceEmphasis	log.sigma.4.0.mm.3D_gldzm_IntensityVariability
0.067036011	0.023475053	6.368421053
0.076740036	0.024111486	12.58536585
0.081652893	0.043366439	4.490909091
0.055839878	0.008654959	26.41226216
0.049130763	0.0186152	3.095238095
0.105650074	0.037643209	7.289855072
0.133333333	0.084432039	4
0.062017747	0.02573111	8.930555556
0.039242601	0.007473392	22.64298094
0.069846189	0.014423383	18.43939394
0.092325142	0.019920791	10.6173913
0.065441052	0.023907207	10.07792208
0.074512382	0.036927244	17.43589744
0.072628116	0.024060814	11.03947368
0.075877802	0.017695152	10.77464789
0.082966131	0.01284447	34.92874109
0.07107438	0.029260765	7.818181818
0.079138592	0.022583554	20.65517241
0.051913516	0.030561065	4.827956989
0.090647867	0.02139297	15.68208092
0.080874089	0.029785115	25.07096774
0.064100556	0.025347036	14.48672566
0.073534272	0.023406584	18.08943089
0.05095332	0.023334628	3.974358974
0.087799168	0.045529828	10.88709677
0.087530846	0.046434743	7.265060241
0.078125977	0.021737367	17.10958904
0.080408487	0.019342324	19.7804878
0.064554419	0.014281496	28.66216216
0.066279192	0.039430738	11.20118343
0.075583696	0.02161315	20.10526316
0.080263388	0.013803947	52.57251908
0.061777481	0.027610641	16.74169742
0.1	0.141638554	1
0.056094675	0.027504866	3.646153846
0.072406648	0.017128341	21.57718121
0.079176638	0.018621498	31.5914787
0.076484816	0.020194847	23.32786885
0.063189422	0.022594717	7.393162393
0.07904	0.032353752	9.88
0.068481445	0.019768854	8.765625
0.068267688	0.01448775	9.489208633
0.076378914	0.022781733	18.02542373
0.066755556	0.030353284	10.01333333
0.065852076	0.018066579	17.91176471
0.115030103	0.03179298	15.75912409
0.056693914	0.009824875	41.8401084
0.087018141	0.045757577	7.30952381
0.091836735	0.083849846	1.285714286
0.050022635	0.00998543	4.70212766
0.070216089	0.022821734	21.97763578
0.101052186	0.03092623	12.02521008
0.069893973	0.01721139	31.3125
0.086507654	0.042302546	4.58490566
0.090044286	0.016089951	47.18320611
0.062142857	0.020703649	8.7
0.062269439	0.013372059	23.28877005
0.052625762	0.009439656	12.26180258
0.0618	0.030052472	6.18
0.082004128	0.033345054	5.98630137
0.087273477	0.030176001	20.16017316
0.053983362	0.007949646	46.4796748
0.093305875	0.02062426	26.96539792
0.105875268	0.02706198	17.25766871
0.091715976	0.065154749	2.384615385
0.067243555	0.018922933	17.34883721
0.063711911	0.019932457	3.631578947
0.072286348	0.027451874	32.45657016
0.095588235	0.023791622	6.5
0.073718642	0.024704107	20.05147059
0.0766	0.01354652	30.64
0.081632653	0.020992515	11.42857143
0.08822328	0.012976931	35.99509804
0.088627401	0.050257733	38.28703704
0.069281595	0.009329925	63.94691224
0.066047322	0.023769744	10.96385542
0.085081124	0.026839039	11.31578947
0.1026459	0.065979165	7.493150685
0.067183365	0.01817015	15.45217391
0.067240482	0.012142252	22.05487805
0.073060942	0.028631554	5.552631579
0.053952153	0.013264297	14.72893773
0.061269338	0.006225215	34.67844523
0.088846881	0.068490374	2.043478261
0.083970214	0.027605996	31.74074074
0.083927883	0.019063464	43.39071567
0.080793082	0.02930973	8.644859813
0.058954082	0.011874442	16.50714286
0.123966942	0.12011423	2.727272727
0.135802469	0.048501436	1.222222222
0.067425773	0.012329163	33.37575758
0.07129923	0.050093124	6.70212766
0.078559028	0.034385504	7.541666667
0.07875	0.032251375	25.2
0.072529654	0.018687774	29.3019802
0.063711911	0.036069721	2.421052632
0.059940512	0.009510512	12.28780488
0.136094675	0.116354386	1.769230769
0.05919864	0.018990115	5.742268041
0.0651341	0.02803164	5.666666667

log.sigma.4.0.mm.3D_gldzm_HighIntensityLargeDistanceEmphasis	log.sigma.4.0.mm.3D_gldzm_SmallDistanceEmphasis	log.sigma.4.0.mm.3D_glcm_SumVariance	log.sigma.4.0.mm.3D_glcm_Homogeneity1
173.2	0.984210526	352.3179261	0.418632812
137.4390244	0.980860434	320.6593135	0.530480223
87.67272727	0.986363636	107.9019825	0.396610182
281.5983087	0.950258398	527.9849131	0.423225013
210.3492063	0.964285714	402.1867594	0.270008469
86.4057971	1	83.67390243	0.435332138
38.03333333	1	45.64070026	0.427108073
160.7916667	0.989583333	213.9344785	0.363100291
1205.52513	0.746184768	1269.875916	0.296579737
215.0151515	0.979587542	422.1276749	0.527578432
113.4956522	0.960869565	130.6533329	0.407861611
148.6038961	0.99512987	275.1298899	0.443753372
119.3290598	0.974358974	116.905475	0.464355931
137.9934211	0.989217836	142.363921	0.411821742
127.8169014	0.98943662	155.196343	0.363399062
181.2874109	0.992544207	508.8508417	0.749874481
124.7363636	0.993181818	170.8315796	0.313180985
136.0153257	0.974137931	167.6643578	0.462248604
173.7526882	1	221.2044739	0.297315742
103.5780347	0.965317919	231.3396014	0.587167043
112.3	0.994713262	279.0761898	0.635865387
134.0707965	0.993362832	168.7387882	0.429028054
144.004065	0.99695122	394.9752548	0.600897544
262.1025641	1	734.1440001	0.314653823
83.71774194	1	162.8700202	0.558427797
96.27710843	0.990963855	147.7440098	0.407839336
123.5068493	1	235.1076055	0.59347728
138.195122	0.990853659	451.0187711	0.675081056
191.2815315	0.98216967	542.6169927	0.591547253
133.0710059	0.981426693	378.7882757	0.553106457
162.1240602	0.979218881	345.3777157	0.620841975
182.9877863	0.981340119	398.1942804	0.659683606
160.5571956	0.974579746	258.7080637	0.429816341
86.9	1	214.4427034	0.28509031
207.2	0.988461538	397.1214009	0.299055163
188.1006711	0.953299776	274.4651763	0.418093001
142.7568922	0.954069201	416.9776986	0.667048878
136.0327869	0.972495446	216.2830548	0.55025433
175.4102564	0.974358974	271.0198902	0.348892427
119	0.964	213.088643	0.409655117
181.8515625	0.994140625	473.4721737	0.477723198
170.0503597	0.973021583	226.5908894	0.394943795
132.9322034	0.980343691	180.7795838	0.402162986
159.04	0.984074074	176.7489647	0.382291063
238.7647059	0.912479575	268.6933707	0.390525591
68.18978102	1	254.7780988	0.742014904
863.5474255	0.835949262	439.3827886	0.379816475
86.66666667	0.982142857	73.47499098	0.403176481
106.5714286	1	182.1369067	0.289473232
289.4680851	0.97606383	535.3248685	0.24852743
132.201278	0.995207668	351.4923598	0.622406353
120.8403361	0.947245565	94.32469556	0.411622202
200.4977679	0.951977927	395.4634225	0.484305877
95.67924528	0.985849057	143.2123881	0.415998516
137.1812977	0.972275233	382.8446435	0.703449597
150.2785714	0.989285714	223.0965153	0.339193427
200.3315508	0.985962567	360.0565283	0.348666347
261.4420601	0.977467811	491.8094266	0.289517487
139.47	0.9925	221.108583	0.407625298
108.5479452	1	160.2677743	0.468225122
139.3030303	0.931818182	206.2110775	0.457916283
262.195122	0.964124403	582.3991027	0.556919745
111.7093426	0.997404844	236.4595569	0.619233809
88.38650307	0.986196319	224.707467	0.569488715
96.5	1	168.7710029	0.321341957
175.6860465	0.950581395	320.4499844	0.425940147
173.2807018	0.986842105	254.186856	0.294338882
152.1915367	0.922976986	291.8458529	0.522248133
93.98529412	0.988970588	96.90272438	0.378935314
123.2867647	0.972426471	265.5817238	0.499384573
172.31	0.986527778	389.8805214	0.497113647
134.4571429	0.973214286	173.8015661	0.418088574
146.0563725	0.977481618	391.8562112	0.645416021
78.125	0.985355581	218.1376517	0.687601688
221.4084507	0.969175093	482.5762137	0.68228905
182.4698795	0.995481928	288.5678436	0.363333101
117.0977444	0.994360902	161.9002666	0.417166054
71.30136986	0.989726027	46.97364484	0.423361742
166.0782609	0.977173913	347.2022167	0.427367667
211.6036585	0.988143631	514.7041159	0.563177913
154.2236842	1	231.7661256	0.312847583
239.2014652	0.991758242	499.9411508	0.393885656
292.8657244	0.953376521	757.9234953	0.567592912
99.52173913	1	208.5863701	0.294749418
130.2936508	0.978799236	237.0092653	0.607249431
128.9651838	0.995647969	201.3123317	0.514309551
120.0093458	0.978971963	150.2369357	0.410694343
256.4857143	0.986607143	469.381633	0.317468461
43.36363636	1	61.89220998	0.40774375
60.77777778	0.833333333	86.22126839	0.354662698
228.9090909	0.953984287	539.7332054	0.542338998
100.5957447	0.992021277	134.3365287	0.399953577
86.96875	0.984375	156.0152389	0.456498854
114.475	0.98828125	309.3698096	0.564529017
135.2227723	0.982948295	322.1690627	0.646341489
192.6842105	1	479.6715279	0.263810798
208.2829268	0.967073171	300.0725348	0.368263431
47.38461538	1	78.25065381	0.357614375
223.5463918	0.992268041	512.8842735	0.314490558
145.6896552	1	358.1428553	0.430581527

log.sigma.4.0.mm.3D_glcm_Homogeneity2	log.sigma.4.0.mm.3D_glcm_ClusterShade	log.sigma.4.0.mm.3D_glcm_MaximumProbability	log.sigma.4.0.mm.3D_glcm_Idmn	log.sigma.4.0.mm.3D_glcm_SumVariance2
0.337972517	4.7493666	0.024347387	0.980690298	53.1154421
0.474924901	-89.91994485	0.095431362	0.985151619	31.95939464
0.309389546	80.73548155	0.037863334	0.965612436	30.6168678
0.346169019	10.12460156	0.037980205	0.98650351	49.39838832
0.171504216	589.2684657	0.023491687	0.957664019	105.103028
0.360157288	43.2548332	0.044249199	0.960503182	17.4533492
0.347723913	20.30561149	0.067699425	0.943131346	11.43340889
0.27514966	203.9636693	0.023898967	0.974528145	43.15139685
0.206711271	2055.461031	0.008062672	0.974167733	280.2719261
0.472362334	-43.71447618	0.048270778	0.991738431	26.98445675
0.326152193	47.64262106	0.052652611	0.970270502	19.05154553
0.369022527	-10.5953076	0.024549476	0.985784372	37.64111424
0.394992016	92.8544705	0.04269854	0.983528013	23.90210386
0.332593887	184.116958	0.029038726	0.974938068	37.25567266
0.274869286	110.8450697	0.021538952	0.968749691	39.34445878
0.733548136	-104.4287496	0.286302476	0.997216148	13.88406049
0.21674205	74.48520976	0.023631456	0.952175183	46.03680511
0.391904174	20.05704824	0.03131892	0.986126765	27.37971318
0.202580116	275.7770315	0.022101021	0.950897645	75.09043592
0.5438091	-58.11609423	0.088244932	0.989789124	28.44080004
0.600807361	-54.12205725	0.191918644	0.994144632	17.55667487
0.353144713	156.2130748	0.035506693	0.977762427	35.92013232
0.561280932	-99.98483368	0.120691293	0.993357948	22.9016504
0.217296925	-488.031255	0.029043988	0.969063063	132.5359087
0.511494418	-48.13557741	0.049994327	0.987682435	19.59788827
0.323203792	-5.095663973	0.03215968	0.967758591	29.21302683
0.551205284	-37.41788054	0.13299397	0.993324538	17.44351892
0.643578891	-123.4108909	0.291602696	0.997133818	23.96454669
0.542392001	-192.0501448	0.239572086	0.99314933	37.44349678
0.499947516	-140.4425315	0.132804014	0.988484247	39.027329
0.582536885	-75.29743333	0.184817928	0.994020979	21.92011535
0.628073427	-84.61378992	0.240504165	0.995605595	18.53590495
0.351361108	8.641250853	0.04188082	0.983681429	44.80522743
0.180110515	97.31554256	0.155128205	0.914734765	43.03939103
0.202436514	78.6394759	0.027382406	0.962336523	67.18517596
0.337593865	32.95393768	0.025350586	0.978705437	32.04737236
0.635524799	-140.5748624	0.200130263	0.993712048	23.56808738
0.498029292	-36.33723079	0.08732166	0.991053034	29.99075805
0.258949678	134.9895538	0.021230841	0.969052676	73.90993098
0.327072902	89.8171869	0.025430001	0.975460422	45.56937138
0.409768522	-117.3072688	0.033484175	0.989067718	41.54760137
0.312450015	181.4798371	0.042687781	0.977723745	32.64303654
0.319156086	22.23132391	0.028046034	0.971677656	24.74852862
0.297088398	258.7753618	0.028562049	0.978781974	44.32203505
0.305373762	398.7166419	0.023340393	0.980972203	56.36919176
0.725253133	-63.96761039	0.238230046	0.994324074	12.70533368
0.29619153	899.2449152	0.015923021	0.990193508	76.28123967
0.320984038	97.47929136	0.0512581	0.952454549	24.82442926
0.18730911	53.29139888	0.111847874	0.908561375	30.17049481
0.153996814	226.9252364	0.021963645	0.947963345	115.2040067
0.582216621	-93.41633397	0.213125073	0.99283142	24.52385023
0.330305851	48.56046708	0.051543931	0.967004503	17.8826512
0.41808012	-87.14566022	0.0632598	0.988902029	38.3804145
0.332408681	43.27766616	0.024331616	0.970339135	39.51080758
0.678535812	-111.5935187	0.274377022	0.995336843	18.57606324
0.246636197	72.91894876	0.01886638	0.959399871	46.84498382
0.258340076	126.8130716	0.021113054	0.977972557	43.5048324
0.195311387	-123.7785505	0.013262694	0.960197089	97.11992424
0.324322763	83.0866949	0.026927255	0.977918429	40.98226572
0.396105642	47.94695029	0.035862362	0.981512363	35.03322996
0.385247849	68.09801326	0.03101697	0.983304182	31.08039095
0.505974014	-141.5979285	0.123128971	0.993667709	40.61284982
0.582260468	-58.39059132	0.129719757	0.993929917	18.81830936
0.519886521	-34.39919188	0.109574402	0.985051883	21.8860435
0.224286613	10.15566791	0.046901752	0.944999659	35.86123811
0.349325989	-63.37805399	0.026302836	0.983450427	42.43661058
0.197038379	-1.596140909	0.035265457	0.939935679	51.6134616
0.463349957	-112.6293658	0.095897086	0.988206832	48.96674704
0.293651312	110.8612296	0.04954037	0.956621794	28.04176026
0.437776089	-43.81529671	0.045266968	0.986388546	32.16675981
0.435322031	-67.7870776	0.050089843	0.985692362	34.02785615
0.336681333	45.12982302	0.028757561	0.97677897	27.54881865
0.610412293	-109.3267079	0.15941843	0.994135491	24.77233159
0.660930069	-92.44112163	0.229698992	0.99172546	19.53608149
0.654640588	-93.83257373	0.24068993	0.996365111	19.43064245
0.277454131	90.53265883	0.032051353	0.970947104	42.58102341
0.337356864	47.30819558	0.037007154	0.976224494	22.0427528
0.345637631	83.74230621	0.051264695	0.969818983	18.66160345
0.348300114	-28.77423957	0.030542117	0.980787664	27.76881487
0.511662054	-75.93016237	0.15244304	0.992187936	27.2374257
0.218527405	81.01663784	0.026084707	0.947071356	44.97793791
0.311394988	78.51921889	0.020109414	0.982868418	50.55507856
0.519011749	-167.2638968	0.127523038	0.993285603	33.35780841
0.198037892	8.938309694	0.068357497	0.912115911	33.04645466
0.56705836	-66.87273051	0.146412023	0.991169822	20.38253505
0.457091728	16.98860421	0.042421534	0.987635398	19.45244049
0.329442744	93.26260957	0.035934372	0.977359539	26.64673078
0.225349122	57.80382333	0.020067183	0.972440921	61.25099526
0.322577448	3.818428071	0.084920236	0.926971186	9.195144345
0.264425246	32.2228701	0.109126984	0.887206572	18.75056689
0.487049861	-199.2293605	0.102883042	0.991583308	45.24175062
0.31683291	264.5666803	0.038945797	0.968759922	42.38221571
0.38465886	10.12256118	0.043174316	0.97497519	25.23004181
0.51606955	-68.13829248	0.080982954	0.98956002	31.80060742
0.611423503	-89.63147458	0.214552773	0.993503335	23.19708805
0.169469365	172.8280355	0.040801187	0.95297956	54.27173823
0.280850462	105.6175471	0.016224604	0.976482025	55.4628452
0.267394823	40.66769133	0.11325416	0.889235891	21.02482747
0.220033056	343.9912885	0.024062944	0.970994951	87.84830682
0.352783217	-106.4687624	0.037119312	0.98272637	40.0313635

log.sigma.4.0.mm.3D_glcm_Contrast	log.sigma.4.0.mm.3D_glcm_DifferenceEntropy	log.sigma.4.0.mm.3D_glcm_InverseVariance	log.sigma.4.0.mm.3D_glcm_Entropy	log.sigma.4.0.mm.3D_glcm_Dissimilarity
9.068929297	2.697799545	0.346912078	6.962263298	2.33995266
5.158711473	2.373607831	0.404851469	6.243832922	1.610276891
9.71878458	2.674900486	0.321362231	6.281174707	2.487156158
12.87785719	2.961415769	0.322532219	7.396673993	2.616656172
34.88411455	3.354867546	0.183866362	6.587744632	4.7592601
8.826991561	2.683015569	0.360370089	6.122682077	2.266530742
8.418171011	2.425215281	0.358377796	5.02303578	2.233582595
17.41409113	3.146405523	0.278819156	7.227205928	3.205526066
40.90966338	3.715914058	0.212426595	8.983632482	4.835605679
4.535258954	2.311148918	0.426492948	6.344825797	1.546910247
10.56402518	2.809200493	0.330722062	6.422785624	2.520999914
7.973106956	2.602547694	0.373307276	6.8776759	2.130606177
7.841106612	2.624061693	0.381100313	6.500115416	2.046585554
12.08596383	2.904174831	0.326637661	6.885359866	2.619510841
15.26119883	3.027457866	0.294186699	7.022229298	3.052973092
1.668315704	1.588947518	0.346562519	4.226242755	0.68418464
21.79418422	3.128166693	0.233976283	6.856643346	3.766607602
7.809247158	2.636008458	0.37452596	6.729607239	2.06105898
29.86552842	3.394396755	0.205098396	7.019612039	4.355003157
3.091435151	2.085119261	0.430403149	5.879391187	1.238002807
2.421560813	1.951840107	0.41365053	5.274876542	1.040115597
10.75150597	2.822573931	0.34858705	6.889280563	2.423377207
3.037222276	2.058916236	0.434436264	5.661973812	1.190182531
22.70842163	3.150136298	0.212188485	6.635329619	3.843871755
3.356811718	2.099798792	0.458313159	5.815519004	1.321082005
10.35807296	2.789333533	0.314144661	6.636494239	2.501496145
3.364434069	2.120770538	0.422513872	5.62583625	1.245875039
2.473457045	1.912887678	0.360378851	4.941938656	0.963681872
5.602782034	2.396783041	0.326315703	5.834098658	1.512343825
4.377180664	2.305393611	0.393120231	6.176614015	1.479466456
3.007009399	2.059256023	0.405765582	5.511731715	1.143668166
3.090380443	2.007083443	0.379365894	5.180953026	1.054615593
10.02111279	2.811536143	0.333131513	7.179820587	2.378761515
31.52051282	1.88775777	0.128871523	3.147435815	4.534615385
26.2635283	3.229353559	0.217433462	6.522393472	4.111402976
10.14375943	2.81148097	0.33562701	7.018242481	2.429483394
2.630524808	1.951931957	0.372026167	4.978860614	0.999543504
4.516129947	2.320068428	0.403775265	6.279694909	1.495482565
19.86919549	3.138281735	0.267073716	7.304042952	3.419608165
9.594115692	2.748536377	0.339036851	7.022539555	2.41902938
6.63642749	2.49419142	0.389127502	6.652601124	1.890938969
13.96132456	2.996705643	0.311737888	6.754153969	2.812926044
11.23717606	2.862451003	0.325096534	6.895655334	2.586570813
15.5421814	3.059304207	0.295784148	7.071274657	2.975834354
12.65309424	2.955006358	0.306773997	7.419824025	2.753948714
1.152709369	1.515807347	0.369473985	4.08192909	0.645413604
18.23733219	3.149229085	0.305895681	7.76804048	3.091412157
12.49619233	2.912798451	0.309355411	6.342962688	2.699981058
30.25276113	2.427378222	0.15172066	3.844684517	4.442582418
46.75324726	3.629191102	0.1650272	7.004280175	5.557015021
3.302384315	2.114448021	0.380058929	5.538437222	1.18384048
10.59038792	2.829220086	0.325108108	6.368026989	2.515949552
6.697511081	2.548266866	0.38074303	6.740741243	1.902149949
9.383052631	2.694296744	0.320100432	6.702218566	2.396485991
2.139110231	1.807018455	0.361116665	4.698113941	0.855599358
20.40682262	3.226120022	0.248942928	7.321778466	3.525140871
18.53709319	3.178934887	0.266993102	7.452612501	3.359889959
32.45314352	3.492375178	0.202416991	7.921832528	4.544338282
10.5059411	2.794226126	0.326371179	6.910555277	2.500124532
5.676464614	2.405266244	0.38472916	6.547026133	1.852080516
7.117808216	2.592102823	0.372842923	6.663329177	2.02311226
6.77435633	2.504166549	0.367152231	6.407258394	1.678690648
2.771549378	1.995737133	0.423857302	5.506988512	1.118283885
3.578967453	2.174755392	0.405352153	5.804936356	1.348941494
18.11082873	2.920552033	0.237282943	5.390592712	3.519294773
11.08656326	2.826255208	0.342123273	7.109950357	2.466271849
27.93238002	3.272634836	0.185273102	6.345292886	4.317231469
6.612260963	2.551499749	0.36614749	6.604225784	1.789371802
14.23768151	2.97519744	0.295436892	6.269135125	2.949483723
5.780589228	2.448373271	0.406961893	6.554129742	1.756008443
6.801837695	2.528901497	0.396152689	6.664452187	1.848111729
10.11061691	2.803288358	0.328800208	6.78082386	2.429486748
2.680875618	1.997684301	0.387432003	5.281098462	1.056386019
1.961997541	1.811414646	0.383173125	4.885387874	0.871181893
3.181159804	1.960885076	0.364765546	4.992771514	1.003676628
19.94740128	3.199940852	0.264136112	7.2289556	3.406934763
10.28562368	2.800927823	0.332612882	6.564011564	2.466073597
9.845764357	2.727689628	0.358526959	5.980805665	2.359549677
9.055364817	2.721828645	0.344758541	6.811112997	2.30863835
5.111554804	2.372647563	0.375419765	6.034087923	1.519497211
24.58115264	3.24421724	0.230736132	6.641996563	3.942535869
14.38694631	3.029126919	0.313546522	7.442895001	2.833440482
5.910594262	2.412493917	0.377993059	6.151349548	1.571397369
25.27594115	2.759302214	0.233389722	4.616898551	4.058655661
3.366039437	2.119290526	0.410586616	5.60952458	1.214878379
5.788818808	2.440631038	0.413091262	6.313132052	1.708519818
9.812537525	2.764535782	0.348016984	6.558044833	2.419797346
25.12776643	3.39076903	0.234827566	7.770755367	3.957507615
9.099001844	2.435601338	0.330162744	4.687056337	2.387458816
17.83333333	2.215647948	0.228089102	3.806100279	3.412698413
6.43221328	2.514309005	0.360511795	6.413842403	1.711972559
12.70150153	2.909976492	0.312517165	6.654086026	2.700973721
7.971697185	2.625120911	0.367851603	6.462255423	2.105600476
3.988533078	2.22160357	0.418944793	6.187155613	1.388967138
2.707983486	2.002049869	0.38603493	5.344541311	1.056165701
41.85109744	3.331242808	0.172909867	5.728228381	5.217318762
15.89683584	3.08622108	0.291845437	7.536525173	3.073719805
17.7791719	2.365393535	0.224597923	3.81135476	3.393899904
21.20817625	3.159748578	0.25200073	6.843995629	3.716534825
8.923143507	2.678021625	0.354011099	6.575676696	2.274135226

log.sigma.4.0.mm.3D_glcm_DifferenceVariance	log.sigma.4.0.mm.3D_glcm_Idn	log.sigma.4.0.mm.3D_glcm_Idm	log.sigma.4.0.mm.3D_glcm_Correlation	log.sigma.4.0.mm.3D_glcm_Autocorrelation
3.480036283	0.905294986	0.337972517	0.708716518	132.4767517
2.462573171	0.923433093	0.474924901	0.721459371	120.6809044
3.294705854	0.873875567	0.309389546	0.516972096	47.95470618
5.828621982	0.924539959	0.346169019	0.586781674	187.0573961
11.40580283	0.859720422	0.171504216	0.501561458	138.3670358
3.583307247	0.871671965	0.360157288	0.328772928	38.63135374
2.989153542	0.84569577	0.347723913	0.186093533	21.88680215
6.944666859	0.893645796	0.27514966	0.4240282	84.43444568
16.82541265	0.894848508	0.206711271	0.747844021	407.7364543
2.094782965	0.940078365	0.472362334	0.70968659	152.6050601
4.04442085	0.885327542	0.326152193	0.283246459	55.50735779
3.204671785	0.919781917	0.369022527	0.647253834	107.4991705
3.539046661	0.916832839	0.394992016	0.502931855	52.13116939
5.060322375	0.896735954	0.332593887	0.509424986	60.70771842
5.665068962	0.881335699	0.274869286	0.444378642	64.68456263
1.182917747	0.973985472	0.733548136	0.783317307	166.4908375
6.388981932	0.852181823	0.21674205	0.380375006	67.91820043
3.446277508	0.922502074	0.391904174	0.555757698	70.8301192
9.838771439	0.852246666	0.202580116	0.437525191	82.51415313
1.511441994	0.936163149	0.5438091	0.803171564	92.21595751
1.31661695	0.953229441	0.600807361	0.756335775	104.226721
4.690441376	0.903721026	0.353144713	0.533575158	70.53561635
1.578253711	0.949237303	0.561280932	0.764494978	141.9029839
7.278998235	0.878589736	0.217296925	0.70958998	244.2101848
1.568274456	0.928232521	0.511494418	0.705531174	68.59388592
3.905813625	0.880401156	0.323203792	0.475089594	62.63381391
1.773208807	0.949309209	0.551205284	0.67559049	91.12698392
1.510496276	0.969377934	0.643578891	0.81143687	154.4329391
3.223757408	0.952042611	0.542392001	0.738518458	185.3761411
2.139260089	0.93222022	0.499947516	0.796843312	139.0963549
1.665123544	0.953343939	0.582536885	0.75702846	125.7121465
1.944425596	0.963355807	0.628073427	0.711059508	139.2151417
4.228377701	0.915066441	0.351361108	0.634103871	102.143612
7.501997863	0.806511767	0.180110515	0.165841967	61.12628205
8.766114018	0.8676198	0.202436514	0.439946673	139.345295
4.093125159	0.902674788	0.337593865	0.520250218	106.149371
1.599622185	0.955587473	0.635524799	0.797972474	144.4651204
2.228804722	0.939923145	0.498029292	0.736539936	87.63490168
7.42414406	0.883946422	0.258949678	0.577259872	103.8080699
3.580220681	0.893791808	0.327072902	0.650923722	86.30971708
2.947377735	0.930821679	0.409768522	0.723952333	169.9534902
5.88060849	0.901958186	0.312450015	0.403337394	88.11487467
4.329513385	0.888099319	0.319156086	0.37529773	73.96990688
6.455739369	0.903823153	0.297088398	0.480929441	71.95628269
4.881762012	0.906318297	0.305373762	0.633900164	104.6458718
0.713400851	0.958949853	0.725253133	0.830131879	91.50014513
8.391251127	0.935023941	0.29619153	0.618744196	159.2043359
5.042908545	0.860343399	0.320984038	0.323229254	33.90328724
9.506121728	0.800918397	0.18730911	0.000959875	56.88722111
14.46329629	0.845547139	0.153996814	0.420856311	179.7046975
1.864035357	0.94996986	0.582216621	0.761599693	127.4348955
4.088149178	0.880132013	0.330305851	0.26141721	42.35928546
2.955353111	0.930502668	0.41808012	0.702506731	145.3694898
3.355036295	0.88438967	0.332408681	0.615834826	61.74514478
1.384047681	0.963407054	0.678535812	0.791698861	132.6238229
7.637868454	0.866672579	0.246636197	0.396383515	87.31238337
6.789803385	0.898998968	0.258340076	0.405404728	132.3603573
10.67270426	0.865481793	0.195311387	0.497574946	172.7501842
4.103010046	0.899977062	0.324322763	0.588992677	88.87858933
2.12739603	0.907111995	0.396105642	0.719207564	68.86654997
2.954076473	0.913367691	0.385247849	0.627152244	83.88709492
3.88013708	0.953146124	0.505974014	0.713273553	201.0988146
1.45657859	0.952165146	0.582260468	0.741543019	91.7763927
1.688736825	0.923065944	0.519886521	0.717375409	88.87726347
5.412043366	0.839422875	0.224286613	0.328083521	63.93057225
4.764869477	0.915705671	0.349325989	0.586331067	121.6771341
8.485066334	0.834862051	0.197038379	0.302438697	92.77698518
3.338803316	0.93250379	0.463349957	0.761148297	112.3995013
5.364468368	0.863258502	0.293651312	0.329786566	42.17862356
2.618468728	0.924077202	0.437776089	0.694884502	103.9801885
3.256659056	0.924435517	0.435322031	0.665095316	143.2749544
4.081947846	0.898518981	0.336681333	0.463369167	72.01956086
1.537675805	0.954921154	0.610412293	0.803805536	138.8226712
1.185854921	0.949188596	0.660930069	0.815781561	83.93777955
2.152460611	0.968629182	0.654640588	0.717661934	162.7213216
7.694702951	0.88850484	0.277454131	0.36833716	108.8502413
4.003478199	0.89713739	0.337356864	0.360827798	66.92561849
4.168918731	0.887058457	0.345637631	0.314259606	23.28028061
3.529399804	0.906691259	0.348300114	0.498442598	128.4879983
2.747306808	0.946118174	0.511662054	0.682873973	178.1925552
8.192672591	0.847651045	0.218527405	0.303197201	87.1717264
6.185580716	0.913646149	0.311394988	0.556318612	178.1569124
3.342585958	0.95174638	0.519011749	0.698483558	250.7517453
8.309873811	0.804699834	0.198037892	0.115848982	70.74862659
1.853793477	0.943821606	0.56705836	0.715119838	91.87818374
2.771709149	0.92943316	0.457091728	0.541403259	81.31358946
3.87401633	0.898529081	0.329442744	0.459352489	63.34363042
9.132023766	0.887135763	0.225349122	0.415810319	166.6285654
3.031292578	0.823703643	0.322577448	0.012336485	26.48813509
5.979339884	0.785966283	0.264425246	0.04087903	30.16666667
3.41813708	0.944011687	0.487049861	0.749421213	188.2781536
5.134197822	0.884587027	0.31683291	0.533501072	56.5811882
3.373381402	0.897614071	0.38465886	0.521665071	65.97316176
1.999457597	0.936061424	0.51606955	0.776047739	118.1288281
1.56828406	0.952955751	0.611423503	0.789741405	117.9754419
12.38707797	0.853487717	0.169469365	0.147353797	156.1381825
6.229191504	0.897259115	0.280850462	0.554834067	114.9238434
5.609685706	0.786768906	0.267394823	0.093994857	26.98637559
6.978378561	0.881889432	0.220033056	0.609156349	178.5106916
3.635519431	0.911630313	0.352783217	0.633874415	132.6436154

log.sigma.4.0.mm.3D_glcm_SumEntropy	log.sigma.4.0.mm.3D_glcm_AverageIntensity	log.sigma.4.0.mm.3D_glcm_Energy	log.sigma.4.0.mm.3D_glcm_SumSquares	log.sigma.4.0.mm.3D_glcm_ClusterProminence
4.745036878	11.02079693	0.01004389	15.54609285	5981.954756
4.361501662	10.67597258	0.023193483	9.279526527	2511.22132
4.282681833	6.533587755	0.015826631	10.08391309	2643.018217
4.801365591	13.3607469	0.009415469	15.83940023	7872.283569
4.750637751	11.04477528	0.011213291	36.0890125	29900.20374
3.940961527	6.038808777	0.018947306	6.57008519	918.8163965
3.352591121	4.587578897	0.035664347	4.962894976	468.9490944
4.594771755	8.831355123	0.008804042	15.141372	7073.523696
5.846888563	18.90471372	0.002573075	81.7678335	165424.5646
4.370273196	12.12392504	0.018988661	7.879928927	2394.521481
4.047539247	7.305198643	0.016614758	7.403892678	1283.00051
4.597817592	10.00391057	0.010976375	11.4035553	3816.605137
4.229126613	6.936154743	0.016140334	7.935802617	2364.13473
4.501090498	7.376097863	0.011566522	12.33540912	5148.440573
4.55474652	7.658198836	0.009296665	13.6514144	4589.32926
3.320664205	12.78422825	0.134961186	3.888094049	1622.098512
4.55964908	7.860933371	0.010067641	16.95774733	5200.992555
4.396371617	8.120163589	0.012828732	8.797240084	2259.155668
4.789058984	8.433988564	0.008819416	26.23899109	13941.53153
4.290105418	9.266865054	0.028447001	7.883058799	1726.473602
3.872899952	10.02200646	0.055780255	4.99455892	951.2941833
4.505467531	8.014308262	0.012781851	11.66790957	5029.341418
4.115067904	11.70186962	0.03533768	6.484718169	2092.013234
4.918132738	14.72613141	0.01121747	38.98662553	35857.66125
4.09787195	8.032968052	0.024851127	5.738674997	1127.61943
4.335269574	7.609872836	0.012228064	9.892774947	1941.093243
3.966409498	9.359742802	0.041165869	5.201988247	1245.821598
3.752964933	12.20905234	0.099892257	6.611826259	1937.871057
4.163559428	13.31976944	0.067077708	10.76352523	4160.275859
4.409568602	11.42025162	0.029428281	10.85112741	3700.920887
4.013850135	10.99914252	0.051099008	6.231781188	1475.303778
3.783677921	11.63430523	0.07728196	5.409348577	1489.295194
4.708619362	9.66655678	0.009775534	13.70658505	4789.913701
2.217948636	7.587820513	0.125080128	18.63997596	4392.995635
4.563159223	11.35799719	0.012046812	23.36217606	11441.98041
4.497758279	10.03355063	0.010230466	10.54778295	2795.119829
3.764744261	11.79953522	0.082180694	6.549653048	2066.706435
4.380632334	9.014596944	0.022858954	8.626722	2082.540549
4.966942976	9.497272862	0.007783164	23.44478162	12750.60333
4.642891095	8.792673943	0.009600897	13.79087177	4427.421524
4.612631837	12.69676706	0.013719897	12.04600722	4965.1349
4.342923741	9.134486273	0.013401264	11.65109027	5148.803272
4.312966064	8.401469108	0.011205139	8.996426171	1861.79909
4.587093003	8.047873775	0.010245108	15.01916353	8003.664003
4.790177998	9.680514929	0.008322891	17.2555715	12876.69704
3.268263368	9.413277355	0.121915771	3.464510761	690.2127688
5.002787667	12.15472908	0.006700008	24.87919564	42167.17662
4.126666182	5.549642389	0.016836503	9.330155398	2055.076126
2.766685634	7.536589799	0.077013585	15.10581398	2529.307092
5.006311627	12.76761033	0.008593425	40.90746467	30213.01083
4.020283358	11.05113944	0.060091051	6.956558636	1811.86744
3.991223025	6.366688804	0.017360968	7.11825978	1078.681235
4.551459709	11.72369351	0.014799072	11.2694814	3478.789006
4.542272219	7.362634024	0.011188173	12.22346505	3592.562043
3.587077767	11.33639774	0.108668392	5.178793368	1558.913095
4.690707158	8.982772043	0.00759948	16.81295161	5528.563146
4.672013208	11.23690774	0.007654996	15.67513945	6298.687584
5.158632408	12.52887935	0.004828502	32.73898131	22138.50716
4.607307082	9.013605286	0.010471271	12.87205171	4605.73371
4.496919388	7.843645371	0.013646197	10.17742364	2850.729563
4.418694431	8.825655601	0.012856987	9.549549791	2450.726956
4.48267015	13.89140781	0.032385279	11.97120476	5613.651962
3.984007723	9.368251689	0.042857904	5.397464684	1058.8576
4.122120349	9.181064973	0.029505098	6.366252737	1045.254925
3.90097394	7.712403148	0.025930017	13.49301671	2956.735163
4.665282811	10.66909578	0.010062485	13.38079346	4593.481986
4.40635747	9.311192946	0.013940433	19.88646041	6534.018739
4.595929303	10.08992784	0.022242444	13.894752	5181.912058
4.148441604	6.22239119	0.016488254	10.56986044	2540.663082
4.459035453	9.867976819	0.015019335	9.486837259	2360.255912
4.500739995	11.6815655	0.014823149	10.20742346	2931.033676
4.357924407	8.225306485	0.011638428	9.41485889	2388.008789
3.931812741	11.54546394	0.06097858	6.863301802	1743.049816
3.74509664	8.918620707	0.083406797	5.374519758	1250.477573
3.671184995	12.59747168	0.093661856	5.674344024	2065.752006
4.63306285	10.1577568	0.009188235	15.63210617	5720.79858
4.172190159	7.998907994	0.014477364	8.08209412	1565.580223
3.860870668	4.590473167	0.021163749	7.126841952	1403.360621
4.383415925	11.12632471	0.011783944	9.206044921	2300.264063
4.2017114	13.13992969	0.035880251	8.087245125	2151.883994
4.450052237	9.057166845	0.011498285	17.38977264	5680.629912
4.810238629	13.02993507	0.007928304	16.61138284	7995.699342
4.316284396	15.62389196	0.036554878	9.879171745	4016.963006
3.353276373	8.284989583	0.043379795	14.58059895	2333.355977
4.003597116	9.360668208	0.042711753	5.937143621	1217.186244
4.167015901	8.825763592	0.019175822	6.310314824	1220.078607
4.262723778	7.689646129	0.013915039	9.114817077	2604.6826
4.905111957	12.58807628	0.006010572	22.09646489	12317.79836
3.03971391	5.140715523	0.04471108	4.573536547	213.0388594
2.734671708	5.464285714	0.074766944	9.145975057	765.7258704
4.489701067	13.36422138	0.029530961	12.93882401	5912.18249
4.434594743	7.01016957	0.013252816	13.77092931	6251.016834
4.268557392	7.851887763	0.014915172	8.30043475	1545.076004
4.427830621	10.54387238	0.02279446	8.947285125	2432.116703
3.955764949	10.62315543	0.066553454	6.476267883	1570.945304
4.128196754	12.39641468	0.020718073	24.58674496	9007.031193
4.856881603	10.24778568	0.006855594	17.83992026	8490.087437
2.736117171	5.078414854	0.078608676	9.700999841	1101.394094
4.826695947	12.73566849	0.009832164	27.55524106	19709.0821
4.514028368	11.17317252	0.013848629	12.23862675	4515.002711

log.sigma.4.0.mm.3D_glcm_SumAverage	log.sigma.4.0.mm.3D_glcm_Imc2	log.sigma.4.0.mm.3D_glcm_Imc1	log.sigma.4.0.mm.3D_glcm_DifferenceAverage	log.sigma.4.0.mm.3D_glcm_Id
22.04159386	0.915339313	-0.233878998	2.33995266	0.418632812
21.35194515	0.87181432	-0.216830746	1.610276891	0.530480223
13.06717551	0.892139978	-0.229606601	2.487156158	0.396610182
26.67744307	0.802770697	-0.138274648	2.616656172	0.423225013
21.97244811	0.991831908	-0.479294606	4.7592601	0.270008469
12.07761755	0.762033264	-0.135389029	2.266530742	0.435332138
9.175157794	0.907880421	-0.307228372	2.233582595	0.427108073
17.66271025	0.835332215	-0.155486807	3.205526066	0.363100291
37.30036358	0.919586796	-0.191528983	4.835605679	0.296579737
24.24785007	0.841492181	-0.185680108	1.546910247	0.527578432
14.61039729	0.724233024	-0.112640831	2.520999914	0.407861611
20.00782114	0.836397648	-0.174355828	2.130606177	0.443753372
13.87230949	0.714819652	-0.112958243	2.046585554	0.464355931
14.75219573	0.805362563	-0.147134824	2.619510841	0.411821742
15.31639767	0.830383223	-0.157451239	3.052973092	0.363399062
25.5684565	0.882999886	-0.313791189	0.68418464	0.749874481
15.72186674	0.926700104	-0.253775274	3.766607602	0.313180985
16.24032718	0.747424148	-0.122962208	2.06105898	0.462248604
16.86797713	0.968048725	-0.329752807	4.355003157	0.297315742
18.53373011	0.906482765	-0.265253842	1.238002807	0.587167043
20.04401291	0.865649047	-0.240305667	1.040115597	0.635865387
16.02861652	0.791627837	-0.142875351	2.423377207	0.429028054
23.40373923	0.883467767	-0.246271451	1.190182531	0.600897544
29.43212763	0.991930251	-0.480676418	3.843871755	0.314653823
16.0659361	0.848528256	-0.206153487	1.321082005	0.558427797
15.21974567	0.832711649	-0.168148963	2.501496145	0.407839336
18.7194856	0.821956978	-0.191682895	1.245875039	0.59347728
24.41773612	0.902286658	-0.302474745	0.963681872	0.675081056
26.63925156	0.883370777	-0.242959709	1.512343825	0.591547253
22.84050324	0.900823378	-0.24730372	1.479466456	0.553106457
21.99828505	0.877824334	-0.245801247	1.143668166	0.620841975
23.26824249	0.866109691	-0.245882827	1.054615593	0.659683606
19.33311356	0.815319672	-0.147925809	2.378761515	0.429816341
15.17564103	0.993831244	-0.846201118	4.534615385	0.28509031
22.71599437	0.986572549	-0.441112694	4.111402976	0.299055163
20.06710126	0.725317367	-0.106812239	2.429483394	0.418093001
23.59907044	0.889920505	-0.282680722	0.999543504	0.667048878
18.02919389	0.864914893	-0.206740949	1.495482565	0.55025433
18.99454572	0.924381711	-0.242044755	3.419608165	0.348892427
17.58534789	0.849038914	-0.171970582	2.41902938	0.409655117
25.39353413	0.901320418	-0.22854998	1.890938969	0.477723198
18.26897255	0.823906244	-0.157441186	2.812926044	0.394943795
16.80293822	0.67187747	-0.089742113	2.586570813	0.402162986
16.09197746	0.830238746	-0.155502728	2.975834354	0.382291063
19.36102986	0.809633432	-0.139781483	2.753948714	0.390525591
18.82655471	0.882458949	-0.328186336	0.645413604	0.742014904
24.05735247	0.803099896	-0.130733352	3.091412157	0.379816475
11.09928478	0.802021947	-0.15333439	2.699981058	0.403176481
15.0731796	0.993439757	-0.736259247	4.442582418	0.289473232
25.4863504	0.987179297	-0.417623339	5.557015021	0.24852743
22.10227888	0.885081688	-0.252244084	1.18384048	0.622406353
12.73337761	0.691220227	-0.099576087	2.515949552	0.411622202
23.44738702	0.832843432	-0.171867339	1.902149949	0.484305877
14.72526805	0.895251952	-0.220907288	2.396485991	0.415998516
22.67279548	0.883404517	-0.288256559	0.855599358	0.703449597
17.96554409	0.863162145	-0.173224454	3.525140871	0.339193427
22.45455085	0.770670515	-0.117784477	3.359889959	0.348666347
25.01374523	0.928791649	-0.226033733	4.544338282	0.289517487
18.02721057	0.881568675	-0.199697206	2.500124532	0.407625298
15.68729074	0.881729603	-0.216195259	1.852080516	0.468225122
17.6513112	0.798148082	-0.146469795	2.02311226	0.457916283
27.75881877	0.884814304	-0.221288571	1.678690648	0.556919745
18.73650338	0.856915784	-0.231429644	1.118283885	0.619233809
18.36212995	0.855351847	-0.213725542	1.348941494	0.569488715
15.4248063	0.982777454	-0.481908182	3.519294773	0.321341957
21.33819157	0.830015897	-0.160183442	2.466271849	0.425940147
18.62238589	0.979107778	-0.406656544	4.317231469	0.294338882
20.17985568	0.891464697	-0.221057864	1.789371802	0.522248133
12.44478238	0.886123459	-0.219964538	2.949483723	0.378935314
19.73595364	0.840196882	-0.178795152	1.756008443	0.499384573
23.363131	0.829765764	-0.172876544	1.848111729	0.497113647
16.45061297	0.757951891	-0.122619897	2.429486748	0.418088574
23.09092789	0.897083124	-0.275790843	1.056386019	0.645416021
17.83724141	0.900857829	-0.300016442	0.871181893	0.687601688
25.19196058	0.865737913	-0.250831379	1.003676628	0.68228905
20.31551361	0.841159182	-0.159590432	3.406934763	0.363333101
15.99781599	0.73704816	-0.117974064	2.466073597	0.417166054
9.180946334	0.784065209	-0.149740739	2.359549677	0.423361742
22.25264943	0.736463433	-0.121488227	2.30863835	0.427367667
26.27985939	0.863150431	-0.211034337	1.519497211	0.563177913
18.11433369	0.962999835	-0.332172724	3.942535869	0.312847583
26.00838644	0.832078003	-0.149959269	2.833440482	0.393885656
31.23379339	0.864948849	-0.212346728	1.571397369	0.567592912
16.56997917	0.995527991	-0.687139023	4.058655661	0.294749418
18.72133642	0.850716464	-0.214856745	1.214878379	0.607249431
17.65152718	0.71441598	-0.119666577	1.708519818	0.514309551
15.37929226	0.801845543	-0.148736812	2.419797346	0.410694343
25.10636334	0.855702007	-0.157590205	3.957507615	0.317468461
10.28143105	0.930783138	-0.364712609	2.387458816	0.40774375
10.92857143	0.981846474	-0.619942234	3.412698413	0.354662698
26.72589252	0.889249796	-0.226324738	1.711972559	0.542338998
14.02033914	0.877158509	-0.200766239	2.700973721	0.399953577
15.70377553	0.816808056	-0.163355615	2.105600476	0.456498854
21.08774476	0.900399242	-0.246872545	1.388967138	0.564529017
21.24631086	0.895022801	-0.269744378	1.056165701	0.646341489
24.72344287	0.988567645	-0.502945372	5.217318762	0.263810798
20.49557136	0.823513611	-0.144131572	3.073719805	0.368263431
10.15682971	0.966427549	-0.55301395	3.393899904	0.357614375
25.44001113	0.979402674	-0.380750107	3.716534825	0.314490558
22.34634504	0.923450921	-0.256024882	2.274135226	0.430581527

log.sigma.4.0.mm.3D_glcm_ClusterTendency	log.sigma.4.0.mm.3D_firstorder_InterquartileRange	log.sigma.4.0.mm.3D_firstorder_Skewness	log.sigma.4.0.mm.3D_firstorder_Uniformity
53.1154421	164.0030136	-0.039111159	0.070264961
31.95939464	119.9012442	-0.529734055	0.099201436
30.6168678	118.072197	0.44813257	0.094449232
49.39838832	137.9638157	0.175688296	0.071722679
105.103028	195.8142929	0.461256959	0.051465543
17.4533492	99.59862518	0.415697937	0.105112873
11.43340889	84.05536652	0.254224771	0.130204082
43.15139685	138.1914978	0.621159682	0.070117723
280.2719261	295.158041	0.601241667	0.038124904
26.98445675	99.25489998	-0.154786483	0.099689629
19.05154553	101.9636078	0.526851994	0.100780896
37.64111424	124.4787407	0.098001727	0.078982775
23.90210386	99.02540207	0.962314755	0.101701219
37.25567266	138.2204247	0.631045601	0.078763487
39.34445878	139.1843786	0.43312428	0.075670209
13.88406049	26.30597556	-1.837924593	0.246262597
46.03680511	155.5135193	0.149668608	0.069336788
27.37971318	104.7246761	0.395487534	0.092213904
75.09043592	211.4244328	0.294760487	0.052107233
28.44080004	111.1441691	-0.399989254	0.107593219
17.55667487	86.95774126	-0.628901904	0.146351603
35.92013232	110.1739769	0.773214602	0.084398649
22.9016504	91.58378601	-0.816688707	0.123482361
132.5359087	231.2273712	-0.363763355	0.05217932
19.59788827	81.54166508	-0.366282278	0.118194444
29.21302683	116.9190941	-0.010882302	0.088526819
17.44351892	72.64614296	-0.251115897	0.142787671
23.96454669	104.8174725	-0.860726754	0.177271938
37.44349678	128.419477	-0.769795841	0.142787683
39.027329	136.7014599	-0.565754227	0.096162964
21.92011535	97.89595032	-0.552645834	0.136083547
18.53590495	73.53532696	-0.756148677	0.172194536
44.80522743	147.42136	0.105937289	0.073009624
43.03939103	188.563879	0.351029581	0.12
67.18517596	171.3011856	-0.000848871	0.061633644
32.04737236	119.675808	0.194252019	0.083660194
23.56808738	82.38628332	-1.179813929	0.176809711
29.99075805	117.4976673	-0.099836098	0.096826463
73.90993098	191.9029427	0.022497117	0.061887986
45.56937138	155.9984055	0.166001874	0.073693774
41.54760137	123.93608	-0.414149062	0.085119077
32.64303654	114.7529602	0.826513871	0.085153726
24.74852862	105.5442009	0.267105696	0.089157791
44.32203505	148.313015	0.688582882	0.073437768
56.36919176	122.0181808	0.711044607	0.075234169
12.70533368	51.72062731	-1.370645274	0.231912615
76.28123967	138.3360775	1.231102197	0.068772747
24.82442926	119.4198914	0.609037153	0.094028932
30.17049481	140.4310074	-0.063281046	0.107438017
115.2040067	235.2517204	-0.082572559	0.048132858
24.52385023	101.2324393	-0.66043985	0.140755839
17.8826512	99.0612793	0.683943948	0.109913859
38.3804145	132.7241735	-0.310925588	0.086854509
39.51080758	142.3767357	0.010069958	0.077362091
18.57606324	47.37003225	-1.346294162	0.212236785
46.84498382	163.7043839	0.183060487	0.06511027
43.5048324	144.4862404	0.405529167	0.071292926
97.11992424	190.0951347	-0.225322493	0.051114586
40.98226572	128.9027786	0.356856199	0.076494622
35.03322996	125.2114115	0.26288907	0.084354105
31.08039095	104.0838013	0.380875814	0.09411084
40.61284982	119.0307502	-0.260500769	0.104105887
18.81830936	72.08501363	-0.619253731	0.136801403
21.8860435	98.7344842	-0.391282455	0.119480753
35.86123811	127.0156555	-0.126430703	0.102883588
42.43661058	128.7797606	-0.122411778	0.07810993
51.6134616	167.297226	-0.194442996	0.067029478
48.96674704	155.7890563	-0.244198516	0.086258846
28.04176026	127.2579346	0.569156832	0.086507898
32.16675981	117.2222524	-0.243233715	0.090110935
34.02785615	124.2665739	-0.252656702	0.087899407
27.54881865	107.5879517	0.486279475	0.090525648
24.77233159	97.85831559	-0.847637077	0.145879976
19.53608149	76.91578756	-1.039530415	0.172865942
19.43064245	48.45610285	-0.740734921	0.197705095
42.58102341	146.35672	0.344034713	0.070482013
22.0427528	107.7771416	0.428091996	0.096241151
18.66160345	95.37240219	1.101461008	0.107621173
27.76881487	106.0176239	-0.105863209	0.089152788
27.2374257	114.3338041	-0.369397369	0.111570411
44.97793791	154.7352142	0.214116389	0.068960858
50.55507856	134.3836441	0.258989716	0.06976912
33.35780841	98.36720276	-0.704895508	0.11818276
33.04645466	149.2588444	-0.184487338	0.090530697
20.38253505	88.60848498	-0.606018989	0.134019353
19.45244049	84.50265884	0.311427381	0.111124608
26.64673078	116.1852417	0.717593443	0.092272471
61.25099526	160.2635956	0.12297448	0.05956111
9.195144345	81.95929241	0.030226815	0.1408
18.75056689	111.0949249	0.012488685	0.147928994
45.24175062	134.4622407	-0.58615818	0.100044472
42.38221571	125.5141144	0.932906737	0.085028566
25.23004181	112.7561817	0.141650204	0.09522929
31.80060742	117.1448395	-0.402375105	0.095856492
23.19708805	92.01734409	-0.693045867	0.148950662
54.27173823	202.3969078	0.230564604	0.073318216
55.4628452	154.0342512	0.230110067	0.065004325
21.02482747	127.0126438	0.247607658	0.149305556
87.84830682	175.0873499	0.175070391	0.059279612
40.0313635	121.1347942	-0.345652761	0.083035714

log.sigma.4.0.mm.3D_firstorder_MeanAbsoluteDeviation	log.sigma.4.0.mm.3D_firstorder_Energy	log.sigma.4.0.mm.3D_firstorder_RobustMeanAbsoluteDeviation	log.sigma.4.0.mm.3D_firstorder_Median
85.0669373	11898397.67	64.95881529	-146.9898224
63.76691143	20460054.65	47.97554705	-82.29793549
67.40583189	5654723.538	47.90037852	-175.1813049
81.98489814	37370127.99	57.0111499	-88.90718079
122.348709	5755030.848	88.04307613	-196.6731873
56.20802895	12798126.35	40.61517497	-227.0264664
47.65105604	970110.5425	34.50812067	-101.246254
84.61421008	23623782.1	58.25402133	-233.1651001
172.4298009	68955412.51	125.0219482	-91.85485077
58.06298863	28880577.52	40.42123631	-80.50794983
61.27767836	18760003.95	42.95451097	-226.3114014
72.04920486	14671515.74	51.89010072	-108.3840408
60.34625589	23010105.93	40.2818313	-117.3953247
77.42100995	14346980.19	55.58119489	-150.4440231
78.14787594	6808711.101	55.95819641	-100.6278801
33.005308	31011008.82	14.39348034	-3.863708258
87.97678694	12033015.2	65.08049483	-197.1562805
61.70677253	34819966.11	43.18164602	-129.0428009
115.4761535	9701639.89	85.75567974	-197.3247375
59.28402646	22291904.92	44.98685273	-41.42212296
47.69640864	38453155	35.3959826	-54.49269104
70.56820642	16709771.43	46.74602566	-108.7862473
51.8200071	27608942.86	36.7906501	-62.38962746
126.1172239	3419478.137	95.28668262	-45.77640152
48.83898914	10769379.99	34.61137067	-82.26128006
64.8587845	12186916.7	47.20720767	-182.7956619
45.43606732	14212834.14	30.18421849	-42.27813721
56.76749924	32062031.29	42.9519926	-33.29100037
70.09526438	49362101.45	52.91645873	-54.12641144
71.24583543	30942832.61	54.61913104	-90.04536438
53.038927	35256153.1	39.29522862	-51.25076294
46.82371255	50718001.43	31.25175826	-26.90398788
79.84504626	30372258.23	59.08590612	-113.3550262
100.4856029	473963.2184	71.73614433	-163.3451233
98.97834684	5070431.497	68.99741738	-173.6560364
68.41346104	40952007.55	49.78144251	-159.705719
52.96194005	41909499.42	37.04065637	-11.61588573
63.98603271	28132865.83	47.64796389	-48.24596596
103.7923937	9553078.222	78.9630503	-115.1697159
80.77216059	18133253.67	62.76010999	-160.6260986
70.46134366	11283042.26	50.34667119	-83.67089844
76.17001674	20715829.16	51.25564918	-234.3133698
64.04696272	35137077.28	45.19663103	-196.2046127
85.6776662	20614225.3	61.46775505	-206.7776031
78.47814081	25877421.66	52.01308732	-125.1634293
37.98902953	19804553.23	24.77943226	-9.524876118
86.8393075	47192281.91	57.68873566	-72.8270874
70.20145202	13418036.27	51.16277351	-249.6187592
80.16772373	1132500.893	51.27603781	-202.2724915
133.1338708	9360657.56	99.53516563	-186.695343
56.01735999	34138113.93	41.44796453	-42.85853767
57.57106997	25753986.19	40.73253829	-257.0495911
70.22224208	50739380.25	52.77806395	-84.85575867
75.07109728	4921821.271	56.22565238	-106.9027405
43.6668846	47974370.52	25.22925223	-7.397426128
89.01214785	19163368.96	66.02311641	-220.7451324
81.5868644	41173675.57	57.34454127	-202.3649979
113.2949155	25166218.05	81.1734603	-175.3867722
75.90678104	11042727.8	53.47711058	-158.6658707
67.77227487	8809058.487	49.60790133	-117.1001358
62.38429966	38045430.81	44.74626036	-153.7805023
70.96951433	81496592.77	49.77999151	-43.86270332
46.97052732	16999136.38	31.99481424	-11.33318281
52.82892321	12906411.83	40.37738467	-48.76030159
76.95170359	3094206.1	55.04192443	-226.7784271
74.16047511	13798674.52	52.97314379	-53.03595543
93.42671169	7406277.97	66.16427716	-231.3578491
80.32874757	56095682.64	62.24693212	-67.52687073
71.68221314	12471329.91	52.58891666	-283.259552
63.95705662	24737570.54	47.69939245	-83.81080627
66.20088549	39912921.3	49.21186518	-90.51725006
63.68725036	25631515.21	44.34812786	-199.584671
55.32771215	49270376.12	41.33255115	-25.00491524
48.97147939	57155467.15	34.36855813	-14.47466087
43.39190224	60535247.54	24.33439062	-2.807829857
85.50938032	25159322	61.91488835	-233.4595566
60.75899889	20653879	42.39636937	-203.6917953
59.55797501	13234635.27	40.55070044	-245.3542328
63.75520145	36740543.54	44.19937723	-181.5780792
60.31945472	34105342.15	45.01938217	-77.71899796
84.86910285	15785803.48	60.05639918	-319.4906616
82.1501153	23803403.66	56.29298897	-133.4272003
62.87255656	41164321.44	42.73430063	-20.43192863
80.06145834	823397.3986	61.8124027	-138.4853516
50.44822929	33162357.87	36.4978972	-40.22842789
51.08884371	27737575.68	35.34687914	-62.10471725
66.46196933	14953566.24	46.80677488	-196.8806915
96.02739436	45593332.51	66.84882652	-256.4101257
44.74509394	436771.6453	32.98397462	-74.61532593
67.6514015	610608.7964	47.68851951	-226.797226
73.6653892	48016828.31	54.75551254	-59.39076233
79.42302892	15603548.99	54.27825107	-227.351387
60.32368031	7138775.983	44.16409313	-110.9867897
63.25901305	41180862.06	47.31068075	-68.28237152
54.91165843	48083872.16	39.66333936	-21.66939735
107.1585166	4588354.395	77.93217418	-254.6497803
88.81326133	16509737.61	63.59506121	-108.4344597
61.84950124	838927.7178	42.38517295	-174.8306656
108.1802785	5174951.879	77.92147732	-123.4692459
71.79500739	5422858.498	49.58656851	-93.32356644

log.sigma.4.0.mm.3D_firstorder_TotalEnergy	log.sigma.4.0.mm.3D_firstorder_Maximum	log.sigma.4.0.mm.3D_firstorder_RootMeanSquared	log.sigma.4.0.mm.3D_firstorder_90Percentile	log.sigma.4.0.mm.3D_firstorder_Minimum
321256737	99.96715546	181.547653	-25.42246246	-402.1663818
552421475.4	80.542099	120.7604045	-6.505636597	-345.3018188
152677535.5	72.58009338	178.7388895	-48.13806763	-322.0889587
1008993456	303.0177002	139.0421151	24.94818993	-415.4494019
155385832.9	198.5997925	238.7058932	2.71758008	-458.2991638
345549411.5	-37.13017273	229.0225699	-123.6348724	-354.6461182
26192984.65	46.5824585	117.7230735	-37.05210381	-216.9684906
637842116.7	171.7423096	242.7183034	-69.12725067	-432.4646606
1861796138	465.4846802	216.1429137	300.0840149	-450.8868408
779775593.1	185.6136475	111.5970109	-1.459043002	-352.4763489
506520106.6	39.51261902	223.368815	-113.3075829	-376.3015747
396130925	220.9015503	139.2161285	7.904181099	-347.7350464
621272860.2	234.7904663	129.7878264	-6.152740479	-271.8363342
387368465.2	191.2911682	164.8402589	0.359280865	-316.8174133
183835199.7	220.3027496	136.7671389	23.75663872	-282.5945435
837297238.3	270.2464905	53.51602202	23.78290348	-322.984436
324891410.5	74.42803192	235.4818103	-70.92007599	-416.6377258
940139085	247.883255	149.7849965	-28.43288116	-312.1322632
261944277	140.9952393	230.8803016	-2.755883002	-423.4924316
601881432.7	127.0044785	89.64382159	21.9028532	-251.6674194
1038235185	196.1796722	87.04582905	-2.488559294	-295.6784058
451163828.6	213.5112457	135.5823987	24.44830742	-292.6655579
745441457.3	168.1192322	97.23742808	-1.031205475	-340.37677
92325909.71	209.6125641	160.3445102	124.9948074	-411.0493164
290773259.8	104.6130829	105.915552	-11.9064661	-271.7014465
329046750.8	33.08845139	200.8831222	-83.37728577	-357.2341309
383746521.7	271.0291138	78.79840878	4.366541195	-271.8487549
865674844.8	362.8189392	90.72809367	0.26056782	-335.1366272
1332776739	298.9993591	115.2547857	-1.43401587	-376.6217041
835456480.6	94.06943512	135.1921749	-9.059690094	-373.4647827
951916133.6	215.397522	91.60962828	-0.64559263	-312.2226868
1369386039	319.9017944	75.77517545	3.16991992	-306.4527588
820050972.2	243.8424225	147.8725012	5.057342529	-349.5751953
12797006.9	97.21743011	177.7569911	15.15165851	-311.4580688
136901650.4	144.2784424	211.8279377	-19.8007431	-458.682312
1105704204	108.4156265	175.2103203	-38.81607018	-398.2301636
1131556484	172.1067657	79.14267438	4.269259453	-306.8951111
759587377.5	263.9648132	96.32574776	29.56667595	-273.06604
257933112	211.1940765	172.5119367	20.72546387	-367.6200256
489597849.1	86.23339081	183.7598915	-27.97503052	-359.7466431
304642141.2	193.5069122	125.6203753	14.45022182	-395.492981
559327387.3	147.6189575	233.1785904	-75.70224762	-448.0059204
948701086.5	58.58573532	206.4995982	-84.03012238	-394.0859375
556584083.2	236.3120575	216.6961768	-47.59790649	-386.2363586
698690384.8	253.595932	152.3431978	21.07962646	-346.6095886
534722937.3	89.75471497	56.87689273	8.862168694	-230.9388428
1274191612	692.9916992	131.0947266	74.70014572	-330.4103699
362286979.4	-2.886508465	242.5923253	-100.0007904	-369.7229614
30577524.12	-1.16582787	226.8861242	-96.06179733	-392.796814
252737754.1	127.4447556	253.2076083	14.64256001	-529.4692383
921729076.1	198.9941864	91.47231705	2.189681435	-311.9682312
695357627.1	5.269738197	252.7957138	-139.658493	-396.766571
1369963267	207.0952454	126.0193247	2.940120888	-368.0584412
132889174.3	107.5919647	143.5039875	-0.580474758	-298.5016785
1295308004	207.0264587	65.14035963	17.78279591	-279.1293945
517410961.9	58.9403801	239.1737578	-74.98406525	-448.4560242
1111689240	200.8855133	217.2959223	-57.78683243	-461.4717102
679487887.3	126.1945724	234.410056	-3.872558498	-508.2070618
298153650.6	145.415329	176.6186466	-27.48610249	-365.0254211
237844579.2	124.9061203	137.4902141	4.566820383	-283.6407471
1027226632	137.9804688	161.7036252	-28.60688171	-332.1477051
2200408005	380.2032776	109.6687838	12.69644022	-388.4277649
458976682.4	258.3064575	64.59559706	39.169133	-236.8457489
348473119.4	93.69272614	85.10379162	14.26923094	-250.240509
83543564.69	-18.49058342	241.6221165	-111.7402496	-406.5330811
372564212.1	288.3032532	109.4441231	52.44294701	-318.0052185
199969505.2	-17.73300552	265.5861078	-90.21833344	-496.0834045
1514583431	246.7929077	127.2002011	10.34474068	-322.1180115
336725907.4	-48.80208969	295.3169437	-164.6295319	-426.1933289
667914404.6	161.9175415	117.492331	8.512089825	-309.5429688
1077648875	144.859436	123.923485	2.5528584	-351.6817627
692050910.8	99.61326599	212.6158371	-96.15321503	-383.5992737
1330300155	199.5443573	83.55700793	7.587287617	-321.4811707
1543197613	117.4398727	72.63308217	9.007706738	-247.8919678
1634451684	383.3588562	64.57074671	24.58987923	-306.4451294
679301694	129.9020538	237.5100753	-64.47983742	-473.3830872
557654733	80.71800232	212.8229177	-98.8900795	-383.7982178
357335152.4	56.97098541	243.0703462	-137.1033417	-349.4286804
991994675.6	70.64757538	200.602989	-81.46591339	-428.9928284
920844238.1	223.7549286	112.3072678	-3.296216249	-389.0079956
426216693.8	-66.57936096	325.4919197	-157.5737335	-543.6517944
642691898.7	227.4596558	165.8866653	11.1918993	-435.5032349
1111436679	285.6509094	91.38629518	30.18842278	-407.1156311
22231729.76	15.23070049	162.9760915	4.902193069	-340.0900269
895383662.6	178.3063965	81.75123475	8.537232018	-267.1477356
748914543.5	242.7722321	88.93370125	20.38674431	-263.7536011
403746288.6	119.0938187	202.4072001	-78.35103149	-357.499054
1231019978	145.9934845	278.2229948	-94.89611359	-567.6629028
11792834.42	29.50790787	93.46353784	-7.762251163	-199.4761353
16486437.5	-82.09343719	216.7254194	-103.4370483	-337.2879333
1296454364	255.4511871	118.3349393	7.684022522	-392.2811279
421295822.7	80.90744781	226.5556527	-50.5683773	-353.3462219
192746951.5	109.7205811	130.9981648	-18.22396469	-278.9379272
1111883276	138.3748016	106.189685	13.71840229	-322.3433838
1298264548	186.6726074	81.5963956	8.392381573	-280.5049744
123885568.7	125.3065643	269.872233	-78.91221466	-529.3670044
445762915.6	228.1257019	155.8172837	28.61146526	-372.095459
22651048.38	-36.20220947	186.9634231	-46.89688034	-284.0093384
139723700.7	198.5099945	178.1801646	72.42335815	-431.6073608
146417179.4	160.4035797	139.1666331	0.308496007	-362.8056335

log.sigma.4.0.mm.3D_firstorder_Entropy	log.sigma.4.0.mm.3D_firstorder_StandardDeviation	log.sigma.4.0.mm.3D_firstorder_Range	log.sigma.4.0.mm.3D_firstorder_Variance	log.sigma.4.0.mm.3D_firstorder_10Percentile
3.979441953	100.8280166	502.1335373	10166.28894	-281.1607056
3.541139554	76.59159553	425.8439178	5866.272505	-202.3039886
3.640388102	83.11362568	394.6690521	6907.874774	-259.2323853
4.0531991	103.3183348	718.4671021	10674.67831	-227.3903656
4.422531102	151.0492959	656.8989563	22815.88979	-375.5834961
3.417281922	68.31910263	317.5159454	4667.499784	-303.9363007
3.086097312	58.41227476	263.5509491	3411.993842	-177.8554794
4.05492177	107.2055016	604.2069702	11493.01958	-345.671051
4.905095752	211.2253759	916.371521	44616.15943	-288.4414063
3.578191404	73.11353088	538.0899963	5345.588398	-179.4139587
3.574163483	75.73719085	415.8141937	5736.122079	-298.7051697
3.836800531	88.88888216	568.6365967	7901.233371	-217.3455719
3.57955867	77.99679567	506.6268005	6083.500135	-189.8006973
3.865797503	95.17015437	508.1085815	9057.358282	-244.5395706
3.903817103	96.00081759	502.8972931	9216.156978	-223.511763
2.621791277	50.81562387	593.2309265	2582.227629	-80.22993469
4.020420544	105.9669374	491.0657578	11228.99182	-352.6344238
3.656359968	77.98796661	560.0155182	6082.122937	-228.425264
4.351561773	137.7275608	564.4876709	18968.88102	-364.6334045
3.412962054	69.90589948	378.6718979	4886.834782	-158.3753708
3.087120435	58.15799854	491.858078	3382.352795	-147.2122162
3.833374456	92.02930589	506.1768036	8469.393142	-206.334787
3.29471499	65.45265325	508.4960022	4284.049818	-154.7272263
4.406666474	148.9451988	620.6618805	22184.67225	-262.256134
3.290502171	60.9603157	376.3145294	3716.160091	-167.7148087
3.650492958	79.11263391	390.3225822	6258.808844	-294.7400238
3.184781685	59.26277977	542.8778687	3512.077066	-128.6958038
3.034846121	67.84360538	697.9555664	4602.75479	-161.2648071
3.395334332	83.98975845	675.6210632	7054.279525	-200.0831604
3.610541773	84.55697477	467.5342178	7149.881983	-227.1170593
3.229670353	64.53318842	527.6202087	4164.532408	-154.2072906
3.061303099	60.58113718	626.3545532	3670.074182	-134.2758636
3.950750054	96.29096839	593.4176178	9271.950594	-236.0218719
3.189898095	117.0925739	408.675499	13710.67086	-266.1769531
4.20312148	122.7809319	602.9607544	15075.15724	-331.3514038
3.769272337	83.63525425	506.6457901	6994.855753	-254.2588242
3.003096636	65.10388744	479.0018768	4238.51616	-146.9424133
3.593991782	76.63653556	537.0308533	5873.158583	-165.4214813
4.192056301	123.1109577	578.8141022	15156.30792	-296.4335938
3.884129146	95.0157022	445.9800339	9027.983665	-278.3480713
3.773419722	87.63343383	588.9998932	7679.618726	-208.2378326
3.859305055	97.18948769	595.6248779	9445.796518	-317.0404053
3.69853391	79.5792432	452.6716728	6332.855948	-293.426767
3.987061912	106.3483611	622.5484161	11309.97392	-311.8531128
3.997282788	101.571652	600.2055206	10316.8005	-233.0124603
2.542688339	48.02133704	320.6935577	2306.048811	-112.1020584
4.137224583	115.9923895	1023.402069	13454.23441	-189.5277557
3.624568684	84.43799772	366.836453	7129.775459	-330.3845825
3.390805482	99.13028578	391.6309861	9826.81356	-299.7457733
4.504568545	158.4525933	656.9139938	25107.22433	-423.1235046
3.259426497	67.78397662	510.9624176	4594.667486	-160.0293076
3.441431846	70.98536505	402.0363092	5038.922051	-319.968927
3.723515691	84.30971978	575.1536865	7108.128849	-210.3627075
3.814106311	89.70573824	406.0936432	8047.119473	-230.2931122
2.832403347	58.11851119	486.1558533	3377.761343	-124.8542366
4.081115432	107.0617831	507.3964043	11462.2254	-352.5712036
4.041722662	101.9498137	662.3572235	10393.76451	-312.5241241
4.461779337	139.7121306	634.4016342	19519.47943	-386.9002167
3.913563049	93.86586057	510.4407501	8810.79978	-265.8610352
3.716868639	82.30123586	408.5468674	6773.493423	-214.1209946
3.615196504	76.82235458	470.1281738	5901.674163	-234.1356201
3.698159494	89.25977147	768.6310425	7967.306803	-189.2415695
3.178952037	59.15358867	495.1522064	3499.147052	-115.7337074
3.255060828	62.15045253	343.9332352	3862.67875	-144.2953674
3.575937201	92.83746291	388.0424976	8618.79452	-365.9725037
3.88459595	91.98584802	606.3084717	8461.396235	-182.5803268
4.087647041	114.8943626	478.350399	13200.71455	-412.7049866
3.787312046	94.5068124	568.9109192	8931.537589	-222.941272
3.696831851	87.06242148	377.3912392	7579.865234	-384.6177551
3.623572299	76.97079533	471.4605103	5924.503333	-194.1119904
3.692496535	80.26063536	496.5411987	6441.769589	-201.8923492
3.683911168	80.10381603	483.2125397	6416.621342	-294.2886963
3.148537012	65.97671955	521.025528	4352.927523	-154.8313232
2.974456583	59.63563713	365.3318405	3556.409216	-137.0699615
2.957023986	60.89136566	689.8039856	3707.758412	-112.1326462
4.042325213	104.4489213	603.285141	10909.57715	-332.413208
3.596281329	76.23931641	464.5162201	5812.433367	-287.9449005
3.457443551	76.07253189	406.3996658	5787.030109	-312.1557648
3.723055371	80.75459078	499.6404037	6521.303932	-290.1529358
3.462244766	73.70441501	612.7629242	5432.340792	-185.4865479
4.036271802	104.3512721	477.0724335	10889.18799	-434.5415527
4.081586985	104.0615981	662.9628906	10828.8162	-258.2152283
3.519546496	79.39729271	692.7665405	6303.930089	-160.380188
3.60663381	94.88617613	355.3207273	9003.38642	-248.6344299
3.214349675	61.77093094	445.4541321	3815.647909	-143.4346039
3.414015721	64.72399657	506.5258331	4189.195732	-142.2647369
3.679615391	82.77218809	476.5928726	6851.235121	-277.149646
4.286331756	120.4601768	713.6563873	14510.6542	-399.0882324
2.962292189	54.00160555	228.9840431	2916.173402	-143.1860428
2.87314068	76.40316956	255.1944962	5837.444318	-290.3079102
3.676116645	89.55268735	647.7323151	8019.683811	-202.4207764
3.803451399	99.70673201	434.2536697	9941.432408	-314.8126892
3.563404544	73.50550335	388.6585083	5403.059023	-205.9130249
3.569634444	75.63676914	460.7181854	5720.920845	-178.8207123
3.207980932	66.25701169	467.1775818	4389.991599	-148.9543396
3.965086972	128.1750036	654.6735687	16428.83155	-392.7145447
4.134760557	110.0764194	600.2211609	12116.8181	-252.2616241
2.919372149	76.77602949	247.8071289	5894.558704	-262.7033325
4.27632263	132.5662255	630.1173553	17573.80414	-289.6107544
3.840894233	90.5452416	523.2092133	8198.440776	-235.6356461

log.sigma.4.0.mm.3D_firstorder_Kurtosis	log.sigma.4.0.mm.3D_firstorder_Mean	log.sigma.4.0.mm.3D_glrlm_ShortRunLowGrayLevelEmphasis	log.sigma.4.0.mm.3D_glrlm_GrayLevelVariance
2.231752022	-150.9743733	0.015397228	16.36800302
2.619970391	-93.36381952	0.015192241	9.412635719
2.784682165	-158.2394257	0.051649389	11.35264831
3.407002367	-93.04854356	0.007909134	18.04646927
2.658792357	-184.8367216	0.027751614	36.58338021
2.563369659	-218.5951457	0.037483053	7.681256948
2.738682943	-102.2092373	0.092512718	5.467354403
3.371847939	-217.7593976	0.02872195	18.75333716
2.40230608	-45.84320809	0.007893007	70.03586389
3.420857627	-84.3107612	0.00872999	9.306670538
3.063668839	-210.1368731	0.026335232	9.628530078
3.026995216	-107.1442815	0.020349738	13.13099734
4.33219863	-103.7370703	0.034422657	10.42476785
2.997733785	-134.5918002	0.033696447	15.0650235
2.99062597	-97.41197718	0.030624424	14.91278848
8.489647016	-16.78502258	0.007122248	6.609234992
2.454987335	-210.2919189	0.046810037	18.18420162
3.588465998	-127.8804998	0.026811277	10.18072665
2.263787094	-185.3020039	0.053228931	29.80533914
2.24924587	-56.11755489	0.017267321	7.907573581
3.206580865	-64.76591357	0.013866896	5.705188281
3.691100493	-99.56502242	0.029116295	14.42800879
3.997220903	-71.91013559	0.010654471	7.281313042
2.209323145	-59.37751838	0.019414278	35.55888154
3.050078149	-86.61376372	0.029827569	6.318082504
2.389426836	-184.64891	0.038270311	10.358189
4.550996216	-51.93372854	0.016858584	6.527797434
3.489484654	-60.2397891	0.009177929	8.070879663
3.16258562	-78.92646005	0.008749982	11.91065535
2.470904835	-105.484796	0.015111157	11.25574677
3.273803631	-65.02147019	0.011398489	7.137955611
4.615840793	-45.51706308	0.009113533	7.314376696
2.683603943	-112.2244449	0.024082942	15.1304543
2.040976263	-133.7418298	0.106294089	22.13739515
2.585244952	-172.6149413	0.02233229	24.35207354
2.675389139	-153.9603864	0.014407204	11.46858977
3.747591814	-45.00051942	0.010108321	7.927203375
2.616037007	-58.35658573	0.019882525	10.07133976
2.370434615	-120.8472605	0.035741346	24.02268872
2.085657373	-157.2886329	0.025738634	14.49058601
3.143212573	-90.00477746	0.010075935	12.50311383
3.774662105	-211.9586245	0.01795209	15.67453528
2.886338206	-190.5498048	0.022567338	10.53874146
3.369628078	-188.8048175	0.035953684	18.55697547
3.612641277	-113.5413996	0.01932965	17.10262516
4.051581283	-30.47838767	0.014237047	4.567691786
6.891940593	-61.08676566	0.010932353	22.37811235
2.583652595	-227.4230877	0.069186331	11.63842368
2.547398619	-204.0845408	0.10208469	15.06993416
2.149702428	-197.5015659	0.023798481	40.30408792
3.150739817	-61.4208214	0.011845654	8.164567682
3.210068989	-242.624712	0.039698377	8.395268821
2.624884305	-93.66291336	0.011701838	11.55428217
2.254147219	-112.0101556	0.047121805	12.53233349
4.54841017	-29.41946821	0.009742937	7.273728401
2.382558256	-213.8734697	0.029469098	18.84380906
3.150174554	-191.8951624	0.01215065	17.15539492
2.452404896	-188.224852	0.020783694	31.24595646
2.863643568	-149.6106498	0.024824875	14.53857609
2.434805964	-110.1365769	0.030557374	11.06563201
2.757480037	-142.2898037	0.020312635	9.272808067
3.943717729	-63.7176219	0.007357235	14.54816592
3.230787067	-25.95080167	0.014648291	6.54789944
2.343557942	-58.13756615	0.016143457	6.233675349
2.338083474	-223.0749933	0.063471039	13.81588828
2.968207717	-59.30109479	0.016712229	13.72002255
2.377166366	-239.4478359	0.038446481	21.21684974
2.422163076	-85.13726309	0.024778508	14.51460224
2.76514665	-282.1918355	0.044421432	12.28239728
2.4703155	-88.76905155	0.016394329	9.645089884
2.642308286	-94.42065737	0.010564426	10.49388572
3.331500714	-196.9489093	0.024540375	10.80290638
2.936157418	-51.27227371	0.009879806	7.55414444
3.203254798	-41.46269901	0.023914205	6.701337401
5.946593084	-21.48541176	0.00713746	8.405169596
2.858679914	-213.3107093	0.020679225	18.09606484
3.055274134	-198.6986687	0.022294791	9.656414031
4.369177271	-230.8596177	0.109323494	9.586875627
3.087513186	-183.6307579	0.014754501	10.87224816
3.158733538	-84.73831248	0.007554688	8.999959343
2.570491739	-308.3112093	0.031032256	17.83706524
3.136214139	-129.1881168	0.009407794	18.03170988
3.883955091	-45.24958407	0.004953336	11.52815979
2.193691862	-132.5059243	0.056182786	14.44988003
3.133041274	-53.55013048	0.018119568	6.729540189
3.442013222	-60.99186409	0.015600803	7.354694972
3.549249476	-184.7090672	0.027063855	11.36509977
2.971285063	-250.7935019	0.01336496	23.52610774
2.420070881	-76.2840711	0.082399458	4.874395453
1.887432783	-202.8113978	0.114533919	9.760672894
3.223051291	-77.35291883	0.009562711	13.47243392
3.296782911	-203.4355706	0.046680799	16.10772963
2.611233804	-108.4318227	0.026594885	9.012643791
2.556619896	-74.5340751	0.015121031	9.203683484
2.920862092	-47.62331547	0.012179562	8.272170256
2.705840275	-237.4914537	0.025506357	27.11432298
2.74545861	-110.2824002	0.019913112	19.6540826
2.059460224	-170.4721762	0.14232867	9.713369662
2.486424964	-119.0561502	0.015765935	27.8416985
2.987756252	-105.6830687	0.01765142	13.70485462

log.sigma.4.0.mm.3D_glrlm_LowGrayLevelRunEmphasis	log.sigma.4.0.mm.3D_glrlm_GrayLevelNonUniformityNormalized	log.sigma.4.0.mm.3D_glrlm_RunVariance	log.sigma.4.0.mm.3D_glrlm_GrayLevelNonUniformity
0.016426617	0.069555523	0.123976713	22.6006454
0.016779675	0.092365454	0.488539646	103.2448737
0.055813647	0.092602036	0.095936711	15.13134416
0.008589686	0.069165281	0.247470631	115.8946416
0.028402743	0.051300415	0.034184581	5.004043104
0.041692195	0.102920957	0.147988291	22.38170221
0.098666987	0.130915081	0.062033641	8.589625925
0.030428721	0.068699471	0.094692146	25.38870827
0.008292576	0.038076653	0.084336663	52.31217715
0.009853193	0.095259884	0.407655393	177.7066591
0.028351035	0.095259176	0.133807575	32.28985674
0.022066313	0.077780172	0.170539098	51.88819613
0.038275054	0.098076313	0.225770019	114.2791406
0.037667692	0.076407472	0.149611556	35.98281316
0.033470897	0.075518938	0.080968029	25.57813412
0.009327302	0.155342732	3.503474593	858.8224824
0.049174872	0.069125763	0.053209406	14.30534946
0.029858716	0.090589552	0.237486062	120.0246975
0.060589583	0.052308799	0.056530861	9.040036594
0.020106646	0.099678661	0.819260596	203.3418915
0.016338446	0.122703166	1.287545453	421.1056689
0.031732745	0.080883454	0.194120869	64.9586682
0.012279331	0.114627161	0.799533617	244.2891004
0.019933056	0.051797724	0.049169252	6.574950555
0.033576116	0.115273586	0.337037544	89.67852371
0.041106164	0.087928604	0.112678416	24.05037296
0.019554948	0.122866828	0.761767636	207.0914135
0.010900342	0.111346509	1.81287237	274.3641359
0.009915468	0.091520173	1.327845514	237.8384519
0.016605214	0.084820162	0.66471826	110.4702937
0.013328077	0.111165395	1.240389375	321.1070305
0.011090635	0.119358483	1.976907108	659.43346
0.02609012	0.071847613	0.226172051	86.80645255
0.107226777	0.118601061	0.030421818	1.725612849
0.022703139	0.061110588	0.041461683	6.628296209
0.015675373	0.082886972	0.166596909	97.55327804
0.012064029	0.120377091	1.86503704	498.2970552
0.023004489	0.088025452	0.627486569	203.5444459
0.038604067	0.061624569	0.092111893	18.4004841
0.027563227	0.073314183	0.133447393	35.48165832
0.010852084	0.083379352	0.217931916	51.41240592
0.019203399	0.080571935	0.124353954	27.85329657
0.024462987	0.087076234	0.150093951	64.54013584
0.038684432	0.07108155	0.113649553	28.42529357
0.020945525	0.073295421	0.135673833	73.50695669
0.018393445	0.164595967	2.576875039	546.7866349
0.011861163	0.067493811	0.133408105	167.1828248
0.077054423	0.08932533	0.123820798	18.3564242
0.106416604	0.105302445	0.036793493	2.230822119
0.024707378	0.04821082	0.032074895	6.812836331
0.01373019	0.102773496	1.416844955	283.3055239
0.043915208	0.104615982	0.141259185	37.58978834
0.012852599	0.083145423	0.374460172	219.7962911
0.055875832	0.078562128	0.124348132	16.90133013
0.012151246	0.131140887	2.8559235	841.5103419
0.031351918	0.064468267	0.086175193	20.12359579
0.01276589	0.070051047	0.084475473	57.05543423
0.022188427	0.05120086	0.059609427	22.31987168
0.026551385	0.075538623	0.107226302	24.27994196
0.034155688	0.083549809	0.214932819	33.6826461
0.022350228	0.094673262	0.225831374	118.0502004
0.008381228	0.082152129	1.001595	407.5917473
0.017500094	0.115081783	1.059126656	327.3836106
0.018405258	0.111539819	0.645835781	152.0257488
0.06566661	0.101720537	0.044781883	5.14851042
0.018099697	0.077804227	0.188316548	78.99767706
0.040566289	0.06670518	0.047315608	6.670991587
0.027706038	0.076291262	0.698731015	204.7589071
0.048862712	0.084330654	0.102441699	11.07483548
0.018142936	0.088870938	0.349560251	131.4620823
0.011787628	0.086473829	0.344934106	185.6638639
0.026782156	0.089159644	0.151971976	44.88940176
0.011860802	0.109900721	1.683143975	498.3207594
0.028469591	0.117177141	2.252074598	751.9314652
0.008935576	0.126828919	2.622948673	1066.972679
0.021909851	0.06825459	0.120351168	27.82231388
0.024144386	0.093505222	0.160004204	38.14994257
0.120126653	0.103493996	0.131642888	20.95361717
0.015815481	0.087054498	0.167876725	70.39489759
0.008545119	0.0970629	0.863175553	195.6746618
0.032320218	0.068392271	0.056688729	9.700775365
0.009984148	0.068439439	0.124059562	53.52963223
0.005682152	0.093585718	0.979881069	336.225916
0.056541706	0.090384986	0.02467296	2.73247483
0.0210546	0.113442572	1.114718222	393.6697511
0.017784956	0.106706015	0.373154758	306.0253325
0.029190675	0.089534103	0.120804084	29.64304288
0.014014964	0.058915541	0.070925612	32.64004872
0.086330964	0.138251925	0.074182829	6.438327947
0.117612754	0.144212431	0.074380165	1.734265734
0.010635504	0.083612237	0.783375901	217.5875671
0.050950375	0.082932355	0.124709919	22.81135677
0.02909061	0.093847526	0.174343136	34.21421366
0.017036033	0.091708834	0.711427323	252.1845098
0.014507496	0.10167489	1.7713257	468.9808673
0.025804961	0.072666723	0.039155069	4.411139938
0.021789961	0.064488913	0.111834724	40.11288607
0.15063443	0.147439533	0.054042685	3.34696463
0.016129285	0.059293926	0.04389499	9.268257174
0.018484275	0.080842929	0.116552847	20.55030378

log.sigma.4.0.mm.3D_glrlm_LongRunEmphasis	log.sigma.4.0.mm.3D_glrlm_ShortRunHighGrayLevelEmphasis	log.sigma.4.0.mm.3D_glrlm_RunLengthNonUniformity	log.sigma.4.0.mm.3D_glrlm_ShortRunEmphasis
1.362675177	138.8692437	266.6703224	0.923097982
2.081118673	99.9236312	790.4842162	0.863341887
1.275476253	61.73286575	140.6666919	0.941253016
1.585560658	188.8676732	1326.366112	0.908739535
1.107705144	182.2994813	90.94445408	0.973073714
1.411874622	52.92240195	176.6032182	0.918875038
1.205034263	32.7690966	57.6451887	0.948741434
1.274025891	112.5907088	316.6931165	0.940690513
1.24030832	358.1343081	1200.054996	0.948003887
1.963303671	132.9812258	1313.658847	0.863025063
1.370369208	72.81208452	280.3403699	0.926014118
1.464475451	108.6787465	532.2912522	0.911750905
1.60711383	60.21044742	879.4209678	0.890648591
1.40895812	80.73244989	383.1614711	0.92036343
1.239230449	88.26958543	295.310692	0.946661491
7.530473185	105.3060071	2359.066866	0.674150479
1.154943908	99.87085667	188.9769657	0.964939334
1.616998885	73.51324189	1005.682788	0.892504747
1.168819302	131.9975191	156.592724	0.96120688
2.709440271	69.13250199	1305.840032	0.823478493
3.512142041	71.53284447	2010.429875	0.790807386
1.488458178	83.47746241	649.8584563	0.916199881
2.721008588	105.8056829	1337.498456	0.815882021
1.149129837	254.5342427	115.91164	0.96475797
1.876246323	59.17030687	542.5708488	0.85806956
1.336438949	71.69656575	226.4699973	0.926668212
2.640870025	75.46232792	1062.455967	0.819559724
4.390745931	98.42707259	1364.866667	0.769347694
3.424935145	132.4784495	1648.720775	0.820351279
2.368718315	104.4573713	899.6514421	0.854049672
3.396532895	89.1034678	1739.975512	0.801257745
4.598463414	99.30300797	3025.199642	0.765840468
1.55134331	103.7144394	958.0000136	0.90975062
1.101437025	84.65786137	13.70667794	0.974640744
1.128252095	179.5322823	99.99878985	0.968728366
1.454897477	109.5079488	937.6007108	0.912108811
4.572326111	89.28171282	2195.204365	0.753567875
2.36422235	75.7244785	1549.93968	0.84297714
1.25731263	134.7552304	262.0407617	0.947806609
1.368035591	92.81156811	399.8525651	0.926117168
1.576573809	157.3427425	476.3521239	0.898215408
1.341485734	113.8191181	288.8838053	0.931064465
1.39280735	85.79586733	614.1727792	0.92658915
1.321541254	97.86226689	336.0294861	0.932962512
1.374267574	110.834383	824.4817508	0.924644443
6.182982356	48.67060767	1443.620608	0.677164111
1.364613483	161.2068192	2052.069113	0.927461238
1.359305244	52.9911262	169.3906019	0.924544391
1.136005171	84.947776	19.76397328	0.965998707
1.100443613	252.4011077	132.3149732	0.974889097
3.646842225	90.77906683	1659.805948	0.801299424
1.406008549	53.63254743	290.5358153	0.917102343
1.849164127	125.1871237	1955.591098	0.881643109
1.363915297	73.29380021	177.0280361	0.923294222
6.07568492	84.82072813	3144.227805	0.72608514
1.24048957	115.0878751	274.1551585	0.949500825
1.2333988	153.7784534	717.6416852	0.951085536
1.166051898	222.1160374	398.369473	0.964690952
1.323199955	100.3308524	267.080017	0.928283238
1.57185479	71.73317347	312.1946954	0.898322551
1.591539302	76.6315982	953.3172817	0.895723999
2.905933515	159.977613	3241.745681	0.833161236
3.217722578	71.89101878	1702.08141	0.793916763
2.379156733	70.3207406	917.0999689	0.843500188
1.143531987	85.40912837	46.14838661	0.964117003
1.487745402	125.1427034	815.6564665	0.913023256
1.152697605	139.2552294	90.68883236	0.961825599
2.385959762	89.13587829	1876.548144	0.859297255
1.291869079	65.44881593	112.0671517	0.938419158
1.829770459	90.42259073	1082.246967	0.877272564
1.825422945	124.7176584	1577.321602	0.878290801
1.425308959	80.04089476	405.3010603	0.915510664
4.179068529	89.28132343	2500.116377	0.769010058
5.169048546	50.08126611	3280.221957	0.741002948
5.682365617	113.3827292	4252.610777	0.737721999
1.321641258	135.5232436	347.1712712	0.937479031
1.417469243	79.7362247	335.0882597	0.922820981
1.360508038	37.84860947	168.5136875	0.928573318
1.453656496	123.644289	650.8135086	0.914122005
2.683729847	139.380349	1356.925397	0.843721406
1.164509486	120.5816391	129.5839459	0.963915525
1.35058147	183.5829951	648.2830755	0.927636464
2.908005074	199.1974388	2336.107348	0.830619128
1.079632437	95.99686495	28.79958318	0.980091891
3.199639972	68.07657028	2122.243911	0.807019187
1.881403081	80.02168504	2064.853187	0.871301281
1.340072537	75.69347519	276.6309316	0.930446648
1.202553115	201.5961188	493.3467328	0.955487783
1.242270473	34.17468369	40.77556813	0.943200685
1.272727273	48.72596154	10.36363636	0.931818182
2.542936916	147.9406972	1791.692737	0.852476988
1.35065561	68.62482046	229.0026076	0.928524268
1.483558272	68.99401394	287.2139174	0.906795427
2.505275184	90.01854806	1814.985109	0.835762521
4.281342335	80.94578019	2594.095056	0.776190283
1.120623911	191.2192548	56.50536511	0.971398024
1.309330948	138.4549795	527.5831066	0.936505103
1.177083786	40.63444346	20.29487903	0.955729054
1.133589461	210.5046889	143.9918346	0.968016662
1.335262441	130.2105895	212.3341081	0.92978777

log.sigma.4.0.mm.3D_glrlm_LongRunHighGrayLevelEmphasis	log.sigma.4.0.mm.3D_glrlm_RunPercentage	log.sigma.4.0.mm.3D_glrlm_LongRunLowGrayLevelEmphasis	log.sigma.4.0.mm.3D_glrlm_RunEntropy
198.8409081	0.900063925	0.021182626	4.429006682
289.3593103	0.796425243	0.026657634	4.392981967
77.22930451	0.923076923	0.074759152	3.987626439
330.9045283	0.866608301	0.012456233	4.678032256
199.2857931	0.965727342	0.031007262	4.543253744
68.29901444	0.891235813	0.063080413	3.892178034
40.45155914	0.937362637	0.123284062	3.328373996
138.7083268	0.921542298	0.038007265	4.4413838
483.1909736	0.930790077	0.010166961	5.247958774
313.8760351	0.804358643	0.01697736	4.456864815
96.00743762	0.901186579	0.038534814	4.049109809
169.7933774	0.881312875	0.030192463	4.403345035
97.470164	0.852911364	0.058539694	4.278723365
103.7695526	0.891899767	0.057421028	4.370901612
104.8849669	0.930473373	0.046619561	4.234377829
1320.476825	0.508176807	0.047983104	4.843809575
113.1292316	0.953562566	0.059527311	4.223094035
126.4142461	0.853637986	0.046118731	4.341456549
148.1411273	0.949704142	0.090174096	4.537306157
282.7993536	0.735011924	0.042313055	4.464346072
412.2869447	0.675846912	0.03961814	4.395377601
120.5997892	0.883219091	0.046690856	4.390679519
425.0792251	0.729109589	0.024725283	4.41617423
304.4622236	0.954308849	0.022067667	4.574949834
136.3058157	0.810336538	0.053539708	4.134507572
99.88975946	0.905756495	0.053478483	4.091256553
261.2612891	0.734885909	0.039646101	4.353615194
760.875262	0.630729732	0.031437071	4.567894108
749.0794637	0.697710524	0.021736214	4.674077841
381.3473009	0.769185333	0.027019493	4.533588323
481.4202672	0.687034223	0.031683628	4.4978641
711.5937275	0.624119343	0.035794769	4.675888739
185.0155294	0.869967326	0.03642623	4.535992496
93.76500423	0.969230769	0.110957527	3.233346984
203.0571533	0.959836624	0.024196306	4.364425672
170.7534424	0.882308846	0.022018751	4.332435043
744.7710666	0.616407804	0.035164023	4.623873178
236.2911953	0.7624315	0.043905614	4.594633625
167.1050236	0.930265996	0.052303112	4.497653625
134.3863418	0.901303538	0.036418637	4.348522952
287.8488148	0.8619688	0.014934907	4.403156263
145.3460779	0.907126994	0.02526665	4.311370527
119.2206686	0.899365198	0.033789968	4.188995045
120.4798377	0.910811284	0.052092741	4.420433001
153.2081546	0.899344602	0.028592902	4.501106093
623.2670115	0.540069862	0.073521157	4.483081154
218.2686245	0.902011317	0.016397237	4.647079935
64.70036132	0.901147099	0.115018281	4.072783139
91.58622946	0.961538462	0.123744261	3.452639845
276.5079455	0.967860906	0.028342964	4.619770814
533.5920574	0.674717195	0.03321786	4.591670966
70.35375495	0.891200611	0.064364375	3.959341845
296.2166246	0.827133743	0.019824969	4.493476288
103.3251597	0.900547152	0.092730072	4.219217706
871.3974231	0.564853243	0.049511842	4.742373025
140.8010815	0.931802526	0.039505675	4.400982425
186.3744727	0.933927311	0.015664364	4.392773545
259.1168487	0.951965066	0.028434027	4.67738827
135.1378465	0.908083442	0.033982214	4.346051641
116.7068066	0.865467151	0.053603665	4.306467917
143.3990971	0.857256146	0.033170986	4.239012663
663.0580218	0.730621651	0.017643882	4.854865165
321.0829609	0.695819644	0.043602571	4.479940392
233.2062485	0.764568765	0.035669726	4.211354093
99.47461882	0.955007257	0.074448896	3.742884955
207.5755348	0.881343483	0.024967581	4.430137631
157.8366807	0.952380952	0.04904552	4.243799424
325.1290115	0.773512902	0.046331433	4.712556416
74.94603568	0.918235611	0.070232814	4.028351261
212.8372436	0.825420673	0.028190597	4.383688991
284.8867581	0.826027762	0.018895736	4.456064119
116.7556183	0.887939221	0.037856705	4.217143146
663.9910658	0.641098309	0.033510969	4.604306187
494.7767956	0.591244089	0.081112227	4.661442023
1012.296571	0.57747143	0.036180857	4.848621222
173.0929618	0.913763367	0.027728525	4.452330215
110.337644	0.894568151	0.033980731	4.094386587
45.38835366	0.903846154	0.179936156	3.905345729
195.2207699	0.885752801	0.021066976	4.279318997
543.6381437	0.745277651	0.017182532	4.480405468
136.4275654	0.95198761	0.037827757	4.237775373
259.1548433	0.904046243	0.01269784	4.567250688
802.0407896	0.727343665	0.01285124	4.702126952
105.2249343	0.975186104	0.057977387	3.665864912
334.1042401	0.698632685	0.046210694	4.466560919
163.1610125	0.81755171	0.031812626	4.257842954
98.36836406	0.906849315	0.03928203	4.130166975
238.5137385	0.94057725	0.016857373	4.587025282
41.11180282	0.930769231	0.103166406	3.230200881
59.03846154	0.923076923	0.129928095	2.984467967
556.4691018	0.758126388	0.01897413	4.687843792
88.95700014	0.9048583	0.072053794	4.238999838
112.7288698	0.876294379	0.040773003	4.123863074
336.2925334	0.752801415	0.030851369	4.561765731
567.279669	0.637262212	0.041461053	4.717736667
217.8188126	0.963369963	0.027027004	4.092116667
178.4076183	0.914705882	0.030975993	4.549785322
47.83303291	0.945512821	0.183857472	3.096216879
245.8285549	0.958942898	0.017594372	4.440667612
187.9306645	0.907692308	0.022214679	4.275475976

log.sigma.4.0.mm.3D_glrlm_HighGrayLevelRunEmphasis	log.sigma.4.0.mm.3D_glrlm_RunLengthNonUniformityNormalized	log.sigma.4.0.mm.3D_glszm_GrayLevelVariance
149.3338907	0.81900665	19.4098615
118.8816128	0.704444573	14.23646639
64.46288419	0.858803972	13.84595041
206.9535353	0.790370867	27.61287093
185.6967436	0.931630837	37.91836735
55.55583571	0.810434641	8.24994749
34.30558911	0.875932717	5.543333333
117.30266	0.856282792	24.16430363
380.2889676	0.872773727	55.87849061
154.5054865	0.702420431	18.89530246
76.71389348	0.825534642	11.85376181
118.2740538	0.795842095	20.13632147
65.87481904	0.753239568	17.42355176
84.63157998	0.812905667	16.24268525
91.37851616	0.870428795	14.8414997
161.4821844	0.423578928	14.41166547
102.3246877	0.912362651	16.54909091
81.40024712	0.757194934	14.2078067
134.7632558	0.904242024	29.49450804
87.5098171	0.635707123	10.18938154
94.82313208	0.583007542	14.26705515
88.88899563	0.806258246	19.88088339
134.1739889	0.623192913	16.86530835
264.2464432	0.912182328	34.78895464
69.51424434	0.694115749	11.60477367
76.58421694	0.826184993	12.98824213
92.74929936	0.62832982	13.78361585
137.2916469	0.551330632	14.60983872
170.7095096	0.632350144	19.35785955
127.7280638	0.688704726	17.30121494
115.9646021	0.59957905	16.48732263
133.8342542	0.545407905	14.78011771
114.0602202	0.791917348	22.80908484
86.47928994	0.939156364	24.49
184.0321283	0.92119186	31.14698225
119.3412612	0.795518702	16.34670736
128.6412118	0.527945162	13.13273158
90.80387225	0.668181047	14.98736899
140.449685	0.874443732	22.83643802
99.63030914	0.82496408	15.76
176.5430592	0.769510677	19.33374023
119.1970308	0.835240352	20.35246623
91.02415647	0.826887507	14.24538567
101.9014131	0.839483874	21.55626667
118.0845892	0.821448254	20.78491295
79.87651465	0.428925831	6.274708296
170.8209905	0.827715122	33.08250711
55.10055544	0.822786133	10.71712018
86.27546669	0.926413015	14.95918367
257.2224753	0.935850209	34.6645541
118.67005	0.600047701	15.91233962
56.60378153	0.806873337	9.16940894
144.6953569	0.737492549	17.3380102
78.46280818	0.8199722	13.09291563
123.7286491	0.48875147	10.9023804
119.4929049	0.877086762	21.35566327
159.4806642	0.880238418	21.50957276
228.6229613	0.912434333	29.3030264
106.5690805	0.829457957	20.8331
78.41038746	0.769756371	13.41865266
86.18329483	0.763100332	13.31987781
197.1834418	0.652010861	28.14004729
92.24659775	0.590547595	10.02420948
86.04904142	0.669678609	7.765967857
88.22222646	0.910436235	14.80621302
137.1273732	0.799781392	19.01940989
142.9715197	0.905368784	22.4124346
107.7730158	0.697598517	17.66185684
67.21608348	0.852105678	10.80968858
105.0816615	0.729109933	14.34212803
144.0400731	0.731610487	14.3321
86.17743204	0.80327797	14.67979592
124.561159	0.549434585	11.34428465
73.91659559	0.509243129	9.810356653
158.0263173	0.504541024	18.43972913
141.7542421	0.850504136	20.60563943
84.56511023	0.819124325	12.69794788
39.11765517	0.830757425	11.18408707
134.9826562	0.801459504	17.49255198
169.576339	0.670673086	18.67340869
123.4159879	0.911482664	18.01021468
196.5134778	0.827986618	26.91067906
245.7066377	0.648240815	21.6145819
97.84247882	0.95065408	15.3610586
87.56824254	0.608686522	12.29859886
90.46712438	0.717715312	12.56193858
79.59380619	0.834528294	15.7405887
208.3599163	0.889924981	24.7248852
35.50953348	0.870285168	6.26446281
50.78846154	0.851239669	11.50617284
179.8704535	0.68643093	19.76330579
72.00829463	0.830787376	17.93571752
75.74696239	0.78568663	12.53081597
112.034504	0.656235966	13.77920898
109.6399528	0.560786437	15.26871753
196.2594461	0.929013447	30.45706371
145.4483207	0.847331668	22.45387269
42.07416135	0.89191463	7.597633136
217.0219508	0.919971664	27.00988415
140.0098521	0.833550203	19.06249174

log.sigma.4.0.mm.3D_glszm_SmallAreaHighGrayLevelEmphasis	log.sigma.4.0.mm.3D_glszm_GrayLevelNonUniformityNormalized	log.sigma.4.0.mm.3D_glszm_SizeZoneNonUniformityNormalized
105.1602567	0.067036011	0.318116343
87.60443309	0.076740036	0.344586556
55.20794885	0.081652893	0.295867769
187.1375685	0.055839878	0.393194445
168.1925309	0.049130763	0.533383724
60.71908649	0.105650074	0.364839319
22.74085658	0.133333333	0.308888889
111.0206374	0.062017747	0.34837963
245.5637983	0.039242601	0.362434633
122.169446	0.069846189	0.342975207
75.36887864	0.092325142	0.355614367
99.29363308	0.065441052	0.346517119
77.63727488	0.074512382	0.322594784
96.16638734	0.072628116	0.380540166
88.50475966	0.075877802	0.352311049
114.9481273	0.082966131	0.349360475
87.67375281	0.07107438	0.392727273
94.62504277	0.079138592	0.396896699
138.3086633	0.051913516	0.422592207
61.82011498	0.090647867	0.306959805
71.04442614	0.080874089	0.314172737
86.60836143	0.064100556	0.30640614
98.24480698	0.073534272	0.344933571
182.8884616	0.05095332	0.459237344
61.97549561	0.087799168	0.391129032
66.91720706	0.087530846	0.318623893
78.50554189	0.078125977	0.304601656
89.7220824	0.080408487	0.324046533
120.9629807	0.064554419	0.354983362
78.58466438	0.066279192	0.341129512
102.8214702	0.075583696	0.376222511
118.7055934	0.080263388	0.408920226
110.1380844	0.061777481	0.343037268
56.30277778	0.1	0.46
159.0534872	0.056094675	0.441420118
108.4056783	0.072406648	0.345389847
82.59943189	0.079176638	0.353081953
88.53148814	0.076484816	0.350067186
104.0831847	0.063189422	0.287018774
64.40522125	0.07904	0.297792
101.7294068	0.068481445	0.302001953
122.5560679	0.068267688	0.360488588
87.08770222	0.076378914	0.365879058
113.1750331	0.066755556	0.396888889
96.67048682	0.065852076	0.31541955
43.52325814	0.115030103	0.317491609
165.9287331	0.056693914	0.362732354
65.33395798	0.087018141	0.413548753
92.36954365	0.091836735	0.469387755
226.1992908	0.050022635	0.505885016
89.9939251	0.070216089	0.370882626
65.80234464	0.101052186	0.395381682
115.7316472	0.069893973	0.380231585
52.5453722	0.086507654	0.238875044
84.09212034	0.090044286	0.360665171
109.5551174	0.062142857	0.392959184
147.1755127	0.062269439	0.414281221
184.8969542	0.052625762	0.406399086
101.1031665	0.0618	0.3704
62.14987197	0.082004128	0.24413586
79.56509331	0.087273477	0.423848878
181.2531901	0.053983362	0.402234659
79.20598976	0.093305875	0.417511764
52.56885347	0.105875268	0.314539501
73.37275908	0.091715976	0.390532544
114.89201	0.067243555	0.363019049
139.0391716	0.063711911	0.456448138
82.65418285	0.072286348	0.353733364
78.96410115	0.095588235	0.539792388
81.07024889	0.073718642	0.359077638
115.0019422	0.0766	0.3843125
95.01560432	0.081632653	0.407040816
93.28356887	0.08822328	0.354503076
46.54147384	0.088627401	0.341424468
132.5009143	0.069281595	0.368090533
117.5420339	0.066047322	0.343010597
84.14703115	0.085081124	0.386059133
58.76716369	0.1026459	0.47607431
112.8456361	0.067183365	0.412400756
135.9738958	0.067240482	0.359031083
115.8253939	0.073060942	0.405124654
162.4997603	0.053952153	0.397818299
180.8523683	0.061269338	0.35605389
80.17391304	0.088846881	0.584120983
76.80633547	0.083970214	0.354763304
89.19019511	0.083927883	0.359352611
84.852198	0.080793082	0.396104463
180.4327909	0.058954082	0.408418367
30.15945707	0.123966942	0.384297521
48.44753086	0.135802469	0.50617284
134.7012046	0.067425773	0.365852464
72.7828291	0.07129923	0.426210955
52.80655751	0.078559028	0.309678819
69.33558033	0.07875	0.307636719
85.59743069	0.072529654	0.358200176
128.51375	0.063711911	0.447368421
144.628186	0.059940512	0.387888162
33.41188034	0.136094675	0.372781065
165.6660699	0.05919864	0.492400893
94.46379827	0.0651341	0.320914256

log.sigma.4.0.mm.3D_glszm_SizeZoneNonUniformity	log.sigma.4.0.mm.3D_glszm_GrayLevelNonUniformity	log.sigma.4.0.mm.3D_glszm_LargeAreaEmphasis	log.sigma.4.0.mm.3D_glszm_ZoneVariance
30.22105263	6.368421053	48.53684211	34.09684211
56.51219512	12.58536585	757.8841463	684.6982079
16.27272727	4.490909091	29	18.64330579
185.9809725	26.41226216	215.2156448	198.5146852
33.6031746	3.095238095	3.857142857	1.286974049
25.17391304	7.289855072	54.31884058	41.81390464
9.266666667	4	8.266666667	2.822222222
50.16666667	8.930555556	27.9375	20.18282215
209.1247834	22.64298094	22.82842288	16.28475741
90.54545455	18.43939394	1401.17803	1324.017777
40.89565217	10.6173913	70.45217391	59.7621172
53.36363636	10.07792208	148.525974	124.3630039
75.48717949	17.43589744	494.4529915	460.3753379
57.84210526	11.03947368	68.42105263	56.35457064
50.02816901	10.77464789	16.73239437	10.1614759
147.0807601	34.92874109	63040.05226	62378.54852
43.2	7.818181818	7.336363636	3.444710744
103.5900383	20.65517241	411.5862069	376.2270078
39.30107527	4.827956989	6.387096774	2.55728986
53.10404624	15.68208092	3755.595376	3498.484346
97.39354839	25.07096774	9789.816129	9521.807544
69.24778761	14.48672566	133.9867257	117.809245
84.85365854	18.08943089	3427.601626	3286.706656
35.82051282	3.974358974	4.474358974	1.566896778
48.5	10.88709677	584.6451613	524.7075963
26.44578313	7.265060241	43.01204819	29.7729714
66.70776256	17.10958904	2481.164384	2371.918934
79.71544715	19.7804878	9218.353659	8967.659214
157.6126126	28.66216216	3371.279279	3301.232936
57.65088757	11.20118343	1246.266272	1145.910927
100.075188	20.10526316	7516.973684	7267.547473
267.8427481	52.57251908	16782.68244	16600.82384
92.96309963	16.74169742	218.4612546	192.1909015
4.6	1	2.7	0.45
28.69230769	3.646153846	4.476923077	1.454674556
102.9261745	21.57718121	196.3959732	176.3568308
140.8796992	31.5914787	17528.27318	17247.05962
106.7704918	23.32786885	2081.816393	1982.993238
33.58119658	7.393162393	17.5982906	10.07100592
37.224	9.88	81.496	63.040384
38.65625	8.765625	176.0390625	144.8363647
50.10791367	9.489208633	39.28776978	31.77464935
86.34745763	18.02542373	98.33898305	86.14823327
59.53333333	10.01333333	44.03333333	35.46795556
85.79411765	17.91176471	135.5625	118.7585289
43.49635036	15.75912409	60276.81752	58279.96718
267.696477	41.8401084	166.501355	152.6565022
34.73809524	7.30952381	30.83333333	23.46598639
6.571428571	1.285714286	3.285714286	0.816326531
47.55319149	4.70212766	3.446808511	1.034404708
116.086262	21.97763578	6348.166134	6178.251161
47.05042017	12.02521008	83.43697479	71.96822258
170.34375	31.3125	999.2566964	948.3956025
12.66037736	4.58490566	47.49056604	27.15557138
188.9885496	47.18320611	47206.33588	46740.79761
55.01428571	8.7	13.72142857	7.995663265
154.9411765	23.28877005	20.95187166	15.51574251
94.69098712	12.26180258	7.313304721	3.449464901
37.04	6.18	40.52	27.9884
17.82191781	5.98630137	156.4109589	115.6610996
97.90909091	20.16017316	506.3766234	466.7029853
346.3240418	46.4796748	3940.139373	3878.203752
120.6608997	26.96539792	6410.788927	6212.066738
51.26993865	17.25766871	1634.699387	1515.179495
10.15384615	2.384615385	7.423076923	3.267751479
93.65891473	17.34883721	170.4651163	150.5278529
26.01754386	3.631578947	5.385964912	1.992613112
158.8262806	32.45657016	1424.069042	1364.445881
36.70588235	6.5	13.13235294	8.709991349
97.66911765	20.05147059	645.9411765	602.5363322
153.725	30.64	799.9725	757.7549938
56.98571429	11.42857143	87.56428571	71.16178571
144.6372549	35.99509804	15038.21814	14739.04685
147.4953704	38.28703704	41753.5	41124.55862
339.7475623	63.94691224	39329.7779	39082.33784
56.93975904	10.96385542	28.53012048	21.3115111
51.34586466	11.31578947	71.47368421	59.71858217
34.75342466	7.493150685	45.78082192	36.3651717
94.85217391	15.45217391	116.4391304	100.6816824
117.7621951	22.05487805	1657.835366	1589.873438
30.78947368	5.552631579	7.223684211	3.380020776
108.6043956	14.72893773	54.58241758	44.54303694
201.5265018	34.67844523	3627.206714	3551.36908
13.43478261	2.043478261	2.217391304	0.400756144
134.1005291	31.74074074	6674.582011	6502.264298
185.7852998	43.39071567	1456.350097	1410.336048
42.38317757	8.644859813	56.27102804	44.63464058
114.3571429	16.50714286	13.875	9.449987245
8.454545455	2.727272727	9.181818182	4.016528926
4.555555556	1.222222222	2.555555556	0.469135802
181.0969697	33.37575758	1397.064646	1349.077539
40.06382979	6.70212766	38.72340426	28.26437302
29.72916667	7.541666667	78.91666667	60.13888889
98.44375	25.2	2855.1	2724.854844
144.7128713	29.3019802	16858.14356	16538.58369
17	2.421052632	3.815789474	1.067174515
79.51707317	12.28780488	52.89756098	41.89458656
4.846153846	1.769230769	4.769230769	1.360946746
47.7628866	5.742268041	4.87628866	2.052502923
27.91954023	5.666666667	33.6091954	23.25115603

log.sigma.4.0.mm.3D_glszm_ZonePercentage	log.sigma.4.0.mm.3D_glszm_LargeAreaLowGrayLevelEmphasis	log.sigma.4.0.mm.3D_glszm_LargeAreaHighGrayLevelEmphasis	log.sigma.4.0.mm.3D_glszm_HighGrayLevelZoneEmphasis
0.263157895	0.630207483	6252.589474	169.5368421
0.116892373	5.799258039	119582.4756	123.7195122
0.310734463	1.398503484	1358.854545	87.18181818
0.244697362	1.286496748	44142.14376	262.6807611
0.623762376	0.102814298	566.047619	208.1587302
0.282786885	2.639885035	1622.811594	86.4057971
0.428571429	0.841271761	244.3333333	38.03333333
0.359102244	0.604895286	2084.201389	160.4375
0.390921409	0.157665305	9878.202773	379.3795494
0.113842173	9.818277171	218811.8598	189.1780303
0.305851064	1.842823282	3185.834783	108.7478261
0.20343461	2.040230887	15252.54545	147.3571429
0.171303075	15.75607188	18864.8547	115.2136752
0.287878788	2.418322988	2948.953947	133.6513158
0.39010989	0.972836792	842.9225352	125.2394366
0.03888068	360.3907567	11152534.17	176.9287411
0.506912442	0.478184981	464.1636364	124.7090909
0.168170103	9.663107724	25212.09195	128.394636
0.510989011	0.741827439	583.7419355	173.7526882
0.062364816	45.4593528	405716.7803	96.79768786
0.061083744	88.59893622	1176852.913	106.3032258
0.248624862	3.224482006	7725.243363	133.4070796
0.084246575	23.53120781	540920.2114	143.5650407
0.586466165	0.070862708	1162.205128	262.1025641
0.129166667	10.1665344	41931.17742	83.71774194
0.274834437	1.252330992	2995.963855	95.95180723
0.095674967	26.20409866	253968.9041	123.5068493
0.063157895	50.81700608	1736383.687	132.5
0.119483315	14.47894674	823527.527	176.0022523
0.0998228	8.587310062	220219.3846	117.7751479
0.063318258	56.65337162	1091866.256	145.6428571
0.074153742	110.2658195	2653493.649	170.1725191
0.195104392	3.12657126	24970.69373	154.0848708
0.666666667	0.201374948	216.4	86.9
0.575221239	0.067911932	719.4615385	207.0153846
0.223388306	2.577461178	17946.58389	155.6342282
0.059632342	105.118981	3034528.381	120.1177945
0.100593668	29.07436871	203670.9475	131.3442623
0.364485981	0.77343219	1739.803419	171.3846154
0.232774674	2.058753168	6479.056	111.8
0.179020979	1.075790274	34309.29688	180.703125
0.364829396	0.638546	2853.302158	164.5251799
0.286407767	1.730655165	6778.762712	122.1059322
0.341685649	1.405323188	2115.106667	153.3466667
0.243946188	2.135200751	10962.55147	156.0110294
0.022378308	609.5767618	6291033.482	68.18978102
0.268754552	1.63326507	20817.82385	238.1775068
0.368421053	2.721375754	748.5833333	84.5952381
0.636363636	0.357644612	203.7857143	106.5714286
0.643835616	0.101867077	706.4042553	286.5957447
0.076715686	45.23690246	971559.7732	131.4920128
0.29528536	3.754438565	2313.378151	88.66386555
0.140219092	6.371310423	188655.5112	160.4821429
0.221757322	2.47146227	3356.245283	94.77358491
0.046347072	323.7832113	7034923.359	124.870229
0.417910448	0.361716532	1220.6	149.2071429
0.428899083	0.274473767	2102.221925	196.328877
0.508733624	0.218271795	1331.570815	258.4420601
0.282485876	0.769148093	3561.44	138.99
0.156652361	4.435503212	9835.424658	108.5479452
0.158762887	8.949963885	36334.24242	108.1082251
0.127066116	17.58009419	946337.2776	248.116144
0.070937653	70.00077729	665886.0381	109.9550173
0.091470258	20.03835887	165688.6135	85.9202454
0.490566038	0.317145053	694.8461538	96.5
0.223958333	1.650684275	24954.12403	164.5116279
0.542857143	0.220878971	595.0877193	173.2280702
0.129506778	12.41588426	226967.4321	124.2249443
0.475524476	0.931122353	385	93.94117647
0.151785714	6.951486458	76114.36029	115.5220588
0.153905348	6.133007165	128270.04	163.905
0.24691358	1.880440745	5845.264286	122.5428571
0.057814936	95.02684987	2547489.228	134.6740196
0.039874469	456.8835718	4157893.095	70.59259259
0.063571871	219.5223865	7193500.16	199.8569881
0.372197309	0.472732287	2449.427711	182.0180723
0.291666667	1.609677405	3943.406015	115.2706767
0.325892857	5.937556252	666.9178082	69.82191781
0.251916758	0.982034	16644.82174	155.0956522
0.121301775	8.547060317	350680.747	198.2987805
0.510067114	0.213442495	579.6578947	154.2236842
0.315606936	0.380491964	9651.278388	233.8278388
0.114830594	12.60908601	1075102.648	269.4823322
0.741935484	0.090637011	211.6956522	99.52173913
0.07617896	70.52002152	710412.7381	111.9285714
0.147419447	21.50728317	109353.3366	128.6344294
0.293150685	1.55597938	2840.102804	114.0654206
0.475382003	0.154586078	2004.728571	250.0464286
0.44	0.696235391	225.5	43.36363636
0.692307692	0.21519125	101.3333333	57.44444444
0.144356955	6.108959413	351523.3111	197.0565657
0.309210526	2.198750254	1544.042553	98.55319149
0.230769231	1.448467185	6303.5625	86.5625
0.08762322	24.85089451	389920.3188	112.346875
0.055940183	130.8584088	2340367.626	128.1856436
0.603174603	0.062248645	731.5789474	192.6842105
0.301470588	1.061586261	5141.531707	203.8487805
0.541666667	0.849303458	163.1538462	47.38461538
0.595092025	0.056406706	1015.329897	222.4329897
0.310714286	0.326327268	4788.413793	145.6896552

log.sigma.4.0.mm.3D_glszm_SmallAreaEmphasis	log.sigma.4.0.mm.3D_glszm_LowGrayLevelZoneEmphasis	log.sigma.4.0.mm.3D_glszm_ZoneEntropy	log.sigma.4.0.mm.3D_glszm_SmallAreaLowGrayLevelEmphasis
0.583363922	0.024047422	5.470870051	0.016527166
0.609697545	0.028847807	5.493272165	0.014754752
0.547527367	0.04488159	5.018193014	0.013107723
0.650388364	0.009832697	6.073634017	0.006614618
0.755925926	0.032173401	5.123717423	0.027503408
0.62489005	0.037643209	4.649898328	0.023983571
0.55832842	0.084432039	4.294739663	0.032248002
0.60504777	0.031264964	5.713322983	0.011722467
0.62258324	0.008627777	6.551136524	0.005540117
0.60751391	0.014547608	5.840921335	0.00922734
0.615309402	0.0290706	5.183668909	0.018027745
0.608877442	0.023983303	5.637896281	0.009580267
0.582157805	0.0453943	5.749280323	0.026233215
0.636291793	0.025366564	5.426975386	0.009465306
0.612909585	0.023020493	5.49897373	0.014354477
0.612433607	0.013192361	5.76985353	0.005205625
0.640332562	0.036078947	5.165698128	0.012846119
0.654392435	0.023766078	5.539308439	0.009127436
0.674334495	0.030561065	5.333641242	0.008371421
0.570745161	0.026296557	5.438074746	0.014030999
0.578906443	0.029816398	5.875173576	0.015287793
0.566076116	0.028733347	5.891942961	0.013895505
0.609143746	0.023491273	5.724019935	0.015462157
0.702854236	0.023334628	5.339907271	0.018351939
0.648819461	0.045529828	5.095024854	0.017971516
0.58021436	0.047438759	5.133368949	0.023963894
0.571273885	0.021737367	5.748171874	0.007362291
0.590954096	0.019406267	5.828711608	0.012146058
0.618326033	0.014453647	6.039741073	0.008595787
0.606359348	0.03968721	5.631290486	0.029441909
0.637078415	0.022102078	5.704442957	0.012831805
0.664334192	0.014488872	5.692159469	0.009289655
0.605033245	0.028229577	5.946352238	0.010599737
0.686111111	0.141638554	3.321928095	0.133648899
0.689239316	0.030389482	5.064449928	0.026774864
0.608311193	0.018092074	5.770042856	0.010046495
0.61753231	0.019399547	5.84239041	0.011335687
0.613638229	0.022400332	5.679820199	0.00938893
0.538314077	0.024632546	5.626578023	0.007087913
0.562536188	0.039881159	5.527204486	0.029240673
0.5596808	0.019888433	5.604334352	0.01477436
0.620034819	0.020797331	5.444234825	0.013135537
0.626664071	0.023078563	5.558974099	0.009543373
0.653449348	0.031523655	5.376100357	0.009928194
0.578683325	0.020312058	6.001243143	0.008406094
0.582716804	0.03179298	5.130065966	0.017339577
0.623497891	0.010880762	6.284782238	0.005835136
0.666839562	0.046931856	4.805135131	0.014709231
0.708829365	0.083849846	3.664497779	0.024676948
0.738956856	0.01840797	5.229021203	0.006806403
0.632935561	0.022966482	5.799751277	0.014988125
0.653141679	0.031508914	4.867640229	0.012567446
0.641050379	0.017915996	5.860086198	0.009447173
0.495562172	0.04318698	5.088782662	0.010460358
0.623851252	0.016835372	5.734808697	0.009386183
0.650970442	0.026170121	5.563221861	0.010259825
0.669031366	0.013716444	5.832158615	0.009842711
0.658919079	0.014527551	5.74808937	0.006260128
0.629246756	0.030521222	5.43617463	0.012670234
0.494038781	0.033345054	5.294152258	0.00908739
0.676307274	0.031177197	5.371293875	0.020730272
0.659172587	0.009415038	6.213450046	0.004739565
0.671835507	0.020639616	5.252298426	0.010346545
0.577908175	0.027377597	5.294371578	0.017068228
0.622582472	0.065154749	4.180832987	0.020220171
0.62216371	0.021646474	5.726014592	0.008181124
0.69715141	0.033090352	4.812908164	0.012149602
0.618109746	0.028667371	5.906168208	0.009192967
0.760168292	0.034821033	4.418859577	0.025153542
0.620238275	0.025463912	5.606299252	0.013936164
0.644266334	0.013659997	5.658764961	0.0064935
0.660861829	0.026507107	5.182796445	0.011360524
0.618008071	0.015108007	5.624877608	0.006296815
0.604465499	0.050927288	5.585628007	0.023321952
0.629468153	0.010796651	6.080036175	0.00643306
0.592315748	0.023950466	5.554345424	0.012933585
0.643556058	0.026908658	5.122624556	0.018331903
0.711590747	0.066264553	4.66688582	0.028109305
0.667495644	0.018503314	5.590011393	0.009247401
0.621718447	0.012235266	5.861769046	0.007754809
0.658309373	0.028631554	4.827554659	0.010826027
0.653877751	0.013423981	5.961143079	0.01033128
0.6191343	0.008170249	6.153991383	0.005505803
0.792270531	0.068490374	4.023136956	0.064631117
0.618005874	0.030006279	5.673194533	0.014188715
0.621330866	0.019302826	5.714419652	0.011215516
0.653543481	0.031220263	5.192034971	0.020127479
0.660513648	0.01276065	5.6913073	0.006260504
0.633616723	0.12011423	3.970573096	0.104181474
0.734567901	0.141094028	2.947702779	0.130285772
0.62842346	0.0127992	6.019434082	0.005824857
0.672990897	0.050217792	5.124393141	0.030823598
0.573226556	0.037206685	5.449611267	0.024145488
0.568334473	0.0325521	5.812954219	0.016724416
0.619937408	0.020767536	5.738852352	0.01173603
0.693669591	0.036069721	4.787143961	0.033527653
0.646274828	0.01537789	5.705257326	0.005666138
0.616324786	0.116354386	3.39274741	0.033120729
0.728532272	0.019204892	5.201738523	0.015626036
0.584750068	0.02803164	5.526292853	0.018979391

log.sigma.4.0.mm.3D_ngtdm_Coarseness	log.sigma.4.0.mm.3D_ngtdm_Complexity	log.sigma.4.0.mm.3D_ngtdm_Strength	log.sigma.4.0.mm.3D_ngtdm_Busyness	log.sigma.4.0.mm.3D_ngtdm_Contrast
0.028861136	0.696021999	4.115764592	0.161897105	0.000321729
0.008538767	0.093044872	0.96122874	0.528231026	4.42E-05
0.048952152	0.789419033	4.037083817	0.230333525	0.000802892
0.005477939	0.303910369	1.648145848	0.393168116	3.70E-05
0.07760658	6.678260601	16.12639424	0.061914179	0.003496345
0.031014199	0.467015324	1.743065574	0.465855301	0.000543079
0.088431365	0.789260835	3.107287671	0.271087105	0.002499744
0.021064939	0.982382925	3.215134386	0.284252224	0.00043813
0.007877299	0.915161298	4.085906579	0.27789309	0.000169851
0.005422942	0.093473633	1.077661497	0.528115287	1.43E-05
0.019313966	0.61613693	1.959123413	0.381736099	0.000298991
0.014303258	0.315274434	2.119425596	0.329111037	0.000109642
0.007296522	0.187665618	1.350532118	0.853962486	4.66E-05
0.017005882	0.573679935	2.699406349	0.421803074	0.00023179
0.023147416	0.893426028	3.472735319	0.270008535	0.000370816
0.001136502	0.011047289	0.424604438	1.831746449	7.70E-07
0.031518466	1.617547473	4.028836298	0.211972069	0.001080851
0.006314245	0.166805496	1.097310072	0.824345532	4.13E-05
0.042915499	2.797250169	6.966673182	0.157771283	0.001831166
0.005109872	0.032462566	0.517869677	1.086228214	1.58E-05
0.002519311	0.023847632	0.457247769	1.394459699	3.77E-06
0.010932833	0.293573681	1.632342827	0.580031609	0.000114643
0.004494469	0.049509666	0.832509138	0.665311565	8.60E-06
0.070532053	3.880151522	15.41833917	0.043209612	0.001991926
0.011678112	0.095046849	1.03379193	0.585839998	4.68E-05
0.025032174	0.534740721	2.054652919	0.347139141	0.000476097
0.005214147	0.060520993	0.929630165	0.73856145	1.04E-05
0.003516319	0.038602895	1.052868086	0.710402558	5.15E-06
0.003099115	0.073581345	0.912820313	0.672228797	1.00E-05
0.008313038	0.07705793	1.080872922	0.4764373	3.54E-05
0.003331043	0.03240472	0.62628313	0.937798579	5.37E-06
0.001401417	0.01975887	0.383894859	1.759351944	1.95E-06
0.007653566	0.262485884	1.558828003	0.513909443	5.94E-05
0.207675806	13.22370227	20.50586905	0.07555226	0.098544506
0.07125079	4.300336923	11.63125283	0.055545928	0.002057607
0.006812046	0.211003394	0.944463793	0.696614817	6.55E-05
0.00183968	0.017347614	0.376070102	1.558654687	3.59E-06
0.004327032	0.060276564	0.731777871	1.006375796	1.43E-05
0.024448505	1.350554378	4.87761651	0.185007849	0.000637467
0.017503649	0.400023395	2.029850776	0.411758695	0.000249182
0.015530412	0.336606811	2.912293845	0.199139663	8.74E-05
0.021568805	1.088953003	3.831623966	0.212772829	0.000314538
0.009573731	0.304006864	1.046761363	0.653680827	0.000131838
0.02018058	0.983171273	4.28849832	0.284643397	0.000319304
0.009039548	0.381733196	2.126932656	0.459933761	8.15E-05
0.00207601	0.006434676	0.244288849	2.528865635	2.97E-06
0.003894187	0.476168247	3.842333641	0.43249817	1.78E-05
0.031357815	0.670690869	2.162404266	0.599910456	0.000942256
0.163929921	9.384080698	11.75492955	0.10736295	0.032177868
0.04433814	6.04009049	10.30503135	0.081001577	0.003038127
0.0033446	0.035185165	0.595083349	0.924028823	6.57E-06
0.016701284	0.45615988	1.505689178	0.648862019	0.000305303
0.003593841	0.082537964	0.685966335	0.851213472	1.68E-05
0.039286821	0.657009908	3.588271213	0.228667307	0.000588855
0.001090345	0.009349812	0.241691338	2.656984885	1.50E-06
0.024235936	1.07753136	3.056687928	0.254707185	0.000607832
0.008770177	0.769348282	2.29131838	0.34340418	0.000123777
0.018657687	1.503145928	4.137997472	0.16045811	0.000512883
0.029045676	0.721863641	4.061585914	0.196335145	0.000296017
0.023717386	0.272912428	2.189822882	0.400294058	0.000203648
0.006773239	0.14895998	0.964625745	0.827322629	4.50E-05
0.001887945	0.067535539	0.84156597	0.854720612	4.41E-06
0.003072139	0.033014867	0.54533407	1.23593572	5.44E-06
0.006255028	0.04512639	0.508295687	1.042700603	2.88E-05
0.094168852	4.099273107	10.15776249	0.073475039	0.004625293
0.008858397	0.312391738	1.741639466	0.372428597	6.59E-05
0.057946643	3.884829373	7.277844448	0.096816188	0.003043965
0.00376781	0.07349261	0.72700009	0.934395864	1.87E-05
0.04705286	1.555649154	4.49871228	0.23698808	0.001316123
0.00622378	0.104270047	0.856282685	0.717409292	3.18E-05
0.004313128	0.082837589	0.640551388	0.838484919	2.31E-05
0.014301496	0.483111165	1.940322349	0.408462639	0.000167563
0.001872486	0.019249468	0.379905397	1.529475789	3.32E-06
0.001242385	0.005025626	0.121108997	4.50455054	2.92E-06
0.000857723	0.015542044	0.38621843	2.135894916	8.13E-07
0.016682493	1.200021854	3.231493157	0.249422616	0.000338405
0.017391903	0.476465055	1.831159365	0.406056957	0.000226862
0.033289874	0.945844237	4.136315046	0.414333307	0.000523606
0.009666271	0.302312307	1.376224296	0.406399609	8.59E-05
0.004471129	0.093539731	1.032323304	0.520311071	1.17E-05
0.044494915	2.495287041	5.144887472	0.150760727	0.001665765
0.011278322	0.623252256	2.658914871	0.23616731	0.000107123
0.002505598	0.073471001	0.963430763	0.598170787	5.02E-06
0.137765161	7.261136299	11.3846937	0.066930579	0.013358356
0.002461247	0.025118154	0.347632635	1.675658262	5.91E-06
0.003057255	0.059901902	0.510008523	1.400530521	1.03E-05
0.02281641	0.727707716	3.253526117	0.287028713	0.000265902
0.012550381	1.299055365	2.908578076	0.241046792	0.000309622
0.101280067	1.029343007	2.639462071	0.359825481	0.004607775
0.224654378	6.597685733	9.22649968	0.139102564	0.070741237
0.003504591	0.101821982	1.043100985	0.567193193	1.20E-05
0.025549397	0.848579784	3.601099063	0.442126924	0.000561512
0.024355106	0.352541779	2.062299215	0.305819077	0.00020717
0.003687654	0.035639736	0.461640031	1.196775115	1.31E-05
0.00189965	0.01659121	0.320419982	1.799596668	3.58E-06
0.062435084	9.24907335	11.85825065	0.080827304	0.00894392
0.01414387	0.684875641	2.845358492	0.273558537	0.000185197
0.130447057	4.162457588	6.349747414	0.220074984	0.028743983
0.048190703	3.072443243	9.944905714	0.077510316	0.001455105
0.032104808	0.946346723	4.94118869	0.122547144	0.000348662

log.sigma.3.5.mm.3D_gldm_GrayLevelVariance	log.sigma.3.5.mm.3D_gldm_HighGrayLevelEmphasis	log.sigma.3.5.mm.3D_gldm_GrayLevelNonUniformityNormalized	log.sigma.3.5.mm.3D_gldm_DependenceEntropy
13.80055402	159.066482	0.07565166	6.284871202
8.913023665	133.6101212	0.113274731	6.700583244
10.1687893	55.29378531	0.092661751	5.526908345
16.4383377	188.1391619	0.075925555	6.937296632
30.38290364	136.2079208	0.060680325	5.432722002
6.048961973	52.38934426	0.12392502	5.65805151
5.266326531	42.12857143	0.135918367	4.495873068
14.66278195	123.2693267	0.078500756	5.955471036
65.22652366	370.7418699	0.04034103	6.960392903
8.070115425	141.1694696	0.107523001	6.880433129
7.736695054	88.78457447	0.104558058	5.783569714
11.11841047	125.1875826	0.085477856	6.556132627
8.463096129	70.17642753	0.104924232	6.461419797
11.96940642	69.85037879	0.088304924	6.157667332
13.48224852	89.03846154	0.079126917	5.777961463
3.759984709	169.8599003	0.270838209	6.66967327
15.62564506	90.04608295	0.0733717	5.610549704
8.726452067	71.86340206	0.095602448	6.714902871
23.67020891	92.50549451	0.065873687	5.491557504
7.064092414	98.65465032	0.121133307	7.005708715
5.027550525	131.7818719	0.161297154	6.622780261
11.93598546	88.52255226	0.089217833	6.467515954
6.396481399	149.8955479	0.135065913	6.851165224
35.6689468	292.1503759	0.05387529	5.726949424
5.753332248	93.50729167	0.123209635	6.601189244
9.812168326	86.05629139	0.087781238	5.984478378
5.458931837	98.61642639	0.159057479	6.780858271
6.387861466	160.7214377	0.202583741	6.692022338
10.28488297	171.9017761	0.161330111	6.787866212
10.21719048	151.7330183	0.114024636	6.735115614
6.148166284	132.0514163	0.149539723	6.804215577
5.273278605	123.450583	0.188372612	6.87383675
13.63227374	123.9654428	0.079463708	6.760093334
21.44888889	87.6	0.12	3.189898095
25.0896703	165.2920354	0.064139713	5.46625225
10.35141406	134.4550225	0.085936042	6.468643739
6.243716026	148.5556718	0.200919171	6.626330343
8.282891788	117.9587731	0.108763123	7.014024018
21.12347512	137.8442368	0.064915907	6.044447422
13.23061772	92.16573557	0.077390427	6.376297244
11.3784811	162.1356643	0.087798914	6.581868233
11.46407093	106.488189	0.091023071	5.93725688
9.945700584	104.2524272	0.089190192	6.277051063
14.61704744	105.7129841	0.078906813	6.124618927
16.99334071	148.0188341	0.074878642	6.50675521
3.36726814	75.08232604	0.253133258	6.502649433
22.41392247	147.3470503	0.070441864	6.773355129
9.564846876	51.41666667	0.099915359	5.586878417
18.17561983	113.6818182	0.08677686	3.732158891
34.20289923	172.1849315	0.052355038	5.563131327
6.663487541	135.3914216	0.159441561	6.840210064
8.165754361	57.93300248	0.109445905	5.78266119
10.93010607	183.998748	0.091299247	6.942798909
11.12585564	79.82845188	0.08292922	5.994013408
5.017504978	114.6257739	0.232651626	6.765977123
15.39491201	90.0238806	0.072719982	6.10997508
17.77724807	164.5149083	0.069488574	6.287776399
28.62672813	207.941048	0.053793787	6.147612195
13.21513614	117.2090395	0.078697054	6.121888429
9.600826134	68.12660944	0.090782663	6.303217166
9.424695504	81.27216495	0.093742398	6.443667023
11.4603434	214.6827037	0.116932818	7.111927298
5.153398264	97.02896416	0.151005852	6.967607778
6.161902099	77.71717172	0.129373924	6.737937798
13.58348167	93.90566038	0.095763617	4.635952474
13.61976454	165.859375	0.078712746	6.625684795
17.73297052	122.1619048	0.068480726	5.191260727
13.13432704	123.4539948	0.097121993	7.06327672
8.555626192	50.21678322	0.114088708	5.355085369
9.142978902	116.2862723	0.095634616	6.875948962
9.80534793	156.8687957	0.092887859	6.865564396
9.066319532	80.55379189	0.0928461	6.165121408
6.404964502	121.5451325	0.163025128	6.827269481
5.043571627	87.19401883	0.19660516	6.78765323
5.506579049	168.7191267	0.209657266	6.930847261
16.01389531	151.9506726	0.07412174	6.226517012
9.230912396	96.875	0.097597338	6.204335172
6.60809949	33.24107143	0.129703444	5.535302013
11.65814948	155.3953998	0.08454849	6.447300439
8.380374325	165.2133876	0.11826934	6.82824796
14.17260484	119.7516779	0.088689699	5.45568975
18.39694744	209.7953757	0.069031374	6.623263213
9.841407965	263.3221749	0.132087655	7.10369293
20.36836629	146.3870968	0.086368366	3.937751955
5.771824268	97.83434099	0.145363529	6.937997078
6.446637947	111.9084688	0.118017499	6.84273563
9.187134547	70.88493151	0.095605179	5.99905082
20.51714367	170.1120543	0.064317236	6.233654052
5.5664	47.04	0.1272	4.54346519
12.55621302	69.38461538	0.159763314	3.238901257
11.96044256	200.287839	0.110270495	7.085659833
13.93312846	66.01973684	0.083578601	5.81454836
9.260147004	84.39903846	0.094801683	6.25513334
8.325945123	126.9879518	0.106877325	7.057784976
6.166564425	102.3433952	0.167860313	6.885521131
26.53968254	133.3174603	0.073318216	4.849985479
17.91566609	154.4617647	0.067634083	6.377838649
13.40104167	57.29166667	0.121527778	3.668295834
27.50415898	244.8404908	0.057623546	5.704634462
12.02096939	122.7214286	0.089005102	6.04793412

log.sigma.3.5.mm.3D_gldm_DependenceNonUniformity	log.sigma.3.5.mm.3D_gldm_GrayLevelNonUniformity	log.sigma.3.5.mm.3D_gldm_SmallDependenceEmphasis
54.00831025	27.31024931	0.238841931
118.8446187	158.9244476	0.118528128
34.84180791	16.40112994	0.247819958
236.4102431	146.7640973	0.24635476
29.73267327	6.128712871	0.433272827
34.98360656	30.23770492	0.252346661
16.54285714	9.514285714	0.333261905
75.51371571	31.47880299	0.348643672
310.0528455	59.54336043	0.327573618
195.9081501	249.3458387	0.12792513
61.89361702	39.31382979	0.26516219
99.55746367	64.70673712	0.193333797
152.3762811	143.3265007	0.168811532
76.42424242	46.625	0.253225037
80.85714286	28.8021978	0.297556759
412.5831178	2932.636129	0.046213522
53.23041475	15.92165899	0.410813453
163.1739691	148.375	0.172573173
43.98901099	11.98901099	0.460265622
174.9343908	336.0237924	0.07530204
286.0723153	818.5830542	0.07252704
120.2717272	81.0990099	0.23047259
179.3410959	394.3924658	0.089426266
38.78947368	7.165413534	0.51823726
79.88333333	118.28125	0.118106332
49.56291391	26.50993377	0.254666753
145.1363041	364.0825688	0.104212013
179.6988447	789.0636714	0.075271952
230.9359526	599.5026911	0.121008736
135.1500295	193.0437094	0.109906408
239.370388	628.2163771	0.077474404
395.3154081	1663.895279	0.075716338
174.4312455	110.37509	0.204686353
7.533333333	1.8	0.722222222
32.34513274	7.247787611	0.515843166
176.3238381	114.6386807	0.201495403
317.1832312	1344.350172	0.067272918
214.2269129	329.7697889	0.119400813
70.16510903	20.83800623	0.342362467
77.89013035	41.55865922	0.225712637
84.61118881	62.77622378	0.176144123
61.70866142	34.67979003	0.3063148
128.7791262	73.49271845	0.251932702
80.19362187	34.64009112	0.261735908
187.5363229	83.4896861	0.251914419
253.3956223	1549.681803	0.031750427
419.6416606	193.4333576	0.252572994
36.55263158	22.78070175	0.304194477
11.09090909	1.909090909	0.659090909
42.75342466	7.643835616	0.509623288
218.879902	650.5215686	0.087460199
61.16377171	44.10669975	0.229829455
304.0691706	291.7010955	0.150544053
41.10041841	19.82008368	0.202653513
461.6831771	2630.359278	0.05451487
63.50149254	24.36119403	0.330755944
180.2201835	60.5940367	0.370101745
117.1659389	24.63755459	0.420427376
59.86440678	27.85875706	0.265726934
63.60085837	42.30472103	0.133398961
173.2419244	136.395189	0.154846654
428.8084416	792.3367769	0.126319211
226.3004418	615.19784	0.081042298
133.5185185	230.5443322	0.099588579
11.26415094	5.075471698	0.382672956
152.1128472	90.67708333	0.228837273
29.72380952	7.19047619	0.436640212
264.4906259	336.7219498	0.135123333
24.06293706	16.31468531	0.295800539
162.1819196	171.3772321	0.147840639
246.1889188	241.4155444	0.142720765
88.61904762	52.64373898	0.211575287
341.7819187	1150.468329	0.068356315
472.6315304	2130.020306	0.052450819
596.5620911	3044.013844	0.068816515
85.05381166	33.05829596	0.341245224
69.37719298	44.50438596	0.272216692
31.52678571	29.05357143	0.249018854
126.2968237	77.19277108	0.224276009
192.0591716	319.8002959	0.137109588
31.93959732	13.2147651	0.331102028
138.583815	59.71213873	0.304006253
306.2854534	651.0600527	0.122249054
12.5483871	2.677419355	0.634408602
283.6509472	721.2938331	0.087849572
301.5086969	413.8873681	0.148202132
57.27945205	34.89589041	0.257760178
121.75382	37.88285229	0.349301788
11.12	6.36	0.378966667
7.461538462	2.076923077	0.769230769
256.8279382	378.117527	0.140171631
59.40789474	25.40789474	0.259421974
51.73557692	39.4375	0.223618893
242.771632	390.3159912	0.097533698
351.2927167	1212.287178	0.069745227
22.65079365	4.619047619	0.544863316
116.9705882	45.99117647	0.280307974
6	2.916666667	0.361550926
43.2208589	9.392638037	0.439110429
46.08571429	24.92142857	0.265215727

log.sigma.3.5.mm.3D_gldm_DependenceNonUniformityNormalized	log.sigma.3.5.mm.3D_gldm_DependenceVariance	log.sigma.3.5.mm.3D_gldm_LargeDependenceEmphasis
0.149607508	3.965316411	17.033241
0.084707497	31.72393745	79.47469708
0.196846372	2.19879345	11.61016949
0.122302247	20.07587551	42.55613037
0.294382904	1.175963141	5.792079208
0.143375437	5.16904058	19.26229508
0.236326531	1.354285714	8.114285714
0.188313506	2.890703416	11.24438903
0.210062903	2.117812736	9.594850949
0.084479582	21.73601616	62.42820181
0.164610684	3.268107741	14.56914894
0.131515804	4.75946734	22.01981506
0.111549254	8.862065343	31.77013177
0.144742883	5.423539371	19.28787879
0.222135008	1.96902548	9.576923077
0.038103354	55.32428722	238.5330624
0.24530145	1.680944594	7.313364055
0.105137867	11.51741683	37.19201031
0.241697863	2.953024997	9.120879121
0.063062145	32.71400308	105.5104542
0.056368929	40.47343623	129.0855172
0.132312131	6.854251762	23.03960396
0.061418184	34.4361869	105.7808219
0.291650178	1.429249816	5.766917293
0.083211806	13.68218316	52.61458333
0.164115609	3.143634051	14.41721854
0.063405987	36.89218805	104.4276977
0.046135775	51.87299368	167.3127086
0.062146381	53.40116547	136.2621098
0.079828724	37.41594607	93.75014767
0.056979383	42.51040148	128.4068079
0.044754377	51.77138116	169.8896185
0.12558045	19.22894428	42.46148308
0.502222222	0.648888889	3
0.286240113	1.588534733	6.061946903
0.13217679	10.06767081	28.65967016
0.047404458	48.06291487	170.7919593
0.070655314	31.28799751	87.08377309
0.218582894	2.2604012	9.45482866
0.145046798	4.253217232	18.22160149
0.118337327	7.286059954	27.90909091
0.161964991	3.877653089	14.65879265
0.156285347	4.043595061	16.22572816
0.182673398	2.916153403	12.92710706
0.168194012	3.68424541	14.95336323
0.041390987	47.11960185	211.2290101
0.15281925	4.398094772	16.95848507
0.16031856	4.067174515	15.23684211
0.504132231	0.247933884	2.363636364
0.292831676	1.298367424	5.520547945
0.053647035	50.49005671	145.1705882
0.151771146	3.805109323	17.45657568
0.095170319	17.39719623	50.09984351
0.171968278	2.826140999	15.32635983
0.040835236	54.5901977	205.0419246
0.189556694	3.406674092	12.12238806
0.206674522	2.679272578	10.0412844
0.255820827	1.606929692	6.943231441
0.169108494	3.452711545	14.33898305
0.136482529	4.62432537	24.35622318
0.119066615	12.34070453	37.20343643
0.063283418	41.25414922	109.824085
0.055547482	37.74077794	120.5802651
0.074926217	28.69695836	82.42536476
0.21253115	2.159487362	9.037735849
0.132042402	5.914339796	21.72048611
0.2830839	0.960544218	5.552380952
0.076288037	37.16457291	90.51312374
0.168272287	5.614162062	17.30769231
0.090503303	17.31106505	51.6171875
0.094724478	16.94488794	49.91342824
0.156294617	3.422381481	16.99647266
0.048431617	49.52125642	161.5234519
0.043624841	49.6074978	184.9601255
0.041088373	52.41280365	188.6889593
0.190703614	3.680890426	12.52017937
0.152142967	5.812538473	18.84210526
0.140744579	4.480867347	18.8125
0.13833168	5.329972684	20.47426068
0.0710278	36.53436723	93.83210059
0.214359714	2.63843971	10.39597315
0.160212503	5.171137024	16.63699422
0.062139471	42.20221221	112.9375127
0.404786681	0.616024974	3.322580645
0.057164641	40.70787757	121.2297461
0.085973395	16.59326993	51.94268606
0.156930006	3.470339651	15.44383562
0.206712767	2.619345615	10.21222411
0.2224	1.7664	8.32
0.573964497	0.213017751	1.923076923
0.074898786	38.69801492	94.33508311
0.195420706	2.038781163	11.19736842
0.124364368	8.141249075	26.40865385
0.06647635	32.53352092	96.20098576
0.048642027	48.59974848	158.7654389
0.359536407	0.703451751	3.920634921
0.172015571	3.102802768	13.26764706
0.25	1.743055556	8.416666667
0.265158644	1.341111822	6.300613497
0.164591837	4.161020408	16.01428571

log.sigma.3.5.mm.3D_gldm_LargeDependenceLowGrayLevelEmphasis	log.sigma.3.5.mm.3D_gldm_SmallDependenceHighGrayLevelEmphasis	log.sigma.3.5.mm.3D_gldm_LargeDependenceHighGrayLevelEmphasis
0.176195475	43.25871195	2439.072022
0.523525209	14.47399719	14277.85959
0.791937634	20.54518323	475.0903955
0.235068617	57.34900315	9474.512675
0.321997776	78.78206271	582.7029703
0.685509478	19.06667386	744.2745902
0.587824458	17.11240476	310.1571429
0.184702699	60.0361375	1014.117207
0.072838695	126.6701907	3379.347561
0.458252136	20.06895651	10300.26736
0.255296171	32.21642029	1054.295213
0.2585874	26.75927915	2738.2893
0.6938444	17.59019787	2214.050512
0.75366341	29.22202394	826.2746212
0.313739337	39.05998712	556.9010989
1.373859834	8.051231335	42356.67852
0.41010416	51.06045472	383.6313364
0.888624094	17.77892522	3201.036082
1.067479915	66.57463909	330.3516484
1.074680546	7.109033241	12963.18241
0.854075786	9.19187512	20727.50522
0.477139642	28.25756403	1744.273927
0.630988197	12.92640309	19667.66678
0.052319622	144.4256767	1839.789474
0.584717145	10.87926391	5743.428125
0.319033575	24.34171619	1263.692053
1.041919895	12.3652914	11699.26824
0.910938771	9.994728147	32224.77638
0.676376879	18.92423212	29948.34957
0.525838457	14.11163686	19540.04135
0.856930859	10.29262142	20926.70555
1.290228097	10.21989721	24146.11423
0.395538353	30.00974612	7039.353492
0.613997974	83.05555556	128
0.093829655	99.54349558	850.2477876
0.239332347	32.75557684	4953.029235
1.011295178	7.850701363	30327.37797
0.740657609	17.28803408	12116.50825
0.260720924	60.19859538	880.4797508
0.480011488	23.45490449	1804.106145
0.203912888	26.7460777	5424.116084
0.293606452	46.26395142	1192.251969
0.246515876	32.52258258	1593.956311
0.255719161	38.34785638	1045.230068
0.182023799	46.66845831	1851.796413
2.591737083	1.649983369	18643.46031
0.263942147	51.37922078	1867.910051
0.964306262	24.40620734	458.7412281
0.390124069	83.61363636	233.9545455
0.431807355	114.8440221	625.9109589
0.949863857	11.5381069	23912.64338
0.747054944	19.00345116	716.5632754
0.259683064	27.59330064	11582.18592
0.557791008	19.91963043	976.7238494
1.71892076	5.528685204	25949.18388
0.520897292	38.87945612	905.1164179
0.112505603	73.43895047	1328.150229
0.110533694	101.6111639	1105.78821
0.199512636	35.86244915	1698.112994
0.836626932	11.77671975	1390.066524
0.543913537	13.03719216	4659.430241
0.461045606	30.08119186	28228.97196
1.270463746	8.417354192	12668.66716
1.061372369	6.904543214	8134.314254
0.392144357	43.25228512	561.0377358
0.176045955	44.77396525	4035.715278
0.220616871	65.67632275	549.8
0.724737124	16.84051428	15683.08249
0.935561769	22.09933713	451.3636364
0.458802445	17.11225691	7671.880022
0.316974065	23.38343687	10128.62409
0.385426189	21.75317549	1317.340388
1.167977632	7.554241941	24444.99617
1.989446435	3.673713081	19154.87392
1.068908504	13.14344616	34573.66423
0.147396587	65.03064616	1461.73991
0.283267856	32.15222659	1603.598684
1.657800647	14.64834087	376.7232143
0.187888227	37.97277534	3684.871851
0.486451536	23.19008975	19777.902
0.195313015	53.99337078	849.5503356
0.101207365	73.53025957	3655.873988
0.389529761	32.2601142	34204.43599
0.086895598	110.2043011	334.1290323
1.143835077	9.23846985	14646.9738
0.536775161	21.6743028	5859.553179
0.420942874	24.70658863	914.1561644
0.166490231	74.04427184	1269.110357
0.455461191	22.16860556	294.76
0.118017019	48.61538462	152.4615385
0.409186238	26.522592	24459.58151
0.515713578	21.74220807	636.5460526
0.43000117	22.09078087	2607.855769
0.679306319	11.05913122	15902.29244
1.436262913	6.896939135	19338.40487
0.112395196	93.92934303	369.2539683
0.197547787	57.17995172	1589.688235
1.10422764	25.83752315	346.4583333
0.072919502	119.1204022	1265.417178
0.261317983	30.84946182	2136.235714

log.sigma.3.5.mm.3D_gldm_SmallDependenceLowGrayLevelEmphasis	log.sigma.3.5.mm.3D_gldm_LowGrayLevelEmphasis	log.sigma.3.5.mm.3D_gldzm_DistanceZoneVariabilityNormalized
0.005034716	0.01301867	0.979383681
0.002518536	0.014166039	0.926035503
0.009630453	0.065279312	1
0.002966826	0.009862152	0.89152436
0.012828872	0.04808617	0.964297521
0.010379715	0.037615327	1
0.018569823	0.074840371	1
0.007080656	0.017838035	0.97515921
0.002540427	0.007373003	0.534545646
0.001665697	0.011763396	0.939927754
0.006407933	0.019786751	0.878798081
0.004407821	0.015810201	0.973159332
0.005739983	0.028750335	0.9504709
0.009245126	0.042197922	0.96162928
0.008209225	0.028671926	0.956226331
0.000470857	0.007194599	0.959838315
0.011972634	0.036447808	0.981310075
0.004818716	0.033938253	0.902529796
0.021379743	0.06666789	0.978949751
0.001682627	0.016113183	0.918751148
0.001128384	0.009677328	0.987786518
0.006788336	0.025290679	0.941960698
0.001443416	0.009395985	0.983674575
0.01358107	0.017476241	1
0.003457578	0.017217914	1
0.007392666	0.028451015	0.955578512
0.001932264	0.015339575	0.974506806
0.001158237	0.008228183	0.948753463
0.002058556	0.010251733	0.928675284
0.001867596	0.011911593	0.934541093
0.001254445	0.010860571	0.959631263
0.001280449	0.012128329	0.952505605
0.004484323	0.019649852	0.91185509
0.042886863	0.149997974	1
0.018840654	0.027428387	0.969704142
0.002913193	0.011967621	0.890792402
0.001243697	0.00932	0.901982386
0.001678299	0.013229347	0.959787068
0.010914329	0.02581431	0.92866941
0.006422623	0.029275681	0.898106509
0.003652584	0.012251852	1
0.005610175	0.023110342	0.928157719
0.005155399	0.018081019	0.949427765
0.007498711	0.02314992	0.97091263
0.003020643	0.013524636	0.747471139
0.001403433	0.01979336	0.967876264
0.003961475	0.01679506	0.666730098
0.019298538	0.062587336	0.875555556
0.03031182	0.10227427	1
0.010553906	0.046287498	1
0.001433399	0.010815238	0.953649323
0.006844549	0.039630711	0.7714
0.001717065	0.008097399	0.846518787
0.008300111	0.03422832	1
0.001292248	0.012949243	0.928263142
0.016178876	0.048373363	0.939907457
0.005868099	0.013143131	0.98933364
0.006460578	0.01435279	0.940982987
0.007427358	0.01938447	1
0.008260696	0.039891679	1
0.00562235	0.026712549	0.885384293
0.001010521	0.006773086	0.918464791
0.001754053	0.01492611	0.958658049
0.003498856	0.02484135	0.987500494
0.024744162	0.042465566	1
0.002686012	0.011731057	0.931122449
0.016664115	0.034695834	0.966111772
0.002885649	0.018177695	0.831345993
0.015107929	0.049934226	0.9608
0.004045223	0.016948673	0.976565795
0.00153209	0.0102054	0.984211526
0.005690331	0.025432632	0.892656488
0.001134088	0.012192578	0.942362445
0.002040237	0.019560632	0.941808349
0.000662043	0.007476199	0.938893969
0.006283027	0.014346627	0.966860245
0.009352002	0.02218398	0.985715025
0.0188012	0.09537565	0.967222222
0.004694405	0.016022	0.981736711
0.001802371	0.008559343	0.967663727
0.009862503	0.021610066	1
0.004000703	0.00910589	0.954139215
0.000941285	0.005021195	0.840541845
0.039893428	0.04556029	1
0.002197331	0.016239724	0.964881611
0.002312956	0.012700202	0.988372491
0.008982897	0.029734791	0.908468935
0.005490956	0.014578825	0.944269014
0.032602554	0.058405112	1
0.097984968	0.101991378	0.702479339
0.001657143	0.008557774	0.878379272
0.011632998	0.051341933	0.95654321
0.009631325	0.030258489	0.980954142
0.002719125	0.013637484	0.946951926
0.001895254	0.015799219	0.959115655
0.039131217	0.049727649	1
0.003443912	0.015400308	0.857988166
0.032873592	0.1415845	1
0.008768512	0.01382927	1
0.013031254	0.025060443	0.97727573

log.sigma.3.5.mm.3D_gldzm_LowIntensityEmphasis	log.sigma.3.5.mm.3D_gldzm_LargeDistanceEmphasis	log.sigma.3.5.mm.3D_gldzm_HighIntensitySmallDistanceEmphasis
0.020950036	1.03125	178.75
0.030145602	1.115384615	113.4022436
0.04738151	1	81.66666667
0.012956474	1.182178218	229.6292079
0.038933421	1.054545455	163.7318182
0.040747117	1	79.65151515
0.070813338	1	47.19354839
0.020201719	1.037735849	166.4040881
0.007790979	2.255136986	220.3639293
0.016860903	1.126712329	156.9020167
0.024517795	1.23853211	116.8305301
0.024778529	1.040816327	139.6870748
0.037219692	1.097046414	110.1413502
0.043292173	1.090909091	113.1127345
0.027425883	1.067164179	126.5690299
0.012604757	1.061503417	176.6679954
0.029443698	1.028301887	119.745283
0.032635607	1.219607843	105.050354
0.049154041	1.031914894	137.875
0.026324514	1.127272727	97.98333333
0.017952343	1.033742331	125.8735515
0.029925188	1.08974359	123.5961538
0.018841426	1.024691358	141.2355967
0.023967904	1	282.3625
0.033500856	1	88.66666667
0.034038046	1.068181818	93.92045455
0.02017729	1.060085837	122.5171674
0.017292652	1.078947368	125.1381579
0.019739366	1.147505423	151.8189323
0.023826484	1.129213483	116.585362
0.021147578	1.078767123	135.9949581
0.019690969	1.151857835	140.5863512
0.026987768	1.177631579	144.541324
0.097497467	1	105.25
0.035004715	1.046153846	190.4769231
0.014978558	1.208633094	159.2244205
0.021268683	1.15503876	111.3456072
0.016654181	1.061583578	151.356305
0.029911099	1.111111111	169.8185185
0.034373681	1.161538462	99.59807692
0.021754881	1	149.8848921
0.022675937	1.111940299	144.4477612
0.021856121	1.099137931	125.1023707
0.029254867	1.080882353	142.3071895
0.014170962	1.47284345	162.2456514
0.063247619	1.089430894	48.75158085
0.017629597	2.126614987	147.4403711
0.068887892	1.2	79.20333333
0.070269687	1	121.1764706
0.025848819	1	212.1954023
0.020281184	1.071216617	130.865727
0.035365491	1.44	75.77027778
0.012706555	1.306382979	165.6218824
0.042593872	1	97.625
0.028465566	1.174168297	100.5807241
0.051293552	1.093023256	114.4844961
0.015508754	1.016085791	195.3116622
0.014433067	1.091304348	237.6913043
0.027971601	1	133.6698113
0.066900928	1	95.1969697
0.047011597	1.183098592	78.72065728
0.009356168	1.133333333	236.7014368
0.02447973	1.080701754	106.1287524
0.050190572	1.018867925	67.21540881
0.057905733	1	109.12
0.01468341	1.107142857	188.4508929
0.036657218	1.051724138	140.6594828
0.023217735	1.278911565	122.5793651
0.051583149	1.06	72.945
0.034482464	1.035573123	112.6057312
0.013346762	1.023872679	163.2871353
0.028494166	1.170731707	100.1910569
0.020512267	1.101234568	111.2897805
0.056464245	1.139908257	67.00447566
0.010490672	1.138678223	202.1397692
0.018036872	1.050561798	184.4676966
0.034266164	1.021582734	117.0791367
0.093804059	1.05	58.55
0.023427027	1.02764977	161.8179724
0.014304061	1.049315068	167.3260274
0.027052031	1	156.9344262
0.012839901	1.086956522	235.3063917
0.008124839	1.277504105	252.2872651
0.055896034	1	163.826087
0.028675628	1.114503817	108.2910305
0.016513691	1.01754386	150.4922027
0.034668491	1.144230769	93.92788462
0.014819973	1.086065574	206.4518443
0.081175275	1	54.86363636
0.1173787	1.545454545	64.95454545
0.014094843	1.195121951	180.9034553
0.056078912	1.066666667	82.08888889
0.048078937	1.028846154	97.01201923
0.033881352	1.096676737	108.6418261
0.035399539	1.090277778	99.39409722
0.065526173	1	160.325
0.015204767	1.230769231	199.2884615
0.12597276	1	65.5
0.017970192	1	265.1818182
0.046432818	1.034482759	114.908046

log.sigma.3.5.mm.3D_gldzm_LowIntensityLargeDistanceEmphasis	log.sigma.3.5.mm.3D_gldzm_HighIntensityEmphasis	log.sigma.3.5.mm.3D_gldzm_DistanceZoneVariability	log.sigma.3.5.mm.3D_gldzm_ZonePercentage
0.02116705	179.875	94.02083333	0.265927978
0.031428703	117.0128205	144.4615385	0.111190306
0.04738151	81.66666667	54	0.305084746
0.020139261	233.0534653	450.219802	0.26125194
0.044994027	163.8545455	53.03636364	0.544554455
0.040747117	79.65151515	66	0.270491803
0.070813338	47.19354839	31	0.442857143
0.020409629	168.1257862	155.0503145	0.396508728
0.011083913	379.9075342	312.1746575	0.395663957
0.018049248	162.6883562	274.4589041	0.125916343
0.029183765	121.3944954	95.78899083	0.289893617
0.0256643	140.1972789	143.0544218	0.194187583
0.043047465	111.4725738	225.2616034	0.173499268
0.064509705	113.7727273	148.0909091	0.291666667
0.041107475	126.6641791	128.1343284	0.368131868
0.014183696	177.9476082	421.3690205	0.040543037
0.03651917	119.7735849	104.0188679	0.488479263
0.046879996	108.3843137	230.145098	0.164304124
0.081068935	137.8829787	92.0212766	0.516483516
0.048417513	99.31515152	151.5939394	0.059480894
0.018169571	126.6380368	322.0184049	0.064236453
0.038216458	124.3076923	220.4188034	0.257425743
0.019286279	141.5843621	239.0329218	0.083219178
0.023967904	282.3625	80	0.601503759
0.033500856	88.66666667	117	0.121875
0.036701398	94.60227273	84.09090909	0.291390728
0.020941258	123.3819742	227.0600858	0.101791175
0.018066897	127.5037594	252.3684211	0.068292683
0.021211869	155.35141	428.1193059	0.124058127
0.024829519	120.3033708	166.3483146	0.105138807
0.023526149	137.2568493	280.2123288	0.06950726
0.021852273	142.0226171	589.6009693	0.070078116
0.030857404	147.6480263	277.2039474	0.218862491
0.097497467	105.25	12	0.8
0.046543176	190.5230769	63.03076923	0.575221239
0.016944593	166.3273381	247.6402878	0.208395802
0.023596774	114.4909561	349.0671835	0.057838888
0.018840891	152.3636364	327.28739	0.112467018
0.035352457	170.8074074	125.3703704	0.420560748
0.036670689	102.4769231	116.7538462	0.242085661
0.021754881	149.8848921	139	0.194405594
0.026623364	146.8432836	124.3731343	0.351706037
0.02258208	128.3146552	220.2672414	0.281553398
0.029756121	144.2058824	132.0441176	0.309794989
0.019012914	189.543131	233.9584665	0.280717489
0.064462652	49.84552846	119.0487805	0.020091473
0.025512826	209.497416	516.0490956	0.28186453
0.121024003	81.25333333	65.66666667	0.328947368
0.070269687	121.1764706	17	0.772727273
0.025848819	212.1954023	87	0.595890411
0.021921421	132.7329377	321.379822	0.082598039
0.039733131	86.61	77.14	0.248138958
0.016440421	176.006383	397.8638298	0.147104851
0.042593872	97.625	56	0.234309623
0.032134325	102.4794521	474.3424658	0.04519724
0.081939546	114.8682171	121.248062	0.385074627
0.015704313	195.924933	369.0214477	0.427752294
0.033119848	238.9043478	216.426087	0.502183406
0.027971601	133.6698113	106	0.299435028
0.066900928	95.1969697	66	0.141630901
0.050407643	83.19248826	188.5868545	0.146391753
0.014808685	240.408046	799.0643678	0.128394333
0.025901611	107.477193	273.2175439	0.069955817
0.050575632	67.44654088	157.0125786	0.089225589
0.057905733	109.12	25	0.471698113
0.017867781	190.4678571	260.7142857	0.243055556
0.088381355	140.6724138	56.03448276	0.552380952
0.026708974	128.9773243	366.6235828	0.127199308
0.052807639	73.68	48.04	0.34965035
0.035452307	113.3201581	247.0711462	0.141183036
0.013528685	164.1087533	371.0477454	0.145055791
0.053979729	105.398374	109.796748	0.216931217
0.022042725	112.9407407	381.6567901	0.057389826
0.060211621	68.5733945	410.6284404	0.040243677
0.01293199	204.6966414	866.5991333	0.063571871
0.01927596	186.0730337	172.1011236	0.399103139
0.035615085	117.1654676	137.0143885	0.304824561
0.094585309	59.35	58.03333333	0.267857143
0.02358912	163.235023	213.0368664	0.237677985
0.014875334	168.6164384	353.1972603	0.134985207
0.027052031	156.9344262	61	0.409395973
0.013449925	238.5317726	285.2876254	0.34566474
0.015548853	263.2151067	511.8899836	0.123554474
0.055896034	163.826087	23	0.741935484
0.029852976	109.6259542	379.1984733	0.079201935
0.016896792	150.8869396	507.0350877	0.146278871
0.042777897	97.80769231	94.48076923	0.284931507
0.016168536	209.5409836	230.4016393	0.41426146
0.081175275	54.86363636	22	0.44
0.420409003	65.63636364	7.727272727	0.846153846
0.015743475	188.1686992	432.1626016	0.143482065
0.056933079	83.45555556	86.08888889	0.296052632
0.051284066	97.07692308	102.0192308	0.25
0.035102334	110.3897281	313.4410876	0.090635268
0.036524882	100.8333333	414.337963	0.059817225
0.065526173	160.325	40	0.634920635
0.039724239	203.1945701	189.6153846	0.325
0.12597276	65.5	12	0.5
0.017970192	265.1818182	88	0.539877301
0.047390673	115.2183908	85.02298851	0.310714286

log.sigma.3.5.mm.3D_gldzm_IntensityVariabilityNormalized	log.sigma.3.5.mm.3D_gldzm_LowIntensitySmallDistanceEmphasis	log.sigma.3.5.mm.3D_gldzm_IntensityVariability
0.071614583	0.020895782	6.875
0.074375411	0.029824826	11.6025641
0.086419753	0.04738151	4.666666667
0.053465347	0.011170556	27
0.057190083	0.03741827	3.145454545
0.116161616	0.040747117	7.666666667
0.123829344	0.070813338	3.838709677
0.06617618	0.020149742	10.52201258
0.039606399	0.007063661	23.13013699
0.069290674	0.016593545	20.23287671
0.090817271	0.02341162	9.899082569
0.064926651	0.024557086	9.544217687
0.076821734	0.035795306	18.20675105
0.07201889	0.03805994	11.09090909
0.072844732	0.024005485	9.76119403
0.083763575	0.012210022	36.77220957
0.077785689	0.02767483	8.245283019
0.080199923	0.029165555	20.45098039
0.059302852	0.041175318	5.574468085
0.091864096	0.020801264	15.15757576
0.080112161	0.017915426	26.11656442
0.065782745	0.02785237	15.39316239
0.072126539	0.018730212	17.52674897
0.049375	0.023967904	3.95
0.084958726	0.033500856	9.94017094
0.087551653	0.033372208	7.704545455
0.089612997	0.020019414	20.87982833
0.083469953	0.017099091	22.20300752
0.065871137	0.019407369	30.36659436
0.069561924	0.023600109	12.38202247
0.075647401	0.02057235	22.0890411
0.076236882	0.019302659	47.19063005
0.062370152	0.026061478	18.96052632
0.111111111	0.097497467	1.333333333
0.05704142	0.032120099	3.707692308
0.082759692	0.014524529	23.00719424
0.081532226	0.020686661	31.55297158
0.075979739	0.016107503	25.90909091
0.064910837	0.028550759	8.762962963
0.076923077	0.033799429	10
0.069613374	0.021754881	9.676258993
0.071508131	0.02168908	9.582089552
0.074576397	0.021695917	17.30172414
0.064554498	0.029177896	8.779411765
0.062683094	0.012991312	19.61980831
0.100535396	0.063018518	12.36585366
0.055211693	0.016076217	42.73385013
0.095466667	0.055853864	7.16
0.08650519	0.070269687	1.470588235
0.052715022	0.025848819	4.586206897
0.070679499	0.019871125	23.8189911
0.1028	0.034330271	10.28
0.06753282	0.011814933	31.74042553
0.086734694	0.042593872	4.857142857
0.083260251	0.027654075	42.54598826
0.062916892	0.043632053	8.11627907
0.060814065	0.015459865	22.68364611
0.0536862	0.009761371	12.34782609
0.062833749	0.027971601	6.660377358
0.081726354	0.066900928	5.393939394
0.082104521	0.046162585	17.48826291
0.055042938	0.008001908	47.88735632
0.089418283	0.02415648	25.48421053
0.107313793	0.050094307	17.06289308
0.0944	0.057905733	2.36
0.064005102	0.013887317	17.92142857
0.063020214	0.023726183	3.655172414
0.071456852	0.022344926	31.51247166
0.1096	0.051277027	5.48
0.071943008	0.034240003	18.20158103
0.073503648	0.013301282	27.71087533
0.084539626	0.022122775	10.39837398
0.086127115	0.020157087	34.88148148
0.087471593	0.055622836	38.13761468
0.069060919	0.009946155	63.7432286
0.059335942	0.0177271	10.56179775
0.074064489	0.033928934	10.29496403
0.092222222	0.093608747	5.533333333
0.062838455	0.023386504	13.6359447
0.067412272	0.014161243	24.60547945
0.080892233	0.027052031	4.93442623
0.052426707	0.012697689	15.67558528
0.061472548	0.006281593	37.43678161
0.088846881	0.055896034	2.043478261
0.080887542	0.028465983	31.78880407
0.080028423	0.016417916	41.0545809
0.082285503	0.03264114	8.557692308
0.061072292	0.014482833	14.90163934
0.107438017	0.081175275	2.363636364
0.140495868	0.041621124	1.545454545
0.066957499	0.013682685	32.94308943
0.072839506	0.05586537	6.555555556
0.073409763	0.047277655	7.634615385
0.078905815	0.033603849	26.11782477
0.070719736	0.035163415	30.55092593
0.07125	0.065526173	2.85
0.063819332	0.009074899	14.1040724
0.097222222	0.12597276	1.166666667
0.059659091	0.017970192	5.25
0.067776457	0.046193355	5.896551724

log.sigma.3.5.mm.3D_gldzm_HighIntensityLargeDistanceEmphasis	log.sigma.3.5.mm.3D_gldzm_SmallDistanceEmphasis	log.sigma.3.5.mm.3D_glcm_SumVariance	log.sigma.3.5.mm.3D_glcm_Homogeneity1
184.375	0.9921875	402.4437734	0.418334403
131.4551282	0.971153846	359.1371521	0.546324765
81.66666667	1	95.55737059	0.411609444
248.7306931	0.956655666	479.5031226	0.428824947
164.3454545	0.986363636	264.6784597	0.288084603
79.65151515	1	91.20863531	0.437054127
47.19354839	1	69.71183391	0.412686145
175.0125786	0.990566038	261.0871892	0.359644736
1212.619863	0.750130803	1205.807707	0.290305123
193.6267123	0.975932268	382.9930083	0.533100992
146.5412844	0.950560652	185.5414144	0.403058774
142.2380952	0.989795918	308.7781586	0.450319702
119.4978903	0.980426629	140.7599688	0.463443078
119.2987013	0.984487734	123.1180979	0.413625239
127.0447761	0.983208955	162.9870965	0.357467281
183.0660592	0.984624146	521.2321984	0.743472183
119.8867925	0.992924528	154.2316105	0.325417289
132.1215686	0.960484749	153.2199907	0.471515615
137.9148936	0.992021277	139.2617272	0.326100732
104.6424242	0.968181818	252.1044638	0.605527992
132.3680982	0.994972733	380.7240907	0.635519469
127.1538462	0.977564103	183.7170338	0.426713513
142.9794239	0.99382716	429.8656507	0.611931605
282.3625	1	845.8941531	0.309616583
88.66666667	1	241.7316865	0.568178265
97.32954545	0.982954545	189.5976903	0.410639269
129.5879828	0.989747258	253.7372989	0.60079615
136.9661654	0.980263158	488.544258	0.679067726
177.6182213	0.971634731	496.8768134	0.600684444
141.5674157	0.973938826	433.1033514	0.567969454
145.2876712	0.984113394	374.663888	0.623801509
157.2617124	0.980939239	344.1883975	0.663461147
168.4013158	0.96484375	293.9117242	0.442519197
105.25	1	210.1461008	0.267434522
190.7076923	0.988461538	349.8345008	0.297245311
202.0611511	0.955835332	335.9713738	0.429118102
127.0723514	0.96124031	441.9978169	0.670956042
156.3929619	0.984604106	296.924402	0.557704238
174.762963	0.972222222	279.2789703	0.343619775
113.9923077	0.959615385	205.143226	0.416513487
149.8848921	1	430.5298226	0.480182022
156.4253731	0.972014925	224.0888246	0.393214156
145.4741379	0.980004789	238.0481576	0.396052813
157.3235294	0.987949346	214.6324428	0.380709366
306.2587859	0.888888889	380.6240514	0.371525752
58.59349593	0.9866757	209.0761721	0.739093202
738.9870801	0.84157874	383.488277	0.372686504
89.45333333	0.95	79.55638282	0.397323388
121.1764706	1	259.1687089	0.256768122
212.1954023	1	315.0509363	0.266781578
140.2017804	0.982195846	380.2907458	0.629962274
138.68	0.901111111	114.0816502	0.403107514
234.8851064	0.936480496	517.7846185	0.491562564
97.625	1	158.1203563	0.426849139
118.8414873	0.971678626	325.137739	0.703611182
116.4031008	0.976744186	166.919139	0.357390529
198.3780161	0.995978552	399.5360085	0.344259105
243.7565217	0.977173913	460.4595747	0.289716858
133.6698113	1	267.6334493	0.405381186
95.1969697	1	141.6677407	0.467873662
101.0798122	0.954225352	196.8770663	0.467169423
255.9701149	0.967943806	616.1321261	0.56546047
114.7578947	0.983723197	246.6623888	0.624123534
68.37106918	0.995283019	189.9191421	0.575788632
109.12	1	193.2516063	0.339804173
198.5357143	0.973214286	411.0874748	0.421196999
140.7241379	0.987068966	223.6828676	0.299133793
154.569161	0.930272109	321.0708424	0.53546478
76.62	0.985	77.65989786	0.413810362
116.1778656	0.991106719	296.1772374	0.508739415
167.3952255	0.99403183	429.3761773	0.505475266
126.2276423	0.957317073	177.7221322	0.413671539
120.6419753	0.977434842	341.6091525	0.652772929
80.43807339	0.976570464	234.6091078	0.689307942
221.75948	0.975675635	495.7891809	0.675357226
192.494382	0.987359551	347.9659771	0.356936812
117.5107914	0.994604317	215.4660556	0.411797554
62.55	0.9875	47.48634079	0.442422621
168.9032258	0.993087558	426.9854126	0.420294986
173.7780822	0.987671233	471.6569389	0.562928526
156.9344262	1	245.9459482	0.354348139
256.7993311	0.98197696	561.9942773	0.385311868
311.1592775	0.934272943	790.7700152	0.574784799
163.826087	1	338.6960457	0.263034647
124.6412214	0.985687023	255.1888292	0.611451657
152.4658869	0.995614035	276.4061933	0.520794496
113.3269231	0.963942308	146.3051304	0.411037226
221.897541	0.978483607	384.2662221	0.330913787
54.86363636	1	86.34633395	0.386864217
68.36363636	0.863636364	133.5481113	0.287019429
217.2296748	0.951219512	575.2288815	0.548270193
88.92222222	0.983333333	135.8540748	0.381984956
97.33653846	0.992788462	185.9847242	0.45106786
119.0060423	0.979187647	339.5636254	0.579792918
110.3402778	0.983940972	274.8895831	0.64923596
160.325	1	287.5617797	0.250905847
218.8190045	0.942307692	341.3206337	0.366935269
65.5	1	116.5908239	0.33830933
265.1818182	1	596.3316326	0.314214878
116.4597701	0.99137931	314.3973006	0.431528439

log.sigma.3.5.mm.3D_glcm_Homogeneity2	log.sigma.3.5.mm.3D_glcm_ClusterShade	log.sigma.3.5.mm.3D_glcm_MaximumProbability	log.sigma.3.5.mm.3D_glcm_Idmn	log.sigma.3.5.mm.3D_glcm_SumVariance2
0.336426252	-9.024914642	0.028621441	0.981163454	43.87874693
0.492004759	-102.579083	0.144176169	0.983466598	29.75625047
0.328968089	72.6328326	0.039328784	0.973298978	27.62966393
0.353213357	-11.4376093	0.063967992	0.986270116	45.46826712
0.190574565	426.6522183	0.031460926	0.955262344	78.03551656
0.361592668	24.6856244	0.051252038	0.957721418	13.20522539
0.329798075	-6.050227542	0.073807175	0.93612392	11.92801345
0.269907478	96.36449093	0.023666184	0.977645444	34.27676508
0.200872293	1734.151946	0.008903455	0.973272306	245.6182757
0.477832314	-48.40319349	0.077103941	0.991432976	24.23455487
0.319033351	17.80411367	0.03524098	0.971834502	16.31506323
0.377377994	-30.46258663	0.029551016	0.986697779	32.22370096
0.393905439	49.39339105	0.036857293	0.981892919	20.64245012
0.335118048	133.6111535	0.033934699	0.975935183	28.14889002
0.26663626	113.809702	0.023485081	0.96994475	33.44343128
0.726621872	-83.07921435	0.300758005	0.997308519	11.64152537
0.231458276	84.9408536	0.023109964	0.956947899	35.33272018
0.402585448	2.691669451	0.032883186	0.984287459	25.00762694
0.236505997	271.8071184	0.035953206	0.949925961	53.13457654
0.564460252	-59.97439321	0.111393031	0.989106721	24.65682246
0.600862514	-51.20988487	0.210920943	0.995004725	15.39454364
0.350112346	79.83666125	0.036533742	0.979082771	29.23443446
0.573475746	-92.60311226	0.147666605	0.994072008	20.30696538
0.214058492	-764.0932509	0.029168471	0.968029085	134.3828292
0.522664522	-45.60929876	0.062486539	0.989319371	17.65431191
0.328512387	0.092170841	0.025280348	0.969589323	28.88994402
0.558855465	-35.44888246	0.180372722	0.993673646	16.13437522
0.648927302	-104.85125	0.303366313	0.99704564	19.77487578
0.554418228	-183.2908693	0.25445573	0.99210292	32.9013301
0.517599532	-147.4883485	0.170841385	0.989525906	33.30666424
0.58611841	-68.83348782	0.193981632	0.994267095	19.01010389
0.632838486	-77.47963825	0.250676949	0.995044502	16.10584951
0.366316781	-24.79641061	0.071996997	0.984718801	40.28902476
0.170275285	117.6536402	0.143589744	0.915721858	38.68809295
0.201425835	103.360963	0.030442608	0.95961045	69.27960364
0.350942895	13.76899885	0.033223723	0.979812597	30.12571189
0.641047448	-125.9187106	0.210544794	0.993040711	20.4023107
0.507048068	-38.91549709	0.111511721	0.992060963	24.86399622
0.254434531	148.3135641	0.02138628	0.967222772	59.67861571
0.334789386	56.93450065	0.025559181	0.976421094	41.88831911
0.415101737	-131.3376942	0.03559139	0.988192487	37.86819116
0.30813818	56.1516377	0.032112375	0.978395504	24.47507866
0.310889637	3.576446771	0.024484914	0.976269949	25.1361793
0.294533961	143.230957	0.02551569	0.98023408	34.92024072
0.284139518	429.296163	0.020534063	0.980346695	58.68621808
0.722820203	-55.23279054	0.234486411	0.994209927	10.60899003
0.28906185	1059.72319	0.016558909	0.988204572	77.70183733
0.315188388	59.94375249	0.044347319	0.953147715	19.96732858
0.158951821	33.71691762	0.083687146	0.907040383	36.0334274
0.17435197	238.7817168	0.023215422	0.936671199	89.53441898
0.591401485	-89.99396424	0.231661432	0.992694986	21.14969052
0.319269409	67.82193868	0.041922677	0.961884125	20.83978101
0.427036852	-95.94424615	0.072381181	0.988801725	35.95608842
0.346845163	58.34714485	0.029360276	0.974678733	34.1393645
0.67977741	-99.6280868	0.279459066	0.995315069	16.28946955
0.2685422	35.57195447	0.023332174	0.955873592	37.41046157
0.253493788	105.3175068	0.020006291	0.976314481	46.33581517
0.195708682	89.97529345	0.01582274	0.961631634	83.46539501
0.322654498	35.84699619	0.025712648	0.979406585	37.16868767
0.396746354	43.64394256	0.030361287	0.979371669	29.91521891
0.395982754	38.9604832	0.034213209	0.980398826	31.29760659
0.516604789	-131.1165163	0.138636137	0.992709328	34.72263506
0.587615161	-54.36853792	0.154851059	0.993268088	16.36666963
0.527880042	-47.28989155	0.131462905	0.984834471	21.10725954
0.240341349	17.60707889	0.060097797	0.943844733	36.30909864
0.344130836	-66.75512972	0.02572177	0.983610691	41.41645267
0.202472676	21.90399605	0.029516125	0.940144014	42.87526559
0.479110476	-125.9987566	0.113887516	0.990136671	43.03922232
0.332986308	77.26713792	0.057759599	0.959880524	16.81309028
0.448661326	-57.02849811	0.072868546	0.986407157	29.41724456
0.445267392	-78.30654253	0.0538871	0.986167492	31.46633432
0.331613952	21.77174882	0.025307185	0.973671488	25.88834972
0.619404119	-99.83554271	0.167807337	0.994197037	21.24632021
0.663913908	-81.16362046	0.243564321	0.992757947	16.20586028
0.648015916	-78.04675622	0.22889062	0.996604108	16.82964695
0.270282993	62.53192599	0.025499598	0.973369928	36.37847391
0.33063068	7.170348278	0.036961068	0.973324212	22.64227017
0.366210377	38.32460054	0.054453686	0.968303502	12.81255778
0.339520443	-63.44868453	0.023137183	0.981452844	31.45123505
0.513067597	-76.90633042	0.156573841	0.991813069	24.98102711
0.265319406	61.52584804	0.037669837	0.953445165	35.33960177
0.302866275	62.63378514	0.020422167	0.980247216	51.57255938
0.527733908	-168.9050936	0.137090277	0.992285487	30.89149833
0.164966055	2.841848834	0.064300016	0.917359631	46.74169053
0.572100006	-64.0975967	0.150885948	0.991309184	18.2825215
0.464561273	5.69118416	0.053515881	0.989574171	17.75925662
0.328192373	44.88528406	0.029946502	0.973584567	23.13696637
0.239590161	59.08885106	0.01810477	0.970630805	52.41179097
0.297886206	-2.023429058	0.071619829	0.92576982	12.4715412
0.192048404	39.23059265	0.109126984	0.866227461	25.32590073
0.494958792	-197.7470177	0.110571041	0.990640257	40.18834203
0.295414952	144.5053386	0.029939255	0.966010374	37.56036263
0.377227548	-13.75854524	0.04101879	0.974616678	25.69369229
0.533356879	-80.6455048	0.110080381	0.989870306	27.86184576
0.61539104	-82.06639131	0.223407494	0.992974987	19.48393119
0.15650766	254.4099858	0.036388818	0.940866736	51.64530017
0.280004467	101.2608549	0.016379432	0.975769224	49.24620689
0.245615795	44.17209459	0.113225983	0.886219161	29.25204263
0.220732246	256.4296308	0.026307696	0.971559188	82.46352321
0.352573788	-103.5857876	0.040025633	0.979601774	36.38453585

log.sigma.3.5.mm.3D_glcm_Contrast	log.sigma.3.5.mm.3D_glcm_DifferenceEntropy	log.sigma.3.5.mm.3D_glcm_InverseVariance	log.sigma.3.5.mm.3D_glcm_Entropy	log.sigma.3.5.mm.3D_glcm_Dissimilarity
8.81898424	2.699547814	0.34127414	6.884197186	2.322397393
5.175916696	2.386495454	0.381754574	6.016245524	1.580096426
8.354689287	2.595075005	0.356039398	6.236010558	2.294959363
14.07357361	3.020455085	0.312468965	7.31345648	2.680991281
32.03494695	3.353307459	0.187520882	6.440040235	4.503347849
8.272443169	2.64591251	0.368558601	5.931232776	2.197592031
9.41574232	2.507002351	0.332480765	4.970315363	2.400062187
16.31924498	3.106271766	0.282292321	7.098333853	3.138861453
44.75784752	3.779188484	0.208395736	8.935425213	5.045331509
4.71554008	2.34459434	0.410715517	6.231217946	1.55752286
9.982377067	2.772757447	0.334235298	6.41355314	2.474345506
7.433930123	2.570280882	0.384115779	6.751393654	2.057935018
7.87954744	2.623065007	0.382093972	6.462970184	2.049307516
11.6516682	2.874118987	0.341231122	6.724506679	2.554854456
16.01871141	3.077182369	0.278518159	6.991622329	3.134843421
1.74858498	1.614511058	0.354057125	4.081509755	0.705346983
21.50933178	3.154866584	0.240343091	6.77735918	3.684569546
7.419681102	2.61556731	0.372240945	6.590701994	2.00598288
25.98765879	3.341908874	0.247336928	6.731583512	3.93017883
2.938436754	2.063475917	0.414620791	5.673827879	1.179291649
2.493124132	1.962730637	0.415921344	5.128637964	1.047478936
11.0791223	2.843567355	0.343989008	6.815782003	2.454292253
2.964600625	2.051834003	0.422219701	5.478877791	1.159806833
23.56860628	3.227014384	0.227548418	6.588209995	3.90980812
3.248245936	2.099404939	0.450549984	5.682486602	1.291119798
9.695911839	2.739785257	0.338090995	6.638406274	2.427756269
3.489475775	2.142521812	0.407640935	5.470123449	1.244682906
2.551632434	1.916460631	0.360177089	4.719090341	0.963903244
5.621642714	2.380334205	0.330844245	5.617508845	1.485295371
4.401211029	2.307366937	0.383830493	5.915667523	1.447291593
3.161689876	2.074756201	0.402198367	5.359873837	1.150436935
2.984176815	1.987135234	0.381297762	5.009352167	1.034145046
10.16751359	2.834524355	0.323963387	7.026210808	2.357255821
35.13269231	1.971091104	0.201261643	3.170512738	4.755769231
28.50151489	3.315140937	0.210540922	6.488598084	4.252452456
9.570415479	2.783164236	0.340195896	6.880248324	2.349436837
2.642297093	1.941315639	0.375914986	4.755567083	0.990241628
4.767314201	2.345674738	0.397107054	6.112478307	1.499478561
21.16610189	3.171373279	0.273107353	7.279867789	3.515824859
9.163798195	2.730243219	0.338107369	6.924040348	2.365807951
6.638066327	2.485560643	0.408161728	6.580413454	1.872196489
12.38280295	2.920702661	0.315273557	6.664129688	2.694002842
11.32071996	2.869479438	0.318236102	6.923843103	2.615031752
14.42573274	3.002809102	0.305687007	7.000182184	2.888668064
15.30763689	3.078566216	0.291209382	7.54467083	3.023017322
1.178633586	1.522625206	0.378432798	3.935335515	0.652723126
22.32712694	3.267373191	0.292265885	7.822340162	3.341514333
12.24115501	2.908519148	0.321977641	6.307186212	2.697332554
38.93186536	2.501606602	0.182113324	4.067086532	5.05541403
43.42051069	3.608300444	0.179240844	6.931448775	5.273270875
3.378222154	2.115022948	0.376020432	5.351101996	1.172646135
10.93089294	2.847200984	0.313079854	6.452370602	2.584604742
6.785004856	2.557686822	0.379779249	6.65608884	1.88561418
7.885206089	2.588955498	0.35404485	6.562323949	2.212543693
2.153302141	1.798458881	0.368945882	4.537772176	0.853334776
18.63428932	3.177314286	0.273262553	7.135864006	3.301660022
20.0666154	3.240900354	0.257327184	7.565477943	3.480750762
33.55517404	3.534799884	0.195686771	7.842041782	4.610431675
10.71276573	2.803164792	0.333658062	6.836210692	2.516544099
5.653991806	2.404707532	0.393390353	6.43350636	1.84803848
6.82829589	2.574579943	0.369829801	6.571239659	1.97595829
6.923561745	2.499948982	0.365583861	6.203195795	1.663891999
2.800890088	2.009410541	0.416596806	5.366746462	1.11211505
3.636362197	2.185855111	0.403258535	5.703972478	1.342255738
16.4235157	2.817443686	0.207022813	5.215437826	3.339141544
11.94816924	2.86367987	0.342112792	7.114064763	2.530519675
25.14938796	3.184661862	0.209898498	6.337454265	4.094818612
6.489096596	2.534143284	0.362861629	6.399833578	1.740606129
10.38406368	2.784574009	0.335832004	5.826939519	2.466566286
5.780829449	2.457296816	0.400298564	6.42977761	1.732860742
6.574186466	2.512168309	0.395872427	6.515172222	1.804178262
9.298170193	2.741610443	0.341210204	6.748069183	2.378208886
2.656141443	1.982409693	0.385551055	5.100462878	1.035729209
1.946264727	1.797571758	0.390054547	4.689680098	0.862122737
3.396751415	1.977429837	0.378750408	4.910550447	1.032168226
19.72118602	3.200073626	0.277887741	7.195806849	3.386757471
10.51910872	2.823359821	0.329905438	6.667213141	2.499923369
8.113789846	2.611345	0.363585016	5.790028555	2.146285985
9.567034751	2.750175395	0.335003311	6.857279768	2.382297088
5.389948643	2.386513804	0.38541032	5.938136536	1.537247149
19.33389696	3.121921672	0.255452421	6.292831091	3.416638826
16.89570149	3.118256116	0.309807882	7.475390134	3.017001976
6.398607509	2.440878821	0.369331366	5.999891268	1.592619516
37.27277455	2.904959146	0.178748691	4.628279691	4.949464104
3.311083502	2.111020051	0.409419066	5.467229534	1.199672901
5.821205585	2.445899711	0.408895557	6.224181973	1.69114782
9.314193043	2.744935899	0.338923907	6.538464817	2.390170749
23.45767951	3.357827821	0.237604705	7.636326557	3.782466205
11.03564389	2.518499915	0.298622028	4.914121708	2.657904405
26.26587302	2.568005041	0.216255354	3.814036787	4.265873016
6.696897519	2.523792577	0.361912187	6.279882593	1.711956797
12.28850648	2.880254591	0.31688808	6.755519982	2.744767266
9.145924942	2.708771637	0.346187129	6.435196234	2.225285571
3.869956615	2.212938782	0.405760374	5.980070696	1.34325088
2.650114971	1.988200272	0.389466922	5.14396908	1.042047316
47.13535806	3.3545484	0.169305141	5.747367455	5.52616359
16.45329938	3.117869794	0.293552481	7.496860255	3.112240078
25.31054949	2.375902969	0.148288671	3.919501079	4.064934425
24.15465083	3.286185032	0.232335714	6.840352815	3.923427693
8.792729855	2.677811506	0.345064475	6.522143687	2.264336025

log.sigma.3.5.mm.3D_glcm_DifferenceVariance	log.sigma.3.5.mm.3D_glcm_Idn	log.sigma.3.5.mm.3D_glcm_Idm	log.sigma.3.5.mm.3D_glcm_Correlation	log.sigma.3.5.mm.3D_glcm_Autocorrelation
3.335375309	0.905748185	0.336426252	0.665101454	147.6739483
2.579959727	0.921349192	0.492004759	0.702188876	131.2262558
2.922875282	0.887919524	0.328968089	0.531856222	43.44228119
6.703329503	0.92545823	0.353213357	0.526927844	170.9654726
11.15579134	0.857904569	0.190574565	0.420383349	94.91965773
3.361664363	0.866957949	0.361592668	0.230577317	40.9477601
3.249408415	0.836230075	0.329798075	0.14078721	30.17448956
6.325263359	0.898551752	0.269907478	0.353804912	99.89416442
18.60097844	0.893475475	0.200872293	0.694798807	387.7352861
2.246142237	0.939871569	0.477832314	0.670809975	139.4425313
3.733548148	0.886664772	0.319033351	0.238176801	73.72447294
3.027647699	0.922171387	0.377377994	0.621419617	117.7515465
3.570338454	0.913197725	0.393905439	0.443661211	60.36770764
4.995313413	0.898996068	0.335118048	0.414826769	53.42623157
5.960262031	0.883209652	0.26663626	0.354930229	66.60294001
1.236148195	0.974199138	0.726621872	0.735476046	168.0569729
6.876272688	0.861074898	0.231458276	0.271786706	61.7874432
3.292993043	0.918145112	0.402585448	0.541977367	65.47607753
9.836642967	0.855363118	0.236505997	0.350525151	54.4944835
1.508009552	0.935843175	0.564460252	0.785854726	98.04890732
1.374929475	0.95687351	0.600862514	0.719391909	133.8831239
4.875857033	0.906467121	0.350112346	0.444149426	75.15288214
1.579963098	0.952575301	0.573475746	0.743237182	151.0198435
7.757263325	0.876914777	0.214058492	0.703251747	276.7563191
1.54095181	0.933447979	0.522664522	0.685684463	93.68445696
3.613429787	0.883202153	0.328512387	0.495132063	77.17845573
1.907300513	0.951515387	0.558855465	0.642694543	95.91277678
1.590381129	0.969444176	0.648927302	0.769126506	163.3282348
3.334532135	0.949806248	0.554418228	0.706146009	170.048061
2.261157925	0.936819139	0.517599532	0.764111076	153.8535273
1.807344704	0.955086779	0.58611841	0.712282804	133.2867546
1.886284048	0.961311478	0.632838486	0.682919515	121.6551475
4.49395151	0.919004385	0.366316781	0.596320843	112.9596715
9.992836538	0.808312325	0.170275285	0.031862162	58.75
9.812882136	0.864268541	0.201425835	0.419247581	123.9653487
3.916822767	0.905552074	0.350942895	0.518219004	125.2775748
1.634149634	0.954010819	0.641047448	0.768816098	149.8759578
2.476181309	0.944543189	0.507048068	0.676004163	112.4372553
8.063884013	0.881443505	0.254434531	0.47827173	106.0483106
3.43404105	0.895782275	0.334789386	0.639790189	83.56799224
3.032830261	0.928945938	0.415101737	0.700818881	156.1435479
5.014288393	0.901504657	0.30813818	0.329213179	87.09501385
4.295168159	0.895892352	0.310889637	0.377937534	92.92861949
5.924261666	0.905981844	0.294533961	0.414811173	84.96473785
5.961206288	0.905033393	0.284139518	0.587299431	140.3385282
0.732525376	0.958521696	0.722820203	0.795064633	76.42059925
10.8347565	0.930740226	0.28906185	0.559125391	140.0326283
4.829469205	0.859932169	0.315188388	0.231444971	36.28248397
12.30952224	0.798928831	0.158951821	-0.049811616	78.09119492
14.40024961	0.833892485	0.17435197	0.346066313	110.2262454
1.971413146	0.950605298	0.591401485	0.722795678	134.6944287
4.09128183	0.870847357	0.319269409	0.316692141	49.62991524
3.113847645	0.931262434	0.427036852	0.681454836	182.0816707
2.789716252	0.891603998	0.346845163	0.623529677	67.21717201
1.404697468	0.96352898	0.67977741	0.763680936	114.1131255
7.498587885	0.863838451	0.2685422	0.337459863	67.8997408
7.593174172	0.896059722	0.253493788	0.392107457	144.7541611
11.2980509	0.8679463	0.195708682	0.422934568	162.2419719
4.245860246	0.903388473	0.322654498	0.548655231	103.7175681
2.133118357	0.902353495	0.396746354	0.679158337	62.01840833
2.857483622	0.907185989	0.395982754	0.641172292	80.57600993
4.088907946	0.950949806	0.516604789	0.666257571	209.1232711
1.510096615	0.950374319	0.587615161	0.705095147	93.96582286
1.768753849	0.923673284	0.527880042	0.703411536	77.0355519
4.818610451	0.838707722	0.240341349	0.377149203	72.1065818
5.296213505	0.916884828	0.344130836	0.551927993	149.7198766
7.613847682	0.835054035	0.202472676	0.266378531	83.0393275
3.398227536	0.939030676	0.479110476	0.736573166	120.7193007
4.213159584	0.869748936	0.332986308	0.237691618	34.93905709
2.710229424	0.92520409	0.448661326	0.67037259	113.1720226
3.21360396	0.926125753	0.445267392	0.651139237	154.5474781
3.526540392	0.890482439	0.331613952	0.472347659	73.35817904
1.558084902	0.955844065	0.619404119	0.776243212	122.3089052
1.18707751	0.952500877	0.663913908	0.782960372	87.7499942
2.310874527	0.969731665	0.648015916	0.662661453	165.3477481
7.74949622	0.892527878	0.270282993	0.300993417	126.88093
4.108640442	0.89129026	0.33063068	0.362390536	85.00639658
3.442932095	0.884099265	0.366210377	0.22738597	24.17270134
3.677018281	0.907792958	0.339520443	0.520417822	153.4053111
2.973268048	0.945713816	0.513067597	0.643339886	164.5449013
7.118583907	0.859592845	0.265319406	0.302213794	92.76886129
7.616496311	0.90927366	0.302866275	0.504850117	196.4618966
3.772762392	0.949889723	0.527733908	0.655526023	258.3297978
12.01632536	0.809876944	0.164966055	0.089157262	106.4798031
1.839496779	0.944502068	0.572100006	0.691012156	96.73078809
2.879193857	0.935611964	0.464561273	0.505018071	104.7521861
3.5259403	0.889892498	0.328192373	0.42411488	61.88846984
8.887367458	0.885016647	0.239590161	0.378705447	139.6158203
3.528764304	0.82151114	0.297886206	0.078621384	36.28253836
7.702317964	0.760145944	0.192048404	0.006211415	42.5952381
3.692594115	0.942359452	0.494958792	0.711725365	197.3927586
4.530175567	0.876521692	0.295414952	0.50301634	57.72627697
4.003309385	0.898153739	0.377227548	0.478108502	75.65676986
2.014421112	0.938193684	0.533356879	0.754281516	126.4835809
1.542353915	0.951369043	0.61539104	0.758787503	101.9216441
13.30991668	0.83755929	0.15650766	0.075699966	95.41770889
6.571588432	0.896308343	0.280004467	0.499452312	127.7423255
8.098460096	0.784763239	0.245615795	0.081077188	36.67255821
8.348096186	0.88422977	0.220732246	0.54459759	203.3692571
3.535216948	0.904408753	0.352573788	0.608232824	118.4548802

log.sigma.3.5.mm.3D_glcm_SumEntropy	log.sigma.3.5.mm.3D_glcm_AverageIntensity	log.sigma.3.5.mm.3D_glcm_Energy	log.sigma.3.5.mm.3D_glcm_SumSquares	log.sigma.3.5.mm.3D_glcm_ClusterProminence
4.636452896	11.7854004	0.010514774	13.17443279	4547.388706
4.219523023	11.18369699	0.034379354	8.733041792	2274.436671
4.187795807	6.212307181	0.016201573	8.996088305	2218.675186
4.710301045	12.79378326	0.011896587	15.15138686	6967.613635
4.608993331	9.130021492	0.012885509	27.51761588	18578.15329
3.772364791	6.301729159	0.021927326	5.36941714	584.8594647
3.282580407	5.429348462	0.037003918	5.335938942	393.1087912
4.475088274	9.769646108	0.009239316	12.69634077	4232.795291
5.75731249	18.66108325	0.002668638	73.79746268	125562.7529
4.259924756	11.59992903	0.022731105	7.237523737	2166.546889
3.978490795	8.493096925	0.015318521	6.574360073	868.1283308
4.494353322	10.56166534	0.012398189	9.914407772	3036.372879
4.163343739	7.561278097	0.01601248	7.13049939	1540.362951
4.298235563	7.021130885	0.013250621	9.950139554	3483.616109
4.397296689	7.889099965	0.009726937	12.36553567	3556.485302
3.161806641	12.86791479	0.146115191	3.347527589	1234.514158
4.371077711	7.635479156	0.010672716	14.21051299	3482.926784
4.308070475	7.815220415	0.013734721	8.106827009	1646.627631
4.532986145	6.903906176	0.011771338	19.78055883	7988.222599
4.16666927	9.623653493	0.037179363	6.898814803	1410.592384
3.747774569	11.43044423	0.064339341	4.471916944	794.5787018
4.377374199	8.402728993	0.013154479	10.07838919	3193.823832
3.985423571	12.11116221	0.04356616	5.817891502	1679.924564
4.890888942	15.83623336	0.01169128	40.21522827	39662.97245
4.01434774	9.490747846	0.027824329	5.225639462	990.7493874
4.340251983	8.506890675	0.011720838	9.646463965	1832.027776
3.847328294	9.630627323	0.053253252	4.905962749	1187.472514
3.569978351	12.61045121	0.111679476	5.583852285	1440.717971
4.012263539	12.77591752	0.076773388	9.631265752	3477.792317
4.223830436	12.10839569	0.041758742	9.426968817	3065.987375
3.885755495	11.37194844	0.057154875	5.54294844	1204.386031
3.647283517	10.87994646	0.085234512	4.772506581	1192.121492
4.61073768	10.26756409	0.013099281	12.61413459	3869.478746
2.161538379	7.568269231	0.120608974	18.45519631	4155.598743
4.584313095	10.66166361	0.012414034	24.44527963	11950.05253
4.433621362	10.96064932	0.011100578	9.924031842	2267.724528
3.586702161	12.05960209	0.094698438	5.761151949	1712.113718
4.233837557	10.3639099	0.028963582	7.407827605	1600.219055
4.820017873	9.816908758	0.007901811	20.2111794	9582.006755
4.588248636	8.682288002	0.010152139	12.76302933	3685.456271
4.543460505	12.17877701	0.014714379	11.12656437	4471.157649
4.210473241	9.168883997	0.013005893	9.214470402	2642.883492
4.327968193	9.4586677	0.010599903	9.114224815	1814.185151
4.46602471	8.935570518	0.010187524	12.37351956	5229.194271
4.816681944	11.39681868	0.00760468	18.84397751	14181.51648
3.124425564	8.605857337	0.130460657	2.946905903	567.4319194
4.980498524	11.37150247	0.006758417	26.57120182	47843.08733
4.00190136	5.859893413	0.016772484	8.052120898	1334.655624
2.838082203	8.866623654	0.063441214	18.74132319	3495.959595
4.853639563	9.928779979	0.009151531	33.23873242	18621.81147
3.874714662	11.4126854	0.070463749	6.131978168	1519.668113
4.078704273	6.866381298	0.015864367	7.942668487	1418.919409
4.491442345	13.22054243	0.016828474	10.68527332	3180.382545
4.441399617	7.787799597	0.012564012	10.50614265	2868.161807
3.457319075	10.51562142	0.117944402	4.610692923	1365.124963
4.520395973	7.949841065	0.008796112	14.01118772	3510.296017
4.723245819	11.76539677	0.007172212	16.83660991	7285.540736
5.058717836	12.25812109	0.005216047	29.68326795	16320.03379
4.526823486	9.853724933	0.010979201	11.97036335	3657.654483
4.38919493	7.479908198	0.014569697	8.892302679	2221.199591
4.391032819	8.628512238	0.013659899	9.53147562	2220.099181
4.325238383	14.22718108	0.039309472	10.50946943	4385.641626
3.85877212	9.517019766	0.051170317	4.79188993	881.8533379
4.057141627	8.523900335	0.036007357	6.185905434	1053.578599
3.860870846	8.192670476	0.0301285	13.18315359	2852.237525
4.635502042	11.93284006	0.010240273	13.37830529	4346.328838
4.287731998	8.860389188	0.013774765	17.00616339	4635.326788
4.452495674	10.56295726	0.028488648	12.38207973	4320.068067
3.746479814	5.77275399	0.023473002	6.79928849	1286.444573
4.381189334	10.35642303	0.018106678	8.799518503	2107.565569
4.411121373	12.17828463	0.017440711	9.510130197	2616.111921
4.317249407	8.318977568	0.011444156	8.796629978	1690.560967
3.79588085	10.8470664	0.070596556	5.975615413	1430.153993
3.572251546	9.175107425	0.094544436	4.538031253	1005.179844
3.569923518	12.7313274	0.095211353	5.099703686	1735.382358
4.505577239	11.08146016	0.009282893	14.10039173	4902.317752
4.223284976	9.053849751	0.013486603	8.290344722	1598.654888
3.704407824	4.795343659	0.023514312	5.231586907	678.0869496
4.440789027	12.1614912	0.011062017	10.25456745	3150.338822
4.135988844	12.63497364	0.038413056	7.592743939	1843.390217
4.330853247	9.420592912	0.015279092	13.66837468	3608.477628
4.815459239	13.73954697	0.007900112	17.61528333	8414.11962
4.19623404	15.88807517	0.044966435	9.388582397	3741.307662
3.331128915	10.19211712	0.042949798	21.00361627	4726.284971
3.894693506	9.642888775	0.048250241	5.39840125	1052.786033
4.094191421	10.08778855	0.021686334	5.895115552	1131.636052
4.190802593	7.643836878	0.013379112	8.112789853	1529.055421
4.794836541	11.51569069	0.00643037	19.15791246	8846.717664
3.395894376	5.991103587	0.037916816	5.876796272	371.8113107
2.694989168	6.537698413	0.073759133	12.89794344	1380.483803
4.366502003	13.74850209	0.034007481	11.72796111	4943.589311
4.427143097	7.168679725	0.011588584	12.46221728	3891.099206
4.253447055	8.456649074	0.015722014	8.709904307	1633.130882
4.298422087	10.97641839	0.029837873	7.932950594	2055.163677
3.788778698	9.884896769	0.075428294	5.53351154	1258.113846
4.073522574	9.736837725	0.020153862	25.29674518	9080.490632
4.776876353	10.9331079	0.007096692	16.42487657	6962.609929
2.666876531	5.933241225	0.07365688	13.64064803	2004.462807
4.8107457	13.76624096	0.010020927	27.11093262	19000.45661
4.452076637	10.56089294	0.014564145	11.29431643	4002.040309

log.sigma.3.5.mm.3D_glcm_SumAverage	log.sigma.3.5.mm.3D_glcm_Imc2	log.sigma.3.5.mm.3D_glcm_Imc1	log.sigma.3.5.mm.3D_glcm_DifferenceAverage	log.sigma.3.5.mm.3D_glcm_Id
23.57080079	0.889629695	-0.20733352	2.322397393	0.418334403
22.36739397	0.855840338	-0.209465299	1.580096426	0.546324765
12.42461436	0.887831894	-0.226053739	2.294959363	0.411609444
25.54295133	0.789516009	-0.13135399	2.680991281	0.428824947
18.26004298	0.985232793	-0.434054139	4.503347849	0.288084603
12.60345832	0.72961984	-0.12161776	2.197592031	0.437054127
10.85869692	0.939148198	-0.369368931	2.400062187	0.412686145
19.53476665	0.801878695	-0.136149957	3.138861453	0.359644736
36.74034016	0.897029063	-0.17027721	5.045331509	0.290305123
23.19985807	0.817194031	-0.170814077	1.55752286	0.533100992
16.98619385	0.690452929	-0.09917786	2.474345506	0.403058774
21.12333068	0.8195083	-0.163213455	2.057935018	0.450319702
15.12255619	0.693936573	-0.105287348	2.049307516	0.463443078
14.04226177	0.76189801	-0.125890642	2.554854456	0.413625239
15.77819993	0.807556335	-0.14304801	3.134843421	0.357467281
25.73582958	0.843300796	-0.273733397	0.705346983	0.743472183
15.27095831	0.898402426	-0.219515534	3.684569546	0.325417289
15.63044083	0.746686353	-0.123841679	2.00598288	0.471515615
13.80781235	0.930352804	-0.260993583	3.93017883	0.326100732
19.24730699	0.896503233	-0.260457462	1.179291649	0.605527992
22.86088846	0.840857917	-0.22172979	1.047478936	0.635519469
16.80545799	0.74166677	-0.118903705	2.454292253	0.426713513
24.22232441	0.867217821	-0.236048662	1.159806833	0.611931605
31.54720337	0.992225334	-0.486533323	3.90980812	0.309616583
18.98149569	0.832226273	-0.198124553	1.291119798	0.568178265
17.01378135	0.829681337	-0.165410403	2.427756269	0.410639269
19.26125465	0.801189264	-0.180606569	1.244682906	0.60079615
25.22052172	0.872497984	-0.274605551	0.963903244	0.679067726
25.55178866	0.866882932	-0.23242757	1.485295371	0.600684444
24.21679139	0.879483191	-0.23254143	1.447291593	0.567969454
22.74389688	0.854241057	-0.227012394	1.150436935	0.623801509
21.75989292	0.843224074	-0.229546991	1.034145046	0.663461147
20.53512817	0.801538068	-0.141954907	2.357255821	0.442519197
15.13653846	0.987093787	-0.784919305	4.755769231	0.267434522
21.32332723	0.983684442	-0.423760125	4.252452456	0.297245311
21.92129864	0.738235815	-0.11337438	2.349436837	0.429118102
24.11920418	0.861371839	-0.259253816	0.990241628	0.670956042
20.7278198	0.838245494	-0.188065834	1.499478561	0.557704238
19.63381752	0.9051837	-0.216934134	3.515824859	0.343619775
17.364576	0.840848245	-0.168801521	2.365807951	0.416513487
24.35755402	0.886615231	-0.215603845	1.872196489	0.480182022
18.33776799	0.77421932	-0.129501344	2.694002842	0.393214156
18.9173354	0.669585672	-0.088039935	2.615031752	0.396052813
17.86835563	0.798789962	-0.137800432	2.888668064	0.380709366
22.75881184	0.795072232	-0.128779135	3.023017322	0.371525752
17.21171467	0.847573787	-0.293227595	0.652723126	0.739093202
22.46625956	0.772879382	-0.115181944	3.341514333	0.372686504
11.71978683	0.774313307	-0.138969204	2.697332554	0.397323388
17.73324731	0.997034309	-0.784905586	5.05541403	0.256768122
19.85755996	0.981711809	-0.388031488	5.273270875	0.266781578
22.8253708	0.861460058	-0.233871642	1.172646135	0.629962274
13.7327626	0.713169711	-0.106080914	2.584604742	0.403107514
26.44108486	0.822372619	-0.166852829	1.88561418	0.491562564
15.57559919	0.883823822	-0.213038769	2.212543693	0.426849139
21.03124285	0.85664519	-0.264991297	0.853334776	0.703611182
15.89968213	0.83204473	-0.154101652	3.301660022	0.357390529
23.50144326	0.774564816	-0.117038605	3.480750762	0.344259105
24.46410252	0.905260865	-0.200407724	4.610431675	0.289716858
19.70744987	0.878114511	-0.19850788	2.516544099	0.405381186
14.9598164	0.86014193	-0.199703516	1.84803848	0.467873662
17.25702448	0.811317689	-0.156520601	1.97595829	0.467169423
28.43744013	0.863814128	-0.206651902	1.663891999	0.56546047
19.03403953	0.831773027	-0.213592491	1.11211505	0.624123534
17.04780067	0.84938439	-0.210629627	1.342255738	0.575788632
16.38534095	0.983117309	-0.497757916	3.339141544	0.339804173
23.86141019	0.822603629	-0.154510044	2.530519675	0.421196999
17.72077838	0.976113976	-0.391533071	4.094818612	0.299133793
21.12591453	0.876000111	-0.211468854	1.740606129	0.53546478
11.54550798	0.817246693	-0.174776641	2.466566286	0.413810362
20.71284606	0.830318444	-0.174316568	1.732860742	0.508739415
24.35656927	0.82321049	-0.170490379	1.804178262	0.505475266
16.63795514	0.745973996	-0.116505851	2.378208886	0.413671539
21.6941328	0.876644897	-0.259365796	1.035729209	0.652772929
18.35021485	0.870514412	-0.270996843	0.862122737	0.689307942
25.45499974	0.835973304	-0.224615516	1.032168226	0.675357226
22.15424352	0.820902384	-0.146919509	3.386757471	0.356936812
18.1076995	0.747258942	-0.118981808	2.499923369	0.411797554
9.590687317	0.731639598	-0.125016832	2.146285985	0.442422621
24.32298239	0.763074662	-0.133392982	2.382297088	0.420294986
25.26994728	0.850479965	-0.202512476	1.537247149	0.562928526
18.84118582	0.925288735	-0.269003884	3.416638826	0.354348139
27.4070303	0.838008726	-0.152738002	3.017001976	0.385311868
31.76184256	0.846273576	-0.19988438	1.592619516	0.574784799
20.38423425	0.995027408	-0.674214819	4.949464104	0.263034647
19.28577755	0.830463711	-0.202133949	1.199672901	0.611451657
20.17557711	0.700981303	-0.113406518	1.69114782	0.520794496
15.28767376	0.762383451	-0.128245151	2.390170749	0.411037226
23.00997025	0.823309178	-0.139344932	3.782466205	0.330913787
11.98220717	0.953838625	-0.409244655	2.657904405	0.386864217
13.07539683	0.984471446	-0.640950148	4.265873016	0.287019429
27.49623726	0.866025038	-0.207927638	1.711956797	0.548270193
14.33735945	0.857631934	-0.182324424	2.744767266	0.381984956
16.91329815	0.841958936	-0.180693277	2.225285571	0.45106786
21.95283678	0.887457283	-0.238993615	1.34325088	0.579792918
19.76979354	0.866139746	-0.244840373	1.042047316	0.64923596
19.39820188	0.987856	-0.496901276	5.52616359	0.250905847
21.8662158	0.800313786	-0.131311526	3.112240078	0.366935269
11.86648245	0.982361054	-0.647949379	4.064934425	0.33830933
27.47635605	0.979981536	-0.382755854	3.923427693	0.314214878
21.12178589	0.908678877	-0.238559594	2.264336025	0.431528439

log.sigma.3.5.mm.3D_glcm_ClusterTendency	log.sigma.3.5.mm.3D_firstorder_InterquartileRange	log.sigma.3.5.mm.3D_firstorder_Skewness	log.sigma.3.5.mm.3D_firstorder_Uniformity
43.87874693	142.7502365	-0.059035528	0.07565166
29.75625047	115.3054247	-0.653930882	0.113274731
27.62966393	116.228035	0.544496478	0.092661751
45.46826712	136.676815	0.167115114	0.075925555
78.03551656	217.0460052	0.536037267	0.060680325
13.20522539	72.22421265	0.491163216	0.12392502
11.92801345	76.66165543	-0.226554202	0.135918367
34.27676508	128.4560242	0.514857673	0.078500756
245.6182757	290.6214046	0.608657947	0.04034103
24.23455487	94.08381844	-0.240922925	0.107523001
16.31506323	89.52767563	0.348035676	0.104558058
32.22370096	113.8007126	-0.009921412	0.085477856
20.64245012	90.66135883	0.739964133	0.104924232
28.14889002	111.3326893	0.777735447	0.088304924
33.44343128	123.8041334	0.570519806	0.079126917
11.64152537	21.79179847	-1.850639699	0.270838209
35.33272018	147.6453247	0.345789467	0.0733717
25.00762694	103.6696291	0.239513097	0.095602448
53.13457654	189.4144268	0.570535581	0.065873687
24.65682246	98.11464904	-0.513700067	0.121133307
15.39454364	80.895401	-0.729140771	0.161297154
29.23443446	104.454319	0.661465751	0.089217833
20.30696538	88.04272103	-0.909712803	0.135065913
134.3828292	209.3676414	-0.466799518	0.05387529
17.65431191	84.34583759	-0.431025622	0.123209635
28.88994402	120.0471992	-0.036161922	0.087781238
16.13437522	68.71266747	-0.194002365	0.159057479
19.77487578	87.92013466	-0.984348734	0.202583741
32.9013301	118.6704797	-0.897430538	0.161330111
33.30666424	127.2706127	-0.729312371	0.114024636
19.01010389	90.51632357	-0.625619772	0.149539723
16.10584951	65.39450097	-0.916602832	0.188372612
40.28902476	143.5442562	0.020075108	0.079463708
38.68809295	158.0194244	0.424400605	0.12
69.27960364	172.3704605	0.06976577	0.064139713
30.12571189	121.2249603	0.063349244	0.085936042
20.4023107	70.91752759	-1.329628448	0.200919171
24.86399622	103.3911347	-0.13952892	0.108763123
59.67861571	175.8063965	0.172631633	0.064915907
41.88831911	150.8794632	0.095955666	0.077390427
37.86819116	113.4086094	-0.511423761	0.087798914
24.47507866	104.178894	0.616235961	0.091023071
25.1361793	106.2925892	0.144863332	0.089190192
34.92024072	130.3872833	0.665227768	0.078906813
58.68621808	123.6858177	0.719770794	0.074878642
10.60899003	40.08886969	-1.553293608	0.253133258
77.70183733	132.1640377	1.44054482	0.070441864
19.96732858	109.644886	0.587113698	0.099915359
36.0334274	137.2052612	-0.31585695	0.08677686
89.53441898	226.3835354	0.002391925	0.052355038
21.14969052	90.41861975	-0.806494579	0.159441561
20.83978101	100.2233734	0.671457747	0.109445905
35.95608842	128.875092	-0.397703639	0.091299247
34.1393645	126.6970711	0.113778829	0.08292922
16.28946955	36.06754959	-1.455077845	0.232651626
37.41046157	150.526268	0.194395201	0.072719982
46.33581517	140.6028404	0.352404912	0.069488574
83.46539501	185.9757404	0.029232094	0.053793787
37.16868767	128.5399113	0.250846213	0.078697054
29.91521891	111.5717659	0.315278013	0.090782663
31.29760659	113.0369606	0.187752622	0.093742398
34.72263506	108.5483962	-0.331147563	0.116932818
16.36666963	63.30953431	-0.718269147	0.151005852
21.10725954	96.36670923	-0.530501268	0.129373924
36.30909864	133.0678253	-0.095671965	0.095763617
41.41645267	129.6302841	-0.165008671	0.078712746
42.87526559	153.224762	-0.127404839	0.068480726
43.03922232	143.5216208	-0.338729462	0.097121993
16.81309028	95.79534149	0.929640731	0.114088708
29.41724456	116.2119682	-0.360943568	0.095634616
31.46633432	123.0242805	-0.376884571	0.092887859
25.88834972	105.7927017	0.244033945	0.0928461
21.24632021	86.08210361	-0.974998645	0.163025128
16.20586028	64.39278059	-1.214247081	0.19660516
16.82964695	41.53049231	-0.716226645	0.209657266
36.37847391	131.5171013	0.401515454	0.07412174
22.64227017	93.88818741	0.180540762	0.097597338
12.81255778	71.68471909	1.157964708	0.129703444
31.45123505	115.7277908	-0.311945147	0.08454849
24.98102711	111.2136033	-0.407034836	0.11826934
35.33960177	126.681778	0.340850552	0.088689699
51.57255938	142.7319183	0.21543156	0.069031374
30.89149833	89.01543903	-0.803646794	0.132087655
46.74169053	160.9379005	-0.227289429	0.086368366
18.2825215	82.1907993	-0.690575962	0.145363529
17.75925662	79.57811356	0.213484381	0.118017499
23.13696637	102.5627213	0.549025305	0.095605179
52.41179097	146.8230286	0.186432274	0.064317236
12.4715412	79.26605606	-0.205554546	0.1272
25.32590073	144.6663818	-0.144265357	0.159763314
40.18834203	125.73745	-0.706151753	0.110270495
37.56036263	132.3285065	0.707438091	0.083578601
25.69369229	110.6497974	-0.019904899	0.094801683
27.86184576	109.6480442	-0.561344975	0.106877325
19.48393119	79.67038712	-0.830961051	0.167860313
51.64530017	178.1279907	0.552370856	0.073318216
49.24620689	146.9853215	0.27568062	0.067634083
29.25204263	172.1690273	0.048733803	0.121527778
82.46352321	167.6420517	0.132180309	0.057623546
36.38453585	110.0364447	-0.370757881	0.089005102

log.sigma.3.5.mm.3D_firstorder_MeanAbsoluteDeviation	log.sigma.3.5.mm.3D_firstorder_Energy	log.sigma.3.5.mm.3D_firstorder_RobustMeanAbsoluteDeviation	log.sigma.3.5.mm.3D_firstorder_Median
77.94702185	9844024.069	58.9395157	-135.0272064
62.12683609	17532698.54	47.08793152	-69.83512115
64.272592	4773729.563	46.05014809	-150.9727936
80.19632254	33702586.33	55.71629258	-77.27587128
110.443338	5211062.222	79.13147326	-187.0727692
46.75159115	9869776.857	30.72831341	-197.1124878
47.33426521	1104337.636	33.8083889	-106.6862717
74.64925708	20080297.61	50.87705417	-207.6476746
166.3326925	63778488.44	122.5368673	-98.88279343
55.15700247	24222670.15	37.9397932	-68.15634918
55.19462675	14985098.38	38.10755975	-192.6993408
66.41544617	12104157.99	46.67506583	-93.10427856
56.49058676	18570138.39	38.3823068	-99.41394806
67.8790026	11779907.96	46.71822416	-132.8495789
73.55174738	6323543.686	51.97762655	-101.5112495
30.07041608	27133078.57	12.17477712	-3.592518687
81.39364486	10491611.39	60.42122423	-194.4516907
59.73880022	28459110.65	43.15687735	-113.3857994
100.2415688	8551845.789	74.5452711	-197.6236877
55.53220172	18653011.7	41.41481617	-31.8156023
45.63011545	31493504.21	33.59154495	-42.39294434
66.16607757	14655658.87	44.38379689	-98.06343842
49.92114148	23001142.85	35.56964532	-50.51668167
123.945929	3493498.791	89.9863497	-41.62876892
48.32077729	9328603.396	34.77697811	-72.45152283
64.74449061	10451750.78	48.02862312	-170.8566284
44.00413324	12536199.33	28.8456494	-34.89712906
52.38859039	25851630.02	38.19199955	-20.99925423
67.06122239	41735758.8	49.97220197	-35.60631561
66.676789	24532403.55	50.63870332	-71.77558899
50.53573589	29511778.68	37.14333326	-40.23314285
43.83258122	43181706.77	28.72631513	-21.20712471
76.46662009	25568583.14	57.14062638	-98.24407959
98.0489308	730073.2445	63.18126786	-200.5856171
102.147442	4909768.337	71.8473717	-157.7610474
66.75374754	32680307.79	49.54000585	-140.7973328
49.66168203	35785462.02	33.54862004	-6.429241657
58.74166113	23214675.04	42.75905545	-37.8708725
95.58214725	8679251.601	71.00321153	-111.4354935
77.88303791	15046030.86	60.57344867	-145.1487274
67.22700566	9739327.644	47.44303658	-74.28351593
66.01468684	16970728.48	44.59196198	-204.4936981
63.17960355	29037668.53	44.78485705	-172.3760452
75.38575187	17253470.64	52.75689251	-183.7635498
78.81552145	23257144	52.15950319	-111.0990906
34.43098359	16586620.31	20.87128583	-7.035186529
86.70008806	47640374.87	56.16680584	-71.59477615
62.41805973	10569280.33	45.59838564	-210.8293686
88.18122872	1537677.464	55.31775379	-236.7105789
124.4852311	8522915.765	95.38251708	-192.8478622
52.75312995	28630064.86	38.15570189	-31.08843517
58.27037735	20035816.01	41.43857949	-228.1013489
68.7731675	44076046.7	51.72079779	-71.52080536
68.82660077	4342626.737	49.98964565	-111.5373688
40.16599979	42019731.48	21.26091058	-5.804852962
80.73188312	16223115.22	58.78862866	-197.2002258
83.92839588	39239930.9	57.93090963	-194.1949844
109.3120929	22664793.4	79.995045	-175.0675735
73.56146117	9124973.82	52.6456819	-136.0818329
62.79533099	7050869.421	44.93579429	-103.3659096
62.97355405	31497970.17	46.60526159	-136.4823608
66.60182623	69360470.11	46.08089925	-32.67551613
43.66405052	14995830.63	28.40652784	-9.064682961
52.38022087	11491977.32	39.64773094	-34.6978569
78.67593431	2867181.795	57.37966113	-223.5356445
74.27503706	13129446.68	53.37167892	-45.66282654
85.91836826	6793938.486	61.05602633	-233.737793
76.05450587	47650842.9	58.59560337	-54.37155914
57.46131602	9727051.198	37.74998047	-262.9091187
62.78979509	21393601.41	46.91352288	-73.17987442
64.67468038	34284686.97	48.56127807	-78.17290497
60.87397772	20718083.61	43.55752579	-179.0600433
51.62136613	41407869.19	37.38685657	-15.62895679
44.69355066	47433430.37	29.76326586	-9.715339184
40.12433593	53780009.55	21.29206511	-2.604987383
78.56392454	21551289.3	53.79528104	-203.5038834
59.25549568	17139891.47	40.15509663	-183.7507935
48.00714617	10681531.71	30.9386673	-219.3205185
67.37315458	30707092.12	46.9796827	-157.5995941
59.1678951	28941716.44	44.04860185	-63.89488983
75.3374126	13018415.35	52.7438328	-298.8262024
84.27724583	21792040.03	57.4220856	-123.3087463
60.75622075	37638061.26	39.8593363	-12.36737347
94.64191335	1164999.798	72.73671267	-152.933197
48.56800679	28881253.67	34.71624962	-30.17945194
48.8874896	24796040.21	33.03176766	-54.61899567
60.91387686	12183995.93	43.61204807	-174.2719269
89.27863615	39235899.3	61.44646413	-238.0256195
48.08680092	460835.0733	34.84204515	-73.3564415
77.35078024	724279.9652	58.63665658	-251.5312805
70.70273911	41943388.37	52.28912884	-47.72273254
76.50814552	12664301.73	55.68035953	-204.0877914
61.46233637	6225396.038	44.28120192	-92.81403351
60.13034793	34395944.13	44.81748018	-54.08770943
50.34313427	40250254.3	35.32953274	-15.75812721
106.3707192	4415170.071	75.77663256	-259.3493042
84.85770038	13798571.92	60.4961697	-97.26994324
73.14004678	1139580.691	52.54901537	-195.8573685
105.7061929	5106805.128	74.56081849	-127.1395493
69.19867419	4846737.614	48.06808752	-86.66793823

log.sigma.3.5.mm.3D_firstorder_TotalEnergy	log.sigma.3.5.mm.3D_firstorder_Maximum	log.sigma.3.5.mm.3D_firstorder_RootMeanSquared	log.sigma.3.5.mm.3D_firstorder_90Percentile	log.sigma.3.5.mm.3D_firstorder_Minimum
265788649.9	99.49091339	165.1325672	-17.80395699	-401.6583252
473382860.6	74.36412048	111.7880929	-2.914870882	-338.625061
128890698.2	114.2257996	164.2261353	-45.9411377	-292.4653931
909969831	355.5653992	132.0430927	24.45160141	-379.2746277
140698680	193.7959442	227.1446136	-31.32030296	-413.664032
266483975.1	-32.37197113	201.1216178	-105.2567169	-336.8889771
29817116.17	13.75006866	125.6035507	-50.54287529	-232.8977509
542168035.4	179.0813751	223.7756804	-83.01308441	-445.7797241
1722019188	481.3143005	207.8710129	281.3283539	-451.1257019
654012094	219.861145	102.2022915	0.462687051	-341.4475708
404597656.3	41.99203491	199.6346289	-96.18101883	-395.0264282
326812265.8	210.9207611	126.4501509	8.759227753	-328.9239807
501393736.4	211.4613342	116.5956158	-1.174134701	-272.987793
318057514.8	203.8704987	149.3667692	-6.816641331	-292.3395081
170735679.5	245.7488861	131.8042972	21.10185966	-282.6936035
732593121.3	277.3093567	50.05822115	18.56500244	-319.5297546
283273507.4	100.4680252	219.882786	-65.90654907	-387.5257263
768395987.6	218.4489441	135.4143856	-20.00410957	-297.4459229
230899836.3	143.0687866	216.7675337	-6.633958721	-366.4386902
503631315.8	108.8409653	82.00139732	21.08896999	-265.1682434
850324613.6	207.9532318	78.77573607	-0.539376557	-301.2451782
395702789.5	243.8366394	126.9757341	25.32410316	-299.1394653
621030856.8	181.6435852	88.75304904	0.622391748	-329.8601379
94324467.37	181.2340698	162.0706889	117.0792847	-445.881073
251872291.7	114.9382095	98.57634201	-7.2625278	-283.9838562
282197270.9	35.95395279	186.0334547	-62.76112823	-372.1486206
338477381.8	299.5104065	74.00482706	5.963722229	-251.9930115
697994010.6	373.8540039	81.46859657	1.371611738	-334.8722534
1126865488	273.22995	105.9781427	0.378368452	-373.1178894
662374895.8	101.237709	120.3764609	-5.478422546	-360.9880066
796818024.4	233.5783997	83.8149233	1.258880377	-315.0909119
1165906083	295.5350037	69.91909314	4.046643829	-291.2424622
690351744.7	254.2457733	135.675743	3.861131954	-345.9328613
19711977.6	73.61043549	220.616295	-41.48680115	-370.6546326
132563745.1	171.6450806	208.4449024	-14.08912544	-442.3978577
882368310.4	104.9645615	156.5183116	-22.46636791	-385.6350708
966207474.5	137.0206604	73.13205873	4.714897633	-300.0158691
626796226	285.4299316	87.50174336	28.02488022	-277.5155029
234339793.2	215.6716766	164.4328647	9.544368744	-362.4481201
406242833.3	109.8708801	167.3878115	-19.70850792	-345.0527039
262961846.4	194.2653503	116.7109132	18.15506287	-368.3234863
458209668.9	134.9138031	211.0511654	-78.94763184	-409.0998535
784017050.2	112.0071182	187.7229048	-67.55093689	-378.9650879
465843707.4	249.2949371	198.2467035	-50.96807098	-388.1793823
627942888	277.0151062	144.4244616	29.98013115	-373.9178162
447838748.3	102.344696	52.05138489	9.076593494	-220.037796
1286290121	723.3890381	131.7156316	77.94772339	-321.0544434
285370568.8	8.894989014	215.3055797	-89.62649765	-336.1948547
41517291.53	-31.18167686	264.3755476	-129.0830215	-469.8963623
230118725.6	108.9538651	241.6115382	-2.967759848	-465.6610107
773011751.2	194.2382507	83.76856666	3.105236077	-314.033783
540967032.3	20.5377121	222.9723414	-107.4566513	-369.0289917
1190053261	198.787262	117.4534792	7.297188568	-377.0577087
117250921.9	106.2870941	134.7960917	14.28495045	-295.3894043
1134532750	241.7138977	60.96381631	16.49997139	-266.5317993
438024110.9	68.93857574	220.0618313	-71.79602814	-398.528656
1059478134	216.2301025	212.1318476	-46.24085503	-458.7094116
611949421.9	182.2888489	222.4554977	2.286352706	-486.1998901
246374293.1	155.1716309	160.5514316	-13.89679613	-364.2463684
190373474.4	124.3482056	123.0065896	12.06238985	-263.4990845
850445194.5	117.2190094	147.1328973	-14.52549801	-321.5344543
1872732693	348.7941895	101.174092	10.7517004	-377.7227783
404887427	226.3771362	60.67010562	39.22956657	-244.8636169
310283387.5	109.1653061	80.30517451	15.67083015	-248.405014
77413908.45	-41.75150299	232.5892726	-103.1090881	-400.5524597
354495060.4	291.2269897	106.7571503	52.31386108	-333.8354187
183436339.1	-10.84143162	254.3701556	-99.79951782	-453.7764587
1286572758	282.199585	117.2352929	9.833872604	-324.9881897
262630382.4	-31.61201286	260.8090049	-157.6378693	-377.3802795
577627238.1	171.4980469	109.2629564	10.43708134	-321.277832
925686548.2	137.5972595	114.8542194	6.363695431	-370.4127808
559388257.6	66.06058502	191.1539432	-69.79705811	-350.3188782
1118012468	207.9102478	76.60046087	7.595047951	-293.6381836
1280702620	147.3457489	66.16797615	8.267940331	-249.4745331
1452060258	440.3815308	60.86142055	24.94970589	-317.474823
581884811	164.0810852	219.82101	-66.3121109	-462.072113
462777069.7	62.67837524	193.8749115	-78.59365845	-376.4370422
288401356.3	44.9973793	218.3698913	-137.2473801	-312.7742615
829091487.1	92.99849701	183.3935077	-54.196492	-432.8208618
781426343.8	237.0592957	103.4567422	-0.680539036	-350.6109924
351497214.5	-62.56342697	295.5874069	-139.8157806	-503.0243835
588385080.8	234.9355164	158.7233753	20.44113808	-434.3139648
1016227654	271.4379578	87.38446131	31.07715378	-411.635376
31454994.56	34.05054092	193.8572636	-19.41964149	-401.9506531
779793849.2	196.6786804	76.29211251	10.34539938	-262.8436584
669493085.6	266.8936768	84.08592054	19.41540985	-280.1654053
328967890	94.24741364	182.7041618	-65.47857819	-327.2046509
1059369281	141.0037384	258.0977141	-82.41860504	-505.7002258
12442546.98	34.60487747	96.0036534	-4.105205011	-221.9127045
19555559.06	-107.0919189	236.0378009	-113.9289124	-377.8106079
1132471486	231.8135834	110.5981884	9.243426704	-381.7159729
341936146.8	81.26267242	204.1050888	-52.94458466	-339.3722534
168085693	127.3762131	122.3310849	-11.99104738	-281.4906921
928690491.4	134.6268311	97.0483672	13.52172327	-319.4515991
1086756866	197.9947205	74.65442799	7.388343477	-267.1699219
119209591.9	156.7742767	264.7301732	-42.27082138	-468.4665833
372561441.9	238.1467743	142.4500528	40.77732925	-361.4795227
30768678.65	-33.90135956	217.9048618	-72.07966461	-335.7790833
137883738.5	209.0068817	177.003087	56.0419426	-455.6506958
130861915.6	134.4878235	131.5666264	-2.615732861	-332.4416199

log.sigma.3.5.mm.3D_firstorder_Entropy	log.sigma.3.5.mm.3D_firstorder_StandardDeviation	log.sigma.3.5.mm.3D_firstorder_Range	log.sigma.3.5.mm.3D_firstorder_Variance	log.sigma.3.5.mm.3D_firstorder_10Percentile
3.882522427	92.77152742	501.1492386	8606.5563	-258.3890686
3.422180423	74.30312072	412.9891815	5520.953749	-192.6274872
3.609021679	79.83010369	406.6911926	6372.845456	-241.6908508
4.006679668	101.250569	734.8400269	10251.67771	-217.4393555
4.197236821	137.4721821	607.4599762	18898.60086	-342.9444885
3.250962228	60.51022	304.5170059	3661.486724	-269.5168976
3.098268869	57.88749408	246.6478195	3350.96197	-199.8373932
3.907991767	95.73761118	624.8610992	9165.690195	-317.234314
4.826801134	201.6334717	932.4400024	40656.05691	-279.9574738
3.500376772	70.38744308	561.3087158	4954.392144	-163.3028503
3.491695781	69.62116716	437.0184631	4847.106916	-268.3016205
3.753123512	83.24517637	539.8447418	6929.759389	-202.812915
3.518751402	72.4542656	484.4491272	5249.620603	-174.2108688
3.742167115	85.91385519	496.2100067	7381.190514	-220.9858414
3.850649855	91.9584558	528.4424896	8456.357592	-211.9197571
2.50178974	47.73932439	596.8391113	2279.043093	-71.93736572
3.922753995	98.50806306	487.9937515	9703.838487	-322.6306763
3.571618561	73.77252556	515.8948669	5442.385528	-209.6978119
4.102135499	121.0488691	509.5074768	14652.8287	-322.2013245
3.305573641	65.99560668	374.0092087	4355.420101	-147.6255951
3.000943965	55.90941453	509.19841	3125.862633	-134.4375854
3.747069412	85.86816413	542.9761047	7373.34161	-196.4805115
3.199142839	62.75288689	511.5037231	3937.924813	-145.4635788
4.393035026	149.6704205	627.1151428	22401.23477	-283.1384216
3.23893027	59.57868154	398.9220657	3549.619294	-153.5406631
3.63583351	78.02061313	408.1025734	6087.216074	-270.5127563
3.102520585	58.06244185	551.503418	3371.247153	-123.2989578
2.876835034	63.44671809	708.7262573	4025.486036	-151.8188995
3.274633012	80.49808391	646.3478394	6479.941514	-187.1204834
3.462359694	79.7962392	462.2257156	6367.43979	-208.6353394
3.132020545	61.84101553	548.6693115	3824.311201	-145.9694824
2.947868446	57.01443366	586.7774658	3250.645646	-125.5737793
3.857901139	92.02154159	600.1786346	8467.964117	-220.5555267
3.189898095	119.3857142	444.2650681	14252.94876	-323.9116577
4.150115941	125.6132463	614.0429382	15778.68764	-320.4772583
3.696738791	80.4835251	490.5996323	6477.597813	-236.7776688
2.857550465	61.75042174	437.0365295	3813.114585	-136.7784271
3.48491603	71.39217901	562.9454346	5096.843224	-149.9534851
4.129172855	115.07074	578.1197968	13241.2752	-264.625824
3.818054476	90.98477593	454.923584	8278.22945	-257.7194641
3.722154116	84.13950662	562.5888367	7079.456574	-194.669342
3.719709787	84.89217132	544.0136566	7206.680751	-288.8978882
3.68466664	78.6039599	490.9722061	6178.582511	-271.5387634
3.889173973	95.2834731	637.4743195	9078.940247	-286.9772827
4.01116866	102.7608398	650.9329224	10559.79019	-222.6922668
2.426211919	45.09316103	322.3824921	2033.393172	-101.9849892
4.126319925	118.1637602	1044.443481	13962.67423	-185.8257828
3.524988777	76.44812362	345.0898438	5844.315605	-289.3414917
3.606936732	109.4496409	438.7146854	11979.2239	-361.5317169
4.396532838	145.8136147	574.6148758	21261.61024	-388.3023682
3.136013849	64.47185529	508.2720337	4156.620124	-149.0595291
3.443991041	71.48632879	389.5667038	5110.295204	-288.7397766
3.675688641	82.34251434	575.8449707	6780.289667	-198.0833038
3.720266389	83.97817084	401.6764984	7052.333177	-216.2277588
2.724881904	55.40969717	508.245697	3070.23454	-115.9853935
3.94410478	98.55556756	467.4672318	9713.199898	-325.9368835
4.085056766	105.417031	674.9395142	11112.75042	-310.6490387
4.376903047	134.0068433	668.488739	17957.83405	-358.9805328
3.864502109	90.41086573	519.4179993	8174.124641	-243.646785
3.626500759	76.90597631	387.84729	5914.529192	-190.7993774
3.583233829	76.19740154	438.7534637	5806.044002	-216.1108093
3.574978546	84.25116576	726.5169678	7098.258931	-174.5287781
3.086648184	56.20408232	471.2407532	3158.898869	-108.9525124
3.212219655	61.87167735	357.5703201	3828.104458	-139.926683
3.590615976	92.78949067	358.8009567	8609.889579	-349.5281799
3.880654969	91.94757852	625.0624084	8454.357195	-179.4507706
4.018894893	105.1671962	442.9350271	11060.13916	-383.1351563
3.672576202	89.81720711	607.1877747	8067.130693	-208.9670593
3.379970351	73.1132086	345.7682667	5345.541272	-326.1786865
3.567221119	75.53206829	492.7758789	5705.09334	-181.5788712
3.619729035	77.94099591	508.0100403	6074.798843	-189.7000671
3.607688094	75.03396606	416.3794632	5630.096063	-270.3960205
3.034618781	62.33082663	501.5484314	3885.131948	-144.8133453
2.83605861	55.78271692	396.820282	3111.711507	-125.9842308
2.876658743	57.9959942	757.8563538	3363.535344	-100.7278702
3.989544818	99.65137682	626.1531982	9930.396903	-317.2941132
3.622035848	75.50623625	439.1154175	5701.191712	-274.6952515
3.220803219	63.50724068	357.7716408	4033.169619	-277.9824158
3.780340997	85.06620936	525.8193588	7236.259975	-270.3998413
3.417529001	72.24630394	587.6702881	5219.528433	-173.8250732
3.702878024	93.28835875	440.4609566	8702.717879	-385.9452209
4.113684992	107.2759412	669.2494812	11508.12756	-250.2468781
3.437734734	77.87521062	683.0733337	6064.548428	-153.2826172
3.65533623	110.8596582	436.001194	12289.86381	-289.5455627
3.129653001	59.7732367	459.5223389	3572.839826	-134.8568726
3.364834131	63.03402588	547.059082	3973.288418	-133.6888123
3.57889375	75.12192555	421.4520645	5643.303698	-254.3710876
4.173008912	112.8662817	646.7039642	12738.79754	-377.2084961
3.166104761	59.262465	256.5175819	3512.039758	-159.4571213
2.81507241	85.43073615	270.718689	7298.410679	-317.2073242
3.578468768	85.89606192	613.5295563	7378.133454	-190.6000092
3.773097704	92.97626834	420.6349258	8644.586474	-289.2602478
3.59779919	75.62028866	408.8669052	5718.428057	-195.4794998
3.459621602	72.00322775	454.0784302	5184.464806	-164.994725
3.071372502	61.76390415	465.1646423	3814.779856	-138.7736725
3.925305425	128.2100259	625.24086	16437.81075	-375.1244995
4.080961221	105.4131575	599.626297	11111.93378	-234.1033875
3.236440502	90.09386395	301.8777237	8116.904321	-318.1648407
4.308099703	130.8189262	664.6575775	17113.59144	-275.1246826
3.754274232	86.22883173	466.9294434	7435.411421	-220.773407

log.sigma.3.5.mm.3D_firstorder_Kurtosis	log.sigma.3.5.mm.3D_firstorder_Mean	log.sigma.3.5.mm.3D_glrlm_ShortRunLowGrayLevelEmphasis	log.sigma.3.5.mm.3D_glrlm_GrayLevelVariance
2.412478821	-136.6096938	0.012505824	13.99848783
2.706159814	-83.52020098	0.014411521	9.087533678
3.080434051	-143.5178667	0.060253596	10.36542392
3.659826361	-84.75671435	0.009641785	17.58710286
2.919627816	-180.8205591	0.045463981	30.55528272
3.093613831	-191.803072	0.03484456	6.309974985
2.463904885	-111.4687847	0.071239192	5.303988059
3.524078222	-202.2618722	0.017031903	15.1322971
2.35365526	-50.54009413	0.006986832	64.56489078
3.851874762	-74.10071688	0.011098899	8.779577734
3.136931355	-187.1012511	0.018716009	8.067884917
3.168558646	-95.18340867	0.015076246	11.55175256
3.997477256	-91.35051732	0.026780821	8.944032856
3.642104177	-122.1852742	0.039196438	12.52542697
3.301853595	-94.42465341	0.026850171	13.71252465
9.061443851	-15.05929652	0.006235819	5.938887445
2.676559002	-196.582301	0.033434609	15.67191646
3.177812813	-113.5547018	0.031374737	8.901777166
2.498064959	-179.8202852	0.059708515	23.74303799
2.412825705	-48.67041258	0.015014512	7.365480266
3.55699863	-55.49553099	0.009046522	5.510764633
3.756646048	-93.5387376	0.02363617	12.6097984
4.026283199	-62.76287837	0.008945294	6.823879663
2.425066354	-62.17454008	0.01764926	35.86079059
3.115059286	-78.53455233	0.01642741	6.084309932
2.31563496	-168.8822969	0.027225293	9.85174249
5.063151644	-45.88537103	0.014404076	6.432174785
3.901213576	-51.10426783	0.007980099	7.429681771
3.354521385	-68.93058265	0.01089176	11.35815182
2.73608724	-90.12797878	0.012128223	10.3054322
3.581027488	-56.57411215	0.010549278	6.766961458
4.769582206	-40.47263197	0.011853129	6.587349382
2.720179119	-99.69926334	0.019296518	13.93648599
2.533804546	-185.5225077	0.141882965	21.41566876
2.597226168	-166.3447916	0.027490662	25.56562174
2.463742744	-134.240024	0.011315637	10.2471483
4.100922499	-39.1814169	0.009229133	7.472472943
2.963382366	-50.59359512	0.012380725	9.11569696
2.681968116	-117.460171	0.024553339	21.24160004
2.117055177	-140.5007117	0.027247959	13.17309708
3.294749127	-80.88251164	0.011982535	11.66935701
3.843807442	-193.2250338	0.021652077	12.00157854
2.882011557	-170.4737706	0.017119583	10.23292534
3.826172609	-173.847103	0.022133068	15.10768898
3.800951315	-101.4821901	0.012712771	17.49265261
4.703285863	-25.9991057	0.019629245	4.224050297
7.731399323	-58.19221054	0.015610964	23.31869391
2.67337202	-201.2763698	0.058340949	9.809586238
2.715950501	-240.6557838	0.097096449	18.05658907
2.040690098	-192.6513045	0.04260738	33.98465825
3.481318347	-53.48413443	0.010682536	7.641251449
2.992421162	-211.2022013	0.035856667	8.32154962
2.662500789	-83.75577659	0.007747434	11.1335896
2.364331961	-105.4402825	0.031382279	11.1802775
5.143396831	-25.42346079	0.012939963	6.924433172
2.502487	-196.7587602	0.045911537	15.73452396
3.199546812	-184.0846825	0.012710189	18.20705204
2.372683405	-177.5629872	0.013741476	28.56538172
2.81450272	-132.6749319	0.01886652	13.50688359
2.62205727	-96.00047856	0.036949704	9.853259906
2.417551751	-125.8651876	0.025859462	8.846943387
4.146664442	-56.0173005	0.006594981	13.24187348
3.470135545	-22.84650622	0.013771074	6.15187016
2.509667218	-51.19391169	0.024630994	6.384629186
2.150477952	-213.2788788	0.040803697	13.56442672
3.001538857	-54.24695325	0.011237456	13.91315371
2.412033082	-231.6118237	0.033259544	17.81980061
2.590471442	-75.34575774	0.01829834	13.46321557
3.680792049	-250.3513447	0.045794245	8.824699089
2.630990598	-78.95125271	0.016800251	9.312005954
2.709557135	-84.36049356	0.009677364	9.799127885
2.722513121	-175.8116434	0.023746936	9.193120675
3.229693075	-44.52525865	0.012098187	7.122946356
3.722579559	-35.58777262	0.020458403	6.153020022
6.75005039	-18.45473293	0.006702779	7.669888419
3.358907926	-195.9359067	0.013865522	16.56119795
3.099594987	-178.5673251	0.021697529	9.706379671
4.937218019	-208.9311844	0.087479401	6.998865024
3.205507422	-162.4712857	0.015706198	11.88512859
3.254610622	-74.05247508	0.008364407	8.884101627
2.660016542	-280.4802974	0.020891974	14.40333556
3.220711471	-116.9828291	0.00891663	19.08011674
4.146948131	-39.64209442	0.00479961	11.54072489
2.274574369	-159.0307356	0.045976694	20.47645282
3.365079199	-47.40935146	0.015901681	6.369458851
3.893547256	-55.65207646	0.011474744	7.054290324
3.173707751	-166.5458107	0.028008649	9.48162997
2.971918221	-232.1112503	0.01367121	20.77463405
2.505757586	-75.52921096	0.056618892	5.623914228
1.803757023	-220.0350717	0.104975105	12.57671762
3.358866812	-69.6694038	0.008692573	12.68590153
2.845888115	-181.6983787	0.0487452	14.09822603
2.746627891	-96.15854754	0.029894287	9.589412553
2.763543139	-65.06858513	0.013792319	8.573228708
3.267612114	-41.93451756	0.016032464	7.42375393
3.020124567	-231.6122921	0.05007768	26.96126143
2.852703788	-95.81275358	0.014466081	18.20257164
1.915978077	-198.4077228	0.134052141	13.5084141
2.725927876	-119.232971	0.013618753	27.48786102
2.980481755	-99.36984338	0.024689423	12.36776025

log.sigma.3.5.mm.3D_glrlm_LowGrayLevelRunEmphasis	log.sigma.3.5.mm.3D_glrlm_GrayLevelNonUniformityNormalized	log.sigma.3.5.mm.3D_glrlm_RunVariance	log.sigma.3.5.mm.3D_glrlm_GrayLevelNonUniformity
0.013323613	0.074960461	0.12323889	24.34143239
0.015859529	0.096277758	0.644236255	104.4809479
0.064578885	0.091593056	0.102485515	14.92177182
0.010355264	0.070751577	0.30739942	117.123625
0.047536531	0.060333845	0.04900815	5.825040186
0.037745597	0.119491844	0.143257836	26.06657659
0.074909799	0.135693014	0.070818569	8.915966543
0.017946772	0.076877767	0.086827421	28.58730945
0.007364678	0.040064989	0.07984531	55.19101263
0.012501851	0.100430464	0.477721311	184.7779771
0.019984216	0.10155739	0.106207868	34.71696934
0.016263304	0.083932871	0.174743218	55.82992863
0.029499575	0.102420834	0.22410489	119.5190055
0.042357754	0.085104295	0.153776241	40.23707196
0.028460377	0.078349904	0.078717484	26.59156845
0.008318688	0.173393291	3.110052668	977.3762754
0.035557357	0.073297483	0.057943573	15.06436408
0.034843268	0.095323309	0.264485781	124.7860952
0.06433927	0.06382788	0.060442694	10.95623807
0.017716132	0.104902032	0.933252598	206.9132471
0.010766494	0.127675755	1.213345882	438.6969269
0.025637313	0.085565958	0.182236299	68.7599122
0.010422359	0.119488981	0.888411025	249.3198921
0.017903194	0.053326702	0.043959189	6.798295455
0.018424347	0.118277188	0.373324107	90.72622232
0.028922979	0.087998121	0.111238399	24.16466055
0.016763725	0.127606603	0.910596256	211.3857404
0.00958252	0.125225964	1.68969911	306.2661161
0.012208633	0.098987569	1.353565041	253.9389313
0.01346977	0.092086513	0.795982204	116.9452186
0.012334725	0.117435232	1.218617907	336.9329854
0.014260253	0.128530852	1.881892211	705.9746476
0.020758137	0.075153467	0.296139029	89.04981003
0.14682802	0.118224295	0.020217737	1.738799662
0.027995474	0.063022875	0.044183373	6.816914402
0.012270056	0.086074184	0.199968687	100.1896231
0.011095953	0.134856682	1.733753247	554.9650075
0.014237014	0.094398851	0.691740885	215.0931407
0.025857027	0.064683681	0.072157771	19.42292712
0.02946712	0.07700043	0.149793786	36.99526995
0.012876424	0.086209084	0.220986131	53.25970867
0.023195338	0.088125544	0.105855048	30.63409197
0.018296346	0.087765307	0.127956517	65.38945808
0.023420809	0.077088135	0.099893663	31.02751658
0.013595685	0.073213307	0.11935082	74.2366224
0.02473562	0.18315804	2.385324	614.9579643
0.016833672	0.068710948	0.131134906	170.2326953
0.062616837	0.096214354	0.110067122	19.96253666
0.100134352	0.087022912	0.017388802	1.881018981
0.044828772	0.052159627	0.043871889	7.306955171
0.012481422	0.110462286	1.430990315	299.8548969
0.039249371	0.105785313	0.129172505	38.21699022
0.00855459	0.085751694	0.403465009	224.327422
0.034119983	0.082871821	0.117125248	17.87141822
0.015832325	0.145197209	2.572592027	935.3177334
0.048512071	0.071635784	0.088520134	22.19708743
0.013271574	0.068215104	0.081955993	55.56260091
0.014309556	0.053588992	0.058518276	23.30451444
0.01985271	0.07804089	0.114938739	25.18193916
0.04065781	0.089555655	0.189463209	36.20165257
0.028115578	0.095318408	0.254547885	117.3861216
0.007535265	0.088310454	1.040820224	431.8396036
0.016423025	0.121537852	1.117581357	342.0815817
0.027603284	0.113885131	0.700392439	153.6280627
0.042771394	0.094238222	0.064858302	4.685337455
0.012055706	0.077491531	0.172620414	79.08145817
0.034580928	0.068367362	0.045232603	6.864595133
0.020381492	0.081070917	0.786204794	213.1433562
0.049590081	0.109414876	0.113653797	14.18987927
0.01835682	0.091624066	0.400921257	133.5380751
0.010745861	0.090316527	0.388456126	191.9536223
0.025647126	0.092170453	0.131105123	46.86143003
0.014406328	0.117750402	1.76800685	526.3863317
0.024404946	0.131344592	2.081615002	843.4294414
0.008454592	0.139462015	2.20832752	1197.785169
0.014549926	0.072222413	0.097816859	29.76945872
0.022904711	0.093920401	0.136035348	38.53271705
0.095501849	0.124662471	0.136581415	24.93859832
0.016672506	0.083943173	0.162078585	68.09804514
0.009424655	0.09926614	0.791315689	200.7186575
0.021864281	0.087032121	0.081915246	12.07756736
0.009370452	0.067551058	0.122420448	53.07895712
0.005524541	0.098154205	1.010133066	346.6999191
0.046316532	0.086361068	0.02467296	2.610640231
0.018454809	0.119790754	1.11882447	412.6381358
0.013044886	0.111848643	0.38574662	317.7419073
0.029944203	0.093952523	0.12304562	31.04886026
0.014484178	0.063565283	0.081324034	34.91037519
0.0590167	0.125631337	0.069783788	5.906662883
0.10551764	0.156270546	0.038194444	1.955128205
0.009633207	0.089071824	0.793581089	229.8556325
0.051613756	0.082552297	0.096612516	23.13967272
0.031851329	0.092383287	0.173686187	33.59982436
0.015518342	0.096583848	0.825115159	258.1785279
0.018845926	0.112912651	1.687281264	519.0353208
0.050619069	0.073170274	0.030355183	4.469311933
0.015426185	0.066945378	0.104015241	41.69194708
0.141307469	0.117300673	0.060303627	2.645995309
0.014005769	0.057620763	0.048962417	8.951934963
0.025944854	0.087095003	0.118139783	22.0961071

log.sigma.3.5.mm.3D_glrlm_LongRunEmphasis	log.sigma.3.5.mm.3D_glrlm_ShortRunHighGrayLevelEmphasis	log.sigma.3.5.mm.3D_glrlm_RunLengthNonUniformity	log.sigma.3.5.mm.3D_glrlm_ShortRunEmphasis
1.361586734	149.3388825	265.1113857	0.922109878
2.337316023	102.6250538	747.2500591	0.853396979
1.292062935	54.01638268	140.045937	0.939472801
1.678060221	169.1461241	1306.110449	0.906920211
1.146416949	134.796513	88.53642317	0.966225695
1.398182675	50.80232209	177.9557417	0.921063093
1.211813829	40.68640126	58.30449973	0.952561459
1.251344925	120.9501282	322.1342452	0.944885995
1.22930052	353.8760024	1208.459658	0.949739666
2.07765642	117.8598223	1280.705873	0.858445054
1.319018641	85.25106136	284.5775105	0.929207737
1.478404846	114.4426992	527.7160286	0.90918807
1.600064835	64.42415871	884.847766	0.892255723
1.405552938	69.33569383	388.0195992	0.923452137
1.231106535	87.96456707	296.9697011	0.94854761
6.960190832	107.282722	2380.596108	0.672119468
1.174649371	89.87231172	185.0083577	0.959337874
1.677560526	63.20818271	978.1335599	0.886387892
1.186543757	94.18991648	152.7371907	0.955405156
2.954828788	71.13118441	1197.601968	0.803845158
3.432967545	92.27174855	1994.317415	0.787757821
1.474769724	84.20494071	647.419757	0.914762491
2.891744967	109.0677296	1274.618794	0.806201068
1.134568375	280.8445722	117.2718907	0.967731474
1.963466559	76.01129284	522.8126806	0.848887567
1.324997378	80.01971652	229.4066531	0.929983184
2.848266896	77.46989382	1038.413278	0.818069246
4.315960952	102.6677302	1303.154083	0.755640517
3.505299055	118.9746789	1589.828445	0.812087507
2.58985944	111.7514355	853.4350237	0.844076587
3.406647267	93.13265454	1694.208372	0.794440734
4.529286021	84.24750399	2934.233961	0.757197342
1.675114288	108.0972108	924.1339298	0.902677101
1.068469992	88.84932375	14.14623838	0.982882502
1.137319687	162.2461878	99.13236464	0.9664764
1.518415726	120.0375563	919.195846	0.908255803
4.469229981	91.35730783	2100.237191	0.74025287
2.479579687	94.53878972	1501.949105	0.836599791
1.219642363	135.4020974	263.9785168	0.949585418
1.405034339	84.60420223	393.6613533	0.922123116
1.574161566	141.38094	480.3502327	0.900294038
1.311108393	103.4429111	291.8115404	0.932386256
1.355241646	98.32620159	619.1705277	0.928373305
1.29139652	102.4632082	340.9384858	0.936343363
1.330674245	141.782819	851.2922663	0.932621613
5.907289482	40.5364507	1446.082781	0.675240025
1.361942363	142.6347308	2050.850932	0.927214932
1.319740054	51.17239087	173.6492931	0.931558165
1.056043956	112.932147	20.8989011	0.985989011
1.132266033	171.4842947	129.2596109	0.96886633
3.730526377	94.17348322	1587.354541	0.790525456
1.376547026	55.92189538	294.0542187	0.920528316
1.91138384	152.4283476	1908.375948	0.875914649
1.349191583	75.94779482	177.4716024	0.924082127
5.769354407	72.38580925	3081.079842	0.717725489
1.260225721	87.31568436	267.3650996	0.942806526
1.230079295	160.086531	716.9052581	0.950828038
1.169856645	203.9717305	394.4071897	0.962017148
1.321401556	110.0736942	272.3918685	0.934087716
1.537633398	63.38175223	312.3243123	0.89751731
1.654131021	67.09315138	928.5961807	0.890152745
3.000158283	163.4170023	3127.314666	0.824966669
3.300647581	73.52524001	1662.842209	0.791677984
2.469801283	59.11491885	899.3035545	0.840033786
1.212137422	92.36384939	43.88788955	0.948953321
1.458822715	150.4908932	825.1314517	0.915767733
1.144469609	120.0012519	91.99834275	0.964832265
2.543943855	92.09370788	1793.593551	0.850098822
1.333840773	50.18879862	107.8799113	0.928107995
1.927277528	95.09342618	1046.922046	0.870102251
1.899544315	129.5151034	1543.206158	0.874003378
1.379765963	75.00262871	414.7993945	0.920540655
4.34774182	77.04963308	2419.181215	0.762142964
5.00796883	52.82177943	3186.666826	0.730516468
5.139736718	116.0832926	4292.043416	0.7338413
1.272567746	147.9147259	356.6946625	0.943693602
1.376002619	91.71782635	338.2748764	0.925022759
1.394472016	33.01651417	161.6709267	0.917577763
1.438769231	140.4015283	657.1859511	0.916549644
2.603139852	126.2142165	1348.622513	0.840464677
1.241868936	117.9414009	121.6447666	0.947433187
1.337766587	196.1371521	659.1632359	0.932095554
2.99811529	201.4747478	2233.872368	0.820512944
1.079632437	146.2554542	28.79958318	0.980091891
3.235243392	70.02609946	2072.741599	0.801246483
1.923201618	98.78588155	2012.718292	0.865102021
1.344448191	67.90377648	274.9807912	0.929333095
1.233235724	166.5433859	481.0819857	0.949018917
1.21024303	46.12469507	41.95198803	0.953367292
1.125	65.71314103	11.58333333	0.96875
2.581362253	152.3754027	1745.605702	0.846253563
1.277634934	63.38330377	240.6960551	0.940588401
1.489875186	75.19052064	284.4178303	0.903941378
2.723068249	92.2131954	1699.279004	0.822029034
4.217796215	68.43283256	2515.814293	0.766264956
1.097326404	132.9037493	57.47232437	0.975668399
1.298062594	149.3375901	528.3901432	0.936667105
1.199105243	56.26311008	19.87799157	0.950223689
1.153032154	239.1971115	141.1864957	0.962874463
1.340516584	112.7427262	210.6812293	0.927753514

log.sigma.3.5.mm.3D_glrlm_LongRunHighGrayLevelEmphasis	log.sigma.3.5.mm.3D_glrlm_RunPercentage	log.sigma.3.5.mm.3D_glrlm_LongRunLowGrayLevelEmphasis	log.sigma.3.5.mm.3D_glrlm_RunEntropy
213.6105895	0.899424675	0.016992689	4.336517544
353.2625401	0.772684906	0.025969797	4.365480025
68.67705823	0.920469361	0.086093136	3.960575476
326.1175621	0.856102511	0.014722291	4.664099619
152.4360856	0.955826352	0.056107463	4.359045168
69.58395789	0.89407314	0.052204619	3.746636223
50.60865432	0.938461538	0.089989658	3.329653836
148.4412883	0.927297142	0.022057559	4.278140021
452.9538951	0.933291641	0.009080977	5.173299638
307.2658005	0.793113743	0.021397413	4.417723131
113.6309749	0.909165303	0.025647691	3.944062915
184.5199253	0.878670867	0.022106494	4.335574519
110.1406554	0.854375493	0.043784317	4.205870525
90.71888546	0.895250583	0.058748495	4.246382571
105.0466409	0.932375317	0.035790175	4.187497016
1219.484854	0.517866784	0.043125826	4.711872771
101.4116828	0.94718185	0.044683102	4.169942081
123.3216511	0.843576527	0.053731824	4.270681379
102.7020614	0.942941674	0.08405208	4.371064105
323.2525442	0.710304476	0.039573038	4.483475526
501.2879007	0.67640773	0.027778994	4.342960188
126.0444684	0.883726834	0.036143303	4.311999074
478.4646696	0.713593256	0.02255605	4.384744666
333.7366156	0.958357432	0.01894065	4.553394103
191.8997292	0.798477564	0.030008133	4.133674948
113.9619368	0.909322466	0.03651026	4.056401377
297.441936	0.722384649	0.036648106	4.325479609
770.8690491	0.62521971	0.028504786	4.487379731
691.1780502	0.688353896	0.02576456	4.629860418
454.1628072	0.749784179	0.02372506	4.474992526
501.9719279	0.681998791	0.029539334	4.457146017
607.7519845	0.620453021	0.042443091	4.598794729
221.5467824	0.853076369	0.02883486	4.50420046
90.45646661	0.979487179	0.16660824	3.234561181
185.3803189	0.957113683	0.030025883	4.327950827
210.7554263	0.872621382	0.017222256	4.268218791
739.4354957	0.612372533	0.032631146	4.548222258
310.7376526	0.751167039	0.028223131	4.544078084
162.7941729	0.935298347	0.031286516	4.431217412
129.7444707	0.894714224	0.040463456	4.304075529
265.3723784	0.863797741	0.017426572	4.340729466
134.1743784	0.91217444	0.02997723	4.161489845
139.3054105	0.904219567	0.023900832	4.16680332
131.8555881	0.91676888	0.029296703	4.308483694
191.7429383	0.909348051	0.01776087	4.474204718
493.0731792	0.545749252	0.088708332	4.380563188
191.793599	0.902151381	0.022677988	4.639941622
63.60620907	0.909919028	0.082470223	3.94782087
119.0894272	0.982517483	0.112285962	3.612904277
189.1770705	0.95943098	0.056354037	4.544922298
562.9324552	0.664215686	0.031490073	4.551106912
75.66748463	0.896354266	0.055452145	3.949648345
376.6067495	0.818514506	0.013772304	4.484562635
104.723682	0.902478275	0.046199435	4.158503271
703.9106953	0.566697057	0.057429798	4.670552044
110.3724592	0.924913892	0.060887103	4.309361131
197.3237464	0.93410374	0.015877614	4.436388385
238.2703928	0.949613705	0.016916718	4.624009518
154.7239232	0.911560191	0.024456573	4.271555876
101.5413016	0.867613074	0.059029303	4.218566995
147.2555888	0.846682527	0.039838557	4.213624611
702.5340145	0.719973209	0.016530254	4.798051035
332.9348233	0.68839923	0.041938958	4.433044627
210.8323875	0.756539757	0.049556905	4.220899638
109.9659056	0.937590711	0.050766413	3.810310265
245.6961426	0.885550214	0.01616309	4.428997272
137.4780351	0.956043956	0.039888944	4.180779575
367.0615531	0.757538107	0.035196542	4.674875582
61.97760592	0.906939215	0.067543828	3.784374805
242.1502955	0.813186813	0.027691018	4.376432281
319.2360376	0.817622162	0.017351776	4.412457994
109.7922462	0.896757563	0.03450287	4.099942651
603.8513462	0.631418886	0.04023124	4.563203725
486.5739024	0.590995584	0.070614528	4.585540578
916.151086	0.589471621	0.032410773	4.747760696
187.2792167	0.924111763	0.017741287	4.369018751
130.1699496	0.89962888	0.028763285	4.12149015
42.9291702	0.892857143	0.131813332	3.732062743
228.1224388	0.888785913	0.021243713	4.310810106
478.3758016	0.747325899	0.01844625	4.46366756
143.1810197	0.931337119	0.026240091	3.99090084
280.7698292	0.908225878	0.011524032	4.578269393
844.6528126	0.714983535	0.012744352	4.705532945
154.8640891	0.975186104	0.047675883	3.705099128
351.2052805	0.693330853	0.041521318	4.422198816
213.4076515	0.809787019	0.023155477	4.247784805
92.14914547	0.905374078	0.039588008	4.044506878
203.1813991	0.932480084	0.018183175	4.512860789
55.65528677	0.94	0.069026226	3.359358417
79.77564103	0.961538462	0.107687778	2.918563809
581.4670172	0.751575925	0.017290407	4.650463431
82.66776698	0.922064777	0.064965012	4.142385834
128.2660926	0.874075444	0.040955129	4.164032941
389.1657379	0.731569635	0.029075052	4.544993087
476.9819866	0.634769827	0.050067924	4.639894107
143.0583508	0.969474969	0.052784624	4.024852916
193.7431658	0.915837104	0.019924774	4.501602921
66.6150679	0.939102564	0.170328778	3.42654644
278.472919	0.952807928	0.015573051	4.491702748
166.7233325	0.906043956	0.031389112	4.188732626

log.sigma.3.5.mm.3D_glrlm_HighGrayLevelRunEmphasis	log.sigma.3.5.mm.3D_glrlm_RunLengthNonUniformityNormalized	log.sigma.3.5.mm.3D_glszm_GrayLevelVariance
160.5825797	0.815698292	18.37326389
124.8842838	0.686699928	14.6918146
56.52241273	0.856176157	12.22222222
186.5378232	0.787899009	28.87483188
138.0392848	0.915786576	34.30876033
53.93641873	0.81453406	7.915748393
42.42157334	0.885012601	5.985431842
125.9065017	0.865637281	20.48036075
371.818632	0.876629453	54.05558032
138.4563961	0.69450465	18.27710405
90.39931168	0.831258072	12.39323289
125.5296586	0.790840028	19.49641353
71.21332973	0.756565117	16.22485713
72.81585469	0.819644637	17.24848204
91.11806796	0.874128796	16.4141791
164.1661557	0.420665642	14.46895772
92.08757465	0.899081405	15.96440014
71.37606237	0.745371115	13.64653595
95.87968458	0.889280684	23.11962426
92.32843787	0.603442316	10.49880624
122.6534191	0.577983296	14.3820618
90.45565285	0.802805898	19.46409526
140.715243	0.607265477	16.01527545
291.0607298	0.919045718	39.39234375
91.16459089	0.678188501	12.06676894
86.05771682	0.833585958	11.75090393
95.88242573	0.625768	13.71703292
146.1175212	0.530856749	13.35140483
155.7694927	0.618354918	18.59842557
139.1478891	0.670609855	15.52859487
122.6145727	0.588293811	16.48277116
115.7599337	0.532407139	14.57712032
120.9204014	0.778782332	21.45532159
89.17075232	0.959564527	19.6875
166.6865925	0.915740929	29.81869822
132.8127322	0.788402463	14.18664924
134.2022991	0.508791983	12.72613158
114.4877353	0.65729771	15.31576096
140.4424886	0.877302325	23.40982167
91.95526698	0.817415192	14.98248521
159.0382983	0.774089246	18.04823767
108.9431107	0.838333397	18.00384273
105.1644673	0.829764424	15.0573536
107.7777254	0.846448399	22.23334775
150.2594818	0.838824494	22.86100705
66.88554247	0.426182436	7.859342984
151.0158556	0.827106825	33.81506353
53.35239032	0.83628598	11.14062222
114.1636031	0.965222397	16.41522491
174.906895	0.921775075	31.02999075
125.0772209	0.582988084	15.87491305
59.5533058	0.813015733	9.3459
177.7427028	0.726927855	17.83562245
81.13170721	0.821070065	12.39253827
106.9695687	0.477428964	11.68950793
91.45586196	0.861676534	20.37173247
166.6930735	0.879325944	23.0694104
210.2369333	0.905707694	27.77081285
117.4845494	0.842840616	20.22721609
69.47363576	0.76826202	13.92401286
77.5428791	0.752735209	13.04203311
203.8887207	0.638167821	26.54263575
94.46231664	0.585949521	11.71208372
73.22225672	0.663681911	8.572287489
95.7824916	0.878770312	15.4176
164.8290894	0.805392007	20.63110969
123.3682135	0.913865278	20.05142687
113.3336268	0.681099042	18.03576699
52.37080705	0.83005224	10.3184
111.867239	0.71581608	15.36493306
151.3963641	0.723397245	14.51703734
81.08092326	0.813791013	12.42487937
109.027717	0.539395954	11.90101509
78.56491229	0.494602176	10.31028533
162.0342938	0.49894859	18.84560333
154.6407343	0.864017125	23.00874258
98.1574309	0.823077377	15.14093473
34.74216992	0.807086587	11.50972222
154.0322552	0.806568867	19.897853
155.3775886	0.664611874	18.83907675
122.3673519	0.8736887	14.89492072
210.0471581	0.837988278	29.29893402
252.1636158	0.630996815	21.61428059
147.9771812	0.95065408	21.90548204
91.24094431	0.599199163	12.50568149
113.0136315	0.706291336	13.68202182
72.13910163	0.831573722	13.66235207
173.1176583	0.875106667	23.73280032
47.74633782	0.888649818	7.754132231
68.52564103	0.923611111	12.74380165
187.0732979	0.674999308	19.64397432
66.77121199	0.856874342	17.12358025
83.62676142	0.779896272	15.73039941
117.7409005	0.632709096	13.56650633
94.38902332	0.545661883	15.4420439
134.9346696	0.939289634	33.199375
157.300676	0.847679279	21.61589648
58.33350164	0.879392745	14.13888889
246.7965947	0.907739991	28.44163223
121.9976976	0.828769356	18.41088651

log.sigma.3.5.mm.3D_glszm_SmallAreaHighGrayLevelEmphasis	log.sigma.3.5.mm.3D_glszm_GrayLevelNonUniformityNormalized	log.sigma.3.5.mm.3D_glszm_SizeZoneNonUniformityNormalized
116.4171311	0.071614583	0.352647569
77.25305278	0.074375411	0.308021039
52.08963509	0.086419753	0.264060357
178.1667268	0.053465347	0.447842368
128.1171743	0.057190083	0.394380165
56.8397216	0.116161616	0.410927456
31.82025986	0.123829344	0.323621228
131.9851987	0.06617618	0.446936434
241.5921005	0.039606399	0.353783543
106.1851401	0.069290674	0.349596547
89.18845256	0.090817271	0.407120613
93.6206429	0.064926651	0.380998658
76.54860754	0.076821734	0.352382987
82.73193781	0.07201889	0.358913813
85.88249185	0.072844732	0.305635999
121.5700936	0.083763575	0.363930241
92.07398039	0.077785689	0.4250623
86.09350706	0.080199923	0.445259516
121.9199701	0.059302852	0.538705297
66.6359947	0.091864096	0.34677686
85.19404391	0.080112161	0.327995032
85.04888608	0.065782745	0.35795164
97.5716566	0.072126539	0.343562126
207.3884531	0.049375	0.530625
57.19919994	0.084958726	0.317700343
62.18814071	0.087551653	0.336002066
86.0755361	0.089612997	0.362485955
87.95547304	0.083469953	0.350952569
106.3439367	0.065871137	0.358858654
79.57481975	0.069561924	0.315301098
90.04474947	0.075647401	0.322879527
98.26069516	0.076236882	0.372274318
101.9594573	0.062370152	0.354289301
103.3356481	0.111111111	0.708333333
156.7647177	0.05704142	0.560236686
115.4273599	0.082759692	0.362584752
76.87126239	0.081532226	0.341706228
109.71413	0.075979739	0.400710348
115.7314911	0.064910837	0.347050754
69.67318982	0.076923077	0.355384615
87.26160401	0.069613374	0.309456032
112.5958597	0.071508131	0.421029182
88.80301779	0.074576397	0.367568371
96.27002409	0.064554498	0.315095156
122.3793921	0.062683094	0.339995305
26.53479997	0.100535396	0.246744663
142.9056848	0.055211693	0.367823114
63.539178	0.095466667	0.464888889
101.7205882	0.08650519	0.584775087
178.7169856	0.052715022	0.502708416
90.75175486	0.070679499	0.344063961
59.75778048	0.1028	0.3846
126.6995703	0.06753282	0.374803078
55.44674871	0.086734694	0.25255102
70.08324653	0.083260251	0.348133624
84.02446781	0.062916892	0.391502914
143.1824316	0.060814065	0.430312875
169.7416156	0.0536862	0.416786389
90.19483055	0.062833749	0.352794589
45.90676915	0.081726354	0.212580349
59.13324963	0.082104521	0.368158875
170.6569205	0.055042938	0.365871317
75.5555102	0.089418283	0.394127424
44.33015365	0.107313793	0.329536015
76.63520278	0.0944	0.4112
142.4121566	0.064005102	0.388061224
104.2404215	0.063020214	0.400713436
92.94268278	0.071456852	0.389750156
50.89221198	0.1096	0.3616
83.28470179	0.071943008	0.405521099
108.6310479	0.073503648	0.335111061
74.94077513	0.084539626	0.369819552
80.83612136	0.086127115	0.361268099
48.04183627	0.087471593	0.366141318
140.6132732	0.069060919	0.381237169
133.8199319	0.059335942	0.403231915
82.86308462	0.074064489	0.396408053
47.05246963	0.092222222	0.442777778
117.3735745	0.062838455	0.382743316
126.4351033	0.067412272	0.421655095
108.4841068	0.080892233	0.351787154
173.9047866	0.052426707	0.422221228
179.8139258	0.061472548	0.364825915
141.3743961	0.088846881	0.584120983
77.80069434	0.080887542	0.358247706
107.7000096	0.080028423	0.396980647
69.93207729	0.082285503	0.406989645
145.6764533	0.061072292	0.390486428
41.77023709	0.107438017	0.433884298
53.36363636	0.140495868	0.702479339
125.0505663	0.066957499	0.354154273
54.09011936	0.072839506	0.309876543
68.22688467	0.073409763	0.372965976
75.3970004	0.078905815	0.353145736
71.43881081	0.070719736	0.372235082
136.6955903	0.07125	0.50125
140.787063	0.063819332	0.363117053
42.54759259	0.097222222	0.333333333
186.0156881	0.059659091	0.416838843
68.10903543	0.067776457	0.342317347

log.sigma.3.5.mm.3D_glszm_SizeZoneNonUniformity	log.sigma.3.5.mm.3D_glszm_GrayLevelNonUniformity	log.sigma.3.5.mm.3D_glszm_LargeAreaEmphasis	log.sigma.3.5.mm.3D_glszm_ZoneVariance
33.85416667	6.875	52.03125	37.89051649
48.05128205	11.6025641	1033.173077	952.2884204
14.25925926	4.666666667	27.90740741	17.16358025
226.160396	27	227.2851485	212.6336869
21.69090909	3.145454545	5.072727273	1.700495868
27.12121212	7.666666667	76.03030303	62.36271809
10.03225806	3.838709677	8	2.901144641
71.06289308	10.52201258	21.94339623	15.58284878
206.609589	23.13013699	19.11643836	12.72870144
102.0821918	20.23287671	1288.565068	1225.493186
44.37614679	9.899082569	72.93577982	61.03644474
56.00680272	9.544217687	186.7823129	160.2633162
83.51476793	18.20675105	502.9451477	469.7247592
55.27272727	11.09090909	79.7012987	67.94619666
40.95522388	9.76119403	19.17910448	11.80017821
159.7653759	36.77220957	67777.59453	67169.225
45.05660377	8.245283019	9.29245283	5.101548594
113.5411765	20.45098039	404.4627451	367.4200077
50.63829787	5.574468085	11.31914894	7.570393843
57.21818182	15.15757576	4630.654545	4348.007126
106.9263804	26.11656442	10611.2546	10368.90802
83.76068376	15.39316239	124.5769231	109.4866864
83.48559671	17.52674897	3726.534979	3582.139647
42.45	3.95	4.5125	1.74859375
37.17094017	9.94017094	655.025641	587.7015122
29.56818182	7.704545455	31.06818182	19.29080579
84.45922747	20.87982833	2818.072961	2721.561311
93.35338346	22.20300752	9793.838346	9579.425081
165.4338395	30.36659436	3783.960954	3718.985465
56.12359551	12.38202247	1424.264045	1333.800436
94.28082192	22.0890411	7506.15411	7299.168735
230.4378029	47.19063005	19618.05977	19414.43287
107.7039474	18.96052632	197.9440789	177.0675965
8.5	1.333333333	1.916666667	0.354166667
36.41538462	3.707692308	5.769230769	2.746982249
100.7985612	23.00719424	225.9064748	202.8802857
132.2403101	31.55297158	20765.41344	20466.48989
136.6422287	25.90909091	2078.152493	1999.093799
46.85185185	8.762962963	12.27407407	6.620246914
46.2	10	71.54615385	54.48289941
43.01438849	9.676258993	168.381295	141.9217432
56.41791045	9.582089552	35.70895522	27.6246937
85.27586207	17.30172414	100.4568966	87.8421522
42.85294118	8.779411765	43.69852941	33.27892517
106.4185304	19.61980831	105.715655	93.02567139
30.3495935	12.36585366	73925.80488	71448.51728
284.6950904	42.73385013	125.7416021	113.1546916
34.86666667	7.16	40.66666667	31.42506667
9.941176471	1.470588235	1.882352941	0.207612457
43.73563218	4.586206897	4.413793103	1.597569032
115.9495549	23.8189911	6520.581602	6374.006393
38.46	10.28	115.67	99.4291
176.1574468	31.74042553	1113.16383	1066.952761
14.14285714	4.857142857	46.375	28.16039541
177.8962818	42.54598826	53601.0998	53111.57335
50.50387597	8.11627907	21.46511628	14.7212307
160.5067024	22.68364611	21.45308311	15.98776675
95.86086957	12.34782609	7.87826087	3.912967864
37.39622642	6.660377358	35.98113208	24.82805269
14.03030303	5.393939394	191.2424242	141.3902663
78.41784038	17.48826291	477.5539906	430.891622
318.308046	47.88735632	4455.512644	4394.851822
112.3263158	25.48421053	7432.091228	7227.751727
52.39622642	17.06289308	2029.572327	1903.963213
10.28	2.36	8.04	3.5456
108.6571429	17.92142857	146.0928571	129.1655102
23.24137931	3.655172414	4.637931034	1.36058264
171.8798186	31.51247166	1764.22449	1702.418509
18.08	5.48	28.54	20.3604
102.5968379	18.20158103	769.6126482	719.4437032
126.33687	27.71087533	840.6923077	793.1664615
45.48780488	10.39837398	97.71544715	76.46559588
146.3135802	34.88148148	17395.42963	17091.80976
159.6376147	38.13761468	48655.55046	48038.09635
351.8819068	63.7432286	42469.94475	42222.50469
71.7752809	10.56179775	24.2247191	17.94659765
55.10071942	10.29496403	64.92086331	54.15868744
26.56666667	5.533333333	68.23333333	54.29555556
83.05529954	13.6359447	118.6958525	100.9938627
153.9041096	24.60547945	1657.654795	1602.773083
21.45901639	4.93442623	13.16393443	7.197527546
126.2441472	15.67558528	44.81270903	36.44339549
222.1789819	37.43678161	3919.417077	3853.91078
13.43478261	2.043478261	2.217391304	0.400756144
140.7913486	31.78880407	7131.73028	6972.31556
203.6510721	41.0545809	1545.908382	1499.173968
42.32692308	8.557692308	60.00961538	47.69221524
95.27868852	14.90163934	18.79098361	12.96390419
9.545454545	2.363636364	9.818181818	4.652892562
7.727272727	1.545454545	1.545454545	0.148760331
174.2439024	32.94308943	1710.563008	1661.988908
27.88888889	6.555555556	36.2	24.79061728
38.78846154	7.634615385	76.55769231	60.55769231
116.8912387	26.11782477	3295.637462	3173.90524
160.8055556	30.55092593	18143.08333	17863.60543
20.05	2.85	3.575	1.094375
80.24886878	14.1040724	40.78733032	31.3198747
4	1.166666667	5.666666667	1.666666667
36.68181818	5.25	5.238636364	1.807722107
29.7816092	5.896551724	27.72413793	17.36609856

log.sigma.3.5.mm.3D_glszm_ZonePercentage	log.sigma.3.5.mm.3D_glszm_LargeAreaLowGrayLevelEmphasis	log.sigma.3.5.mm.3D_glszm_LargeAreaHighGrayLevelEmphasis	log.sigma.3.5.mm.3D_glszm_HighGrayLevelZoneEmphasis
0.265927978	0.469850377	7936.697917	179.875
0.111190306	6.752899369	176404.5321	117.0128205
0.305084746	1.734076786	1020.87037	81.66666667
0.26125194	1.275891159	46527.36436	233.0534653
0.544554455	0.258382625	583.8727273	163.8545455
0.270491803	2.525704525	2893.19697	79.65151515
0.442857143	0.530594032	333.7741935	47.19354839
0.396508728	0.326925537	1988.72327	168.1257862
0.395663957	0.124339897	7586.92637	379.9075342
0.125916343	9.4072033	191616.3425	162.6883562
0.289893617	1.187576128	5030.449541	121.3944954
0.194187583	1.848058659	23067.19048	140.1972789
0.173499268	11.26661658	26378.55274	111.4725738
0.291666667	2.737961101	3072.746753	113.7727273
0.368131868	0.630093896	1021	126.6641791
0.040543037	387.377048	11964124.21	177.9476082
0.488479263	0.459684362	489.8301887	119.7735849
0.164304124	9.510674917	26633.58431	108.3843137
0.516483516	1.132023356	402.4042553	137.8829787
0.059480894	49.2741979	527537.2727	99.31515152
0.064236453	73.10487946	1613529.948	126.6380368
0.257425743	2.077046599	9568.918803	124.3076923
0.083219178	22.76473803	643793.6337	141.5843621
0.601503759	0.047793083	1339.175	282.3625
0.121875	7.309836669	65965.51282	88.66666667
0.291390728	0.660404677	2677.738636	94.60227273
0.101791175	27.0730352	309913.2833	123.3819742
0.068292683	51.90468615	1885056.248	127.5037594
0.124058127	17.81499393	831578.1063	155.35141
0.105138807	8.072768694	278837.2472	120.3033708
0.06950726	51.30886586	1160030.445	137.2568493
0.070078116	143.7629893	2751426.777	142.0226171
0.218862491	1.880796912	28895.28289	147.6480263
0.8	0.374164134	122.1666667	105.25
0.575221239	0.10281665	808.6153846	190.5230769
0.208395802	2.212104541	27826.65827	166.3273381
0.057838888	120.0373618	3666095.581	114.4909561
0.112467018	18.95151328	264655.5103	152.3636364
0.420560748	0.32146546	1184.992593	170.8074074
0.242085661	1.684622125	6435.592308	102.4769231
0.194405594	1.113397142	30489.31655	149.8848921
0.351706037	0.580153412	2860.059701	146.8432836
0.281553398	1.255454172	9440.025862	128.3146552
0.309794989	0.803867978	3233.617647	144.2058824
0.280717489	1.052659099	12482.31629	189.543131
0.020091473	885.4096262	6426013.48	49.84552846
0.28186453	1.494473212	13197.75065	209.497416
0.328947368	2.69496024	1034.013333	81.25333333
0.772727273	0.256525439	199	121.1764706
0.595890411	0.312348222	553.5977011	212.1954023
0.082598039	42.99170236	1036472.258	132.7329377
0.248138958	4.275027883	3865.03	86.61
0.147104851	5.30560571	256422.234	176.006383
0.234309623	1.351366713	3124.446429	97.625
0.04519724	431.863257	6786677.795	102.4794521
0.385074627	0.700934332	1569.224806	114.8682171
0.427752294	0.21571892	2761.895442	195.924933
0.502183406	0.139704828	1211.334783	238.9043478
0.299435028	0.479647901	4044.415094	133.6698113
0.141630901	5.969525468	10187.86364	95.1969697
0.146391753	7.879472948	40126.52113	83.19248826
0.128394333	18.26597961	1121951.087	240.408046
0.069955817	77.17029295	777357.9088	107.477193
0.089225589	27.36593894	184681.6604	67.44654088
0.471698113	0.260788975	640.2	109.12
0.243055556	0.950976803	27881.4	190.4678571
0.552380952	0.158197372	495.4310345	140.6724138
0.127199308	12.8448399	297713.9524	128.9773243
0.34965035	1.43330945	782.78	73.68
0.141183036	6.993015851	102500.3676	113.3201581
0.145055791	5.108615002	158323.5703	164.1087533
0.216931217	1.889724179	7016.764228	105.398374
0.057389826	124.9919267	2565532.237	112.9407407
0.040243677	500.2111026	4973045.509	68.5733945
0.063571871	236.1176524	7762718.155	204.6966414
0.399103139	0.255769114	2884.404494	186.0730337
0.304824561	0.926298695	5278.438849	117.1654676
0.267857143	5.106155762	1328.2	59.35
0.237677985	0.86441124	20827.70507	163.235023
0.134985207	8.957869229	327211.8712	168.6164384
0.409395973	0.256066454	1088.344262	156.9344262
0.34566474	0.264355393	9306.076923	238.5317726
0.123554474	13.14550574	1186906.658	263.2151067
0.741935484	0.076204128	272.9565217	163.826087
0.079201935	66.30335763	821681.3791	109.6259542
0.146278871	16.48434369	155802.3372	150.8869396
0.284931507	1.517955537	3263.125	97.80769231
0.41426146	0.252133731	2570.393443	209.5409836
0.44	0.427386988	417.6363636	54.86363636
0.846153846	0.126848397	114.7272727	65.63636364
0.143482065	7.018899804	441260.8272	188.1686992
0.296052632	1.52079883	1713.266667	83.45555556
0.25	1.310994151	6537.5	97.07692308
0.090635268	24.75766561	490740.9668	110.3897281
0.059817225	159.1480293	2174260.014	100.8333333
0.634920635	0.120101377	372.325	160.325
0.325	0.53908532	4825.176471	203.1945701
0.5	0.744636309	280.4166667	65.5
0.539877301	0.056475746	1149.579545	265.1818182
0.310714286	0.353062635	3653.83908	115.2183908

log.sigma.3.5.mm.3D_glszm_SmallAreaEmphasis	log.sigma.3.5.mm.3D_glszm_LowGrayLevelZoneEmphasis	log.sigma.3.5.mm.3D_glszm_ZoneEntropy	log.sigma.3.5.mm.3D_glszm_SmallAreaLowGrayLevelEmphasis
0.614168571	0.020950036	5.294874257	0.016125217
0.573821538	0.030145602	5.594992905	0.012552407
0.513904242	0.04738151	4.868326336	0.009569149
0.694916143	0.012956474	5.944979429	0.008683373
0.63989126	0.038933421	5.149452668	0.014074331
0.663853261	0.040747117	4.545125795	0.028982217
0.574715502	0.070813338	4.103727666	0.025979544
0.693639778	0.020201719	5.301565693	0.014845841
0.614186118	0.007790979	6.552646744	0.005007509
0.614269677	0.016860903	5.866587954	0.007549316
0.65752249	0.024517795	5.067439656	0.017639159
0.641343531	0.024778529	5.556678233	0.017170433
0.61416123	0.037219692	5.634987874	0.022027607
0.618015622	0.043292173	5.52876749	0.020619698
0.556743097	0.027425883	5.548198545	0.0157834
0.625161486	0.012604757	5.735836636	0.005722355
0.675661613	0.029443698	5.113066418	0.019324393
0.692602717	0.032635607	5.372008635	0.014996636
0.760344568	0.049154041	4.956133071	0.032338259
0.611601164	0.026324514	5.342270605	0.015743722
0.593362088	0.017952343	5.832500847	0.010952384
0.61973777	0.029925188	5.811377084	0.020115736
0.608198094	0.018841426	5.737977888	0.011952565
0.75372201	0.023967904	5.290311532	0.02184599
0.579987439	0.033500856	5.386988067	0.020339852
0.59908418	0.034038046	5.186078068	0.016601593
0.624769317	0.02017729	5.518557642	0.011406422
0.615420877	0.017292652	5.749373099	0.011548667
0.622331154	0.019739366	6.051907437	0.011230507
0.581902395	0.023826484	5.777409984	0.009836775
0.586618651	0.021147578	5.849706055	0.009717394
0.632934717	0.019690969	5.885201041	0.010030861
0.615108507	0.026987768	5.908385202	0.013296879
0.863425926	0.097497467	3.251629167	0.032034504
0.772425083	0.035004715	4.926686568	0.030166878
0.624784149	0.014978558	5.614251623	0.009795102
0.607202601	0.021268683	5.898267414	0.013821323
0.658041952	0.016654181	5.587616848	0.008451226
0.606618897	0.029911099	5.632490417	0.02172805
0.618116463	0.034373681	5.470477837	0.017180056
0.571875873	0.021754881	5.620423494	0.014602282
0.673625421	0.022675937	5.199001099	0.011404149
0.62718643	0.021856121	5.609416972	0.013723616
0.572164536	0.029254867	5.783666978	0.018527318
0.600011803	0.014170962	6.001844031	0.00636019
0.513337049	0.063247619	5.558800506	0.031480978
0.629092028	0.017629597	6.327074532	0.009700218
0.70663236	0.068887892	4.631453974	0.046229779
0.779411765	0.070269687	3.734521665	0.023705749
0.735569826	0.025848819	5.086469146	0.008294153
0.608602609	0.020281184	5.785759525	0.010518532
0.643594347	0.035365491	4.967438961	0.016958367
0.636222133	0.012706555	5.974843933	0.008119323
0.516043896	0.042593872	5.08655978	0.025842048
0.612916758	0.028465566	5.821667811	0.015954907
0.649917146	0.051293552	5.497797919	0.032233954
0.681847354	0.015508754	5.773794866	0.011638823
0.670327488	0.014433067	5.777919014	0.010783529
0.611291757	0.027971601	5.552022314	0.019740997
0.452183219	0.066900928	5.320539006	0.040680584
0.630228009	0.047011597	5.481641301	0.025761587
0.627910697	0.009356168	6.315660382	0.004560832
0.651805526	0.02447973	5.344499179	0.015503642
0.59368943	0.050190572	5.244842433	0.021713792
0.657436111	0.057905733	4.13366069	0.049754388
0.647271235	0.01468341	5.783603058	0.006895336
0.648558429	0.036657218	5.058592582	0.026813992
0.648427758	0.023217735	5.882371048	0.012916335
0.616177319	0.051583149	4.550435501	0.033817592
0.662009104	0.034482464	5.547364826	0.020488352
0.599111362	0.013346762	5.855974436	0.005838354
0.6308508	0.028494166	5.314318474	0.017134981
0.623672972	0.020512267	5.61132259	0.008887971
0.627963585	0.056464245	5.519669241	0.028054677
0.641343502	0.010490672	6.037858923	0.00591596
0.658188044	0.018036872	5.652555022	0.013426577
0.653721042	0.034266164	5.391412998	0.026068772
0.688543479	0.093804059	4.720333106	0.046806513
0.642756865	0.023427027	5.81635291	0.013402566
0.675097286	0.014304061	5.735842719	0.009780786
0.600902648	0.027052031	4.838455526	0.0206627
0.673121504	0.012839901	5.889693839	0.00993297
0.627960997	0.008124839	6.245866391	0.005451066
0.792270531	0.055896034	3.914866304	0.052915763
0.622178237	0.028675628	5.723899709	0.017265159
0.65497056	0.016513691	5.644773165	0.01125514
0.661578579	0.034668491	5.122986714	0.025156204
0.646214972	0.014819973	5.760919214	0.011021634
0.672909652	0.081175275	3.970573096	0.068093526
0.863636364	0.1173787	3.277613437	0.115011276
0.615735394	0.014094843	6.05096676	0.007225644
0.573696017	0.056078912	5.355866086	0.021923321
0.632606858	0.048078937	5.421655551	0.029359738
0.61631997	0.033881352	5.632442873	0.021695464
0.633209481	0.035399539	5.755093802	0.018037517
0.734118056	0.065526173	4.53418372	0.05884053
0.62183866	0.015204767	5.681997053	0.006254765
0.580300926	0.12597276	3.418295834	0.039397977
0.66911133	0.017970192	5.301111626	0.015182504
0.603215132	0.046432818	5.468299961	0.038523811

log.sigma.3.5.mm.3D_ngtdm_Coarseness	log.sigma.3.5.mm.3D_ngtdm_Complexity	log.sigma.3.5.mm.3D_ngtdm_Strength	log.sigma.3.5.mm.3D_ngtdm_Busyness	log.sigma.3.5.mm.3D_ngtdm_Contrast
0.025831362	0.661471762	3.486632227	0.178251318	0.000309548
0.008017363	0.085481007	0.900672765	0.559511913	4.73E-05
0.050342246	0.808304761	4.650761824	0.243249874	0.000687052
0.005054873	0.353530521	1.681675392	0.407892478	3.55E-05
0.063802694	5.650425418	12.70415609	0.090364496	0.003828753
0.02966944	0.399251095	1.350953466	0.466316071	0.000503541
0.080371487	0.982680024	3.044779688	0.195632444	0.002156232
0.018665844	1.197345445	3.509972503	0.227343911	0.000319861
0.006794955	1.063477283	3.690353692	0.303694594	0.000171421
0.004972849	0.089663067	0.941331939	0.620004024	1.53E-05
0.018577802	0.546070283	1.658053393	0.36440778	0.000277359
0.013716803	0.284456107	1.86872358	0.335035916	0.000104147
0.006744256	0.171104338	1.010455413	0.883193344	4.60E-05
0.015939847	0.611380042	2.667535134	0.427082686	0.000193599
0.021556424	0.97255961	3.208605945	0.283968753	0.000359593
0.000977888	0.013123735	0.445526748	1.987327696	6.72E-07
0.029782127	1.987298061	4.455311684	0.20942312	0.000862848
0.006011432	0.152150806	0.941703995	0.946929858	4.03E-05
0.03633886	2.408408081	5.586364115	0.274223198	0.001730588
0.004750475	0.028369376	0.453360479	1.187346228	1.59E-05
0.002294141	0.025449592	0.451285978	1.327307354	3.67E-06
0.009455879	0.374952908	1.675165452	0.531878602	9.81E-05
0.004174578	0.051338885	0.841137981	0.672600172	8.01E-06
0.066810122	4.494415826	16.85662566	0.037276539	0.001880373
0.0112284	0.097987881	1.086575914	0.508242023	4.39E-05
0.026791359	0.588786035	2.390821678	0.274880295	0.000381189
0.004787649	0.071158552	1.018981622	0.717332844	9.47E-06
0.002982638	0.035020087	0.85255745	0.888690083	5.77E-06
0.002822079	0.062073067	0.705564267	0.83420747	1.11E-05
0.007281622	0.082931442	1.089722869	0.480231527	3.24E-05
0.002970926	0.038259954	0.666541456	0.93054475	4.73E-06
0.001274054	0.01820506	0.321774566	2.139476684	1.93E-06
0.006879914	0.23004519	1.226473085	0.593492953	6.74E-05
0.189524253	14.71734628	18.53590627	0.159567628	0.105235254
0.063935845	4.248119094	10.54137477	0.068588819	0.002436861
0.006451449	0.218411128	0.924672116	0.64479442	5.92E-05
0.001572522	0.016402837	0.332083549	1.866336381	3.73E-06
0.003850674	0.071361609	0.755226888	0.88069347	1.20E-05
0.022193775	1.482086959	4.263078858	0.201340767	0.000608575
0.017007563	0.405455331	2.047733927	0.394736573	0.000221442
0.015112754	0.310284669	2.499115002	0.22631227	9.18E-05
0.019855416	1.035930673	3.450238158	0.226175376	0.000256692
0.008918063	0.364442709	1.165145161	0.561832625	0.000118829
0.018849673	0.980596978	3.783126778	0.249577015	0.000257766
0.008184089	0.468098749	2.104407375	0.41077953	8.66E-05
0.001805073	0.006718815	0.205212568	3.103486449	2.77E-06
0.003512721	0.507960659	3.337133126	0.544705773	2.19E-05
0.027268978	0.730327394	1.966267354	0.564942527	0.000817444
0.148510481	13.14755593	12.70806538	0.084941338	0.038109088
0.041216136	4.915040953	7.65405022	0.113615432	0.003014647
0.002944898	0.033630103	0.526138071	1.053471048	6.78E-06
0.015804659	0.453329623	1.352100402	0.666356838	0.00031241
0.00345291	0.092136816	0.739741832	0.725041434	1.49E-05
0.03868398	0.567265432	3.121253715	0.266006547	0.000556587
0.000950053	0.008844156	0.20420664	3.4516843	1.59E-06
0.022584921	0.859738704	2.353259337	0.338527148	0.000612049
0.008580001	0.730182233	1.957007948	0.369723046	0.00014955
0.01659599	1.701537968	3.75112284	0.19090363	0.000507714
0.027723617	0.760487639	3.851356488	0.180402606	0.000282583
0.021713417	0.246038992	1.782834542	0.475293115	0.00020879
0.006786717	0.114153141	0.727746783	1.027270782	5.38E-05
0.001713983	0.065113165	0.689952384	0.934778584	4.39E-06
0.002823426	0.030104684	0.445575045	1.390189797	5.63E-06
0.005990705	0.04519873	0.478957747	1.118391512	2.90E-05
0.110766442	3.371762529	9.238708231	0.078594657	0.004971947
0.008435564	0.328556215	1.623043299	0.351557616	7.00E-05
0.058422791	3.343110145	6.263881996	0.110071278	0.002726476
0.003430785	0.085007048	0.802376064	0.879651413	1.57E-05
0.046843024	0.958646383	3.054034693	0.33276097	0.001218967
0.005920684	0.096145362	0.761140843	0.761420539	3.42E-05
0.004003498	0.085904003	0.632741344	0.831202363	2.19E-05
0.014234257	0.36631822	1.442546083	0.482455787	0.00017054
0.001692349	0.017906422	0.330874719	1.871958393	3.33E-06
0.001065481	0.006170661	0.132058661	4.498605749	2.31E-06
0.000741224	0.018576811	0.410321068	2.319843119	7.39E-07
0.016220746	1.253308204	3.037015884	0.227839355	0.000317719
0.016893255	0.534506233	1.87401376	0.323672857	0.000208662
0.031953562	0.543240627	2.354967386	0.510053333	0.000510446
0.009449009	0.349989836	1.533292462	0.364971039	9.06E-05
0.004126864	0.107321916	1.047316276	0.547340456	1.11E-05
0.039035342	1.829514835	3.964732027	0.185943772	0.00169554
0.010901436	0.723233035	2.675779142	0.22028568	0.000114473
0.002256988	0.072568979	0.832795279	0.675775664	5.56E-06
0.136147633	9.500092445	13.60583075	0.07559562	0.024036619
0.002229247	0.026035729	0.332214307	1.753195132	5.64E-06
0.002909502	0.068202337	0.55690262	1.167829385	9.03E-06
0.021821875	0.577720936	2.334902949	0.353428043	0.000269703
0.01212272	1.214538011	2.615688456	0.271651256	0.000291202
0.114979971	1.560795754	3.845715104	0.149951154	0.003649837
0.165376868	9.594723866	10.22354373	0.120564928	0.110406403
0.003143023	0.096976627	0.895797264	0.63471231	1.24E-05
0.024775443	0.780177864	2.923883741	0.486990578	0.000558843
0.023282057	0.373149802	1.929507529	0.296985523	0.000238561
0.003410235	0.036667637	0.455562508	1.162433192	1.19E-05
0.00165029	0.01553829	0.265295687	2.289248443	3.59E-06
0.065858363	8.857743112	11.01777328	0.107410547	0.01045131
0.012956371	0.732123072	2.573903151	0.274233123	0.000179743
0.125107863	7.286778883	8.585086495	0.137812115	0.03140741
0.045315985	3.713426116	9.888772058	0.074489827	0.001458085
0.031049061	0.736138101	3.869909716	0.154428149	0.000380693

log.sigma.3.0.mm.3D_gldm_GrayLevelVariance	log.sigma.3.0.mm.3D_gldm_HighGrayLevelEmphasis	log.sigma.3.0.mm.3D_gldm_GrayLevelNonUniformityNormalized	log.sigma.3.0.mm.3D_gldm_DependenceEntropy
12.17415459	148.8891967	0.080992319	6.087181495
8.280860329	166.9187455	0.131523987	6.624553128
9.05071978	62.99435028	0.098662581	5.715259011
15.78628707	171.1645111	0.081601471	6.929788292
23.12361533	117.0792079	0.06440545	5.404069993
5.281963854	53.88114754	0.133834991	5.649744437
6.47122449	55.27142857	0.115510204	4.470154939
12.64228456	116.9775561	0.082679834	6.011461425
57.21693436	295.0785908	0.042853681	7.004576932
7.33226114	150.2785683	0.120583083	6.744294151
7.75763213	152.2047872	0.10246435	5.938660602
9.552537392	114.0673712	0.093539994	6.578250904
7.623970233	77.77379209	0.107215819	6.478609027
9.914912621	77.5094697	0.099510732	6.06541525
13.56994928	93.87912088	0.082674194	5.860758168
3.264440655	171.2167529	0.298192075	6.480728229
13.43090743	83.46543779	0.084244728	5.599889063
7.914264268	65.83891753	0.099871134	6.528463203
17.14092501	78.18681319	0.085738437	5.743114509
6.094292116	103.3377794	0.1386575	6.846403477
4.524931932	139.5424631	0.183895945	6.488405744
10.6555155	93.36743674	0.091422894	6.434705692
5.662457192	135.0530822	0.153182117	6.712363335
34.36791226	322.6015038	0.059641585	5.699058402
5.835828993	120.28125	0.125540365	6.48597944
9.558089557	100.8807947	0.089689049	5.994831153
5.189034265	84.63171691	0.171294475	6.657940176
5.448255869	144.6094994	0.232996057	6.517464896
9.291419165	181.8291173	0.180159315	6.655779293
8.69298585	164.8470171	0.138891283	6.58065215
5.532731266	117.3503928	0.166887017	6.667940124
4.541415705	127.7682554	0.206469841	6.724335065
12.4095192	134.2822174	0.088276134	6.667231843
19.97333333	108.3333333	0.111111111	3.240223929
24.10055603	130.0265487	0.061007127	5.355373268
9.520114568	108.547976	0.090836141	6.45848113
5.514662142	153.7710357	0.230040214	6.455232875
7.165876103	123.5168206	0.125933099	6.858362152
18.62270358	140.0342679	0.067672092	6.012016353
12.14238701	104.9757914	0.080379652	6.366430868
10.02484229	123.9020979	0.095994914	6.555083369
9.702289182	103.4514436	0.09492219	5.88954784
10.29303981	101.0279126	0.08761429	6.258359319
12.53324754	118.1685649	0.083535266	6.157878215
17.90197752	186.0170404	0.073524101	6.496830073
2.879164412	61.4059131	0.282953142	6.324602394
22.90988269	200.9369993	0.073229206	6.799026606
8.710584026	51.82017544	0.098684211	5.609944635
20.90082645	143.9090909	0.090909091	3.823067982
26.89116157	132.1643836	0.059954963	5.3960752
6.048148549	142.3308824	0.177422145	6.726747632
8.92285526	63.23573201	0.105283574	5.816287159
10.14032979	194.657903	0.099819799	6.892491771
9.514084137	84.45606695	0.093783372	5.934456913
4.420888123	117.4953122	0.255392666	6.588258668
13.91324571	85.32835821	0.080561372	6.081704691
18.72673675	178.8130734	0.067844668	6.362867719
27.73179859	221.9628821	0.053193112	6.14250381
12.23349772	132.5508475	0.082256057	6.177295205
8.279633075	77.03433476	0.098970325	6.275733003
9.068430463	93.61993127	0.095274737	6.378500727
10.06838489	194.6127509	0.135748358	6.940985344
4.552586188	98.78645066	0.170650887	6.799689153
5.994581052	82.63075196	0.144269733	6.725308837
12.84371663	73.56603774	0.092915628	4.538872341
13.44957969	170.7647569	0.079717942	6.664126293
14.57269841	111.9238095	0.075555556	5.33202948
11.43805674	130.6048457	0.115682421	6.921584736
6.879358404	42.37762238	0.135801262	5.156336979
8.777282715	147.0234375	0.102113685	6.781842113
8.944424862	167.5648326	0.101960813	6.855762589
8.571316592	79.43386243	0.093586406	6.079286683
5.4870566	126.8427094	0.186848346	6.72178833
4.312447849	110.4020676	0.227000859	6.55669088
4.980077183	171.217577	0.222321444	6.774346204
15.28907177	126.293722	0.077017434	6.056604996
9.576711103	114.7741228	0.095519775	6.133302663
5.612224968	32.75446429	0.129105548	5.407327925
12.26884397	154.0952903	0.082329117	6.389186344
7.976543489	151.2585059	0.131895876	6.719285554
11.01878294	70.17449664	0.088059096	5.448021944
19.64486084	198.8023121	0.067066725	6.667337012
9.304350711	239.046054	0.150657371	6.947230759
23.50884495	151.7096774	0.090530697	4.180002762
5.292477565	123.401451	0.162365457	6.810672081
6.074703012	138.9714856	0.126784193	6.767784934
8.074580597	53.42465753	0.100108838	5.8907828
17.76442475	167.5670628	0.068698637	6.21879771
7.64	65.4	0.12	4.55326969
15.01775148	87.92307692	0.195266272	3.085055103
10.95386315	268.7862351	0.121989624	7.029512558
12.0706263	64.49013158	0.085634522	5.848375906
9.739275148	113.2307692	0.10034902	6.254699525
7.399880514	134.4742607	0.121810852	6.871056812
5.215051689	106.12545	0.191076667	6.677232512
26.16376921	142.6666667	0.069286974	4.869749169
16.27385597	143.5985294	0.071051038	6.394315992
14.88888889	61.58333333	0.170138889	3.501629167
24.83924875	247.2699387	0.05965599	5.739721333
11.09586735	108.4	0.090739796	5.939234771

log.sigma.3.0.mm.3D_gldm_DependenceNonUniformity	log.sigma.3.0.mm.3D_gldm_GrayLevelNonUniformity	log.sigma.3.0.mm.3D_gldm_SmallDependenceEmphasis
59.8199446	29.23822715	0.236455217
110.6421953	184.528154	0.124880736
28.5819209	17.46327684	0.245531535
224.4361097	157.7356441	0.228362765
27.35643564	6.504950495	0.416135864
33.86885246	32.6557377	0.210290531
18.94285714	8.085714286	0.428746032
74.70573566	33.15461347	0.339944177
299.8089431	63.25203252	0.327403312
190.5437689	279.632169	0.130907526
59.39361702	38.52659574	0.269494302
90.77807133	70.80977543	0.184987349
144.1918009	146.4568082	0.158179159
74.59469697	52.54166667	0.257413582
76.03296703	30.09340659	0.383882675
436.7319911	3228.82379	0.049554901
46.28110599	18.28110599	0.420595689
165.9342784	155	0.161048553
35.23076923	15.6043956	0.399179196
168.8284066	384.6359048	0.068839602
267.6388177	933.2719212	0.08108839
121.589659	83.10341034	0.222864362
169.8143836	447.2917808	0.084980026
34.09774436	7.932330827	0.450871449
82.61875	120.51875	0.124767984
48.01324503	27.08609272	0.270511282
142.5771079	392.0930537	0.106756187
179.4287548	907.5196406	0.071533375
212.3697524	669.4720129	0.117894634
118.6207915	235.1429415	0.111947056
230.2558915	701.092359	0.079598791
406.7869354	1823.748104	0.072321204
171.3470122	122.6155508	0.204062215
8.333333333	1.666666667	0.5
32.57522124	6.89380531	0.426224189
165.4947526	121.1754123	0.193248787
318.8272306	1539.199073	0.068491103
210.7117414	381.8291557	0.117153578
69.03115265	21.72274143	0.370273915
76.10614525	43.16387337	0.204017454
75.4	68.63636364	0.182025876
65.73490814	36.16535433	0.268198825
131.2961165	72.19417476	0.254026956
73.38724374	36.67198178	0.26729889
197.5363229	81.9793722	0.278673919
265.4439726	1732.239138	0.036213275
407.583394	201.0873999	0.272940143
39.74561404	22.5	0.300411495
11.09090909	2	0.659090909
43.83561644	8.753424658	0.540359055
213.2102941	723.8823529	0.093149464
61.24317618	42.4292804	0.221539824
282.6413146	318.9242567	0.145183558
38.80753138	22.41422594	0.216486643
480.6509818	2887.469485	0.054308746
62.2119403	26.9880597	0.315919881
177.3165138	59.16055046	0.365183224
121.9388646	24.36244541	0.442003962
53.55932203	29.11864407	0.250693926
59.44206009	46.12017167	0.154347417
164.2687285	138.6247423	0.14459498
409.5903188	919.8308737	0.127116949
228.9828179	695.2317133	0.079500689
127.9393939	257.0886644	0.092228048
12.09433962	4.924528302	0.353873166
151.3107639	91.83506944	0.230860806
26.61904762	7.933333333	0.39462963
244.7011826	401.0709547	0.123963703
22.81818182	19.41958042	0.282107695
152.6707589	182.9877232	0.139884493
228.9538284	264.9961524	0.155568107
92.08289242	53.06349206	0.193694039
326.8874876	1318.588777	0.066537061
487.6392837	2459.327303	0.054069879
631.5084372	3227.885047	0.072365184
90.69955157	34.34977578	0.350990218
69.13157895	43.55701754	0.259180696
36.875	28.91964286	0.209976331
127.1927711	75.16648412	0.225081893
177.8217456	356.6464497	0.134952383
32.42281879	13.12080537	0.409512377
138.8543353	58.01271676	0.310169702
293.3436803	742.5901806	0.13133743
17.70967742	2.806451613	0.777777778
270.2607819	805.6573962	0.086461096
293.1579698	444.6321642	0.151314143
53.96986301	36.53972603	0.231799693
120.6536503	40.46349745	0.371820344
12.08	6	0.425316667
7.461538462	2.538461538	0.769230769
233.2945465	418.3024205	0.142959096
57.51315789	26.03289474	0.233807454
52.22596154	41.74519231	0.245841301
236.213034	444.8532311	0.092841891
354.1002492	1379.955691	0.069655316
23.06349206	4.365079365	0.568425926
117.0911765	48.31470588	0.273106618
7	4.083333333	0.304976852
41.99386503	9.72392638	0.47933197
44.17857143	25.40714286	0.255227832

log.sigma.3.0.mm.3D_gldm_DependenceNonUniformityNormalized	log.sigma.3.0.mm.3D_gldm_DependenceVariance	log.sigma.3.0.mm.3D_gldm_LargeDependenceEmphasis
0.165706218	2.751528917	14.64542936
0.078861151	37.21915821	91.96650036
0.161479779	3.418430208	15.10169492
0.116107661	23.86481801	50.15364718
0.270855798	1.334771101	6.386138614
0.138806772	5.234412792	20.77868852
0.270612245	1.233469388	6.2
0.186298593	2.737545165	11.29426434
0.20312259	2.472462746	10.35501355
0.082166351	28.99820985	74.26865028
0.157961747	4.111673834	16.39893617
0.119918192	8.27053882	28.74108322
0.105557687	10.58154855	36.66178624
0.141277835	4.843993916	18.70833333
0.208881778	2.598206738	9.758241758
0.040333579	48.08371829	217.6701145
0.213276986	2.912952069	10.03225806
0.106916416	10.49890896	36.10824742
0.193575655	5.882381355	15.41758242
0.060860997	34.73528579	113.8644557
0.052736713	41.94856052	135.0149754
0.133762001	6.758858064	23.05060506
0.058155611	38.49951961	117.319863
0.256374018	1.912487987	7.240601504
0.086061198	15.70618056	53.63125
0.158984255	3.706723389	15.38410596
0.062287946	37.21742579	105.6028834
0.046066433	49.96004186	169.1817715
0.057150095	48.16710678	133.386437
0.070065441	43.75918003	110.5581807
0.054809781	39.74780004	127.0085694
0.046053089	47.71134449	163.0749462
0.12335998	23.51271043	50.12167027
0.555555556	0.222222222	3
0.288276294	1.048163521	5.672566372
0.124059035	12.93964787	34.65217391
0.047650162	43.49329049	165.8252877
0.069495957	33.02801211	92.56002639
0.21505032	2.291320931	9.286604361
0.141724665	7.063997864	23.89385475
0.105454545	11.7731449	36.60559441
0.172532567	3.819910306	14.60104987
0.159339947	3.733080403	15.64563107
0.16716912	3.528696925	14.45330296
0.177162621	3.543003077	13.70852018
0.043359029	41.03330259	196.2437112
0.148428039	5.485564118	18.44136926
0.174322869	2.897199138	12.64912281
0.504132231	0.247933884	2.363636364
0.300243948	2.131919685	6.698630137
0.052257425	47.87957877	141.7509804
0.151968179	3.659624774	16.91066998
0.088463635	19.27427098	55.78309859
0.162374608	3.1296721	15.9874477
0.042512912	47.86021768	191.5122944
0.185707284	3.622134106	12.87462687
0.203344626	3.06186348	10.76376147
0.266242063	1.806773326	6.943231441
0.15129752	6.109802419	19.97175141
0.127558069	5.739155262	26.08583691
0.11289947	15.84036372	43.97044674
0.060447214	39.78114045	112.237013
0.056205895	38.35994389	122.473245
0.071795395	34.24015942	95.43434343
0.228195087	1.714489142	8.396226415
0.131346149	7.614724995	24.18923611
0.253514739	1.485351474	7.019047619
0.070580093	37.69201293	97.49437554
0.159567705	3.479778962	14.88811189
0.085195736	20.78693873	60.11495536
0.088093047	20.59290244	56.64370912
0.16240369	3.608017693	17.20811287
0.046321027	47.70760822	165.5917529
0.045010087	43.39190623	173.2265091
0.043495312	44.22864177	168.738825
0.203362223	3.010396348	10.86547085
0.15160434	5.912203755	19.42105263
0.164620536	2.924027423	16
0.139313002	6.163564144	21.82365827
0.06576248	37.00385961	99.65014793
0.217602811	2.633755236	9.590604027
0.160525243	6.462614855	18.37109827
0.059513832	39.19375728	110.2091702
0.571279917	0.283038502	2.032258065
0.054466099	41.02548543	126.802499
0.083592235	18.87843345	56.07556316
0.147862638	4.233019328	18.09589041
0.204844907	2.41325835	9.709677419
0.2416	1.6464	7.6
0.573964497	0.213017751	1.923076923
0.068035738	36.95659932	96.20034996
0.189188019	2.132920706	12.23026316
0.125543177	13.64571006	33.37980769
0.064680458	32.18933256	98.87185104
0.049030774	41.25833523	146.8964276
0.366087176	1.096497858	4.428571429
0.172192907	3.362491349	13.90588235
0.291666667	1.083333333	7.333333333
0.257631074	1.812563514	7.049079755
0.157780612	3.703010204	15.50714286

log.sigma.3.0.mm.3D_gldm_LargeDependenceLowGrayLevelEmphasis	log.sigma.3.0.mm.3D_gldm_SmallDependenceHighGrayLevelEmphasis	log.sigma.3.0.mm.3D_gldm_LargeDependenceHighGrayLevelEmphasis
0.14690207	33.75840709	2189.277008
0.457766982	17.49406021	19790.45688
0.584784158	19.39705461	798.9717514
0.296631272	46.16656021	10293.95292
0.206201713	62.22979098	570.5049505
0.565141751	15.46307475	985.8811475
0.441422004	26.77886905	268.6142857
0.181427734	51.00643038	1031.650873
0.103877246	103.534105	2747.5271
0.461317544	21.89033963	13111.44718
0.134552459	49.23897233	2298.731383
0.312602297	24.60847692	3602.735799
0.589427918	16.56721002	3154.234993
0.450518125	30.4915298	1090.043561
0.273239292	49.72279147	602.543956
1.248395242	8.490981055	38623.65432
0.371424035	50.95663158	460.8294931
0.786110798	12.92100747	3102.421392
0.752336846	50.98370156	495.2637363
1.026750219	6.270677455	14264.98378
0.847580192	10.18837243	22296.05517
0.405130261	27.66279448	2071.633663
0.756945199	10.67003033	19530.68459
0.056426478	144.1490226	2466.315789
0.40654626	13.75892543	7911.0125
0.221742993	27.42989708	1862.344371
1.235241608	11.25290121	10041.91699
1.038660104	7.982206506	28570.83543
0.634102339	19.24668626	29787.65743
0.550186223	15.08276844	23849.65623
0.96219909	9.404015208	18070.78577
1.182850657	9.809933376	23588.35243
0.353985217	30.51750459	8917.900648
0.108999713	55.33333333	320.3333333
0.256841862	71.50650688	581.1061947
0.328409059	24.18871132	5244.458021
0.962471942	7.816368856	29626.99567
0.708659749	16.71767328	13245.53991
0.167753726	64.14017843	1003.922118
0.303276129	22.18112914	3223.124767
0.465277665	21.53673831	5504.397203
0.211900355	34.73591893	1284.165354
0.237019565	29.36571891	1614.413835
0.220889538	42.72382947	1452.519362
0.117933572	59.50513573	2316.063677
3.123925291	1.497313355	13937.78651
0.141184529	73.66288941	3004.140932
0.734554664	21.20960708	436.1184211
0.077891764	92.70454545	348.7272727
0.37014382	99.36439685	367.6164384
0.887864568	12.38025598	23750.33088
0.554058683	17.43402423	996.5012407
0.263998865	26.57652431	13371.99437
0.314591591	21.40654078	1228.723849
1.574249701	5.639193458	24321.67663
0.38667334	34.4276673	986.5343284
0.104632236	78.20523437	1626.65711
0.078156086	108.7946705	1166.530568
0.178731998	37.53921053	3107.30226
0.514320575	15.19594616	1913.502146
0.4355406	12.78023588	6502.672852
0.522683243	26.67104325	25668.41057
1.246349276	8.27631254	12844.892
1.113403807	6.440190175	9724.088664
0.362031245	31.13314465	571.1132075
0.168175275	43.92173145	4641.297743
0.203527107	56.02454233	551.4857143
0.67197021	15.71586596	17189.54802
0.742882884	18.30577469	408.5594406
0.374188455	19.86622346	11113.2779
0.307467762	25.41167447	12224.17661
0.329844445	16.27797125	1581.222222
1.157058009	7.161342299	25156.07014
1.450326665	4.89458069	21745.54938
0.95177113	14.10612831	30783.40065
0.158757204	56.52966454	1201.580717
0.211775619	33.79529597	2291.212719
1.099977997	10.60650027	395.65625
0.213826363	35.67364329	4256.815991
0.563895082	20.01903154	18938.70673
0.47379183	37.96412483	503.0604027
0.120358505	68.68084718	4064.712139
0.425331339	31.85475801	29827.97606
0.067346628	124.6451613	265.1290323
0.920992767	10.76431246	18614.54696
0.426452091	25.88651622	8048.285429
0.660751133	16.91395611	924.230137
0.102933297	75.37803595	1391.767402
0.25803898	33.09804444	436.4
0.109956336	64.26923077	182.5384615
0.310320382	35.26646775	31596.8014
0.570301627	16.9616791	779.3453947
0.292119748	30.34809877	4727.139423
0.647572699	10.68012466	16688.49343
1.278368309	6.968040584	18118.68707
0.083959074	91.57149912	483.3650794
0.182100337	51.51082615	1779.436765
2.800120692	25.27951389	364.3333333
0.060405848	132.1634526	1442.165644
0.219095817	26.02233015	1983.292857

log.sigma.3.0.mm.3D_gldm_SmallDependenceLowGrayLevelEmphasis	log.sigma.3.0.mm.3D_gldm_LowGrayLevelEmphasis	log.sigma.3.0.mm.3D_gldzm_DistanceZoneVariabilityNormalized
0.006269491	0.013555379	0.938836565
0.002177346	0.008815245	0.933412604
0.011534424	0.038987577	1
0.005095136	0.01346887	0.87600787
0.019837534	0.037790278	1
0.010078769	0.035394074	0.935555556
0.024060791	0.053524391	1
0.007708709	0.017514584	0.947059096
0.004069687	0.010739523	0.530229819
0.002231856	0.010596155	0.875306343
0.00462871	0.010815545	0.915512465
0.003838477	0.017313413	0.973338203
0.005733842	0.024210359	0.937028464
0.005741531	0.027663783	0.960622222
0.012207702	0.028139395	0.950648934
0.000492595	0.006866724	0.96748856
0.01098753	0.034538759	0.980771043
0.007285275	0.038303319	0.895061728
0.014172659	0.040497001	1
0.00165242	0.014173165	0.972608025
0.001276113	0.008844297	0.978439219
0.005547479	0.02479006	0.947664222
0.001523804	0.010313299	1
0.010133888	0.013924752	1
0.003176398	0.012131806	1
0.006466467	0.022465121	0.958352196
0.003905914	0.019301555	0.966676242
0.001314251	0.009074637	0.978650173
0.001864772	0.008913603	0.930674254
0.002290868	0.009393735	0.946848219
0.001522429	0.012528116	0.967435639
0.001159961	0.01042404	0.936755254
0.003982538	0.014129469	0.910161704
0.079299879	0.085239846	1
0.013398314	0.04498701	1
0.003661146	0.016035023	0.869119429
0.001366841	0.008691081	0.891026274
0.002199388	0.01187808	0.976747405
0.009023082	0.019316799	0.917959184
0.006426951	0.020908972	0.856866825
0.003917913	0.020970704	1
0.007062195	0.019052312	0.891830153
0.009203028	0.023471889	0.956001528
0.004332719	0.018475477	0.956323731
0.002770038	0.010102765	0.712869865
0.002462519	0.027699462	0.927626249
0.0026314	0.00862639	0.655905279
0.013664619	0.050237737	0.834710744
0.062619926	0.065674294	1
0.014848054	0.036125951	1
0.001851873	0.009878537	0.951642647
0.00891267	0.032599902	0.738548199
0.001653327	0.007291116	0.838704012
0.01071822	0.026947715	1
0.001266744	0.011843331	0.968879376
0.023662283	0.054292866	0.952783559
0.00509127	0.011999853	0.973688889
0.006469069	0.011548738	0.936576189
0.007147729	0.015714144	0.979800083
0.006311461	0.025473296	0.972227733
0.003855664	0.020208913	0.905891579
0.001350735	0.007338537	0.910183184
0.001628811	0.013843338	0.944116854
0.003389616	0.021836468	0.947406866
0.02700876	0.057856878	1
0.003357467	0.010900692	0.938418509
0.014252678	0.02767417	1
0.002452091	0.014387291	0.804943311
0.023265894	0.05901266	0.953514739
0.00309639	0.011121661	0.944489796
0.00171625	0.008923648	0.971569896
0.00633105	0.025025812	0.795369169
0.001255822	0.010658613	0.946776349
0.001166797	0.01215249	0.941960698
0.000716048	0.007170424	0.904617118
0.007621883	0.021470441	0.977903311
0.006854822	0.016946916	1
0.028676747	0.081421751	0.8872
0.004384883	0.018517547	0.981568415
0.002277114	0.00958845	0.961970826
0.012533209	0.050994634	1
0.004747135	0.010940144	0.942542417
0.001121864	0.005706222	0.882303408
0.047364752	0.050564632	1
0.001392173	0.010807421	0.939934254
0.001537326	0.009600317	0.98510326
0.022110075	0.054256763	0.880488909
0.005890562	0.012992586	0.968387607
0.028516411	0.046771926	1
0.090516949	0.094404826	0.702479339
0.001411522	0.005278033	0.898786393
0.014048364	0.058613487	0.934142562
0.009967067	0.01992944	0.932854442
0.00226233	0.011870156	0.980584391
0.001696224	0.013685542	0.946592023
0.026069903	0.032705893	1
0.003795248	0.015661052	0.890779248
0.018621337	0.192438588	1
0.009275807	0.013082521	1
0.01148757	0.029574595	1

log.sigma.3.0.mm.3D_gldzm_LowIntensityEmphasis	log.sigma.3.0.mm.3D_gldzm_LargeDistanceEmphasis	log.sigma.3.0.mm.3D_gldzm_HighIntensitySmallDistanceEmphasis
0.025075711	1.094736842	141.1342105
0.019033736	1.103448276	126.625
0.046482299	1	78.83333333
0.023272318	1.209109731	197.6442949
0.045348359	1	140.8235294
0.048015769	1.1	72.55833333
0.056316459	1	59.56756757
0.022131225	1.081632653	146.8112245
0.012112787	2.280487805	178.7021027
0.019512206	1.264026403	151.8969564
0.016256124	1.175438596	174.8540448
0.026448987	1.040540541	127.0405405
0.040330575	1.12037037	109.3414352
0.025563485	1.093333333	118.9683333
0.03135974	1.075949367	124.5332278
0.012155368	1.049586777	172.3264463
0.029965537	1.029126214	117.7014563
0.055672907	1.166666667	80.90918803
0.035231902	1	124.175
0.028815135	1.041666667	91.28819444
0.017637559	1.032697548	121.4046322
0.030613479	1.102678571	122.0558036
0.02200267	1	121.0714286
0.019994918	1	318.6527778
0.027396771	1	104.7948718
0.028472027	1.063829787	97.86702128
0.039380905	1.050847458	106.9830508
0.020215005	1.032374101	102.0773381
0.018513737	1.118834081	154.743149
0.02282914	1.081967213	122.7062842
0.025119994	1.049668874	116.5347682
0.017420742	1.169520548	139.8531678
0.020990012	1.157718121	145.3926174
0.121919802	1	109.5
0.039015814	1	159.3220339
0.020581384	1.228782288	118.7979705
0.022360102	1.173493976	103.1572289
0.020155607	1.035294118	150.1044118
0.023314231	1.128571429	166.8803571
0.032649869	1.232758621	102.0625
0.02794423	1	114.3445946
0.026217129	1.172131148	122.2459016
0.039287656	1.089686099	111.5358744
0.01967243	1.103703704	154.3722222
0.011253456	1.54519774	176.9073289
0.098287838	1.236024845	35.37025535
0.009591198	2.164053076	191.9595815
0.045675034	1.272727273	67.90584416
0.082355122	1	142.0588235
0.027739149	1	176.2045455
0.022311693	1.074380165	126.9703857
0.038094908	1.463917526	69.68556701
0.01229861	1.337690632	161.6220044
0.047962716	1	99.15517241
0.027455332	1.04743083	101.7643281
0.080252729	1.072580645	103.4798387
0.013944351	1.04	206.906
0.013467318	1.098360656	242.9815574
0.027853082	1.030612245	146.6836735
0.044295252	1.042253521	103.5774648
0.034224882	1.148514851	81.08663366
0.011595509	1.158075601	208.2505727
0.022607701	1.103942652	107.6353047
0.048668628	1.081081081	65.47635135
0.075414982	1	80.17391304
0.015923722	1.09540636	181.3507067
0.03377394	1	134.5294118
0.02175236	1.328571429	121.6845238
0.084735231	1.071428571	64.38690476
0.024573215	1.085714286	135.0418367
0.012799198	1.043269231	158.9188702
0.036253718	1.347107438	72.39876033
0.02200824	1.094292804	104.3083816
0.026981874	1.08974359	86.05395299
0.011132693	1.271991911	196.4994944
0.025405216	1.033519553	154.8226257
0.026058428	1	128.0217391
0.131566284	1.18	48.305
0.024663557	1.027906977	149.7174419
0.018053609	1.058171745	145.0152355
0.041226954	1	89.64285714
0.015491895	1.088815789	214.5148026
0.009183639	1.195522388	231.7533582
0.057309841	1	158.8076923
0.018257726	1.215384615	123.2871795
0.011656237	1.022514071	173.4924953
0.094804213	1.191489362	65.3856383
0.015890519	1.048192771	195.7560241
0.062222495	1	74.48
0.108506164	1.545454545	84.11363636
0.010176631	1.16039604	230.609901
0.072004505	1.102272727	69.46875
0.039176375	1.104347826	118.5804348
0.030587888	1.029411765	110.0923203
0.0300114	1.093607306	99.17865297
0.041613408	1	154.7619048
0.015942845	1.173913043	186.5362319
0.126598839	1	71.3
0.017689288	1	269.8131868
0.049816523	1	99.25882353

log.sigma.3.0.mm.3D_gldzm_LowIntensityLargeDistanceEmphasis	log.sigma.3.0.mm.3D_gldzm_HighIntensityEmphasis	log.sigma.3.0.mm.3D_gldzm_DistanceZoneVariability	log.sigma.3.0.mm.3D_gldzm_ZonePercentage
0.025784535	144.3789474	89.18947368	0.263157895
0.021623816	129.8965517	162.4137931	0.124019957
0.046482299	78.83333333	54	0.305084746
0.034143579	202.610766	423.1118012	0.249870667
0.045348359	140.8235294	51	0.504950495
0.049046277	75.08333333	56.13333333	0.245901639
0.056316459	59.56756757	37	0.528571429
0.022609853	150.7755102	139.2176871	0.366583541
0.017677074	317.0853659	304.3519164	0.388888889
0.020488097	167.0891089	265.2178218	0.130659767
0.017312634	181.6403509	104.3684211	0.303191489
0.027214752	127.7297297	144.0540541	0.195508587
0.045625587	111.3657407	202.3981481	0.158125915
0.026299145	120.9266667	144.0933333	0.284090909
0.039461592	126.2088608	150.2025316	0.434065934
0.012638009	173.7272727	468.2644628	0.044698929
0.033201783	117.7669903	101.0194175	0.474654378
0.061238829	83.72649573	209.4444444	0.150773196
0.035231902	124.175	80	0.43956044
0.050073638	91.54861111	140.0555556	0.051910598
0.017981256	122.8065395	359.0871935	0.072315271
0.033413465	123.3705357	212.2767857	0.246424642
0.02200267	121.0714286	224	0.076712329
0.019994918	318.6527778	72	0.541353383
0.027396771	104.7948718	117	0.121875
0.029129797	99.4787234	90.08510638	0.311258278
0.039925662	108.4449153	228.1355932	0.103101791
0.020501288	103	272.0647482	0.071373556
0.019516133	158.441704	415.0807175	0.120021529
0.023281903	126.6775956	173.273224	0.108092144
0.025574089	118.0198675	292.1655629	0.071887646
0.020984975	141.8972603	547.0650685	0.066115702
0.023312769	148.9530201	271.2281879	0.214542837
0.121919802	109.5	10	0.666666667
0.039015814	159.3220339	59	0.522123894
0.022763003	125.195572	235.5313653	0.203148426
0.024597242	107.3662651	369.7759036	0.062023614
0.020863668	150.6205882	332.0941176	0.112137203
0.026331757	169.05	128.5142857	0.436137072
0.035135504	108.5862069	99.39655172	0.216014898
0.02794423	114.3445946	148	0.206993007
0.029932098	128.147541	108.8032787	0.320209974
0.039799942	114.5515695	213.1883408	0.270631068
0.020180675	157.8814815	129.1037037	0.307517084
0.015196908	216.4519774	252.3559322	0.317488789
0.10207032	37.47204969	149.3478261	0.026298595
0.013825959	272.8564536	543.7454765	0.301893664
0.091347533	71.83116883	64.27272727	0.337719298
0.082355122	142.0588235	17	0.772727273
0.027739149	176.2045455	88	0.602739726
0.022830757	130.1212121	345.446281	0.088970588
0.042728337	82.96907216	71.63917526	0.240694789
0.014215456	176.4705882	384.9651416	0.143661972
0.047962716	99.15517241	58	0.242677824
0.027997065	102.9664032	490.2529644	0.044754997
0.130151923	103.5887097	118.1451613	0.370149254
0.01418627	208.896	365.1333333	0.430045872
0.026757969	245.5081967	228.5245902	0.532751092
0.028703422	146.9591837	96.02040816	0.276836158
0.044955463	104.2535211	69.02816901	0.152360515
0.036854884	84.65841584	182.990099	0.138831615
0.018203936	211.6861397	794.5899198	0.128837072
0.024114972	109.7741935	263.4086022	0.068483063
0.050129432	66.62162162	140.2162162	0.08305275
0.075414982	80.17391304	23	0.433962264
0.018499615	183.3674912	265.5724382	0.245659722
0.03377394	134.5294118	51	0.485714286
0.027036035	129.4666667	338.0761905	0.121142198
0.085617065	65.83333333	40.04761905	0.293706294
0.025753019	136.9795918	231.4	0.13671875
0.013079565	160.6153846	404.1730769	0.160061562
0.04024425	82.12396694	96.23966942	0.21340388
0.023425726	106.1141439	381.5508685	0.057106419
0.028079136	87.98504274	440.8376068	0.043197342
0.013339677	202.4651163	894.6663296	0.068117639
0.025565213	156.5865922	175.0446927	0.401345291
0.026058428	128.0217391	138	0.302631579
0.133844525	51.98	44.36	0.223214286
0.024817313	150.9906977	211.0372093	0.235487404
0.018499643	147.4709141	347.2714681	0.133505917
0.041226954	89.64285714	70	0.469798658
0.016223702	218.3486842	286.5328947	0.351445087
0.015795451	238.4537313	591.1432836	0.135930209
0.057309841	158.8076923	26	0.838709677
0.020002811	126.2410256	366.574359	0.07859734
0.011846824	174.5140713	525.0600375	0.151981751
0.09653023	71.10638298	82.76595745	0.257534247
0.016409468	197.0843373	241.1285141	0.422750424
0.062222495	74.48	25	0.5
0.398278891	85.27272727	7.727272727	0.846153846
0.01096697	239.829703	453.8871287	0.147273258
0.074179878	70.73863636	82.20454545	0.289473684
0.04249441	119.9043478	107.2782609	0.276442308
0.030856983	111.0163399	300.0588235	0.083789704
0.031050154	101.260274	414.6073059	0.06064802
0.041613408	154.7619048	42	0.666666667
0.035736301	189.2028986	184.3913043	0.304411765
0.126598839	71.3	10	0.416666667
0.017689288	269.8131868	91	0.558282209
0.049816523	99.25882353	85	0.303571429

log.sigma.3.0.mm.3D_gldzm_IntensityVariabilityNormalized	log.sigma.3.0.mm.3D_gldzm_LowIntensitySmallDistanceEmphasis	log.sigma.3.0.mm.3D_gldzm_IntensityVariability
0.082770083	0.024898506	7.863157895
0.075307174	0.018386216	13.10344828
0.086419753	0.046482299	4.666666667
0.0518027	0.020563489	25.02070393
0.058054594	0.045348359	2.960784314
0.100555556	0.047758143	6.033333333
0.120525931	0.056316459	4.459459459
0.068443704	0.022011569	10.06122449
0.039140939	0.010857003	22.46689895
0.066496749	0.019324704	20.14851485
0.087257618	0.016025722	9.947368421
0.070124178	0.026257545	10.37837838
0.075617284	0.039070329	16.33333333
0.078222222	0.025431011	11.73333333
0.070501522	0.029334277	11.13924051
0.077837921	0.012034708	37.67355372
0.075878971	0.029156475	7.815533981
0.078311053	0.054281426	18.32478632
0.0665625	0.035231902	5.325
0.08757716	0.023500509	12.61111111
0.077935095	0.017551634	28.60217984
0.067004145	0.02994793	15.00892857
0.074019452	0.02200267	16.58035714
0.053626543	0.019994918	3.861111111
0.083205493	0.027396771	9.735042735
0.08669081	0.028307584	8.14893617
0.072033898	0.039244715	17
0.091040836	0.020143434	25.30935252
0.064107462	0.01827287	28.59192825
0.074322912	0.022715949	13.6010929
0.072014385	0.025006471	21.74834437
0.079676065	0.016671198	46.53082192
0.064321427	0.020425894	19.16778523
0.1	0.121919802	1
0.056018386	0.039015814	3.305084746
0.081984178	0.020054201	22.21771218
0.089052112	0.021800817	36.95662651
0.073148789	0.019978592	24.87058824
0.062244898	0.022559849	8.714285714
0.079072533	0.032028461	9.172413793
0.073228634	0.02794423	10.83783784
0.077936039	0.025288387	9.508196721
0.068229806	0.039181729	15.21524664
0.072702332	0.019581948	9.814814815
0.05996042	0.010293804	21.2259887
0.110836773	0.09776193	17.8447205
0.051220751	0.0089172	42.46200241
0.100354191	0.03425691	7.727272727
0.08650519	0.082355122	1.470588235
0.061983471	0.027739149	5.454545455
0.070115126	0.022181927	25.45179063
0.095759379	0.03693655	9.288659794
0.071458746	0.011866449	32.79956427
0.07372176	0.047962716	4.275862069
0.082574325	0.027319898	41.7826087
0.061524454	0.067777931	7.629032258
0.057578667	0.013883872	21.592
0.052875571	0.010144655	12.90163934
0.065597668	0.027640497	6.428571429
0.077563975	0.044130199	5.507042254
0.081168513	0.033567382	16.3960396
0.054986754	0.009963436	48.00343643
0.091275806	0.022258539	25.46594982
0.095416362	0.048303427	14.12162162
0.088846881	0.075414982	2.043478261
0.063491865	0.015279749	17.96819788
0.07420223	0.03377394	3.784313725
0.070419501	0.020431441	29.57619048
0.099773243	0.084514772	4.19047619
0.069154519	0.024278264	16.94285714
0.075859837	0.012729106	31.55769231
0.085035175	0.035256085	10.2892562
0.079287478	0.021670183	31.9528536
0.084383447	0.026707559	39.49145299
0.065064547	0.010734975	64.34883721
0.061764614	0.025365217	11.05586592
0.071938668	0.026058428	9.927536232
0.0872	0.130996724	4.36
0.064705246	0.024625117	13.91162791
0.064954996	0.0179421	23.44875346
0.08122449	0.041226954	5.685714286
0.052263677	0.015308943	15.88815789
0.060209401	0.007538056	40.34029851
0.085798817	0.057309841	2.230769231
0.076831032	0.017968629	29.96410256
0.078549328	0.011608591	41.86679174
0.077184246	0.094372708	7.255319149
0.057692618	0.015760782	14.36546185
0.1008	0.062222495	2.52
0.173553719	0.036062982	1.909090909
0.064123125	0.009979047	32.38217822
0.078254132	0.071460662	6.886363636
0.065557656	0.038346866	7.539130435
0.073988637	0.030520614	22.64052288
0.072767457	0.029769328	31.87214612
0.06122449	0.041613408	2.571428571
0.061541693	0.010994481	12.73913043
0.14	0.126598839	1.4
0.056394155	0.017689288	5.131868132
0.072387543	0.049816523	6.152941176

log.sigma.3.0.mm.3D_gldzm_HighIntensityLargeDistanceEmphasis	log.sigma.3.0.mm.3D_gldzm_SmallDistanceEmphasis	log.sigma.3.0.mm.3D_glcm_SumVariance	log.sigma.3.0.mm.3D_glcm_Homogeneity1
157.3578947	0.976315789	386.5276801	0.410830111
142.9827586	0.974137931	482.3773263	0.560639818
78.83333333	1	125.1969518	0.432597889
224.8322981	0.950023004	436.3696017	0.439596371
140.8235294	1	228.1566716	0.306603236
85.18333333	0.975	105.3155465	0.443972114
59.56756757	1	106.4036123	0.375192826
166.6326531	0.979591837	266.9685614	0.362741915
1023.108014	0.746842334	912.8655189	0.294026657
244.8580858	0.948661533	423.8859934	0.541445302
220.0526316	0.96588694	397.7993639	0.406952356
130.4864865	0.989864865	284.4277862	0.464594104
121.1296296	0.97505144	170.2273467	0.474133529
133.0266667	0.984074074	153.9027671	0.417327596
132.9113924	0.981012658	183.0567861	0.348161161
179.3305785	0.987603306	535.0799897	0.732379996
118.0291262	0.992718447	150.8545652	0.348509975
94.9957265	0.958333333	144.0343457	0.473565394
124.175	1	123.2359712	0.375533195
92.59027778	0.989583333	273.4257458	0.619412794
128.4141689	0.991825613	415.7152577	0.641873284
131.4866071	0.979290675	208.3622885	0.425970737
121.0714286	1	389.1603235	0.623161943
318.6527778	1	973.5597476	0.307763261
104.7948718	1	338.5632092	0.562005224
105.9255319	0.984042553	249.9767197	0.414376569
114.2923729	0.987288136	214.3863666	0.602585463
106.6906475	0.991906475	441.8603188	0.684992445
175.7869955	0.97278276	541.4266437	0.60833345
142.5628415	0.979508197	490.4100879	0.587312184
123.9602649	0.987582781	333.4279177	0.626263799
158.3613014	0.974743151	367.2007367	0.662221374
166.5503356	0.964299031	336.1958931	0.456222817
109.5	1	268.8709302	0.288770394
159.3220339	1	262.2310918	0.306135654
154.4760148	0.946904469	266.4114364	0.442245292
124.2024096	0.956626506	470.0693915	0.673393205
152.6852941	0.991176471	322.6433176	0.565562878
177.7285714	0.967857143	301.1837254	0.332717913
134.6810345	0.941810345	254.9283133	0.430308504
114.3445946	1	319.1909976	0.49627824
151.7540984	0.956967213	236.2653867	0.393740423
131.0986547	0.982561036	241.2139278	0.393697488
179.3259259	0.982304527	273.4288818	0.386763488
380.6694915	0.869978029	513.1537322	0.353620395
54.10559006	0.968599034	168.7781581	0.732172253
927.6393245	0.835477818	557.1061611	0.367765809
87.53246753	0.931818182	90.38094337	0.382642315
142.0588235	1	343.9266473	0.243221071
176.2045455	1	230.6992912	0.290453393
142.7245179	0.981404959	411.4854186	0.629660403
136.1030928	0.884020619	139.3139881	0.400398978
260.3529412	0.932522392	565.9495979	0.504855537
99.15517241	1	182.1778766	0.436342985
107.7747036	0.988142292	341.1818818	0.699113135
104.0241935	0.981854839	170.0380172	0.363782546
216.856	0.99	462.4921196	0.345718885
255.6147541	0.975409836	526.3894115	0.286872013
148.0612245	0.992346939	327.1501879	0.419454817
106.9577465	0.98943662	173.3922021	0.472911634
98.94554455	0.962871287	243.6640221	0.481395749
228.4524628	0.964299351	560.7159778	0.574228253
120.6236559	0.977996814	257.7742631	0.626399988
71.2027027	0.97972973	209.9533708	0.591742114
80.17391304	1	150.5685327	0.362151778
191.434629	0.97614841	432.767613	0.423338757
134.5294118	1	217.811903	0.316893004
160.5952381	0.917857143	354.8919992	0.551139214
71.61904762	0.982142857	69.80552969	0.421621097
144.7306122	0.978571429	403.3274665	0.521955536
167.4014423	0.989182692	476.9467142	0.514318182
121.0247934	0.91322314	188.0550078	0.415141093
115.2009926	0.979183899	366.9574681	0.662043733
95.70940171	0.977564103	318.9072404	0.685690051
248.132457	0.960299405	509.9609952	0.663855521
163.6424581	0.991620112	286.7675201	0.349360932
128.0217391	1	283.8261752	0.410735992
66.68	0.955	57.06486385	0.434791072
156.0837209	0.993023256	435.3997685	0.423732611
157.2936288	0.985457064	433.6028872	0.573964735
89.64285714	1	132.2746798	0.381294458
233.6842105	0.977796053	538.1623242	0.380908388
266.7477612	0.952777778	718.3031187	0.576881761
158.8076923	1	338.8128689	0.242768984
160.6717949	0.975	345.831394	0.619890857
178.6003752	0.994371482	369.9873929	0.52523538
93.9893617	0.95212766	108.9647964	0.419211501
202.3975904	0.987951807	401.9124252	0.338707728
74.48	1	131.418237	0.359763343
89.90909091	0.863636364	184.7415186	0.276378835
276.7089109	0.95990099	825.9799257	0.556793084
75.81818182	0.974431818	144.6576287	0.381373119
125.2	0.973913043	279.5366873	0.454994103
114.7124183	0.992647059	372.7753071	0.588010522
111.0479452	0.979134957	295.4889667	0.64760881
154.7619048	1	323.7305054	0.251823333
199.8695652	0.956521739	318.680394	0.367171021
71.3	1	122.6105431	0.326627324
269.8131868	1	607.2297431	0.315015572
99.25882353	1	279.303031	0.432438409

log.sigma.3.0.mm.3D_glcm_Homogeneity2	log.sigma.3.0.mm.3D_glcm_ClusterShade	log.sigma.3.0.mm.3D_glcm_MaximumProbability	log.sigma.3.0.mm.3D_glcm_Idmn	log.sigma.3.0.mm.3D_glcm_SumVariance2
0.327038055	-10.82585076	0.023208826	0.979370001	37.77257154
0.508561266	-108.2177228	0.17890803	0.986615971	26.21937938
0.352727558	44.05623273	0.036641933	0.978973777	23.89180891
0.367003496	-31.99976764	0.083393163	0.985142014	41.13128156
0.212873952	245.173717	0.030592654	0.954643177	53.79705626
0.369223793	5.410584071	0.056849613	0.958108888	11.3251881
0.287823003	-22.42157593	0.062952326	0.919275254	15.74113077
0.272360975	48.17567859	0.024804212	0.977120033	30.68028853
0.206400812	1340.47526	0.009058333	0.96955972	201.7196669
0.486807789	-46.39030305	0.11578594	0.991832478	20.86806338
0.322897276	-0.195080489	0.033598031	0.976342104	18.12410534
0.393859876	-37.04321422	0.033792666	0.985135032	27.20052869
0.407506044	18.33655835	0.039231721	0.979758111	18.51325241
0.338690039	82.65006185	0.034624601	0.977028012	21.79995014
0.256609641	129.7473258	0.028315743	0.971148454	31.88432218
0.7158164	-62.28627529	0.299316376	0.99697879	9.407442964
0.260289184	90.16519128	0.038168591	0.959389925	27.2171723
0.405073848	-11.65994475	0.045669055	0.980001361	23.13853273
0.294912672	194.1777379	0.049386976	0.955889791	33.01345343
0.581811554	-56.32266905	0.135733029	0.988603705	20.17755428
0.608700942	-45.50154003	0.236690294	0.995281734	12.77597022
0.348947099	20.5413811	0.0366402	0.97895169	25.02355833
0.585616771	-78.2361489	0.174598909	0.993613514	16.98446207
0.213764348	-785.7127747	0.030109718	0.968188302	124.4980165
0.514447658	-49.14665238	0.093387267	0.991810289	16.5784108
0.332743067	-2.300199742	0.028860572	0.967450785	28.92026597
0.561594583	-25.86449548	0.202424448	0.993428966	14.36709606
0.656932252	-85.51716979	0.317640436	0.996740867	15.48851127
0.565551923	-166.9435683	0.254156817	0.991521752	28.01084211
0.539535731	-130.163083	0.212916155	0.989724054	26.6044919
0.589727858	-61.48601321	0.202529405	0.993554464	15.84788946
0.63239031	-64.15047976	0.248902973	0.994821765	13.27586043
0.384046763	-45.40000333	0.094875613	0.984757951	35.27707208
0.191060642	83.42059704	0.166346154	0.916136309	34.23554487
0.211835091	125.4283596	0.030872185	0.957804261	64.67557862
0.366639688	-2.983021617	0.053455128	0.976749064	27.77162892
0.646016179	-108.4161926	0.21715499	0.993038959	16.86113502
0.517173411	-37.93758505	0.131929547	0.991809397	20.10946131
0.241930054	144.4778244	0.02049266	0.966438868	48.38466689
0.35125669	6.797523106	0.037957215	0.976949341	37.73591069
0.433761871	-118.3978092	0.063541522	0.986481838	31.81283327
0.307776696	8.395226984	0.036743756	0.975607137	21.90578894
0.308313026	-2.821718006	0.023898592	0.979636978	26.16324396
0.299982908	71.15385264	0.028195067	0.981549188	30.34606695
0.264555713	396.9765701	0.017110141	0.97726946	59.15727509
0.716871256	-44.44131187	0.23348423	0.993178359	8.348631296
0.285048031	1086.97208	0.01958512	0.987124921	73.75565747
0.295815184	36.51377614	0.03504394	0.952056063	19.17270356
0.145684747	25.24485574	0.084863747	0.901620692	41.1150964
0.201802957	229.3313792	0.02757306	0.935915665	64.64577799
0.59253447	-82.90246088	0.236043806	0.992285538	17.92145682
0.316948222	86.70643282	0.038184339	0.960724733	25.36683662
0.444539733	-95.689992	0.083862525	0.990429177	32.11607012
0.357857536	42.0120369	0.037841635	0.975002616	28.3780819
0.676433422	-81.48168672	0.276498658	0.99607632	13.44900572
0.275905297	6.633727725	0.029796808	0.956234256	32.55651218
0.256958493	90.86644105	0.020507647	0.974794866	48.90194287
0.193343368	213.6663302	0.017287647	0.961961497	75.1305388
0.339232397	-14.54308547	0.03229342	0.979109648	33.21328142
0.404368752	30.14387475	0.037589041	0.981751226	24.5971151
0.413912234	-4.580006943	0.046773817	0.97945396	29.36046225
0.528115878	-110.5782223	0.155335598	0.993003286	28.75008431
0.590270655	-45.26863469	0.177606692	0.991749833	13.55884697
0.546818507	-56.05349801	0.150186504	0.98522151	19.63903373
0.267466821	34.23970817	0.058965107	0.947308317	35.93272715
0.346710921	-72.48904151	0.033467262	0.985060674	39.09401934
0.21955414	48.33322252	0.033070443	0.942831671	33.65956178
0.498587117	-126.6646151	0.130785243	0.991225203	35.80639264
0.342877111	40.98835997	0.067900434	0.958964099	12.75354332
0.464683515	-71.21660269	0.089014979	0.988625213	26.55293261
0.455589633	-87.53125855	0.07447624	0.987780333	27.79696832
0.333566022	14.68062145	0.024058445	0.964126532	26.10611172
0.631034895	-82.96032371	0.184000684	0.994401271	17.08037328
0.661542337	-67.09456675	0.251895367	0.994789266	12.89804842
0.636712724	-57.95209775	0.205556544	0.996709918	14.12997746
0.259468055	32.08219457	0.025079119	0.971651263	32.79275448
0.328277861	-24.76784218	0.0414564	0.972307187	24.19809063
0.357052185	20.74840084	0.047231702	0.965085546	12.38113128
0.344323407	-81.07459457	0.027931072	0.981203172	33.15313417
0.526878268	-71.77905179	0.16920067	0.99211755	21.91286376
0.296830933	31.72850098	0.034954437	0.950724458	27.20747798
0.298667254	31.26139536	0.026307468	0.97815075	51.53358482
0.532271143	-158.0292937	0.149002717	0.993136186	27.41019296
0.15054385	8.384993047	0.058103435	0.91641608	50.69318827
0.582094147	-58.93133402	0.162927109	0.993505507	15.79783702
0.469517156	-4.297183731	0.07282324	0.99024735	15.88143046
0.336546538	29.21708879	0.033431222	0.971671929	21.4175402
0.247413216	54.57925192	0.019323551	0.973309292	44.54975976
0.266219982	-0.641738989	0.077420062	0.926897059	18.78292974
0.183504861	31.33759853	0.126984127	0.863723839	30.28798186
0.506759137	-192.5422406	0.118261279	0.990758865	34.68803554
0.292159092	53.01493278	0.026107587	0.962460788	33.44353214
0.382953582	-39.05162736	0.07489125	0.976792077	25.4825081
0.544107822	-84.19153492	0.131296947	0.989282885	23.4381041
0.61580421	-68.47412679	0.218954042	0.993874296	15.59109294
0.156335658	255.9790962	0.044041652	0.944564123	50.54209813
0.279819265	90.26927646	0.019189518	0.975376604	42.58530726
0.231934092	40.07351319	0.127052862	0.887596997	32.46252324
0.222109212	142.457825	0.026093244	0.970467519	69.17230754
0.355088268	-89.22971938	0.037082107	0.972767748	33.67555865

log.sigma.3.0.mm.3D_glcm_Contrast	log.sigma.3.0.mm.3D_glcm_DifferenceEntropy	log.sigma.3.0.mm.3D_glcm_InverseVariance	log.sigma.3.0.mm.3D_glcm_Entropy	log.sigma.3.0.mm.3D_glcm_Dissimilarity
8.7877588	2.698553912	0.340173762	6.811308642	2.345659435
5.177192171	2.38361539	0.370635017	5.776044803	1.545597937
7.257767831	2.526460186	0.357402001	6.114856477	2.130687781
15.41791971	3.068149327	0.306677301	7.184507488	2.727631962
27.65326712	3.291589821	0.22222112	6.331487685	4.145819316
8.251595327	2.649465159	0.363122657	5.8752032	2.172828111
12.16730758	2.620696243	0.312934317	5.195411509	2.790570531
15.44548957	3.077162001	0.28089693	7.062274876	3.067380285
49.22398938	3.835372284	0.208554483	8.890183046	5.20119461
4.909568162	2.368907283	0.396554147	6.038061186	1.555353264
10.28195993	2.799646485	0.324953561	6.507049444	2.483154492
6.965756019	2.554645731	0.380413128	6.60122416	1.980030333
8.063001317	2.639102697	0.382120954	6.395503005	2.03166036
11.14987416	2.841618504	0.344900974	6.554524987	2.488182606
18.30008179	3.169164362	0.267727812	6.995504563	3.325136501
1.818112351	1.631355125	0.377558613	3.907117454	0.731539287
20.35951125	3.172680993	0.264823067	6.530597557	3.491810621
7.024277205	2.581215607	0.377640392	6.453360159	1.965059865
20.88130475	3.17714994	0.290686169	6.30973856	3.365597509
2.713475771	2.01491657	0.418666724	5.412046996	1.117147398
2.581100254	1.966734385	0.412735746	4.908639573	1.040765259
11.17824062	2.856616466	0.339427793	6.785914003	2.468063785
2.919150681	2.047197643	0.407199828	5.240960708	1.132437062
27.14850743	3.315738196	0.204846717	6.612037787	4.180288589
3.395659943	2.135049315	0.44216455	5.608225704	1.3307155
9.210549698	2.710790348	0.34109765	6.55690701	2.377089035
3.654787168	2.151827187	0.409662421	5.340396632	1.250571035
2.634884928	1.908654989	0.362310042	4.467892451	0.956327667
5.627364446	2.352031024	0.344495043	5.395958626	1.454142305
4.330097004	2.288813164	0.366851095	5.571933405	1.394564143
3.283404631	2.080650883	0.404193755	5.162155272	1.153487218
2.866269356	1.968982866	0.39203537	4.827324459	1.022417067
10.18851588	2.835180367	0.33096956	6.832611818	2.309361811
38.83846154	1.894168027	0.126576437	3.1570512	4.951282051
27.70778809	3.304491438	0.221631114	6.512481719	4.14452813
9.128276902	2.763768918	0.341081654	6.726556792	2.274735045
2.647348468	1.916786054	0.390276841	4.502323372	0.979296479
4.95501887	2.350261922	0.397612733	5.883626526	1.490064709
23.39743003	3.244073625	0.252891562	7.291010679	3.730144606
8.95291624	2.739523475	0.33500399	6.838349938	2.310416704
6.410235606	2.479582524	0.399936373	6.386984774	1.810452313
11.71794494	2.896273071	0.312581448	6.657793892	2.650331337
11.54011385	2.880958104	0.317975693	6.933497859	2.639330192
13.41600988	2.97351325	0.300326749	6.90829786	2.798284924
19.33433322	3.230178437	0.270924112	7.652056265	3.363947424
1.208572449	1.524468188	0.399251898	3.739327847	0.66602144
27.11105329	3.378345269	0.283432985	7.821961078	3.593396464
12.43680008	2.906865408	0.30608878	6.412157795	2.775588277
45.99739982	2.655261906	0.160065079	4.054687819	5.527730603
37.30682826	3.527104807	0.220265727	6.793816691	4.792657699
3.589833384	2.130817247	0.384040412	5.211322537	1.189563875
11.33054637	2.865475252	0.315705969	6.545859662	2.623352486
6.79732577	2.547933369	0.388964655	6.494095914	1.837827907
6.905486895	2.509742323	0.369714975	6.35930022	2.07653978
2.154325725	1.787880609	0.387553966	4.351744019	0.856380932
18.77168917	3.171031938	0.276213772	6.966452549	3.260188189
21.54581424	3.301597724	0.259364189	7.627959012	3.564307811
35.74265587	3.590008528	0.19532283	7.848959648	4.729142935
10.90978472	2.842383266	0.321573694	6.754691368	2.500324941
5.61276791	2.41360897	0.404499347	6.306027648	1.825031285
6.409345967	2.53965781	0.378477806	6.40847601	1.89354151
7.124991907	2.483096541	0.370733001	5.939089965	1.641936156
2.808852625	2.013665542	0.415073384	5.192217187	1.106808894
3.551409939	2.169366627	0.395865007	5.515230394	1.294711403
13.50304036	2.542839995	0.25175678	5.252843517	2.99199038
12.64972056	2.894461305	0.338430269	7.099455298	2.560573525
21.50499468	3.108285955	0.212655563	6.257160499	3.770038561
6.22774226	2.49529511	0.366885565	6.090040158	1.669536541
9.372537956	2.695806842	0.357913772	5.64488843	2.335167629
5.817989111	2.463357181	0.395884455	6.270438962	1.69995545
6.319572338	2.498852798	0.393110307	6.309487076	1.760890972
9.020149151	2.722268593	0.344522746	6.715462257	2.353551566
2.565158616	1.949129991	0.387181498	4.859337	1.001028927
1.953940132	1.786856144	0.407302404	4.459060494	0.864477789
3.743364153	1.999877706	0.400547149	4.818208291	1.076451682
19.69458426	3.200915288	0.271601472	7.139899647	3.391166307
10.99405883	2.866432981	0.31712888	6.704896504	2.541775579
7.74138384	2.595003452	0.36578286	5.888855886	2.152913114
9.746406183	2.760511446	0.339557473	6.837146818	2.376682788
5.646792208	2.384158093	0.383933448	5.72829043	1.521198799
13.05296455	2.852833049	0.330087646	6.282414674	2.790023814
20.39466231	3.229680525	0.294907915	7.533122847	3.240243609
6.993594945	2.44953541	0.38065223	5.810643063	1.615586011
46.42490864	2.978839658	0.2031359	4.669030607	5.508364285
3.284038269	2.099792558	0.405167533	5.258054398	1.175770345
5.928124133	2.457449975	0.403661561	6.109144285	1.686569843
8.941406834	2.726384704	0.329389683	6.529377756	2.341169459
21.30327099	3.289600559	0.2552996	7.518080382	3.570359713
15.1324887	2.733436096	0.251080337	4.970950453	3.109675841
31.94444444	2.507353404	0.202780397	3.631497105	4.674603175
7.17816468	2.530189513	0.367190345	6.098487049	1.715486572
12.08807258	2.883498186	0.291992221	6.748513488	2.762810566
10.39277658	2.787107603	0.340198743	6.446243678	2.301835077
3.692617102	2.183553654	0.412625669	5.75134463	1.300832499
2.54687559	1.958454511	0.410930493	4.898179932	1.02715207
47.7849529	3.377199444	0.165144598	5.750446752	5.511818759
16.75573203	3.132759697	0.287289911	7.414688829	3.133839283
28.98067509	2.542622874	0.121199909	3.734075818	4.358171956
25.20610735	3.299917429	0.228287186	6.814459575	3.984911878
8.641190771	2.652710024	0.355538263	6.436227976	2.252402179

log.sigma.3.0.mm.3D_glcm_DifferenceVariance	log.sigma.3.0.mm.3D_glcm_Idn	log.sigma.3.0.mm.3D_glcm_Idm	log.sigma.3.0.mm.3D_glcm_Correlation	log.sigma.3.0.mm.3D_glcm_Autocorrelation
3.2070134	0.900575909	0.327038055	0.621472571	142.1891057
2.696702642	0.930280787	0.508561266	0.667372896	166.7540779
2.616965742	0.899827587	0.352727558	0.527507724	54.47044639
7.816855638	0.924879556	0.367003496	0.453267428	156.3055282
10.07166877	0.858324656	0.212873952	0.322474459	83.77585297
3.45506379	0.868598544	0.369223793	0.159258739	45.57347797
4.023164533	0.814972691	0.287823003	0.141910567	42.98628832
5.938993409	0.897029982	0.272360975	0.329760178	101.6404962
21.47300009	0.889036645	0.206400812	0.610673846	299.2414423
2.451220859	0.942427794	0.486807789	0.614723754	150.1953256
4.012461714	0.896222915	0.322897276	0.274658175	140.5587617
2.91643184	0.918679479	0.393859876	0.588020371	109.1735974
3.828351932	0.910508937	0.407506044	0.388064507	69.98743716
4.850938053	0.901275227	0.338690039	0.324819778	63.6975306
7.049312318	0.886140557	0.256609641	0.271699696	72.86378488
1.269695958	0.972219425	0.7158164	0.671259615	169.7423078
7.409980861	0.868198546	0.260289184	0.16920661	60.25646527
3.066710997	0.908096697	0.405073848	0.533621267	62.08031974
9.176244861	0.869273639	0.294912672	0.230037019	49.56645373
1.434690259	0.935484306	0.581811554	0.761140768	103.4578003
1.47984969	0.9589839	0.608700942	0.66125945	142.2544393
4.921899453	0.905976461	0.348947099	0.376713144	82.98624751
1.600597304	0.951755958	0.585616771	0.703734786	136.9050973
9.188139242	0.877852208	0.213764348	0.644677088	313.0826507
1.578954255	0.940742509	0.514447658	0.65429646	122.9065573
3.39524862	0.879210047	0.332743067	0.514252813	96.88816095
2.062889704	0.951459751	0.561594583	0.591885274	82.61248419
1.69358131	0.968786534	0.656932252	0.705855822	147.2676723
3.441802281	0.949243877	0.565551923	0.662134454	181.2189344
2.344991641	0.939267374	0.539535731	0.716057665	168.4425195
1.925557989	0.953278363	0.589727858	0.653108805	119.0118734
1.797061426	0.960142817	0.63239031	0.638729011	126.9367662
4.761894884	0.920865624	0.384046763	0.550591358	125.3787097
11.74387821	0.811468937	0.191060642	-0.063099909	74.78910256
10.02763578	0.863181136	0.211835091	0.399323147	95.56866779
3.846136283	0.900260642	0.366639688	0.504657378	102.7022262
1.665546824	0.954574593	0.646016179	0.725784893	155.5951093
2.700191699	0.945130718	0.517173411	0.601737401	118.7727669
8.471841958	0.879359248	0.241930054	0.352873068	112.1096294
3.511497204	0.898238008	0.35125669	0.614758101	99.8557634
3.050188238	0.925580999	0.433761871	0.663154361	120.3615093
4.60658071	0.894783616	0.307776696	0.302885546	91.16333626
4.393392133	0.902855849	0.308313026	0.385016263	93.97661182
5.478464403	0.908379412	0.299982908	0.384287201	104.0176512
7.774566101	0.899417573	0.264555713	0.509693664	180.7532091
0.747627702	0.954793151	0.716871256	0.740369565	62.561551
13.85228526	0.929529815	0.285048031	0.468688155	192.4515832
4.573689511	0.85560982	0.295815184	0.208409326	40.42032838
14.33251012	0.793355463	0.145684747	-0.073158047	100.9349123
13.54557412	0.836416279	0.201802957	0.264350864	83.39751053
2.14660246	0.950132895	0.59253447	0.663532758	142.377384
4.284522031	0.869405163	0.316948222	0.385931455	58.70347945
3.322924355	0.9377396	0.444539733	0.64855913	195.2788995
2.480272177	0.891726794	0.357857536	0.607403796	75.22823901
1.403135638	0.966272013	0.676433422	0.719649629	117.2256608
8.008844022	0.865866757	0.275905297	0.269623553	68.49722834
8.536480572	0.894333047	0.256958493	0.38110702	164.2005982
12.49954774	0.869245717	0.193343368	0.351526134	181.7931923
4.55079312	0.904364982	0.339232397	0.501040451	122.2231206
2.197051182	0.908486767	0.404368752	0.62453945	72.48941127
2.762691357	0.90634668	0.413912234	0.640261904	95.60718597
4.373529374	0.953179877	0.528115878	0.600972446	190.2038647
1.540027323	0.945783889	0.590270655	0.652936631	96.06300248
1.814746936	0.926491768	0.546818507	0.689810528	82.75344615
3.36171092	0.845033133	0.267466821	0.461155375	58.6484652
5.850734094	0.921532314	0.346710921	0.509308174	155.7827746
6.75236766	0.838919687	0.21955414	0.221914986	82.02538067
3.389258052	0.943503545	0.498587117	0.70153839	129.5398147
3.872814131	0.868279156	0.342877111	0.152720816	32.16481292
2.870384805	0.932682276	0.464683515	0.638665238	145.4957625
3.133751541	0.930631864	0.455589633	0.624348278	167.4310137
3.364021868	0.873659674	0.333566022	0.487114742	76.90322755
1.541344217	0.95731209	0.631034895	0.736485417	128.1282608
1.192198541	0.959142742	0.661542337	0.732992331	111.4691955
2.564826559	0.970308944	0.636712724	0.579069711	167.9346221
7.805143999	0.888640774	0.259468055	0.249467047	107.0137238
4.405622185	0.889938171	0.328277861	0.371214016	107.0969916
3.042106315	0.876096639	0.357052185	0.232535295	28.18501179
3.879002119	0.908242085	0.344323407	0.530233296	155.9659857
3.285469566	0.948414131	0.526878268	0.587373786	151.5118096
5.0834429	0.855876269	0.296830933	0.352363004	55.48647102
9.711435884	0.907032538	0.298667254	0.430392331	188.1956073
4.309966965	0.953909623	0.532271143	0.591323224	234.9457082
15.41492538	0.809048799	0.15054385	0.02145808	104.5299652
1.875434362	0.952316166	0.582094147	0.652219269	123.0879411
3.021573983	0.938286509	0.469517156	0.453362679	132.567
3.383063423	0.886554179	0.336546538	0.410256786	48.88925295
8.387441518	0.890642558	0.247413216	0.349583063	144.9290471
4.810860818	0.822925124	0.266219982	0.129319429	50.43324001
9.652809272	0.758938107	0.183504861	0.002506394	55.77777778
4.170719025	0.944540995	0.506759137	0.65291545	268.2531742
4.250942254	0.869425282	0.292159092	0.467768011	60.99745782
4.916994239	0.904865504	0.382953582	0.424064919	105.4464871
1.95982759	0.937115276	0.544107822	0.724882396	135.1372493
1.473405746	0.95408591	0.61580421	0.716696297	106.4618545
14.03938784	0.842981525	0.156335658	0.059218697	106.0697121
6.75811826	0.89576959	0.279819265	0.434970439	119.7924998
9.487656814	0.785045883	0.231934092	0.059323081	38.23041424
8.916653497	0.882933946	0.222109212	0.462372298	205.619571
3.347289931	0.890832447	0.355088268	0.591024867	107.0246939

log.sigma.3.0.mm.3D_glcm_SumEntropy	log.sigma.3.0.mm.3D_glcm_AverageIntensity	log.sigma.3.0.mm.3D_glcm_Energy	log.sigma.3.0.mm.3D_glcm_SumSquares	log.sigma.3.0.mm.3D_glcm_ClusterProminence
4.558725693	11.61548194	0.010827724	11.64008258	3575.409599
4.058623737	12.70770524	0.046338437	7.849142887	1947.191571
4.120770172	7.091705081	0.017105111	7.787394186	1690.387597
4.605036472	12.26367135	0.015208885	14.39175319	5920.472863
4.37809657	8.785838249	0.013892211	20.36258085	9493.734756
3.693701917	6.693510545	0.023213242	4.894195856	409.4234015
3.464440268	6.484269836	0.03147057	6.977109589	632.2167678
4.41121201	9.890714231	0.009462227	11.53144453	2906.807475
5.651961239	16.41561283	0.002808026	63.85130356	87323.29357
4.108526484	12.09133659	0.030259811	6.444407886	1846.75085
4.06097373	11.77248726	0.013992793	7.101516317	991.9390016
4.36976758	10.20330762	0.014263911	8.541571177	2206.059033
4.099732967	8.208033292	0.01681288	6.644063432	1106.386722
4.132225156	7.81217599	0.014785525	8.237456075	2289.720221
4.374438234	8.33441228	0.010124551	12.54610099	3796.977272
2.983549379	12.95547703	0.154287823	2.806388829	874.3558099
4.185103749	7.650081607	0.013621455	11.89417089	2441.991996
4.244023958	7.618922439	0.015179283	7.540702484	1249.75922
4.144724999	6.819951968	0.017111298	13.47368954	4055.661769
3.995438462	9.954230396	0.048054023	5.722757512	1071.883811
3.56637048	11.81959679	0.078713613	3.839267619	622.6917684
4.295818745	8.917391552	0.013050922	9.050449737	2064.137316
3.807363353	11.54921659	0.055187917	4.975903189	1212.762225
4.849227604	17.1739566	0.011559203	39.45597845	37511.36397
3.930792069	10.93608974	0.031045113	4.993517684	939.1528914
4.313455326	9.588761842	0.012695958	9.532703916	1836.493044
3.738708979	8.940457463	0.061057356	4.505470806	1022.518875
3.356058467	12.00227115	0.126962108	4.532161982	1046.714398
3.846201134	13.25206818	0.082942216	8.409551639	2824.177438
3.989132825	12.76166214	0.059126768	7.733647226	2233.561002
3.708393735	10.76415112	0.06543968	4.782823523	932.0834547
3.488588731	11.15044605	0.090787881	4.035532447	884.2976416
4.480801856	10.9132171	0.017546716	11.36639699	3012.200477
2.141025559	8.687820513	0.131423611	18.2685016	3356.815265
4.529904444	9.286368851	0.01218578	23.09584168	10379.07368
4.355987531	9.90130683	0.013243809	9.224976456	1800.133981
3.37255707	12.33043481	0.108687028	4.877120871	1374.820708
4.052793092	10.72298022	0.03732692	6.266120046	1208.240653
4.678459412	10.28817636	0.007776973	17.94552423	7597.097321
4.515127158	9.625615658	0.011148062	11.67220673	2941.227072
4.403544411	10.67711162	0.018266887	9.555767219	3312.24989
4.187544112	9.413132137	0.013288268	8.405933471	1660.095466
4.345439705	9.502825599	0.010452296	9.425839452	1908.737454
4.389600933	9.989355255	0.01060966	10.97737674	3649.841217
4.831200262	13.11632177	0.006931924	20.35445878	13743.83056
2.926244092	7.795802069	0.141737776	2.389300936	431.5483982
4.906960867	13.62647223	0.007228617	26.75394981	47604.12931
4.00969546	6.22315658	0.014412925	7.902375909	996.4364731
2.828730443	10.09352453	0.063480298	21.77812406	4444.43226
4.61533058	8.744740259	0.010450279	25.48815156	10156.82884
3.724022827	11.78100622	0.077091357	5.377822551	1278.992422
4.185598154	7.428343944	0.01402935	9.174345747	1832.550444
4.387487043	13.74715749	0.02090159	9.750074043	2716.874813
4.315460323	8.357927666	0.015256488	8.820892199	2177.707001
3.288513515	10.69584763	0.125920874	3.900832862	1093.596664
4.406558555	8.065014669	0.010270568	12.83205034	2758.434759
4.760288698	12.56476007	0.006906547	18.01702866	8183.847476
4.983453091	13.14963773	0.005336906	28.24608317	14303.58532
4.456170599	10.80017769	0.011835126	11.03076654	2895.08097
4.263631214	8.230312428	0.016724003	7.552470753	1617.59539
4.321784905	9.479422577	0.01624837	8.942452055	1851.573658
4.123897815	13.60376901	0.049189953	9.070551309	3291.428438
3.699010324	9.663056351	0.061353221	4.091924899	675.4393918
3.951525743	8.872357694	0.04611891	5.797610916	1019.842426
3.861243034	7.281402065	0.029617421	12.35894188	2700.851852
4.586271174	12.21805402	0.010630837	13.01404696	4074.945192
4.206309147	8.884299952	0.014655217	13.79113912	2947.478656
4.241302265	11.05178421	0.038511223	10.51192998	3349.963028
3.640668587	5.595656751	0.026591722	5.53152032	734.4067918
4.280440506	11.84502104	0.022289038	8.092730429	1903.51967
4.268945001	12.72967825	0.021993629	8.529135165	2166.413784
4.320140914	8.521840986	0.01131798	8.781565217	1610.801726
3.611102374	11.15781368	0.083098465	4.911382973	1066.210162
3.362181376	10.42740645	0.10766323	3.712997138	770.635922
3.449595906	12.86655193	0.096440432	4.537035445	1435.699481
4.4379931	10.18399872	0.009494204	13.12183468	4158.345785
4.265197615	10.18749231	0.013331239	8.798037365	1808.473825
3.716111692	5.197718104	0.021204537	5.030628779	444.659469
4.452444414	12.2500856	0.011495589	10.72488509	3483.352608
3.99585092	12.14275656	0.046042254	6.895188328	1549.950413
4.165486502	7.206808244	0.015184498	10.06511063	2056.105066
4.804830418	13.49430938	0.00777658	18.67359725	8424.991445
4.036385141	15.17573952	0.054326844	8.700297571	3365.913749
3.385823351	10.15607288	0.041148776	24.27952423	5801.279246
3.738825463	10.9524613	0.05834985	4.770468822	881.6321017
3.993459802	11.4050019	0.025079629	5.452388648	1053.455616
4.175893802	6.764741048	0.013582939	7.589736758	1227.651007
4.686911912	11.80469972	0.007106947	16.64272936	6237.44567
3.468992056	7.034818521	0.036778586	8.47885461	849.915298
2.568005041	7.488095238	0.085034014	15.55810658	1883.732704
4.204936113	16.17070076	0.040313496	10.50851544	4219.372279
4.377267956	7.459201044	0.011206375	11.38290118	2492.292028
4.228256489	10.08314622	0.018568009	8.96882117	1770.530678
4.130982461	11.41034077	0.037999003	6.7826803	1659.59883
3.587701529	10.15867252	0.083359314	4.534492133	934.2851264
4.015664756	10.29753083	0.020536426	25.20338796	9125.147464
4.675677693	10.64549373	0.007674506	14.83525982	5586.642294
2.769005483	6.077134938	0.083069379	15.36079958	2432.486041
4.70508116	13.98054149	0.010211209	24.06445697	14698.42481
4.406004824	10.03724712	0.01488101	10.57918735	3247.937866

log.sigma.3.0.mm.3D_glcm_SumAverage	log.sigma.3.0.mm.3D_glcm_Imc2	log.sigma.3.0.mm.3D_glcm_Imc1	log.sigma.3.0.mm.3D_glcm_DifferenceAverage	log.sigma.3.0.mm.3D_glcm_Id
23.23096387	0.86125144	-0.183974622	2.345659435	0.410830111
25.41541048	0.83054726	-0.196508872	1.545597937	0.560639818
14.18341016	0.863973204	-0.20799415	2.130687781	0.432597889
24.48424777	0.780113892	-0.127487422	2.727631962	0.439596371
17.5716765	0.979608771	-0.405866549	4.145819316	0.306603236
13.38702109	0.727393807	-0.122234647	2.172828111	0.443972114
12.96853967	0.945621675	-0.376246279	2.790570531	0.375192826
19.78142846	0.781431775	-0.126587509	3.067380285	0.362741915
32.31514052	0.864294735	-0.145720854	5.20119461	0.294026657
24.18267317	0.784620115	-0.154708876	1.555353264	0.541445302
23.54497451	0.696448541	-0.099346382	2.483154492	0.406952356
20.40661523	0.793265639	-0.150447121	1.980030333	0.464594104
16.41606658	0.688184249	-0.103644062	2.03166036	0.474133529
15.62435198	0.730460641	-0.113281931	2.488182606	0.417327596
16.66882456	0.814766107	-0.145913767	3.325136501	0.348161161
25.91095407	0.784474262	-0.227028862	0.731539287	0.732379996
15.30016321	0.872576877	-0.20080719	3.491810621	0.348509975
15.23784488	0.730207342	-0.119385087	1.965059865	0.473565394
13.63990394	0.893003079	-0.226148608	3.365597509	0.375533195
19.90846079	0.880212316	-0.251644621	1.117147398	0.619412794
23.63919359	0.803996394	-0.198994957	1.040765259	0.641873284
17.8347831	0.705995101	-0.103695171	2.468063785	0.425970737
23.09843318	0.840954538	-0.219839494	1.132437062	0.623161943
33.97079943	0.990259136	-0.462655983	4.180288589	0.307763261
21.87217949	0.805258285	-0.181493017	1.3307155	0.562005224
19.17752368	0.830754072	-0.167833829	2.377089035	0.414376569
17.88091493	0.773280618	-0.165614245	1.250571035	0.602585463
24.0042458	0.824841223	-0.235915455	0.956327667	0.684992445
26.50413636	0.837887847	-0.213191619	1.454142305	0.60833345
25.52332428	0.849292096	-0.215523846	1.394564143	0.587312184
21.52830224	0.817636137	-0.201881079	1.153487218	0.626263799
22.3008921	0.797483405	-0.198561668	1.022417067	0.662221374
21.82643419	0.788258091	-0.137600093	2.309361811	0.456222817
17.37564103	0.993470634	-0.850783994	4.951282051	0.288770394
18.5727377	0.983365971	-0.418069796	4.14452813	0.306135654
19.80261366	0.73161821	-0.112470802	2.274735045	0.442245292
24.66086962	0.818199136	-0.228151911	0.979296479	0.673393205
21.44596045	0.805494039	-0.169376969	1.490064709	0.565562878
20.57635273	0.887355659	-0.197293617	3.730144606	0.332717913
19.25123132	0.823156639	-0.158650193	2.310416704	0.430308504
21.35422324	0.862150198	-0.198894902	1.810452313	0.49627824
18.82626427	0.751236397	-0.119181592	2.650331337	0.393740423
19.0056512	0.69854025	-0.096775538	2.639330192	0.393697488
19.97550541	0.783627719	-0.131141671	2.798284924	0.386763488
26.13748385	0.776781179	-0.117019872	3.363947424	0.353620395
15.59160414	0.782225255	-0.240135717	0.66602144	0.732172253
26.89117128	0.732299153	-0.097293737	3.593396464	0.367765809
12.44631316	0.753361717	-0.124559314	2.775588277	0.382642315
20.18704906	0.993905281	-0.776384974	5.527730603	0.243221071
17.48948052	0.966268832	-0.335547385	4.792657699	0.290453393
23.56201245	0.823161089	-0.204690141	1.189563875	0.629660403
14.85668789	0.728628569	-0.112444033	2.623352486	0.400398978
27.49113522	0.806731616	-0.159983846	1.837827907	0.504855537
16.71585533	0.884246384	-0.21764105	2.07653978	0.436342985
21.39169526	0.814525654	-0.232584584	0.856380932	0.699113135
16.13002934	0.832072062	-0.156447571	3.260188189	0.363782546
25.0764245	0.791133373	-0.123561154	3.564307811	0.345718885
26.21522912	0.894385646	-0.188018273	4.729142935	0.286872013
21.60035538	0.860570563	-0.18537027	2.500324941	0.419454817
16.46062486	0.832336926	-0.180145786	1.825031285	0.472911634
18.95884515	0.808360987	-0.158971809	1.89354151	0.481395749
27.187911	0.836355697	-0.190584841	1.641936156	0.574228253
19.3261127	0.789576468	-0.186764164	1.106808894	0.626399988
17.74471539	0.841361759	-0.20994743	1.294711403	0.591742114
14.56280413	0.984463741	-0.503424271	2.99199038	0.362151778
24.42616219	0.805416926	-0.14533757	2.560573525	0.423338757
17.7685999	0.962119092	-0.346103565	3.770038561	0.316893004
22.10324216	0.851300269	-0.198473943	1.669536541	0.551139214
11.1913135	0.776907715	-0.154463304	2.335167629	0.421621097
23.69004208	0.816757112	-0.16886556	1.69995545	0.521955536
25.45935651	0.802915598	-0.16131858	1.760890972	0.514318182
17.04368197	0.718660434	-0.106681032	2.353551566	0.415141093
22.31562736	0.844291689	-0.236026134	1.001028927	0.662043733
20.8548129	0.821843063	-0.23222602	0.864477789	0.685690051
25.7167416	0.796091415	-0.194738915	1.076451682	0.663855521
20.36799745	0.810633324	-0.141238	3.391166307	0.349360932
20.37498462	0.764820745	-0.124950673	2.541775579	0.410735992
10.39543621	0.724577247	-0.119932555	2.152913114	0.434791072
24.5001712	0.783440542	-0.143764574	2.376682788	0.423732611
24.2849573	0.833326438	-0.194617088	1.521198799	0.573964735
14.41361649	0.916335619	-0.256163314	2.790023814	0.381294458
26.86264392	0.829120398	-0.145395075	3.240243609	0.380908388
30.32085644	0.817114019	-0.181352712	1.615586011	0.576881761
20.31214576	0.99690396	-0.716697002	5.508364285	0.242768984
21.90492259	0.802922316	-0.187254873	1.175770345	0.619890857
22.8100038	0.670299476	-0.101907843	1.686569843	0.52523538
13.5294821	0.713884772	-0.106860331	2.341169459	0.419211501
23.58819205	0.80207581	-0.130136294	3.570359713	0.338707728
14.06963704	0.971565698	-0.474245196	3.109675841	0.359763343
14.97619048	0.982187232	-0.640560719	4.674603175	0.276378835
32.33355279	0.840230832	-0.190762121	1.715486572	0.556793084
14.91840209	0.836500308	-0.165844167	2.762810566	0.381373119
20.16629245	0.83303996	-0.172535245	2.301835077	0.454994103
22.82068154	0.862516147	-0.221059969	1.300832499	0.588010522
20.31734504	0.825519305	-0.215719221	1.02715207	0.64760881
20.50979552	0.992002096	-0.53795564	5.511818759	0.251823333
21.29098746	0.775554894	-0.119237292	3.133839283	0.367171021
12.15426988	0.950214811	-0.49862022	4.358171956	0.326627324
27.89870776	0.975588679	-0.364348075	3.984911878	0.315015572
20.07449425	0.890841443	-0.223491156	2.252402179	0.432438409

log.sigma.3.0.mm.3D_glcm_ClusterTendency	log.sigma.3.0.mm.3D_firstorder_InterquartileRange	log.sigma.3.0.mm.3D_firstorder_Skewness	log.sigma.3.0.mm.3D_firstorder_Uniformity
37.77257154	132.8209534	-0.076469574	0.080992319
26.21937938	108.0122023	-0.8019818	0.131523987
23.89180891	105.8254776	0.574604799	0.098662581
41.13128156	136.5901299	0.133827801	0.081601471
53.79705626	170.2695618	0.531558817	0.06440545
11.3251881	60.97650528	0.463530984	0.133834991
15.74113077	86.38582039	-0.442455334	0.115510204
30.68028853	117.1855545	0.476558513	0.082679834
201.7196669	273.823864	0.623223057	0.042853681
20.86806338	85.24076843	-0.268855995	0.120583083
18.12410534	93.94237328	0.105340097	0.10246435
27.20052869	103.1457787	-0.094971131	0.093539994
18.51325241	92.33106709	0.452476223	0.107215819
21.79995014	93.97362709	0.828609844	0.099510732
31.88432218	116.6759577	0.681154516	0.082674194
9.407442964	17.94341475	-1.833277181	0.298192075
27.2171723	132.4265976	0.633632508	0.084244728
23.13853273	103.4732389	0.056739998	0.099871134
33.01345343	149.0991096	0.860226202	0.085738437
20.17755428	86.25443977	-0.636551369	0.1386575
12.77597022	73.79281569	-0.829337981	0.183895945
25.02355833	101.9511566	0.445881123	0.091422894
16.98446207	79.56564343	-1.004359606	0.153182117
124.4980165	199.6403999	-0.437552539	0.059641585
16.5784108	85.80105495	-0.575876457	0.125540365
28.92026597	117.539114	-0.090672987	0.089689049
14.36709606	63.9113102	-0.099922644	0.171294475
15.48851127	68.40152848	-1.179037885	0.232996057
28.01084211	105.8913832	-1.024537449	0.180159315
26.6044919	110.0452909	-0.916699291	0.138891283
15.84788946	79.49500847	-0.712853073	0.166887017
13.27586043	56.77118814	-1.080963615	0.206469841
35.27707208	138.8703651	-0.026332643	0.088276134
34.23554487	126.6737404	0.165984885	0.111111111
64.67557862	174.8149948	0.221976278	0.061007127
27.77162892	118.981636	-0.045653114	0.090836141
16.86113502	55.17782813	-1.512331781	0.230040214
20.10946131	89.11133616	-0.166621555	0.125933099
48.38466689	156.1919174	0.314324529	0.067672092
37.73591069	144.0856285	-0.007389388	0.080379652
31.81283327	107.4913015	-0.567315551	0.095994914
21.90578894	100.1002274	0.322475418	0.09492219
26.16324396	111.4125957	0.077629313	0.08761429
30.34606695	119.5703163	0.536267418	0.083535266
59.15727509	124.8814964	0.720936335	0.073524101
8.348631296	30.80624115	-1.758137697	0.282953142
73.75565747	125.050964	1.568947224	0.073229206
19.17270356	109.8635921	0.478686226	0.098684211
41.1150964	143.5847054	-0.554748573	0.090909091
64.64577799	221.6173649	0.195457126	0.059954963
17.92145682	77.65230864	-0.952536757	0.177422145
25.36683662	102.9405518	0.627183375	0.105283574
32.11607012	121.7283883	-0.470183908	0.099819799
28.3780819	105.4467659	0.183470543	0.093783372
13.44900572	27.67742038	-1.553755171	0.255392666
32.55651218	127.698616	0.174632472	0.080561372
48.90194287	137.6061859	0.299000921	0.067844668
75.1305388	202.778656	0.232513529	0.053193112
33.21328142	118.8595657	0.043936734	0.082256057
24.5971151	102.1731548	0.29901797	0.098970325
29.36046225	121.595808	-0.025358024	0.095274737
28.75008431	95.82584681	-0.376919041	0.135748358
13.55884697	52.01019597	-0.781806738	0.170650887
19.63903373	90.85042068	-0.650913265	0.144269733
35.93272715	150.3753738	0.030935907	0.092915628
39.09401934	126.2758398	-0.210168654	0.079717942
33.65956178	133.0580444	0.0648788	0.075555556
35.80639264	128.7298322	-0.457604541	0.115682421
12.75354332	75.11357117	1.093280758	0.135801262
26.55293261	111.0250504	-0.484337051	0.102113685
27.79696832	117.0631571	-0.498079252	0.101960813
26.10611172	103.8064308	0.087406679	0.093586406
17.08037328	71.43000615	-1.126918083	0.186848346
12.89804842	50.88595173	-1.404576823	0.227000859
14.12997746	33.96167278	-0.597121895	0.222321444
32.79275448	121.7078457	0.421881063	0.077017434
24.19809063	102.6510544	-0.039517504	0.095519775
12.38113128	72.76678467	0.83886505	0.129105548
33.15313417	119.9859238	-0.412605607	0.082329117
21.91286376	104.0168867	-0.439212137	0.131895876
27.20747798	108.3263397	0.314098938	0.088059096
51.53358482	144.4638081	0.201794736	0.067066725
27.41019296	76.39880657	-0.860259143	0.150657371
50.69318827	172.8514366	-0.215061885	0.090530697
15.79783702	74.17994577	-0.760967181	0.162365457
15.88143046	72.66096163	0.097240041	0.126784193
21.4175402	99.50654602	0.429669014	0.100108838
44.54975976	134.0744476	0.261620189	0.068698637
18.78292974	98.92489147	-0.284083011	0.12
30.28798186	166.2388229	-0.23920969	0.195266272
34.68803554	114.87283	-0.890470286	0.121989624
33.44353214	131.3260021	0.435957762	0.085634522
25.4825081	114.7655144	-0.209704854	0.10034902
23.4381041	99.20663106	-0.726195731	0.121810852
15.59109294	67.70561685	-0.965941509	0.191076667
50.54209813	151.8783417	0.731116621	0.069286974
42.58530726	137.8259401	0.314898471	0.071051038
32.46252324	206.2868252	-0.091836711	0.170138889
69.17230754	163.9467754	0.084513315	0.05965599
33.67555865	111.4781218	-0.375074703	0.090739796

log.sigma.3.0.mm.3D_firstorder_MeanAbsoluteDeviation	log.sigma.3.0.mm.3D_firstorder_Energy	log.sigma.3.0.mm.3D_firstorder_RobustMeanAbsoluteDeviation	log.sigma.3.0.mm.3D_firstorder_Median
71.91896037	7894450.476	53.48936893	-118.4529572
60.01812003	14631388.33	45.20824304	-54.08587646
59.50186715	3895843.358	42.34094164	-130.2603912
78.40181533	30162084.91	54.13215753	-64.42247772
97.64694517	4380177.419	71.09267436	-172.6910706
44.14092016	7305564.971	28.48826156	-169.3287888
51.52492929	1192831.173	37.3782516	-100.6610107
69.5745034	16429748.42	48.57837789	-188.4032593
156.7937614	56645939.71	116.5138188	-98.87498474
52.33990226	19849140.57	35.69822653	-54.93688583
54.73631501	11583799.58	38.4689755	-165.8386765
60.89524938	9549961.837	42.27274195	-77.72062683
54.17053445	14716730.15	37.7346206	-82.71717834
59.7281128	9275471.843	39.65907111	-113.7239304
71.48581868	5870649.836	48.563724	-100.7890358
26.85448583	23091584.68	10.11929695	-3.259835482
73.94951352	8705424.339	53.46069128	-188.5106354
57.76435721	22325157.32	43.14622615	-96.26371765
85.11067276	7034911.774	61.05920305	-196.4901047
50.77347628	15018528.71	36.76203932	-20.28823376
42.66411419	25154367.24	30.68901963	-30.03064346
63.49646736	12574924.54	43.85858575	-85.52872467
46.82062886	18285080.58	33.05743219	-37.55144882
118.0240556	3407245.151	82.2977328	-57.99367905
48.25681831	8043310.373	35.13623783	-61.9727478
64.75119595	8459562.292	49.03275115	-150.8555984
42.2975224	10869290.47	27.35301208	-27.54104805
46.58555121	19991844.31	31.58501287	-12.45693684
62.8590476	34270464.89	45.59690799	-21.50754642
61.06052128	18649562.56	45.38831256	-54.54629135
47.00142314	23866208.15	33.80252351	-28.72140312
40.24782931	35361104.17	25.69014972	-15.36882401
73.14045465	21072067.99	54.78592696	-83.09326172
90.44458072	965771.7395	55.29813177	-222.6554871
100.5420216	4419304.447	71.40913396	-152.0487671
64.20768991	25033907.31	48.42786058	-116.4776497
45.3452288	29774886.99	28.48693936	-4.526154518
53.39664758	18927670.25	37.60058277	-28.21927547
87.12910223	7779769.692	61.77917818	-115.6661987
73.98990439	12072342	57.62897188	-123.502243
63.25746069	8050632.374	44.22653391	-59.92344284
61.14700349	13306645.01	42.66360051	-175.6071625
64.15552062	23390074.19	45.8249967	-150.298912
70.08718307	14010600.89	49.52734007	-165.6564026
80.95052764	21019571.5	53.95523585	-100.3773193
30.32532516	13338549.51	16.59731706	-5.523535013
85.63287298	47237664.05	53.54286854	-69.20827866
60.64724022	8047985.657	45.1166863	-181.462944
91.05363915	1803285.403	54.68785286	-259.1613693
112.5371853	7153859.465	88.48521373	-189.3723602
48.84236874	23596834.98	33.87038412	-21.26263237
60.86896864	15045985.39	43.60728132	-193.0699921
66.32027506	37117542.7	49.64893218	-56.42184448
62.29164152	3651286.773	43.9562969	-104.4146652
36.09224542	35842039.95	17.13625901	-4.727232218
75.47495104	13347273.87	53.37872361	-167.8778381
84.73492705	35818858.7	57.86941455	-173.3194275
108.4205974	20156307.26	79.98770324	-169.9633026
71.30653124	7285276.177	51.77194349	-113.6643982
58.44160745	5426018.413	41.6614511	-87.94731522
63.04896371	25068497.3	48.55338195	-115.2438812
61.65142211	57700661.22	41.66921014	-21.16609192
39.86954806	12974765.71	24.49416157	-7.190887213
50.88603119	9903642.979	37.7174458	-22.63231564
73.98557132	2401313.086	54.88622408	-198.1444397
73.62870341	12334237.36	52.67331085	-38.74687386
78.24961996	5836020.791	55.98898287	-218.2462769
70.81516285	39005905.78	53.84817853	-37.23746872
49.58769165	7134339.812	31.81922215	-230.3413849
61.29038371	18133271.4	45.80765208	-58.21994019
62.01387895	28287001.21	46.58989219	-62.75670624
59.86366294	16095735.93	43.66598933	-153.3619385
46.90047841	33470252.54	32.3373343	-9.413175583
39.65210606	38034337.08	24.39360279	-7.489212751
36.61109648	47512260.13	18.28011366	-2.545796394
75.90544215	17812976.34	50.7456029	-177.853157
60.64831552	13837646.72	41.26222736	-152.8179092
45.51775504	8181885.03	30.71382555	-188.874527
69.69422822	24738335.5	49.26416749	-134.3815002
57.33261994	24190453.48	42.29005751	-47.42078018
66.03668618	10021376.75	46.74108481	-250.3546906
87.18491962	19748597.68	59.65599445	-107.4232407
57.36742806	33861411.93	35.59703861	-7.844558239
103.0127881	1413723.376	77.30239155	-158.0986633
45.5945863	24368232.5	31.65866481	-22.35145664
46.32054245	21566651.45	30.42710971	-48.19223785
56.8006577	9480030.724	40.22120244	-148.0700684
82.99902697	31721959.11	57.61527579	-213.1401825
56.2579111	505230.4109	40.3624823	-65.03852463
87.21265533	780696.5108	68.48444753	-255.3158875
67.08062524	36030565.7	48.65650712	-32.31160736
72.3987276	9736051.591	54.34070461	-169.1742859
63.19264013	5479903.812	44.49538262	-77.43403625
56.04715965	27903899.83	41.23437455	-38.82409096
45.18638131	32487265.36	30.39920558	-11.34253263
101.3116434	3990541.917	67.53943713	-244.3285675
81.25813082	11353166.7	57.86638196	-82.18761063
80.10118793	1342330.515	58.77032452	-209.7864761
99.63748524	4659362.7	68.89651353	-123.9826431
66.34761624	4195655.142	46.51775895	-81.55436325

log.sigma.3.0.mm.3D_firstorder_TotalEnergy	log.sigma.3.0.mm.3D_firstorder_Maximum	log.sigma.3.0.mm.3D_firstorder_RootMeanSquared	log.sigma.3.0.mm.3D_firstorder_90Percentile	log.sigma.3.0.mm.3D_firstorder_Minimum
213150162.9	94.75663757	147.8792882	-10.44258595	-375.2705688
395047484.9	77.34020233	102.1207352	-0.237486041	-363.0544128
105187770.7	145.4339905	148.3590727	-40.3856369	-281.47229
814376292.7	392.3302917	124.915046	26.64714317	-373.3213196
118264790.3	170.9084015	208.2500738	-25.4032402	-386.4951172
197250254.2	-5.371824265	173.0342164	-87.07075119	-301.4017334
32206441.68	-2.349281788	130.5390567	-35.89898415	-261.9191895
443603207.4	180.7710266	202.4152692	-66.06915283	-418.9447937
1529440372	507.6069336	195.9030575	255.7385712	-403.5960083
535926795.4	245.3309631	92.5167737	3.250662947	-334.9717407
312762588.7	30.20765686	175.5220146	-71.52648163	-425.3687744
257848969.6	195.0817871	112.3189095	10.26373711	-306.81604
397351714	182.7462769	103.7959274	1.076394916	-261.0803528
250437739.8	211.0529785	132.5412446	-8.728404474	-297.5476379
158507545.6	277.1981506	126.9966885	30.82763481	-296.7138062
623472786.3	263.6808167	46.17987418	13.5230731	-311.8524475
235046457.2	131.2364502	200.292693	-53.76794586	-356.7715454
602779247.7	167.7923584	119.9365095	-9.468568516	-273.7210388
189942617.9	140.6626282	196.6045813	-9.555063629	-343.5729065
405500275.3	92.91000366	73.58011567	18.45830765	-263.8042603
679167915.5	244.2909088	70.40259629	1.135073209	-304.0454407
339522962.5	245.68013	117.6171772	14.04826851	-298.337738
493697175.5	192.7559662	79.13288757	1.897445834	-313.7893066
91995619.07	223.1004028	160.0574415	119.120813	-456.2712402
217169380.1	159.682785	91.53386425	-2.161919141	-304.1868591
228408181.9	23.97169495	167.3672479	-45.91347923	-360.1247253
293470842.8	310.8697205	68.90927826	7.496220016	-231.8415375
539779796.4	366.5869446	71.64282451	2.623770237	-309.3032227
925302552	229.3332977	96.03336466	2.136463404	-368.8323059
503538189	105.8291702	104.9556556	-2.597249079	-355.7527466
644387620.1	247.2259521	75.37292534	2.903080225	-294.2701721
954749812.5	254.2628937	63.2715966	5.1623312	-282.5793762
568945835.6	269.0553894	123.1692953	10.11415749	-328.005249
26075836.97	24.46162796	253.7415666	-108.2505005	-441.850769
119321220.1	194.3720703	197.7596746	5.963183689	-396.5437317
675915497.3	104.1882172	136.9892239	-9.689544773	-335.4969177
803921948.6	143.2175293	66.70824973	5.283806324	-313.9873352
511047096.6	286.2223206	79.01034936	21.27547741	-296.1187439
210053781.7	239.1975098	155.6792942	12.49653625	-374.8518372
325953234	112.9795456	149.9369327	-8.651756859	-336.1307983
217367074.1	183.9888458	106.1113835	22.66435661	-323.3806458
359279415.4	117.5825806	186.8838609	-68.41262817	-386.213562
631532003.2	180.4984131	168.4814897	-44.9533474	-369.8985901
378286224	232.0005798	178.6471618	-45.368013	-394.7705994
567528430.6	289.332489	137.3012514	44.7926506	-392.7049255
360140836.7	113.4698868	46.67750562	9.68278904	-195.4851074
1275416929	701.1326294	131.1577447	86.25748444	-350.1347961
217295612.7	29.76383972	187.878106	-70.00488281	-305.79245
48688705.88	-65.19373322	286.2997002	-124.407782	-519.1328125
193154205.6	123.3175507	221.357262	-16.99244356	-403.0265808
637114544.3	192.53125	76.04957583	4.499289608	-321.762085
406241605.6	38.25744629	193.2225436	-64.44386444	-341.662262
1002173653	244.6727753	107.7839703	10.48190422	-389.4977722
98584742.86	97.69733429	123.6015797	13.72133713	-277.6338806
967735078.7	293.6021423	56.30433964	14.72089195	-268.989624
360376394.6	81.63031769	199.6061336	-71.48513794	-372.3058472
967109184.8	221.1567993	202.6738089	-30.80516491	-455.0783386
544220296.1	208.8562469	209.7841855	17.38317299	-475.3536072
196702456.8	152.7743225	143.4568793	-10.56055984	-355.2418213
146502497.2	130.1920166	107.9065167	17.17012978	-254.0222931
676849427	94.37535858	131.2600768	-2.583563089	-308.0358887
1557917853	388	92.27917355	8.0773592	-355.9866028
350318674.3	190.4399567	56.43379418	38.19011078	-238.9967041
267398360.4	109.4869537	74.54931171	17.05618477	-242.9343719
64835453.33	-13.41598988	212.8562756	-87.93459778	-357.789917
333024408.6	325.3478088	103.4736839	57.18975792	-330.1943054
157572561.4	-6.528203964	235.756549	-90.37477722	-432.1954956
1053159456	320.6781921	106.0689483	10.17506046	-308.5626831
192627174.9	-15.10867023	223.3617877	-136.9280884	-333.2371216
489598327.8	177.2702484	100.5933052	13.40665293	-343.8257141
763749032.7	151.3070221	104.3254616	9.007419968	-357.6520386
434584870.2	23.68082428	168.4860271	-48.98309174	-341.3824463
903696818.7	201.365921	68.86831162	7.195456505	-277.8923035
1026927101	179.0601807	59.25070332	8.136774731	-257.4667053
1282831023	489.1898193	57.20506618	25.63788109	-324.7773438
480950361.2	198.0173035	199.8484647	-55.79509735	-422.214325
373616461.4	65.29403687	174.2002195	-59.61147881	-389.0979614
220910895.8	29.93749619	191.1184775	-101.2053673	-283.2837524
667935058.4	114.0763321	164.6075871	-30.6044117	-420.2586975
653142244	295.127655	94.58421446	1.173209858	-345.2749329
270577172.1	-55.3045311	259.3406294	-127.3199677	-420.5499573
533212137.5	297.4196777	151.0984737	22.65402145	-400.5813293
914258122.2	361.4390259	82.88446193	30.34394531	-384.6521301
38170531.17	59.06024551	213.5508836	-53.31864929	-439.2627869
657942277.4	225.5117798	70.07831241	11.23830023	-279.6495056
582299589.1	268.822876	78.41939308	18.87476501	-310.9753723
255960829.6	110.3699341	161.1604384	-49.74263611	-290.9512024
856492896	173.7345123	232.0717906	-71.38036346	-484.6695251
13641221.1	60.16775131	100.5216803	19.70175457	-248.1019745
21078805.79	-108.284462	245.058315	-115.2227539	-410.4454041
972825274	220.9664307	102.5065927	10.17634544	-426.8202515
262873393	81.45492554	178.9594521	-32.92383842	-311.0045166
147957402.9	160.5567169	114.7730182	-3.552403092	-314.3697205
753405295.4	118.8815384	87.41119704	12.2923172	-305.135376
877156164.7	206.9189758	67.06992734	7.40703721	-260.9441833
107744631.7	179.9158325	251.6782374	-22.40084763	-459.0455627
306535500.9	263.1475525	129.2123577	51.98113594	-340.871582
36242923.91	-35.40023422	236.4961694	-103.3835091	-371.9241638
125802792.9	208.3914948	169.0711307	38.05074844	-455.5527954
113282688.8	99.85998535	122.4111214	3.493069363	-313.4880371

log.sigma.3.0.mm.3D_firstorder_Entropy	log.sigma.3.0.mm.3D_firstorder_StandardDeviation	log.sigma.3.0.mm.3D_firstorder_Range	log.sigma.3.0.mm.3D_firstorder_Variance	log.sigma.3.0.mm.3D_firstorder_10Percentile
3.790643736	86.6292168	470.0272064	7504.621203	-230.6427917
3.297054072	71.77289036	440.3946152	5151.347791	-180.9632721
3.514142063	74.6909731	426.9062805	5578.741463	-221.4795532
3.944299917	99.37094694	765.6516113	9874.585095	-203.5636749
4.136011885	120.7910731	557.4035187	14590.48334	-309.8134766
3.179313991	57.61507934	296.0299091	3319.497367	-229.2058197
3.247529097	62.86999726	259.5699077	3952.636556	-205.7483887
3.825020937	88.02114146	599.7158203	7747.721344	-290.4344788
4.743459559	188.9276064	911.2029419	35693.64046	-261.4326782
3.388709728	67.38740239	580.3027039	4541.062001	-150.0045135
3.492603972	68.22601241	455.5764313	4654.788769	-244.0840988
3.651221342	76.82640408	501.8978271	5902.296364	-187.3333008
3.466145998	68.40263185	443.8266296	4678.920044	-160.222023
3.613160358	78.06955122	508.6006165	6094.854827	-193.9073975
3.84426174	91.51840284	573.9119568	8375.618059	-198.7173645
2.359014824	44.26879244	575.5332642	1959.725984	-61.85179634
3.779330231	90.98448527	488.0079956	8278.176559	-285.6152832
3.483183932	69.78256249	441.5133972	4869.606027	-190.629422
3.813534091	103.2357682	484.2355347	10657.62384	-276.2513885
3.174122613	61.0914124	356.7142639	3732.160669	-133.841832
2.878531146	52.96114287	548.3363495	2804.882654	-121.7426834
3.695895499	80.80664002	544.017868	6529.713071	-188.6829468
3.070229761	58.77214473	506.5452728	3454.164997	-134.4386749
4.320761459	146.9127744	679.3716431	21583.36328	-272.1747131
3.220327542	59.48479012	463.8696442	3538.440256	-150.2710617
3.592168236	77.27418269	384.0964203	5971.29931	-248.0113647
3.031250089	56.54324088	542.7112579	3197.138089	-116.0299149
2.704106472	58.32660462	675.8901672	3401.992807	-136.4215546
3.138852705	76.21047096	598.1656036	5808.035885	-173.0821991
3.274973703	73.95054919	461.5819168	5468.683726	-187.7452637
3.01164979	58.30738794	541.4961243	3399.751488	-132.0135803
2.810730261	52.82365525	536.8422699	2790.338554	-114.1256287
3.76394797	87.85494369	597.0606384	7718.49113	-201.8291107
3.240223929	114.9701452	466.312397	13218.13428	-368.574823
4.204566832	122.4389591	590.915802	14991.29871	-319.6028931
3.612642697	76.95781695	439.6851349	5922.505589	-217.0641266
2.690819881	57.91706676	457.2048645	3354.386622	-129.2490082
3.350616048	66.4312585	582.3410645	4413.112106	-136.1669296
4.081989776	108.6061142	614.0493469	11795.28805	-248.1318054
3.760964209	86.7798952	449.1103439	7530.750211	-236.4413544
3.610885073	79.22410453	507.3694916	6276.458738	-180.6446747
3.634368694	77.20911624	503.7961426	5961.24763	-261.816864
3.709424761	79.48014029	550.3970032	6317.0927	-252.706398
3.790939944	87.8835304	626.7711792	7723.514915	-259.5658447
4.035522392	105.2133711	682.0374146	11069.85347	-210.0050842
2.266786038	41.45128018	308.9549942	1718.208629	-89.79576416
4.110650408	119.531324	1051.267426	14287.73742	-179.0228729
3.49424594	73.01098971	335.5562897	5330.604619	-256.814035
3.572623664	114.5438221	453.9390793	13120.28718	-404.9629608
4.204376625	128.8490252	526.3441315	16602.07131	-348.6982269
3.030564281	61.07780692	514.293335	3730.498499	-134.7696915
3.467604096	74.22265288	379.9197083	5509.0022	-263.166687
3.590378069	79.34816324	634.1705475	6296.131009	-183.8805267
3.619172155	77.2884071	375.3312149	5973.497873	-193.7032013
2.598226798	52.08672675	562.5917664	2713.027103	-102.5659676
3.839859007	93.19110599	453.9361649	8684.582235	-306.3973938
4.130106894	108.0995739	676.2351379	11685.51787	-305.8824677
4.383687992	131.3680024	684.2098541	17257.55206	-325.4800293
3.807705882	87.15182982	508.0161438	7595.441441	-225.2184967
3.538883909	71.74994395	384.2143097	5148.054457	-168.4775848
3.526149569	74.75480717	402.4112473	5588.281196	-201.255481
3.42619827	78.90581398	743.9866028	6226.12748	-160.2072067
2.973817254	52.93498828	429.4366608	2802.112985	-100.1936295
3.137404055	60.46247711	352.4213257	3655.711138	-132.437149
3.616782428	88.02775229	344.3739271	7748.885173	-307.4844971
3.86734777	91.30754443	655.5421143	8337.067669	-172.9837509
3.869860154	95.11599062	425.6672916	9047.051673	-348.8748474
3.503150902	83.90792455	629.2408752	7040.539802	-192.1064392
3.178851053	64.3741935	318.1284513	4144.036789	-281.760199
3.505770062	73.67905768	521.0959625	5428.60354	-171.1782288
3.513438873	74.523888	508.9590607	5553.809883	-174.4899017
3.542128628	73.1288346	365.0632706	5347.82645	-245.9890564
2.887878209	57.77799718	479.2582245	3338.296959	-130.209314
2.673510402	51.25531205	436.526886	2627.107014	-112.7512589
2.791809124	55.08650844	813.9671631	3034.523412	-90.01386108
3.953153931	98.07500299	620.2316284	9618.706212	-298.5098572
3.646686988	76.98866358	454.3919983	5927.25432	-251.6628952
3.188486636	58.09728106	313.2212486	3375.294066	-250.1971924
3.805192969	87.27695777	534.3350296	7617.267357	-250.3402588
3.324584857	70.47608343	640.4025879	4966.878336	-164.0017929
3.672508209	81.92855789	365.2454262	6712.288597	-348.9725586
4.146070568	110.4794832	698.0010071	12205.7162	-244.7518005
3.326312101	75.71323322	746.091156	5732.493684	-145.6252228
3.735666068	120.6441884	498.3230324	14555.02019	-307.4558411
3.021279856	57.00047142	505.1612854	3249.053742	-124.9998642
3.301477029	60.97543583	579.7982483	3718.003775	-122.615863
3.500852593	70.51982929	401.3211365	4973.046324	-230.0289795
4.086060506	104.945401	658.4040375	11013.53719	-345.3335083
3.284974925	69.43043951	308.2697258	4820.585931	-161.6239883
2.565448372	95.79938287	302.1609421	9177.521758	-329.2218628
3.475894594	82.23724568	647.7866821	6762.964577	-175.7127869
3.71009699	86.3114585	392.4594421	7449.667868	-260.9487061
3.599162456	78.68930441	474.9264374	6192.006629	-190.4549103
3.333498483	67.56128403	424.0169144	4564.527099	-150.7690948
2.908091542	56.5557439	467.8631592	3198.552168	-125.8027611
4.050548527	127.6993566	638.9613953	16307.12566	-361.4180847
4.014856583	101.0910567	604.0191345	10219.40175	-209.4705078
2.725412897	97.86988659	336.5239296	9578.514701	-357.2777313
4.268692502	124.577534	663.9442902	15519.56197	-260.439502
3.674885218	82.84871424	413.3480225	6863.90945	-195.2870224

log.sigma.3.0.mm.3D_firstorder_Kurtosis	log.sigma.3.0.mm.3D_firstorder_Mean	log.sigma.3.0.mm.3D_glrlm_ShortRunLowGrayLevelEmphasis	log.sigma.3.0.mm.3D_glrlm_GrayLevelVariance
2.529294765	-119.8484988	0.01325088	12.22433965
2.863379914	-72.64500517	0.008832318	8.690479995
3.626346834	-128.1860873	0.036357609	9.227586481
3.886636085	-75.6913708	0.013741562	17.14732141
3.01271365	-169.6396473	0.037081123	23.38584569
3.359506238	-163.1604814	0.033595073	5.618590049
2.432445791	-114.4019614	0.050337879	6.378572849
3.53844014	-182.2751213	0.016823744	12.99088817
2.362872026	-51.81088186	0.010277493	57.17008721
4.361824297	-63.3899946	0.01039389	8.217030775
3.139287466	-161.7194758	0.010486558	8.08804958
3.120108742	-81.9343705	0.016725539	10.02098467
3.499897632	-78.06839631	0.023087131	8.067939414
4.234955369	-107.1089478	0.025853635	10.46765014
3.748383781	-88.04851408	0.026888919	13.90676599
9.713036232	-13.14742542	0.005696699	5.105961069
3.150683987	-178.4348237	0.032516316	13.71563742
2.734350368	-97.54568308	0.037049702	7.965388118
3.014098346	-167.3192683	0.036806943	17.64865315
2.710950528	-41.01064196	0.013487224	6.685398828
4.202007499	-46.38580506	0.008284903	5.232115185
3.640389676	-85.46395321	0.023521467	11.21135346
4.090525167	-52.98913945	0.009975806	6.206923516
2.603104261	-63.52181759	0.014028422	34.34290367
3.331565759	-69.57016637	0.01176607	6.144955593
2.26057754	-148.4604202	0.021893807	9.508392599
5.706931959	-39.38718753	0.018886316	6.242354567
4.596536458	-41.60170064	0.008759363	6.727442414
3.649532859	-58.4326214	0.009310327	10.69805191
3.145440861	-74.47822441	0.009674219	9.111899804
3.992482909	-47.76323258	0.012301658	6.319408975
5.02913153	-34.82752333	0.009765812	5.746231298
2.818711557	-86.32603411	0.014018611	12.87016784
2.852310811	-226.2004606	0.087010449	20.33484563
2.597352359	-155.2983909	0.04300211	24.37480167
2.317585151	-113.3293513	0.015416655	9.387518543
4.644756449	-33.09990877	0.008470273	6.875312903
3.521392175	-42.77292601	0.011389299	8.209667908
3.07303043	-111.5381307	0.01863228	18.91692242
2.090171439	-122.2715567	0.020305264	12.13904317
3.308966255	-70.59155021	0.019772779	10.3310801
3.448662381	-170.1891002	0.018441552	10.13410996
2.990709045	-148.5561162	0.023078989	10.56250441
3.87658708	-155.5355056	0.017517839	12.95780961
3.889530787	-88.21439886	0.009609255	18.27826461
5.579201915	-21.46114867	0.028214249	3.729279056
8.204857684	-53.98718896	0.008115508	24.2163661
2.47909172	-173.1114615	0.046149455	8.826652481
2.856948658	-262.387559	0.066611269	21.05601041
2.009216167	-179.9915724	0.033462005	26.74030158
4.021705178	-45.31047876	0.009757226	7.253948704
2.634519823	-178.398288	0.030168736	8.869930196
2.851069578	-72.94692075	0.006987355	10.53745261
2.585196219	-96.45648055	0.026212457	9.79085499
6.010972056	-21.38110284	0.01138282	6.235313095
2.824421922	-176.5163628	0.054024989	14.35423252
3.246752593	-171.4384874	0.01161223	19.23684764
2.437077359	-163.5599353	0.011239587	27.73056052
2.782886059	-113.949264	0.015583956	12.6034981
2.773511326	-80.59628949	0.024036523	8.660639067
2.193952297	-107.8931257	0.019914633	8.467634575
4.432200351	-47.84682217	0.007138974	11.94334278
3.791177183	-19.56170088	0.012653064	5.630610434
2.691374536	-43.61064937	0.021777078	6.456533747
2.050511974	-193.8012098	0.056484171	12.96409529
3.141565888	-48.67993007	0.010589726	13.95779876
2.400810812	-215.7176366	0.026663231	14.59660397
2.842906899	-64.88514467	0.01457288	12.06421825
4.371043199	-213.8842009	0.056758915	7.224702781
2.87499971	-68.48656451	0.011050903	9.121378858
2.811166589	-73.00679451	0.00859796	9.057396227
2.351945501	-151.7883885	0.023890377	8.525186607
3.642994146	-37.47729161	0.010291511	6.446138178
4.477324738	-29.72438108	0.011499725	5.512360163
8.036838712	-15.42388358	0.006291027	6.975576063
3.759136869	-174.1284084	0.020975402	15.88311895
3.103228579	-156.2640783	0.016589392	10.02860915
3.755120015	-182.0741014	0.077714417	5.877600299
3.216129333	-139.5650041	0.018173438	12.39133781
3.691798645	-63.08165573	0.009515145	8.760143106
2.586794615	-246.0594917	0.047793647	11.26101847
3.316438327	-103.0777986	0.010756532	20.36565417
4.591045754	-33.72447695	0.005480275	11.22579544
2.421285828	-176.20715	0.050865374	23.5909986
3.770088625	-40.76660555	0.010201549	6.08036454
4.46144738	-49.31123033	0.00872754	6.813250977
2.905852252	-144.9125274	0.053052313	8.332384048
3.108405968	-206.987388	0.012608084	18.22996078
2.521250157	-72.69128068	0.046237344	7.743267388
1.841691165	-225.5572122	0.0971099	15.0488063
3.870283903	-61.19343894	0.005234969	11.98663284
2.501376012	-156.7699513	0.055939847	12.17638
3.094671513	-83.55141575	0.020410184	10.26356132
3.039508796	-55.46341379	0.011950366	7.928451349
3.721992176	-36.05305792	0.01334406	6.372783533
3.339989611	-216.8751012	0.032934783	26.5813957
2.962247721	-80.47627993	0.014854981	16.72928097
1.8834	-215.2949684	0.165584982	14.66825596
2.95239229	-114.3043537	0.013064773	25.12247377
2.84461559	-90.11422307	0.029661858	11.35839147

log.sigma.3.0.mm.3D_glrlm_LowGrayLevelRunEmphasis	log.sigma.3.0.mm.3D_glrlm_GrayLevelNonUniformityNormalized	log.sigma.3.0.mm.3D_glrlm_RunVariance	log.sigma.3.0.mm.3D_glrlm_GrayLevelNonUniformity
0.013959894	0.080783208	0.112476761	26.41805055
0.009832076	0.102576209	0.731774635	108.6527047
0.03910014	0.097808944	0.112633682	15.70177577
0.014601084	0.07294493	0.368854059	118.6734712
0.038176888	0.063590245	0.056257019	6.115419733
0.03619781	0.128096065	0.145978185	27.72767449
0.052868382	0.116600359	0.046948651	7.774800599
0.017679035	0.081501928	0.088497757	30.26050001
0.010796765	0.042464394	0.086154223	58.3194989
0.01154546	0.10708211	0.573246712	193.6950372
0.011091543	0.100069375	0.121328152	34.00568375
0.01806398	0.091035217	0.201960283	59.57642462
0.025311181	0.104782607	0.2608926	120.509856
0.027873835	0.095722106	0.139495316	45.25350876
0.028210618	0.081187941	0.075898256	27.65126872
0.007702252	0.203172468	2.513752261	1188.754938
0.034316069	0.0821292	0.07990976	16.68073144
0.040318111	0.099063477	0.270742244	129.7226261
0.039798134	0.081033792	0.095424016	13.56606712
0.015821121	0.113003922	1.031751797	218.6831602
0.009866103	0.135931905	1.291526522	461.1299648
0.025384047	0.08809709	0.171631942	70.7459559
0.011622237	0.127567905	1.008445626	260.0055202
0.014303507	0.058638087	0.052495473	7.408123714
0.013052599	0.118104939	0.395283334	90.95805376
0.023088165	0.089791491	0.119937052	24.59549533
0.021575657	0.132381337	0.92318565	218.6094489
0.010596087	0.144343167	1.630202936	349.3809796
0.010539549	0.112132966	1.231293476	285.7833151
0.010778129	0.10201716	0.963191689	125.1590762
0.014359918	0.126719833	1.12012348	362.1293219
0.011915021	0.143208079	1.662510479	792.706492
0.015078796	0.079663184	0.350304852	92.9597338
0.087255228	0.111547183	0.025510204	1.63003663
0.044746923	0.060475964	0.048385772	6.530602007
0.01669733	0.089870686	0.249271315	103.0105982
0.010257022	0.157660996	1.592761615	649.7939514
0.012985933	0.103788733	0.732515923	233.6093137
0.019454569	0.067116508	0.080253039	20.18287224
0.021706305	0.079456918	0.164203573	37.5769176
0.021843408	0.092221053	0.264612166	55.85851136
0.019451293	0.091661438	0.105363379	31.86298362
0.02424626	0.08655626	0.126726652	64.58139112
0.018670126	0.082027545	0.10432459	32.81693609
0.010195926	0.072378125	0.107741391	73.90889276
0.035092496	0.212152919	2.030695669	729.76915
0.008701487	0.070534908	0.141535228	174.3532732
0.049661517	0.096797747	0.097970609	20.26886768
0.066735515	0.090966018	0.017388802	1.966300366
0.035262473	0.059191994	0.048058566	8.264476529
0.011378449	0.121086205	1.347381055	329.6097754
0.032638857	0.10304755	0.128093458	37.31611549
0.007754719	0.090646734	0.440052141	233.6382587
0.027753187	0.091752533	0.119836415	19.74595901
0.014097924	0.167679509	2.163231775	1100.758891
0.056098939	0.078860247	0.096519725	24.34977182
0.012136894	0.066508651	0.084875604	54.03781313
0.011611532	0.052941054	0.057878518	23.06400198
0.016393216	0.080829899	0.136642888	25.61592406
0.026223565	0.096418248	0.197908534	38.87980478
0.02160199	0.096244567	0.304674647	116.8230539
0.008204583	0.098415373	0.995422368	475.435764
0.01514869	0.13163625	1.085312362	368.7817578
0.024536511	0.119131984	0.814640489	156.7884318
0.058726185	0.090852073	0.066965899	4.525814519
0.011307109	0.077509318	0.184856619	78.79001009
0.027753296	0.075593592	0.05323152	7.523224867
0.016298618	0.091159205	0.823260684	234.5501709
0.059995316	0.128963458	0.12509749	16.76471274
0.0121305	0.094823844	0.453567164	135.5349491
0.009510287	0.096379099	0.436990897	202.3478236
0.025546032	0.093625683	0.12985605	47.58885078
0.012440979	0.131285818	1.744009262	577.3213247
0.014201099	0.153826362	1.798321696	1004.456849
0.007959945	0.155702509	1.725254652	1382.173761
0.021849117	0.074948469	0.087689859	31.11323494
0.017535558	0.091984679	0.140340069	37.63524881
0.082977587	0.125661268	0.123293116	25.31483938
0.019281184	0.082096624	0.175891162	66.38345192
0.01072911	0.104853949	0.819261606	208.3639607
0.05058531	0.086781526	0.079847717	12.11566508
0.011303059	0.065474743	0.126005274	51.30423961
0.006308688	0.108902389	0.954548242	384.3796127
0.051014312	0.090241113	0.012393162	2.762944582
0.012064037	0.128160251	1.145663076	434.5776017
0.009922288	0.117286858	0.407041012	330.7899757
0.056138401	0.09813059	0.1350663	32.07036332
0.013148146	0.067459075	0.076930651	37.13610096
0.047643808	0.117228484	0.065263178	5.538734038
0.097636383	0.191383136	0.038194444	2.394230769
0.005869416	0.095992252	0.792818269	244.7349581
0.05934024	0.084799395	0.095346772	23.62323024
0.021426627	0.094488325	0.219140065	34.11930342
0.013506785	0.10341746	0.834835095	273.9244958
0.01589357	0.132620854	1.430832843	617.8419384
0.033306475	0.068644155	0.034293513	4.187667749
0.015797782	0.069837048	0.107606213	43.38854793
0.182287904	0.169395572	0.057160035	3.830622276
0.013367892	0.059182535	0.051285048	9.169832104
0.030928665	0.089178318	0.117134479	22.63149395

log.sigma.3.0.mm.3D_glrlm_LongRunEmphasis	log.sigma.3.0.mm.3D_glrlm_ShortRunHighGrayLevelEmphasis	log.sigma.3.0.mm.3D_glrlm_RunLengthNonUniformity	log.sigma.3.0.mm.3D_glrlm_ShortRunEmphasis
1.333985319	137.8299874	270.5793128	0.926883364
2.51086909	125.3960372	707.3951204	0.841534526
1.332597578	59.97201645	133.1116152	0.927513409
1.788228626	150.7165499	1258.428863	0.899134785
1.163458964	115.3179646	87.6299784	0.963474245
1.422143386	50.75889765	172.7265442	0.912668785
1.152341527	54.14253684	60.57080972	0.961914618
1.25596196	113.6879233	321.102079	0.944245478
1.242501101	282.1935821	1198.434776	0.947832871
2.227874054	123.8757844	1247.270686	0.854700525
1.348035615	143.3051998	280.6251224	0.926501987
1.548240831	101.6384535	503.9311267	0.897880242
1.679110087	69.00775124	855.5979143	0.884579934
1.38924229	75.18157762	385.3003276	0.921417602
1.219611772	92.94292572	300.2921436	0.951670355
6.077776812	109.6844013	2478.4178	0.67266543
1.224036698	83.6957524	179.7256525	0.952603944
1.685564117	55.94008437	981.6602186	0.887058981
1.27952791	79.93431597	142.8237882	0.938941457
3.129694532	73.60743929	1146.016208	0.795715315
3.571418049	97.31806489	1940.838388	0.782612184
1.461510599	87.08226705	643.7247007	0.913265887
3.109050444	95.66883918	1215.0265	0.796909188
1.163747633	308.4041268	114.1043321	0.960420102
1.973485151	96.75943297	533.7639147	0.855128926
1.340668346	91.95871803	229.1964401	0.930164875
2.871502492	66.6943975	1040.254869	0.819312604
4.310032616	91.39517552	1240.268318	0.742104058
3.415670004	125.4517337	1521.339105	0.798351856
2.887089495	118.2873656	792.9317112	0.829182373
3.322777743	81.48211299	1639.417324	0.783899972
4.263488507	87.06634775	2914.820601	0.751922982
1.772374934	114.3753299	896.9833966	0.896787647
1.082417582	106.21337	13.9010989	0.979395604
1.144647455	128.2821283	98.96856232	0.966234633
1.610320921	93.40089168	890.6856713	0.901656348
4.315926954	95.72825455	2055.29435	0.732205992
2.566337524	97.56968186	1457.113192	0.830020397
1.225007145	136.7573471	266.813447	0.952731269
1.456733004	92.74208333	374.5442883	0.909992822
1.675042275	105.7603036	456.8186985	0.888039621
1.309252568	98.76934994	291.6079462	0.93260885
1.351839002	94.16371455	621.5758474	0.928859378
1.310817362	113.0336638	334.7093239	0.931187674
1.302209268	176.8847307	866.7354539	0.936888133
5.36268964	34.05076258	1485.134932	0.675153203
1.37857537	193.1979803	2046.979025	0.927100218
1.286019357	50.89167409	178.2262961	0.937845696
1.056043956	141.6279512	20.8989011	0.985989011
1.142550649	134.09349	128.0393033	0.966839992
3.633419083	100.0621766	1570.163714	0.786147141
1.371363174	59.33956091	296.6902868	0.922565891
1.995138492	158.8167037	1832.383402	0.866201444
1.356990659	79.21875773	176.4814321	0.922971754
5.238745604	75.57156881	3086.526002	0.711473691
1.277181996	81.77083931	264.8774044	0.940601182
1.23861843	173.2232952	712.2623209	0.949243885
1.16472102	217.7122349	396.8598988	0.963853647
1.386723042	120.5968048	257.8333919	0.920546896
1.547814423	70.4828913	310.3762862	0.896907027
1.745760859	74.29962981	901.2949896	0.884161726
3.001574343	146.0244442	2996.089383	0.813701615
3.264861423	75.3698942	1633.981921	0.78827321
2.676332181	61.97143106	853.2792094	0.82959342
1.208642487	70.79301277	44.16120832	0.951638049
1.482021547	153.9009765	817.4396607	0.913701539
1.169666472	110.3151306	89.57847697	0.958464517
2.660815964	95.14077741	1691.189367	0.836100865
1.338689852	41.85191545	109.6299644	0.934010606
2.039841249	117.8605451	1000.933933	0.860466151
1.987293231	136.0093942	1504.285097	0.868738416
1.377765851	72.14909151	414.1082897	0.920532205
4.411631675	80.36291458	2295.764375	0.748841381
4.639236305	69.58370445	3193.028326	0.72511784
4.465998874	120.1130619	4444.386759	0.734274056
1.244561862	122.2540541	363.4119489	0.948561741
1.387713618	106.3427655	335.6648212	0.922837825
1.363043148	31.53019796	164.7713605	0.922323754
1.464027924	136.8094322	655.5826833	0.915852478
2.698498555	112.4686277	1281.736702	0.828132562
1.223686369	69.08852695	123.9594355	0.953206769
1.347701983	184.283204	653.4786509	0.929913208
2.937029419	182.8466484	2196.378973	0.815402966
1.038461538	151.0790323	29.87179487	0.990384615
3.325322806	87.8010357	1972.009361	0.789523048
1.96495914	120.788031	1976.756641	0.86116136
1.385950156	50.0119207	266.2952142	0.920694523
1.22285607	162.4379376	483.8432562	0.950669462
1.190087185	63.65304302	42.49316776	0.958219376
1.125	84.11258013	11.58333333	0.96875
2.623645685	205.2534864	1673.705174	0.835500553
1.290774216	60.54855679	235.0192739	0.934058043
1.555155638	99.48536751	284.0572174	0.905896085
2.769988309	97.21716737	1649.025036	0.814528821
3.895841039	71.67408583	2511.791863	0.760883649
1.10497854	141.1012188	57.38672105	0.975309367
1.307604952	137.3044136	524.0997905	0.934652109
1.188941493	60.02951939	20.09998697	0.952764627
1.161998748	241.361154	140.2052367	0.960663225
1.341615215	98.33934756	211.3845492	0.927739816

log.sigma.3.0.mm.3D_glrlm_LongRunHighGrayLevelEmphasis	log.sigma.3.0.mm.3D_glrlm_RunPercentage	log.sigma.3.0.mm.3D_glrlm_LongRunLowGrayLevelEmphasis	log.sigma.3.0.mm.3D_glrlm_RunEntropy
198.5181231	0.905817175	0.017204785	4.236325062
469.1526037	0.75387905	0.017951951	4.337182634
81.70987317	0.906996958	0.051753375	3.927530821
320.938775	0.841259103	0.019976697	4.665711513
133.0489218	0.952018279	0.043213352	4.316768178
74.56685417	0.886822194	0.048282047	3.720767836
62.57597185	0.952747253	0.062990397	3.423694763
142.4792086	0.925954345	0.021661171	4.200652815
361.8704474	0.930477382	0.013213254	5.105643987
354.5513895	0.77967957	0.019880698	4.356700056
201.8564474	0.903641571	0.013964559	3.967536921
178.6331546	0.86444467	0.024737351	4.290885852
131.6523677	0.842043023	0.037406698	4.201462054
102.1207434	0.895250583	0.037972654	4.135241606
109.8345801	0.935545224	0.034127108	4.176739322
1065.68725	0.53759484	0.037151567	4.508869639
96.34405027	0.935838355	0.042730688	4.085257363
117.1646126	0.843824346	0.058388255	4.185144086
91.54993796	0.919695689	0.053233254	4.192731612
356.0610953	0.696328545	0.036590123	4.434556689
545.2234123	0.667419477	0.026675663	4.290767647
134.3916853	0.883219091	0.034390309	4.271783348
465.2921507	0.696996839	0.026164631	4.332669467
377.0812567	0.949681897	0.015421172	4.530479495
252.5363188	0.801602564	0.021018379	4.096245541
138.5971862	0.907030056	0.02854694	4.012706831
256.1616166	0.720401922	0.04502302	4.270388157
681.9976561	0.618504987	0.031752083	4.389212523
693.419006	0.683406475	0.022853416	4.560908116
543.9316257	0.724112863	0.02091378	4.407253571
430.0280999	0.67917895	0.033306317	4.392567842
584.6199261	0.625355964	0.036577775	4.470532418
257.3005152	0.840062026	0.021554746	4.46719719
117.1336996	0.974358974	0.088234343	3.238571014
145.691683	0.955752212	0.051824735	4.371375997
186.7907564	0.859243455	0.023596243	4.228414898
725.8305783	0.613062323	0.030179803	4.426643753
335.9251874	0.741703877	0.025948144	4.479627297
166.9323906	0.936736161	0.023337401	4.386456889
158.3258741	0.880676121	0.028417507	4.306011605
217.9620814	0.846799354	0.031692651	4.29495334
132.2568195	0.91217444	0.024053107	4.081163046
136.5144383	0.905713219	0.029854987	4.182827618
150.7909116	0.911336955	0.023732814	4.228237081
238.8301074	0.915833046	0.012893536	4.472326108
360.7656733	0.559294348	0.109608412	4.204324997
266.7561059	0.90002241	0.011682309	4.640604124
63.61639592	0.918353576	0.065892212	3.885231
152.2769897	0.982517483	0.067232499	3.588041358
143.1572029	0.956269758	0.04315021	4.391393656
565.353209	0.66581825	0.028610027	4.494972566
84.76733235	0.898453903	0.044722575	3.948232744
415.8646386	0.806067172	0.012854379	4.462685143
113.3589654	0.900547152	0.034730708	4.07488119
645.5331941	0.577480984	0.050079433	4.534836599
107.3398691	0.921469575	0.064981958	4.232112696
216.6905297	0.931721948	0.014520692	4.49283496
253.7088968	0.951293248	0.013318407	4.621529339
186.8123853	0.895262929	0.020156923	4.285042835
117.5567741	0.864971938	0.037427016	4.159957563
184.5175663	0.834469997	0.030757792	4.193074394
631.4847673	0.711073018	0.018077549	4.722960477
332.2515675	0.685718062	0.03916831	4.358281391
242.1140702	0.737589571	0.04659525	4.244737323
88.1928286	0.93904209	0.067875737	3.839086796
258.3152296	0.881877671	0.014980377	4.436876685
127.2766232	0.947985348	0.032211462	4.072169232
399.9186039	0.741030818	0.029479985	4.604945688
53.24116193	0.908552985	0.076810518	3.60345184
324.3058262	0.797261332	0.019212886	4.37854433
360.0830576	0.807529523	0.015610336	4.349346157
111.2195724	0.896621897	0.033234224	4.033647429
622.8693927	0.620867442	0.037494566	4.5077015
552.7308051	0.600211585	0.045947101	4.447211111
794.8697203	0.609291803	0.027891981	4.604929712
154.0873329	0.930665747	0.025754504	4.311818015
159.1863907	0.897098516	0.02201953	4.148200682
42.73215814	0.899381868	0.107498861	3.681087702
234.6109186	0.88625832	0.024989348	4.321636347
451.9342739	0.734040737	0.0211106	4.453549749
83.09649708	0.937016004	0.062832151	3.949288841
270.2738469	0.905735883	0.013815201	4.614663001
744.4875999	0.71434368	0.014143421	4.640562195
156.598263	0.987593052	0.051610064	3.76987329
448.0156477	0.682246613	0.029949545	4.392088013
274.0627642	0.803886732	0.017680392	4.221853512
72.71977155	0.895258166	0.070729311	4.003963179
201.1828149	0.934569675	0.015520899	4.43240192
76.95479875	0.944615385	0.053571293	3.489526486
100.1282051	0.961538462	0.099742317	2.684478613
772.2545388	0.742423223	0.011417252	4.614120606
82.57057229	0.916244939	0.07392213	4.116757259
183.2102563	0.86760355	0.026893088	4.192979196
413.3035255	0.724387059	0.026126718	4.488705526
448.6545681	0.643152334	0.042596386	4.49031315
155.110051	0.968253968	0.034890367	4.148478721
183.8087608	0.913574661	0.020122754	4.450649077
71.67029709	0.942307692	0.249099591	2.921828878
282.5774415	0.950448325	0.014622874	4.480925319
148.9388689	0.906318681	0.036433744	4.105523192

log.sigma.3.0.mm.3D_glrlm_HighGrayLevelRunEmphasis	log.sigma.3.0.mm.3D_glrlm_RunLengthNonUniformityNormalized	log.sigma.3.0.mm.3D_glszm_GrayLevelVariance
148.81823	0.826332759	15.61617729
155.6670147	0.666196683	13.40546968
63.92716537	0.827677067	12.74245542
168.3945927	0.772508703	29.71276828
118.5349309	0.909604792	27.04575163
54.81807737	0.796758217	9.743055556
55.82922384	0.906102697	6.317019722
118.9187488	0.864287614	18.91637744
296.7073843	0.872083845	54.8598168
146.5203369	0.688269389	20.38344825
153.5896332	0.825192569	11.95544783
113.5048569	0.767817304	18.32943755
77.75031799	0.74201295	16.57388117
79.80465555	0.814499919	14.83666667
96.00748191	0.88125347	18.07518827
166.8755957	0.421810367	14.67556861
85.98398194	0.884014715	15.81581676
64.10472926	0.747157943	13.56797429
82.04784981	0.852505219	19.624375
96.78071449	0.590048742	10.17317708
130.3615606	0.570119319	14.57851792
94.4086044	0.79940672	18.76512675
125.789585	0.59267047	15.45910395
321.7822327	0.901984093	35.75906636
116.0555086	0.689503128	13.32281394
99.66004246	0.8347165	10.83850158
82.39680159	0.628378165	17.14527076
132.5615427	0.511098151	11.85932405
166.9557892	0.595587213	19.29693438
150.8247932	0.645239183	14.98850369
109.2488179	0.571688079	17.0905662
120.5858603	0.524766807	13.86648527
129.7152529	0.767656639	20.7619927
108.3974359	0.948979592	26.69
131.5539213	0.915563239	28.04481471
105.3021924	0.775698958	13.66326711
141.0367687	0.497564829	11.42069967
119.619122	0.645937493	17.35393599
142.0250519	0.885177941	24.19158163
103.1304907	0.791020827	14.69678954
120.9286411	0.750429254	15.28830351
104.8330723	0.838294668	15.99596883
101.1951787	0.831088214	17.31251382
119.9640961	0.835545816	18.81624143
187.5577297	0.847958733	23.75993488
55.62028331	0.42737448	6.982446665
205.1611203	0.827365205	38.53385057
53.1804208	0.850215946	8.865913307
143.7577589	0.965222397	23.63321799
135.8530979	0.916580583	22.78254132
133.0743944	0.575608849	16.46094301
63.906566	0.817825222	10.45594643
187.6784178	0.708758566	17.34802854
85.1545773	0.818365747	15.33799049
111.4698824	0.469340929	12.64128873
86.18510326	0.856799759	21.27699011
180.8991788	0.875881193	24.805376
224.1903178	0.910024653	28.79118517
131.5903394	0.812745599	21.8846314
77.84215949	0.766663526	16.26383654
87.63669478	0.741413809	12.57663464
185.0841377	0.619096082	26.18985237
96.81978684	0.579689269	11.41614316
77.80604153	0.646072984	9.148831264
73.99187432	0.88384598	14.77504726
169.2840561	0.801200856	21.99670367
113.6820524	0.897951719	16.10380623
119.8360444	0.656483862	18.61585034
43.78410266	0.842983275	11.70578231
140.7977364	0.69830458	16.78640566
160.7063446	0.713854424	13.71632304
78.7978104	0.813085349	11.20251349
115.4008143	0.520277236	12.65370761
102.4127974	0.487625116	11.1980194
166.4988682	0.499730276	21.12852909
127.7373349	0.874530817	24.52925939
114.9734267	0.818772649	16.244749
33.55823081	0.816609505	11.7844
151.248384	0.80592185	19.75502434
141.614369	0.643069248	19.92486246
71.46082468	0.885966717	12.45408163
198.2858079	0.833116636	30.73078255
229.9822037	0.621663902	22.58307864
152.1828784	0.975213675	24.78254438
115.9419788	0.580089796	13.88102564
139.4467806	0.699073609	14.70273048
53.94441651	0.813762949	14.37664101
169.3769037	0.878549064	24.40996113
65.93086805	0.897916464	9.1936
87.31570513	0.923611111	15.32231405
254.2108375	0.655483312	21.02409568
64.703805	0.842037312	14.31805269
111.0922718	0.784913944	18.68325142
125.3328856	0.620087327	14.45039301
99.14447898	0.537527303	14.48977294
143.8234203	0.93873656	31.65759637
145.4122125	0.842914471	21.70678429
62.35767493	0.88567993	15.05
249.5016765	0.903068201	28.71078372
107.0105883	0.830183764	15.69771626

log.sigma.3.0.mm.3D_glszm_SmallAreaHighGrayLevelEmphasis	log.sigma.3.0.mm.3D_glszm_GrayLevelNonUniformityNormalized	log.sigma.3.0.mm.3D_glszm_SizeZoneNonUniformityNormalized
84.88407321	0.082770083	0.352243767
85.72229495	0.075307174	0.332804862
47.91628195	0.086419753	0.287379973
144.9713008	0.0518027	0.390515627
107.0009259	0.058054594	0.402537486
47.47924402	0.100555556	0.311111111
41.49906922	0.120525931	0.399561724
116.9902614	0.068443704	0.480031468
204.0381529	0.039140939	0.367492625
110.9351598	0.066496749	0.332200547
128.4201994	0.087257618	0.393351801
92.04333545	0.070124178	0.352903579
75.93982458	0.075617284	0.361325446
90.07288854	0.078222222	0.412355556
100.3212507	0.070501522	0.460903701
117.0442454	0.077837921	0.367179496
98.8614236	0.075878971	0.525685739
63.95320112	0.078311053	0.409051063
107.3776986	0.0665625	0.538125
59.55770993	0.08757716	0.335455247
86.51368532	0.077935095	0.378553557
86.15643902	0.067004145	0.35678412
83.67089407	0.074019452	0.337531888
222.6422522	0.053626543	0.445987654
70.67277543	0.083205493	0.349696837
68.46892524	0.08669081	0.385468538
79.52517397	0.072033898	0.375610457
62.93261901	0.091040836	0.284690233
112.82359	0.064107462	0.368808944
81.73212121	0.074322912	0.317208636
84.03416748	0.072014385	0.335029165
96.61743434	0.079676065	0.348499953
105.1799199	0.064321427	0.362055763
69.75	0.1	0.5
119.5712047	0.056018386	0.397299627
89.16193745	0.081984178	0.35913182
68.49999508	0.089052112	0.325951517
104.6509555	0.073148789	0.384653979
123.7092176	0.062244898	0.413673469
72.45660342	0.079072533	0.360285375
71.88608895	0.073228634	0.330168006
83.93836095	0.077936039	0.331631282
81.57507243	0.068229806	0.399264011
110.7928169	0.072702332	0.350562414
140.1513797	0.05996042	0.356267356
23.33511349	0.110836773	0.305505189
197.8507001	0.051220751	0.408975891
50.22777417	0.100354191	0.410693203
108.9264706	0.08650519	0.584775087
155.5235035	0.061983471	0.58393595
89.67631509	0.070115126	0.362945761
52.49799862	0.095759379	0.336805187
120.0566588	0.071458746	0.363891381
61.72758564	0.07372176	0.319262782
70.86902069	0.082574325	0.349724258
77.11252674	0.061524454	0.381633715
152.1721439	0.057578667	0.420714667
172.1318896	0.052875571	0.433586402
103.4197075	0.065597668	0.383173678
64.44110778	0.077563975	0.302519341
58.25623093	0.081168513	0.311047936
153.4828554	0.054986754	0.392955786
72.63530889	0.091275806	0.377243355
39.95289508	0.095416362	0.28944485
58.60296855	0.088846881	0.364839319
136.1704886	0.063491865	0.393849343
96.31452751	0.07420223	0.374855825
88.5423032	0.070419501	0.342653061
51.38705586	0.099773243	0.469387755
98.36510775	0.069154519	0.38485631
110.3365803	0.075859837	0.369845599
48.65898492	0.085035175	0.269039
72.68406947	0.079287478	0.344777691
59.20319889	0.084383447	0.346016875
144.9379918	0.065064547	0.396690185
119.1199491	0.061764614	0.422989295
86.06857522	0.071938668	0.361478681
36.69472815	0.0872	0.34
111.4240515	0.064705246	0.401449432
109.7332736	0.064954996	0.426032642
72.3038688	0.08122449	0.492653061
157.8277021	0.052263677	0.425207756
171.3701343	0.060209401	0.393432836
143.0961538	0.085798817	0.730769231
89.01081897	0.076831032	0.368139382
124.2256119	0.078549328	0.400124609
51.92316891	0.077184246	0.35174287
150.8160149	0.057692618	0.455686199
58.3434	0.1008	0.4912
71.29545455	0.173553719	0.702479339
165.0521551	0.064123125	0.382170375
39.07265389	0.078254132	0.260330579
86.37985423	0.065557656	0.392362949
74.5519269	0.073988637	0.341407151
70.39958743	0.072767457	0.364483643
124.1106151	0.06122449	0.526077098
133.4007786	0.061541693	0.368853415
45.27913889	0.14	0.28
209.8335926	0.056394155	0.517449583
60.03729117	0.072387543	0.323737024

log.sigma.3.0.mm.3D_glszm_SizeZoneNonUniformity	log.sigma.3.0.mm.3D_glszm_GrayLevelNonUniformity	log.sigma.3.0.mm.3D_glszm_LargeAreaEmphasis	log.sigma.3.0.mm.3D_glszm_ZoneVariance
33.46315789	7.863157895	50.97894737	36.53894737
57.90804598	13.10344828	1111.005747	1045.990256
15.51851852	4.666666667	27.90740741	17.16358025
188.6190476	25.02070393	274.2380952	258.2215278
20.52941176	2.960784314	7.392156863	3.470203768
18.66666667	6.033333333	74	57.46222222
14.78378378	4.459459459	5.513513514	1.934258583
70.56462585	10.06122449	25.96598639	18.52459623
210.9407666	22.46689895	22.68641115	16.07416625
100.6567657	20.14851485	1444.313531	1385.738
44.84210526	9.947368421	44.21052632	33.33210219
52.22972973	10.37837838	190.4662162	164.3043736
78.0462963	16.33333333	617.9537037	577.9597908
61.85333333	11.73333333	92.57333333	80.18293333
72.82278481	11.13924051	15.86075949	10.55327672
177.714876	37.67355372	68424.07438	67923.57243
54.14563107	7.815533981	15.19417476	10.75558488
95.71794872	18.32478632	571.6923077	527.7025349
43.05	5.325	18.325	13.149375
48.30555556	12.61111111	6291.277778	5920.180363
138.9291553	28.60217984	11285.49591	11094.27298
79.91964286	15.00892857	137.71875	121.2510961
75.60714286	16.58035714	4806.723214	4636.793367
32.11111111	3.861111111	5.930555556	2.518325617
40.91452991	9.735042735	721.8632479	654.539119
36.23404255	8.14893617	34.72340426	24.40153916
88.6440678	17	3012.944915	2918.871355
79.14388489	25.30935252	11419.57194	11223.26967
164.4887892	28.59192825	4538.7713	4469.351767
58.04918033	13.6010929	1658.442623	1572.854848
101.1788079	21.74834437	8217.764901	8024.260219
203.5239726	46.53082192	23515.42295	23286.65732
107.8926174	19.16778523	250.238255	228.5126458
5	1	2.5	0.25
23.44067797	3.305084746	6.118644068	2.450445274
97.32472325	22.21771218	271.6752768	247.4441797
135.2698795	36.95662651	22853.58313	22593.6355
130.7823529	24.87058824	2535.517647	2455.993218
57.91428571	8.714285714	13.33571429	8.078520408
41.79310345	9.172413793	93.50862069	72.07810642
48.86486486	10.83783784	173.1824324	149.843088
40.45901639	9.508196721	37.92622951	28.17340769
89.03587444	15.21524664	102.941704	89.28818195
47.32592593	9.814814815	47.22222222	36.64768176
126.1186441	21.2259887	67.1779661	57.25724568
49.1863354	17.8447205	63412.7205	61966.83168
339.0410133	42.46200241	189.4161641	178.4440073
31.62337662	7.727272727	34.88311688	26.11536515
9.941176471	1.470588235	1.882352941	0.207612457
51.38636364	5.454545455	5.75	2.997417355
131.7493113	25.45179063	6823.856749	6697.526581
32.67010309	9.288659794	87.86597938	70.6049527
167.0261438	32.79956427	1355.488017	1307.035499
18.51724138	4.275862069	54.67241379	37.69233056
176.9604743	41.7826087	60482.27273	59983.02404
47.32258065	7.629032258	25.18548387	17.88677159
157.768	21.592	19.712	14.30482489
105.795082	12.90163934	8.327868852	4.804555227
37.55102041	6.428571429	51.08163265	38.03331945
21.47887324	5.507042254	223.0140845	179.9361238
62.83168317	16.3960396	495.2029703	443.3201892
343.0504009	48.00343643	5451.074456	5390.829831
105.2508961	25.46594982	8588.351254	8375.128454
42.83783784	14.12162162	2382.243243	2237.268627
8.391304348	2.043478261	9.608695652	4.298676749
111.459364	17.96819788	165.4416961	148.8713306
19.11764706	3.784313725	7.039215686	2.800461361
143.9142857	29.57619048	2373.088095	2304.947001
19.71428571	4.19047619	51.54761905	39.95521542
94.28979592	16.94285714	816.7918367	763.2930612
153.8557692	31.55769231	852.8629808	813.8305231
32.55371901	10.2892562	84.91735537	62.9592241
138.9454094	31.9528536	20328.43921	20021.79826
161.9358974	39.49145299	52495.11538	51959.21267
392.3265925	64.34883721	42635.08696	42419.57031
75.7150838	11.05586592	28.67039106	22.46222028
49.88405797	9.927536232	50.55072464	39.63201008
17	4.36	72.28	52.2096
86.31162791	13.91162791	132.8511628	114.8183018
153.7977839	23.44875346	1907.722992	1851.618327
34.48571429	5.685714286	10.78571429	6.254897959
129.2631579	15.88815789	48.57565789	40.47938669
263.6	40.34029851	4493.347761	4439.226485
19	2.230769231	1.653846154	0.232248521
143.574359	29.96410256	8607.482051	8445.605365
213.2664165	41.86679174	1829.750469	1786.457526
33.06382979	7.255319149	53.0106383	37.93311453
113.4658635	14.36546185	22.18875502	16.59334527
12.28	2.52	6.72	2.72
7.727272727	1.909090909	1.545454545	0.148760331
192.9960396	32.38217822	2004.980198	1958.874753
22.90909091	6.886363636	31.47727273	19.54338843
45.12173913	7.539130435	72.4	59.31448015
104.4705882	22.64052288	4199.143791	4056.708146
159.6438356	31.87214612	20495.00913	20223.13571
22.0952381	2.571428571	3.214285714	0.964285714
76.352657	12.73913043	46.85990338	36.06851969
2.8	1.4	7.6	1.84
47.08791209	5.131868132	5.989010989	2.780582055
27.51764706	6.152941176	34.51764706	23.66643599

log.sigma.3.0.mm.3D_glszm_ZonePercentage	log.sigma.3.0.mm.3D_glszm_LargeAreaLowGrayLevelEmphasis	log.sigma.3.0.mm.3D_glszm_LargeAreaHighGrayLevelEmphasis	log.sigma.3.0.mm.3D_glszm_HighGrayLevelZoneEmphasis
0.263157895	0.487276819	7492.494737	144.3789474
0.124019957	5.482163698	236105.7759	129.8965517
0.305084746	0.890840802	1486.444444	78.83333333
0.249870667	1.550077847	54528.91718	202.610766
0.504950495	0.21888025	728.254902	140.8235294
0.245901639	1.889202624	3402.516667	75.08333333
0.528571429	0.279477822	293.4864865	59.56756757
0.366583541	0.365598124	2459.632653	150.7755102
0.388888889	0.244077156	5092.662021	317.0853659
0.130659767	9.208296739	238290.8317	167.0891089
0.303191489	0.358274719	6130.04386	181.6403509
0.195508587	1.888720877	22537.91216	127.7297297
0.158125915	11.23257606	39675.60648	111.3657407
0.284090909	2.023955103	5139.886667	120.9266667
0.434065934	0.392230319	1009.291139	126.2088608
0.044698929	389.2469538	12108448.73	173.7272727
0.474654378	0.558409367	647.2815534	117.7669903
0.150773196	12.1708887	39699	83.72649573
0.43956044	0.847490309	631.325	124.175
0.051910598	57.55948848	758806.7847	91.54861111
0.072315271	72.51431194	1803695.117	122.8065395
0.246424642	2.015213237	11920.56696	123.3705357
0.076712329	31.73792518	757744.5045	121.0714286
0.541353383	0.050737743	1969.361111	318.6527778
0.121875	5.608740539	100341.265	104.7948718
0.311258278	0.489996474	3820.510638	99.4787234
0.103101791	33.89572593	280402.0254	108.4449153
0.071373556	67.94508391	1939903.41	103
0.120021529	20.57935184	1017300.935	158.441704
0.108092144	8.230071138	351124.3825	126.6775956
0.071887646	61.70024329	1138324.546	118.0198675
0.066115702	168.2416693	3352733.45	141.8972603
0.214542837	1.871126445	41364.54362	148.9530201
0.666666667	0.139739702	268.5	109.5
0.522123894	0.249352479	626.1186441	159.3220339
0.203148426	3.063379012	30186.79336	125.195572
0.062023614	129.8926496	4071029.561	107.3662651
0.112137203	20.87701709	337846.0676	150.6205882
0.436137072	0.22172024	1420.421429	169.05
0.216014898	1.229061563	11467.37931	108.5862069
0.206993007	1.631869074	24520.08108	114.3445946
0.320209974	0.532295695	3203.008197	128.147541
0.270631068	1.324240846	9706.982063	114.5515695
0.307517084	0.641775114	4519.585185	157.8814815
0.317488789	0.508429524	10437.12712	216.4519774
0.026298595	925.0788099	4478651.484	37.47204969
0.301893664	1.358225253	28686.99276	272.8564536
0.337719298	2.045554527	1007.441558	71.83116883
0.772727273	0.090260544	274.5882353	142.0588235
0.602739726	0.285324362	387.4318182	176.2045455
0.088970588	41.89452806	1135188.606	130.1212121
0.240694789	2.957779254	3590.350515	82.96907216
0.143661972	5.873130745	329019.4161	176.4705882
0.242677824	1.060643919	3972.982759	99.15517241
0.044754997	481.5951836	7697708.111	102.9664032
0.370149254	0.589176009	2145.379032	103.5887097
0.430045872	0.172211543	3026.098667	208.896
0.532751092	0.096850271	1275.094262	245.5081967
0.276836158	0.496256518	6876.377551	146.9591837
0.152360515	4.424295154	14434.84507	104.2535211
0.138831615	5.966284762	55150.75743	84.65841584
0.128837072	24.56954561	1234644.582	211.6861397
0.068483063	86.24744423	900079.233	109.7741935
0.08305275	26.63781202	239493.7162	66.62162162
0.433962264	0.377821472	713.0869565	80.17391304
0.245659722	0.97247801	33110.30742	183.3674912
0.485714286	0.167423929	645.3333333	134.5294118
0.121142198	15.00036302	413255.4357	129.4666667
0.293706294	2.463028454	1366.047619	65.83333333
0.13671875	5.228123914	143660.0163	136.9795918
0.160061562	4.32157153	182187.8678	160.6153846
0.21340388	1.38934208	7103.991736	82.12396694
0.057106419	138.8549151	3058122.524	106.1141439
0.043197342	427.3161829	6576628.688	87.98504274
0.068117639	236.6401186	7766635.422	202.4651163
0.401345291	0.363262435	2983.413408	156.5865922
0.302631579	0.517637292	5852.92029	128.0217391
0.223214286	4.141154351	1814.42	51.98
0.235487404	1.027814531	23279.42791	150.9906977
0.133505917	10.87430003	349830.7562	147.4709141
0.469798658	0.457087853	590.2285714	89.64285714
0.351445087	0.302182287	9877.828947	218.3486842
0.135930209	16.76797524	1217739.227	238.4537313
0.838709677	0.068382868	227.8076923	158.8076923
0.07859734	63.06748135	1222909.128	126.2410256
0.151981751	14.20498587	245814.2458	174.5140713
0.257534247	1.825570237	2457.340426	71.10638298
0.422750424	0.200793833	3304.46988	197.0843373
0.5	0.233642396	399.92	74.48
0.846153846	0.117695693	141.1818182	85.27272727
0.147273258	6.247617528	660740.6693	239.829703
0.289473684	1.299847967	1787.193182	70.73863636
0.276442308	0.696782901	9270.13913	119.9043478
0.083789704	28.35000297	667773.9935	111.0163399
0.06064802	171.2443313	2519111.603	101.260274
0.666666667	0.0736793	397.4761905	154.7619048
0.304411765	0.565880888	5804.439614	189.2028986
0.416666667	1.772662999	468	71.3
0.558282209	0.052280185	1328.406593	269.8131868
0.303571429	0.437817778	4312.776471	99.25882353

log.sigma.3.0.mm.3D_glszm_SmallAreaEmphasis	log.sigma.3.0.mm.3D_glszm_LowGrayLevelZoneEmphasis	log.sigma.3.0.mm.3D_glszm_ZoneEntropy	log.sigma.3.0.mm.3D_glszm_SmallAreaLowGrayLevelEmphasis
0.616210849	0.025075711	5.277361794	0.0212572
0.598277243	0.019033736	5.643178723	0.013499982
0.547569598	0.046482299	5.039532123	0.029346726
0.648857437	0.023272318	6.15089589	0.01667173
0.655359488	0.045348359	4.970965783	0.033790057
0.568612711	0.048015769	4.850489296	0.03133378
0.653847368	0.056316459	4.141621609	0.040649132
0.717220257	0.022131225	5.275565086	0.017934537
0.628884316	0.012112787	6.595594755	0.008436602
0.59667179	0.019512206	6.011274114	0.011330308
0.650476613	0.016256124	5.076801611	0.013188457
0.615332323	0.026448987	5.553591289	0.012417422
0.621059652	0.040330575	5.625746526	0.026597412
0.665737077	0.025563485	5.205848049	0.011801362
0.705565875	0.03135974	5.288383635	0.023891814
0.629677662	0.012155368	5.847632889	0.006044329
0.75125611	0.029965537	4.955058475	0.017305349
0.664257294	0.055672907	5.416007153	0.028829006
0.754894446	0.035231902	4.946762239	0.026619184
0.597658724	0.028815135	5.332369289	0.018576256
0.63928038	0.017637559	5.77114118	0.012263013
0.615007593	0.030613479	5.768452682	0.015151608
0.599468338	0.02200267	5.72642035	0.012818652
0.694304377	0.019994918	5.255004127	0.017798138
0.613720272	0.027396771	5.420101768	0.020753336
0.644605247	0.028472027	5.049641809	0.014373867
0.636439915	0.039380905	5.708349069	0.028992756
0.551724283	0.020215005	5.958588926	0.01281797
0.631061171	0.018513737	6.068815653	0.010874054
0.583330923	0.02282914	5.723052867	0.016080618
0.600194417	0.025119994	5.880931607	0.010871316
0.61270246	0.017420742	5.85106594	0.010738602
0.624777002	0.020990012	5.940947288	0.013533059
0.625	0.121919802	3.321928095	0.117464827
0.647938742	0.039015814	5.03731588	0.016161773
0.621925613	0.020581384	5.515283579	0.012225503
0.593248553	0.022360102	5.957083803	0.015790507
0.643814019	0.020155607	5.720070882	0.01405621
0.667517533	0.023314231	5.493500404	0.017962618
0.62263719	0.032649869	5.384482048	0.022928629
0.594050891	0.02794423	5.453700573	0.013267794
0.588401867	0.026217129	5.346732732	0.017733759
0.656541184	0.039287656	5.596329606	0.027918598
0.607634856	0.01967243	5.469527454	0.008547264
0.617052638	0.011253456	6.012115938	0.005885692
0.570512088	0.098287838	5.402419851	0.048194139
0.664145069	0.009591198	6.250995285	0.006705441
0.663627063	0.045675034	4.687288109	0.030822367
0.779411765	0.082355122	3.852168724	0.080378767
0.790403768	0.027739149	4.80763524	0.020445253
0.625196262	0.022311693	5.813371646	0.015517992
0.599573403	0.038094908	5.058287159	0.028207632
0.626888262	0.01229861	6.022204495	0.008475257
0.579223257	0.047962716	5.034283797	0.037544873
0.614669677	0.027455332	5.862329026	0.016812684
0.637682506	0.080252729	5.442984445	0.052867889
0.673941518	0.013944351	5.841438961	0.009882718
0.682840607	0.013467318	5.742610022	0.010988151
0.641697703	0.027853082	5.256021677	0.022690492
0.569202661	0.044295252	5.21961895	0.030198727
0.577079912	0.034224882	5.619740938	0.015697661
0.65165615	0.011595509	6.218800944	0.007320588
0.637122674	0.022607701	5.404982351	0.014656161
0.555888824	0.048668628	5.551467629	0.025119727
0.622871007	0.075414982	3.96900163	0.053906243
0.652089544	0.015923722	5.809798586	0.01040379
0.632321623	0.03377394	4.853318724	0.02630387
0.608339834	0.02175236	6.108466438	0.012468383
0.707757802	0.084735231	4.252453066	0.067416824
0.642889608	0.024573215	5.64127851	0.017960185
0.631238169	0.012799198	5.770488329	0.006880829
0.531644871	0.036253718	5.466644223	0.020132665
0.609281933	0.02200824	5.80025855	0.013635003
0.610373315	0.026981874	5.728370647	0.015032184
0.654921519	0.011132693	6.074973255	0.006556466
0.673012434	0.025405216	5.566322942	0.01408416
0.621895126	0.026058428	5.427809249	0.019278667
0.593351175	0.131566284	4.75856294	0.105239128
0.658623662	0.024663557	5.673943629	0.011090671
0.678350616	0.018053609	5.732087236	0.013318043
0.726204115	0.041226954	4.666374046	0.018046124
0.677517678	0.015491895	5.938669549	0.011540554
0.652060434	0.009183639	6.103184199	0.006006726
0.879273504	0.057309841	4.085055103	0.055728668
0.628071459	0.018257726	5.688501437	0.011091218
0.65761175	0.011656237	5.697079528	0.006348942
0.612977632	0.094804213	5.285095076	0.073585616
0.701738403	0.015890519	5.673453045	0.011951409
0.710911111	0.062222495	3.94346519	0.052975611
0.863636364	0.108506164	3.095795255	0.106208782
0.64206689	0.010176631	6.047557958	0.007669207
0.512071302	0.072004505	5.44370346	0.03113822
0.650627808	0.039176375	5.500609918	0.032249115
0.606335576	0.030587888	5.749520099	0.018652021
0.623481026	0.0300114	5.743378887	0.017181316
0.751488095	0.041613408	4.67173022	0.037609092
0.628843589	0.015942845	5.741427458	0.007724592
0.468583333	0.126598839	3.121928095	0.017378914
0.745777532	0.017689288	5.169897871	0.015879999
0.587129711	0.049816523	5.286765313	0.030166249

log.sigma.3.0.mm.3D_ngtdm_Coarseness	log.sigma.3.0.mm.3D_ngtdm_Complexity	log.sigma.3.0.mm.3D_ngtdm_Strength	log.sigma.3.0.mm.3D_ngtdm_Busyness	log.sigma.3.0.mm.3D_ngtdm_Contrast
0.023863253	0.686255297	3.372465875	0.188246464	0.000273186
0.007148278	0.101971688	1.019424281	0.481545133	3.96E-05
0.044468431	0.883720502	4.517648519	0.217505565	0.000617534
0.004653577	0.363115936	1.538899303	0.475184201	3.91E-05
0.064681349	5.071418831	10.53077201	0.095668492	0.002823988
0.030514807	0.388096215	1.315303674	0.388161071	0.000433549
0.070612862	1.268636279	2.808357248	0.219124849	0.003281071
0.018426035	1.046379658	3.035214384	0.244755554	0.000292057
0.0060145	1.114512002	3.14216824	0.395251531	0.000172003
0.004568824	0.110990412	1.108628958	0.547292973	1.19E-05
0.01841612	0.565689179	1.758052605	0.274952803	0.000274022
0.012801525	0.2827524	1.787089115	0.347979673	8.80E-05
0.006240597	0.156701481	0.761361935	0.922991669	4.80E-05
0.014962933	0.610899386	2.392215556	0.367958273	0.000161458
0.020233191	1.192052823	3.399398794	0.262723322	0.000360484
0.000794254	0.013143446	0.365710955	2.530677478	6.91E-07
0.027786602	1.814172364	3.831565675	0.252441141	0.000821771
0.005668589	0.119194613	0.663583586	1.166173169	4.47E-05
0.032935624	2.201377165	4.780235764	0.287629163	0.001312942
0.00440015	0.024834272	0.417677808	1.300709421	1.53E-05
0.002074609	0.029600325	0.487749067	1.296492941	3.12E-06
0.008731915	0.395153053	1.527398401	0.516501492	9.12E-05
0.003740255	0.043372648	0.65152456	0.871487031	8.92E-06
0.053537971	4.927226191	15.05152162	0.043339466	0.001964232
0.010802845	0.136050647	1.48528063	0.37434068	3.61E-05
0.027000299	0.432527945	1.900687486	0.331664728	0.000466437
0.004547775	0.084972519	1.182255238	0.754738487	8.52E-06
0.002490438	0.036632354	0.736581668	1.078783635	5.19E-06
0.002483067	0.063814138	0.672497633	0.886433039	1.03E-05
0.006375296	0.087656217	1.095639063	0.489862255	2.81E-05
0.002591039	0.041301078	0.613699837	1.092990533	4.54E-06
0.001104075	0.017180393	0.269447891	2.495804532	1.87E-06
0.006323501	0.283801125	1.383930192	0.499011298	5.22E-05
0.192778155	15.56027115	16.78416744	0.082600473	0.109900151
0.072004787	3.813872259	10.58589719	0.080685866	0.002313447
0.006189775	0.174605296	0.707726731	0.817503094	6.64E-05
0.001310433	0.01745207	0.323012441	2.138147193	3.41E-06
0.003421681	0.081148799	0.769600866	0.855494398	9.90E-06
0.021363613	1.776132247	4.184009162	0.188417584	0.0005326
0.01687712	0.400505147	1.983561993	0.341499864	0.000199428
0.013786814	0.261174731	1.887612111	0.314249037	0.000102022
0.018609917	0.754281946	2.239066479	0.291853736	0.000291457
0.008936466	0.3729505	1.23993047	0.576009639	0.000123768
0.017492536	0.917866309	3.324566436	0.252977881	0.000241111
0.007244197	0.563186371	1.922256851	0.385125816	9.63E-05
0.001465611	0.005452633	0.134699259	5.026554303	3.51E-06
0.003188916	0.659114038	3.525975099	0.442027082	2.16E-05
0.026091944	0.77841107	1.870053531	0.55928887	0.000753543
0.13842987	15.73567798	14.64262952	0.06492044	0.049125247
0.035792373	4.173414455	5.970747767	0.16557401	0.002763974
0.002575783	0.039269533	0.527471182	1.064425467	5.95E-06
0.015385809	0.46801055	1.339539743	0.702450331	0.00034398
0.00327825	0.111507466	0.899638027	0.632568117	1.17E-05
0.037412155	0.532839434	3.039988408	0.236446002	0.000449841
0.000805164	0.012046537	0.267499281	3.474017081	1.19E-06
0.021250231	0.908224836	2.280547882	0.316226728	0.000559492
0.00823152	0.90685194	2.22215936	0.32412253	0.000143487
0.015973013	1.994761523	3.766571545	0.197265734	0.000490558
0.026166831	0.792710006	3.640864265	0.162540577	0.000267612
0.020713935	0.289305028	1.977171519	0.368995749	0.000158037
0.006727412	0.097479835	0.6286313	0.970802733	5.64E-05
0.00150482	0.064251769	0.613853396	1.131095154	4.30E-06
0.002480755	0.027209392	0.353839637	1.640161991	5.75E-06
0.00571986	0.044295829	0.494211909	1.085450838	2.75E-05
0.120523352	2.899891912	9.766530804	0.080422239	0.004220633
0.008177331	0.420214694	1.981382781	0.307994422	6.12E-05
0.057720623	2.504518689	4.945813503	0.152814784	0.002644127
0.002986941	0.09565461	0.869334956	0.868918943	1.26E-05
0.040761713	0.816595459	2.468688366	0.381989776	0.00109617
0.005653204	0.134989401	1.037229348	0.530734139	2.46E-05
0.003649342	0.098932312	0.689034068	0.765901933	1.84E-05
0.013992428	0.237348964	0.917348487	0.722676486	0.000227631
0.001471104	0.018265482	0.321045095	2.039954206	2.89E-06
0.000866781	0.00894005	0.180323597	4.057743522	1.64E-06
0.000640075	0.023891726	0.477859025	2.430576334	6.32E-07
0.015624016	1.147064092	2.719762837	0.270935811	0.000338828
0.016784936	0.537464403	1.857192387	0.295026461	0.000218463
0.03011426	0.556847156	2.172165584	0.508987034	0.000429484
0.009631784	0.345740208	1.559122662	0.362262506	9.47E-05
0.003644169	0.104691015	0.918348631	0.664956217	1.21E-05
0.044328867	1.072926845	2.916444903	0.291066926	0.001448022
0.010236758	0.793590282	2.50672165	0.24368551	0.000135336
0.002003758	0.083847731	0.857783721	0.746443443	5.19E-06
0.100114884	14.95035783	14.67965421	0.060359506	0.024255795
0.00204201	0.033964835	0.440055738	1.440408991	4.29E-06
0.002669184	0.070914097	0.521734092	1.100068595	8.88E-06
0.021419493	0.446333136	1.859839476	0.485596509	0.000292538
0.011912914	1.195527207	2.619077939	0.260328683	0.000243124
0.102662927	2.209153647	4.847359367	0.124751723	0.004718795
0.109442963	11.09249671	10.26809182	0.160518018	0.185764804
0.002826252	0.111172288	0.993182827	0.561203374	1.09E-05
0.023552947	0.684512041	2.380404608	0.46636398	0.00053728
0.02165881	0.574813569	2.647937756	0.194009744	0.000192831
0.003113753	0.03302493	0.417104098	1.25844546	1.17E-05
0.0013744	0.01651629	0.257237014	2.577166565	3.13E-06
0.075964254	9.918186786	12.24169915	0.08370382	0.008353925
0.011930542	0.725754079	2.293106175	0.319806611	0.000186623
0.078082071	6.441089401	6.837809137	0.258728026	0.073625261
0.042079324	3.969604331	9.054960698	0.075882235	0.001391193
0.030907158	0.557752295	2.983509045	0.194658145	0.00042507

log.sigma.2.5.mm.3D_gldm_GrayLevelVariance	log.sigma.2.5.mm.3D_gldm_HighGrayLevelEmphasis	log.sigma.2.5.mm.3D_gldm_GrayLevelNonUniformityNormalized	log.sigma.2.5.mm.3D_gldm_DependenceEntropy
10.93199101	119.7036011	0.084997813	6.166006692
7.675343894	177.9750535	0.154940868	6.520546657
7.756264164	88.24858757	0.108302212	5.702447873
15.56211459	236.1914123	0.089124041	6.891644484
17.65395549	126.970297	0.073032056	5.169322409
5.885581833	57.09016393	0.125436711	5.767527583
10.14714286	112.1571429	0.091428571	4.896930695
11.93740089	78.2319202	0.08511763	5.934708991
48.34523469	259.5	0.046490001	6.986101008
6.76438131	160.4217335	0.13654586	6.560089797
8.007858477	131.3005319	0.100398936	6.003105625
8.100975658	103.996037	0.103074955	6.380005194
6.986068267	86.69985359	0.110760382	6.471027224
8.337978507	70.86174242	0.109396522	6.00301356
14.62343769	122.1785714	0.081798696	5.927198598
2.732226094	124.7135205	0.323695228	6.231759302
11.80165219	80.22580645	0.095457538	5.629736029
6.966620639	75.50837629	0.110040088	6.487366528
13.55259027	70.87912088	0.092017872	5.810149935
5.015277952	89.0739005	0.166814727	6.670549003
3.970181271	124.4072906	0.209327982	6.327259385
9.528291223	119.6512651	0.095586126	6.402041511
4.685057117	121.0071918	0.180693141	6.550319385
33.00548363	256.6541353	0.053309967	5.424207601
5.635589193	129.1927083	0.14671441	6.365225626
9.089480725	82.01655629	0.094930047	5.854140109
4.897082386	72.00961118	0.182133634	6.481506262
4.452504033	108.3145058	0.267056379	6.342756568
8.205351774	192.3347686	0.203239765	6.532004731
7.156203805	154.1772002	0.175028408	6.3910415
4.817969288	103.6786479	0.188234334	6.501690439
3.785879729	110.7946338	0.228282848	6.532161484
11.50274319	103.3671706	0.096414749	6.662342178
16.64	108.8	0.12	3.373557262
21.41091706	101.1415929	0.068212076	5.517891215
8.678423719	86.3005997	0.100791433	6.425997009
4.752023058	158.9593484	0.263710383	6.238826866
6.288363002	152.6728232	0.14664054	6.693490264
17.61791908	147.6915888	0.072252793	6.052425973
10.86639687	80.93296089	0.087398437	6.382686217
8.679904152	111.0433566	0.105939655	6.467070891
9.113412005	85.96325459	0.09742975	5.955511017
10.77912474	143.0012136	0.088309454	6.26556733
11.43321174	134.7494305	0.085776848	6.13137721
18.90999135	176.2493274	0.070726538	6.493072537
2.328713096	63.57301535	0.312399964	6.08710934
23.42418954	235.399126	0.07728304	6.787830271
8.367247615	53.81140351	0.098145583	5.627420447
20.58884298	142.5909091	0.107438017	3.6412498
20.49620004	85.48630137	0.072433853	5.628841108
5.549182466	126.7487745	0.199149846	6.544732185
9.43368902	53.8560794	0.098572123	5.844540553
9.285890268	266.7402191	0.118417911	6.736933756
8.191593284	73.33472803	0.098685247	5.973720838
3.84525371	120.4932779	0.278591205	6.357590024
13.80328804	121.3432836	0.081773223	6.049055015
19.29100954	172.1479358	0.066955643	6.39783333
28.63282546	188.3406114	0.053965409	6.127282317
11.4168103	128.1666667	0.088256887	6.131950394
7.21291606	69.98283262	0.108677633	6.171184209
8.466445129	128.1395189	0.102386604	6.387591626
8.870734128	175.8813459	0.160381665	6.692696401
4.032268762	82.34216986	0.194528011	6.627748999
5.419841576	87.82996633	0.165920963	6.578642265
10.69419722	57.60377358	0.100747597	4.727551586
13.04606044	176.7838542	0.083432798	6.661869384
12.90285714	90.34285714	0.081723356	5.168495398
9.615038957	138.2382463	0.139794888	6.71369863
6.613624138	36.9020979	0.125238398	5.130884212
8.265424456	156.9910714	0.115469796	6.682742509
8.120705851	130.9730666	0.114940322	6.709099399
8.555303603	80.13227513	0.094214732	6.145074664
4.525618625	110.4956781	0.21868068	6.518806099
3.528425356	114.5220602	0.256737289	6.251540648
4.597133687	173.697431	0.238029739	6.527463873
16.38587142	149.9282511	0.075599751	6.119955647
9.947887812	113.9342105	0.091229994	6.200597896
5.886399872	60.9375	0.123804209	5.464252928
11.8956703	153.0186199	0.087566836	6.45146444
7.554518727	161.2448225	0.152622348	6.576405713
9.205981713	76.79865772	0.093914689	5.491674361
21.02074376	190.2312139	0.068087808	6.541818361
8.827072323	385.1744776	0.172367645	6.717891749
22.83662851	149.5806452	0.088449532	4.155651552
4.682812917	128.8242644	0.184786481	6.637633631
5.680745072	169.932706	0.137340294	6.697032791
7.524008257	65.86849315	0.106203791	5.884411256
15.48817742	170.8692699	0.073575829	6.237916961
9.9744	71.44	0.096	4.44836744
17.86982249	94.76923077	0.147928994	3.39274741
10.2543308	427.6097988	0.140760778	6.801331475
10.65372879	97.99013158	0.085850935	5.828875229
10.99171944	171.4158654	0.107456546	6.279336214
6.349986924	119.9370208	0.142654057	6.706593697
4.250154283	110.2414844	0.2164059	6.379660953
23.51473923	139.3333333	0.07079869	4.792689598
15.19982483	113.4720588	0.074273356	6.338600151
15.38888889	74.16666667	0.09375	3.834962501
21.20027099	199.7423313	0.066204976	5.642885403
9.986109694	98.68214286	0.093979592	5.8660149

log.sigma.2.5.mm.3D_gldm_DependenceNonUniformity	log.sigma.2.5.mm.3D_gldm_GrayLevelNonUniformity	log.sigma.2.5.mm.3D_gldm_SmallDependenceEmphasis
54.1468144	30.68421053	0.219229557
99.79258731	217.3820385	0.128271027
27.15819209	19.16949153	0.224949557
209.4956027	172.2767719	0.253152006
28.5049505	7.376237624	0.520647002
32.8442623	30.60655738	0.246047059
20.45714286	6.4	0.492710317
74.71072319	34.13216958	0.312260498
286.1910569	68.61924119	0.314257546
186.992238	316.6498491	0.132966411
55.49468085	37.75	0.267007866
89.19550859	78.02774108	0.189102947
133.0732064	151.2986823	0.176151881
70.73106061	57.76136364	0.243279849
76.01098901	29.77472527	0.385944442
485.9586258	3504.971925	0.051701384
43.21198157	20.71428571	0.385061379
143.3530928	170.7822165	0.147382612
29.48351648	16.74725275	0.337017584
157.8940159	462.7440519	0.072224095
259.9229557	1062.339507	0.079146734
120.4653465	86.88778878	0.250388902
162.6041096	527.6239726	0.090975154
41.34586466	7.090225564	0.512754804
76.85833333	140.8458333	0.125602128
47.28476821	28.66887417	0.209275122
142.2442114	416.9038882	0.107415149
179.9530167	1040.184596	0.081431428
207.0543595	755.2389666	0.113379487
110.2900177	296.3230951	0.114759198
224.6619852	790.7724351	0.08962258
422.0933998	2016.422393	0.073867862
146.7408207	133.9200864	0.194161746
7.8	1.8	0.7
27.14159292	7.707964602	0.410786627
151.6041979	134.4557721	0.190881374
337.6005081	1764.486175	0.065281727
194.6424802	444.6141161	0.124898717
67.25545171	23.19314642	0.375892937
72.00931099	46.93296089	0.194319369
72.57762238	75.74685315	0.178869347
60.71128609	37.12073491	0.269403863
124.5097087	72.76699029	0.229979161
70.77220957	37.65603645	0.263757442
202.9174888	78.86008969	0.302042748
288.5213982	1912.512578	0.037918242
398.5440641	212.219228	0.28798673
43.36842105	22.37719298	0.283411461
10.63636364	2.363636364	0.686868687
30.69863014	10.57534247	0.383208793
219.9965686	812.5313725	0.10070623
62.6674938	39.72456576	0.225717001
267.1302034	378.3452269	0.145798828
34.0209205	23.58577406	0.201100875
533.0031842	3149.752167	0.055195161
61.4	27.39402985	0.350844253
174.4472477	58.3853211	0.375259758
131.2838428	24.71615721	0.471041104
52.86440678	31.24293785	0.248333155
57.36051502	50.64377682	0.145210106
146.0075601	148.9725086	0.144051585
405.9808146	1086.746163	0.129169372
224.5483554	792.5071183	0.084927971
118.0942761	295.671156	0.087404969
12.54716981	5.339622642	0.332106597
148.2395833	96.11458333	0.222059965
26.65714286	8.580952381	0.43366394
235.274589	484.668878	0.118631777
25.25174825	17.90909091	0.21992238
149.3828125	206.921875	0.149213831
218.4220854	298.7298961	0.157903742
83.97707231	53.41975309	0.183935164
330.3158566	1543.229559	0.063714885
537.2095256	2781.491785	0.054044961
704.9607411	3455.953785	0.079380562
88.69955157	33.71748879	0.335107208
68.64035088	41.60087719	0.25166438
38.49107143	27.73214286	0.196010406
118.0865279	79.94852136	0.20947182
168.1501479	412.6908284	0.13147665
28.34228188	13.99328859	0.31811354
138.4219653	58.89595376	0.31794436
299.8094948	849.6001217	0.132370911
12.93548387	2.741935484	0.575268817
258.8625554	916.91052	0.087274799
278.9806102	481.6524095	0.154670143
54.37534247	38.76438356	0.226194799
113.7470289	43.33616299	0.374096422
16.32	4.8	0.435905556
7.461538462	1.923076923	0.769230769
233.1463984	482.6687081	0.148345859
56.78947368	26.09868421	0.217721313
50.19711538	44.70192308	0.257117262
223.3127054	520.9726177	0.092688276
374.9520908	1562.883412	0.067178939
18.17460317	4.46031746	0.519982363
114.5852941	50.50588235	0.295410282
12.33333333	2.25	0.5625
41.80981595	10.79141104	0.451029968
44.25	26.31428571	0.264800181

log.sigma.2.5.mm.3D_gldm_DependenceNonUniformityNormalized	log.sigma.2.5.mm.3D_gldm_DependenceVariance	log.sigma.2.5.mm.3D_gldm_LargeDependenceEmphasis
0.149991176	5.305530191	19.89750693
0.071128002	39.38818406	100.8032787
0.153436114	3.548597146	16.5819209
0.10837848	26.38688137	55.79772375
0.282227233	2.014116263	6.801980198
0.134607632	5.32094867	20.35245902
0.292244898	1.196734694	5.428571429
0.18631103	2.523292766	11.40399002
0.19389638	3.332439171	12.0501355
0.080634859	31.31606109	78.7352307
0.147592236	4.312782933	17.12765957
0.11782762	10.18120614	32.13606341
0.09741816	16.54550482	46.17862372
0.133960342	4.988980716	19.91666667
0.208821398	3.006943606	10.43406593
0.044879814	39.0594288	191.0445142
0.199133556	3.575357302	11.76497696
0.092366683	14.99948022	47.57474227
0.161997343	4.818017148	15.97802198
0.056919256	33.17942331	117.1917808
0.051216346	40.24101127	136.6366502
0.132525134	7.60943069	23.65346535
0.055686339	38.50681695	121.080137
0.310871163	1.013624286	4.864661654
0.080060764	21.63604167	64.86666667
0.15657208	4.95276523	18.85430464
0.062142513	31.27261671	96.09305374
0.046201031	43.35523935	157.0143774
0.055719688	44.31027929	132.1227126
0.065144724	44.04725689	118.1210868
0.053478216	34.53361446	118.2815996
0.047785962	39.79549583	147.320163
0.105644939	23.64435156	55.20302376
0.52	0.24	2.2
0.240191088	1.962408959	7.884955752
0.113646325	20.11319003	47.85307346
0.050455912	35.65764102	154.947392
0.064196069	32.15857546	94.09630607
0.209518541	2.139944294	9.168224299
0.134095551	8.668712656	27.09310987
0.101507164	15.4906235	43.42517483
0.159347208	3.540882193	14.9160105
0.151104015	7.298802903	21.9684466
0.161212322	3.597335008	15.00911162
0.181988779	3.966297332	13.73542601
0.047128618	33.99810731	176.5122509
0.145136221	6.5247434	20.02621996
0.190212373	2.359649123	11.35964912
0.483471074	0.338842975	2.454545455
0.21026459	3.233627322	10.73972603
0.053920728	42.09170031	130.2122549
0.155502466	6.246993701	20.45409429
0.083608827	24.5834345	66.64444444
0.142346948	4.398137288	19.70292887
0.047143391	37.38325661	165.3456572
0.183283582	3.480026732	12.33731343
0.200054183	2.803194176	10.42889908
0.286645945	1.825384718	6.554585153
0.149334482	8.822337132	24.10734463
0.123091234	7.684834865	29.8111588
0.100348839	20.05559878	53.95120275
0.059914524	35.87027135	108.3311688
0.055117417	35.22653661	118.1732941
0.066270638	37.1239216	107.4691358
0.236739053	2.437166251	9.716981132
0.128680194	9.177803699	27.13715278
0.253877551	2.352290249	8.066666667
0.067861145	33.27840867	95.73262186
0.176585652	2.808450291	14.21678322
0.083360944	25.41776821	68.38169643
0.084040818	22.06304997	60.87225856
0.148107711	4.396094423	19.87830688
0.046806838	42.71192307	162.6046479
0.04958552	33.77405278	150.9425881
0.048554359	34.4430234	141.590261
0.198877918	2.582175391	10.53811659
0.150527085	4.449830717	17.5745614
0.17183514	2.820153061	15.32142857
0.129339023	16.44042185	38.24205915
0.062185706	37.01127794	105.8202663
0.190216657	2.708526643	11.38926174
0.160025393	8.025019212	20.66242775
0.060825623	32.04485417	98.90342869
0.417273673	0.422476587	3.129032258
0.052168995	37.95044986	125.6267634
0.079549646	21.6187141	61.88166524
0.148973541	3.887363483	17.34520548
0.193118895	3.046439968	11.0950764
0.3264	1.0336	5.36
0.573964497	0.213017751	1.923076923
0.067992534	32.76267671	90.5608049
0.186807479	2.580289127	13.61842105
0.120666143	17.751826	40.08653846
0.061148057	30.3896666	99.77327492
0.051918041	31.59103453	129.9747992
0.288485765	1.719324767	6.587301587
0.168507785	6.118546713	17.47941176
0.513888889	0.243055556	2.75
0.256501938	2.914599721	8.668711656
0.158035714	5.355867347	17.85714286

log.sigma.2.5.mm.3D_gldm_LargeDependenceLowGrayLevelEmphasis	log.sigma.2.5.mm.3D_gldm_SmallDependenceHighGrayLevelEmphasis	log.sigma.2.5.mm.3D_gldm_LargeDependenceHighGrayLevelEmphasis
0.192102472	26.11692094	2898.152355
0.477253648	18.74541816	22235.49608
0.242859199	21.02147927	1539.887006
0.220519944	68.36594011	15406.75944
0.115425645	79.21183168	643.029703
0.514159318	18.7731816	1006.696721
0.106430903	53.63293651	550.9
0.305590096	29.34579672	828.7705736
0.122323159	86.02889654	2853.902439
0.447870105	23.40442487	14557.86201
0.159843003	38.75154844	2251.912234
0.333030533	21.73547451	3883.219287
0.554797293	18.37316994	4695.657394
0.460769134	26.14384005	1201.486742
0.139291557	59.30575921	1016.03022
1.522034502	6.680136104	24507.51478
0.280336638	42.737075	631.9677419
0.662849193	11.5879589	4819.019974
0.548867355	35.14052413	691.8681319
1.231818397	5.37358125	12310.85761
0.980010926	8.693652551	19611.66246
0.233397716	35.91275787	2937.864686
0.879379627	9.700354712	17501.45856
0.055209066	145.4973851	1249.210526
0.440644253	13.69008615	10330.16979
0.317354732	15.89097185	2103.990066
1.346230059	9.260393003	7623.44692
1.320283677	6.722520761	19339.50886
0.605504232	19.29176083	30014.22928
0.641418234	13.84264175	22709.91613
1.02282652	8.553191635	14498.15092
1.248141191	8.290786112	18183.87762
0.544253118	22.84477465	7463.660907
0.112594234	75.9	240.4
0.472647854	56.37888397	524.6814159
0.52895901	18.41945602	5976.153673
0.891539719	7.670143792	27690.35764
0.578219754	21.81580156	16050.79947
0.112927529	63.85011855	1241.049844
0.508256685	14.18735176	3010.590317
0.433291265	17.56136315	5807.071329
0.263187852	28.05905341	1229.328084
0.157685892	34.4083116	4002.078883
0.156664485	44.63318029	2008.585421
0.135857451	62.56747727	2233.851121
2.624455729	1.681735952	12586.59
0.116118358	90.21214239	4012.003642
0.563222573	19.31433166	502.4429825
0.081673828	105.7777778	302.7727273
0.778235255	49.13656709	408.9794521
0.938480368	12.27956052	18857.41152
0.684432067	13.8783881	1723.498759
0.222367739	36.54222801	20999.20094
0.404949221	18.25777258	1436.958159
1.338425624	5.993435425	21163.16407
0.131321681	47.3887945	1612.955224
0.123583888	79.43622583	1548.700688
0.095346827	96.34643572	949.9563319
0.209786167	33.81693546	3855.087571
0.574364232	12.47368414	2228.566524
0.351604858	15.77844345	10005.54845
0.564273538	25.25582633	21822.12485
1.472615931	7.750885813	10125.56112
1.13509551	6.274722902	11199.06678
0.643866675	19.2068577	432.9245283
0.177401758	42.59860921	5155.261285
0.205250947	50.48155264	492.5142857
0.608100794	15.42120344	16877.53158
0.834813597	12.7429275	374.2027972
0.385328631	20.30737902	13105.02567
0.417015195	19.72426754	10339.77107
0.330345997	13.14343615	1984.809524
1.323031519	5.754647324	20927.9966
1.231495778	4.844245737	19112.37733
0.797969005	15.93540526	25714.64846
0.141905645	58.94731322	1475.986547
0.203511792	33.07483752	2143.114035
0.335920871	12.28887162	969.6964286
0.249712212	31.57616058	8230.431544
0.571982916	21.17897285	20545.99038
0.240694187	28.79721396	832.2751678
0.128559936	68.2411255	4632.8
0.241158301	50.04599634	41288.57963
0.072143951	95.27598566	434.6129032
0.882725788	11.51673765	18688.28416
0.368770714	30.62674647	10935.92187
0.40115844	16.7100147	1210.761644
0.095041714	74.90471378	1754.560272
0.289965766	32.80402778	358.08
0.110278896	68	201.8461538
0.18695686	56.88195489	44883.49723
0.175446813	18.62274066	1667.240132
0.214602068	46.91575909	8283.545673
0.742397897	9.189282496	14599.09967
1.094367035	6.884979326	16246.21241
0.103065192	88.72683862	721.047619
0.288122868	43.55004346	1915.527941
0.414259519	42.16666667	202.1666667
0.077679134	94.37902798	1456.257669
0.206910008	20.71162127	2277.939286

log.sigma.2.5.mm.3D_gldm_SmallDependenceLowGrayLevelEmphasis	log.sigma.2.5.mm.3D_gldm_LowGrayLevelEmphasis	log.sigma.2.5.mm.3D_gldzm_DistanceZoneVariabilityNormalized
0.007240835	0.016804646	0.95240928
0.00204426	0.007979218	0.945694579
0.009905536	0.022489231	1
0.002436423	0.006783031	0.92056504
0.018971247	0.026915127	1
0.012262602	0.035389629	0.865574459
0.020849184	0.027632626	1
0.015833715	0.038349186	0.956855536
0.003800354	0.011868843	0.565528121
0.002175676	0.00877	0.860621485
0.005657727	0.012750818	0.948753463
0.004859708	0.016813156	0.972608025
0.00409768	0.019324532	0.939223376
0.00780926	0.031675195	0.957559133
0.009893054	0.018229738	0.97515921
0.001009241	0.009883357	0.943338675
0.014771253	0.029104389	0.979168975
0.006525453	0.025076847	0.91322314
0.022509088	0.045354689	1
0.002650304	0.01725361	0.98795225
0.00166087	0.010109185	0.973619492
0.004919039	0.014044371	0.953175346
0.001970401	0.011078413	0.983936796
0.011649779	0.018064504	1
0.002906268	0.011207104	0.983607677
0.007463348	0.030806089	0.947406866
0.005113082	0.024547478	0.98333451
0.002390195	0.013098043	0.982091015
0.001414033	0.007607751	0.921357818
0.002398378	0.009653362	0.98984798
0.002743707	0.014191236	0.982710249
0.001428906	0.012143787	0.888340175
0.008178452	0.024495692	0.885561594
0.074913432	0.082449592	1
0.01512333	0.058088693	1
0.006067584	0.022686008	0.936013192
0.001069378	0.007922703	0.920880856
0.001584382	0.008490155	0.968093499
0.008833493	0.01676546	0.945248959
0.010144938	0.038759842	0.869498307
0.006873649	0.018855188	0.985294925
0.014453095	0.02952629	0.948753463
0.004095199	0.011749557	0.908472791
0.004778855	0.013687991	0.911303407
0.003212437	0.012020943	0.714895874
0.002450816	0.023520578	0.924506173
0.002277373	0.006880258	0.655384183
0.013037613	0.046489554	0.828707085
0.062590474	0.065775832	1
0.015321373	0.05641964	0.971020761
0.002597203	0.012041046	0.92207659
0.01587681	0.05735457	0.763168012
0.001120946	0.004806697	0.744948476
0.010035216	0.030686161	1
0.001146797	0.010894155	0.927769115
0.011098638	0.020142159	0.984849368
0.004686802	0.014810303	0.963929706
0.014425057	0.021094753	0.953694012
0.007455114	0.018422254	1
0.007531643	0.030411098	0.918270184
0.002532522	0.01236317	0.934065416
0.001565008	0.0080538	0.934924792
0.002252805	0.017599633	0.933463379
0.003290382	0.018328193	0.9608
0.033064645	0.065850666	1
0.003367728	0.010409918	0.913221787
0.020949018	0.033863992	1
0.002393664	0.01195942	0.8828125
0.025887696	0.072613231	0.900277008
0.003864671	0.010456598	0.954763314
0.003146055	0.012247974	0.940561938
0.008318534	0.025538067	0.91322314
0.001607934	0.012554633	0.975004006
0.001332347	0.011086574	0.940884348
0.000856401	0.00699319	0.816973644
0.005998931	0.018842007	0.943867152
0.008477075	0.019200446	0.968512
0.009635936	0.029109877	0.926035503
0.00522533	0.016528182	0.96280407
0.002058182	0.008654704	0.95571196
0.021366038	0.037685321	1
0.007077423	0.013268241	0.961071355
0.000672718	0.003137866	0.875393599
0.045870885	0.05047891	1
0.001311345	0.009843117	0.926925
0.001427045	0.007291654	0.94506112
0.013381299	0.032839708	0.89721172
0.005043005	0.01097357	0.952029024
0.02437511	0.06601855	1
0.09130977	0.095103595	0.702479339
0.000927744	0.003068569	0.868255286
0.00904321	0.021221424	0.948088889
0.005594119	0.011253846	1
0.002409117	0.01277953	0.967329279
0.0013443	0.011758732	0.929834847
0.02711018	0.034124284	1
0.008099027	0.024707516	0.903756716
0.042510233	0.116860091	1
0.011077289	0.016083519	1
0.014336179	0.027899182	1

log.sigma.2.5.mm.3D_gldzm_LowIntensityEmphasis	log.sigma.2.5.mm.3D_gldzm_LargeDistanceEmphasis	log.sigma.2.5.mm.3D_gldzm_HighIntensitySmallDistanceEmphasis
0.033494329	1.073170732	116.4329268
0.017044704	1.083798883	133.7206704
0.041392132	1	91.21276596
0.009695018	1.133458647	256.7744361
0.03407183	1	147.8666667
0.053028045	1.217391304	67.22463768
0.037650034	1	110
0.055398616	1.066176471	89.98161765
0.013134966	2.107407407	168.8492284
0.016926811	1.28802589	159.01726
0.02007601	1.078947368	138.247807
0.028393784	1.041666667	112.6909722
0.026910461	1.094117647	105.65
0.039300178	1.100719424	105.7284173
0.024557475	1.037735849	147.918239
0.022719114	1.174757282	128.1656132
0.036351281	1.031578947	108.5789474
0.046885263	1.136363636	77.43636364
0.06410282	1	101.4929577
0.045979583	1.018181818	71.44848485
0.023330375	1.040106952	104.1871658
0.019766606	1.091633466	137.1525011
0.024048421	1.024291498	103.2105263
0.021734759	1	278.6125
0.024969034	1.024793388	100.9669421
0.045739882	1.081081081	70.69256757
0.054220536	1.025210084	86.85294118
0.032347466	1.027108434	74.4314759
0.014304187	1.123006834	158.7306378
0.022777756	1.015306122	111.3278061
0.036387917	1.026162791	91.76889535
0.023232775	1.387959866	109.8266328
0.046029213	1.182795699	112.6487455
0.096781856	1	108.5833333
0.042963684	1	133
0.034007394	1.099264706	91.54044118
0.018049007	1.123786408	105.9429612
0.013096039	1.048648649	179.1101351
0.021906803	1.084507042	165.3978873
0.065746866	1.210526316	65.61842105
0.040511922	1.022222222	94.34814815
0.053992686	1.078947368	95.89692982
0.018427602	1.167464115	138.7134503
0.018439691	1.139534884	158.1724806
0.012491687	1.614173228	164.5528762
0.086203732	1.227777778	37.21867284
0.008019137	2.165492958	219.5808881
0.047174159	1.283783784	64.56756757
0.081999058	1	151.2352941
0.043212834	1.044117647	121.9301471
0.030356306	1.134177215	113.8912096
0.075259142	1.411764706	52.51960784
0.008015797	1.797894737	203.8351316
0.054031467	1	89.78431373
0.024929642	1.112570356	103.2818949
0.03166057	1.022900763	126.6412214
0.014196253	1.05511811	200.4750656
0.028134402	1.071146245	201.4110672
0.031139827	1	131.4803922
0.058162188	1.197183099	86.47809077
0.021720458	1.126213592	97.40264293
0.013235763	1.106502242	190.4804123
0.032506443	1.133956386	89.83610246
0.041485036	1.06	66.65666667
0.08873858	1	58.86956522
0.016591101	1.154716981	183.4113208
0.043813165	1	111.3518519
0.022319291	1.1875	120.8575
0.109224886	1.157894737	54.11184211
0.027304716	1.088461538	129.2769231
0.020822929	1.12	117.2419118
0.048142696	1.136363636	63.71363636
0.031064109	1.037974684	86.29240506
0.029801521	1.101419878	81.08677034
0.012001993	1.853432282	193.7643709
0.019823855	1.086705202	167.9783237
0.033724648	1.048	127.882
0.047432429	1.115384615	57.60096154
0.029697774	1.056872038	139.7381517
0.016927676	1.081920904	154.1031073
0.061280271	1	85.75438596
0.021811801	1.075907591	205.1435644
0.005146301	1.242647059	355.183701
0.069544131	1	158.1904762
0.016755529	1.325	127.4239056
0.009389119	1.084805654	197.3334806
0.05783243	1.163043478	68.41032609
0.013547001	1.073770492	192.7008197
0.065710026	1	72.67857143
0.109406084	1.545454545	89.75
0.006316051	1.235181644	352.6405354
0.039377448	1.08	81.42666667
0.021143403	1	178.1610169
0.032080898	1.049833887	91.87043189
0.023154572	1.121065375	98.82048695
0.046473584	1	160.8205128
0.02939393	1.152073733	146.3110599
0.095002615	1	74.58823529
0.021744405	1	206.3695652
0.052427072	1	77.05681818

log.sigma.2.5.mm.3D_gldzm_LowIntensityLargeDistanceEmphasis	log.sigma.2.5.mm.3D_gldzm_HighIntensityEmphasis	log.sigma.2.5.mm.3D_gldzm_DistanceZoneVariability	log.sigma.2.5.mm.3D_gldzm_ZonePercentage
0.033959289	119.597561	78.09756098	0.227146814
0.017566234	137.1731844	169.2793296	0.127583749
0.041392132	91.21276596	47	0.265536723
0.010250612	265.424812	489.7406015	0.275219865
0.03407183	147.8666667	60	0.594059406
0.055340201	72.76811594	59.72463768	0.282786885
0.037650034	110	41	0.585714286
0.055823991	92.66176471	130.1323529	0.33915212
0.018048769	276.6574074	305.3851852	0.365853659
0.017857344	177.7702265	265.9320388	0.133247089
0.020478514	142.3596491	108.1578947	0.303191489
0.02899113	113.5763889	140.0555556	0.190224571
0.028918645	107.7294118	239.5019608	0.186676428
0.040094056	107.8417266	133.1007194	0.263257576
0.025775703	150.2767296	155.0503145	0.436813187
0.024714547	130.4194175	485.8194175	0.047561877
0.038324966	108.7052632	93.02105263	0.437788018
0.053404707	79.57727273	200.9090909	0.141752577
0.06410282	101.4929577	71	0.39010989
0.046263674	71.73939394	163.0121212	0.059480894
0.0235812	106.1122995	364.1336898	0.073694581
0.02025089	141.0318725	239.247012	0.276127613
0.024291336	103.8178138	243.0323887	0.084589041
0.021734759	278.6125	80	0.601503759
0.025095531	102.1818182	119.0165289	0.126041667
0.046281767	73.90540541	70.10810811	0.245033113
0.055095886	87.35714286	234.0336134	0.103975535
0.032612136	75.16566265	326.0542169	0.085237484
0.015148692	163.5330296	404.476082	0.118137783
0.022904253	111.7908163	194.0102041	0.115770821
0.036522774	93.05523256	338.0523256	0.081885265
0.028669718	114.1153846	531.2274247	0.067700668
0.04877075	116.4659498	247.0716846	0.200863931
0.096781856	108.5833333	12	0.8
0.042963684	133	55	0.486725664
0.035200616	93.87867647	254.5955882	0.203898051
0.019214975	109.368932	379.4029126	0.06157525
0.013508913	180.6567568	358.1945946	0.122031662
0.023088177	167.4788732	134.2253521	0.442367601
0.06824198	70.5	99.12280702	0.212290503
0.040734144	94.9037037	133.0148148	0.188811189
0.054607594	98.64035088	108.1578947	0.299212598
0.019278992	146.215311	189.8708134	0.253640777
0.019273164	164.2945736	117.5581395	0.293849658
0.016367494	209.3543307	272.3753281	0.341704036
0.08958015	39.72777778	166.4111111	0.029402156
0.011595178	314.9131455	558.3873239	0.310269483
0.052815038	70.24324324	61.32432432	0.324561404
0.081999058	151.2352941	17	0.772727273
0.087330481	121.9411765	66.02941176	0.465753425
0.031451958	119.0050633	364.2202532	0.096813725
0.079716948	63.10784314	77.84313725	0.253101737
0.010515784	244.1347368	353.8505263	0.148669797
0.054031467	89.78431373	51	0.213389121
0.026352652	105.7598499	494.5009381	0.04714311
0.03177741	127.7633588	129.0152672	0.391044776
0.014404682	204.4908136	367.2572178	0.436926606
0.04045276	203.6996047	241.284585	0.552401747
0.031139827	131.4803922	102	0.288135593
0.0597918	90.54929577	65.1971831	0.152360515
0.022483216	101.6601942	192.4174757	0.141580756
0.015410452	193.7253363	833.9529148	0.131641086
0.033848257	92.79127726	299.6417445	0.078792342
0.042209739	67.98666667	144.12	0.084175084
0.08873858	58.86956522	23	0.433962264
0.019334143	186.509434	242.0037736	0.230034722
0.043813165	111.3518519	54	0.514285714
0.02420758	125.9575	353.125	0.115373522
0.110989019	57.68421053	34.21052632	0.265734266
0.027812959	132.4461538	248.2384615	0.145089286
0.021634698	120.7294118	399.7388235	0.163524432
0.049130735	68.69090909	100.4545455	0.194003527
0.031475795	87.26835443	385.1265823	0.055972793
0.030909762	83.32048682	463.8559838	0.045504892
0.017386443	207.6131725	880.6975881	0.074247538
0.02028092	172.4393064	163.2890173	0.387892377
0.03410666	129.496	121.064	0.274122807
0.048174445	62.11538462	48.15384615	0.232142857
0.030047341	142.3080569	203.1516588	0.231106243
0.017430075	156.9265537	338.3220339	0.13091716
0.061280271	85.75438596	57	0.382550336
0.022303769	207.970297	291.2046205	0.350289017
0.005909646	371.9279412	595.2676471	0.137959018
0.069544131	158.1904762	21	0.677419355
0.019285816	131.6425	370.77	0.080612656
0.009974392	201.4823322	534.9045936	0.161391503
0.059116904	73.75	82.54347826	0.252054795
0.013910746	196.9918033	232.295082	0.41426146
0.065710026	72.67857143	28	0.56
0.399178811	90.90909091	7.727272727	0.846153846
0.007001195	374.2198853	454.0975143	0.152522601
0.039933004	84.30666667	71.10666667	0.246710526
0.021143403	178.1610169	118	0.283653846
0.032672753	92.9269103	291.166113	0.082420591
0.024374723	102.3535109	384.0217918	0.057186375
0.046473584	160.8205128	39	0.619047619
0.038100522	147.9631336	196.1152074	0.319117647
0.095002615	74.58823529	17	0.708333333
0.021744405	206.3695652	92	0.564417178
0.052427072	77.05681818	88	0.314285714

log.sigma.2.5.mm.3D_gldzm_IntensityVariabilityNormalized	log.sigma.2.5.mm.3D_gldzm_LowIntensitySmallDistanceEmphasis	log.sigma.2.5.mm.3D_gldzm_IntensityVariability
0.06543724	0.033378089	5.365853659
0.079679161	0.016914321	14.26256983
0.086464464	0.041392132	4.063829787
0.052970773	0.009562566	28.18045113
0.067222222	0.03407183	4.033333333
0.081705524	0.052450006	5.637681159
0.088637716	0.037650034	3.634146341
0.068014706	0.055292272	9.25
0.042640604	0.012033797	23.02592593
0.06842199	0.016730269	21.14239482
0.082179132	0.019975384	9.368421053
0.071566358	0.028244447	10.30555556
0.080107651	0.026408415	20.42745098
0.072511775	0.03915722	10.07913669
0.0582651	0.024252918	9.264150943
0.077975304	0.022434244	40.15728155
0.071024931	0.03585786	6.747368421
0.089586777	0.045255402	19.70909091
0.069629042	0.06410282	4.943661972
0.086280992	0.045908561	14.23636364
0.083988676	0.023267669	31.41176471
0.067887811	0.019668121	17.03984064
0.076693603	0.023987692	18.94331984
0.053125	0.021734759	4.25
0.086264599	0.02493741	10.43801653
0.096055515	0.045604411	7.108108108
0.072840901	0.054001698	17.33613445
0.09529685	0.032281298	31.63855422
0.067761168	0.014093061	29.74715262
0.077259475	0.022746132	15.14285714
0.077119389	0.036354202	26.52906977
0.081626604	0.022379351	48.81270903
0.061342994	0.045343829	17.11469534
0.111111111	0.096781856	1.333333333
0.060495868	0.042963684	3.327272727
0.075448746	0.033709089	20.52205882
0.088898577	0.017757515	36.62621359
0.070489408	0.012992821	26.08108108
0.062685975	0.02161146	8.901408451
0.078177901	0.065123087	8.912280702
0.076323731	0.040456366	10.3037037
0.07463835	0.053838959	8.50877193
0.067374831	0.01823315	14.08133971
0.075536326	0.018231323	9.744186047
0.055889667	0.01179162	21.29396325
0.110864198	0.085735038	19.95555556
0.047347859	0.007454832	40.34037559
0.094594595	0.04576394	7
0.093425606	0.081999058	1.588235294
0.066176471	0.032183422	4.5
0.068956898	0.030099038	27.23797468
0.0911188	0.07414469	9.294117647
0.069127978	0.007580277	32.83578947
0.088811995	0.054031467	4.529411765
0.076085311	0.02457389	40.55347092
0.062292407	0.031631359	8.160305344
0.056743891	0.014144146	21.61942257
0.050602259	0.025054812	12.80237154
0.064590542	0.031139827	6.588235294
0.077563975	0.057884119	5.507042254
0.088179847	0.021550838	18.16504854
0.054128376	0.012699461	48.28251121
0.089546879	0.032228203	28.74454829
0.103022222	0.04130386	15.45333333
0.096408318	0.08873858	2.217391304
0.059793521	0.015934458	15.84528302
0.072702332	0.043813165	3.925925926
0.0657375	0.021847218	26.295
0.090027701	0.108783853	3.421052632
0.068343195	0.027196648	17.76923077
0.075620761	0.020649398	32.13882353
0.08231405	0.047895686	9.054545455
0.079314212	0.030961188	31.32911392
0.083493452	0.029537797	41.16227181
0.063095267	0.011393818	68.01669759
0.059039727	0.019709589	10.21387283
0.067904	0.033629145	8.488
0.112426036	0.047246925	5.846153846
0.066687631	0.029610382	14.07109005
0.063280028	0.016816027	22.40112994
0.08341028	0.061280271	4.754385965
0.047435437	0.021700127	14.37293729
0.05866782	0.004979172	39.89411765
0.070294785	0.069544131	1.476190476
0.072475	0.016482615	28.99
0.081209654	0.009242801	45.96466431
0.087192817	0.057511312	8.02173913
0.060299651	0.013456064	14.71311475
0.091836735	0.065710026	2.571428571
0.123966942	0.036962902	1.363636364
0.060903963	0.006155876	31.85277247
0.086222222	0.03923856	6.466666667
0.061907498	0.021143403	7.305084746
0.075330294	0.031932935	22.6744186
0.07537712	0.022876438	31.13075061
0.057199211	0.046473584	2.230769231
0.058761069	0.027217282	12.75115207
0.093425606	0.095002615	1.588235294
0.062145558	0.021744405	5.717391304
0.080578512	0.052427072	7.090909091

log.sigma.2.5.mm.3D_gldzm_HighIntensityLargeDistanceEmphasis	log.sigma.2.5.mm.3D_gldzm_SmallDistanceEmphasis	log.sigma.2.5.mm.3D_glcm_SumVariance	log.sigma.2.5.mm.3D_glcm_Homogeneity1
132.2560976	0.981707317	309.1295572	0.428280649
150.9832402	0.979050279	535.3358003	0.577512724
91.21276596	1	216.9018963	0.453439956
302.7330827	0.968723893	667.3422417	0.445400905
147.8666667	1	278.2310137	0.313931223
94.94202899	0.945652174	121.2387894	0.438053791
110	1	282.2503824	0.318894383
103.3823529	0.983455882	173.3515973	0.368518525
811.8407407	0.775681584	784.2558134	0.299565171
278.197411	0.942376843	469.684958	0.544753392
158.8070175	0.980263158	348.3218975	0.408251846
117.1180556	0.989583333	263.3540845	0.472349794
116.0470588	0.976470588	206.5870244	0.484802907
120.8992806	0.982813749	146.4541526	0.429528509
159.7106918	0.990566038	275.9558646	0.343921565
147.9941748	0.97770953	380.898534	0.717654107
109.2105263	0.992105263	160.0820436	0.367935449
88.14090909	0.965909091	181.8780882	0.497945836
101.4929577	1	122.7591357	0.389723143
72.9030303	0.995454545	235.5802967	0.630955447
113.8128342	0.989973262	372.4035981	0.64804021
160.0199203	0.981518371	303.929727	0.424195399
106.2469636	0.993927126	351.3330049	0.631196262
278.6125	1	745.4434753	0.282302946
107.0413223	0.993801653	381.1469759	0.573850609
86.75675676	0.97972973	204.5675487	0.431440722
89.37394958	0.993697479	179.3849029	0.597648455
78.10240964	0.993222892	324.6232756	0.681620052
182.7425968	0.969248292	590.4754314	0.616236102
113.6428571	0.996173469	466.9939442	0.602971319
98.2005814	0.993459302	295.2922389	0.624974581
152.8662207	0.953409513	318.485066	0.655869621
131.734767	0.954301075	250.4779083	0.474615491
108.5833333	1	271.2555618	0.253442404
133	1	198.8551846	0.328218596
103.2316176	0.975183824	207.7140461	0.462878718
123.0728155	0.969053398	499.7617572	0.673858646
186.8432432	0.987837838	429.9289193	0.572040943
175.8028169	0.978873239	344.1139738	0.322972771
90.02631579	0.947368421	194.7522884	0.443827863
97.12592593	0.994444444	288.4684041	0.508723445
109.6140351	0.980263158	201.3770736	0.400627824
182.3684211	0.963450292	390.8562937	0.406629591
188.7829457	0.965116279	351.0376208	0.395857052
405.6850394	0.871336395	486.2970871	0.341139854
57.12222222	0.967746914	181.0717251	0.721494513
1049.360329	0.834922731	679.3100143	0.365982102
92.94594595	0.929054054	108.3956216	0.378660466
151.2352941	1	340.068599	0.241133363
121.9852941	0.988970588	135.4299342	0.331377587
141.3620253	0.969268636	366.9011039	0.621944675
105.4607843	0.897058824	120.5775867	0.404142781
559.9621053	0.885467836	834.9291334	0.513958201
89.78431373	1	162.766237	0.452636968
115.6716698	0.971857411	359.2783326	0.686386911
132.2519084	0.994274809	295.0415407	0.362940021
220.5538058	0.986220472	462.3414788	0.343625488
212.8537549	0.982213439	446.2862228	0.27838047
131.4803922	1	327.4802441	0.42792688
114.4084507	0.966353678	157.7583712	0.476783403
124.2135922	0.973840345	367.8890337	0.50018141
207.5470852	0.97462008	509.7809473	0.578203489
107.9626168	0.973433714	210.8352185	0.625862253
73.30666667	0.985	232.9515125	0.611471512
58.86956522	1	119.5365698	0.383081263
201.3169811	0.965513627	459.9958895	0.429829954
111.3518519	1	184.2836191	0.334551648
146.3575	0.953125	392.1668164	0.560924362
71.97368421	0.960526316	65.77433077	0.416330323
148.9692308	0.98215812	447.8727068	0.531286793
140.6352941	0.976617647	365.1236979	0.523204164
88.6	0.965909091	201.9518485	0.423604943
91.1721519	0.990506329	320.9085669	0.668199053
93.77890467	0.976898805	342.1864891	0.675860112
363.7115028	0.919454687	525.4656569	0.645491155
190.283237	0.978323699	374.8551444	0.348384738
135.952	0.988	296.7270637	0.410946339
80.17307692	0.971153846	148.8732353	0.423293401
152.5876777	0.985781991	446.2281141	0.45051092
171.0451977	0.982658506	478.6043326	0.587515553
85.75438596	1	174.2749418	0.404768412
224.029703	0.984690136	519.7891584	0.381065238
455.4735294	0.949142157	1237.260995	0.563363016
158.1904762	1	334.350797	0.252766712
177.95	0.968936806	372.897778	0.625370384
218.0777385	0.978798587	483.0088413	0.532881152
95.10869565	0.95923913	155.6095277	0.42074608
214.1557377	0.981557377	441.8644599	0.351721706
72.67857143	1	141.9031453	0.325017982
95.54545455	0.863636364	202.1658182	0.257524365
470.9483748	0.946582218	1400.262203	0.542028738
95.82666667	0.98	265.2947647	0.392828893
178.1610169	1	474.0646948	0.458887864
97.15282392	0.987541528	335.1725559	0.596695372
117.5617433	0.972423998	319.7049263	0.644668426
160.8205128	1	328.2050305	0.293861207
154.5714286	0.961981567	239.0105868	0.371141323
74.58823529	1	158.3531812	0.277968097
206.3695652	1	470.5458898	0.322429571
77.05681818	1	258.9082217	0.439515675

log.sigma.2.5.mm.3D_glcm_Homogeneity2	log.sigma.2.5.mm.3D_glcm_ClusterShade	log.sigma.2.5.mm.3D_glcm_MaximumProbability	log.sigma.2.5.mm.3D_glcm_Idmn	log.sigma.2.5.mm.3D_glcm_SumVariance2
0.349256807	-17.51647921	0.031829083	0.977560819	31.56807707
0.529750643	-109.0251438	0.208494613	0.987021899	22.49910372
0.380413765	8.611384006	0.040759562	0.983566467	19.87860981
0.375824439	-62.66605559	0.099129327	0.985465537	36.83803313
0.218560205	96.40790912	0.035552548	0.957347477	36.7084645
0.363768174	2.792415711	0.047253337	0.959816499	12.78222456
0.222717466	-36.14446867	0.049411421	0.929310545	23.28780547
0.27914155	37.03384212	0.023892345	0.969289286	29.00705465
0.214222917	873.5489252	0.011117753	0.962355705	158.5213497
0.491096345	-31.09215202	0.152104182	0.992377431	17.81444231
0.323816825	-7.205763909	0.031548133	0.974405484	19.7174983
0.403676092	-32.73778172	0.059926658	0.98250412	21.66842844
0.420950046	-0.980529766	0.065684599	0.977211092	16.22630963
0.353977281	41.27333714	0.041732196	0.976694904	17.37404518
0.25385935	151.3918199	0.026539712	0.97069452	33.18331048
0.70143209	-42.316422	0.288304243	0.995987342	7.107398383
0.283167415	81.16963446	0.041007622	0.96260833	20.96563765
0.434539579	-24.29820234	0.077256793	0.981656132	19.74062905
0.30921668	99.93305969	0.048262437	0.957040808	22.32583073
0.596811114	-47.3817338	0.159179499	0.989442529	15.2599484
0.616898273	-35.82850678	0.252077366	0.996458767	10.05141323
0.347215531	-1.655816301	0.034533111	0.981718102	21.48048375
0.595870168	-57.29393738	0.202578609	0.993157773	12.89912812
0.186922951	-476.8216819	0.024091045	0.963323225	114.6518463
0.526679061	-60.21630121	0.150216639	0.992714674	14.72855061
0.353159168	-16.20037476	0.034518484	0.966026264	27.32529644
0.557999587	-18.45873012	0.198577783	0.991529262	12.24826148
0.655882465	-64.41592166	0.307987006	0.995922128	11.29970809
0.576535837	-141.1445692	0.250998673	0.990148747	22.56949024
0.559024929	-106.7505381	0.24694584	0.990068926	19.97844923
0.590731707	-48.48667022	0.196139762	0.993416369	12.39861156
0.626900636	-48.33464762	0.238144935	0.992868885	10.33802679
0.407062471	-55.65477344	0.108792535	0.983464848	30.26906145
0.156791739	21.32831668	0.1375	0.896091153	27.04396902
0.236199394	149.4705917	0.032729909	0.958661424	53.35615641
0.39099174	-21.88732462	0.089297024	0.975156507	24.16642218
0.649131258	-87.96266627	0.22475256	0.993757074	13.18299467
0.526456227	-33.70535896	0.145447025	0.991972389	15.82111301
0.231792378	134.0873191	0.022264872	0.965473028	42.85183294
0.368918856	-32.99632306	0.04958646	0.970243983	32.02606693
0.449135051	-89.18100311	0.099260238	0.983769186	25.00754235
0.316322138	-6.21450644	0.037788674	0.971598447	21.3157874
0.324742231	-10.1629155	0.031455362	0.983138333	26.42992115
0.311911423	21.49726699	0.026705238	0.982616469	27.841954
0.253049445	331.6146061	0.017320636	0.975058093	56.92438579
0.706939865	-31.26269283	0.232354828	0.993986098	6.041620117
0.285634156	941.3335921	0.026501428	0.983863906	67.69998637
0.291029327	30.09486798	0.028299425	0.940366839	20.50866512
0.143133538	17.86981388	0.084881785	0.892736044	40.44898296
0.245687772	188.9792938	0.045447384	0.93716243	42.8366584
0.585619582	-68.37821133	0.236186192	0.991233664	14.55549158
0.321353792	56.28409353	0.033840609	0.956238735	27.17968184
0.455039038	-91.68847188	0.122694709	0.992701265	27.40219029
0.378121694	20.39919399	0.037040941	0.976215561	22.97741487
0.665145502	-63.17149312	0.251350593	0.996836255	10.60680722
0.275508034	-2.47149287	0.031396717	0.968094751	31.15697739
0.255125601	101.239057	0.020634197	0.972806586	49.61587259
0.185331974	238.0233121	0.016192534	0.949370015	71.17103904
0.349969092	-62.1625729	0.057296813	0.975364077	29.1407339
0.407288195	11.91250337	0.050934823	0.979528327	19.87822668
0.436578745	-38.89513529	0.07269185	0.982442583	25.5552756
0.53495331	-88.81377108	0.165563646	0.992660123	22.94321394
0.590565834	-34.13452086	0.18931994	0.991550573	10.85154165
0.570072091	-52.53691081	0.17950024	0.984365186	16.37529995
0.292149997	46.14252313	0.063416795	0.952401279	29.05246416
0.355503165	-83.43323671	0.047086969	0.985358564	35.0320276
0.239696141	50.99426162	0.042343204	0.942949972	26.42146278
0.512066858	-114.9731022	0.125030315	0.992188646	27.84524703
0.336336634	26.47795778	0.055468365	0.956948266	13.38220341
0.476008533	-75.00489882	0.120947743	0.988453278	22.51773592
0.466822718	-87.99821133	0.091061477	0.985607667	23.44577287
0.342878848	-1.004171345	0.026589696	0.960289038	26.41654603
0.640131543	-64.65713893	0.206108385	0.992769264	12.80571182
0.653512523	-50.17243383	0.238143801	0.99613026	9.590981694
0.61815666	-38.06303833	0.181671927	0.99644856	11.61017921
0.259872316	13.91941998	0.024025496	0.972549689	33.7862404
0.329685792	-41.06412578	0.028264454	0.974331639	25.13086101
0.342136204	19.50446754	0.044459852	0.96724509	15.22412845
0.375462147	-89.50505351	0.075705652	0.981917671	30.81364393
0.544123922	-61.5048225	0.193120971	0.992889109	18.57303529
0.320343944	-6.572996742	0.042963178	0.960959276	22.7783037
0.301961853	4.032366329	0.037188199	0.9771078	49.69550463
0.518901347	-124.1407695	0.134185813	0.994651666	23.37538194
0.160066774	9.240558939	0.067282877	0.92207807	47.19663625
0.59000441	-48.60591576	0.174590258	0.994081436	12.71585524
0.479318048	-8.88633721	0.098525193	0.990569952	13.73978981
0.33932255	22.32695443	0.03571733	0.975272226	20.28539381
0.262538069	52.74092222	0.019246846	0.976794756	38.04641667
0.2287541	3.449826746	0.050629814	0.91084518	24.44053587
0.161605504	53.750025	0.109126984	0.864236679	37.81229529
0.49127742	-182.0975094	0.117403166	0.992175758	28.53448875
0.306460484	6.138926021	0.023678081	0.968165719	29.94358872
0.388306095	-60.65630734	0.109363074	0.981460768	25.84672845
0.555881318	-74.084802	0.143959938	0.98982437	18.4169289
0.615827764	-51.83685638	0.203249478	0.994248904	11.75978872
0.202295701	157.1471463	0.041428436	0.951118202	45.86084532
0.286061814	77.04126854	0.03019797	0.969677002	36.83920129
0.177715382	39.86139033	0.096319813	0.897713043	34.49965502
0.229818522	35.57639955	0.033825457	0.964261958	53.12189397
0.363841536	-76.30675546	0.041990836	0.97382392	29.1245753

log.sigma.2.5.mm.3D_glcm_Contrast	log.sigma.2.5.mm.3D_glcm_DifferenceEntropy	log.sigma.2.5.mm.3D_glcm_InverseVariance	log.sigma.2.5.mm.3D_glcm_Entropy	log.sigma.2.5.mm.3D_glcm_Dissimilarity
8.76204593	2.704494185	0.34827372	6.66626713	2.277994212
5.04810338	2.34649676	0.374782077	5.482303022	1.480768952
6.280706921	2.43510484	0.394660676	5.933591967	1.948316143
18.28006967	3.13616152	0.305048511	7.055900036	2.858575975
23.49874413	3.200282182	0.236448706	6.260703806	3.840983904
9.206546269	2.711653132	0.36362037	6.071732801	2.269736602
21.71394476	2.989308343	0.214274814	5.431340852	3.784707842
15.1020254	3.054369538	0.288188543	6.978417444	3.00385115
53.34877924	3.880545803	0.212671946	8.793155524	5.319418235
5.406416321	2.407023514	0.391710059	5.853110629	1.585342259
10.06402807	2.788856752	0.320838691	6.516501442	2.467852565
6.80029218	2.542356722	0.384591248	6.364323534	1.940032012
8.279787296	2.656886226	0.380106654	6.292840258	2.019137334
10.36686961	2.781831973	0.363333432	6.388715678	2.363307437
22.12718416	3.283427113	0.260919017	7.068104457	3.5652761
1.878542551	1.641566401	0.409474713	3.729052646	0.761855668
18.89260875	3.14311109	0.286075006	6.361647742	3.273423487
6.403274446	2.535453042	0.379408636	6.155811735	1.842262434
18.41182985	3.114909782	0.291606541	6.241459853	3.145589561
2.510305022	1.96212937	0.427043817	5.088175784	1.061461072
2.66932472	1.956859561	0.415570311	4.663991032	1.02803405
11.4385032	2.876644946	0.339633738	6.727387456	2.493120823
2.852160848	2.023972355	0.409944405	4.960084606	1.102571477
34.29685423	3.443289845	0.194243571	6.653321339	4.672033343
3.318500539	2.130786786	0.422598118	5.307772212	1.300011641
8.456709967	2.667602163	0.345456346	6.421203025	2.263071584
4.004178022	2.177074466	0.424750709	5.217726541	1.287016253
2.653272506	1.886872316	0.387797385	4.198701384	0.954390462
5.646868427	2.319068755	0.356089463	5.113945383	1.418897077
4.198037866	2.254020376	0.368826845	5.195256776	1.336272625
3.39303057	2.062905752	0.425044542	4.916211902	1.151919193
2.727189684	1.948646458	0.413984601	4.630022016	1.018031334
10.36556141	2.826105761	0.336417856	6.593130326	2.246437284
39.40576923	2.057692226	0.19119755	3.179487097	5.113461538
25.1956474	3.263420084	0.237457772	6.383287581	3.855594754
8.847532542	2.756403541	0.335753399	6.477965822	2.190207233
2.632271365	1.880224688	0.41120129	4.20700845	0.96561305
5.316588762	2.355067633	0.404397815	5.628703378	1.490584765
25.94376348	3.306354127	0.235893459	7.239700936	3.96254504
8.427678991	2.697639614	0.353428366	6.619598747	2.208001706
6.431801363	2.485545977	0.398866965	6.179411241	1.775020544
11.44702027	2.862049553	0.320537852	6.604782597	2.590693207
12.18843611	2.923594539	0.316564767	6.851293985	2.637659426
12.59714428	2.936494329	0.317467345	6.825915745	2.696296916
24.68756159	3.394214945	0.257417349	7.790871928	3.742678888
1.230418947	1.524599669	0.426402317	3.547490202	0.685102445
33.3086516	3.47559064	0.286300952	7.767356358	3.852770311
11.90353083	2.87304022	0.311258921	6.484238482	2.751519703
45.8472139	2.622555365	0.159572192	4.051293991	5.535364635
30.5900272	3.429334686	0.229206761	6.43205826	4.219805807
4.167744511	2.171463755	0.39859777	5.048714567	1.248512387
11.29551237	2.861445533	0.313632779	6.567870679	2.610521279
7.339164909	2.584513651	0.376529946	6.306335028	1.853729558
6.581213626	2.509633196	0.376761782	6.253808365	1.991939712
2.218729668	1.776043727	0.424623203	4.148575242	0.877465238
19.37828489	3.186059074	0.282763639	6.967672532	3.276092071
23.70718384	3.353384079	0.258723279	7.631531974	3.667294017
39.82239437	3.685278501	0.190378666	7.874052302	4.976843544
10.82028286	2.847585109	0.325476557	6.620307789	2.463008781
5.636141297	2.420313748	0.389988597	6.163318438	1.819900932
6.093614209	2.516615566	0.378264426	6.156420952	1.809691575
7.56637947	2.465155183	0.386230977	5.643862066	1.638496477
2.895817588	2.020998792	0.421486729	4.990243733	1.112687826
3.313460452	2.119339603	0.389872653	5.215947717	1.216981528
10.47074371	2.398299032	0.292527902	5.190068623	2.639640845
13.37281377	2.922486854	0.340486999	7.021221484	2.577241277
19.49414138	3.067380647	0.238227227	6.115499335	3.510920632
5.961285518	2.448158823	0.381884015	5.749993077	1.609620134
9.837317095	2.72795342	0.348112626	5.845232232	2.402449863
5.960870221	2.465077634	0.393399802	6.044097192	1.679776096
6.234028623	2.490341284	0.39576593	6.090032241	1.726267277
8.823991766	2.713807443	0.338522769	6.658781279	2.31273764
2.473187462	1.907141257	0.400028534	4.544932471	0.969055488
1.931932696	1.759756392	0.442280997	4.200299982	0.872643167
4.341723801	2.033868414	0.431477569	4.716966046	1.149183655
22.48031732	3.282929556	0.271155875	7.193971471	3.534557784
11.27914755	2.880661124	0.325817459	6.753569963	2.550800022
8.139611294	2.622761259	0.346741223	6.05343314	2.250689088
9.405708614	2.763523135	0.329744043	6.62744818	2.266832693
5.952590947	2.359680727	0.386861831	5.430402236	1.491451443
9.945330437	2.728516324	0.328733791	6.149537284	2.467640828
24.78295216	3.322859107	0.296074186	7.44792963	3.463499064
8.589941118	2.517653626	0.394592422	5.670456317	1.746973054
46.98904952	2.986361491	0.176124876	4.613063711	5.515722419
3.275626246	2.077119114	0.41428496	5.012626181	1.15367028
6.236670849	2.47649072	0.401641049	5.95987882	1.689221287
8.669206811	2.698321095	0.342234461	6.433298688	2.303072055
19.82383506	3.23769008	0.271925195	7.382897286	3.392489829
19.35565614	2.824070004	0.244028617	5.261447361	3.538379718
37.18253968	2.510193831	0.177213824	3.806100279	5.055555556
9.085297553	2.623298158	0.372317737	5.966688629	1.881173402
11.3185589	2.848850445	0.305841657	6.710384259	2.654062072
12.91048969	2.886340525	0.321986619	6.360417771	2.458520741
3.509404034	2.142574673	0.423570237	5.458709374	1.253283845
2.389747335	1.903276134	0.444121769	4.604017901	1.00354128
39.42285843	3.31400885	0.200129751	5.815577209	4.798262898
17.89609127	3.180826274	0.28949178	7.330543235	3.190191624
29.43774046	2.516218897	0.171520167	4.07791318	4.496838631
24.88860228	3.31558777	0.228230376	6.718055661	3.915078772
8.256256359	2.61865632	0.359946253	6.346585217	2.199336175

log.sigma.2.5.mm.3D_glcm_DifferenceVariance	log.sigma.2.5.mm.3D_glcm_Idn	log.sigma.2.5.mm.3D_glcm_Idm	log.sigma.2.5.mm.3D_glcm_Correlation	log.sigma.2.5.mm.3D_glcm_Autocorrelation
3.50628682	0.89945184	0.349256807	0.56341701	116.8928151
2.767714459	0.933324659	0.529750643	0.628935896	180.0538215
2.411173563	0.911790265	0.380413765	0.510854005	84.76574927
9.970849007	0.928478159	0.375824439	0.335014336	223.7324983
8.475131779	0.861654464	0.218560205	0.215145636	100.9331698
3.975427716	0.872209927	0.363768174	0.165078562	51.39033462
7.01258944	0.823283627	0.222717466	0.036843103	97.94663419
5.986538192	0.883246024	0.27914155	0.315250544	70.55709533
24.41721744	0.879970793	0.214222917	0.500234281	259.3127266
2.855487578	0.945770952	0.491096345	0.529575435	161.8986332
3.874234479	0.892107142	0.323816825	0.321739392	126.0125838
2.934746516	0.913074975	0.403676092	0.517732782	101.1492017
4.097432387	0.90739306	0.420950046	0.316394116	81.04232863
4.690716396	0.901875588	0.353977281	0.254732421	60.77521373
9.231902099	0.88793832	0.25385935	0.200662716	102.4393738
1.287437133	0.967284015	0.70143209	0.575339786	123.5592059
7.824524791	0.875959618	0.283167415	0.065656694	62.61713388
2.929499688	0.913501053	0.434539579	0.508747785	74.05438304
8.30926065	0.871100104	0.30921668	0.094436083	50.02362571
1.358413053	0.938476812	0.596811114	0.714909615	89.57220292
1.598593567	0.965122284	0.616898273	0.577005996	127.2067422
5.086696894	0.911954498	0.347215531	0.300139418	112.7430899
1.607940243	0.950933643	0.595870168	0.633805259	123.0627713
12.0656651	0.870102839	0.186922951	0.539438408	244.5383176
1.580988293	0.944594541	0.526679061	0.623857466	133.7302146
3.207669556	0.878044736	0.353159168	0.526099823	81.98465438
2.325354275	0.946157541	0.557999587	0.504003442	70.37835912
1.721575516	0.965467734	0.655882465	0.615458021	110.3748383
3.575575557	0.946868042	0.576535837	0.594586352	192.7462365
2.375878609	0.941867689	0.559024929	0.646569405	158.3594151
2.044544464	0.953457661	0.590731707	0.565457089	105.4467388
1.673047892	0.952694202	0.626900636	0.574784289	110.6339869
5.24726982	0.920696908	0.407062471	0.487743475	96.82917031
11.5234562	0.788445418	0.156791739	-0.229331018	75.64294872
9.894538197	0.867533815	0.236199394	0.35550158	74.92647998
3.964694072	0.899612345	0.39099174	0.462172424	82.70988072
1.68026118	0.957214893	0.649131258	0.663060034	161.1095498
3.068789651	0.947427866	0.526456227	0.493293685	148.7617496
8.991785931	0.876952442	0.231792378	0.251153852	124.6174205
3.464579161	0.887420202	0.368918856	0.580509124	79.36467818
3.222712342	0.920663833	0.449135051	0.589105072	109.1802942
4.667701314	0.888054011	0.316322138	0.300381043	79.78827264
5.042245181	0.913204736	0.324742231	0.364038642	140.6066877
5.246192451	0.911392299	0.311911423	0.370145742	128.2477206
10.41238749	0.896639805	0.253049445	0.397584842	171.2410908
0.748807277	0.956403747	0.706939865	0.653313671	64.74390949
18.15832	0.924324737	0.285634156	0.348173331	226.9855787
4.17797712	0.838843928	0.291029327	0.265025747	47.32199938
14.25028659	0.784699442	0.143133538	-0.078717497	99.87062937
12.37103678	0.842331028	0.245687772	0.159196826	51.8090942
2.583546311	0.94820442	0.585619582	0.550785739	126.9907815
4.319250185	0.863156286	0.321353792	0.414339465	52.32278303
3.819824788	0.946670942	0.455039038	0.574660844	271.7290684
2.55409427	0.895981607	0.378121694	0.552964311	68.12137693
1.433308664	0.969096561	0.665145502	0.648021316	120.4218924
8.53817451	0.884919776	0.275508034	0.233024	109.4113445
9.94542752	0.892329214	0.255125601	0.346288995	163.5117045
14.29482604	0.851511038	0.185331974	0.275834959	155.5228871
4.666998021	0.897998777	0.349969092	0.452575258	121.2407084
2.243981801	0.903971485	0.407288195	0.553918625	66.61133489
2.764147813	0.914662786	0.436578745	0.612896688	133.3447212
4.836722964	0.953560435	0.53495331	0.501103723	172.1495914
1.623282743	0.945621324	0.590565834	0.573649868	79.91952711
1.77741972	0.92658419	0.570072091	0.658413211	88.63624189
2.195105737	0.851693134	0.292149997	0.490356265	49.19650413
6.524547817	0.92384339	0.355503165	0.444003723	163.2078755
6.960726588	0.841906146	0.239696141	0.143119731	70.31039688
3.327547023	0.947270412	0.512066858	0.643995012	138.7172032
4.016903464	0.865127394	0.336336634	0.152180648	30.93772729
3.088912728	0.933729643	0.476008533	0.578679834	157.0594122
3.187864011	0.926184241	0.466822718	0.57271325	131.687641
3.360570436	0.868596691	0.342878848	0.499315309	81.49996011
1.516148617	0.952530798	0.640131543	0.672605185	111.8862096
1.158064558	0.963817355	0.653512523	0.659257316	115.8536493
3.004379979	0.969345486	0.61815666	0.452882575	170.3642844
9.717343464	0.892630591	0.259872316	0.19417014	134.0629533
4.683636607	0.894482959	0.329685792	0.375794566	111.1483997
2.973772208	0.877940395	0.342136204	0.304958839	61.31153302
4.099248472	0.9126032	0.375462147	0.513815354	158.16542
3.68802266	0.952971952	0.544123922	0.510382361	162.4857764
3.746543011	0.868728068	0.320343944	0.387485357	70.48361437
12.60598931	0.907889877	0.301961853	0.331579643	180.8987898
5.465978652	0.959923434	0.518901347	0.461935283	378.0512906
16.05253686	0.815760556	0.160066774	-0.023970968	102.9111359
1.923753042	0.955095304	0.59000441	0.585370061	129.1400193
3.332849132	0.940697823	0.479318048	0.370866268	164.6609872
3.291299014	0.893323116	0.33932255	0.401343188	65.00339235
8.189688779	0.898791065	0.262538069	0.311054342	156.862796
5.815251708	0.804274431	0.2287541	0.134128718	53.80470983
10.91962711	0.75744765	0.161605504	0.030327408	60.17857143
5.470599797	0.949849993	0.49127742	0.51507655	426.8445228
4.073926434	0.879720326	0.306460484	0.452165709	101.4625799
6.688647886	0.917648872	0.388306095	0.336540817	164.090849
1.905753769	0.939289216	0.555881318	0.675426224	121.4421428
1.367755525	0.954943782	0.615827764	0.658705906	111.3606011
14.60346734	0.857186332	0.202295701	0.099214125	110.8255326
7.567725229	0.887164561	0.286061814	0.344889385	92.95900715
8.896774855	0.787445423	0.177715382	0.075523299	50.1969253
9.208817939	0.87380501	0.229818522	0.355157253	162.6403589
3.093577261	0.893056256	0.363841536	0.561418908	99.87449577

log.sigma.2.5.mm.3D_glcm_SumEntropy	log.sigma.2.5.mm.3D_glcm_AverageIntensity	log.sigma.2.5.mm.3D_glcm_Energy	log.sigma.2.5.mm.3D_glcm_SumSquares	log.sigma.2.5.mm.3D_glcm_ClusterProminence
4.430734177	10.54326976	0.012450985	10.08253075	2470.544945
3.86434843	13.25454369	0.061006333	6.886801775	1664.917629
4.004631388	9.019325142	0.019572405	6.539829182	1118.512
4.494632098	14.88442734	0.019255257	14.2308309	5011.434672
4.227194269	9.878016821	0.014879864	15.05180216	4245.645307
3.799451691	7.105771075	0.020370597	5.497192707	483.5091477
3.67063783	9.873909682	0.026094195	11.25043756	1539.120304
4.367897783	8.189412117	0.009969437	11.02727001	2371.852632
5.516440608	15.55137685	0.003096927	54.55012573	54926.44574
3.946358325	12.6040333	0.039758387	5.846656503	1512.90624
4.108996952	11.11692508	0.013563607	7.445381591	1106.814193
4.196374347	9.870348789	0.017922848	7.117180154	1367.982129
3.986701443	8.891162277	0.019690561	6.126524232	833.2829983
4.005233797	7.682237271	0.016884535	6.935228698	1401.737609
4.387100948	9.997379983	0.009885261	14.05763859	4960.684866
2.780031967	11.0567349	0.160563285	2.246485234	553.975653
3.969197888	7.878688699	0.015834285	9.964561598	1792.784139
4.086749285	8.409177433	0.021025754	6.535975873	895.4855576
3.987233217	7.002149396	0.01763399	10.18441514	2111.716201
3.745863695	9.294086788	0.063041277	4.442563354	753.4887357
3.357797354	11.19681081	0.092632472	3.187676905	468.6776874
4.193261867	10.49891692	0.013564098	8.229746738	1492.562012
3.563058146	10.97940309	0.070109757	3.937822242	743.0836329
4.851705022	15.11233387	0.011025707	38.68828037	32304.47809
3.739647646	11.43958525	0.045583334	4.511762787	930.8481692
4.269271745	8.789242091	0.014295992	8.945501602	1608.594613
3.603255833	8.26527076	0.064220047	4.063109876	846.3384424
3.10455757	10.40243999	0.13642229	3.488245148	726.6021192
3.629814591	13.7298679	0.092077645	7.054089667	2152.046337
3.710992707	12.42585626	0.078718741	6.044121774	1541.332676
3.49824274	10.15834507	0.073819194	3.947910532	671.5242987
3.30131697	10.4273293	0.096122255	3.266304119	591.0092054
4.329601516	9.583476869	0.022906146	10.15865572	2270.034901
2.153846072	8.851602564	0.120005342	16.61243456	1956.043892
4.419778931	8.235192937	0.013639738	19.63795095	7717.311912
4.21604895	8.881032559	0.018857284	8.25348868	1288.723274
3.118865684	12.58848725	0.125234248	3.953816509	1050.037764
3.827947003	12.08859712	0.047560799	5.284425444	922.4350967
4.589700366	10.97324393	0.0082329	17.2353076	6670.806624
4.387004892	8.57055092	0.013804424	10.11343648	2130.967884
4.21785358	10.22390522	0.024910339	7.859835929	2050.081772
4.170317111	8.792274413	0.013639022	8.190701917	1378.64107
4.326318207	11.70717958	0.011605178	9.678689332	1969.640265
4.336523512	11.15607702	0.011096461	10.1575609	2792.767503
4.828114944	12.85036176	0.006389052	21.36201425	12346.47088
2.713163732	7.971172032	0.151826257	1.818009766	286.3133544
4.825756355	15.02991356	0.008406692	26.99457891	39418.10731
4.069093354	6.720185213	0.013368599	8.103048988	1044.436482
2.840888626	10.04493839	0.063743162	21.57404921	4097.376517
4.341940468	6.976797366	0.014714496	18.3566714	5283.073949
3.535944649	11.1530989	0.085059792	4.680809021	1042.811856
4.24558942	6.951956146	0.013108672	9.618798552	1717.101533
4.246210936	16.35561323	0.02985564	8.848130293	2324.767099
4.181010019	8.000860059	0.016407182	7.389657123	1496.579086
3.082928793	10.87797361	0.131263883	3.210597811	837.7933956
4.393474596	10.31754512	0.010407163	12.63381557	2753.250805
4.758033106	12.56542048	0.00687408	18.93175978	8880.741578
4.942847777	12.15732543	0.005220776	27.96589839	14430.30593
4.330085525	10.80080719	0.014446375	9.990254192	2490.021125
4.139432696	7.940067587	0.019283298	6.378591996	1118.6565
4.168301784	11.33441535	0.022173023	7.912222452	1440.530338
3.88249685	12.98339188	0.061047036	7.726461106	2390.411956
3.514425992	8.827778984	0.073012221	3.43683981	501.0686435
3.76339776	9.238999514	0.06170694	4.9221901	811.6622222
3.839831725	6.67288777	0.031365948	9.880801967	1931.036912
4.509217814	12.57011505	0.012086576	12.21302882	3577.008181
4.00204188	8.279908186	0.016842184	11.47890104	1887.127506
3.999776229	11.54388286	0.049932195	8.467972222	2445.312983
3.727032649	5.48102762	0.022193832	5.804880127	574.5268842
4.108578575	12.36580658	0.031437876	7.119651535	1560.084214
4.090409157	11.28570074	0.028638665	7.419950372	1662.946687
4.314066672	8.779927259	0.011844003	8.810134448	1595.163453
3.357101445	10.45469274	0.099768404	3.819724821	743.8029789
3.111594297	10.67412315	0.118417223	2.880728597	539.3447078
3.296936663	12.99465213	0.099211071	4.085689974	1203.254011
4.448526724	11.46084657	0.008996692	14.17077109	4396.945307
4.276908928	10.37611318	0.012196768	9.102502139	1915.501538
3.87460023	7.715104244	0.018562607	5.840934936	602.1901409
4.346805192	12.35935563	0.016382072	10.05483814	3034.996397
3.797555831	12.62476166	0.060037339	6.160418395	1346.877127
4.097997897	8.201336179	0.017100488	8.180908535	1325.53585
4.752457846	13.30955321	0.009263053	19.5228895	8184.419325
3.855800919	19.43023024	0.060748032	7.699422445	2827.760745
3.347695026	10.12586169	0.043499346	23.54642144	5306.350386
3.541405241	11.25952441	0.069989338	3.997870372	664.6414819
3.855653988	12.75855186	0.030572426	4.994115164	923.7929671
4.129408046	7.879495509	0.014570199	7.238650156	1091.701
4.58926412	12.3568287	0.007663186	14.71222302	4528.175646
3.669034535	7.245414229	0.028398246	10.949048	1463.985993
2.679116152	7.742063492	0.074766944	18.74870874	2982.783566
4.047994726	20.6432508	0.045133381	9.043884596	3790.65718
4.33879273	9.837998298	0.011207498	10.31553691	1935.195864
4.195269483	12.68271341	0.024267415	9.689304535	2156.240722
3.902367036	10.84940571	0.049039113	5.481583233	1177.676295
3.334399377	10.44107246	0.093087647	3.537384015	629.0481148
4.104563352	10.48516867	0.019318996	21.83698193	6953.460147
4.566989969	9.392373336	0.008534526	13.68382314	4476.698817
2.892997003	6.973037219	0.065458247	15.98434887	2748.407354
4.514611066	12.47196126	0.011315358	19.50262406	9546.14068
4.302242556	9.728300066	0.016275778	9.345207915	2369.867598

log.sigma.2.5.mm.3D_glcm_SumAverage	log.sigma.2.5.mm.3D_glcm_Imc2	log.sigma.2.5.mm.3D_glcm_Imc1	log.sigma.2.5.mm.3D_glcm_DifferenceAverage	log.sigma.2.5.mm.3D_glcm_Id
21.08653951	0.843905236	-0.174874837	2.277994212	0.428280649
26.50908738	0.795885871	-0.182938077	1.480768952	0.577512724
18.03865028	0.829717038	-0.187172132	1.948316143	0.453439956
29.60291768	0.781344722	-0.128334772	2.858575975	0.445400905
19.75603364	0.966004467	-0.357522913	3.840983904	0.313931223
14.21154215	0.730138235	-0.119475077	2.269736602	0.438053791
19.74781936	0.972273677	-0.445601	3.784707842	0.318894383
16.37882423	0.782676258	-0.127640933	3.00385115	0.368518525
30.52700114	0.823444996	-0.123358926	5.319418235	0.299565171
25.20248276	0.73477536	-0.131618766	1.585342259	0.544753392
22.23385015	0.73005296	-0.113278947	2.467852565	0.408251846
19.74069758	0.756941333	-0.134583037	1.940032012	0.472349794
17.78232455	0.686395376	-0.102967685	2.019137334	0.484802907
15.36447454	0.691935161	-0.100475923	2.363307437	0.429528509
19.96700411	0.832359058	-0.154719392	3.5652761	0.343921565
22.11346981	0.701686855	-0.174503231	0.761855668	0.717654107
15.7573774	0.855273532	-0.188707039	3.273423487	0.367935449
16.81835487	0.722365965	-0.122031969	1.842262434	0.497945836
14.00429879	0.869245862	-0.20382488	3.145589561	0.389723143
18.58817358	0.839520051	-0.22393751	1.061461072	0.630955447
22.39273619	0.750399236	-0.1695666	1.02803405	0.64804021
20.99783383	0.680748927	-0.093211561	2.493120823	0.424195399
21.95880618	0.77969987	-0.182771003	1.102571477	0.631196262
29.94755385	0.992849429	-0.486650603	4.672033343	0.282302946
22.87917051	0.766396628	-0.165640108	1.300011641	0.573850609
17.57848418	0.819470577	-0.16383008	2.263071584	0.431440722
16.53054152	0.7242694	-0.139658913	1.287016253	0.597648455
20.80487998	0.74970936	-0.187943468	0.954390462	0.681620052
27.45973581	0.796111262	-0.189847577	1.418897077	0.616236102
24.85171252	0.787092721	-0.17996087	1.336272625	0.602971319
20.31669013	0.762380476	-0.170551852	1.151919193	0.624974581
20.85465861	0.733664209	-0.161824566	1.018031334	0.655869621
19.16695374	0.780428113	-0.137890715	2.246437284	0.474615491
17.70320513	0.994622433	-0.862181325	5.113461538	0.253442404
16.47038587	0.976524491	-0.390268828	3.855594754	0.328218596
17.76206512	0.713589302	-0.108502378	2.190207233	0.462878718
25.17697449	0.759200606	-0.193603534	0.96561305	0.673858646
24.17719423	0.758112216	-0.146003567	1.490584765	0.572040943
21.94354301	0.875452473	-0.188616396	3.96254504	0.322972771
17.14110184	0.793737625	-0.1457252	2.208001706	0.443827863
20.44781043	0.834205171	-0.181736302	1.775020544	0.508723445
17.58454883	0.756378269	-0.122663844	2.590693207	0.400627824
23.41092697	0.716754746	-0.105633995	2.637659426	0.406629591
22.30631158	0.779909642	-0.131680606	2.696296916	0.395857052
25.54785893	0.751385834	-0.102964822	3.742678888	0.341139854
15.94234406	0.692760445	-0.181634842	0.685102445	0.721494513
29.55538518	0.716348334	-0.08985825	3.852770311	0.365982102
13.44037043	0.743828082	-0.119331177	2.751519703	0.378660466
20.08987679	0.993993178	-0.737881615	5.535364635	0.241133363
13.95359473	0.937230307	-0.285315091	4.219805807	0.331377587
22.30619779	0.766037486	-0.167736355	1.248512387	0.621944675
13.90391229	0.739397084	-0.117123431	2.610521279	0.404142781
32.66208419	0.766330294	-0.139511737	1.853729558	0.513958201
16.00172012	0.851408309	-0.190059126	1.991939712	0.452636968
21.75537387	0.744049736	-0.186586635	0.877465238	0.686386911
20.63509023	0.841037295	-0.162625876	3.276092071	0.362940021
25.04698325	0.798198784	-0.126943319	3.667294017	0.343625488
24.29005502	0.889179705	-0.182840348	4.976843544	0.27838047
21.60161437	0.83744669	-0.170767924	2.463008781	0.42792688
15.88013517	0.784487272	-0.150795398	1.819900932	0.476783403
22.6688307	0.789848811	-0.153383818	1.809691575	0.50018141
25.94633702	0.796231657	-0.169252922	1.638496477	0.578203489
17.65555797	0.729856573	-0.154526419	1.112687826	0.625862253
18.47799903	0.818582696	-0.202113024	1.216981528	0.611471512
13.34577554	0.984395569	-0.507019161	2.639640845	0.383081263
25.12214688	0.785987493	-0.135838758	2.577241277	0.429829954
16.55981637	0.948658322	-0.322527374	3.510920632	0.334551648
23.08586992	0.808614547	-0.175749061	1.609620134	0.560924362
10.96205524	0.797507488	-0.160371068	2.402449863	0.416330323
24.73161317	0.79190381	-0.158434829	1.679776096	0.531286793
22.57140149	0.758058379	-0.140303215	1.726267277	0.523204164
17.55985452	0.729623595	-0.112512492	2.31273764	0.423604943
20.90938548	0.789594042	-0.2024931	0.969055488	0.668199053
21.3482463	0.751098278	-0.187431282	0.872643167	0.675860112
25.96512112	0.735733397	-0.156809773	1.149183655	0.645491155
22.90789094	0.828821316	-0.150403501	3.534557784	0.348384738
20.75222636	0.764778646	-0.124372251	2.550800022	0.410946339
15.43020849	0.735383299	-0.122401349	2.250689088	0.423293401
24.71871126	0.781025118	-0.14568187	2.266832693	0.45051092
25.24480846	0.813932451	-0.188292778	1.491451443	0.587515553
16.40267236	0.900994462	-0.245655195	2.467640828	0.404768412
26.43139353	0.837835166	-0.152105158	3.463499064	0.381065238
38.69642815	0.770761651	-0.153690487	1.746973054	0.563363016
20.25172338	0.997338499	-0.733962758	5.515722419	0.252766712
22.51904882	0.757011579	-0.163891417	1.15367028	0.625370384
25.51710372	0.638222519	-0.091221744	1.689221287	0.532881152
15.75899102	0.732249898	-0.115393418	2.303072055	0.42074608
24.68047214	0.781694939	-0.121385856	3.392489829	0.351721706
14.49082846	0.982397442	-0.498559482	3.538379718	0.325017982
15.48412698	0.990111638	-0.697470507	5.055555556	0.257524365
41.08401868	0.786777886	-0.155808719	1.881173402	0.542028738
19.6759966	0.817905735	-0.156006071	2.654062072	0.392828893
25.36542681	0.842098003	-0.180581334	2.458520741	0.458887864
21.69881143	0.822137316	-0.196665167	1.253283845	0.596695372
20.88214493	0.767985482	-0.182534461	1.00354128	0.644668426
20.88529576	0.991155597	-0.527892887	4.798262898	0.293861207
18.78474667	0.74524773	-0.107802702	3.190191624	0.371141323
13.94607444	0.995323947	-0.752901489	4.496838631	0.277968097
24.94392253	0.965015083	-0.334639085	3.915078772	0.322429571
19.45660013	0.878951469	-0.214674309	2.199336175	0.439515675

log.sigma.2.5.mm.3D_glcm_ClusterTendency	log.sigma.2.5.mm.3D_firstorder_InterquartileRange	log.sigma.2.5.mm.3D_firstorder_Skewness	log.sigma.2.5.mm.3D_firstorder_Uniformity
31.56807707	127.2420311	-0.037765396	0.084997813
22.49910372	97.839818	-0.971040442	0.154940868
19.87860981	98.6747818	0.405345457	0.108302212
36.83803313	126.9287949	0.116100608	0.089124041
36.7084645	158.7226105	0.313584404	0.073032056
12.78222456	69.73708725	0.359426314	0.125436711
23.28780547	123.4570065	-0.351415107	0.091428571
29.00705465	113.1096573	0.567327283	0.08511763
158.5213497	250.06637	0.621848578	0.046490001
17.81444231	79.9270792	-0.160530113	0.13654586
19.7174983	99.55935097	-0.001856074	0.100398936
21.66842844	96.24591637	-0.124262808	0.103074955
16.22630963	91.94290018	0.19152552	0.110760382
17.37404518	87.14030361	0.803296247	0.109396522
33.18331048	113.4148984	0.74261613	0.081798696
7.107398383	16.44295847	-1.776586229	0.323695228
20.96563765	108.6936722	0.902553438	0.095457538
19.74062905	105.7491226	-0.133152963	0.110040088
22.32583073	107.6215744	0.865197309	0.092017872
15.2599484	69.66723764	-0.787386619	0.166814727
10.05141323	61.68110049	-0.86056716	0.209327982
21.48048375	105.3062916	0.317141476	0.095586126
12.89912812	68.59010559	-1.076145674	0.180693141
114.6518463	191.4268875	-0.216499177	0.053309967
14.72855061	83.98842716	-0.805873225	0.14671441
27.32529644	126.1661863	-0.168009775	0.094930047
12.24826148	58.16785383	0.028414194	0.182133634
11.29970809	46.57565907	-1.40847314	0.267056379
22.56949024	86.05815229	-1.140756982	0.203239765
19.97844923	88.28178501	-1.114194608	0.175028408
12.39861156	67.24105167	-0.769841468	0.188234334
10.33802679	47.46323673	-1.228609693	0.228282848
30.26906145	130.9510427	-0.011798951	0.096414749
27.04396902	131.8059921	-0.496833329	0.12
53.35615641	164.5987587	0.45889478	0.068212076
24.16642218	123.6115518	-0.123793913	0.100791433
13.18299467	33.45516348	-1.730351006	0.263710383
15.82111301	74.9167145	-0.141272843	0.14664054
42.85183294	138.9962273	0.401426573	0.072252793
32.02606693	142.6193504	-0.143931324	0.087398437
25.00754235	100.8352551	-0.557876189	0.105939655
21.3157874	101.5140457	0.209004369	0.09742975
26.42992115	112.3994656	0.119732485	0.088309454
27.841954	120.7107086	0.368647018	0.085776848
56.92438579	129.5487499	0.729863876	0.070726538
6.041620117	24.26689363	-1.971253209	0.312399964
67.69998637	112.4620583	1.621540716	0.07728304
20.50866512	111.2254677	0.392648055	0.098145583
40.44898296	150.2246895	-0.552273355	0.107438017
42.8366584	189.2908859	0.460123449	0.072433853
14.55549158	63.24222544	-0.989233079	0.199149846
27.17968184	119.4245682	0.44353043	0.098572123
27.40219029	110.8114419	-0.526341758	0.118417911
22.97741487	94.73755646	0.169155757	0.098685247
10.60680722	22.08048981	-1.606020061	0.278591205
31.15697739	119.9612389	0.193907223	0.081773223
49.61587259	137.1764164	0.358144747	0.066955643
71.17103904	194.2397623	0.254775156	0.053965409
29.1407339	123.1967163	-0.291998342	0.088256887
19.87822668	94.05020618	0.230893737	0.108677633
25.5552756	125.1353388	-0.254228947	0.102386604
22.94321394	80.6340439	-0.360526296	0.160381665
10.85154165	41.20424342	-0.770605175	0.194528011
16.37529995	78.36924893	-0.784468235	0.165920963
29.05246416	133.4313126	0.190084366	0.100747597
35.0320276	124.2150073	-0.253851721	0.083432798
26.42146278	128.6304016	0.327398912	0.081723356
27.84524703	109.905956	-0.617599401	0.139794888
13.38220341	81.09970856	0.927497441	0.125238398
22.51773592	102.8177193	-0.598501239	0.115469796
23.44577287	106.8902596	-0.598354906	0.114940322
26.41654603	113.9129944	-0.013029502	0.094214732
12.80571182	54.6932621	-1.315783617	0.21868068
9.590981694	36.15162516	-1.593622946	0.256737289
11.61017921	29.91754007	-0.284292609	0.238029739
33.7862404	116.6472244	0.400414143	0.075599751
25.13086101	103.8491402	-0.075599994	0.091229994
15.22412845	75.12268448	0.51411328	0.123804209
30.81364393	121.89785	-0.420134518	0.087566836
18.57303529	91.69692439	-0.430235429	0.152622348
22.7783037	111.2086945	-0.002007369	0.093914689
49.69550463	151.2132616	0.236087308	0.068087808
23.37538194	58.33362484	-0.850827151	0.172367645
47.19663625	159.5610962	-0.075653643	0.088449532
12.71585524	62.78777415	-0.779244478	0.184786481
13.73978981	68.48136377	0.037574995	0.137340294
20.28539381	90.79563141	0.336775873	0.106203791
38.04641667	134.2536163	0.356346427	0.073575829
24.44053587	112.5937765	-0.181812769	0.096
37.81229529	176.8310776	-0.258902669	0.147928994
28.53448875	98.3626045	-1.21094276	0.140760778
29.94358872	123.6157837	0.165767244	0.085850935
25.84672845	109.9143028	-0.260524688	0.107456546
18.4169289	86.36247586	-0.880002767	0.142654057
11.75978872	53.84837785	-1.073571724	0.2164059
45.86084532	135.9963684	0.682814627	0.07079869
36.83920129	136.3482617	0.342346169	0.074273356
34.49965502	136.6934662	-0.138251801	0.09375
53.12189397	161.1522493	0.021528589	0.066204976
29.1245753	106.608171	-0.455394542	0.093979592

log.sigma.2.5.mm.3D_firstorder_MeanAbsoluteDeviation	log.sigma.2.5.mm.3D_firstorder_Energy	log.sigma.2.5.mm.3D_firstorder_RobustMeanAbsoluteDeviation	log.sigma.2.5.mm.3D_firstorder_Median
67.96774328	6181262.832	49.90421671	-103.5587234
56.95914101	11815458.38	41.56173915	-35.68035507
55.50124225	3101181.755	39.67954083	-114.9447174
76.94289475	27191908.95	52.4442881	-54.3263855
85.3824543	3423877.92	63.22590005	-148.4175415
46.06462063	5228174.828	29.90427889	-137.2110672
66.19507205	1313147.312	49.839102	-107.9001274
67.69423652	13053877.41	46.99591069	-165.6460571
144.2652198	48266893.73	106.7436669	-88.64467621
49.37380059	16037979.39	33.24009597	-41.85123062
56.40917748	8694562.443	40.41698495	-137.7984085
56.95194382	7317898.977	40.20022465	-62.9065361
52.39682675	11560481.39	36.48394158	-65.67851257
55.05759172	6993041.037	36.32430613	-99.53886032
73.01192109	5566776.384	48.18281281	-90.63272095
23.49773649	18998452.04	8.522235655	-3.032380939
67.03370105	6877149.329	44.95282622	-174.289505
55.47276691	16681263.52	42.71358782	-75.80039597
72.05900036	5533017.813	48.61407341	-165.7798386
44.7394884	11550372.78	30.79369542	-10.64873934
38.84953265	19573204.81	26.70214161	-18.47066879
61.0594708	10630834.85	42.47508582	-72.4072113
42.38372105	13711410.63	29.37656508	-24.20511246
115.0971274	3258898.821	80.18378553	-59.89230728
47.59771226	6751038.257	34.16819579	-46.538517
62.68897856	6345921.468	47.79226249	-118.6853027
40.21967424	9325513.741	25.22232051	-19.55070877
39.59172559	14687499.34	23.66154732	-7.878685951
57.30726568	27403649.02	39.23320369	-11.07705164
54.40578241	13442744.6	39.30624763	-32.364254
42.4767025	18607082.29	29.26537043	-18.07520103
35.91038389	27633744.4	21.96418558	-11.02789879
70.10727044	17215096.47	52.14224753	-63.79024887
83.54719971	1094701.276	54.02294821	-239.6721497
92.30778724	3717180.94	65.14929043	-143.1655884
61.67823418	18453563.23	47.37628684	-88.73259735
40.0139459	24080406.62	21.93520342	-3.445036411
48.3268101	15511063.24	32.40992255	-18.25615406
83.71968904	6870326.856	58.81487162	-106.4214554
70.59384827	9385976.144	55.75006615	-103.7941895
59.02858186	6440106.88	40.81562916	-48.45129395
59.51416949	10026659.28	41.50781817	-146.193512
65.1533915	18449250.03	46.45929438	-128.604599
67.72914389	11118827.88	48.16038132	-139.2831268
83.75586386	19241926.28	55.92476032	-85.54746246
25.73329746	10207430.39	12.78798708	-4.606261969
83.92342371	46553733.16	49.93153188	-64.18115616
60.51680591	5929183.152	44.94661004	-153.8078308
91.08069497	1789202.73	55.96569347	-237.1147995
96.82511987	5618515.641	75.00793722	-177.2716522
44.79110882	19534550.57	28.78220637	-14.90459442
64.43696209	10901742.56	48.76082077	-159.3627777
62.63941756	30540703.23	45.81495301	-40.06354523
57.09773279	2939663.14	39.98355983	-86.96616364
31.63864294	29748234	13.43093825	-3.922800064
72.96630382	10795977.6	49.77732473	-148.5203705
86.03295391	31073803.38	58.79093489	-158.1545486
110.0760372	18005292.46	80.47274395	-160.8354187
68.40184086	5618018.399	48.98534025	-86.33214951
54.45885484	4021372.86	38.77340016	-69.13033295
61.53268314	19075441.32	48.44263973	-90.38075256
56.40974144	47557106.01	36.63291718	-10.59038925
35.83237218	11082585.89	20.58701109	-6.43525672
47.52899733	8241423.129	33.68950248	-14.07772827
68.31790541	1799067.451	50.93930849	-164.8501587
72.11057123	11478686.07	50.76733638	-29.57244873
73.35357354	4763887.145	52.54832379	-199.8804169
64.05512295	30658688.25	47.27440169	-20.91603661
49.54607145	4963985.008	32.31200957	-187.2451324
58.77634608	15145348.99	42.80834002	-43.03917122
58.4990741	22443459.44	43.43291154	-44.5836525
60.14862035	11944947.62	44.82869826	-129.0065308
41.22577419	25876451.43	26.4136684	-5.952487946
34.01431643	29384506.3	18.83008906	-6.383485794
33.34036039	42988964.08	15.73085792	-2.878696442
77.23309521	14398638.81	50.90815932	-153.7472229
63.11473965	10829235.91	44.12654365	-128.9749756
47.40659529	5961620.899	32.81523081	-158.7002106
69.60707435	18949220.71	50.22473906	-109.3633652
54.69491779	20034974.55	39.10996415	-30.69039345
62.58292922	7253802.806	45.81775607	-214.5071564
89.3559609	17851874.36	60.75451522	-85.51780701
53.14884276	30621335.88	30.23178883	-5.983679771
100.619962	1451633.098	74.42476548	-158.002182
41.89699422	19995511.28	27.86443164	-15.34883451
44.09190114	18503653.54	28.23841429	-39.46011734
54.61039603	7010808.307	38.54161948	-128.55896
77.91885106	24004445.45	54.5421856	-181.0720062
66.27308532	539329.948	49.30624276	-61.6375103
93.04203002	765282.0323	72.62832792	-222.8825378
62.79421423	31140050.13	43.25786304	-18.54283905
67.57468155	7099056.129	50.79582413	-129.0804596
65.2991014	4942459.037	44.61071046	-59.23304558
51.33470368	22092659.05	36.57854204	-25.05211544
39.44932766	25233723.98	25.16508323	-9.698468208
94.1323733	3302366.669	60.12260811	-210.2611542
77.96447223	9292552.926	55.20827636	-64.24486542
80.60953474	1366070.849	58.07310443	-210.3715515
91.43451134	3911234.312	62.98830476	-107.8926849
63.993787	3430901.012	45.47204411	-65.28078461

log.sigma.2.5.mm.3D_firstorder_TotalEnergy	log.sigma.2.5.mm.3D_firstorder_Maximum	log.sigma.2.5.mm.3D_firstorder_RootMeanSquared	log.sigma.2.5.mm.3D_firstorder_90Percentile	log.sigma.2.5.mm.3D_firstorder_Minimum
166894096.5	110.8198776	130.8533978	0.200942367	-344.4633789
319017376.2	98.45578003	91.76909563	2.587829638	-350.2727051
83731907.39	149.2244568	132.3661621	-32.27375793	-314.1853943
734181541.6	411.5481567	118.6052523	34.50134888	-408.5057373
92444703.83	131.5407715	184.1189327	-17.0512085	-396.6569824
141160720.3	36.17888641	146.379459	-60.83515129	-278.7085876
35454977.42	30.79515457	136.9644016	-14.50019455	-326.6925964
352454690.1	173.5043182	180.4253592	-49.75250244	-349.5323486
1303206131	461.3511353	180.8345861	230.9018631	-385.6842651
433025443.5	299.8927307	83.16190921	5.77579031	-344.5310669
234753186	66.16055298	152.0652369	-43.38290787	-398.7408142
197583272.4	171.5425568	98.32076866	12.59249039	-288.458313
312132997.6	154.6186218	91.99465814	8.512476444	-260.2470093
188812108	211.4492188	115.0842991	-0.858133852	-271.550415
150302962.4	321.4362488	123.6662513	44.72440071	-304.8279724
512958205.1	239.2116547	41.88755247	11.81358624	-268.723053
185683031.9	155.9485931	178.0222806	-30.65663147	-341.2581787
450394115	160.3909149	103.6737035	-1.955418229	-264.6266174
149391480.9	137.855896	174.3593897	-13.38860092	-320.0232849
311860065.1	101.2644424	64.52749	13.39793053	-226.893692
528476529.9	372.1439819	62.10305246	2.731272602	-294.5038757
287032540.9	266.4953918	108.1438294	16.26465912	-310.4338684
370208086.9	199.0894623	68.52509446	2.996429229	-281.0853271
87990268.18	279.8650208	156.534337	111.1243805	-422.0793762
182278033	195.6124725	83.85899784	-0.0134649	-308.3280334
171339879.6	32.14542007	144.958563	-24.20776253	-320.860321
251788871	287.4339294	63.82833075	11.74164639	-224.2667694
396562482.2	343.1583862	61.4073278	4.0750741	-268.2900085
739898523.6	197.453476	85.87492003	3.843833685	-354.1726074
362954104.3	145.8081665	89.1077535	0.277012002	-335.4116821
502391221.9	257.2676086	66.55225854	5.353443146	-263.8027954
746111098.8	195.6462402	55.93269596	6.793379211	-274.2567749
464807604.6	295.4417725	111.327795	14.12156849	-291.6915894
29556934.45	-65.5584259	270.1482649	-157.4366425	-471.0610962
100363885.4	204.5767212	181.3709088	16.8958457	-339.1393738
498246207.2	134.1085205	117.6148621	-3.170301819	-284.0224915
650170978.8	163.5817871	59.99104031	6.208049774	-309.5223389
418798707.6	296.6286926	71.52472321	17.29784184	-301.1930237
185498825.1	250.9441071	146.297257	24.36995506	-359.8476868
253421355.9	92.38227081	132.2064306	-1.028089833	-299.8352356
173882885.8	162.5203247	94.90596656	20.46865044	-295.3945923
270719800.6	115.0027847	162.2241999	-46.78823853	-331.0897522
498129750.8	240.5503693	149.6324381	-24.03058605	-380.9173584
300208352.8	247.3114624	159.1465556	-30.8285141	-397.559021
519532009.6	372.9947815	131.3671686	66.30543213	-370.87146
275600620.5	129.2612762	40.83302464	10.97344036	-199.9852753
1256950795	669.4597168	130.2047986	83.59658051	-377.8041992
160087945.1	24.34959984	161.2612454	-36.08727226	-281.1350098
48308473.7	-93.51983643	285.1795869	-141.379068	-505.9114075
151699922.3	135.7476959	196.1708027	0.990899205	-333.7770081
527432865.5	212.8243408	69.19450876	7.2448318	-297.3313293
294347049	59.56026459	164.473312	-25.75303497	-293.098053
824598987.1	317.8479309	97.76965556	12.64286327	-435.1495972
79370904.79	124.4923706	110.9046698	3.07374115	-260.4092102
803202317.9	371.7075806	51.29512635	13.47442913	-259.6225586
291491395.2	165.4970551	179.5182409	-44.10214615	-384.832428
838992691.2	227.7578278	188.7726029	-22.88242474	-445.8825378
486142896.4	197.8732452	198.2747226	39.35680275	-446.9374084
151686496.8	135.5168152	125.9766188	0.120142832	-349.8394165
108577067.2	136.509964	92.89540083	20.34505463	-237.2475891
515036915.6	80.23366547	114.5000827	1.769207311	-328.658844
1284041862	409.6119385	83.77626816	8.627922058	-325.4385376
299229819	201.7386932	52.15669221	36.62582741	-213.4359131
222518424.5	92.31459808	68.00600393	19.24675274	-237.8966217
48574821.19	9.674722672	184.2407905	-60.44733887	-311.5491028
309924524	352.0287781	99.82052503	56.7882225	-329.1846008
128624952.9	16.52271652	213.0031778	-76.46991119	-384.1813354
827784582.8	349.1385498	94.03723779	11.08323288	-300.2121887
134027595.2	30.61837578	186.3147387	-93.81509552	-299.8276672
408924422.7	187.1525574	91.93283222	15.95493793	-328.7214355
605973404.8	166.2315521	92.92696648	12.99393005	-312.1860046
322513585.8	15.13704109	145.1445039	-20.93297043	-307.5350647
698664188.5	170.3382874	60.5539253	7.594463444	-257.8631287
793381670.2	270.5696106	52.07925747	9.663876534	-260.2116394
1160702030	515.0124512	54.41393548	24.35678406	-309.6471558
388763247.8	235.8281555	179.6773317	-19.90136719	-410.4929199
292389369.5	108.8949432	154.1049135	-36.52198219	-373.4078979
160963764.3	28.25025749	163.13914	-69.68147888	-302.0865479
511628959.1	141.6968689	144.0655987	-11.82740364	-395.0143433
540944312.9	349.3135681	86.07777924	2.889789391	-337.635376
195852675.7	-37.99495316	220.6427889	-101.4739594	-384.6509094
482000607.7	351.4193726	143.6593368	28.5016922	-384.5619507
826776068.8	451.155304	78.81931524	30.25030594	-493.9676819
39194093.64	82.31555176	216.3951805	-60.13547897	-432.4515381
539878804.6	272.7512207	63.48014013	12.82989817	-287.3373108
499598645.6	263.7562866	72.63749544	20.23615227	-332.3151855
189291824.3	132.4358826	138.5918251	-22.56441689	-292.5999756
648120027	210.564209	201.8776271	-51.56738892	-473.1686401
14561908.6	72.78987122	103.8585527	34.66209259	-244.3838959
20662614.87	-93.5675354	242.6269743	-95.10683136	-423.6705322
840781353.6	264.5584717	95.29627196	11.83613052	-527.8508911
191674515.5	90.1492157	152.81413	-17.13228359	-329.0393982
133446394	225.103653	108.9995924	7.384688854	-351.5047607
596501794.3	138.8247528	77.7783294	11.45036116	-283.7271423
681310547.5	207.0292969	59.11013239	8.553221416	-257.1109619
89163900.06	196.2267609	228.9509086	-29.64456787	-438.0432129
250898929	262.3006897	116.8996108	60.25379906	-289.6533203
36883912.91	-49.31163788	238.5783282	-100.6868958	-382.1497498
105603326.4	192.9023438	154.9041721	32.3207222	-412.6592407
92634327.33	102.2509155	110.6942541	13.35880871	-290.4631958

log.sigma.2.5.mm.3D_firstorder_Entropy	log.sigma.2.5.mm.3D_firstorder_StandardDeviation	log.sigma.2.5.mm.3D_firstorder_Range	log.sigma.2.5.mm.3D_firstorder_Variance	log.sigma.2.5.mm.3D_firstorder_10Percentile
3.725080834	82.45150455	455.2832565	6798.250602	-206.4056244
3.161578423	68.64234283	448.7284851	4711.771229	-168.4821136
3.381116032	70.0117401	463.4098511	4901.643752	-195.2315613
3.893135963	98.54449937	820.053894	9711.018356	-195.9112762
3.982131044	104.687339	528.1977539	10959.43895	-278.570343
3.275509826	59.80638859	314.8874741	3576.804116	-205.1941055
3.572869225	79.62978662	357.487751	6340.902917	-225.1765152
3.768638776	85.66535129	523.0366669	7338.552411	-260.8040771
4.636509232	173.9316469	847.0354004	30252.2178	-241.0230865
3.280805677	64.48081656	644.4237976	4157.775705	-136.7482239
3.511207612	69.79907505	464.9013672	4871.910878	-222.757843
3.518830382	71.03413031	460.0008698	5045.847668	-167.361264
3.420171076	65.7181463	414.8656311	4318.874753	-144.4023514
3.491528202	72.01111087	482.9996338	5185.600089	-170.6319107
3.89695086	95.51127484	626.2642212	9122.403622	-194.1062286
2.214483151	40.38937112	507.9347076	1631.301299	-51.32446785
3.658099067	85.51957343	497.2067719	7313.59744	-249.2052277
3.360350138	65.69965246	425.0175323	4316.444333	-169.8092148
3.710925917	91.92952788	457.8791809	8451.038096	-245.3726517
2.993539275	55.24214009	328.1581345	3051.694041	-116.7160568
2.738363004	49.49598782	666.6478577	2449.85281	-108.7414413
3.636099283	77.56518945	576.9292603	6016.358614	-170.1189209
2.889337506	53.56732081	480.1747894	2869.457859	-120.4664436
4.430875767	142.8788603	701.944397	20414.36872	-251.4862244
3.0929162	59.23843549	503.940506	3509.19224	-140.4976517
3.526532318	74.78297854	353.0057411	5592.493879	-220.5140213
2.958157985	54.95840177	511.7006989	3020.425925	-109.1833191
2.51293343	52.42426875	611.4483948	2748.303954	-116.9195862
2.981766014	71.44950301	551.6260834	5105.03148	-159.3018036
3.049934338	66.94791521	481.2198486	4482.02335	-161.3156738
2.86305853	54.11049476	521.0704041	2927.945643	-119.2033997
2.663045898	48.01810877	469.9030151	2305.73877	-99.16253967
3.678641665	84.58246823	587.1333618	7154.193932	-185.638504
3.189898095	105.1727267	405.5026703	11061.30245	-395.0348572
4.0811617	115.3603167	543.716095	13308.00266	-290.7123108
3.4999976	73.40644418	418.131012	5388.506047	-192.5276443
2.511465001	53.62562772	473.104126	2875.707949	-117.1088257
3.20833721	62.34168125	597.8217163	3886.485221	-121.4525627
4.025805878	104.4278791	610.7917938	10905.18193	-229.9807281
3.642008108	82.31961712	392.2175064	6776.519362	-217.0833679
3.5122574	73.8530418	457.914917	5454.271783	-160.03367
3.608766255	75.09865423	446.0925369	5639.807867	-236.8944244
3.714705526	81.66065476	621.4677277	6668.462537	-230.2459854
3.75161833	85.07620934	644.8704834	7237.961395	-236.3081329
4.089217026	108.5833872	743.8662415	11790.35198	-199.2395599
2.113784534	37.1642997	329.2465515	1381.185172	-72.58570786
4.098655205	120.8181804	1047.263916	14597.03272	-174.325592
3.469164094	72.77107615	305.4846096	5295.629525	-235.3323792
3.390805482	111.2652927	412.391571	12379.96536	-416.6533569
3.956504318	112.7613511	469.524704	12715.1223	-297.3371429
2.914822651	58.44811591	510.1556702	3416.182254	-121.5520363
3.491228626	76.97026013	352.6583176	5924.420944	-233.8147522
3.470698573	75.99360018	752.9975281	5775.027268	-169.5595764
3.536052429	71.09995591	384.9015808	5055.203731	-177.5525116
2.459458764	48.26124214	631.3301392	2329.147493	-86.039814
3.871159945	93.40671782	550.329483	8724.814933	-273.7109009
4.145789191	109.6588669	673.6403656	12025.06709	-290.4301819
4.378597898	133.6556443	644.8106537	17863.83126	-308.4263031
3.740457272	84.25761024	485.3562317	7099.344883	-203.074263
3.427243356	66.74299201	373.7575531	4454.626983	-145.9237671
3.432713164	72.05206881	408.8925095	5191.50062	-186.6357574
3.258605512	74.0007392	735.0504761	5476.109402	-145.8844604
2.851060429	49.5908607	415.1746063	2459.253465	-88.89905624
3.012939659	57.8801523	330.2112198	3350.11203	-124.404303
3.508568513	80.85427576	321.2238255	6537.413909	-272.6527466
3.825615754	90.13259788	681.2133789	8123.885201	-168.6859863
3.773244879	89.70692484	400.704052	8047.332364	-304.0185852
3.316783168	76.99925126	649.3507385	5928.884695	-172.3208679
3.251245027	63.60374682	330.446043	4045.436609	-242.693985
3.430203262	71.59448628	515.8739929	5125.770466	-159.3183685
3.399437772	70.47246956	478.4175568	4966.368965	-162.3837097
3.523062711	72.38681353	322.6721058	5239.850774	-219.1249207
2.701383384	52.44834993	428.201416	2750.829411	-115.1323456
2.493981727	46.25264155	530.78125	2139.30685	-97.63656769
2.701587285	52.97399251	824.6596069	2806.243882	-78.92291107
4.006788054	100.6494187	646.3210754	10130.30548	-272.8737335
3.674529118	78.91586959	482.3028412	6227.714473	-240.2909546
3.231207092	59.49757707	330.3368053	3539.961677	-217.283638
3.742009142	86.17587911	536.7112122	7426.28214	-224.3626709
3.20585772	68.65039588	686.9489441	4712.876854	-151.3813995
3.576332765	76.42409638	346.6559563	5840.642508	-308.7506287
4.15692656	113.9908199	735.9813232	12993.90702	-234.9651154
3.205634473	73.7862278	945.1229858	5444.407413	-136.5991547
3.800182197	119.21009	514.7670898	14211.04555	-316.0050354
2.887461582	53.73687074	560.0885315	2887.651277	-112.3902794
3.226956451	59.2634141	596.0714722	3512.152251	-111.2078308
3.440410543	67.9657797	425.0358582	4619.34721	-200.2840088
3.985182597	98.25423812	683.7328491	9653.895309	-294.3322388
3.507656191	79.2781188	317.1737671	6285.02012	-162.5742416
2.968918564	104.0333961	330.1029968	10822.94751	-322.334552
3.354305738	79.78678456	792.4093628	6365.930991	-163.6016235
3.687905949	80.92898163	419.1886139	6549.500068	-236.1598953
3.609249558	82.9689573	576.6084137	6883.847875	-186.0799561
3.180310725	62.79020897	422.5518951	3942.610343	-138.2620361
2.720103178	50.89622022	464.1402588	2590.425233	-111.6990257
4.099852448	122.2874928	634.2699738	14954.23088	-325.5137451
3.96270563	97.49663943	551.95401	9505.594701	-189.2521896
3.553508855	95.12356965	332.8381119	9048.493502	-353.2375732
4.118532376	114.3965737	605.5615845	13086.57607	-238.1487396
3.631942226	79.17754427	392.7141113	6269.083517	-188.5440414

log.sigma.2.5.mm.3D_firstorder_Kurtosis	log.sigma.2.5.mm.3D_firstorder_Mean	log.sigma.2.5.mm.3D_glrlm_ShortRunLowGrayLevelEmphasis	log.sigma.2.5.mm.3D_glrlm_GrayLevelVariance
2.484022446	-101.6088634	0.0166819	11.10965921
3.146087516	-60.9080921	0.008019495	8.415609659
3.998360086	-112.3350218	0.022139517	7.997448695
4.112522336	-66.00142056	0.006719431	17.2210362
2.758886875	-151.460696	0.026915957	18.03302193
3.320634186	-133.6044232	0.03422943	6.29159013
2.459744434	-111.4376256	0.027620125	10.13313179
3.429187458	-158.7915547	0.037538919	12.26465175
2.413631069	-49.48666201	0.011348705	48.49862778
5.124088227	-52.51787732	0.008519903	7.746733417
2.989906728	-135.0996869	0.012433874	8.278687586
3.005395204	-67.97886349	0.01633795	8.523591036
3.210732526	-64.37501357	0.018503687	7.506673574
4.493860772	-89.770796	0.03036414	8.899263794
4.203482946	-78.55531868	0.01793097	15.25486682
10.54921859	-11.10251111	0.008483747	4.17470969
3.812026349	-156.13563	0.02829469	12.36012875
2.460548075	-80.19845678	0.024532945	6.995038244
3.402883795	-148.1558594	0.04412083	14.24354863
3.217853649	-33.34820722	0.016938478	5.775822589
5.649098242	-37.50914975	0.00954497	4.838440598
3.734592677	-75.35734354	0.013543959	10.11375964
4.274244576	-42.73442069	0.010690198	5.392057602
2.63696876	-63.94239548	0.018039541	33.1829425
3.827649934	-59.35603827	0.01098532	6.007133186
2.210544452	-124.1792701	0.030273488	8.934437586
6.425966903	-32.45966545	0.02447773	5.951314708
5.821139695	-31.97742882	0.013104758	5.80960389
4.211414359	-47.63895895	0.007679136	9.862837889
3.659439147	-58.80619341	0.00993857	8.002349433
4.78363976	-38.74606397	0.014100437	5.736313233
5.47668615	-28.68323044	0.011405035	4.830164602
3.062162582	-72.38566166	0.025175295	12.19497427
2.578177744	-248.8348501	0.083103111	16.6382752
2.859283237	-139.9550067	0.054879153	21.76053648
2.268156076	-91.8953195	0.022427712	8.636042602
5.517269838	-26.89269359	0.007347917	6.16833139
4.386227425	-35.0613863	0.007899068	7.473356217
3.184415164	-102.4583109	0.016478629	17.96134713
2.015822507	-103.4505725	0.03834705	10.82633952
3.23678197	-59.60596201	0.018864369	9.166266327
3.122129903	-143.7945867	0.029424792	9.463753593
3.295629931	-125.3850231	0.011482753	10.9447389
3.794843916	-134.4978244	0.013177236	11.77240322
3.873575753	-73.93903579	0.011406634	19.47585276
6.831708843	-16.91599034	0.023267649	3.116514612
8.154559184	-48.54129015	0.00650851	25.10704831
2.290041369	-143.9081642	0.043311228	8.404613272
2.653170706	-262.5784291	0.066676415	20.87000044
2.213775134	-160.5237102	0.051431714	20.69150797
4.876286406	-37.03643866	0.012233545	6.927654196
2.229504296	-145.3514686	0.055536289	9.054250338
3.3929385	-61.51323663	0.004580109	10.06490986
2.814768661	-85.11546302	0.029934722	8.48605656
7.362616334	-17.37936978	0.009956475	5.390726312
3.243097922	-153.3035677	0.020233444	14.37592526
3.450094566	-153.6555516	0.014240475	19.84245613
2.44844552	-146.4548885	0.021024722	28.8380354
2.953203109	-93.65235496	0.018479433	11.78779445
2.916631421	-64.61368672	0.029463795	7.573390325
2.186053794	-88.98746163	0.012183872	8.060051053
4.943497951	-39.27281126	0.007838839	10.8839473
4.305603415	-16.15757028	0.016367284	5.100625918
2.989698841	-35.70300464	0.018341258	6.146709772
2.139512029	-165.5513666	0.063420858	10.67563551
3.457202093	-42.89815866	0.010153649	13.80418668
2.580345467	-193.1916701	0.033461879	13.21089657
3.243287255	-53.98256567	0.012084325	10.57505487
3.854760523	-175.1220867	0.070057435	6.926830297
3.221331923	-57.6704012	0.010638089	8.992030733
2.916788254	-60.57270122	0.012066192	8.435391569
2.251013856	-125.8057083	0.024883677	8.485155447
4.303746086	-30.26464041	0.01233877	5.643089446
5.697494747	-23.93621125	0.010202919	4.608265109
10.21304806	-12.43513139	0.006094806	6.372974195
3.830566872	-148.8409824	0.018258124	16.93333002
3.021488954	-132.3654407	0.018998994	10.29476862
2.960650208	-151.9026574	0.028164621	5.986398189
3.074620972	-115.4496192	0.016803724	11.9113592
4.522370975	-51.92790411	0.00854242	8.735080424
2.368340916	-206.9845352	0.037816851	9.495216141
3.435998601	-87.43053253	0.013391801	22.03916076
5.733816068	-27.71420289	0.002885525	10.90082933
2.536798041	-180.5985287	0.05132386	23.21198001
4.41708604	-33.79462847	0.009118551	5.603817418
4.96445035	-42.00063681	0.006601606	6.495083208
2.900803566	-120.7822288	0.031995182	7.606213095
3.301210421	-176.3538517	0.010680467	15.95461049
2.310546615	-67.09380627	0.064430422	10.00194322
1.896434067	-219.1914714	0.097867502	17.82380013
5.528011999	-52.10996506	0.002996817	11.65153822
2.308033169	-129.625068	0.021042559	10.65578417
3.72232687	-70.68990925	0.011492818	11.88856028
3.384685153	-45.90052484	0.012711676	7.090416707
4.320166832	-30.05964935	0.010834175	5.234660085
3.606565612	-193.5569365	0.034690031	24.27537495
3.060808266	-64.4974752	0.023954885	15.75591272
1.958936342	-218.7947102	0.112633417	15.30904949
3.012247287	-104.4448489	0.016186829	21.58940775
2.757880807	-77.35718702	0.02850935	10.2475047

log.sigma.2.5.mm.3D_glrlm_LowGrayLevelRunEmphasis	log.sigma.2.5.mm.3D_glrlm_GrayLevelNonUniformityNormalized	log.sigma.2.5.mm.3D_glrlm_RunVariance	log.sigma.2.5.mm.3D_glrlm_GrayLevelNonUniformity
0.017573199	0.083886948	0.142122892	27.00002605
0.008966907	0.111479022	0.813202218	115.5319107
0.023337324	0.106685063	0.117545526	16.98685839
0.007243333	0.075175724	0.411447867	120.7044483
0.027459319	0.071633233	0.050305245	6.904647777
0.036510716	0.119792921	0.146179184	26.00332048
0.028137523	0.091178144	0.04046902	6.122551998
0.039195102	0.084032315	0.091551649	31.13012065
0.011962349	0.045912154	0.090428881	62.67500085
0.009497135	0.113393798	0.631832264	203.5496657
0.013133743	0.099010041	0.130986288	33.53924526
0.017631342	0.098835063	0.219787165	64.22370045
0.020363385	0.10610868	0.309922838	120.1814697
0.032487	0.105274197	0.153886814	49.4777802
0.018596151	0.079333526	0.077780545	26.96273232
0.011190533	0.238244906	1.956103215	1462.196997
0.029546312	0.091577407	0.087956754	18.45682862
0.026893529	0.106396743	0.346258199	135.2696974
0.046499388	0.087931622	0.107843818	14.57053555
0.01972243	0.130447459	1.018522061	248.8711424
0.011381282	0.14984424	1.255148483	503.4369735
0.01447942	0.092050806	0.184074519	74.02424559
0.012488333	0.141219855	1.039458078	284.6804114
0.018343468	0.052968832	0.040606032	6.78519539
0.012202392	0.128008762	0.47887224	96.68872959
0.032001504	0.094550149	0.128653792	25.55767972
0.027727364	0.141232847	0.762282227	235.9140998
0.015569316	0.17837155	1.422095339	439.1933287
0.008834526	0.130998629	1.168522998	331.2040382
0.011157756	0.117985148	1.011213801	141.5834996
0.0163195	0.143963651	1.005209738	415.8617445
0.013833626	0.165538288	1.376336312	937.1033816
0.026943361	0.083514247	0.38393562	95.42697711
0.083435355	0.11960577	0.015115696	1.76737109
0.057696951	0.066530104	0.059290123	7.106132758
0.024256971	0.094751352	0.335207182	105.6836272
0.009084257	0.190452666	1.40882574	791.314895
0.009144059	0.116582908	0.730121321	260.3539744
0.017074729	0.07138438	0.078357822	21.46357031
0.040807113	0.085127767	0.183624548	39.92266224
0.020310333	0.09751737	0.300983887	58.25541574
0.030685276	0.094894807	0.117110014	32.85450072
0.01217678	0.087847384	0.160882079	64.49844719
0.013956292	0.084730979	0.11227864	33.79389595
0.012107793	0.069217042	0.099307013	70.86853269
0.02885002	0.246210021	1.67590309	876.4775351
0.006971419	0.073241279	0.149138693	180.4616902
0.046259543	0.097317992	0.090418342	20.48002227
0.066827396	0.10661201	0.017334729	2.305727606
0.055192878	0.069531394	0.075558161	9.473268457
0.014036388	0.137041795	1.172021575	379.6069266
0.059118083	0.098805964	0.133811037	35.57715404
0.005122174	0.097768262	0.507892584	246.6970985
0.031809368	0.096880575	0.142889685	20.56277022
0.012471075	0.200887437	1.656679686	1376.436571
0.020885304	0.079462494	0.090945678	24.5999749
0.01492709	0.065817695	0.084805657	53.5024959
0.021475455	0.05345212	0.052567413	23.37319845
0.019414703	0.084880548	0.149256105	26.68361631
0.031864921	0.105001373	0.221775636	42.00272208
0.013325403	0.098760528	0.364977979	117.0346847
0.009006361	0.114437592	0.938880799	552.8369604
0.019469611	0.147851839	0.992892253	415.4859862
0.020805726	0.128232296	0.92642808	163.911653
0.066470937	0.0988854	0.081652355	4.901038953
0.010849102	0.079839054	0.20301993	80.55536157
0.034444481	0.080255548	0.058009796	7.977498907
0.013556334	0.106313326	0.779787141	271.2363063
0.074088627	0.121278501	0.114200967	15.76377239
0.011604627	0.098592669	0.500473566	139.0574988
0.013287541	0.103337621	0.471667099	214.7235628
0.02648778	0.094180325	0.144760906	47.36119999
0.014859675	0.153667616	1.64163138	672.3308224
0.012615772	0.187887204	1.462353219	1271.516429
0.00763088	0.177129281	1.295602568	1651.162437
0.01910278	0.074008835	0.084167194	30.68986226
0.019971452	0.089753004	0.128084025	36.80072713
0.029845055	0.122379981	0.116161246	24.74515083
0.017782382	0.084633831	0.267829187	66.35435055
0.009716411	0.112847225	0.874231162	219.9378787
0.039038373	0.091998489	0.094984784	12.68760285
0.013931544	0.064877137	0.138216335	50.61476529
0.003371766	0.126621574	0.79810973	453.0621766
0.051527928	0.087130777	0.024732055	2.635446793
0.010897686	0.142541423	1.099001326	480.7176108
0.007536911	0.123519377	0.458320292	344.2769421
0.033889644	0.105033504	0.127462036	34.40641676
0.011142812	0.0723786	0.088954522	39.62005745
0.066363048	0.095316662	0.044616688	4.569887586
0.098381249	0.143408941	0.038194444	1.794871795
0.003374506	0.108672174	0.721997009	278.5158953
0.021975945	0.086147549	0.107155861	23.84781025
0.012134445	0.09378166	0.25572003	33.44823907
0.01448669	0.115039043	0.839336794	302.1656904
0.013158171	0.160639714	1.174510671	764.5191787
0.035168868	0.069050386	0.048695998	4.149739463
0.025227297	0.072404556	0.124642875	44.75226696
0.115617358	0.094308928	0.022393486	2.212374582
0.016532763	0.065399298	0.060514141	10.08723421
0.029502105	0.091634942	0.130261848	23.15946507

log.sigma.2.5.mm.3D_glrlm_LongRunEmphasis	log.sigma.2.5.mm.3D_glrlm_ShortRunHighGrayLevelEmphasis	log.sigma.2.5.mm.3D_glrlm_RunLengthNonUniformity	log.sigma.2.5.mm.3D_glrlm_ShortRunEmphasis
1.404391879	107.3011218	260.3638402	0.917717098
2.678620538	132.1793293	676.010356	0.831920212
1.3575255	80.99128701	129.8643595	0.920832387
1.871483648	205.6378207	1223.095733	0.893112337
1.149571987	125.2557338	88.08627163	0.965259967
1.416443924	53.89228863	175.0047514	0.915796273
1.129712048	108.9938853	61.8224597	0.967571988
1.264728947	74.68496088	318.6999379	0.942284592
1.260349582	247.0537736	1176.741203	0.943192689
2.311678377	131.6013505	1233.082141	0.853480799
1.368724474	121.6705439	279.2138969	0.924788534
1.584309425	91.19027972	497.1326527	0.895604771
1.773550611	74.7869312	829.3328349	0.878087559
1.42082797	67.41515288	379.18152	0.916970628
1.226864903	119.0526275	298.2892072	0.949653737
5.181364803	82.07210691	2668.426586	0.681081269
1.251352432	79.33140788	175.6667499	0.946425328
1.849664243	61.01156236	910.7908152	0.869403994
1.318923374	70.26160587	138.2669624	0.930079643
3.181201543	62.65755922	1093.470233	0.783624295
3.577550552	86.01228329	1863.812994	0.77172822
1.471105616	110.3008273	648.9725587	0.916072766
3.180254062	85.27915824	1172.772275	0.789287349
1.120089937	250.1822286	119.0964221	0.972029058
2.124171658	101.2648601	514.1851933	0.848250515
1.379972969	71.97736906	218.7951424	0.918400211
2.663344854	56.50626971	1038.627724	0.814866491
4.033106889	69.95646409	1260.330473	0.741373745
3.381595843	133.2515659	1464.897621	0.787680628
3.033002712	108.9039754	745.4058055	0.814014944
3.15646378	72.75738634	1658.022284	0.784296332
3.856260858	76.50850613	2986.457532	0.752566155
1.867766799	85.3996832	843.6898166	0.881776771
1.051986475	107.3850803	14.3660186	0.987003381
1.182478091	100.3614823	95.59115927	0.956888665
1.776077636	70.94160559	840.4166342	0.888833635
4.083481632	101.5385106	2043.103449	0.726061495
2.592174824	120.6538584	1415.350864	0.822344112
1.224369159	142.3409552	266.535838	0.952144424
1.499510289	69.50955287	368.7907779	0.906496714
1.745045043	93.39917408	442.5541413	0.882223434
1.330280426	80.8661653	290.1148914	0.931721877
1.425909623	128.426956	597.8788422	0.91985028
1.325490434	126.6923769	332.5117988	0.930178733
1.286763268	167.4676192	870.8936209	0.937884773
4.756697901	37.53334295	1568.886606	0.683044881
1.393755862	225.1531162	2030.055054	0.925188444
1.266909365	51.87140518	180.5952785	0.940800781
1.057142857	141.5178238	20.91501832	0.985714286
1.225796782	86.3916958	119.2278202	0.948609379
3.379232442	91.27510335	1606.391332	0.788496567
1.391440468	46.79864145	291.5746654	0.91776443
2.128154889	216.1001068	1753.547804	0.857486627
1.414354368	67.52133078	170.1632887	0.91438316
4.467182152	80.85047336	3293.110057	0.718531111
1.263904057	113.8582882	266.4046698	0.94229105
1.238170928	166.6534392	713.578675	0.949430351
1.150617101	184.808496	400.6280181	0.966350376
1.419785757	114.1762823	252.4981653	0.915492866
1.597985459	62.48815789	304.9504153	0.892802726
1.879660052	99.78942597	851.7476512	0.870810262
2.939695423	132.6670893	2961.782785	0.809970363
3.14262229	63.40653066	1617.297324	0.784228488
2.906098903	64.51497843	793.9192845	0.813473238
1.241574091	55.70749742	43.8685864	0.948748476
1.517625728	159.5538718	803.5289919	0.910263361
1.176002058	89.33589047	89.26194975	0.958671012
2.650170002	100.6608953	1625.777041	0.824756601
1.331292884	35.86287295	108.6577408	0.929728226
2.131077172	124.7080396	973.1747883	0.854663077
2.056328176	104.128748	1464.633456	0.862663349
1.419971329	70.79412248	402.0359636	0.913113293
4.336439141	69.70314033	2207.935677	0.736751951
4.104386296	75.49435494	3366.531748	0.731007626
3.767510379	126.3891731	4830.852245	0.746776582
1.242993012	143.6049323	361.602246	0.946970519
1.369443117	105.0943396	336.0305079	0.922812093
1.349212359	55.96056182	166.3407148	0.923792258
1.638060922	130.1669461	611.5695551	0.901445344
2.830383248	118.93367	1212.050988	0.815096547
1.268253345	73.1195293	119.303	0.943329948
1.370380399	174.4082236	648.5532937	0.928545322
2.726198661	300.0383155	2207.980603	0.81288811
1.078514589	147.4551917	28.77648099	0.980371353
3.304397744	91.77568434	1916.790654	0.780758366
2.053715833	145.9724257	1932.484352	0.856908743
1.374042915	60.45797341	266.7523941	0.920305373
1.248929419	164.1975669	478.7455621	0.948102218
1.136525081	69.48615066	44.17848376	0.967726775
1.125	90.25961538	11.58333333	0.96875
2.535598793	332.1001834	1670.271195	0.83289468
1.318762278	88.35577644	231.7693291	0.930582549
1.626109389	149.4444056	275.694827	0.89935598
2.805275511	86.59052185	1602.155146	0.807351799
3.536400884	75.93889247	2554.426996	0.759026611
1.151444536	137.2761857	54.77605511	0.963563368
1.337401932	108.046695	519.8186061	0.933368467
1.070234114	72.90635452	22.43143813	0.982441472
1.180991442	193.4610928	138.9073264	0.958696412
1.362467955	87.71034547	209.4657031	0.926790417

log.sigma.2.5.mm.3D_glrlm_LongRunHighGrayLevelEmphasis	log.sigma.2.5.mm.3D_glrlm_RunPercentage	log.sigma.2.5.mm.3D_glrlm_LongRunLowGrayLevelEmphasis	log.sigma.2.5.mm.3D_glrlm_RunEntropy
172.8441704	0.891540592	0.021750035	4.227419605
526.723975	0.737046987	0.017390148	4.301818185
120.4583249	0.899608866	0.028645245	3.847791729
462.0265182	0.82987783	0.011009753	4.675474231
142.4741971	0.954303123	0.029798578	4.180668264
78.71030786	0.889344262	0.047350475	3.815964102
126.1638178	0.959340659	0.030207117	3.732670337
97.47319585	0.923844236	0.046533499	4.153284574
323.3378504	0.92490098	0.014736884	5.026908748
391.2386103	0.773609314	0.017398954	4.290927543
178.8876947	0.900777414	0.016580423	3.993352357
169.1243225	0.858246113	0.024383946	4.17786292
158.4137994	0.829091114	0.030956984	4.191921747
96.87470718	0.88986014	0.042945561	4.037887156
146.0342043	0.93364328	0.021552823	4.246942137
657.5507394	0.564299111	0.044945436	4.249092041
95.74550755	0.928394186	0.035449468	4.013399904
151.3729108	0.818943299	0.040515887	4.145007733
87.78115823	0.909974641	0.05729121	4.127931325
307.1943707	0.685929788	0.044456506	4.348717001
480.0240405	0.660841228	0.030513429	4.218057923
176.2328339	0.884403825	0.019412301	4.205404318
420.8763708	0.688962065	0.029166862	4.235588421
286.17888	0.962984384	0.019650191	4.57194965
294.9858505	0.785576923	0.020476089	4.038108824
117.8240481	0.895058584	0.039747833	4.000501284
199.4031791	0.728803307	0.052876311	4.190929425
469.67393	0.628419078	0.042071512	4.209068264
710.1337869	0.678045044	0.020357454	4.467251554
522.9443979	0.707437866	0.022859698	4.316664006
355.5607093	0.686484903	0.036019417	4.260530263
452.8667417	0.639638071	0.038983322	4.293687421
209.1972758	0.822395747	0.037363566	4.462257539
114.4571429	0.984615385	0.08476433	3.20489479
115.2436544	0.944860449	0.069407578	4.316245604
169.1260104	0.835889747	0.034795478	4.20379896
694.2781597	0.618385202	0.027325904	4.269064454
413.3374821	0.735767201	0.019435536	4.400070148
178.5174091	0.936496525	0.019815197	4.337470203
128.6717261	0.873370577	0.052617301	4.21115005
203.6920033	0.835180204	0.028538887	4.237529433
113.2336789	0.908742176	0.036550742	4.05850934
211.4291847	0.891150112	0.015572692	4.216136623
177.1054209	0.90853338	0.017518568	4.198157565
223.8198435	0.918247672	0.015219525	4.520588559
323.6620928	0.579310431	0.087510672	3.99009437
317.4890373	0.897137095	0.00933977	4.649473123
66.37969773	0.923076923	0.059301373	3.84105599
149.6373293	0.982517483	0.067431323	3.421203831
97.94002206	0.93308746	0.071803495	4.236438813
459.2928398	0.677413273	0.03196813	4.381190285
78.81191681	0.893491124	0.07564395	3.95085893
602.2411195	0.789021307	0.009097524	4.435315546
102.9702323	0.88799485	0.040878892	4.047866104
557.5321071	0.603382819	0.041268618	4.311780547
154.0180279	0.923995408	0.023807578	4.250601207
208.7561225	0.932251235	0.018005224	4.503972582
213.2847126	0.954820289	0.023415037	4.606380899
187.6961791	0.888092134	0.023709675	4.255365888
111.9889427	0.857543744	0.044680724	4.074256171
269.6009818	0.814538726	0.020093295	4.191209047
550.7563109	0.711061666	0.019642851	4.604619511
263.4028465	0.688172652	0.04734795	4.263719484
276.1921771	0.715876716	0.042744358	4.232538897
70.84633134	0.934687954	0.07988005	3.718672706
271.9224642	0.875467415	0.014517727	4.42808563
102.8621889	0.946520147	0.038602915	4.016235461
409.2687239	0.734507777	0.025163162	4.506069149
46.73984452	0.908552985	0.093599392	3.666547876
359.5809593	0.786358173	0.018414608	4.372658882
294.4768518	0.798857549	0.021377888	4.296008158
117.7066612	0.88712522	0.034009312	4.043609393
523.6844802	0.61732486	0.043275423	4.393410847
497.5152195	0.622136863	0.0386717	4.211899681
670.100009	0.640338654	0.023521536	4.402089495
184.2838599	0.929975854	0.022748656	4.366289397
156.5716055	0.899122807	0.024495848	4.166735007
82.22253836	0.902472527	0.037768062	3.676261566
271.311614	0.858876064	0.023108661	4.34874327
497.0391473	0.719418525	0.02019717	4.438190292
96.58600524	0.925141972	0.044661076	3.904527773
265.711148	0.901734104	0.016508579	4.645330803
1088.976625	0.72397272	0.007665417	4.529427141
160.3984941	0.975186104	0.052344204	3.864970817
457.5597929	0.678324497	0.027816778	4.314680758
351.7191511	0.794411178	0.014069797	4.188476014
90.22786647	0.897365648	0.042790705	3.928632919
210.7215484	0.929345697	0.013301435	4.35236592
81.11877142	0.958461538	0.074145168	3.649174689
108.3846154	0.961538462	0.100436238	3.07882022
1156.445021	0.746057384	0.006639362	4.523766535
132.2103327	0.910678138	0.026395013	4.093029831
290.4787936	0.856693787	0.015780398	4.267204355
369.009647	0.71808914	0.028939678	4.406618565
416.025815	0.65696696	0.035454696	4.278627129
156.8474573	0.953601954	0.037141194	4.272503
149.0561811	0.908823529	0.031898067	4.414423281
79.3277592	0.977564103	0.127553121	3.565713618
233.1168375	0.946201038	0.018035273	4.337276436
140.105959	0.902472527	0.03419743	4.066976564

log.sigma.2.5.mm.3D_glrlm_HighGrayLevelRunEmphasis	log.sigma.2.5.mm.3D_glrlm_RunLengthNonUniformityNormalized	log.sigma.2.5.mm.3D_glszm_GrayLevelVariance
118.0309914	0.807576567	18.86451517
166.2906029	0.65054121	13.0810524
88.05481621	0.8138405	13.24762336
231.9576374	0.761024199	30.15995817
128.5635947	0.913216542	21.67888889
58.05149033	0.804347649	11.74711195
112.4278718	0.919061959	10.97323022
78.8296224	0.859661572	20.16154844
260.9263623	0.861566001	47.91979081
156.2891476	0.686047243	19.85724909
131.4946069	0.822555838	13.71629732
102.6551673	0.763324607	16.19786844
85.61912297	0.73033023	14.58798923
72.35712873	0.805738344	16.56363542
123.958573	0.876815224	24.10308137
122.4987022	0.432833672	14.86735413
82.26716897	0.870475693	18.75501385
72.26959036	0.71401517	12.11345041
73.44121943	0.833412154	21.25173577
83.78229189	0.571331697	10.52062443
116.9329275	0.553282461	14.58777775
119.9589939	0.804989141	18.09590324
113.2961718	0.580002753	13.37540363
257.1213755	0.929302952	34.86234375
123.4472926	0.677531578	11.53882931
79.99663492	0.808296024	10.12143901
70.34461994	0.620340206	17.51530612
100.7519851	0.510352627	11.21384635
178.9403473	0.578271602	17.94324438
141.8867028	0.620172608	13.31557164
97.33878049	0.572215172	17.15264163
105.526828	0.525760151	12.73944643
99.20434756	0.737730471	21.56064285
108.7994928	0.969768608	16.74305556
103.2213963	0.89374702	24.4614876
82.12904619	0.751649828	13.47316987
148.7615689	0.489984826	10.72353662
148.8870486	0.632595093	17.99086194
148.7127361	0.884100391	22.80202341
78.3945193	0.784900931	13.19244383
107.9722675	0.738605961	14.56899863
86.43343557	0.837040049	16.84895352
140.8523162	0.812456785	19.03303496
135.5200771	0.833011354	17.67814434
177.5491855	0.849965697	27.4938861
59.1420979	0.437112053	7.301080247
239.8462272	0.823250567	44.98212987
54.56483777	0.856855869	9.234477721
143.1417249	0.965330542	25.00346021
88.6167262	0.874346782	20.16868512
120.1813962	0.578898679	17.63330236
52.24441007	0.80792108	10.04661669
257.7706189	0.693310236	18.81883657
73.62140898	0.80051578	14.33294887
116.2726853	0.479203221	14.41933338
121.0518256	0.859908135	23.59874133
174.0645226	0.876642997	26.08255661
189.9692674	0.915572611	31.08362886
126.12206	0.802346893	19.16195694
69.96993477	0.759189133	14.54036897
119.9032852	0.717075992	11.68668112
168.5189972	0.612547161	27.13596066
81.41027086	0.57288948	12.4069254
82.83023433	0.619020409	7.769066667
58.15566584	0.879646582	11.67863894
175.7999737	0.793844176	24.11528658
91.93530899	0.897295676	16.83024691
128.3924253	0.636617348	19.60984375
37.86114107	0.834091715	13.00554017
150.3107435	0.688311077	17.95615385
124.6361584	0.702564558	13.64343806
78.62953687	0.797718013	11.48231405
101.6214177	0.50338879	12.43490466
108.4712823	0.49602618	11.3977017
170.9580348	0.517228343	23.45467711
150.8817572	0.870754611	25.09058104
113.849218	0.81804789	17.136
60.79285685	0.820749991	8.713017751
147.5208105	0.776609401	19.21376429
151.9854909	0.620786212	22.26167449
77.18693524	0.863419078	13.05632502
188.6861563	0.830607084	36.73191081
375.7275157	0.616818065	25.03731618
150.0438521	0.95053589	29.74603175
122.136932	0.566774415	15.16444375
169.9093603	0.69164209	14.14072157
65.82488179	0.81270758	11.99090265
172.1457539	0.873718424	22.62194303
71.59862799	0.920145714	10.38137755
93.88461538	0.923611111	17.88429752
410.1685176	0.650702375	24.60854242
96.39519158	0.835068687	13.52248889
168.0837723	0.771777168	22.75732548
112.4154972	0.608127762	13.82922926
104.4750532	0.534973185	14.36638545
141.07648	0.910043902	31.21104536
114.727104	0.840521859	22.97691605
74.19063545	0.955213029	14.29757785
200.9355869	0.898796986	24.70604915
96.39223906	0.827277023	13.23850723

log.sigma.2.5.mm.3D_glszm_SmallAreaHighGrayLevelEmphasis	log.sigma.2.5.mm.3D_glszm_GrayLevelNonUniformityNormalized	log.sigma.2.5.mm.3D_glszm_SizeZoneNonUniformityNormalized
76.45503723	0.06543724	0.358715051
90.78394553	0.079679161	0.348022846
54.69172234	0.086464464	0.29651426
206.6758908	0.052970773	0.471069591
123.4412058	0.067222222	0.562222222
54.61884882	0.081705524	0.395925226
75.78140921	0.088637716	0.47769185
70.01221455	0.068014706	0.444636678
174.1694019	0.042640604	0.365925926
118.708389	0.06842199	0.334652968
99.8962253	0.082179132	0.392582333
83.54336788	0.071566358	0.401234568
73.05390765	0.080107651	0.379992311
84.46569066	0.072511775	0.431809948
116.5341434	0.0582651	0.4584075
88.77166171	0.077975304	0.365240833
86.78703651	0.071024931	0.486537396
57.56790188	0.089586777	0.394958678
79.70303166	0.069629042	0.463201746
49.8402744	0.086280992	0.377998163
69.62741336	0.083988676	0.327118305
102.1334201	0.067887811	0.401787273
71.06270483	0.076693603	0.36678195
215.5451285	0.053125	0.4890625
62.68522113	0.086264599	0.319581996
39.7279797	0.096055515	0.254565376
64.17974503	0.072840901	0.368688652
50.20577996	0.09529685	0.326970533
111.9611243	0.067761168	0.338245443
73.13347616	0.077259475	0.333610995
69.11320133	0.077119389	0.387844781
79.93287826	0.081626604	0.362272234
87.42952083	0.061342994	0.383589625
88.02083333	0.111111111	0.625
102.6409938	0.060495868	0.434710744
68.11787161	0.075448746	0.358509948
68.07239036	0.088898577	0.309159676
129.8963635	0.070489408	0.41100073
121.777377	0.062685975	0.433743305
41.6825862	0.078177901	0.30378578
60.04735963	0.076323731	0.360438957
74.17746033	0.07463835	0.390120037
95.5673838	0.067374831	0.341681738
119.9237799	0.075536326	0.368547563
145.5224396	0.055889667	0.388031221
23.74826953	0.110864198	0.268024691
241.9366817	0.047347859	0.459906544
45.58710454	0.094594595	0.350255661
128.5653595	0.093425606	0.619377163
93.50924378	0.066176471	0.406574394
84.81937323	0.068956898	0.373485018
41.32838196	0.0911188	0.314109958
157.9778885	0.069127978	0.341331856
61.40173702	0.088811995	0.321030373
69.85321082	0.076085311	0.332885117
101.6212753	0.062292407	0.463084902
157.3986604	0.056743891	0.450210456
147.2981374	0.050602259	0.465606399
87.6788078	0.064590542	0.338139177
51.33706723	0.077563975	0.252926007
66.80315856	0.088179847	0.320105571
148.3632782	0.054128376	0.411869835
63.33248465	0.089546879	0.369280189
36.36855641	0.103022222	0.240355556
31.78872252	0.096408318	0.334593573
136.2498544	0.059793521	0.404286223
86.58142994	0.072702332	0.444444444
90.64494033	0.0657375	0.348475
37.51173529	0.090027701	0.271468144
91.76807006	0.068343195	0.421952663
86.10191468	0.075620761	0.380916263
37.72658846	0.08231405	0.283140496
59.28571422	0.079314212	0.332209582
51.78954763	0.083493452	0.310994902
155.4533104	0.063095267	0.436491682
122.7524737	0.059039727	0.394433493
93.70562345	0.067904	0.40416
32.39196798	0.112426036	0.24704142
97.11203929	0.066687631	0.347543856
122.1407835	0.063280028	0.442098375
61.10912557	0.08341028	0.369652201
161.420044	0.047435437	0.45737346
258.4496372	0.05866782	0.38942474
124.9907407	0.070294785	0.501133787
97.11346265	0.072475	0.3817
139.797282	0.081209654	0.400885265
47.02768211	0.087192817	0.340028355
153.7271912	0.060299651	0.491165009
47.64519841	0.091836735	0.397959184
75.09090909	0.123966942	0.702479339
255.6475745	0.060903963	0.400078237
42.40938935	0.086222222	0.2544
131.8420731	0.061907498	0.431054295
64.56657701	0.075330294	0.366761956
72.82122853	0.07537712	0.373156904
134.6785897	0.057199211	0.541091387
110.4530154	0.058761069	0.418208074
52	0.093425606	0.515570934
144.296875	0.062145558	0.435018904
42.48043439	0.080578512	0.338068182

log.sigma.2.5.mm.3D_glszm_SizeZoneNonUniformity	log.sigma.2.5.mm.3D_glszm_GrayLevelNonUniformity	log.sigma.2.5.mm.3D_glszm_LargeAreaEmphasis	log.sigma.2.5.mm.3D_glszm_ZoneVariance
29.41463415	5.365853659	71.64634146	52.2648721
62.29608939	14.26256983	1399.435754	1338.001685
13.93617021	4.063829787	38.91489362	24.73245813
250.6090226	28.18045113	329.3552632	316.1532414
33.73333333	4.033333333	5.716666667	2.883055556
27.31884058	5.637681159	64.55072464	52.0457887
19.58536585	3.634146341	4.536585366	1.621653778
60.47058824	9.25	36.83088235	28.13705666
197.6	23.02592593	24.63333333	17.16222222
103.407767	21.14239482	1646.728155	1590.405316
44.75438596	9.368421053	47.10526316	36.22683903
57.77777778	10.30555556	248.8680556	221.2325907
96.89803922	20.42745098	462.7843137	434.0883353
60.02158273	10.07913669	123.7841727	109.3551058
72.88679245	9.264150943	16.86792453	11.6269926
188.0990291	40.15728155	70152.7068	69710.64596
46.22105263	6.747368421	19.63157895	14.41396122
86.89090909	19.70909091	726.3363636	676.5697521
32.88732394	4.943661972	22.3943662	15.82344773
62.36969697	14.23636364	6575.066667	6292.419247
122.342246	31.41176471	12603.81551	12419.68367
100.8486056	17.03984064	123.1832669	110.0679037
90.5951417	18.94331984	5142.59919	5002.842761
39.125	4.25	4.1625	1.39859375
38.66942149	10.43801653	844.8760331	781.929513
18.83783784	7.108108108	52.02702703	35.37180424
87.74789916	17.33613445	3448.147059	3355.647924
108.5542169	31.63855422	11078.0512	10940.41308
148.4897494	29.74715262	5474.90205	5403.251031
65.3877551	15.14285714	2118.158163	2043.54735
133.4186047	26.52906977	8541.584302	8392.446246
216.638796	48.81270903	25988.2592	25770.07962
107.0215054	17.11469534	340.2903226	315.5049139
7.5	1.333333333	1.75	0.1875
23.90909091	3.327272727	9.145454545	4.924297521
97.51470588	20.52205882	279.3382353	255.2849805
127.3737864	36.62621359	27139.8568	26876.10974
152.0702703	26.08108108	2772.47027	2705.31889
61.5915493	8.901408451	13.06338028	7.953233485
34.63157895	8.912280702	103.2192982	81.03024007
48.65925926	10.3037037	266.7037037	238.6529492
44.47368421	8.50877193	61.88596491	50.71629732
71.41148325	14.08133971	97.47368421	81.92971773
47.54263566	9.744186047	50.76744186	39.18634697
147.839895	21.29396325	56.93700787	48.37255186
48.24444444	19.95555556	63307.58889	62150.83321
391.8403756	40.34037559	219.8098592	209.4220999
25.91891892	7	35.7027027	26.20964207
10.52941176	1.588235294	2	0.325259516
27.64705882	4.5	11.67647059	7.066608997
147.5265823	27.23797468	7292.496203	7185.805608
32.03921569	9.294117647	52.06862745	36.45838139
162.1326316	32.83578947	1599.353684	1554.11036
16.37254902	4.529411765	73.23529412	51.27412534
177.4277674	40.55347092	63524.43527	63074.48601
60.66412214	8.160305344	31.80916031	25.26962298
171.5301837	21.61942257	20.23097113	14.99275976
117.798419	12.80237154	7.786561265	4.509459607
34.49019608	6.588235294	45.78431373	33.73933103
17.95774648	5.507042254	221.1830986	178.1051379
65.94174757	18.16504854	580.538835	530.6513573
367.3878924	48.28251121	6665.008969	6607.303485
118.5389408	28.74454829	8609.339564	8448.263138
36.05333333	15.45333333	2888.386667	2747.252267
7.695652174	2.217391304	9.173913043	3.86389414
107.1358491	15.84528302	193.954717	175.0568316
24	3.925925926	7.611111111	3.830246914
139.39	26.295	3295.2775	3220.151944
10.31578947	3.421052632	43.39473684	29.2333795
109.7076923	17.76923077	1028.115385	980.6114793
161.8894118	32.13882353	1110.209412	1072.81259
31.14545455	9.054545455	101.9727273	75.40338843
131.2227848	31.32911392	24778.67089	24459.48326
153.3204868	41.16227181	57444.18256	56961.25296
470.5380334	68.01669759	42626.86364	42445.46422
68.23699422	10.21387283	26.50867052	19.8624077
50.52	8.488	68.304	54.996096
12.84615385	5.846153846	56.42307692	37.86686391
73.33175355	14.07109005	138.3175355	119.5944835
156.5028249	22.40112994	2571.423729	2513.078298
21.07017544	4.754385965	16.89473684	10.0615574
138.5841584	14.37293729	63.40264026	55.25284014
264.8088235	39.89411765	5312.460294	5259.919115
10.52380952	1.476190476	2.714285714	0.535147392
152.68	28.99	9752.94	9599.055975
226.9010601	45.96466431	1964.991166	1926.599349
31.2826087	8.02173913	72.85869565	57.11850189
119.8442623	14.71311475	21.43852459	15.61144518
11.14285714	2.571428571	4.642857143	1.454081633
7.727272727	1.363636364	1.545454545	0.148760331
209.2409178	31.85277247	2389.026769	2346.040317
19.08	6.466666667	44.16	27.73048889
50.86440678	7.305084746	94.57627119	82.14765872
110.3953488	22.6744186	5271.694352	5124.487324
154.1138015	31.13075061	24913.32688	24607.5428
21.1025641	2.230769231	3.974358974	1.364891519
90.75115207	12.75115207	51.59447005	41.77476693
8.764705882	1.588235294	2.235294118	0.242214533
40.02173913	5.717391304	5.380434783	2.241375236
29.75	7.090909091	41.36363636	31.23966942

log.sigma.2.5.mm.3D_glszm_ZonePercentage	log.sigma.2.5.mm.3D_glszm_LargeAreaLowGrayLevelEmphasis	log.sigma.2.5.mm.3D_glszm_LargeAreaHighGrayLevelEmphasis	log.sigma.2.5.mm.3D_glszm_HighGrayLevelZoneEmphasis
0.227146814	0.72741587	9146.085366	119.597561
0.127583749	6.594964492	305650.9665	137.1731844
0.265536723	0.547847084	3634.12766	91.21276596
0.275219865	1.273765878	89140.67293	265.424812
0.594059406	0.104926581	583.1666667	147.8666667
0.282786885	1.465435039	3301.710145	72.76811594
0.585714286	0.087922092	505.097561	110
0.33915212	0.782636147	2582.375	92.66176471
0.365853659	0.26538114	4892.866667	276.6574074
0.133247089	9.455215575	296293.178	177.7702265
0.303191489	0.421177925	6014.657895	142.3596491
0.190224571	2.535384345	27873.67361	113.5763889
0.186676428	6.545505408	37391.31765	107.7294118
0.263257576	2.703736902	6722.007194	107.8417266
0.436813187	0.217922786	1668.320755	150.2767296
0.047561877	552.2540631	8983487.049	130.4194175
0.437788018	0.472445435	1039.905263	108.7052632
0.141752577	10.29616655	66072.54091	79.57727273
0.39010989	0.734518054	984.2253521	101.4929577
0.059480894	67.3755761	682245.4121	71.73939394
0.073694581	90.03777052	1789583.848	106.1122995
0.276127613	1.164859714	14774.08367	141.0318725
0.084589041	37.54785266	721418.8138	103.8178138
0.601503759	0.056612389	962.1875	278.6125
0.126041667	5.723681925	130679.2975	102.1818182
0.245033113	0.903730897	5145.5	73.90540541
0.103975535	45.64554667	271197.5378	87.35714286
0.085237484	90.13244688	1374023.581	75.16566265
0.118137783	24.15780932	1252150.456	163.5330296
0.115770821	11.42869643	402708.5408	111.7908163
0.081885265	72.73290444	1029366.61	93.05523256
0.067700668	214.244312	3199457.898	114.1153846
0.200863931	3.052774307	46152.09677	116.4659498
0.8	0.115622257	190.8333333	108.5833333
0.486725664	0.541874359	624.7090909	133
0.203898051	3.274802409	30836.78309	93.87867647
0.06157525	153.3727154	4848746.328	109.368932
0.122031662	17.22243186	461148.2811	180.6567568
0.442367601	0.167016077	1626.697183	167.4788732
0.212290503	1.704571135	10501.60526	70.5
0.188811189	2.335568165	34993.74815	94.9037037
0.299212598	0.919585296	5025.140351	98.64035088
0.253640777	0.75471229	14788.44498	146.215311
0.293849658	0.482809617	6486.100775	164.2945736
0.341704036	0.436393354	9029.564304	209.3543307
0.029402156	910.3837643	4495853.811	39.72777778
0.310269483	1.17080908	43223.03991	314.9131455
0.324561404	1.490676894	1255.175676	70.24324324
0.772727273	0.093205114	258.6470588	151.2352941
0.465753425	0.781316607	469.2794118	121.9411765
0.096813725	51.31246766	1053307.785	119.0050633
0.253101737	2.354936338	2828.588235	63.10784314
0.148669797	5.052751092	515413.3579	244.1347368
0.213389121	1.528062508	4875.784314	89.78431373
0.04714311	499.2241562	8171548.737	105.7598499
0.391044776	0.2994805	4158.916031	127.7633588
0.436926606	0.205242393	2972.401575	204.4908136
0.552401747	0.114870968	1079.905138	203.6996047
0.288135593	0.416683089	6818.186275	131.4803922
0.152360515	4.378651807	14250.87324	90.54929577
0.141580756	4.040366272	97666.76214	101.6601942
0.131641086	33.56087661	1341528.93	193.7253363
0.078792342	103.3103496	741909.8692	92.79127726
0.084175084	29.94811768	298836.0333	67.98666667
0.433962264	0.483475255	520.826087	58.86956522
0.230034722	1.143911168	37933.55849	186.509434
0.514285714	0.201754321	505.8888889	111.3518519
0.115373522	19.19383122	585232.46	125.9575
0.265734266	2.513823822	1055.710526	57.68421053
0.145089286	5.838096634	191602.7154	132.4461538
0.163524432	7.050594896	186433.8753	120.7294118
0.194003527	1.635794592	9231.481818	68.69090909
0.055972793	196.8679477	3178921.949	87.26835443
0.045504892	457.2200106	7300306.832	83.32048682
0.074247538	237.0859546	7732832.175	207.6131725
0.387892377	0.29368919	3536.65896	172.4393064
0.274122807	0.724368583	7970.816	129.496
0.232142857	1.269305374	3217.269231	62.11538462
0.231106243	0.893769622	27157.45972	142.3080569
0.13091716	14.02894482	487923.7853	156.9265537
0.382550336	0.35929699	1258.298246	85.75438596
0.350289017	0.359227594	13875.85479	207.970297
0.137959018	12.75013897	2223598.034	371.9279412
0.677419355	0.086608793	391.0952381	158.1904762
0.080612656	67.89057225	1431486.5	131.6425
0.161391503	11.87509441	334126.0636	201.4823322
0.252054795	1.687415472	4008.717391	73.75
0.41426146	0.17079885	3423.07377	196.9918033
0.56	0.255796275	327.2857143	72.67857143
0.846153846	0.118373307	154.1818182	90.90909091
0.152522601	4.832652118	1190883.962	374.2198853
0.246710526	0.571627429	5086.333333	84.30666667
0.283653846	0.536436002	18546.5339	178.1610169
0.082420591	38.57936672	753254.0266	92.9269103
0.057186375	201.3924263	3134285.845	102.3535109
0.619047619	0.084784736	467.8974359	160.8205128
0.319117647	0.762452789	5385.903226	147.9631336
0.708333333	0.304931624	164.9411765	74.58823529
0.564417178	0.057516009	967.4565217	206.3695652
0.314285714	0.467383261	4955.897727	77.05681818

log.sigma.2.5.mm.3D_glszm_SmallAreaEmphasis	log.sigma.2.5.mm.3D_glszm_LowGrayLevelZoneEmphasis	log.sigma.2.5.mm.3D_glszm_ZoneEntropy	log.sigma.2.5.mm.3D_glszm_SmallAreaLowGrayLevelEmphasis
0.619392649	0.033494329	5.374269194	0.027120787
0.611193364	0.017044704	5.525676432	0.012331152
0.560081961	0.041392132	5.027889118	0.032452147
0.713055963	0.009695018	5.881633089	0.007385818
0.774446502	0.03407183	4.742749405	0.030065547
0.651944476	0.053028045	4.79122224	0.035444028
0.715704607	0.037650034	4.461091334	0.03423351
0.692479599	0.055398616	5.365467739	0.038469356
0.627837387	0.013134966	6.508602324	0.007616003
0.599241474	0.016926811	6.001790919	0.012529576
0.649807315	0.02007601	5.195406513	0.016302472
0.657811583	0.028393784	5.45539008	0.019427209
0.639363012	0.026910461	5.450158388	0.014929629
0.680136403	0.039300178	5.315839561	0.019728147
0.703412865	0.024557475	5.457902513	0.020170328
0.627924481	0.022719114	5.905881473	0.014463607
0.721666208	0.036351281	5.019158373	0.030107555
0.651461561	0.046885263	5.413006134	0.035578962
0.705381347	0.06410282	5.054696331	0.05242897
0.638316133	0.045979583	5.30958774	0.030269873
0.591071336	0.023330375	5.794209678	0.016444978
0.657980427	0.019766606	5.632352119	0.014544746
0.627989285	0.024048421	5.690950965	0.016905475
0.725131944	0.021734759	5.080875438	0.017490418
0.582202296	0.024969034	5.39836438	0.018191132
0.510679521	0.045739882	5.11986971	0.018045485
0.629690335	0.054220536	5.660283938	0.035377747
0.591422334	0.032347466	5.706117814	0.020591668
0.603297295	0.014304187	6.027724059	0.007790345
0.598126183	0.022777756	5.700064818	0.015890515
0.64730314	0.036387917	5.778818506	0.024592753
0.625325952	0.023232775	5.787991065	0.012568348
0.64370848	0.046029213	5.933708597	0.032415339
0.8125	0.096781856	3.418295834	0.092071756
0.680645212	0.042963684	4.990272895	0.016248378
0.621963472	0.034007394	5.692977751	0.022234153
0.576087816	0.018049007	5.931218004	0.011720316
0.665971118	0.013096039	5.67741953	0.009571365
0.683604646	0.021906803	5.498820752	0.017859774
0.565715181	0.065746866	5.472457468	0.032991221
0.621507823	0.040511922	5.411768009	0.03074682
0.648261005	0.053992686	5.332602133	0.042618454
0.603900326	0.018427602	5.805492174	0.012750316
0.630666882	0.018439691	5.431208071	0.012824375
0.646075323	0.012491687	5.971925138	0.006460572
0.532324557	0.086203732	5.563342765	0.045866654
0.705204434	0.008019137	6.23196228	0.005863404
0.610331759	0.047174159	4.815419372	0.029514947
0.815359477	0.081999058	3.616874606	0.080218786
0.660342785	0.043212834	5.037696989	0.024328217
0.63533102	0.030356306	5.875934961	0.020128842
0.578192494	0.075259142	5.156069553	0.04523418
0.606108041	0.008015797	6.096350162	0.005600478
0.582669971	0.054031467	4.834094214	0.036147366
0.599300248	0.024929642	6.04200118	0.014804232
0.706591609	0.03166057	5.376882377	0.025211543
0.696719096	0.014196253	5.799255983	0.007376271
0.708603196	0.028134402	5.754000839	0.024459179
0.598079679	0.031139827	5.610281259	0.021175554
0.511606414	0.058162188	5.362785106	0.035170329
0.585143984	0.021720458	5.636439384	0.011274799
0.667398696	0.013235763	6.110429197	0.008530178
0.62940391	0.032506443	5.496539924	0.017690437
0.50314664	0.041485036	5.623436984	0.026222059
0.564223664	0.08873858	4.229871195	0.067818402
0.660873354	0.016591101	5.853237293	0.011424394
0.689768204	0.043813165	4.712949308	0.03723699
0.612553052	0.022319291	6.119570013	0.014156863
0.521639254	0.109224886	4.754377842	0.077906862
0.674621052	0.027304716	5.666275123	0.023243319
0.641516139	0.020822929	5.793183705	0.014731552
0.544055639	0.048142696	5.452468209	0.033368208
0.596414062	0.031064109	5.876303113	0.018051694
0.577199144	0.029801521	5.917987508	0.019416821
0.687191703	0.012001993	5.998608286	0.007960259
0.650734505	0.019823855	5.718309535	0.01004975
0.659528362	0.033724648	5.427745538	0.026743305
0.488788824	0.047432429	4.931705709	0.032095812
0.611216761	0.029697774	5.78040057	0.016451194
0.690523129	0.016927676	5.69127261	0.012414925
0.626095134	0.061280271	4.872731138	0.050590551
0.702241606	0.021811801	5.91706931	0.018089941
0.648532052	0.005146301	6.15489201	0.003776278
0.73015873	0.069544131	4.070656113	0.066471074
0.641839753	0.016755529	5.703495393	0.010626211
0.657840196	0.009389119	5.67158898	0.006569922
0.602673785	0.05783243	5.1676373	0.045859518
0.726818054	0.013547001	5.552000694	0.010420506
0.626428571	0.065710026	4.182005815	0.031659523
0.863636364	0.109406084	3.459431619	0.107164278
0.657593768	0.006316051	6.054176444	0.005122023
0.513709612	0.039377448	5.346965817	0.030274426
0.681907848	0.021143403	5.462109923	0.01767229
0.628243607	0.032080898	5.689045939	0.020615508
0.634684175	0.023154572	5.767768229	0.014672713
0.759707977	0.046473584	4.701943565	0.042470712
0.672192563	0.02939393	5.642816042	0.018240123
0.691176471	0.095002615	3.852168724	0.042520363
0.683556763	0.021744405	5.308903196	0.018496121
0.597053704	0.052427072	5.247078813	0.039796192

log.sigma.2.5.mm.3D_ngtdm_Coarseness	log.sigma.2.5.mm.3D_ngtdm_Complexity	log.sigma.2.5.mm.3D_ngtdm_Strength	log.sigma.2.5.mm.3D_ngtdm_Busyness	log.sigma.2.5.mm.3D_ngtdm_Contrast
0.025170289	0.581793564	2.902238278	0.206732969	0.000264091
0.006546198	0.090633951	0.920879347	0.526054132	4.07E-05
0.047760162	0.718104892	4.454729551	0.166907656	0.000537378
0.004475824	0.404134831	1.467552759	0.388296525	3.85E-05
0.061630497	4.571264288	8.783494801	0.086180009	0.002295523
0.030122292	0.471600235	1.540335989	0.349452679	0.000439462
0.064481883	2.921619389	4.294353109	0.14242669	0.004313531
0.01774664	0.863290965	2.469713836	0.37903558	0.000336145
0.005159249	1.096050331	2.371264319	0.501581718	0.000178776
0.004174756	0.141324828	1.313781303	0.503408994	9.84E-06
0.018207317	0.551007053	1.729091646	0.300544862	0.000279762
0.012077222	0.253538888	1.437816999	0.389519871	8.33E-05
0.006064284	0.141937754	0.593494863	0.921990394	5.08E-05
0.014358206	0.542483935	2.071132464	0.399702154	0.00014687
0.019585359	1.591086093	3.805880964	0.201222495	0.00036203
0.000638753	0.011742231	0.23916153	4.052371127	7.68E-07
0.027895399	1.633514453	3.410811843	0.260832239	0.00078923
0.005340076	0.116194133	0.631862855	1.047619003	3.81E-05
0.031712698	1.967212804	3.930085646	0.26943784	0.001144027
0.003709655	0.024650619	0.373298103	1.521297139	1.25E-05
0.001827359	0.030475816	0.502377762	1.607750823	3.17E-06
0.008137547	0.45320982	1.551588301	0.425995069	7.69E-05
0.003259244	0.044605021	0.583323313	1.003082484	7.58E-06
0.055129587	5.768648388	13.33458824	0.050483336	0.002059608
0.009735564	0.123935532	1.36845182	0.407092361	3.91E-05
0.026530907	0.415406021	1.894064386	0.323379275	0.000421654
0.004043764	0.086193758	0.943602356	0.966528084	9.80E-06
0.002026936	0.041764963	0.674827633	1.395824173	4.06E-06
0.002148995	0.065688482	0.629166608	0.964931596	9.39E-06
0.005155709	0.091131972	0.996694064	0.584632189	2.36E-05
0.002175768	0.045130089	0.585909817	1.339075056	4.21E-06
0.000953988	0.014054309	0.186740606	3.424610414	1.96E-06
0.006118099	0.273824858	1.242092055	0.601350078	5.46E-05
0.135754557	15.83523144	12.07748196	0.100448662	0.096777331
0.065727301	3.593918077	9.648222855	0.112928581	0.002292582
0.00563714	0.157335332	0.61624211	0.998290797	6.72E-05
0.00107371	0.02043261	0.338096455	2.34215913	2.75E-06
0.002989201	0.095001352	0.788915422	0.791602207	8.50E-06
0.019635762	1.931793505	3.837724427	0.191170132	0.000542434
0.015875425	0.266543416	1.302934545	0.503166414	0.00024782
0.013487726	0.255130327	1.608337637	0.321497996	9.08E-05
0.018975867	0.70166526	2.108863712	0.306106447	0.000268827
0.008740706	0.475547747	1.572902478	0.411059483	0.000108595
0.016806893	0.905135931	3.14389173	0.229580008	0.00021615
0.006606386	0.794870827	2.136901448	0.39374787	0.000103332
0.001189557	0.007305051	0.14604396	5.337293169	2.64E-06
0.002910145	0.717613363	2.816852941	0.424878512	2.39E-05
0.026212211	0.532055436	1.274436268	0.824008729	0.000944462
0.119485761	14.88576554	13.20689596	0.075895449	0.058083824
0.032451872	3.135827618	4.361542935	0.329257179	0.002716908
0.00215966	0.043719736	0.459778578	1.313131318	6.12E-06
0.015615161	0.34207667	1.038916123	0.990795869	0.000473089
0.002864416	0.121782342	0.977706861	0.594718794	1.11E-05
0.037091261	0.537157739	2.967550581	0.232368058	0.000381777
0.000653342	0.01362667	0.283186304	4.157589712	1.11E-06
0.022413009	1.25671139	3.202603858	0.188397154	0.000412819
0.008332853	0.917347571	2.205908724	0.33210207	0.000151822
0.013963539	1.851878682	2.808571242	0.290108869	0.00066585
0.02426163	0.721940222	2.999327251	0.17519921	0.000286226
0.018462104	0.265439134	1.582526187	0.43431918	0.000161173
0.006373045	0.100701427	0.66107511	0.779848453	5.21E-05
0.001262351	0.069361362	0.586336663	1.375682216	4.03E-06
0.002154546	0.029199554	0.332007185	2.013774301	5.40E-06
0.005452793	0.037160946	0.457283505	1.177667261	2.77E-05
0.127379736	2.261932844	8.76731479	0.104594957	0.003656692
0.007698777	0.445173575	1.893942032	0.31211104	6.23E-05
0.054689707	2.553749834	4.645773678	0.168710238	0.002326589
0.002528855	0.09915107	0.857699626	0.943847588	1.07E-05
0.042447316	0.952899731	2.950887453	0.374403428	0.000905462
0.005358852	0.136675011	1.006202858	0.519538577	2.39E-05
0.003299979	0.081873875	0.505082208	1.044035452	2.09E-05
0.01398097	0.195899261	0.83138974	0.819924887	0.000253384
0.001233977	0.014874933	0.225720747	2.874729139	3.04E-06
0.000709038	0.010042409	0.183918983	4.713909864	1.44E-06
0.000537268	0.03098404	0.523005364	2.642991555	5.75E-07
0.015084906	1.629626553	3.418109318	0.206969291	0.000307061
0.017155624	0.61088161	2.13515131	0.267783642	0.000204203
0.027236704	0.570918621	1.886222193	0.396614403	0.000462148
0.009318701	0.339211417	1.579172531	0.349631098	9.00E-05
0.003360848	0.119664654	0.993221676	0.64036237	1.10E-05
0.046451382	0.989954393	3.138265988	0.210365612	0.0010541
0.009617106	0.969538345	2.601811319	0.247683814	0.000151789
0.001649861	0.110077371	0.995274506	0.645921443	4.77E-06
0.110695033	15.48801709	16.09745162	0.055214649	0.020620179
0.001791625	0.039076928	0.478009578	1.470475594	3.56E-06
0.00246325	0.090967021	0.611065845	0.922520002	7.34E-06
0.020674783	0.387543889	1.548619864	0.49724981	0.000311193
0.01156651	1.088667263	2.444176858	0.272962972	0.000222375
0.103940709	2.940031353	5.666677321	0.120501873	0.005792182
0.138851802	15.49615385	13.40799957	0.104027778	0.141547753
0.002405655	0.175832133	1.530244485	0.406580492	7.82E-06
0.025110835	0.713680041	2.600512679	0.273836868	0.000392129
0.019657454	0.897628708	3.587336844	0.13756862	0.000184801
0.002820137	0.033226656	0.385094554	1.372298863	1.01E-05
0.001139824	0.019141888	0.275183959	2.731812548	2.34E-06
0.102889539	9.281007867	14.63475399	0.050721268	0.005330148
0.010882272	0.706121774	1.854279002	0.415703925	0.000204847
0.147803313	9.583411759	11.2703365	0.078977606	0.026167151
0.037648979	3.489528367	6.908195564	0.096752183	0.001432535
0.032312234	0.543350494	2.947591839	0.18877328	0.000369045

log.sigma.2.0.mm.3D_gldm_GrayLevelVariance	log.sigma.2.0.mm.3D_gldm_HighGrayLevelEmphasis	log.sigma.2.0.mm.3D_gldm_GrayLevelNonUniformityNormalized	log.sigma.2.0.mm.3D_gldm_DependenceEntropy
11.56421452	95.48199446	0.088435479	6.096039294
7.135049677	190.4739843	0.190659055	6.28274923
8.197708194	101.7514124	0.108302212	5.651071692
17.67868606	219.9715468	0.100031607	6.73717564
14.9718655	168.6336634	0.07930595	5.170539766
7.19853534	61.01639344	0.111058855	5.632883902
21.45387755	227.1714286	0.066122449	4.766653331
14.02481328	116.2817955	0.078612695	5.962391904
42.65289024	289.8624661	0.046714918	6.920989755
6.817056392	146.0957309	0.151623138	6.388038196
9.181841331	92.39893617	0.094273427	5.904553156
8.038443484	95.21532365	0.112215535	6.248851257
7.305917181	96.9033675	0.116941664	6.404378656
7.997575184	82.51893939	0.111828512	6.08672065
18.66147657	209.9972527	0.075730588	5.871344729
2.395841629	105.0115441	0.344358624	5.882518345
12.05075495	79.86635945	0.094013464	5.610549875
6.553469002	106.4890464	0.129751933	6.317220616
14.83238739	125.1978022	0.083081753	5.737106713
4.11445605	113.3900505	0.210572059	6.380541496
3.75305146	131.9757635	0.239298367	6.021695856
10.71864656	130.5115512	0.09540701	6.311652805
3.9356535	88.16027397	0.210924423	6.258282393
33.45299339	256.2030075	0.054666742	5.615453774
5.645763889	117.8291667	0.166671007	6.131872758
8.701153458	120.5562914	0.103767379	5.731146656
5.144708075	95.34906073	0.188774699	6.252999594
3.765903359	137.56457	0.303699651	6.014727248
7.78926863	232.6520452	0.227687329	6.331915949
6.003406543	143.9444773	0.198860253	6.174812981
4.386283721	132.1930493	0.215496067	6.129646942
3.22380495	115.5602853	0.243416062	6.23627305
12.06905227	159.562995	0.108685387	6.53237448
16.35555556	85.8	0.111111111	3.506890596
21.74218811	134.3274336	0.075103767	5.490285768
8.816702593	101.6341829	0.108604618	6.346931957
4.264097008	140.1358541	0.300728637	5.90370779
6.438173881	159.9218338	0.170369402	6.436863985
17.79051057	137.423676	0.068894906	5.984674
10.81831265	111.9013035	0.09463916	6.212514106
8.611308132	250.3594406	0.1183608	6.408361839
10.87709509	128.9501312	0.090141291	5.846814201
12.78067561	142.4199029	0.085107574	6.353542572
13.33476891	134.5353075	0.083556021	6.099917067
22.04005389	169.1345291	0.06654065	6.545012674
1.907531185	82.97811173	0.326765386	5.75006065
26.6528662	217.3663511	0.0778334	6.767327444
9.711507387	88.12719298	0.089873807	5.633451042
16.5392562	76.95454545	0.095041322	3.788754914
19.21115594	100.8287671	0.072340026	5.510326491
5.878104575	134.322549	0.210168084	6.282979529
11.12728974	73.82382134	0.087070298	5.880035107
9.502552159	429.5280125	0.133989974	6.529318902
7.931513804	99.9790795	0.103902243	5.819268823
3.471228604	146.4075712	0.301234504	5.965944856
17.08100691	145.8865672	0.073272444	5.960984613
21.53265718	223.2247706	0.06607451	6.404765155
33.97700749	241.19869	0.048282832	6.335941445
11.67592327	197.220339	0.103641993	6.139940264
7.192543609	64.05579399	0.116008768	6.158983072
8.136273308	168.737457	0.115227265	6.199695963
8.743313089	212.3866588	0.187034436	6.382544089
3.844638305	103.5405007	0.212951295	6.314203541
5.178201771	158.2042649	0.204358084	6.336386391
9.425418298	63.01886792	0.114275543	4.878494983
13.85069143	182.8958333	0.090838397	6.591239652
17.24571429	94.68571429	0.079727891	5.253292276
8.374679921	197.9431785	0.165015999	6.447322599
8.524426622	72.63636364	0.099418064	5.186554842
8.455671038	167.4520089	0.135380959	6.504720684
7.72043588	166.3989996	0.130321974	6.543541405
8.923627247	81.33862434	0.093424658	6.108146326
3.907855925	137.7711492	0.253000904	6.230863977
3.004514943	141.3154883	0.284276941	5.906831196
4.955003085	264.5335078	0.237658764	6.237463799
21.05596332	206.0403587	0.065776509	6.182648534
11.36691578	114.5942982	0.086536242	6.15786629
7.258430325	66.01339286	0.106226084	5.590416327
11.80137217	151.9890471	0.092456653	6.382790954
7.843067114	172.3635355	0.182653192	6.309499097
11.79892798	129.7181208	0.08661772	5.434974926
24.83324401	302.7780347	0.071311437	6.497168671
9.768286293	1073.687361	0.187892048	6.462137715
17.98959417	91.06451613	0.078043704	4.220167681
4.497333625	113.0483676	0.204618624	6.341695013
5.997759502	178.7399487	0.141558668	6.455363441
7.88167386	101.0438356	0.101099643	5.800163193
15.82846815	131.9117148	0.073645009	6.095725677
11.8944	95.8	0.0904	4.75856294
20.36686391	89.38461538	0.147928994	3.085055103
11.22959105	728.5132692	0.161438542	6.562446024
11.67239439	120.4078947	0.08576437	5.943512754
13.49037884	187.4639423	0.105931028	6.252727724
6.010037861	175.5087623	0.177611721	6.385698756
3.667770277	114.6546663	0.240435296	6.010003143
22.83497103	142.0952381	0.073318216	4.957205915
15.5500346	173.7176471	0.075307093	6.488545431
12.33333333	61.33333333	0.100694444	3.688721876
17.81504761	209.3742331	0.072452859	5.489078677
9.577755102	110.15	0.104081633	5.878269935

log.sigma.2.0.mm.3D_gldm_DependenceNonUniformity	log.sigma.2.0.mm.3D_gldm_GrayLevelNonUniformity	log.sigma.2.0.mm.3D_gldm_SmallDependenceEmphasis
51.07756233	31.92520776	0.266550173
93.85673557	267.4946543	0.13674414
25.65536723	19.16949153	0.2502767
210.5312985	193.3610967	0.267352061
27.01980198	8.00990099	0.439169417
39.8852459	27.09836066	0.267654688
28.28571429	4.628571429	0.587003968
71.92269327	31.52369077	0.340043193
295.3224932	68.95121951	0.356376569
179.1586891	351.6140578	0.154257202
61.01595745	35.44680851	0.270954078
89.48084544	84.94715984	0.202106775
132.0512445	159.7423133	0.211782613
67.69318182	59.04545455	0.24356628
85.42857143	27.56593407	0.437318376
588.0821943	3728.715183	0.06344522
43.61751152	20.40092166	0.360913899
140.3157216	201.375	0.15834615
39.23076923	15.12087912	0.402786748
150.5270368	584.1268926	0.074957145
276.3024631	1214.439212	0.095206568
127.4422442	86.7249725	0.290915896
162.7972603	615.8993151	0.108768055
42.87969925	7.270676692	0.532946951
74.00625	160.0041667	0.151963335
42.66887417	31.33774834	0.217890989
150.5089559	432.1052862	0.128552488
197.6700899	1182.910141	0.086994566
206.2432723	846.0861141	0.118814226
107.0189014	336.6704076	0.121982652
242.4182338	905.2989764	0.104998832
473.7241028	2150.094079	0.083883122
134.5608351	150.9640029	0.219907651
11.53333333	1.666666667	0.9
26.23893805	8.486725664	0.384055821
139.826087	144.8785607	0.204326509
388.2527275	2012.17531	0.07335307
190.6398417	516.5600264	0.144766912
72.32087227	22.1152648	0.370263014
67.283054	50.82122905	0.214165359
68.60559441	84.62797203	0.205690194
66.95800525	34.34383202	0.288386479
117.9854369	70.12864078	0.250487733
75.29612756	36.68109339	0.318113978
213.7103139	74.19282511	0.370556739
345.8970925	2000.457694	0.059412526
408.7137655	213.7305171	0.305096606
46.04385965	20.49122807	0.314185743
10.63636364	2.090909091	0.686868687
41.19178082	10.56164384	0.492438884
239.3647059	857.4857843	0.118926594
67.1439206	35.08933002	0.274290306
265.1577465	428.0979656	0.175167187
33.63598326	24.83263598	0.225417683
639.9975234	3405.757297	0.070398859
71.39402985	24.54626866	0.361028527
178.6972477	57.61697248	0.37245572
130.6637555	22.11353712	0.496771706
48.23163842	36.68926554	0.300989312
54.04291845	54.06008584	0.185079496
136.0350515	167.6556701	0.171047115
429.5191854	1267.345336	0.144436555
241.1561119	867.5635739	0.113388191
111.8125701	364.1661055	0.104478784
8.962264151	6.056603774	0.286343858
148.6493056	104.6458333	0.24965843
30.6	8.371428571	0.460402116
233.1511393	572.1104701	0.12778071
32.04895105	14.21678322	0.280135214
140.4162946	242.6026786	0.176364923
218.6791073	338.7068103	0.18365176
80.58024691	52.97178131	0.217315004
359.8842284	1785.427377	0.074974707
629.1299612	3079.856378	0.071104604
855.7989531	3450.567601	0.107356051
97.1838565	29.33632287	0.401638005
69.32894737	39.46052632	0.291290453
38.01785714	23.79464286	0.213594313
106.4260679	84.41292442	0.226193672
169.9785503	493.8942308	0.14924207
33.55033557	12.90604027	0.321057047
144.2439306	61.68439306	0.36222431
330.991479	926.1199026	0.156402834
16.16129032	2.419354839	0.729390681
277.4796453	1015.317614	0.106341597
304.6361563	496.4462504	0.175623909
54.54520548	36.90136986	0.232819676
121.9813243	43.37691002	0.362777829
13.16	4.52	0.397188889
9.615384615	1.923076923	0.884615385
248.5870516	553.5727617	0.172401144
51.20394737	26.07236842	0.319236161
53.0625	44.06730769	0.288641823
223.9003286	648.6380066	0.113530075
426.6892827	1736.423705	0.082977191
19.53968254	4.619047619	0.445163139
107.6794118	51.20882353	0.299384635
9	2.416666667	0.402777778
47.93251534	11.80981595	0.470612593
41.89285714	29.14285714	0.245742465

log.sigma.2.0.mm.3D_gldm_DependenceNonUniformityNormalized	log.sigma.2.0.mm.3D_gldm_DependenceVariance	log.sigma.2.0.mm.3D_gldm_LargeDependenceEmphasis
0.141489092	10.30021255	26.63434903
0.066897174	38.20468409	104.2159658
0.144945578	4.394969517	18.00564972
0.108914278	24.64030698	54.58096223
0.267522792	1.313204588	6.188118812
0.163464123	3.152915883	14.55737705
0.404081633	0.511020408	3.257142857
0.179358337	5.220974994	15.10972569
0.20008299	4.413128576	12.75880759
0.077256873	27.65202916	73.69167745
0.162276483	7.18741512	19.88829787
0.118204551	13.30108246	36.08058124
0.096670018	17.52014946	46.4875549
0.128206784	7.397368572	25.29924242
0.234693878	2.291510687	8.296703297
0.054311248	28.72646598	155.1248615
0.201002357	3.948777846	12.24423963
0.090409614	21.08278643	57.92268041
0.215553677	2.464798937	9.362637363
0.054263532	31.20499551	118.4866619
0.054443835	32.85070155	120.4344828
0.140200489	8.86124454	23.95049505
0.055752486	34.00305874	113.0424658
0.322403754	0.91525807	4.533834586
0.077089844	19.27979167	60.56041667
0.141287663	7.039691242	23.09271523
0.065753148	24.49192512	79.56531236
0.050749702	33.0561405	133.9258023
0.055501419	35.01870711	117.146394
0.063212582	32.15225683	97.33904312
0.057704888	26.59518423	101.2380386
0.053631168	29.60886579	119.5010755
0.096876051	22.67758553	55.85817135
0.768888889	0.115555556	1.4
0.232202992	2.545226721	9.17699115
0.104817157	20.40186204	49.95502249
0.058026114	28.66722324	138.1762068
0.062875937	28.90909411	90.72229551
0.225298668	1.812346542	8.258566978
0.125294328	14.41772174	36.24767225
0.09595188	17.95538951	47.87552448
0.175742796	4.593809632	15.40944882
0.14318621	9.088297672	24.77912621
0.171517375	4.662885726	15.02277904
0.191668443	4.044489131	12.67174888
0.056500668	24.36391436	131.9647174
0.148839682	7.748145618	21.35688274
0.201946753	2.346183441	10.47368421
0.483471074	0.338842975	2.454545455
0.282135485	2.295364984	7.342465753
0.05866782	29.80928105	100.9303922
0.166610225	9.428085882	23.33746898
0.08299147	21.00825929	59.34741784
0.140736332	5.374240647	20.28870293
0.056606892	27.20576411	133.843446
0.213116507	3.784397416	11.67462687
0.204928036	3.174459221	10.85091743
0.285292042	1.558151065	6.043668122
0.136247566	10.17964187	26.4519774
0.115971928	7.726334985	29.01287554
0.093494881	17.81937861	51.03024055
0.06338831	31.05502614	100.7883707
0.05919394	25.96530798	92.93667158
0.06274555	33.54248307	104.1515152
0.169099324	3.269490922	13.67924528
0.129035856	11.10373264	29.34375
0.291428571	1.087891156	5.438095238
0.06724867	28.32067034	88.06951255
0.224118539	2.431708152	10.69230769
0.078357307	23.25334821	65.50334821
0.08413971	18.97075073	54.93843786
0.142116838	6.010121653	21.40917108
0.050996773	34.48947467	142.5145246
0.058069961	25.02162174	123.4696326
0.058943381	25.56227131	104.7108616
0.217901024	2.845643387	9.798206278
0.152037165	5.21421976	17.64912281
0.169722577	4.263950893	16.95535714
0.116567435	13.18292307	34.86199343
0.06286189	34.78659273	102.7758876
0.225170037	1.492815639	8.44966443
0.166755989	8.529447693	20.90520231
0.067151852	25.10526712	81.02556299
0.521331946	0.301768991	2.225806452
0.055920928	30.22955632	106.1813785
0.086865171	16.45609543	49.59652124
0.149438919	6.741632576	22.11232877
0.207099023	2.382698078	9.679117148
0.2632	1.1216	6.32
0.73964497	0.130177515	1.461538462
0.072495495	27.54605959	80.34033246
0.168434037	5.667893006	16.88157895
0.127554087	15.84594582	36.22596154
0.061308962	29.38449367	97.37020811
0.059081872	24.52314434	108.9329826
0.310153691	1.14638448	5.603174603
0.158352076	8.720406574	22
0.375	0.5	4.5
0.294064511	1.214498099	5.539877301
0.149617347	7.080153061	21.79285714

log.sigma.2.0.mm.3D_gldm_LargeDependenceLowGrayLevelEmphasis	log.sigma.2.0.mm.3D_gldm_SmallDependenceHighGrayLevelEmphasis	log.sigma.2.0.mm.3D_gldm_LargeDependenceHighGrayLevelEmphasis
0.318753657	23.99024702	3329.603878
0.476282623	21.40176504	23389.04277
0.185675325	21.86260609	2169.774011
0.237067334	68.46997386	13662.03777
0.065883421	84.46171892	868.3960396
0.357647084	17.07676976	878.3852459
0.035023822	124.8952381	762.3714286
0.143095724	42.52591485	2307.129676
0.076783497	111.1673526	3548.73103
0.468869158	24.17030011	12036.49677
0.220437691	26.8225083	2522.734043
0.378900453	19.46114778	4081.809775
0.47150551	24.06095822	5133.654466
0.371798856	26.92452819	2159.102273
0.054580199	107.576456	1551.211538
1.474983508	6.966536493	16689.50656
0.244270387	41.90081391	832.1981567
0.472031711	16.21919185	8000.902706
0.119271875	61.13691781	1000.593407
0.973188904	7.350191933	15028.22206
0.835654826	10.70751631	17661.89025
0.193719269	43.8142687	3404.051705
1.165043217	8.605847745	11568.97671
0.039832928	138.8346909	1230.172932
0.459805022	13.66477203	8630.03125
0.179628266	20.82523991	3784.735099
0.823061027	13.50823057	8066.006116
0.914687391	9.160860852	19922.82978
0.458576632	23.47196903	30682.64505
0.591755587	13.14634473	16519.5381
0.698067115	12.6106394	15065.199
0.975954833	9.092633531	15058.88305
0.316237773	37.78359037	10757.65947
0.10660695	75.8	125.8
0.134830106	65.08090189	1026.522124
0.429318427	21.27545914	6786.629685
0.920420948	7.219343101	21241.45584
0.542399099	26.34318003	15643.20811
0.120542934	59.60176046	976.9252336
0.289598981	20.50555243	5694.94041
0.177268179	47.1164129	13570.97203
0.132584522	40.36585565	2431.034121
0.173084991	38.11342303	4598.456311
0.148913189	47.65070607	2324.763098
0.125281823	76.92495027	1884.289686
1.514070817	3.902864563	11794.33551
0.135895135	86.66568607	4101.289148
0.172501371	30.20308892	1028.109649
0.430627486	61.8989899	142.2272727
0.205680177	65.48394193	442.6917808
0.710383295	16.10259069	14902.78824
0.361490168	17.3735636	2825.486352
0.125848531	69.98546479	28595.7252
0.221353037	23.32860933	2255.401674
0.890064683	9.193145833	20507.0092
0.098352031	54.73889749	2138.8
0.071605953	97.13817576	2152.731651
0.054388424	125.5814105	1342.722707
0.127625539	52.16208789	6374.214689
0.6477494	15.72499367	1869.416309
0.247459408	21.91391847	11389.79175
0.442986075	32.88009054	23521.45927
0.90655655	13.32595665	9855.749632
0.608759093	13.7871531	18310.33782
0.35579773	17.87264055	864.9811321
0.185650468	50.10173851	5613.309028
0.120185879	44.59505291	473.752381
0.389804333	22.14192178	20739.20248
0.224876335	21.66832537	799.1118881
0.348729935	25.61425839	12950.30134
0.296774398	27.55717424	10952.86533
0.298979993	14.94904502	2412.104056
0.953207397	8.244187041	21782.12994
0.828970176	8.359584693	18725.36478
0.392780496	31.58994714	28237.74172
0.07089889	96.29898226	2012.269058
0.201127906	36.77425307	2311.563596
0.289337384	13.16369831	1529.852679
0.212858263	30.64482458	7016.03943
0.534207872	24.94210226	20316.3983
0.083683879	36.27931022	1228.348993
0.069105943	119.114637	7244.523699
0.073108359	163.7585344	90439.7186
0.102532136	72.37455197	180.1612903
0.860988907	11.94998475	13591.07416
0.280234754	35.57979722	9173.266895
0.205185093	21.9255555	3099.115068
0.126189449	57.09550134	1185.185059
0.134537289	40.68152222	617.84
0.116747803	80.03846154	126.7692308
0.101398593	112.1446068	64460.0974
0.139405628	29.33395202	2862.532895
0.185775896	59.2535166	7735.84375
0.497154622	16.00377088	19622.53176
0.881940333	8.580464126	13897.19164
0.07784418	71.69366843	764.6031746
0.147658049	62.85642727	3894.661765
0.456414724	26.58333333	300.3333333
0.045166623	106.0501715	1094.331288
0.197529998	19.50001076	3057.728571

log.sigma.2.0.mm.3D_gldm_SmallDependenceLowGrayLevelEmphasis	log.sigma.2.0.mm.3D_gldm_LowGrayLevelEmphasis	log.sigma.2.0.mm.3D_gldzm_DistanceZoneVariabilityNormalized
0.016228327	0.030587589	0.928028649
0.002146577	0.007388137	1
0.012902447	0.021737825	1
0.003519691	0.00852029	0.924644368
0.01375159	0.018625001	1
0.025414858	0.048802399	0.944489796
0.018769409	0.021074621	1
0.009407335	0.018901181	0.92648789
0.003574472	0.008308149	0.580175821
0.002522898	0.009446691	0.835542857
0.009111239	0.022540449	0.966532606
0.006722218	0.020859231	0.97484685
0.00574935	0.017189853	0.925348595
0.009982706	0.02393565	0.985075471
0.00652153	0.010260521	0.936576189
0.001966224	0.012296548	0.885237689
0.013554936	0.029488653	0.978024691
0.004142619	0.014796907	0.961846508
0.013439074	0.022008872	1
0.001893826	0.011531764	0.978024691
0.001803896	0.009259332	0.95819828
0.005797131	0.013052392	0.907503858
0.004976898	0.017824305	0.96105434
0.01227647	0.016123044	1
0.005535371	0.015011971	1
0.007646499	0.016766788	1
0.003452001	0.01568266	0.946781434
0.002123503	0.009389632	0.982907253
0.001403227	0.006151043	0.922575178
0.003332943	0.010525006	1
0.002247597	0.009856535	0.93529277
0.001631658	0.010762362	0.864555102
0.003871427	0.010730136	0.953106259
0.10160695	0.10260695	1
0.018746378	0.029048027	1
0.006284518	0.017901765	0.953668486
0.002113011	0.009838652	0.953424582
0.001944153	0.00822018	0.94577332
0.011209912	0.020198769	0.958922197
0.009949338	0.020464468	0.924926758
0.002666438	0.006144944	1
0.00687144	0.014929641	0.952029024
0.00620001	0.014423795	0.942200091
0.0095094	0.018854591	0.914803846
0.004985895	0.013639252	0.708039165
0.001973644	0.014757905	0.662240719
0.004001582	0.010035489	0.660668656
0.011012832	0.023796944	0.932666306
0.048083294	0.12297597	1
0.027495476	0.041587908	0.976193932
0.002877624	0.011417609	0.87651169
0.017960379	0.043255007	0.954911243
0.000880318	0.002923023	0.749537462
0.009135333	0.019536471	0.967750605
0.001334756	0.008490474	0.862994821
0.01124765	0.019157693	0.957466919
0.004472812	0.009289668	0.963648834
0.008742312	0.013424576	0.933901025
0.006280785	0.011325694	0.983194484
0.011330494	0.039048889	0.935061728
0.002872039	0.008538711	0.946502686
0.001442631	0.00624738	0.932092667
0.002356908	0.012548506	0.861310212
0.001754155	0.008247672	0.98984798
0.035130618	0.053123063	1
0.004086783	0.011873438	0.927158736
0.035720743	0.045836309	1
0.001545473	0.006879153	0.922773241
0.016376004	0.032254686	1
0.003377959	0.009419825	0.951113281
0.002551093	0.008465417	0.940351998
0.012125516	0.028849699	0.874693696
0.001499319	0.009090916	0.968999938
0.001151391	0.008316254	0.766346475
0.000625774	0.004248686	0.629366728
0.008569822	0.013388093	0.933094795
0.00906554	0.021996969	0.919604871
0.012578321	0.030546698	0.931122449
0.005541946	0.01417857	0.973688889
0.002925986	0.008755991	0.952522185
0.013839527	0.020307784	0.965527855
0.003731282	0.00653785	0.931515003
0.000372562	0.001173468	0.790743708
0.054068691	0.063474642	1
0.002588437	0.012144927	0.857085799
0.001551789	0.00692915	0.910690284
0.008277071	0.017979302	0.977780583
0.00726813	0.01821376	0.928386039
0.026897078	0.038529506	1
0.109625296	0.111049798	0.722222222
0.000745091	0.001906912	0.919784476
0.010303166	0.019249752	0.981983471
0.006028259	0.011003973	0.955578512
0.00164019	0.007347282	0.962366797
0.001673119	0.010924771	0.769599105
0.028395213	0.034611602	1
0.004392171	0.010616306	0.914335699
0.031115548	0.112317358	1
0.009382001	0.013527139	1
0.010228994	0.020286314	1

log.sigma.2.0.mm.3D_gldzm_LowIntensityEmphasis	log.sigma.2.0.mm.3D_gldzm_LargeDistanceEmphasis	log.sigma.2.0.mm.3D_gldzm_HighIntensitySmallDistanceEmphasis
0.060075697	1.112149533	84.0911215
0.0165342	1	143.8820513
0.047105696	1	89.16
0.013569375	1.117647059	237.5
0.027978362	1	190.1730769
0.097995619	1.085714286	62.575
0.028198792	1	217.1666667
0.027822242	1.114649682	111.8200637
0.009920284	2.040650407	198.1620596
0.017305023	1.38	136.4127778
0.035094189	1.093220339	90.36464218
0.038589514	1.038216561	92.88535032
0.027727214	1.15483871	111.3985887
0.041353874	1.022556391	110.6842105
0.013871359	1.098360656	233.8879781
0.033927047	1.530046225	104.6166102
0.037312311	1.033333333	106.8694444
0.027802256	1.058365759	94.42120623
0.03281343	1	149.7875
0.02915082	1.033333333	91.94027778
0.02047407	1.134042553	102.8305106
0.019638897	1.145833333	137.8003472
0.05113074	1.059602649	73.35844371
0.021348921	1	255.6463415
0.036380633	1	83.89677419
0.033509095	1	94.1025641
0.031266778	1.115646259	101.4889456
0.025477351	1.025862069	95.29454023
0.013413549	1.121019108	180.7584926
0.028175351	1	97.89908257
0.023271062	1.286052009	109.2516246
0.021817659	1.551428571	98.74452381
0.018084173	1.072072072	160.3235736
0.109221732	1	84.78571429
0.043012315	1	161.8703704
0.031651544	1.071186441	97.56949153
0.030590327	1.071570577	87.31908549
0.014004085	1.138888889	181.9389468
0.027694482	1.062937063	156.4073427
0.044287533	1.1171875	89.35351563
0.012821104	1	218.4230769
0.024236164	1.073770492	125.9836066
0.024874114	1.089361702	136.5170213
0.029843258	1.133757962	138.5477707
0.015032793	1.640086207	156.1748084
0.038121506	2.454022989	45.18615262
0.013479872	2.094978166	199.500326
0.033468558	1.104651163	86.83139535
0.093573478	1	86
0.052506903	1.036144578	126.9909639
0.027152767	1.237704918	119.1347905
0.06796893	1.069230769	57.85192308
0.005057427	1.793277311	318.2851657
0.038630319	1.049180328	100.0491803
0.020690074	1.568854569	115.7790548
0.030497727	1.065217391	139.7934783
0.011974873	1.055555556	247.598545
0.016519164	1.102661597	247.2927757
0.020863995	1.025423729	169.3220339
0.066659524	1.155555556	81.93271605
0.017358082	1.1796875	114.6203342
0.010518909	1.110351563	217.0832791
0.022296987	1.354120267	110.8970552
0.017407926	1.015306122	122.8010204
0.101725972	1	66.78947368
0.018480741	1.128930818	190.1287561
0.069280405	1	98.38596491
0.013016373	1.195121951	158.0660415
0.054587465	1	75.02083333
0.020502066	1.090625	134.2180556
0.014278327	1.142566191	141.1559176
0.056781325	1.201492537	57.88619403
0.022024562	1.047244094	101.4995079
0.01809898	2.771587744	93.52549867
0.006020556	4.148963731	245.0196701
0.020273642	1.103960396	222.5779703
0.035127747	1.125874126	113.8234266
0.056391231	1.107142857	54.14732143
0.026841685	1.04	126.6677778
0.020480052	1.072992701	156.2493917
0.037939711	1.052631579	111.6535088
0.010188194	1.106508876	311.3306213
0.002315695	1.701456311	932.3037625
0.070770007	1	95.84
0.026413225	1.834615385	100.8679204
0.009169988	1.140549273	196.0076737
0.035331826	1.033707865	91.15730337
0.022224994	1.111570248	144.463843
0.055809152	1	101.2692308
0.119275141	1.5	88.45833333
0.004233633	1.149038462	615.1681802
0.032005613	1.027272727	89.23636364
0.020195561	1.068181818	192.6382576
0.015413679	1.057553957	130.3699041
0.022356346	2.598930481	85.45714198
0.053808223	1	151.5428571
0.014679967	1.134529148	202.5526906
0.099190827	1	62.57142857
0.017977284	1	219.9784946
0.038151565	1	80.35294118

log.sigma.2.0.mm.3D_gldzm_LowIntensityLargeDistanceEmphasis	log.sigma.2.0.mm.3D_gldzm_HighIntensityEmphasis	log.sigma.2.0.mm.3D_gldzm_DistanceZoneVariability	log.sigma.2.0.mm.3D_gldzm_ZonePercentage
0.061051213	87.3364486	99.29906542	0.296398892
0.0165342	143.8820513	195	0.138987883
0.047105696	89.16	50	0.282485876
0.014104343	246.4705882	518.7254902	0.290222452
0.027978362	190.1730769	52	0.514851485
0.098647429	65.41428571	66.11428571	0.286885246
0.028198792	217.1666667	48	0.685714286
0.028460134	117.4140127	145.4585987	0.391521197
0.013769987	313.4699187	356.8081301	0.416666667
0.018875183	157.2314286	292.44	0.150927124
0.035650204	92.61016949	114.0508475	0.313829787
0.038854907	94.2611465	153.0509554	0.207397622
0.030224553	113.9612903	286.8580645	0.226939971
0.041510516	111.4962406	131.0150376	0.251893939
0.014675494	239.568306	171.3934426	0.502747253
0.038897377	110.385208	574.5192604	0.0599372
0.037587794	107.8777778	88.02222222	0.414746544
0.028352499	96.09338521	247.1945525	0.165592784
0.03281343	149.7875	80	0.43956044
0.029984153	92.46111111	176.0444444	0.064888248
0.021211797	106.4446809	450.3531915	0.092610837
0.020356566	147.7222222	261.3611111	0.316831683
0.051568591	75.46688742	290.2384106	0.103424658
0.021348921	255.6463415	82	0.616541353
0.036380633	83.89677419	155	0.161458333
0.033509095	94.1025641	78	0.258278146
0.032357221	104.1360544	278.3537415	0.128440367
0.025663825	96.23850575	342.0517241	0.089345315
0.014024456	187.2186837	434.5329087	0.126749193
0.028175351	97.89908257	218	0.128765505
0.025059116	114.1347518	395.6288416	0.100690312
0.02825845	105.4285714	605.1885714	0.079248274
0.018592316	163.006006	317.3843844	0.239740821
0.109221732	84.78571429	14	0.933333333
0.043012315	161.8703704	54	0.477876106
0.032491142	99.30847458	281.3322034	0.22113943
0.031359706	89.0695825	479.5725646	0.075175609
0.01486479	185.6828704	408.5740741	0.142480211
0.029444191	157.0629371	137.1258741	0.445482866
0.045203887	93.1796875	118.390625	0.238361266
0.012821104	218.4230769	156	0.218181818
0.024583139	129.9672131	116.147541	0.320209974
0.025224212	143.1617021	221.4170213	0.285194175
0.030502415	145.7515924	143.6242038	0.357630979
0.018785993	202.8168103	328.5301724	0.416143498
0.055826767	60.4683908	230.4597701	0.056844169
0.020097088	284.308952	605.1724891	0.333576111
0.034260729	90.72093023	80.20930233	0.377192982
0.093573478	86	17	0.772727273
0.052805619	128.0843373	81.02409639	0.568493151
0.028378509	131.432377	427.7377049	0.119607843
0.068486917	60.22307692	124.1384615	0.322580645
0.006611579	384.4369748	445.9747899	0.186228482
0.038881239	102.4590164	59.03278689	0.255230126
0.024928057	124.3513514	670.5469755	0.068724571
0.030852549	143.0652174	132.1304348	0.411940299
0.01212236	253.1402116	364.2592593	0.433486239
0.016968268	253.8859316	245.6159696	0.574235808
0.021040548	170.2372881	116.0169492	0.333333333
0.067945107	85.14444444	84.15555556	0.193133047
0.017985066	121.234375	242.3046875	0.175945017
0.0112748	222.0126953	954.4628906	0.151121606
0.025266365	118.8374165	386.7282851	0.110211095
0.017486019	123.5510204	194.0102041	0.109988777
0.101725972	66.78947368	19	0.358490566
0.019287191	195.3301887	294.836478	0.276041667
0.069280405	98.38596491	57	0.542857143
0.013876683	165.5432373	416.1707317	0.130083646
0.054587465	75.02083333	48	0.335664336
0.021022973	138.278125	304.35625	0.178571429
0.014973949	146.2912424	461.712831	0.188918815
0.058014999	66.47761194	117.2089552	0.23633157
0.022431876	102.992126	492.2519685	0.071985263
0.029745441	110.9164345	550.2367688	0.066272845
0.017860119	296.5569948	971.742228	0.106343412
0.020640868	230.460396	188.4851485	0.452914798
0.035931439	119.8391608	131.5034965	0.313596491
0.056981545	59.03571429	52.14285714	0.25
0.026981057	129.5644444	219.08	0.246440307
0.020954074	159.7347932	391.486618	0.151997041
0.038121827	115.4561404	55.03508772	0.382550336
0.010557484	319.9556213	314.852071	0.390751445
0.002952472	1034.118932	651.5728155	0.167173869
0.070770007	95.84	25	0.806451613
0.032710992	110.1673077	445.6846154	0.104796453
0.009777969	205.2487884	563.7172859	0.176504135
0.035503805	92.80898876	87.02247191	0.243835616
0.022703367	151.6942149	224.6694215	0.410865874
0.055809152	101.2692308	26	0.52
0.379275141	90.08333333	8.666666667	0.923076923
0.004499366	637.6025641	573.9455128	0.181977253
0.032112147	90.98181818	108.0181818	0.361842105
0.020433126	197.5757576	126.1363636	0.317307692
0.015710718	133.4148681	401.3069544	0.114184009
0.03520326	100.6613191	431.745098	0.077679313
0.053808223	151.5428571	35	0.555555556
0.015987673	207.3991031	203.896861	0.327941176
0.099190827	62.57142857	14	0.583333333
0.017977284	219.9784946	93	0.570552147
0.038151565	80.35294118	85	0.303571429

log.sigma.2.0.mm.3D_gldzm_IntensityVariabilityNormalized	log.sigma.2.0.mm.3D_gldzm_LowIntensitySmallDistanceEmphasis	log.sigma.2.0.mm.3D_gldzm_IntensityVariability
0.074329636	0.059831818	7.953271028
0.07634451	0.0165342	14.88717949
0.0848	0.047105696	4.24
0.045815182	0.013435633	25.70231729
0.068786982	0.027978362	3.576923077
0.095918367	0.097832666	6.714285714
0.063368056	0.028198792	3.041666667
0.065763317	0.027662769	10.32484076
0.041253222	0.009053177	25.37073171
0.066628571	0.017019955	23.32
0.075840276	0.035003228	8.949152542
0.074932046	0.038523166	11.76433121
0.067866805	0.027156563	21.03870968
0.069817401	0.041314714	9.285714286
0.053121921	0.013670325	9.721311475
0.080519752	0.033454741	52.25731895
0.067160494	0.037243441	6.044444444
0.08322609	0.027664696	21.38910506
0.0590625	0.03281343	4.725
0.083395062	0.028942487	15.01111111
0.093861476	0.02037488	44.11489362
0.063102816	0.01945948	18.17361111
0.085588351	0.051021277	25.84768212
0.049672814	0.021348921	4.073170732
0.094027055	0.036380633	14.57419355
0.099605523	0.033509095	7.769230769
0.072585497	0.03106707	21.34013605
0.090401638	0.025430732	31.45977011
0.063464373	0.013260823	29.89171975
0.085472603	0.028175351	18.63302752
0.079422564	0.023097696	33.59574468
0.081926531	0.021001542	57.34857143
0.06061016	0.017957137	20.18318318
0.102040816	0.109221732	1.428571429
0.059670782	0.043012315	3.222222222
0.069209997	0.031441644	20.41694915
0.085566126	0.030397982	43.03976143
0.062917953	0.013846779	27.18055556
0.05853587	0.027257055	8.370629371
0.076416016	0.044058444	9.78125
0.077498356	0.012821104	12.08974359
0.074173609	0.02414942	9.049180328
0.066038932	0.02478659	15.51914894
0.065114203	0.029678468	10.22292994
0.050627973	0.014389898	23.49137931
0.136725459	0.03624574	47.58045977
0.044051887	0.012556252	40.35152838
0.083288264	0.033270515	7.162790698
0.093425606	0.093573478	1.588235294
0.062273189	0.052432224	5.168674699
0.063507794	0.026901754	30.99180328
0.089349112	0.067839433	11.61538462
0.066020761	0.004798626	39.28235294
0.074442354	0.038567589	4.540983607
0.077765024	0.020247598	60.42342342
0.057760975	0.030409021	7.971014493
0.049116766	0.011938001	18.56613757
0.044225014	0.016406887	11.63117871
0.065211146	0.020819856	7.694915254
0.081975309	0.066440159	7.377777778
0.088348389	0.017276428	22.6171875
0.053062439	0.010335472	54.3359375
0.087871588	0.021792231	39.45434298
0.095793419	0.017388403	18.7755102
0.08033241	0.101725972	1.526315789
0.05672244	0.018292777	18.03773585
0.066174207	0.069280405	3.771929825
0.06689741	0.012871741	30.17073171
0.078993056	0.054587465	3.791666667
0.066953125	0.020392385	21.425
0.074062245	0.01415317	36.36456212
0.085542437	0.056472907	11.46268657
0.081413913	0.021922734	41.35826772
0.088430413	0.017364676	63.49303621
0.074897145	0.005299976	115.6411917
0.050142143	0.020181836	10.12871287
0.064893149	0.034926824	9.27972028
0.106505102	0.056243653	5.964285714
0.069985185	0.026806843	15.74666667
0.063372819	0.020361546	26.04622871
0.071714374	0.037894182	4.087719298
0.042523021	0.010095871	14.37278107
0.05766625	0.002228489	47.51699029
0.0752	0.070770007	1.88
0.075118343	0.025931589	39.06153846
0.076043752	0.009017993	47.07108239
0.076884232	0.035288831	6.842696629
0.059661225	0.0221054	14.43801653
0.091715976	0.055809152	2.384615385
0.138888889	0.054275141	1.666666667
0.057928583	0.004177523	36.1474359
0.08231405	0.031978979	9.054545455
0.05785124	0.02013617	7.636363636
0.079228703	0.015339419	33.0383693
0.083156192	0.021484263	46.65062389
0.057959184	0.053808223	2.028571429
0.055279616	0.014353041	12.32735426
0.091836735	0.099190827	1.285714286
0.067175396	0.017977284	6.247311828
0.088442907	0.038151565	7.517647059

log.sigma.2.0.mm.3D_gldzm_HighIntensityLargeDistanceEmphasis	log.sigma.2.0.mm.3D_gldzm_SmallDistanceEmphasis	log.sigma.2.0.mm.3D_glcm_SumVariance	log.sigma.2.0.mm.3D_glcm_Homogeneity1
100.317757	0.971962617	242.1642926	0.428765488
143.8820513	1	596.9445915	0.58926445
89.16	1	276.923808	0.444242764
282.3529412	0.970588235	626.0543594	0.44410213
190.1730769	1	443.0896051	0.313277847
76.77142857	0.978571429	138.3574167	0.413226829
217.1666667	1	660.4695802	0.248184658
139.7898089	0.97133758	308.2208367	0.370862162
884.5333333	0.786811201	869.8811679	0.289359685
274.8171429	0.930396825	430.0837288	0.53887755
108.9745763	0.986111111	236.4016967	0.398463248
99.76433121	0.99044586	242.901575	0.472557274
130.3451613	0.970362903	246.038891	0.478692756
114.7443609	0.994360902	190.5699471	0.441738946
262.2896175	0.975409836	572.2279714	0.31607121
173.4067797	0.951468699	320.4129973	0.694156039
111.9111111	0.991666667	172.6382898	0.367385287
102.7821012	0.98540856	292.0063385	0.511057275
149.7875	1	299.9783678	0.346531921
94.54444444	0.991666667	327.6366331	0.638305697
134	0.983196809	407.7523622	0.637663979
187.4097222	0.963541667	349.96066	0.409833763
83.90066225	0.985099338	248.4576724	0.628376397
255.6463415	1	754.4033825	0.260456508
83.89677419	1	353.372719	0.570381951
94.1025641	1	343.7927822	0.447456004
118.1258503	0.978647014	258.7935161	0.575117071
100.0143678	0.993534483	437.3180371	0.671003098
213.059448	0.969745223	747.8936112	0.61338348
97.89908257	1	443.3660749	0.595721649
163.2009456	0.972251116	402.5197472	0.613082685
168.1114286	0.941912302	343.1795922	0.635659196
173.7357357	0.981981982	441.8527725	0.480290381
84.78571429	1	211.0575847	0.210160045
161.8703704	1	313.9271864	0.332571457
106.2644068	0.98220339	263.4225172	0.468931734
96.07157058	0.982107356	443.9242144	0.667356365
212.3773148	0.978298611	465.5957637	0.570550216
159.6853147	0.984265734	332.5268555	0.306710226
108.484375	0.970703125	307.9185611	0.455091858
218.4230769	1	777.5821385	0.511343165
145.9016393	0.981557377	352.2533616	0.388258275
169.7404255	0.977659574	401.302987	0.404614999
174.566879	0.96656051	370.5234153	0.383302862
412.0775862	0.867591595	461.9813163	0.31654393
192.5431034	0.839131545	251.32534	0.683249127
877.6954148	0.838875243	622.8554863	0.35833842
106.2790698	0.973837209	220.6954081	0.36518879
86	1	157.2066738	0.261990081
132.4578313	0.990963855	192.6459844	0.30393244
187.4897541	0.949681239	397.6925717	0.594313204
69.70769231	0.982692308	187.066475	0.399723929
887.5327731	0.887173203	1418.80759	0.497037156
112.0983607	0.987704918	258.6204887	0.447494391
204.9047619	0.940434005	459.4107549	0.66430101
156.1521739	0.983695652	390.1474988	0.344625774
275.3068783	0.986111111	657.7129645	0.334546528
280.2585551	0.974334601	646.7986875	0.257053406
173.8983051	0.993644068	572.4956466	0.432129636
103.9666667	0.97345679	144.5135702	0.467257068
172.7773438	0.976779514	520.0200546	0.500898082
242.5810547	0.973497179	648.9266539	0.574594127
166.8819599	0.941165553	285.8830117	0.599848624
126.5510204	0.996173469	481.350453	0.614230791
66.78947368	1	153.0727362	0.402617781
219.7138365	0.971261356	488.0740909	0.427256112
98.38596491	1	212.117363	0.308232655
213.2461197	0.968619118	613.9582343	0.560881125
75.02083333	1	178.8445552	0.371337127
156.865625	0.980815972	495.483236	0.529267782
177.6313646	0.975673229	494.9465084	0.515415602
100.8432836	0.949626866	214.52504	0.4205503
108.9625984	0.988188976	425.3656302	0.658743241
396.4387187	0.890310412	445.6568545	0.654582774
1173.106865	0.814936422	852.8164674	0.596962922
261.990099	0.974009901	569.7608149	0.316501252
143.9020979	0.968531469	310.2340302	0.39859449
78.58928571	0.973214286	174.3025509	0.412716633
141.1511111	0.99	453.5311525	0.450837286
173.676399	0.981751825	528.1853387	0.586588713
130.6666667	0.986842105	366.1638535	0.353848596
354.4556213	0.973372781	909.0746255	0.361962089
1825.557039	0.907493258	3828.494712	0.547446722
95.84	1	181.2289846	0.256183359
228.4288462	0.935340278	327.0529007	0.608426356
242.2132472	0.964862682	520.6524874	0.508693459
99.41573034	0.991573034	279.8850562	0.427290954
180.6157025	0.972107438	336.3950781	0.340125571
101.2692308	1	209.6851528	0.319780231
96.58333333	0.875	195.5737175	0.228610009
740.5096154	0.968082265	2547.0409	0.538446504
97.96363636	0.993181818	352.6655954	0.389621182
217.3257576	0.982954545	534.1831614	0.445277722
145.5947242	0.985611511	539.5934429	0.592840211
315.8431373	0.890718196	344.5969664	0.627114366
151.5428571	1	366.2236174	0.31341185
226.7847534	0.966367713	429.9959157	0.377895406
62.57142857	1	135.4924151	0.332516611
219.9784946	1	527.3680381	0.3038107
80.35294118	1	307.2384958	0.449176714

log.sigma.2.0.mm.3D_glcm_Homogeneity2	log.sigma.2.0.mm.3D_glcm_ClusterShade	log.sigma.2.0.mm.3D_glcm_MaximumProbability	log.sigma.2.0.mm.3D_glcm_Idmn	log.sigma.2.0.mm.3D_glcm_SumVariance2
0.351404791	-36.18974234	0.071986532	0.979355452	28.57685547
0.545810073	-107.6152938	0.2360853	0.98768759	17.98041574
0.368936244	-33.33189609	0.052558117	0.978143602	19.00492054
0.379216276	-71.41743379	0.112672915	0.98167409	36.23076042
0.218777599	8.787633022	0.034421883	0.949517585	26.97729523
0.333531953	-6.279765826	0.041779652	0.954959477	14.56737243
0.155688081	-75.80863959	0.039234042	0.919395645	43.51188336
0.284643555	20.93561358	0.025527686	0.970106475	30.9405178
0.204877999	560.4034455	0.013468193	0.956258541	121.378122
0.487242403	-7.460583489	0.162351612	0.993280241	15.64702894
0.312990306	-29.0095094	0.037507892	0.967813956	21.49470091
0.405444286	-31.88208022	0.087366405	0.984699177	18.3559758
0.415101366	-9.786469494	0.085887773	0.976250524	15.11871964
0.368428649	11.37074463	0.038489023	0.97770573	15.17811848
0.226852626	172.8408887	0.02315321	0.972974905	39.49807454
0.678545056	-28.67029194	0.251349796	0.995869595	5.325356049
0.281976264	57.69758699	0.040778933	0.958271763	20.13400555
0.451796537	-39.5544105	0.119755257	0.984706247	16.68260808
0.258217584	15.38102323	0.034956157	0.958012084	22.37801018
0.607380728	-36.90314057	0.194481643	0.991444322	10.67851634
0.608147339	-26.22004802	0.243368233	0.997558155	7.891619129
0.332154259	24.48204707	0.042688013	0.983753208	22.56120335
0.595093887	-38.7941955	0.216917313	0.991976264	9.341526992
0.168118281	-97.82176372	0.030605973	0.956868897	97.40521361
0.525401949	-74.90803715	0.152155086	0.991929194	13.20461967
0.373878488	-44.77289033	0.059002469	0.976624963	23.75493797
0.533024695	-12.15826461	0.179825733	0.992086603	10.78830158
0.648667036	-45.66265117	0.280982123	0.996609081	7.923035583
0.578683574	-125.8805539	0.228285332	0.993014266	18.03262162
0.55613737	-82.093951	0.218622921	0.990590322	14.32216334
0.580008369	-35.16116394	0.184929381	0.994769347	9.519274782
0.606367777	-33.44791122	0.203782131	0.992551035	7.734890646
0.417427992	-68.55769829	0.115587704	0.985404056	27.14788648
0.106396759	14.16278748	0.118269231	0.85940434	26.49582799
0.243934989	133.2956742	0.047174658	0.961719776	46.48037023
0.40142918	-44.34511819	0.102405204	0.977219165	21.3615323
0.645869151	-72.0169277	0.222242938	0.994320261	9.971860985
0.527930429	-25.83506239	0.163551373	0.992219736	13.3356282
0.212796226	107.3462803	0.019087349	0.9560071	39.59790571
0.384061796	-74.0467072	0.078859561	0.97376039	27.87330839
0.454283054	-74.74431087	0.113590592	0.990244538	20.53721479
0.302033165	-28.15042764	0.032481677	0.974411804	23.39831889
0.324354141	-16.05874977	0.04984796	0.979920524	28.53083183
0.298853324	11.20888728	0.026313259	0.982354957	30.82419285
0.228473067	304.577024	0.016001327	0.973195975	56.65397905
0.66620712	-19.91970386	0.205685517	0.994620874	4.158307765
0.280144506	889.6860839	0.029950169	0.976633621	67.90083868
0.274248561	6.680105057	0.026022094	0.959256089	22.94569537
0.165644846	49.28792746	0.095533633	0.886368346	30.94059871
0.210929288	113.1388971	0.033224595	0.936710257	32.95468439
0.557541164	-51.40389694	0.19669053	0.991699938	12.80823362
0.319019236	-18.17976335	0.056686029	0.955551068	28.29304018
0.438385729	-91.03111214	0.121212985	0.99357968	24.86205881
0.373932146	-11.33956293	0.04290841	0.978393399	18.63513496
0.643093904	-48.08435518	0.220147253	0.997576595	8.045569708
0.255753128	-36.41721072	0.025470569	0.968808591	35.97462479
0.247898053	127.131525	0.019762133	0.972347717	50.23497403
0.166047911	124.4567759	0.012917763	0.948459039	76.72908522
0.357757714	-114.55337	0.076032599	0.977176606	26.39839311
0.397151617	16.15164511	0.056988462	0.985079779	16.63748639
0.440326196	-64.96341074	0.081227863	0.985916971	21.0347983
0.534802622	-73.12885731	0.167238807	0.99085395	18.98751
0.56249105	-23.82963733	0.167389181	0.992472221	8.651348363
0.576732805	-55.35795624	0.202578646	0.989114446	13.38711487
0.313816905	11.05182945	0.081011585	0.953842595	24.20353121
0.353874799	-98.24683436	0.065551547	0.982540907	31.82340809
0.21591018	70.0313606	0.037807836	0.941533825	30.01358976
0.515613832	-105.766223	0.12957302	0.993656467	21.12330602
0.281536148	8.185767777	0.034248752	0.955014206	18.63015655
0.476565561	-82.57987128	0.126824733	0.988255533	19.41353727
0.459476655	-84.19377532	0.089860743	0.987669789	19.06882768
0.340169481	-36.52964942	0.030007779	0.957309448	24.70480707
0.633326226	-49.50435502	0.209181978	0.994177269	9.29477382
0.63140562	-35.87538753	0.212223255	0.997758744	6.947513797
0.5629117	-20.46571936	0.15052049	0.995585917	10.16845755
0.227524383	-14.11541402	0.020117632	0.966997805	40.16103516
0.316150695	-49.66846311	0.031363355	0.971979396	26.58623983
0.329882829	2.922308962	0.035612898	0.961295635	18.50410438
0.379004975	-87.90312782	0.080625407	0.984343574	27.79645717
0.546065976	-59.58004245	0.213109493	0.991940182	16.04710847
0.260749009	-38.67414768	0.035308219	0.952401823	27.35679849
0.282521452	-51.06888126	0.038745625	0.975461505	45.15341969
0.503477345	-132.144761	0.133053188	0.995995618	21.8615239
0.155992026	47.95798067	0.066298445	0.92867795	32.74124727
0.572630789	-41.68718203	0.160465366	0.994510376	10.36181475
0.451672241	-10.16804741	0.102410675	0.988369915	12.79941973
0.348721549	-8.048362997	0.035619112	0.977870863	19.43882996
0.249961502	39.1816113	0.019110841	0.970175272	34.92583884
0.22183035	-0.486459382	0.056802933	0.929058499	26.42187881
0.120728956	66.07301027	0.109126984	0.886983127	44.14487276
0.491674468	-260.3240647	0.123387009	0.993317407	27.60881727
0.305378769	-60.83215363	0.038436567	0.96380682	28.48520554
0.377638436	-55.69018545	0.103644153	0.980130805	27.54011121
0.553077327	-70.86097216	0.170242	0.992492159	14.65499473
0.597592417	-38.23271478	0.180357166	0.994679486	8.690056984
0.222687182	8.053231739	0.050390549	0.957593203	44.79749101
0.297599702	61.33199305	0.042867639	0.972112067	33.32320776
0.240016832	37.44279096	0.099718657	0.90224837	26.03422203
0.207166414	-28.20264229	0.025607817	0.960669515	40.14538971
0.376967761	-85.80253565	0.069362761	0.968327961	24.65314946

log.sigma.2.0.mm.3D_glcm_Contrast	log.sigma.2.0.mm.3D_glcm_DifferenceEntropy	log.sigma.2.0.mm.3D_glcm_InverseVariance	log.sigma.2.0.mm.3D_glcm_Entropy	log.sigma.2.0.mm.3D_glcm_Dissimilarity
11.02053006	2.824314895	0.333492806	6.512813667	2.446796544
5.374520153	2.331242499	0.383120025	5.104056349	1.463767861
7.679984532	2.561102987	0.367317509	5.896832909	2.119736433
25.39675772	3.258489625	0.313744521	6.946516186	3.190081378
23.34731496	3.209492095	0.238263504	6.26614954	3.856419043
10.47236905	2.781943048	0.351678491	6.230562432	2.450326953
47.92438083	3.388622784	0.169997723	5.666316	5.642093317
17.82234175	3.159125052	0.285561938	7.030741379	3.169930762
63.77024243	3.9988694	0.204040683	8.818894886	5.773512857
7.329160838	2.513007364	0.398238013	5.707793437	1.732587823
11.59009207	2.89661224	0.304372861	6.57538644	2.643340033
8.078361482	2.6210267	0.381741594	6.191034053	2.031898475
9.735919876	2.745064779	0.375911494	6.231182905	2.148931329
11.06244277	2.816161159	0.354444183	6.285153288	2.365063758
32.93945626	3.520177254	0.246064076	7.27742126	4.264048862
2.149403217	1.658456052	0.458285854	3.57025468	0.825139005
19.74431233	3.168978107	0.284228454	6.422881054	3.300303019
6.538301509	2.540787428	0.383383653	5.833648093	1.816097704
21.64852385	3.236927139	0.270965798	6.537768029	3.529069706
2.611446472	1.952012291	0.435305872	4.677235492	1.04815528
3.310211794	1.989257439	0.441707639	4.428918696	1.090158883
16.06396687	3.061883258	0.322755687	6.82696148	2.831033053
3.081516539	2.026998269	0.428861692	4.686696483	1.119489414
50.48980812	3.708588671	0.176174311	6.666087859	5.570679398
3.71360463	2.177553789	0.438516662	5.100367655	1.341704923
8.180840798	2.652753659	0.359369953	6.153960904	2.176816297
5.339745273	2.293472772	0.432986853	5.2040475	1.449415633
3.016860205	1.860653999	0.434612273	3.904327003	0.980939632
6.803038942	2.307306437	0.394854626	4.84404389	1.471659882
4.434843717	2.231723709	0.417250378	4.895809169	1.346469707
4.090209711	2.09554158	0.450701951	4.679049917	1.221082648
2.850761854	1.964533801	0.450019795	4.484786335	1.068868261
12.61044138	2.883697933	0.348854474	6.386158289	2.346761417
43.14038462	1.866666585	0.148265986	3.253205046	5.625
30.17661843	3.334700662	0.233783996	6.334282209	4.082786311
10.07230881	2.82147524	0.344568888	6.325007203	2.258085959
2.937180623	1.851579937	0.447395732	3.902531606	0.987107749
7.020166171	2.428783836	0.412132418	5.391012856	1.612077072
28.80740163	3.41032816	0.209352669	7.278818389	4.238655345
9.424429673	2.764260149	0.348932497	6.381983282	2.25469127
8.285814593	2.5816048	0.394752512	5.97432286	1.893426102
13.91681666	2.96737045	0.303120362	6.652082519	2.804865078
16.46635709	3.070360328	0.304894347	6.894927589	2.885727321
16.23007164	3.060361452	0.309439777	6.867189656	2.955664473
36.67552689	3.65513387	0.231147937	8.005938141	4.473574454
1.429787809	1.599308308	0.481023759	3.566034596	0.78232396
48.47543267	3.65854375	0.274401582	7.838298503	4.457442897
13.21292105	2.92996348	0.291253306	6.637145426	2.909924259
33.64360084	2.481222296	0.199130784	3.997328512	4.802258852
30.82242586	3.45849926	0.218579645	6.594173818	4.32785066
6.202391614	2.293306444	0.428654898	5.03264785	1.452212984
13.25584385	2.970251804	0.315722907	6.620829483	2.768918987
10.29744529	2.730595267	0.381150603	6.209725322	2.099731703
7.569771015	2.600414497	0.376491754	6.155321268	2.097315067
2.586909116	1.80319589	0.465422964	3.975493296	0.945539884
24.39893967	3.309522951	0.264347334	7.070640421	3.64217923
32.19936205	3.524184491	0.251797562	7.709288154	4.1062909
54.10720008	3.899230352	0.166345913	8.042573694	5.796137195
12.16089785	2.918111988	0.330542993	6.36141141	2.544223416
7.040135627	2.551612827	0.381101713	6.085402252	1.97150711
6.623936448	2.550485077	0.394100283	5.954874317	1.841371191
9.708600554	2.507324182	0.405181521	5.364480099	1.764454724
3.518324423	2.105063775	0.446857808	4.899358016	1.228252495
3.7957047	2.142601184	0.40627558	4.888660255	1.246012595
10.09584572	2.529102261	0.273581852	5.067188848	2.567401908
16.49158	3.027231336	0.334319646	6.946245408	2.753016597
28.34348985	3.319739283	0.241528484	6.18893846	4.128425545
6.353854421	2.438813518	0.407493897	5.459254389	1.623117644
13.17651251	2.925053721	0.292639645	6.255716796	2.877299478
7.370696485	2.536775424	0.402550564	5.839794969	1.779868936
7.026752988	2.543940261	0.40397913	5.928047606	1.80678613
9.64895556	2.780706993	0.335519804	6.628479815	2.390499666
2.712400201	1.899381148	0.436859177	4.265003218	0.997563971
2.213401361	1.797454876	0.474959552	4.037707949	0.938147646
6.515754699	2.219825927	0.455597801	4.915877537	1.411844362
33.84918869	3.562333083	0.235019895	7.488304808	4.341081548
13.79297091	3.012110108	0.309012106	6.867194146	2.784013308
9.77448067	2.751636471	0.321068879	6.23879386	2.451421564
10.50565177	2.827713264	0.342605795	6.526257873	2.337928503
7.407359362	2.404212646	0.39870411	5.14398356	1.579736823
15.7625473	3.031854831	0.258092605	6.350691029	3.169358664
34.48800486	3.515321912	0.279560499	7.40134255	3.999922554
12.40959272	2.596431028	0.416639685	5.586261239	1.94817345
35.09112992	2.830803824	0.195982292	4.603454196	4.804296878
3.922938022	2.131669526	0.436678628	4.878161622	1.239574592
7.823238009	2.594497136	0.401284132	5.985304449	1.881814295
9.534937964	2.776956061	0.336712743	6.354940374	2.365170599
22.56036384	3.316079257	0.262046981	7.388918054	3.606799285
22.06147959	2.747708158	0.204732659	5.25765567	3.783372991
39.63095238	2.364496261	0.140728407	3.722766946	5.218253968
13.45835865	2.695283217	0.393838444	5.804151141	2.078164246
13.11567238	2.954231838	0.304515551	6.603139111	2.808256585
19.08988539	3.06320312	0.329799597	6.376893639	2.842653295
4.206711207	2.205367839	0.427714948	5.151718912	1.323720218
2.685347218	1.939089958	0.469495536	4.43240956	1.06565242
34.62497208	3.133524741	0.241590564	5.713593567	4.315858729
21.06274443	3.283604257	0.29117809	7.28551705	3.357295334
21.8934051	2.485862675	0.232425649	3.968804126	3.700552652
25.32755059	3.305685389	0.217129239	6.706321688	4.036912057
9.092951633	2.678224437	0.354751289	6.109137464	2.245946263

log.sigma.2.0.mm.3D_glcm_DifferenceVariance	log.sigma.2.0.mm.3D_glcm_Idn	log.sigma.2.0.mm.3D_glcm_Idm	log.sigma.2.0.mm.3D_glcm_Correlation	log.sigma.2.0.mm.3D_glcm_Autocorrelation
4.952481648	0.906650643	0.351404791	0.441056214	93.924207
3.159365657	0.937489598	0.545810073	0.533896172	194.09734
3.089051478	0.901112186	0.368936244	0.41710137	103.1081592
15.10951561	0.924565087	0.379216276	0.173797388	208.3368594
8.136951636	0.849970479	0.218777599	0.061389653	150.4354405
4.412378862	0.863474102	0.333531953	0.16256685	57.30221338
15.33582618	0.811879896	0.155688081	-0.053937868	205.8026802
7.679184179	0.88777808	0.284643555	0.268689427	114.1647931
29.8700013	0.872222534	0.204877999	0.316133207	282.5373611
4.288293479	0.951791489	0.487242403	0.359778735	147.989959
4.493820246	0.88064544	0.312990306	0.295481628	90.79601285
3.852841343	0.920970975	0.405444286	0.38551624	93.06194008
5.026559386	0.907272282	0.415101366	0.205905535	92.57838002
5.387654166	0.906579118	0.368428649	0.158942157	74.69567023
14.55886896	0.893932924	0.226852626	0.094755677	191.9647988
1.460862741	0.966158884	0.678545056	0.417615657	104.0145365
8.660609771	0.8706411	0.281976264	0.013645558	66.9005179
3.174316216	0.922454396	0.451796537	0.434558641	107.2267091
9.079328615	0.867774756	0.258217584	0.01013745	107.7467399
1.4929926	0.945859723	0.607380728	0.603112158	114.4690231
2.112272782	0.971934885	0.608147339	0.406237162	134.789147
7.925903037	0.919728149	0.332154259	0.165546718	125.8333955
1.806264124	0.9481366	0.595093887	0.499089926	89.61174415
19.05459103	0.862533446	0.168118281	0.318442637	242.7107966
1.872895832	0.94325523	0.525401949	0.549045361	122.951554
3.314178449	0.899148204	0.373878488	0.488718291	125.0538091
3.22186249	0.94896538	0.533024695	0.334899813	93.65949442
2.041596155	0.969205522	0.648667036	0.444239428	139.6624059
4.583670409	0.957043041	0.578683574	0.444254312	233.7917486
2.597789723	0.944104754	0.55613737	0.51886576	148.0119526
2.584446349	0.959411826	0.580008369	0.393607813	134.4201078
1.696471748	0.950296705	0.606367777	0.453212604	115.8541943
7.042476571	0.928820114	0.417427992	0.362999521	154.259069
9.777537393	0.746571665	0.106396759	-0.271849406	56.71538462
13.08974141	0.875663859	0.243934989	0.213024262	111.4097242
4.917326257	0.906635879	0.40142918	0.356174091	99.22546586
1.948052525	0.960126285	0.645869151	0.538961947	141.8923524
4.405211093	0.951208861	0.527930429	0.306987427	156.2333826
9.734620854	0.860679596	0.212796226	0.164101468	119.725698
4.245236715	0.897207224	0.384061796	0.490727156	114.3939882
4.656424197	0.94092306	0.454283054	0.423406268	251.6114745
5.955749724	0.894593847	0.302033165	0.25398161	127.1326451
7.949244763	0.910520609	0.324354141	0.264900606	142.9207034
7.406174588	0.913378302	0.298853324	0.30058245	133.5894349
16.42600766	0.895130811	0.228473067	0.217592238	160.9372242
0.811227675	0.955915925	0.66620712	0.47952778	84.19119758
28.33720765	0.913907732	0.280144506	0.174171748	206.0471981
4.624120617	0.863449056	0.274248561	0.269767795	85.57944082
9.793349327	0.777308157	0.165644846	-0.050227971	48.05862748
11.8868549	0.837434469	0.210929288	0.027890583	71.7362815
4.07012858	0.951391545	0.557541164	0.342827631	134.1292604
5.423767825	0.864761919	0.319019236	0.360067287	74.83564026
5.808075065	0.95184553	0.438385729	0.41310487	432.8925979
3.135884379	0.902118292	0.373932146	0.41960415	98.00443111
1.681646142	0.972644619	0.643093904	0.506371684	146.4519402
10.97033379	0.88673202	0.255753128	0.190350619	138.3579333
14.97741452	0.895367912	0.247898053	0.219169126	220.7193244
19.80201722	0.850488714	0.166047911	0.167602717	214.3202924
5.627091955	0.903964633	0.357757714	0.362520151	193.0607838
3.064933508	0.919137085	0.397151617	0.399896775	60.70815078
3.19167879	0.924571056	0.440326196	0.518481785	176.603341
6.557359748	0.951000177	0.534802622	0.319641767	208.5633584
1.986447505	0.948156161	0.56249105	0.41661094	101.0015388
2.198006618	0.940265781	0.576732805	0.551778954	160.4303388
2.785286609	0.855614386	0.313816905	0.423980244	59.9407163
8.76988571	0.920161532	0.353874799	0.313307723	169.8801489
11.13139183	0.843388405	0.21591018	0.022458124	77.79378296
3.686295015	0.953253693	0.515613832	0.532472215	200.621485
4.815325428	0.858391877	0.281536148	0.172142307	70.46676663
4.160943069	0.936062026	0.476565561	0.446124443	168.4161634
3.710654927	0.932181183	0.459476655	0.45266845	168.2366156
3.815288475	0.865579261	0.340169481	0.437710245	84.96907145
1.704331941	0.957641571	0.633326226	0.542949132	139.4999521
1.324995183	0.971786729	0.63140562	0.51035026	142.9440681
4.508142627	0.965721786	0.5629117	0.217497803	261.0831615
14.78041912	0.883470049	0.227524383	0.075932728	191.9337721
5.976111016	0.891431584	0.316150695	0.312737423	114.9994563
3.611444795	0.869484807	0.329882829	0.309940381	70.19641255
4.912816904	0.9200632	0.379004975	0.432472085	158.9704044
4.873726445	0.952543358	0.546065976	0.363770686	173.6547256
5.518995833	0.853953374	0.260749009	0.26567421	130.4724012
18.31660525	0.906733151	0.282521452	0.134952747	292.0782765
8.563017494	0.967454994	0.503477345	0.273338289	1070.805604
11.64015362	0.820649602	0.155992026	-0.048108655	57.58428441
2.369584766	0.957198021	0.572630789	0.444804282	113.3172212
4.238554752	0.934846503	0.451672241	0.235908405	174.3091284
3.881019344	0.900805354	0.348721549	0.341531889	104.4814333
9.422363017	0.886783371	0.249961502	0.211485223	122.811079
6.172461797	0.823782539	0.22183035	0.102211642	75.05273669
11.98992189	0.772659371	0.120728956	0.063139593	56.67857143
9.079066425	0.957505123	0.491674468	0.339503533	734.0337998
5.044785894	0.874759066	0.305378769	0.366940657	127.8061964
10.80580086	0.919053127	0.377638436	0.183803623	180.1115683
2.425883026	0.949062915	0.553077327	0.547298033	178.4599166
1.539591364	0.95630563	0.597592417	0.523243687	116.2431507
15.60196382	0.870404274	0.222687182	0.139628602	122.8905186
9.683760841	0.894679365	0.297599702	0.223034265	151.6842796
7.959020616	0.79969187	0.240016832	0.069513785	44.60596604
8.759118134	0.865228506	0.207166414	0.222188194	178.939556
3.735224867	0.886623362	0.376967761	0.46337215	113.3319388

log.sigma.2.0.mm.3D_glcm_SumEntropy	log.sigma.2.0.mm.3D_glcm_AverageIntensity	log.sigma.2.0.mm.3D_glcm_Energy	log.sigma.2.0.mm.3D_glcm_SumSquares	log.sigma.2.0.mm.3D_glcm_ClusterProminence
4.311820235	9.460572337	0.017096077	9.899346382	2073.421392
3.575507962	13.81808145	0.081891882	5.838733972	1487.614609
3.969575418	10.01270869	0.02131239	6.671226268	1039.049678
4.394328186	14.46615456	0.025274105	16.09503997	5797.707954
4.062977641	12.22561973	0.014690432	12.58115255	2199.279843
3.87936227	7.501414428	0.017169494	6.259935371	634.8868819
3.939853694	14.37922792	0.021293474	22.85906605	5960.395225
4.410389523	10.52920615	0.009519482	12.19071489	2662.439175
5.392069988	16.72491397	0.003096591	47.69277128	36726.14023
3.801654095	12.09215052	0.047201964	5.912690832	1528.913307
4.139300438	9.396498657	0.01342909	8.271198243	1208.959107
4.041880886	9.512358491	0.023512624	6.608584321	1102.745284
3.90887452	9.551407134	0.023956915	6.213659879	830.4871367
3.92352719	8.582438045	0.01817894	6.560140314	973.2670671
4.511353523	13.96119657	0.008493993	19.25935988	7328.987889
2.569077554	10.15972499	0.163453802	1.868689817	379.0348282
4.003574549	8.170282396	0.015271178	9.96957947	1653.282385
3.871612362	10.23155742	0.032835294	5.805227397	752.563892
4.084607212	10.37018606	0.013482895	11.00663351	1756.333018
3.405757675	10.60417658	0.087131252	3.322490702	511.583118
3.124751125	11.56401485	0.104035409	2.835617135	420.1280391
4.197139748	11.16514942	0.013929121	9.905310155	2176.042926
3.303941696	9.383181868	0.085101355	3.105760883	453.3484385
4.769677272	15.45907985	0.011075905	38.66596405	24718.83015
3.519505787	10.98029029	0.054790105	4.229556076	1111.881054
4.126109702	11.00666897	0.01894037	7.983944692	1369.907216
3.466631271	9.607038312	0.065938874	4.032011714	823.9256097
2.810286825	11.7671624	0.146317177	2.751381332	582.1211458
3.380948093	15.20774205	0.100443832	6.263543236	1978.995914
3.415802378	12.06361378	0.082748039	4.689251764	1043.173467
3.246846508	11.53700551	0.085856841	3.425448744	555.9270045
3.098793616	10.70660129	0.098370603	2.646413125	384.2527472
4.182417742	12.28118919	0.03022081	10.0585146	2245.608408
2.077356769	7.756089744	0.1140625	17.40905315	1883.706714
4.36759789	10.40843402	0.014893838	19.89576829	7127.698685
4.079856544	9.818101846	0.024449199	7.858460279	1146.749761
2.844371918	11.83774477	0.143645894	3.227260402	877.5473612
3.605970025	12.44028606	0.062606476	5.141659793	952.3498064
4.526066593	10.81622728	0.007767815	17.10132683	5153.827724
4.222089263	10.47711386	0.020191939	9.324434515	1855.795089
4.016812048	15.78324914	0.032349845	7.3486415	1574.261289
4.206892193	11.16874779	0.012636348	9.328783886	1583.377169
4.352286766	11.84704209	0.012254232	11.48866687	2654.784653
4.372252096	11.41659747	0.011019009	12.03331763	4011.509893
4.843815134	12.66463319	0.005577777	24.83284637	12622.34617
2.555618805	9.138278502	0.144840914	1.397023893	179.3479401
4.81149663	14.48936456	0.008761531	31.60879097	36656.84182
4.176267931	9.11773941	0.011649392	9.039654104	1259.83186
2.751297217	6.964144189	0.06646456	16.14604989	2392.322844
4.241433054	8.433617383	0.0124225	15.94427756	3677.766207
3.401811912	11.51196539	0.082997759	4.777087649	1101.1567
4.264259049	8.429077791	0.013952563	10.38722101	1662.190492
4.101583409	20.80046889	0.035026123	8.317147402	2538.463105
4.027690418	9.758491611	0.018327332	6.551226493	1096.694619
2.845325601	12.04605644	0.137316572	2.669094099	678.7498547
4.460171958	11.65682939	0.009835005	15.29618141	4089.845318
4.768533623	14.86366102	0.006598703	21.95492985	9727.17281
5.022711815	14.64255943	0.004562036	34.10192382	16774.91608
4.163815698	13.76576828	0.020012925	9.63982274	2710.140916
3.96522077	7.635649506	0.021546604	5.919405505	1165.422057
3.96868592	13.15265513	0.029734564	6.914683686	1214.99551
3.624147448	14.38509546	0.076028602	7.349349368	2117.546065
3.321907731	9.98585488	0.077206759	3.042418196	405.8376229
3.510124515	12.5707433	0.082522591	4.295704893	765.4893077
3.672990665	7.508676561	0.035224207	8.574844231	1303.119779
4.412731255	12.90368683	0.01560617	12.30811413	3753.129897
4.100759013	8.795729288	0.015862638	14.5892699	2796.594574
3.718617711	14.03686674	0.062908745	6.904714708	1879.436758
3.969848133	8.312250781	0.015183635	7.951667267	859.7168153
3.903674802	12.86077661	0.041600625	6.69605844	1488.766171
3.895121382	12.85344595	0.034680448	6.523895166	1338.192186
4.247142429	9.010561094	0.012901359	8.588440657	1449.628183
3.084835265	11.74105788	0.11603983	3.001793505	556.4725317
2.86762523	11.90650574	0.125410032	2.293518192	396.0250593
3.231341151	16.19359201	0.084347552	4.345038601	1267.678799
4.584354543	13.89634775	0.007131929	19.2325378	5763.09991
4.308360589	10.57228761	0.011781532	10.09480268	2160.963601
4.015079885	8.245710857	0.015933869	7.069646262	812.6204952
4.243259516	12.43331988	0.019269201	9.575527235	2450.459389
3.561383044	13.10311492	0.078946235	5.934714445	1395.436183
4.186164255	11.29341148	0.014388055	10.77983645	2065.262751
4.632817183	17.28474947	0.010918716	20.55365296	8152.122134
3.676300062	32.76265177	0.064840898	8.604253339	4021.688762
3.084377646	7.613102122	0.043390237	16.9580943	2784.234414
3.343198714	10.57001102	0.076695601	3.584668166	588.2078106
3.776073211	13.15526576	0.031576267	5.155664433	874.8096947
4.06241084	10.09910479	0.01525585	7.243441981	971.4991413
4.522983734	10.94657193	0.007652237	14.4703877	3835.518149
3.666725921	8.59778235	0.029094189	12.1208396	1707.942045
2.596600329	7.450396825	0.079396573	20.94395629	4250.937354
3.861843632	27.10652572	0.056241707	9.846456014	6162.234532
4.264878429	11.13292737	0.013447476	10.40021948	1941.285287
4.175301743	13.36489833	0.024987028	11.81306801	3205.409568
3.610568033	13.26056539	0.068495145	4.715426484	1004.874278
3.09626845	10.71168831	0.099723781	2.84385105	451.4751106
4.018085516	11.00763454	0.021574382	20.36574197	5836.292836
4.4656402	12.19303912	0.01016068	13.63478792	4258.317852
2.749781787	6.57862362	0.070314331	11.98190678	1589.2666
4.401757215	13.23681888	0.011048342	16.36823508	5354.446298
4.114386019	10.46082439	0.021482863	8.436525272	1784.40437

log.sigma.2.0.mm.3D_glcm_SumAverage	log.sigma.2.0.mm.3D_glcm_Imc2	log.sigma.2.0.mm.3D_glcm_Imc1	log.sigma.2.0.mm.3D_glcm_DifferenceAverage	log.sigma.2.0.mm.3D_glcm_Id
18.92114467	0.839340018	-0.174458947	2.446796544	0.428765488
27.6361629	0.748934778	-0.162000973	1.463767861	0.58926445
20.02541737	0.858104118	-0.207198124	2.119736433	0.444242764
28.67863512	0.793574884	-0.134579409	3.190081378	0.44410213
24.45123947	0.947306035	-0.309146461	3.856419043	0.313277847
15.00282886	0.735134174	-0.118177404	2.450326953	0.413226829
28.75845583	0.992771472	-0.56366885	5.642093317	0.248184658
21.0584123	0.792732733	-0.132545472	3.169930762	0.370862162
32.74762738	0.802407779	-0.11144048	5.773512857	0.289359685
24.15832917	0.671935178	-0.105473167	1.732587823	0.53887755
18.79299731	0.743505654	-0.117799478	2.643340033	0.398463248
19.02471698	0.720551251	-0.118543798	2.031898475	0.472557274
19.10281427	0.673896284	-0.096922727	2.148931329	0.478692756
17.16487609	0.706681598	-0.107248644	2.365063758	0.441738946
27.59105224	0.867496224	-0.174028234	4.264048862	0.31607121
20.31944997	0.601868173	-0.123325721	0.825139005	0.694156039
16.34056479	0.861607982	-0.192290926	3.300303019	0.367385287
20.46311484	0.679133633	-0.109571162	1.816097704	0.511057275
20.74037212	0.889333045	-0.215285555	3.529069706	0.346531921
21.20835316	0.763040999	-0.179054343	1.04815528	0.638305697
23.12072431	0.664940342	-0.127792298	1.090158883	0.637663979
22.28917715	0.693632604	-0.09458267	2.831033053	0.409833763
18.76636374	0.703535718	-0.144316734	1.119489414	0.628376397
30.38122426	0.990399304	-0.460620235	5.570679398	0.260456508
21.96058059	0.700106254	-0.133499691	1.341704923	0.570381951
22.01333795	0.803461218	-0.159087168	2.176816297	0.447456004
19.21407662	0.66647728	-0.110958482	1.449415633	0.575117071
23.53163475	0.636505197	-0.130475929	0.980939632	0.671003098
30.39593369	0.751668118	-0.167947421	1.471659882	0.61338348
24.12722755	0.705024979	-0.139949208	1.346469707	0.595721649
23.07036679	0.668489349	-0.125072147	1.221082648	0.613082685
21.41320258	0.638434268	-0.11623393	1.068868261	0.635659196
24.54487384	0.770766454	-0.136609766	2.346761417	0.480290381
15.51217949	0.99595394	-0.881159876	5.625	0.210160045
20.71452424	0.975951443	-0.390943963	4.082786311	0.332571457
19.63620369	0.702635497	-0.105852284	2.258085959	0.468931734
23.67548954	0.672772625	-0.15029972	0.987107749	0.667356365
24.8719251	0.722870999	-0.131484879	1.612077072	0.570550216
21.63245455	0.867056944	-0.176547053	4.238655345	0.306710226
20.95422771	0.773921114	-0.1395604	2.25469127	0.455091858
31.5303809	0.812291469	-0.170081645	1.893426102	0.511343165
22.33749557	0.78013735	-0.133932045	2.804865078	0.388258275
23.65330819	0.736464174	-0.110402955	2.885727321	0.404614999
22.79340323	0.792410417	-0.137371122	2.955664473	0.383302862
24.9746365	0.73583433	-0.092486209	4.473574454	0.31654393
18.276557	0.534571706	-0.098210725	0.78232396	0.683249127
28.36780069	0.714354993	-0.087026352	4.457442897	0.35833842
18.23547882	0.785899071	-0.135964566	2.909924259	0.36518879
13.92828838	0.995037423	-0.758593854	4.802258852	0.261990081
16.86723477	0.942789697	-0.287509427	4.32785066	0.30393244
23.01965154	0.699586194	-0.12932553	1.452212984	0.594313204
16.85815558	0.760810976	-0.125013997	2.768918987	0.399723929
41.4363644	0.710925562	-0.113642766	2.099731703	0.497037156
19.51698322	0.803453677	-0.156912382	2.097315067	0.447494391
24.09038213	0.634417814	-0.12870121	0.945539884	0.66430101
23.27554227	0.878332157	-0.189638727	3.64217923	0.344625774
29.39653121	0.820257959	-0.135724432	4.1062909	0.334546528
28.8729016	0.91698282	-0.204969319	5.796137195	0.257053406
27.53153655	0.820179086	-0.164346974	2.544223416	0.432129636
15.27129901	0.719959572	-0.120386692	1.97150711	0.467257068
26.30531026	0.730461595	-0.125622859	1.841371191	0.500898082
28.72220767	0.754410562	-0.14954302	1.764454724	0.574594127
19.97170976	0.622245937	-0.103145	1.228252495	0.599848624
25.1414866	0.745979229	-0.163113997	1.246012595	0.614230791
15.01735312	0.972113941	-0.451558204	2.567401908	0.402617781
25.77089592	0.766097187	-0.125364603	2.753016597	0.427256112
17.59145858	0.962677544	-0.351831683	4.128425545	0.308232655
28.06586126	0.738220019	-0.140455724	1.623117644	0.560881125
16.62450156	0.864678346	-0.199505549	2.877299478	0.371337127
25.72155322	0.739384501	-0.133139705	1.779868936	0.529267782
25.70689189	0.678208139	-0.106112603	1.80678613	0.515415602
18.02112219	0.68789465	-0.098065223	2.390499666	0.4205503
23.48211576	0.704395808	-0.156064974	0.997563971	0.658743241
23.81253463	0.623434767	-0.120167413	0.938147646	0.654582774
32.2595523	0.631700884	-0.099178666	1.411844362	0.596962922
27.59010434	0.852942238	-0.159733156	4.341081548	0.316501252
21.14457523	0.776914673	-0.12736344	2.784013308	0.39859449
16.49142171	0.784842272	-0.145184765	2.451421564	0.412716633
24.86663977	0.75775282	-0.131939365	2.337928503	0.450837286
26.19082367	0.772271252	-0.167834516	1.579736823	0.586588713
22.58682296	0.905441898	-0.23892765	3.169358664	0.353848596
34.02325883	0.826906037	-0.146229705	3.999922554	0.361962089
65.37400707	0.725557942	-0.128864726	1.94817345	0.547446722
15.22620424	0.997067328	-0.724866466	4.804296878	0.256183359
21.13810154	0.66961509	-0.120889135	1.239574592	0.608426356
26.31053152	0.572494467	-0.068706944	1.881814295	0.508693459
20.19820959	0.741318489	-0.120828202	2.365170599	0.427290954
21.88051246	0.765324895	-0.113692933	3.606799285	0.340125571
17.1955647	0.98653989	-0.534969148	3.783372991	0.319780231
14.90079365	0.987894752	-0.673674522	5.218253968	0.228610009
54.0550236	0.749594195	-0.136427421	2.078164246	0.538446504
22.26585473	0.789621461	-0.141656457	2.808256585	0.389621182
26.68273337	0.871601724	-0.204217561	2.842653295	0.445277722
26.52113078	0.735452714	-0.149182978	1.323720218	0.592840211
21.42337662	0.653319583	-0.122947096	1.06565242	0.627114366
21.92904077	0.991171943	-0.536908886	4.315858729	0.31341185
24.38138345	0.764499033	-0.114950855	3.357295334	0.377895406
13.15724724	0.993301363	-0.728562572	3.700552652	0.332516611
26.47363776	0.950217516	-0.298926659	4.036912057	0.3038107
20.92164879	0.840326782	-0.185795362	2.245946263	0.449176714

log.sigma.2.0.mm.3D_glcm_ClusterTendency	log.sigma.2.0.mm.3D_firstorder_InterquartileRange	log.sigma.2.0.mm.3D_firstorder_Skewness	log.sigma.2.0.mm.3D_firstorder_Uniformity
28.57685547	129.0966148	-0.004711246	0.088435479
17.98041574	79.39888704	-1.240969587	0.190659055
19.00492054	102.0721779	-0.207864513	0.108302212
36.23076042	118.7021477	0.234871508	0.100031607
26.97729523	144.6638107	-0.111898281	0.07930595
14.56737243	86.63748932	0.103987362	0.111058855
43.51188336	176.8060117	-0.339876653	0.066122449
30.9405178	120.5517731	0.407316733	0.078612695
121.378122	212.9050112	0.577537765	0.046714918
15.64702894	73.24947667	0.164423607	0.151623138
21.49470091	111.5629959	-0.053763344	0.094273427
18.3559758	94.16978073	-0.07053246	0.112215535
15.11871964	86.81847334	0.085694905	0.116941664
15.17811848	87.7871623	0.611147363	0.111828512
39.49807454	113.5881991	0.59580839	0.075730588
5.325356049	17.04953289	-1.57353277	0.344358624
20.13400555	104.5729218	0.937855372	0.094013464
16.68260808	101.1592026	-0.424224387	0.129751933
22.37801018	105.934164	0.352991476	0.083081753
10.67851634	50.04592955	-1.088774736	0.210572059
7.891619129	46.88023502	-0.610200508	0.239298367
22.56120335	101.1822491	0.584431354	0.09540701
9.341526992	55.75338399	-1.066401442	0.210924423
97.40521361	239.3294601	0.058925661	0.054666742
13.20461967	75.230232	-1.17991195	0.166671007
23.75493797	118.9189625	-0.410935539	0.103767379
10.78830158	52.51025021	0.203312348	0.188774699
7.923035583	25.82876194	-1.534362175	0.303699651
18.03262162	57.84580456	-1.31398311	0.227687329
14.32216334	66.94852185	-1.327091883	0.198860253
9.519274782	52.43378854	-0.653838646	0.215496067
7.734890646	38.37705207	-1.317179806	0.243416062
27.14788648	117.587676	0.015291386	0.108685387
26.49582799	145.2484741	-0.399485255	0.111111111
46.48037023	129.9595871	0.584354276	0.075103767
21.3615323	114.4335151	-0.23832728	0.108604618
9.971860985	17.71028149	-2.072118187	0.300728637
13.3356282	61.84568977	0.030951961	0.170369402
39.59790571	150.1953354	0.364837746	0.068894906
27.87330839	139.2302589	-0.353361822	0.09463916
20.53721479	90.01722997	-0.574727523	0.1183608
23.39831889	104.9394379	0.152231631	0.090141291
28.53083183	126.7564116	0.223248767	0.085107574
30.82419285	120.9077797	0.274909175	0.083556021
56.65397905	136.7431951	0.802393324	0.06654065
4.158307765	23.36948013	-2.153943419	0.326765386
67.90083868	110.3198832	1.607782223	0.0778334
22.94569537	119.3683767	0.168097826	0.089873807
30.94059871	163.0476875	-0.091684191	0.095041322
32.95468439	152.4241142	0.500559097	0.072340026
12.80823362	49.25906518	-0.657011935	0.210168084
28.29304018	137.987051	-0.006822553	0.087070298
24.86205881	89.23669875	-0.665024133	0.133989974
18.63513496	96.89663601	-0.046230895	0.103902243
8.045569708	21.60933506	-1.646815247	0.301234504
35.97462479	143.1962547	0.129733445	0.073272444
50.23497403	143.6796169	0.538146139	0.06607451
76.72908522	203.1076598	0.143405451	0.048282832
26.39839311	110.2839518	-0.77578778	0.103641993
16.63748639	82.82901382	0.545160035	0.116008768
21.0347983	113.9216866	-0.586173979	0.115227265
18.98751	61.02942687	-0.259509566	0.187034436
8.651348363	35.56925607	-0.57029772	0.212951295
13.38711487	53.61911446	-1.13306018	0.204358084
24.20353121	119.7794189	0.133117147	0.114275543
31.82340809	110.1851759	-0.260373239	0.090838397
30.01358976	110.7987442	0.537330665	0.079727891
21.12330602	87.6758194	-0.900166091	0.165015999
18.63015655	97.37340927	0.275780083	0.099418064
19.41353727	90.21824607	-0.736088639	0.135380959
19.06882768	92.13508624	-0.73902417	0.130321974
24.70480707	115.7009335	-0.202127956	0.093424658
9.29477382	35.49138737	-1.577443616	0.253000904
6.947513797	27.22035027	-1.667123879	0.284276941
10.16845755	31.99940872	0.225879714	0.237658764
40.16103516	145.3372536	0.235873808	0.065776509
26.58623983	113.2991409	-0.008694026	0.086536242
18.50410438	93.58033371	0.236867474	0.106226084
27.79645717	125.4514207	-0.356144207	0.092456653
16.04710847	75.45746115	-0.399773851	0.182653192
27.35679849	113.3743591	-0.1382912	0.08661772
45.15341969	153.1228623	0.236658041	0.071311437
21.8615239	43.91798496	-0.845218959	0.187892048
32.74124727	174.1662788	0.328172412	0.078043704
10.36181475	50.74378794	-0.748004898	0.204618624
12.79941973	65.81185758	0.125897387	0.141558668
19.43882996	102.2047195	-0.03237343	0.101099643
34.92583884	133.1806793	0.449670148	0.073645009
26.42187881	138.5795999	-0.143805428	0.0904
44.14487276	178.271347	0.063580542	0.147928994
27.60881727	80.5452373	-1.728311304	0.161438542
28.48520554	138.8508563	-0.348717753	0.08576437
27.54011121	101.6984856	0.04437726	0.105931028
14.65499473	69.10745472	-1.05834701	0.177611721
8.690056984	40.19620705	-1.126776886	0.240435296
44.79749101	153.5271149	0.494730235	0.073318216
33.32320776	125.2088864	0.312413357	0.075307093
26.03422203	135.5383778	0.03602696	0.100694444
40.14538971	142.051846	-0.126187561	0.072452859
24.65314946	105.9641697	-0.631813637	0.104081633

log.sigma.2.0.mm.3D_firstorder_MeanAbsoluteDeviation	log.sigma.2.0.mm.3D_firstorder_Energy	log.sigma.2.0.mm.3D_firstorder_RobustMeanAbsoluteDeviation	log.sigma.2.0.mm.3D_firstorder_Median
69.70941339	5073898.664	51.91768246	-80.43557739
53.0429709	9587954.789	35.90142904	-19.52921867
56.68638087	2542863.765	40.53170491	-94.99291229
78.43287398	27436206.12	50.44596587	-37.69140625
78.65606388	2601688.416	58.47816061	-138.1450958
52.20524258	3785076.682	35.07991064	-106.1506157
97.32379459	1693800.097	73.95586606	-96.28783417
73.81254075	10735197.36	51.38462711	-139.0069733
132.9025541	42003495.18	95.96322576	-74.716465
47.88287162	13929312.66	30.70098844	-29.3043251
62.30438244	6606507.255	45.80689024	-108.1124153
56.57220841	6060331.943	39.6904386	-44.95415115
52.47972162	9747393.837	35.33442214	-48.22613716
53.25593252	5310996.481	34.72002514	-74.0848465
79.95964442	5861822.713	50.43517457	-73.00704956
20.95550324	16246030.74	8.015387149	-2.794410944
65.73434717	5356275.6	43.12858013	-138.7229004
53.25665208	12316605.22	40.68821342	-49.92425537
73.45095741	4471899.909	46.59183532	-130.1890182
38.35938251	8888981.449	23.67966153	-6.380284786
35.07610165	16003316.87	21.62194952	-11.58605766
62.55380158	9840146.055	41.87373523	-62.72003937
37.73252531	10143365.11	24.64668526	-13.04436684
120.1503505	3329802.125	89.00582251	-51.94002533
45.85992493	5530511.952	31.14450797	-29.22180748
61.4602856	4503470.808	46.90927647	-93.6001091
39.30975049	8705772.631	23.21489493	-14.19708824
32.0264998	10905705.16	15.05403621	-5.795166016
51.20366288	22938049.64	30.06903967	-6.79212451
47.27763165	9516838.631	30.68990515	-18.01799202
38.02101476	15013771.18	23.59660654	-12.43981934
31.65267774	21524998.9	18.12178572	-8.240486145
69.64178326	15268058	49.63164159	-38.30501938
86.41491706	1115288.475	60.33166807	-212.3885193
86.8691315	3207474.634	54.23142842	-123.4066391
61.86477337	14054132.38	46.76304582	-59.91432762
33.98170023	20122580.71	13.96125223	-3.181988478
45.44029164	14373679.37	27.6261372	-12.935606
85.30669654	6206281.828	61.33233923	-98.27635956
70.52554446	7536398.835	55.34896581	-74.18417358
56.58802226	5462861.163	38.04657122	-33.02180862
65.21766906	7668386.282	46.59869797	-120.8461304
70.90996969	15224130.19	51.14035902	-102.677784
70.7853616	9149882.927	49.52091184	-113.0165634
89.78380405	19278530.35	59.23771026	-73.17155457
21.98684937	7901673.325	10.83203339	-4.053601265
86.60647876	50584562.38	48.99335205	-52.63464165
64.04946301	4400137.12	47.99229651	-117.7204857
89.32610857	1508156.108	65.98012972	-230.0928879
90.61563733	4473053.289	65.86709014	-160.2775116
42.11168914	18181127.82	23.82463398	-11.41377783
71.07172783	8018517.782	56.11438419	-116.4001007
59.55755463	26790047	40.17810837	-25.64522552
56.60274624	2436756.534	39.96153882	-67.26745605
27.70798985	25889442.69	10.9607791	-3.573417783
81.75335396	9112836.218	56.85446641	-120.9257736
89.01777704	27034687.64	59.08777281	-136.2834778
118.5096638	17183242.36	85.45434141	-137.2420731
66.79112286	4420452.661	45.84355187	-53.28954124
51.4827922	3206650.013	34.79370448	-49.44378662
60.0636256	14597313.78	46.33053639	-57.65158844
51.90876536	42928650.31	29.98221586	-7.665413857
33.03563695	10277961.66	17.47659602	-5.805140734
43.01302406	7089904.56	26.20512735	-8.976556778
66.74983292	1228461.943	50.24159494	-146.9570923
71.35853711	11577875.25	46.93290421	-19.64777184
80.37991495	4049066.238	53.40169255	-177.8953552
57.18715673	24439565.55	38.69133581	-12.62464142
59.03169888	3434128.89	41.73077103	-144.5775452
57.0802424	13440668.89	38.76805134	-23.62544918
55.30370894	18332381.38	38.63012822	-27.61040878
61.98258347	8805060.698	46.6040078	-100.5871201
35.28400954	20459802.01	19.17698034	-4.545744896
28.90153279	23467012.05	14.31800848	-5.903087378
32.70659292	45496906.7	15.1293395	-3.403990269
88.87579565	12495361.8	59.66596645	-121.8580551
67.28517585	8576181.852	47.06529163	-106.4093208
55.07702099	4286539.675	40.37776299	-127.7928848
69.45633315	14309204.57	50.73531727	-83.39836121
52.3665504	17655695.89	34.13707626	-14.18565321
67.89759922	5166178.589	47.99935614	-168.0986481
94.85789286	17681182.05	61.61057964	-60.80136108
50.33159453	32211370.12	24.47735163	-5.353724957
88.79265171	1287332.201	66.36067378	-176.415741
38.47578904	17299192.67	23.29544637	-10.99889469
44.39444303	17096777.93	27.56554355	-29.56267738
56.55092176	5147080.427	41.13259424	-98.46124268
79.0900944	17839325.11	55.46999403	-151.7008972
74.26246782	556530.5096	58.01284402	-49.95654297
95.77141162	696759.9904	63.30088147	-187.5737762
60.59056699	30083121.96	37.06585047	-11.33656502
72.42803225	5346141.042	56.08565106	-91.83299637
68.76256802	4908873.181	44.2182445	-45.59113884
47.35398848	18451929.65	30.93241585	-13.4839263
34.46512857	20384718.77	20.02768395	-9.025682449
92.38488332	2624548.584	60.77242656	-160.0896759
77.69182961	8214162.451	53.58342371	-43.699049
73.39913114	1255913.416	52.7920147	-229.5172958
84.54703331	3190811.893	58.27598498	-88.52725983
62.98846576	2706217.237	45.24434329	-44.94071198

log.sigma.2.0.mm.3D_firstorder_TotalEnergy	log.sigma.2.0.mm.3D_firstorder_Maximum	log.sigma.2.0.mm.3D_firstorder_RootMeanSquared	log.sigma.2.0.mm.3D_firstorder_90Percentile	log.sigma.2.0.mm.3D_firstorder_Minimum
136995263.9	230.7053833	118.5542954	8.085170746	-286.8728943
258874779.3	118.5018616	82.66737612	5.715514088	-357.0541382
68657321.65	120.5872345	119.8601767	-16.81744423	-318.8163757
740777565.1	468.223877	119.1368468	32.265522	-398.7088013
70245587.24	53.27017212	160.4970132	6.592370033	-415.1544189
102197070.4	72.56473541	124.5496261	-19.08861675	-273.6762695
45732602.62	97.51000214	155.5543128	43.32451057	-426.5864258
289850328.8	192.1485291	163.6186595	-15.49801922	-359.5439453
1134094370	442.3201904	168.6939613	213.1631241	-401.0517578
376091441.7	453.86026	77.50227822	9.821013069	-324.1287537
178375695.9	114.5837326	132.5537552	-12.21122551	-314.7621765
163628962.5	272.1143188	89.47470321	17.99974747	-262.5253906
263179633.6	191.3247375	84.47319239	18.24030018	-263.9008179
143396905	230.4207306	100.2930978	7.351511049	-254.9894562
158269213.3	423.2086487	126.9011763	72.14752274	-398.8941956
438642830.1	279.989563	38.73463891	12.90256414	-238.2383881
144619441.2	162.8454285	157.1091904	-11.66727715	-305.4173889
332548341.1	178.1066284	89.08398832	2.276199746	-288.7756653
120741297.5	141.2758179	156.7510084	10.63251791	-354.5949402
240002499.1	138.5820313	56.60734311	10.32020731	-261.9430237
432089555.4	578.8603516	56.15481217	5.644168091	-294.6593628
265683943.5	403.8762207	104.0444289	31.1511795	-318.5031433
273870857.9	212.2946777	58.93857119	4.857972383	-240.6537628
89904657.37	368.0626526	158.2280196	109.6347565	-406.3163452
149323822.7	205.1803131	75.90092193	4.684938908	-296.5073853
121593711.8	75.30682373	122.1153348	-8.384539223	-326.9670105
235055861	352.3047791	61.67096781	18.49566536	-241.5617065
294454039.4	401.0083923	52.91431096	6.825026798	-277.0021667
619327340.4	332.6647644	78.56704	5.797228098	-392.5600281
256954643	189.9677734	74.97524155	3.77964282	-311.7755127
405371821.9	359.4894409	59.78174058	9.125415802	-288.3928833
581174970.2	193.3851013	49.36480817	10.43690929	-252.8720551
412237566.1	341.0654297	104.8433231	15.590065	-329.8876038
30112788.81	-82.58989716	272.6766674	-147.9317413	-441.5003357
86601815.12	254.2280121	168.4776878	37.14690285	-368.0467529
379461574.3	191.9998169	102.6417635	3.572986054	-277.7434082
543309679.3	240.0463715	54.83985832	8.431943893	-292.8451538
388089343.1	387.1689453	68.85244676	16.70784302	-320.8723145
167569609.3	231.4759827	139.0475145	51.3540802	-340.3761597
203482768.5	103.0171738	118.4662917	6.562450123	-319.6439819
147497251.4	255.6685181	87.40918295	18.28032837	-409.0065918
207046429.6	159.4411926	141.8696518	-13.69845295	-352.1186218
411051515	282.3873291	135.9260319	-2.463856363	-373.6331177
247046839	330.2213135	144.3695187	-8.764947891	-364.4315186
520520319.6	505.4225464	131.4920597	97.46212769	-333.2402954
213345179.8	164.6638336	35.92633149	14.21092491	-207.830307
1365783184	670.2233887	135.7246505	91.834095	-365.3416138
118803702.2	83.03346252	138.9202902	-8.423850012	-303.9407349
40720214.9	-61.90565491	261.8254196	-128.5051895	-407.6397705
120772438.8	148.9728546	175.0352859	21.97564316	-343.3669128
490890451.2	332.1611023	66.75446637	11.56207247	-297.0424194
216499980.1	90.35498047	141.0569617	-4.046276665	-296.2311707
723331269	407.1193237	91.56959777	15.76664009	-538.3151855
65792426.43	144.4259338	100.9734323	11.5590992	-283.5155945
699014952.5	489.6340332	47.85274553	15.59423113	-281.8587036
246046577.9	241.2664032	164.9317925	-11.46555729	-375.4981689
729936566.2	302.0599365	176.0769211	10.1969491	-452.246521
463947543.7	267.7810059	193.6956283	73.89365692	-455.3840942
119352221.8	139.8733063	111.7459369	9.175379562	-386.1238403
86579550.34	286.0875244	82.9531386	22.54754257	-214.4423981
394127472	145.286499	100.1624583	6.806880093	-359.4990234
1159073558	398.313385	79.59520737	11.59137726	-362.2944031
277504964.9	256.7940674	50.22766447	32.0154995	-248.0341492
191427423.1	116.6783066	63.07631955	19.32360783	-311.6794128
33168472.45	29.18938255	152.2449579	-41.18957214	-291.1013794
312602631.8	365.9476013	100.25088	62.48499107	-349.1213989
109324788.4	101.2494583	196.3734595	-5.21193186	-359.4885254
659868269.8	376.5992737	83.95949541	13.39870758	-372.3438416
92721480.03	60.34157181	154.9673751	-42.82393799	-302.0604858
362898059.9	248.3439026	86.60469538	16.90693321	-347.9162292
494974297.3	214.521698	83.98588483	17.4135746	-328.4181519
237736638.9	44.73760605	124.6162412	-2.199626923	-296.1833191
552414654.2	221.4449615	53.84441397	8.69680233	-276.573822
633609325.3	459.8247986	46.5408684	13.41031027	-295.3927307
1228416481	518.8522339	55.97867086	27.68213921	-380.2661438
337374768.6	285.6757507	167.38132	5.325317621	-434.4848938
231556910	157.0525055	137.140134	-9.592456818	-339.6239014
115736571.2	60.75691605	138.3341528	-23.55054913	-286.7973633
386348523.5	234.204895	125.1907835	0.379428402	-355.8464355
476703789	351.8539734	80.80515659	5.415753126	-341.3869324
139486821.9	-5.800123215	186.2051007	-43.1269516	-400.6397705
477391915.4	399.8104858	142.9708813	46.52129974	-454.6028442
869706993.1	500.7156067	80.83979247	30.84810753	-823.961853
34757969.44	103.4761047	203.7813662	-50.4007988	-351.624115
467078202	353.5057373	59.04519182	14.74942093	-264.0801086
461613004	272.5036621	69.82151249	24.83377266	-328.832428
138971171.5	151.8598328	118.7501167	-2.207553291	-322.2052307
481661777.9	225.557663	174.0329824	-22.42497597	-390.230957
15026323.76	116.1582642	105.5017071	58.35946083	-265.0457764
18812519.74	-12.36628437	231.5100912	-61.16838531	-392.1717529
812244292.9	358.3546753	93.66508026	16.3236908	-676.8798828
144345808.1	84.14199066	132.6121799	1.94577179	-332.0498962
132539575.9	342.2135925	108.6286146	15.74888277	-362.1434631
498202100.7	208.4464722	71.08132434	11.56402979	-329.1087646
550387406.9	268.4611206	53.1280187	12.75631285	-261.9481506
70862811.78	219.4432983	204.1065931	-37.26505051	-404.317749
221782386.2	290.4060669	109.9074641	79.05790253	-337.7197876
33909662.25	-68.04516602	228.756914	-91.5502594	-364.7818298
86151921.11	162.3013763	139.9125909	29.12310791	-420.1919861
73067865.39	99.52011871	98.31104495	22.97349453	-295.7988892

log.sigma.2.0.mm.3D_firstorder_Entropy	log.sigma.2.0.mm.3D_firstorder_StandardDeviation	log.sigma.2.0.mm.3D_firstorder_Range	log.sigma.2.0.mm.3D_firstorder_Variance	log.sigma.2.0.mm.3D_firstorder_10Percentile
3.719438628	84.09838293	517.5782776	7072.538011	-196.4446716
2.997632211	66.53782274	475.5559998	4427.281856	-155.4176453
3.436850118	71.32896284	439.4036102	5087.82094	-188.2375061
3.879465633	104.9074393	866.9326782	11005.57082	-195.5869324
3.819788222	96.14968158	468.4245911	9244.761268	-244.3792725
3.397053638	67.09486073	346.2410049	4501.720336	-188.1592896
4.052736639	115.9322009	524.0964279	13440.27521	-259.1826263
3.866121825	93.44569711	551.6924744	8732.098308	-253.451004
4.623525272	162.8064939	843.3719482	26505.95447	-231.86026
3.214044055	65.01230991	777.9890137	4226.600439	-125.1416702
3.593569656	75.53437946	429.3459091	5705.44248	-209.3932037
3.457932678	70.93548489	534.6397095	5031.843017	-149.0775421
3.414845113	67.28775489	455.2255554	4527.641958	-137.4964752
3.469077644	70.45382067	485.4101868	4963.740846	-154.0018921
4.044212782	107.7513924	822.1028442	11610.36256	-193.6648865
2.07938927	37.65653873	518.227951	1418.014909	-39.82667503
3.684611818	86.39380917	468.2628174	7463.890263	-226.406427
3.234222653	63.51887745	466.8822937	4034.647793	-154.4468872
3.89606098	95.98430119	495.8707581	9212.986075	-237.8730347
2.763452579	50.23669338	400.5250549	2523.725362	-101.7600365
2.59707332	48.00129953	873.5197144	2304.124757	-97.40944672
3.682503234	81.97522234	722.379364	6719.937078	-163.2529602
2.728540624	49.17086056	452.9484406	2417.773528	-102.3093964
4.396391769	144.6408345	774.3789978	20920.97102	-241.4142517
2.979877722	58.70875507	501.6876984	3446.717922	-129.8350845
3.441465898	72.97905216	402.2738342	5325.942054	-196.8109314
2.950053452	56.18901027	593.8664856	3157.204876	-99.07802887
2.309596032	47.80226854	678.0105591	2285.056877	-88.4204895
2.844106911	69.26744903	725.2247925	4797.979494	-140.2493362
2.877881383	60.85403723	501.7432861	3703.213848	-135.2339172
2.718464381	51.77498294	647.8823242	2680.648858	-103.1904755
2.549992594	44.01773234	446.2571564	1937.560761	-85.03714294
3.636548929	86.79839124	670.9530334	7533.960722	-178.6705078
3.323231429	102.9835848	358.9104385	10605.61874	-400.5237183
4.071034656	116.2151672	622.274765	13505.96508	-267.6572693
3.480969537	73.80514327	469.7432251	5447.199173	-176.794249
2.332441794	50.71286735	532.8915253	2571.794915	-97.3551712
3.112937517	62.99022253	708.0412598	3967.768134	-111.7825615
4.037485308	105.5212017	571.8521423	11134.72401	-220.6486664
3.607711504	81.76438931	422.6611557	6685.415359	-196.4955963
3.449979757	72.65522282	664.6751099	5278.781403	-152.9572174
3.69458021	81.28604	511.5598145	6607.420299	-222.6373596
3.776939655	89.11871263	656.0204468	7942.14494	-213.4848267
3.815126124	90.58747145	694.652832	8206.089983	-218.804892
4.191517881	117.3338899	838.6628418	13767.24173	-194.5812256
2.046722594	33.67407059	372.4941406	1133.94303	-50.95851212
4.13521853	128.9455597	1035.565002	16626.95738	-176.6258926
3.608799883	77.20996596	386.9741974	5961.378843	-214.0989151
3.538310596	100.8590809	345.7341156	10172.55421	-381.3328735
3.996160739	109.2002749	492.3397675	11924.70003	-260.6742554
2.880819104	60.07338439	629.2035217	3608.811512	-112.6685547
3.621265904	82.92254496	386.5861511	6876.148463	-225.9150726
3.404482811	76.57705658	945.4345093	5864.045595	-164.1152802
3.483023701	70.33310624	427.9415283	4946.745834	-160.6600586
2.333561277	45.87369461	771.4927368	2104.395857	-64.81692886
4.022813213	103.2830291	616.7645721	10667.38409	-267.2862366
4.208540518	116.0906721	754.3064575	13477.04415	-268.5408081
4.534749163	145.6619998	723.1651001	21217.41819	-322.3466888
3.635735362	84.66484078	525.9971466	7168.135264	-187.4949448
3.381362627	66.70281748	500.5299225	4449.26586	-130.6800156
3.356566018	71.11078994	504.7855225	5056.744446	-171.575351
3.117919466	73.45305952	760.6077881	5395.351952	-134.6346207
2.779610236	48.57255048	504.8282166	2359.29266	-78.30654907
2.854091719	56.50264045	428.3577194	3192.548378	-117.8803474
3.420130551	76.79900842	320.2907619	5898.087694	-217.367334
3.82723566	92.95034074	715.0690002	8639.765843	-162.278775
3.914816042	102.9100395	460.7379837	10590.47622	-297.8619934
3.160443973	71.93210306	748.9431152	5174.227451	-153.0307159
3.52775212	71.99498832	362.4020576	5183.278344	-224.6166229
3.349119405	72.54957228	596.2601318	5263.440438	-151.0746704
3.317276373	69.05880706	542.9398499	4769.118832	-149.5186218
3.543808159	74.37599803	340.9209251	5531.789083	-200.7906372
2.545553419	48.56100225	498.0187836	2358.170939	-96.90482025
2.347022937	42.73759606	755.2175293	1826.502117	-78.02826614
2.712340337	55.14899867	899.1183777	3041.412055	-68.28853149
4.179526153	114.2734116	720.1606445	13058.4126	-270.5170441
3.767443277	84.10929714	496.6764069	7074.373866	-222.3284836
3.400309707	66.84572879	347.5542793	4468.351458	-201.4784927
3.70225887	85.30047263	590.0513306	7276.170631	-209.5327576
3.088540514	69.6219375	693.2409058	4847.214181	-140.5429459
3.708630545	84.76613957	394.8396473	7185.298418	-274.3910706
4.201797901	124.3259156	854.4133301	15456.9333	-237.6719574
3.151238896	77.76008262	1324.67746	6046.63045	-127.3545303
3.824533407	107.5583839	455.1002197	11568.80595	-317.2218018
2.797104203	52.5355629	617.5858459	2759.98537	-102.2800003
3.228088464	60.92346515	601.3360901	3711.668605	-106.3248734
3.474447377	69.60393704	474.0650635	4844.708051	-184.2368134
3.983229636	99.03331285	615.78862	9807.597054	-259.0547852
3.598759322	87.01429114	381.2040405	7571.486862	-168.5304733
2.87314068	112.5018476	379.8054686	12656.66571	-341.4766052
3.281526636	83.19680363	1035.234558	6921.708134	-154.0582764
3.68189226	84.70497684	416.1918869	7174.933102	-214.9573105
3.705969936	91.76329904	704.3570557	8420.503051	-177.4590988
3.017902164	60.72628955	537.5552368	3687.682242	-127.486657
2.574825221	47.27109705	530.4092712	2234.556616	-96.38411484
4.008561179	119.7813567	623.7610474	14347.57341	-311.6686279
3.978053066	98.68113781	628.1258545	9737.966959	-173.5645325
3.438721876	86.62538014	296.7366638	7503.956484	-320.2938171
4.004972728	105.886754	582.4933624	11212.00467	-222.9943542
3.519970785	76.81724384	395.3190079	5900.888951	-182.4315018

log.sigma.2.0.mm.3D_firstorder_Kurtosis	log.sigma.2.0.mm.3D_firstorder_Mean	log.sigma.2.0.mm.3D_glrlm_ShortRunLowGrayLevelEmphasis	log.sigma.2.0.mm.3D_glrlm_GrayLevelVariance
2.777211339	-83.56185099	0.031464779	11.93466857
4.071078794	-49.0572443	0.007448702	8.368171668
3.427365538	-96.32570274	0.022243375	8.555003534
4.868795244	-56.46253131	0.008685417	20.08109071
2.56243767	-128.5088712	0.018799138	15.29597573
3.087136308	-104.9327833	0.049377547	7.553476646
2.466066998	-103.7153269	0.021358461	21.54338672
3.266497418	-134.3092232	0.018928818	14.35784024
2.617996058	-44.17802725	0.008075363	43.48791058
7.092976155	-42.19007809	0.009140387	8.050031365
2.584135306	-108.9268357	0.022499738	9.303623874
3.767480282	-54.53328797	0.020908597	8.650032767
3.607964728	-51.0693477	0.016945856	8.132136572
4.457138423	-71.3790208	0.023505684	8.638650436
4.551868798	-67.03391672	0.010230281	19.4237626
12.67626737	-9.075094639	0.011027947	3.527915357
3.944887709	-131.2227398	0.028960743	12.695509
2.830772638	-62.46046094	0.014715729	6.826450092
3.162721487	-123.9269646	0.022063876	15.47444088
4.502436542	-26.08957515	0.010834336	5.059410903
11.12168902	-29.14169131	0.008601152	4.81057331
5.078474349	-64.07266271	0.012853438	11.51682029
5.086404135	-32.4958712	0.01823819	4.766852611
2.697732659	-64.14931943	0.016251485	33.6544594
4.889200997	-48.10646555	0.015455544	6.267779065
2.603185204	-97.90920763	0.016998556	8.701316444
7.707210318	-25.41856398	0.01521765	6.313120107
10.26568295	-22.69069031	0.008937445	5.132638974
6.210433662	-37.07829932	0.006138306	9.856665794
4.622924383	-43.79581028	0.010823155	7.0800126
7.348029162	-29.88657973	0.009411416	5.435114079
6.400661983	-22.34554822	0.009799062	4.109425486
3.949612012	-58.80613633	0.010855557	13.43048077
2.08340812	-252.481576	0.103074571	16.42906855
3.685086619	-121.9785481	0.029290176	22.56708145
2.658579133	-71.33114641	0.018159123	9.273337487
7.989675628	-20.87139539	0.009629205	5.797839566
5.842343477	-27.80092246	0.007739925	8.011411094
2.814409175	-90.55102038	0.019936476	18.14609055
2.204793596	-85.72541574	0.021215598	10.99572875
4.534668836	-48.59613009	0.006142678	9.479265052
3.098573859	-116.2737194	0.014806678	11.16547607
4.062893687	-102.6340158	0.014490975	13.1964548
4.607962079	-112.4120453	0.018860317	13.78982557
4.109620113	-59.35419128	0.013231122	22.93328573
9.023954085	-12.52031407	0.013118537	2.532718986
7.879378034	-42.35827414	0.00983415	28.78788929
2.268913281	-115.4879568	0.023385617	9.814384545
1.818427748	-241.6195276	0.118112622	16.53194102
2.512706408	-136.7941931	0.041378987	19.70466653
6.978206679	-29.10922995	0.01143639	7.445295869
2.024271324	-114.1092371	0.044061741	10.86674353
5.117849846	-50.20901952	0.002797646	10.69743884
3.188500291	-72.44921119	0.019534648	8.371097377
10.75476943	-13.61944923	0.007637364	4.834265791
3.382888246	-128.5889267	0.019416331	17.59134455
3.998095482	-132.3859434	0.009125674	22.40126566
2.444441959	-127.6737178	0.01337239	34.41175082
3.866966507	-72.93160594	0.011647029	12.3263761
4.665485318	-49.31487953	0.038406203	7.765572836
2.782078	-70.53916369	0.008509637	8.285485228
6.265662134	-30.66015465	0.005972691	11.12944964
5.57785862	-12.78771355	0.01144397	4.862993019
4.304685317	-28.03700608	0.007876762	6.283321425
2.154867749	-131.4550856	0.054315251	9.744699892
4.216538696	-37.55626567	0.0118213	15.05605033
2.90141167	-167.2484959	0.046519548	17.61305899
4.165901417	-43.30091706	0.006716314	9.632018892
2.699601748	-137.2283098	0.032064481	8.66748647
4.245832556	-47.2962242	0.009538194	9.603289204
3.587970981	-47.79654818	0.008238758	8.39682367
2.23909066	-99.98709166	0.029030115	8.915215307
6.123373371	-23.26048101	0.008486838	5.146276606
8.921089365	-18.42689108	0.007317048	3.959154621
13.44722555	-9.602058976	0.00360301	6.63218564
3.52834149	-122.3032856	0.013469846	21.77117963
3.014359401	-108.3191695	0.021920298	11.84898846
2.474768555	-121.1114626	0.030352218	7.230903092
3.058138641	-91.63275425	0.014476259	12.2241086
5.746476652	-41.01535262	0.008966743	9.684753114
2.635506566	-165.7921624	0.020661857	12.02938878
3.965760111	-70.59560603	0.006598699	26.46494827
10.10164797	-22.10071484	0.001104482	12.19533257
2.680923571	-173.0839081	0.063553117	18.1038947
6.035414792	-26.9508684	0.011734013	5.562353879
5.306443013	-34.10828346	0.006361346	6.83490576
2.86774492	-96.2126923	0.018015353	7.863626918
3.334571189	-143.107938	0.017814281	16.30556183
2.202784521	-59.6583886	0.038839077	11.89560274
1.951661353	-202.3369878	0.112913232	20.78035626
9.693141079	-43.0283526	0.001893578	13.34335522
2.232613976	-102.0345881	0.019591482	11.68761381
4.564545719	-58.13495389	0.011303022	14.87010666
4.375100797	-36.94417989	0.00703335	7.162193265
5.674588908	-24.24932485	0.010040822	4.572276222
3.936333356	-165.263208	0.035398941	23.55987171
3.271773664	-48.39094651	0.01041758	16.52144957
1.97742114	-211.7209702	0.109101597	12.27475445
2.910323266	-91.45232863	0.01356389	18.05122964
2.738045392	-61.3528533	0.020713297	9.863159147

log.sigma.2.0.mm.3D_glrlm_LowGrayLevelRunEmphasis	log.sigma.2.0.mm.3D_glrlm_GrayLevelNonUniformityNormalized	log.sigma.2.0.mm.3D_glrlm_RunVariance	log.sigma.2.0.mm.3D_glrlm_GrayLevelNonUniformity
0.032736632	0.083002045	0.183846303	26.46504994
0.008353554	0.130232277	0.842462909	133.0222208
0.023078472	0.105597318	0.131216716	16.76645642
0.009266621	0.078799562	0.41073653	126.2382983
0.019101437	0.077886309	0.056196319	7.502029123
0.051158314	0.107582541	0.112889932	23.85003883
0.021476745	0.065959295	0.025187393	4.50085588
0.019623918	0.078013984	0.101254139	28.70301134
0.008437492	0.046009014	0.092021361	62.97212648
0.010189896	0.120705061	0.577286211	217.7701199
0.023532013	0.093035488	0.133238044	31.52871248
0.022292122	0.102277504	0.238361588	66.21315148
0.018349348	0.105998329	0.336718519	120.4338774
0.025044732	0.10741298	0.172336412	49.66857487
0.010494686	0.073377763	0.065984244	25.22211479
0.013967602	0.27634528	1.379682786	1818.406973
0.030114439	0.091132238	0.100333514	18.35632823
0.016089703	0.114172171	0.410980975	142.7355722
0.022645265	0.080275982	0.077762843	13.69607033
0.012864627	0.159723714	1.002979432	301.7924292
0.010244814	0.174917619	0.999166704	602.9523105
0.013583953	0.090709961	0.166843107	73.33840096
0.020731092	0.160414541	0.931246296	326.8962287
0.016445132	0.054216133	0.037068259	6.960903896
0.016786623	0.141762049	0.430131925	107.8505894
0.01781171	0.100580328	0.152803209	26.86469393
0.017202226	0.148552312	0.599143278	256.3570354
0.010694344	0.227036831	1.155366943	579.3079431
0.007063898	0.1598085	0.963679943	411.1304471
0.012054212	0.145041651	0.782658738	179.061705
0.01095979	0.168713631	0.793067028	501.0034194
0.011842203	0.189889304	1.003632268	1131.848632
0.011699956	0.088753094	0.384137478	100.779144
0.103115779	0.110413396	0.005102041	1.648351648
0.029991524	0.071614268	0.064589851	7.605089256
0.019453423	0.096039386	0.344718515	106.3026613
0.011522198	0.230801781	1.181257594	984.3170967
0.008908865	0.129726389	0.704753206	289.8118971
0.020587301	0.068070896	0.066570089	20.55777909
0.022254489	0.08721749	0.231311774	40.23508324
0.006620069	0.103851576	0.328766868	61.53410888
0.015455764	0.088335519	0.11353787	30.69847533
0.015219267	0.082594699	0.174236354	60.31341243
0.01958274	0.081994442	0.108545553	32.92705965
0.013854954	0.064734532	0.09150789	66.80182028
0.016400162	0.272333956	1.096840377	1067.542805
0.01038046	0.072570991	0.148395852	178.6916336
0.024327855	0.089450142	0.078701246	18.94196641
0.121181494	0.094873366	0.017388802	2.051215451
0.042333788	0.070369122	0.055552103	9.783800446
0.012980142	0.156710669	0.844035321	457.2925913
0.045820445	0.08512082	0.152837543	30.70371613
0.003103043	0.108140326	0.447601053	276.7383947
0.020525347	0.100166922	0.137498137	21.3090278
0.009437847	0.234898674	1.18382782	1706.900834
0.019904166	0.071996581	0.09429877	22.44599289
0.009474797	0.064419961	0.084974877	52.35916918
0.013638599	0.047895124	0.050292862	20.99229441
0.012121583	0.094730335	0.164247618	29.62635805
0.041202351	0.110192714	0.226511258	44.25166138
0.009238023	0.103987366	0.354196154	123.6196219
0.006887692	0.134088762	0.819256457	652.7894826
0.013470642	0.168350832	0.718470136	496.9802997
0.00909825	0.151879956	0.819606197	194.0604659
0.055958343	0.108763277	0.110981185	5.279169532
0.012525088	0.082171627	0.204715426	82.82094849
0.047090524	0.078490743	0.04472429	7.899461237
0.007604899	0.125107089	0.687360101	321.8977192
0.03320322	0.098288068	0.078534128	13.04434742
0.010408574	0.109608376	0.477163566	155.0217071
0.009074184	0.112861721	0.398940417	237.1990748
0.030452414	0.092722572	0.163121874	46.65533908
0.010304905	0.186091652	1.317394769	841.3011212
0.009066982	0.224168847	1.074782751	1598.428798
0.004437127	0.186364341	0.843142182	1885.637663
0.013809817	0.06430822	0.077986842	26.87721271
0.022940555	0.084119191	0.127674619	34.63801479
0.031818409	0.106645645	0.118084893	21.53687906
0.015294937	0.085336552	0.243524868	66.97605476
0.010023756	0.126807154	0.838037823	247.843
0.021097277	0.08536172	0.07283324	11.92235194
0.006855588	0.065849671	0.139530315	51.45102462
0.001261357	0.143530294	0.629242649	532.0014459
0.06394281	0.077915814	0.014853705	2.379373093
0.013616437	0.159968184	0.844910458	558.8828638
0.007147972	0.125764526	0.356264042	360.4620926
0.018930667	0.101004857	0.145542917	32.72246771
0.018514266	0.072455009	0.076319113	39.88762744
0.039583826	0.090309648	0.04885817	4.292720258
0.113106133	0.145668968	0.019097222	1.858974359
0.002104532	0.122983188	0.627174032	320.5830283
0.020241816	0.083676143	0.11657416	23.14454093
0.0118545	0.09152339	0.233281889	32.95888727
0.008107818	0.133941178	0.788525193	353.4998817
0.012034121	0.188570098	0.897552897	934.1720423
0.035731512	0.071932505	0.047828974	4.339258448
0.010971779	0.071520116	0.159670281	43.70182879
0.112486928	0.100548353	0.038129057	2.321718137
0.013797458	0.071987562	0.045401869	11.24651632
0.021549888	0.097776909	0.151862082	24.40222389

log.sigma.2.0.mm.3D_glrlm_LongRunEmphasis	log.sigma.2.0.mm.3D_glrlm_ShortRunHighGrayLevelEmphasis	log.sigma.2.0.mm.3D_glrlm_RunLengthNonUniformity	log.sigma.2.0.mm.3D_glrlm_ShortRunEmphasis
1.470635121	83.81391629	257.9289773	0.91753749
2.762503225	141.8478446	649.42552	0.822802294
1.385665871	90.76586187	130.0524465	0.920148283
1.874720719	191.2242596	1212.521123	0.891015124
1.157542829	165.1143708	88.09806256	0.965698326
1.326356682	56.92262031	184.9037593	0.930217052
1.079693497	221.9531351	64.87287603	0.980076626
1.29094315	106.3591558	312.8451974	0.937708329
1.255995019	276.7707097	1191.068416	0.946581462
2.23958316	120.4435384	1239.265042	0.853409073
1.368596892	82.69502976	281.2222282	0.927166106
1.612319642	82.38175916	494.6427368	0.895096079
1.791030248	84.24730218	846.8573676	0.884911083
1.478680529	75.71169855	362.9106165	0.906639009
1.18846612	204.0696999	308.2006941	0.958323637
4.155635941	72.22505045	3065.101842	0.705573087
1.266773599	77.90903973	176.1442551	0.94755404
1.965360574	84.60579339	884.7703679	0.864579467
1.216655211	121.6568184	151.329619	0.954001022
3.206733396	81.14915698	1060.027983	0.775757534
3.187922235	94.46260522	1935.840556	0.777024216
1.436872283	119.6591223	657.5271159	0.919498776
3.019516286	63.10236528	1190.501181	0.791390917
1.111560027	248.8032903	119.8524762	0.973477515
2.04672963	92.40834474	518.4973299	0.849259245
1.436883775	103.7059206	212.8438047	0.911592962
2.373833344	77.08397166	1107.595381	0.82787086
3.566824729	95.69782758	1347.075639	0.7531188
3.101250238	167.0556501	1484.871725	0.786234307
2.687825075	106.1771189	773.4667739	0.818114124
2.813530701	96.91474348	1735.652603	0.791604848
3.231371519	83.38857719	3280.044913	0.768771399
1.887729763	132.5433695	826.5865173	0.876545057
1.016483516	85.30989011	14.78021978	0.995879121
1.199927184	130.8305313	93.87354585	0.952453401
1.804814218	83.1469007	821.616992	0.883839964
3.689365061	93.26900992	2143.697274	0.734856059
2.559359141	127.5380875	1406.728528	0.820645903
1.200167631	133.1805751	268.8628273	0.954336289
1.592602561	92.77399973	355.6132737	0.898751415
1.796381009	212.5180804	436.1919431	0.880205563
1.319454321	118.4593257	293.2813861	0.933991811
1.453254341	126.0706586	589.4017218	0.916593846
1.30602791	124.7701318	339.9622858	0.935889763
1.259638723	162.9826344	892.8119756	0.944595666
3.575522	56.52020335	1953.367649	0.730478266
1.393688681	206.9740206	2026.568555	0.925003266
1.239608601	82.57362482	183.3310673	0.944575485
1.056043956	76.85722194	20.8989011	0.985989011
1.160170184	100.8297293	126.7462008	0.964289941
2.822252768	102.5875964	1770.190592	0.806011034
1.405206382	62.54611877	297.0317884	0.92432416
2.017679393	356.4563488	1807.327052	0.864281601
1.403167985	90.24132396	170.8301005	0.915368953
3.649373689	103.4818929	3702.645527	0.740201711
1.252001987	136.2531795	274.0282539	0.949997216
1.239316104	215.1089808	713.0688886	0.949248072
1.142899859	234.9311222	403.5621935	0.96830433
1.447986765	173.3231593	249.9883954	0.913733705
1.590432121	58.37647868	310.1326151	0.898280183
1.85868027	134.1996667	856.4293978	0.872228904
2.776856113	163.3818284	2979.239797	0.809605182
2.646370578	82.83681287	1790.324284	0.805523627
2.796964477	118.9417406	774.8350351	0.805552255
1.326715326	58.86939044	41.39716886	0.933229826
1.520194596	165.9335975	800.7833225	0.910240735
1.136011558	92.14088234	92.6226933	0.967750758
2.519357463	148.981233	1636.508397	0.824371117
1.242045176	68.16486769	114.5653581	0.943400217
2.095638802	133.9758441	975.7231032	0.855218616
1.944247055	135.6420538	1487.163369	0.864856319
1.437979295	70.67626102	406.4413954	0.916865374
3.812887134	93.3805158	2349.870844	0.747896058
3.423393369	99.5970137	3739.85992	0.750923547
2.916897425	206.0233172	5786.401944	0.784217817
1.219294999	197.8420333	370.681108	0.953481079
1.358132434	105.017658	341.0031372	0.926822655
1.354552571	58.31289192	165.6948045	0.922571536
1.61335061	128.6631109	609.5721122	0.899102676
2.77681925	129.545078	1215.742351	0.81563523
1.217590262	121.8283787	123.5201398	0.951499466
1.366834195	278.2914343	652.0760927	0.930331024
2.415077542	874.0380472	2385.577368	0.829016158
1.04668435	90.5487208	29.6571176	0.988328912
2.884634071	83.11110748	2044.287072	0.792148327
1.860272754	155.5591509	2055.685925	0.870702515
1.417712358	87.80116709	259.5912289	0.914438822
1.223116356	126.8881539	484.0942242	0.950447068
1.159409233	92.10055046	43.0059488	0.960147692
1.0625	88.29366987	12.29166667	0.984375
2.374391099	583.5136415	1721.960792	0.838774989
1.32896577	106.0598551	232.3302769	0.932269722
1.576300746	166.1636637	281.7771376	0.904553324
2.730875306	130.3742618	1608.171581	0.807944723
3.048117629	82.30818528	2763.2475	0.774145184
1.142068149	138.2900976	55.5261843	0.967431083
1.401705501	162.5808073	508.0228705	0.928467244
1.125092299	59.11718716	21.38609217	0.968726925
1.134738866	203.8936719	143.8941697	0.968528355
1.418251159	96.11211841	202.2367563	0.918100022

log.sigma.2.0.mm.3D_glrlm_LongRunHighGrayLevelEmphasis	log.sigma.2.0.mm.3D_glrlm_RunPercentage	log.sigma.2.0.mm.3D_glrlm_LongRunLowGrayLevelEmphasis	log.sigma.2.0.mm.3D_glrlm_RunEntropy
147.619682	0.88301726	0.03925912	4.220954852
569.6681499	0.725971819	0.016679478	4.246628283
145.0031721	0.89656671	0.027082542	3.892276335
428.2012941	0.828007481	0.013368807	4.712401923
191.852613	0.953541508	0.02052382	4.016627003
80.53432007	0.908575032	0.059503736	3.841988646
246.0666823	0.974725275	0.021949883	4.134902466
153.90354	0.917513908	0.022746608	4.252845353
361.2132415	0.927350427	0.010120023	5.004304103
340.8324495	0.777490298	0.018437445	4.244610614
131.7311341	0.901391162	0.028384182	4.048728054
159.4034001	0.854892795	0.029631327	4.143085873
179.0331564	0.831456245	0.027075949	4.201360412
121.1071694	0.875728438	0.032667693	4.078863376
245.9226043	0.944209637	0.011680856	4.349828876
442.3356771	0.606049842	0.044370999	3.924272261
98.14544495	0.927685218	0.03577785	4.028969144
227.6561475	0.80501586	0.024838825	4.098474478
149.2362999	0.937447168	0.025335987	4.197538873
384.2278539	0.679135933	0.031716472	4.208553185
443.3019741	0.678514589	0.025754454	4.064857802
189.0403593	0.889058137	0.017365388	4.236461728
286.2236477	0.696522655	0.042524372	4.092025827
285.5948529	0.96529786	0.017252719	4.531135221
257.8372573	0.791346154	0.025268235	3.94630056
183.3921117	0.884360672	0.021578143	3.963744636
231.572425	0.7530329	0.032026339	4.115252979
512.0172294	0.652177348	0.028485137	3.935467885
765.6549373	0.689906434	0.015574994	4.334189976
417.7365104	0.727929483	0.022689053	4.127236574
392.4935012	0.706168861	0.023821409	4.0797579
389.764783	0.673801914	0.030542656	4.055634268
319.4581797	0.816913108	0.017157338	4.489005526
87.37032967	0.994871795	0.103280614	3.338493676
157.8015654	0.939414568	0.032877419	4.345094825
199.3518534	0.829373775	0.02740689	4.250205174
544.1277921	0.635974846	0.029834661	4.05038602
421.44542	0.736071646	0.018534025	4.350678726
162.2583686	0.940809969	0.023399804	4.333546252
193.160006	0.85875949	0.027731978	4.240471707
466.642304	0.828079613	0.00965388	4.235018189
173.9016604	0.911972542	0.018429934	4.119625093
216.8115098	0.886109037	0.018808659	4.316156066
177.3199455	0.914666199	0.022935381	4.238991034
208.9676223	0.925491549	0.016646176	4.595820413
308.3981398	0.639496896	0.046521962	3.686374855
294.7985246	0.896576839	0.013086052	4.696031538
110.1099798	0.928812416	0.028366749	3.953420436
80.1463703	0.982517483	0.133456983	3.563682997
112.2598518	0.952054795	0.046662042	4.21418104
396.1005335	0.714102564	0.026290853	4.244649928
113.4757097	0.895018133	0.054096449	4.071257708
900.9409652	0.800312989	0.005320089	4.363700153
141.9069585	0.889925974	0.02516938	4.007842771
547.5972891	0.641286451	0.027315918	4.03427046
186.9850563	0.930424799	0.022148666	4.358553722
272.4410756	0.931898377	0.011081095	4.57661255
273.5102124	0.957003695	0.014908679	4.750904531
296.6156849	0.883311604	0.014453782	4.193814551
101.0347332	0.861010234	0.055919089	4.031657282
342.0306963	0.816812054	0.013754698	4.169879515
615.1215746	0.717282717	0.014936549	4.488769058
276.4841043	0.723707564	0.02990868	4.085420643
464.2660375	0.715272382	0.019965829	4.154099532
83.38025524	0.91436865	0.064036266	3.736404373
280.0276674	0.874198718	0.016208361	4.454935042
106.9654784	0.958241758	0.049452672	4.092705832
537.8094867	0.741163941	0.014673409	4.374520674
90.46866524	0.927918236	0.038084424	3.849201835
372.9383812	0.788461538	0.016544223	4.340962725
343.1064653	0.807795898	0.01450362	4.22484776
124.2499602	0.887532221	0.037322753	4.056535856
556.8127504	0.638711154	0.029528876	4.208480121
501.9406209	0.656842419	0.0255814	3.945565779
777.8451843	0.696286563	0.011697296	4.182020922
249.7815735	0.937047258	0.015362192	4.504816334
158.0479981	0.902834008	0.02753579	4.241450121
92.68224052	0.901442308	0.038680988	3.815651613
262.6162081	0.859381582	0.019650127	4.346773839
510.416352	0.721324533	0.019562305	4.387265819
160.2174721	0.937016004	0.023008152	3.998383744
420.9963094	0.903156959	0.008141017	4.700271575
2634.1693	0.750846638	0.002544107	4.396413103
94.69063062	0.985111663	0.065501578	3.854875578
345.34959	0.703267913	0.029528271	4.167691286
334.4458935	0.817047224	0.012187957	4.115344023
150.4072397	0.887671233	0.023262401	3.978100171
159.3040371	0.934569675	0.021602376	4.333744066
110.9360884	0.950769231	0.042562824	3.753377505
94.62179487	0.980769231	0.113877738	2.926096135
1800.35326	0.759001279	0.003859429	4.451508611
168.8580507	0.909665992	0.023269966	4.091776921
303.110253	0.864829882	0.015014821	4.352080554
510.8863044	0.721332884	0.017233341	4.301068955
368.0034264	0.68509682	0.029115775	4.039949503
161.4549872	0.957264957	0.037140928	4.155094705
242.3584815	0.898303167	0.013903181	4.473347093
69.34630587	0.961538462	0.126028254	3.497768384
236.3776896	0.958470977	0.01477449	4.195059475
163.5801278	0.890934066	0.025769909	4.016432761

log.sigma.2.0.mm.3D_glrlm_HighGrayLevelRunEmphasis	log.sigma.2.0.mm.3D_glrlm_RunLengthNonUniformityNormalized	log.sigma.2.0.mm.3D_glszm_GrayLevelVariance
92.99841613	0.808098582	18.30675168
179.7416061	0.634778135	15.0886522
100.0290276	0.815482427	15.2
216.34106	0.756575274	38.82300196
170.0175994	0.914127074	20.6727071
61.13292209	0.833258646	14.18959184
226.7758445	0.949625213	23.49305556
114.6535525	0.849648118	21.69832448
291.2492645	0.869722313	49.60555225
142.9490428	0.68591292	23.53176327
90.47675147	0.828140432	14.63228957
93.31046078	0.762722152	17.5592519
95.69654828	0.743915971	17.92129032
83.01407957	0.78416239	19.018373
211.6588732	0.896371036	29.39753352
103.6366005	0.463812656	15.37528164
81.19183412	0.873352011	20.97395062
101.3049827	0.705840562	12.19633908
126.5295741	0.886593037	24.62734375
108.4757245	0.559577959	12.35861111
126.2213286	0.560664382	15.70892259
130.1963012	0.812075408	22.4066358
83.32730695	0.582911942	13.20329152
255.8421499	0.932834559	36.82287329
112.7073478	0.678902951	10.93211238
116.6591739	0.795074955	10.77843524
94.00584393	0.641093031	19.79619603
132.0055354	0.526523441	13.94024145
221.4061671	0.575798072	22.44248809
135.8064046	0.625688918	12.54860702
126.7422193	0.583172205	17.74227766
111.5987534	0.548845919	13.32342041
154.4230555	0.727657875	26.33375718
85.72197802	0.989795918	17.31122449
135.9890111	0.883127505	32.16358025
97.02575191	0.741421459	17.26786556
133.2474789	0.501212745	12.89798387
157.1210143	0.62924937	23.06133723
138.6302894	0.88844376	23.92684239
106.8002009	0.769775354	15.3817749
245.0855611	0.734745536	18.09171598
127.6465883	0.842591112	19.54501478
139.4259842	0.805639102	22.9094794
133.852242	0.845815968	22.78185728
171.0241006	0.864922614	32.97524803
80.04062758	0.496229002	7.905890805
220.908982	0.822679565	52.67783199
87.73161543	0.864844137	13.03515414
77.51505162	0.965222397	16.2283737
102.9647875	0.911112483	24.92727537
130.1852301	0.605737209	23.02502268
70.19310204	0.822186699	11.76005917
419.4619329	0.705198822	22.09455264
99.23067668	0.802122969	16.36495566
142.9747932	0.508242293	16.23953131
144.3680069	0.878205737	27.58060281
225.1204901	0.876290238	34.05322499
242.0582781	0.920340822	39.36925501
192.9147272	0.798907451	19.65527147
64.52854269	0.769582391	16.99024691
159.9486859	0.719390645	12.12982178
206.1625208	0.611482756	30.39031887
103.061754	0.604956815	14.69743702
152.2917466	0.605441659	10.28061224
63.05650515	0.847511933	14.79778393
182.4428101	0.793293387	27.88007792
94.97863242	0.919385022	23.2748538
187.8488221	0.635476686	19.94236017
72.45128376	0.862342798	13.98567708
161.1075171	0.68886646	20.48780273
160.7486929	0.705992563	15.04645327
78.81983206	0.806076808	11.89418579
130.6749425	0.518655462	13.78633207
136.6949111	0.523216319	12.35965736
263.3441994	0.571218587	24.81970657
206.8180743	0.88592769	33.21088619
113.7861259	0.8269162	20.81578561
64.4837069	0.818241669	11.03061224
146.5815022	0.77275103	19.73404444
164.4403995	0.62160809	25.77247352
128.7468991	0.882287671	16.50600185
300.4891335	0.834220032	46.33704177
1062.480684	0.643099924	33.13377203
91.37710276	0.97029259	19.8016
108.3814367	0.583876358	17.00768861
178.538965	0.716117294	16.73012128
98.19253454	0.800210043	14.01843202
132.8139023	0.878554012	24.24690936
95.86765806	0.902283661	12.47485207
89.55929487	0.961805556	22.02083333
709.3723131	0.660163263	30.10646778
116.6581883	0.838651196	11.93355372
185.1592827	0.781709371	29.36271809
167.4188877	0.60854724	15.61169482
110.0221932	0.55654741	13.84941583
142.5507787	0.919153863	34.90285714
174.4319452	0.830831343	26.41818657
61.1630109	0.923741885	11.55102041
210.000089	0.92060329	20.95617991
107.0992537	0.80865334	10.58131488

log.sigma.2.0.mm.3D_glszm_SmallAreaHighGrayLevelEmphasis	log.sigma.2.0.mm.3D_glszm_GrayLevelNonUniformityNormalized	log.sigma.2.0.mm.3D_glszm_SizeZoneNonUniformityNormalized
60.02947371	0.074329636	0.405013538
102.4056674	0.07634451	0.381617357
54.20243891	0.0848	0.3816
202.3462442	0.045815182	0.487755822
138.8768301	0.068786982	0.443047337
44.22511168	0.095918367	0.428979592
154.7303241	0.063368056	0.521701389
90.51994177	0.065763317	0.431133109
212.9778578	0.041253222	0.417225197
116.0761886	0.066628571	0.425812245
65.95506826	0.075840276	0.356650388
66.41676279	0.074932046	0.38772364
85.70115658	0.067866805	0.444432882
91.17714823	0.069817401	0.518796992
188.6161406	0.053121921	0.492251187
81.99519893	0.080519752	0.437855086
89.78073912	0.067160494	0.422469136
68.42512643	0.08322609	0.34563733
119.7664194	0.0590625	0.5115625
63.43026168	0.083395062	0.362160494
75.17236484	0.093861476	0.390828429
114.5364788	0.063102816	0.466049383
59.50365333	0.085588351	0.457721153
198.0245855	0.049672814	0.524092802
54.91648697	0.094027055	0.373818939
48.49860883	0.099605523	0.286653517
76.78608872	0.072585497	0.405733722
64.86046706	0.090401638	0.368047298
131.5557456	0.063464373	0.38404533
61.95155061	0.085472603	0.326193081
88.99594761	0.079422564	0.437922304
75.43511662	0.081926531	0.394559184
128.0925095	0.06061016	0.420663907
79.42857143	0.102040816	0.867346939
117.8804952	0.059670782	0.386831276
74.50808555	0.069209997	0.387118644
56.2417514	0.085566126	0.329549542
144.6744642	0.062917953	0.486850566
110.1486899	0.05853587	0.385104406
59.05557937	0.076416016	0.335083008
154.4395879	0.077498356	0.424720579
99.1030317	0.074173609	0.410373555
103.0580436	0.066038932	0.360434586
109.1635073	0.065114203	0.437705384
158.7292413	0.050627973	0.47235434
40.40975324	0.136725459	0.356833796
220.3733967	0.044051887	0.465368223
62.63237118	0.083288264	0.342076798
76.70751634	0.093425606	0.619377163
105.9235653	0.062273189	0.520104514
98.27309537	0.063507794	0.41411079
39.40272395	0.089349112	0.334792899
265.6156265	0.066020761	0.395522915
65.45697365	0.074442354	0.346949745
84.47555009	0.077765024	0.364032538
106.9151821	0.057760975	0.423020374
187.9876859	0.049116766	0.432700652
188.2585616	0.044225014	0.504344432
122.7821988	0.065211146	0.482619937
62.37569564	0.081975309	0.386419753
79.31165759	0.088348389	0.391357422
172.5272629	0.053062439	0.437961578
87.00054122	0.087871588	0.425488961
68.53558268	0.095793419	0.269887547
36.47404971	0.08033241	0.329639889
140.2242615	0.05672244	0.401151062
67.39047609	0.066174207	0.450907972
113.6669552	0.06689741	0.355430898
45.86189133	0.078993056	0.282118056
103.4963873	0.066953125	0.457460938
106.8971537	0.074062245	0.44737246
40.40726707	0.085542437	0.337046113
68.47599061	0.081413913	0.3499907
77.40933145	0.088430413	0.390984707
221.8737939	0.074897145	0.456551686
182.9279345	0.050142143	0.478580531
94.60534057	0.064893149	0.459533474
32.75652095	0.106505102	0.256377551
91.15939822	0.069985185	0.400967901
127.6972369	0.063372819	0.457403165
63.32291177	0.071714374	0.340104648
263.0849859	0.042523021	0.543538392
728.3050755	0.05766625	0.436672283
85.47444444	0.0752	0.6672
82.7382802	0.075118343	0.405451183
150.2439384	0.076043752	0.446577287
64.84736461	0.076884232	0.416487817
116.3482687	0.059661225	0.438836145
65.42680556	0.091715976	0.360946746
85.02083333	0.138888889	0.847222222
446.3030974	0.057928583	0.440679446
57.12273349	0.08231405	0.414049587
157.5797364	0.05785124	0.485766758
87.56219745	0.079228703	0.365445773
73.48708918	0.083156192	0.399741358
110.5375873	0.057959184	0.415510204
156.3529847	0.055279616	0.43155905
38.5	0.091836735	0.387755102
158.6779361	0.067175396	0.436466644
39.20763034	0.088442907	0.303252595

log.sigma.2.0.mm.3D_glszm_SizeZoneNonUniformity	log.sigma.2.0.mm.3D_glszm_GrayLevelNonUniformity	log.sigma.2.0.mm.3D_glszm_LargeAreaEmphasis	log.sigma.2.0.mm.3D_glszm_ZoneVariance
43.3364486	7.953271028	55.82242991	44.43968906
74.41538462	14.88717949	1711.451282	1659.685102
19.08	4.24	33.7	21.1684
273.631016	25.70231729	397.1105169	385.2381316
23.03846154	3.576923077	7.442307692	3.669748521
30.02857143	6.714285714	64	51.84979592
25.04166667	3.041666667	2.75	0.623263889
67.68789809	10.32484076	23.66242038	17.13878859
256.5934959	25.37073171	25.99674797	20.23674797
149.0342857	23.32	1856.225714	1812.325624
42.08474576	8.949152542	36	25.84659581
60.87261146	11.76433121	248.7452229	225.4968559
137.7741935	21.03870968	421.9935484	402.5767326
69	9.285714286	154.3909774	138.6306744
90.08196721	9.721311475	10.83060109	6.874197498
284.1679507	52.25731895	60304.64715	60026.28697
38.02222222	6.044444444	16.96666667	11.15320988
88.82879377	21.38910506	749.9688716	713.5004315
40.925	4.725	17.525	12.349375
65.18888889	15.01111111	8204.6	7967.097654
183.6893617	44.11489362	11907.15106	11790.55702
134.2222222	18.17361111	107.0381944	97.07628038
138.2317881	25.84768212	5182.980132	5089.493005
42.97560976	4.073170732	4.451219512	1.820493754
57.94193548	14.57419355	776.5935484	738.2335068
22.35897436	7.769230769	66.8974359	51.90664037
119.2857143	21.34013605	2876.411565	2815.794218
128.0804598	31.45977011	12460.16954	12334.89684
180.8853503	29.89171975	6094.670913	6032.425178
71.11009174	18.63302752	2242.068807	2181.757196
185.2411348	33.59574468	8190.579196	8091.945654
276.1914286	57.34857143	24748.41	24589.18166
140.0810811	20.18318318	399.1381381	381.7394692
12.14285714	1.428571429	1.214285714	0.066326531
20.88888889	3.222222222	10.01851852	5.63957476
114.2	20.41694915	340.6711864	320.2223959
165.7634195	43.03976143	25912.53082	25735.58264
210.3194444	27.18055556	3054.00463	3004.745027
55.06993007	8.370629371	11.1958042	6.156878087
42.890625	9.78125	122.4765625	104.8759155
66.25641026	12.08974359	274.0320513	253.0251068
50.06557377	9.049180328	44.7295082	34.97668637
84.70212766	15.51914894	81.96595745	69.67123585
68.71974522	10.22292994	37.47133758	29.65272425
219.1724138	23.49137931	32.91594828	27.14145641
124.1781609	47.58045977	34297.61494	33988.1381
426.2772926	40.35152838	235.0545852	226.0676808
29.41860465	7.162790698	15.72093023	8.69226609
10.52941176	1.588235294	2	0.325259516
43.1686747	5.168674699	6.795180723	3.700972565
202.0860656	30.99180328	6485.729508	6415.828944
43.52307692	11.61538462	40.26923077	30.65923077
235.3361345	39.28235294	1756.536134	1727.701942
21.16393443	4.540983607	63.42622951	48.07524859
282.8532819	60.42342342	47671.82497	47460.09812
58.37681159	7.971014493	22.77536232	16.88243016
163.5608466	18.56613757	21.16931217	15.84762465
132.6425856	11.63117871	5.771863118	2.739232893
56.94915254	7.694915254	66.69491525	57.69491525
34.77777778	7.377777778	185.0222222	158.2128395
100.1875	22.6171875	616.4414063	584.1381683
448.4726563	54.3359375	7299.859375	7256.072205
191.0445434	39.45434298	7062.47216	6980.143819
52.89795918	18.7755102	2885.295918	2802.634423
6.263157895	1.526315789	15.73684211	7.95567867
127.5660377	18.03773585	188.4339623	175.3104308
25.70175439	3.771929825	6.333333333	2.939981533
160.2993348	30.17073171	3815.660754	3756.565228
13.54166667	3.791666667	18.39583333	9.520399306
146.3875	21.425	1052.7125	1021.3525
219.6598778	36.36456212	1209.448065	1181.429262
45.1641791	11.46268657	103.6940299	85.78976387
177.7952756	41.35826772	23127.08465	22934.10442
280.7270195	63.49303621	44456.07799	44228.39595
704.9158031	115.6411917	29750.92034	29662.49457
96.67326733	10.12871287	15.82178218	10.94686795
65.71328671	9.27972028	54.76923077	44.60071397
14.35714286	5.964285714	47.42857143	31.42857143
90.21777778	15.74666667	169.8622222	153.3966617
187.9927007	26.04622871	2822.49635	2779.212117
19.38596491	4.087719298	15.24561404	8.412434595
183.7159763	14.37278107	70.00887574	63.45951647
359.8179612	47.51699029	4883.707524	4847.925639
16.68	1.88	1.8	0.2624
210.8346154	39.06153846	8684.411538	8593.355902
276.4313409	47.07108239	1974.450727	1942.351821
37.06741573	6.842696629	80.3258427	63.50662795
106.1983471	14.43801653	20.45867769	14.53488491
9.384615385	2.384615385	5.307692308	1.609467456
10.16666667	1.666666667	1.25	0.076388889
274.9839744	36.1474359	2468.921474	2438.724336
45.54545455	9.054545455	30.4	22.76231405
64.12121212	7.636363636	82.77272727	72.84067952
152.3908873	33.0383693	4934.081535	4857.382583
224.254902	46.65062389	20913.03387	20747.3084
14.54285714	2.028571429	5.057142857	1.817142857
96.23766816	12.32735426	64.0896861	54.79128879
5.428571429	1.285714286	3.428571429	0.489795918
40.59139785	6.247311828	4.806451613	1.734535784
25.77647059	7.517647059	51.45882353	40.60761246

log.sigma.2.0.mm.3D_glszm_ZonePercentage	log.sigma.2.0.mm.3D_glszm_LargeAreaLowGrayLevelEmphasis	log.sigma.2.0.mm.3D_glszm_LargeAreaHighGrayLevelEmphasis	log.sigma.2.0.mm.3D_glszm_HighGrayLevelZoneEmphasis
0.296398892	0.675482373	6662.831776	87.3364486
0.138987883	7.668954047	386228.2718	143.8820513
0.282485876	0.375133083	3969.56	89.16
0.290222452	1.610622091	100668.6114	246.4705882
0.514851485	0.083947588	1064.153846	190.1730769
0.286885246	1.373458124	3712.8	65.41428571
0.685714286	0.037785244	658.625	217.1666667
0.391521197	0.239440591	3149.394904	117.4140127
0.416666667	0.171037464	5693.835772	313.4699187
0.150927124	11.68187156	300441.5143	157.2314286
0.313829787	0.449375507	3875.457627	92.61016949
0.207397622	2.480229706	27558.71338	94.2611465
0.226939971	4.562278859	42153.6	113.9612903
0.251893939	2.341219359	11565.95489	111.4962406
0.502747253	0.073960261	2021.404372	239.568306
0.0599372	564.7523246	6500660.929	110.385208
0.414746544	0.337903967	1174.722222	107.8777778
0.165592784	5.649240742	104658.4241	96.09338521
0.43956044	0.217822249	1851.8	149.7875
0.064888248	66.56981682	1032781.456	92.46111111
0.092610837	82.23135915	1739616.266	106.4446809
0.316831683	0.817137882	15180.06597	147.7222222
0.103424658	52.37112138	522444.1523	75.46688742
0.616541353	0.043125633	1225.865854	255.6463415
0.161458333	5.676967772	110058.7484	83.89677419
0.258278146	0.566426258	9963.487179	94.1025641
0.128440367	28.63289771	294372.8367	104.1360544
0.089345315	83.66190798	1866126.948	96.23850575
0.126749193	23.27272544	1604869.013	187.2186837
0.128765505	13.24458761	383874.6239	97.89908257
0.100690312	55.88806068	1215034.778	114.1347518
0.079248274	198.6878956	3119020.543	105.4285714
0.239740821	2.135885806	77755.39039	163.006006
0.933333333	0.111364589	106.2142857	84.78571429
0.477876106	0.155927852	1159.611111	161.8703704
0.22113943	2.786660125	45659.2	99.30847458
0.075175609	169.6416395	3986003.958	89.0695825
0.142480211	18.0424125	524341.9514	185.6828704
0.445482866	0.155439059	1362.72028	157.0629371
0.238361266	1.119604539	18045.77344	93.1796875
0.218181818	0.987134342	78278.40385	218.4230769
0.320209974	0.370253278	6504	129.9672131
0.285194175	0.530593863	15180.99574	143.1617021
0.357630979	0.356219912	5258.821656	145.7515924
0.416143498	0.281208331	4919.032328	202.8168103
0.056844169	389.2985266	3059745.546	60.4683908
0.333576111	1.293416474	45093.75328	284.308952
0.377192982	0.24563073	1534.104651	90.72093023
0.772727273	0.294995047	129.7058824	86
0.568493151	0.191630358	457.0481928	128.0843373
0.119607843	44.26383468	960445.6434	131.432377
0.322580645	0.669191296	4473.915385	60.22307692
0.186228482	3.624850632	855994.1277	384.4369748
0.255230126	0.743410907	6371.032787	102.4590164
0.068724571	312.1592203	7328996.409	124.3513514
0.411940299	0.18651587	3855.724638	143.0652174
0.433486239	0.133451295	4187.862434	253.1402116
0.574235808	0.057043281	1217.547529	253.8859316
0.333333333	0.317952221	15708.64407	170.2372881
0.193133047	3.523478335	11960.02222	85.14444444
0.175945017	2.919869454	136018.6836	121.234375
0.151121606	31.38365282	1708583.975	222.0126953
0.110211095	67.05068037	753893.2205	118.8374165
0.109988777	16.57091313	509658.2755	123.5510204
0.358490566	0.485601368	941.1578947	66.78947368
0.276041667	1.009552454	37986.02516	195.3301887
0.542857143	0.154480635	547.3333333	98.38596491
0.130083646	16.34728926	900970.4146	165.5432373
0.335664336	0.378230228	1351.8125	75.02083333
0.178571429	5.417662932	209275.7938	138.278125
0.188918815	6.159641758	243110.2098	146.2912424
0.23633157	1.391975159	10386.21642	66.47761194
0.071985263	152.7800111	3531881.451	102.992126
0.066272845	293.5413905	6781600.54	110.9164345
0.106343412	110.6304732	8038359.883	296.5569948
0.452914798	0.108656313	3236.366337	230.460396
0.313596491	0.525818102	7241.755245	119.8391608
0.25	0.91866369	3386.375	59.03571429
0.246440307	0.98278324	33596.71111	129.5644444
0.151997041	14.38395836	559511.6715	159.7347932
0.382550336	0.163574831	2047.403509	115.4561404
0.390751445	0.216883277	24518.24556	319.9556213
0.167173869	4.370786014	5464496.271	1034.118932
0.806451613	0.095385653	155.08	95.84
0.104796453	69.96079416	1096438.581	110.1673077
0.176504135	11.07519594	358587.2423	205.2487884
0.243835616	0.886299611	8762.213483	92.80898876
0.410865874	0.232201584	2628.768595	151.6942149
0.52	0.144188861	487.3076923	101.2692308
0.923076923	0.122361561	110.3333333	90.08333333
0.181977253	3.068477719	1992612.59	637.6025641
0.361842105	0.250987256	5140.945455	90.98181818
0.317307692	0.427347433	17428.06818	197.5757576
0.114184009	24.73187327	993935.4053	133.4148681
0.077679313	165.0056037	2680267.412	100.6613191
0.555555556	0.090834016	726.4571429	151.5428571
0.327941176	0.430538003	10878.26906	207.3991031
0.583333333	0.384210791	216	62.57142857
0.570552147	0.044321529	969.4193548	219.9784946
0.303571429	0.447544166	7284.282353	80.35294118

log.sigma.2.0.mm.3D_glszm_SmallAreaEmphasis	log.sigma.2.0.mm.3D_glszm_LowGrayLevelZoneEmphasis	log.sigma.2.0.mm.3D_glszm_ZoneEntropy	log.sigma.2.0.mm.3D_glszm_SmallAreaLowGrayLevelEmphasis
0.65862206	0.060075697	5.273695259	0.049495385
0.640278154	0.0165342	5.477159062	0.012471043
0.628785987	0.047105696	4.719079571	0.042058101
0.725224366	0.013569375	6.030254019	0.010314447
0.690889271	0.027978362	4.794297113	0.025337852
0.678365782	0.097995619	4.737484132	0.078354217
0.749131944	0.028198792	4.678508855	0.026833422
0.681643753	0.027822242	5.347093938	0.020813255
0.670700521	0.009920284	6.36881949	0.007196452
0.678335926	0.017305023	5.777009218	0.013179926
0.615251097	0.035094189	5.316148507	0.023565698
0.645812473	0.038589514	5.483239913	0.024397082
0.691947979	0.027727214	5.439741386	0.020759905
0.745698214	0.041353874	5.16163834	0.035099406
0.728939758	0.013871359	5.582931463	0.011972934
0.68821777	0.033927047	5.619207112	0.026157436
0.675259329	0.037312311	5.193032508	0.027444926
0.609063282	0.027802256	5.692831427	0.019221421
0.73937768	0.03281343	5.070513921	0.027789792
0.624543963	0.02915082	5.382313533	0.021349609
0.64933692	0.02047407	5.552660433	0.015178134
0.709158452	0.019638897	5.602471847	0.015884488
0.701009451	0.05113074	5.323314987	0.04030827
0.750608806	0.021348921	5.278241109	0.018906611
0.634847025	0.036380633	5.308384104	0.028688651
0.547254461	0.033509095	5.094455386	0.025331902
0.660738912	0.031266778	5.633443424	0.019593205
0.629717009	0.025477351	5.64814705	0.019588122
0.64258119	0.013413549	5.997383762	0.008348561
0.588319538	0.028175351	5.664330633	0.021485047
0.68781654	0.023271062	5.617111781	0.017881054
0.651859027	0.021817659	5.606780149	0.014665341
0.674068591	0.018084173	5.809778362	0.013162987
0.946428571	0.109221732	3.521640636	0.108686018
0.640629922	0.043012315	5.106377317	0.036141332
0.645373142	0.031651544	5.641583541	0.022990733
0.595102711	0.030590327	5.89353184	0.022512484
0.724851004	0.014004085	5.645705668	0.010887654
0.644330647	0.027694482	5.691882943	0.022462874
0.599865025	0.044287533	5.463278786	0.036545278
0.676412612	0.012821104	5.416772689	0.010891439
0.664950839	0.024236164	5.190967384	0.01859283
0.622580647	0.024874114	5.888493945	0.018323888
0.686416619	0.029843258	5.417152577	0.023496881
0.714501917	0.015032793	5.905399201	0.009149078
0.618546957	0.038121506	5.080468587	0.025125171
0.709295238	0.013479872	6.27896484	0.009754587
0.603790652	0.033468558	5.180235505	0.024638823
0.815359477	0.093573478	3.690116518	0.045832465
0.745005193	0.052506903	4.988369745	0.045037253
0.668929424	0.027152767	5.825366125	0.019053563
0.598264619	0.06796893	5.313059426	0.044097903
0.652826471	0.005057427	5.940656559	0.003887069
0.604586496	0.038630319	5.028777501	0.031544955
0.627240675	0.020690074	5.965113635	0.014689964
0.674580565	0.030497727	5.581102947	0.024329085
0.683103793	0.011974873	6.008642824	0.00885006
0.736111256	0.016519164	5.842895029	0.013952267
0.721209109	0.020863995	5.258229784	0.017127136
0.644216755	0.066659524	5.186410937	0.042694072
0.65004281	0.017358082	5.398511219	0.013529824
0.688180479	0.010518909	6.115756554	0.007382895
0.67814881	0.022296987	5.510057258	0.01620407
0.531787026	0.017407926	5.738306896	0.011920166
0.580730994	0.101725972	4.037401198	0.091927049
0.65843513	0.018480741	5.911352702	0.011465143
0.694965616	0.069280405	4.936058164	0.062872665
0.61927673	0.013016373	6.006415009	0.008710172
0.541630657	0.054587465	4.970175521	0.04181726
0.702783894	0.020502066	5.610668688	0.015979702
0.69497145	0.014278327	5.627272832	0.010901963
0.600423003	0.056781325	5.354987236	0.042792065
0.613780819	0.022024562	5.847167702	0.015398145
0.649959841	0.01809898	5.657534033	0.012641818
0.702573777	0.006020556	5.86178764	0.00440148
0.71783554	0.020273642	5.683091989	0.017444676
0.702159557	0.035127747	5.413626894	0.023760583
0.511522576	0.056391231	4.943702637	0.041446751
0.658321852	0.026841685	5.657515606	0.01828919
0.703093772	0.020480052	5.743642318	0.016460377
0.601821503	0.037939711	5.019477243	0.033739055
0.764273499	0.010188194	5.865972017	0.008708877
0.687201271	0.002315695	6.128799225	0.001969839
0.844444444	0.070770007	4.13366069	0.06506054
0.661719145	0.026413225	5.673380094	0.019007755
0.694980791	0.009169988	5.583074686	0.006643342
0.6657984	0.035331826	5.14974165	0.029697175
0.68782927	0.022224994	5.651323539	0.01391667
0.61258547	0.055809152	4.440636353	0.048550279
0.9375	0.119275141	3.084962501	0.118503536
0.689837112	0.004233633	6.057574291	0.003673424
0.667969291	0.032005613	5.109103606	0.02498016
0.722613947	0.020195561	5.463012874	0.017359562
0.627848424	0.015413679	5.809474326	0.011070113
0.657163629	0.022356346	5.6576304	0.015262699
0.652075154	0.053808223	4.76485766	0.049356644
0.682844966	0.014679967	5.715328452	0.011101618
0.551587302	0.099190827	3.807354922	0.030691568
0.685282092	0.017977284	5.155253172	0.015309269
0.56180468	0.038151565	5.127111895	0.029542856

log.sigma.2.0.mm.3D_ngtdm_Coarseness	log.sigma.2.0.mm.3D_ngtdm_Complexity	log.sigma.2.0.mm.3D_ngtdm_Strength	log.sigma.2.0.mm.3D_ngtdm_Busyness	log.sigma.2.0.mm.3D_ngtdm_Contrast
0.022686044	0.699866725	2.772784087	0.245918078	0.000326068
0.005763887	0.106847045	0.962467971	0.500368248	3.61E-05
0.043676264	0.979613617	4.354311266	0.126058991	0.00051641
0.004018634	0.615022252	1.802132192	0.374007821	4.01E-05
0.0501825	3.289951059	4.839767446	0.133606864	0.003231296
0.028825804	0.526908954	1.455300416	0.385246596	0.000600125
0.060420684	8.11829572	7.542394926	0.08172571	0.007352582
0.017738525	1.055360696	2.588979065	0.293693345	0.000393002
0.004705685	1.235702653	1.87829266	0.483791279	0.000183745
0.003648782	0.189364418	1.491245138	0.569177183	1.08E-05
0.017501703	0.661908163	1.760320048	0.341943467	0.000314612
0.010758921	0.30002861	1.491468194	0.437857602	9.20E-05
0.005630042	0.17503932	0.616933333	0.831062284	5.30E-05
0.013711727	0.646017526	2.123830716	0.335978633	0.0001358
0.018667416	2.412354676	4.562767306	0.135242178	0.000435158
0.000531953	0.015019279	0.249603466	4.963993716	6.94E-07
0.028058426	1.850797118	3.608025078	0.236004336	0.000753058
0.004689649	0.136030517	0.680645415	0.844988987	3.34E-05
0.036228824	2.507979162	4.084848137	0.13453734	0.001134433
0.002900308	0.036308201	0.46527623	1.334683584	8.68E-06
0.001492998	0.047147022	0.728559093	1.565785496	2.57E-06
0.007968228	0.586357749	1.799671529	0.397548246	9.08E-05
0.002793292	0.043853723	0.460993588	1.423740748	7.76E-06
0.041887781	7.85835353	11.28659576	0.065467016	0.002644942
0.008924017	0.147545926	1.426441081	0.405820418	3.71E-05
0.025182874	0.527362082	2.57245295	0.221374085	0.00035472
0.003521133	0.138508133	1.185531162	0.807578385	9.45E-06
0.001621679	0.065537185	1.003601711	1.23362656	2.63E-06
0.00183898	0.119684715	1.032042115	0.753979264	6.15E-06
0.004444706	0.110077876	0.941368784	0.623339132	1.95E-05
0.001757655	0.075154129	0.843146524	1.168419518	3.14E-06
0.000803061	0.015620618	0.168176779	3.914201022	1.83E-06
0.005555445	0.384230735	1.499073589	0.427499826	4.98E-05
0.167475758	16.91039307	13.25073253	0.076682536	0.082280905
0.057738037	5.239686199	9.87192768	0.080170218	0.002297985
0.005234262	0.21824645	0.736002774	0.798411221	6.07E-05
0.000888865	0.025442525	0.33717989	2.771748494	2.48E-06
0.002626308	0.131243124	0.863458213	0.779093913	8.75E-06
0.018620423	1.942228019	3.216542717	0.246766907	0.00065256
0.014749256	0.379596457	1.582719379	0.337473036	0.000212168
0.012472375	0.416760291	2.412205892	0.178488848	7.50E-05
0.017831085	0.993929321	2.53916164	0.227368471	0.000293458
0.008229335	0.786212793	2.210255886	0.331761466	0.000104591
0.015488523	1.171427948	3.564765172	0.229116686	0.000262231
0.005973694	1.151873286	2.273292289	0.424899748	0.000130985
0.000977702	0.0128025	0.188496374	4.35886917	1.69E-06
0.002570595	0.830077612	2.166112487	0.535844083	3.59E-05
0.027547533	0.915146004	2.150238026	0.327838198	0.000748266
0.13424155	12.31035122	11.39757161	0.087358041	0.035087139
0.033082186	3.110069914	3.740474097	0.284799384	0.002743875
0.001793971	0.080622024	0.669904562	1.236903212	5.61E-06
0.015540755	0.440450795	1.103862626	0.644236697	0.000546399
0.002451154	0.21833622	1.441466329	0.40492133	8.77E-06
0.031177985	0.659947366	2.642054101	0.211630094	0.000437127
0.000537322	0.019158948	0.351665897	4.04989853	9.53E-07
0.02126162	1.988648907	4.203097627	0.153759956	0.000457807
0.008169742	1.348575109	2.830978208	0.242474472	0.000144642
0.013276024	3.012503783	3.509347384	0.208270125	0.000701025
0.019244869	1.051354207	3.688065668	0.137121043	0.000271525
0.016578445	0.442750674	2.303228896	0.389944905	0.000145635
0.00569393	0.15986028	0.928404555	0.527257344	3.91E-05
0.001088506	0.089352404	0.581069431	1.31147866	4.01E-06
0.001816366	0.041939904	0.389591352	1.830809795	4.81E-06
0.004325005	0.057799743	0.632024903	0.853706426	2.24E-05
0.109437489	2.222318733	8.41888096	0.105373094	0.003152621
0.007236971	0.539726685	1.9248205	0.305488288	6.95E-05
0.046426768	4.731504989	6.058284705	0.149499359	0.002901913
0.002115271	0.104238562	0.78116036	0.906028104	1.00E-05
0.043515657	1.401592264	3.198876041	0.202475265	0.001043131
0.004633137	0.183518274	1.112994024	0.495121453	2.32E-05
0.002938372	0.123266216	0.656213199	0.824090972	1.65E-05
0.013519484	0.20463952	0.782352602	0.775077989	0.000285941
0.001014326	0.020646246	0.276413265	2.669859095	2.34E-06
0.00057178	0.017411912	0.292120795	4.038787982	9.24E-07
0.000467918	0.044784641	0.534539365	2.221099017	6.37E-07
0.013707159	2.383061532	3.412043164	0.183129635	0.00042809
0.017078642	0.737168157	2.270929516	0.258490639	0.000234648
0.027886004	0.6870435	1.991875283	0.357836038	0.00054027
0.008813711	0.414498074	1.696467512	0.334986674	8.79E-05
0.002927167	0.162758867	1.155215272	0.631333732	1.10E-05
0.041588389	1.695894114	4.077718876	0.146737008	0.001279052
0.008443585	1.520785226	2.96677815	0.179883969	0.000169545
0.001446046	0.187280901	1.457945867	0.364712925	4.73E-06
0.130045255	11.72584881	12.59148132	0.074539886	0.01743107
0.001534102	0.049682468	0.511944269	1.712771415	3.55E-06
0.002225874	0.103094028	0.518111668	0.995455499	9.01E-06
0.019487458	0.566564428	1.905005977	0.310243084	0.00027507
0.010632642	1.076395758	2.012311233	0.375063986	0.000272451
0.093744547	3.970102328	6.60514967	0.10507572	0.00677757
0.146286572	15.18817883	14.65651131	0.133835341	0.222054196
0.001975441	0.314838888	2.342084881	0.304006866	7.35E-06
0.022202311	0.725934757	2.189638975	0.259973202	0.000555803
0.01891205	1.37605706	4.186420253	0.119089567	0.000217903
0.002222001	0.059734437	0.563368692	1.042497329	7.25E-06
0.000923444	0.026994482	0.318575006	2.867152432	1.89E-06
0.100444128	7.805211512	14.93333656	0.053081784	0.005788992
0.01066575	0.93783662	2.00980492	0.285024766	0.000196502
0.18061351	6.281019621	9.97886431	0.094644178	0.019831829
0.033564916	3.011305865	5.037572403	0.115295849	0.001537277
0.027999615	0.530005064	2.489472987	0.208771141	0.00043399

log.sigma.1.5.mm.3D_gldm_GrayLevelVariance	log.sigma.1.5.mm.3D_gldm_HighGrayLevelEmphasis	log.sigma.1.5.mm.3D_gldm_GrayLevelNonUniformityNormalized	log.sigma.1.5.mm.3D_gldm_DependenceEntropy
11.86146515	111.7506925	0.112683297	6.041348498
6.287130368	232.4647185	0.230184377	5.942748355
8.247119282	119.6271186	0.110791918	5.594010305
19.69111591	370.250388	0.11739229	6.636604751
11.95745515	124.8217822	0.08440349	5.143677949
8.585998388	80.69672131	0.103130879	5.893886867
32.06959184	295.9	0.066938776	4.73287436
15.92614474	194.925187	0.077729616	6.029970939
34.58642159	363.0406504	0.052566447	6.744819433
6.984421212	112.1552393	0.167110253	6.176195831
8.792213671	96.63297872	0.102011657	6.0312339
8.969295819	128.1453104	0.12765924	6.182132007
7.129131126	152.8030747	0.132222839	6.266316503
7.272368572	117.125	0.123199323	6.050415008
21.18399801	293.1620879	0.071670088	5.970598234
2.079022644	177.1939416	0.367938566	5.55691885
11.6528701	69.18894009	0.090063497	5.567456749
5.853839658	168.7944588	0.159631071	6.076267915
15.35216157	133.5879121	0.079217486	5.802349229
3.218959891	141.5908435	0.263565168	6.024177989
3.608054551	164.4104433	0.272757621	5.75259617
11.61126784	145.8294829	0.098865882	6.164307263
3.096209303	95.49965753	0.255783918	5.952147647
31.53609588	263.8120301	0.057154164	5.614508684
4.573566623	128.084375	0.204819878	5.830632149
7.725944038	123.7516556	0.119994737	5.704223072
5.246565096	100.9768458	0.201917122	6.092763195
3.039028279	145.3250321	0.337801368	5.677411487
6.641287827	277.5696986	0.270423711	6.045158039
4.918054921	133.1305375	0.244015436	5.865239355
3.804034371	118.0511783	0.243396838	5.822171442
2.548660082	168.4983584	0.270790893	5.920177545
12.68683749	230.6796256	0.139828986	6.349342206
21.58222222	83.46666667	0.093333333	3.506890596
22.86161798	159.9380531	0.070874775	5.515777666
8.786569234	140.7436282	0.130126841	6.226011486
3.563231256	170.4752653	0.347480441	5.507390052
6.91992711	194.7328496	0.193622904	6.14212629
16.08870256	130.7320872	0.072738036	5.906601674
10.17001134	151.5009311	0.118122961	6.02860169
8.37530246	300.9048951	0.156930901	6.206475236
11.62445836	183.6404199	0.090554626	5.912763969
13.77600976	144.7451456	0.089914813	6.26842353
14.60546593	138.6309795	0.088464672	6.014403412
24.1612548	214.626009	0.066297734	6.417902992
1.628431012	126.4274747	0.316882221	5.465927545
28.82938674	359.4705025	0.077093663	6.578510359
10.35524392	113.627193	0.092797784	5.80410907
15.84504132	62.95454545	0.123966942	3.913977073
19.49150873	107.1575342	0.065959842	5.521988107
6.009468474	166.5284314	0.22212959	6.073268215
11.06745316	96.68734491	0.10629337	5.996793237
9.427579184	586.8707355	0.157366484	6.289809897
8.289035556	114.1464435	0.109644439	5.815740918
3.125264544	175.0074297	0.31759418	5.64195998
20.12335932	205.4895522	0.073504121	6.058253541
22.54513509	261.0917431	0.067534298	6.266373929
35.65610877	377.8253275	0.047872848	6.041438639
10.68490057	220.6073446	0.119314373	5.93987683
6.840649119	89.93991416	0.127981727	5.799283413
7.739181162	163.2597938	0.140293572	6.07939935
8.194828347	253.1385773	0.229466472	6.041130262
3.667944933	150.9381443	0.229228227	6.015634833
4.53638801	192.46633	0.247229749	5.96456781
8.354574582	69.96226415	0.115699537	4.499252238
13.975332	218.2022569	0.10612582	6.406122423
24.3261678	177.1428571	0.068117914	5.361492184
7.050569426	209.5483127	0.200780626	6.076339804
9.928994083	102.3846154	0.091887134	5.37110281
8.319623675	267.0770089	0.168701172	6.277812599
6.994248979	180.1612159	0.155863215	6.274904703
8.741306857	101.7089947	0.102824669	5.966179936
3.166271071	143.6813093	0.30334446	5.742097609
2.568905437	146.4094517	0.296209562	5.602064565
5.33904441	268.7779461	0.227780555	5.97409484
25.6393905	345.5358744	0.063634901	6.178228796
12.15625962	189.5833333	0.085526316	6.04299844
8.070711097	89.55357143	0.102200255	5.507702956
10.3949667	176.5027382	0.105225842	6.251163382
7.385621865	185.0417899	0.221647581	6.091150774
13.69631999	131.4697987	0.081212558	5.227765354
26.16907481	368.3976879	0.077004912	6.489273055
10.98549445	2113.23311	0.203622418	6.198019816
15.42559834	112.2258065	0.115504683	4.171465262
4.237510846	141.7629988	0.235450122	6.018654457
6.117945867	138.778158	0.149842724	6.262011147
7.846395196	145.1534247	0.113004316	5.760515039
16.13859063	167.8590832	0.074186919	5.976299181
12.2356	104.78	0.0968	4.66346519
22.63905325	127.3076923	0.100591716	3.39274741
11.36231996	1182.263342	0.180592754	6.321903105
12.63130843	198.7203947	0.101086392	5.9070104
14.20374908	152.8317308	0.122434357	6.197190301
5.41849535	159.5399781	0.215882998	6.08302429
3.233420973	119.7310994	0.25206926	5.768803828
25.35046611	155.2380952	0.076845553	4.845784487
15.4532526	217.9294118	0.086946367	6.33109213
12.55555556	82	0.125	3.970175521
16.2970379	260.2699387	0.072227032	5.612630612
8.50872449	105.25	0.123163265	5.777882061

log.sigma.1.5.mm.3D_gldm_DependenceNonUniformity	log.sigma.1.5.mm.3D_gldm_GrayLevelNonUniformity	log.sigma.1.5.mm.3D_gldm_SmallDependenceEmphasis
44.08587258	40.67867036	0.2839785
89.79686386	322.9486814	0.14747438
27.57627119	19.61016949	0.253358127
211.8608381	226.9192964	0.288040736
28.94059406	8.524752475	0.355426293
34.39344262	25.16393443	0.300460273
31.37142857	4.685714286	0.665277778
70.0872818	31.16957606	0.335821191
309.3279133	77.58807588	0.388086651
180.1099612	387.5286762	0.186222503
55.77659574	38.35638298	0.248623535
81.71598415	96.63804491	0.249152668
129.0673499	180.6163982	0.230674103
57.76893939	65.04924242	0.245695431
85.52197802	26.08791209	0.41981282
703.9444034	3984.038788	0.071019456
44.61290323	19.5437788	0.362295709
123.7152062	247.7474227	0.174837051
39.30769231	14.41758242	0.440692672
157.8125451	731.1297765	0.089311974
310.0746798	1384.244926	0.114746157
131.2024202	89.86908691	0.304740375
165.4321918	746.8890411	0.113996076
44.11278195	7.601503759	0.572552214
72.66041667	196.6270833	0.169470436
37.70860927	36.2384106	0.24706491
160.7413718	462.1882918	0.163506943
227.7573813	1315.736329	0.091015129
220.6948332	1004.89451	0.127770578
108.746013	413.1181335	0.132653838
269.0471316	1022.510117	0.123461292
520.8000679	2391.895958	0.09191382
126.4283657	194.2224622	0.233239135
9.133333333	1.4	0.8
27.9380531	8.008849558	0.446825146
129.0224888	173.5892054	0.228041699
469.4304289	2324.991631	0.079674256
211.9129288	587.0646438	0.17388293
75.09968847	23.34890966	0.382156999
59.09683426	63.4320298	0.23395529
66.4965035	112.2055944	0.224721667
56.7847769	34.50131234	0.291516477
116.1092233	74.08980583	0.282819335
73.11845103	38.83599089	0.311760895
224.5443946	73.92197309	0.386250667
404.305129	1939.952957	0.084246673
445.2483613	211.6991988	0.337790598
38.33333333	21.15789474	0.299770221
6.727272727	2.727272727	0.480429293
39.52054795	9.630136986	0.42555175
270.6093137	906.2887255	0.147824994
51.87344913	42.83622829	0.26655554
264.5073552	502.7859155	0.196597723
34.58995816	26.20502092	0.284733255
743.9386167	3590.719795	0.093107798
64.02089552	24.6238806	0.394320619
193.0091743	58.88990826	0.412337323
159.2183406	21.92576419	0.584808345
50.31073446	42.23728814	0.289576981
60.20600858	59.63948498	0.215876282
127.7037801	204.1271478	0.190785209
476.8190673	1554.864817	0.156524194
264.9376534	933.8757977	0.13471341
119.006734	440.5634119	0.115890654
11.98113208	6.132075472	0.41514643
141.4982639	122.2569444	0.264833186
33.60952381	7.152380952	0.567547619
241.4234208	696.1064321	0.15272571
29.93706294	13.13986014	0.377274642
139.5145089	302.3125	0.186690253
223.3439785	405.0884956	0.205563934
76.31216931	58.3015873	0.249187079
437.3769307	2140.701856	0.080871446
727.881115	3209.134392	0.095151052
1001.627178	3307.145878	0.130825827
104.6547085	28.38116592	0.433214485
80.26315789	39	0.357118248
34.80357143	22.89285714	0.276082526
107.3066813	96.07119387	0.255238936
170.0717456	599.3350592	0.155368318
41.2147651	12.10067114	0.377995899
146.8196532	66.60924855	0.366961269
361.2511666	1003.6549	0.175615844
8.741935484	3.580645161	0.507455197
304.2648126	1168.303507	0.132129223
326.7673225	525.4984317	0.20829112
49	41.24657534	0.28308589
128.663837	43.69609508	0.390175265
12.88	4.84	0.410513719
9.615384615	1.307692308	0.884615385
263.7524059	619.2525518	0.196962752
45.83552632	30.73026316	0.289975016
54.52884615	50.93269231	0.315201061
231.9704272	788.4047097	0.13273796
473.2295763	1820.444198	0.112711433
18.11111111	4.841269841	0.49680776
101.9823529	59.12352941	0.320939336
6.583333333	3	0.455439815
44.79141104	11.77300613	0.476749266
38.53571429	34.48571429	0.248141222

log.sigma.1.5.mm.3D_gldm_DependenceNonUniformityNormalized	log.sigma.1.5.mm.3D_gldm_DependenceVariance	log.sigma.1.5.mm.3D_gldm_LargeDependenceEmphasis
0.122121531	14.20668964	35.7867036
0.064003467	30.2920897	93.8253742
0.155798142	5.962335217	19.40677966
0.109602089	25.26010916	58.13398862
0.286540535	1.345946476	7.03960396
0.140956732	6.607565171	20.63934426
0.448163265	0.533877551	2.914285714
0.174781251	6.418517298	17.13965087
0.209571757	6.472486615	15.35094851
0.077667081	23.96530601	67.23889608
0.14834201	11.60083748	28.20212766
0.107947139	14.88962026	39.31175694
0.094485615	19.32368395	50.49048316
0.10941087	12.80060549	35.83333333
0.234950489	1.9034235	7.774725275
0.065011489	23.15851644	141.1461027
0.205589416	2.290895963	9.608294931
0.079713406	21.22285843	62.80541237
0.215976331	4.518536409	12.49450549
0.056889886	27.00213487	116.1391492
0.061098459	26.53662856	109.448867
0.144337096	9.808014465	25
0.05665486	29.7777402	111.1109589
0.331675052	1.2187235	4.894736842
0.075687934	20.50551649	64.86666667
0.124862945	10.40375422	29.45033113
0.070223404	22.3563425	72.81039755
0.058474296	24.63810613	119.9247754
0.059390429	29.60868342	114.0645856
0.064232731	26.53745156	94.34435913
0.064043592	22.65084208	91.0623661
0.058960723	25.03539029	110.7314616
0.091021142	23.71991286	62.17062635
0.608888889	0.195555556	1.8
0.247239408	1.712741797	7.212389381
0.096718507	21.00360539	53.56371814
0.070158486	22.7655001	132.2557166
0.069892127	25.63765394	83.80343008
0.233955416	2.369697499	8.975077882
0.110049971	18.65518832	46.60521415
0.093002103	22.17895056	56.9972028
0.149041409	8.92975386	23.39370079
0.140909252	8.6390977	23.8907767
0.166556836	9.945226519	23.14578588
0.201385107	3.394220676	11.19910314
0.066041347	19.87845166	100.0163345
0.162144341	6.31999773	18.01310998
0.168128655	4.970683287	16.14035088
0.305785124	0.909090909	4.909090909
0.270688685	1.176956277	6.04109589
0.066325812	24.47688005	87.35245098
0.128718236	15.53539521	37.36724566
0.082787905	18.60729573	56.49045383
0.144727858	9.114581327	24.28870293
0.065800338	23.10874585	117.5075181
0.191107151	6.003653375	15.73432836
0.221340796	2.618797871	9.229357798
0.347638298	0.965122709	4.296943231
0.142120719	8.697787992	23.76271186
0.129197443	5.84308055	23.10300429
0.087768921	19.57692233	55.33814433
0.070368812	27.75953109	100.255608
0.065031334	21.3187675	80.61708395
0.066782679	25.0142049	90.74971942
0.226059096	2.876468494	9.754716981
0.122828354	11.64264745	31.16493056
0.320090703	1.684897959	5.8
0.069634676	25.07998102	84.56158062
0.20935009	3.706098098	11.72727273
0.077854079	23.03260523	67.99441964
0.085934582	19.10296158	54.50981147
0.134589364	7.62431063	23.66666667
0.061977743	26.28077677	130.5683718
0.067184892	21.59276942	105.2593687
0.068987339	19.65297364	78.40822371
0.234651813	2.819461481	9.103139013
0.176015697	4.620652508	14.48245614
0.155373087	5.799665179	18.49107143
0.117531962	12.66625318	33.95947426
0.062896356	30.50064719	100.3195266
0.276609162	1.045088059	6.22147651
0.169733703	8.696066023	21.03930636
0.073290965	22.94188761	74.94035301
0.281997919	2.399583767	7.64516129
0.061318987	25.44867669	95.30350665
0.093175741	14.21907011	42.99600798
0.134246575	11.36411334	29.14246575
0.218444545	2.313362408	9.016977929
0.2576	1.6864	7.64
0.73964497	0.130177515	1.461538462
0.07691817	25.6921778	76.64654418
0.150774758	7.835483033	21.84868421
0.131078957	17.9852071	40.36538462
0.063518737	26.54713287	94.51478642
0.065526111	22.03080973	93.93519801
0.287477954	1.477450239	6.206349206
0.149974048	12.31467128	28.36176471
0.274305556	1.472222222	6.916666667
0.274793933	1.565057021	6.36196319
0.137627551	8.239591837	24.7

log.sigma.1.5.mm.3D_gldm_LargeDependenceLowGrayLevelEmphasis	log.sigma.1.5.mm.3D_gldm_SmallDependenceHighGrayLevelEmphasis	log.sigma.1.5.mm.3D_gldm_LargeDependenceHighGrayLevelEmphasis
0.311866985	28.04419889	5066.254848
0.367075745	26.77840833	24309.26871
0.155119078	25.69122958	2937.084746
0.148356992	112.3452976	23418.7822
0.091297736	45.82410066	783.8712871
0.301996264	25.95737311	1814.172131
0.033821993	207.3414683	733.7571429
0.084837237	65.67777838	4197.86783
0.050479699	152.0657232	5541.463415
0.582792714	20.99112784	8190.557999
0.272695252	22.87515966	3599.707447
0.29877397	30.15157756	5701.313078
0.315425357	37.53198126	8387.308199
0.300840162	32.73940829	4626.352273
0.038105797	134.3386058	2134.634615
0.790798464	12.8890161	25406.9348
0.256219643	30.300932	588.8663594
0.326320066	26.15087357	12526.7674
0.124352752	63.32705488	1626.313187
0.777410354	10.76645173	17653.67015
0.628755763	16.44478286	19260.71586
0.176385657	49.0316723	3945.345435
1.085741124	9.773926148	11626.37568
0.035767494	145.0927047	1333.736842
0.453913178	16.3946201	9619.813542
0.199335393	21.27233567	4856.63245
0.716834293	18.27169214	7642.924421
0.797167844	10.42676782	18253.68395
0.38400837	29.22082835	34246.65689
0.646187311	13.35140564	14102.50916
0.721705686	13.4363195	11742.87812
0.629213098	14.3514247	19721.41356
0.247157535	53.80542729	16191.66451
0.158916835	72.56666667	127.0666667
0.08310392	76.47639461	1128.557522
0.335722523	30.39387313	9084.743628
0.740307891	9.860721694	23917.18488
0.419062184	38.36321867	17082.79354
0.114504439	54.35774032	1064.364486
0.258622943	27.41008611	9102.271881
0.178283906	62.5227301	18613.80839
0.11633909	53.80274885	5300.76378
0.159010935	38.97646583	4371.492718
0.183482289	45.76438842	4040.881549
0.072891562	98.97997171	2167.506726
0.76971195	9.226194467	13149.7999
0.057421567	143.284351	6151.589221
0.152331629	34.2423971	2304.723684
0.951724815	36.71717172	211.1363636
0.168691708	54.11123858	567.1027397
0.505389953	24.96504824	15407.34363
0.344249802	19.62273361	5258.900744
0.090221916	106.8135413	35821.55618
0.208688208	31.78828404	3214.330544
0.659510974	14.91805848	21166.61419
0.083754362	83.07606334	3954.253731
0.043889972	116.0600087	2418.566514
0.020307039	234.8248563	1522.030568
0.10530736	59.64245754	6033.375706
0.29506119	23.01835376	2116.030043
0.286543301	24.14021701	11226.72784
0.379082344	39.72969569	26873.03114
0.537802811	22.24921371	12286.18655
0.446414887	19.05797224	18748.28339
0.174434718	27.81929031	892.6792453
0.153450195	61.01280733	7132.44184
0.06352996	108.9882143	844.6857143
0.36308143	26.8648577	20123.10355
0.120591467	31.33856921	1506.622378
0.234273435	44.97624428	20189.71875
0.278157708	32.80415793	11180.38361
0.224672641	18.93320701	3190.647266
0.861856843	9.718448553	20059.21255
0.69302795	12.49228684	16186.05649
0.292960656	38.54963644	21211.88794
0.032342281	156.2178815	3361.91704
0.078151475	65.75368304	3146.434211
0.192381562	18.85965421	2283.705357
0.171510735	40.00949429	7397.998905
0.503716627	26.91843594	20327.15717
0.068000301	38.11225019	948.4899329
0.057314784	139.6341773	8512.448555
0.034960801	365.2061695	161815.8933
0.141693833	66.58645161	641.5806452
0.631972972	18.13364868	14653.05522
0.313344274	31.51376013	6149.452238
0.168122468	35.49033175	5590.643836
0.066727792	70.31628792	1576.619694
0.145552511	50.69841995	800.5
0.096069297	119.9230769	156.8461538
0.061632324	215.736844	96133.59784
0.101633769	43.11710662	5572.003289
0.271974331	53.87552452	6690.206731
0.547050162	18.68252196	16716.43921
0.742774558	12.44505139	12140.13043
0.088021126	74.83186067	1039.301587
0.141528375	80.39413875	6135.147059
0.196735213	40.03385417	479.5
0.036428444	126.2558668	1579.582822
0.232123785	18.76106134	3250.221429

log.sigma.1.5.mm.3D_gldm_SmallDependenceLowGrayLevelEmphasis	log.sigma.1.5.mm.3D_gldm_LowGrayLevelEmphasis	log.sigma.1.5.mm.3D_gldzm_DistanceZoneVariabilityNormalized
0.014277831	0.025703187	1
0.001979057	0.005881875	0.982340741
0.010589958	0.018899099	1
0.001851486	0.004123103	0.930360412
0.015920331	0.023320153	1
0.018593383	0.031934401	0.9753125
0.018236775	0.020149442	1
0.00639062	0.010651902	0.973514707
0.002706313	0.005194382	0.572954391
0.007491704	0.017970405	0.857065294
0.009561821	0.021422868	1
0.007304583	0.016133911	0.961367662
0.003260258	0.009078668	0.917813058
0.005628871	0.013766251	1
0.008369991	0.010813625	0.976881776
0.000704715	0.00616168	0.819764391
0.029099537	0.046606268	0.978725864
0.002432815	0.007828978	0.979023337
0.014494725	0.021266915	0.978497164
0.001541237	0.00847861	1
0.001561958	0.007154912	0.916284185
0.006637648	0.01300385	0.874817544
0.003630329	0.013933803	0.987879239
0.013262337	0.01544601	1
0.004279334	0.011521899	0.976611463
0.008661274	0.015526226	0.954048443
0.005350647	0.015175692	0.884006664
0.002131744	0.008628911	0.944521565
0.001301945	0.00460578	0.954048443
0.004007504	0.011286483	0.95865405
0.003266189	0.011386703	0.787370606
0.000969355	0.006587587	0.787911883
0.002919305	0.006812787	0.977656484
0.098361279	0.110472391	1
0.026068974	0.031376487	1
0.005078447	0.011908619	0.976542895
0.00182479	0.007547322	0.974705632
0.00171593	0.006558084	0.885588383
0.014250613	0.021923347	0.918515165
0.008358761	0.014195658	0.945620813
0.002533243	0.00523068	0.988095665
0.005575497	0.010208742	0.98449707
0.00885083	0.015967422	0.926845841
0.011936275	0.022649955	0.962278107
0.004758978	0.009483766	0.6743012
0.00127945	0.008755437	0.552996504
0.002054558	0.004453395	0.643561837
0.009392448	0.01856822	0.926035503
0.050122662	0.171800416	1
0.025800577	0.042496985	0.974363299
0.002182424	0.007966142	0.768310148
0.020607468	0.035888405	0.968008461
0.000780427	0.002182735	0.734180564
0.011289618	0.01880875	0.948753463
0.001342241	0.006844943	0.687031185
0.008403364	0.012848486	0.986395196
0.004719416	0.007653428	0.95695865
0.005178557	0.006948277	0.904396198
0.008873246	0.012348677	0.982302296
0.008453402	0.02036132	0.8872
0.00359372	0.009359456	0.863335353
0.001313481	0.004991163	0.919011007
0.001548427	0.007785395	0.782200981
0.001692032	0.006635905	1
0.031596436	0.044681028	1
0.003512764	0.00922486	0.941410219
0.018730293	0.022485313	0.971020761
0.001894708	0.006363653	0.822187259
0.018184134	0.025393406	1
0.001832851	0.005188509	0.92831227
0.002875554	0.008389187	0.888760331
0.012559115	0.022897133	0.914283366
0.001512809	0.008421269	0.975946823
0.00144917	0.007924066	0.572590237
0.000874285	0.004314855	0.606258082
0.004857479	0.007082852	0.946942149
0.005374335	0.009450828	0.945987654
0.013470841	0.023522963	0.972227733
0.005153852	0.010171278	0.953152
0.002567212	0.007628878	0.97803354
0.022534938	0.027782542	0.970594787
0.0038047	0.005832926	0.948753463
0.000294171	0.00068653	0.782110951
0.042236142	0.048447475	1
0.002131836	0.008915703	0.76946054
0.002938785	0.009604726	0.89400612
0.006537091	0.012199723	0.947406866
0.007143895	0.012222528	0.925761393
0.024955671	0.036387475	1
0.087054874	0.088857759	0.847222222
0.000517437	0.00119743	0.860159045
0.010522327	0.014598177	0.980771043
0.010380866	0.017460886	0.959476813
0.002410598	0.008573943	0.930862112
0.002343604	0.010496753	0.602660633
0.04120714	0.045668496	1
0.004175849	0.008283427	0.898333447
0.052981701	0.064093372	1
0.008796354	0.011243516	1
0.015128404	0.025873621	1

log.sigma.1.5.mm.3D_gldzm_LowIntensityEmphasis	log.sigma.1.5.mm.3D_gldzm_LargeDistanceEmphasis	log.sigma.1.5.mm.3D_gldzm_HighIntensitySmallDistanceEmphasis
0.048044233	1	96.43103448
0.013367251	1.048888889	169.7033333
0.038150201	1	98.85185185
0.006357516	1.108374384	368.1502463
0.03780119	1	130.4130435
0.060734434	1.0375	83.990625
0.024737965	1	306.245283
0.018273478	1.040268456	184.385906
0.006648525	2.034431138	262.6006737
0.043679232	1.31838565	97.19768311
0.038793136	1	87.20560748
0.03110634	1.0591133	116.0307882
0.014305646	1.179878049	156.9544588
0.022971498	1	133.2919708
0.018331493	1.035087719	311.1769006
0.010437229	1.908847185	164.2903134
0.073614694	1.032258065	79.19354839
0.014255981	1.03180212	140.6934629
0.031192193	1.032608696	137.5788043
0.018809289	1	113.3122363
0.014218438	1.344827586	128.7360086
0.0210904	1.201277955	144.2891374
0.034058359	1.018292683	79.90320122
0.021031089	1	257.6976744
0.025569591	1.035502959	89.23668639
0.033047148	1.070588235	81.61764706
0.033201365	1.234693878	102.723852
0.024598286	1.133522727	104.3316959
0.010214578	1.070588235	215.5387255
0.030746991	1.105042017	90.32971522
0.027929867	1.851272016	90.01469504
0.011060541	1.870063694	136.5315384
0.012402619	1.033898305	218.8658192
0.118151904	1	87.92307692
0.053707139	1	169.3571429
0.02208573	1.035608309	125.8998516
0.023685972	1.069343066	112.22776
0.010243962	1.417910448	207.2439293
0.034179203	1.127659574	140.3741135
0.032990095	1.083916084	109.1276224
0.011191979	1.017964072	267.9835329
0.01796783	1.0234375	174.3535156
0.030308508	1.114068441	123.3631179
0.039099105	1.057692308	135.1121795
0.012264321	1.731808732	190.9121766
0.015815071	3.011741683	76.30367852
0.006034832	2.203389831	309.4463623
0.030699353	1.115384615	102.2884615
0.119093822	1	73.07692308
0.056221846	1.038961039	119.9383117
0.015311464	1.83891547	140.1030848
0.076229825	1.048780488	67.68495935
0.003932102	1.891729323	438.6182435
0.037338409	1.078947368	102.8848684
0.015123923	2.730696798	128.2462994
0.020531878	1.020547945	201.4880137
0.010786539	1.06601467	269.7194377
0.008408453	1.151006711	380.8825503
0.028702563	1.026785714	200.1428571
0.040670154	1.18	100.5525
0.019023663	1.355263158	107.5788788
0.008573451	1.320603908	240.250706
0.011823626	1.602540835	149.8229104
0.014311657	1	157.9267241
0.071165308	1	66.8
0.014767822	1.105421687	221.3113286
0.030041513	1.044117647	187.2095588
0.01254904	1.65625	149.5574624
0.044162357	1	83.75806452
0.010069922	1.13960114	221.2404242
0.015362965	1.3	143.5873485
0.048714018	1.134615385	67.5849359
0.020158908	1.074523397	111.1225327
0.015930051	4.416179337	91.58006028
0.00696258	4.563824009	239.2111571
0.010532977	1.081818182	342.1352273
0.014487964	1.083333333	175.6666667
0.044999824	1.042253521	67.3943662
0.019992573	1.072	145.841
0.016640895	1.044345898	163.0442227
0.051741787	1.044776119	103.7350746
0.010018858	1.078947368	362.7368421
0.001609978	1.74864572	1845.293172
0.070976232	1	125.7368421
0.017039845	2.438914027	114.2879952
0.014561324	1.168449198	142.1948529
0.022804157	1.081081081	115.3153153
0.017754735	1.115830116	162.5202703
0.05232911	1	113.9615385
0.094960488	1.25	130.3333333
0.002525008	1.389270977	1008.151798
0.032479462	1.029126214	143.776699
0.032613418	1.062068966	157.9137931
0.018818794	1.307086614	127.1656201
0.021987121	3.669987547	79.42599163
0.073384003	1	152.25
0.012510187	1.161157025	240.5702479
0.100406672	1	88.92307692
0.016886191	1	262.8295455
0.057032961	1	72.95061728

log.sigma.1.5.mm.3D_gldzm_LowIntensityLargeDistanceEmphasis	log.sigma.1.5.mm.3D_gldzm_HighIntensityEmphasis	log.sigma.1.5.mm.3D_gldzm_DistanceZoneVariability	log.sigma.1.5.mm.3D_gldzm_ZonePercentage
0.048044233	96.43103448	116	0.32132964
0.01353625	171.7333333	221.0266667	0.160370634
0.038150201	98.85185185	54	0.305084746
0.006673614	380.3743842	566.589491	0.31505432
0.03780119	130.4130435	46	0.455445545
0.060956327	85.575	78.025	0.327868852
0.024737965	306.245283	53	0.757142857
0.018418737	187.3355705	145.0536913	0.371571072
0.009920604	389.6601796	382.7335329	0.452574526
0.045445262	111.4663677	382.2511211	0.192324278
0.038793136	87.20560748	107	0.284574468
0.031454674	118.6502463	195.1576355	0.268163804
0.015610388	162.679878	301.0426829	0.24011713
0.022971498	133.2919708	137	0.259469697
0.018396213	316.619883	167.0467836	0.46978022
0.015610823	179.6621984	611.5442359	0.068895456
0.073838708	80.35483871	91.02150538	0.428571429
0.014401526	142.4982332	277.0636042	0.182345361
0.03133712	139.4130435	90.02173913	0.505494505
0.018809289	113.3122363	237	0.085436193
0.01608	136.9982759	531.4448276	0.114285714
0.02211188	157.4153355	273.8178914	0.344334433
0.034162526	80.74695122	324.0243902	0.112328767
0.021031089	257.6976744	86	0.646616541
0.025750728	90.97633136	165.0473373	0.176041667
0.033360874	85.58823529	81.09411765	0.281456954
0.035094502	109.1964286	346.5306122	0.171253823
0.025608762	107.6164773	332.4715909	0.090372272
0.010503571	220.4196078	486.5647059	0.137244349
0.031429082	93.1302521	228.1596639	0.140578854
0.034097754	104.5949119	402.3463796	0.121637705
0.016650175	153.4585987	618.510828	0.088871278
0.012530261	221.1920904	346.0903955	0.254859611
0.118151904	87.92307692	13	0.866666667
0.053707139	169.3571429	56	0.495575221
0.022442431	126.9881306	329.0949555	0.252623688
0.024021536	114.2956204	534.1386861	0.081901061
0.012214569	219.1100746	474.6753731	0.176781003
0.036256954	144.5815603	129.5106383	0.439252336
0.033329183	114.3356643	135.2237762	0.266294227
0.011254138	269.2814371	165.011976	0.233566434
0.018071997	175.671875	126.015625	0.335958005
0.030713191	132.6539924	243.7604563	0.319174757
0.039294038	139.4679487	150.1153846	0.355353075
0.016432517	251.5051975	324.3388773	0.431390135
0.032202047	107.62818	282.5812133	0.083469454
0.030485825	420.1994018	645.4925224	0.365258558
0.031425849	107.5769231	72.23076923	0.342105263
0.119093822	73.07692308	13	0.590909091
0.056702846	120.7272727	75.02597403	0.52739726
0.019781019	164.4210526	481.7304625	0.153676471
0.076555838	69.61788618	119.0650407	0.305210918
0.005468246	529.7924812	488.2300752	0.208137715
0.037715244	107.0394737	72.10526316	0.317991632
0.024860307	156.5047081	729.6271186	0.093932425
0.020623202	202.6438356	144.0136986	0.435820896
0.010972057	276.4254279	391.396088	0.469036697
0.008949706	396.0436242	269.5100671	0.650655022
0.028785235	202.3125	110.0178571	0.316384181
0.042149752	106.62	88.72	0.214592275
0.020752086	120.5394737	262.4539474	0.208934708
0.009841858	249.19627	1034.806394	0.166174734
0.015642114	166.8076225	430.9927405	0.135247914
0.014311657	157.9267241	232	0.130190797
0.071165308	66.8	25	0.471698113
0.015283604	226.5421687	312.5481928	0.288194444
0.030194169	190.3970588	66.02941176	0.647619048
0.015324982	169.5520833	473.5798611	0.166137871
0.044162357	83.75806452	62	0.433566434
0.01050564	232.0968661	325.8376068	0.195870536
0.016878761	153.6163636	488.8181818	0.211619854
0.049388185	74.32051282	142.6282051	0.275132275
0.020731081	112.6239168	563.1213172	0.081762789
0.039462976	126.6052632	587.4775828	0.094701865
0.020582761	295.172189	1116.12113	0.126799366
0.01070489	352.2772727	208.3272727	0.493273543
0.014806125	182.05	170.2777778	0.394736842
0.045215403	69.46478873	69.02816901	0.316964286
0.020237223	151.148	238.288	0.273822563
0.016924171	164.556541	441.0931264	0.166789941
0.051896722	106.9701493	65.02985075	0.44966443
0.010181861	372.5350877	324.4736842	0.395375723
0.001959042	2055.445287	721.8884074	0.187259079
0.070976232	125.7368421	19	0.612903226
0.02620225	132.5882353	510.1523379	0.133615478
0.015604323	151.578877	668.7165775	0.21328771
0.023153198	120.0990991	105.1621622	0.304109589
0.018106327	172.5714286	239.7722008	0.439728353
0.05232911	113.9615385	26	0.52
0.101904933	132.5833333	10.16666667	0.923076923
0.002852147	1080.848693	625.3356259	0.212015165
0.032569358	146.1359223	101.0194175	0.338815789
0.032941004	160.9862069	139.1241379	0.348557692
0.020343012	133.6791339	472.8779528	0.139101862
0.043490431	107.5902864	483.9364882	0.111188037
0.073384003	152.25	36	0.571428571
0.013488108	248.8884298	217.3966942	0.355882353
0.100406672	88.92307692	13	0.541666667
0.016886191	262.8295455	88	0.539877301
0.057032961	72.95061728	81	0.289285714

log.sigma.1.5.mm.3D_gldzm_IntensityVariabilityNormalized	log.sigma.1.5.mm.3D_gldzm_LowIntensitySmallDistanceEmphasis	log.sigma.1.5.mm.3D_gldzm_IntensityVariability
0.075802616	0.048044233	8.793103448
0.076464198	0.013340243	17.20444444
0.092592593	0.038150201	5
0.045127682	0.006278492	27.48275862
0.074669187	0.03780119	3.434782609
0.0765625	0.060678961	6.125
0.065147739	0.024737965	3.452830189
0.058600964	0.018237163	8.731543624
0.041490373	0.005892736	27.71556886
0.075971767	0.043318151	33.88340807
0.078172766	0.038793136	8.364485981
0.071076707	0.031019256	14.42857143
0.069545657	0.014032626	22.81097561
0.069636102	0.022971498	9.540145985
0.050784857	0.018315313	8.684210526
0.08921217	0.009989165	66.55227882
0.073881374	0.07355869	6.870967742
0.082620585	0.014219595	23.38162544
0.065689981	0.031155961	6.043478261
0.090138689	0.018809289	21.3628692
0.090041617	0.014036433	52.22413793
0.066092335	0.02083503	20.68690096
0.092708953	0.034032318	30.40853659
0.050838291	0.021031089	4.372093023
0.107594272	0.025524306	18.18343195
0.100069204	0.032968717	8.505882353
0.075853811	0.032822125	29.73469388
0.095848399	0.024437789	33.73863636
0.066658977	0.01014233	33.99607843
0.090247864	0.03063887	21.4789916
0.089785961	0.02724727	45.88062622
0.089997972	0.010456135	70.64840764
0.055140605	0.012370709	19.51977401
0.088757396	0.118151904	1.153846154
0.054846939	0.053707139	3.071428571
0.071331085	0.021996555	24.03857567
0.082849379	0.023633903	45.40145985
0.063509412	0.010020222	34.04104478
0.057290881	0.033659765	8.078014184
0.078781359	0.032905323	11.26573427
0.0755495	0.011176439	12.61676647
0.067016602	0.017941788	8.578125
0.061255765	0.030207337	16.11026616
0.059664694	0.039050372	9.307692308
0.047376178	0.011416981	22.78794179
0.187365244	0.014023555	95.74363992
0.043447921	0.004170058	43.5782652
0.078238001	0.030517729	6.102564103
0.100591716	0.119093822	1.307692308
0.059537865	0.056101596	4.584415584
0.06708485	0.014779919	42.06220096
0.078722982	0.076148322	9.682926829
0.067782238	0.003680651	45.07518797
0.074792244	0.037244201	5.684210526
0.098692727	0.014296735	104.8116761
0.053762432	0.020509047	7.849315068
0.050101327	0.01074016	20.49144254
0.044074591	0.00827314	13.13422819
0.064732143	0.028681895	7.25
0.0854	0.040300254	8.54
0.085634522	0.018754013	26.03289474
0.056161644	0.008426284	63.23801066
0.088948982	0.011229748	49.01088929
0.091520511	0.014311657	21.23275862
0.0944	0.071165308	2.36
0.052892292	0.014650456	17.56024096
0.056228374	0.030003349	3.823529412
0.073067371	0.012232253	42.08680556
0.08792924	0.044162357	5.451612903
0.06702056	0.009985723	23.52421652
0.072700826	0.015135868	39.98545455
0.086538462	0.048545476	13.5
0.082549132	0.020087978	47.63084922
0.121444775	0.014472895	124.6023392
0.071194746	0.006197684	131.0695274
0.048595041	0.010489999	10.69090909
0.064753086	0.014408424	11.65555556
0.096607816	0.044945929	6.85915493
0.072	0.019931411	18
0.067507043	0.0165797	30.44567627
0.07017153	0.051703054	4.701492537
0.042628501	0.009978108	14.57894737
0.057048181	0.00156426	52.65547129
0.096952909	0.070976232	1.842105263
0.082787913	0.016379572	54.88838612
0.071484744	0.014300574	53.47058824
0.08611314	0.022716896	9.558558559
0.058720055	0.017666837	15.20849421
0.085798817	0.05232911	2.230769231
0.097222222	0.093224377	1.166666667
0.060554861	0.002473795	44.02338377
0.078518239	0.032456988	8.087378641
0.057027348	0.032531521	8.268965517
0.078794408	0.01867867	40.02755906
0.108777948	0.020434153	87.3486924
0.064814815	0.073384003	2.333333333
0.055597295	0.012265706	13.45454545
0.088757396	0.100406672	1.153846154
0.066632231	0.016886191	5.863636364
0.088248743	0.057032961	7.148148148

log.sigma.1.5.mm.3D_gldzm_HighIntensityLargeDistanceEmphasis	log.sigma.1.5.mm.3D_gldzm_SmallDistanceEmphasis	log.sigma.1.5.mm.3D_glcm_SumVariance	log.sigma.1.5.mm.3D_glcm_Homogeneity1
96.43103448	1	303.6217127	0.447142995
186.2533333	0.992716049	765.4358991	0.593570484
98.85185185	1	351.0875873	0.436812857
429.270936	0.972906404	1149.400062	0.446047287
130.4130435	1	331.446703	0.326185874
91.9125	0.990625	202.3577915	0.411477755
306.245283	1	901.4613616	0.210539283
199.1342282	0.989932886	592.1066085	0.370407605
1002.868263	0.779649035	1101.196514	0.288846134
195.5067265	0.940333832	318.5476285	0.527310739
87.20560748	1	259.2436096	0.419100888
129.1280788	0.985221675	357.3512746	0.472907538
196.347561	0.966992717	440.797326	0.485160138
133.2919708	1	312.1714068	0.465649186
338.3918129	0.99122807	864.825511	0.299864232
349.0495979	0.920115605	587.6183729	0.686663498
85	0.991935484	150.3753363	0.359155119
149.7173145	0.99204947	518.4210285	0.529469665
146.75	0.991847826	343.3095651	0.341793811
113.3122363	1	440.831661	0.646019028
207.3948276	0.963978927	531.1486768	0.630095869
209.9201278	0.949680511	408.8297057	0.401657844
84.12195122	0.995426829	282.629823	0.63696949
257.6976744	1	782.0852324	0.255266304
97.93491124	0.99112426	399.1820448	0.582693947
101.4705882	0.982352941	365.6251801	0.462335595
140.9030612	0.952664399	280.6008886	0.561220384
126.1505682	0.977765941	474.0684985	0.670072496
239.9431373	0.982352941	927.0405006	0.621882486
110.2521008	0.983076564	415.5409247	0.606724501
230.9765166	0.904425962	358.2551359	0.602729896
304.1044586	0.906156925	539.8454075	0.633744872
230.4971751	0.991525424	701.1563274	0.489792357
87.92307692	1	207.0729403	0.200255263
169.3571429	1	414.5123334	0.315847365
131.3412463	0.991097923	402.3758041	0.482760067
129.0109489	0.989824108	565.6535984	0.673399492
314.2126866	0.950919362	595.7096514	0.559438519
161.4113475	0.968085106	327.2801268	0.312751733
135.1678322	0.979020979	457.180522	0.476538252
274.4730539	0.995508982	976.3731937	0.522505121
180.9453125	0.994140625	549.0425641	0.411670087
169.8174905	0.97148289	416.6372407	0.402609071
156.8910256	0.985576923	396.2724177	0.391986481
526.2869023	0.848305036	610.3733518	0.300672645
373.6614481	0.771595456	405.2602343	0.641686827
1208.437687	0.828798327	1099.64683	0.336720327
128.7307692	0.971153846	313.291907	0.386973756
73.07692308	1	127.2670689	0.285051372
123.8831169	0.99025974	230.9124528	0.28994013
344.5550239	0.896345915	514.2350868	0.575971886
77.3495935	0.987804878	268.5727655	0.434960602
1233.085714	0.878832498	2035.938409	0.500109351
123.6578947	0.980263158	315.1268742	0.435694157
479.9171375	0.850953219	569.7963931	0.648495948
207.2671233	0.994863014	607.3966115	0.345757525
303.2493888	0.983496333	800.3539634	0.315312068
456.6879195	0.962248322	1109.225108	0.234293739
210.9910714	0.993303571	669.4609581	0.430777968
130.89	0.955	231.1974197	0.449285882
197.2763158	0.94236568	508.3484162	0.506667848
336.6944938	0.965964081	808.4690596	0.57977136
270.0471869	0.903937286	454.8462379	0.583851564
157.9267241	1	615.3643371	0.611192432
66.8	1	185.2421979	0.382377592
251.3343373	0.976991299	623.0586693	0.430475541
203.1470588	0.988970588	492.2039326	0.27584063
331.7395833	0.922366898	669.4122402	0.565007589
83.75806452	1	283.324198	0.361581189
282.005698	0.971430833	863.904407	0.531550539
215.7490909	0.953409091	551.7243763	0.514438223
101.2628205	0.966346154	288.1417366	0.424620477
122.8526863	0.990094839	458.8059638	0.659210777
652.4639376	0.780885402	468.7965809	0.632566777
1278.10755	0.801977413	874.8992615	0.548909671
392.8454545	0.979545455	1037.75154	0.288885653
207.5833333	0.979166667	572.7553361	0.375072332
77.74647887	0.98943662	255.3880455	0.412085562
172.376	0.982	545.7838006	0.452924353
173.1286031	0.991377186	584.030029	0.592789379
119.9104478	0.98880597	382.4799677	0.319444042
411.7280702	0.980263158	1114.963751	0.353716591
3683.855905	0.903109125	7853.520645	0.527235372
125.7368421	1	258.1700661	0.323525826
371.3936652	0.892845625	434.5052663	0.598964965
189.1149733	0.957887701	390.6476052	0.489376795
139.2342342	0.97972973	438.0021596	0.440847605
212.7760618	0.971042471	470.0710074	0.326767769
113.9615385	1	243.6638624	0.338581995
141.5833333	0.9375	304.8107094	0.229941798
1567.489684	0.941184854	4283.028424	0.530655153
155.5728155	0.992718447	632.9544725	0.41081823
173.2758621	0.984482759	423.2769891	0.453141439
198.4685039	0.97062664	496.5816057	0.59315122
466.1818182	0.800422948	367.1764159	0.604973244
152.25	1	428.6192862	0.290073821
282.161157	0.959710744	592.3507291	0.381474796
88.92307692	1	212.7030554	0.357222788
262.8295455	1	725.3563096	0.304352107
72.95061728	1	298.8360785	0.46304745

log.sigma.1.5.mm.3D_glcm_Homogeneity2	log.sigma.1.5.mm.3D_glcm_ClusterShade	log.sigma.1.5.mm.3D_glcm_MaximumProbability	log.sigma.1.5.mm.3D_glcm_Idmn	log.sigma.1.5.mm.3D_glcm_SumVariance2
0.377814836	-70.31573544	0.102949649	0.978767411	24.33125192
0.556779734	-94.38786163	0.231795147	0.989090394	13.36388427
0.362980925	-54.60938331	0.060227626	0.974090502	16.88204216
0.385580536	-22.62426167	0.119589118	0.985079468	36.35553344
0.232206734	-11.598109	0.039022131	0.939693	20.7727956
0.330257822	-20.18176687	0.043362864	0.958287712	15.88906387
0.119683697	-109.2378504	0.038980545	0.913364673	61.17517239
0.286621109	-23.64892453	0.042035246	0.975490961	31.84139645
0.205515648	322.3643444	0.0217533	0.957540071	83.70251833
0.475042545	5.071537912	0.16069428	0.992038499	13.8788566
0.339358343	-38.7440565	0.069271481	0.963686587	18.45736332
0.408477865	-27.11894906	0.097813251	0.988761444	16.83637693
0.423654101	-11.65557974	0.109044466	0.982461987	12.83308806
0.398032865	-9.857327226	0.068100812	0.978710454	12.48436095
0.208540561	120.1999951	0.02350167	0.973416169	38.40820518
0.671991328	-15.90336873	0.234329872	0.996705281	3.853749393
0.271618852	20.92677048	0.036414781	0.95077205	19.49094215
0.476402954	-42.60309041	0.13103258	0.988280945	12.56416109
0.250621732	-25.76822156	0.03838031	0.958595829	23.28559846
0.619786896	-27.12814664	0.208005671	0.992862128	6.845333974
0.601642779	-16.549932	0.22127904	0.997852727	6.241538299
0.324811699	38.38102022	0.057200176	0.984947316	22.35532802
0.607976813	-21.79947427	0.227135882	0.993884429	6.118852612
0.163883068	68.45665814	0.027512264	0.958039613	78.67968449
0.541863177	-55.63541505	0.17771434	0.990095208	9.577302764
0.39309683	-56.07605325	0.085437419	0.975939103	18.38975298
0.517279365	-4.85843583	0.159791505	0.99097778	9.350406955
0.651113373	-24.79551152	0.243590835	0.997243067	5.232424372
0.591896435	-90.32606433	0.214770098	0.994053903	12.86818058
0.572356208	-54.81927923	0.212637397	0.991165137	9.552450552
0.568612861	-19.67918087	0.167306192	0.99458061	7.080783086
0.605993643	-18.12578266	0.186885596	0.994552377	5.214764119
0.433439462	-83.84036926	0.133475638	0.987750003	23.80403005
0.100467968	28.38646278	0.124679487	0.867634066	31.06169872
0.226759188	91.64017404	0.034631377	0.958075014	42.4399157
0.422345008	-54.96979598	0.117950422	0.985609896	17.68502758
0.65626763	-50.18878108	0.217868834	0.995646263	6.88679676
0.51766388	-13.6696088	0.15643913	0.990830186	12.19559795
0.221071713	43.70240908	0.023235113	0.94090442	33.07156159
0.411048222	-89.16890304	0.117215984	0.977074594	21.85465451
0.470023052	-53.68637693	0.159262247	0.990148598	16.04106239
0.333004215	-60.36622775	0.061344967	0.978691602	21.57428951
0.325703417	-29.03313618	0.050246883	0.980611315	27.58287255
0.311985051	28.20961322	0.067213922	0.981912239	31.28618229
0.213573914	290.9579417	0.018267777	0.969627001	53.83567167
0.617155061	-11.10473558	0.177189207	0.995641691	2.973586937
0.256998134	780.0767709	0.033701967	0.978742417	67.8514317
0.302182473	-27.04130567	0.035835997	0.97342169	21.39364894
0.184135446	10.89450278	0.103303641	0.88471102	29.86573059
0.193893236	29.52365563	0.026612357	0.93176667	32.32305153
0.537651678	-25.83157699	0.16998154	0.991622316	11.34778174
0.362582865	-74.81053831	0.0949036	0.966204563	24.10322791
0.445118633	-98.59274778	0.117789969	0.993599529	20.83056879
0.360236495	-31.73731853	0.075943162	0.979588989	15.90365677
0.625933373	-33.7059544	0.201400407	0.997447627	5.894230082
0.260760697	-97.95280784	0.043752514	0.974946571	38.51806664
0.227259423	119.8111267	0.020350967	0.97327052	45.98615506
0.142227672	27.78126158	0.014980857	0.958362124	68.91308379
0.358965607	-114.896537	0.067716657	0.976263312	21.06945702
0.377919556	10.99085574	0.051188918	0.986068064	13.32580767
0.449612582	-72.17619471	0.101581852	0.986218108	16.25783286
0.544036837	-51.12172518	0.172684172	0.991624682	14.75749109
0.544781655	-11.0034175	0.154578795	0.993043525	6.83850318
0.57739125	-43.12231534	0.187153129	0.990392239	9.863145298
0.291725413	-27.20648728	0.06300224	0.946051778	18.10959069
0.360268438	-95.86913262	0.073807794	0.981482621	27.51294743
0.182259453	107.805889	0.036914733	0.946533834	41.39763619
0.522888653	-81.95304877	0.1399535	0.992851003	14.90456484
0.271270626	-26.83819205	0.040580992	0.953589828	20.50933485
0.482045217	-73.69734238	0.136673939	0.990739577	16.20683251
0.459578347	-70.71574142	0.102327254	0.989906718	14.42878967
0.346727962	-67.5329523	0.05496249	0.964706944	20.34014805
0.637263432	-31.04282215	0.214194824	0.994836393	6.140071862
0.605977387	-20.81170096	0.186116446	0.998102909	4.945431632
0.506772326	-7.960922902	0.172789961	0.995188322	8.592926456
0.19780292	-30.97320879	0.022787545	0.972782367	41.87484729
0.290501042	-45.89983017	0.034260148	0.97561118	25.4052866
0.331542466	-30.09211976	0.048326078	0.965834564	18.12274129
0.383764843	-61.14097232	0.078059235	0.98443361	21.28087437
0.556936002	-48.97722151	0.202930776	0.992016363	12.6946483
0.223926222	-71.05452769	0.031276258	0.952764533	29.09555315
0.274676428	-12.29613615	0.051336973	0.97738877	43.35647182
0.482472256	-121.3276726	0.171104609	0.996752491	19.28258029
0.23360801	3.284825906	0.093832023	0.942145528	25.19220117
0.563530861	-32.38028349	0.158249075	0.99441696	8.082736796
0.428811546	-4.879267791	0.098752751	0.988159471	11.69584132
0.368597624	-37.534896	0.068512084	0.976692522	16.3932513
0.234584587	4.465491427	0.020870155	0.968644863	32.41787405
0.250348248	-44.66789642	0.061366727	0.942745711	23.79570898
0.127860175	5.759641187	0.077380952	0.905130012	42.25270849
0.484495618	-261.2933216	0.125181918	0.994239338	23.84698454
0.334521123	-118.261759	0.063486036	0.966759394	25.67560393
0.390312548	-21.51788281	0.123303482	0.975884312	26.32129905
0.5556738	-56.2193664	0.174596838	0.990564395	10.87120195
0.571283162	-25.23946306	0.164946818	0.995017095	6.291742765
0.197367709	-52.19205898	0.044217949	0.950599011	49.93513747
0.303159197	38.30559859	0.069736685	0.980158132	29.02516635
0.272393874	7.677113961	0.104846863	0.932826152	22.05504574
0.210856627	-38.0029423	0.028542829	0.960857768	33.18978919
0.395856452	-75.51380787	0.086388365	0.963243961	17.78846979

log.sigma.1.5.mm.3D_glcm_Contrast	log.sigma.1.5.mm.3D_glcm_DifferenceEntropy	log.sigma.1.5.mm.3D_glcm_InverseVariance	log.sigma.1.5.mm.3D_glcm_Entropy	log.sigma.1.5.mm.3D_glcm_Dissimilarity
13.72082442	2.950600063	0.331103993	6.098254644	2.583664614
5.760150788	2.291271296	0.424455584	4.707581158	1.457442636
9.202800766	2.714076177	0.363423002	5.882048345	2.290569521
32.35782014	3.354984812	0.315100453	6.741750995	3.481696365
20.53274319	3.135836452	0.241149687	6.122639104	3.637094683
12.43583071	2.932593452	0.307440396	6.311951709	2.654398969
72.61945662	3.603902874	0.125061864	5.645954224	7.0271191
21.37289898	3.269851381	0.278679558	6.946816082	3.387155802
64.90788109	4.03522955	0.200181067	8.653228829	5.861754002
9.470579279	2.626568844	0.393596997	5.627958741	1.908645128
11.93093929	2.929166585	0.305733129	6.334027052	2.605694874
11.12217217	2.770917394	0.373295863	5.988952772	2.228531161
10.26904277	2.775359952	0.374765243	6.035356212	2.164788569
10.61533676	2.789693017	0.359710749	6.020710268	2.253792662
38.68603791	3.629280803	0.218751605	7.312226686	4.637695723
2.375539714	1.656411113	0.478109078	3.383515103	0.85363301
19.21853038	3.165615068	0.281640446	6.49239908	3.310445128
6.603300293	2.516170206	0.399761743	5.432712221	1.748647634
23.12319812	3.306589528	0.232652548	6.594102798	3.690933459
2.733854373	1.910810357	0.455192889	4.205183482	1.027892628
4.184713674	2.037864966	0.454497038	4.214264803	1.168363572
20.32727906	3.187625595	0.31251579	6.801253262	3.106384747
3.144837852	1.983638288	0.445540297	4.275347831	1.090543748
59.20928136	3.788117378	0.165677504	6.63330936	5.947025702
3.800330833	2.166078079	0.445736761	4.727077594	1.310221007
8.514225027	2.697072839	0.361914713	5.845330056	2.154869321
6.676066986	2.39903452	0.429271232	5.142173975	1.589842483
3.213212519	1.799606745	0.466279926	3.604025685	0.967715381
7.453470945	2.253785386	0.415689806	4.452680763	1.460678851
4.630440098	2.180286139	0.437521047	4.468026473	1.31199444
4.621265162	2.134498629	0.458596271	4.529241779	1.279036556
2.762162687	1.929770298	0.468895257	4.22594075	1.054350422
16.42902464	2.966173399	0.353138362	6.063914067	2.539653549
50.54487179	1.935897354	0.121948364	3.240384533	6.166666667
36.21710193	3.436274703	0.228739526	6.34519945	4.450363886
11.35520183	2.838987732	0.365692594	6.023339313	2.280514617
3.15369311	1.77668257	0.470284003	3.515135876	0.967416715
9.622867812	2.532937281	0.417161941	5.250163615	1.810138961
28.24087124	3.450626859	0.220805177	7.276408986	4.173434328
10.20067086	2.805423794	0.344690976	5.979248464	2.242588209
10.345434	2.649622835	0.390684523	5.603378542	1.994479443
14.88948551	3.019079375	0.312564845	6.484540355	2.77993707
19.98471466	3.161728719	0.315366232	6.79614354	3.062651323
20.91684753	3.183590559	0.297626739	6.732587734	3.175729108
47.54915837	3.819168605	0.221090143	8.077702065	5.019388597
1.794305847	1.737063085	0.500208101	3.767639049	0.918322763
61.38882416	3.832623379	0.252706942	7.968415261	5.046550941
14.08883866	3.006905857	0.296753332	6.428779254	2.881796063
29.26863969	2.471178333	0.14370101	3.927142263	4.516258741
33.36409382	3.486919103	0.187391999	6.682436013	4.580413714
8.453430914	2.407104747	0.433700956	5.02756301	1.638926183
13.86888089	3.006442701	0.311861805	6.215533632	2.697919559
13.30213016	2.797482322	0.390254095	5.972303973	2.229717233
9.737032203	2.760975945	0.348179304	6.05469538	2.335588177
3.099806997	1.858142878	0.481391133	3.882018681	1.017053292
30.94596195	3.466334334	0.24683349	7.015882619	4.011228312
39.22790214	3.650649045	0.238727707	7.730525198	4.524233618
60.78167129	3.973671582	0.15703701	8.075599469	6.237431708
12.79532624	2.920301686	0.351148279	6.171895244	2.571346406
8.636625495	2.657518649	0.386179095	5.979088312	2.142864492
7.855330054	2.624512025	0.395154227	5.721826891	1.918058841
11.28989274	2.504146483	0.417009571	5.010679408	1.830865975
4.24639518	2.190302319	0.453459091	4.796637135	1.332681656
4.17535575	2.132088646	0.437415495	4.510586292	1.267272688
10.50119595	2.613796899	0.302332624	5.091357512	2.623528408
20.50891104	3.115879692	0.336995242	6.740913974	2.925132365
43.40573036	3.569169046	0.18792245	6.366405781	5.097347406
6.806333239	2.425101339	0.424291307	5.130221547	1.622026432
15.49407855	3.031118481	0.278083236	6.33943603	3.090606539
9.681898583	2.623589824	0.401869133	5.556394632	1.921832304
7.902295223	2.584795493	0.407750569	5.681174921	1.861785559
10.27622872	2.829407855	0.341519756	6.39514032	2.418614713
2.912327111	1.85845162	0.462215806	3.856345375	0.996440129
2.686552255	1.877660862	0.486689932	4.031630857	1.027630781
7.950262496	2.330426854	0.476836199	4.946266584	1.619427119
43.8277394	3.746462169	0.203162204	7.55505604	4.979630812
16.72975763	3.128377416	0.299539797	6.894894303	3.084439372
11.07550468	2.84144631	0.32546467	6.18615915	2.547936502
11.44409673	2.855440438	0.356704856	6.332065924	2.370562296
8.434356473	2.392819906	0.415093399	4.781479758	1.616303155
21.69630522	3.185217094	0.23400269	6.43822916	3.724863671
41.21579877	3.633626472	0.264241854	7.379266162	4.343851256
16.01300441	2.6348666	0.442850788	5.345822777	2.120043483
27.41292895	2.817248482	0.218820162	4.389910782	4.097920029
4.681179392	2.179031768	0.449641263	4.673590703	1.314595902
9.326658214	2.702894239	0.391771519	5.967402127	2.055851159
10.14256747	2.816281513	0.350963657	6.049534326	2.369770293
25.71916766	3.416774664	0.24290575	7.408695112	3.858829044
22.22000211	2.80677609	0.253625432	5.150023601	3.680102024
44.84920635	2.568005041	0.154157896	3.960862184	5.484126984
16.991729	2.770895081	0.396521909	5.682127338	2.257794648
15.21373985	3.042754642	0.32097785	6.314805705	2.838603942
22.39741989	3.121166008	0.334606683	6.177104957	2.982274206
4.945408728	2.2474158	0.434393772	4.83670959	1.380201525
3.263677136	2.03507855	0.472799839	4.432136595	1.171917074
43.57259784	3.415945748	0.187553799	5.784019708	4.978184874
24.99182882	3.389618217	0.275129464	7.098948637	3.570368362
21.82262396	2.4531531	0.271700893	3.952353483	3.52443454
27.29252589	3.381227103	0.216776179	6.688305422	4.182110624
9.604259912	2.744050608	0.364587045	5.808403001	2.231225451

log.sigma.1.5.mm.3D_glcm_DifferenceVariance	log.sigma.1.5.mm.3D_glcm_Idn	log.sigma.1.5.mm.3D_glcm_Idm	log.sigma.1.5.mm.3D_glcm_Correlation	log.sigma.1.5.mm.3D_glcm_Autocorrelation
6.996112694	0.910670045	0.377814836	0.276508288	110.6142431
3.602384894	0.943193684	0.556779734	0.39216452	236.9373892
3.877506571	0.894943323	0.362980925	0.288391224	124.0403453
20.1944251	0.933701545	0.385580536	0.058897872	355.2176453
7.054664396	0.837079446	0.232206734	-0.003944707	115.8738672
5.367608419	0.869652431	0.330257822	0.119450858	78.04824786
22.04378443	0.803277348	0.119683697	-0.092009247	270.1348929
9.856332074	0.900061111	0.286621109	0.196572315	199.4519222
30.27573119	0.873251118	0.205515648	0.131746726	348.1537013
5.807862015	0.949161225	0.475042545	0.189121274	112.4003
5.070891144	0.877671602	0.339358343	0.211828757	96.70741797
6.09267814	0.935569309	0.408477865	0.204351841	125.6993118
5.552340317	0.920984722	0.423654101	0.103456203	149.8920192
5.47700781	0.911030243	0.398032865	0.080647497	111.4400551
17.10659299	0.894004307	0.208540561	0.001128944	275.968505
1.643032955	0.97009595	0.671991328	0.232469162	176.4203408
8.12427158	0.858186552	0.271618852	0.008756767	59.78450875
3.513291349	0.934184596	0.476402954	0.308165425	171.2397993
9.409449666	0.867495496	0.250621732	0.000508913	121.1470562
1.665673259	0.952175257	0.619786896	0.424704459	142.9468557
2.816404914	0.974776574	0.601642779	0.198247097	166.9372086
10.57246047	0.924320409	0.324811699	0.048929302	142.2596462
1.943183843	0.955834954	0.607976813	0.317121674	96.70636001
23.55275939	0.865785412	0.163883068	0.146021628	247.968791
2.069063376	0.939717388	0.541863177	0.420749063	133.1131664
3.788075945	0.900896982	0.39309683	0.36809114	129.1175696
4.144077883	0.946724476	0.517279365	0.16628939	98.65183696
2.273539477	0.973138134	0.651113373	0.237303891	146.6375764
5.283259302	0.962226735	0.591896435	0.259764105	278.6887783
2.900592983	0.948068401	0.572356208	0.339374616	135.9598952
2.981166712	0.959119026	0.568612861	0.207413899	119.1176042
1.644841124	0.956912136	0.605993643	0.301418991	169.2762553
9.943632073	0.938037097	0.433439462	0.183037677	226.6579165
10.0713141	0.750785261	0.100467968	-0.24038406	53.26282051
16.09791272	0.870978714	0.226759188	0.083730611	140.3458683
6.135731977	0.927995299	0.422345008	0.216017945	139.0713998
2.212735896	0.966622888	0.65626763	0.365879769	172.0258831
6.340613198	0.949547357	0.51766388	0.117629504	191.1732388
10.36907052	0.841499673	0.221071713	0.080957836	117.7597262
5.114260927	0.907540698	0.411048222	0.360556304	157.0062477
6.327504025	0.944234628	0.470023052	0.218553344	303.0219792
7.085114935	0.907222247	0.333004215	0.184831392	184.6270467
10.49004567	0.914747163	0.325703417	0.158369504	145.5527399
10.77596515	0.916463965	0.311985051	0.193970292	139.4166179
22.25614138	0.890319342	0.213573914	0.065741476	203.250809
0.950167936	0.958022734	0.617155061	0.242916446	127.5654323
35.79128451	0.917281914	0.256998134	0.055440373	342.8043854
5.739269381	0.89179865	0.302182473	0.206171518	113.65409
8.190658358	0.775694551	0.184135446	0.00302982	40.21392219
12.25099806	0.829030452	0.193893236	-0.016629241	83.87684785
5.75732429	0.952666461	0.537651678	0.144281458	166.0376494
6.49570769	0.886741278	0.362582865	0.265982727	99.31166913
8.295808468	0.954957494	0.445118633	0.219531928	596.3671121
4.267472348	0.906387706	0.360236495	0.238140444	113.4806056
2.061672835	0.972382185	0.625933373	0.306042954	174.9131287
14.69028536	0.900028909	0.260760697	0.110597905	201.9737564
18.4514339	0.897212994	0.227259423	0.086296181	260.326153
21.48500715	0.862083858	0.142227672	0.061821721	348.0254354
6.155250305	0.903491552	0.358965607	0.240386877	218.2233773
3.999843473	0.922874072	0.377919556	0.209462986	87.03481906
4.160951811	0.928558801	0.449612582	0.346550572	169.7427436
7.921025623	0.954928994	0.544036837	0.131587259	249.0175896
2.460855729	0.950610406	0.544781655	0.230899874	148.2156868
2.5443165	0.945029956	0.57739125	0.399661047	194.7097438
3.487883579	0.843816741	0.291725413	0.266547783	70.00440827
11.87695153	0.921623138	0.360268438	0.144896563	206.6525877
17.25286942	0.849551076	0.182259453	-0.023905154	162.8868641
4.164807289	0.952204918	0.522888653	0.368632761	212.381714
5.8869018	0.857634647	0.271270626	0.139649504	103.4613891
5.972055988	0.946076994	0.482045217	0.250351972	270.0241778
4.412701197	0.93986065	0.459578347	0.28508773	181.5055133
4.357661753	0.878930715	0.346727962	0.328276341	106.451277
1.915937163	0.961234703	0.637263432	0.35224736	144.9921272
1.628665628	0.973965424	0.605977387	0.291733286	147.56732
5.323828491	0.962704535	0.506772326	0.040937943	264.5141993
18.93007714	0.892280402	0.19780292	-0.024326248	326.328426
7.191022373	0.897563464	0.290501042	0.204017965	193.4787989
4.480857826	0.879243809	0.331542466	0.24189183	95.47663908
5.770873315	0.922311205	0.383764843	0.288001673	183.8847512
5.801369065	0.954788994	0.556936002	0.200135082	185.6965156
7.562409161	0.853875517	0.223926222	0.144435346	133.7942098
22.2655035	0.910795488	0.274676428	0.029702512	348.4256761
11.49828823	0.971692753	0.482472256	0.095206984	2112.796125
9.956742846	0.844100708	0.23360801	-0.028775383	84.44738125
2.94681178	0.95798772	0.563530861	0.262009783	142.0046849
5.079461819	0.934159039	0.428811546	0.109752585	134.4463632
4.501934897	0.901511944	0.368597624	0.234237958	150.4630727
10.75353167	0.883880933	0.234584587	0.113363213	162.8601749
7.188090548	0.844293764	0.250348248	0.045800717	85.33496809
14.54925674	0.793312652	0.127860175	-0.024905345	90.36507937
11.86613999	0.961623839	0.484495618	0.164541131	1189.989758
7.051943704	0.886403597	0.334521123	0.250391929	208.8189591
13.39033852	0.91421159	0.390312548	0.083225968	144.5196976
3.028861976	0.945486755	0.5556738	0.369553579	161.786498
1.88759003	0.957570892	0.571283162	0.3137138	121.0077128
18.33384311	0.857899008	0.197367709	0.073603825	141.0138138
12.20303159	0.912037994	0.303159197	0.073992381	197.7172293
8.885951112	0.837429178	0.272393874	-0.003859312	68.50850923
9.702375709	0.865985038	0.210856627	0.098270321	235.5608857
4.513997757	0.882547684	0.395856452	0.292993957	107.8716821

log.sigma.1.5.mm.3D_glcm_SumEntropy	log.sigma.1.5.mm.3D_glcm_AverageIntensity	log.sigma.1.5.mm.3D_glcm_Energy	log.sigma.1.5.mm.3D_glcm_SumSquares	log.sigma.1.5.mm.3D_glcm_ClusterProminence
4.070090927	10.38928493	0.029740885	9.513019086	1874.654371
3.238115313	15.33079589	0.097967655	4.781008763	1285.939302
3.82231703	11.05007489	0.022943696	6.521210732	982.1757099
4.279483792	19.02315579	0.033519265	17.20137508	7395.537612
3.904505021	10.75900523	0.016561227	10.3263847	1351.017202
3.916444731	8.78512906	0.017050046	7.081223645	764.1904554
4.066044233	16.60780037	0.021877278	33.82112077	10982.23068
4.390417856	14.06423584	0.011451434	13.63589315	3309.694906
5.167381089	18.90632045	0.004001612	37.43364003	21827.27139
3.645155127	10.56459313	0.052610456	6.054494347	1650.986939
3.986591283	9.749476651	0.018358266	7.597075654	950.3313035
3.842985706	11.15634669	0.032162669	7.118429592	1532.121677
3.747253962	12.21671672	0.032353065	5.775532707	717.0971936
3.758069393	10.53400887	0.024827478	5.774924427	658.7993607
4.481598681	16.88924268	0.008106534	20.08647839	7502.098651
2.375719147	13.26843767	0.172505907	1.557565002	253.14122
4.022300466	7.723933591	0.013873222	9.677368132	1283.945963
3.566760237	13.02866641	0.049834337	4.791865346	612.0495377
4.122015159	11.00371843	0.013042048	11.60219915	1743.908978
2.99385282	11.91289015	0.115487634	2.394797087	327.3472892
2.890263851	12.91592614	0.117510884	2.685083588	436.7974377
4.154012463	11.98634165	0.015665175	11.3222901	2706.573578
2.963870742	9.795998382	0.109446985	2.315922616	257.6958446
4.664662419	15.97839975	0.011336355	36.17392297	18577.39946
3.211842442	11.47441968	0.077043127	3.344408399	727.5206692
3.874176965	11.25349229	0.027894935	6.725994502	981.5276279
3.327802623	9.899547017	0.068592342	4.021907889	746.5300817
2.524685332	12.09303456	0.157216013	2.158522479	442.4000613
3.071792743	16.68110793	0.120061235	5.143659254	1465.931761
3.066252759	11.60708997	0.104529308	3.545722662	656.5586819
3.032345122	10.88823991	0.092874975	2.958437109	452.7464447
2.852282429	12.9869946	0.109971355	1.994231701	215.1565771
3.961994258	15.07190741	0.046373651	10.43386512	2451.870804
2.073655206	7.53525641	0.115130876	20.40164263	3079.844412
4.27415558	11.86124334	0.014240118	20.7281817	6808.569238
3.838179658	11.72655926	0.037309798	7.275947706	1156.180225
2.522303612	13.08076698	0.167053359	2.516143702	655.6208456
3.450609527	13.83736151	0.072704762	5.710167583	1053.790982
4.444413485	10.79388523	0.008077791	15.32810821	3188.994125
3.962971487	12.41314573	0.03428179	8.013831341	1551.078707
3.744234932	17.42470479	0.052216793	6.895781272	1323.005147
4.086687171	13.52555172	0.016403367	9.115943754	1646.942899
4.248823827	12.01090993	0.015296072	12.25658668	3265.802106
4.29315016	11.72974788	0.015434902	13.55556617	5268.598467
4.812301719	14.49366891	0.005528553	27.12250463	13176.38177
2.505876597	11.28141084	0.121233092	1.191973196	103.7128348
4.821177647	18.81965536	0.008490861	34.52631311	33804.88034
4.065499433	10.5743956	0.014644212	8.870621899	1238.58016
2.806306664	6.315938228	0.070491841	14.78359257	2073.724607
4.255647837	9.168969103	0.011114812	16.42178634	3207.78703
3.289911384	12.87518042	0.083785581	5.125101391	1138.250816
4.038887349	9.835394183	0.026191542	9.493027198	1481.984812
3.874325072	24.43390528	0.043615626	8.568759964	3159.405758
3.86275102	10.57979537	0.022807388	6.410172244	930.9433256
2.651543651	13.20256181	0.136956385	2.272762601	553.1539443
4.440017263	14.25239303	0.011434429	18.05726029	5440.423123
4.702267854	16.34858858	0.006692615	22.53209544	8973.037812
4.954110864	19.01577859	0.004498136	32.17451973	15136.25951
3.941412595	14.70202794	0.024187188	8.466195815	2344.519044
3.772966966	9.266002335	0.023537405	5.490608291	953.3398415
3.712579824	12.9475237	0.041300835	6.028290728	1027.877236
3.33284435	15.80549608	0.096282552	6.70979971	1775.162025
3.129474093	12.14770949	0.080390785	2.77122459	345.21056
3.19885433	13.90263776	0.102756267	3.509625262	595.3100327
3.578017975	8.249524929	0.033063409	7.15269666	813.6723277
4.225473899	14.38694417	0.021121227	12.58541227	3828.26114
4.333446978	12.8210188	0.013892921	21.69113255	5519.502136
3.42433123	14.50845567	0.076841432	5.464051554	1344.812381
4.00902269	10.10944039	0.01513615	9.00085335	1076.95492
3.650425087	16.41793661	0.057056183	6.675343586	1508.617912
3.644801636	13.41309273	0.044195445	5.598897014	1080.023357
4.026377385	10.1944578	0.018049943	7.654094193	1264.401131
2.739670182	12.00767368	0.141251862	2.263099743	360.921413
2.711895074	12.12729217	0.119176292	1.929454352	286.6704117
3.084880658	16.38042932	0.077382727	4.302053235	1177.339517
4.599982914	18.43507202	0.00715483	21.05006187	6945.02096
4.268773636	13.83077363	0.01192048	10.53376106	2153.966844
3.95971839	9.679923459	0.017511777	7.299561492	842.8218882
4.040175037	13.47210576	0.023968699	8.215789603	1602.667367
3.273488392	13.60757361	0.097612703	5.42170295	1191.660985
4.178698827	11.48523463	0.013591369	12.69796459	2499.694086
4.568667551	18.97342685	0.011926319	20.76299484	8942.197782
3.401358374	46.1203983	0.073336564	8.735332613	5502.134541
3.182048087	9.213431761	0.052840091	13.15128253	1885.535532
3.11194476	11.88343425	0.088037089	3.224024785	533.2524966
3.672925919	11.57139433	0.033955808	5.283696245	795.6755424
3.872427224	12.20217485	0.023115483	6.633954695	774.9684258
4.473003766	12.72141959	0.007785436	14.83434191	3429.981107
3.609931795	9.214401253	0.03158697	11.50392777	1456.018863
2.877528851	9.53968254	0.065035273	21.77547871	4632.345582
3.680518091	34.58662911	0.063102024	9.634390691	7206.069854
4.077793533	14.35916103	0.020321711	10.22233595	2274.010376
4.038429834	12.00111574	0.033374961	12.3930698	3582.846278
3.283807162	12.66104438	0.08738508	3.95415267	778.0937119
2.935051737	10.96584677	0.095585212	2.388854975	327.268107
4.159131438	11.90459525	0.020045198	24.63298355	6922.572183
4.316392001	14.12565271	0.014602301	13.98187475	3936.472273
2.74202402	8.233708386	0.069857971	10.96941742	1009.530851
4.290612076	15.2993309	0.01126932	15.12057877	3564.458803
3.811035691	10.28672865	0.029027145	6.848182425	1164.460217

log.sigma.1.5.mm.3D_glcm_SumAverage	log.sigma.1.5.mm.3D_glcm_Imc2	log.sigma.1.5.mm.3D_glcm_Imc1	log.sigma.1.5.mm.3D_glcm_DifferenceAverage	log.sigma.1.5.mm.3D_glcm_Id
20.77856987	0.812359358	-0.164380989	2.583664614	0.447142995
30.66159178	0.637268958	-0.11257075	1.457442636	0.593570484
22.10014979	0.806625061	-0.165835602	2.290569521	0.436812857
37.64087409	0.78199734	-0.13213997	3.481696365	0.446047287
21.51801046	0.942561475	-0.307151519	3.637094683	0.326185874
17.57025812	0.779515364	-0.138670934	2.654398969	0.411477755
33.03422224	0.993334856	-0.56431789	7.0271191	0.210539283
28.05852442	0.828367943	-0.154058522	3.387155802	0.370407605
37.06427674	0.771432293	-0.099572168	5.861754002	0.288846134
21.09940006	0.589448875	-0.075288518	1.908645128	0.527310739
19.4989533	0.732638392	-0.115900416	2.605694874	0.419100888
22.29496395	0.693512536	-0.106194274	2.228531161	0.472907538
24.43343344	0.617333141	-0.078028383	2.164788569	0.485160138
21.06801773	0.700899385	-0.108613313	2.253792662	0.465649186
33.22830197	0.877806114	-0.181654169	4.637695723	0.299864232
26.53679974	0.488412685	-0.080208586	0.85363301	0.686663498
15.44786718	0.846990431	-0.17916555	3.310445128	0.359155119
26.05733282	0.615368749	-0.089004526	1.748647634	0.529469665
22.00743685	0.897433968	-0.222103593	3.690933459	0.341793811
23.8257803	0.626224604	-0.117807635	1.027892628	0.646019028
25.80094568	0.537088385	-0.079303436	1.168363572	0.630095869
23.81136975	0.709793512	-0.098831673	3.106384747	0.401657844
19.59199676	0.579410419	-0.098338253	1.090543748	0.63696949
31.17338783	0.988873259	-0.451856297	5.947025702	0.255266304
22.94883936	0.609419318	-0.098860183	1.310221007	0.582693947
22.50698459	0.753737223	-0.136457179	2.154869321	0.462335595
19.79717342	0.581739016	-0.078868299	1.589842483	0.561220384
24.1770305	0.50817606	-0.082519896	0.967715381	0.670072496
33.30649926	0.664456761	-0.12949107	1.460678851	0.621882486
23.21417994	0.597867628	-0.098817431	1.31199444	0.606724501
21.7716956	0.520966256	-0.070304529	1.279036556	0.602729896
25.97398921	0.52983196	-0.078560397	1.054350422	0.633744872
29.9869013	0.735331774	-0.122443632	2.539653549	0.489792357
15.07051282	0.990438455	-0.837657396	6.166666667	0.200255263
23.55863156	0.973185085	-0.381385524	4.450363886	0.315847365
23.45069422	0.667268273	-0.09532792	2.280514617	0.482760067
26.16041312	0.538690997	-0.097646244	0.967416715	0.673399492
27.60643854	0.658146799	-0.103840576	1.810138961	0.559438519
21.58777046	0.835779161	-0.153432665	4.173434328	0.312751733
24.82629147	0.722862584	-0.120534283	2.242588209	0.476538252
34.73282461	0.753293596	-0.140398851	1.994479443	0.522505121
27.05110344	0.785205465	-0.13940676	2.77993707	0.411670087
23.96914914	0.73281918	-0.108435218	3.062651323	0.402609071
23.39066249	0.795550525	-0.139545672	3.175729108	0.391986481
28.40227071	0.744675378	-0.094170769	5.019388597	0.300672645
22.56282168	0.339779385	-0.034736171	0.918322763	0.641686827
36.94232652	0.721648998	-0.088428853	5.046550941	0.336720327
21.1487912	0.78818555	-0.141607138	2.881796063	0.386973756
12.63187646	0.986007615	-0.648165737	4.516258741	0.285051372
18.33793821	0.954590993	-0.30897867	4.580413714	0.28994013
25.71483275	0.626370854	-0.095957626	1.638926183	0.575971886
19.67078837	0.72160836	-0.114045972	2.697919559	0.434960602
48.76389768	0.61675113	-0.080519757	2.229717233	0.500109351
21.15959074	0.775140115	-0.141633506	2.335588177	0.435694157
26.39804029	0.473871773	-0.066640633	1.017053292	0.648495948
28.28782605	0.882671332	-0.194463601	4.011228312	0.345757525
32.15812108	0.806002196	-0.126264323	4.524233618	0.315312068
37.18927204	0.906546651	-0.193738279	6.237431708	0.234293739
29.40405588	0.771263166	-0.137366273	2.571346406	0.430777968
18.53200467	0.676975329	-0.099590866	2.142864492	0.449285882
25.8950474	0.611379946	-0.080846285	1.918058841	0.506667848
31.5054804	0.66650128	-0.112860025	1.830865975	0.57977136
24.29541897	0.498481424	-0.060923212	1.332681656	0.583851564
27.80527551	0.617033031	-0.108568098	1.267272688	0.611192432
16.49904986	0.943410981	-0.361271909	2.623528408	0.382377592
28.62810976	0.75525807	-0.120303018	2.925132365	0.430475541
25.56240391	0.97868359	-0.400472204	5.097347406	0.27584063
29.00717387	0.614543866	-0.091482331	1.622026432	0.565007589
20.21888078	0.867825862	-0.200486672	3.090606539	0.361581189
32.76517424	0.671160722	-0.104567642	1.921832304	0.531550539
26.82273532	0.567886471	-0.069940536	1.861785559	0.514438223
20.3889156	0.657986684	-0.087241533	2.418614713	0.424620477
24.01534735	0.558620699	-0.096973486	0.996440129	0.659210777
24.24887425	0.445306731	-0.05547753	1.027630781	0.632566777
32.51789289	0.530754069	-0.065031356	1.619427119	0.548909671
36.1541624	0.866403532	-0.167852319	4.979630812	0.288885653
27.66154726	0.758531467	-0.117399948	3.084439372	0.375072332
19.35984692	0.773389429	-0.138268992	2.547936502	0.412085562
26.93625672	0.707315095	-0.109367918	2.370562296	0.452924353
27.17562584	0.678080123	-0.125172461	1.616303155	0.592789379
22.97046926	0.935495809	-0.280771469	3.724863671	0.319444042
37.3032375	0.833613686	-0.148198121	4.343851256	0.353716591
91.91249981	0.651493769	-0.099880339	2.120043483	0.527235372
18.42686352	0.984058593	-0.584806407	4.097920029	0.323525826
23.76154289	0.541899816	-0.075070689	1.314595902	0.598964965
23.13885509	0.506724882	-0.050135777	2.055851159	0.489376795
24.40434971	0.692564217	-0.103860468	2.369770293	0.440847605
25.39024483	0.759325617	-0.109936893	3.858829044	0.326767769
18.42880251	0.984100637	-0.512008704	3.680102024	0.338581995
19.07936508	0.995866973	-0.759603126	5.484126984	0.229941798
68.94257599	0.686349048	-0.107974877	2.257794648	0.530655153
28.71832207	0.784508679	-0.142146942	2.838603942	0.41081823
23.96102012	0.858348091	-0.196396164	2.982274206	0.453141439
25.32208876	0.601825323	-0.093910352	1.380201525	0.59315122
21.93169354	0.494731033	-0.063619009	1.171917074	0.604973244
23.60702012	0.989575489	-0.510617947	4.978184874	0.290073821
28.05024991	0.770170387	-0.119354137	3.570368362	0.381474796
16.46741677	0.982884743	-0.633273286	3.52443454	0.357222788
30.5986618	0.946583806	-0.291304315	4.182110624	0.304352107
20.5734573	0.772978695	-0.147652719	2.231225451	0.46304745

log.sigma.1.5.mm.3D_glcm_ClusterTendency	log.sigma.1.5.mm.3D_firstorder_InterquartileRange	log.sigma.1.5.mm.3D_firstorder_Skewness	log.sigma.1.5.mm.3D_firstorder_Uniformity
24.33125192	114.9355226	-0.236408409	0.112683297
13.36388427	47.93356711	-1.692643651	0.230184377
16.88204216	87.34246445	-0.698530822	0.110791918
36.35553344	101.3941087	0.365615379	0.11739229
20.7727956	117.1300201	-0.212862677	0.08440349
15.88906387	94.91415787	-0.029688579	0.103130879
61.17517239	238.7883244	-0.365893205	0.066938776
31.84139645	136.714529	0.108797643	0.077729616
83.70251833	180.2624102	0.504914625	0.052566447
13.8788566	58.94411877	0.360174602	0.167110253
18.45736332	112.9103184	-0.173758723	0.102011657
16.83637693	81.02218759	0.064939676	0.12765924
12.83308806	73.86087468	0.05950711	0.132222839
12.48436095	80.05364299	0.19309361	0.123199323
38.40820518	126.57837	0.34790228	0.071670088
3.853749393	17.53801894	-1.17532811	0.367938566
19.49094215	103.6032524	0.700929771	0.090063497
12.56416109	76.63885543	-0.758007678	0.159631071
23.28559846	121.1977148	0.075254456	0.079217486
6.845333974	28.7208066	-1.469263134	0.263565168
6.241538299	32.33052611	-0.15033277	0.272757621
22.35532802	100.1662562	0.763414875	0.098865882
6.118852612	39.07627742	-1.024932771	0.255783918
78.67968449	206.6575279	0.153621168	0.057154164
9.577302764	55.5122408	-1.50212811	0.204819878
18.38975298	96.85620141	-0.719559403	0.119994737
9.350406955	42.00802708	0.322991983	0.201917122
5.232424372	18.17304826	-1.349112887	0.337801368
12.86818058	28.12116516	-1.416059599	0.270423711
9.552450552	40.46565962	-1.485919812	0.244015436
7.080783086	38.56392384	-0.427229729	0.243396838
5.214764119	29.78453636	-1.279306185	0.270790893
23.80403005	97.12508416	-0.07424444	0.139828986
31.06169872	185.4964142	0.205809073	0.093333333
42.4399157	134.1787605	0.498268564	0.070874775
17.68502758	92.95034042	-0.355268964	0.130126841
6.88679676	16.17204809	-2.389742131	0.347480441
12.19559795	47.35997069	0.279010542	0.193622904
33.07156159	134.6041927	0.215325889	0.072738036
21.85465451	122.1877997	-0.685070843	0.118122961
16.04106239	73.97822917	-0.452580775	0.156930901
21.57428951	120.5280037	-0.036729601	0.090554626
27.58287255	127.1181319	0.174143813	0.089914813
31.28618229	122.9428625	0.382299665	0.088464672
53.83567167	136.3453236	0.879120477	0.066297734
2.973586937	26.09619665	-2.165471049	0.316882221
67.8514317	111.398144	1.464916403	0.077093663
21.39364894	125.6709852	-0.021833978	0.092797784
29.86573059	164.1067905	-0.018760567	0.123966942
32.32305153	153.6018205	0.254324329	0.065959842
11.34778174	38.5970248	-0.091766102	0.22212959
24.10322791	134.085299	-0.454941649	0.10629337
20.83056879	65.7749052	-0.860639063	0.157366484
15.90365677	91.14051056	-0.329361476	0.109644439
5.894230082	22.49202228	-1.707189468	0.31759418
38.51806664	148.2636585	-0.03875381	0.073504121
45.98615506	136.711462	0.561402066	0.067534298
68.91308379	210.2680022	0.002371254	0.047872848
21.06945702	95.09685189	-1.117431139	0.119314373
13.32580767	67.2363006	0.627119542	0.127981727
16.25783286	94.0075278	-0.9223549	0.140293572
14.75749109	37.45832801	-0.06884674	0.229466472
6.83850318	33.71210337	-0.167824231	0.229228227
9.863145298	29.61980176	-1.474642463	0.247229749
18.10959069	115.352766	-0.257846108	0.115699537
27.51294743	88.40611386	-0.172463422	0.10612582
41.39763619	140.6132774	0.403402389	0.068117914
14.90456484	57.95268464	-1.200811485	0.200780626
20.50933485	113.234724	-0.259444459	0.091887134
16.20683251	66.25800025	-0.900571017	0.168701172
14.42878967	69.21988845	-0.907320829	0.155863215
20.34014805	108.4143286	-0.545574992	0.102824669
6.140071862	19.7829411	-1.676446931	0.30334446
4.945431632	26.46215773	-1.311758394	0.296209562
8.592926456	35.32611847	0.46980817	0.227780555
41.87484729	149.6670339	0.078699721	0.063634901
25.4052866	115.7844524	-0.045674699	0.085526316
18.12274129	108.1574802	-0.156089045	0.102200255
21.28087437	115.6085815	-0.346040156	0.105225842
12.6946483	49.87093231	-0.389101942	0.221647581
29.09555315	123.2405396	-0.252961912	0.081212558
43.35647182	135.4673615	0.210620925	0.077004912
19.28258029	38.18431568	-0.921871642	0.203622418
25.19220117	125.7104149	0.190884243	0.115504683
8.082736796	36.76475906	-0.718917175	0.235450122
11.69584132	59.29763246	0.19207508	0.149842724
16.3932513	106.9014397	-0.595051209	0.113004316
32.41787405	124.4850845	0.320062299	0.074186919
23.79570898	131.749074	-0.261468602	0.0968
42.25270849	155.7702789	0.132815445	0.100591716
23.84698454	57.42430305	-2.071438205	0.180592754
25.67560393	124.8241189	-0.94921099	0.101086392
26.32129905	95.5789075	0.314273357	0.122434357
10.87120195	48.12278271	-1.219672139	0.215882998
6.291742765	33.4521848	-1.121434511	0.25206926
49.93513747	178.6316376	0.432351734	0.076845553
29.02516635	102.3807557	0.323326112	0.086946367
22.05504574	140.2845116	-0.066080918	0.125
33.18978919	133.9539324	-0.308721999	0.072227032
17.78846979	90.7371583	-0.968357055	0.123163265

log.sigma.1.5.mm.3D_firstorder_MeanAbsoluteDeviation	log.sigma.1.5.mm.3D_firstorder_Energy	log.sigma.1.5.mm.3D_firstorder_RobustMeanAbsoluteDeviation	log.sigma.1.5.mm.3D_firstorder_Median
68.71333663	4043292.635	48.81004648	-41.53481293
45.60965045	7256878.342	25.54999825	-10.76523685
55.58906694	1835663.694	38.40609543	-65.41701508
77.79865348	27376681.64	45.90684559	-19.86985397
68.97475305	1692521.279	48.76816168	-99.51608276
58.03775307	2639933.999	39.8449758	-70.36136627
117.545315	1874381.305	89.19309419	-64.1086235
78.90560081	8227279.65	55.4257677	-101.6330032
114.962165	33492063.48	78.19422135	-44.4228878
45.30680575	12162283.29	26.58878217	-18.85665894
62.0019995	4366482.749	46.18375043	-73.81245422
55.16110766	5429338.5	35.22038068	-25.09353256
49.34765722	7785411.115	31.07535282	-27.8444643
50.05489959	3697012.15	32.09598998	-45.05847168
84.8883524	5719719.905	53.38885095	-58.95483208
18.58414641	13789174.64	7.637842636	-2.261470079
65.79718101	3640563.576	43.94373988	-105.8207855
47.64262768	8474811.398	33.4508904	-23.34545422
76.56444943	3260923.927	50.93509104	-89.80603409
30.45446848	6308579.902	15.36540102	-3.86757338
30.72715548	13220595.64	16.08358799	-7.64626503
62.85391794	8719742.154	40.43969701	-45.06249237
30.92980642	6881186.651	17.85179088	-6.483213902
113.809934	2979143.543	83.41079173	-49.66387558
40.25761455	3876774.276	25.10106989	-15.18933344
56.20845054	2797508.625	42.08361501	-55.4101696
37.11922881	8123360.069	19.74693491	-10.60738087
24.61016854	7956110.093	9.47831256	-3.427135944
42.16787225	17543582.26	19.48734699	-3.886687279
38.64745041	6458167.676	20.82257374	-9.988140106
32.27392648	11475069.86	17.72847106	-8.824678421
26.35628408	15602141.66	13.99918858	-5.813669682
66.48412836	13636095.92	42.46812338	-15.65407562
99.43750451	905274.8926	71.62437981	-227.7945404
89.70124621	2588950.978	56.22419186	-93.70742035
58.28380526	10565552.29	40.37455901	-28.80639839
26.70629678	15771410.97	8.506207813	-2.504057407
42.86206284	14150337.28	22.883252	-8.406905651
81.64771572	4791674.603	58.6631247	-67.73803711
66.62618337	5674191.028	49.65241629	-42.76649857
52.54310549	4594007.73	32.26827114	-16.201828
67.55770099	5500540.668	47.60644675	-82.54584503
72.32685867	11836589.1	51.05594963	-63.74797821
73.09291176	7054763.506	50.72501325	-78.98743439
91.31403856	18754641.29	58.4656125	-53.89595795
20.27194964	6342423.58	11.11847587	-3.185165286
88.6674737	52040250.57	49.2417208	-39.62834549
67.30894414	3063512.68	50.74451519	-75.57144165
89.19635357	1010990.075	69.21228027	-180.9631882
90.53347862	3349167.832	65.32733753	-110.0954971
38.92952013	16901223.02	19.42857907	-8.184107304
69.92994673	5375719.745	54.2994534	-67.02772522
54.23487729	23044775.27	31.72599303	-16.24258804
55.76945308	1958939.879	37.94178476	-46.20982361
24.24470792	22502372.82	10.0329037	-2.879429102
89.18641074	7366051.442	62.45200458	-81.23734283
88.33889966	21295031.34	55.88933	-108.8498421
121.4651016	14841501.49	86.5967006	-95.04043961
62.35527191	3256261.898	40.72671046	-30.52800751
47.35743556	2514092.005	29.67599664	-35.06524277
55.84844105	10709390.14	39.36693523	-26.62010002
45.25399317	37447128.96	21.68070582	-5.295176983
30.25919345	9458600.435	15.28678034	-4.932786703
35.92766707	5604508.776	17.6826147	-4.85735321
61.91035328	726992.8608	45.04705814	-88.00792694
66.71054453	11023379.08	39.56662075	-14.74147081
97.39267591	3336332.182	66.19673839	-150.7696381
48.21907	18474220.98	27.99232209	-8.81554985
65.13405386	2251669.265	47.85115719	-92.66246033
52.22901904	11425927.78	31.20503119	-13.55500269
49.2276711	14121526.25	30.78499585	-14.62669659
60.80794778	5995538.986	45.11765477	-61.66435623
27.9809723	15239020.62	12.02502033	-3.373637676
24.76141738	18595166.97	12.05504726	-4.60090661
32.87323208	48136775.05	15.47178722	-2.952002287
96.10431062	10704183.75	62.82439058	-86.11502838
68.22676447	6266767.239	47.3534963	-75.07165909
58.63686633	2810759.327	43.77676712	-88.90461731
64.2316194	9758852.809	45.72430231	-52.05826569
46.36477474	14562884.1	25.61073446	-7.311211348
73.8295444	3291656.445	52.10574153	-114.1477585
92.9868658	16218192.94	56.90885131	-34.55543137
47.30210614	34872593.42	19.47617076	-4.004946232
78.44866878	945666.1559	53.32644177	-158.2300568
33.82649301	14684167.48	17.84975251	-7.428314447
43.31756362	15334443.54	25.74935182	-19.28467941
57.61355206	3483376.218	43.35434437	-57.02641296
78.59737397	12314262.39	54.18467188	-102.3832474
73.07325594	497204.3418	55.1117185	-33.12671089
98.08810237	507186.4011	60.79886439	-183.9051971
56.02595277	27663507.76	29.37096855	-7.389399052
72.83134699	3940351.742	52.83530542	-48.36274147
66.68653213	4431747.456	40.15230115	-26.82563972
41.35832495	14832161.27	23.02071551	-8.465019226
29.96230728	16352389.61	16.07797475	-7.340236902
95.77302915	1990071.864	64.37665717	-122.9331055
73.63638379	7260921.509	47.49775733	-22.795228
74.97247847	969894.6896	52.5881424	-164.5631409
79.8227274	2469921.969	55.57466899	-66.80532074
57.97858349	1970643.665	39.92643881	-20.99773502

log.sigma.1.5.mm.3D_firstorder_TotalEnergy	log.sigma.1.5.mm.3D_firstorder_Maximum	log.sigma.1.5.mm.3D_firstorder_RootMeanSquared	log.sigma.1.5.mm.3D_firstorder_90Percentile	log.sigma.1.5.mm.3D_firstorder_Minimum
109168901.2	280.1752319	105.8312648	9.693327904	-291.7525024
195935715.2	127.7494812	71.91940499	9.748914909	-390.1929932
49562919.74	112.9029083	101.8380151	3.641631794	-309.7500916
739170404.4	575.4487915	119.0075394	37.2151062	-486.7497253
45698074.54	72.8592453	129.4512898	21.0963459	-343.6558838
71278217.96	121.9111938	104.0163527	10.7720439	-266.1072388
50608295.24	152.4246979	163.6364134	97.48179703	-472.3294983
222136550.5	258.3786011	143.2372398	2.443171501	-420.2160645
904285714.1	407.7524109	150.6356529	186.1841354	-452.4830933
328381648.8	532.399353	72.419777	18.59515266	-273.1321716
117895034.2	123.8645172	107.7635654	2.51530242	-275.0602722
146592139.5	437.8490601	84.68871044	24.81406059	-294.5452271
210206100.1	230.769928	75.49451957	26.76390362	-323.2596741
99819328.06	209.2775269	83.67745785	15.79608946	-288.0805969
154432437.4	440.7718201	125.3535649	90.33551331	-446.7495117
372307715.4	306.5484314	35.68576511	13.90264273	-305.1903076
98295216.55	153.6346436	129.5252511	6.993248653	-263.6416931
228819907.8	205.3639526	73.89569916	8.258571529	-333.9610291
88044946.04	192.6413879	133.8550127	41.85405807	-328.4542236
170331657.4	174.5860596	47.68838068	8.614799023	-285.4462891
356956082.3	726.6950684	51.03962653	10.53932915	-323.4330139
235433038.2	540.8240967	97.9422056	35.71580887	-310.0993958
185792039.6	289.0908508	48.54452359	6.875197124	-228.7698822
80436875.67	440.4619751	149.6648774	110.1917877	-402.7805176
104672905.4	157.6823883	63.54767138	9.446293068	-277.258728
75532732.87	103.6377869	96.24590169	3.408412147	-301.6131287
219330721.9	398.517334	59.57238061	23.53479691	-244.5147247
214814972.5	521.003479	45.19565253	10.3676899	-280.7487793
473676721	405.1917725	68.71021162	7.72061038	-420.6776428
174370527.2	230.2278748	61.76268663	8.979111481	-282.5117493
309826886.1	406.422821	52.26384152	14.73835278	-265.9154053
421257824.9	206.8584442	42.02792932	13.93725243	-306.5940857
368174589.9	461.6405029	99.08180301	17.14480476	-381.3121948
24442422.1	5.649767399	245.6657475	-74.48469086	-393.7287598
69901676.4	293.6661987	151.3640334	46.15430946	-360.9869995
285269911.7	349.3604126	88.99552861	10.64685001	-306.109375
425828096.1	321.6683655	48.5500587	11.02149487	-319.6545105
382059106.6	400.1789856	68.3154286	19.79034443	-330.6697083
129375214.3	171.3666534	122.1774742	68.87511444	-315.1099854
153203157.7	140.0153046	102.7933059	11.52456436	-340.2106934
124038208.7	323.8783875	80.15725567	24.75436478	-446.0144958
148514598	224.6965332	120.1545447	6.877381802	-380.6743164
319587905.7	386.5418701	119.8532127	10.083144	-348.496582
190478614.7	425.0353088	126.7678048	6.424252224	-344.4718018
506375314.7	531.0775146	129.6931227	113.3250427	-351.1627197
171245436.7	203.6575928	32.18703401	19.95284843	-265.8647461
1405086766	756.6881104	137.6636953	102.8269615	-454.9376526
82714842.35	207.7891541	115.9157415	11.8676652	-323.0480347
27296732.03	-14.74705887	214.369061	-76.18274155	-349.6468506
90427531.45	151.8445129	151.4579338	57.82501221	-321.8282166
456333021.5	421.7823181	64.36191904	17.08863773	-317.6304932
145144433.1	163.6290283	115.4956923	3.931037045	-290.003418
622208932.4	453.0219421	84.92798252	20.6789341	-621.7017212
52891376.73	224.7669678	90.53397797	24.99786797	-276.2839966
607564066	523.7248535	44.6128191	18.52927113	-321.1126099
198883388.9	377.993103	148.2842313	11.21415844	-414.6557007
574965846.2	409.7767944	156.2719025	39.29341774	-459.5512085
400720540.2	329.3909302	180.013958	98.69114838	-529.8852539
87919071.24	133.4919281	95.90870627	21.80339813	-381.2322388
67880484.13	327.9934692	73.45098516	31.41202736	-235.2261658
289153533.8	219.7592163	85.79280619	14.73033333	-340.3274841
1011072482	472.1774597	74.34000132	15.67483044	-375.83255
255382211.7	277.6735535	48.18400858	29.17589493	-277.8047791
151321736.9	144.8282318	56.08089383	17.38483696	-346.3240051
19628807.24	47.1901741	117.1189414	-8.135972977	-262.1440125
297631235.2	397.8205872	97.82078232	59.87292366	-363.59198
90080968.91	207.9087372	178.2542909	38.99693298	-411.0783386
498803966.5	352.6384277	72.99719064	17.47943535	-360.8720703
60795070.14	76.53442383	125.4828232	-4.419687748	-305.3452759
308500049.9	341.1202087	79.85033355	19.20814114	-400.6936951
381281208.8	316.0644531	73.71191246	24.17309647	-345.1964111
161879552.6	99.81658936	102.8306512	11.65523167	-288.6774597
411453556.8	257.6625061	46.46955029	10.0947546	-287.3461304
502069508.2	615.0422363	41.42911209	17.37815475	-285.8039856
1299692926	554.2739258	57.57979879	31.46523857	-394.6239929
289012961.2	404.5932922	154.9206635	52.29741287	-513.8118286
169202715.5	213.1628876	117.2301609	11.59555674	-395.0916138
75890501.83	124.1464157	112.0180017	1.489367527	-299.3323669
263489025.8	254.1820221	103.3865385	13.4700552	-358.6390076
393197870.8	414.2337036	73.38720591	9.407172012	-333.1385193
88874724.01	114.2781143	148.6326142	0.144527078	-360.2284546
437891209.4	495.79599	136.9282878	74.32645416	-479.3074341
941560022.4	528.9296265	84.11292083	33.42300949	-1141.107056
25532986.21	124.9293747	174.6578366	-39.66053009	-363.3070679
396472521.9	383.940094	54.39967243	17.60319996	-278.3467102
414029975.6	371.0490723	66.12506736	31.90957489	-293.8180847
94051157.9	134.5475006	97.6908209	6.576297569	-343.8905945
332485084.4	259.9497375	144.5927622	15.64993706	-376.1491394
13424517.23	152.4190521	99.7200423	47.68291855	-273.2415161
13694032.83	69.74263	197.5204763	-15.93919907	-376.9016113
746914709.4	418.361969	89.81934455	23.00053177	-855.0222168
106389497	79.19584656	113.8493889	19.08199463	-396.3539429
119657181.3	361.0267029	103.2145294	22.6851511	-315.020813
400468354.4	216.8279114	63.72896056	14.63924608	-302.0658569
441514519.6	336.0252686	47.58410126	17.65105591	-273.7569275
53731940.32	269.2703247	177.7313768	-8.815069008	-386.2312317
196044880.7	450.5599365	103.3335655	92.46114731	-352.6257324
26187156.62	18.60687256	201.0280546	-77.19049759	-356.9538269
66687893.16	150.7757416	123.0970979	58.17506638	-425.9536438
53207378.94	95.09558868	83.89286673	32.81602402	-269.9129639

log.sigma.1.5.mm.3D_firstorder_Entropy	log.sigma.1.5.mm.3D_firstorder_StandardDeviation	log.sigma.1.5.mm.3D_firstorder_Range	log.sigma.1.5.mm.3D_firstorder_Variance	log.sigma.1.5.mm.3D_firstorder_10Percentile
3.566190037	85.23256682	571.9277344	7264.590447	-185.532074
2.772553547	62.40356705	517.9424744	3894.20518	-132.2422119
3.406974041	70.58075816	422.6529999	4981.643422	-186.8512665
3.806854796	110.5288619	1062.198517	12216.62932	-187.4662567
3.726894176	86.59140705	416.5151291	7498.071775	-200.8840179
3.535182283	72.48956239	388.0184326	5254.736655	-172.2824951
4.13204784	141.5802259	624.7541962	20044.96035	-263.3778381
3.952993275	99.71593971	678.5946655	9943.268631	-235.5888519
4.511053345	146.6703195	860.2355042	21512.18263	-205.3778229
3.150395411	65.45117602	805.5315247	4283.856443	-115.0747589
3.518331655	74.35018309	398.9247894	5527.949725	-173.8367767
3.419154433	74.68013464	732.3942871	5577.12251	-141.9976898
3.33163706	66.35324631	554.0296021	4402.753296	-117.4066544
3.38021612	66.80725901	497.3581238	4463.209856	-128.9781143
4.12698656	114.5734763	887.5213318	13127.08148	-175.0689438
1.934679591	35.05717165	611.738739	1229.005284	-29.37256584
3.690937805	84.76412697	417.2763367	7184.957221	-196.1002991
3.059678879	59.74961568	539.3249817	3570.016574	-129.6829483
3.919473289	98.23167169	521.0956116	9649.461322	-216.7660187
2.477423708	43.98806345	460.0323486	1934.949726	-81.23079071
2.435466466	46.79999983	1050.128082	2190.239984	-81.52092896
3.68009345	85.30805002	850.9234924	7277.463397	-148.3589172
2.486788088	43.27667263	517.860733	1872.870394	-81.23381348
4.327504031	138.8817977	843.2424927	19288.15373	-236.7048065
2.765138175	53.41625755	434.9411163	2853.29657	-110.2294952
3.302531433	67.97573862	405.2509155	4620.701041	-162.7690628
2.900563337	56.86080896	643.0320587	3233.151596	-87.53071289
2.095162242	42.92608104	801.7522583	1842.648433	-55.67430038
2.594037529	63.70230015	825.8694153	4057.983045	-113.4215088
2.638707746	54.45021957	512.739624	2964.826411	-107.790625
2.568037617	48.12684016	672.3382263	2316.192744	-81.41680908
2.379081195	39.09692117	513.4525299	1528.569245	-63.97530975
3.492879028	88.918531	842.9526978	7906.505155	-169.6595184
3.506890596	115.2949856	399.3785272	13292.9337	-352.7823669
4.102801803	118.9755242	654.6531982	14155.17537	-238.6451813
3.388012566	73.55764891	655.4697876	5410.727713	-159.433519
2.081114305	46.34670395	641.322876	2148.016967	-59.77189636
3.011767906	65.38193226	730.8486938	4274.797066	-104.7131767
3.975106295	100.5017657	486.4766388	10100.60491	-188.9786072
3.474341848	79.68990228	480.2259979	6350.480526	-178.9105225
3.261656029	72.14516041	769.8928833	5204.924171	-128.045459
3.723227909	85.23528701	605.3708496	7265.054152	-194.6329956
3.775664656	92.37154563	735.0384521	8532.502442	-197.3296143
3.800665049	94.71960332	769.5071106	8971.803254	-195.6255096
4.236201664	122.5172965	882.2402344	15010.48793	-182.9253204
2.06262297	31.11269603	469.5223389	967.999854	-36.33592148
4.18635625	133.678393	1211.625763	17869.91276	-173.3815079
3.607114402	80.6637104	530.8371887	6506.634175	-191.230867
3.2089873	98.84495368	334.8997917	9770.324867	-326.2941193
4.080394467	110.9285572	473.6727295	12305.14481	-247.0947037
2.829525975	60.8978008	739.4128113	3708.542142	-96.19594193
3.519528976	82.40276485	453.6324463	6790.215655	-202.326236
3.276645357	76.48818506	1074.723663	5850.442454	-147.4519836
3.478363555	71.33725515	501.0509644	5089.003972	-148.1570099
2.222663196	43.56300064	844.8374634	1897.735025	-40.16517448
4.050312388	112.2226304	792.6488037	12593.91877	-255.6637451
4.212018632	118.4838066	869.3280029	14038.41242	-241.2032028
4.556559707	149.750065	859.2761841	22425.08196	-294.7763184
3.500908815	81.21786902	514.7241669	6596.342248	-150.4736679
3.303918763	64.87154046	563.219635	4208.316762	-111.7355919
3.236189742	69.19716065	560.0867004	4788.247042	-153.1329803
2.888035055	71.22936829	848.0100098	5073.622907	-116.0188217
2.688853449	47.31250071	555.4783325	2238.472724	-59.81102524
2.64226953	52.50628044	491.1522369	2756.909486	-101.4435104
3.302536729	73.65947171	309.3341866	5425.717772	-195.7727631
3.743598452	93.10573268	761.4125671	8668.677458	-149.3270905
4.172625459	122.944916	618.9870758	15115.45237	-278.8074646
2.95075352	65.88174761	713.510498	4340.404669	-129.7624908
3.635756849	78.52132476	381.8796997	6165.598443	-203.3522736
3.178713774	71.59331635	741.8139038	5125.602946	-139.3231705
3.167604255	65.64524644	661.2608643	4309.298381	-126.0123581
3.493531232	73.49988533	388.4940491	5402.233144	-178.1357788
2.285780963	43.61101789	545.0086365	1901.920881	-73.98590851
2.26235538	39.42399243	900.8462219	1554.25118	-55.67066154
2.74832258	57.19457312	948.8979187	3271.219194	-55.97860794
4.276513071	125.9230634	918.4051208	15856.6179	-245.9758377
3.792145628	86.21591595	608.2545013	7433.184163	-196.3424759
3.454798231	71.01736466	423.4787827	5043.466083	-182.2127487
3.573025406	80.21526688	612.8210297	6434.489041	-173.9506744
2.866289224	67.45769478	747.3722229	4550.540585	-122.6422005
3.834229471	92.03570058	474.5065689	8470.570182	-236.4700073
4.191505926	127.5414287	975.1034241	16266.81604	-212.1505707
3.064446281	82.53579413	1670.036682	6812.157312	-104.6072235
3.428899132	99.57769501	488.2364426	9915.717343	-264.9891663
2.654836995	50.82400794	662.2868042	2583.079783	-87.70914001
3.198423248	61.3994868	664.867157	3769.896979	-99.0281189
3.373051105	69.56109656	478.4380951	4838.746155	-157.3154602
4.004137566	100.2586605	636.098877	10051.799	-228.4324097
3.56359082	87.34163425	425.6605682	7628.561073	-170.3091064
3.39274741	118.9262858	446.6442413	14143.46146	-285.2521973
3.197306008	83.94273402	1273.384186	7046.382595	-139.1514832
3.618409972	88.88871486	475.5497894	7901.20363	-193.8158508
3.614663592	93.79893945	676.0475159	8798.241041	-170.402153
2.818188311	57.65601055	518.8937683	3324.215553	-111.0009003
2.506540432	44.19533428	609.782196	1953.227572	-78.14314194
3.95280691	124.5030935	655.5015564	15501.02028	-266.3805298
3.898457553	98.28748283	803.1856689	9660.42928	-152.9469193
3.267894148	89.9012409	375.5606995	8082.233115	-286.5705933
3.977875405	100.0914191	576.7293854	10018.29219	-202.3711273
3.350880423	72.85998036	365.0085526	5308.576738	-147.1011032

log.sigma.1.5.mm.3D_firstorder_Kurtosis	log.sigma.1.5.mm.3D_firstorder_Mean	log.sigma.1.5.mm.3D_glrlm_ShortRunLowGrayLevelEmphasis	log.sigma.1.5.mm.3D_glrlm_GrayLevelVariance
3.591636156	-62.73488791	0.027158937	12.74931233
6.149912337	-35.75186196	0.005836852	7.744593191
3.341272084	-73.41211003	0.019425506	8.518868071
6.284428774	-44.11536144	0.004122556	23.10590955
2.767081424	-96.22663173	0.023531689	12.26854016
2.962545257	-74.59668209	0.032671243	9.15198976
2.45567588	-82.04825064	0.020339405	32.11958188
3.593589238	-102.8281976	0.010820303	16.53281487
3.035509719	-34.33536506	0.005131638	36.12587566
9.737680561	-30.99625231	0.018907778	8.403833308
2.659748837	-78.00664274	0.021728184	9.113396847
6.257178424	-39.93814174	0.016569377	10.03583648
4.609045898	-36.00929309	0.00880368	8.199691773
4.655931851	-50.38558421	0.013592488	8.072608289
4.854978563	-50.85700288	0.011035177	22.00215722
16.71011696	-6.66847413	0.004851655	3.055144447
3.485952565	-97.9379061	0.046838281	12.13498759
4.2231356	-43.48054486	0.007611398	6.514690274
2.984876352	-90.92691083	0.021601131	16.16173277
6.863526335	-18.41824979	0.007629701	4.21086332
20.98372415	-20.36672512	0.006445185	4.781148153
6.874349822	-48.11665241	0.013071639	12.62298415
7.329225785	-21.99318933	0.013415416	3.997027419
3.255680437	-55.78011999	0.015682935	32.00467178
5.846512002	-34.42397372	0.011513725	5.23229894
2.965782423	-68.13642602	0.016053722	7.958942379
8.69061216	-17.76842524	0.015002388	6.50861335
19.69523792	-14.1420852	0.007944748	4.2376754
9.366259583	-25.75092494	0.004373651	8.851936542
6.669794072	-29.15138159	0.011516122	6.118194908
10.78088195	-20.37931271	0.010965834	4.820790481
8.208484136	-15.42003884	0.005620243	3.324627579
5.454111131	-43.71153774	0.006891761	14.86436743
2.000140128	-216.9302326	0.110521843	21.65316763
3.849403085	-93.57294069	0.032280287	23.68692738
4.038808038	-50.09467436	0.012143711	9.791543499
12.5838825	-14.4599873	0.007072609	5.074827486
7.307555806	-19.80405812	0.006111124	8.79425905
2.621513737	-69.47467384	0.022013524	16.55084759
3.08945441	-64.93060305	0.015108006	10.87933233
6.857448458	-34.93224106	0.005304449	9.757100138
3.57714786	-84.68801841	0.010356156	12.2298872
5.009910428	-76.36943206	0.016425569	14.50487212
5.884463153	-84.25124966	0.023334997	15.38490599
4.757120051	-42.54195758	0.009336492	25.32612216
11.47088966	-8.246532878	0.007423746	2.10868934
8.027610911	-32.88434648	0.004346604	31.057751
2.968326011	-83.24557014	0.018650247	10.59659682
1.676188813	-190.2203182	0.163697103	15.92742158
2.37281079	-103.1230376	0.042310481	19.88309108
9.716656077	-20.83061402	0.0075653	7.686285957
2.37753282	-80.9261348	0.03867154	11.34118801
8.013933572	-36.9096161	0.002094152	10.98437092
4.043851556	-55.74403283	0.019279858	8.871377572
15.9219686	-9.621257873	0.006046857	4.369057757
3.71428089	-96.92416876	0.013134692	20.91085677
4.439775838	-101.8945292	0.007637442	23.38865401
2.638052104	-99.89966526	0.006940097	36.0845588
5.110813218	-51.0111526	0.012988406	11.4689183
5.897411689	-34.4489544	0.020087374	7.410856896
3.830183086	-50.71645248	0.009482519	8.441349972
8.528997815	-21.27940058	0.004663676	10.85265869
7.784799436	-9.122826287	0.006878102	4.654094784
6.636874675	-19.70170468	0.006247429	5.680491483
2.24081736	-91.05563496	0.045664103	8.472654634
5.32241115	-30.00379969	0.009255257	15.52060043
3.010332414	-129.0702903	0.022803288	25.00619202
5.771435403	-31.43541272	0.006195201	8.521908667
2.489072302	-97.87921368	0.026033721	10.19777315
6.243463754	-35.36202512	0.005097246	9.838644472
5.227078952	-33.52831128	0.008333041	7.968463913
2.616868447	-71.9159905	0.023578187	8.968224013
9.356785929	-16.04675118	0.007598477	4.363114183
16.37943364	-12.73264108	0.006961705	3.400786486
15.12547529	-6.649363506	0.003802974	6.865915984
4.008560771	-90.24297252	0.007157016	26.52993729
3.380647196	-79.43378657	0.009457764	12.67775919
2.642849534	-86.6289018	0.024009332	8.141860571
3.804821053	-65.22489794	0.010388577	11.06336546
7.368295861	-28.89881323	0.007591413	9.539461964
2.802122899	-116.709399	0.02852891	13.97237698
4.789133087	-49.82509373	0.005967139	28.2423339
17.36460518	-16.21191344	0.000678257	13.92474162
3.44961579	-143.4909144	0.049812481	16.02860374
8.849864626	-19.39702497	0.008338731	5.412999505
5.842045091	-24.54847357	0.009086842	7.009983804
3.313163231	-68.59118263	0.012454188	8.050598511
3.5005401	-104.1886168	0.012198956	16.71642039
2.598137574	-48.11991026	0.036738318	12.44826232
2.388235435	-157.7050319	0.0901393	22.99838716
14.38014564	-31.95515704	0.001188481	13.95106956
3.563645364	-71.13704894	0.01541273	13.08520221
5.292381361	-43.06968813	0.018365079	15.98587381
6.005989436	-27.15078012	0.008362106	6.761310334
8.161177775	-17.6357342	0.009736511	4.093699916
4.028644285	-126.8362015	0.047143326	26.05057218
4.28921417	-31.89665294	0.008248515	16.9249537
2.431288241	-179.8055773	0.065633148	12.88651391
3.143144563	-71.65614647	0.01139551	16.63689841
3.453799407	-41.5864924	0.027015818	8.962167793

log.sigma.1.5.mm.3D_glrlm_LowGrayLevelRunEmphasis	log.sigma.1.5.mm.3D_glrlm_GrayLevelNonUniformityNormalized	log.sigma.1.5.mm.3D_glrlm_RunVariance	log.sigma.1.5.mm.3D_glrlm_GrayLevelNonUniformity
0.028244887	0.095997944	0.226391438	29.81488378
0.006575201	0.167224864	0.728424878	171.9642172
0.020097457	0.106611785	0.136735328	16.93961375
0.004446662	0.088032582	0.404649933	139.2236518
0.024003389	0.083389226	0.058080859	7.972576589
0.033903434	0.097981413	0.139486774	21.38705989
0.020445836	0.066848261	0.020835495	4.582270425
0.011167472	0.075004252	0.117037152	27.44909153
0.00533091	0.050443516	0.101286052	68.78763906
0.020332338	0.130695386	0.489136141	238.184634
0.022751022	0.096785534	0.177987491	32.09688417
0.017523451	0.110867117	0.249924296	71.24492128
0.00961244	0.113536975	0.3681598	127.7913812
0.014570798	0.113409377	0.241800005	51.13977014
0.011204424	0.069769745	0.062143166	24.00656554
0.00648091	0.309241099	1.180153662	2080.489502
0.048097141	0.088243981	0.080688165	17.89801689
0.008445095	0.1330153	0.438493037	163.2436967
0.022129255	0.075859282	0.087252128	12.839269
0.009260906	0.205555094	0.930400503	385.9496551
0.007755557	0.210760169	0.857266429	736.2520604
0.013692703	0.092228529	0.172651726	74.52384624
0.015656895	0.197201638	0.887859504	398.7514472
0.01582406	0.056432293	0.037632859	7.240934259
0.01269607	0.168275711	0.440409003	126.4699237
0.016766019	0.110886782	0.190998793	29.17108868
0.016711076	0.161525929	0.526489555	283.0562711
0.009619768	0.277771658	0.951350156	718.2248222
0.005154957	0.203533246	0.904371703	519.3938337
0.012920551	0.185743535	0.722237233	227.1984231
0.012639834	0.196302232	0.67147519	594.0980141
0.006963126	0.219777878	0.898428773	1326.453024
0.007471104	0.105936871	0.434301929	117.7492359
0.111020927	0.093103087	0.010204082	1.382417582
0.032621216	0.069086198	0.058785541	7.403771773
0.0130323	0.106835785	0.362912219	116.7575457
0.008620777	0.285036244	1.09092234	1213.077476
0.007033716	0.148145175	0.628425543	334.7394945
0.022564574	0.071317315	0.069866872	21.51205413
0.015825551	0.098533733	0.29606274	44.2131546
0.005710809	0.123299858	0.397676429	71.59443325
0.010794783	0.085711968	0.148042916	29.13871711
0.017051695	0.08418153	0.165458411	61.6332092
0.024097911	0.082499335	0.148315933	32.55975009
0.009687367	0.063990347	0.085853996	66.4311426
0.009187746	0.27005916	0.767655299	1147.878872
0.004572854	0.071464901	0.12674655	177.9958481
0.019380169	0.090707994	0.110228312	18.82128339
0.170190618	0.122293334	0.038266178	2.588611389
0.043279487	0.065397642	0.047354189	9.105704928
0.008697766	0.172259577	0.66788347	515.6411556
0.039925718	0.092637108	0.227424421	32.08217937
0.002321493	0.127215136	0.40188796	326.0046381
0.020043022	0.102206938	0.153556789	21.71710669
0.007439851	0.257931975	0.967456685	1939.37755
0.013484501	0.069513489	0.113119934	21.39632773
0.007848563	0.065966671	0.073850194	54.05654787
0.00704106	0.047506375	0.033091406	21.06717645
0.013349824	0.109111382	0.155747872	34.35752347
0.02133194	0.121552058	0.171006337	49.79403728
0.010253202	0.117365003	0.387601259	138.1083679
0.005446965	0.168630703	0.790509021	812.5455526
0.008133871	0.18674505	0.620073351	565.0258303
0.007240889	0.193965312	0.658808287	251.2757594
0.046524482	0.113348547	0.068588098	5.635990219
0.009795123	0.092658497	0.207866749	92.77652594
0.023065747	0.066215802	0.042501425	6.677125573
0.007023519	0.153519789	0.629210561	394.9760866
0.026576749	0.089093783	0.080402379	11.84489641
0.005646021	0.13201302	0.490034942	184.7888234
0.009081222	0.130863401	0.389287727	275.4787891
0.024519722	0.097311108	0.166811575	48.80666977
0.009349642	0.237521253	1.106704185	1082.788755
0.008496479	0.242264809	0.844024515	1802.708425
0.004499289	0.186652963	0.592627132	2015.421089
0.007307944	0.061946348	0.070577427	26.02712508
0.009800623	0.082657928	0.108639635	34.59385116
0.024873828	0.100533366	0.124487752	20.29947713
0.010943664	0.095478821	0.243723173	75.10802241
0.008606449	0.161681872	0.774821582	313.6638374
0.028831515	0.080227153	0.054462448	11.36963331
0.006160528	0.069035854	0.144987504	53.95930228
0.000755888	0.159050112	0.555744514	596.919338
0.05032647	0.111805409	0.054914294	3.296201314
0.009731886	0.186698216	0.733088898	664.3319317
0.010013747	0.131701214	0.298920964	384.4486136
0.013038277	0.106560521	0.179925829	34.09037519
0.012540607	0.072481823	0.070392246	40.08530236
0.037538865	0.09487178	0.058238158	4.485893574
0.090383441	0.099749343	0.019097222	1.272435897
0.001317608	0.137087603	0.58249509	359.1827953
0.015779835	0.092724751	0.144578334	25.22095096
0.019158932	0.101477986	0.233229406	36.18414905
0.009564329	0.164123002	0.724942565	432.4631453
0.011446874	0.202854422	0.723061046	1043.637116
0.047441863	0.075226745	0.04762675	4.52644835
0.008678967	0.077061399	0.191857022	46.36588482
0.06646952	0.121043942	0.058032427	2.760312152
0.011561707	0.071670454	0.049658803	11.14734913
0.027968288	0.112937781	0.159629411	27.92042936

log.sigma.1.5.mm.3D_glrlm_LongRunEmphasis	log.sigma.1.5.mm.3D_glrlm_ShortRunHighGrayLevelEmphasis	log.sigma.1.5.mm.3D_glrlm_RunLengthNonUniformity	log.sigma.1.5.mm.3D_glrlm_ShortRunEmphasis
1.583176045	94.53606708	239.9023888	0.899604249
2.606903555	177.4110616	645.1745576	0.818875408
1.384755392	105.9034758	130.4698828	0.922862576
1.900962435	319.3135975	1168.802909	0.882037979
1.176734927	120.7241827	85.94813569	0.958668026
1.391343748	73.70777008	177.8293018	0.920968742
1.065257566	293.0303538	65.73766076	0.983685609
1.319048346	177.768984	309.787815	0.935934442
1.273539578	344.9317788	1180.889983	0.944778011
2.114276979	92.94601785	1252.879088	0.854437512
1.467654346	84.49707956	266.6192231	0.915180805
1.642888219	110.671621	485.0554829	0.890957758
1.845612998	132.2228713	827.7185517	0.880560228
1.617435313	103.652338	344.5379887	0.895345831
1.182056286	283.0914087	308.8796575	0.958728628
3.797362589	124.3217884	3188.006399	0.712861245
1.228973388	66.85425811	178.7673143	0.951052932
2.045910659	134.6338625	841.0008032	0.852912577
1.245648418	127.252005	147.7273756	0.947607951
3.151967929	102.4441994	1025.65989	0.766142984
2.970396024	121.0512381	1959.010052	0.777473375
1.443946626	133.376236	657.8420204	0.920042158
2.996732686	69.8663875	1153.997718	0.782969679
1.11315004	255.9534371	119.6171165	0.973070527
2.083353093	100.6758624	502.1602382	0.843062348
1.51668165	104.1955776	205.8285641	0.90456384
2.237317362	83.1872481	1145.370115	0.835516683
3.249492273	104.2392216	1358.366273	0.751532054
3.062144583	201.4059552	1426.454186	0.77482143
2.651613468	98.90846055	740.537847	0.805745284
2.60149675	89.3030427	1795.513544	0.798586359
3.064018568	124.8924824	3335.494906	0.770951901
1.999154089	191.2772468	786.8609399	0.865761786
1.032967033	83.33580586	14.56043956	0.991758242
1.172247593	154.2144732	96.83968596	0.960978254
1.856758123	115.9386044	792.7589074	0.8753923
3.56767882	116.6451539	2103.62934	0.729988746
2.430792964	158.1402105	1437.727554	0.825125747
1.204171458	126.1503874	267.8491803	0.954286058
1.733781709	123.2213857	332.9076915	0.884084372
1.92231997	255.2542206	420.3411396	0.873739495
1.405811232	163.911359	276.5854202	0.920530615
1.437273023	127.9719903	594.4730382	0.91905007
1.388643272	125.1746377	327.5725235	0.927793101
1.240035105	206.8802288	908.0080034	0.948871135
2.846748152	95.15743345	2367.863376	0.774711245
1.343173196	339.4595471	2086.046297	0.932010219
1.319648258	102.9651381	173.5010213	0.931452641
1.123076923	62.46934732	19.57435897	0.969230769
1.147779914	104.5783974	126.5464948	0.963688133
2.530213856	131.4930648	1848.089375	0.81366743
1.587827903	78.08227051	267.7845559	0.899823614
1.961351084	490.7810035	1791.337167	0.861213906
1.424134787	102.4056637	171.6757485	0.917261619
3.237521693	128.8256567	3985.163931	0.755901562
1.300942147	189.4271229	264.4471736	0.941181768
1.208521086	250.0003548	728.8971682	0.95485718
1.100240744	370.5031389	416.0335125	0.975716863
1.42329402	197.8037098	254.994902	0.918312687
1.473482615	82.12667818	324.8145091	0.909388282
1.92061745	130.5161899	835.7701428	0.86737787
2.77132681	195.7114841	2876.178574	0.800773888
2.442465419	123.2444793	1885.605381	0.816982532
2.560426554	148.1755912	783.287285	0.805037177
1.211169008	64.75156176	43.86586336	0.951006418
1.538161888	196.1957847	785.0430594	0.9054311
1.128784407	174.0186565	93.28906862	0.969530743
2.44961469	159.7971041	1612.432467	0.819440242
1.239163079	94.06900195	115.4067127	0.945713723
2.136803828	216.4810661	949.8864002	0.849275108
1.921330194	149.4270151	1499.147364	0.867830586
1.449647846	87.93748485	402.7515135	0.914904065
3.5150409	100.1689896	2339.969511	0.743975233
2.970302511	108.7833351	4116.849215	0.772045622
2.402065582	219.6787662	6721.426074	0.817116571
1.198238163	329.8826564	375.465927	0.956889083
1.297786954	175.437285	357.6419981	0.939447309
1.359762804	78.33431244	166.3981044	0.924727749
1.603475635	151.6157532	612.4139053	0.901788232
2.724664001	140.5018718	1177.003861	0.806787532
1.164505764	124.4457477	128.7654352	0.962101101
1.371491119	340.050416	655.0272526	0.93187629
2.284888612	1744.638423	2442.314282	0.833908412
1.166733331	110.3676464	26.73831979	0.961482226
2.681587686	108.2148818	2122.895772	0.800594742
1.746400438	121.77387	2140.937724	0.87985386
1.484612308	124.4630541	253.2092509	0.909702351
1.20565694	159.9059063	489.7968754	0.9536877
1.187342246	100.7110773	42.36877255	0.955152115
1.0625	127.6266026	12.29166667	0.984375
2.299287666	967.5812262	1742.556378	0.841613849
1.397849564	174.3180597	222.4318098	0.922060977
1.600415691	135.3402199	273.6606481	0.897223144
2.650928761	120.2277236	1589.870023	0.804774367
2.697092602	90.6060876	3016.63208	0.794063528
1.145734204	149.0207243	54.91092782	0.965015095
1.472769062	202.2390267	489.238722	0.9199758
1.176330915	79.79228421	20.66012249	0.959987275
1.149771417	252.0240744	142.0021035	0.96483022
1.44827621	91.06008142	196.3170347	0.910670044

log.sigma.1.5.mm.3D_glrlm_LongRunHighGrayLevelEmphasis	log.sigma.1.5.mm.3D_glrlm_RunPercentage	log.sigma.1.5.mm.3D_glrlm_LongRunLowGrayLevelEmphasis	log.sigma.1.5.mm.3D_glrlm_RunEntropy
187.3911334	0.859791178	0.033970827	4.21625613
637.6440116	0.731893196	0.012937657	4.046068146
172.3075156	0.897435897	0.023361106	3.862281287
722.1757026	0.817939433	0.006811293	4.722317192
145.5803231	0.946686976	0.026008859	3.947247136
113.2379933	0.894388398	0.039832923	4.031468673
312.9722483	0.979120879	0.020871559	4.200950999
264.8190327	0.912526376	0.012824457	4.375747889
460.4185834	0.923858662	0.006284804	4.910779537
244.0631231	0.785451289	0.030446884	4.18535198
148.6800023	0.881751227	0.028023414	4.056444301
217.1094158	0.848389391	0.022770462	4.158206129
288.4514449	0.823741412	0.015078819	4.170971318
192.5357958	0.853875291	0.02007049	4.072525528
344.0154318	0.945266272	0.011948171	4.427730483
678.4294437	0.620684266	0.022215949	3.702672906
83.53426316	0.934420418	0.053944189	4.012099579
366.1094083	0.790444092	0.014106023	4.029371039
165.4911704	0.929839391	0.024535024	4.255121692
462.0180357	0.67533692	0.023905192	3.98423443
504.6983448	0.688245548	0.019176336	3.903222303
212.7037787	0.888550394	0.01698301	4.236249482
299.0620892	0.691596417	0.035554857	3.911317893
294.5544663	0.964719491	0.016409129	4.484127944
281.7719125	0.782291667	0.020500906	3.792130147
200.1145546	0.870606215	0.020468043	3.890773344
228.8838144	0.765265316	0.029145386	4.043849441
483.7603983	0.66301965	0.024638754	3.679478388
884.1476365	0.685000414	0.012167664	4.134875641
372.4840914	0.721750193	0.024444338	3.959318988
319.0724782	0.720341311	0.025558975	3.892094464
530.456334	0.682414721	0.018804062	3.856947274
480.7043327	0.799966772	0.011570479	4.477080268
85.58168498	0.98974359	0.113017264	3.509159833
186.7557063	0.948264125	0.034127555	4.33189194
277.0724069	0.818994349	0.018722544	4.245771573
628.1387523	0.636009335	0.023098425	3.804237226
481.3619085	0.745128882	0.014329302	4.250390935
155.6289748	0.93961179	0.025012548	4.2812234
282.8615629	0.835123908	0.020114092	4.225639532
594.5685677	0.811511565	0.008618544	4.172208841
267.0807026	0.892186554	0.012933981	4.232434382
217.1737304	0.888256161	0.02020124	4.329714044
199.3171148	0.898720869	0.027742669	4.28692683
262.0594681	0.931010693	0.011282044	4.619215032
366.6633901	0.694154751	0.023478741	3.464908177
476.9696217	0.906941565	0.005692452	4.704337103
154.2445556	0.909919028	0.022648233	4.033970482
69.04352314	0.961538462	0.196164678	3.318213496
121.5889767	0.953635406	0.047170496	4.286942436
431.539053	0.733484163	0.017700591	4.134576513
169.4989098	0.85875167	0.04632253	4.159061607
1181.013764	0.801733478	0.003873139	4.267471143
166.4072114	0.888638558	0.023828146	4.002756581
575.043032	0.664772959	0.020072494	3.816648662
274.3252867	0.918484501	0.015161839	4.446039147
314.7820512	0.939573042	0.008811864	4.545727335
413.6878473	0.968256634	0.007453244	4.729508657
321.6999732	0.889178618	0.015180741	4.069010128
132.4813266	0.878672829	0.027588321	3.906451692
337.455992	0.808458895	0.015272355	4.1322526
719.9033326	0.7109822	0.012237613	4.327301803
369.611534	0.742286923	0.017854662	3.931622634
508.2294622	0.726625227	0.015034121	3.953899609
86.75573837	0.937590711	0.050253011	3.549139702
338.3806288	0.86852297	0.012666351	4.422221165
197.0782107	0.96043956	0.024178724	4.34447899
542.1114303	0.741829558	0.013394871	4.201192904
130.6667193	0.929532006	0.028911716	3.959187037
593.6066661	0.780563187	0.009649933	4.249199527
361.3167581	0.809601326	0.01399423	4.098552302
156.254812	0.884411884	0.029123091	4.047012419
523.3093572	0.645687315	0.026139857	3.950296391
445.3502845	0.686655969	0.021566925	3.732889293
646.6837307	0.743646257	0.009773155	4.00799993
415.8399625	0.942048982	0.007978815	4.586754818
250.2568469	0.917678812	0.011438716	4.209607271
128.2606077	0.901442308	0.028896983	3.89184638
298.1942269	0.860982391	0.014163187	4.234440013
525.5833087	0.717085799	0.017407445	4.237775049
155.7123056	0.950955085	0.030135833	4.059039679
512.1432887	0.903334815	0.007176373	4.706728888
4869.469595	0.761115533	0.001353558	4.282192921
127.7502356	0.950372208	0.052580271	3.592076354
394.1140287	0.717003069	0.021103277	4.002397605
243.7260994	0.832137922	0.015603409	4.042541908
228.3129324	0.876290832	0.016025353	3.939649218
202.6064587	0.938879457	0.014050797	4.338217794
123.8787711	0.944615385	0.040796265	3.753280704
132.6266026	0.980769231	0.091360004	3.399134516
2784.445364	0.763914126	0.002364409	4.377578919
291.3511627	0.894483806	0.017621587	4.121756174
248.635147	0.856508876	0.023462795	4.330306356
443.0171108	0.721375011	0.019343854	4.14859168
334.0908162	0.712321326	0.025104823	3.862707469
178.7207792	0.954822955	0.048676249	4.132562871
318.9237689	0.88438914	0.011131737	4.464381395
94.66907006	0.948717949	0.07046621	3.428557661
298.3461925	0.95422369	0.012260303	4.185553093
159.619399	0.882417582	0.032465158	3.915763947

log.sigma.1.5.mm.3D_glrlm_HighGrayLevelRunEmphasis	log.sigma.1.5.mm.3D_glrlm_RunLengthNonUniformityNormalized	log.sigma.1.5.mm.3D_glszm_GrayLevelVariance
107.9169146	0.77173499	19.7175981
223.5497751	0.626835746	14.87162469
116.8785994	0.819139806	11.74074074
365.1221591	0.738980138	45.8869233
125.4398437	0.897613184	17.40689981
80.31479294	0.814184208	17.5125
297.0187327	0.958329011	33.68387326
191.9822266	0.84614583	28.30485113
364.1206496	0.86572523	47.79779438
110.1621369	0.687080366	22.53216433
94.10591828	0.803149919	15.03502489
125.8608552	0.754252913	21.19541848
151.1230052	0.734883345	19.4836314
116.2546312	0.763126267	18.31722521
294.3471707	0.897317261	36.10635751
175.7672838	0.47286052	15.78101618
69.82592372	0.880422468	19.91675338
162.4831824	0.684191064	13.59952053
133.9826854	0.872174065	24.08412098
137.3672219	0.545026796	12.72064662
159.7539337	0.560669759	16.33941439
145.2490904	0.81310945	24.46633119
92.05506836	0.569748266	12.7347468
263.3771254	0.93170297	38.24445646
123.2489805	0.666988476	9.122019537
118.9073891	0.781259174	10.00055363
100.1431537	0.653409285	19.86499766
141.9345956	0.524042072	16.22939534
268.1251388	0.557926393	23.32003076
127.4324464	0.604885849	13.56085375
114.6196818	0.592952472	16.80057904
164.9117047	0.551752611	12.28787537
224.876449	0.707733834	32.09074659
83.78498168	0.979591837	22.68639053
160.2518597	0.903042035	37.10714286
135.8523839	0.724989475	18.815786
165.0492174	0.49393975	15.89501305
192.8167082	0.636172385	25.55760957
131.5571893	0.887271125	23.42548162
144.5262201	0.7414828	16.56090762
295.7736196	0.723351251	21.26609057
180.3478301	0.813149122	21.75683594
141.4914237	0.81091342	24.7469242
136.338224	0.829462274	27.46100427
216.515441	0.874494307	40.80277143
124.5113122	0.556758118	7.706442607
362.0989117	0.837356969	59.76747325
111.8596196	0.83576582	15.85946746
63.78418248	0.923467643	16.24852071
107.8949165	0.90845718	26.35992579
163.713123	0.617092663	23.46697394
90.92222434	0.772519898	14.03661841
577.1881595	0.698605152	25.1990186
112.6957092	0.806940223	16.53930055
172.6305089	0.529554615	17.47795972
202.9517735	0.858430797	31.14148996
261.4836888	0.888763111	36.13752907
378.8701997	0.937855407	42.27549435
217.6137122	0.809143716	22.70463967
90.0964512	0.791150569	18.26
155.8148458	0.709960621	14.25965201
248.2152639	0.596688734	31.25371961
150.8115688	0.62255125	15.70953982
187.877994	0.603795867	12.78654504
68.94574941	0.880601581	11.4464
217.2868389	0.783234804	32.56103934
178.4780608	0.923849153	32.30341696
201.3457692	0.626493231	18.80825617
100.7307691	0.867518546	13.14386056
260.0943473	0.678051406	22.7959351
175.5015505	0.711180944	16.98151736
98.46270813	0.801851008	11.34204471
138.9222611	0.512773683	15.10401317
143.4800146	0.55285384	12.39041927
268.7134849	0.622351627	28.06900579
344.9998161	0.893209738	39.14816116
187.9306482	0.853972159	20.05367284
86.83269913	0.822408433	10.32652252
171.5377643	0.7765861	19.265744
178.9795931	0.606437356	26.50706732
130.2667599	0.90675238	18.27311205
366.2034096	0.837928717	52.20882494
2101.226477	0.650629928	42.22658461
113.6820876	0.905366094	22.55401662
138.0410822	0.596241954	17.32550294
138.4933401	0.732795387	18.12696067
140.3295627	0.791023321	12.20128236
167.7542543	0.885342178	26.15208479
105.1156358	0.892759445	14.72928994
128.6266026	0.961805556	24.07638889
1163.427182	0.664721578	33.92122287
193.2245478	0.816832803	15.33339617
151.5895154	0.766871869	30.39648038
153.8984738	0.602938499	17.35356733
116.6161757	0.585958784	13.11575986
154.7938513	0.912171422	36.6875
218.9446214	0.812991859	29.43236459
82.68624129	0.903820024	17.32544379
260.7714914	0.912044055	21.52156508
102.3143553	0.793088278	11.10501448

log.sigma.1.5.mm.3D_glszm_SmallAreaHighGrayLevelEmphasis	log.sigma.1.5.mm.3D_glszm_GrayLevelNonUniformityNormalized	log.sigma.1.5.mm.3D_glszm_SizeZoneNonUniformityNormalized
71.37293842	0.075802616	0.45793698
114.3578616	0.076464198	0.393580247
57.24495401	0.092592593	0.320987654
306.5256178	0.045127682	0.527427935
72.43474503	0.074669187	0.328922495
65.82212374	0.0765625	0.4965625
253.4167977	0.065147739	0.606977572
141.7788032	0.058600964	0.449033827
279.1701515	0.041490373	0.44223529
83.56799323	0.075971767	0.462476623
57.05912641	0.078172766	0.345619705
87.44371801	0.071076707	0.470892281
125.9167978	0.069545657	0.50747323
103.8897714	0.069636102	0.48947733
238.9991145	0.050784857	0.48435416
140.1825139	0.08921217	0.501775331
59.73262545	0.073881374	0.414961267
104.3687724	0.082620585	0.427412004
110.5370614	0.065689981	0.531899811
80.22421035	0.090138689	0.414766152
106.5247168	0.090041617	0.496397146
118.7163476	0.066092335	0.454480499
64.08188524	0.092708953	0.479067519
199.2528779	0.050838291	0.58815576
63.30401549	0.107594272	0.446728056
48.16009983	0.100069204	0.379377163
84.76789613	0.075853811	0.458780196
80.88050021	0.095848399	0.497933884
159.103835	0.066658977	0.454371396
65.5049547	0.090247864	0.414236283
83.14366957	0.089785961	0.495153588
109.4225612	0.089997972	0.434453325
173.5607543	0.055140605	0.477640525
81.63461538	0.088757396	0.73964497
131.4639734	0.054846939	0.524872449
95.23472427	0.071331085	0.431165195
80.6149023	0.082849379	0.438016144
181.0635141	0.063509412	0.5423953
98.78670346	0.057290881	0.420552286
72.19644616	0.078781359	0.385300015
200.1538037	0.0755495	0.484814802
129.9569204	0.067016602	0.420043945
95.10873613	0.061255765	0.416877503
103.6897233	0.059664694	0.411653517
197.9851899	0.047376178	0.487969018
67.48502038	0.187365244	0.381060887
333.1522059	0.043447921	0.504127697
77.50344176	0.078238001	0.390861275
54.34401709	0.100591716	0.431952663
88.50366088	0.059537865	0.394501602
127.0034998	0.06708485	0.472971976
47.78715125	0.078722982	0.39480468
385.8534795	0.067782238	0.463422466
77.00637288	0.074792244	0.435249307
113.6279735	0.098692727	0.462757261
163.1882187	0.053762432	0.525426909
209.2793308	0.050101327	0.480096365
327.3656161	0.044074591	0.599027071
143.285937	0.064732143	0.452646684
83.07185931	0.0854	0.4692
79.76620423	0.085634522	0.395061461
193.5214503	0.056161644	0.491103231
124.2543525	0.088948982	0.476207259
88.87134684	0.091520511	0.298788644
48.97998662	0.0944	0.4848
166.740653	0.052892292	0.445256931
154.8759253	0.056228374	0.595155709
117.747548	0.073067371	0.409891011
51.79652774	0.08792924	0.430280957
171.6431103	0.06702056	0.463559549
116.6337108	0.072700826	0.500859504
45.61088314	0.086538462	0.400641026
77.99002614	0.082549132	0.377314683
91.95711781	0.121444775	0.442023947
227.3521201	0.071194746	0.496230616
269.4288791	0.048595041	0.496735537
135.5675111	0.064753086	0.495802469
39.72180658	0.096607816	0.367982543
110.5770702	0.072	0.461664
128.2296237	0.067507043	0.458370411
51.64157021	0.07017153	0.357540655
297.3760861	0.042628501	0.53956773
1494.652624	0.057048181	0.471934868
100.1081871	0.096952909	0.506925208
105.3425412	0.082787913	0.489754737
114.8026077	0.071484744	0.483145786
88.24637773	0.08611314	0.463517572
132.0770333	0.058720055	0.461486859
86.6025641	0.085798817	0.423076923
128.5833333	0.097222222	0.847222222
792.9757737	0.060554861	0.490627761
87.12630235	0.078518239	0.392402677
134.8951316	0.057027348	0.515719382
98.04645074	0.078794408	0.420880092
80.47118209	0.108777948	0.456207962
114.1018519	0.064814815	0.544753086
188.9318493	0.055597295	0.472986818
64.27403846	0.088757396	0.514792899
195.4961405	0.066632231	0.507231405
40.53791056	0.088248743	0.351623228

log.sigma.1.5.mm.3D_glszm_SizeZoneNonUniformity	log.sigma.1.5.mm.3D_glszm_GrayLevelNonUniformity	log.sigma.1.5.mm.3D_glszm_LargeAreaEmphasis	log.sigma.1.5.mm.3D_glszm_ZoneVariance
53.12068966	8.793103448	87.99137931	78.30640606
88.55555556	17.20444444	1807.097778	1768.215625
17.33333333	5	35.61111111	24.86728395
321.2036125	27.48275862	516.090312	506.0156816
15.13043478	3.434782609	8.630434783	3.809546314
39.725	6.125	42.525	33.2225
32.16981132	3.452830189	2.150943396	0.406550374
66.90604027	8.731543624	32.06040268	24.81744066
295.4131737	27.71556886	24.16167665	19.27942917
206.264574	33.88340807	1713.073991	1686.038655
36.98130841	8.364485981	59.53271028	47.18438292
95.591133	14.42857143	247.4137931	233.5078745
166.4512195	22.81097561	516.1158537	498.7716761
67.05839416	9.540145985	145.4452555	130.5918269
82.8245614	8.684210526	12.44444444	7.91327246
374.3243968	66.55227882	56635.34853	56424.67069
38.59139785	6.870967742	15.92473118	10.48028674
120.9575972	23.38162544	984.2897527	954.2144115
48.93478261	6.043478261	12.86956522	8.956049149
98.29957806	21.3628692	8069.367089	7932.368459
287.9103448	52.22413793	11389.94655	11313.38405
142.2523962	20.68690096	113.2428115	104.8087048
157.1341463	30.40853659	6009.908537	5930.655116
50.58139535	4.372093023	3.872093023	1.480394808
75.49704142	18.18343195	966.9585799	934.6908021
32.24705882	8.505882353	73.50588235	60.88249135
179.8418367	29.73469388	2350.058673	2315.961416
175.2727273	33.73863636	14252.61648	14130.17471
231.7294118	33.99607843	6947.176471	6894.086674
98.58823529	21.4789916	2724.852941	2674.251836
253.0234834	45.88062622	7680.279843	7612.692782
341.0458599	70.64840764	25024.85987	24898.24721
169.0847458	19.51977401	597.9745763	582.5789285
9.615384615	1.153846154	1.461538462	0.130177515
29.39285714	3.071428571	8.089285714	4.017538265
145.3026706	24.03857567	452.7181009	437.0487193
240.0328467	45.40145985	27969.7719	27820.69136
290.7238806	34.04104478	2942.05597	2910.05753
59.29787234	8.078014184	16.34751773	11.16462955
55.0979021	11.26573427	165.3076923	151.2058291
80.96407186	12.61676647	404.5209581	386.1902542
53.765625	8.578125	47.2734375	38.41351318
109.6387833	16.11026616	94.58555133	84.769362
64.21794872	9.307692308	42.44230769	34.52313445
234.7130977	22.78794179	45.31185031	39.9383215
194.7221135	95.74363992	22317.93346	22174.40275
505.6400798	43.5782652	254.7298106	247.2343349
30.48717949	6.102564103	27.97435897	19.42998028
5.615384615	1.307692308	3.846153846	0.982248521
30.37662338	4.584415584	5.688311688	2.093101703
296.553429	42.06220096	5314.749601	5272.406248
48.56097561	9.682926829	86.33333333	75.5983872
308.1759398	45.07518797	2054.488722	2031.405393
33.07894737	5.684210526	48.67105263	38.78168283
491.4482109	104.8116761	37002.72881	36889.39254
76.71232877	7.849315068	23.03424658	17.76942203
196.3594132	20.49144254	15.63325183	11.08770273
178.5100671	13.13422819	4.295302013	1.933201207
50.69642857	7.25	79.875	69.8848852
46.92	8.54	217.14	195.4244
120.0986842	26.03289474	719.7269737	696.8194144
552.982238	63.23801066	8628.902309	8592.68885
262.3901996	49.01088929	6330.14882	6275.480107
69.31896552	21.23275862	3192.310345	3133.312054
12.12	2.36	8.76	4.2656
147.8253012	17.56024096	249.9096386	237.8695747
40.47058824	3.823529412	5.044117647	2.659818339
236.0972222	42.08680556	3767.960069	3731.730538
26.67741935	5.451612903	14.20967742	8.889958377
162.7094017	23.52421652	1324.08547	1298.020227
275.4727273	39.98545455	1421.565455	1399.235534
62.5	13.5	117.0064103	103.7959813
217.7105719	47.63084922	25188.27556	25038.69037
453.5165692	124.6023392	32394.02534	32282.52327
913.5605649	131.0695274	23227.89951	23165.70303
109.2818182	10.69090909	13.96363636	9.853801653
89.24444444	11.65555556	33.92222222	27.50444444
26.12676056	6.85915493	32.73239437	22.77881373
115.416	18	204.732	191.394896
206.7250554	30.44567627	3281.343681	3245.396876
23.95522388	4.701492537	8.970149254	4.024504344
184.5321637	14.57894737	93.90350877	87.50645498
435.595883	52.65547129	4725.342362	4696.824679
9.631578947	1.842105263	4.157894737	1.495844875
324.7073906	54.88838612	8130.720965	8074.708271
361.3930481	53.47058824	1730.164439	1708.182381
51.45045045	9.558558559	67.09009009	56.27725022
119.5250965	15.20849421	19.14671815	13.97504509
11	2.230769231	6.153846154	2.455621302
10.16666667	1.166666667	1.25	0.076388889
356.6863824	44.02338377	2541.651994	2519.405266
40.41747573	8.087378641	57.32038835	48.609294
74.77931034	8.268965517	97.2	88.96903686
213.8070866	40.02755906	5169.925197	5118.243815
366.3349938	87.3486924	15121.16812	15040.28016
19.61111111	2.333333333	5.694444444	2.631944444
114.4628099	13.45454545	77.49586777	69.60023222
6.692307692	1.153846154	5.384615385	1.976331361
44.63636364	5.863636364	6.852272727	3.421358471
28.48148148	7.148148148	68.88888889	56.93949093

log.sigma.1.5.mm.3D_glszm_ZonePercentage	log.sigma.1.5.mm.3D_glszm_LargeAreaLowGrayLevelEmphasis	log.sigma.1.5.mm.3D_glszm_LargeAreaHighGrayLevelEmphasis	log.sigma.1.5.mm.3D_glszm_HighGrayLevelZoneEmphasis
0.32132964	0.736026023	12559.51724	96.43103448
0.160370634	6.9429496	472778.72	171.7333333
0.305084746	0.30649479	5157.925926	98.85185185
0.31505432	1.285546781	209000.7323	380.3743842
0.455445545	0.121635811	966.1956522	130.4130435
0.327868852	0.639995745	3572.85	85.575
0.757142857	0.03204791	597.2264151	306.245283
0.371571072	0.160478607	7744.114094	187.3355705
0.452574526	0.08047171	8542.362275	389.6601796
0.192324278	14.33740883	207971.4529	111.4663677
0.284574468	0.589497293	7309.981308	87.20560748
0.268163804	1.761124723	36351.73399	118.6502463
0.24011713	3.294494435	83198.89329	162.679878
0.259469697	1.241615105	18003.9562	133.2919708
0.46978022	0.064257599	3360.567251	316.619883
0.068895456	316.0717112	10203424.26	179.6621984
0.428571429	0.382167349	1024.107527	80.35483871
0.182345361	4.927121949	199181.2686	142.4982332
0.505494505	0.134982569	1679.206522	139.4130435
0.085436193	53.44748155	1227838.19	113.3122363
0.114285714	64.91360788	2009667.864	136.9982759
0.344334433	0.756996255	17849.68371	157.4153355
0.112328767	57.77948371	631082.503	80.74695122
0.646616541	0.036237184	1044.77907	257.6976744
0.176041667	6.600317435	143759.2722	90.97633136
0.281456954	0.506381846	11889.11765	85.58823529
0.171253823	22.46342915	249015.2398	109.1964286
0.090372272	93.75327767	2180648.486	107.6164773
0.137244349	23.14156249	2093163.767	220.4196078
0.140578854	18.25501778	410109.7731	93.1302521
0.121637705	59.96672572	992853.6419	104.5949119
0.088871278	141.6052647	4452897.029	153.4585987
0.254859611	2.296443927	157607.1808	221.1920904
0.866666667	0.146100622	113.0769231	87.92307692
0.495575221	0.112469616	1286.053571	169.3571429
0.252623688	2.687353951	77966.17211	126.9881306
0.081901061	155.5840802	5056791.467	114.2956204
0.176781003	14.58410719	598319.7612	219.1100746
0.439252336	0.198088911	1982.815603	144.5815603
0.266294227	0.894065807	32498.05594	114.3356643
0.233566434	1.245895417	132945.8743	269.2814371
0.335958005	0.23938612	10556.08594	175.671875
0.319174757	0.561285469	17977.59696	132.6539924
0.355353075	0.331971868	7357.224359	139.4679487
0.431390135	0.257171335	8825.60499	251.5051975
0.083469454	170.9712045	2936434.769	107.62818
0.365258558	0.756712434	87388.9671	420.1994018
0.342105263	0.264541864	3960.115385	107.5769231
0.590909091	0.818260621	196.3846154	73.07692308
0.52739726	0.162795658	557.3636364	120.7272727
0.153676471	30.08947493	945268.9346	164.4210526
0.305210918	0.778757474	12114.5935	69.61788618
0.208137715	3.237498222	1307866.605	529.7924812
0.317991632	0.423602735	6376.986842	107.0394737
0.093932425	206.1052934	6680011.556	156.5047081
0.435820896	0.126334546	5714.321918	202.6438356
0.469036697	0.070490478	4315.012225	276.4254279
0.650655022	0.021397679	1516.483221	396.0436242
0.316384181	0.343502215	20578.38393	202.3125
0.214592275	2.687230594	18985.12	106.62
0.208934708	3.594017637	146829.4112	120.5394737
0.166174734	32.25945554	2317475.383	249.19627
0.135247914	41.77778525	967362.2105	166.8076225
0.130190797	15.50946981	661097.2457	157.9267241
0.471698113	0.20894141	667.36	66.8
0.288194444	1.116248801	57967.41265	226.5421687
0.647619048	0.063907069	743.2647059	190.3970588
0.166137871	15.86319091	900345.8472	169.5520833
0.433566434	0.161074608	1826.048387	83.75806452
0.195870536	4.448146213	396988.2849	232.0968661
0.211619854	6.943067511	294256.0418	153.6163636
0.275132275	1.008329349	15840.67308	74.32051282
0.081762789	164.8851734	3873434.095	112.6239168
0.094701865	211.2367176	5001177.739	126.6052632
0.126799366	85.91616898	6305808.437	295.172189
0.493273543	0.048560386	5246.259091	352.2772727
0.394736842	0.172772924	7592.022222	182.05
0.316964286	0.373360113	3816.985915	69.46478873
0.273822563	0.990531905	45136.06	151.148
0.166789941	16.13139475	671359.1729	164.556541
0.44966443	0.110773516	1403.507463	106.9701493
0.395375723	0.24130369	38380.74854	372.5350877
0.187259079	2.189139776	10210216.11	2055.445287
0.612903226	0.113554948	391.5263158	125.7368421
0.133615478	53.63843586	1243424.284	132.5882353
0.21328771	12.2071848	249006.8195	151.578877
0.304109589	0.39520733	12570.11712	120.0990991
0.439728353	0.126479682	3574.158301	172.5714286
0.52	0.138015746	664.8076923	113.9615385
0.923076923	0.098866738	148.5833333	132.5833333
0.212015165	2.025295727	3195389.495	1080.848693
0.338815789	0.258785417	14799.81553	146.1359223
0.348557692	0.621047661	16546.5931	160.9862069
0.139101862	29.26681586	920332.563	133.6791339
0.111188037	117.3288763	1965327.599	107.5902864
0.571428571	0.110778867	905.1666667	152.25
0.355882353	0.379656341	16877.54545	248.8884298
0.541666667	0.2014018	382.9230769	88.92307692
0.539877301	0.043548256	1744.477273	262.8295455
0.289285714	0.60789238	9343.012346	72.95061728

log.sigma.1.5.mm.3D_glszm_SmallAreaEmphasis	log.sigma.1.5.mm.3D_glszm_LowGrayLevelZoneEmphasis	log.sigma.1.5.mm.3D_glszm_ZoneEntropy	log.sigma.1.5.mm.3D_glszm_SmallAreaLowGrayLevelEmphasis
0.701314763	0.048044233	5.211646538	0.039533696
0.651102963	0.013367251	5.444041632	0.010657348
0.573699038	0.038150201	4.718328827	0.030989198
0.754129405	0.006357516	5.994477974	0.005218296
0.566457998	0.03780119	4.898455706	0.032613303
0.728835117	0.060734434	4.832769546	0.052108842
0.80725891	0.024737965	4.591338738	0.023726061
0.695896802	0.018273478	5.492560639	0.015647629
0.691184583	0.006648525	6.221733068	0.005237849
0.706640685	0.043679232	5.533039365	0.033622164
0.609306833	0.038793136	5.469923879	0.028647745
0.712745708	0.03110634	5.440358087	0.02356854
0.739547431	0.014305646	5.321450819	0.011592823
0.724082959	0.022971498	5.180394654	0.018741084
0.722353552	0.018331493	5.727263773	0.017086733
0.736023373	0.010437229	5.323312799	0.008024508
0.668435073	0.073614694	5.191220677	0.062918262
0.679434034	0.014255981	5.466737927	0.011007026
0.75612598	0.031192193	5.078258837	0.02707047
0.669072978	0.018809289	5.302632911	0.014209598
0.732002662	0.014218438	5.272088461	0.011426132
0.699129782	0.0210904	5.557737744	0.017217697
0.715497789	0.034058359	5.20512867	0.027283892
0.79219396	0.021031089	5.227502151	0.020014974
0.693986684	0.025569591	4.881112738	0.02103409
0.638613013	0.033047148	4.879602408	0.028116286
0.704246236	0.033201365	5.521961345	0.027287679
0.733130644	0.024598286	5.111046507	0.0208828
0.700379418	0.010214578	5.770650316	0.008276839
0.668637445	0.030746991	5.25866567	0.025078458
0.730482407	0.027929867	5.302509921	0.023136027
0.685113069	0.011060541	5.433677244	0.008229166
0.717725305	0.012402619	5.789485521	0.010134021
0.884615385	0.118151904	3.546593564	0.111164724
0.740906346	0.053707139	5.03736536	0.050825117
0.682837099	0.02208573	5.568378543	0.017471306
0.687998072	0.023685972	5.47394774	0.019466562
0.763872053	0.010243962	5.495949986	0.008012091
0.672167638	0.034179203	5.489491272	0.029896904
0.644371888	0.032990095	5.301348368	0.029091446
0.723149517	0.011191979	5.290357972	0.009895442
0.672779977	0.01796783	5.330619021	0.015052558
0.670211566	0.030308508	5.742507182	0.025002952
0.665661695	0.039099105	5.64481568	0.02929096
0.725810176	0.012264321	5.934412223	0.009552069
0.640507088	0.015815071	4.766093093	0.011676696
0.737867153	0.006034832	6.212320989	0.004878932
0.648714166	0.030699353	5.118279623	0.024019048
0.675747863	0.119093822	3.700439718	0.044148543
0.649436217	0.056221846	5.29250667	0.044287668
0.715056454	0.015311464	5.670913795	0.011883581
0.651209794	0.076229825	5.223377609	0.06053733
0.707768788	0.003932102	5.743607389	0.003274675
0.681816591	0.037338409	5.114520073	0.032934056
0.70742311	0.015123923	5.457695297	0.01193914
0.7502355	0.020531878	5.399619181	0.017961018
0.720004934	0.010786539	5.957251312	0.009227432
0.800722195	0.008408453	5.634887397	0.007520893
0.697921892	0.028702563	5.270487544	0.026859668
0.709859052	0.040670154	4.987397875	0.034055492
0.651687087	0.019023663	5.392571307	0.014652643
0.728456177	0.008573451	5.907317708	0.006616312
0.717427827	0.011823626	5.348708043	0.009120731
0.555794377	0.014311657	5.632036969	0.010588867
0.716652438	0.071165308	4.05366069	0.062804019
0.693662389	0.014767822	5.90764392	0.009744969
0.797934239	0.030041513	5.002544643	0.028256358
0.665011419	0.01254904	5.696953076	0.009516951
0.679225864	0.044162357	4.745556037	0.039521755
0.707533362	0.010069922	5.642195556	0.008066176
0.734045896	0.015362965	5.491327652	0.01126208
0.655891827	0.048714018	5.197435615	0.041261544
0.637717658	0.020158908	5.710746697	0.014586086
0.691309762	0.015930051	5.170305383	0.012372185
0.731876428	0.00696258	5.867903362	0.005552635
0.731686046	0.010532977	5.731997784	0.009232074
0.73026192	0.014487964	5.371579314	0.012380928
0.628454788	0.044999824	4.782595678	0.038547802
0.705623595	0.019992573	5.482758408	0.016927495
0.703769807	0.016640895	5.694864075	0.013425503
0.614887806	0.051741787	5.022564609	0.048158573
0.761955675	0.010018858	5.915192346	0.009017399
0.714299683	0.001609978	6.066708496	0.001458611
0.736842105	0.070976232	3.997670276	0.067795755
0.727049632	0.017039845	5.39549545	0.013045134
0.722361167	0.014561324	5.577480997	0.011280648
0.704871838	0.022804157	5.011852949	0.019314196
0.704793205	0.017754735	5.630827988	0.014585943
0.66673344	0.05232911	4.286790199	0.045359089
0.9375	0.094960488	3.418295834	0.093983926
0.72776604	0.002525008	5.818168133	0.002253011
0.646930877	0.032479462	5.154805203	0.029592169
0.74549987	0.032613418	5.43995086	0.027541632
0.673609544	0.018818794	5.539916123	0.014406765
0.701134315	0.021987121	5.323602585	0.017085603
0.761236497	0.073384003	4.537844793	0.071137464
0.71425956	0.012510187	5.589766713	0.010463652
0.724230769	0.100406672	3.546593564	0.095499984
0.739257049	0.016886191	5.100049199	0.015638045
0.613197157	0.057032961	5.127741244	0.047314289

log.sigma.1.5.mm.3D_ngtdm_Coarseness	log.sigma.1.5.mm.3D_ngtdm_Complexity	log.sigma.1.5.mm.3D_ngtdm_Strength	log.sigma.1.5.mm.3D_ngtdm_Busyness	log.sigma.1.5.mm.3D_ngtdm_Contrast
0.018364428	0.953835061	2.941239211	0.221070276	0.000347374
0.004550807	0.151067416	1.196562929	0.473859767	2.80E-05
0.040282194	0.888406316	3.255990943	0.148546349	0.000769856
0.003690788	0.918300376	2.42914009	0.255113797	3.76E-05
0.053111028	2.65025532	4.060230487	0.146756954	0.002741545
0.027328412	0.769377306	1.88096441	0.280415732	0.000610015
0.045600511	14.98997252	9.821597957	0.067809459	0.010197887
0.016691046	1.769804801	3.714778139	0.152054137	0.000321787
0.004554229	1.208901014	1.584597312	0.382013774	0.000165653
0.003274314	0.221295638	1.429486976	0.755475196	1.39E-05
0.01633953	0.587729964	1.478084142	0.362960221	0.000346391
0.00991967	0.533429773	2.248261628	0.294594815	8.18E-05
0.005507024	0.23086035	0.803488482	0.547584114	4.36E-05
0.013526687	0.566464326	1.704607953	0.284197432	0.000136791
0.017811313	3.322822175	5.311203326	0.100623588	0.000445598
0.000467033	0.019324574	0.294569608	3.889455411	5.45E-07
0.026671251	1.501152839	2.628677897	0.320773585	0.000935552
0.004264446	0.174707985	0.876197711	0.595563601	2.48E-05
0.032765229	3.025378165	4.40700439	0.125859179	0.001121681
0.002325965	0.044836003	0.499196919	1.303461656	6.48E-06
0.00122015	0.069226451	0.939465638	1.461694796	2.19E-06
0.007738126	0.827495932	2.359515391	0.318880041	8.42E-05
0.00233236	0.047094484	0.461548439	1.573985026	6.41E-06
0.040169543	8.043197984	10.01647259	0.070780415	0.002890849
0.007328305	0.142131682	1.192834554	0.484879452	3.61E-05
0.022531767	0.567430555	2.40556299	0.214679476	0.000335175
0.003176444	0.166953586	1.137908832	0.826532238	1.01E-05
0.001371821	0.06423403	0.940876149	1.46714488	2.43E-06
0.001457283	0.136777605	1.138948633	0.81921317	5.11E-06
0.003675315	0.143009742	1.060580351	0.671934724	1.39E-05
0.001486112	0.086031472	0.852709566	1.445091308	2.99E-06
0.000708401	0.020495571	0.227151483	3.181056648	1.21E-06
0.004549819	0.632615714	2.244537581	0.32555458	4.08E-05
0.168150157	23.08164283	15.53914445	0.093311698	0.10357937
0.053325905	6.816485359	9.584723635	0.070980276	0.002564839
0.004909614	0.302879967	1.020100627	0.592315697	5.33E-05
0.000734728	0.035457571	0.460588318	2.544585084	1.67E-06
0.002315687	0.173813629	0.94821731	0.721815857	9.24E-06
0.018762484	1.336043452	2.042719722	0.321023165	0.000794646
0.012738892	0.508365449	1.962208513	0.258317854	0.000171587
0.010148933	0.5737101	2.951579478	0.168247041	6.76E-05
0.018198597	1.290697629	3.110257207	0.154762705	0.000277019
0.007928723	0.892197493	2.256400078	0.328360019	0.000123822
0.014386307	1.460438443	4.069431406	0.21792473	0.000289829
0.005949803	1.526105835	2.528824493	0.320122788	0.000133768
0.000913318	0.016410496	0.20098876	3.792883829	1.71E-06
0.002509782	1.226263721	2.458175671	0.33838701	3.39E-05
0.025491431	1.32227743	2.941233053	0.206096643	0.000666024
0.097877386	10.71558973	8.933222318	0.148658082	0.048574094
0.035358286	3.593784359	3.984270802	0.214101393	0.002610757
0.001654506	0.114235665	0.791955456	1.051341295	5.20E-06
0.013723077	0.64088335	1.488657443	0.39554161	0.00043536
0.002100408	0.305051683	1.767789974	0.353611067	8.27E-06
0.029090181	1.012129088	3.14117796	0.16055952	0.000422821
0.000474641	0.028796242	0.437120701	3.436563837	6.97E-07
0.019232703	2.500220367	4.399588383	0.130499184	0.000565322
0.007704688	1.730412424	3.028539231	0.211028285	0.000148713
0.012288069	3.658583107	3.687079892	0.165567651	0.000756103
0.016712818	1.16032861	3.672579588	0.141592226	0.000264742
0.014859648	0.559163174	2.228203601	0.320445689	0.000141777
0.00460415	0.226847168	1.011310831	0.546922049	3.54E-05
0.000879111	0.109332579	0.653309907	1.346114925	3.55E-06
0.001605314	0.0529879	0.413123246	1.589146235	4.72E-06
0.003348337	0.094802602	0.926600743	0.757728739	1.52E-05
0.106172291	1.846025882	5.332452816	0.116415258	0.004350765
0.006770678	0.654372795	1.994437268	0.261878253	6.68E-05
0.046774142	8.492421084	8.376885483	0.085225135	0.00352279
0.001738559	0.116696663	0.769304959	0.981246681	8.30E-06
0.042920055	1.717171037	3.528633501	0.144935434	0.001180207
0.003716097	0.298613762	1.602137982	0.385779885	1.88E-05
0.002571412	0.160461513	0.779676736	0.777519508	1.35E-05
0.011941771	0.309353064	0.980965962	0.499404629	0.000231316
0.00078897	0.030242408	0.34968067	2.756559734	1.56E-06
0.000517162	0.02105918	0.33850668	4.356613968	8.89E-07
0.000442456	0.060242041	0.552321653	2.130999089	6.81E-07
0.013985902	3.280761518	4.36087152	0.117284751	0.000471722
0.01522089	0.979211195	2.403091748	0.206527141	0.000262966
0.028089388	0.871043988	2.360429858	0.241126685	0.000569526
0.007912721	0.499409608	1.819817765	0.306834333	7.55E-05
0.002284183	0.199637092	1.226274784	0.695097584	9.71E-06
0.038778925	2.369203598	4.418090467	0.130654693	0.001406694
0.007976802	2.261330682	3.811094561	0.135944235	0.0001484
0.001284132	0.288737078	2.029553846	0.241870612	4.46E-06
0.103355027	10.09724765	11.4511536	0.076319855	0.018701212
0.001289819	0.077145538	0.701390919	1.470689012	2.69E-06
0.002097613	0.125678824	0.555893295	1.128716684	9.34E-06
0.015949943	0.707553793	1.995178898	0.243816134	0.000265645
0.010561223	1.349085189	2.219936601	0.275443047	0.000251055
0.090934969	4.223936347	6.707221019	0.102851381	0.007361121
0.183897206	24.46025641	18.03514515	0.095529279	0.145000355
0.001815846	0.383557781	2.546353417	0.250898284	7.89E-06
0.019860671	1.159203987	3.398901531	0.150569877	0.000471969
0.01701384	1.674674362	4.397803387	0.138176923	0.000232541
0.001794106	0.065156943	0.491631569	1.310234262	7.32E-06
0.000846146	0.034153496	0.331592621	2.881356254	1.73E-06
0.074700185	9.85001611	13.20717393	0.064047038	0.007546815
0.01020132	1.175645325	2.467925326	0.230797603	0.000191958
0.151640375	7.462058541	11.39643047	0.077888383	0.021387353
0.033878457	3.344025487	4.73223073	0.102359738	0.001507598
0.022808888	0.515062061	1.918548837	0.256689453	0.000493686

log.sigma.1.0.mm.3D_gldm_GrayLevelVariance	log.sigma.1.0.mm.3D_gldm_HighGrayLevelEmphasis	log.sigma.1.0.mm.3D_gldm_GrayLevelNonUniformityNormalized	log.sigma.1.0.mm.3D_gldm_DependenceEntropy
4.84015623	71.7534626	0.179564307	5.700979904
2.461288279	97.52031361	0.312162259	5.567901278
3.248300297	52.48587571	0.190270995	5.359148495
8.395482497	192.2483187	0.165161198	6.201189811
4.408195275	34.82178218	0.141848838	5.125853503
3.704850847	39.60655738	0.151941682	5.498468904
14.08265306	130.4142857	0.091836735	4.859370831
6.745244122	94.43142145	0.121920884	5.744550082
12.17230108	175.5304878	0.09203902	6.526227093
2.900082767	64.77231565	0.237999978	5.82122526
3.278059925	38.8643617	0.162842349	5.646231646
3.982067851	63.09114927	0.207530246	5.765016841
2.731537078	79.98096633	0.217250568	5.829229397
2.553030303	44.8030303	0.212680785	5.773467749
8.683794228	140.5549451	0.109784446	5.629496985
0.887440469	70.80162542	0.415900594	5.197697964
4.360551296	34.68663594	0.142899616	5.391475714
2.195715206	76.58634021	0.240633137	5.8375498
5.808748943	50.08241758	0.128426519	5.435898916
1.185834292	51.87058399	0.346200609	5.546307491
1.538574816	84.27507389	0.336330763	5.359914639
4.630550624	77.03960396	0.15895924	5.99285702
1.132190725	50.82020548	0.34699498	5.517352739
11.1727062	97.67669173	0.088473062	5.357824733
1.629547526	48.08229167	0.294461806	5.496516729
2.76031753	41.01986755	0.201942897	5.475707904
2.192311091	53.30013106	0.277884371	5.689534745
1.157767125	68.90397946	0.400295629	5.27399022
2.318906272	122.8162002	0.327075133	5.664575687
1.847002127	64.89604253	0.322594094	5.438670301
1.446289553	51.94049036	0.333375641	5.398214817
0.988115861	85.56854976	0.361568418	5.437391076
5.264083063	97.08711303	0.20260133	5.96800491
9.093333333	42.73333333	0.12	3.373557262
9.221082309	82.30088496	0.108309186	5.223915277
3.539319358	75.6874063	0.195468982	5.901398164
1.398396108	86.30204753	0.401306082	5.124436909
3.000090395	85.88093668	0.26530987	5.712103355
5.916460438	56.45482866	0.117923933	5.684811917
3.910746301	69.74115456	0.18259591	5.81202529
3.532581544	120.6867133	0.224118539	5.826349911
4.899580466	100.2309711	0.144811623	5.705628421
5.491463616	68.52427184	0.151377957	5.990991632
6.160989202	66.60364465	0.132943478	5.913987749
10.07442418	103.2358744	0.104425989	6.186995628
0.721049138	70.28079059	0.4026804	5.103571731
11.67214339	261.5331391	0.122118699	6.288966077
4.349415205	65.71052632	0.14881502	5.575671118
7.969008264	39.22727273	0.107438017	3.6412498
7.759992494	62.47945205	0.10189529	5.156855728
2.509512627	67.63995098	0.292174765	5.700156274
4.143058574	65.86104218	0.172669002	5.694750209
3.857491141	355.3818466	0.240736969	5.845201959
3.392342571	43.78661088	0.176414979	5.322914037
1.299527741	87.88200955	0.394289546	5.179574416
8.643653375	118.4865672	0.118823791	5.836098152
8.937058118	116.6490826	0.107069586	5.9705437
14.0803236	168.8056769	0.075008104	5.931948989
4.022662709	93.57627119	0.184477641	5.741620731
2.593600914	48.92703863	0.20127466	5.430535013
3.094731522	74.91890034	0.218004511	5.750087452
3.160686146	125.7960449	0.290436879	5.671467662
1.510763685	54.97938144	0.326227434	5.514569201
1.710446843	125.3294052	0.319483651	5.578326373
3.727305091	35.98113208	0.178355287	4.506617512
5.463493441	102.8420139	0.174691659	6.092484774
11.37324263	107.6	0.093696145	5.282781113
2.643958127	99.7747332	0.277897027	5.664328168
4.403736124	46.79020979	0.13805076	5.226237164
3.301236834	119.2840402	0.249715377	5.7697609
2.606186918	99.38514813	0.243485337	5.849428933
3.35795626	53.2010582	0.174746259	5.666204984
1.228475615	67.94105144	0.366693675	5.344564633
1.038685602	69.11260845	0.381206237	5.141958252
2.247772575	131.5308906	0.317379246	5.501539847
10.91664823	197.367713	0.099991956	5.866801194
4.839085103	102.3114035	0.138715759	5.829513992
3.607142857	63.66964286	0.158920599	5.386998451
3.87284316	88.44030668	0.176785605	5.960837765
2.82699406	100.9204882	0.289691089	5.680878168
5.642808882	57.98657718	0.127156434	5.201780629
10.23750075	193.3156069	0.125383407	6.101751123
4.6630428	1039.664232	0.285254715	5.765796047
7.150884495	62.67741935	0.138397503	4.066784213
1.718646559	67.39983877	0.322506592	5.569127419
2.500595453	66.67408041	0.2293905	5.809870557
3.210613624	86.74520548	0.179358229	5.549907033
6.554858311	94.54499151	0.121062144	5.635771587
4.8064	46.28	0.1672	4.67856294
9.905325444	63.30769231	0.171597633	3.085055103
4.493482375	573.0565763	0.247749009	5.889276174
4.9915058	107.1743421	0.163608033	5.746401924
5.617580436	79.75961538	0.173943695	5.932651973
2.120325672	81.91237678	0.294056041	5.608771926
1.275658832	52.28080864	0.342686019	5.301505458
10.49332326	88.38095238	0.110103301	4.643325955
6.257592993	104.1220588	0.146786332	6.098246492
7.166666667	63.41666667	0.152777778	3.855388542
6.226052919	86.67484663	0.116037487	5.311854784
3.427946429	63.10357143	0.206734694	5.48493851

log.sigma.1.0.mm.3D_gldm_DependenceNonUniformity	log.sigma.1.0.mm.3D_gldm_GrayLevelNonUniformity	log.sigma.1.0.mm.3D_gldm_SmallDependenceEmphasis
33.07756233	64.82271468	0.198501686
82.87598004	437.9636493	0.091628817
18.01694915	33.6779661	0.190479246
163.24418	319.256596	0.229726564
19.11881188	14.32673267	0.253666765
28.55737705	37.07377049	0.247900445
23.57142857	6.428571429	0.538194444
50.98503741	48.89027431	0.272295793
206.6287263	135.8495935	0.264154656
149.3354894	551.9219491	0.137614773
38.69680851	61.2287234	0.195976945
58.35006605	157.1003963	0.194399117
96.6090776	296.7642753	0.159620286
39.16666667	112.2954545	0.176295912
64.01098901	39.96153846	0.323629202
824.5916143	4503.371629	0.043140238
30.29032258	31.00921659	0.27024107
98.18170103	373.4626289	0.112843097
27.3956044	23.37362637	0.302991642
163.3439077	960.3604903	0.058536617
346.7710345	1706.878621	0.077752428
89.08360836	144.4939494	0.205342842
165.5212329	1013.225342	0.072715084
33.57142857	11.76691729	0.480071011
62.99166667	282.6833333	0.101200499
26.21854305	60.98675497	0.168493296
147.2743556	636.0773263	0.115571536
266.5178434	1559.151476	0.060957588
224.4020452	1215.411195	0.083891893
110.4731246	546.1518015	0.086224006
281.9350155	1400.511069	0.078950449
589.431337	3193.733839	0.054169312
103.4982001	281.4132469	0.18079021
6.733333333	1.8	0.540740741
22.39823009	12.23893805	0.321539153
97.83658171	260.7556222	0.148350412
546.9494844	2685.138993	0.05432287
203.762533	804.4195251	0.118449335
52.21495327	37.85358255	0.27006845
40.17690875	98.05400372	0.175563509
52.53566434	160.2447552	0.165065383
40.6167979	55.17322835	0.212430247
79.0631068	124.7354369	0.223560454
50.91343964	58.36218679	0.234234613
158.2681614	116.4349776	0.282270068
466.854296	2465.209409	0.057139784
323.4610342	335.3379461	0.265552307
25.77192982	33.92982456	0.196856066
11.81818182	2.363636364	0.727272727
33.61643836	14.87671233	0.328928668
258.7088235	1192.073039	0.103347735
34.77667494	69.58560794	0.178872179
211.0525822	769.1546166	0.133777907
25.23430962	42.16317992	0.190706096
905.7502211	4457.837608	0.059719739
48.54626866	39.80597015	0.289038188
146.837156	93.3646789	0.298938064
102.0873362	34.35371179	0.403381457
32.71751412	65.30508475	0.202023394
42.4248927	93.79399142	0.149109825
97.74501718	317.1965636	0.14211134
465.6986423	1968.000295	0.106073315
262.8600884	1329.050565	0.089000554
112.9057239	569.3198653	0.074017262
8.58490566	9.452830189	0.237488927
97.44791667	201.2447917	0.191493622
23.28571429	9.838095238	0.415593834
228.8978944	963.4689934	0.093520458
19.1958042	19.74125874	0.285055082
118.2645089	447.4899554	0.137553529
168.0888803	632.8183917	0.126681558
51.23985891	99.08112875	0.157327371
496.1801049	2587.757262	0.054304078
873.3651468	4129.98837	0.056006269
1076.612783	4608.029272	0.097516725
80.83408072	44.59641256	0.361848345
54.44736842	63.25438596	0.226753852
24.16964286	35.59821429	0.169156701
72.55312158	161.4052574	0.169795574
165.6323964	783.3247041	0.110410814
25.05369128	18.94630872	0.307917313
102.9052023	108.4566474	0.288350706
330.5418949	1406.020491	0.127207878
8.548387097	4.290322581	0.485936106
306.5997582	1600.277711	0.085961233
244.9201597	804.4724836	0.14036483
33.30136986	65.46575342	0.188344962
89.34634975	71.30560272	0.269185738
7.36	8.36	0.25213665
4.692307692	2.230769231	0.603632479
229.5855935	849.5313502	0.135213491
31.35526316	49.73684211	0.255336789
40.80769231	72.36057692	0.24606193
234.4211391	1073.892662	0.084416868
520.0013847	2474.878427	0.069806739
14.77777778	6.936507937	0.368386806
69.40294118	99.81470588	0.227772232
5.333333333	3.666666667	0.383252315
32.7791411	18.91411043	0.348169867
27.09285714	57.88571429	0.204026733

log.sigma.1.0.mm.3D_gldm_DependenceNonUniformityNormalized	log.sigma.1.0.mm.3D_gldm_DependenceVariance	log.sigma.1.0.mm.3D_gldm_LargeDependenceEmphasis
0.091627596	18.01755665	52.99445983
0.059070549	25.50587708	109.300784
0.101790673	15.18573845	44.17514124
0.084451205	25.17511493	68.60682876
0.189295167	3.721399863	14.72277228
0.117038431	11.71177103	32.63114754
0.336734694	0.836734694	4.285714286
0.127144732	10.11314606	28.36159601
0.139992362	9.513520759	25.55691057
0.064396503	23.36958857	82.50495903
0.102917044	12.64460729	38.88297872
0.077080668	20.87968045	64.41875826
0.070724069	24.90061073	76.77013177
0.074179293	24.15414084	70.1780303
0.175854365	3.596667069	13.13186813
0.07615364	17.8971613	149.0999261
0.13958674	5.142984561	19.11059908
0.063261405	20.61988522	85.61340206
0.150525299	7.31191885	20.94505495
0.058883889	23.83018595	135.6070656
0.068329268	21.70872708	119.8299507
0.098001769	15.73349986	43.90869087
0.056685354	25.79735175	130.4390411
0.252416756	2.063090056	7.601503759
0.065616319	18.80166667	83.60416667
0.086816368	15.46414631	50.12582781
0.064340042	21.64062936	92.6225426
0.068425634	18.74491209	125.7173299
0.060388064	24.10210784	120.1695371
0.065252879	21.59540152	107.5599527
0.067111406	20.87114113	117.0380862
0.066730594	21.18636535	134.8442205
0.074512743	25.52828793	79.12670986
0.448888889	0.355555556	3.133333333
0.198214426	3.256323909	12.15044248
0.073340766	20.77153852	69.43628186
0.081744057	17.84776299	140.6099238
0.067204002	24.52759832	101.9815303
0.162663406	4.180995914	15.64797508
0.074817335	21.82681217	66.29608939
0.073476454	21.65031835	70.57202797
0.106605769	13.36992718	39.11023622
0.095950372	16.08233575	44.90776699
0.115975945	12.10645441	33.51025057
0.141944539	6.076322468	19.8161435
0.076258461	17.04259038	128.5481869
0.11779353	10.5640362	30.34158776
0.11303478	8.967143737	30.5
0.537190083	0.231404959	2.090909091
0.230249578	2.402326891	9.684931507
0.063409025	23.9685544	107.1715686
0.086294479	18.99957515	56.23076923
0.066057146	22.52681434	84.54428795
0.105582885	12.0834019	37.25104603
0.080112349	18.63210647	139.8774102
0.144914235	13.89111161	32.75522388
0.168391234	4.51115226	15.26834862
0.222898114	2.066207738	8.414847162
0.092422356	13.39458649	43.11864407
0.091040542	10.44136013	43.86266094
0.067178706	22.69150057	79.44261168
0.068727663	24.44345589	114.4468713
0.064521377	21.4244884	110.2484045
0.063358992	20.89674019	103.5005612
0.161979352	9.963688145	27.03773585
0.084590205	16.74911989	51.96180556
0.221768707	2.15292517	8.619047619
0.066021891	23.05793443	107.7435823
0.134236393	8.579197027	24.86013986
0.06599582	23.70232656	88.86830357
0.064674444	21.57349447	81.43786072
0.090370122	14.4758421	47.71604938
0.070310345	21.20445726	142.1047187
0.08061336	17.77178298	133.4980617
0.074151993	21.72101017	115.8760934
0.181242333	7.512397193	18.43497758
0.119402124	9.426515851	28.89473684
0.107900191	8.286670918	32.40178571
0.079466727	18.18838512	57.56297919
0.061254584	26.03179543	111.0887574
0.168145579	3.261474708	13.51006711
0.118965552	15.18611915	37.66358382
0.06706064	24.25472145	104.3515926
0.275754422	2.680541103	8.225806452
0.061789552	24.39991464	123.3546957
0.069837513	18.17724216	67.02566296
0.09123663	16.89918559	49.09315068
0.151691595	4.643506735	17.03056027
0.1472	5.2144	19.96
0.360946746	1.301775148	5
0.066954095	24.62984438	93.71507728
0.103142313	13.48991517	39.08552632
0.098095414	19.21928162	49.94711538
0.064189797	23.31771545	111.7250821
0.072002407	20.53585336	125.7587926
0.234567901	4.308390023	12.74603175
0.102063149	19.23231834	48.45588235
0.222222222	1.743055556	8.416666667
0.201099025	2.984530844	10.91411043
0.096760204	17.21918367	48.9

log.sigma.1.0.mm.3D_gldm_LargeDependenceLowGrayLevelEmphasis	log.sigma.1.0.mm.3D_gldm_SmallDependenceHighGrayLevelEmphasis	log.sigma.1.0.mm.3D_gldm_LargeDependenceHighGrayLevelEmphasis
0.663074552	11.96424557	4487.903047
1.0655831	6.918084362	11422.91162
0.739157699	7.622074269	2833.785311
0.345120166	49.34053586	13939.08536
0.687775601	9.015899028	449.6534653
0.847067356	11.21361735	1437.97541
0.059083576	63.13343254	628.7857143
0.273138764	23.18303338	3273.942643
0.162814175	49.56481383	4420.609079
1.254824488	9.343748009	5597.294092
0.976947104	7.697251635	1795.720745
0.988770436	12.90060642	4425.247028
0.944392616	14.4007021	6384.392387
1.538113382	8.762097452	3353.625
0.116438646	52.67610504	1654.763736
2.124816031	3.352827382	10637.68175
0.704949371	11.43707611	654.2534562
1.042679605	7.744200844	7253.778351
0.474439091	16.78646764	1140.895604
2.583975851	2.728953607	7301.182769
1.386164042	5.925946835	10511.53734
0.580980901	18.07760122	3506.722772
2.531439865	3.480619061	6868.035274
0.113085619	47.90222013	763.556391
1.619637345	3.445324281	4480.011458
1.060000161	4.507551855	2581.880795
1.767893711	7.097426751	5013.452163
1.805062793	3.357356678	8905.451348
0.941008054	8.95044614	15544.2979
1.569753661	4.362043605	7548.77909
2.187886993	3.783250642	6430.404666
1.5565001	4.439683459	11852.24058
0.775281281	18.34230104	8356.211663
0.276190504	24.82962963	128.8666667
0.185864653	29.66892085	1001.292035
0.851738751	10.91152678	5909.768366
1.592615258	3.652748698	12602.22792
1.189080367	12.05496028	8936.233179
0.366425477	16.74474153	864.1246106
0.827799805	8.845315143	5603.413408
0.568669947	18.94935588	8932.379021
0.359778721	20.93533581	4575.343832
0.60745058	15.03799184	3660.274272
0.50735729	17.73004408	2590.498861
0.242474627	35.7314614	1860.87713
1.815331307	3.490949439	9247.439562
0.121508486	79.65041285	7830.364894
0.428595004	12.34941912	2450.921053
0.181885901	28.72727273	81.22727273
0.249730214	26.34174003	584.1643836
1.573825074	7.849413994	7498.3875
0.727785829	8.748765001	4659.69727
0.22839438	44.17588491	31517.63787
0.834752121	8.346368164	1857.92887
1.592933081	4.90253496	12459.71272
0.248626328	30.61325182	4665.680597
0.15010296	36.35169504	1790.020642
0.064638423	70.79238545	1407.740175
0.434732473	17.37788475	4511.333333
0.914515657	8.028827591	2251.978541
0.934014573	8.075928516	6993.398625
0.894477848	14.4350865	14866.85921
2.076530009	5.768858001	5998.805106
0.801018222	7.875603395	13545.15544
0.719991015	7.225568555	1221.226415
0.511169161	21.38496202	5526.331597
0.115170464	47.72984397	859.0666667
1.007183227	7.748364844	11766.78194
0.492728359	10.78340443	1478.678322
0.704493926	15.41347403	11424.17132
0.76909207	11.51420653	8837.485956
0.781735018	6.258197565	3199.271605
2.057200304	3.269378823	10003.40541
1.910681575	3.495864006	9490.505077
0.890810532	14.46791588	15244.35037
0.0930807	73.34666266	3983.443946
0.275609316	22.70921618	3253.030702
0.463269988	8.465403467	2510.598214
0.59460191	13.46893984	5837.631982
1.059965497	10.71480921	11828.6091
0.26543861	15.75166406	925.4765101
0.188490227	61.16475199	7835.879769
0.09914933	131.0295119	110228.9533
0.207374694	34.59581541	432.6129032
1.773758355	5.750158668	8763.448609
1.028793398	10.69426044	4509.096664
0.478881423	12.99326354	5291.063014
0.191460269	26.3122891	1705.298812
0.570943368	14.68180624	1019.4
0.208445585	47.53846154	213.3076923
0.157794295	71.7137693	55964.27851
0.320370641	20.41768151	5039.009868
0.633234266	22.35802783	4148.201923
1.306224717	6.273153621	9763.410734
2.350417471	3.421278293	6905.296871
0.15280692	30.28461361	1536.634921
0.498486006	28.0301788	4930.866176
0.230533298	25.53248843	501.3333333
0.155123953	30.01938192	918.3680982
0.712609375	9.743326362	3621.175

log.sigma.1.0.mm.3D_gldm_SmallDependenceLowGrayLevelEmphasis	log.sigma.1.0.mm.3D_gldm_LowGrayLevelEmphasis	log.sigma.1.0.mm.3D_gldzm_DistanceZoneVariabilityNormalized
0.010979954	0.024568375	1
0.003387625	0.013594105	0.985075471
0.014801967	0.033763728	1
0.002436291	0.00706593	0.953612696
0.039303325	0.085289939	1
0.026139706	0.052346712	0.936576189
0.02309604	0.027646707	1
0.010022559	0.018884286	1
0.003189899	0.008633663	0.63017301
0.005735742	0.021276248	0.856890746
0.021959057	0.050877671	1
0.009707947	0.027421497	1
0.003741945	0.015527757	0.963482964
0.012419625	0.034076414	1
0.007578698	0.013445821	0.969931822
0.001235509	0.01543465	0.923936519
0.032334685	0.063798499	1
0.003349608	0.015949404	1
0.026674468	0.049736204	0.937565036
0.002714255	0.022561515	1
0.001939354	0.013330289	0.968676343
0.00708699	0.019644339	0.89176072
0.003423937	0.022965916	0.989529086
0.018846715	0.027374923	1
0.008062113	0.028971431	1
0.018319936	0.042829184	0.965527855
0.004891648	0.024341993	0.909138818
0.002678893	0.01683352	1
0.001615743	0.009458679	0.967541309
0.005135445	0.020453888	1
0.004229356	0.023609932	0.877317232
0.001028095	0.012499643	0.913203097
0.00686796	0.016858739	1
0.091469856	0.127204613	1
0.019196856	0.031155902	1
0.004734225	0.01809648	0.990000253
0.002015691	0.013545947	1
0.002792644	0.014459132	0.945703302
0.020869392	0.039837156	0.91933518
0.013498147	0.026932281	1
0.003774859	0.011147651	1
0.006498975	0.0153383	1
0.011825921	0.027464008	0.930092083
0.01475534	0.031734712	0.929298881
0.00723672	0.017314366	0.693819946
0.001488435	0.015221739	0.55566778
0.001759709	0.00498664	0.647942366
0.010587569	0.025703418	1
0.134658686	0.144104129	1
0.015610905	0.037898058	1
0.004102138	0.019401757	0.804941559
0.010058978	0.024978671	1
0.000806857	0.003282144	0.726862778
0.022719141	0.048041728	0.961553249
0.001647967	0.012913123	0.888817668
0.008737959	0.015910984	0.982144306
0.007229738	0.014953789	0.972513931
0.007459615	0.012169802	0.944745176
0.008568101	0.017982152	0.973688889
0.007887214	0.028498935	0.863754325
0.005688809	0.018338838	0.935401271
0.001556711	0.009274771	0.978757125
0.003211031	0.021490668	0.884175883
0.001469023	0.009101153	1
0.033961759	0.067071871	1
0.005652199	0.015133086	0.966428248
0.019057625	0.026182667	0.96001666
0.002400187	0.012265066	0.969196728
0.029166495	0.048788835	1
0.002848604	0.010750737	0.938523558
0.002708752	0.012205935	0.912815692
0.012304654	0.031638383	0.955079931
0.001839461	0.016759606	0.988858288
0.001778742	0.016046891	0.755907154
0.001135418	0.008400115	0.594330463
0.005632272	0.009366227	0.977530992
0.005794928	0.01435674	0.963648834
0.011147223	0.025857316	0.95125
0.005460537	0.015856255	0.974526535
0.00261103	0.012091357	1
0.027584204	0.043577969	0.921699292
0.003982943	0.008235334	0.956226331
0.0003372	0.001182486	0.848585859
0.050696676	0.061206375	1
0.002769351	0.017693817	0.901981376
0.004539836	0.019202795	0.920765225
0.006806493	0.01664878	1
0.007867402	0.017225629	0.953783131
0.026441735	0.054745976	1
0.090575966	0.100735835	1
0.000604628	0.00212043	0.939836327
0.010141022	0.01717938	0.976473923
0.013214774	0.024817124	0.982144306
0.002759926	0.014983408	0.97366475
0.003586917	0.023004614	0.748975039
0.028355657	0.037796932	1
0.005374278	0.014621317	0.965127271
0.049020676	0.062915054	1
0.014373659	0.023572989	1
0.012330059	0.028365588	1

log.sigma.1.0.mm.3D_gldzm_LowIntensityEmphasis	log.sigma.1.0.mm.3D_gldzm_LargeDistanceEmphasis	log.sigma.1.0.mm.3D_gldzm_HighIntensitySmallDistanceEmphasis
0.054608082	1	57.27848101
0.038335885	1.022556391	68.32518797
0.071552349	1	38.65853659
0.01082262	1.071274298	203.8439525
0.157942051	1	36.4
0.108022765	1.098360656	43.74590164
0.038133644	1	120.5454545
0.03609734	1	82.69230769
0.011864379	1.823529412	143.515817
0.044182051	1.308917197	57.95789101
0.117641281	1	38.31081081
0.055092519	1	63.56493506
0.024534656	1.055813953	88.1872093
0.072896091	1	49.66304348
0.022230262	1.045801527	155.8473282
0.031851655	1.24137931	76.42330323
0.113233027	1	40.3880597
0.031544475	1	65.38823529
0.087436038	1.096774194	51.63306452
0.052507982	1	43.53642384
0.026757444	1.047745358	70.7035809
0.033784192	1.172248804	80.08492823
0.052431333	1.015789474	45.69473684
0.037960704	1	97.95774648
0.087718345	1	29.76344086
0.104400814	1.052631579	24.3377193
0.046079366	1.200787402	59.9753937
0.048558133	1	50.02678571
0.020713658	1.04950495	99.96287129
0.063225934	1	45.9047619
0.059129065	1.418006431	42.45495177
0.020433284	1.341981132	78.38829927
0.037975595	1	97.99622642
0.154240719	1	44.2
0.052566133	1	90.6
0.033870023	1.015075377	71.3919598
0.040327288	1	61.34269663
0.024767814	1.177285319	99.83475685
0.072994725	1.126315789	61.75789474
0.078283584	1	48.29473684
0.023090451	1	113.0082645
0.030185634	1	94.87209302
0.054997829	1.10880829	59.59585492
0.064341909	1.110091743	68.40366972
0.025908508	1.675675676	97.57248916
0.027845291	2.990825688	41.04454893
0.006659183	2.195478723	226.5948397
0.055049421	1	59.5106383
0.168430982	1	39.38888889
0.04759573	1	75.25423729
0.042837686	1.453883495	64.89050701
0.054474885	1	46.06097561
0.006047151	1.893424036	267.7108844
0.115455704	1.058823529	40.00490196
0.030006175	1.383206107	74.59981976
0.028514553	1.027027027	104.1261261
0.023658634	1.041811847	119.6245645
0.01736997	1.085308057	172.0462085
0.041897332	1.04	83.2
0.055522549	1.220588235	50.5625
0.04180157	1.146919431	49.82372301
0.015198803	1.048257373	132.5017594
0.038202261	1.312138728	62.9059088
0.020816103	1	102.8492063
0.124082652	1	32.17647059
0.02934062	1.072340426	108.7765957
0.042320406	1.06122449	113.3928571
0.027266778	1.099688474	78.29614486
0.100088887	1	35.88636364
0.021694348	1.095238095	103.2668651
0.022473877	1.181268882	84.03700906
0.081015266	1.068965517	36.43678161
0.038049419	1.016806723	56.75070028
0.034936324	2.620938628	50.43331645
0.011957304	4.508858965	121.3896581
0.015034121	1.034090909	196.1392045
0.025576343	1.055555556	94.82638889
0.064249236	1.075	47.05625
0.032733252	1.038709677	77.54354839
0.024410182	1	92.51282051
0.086484834	1.12244898	46.35714286
0.013561486	1.067164179	202.4235075
0.002584765	1.516666667	949.4465231
0.090866918	1	68.55555556
0.035587669	1.54589372	61.53807703
0.033674052	1.134020619	73.27857961
0.036864282	1	65.82608696
0.02923934	1.071005917	90.04585799
0.096451156	1	53.93333333
0.133980484	1	74
0.004401929	1.113402062	510.2943299
0.038203577	1.035714286	75.49107143
0.051823453	1.027027027	87.53378378
0.034840845	1.096345515	69.84775286
0.057486975	2.552401747	39.66558529
0.063240292	1	81.56666667
0.022935066	1.053254438	121.5754438
0.10256741	1	67.25
0.038830569	1	84.828125
0.059900443	1	46.6

log.sigma.1.0.mm.3D_gldzm_LowIntensityLargeDistanceEmphasis	log.sigma.1.0.mm.3D_gldzm_HighIntensityEmphasis	log.sigma.1.0.mm.3D_gldzm_DistanceZoneVariability	log.sigma.1.0.mm.3D_gldzm_ZonePercentage
0.054608082	57.27848101	79	0.218836565
0.038614359	68.78195489	131.0150376	0.094796864
0.071552349	38.65853659	41	0.231638418
0.011152238	209.6868251	441.5226782	0.239524056
0.157942051	36.4	30	0.297029703
0.110597142	45.04918033	57.13114754	0.25
0.038133644	120.5454545	44	0.628571429
0.03609734	82.69230769	117	0.291770574
0.017921168	191.3670588	267.8235294	0.287940379
0.04667867	67.05414013	269.0636943	0.135403191
0.117641281	38.31081081	74	0.196808511
0.055092519	63.56493506	154	0.20343461
0.025071679	89.85116279	207.1488372	0.157393851
0.072896091	49.66304348	92	0.174242424
0.022417784	158.8244275	127.0610687	0.35989011
0.035554808	79.33743842	375.1182266	0.037495382
0.113233027	40.3880597	67	0.30875576
0.031544475	65.38823529	170	0.109536082
0.08851728	53.82258065	58.12903226	0.340659341
0.052507982	43.53642384	151	0.05443403
0.027035544	72.92572944	365.1909814	0.074285714
0.035258499	86.08133971	186.3779904	0.229922992
0.052589228	46.08947368	188.0105263	0.065068493
0.037960704	97.95774648	71	0.533834586
0.087718345	29.76344086	93	0.096875
0.105050587	25.40350877	55.03508772	0.188741722
0.049684887	62.58661417	230.9212598	0.110965487
0.048558133	50.02678571	224	0.057509628
0.021286603	101.3267327	293.1650165	0.08153929
0.063225934	45.9047619	147	0.086828116
0.068010923	45.94533762	272.8456592	0.074029993
0.025930279	81.64150943	387.1981132	0.048001811
0.037975595	97.99622642	265	0.190784737
0.154240719	44.2	10	0.666666667
0.052566133	90.6	45	0.398230088
0.034288783	71.52763819	197.0100503	0.149175412
0.040327288	61.34269663	356	0.053205799
0.026680751	102.4155125	341.398892	0.119063325
0.07638069	64.09473684	87.33684211	0.295950156
0.078283584	48.29473684	95	0.176908752
0.023090451	113.0082645	121	0.169230769
0.030185634	94.87209302	86	0.225721785
0.056061627	63.93264249	179.507772	0.234223301
0.066060608	71.40366972	101.293578	0.248291572
0.0350965	126.6276276	231.042042	0.298654709
0.057767791	58.58715596	181.7033639	0.053413917
0.033972938	301.6555851	487.2526596	0.273852877
0.055049421	59.5106383	47	0.206140351
0.168430982	39.38888889	18	0.818181818
0.04759573	75.25423729	59	0.404109589
0.048467741	74.73543689	331.6359223	0.100980392
0.054474885	46.06097561	82	0.203473945
0.008570571	323.3968254	320.5464853	0.138028169
0.11618192	41.19607843	49.03921569	0.213389121
0.03466775	78.96946565	582.1755725	0.05793384
0.028784823	104.8018018	109.018018	0.331343284
0.023925255	121.5296167	279.1114983	0.32912844
0.018482519	175.4834123	199.3412322	0.46069869
0.04217511	84.64	73.02666667	0.211864407
0.059465904	54.41176471	58.73529412	0.145922747
0.043294853	52.7535545	197.3696682	0.145017182
0.015560815	133.847185	730.152815	0.110094451
0.043317971	66.6416185	305.9248555	0.084928817
0.020816103	102.8492063	126	0.070707071
0.124082652	32.17647059	17	0.320754717
0.029898822	110.5787234	227.1106383	0.203993056
0.042682681	115.9795918	47.04081633	0.466666667
0.02829258	79.75389408	311.1121495	0.092587251
0.100088887	35.88636364	44	0.307692308
0.022360255	107.484127	236.5079365	0.140625
0.024369088	87.89123867	302.141994	0.127356676
0.081704922	38.16091954	83.09195402	0.153439153
0.038217487	57.17086835	353.022409	0.050588069
0.056950986	59.55234657	418.7725632	0.051135315
0.0399424	149.7051736	838.6002835	0.097183002
0.015152083	198.6022727	172.0454545	0.394618834
0.025841524	97.75	104.0740741	0.236842105
0.064869071	49.325	38.05	0.178571429
0.033106609	78.63225806	151.0516129	0.169769989
0.024410182	92.51282051	312	0.115384615
0.087496809	50.06122449	45.16326531	0.32885906
0.01381386	206.9067164	256.2686567	0.30982659
0.003075403	1019.634848	560.0666667	0.1339014
0.090866918	68.55555556	18	0.580645161
0.045097347	64.28502415	373.4202899	0.083434099
0.035475701	77.0185567	446.571134	0.138294839
0.036864282	65.82608696	69	0.189041096
0.029601409	93.69822485	161.1893491	0.286926995
0.096451156	53.93333333	15	0.3
0.133980484	74	9	0.692307692
0.004657086	523.2185567	455.8206186	0.141440653
0.038414904	77	82.02380952	0.276315789
0.052046817	88.35135135	109.018018	0.266826923
0.035794645	71.27574751	293.0730897	0.082420591
0.087792399	47.37336245	343.0305677	0.063417336
0.063240292	81.56666667	30	0.476190476
0.023440745	123.2485207	163.1065089	0.248529412
0.10256741	67.25	12	0.5
0.038830569	84.828125	64	0.392638037
0.059900443	46.6	65	0.232142857

log.sigma.1.0.mm.3D_gldzm_IntensityVariabilityNormalized	log.sigma.1.0.mm.3D_gldzm_LowIntensitySmallDistanceEmphasis	log.sigma.1.0.mm.3D_gldzm_IntensityVariability
0.108155744	0.054608082	8.544303797
0.126236644	0.038266267	16.78947368
0.135038667	0.071552349	5.536585366
0.064221039	0.010740215	29.73434125
0.104444444	0.157942051	3.133333333
0.109916689	0.10737917	6.704918033
0.079545455	0.038133644	3.5
0.092409964	0.03609734	10.81196581
0.060794464	0.010464798	25.83764706
0.108523672	0.043680733	34.07643312
0.127830533	0.117641281	9.459459459
0.105161073	0.055092519	16.19480519
0.108015143	0.024400401	23.22325581
0.115311909	0.072896091	10.60869565
0.07219859	0.022183381	9.458015267
0.111735301	0.03150719	45.36453202
0.107150813	0.113233027	7.179104478
0.1216609	0.031544475	20.68235294
0.095213319	0.087165727	5.903225806
0.158019385	0.052507982	23.86092715
0.130578559	0.026687919	49.22811671
0.090725945	0.033415615	18.96172249
0.147423823	0.052391859	28.01052632
0.075976989	0.037960704	5.394366197
0.161752804	0.087718345	15.04301075
0.151123423	0.104238371	8.614035088
0.107415215	0.045475016	27.28346457
0.137555804	0.048558133	30.8125
0.093814332	0.020570422	28.42574257
0.130640011	0.063225934	19.20408163
0.13822231	0.058052158	42.98713826
0.132142221	0.019920651	56.02830189
0.080185119	0.037975595	21.2490566
0.1	0.154240719	1
0.079506173	0.052566133	3.577777778
0.108254842	0.033765332	21.54271357
0.123658629	0.040327288	44.02247191
0.08211263	0.024514537	29.64265928
0.093407202	0.072148234	8.873684211
0.114903047	0.078283584	10.91578947
0.103476538	0.023090451	12.52066116
0.099783667	0.030185634	8.581395349
0.094042793	0.054731879	18.15025907
0.086103863	0.063912234	9.385321101
0.067211355	0.023946645	22.38138138
0.239944262	0.024540022	78.4617737
0.060307266	0.004529742	45.35106383
0.104572205	0.055049421	4.914893617
0.104938272	0.168430982	1.888888889
0.093363976	0.04759573	5.508474576
0.096156565	0.041817494	39.61650485
0.127305175	0.054474885	10.43902439
0.098451777	0.005645235	43.41723356
0.114955786	0.11527415	5.862745098
0.123435697	0.029443084	80.85038168
0.078808538	0.028446985	8.747747748
0.070184171	0.023591979	20.14285714
0.060623077	0.017091833	12.79146919
0.092977778	0.041827887	6.973333333
0.144463668	0.05453671	9.823529412
0.131061746	0.041554049	27.65402844
0.080583487	0.015130609	60.1152815
0.127819172	0.037415711	44.22543353
0.133786848	0.020816103	16.85714286
0.128027682	0.124082652	2.176470588
0.07983703	0.029233904	18.76170213
0.077051229	0.042229837	3.775510204
0.103327802	0.027142534	33.1682243
0.132231405	0.100088887	5.818181818
0.094954649	0.021527871	23.92857143
0.114666715	0.022124402	37.95468278
0.120095125	0.080842853	10.44827586
0.124889171	0.038007403	44.58543417
0.151846108	0.033361195	84.12274368
0.116522454	0.010307144	164.4131821
0.069537707	0.015004631	12.23863636
0.099108368	0.025510048	10.7037037
0.12125	0.064094277	4.85
0.092112383	0.032639913	14.27741935
0.098886423	0.024410182	30.8525641
0.10787172	0.08623184	5.285714286
0.062764536	0.013498393	16.82089552
0.08369146	0.002521151	55.23636364
0.111111111	0.090866918	2
0.118870919	0.034806379	49.21256039
0.105435222	0.03324655	51.13608247
0.140516698	0.036864282	9.695652174
0.085956374	0.029148823	14.52662722
0.111111111	0.096451156	1.666666667
0.135802469	0.133980484	1.222222222
0.082010841	0.004350405	39.77525773
0.115362812	0.038150745	9.69047619
0.081081081	0.051767612	9
0.110274721	0.034737295	33.19269103
0.139699853	0.055229035	63.98253275
0.106666667	0.063240292	3.2
0.074262106	0.022808646	12.55029586
0.111111111	0.10256741	1.333333333
0.099121094	0.038830569	6.34375
0.125207101	0.059900443	8.138461538

log.sigma.1.0.mm.3D_gldzm_HighIntensityLargeDistanceEmphasis	log.sigma.1.0.mm.3D_gldzm_SmallDistanceEmphasis	log.sigma.1.0.mm.3D_glcm_SumVariance	log.sigma.1.0.mm.3D_glcm_Homogeneity1
57.27848101	1	190.7633157	0.531434049
70.60902256	0.994360902	300.88043	0.651195843
38.65853659	1	138.3287229	0.544601955
233.0583153	0.982181425	580.707742	0.516581758
36.4	1	71.42267319	0.426976317
50.26229508	0.975409836	88.51504178	0.486685462
120.5454545	1	354.9370043	0.28129417
82.69230769	1	262.6512167	0.457478618
412.6282353	0.820261438	494.1833646	0.385188783
116.9140127	0.940463553	175.922516	0.590902148
38.31081081	1	89.29561996	0.513010774
63.56493506	1	163.044228	0.56205936
96.50697674	0.986046512	221.4139731	0.573589292
49.66304348	1	106.4374326	0.577703252
170.7328244	0.988549618	383.9585988	0.389642264
97.408867	0.969535762	218.7989759	0.711854622
40.3880597	1	69.03698782	0.456267255
65.38823529	1	220.0190098	0.613432695
62.58064516	0.975806452	109.9550487	0.434098622
43.53642384	1	146.4495527	0.694783064
81.81432361	0.98806366	259.5671636	0.671542309
110.0669856	0.956937799	201.536228	0.500328505
47.66842105	0.996052632	144.2507263	0.691422605
97.95774648	1	241.7185119	0.345755518
29.76344086	1	135.34405	0.655950018
29.66666667	0.986842105	101.9648065	0.562401917
75.70866142	0.962926509	140.3370178	0.625022771
50.02678571	1	213.1927361	0.701797893
106.7821782	0.987623762	388.7365461	0.665546612
45.9047619	1	191.1567366	0.661087752
70.97749196	0.947654519	146.2958954	0.666684714
105.870283	0.963230477	264.7931967	0.689116935
97.99622642	1	269.0132045	0.562909143
44.2	1	105.0488945	0.281906543
90.6	1	194.2879422	0.395763398
72.07035176	0.996231156	205.5149358	0.558425873
61.34269663	1	273.5478344	0.702046956
121.8559557	0.977416128	243.3458887	0.618928346
73.44210526	0.968421053	125.183581	0.405469033
48.29473684	1	194.3356268	0.562406849
113.0082645	1	359.943722	0.596522586
94.87209302	1	285.7686814	0.505550347
81.27979275	0.972797927	179.0790204	0.497896312
83.40366972	0.972477064	169.4899743	0.471826722
257.1891892	0.859547047	258.8158209	0.389397232
208.9266055	0.774115698	219.3721249	0.698263401
834.1462766	0.831985077	808.218453	0.411369238
59.5106383	1	171.8039045	0.486636761
39.38888889	1	79.3744516	0.304401485
75.25423729	1	132.9427436	0.368865147
125.9490291	0.916194714	189.3225271	0.630643014
46.06097561	1	179.9191978	0.532933356
736.8321995	0.87569287	1216.980129	0.577234037
45.96078431	0.985294118	105.614442	0.524757544
113.7633588	0.952809584	275.8499869	0.696338299
107.5045045	0.993243243	331.2108165	0.440855536
129.1498258	0.989547038	318.4119448	0.401567475
189.2322275	0.978672986	461.1571671	0.32793815
90.4	0.99	261.8565867	0.531577766
69.80882353	0.944852941	120.3796856	0.544079054
68.36966825	0.973802001	215.8653388	0.585832934
141.7774799	0.991705764	384.5508657	0.634217341
89.7283237	0.950867052	149.9314721	0.655256141
102.8492063	1	397.4471669	0.663715853
32.17647059	1	85.80003086	0.497544856
120.5106383	0.986643026	275.6710193	0.528827363
126.3265306	0.984693878	285.5535539	0.345156483
90.41744548	0.987300969	302.9517014	0.633638616
35.88636364	1	113.9440053	0.460890559
124.3531746	0.976190476	363.5084393	0.604359117
106.570997	0.964753273	294.9993004	0.602906888
45.05747126	0.982758621	139.9099094	0.532587699
58.85154062	0.995798319	204.7341114	0.694613872
193.200361	0.886998914	211.0904317	0.691194625
624.794472	0.794225838	409.7406295	0.641686972
208.4545455	0.991477273	578.0870012	0.382825176
109.4444444	0.986111111	290.6811928	0.469739826
58.4	0.98125	175.7385882	0.507638711
82.98709677	0.990322581	255.6483791	0.551823098
92.51282051	1	305.9979394	0.64272303
64.87755102	0.969387755	147.3336535	0.416312754
224.8395522	0.983208955	570.5995063	0.459706363
1576.683333	0.934446128	3790.146961	0.613326252
68.55555556	1	141.546532	0.388086278
99.19323671	0.956151684	195.5398333	0.663749886
92.89484536	0.968785796	175.8021804	0.574691391
65.82608696	1	252.9444159	0.533621422
108.3076923	0.982248521	251.0072028	0.418263792
53.93333333	1	96.62356754	0.457886665
74	1	140.2926172	0.381700244
581.9257732	0.976231386	2022.586801	0.599852736
83.03571429	0.991071429	319.609251	0.510505608
91.62162162	0.993243243	206.2679733	0.522039791
82.4551495	0.988948874	243.4113486	0.648606937
142.7641921	0.882398999	149.3643212	0.672339308
81.56666667	1	232.1463098	0.36932654
129.9408284	0.986686391	264.2292818	0.478439725
67.25	1	165.6762994	0.402083193
84.828125	1	208.5106508	0.392583877
46.6	1	172.033984	0.566911858

log.sigma.1.0.mm.3D_glcm_Homogeneity2	log.sigma.1.0.mm.3D_glcm_ClusterShade	log.sigma.1.0.mm.3D_glcm_MaximumProbability	log.sigma.1.0.mm.3D_glcm_Idmn	log.sigma.1.0.mm.3D_glcm_SumVariance2
0.47822641	-21.16658748	0.14398914	0.978201093	9.060373207
0.625901651	-22.29534913	0.25718047	0.989127051	4.802482774
0.492427162	-13.66769768	0.166778151	0.96951275	6.109088653
0.465220176	-5.532403446	0.141305386	0.987305489	14.70222463
0.345917361	0.529107894	0.066240058	0.934675194	7.498831139
0.420627966	-4.340105333	0.092648023	0.965321685	6.634185015
0.182639039	-26.00547787	0.049849249	0.923086587	26.1117516
0.38862865	-10.55852138	0.080228819	0.979216049	12.58798298
0.307040766	55.56718568	0.042732363	0.963691711	28.8075248
0.552586171	-0.395961442	0.186881011	0.992004155	5.252322138
0.454912129	-9.896066399	0.107676865	0.966137454	6.477088752
0.516717721	-7.212128942	0.160676003	0.989166955	6.726329262
0.530807543	-2.880920258	0.18164407	0.985349866	4.626511096
0.534439707	-3.511079528	0.186890976	0.980191939	4.275285334
0.306366813	22.75133104	0.038874447	0.975230513	15.44036372
0.702976335	-2.816958798	0.24738608	0.995511651	1.571013068
0.383889508	0.993861446	0.065326665	0.95679466	7.146649335
0.5798324	-9.33596168	0.175816374	0.988634844	4.34850969
0.353946294	-5.800398596	0.074831975	0.960370196	8.843284591
0.680095901	-4.84841759	0.256690307	0.991658747	2.324123615
0.653571181	-3.040687987	0.232361988	0.99777172	2.47105266
0.440240058	6.712307872	0.110308027	0.987943172	8.129467046
0.675370973	-3.387780969	0.279941424	0.994132754	2.090804732
0.256674763	38.03761433	0.037700432	0.963025123	26.4875842
0.633887399	-9.825525993	0.214317207	0.987078321	3.158125905
0.515107639	-12.86101922	0.148639177	0.969051444	6.011784093
0.595855371	-0.983608413	0.194607626	0.990373082	3.727789521
0.691060562	-4.414431884	0.247347401	0.996930405	1.860847911
0.645020071	-15.94300842	0.227319305	0.99426209	4.180845004
0.640064031	-10.64799034	0.223139164	0.991457565	3.229441675
0.647441852	-3.731943893	0.214203319	0.994293949	2.556051816
0.674442598	-2.856654206	0.238838888	0.994558146	1.8964708
0.518542854	-22.55097377	0.169600824	0.988468586	8.959229741
0.171217396	-1.5476664	0.158333333	0.87144345	12.70330662
0.313902983	12.46551966	0.051396503	0.961697652	15.18433748
0.512454992	-12.50181563	0.146307267	0.989499576	6.506024499
0.691033599	-9.811795087	0.238839618	0.99555268	2.481958086
0.588781936	-3.553847624	0.193749062	0.989407172	4.749372438
0.322187396	4.842083406	0.044374961	0.952718023	11.72185618
0.514131091	-22.0824175	0.155167102	0.979959404	7.51271596
0.56090196	-10.49205464	0.186592998	0.989786586	5.985011881
0.445262391	-19.07939666	0.108174532	0.98225896	8.421161389
0.437902714	-11.22966105	0.107859374	0.982166912	10.31399664
0.405275629	5.090541624	0.090310534	0.98285038	12.22745157
0.310082099	71.28577751	0.038755816	0.975003948	21.08708976
0.687083012	-2.164775438	0.230449701	0.994303021	1.253176552
0.339697449	144.3858374	0.063211716	0.983209485	24.71955719
0.421361891	-10.82023165	0.075981776	0.978217312	8.120913664
0.204811077	-5.447639023	0.095533633	0.882125089	15.46861729
0.279994001	2.750483829	0.041843007	0.932689506	13.13764355
0.60289171	-4.560146492	0.201968902	0.991587517	4.391710198
0.478903381	-20.25330767	0.14397677	0.978235485	8.198856415
0.536535	-22.97726671	0.17078591	0.994169334	7.720801354
0.47124558	-9.61201938	0.115473346	0.97970779	5.939658674
0.684325119	-6.583484988	0.237838942	0.997025954	2.228650464
0.366653263	-31.69985811	0.104074208	0.978686659	15.36270046
0.322128407	30.19625033	0.03599566	0.976438003	17.78943209
0.236576885	-8.762236738	0.021756054	0.961240751	27.85609133
0.477914475	-24.72428335	0.115426255	0.978917061	7.443743484
0.495457412	-0.244559338	0.111578917	0.984678806	4.766130028
0.546094558	-17.29759159	0.147566787	0.986941214	5.780582982
0.607784568	-9.707067936	0.203994033	0.993643282	5.2604903
0.632999741	-0.900697637	0.216016195	0.991705663	2.608505596
0.64214613	-7.534370872	0.208210583	0.993774662	3.375851711
0.428189837	-11.14125563	0.15413274	0.947673651	7.196772953
0.4786812	-22.32851596	0.117685254	0.985611715	9.790757078
0.25297581	28.28913144	0.041167245	0.955985915	19.10185725
0.605953659	-17.84308351	0.178946093	0.991616545	5.034273788
0.389742523	-12.42121538	0.086022005	0.951094796	8.437950465
0.569922833	-17.41614806	0.182827694	0.991508603	5.877614304
0.567944754	-14.41256673	0.154102099	0.991458131	4.93486461
0.479887946	-17.9169736	0.114314206	0.973442177	7.055247698
0.681063845	-5.566304868	0.244596456	0.994717626	2.191517944
0.678345563	-3.527229282	0.229481169	0.998133588	1.842066374
0.617921915	-1.897858827	0.189094943	0.995106963	3.66956123
0.299430698	-10.30201068	0.056725599	0.97926662	18.66948089
0.401259797	-9.981238383	0.065003061	0.977367514	9.53215699
0.445579607	-13.28670287	0.08130947	0.97697767	7.260986271
0.504443247	-12.39258496	0.138529024	0.984894772	7.144422615
0.616857308	-10.27597651	0.224223161	0.992899858	4.469887281
0.332974561	-20.87069167	0.06220965	0.958692719	11.56389557
0.396014676	-8.38142509	0.091137761	0.982351572	16.60916873
0.584407181	-29.66525774	0.177075963	0.996659795	8.416988783
0.305237383	-11.00372141	0.103097757	0.944140423	12.00199601
0.642966949	-6.76361479	0.210740345	0.993856461	3.003149452
0.532759661	-1.077381823	0.162171135	0.988417265	4.598113451
0.479151677	-11.81919462	0.110039529	0.976455313	6.114449513
0.339259691	-5.052469804	0.05787634	0.975916031	12.40062515
0.381051334	-14.55103563	0.1181243	0.941132033	9.051768135
0.294386533	-13.65974197	0.154761905	0.908225645	16.75875535
0.566015753	-58.47402518	0.167887988	0.99505602	8.756195627
0.453552601	-30.80444047	0.103622916	0.975501971	9.119947217
0.470208351	-1.97109104	0.15109643	0.978176595	10.00760532
0.624263577	-11.83575006	0.211301066	0.990524181	3.882750333
0.655058089	-4.743139892	0.217013554	0.994897134	2.287606488
0.280159658	-21.99731125	0.070012782	0.956039948	19.58939706
0.412543883	9.229520015	0.126187595	0.984272193	10.99892061
0.314805622	-5.77381621	0.139335451	0.933646484	11.96101577
0.305308242	-5.017240039	0.048591694	0.956574043	12.45644456
0.520897572	-19.47860821	0.163420795	0.969375558	6.273002819

log.sigma.1.0.mm.3D_glcm_Contrast	log.sigma.1.0.mm.3D_glcm_DifferenceEntropy	log.sigma.1.0.mm.3D_glcm_InverseVariance	log.sigma.1.0.mm.3D_glcm_Entropy	log.sigma.1.0.mm.3D_glcm_Dissimilarity
6.253692485	2.468631432	0.400375512	5.027554739	1.713019097
2.658612991	1.892425397	0.452602963	3.757120284	1.010415899
4.12322512	2.253158755	0.419487351	4.723868647	1.476186055
13.5576621	2.810432876	0.377552488	5.703269025	2.235964019
7.805113754	2.580147617	0.342419554	5.346030169	2.221029727
5.669531716	2.446960387	0.396651964	5.386797586	1.792676402
30.21807014	3.169892073	0.174483163	5.375843509	4.519508966
9.107898123	2.732041082	0.374561232	5.885144253	2.188867884
22.30955778	3.33207086	0.289087802	7.334420364	3.398549268
4.179646426	2.172951372	0.447877347	4.602543362	1.304756704
4.650960249	2.323797046	0.428065531	5.199352186	1.604481168
5.207723695	2.319279256	0.428168056	4.817847203	1.495982621
4.080785059	2.222702073	0.442672965	4.789113913	1.365111618
3.747904844	2.172447008	0.438423011	4.65564117	1.320582674
14.65697927	3.005430298	0.32017037	6.401676896	2.837022665
1.214545812	1.41143043	0.491836312	2.855495272	0.679107519
7.290252306	2.569418165	0.376201642	5.508341739	2.040862761
2.721599986	1.987441021	0.461817014	4.294667253	1.11644767
8.889748794	2.700640859	0.325442576	5.58604815	2.283146501
1.267171203	1.536302605	0.479339945	3.337629405	0.736598368
1.963583167	1.677904131	0.488639552	3.462327736	0.855862516
7.541040819	2.582300994	0.395645667	5.585541856	1.88730565
1.385302352	1.577116257	0.473555391	3.360677996	0.758197187
20.42920345	3.138909892	0.274558685	6.303237669	3.461441571
1.684518487	1.703967456	0.49144071	3.647091056	0.877101417
3.471098333	2.157401182	0.437972776	4.619587804	1.348777153
3.018194339	1.971775169	0.47077249	4.16639705	1.1008659
1.422615172	1.475768389	0.494051966	2.933821836	0.723498144
2.714286389	1.811371243	0.463650598	3.651397495	0.961984216
2.063333186	1.754800869	0.480200557	3.572262069	0.908624544
1.983815505	1.705904965	0.487563914	3.524022903	0.874166179
1.271120667	1.533991325	0.491426226	3.296353641	0.745438297
6.649033999	2.439653169	0.410520398	4.960934197	1.627654411
20.25576923	1.655725759	0.157636539	3.12179479	3.894230769
14.48550311	2.930112654	0.313350382	5.787553033	2.83434673
4.937054477	2.327296437	0.429459493	4.935306209	1.498318619
1.527407788	1.499083475	0.487925166	2.937318954	0.739520979
4.208881519	2.093819824	0.45456542	4.282677215	1.231831544
10.79236045	2.846952305	0.321905759	6.251641581	2.544607525
4.325724076	2.28546269	0.413609195	4.837171974	1.440855725
4.485738517	2.15832371	0.453185763	4.558574616	1.313969819
6.348988835	2.478621016	0.400474859	5.35466175	1.777556265
8.011401063	2.610148276	0.394360399	5.556614532	1.933164534
9.276222721	2.69705424	0.375914757	5.801819955	2.116221536
19.08272702	3.204079294	0.31152489	6.938590043	3.126824511
0.993011978	1.413180922	0.50719026	2.94099842	0.683688122
25.7564063	3.294769135	0.321628641	6.839743687	3.299655366
6.067259184	2.479745059	0.394442002	5.31976848	1.833057902
15.36435231	2.204005482	0.288274184	3.992833007	3.324497724
13.81122915	2.960195986	0.292815297	6.096664463	2.945584241
3.703783777	2.011892153	0.461118773	4.12680794	1.143582627
5.369560449	2.410451249	0.401992033	5.011406166	1.639952786
6.03156861	2.327811821	0.431595888	4.813492268	1.490402197
4.32439994	2.255469177	0.449252877	4.934189929	1.525868176
1.500870113	1.517974641	0.493087672	3.052115221	0.747447222
12.52439307	2.92660266	0.31800835	6.002928395	2.527647274
15.15116566	3.042844763	0.327402713	6.657453598	2.819573791
23.04206907	3.353706018	0.243204636	7.26108256	3.772928256
5.227591919	2.392701927	0.415090834	5.060162151	1.612272386
3.706423437	2.164009637	0.46493087	4.83939681	1.396318014
3.590270457	2.152224868	0.45003833	4.560008149	1.285297276
4.265770685	2.040808425	0.45429834	4.08865824	1.187967947
1.988684956	1.762524713	0.484425494	3.706988954	0.913677488
1.912115712	1.728302565	0.475047916	3.637862202	0.885117813
4.94403109	2.277652626	0.361040697	4.450811604	1.713351247
7.839702669	2.520113036	0.424197994	5.419980236	1.788571772
20.51983209	3.122017462	0.246420229	5.97301399	3.492938602
2.893436104	1.952424804	0.464643369	4.041780371	1.075478042
6.911413614	2.549006347	0.375934923	5.430444426	2.012397757
4.022165367	2.132404681	0.451380068	4.414747369	1.261020165
3.273463229	2.065356548	0.4630958	4.423290101	1.193113247
4.25348489	2.276712798	0.439942263	5.01698395	1.505157357
1.428160561	1.542829046	0.4856	3.192683992	0.749448406
1.301255667	1.499788936	0.502130219	3.117336104	0.734587893
3.302599506	1.891952771	0.483158216	3.932686226	1.051446396
17.864726	3.155202864	0.295515021	6.595780584	3.097891063
7.133731592	2.603109403	0.381791816	5.760117404	1.988954334
4.930401237	2.353688269	0.403073394	5.127301725	1.654298058
4.749532346	2.30453056	0.436956139	5.041576629	1.491948181
3.384267757	1.960844908	0.455858657	3.929974249	1.08838254
9.222559257	2.68280437	0.326893721	5.634564548	2.372028832
15.53481469	2.975415981	0.360612502	6.139439552	2.579272843
7.566376432	2.202349201	0.45366089	4.376495888	1.429746782
12.90620661	2.42303825	0.319498159	4.277339448	2.794965718
2.125767266	1.757523448	0.476935154	3.650877754	0.904650678
4.040799629	2.205646206	0.449664367	4.775390378	1.350402377
4.348622465	2.299030772	0.419930693	4.868571164	1.525821972
10.57662119	2.843512474	0.343720039	6.241801566	2.451955133
8.631257715	2.442460834	0.303756029	4.492800734	2.217063344
20.25	2.348623245	0.182515577	3.679116152	3.488095238
6.946256684	2.270663876	0.439499136	4.612450876	1.460071367
6.211356056	2.475282422	0.414996681	5.192053602	1.757013181
9.217213728	2.607339828	0.402695838	5.331600312	1.934764222
2.287506612	1.835333001	0.473949612	3.879631081	0.969234279
1.540182864	1.618636336	0.499501797	3.415957638	0.813447206
18.53730385	2.955865536	0.247518735	5.288573449	3.274487225
9.744411433	2.787679999	0.347035383	5.850776568	2.193409796
13.91663977	2.307018844	0.248849179	3.745712638	2.853986612
11.03864157	2.823086241	0.301186303	5.95225058	2.638302498
4.287148124	2.233786706	0.422963829	4.621316821	1.42067407

log.sigma.1.0.mm.3D_glcm_DifferenceVariance	log.sigma.1.0.mm.3D_glcm_Idn	log.sigma.1.0.mm.3D_glcm_Idm	log.sigma.1.0.mm.3D_glcm_Correlation	log.sigma.1.0.mm.3D_glcm_Autocorrelation
3.311096386	0.911670326	0.47822641	0.180201127	71.69727359
1.630501012	0.942076802	0.625901651	0.282438219	98.92967084
1.933508701	0.891771564	0.492427162	0.189705234	53.9884542
8.551276044	0.938827816	0.465220176	0.040494315	188.3758283
2.813506971	0.833198427	0.345917361	-0.023258401	31.80146435
2.447858393	0.880227131	0.420627966	0.078348688	38.49319937
9.550814718	0.814079205	0.182639039	-0.075570064	118.3197121
4.30701169	0.908441642	0.38862865	0.160003531	96.20066302
10.72204426	0.88314474	0.307040766	0.127346132	170.4303463
2.473563403	0.947734561	0.552586171	0.114181663	64.75019519
2.067531668	0.883045037	0.454912129	0.16327167	38.75887573
2.953710424	0.938248064	0.516717721	0.12847736	61.70554765
2.213480591	0.927368521	0.530807543	0.059783271	79.01726358
1.991060598	0.915516829	0.534439707	0.065465156	43.0352244
6.59639733	0.897959481	0.306366813	0.027250213	133.4172982
0.751765388	0.96157097	0.702976335	0.126098017	70.38655966
3.077472117	0.866800874	0.383889508	-0.006719945	31.47006173
1.468835206	0.93549465	0.5798324	0.227922878	77.59833665
3.64458103	0.870678803	0.353946294	-0.003162126	45.83980037
0.720141984	0.945981489	0.680095901	0.290631172	52.14304869
1.22959357	0.97252318	0.653571181	0.116026091	84.96913075
3.951373228	0.931910226	0.440240058	0.036114961	75.36206251
0.806437203	0.954688334	0.675370973	0.200856525	51.24216569
8.366498954	0.874198709	0.256674763	0.127815641	89.46834904
0.912127626	0.93153566	0.633887399	0.299377576	49.60995208
1.638601158	0.891484762	0.515107639	0.267382542	42.22102996
1.805659182	0.943654373	0.595855371	0.105507499	52.28798727
0.898808191	0.968317988	0.691060562	0.132755576	69.22731991
1.778202655	0.959244212	0.645020071	0.206407056	123.1550429
1.236371349	0.946918176	0.640064031	0.21670594	65.8405575
1.219082175	0.956507003	0.647441852	0.125293509	52.13589346
0.712045338	0.955179821	0.674442598	0.194022685	85.82767378
3.992151193	0.939238226	0.518542854	0.147475613	94.80204887
3.951682692	0.755722331	0.171217396	-0.231478249	30.96858974
6.378765499	0.875286882	0.313902983	0.024992048	73.55230756
2.689992528	0.937843504	0.512454992	0.136866345	75.03459988
0.979608479	0.962874608	0.691033599	0.235793894	86.76389864
2.688297421	0.944338524	0.588781936	0.061021384	83.83873684
4.255506659	0.858422796	0.322187396	0.041545578	52.06464388
2.239466675	0.914324595	0.514131091	0.267528084	72.13445973
2.745474566	0.943192723	0.56090196	0.147212634	120.0871003
3.163886826	0.916626967	0.445262391	0.143334751	101.2132716
4.245844469	0.918801796	0.437902714	0.124599306	68.83950725
4.781792624	0.918708276	0.405275629	0.135249931	66.34613274
9.286394707	0.901193595	0.310082099	0.049186269	96.1057188
0.523949117	0.952539052	0.687083012	0.114926612	70.62915419
14.84925797	0.925254579	0.339697449	-0.016724603	257.670599
2.697854003	0.904195893	0.421361891	0.145264198	66.03061503
3.796413536	0.768313567	0.204811077	-0.008946121	28.60841936
5.088429301	0.830594656	0.279994001	-0.025621037	53.68311399
2.393130554	0.950172532	0.60289171	0.084586234	66.96174121
2.667120142	0.909345386	0.478903381	0.205941657	67.90162462
3.805455699	0.957078987	0.536535	0.123498015	360.6853143
1.993237255	0.908504292	0.47124558	0.155485969	43.43843678
0.940959072	0.968709385	0.684325119	0.191952627	87.64485448
6.099356782	0.908479716	0.366653263	0.102140885	117.0793056
7.119819406	0.902750007	0.322128407	0.082260276	114.8718614
8.699199316	0.868897632	0.236576885	0.088501824	159.3946366
2.620656053	0.910631537	0.477914475	0.174439873	92.71770266
1.748789337	0.920243042	0.495457412	0.123167896	48.01579839
1.936195738	0.93101612	0.546094558	0.232915956	77.15566752
2.85189189	0.958040387	0.607784568	0.103599614	123.9256632
1.151849665	0.94638669	0.632999741	0.133806314	53.76571347
1.124933874	0.953478355	0.64214613	0.274141872	126.427786
1.973851613	0.853954424	0.428189837	0.183952403	36.15778121
4.629504155	0.931169159	0.4786812	0.1096295	97.92615008
8.257488116	0.862872872	0.25297581	-0.037228712	101.6516893
1.735708338	0.947591015	0.605953659	0.268090203	100.8255479
2.84464584	0.858543249	0.389742523	0.098639075	47.14969376
2.429780921	0.947383283	0.569922833	0.186218935	120.0329774
1.846794627	0.944711653	0.567944754	0.198208767	99.92454722
1.975980423	0.897312857	0.479887946	0.247460002	55.2422133
0.865777876	0.957869203	0.681063845	0.209869668	68.30151597
0.761174754	0.973470663	0.678345563	0.170510675	69.36943856
2.195246226	0.962258304	0.617921915	0.052697203	129.3290321
8.248395726	0.907232157	0.299430698	0.017158722	191.6420394
3.172078849	0.902463917	0.401259797	0.143444229	104.2303461
2.168443088	0.90165106	0.445579607	0.191417175	66.9336397
2.515142507	0.925319604	0.504443247	0.193724598	91.25360057
2.195627073	0.954389319	0.616857308	0.138836509	101.0552341
3.532637061	0.865709967	0.332974561	0.112034297	58.70774212
8.868329994	0.923067224	0.396014676	0.03380369	187.5862063
5.516389546	0.971868999	0.584407181	0.054642759	1037.017647
4.761297125	0.847599852	0.305237383	-0.025098678	51.06494446
1.306248511	0.955183294	0.642966949	0.169217735	67.32760603
2.213851537	0.93507815	0.532759661	0.063551834	64.88911542
2.015758127	0.902831321	0.479151677	0.167899944	89.47087502
4.548738139	0.898101387	0.339259691	0.078997067	93.13489093
3.32758755	0.848537994	0.381051334	0.031138423	39.2306488
7.695011338	0.810606179	0.294386533	-0.080301293	46.67063492
4.81058165	0.963792652	0.566015753	0.111949606	574.8956224
3.111728764	0.903890877	0.453552601	0.185510362	111.1549871
5.45549717	0.916963155	0.470208351	0.044698108	76.13018186
1.345849938	0.943674113	0.624263577	0.256338061	82.77928886
0.878062728	0.956721913	0.655058089	0.194547846	52.59774776
7.623568677	0.865103416	0.280159658	0.031256321	83.19619333
4.926852768	0.922212803	0.412543883	0.059908787	95.59768512
5.468318296	0.83716279	0.314805622	-0.068335865	56.2984556
4.055314142	0.86094212	0.305308242	0.060722886	78.59750748
2.251622696	0.897000888	0.520897572	0.181308362	64.14488328

log.sigma.1.0.mm.3D_glcm_SumEntropy	log.sigma.1.0.mm.3D_glcm_AverageIntensity	log.sigma.1.0.mm.3D_glcm_Energy	log.sigma.1.0.mm.3D_glcm_SumSquares	log.sigma.1.0.mm.3D_glcm_ClusterProminence
3.372663355	8.425454303	0.059321395	3.828516423	311.0352467
2.63191439	9.919327339	0.14341948	1.865273941	203.9443532
3.130603361	7.313549153	0.06151705	2.558078443	137.8727012
3.638440899	13.75307213	0.056783864	7.410354518	1407.212654
3.302136085	5.644547134	0.029952943	3.825986223	180.4301693
3.321157835	6.18465876	0.033907571	3.075929183	141.5785228
3.722043516	10.92110734	0.027083726	14.08245544	1871.459856
3.714449761	9.76355619	0.026650282	5.423970275	588.0724102
4.412646805	12.99238136	0.01130382	12.77927064	2816.351898
2.996150841	8.03004606	0.086519396	2.357992141	244.2279684
3.278760325	6.188410451	0.040971404	2.78201225	136.7554058
3.159755317	7.831019158	0.073340864	2.983513239	287.8167472
3.039671963	8.881443134	0.071973539	2.176824039	101.0472014
2.993236057	6.549891409	0.069685632	2.005797545	83.70025673
3.887586752	11.54204992	0.016462218	7.524335746	1153.098615
2.030127205	8.384350696	0.201491785	0.69638972	35.30623675
3.363194296	5.611441159	0.029139799	3.60922541	160.0807775
2.886316707	8.785829182	0.088979963	1.767527419	86.27258168
3.488716727	6.770782933	0.029560562	4.433258346	261.6894241
2.4004228	7.202660228	0.162014745	0.897823704	39.28491309
2.387768227	9.211672289	0.15467382	1.11996355	77.04918952
3.438901412	8.672551038	0.037871706	3.917626966	416.6906916
2.369196484	7.146015242	0.160580801	0.869026771	30.43980651
4.088372552	9.375594201	0.014753627	11.72919691	2335.089897
2.537703182	7.017129234	0.129800765	1.210661098	74.83443633
3.102310034	6.448559679	0.065669654	2.370720606	116.4142925
2.74957906	7.218745185	0.111745925	1.686495965	114.2968358
2.090273541	8.313701149	0.193719507	0.820865771	51.86824099
2.552137655	11.08094435	0.150625728	1.723782848	167.0749348
2.484139938	8.096164181	0.146098775	1.323193715	86.48908064
2.432112691	7.210590767	0.146991817	1.13496683	61.07030495
2.297876616	9.255865772	0.161992241	0.791897867	27.461069
3.2879238	9.70679447	0.079354749	3.902065935	414.187528
1.9285135	5.679807692	0.126014957	8.239768964	508.0673481
3.751031921	8.565249103	0.022306815	7.417460145	870.3681244
3.172268811	8.63950915	0.066104739	2.860769744	194.1089585
2.139727639	9.301885452	0.196843527	1.002341468	90.13091005
2.851402206	9.148950059	0.112461716	2.239563489	171.2392837
3.750181825	7.198533486	0.017473899	5.628554157	410.3194888
3.224437331	8.446082492	0.066095678	2.959610009	224.9270181
3.068944458	10.94124913	0.088305048	2.617687599	224.6247008
3.416113032	10.0344843	0.038140413	3.692537556	302.0122253
3.534520426	8.261834453	0.039643031	4.581349425	514.3285515
3.661110132	8.099231661	0.029803122	5.375918573	842.0868489
4.13733965	9.782274303	0.013865215	10.11327288	2139.863132
2.031743905	8.400239131	0.18447185	0.561547133	14.64625805
4.129320228	16.26481519	0.02005598	13.63257843	4503.36509
3.394931057	8.094086566	0.034661359	3.547043212	216.7790812
2.706063338	5.335664336	0.06643699	7.708242401	529.0748057
3.733051943	7.336902015	0.017666451	6.737218175	517.06583
2.74615858	8.172486788	0.122743371	2.023873494	174.1201581
3.290764015	8.196961156	0.055453706	3.392104216	213.2521665
3.186806859	19.02677167	0.083748795	3.551477879	573.5112864
3.136935307	6.559947683	0.050402243	2.566014654	147.4530098
2.162856005	9.352155998	0.189200742	0.932380144	83.58447786
3.803299807	10.78704104	0.029009064	6.971773381	935.9539326
4.037618997	10.68583739	0.014998994	8.235149436	1378.153842
4.34402667	12.57376609	0.008245344	12.7245401	2396.047399
3.250390626	9.600106928	0.052382234	3.167833851	302.0011399
3.068704336	6.910131206	0.054924172	2.118138366	109.6411172
3.011052734	8.752560658	0.082290509	2.34271336	155.0374665
2.766694896	11.12099618	0.130779192	2.381565246	249.7887873
2.506279865	7.321918407	0.138549553	1.149297638	56.88495614
2.604297911	11.22768526	0.142368938	1.321991856	81.72162259
3.069978264	5.964078564	0.0617653	3.035201011	152.8062431
3.436604845	9.870958793	0.052809939	4.407614937	596.5541984
3.876974953	10.09926856	0.018578458	9.905422335	1230.450197
2.76881704	10.01447122	0.122124018	1.981927473	184.664949
3.406718066	6.838538826	0.031432454	3.83734102	193.3903692
2.958515296	10.93472511	0.101380917	2.474944918	234.4620935
2.920073466	9.97531655	0.091840539	2.05208196	144.6135531
3.244903047	7.385124377	0.050568966	2.827183147	184.8056173
2.274167598	8.252906251	0.175162156	0.904919626	47.8008765
2.17606632	8.320862383	0.177756812	0.788246857	43.17363466
2.585328952	11.36825585	0.131480001	1.743040184	194.6046013
4.02135433	13.84717058	0.015945769	9.251312632	1458.681517
3.592870797	10.17968118	0.027824105	4.166472146	330.522974
3.312004799	8.145403078	0.038243801	3.047846877	176.4290117
3.280657902	9.520834591	0.056852533	2.97348874	214.2044432
2.713764503	10.03907993	0.131895272	1.96353876	162.1358061
3.598643251	7.62308114	0.026298274	5.196613708	401.3245993
3.836048917	13.70538237	0.033040277	8.231839736	1493.038532
2.903160575	32.24624219	0.118599755	3.878956997	1144.154378
2.944391484	7.159008833	0.058549429	6.227050656	446.7291312
2.508239183	8.191939863	0.142276146	1.28222918	82.12726243
3.009226756	8.046685598	0.072786856	2.15972827	129.4546201
3.160564395	9.435445904	0.052556144	2.615767994	122.7100722
3.807679049	9.626683786	0.019669933	5.744311585	533.3959071
3.153307963	6.253740894	0.056056806	4.420756463	221.1738987
2.679116152	6.89484127	0.086041824	9.252188838	829.375002
3.059445629	23.99372924	0.102614692	3.658540242	1087.833617
3.396746828	10.50822991	0.046272415	3.832825818	365.2622117
3.418776604	8.713827859	0.054263431	4.806204761	579.8431696
2.67615163	9.076332348	0.129621454	1.542564236	111.4905595
2.351618387	7.239523482	0.15507997	0.956947338	45.76403366
3.63669722	9.105027521	0.030116347	9.531675228	1105.015156
3.609619463	9.761272783	0.037961827	5.18583301	660.7321829
2.670906938	7.503332778	0.085396153	6.469413886	325.3746452
3.689190626	8.845103237	0.019999535	5.873771532	478.7917523
3.082087951	7.977804541	0.070066292	2.640037736	187.8920225

log.sigma.1.0.mm.3D_glcm_SumAverage	log.sigma.1.0.mm.3D_glcm_Imc2	log.sigma.1.0.mm.3D_glcm_Imc1	log.sigma.1.0.mm.3D_glcm_DifferenceAverage	log.sigma.1.0.mm.3D_glcm_Id
16.85090861	0.653592078	-0.107494516	1.713019097	0.531434049
19.83865468	0.490214987	-0.07511321	1.010415899	0.651195843
14.62709831	0.610496261	-0.095576516	1.476186055	0.544601955
27.4289425	0.670012873	-0.100525585	2.235964019	0.516581758
11.28909427	0.801072521	-0.178045091	2.221029727	0.426976317
12.36931752	0.627110055	-0.089156365	1.792676402	0.486685462
21.84221467	0.966560844	-0.412895186	4.519508966	0.28129417
19.52711238	0.676384688	-0.099632783	2.188867884	0.457478618
25.98476271	0.599569898	-0.059609771	3.398549268	0.385188783
16.06009212	0.442330221	-0.047305752	1.304756704	0.590902148
12.3768209	0.579842433	-0.077037081	1.604481168	0.513010774
15.66203832	0.548579445	-0.073772151	1.495982621	0.56205936
17.76288627	0.469569238	-0.051419102	1.365111618	0.573589292
13.09978282	0.53861923	-0.072907628	1.320582674	0.577703252
23.08409985	0.734280904	-0.114606522	2.837022665	0.389642264
16.76870139	0.386295622	-0.056991383	0.679107519	0.711854622
11.22288232	0.688043498	-0.111333103	2.040862761	0.456267255
17.57165836	0.480039511	-0.063172756	1.11644767	0.613432695
13.54156587	0.754898828	-0.142891587	2.283146501	0.434098622
14.40532046	0.446446721	-0.068877238	0.736598368	0.694783064
18.42195532	0.408520954	-0.052496724	0.855862516	0.671542309
17.34510208	0.558949451	-0.066715332	1.88730565	0.500328505
14.29203048	0.421806038	-0.061182398	0.758197187	0.691422605
18.7511884	0.931059485	-0.278810315	3.461441571	0.345755518
14.03425847	0.468839243	-0.069037343	0.877101417	0.655950018
12.89711936	0.571247923	-0.084041676	1.348777153	0.562401917
14.43749037	0.470130886	-0.059075138	1.1008659	0.625022771
16.6274023	0.36214251	-0.048499955	0.723498144	0.701797893
22.16188869	0.515295853	-0.087472928	0.961984216	0.665546612
16.19232836	0.439615797	-0.060869557	0.908624544	0.661087752
14.42118153	0.383446614	-0.045834753	0.874166179	0.666684714
18.51173154	0.385598061	-0.050627172	0.745438297	0.689116935
19.41358894	0.583513924	-0.082137949	1.627654411	0.562909143
11.35961538	0.995201073	-0.876774949	3.894230769	0.281906543
17.13049821	0.896874936	-0.253107146	2.83434673	0.395763398
17.2790183	0.528592909	-0.065669341	1.498318619	0.558425873
18.6037709	0.396099384	-0.059644081	0.739520979	0.702046956
18.29790012	0.52942999	-0.075211887	1.231831544	0.618928346
14.39706697	0.655810767	-0.086597221	2.544607525	0.405469033
16.89216498	0.582697547	-0.084677385	1.440855725	0.562406849
21.88249827	0.598688829	-0.094476632	1.313969819	0.596522586
20.0689686	0.671888973	-0.107440515	1.777556265	0.505550347
16.52366891	0.577423095	-0.071684901	1.933164534	0.497896312
16.19846332	0.654833283	-0.093021385	2.116221536	0.471826722
19.55531952	0.597457793	-0.061865426	3.126824511	0.389397232
16.80047826	0.250514893	-0.024008574	0.683688122	0.698263401
32.12027082	0.577508515	-0.056928497	3.299655366	0.411369238
16.18817313	0.652777772	-0.099763954	1.833057902	0.486636761
10.67132867	0.992124743	-0.694572164	3.324497724	0.304401485
14.67380403	0.827449208	-0.175798522	2.945584241	0.368865147
16.34497358	0.500018551	-0.068581926	1.143582627	0.630643014
16.39392231	0.563047226	-0.076203293	1.639952786	0.532933356
37.96111644	0.481765184	-0.054471205	1.490402197	0.577234037
13.11989537	0.610062714	-0.091348178	1.525868176	0.524757544
18.704312	0.353623283	-0.045677028	0.747447222	0.696338299
21.57408208	0.767158642	-0.138728345	2.527647274	0.440855536
21.37167479	0.63735941	-0.075749122	2.819573791	0.401567475
25.14753219	0.775142384	-0.119875575	3.772928256	0.32793815
19.20021386	0.606680013	-0.08730759	1.612272386	0.531577766
13.82026241	0.52111154	-0.064626409	1.396318014	0.544079054
17.50512132	0.459894906	-0.051981496	1.285297276	0.585832934
22.24199236	0.542523619	-0.083797215	1.187967947	0.634217341
14.64383681	0.366060495	-0.040185985	0.913677488	0.655256141
22.45537053	0.481472212	-0.075806455	0.885117813	0.663715853
11.92815713	0.86876083	-0.280112357	1.713351247	0.497544856
19.74191759	0.621634173	-0.08924495	1.788571772	0.528827363
20.19853713	0.928476438	-0.289448787	3.492938602	0.345156483
20.02894244	0.485684066	-0.066550266	1.075478042	0.633638616
13.67707765	0.696307186	-0.117599509	2.012397757	0.460890559
21.86945021	0.527790092	-0.073148331	1.261020165	0.604359117
19.9506331	0.441271437	-0.050406271	1.193113247	0.602906888
14.77024875	0.521436316	-0.062642815	1.505157357	0.532587699
16.5058125	0.405051942	-0.056757113	0.749448406	0.694613872
16.64140661	0.340846502	-0.040154109	0.734587893	0.691194625
22.7365117	0.430759578	-0.051446085	1.051446396	0.641686972
27.67153447	0.738031804	-0.112989528	3.097891063	0.382825176
20.35936236	0.587604325	-0.071729452	1.988954334	0.469739826
16.29080616	0.642177812	-0.099633544	1.654298058	0.507638711
19.04166918	0.574199809	-0.080263766	1.491948181	0.551823098
20.07815987	0.519995428	-0.079730128	1.08838254	0.64272303
15.24616228	0.828531423	-0.189088614	2.372028832	0.416312754
27.37255904	0.709431462	-0.109110593	2.579272843	0.459706363
64.39889338	0.561268848	-0.084441863	1.429746782	0.613326252
14.31801767	0.97586989	-0.540107469	2.794965718	0.388086278
16.38387973	0.414169195	-0.052604347	0.904650678	0.663749886
16.0933712	0.405299549	-0.03799888	1.350402377	0.574691391
18.87089181	0.53101862	-0.067087597	1.525821972	0.533621422
19.25336757	0.612927901	-0.07309602	2.451955133	0.418263792
12.50748179	0.927654899	-0.370031966	2.217063344	0.457886665
13.78968254	0.960938412	-0.541675462	3.488095238	0.381700244
47.93501918	0.578619192	-0.086318768	1.460071367	0.599852736
21.01645983	0.595338082	-0.082442704	1.757013181	0.510505608
17.42765572	0.756428823	-0.148424044	1.934764222	0.522039791
18.1526647	0.457050468	-0.061352518	0.969234279	0.648606937
14.47904696	0.371520699	-0.043750149	0.813447206	0.672339308
18.21005504	0.947007324	-0.363764327	3.274487225	0.36932654
19.52254557	0.635221656	-0.085157663	2.193409796	0.478439725
15.00666556	0.977552831	-0.614612268	2.853986612	0.402083193
17.69020647	0.816084911	-0.17251689	2.638302498	0.392583877
15.95560908	0.605093669	-0.097079923	1.42067407	0.566911858

log.sigma.1.0.mm.3D_glcm_ClusterTendency	log.sigma.1.0.mm.3D_firstorder_InterquartileRange	log.sigma.1.0.mm.3D_firstorder_Skewness	log.sigma.1.0.mm.3D_firstorder_Uniformity
9.060373207	67.0320394	-0.504522103	0.179564307
4.802482774	21.66878486	-2.024259742	0.312162259
6.109088653	56.45973444	-0.898173014	0.190270995
14.70222463	57.34714842	0.317981652	0.165161198
7.498831139	71.40505886	-0.10478584	0.141848838
6.634185015	62.32060802	0.009590528	0.151941682
26.1117516	154.968153	-0.296761357	0.091836735
12.58798298	85.46207619	-0.02150943	0.121920884
28.8075248	94.14187193	0.432805508	0.09203902
5.252322138	35.0584451	0.257067627	0.237999978
6.477088752	64.13799822	-0.277620232	0.162842349
6.726329262	46.53167009	0.103634353	0.207530246
4.626511096	39.79353893	-0.008579556	0.217250568
4.275285334	43.8645243	-0.003207508	0.212680785
15.44036372	85.58975148	0.278671562	0.109784446
1.571013068	10.95840764	-0.990099167	0.415900594
7.146649335	70.19496155	0.523505196	0.142899616
4.34850969	40.43688485	-0.854618411	0.240633137
8.843284591	71.98705935	0.084749884	0.128426519
2.324123615	12.45292085	-1.61996901	0.346200609
2.47105266	17.35558462	-0.066624223	0.336330763
8.129467046	57.39694047	0.702982888	0.15895924
2.090804732	17.93736428	-1.069421962	0.34699498
26.4875842	119.504118	0.172229267	0.088473062
3.158125905	28.98056763	-1.588047868	0.294461806
6.011784093	58.0343968	-0.864956044	0.201942897
3.727789521	24.84356737	0.31519607	0.277884371
1.860847911	11.40162969	-1.384424234	0.400295629
4.180845004	11.7591064	-1.404565948	0.327075133
3.229441675	20.57980371	-1.621405442	0.322594094
2.556051816	21.5420413	-0.39547964	0.333375641
1.8964708	16.20004249	-1.175423609	0.361568418
8.959229741	53.52275443	-0.158687471	0.20260133
12.70330662	115.6016293	0.05123797	0.12
15.18433748	90.12644815	0.389860166	0.108309186
6.506024499	50.36990014	-0.349775613	0.195468982
2.481958086	10.69804025	-2.476792434	0.401306082
4.749372438	25.97566473	0.375090361	0.26530987
11.72185618	77.73737907	0.135081182	0.117923933
7.51271596	66.07071972	-0.916644567	0.18259591
5.985011881	37.74050272	-0.246347016	0.224118539
8.421161389	73.42467928	-0.105839467	0.144811623
10.31399664	71.84405065	0.06132756	0.151377957
12.22745157	76.49543905	0.452785514	0.132943478
21.08708976	84.08978748	0.835371167	0.104425989
1.253176552	18.13051915	-2.018784306	0.4026804
24.71955719	70.82635498	1.270179438	0.122118699
8.120913664	73.25737441	-0.035181833	0.14881502
15.46861729	119.0488367	0.027796104	0.107438017
13.13764355	102.1180922	0.115682237	0.10189529
4.391710198	23.00298178	0.10053387	0.292174765
8.198856415	74.34771734	-0.732571717	0.172669002
7.720801354	34.7037766	-0.913977174	0.240736969
5.939658674	53.3867017	-0.438355105	0.176414979
2.228650464	15.06904447	-1.531842907	0.394289546
15.36270046	92.27559376	-0.160679296	0.118823791
17.78943209	84.55431628	0.50244319	0.107069586
27.85609133	130.0145388	-0.075242911	0.075008104
7.443743484	51.23001361	-1.092336318	0.184477641
4.766130028	39.55907512	0.267906145	0.20127466
5.780582982	50.19282258	-1.035030905	0.218004511
5.2604903	18.20787632	0.056536861	0.290436879
2.608505596	20.76337826	0.200375901	0.326227434
3.375851711	16.62414384	-1.332365953	0.319483651
7.196772953	66.65495205	-0.396524364	0.178355287
9.790757078	50.29842377	-0.114720373	0.174691659
19.10185725	105.059494	0.261151743	0.093696145
5.034273788	28.15918159	-1.333557871	0.277897027
8.437950465	75.99040413	-0.44512965	0.13805076
5.877614304	33.5146383	-1.02267678	0.249715377
4.93486461	36.76022601	-0.934487924	0.243485337
7.055247698	63.17676842	-0.783448922	0.174746259
2.191517944	11.10191894	-1.622421986	0.366693675
1.842066374	17.02736402	-0.945171614	0.381206237
3.66956123	23.50508881	0.460144718	0.317379246
18.66948089	95.73800129	0.071129798	0.099991956
9.53215699	70.90368426	-0.092094874	0.138715759
7.260986271	64.08163714	-0.450007961	0.158920599
7.144422615	61.04568613	-0.433703063	0.176785605
4.469887281	22.38033283	-0.41241077	0.289691089
11.56389557	78.3718586	-0.360026871	0.127156434
16.60916873	78.74764252	0.190003713	0.125383407
8.416988783	23.54359531	-0.927269294	0.285254715
12.00199601	60.0285778	-0.220547284	0.138397503
3.003149452	20.87057447	-0.71429495	0.322506592
4.598113451	36.00379825	0.135282086	0.2293905
6.114449513	64.42511651	-0.861333063	0.179358229
12.40062515	81.9483881	0.167209732	0.121062144
9.051768135	70.66164923	-0.355666903	0.1672
16.75875535	105.2202597	-0.12811799	0.171597633
8.756195627	29.309515	-2.055684992	0.247749009
9.119947217	75.05934966	-1.145116593	0.163608033
10.00760532	53.38796747	0.33717518	0.173943695
3.882750333	23.31099218	-1.309457634	0.294056041
2.287606488	20.38809609	-1.094504994	0.342686019
19.58939706	114.5660076	0.236367719	0.110103301
10.99892061	60.88790751	0.396429518	0.146786332
11.96101577	83.85553646	-0.269256332	0.152777778
12.45644456	84.89383745	-0.318199248	0.116037487
6.273002819	49.64845037	-1.27556827	0.206734694

log.sigma.1.0.mm.3D_firstorder_MeanAbsoluteDeviation	log.sigma.1.0.mm.3D_firstorder_Energy	log.sigma.1.0.mm.3D_firstorder_RobustMeanAbsoluteDeviation	log.sigma.1.0.mm.3D_firstorder_Median
42.591105	1444120.198	28.92367553	-12.19043064
26.67530703	2555630.206	13.16619787	-4.581737518
35.53320877	596337.8723	24.44315355	-24.7205143
48.9795502	11057036.41	27.19581678	-7.175714016
39.73902374	497480.2032	27.649637	-51.57009506
37.74390947	902340.2398	25.85164569	-36.24185753
79.17480388	740870.6932	60.86248489	-30.63177109
50.68249687	2816292.816	35.68606446	-47.74525833
66.12368659	11634676.24	43.36325315	-16.97162437
28.18374315	4693099.628	15.62367904	-8.301012039
36.2820964	1311452.774	25.99701576	-35.72412109
34.66781393	2161231.334	20.38144963	-10.52458954
29.30934993	2701185.962	17.57958603	-12.12039518
29.20126163	1149536.631	18.43195604	-20.39272213
54.71038707	2190595.075	35.43457989	-29.73742867
11.14298157	5078319.577	4.757870309	-1.12240839
40.96395627	1130195.135	27.92905984	-50.79096222
27.60932307	2749087.819	18.03675118	-9.008279324
47.17714628	1039083.132	31.45060845	-44.15475082
16.48111732	2026115.68	7.24341156	-1.880185902
18.30548649	4967215.403	8.894937483	-3.724141359
38.22361567	3086512.857	23.94078933	-21.49749374
16.91117341	2146006.545	8.845282908	-2.809009671
66.65027202	1038896.836	47.36719335	-27.81265259
22.85515221	1210606.89	13.47603234	-6.937596321
33.31178461	843119.7548	23.82063113	-20.71987247
23.03440404	3187435.054	11.68494403	-5.150262833
13.55597912	2660356.494	5.187024307	-1.655387402
22.95477331	5630006.979	9.033901693	-1.739352226
21.95379385	2180621.34	10.67425312	-4.289423466
18.70141911	3933128.111	9.883117237	-4.131412506
14.8716445	5180880.183	7.58201064	-2.947394371
40.7067622	5208164.195	24.18061203	-6.348749161
66.17907539	296687.5369	46.85834005	-120.2946854
57.11198748	917752.3533	35.2750744	-48.30250168
34.97280228	3686476.306	22.53359042	-11.2883935
14.69121224	5502565.545	4.73701914	-1.206338167
26.49253885	5804694.309	12.98568115	-4.309233427
48.11691107	1563669.103	33.53019813	-36.16975784
39.72277133	1899052.13	28.1681852	-14.2585659
31.78457482	1745231.838	17.64558279	-7.103190899
42.32728089	1833197.091	29.31007155	-38.70020676
44.64775184	4045240.629	30.76953918	-28.33663177
46.76672887	2481381.788	31.97189394	-35.17102432
58.20037333	7481654.688	36.4802864	-29.26804543
13.20580383	2498855.451	7.742446325	-1.73465395
56.18883851	20669407.88	31.23716105	-18.03989315
41.9474484	1011000.415	30.45422221	-35.53694916
64.10874976	324273.1244	49.22413176	-102.1506577
57.42980816	1121512.433	41.88810408	-50.54623985
23.87389957	6518467.933	11.40128123	-4.006652832
41.24789065	1671338.839	30.38968861	-24.07575607
32.36087248	8586111.666	17.46778651	-7.703852177
34.53563243	698395.6128	22.35277839	-23.45440292
14.77904342	8649882.574	6.467758934	-1.401394248
57.94590337	2661342.225	39.70359956	-35.98176575
55.52102716	7270678.922	35.34961675	-56.9708004
75.54134643	5258534.163	53.68249587	-47.53767014
37.18014091	1120306.562	23.17504057	-14.35191727
28.66347071	858045.6509	17.77544212	-16.57801723
33.8160861	3694436.356	22.21745151	-10.37123013
25.97339531	13536310.03	11.21287699	-2.816745758
18.41357534	3689425.861	9.192498082	-2.788934588
19.99126647	1916511.318	9.097425193	-2.231265068
38.61828859	216762.831	27.84099012	-36.33971405
39.84424728	4185777.796	22.14824025	-7.423553705
66.40100325	1206398.473	44.93531713	-75.69158173
27.56639032	6380095.157	14.58352739	-4.51909256
42.85244917	737717.6877	31.51680497	-42.12665176
31.08168397	4203894.741	17.05172563	-5.943150997
28.50238683	4791341.719	16.85739322	-6.600139141
36.94672063	1921200.09	26.40164352	-23.82049942
15.39537506	5120681.109	6.079351598	-1.703623414
14.83781295	6727611.247	7.466261029	-2.329545259
21.0602138	19714948.59	10.07359653	-1.61814189
61.40002991	3949443.121	39.57662479	-43.56592369
42.56750984	2092856.602	29.00241099	-34.17489243
37.08709626	900730.5796	26.87956071	-37.90632057
37.67958717	3107709.529	25.75268729	-21.43159485
26.57574084	5106467.069	12.92732598	-3.307350516
47.40088933	993219.0666	33.74455004	-54.40280533
56.43480838	5967925.22	33.29779642	-17.03484344
29.13593669	14455253.13	11.31710869	-1.763656497
48.76025587	320310.9704	30.55335463	-73.66008759
20.04976026	5453785.883	9.910411007	-3.444619298
27.02629257	5837894.109	15.73505025	-8.694012642
35.36145982	1119260.351	25.76953696	-24.49214363
49.07498924	4030166.809	33.27780209	-47.32416153
43.19972688	179751.9903	30.41493716	-12.72544575
66.6146484	166755.6913	40.96828927	-96.43680573
33.25564697	10306852.13	16.15676601	-3.855182409
43.78947117	1309774.14	29.62619766	-16.75397205
41.10209439	1643595.895	24.10185802	-11.73935127
23.97224883	5268090.104	12.08779778	-4.311468124
17.78348016	5885667.94	9.321111341	-3.700865626
64.23313745	697400.3649	44.84716061	-58.69029236
44.40898511	2767799.717	27.04908626	-10.57975674
50.1947361	326489.3557	33.4982412	-85.58858871
49.58369028	880751.6366	33.97285217	-36.00175476
34.49905737	686072.1832	21.60939575	-7.287879229

log.sigma.1.0.mm.3D_firstorder_TotalEnergy	log.sigma.1.0.mm.3D_firstorder_Maximum	log.sigma.1.0.mm.3D_firstorder_RootMeanSquared	log.sigma.1.0.mm.3D_firstorder_90Percentile	log.sigma.1.0.mm.3D_firstorder_Minimum
38991245.34	158.4543152	63.24818541	7.260497093	-216.0262299
69002015.56	102.6105652	42.67958332	7.625699997	-235.8136749
16101122.55	68.47329712	58.04429792	6.605453014	-179.1622925
298539983	407.2182617	75.63162694	32.60836525	-348.4222412
13431965.49	61.48260117	70.18223825	16.23670959	-168.986908
24363186.48	122.6811142	60.81213478	17.54797363	-166.7705994
20003508.72	128.3427887	102.877923	78.35956421	-295.3369141
76039906.02	201.2896271	83.80437998	7.281289577	-270.1037598
314136258.4	244.0461884	88.78384975	104.2836037	-306.8320313
126713690	332.9970093	44.98622101	13.70885201	-189.4586487
35409224.91	92.77193451	59.05849909	6.824885845	-162.1422424
58353246.01	323.0783081	53.43215471	17.93208618	-194.8368835
72932020.98	159.5918884	44.46843981	20.26851654	-221.6841583
31037489.04	128.357132	46.65996904	12.74702215	-171.8254547
59146067.04	259.9291382	77.57653214	60.06785774	-284.169281
137114628.6	184.9375458	21.65638134	9.206895828	-191.7471161
30515268.65	108.1265945	72.16836227	11.28296165	-161.4265594
74225371.11	141.8970032	42.08704635	7.65840683	-208.1554108
28055244.56	138.8237305	75.55956576	34.53524075	-181.1643982
54705123.35	117.9264374	27.02582065	5.206688023	-173.9118347
134114815.9	478.6517639	31.28516671	9.058429718	-213.1268005
83335847.14	360.4595032	58.27095068	29.53682022	-222.0802612
57942176.72	191.9273071	27.10966153	4.725716448	-152.0203857
28050214.58	282.9072571	88.38130123	66.52077637	-247.4643402
32686386.04	79.09268188	35.51124954	9.151495171	-164.9231415
22764233.38	66.87232208	52.83736621	6.767645454	-163.4876251
86060746.45	242.0834656	37.31623126	17.26132507	-155.9228668
71829625.33	323.8140259	26.13461986	8.200212479	-184.7286682
152010188.4	249.3969574	38.92392382	5.459251642	-261.9757385
58876776.18	159.452774	35.88902416	8.262225723	-189.4216003
106194459	263.469574	30.59797642	11.56686592	-166.5478668
139883764.9	131.7453613	24.21852331	9.446527863	-209.220108
140620433.3	324.752594	61.23379993	13.65583076	-240.9928894
8010563.496	18.0953064	140.638434	-21.64355106	-248.8558502
24779313.54	189.453537	90.12048736	33.76257095	-239.1064911
99534860.27	289.1698608	52.56876917	10.77646847	-215.3103638
148569269.7	209.7963409	28.67722325	8.116586685	-215.2579803
156726746.4	237.9246979	43.75473706	15.26737146	-212.9771271
42219065.78	128.0822296	69.79429283	45.91771317	-192.5306549
51274407.5	112.1439667	59.46772171	9.194534111	-215.0191193
47121259.63	207.390213	49.40530022	17.34391899	-250.0880127
49496321.47	172.6005249	69.36527173	9.752400398	-250.3433685
109221497	255.5943756	70.06620161	10.81073523	-210.9342041
66997308.27	321.9518127	75.18211432	11.30714436	-216.5258789
202004676.6	375.7927856	81.91461533	81.08783112	-238.4479828
67469097.19	120.6674728	20.20337406	15.16285648	-197.5487976
558074012.7	509.7026672	86.75884291	66.75270462	-379.8085632
27297011.22	173.7594147	66.58988173	14.27767162	-201.8339386
8755374.36	17.3640976	121.4071146	-8.34361763	-205.6280212
30280835.69	91.7974472	87.64469183	47.83855629	-211.6243286
175998634.2	290.2727661	39.9707857	13.91077108	-198.9639282
45126148.64	130.0081177	64.39908987	6.244643307	-205.7606201
231825015	286.3486328	51.83974242	15.16230202	-452.410614
18856681.55	154.5970917	54.05698267	15.59009514	-168.5730438
233546829.5	313.1870117	27.65990062	12.90735626	-203.9248505
71856240.06	271.5232544	89.13083164	19.73354607	-278.3700562
196308330.9	287.7286987	91.31229131	29.22427235	-284.5239258
141980422.4	202.4579163	107.1518345	66.37961121	-341.0227051
30248277.19	100.8742218	56.25573574	19.50339203	-225.0483093
23167232.57	180.5582123	42.91036798	22.75564384	-150.6047058
99749781.62	150.3295288	50.38979576	11.68192291	-202.1241302
365480370.8	349.4986877	44.69546374	12.33278561	-250.2486267
99614498.24	199.6321564	30.09323534	17.70078373	-168.7138672
51745805.58	141.7415466	32.79456271	9.914918518	-260.0766907
5852596.436	47.19764709	63.95205028	1.283036053	-163.0601044
113016000.5	294.9257813	60.27841795	36.15041084	-230.2824402
32572758.77	189.1946106	107.1891285	41.65663147	-279.6328125
172262569.2	197.8757782	42.89796171	12.75081692	-245.2995758
19918377.57	51.88991928	71.82523894	9.134943771	-196.6370239
113505158	232.47789	48.43473345	14.05019808	-259.0903625
129366226.4	223.003479	42.93638072	17.27338219	-237.9902191
51872402.42	76.96955872	58.20962074	11.32747421	-189.8110809
138258389.9	181.804306	26.9372841	7.146495819	-198.9846039
181645503.7	425.9081116	24.91931089	12.68508663	-189.1647644
532303612	348.7522583	36.84931948	21.5278183	-260.2962341
106634964.3	312.6585693	94.10235723	43.54052734	-354.9748535
56507128.26	148.6257935	67.74657048	18.15027523	-255.8598938
24319725.65	109.5740814	63.41229107	9.25243721	-201.3697815
83908157.29	163.7418671	58.34247126	11.62497826	-230.0963287
137874610.9	271.4779968	43.45671803	7.70237608	-249.5743866
26816914.8	108.1647568	81.64496173	10.80236454	-215.5633087
161133980.9	336.1376343	83.0622388	47.30883713	-337.1057129
390291834.4	346.4327698	54.15436172	22.60103188	-798.3779907
8648396.201	88.84125519	101.6494562	2.288364887	-235.3846588
147252218.8	243.3203583	33.1528342	12.40271482	-192.993988
157623140.9	245.9292297	40.80000569	22.91961365	-182.8211975
30220029.47	70.6116333	55.37568703	8.921713638	-233.0799866
108814503.8	204.1430817	82.71873107	17.63647461	-257.6651306
4853303.739	96.48456573	59.95865081	23.94493122	-166.5001678
4502403.666	65.08698273	113.2579396	9.411416245	-234.4225464
278285007.5	282.2964172	54.82508076	17.91154861	-575.2572021
35363901.79	82.22016907	65.63891809	18.30305157	-251.0516815
44377089.18	232.6638031	62.85659609	29.85060406	-218.4244385
142238432.8	148.4939728	37.98054679	10.90853977	-219.293457
158913034.4	225.9603424	28.54756931	12.99433126	-171.9805756
18829809.85	199.8986359	105.2133407	10.2972435	-265.1689453
74730592.37	330.5661316	63.79885345	63.58846817	-232.0022583
8815212.604	32.88705063	116.6349997	-24.07678299	-252.6218872
23780294.19	109.1983871	73.50771574	43.44807587	-248.0656586
18523948.95	63.24974442	49.50007876	24.10494137	-194.2241211

log.sigma.1.0.mm.3D_firstorder_Entropy	log.sigma.1.0.mm.3D_firstorder_StandardDeviation	log.sigma.1.0.mm.3D_firstorder_Range	log.sigma.1.0.mm.3D_firstorder_Variance	log.sigma.1.0.mm.3D_firstorder_10Percentile
2.9130188	54.07908448	374.480545	2924.547378	-105.8101349
2.179397109	38.53818026	338.4242401	1485.191338	-70.46418915
2.722676143	44.25490864	247.6355896	1958.496939	-100.0854752
3.235833164	71.89770409	755.6405029	5169.279854	-115.2533157
3.026509243	50.16174091	230.4695091	2516.200251	-108.1753693
2.963718342	47.73815576	289.4517136	2278.931515	-97.14893188
3.614807753	93.98014756	423.6797028	8832.268135	-158.2498795
3.315547627	64.20195683	471.3933868	4121.89126	-135.7973022
3.76065623	86.96772736	550.8782196	7563.385603	-119.4557686
2.561500304	42.03618496	522.455658	1767.040846	-67.29613953
2.86329714	44.62445134	254.9141769	1991.341657	-98.20324707
2.771780027	49.31862796	517.9151917	2432.327064	-86.40498657
2.648063379	40.76717548	381.2760468	1661.962597	-69.54963684
2.620564163	39.38535486	300.1825867	1551.206177	-73.34642029
3.507050525	72.9877052	544.0984192	5327.205111	-106.9172913
1.599003218	21.38665585	376.6846619	457.3890486	-16.10335674
3.04905814	52.15880312	269.553154	2720.540743	-113.4519073
2.415731574	36.07213794	350.0524139	1301.199136	-72.84452133
3.237054817	60.00760796	319.9881287	3600.913013	-129.0819122
1.929456638	25.4107495	291.8382721	645.7061901	-42.17715263
1.989372456	29.60940682	691.7785645	876.7169724	-45.76661453
3.029192971	52.93802611	582.5397644	2802.434609	-85.23514099
1.920464919	24.88334306	343.9476929	619.180762	-43.19118233
3.683373172	83.08533618	530.3715973	6903.173088	-130.6044586
2.121565223	31.10453153	244.0158234	967.4918815	-62.34670029
2.573362416	40.80660566	230.3599472	1665.179066	-97.04971619
2.343267718	36.26322734	398.0063324	1315.021657	-50.8240242
1.672902932	25.28637139	508.5426941	639.4005781	-25.47002945
2.11414838	36.79720844	511.3726959	1354.034549	-59.06938362
2.081310742	32.96874214	348.8743744	1086.937958	-57.93504868
1.981502803	28.96111674	430.0174408	838.7462826	-44.04548645
1.832117343	23.00237826	340.9654694	529.1094056	-33.81665344
2.889345258	56.83959288	565.7454834	3230.739319	-100.2873657
3.189898095	76.92953453	266.9511566	5918.153283	-210.9772461
3.490863218	76.28008314	428.5600281	5818.651084	-136.946759
2.769585887	46.17639804	504.4802246	2132.259736	-91.25591888
1.694794406	27.78033518	425.0543213	771.7470225	-21.76391602
2.467295008	42.62482017	450.901825	1816.875294	-62.25331192
3.308651181	60.30981019	320.6128845	3637.273205	-106.0761795
2.814116275	48.88660508	327.1630859	2389.900157	-105.2555161
2.666224771	46.32247565	457.4782257	2145.77175	-80.6407486
3.092571476	54.24525812	422.9438934	2942.548028	-115.3883209
3.072454282	57.87253806	466.5285797	3349.230661	-121.4447845
3.214224221	61.36416274	538.4776917	3765.560468	-123.7738724
3.613256987	79.08565273	614.2407684	6254.540467	-109.3143143
1.602601209	19.80515238	318.2162704	392.244061	-22.61896324
3.545125378	85.05129202	889.5112305	7233.722274	-109.0188637
2.986105716	51.82863956	375.5933533	2686.207879	-107.7985718
3.265583323	71.13788726	222.9921188	5060.599004	-198.1192184
3.433009938	69.93791072	303.4217758	4891.311355	-147.3380585
2.306878959	38.53453069	489.2366943	1484.910055	-57.10557404
2.844278717	49.97486242	335.7687378	2497.486874	-113.6670624
2.619984318	48.30418991	738.7592468	2333.294763	-83.71974182
2.798184469	45.3356925	323.1701355	2055.325014	-90.32016907
1.745835106	27.24246424	517.1118622	742.1518579	-21.56629467
3.448960265	73.67652987	549.8933105	5428.231053	-154.5590057
3.544313664	74.26846338	572.2526245	5515.804653	-137.9691452
3.938628857	93.48051188	543.4806213	8738.606101	-176.198526
2.837936872	50.08855256	325.9225311	2508.863097	-90.27770157
2.653858991	39.18872549	331.1629181	1535.756206	-65.91938019
2.591758254	43.26585254	352.4536591	1871.933996	-90.61299133
2.371651946	43.44987337	599.7473145	1887.891496	-65.05606461
2.076334796	29.73882983	368.3460236	884.3979997	-33.61495628
2.102409242	31.31727363	401.8182373	980.7716277	-53.02169075
2.797064988	45.9656043	210.2577515	2112.836779	-107.6805649
3.038782369	58.10555864	525.2082214	3376.255945	-88.57851486
3.644830179	83.79897707	468.8274231	7022.268558	-162.0449127
2.342502344	39.78596551	443.175354	1582.923051	-72.40723724
3.030925709	51.55324738	248.5269432	2657.737315	-124.7744827
2.540300431	44.81429038	491.5682526	2008.320622	-82.80598755
2.484107912	39.62410855	460.9936981	1570.069978	-71.65791473
2.780498507	45.28510754	266.7806396	2050.740965	-101.1912094
1.843054237	25.73748451	380.7889099	662.4181088	-37.91738663
1.7477531	24.11137833	615.072876	581.358565	-29.09137707
2.181942554	36.70274934	609.0484924	1347.091809	-32.26073151
3.661641729	82.00787297	667.6334229	6725.291228	-146.1297455
3.118802892	54.46337544	404.4856873	2966.259264	-113.4155922
2.870852907	46.01315683	310.9438629	2117.210602	-106.6448608
2.860026151	48.42002564	393.8381958	2344.498883	-95.36697388
2.326081449	41.03308752	521.0523834	1683.714271	-69.98790817
3.201057315	58.50364165	323.7280655	3422.676087	-136.371817
3.504851725	79.43882395	673.2433472	6310.526751	-120.7113312
2.46747447	53.49951291	1144.81076	2862.197882	-56.92498703
3.163041877	66.24084016	324.225914	4387.848906	-170.5622253
2.083621967	31.66350187	436.3143463	1002.57735	-47.39754944
2.561529183	38.91487657	428.7504272	1514.367618	-58.95173798
2.728761959	43.39605927	303.6916199	1883.21796	-91.91806793
3.353654128	63.34190344	461.8082123	4012.196731	-134.1177979
2.871671994	54.01327053	262.9847336	2917.433393	-103.8987801
2.777362795	81.12306076	299.5095291	6580.950987	-181.974054
2.623343969	52.32839073	857.5536194	2738.260476	-81.31777649
2.962145427	55.60926781	333.2718506	3092.390666	-114.7436852
3.045021866	58.88782475	451.0882416	3467.775904	-97.79563904
2.244841275	35.31193747	367.7874298	1246.932928	-63.36864281
1.939150143	27.10979264	397.940918	734.9408572	-41.947052
3.389867433	81.49083596	465.0675812	6640.756346	-156.6556
3.228973513	61.95019899	562.5683899	3837.827155	-91.28030243
2.975412897	62.49618464	285.5089378	3905.773094	-168.6789795
3.326882505	62.98570769	357.2640457	3967.199373	-114.2329865
2.66417451	45.36630141	257.4738655	2058.101304	-91.8412323

log.sigma.1.0.mm.3D_firstorder_Kurtosis	log.sigma.1.0.mm.3D_firstorder_Mean	log.sigma.1.0.mm.3D_glrlm_ShortRunLowGrayLevelEmphasis	log.sigma.1.0.mm.3D_glrlm_GrayLevelVariance
4.183389104	-32.79917041	0.025357581	5.490976565
7.841707303	-18.33999712	0.012964776	3.189253703
3.4433317	-37.55853542	0.034676934	3.537144108
6.973840176	-23.47047378	0.006799826	10.25290499
2.750817557	-49.08509259	0.0855237	4.641911747
3.31874004	-37.67206155	0.053152798	4.072120812
2.434753338	-41.8521076	0.028021546	14.13584763
3.943609533	-53.86355766	0.019159564	7.200447387
3.382024709	-17.86578782	0.008327191	13.07150162
10.33799287	-16.02246033	0.02028501	3.652599932
2.966263953	-38.68545796	0.051783442	3.566882218
7.694646411	-20.55889327	0.027824785	4.78997297
5.282093198	-17.76174379	0.014300556	3.366724595
4.810775061	-25.0189235	0.033449212	3.073651423
4.745367828	-26.28522833	0.013350495	9.229220833
19.29145421	-3.407316258	0.011616631	1.262387225
3.386208647	-49.87716682	0.063685254	4.679068902
5.192511179	-21.68225853	0.014603452	2.626571648
3.075469357	-45.91660881	0.050617367	6.2325108
8.566898645	-9.202651346	0.01894208	1.609472785
24.22632966	-10.10171685	0.011196586	2.097021064
7.727777001	-24.35300977	0.019089906	5.292203556
9.10367287	-10.75885618	0.019590429	1.532398528
3.591789874	-30.13438766	0.027565726	11.48018091
6.144131441	-17.1335041	0.028181507	1.953108365
3.030757545	-33.56498476	0.044217432	2.98685841
8.56336317	-8.802241694	0.022090976	2.848377941
23.75434634	-6.604375608	0.014181088	1.606364223
10.989683	-12.69004716	0.008217447	3.157570554
8.590958752	-14.18041245	0.019494389	2.399834526
12.05588451	-9.873696279	0.021012242	1.931871673
9.755056843	-7.578091156	0.009704212	1.33043676
6.391962065	-22.77803625	0.017242865	6.50178822
1.949674991	-117.7328155	0.127235433	9.168219093
3.953775246	-47.9901152	0.031593725	9.751543303
5.565707948	-25.12400757	0.017431391	4.163705905
16.21857867	-7.115905474	0.011469623	2.006266792
7.949079759	-9.879358311	0.012948012	4.011748339
2.899959921	-35.12791065	0.039753495	6.211067183
3.796974415	-33.86015015	0.028649521	4.419698217
7.446756854	-17.17882241	0.010663499	4.318316747
4.248372137	-43.23185046	0.015221588	5.350777653
5.303078843	-39.49736633	0.028158728	6.119858425
6.479949771	-43.43719426	0.032280479	6.712627619
5.046784685	-21.3415964	0.016859223	10.85543942
12.10089456	-3.991523815	0.011809812	0.953074049
8.047179118	-17.12817999	0.004721715	13.09540256
3.822931198	-41.80914337	0.025750186	4.629121959
1.675149714	-98.38235853	0.145327512	8.044827107
2.322619876	-52.82310717	0.037207976	7.995149456
10.5367521	-10.61855234	0.017915326	3.371341232
3.181976103	-40.61718727	0.025567024	4.50286004
10.36567433	-18.8165919	0.002961372	4.855259105
4.342197657	-29.44201694	0.049511238	3.757182288
18.12707998	-4.787300298	0.010459163	1.883827354
4.031531091	-50.1604834	0.016313745	9.344252449
4.628516861	-53.12372248	0.014779314	9.45604842
2.825941452	-52.37279388	0.012148147	14.55124605
5.239021434	-25.60946517	0.018179191	4.525102199
5.121011823	-17.47980189	0.026976878	2.959331225
4.35118472	-25.83016687	0.018096307	3.606279513
10.17903464	-10.47821467	0.008034284	4.348238036
9.702717003	-4.604868452	0.018077553	2.042716204
9.389891636	-9.731994427	0.007848924	2.205507945
2.659618654	-44.46378252	0.069373565	3.947789954
6.326021003	-16.03844521	0.014867608	6.44144095
3.3300434	-66.83741996	0.026547132	11.82831187
6.966261213	-16.04094971	0.011220857	3.396508893
2.53227974	-50.01127506	0.050530753	4.638086701
7.425640994	-18.37397024	0.010135056	4.160957571
6.423607983	-16.53671101	0.011212581	3.15906135
3.142008365	-36.57347374	0.032193802	3.604734105
11.89653889	-7.949790317	0.013807358	1.721125551
22.5174052	-6.293924871	0.012870016	1.432614917
15.09021108	-3.283372784	0.00681271	3.147761787
4.687120194	-46.15151576	0.009441306	11.65853322
3.6835441	-40.2906757	0.014066921	5.250675125
3.393448573	-43.6337949	0.025975778	3.827558574
4.62154435	-32.54758164	0.015494547	4.403261062
8.888079444	-14.30985921	0.010988678	3.800773267
2.90475305	-56.94930806	0.044565656	5.861684931
5.218678977	-24.26538199	0.00821121	11.58714863
21.15351744	-8.396249805	0.001075421	6.397702503
3.637475231	-77.10228948	0.062696332	7.509986242
10.59645644	-9.825124187	0.015494022	2.340511002
6.480049938	-12.25858256	0.017599433	3.041921008
3.942997311	-34.39838301	0.016608525	3.424310352
3.743541597	-53.19954641	0.017002516	6.97342171
2.933701419	-26.03087424	0.054914328	5.056823228
2.388098589	-79.03423236	0.108649787	11.04229167
16.17691807	-16.3563139	0.001950661	5.82442952
4.437482933	-34.87229418	0.017860152	5.448748665
5.920051373	-21.98125948	0.025651941	6.566541945
7.239131025	-13.98531395	0.013549166	2.77803844
9.554383893	-8.94554953	0.020090629	1.720017878
4.012859995	-66.55141408	0.039215949	10.74976777
5.202025874	-15.24685369	0.014148547	7.225672293
3.00757601	-98.47817047	0.064481153	7.34695972
3.256559217	-37.89703023	0.023633422	6.484036373
4.453631246	-19.80294153	0.028968589	3.833167783

log.sigma.1.0.mm.3D_glrlm_LowGrayLevelRunEmphasis	log.sigma.1.0.mm.3D_glrlm_GrayLevelNonUniformityNormalized	log.sigma.1.0.mm.3D_glrlm_RunVariance	log.sigma.1.0.mm.3D_glrlm_GrayLevelNonUniformity
0.027219014	0.150619752	0.330257905	44.13109989
0.015267696	0.248600215	0.814734241	239.4730263
0.037001686	0.166280645	0.261151961	24.49119108
0.007573403	0.12714018	0.491522108	192.972472
0.088854811	0.136354211	0.121187668	12.54875946
0.056150845	0.14213683	0.206654431	29.92076918
0.028269622	0.09114249	0.035232882	6.169587587
0.020125884	0.114147061	0.190787517	40.02241939
0.008885496	0.085730442	0.176006794	111.925175
0.023209361	0.193437209	0.59573044	333.2343593
0.055181792	0.150974062	0.253834895	47.77190582
0.030448528	0.170348648	0.420418209	101.2236515
0.016420132	0.177389133	0.55374824	184.5306825
0.037205568	0.17973357	0.46373755	73.81258633
0.013877377	0.105238266	0.095343764	35.23407875
0.016173154	0.372291939	1.235240746	2411.032397
0.066832944	0.13716468	0.130855967	26.64363139
0.017187289	0.205538851	0.60689982	232.4500818
0.052614545	0.119775424	0.135383951	19.54536632
0.024416662	0.286951674	1.103320883	503.515092
0.014196632	0.280549644	0.942916913	936.4776661
0.020781041	0.142328894	0.296088035	107.9762975
0.024805753	0.283619672	1.06359257	534.6323694
0.028117376	0.086914939	0.062863821	10.96047402
0.032310338	0.254368402	0.591098993	178.1099168
0.047483018	0.179590934	0.327958057	44.07609113
0.026243365	0.22855534	0.678037316	373.8447393
0.018195324	0.356107026	0.968979166	889.0963965
0.010242976	0.269013764	0.939215012	662.0126554
0.023046795	0.2682868	0.822017361	309.817645
0.0259274	0.282351639	0.90186209	784.4784838
0.013005072	0.309511763	1.147159214	1720.034828
0.018997054	0.156624458	0.569738714	164.6832222
0.128766007	0.11932301	0.025319777	1.744040575
0.032490003	0.104458486	0.090866323	10.90908321
0.019616344	0.16297861	0.483558207	167.4755526
0.01488602	0.352467575	1.185599123	1444.240411
0.015540945	0.210367107	0.780746249	446.6215844
0.041459362	0.114164817	0.110275873	33.29013931
0.030777856	0.152744912	0.429181733	64.17715923
0.012039038	0.183904777	0.469345812	101.2042622
0.016373155	0.133072853	0.248012502	42.76681145
0.030003092	0.133583935	0.30833984	91.62887688
0.034118225	0.122589948	0.21152679	46.31749535
0.017852143	0.098315692	0.139968275	98.2158432
0.015822464	0.360777838	1.049860496	1397.140515
0.00512007	0.109148014	0.205738265	260.0189052
0.027407827	0.141210259	0.189059092	27.69129644
0.145708098	0.107521627	0.0139543	2.332334332
0.038600212	0.100162841	0.078744322	13.66899857
0.021370271	0.235960823	0.847784171	662.0847148
0.027656918	0.147875506	0.34980914	47.93241028
0.003482441	0.192034742	0.608354811	451.3416124
0.052388895	0.161373474	0.235746507	32.61616812
0.013836214	0.342625231	1.183432797	2382.802448
0.017068928	0.104274108	0.21270504	30.4514934
0.015432924	0.103161058	0.118315362	82.09554296
0.012471051	0.073544291	0.069657147	31.71647824
0.019544482	0.164985333	0.264641614	48.41829332
0.030034848	0.184060021	0.304493123	70.05443879
0.020349969	0.180678729	0.575625167	196.8721105
0.009914735	0.228710034	0.934734792	1044.353858
0.022675669	0.272693404	0.860283685	751.2236202
0.009672484	0.270576186	0.773501191	332.2112687
0.072172979	0.158466025	0.165619961	7.398379812
0.016290331	0.148041535	0.349638749	138.3036177
0.027094	0.091419366	0.065276513	9.029962939
0.013477494	0.224724494	0.838352685	533.3513912
0.052442238	0.130446144	0.150010569	16.47746819
0.011734537	0.19948128	0.653714005	260.3330039
0.013063688	0.204012603	0.605971474	392.962206
0.034717888	0.158034307	0.318586942	73.22839712
0.018081105	0.309164585	1.223272718	1343.667551
0.017025266	0.331508607	1.118937354	2244.022119
0.008726964	0.260391323	0.939411612	2515.653986
0.009776177	0.093722249	0.124019175	38.10121166
0.015024208	0.13073335	0.206478801	51.79829081
0.027776983	0.152889586	0.213564146	29.10626364
0.017135259	0.154514091	0.413803585	112.5760925
0.013201564	0.228551126	0.87613791	422.710934
0.04585077	0.123241515	0.10818592	16.81600384
0.008741978	0.107579147	0.255960061	79.70018552
0.001284208	0.226245689	0.813411775	774.3232777
0.063529022	0.133076118	0.057432941	3.913021481
0.019269604	0.265502804	0.98613018	864.1101778
0.020246797	0.195882747	0.482469289	528.8770461
0.018012206	0.165051123	0.305328874	49.42923012
0.017839861	0.114499472	0.128805976	60.91379009
0.057740151	0.156533776	0.142980181	6.993999905
0.110951808	0.149583333	0.159791667	1.725
0.002287083	0.195575417	0.72164704	482.0946358
0.018813021	0.146213167	0.252080729	37.5202206
0.027304567	0.144596528	0.311536799	49.67365902
0.016426281	0.23770144	0.872786148	587.6400447
0.025200122	0.28768406	1.042255092	1337.938988
0.039893293	0.10715486	0.080586488	6.257882353
0.015416758	0.122382451	0.328001607	69.13036954
0.065625265	0.148410039	0.060125572	3.34956348
0.024332552	0.113150124	0.085834815	17.15785674
0.031122403	0.179081295	0.29828863	41.26669214

log.sigma.1.0.mm.3D_glrlm_LongRunEmphasis	log.sigma.1.0.mm.3D_glrlm_ShortRunHighGrayLevelEmphasis	log.sigma.1.0.mm.3D_glrlm_RunLengthNonUniformity	log.sigma.1.0.mm.3D_glrlm_ShortRunEmphasis
1.855738194	57.9094852	205.8078586	0.862706699
2.942825466	70.67884659	532.1309643	0.771417411
1.715993945	42.5702716	106.5587278	0.873108042
2.115941533	161.7662123	1044.10001	0.85496049
1.330497394	33.02524568	77.59086612	0.933941729
1.553462944	35.1597927	161.897307	0.898243566
1.106261655	126.0520567	63.43540584	0.974749511
1.502097439	82.48997051	277.190513	0.908982728
1.45568024	162.1547775	1055.362777	0.917846476
2.411480618	51.71473737	1073.562747	0.817250802
1.67082589	33.01425017	234.6860961	0.883891909
2.047874241	51.61755967	398.5900249	0.845369055
2.281375503	66.08318065	681.1568363	0.835843969
2.123437567	37.19488931	274.095308	0.842617568
1.278823989	133.9963837	285.1486361	0.938564728
4.04732695	48.03246042	2846.332582	0.683621643
1.386549719	32.16535774	157.8047153	0.918556556
2.502909596	57.787735	675.2870996	0.799709315
1.381557629	45.95933634	133.3917372	0.922426887
3.642353542	35.95630503	853.3943496	0.721384244
3.260617175	60.35791383	1716.031897	0.74450526
1.738601791	67.89157815	560.3014788	0.88200597
3.490469941	36.02914037	952.5922443	0.736639988
1.179779921	93.79809331	114.4401719	0.961297394
2.484864957	35.68000546	415.4107831	0.797300158
1.852574088	32.21570455	171.8901115	0.86179218
2.639646246	41.97376975	951.8546929	0.791397058
3.419533808	48.22224752	1215.700617	0.722861717
3.250724251	87.49488247	1281.655495	0.74736601
2.977821174	46.46508768	627.3283918	0.765093637
3.190740371	36.9619904	1434.726261	0.746314959
3.714497649	59.92415584	2685.838298	0.718798411
2.316321358	77.79227071	682.5514588	0.832762898
1.084953508	41.95824176	13.92645816	0.978761623
1.265770335	77.81030296	89.75408668	0.941698988
2.17176493	60.1497079	673.2485617	0.836522654
3.855830171	57.94780045	1886.488686	0.703232559
2.824349321	67.07646472	1218.788885	0.786246215
1.326462209	52.84038074	243.1654388	0.929247333
2.07088717	53.57047906	280.4902268	0.842808903
2.162463203	98.331124	358.2066089	0.833559375
1.656869017	85.41068128	238.6910916	0.884820262
1.759117756	57.57326672	506.8655607	0.882285135
1.564792496	58.31956496	289.3727228	0.896909887
1.386473102	97.22696603	816.2396177	0.922554866
3.563686683	49.27119543	1861.947436	0.718404892
1.53560646	238.7340647	1858.179303	0.904333069
1.544972418	56.06867426	148.6469213	0.892745943
1.043956044	38.81485181	21.10622711	0.989010989
1.224574424	60.09180688	120.0333707	0.950921415
2.965217609	51.3030448	1563.772483	0.775016485
1.904124515	51.2452097	224.2716625	0.856959126
2.458586344	279.7573623	1435.886247	0.809863192
1.637334601	37.48987652	150.2439902	0.885255145
3.835217192	60.85804952	3226.078946	0.705767206
1.532633828	103.479025	231.6369007	0.9097717
1.323299571	109.3289946	674.2592666	0.935615297
1.198682612	161.6970272	384.8263012	0.956333112
1.723556156	78.80378705	210.3039366	0.870879878
1.816847019	41.77105725	267.0788179	0.86178638
2.362338327	56.29895405	688.3319299	0.822356359
3.142415904	94.39781147	2487.424167	0.766635474
3.056067291	42.22957473	1476.435273	0.759922142
2.885623169	93.32157624	674.3720997	0.76882512
1.466080317	31.21973451	36.98276074	0.909574521
1.879479251	88.15506776	657.0916972	0.863316155
1.197435607	103.5536552	87.72237204	0.954139953
2.974380233	71.8366404	1307.360071	0.77063045
1.432952954	40.43736162	100.3439613	0.91145978
2.54481718	91.90445094	790.1008944	0.806247673
2.435300085	77.30017551	1191.395807	0.813884102
1.825150596	42.48547374	328.050431	0.865686416
3.871473128	46.25891652	2039.882313	0.710606808
3.685259014	47.74765636	3203.75221	0.713394946
3.201138179	99.66057904	5096.40839	0.754522602
1.32982994	183.215722	344.7759396	0.936316701
1.53307334	91.16253309	310.735931	0.905952779
1.609879338	53.3599485	141.8622855	0.884140098
2.008861651	71.87935298	503.1774885	0.853905675
3.019217881	74.79179237	1028.636486	0.774156135
1.310801805	52.99012976	116.22703	0.935939086
1.622767959	172.4535579	573.8878955	0.900865118
2.890695136	803.5341219	1938.18983	0.781157909
1.176025301	61.14648335	26.52148541	0.959280986
3.313294602	48.18529297	1682.858176	0.746896829
2.174177693	55.63375615	1756.354398	0.833795875
1.797203885	70.37729827	213.7857792	0.86901432
1.355936874	87.1197765	441.1925332	0.928100605
1.428980186	42.00719983	36.40828499	0.914088321
1.4625	63.33125	9.275	0.912152778
2.659524027	448.3915347	1477.948383	0.802522135
1.659118461	89.83830872	192.182784	0.887930844
1.784499403	69.31643093	249.5920493	0.875804199
3.060000697	59.2861908	1335.974619	0.763188292
3.455589049	36.70708015	2331.654176	0.735393165
1.247875323	80.13195207	50.35506063	0.942477889
1.779133637	93.27631014	419.0828864	0.884246076
1.201971941	60.6209117	19.92385875	0.949507015
1.242874598	82.28771974	132.4837119	0.948159414
1.786018754	51.59267673	163.5837473	0.867222078

log.sigma.1.0.mm.3D_glrlm_LongRunHighGrayLevelEmphasis	log.sigma.1.0.mm.3D_glrlm_RunPercentage	log.sigma.1.0.mm.3D_glrlm_LongRunLowGrayLevelEmphasis	log.sigma.1.0.mm.3D_glrlm_RunEntropy
140.2110231	0.810995099	0.038408168	3.781341729
297.0018724	0.686386315	0.034436253	3.619879597
95.19214113	0.831377662	0.049698821	3.468100687
414.3773328	0.784989454	0.013306975	4.289557684
45.41204882	0.910891089	0.104881876	3.438030368
62.52805594	0.862547289	0.071589924	3.586960417
145.8953154	0.967032967	0.029282472	3.748738459
147.9606452	0.874160752	0.025184391	3.905941397
254.095704	0.884406921	0.01191456	4.350092169
159.0304558	0.742694132	0.045115543	3.768700812
67.48236611	0.841448445	0.073569562	3.581847
132.8044776	0.784676354	0.047294627	3.794944774
184.7560489	0.761459624	0.032493014	3.754012439
96.86421878	0.777534965	0.062747217	3.625674054
177.2246515	0.919695689	0.016243104	3.929104382
287.5717991	0.597908556	0.059777445	3.39087903
47.69267389	0.894718185	0.081619492	3.558134984
199.6204302	0.728390167	0.035965167	3.645951442
69.83367817	0.896449704	0.061899311	3.731112782
192.5597535	0.631828518	0.075363932	3.578542477
280.3783273	0.657476317	0.040492795	3.547452248
134.8061414	0.834306508	0.030898081	3.825839008
180.636794	0.645021075	0.073539897	3.505781138
115.4344635	0.94794679	0.03065109	3.920521261
125.0296257	0.728846154	0.061786873	3.350592891
81.76242773	0.812022415	0.066586662	3.415047687
141.4575438	0.714420136	0.058038044	3.683709316
239.3534818	0.640663573	0.052976647	3.290413655
409.9882007	0.66148464	0.028011996	3.69133692
200.7771496	0.681857422	0.052264853	3.537300728
170.4446716	0.661289437	0.067343758	3.516038627
322.3664986	0.628421392	0.044504685	3.493320348
232.4569417	0.756880988	0.032193854	4.048238935
46.21225697	0.974358974	0.134888304	3.217652973
103.8505003	0.923757658	0.036479608	3.845657623
171.2950134	0.770153385	0.034596068	3.833688789
339.9303832	0.61231505	0.047386841	3.447625539
244.3938819	0.699969556	0.037105314	3.883889936
74.40795159	0.908219506	0.049246246	3.764685057
154.5527429	0.781979659	0.044709604	3.789226933
265.4934628	0.769445939	0.02149845	3.753782162
172.2364878	0.843327276	0.022701806	3.811118524
126.5258914	0.831964152	0.041027399	3.868733849
107.267499	0.860522166	0.043464301	3.874247345
140.2966511	0.895895136	0.022718174	4.16351161
253.564009	0.632322268	0.052129573	3.224552274
399.1034214	0.867415541	0.007304447	4.245706565
105.4540346	0.859986505	0.035690698	3.628391371
40.93023643	0.986013986	0.14723044	3.287018037
75.73211916	0.934668072	0.044764679	3.731143053
203.5735615	0.687631976	0.050666673	3.778357323
135.2508664	0.803779347	0.040080248	3.71849569
891.1756556	0.735572409	0.007445366	3.887773572
73.85805151	0.845510138	0.067583569	3.505738505
339.4832799	0.61495598	0.046242412	3.500096327
189.512654	0.87141217	0.021288729	4.049902625
154.2255987	0.912314749	0.018641506	4.010716173
201.9542941	0.941551898	0.013921088	4.252608137
165.9113026	0.8287701	0.027038255	3.655460786
89.88150612	0.816110928	0.047610055	3.505915541
189.0915394	0.748717949	0.036881208	3.735452277
401.4270018	0.673576424	0.0268234	3.925415519
166.9647502	0.675975228	0.061659892	3.573498014
368.9006131	0.688897522	0.024385518	3.528615117
54.91475485	0.879535559	0.086164008	3.281689557
194.8077085	0.810229701	0.025176389	3.9755301
127.7854877	0.940659341	0.029432425	3.922057346
310.0061308	0.68451998	0.032243186	3.806697146
70.15148694	0.883270576	0.06151953	3.54469579
313.543389	0.727979052	0.024158684	3.828391645
250.6713757	0.740876669	0.026910514	3.695019708
104.2050602	0.81671415	0.049155694	3.608453938
268.0981955	0.615559019	0.059972893	3.572805791
258.6459674	0.624707119	0.055616325	3.431228057
420.8247715	0.665255607	0.025718954	3.759200196
265.5275038	0.911348741	0.011462325	4.11415948
160.0473657	0.868758435	0.020241929	3.752254873
107.3972316	0.849587912	0.036895319	3.539786692
186.0686651	0.797118544	0.027941354	3.794680935
312.5568761	0.683744879	0.032666714	3.803167406
77.80315107	0.915332989	0.05189216	3.590884725
318.5334475	0.856113828	0.011945075	4.226336528
3027.262761	0.694242864	0.003081093	3.960943264
72.55367349	0.947890819	0.067065239	3.355505993
229.3900469	0.65586147	0.052916339	3.661455347
145.1973344	0.769647518	0.038542507	3.660512454
165.6308607	0.820231823	0.026214621	3.518197492
129.1872026	0.903095207	0.021903906	3.860888308
66.68699159	0.890769231	0.071037758	3.31782824
84.3625	0.884615385	0.123246309	3.094203899
1552.362421	0.718778742	0.00509024	3.977768574
186.9495321	0.843876518	0.024289586	3.691212385
143.3732259	0.825258876	0.037524638	3.884323868
258.6198252	0.676826186	0.0408514	3.736701731
185.3459631	0.643929872	0.071548795	3.547771402
114.5063537	0.926739927	0.042726192	3.684861503
183.4295887	0.830542986	0.023654076	4.045217897
75.76066325	0.939102564	0.07020171	3.167787344
107.3740271	0.930155734	0.027616086	3.665256096
118.2476799	0.821978022	0.043230691	3.491265857

log.sigma.1.0.mm.3D_glrlm_HighGrayLevelRunEmphasis	log.sigma.1.0.mm.3D_glrlm_RunLengthNonUniformityNormalized	log.sigma.1.0.mm.3D_glszm_GrayLevelVariance
69.18873691	0.701753581	11.07290498
94.44972108	0.552010913	7.165357002
50.46105114	0.721772775	5.244497323
190.3312708	0.687833694	25.53664942
35.14407702	0.842088907	7.955555556
39.31452916	0.768353306	9.441547971
129.751412	0.936197869	15.01652893
92.26325859	0.789916547	13.53247133
176.1535752	0.8080806	21.26408858
63.93181945	0.622928735	12.69874031
37.98825478	0.740633858	8.357377648
61.93740987	0.670404627	12.82244055
79.40191859	0.654398404	10.43405084
44.37836054	0.666547808	9.230033081
141.7580206	0.851243117	18.46524095
70.53209244	0.438607025	9.495601689
34.86742936	0.810274345	9.560703943
74.21509376	0.595609869	7.806920415
49.90291329	0.816999131	11.57258065
50.85421838	0.484421724	6.576465944
82.64096276	0.513903829	9.982339987
76.9271986	0.737454516	12.97474875
49.86301664	0.503992328	6.928227147
97.60952334	0.905731403	15.42947828
46.31347081	0.592040432	4.222915944
38.98993364	0.69944792	3.951985226
53.1306075	0.581781692	10.96182342
67.82305902	0.486004069	8.239795918
119.841854	0.519123144	12.4821096
62.71668839	0.542997473	7.751029664
50.6712091	0.516261545	8.895999835
84.43069666	0.481364499	6.802687789
94.95162346	0.649120233	17.13201851
42.8090448	0.949360445	10.56
82.4728226	0.858711163	17.40246914
73.54539372	0.654943218	11.17269766
84.38648986	0.460193895	9.064922358
85.50562755	0.5739984	14.95933886
56.67795159	0.832393987	11.18825485
66.32702621	0.666650095	8.139168975
119.1770667	0.650358641	12.01407008
98.09190141	0.742089	12.82220119
66.59792515	0.738143028	13.61824479
65.63874726	0.765199623	14.94705833
104.4362503	0.816875541	21.82660138
69.4278206	0.479959147	4.629819787
262.8041602	0.779881511	31.11433164
64.10538384	0.756967362	10.5106383
39.23792874	0.972091401	9.138888889
62.87026335	0.879157466	11.25423729
66.83767214	0.557131529	14.22063225
62.46068085	0.691166738	6.922218917
349.9673487	0.610708718	15.0400399
42.97825067	0.742747356	8.638985006
87.10255841	0.463441303	10.42117825
115.7017786	0.792476291	15.49078809
116.7475093	0.846207453	17.37585742
168.9653695	0.891896145	22.12277352
91.86543719	0.715919895	10.90915556
48.64326946	0.699957694	9.442906574
71.31221293	0.631420787	7.653736439
124.3024348	0.544695317	16.76666259
55.27666412	0.535486456	9.302582779
123.2509951	0.548677013	7.399407911
35.06735689	0.791603748	5.98615917
102.3963083	0.702568883	17.2713445
108.1131704	0.887117091	18.81632653
96.22042847	0.550682552	10.82768995
45.45259013	0.793998847	6.873450413
116.3581562	0.605152589	12.65362812
96.90553009	0.617632171	9.426967625
50.83987589	0.706831697	6.697846479
66.5354008	0.469022397	8.366750622
68.03339263	0.473087088	7.785100809
131.7573657	0.527449156	13.81997076
196.3190256	0.847557674	21.22830579
101.2793385	0.783821913	11.50094307
61.81205607	0.742250001	8.044375
85.95699145	0.687412916	11.28640999
98.63328632	0.556014917	13.69029421
57.21735561	0.848756259	9.257809246
192.0587882	0.774315749	25.56230508
1033.656017	0.566146064	23.65862948
63.25961105	0.900154601	11.4691358
65.85976945	0.516855737	10.01012859
66.67054993	0.650143891	10.08254225
83.44876144	0.712937455	6.825876917
94.15449017	0.82900286	14.39340359
46.08262646	0.808190295	8.595555556
66.7375	0.800416667	13.50617284
565.1226692	0.599339017	18.77621851
103.776238	0.7485205	8.346938776
79.40393431	0.726082847	15.56432108
79.67433374	0.540024889	10.11399433
51.04547964	0.501240919	8.023764802
86.49457032	0.861031437	14.87222222
104.8579668	0.741755804	15.65393369
63.64886201	0.879748857	9.743055556
86.7646933	0.873320564	10.16772461
61.11200135	0.709245916	6.228639053

log.sigma.1.0.mm.3D_glszm_SmallAreaHighGrayLevelEmphasis	log.sigma.1.0.mm.3D_glszm_GrayLevelNonUniformityNormalized	log.sigma.1.0.mm.3D_glszm_SizeZoneNonUniformityNormalized
39.70900646	0.108155744	0.394648294
44.61169291	0.126236644	0.343433772
21.80894343	0.135038667	0.306365259
176.1309891	0.064221039	0.55138103
18.59900388	0.104444444	0.335555556
36.58040446	0.109916689	0.536683687
82.93048611	0.079545455	0.5
61.54623678	0.092409964	0.49053985
127.2857125	0.060794464	0.400193772
52.51467938	0.108523672	0.484826971
29.38898469	0.127830533	0.46311176
52.55871751	0.105161073	0.509613763
71.03508125	0.108015143	0.493391022
39.61893474	0.115311909	0.534499055
117.9726297	0.07219859	0.439426607
61.27863667	0.111735301	0.475648523
30.85082839	0.107150813	0.417687681
51.36521737	0.1216609	0.488650519
40.98583392	0.095213319	0.451612903
31.3065865	0.158019385	0.405201526
55.07941319	0.130578559	0.45674704
61.20837193	0.090725945	0.392962615
36.80701737	0.147423823	0.493684211
78.50905052	0.075976989	0.574290815
22.17178732	0.161752804	0.455659614
14.53239436	0.151123423	0.34502924
49.92641329	0.107415215	0.513361027
38.47940584	0.137555804	0.497090242
76.07400504	0.093814332	0.456915989
32.90308388	0.130640011	0.39987968
37.41453311	0.13822231	0.510385542
57.14508729	0.132142221	0.417074582
77.55189826	0.080185119	0.470188679
32.26944444	0.1	0.46
59.41286854	0.079506173	0.349135802
53.16250979	0.108254842	0.426984167
44.87669084	0.123658629	0.431495392
79.91982383	0.08211263	0.469694063
42.12013338	0.093407202	0.386814404
35.13654881	0.114903047	0.509141274
81.48771373	0.103476538	0.464517451
70.66313695	0.099783667	0.442942131
51.14009946	0.094042793	0.475100003
56.62536203	0.086103863	0.425974245
97.44485038	0.067211355	0.47682818
43.98585569	0.239944262	0.504026036
237.4513695	0.060307266	0.520265109
41.75073332	0.104572205	0.372566772
32.97222222	0.104938272	0.654320988
53.45742103	0.093363976	0.343866705
60.67348058	0.096156565	0.478332077
29.37582766	0.127305175	0.360202261
228.327016	0.098451777	0.436356251
28.31915958	0.114955786	0.364090734
59.69069879	0.123435697	0.474557427
68.70545842	0.078808538	0.401022644
82.43933149	0.070184171	0.408903835
126.8888849	0.060623077	0.463399295
61.47042088	0.092977778	0.480888889
40.51784712	0.144463668	0.457179931
38.57335957	0.131061746	0.444666562
103.1220344	0.080583487	0.460518655
51.04637891	0.127819172	0.495372381
58.66899605	0.133786848	0.328168304
14.89232648	0.128027682	0.307958478
81.53551389	0.07983703	0.441303757
86.0521227	0.077051229	0.50603915
55.74357772	0.103327802	0.4103124
26.47135986	0.132231405	0.488636364
83.8823642	0.094954649	0.481135047
63.78785377	0.114666715	0.440092734
24.53946113	0.120095125	0.390408244
41.41951659	0.124889171	0.407292329
46.49429589	0.151846108	0.487351588
116.2842238	0.116522454	0.507502307
152.7586403	0.069537707	0.501033058
69.60976149	0.099108368	0.429526749
29.51283768	0.12125	0.3625
58.00461565	0.092112383	0.488033299
70.70923932	0.098886423	0.440910585
35.83047009	0.10787172	0.478550604
166.7225681	0.062764536	0.518378258
756.5216368	0.08369146	0.487268136
53.26388889	0.111111111	0.5
52.14097152	0.118870919	0.480232911
58.43696982	0.105435222	0.470549474
46.81960148	0.140516698	0.479941189
68.61128141	0.085956374	0.422289136
41.31498148	0.111111111	0.395555556
66.16666667	0.135802469	0.62962963
367.6536071	0.082010841	0.451308322
56.26220664	0.115362812	0.528061224
70.66075826	0.081081081	0.484944404
50.91543496	0.110274721	0.386099491
38.7376132	0.139699853	0.505425144
52.03030556	0.106666667	0.364444444
89.09951644	0.074262106	0.422499212
43.85453704	0.111111111	0.402777778
57.31373344	0.099121094	0.419433594
28.76857111	0.125207101	0.396923077

log.sigma.1.0.mm.3D_glszm_SizeZoneNonUniformity	log.sigma.1.0.mm.3D_glszm_GrayLevelNonUniformity	log.sigma.1.0.mm.3D_glszm_LargeAreaEmphasis	log.sigma.1.0.mm.3D_glszm_ZoneVariance
31.17721519	8.544303797	247.4810127	226.5995834
45.67669173	16.78947368	4428.593985	4317.315281
12.56097561	5.536585366	113	94.36287924
255.2894168	29.73434125	1092.658747	1075.228573
10.06666667	3.133333333	34.03333333	22.69888889
32.73770492	6.704918033	102.8196721	86.81967213
22	3.5	3.590909091	1.059917355
57.39316239	10.81196581	94.69230769	82.94557674
170.0823529	25.83764706	155.7035294	143.642209
152.2356688	34.07643312	3599.894904	3545.351505
34.27027027	9.459459459	234.2432432	208.4258583
78.48051948	16.19480519	676.1103896	651.9474195
106.0790698	23.22325581	1663.813953	1623.447139
49.17391304	10.60869565	558.6086957	525.6710775
57.5648855	9.458015267	37.86259542	30.14183323
193.1133005	45.36453202	118599.7438	117888.4576
27.98507463	7.179104478	48.97014925	38.48028514
83.07058824	20.68235294	2870.447059	2787.1009
28	5.903225806	47.09677419	38.47970864
61.18543046	23.86092715	17169.80132	16832.31279
172.193634	49.22811671	22153.75332	21972.5403
82.1291866	18.96172249	408.3779904	389.4617339
93.8	28.01052632	14880.69474	14644.50637
40.77464789	5.394366197	8.887323944	5.378297957
42.37634409	15.04301075	2740.666667	2634.110995
19.66666667	8.614035088	230.1754386	202.104032
130.3937008	27.28346457	5423.153543	5341.940805
111.3482143	30.8125	26730.77232	26428.41612
138.4455446	28.42574257	14391.86139	14241.45504
58.78231293	19.20408163	6075.897959	5943.256513
158.7299035	42.98713826	18241.07074	18058.60363
176.8396226	56.02830189	64725.54953	64291.55451
124.6	21.2490566	1289.384906	1261.911485
4.6	1	2.7	0.45
15.71111111	3.577777778	14.15555556	7.849876543
84.96984925	21.54271357	1521.658291	1476.721143
153.6123596	44.02247191	50127.75562	49774.5057
169.5595568	29.64265928	6175.861496	6105.32011
36.74736842	8.873684211	59.31578947	47.89850416
48.36842105	10.91578947	440.3684211	408.4161773
56.20661157	12.52066116	818.5371901	783.6198347
38.09302326	8.581395349	165.244186	145.6172255
91.69430052	18.15025907	349.2331606	331.0051277
46.43119266	9.385321101	153.733945	137.513004
158.7837838	22.38138138	219.996997	208.7855603
164.8165138	78.4617737	45762.9052	45412.40268
391.2393617	45.35106383	838.7473404	825.4131889
17.5106383	4.914893617	113.8723404	90.33952014
11.77777778	1.888888889	1.666666667	0.172839506
20.28813559	5.508474576	16.13559322	10.0120655
197.0728155	39.61650485	11271.16505	11173.09737
29.53658537	10.43902439	258.3292683	234.1756395
192.4331066	43.41723356	5139.421769	5086.933222
18.56862745	5.862745098	158.7647059	136.8035371
310.8351145	80.85038168	76163.45649	75865.512
44.51351351	8.747747748	72.4954955	63.38706274
117.3554007	20.14285714	61.50522648	52.27379232
97.77725118	12.79146919	15.38388626	10.67231194
36.06666667	6.973333333	243.6266667	221.3482667
31.08823529	9.823529412	594	547.0371972
93.82464455	27.65402844	1932.336493	1884.785337
343.5469169	60.1152815	16906.0563	16823.55341
171.3988439	44.22543353	15152.95954	15014.31912
41.34920635	16.85714286	7688.920635	7488.900227
5.235294118	2.176470588	26.17647059	16.4567474
103.706383	18.76170213	797.906383	773.8755274
24.79591837	3.775510204	11.20408163	6.612244898
131.7102804	33.1682243	9862.327103	9745.673644
21.5	5.818181818	45.02272727	34.46022727
121.2460317	23.92857143	2933.84127	2883.273369
145.6706949	37.95468278	4472.752266	4411.098931
33.96551724	10.44827586	501.5517241	459.0772889
145.4033613	44.58543417	49875.63866	49484.88432
269.9927798	84.12274368	79652.52708	79270.09163
716.0857548	164.4131821	45876.76612	45770.88479
88.18181818	12.23863636	48.77272727	42.35111054
46.38888889	10.7037037	176.6481481	158.8209877
14.5	4.85	148.85	117.49
75.64516129	14.27741935	677.6709677	642.9750676
137.5641026	30.8525641	6410.685897	6335.574786
23.44897959	5.285714286	37.97959184	28.73302791
138.9253731	16.82089552	237.2126866	226.7952077
321.5969697	55.23636364	10152.45606	10096.68232
9	2	5.166666667	2.200617284
198.8164251	49.21256039	18563.99517	18420.34279
228.2164948	51.13608247	4967.028866	4914.742549
33.11594203	9.695652174	267.8405797	239.858013
71.36686391	14.52662722	109.7337278	97.58705928
5.933333333	1.666666667	25.2	14.08888889
5.666666667	1.222222222	3	0.913580247
218.8845361	39.77525773	5514.96701	5464.980653
44.35714286	9.69047619	132.452381	119.3548753
53.82882883	9	197.2072072	183.161594
116.2159468	33.19269103	12459.24252	12312.0355
231.4847162	63.98253275	38053.80349	37805.15604
10.93333333	3.2	8.1	3.69
71.40236686	12.55029586	279.9763314	263.786422
4.833333333	1.333333333	6.166666667	2.166666667
26.84375	6.34375	18.734375	12.24780273
25.8	8.138461538	192.0307692	173.4745562

log.sigma.1.0.mm.3D_glszm_ZonePercentage	log.sigma.1.0.mm.3D_glszm_LargeAreaLowGrayLevelEmphasis	log.sigma.1.0.mm.3D_glszm_LargeAreaHighGrayLevelEmphasis	log.sigma.1.0.mm.3D_glszm_HighGrayLevelZoneEmphasis
0.218836565	3.060739841	20972.46835	57.27848101
0.094796864	42.17274693	469564.7895	68.78195489
0.231638418	1.9203782	7176.634146	38.65853659
0.239524056	5.396774877	222950.635	209.6868251
0.297029703	1.585757443	1031.9	36.4
0.25	2.961465044	4134.557377	45.04918033
0.628571429	0.06305339	517.6590909	120.5454545
0.291770574	0.900123067	10845.08547	82.69230769
0.287940379	0.951636609	26813.60706	191.3670588
0.135403191	53.87911596	244566.7803	67.05414013
0.196808511	6.055981554	10080.55405	38.31081081
0.20343461	10.23261403	45863.14286	63.56493506
0.157393851	20.76543386	135610.8558	89.85116279
0.174242424	12.644668	25734	49.66304348
0.35989011	0.328231035	4802.503817	158.8244275
0.037495382	1687.990475	8447861.847	79.33743842
0.30875576	1.638337141	1778.492537	40.3880597
0.109536082	34.01659157	245990.8588	65.38823529
0.340659341	1.096110813	2429.532258	53.82258065
0.05443403	324.7090927	924293.0265	43.53642384
0.074285714	254.9968525	1945999.066	72.92572944
0.229922992	5.385549939	31903.48804	86.08133971
0.065068493	287.0472215	783669.3947	46.08947368
0.533834586	0.139365803	869.5633803	97.95774648
0.096875	52.75788	145858.1398	29.76344086
0.188741722	4.790266843	11859.19298	25.40350877
0.110965487	102.6789756	292377.4173	62.58661417
0.057509628	381.7169392	1897573.219	50.02678571
0.08153929	111.8577421	1866104.234	101.3267327
0.086828116	88.2615129	424246.8095	45.9047619
0.074029993	339.4005951	998794.9646	45.94533762
0.048001811	748.4589013	5660324.224	81.64150943
0.190784737	12.26213763	137524.6189	97.99622642
0.666666667	0.268020954	113.7	44.2
0.398230088	0.24396631	1134.8	90.6
0.149175412	18.4756093	128254.5678	71.52763819
0.053205799	566.4996849	4485118.031	61.34269663
0.119063325	71.40738438	540629.3767	102.4155125
0.295950156	1.30708037	3245.578947	64.09473684
0.176908752	5.484917929	37025.37895	48.29473684
0.169230769	6.529888447	103870.1901	113.0082645
0.225721785	1.500963411	19094.09302	94.87209302
0.234223301	4.412208286	28956.46632	63.93264249
0.248291572	2.213002181	11700.90826	71.40366972
0.298654709	2.580888574	19797.87988	126.6276276
0.053413917	645.7713537	3288205.257	58.58715596
0.273852877	3.303805327	215477.2952	301.6555851
0.206140351	1.697854159	8734.170213	59.5106383
0.818181818	0.191519843	65.05555556	39.38888889
0.404109589	0.415035347	925.8983051	75.25423729
0.100980392	163.0739016	790648.5121	74.73543689
0.203473945	3.264071317	21496.63415	46.06097561
0.138028169	13.77455805	1923302.791	323.3968254
0.213389121	3.611304099	7708.294118	41.19607843
0.05793384	862.0087982	6803832.305	78.96946565
0.331343284	0.547210832	10347.73874	104.8018018
0.32912844	0.567127075	7343.505226	121.5296167
0.46069869	0.118515169	2466.895735	175.4834123
0.211864407	2.438494175	25260.82667	84.64
0.145922747	12.94437968	28659.08824	54.41176471
0.145017182	22.43593505	169424.9716	52.7535545
0.110094451	131.091898	2196970.657	133.847185
0.084928817	282.1688136	828381.7168	66.6416185
0.070707071	59.35658938	1004305.817	102.8492063
0.320754717	0.862007766	1079.705882	32.17647059
0.203993056	7.520527472	86198.24681	110.5787234
0.466666667	0.160889435	1108.857143	115.9795918
0.092587251	91.3096127	1076624.502	79.75389408
0.307692308	0.963754268	2578.795455	35.88636364
0.140625	22.95434852	378550.8135	107.484127
0.127356676	41.70914153	485663.0514	87.89123867
0.153439153	8.144613295	32901.5977	38.16091954
0.050588069	717.3822958	3514730.353	57.17086835
0.051135315	1133.087443	5677084.718	59.55234657
0.097183002	351.5744354	6033872.851	149.7051736
0.394618834	0.240862133	10589.85227	198.6022727
0.236842105	1.650070906	19796.40741	97.75
0.178571429	2.266605855	10870.95	49.325
0.169769989	6.946630354	68723.92258	78.63225806
0.115384615	60.49469791	685481.6442	92.51282051
0.32885906	0.734474654	2570.44898	50.06122449
0.30982659	1.163080553	49337.37313	206.9067164
0.1339014	9.623200037	10723003.5	1019.634848
0.580645161	0.173721042	289.7222222	68.55555556
0.083434099	267.0046381	1309791.155	64.28502415
0.138294839	75.4748944	334025.9093	77.0185567
0.189041096	2.787167259	27134.98551	65.82608696
0.286926995	1.134454293	11269.3432	93.69822485
0.3	0.814171016	1230.8	53.93333333
0.692307692	0.187079501	150.3333333	74
0.141440653	9.250079083	3294858.616	523.2185567
0.276315789	1.074414469	17055.86905	77
0.266826923	2.463830433	16454.91892	88.35135135
0.082420591	143.6454506	1093726.664	71.27574751
0.063417336	707.0013363	2086575.904	47.37336245
0.476190476	0.148781882	867.2666667	81.56666667
0.248529412	2.788202382	28966.28994	123.2485207
0.5	0.229357916	355.1666667	67.25
0.392638037	0.264778997	1638	84.828125
0.232142857	2.68660687	14425.09231	46.6

log.sigma.1.0.mm.3D_glszm_SmallAreaEmphasis	log.sigma.1.0.mm.3D_glszm_LowGrayLevelZoneEmphasis	log.sigma.1.0.mm.3D_glszm_ZoneEntropy	log.sigma.1.0.mm.3D_glszm_SmallAreaLowGrayLevelEmphasis
0.652008463	0.054608082	4.772012003	0.043940279
0.606947691	0.038335885	5.09112917	0.029749099
0.56048776	0.071552349	4.375487065	0.054838787
0.769424338	0.01082262	5.41593849	0.008701049
0.5559063	0.157942051	4.440223929	0.111818487
0.754431599	0.108022765	4.374837904	0.094637357
0.734337121	0.038133644	4.55546713	0.035498073
0.726323716	0.03609734	4.830931229	0.03142044
0.657773453	0.011864379	5.884235395	0.008972462
0.723120864	0.044182051	4.987697173	0.034901428
0.704295332	0.117641281	4.331727563	0.097193684
0.741128905	0.055092519	4.662252604	0.039890224
0.729128354	0.024534656	4.718694018	0.018991948
0.755465147	0.072896091	4.375486859	0.063334399
0.688167192	0.022230262	5.316431568	0.019216876
0.716152848	0.031851655	4.905143059	0.024594744
0.667465895	0.113233027	4.608020766	0.094553041
0.722435823	0.031544475	4.802559001	0.024751579
0.697810521	0.087436038	4.672197222	0.069979251
0.661576092	0.052507982	4.573836919	0.037588451
0.701843165	0.026757444	4.851218438	0.020596447
0.650126932	0.033784192	5.274128866	0.026396346
0.728655433	0.052431333	4.507262249	0.041648108
0.782754289	0.037960704	4.734204958	0.033195895
0.700535503	0.087718345	4.426669731	0.070980432
0.601701243	0.104400814	4.396217814	0.084506296
0.742790003	0.046079366	4.845193421	0.034722698
0.730574245	0.048558133	4.568488881	0.03979798
0.702680448	0.020713658	5.173659577	0.015994657
0.656227754	0.063225934	4.834611606	0.050688768
0.74117819	0.059129065	4.651077827	0.046821312
0.671411407	0.020433284	4.90507561	0.014683312
0.711522743	0.037975595	5.310282572	0.031998699
0.686111111	0.154240719	3.321928095	0.128063235
0.602395134	0.052566133	4.924969207	0.044077397
0.677877436	0.033870023	5.029935307	0.024931418
0.683054732	0.040327288	5.00861038	0.03142019
0.711638027	0.024767814	5.292674439	0.018421876
0.644567945	0.072994725	5.059358641	0.062882212
0.733318727	0.078283584	4.575164606	0.070251794
0.706418719	0.023090451	4.828886867	0.01952593
0.684289094	0.030185634	4.7247017	0.025423011
0.715669803	0.054997829	4.993886716	0.043444742
0.676880334	0.064341909	5.092328449	0.051986028
0.717439909	0.025908508	5.512273138	0.020663143
0.737439445	0.027845291	3.79173942	0.022090891
0.749290652	0.006659183	5.694032863	0.005374474
0.628485678	0.055049421	4.552048097	0.043664051
0.833333333	0.168430982	3.614369446	0.162658767
0.589288947	0.04759573	4.803850838	0.030474282
0.718408528	0.042837686	5.153858021	0.032863348
0.618555773	0.054474885	4.682682188	0.042833463
0.686861369	0.006047151	5.359399487	0.004923152
0.608710925	0.115455704	4.343514803	0.094073477
0.716186753	0.030006175	4.985738233	0.023177995
0.655628391	0.028514553	5.124377498	0.023735345
0.664573328	0.023658634	5.616871067	0.01907835
0.706412112	0.01736997	5.457524085	0.014740914
0.715637479	0.041897332	4.803567317	0.03709907
0.697866936	0.055522549	4.283621439	0.044566551
0.692947751	0.04180157	4.751783748	0.033278538
0.705702979	0.015198803	5.490123873	0.011204528
0.731106581	0.038202261	4.747092225	0.02985693
0.592775023	0.020816103	5.102987632	0.016323686
0.511004195	0.124082652	3.616874606	0.092901117
0.690565872	0.02934062	5.349309881	0.023935487
0.73585854	0.042320406	4.655127191	0.038935385
0.665399733	0.027266778	5.269671863	0.020542121
0.71803566	0.100088887	4.013394053	0.08687559
0.719639487	0.021694348	5.058942348	0.017030708
0.68988973	0.022473877	5.055256273	0.017074325
0.644561758	0.081015266	4.827030422	0.067815388
0.663507104	0.038049419	5.048712926	0.026792909
0.725214934	0.034936324	4.59829559	0.027246326
0.740321738	0.011957304	5.124727858	0.009155006
0.735112433	0.015034121	5.343037997	0.013099203
0.680480695	0.025576343	4.875193179	0.020934952
0.618562172	0.064249236	4.37507052	0.055272264
0.724135619	0.032733252	5.023466568	0.027854911
0.688897649	0.024410182	5.128266105	0.018721175
0.713546265	0.086484834	4.476456017	0.077532396
0.747222768	0.013561486	5.428596969	0.011574514
0.725601392	0.002584765	5.486086783	0.002284615
0.726828231	0.090866918	3.683542362	0.085089587
0.720131793	0.035587669	4.798878164	0.025449711
0.713169518	0.033674052	5.018839353	0.026695864
0.715571972	0.036864282	4.228384102	0.031973599
0.675286395	0.02923934	5.15672331	0.023676786
0.626277778	0.096451156	3.640223929	0.079179953
0.8125	0.133980484	2.947702779	0.129386285
0.69859403	0.004401929	5.471268873	0.003779578
0.750822451	0.038203577	4.441102492	0.034749967
0.722802074	0.051823453	5.021101306	0.045588666
0.645586684	0.034840845	5.188601277	0.026194945
0.737839706	0.057486975	4.61337119	0.045128123
0.612083333	0.063240292	4.298068512	0.056447835
0.675211	0.022935066	5.374373037	0.01840078
0.640925926	0.10256741	3.584962501	0.094997512
0.671712749	0.038830569	4.686889062	0.033500771
0.65293893	0.059900443	4.386636753	0.047231722

log.sigma.1.0.mm.3D_ngtdm_Coarseness	log.sigma.1.0.mm.3D_ngtdm_Complexity	log.sigma.1.0.mm.3D_ngtdm_Strength	log.sigma.1.0.mm.3D_ngtdm_Busyness	log.sigma.1.0.mm.3D_ngtdm_Contrast
0.017098741	0.367727483	1.564465919	0.358619814	0.000176809
0.004012481	0.064124641	0.593779252	1.074577602	1.46E-05
0.036130613	0.316325277	1.502910044	0.29278585	0.000394131
0.003839365	0.3004512	1.20710916	0.482495594	2.27E-05
0.05154409	0.602429639	1.413746087	0.447985252	0.001719664
0.028277369	0.313904964	1.110078473	0.465616681	0.000323705
0.050358045	4.71839823	4.811546	0.144076081	0.006555842
0.016307966	0.587273804	1.821920332	0.299131527	0.00019406
0.004539297	0.315789761	0.648849982	0.748627704	8.78E-05
0.003048019	0.09152427	0.783960272	1.324168297	6.97E-06
0.018360926	0.14981466	0.620790539	0.769744041	0.000198395
0.009327753	0.187262846	1.115295144	0.620930847	5.42E-05
0.00548857	0.078665388	0.434245091	0.991493995	2.16E-05
0.013471866	0.144882167	0.731625169	0.665457544	7.01E-05
0.017410694	1.087118013	2.588411415	0.201677445	0.000249322
0.000433741	0.007072054	0.101778525	10.26900229	4.39E-07
0.027243453	0.408318467	1.144619914	0.606088611	0.000507873
0.004311924	0.058613651	0.452930539	1.187831969	1.22E-05
0.031924632	0.783175461	1.817814558	0.322706162	0.000676021
0.002168885	0.015101577	0.180896777	3.521684682	4.18E-06
0.001137964	0.025983149	0.426219864	3.218302786	1.52E-06
0.007869693	0.302427705	1.388305939	0.53530211	4.01E-05
0.002162995	0.018304668	0.230745608	3.198489228	3.31E-06
0.044071539	2.132434895	4.249814493	0.15488712	0.001533144
0.006668401	0.04142608	0.383037246	1.362920338	2.27E-05
0.020954795	0.140548363	0.731951484	0.622924272	0.000210973
0.002821731	0.055084603	0.463632461	1.886468874	6.68E-06
0.001248322	0.025338009	0.398464197	3.365261385	1.48E-06
0.001443935	0.042706453	0.456334404	1.882884837	2.85E-06
0.003375867	0.048245193	0.452316359	1.552883984	8.35E-06
0.001355789	0.026117747	0.320574414	3.697782745	1.93E-06
0.00063335	0.007067608	0.095820571	7.611898552	8.04E-07
0.004581718	0.200560782	1.032747728	0.726845376	2.56E-05
0.190890699	8.548937096	8.512796103	0.116586052	0.041468857
0.052576213	2.266507187	4.603376885	0.129535977	0.00138885
0.004870677	0.126700978	0.65947985	0.982493707	2.47E-05
0.000702951	0.015787677	0.222455556	5.022122224	9.38E-07
0.002158993	0.056102013	0.385857943	1.730665022	6.35E-06
0.019821258	0.415097119	1.043727332	0.544251049	0.0003778
0.013003103	0.176028741	0.916805853	0.501150827	9.35E-05
0.010390162	0.223849042	1.544465754	0.331923756	3.61E-05
0.018144919	0.47328152	1.756716177	0.258158915	0.000143754
0.007705263	0.257000574	1.02005683	0.721747567	7.65E-05
0.015643045	0.433855688	1.77214029	0.430938704	0.000190634
0.006157558	0.562345634	1.459019523	0.543530521	6.90E-05
0.000785351	0.007209342	0.091137176	7.978282758	1.11E-06
0.002558039	0.375801045	1.132844076	0.541851091	1.96E-05
0.025989629	0.470769422	1.650577998	0.331296449	0.000364238
0.165528442	4.347442732	5.682211337	0.156361968	0.020897992
0.036714632	0.984634853	1.663437042	0.395289881	0.001598914
0.001473265	0.045631811	0.41646641	2.593202269	3.17E-06
0.014447348	0.252479426	1.026044731	0.525199439	0.000178022
0.001919743	0.122788923	1.036270961	0.618705223	3.97E-06
0.027928634	0.266321344	1.234333764	0.442844422	0.000324679
0.000419412	0.012343354	0.204882766	7.439024366	4.23E-07
0.01948326	0.90134207	2.403787685	0.213359902	0.000308584
0.007457688	0.498578866	1.318192015	0.489649396	9.46E-05
0.012515822	1.324246695	1.998509	0.274785097	0.00035586
0.017482237	0.349396386	1.584990636	0.296195732	0.000143681
0.014588783	0.172951791	0.953294061	0.606623163	7.87E-05
0.004210012	0.074768138	0.434864331	1.274184624	2.20E-05
0.00085586	0.041794892	0.358275967	2.655332642	1.81E-06
0.001386089	0.019528715	0.190355657	4.222727914	2.82E-06
0.003163411	0.044746766	0.538917995	1.212370722	6.87E-06
0.11093433	0.678975904	2.876686211	0.183753896	0.002503597
0.006392246	0.236471588	1.131465626	0.523318501	3.31E-05
0.048406124	3.056734049	4.568060043	0.137287786	0.002251649
0.001615417	0.039360766	0.329280311	2.187133337	4.67E-06
0.042014951	0.493320096	1.430268471	0.336186324	0.00088319
0.003453897	0.105558715	0.785443489	0.847654761	1.04E-05
0.002351954	0.056776956	0.420570272	1.537637892	6.61E-06
0.011244703	0.123463012	0.554129776	0.836853016	0.000106846
0.000755853	0.011576683	0.149824008	6.108225676	9.92E-07
0.000461458	0.009136804	0.189447911	9.834437695	5.31E-07
0.000389317	0.018503811	0.210425435	5.30564963	4.81E-07
0.014360258	1.205983528	2.597113111	0.183992527	0.000239234
0.015164213	0.306053203	1.123614212	0.38568129	0.000151618
0.029140156	0.402401397	1.729344567	0.285804097	0.000264032
0.007706729	0.16157607	0.855822333	0.587580065	3.79E-05
0.00219286	0.060029936	0.488804889	1.508171134	6.15E-06
0.040019555	0.752804829	2.083821519	0.255853478	0.00084006
0.008171999	0.640247289	1.744027636	0.270874937	8.69E-05
0.001157993	0.103431467	1.063994247	0.529444758	2.61E-06
0.157782568	4.133841018	7.526693087	0.070420397	0.007845228
0.001163995	0.021891675	0.235256417	3.835922516	2.05E-06
0.001997881	0.034721835	0.221836548	2.675689849	6.41E-06
0.015830825	0.217802667	0.994184382	0.466009465	0.000168264
0.010963521	0.480407797	1.282954691	0.421374912	0.000124377
0.095531008	1.311917877	3.311169794	0.170596567	0.003625187
0.18254154	8.668336621	9.799379989	0.135392902	0.083538679
0.001747184	0.139038833	1.332016477	0.514625497	4.21E-06
0.02026485	0.440454543	1.947388761	0.246700186	0.000222707
0.017844393	0.551583083	2.178957006	0.249996198	0.000124305
0.001644198	0.022696325	0.212035315	2.933782722	4.64E-06
0.00075429	0.011033951	0.132030329	7.646810691	1.22E-06
0.071568133	3.45189851	6.783546821	0.117058518	0.004448055
0.009908451	0.451125235	1.468264541	0.392210954	8.68E-05
0.166881002	3.883324691	7.203378044	0.093144464	0.014291503
0.036500317	1.065263879	2.411723494	0.198299937	0.000717142
0.02208225	0.201575328	1.102295702	0.418864536	0.000260266

log.sigma.0.5.mm.3D_gldm_GrayLevelVariance	log.sigma.0.5.mm.3D_gldm_HighGrayLevelEmphasis	log.sigma.0.5.mm.3D_gldm_GrayLevelNonUniformityNormalized	log.sigma.0.5.mm.3D_gldm_DependenceEntropy
0.405844031	10.29085873	0.460931086	4.867145258
0.322131224	10.96935139	0.48850366	4.886166594
0.277889495	4.553672316	0.557023844	4.941633611
0.647371904	18.58147957	0.395590888	5.27034753
0.341731203	4.623762376	0.489461817	4.595320741
0.303329078	4.758196721	0.547198334	4.820482568
0.87122449	9.785714286	0.295102041	4.223338691
0.496738204	9.229426434	0.433423921	5.043441885
0.877381831	19.47357724	0.317475268	5.689297623
0.32504308	5.579991376	0.503610146	4.90518946
0.267138694	4.603723404	0.568087936	4.881432593
0.385380657	5.494055482	0.47041527	4.937811404
0.289380886	10.90190337	0.537493917	4.942280952
0.232308884	4.943181818	0.618485996	4.794109044
0.599467154	11.09065934	0.396947832	5.043296024
0.267899582	6.224510528	0.495206523	4.81761379
0.331712289	4.294930876	0.528170061	4.889787629
0.277934939	10.77770619	0.537280629	4.85496435
0.390079701	4.412087912	0.47343316	4.773326443
0.251438842	5.787671233	0.522903477	5.01848703
0.276966837	11.54935961	0.509682254	4.763532711
0.378433003	10.7469747	0.51422821	5.081983409
0.232459068	5.577739726	0.555109542	4.922993357
0.756967607	11.11278195	0.322516818	5.059866862
0.259565972	5.454166667	0.534826389	4.90591435
0.296138327	4.857615894	0.528507522	4.753384054
0.299425463	5.94757536	0.496856297	4.871914132
0.280558235	6.052888318	0.493493683	4.874009989
0.326015798	11.46824543	0.477681825	5.084218192
0.288514361	5.641464855	0.504550372	4.785264339
0.272728957	5.874553678	0.509368582	4.765703264
0.251029375	11.65742103	0.518514836	4.859956754
0.471732801	10.98632109	0.42722958	5.108816572
0.755555556	6.2	0.315555556	3.323231429
0.619155768	9.513274336	0.394627614	4.647291246
0.353679794	10.66191904	0.482057322	4.96000419
0.294236022	11.94649529	0.481948518	4.758053367
0.349294313	11.70877309	0.477743341	4.930300699
0.415950932	5.049844237	0.447695577	5.106420973
0.377356789	10.141527	0.4757897	4.949023511
0.371139909	11.15524476	0.474364517	5.134065967
0.372290078	9.706036745	0.501381225	4.964848442
0.437759214	9.924757282	0.45645796	5.081881413
0.486724332	9.651480638	0.452825587	5.170679869
0.705933359	11.4161435	0.380445213	5.342273281
0.258099574	12.14831754	0.498934173	4.657536725
0.835638304	19.43918427	0.380523296	5.397549712
0.355551708	9.916666667	0.496999077	4.76367269
0.613636364	6.863636364	0.351239669	3.243300369
0.517545506	9.191780822	0.372490148	4.614892402
0.332520185	5.896568627	0.480299765	4.906718398
0.370632169	9.610421836	0.475915744	4.888304089
0.399326412	28.27574335	0.459080087	5.073466553
0.328565676	5.117154812	0.497102642	4.756008305
0.291698959	12.07810012	0.483172624	4.673022849
0.617188683	9.456716418	0.377981733	5.121306484
0.644031174	9.404816514	0.397706948	5.2664901
0.940242368	16.7139738	0.287532656	5.341481486
0.379847745	10.80508475	0.473219701	5.013757297
0.257883733	5.461373391	0.545202527	4.60705889
0.348274583	10.72508591	0.472970796	4.889206855
0.37509313	11.69510035	0.457521964	4.994422642
0.280454932	6.137947963	0.495322662	4.82037103
0.278292339	11.7362514	0.497677234	4.973567926
0.301886792	4.301886792	0.532929868	4.190487559
0.477430556	11.58854167	0.418924214	5.287043292
0.807619048	8.647619048	0.325351474	4.567558136
0.335689361	11.42053649	0.467823574	4.856396454
0.370189251	4.286713287	0.465010514	4.458025119
0.372348085	11.234375	0.46042057	4.96272468
0.326548184	11.40092343	0.477512365	5.126497022
0.313136686	4.784832451	0.513700313	4.823945379
0.267675094	11.70964999	0.50520743	4.773524461
0.265482057	6.103009046	0.500769308	4.572884921
0.336845842	12.29761003	0.467511597	4.796151694
0.741906131	16.867713	0.35743731	5.118019892
0.382656202	9.916666667	0.475752155	5.023782867
0.351702009	9.540178571	0.50518176	4.725427775
0.327737716	10.27820372	0.525023123	4.993994988
0.338925895	11.26997041	0.484458155	5.004887349
0.405747489	9.046979866	0.463177334	4.703253717
0.751185806	18.96878613	0.360197801	5.339209118
0.484307971	89.23148712	0.426922144	5.055737471
0.543184183	8.419354839	0.433922997	3.65533623
0.294288182	5.924627166	0.492681177	4.959660849
0.291129989	5.742229826	0.512295625	4.916968041
0.299793582	10.4	0.526170013	4.850533307
0.512329896	9.239388795	0.419003174	5.048729924
0.4516	5.38	0.3992	4.28107688
0.698224852	7.538461538	0.325443787	2.87314068
0.44198349	40.48293963	0.441875904	5.111290627
0.444208795	10.375	0.436764197	4.971626827
0.469483866	10.89182692	0.447600777	5.12557868
0.302817988	11.36226725	0.492661825	4.905779501
0.26815478	5.935613403	0.505981331	4.624968174
0.895439657	8.968253968	0.314184933	4.41726921
0.504628028	11.45294118	0.415536332	5.056243994
0.409722222	7.083333333	0.4375	3.886842188
0.473785238	10.11042945	0.416989725	4.599172654
0.384642857	5.907142857	0.432755102	4.906333168

log.sigma.0.5.mm.3D_gldm_DependenceNonUniformity	log.sigma.0.5.mm.3D_gldm_GrayLevelNonUniformity	log.sigma.0.5.mm.3D_gldm_SmallDependenceEmphasis
21.23822715	166.3961219	0.049907704
90.08339273	685.3706344	0.02578203
9.124293785	98.59322034	0.062077626
108.0977755	764.6771857	0.067131935
6.445544554	49.43564356	0.06143361
11.49180328	133.5163934	0.066247263
11.54285714	20.65714286	0.125015387
21.77306733	173.8029925	0.062373649
85.28184282	468.5934959	0.053351137
143.9064252	1167.871928	0.034020111
18.09042553	213.6010638	0.055762237
46.24702774	356.1043593	0.047675233
68.67789165	734.2166911	0.042242831
25.37878788	326.5606061	0.04890258
21.52197802	144.489011	0.091968183
911.7085334	5362.096232	0.014506607
11.33179724	114.6129032	0.089567778
97.97293814	833.8595361	0.035672388
10.52747253	86.16483516	0.080063583
176.813266	1450.534247	0.020292726
392.8092611	2586.637438	0.02392723
43.55225523	467.4334433	0.066741624
174.5582192	1620.919863	0.022680297
10.95488722	42.89473684	0.138508395
63.6375	513.4333333	0.027396293
19.40397351	159.6092715	0.035117724
160.2887724	1137.304063	0.029480639
298.5306804	1922.157895	0.023609187
242.3988159	1775.065662	0.025668147
130.6266982	854.2037803	0.029357262
342.2539871	2139.857415	0.019938786
650.7722178	4580.041549	0.01478457
82.09575234	593.4218862	0.055360405
4.066666667	4.733333333	0.191444444
8.663716814	44.59292035	0.120482244
75.42578711	643.0644678	0.040057089
615.4872216	3224.717531	0.016817134
211.5217678	1448.51781	0.037434512
17.91588785	143.7102804	0.056607493
30.47113594	255.4990689	0.059108952
40.07132867	339.1706294	0.05590769
19.48818898	191.0262467	0.094208567
42.84951456	376.1213592	0.057070751
21.6332574	198.7904328	0.070716933
58.64125561	424.1964126	0.080440093
567.9754982	3054.475008	0.014102385
144.7552804	1044.91697	0.07077053
14.22807018	113.3157895	0.065780972
4.363636364	7.727272727	0.225855043
13.02739726	54.38356164	0.057989347
290.8936275	1959.623039	0.030770552
22.83622829	191.7940447	0.048749908
201.2472613	1466.760876	0.034162316
13.76987448	118.8075314	0.048499175
1092.499027	5462.74969	0.016256447
19.8358209	126.6238806	0.07846982
49.78899083	346.8004587	0.081284929
37.70742358	131.6899563	0.101995544
21.16384181	167.519774	0.052830524
32.19313305	254.0643777	0.035762413
101.5649485	688.1725086	0.037356683
492.770366	3100.168831	0.032861515
304.55081	2017.944526	0.023694675
126.3468013	886.8608305	0.021571937
3.79245283	28.24528302	0.049870021
65.27430556	482.6006944	0.04557926
9.933333333	34.16190476	0.14671175
281.5653303	1621.944332	0.02560667
9.461538462	66.4965035	0.046571527
121.53125	825.0736607	0.037015562
166.1873798	1241.054636	0.034835635
35.76190476	291.2680776	0.039063666
584.7982145	3565.248831	0.017272406
1066.308289	5425.334687	0.015192565
1337.570012	6787.800882	0.021349522
27.97757848	159.4170404	0.096175281
23.81578947	216.9429825	0.051655014
14.08035714	113.1607143	0.048592516
49.27820372	479.3461117	0.052676032
170.0022189	1309.974852	0.03919206
10.39597315	69.01342282	0.084521111
52.73641618	311.5710983	0.095314812
388.0184622	2104.299249	0.032454476
3.516129032	13.4516129	0.183111749
355.1414752	2444.683998	0.023025689
200.3943541	1796.620758	0.033767338
23.03835616	192.0520548	0.061880246
33.4516129	246.7928693	0.082567618
5.72	19.96	0.083032497
3.615384615	4.230769231	0.473824786
245.1131525	1515.192476	0.041572393
20.42763158	132.7763158	0.060145928
23.29326923	186.2019231	0.086017638
258.6719606	1799.200986	0.025290196
649.133481	3654.197175	0.017841168
6.80952381	19.79365079	0.086138596
36.17058824	282.5647059	0.060125795
2.75	10.5	0.139459126
13.96932515	67.96932515	0.074797447
19.72857143	121.1714286	0.054884901

log.sigma.0.5.mm.3D_gldm_DependenceNonUniformityNormalized	log.sigma.0.5.mm.3D_gldm_DependenceVariance	log.sigma.0.5.mm.3D_gldm_LargeDependenceEmphasis
0.058831654	24.45891299	135.5540166
0.064207693	19.74418325	158.0035638
0.051549682	33.94337515	155.1920904
0.055922284	28.13798729	141.809105
0.063817273	33.75374963	124.6633663
0.047097554	41.13517872	173.5737705
0.164897959	3.084897959	20.48571429
0.054296926	32.89529294	135.3541147
0.057779026	24.87357063	111.6056911
0.06205538	21.73084635	168.3402329
0.048112834	39.3256847	189.6489362
0.061092507	22.83253614	141.346103
0.050276641	33.82420593	203.8535871
0.048065886	46.28644399	245.9090909
0.059126313	24.63576259	95.76923077
0.084199163	12.96874475	171.6477651
0.052220264	43.07795876	154.1520737
0.063126893	22.9207524	187.8247423
0.057843256	44.88745321	151.3626374
0.063739461	20.84868298	204.0245133
0.07740084	15.59366997	173.3720197
0.047912272	34.9040508	165.3762376
0.059780212	22.71836695	203.3630137
0.082367573	12.44784895	52.05263158
0.066289063	17.75123264	143.0979167
0.064251568	22.24713828	137.205298
0.070025676	17.56171833	159.9619921
0.076644591	14.34392864	149.9961489
0.065231113	19.96559144	168.5220667
0.077156939	16.10986833	160.6916716
0.081469647	14.63256654	175.0066651
0.073675107	16.43585192	196.2168006
0.059104213	26.08591209	151.2390209
0.271111111	1.2	10.2
0.07667006	26.05137442	87.38938053
0.05654107	27.39098741	169.4902549
0.091987329	12.30765123	164.7605739
0.069763116	21.22710916	171.9043536
0.055812735	30.39865685	121.211838
0.05674327	27.09094251	154.4823091
0.056043816	26.03674312	145.0755245
0.051150102	37.53563285	166.8740157
0.052001838	31.55629654	150.7475728
0.049278491	35.70954904	147.0865604
0.052593054	31.35163788	123.1847534
0.092776135	10.42136121	158.2332571
0.05271496	29.2282316	123.5571741
0.062403817	27.1446599	137.5789474
0.198347107	3.785123967	21.27272727
0.089228748	20.34359167	75.87671233
0.071297458	18.2466299	165.2622549
0.056665579	28.65142942	153.3920596
0.062988188	21.27855584	162.4754304
0.057614538	25.81723009	134.4476987
0.096630022	11.07530568	164.2120998
0.059211406	29.68598797	111.7641791
0.057097467	29.24595994	106.6536697
0.082330619	15.65151313	57.70305677
0.059784864	22.49072744	121.9265537
0.069083976	18.29836615	157.4978541
0.069804088	20.4394497	161.2735395
0.072722899	19.48298286	168.066706
0.07475474	15.67119603	165.5743741
0.070901684	16.1323095	159.8630752
0.071555714	24.25133499	107.3018868
0.056661724	24.49064128	134.1944444
0.094603175	17.23664399	56.86666667
0.081212959	15.88996171	168.5517739
0.066164605	27.50599051	116.7622378
0.067818778	20.08851717	154.4776786
0.063942816	20.08991619	157.9242016
0.063072142	23.6692764	162.9259259
0.082867821	14.60330358	189.9321241
0.098422401	10.61616519	171.2929666
0.092125492	13.38295608	168.9578483
0.062729997	25.40909731	91.50672646
0.052227608	31.12711604	141.5614035
0.062858737	22.6644611	129.75
0.053973936	29.53681819	171.0503834
0.062870643	22.88422368	174.1087278
0.069771632	20.07269943	83.53691275
0.060966955	23.63448963	104.8843931
0.078721538	15.59535775	144.6429296
0.113423517	15.63579605	52.80645161
0.071572244	17.64149089	182.2273277
0.057141247	23.67533734	163.7402338
0.063118784	24.70096453	152.9178082
0.05679391	32.47573367	120.9762309
0.1144	12.5136	60.4
0.278106509	1.408284024	6.384615385
0.071482401	19.3790947	149.6211724
0.067196157	18.67724204	114.125
0.055993436	27.42418639	120.25
0.070830219	18.87496266	182.0175246
0.089882786	12.61158744	181.0720022
0.10808768	6.671705719	40.23809524
0.053192042	32.71820069	138.9294118
0.114583333	8.020833333	41.08333333
0.085701381	16.23945199	67.14723926
0.070459184	16.97142857	97.97142857

log.sigma.0.5.mm.3D_gldm_LargeDependenceLowGrayLevelEmphasis	log.sigma.0.5.mm.3D_gldm_SmallDependenceHighGrayLevelEmphasis	log.sigma.0.5.mm.3D_gldm_LargeDependenceHighGrayLevelEmphasis
14.41905894	0.550665302	1334.412742
16.24883563	0.275187795	1626.832502
38.34761456	0.20578495	658
8.403356189	1.822081792	2488.877393
31.67381738	0.329308	515.0594059
43.08145492	0.350410571	717.2745902
2.53043254	1.116939363	222.4142857
15.31815877	0.707651941	1245.114713
6.462295788	1.218903634	2071.399051
38.27275837	0.239192659	821.2031048
47.47983156	0.291949167	782.9707447
32.28436445	0.323523325	690.7278732
21.67637648	0.565451193	1977.434846
60.70025779	0.373975942	1016.890152
10.2375	1.218112364	939.3379121
33.65179909	0.108815946	1021.635851
40.17581925	0.62313362	616.202765
19.53659507	0.413367737	1888.218428
39.16231685	0.560263488	609.2252747
43.97524884	0.125569056	1071.677361
17.03843455	0.277862255	1882.080591
17.84656261	0.830312443	1571.937294
45.91120148	0.148715945	991.4599315
5.31012949	1.56461164	605.0977444
32.01765046	0.143337164	717.1875
32.64449963	0.143590379	627.6192053
34.16957332	0.217144006	851.8034076
30.31693767	0.138338944	860.6767651
16.45394802	0.324446773	1853.338536
34.65131341	0.170825035	847.783225
36.75309424	0.129015811	953.9521542
19.13843667	0.176972109	2150.027397
15.44981031	0.768571518	1580.2491
2.720833333	1.672166667	62.2
10.22012045	1.653245436	777.9380531
17.80748084	0.486953578	1688.658921
15.12499074	0.184405915	1944.693768
17.09323833	0.579388929	1840.842018
30.23604621	0.371792516	533.2679128
16.25317815	0.49224709	1538.175047
14.85774242	0.749199737	1494.928671
18.50071158	1.089462117	1542.020997
16.54280508	0.728214491	1429.597087
16.60666949	0.932743468	1349.833713
13.37662641	1.341473906	1193.658296
14.28778808	0.14083364	1898.510944
7.421300581	1.759318697	2127.662054
14.86238304	0.660000266	1315.171053
4.340277778	1.870402494	122.9545455
9.2019121	0.523233005	673.4520548
35.29443811	0.216492906	885.4872549
16.6885987	0.503141443	1469.362283
6.044128755	0.994139201	4484.345853
32.43041027	0.276037266	597.2175732
15.09024192	0.201512587	1936.283478
12.77394693	0.853024412	1035.462687
12.4891912	0.946049125	962.0607798
3.953678717	2.030603402	929.0240175
12.62115035	0.463337231	1241.70904
36.07615045	0.193114698	749.9377682
16.24303456	0.297430455	1705.963574
16.39197752	0.527075775	1853.298701
34.45605902	0.185917367	912.7250859
14.99748098	0.229687731	1838.621773
27.17348008	0.196635989	440.2264151
13.45492115	0.715996615	1442.668403
7.590074074	1.48053726	474.3428571
16.1630981	0.248554227	1896.953851
30.07264957	0.180552296	483.6293706
15.30340805	0.418143531	1668.840402
15.39582361	0.399747131	1742.858407
39.44228885	0.185799983	743.4056437
18.30623786	0.205555907	2113.581834
34.33496494	0.091734459	991.5887022
15.44105109	0.339461091	2005.959983
5.770873019	2.048400415	1501.486547
15.63927875	0.596022064	1322.763158
14.20817088	0.38044952	1231.415179
18.26736278	0.519438316	1657.447974
17.5607833	0.481098238	1830.39645
9.551815809	0.794350717	762.0268456
6.008701252	2.157113187	1922.848555
1.631859096	2.913422944	12978.02678
6.341397849	2.300111472	465.0645161
37.88268239	0.17895428	1011.342201
37.41166096	0.256295179	785.7735957
15.6651446	0.513548752	1570.821918
13.88470147	1.048299513	1098.83871
16.28166667	0.632989752	285.94
1.422008547	4.514957265	34.46153846
3.770102573	1.621286544	6078.29513
11.30676626	0.304916185	1245.131579
12.7150414	1.235517704	1191.021635
18.06843153	0.273404963	1955.444414
37.65618054	0.110353526	1000.101495
5.196732804	1.264589433	391.7619048
14.83063786	0.954093954	1356.267647
5.971354167	1.049406512	320.3333333
7.444988071	0.751744634	638.4785276
17.43611111	0.159235559	656.5571429

log.sigma.0.5.mm.3D_gldm_SmallDependenceLowGrayLevelEmphasis	log.sigma.0.5.mm.3D_gldm_LowGrayLevelEmphasis	log.sigma.0.5.mm.3D_gldzm_DistanceZoneVariabilityNormalized
0.009787053	0.117416898	1
0.005048784	0.105185119	1
0.042484308	0.306183302	1
0.005112395	0.063366535	0.962278107
0.0278884	0.325082508	1
0.038551473	0.292975865	1
0.047079316	0.180630952	1
0.01534508	0.142847049	1
0.004513915	0.063061916	0.7256
0.016609426	0.236474294	0.873888889
0.027639844	0.297059693	1
0.023540284	0.260127697	1
0.005823198	0.10268932	1
0.014765696	0.250157828	1
0.019421488	0.121663614	1
0.003829539	0.193522196	1
0.033861645	0.350678443	1
0.005375223	0.10382016	1
0.032586866	0.361034799	1
0.007367664	0.207845169	1
0.003535319	0.09555858	0.952968501
0.01124509	0.108686429	0.966676242
0.007570572	0.211396499	1
0.032715636	0.131319967	1
0.011596396	0.229050926	1
0.020493122	0.285779985	1
0.011637474	0.210115771	1
0.01083208	0.204400228	1
0.003691559	0.099417011	0.971434573
0.014018845	0.227279812	1
0.008461102	0.20852059	0.962976148
0.001767065	0.09347315	0.970156107
0.012382201	0.11429555	1
0.059566358	0.331944444	1
0.028472584	0.147111603	1
0.006205251	0.109132101	1
0.002519986	0.093342508	1
0.004655443	0.096976418	1
0.026149129	0.308259778	1
0.015971502	0.120910408	1
0.009164955	0.105153458	1
0.017068333	0.124946048	1
0.008944101	0.12416633	0.895061728
0.015026333	0.131726442	1
0.013155459	0.116678726	0.641760837
0.002055368	0.089883889	1
0.005777322	0.06202355	0.591150107
0.013545037	0.120911306	1
0.103466777	0.244318182	1
0.014264351	0.143312405	1
0.014684245	0.222502383	0.901371028
0.009146125	0.12532327	1
0.00187125	0.037902524	0.74744898
0.023972264	0.275366109	1
0.002185005	0.091953006	1
0.026738828	0.153742952	1
0.023756842	0.153884748	0.968757874
0.013056742	0.080976146	1
0.014152763	0.114872881	1
0.019620723	0.225768956	1
0.007436285	0.108476709	1
0.003834477	0.098792468	1
0.008386607	0.197883871	0.968262227
0.00339518	0.094420439	1
0.03071562	0.342243187	1
0.00791353	0.10733724	1
0.057372774	0.189867725	1
0.004720978	0.10047239	1
0.029585112	0.372571873	1
0.007073196	0.105556486	0.967750605
0.005323278	0.10021846	1
0.020970054	0.298745836	1
0.002284361	0.093860863	1
0.006353493	0.197740049	1
0.00285665	0.091770685	0.945497423
0.012577101	0.075686173	1
0.008677644	0.121086745	1
0.012560486	0.126139633	1
0.011051712	0.113652184	1
0.006192417	0.101605132	1
0.018673738	0.138318419	1
0.010266031	0.065132589	1
0.000610199	0.011652584	0.966676242
0.031801594	0.1890681	1
0.008352622	0.21297875	1
0.012714314	0.218142128	0.929506246
0.01291626	0.110806697	1
0.019345718	0.14337625	0.95240928
0.016473017	0.305	1
0.119405864	0.22542735	1
0.001845605	0.026360597	0.964613262
0.025218774	0.126576206	1
0.015968365	0.114614049	1
0.004103354	0.099081249	0.970156107
0.007805833	0.205437821	0.975004006
0.020799641	0.196291887	1
0.00909006	0.107464653	1
0.055064226	0.198206019	1
0.020362297	0.129207566	1
0.037568957	0.249404762	1

log.sigma.0.5.mm.3D_gldzm_LowIntensityEmphasis	log.sigma.0.5.mm.3D_gldzm_LargeDistanceEmphasis	log.sigma.0.5.mm.3D_gldzm_HighIntensitySmallDistanceEmphasis
0.212256944	1	10.875
0.271415344	1	9.047619048
0.747222222	1	2.9
0.081105893	1.057692308	29.16346154
0.461805556	1	5.375
0.674707602	1	4.421052632
0.440401235	1	7.222222222
0.285944444	1	12.15
0.091220522	1.58	22.84888889
0.631888889	1.283333333	5.816666667
0.602272727	1	4.409090909
0.58733165	1	6.787878788
0.17185941	1	12
0.384348291	1	7.153846154
0.238680556	1	13.34375
0.442060531	1	8.358208955
0.436237374	1	6.5
0.180772222	1	10.16
0.513194444	1	5.85
0.533371914	1	5.277777778
0.195713688	1.072289157	9.798192771
0.187916523	1.050847458	11.51271186
0.488715278	1	6
0.23	1	11.73684211
0.647660819	1	3.578947368
0.652777778	1	3.5
0.525608974	1	7.692307692
0.724242424	1	4.709090909
0.184041868	1.043478261	12.01449275
0.6640625	1	4.75
0.637646751	1.056603774	5.905660377
0.166645623	1.045454545	10.24621212
0.250786902	1	14.47619048
0.355902778	1	7.5
0.222123016	1	14.35714286
0.188927649	1	10.86046512
0.219166667	1	8.9
0.140224116	1	16.42045455
0.496527778	1	6.9375
0.29780754	1	7.5
0.185826023	1	12.63157895
0.184766082	1	11.23684211
0.174205247	1.166666667	12.42361111
0.231727438	1	13.8
0.181621242	1.857142857	13.5021645
0.256890681	1	5.129032258
0.088405589	2.275280899	18.69011392
0.228194444	1	8.4375
0.414351852	1	7.833333333
0.232638889	1	10.25
0.616718213	1.206185567	6.567296678
0.196388889	1	10.29411765
0.063081639	1.607142857	23.56646825
0.604166667	1	4.818181818
0.192003229	1	11.57608696
0.423625731	1	11.78947368
0.336186067	1.047619048	12.24206349
0.138756904	1	21.74358974
0.32874183	1	7.117647059
0.685515873	1	4.5
0.234674941	1	6.255319149
0.13531225	1	17.05202312
0.494036738	1.048387097	8.193548387
0.215050505	1	9.136363636
0.590277778	1	3.75
0.21	1	18.65714286
0.351013072	1	11.64705882
0.230057319	1	8.317460317
0.578703704	1	4.166666667
0.227727687	1.049180328	9.524590164
0.190543118	1	10.59701493
0.606944444	1	4.35
0.175244883	1	10.61842105
0.748950701	1	4.722891566
0.156464016	1.08411215	17.78037383
0.142270081	1	21.23076923
0.183430556	1	11.45
0.252175926	1	7.916666667
0.239969807	1	8.47826087
0.182777778	1	11.79775281
0.223162393	1	9.461538462
0.115034405	1	23.18421053
0.021257364	1.050847458	91.86016949
0.23015873	1	11.14285714
0.498174074	1	8.093333333
0.517510163	1.109756098	7.283536585
0.235809179	1	6.695652174
0.237987805	1.073170732	13.71341463
0.282407407	1	6.833333333
0.244047619	1	9.142857143
0.050678321	1.054054054	37.68693694
0.440200617	1	4.111111111
0.197664931	1	14.5625
0.201531987	1.045454545	9.382575758
0.699082278	1.037974684	4.863924051
0.248769841	1	13.57142857
0.162144358	1	17.23529412
0.355902778	1	7.5
0.27017094	1	9.615384615
0.840740741	1	1.933333333

log.sigma.0.5.mm.3D_gldzm_LowIntensityLargeDistanceEmphasis	log.sigma.0.5.mm.3D_gldzm_HighIntensityEmphasis	log.sigma.0.5.mm.3D_gldzm_DistanceZoneVariability	log.sigma.0.5.mm.3D_gldzm_ZonePercentage
0.212256944	10.875	16	0.04432133
0.271415344	9.047619048	21	0.014967926
0.747222222	2.9	10	0.056497175
0.082357897	29.88461538	100.0769231	0.05380238
0.461805556	5.375	8	0.079207921
0.674707602	4.421052632	19	0.077868852
0.440401235	7.222222222	9	0.128571429
0.285944444	12.15	20	0.049875312
0.145559297	26.48	36.28	0.033875339
0.644967593	6.95	52.43333333	0.025873221
0.602272727	4.409090909	22	0.058510638
0.58733165	6.787878788	33	0.043593131
0.17185941	12	49	0.035871157
0.384348291	7.153846154	26	0.049242424
0.238680556	13.34375	32	0.087912088
0.442060531	8.358208955	67	0.006187662
0.436237374	6.5	22	0.101382488
0.180772222	10.16	50	0.032216495
0.513194444	5.85	20	0.10989011
0.533371914	5.277777778	36	0.01297765
0.206195616	10.06024096	79.09638554	0.01635468
0.189950421	11.83050847	57.03389831	0.064906491
0.488715278	6	40	0.01369863
0.23	11.73684211	19	0.142857143
0.647660819	3.578947368	19	0.019791667
0.652777778	3.5	12	0.039735099
0.525608974	7.692307692	52	0.022717344
0.724242424	4.709090909	55	0.014120668
0.194911433	12.05797101	67.02898551	0.018568353
0.6640625	4.75	40	0.023626698
0.641184486	6.132075472	51.03773585	0.012616044
0.168463805	10.53030303	64.03030303	0.00747198
0.250786902	14.47619048	63	0.045356371
0.355902778	7.5	4	0.266666667
0.222123016	14.35714286	14	0.123893805
0.188927649	10.86046512	43	0.032233883
0.219166667	8.9	60	0.008967269
0.140224116	16.42045455	88	0.029023747
0.496527778	6.9375	16	0.049844237
0.29780754	7.5	28	0.052141527
0.185826023	12.63157895	38	0.053146853
0.184766082	11.23684211	38	0.099737533
0.179853395	13.69444444	32.22222222	0.04368932
0.231727438	13.8	25	0.056947608
0.309878155	17.54545455	49.41558442	0.069058296
0.256890681	5.129032258	31	0.005063705
0.283154356	26.7752809	105.2247191	0.064821559
0.228194444	8.4375	16	0.070175439
0.414351852	7.833333333	6	0.272727273
0.232638889	10.25	8	0.054794521
0.628908935	7.278350515	87.43298969	0.02377451
0.196388889	10.29411765	17	0.042183623
0.078137215	28.73809524	62.78571429	0.02629108
0.604166667	4.818181818	11	0.046025105
0.192003229	11.57608696	92	0.008137272
0.423625731	11.78947368	19	0.056716418
0.338090829	12.53968254	61.03174603	0.072247706
0.138756904	21.74358974	39	0.085152838
0.32874183	7.117647059	17	0.048022599
0.685515873	4.5	14	0.030042918
0.234674941	6.255319149	47	0.032302405
0.13531225	17.05202312	173	0.025531287
0.497060932	8.387096774	60.03225806	0.015218459
0.215050505	9.136363636	22	0.012345679
0.590277778	3.75	4	0.075471698
0.21	18.65714286	35	0.030381944
0.351013072	11.64705882	17	0.161904762
0.230057319	8.317460317	63	0.01817133
0.578703704	4.166666667	6	0.041958042
0.229093807	9.967213115	59.03278689	0.034040179
0.190543118	10.59701493	67	0.025779146
0.606944444	4.35	20	0.035273369
0.175244883	10.61842105	76	0.010769449
0.748950701	4.722891566	83	0.007661067
0.162601087	18.27102804	202.3364486	0.014739307
0.142270081	21.23076923	39	0.087443946
0.183430556	11.45	20	0.043859649
0.252175926	7.916666667	12	0.053571429
0.239969807	8.47826087	46	0.050383352
0.182777778	11.79775281	89	0.032914201
0.223162393	9.461538462	13	0.087248322
0.115034405	23.18421053	76	0.087861272
0.021986329	92.94067797	114.0677966	0.023939947
0.23015873	11.14285714	7	0.225806452
0.498174074	8.093333333	75	0.015114873
0.523546748	7.804878049	76.2195122	0.023381808
0.235809179	6.695652174	23	0.063013699
0.240914634	14.17073171	39.04878049	0.069609508
0.282407407	6.833333333	6	0.12
0.244047619	9.142857143	7	0.538461538
0.053448592	37.96396396	107.0720721	0.032370954
0.440200617	4.111111111	18	0.059210526
0.197664931	14.5625	32	0.076923077
0.203350168	9.666666667	64.03030303	0.018072289
0.700601266	5.101265823	77.02531646	0.010938798
0.248769841	13.57142857	7	0.111111111
0.162144358	17.23529412	34	0.05
0.355902778	7.5	4	0.166666667
0.27017094	9.615384615	13	0.079754601
0.840740741	1.933333333	15	0.053571429

log.sigma.0.5.mm.3D_gldzm_IntensityVariabilityNormalized	log.sigma.0.5.mm.3D_gldzm_LowIntensitySmallDistanceEmphasis	log.sigma.0.5.mm.3D_gldzm_IntensityVariability
0.3359375	0.212256944	5.375
0.287981859	0.271415344	6.047619048
0.54	0.747222222	5.4
0.225036982	0.080792893	23.40384615
0.40625	0.461805556	3.25
0.457063712	0.674707602	8.684210526
0.259259259	0.440401235	2.333333333
0.255	0.285944444	5.1
0.2048	0.078089342	10.24
0.416111111	0.629133616	24.96666667
0.466942149	0.602272727	10.27272727
0.377410468	0.58733165	12.45454545
0.369429404	0.17185941	18.10204082
0.399408284	0.384348291	10.38461538
0.236328125	0.238680556	7.5625
0.334372912	0.442060531	22.40298507
0.392561983	0.436237374	8.636363636
0.3968	0.180772222	19.84
0.375	0.513194444	7.5
0.447530864	0.533371914	16.11111111
0.504717666	0.193093206	41.89156627
0.349037633	0.187408048	20.59322034
0.4125	0.488715278	16.5
0.229916898	0.23	4.368421053
0.429362881	0.647660819	8.157894737
0.430555556	0.652777778	5.166666667
0.36612426	0.525608974	19.03846154
0.510082645	0.724242424	28.05454545
0.301407267	0.181324477	20.79710145
0.4575	0.6640625	18.3
0.426842293	0.636762317	22.62264151
0.408631772	0.166191077	26.96969697
0.234567901	0.250786902	14.77777778
0.25	0.355902778	1
0.234693878	0.222123016	3.285714286
0.402920498	0.188927649	17.3255814
0.501666667	0.219166667	30.1
0.369059917	0.140224116	32.47727273
0.3203125	0.496527778	5.125
0.37244898	0.29780754	10.42857143
0.293628809	0.185826023	11.15789474
0.347645429	0.184766082	13.21052632
0.236111111	0.17279321	8.5
0.2032	0.231727438	5.08
0.237645471	0.157349221	18.2987013
0.762747138	0.256890681	23.64516129
0.205529605	0.067696296	36.58426966
0.390625	0.228194444	6.25
0.277777778	0.414351852	1.666666667
0.34375	0.232638889	2.75
0.426293974	0.614386455	41.35051546
0.349480969	0.196388889	5.941176471
0.31462585	0.060127593	26.42857143
0.388429752	0.604166667	4.272727273
0.379017013	0.192003229	34.86956522
0.24099723	0.423625731	4.578947368
0.217435122	0.335709877	13.6984127
0.230769231	0.138756904	9
0.349480969	0.32874183	5.941176471
0.459183673	0.685515873	6.428571429
0.664101403	0.234674941	31.21276596
0.305756958	0.13531225	52.89595376
0.379812695	0.49328069	23.5483871
0.404958678	0.215050505	8.909090909
0.375	0.590277778	1.5
0.25877551	0.21	9.057142857
0.259515571	0.351013072	4.411764706
0.483497103	0.230057319	30.46031746
0.388888889	0.578703704	2.333333333
0.388336469	0.227386157	23.68852459
0.383381599	0.190543118	25.68656716
0.465	0.606944444	9.3
0.397506925	0.175244883	30.21052632
0.547394397	0.748950701	45.43373494
0.282164381	0.154929748	60.38317757
0.216305062	0.142270081	8.435897436
0.31	0.183430556	6.2
0.388888889	0.252175926	4.666666667
0.442344045	0.239969807	20.34782609
0.339224845	0.182777778	30.19101124
0.325443787	0.223162393	4.230769231
0.189058172	0.115034405	14.36842105
0.196782534	0.021075123	23.22033898
0.387755102	0.23015873	2.714285714
0.336177778	0.498174074	25.21333333
0.372992267	0.516001016	30.58536585
0.534971645	0.235809179	12.30434783
0.294467579	0.237256098	12.07317073
0.5	0.282407407	3
0.306122449	0.244047619	2.142857143
0.240321402	0.049985754	26.67567568
0.456790123	0.440200617	8.222222222
0.22265625	0.197664931	7.125
0.429292929	0.201077441	28.33333333
0.499439192	0.698702532	39.4556962
0.183673469	0.248769841	1.285714286
0.346020761	0.162144358	11.76470588
0.25	0.355902778	1
0.24260355	0.27017094	3.153846154
0.662222222	0.840740741	9.933333333

log.sigma.0.5.mm.3D_gldzm_HighIntensityLargeDistanceEmphasis	log.sigma.0.5.mm.3D_gldzm_SmallDistanceEmphasis	log.sigma.0.5.mm.3D_glcm_SumVariance	log.sigma.0.5.mm.3D_glcm_Homogeneity1
10.875	1	21.29378521	0.744746139
9.047619048	1	24.63437822	0.754356438
2.9	1	7.606091216	0.798276914
32.76923077	0.985576923	42.50635747	0.707961398
5.375	1	7.191763509	0.761831953
4.421052632	1	7.95634152	0.785064357
7.222222222	1	16.52098488	0.585765239
12.15	1	17.99424547	0.738118052
45.36	0.877222222	40.34659678	0.656753186
14.15	0.947685185	9.459606811	0.755755688
4.409090909	1	7.770264557	0.795784921
6.787878788	1	8.53642701	0.746377229
12	1	25.2778939	0.780345144
7.153846154	1	9.412053698	0.835232035
13.34375	1	21.08252851	0.695365063
8.358208955	1	11.67720778	0.750727753
6.5	1	6.466488022	0.795277592
10.16	1	24.80794751	0.779411614
5.85	1	6.270123805	0.773386883
5.277777778	1	10.69680414	0.78379854
11.10843373	0.981927711	27.58140417	0.759322125
13.10169492	0.987288136	23.49511412	0.757904632
6	1	10.65250524	0.790358831
11.73684211	1	18.62165675	0.658522066
3.578947368	1	10.06870372	0.772735011
3.5	1	7.834104697	0.773557865
7.692307692	1	10.55941527	0.754191377
4.709090909	1	11.23778693	0.749454878
12.23188406	0.989130435	25.86867819	0.754976418
4.75	1	10.08108386	0.759252997
7.037735849	0.985849057	10.806312	0.760319906
11.66666667	0.988636364	28.14735026	0.770696237
14.47619048	1	22.13335847	0.730691364
7.5	1	10.67601003	0.566987179
14.35714286	1	16.52252064	0.70827239
10.86046512	1	22.90769157	0.751501923
8.9	1	28.00314945	0.748145724
16.42045455	1	26.16731862	0.747517154
6.9375	1	6.823408866	0.727346215
7.5	1	21.81986574	0.7577012
12.63157895	1	24.10085036	0.759142385
11.23684211	1	20.84800865	0.77733382
18.77777778	0.958333333	20.26504585	0.740075704
13.8	1	19.06257823	0.743540252
35.62337662	0.829004329	20.36805836	0.677465794
5.129032258	1	29.77814322	0.749619215
75.30898876	0.796855493	42.51721504	0.678755595
8.4375	1	21.33642727	0.755763646
7.833333333	1	10.14196874	0.619505957
10.25	1	16.26433485	0.68628718
10.8556701	0.959908362	10.00817477	0.743456229
10.29411765	1	20.07242347	0.752540258
57.20238095	0.887896825	78.10861261	0.7407934
4.818181818	1	8.349909031	0.760782379
11.57608696	1	28.43352363	0.744679647
11.78947368	1	16.99031098	0.70475162
13.73015873	0.988095238	16.40682175	0.706423898
21.74358974	1	32.79473418	0.623968499
7.117647059	1	23.22451614	0.746796005
4.5	1	10.01472463	0.772015837
6.255319149	1	23.81675729	0.750665879
17.05202312	1	25.4863059	0.738981461
9.161290323	0.987903226	11.15237461	0.753056019
9.136363636	1	27.55287826	0.767476762
3.75	1	6.874891839	0.786019815
18.65714286	1	23.22276711	0.728393817
11.64705882	1	13.80090151	0.64419909
8.317460317	1	25.54276973	0.748449153
4.166666667	1	6.157373091	0.738137518
11.73770492	0.987704918	24.51624969	0.743156598
10.59701493	1	25.62503451	0.753785861
4.35	1	7.6014704	0.768345348
10.61842105	1	27.89299641	0.764015515
4.722891566	1	11.51520909	0.75165367
20.23364486	0.978971963	27.95592545	0.737531229
21.23076923	1	36.25497819	0.670117372
11.45	1	21.05002566	0.74967545
7.916666667	1	20.61055547	0.757660169
8.47826087	1	23.26560983	0.773627211
11.79775281	1	25.38227038	0.756611929
9.461538462	1	18.3323256	0.719967836
23.18421053	1	42.6370519	0.688007851
97.26271186	0.987288136	289.5676687	0.720090404
11.14285714	1	14.87199086	0.698071942
8.093333333	1	10.48417154	0.759888933
9.890243902	0.972560976	9.978261788	0.759926939
6.695652174	1	23.78933459	0.771966273
16	0.981707317	17.10221499	0.718826657
6.833333333	1	6.742624806	0.704256396
9.142857143	1	10.77848617	0.579695767
39.07207207	0.986486486	118.9126541	0.731039672
4.111111111	1	21.99629651	0.735340209
14.5625	1	22.06144949	0.726074407
10.8030303	0.988636364	26.02960854	0.762717087
6.050632911	0.990506329	11.01978417	0.756433903
13.57142857	1	14.57544508	0.626227605
17.23529412	1	22.11417551	0.714798666
7.5	1	13.68330606	0.708467067
9.615384615	1	18.58032874	0.697015161
1.933333333	1	9.932159674	0.747101619

log.sigma.0.5.mm.3D_glcm_Homogeneity2	log.sigma.0.5.mm.3D_glcm_ClusterShade	log.sigma.0.5.mm.3D_glcm_MaximumProbability	log.sigma.0.5.mm.3D_glcm_Idmn	log.sigma.0.5.mm.3D_glcm_SumVariance2
0.738830933	-0.032270884	0.402799127	0.976173098	0.799834507
0.752079652	-0.122754576	0.38217298	0.979549697	0.651111434
0.79584143	0.06599619	0.5397291	0.956470519	0.567250742
0.694681593	0.16980819	0.332649772	0.987700236	1.146339107
0.755496188	0.001091702	0.480888456	0.94408116	0.602695966
0.780606504	0.114798479	0.537339946	0.970527261	0.53805503
0.550194761	-0.502817252	0.163122882	0.941235349	1.498485082
0.729229891	0.148005247	0.421115427	0.980690969	0.932397863
0.629960901	1.013287084	0.241441041	0.970736777	2.076848653
0.752155217	0.219104783	0.398390243	0.984555273	0.647632984
0.793553735	0.011255555	0.550014482	0.956239279	0.516271844
0.741778583	0.11912694	0.385801679	0.98352233	0.723654684
0.777196246	0.177038486	0.47905674	0.980930372	0.55197932
0.832933134	0.205863363	0.633907489	0.978315732	0.412490882
0.680969144	0.385421457	0.33713604	0.974208896	1.074229343
0.750062964	0.024095823	0.296019339	0.970035571	0.532859794
0.789121407	0.011968914	0.565363869	0.969903968	0.551231158
0.777687484	0.078050616	0.449260691	0.981851393	0.59038447
0.765547323	0.043725387	0.521838873	0.96593884	0.593840624
0.783445969	0.108344359	0.400770861	0.974238882	0.551555558
0.758175169	0.078869166	0.361084936	0.992200941	0.543739307
0.750295909	0.346682571	0.465309926	0.986729209	0.697210011
0.789991643	0.164872686	0.45023337	0.974992744	0.504436533
0.632530969	0.198574232	0.187969123	0.966130119	1.770276707
0.772082612	0.0500424	0.410007912	0.953693866	0.552959641
0.771542546	0.015859626	0.456330935	0.952076157	0.657413156
0.751986075	0.120470118	0.355117523	0.979480629	0.581614498
0.748568803	0.022293859	0.31173981	0.9859513	0.54449518
0.752630747	-0.056281396	0.345156406	0.985442632	0.651142359
0.757992063	0.01436105	0.36800175	0.970468794	0.583929338
0.759078987	0.075305586	0.35352798	0.980610975	0.555687727
0.770385737	0.095004956	0.368509914	0.982154131	0.531494512
0.723476112	-0.144603274	0.346750527	0.985837402	0.863963574
0.534358974	-0.103669516	0.254166667	0.916711916	1.09542735
0.693353438	0.226807549	0.386884489	0.965292124	1.053359125
0.747193243	0.00478218	0.40698085	0.984284314	0.696748326
0.747177184	-0.064925265	0.288677303	0.985875808	0.587164826
0.743080824	0.103783966	0.357789424	0.983884219	0.646575591
0.715927537	0.126125803	0.396449677	0.956964934	0.815758874
0.753768236	-0.100161786	0.420358548	0.978587046	0.746290266
0.754583952	0.052346479	0.398155244	0.98429421	0.71040452
0.772418312	-0.095336933	0.50580251	0.979023244	0.668120813
0.732621896	0.048144284	0.415365716	0.981176417	0.847509125
0.734106533	0.480721016	0.437808795	0.984542286	0.973380408
0.655950962	1.172505167	0.327210649	0.975318004	1.451892518
0.749430884	0.014977065	0.287079573	0.980616015	0.510571022
0.657910437	2.51657389	0.324060101	0.984977961	1.737928701
0.750647511	-0.053476636	0.44818815	0.977786914	0.70282153
0.59950383	-0.120906656	0.245787546	0.936133376	1.075333797
0.671272528	-0.001150304	0.306830243	0.949286098	0.907811625
0.739986809	0.073726535	0.338301958	0.97741467	0.641095135
0.747945563	-0.067298997	0.428044818	0.977900658	0.769252986
0.73538534	-0.167778136	0.34826765	0.989759533	0.810010314
0.757769374	0.01335444	0.443850229	0.969109598	0.614454616
0.743659773	-0.033917161	0.283974926	0.989363024	0.566676369
0.691178377	-0.155006525	0.35447159	0.975529767	1.107465123
0.689248179	0.518705402	0.37879464	0.97255704	1.286037872
0.593978177	-0.004049298	0.18693004	0.970412033	1.875996316
0.742360134	-0.225667026	0.384492901	0.977405633	0.740229678
0.770362718	0.133658905	0.436199772	0.971671078	0.529027754
0.747707841	-0.074477297	0.368785068	0.978821406	0.703263184
0.734390042	-0.012785356	0.323445833	0.987479125	0.706242118
0.751825152	0.094254077	0.332264097	0.980153304	0.549449629
0.766634942	0.019536832	0.334829917	0.98145182	0.596363711
0.783958218	0.006904393	0.529303997	0.954508028	0.58067661
0.720390126	-0.190577089	0.318519288	0.980031009	0.893362378
0.618230561	0.583249854	0.250675088	0.950194177	1.399654168
0.74610967	-0.107116824	0.322655706	0.979076096	0.685922672
0.731862387	-0.009685224	0.430206428	0.939421564	0.698915509
0.739138006	-0.089082898	0.343365507	0.983890425	0.719428604
0.75118918	-0.071513165	0.341947483	0.985326154	0.666980864
0.765576002	-0.045541305	0.445021219	0.950047617	0.686064651
0.763330086	0.048438916	0.348725727	0.981377805	0.550666
0.751207827	0.040099248	0.309900614	0.989859249	0.527258135
0.734449512	0.026678998	0.274320627	0.987915647	0.628814453
0.650475756	-0.042310028	0.308764902	0.981606321	1.296584546
0.742458028	-0.050214729	0.440896813	0.975761031	0.731488509
0.752486761	-0.112447456	0.445185564	0.977899138	0.742096178
0.769756603	0.057144088	0.470797624	0.979943136	0.641178815
0.75346599	0.03151577	0.380504173	0.985076323	0.639006571
0.711191253	-0.155702303	0.392468992	0.97229189	0.8372168
0.671895485	-0.11686872	0.281106497	0.98292433	1.266329972
0.713626573	-0.379676871	0.23972251	0.995303801	0.876127934
0.681607367	-0.6182813	0.395918078	0.948428772	0.987969754
0.758407964	0.025785652	0.343525455	0.980409085	0.596908098
0.756964142	0.157081758	0.407384348	0.979413672	0.572210408
0.769583979	0.062074093	0.43688311	0.970961663	0.633419658
0.706461832	0.049845441	0.394866885	0.977865077	0.96980461
0.692868473	-0.185962582	0.305959936	0.925959379	0.945396996
0.546428571	-0.177934548	0.242063492	0.916470588	1.111992945
0.725013279	-0.619095042	0.293754069	0.991183588	0.855956203
0.728767315	-0.328437325	0.333970096	0.962350729	0.887974797
0.716083063	0.135234201	0.377971742	0.978711425	0.877562715
0.761290237	-0.001862326	0.369772022	0.980774639	0.616518316
0.755732763	0.051392773	0.339640833	0.980774864	0.540202026
0.598463041	-0.266097314	0.195455794	0.959755801	1.709304982
0.703387695	0.317532747	0.361122166	0.983667691	0.897155578
0.696319983	-0.183851732	0.360164195	0.954626726	0.642475519
0.684869899	-0.14070133	0.335023321	0.968162728	0.950252874
0.742834952	-0.270131383	0.258252106	0.943840835	0.798645317

log.sigma.0.5.mm.3D_glcm_Contrast	log.sigma.0.5.mm.3D_glcm_DifferenceEntropy	log.sigma.0.5.mm.3D_glcm_InverseVariance	log.sigma.0.5.mm.3D_glcm_Entropy	log.sigma.0.5.mm.3D_glcm_Dissimilarity
0.643104337	1.225190445	0.461906292	2.58472001	0.542465835
0.539905532	1.106565438	0.472671684	2.291970573	0.503184835
0.452155859	1.071045364	0.38365786	2.110186985	0.415623593
1.066118587	1.477452817	0.468941762	3.063368171	0.682626508
0.603051388	1.199813203	0.424858006	2.321948648	0.508014918
0.523084334	1.144249842	0.393497877	2.157821681	0.452836549
1.701959735	1.616316523	0.533500877	3.396663896	1.032491088
0.748076311	1.313670016	0.449813914	2.746460019	0.575831981
1.607098619	1.737352963	0.455017931	3.861303887	0.881058841
0.589419302	1.164911746	0.458221352	2.315751635	0.510921701
0.453053878	1.067145761	0.390301771	2.055099328	0.419586088
0.632878062	1.193876999	0.46878376	2.467472896	0.535125357
0.507494359	1.122228985	0.413528055	2.141871255	0.455921984
0.382305443	1.004537182	0.310600578	1.752682657	0.342162351
1.029517998	1.462998231	0.487567583	3.026731608	0.702039571
0.512911981	1.039254241	0.493102559	2.099683995	0.502047058
0.545836873	1.173728273	0.358924152	2.185814189	0.442437134
0.47811672	1.072076248	0.427075309	2.162965656	0.45020698
0.618449392	1.233930603	0.389211006	2.335541877	0.493829361
0.43959403	1.004982957	0.429533	2.011422626	0.434189057
0.510318633	1.061610078	0.471862932	2.115700112	0.48790022
0.692733795	1.276075409	0.420460435	2.436036261	0.530696586
0.426919418	1.000603468	0.416287846	1.92453892	0.421167164
1.360561456	1.619157024	0.466020937	3.532408782	0.838171253
0.467577947	1.029187355	0.449229241	2.071692631	0.457791971
0.493190633	1.077983677	0.436509811	2.259087965	0.462960861
0.543030428	1.108984042	0.473422897	2.214965803	0.503861613
0.52347066	1.050849282	0.49373203	2.128213368	0.506216072
0.546998584	1.110402847	0.470621751	2.304134131	0.503385322
0.508657804	1.065018905	0.471175371	2.18230111	0.488122862
0.510549616	1.068015919	0.469046766	2.133366589	0.486577205
0.465036664	1.010608714	0.456076429	2.017187052	0.460196549
0.732630997	1.282184074	0.478187332	2.714820183	0.582642712
1.605128205	1.104223924	0.597649573	2.507152402	1.043589744
0.990646214	1.439646978	0.458205469	2.952693721	0.675783094
0.594583727	1.179851361	0.461565879	2.440784179	0.520395591
0.525454532	1.055287458	0.495749989	2.192421211	0.508944013
0.611465584	1.189124981	0.468258143	2.378752029	0.530061696
0.791914543	1.3444275	0.451841431	2.742391469	0.605439862
0.57224098	1.161538994	0.452302589	2.446631891	0.505759769
0.599182742	1.188450491	0.443703162	2.422269897	0.508793332
0.570980489	1.183276865	0.404372121	2.356662403	0.47440708
0.734382415	1.29100391	0.45693639	2.66884503	0.567405492
0.82417826	1.35172191	0.432101067	2.716882659	0.578459349
1.342972052	1.634152083	0.460915907	3.392283005	0.793953088
0.504591223	1.009226409	0.499228995	2.034271663	0.50171373
1.645230572	1.701874685	0.45443476	3.441192301	0.830555702
0.594939259	1.189190097	0.446747437	2.437188762	0.514744024
1.18987679	1.208540639	0.597380166	2.788104225	0.865806416
0.931608061	1.38274952	0.505284535	2.896447224	0.703147131
0.608146188	1.163724857	0.483957784	2.357681102	0.534564436
0.590019323	1.17769138	0.457466374	2.503399364	0.518427282
0.687924072	1.237136065	0.472443241	2.575491	0.554533822
0.538695353	1.121220393	0.453954572	2.269076604	0.49350027
0.534516219	1.05765798	0.502232295	2.176694796	0.516277248
0.971852529	1.437979375	0.476297894	3.063268998	0.675558095
1.111166876	1.532234926	0.441075456	3.184483041	0.701675657
1.586001064	1.692695473	0.499763737	3.813021417	0.939919581
0.604755722	1.179290354	0.470002427	2.437830462	0.530192397
0.489030701	1.064264852	0.442536736	2.07942017	0.46423392
0.560980643	1.133001728	0.474515025	2.399585839	0.513983705
0.640269485	1.199871476	0.483767291	2.477963706	0.549232593
0.520604857	1.066380711	0.483807791	2.139061137	0.500392222
0.486686653	1.045516731	0.458027411	2.148477618	0.470044543
0.469192313	1.050507191	0.411209894	2.104108497	0.438268356
0.783031741	1.310180964	0.475347105	2.739725832	0.595688139
1.466198051	1.638683306	0.491888059	3.378679959	0.879781754
0.552181038	1.111364494	0.484009195	2.362206277	0.515168926
0.649227571	1.212860556	0.472739531	2.483266445	0.555100616
0.609789059	1.17641337	0.480453588	2.463480001	0.53633882
0.550643932	1.121188033	0.471097525	2.334007523	0.506434791
0.518696226	1.108692253	0.440808367	2.325447159	0.477156035
0.486667837	1.037651521	0.466362422	2.091026381	0.475561162
0.508834607	1.029475507	0.492967942	2.076839464	0.499384385
0.617344272	1.152488417	0.498796837	2.344094768	0.545188403
1.268749164	1.575687524	0.492597982	3.327578349	0.791402639
0.657029509	1.253412259	0.44155263	2.539074058	0.538741539
0.592145544	1.182095819	0.44249457	2.456568408	0.511212989
0.535424231	1.141674597	0.421097767	2.287998369	0.472976366
0.56321764	1.143196932	0.460711737	2.322742772	0.504721672
0.754640538	1.287439786	0.488034543	2.694925709	0.607121334
1.186574938	1.53556668	0.484842355	3.226140184	0.740985175
0.821679201	1.285383576	0.503349523	2.669693445	0.610337744
0.967763006	1.331973904	0.46877268	2.573076558	0.691948223
0.516671307	1.078736682	0.467939306	2.201282756	0.488724103
0.547507014	1.132656172	0.455770692	2.213798817	0.496292366
0.505004953	1.09676218	0.436662481	2.246310033	0.468194194
0.862888316	1.391231196	0.459920525	2.910289047	0.632790162
0.819245674	1.257178747	0.49896033	2.722105426	0.648426824
1.630952381	1.441619419	0.56558642	2.919807885	1.027777778
0.748590057	1.265044492	0.486028164	2.611640218	0.581199589
0.678107215	1.243282516	0.475259601	2.627442491	0.565072344
0.841804213	1.35709956	0.463365606	2.757776256	0.61235521
0.50502023	1.071711196	0.462901341	2.220288683	0.48201384
0.502536798	1.04175416	0.48138319	2.10130873	0.490868195
1.668756693	1.646779282	0.508779348	3.519247551	0.944084587
0.847310518	1.375805635	0.475880893	2.834039806	0.635406897
0.826007539	1.264293958	0.484370811	2.333146994	0.643801284
0.875083486	1.358629626	0.506298509	2.895540947	0.671064083
0.591130119	1.162907739	0.471130084	2.453530658	0.527130101

log.sigma.0.5.mm.3D_glcm_DifferenceVariance	log.sigma.0.5.mm.3D_glcm_Idn	log.sigma.0.5.mm.3D_glcm_Idm	log.sigma.0.5.mm.3D_glcm_Correlation	log.sigma.0.5.mm.3D_glcm_Autocorrelation
0.347951713	0.911928466	0.738830933	0.108417753	10.25502236
0.286109737	0.91699793	0.752079652	0.092651543	10.92339117
0.27827288	0.897920715	0.79584143	0.110376922	4.545320444
0.599492438	0.934914981	0.694681593	0.036301678	18.20127008
0.340536382	0.877748094	0.755496188	-0.003261369	4.409126422
0.317483517	0.911748531	0.780606504	0.012836081	4.601442138
0.617803812	0.84315172	0.550194761	-0.069228527	9.081014156
0.4159977	0.920684128	0.729229891	0.108674011	9.327192848
0.829103484	0.89910404	0.629960901	0.127672988	19.16964434
0.328010634	0.928316072	0.752155217	0.047206099	5.450496511
0.276156331	0.896776868	0.793553735	0.06611275	4.510108861
0.345582367	0.925170859	0.741778583	0.067751895	5.281847984
0.299216159	0.925220255	0.777196246	0.041652079	10.73594504
0.264193271	0.932862512	0.832933134	0.038298194	4.685849805
0.534983848	0.905139889	0.680969144	0.022995308	10.6519597
0.26013763	0.899945948	0.750062964	0.019099021	6.007116555
0.349140801	0.914874988	0.789121407	0.00462391	4.01664987
0.2746087	0.925620269	0.777687484	0.10559907	10.78181611
0.372788881	0.905334593	0.765547323	-0.020063861	4.028588983
0.249646973	0.913341467	0.783445969	0.112910729	5.649153061
0.271521789	0.94602365	0.758175169	0.031899987	11.4799491
0.408927969	0.935772213	0.750295909	0.003181777	10.55656588
0.248190055	0.915956447	0.789991643	0.083296497	5.467653575
0.652426834	0.889118302	0.632530969	0.131266153	10.62620523
0.257031296	0.886041306	0.772082612	0.083617484	5.463287522
0.27737975	0.885771273	0.771542546	0.142817682	4.875154464
0.288980947	0.916926659	0.751986075	0.034230291	5.738714653
0.266560494	0.927974943	0.748568803	0.019511345	5.886877952
0.291457155	0.928836779	0.752630747	0.086215881	11.34524962
0.269731052	0.903047576	0.757992063	0.068704246	5.579729493
0.273461702	0.919445602	0.759078987	0.042320829	5.755499575
0.251355349	0.923414946	0.770385737	0.066547854	11.55384403
0.392388934	0.929147871	0.723476112	0.082660732	10.71263318
0.393461538	0.809450549	0.534358974	-0.196121688	5.054487179
0.529760434	0.894404425	0.693353438	0.029594579	8.863004804
0.323437997	0.926949959	0.747193243	0.07927429	10.54912745
0.265945718	0.927582098	0.747177184	0.055516155	11.82350163
0.329848708	0.925681034	0.743080824	0.028075283	11.43220027
0.422603873	0.885012083	0.715927537	0.015849508	4.684063512
0.315465529	0.91725394	0.753768236	0.131613098	10.28806607
0.33897027	0.928854708	0.754583952	0.086019518	10.95025227
0.344937999	0.923118624	0.772418312	0.07970554	9.67241155
0.410850802	0.921718314	0.732621896	0.071642238	9.98281855
0.488473156	0.930798481	0.734106533	0.081970447	9.621399683
0.711196754	0.907756511	0.655950962	0.038430678	10.9125161
0.251225893	0.916449307	0.749430884	0.005919065	12.0109841
0.954564358	0.92995554	0.657910437	0.027742351	18.91494628
0.328973483	0.916102296	0.750647511	0.083177714	9.991734738
0.396895776	0.837457912	0.59950383	-0.062082465	5.728432678
0.432513398	0.866961244	0.671272528	-0.012630227	8.593903323
0.32171622	0.912544923	0.739986809	0.026414046	5.694314061
0.320225249	0.915287303	0.747945563	0.132012255	9.757189001
0.380171354	0.939749782	0.73538534	0.081382945	28.40717981
0.294337696	0.902806449	0.757769374	0.064693849	4.9691755
0.267353411	0.935710576	0.743659773	0.029014441	11.87963275
0.513111409	0.908432996	0.691178377	0.066713076	9.257635367
0.614095709	0.906520045	0.689248179	0.074871257	9.236197938
0.697035966	0.891007746	0.593978177	0.078513582	16.18295582
0.322596434	0.913371702	0.742360134	0.100964171	10.69250453
0.270021069	0.907979775	0.770362718	0.040640089	5.357406971
0.296545524	0.915442517	0.747707841	0.112479404	10.87198689
0.337930542	0.932555265	0.734390042	0.049228139	11.42149768
0.269738093	0.917074218	0.751825152	0.026960971	5.870392958
0.265309605	0.922035551	0.766634942	0.101302781	11.69237928
0.27384173	0.891979109	0.783958218	0.103711197	4.24649945
0.427222414	0.917992145	0.720390126	0.065627678	11.11163839
0.687630294	0.866333885	0.618230561	-0.025049297	8.187670678
0.286604432	0.915009787	0.74610967	0.107969099	11.38963083
0.33939694	0.865931194	0.731862387	0.03530125	4.198949239
0.321875195	0.924646907	0.739138006	0.082208753	11.14607437
0.293485691	0.92842376	0.75118918	0.095150413	11.33337204
0.289359052	0.882788001	0.765576002	0.139729884	4.835181205
0.259942776	0.921000321	0.763330086	0.061731022	11.60252426
0.259077902	0.937699161	0.751207827	0.01771997	5.932679334
0.319503693	0.932785642	0.734449512	0.009445786	11.95652093
0.640547603	0.917003041	0.650475756	0.008515239	16.45020764
0.366252502	0.912978378	0.742458028	0.053625398	9.956972459
0.328424739	0.916708937	0.752486761	0.113444529	9.816714698
0.309517937	0.922636561	0.769756603	0.086433335	10.40797539
0.30788755	0.928903728	0.75346599	0.063618983	11.15969461
0.383094966	0.902249899	0.711191253	0.051032687	9.220189636
0.636606648	0.922188423	0.671895485	0.032583051	18.54151792
0.448723445	0.957313982	0.713626573	0.032300215	89.02824096
0.456304614	0.870584401	0.681607367	0.013432577	7.568272753
0.277578454	0.919181218	0.758407964	0.07195827	5.760650109
0.300519063	0.918470454	0.756964142	0.022064013	5.481022768
0.28476715	0.907579986	0.769583979	0.113026342	10.60479105
0.461475622	0.913556193	0.706461832	0.058152942	9.091520351
0.366121129	0.846434237	0.692868473	0.076799264	4.651575765
0.572310406	0.813756614	0.546428571	-0.189008379	6.051587302
0.410436616	0.943266249	0.725013279	0.06578589	40.54447704
0.358023702	0.89064333	0.728767315	0.131811476	10.64501353
0.465126891	0.916308407	0.716083063	0.022754987	10.61707222
0.272357169	0.920204089	0.761290237	0.099310907	11.29861835
0.261359511	0.918460975	0.755732763	0.036133762	5.798297137
0.759457274	0.876933325	0.598463041	0.015861408	8.695275417
0.442967585	0.92342085	0.703387695	0.028520251	10.7021697
0.394051124	0.877313285	0.696319983	-0.127677034	6.566762724
0.422037624	0.892909589	0.684869899	0.042378754	9.564350795
0.311106423	0.871417476	0.742834952	0.148428574	5.912383688

log.sigma.0.5.mm.3D_glcm_SumEntropy	log.sigma.0.5.mm.3D_glcm_AverageIntensity	log.sigma.0.5.mm.3D_glcm_Energy	log.sigma.0.5.mm.3D_glcm_SumSquares	log.sigma.0.5.mm.3D_glcm_ClusterProminence
1.866039468	3.19613492	0.233396167	0.360734711	1.98409358
1.704623996	3.300838824	0.251797605	0.297754241	1.445485036
1.598580026	2.125168284	0.338807842	0.25485165	1.097788898
2.096924591	4.263922177	0.185885914	0.553114423	6.378974729
1.638673396	2.099507449	0.285582581	0.301436838	1.182815943
1.565513751	2.144201784	0.337873054	0.265284841	1.042362293
2.175775333	3.021188081	0.110593327	0.800111204	6.108927443
1.962871645	3.046459512	0.228540337	0.420118543	3.401813491
2.543636867	4.364853821	0.106033737	0.920986818	13.90739788
1.694637463	2.331504736	0.259750939	0.309263071	1.73923545
1.546989345	2.119927358	0.351234803	0.24233143	0.946056294
1.791891195	2.293269464	0.240268222	0.339133186	1.917503162
1.577472633	3.274871919	0.306855825	0.26486842	0.940525235
1.32632885	2.162918295	0.440710083	0.198699081	0.739944877
2.051185889	3.26199075	0.186340177	0.525936835	5.015164149
1.561673086	2.449922128	0.24828971	0.261442944	0.657970362
1.578518229	2.003565734	0.352808429	0.274267008	1.172549709
1.637594335	3.279277317	0.287622049	0.267125298	1.011631196
1.636523069	2.00855243	0.310811895	0.303072504	1.304393582
1.556772393	2.370889236	0.277549816	0.247787397	0.683772872
1.574224999	3.386971998	0.260626128	0.263514485	0.783616158
1.723496074	3.248904168	0.282037993	0.347485952	2.363341942
1.482819677	2.334147435	0.304725472	0.232838988	0.589343505
2.385536432	3.243540052	0.11087935	0.782709541	9.127960774
1.581205773	2.332771851	0.278101583	0.255134397	0.840350157
1.719246688	2.198611565	0.279281189	0.287650947	1.290723278
1.628380556	2.393541893	0.252584044	0.281161232	0.989310359
1.580466262	2.425202147	0.248682852	0.26699146	0.751008454
1.707403	3.364393233	0.241596099	0.299535236	1.252424157
1.634756945	2.358139239	0.256387827	0.273146786	0.906560145
1.591801309	2.396703903	0.256820464	0.266559336	0.776073278
1.538325642	3.396644647	0.268490541	0.249132794	0.623334389
1.924390724	3.267972057	0.208750429	0.399148643	2.516932554
1.521418828	2.270512821	0.202548077	0.675138889	3.37644042
2.018782576	2.974217206	0.202659374	0.511001335	3.817017731
1.775248085	3.243995818	0.247776798	0.322833013	1.505273396
1.636585196	3.436285523	0.239775842	0.278154839	0.922101499
1.707976206	3.379852195	0.241904695	0.314510294	1.318736853
1.876459466	2.162761878	0.217174692	0.401918354	2.145467351
1.811300412	3.20069479	0.250253391	0.329632812	1.822981707
1.773530727	3.304898259	0.248325062	0.327396815	1.591475423
1.720352427	3.106110661	0.303466229	0.309775326	1.743001366
1.904429781	3.155030261	0.235361157	0.395472885	2.74310021
1.939961144	3.095718375	0.242144832	0.449389667	5.518859195
2.249773617	3.29926783	0.160387454	0.698716143	9.301228597
1.521169766	3.465469446	0.251088894	0.253790561	0.550968029
2.307089929	4.346457612	0.164710858	0.845789818	20.0178808
1.771392966	3.156671114	0.264251205	0.324440197	1.646115681
1.79599574	2.397460872	0.166854007	0.566302647	2.840626715
1.947900342	2.932311846	0.166194245	0.459854922	2.29504882
1.708739057	2.384546049	0.238517294	0.312310331	1.327313369
1.83971585	3.116429895	0.245187117	0.339818077	1.785953064
1.862045309	5.326967425	0.224090454	0.374483597	3.015597049
1.66914743	2.224887835	0.276392375	0.288287492	1.217890215
1.612214661	3.445516532	0.241641425	0.275298147	0.893487208
2.089504209	3.036958228	0.181758215	0.519829413	4.465570079
2.177202599	3.031655697	0.188393223	0.599301187	6.94536218
2.472168328	4.013094328	0.094739184	0.865499345	10.88871654
1.788025809	3.264735108	0.241842056	0.33624635	2.087986975
1.545584441	2.312417546	0.291870706	0.254514614	0.754905119
1.776192219	3.291861904	0.235914232	0.316060957	1.423106613
1.776374349	3.377124865	0.225326598	0.336627901	1.706986268
1.586722678	2.421392042	0.252097743	0.267513621	0.751682765
1.638917213	3.415386903	0.249781877	0.270762591	0.843463544
1.602727673	2.053517951	0.329184494	0.262467231	1.105433058
1.933471857	3.329247691	0.20365771	0.41909853	3.047593286
2.20828236	2.864143655	0.133426886	0.716463055	6.165123911
1.754185913	3.369887689	0.230023597	0.309525928	1.384773465
1.757625981	2.046064071	0.245258202	0.33703577	1.409627404
1.790878225	3.334454784	0.228257014	0.332304416	1.666895459
1.728850952	3.362159913	0.237795413	0.304406199	1.31464446
1.749402497	2.189339615	0.269549917	0.301190219	1.473535623
1.578843192	3.403894838	0.25769666	0.259333459	0.707723095
1.554785688	2.434761186	0.251496521	0.259023186	0.664056802
1.687349028	3.457404945	0.230202456	0.311539681	1.380829905
2.19865115	4.054934827	0.153132914	0.641333427	6.554668655
1.79781232	3.15247785	0.254722112	0.347129504	1.866618262
1.797572904	3.127129309	0.260295136	0.333560431	1.84816994
1.688469959	3.221984425	0.29196229	0.294150762	1.404759904
1.701349225	3.337768333	0.250973806	0.300556053	1.241252443
1.885180364	3.032930928	0.216819988	0.397964335	2.108498382
2.175644016	4.303640045	0.16317059	0.613226227	6.880018449
1.878612194	9.434754806	0.203690023	0.424451784	5.21882511
1.781260482	2.749732426	0.233760023	0.48893319	3.218567677
1.647607385	2.395945767	0.248677868	0.278394851	0.940673495
1.612989358	2.339830613	0.270900947	0.279929355	1.013240389
1.691194347	3.251547293	0.272362852	0.284606153	1.100288318
2.005703805	3.010710383	0.207007871	0.458173231	3.333812026
1.899204857	2.149137215	0.181950048	0.441160668	2.378665681
1.866197667	2.486111111	0.156840514	0.685736332	2.995329545
1.865404667	6.365337618	0.210950429	0.401136565	4.369781235
1.915856289	3.254549606	0.206848882	0.391520503	2.530888054
1.911604401	3.256997774	0.21906852	0.429841732	3.15235975
1.673340819	3.357185277	0.253549486	0.280384637	1.069415899
1.574825871	2.406005343	0.255528746	0.260684706	0.70622872
2.312707523	2.946631607	0.108015682	0.844515419	8.6200399
1.933021074	3.269499613	0.205880634	0.436116524	3.466840302
1.548220751	2.564210469	0.238882201	0.367120765	1.166275991
1.980687739	3.089487715	0.182735257	0.45633409	2.668052038
1.820545814	2.420814353	0.209544853	0.347443859	1.657854468

log.sigma.0.5.mm.3D_glcm_SumAverage	log.sigma.0.5.mm.3D_glcm_Imc2	log.sigma.0.5.mm.3D_glcm_Imc1	log.sigma.0.5.mm.3D_glcm_DifferenceAverage	log.sigma.0.5.mm.3D_glcm_Id
6.39226984	0.259325496	-0.027575869	0.542465835	0.744746139
6.601677649	0.200679713	-0.022718991	0.503184835	0.754356438
4.250336568	0.217940392	-0.024524904	0.415623593	0.798276914
8.527844353	0.282570821	-0.027436065	0.682626508	0.707961398
4.199014897	0.265277118	-0.034129551	0.508014918	0.761831953
4.288403568	0.202480225	-0.020479917	0.452836549	0.785064357
6.042376162	0.558899424	-0.112060036	1.032491088	0.585765239
6.092919024	0.305050125	-0.036423698	0.575831981	0.738118052
8.729707641	0.336784285	-0.031889865	0.881058841	0.656753186
4.663009472	0.156302264	-0.011581017	0.510921701	0.755755688
4.239854715	0.16857828	-0.016836355	0.419586088	0.795784921
4.586538927	0.214596359	-0.020760268	0.535125357	0.746377229
6.549743838	0.133525657	-0.009055094	0.455921984	0.780345144
4.325836591	0.203972199	-0.029218542	0.342162351	0.835232035
6.523981499	0.294055791	-0.030357591	0.702039571	0.695365063
4.899844255	0.102551777	-0.005963713	0.502047058	0.750727753
4.007131469	0.248845932	-0.03053361	0.442437134	0.795277592
6.558554634	0.179864153	-0.016635278	0.45020698	0.779411614
4.01710486	0.234371451	-0.026062201	0.493829361	0.773386883
4.741778471	0.156804255	-0.017200098	0.434189057	0.78379854
6.773943997	0.124918703	-0.007868835	0.48790022	0.759322125
6.497808336	0.230265466	-0.025233271	0.530696586	0.757904632
4.66829487	0.135430843	-0.012887974	0.421167164	0.790358831
6.487080105	0.480576864	-0.073170013	0.838171253	0.658522066
4.665543702	0.168120643	-0.015936526	0.457791971	0.772735011
4.39722313	0.230231808	-0.026878858	0.462960861	0.773557865
4.787083785	0.129088421	-0.007752315	0.503861613	0.754191377
4.850404294	0.116933111	-0.007369762	0.506216072	0.749454878
6.728786467	0.203481587	-0.023011629	0.503385322	0.754976418
4.716278478	0.159799178	-0.012615261	0.488122862	0.759252997
4.793407807	0.122576453	-0.007431614	0.486577205	0.760319906
6.793289294	0.121964505	-0.011499077	0.460196549	0.770696237
6.535944114	0.245152978	-0.023040829	0.582642712	0.730691364
4.541025641	0.852354955	-0.480202859	1.043589744	0.566987179
5.948434413	0.398559179	-0.058800463	0.675783094	0.70827239
6.487991635	0.167995245	-0.012227666	0.520395591	0.751501923
6.872571046	0.162859684	-0.012775818	0.508944013	0.748145724
6.759704389	0.170955278	-0.012645956	0.530061696	0.747517154
4.325523757	0.21586224	-0.018920752	0.605439862	0.727346215
6.40138958	0.23619069	-0.025099822	0.505759769	0.7577012
6.609796519	0.245168586	-0.026936671	0.508793332	0.759142385
6.212221323	0.26890233	-0.032962738	0.47440708	0.77733382
6.310060523	0.249253557	-0.024890558	0.567405492	0.740075704
6.191436751	0.30801196	-0.03797443	0.578459349	0.743540252
6.598535659	0.225312123	-0.01602124	0.793953088	0.677465794
6.930938892	0.096332652	-0.005736282	0.50171373	0.749619215
8.692915224	0.276822339	-0.023837564	0.830555702	0.678755595
6.313342227	0.218817579	-0.022444547	0.514744024	0.755763646
4.794921745	0.704201888	-0.239340263	0.865806416	0.619505957
5.864623692	0.249769709	-0.023963955	0.703147131	0.68628718
4.769092099	0.165937683	-0.012202692	0.534564436	0.743456229
6.232859789	0.22687472	-0.023307359	0.518427282	0.752540258
10.65393485	0.219773761	-0.020185493	0.554533822	0.7407934
4.449775671	0.229071934	-0.025281117	0.49350027	0.760782379
6.891033064	0.145381019	-0.011565408	0.516277248	0.744679647
6.073916456	0.317020082	-0.034910785	0.675558095	0.70475162
6.063311395	0.270032398	-0.025793208	0.701675657	0.706423898
8.026188656	0.326528838	-0.030265818	0.939919581	0.623968499
6.529470216	0.23147174	-0.023616119	0.530192397	0.746796005
4.624835093	0.173967141	-0.018219144	0.46423392	0.772015837
6.583723809	0.181790604	-0.014518712	0.513983705	0.750665879
6.754249731	0.195128999	-0.016035981	0.549232593	0.738981461
4.842784084	0.09846257	-0.005275443	0.500392222	0.753056019
6.830773806	0.176179099	-0.01522051	0.470044543	0.767476762
4.107035901	0.359337499	-0.067496674	0.438268356	0.786019815
6.658495382	0.288531421	-0.032869885	0.595688139	0.728393817
5.72828731	0.455307196	-0.067549498	0.879781754	0.64419909
6.739775377	0.186173644	-0.015257826	0.515168926	0.748449153
4.092128142	0.234481603	-0.026175986	0.555100616	0.738137518
6.668909569	0.196153715	-0.016584533	0.53633882	0.743156598
6.724319826	0.184141294	-0.016280144	0.506434791	0.753785861
4.378679229	0.222616791	-0.024929169	0.477156035	0.768345348
6.807789675	0.133626786	-0.009153666	0.475561162	0.764015515
4.869522372	0.089395669	-0.004091169	0.499384385	0.75165367
6.91480989	0.191791468	-0.016201009	0.545188403	0.737531229
8.109869655	0.322049929	-0.032947467	0.791402639	0.670117372
6.3049557	0.210646153	-0.01902022	0.538741539	0.74967545
6.254258617	0.278619804	-0.036039449	0.511212989	0.757660169
6.443968851	0.225721255	-0.025388777	0.472976366	0.773627211
6.675536665	0.201543851	-0.01828753	0.504721672	0.756611929
6.065861855	0.340016781	-0.046503776	0.607121334	0.719967836
8.607280089	0.307914903	-0.031339912	0.740985175	0.688007851
18.86950961	0.295172029	-0.034681093	0.610337744	0.720090404
5.499464851	0.619122438	-0.19012516	0.691948223	0.698071942
4.791891535	0.158198843	-0.01269398	0.488724103	0.759888933
4.679661225	0.118295923	-0.006843966	0.496292366	0.759926939
6.503094585	0.184299597	-0.016604482	0.468194194	0.771966273
6.021420767	0.226322543	-0.018801624	0.632790162	0.718826657
4.298274431	0.413427104	-0.083086158	0.648426824	0.704256396
4.972222222	0.772921729	-0.275655534	1.027777778	0.579695767
12.73067524	0.220783246	-0.01978727	0.581199589	0.731039672
6.509099212	0.234244112	-0.024390577	0.565072344	0.735340209
6.513995548	0.295435986	-0.033489313	0.61235521	0.726074407
6.714370554	0.185657802	-0.016618611	0.48201384	0.762717087
4.812010685	0.105467399	-0.005645998	0.490868195	0.756433903
5.893263213	0.594106391	-0.12151722	0.944084587	0.626227605
6.538999227	0.20914641	-0.016059902	0.635406897	0.714798666
5.128420938	0.55638509	-0.161396228	0.643801284	0.708467067
6.17897543	0.296343503	-0.033131193	0.671064083	0.697015161
4.841628707	0.220598865	-0.024432397	0.527130101	0.747101619

log.sigma.0.5.mm.3D_glcm_ClusterTendency	log.sigma.0.5.mm.3D_firstorder_InterquartileRange	log.sigma.0.5.mm.3D_firstorder_Skewness	log.sigma.0.5.mm.3D_firstorder_Uniformity
0.799834507	16.8581128	-0.519156405	0.460931086
0.651111434	5.296886548	-2.041667393	0.48850366
0.567250742	13.9847517	-0.907650915	0.557023844
1.146339107	14.10954416	0.311987756	0.395590888
0.602695966	17.42427635	-0.087330494	0.489461817
0.53805503	15.4756259	0.02165422	0.547198334
1.498485082	38.52377653	-0.289324169	0.295102041
0.932397863	21.22642589	-0.021248259	0.433423921
2.076848653	23.52155268	0.427593731	0.317475268
0.647632984	8.703526407	0.244569887	0.503610146
0.516271844	15.94657181	-0.282408998	0.568087936
0.723654684	11.63462722	0.104843198	0.47041527
0.55197932	9.93390625	-0.014032398	0.537493917
0.412490882	10.69472669	-5.22E-05	0.618485996
1.074229343	21.34408003	0.283994662	0.396947832
0.532859794	2.756673992	-0.999156831	0.495206523
0.551231158	17.35916328	0.518822303	0.528170061
0.59038447	10.05976066	-0.850461677	0.537280629
0.593840624	18.01087117	0.093275653	0.47343316
0.551555558	3.126861244	-1.614635938	0.522903477
0.543739307	4.351289183	-0.069208302	0.509682254
0.697210011	13.9688381	0.697941207	0.51422821
0.504436533	4.491942063	-1.077062066	0.555109542
1.770276707	29.77973318	0.174046089	0.322516818
0.552959641	7.154539689	-1.584048763	0.534826389
0.657413156	14.45285553	-0.869884737	0.528507522
0.581614498	6.233169436	0.315193165	0.496856297
0.54449518	2.870967865	-1.409671203	0.493493683
0.651142359	2.877686486	-1.397085245	0.477681825
0.583929338	5.164206684	-1.633443487	0.504550372
0.555687727	5.393605709	-0.395616415	0.509368582
0.531494512	4.038683772	-1.173812501	0.518514836
0.863963574	13.2040267	-0.162687755	0.42722958
1.09542735	28.95500374	0.027639121	0.315555556
1.053359125	22.82997918	0.392424754	0.394627614
0.696748326	12.54328608	-0.342185694	0.482057322
0.587164826	2.67858386	-2.47980079	0.481948518
0.646575591	6.442180246	0.378856049	0.477743341
0.815758874	19.4346596	0.132661733	0.447695577
0.746290266	16.22601215	-0.926923977	0.4757897
0.71040452	9.309906512	-0.229271489	0.474364517
0.668120813	18.13394809	-0.103462139	0.501381225
0.847509125	17.93621795	0.049908648	0.45645796
0.973380408	19.24528599	0.4571538	0.452825587
1.451892518	21.24172962	0.830502598	0.380445213
0.510571022	4.566674173	-2.00866394	0.498934173
1.737928701	17.72202951	1.258255034	0.380523296
0.70282153	18.41500109	-0.024938193	0.496999077
1.075333797	29.81644011	0.043502364	0.351239669
0.907811625	25.72765182	0.113964078	0.372490148
0.641095135	5.769051135	0.098916401	0.480299765
0.769252986	18.33202639	-0.747221546	0.475915744
0.810010314	8.633635879	-0.917346264	0.459080087
0.614454616	13.2881285	-0.442619887	0.497102642
0.566676369	3.804226756	-1.508345261	0.483172624
1.107465123	22.75985324	-0.167662949	0.377981733
1.286037872	21.35907441	0.501657227	0.397706948
1.875996316	32.17223251	-0.078989618	0.287532656
0.740229678	12.85061839	-1.076760648	0.473219701
0.529027754	10.03688827	0.222969273	0.545202527
0.703263184	12.39579737	-1.035835248	0.472970796
0.706242118	4.554179698	0.063857994	0.457521964
0.549449629	5.223070681	0.222204318	0.495322662
0.596363711	4.217749834	-1.302758168	0.497677234
0.58067661	16.59069657	-0.3929592	0.532929868
0.893362378	12.53175223	-0.11514224	0.418924214
1.399654168	26.29059172	0.255721933	0.325351474
0.685922672	6.947542727	-1.33688044	0.467823574
0.698915509	18.84726524	-0.448615839	0.465010514
0.719428604	8.442586988	-1.031201174	0.46042057
0.666980864	9.17036587	-0.93031854	0.477512365
0.686064651	15.87055594	-0.793654567	0.513700313
0.550666	2.784921587	-1.619263954	0.50520743
0.527258135	4.293280393	-0.9316029	0.500769308
0.628814453	5.901175141	0.455946133	0.467511597
1.296584546	23.98074007	0.076749512	0.35743731
0.731488509	17.67825155	-0.095005792	0.475752155
0.742096178	16.2140632	-0.45786392	0.50518176
0.641178815	15.02815202	-0.442948934	0.525023123
0.639006571	5.48994863	-0.411765366	0.484458155
0.8372168	19.55264235	-0.368372902	0.463177334
1.266329972	19.56605434	0.191299745	0.360197801
0.876127934	5.863369465	-0.921862992	0.426922144
0.987969754	15.05552769	-0.227942304	0.433922997
0.596908098	5.237945169	-0.713643657	0.492681177
0.572210408	9.001866162	0.131262174	0.512295625
0.633419658	15.80156508	-0.865882614	0.526170013
0.96980461	20.38022757	0.161741874	0.419003174
0.945396996	17.21532154	-0.366006545	0.3992
1.111992945	26.38399506	-0.138887958	0.325443787
0.855956203	7.231724143	-2.050938569	0.441875904
0.887974797	18.32793486	-1.146248027	0.436764197
0.877562715	13.2196611	0.34085263	0.447600777
0.616518316	5.731372371	-1.314630657	0.492661825
0.540202026	5.10254544	-1.088933534	0.505981331
1.709304982	28.94385099	0.229525981	0.314184933
0.897155578	15.39855039	0.402546515	0.415536332
0.642475519	21.14693522	-0.277384356	0.4375
0.950252874	21.48998034	-0.313279874	0.416989725
0.798645317	12.06464642	-1.291118008	0.432755102

log.sigma.0.5.mm.3D_firstorder_MeanAbsoluteDeviation	log.sigma.0.5.mm.3D_firstorder_Energy	log.sigma.0.5.mm.3D_firstorder_RobustMeanAbsoluteDeviation	log.sigma.0.5.mm.3D_firstorder_Median
10.66316212	90652.36727	7.216339996	-3.007504463
6.677834947	160640.2472	3.281998409	-1.138796329
8.910202425	37218.64925	6.117896561	-5.890553951
12.2846428	696678.3978	6.801191691	-1.784407854
9.874608137	30783.56135	6.849382505	-12.90151024
9.484264465	56730.03886	6.49249742	-8.955046654
19.90645483	46464.00103	15.31129453	-7.723807573
12.69811755	176352.4292	8.925086961	-11.88749027
16.53250855	728544.489	10.82773253	-4.228366137
7.069166677	295886.7102	3.907699868	-2.057159901
9.036823999	81351.80284	6.453378174	-8.77314949
8.695935112	136170.8569	5.089615995	-2.572307825
7.311928457	168405.5183	4.374130174	-3.038632154
7.275859798	71387.85192	4.592335536	-4.987263203
13.73080718	137381.3446	8.919408846	-7.510146379
2.789519965	318556.4759	1.191664264	-0.276787251
10.25797558	70439.43602	7.007275979	-12.53361702
6.901442372	171968.4645	4.499513737	-2.226110816
11.75634848	64628.19483	7.823556989	-11.13437748
4.104424004	126136.9968	1.800348632	-0.462021202
4.585722934	312520.5351	2.225338034	-0.918214202
9.52894091	192154.9374	5.95601943	-5.297622204
4.211731103	133425.7657	2.196679792	-0.696029335
16.63103782	64771.39277	11.80445835	-6.928987503
5.693823212	75252.8055	3.352577577	-1.706637323
8.334749928	52537.64359	5.947472957	-5.065522909
5.77793077	200695.3513	2.927039628	-1.288216114
3.38066384	165735.417	1.295514304	-0.419795662
5.706388125	348808.6894	2.240011137	-0.445490271
5.489164278	136837.0764	2.662088034	-1.091191053
4.672683694	245587.4192	2.470361894	-1.032278657
3.714558678	323845.856	1.891623442	-0.747606277
10.1926986	327618.3542	6.028184902	-1.562948108
16.61972992	18501.42493	11.77409681	-29.58858871
14.30958489	57474.95125	8.820405018	-12.05822277
8.743123791	230963.5828	5.611599322	-2.707286239
3.665554481	344127.2108	1.18869267	-0.304838628
6.647990928	366387.5536	3.2497537	-1.085484087
11.97803603	96995.71622	8.322679081	-8.786998749
9.926933849	118513.5649	7.014361481	-3.431704521
7.963126259	109855.8839	4.401518372	-1.751423597
10.5975187	114787.9046	7.33306031	-9.664346695
11.1646924	252748.086	7.67984991	-7.082167864
11.74455517	155827.9544	8.016835913	-8.598047256
14.61974036	471886.2594	9.150459489	-7.409464836
3.327860521	158113.9027	1.955791417	-0.443131685
14.09332651	1298852.375	7.838459045	-4.39848876
10.49387573	63278.26486	7.584189504	-8.771320343
16.23419963	20344.46278	12.42422482	-25.63783932
14.38237723	69974.31936	10.48276582	-12.48960447
5.986366306	409637.6058	2.857845833	-1.008434057
10.27613926	104020.2669	7.537531199	-5.907015324
8.090684323	538944.2527	4.354268473	-1.924267054
8.64447094	43785.03534	5.577888154	-5.554978371
3.708019596	544917.0095	1.627441074	-0.356639922
14.54222494	167276.5469	9.936963252	-8.743210793
13.89849009	454343.6746	8.856604319	-14.16260958
18.90706563	328878.6445	13.43353979	-11.88103437
9.299656041	70247.19821	5.782123476	-3.431462407
7.16555443	53464.0497	4.446474086	-4.190906525
8.463169862	231680.5247	5.546759497	-2.650827169
6.482543258	846152.8502	2.79286033	-0.70136109
4.611656849	231887.2475	2.302385582	-0.717326015
4.977104906	119554.8562	2.263274184	-0.590783477
9.673481217	13546.27406	6.952103384	-8.933653831
9.957970193	262400.7026	5.519160321	-1.85301733
16.6836582	75640.8444	11.28460879	-18.29009247
6.885490921	399459.9033	3.632707587	-1.118786931
10.77795096	46365.95758	7.917659809	-10.59426212
7.774291123	263735.939	4.252955284	-1.475990653
7.106376563	298813.2287	4.197840252	-1.633402109
9.231582714	119956.7795	6.580315594	-5.899315357
3.840088254	319609.1633	1.516284009	-0.439772546
3.71617597	422198.3777	1.873922452	-0.587664068
5.288029838	1241712.717	2.53170052	-0.395116061
15.38638168	247780.965	9.913342418	-10.78298807
10.65846867	130865.9987	7.248871006	-8.612975597
9.277934337	56368.2386	6.70311105	-9.373478889
9.39405653	193677.7717	6.398293161	-5.26887989
6.624533554	319102.5963	3.206938239	-0.834213048
11.83782018	61732.86275	8.415785756	-13.32448959
14.11051947	373517.3953	8.311663094	-4.214984417
7.298751123	910103.8826	2.831099835	-0.423803627
12.29551097	20105.19466	7.681714944	-18.32970428
5.016998231	342564.2609	2.47690894	-0.863927484
6.775582834	367089.9326	3.939938603	-2.156224728
8.84466968	69892.91492	6.423467594	-5.921732426
12.2894909	252206.9596	8.317699998	-11.79012108
10.76021668	11225.62493	7.544153335	-3.483349562
16.8244411	10429.40475	10.39343896	-23.61711311
8.308616937	645511.5524	4.025995861	-0.948003411
10.90771867	81475.92862	7.335762209	-4.222060919
10.29592189	103109.3024	6.029158908	-2.862813473
5.989331096	329939.5172	3.011766358	-1.087625504
4.449391808	368707.7871	2.333004371	-0.920699
16.09534939	43622.08972	11.23581806	-14.53639412
11.11853783	174184.5587	6.748807392	-2.67329061
12.63729104	20410.83767	8.455180772	-21.44767189
12.44168488	55325.43897	8.506127578	-8.78840065
8.631764359	43031.77709	5.374054001	-1.756110966

log.sigma.0.5.mm.3D_firstorder_TotalEnergy	log.sigma.0.5.mm.3D_firstorder_Maximum	log.sigma.0.5.mm.3D_firstorder_RootMeanSquared	log.sigma.0.5.mm.3D_firstorder_90Percentile	log.sigma.0.5.mm.3D_firstorder_Minimum
2447613.916	39.78419113	15.84659556	1.789785624	-54.83870697
4337286.675	26.01974297	10.70035894	1.945739031	-58.67136002
1004903.53	17.2374649	14.50085703	1.527780032	-44.48679352
18810316.74	102.3444061	18.98454728	8.528258324	-87.91186523
831156.1563	15.86065197	17.45817104	4.209029675	-41.62080383
1531711.049	31.6008606	15.2479559	4.578016663	-41.65124512
1254528.028	32.52916718	25.76376221	20.0289711	-73.83895874
4761515.59	50.94273758	20.97097087	1.762871385	-67.47808838
19670701.2	62.34838486	22.2169716	26.13574219	-77.62229919
7988941.176	83.21783447	11.29567934	3.48618021	-48.07194138
2196498.677	23.30981445	14.70922084	1.637142718	-40.28820419
3676613.137	81.37725067	13.41201855	4.551483631	-48.91231918
4546948.995	39.8504715	11.1033188	5.180096865	-55.70695496
1927472.002	31.94349098	11.62773688	3.144096422	-42.62893677
3709296.305	64.81364441	19.42733323	15.2508316	-71.10142517
8601024.848	46.74681854	5.42399269	2.313941765	-47.71233368
1901864.772	27.62901115	18.01681703	2.785517311	-40.24297714
4643148.541	35.66634369	10.52636799	1.984111357	-51.94397736
1744961.26	34.9510994	18.84409647	8.695121241	-45.01713562
3405698.915	29.70088959	6.743230653	1.293495166	-43.3874054
8438054.448	120.0184174	7.847318077	2.287099791	-54.4677887
5188183.31	90.24645996	14.5393112	7.545985413	-56.05569458
3602495.673	48.00703049	6.759715627	1.159741116	-38.31001282
1748827.605	70.86416626	22.0681434	16.74522781	-61.60240555
2031825.748	20.24432945	8.853718939	2.3447052	-41.19609833
1418516.377	16.63170815	13.18960603	1.667249668	-41.05822754
5418774.484	60.4205513	9.363662681	4.333812809	-39.3011055
4474856.26	80.42755127	6.523098428	2.064890242	-46.55932617
9417834.615	62.15829849	9.688483301	1.339551032	-65.03394318
3694601.062	39.88352585	8.990284063	2.142070246	-48.41574478
6630860.318	65.85990143	7.645866607	2.929553747	-42.11186218
8743838.113	32.98021317	6.055012679	2.372006226	-52.6608429
8845695.564	81.77467346	15.3579408	3.558450031	-60.9741478
499538.473	4.280409336	35.1201983	-5.179057932	-62.39176559
1551823.684	48.09283447	22.55278003	9.012852669	-60.049263
6236016.735	73.58194733	13.15811989	2.717691612	-54.26248932
9291434.692	52.51231384	7.17156588	2.035864115	-54.2977066
9892463.947	59.4444313	10.99273497	3.969345617	-53.0895462
2618884.338	32.70804977	17.3829612	11.45253754	-48.16469955
3199866.251	28.19750786	14.85582887	2.245110559	-54.03264618
2966108.864	52.26277542	12.39534561	4.432803154	-62.85985947
3099273.423	43.61704636	17.35743614	2.479039192	-62.87542725
6824198.323	63.69972992	17.51379782	2.745806718	-53.04230881
4207354.768	81.44208527	18.84041326	2.90674243	-54.11424255
12740929	95.14427185	20.57222341	20.27836456	-60.31531906
4269075.372	29.84712982	5.082043365	3.823411202	-50.06461334
35069014.13	127.9463501	21.74851608	16.58729744	-96.5258255
1708513.151	44.28671265	16.65941924	3.493030524	-50.66161346
549300.495	5.6129632	30.40967458	-1.521079028	-51.7174263
1889306.623	23.12362671	21.89237675	11.9219346	-52.95951462
11060215.36	73.06874847	10.0200486	3.537431097	-49.4389534
2808547.207	33.0234108	16.06595177	1.64870491	-51.89702225
14551494.82	71.77534485	12.98782688	3.783789158	-114.5437775
1182195.954	38.6736145	13.53517583	4.065747166	-41.87966156
14712759.26	77.99381256	6.942416927	3.257881284	-51.48703003
4516466.766	68.31609344	22.34575968	4.79665556	-69.98690033
12267279.21	72.21242523	22.8262201	6.89679265	-71.13561249
8879723.401	50.35080338	26.79693346	16.99370899	-85.35222626
1896674.352	25.39934921	14.08681699	4.920760727	-56.04377365
1443529.342	44.11997986	10.71119603	5.767564058	-37.74299622
6255374.167	37.58851624	12.61866083	2.926181984	-50.52471542
22846126.96	88.23954773	11.17474733	3.106636882	-63.0185585
6260955.683	50.28657913	7.544455834	4.435035419	-41.88373566
3227981.117	36.74396133	8.190864864	2.445517254	-65.8527832
365749.3996	12.44716167	15.98718476	0.299911636	-40.96441269
7084818.969	74.98860931	15.09232877	9.03494978	-58.08041763
2042302.799	48.19132996	26.84006323	10.59166508	-70.58345032
10785417.39	49.07104111	10.73395266	3.232939434	-61.22169495
1251880.855	13.12132549	18.00659506	2.23620429	-49.65377045
7120870.353	58.15493774	12.13153218	3.523573923	-65.29673004
8067957.174	56.1000824	10.72251766	4.331388474	-59.97145462
3238833.046	19.37915421	14.54523925	2.724425125	-47.81439972
8629447.41	45.67942047	6.729759627	1.783965826	-50.10322189
11399356.2	107.1867523	6.242576375	3.174804974	-48.21606064
33526243.37	87.44764709	9.247880557	5.433433342	-65.78746033
6690086.056	79.04354095	23.57037764	10.75406933	-89.59329224
3533381.965	37.39666367	16.94068591	4.522584677	-64.20903778
1521942.442	28.17419434	15.86328851	2.318371677	-50.68227005
5229299.836	41.11824036	14.56479919	2.838112593	-57.83019638
8615770.1	68.1146698	10.8633013	1.914849722	-63.38186264
1666787.294	27.46323395	20.35471727	2.732631636	-53.84739304
10084969.67	84.39937592	20.78008705	12.17834396	-85.15961456
24572804.83	86.53510284	13.58832966	5.658820915	-200.8304901
542840.2559	22.5484848	25.46673647	0.987012565	-59.25199509
9249235.043	61.63721848	8.308883045	3.115786719	-49.54650879
9911428.179	61.48769379	10.23100635	5.765807343	-46.31689072
1887108.703	17.47072411	13.83789862	2.259296465	-58.40779114
6809587.908	51.47809219	20.69287746	4.132536507	-65.23464966
303091.8731	24.00700188	14.98374114	5.948103237	-41.63122559
281593.9283	16.4903183	28.32422822	2.833814025	-58.54723358
17428811.91	70.49519348	13.7204472	4.473382378	-144.3312531
2199850.073	21.55959129	16.37110024	4.403925276	-62.51430893
2783951.164	58.88351822	15.74353518	7.659902096	-54.82836914
8908366.966	37.4458046	9.504992596	2.735027409	-55.41107178
9955110.252	56.81300354	7.14516748	3.262399364	-42.9209938
1177796.422	50.26392365	26.31376299	2.69960413	-66.5762558
4702983.085	83.26795959	16.00480437	15.71934299	-58.31973648
551092.6171	8.30766201	29.1625028	-5.66227684	-63.62779617
1493786.852	27.45644379	18.42335124	10.837006	-61.77073669
1161857.982	15.95995998	12.39697214	5.842931795	-48.8089447

log.sigma.0.5.mm.3D_firstorder_Entropy	log.sigma.0.5.mm.3D_firstorder_StandardDeviation	log.sigma.0.5.mm.3D_firstorder_Range	log.sigma.0.5.mm.3D_firstorder_Variance	log.sigma.0.5.mm.3D_firstorder_10Percentile
1.394536808	13.56675214	94.6228981	184.0567637	-26.70902824
1.218293735	9.672244251	84.69110298	93.55230886	-17.68157349
1.129449259	11.09277382	61.72425842	123.049631	-25.09772072
1.671760439	18.05895858	190.2562714	326.1259849	-28.79163933
1.2674031	12.47479739	57.4814558	155.6205698	-26.85370445
1.183538738	12.00948826	73.25210571	144.2278083	-24.27885494
1.884430516	23.58222037	106.3681259	556.1211177	-39.86605835
1.521666108	16.10590047	118.420826	259.4000301	-33.75926208
1.925887577	21.76664878	139.9706841	473.7869992	-30.14734936
1.207925156	10.56368062	131.2897758	111.5913483	-16.89182663
1.102812322	11.13441188	63.59801865	123.975128	-24.45090389
1.333968185	12.39220527	130.2895699	153.5667514	-21.51759796
1.141597278	10.18682518	95.55742645	103.7714072	-17.29591274
0.989945773	9.817124818	74.57242775	96.3759397	-18.32121277
1.645491974	18.29076807	135.9150696	334.5521966	-26.50299549
1.076073477	5.357069767	94.45915222	28.69819649	-4.048498487
1.238945379	13.05964358	67.8719883	170.5542905	-28.22071419
1.122300516	9.028903	87.61032104	81.52108938	-18.07347431
1.356671585	14.97603225	79.96823502	224.2815419	-31.64464302
1.031002123	6.341642997	73.08829498	40.2164359	-10.51217833
1.100827778	7.432977334	174.4862061	55.24915205	-11.41045418
1.291944396	13.22196926	146.3021545	174.820471	-21.33954811
0.974878353	6.204355078	86.3170433	38.49402193	-10.68684082
1.816842601	20.75932846	132.4665718	430.9497182	-32.29993896
1.065642998	7.758840249	61.44042778	60.19960201	-15.49734688
1.169261574	10.20138578	57.68993568	104.0682718	-24.38780708
1.161128384	9.103415965	99.7216568	82.87218223	-12.69775009
1.109436105	6.31147207	126.9868774	39.83467969	-6.240877724
1.229675612	9.158731736	127.1922417	83.88236701	-14.55193329
1.144966676	8.264995007	88.29927063	68.31014246	-14.54605045
1.091491592	7.240432565	107.9717636	52.42386373	-10.90517139
1.025346192	5.753029141	85.64105606	33.0973443	-8.480766106
1.488378034	14.26561602	142.7488213	203.5078005	-25.19491272
1.781937064	19.32280385	66.67217493	373.3707485	-53.03007584
1.659179728	19.14011916	108.1420975	366.3441616	-33.98158569
1.294458989	11.57146489	127.8444366	133.8987998	-22.75259705
1.147912241	6.949275574	106.8100204	48.292431	-5.350251675
1.270179486	10.71426682	112.5339775	114.7955134	-15.49686289
1.409130635	15.04455851	80.87274933	226.3387406	-26.67252731
1.333241802	12.22418182	82.23015404	149.4306213	-26.42194862
1.319690275	11.63882012	115.1226349	135.4621338	-20.26510086
1.326295047	13.60143531	106.4924736	184.9990426	-28.87873459
1.429675724	14.48159923	116.7420387	209.7167163	-30.20705433
1.463927224	15.41890902	135.5563278	237.7427554	-31.33757324
1.733052106	19.87354205	155.4595909	394.9576734	-27.42139816
1.037157594	4.98341267	79.91174316	24.83440184	-5.71338563
1.780122249	21.3265258	224.4721756	454.8207028	-27.20203686
1.300173882	13.00987934	94.94832611	169.2569603	-27.02389679
1.684038436	18.06366013	57.3303895	326.2958171	-49.5231514
1.542886989	17.50453095	76.08314133	306.4086037	-36.879282
1.234201841	9.662986867	122.5077019	93.3733152	-14.28870211
1.332252255	12.47847733	84.92043304	155.7123964	-28.32444153
1.35624341	12.11295591	186.3191223	146.7237009	-20.85969276
1.24477275	11.36540674	80.55327606	129.1724703	-22.60247726
1.141940874	6.839346139	129.4808426	46.77665561	-5.410728455
1.687492457	18.52074634	138.3029938	343.0180452	-38.69366226
1.681664123	18.59580112	143.3480377	345.8038194	-34.32216148
1.9999478	23.40806548	135.7030296	547.9375294	-43.99512291
1.312105002	12.55226154	81.44312286	157.5592699	-22.60368805
1.06315471	9.779422732	81.86297607	95.63710897	-16.62464714
1.282936729	10.84894687	88.11323166	117.6996482	-22.76356812
1.32504869	10.86582663	151.2581062	118.0661884	-16.15383434
1.112142059	7.456503993	92.17031479	55.5994518	-8.425981998
1.108501764	7.824944765	102.5967445	61.22976058	-13.07945366
1.185471879	11.5384784	53.41157436	133.1364837	-26.75600891
1.489273932	14.55324598	133.0690269	211.7969686	-22.29020863
1.85585499	21.06920216	118.7747803	443.9112796	-40.16018524
1.251793823	9.960779727	110.2927361	99.21713277	-18.04078445
1.320926433	12.96555322	62.77509594	168.1055703	-31.14036598
1.323468694	11.2326048	123.4516678	126.1714106	-20.4935894
1.23109677	9.901732252	116.071537	98.04430159	-17.85182304
1.207673561	11.3295667	67.19355392	128.3590816	-25.35510025
1.079975642	6.431156536	95.78264236	41.35977439	-9.490080452
1.062270476	6.042251539	155.402813	36.50880366	-7.249284029
1.236667497	9.211660481	153.2351074	84.85468882	-8.040195465
1.801516063	20.57347464	168.6368332	423.2678589	-36.22688103
1.353985782	13.6519468	101.6057014	186.3756514	-28.56171322
1.293382591	11.54940416	78.85646439	133.3887365	-26.54750175
1.235777636	12.10498205	98.94843674	146.5305904	-23.65352936
1.2608085	10.26285604	131.4965324	105.3262142	-17.33693657
1.394880378	14.63560861	81.31062698	214.2010393	-34.12066345
1.7964638	19.88714834	169.5589905	395.498669	-30.0665863
1.446437993	13.42575612	287.365593	180.2509275	-14.21662273
1.527731867	16.71805115	81.80047989	279.4932342	-42.7008934
1.153261711	7.939731762	111.1837273	63.03934046	-11.84369001
1.14285363	9.765585494	107.8045845	95.36666005	-14.80171776
1.170627144	10.8649476	75.87851524	118.0470864	-23.0190239
1.550355417	15.87772416	116.7127419	252.1021246	-33.55048752
1.427725405	13.50153653	65.63822746	182.2914887	-25.834375
1.76049503	20.47934698	75.03755188	419.4036529	-46.02935867
1.3938596	13.10178912	214.8264465	171.6568782	-20.20717888
1.418384408	13.88472018	84.07390022	192.7854545	-28.34728336
1.470052986	14.76080948	113.7118874	217.8814966	-24.34214306
1.179360753	8.841753618	92.85687637	78.17660705	-15.89449368
1.081475967	6.788621901	99.73399734	46.08538732	-10.40691538
1.918258723	20.41337529	116.8401794	416.7058908	-39.16321106
1.531812328	15.54732628	141.5876961	241.7193544	-22.74208183
1.391835778	15.73291815	71.93545818	247.5247135	-42.20059929
1.498971995	15.81472608	89.22718048	250.1055608	-28.58747482
1.315316081	11.37760785	64.76890469	129.4499605	-23.19960709

log.sigma.0.5.mm.3D_firstorder_Kurtosis	log.sigma.0.5.mm.3D_firstorder_Mean	log.sigma.0.5.mm.3D_glrlm_ShortRunLowGrayLevelEmphasis	log.sigma.0.5.mm.3D_glrlm_GrayLevelVariance
4.219285483	-8.18888436	0.092873257	0.535947271
7.933983607	-4.576611485	0.074543334	0.401826204
3.442273864	-9.339444497	0.265565449	0.391397096
6.997448358	-5.855514558	0.049228099	0.883891947
2.772570523	-12.21340109	0.283207929	0.454221487
3.359515932	-9.395336659	0.246717574	0.457311829
2.43014849	-10.37546749	0.174415994	0.903166551
3.967588062	-13.43062132	0.124659128	0.671183902
3.40428321	-4.450486277	0.050872845	1.065051038
10.32620359	-4.00012794	0.172428341	0.420958249
3.000029645	-9.611766217	0.243688562	0.412425612
7.733274904	-5.129862592	0.211758836	0.500268863
5.30726179	-4.417270781	0.0657965	0.405066723
4.806595941	-6.231237863	0.160824606	0.38116378
4.724328581	-6.547448348	0.104883795	0.755583719
19.36961509	-0.849411685	0.122845889	0.285301677
3.402259377	-12.41174466	0.312166392	0.487124274
5.219171467	-5.411407729	0.067572828	0.36870168
3.097804732	-11.43758846	0.328322067	0.564407715
8.625044642	-2.292318421	0.123992937	0.283821391
24.20381547	-2.516197322	0.060821946	0.322595102
7.798017779	-6.047404343	0.0790084	0.537417308
9.147441306	-2.6832319	0.121953118	0.271721355
3.597573095	-7.486870842	0.120761321	0.835821838
6.134135553	-4.264825559	0.164819483	0.300580405
3.030666689	-8.360468617	0.239383165	0.382263383
8.548941302	-2.192258325	0.142429227	0.352639294
23.72017792	-1.648069603	0.142845251	0.309638454
11.00419815	-3.15980089	0.068199165	0.394082347
8.705180856	-3.537663788	0.165681021	0.343165477
12.06959786	-2.456707643	0.13623728	0.311292578
9.831804478	-1.888341664	0.05447668	0.274625707
6.440591941	-5.688457193	0.091574729	0.628222611
1.935954622	-29.32673831	0.318841282	0.778693271
3.981187367	-11.92827421	0.13069559	0.790179819
5.68511787	-6.263969925	0.079182165	0.479434984
16.43356399	-1.77170149	0.062793442	0.327971685
7.972449343	-2.45859894	0.063631179	0.441472713
2.927481074	-8.707961851	0.267084952	0.535070415
3.827829357	-8.441861775	0.099167151	0.509215141
7.441805399	-4.264089468	0.07869875	0.476239219
4.297441415	-10.78339218	0.099092863	0.549763447
5.295279765	-9.849690243	0.099630366	0.606007128
6.50766525	-10.82674542	0.109032336	0.684455583
5.051660253	-5.315891521	0.09476904	0.92312397
12.10076947	-0.996374888	0.0587473	0.266844363
8.042436611	-4.263478492	0.04891788	1.106334582
3.899398866	-10.40573347	0.096894115	0.483681112
1.69094625	-24.46328864	0.231033919	0.665250895
2.327390811	-13.14791072	0.12342951	0.6059833
10.5124253	-2.651048619	0.161767435	0.409282757
3.255134362	-10.11940759	0.100085116	0.501366833
10.54251168	-4.68614407	0.026625842	0.520519117
4.334781809	-7.350409133	0.225779439	0.430990626
18.19963315	-1.191846125	0.060382613	0.322834987
4.049612531	-12.50259695	0.140393927	0.797888679
4.64171695	-13.23754149	0.139204947	0.836689256
2.836972043	-13.04370015	0.072684497	1.061020073
5.202957258	-6.393679937	0.093292026	0.477812128
5.010046408	-4.369509289	0.153697691	0.319643603
4.366966734	-6.44445133	0.080761866	0.443704122
10.25414368	-2.609365721	0.068489718	0.4731736
9.841923247	-1.148634851	0.125911451	0.312943189
9.674046087	-2.42084833	0.063694118	0.307788163
2.698421659	-11.06587515	0.306343358	0.401724847
6.393726729	-3.9976767	0.083700606	0.601246634
3.350951833	-16.62761903	0.178720146	0.944917324
7.021935498	-4.000075855	0.070801548	0.405570523
2.533472107	-12.49527492	0.345371474	0.486838848
7.463364287	-4.582866192	0.079184761	0.471539345
6.495125335	-4.114375216	0.071158928	0.390142273
3.173796306	-9.121672182	0.249015372	0.419069843
12.02953944	-1.982395079	0.057189903	0.300599962
22.83712241	-1.56874349	0.127252947	0.286466363
15.04424749	-0.817683307	0.060527368	0.398233355
4.739989821	-11.50194953	0.066780304	0.937303194
3.702394635	-10.03051284	0.095542943	0.520738744
3.45434286	-10.87452003	0.102793067	0.463704799
4.672536191	-8.099554617	0.084809017	0.465889013
9.000446214	-3.561614945	0.070516453	0.437456206
2.921319709	-14.14614703	0.120707947	0.496556375
5.234980963	-6.026055816	0.055214714	0.959800955
21.29633003	-2.095656327	0.008337991	0.610259095
3.640530091	-19.21097166	0.181017536	0.647307091
10.6841959	-2.449121678	0.144678525	0.344906836
6.518323037	-3.050709892	0.147825188	0.355681421
3.957144862	-8.569734636	0.083878647	0.388819599
3.767694363	-13.27000576	0.125063899	0.679709503
2.943883862	-6.497769608	0.249956076	0.489768619
2.36520194	-19.56676394	0.204777813	0.76520253
16.23240991	-4.073547996	0.019310006	0.561889087
4.47363406	-8.673377001	0.112305313	0.536183454
5.960590121	-5.475162405	0.093152918	0.610099662
7.303559512	-3.488305777	0.065325261	0.370483999
9.602190395	-2.228908028	0.131581183	0.300999484
4.023161754	-16.60446421	0.185803861	0.984650386
5.238019351	-3.799264192	0.081982776	0.662493875
3.024773711	-24.55456895	0.184210166	0.451473291
3.253820264	-9.450624854	0.11377359	0.557274067
4.506732044	-4.922901352	0.226985343	0.420867453

log.sigma.0.5.mm.3D_glrlm_LowGrayLevelRunEmphasis	log.sigma.0.5.mm.3D_glrlm_GrayLevelNonUniformityNormalized	log.sigma.0.5.mm.3D_glrlm_RunVariance	log.sigma.0.5.mm.3D_glrlm_GrayLevelNonUniformity
0.123763	0.381136426	0.914015544	87.21447471
0.107645215	0.431972202	1.267177985	355.3297849
0.342183256	0.446979017	1.045210085	48.90090519
0.065601216	0.322354308	1.168741941	391.6419889
0.353926438	0.400661406	0.72494149	27.22823706
0.323385996	0.414126243	1.196738238	60.35233941
0.187887049	0.289948547	0.149421268	17.83343745
0.156375915	0.346259764	0.988414713	90.2336178
0.065030407	0.274897704	0.826549672	276.1693315
0.246200277	0.43685714	1.427808611	580.4189153
0.329809419	0.428602095	1.542421101	91.77875332
0.279731018	0.401924037	1.062758627	188.7602816
0.101559368	0.435712976	1.972099046	319.8529226
0.255574257	0.453842971	2.229659867	119.0076603
0.127860982	0.336724184	0.630551357	87.59036379
0.193285231	0.483434829	1.471724731	2899.935553
0.392837263	0.404298182	1.111364867	55.8440556
0.105002328	0.455601677	1.622402093	385.8451748
0.408098562	0.361652079	0.990353378	42.33968682
0.205583775	0.487242028	1.906201777	700.2615941
0.094878016	0.473604891	1.496862779	1334.834136
0.110469375	0.405482036	1.367411864	221.2408435
0.204435279	0.500360417	1.900174243	762.3738904
0.138103936	0.307235444	0.331479068	32.59963387
0.234442759	0.491704486	1.110469367	287.1620739
0.313034522	0.449386744	0.948126005	85.20059758
0.211252449	0.458663191	1.313013927	608.5107915
0.208211339	0.474567157	1.204361204	1091.70947
0.101328583	0.436980718	1.445366662	925.101855
0.237617763	0.461190801	1.33102633	449.9129038
0.210477881	0.478533127	1.48359832	1108.583406
0.091498077	0.492115457	1.885091199	2272.796911
0.121966557	0.360148584	1.216609203	304.3177672
0.336097707	0.313496914	0.099350266	4.341786419
0.15595324	0.325695474	0.514948767	27.24201426
0.112663231	0.398182493	1.4066066	308.2567897
0.094856804	0.46362748	1.446307236	1748.291966
0.096452339	0.422745512	1.479392084	726.0222958
0.332565783	0.375198949	0.866056721	81.16642594
0.131357084	0.389903288	1.14335093	126.655527
0.107892764	0.411986608	1.094540209	182.4191598
0.133585057	0.378647508	1.226309833	86.91729017
0.131976572	0.362437859	1.190769356	185.0436118
0.140840111	0.355768771	1.122294981	98.92462865
0.120703932	0.305496143	0.946116644	228.2755548
0.089957372	0.493351493	1.361233358	1724.255132
0.063279111	0.305835686	0.997041206	558.6258784
0.128350952	0.402350867	0.900883351	58.30835303
0.251481087	0.337118823	0.12605585	6.517360039
0.148279437	0.340179404	0.487120592	37.34886313
0.231694952	0.435726041	1.452546282	1017.169821
0.134777163	0.384049506	1.063425798	94.38982964
0.038605007	0.400217609	1.382012819	743.5832837
0.294974852	0.416503816	0.893309227	63.51973665
0.092512288	0.466940133	1.450917338	2969.592373
0.168997038	0.312479727	0.757145549	72.30476353
0.167202806	0.323705221	0.808370751	198.2640088
0.083702936	0.26837633	0.425213999	97.02471589
0.121610976	0.416427864	0.761227172	96.63033723
0.228613189	0.480959464	1.231176929	131.5048085
0.114235425	0.409613252	1.273912397	347.0634186
0.100403616	0.41045027	1.531937873	1584.543209
0.194661706	0.471463032	1.4201821	1090.346318
0.09531676	0.478122702	1.350337654	492.0934511
0.378808752	0.439661478	0.530912083	16.07613278
0.111670928	0.374087299	1.022281772	274.2668063
0.201586742	0.29468951	0.350631986	24.68328593
0.10420151	0.427880722	1.451145458	835.6637539
0.419277597	0.382384353	0.669476421	36.92646423
0.110498399	0.40785686	1.295064164	433.3104158
0.102558061	0.43905272	1.354056218	670.3254818
0.33459705	0.42325407	1.251926304	140.6918964
0.093044459	0.478060623	1.781146239	1785.584127
0.198358573	0.485796407	1.5445452	2899.710282
0.092550948	0.443581832	1.508706848	3598.704949
0.07992456	0.29969153	0.633612805	97.09934771
0.126634603	0.382097794	1.090234858	110.7857917
0.135027512	0.418492507	0.85253606	60.24493743
0.119541507	0.419314873	1.51058259	223.5238538
0.103408973	0.421298787	1.551603963	644.3363943
0.145906695	0.401196474	0.559878041	43.88595062
0.068466415	0.30770769	0.785924325	184.2518954
0.011874573	0.394467755	1.200213847	1169.618949
0.203273478	0.373284883	0.27059544	9.352497079
0.219028637	0.459082072	1.630403882	1241.697491
0.216275258	0.455696736	1.490057151	932.6686427
0.116678903	0.452158024	1.049843702	99.79626394
0.154448057	0.340350619	0.875199697	135.8628039
0.309338491	0.384850197	0.310015178	14.85741502
0.227570847	0.313328997	0.303779589	3.452020202
0.027129305	0.401313584	1.263327214	825.2158622
0.13996106	0.390953533	0.729908804	78.81869046
0.119508125	0.377359852	0.815910722	104.9490475
0.100193924	0.444970786	1.655179612	889.3949036
0.206802542	0.478814787	1.643406601	1864.855394
0.208386064	0.300774327	0.238471522	15.45706587
0.108908076	0.349006237	1.061512725	152.479459
0.208994732	0.417099544	0.228811273	8.208223063
0.134627085	0.37337595	0.400880783	46.54250571
0.277010704	0.414656148	0.59568473	80.42680947

log.sigma.0.5.mm.3D_glrlm_LongRunEmphasis	log.sigma.0.5.mm.3D_glrlm_ShortRunHighGrayLevelEmphasis	log.sigma.0.5.mm.3D_glrlm_RunLengthNonUniformity	log.sigma.0.5.mm.3D_glrlm_ShortRunEmphasis
3.429892073	7.456089433	108.1952748	0.710542318
4.195390328	7.675138343	350.1642585	0.671670836
3.797393704	3.256048159	50.41907883	0.694136568
3.707741036	14.16205344	594.9271604	0.726503457
3.035552018	3.627463746	35.21689399	0.735095514
4.062051048	3.692238214	65.27752793	0.690952886
1.4746949	8.639853163	48.38332291	0.899562042
3.381405965	6.798772555	132.2075715	0.738007675
2.999670416	15.27309363	542.2214386	0.762017845
4.489970068	4.122400196	551.5995827	0.66387851
4.758070937	3.420151761	90.14338917	0.667940216
3.694748846	4.171837241	217.0421609	0.701338223
5.473157739	7.936609517	289.6309447	0.647655564
6.436693888	3.859536616	92.60013887	0.606135192
2.600170579	9.192018888	148.6383703	0.783526375
4.753380703	4.111043383	2319.213179	0.632663488
3.698503648	3.377123423	68.30938131	0.72611662
5.058398742	7.208196223	323.5200691	0.629036106
3.477904729	3.507732136	58.20310644	0.728074135
5.731911838	3.833069757	512.8016508	0.602131845
4.748426187	7.784398171	1101.398793	0.640551327
4.215868203	8.292918815	249.8309371	0.699325195
5.653757455	3.899580244	553.2162142	0.610940933
1.939706421	9.166285908	70.86885232	0.837016271
3.855211884	3.923924367	257.9560185	0.681911405
3.559902573	3.449948667	87.07199846	0.694820182
4.29981383	4.331178559	552.0227429	0.663965883
4.09789586	4.197580222	974.0755957	0.668246279
4.596498953	7.740002899	869.4308861	0.655008695
4.365065674	3.906318877	402.0810534	0.65989561
4.780871234	3.982692778	889.1712609	0.634157773
5.646453417	7.51475817	1686.114903	0.605873354
3.928867513	8.008466096	389.2095008	0.703589926
1.292828966	5.861406578	12.10777684	0.940489888
2.389251243	7.969856217	49.99857592	0.797354345
4.395365098	7.562314589	331.0228974	0.676067464
4.59999214	7.771113631	1503.398584	0.64805501
4.606408701	8.407808939	702.6001994	0.659190092
3.147798574	4.089919931	115.6342807	0.752677428
3.911944156	6.91675229	146.1430861	0.691665999
3.73453696	8.282294872	207.42775	0.706591158
4.028653307	7.048256774	103.6663227	0.695069373
3.852679159	7.288749871	242.7170128	0.713215028
3.650781404	7.353367135	138.1730142	0.729884698
3.182641923	9.546781368	401.7498828	0.760587416
4.464168445	7.97350912	1415.151425	0.650927161
3.266455386	15.82691973	971.7847067	0.756931964
3.426280725	7.09619966	68.38556908	0.707492049
1.440940587	6.350949107	14.99209487	0.894263279
2.267505478	7.609007301	67.61205708	0.810472904
4.516412885	4.218658842	965.5139507	0.662473915
3.797991885	6.516287506	109.8655077	0.689732381
4.346737582	19.24263733	789.1173367	0.673162181
3.37638064	3.972691551	72.78058715	0.71341281
4.628461066	7.929395201	2524.954237	0.64514918
2.872677828	7.339543994	126.8901444	0.766814686
2.904079907	7.564554346	349.7243131	0.779498169
2.050837221	14.36535824	244.4582819	0.846444876
3.116992416	7.945161426	113.3882539	0.720286547
4.338908126	3.98559089	114.2403529	0.65492792
4.238566492	7.065376751	354.2515889	0.665874985
4.624563125	8.21519214	1611.458953	0.667073909
4.54958588	4.386356772	932.8538955	0.651717154
4.371231915	7.833371899	424.8686676	0.658534476
2.691381863	3.217847809	18.80333654	0.730507795
3.539850788	8.717648384	354.5286673	0.71834072
1.948143761	7.570447772	56.65608027	0.845937236
4.610643382	7.417251381	780.5855831	0.649423502
2.875437374	3.191822308	49.75493386	0.740127061
4.159522446	7.790153262	461.4781717	0.68020329
4.30832153	7.827523006	655.3505215	0.673870993
4.258056281	3.252458402	139.9821796	0.664717344
5.372845645	7.557811988	1366.963972	0.615991094
4.848782584	4.0386741	2293.489274	0.633657576
4.720171787	8.241589554	3211.241496	0.64571791
2.550954903	13.6994265	193.4485753	0.798917785
3.618053574	7.468890621	142.0387453	0.724627479
3.35431348	6.637869844	68.39372088	0.709389711
4.656695476	7.167519042	229.999165	0.670277844
4.689280155	7.888867497	632.452087	0.663595478
2.497746707	7.099076293	65.32743959	0.791905992
2.887930203	14.98264695	332.2747147	0.772296368
3.969778967	61.10494975	1312.992219	0.687934252
1.852703885	7.018746277	16.8221063	0.836988919
5.005800699	3.993949682	1035.467429	0.634072698
4.455900701	4.462022744	888.8383874	0.68019791
3.858101983	7.019333741	95.84496681	0.676563322
3.087265457	7.192132054	216.4673841	0.762723442
2.089353611	4.521218277	24.52393255	0.796196927
1.761363636	7.349116162	8.431818182	0.887784091
4.054591486	27.71328545	914.7901875	0.690009832
3.024171784	7.141257213	100.1063232	0.727699126
3.073443416	8.576632908	144.5018547	0.746659571
5.009071344	7.554712015	773.5739454	0.638008154
5.087891462	3.962109069	1457.056952	0.625129903
1.760769853	7.411936647	34.93312435	0.845385852
3.503454891	9.345367378	221.5388043	0.739052588
1.777717312	5.794483692	13.5054428	0.839866286
2.131773601	8.388166961	77.93734024	0.815414137
2.72267468	3.990314407	101.6150322	0.741994089

log.sigma.0.5.mm.3D_glrlm_LongRunHighGrayLevelEmphasis	log.sigma.0.5.mm.3D_glrlm_RunPercentage	log.sigma.0.5.mm.3D_glrlm_LongRunLowGrayLevelEmphasis	log.sigma.0.5.mm.3D_glrlm_RunEntropy
34.6395307	0.633070531	0.382900428	2.927105313
44.54824043	0.58621635	0.43522541	2.931943066
16.67516121	0.614950022	1.043097825	2.674605282
66.74977692	0.628397469	0.226862564	3.280168183
13.35026439	0.671744097	0.905141964	2.53392043
17.97541789	0.595838588	1.086197248	2.815491064
14.72191509	0.878021978	0.249048078	2.450248771
31.11562921	0.649146365	0.432998183	2.974657034
57.18741832	0.680268918	0.182234801	3.307546315
23.22494184	0.572760142	1.037731783	3.003368148
20.57659592	0.566898527	1.281288478	2.85747899
19.102277	0.61975409	0.898942312	2.934983211
55.47526282	0.536941097	0.57446469	3.054192416
28.38191493	0.49504662	1.599115705	2.98797732
27.53786338	0.714074387	0.29833724	2.883966735
28.78933051	0.553969765	0.928006614	2.896190025
15.2542632	0.633108827	1.126025476	2.719199723
52.20931961	0.544557891	0.526120097	3.025959245
14.64401278	0.64158918	1.077936508	2.739113315
31.18768746	0.517913593	1.220255203	2.989112303
52.88520364	0.555346722	0.461239559	2.942389439
42.45315311	0.599136837	0.454818508	2.994043046
29.01525519	0.521522655	1.246221082	2.927273314
21.86921737	0.796414112	0.236281059	2.691472854
20.13682931	0.607852564	0.870925089	2.701466688
16.80608191	0.626082527	0.930980321	2.718370813
24.0722591	0.579493901	0.910397295	2.914466445
24.1493162	0.590500642	0.830035363	2.801158123
51.43171378	0.5696779	0.452763981	3.03017175
23.74246812	0.57599164	0.961461365	2.907488079
26.89719166	0.551388863	1.000120245	2.94450988
63.29782626	0.52275993	0.542452764	2.961756377
42.00487138	0.608185191	0.422516392	3.148010347
7.907650042	0.923076923	0.41882054	2.036768445
22.03624427	0.737236215	0.324747997	2.760641006
45.10097156	0.579979241	0.469538028	3.042798656
54.59407463	0.563569893	0.425076568	2.949053977
51.29473694	0.566343617	0.452706692	3.093745338
14.89694005	0.671938653	0.884823562	2.775318664
39.2516302	0.604354677	0.439140915	2.979267968
40.01383021	0.618827327	0.386419651	2.935392777
38.07397192	0.601049869	0.47140328	2.967269292
37.3197329	0.618558626	0.449087934	3.028578954
34.21282713	0.63255651	0.446757852	2.997570074
33.5901533	0.669886168	0.358283909	3.159932627
53.8378959	0.57085417	0.402282193	2.77962547
59.7907258	0.6649112	0.199264954	3.260696291
33.33666066	0.634278003	0.392040232	2.807965988
9.615320679	0.877622378	0.337768181	2.195648798
20.55392705	0.751844046	0.308082645	2.547221529
25.19238056	0.572115385	0.981480449	3.030564035
36.42147345	0.608894827	0.443512431	2.923627244
121.0546035	0.581437342	0.163143437	3.133951886
16.25802185	0.637592533	0.868050163	2.7199109
55.04079555	0.562505953	0.426149116	2.947417244
26.88297598	0.690011481	0.390098592	2.983062571
26.73729372	0.700070572	0.397866213	2.995563839
33.85635318	0.789049379	0.157391846	2.935046963
32.92075932	0.654932638	0.339464052	2.763421728
22.11194086	0.584681413	0.981838324	2.771693512
45.14017686	0.582024848	0.440870684	3.020153417
52.26144782	0.569634911	0.453593696	3.141355908
26.28359859	0.567557872	0.926147128	2.887825175
50.57917275	0.577354744	0.41200769	2.844374919
11.43999352	0.687953556	0.819029563	2.381287428
39.45843002	0.635616987	0.367095367	3.040673357
16.4891372	0.796336996	0.337167225	2.707977752
52.20172615	0.563244658	0.451082231	3.07048395
12.16840586	0.675094137	0.92763233	2.577739081
45.68764558	0.592591003	0.423739891	3.039836763
48.03315989	0.586912126	0.426368306	2.962306719
19.90297936	0.584588251	1.136889088	2.92063076
60.94214847	0.529185424	0.512838641	3.009222313
28.68106751	0.55092941	0.966159255	2.896387875
56.90294842	0.558732059	0.431701442	3.086063031
42.50051649	0.725767506	0.179333558	2.996374159
34.88941615	0.634278003	0.417285412	2.882951687
31.96921432	0.640453297	0.395189766	2.788825319
46.37612843	0.580925099	0.508832525	3.010794654
50.69708311	0.565487028	0.474426642	3.088506316
22.72750477	0.732059886	0.320809164	2.540882823
53.92283767	0.691774122	0.177090016	3.155387686
354.9650281	0.601541895	0.045464959	3.158588123
15.70279461	0.803970223	0.314592752	2.311555765
28.49931427	0.54503457	1.051824789	3.038476441
23.13924248	0.583273014	0.997577725	2.872537111
39.74371435	0.602950474	0.410177831	2.806637676
28.1929017	0.676635758	0.401601278	2.908038223
10.82401159	0.772307692	0.626519572	2.292779714
11.79040404	0.846153846	0.396867985	2.23541018
164.5118864	0.599524418	0.104147951	3.103093688
32.14454417	0.662702429	0.342480453	2.825261234
32.07349059	0.667899408	0.338223436	2.904634451
54.95800392	0.547202797	0.497290711	3.059909384
28.94995209	0.53926038	1.051664795	2.974597135
15.99380067	0.815628816	0.317096188	2.672373
37.16489767	0.642081448	0.37424988	3.005806447
12.86975758	0.817307692	0.326343206	2.092216254
21.21889617	0.764039641	0.260566379	2.535013085
17.0264621	0.692307692	0.593536257	2.576276337

log.sigma.0.5.mm.3D_glrlm_HighGrayLevelRunEmphasis	log.sigma.0.5.mm.3D_glrlm_RunLengthNonUniformityNormalized	log.sigma.0.5.mm.3D_glszm_GrayLevelVariance
10.41395464	0.471639159	1.875
11.23047582	0.424563576	1.419501134
4.654716085	0.455956653	0.65
19.06610422	0.489355684	3.520340237
4.795835208	0.511162443	0.859375
5.073001373	0.446563965	1.218836565
9.681011643	0.77857532	1.777777778
9.229347434	0.506314135	2.2275
19.77622995	0.53889754	3.0544
5.919834175	0.414544507	2.3275
4.877815861	0.41884602	0.935950413
5.744288318	0.460345093	2.027548209
11.623725	0.393927724	1.86422324
5.627058677	0.351026869	1.094674556
11.44456947	0.571054082	2.577148438
6.365008239	0.385491951	1.767877033
4.473324304	0.49233418	1.126033058
11.14721005	0.379934651	1.3984
4.614314368	0.494840845	1.2275
6.090901822	0.353700673	1.052469136
11.8789935	0.390249672	1.906807955
11.34720627	0.455752279	2.104567653
5.994531698	0.36059213	1.16
11.02319481	0.662437318	2.094182825
5.619570593	0.439455729	0.742382271
4.933545499	0.456125075	0.722222222
6.246367628	0.415576593	2.00591716
6.17588526	0.421947047	1.598677686
11.67044234	0.407470228	1.892039488
5.796221448	0.411258969	1.3275
6.086650911	0.38348524	1.90245639
12.04229004	0.361745045	1.255509642
11.20743855	0.460270555	2.721088435
6.226824458	0.866122712	1.25
9.761646329	0.595820647	2.107142857
10.96377053	0.42721413	1.860465116
11.98210679	0.398353702	1.61
12.24179785	0.4086753	2.27259814
5.274010095	0.529975095	1.58984375
10.12431284	0.447922256	1.25
11.477657	0.466409996	1.983379501
9.936619955	0.450986372	1.430055402
10.09781736	0.473291991	2.020833333
9.884464226	0.495286439	2.7776
12.05717422	0.537075824	3.06594704
12.20655563	0.40291213	0.4578564
20.26712332	0.531450992	4.079156672
10.01062504	0.470072737	1.21484375
6.989428459	0.769622799	1.583333333
9.2948032	0.614282577	1.25
6.150750662	0.413195834	2.31969391
9.557717456	0.445748745	1.294117647
28.4971485	0.424481407	2.776643991
5.364989076	0.475939382	1.173553719
12.20125822	0.396064728	2.113775992
9.525111661	0.547300846	3.102493075
9.565889111	0.567073784	2.860670194
16.86076455	0.673459142	3.187376726
10.95233743	0.486476377	1.301038062
5.784104027	0.4113701	1.31122449
10.70758975	0.417717085	0.777727478
12.02629242	0.416859766	2.32062548
6.445711159	0.402290281	2.056191467
11.83055677	0.411591386	1.448347107
4.349989529	0.511391332	0.6875
11.89999198	0.481134036	2.884897959
8.799590911	0.673721578	2.290657439
11.44923801	0.399356757	1.374653565
4.312010127	0.513907191	0.805555556
11.37511489	0.433633256	1.830690675
11.53679302	0.426889893	1.686344397
4.853784364	0.418388023	0.9275
12.00893346	0.365181599	1.539300554
6.249773611	0.3837944	1.712875599
12.52857855	0.395377728	2.863568871
17.01938153	0.595188953	3.547666009
10.13092286	0.48851229	1.5275
9.481586904	0.472405698	1.243055556
10.51560981	0.425452762	1.444234405
11.62061064	0.413049626	1.900012625
9.004483238	0.590098018	1.360946746
19.19215352	0.554051245	3.638504155
89.03165522	0.442391027	4.6125395
8.394368736	0.667000744	1.265306122
6.122748808	0.382440334	2.008888889
6.189665209	0.43311842	1.974419988
10.41661309	0.432237276	0.767485822
9.336449344	0.54073573	2.00594884
5.532976335	0.615237232	0.583333333
7.987373737	0.756638353	0.979591837
40.34185911	0.44445643	3.249573898
10.10267744	0.495442394	0.543209877
11.2271066	0.518702545	2.3125
11.64108426	0.386468254	1.552800735
6.145158169	0.373880186	1.638839929
8.868319285	0.675221311	2.775510204
12.12502128	0.506377068	2.615916955
6.983977572	0.677482511	1.25
10.21189107	0.622368206	1.514792899
5.640104837	0.519541243	0.328888889

log.sigma.0.5.mm.3D_glszm_SmallAreaHighGrayLevelEmphasis	log.sigma.0.5.mm.3D_glszm_GrayLevelNonUniformityNormalized	log.sigma.0.5.mm.3D_glszm_SizeZoneNonUniformityNormalized
6.995471876	0.3359375	0.2890625
6.908701551	0.287981859	0.428571429
1.405365078	0.54	0.4
25.46429276	0.225036982	0.579696746
2.440993469	0.40625	0.21875
2.660025309	0.457063712	0.279778393
3.100089891	0.259259259	0.185185185
8.877164797	0.255	0.38
18.12595258	0.2048	0.432
5.317863322	0.416111111	0.449444444
2.863065339	0.466942149	0.29338843
4.422055705	0.377410468	0.31496786
9.624152338	0.369429404	0.421907539
4.802905433	0.399408284	0.310650888
9.15153031	0.236328125	0.3671875
6.273319023	0.334372912	0.412341279
4.913437367	0.392561983	0.334710744
8.205951368	0.3968	0.4376
3.340661312	0.375	0.19
4.386709161	0.447530864	0.575617284
8.361030857	0.504717666	0.563071563
8.511984835	0.349037633	0.434645217
4.959028078	0.4125	0.625
8.014373328	0.229916898	0.434903047
2.27111733	0.429362881	0.307479224
1.036442251	0.430555556	0.166666667
5.146100714	0.36612426	0.434911243
4.305244747	0.510082645	0.598016529
8.093740591	0.301407267	0.304767906
4.166146616	0.4575	0.4525
4.534136612	0.426842293	0.459594162
7.02628739	0.408631772	0.426538108
11.13474716	0.234567901	0.434618292
4.130277778	0.25	0.25
10.21049927	0.234693878	0.43877551
8.136767376	0.402920498	0.342347215
6.528112636	0.501666667	0.363888889
12.33080428	0.369059917	0.491993802
3.758918961	0.3203125	0.2265625
5.668037984	0.37244898	0.474489796
9.530127573	0.293628809	0.38365651
8.906854857	0.347645429	0.491689751
10.98646075	0.236111111	0.464506173
11.7646153	0.2032	0.5872
13.89836849	0.237645471	0.490976556
4.129032352	0.762747138	0.764828304
16.53672016	0.205529605	0.338277995
5.511421275	0.390625	0.3046875
5.693415638	0.277777778	0.555555556
1.850738172	0.34375	0.1875
5.117244644	0.426293974	0.438410033
6.898871202	0.349480969	0.314878893
20.46987265	0.31462585	0.390306122
2.582913546	0.388429752	0.20661157
8.90442163	0.379017013	0.450378072
8.298528147	0.24099723	0.501385042
8.016255712	0.217435122	0.361048123
13.58719672	0.230769231	0.310979619
3.877564035	0.349480969	0.314878893
3.464322845	0.459183673	0.540816327
3.911953839	0.664101403	0.324581258
13.51400269	0.305756958	0.433191887
6.625448093	0.379812695	0.596253902
6.486365472	0.404958678	0.483471074
0.105331155	0.375	0.25
14.02221161	0.25877551	0.425306122
6.219407763	0.259515571	0.39100346
5.835890561	0.483497103	0.408919123
0.399167843	0.388888889	0.222222222
6.817508939	0.388336469	0.301800591
8.792175968	0.383381599	0.533971931
3.091152299	0.465	0.345
7.965761631	0.397506925	0.460526316
3.999897746	0.547394397	0.526781826
12.54590767	0.282164381	0.392960084
16.00869967	0.216305062	0.376725838
7.524293314	0.31	0.29
3.492725802	0.388888889	0.236111111
6.00381059	0.442344045	0.388468809
8.873874292	0.339224845	0.450574422
5.71317207	0.325443787	0.325443787
17.65189028	0.189058172	0.483033241
63.38195841	0.196782534	0.457770756
9.19800598	0.387755102	0.387755102
7.060890175	0.336177778	0.507911111
6.027210269	0.372992267	0.496728138
4.541159047	0.534971645	0.417769376
10.95486874	0.294467579	0.514574658
3.387121088	0.5	0.222222222
7.451428571	0.306122449	0.551020408
29.0339741	0.240321402	0.509617726
1.721486331	0.456790123	0.401234568
12.88574911	0.22265625	0.619140625
7.00116979	0.429292929	0.444903581
4.28146539	0.499439192	0.498157347
8.763585698	0.183673469	0.183673469
12.43196081	0.346020761	0.411764706
4.275659288	0.25	0.375
3.967609668	0.24260355	0.195266272
0.784204023	0.662222222	0.333333333

log.sigma.0.5.mm.3D_glszm_SizeZoneNonUniformity	log.sigma.0.5.mm.3D_glszm_GrayLevelNonUniformity	log.sigma.0.5.mm.3D_glszm_LargeAreaEmphasis	log.sigma.0.5.mm.3D_glszm_ZoneVariance
4.625	5.375	3663.6875	3154.621094
9	6.047619048	45337.19048	40873.678
4	5.4	1713.5	1400.21
60.28846154	23.40384615	13891.35577	13545.89636
1.75	3.25	586.125	426.734375
5.315789474	8.684210526	1663.157895	1498.238227
1.666666667	2.333333333	137.5555556	77.0617284
7.6	5.1	3224.15	2822.1475
21.6	10.24	13303.8	12432.3696
26.96666667	24.96666667	44798.68333	43304.86083
6.454545455	10.27272727	3538.909091	3246.809917
10.39393939	12.45454545	8004.818182	7478.602388
20.67346939	18.10204082	20282.40816	19505.25031
8.076923077	10.38461538	6444.384615	6031.982249
11.75	7.5625	1568.5	1439.109375
27.62686567	22.40298507	863710.1493	837591.73
7.363636364	8.636363636	1058.681818	961.3904959
21.88	19.84	25534.6	24571.1184
3.8	7.5	706.3	623.49
20.72222222	16.11111111	110694.6667	104757.108
46.73493976	41.89156627	157799.3373	154060.6779
25.6440678	20.59322034	7011.40678	6774.03792
25	16.5	117401.25	112072.25
8.263157895	4.368421053	249	200
5.842105263	8.157894737	24931.36842	22378.45983
2	5.166666667	3628	2994.638889
22.61538462	19.03846154	49771.86538	47834.17271
32.89090909	28.05454545	135657.1091	130641.8942
21.02898551	20.79710145	93771.13043	90870.7616
18.1	18.3	35848.325	34056.91938
24.35849057	22.62264151	169129.4151	162846.6095
28.15151515	26.96969697	608250.7424	590339.3813
27.38095238	14.77777778	12522.84127	12036.74376
1	1	17.75	3.6875
6.142857143	3.285714286	334.2142857	269.0663265
14.72093023	17.3255814	19512.88372	18550.44132
21.83333333	30.1	358843.4833	346407.5164
43.29545455	32.47727273	49628.47727	48441.36157
3.625	5.125	2724.6875	2322.183594
13.28571429	10.42857143	4739.964286	4372.146684
14.57894737	11.15789474	6191.236842	5837.202909
18.68421053	13.21052632	1798.5	1697.972992
16.72222222	8.5	8087.666667	7563.765432
14.68	5.08	3349.64	3041.2864
37.80519481	18.2987013	5852.324675	5642.639231
23.70967742	23.64516129	603183.4839	564183.6046
60.21348315	36.58426966	15261.04494	15023.05365
4.875	6.25	1558.625	1355.5625
3.333333333	1.666666667	27.66666667	14.22222222
1.5	2.75	933.5	600.4375
42.5257732	41.35051546	82036.84536	80267.64566
5.352941176	5.941176471	4317.588235	3755.619377
32.78571429	26.42857143	55148.65476	53701.9392
2.272727273	4.272727273	2510.636364	2038.561983
41.43478261	34.86956522	668675.6739	653573.4012
9.526315789	4.578947368	2187.526316	1876.65374
22.74603175	13.6984127	4443.079365	4251.498614
12.12820513	9	1210	1072.0881
5.352941176	5.941176471	3400.705882	2967.086505
7.571428571	6.428571429	8429.428571	7321.489796
15.25531915	31.21276596	20938.87234	19980.50883
74.94219653	52.89595376	119924.7514	118390.6482
36.96774194	23.5483871	132235.5484	127917.7867
10.63636364	8.909090909	71608.27273	65047.27273
1	1.5	368.25	192.6875
14.88571429	9.057142857	15650.85714	14567.50694
6.647058824	4.411764706	195.1176471	156.9688581
25.76190476	30.46031746	88500.33333	85471.84026
1.333333333	2.333333333	1510.833333	942.8055556
18.40983607	23.68852459	23787.5082	22924.49718
35.7761194	25.68656716	47324.91045	45820.16529
6.9	9.3	8034.45	7230.7275
35	30.21052632	328704.1184	320082.0185
43.72289157	45.43373494	706924.1687	689886.0563
84.09345794	60.38317757	458407.1822	453804.1304
14.69230769	8.435897436	1618.564103	1487.784352
5.8	6.2	4747.6	4227.76
2.833333333	4.666666667	2043	1694.555556
17.86956522	20.34782609	9270.717391	8876.781191
40.1011236	30.19101124	39196.67416	38273.60687
4.230769231	4.230769231	727.7692308	596.4023669
36.71052632	14.36842105	3332.75	3203.209661
54.01694915	23.22033898	87220.72034	85475.88832
2.714285714	2.714285714	57.85714286	38.24489796
38.09333333	25.21333333	160771.2533	156394.1077
40.73170732	30.58536585	76297.84146	74468.7146
9.608695652	12.30434783	2991.434783	2739.591682
21.09756098	12.07317073	2294.853659	2088.475907
1.333333333	3	146	76.55555556
3.857142857	2.142857143	5.571428571	2.12244898
56.56756757	26.67567568	46045.79279	45091.48381
7.222222222	8.222222222	2192.666667	1907.432099
19.8125	7.125	2320.625	2151.625
29.36363636	28.33333333	98644.51515	95582.73737
39.35443038	39.4556962	333306.3544	324949.1546
1.285714286	1.285714286	157.5714286	76.57142857
14	11.76470588	5533.117647	5133.117647
1.5	1	63	27
2.538461538	3.153846154	491.3076923	334.0946746
5	9.933333333	2215.2	1866.755556

log.sigma.0.5.mm.3D_glszm_ZonePercentage	log.sigma.0.5.mm.3D_glszm_LargeAreaLowGrayLevelEmphasis	log.sigma.0.5.mm.3D_glszm_LargeAreaHighGrayLevelEmphasis	log.sigma.0.5.mm.3D_glszm_HighGrayLevelZoneEmphasis
0.04432133	389.7401563	36031.8125	10.875
0.014967926	4601.747606	473678.381	9.047619048
0.056497175	423.7361111	7273	2.9
0.05380238	826.3886139	243704	29.88461538
0.079207921	152.9722222	2442.625	5.375
0.077868852	406.1425439	7069.947368	4.421052632
0.128571429	14.95583333	1575.888889	7.222222222
0.049875312	370.8964444	29384.65	12.15
0.033875339	803.0245873	237019.34	26.48
0.025873221	10028.78319	221757.5333	6.95
0.058510638	870.6729798	14849.22727	4.409090909
0.043593131	1809.579125	39196.51515	6.787878788
0.035871157	2122.497012	201463.9592	12
0.049242424	1581.420406	26904.30769	7.153846154
0.087912088	160.4634722	16304.46875	13.34375
0.006187662	169048.2392	5142842.433	8.358208955
0.101382488	272.0372475	4252.545455	6.5
0.032216495	2672.847022	253554.62	10.16
0.10989011	179.8909722	2877.95	5.85
0.01297765	23902.08816	578624.3056	5.277777778
0.01635468	15531.23727	1708522.614	10.06024096
0.064906491	747.4993384	67709	11.83050847
0.01369863	26507.00174	571985.375	6
0.142857143	25.46950292	2864.736842	11.73684211
0.019791667	5616.406433	122136.9474	3.578947368
0.039735099	868.5393519	16186.58333	3.5
0.022717344	10559.5394	266940.6154	7.692307692
0.014120668	27387.48131	777680.6909	4.709090909
0.018568353	9155.835004	1026256.812	12.05797101
0.023626698	7837.861285	183992.475	4.75
0.012616044	35915.99801	905803.9057	6.132075472
0.00747198	59445.71371	6646128.333	10.53030303
0.045356371	1273.235456	130652.5079	14.47619048
0.266666667	4.828125	112.25	7.5
0.123893805	42.52668651	2868.928571	14.35714286
0.032233883	2025.294548	196675.8837	10.86046512
0.008967269	33108.41111	4203877.4	8.9
0.029023747	4891.161588	536528.3523	16.42045455
0.049844237	659.9665799	12397.9375	6.9375
0.052141527	501.9473611	46531.21429	7.5
0.053146853	631.0848173	63986.26316	12.63157895
0.099737533	198.564152	16558.15789	11.23684211
0.04368932	881.0403318	77168.55556	13.69444444
0.056947608	385.434783	30740.36	13.8
0.069058296	616.3647562	58981.7013	17.54545455
0.005063705	54962.60815	7164991.548	5.129032258
0.064821559	894.8103994	268647.2697	26.7752809
0.070175439	167.9990278	14881.9375	8.4375
0.272727273	5.229166667	181.1666667	7.833333333
0.054794521	124.7092014	8103.75	10.25
0.02377451	17340.62107	442556.9278	7.278350515
0.042183623	475.6277614	40569.35294	10.29411765
0.02629108	2049.820554	1520791.488	28.73809524
0.046025105	590.3819444	11603.18182	4.818181818
0.008137272	61169.51843	7909945.315	11.57608696
0.056716418	271.0194152	19741.05263	11.78947368
0.072247706	539.7635053	39046.1746	12.53968254
0.085152838	88.63728967	18912.33333	21.74358974
0.048022599	347.4655882	35119.70588	7.117647059
0.030042918	1921.923611	40419.78571	4.5
0.032302405	2133.82529	216526.9149	6.255319149
0.025531287	11564.02452	1333472	17.05202312
0.015218459	26834.23228	753057.4194	8.387096774
0.012345679	6852.99702	803487.2727	9.136363636
0.075471698	97.34027778	1523	3.75
0.030381944	1505.896849	175864.5143	18.65714286
0.161904762	31.76300654	1423.294118	11.64705882
0.01817133	8562.242928	980517.9841	8.317460317
0.041958042	419.3703704	6271.5	4.166666667
0.034040179	2349.025583	256607.6393	9.967213115
0.025779146	4640.906235	515205.7463	10.59701493
0.035273369	1953.455556	35402.65	4.35
0.010769449	31752.89896	3645275.75	10.61842105
0.007661067	141922.6982	4080867.88	4.722891566
0.014739307	41444.19642	5492736.547	18.27102804
0.087443946	99.94377293	27141.76923	21.23076923
0.043859649	516.3571389	45216.8	11.45
0.053571429	226.503912	18978.91667	7.916666667
0.050383352	993.6622343	88801.34783	8.47826087
0.032914201	3943.975265	412133.3708	11.79775281
0.087248322	82.82252137	6615.538462	9.461538462
0.087861272	191.7971412	60475.34211	23.18421053
0.023939947	985.3009578	7809516.661	92.94067797
0.225806452	7.623015873	494.4285714	11.14285714
0.015114873	33610.12308	880655.04	8.093333333
0.023381808	16805.61916	386957.3415	7.804878049
0.063013699	316.3164251	29270.86957	6.695652174
0.069609508	283.3775678	20211.12195	14.17073171
0.12	37.94907407	727.8333333	6.833333333
0.538461538	1.228174603	33.14285714	9.142857143
0.032370954	1168.733727	1852720.631	37.96396396
0.059210526	224.1284722	22753	4.111111111
0.076923077	242.963941	23274.65625	14.5625
0.018072289	9756.322832	1061486.409	9.666666667
0.010938798	69605.01237	1827297.658	5.101265823
0.111111111	22.24876984	1398.428571	13.57142857
0.05	551.2855676	59309.47059	17.23529412
0.166666667	10.02256944	465.5	7.5
0.079754601	52.83480769	4931.076923	9.615384615
0.053571429	423.3666667	13649.2	1.933333333

log.sigma.0.5.mm.3D_glszm_SmallAreaEmphasis	log.sigma.0.5.mm.3D_glszm_LowGrayLevelZoneEmphasis	log.sigma.0.5.mm.3D_glszm_ZoneEntropy	log.sigma.0.5.mm.3D_glszm_SmallAreaLowGrayLevelEmphasis
0.541809824	0.212256944	3.405639062	0.1279507
0.667650784	0.271415344	2.951143018	0.203870314
0.604457589	0.747222222	2.160964047	0.515465285
0.786092705	0.081105893	3.533081237	0.060189123
0.39580534	0.461805556	2.75	0.168550258
0.507710092	0.674707602	3.005314857	0.34531761
0.378317893	0.440401235	2.947702779	0.258679966
0.618115545	0.285944444	3.101609497	0.188744557
0.677268298	0.091220522	3.749360544	0.069404703
0.695293594	0.631888889	3.230703096	0.428975562
0.549816241	0.602272727	3.044912733	0.292788309
0.576090587	0.58733165	3.467451695	0.322459865
0.664966164	0.17185941	2.858105696	0.103715999
0.564582217	0.384348291	3.112585391	0.156758278
0.623809901	0.238680556	3.608458593	0.157227833
0.655980539	0.442060531	3.133691515	0.241207805
0.575717513	0.436237374	2.970573096	0.160035688
0.686487466	0.180772222	3.063864803	0.114546842
0.36964341	0.513194444	3.446439345	0.112445454
0.778684215	0.533371914	2.43874392	0.39804718
0.777506698	0.195713688	2.540971444	0.150066935
0.683520972	0.187916523	3.351739447	0.134960755
0.815277815	0.488715278	2.300412999	0.395876741
0.677095485	0.23	3.321104331	0.188632607
0.546122859	0.647660819	2.794788541	0.348793056
0.292445953	0.652777778	3.022055209	0.209350143
0.681356875	0.525608974	3.002008751	0.362305027
0.796153771	0.724242424	2.510600849	0.550194166
0.56739144	0.184041868	4.118369042	0.10064066
0.691145933	0.6640625	2.688803163	0.39618057
0.699230881	0.637646751	2.953057202	0.433763104
0.677316792	0.166645623	3.045281352	0.118168939
0.68367324	0.250786902	3.610050909	0.199223929
0.294722222	0.355902778	2	0.046674383
0.656114227	0.222123016	2.835237745	0.183789202
0.59766026	0.188927649	3.377593875	0.107167369
0.621264263	0.219166667	3.480924306	0.135658658
0.724002442	0.140224116	3.090449554	0.102858473
0.441939843	0.496527778	3.280639062	0.221497902
0.693788078	0.29780754	3.039148672	0.232910841
0.639657157	0.185826023	3.439268146	0.120946689
0.721744736	0.184766082	3.043337053	0.135816388
0.701087107	0.174205247	3.471001128	0.105859961
0.773629348	0.231727438	3.399079571	0.195220018
0.722258682	0.181621242	3.687477592	0.131873016
0.887096781	0.256890681	1.144634867	0.239193549
0.601296714	0.088405589	4.601771936	0.05518476
0.5340093	0.228194444	3.108458593	0.132089907
0.670781893	0.414351852	1.918295834	0.354909694
0.326683822	0.232638889	2.75	0.16106011
0.686617453	0.616718213	3.099703406	0.419107604
0.558015031	0.196388889	3.286895755	0.114893605
0.648719497	0.063081639	3.814788485	0.042321525
0.410976096	0.604166667	3.277613437	0.235936015
0.695660271	0.192003229	3.40602077	0.134343396
0.701780578	0.423625731	3.076103171	0.349755135
0.622223621	0.336186067	4.067463887	0.234392058
0.572646139	0.138756904	3.879532439	0.098275582
0.572314603	0.32874183	3.286895755	0.21827049
0.750004526	0.685515873	2.117963175	0.552579968
0.588360429	0.234674941	3.121352528	0.142060532
0.684059092	0.13531225	3.620000333	0.07460421
0.798499113	0.494036738	2.588455655	0.405390066
0.709659224	0.215050505	2.855603777	0.164460238
0.073533309	0.590277778	2	0.069924126
0.671028513	0.21	3.413461651	0.146481294
0.640599944	0.351013072	3.00719649	0.316657389
0.661766919	0.230057319	3.222901059	0.159267785
0.063252303	0.578703704	2.251629167	0.02591978
0.566142794	0.227727687	3.748499527	0.128377673
0.754325026	0.190543118	2.97833981	0.135006418
0.591122051	0.606944444	2.765957321	0.313340769
0.703628237	0.175244883	3.112344875	0.122328749
0.752909788	0.748950701	2.5833597	0.546581037
0.649521351	0.156464016	3.946901117	0.112333402
0.634170467	0.142270081	3.630804142	0.104893876
0.540428663	0.183430556	3.341446071	0.099762151
0.470206203	0.252175926	3.022055209	0.160802876
0.6399045	0.239969807	3.017537231	0.165141591
0.696581819	0.182777778	3.507518024	0.127488928
0.562185269	0.223162393	3.180832987	0.149587131
0.720708435	0.115034405	3.861727135	0.090879419
0.701772472	0.021257364	4.009598177	0.017771517
0.611506837	0.23015873	1.664497779	0.072464604
0.739038288	0.498174074	3.072655768	0.306613078
0.730800975	0.517510163	2.91079715	0.364140039
0.646141593	0.235809179	2.277845241	0.161534247
0.735518284	0.237987805	3.030510901	0.205987938
0.379518367	0.282407407	2.251629167	0.045229176
0.735873016	0.244047619	2.235926351	0.195652557
0.737117216	0.050678321	3.618860776	0.041238907
0.638681658	0.440200617	2.552179697	0.368002187
0.796949143	0.197664931	3.171765829	0.162309611
0.688815143	0.201531987	3.251044616	0.142828784
0.732942169	0.699082278	2.67047895	0.482305739
0.300966028	0.248769841	2.807354922	0.019945227
0.661909618	0.162144358	3.273833774	0.116731092
0.50456571	0.355902778	2	0.26656097
0.410994238	0.27017094	3.546593564	0.183693929
0.58415633	0.840740741	2.330124965	0.534149551

log.sigma.0.5.mm.3D_ngtdm_Coarseness	log.sigma.0.5.mm.3D_ngtdm_Complexity	log.sigma.0.5.mm.3D_ngtdm_Strength	log.sigma.0.5.mm.3D_ngtdm_Busyness	log.sigma.0.5.mm.3D_ngtdm_Contrast
0.020277249	0.012747872	0.148693308	2.663555414	5.08E-05
0.003798741	0.003683582	0.036929679	13.19611652	1.06E-05
0.050895622	0.005254306	0.092912353	3.699687059	0.000181225
0.003260247	0.01143012	0.121966711	5.560661136	5.61E-06
0.077723899	0.011367018	0.139214753	2.753117797	0.000487621
0.0356939	0.009483617	0.144963712	3.027414492	7.99E-05
0.067598486	0.111276651	0.497648258	1.065356118	0.001025911
0.020053889	0.018631585	0.229358997	2.236202374	4.00E-05
0.005024203	0.008913619	0.072745148	5.792151849	1.84E-05
0.002257114	0.003727237	0.052001661	24.25673177	4.37E-06
0.021926068	0.002682034	0.04130825	8.406145778	8.61E-05
0.007955222	0.008203618	0.117504505	9.623546012	2.42E-05
0.004270745	0.003469508	0.040178529	11.45104174	9.09E-06
0.015609959	0.003863138	0.072237884	6.38199265	2.45E-05
0.017147552	0.023478708	0.196639914	2.533721683	6.60E-05
0.000388719	0.000311956	0.002272641	255.7954739	2.04E-06
0.0467526	0.011005107	0.174361637	2.484717929	9.92E-05
0.003933084	0.002875144	0.037706747	12.34363328	7.15E-06
0.055172262	0.014308073	0.19183872	2.244053775	0.000154254
0.001833783	0.001021974	0.010653818	56.50802182	6.23E-06
0.000913661	0.001906651	0.028436692	40.27215629	1.71E-06
0.007092282	0.011466441	0.164623932	4.377304833	9.25E-06
0.001764071	0.000946	0.010329259	57.00330089	5.34E-06
0.042833255	0.074009174	0.507307816	1.084170403	0.000253272
0.005373353	0.001573364	0.014838839	35.904222	3.81E-05
0.023778071	0.003675857	0.050620193	8.204637025	0.000125881
0.002031509	0.002427859	0.024207067	35.35230422	6.35E-06
0.001117657	0.001747417	0.020217394	62.01895302	3.55E-06
0.001356759	0.002087588	0.022221165	27.88743162	2.78E-06
0.002970281	0.001771851	0.016362407	35.91555355	1.29E-05
0.001104556	0.001297553	0.01371531	64.08965451	3.01E-06
0.000525267	0.000595747	0.006103958	91.81752855	1.26E-06
0.004494509	0.008393584	0.097880095	7.190409124	8.40E-06
0.26431121	0.320734482	1.216268773	0.556385118	0.00748517
0.06727874	0.045719545	0.401411292	0.992659349	0.000316422
0.004807086	0.005446062	0.066557587	8.052550777	8.01E-06
0.000665152	0.001221438	0.011800603	55.42469674	1.43E-06
0.001612946	0.002681958	0.025553652	23.56157497	3.90E-06
0.023844686	0.008668709	0.09343186	5.142138609	0.000102921
0.013606264	0.008329981	0.10395029	3.852705694	3.01E-05
0.00831152	0.01021098	0.120017753	4.653027805	1.61E-05
0.023270445	0.010862099	0.149394074	2.294380539	4.08E-05
0.00899369	0.008907194	0.108463362	4.619795899	1.68E-05
0.017980766	0.024270927	0.369509977	1.897340514	2.48E-05
0.005875499	0.011771449	0.104031676	6.017602153	1.90E-05
0.000673036	0.000960986	0.008127562	70.77354648	2.08E-06
0.002191128	0.011374268	0.112622673	7.312956646	4.59E-06
0.035716464	0.018789119	0.2490942	1.462788864	6.49E-05
0.223097176	0.169427637	0.944266527	0.58006904	0.003336346
0.045653102	0.024534723	0.192355282	2.258495939	0.000330831
0.001161772	0.001399553	0.013329293	63.49213702	4.11E-06
0.020510766	0.010801333	0.140255285	2.636636914	3.86E-05
0.001733552	0.004752121	0.06382044	9.216934682	2.19E-06
0.026017281	0.011831122	0.125287807	4.225483875	0.00010258
0.000379701	0.001054514	0.011370591	76.96216948	6.17E-07
0.024606222	0.024332855	0.247733291	1.909994773	6.77E-05
0.008623508	0.009253329	0.094076292	5.56883725	2.77E-05
0.012859047	0.033191742	0.199884824	2.49838288	7.39E-05
0.016054586	0.014506285	0.143292897	3.199328929	5.14E-05
0.011549066	0.005939111	0.063638122	8.891490493	3.87E-05
0.004158043	0.003377419	0.036466644	12.35957435	1.08E-05
0.000729893	0.001756341	0.019032996	42.1864495	1.37E-06
0.001082494	0.001391958	0.013199424	64.23287053	3.35E-06
0.002753712	0.002864852	0.031008316	17.89619627	6.86E-06
0.196704416	0.01665007	0.320030094	1.020605332	0.000650512
0.004741906	0.007171881	0.065295386	8.605138344	1.51E-05
0.059509373	0.059374475	0.371720058	1.418350432	0.000561169
0.001493402	0.001517205	0.014995638	34.18864489	4.55E-06
0.060780098	0.007892877	0.09918019	3.869644426	0.000378669
0.003040728	0.00433696	0.045957515	12.73076604	6.72E-06
0.001956035	0.002972953	0.03202159	19.35030816	3.95E-06
0.012993391	0.001957846	0.026573931	15.4962959	7.28E-05
0.000656585	0.000759821	0.00747892	74.46957495	1.73E-06
0.00039556	0.000935996	0.013078156	129.8477626	7.91E-07
0.00029981	0.000847209	0.008400223	98.0580277	5.87E-07
0.01631657	0.039923239	0.344704236	1.419662216	3.85E-05
0.016167595	0.010279324	0.115086367	3.326010104	3.82E-05
0.038421255	0.018127686	0.263574706	1.381541783	6.26E-05
0.007489036	0.004734019	0.062246525	6.74289579	1.49E-05
0.001968899	0.002833008	0.030626611	19.34304365	3.99E-06
0.049786354	0.031816968	0.343358298	1.159995327	0.000128246
0.007020141	0.02089421	0.167286449	3.13976399	2.08E-05
0.00096679	0.005318686	0.082897952	8.220860499	1.57E-06
0.203864499	0.101293017	0.80728693	0.449886944	0.001761687
0.000962356	0.001097468	0.011572086	74.79538859	2.78E-06
0.001390296	0.001539306	0.016522163	51.30337554	3.92E-06
0.019056878	0.008046202	0.11038625	3.773283968	5.46E-05
0.01260232	0.01381788	0.143518065	3.608510441	3.07E-05
0.118489809	0.03061547	0.271367149	2.244559676	0.001553222
0.324526732	0.361801446	1.359622327	0.455208333	0.007947415
0.001485177	0.006403236	0.087554961	7.778922444	1.90E-06
0.021828616	0.010286823	0.116973797	3.72769622	0.000116065
0.014945997	0.019089936	0.186571543	2.722905435	4.02E-05
0.001402119	0.001402866	0.014622884	35.47380587	3.74E-06
0.000623092	0.000771808	0.007796335	113.4597275	1.75E-06
0.100868598	0.167205356	1.102753916	0.570909466	0.000694944
0.008875433	0.018151653	0.183434767	3.674979927	1.98E-05
0.20275578	0.138174531	0.970224739	0.462378927	0.001629229
0.042612665	0.032121391	0.292747718	1.326336295	0.000151349
0.019685802	0.005239149	0.053089305	10.52030255	0.000216951

original_gldm_GrayLevelVariance	original_gldm_HighGrayLevelEmphasis	original_gldm_GrayLevelNonUniformityNormalized	original_gldm_DependenceEntropy	original_gldm_DependenceNonUniformity
14.25523131	596.3878116	0.275573392	5.694047013	26.70083102
8.302931962	523.8809694	0.281777314	6.176825843	71.53171775
6.885441604	253.5706215	0.167033739	5.308771232	19.59887006
53.55762696	1383.2597	0.238069749	6.355721454	172.1536472
53.89334379	360.6633663	0.046564062	5.501835369	32.98019802
26.03592784	400.0778689	0.094766192	5.975776533	41.76229508
42.67122449	186.3285714	0.055102041	4.868728289	34.37142857
28.55281995	656.4239401	0.12984372	5.804862518	56.42643392
240.5515286	1065.167344	0.052557267	7.103833451	242.1178862
10.85788007	880.4083657	0.230376359	6.093272059	145.0526089
30.24229714	464.4547872	0.150916704	6.071056084	44.20744681
14.97244738	949.1347424	0.197018056	5.974359805	61.69484808
39.88244578	648.1222548	0.118652315	6.444773985	153.0995608
36.064046	631.9602273	0.080915691	6.271541886	88.28030303
60.5152382	713.9917582	0.041359739	6.257021316	111.3681319
8.538799201	1069.903491	0.305143672	6.248671123	520.8328408
46.80315997	609.1705069	0.055872921	5.719877602	63.02764977
18.38804436	601.2744845	0.199910823	6.481754609	104.2280928
53.62314334	683.3021978	0.057058326	5.885883671	43.48351648
6.655286186	496.6535689	0.400534316	5.934617921	113.5414564
6.45796792	1634.560591	0.406539426	5.605968071	234.35133
34.17413204	1244.586359	0.089321913	6.5768656	134.049505
6.375819103	629.3910959	0.362503753	5.984543798	127.109589
122.1654135	606.1654135	0.03454124	5.73679938	52.66917293
4.16124566	380.6020833	0.220492622	6.016124256	68.98541667
7.689882023	181.3708609	0.201438533	5.676839558	28.66225166
10.80215806	729.201398	0.248567951	5.971252209	143.2315422
3.497487876	1716.075995	0.393964482	5.829352025	157.8569961
19.76588656	1358.900431	0.368662816	6.143903994	159.1280947
7.981121668	758.0874188	0.323977086	5.71431077	88.60543414
6.656277586	1074.068079	0.301557461	5.784906624	237.1204475
12.23629223	902.5421714	0.252838008	6.387930079	446.7864825
33.21749569	916.1929446	0.259019106	6.171627496	97.99784017
38.64888889	141.3333333	0.102222222	3.373557262	9.133333333
61.86780484	777.5486726	0.09092333	5.536474095	29.61946903
40.01908791	840.2218891	0.211806765	6.231153971	115.9895052
4.143030606	941.2294126	0.492577142	5.410786196	291.9675684
24.36964899	1183.691293	0.249815077	6.230395211	182.6728232
61.4801875	718.1806854	0.043079939	6.310301285	91.11838006
10.42866605	430.2588454	0.291588208	5.824327346	34.1396648
19.86833586	980.0013986	0.272312583	5.885267187	51.24895105
33.60534854	613.3910761	0.143075619	5.856705505	55.81364829
34.15761235	706.0618932	0.106681238	6.423130009	107.6820388
42.97115519	764.8542141	0.099454652	6.203091863	71.92938497
87.5703513	1187.251121	0.04419554	6.750455417	272.2914798
1.611579817	211.6247958	0.260038639	5.966148026	387.9032996
73.3241493	1774.721413	0.083892667	6.695157153	410.7844137
29.62671207	492.8201754	0.126231148	5.639877772	36.24561404
48.96694215	250.0909091	0.082644628	4.004886164	12.18181818
76.25112591	603.0479452	0.060424095	5.674356196	44.94520548
14.23590903	668.0156863	0.257748462	6.119358756	229.4544118
21.5502466	621.7394541	0.229303795	5.795611993	34.5235732
15.79530947	1045.230986	0.24184884	6.029444704	213.3574335
14.40506994	221.4518828	0.131597836	5.694838691	29.56903766
4.427381758	1083.892181	0.319116785	5.944319973	568.4303909
65.67190911	1175.456716	0.109841836	6.086843559	51.4
50.29531921	897.0252294	0.068549575	6.380419477	192.0068807
118.599078	886.4213974	0.028565435	6.502829023	177.0742358
34.61796578	557.5282486	0.110536564	6.020151558	51.74011299
9.897534491	320.8218884	0.119315147	6.031861731	54.9527897
10.00844109	556.057732	0.195692068	6.05135859	109.4618557
33.47888267	1318.504575	0.357959947	5.857060024	351.4817001
7.670033444	538.4781541	0.220117203	6.192144387	247.8271969
3.648753403	273.7732884	0.344121474	5.97461636	80.42199776
4.224279103	44.41509434	0.223923104	4.418786908	6.471698113
46.21809594	1282.302083	0.129157926	6.46026535	126.5190972
47.90439909	433.9333333	0.056689342	5.264597904	32.33333333
11.98662339	823.2094029	0.248818041	6.205230149	202.2327661
22.87397917	331.6993007	0.128759353	5.515020792	20.56643357
13.84254393	663.608817	0.232578823	6.07268623	113.3705357
15.71753513	756.9438245	0.153786174	6.498441415	217.9572913
14.31608546	655.3791887	0.150509038	6.035972635	56.14638448
7.113078027	905.5153748	0.356773482	6.146553058	299.4744226
3.686000048	1077.019476	0.300404081	5.84245142	594.5816873
19.4552066	1461.37785	0.190645368	6.503835804	970.2718507
64.31056828	943.8452915	0.060337027	6.364021862	107.9910314
30.86264524	637.0372807	0.081698215	6.155440765	79.23245614
10.26098135	380.3080357	0.171117666	5.445340569	24.55357143
22.48378479	723.4282585	0.152917155	6.18001501	93.55202629
19.13502624	1006.126479	0.332725898	6.071082121	127.6198225
24.45205171	306.8724832	0.082834107	5.708319484	34.11409396
77.54653213	1203.401156	0.075096395	6.563578617	151.0924855
30.13701068	1684.591398	0.204731945	6.312373019	332.2408196
41.22164412	268.1612903	0.069719043	4.28468381	17.70967742
8.657737702	776.1777509	0.219901806	6.339172583	305.6352277
27.29285768	765.4833191	0.147760449	6.482426632	319.4927288
14.64382811	414.7534247	0.18660912	5.981192523	33.49863014
48.82128208	933.2020374	0.066294632	6.218902988	127.0747029
33.12	525.96	0.1312	4.40637013	9.44
46.85207101	181.7692308	0.112426036	3.238901257	13
21.48944658	857.6757072	0.234570453	6.332952851	226.1146107
5.988919668	199.0526316	0.16984072	5.892517935	29.05921053
43.41974852	931.9182692	0.124341254	6.370232271	49.55769231
7.482863746	474.5492881	0.27613206	6.219269	190.7190581
5.225670033	749.8762116	0.285478822	5.746979299	400.5350318
52.0382968	942.5396825	0.0909549	5.103953733	15.03174603
81.6707526	831.3029412	0.08490917	6.603793446	108.2411765
22.984375	201.875	0.121527778	4.053508855	8
45.91825059	317.4601227	0.046407467	5.61375772	69.36809816
3.915255102	80.97857143	0.171632653	5.700898337	31.49285714

original_gldm_GrayLevelNonUniformity	original_gldm_SmallDependenceEmphasis	original_gldm_DependenceNonUniformityNormalized	original_gldm_DependenceVariance	original_gldm_LargeDependenceEmphasis
99.48199446	0.230529865	0.073963521	42.56899502	117.5041551
395.3335709	0.131372851	0.050984831	40.08448447	141.6443336
29.56497175	0.239769579	0.110728079	9.843403875	31.77966102
460.1888257	0.281566291	0.089060345	62.62835887	144.5809622
4.702970297	0.530924092	0.326536614	0.869326537	4.445544554
23.12295082	0.385663513	0.171156947	19.12321956	36.59836066
3.857142857	0.724305556	0.491020408	0.564081633	2.771428571
52.06733167	0.355816451	0.140714299	16.44007189	39.77306733
77.57452575	0.385559586	0.164036508	17.49862663	34.57859079
534.2427771	0.167826578	0.062549637	34.53942524	98.6291505
56.74468085	0.297494257	0.117572997	44.48528746	87.56914894
149.1426684	0.204067564	0.081499139	18.2215657	57.94319683
162.079063	0.290634569	0.112078741	26.99249714	61.4114202
42.72348485	0.394260214	0.167197544	12.86690771	29.04924242
15.05494505	0.551024068	0.305956406	1.88787586	6.247252747
3304.095678	0.07366672	0.048100558	33.51342314	157.7737348
12.12442396	0.518608081	0.290449999	2.343222409	7.175115207
310.2615979	0.195237895	0.067157276	35.6480896	99.48324742
10.38461538	0.469695236	0.23892042	4.469870789	11.65934066
1111.082192	0.108895816	0.04093059	70.72943009	250.2739726
2063.187586	0.110761738	0.046177602	52.29074604	192.4250246
81.19361936	0.357148902	0.147469202	15.73517	33.73927393
1058.510959	0.128317783	0.043530681	73.43257225	228.0260274
4.593984962	0.63053467	0.396008819	0.690033354	3.45112782
211.6729167	0.143402809	0.071859809	19.51581597	66.03958333
60.83443709	0.225062132	0.094908118	18.2310425	51.50331126
568.9720402	0.155331965	0.06257385	27.57897487	86.55788554
1534.491656	0.077219179	0.040528112	49.99850808	191.3794608
1369.951023	0.133187133	0.042822415	68.44623372	231.0979548
548.4932073	0.130391055	0.052336346	36.15644349	129.3650325
1266.842895	0.108612452	0.05644381	25.86833617	110.8143299
2233.318125	0.133991393	0.050581511	41.02594194	135.6061361
359.7775378	0.223716898	0.070552801	46.62083707	130.1807055
1.533333333	0.8	0.608888889	0.195555556	1.8
10.27433628	0.515160335	0.262119195	8.370428381	17.15929204
282.5502249	0.270078696	0.086948655	59.10999598	132.5757121
3295.833657	0.067493982	0.043635864	56.53108567	250.7399492
757.439314	0.184894228	0.060248293	42.7665569	125.8937995
13.82866044	0.507649573	0.283857882	2.198872294	7.049844237
156.5828678	0.179348873	0.063574795	32.61781953	106.027933
194.7034965	0.223635184	0.071676855	43.50762189	110.8853147
54.51181102	0.377603257	0.146492515	27.34740047	56.49606299
87.90533981	0.331503423	0.130682086	16.24782614	37.98786408
43.66059226	0.384180022	0.163848257	17.56817368	36.39863326
49.27802691	0.456475981	0.244207605	3.672829938	10.1103139
1591.95655	0.070738189	0.063362185	18.27477179	93.64848089
230.3692644	0.34926702	0.149593741	8.622897425	23.12163146
28.78070175	0.380053259	0.158971991	11.45129271	29.85087719
1.818181818	0.765782828	0.553719008	0.975206612	3.363636364
8.821917808	0.567804243	0.307843873	5.783824357	12.1369863
1051.613725	0.149192208	0.056238826	36.77081507	113.7284314
92.40942928	0.246807286	0.085666435	27.76939702	81.09925558
772.7070423	0.172816521	0.066778539	23.8154883	80.83098592
31.45188285	0.299501884	0.123719823	11.04735561	30.60669456
3607.934371	0.08101202	0.050276879	30.4329958	136.4650628
36.79701493	0.37246501	0.153432836	21.68417019	44.69552239
59.77522936	0.442218996	0.220191377	4.032799217	11.41972477
13.08296943	0.614139962	0.386624969	1.280315021	4.423580786
39.1299435	0.346755124	0.146158511	10.52529605	26.43502825
55.60085837	0.245131422	0.117924441	8.497872497	27.69957082
284.7319588	0.18132338	0.075231516	18.52920206	62.05292096
2425.5366	0.152574132	0.051871561	56.58171698	186.6009445
896.7574865	0.133345827	0.060831418	24.92935408	87.52331861
613.2244669	0.114049154	0.04513019	47.49628975	167.2648709
11.86792453	0.255479286	0.122107512	13.73798505	40.66037736
148.7899306	0.279002793	0.109825605	14.33361967	38.52430556
5.952380952	0.534259921	0.307936508	1.814603175	6.085714286
862.6521488	0.143165617	0.058330766	28.52949175	106.0781656
18.41258741	0.318523355	0.143821214	7.59773094	22.55244755
416.78125	0.167507942	0.063264808	31.63466473	93.24665179
399.6902655	0.191498613	0.083861982	15.37263111	49.67102732
85.33862434	0.240049165	0.099023606	12.85125152	39.82892416
2517.750461	0.084216371	0.042436506	48.26074326	218.9005243
3254.577811	0.084810916	0.054881086	25.31656244	114.6586672
2767.980095	0.136186722	0.066827733	19.03505176	75.71409877
26.9103139	0.495778129	0.242132357	6.662008084	15.05381166
37.25438596	0.37821095	0.173755386	7.180805633	18.43859649
38.33035714	0.261619352	0.109614158	10.69515306	33.59821429
139.6133625	0.270903373	0.102466622	19.35344285	48.8313253
899.6908284	0.146685518	0.04719668	60.9169103	202.3720414
12.34228188	0.479140914	0.228953651	6.250529255	15.65771812
64.9583815	0.392590627	0.174673394	6.746941094	18.18150289
1009.123757	0.157957432	0.067405319	20.90003832	73.64434977
2.161290323	0.777777778	0.571279917	0.283038502	2.032258065
1091.152761	0.123929692	0.061595169	22.51198248	93.36074164
518.1958939	0.231270459	0.091101434	17.11223575	49.26860565
68.11232877	0.257976371	0.091777069	22.06782511	60.64383562
39.0475382	0.444289674	0.215746524	4.263679627	11.98811545
6.56	0.29747483	0.1888	2.8416	13.6
1.461538462	1	1	0	1
804.3420822	0.191568272	0.065941852	31.89199544	98.3175853
51.63157895	0.231088432	0.095589508	14.34552458	42.95394737
51.72596154	0.301998753	0.119129068	14.11184819	36.35576923
1008.434283	0.134829894	0.052223181	36.67037762	133.8860898
2061.728053	0.107941995	0.055460403	27.00751987	105.2400997
5.73015873	0.428726582	0.238599143	3.020912069	10.04761905
57.73823529	0.375898998	0.159178201	18.02228374	38.59117647
2.916666667	0.524525463	0.333333333	1.333333333	5.333333333
7.564417178	0.667658487	0.425571154	0.9636042	3.687116564
48.05714286	0.212865924	0.11247449	9.817295918	31.30714286

original_gldm_LargeDependenceLowGrayLevelEmphasis	original_gldm_SmallDependenceHighGrayLevelEmphasis	original_gldm_LargeDependenceHighGrayLevelEmphasis	original_gldm_SmallDependenceLowGrayLevelEmphasis
0.179639975	100.2561951	78478.74238	0.0037154
0.256016287	49.63261445	79043.18104	0.001716527
0.115272155	43.78769624	9380.135593	0.00736101
0.091825408	268.0915457	231379.628	0.001450643
0.055377499	154.9918757	2011.574257	0.014622708
0.073610469	110.2706094	20321.43033	0.007094066
0.05701061	134.2925595	514.4428571	0.036820297
0.055223292	160.3532202	32040.20449	0.006315349
4.929080319	377.9238323	22271.8435	0.007130279
0.103778281	114.1220947	94423.85123	0.000752612
0.148980177	89.24202834	54083.88032	0.005297998
0.058552677	148.7236025	59252.89168	0.002012852
0.082207364	123.1313286	51734.45315	0.002405598
0.038772171	170.6356474	24254.13826	0.004116765
0.017128387	325.6792611	5846.876374	0.005284455
0.144012147	61.87947812	173746.4504	0.000323164
0.017642175	251.6192699	5801.465438	0.008483169
0.150286618	79.11527297	67000.76546	0.001812275
0.024727662	231.7254177	11220.23626	0.01262225
0.475975992	37.21449712	131918.3544	0.000922209
0.114634991	149.9525481	323672.4203	0.000288698
0.024513367	362.1337403	50085.63586	0.001811342
0.339552955	60.2482092	153466.639	0.000735866
0.04610964	347.9569758	2498.255639	0.01446492
0.166351851	46.73276189	26588.58958	0.001809478
0.269358083	29.73529404	10498.55629	0.007310513
0.11201327	88.81682223	67561.9799	0.000947955
0.109497599	116.9748774	335416.8703	0.000314145
0.161072868	126.2917452	332945.246	0.000701616
0.168590324	75.34266005	102331.1353	0.000676467
0.100583684	95.81903774	122654.3033	0.000424951
0.141511329	89.53900522	130480.5866	0.000453796
0.129573632	134.7747069	134290.3542	0.002847514
0.137324698	104.3333333	289.3333333	0.080384101
0.027366411	291.7384391	18114.30973	0.011730719
0.13220833	143.2543028	135106.2819	0.001847146
0.261477507	51.87337958	240691.2378	0.000285726
0.097176354	153.0513966	164309.8305	0.000665097
0.013418237	307.095306	6826.616822	0.004961573
0.228504688	52.52872191	50140.71322	0.004232647
0.104243041	167.7241793	120020.1469	0.001954657
0.079588777	159.8812317	43927.5958	0.007266713
0.047846057	166.506158	33118.9551	0.003916809
0.040882227	218.4747365	36115.37358	0.003507947
0.010414105	431.9443381	15830.1139	0.002331495
0.445139505	13.83033666	19948.31379	0.000889365
0.015618185	488.0725244	48725.55972	0.000883262
0.05690615	127.8691438	18196.84649	0.008371761
0.061177338	134.2556818	1342.636364	0.055514095
0.024631104	239.6286217	11498.43151	0.011707686
0.15740062	70.55336555	82967.76324	0.001011922
0.118222517	100.103832	57587.91811	0.004233293
0.075331571	155.5846317	87353.15837	0.000584648
0.124563735	43.42233758	8562.782427	0.00958527
0.124503669	76.39163812	150133.4505	0.000388032
0.034910427	321.4697484	66434.23881	0.004583514
0.013667242	322.4163402	12260.12156	0.002864797
0.010064324	483.6796751	5446.438865	0.004206326
0.066450967	143.3349594	17477.12147	0.003954617
0.081765567	61.53943701	9914.637339	0.003505156
0.110837992	76.21936833	36810.94158	0.001067615
0.130476805	129.8565892	268575.3101	0.000609874
0.154426906	54.46028593	50203.51939	0.001519251
0.590525957	26.51677891	47615.73176	0.001776857
0.760389983	5.595185175	2415.924528	0.05898504
0.027922621	266.4392231	56929.90451	0.001891663
0.027183984	185.6065668	3633.8	0.014654316
0.123340837	86.78670621	91875.35016	0.000774758
0.115330504	73.76504403	9265.517483	0.008019424
0.130970181	78.21731938	67279.1144	0.001460595
0.064425377	117.7235788	39382.80531	0.001589578
0.057547884	121.4948139	28792.04586	0.002442353
0.234815629	56.58656663	204604.2485	0.000352069
0.104739553	79.2306756	125807.5408	0.000202062
0.050033829	155.9498964	115677.3117	0.00029865
0.016861396	367.2959686	18150.66592	0.004634024
0.0271098	183.8552941	14680.52412	0.003788545
0.089099516	73.75518314	14239.40625	0.010087439
0.061720874	144.8789872	40473.92333	0.001924979
0.187958267	98.93055098	219158.3225	0.000970146
0.072252814	127.0717451	5289.221477	0.026161909
0.017111695	361.2640916	25954.51098	0.004283633
0.044015555	209.6225612	129510.2635	0.000589138
0.051154117	188.9032258	631.6451613	0.047577206
0.115297615	70.61899517	76122.98509	0.000581752
0.057917303	126.7425094	43523.92757	0.001398707
0.139526802	75.06697128	27823.83836	0.007495655
0.012918413	313.099567	14019.32428	0.002904858
0.043788644	105.2999965	8189.24	0.021631053
0.100856464	181.7692308	181.7692308	0.100856464
0.113118313	153.9993669	86401.18402	0.000999824
0.215138685	40.8379342	8918.467105	0.006064392
0.038007543	207.4736452	39393.10096	0.003721537
0.264973707	44.28589763	68161.76013	0.001260663
0.135608799	64.20889365	82021.72002	0.000538823
0.027690013	333.6875062	10302.57143	0.018255768
0.037836377	187.9861185	44962.09412	0.003751543
0.069616602	82.77072917	1354.125	0.050267693
0.027928094	215.4537986	1224.417178	0.012451498
0.412673371	18.07705292	2555.8	0.008456929

original_gldm_LowGrayLevelEmphasis	original_gldzm_DistanceZoneVariabilityNormalized	original_gldzm_LowIntensityEmphasis	original_gldzm_LargeDistanceEmphasis	original_gldzm_HighIntensitySmallDistanceEmphasis
0.004970436	0.976747405	0.015912566	1.035294118	424.85
0.003356645	0.957691746	0.013163746	1.064864865	354.3878378
0.01018692	1	0.029770481	1	185.3181818
0.001964693	0.91989283	0.004966026	1.150779896	909.4783844
0.019092755	0.968262227	0.025504629	1.048387097	289.0040323
0.008577232	1	0.016833225	1	292.1047619
0.040062375	1	0.047265214	1	185.625
0.007168097	1	0.016280607	1	466.9936306
0.048573687	0.57319247	0.021327302	1.998384491	852.6467421
0.001677911	0.811860812	0.004470554	1.409547739	604.2305276
0.006964318	0.969704142	0.015915254	1.046153846	298.3923077
0.002815095	0.962513692	0.009750082	1.057324841	701.089172
0.004757077	0.911056533	0.008848089	1.17218543	397.1482708
0.00497471	1	0.009827683	1	437.1688889
0.006961891	0.973573023	0.009343972	1.040178571	597.0245536
0.001215923	0.76646631	0.004683913	2.127107652	685.926217
0.009285422	1	0.014741665	1	499.0472441
0.003208619	0.941238214	0.008914712	1.105740181	381.5891239
0.013336602	1	0.024118012	1	514.1666667
0.002697339	0.93191232	0.008390796	1.234920635	314.3447478
0.000823597	0.87036844	0.002652645	1.366425993	1232.460038
0.002311143	0.912033154	0.004828474	1.13832853	965.1592219
0.002087508	0.936276862	0.005546714	1.173366834	447.6445422
0.018077895	1	0.021317663	1	566.8333333
0.004057422	0.986577794	0.012038818	1.02027027	316.9527027
0.013021483	1	0.030733431	1	132.6455696
0.002111622	0.897028571	0.006299372	1.177142857	514.4892857
0.000847657	0.88863258	0.004204636	1.227586207	1411.692816
0.001321087	0.942409635	0.005184277	1.118110236	900.8772419
0.002676619	0.914793641	0.007346164	1.17699115	540.0752212
0.001250197	0.780154709	0.00408819	1.673515982	747.1643239
0.001402209	0.823938982	0.003373945	1.882209586	572.6337209
0.003771306	0.95837935	0.012246831	1.108761329	582.2301108
0.09177222	1	0.097130932	1	134.6153846
0.012226487	1	0.02081498	1	597.140625
0.002712643	0.989677575	0.006596295	1.046632124	515.4353951
0.00126717	0.799935899	0.00451282	2.014354067	653.4305635
0.001340542	0.86852054	0.003522509	1.345762712	743.8020833
0.005832743	0.885839871	0.009068557	1.182320442	551.3176796
0.00605621	0.9802	0.022918757	1.03	286.05
0.00271008	0.953243214	0.008440678	1.071856287	725.0958084
0.008176487	1	0.018357144	1	422.7631579
0.004879501	0.972794293	0.011348886	1.04137931	485.1612069
0.004389758	0.93723886	0.008565931	1.097297297	543.5824324
0.002983734	0.680757156	0.004752402	1.692982456	806.4011696
0.005347856	0.674485792	0.014467847	2.376770538	154.8118783
0.001488245	0.661597497	0.002558169	2.056751468	1174.103107
0.009489838	0.957010023	0.021132797	1.065934066	326.2335165
0.055999684	1	0.06797724	1	197.4444444
0.012414904	0.95654321	0.01921544	1.066666667	415.8694444
0.002319989	0.787829519	0.006999689	1.57635468	384.7784145
0.005345984	0.981133787	0.016378245	1.028571429	404.0214286
0.001365884	0.70337177	0.003438152	2.022018349	741.0134811
0.013468532	1	0.03163802	1	139.6184211
0.001234671	0.723543851	0.005229796	2.653799759	771.996438
0.005141911	1	0.011200069	1	874.676259
0.003496069	0.959201389	0.005950833	1.0625	730.2847222
0.005074285	0.933613281	0.006349494	1.103125	761.2140625
0.009596752	0.969704142	0.015062389	1.046153846	403.5634615
0.00587883	0.96748856	0.013941494	1.049586777	237.7107438
0.003520758	0.926845841	0.007852865	1.114068441	390.1140684
0.001264697	0.896224073	0.004088906	1.510086455	745.2545608
0.003111689	0.837684711	0.011580304	1.395176252	352.7411358
0.005018573	0.935850498	0.015313607	1.099526066	222.8293839
0.076251393	1	0.191783723	1	24.88235294
0.002433977	0.92518601	0.006581992	1.131343284	892.7010779
0.016181806	1	0.023628676	1	368.0895522
0.001791079	0.905545728	0.005537281	1.280163599	551.5423341
0.020437365	1	0.032737065	1	231.3921569
0.00273155	0.948823102	0.008770456	1.111111111	440.4277415
0.002658649	0.922574014	0.008336079	1.203592814	578.4635867
0.003572482	0.932057132	0.009902688	1.105633803	475.6320423
0.001362084	0.873407974	0.004241569	1.512437811	596.9310802
0.001046185	0.684556935	0.002545477	3.122911695	732.5510135
0.000904448	0.695269819	0.002367264	3.328541001	874.7947562
0.005121731	0.943596012	0.008688055	1.087136929	728.8340249
0.004726992	0.9788384	0.009418376	1.032085561	479.9692513
0.01192569	1	0.03667819	1	281.483871
0.003081854	0.962560457	0.007075297	1.057251908	513.9704198
0.001808693	0.9365875	0.006634398	1.185	631.3450694
0.02787355	1	0.050951645	1	271.1818182
0.004793249	0.978024691	0.010397329	1.033333333	907.7097222
0.001390629	0.842199351	0.004491688	1.531496063	1172.143657
0.048204089	1	0.056927452	1	251.6538462
0.00168405	0.87860123	0.004830961	1.469115192	504.6642641
0.002480776	0.932286857	0.006017964	1.105263158	508.4590643
0.009160582	1	0.028604981	1	288.9895833
0.003441202	0.972131004	0.00615097	1.042402827	700.4257951
0.022832249	1	0.060416957	1	389.3333333
0.100856464	0.73964497	0.100856464	1.461538462	135.1538462
0.001989462	0.84763611	0.005183462	1.486008837	728.3344723
0.010039553	0.947406866	0.02541455	1.081081081	172.2297297
0.004551935	0.956855536	0.011615043	1.066176471	672.8860294
0.00308741	0.909148104	0.009350057	1.278978389	299.3838409
0.001712196	0.772952608	0.005256122	2.365853659	486.4864201
0.018807094	1	0.035020381	1	838.969697
0.004605417	0.931592978	0.009353709	1.106382979	473.7952128
0.052325775	1	0.081110117	1	173.0666667
0.014576538	0.983607677	0.017513096	1.024793388	315.7768595
0.020261387	1	0.037817102	1	82.73913043

original_gldzm_LowIntensityLargeDistanceEmphasis	original_gldzm_HighIntensityEmphasis	original_gldzm_DistanceZoneVariability	original_gldzm_ZonePercentage	original_gldzm_IntensityVariabilityNormalized
0.015960981	431.2823529	83.02352941	0.235457064	0.078477509
0.013279698	363.5621622	177.172973	0.131860299	0.084353543
0.029770481	185.3181818	44	0.248587571	0.099173554
0.005112107	951.1750433	530.7781629	0.298499741	0.038557771
0.025562164	299.1774194	60.03225806	0.613861386	0.044745057
0.016833225	292.1047619	105	0.430327869	0.060136054
0.047265214	185.625	56	0.8	0.052933673
0.016280607	466.9936306	157	0.391521197	0.06040813
0.088741934	977.6090468	354.8061389	0.419376694	0.032328447
0.005724824	656.8668342	323.120603	0.171625701	0.075414762
0.015989455	305.6153846	126.0615385	0.345744681	0.057159763
0.009799671	717.656051	151.1146497	0.207397622	0.064546229
0.009113972	421.9381898	412.7086093	0.331625183	0.04647457
0.009827683	437.1688889	225	0.426136364	0.054024691
0.009433019	606.3660714	218.0803571	0.615384615	0.036949936
0.005737736	800.6498054	590.9455253	0.071204285	0.073051825
0.014741665	499.0472441	127	0.585253456	0.0499101
0.009070518	397.4048338	311.5498489	0.213273196	0.057347049
0.024118012	514.1666667	96	0.527472527	0.042317708
0.008825825	329.6730159	293.552381	0.113554434	0.069166037
0.002890955	1316.129964	482.1841155	0.109162562	0.080523661
0.004969903	1002.804035	316.4755043	0.381738174	0.048227292
0.005804601	465.9045226	372.638191	0.13630137	0.080768162
0.021317663	566.8333333	96	0.721804511	0.032986111
0.012080699	319.4054054	146.0135135	0.154166667	0.107925493
0.030733431	132.6455696	79	0.261589404	0.098221439
0.006551083	544.4028571	313.96	0.152905199	0.070204082
0.004331846	1493.2	257.7034483	0.074454429	0.129916766
0.005264329	935.9822835	478.7440945	0.136706136	0.046701593
0.007579725	565.8362832	206.7433628	0.133490845	0.086302765
0.004700943	855.6004566	341.7077626	0.10426089	0.091126123
0.004286778	647.757108	1014.268887	0.13936375	0.056013716
0.012348339	600.6132931	317.223565	0.238300936	0.048803863
0.097130932	134.6153846	13	0.866666667	0.100591716
0.02081498	597.140625	64	0.566371681	0.045410156
0.006648229	519.0829016	382.015544	0.289355322	0.045652232
0.005536792	742.9832536	334.3732057	0.062471977	0.100879101
0.003818547	808.9288136	512.4271186	0.194591029	0.051732261
0.009249325	600.3867403	160.3370166	0.563862928	0.039894997
0.022993757	289.05	98.02	0.186219739	0.0748
0.00850484	745.2694611	159.1916168	0.233566434	0.061995769
0.018357144	422.7631579	152	0.398950131	0.056440443
0.011455221	493.1344828	282.1103448	0.351941748	0.046848989
0.008682146	565	173.3891892	0.421412301	0.050577064
0.011581888	949.345614	388.0315789	0.511210762	0.031388119
0.020990458	189.0028329	238.0934844	0.057660895	0.138762048
0.0228808	1372.124266	676.1526419	0.372177713	0.03585311
0.021226787	337.8131868	87.08791209	0.399122807	0.059533873
0.06797724	197.4444444	18	0.818181818	0.067901235
0.019274884	434.5777778	86.08888889	0.616438356	0.039753086
0.008065857	444.001642	479.7881773	0.149264706	0.05666238
0.01642396	408.4857143	103.0190476	0.260545906	0.066666667
0.00477828	890.6954128	383.3376147	0.17057903	0.063082232
0.03163802	139.6184211	76	0.317991632	0.077908587
0.006710981	920.0506634	599.8178528	0.073323899	0.090822579
0.011200069	874.676259	139	0.414925373	0.043631282
0.006028281	744.0138889	414.375	0.495412844	0.037390689
0.006439398	800.5375	298.75625	0.698689956	0.027441406
0.015135447	411.1384615	126.0615385	0.367231638	0.051597633
0.014089569	242.2975207	117.0661157	0.259656652	0.075336384
0.008026205	409.1634981	243.7604563	0.180756014	0.070277147
0.00443942	809.70317	932.9692603	0.15363046	0.039889045
0.012337921	392.1651206	451.5120594	0.132302405	0.076011717
0.015644099	230.4075829	197.464455	0.118406285	0.089283709
0.191783723	24.88235294	17	0.320754717	0.134948097
0.006673899	935.8149254	309.9373134	0.290798611	0.043207841
0.023628676	368.0895522	67	0.638095238	0.051904656
0.005885044	583.0899796	442.8118609	0.14104413	0.058535219
0.032737065	231.3921569	51	0.356643357	0.062668205
0.008925554	455.4705882	290.3398693	0.170758929	0.059763339
0.008598062	604.1497006	462.2095808	0.192766449	0.051645213
0.010042363	496.1830986	132.3521127	0.250440917	0.064768895
0.004803309	648.3482587	526.6650083	0.085447074	0.073235261
0.004427538	904.8639618	573.6587112	0.077349086	0.09806563
0.003962821	1089.774228	1305.71672	0.129347751	0.047719176
0.008762076	755.0124481	227.406639	0.540358744	0.037103356
0.00946187	486.0855615	183.0427807	0.410087719	0.055105951
0.03667819	281.483871	62	0.276785714	0.098335068
0.007142457	526.3969466	252.1908397	0.286966046	0.059000058
0.00683049	658.0925	374.635	0.147928994	0.055275
0.050951645	271.1818182	77	0.516778523	0.05515264
0.010420243	920.1388889	352.0888889	0.416184971	0.031805556
0.004811248	1290.812336	641.7559055	0.154595253	0.050743657
0.056927452	251.6538462	26	0.838709677	0.059171598
0.005437294	547.2954925	526.2821369	0.120717453	0.066050541
0.006206698	527.425731	797.1052632	0.243798118	0.047931329
0.028604981	288.9895833	96	0.263013699	0.0625
0.006196055	711.1378092	275.1130742	0.480475382	0.042864813
0.060416957	389.3333333	18	0.36	0.080246914
0.102045511	181.7692308	9.615384615	1	0.112426036
0.005699845	797.5360825	575.544919	0.198016915	0.049373049
0.025854443	176.2837838	70.10810811	0.243421053	0.093864134
0.01170158	687.6102941	130.1323529	0.326923077	0.048442907
0.009896156	319.1827112	462.7563851	0.139375685	0.075277616
0.007091416	567.2859079	570.4390244	0.10218776	0.092221708
0.035020381	838.969697	33	0.523809524	0.061524334
0.009451606	503.4893617	262.7092199	0.414705882	0.034077763
0.081110117	173.0666667	15	0.625	0.093333333
0.017549772	319.9669421	119.0165289	0.742331288	0.043371354
0.037817102	82.73913043	69	0.246428571	0.098508717

original_gldzm_LowIntensitySmallDistanceEmphasis	original_gldzm_IntensityVariability	original_gldzm_HighIntensityLargeDistanceEmphasis	original_gldzm_SmallDistanceEmphasis	original_glcm_SumVariance
0.015900463	6.670588235	457.0117647	0.991176471	2136.499733
0.013134758	15.60540541	400.2594595	0.983783784	1879.653018
0.029770481	4.363636364	185.3181818	1	854.4478634
0.004933183	22.24783362	1154.341421	0.968082034	5068.98179
0.025490245	2.774193548	339.8709677	0.987903226	1204.732989
0.016833225	6.314285714	292.1047619	1	1393.235763
0.047265214	2.964285714	185.625	1	548.0187433
0.016280607	9.484076433	466.9936306	1	2312.087395
0.006616041	20.01130856	1510.048465	0.777329025	3267.863055
0.004270516	30.01507538	917.6633166	0.919946957	3161.595678
0.015896703	7.430769231	334.5076923	0.988461538	1682.709472
0.009737685	10.13375796	783.9235669	0.98566879	3436.572482
0.008789851	21.05298013	547.6467991	0.964311994	2277.593587
0.009827683	12.15555556	437.1688889	1	2226.350152
0.00932171	8.276785714	643.7321429	0.989955357	2454.97027
0.004583941	56.3229572	2029.042802	0.894671231	3937.137948
0.014741665	6.338582677	499.0472441	1	2123.388798
0.008880366	18.98187311	470.4561934	0.976921786	2115.208227
0.024118012	4.0625	514.1666667	1	2448.592091
0.00833664	21.78730159	457.7619048	0.971379189	1790.945412
0.00261646	44.6101083	1890.341155	0.945820297	6186.652872
0.004793117	16.73487032	1153.383285	0.965417867	4440.016033
0.005507768	32.14572864	583.1432161	0.97340871	2282.576482
0.021317663	3.166666667	566.8333333	1	1996.317924
0.012028348	15.97297297	329.2162162	0.994932432	1318.934001
0.030733431	7.759493671	132.6455696	1	574.8271706
0.006239972	24.57142857	675.4857143	0.958888889	2603.925015
0.004179258	37.67586207	1901.665517	0.954597701	6521.050655
0.005168854	23.72440945	1114.425197	0.977034121	5084.80377
0.007299981	19.50442478	700.9159292	0.965585054	2789.461446
0.00399994	39.91324201	1606.883562	0.903138635	3971.883704
0.003290334	68.95288383	1522.90658	0.92242856	3274.33743
0.012230754	16.15407855	718.0936556	0.982880161	3360.278906
0.097130932	1.307692308	134.6153846	1	448.6882336
0.02081498	2.90625	597.140625	1	2768.380417
0.006590893	17.62176166	561.6813472	0.995628238	3085.39134
0.004420534	42.16746411	1751	0.909261696	3525.632017
0.003472363	30.5220339	1234.211864	0.944809322	4383.086574
0.009023364	7.220994475	796.6629834	0.95441989	2533.080166
0.022900007	7.48	301.05	0.9925	1526.693209
0.008424637	10.35329341	825.9640719	0.982035928	3590.85442
0.018357144	8.578947368	422.7631579	1	2199.775378
0.011322303	13.5862069	525.0275862	0.989655172	2486.011279
0.008536877	9.356756757	650.6702703	0.975675676	2722.556875
0.003072219	17.89122807	1646.082456	0.852095517	4154.153264
0.013711087	48.98300283	502.7648725	0.845708215	691.9061595
0.000936353	36.64187867	2566.535225	0.840080724	6273.411959
0.0211093	5.417582418	384.1318681	0.983516484	1742.991037
0.06797724	1.222222222	197.4444444	1	897.8553747
0.019200579	3.577777778	509.4111111	0.983333333	2165.624722
0.006808059	34.50738916	827.270936	0.907119595	2360.216073
0.016366817	7	426.3428571	0.992857143	2246.961327
0.003274217	34.37981651	1850.994495	0.862550968	3787.269857
0.03163802	5.921052632	139.6184211	1	732.6337909
0.005112691	75.29191797	2781.925211	0.868919601	4013.029435
0.011200069	6.064748201	874.676259	1	4273.144126
0.005931471	16.15277778	798.9305556	0.984375	3010.56986
0.006327018	8.78125	957.83125	0.97421875	3127.186988
0.015044124	6.707692308	441.4384615	0.988461538	1903.548545
0.013904475	9.115702479	260.6446281	0.987603306	1069.581864
0.00780953	18.48288973	485.3612167	0.97148289	1956.081432
0.004055633	41.52449568	1565.872238	0.954738232	4937.823383
0.011450884	40.9703154	614.0055659	0.931161101	1883.320567
0.015230984	18.83886256	260.7203791	0.975118483	937.307646
0.191783723	2.294117647	24.88235294	1	119.935847
0.006561438	14.47462687	1126.432836	0.970480929	4664.168846
0.023628676	3.47761194	368.0895522	1	1501.2692
0.005486017	28.62372188	817.196319	0.96085776	2982.82818
0.032737065	3.196078431	231.3921569	1	1145.83716
0.008741808	18.2875817	536.9346405	0.979484386	2359.061969
0.008295309	25.8742515	765.7345309	0.968022289	2647.76622
0.009867769	9.197183099	578.3873239	0.973591549	2329.574914
0.004181617	44.16086235	1122.026534	0.944988944	3347.458165
0.002410714	82.17899761	3324.057279	0.848426044	3981.15144
0.002259404	89.61661342	4600.567625	0.854472023	5357.974308
0.008669549	8.941908714	859.7261411	0.978215768	3367.221111
0.009407502	10.30481283	510.5508021	0.99197861	2178.162099
0.03667819	6.096774194	281.483871	1	1311.869828
0.007058508	15.45801527	576.1030534	0.985687023	2569.050955
0.006603744	22.11	847.66	0.973194444	3740.143268
0.050951645	4.246753247	271.1818182	1	992.1752743
0.0103916	11.45	969.8555556	0.991666667	4306.881867
0.004449745	38.66666667	2209.149606	0.931754156	6231.04801
0.056927452	1.538461538	251.6538462	1	939.4339113
0.004763804	39.56427379	919.1101836	0.947140141	2776.070224
0.00597078	40.98128655	603.2923977	0.973684211	2689.046898
0.028604981	6	288.9895833	1	1427.048916
0.006139699	12.13074205	753.9858657	0.989399293	3388.767369
0.060416957	1.444444444	389.3333333	1	1824.987992
0.100559202	1.461538462	368.2307692	0.884615385	648.0418617
0.00511639	33.52430044	1285.396171	0.934552037	3063.588781
0.025304576	6.945945946	192.5	0.97972973	621.1074
0.011593408	6.588235294	746.5073529	0.983455882	3322.046608
0.00927495	38.31630648	466.1237721	0.962248963	1674.490749
0.005104968	68.0596206	1632.929539	0.895396295	2733.59603
0.035020381	2.03030303	838.969697	1	3300.615649
0.009329234	9.609929078	622.2659574	0.973404255	3038.421831
0.081110117	1.4	173.0666667	1	694.6231868
0.017503926	5.247933884	336.7272727	0.993801653	1044.78483
0.037817102	6.797101449	82.73913043	1	212.4684421

original_glcm_Homogeneity1	original_glcm_Homogeneity2	original_glcm_ClusterShade	original_glcm_MaximumProbability	original_glcm_Idmn	original_glcm_SumVariance2	original_glcm_Contrast	original_glcm_DifferenceEntropy
0.602391894	0.567962411	-353.9883929	0.297856216	0.978748441	24.76915804	19.93696743	2.595934863
0.650492641	0.627415729	-180.587258	0.217332638	0.991439274	12.48011931	7.491183048	2.054657954
0.503510228	0.445507797	-69.2114179	0.09419992	0.980001896	11.22349233	8.592852066	2.496365873
0.535639236	0.497082787	-2420.464598	0.308792233	0.980517983	108.4985233	63.20673467	3.332713275
0.234443199	0.147948719	-463.463506	0.028266896	0.92646638	116.5701809	76.30977505	3.886903532
0.401139101	0.329643755	-470.6586237	0.116365596	0.958638835	52.72497975	31.65869061	3.437585814
0.199346932	0.114999604	166.8047138	0.04290418	0.912964448	84.67498132	98.32996939	3.659348834
0.430839003	0.371941676	-662.7704031	0.097846043	0.963339501	55.00641248	41.04323534	3.430368757
0.314642382	0.251528791	-16059.42634	0.043011626	0.936183732	861.5861568	183.179579	4.467634877
0.575201776	0.536108343	-334.5352388	0.23991923	0.98941796	25.52999355	13.29115676	2.572768408
0.470052644	0.411254813	-568.6530862	0.229602192	0.962209043	52.54522177	30.81654845	3.335710472
0.540025077	0.497477984	-450.3242409	0.146236634	0.988257869	28.109262	16.9785214	2.614874755
0.44744409	0.390775222	-1385.965208	0.135745242	0.969953602	96.31611439	34.24714402	3.377353353
0.380141508	0.310327133	-807.3138171	0.065441551	0.966689843	76.32787859	40.42965693	3.569128642
0.239762811	0.151837322	-1190.717706	0.015270135	0.954532948	135.5357934	81.88375424	4.125166661
0.673281827	0.655691296	-446.5818177	0.229247662	0.996630227	17.46700183	5.904324928	1.801666121
0.268557178	0.182675008	-734.7544395	0.025524721	0.948027985	92.03263051	68.73434071	3.923925205
0.557411767	0.518118509	-495.9651014	0.196682696	0.980813531	34.38996504	19.15099654	2.764114024
0.302565127	0.225199525	-989.6129461	0.037902102	0.950249978	111.5629297	69.90555318	3.872840858
0.727291595	0.708383266	-166.6570829	0.463316775	0.994556509	12.15458003	4.71812212	1.857132132
0.702973955	0.685408639	-218.8822929	0.425203827	0.996827978	12.96365954	6.445738358	1.892649322
0.387262954	0.318726697	-1021.238621	0.083334851	0.980756817	81.22090997	41.52240428	3.587596332
0.710822227	0.686624267	-136.5745535	0.454178737	0.994456435	12.34239216	6.188386976	2.035227012
0.192554845	0.112345326	-1292.704001	0.02129329	0.925937849	351.5370165	139.6857513	4.215962995
0.595039808	0.558602053	-48.7095013	0.171551743	0.993170842	7.377114179	3.895347711	2.09394879
0.538175635	0.491199026	-101.0344943	0.156733573	0.974940463	13.65672785	9.811822345	2.557642541
0.575353951	0.538268771	-337.6967818	0.216098998	0.992221714	22.99400064	11.84838554	2.456502618
0.721687533	0.707002547	-91.64817588	0.382421871	0.998383811	5.67441283	3.648571569	1.649360953
0.711944646	0.692250577	-950.9461872	0.450392436	0.992652518	37.65441986	18.22578544	2.131697361
0.655757219	0.633660745	-196.1268782	0.305027026	0.993136582	12.52408121	7.72213006	2.015330442
0.637431416	0.613517667	-225.5159459	0.221386389	0.995271892	12.41713711	7.020373135	1.994571761
0.618081511	0.587230952	-399.1178412	0.25874035	0.996199278	25.67106002	9.222448202	2.323708494
0.594382895	0.563754075	-1296.642677	0.291060004	0.978929839	61.01259061	34.59645334	2.750346598
0.17752123	0.0867785	-38.00667735	0.132692308	0.843885072	62.44925214	97.80897436	2.019230687
0.328460237	0.257669609	-2089.589136	0.090837322	0.950646145	135.8842606	80.04986873	3.733732546
0.547994993	0.50802094	-1529.196585	0.302452546	0.974497795	84.73909471	35.36704692	3.083812072
0.747468523	0.738308268	-123.7877468	0.478469053	0.998021437	6.698483089	3.90784611	1.505562134
0.589185725	0.556405219	-995.8686649	0.255757484	0.988095111	50.18639661	19.70902019	2.626850709
0.246303801	0.16176299	-1364.579894	0.018076881	0.95071079	145.4343851	83.13391442	4.096894727
0.627518841	0.599989886	-203.0692251	0.263871199	0.985852414	15.49727869	10.91670726	2.27407077
0.596754259	0.563344439	-659.3142964	0.309087143	0.990505358	36.76764438	19.71991626	2.606598064
0.446982101	0.391168863	-717.1518356	0.179061052	0.964455368	58.33099967	39.57125919	3.402842537
0.414659544	0.352113974	-892.9601026	0.091815301	0.971860932	72.4484373	40.70085706	3.481419713
0.399256116	0.335015526	-1566.767772	0.103966311	0.96654658	102.4231271	46.23050394	3.576415986
0.259190952	0.177299631	-5230.817599	0.025620498	0.963102498	266.3459901	89.9981056	4.239866686
0.634795399	0.608606281	-6.535536788	0.143888911	0.994842509	3.338109282	1.753268634	1.711569836
0.34712574	0.278253573	-5751.972865	0.045974639	0.968718612	211.8530338	105.5601141	4.037442515
0.416283131	0.353493064	-628.1245028	0.089336315	0.962870789	49.62891665	33.1880417	3.292362144
0.206771445	0.120714698	-391.4957461	0.082484182	0.888525456	93.5592361	92.3504773	2.698666514
0.268128132	0.1978361	-1194.732508	0.046475319	0.923389113	150.0811281	111.9168986	4.125982536
0.589554595	0.556009459	-474.2398024	0.253650583	0.986743957	31.96831188	14.20121704	2.500319601
0.552307471	0.516331034	-455.5915699	0.184964753	0.975903258	33.15449435	25.23084905	2.754789356
0.551650098	0.51287943	231.3248808	0.185075914	0.996210971	38.24283291	19.60663622	2.676299752
0.459672662	0.397961734	-214.6588489	0.106960606	0.964253778	27.13995878	15.78350187	2.923596797
0.66646955	0.646638755	-116.0316635	0.258899873	0.998362915	7.330223585	4.105483647	1.771020042
0.410181726	0.354942501	-3117.662728	0.118074742	0.964151231	150.5203941	73.74082336	3.694671543
0.29561656	0.216260937	-1252.473073	0.032251185	0.966253854	108.0522996	71.78741338	4.042657085
0.184090529	0.106487453	-1638.706091	0.010923158	0.940968152	303.6198575	166.4840133	4.625032371
0.413288709	0.349443446	-1069.292355	0.075887607	0.967386799	68.37978851	35.69936175	3.312367083
0.44690382	0.376845143	-105.9259907	0.054464203	0.979822533	18.26346526	11.9719932	2.835876346
0.532234399	0.485251329	-170.701565	0.124226516	0.989014786	15.61912661	11.20811597	2.579684671
0.637812386	0.616521361	-1831.629429	0.358296033	0.990863891	66.24793435	27.1647495	2.394971317
0.575744619	0.536259515	-172.9237563	0.181617825	0.991467452	14.84096161	8.097210274	2.374285873
0.708412936	0.690911132	-28.67501243	0.361278775	0.996957522	6.160227717	3.833941207	1.770222607
0.551029165	0.501146274	-23.25515264	0.196738649	0.943653684	7.17788744	5.863759465	2.253509091
0.447073834	0.389492544	-2136.553977	0.094694267	0.981282842	96.30553142	44.8619014	3.309385901
0.255344884	0.171044701	-706.7335003	0.037217812	0.929639174	95.31723518	74.10925477	3.87473097
0.607533171	0.578966616	-357.8601906	0.157776996	0.992091816	20.81547742	11.73242157	2.276420938
0.42627028	0.362076331	-437.8325627	0.08727295	0.959998397	38.45714203	26.90849284	3.104377523
0.572430783	0.535525428	-404.8506027	0.215397816	0.9857479	26.57637788	15.3775304	2.555461779
0.505931784	0.453707633	-473.4068846	0.0911216	0.989976544	30.53792174	17.29183167	2.742946702
0.477809729	0.419515073	-252.6097679	0.083259529	0.984258357	22.96965185	16.21834468	2.854235298
0.729931377	0.717147324	-280.5480244	0.285302797	0.996121238	13.83515856	5.07373929	1.702394164
0.641153523	0.617083477	-80.93036539	0.228315956	0.998283898	6.701151899	3.836685656	1.859031499
0.554535194	0.513837087	-1078.174528	0.111010632	0.995802087	41.93057515	15.997586	2.466521091
0.299784879	0.224765943	-1633.566987	0.033461295	0.959552929	118.2018192	85.28483873	4.085262707
0.35415566	0.276547943	-707.3980647	0.051799264	0.967502959	68.63195846	38.71424022	3.59080843
0.49417098	0.435185401	-150.9134001	0.104835457	0.98180568	16.09218985	11.64526971	2.609550998
0.473941125	0.420228299	-661.5715931	0.131991945	0.978086363	45.57549388	28.13953571	3.103943974
0.69629924	0.678260945	-767.502874	0.399094534	0.988930934	33.8800069	17.97655889	2.112069984
0.368734448	0.286141914	-313.8200135	0.05503805	0.967396955	46.2178201	27.78799172	3.281326704
0.348563791	0.27660261	-3872.56565	0.041001698	0.969601725	167.1250085	81.25248978	3.854228337
0.534894417	0.492509318	-1791.650544	0.131166593	0.996906856	59.53552904	28.03444761	2.599080866
0.224561954	0.141688128	-333.6635734	0.070494249	0.900367436	63.3027485	77.1089719	2.897171489
0.598533023	0.5665351	-202.0221476	0.137658118	0.993252812	15.9998762	8.807298795	2.24870784
0.480514263	0.425796422	-1108.373903	0.114439771	0.987834354	64.87056326	22.57680308	3.045853656
0.534954893	0.491215717	-260.8205217	0.134530563	0.979015078	23.46752724	15.79845659	2.707248798
0.299293341	0.218124146	-1104.508822	0.029784359	0.961459355	88.4586737	65.29385919	3.972236009
0.387735781	0.319323551	-680.9404675	0.079992775	0.94914029	54.6956299	53.83275959	2.947465011
0.15527397	0.057297517	-264.7590633	0.091269841	0.858014051	92.30398085	97.15873016	2.581622402
0.564505937	0.528969941	-13.68540216	0.176381567	0.994650866	44.13250391	26.07398675	2.701711249
0.520610733	0.46590866	-46.48912537	0.09657233	0.98722023	10.33970009	7.433766929	2.490554016
0.442561381	0.38675369	-1802.370834	0.091972215	0.973605528	90.88389695	47.07962425	3.248162312
0.638984632	0.612768854	-151.075792	0.19404706	0.992344681	13.40921869	7.190187172	2.157732495
0.615446472	0.586073761	-116.9472065	0.228290311	0.995134842	9.247781383	5.176699483	2.039544762
0.315142301	0.231782949	-1516.530101	0.06462068	0.958679911	91.42165156	77.52262836	3.450852217
0.372443197	0.312181951	-2932.424962	0.078356317	0.954668527	183.0537849	76.29403949	3.861165906
0.339987481	0.25847403	-345.6900929	0.119240802	0.926273061	43.47840595	34.93874352	2.493236673
0.211959946	0.121707464	3.946764643	0.02577761	0.946892229	89.06495126	74.54764928	3.950147097
0.516256395	0.459683476	-15.35143558	0.111250421	0.976141721	7.823634207	5.221035303	2.362331246

original_glcm_InverseVariance	original_glcm_Entropy	original_glcm_Dissimilarity	original_glcm_DifferenceVariance	original_glcm_Idn	original_glcm_Idm	original_glcm_Correlation	original_glcm_Autocorrelation
0.342419478	4.307337343	2.202901999	15.03792241	0.93684022	0.567962411	0.113852694	604.4395153
0.437631151	4.068681853	1.293607382	5.807809058	0.95952185	0.627415729	0.246766971	534.0247269
0.40922932	5.055061781	1.90400513	4.941330665	0.917224522	0.445507797	0.134222371	262.9172609
0.265006241	5.574405829	4.260791734	44.84958521	0.936142283	0.497082787	0.270528741	1395.984545
0.159941527	6.555749897	6.876877044	27.8151415	0.825312788	0.147948719	0.219130735	365.765475
0.270116443	6.299918981	3.803516318	17.04179675	0.883249927	0.329643755	0.247471612	424.1881621
0.134045246	5.704357314	8.050026111	32.48155913	0.805757083	0.114999604	-0.077163341	161.6166574
0.313402512	6.011918288	3.975297053	25.1695392	0.89888739	0.371941676	0.150111947	670.7738563
0.211600618	8.53839601	8.761758729	104.7421896	0.864943408	0.251528791	0.654364575	924.4029891
0.395857647	5.054330253	1.925665323	9.56710923	0.950787277	0.536108343	0.315109072	887.6156587
0.249893359	5.658920456	3.547677612	18.02682446	0.895674894	0.411254813	0.256974971	498.8096969
0.427461997	5.116339873	2.162870257	12.27295922	0.948346549	0.497477984	0.248169993	960.066323
0.315377251	6.618560982	3.668393324	20.5409526	0.907240527	0.390775222	0.476264815	670.6783948
0.288638781	6.95263686	4.252913528	22.14205012	0.895511802	0.310327133	0.315294643	660.3362393
0.15631028	8.015489336	6.859759415	34.27607828	0.862905501	0.151837322	0.253101543	727.0913069
0.467211861	3.974634064	1.055754764	4.775744437	0.975865648	0.655691296	0.500670718	1074.705832
0.188145135	7.121878025	6.151949408	28.77472991	0.85788735	0.182675008	0.178628884	628.9976247
0.373023923	5.324221804	2.378177832	13.36586283	0.934382417	0.518118509	0.28777757	611.6539016
0.220778524	6.874278052	5.969815013	33.7822048	0.866744948	0.225199525	0.232759804	717.1227879
0.332084977	3.494246941	0.98592795	3.737381068	0.969495105	0.708383266	0.431597298	503.9097488
0.387234397	3.489586425	1.096152665	5.241374646	0.977281569	0.685408639	0.326309787	1646.811095
0.284508673	7.1762633	4.261555536	23.09450355	0.9196704	0.318726697	0.325342231	1260.782507
0.302543247	3.759231228	1.160663371	4.830626149	0.968531577	0.686624267	0.333183365	636.7946044
0.113124653	6.861832643	9.363832049	48.83081091	0.822975941	0.112345326	0.438130843	577.1466277
0.463752118	4.6623311	1.249463978	2.328056528	0.951705969	0.558602053	0.300520484	387.6622125
0.410351526	4.938058805	1.909095413	6.136469223	0.915467373	0.491199026	0.161763986	184.5944271
0.430164055	4.819837228	1.809588935	8.557268596	0.957802351	0.538268771	0.324187559	734.0992707
0.407925088	3.293016479	0.824022244	2.966729397	0.983124993	0.707002547	0.211365875	1726.047294
0.301049362	3.901375276	1.627884903	15.52236121	0.970889892	0.692250577	0.350381301	1372.531725
0.439263495	3.818259842	1.273811298	6.089298242	0.964840703	0.633660745	0.227877574	768.5357851
0.47236761	4.063849661	1.248085865	5.459301529	0.969443178	0.613517667	0.272192556	1081.491728
0.40781273	4.859328328	1.568009753	6.733788359	0.970673474	0.587230952	0.456727626	915.1797636
0.353682869	4.800427534	2.757483355	26.90656109	0.941461138	0.563754075	0.283181067	935.176025
0.066137512	3.224358892	8.393589744	23.5307906	0.736964085	0.0867785	-0.261182537	110.4532051
0.233255291	5.966385864	6.07699074	41.97503788	0.873417577	0.257669609	0.251710285	790.4456455
0.297811252	5.440831761	3.249208051	24.67447443	0.926086445	0.50802094	0.410950329	872.4507852
0.402398213	2.826639321	0.758639659	3.329579788	0.983656072	0.738308268	0.266771664	943.7350653
0.386015014	4.947976026	2.208643937	14.8035491	0.952034499	0.556405219	0.431450152	1208.468267
0.166129139	7.859704271	6.958573644	32.18552773	0.8585658	0.16176299	0.284601993	747.8144199
0.409807123	4.080596263	1.610833721	8.29809135	0.947808279	0.599989886	0.172833378	438.757964
0.359117058	4.531160891	2.214009741	14.75714724	0.956667681	0.563344439	0.304036401	994.047078
0.30219715	5.834464358	3.896474205	24.27944342	0.900580214	0.391168863	0.1975041	639.927575
0.314061853	6.692627153	4.02176285	24.23270163	0.909076022	0.352113974	0.284265452	725.6730001
0.289704738	6.578834384	4.389883464	26.76891677	0.899379574	0.335015526	0.378517407	790.3666181
0.187421604	8.670262217	6.945510564	41.15479521	0.88038177	0.177299631	0.502108117	1198.176217
0.505179724	4.161849718	0.922098058	0.902030849	0.95343827	0.608606281	0.308206478	211.7955519
0.266756909	7.984407019	6.202416831	66.65891304	0.907629704	0.278253573	0.342215315	1748.325461
0.334867205	5.824672249	3.7041374	19.25765413	0.89452657	0.353493064	0.189644386	514.7310218
0.116977417	4.069431409	7.844105894	28.86516823	0.782095432	0.120714698	-0.001246747	244.9733516
0.189051547	6.648278204	7.89621931	48.15857704	0.832888468	0.1978361	0.150712677	627.9868873
0.413138932	4.930389737	1.905863049	10.53923031	0.947860681	0.556009459	0.387658942	671.5111215
0.389175587	4.712052395	2.644754737	18.16088769	0.929452037	0.516331034	0.137184599	638.2504403
0.418787483	5.138717453	2.264111858	14.45393913	0.970984435	0.51287943	0.323120816	1053.916036
0.372271776	5.658768619	2.606381925	8.929261817	0.893437218	0.397961734	0.259986003	233.4782567
0.474899008	3.857046551	0.975957321	3.151658602	0.981120927	0.646638755	0.265101968	1089.271213
0.284801134	6.29938686	5.291891878	45.3464554	0.901496076	0.354942501	0.350007688	1200.330048
0.212899837	7.869845941	6.041124764	34.57967331	0.888209543	0.216260937	0.212996626	878.2649947
0.108360027	8.578143018	10.25560364	58.29908402	0.840560716	0.106487453	0.304016288	903.610015
0.340986887	6.28574306	3.75292618	21.33897007	0.902734304	0.349443446	0.309146686	562.8987388
0.363886725	6.109046292	2.40011685	6.110025241	0.913021265	0.376845143	0.201994881	331.2912244
0.427896075	5.270379767	1.941146349	7.428788394	0.944796622	0.485251329	0.152553981	565.274028
0.394055572	4.210102797	2.212531594	22.23035667	0.964372325	0.616521361	0.421352945	1335.317277
0.434571234	5.008946637	1.599359906	5.513327728	0.952272039	0.536259515	0.300234278	543.9619657
0.400562817	3.61515099	0.915041629	2.993434374	0.975752797	0.690911132	0.231835982	276.0452972
0.388969667	3.995015368	1.642612222	3.091626966	0.865956304	0.501146274	0.102690584	45.41565046
0.353173336	6.473970988	3.740543081	30.70105534	0.932872432	0.389492544	0.361840654	1306.724211
0.173938048	6.402336619	6.578146393	30.2785885	0.833175111	0.171044701	0.126080091	448.2832143
0.450199858	4.619247498	1.647908441	9.009489227	0.961017506	0.578966616	0.272384178	832.448676
0.350929684	5.54303594	3.306219816	15.9125179	0.890713541	0.362076331	0.172623488	346.8726338
0.415714405	4.957705509	2.009528056	11.32061419	0.94525774	0.535525428	0.267986307	670.1188752
0.412282736	5.920039454	2.301612499	11.93109828	0.949615908	0.453707633	0.280429464	760.7774765
0.389048083	5.716483058	2.468442275	10.07540533	0.931274382	0.419515073	0.167624916	671.939694
0.387464805	3.465567596	0.924981068	4.210468721	0.976151841	0.717147324	0.453865628	913.5315836
0.486733734	4.015644697	1.053990398	2.723540685	0.978625513	0.617083477	0.26651446	1082.766826
0.450622088	5.449749321	1.938212205	12.225205	0.970789402	0.513837087	0.445123967	1471.455939
0.222382297	7.616904649	6.428744848	43.23096653	0.881384632	0.224765943	0.161384184	969.7645857
0.268898388	7.044030354	4.320349771	19.78686926	0.892670588	0.276547943	0.280765072	647.0813577
0.397517732	5.231285467	2.108459173	7.166890152	0.924226885	0.435185401	0.16399585	390.5763457
0.366870976	5.913396397	3.100224647	18.25145461	0.924733436	0.420228299	0.232218812	737.5705979
0.349331744	3.908450134	1.637062843	15.26547981	0.964024062	0.678260945	0.305711503	1022.991733
0.257821723	6.222584979	3.729120898	13.72777878	0.889773746	0.286141914	0.246260264	311.6971306
0.279626037	7.517441358	5.571819583	49.95969642	0.907364559	0.27660261	0.349375547	1222.822309
0.450183525	5.310954028	2.378640334	22.3203588	0.976833715	0.492509318	0.362821731	1691.768846
0.204302195	4.617888239	6.901890097	27.89401197	0.798669551	0.141688128	-0.101972183	267.0869623
0.456792733	4.912338033	1.523146431	6.474754514	0.961048706	0.5665351	0.289915879	783.9466651
0.371431048	6.301950821	2.811247408	14.57507888	0.941885554	0.425796422	0.482164754	777.5488531
0.400757384	5.106648596	2.258745241	10.67128631	0.927663284	0.491215717	0.192987461	422.9213988
0.220180168	7.458146548	5.746619191	31.85207228	0.881515818	0.218124146	0.153502747	975.6260709
0.296199004	4.892022484	4.569418253	30.50547384	0.881070368	0.319323551	-0.010717927	521.3155155
0.077446142	3.877528851	8.30952381	27.69501134	0.746788061	0.057297517	-0.021606218	172.9603175
0.409448473	5.222859045	2.435857522	20.11336979	0.968272663	0.528969941	0.254914493	861.7450378
0.410035649	5.201095914	1.788566251	4.210195818	0.933320561	0.46590866	0.163331015	198.5221149
0.367841422	6.160429455	3.862461599	31.83293594	0.920918881	0.38675369	0.326037317	945.9337187
0.424090203	4.351569288	1.365274772	5.314367798	0.959522648	0.612768854	0.295540253	482.3490524
0.477246404	4.215229124	1.237612749	3.641476641	0.964345681	0.586073761	0.27712799	757.6974448
0.208181884	5.412330953	5.747911666	42.7335375	0.884723538	0.231782949	0.114251113	920.6869495
0.280815513	7.139262798	5.678365618	43.43142834	0.884998707	0.312181951	0.414989824	877.2274508
0.244535149	3.861294033	4.334964822	15.31397994	0.835490841	0.25847403	0.08763625	201.7925254
0.132486167	7.085393309	7.054111225	23.91336222	0.843351088	0.121707464	0.094660779	323.5418037
0.425020942	5.216469459	1.644470326	2.489073646	0.903053236	0.459683476	0.194815443	78.80622198

original_glcm_SumEntropy	original_glcm_AverageIntensity	original_glcm_Energy	original_glcm_SumSquares	original_glcm_ClusterProminence	original_glcm_SumAverage	original_glcm_Imc2	original_glcm_Imc1
3.168119306	24.70355884	0.127157698	10.04884323	8055.810973	49.1213782	0.666695429	-0.125539075
2.953372944	23.14514918	0.120680898	4.914373363	4177.259176	46.16381821	0.54279552	-0.083618787
3.351328839	16.19418451	0.044241786	4.954086099	1054.423377	32.38836902	0.717444404	-0.135152037
3.991609134	37.59132252	0.112701597	37.12795006	94066.14339	74.42153907	0.729189781	-0.126065344
4.733135265	19.44804763	0.011805938	47.96417583	34466.78134	37.70804483	0.994232398	-0.506497835
4.322930734	20.46680933	0.027009412	21.09591759	12066.69468	40.93361867	0.877988057	-0.210966796
4.166756489	13.09876905	0.021079468	47.40016776	20018.42471	25.68259323	0.997079188	-0.631994135
4.155129633	26.09092502	0.036768701	21.05425838	17236.53913	51.66323566	0.816708085	-0.164724386
5.89540034	28.21959598	0.007307121	257.716106	1410618.872	54.94019303	0.921740176	-0.199549324
3.48252318	29.79635478	0.086639468	8.817272517	7519.363118	59.48283363	0.593168533	-0.083126644
4.049868253	22.38716437	0.067115139	20.38339474	13578.34696	44.42250509	0.795974218	-0.159658774
3.49809272	30.98044943	0.05943683	10.49764262	11926.7633	61.87990049	0.661096682	-0.107297079
4.488591146	25.84666185	0.038654053	29.61275179	43738.83722	51.19126402	0.804275986	-0.147057998
4.675627347	25.78351679	0.016662687	26.25711459	25148.17667	51.04300905	0.863630619	-0.178741831
5.269488711	27.2655001	0.004658801	51.34817608	58847.12198	53.42845217	0.960736911	-0.273705978
2.869957078	32.80799843	0.115488042	5.18224898	15766.17106	65.47714981	0.569189623	-0.095170628
4.857260815	25.33901983	0.008676566	36.35003001	28764.36935	49.92311951	0.961973437	-0.305845069
3.693152764	24.8185023	0.069674472	11.83809562	11948.1196	49.30883774	0.671807389	-0.109438752
4.825715537	26.86918371	0.01157028	41.44695539	39358.84235	53.16549173	0.973686175	-0.352575161
2.637409042	22.44284949	0.241601853	4.177232219	3392.690003	44.81284478	0.575462082	-0.109947602
2.548903841	40.58988916	0.216790058	4.327779049	5442.102617	81.12161849	0.4551297	-0.065903833
4.714120308	35.63467887	0.017598909	27.54366377	33723.2094	70.73438195	0.814196167	-0.140802103
2.761703864	25.24982613	0.228163963	4.178645182	2480.817677	50.40852025	0.558658017	-0.098122633
5.242013963	23.27155653	0.009333904	121.6488633	247526.5786	45.77133205	0.998506678	-0.59815042
3.119100077	19.66692883	0.071505534	2.818115473	799.911549	39.33385765	0.542668836	-0.074555145
3.41337372	13.55103205	0.063501423	5.86713755	1550.744371	27.1020641	0.698248921	-0.12821337
3.282861423	27.11030759	0.085714202	7.68577521	7834.724559	54.08549987	0.571221008	-0.079450146
2.361459233	41.5712344	0.198266812	2.128665754	2629.463746	83.07924013	0.427444473	-0.061455774
2.921039293	37.06281874	0.224349559	12.35333326	33642.26696	73.96392996	0.65250115	-0.137457298
2.70531634	27.74087592	0.150478057	4.482683151	4804.844715	55.40141489	0.492172409	-0.07302063
2.806829789	32.88254656	0.114742723	4.505261586	6387.266788	65.7310005	0.431528801	-0.050948146
3.371097248	30.25653078	0.103424006	8.007504295	10086.10809	60.36743847	0.628612558	-0.102820547
3.505935265	30.58829134	0.118771731	21.43588509	40731.50937	60.94469454	0.696482395	-0.130067498
2.160256328	10.83653846	0.118803419	40.06455662	10168.68294	21.67307692	0.996331445	-0.892579731
4.426436871	28.22585668	0.02437304	47.08409806	76984.05131	55.72192577	0.981796687	-0.435745712
3.87741319	29.45894654	0.108692697	27.80080715	46634.33438	58.65423674	0.755029615	-0.145944841
2.097252018	30.76003969	0.272554015	2.358598371	3243.452274	61.4178189	0.363308029	-0.048585958
3.482211104	34.75742042	0.106081906	16.40308757	30249.39343	69.30582306	0.652847325	-0.106373664
5.257437324	27.56313248	0.005227201	54.85021714	68493.52619	54.11739353	0.963854027	-0.286027807
2.964391396	20.91915059	0.126088966	6.603496487	4071.276128	41.83830117	0.569023665	-0.094622196
3.306163178	31.58926899	0.127125883	12.69561332	18897.08942	62.92099941	0.724468587	-0.149798178
4.132580059	25.48887244	0.05055126	21.61843889	19126.69841	50.40694746	0.844821335	-0.191063145
4.454009308	27.00363294	0.024565597	25.33794228	25071.26579	53.57968582	0.804234194	-0.145334727
4.538944468	28.03941368	0.026684007	34.62337999	52202.39199	55.72257941	0.893886428	-0.216963067
5.591855981	34.56076268	0.004503122	83.591427	279141.4165	67.94291757	0.905110766	-0.178226535
2.838673073	14.53956925	0.080358007	1.272844479	103.2580986	29.0791385	0.420670886	-0.047164069
5.132293113	41.96240955	0.012096059	71.38056609	318788.7652	82.98727358	0.783984828	-0.112578304
4.044376851	22.79307251	0.031392078	19.81930481	16189.4253	45.19191928	0.855397987	-0.201280662
2.923735438	15.62661783	0.062373639	46.47742835	23511.64906	31.25323565	0.996298561	-0.787712379
4.843961302	25.38310576	0.013101367	60.79642166	60800.52253	49.72980476	0.983860257	-0.412455426
3.403601957	25.92318177	0.099831342	10.40254039	11028.132	51.65522269	0.631275332	-0.098355986
3.398051833	25.36981618	0.089825744	13.00281354	10401.8783	50.44825371	0.723071997	-0.145615131
3.555327652	32.52140454	0.074937424	14.19880947	30669.41828	64.78435701	0.619085211	-0.089984811
3.813564206	15.18612712	0.036000189	10.73086516	3755.448037	30.37225424	0.805814707	-0.173015542
2.693337008	33.00653576	0.127335213	2.640906864	3418.976158	65.98373242	0.427118857	-0.053510746
4.528254907	34.71717169	0.033255302	50.69164211	126484.5611	68.73366954	0.935567791	-0.280112913
5.090553554	29.96467351	0.007541045	41.02178856	47731.54111	58.95898242	0.869539993	-0.161640557
5.835096901	30.47365584	0.003084871	111.5866469	206893.5587	58.95399641	0.98578007	-0.343011482
4.266976336	23.79269297	0.024452216	23.78035724	31736.85504	47.10473768	0.883553978	-0.213304694
3.893103338	18.1579192	0.022545944	7.558864614	2047.815154	36.3158384	0.680161878	-0.101381268
3.454179364	23.83588472	0.055395061	6.173015623	3716.807714	47.50434657	0.537951304	-0.063631411
3.019518039	36.62362028	0.172808863	20.16977066	68236.57307	72.8159435	0.578033275	-0.090014337
3.347801271	23.36268583	0.06957462	5.240361803	3654.05801	46.57361612	0.511615519	-0.060145637
2.679559472	16.60552996	0.175679612	2.595563476	956.338419	33.19418121	0.523752902	-0.086640027
2.811778998	6.714215273	0.093686067	3.260411726	201.4999065	13.42843055	0.84709518	-0.276489199
4.355061491	36.06803914	0.029644774	33.40712586	79420.59183	71.93979888	0.796559259	-0.147005058
4.600814067	21.36094014	0.013602024	41.67245087	26833.5733	42.08829125	0.988854688	-0.456393274
3.201335776	28.8461201	0.086701746	7.570647327	9352.348901	57.62553801	0.543092794	-0.074324699
3.817078674	18.54645002	0.03476004	16.34140872	9901.426294	37.09290004	0.887321659	-0.246550264
3.369448058	25.91581288	0.082826517	9.542468873	9783.426858	51.66489366	0.596897231	-0.085411196
3.885479551	27.60209039	0.035614464	10.78921745	13694.95038	55.04413785	0.63512763	-0.086776826
3.751717984	25.98630128	0.034331116	9.015108578	5661.986524	51.77820406	0.680439722	-0.104794114
2.639410279	30.21373128	0.17185304	4.233341292	8100.946066	60.37679353	0.572368544	-0.113033333
2.745909188	32.91365254	0.109306746	2.40192272	1869.192252	65.78905702	0.380984961	-0.03995824
3.638873141	38.32878397	0.048937269	13.43147765	40145.37773	76.5497337	0.592496365	-0.077800776
5.018888919	31.4710556	0.008338718	46.48401569	65041.25053	62.01315669	0.928816662	-0.227730605
4.651536295	25.58806343	0.013184671	23.71652259	21450.08471	50.57965816	0.867047383	-0.178121282
3.472938553	19.7348051	0.04340708	6.934364891	2939.587187	39.4696102	0.718078237	-0.131319337
3.922520673	27.22817621	0.043775938	16.28253917	17277.34403	54.15504807	0.743620208	-0.130922279
2.965377271	31.98800282	0.190682496	11.47712242	24090.98932	63.8435514	0.648800285	-0.132965206
4.291971487	17.56940588	0.017743911	18.95732079	9954.909327	35.04564122	0.953386321	-0.324106373
4.981600872	35.05155633	0.011490202	56.03835028	173284.776	69.31793651	0.897457103	-0.196147747
3.511870995	41.22599457	0.059021377	19.69294407	84255.79002	82.07023274	0.616817934	-0.086427163
3.298058418	16.43020393	0.044177549	35.1029301	11020.19512	32.86040786	0.998183819	-0.76659682
3.39753377	28.00022348	0.067451251	5.693790273	4490.105314	55.93380021	0.561997695	-0.076993888
4.162129167	27.76644869	0.037660804	20.79505372	32473.22916	55.38835721	0.684122359	-0.098587236
3.572517299	20.51832313	0.05983168	9.816495959	5170.62091	41.03664625	0.717044321	-0.134279342
4.836638436	31.56744664	0.009103804	35.98924831	37274.89549	62.28319188	0.840245016	-0.148718582
3.583317717	22.98323182	0.039191423	25.76246903	18477.48558	45.63916996	0.975285019	-0.480124822
2.821973295	13.19444444	0.069664903	47.36567775	20090.00559	26.38888889	0.994302246	-0.732640828
3.607696509	29.45197018	0.081123123	17.20824293	32730.04595	58.55666543	0.668999161	-0.107076739
3.414515496	14.06388072	0.041941474	4.443366755	875.0490078	28.12776143	0.655678133	-0.104055396
4.312799004	30.87682325	0.029317853	30.32127193	61317.93047	61.15308512	0.877147393	-0.211750142
3.097821077	21.95714923	0.109913452	5.028336646	2834.126661	43.85392123	0.511583081	-0.070346147
2.820453563	27.53084273	0.103671645	3.303751205	2452.700659	55.01557832	0.385885221	-0.03912946
3.92383662	30.61389852	0.027770505	38.69935651	53978.21158	60.55770264	0.979385386	-0.457604307
4.892789949	29.54608034	0.021813206	60.4971896	115916.3854	58.32655719	0.900149107	-0.209737758
2.753338055	14.11738294	0.0769216	19.60428737	7667.211459	28.23476588	0.991445372	-0.703786207
4.859747725	18.501186	0.008465515	40.3942979	23851.04267	35.7695695	0.989525411	-0.432963316
3.376712559	8.84040148	0.04404416	3.261167377	255.0498886	17.68080296	0.682572667	-0.115551769

original_glcm_DifferenceAverage	original_glcm_Id	original_glcm_ClusterTendency	original_firstorder_InterquartileRange	original_firstorder_Skewness	original_firstorder_Uniformity	original_firstorder_MeanAbsoluteDeviation
2.202901999	0.602391894	24.76915804	37	-3.159468765	0.275573392	58.46543535
1.293607382	0.650492641	12.48011931	30	-4.107195645	0.281777314	38.7454467
1.90400513	0.503510228	11.22349233	62	-2.265283404	0.167033739	45.15560663
4.260791734	0.535639236	108.4985233	92	-2.235767362	0.238069749	130.1100086
6.876877044	0.234443199	116.5701809	305	-0.225008837	0.046564062	156.0617587
3.803516318	0.401139101	52.72497975	172.25	-1.196491921	0.094766192	102.3373421
8.050026111	0.199346932	84.67498132	224	0.3178995	0.055102041	135.3428571
3.975297053	0.430839003	55.00641248	129	-1.872713235	0.12984372	99.14478144
8.761758729	0.314642382	861.5861568	675.25	-0.776353805	0.052557267	336.3288543
1.925665323	0.575201776	25.52999355	46	-2.953676822	0.230376359	53.01321386
3.547677612	0.470052644	52.54522177	169.25	-1.26817097	0.150916704	112.3830353
2.162870257	0.540025077	28.109262	46	-3.353448058	0.197018056	57.57144328
3.668393324	0.44744409	96.31611439	186	-1.422450994	0.118652315	125.8076911
4.252913528	0.380141508	76.32787859	202	-1.304627626	0.080915691	120.3686582
6.859759415	0.239762811	135.5357934	289.25	-0.750324928	0.041359739	158.851754
1.055754764	0.673281827	17.46700183	27	-5.951801958	0.305143672	31.3742709
6.151949408	0.268557178	92.03263051	219	-1.038586188	0.055872921	137.8637898
2.378177832	0.557411767	34.38996504	50	-2.473777221	0.199910823	72.15529679
5.969815013	0.302565127	111.5629297	264.25	-0.956995816	0.057058326	150.5739645
0.98592795	0.727291595	12.15458003	21	-3.636721116	0.400534316	35.45930904
1.096152665	0.702973955	12.96365954	21	-5.045356937	0.406539426	31.48645315
4.261555536	0.387262954	81.22090997	170	-1.609287411	0.089321913	113.0707955
1.160663371	0.710822227	12.34239216	25	-3.518519188	0.362503753	37.41447739
9.363832049	0.192554845	351.5370165	492	-0.110331205	0.03454124	240.8305727
1.249463978	0.595039808	7.377114179	39.25	-3.023914743	0.220492622	32.43356771
1.909095413	0.538175635	13.65672785	54	-2.07673295	0.201438533	47.79917547
1.809588935	0.575353951	22.99400064	36	-3.312875238	0.248567951	47.5383452
0.824022244	0.721687533	5.67441283	20	-8.66860141	0.393964482	20.45510188
1.627884903	0.711944646	37.65441986	25	-4.164243071	0.368662816	57.30535383
1.273811298	0.655757219	12.52408121	25	-4.549201435	0.323977086	36.23919066
1.248085865	0.637431416	12.41713711	28	-5.361886824	0.301557461	30.72251339
1.568009753	0.618081511	25.67106002	34	-3.023846833	0.252838008	53.16872533
2.757483355	0.594382895	61.01259061	46	-2.72143184	0.259019106	92.87660996
8.393589744	0.17752123	62.44925214	235	0.322170169	0.102222222	133.6
6.07699074	0.328460237	135.8842606	239	-1.411560503	0.09092333	155.9827708
3.249208051	0.547994993	84.73909471	127.75	-1.746939623	0.211806765	121.7057446
0.758639659	0.747468523	6.698483089	17	-7.146890263	0.492577142	19.35514629
2.208643937	0.589185725	50.18639661	43.25	-2.648823651	0.249815077	81.58731316
6.958573644	0.246303801	145.4343851	328	-0.611645338	0.043079939	163.1936996
1.610833721	0.627518841	15.49727869	31	-3.312977073	0.291588208	47.92986763
2.214009741	0.596754259	36.76764438	38.5	-3.14080438	0.272312583	68.55499242
3.896474205	0.446982101	58.33099967	159	-1.667822034	0.143075619	112.0330254
4.02176285	0.414659544	72.4484373	160.25	-1.568931504	0.106681238	113.9883766
4.389883464	0.399256116	102.4231271	188	-1.376554402	0.099454652	130.8331111
6.945510564	0.259190952	266.3459901	337.5	-1.032512223	0.04419554	191.2343108
0.922098058	0.634795399	3.338109282	35	-2.186585281	0.260038639	22.07754708
6.202416831	0.34712574	211.8530338	207	-1.952240927	0.083892933	159.6876052
3.7041374	0.416283131	49.62891665	148.25	-1.66717814	0.126231148	105.5254309
7.844105894	0.206771445	93.5592361	279.25	-0.092783455	0.082644628	153.3181818
7.89621931	0.268128132	150.0811281	372.5	-0.581338484	0.060424095	191.5773128
1.905863049	0.589554595	31.96831188	37.25	-2.998683341	0.257748462	58.24806264
2.644754737	0.552307471	33.15449435	47.5	-2.489109105	0.229303795	78.39354962
2.264111858	0.551650098	38.24283291	35	-0.344974803	0.24184884	49.41093111
2.606381925	0.459672662	27.13995878	95.5	-1.511316716	0.131597836	72.27891669
0.975957321	0.66646955	7.330223585	26	-6.543292335	0.319116785	24.24247656
5.291891878	0.410181726	150.5203941	211	-1.678872613	0.109841836	157.2017821
6.041124764	0.29561656	108.0522996	197.25	-1.288383231	0.068549575	139.7168852
10.25560364	0.184090529	303.6198575	461.25	-0.218567373	0.028565435	235.0321313
3.75292618	0.413288709	68.37978851	101	-1.825692869	0.110632321	107.0980561
2.40011685	0.44690382	18.26346526	77	-1.474190047	0.119315147	57.51114406
1.941146349	0.532234399	15.61912661	44	-2.963250738	0.195692068	48.38922072
2.212531594	0.637812386	66.24793435	22	-3.17390162	0.357959947	84.65102238
1.599359906	0.575744619	14.84096161	41	-3.312326489	0.220117203	41.44029201
0.915041629	0.708412936	6.160227717	24	-2.386555173	0.344121474	24.16728578
1.642612222	0.551029165	7.17788744	55	-1.288883837	0.223923104	41.89320043
3.740543081	0.447073834	96.30553142	100	-2.321147051	0.129157926	116.2001848
6.578146393	0.255344884	95.31723518	221	-0.811407876	0.056689342	142.0351927
1.647908441	0.607533171	20.81547742	33	-3.58817887	0.248818041	47.26183242
3.306219816	0.42627028	38.45714203	115	-1.668396286	0.128759353	91.41933591
2.009528056	0.572430783	26.57637788	39.25	-3.163393686	0.232578823	55.14892827
2.301612499	0.505931784	30.53792174	53	-2.956995123	0.153786174	57.39940496
2.468442275	0.477809729	22.96965185	60.5	-2.48336368	0.150509038	62.76193587
0.924981068	0.729931377	13.83515856	22	-5.225671785	0.356773482	30.35394096
1.053990398	0.641153523	6.701151899	29	-5.339988188	0.300404081	24.51449786
1.938212205	0.554535194	41.93057515	45	-3.881245839	0.190645368	56.68710388
6.428744848	0.299784879	118.2018192	243.5	-1.301396019	0.060337027	160.2430775
4.320349771	0.35415566	68.63195846	161.25	-1.45718483	0.081698215	106.7386696
2.108459173	0.49417098	16.09218985	55.25	-2.759706073	0.171117666	52.10100446
3.100224647	0.473941125	45.57549388	77	-2.172096819	0.152917155	82.72294195
1.637062843	0.69629924	33.8800069	28	-3.789954458	0.332725898	58.90103337
3.729120898	0.368734448	46.2178201	117	-1.175743777	0.082834107	90.97932526
5.571819583	0.348563791	167.1250085	169	-1.754618957	0.075096395	162.8750496
2.378640334	0.534894417	59.53552904	40	-3.617699157	0.204731945	67.34438349
6.901890097	0.224561954	63.3027485	216	-0.696857085	0.069719043	133.8272633
1.523146431	0.598533023	15.9998762	39	-3.637821167	0.219901806	41.14683119
2.811247408	0.480514263	64.87056326	87	-2.08586242	0.147760449	92.77233972
2.258745241	0.534954893	23.46752724	48	-2.309475466	0.18660912	64.8545243
5.746619191	0.299293341	88.4586737	220	-1.258681268	0.066294632	139.6142811
4.569418253	0.387735781	54.6956299	61	-2.120171131	0.1312	98.0104
8.30952381	0.15527397	92.30398085	333	-0.081062685	0.112426036	147.2544379
2.435857522	0.564505937	44.13250391	32	-0.678995934	0.234570453	55.40747613
1.788566251	0.520610733	10.33970009	52	-1.586641238	0.169862361	40.24536877
3.862461599	0.442561381	90.88389695	87.5	-2.192408766	0.124341254	113.3537583
1.365274772	0.638984632	13.40921869	30	-3.183532115	0.27613206	40.1573547
1.237612749	0.615446472	9.247781383	29	-4.308004373	0.285478822	30.17357629
5.747911666	0.315142301	91.42165156	138	-2.048056066	0.0909549	126.0831444
5.678365618	0.372443197	183.0537849	340	-1.000446902	0.08490917	193.4231142
4.334964822	0.339987481	43.47840595	134.75	-1.118131167	0.121527778	97.25
7.054111225	0.211959946	89.06495126	256.5	0.100607873	0.046407467	140.3946705
1.644470326	0.516256395	7.823634207	51	-0.714151594	0.171632653	35.6569898

original_firstorder_Energy	original_firstorder_RobustMeanAbsoluteDeviation	original_firstorder_Median	original_firstorder_TotalEnergy	original_firstorder_Maximum	original_firstorder_RootMeanSquared
3216081	22.74099268	24	86834187	67	94.38650134
7297678	14.39810711	23	197037306	117	72.12129444
768588	26.42291635	23	20751876	82	65.89616897
69862678	63.54730776	34	1886292306	351	190.1107554
7388699	122.3694559	-199	199494873	91	270.4726153
5555643	72.10756082	-42.5	150002361	69	150.8940976
18687092	102.067602	-487.5	504551484	-45	516.6802272
7676863	59.40770064	21	207275301	90	138.3629168
372922781	279.3002106	-148	10068915087	118	502.6506846
15593874	24.63791721	28	421034598	92	82.00241375
8786256	80.05755556	3	237228912	65	152.8649801
7005025	22.5786079	30	189135675	116	96.19597593
41022655	86.47552204	6	1107611685	90	173.2951996
15012255	83.54625907	-19	405330885	106	168.6188042
28197763	116.329377	-167.5	761339601	138	278.3278316
64579621	11.94129072	40	1743649767	199	77.22779275
9531291	97.48197401	-75	257344857	113	209.5781477
18011709	32.07130208	30	486316143	108	107.7287455
9403576	110.5848765	-91.5	253896552	94	227.3059612
12121641	10.78092687	34	327284307	163	66.10396053
22611240	9.35600187	37	610503480	95	66.74890822
23647491	75.03475874	-9	638482257	165	161.2911702
11904846	13.96139877	30	321430842	176	63.85140605
27765230	191.6587755	-345	749661210	58	456.9038496
2890126	17.79546432	33	78033402	123	54.86846013
1520313	24.29897204	37	41048451	143	70.95173716
15509075	17.69961925	31	418745025	264	82.3133073
10135449	9.209520569	29	273657123	101	51.01146137
46014033	12.7125471	34	1242378891	241	111.2774839
9202944	12.1610294	40	248479488	104	73.72841721
20728622	11.94725594	42	559672794	107	70.24394193
67984819	19.41275106	36	1835590113	454	87.73075313
29370662	34.04724781	36	793007874	142	145.4138406
1202227	100.3801653	-257	32460129	29	283.1050453
5994786	102.722131	-33	161859222	123	230.3284688
37531725	73.01797031	25	1013356575	81	167.7340943
22035848	7.198333489	35	594967896	309	57.38777264
46545133	32.19487725	37	1256718591	97	123.9003517
24649155	123.7086103	-155	665527185	124	277.1076413
3513150	17.11299247	25	94855050	124	80.88373613
9000292	25.20137931	26	243007884	276	112.1954588
9395089	72.50109631	9	253667403	80	157.0319275
21084544	73.36741161	0	569282688	153	159.962617
15481844	88.73916625	-17	418009788	82	187.7928632
98721443	138.0370836	-111	2665478961	130	297.5557272
20808933	14.61346811	51	561841191	144	58.30130061
134876451	95.7206795	39	3641664177	200	221.6245151
4828327	67.94858013	10.5	130364829	87	145.522757
1856159	116.125	-250.5	50116293	34	290.4666308
12177821	156.5172036	-135	328801167	79	288.8074322
36095637	21.70426161	33	974582199	108	94.05833218
5671902	32.75422954	19	153141354	84	118.6347273
32167736	16.54552612	30	868528872	953	100.3401663
2355986	45.61640306	10	63611622	91	99.28586006
43661446	11.12142795	41	1178859042	406	62.14333648
15902957	97.50473321	22	429379839	119	217.8795847
30540157	92.08437944	8	824584239	278	187.1446388
80875729	187.1126041	-305	2183644683	213	420.2196582
7703672	58.42105263	66	207999144	175	147.5187626
2868645	35.32147863	19	77453415	134	78.45948354
12163876	21.40053713	67	328424652	204	91.43332124
143181543	16.89043358	31	3865901661	351	145.3639851
25188631	19.00941476	56	680093037	196	78.63063262
4012903	10.35660115	4	108348381	447	47.45429013
146331	25.98572278	17	3950937	51	52.54485954
37359489	54.81933791	8	1008706203	180	180.083625
5442210	102.5487008	-101	146939670	89	227.6632852
27812572	15.4432561	46	750939444	205	89.56609824
2049938	53.8916125	53	55348326	128	119.7298241
15469538	20.21915467	27	417677526	105	92.91154227
30200123	24.31877185	63	815403321	343	107.7956452
5322895	30.96659503	48	143718165	134	96.89077288
32014328	9.534585407	25	864386856	108	67.35380518
34983764	12.12397383	39	944561628	354	56.82492245
195257446	19.63237108	60	5271951042	565	115.9672743
20236798	107.5963262	13	546393546	233	213.0117052
11162201	70.76547732	-30	301379427	116	156.4560992
1453681	26.22446661	13	39249387	95	80.55834731
13410900	41.62176046	19	362094300	138	121.1974754
32385255	14.73806102	30	874401885	101	109.4385466
2457661	53.59508509	60	66356847	280	128.4304058
44010246	89.83933183	37	1188276642	286	225.5635101
96848460	17.71667469	59	2614908420	1311	140.1738335
1863576	100.416	-121	50316552	34	245.1843283
41371684	17.24197208	71	1117035468	208	91.31102532
63601021	47.87840476	18	1717227567	324	134.6679226
3363200	29.7490641	44	90806400	136	95.99086715
20738939	97.09038455	-2	559951353	143	187.6444051
1932055	42.2125	-85	52165485	8	196.573396
1204880	103.8024691	-250	32531760	5	304.4389543
46322810	16.03378515	25	1250715870	960	116.2287871
2213284	22.28512397	66.5	59758668	275	85.32607734
12012474	52.21821019	18.5	324336798	153	169.9298073
16920235	15.03971017	21	456846345	184	68.06718823
37325092	12.78556822	56	1007777484	148	71.89053265
2069230	62.5214494	30	55869210	199	181.2316767
50826800	148.7694991	-31.5	1372323600	92	273.3958561
415110	61.45679012	-6	11207970	91	131.5152082
24763723	105.1993474	-335	668620521	121	389.7751672
1563749	21.64835556	63.5	42221223	194	74.73163893

original_firstorder_90Percentile	original_firstorder_Minimum	original_firstorder_Entropy	original_firstorder_StandardDeviation	original_firstorder_Range	original_firstorder_Variance	original_firstorder_10Percentile
44	-586	2.612933498	93.95164523	653	8826.911641	-109
47	-534	2.441188325	71.962165	651	5178.553192	-46
60.8	-357	2.938645524	65.72586966	439	4319.889942	-63.4
53	-940	3.291890592	183.1332135	1291	33537.7739	-331.8
43	-608	4.560384787	183.4365106	699	33648.95344	-436
41	-539	3.85372135	127.8722469	608	16351.31153	-270
-259.7	-773	4.322090253	164.5673263	728	27082.4049	-698.7
64	-637	3.59231943	133.4231665	727	17801.74137	-247
38	-1023	4.76745905	387.6985557	1141	150310.1701	-976
53	-713	2.819707563	81.99916773	805	6723.863508	-98
46.5	-564	3.572505052	137.6116227	629	18936.95869	-294.5
62	-731	2.9686441	96.18387518	847	9251.337844	-100.8
53	-670	3.855057936	157.6069474	760	24839.94986	-318
58.3	-667	4.094985687	149.8306378	773	22449.22004	-284
20	-808	4.786136356	194.4187645	946	37798.65598	-468.8
63	-753	2.22005085	72.59649666	952	5270.251327	4
51.4	-685	4.421602049	171.5030576	798	29413.29877	-363.4
59	-583	3.080191308	107.2997022	691	11513.22608	-157
55.9	-740	4.436656811	182.5978677	834	33341.98128	-400.9
49	-503	2.132663046	64.18424422	666	4119.617206	-34
53	-968	2.004053546	63.35818411	1063	4014.259493	-7.6
55.2	-922	4.042033803	146.0821106	1087	21339.98303	-275.6
47	-583	2.246183136	62.90869743	759	3957.504213	-50.1
21.8	-886	5.020570951	276.8340242	944	76637.07694	-734.2
64	-440	2.63531708	50.3392034	563	2534.035399	-34
68	-293	2.897050018	69.03647943	436	4766.035492	-67
56	-640	2.697680138	82.00002158	904	6724.003539	-62
46.6	-994	1.835959571	46.3155671	1095	2145.131756	0
50	-877	2.35080248	111.257507	1118	12378.23286	-46.5
59	-643	2.258046372	70.41118719	747	4957.735281	-17.8
64	-756	2.255193803	64.09166299	863	4107.741264	1
59	-714	2.76389735	87.35333563	1168	7630.605245	-86
57.2	-735	2.944756049	144.0609238	877	20753.54976	-206
-19.4	-471	3.373557262	154.8530486	500	23979.46667	-408
39.8	-774	4.13441191	197.0294745	897	38820.61383	-405.6
48	-750	3.338952767	158.2360468	831	25038.64651	-317.8
50	-706	1.590347397	50.43466937	1015	2543.655875	16
58	-839	2.902276113	123.3509227	936	15215.45014	-182.9
30	-806	4.737946015	194.5998432	930	37869.09896	-473
46	-490	2.497117237	80.88287866	614	6542.04006	-85.8
47	-751	2.744634664	111.4281351	1027	12416.2293	-146
48	-633	3.637097797	144.6909262	713	20935.46413	-272
51.7	-700	3.883668921	146.1275184	853	21353.25162	-294.1
43	-732	3.987049058	164.1616637	814	26949.05184	-361.2
45.6	-981	4.874524224	233.6986457	1111	54615.05701	-516.2
83	-297	2.22916402	31.10748167	441	967.675416	16
102	-1051	4.29957729	214.175063	1251	45870.95763	-345.5
55.3	-553	3.618588932	136.3506771	640	18591.50714	-269
-37.9	-553	3.823067982	172.5215102	587	29763.67149	-456.6
33	-740	4.489185626	217.7052416	819	47395.5722	-525.5
56	-608	2.740045037	94.04990483	716	8845.384599	-99.1
44	-616	2.939167004	115.7338412	700	13394.322	-207.2
59	-753	2.822530435	99.34230219	1706	9868.893005	-58
51.2	-354	3.409303891	95.10684916	445	9045.312757	-173.4
64	-773	2.142124065	52.31283484	1179	2736.632689	7
68	-888	4.006188346	202.4303344	1007	40978.04028	-412
96	-756	4.426778971	177.1007326	1034	31364.6695	-337
23.3	-983	5.255472999	271.8879997	1196	73923.08439	-706.1
120	-535	3.745599365	147.2313925	710	21677.08293	-210.1
81	-420	3.427171832	78.41700187	554	6149.226183	-109
101	-524	2.965162158	78.99930089	728	6240.889541	-36
48	-881	2.450312388	144.3894672	1232	20848.31823	-162
86	-522	2.762929496	68.87653018	718	4743.97641	-28
26	-381	2.143406805	47.373872	828	2244.283748	-29.9
40.8	-149	2.562108583	52.3411258	200	2739.59345	-83.2
51	-906	3.73905226	170.1933845	1086	28965.78814	-312.9
37.6	-610	4.37960254	173.544943	699	30117.84726	-428.8
74	-655	2.655255353	86.25433958	860	7439.811097	-38.8
93.8	-421	3.532484535	119.7269805	549	14334.54986	-182.4
56	-618	2.808069144	92.90788142	723	8631.874431	-90
115	-620	3.298212017	99.0761667	963	9816.086808	-33
92.4	-584	3.273121217	94.74302142	718	8976.240108	-102.4
45	-722	2.033887517	66.34780788	830	4402.031611	-6
63	-769	2.191927113	47.57890323	1123	2263.752033	3
101	-887	2.993000577	110.1440742	1452	12131.71709	-9
108.5	-782	4.55267915	200.6044854	1015	40242.15954	-373
52	-657	4.050910963	138.491459	773	19179.88423	-271
55.8	-469	3.033077887	80.1755755	564	6428.122907	-92.1
56.8	-652	3.430619062	118.3264944	790	14001.15928	-208.8
48	-754	2.429539073	109.4055975	855	11969.58475	-69
155.6	-372	4.00939831	124.2035712	652	15426.52709	-133.2
118.2	-858	4.417785424	220.2198449	1144	48496.78007	-387.6
98	-973	2.990961864	137.1797934	2284	18818.29571	-6
-16	-532	4.091135423	159.5974254	566	25471.33819	-449
104	-615	2.749986114	73.17896644	823	5355.16113	3
57	-691	3.512494519	130.3492192	1015	16990.91894	-235
80	-454	3.081398751	95.23507793	590	9069.720068	-119.6
91.4	-789	4.336598747	174.1976674	932	30344.82734	-341.2
-37	-673	3.311037605	143.505625	681	20593.8644	-323.3
-28.4	-520	3.238901257	173.289169	525	30029.13609	-470.4
56	-691	2.980609855	115.7989094	1651	13409.38741	-63
118.7	-254	2.949521916	60.89431855	529	3708.118032	0.6
63.5	-772	3.711000399	164.3779301	925	27020.1039	-303.5
45	-519	2.497333369	68.03333501	703	4628.534673	-69
81	-625	2.338485286	56.64092484	773	3208.194367	12
152.4	-738	3.819071181	180.3450585	937	32524.34014	-259.4
36.1	-801	4.316905857	225.9828114	893	51068.23106	-524
64.2	-363	3.288320189	120.6543502	454	14557.47222	-219.5
-125	-729	4.632540479	169.4043658	850	28697.83914	-574.4
104	-149	2.92952887	49.00860386	343	2401.843253	-2.2

original_firstorder_Kurtosis	original_firstorder_Mean	original_glrlm_ShortRunLowGrayLevelEmphasis	original_glrlm_GrayLevelVariance	original_glrlm_LowGrayLevelRunEmphasis	original_glrlm_GrayLevelNonUniformityNormalized
14.35966067	-9.049861496	0.006108954	18.47164336	0.006418769	0.19314459
23.39737425	4.788310763	0.003746971	11.85136754	0.004193437	0.214710468
9.895367133	4.734463277	0.01093038	7.538986311	0.011308031	0.155324754
7.662828951	-51.03259183	0.002451304	69.01869706	0.002563066	0.119835498
2.042691587	-198.7623762	0.019122387	53.53675134	0.01940363	0.046261914
3.872437346	-80.11065574	0.009325392	26.84393726	0.009498795	0.077536707
2.426697422	-489.7714286	0.040504176	43.100454	0.040653489	0.054788332
6.306234339	-36.64089776	0.008066569	31.18635664	0.00819523	0.104786451
1.979740355	-319.9180217	0.032558506	213.8865027	0.040145691	0.046274593
14.28375696	-0.729624838	0.001714125	13.57257328	0.001907096	0.164980843
3.670592219	-66.56382979	0.008145371	32.7021019	0.008389328	0.090232446
17.56814476	1.525759577	0.003142343	18.00551569	0.00328753	0.166103453
4.24211499	-72.05051245	0.005265643	43.15398806	0.005484919	0.082680109
4.216654541	-77.35037879	0.005364049	37.5762961	0.005467317	0.069663254
3.048972464	-199.1675824	0.00707611	60.951541	0.007157237	0.040719264
45.70494118	26.34161433	0.001144177	13.17549576	0.001408002	0.250582667
3.597257943	-120.4562212	0.009614666	47.19968581	0.009666941	0.054577538
9.250224661	-9.605025773	0.003555674	23.11289404	0.003834275	0.145639189
3.27659024	-135.3736264	0.014138005	54.22910163	0.014194191	0.053523935
18.70773604	15.81506849	0.002873887	11.39267977	0.003421764	0.210211841
39.28686679	21.00374384	0.000826681	10.36423836	0.000987314	0.253118194
6.204981142	-68.37293729	0.002480207	36.30511489	0.002541044	0.07520569
19.24264233	10.93150685	0.002172917	9.979973526	0.002555094	0.196867227
1.787002253	-363.4887218	0.018051132	121.2986387	0.018265987	0.034299576
19.93509719	21.82916667	0.004086691	4.999437396	0.004502348	0.190053959
7.791969081	16.37417219	0.013977667	8.84805414	0.014735036	0.167183844
16.79030735	7.174748799	0.002159415	13.66464445	0.002392987	0.193931834
125.0750929	21.37843389	0.000886578	5.651390196	0.001048239	0.294667337
22.76284829	2.108449946	0.001649656	33.20542611	0.001841395	0.198646599
29.03100313	21.86650916	0.002971943	11.3406231	0.003316209	0.239222034
41.31705655	28.74839324	0.00119958	9.258612084	0.00140916	0.24677039
14.15296994	8.128948262	0.001349803	16.95029357	0.001582502	0.172209564
10.17741529	-19.78977682	0.004835115	44.20443579	0.00504086	0.160514059
1.834010308	-237	0.091685695	38.31122623	0.092154985	0.102194314
4.30137623	-119.2920354	0.01310107	63.88747409	0.013153122	0.07680863
5.153649016	-55.64242879	0.003244851	48.4892955	0.003413967	0.107522214
66.57149004	27.38066059	0.001156616	7.866954531	0.001489052	0.307490887
10.16720883	-11.65534301	0.00144243	32.04954816	0.001598026	0.162934125
2.527003693	-197.2803738	0.005997985	61.6634941	0.00604669	0.04232101
15.77834865	-0.372439479	0.007237287	13.72725378	0.007674829	0.221388537
14.73048448	-13.0993007	0.003228249	25.52646111	0.003394629	0.187269379
5.36052558	-61.02099738	0.009430676	36.80785024	0.009579756	0.100782209
5.016420393	-65.07524272	0.005360785	36.87449464	0.005483899	0.087109314
4.028968802	-91.19817768	0.004772087	45.26392414	0.004874102	0.079568957
3.352760926	-184.1856502	0.003067824	88.54597735	0.003110903	0.041629029
19.00096839	49.30888598	0.004535909	1.985773332	0.005599127	0.235533454
6.885505957	-56.97778587	0.001517127	77.12668141	0.001578253	0.072782044
5.308427154	-50.84649123	0.01047665	32.04612322	0.010632238	0.106443174
1.781718693	-233.6818182	0.057166784	48.65876189	0.057202859	0.079652058
2.083760285	-189.7739726	0.013058919	76.37794843	0.013113212	0.054743128
12.79992959	1.259068627	0.002434868	18.64993095	0.002713606	0.179469586
9.249885448	-26.07444169	0.006361449	26.42439636	0.006608235	0.174188344
20.74271792	14.11580595	0.001346763	20.5317997	0.001513161	0.192549129
4.648464737	-28.50209205	0.0144675	15.40675164	0.014887708	0.114229099
68.58947753	33.54342827	0.00118312	6.567622445	0.001415818	0.255996931
5.214269234	-80.58208955	0.005827222	70.81740362	0.005901901	0.083802804
4.196908471	-60.48509174	0.003621062	52.11857282	0.00367168	0.063489673
1.9309551	-320.4082969	0.005134244	117.9575386	0.00517535	0.028267331
5.670802407	9.203389831	0.010053254	37.40131549	0.010380824	0.097453451
5.864295936	2.581545064	0.006042917	10.79563496	0.006327634	0.110220952
14.59005669	46.03436426	0.00363881	12.15345023	0.003954323	0.162572231
12.85464382	-16.80386659	0.001463655	50.51301464	0.001638311	0.206713151
17.81947116	37.93151694	0.00326667	9.78787396	0.0036014	0.176557662
29.17805662	-2.761503928	0.005009355	5.589419262	0.0059334	0.246704773
3.622821465	-4.622641509	0.085358716	4.58660146	0.087842913	0.192249589
8.339569099	-58.85850694	0.002667978	51.78567834	0.002743748	0.108177998
2.735920911	-147.352381	0.016689947	48.01684591	0.0167716	0.055295704
18.17239544	24.13037208	0.001816272	16.26540154	0.002063075	0.199342562
5.153551086	0.825174825	0.021403161	24.34224508	0.022066215	0.114131329
14.24750952	-0.824776786	0.002950673	17.70269894	0.003205019	0.176247573
14.47630663	42.47133513	0.002806643	18.68257002	0.002978934	0.134885067
10.69499589	20.28747795	0.003826072	16.26759678	0.00399376	0.13236516
36.22931865	11.5975627	0.001268086	12.33675724	0.001613712	0.270006698
48.93919278	31.06959572	0.000894573	5.141447865	0.00111023	0.248829848
20.92042665	36.28624561	0.000863431	24.84880316	0.000985211	0.164885186
4.158632506	-71.6367713	0.005411955	66.67518676	0.005457853	0.054849215
4.961875949	-72.79166667	0.004973029	32.12278032	0.005063739	0.074111616
13.42302627	-7.84375	0.013292611	11.51105174	0.013558477	0.150866361
7.92939218	-26.22343921	0.003297641	25.46044537	0.003454563	0.123256867
18.47534766	-2.685281065	0.002199031	30.07443565	0.002444291	0.19775033
4.837345918	32.67785235	0.029852033	26.06254449	0.030038193	0.075801991
5.424119526	-48.80693642	0.005151958	82.61417247	0.005202503	0.066976531
20.70844981	28.81679854	0.001515426	38.49766237	0.00163015	0.170441905
2.402382668	-186.1290323	0.048765931	41.56074961	0.048793652	0.068841234
20.60323825	54.61265619	0.001603757	11.39057831	0.001864145	0.185587181
7.218612283	-33.83090961	0.002607388	31.00068707	0.002762464	0.120391168
8.702916734	12.02191781	0.010607162	17.46961485	0.010926075	0.152397088
4.053117716	-69.75382003	0.003583444	50.2898548	0.003629085	0.06168676
6.833975788	-134.34	0.024893413	35.62742717	0.025008813	0.125049435
1.683627034	-250.3076923	0.100856464	46.85207101	0.100856464	0.112426036
17.14418191	9.987168271	0.002091149	29.04103254	0.002307333	0.176643929
9.280942125	59.76973684	0.010441535	6.928915304	0.011070277	0.153427038
7.396456854	-43.08173077	0.005046216	48.07407024	0.005151982	0.107429558
15.2480883	2.146495071	0.003182297	10.4111444	0.003657551	0.211904997
28.81717705	44.27250069	0.001621441	7.066353081	0.001894608	0.228275476
7.847439705	-17.9047619	0.01998351	54.77847605	0.020031935	0.085989627
2.643030833	-153.8735294	0.00508599	85.58005078	0.005180007	0.065451099
3.362920843	-52.33333333	0.054175901	23.47937091	0.054303458	0.11813301
2.401302787	-351.0368098	0.014702735	46.32145285	0.014809531	0.045905069
4.829962531	56.41785714	0.019928495	4.334467936	0.021371539	0.157078088

original_glrlm_RunVariance	original_glrlm_GrayLevelNonUniformity	original_glrlm_LongRunEmphasis	original_glrlm_ShortRunHighGrayLevelEmphasis	original_glrlm_RunLengthNonUniformity	original_glrlm_ShortRunEmphasis
0.770899333	49.28610561	2.791424354	436.0016826	151.817936	0.798562203
1.114130747	196.4119616	3.495849812	370.2567797	483.3451813	0.754506476
0.216399452	23.63785574	1.58438902	216.910448	115.8634368	0.893265379
1.334969015	160.1528963	3.439084349	1010.806734	858.951889	0.826704431
0.034177727	4.513113468	1.107768628	344.646791	90.94872867	0.973057843
0.19687667	16.61348961	1.496604969	336.6432478	171.9932377	0.914984113
0.020840729	3.764521639	1.061741754	183.7121351	66.38429842	0.985821475
0.257021699	35.85941109	1.635928722	549.9135451	263.685881	0.898062233
0.220537993	60.09943113	1.515095286	1014.641333	1057.129046	0.920017838
0.749478691	279.6451744	2.627344294	683.3533288	1062.571858	0.819130716
0.56237544	26.7307769	2.189461626	348.4259809	211.7622651	0.868394095
0.398916055	100.2028061	1.984311044	781.3085513	413.0750226	0.852991887
0.422195163	91.87707068	1.939188524	507.1346007	810.9174249	0.877403412
0.187099005	32.53119504	1.470670109	540.1958307	378.5024572	0.918649712
0.047058713	14.20291317	1.137063458	674.6160016	321.1415408	0.968467989
1.398727651	1655.65376	4.113407911	737.684129	3140.312731	0.714661592
0.05051699	11.29993184	1.151068753	571.6282537	188.8996599	0.964802169
0.748187743	165.6181955	2.635698603	454.8244492	716.1375132	0.820065961
0.084844765	9.120295523	1.232091567	620.6173969	150.7371856	0.952249741
3.459475101	305.9884939	7.157146462	321.3390089	690.4813484	0.715778589
2.069775831	749.6093757	5.019122025	1147.604725	1462.440252	0.730363135
0.21843828	59.85648209	1.526358151	1091.442191	639.7065811	0.91500969
3.022523779	323.0582423	6.246614814	430.1502875	852.1790696	0.747666833
0.02784205	4.446565016	1.082118044	588.3090013	123.4005341	0.980832584
0.443481298	141.6990863	2.111007778	309.7622859	489.0926497	0.83621481
0.322844418	41.30092542	1.834251141	149.1437147	175.163113	0.866161188
0.646510591	330.0543802	2.462999592	574.6750707	1073.377263	0.821699295
2.094144792	668.1078345	5.105152735	1205.614665	1091.223199	0.719170086
3.116051137	405.9180348	6.493621531	900.9319719	998.3442477	0.72641523
1.073778193	270.4706378	3.335779392	558.1147509	621.8103565	0.769089373
0.835865	709.2231325	2.978096713	803.9402624	1578.194784	0.769276816
1.258687158	1012.068649	3.540668732	661.8552798	3319.782434	0.779209273
1.025656313	153.3235031	3.152259093	655.4047271	565.7587739	0.797322967
0.010204082	1.517216117	1.032967033	139.3287546	14.56043956	0.991758242
0.10324501	8.032015809	1.275092704	698.5808789	90.93820183	0.94617947
1.1580682	101.8267667	3.15377455	607.1240102	626.2512512	0.837290466
3.665690469	1034.399174	7.646862908	620.1512667	1451.59895	0.682495835
1.101149637	339.9630961	3.218807168	870.4276957	1246.467865	0.800530358
0.052199393	12.95690133	1.152634588	674.4813975	279.6571952	0.965238475
0.75324153	84.38277233	2.754134276	318.9593856	225.8501592	0.796993016
0.833442689	96.8918244	2.754779916	754.2296854	328.4295333	0.82312774
0.356462787	31.94541022	1.80863384	495.4924845	237.7650665	0.8881647
0.24195793	61.72710525	1.601421658	596.5518573	549.2040869	0.901195235
0.220034885	30.47007753	1.539417343	649.3626556	305.0116794	0.911667805
0.073308709	43.6808995	1.203599679	1100.62733	937.3323037	0.956821063
0.691639154	1015.914133	2.713305292	163.0683206	2418.755745	0.776234814
0.160843291	178.3012794	1.418342972	1577.613262	2003.843562	0.922708358
0.18597025	21.21768851	1.501397503	419.6385569	157.1000855	0.907420051
0.020877377	1.717782218	1.067032967	237.1963287	20.67545788	0.983241758
0.071841238	7.529108766	1.201236833	542.4483155	123.0625991	0.957279514
0.983709066	513.6178764	3.023900532	501.1999035	1722.626082	0.803526775
0.521248789	53.25148451	2.270396026	472.7346778	195.5980195	0.827351675
0.58750754	460.3618042	2.378250299	840.769881	1505.712994	0.821370665
0.189216388	23.72020842	1.5187863	186.7563581	161.0570609	0.901777035
1.213125041	1860.016767	3.649998722	793.5486047	3741.04572	0.744721962
0.259411018	24.00357755	1.634193708	979.3134014	220.7595013	0.899065389
0.082682867	51.72558944	1.230689852	832.6824633	717.6378342	0.951291564
0.031094751	12.5617865	1.093777047	849.4321076	418.6533305	0.977340087
0.170507594	30.54227815	1.448906177	490.3251081	252.9012803	0.917289724
0.188252148	44.71537013	1.516262981	281.0035983	315.9692677	0.902682337
0.436890217	185.7339626	2.06601044	453.8296372	765.3202573	0.84484249
2.099962353	840.8588949	4.884844938	893.9419072	2160.289806	0.756516175
0.654246904	528.3090381	2.517664781	419.5974964	1839.641064	0.81159011
1.591239226	271.8089774	4.23656655	195.5067679	563.0438836	0.74218868
0.224361503	8.59654185	1.679399824	34.21014786	32.40450499	0.868726006
0.254261912	105.8793203	1.643553837	1092.426658	740.0987072	0.8918486
0.044064555	5.569844255	1.133046636	408.5432418	92.78752439	0.968509789
0.822915438	483.8654658	2.871090995	620.0744077	1398.409697	0.787350262
0.143628931	14.53059048	1.40944248	288.9914708	102.6121978	0.916638458
0.712484255	232.7530196	2.561363007	516.8509564	834.1856712	0.821920726
0.347588196	285.2552063	1.866864809	640.7951182	1495.78491	0.865220718
0.274422172	63.01476266	1.705076373	557.8131067	351.3705419	0.881666619
2.465264269	1023.464096	5.992630869	580.7020327	1622.776607	0.679245325
0.939407906	1819.737269	3.142515452	809.0581829	3966.866665	0.764205528
0.550326244	1792.998314	2.335464695	1160.811535	6776.066307	0.817433252
0.097226338	22.68455221	1.262046754	860.823723	361.1809639	0.947779127
0.120150551	30.74036514	1.330423205	571.5482114	349.3287661	0.933894688
0.210404619	28.8868441	1.583563763	326.4185607	143.728481	0.889359007
0.331993662	93.49792659	1.796329464	605.6072051	560.6730679	0.881393177
2.348944103	311.2276939	5.332914009	674.0253861	798.6733735	0.74007293
0.097018086	10.402795	1.280363904	283.1622622	117.4676872	0.939829559
0.125630407	52.64217551	1.338594066	1085.607523	660.4473558	0.933147252
0.525191812	637.9445034	2.264534126	1356.840613	2395.801871	0.827400051
0.012375072	2.108656342	1.038992042	263.5983778	29.88275862	0.990251989
0.671717396	658.481037	2.638769358	590.1879954	2059.428593	0.789665606
0.339789993	346.6233413	1.830005924	631.6014924	2075.71668	0.873157827
0.367279909	44.54099038	1.941013649	335.6660699	200.9911442	0.854182075
0.084115318	33.85196432	1.237441696	856.743037	480.6799373	0.949224551
0.11529173	5.724015194	1.339525128	476.0669065	38.86475617	0.931236937
0	1.461538462	1	181.7692308	13	1
0.757371411	439.3562906	2.664413097	686.9664078	1536.226878	0.813635059
0.255701295	38.87292213	1.703928055	171.2479266	182.946762	0.873614286
0.2305867	38.3756742	1.599672575	795.6577972	272.8427728	0.895627016
1.121099789	510.6077096	3.435452593	337.842454	1302.412034	0.763178623
0.870214053	1151.261208	2.92486119	574.620151	2920.296293	0.789130667
0.068355935	5.075138293	1.211371022	882.613893	51.88597455	0.950135747
0.246615313	38.50116914	1.590053006	677.6991649	463.8196155	0.907780056
0.038266253	2.728387554	1.123137732	191.5565521	21.35481909	0.969215567
0.02596112	7.295628991	1.078492175	311.0871551	151.1775313	0.980921352
0.21416772	37.8709153	1.579558618	72.49176764	184.523099	0.894697434

original_glrlm_LongRunHighGrayLevelEmphasis	original_glrlm_RunPercentage	original_glrlm_LongRunLowGrayLevelEmphasis	original_glrlm_RunEntropy	original_glrlm_HighGrayLevelRunEmphasis
1759.469581	0.705518858	0.009130944	3.97731802	568.6620786
1897.397301	0.650858051	0.008694924	4.077361656	506.4078041
416.984722	0.858322468	0.013355238	3.580551613	247.6051392
5187.898797	0.690278165	0.004100037	4.892207961	1286.83405
406.7310212	0.965727342	0.020528604	4.685238137	357.0636371
649.4602146	0.877679697	0.010467375	4.400931808	381.3323629
197.9218081	0.981318681	0.041273086	4.38101631	186.3278254
1141.5689	0.852676002	0.008992431	4.321651417	631.3206935
1499.464321	0.879508026	0.10286444	5.338058916	1092.077685
2406.919914	0.730553621	0.003620383	4.114538417	854.3787125
1151.118639	0.786006547	0.010440991	4.537426781	426.3910183
1935.829128	0.796057311	0.004255154	3.982904136	930.9279008
1389.27979	0.812816759	0.006889192	4.787795363	607.0422202
995.1408304	0.883741259	0.006054144	4.653458874	606.7478271
832.4744817	0.958157227	0.007494728	4.99242857	704.1846459
4476.241992	0.609722656	0.004255759	4.031109297	1050.894006
721.6362779	0.953917051	0.009887196	4.62947972	599.7062906
1675.065923	0.731165741	0.006308144	4.394625498	575.253764
885.3781575	0.935333897	0.014459437	4.726672099	665.1386186
3715.009408	0.523099107	0.015125249	4.38173543	469.9656613
8359.870205	0.583160288	0.003378214	3.948696777	1600.834661
1985.47676	0.87526445	0.002909206	4.675496855	1214.229073
4126.083629	0.560247629	0.010365859	4.260818878	597.5766125
662.9192461	0.974551764	0.019155408	5.10186833	602.6206582
820.342511	0.776121795	0.0072972	3.6976054	375.0280205
346.2514247	0.816607234	0.019232297	3.779624228	176.3362085
1851.245427	0.743085661	0.004287412	3.956366343	712.7428138
8884.747677	0.581139528	0.003392833	3.697136905	1693.916514
9207.552366	0.54794237	0.005658257	4.573733447	1292.952727
2589.267713	0.667136172	0.006480575	3.820881492	741.0690273
3247.305851	0.683976343	0.003202035	3.740244606	1059.119384
3315.097408	0.664414042	0.004236241	4.401737633	873.3047673
3082.462981	0.686880434	0.00716441	4.527618375	863.9250423
146.952381	0.98974359	0.094032148	3.374361298	140.8534799
1045.365017	0.924438393	0.013416441	4.515826596	754.9528901
2957.982708	0.708799446	0.005560782	4.770225215	769.9309176
7303.412426	0.502465999	0.008416992	3.823764047	923.8668162
4020.627617	0.68779176	0.003310274	4.4616122	1129.715695
850.8320361	0.9537503	0.006274153	4.961940274	707.2655576
1241.576436	0.708924223	0.011407912	3.900242778	413.825699
2836.880527	0.722754169	0.005024714	4.092068039	943.2935602
1207.137866	0.83080961	0.010636949	4.466837155	581.1618606
1200.310225	0.859129948	0.006209548	4.608706619	680.7679025
1261.592397	0.871561241	0.005467065	4.624592958	734.3090843
1478.480203	0.940876164	0.003302932	5.201263626	1165.941169
576.0268243	0.704546026	0.013740498	3.554384238	210.8825089
2612.595515	0.892010757	0.001876245	4.907020893	1737.491769
781.2119164	0.873481781	0.011471098	4.256161069	475.9146994
276.8020979	0.979020979	0.057347158	3.896302471	245.1174825
771.5884928	0.941517387	0.013360673	4.765932529	582.1870911
2115.917942	0.701055807	0.005524313	4.202815493	643.2185443
1494.608644	0.757587326	0.008408666	4.144961714	594.3377841
2522.385756	0.748043818	0.002793075	4.100104967	1033.549181
356.4274851	0.868361764	0.017007737	4.044702128	213.4403949
3988.927016	0.642415872	0.003829933	3.790147669	1074.586303
2059.298979	0.853960964	0.006353706	4.734861887	1125.893099
1132.455687	0.933927311	0.003907627	4.80344647	884.64019
990.3103728	0.970272086	0.005341717	5.395773828	876.5754793
840.1263286	0.885049978	0.01187039	4.347118001	544.5384314
499.0397847	0.869924067	0.007828301	4.063287546	315.5914931
1177.594869	0.784721121	0.005970733	4.033361094	545.857069
6828.284	0.5999001	0.004356649	4.448336062	1241.264987
1394.935841	0.734167894	0.006271768	4.040398594	526.7563011
1188.188696	0.617542951	0.017360544	3.904517789	268.0137694
81.18748366	0.838896952	0.100361314	3.203628943	41.7375371
2194.188474	0.849292201	0.003189685	4.545214058	1250.091178
506.3366088	0.958974359	0.017107931	4.533379517	427.0688066
2425.277322	0.699762597	0.00423121	4.114470756	804.4557788
488.6193079	0.889725659	0.024917979	4.073702697	322.619618
1768.443084	0.736564217	0.005395173	4.090470152	644.1308139
1436.775608	0.813212182	0.004083543	4.252034665	748.0776855
1151.218907	0.838692172	0.004984375	4.080243781	642.7979299
5546.882404	0.536041683	0.006968244	4.206402051	880.2581577
3417.326376	0.674919413	0.003066926	3.690851174	1067.525756
3478.358853	0.748901969	0.001869318	4.238567026	1439.450498
1239.161927	0.927043808	0.005684758	4.942947333	923.4016701
881.3517793	0.909412955	0.005509083	4.516760676	622.7983796
619.941377	0.854395604	0.01495348	3.744781251	373.0976985
1359.587901	0.829640239	0.004469353	4.295307264	702.7625167
5645.331688	0.581019572	0.006458497	4.441255381	953.0116251
399.6311365	0.920495612	0.030901713	4.393948668	303.7829574
1664.899681	0.908403735	0.005453809	4.934877703	1180.769749
3885.310186	0.759133542	0.002415863	4.212113522	1660.639236
280.8447306	0.987593052	0.048904537	4.129327714	267.0476484
2095.840754	0.714724212	0.003885459	4.117193386	761.1217036
1469.515856	0.820359281	0.003746823	4.457296869	741.7627886
836.2786896	0.799578504	0.012985421	4.057086443	403.0195839
1193.786609	0.931565887	0.003840645	4.712300216	915.8839886
723.7268606	0.912307692	0.025576067	3.649997518	517.7072192
181.7692308	1	0.100856464	3.238901257	181.7692308
2312.046299	0.724992709	0.004220147	4.401221534	850.2406779
343.4273486	0.832742915	0.014544658	3.749020223	197.3936935
1554.663865	0.857063609	0.005745992	4.441667458	907.5463426
1695.803426	0.659238352	0.008481633	4.122843314	457.5476075
2236.552399	0.698272373	0.004373093	3.749721878	738.0924785
1157.408154	0.936507937	0.020236945	4.090320116	935.0404479
1461.4444	0.864027149	0.005742613	5.01019273	781.9137547
231.423533	0.961538462	0.054813685	3.422571451	199.5299483
342.7483418	0.974988202	0.015243437	4.740826295	317.2782851
127.8915783	0.860164835	0.029195607	3.603403719	80.99401235

original_glrlm_RunLengthNonUniformityNormalized	original_glszm_GrayLevelVariance	original_glszm_SmallAreaHighGrayLevelEmphasis	original_glszm_GrayLevelNonUniformityNormalized
0.595021803	31.75280277	360.1123553	0.078477509
0.527845974	29.56184076	268.3674429	0.084353543
0.759946137	13.94628099	118.0113153	0.099173554
0.643629559	89.03517567	762.2177489	0.038557771
0.931644545	47.80150884	197.5795968	0.044745057
0.802882627	30.28154195	201.068932	0.060136054
0.965006059	47.14253827	158.5988343	0.052933673
0.770034181	37.9496937	336.5619102	0.06040813
0.813894437	133.1385919	742.5093726	0.032328447
0.626656372	25.19080958	465.6733958	0.075414762
0.715601057	32.60331361	195.5956572	0.057159763
0.684072395	39.00377297	527.5769607	0.064546229
0.729784364	43.88038536	285.1024531	0.04647457
0.810362341	38.41236543	323.3839289	0.054024691
0.920393129	63.72568559	449.9267184	0.036949936
0.474083868	58.42303273	548.9256279	0.073051825
0.911788932	49.40690681	352.1260731	0.0499101
0.62916217	35.25588485	275.1234099	0.057347049
0.883821629	55.49305556	348.6032179	0.042317708
0.474967028	23.3674578	234.8736634	0.069166037
0.493714612	27.65690938	1008.784315	0.080523661
0.80328866	44.66069812	776.0148191	0.048227292
0.519874725	21.09734603	328.3107655	0.080768162
0.951290013	113.4982639	432.0991348	0.032986111
0.655036698	13.62819576	194.3586299	0.107925493
0.708097348	13.31068739	80.83761349	0.098221439
0.630441545	32.4799102	406.9279568	0.070204082
0.479491217	28.68627824	1055.662148	0.129916766
0.488925824	72.3397723	723.3465761	0.046701593
0.549051082	29.68840551	404.3176741	0.086302765
0.548761389	34.21532599	645.068088	0.091126123
0.564238163	35.76748379	457.4798604	0.056013716
0.59254284	67.71489855	455.4102943	0.048803863
0.979591837	34.61538462	113.2692308	0.100591716
0.869877416	71.72827148	435.5398455	0.045410156
0.662044149	50.01259094	398.3828844	0.045652232
0.430975025	39.972282	535.3247649	0.100879101
0.597320313	47.73006607	606.2755369	0.051732261
0.912902071	62.96675926	446.1572942	0.039894997
0.59201063	27.5811	221.3476157	0.0748
0.63501341	49.19444942	574.2173098	0.061995769
0.750505132	41.48614958	338.0489263	0.056440443
0.774723812	42.67045184	368.8611216	0.046848989
0.796339738	47.86781592	419.3971687	0.050577064
0.893185449	89.82723607	667.7050796	0.031388119
0.559872223	10.06484283	133.3367941	0.138762048
0.817844329	90.38618208	1036.279811	0.03585311
0.787401729	38.2569738	271.1669513	0.059533873
0.958245247	42.58024691	148.4340278	0.067901235
0.894712713	68.49333333	332.7404466	0.039753086
0.601619259	35.88203224	325.881556	0.05666238
0.639849436	39.4799093	315.6822342	0.066666667
0.6295355	64.95037118	711.664482	0.063082232
0.775028971	17.74844183	99.91795462	0.077908587
0.513976677	37.62386412	676.7244981	0.090822579
0.770934728	80.16645101	647.6742063	0.043631282
0.88055797	66.08659122	524.4546418	0.037390689
0.941844511	111.8030859	613.4153544	0.027441406
0.806748682	51.30674556	300.4213685	0.051597633
0.77745117	17.54538624	161.3829803	0.075336384
0.669315749	26.61154563	297.639377	0.070277147
0.531178448	87.63760922	647.5331722	0.039889045
0.613968265	26.63896242	271.9535445	0.076011717
0.510295686	20.91282765	167.0949957	0.089283709
0.721015716	5.418685121	11.99572267	0.134948097
0.755693019	85.96189797	694.2960187	0.043207841
0.9206927	47.30541323	246.123835	0.051904656
0.575782281	41.40957089	454.1390055	0.058535219
0.805467364	30.41983852	151.8682989	0.062668205
0.631255156	38.029604	311.6439954	0.059763339
0.706100275	50.06884435	429.500088	0.051645213
0.736772168	29.37968657	361.9595177	0.064768895
0.427610641	37.1189877	482.2891079	0.073235261
0.541691025	24.8000524	659.3752631	0.09806563
0.622842876	77.93810174	831.5861871	0.047719176
0.873071112	78.14910212	583.5692855	0.037103356
0.841927529	39.45271526	343.3266788	0.055105951
0.750121894	19.2528616	192.0442525	0.098335068
0.737586614	36.33226502	385.7370807	0.059000058
0.507470153	59.67859375	523.001041	0.055275
0.855222652	37.52875696	216.130686	0.05515264
0.840079172	117.9906173	687.2216047	0.031805556
0.639597028	118.7834008	950.2054783	0.050743657
0.975249855	45.84171598	208.2115385	0.059171598
0.579562335	30.587189	399.3215265	0.066050541
0.720549831	46.76498068	376.9203124	0.047931329
0.686873457	29.98079427	221.5321273	0.0625
0.875596944	55.1987414	516.7177189	0.042864813
0.843464953	61.32098765	188.8740177	0.080246914
1	46.85207101	181.7692308	0.112426036
0.617477132	88.91011428	638.1714073	0.049373049
0.721181384	16.6371439	126.9274047	0.093864134
0.763391358	71.03757569	486.0544805	0.048442907
0.53997524	21.23034881	234.1081952	0.075277616
0.578574987	23.15758734	414.3301444	0.092221708
0.878047612	81.88613407	523.8803092	0.061524334
0.788570921	78.13530507	351.5329007	0.034077763
0.923467493	29.06666667	105.7873333	0.093333333
0.950923873	50.57304829	271.5984022	0.043371354
0.762689426	9.376181474	54.2434234	0.098508717

original_glszm_SizeZoneNonUniformityNormalized	original_glszm_SizeZoneNonUniformity	original_glszm_GrayLevelNonUniformity	original_glszm_LargeAreaEmphasis	original_glszm_ZoneVariance	original_glszm_ZonePercentage
0.723737024	61.51764706	6.670588235	407.1882353	389.1507266	0.235457064
0.566281958	104.7621622	15.60540541	2785.691892	2728.178115	0.131860299
0.510330579	22.45454545	4.363636364	92.97727273	76.79493802	0.248587571
0.672668947	388.1299827	22.24783362	1434.181976	1422.958895	0.298499741
0.514568158	31.90322581	2.774193548	4.016129032	1.362382934	0.613861386
0.535782313	56.25714286	6.314285714	36.99047619	31.59038549	0.430327869
0.691964286	38.75	2.964285714	1.928571429	0.366071429	0.8
0.605744655	95.10191083	9.484076433	108.5159236	101.9922918	0.391521197
0.586669833	363.1486268	20.01130856	86.68820679	81.00240891	0.419376694
0.510290144	203.0954774	30.01507538	2737.198492	2703.248838	0.171625701
0.475502959	61.81538462	7.430769231	144.6307692	136.2653254	0.345744681
0.580916062	91.20382166	10.13375796	599.6624204	576.4140533	0.207397622
0.491932615	222.8454746	21.05298013	402.9536424	393.8606884	0.331625183
0.613985185	138.1466667	12.15555556	72.41777778	66.91093333	0.426136364
0.594985651	133.2767857	8.276785714	5.214285714	2.573660714	0.615384615
0.480782786	370.6835279	56.3229572	43889.58236	43692.34566	0.071204285
0.561907124	71.36220472	6.338582677	7.393700787	4.474176948	0.585253456
0.51824098	171.5377644	18.98187311	1267.933535	1245.948485	0.213273196
0.553385417	53.125	4.0625	9.958333333	6.364149306	0.527472527
0.534210128	168.2761905	21.78730159	9570.406349	9492.854563	0.113554434
0.574059352	318.0288809	44.6101083	18145.69856	18061.78105	0.109162562
0.598933635	207.8299712	16.73487032	123.9337176	117.0714315	0.381738174
0.519342946	206.6984925	32.14572864	7486.547739	7432.720815	0.13630137
0.591362847	56.77083333	3.166666667	2.53125	0.61187066	0.721804511
0.371530314	54.98648649	15.97297297	993.8243243	951.7498174	0.154166667
0.427014901	33.73417722	7.759493671	183.6708861	169.0572024	0.261589404
0.547216327	191.5257143	24.57142857	3448.242857	3405.471257	0.152905199
0.498668252	144.6137931	37.67586207	20108.16897	19927.77628	0.074454429
0.637810776	324.007874	23.72440945	9543.031496	9489.522847	0.136706136
0.539274806	121.8761062	19.50442478	3798.756637	3742.639302	0.133490845
0.567513188	248.5707763	39.91324201	11614.48174	11522.48824	0.10426089
0.509528093	627.229082	68.95288383	14854.09829	14802.61097	0.13936375
0.624720475	206.7824773	16.15407855	1417.483384	1399.873824	0.238300936
0.73964497	9.615384615	1.307692308	1.461538462	0.130177515	0.866666667
0.645996094	41.34375	2.90625	13.234375	10.11694336	0.566371681
0.618459556	238.7253886	17.62176166	888.2901554	876.3465059	0.289355322
0.543211007	227.062201	42.16746411	51248.70096	50992.47124	0.062471977
0.577247917	340.5762712	30.5220339	3678.59322	3652.184074	0.194591029
0.55361558	100.2044199	7.220994475	7.464088398	4.318854736	0.563862928
0.6318	63.18	7.48	815.83	786.9931	0.186219739
0.614471655	102.6167665	10.35329341	760.6047904	742.2740866	0.233566434
0.680315097	103.4078947	8.578947368	114.0723684	107.7894304	0.398950131
0.594435196	172.3862069	13.5862069	186.9103448	178.8369084	0.351941748
0.573177502	106.0378378	9.356756757	79.18378378	73.55278305	0.421412301
0.529024315	301.5438596	17.89122807	35.23684211	31.41035703	0.511210762
0.471290196	166.3654391	48.98300283	25424.30595	25123.53406	0.057660895
0.578905948	591.6418787	36.64187867	360.3933464	353.1739768	0.372177713
0.706315662	64.27472527	5.417582418	59.93406593	53.65656322	0.399122807
0.796296296	14.33333333	1.222222222	2	0.50617284	0.818181818
0.676296296	60.86666667	3.577777778	9.555555556	6.923950617	0.616438356
0.53937786	328.4811166	34.50738916	6572.220033	6527.336634	0.149264706
0.668208617	70.16190476	7	341.952381	327.2214059	0.260545906
0.636461577	346.8715596	34.37981651	4235.906422	4201.53886	0.17057903
0.569598338	43.28947368	5.921052632	83.06578947	73.17641967	0.317991632
0.54122935	448.6791315	75.29191797	47478.21472	47292.21645	0.073323899
0.580663527	80.71223022	6.064748201	74.95683453	69.14838776	0.414925373
0.545010288	235.4444444	16.15277778	31.12962963	27.05521262	0.495412844
0.587558594	188.01875	8.78125	3.31875	1.270273438	0.698689956
0.589704142	76.66153846	6.707692308	79.03076923	71.6156213	0.367231638
0.469708353	56.83471074	9.115702479	160.1157025	145.2836555	0.259656652
0.570703639	150.095057	18.48288973	1422.688213	1392.081655	0.180756014
0.656424723	683.3381364	41.52449568	15193.11239	15150.74367	0.15363046
0.505763783	272.606679	40.9703154	6150.085343	6092.955304	0.132302405
0.519732261	109.6635071	18.83886256	4823.109005	4751.782575	0.118406285
0.439446367	7.470588235	2.294117647	34.52941176	24.80968858	0.320754717
0.582659835	195.1910448	14.47462687	409.5761194	397.7507329	0.290798611
0.515705057	34.55223881	3.47761194	4.104477612	1.648474048	0.638095238
0.624641918	305.4498978	28.62372188	5776.836401	5726.568591	0.14104413
0.497885429	25.39215686	3.196078431	42.52941176	34.6674356	0.356643357
0.535755479	163.9411765	18.2875817	2227.039216	2192.74403	0.170758929
0.533547675	267.3073852	25.8742515	1648.796407	1621.884957	0.192766449
0.556635588	79.04225352	9.197183099	281.7816901	265.8379786	0.250440917
0.55919683	337.1956882	44.16086235	28478.60531	28341.64157	0.085447074
0.522826824	438.1288783	82.17899761	40513.05967	40345.91611	0.077349086
0.586315853	1101.101171	89.61661342	19446.27849	19386.50863	0.129347751
0.64435874	155.2904564	8.941908714	19.69294606	16.26814277	0.540358744
0.54902914	102.6684492	10.30481283	56.64171123	50.69541594	0.410087719
0.544224766	33.74193548	6.096774194	119.1612903	106.1082206	0.276785714
0.5676534	148.7251908	15.45801527	384.4694656	372.3261028	0.286966046
0.674125	269.65	22.11	5846.83	5801.1324	0.147928994
0.677179963	52.14285714	4.246753247	14.58441558	10.83993928	0.516778523
0.612592593	220.5333333	11.45	87.83611111	82.06277006	0.416184971
0.574582705	437.832021	38.66666667	6005.300525	5963.458982	0.154595253
0.730769231	19	1.538461538	1.653846154	0.232248521	0.838709677
0.573992269	343.8213689	39.56427379	8468.193656	8399.572209	0.120717453
0.528005198	451.4444444	40.98128655	1796.789474	1779.965085	0.243798118
0.666883681	64.02083333	6	223.3854167	208.929579	0.263013699
0.595500006	168.5265018	12.13074205	30.08127208	25.74957859	0.480475382
0.401234568	7.222222222	1.444444444	16.88888889	9.172839506	0.36
1	13	1.461538462	1	0	1
0.601749085	408.5876289	33.52430044	3733.671576	3708.168332	0.198016915
0.526661797	38.97297297	6.945945946	128.2972973	111.4207451	0.243421053
0.554606401	75.42647059	6.588235294	107.5	98.14359862	0.326923077
0.566923086	288.5638507	38.31630648	6967.662083	6916.183572	0.139375685
0.542952828	400.699187	68.0596206	19445.01084	19349.24685	0.10218776
0.478420569	15.78787879	2.03030303	7.303030303	3.658402204	0.523809524
0.57693275	162.6950355	9.609929078	112.0851064	106.2705095	0.414705882
0.493333333	7.4	1.4	3.733333333	1.173333333	0.625
0.653438973	79.0661157	5.247933884	2.884297521	1.069599071	0.742331288
0.36399916	25.11594203	6.797101449	115.5942029	99.12707414	0.246428571

original_glszm_LargeAreaLowGrayLevelEmphasis	original_glszm_LargeAreaHighGrayLevelEmphasis	original_glszm_HighGrayLevelZoneEmphasis	original_glszm_SmallAreaEmphasis	original_glszm_LowGrayLevelZoneEmphasis
0.629829081	269735.9765	431.2823529	0.868139916	0.015912566
5.018841631	1552735.746	363.5621622	0.779345489	0.013163746
0.352540642	26673.13636	185.3181818	0.733957609	0.029770481
0.898753163	2300296.276	951.1750433	0.844959262	0.004966026
0.048811212	1806.822581	299.1774194	0.743060036	0.025504629
0.08407054	20075.30476	292.1047619	0.759300868	0.016833225
0.056686157	361.9464286	185.625	0.857762897	0.047265214
0.149257416	87581.23567	466.9936306	0.805200971	0.016280607
8.253923988	83510.49596	977.6090468	0.792048604	0.021327302
2.861432486	2623430.279	656.8668342	0.741829615	0.004470554
0.252543415	88805.94615	305.6153846	0.715052454	0.015915254
0.600175781	608708.8981	717.656051	0.788729886	0.009750082
0.489768872	343050.2141	421.9381898	0.729160801	0.008848089
0.095735916	60273.66667	437.1688889	0.810302416	0.009827683
0.017726659	4565.40625	606.3660714	0.797362202	0.009343972
39.80509393	48446547.33	800.6498054	0.720830304	0.004683913
0.023207977	5834.708661	499.0472441	0.776282525	0.014741665
1.876317496	861888.5438	397.4048338	0.747909838	0.008914712
0.034009618	9174.072917	514.1666667	0.770604349	0.024118012
18.16915475	5045520.397	329.6730159	0.757465214	0.008390796
10.78171397	30549734.7	1316.129964	0.785697062	0.002652645
0.087325455	185079.2651	1002.804035	0.799200797	0.004828474
11.15464161	5028958.854	465.9045226	0.747931396	0.005546714
0.035737943	1719.416667	566.8333333	0.797725694	0.021317663
2.469907668	402621.4459	319.4054054	0.632290451	0.012038818
0.935293515	37901.11392	132.6455696	0.672849923	0.030733431
4.447042851	2680477.2	544.4028571	0.767079829	0.006299372
11.52289161	35114716.85	1493.2	0.732495339	0.004204636
6.632880031	13741557.89	935.9822835	0.823463477	0.005184277
4.808267434	3015412.991	565.8362832	0.761122399	0.007346164
10.49635464	12868015.23	855.6004566	0.781080998	0.00408819
15.3757869	14363499.32	647.757108	0.741698603	0.003373945
1.38641915	1462718.508	600.6132931	0.817055902	0.012246831
0.123411207	220	134.6153846	0.884615385	0.097130932
0.032330284	13682.95313	597.140625	0.825192385	0.02081498
0.875136396	908071.6891	519.0829016	0.813755318	0.006596295
53.46099288	49141764.04	742.9832536	0.763823456	0.00451282
2.812680765	4818485.254	808.9288136	0.786838345	0.003522509
0.017052944	7113.20442	600.3867403	0.771309616	0.009068557
1.752827087	384877.27	289.05	0.819982191	0.022918757
0.713606389	819593.8623	745.2694611	0.80956607	0.008440678
0.164120618	88424.25	422.7631579	0.848767507	0.018357144
0.223291789	164588.1621	493.1344828	0.797384307	0.011348886
0.088556592	78308.08108	565	0.784752928	0.008565931
0.027002364	58534.84035	949.345614	0.755592368	0.004752402
119.3122734	5461253.618	189.0028329	0.712982291	0.014467847
0.172391634	777317.2006	1372.124266	0.78859028	0.002558169
0.11780955	36482.53846	337.8131868	0.860281997	0.021132797
0.070133551	669.1111111	197.4444444	0.90625	0.06797724
0.028795348	8646.833333	434.5777778	0.846884126	0.01921544
8.998659753	4808776.016	444.001642	0.762478813	0.006999689
0.499665837	241379.019	408.4857143	0.84079328	0.016378245
3.941590438	4559847.815	890.6954128	0.823809215	0.003438152
0.335817347	23026.85526	139.6184211	0.780304106	0.03163802
43.12618179	52322615.38	920.0506634	0.763103033	0.005229796
0.061784369	110462.2302	874.676259	0.789648121	0.011200069
0.034029388	33683.14815	744.0138889	0.765960863	0.005950833
0.009934882	3819.284375	800.5375	0.794873065	0.006349494
0.157054126	52742.60769	411.1384615	0.794333876	0.015062389
0.463070124	57379.42975	242.2975207	0.710247121	0.013941494
2.415237207	846184.8517	409.1634981	0.782239605	0.007852865
10.57384093	21843716.85	809.70317	0.836301111	0.004088906
10.70858458	3543480.968	392.1651206	0.738725634	0.011580304
17.06247975	1367378.161	230.4075829	0.748401945	0.015313607
0.798132262	1967.294118	24.88235294	0.662227772	0.191783723
0.281663549	609043.0328	935.8149254	0.790294138	0.006581992
0.030907908	2281.895522	368.0895522	0.745381426	0.023628676
6.650408826	5028405.773	583.0899796	0.816894348	0.005537281
0.193770482	17355.47059	231.3921569	0.729825469	0.032737065
3.116969888	1597359.905	455.4705882	0.759556649	0.008770456
2.094975924	1305958.525	604.1497006	0.757613006	0.008336079
0.399044659	203879.662	496.1830986	0.772966978	0.009902688
30.4106858	26699959.69	648.3482587	0.775720537	0.004241569
36.98293693	44430510.94	904.8639618	0.751018581	0.002545477
12.71522467	29788387.61	1089.774228	0.793482509	0.002367264
0.024162341	23696.07884	755.0124481	0.828096769	0.008688055
0.07912817	45549.28877	486.0855615	0.767103056	0.009418376
0.317619393	50217.85484	281.483871	0.760925024	0.03667819
0.472757744	318019.6107	526.3969466	0.780754258	0.007075297
5.417069143	6320752.19	658.0925	0.843283369	0.006634398
0.09169013	4927.142857	271.1818182	0.842118996	0.050951645
0.070427916	127582.5111	920.1388889	0.808112321	0.010397329
3.414635481	10596272.24	1290.812336	0.784845987	0.004491688
0.058804831	480.8076923	251.6538462	0.879273504	0.056927452
10.41045168	6904831.783	547.2954925	0.784060211	0.004830961
2.036109309	1592935.071	527.425731	0.754653385	0.006017964
0.511257276	103103.9271	288.9895833	0.834704645	0.028604981
0.030427089	35944.99647	711.1378092	0.797941005	0.00615097
0.086714198	10081.66667	389.3333333	0.649562745	0.060416957
0.100856464	181.7692308	181.7692308	1	0.100856464
4.266811365	3274430.975	797.5360825	0.80254779	0.005183462
0.637982842	27000.27027	176.2837838	0.747360852	0.02541455
0.111395941	116042.9118	687.6102941	0.771646013	0.011615043
13.7840039	3531396.263	319.1827112	0.780619905	0.009350057
25.06131476	15111626.65	567.2859079	0.764894066	0.005256122
0.041287084	7289.818182	838.969697	0.709585455	0.035020381
0.105407065	131073.9929	503.4893617	0.787533687	0.009353709
0.092637576	873.2666667	173.0666667	0.726740741	0.081110117
0.026766712	931.1239669	319.9669421	0.835482094	0.017513096
1.444061878	9716.521739	82.73913043	0.624475579	0.037817102

original_glszm_ZoneEntropy	original_glszm_SmallAreaLowGrayLevelEmphasis	original_ngtdm_Coarseness	original_ngtdm_Complexity	original_ngtdm_Strength	original_ngtdm_Busyness	original_ngtdm_Contrast
4.479363072	0.015551135	0.009573925	1.319801661	8.407797377	0.128295582	0.000352697
5.012579818	0.01252441	0.00406097	0.315426081	4.805276834	0.233300784	1.96E-05
4.356492414	0.028664545	0.033914123	0.835081541	4.51590266	0.10553799	0.000668192
5.804063146	0.004761253	0.001415954	1.775485374	5.332357191	0.249303424	9.13E-05
5.195816472	0.02300018	0.052572885	12.50021552	14.3587628	0.046738576	0.006908499
5.158300195	0.016081	0.019994972	2.87912809	6.885304073	0.090070179	0.001272054
4.722907494	0.045414574	0.049169121	19.04655229	11.61171455	0.096742167	0.014091859
5.061087486	0.015978227	0.01013661	2.297297912	5.903529342	0.111632913	0.00074137
6.237143178	0.012559382	0.002676204	4.006478793	5.007086688	0.207151253	0.000870342
5.437193064	0.004089796	0.002313372	0.247448551	2.54313171	0.34591625	2.11E-05
5.34772279	0.014841616	0.009587342	2.341940259	5.875020494	0.141650091	0.000817454
5.156551978	0.009496826	0.006704514	0.999496953	8.058755702	0.107648236	9.18E-05
5.868540467	0.006321503	0.004140612	0.830331071	3.388491015	0.217488374	0.000163935
5.247670762	0.009470927	0.011468199	2.243563479	6.19691395	0.09041598	0.000481733
5.766845353	0.008154564	0.01652878	7.196001742	9.05229186	0.061456141	0.001178686
5.823906902	0.004155111	0.00062521	0.066029308	2.584993324	0.70991441	9.14E-07
5.319641258	0.014299875	0.024124912	7.498870341	10.30300884	0.052270121	0.002174219
5.572857541	0.008095891	0.003596402	0.489798426	3.484041425	0.244536533	5.38E-05
5.414091376	0.023742555	0.026369765	8.656374545	10.79330422	0.047537288	0.003391737
5.255317331	0.007624875	0.002311262	0.110774463	2.715206844	0.41202484	5.87E-06
5.115845565	0.002498228	0.001105323	0.103404826	3.070566392	0.433830512	2.89E-06
5.623431151	0.004626734	0.006005398	1.894004011	5.440485853	0.102804792	0.000205984
5.239512697	0.005056013	0.002107179	0.114788123	2.245587634	0.421844949	6.43E-06
5.573571001	0.019478875	0.036166993	26.91898701	24.33735679	0.035787556	0.00784174
5.21617927	0.010881944	0.007279997	0.202240544	2.655462655	0.258676639	2.89E-05
4.826197761	0.025744426	0.018321345	0.737590132	3.545584617	0.155581098	0.000269362
5.424280593	0.005793572	0.002369198	0.35174794	3.531434488	0.267592646	1.19E-05
5.004861923	0.00403777	0.00163951	0.098506418	4.93164238	0.303605052	1.79E-06
5.720786894	0.005007718	0.001354382	0.348285253	6.161322141	0.258904027	8.20E-06
5.115562791	0.003665408	0.003310389	0.25710778	4.571883829	0.244069041	1.61E-05
5.118364228	0.003809403	0.001309569	0.156632504	3.551078452	0.424827283	3.52E-06
5.863683637	0.002969229	0.000788662	0.104006147	1.70476684	0.641822281	2.36E-06
5.565340471	0.011710016	0.002463889	1.00745002	5.279588031	0.229559066	9.83E-05
3.39274741	0.090560863	0.104496897	38.50548474	18.43268335	0.107766466	0.292128609
5.132659766	0.020619449	0.032319915	15.03079416	23.2715249	0.030806544	0.005857131
5.585041001	0.006184808	0.002860185	1.029737154	4.397870854	0.235332401	0.00015222
5.257467732	0.00422656	0.001014433	0.063780397	3.357418031	0.509030841	7.05E-07
5.691036402	0.003246417	0.001612092	0.37237777	3.52503061	0.31828342	2.60E-05
5.668358972	0.008542524	0.017406451	7.248195886	8.779475153	0.062524373	0.001529652
4.7896535	0.022361448	0.007181988	0.715675053	5.181325208	0.187166465	0.000133192
5.232721161	0.008208607	0.005963401	1.282560741	10.15404721	0.103201292	0.000107873
5.05777519	0.018042072	0.01024224	3.050050881	8.304471809	0.094278478	0.00067872
5.583049384	0.01086961	0.005953982	1.836164533	4.979959862	0.134658267	0.000242886
5.416679724	0.008082903	0.011168203	3.200410854	8.329968754	0.077305567	0.000681796
6.329299757	0.004369924	0.005384089	3.735704393	5.94555383	0.100775122	0.000372143
4.857159758	0.012822494	0.001148654	0.023124472	0.435695753	2.072115107	1.24E-06
6.21291458	0.002213357	0.001666791	1.946056444	4.405826513	0.186349085	8.73E-05
4.787086536	0.020778601	0.017326742	4.023217725	10.10079541	0.075317381	0.001340117
3.947702779	0.067792471	0.092719867	42.75977642	23.4119974	0.049931366	0.114469867
5.232030008	0.018844304	0.021601781	17.43640474	12.4724576	0.056299997	0.00691085
5.667638497	0.006302658	0.001348164	0.170733501	1.919652441	0.569675823	1.17E-05
4.829367625	0.016068329	0.008967366	2.056713807	9.307791989	0.104054524	0.000362289
5.556081329	0.00323735	0.001449975	0.746865278	6.143702027	0.2242382	5.85E-06
4.726573448	0.028899482	0.023316231	1.345766037	4.699329564	0.116238436	0.000759281
5.415473284	0.004955947	0.000566285	0.069299092	2.23414332	0.694520534	3.72E-07
5.504616865	0.010913798	0.01205059	7.359954935	16.11712711	0.047406361	0.001252903
6.027916938	0.005631676	0.005615255	3.450172389	5.396551816	0.110119088	0.000299046
6.13660294	0.005807481	0.012026129	11.35255815	8.567797346	0.076248614	0.001791921
5.235760048	0.008427135	0.016512497	2.673097254	8.727823452	0.065889823	0.000701451
5.043310425	0.012676591	0.014428085	0.668182917	2.549961881	0.166282863	0.000240445
5.203683225	0.004541224	0.004209582	0.417122624	3.051836116	0.223765026	2.91E-05
5.826620279	0.00379653	0.000567149	0.231433753	2.489790961	0.670282975	1.09E-05
5.470954417	0.010924184	0.001651605	0.118259416	1.523760756	0.5623472	5.90E-06
5.04753966	0.014118481	0.003697763	0.128743442	2.639120069	0.395853005	5.91E-06
3.130717988	0.179329659	0.089424145	0.819225382	3.094631414	0.157293285	0.00308227
5.942930449	0.006359743	0.00422	2.297941561	9.79249047	0.094537132	0.000121784
4.975140177	0.022611669	0.040583504	13.01743504	12.78713064	0.046404888	0.007014174
5.43769495	0.005229376	0.001525672	0.26259077	2.898283979	0.378055401	8.43E-06
4.920179695	0.017039375	0.03134747	4.153902906	9.66771182	0.058890444	0.002020845
5.412150443	0.008136726	0.002963539	0.370014055	3.378892284	0.283839744	3.27E-05
5.84002218	0.007902161	0.002546061	0.463818864	3.186375289	0.229937727	1.55E-05
5.109545136	0.009444046	0.009855525	1.133865096	5.798708006	0.10736955	0.000171707
5.403035556	0.0038359	0.000829843	0.065843515	2.321685769	0.721571105	1.75E-06
5.210298422	0.002277973	0.000576706	0.05196326	1.42148923	0.894217195	5.50E-07
6.039475493	0.002087906	0.000424368	0.154725275	1.784679422	0.629175825	1.36E-06
5.72081538	0.008479821	0.011339952	7.836990036	10.27655722	0.054884138	0.000880361
5.40724826	0.008980465	0.013155415	2.61298804	7.209626806	0.077192873	0.000456906
4.281767529	0.035956043	0.024040027	1.039406622	5.090326345	0.101822675	0.000690847
5.382523438	0.006347527	0.005408776	1.037727615	4.2428919	0.158366553	0.000147191
5.415706688	0.006362212	0.001798573	0.345156742	4.704011169	0.284461248	1.81E-05
4.850196847	0.050320232	0.044448237	4.317191577	9.424379091	0.049812975	0.001685783
6.119405019	0.010165267	0.006453357	4.495314556	9.045616069	0.066828656	0.000351519
6.037249493	0.003327224	0.001112786	0.527704972	5.077932684	0.250180288	9.55E-06
4.286790199	0.056590005	0.119861527	30.5159211	22.55894208	0.028154995	0.038132154
5.408458056	0.004486924	0.001263697	0.135666837	1.905977972	0.523939059	4.36E-06
5.94583329	0.005373432	0.001680393	0.387798812	2.13825316	0.367048663	2.45E-05
4.81718545	0.028140565	0.013111907	1.238915851	5.70566654	0.115095773	0.000351413
5.626315357	0.005914138	0.007846318	3.754531964	5.479039882	0.103341784	0.000706133
3.947702779	0.059774576	0.084275277	9.527746515	22.79652359	0.030189996	0.020460599
3.238901257	0.100856464	0.105220558	45.96015779	21.32878756	0.108567663	0.556002552
6.013903876	0.004802545	0.001422704	0.815703334	5.919624387	0.239212579	7.18E-06
4.71886198	0.023772527	0.022405761	0.685901305	3.788229704	0.138194532	0.000210616
5.563525548	0.011154183	0.010997824	4.025139059	13.79538473	0.0564488	0.000512557
5.196790648	0.008533516	0.001464975	0.115994245	1.601379003	0.719101515	7.34E-06
5.14484471	0.004859418	0.000858534	0.06309748	1.244201505	0.91320011	2.06E-06
4.741363816	0.034742692	0.066666023	16.5297024	28.05983943	0.02291709	0.017576163
5.842780993	0.008812029	0.006456466	3.281695201	5.680512131	0.12099272	0.000881674
3.906890596	0.079905286	0.128065545	11.86066017	17.84672767	0.049342822	0.049376039
5.291376643	0.016325433	0.031171848	11.00193205	10.29776604	0.078199994	0.00293931
4.945036414	0.030211895	0.025650041	0.349830095	1.720818046	0.253746206	0.000194662

wavelet.LHL_gldm_GrayLevelVariance	wavelet.LHL_gldm_HighGrayLevelEmphasis	wavelet.LHL_gldm_GrayLevelNonUniformityNormalized	wavelet.LHL_gldm_DependenceEntropy	wavelet.LHL_gldm_DependenceNonUniformity
31.95394449	571.4542936	0.066428281	6.395055314	67.87534626
20.10616391	281.3328582	0.101971186	6.71216005	156.6265146
63.35350634	393.6892655	0.047655527	5.818996971	59.75706215
51.49114423	610.9249871	0.061738975	7.075357391	337.2690119
41.01970395	364.6633663	0.04950495	5.244409626	41.97029703
34.59827667	335.8483607	0.056066917	6.049744576	57.69672131
56.8277551	318.3428571	0.046122449	5.200432303	30.34285714
94.40942531	617.0049875	0.033264719	6.416676197	142.8753117
115.8890592	1623.338076	0.028054105	7.142148095	452.8252033
26.1101239	755.9952566	0.068487053	6.705499995	417.3510134
43.5068965	347.6196809	0.051564622	6.305157848	96.30319149
59.7225089	662.01321	0.050046331	6.865596801	156.8388375
89.44935036	529.9575403	0.052661478	7.028236443	254.3455344
22.46780303	270.530303	0.066101354	6.573134404	90.14015152
55.45601377	490.2472527	0.04532967	6.240661291	116.1593407
12.33048119	1007.818064	0.175561341	6.657103162	755.6970816
71.03089894	798.0414747	0.042961201	6.038506648	77.94930876
64.1190578	1482.913015	0.063087868	7.113763413	244.681701
65.52617438	423.4010989	0.039548364	5.888880938	66.75824176
19.21955131	375.0872386	0.099140021	7.066496116	280.6798846
22.05064036	770.6094581	0.152039137	6.754600289	414.2449261
88.17456773	712.239824	0.042926075	6.856689342	239.1628163
14.21892991	567.209589	0.184448536	6.443408811	223.5890411
113.3073662	1411.473684	0.038498502	5.785403842	62.12781955
46.4503635	1068.784375	0.110737847	6.562467076	129.575
47.72695277	676.2086093	0.051993334	6.273950171	77.97350993
19.35032954	245.768021	0.079521964	6.678600855	349.9445173
29.14804148	506.8089859	0.091174657	7.139542321	463.5910141
28.66493278	504.4354144	0.141491105	7.07394758	296.0871905
10.82223771	394.523922	0.182198057	6.286624324	130.5180154
23.42125102	646.4827422	0.067964854	6.888360712	654.290169
6.86683833	331.6539115	0.215722475	6.25822257	570.5668516
32.73006928	441.3578114	0.080298198	6.785845304	211.0071994
153.1288889	1304.6	0.093333333	3.506890596	7.8
51.10376694	343.7256637	0.050199702	5.454887538	42.92920354
30.12898049	440.814093	0.087589264	6.856691453	163.3883058
13.72460096	971.1974294	0.288400239	6.081941257	418.6078314
13.35504389	418.1052111	0.159105208	6.505841755	255.5890501
25.26724314	173.788162	0.057481973	6.121795534	81.4423676
28.50415267	499.4022346	0.075233468	6.480422388	91.96461825
37.98524329	432.2993007	0.060714949	6.426003835	158.6671329
30.12541936	327.3937008	0.058314561	6.342192752	85.88713911
55.46421523	519.5424757	0.052152064	6.689768738	191.6893204
38.39520343	333.3895216	0.051841782	6.241480821	119.5831435
85.23193629	1089.013453	0.0328782	6.795945348	360.9892377
12.93072379	342.5352826	0.123933626	6.717342592	604.1483175
87.18639361	1224.099053	0.033908024	6.990802853	833.4246176
32.71058403	213.1885965	0.059325946	6.02146862	57.07017544
83.10743802	300	0.082644628	4.004886164	11.90909091
41.6194408	281.2328767	0.046537812	5.634805254	51.32876712
14.78656863	360.7465686	0.151560097	6.5787408	350.9887255
42.3422101	298.8188586	0.051493452	6.344937923	96.8808933
30.30345733	547.5633803	0.103806858	6.690417728	387.3981221
24.33318744	182.9456067	0.060905796	5.804606713	67.12552301
16.49311217	982.23147	0.257842457	6.155261252	723.163453
41.27342393	848.0029851	0.052777902	6.199748488	87.21492537
133.3620697	1560.676606	0.025552879	6.765483001	374.4174312
59.18870731	555.1746725	0.039663622	6.438066732	142.7860262
18.68878675	425.4971751	0.099221169	6.104499433	60.0960452
89.86984472	2550.540773	0.096253385	6.512063181	65.3776824
23.07626315	511.9876289	0.102915648	6.592931078	173.6735395
17.95853891	789.594451	0.166910193	6.628793291	530.7252066
17.31894898	712.8851252	0.134780614	6.766365101	359.191458
42.02882926	593.8832772	0.156121635	6.617772218	167.4904602
29.749377	146.2264151	0.0751157	4.779530596	14.66037736
43.09872248	977.8550347	0.048695505	6.70047727	277.0017361
96.58285714	607.3428571	0.035102041	5.456730279	53.41904762
18.16774818	475.8586674	0.146879029	6.688736295	299.1072974
37.59332975	244.5104895	0.053156634	5.496502526	58.37062937
33.1786035	600.5061384	0.064101239	6.716751261	327.0457589
34.87774547	548.0161601	0.106504248	6.824323876	302.7599077
24.32577164	245.1128748	0.070493858	6.487239903	94.66137566
14.77464298	750.7545699	0.116754722	6.965516787	634.7446507
11.93807627	485.0347056	0.19667848	6.465801867	719.7297397
12.33554801	653.5597493	0.172057879	6.411543344	1084.634961
37.887269	392.6098655	0.051287981	6.352603277	118.9506726
39.96912512	460.7587719	0.051371576	6.347312181	114.2982456
93.53667092	900.7053571	0.038464605	6.227440458	73.875
41.59622299	580.4665936	0.055029638	6.717617224	198.1412924
21.10107697	535.8069527	0.152621801	6.631993593	235.8321006
48.74257916	290.9731544	0.059231566	5.772034903	39.53691275
111.2460557	1402.256647	0.028939156	6.769122181	333.5179191
29.54001634	1586.321972	0.07626153	6.970898326	684.7261108
90.04786681	374.6774194	0.071800208	4.155651552	12.29032258
21.32255671	539.3849254	0.169454805	6.585376861	367.8585248
24.93980697	525.4767608	0.075287691	6.72381673	570.4262903
34.03763558	342.5452055	0.067622443	6.380600439	69.63013699
84.729117	607.8879457	0.031404844	6.563762263	212.3955857
13.1744	137.72	0.1136	4.66875844	14.08
80.82840237	380.3846154	0.100591716	3.39274741	7.461538462
30.41468762	1544.757072	0.074766962	7.024158783	498.8145232
66.51314707	446.6348684	0.041248269	6.25460085	86.84210526
23.68380178	317.2788462	0.086503791	6.213232108	75.80288462
38.38355793	866.7261774	0.118414162	6.942719288	360.6325301
10.97414474	602.0659097	0.205582944	6.200715717	494.2633619
217.5273369	836.984127	0.041068279	5.298630916	29.73015873
81.20584775	1176.426471	0.042011246	6.872319988	166.6529412
107.6232639	364.9583333	0.0625	4.136842188	18.75
102.1847266	761.0674847	0.031427604	5.953493933	68.00613497
30.32836735	322.25	0.067091837	5.991549072	65.22857143

wavelet.LHL_gldm_GrayLevelNonUniformity	wavelet.LHL_gldm_SmallDependenceEmphasis	wavelet.LHL_gldm_DependenceNonUniformityNormalized	wavelet.LHL_gldm_DependenceVariance
23.98060942	0.389028084	0.18802035	5.031591225
143.0655738	0.269769242	0.11163686	17.8745464
8.435028249	0.576124403	0.337610521	1.376998947
119.3414382	0.377552524	0.174479572	16.10789701
5	0.626031353	0.415547495	0.647975689
13.68032787	0.417809195	0.236461973	2.986025262
3.228571429	0.633829365	0.433469388	0.473469388
13.33915212	0.585182509	0.356297535	1.123264159
41.40785908	0.511390765	0.306792143	1.238475041
158.8214748	0.340487798	0.179970252	4.664071907
19.38829787	0.465328248	0.256125509	2.167468311
37.88507266	0.404986886	0.207184726	5.842400912
71.93557833	0.38118823	0.186197317	9.762412404
34.90151515	0.36524459	0.170719984	7.286099633
16.5	0.539626068	0.319119068	1.24480739
1900.978205	0.17033867	0.069791012	24.31388314
9.322580645	0.615586441	0.359213404	2.049650662
97.91237113	0.349603056	0.157655735	16.88533585
7.197802198	0.60245116	0.366803526	1.099263374
275.0144196	0.23478907	0.101182366	22.20452092
771.5986207	0.22223232	0.081624616	29.84801627
39.01980198	0.492473763	0.263105408	2.778703613
538.589726	0.215023855	0.076571589	28.58408285
5.120300752	0.704156224	0.467126463	0.803211035
106.3083333	0.342604943	0.134973958	15.4975651
15.70198675	0.475818504	0.25819043	1.882899873
182.0257754	0.288977487	0.15288096	5.06759721
355.1252888	0.272747383	0.119022083	17.6404827
525.7809473	0.202248892	0.079679007	38.14386657
308.4613113	0.197090118	0.077092744	24.35870924
285.5203523	0.282831631	0.155746291	4.469103915
1905.476622	0.141349689	0.064594911	25.02503676
111.5341973	0.346823262	0.151913031	17.63775857
1.4	0.7	0.52	0.24
5.672566372	0.631978859	0.379904456	1.431905396
116.844078	0.281412776	0.122479989	17.07411736
1929.685996	0.132328847	0.06256282	28.87811967
482.4069921	0.225048422	0.08429718	26.86599404
18.4517134	0.461715886	0.253714541	1.783387195
40.40037244	0.383017157	0.171256272	9.064039477
43.41118881	0.400871181	0.221912074	2.421810357
22.21784777	0.445283262	0.225425562	3.434531314
42.97330097	0.476304374	0.23263267	7.064726883
22.75854214	0.486667765	0.27239896	2.234338759
36.65919283	0.543993757	0.323757164	1.241685133
758.7216596	0.209950201	0.098684795	14.63188445
93.11143481	0.516345803	0.303504959	1.263552256
13.52631579	0.459777544	0.250307787	1.956063404
1.818181818	0.755050505	0.541322314	0.32231405
6.794520548	0.563926941	0.351566898	0.779696003
618.3651961	0.224564681	0.086026648	26.35261222
20.75186104	0.42805474	0.240399239	1.955999975
331.6629108	0.295004019	0.121251368	13.29136772
14.55648536	0.475122036	0.280859929	1.255020045
2915.166814	0.138731782	0.063962803	26.1031927
17.68059701	0.486436995	0.260343061	2.724562263
22.28211009	0.66834941	0.429377788	0.863100749
18.16593886	0.534074441	0.311759883	1.419213974
35.12429379	0.385437477	0.16976284	12.06824348
44.85407725	0.338548416	0.140295456	10.05840962
149.742268	0.29655708	0.119363257	15.36254225
1130.983471	0.203594724	0.078324263	30.2464803
549.0962199	0.208865606	0.088166779	20.05378673
278.2087542	0.25732315	0.093990157	23.31142955
3.981132075	0.491813417	0.276610894	1.35991456
56.09722222	0.444119192	0.240452896	2.218964
3.685714286	0.732275132	0.508752834	0.395827664
509.2295933	0.218983489	0.086272656	26.53463947
7.601398601	0.652534965	0.408186219	1.04944007
114.8694196	0.356775873	0.182503214	3.660563566
276.8045402	0.293664636	0.116490923	17.80838547
39.97001764	0.350394199	0.16695128	6.335488928
823.9380757	0.19302105	0.089945395	19.07261948
2130.814658	0.154147938	0.066432503	25.57171628
2498.108341	0.167382303	0.074704522	18.66166589
22.87443946	0.470986763	0.266705544	1.860644694
23.4254386	0.459873866	0.250654047	2.165031548
8.616071429	0.573282338	0.329799107	1.812420281
50.24205915	0.430289726	0.217022226	5.227317715
412.6893491	0.243081437	0.087216014	28.15325039
8.825503356	0.482705423	0.265348408	2.045583532
25.03236994	0.622332842	0.385569849	0.857995924
375.8930818	0.307665871	0.138917856	9.513969044
2.225806452	0.638888889	0.396462019	1.232049948
840.8347441	0.19866379	0.074135132	24.29337207
264.0339321	0.326025875	0.162653633	6.327109356
24.68219178	0.40663332	0.190767499	11.42650403
18.49745331	0.580248066	0.360603711	0.914213899
5.68	0.500522222	0.2816	1.6
1.307692308	0.769230769	0.573964497	0.213017751
256.3759113	0.319997523	0.145469386	10.82808965
12.53947368	0.506132985	0.28566482	1.488919668
35.98557692	0.386384919	0.182218473	8.918061206
432.4485214	0.259645785	0.098749324	24.07866835
1484.720022	0.15361682	0.068438571	21.94536372
2.587301587	0.705828924	0.471907281	1.064751827
28.56764706	0.456039115	0.245077855	4.442871972
1.5	0.888888889	0.78125	0.4375
5.122699387	0.653790048	0.417215552	0.967894915
18.78571429	0.461876832	0.232959184	3.059183673

wavelet.LHL_gldm_LargeDependenceEmphasis	wavelet.LHL_gldm_LargeDependenceLowGrayLevelEmphasis	wavelet.LHL_gldm_SmallDependenceHighGrayLevelEmphasis	wavelet.LHL_gldm_LargeDependenceHighGrayLevelEmphasis
14.43490305	0.029325887	227.0338234	8371.759003
42.84604419	0.156350935	88.4268121	12750.09408
5.04519774	0.029118557	250.3419068	1688.536723
31.9881014	0.058112833	256.9250002	18843.09157
3.316831683	0.02475606	225.552049	1162.386139
9.442622951	0.039541291	161.4097412	3040.389344
2.942857143	0.14246131	223.6385913	886.6857143
4.44638404	0.01573691	380.0987395	2534.870324
5.249322493	0.005070173	869.3111457	7968.114499
14.28503665	0.021132913	274.7283825	10311.7003
7.521276596	0.03967896	185.6335612	2084.598404
14.45046235	0.026990368	291.6905374	9344.285337
21.22108346	0.058780429	276.651517	9426.17716
18.54924242	0.082737627	103.3452522	4848.049242
5.049450549	0.037329818	289.1699649	2196.005495
73.96509051	0.074973426	177.5367553	73388.82508
5.921658986	0.014754112	488.5076925	4557.221198
33.80541237	0.022884832	539.4099785	52593.85116
4.307692308	0.031095294	275.4227335	1526.642857
52.37130497	0.146493979	97.21706062	19520.52992
77.90364532	0.098033541	176.5813973	62756.80729
8.284928493	0.020145444	417.0560341	4424.332233
80.76986301	0.137222996	117.3178844	48044.77534
3.22556391	0.010798101	1021.413743	4519.804511
36.48541667	0.043476415	385.6542373	40512.55521
7.072847682	0.015055322	310.9861727	5040.056291
17.21231979	0.094134627	80.77214633	4020.859327
40.56970475	0.085326228	148.3144604	20318.19949
92.15608181	0.175131157	103.4563868	50750.33262
71.49143532	0.174378697	75.7438626	29791.11341
16.20066651	0.026994991	187.0266634	10524.16615
88.14593004	0.262363425	50.08965094	29927.80131
36.57811375	0.085182052	163.5017986	16924.2131
2.2	0.068534266	872.1	3034.6
4.787610619	0.044269746	237.8585029	1338.504425
40.19190405	0.094665054	134.6323769	18217.32384
118.86848	0.120572891	123.8898691	118093.3684
72.09960422	0.174758962	102.7617391	30140.3783
7.112149533	0.089228811	83.14978013	1228.087227
21.59590317	0.047420828	206.5494369	10651.09683
9.008391608	0.032370119	190.7836383	3558.358042
10.3648294	0.039810236	152.2826347	3487.141732
15.61893204	0.047693104	254.8928946	8292.025485
7.350797267	0.033822188	178.8246262	2204.769932
4.99103139	0.007535686	612.0334613	5117.465471
41.57954917	0.127981509	78.991144	14042.10568
5.304442826	0.006058099	661.0572647	6114.062637
7.359649123	0.071101225	126.8398631	1205.583333
2.181818182	0.246556876	226.2020202	596
4	0.036018377	169.3974981	1107.616438
69.67352941	0.190210463	86.86231891	26074.34265
7.689826303	0.050750322	150.1319692	1858.580645
34.40532081	0.066606198	177.0116839	18841.5928
5.719665272	0.059170287	97.5832357	898.6778243
101.0130904	0.102644883	141.9586524	100023.517
8.271641791	0.0134242	429.9984523	7038.820896
3.5	0.004628097	1083.026224	5010.678899
5.419213974	0.019162919	315.5264656	2654.69869
26.52542373	0.063437344	167.9842288	11931.07345
26.68240343	0.013578996	889.4631143	64975.39056
38.22474227	0.073130043	157.6241377	20786.9567
85.61009445	0.106386836	164.8064205	69588.73937
54.38291605	0.079593485	166.5868041	37651.11144
61.91582492	0.117561214	166.3893217	36865.87205
5.905660377	0.124589823	84.63720126	620.7924528
8.017361111	0.009833101	435.2977497	7865.622396
2.40952381	0.021173207	469.5925926	1313.095238
69.37727142	0.139893019	103.3311648	35218.97029
3.937062937	0.03879844	177.6363636	765.2937063
12.58705357	0.024013649	229.7307629	7366.046317
42.79299731	0.081738499	173.7088141	23603.19738
17.69488536	0.090244425	100.4502874	4367.663139
52.09479949	0.070423167	149.1487269	39162.58368
84.98430866	0.172010247	77.34775946	42630.57763
61.09945589	0.094703415	115.4135702	39772.2423
6.887892377	0.026720586	205.5298199	2480.201794
7.671052632	0.026012496	235.3602111	3206.881579
5.776785714	0.013336372	551.8378029	4636.857143
13.18181818	0.025322927	261.2870755	7963.504929
74.04585799	0.136801989	137.8450008	40844.72707
7.161073826	0.061388606	179.6277113	1694.208054
3.78150289	0.004818616	901.091251	5022.124855
25.22154595	0.016398252	501.53715	39789.92879
4.612903226	0.067987001	273.7777778	1293.903226
71.38250705	0.132517429	113.5835939	39292.78154
17.98659823	0.039253351	182.6339401	9454.242087
23.4	0.092869793	157.3203031	8743.739726
4.110356537	0.018677113	364.7287974	2358.064516
6.44	0.07949599	72.8077	875.96
1.923076923	0.618599927	269.3846154	824.3846154
26.33041703	0.017588653	505.4572217	40707.34267
5.921052632	0.034285226	271.3695587	2276.200658
21.06730769	0.073006351	131.7391684	6874.072115
58.91840088	0.069481973	251.5230731	51017.84228
76.40902797	0.128805347	101.8351339	45776.39103
3.634920635	0.033317859	631.087522	2519.904762
11.02058824	0.012199093	559.4367544	12546.87647
2	0.118101573	363.1805556	380.9583333
3.773006135	0.015152877	486.4270041	3228.834356
9.671428571	0.052193344	158.1101348	3036.557143

wavelet.LHL_gldm_SmallDependenceLowGrayLevelEmphasis	wavelet.LHL_gldm_LowGrayLevelEmphasis	wavelet.LHL_gldzm_DistanceZoneVariabilityNormalized	wavelet.LHL_gldzm_LowIntensityEmphasis
0.003867835	0.005113334	0.975004006	0.009155103
0.003051101	0.006719914	0.867010285	0.011382339
0.011970845	0.013694727	0.931709609	0.019210269
0.001919646	0.003357365	0.742183191	0.004868156
0.014318519	0.015784512	0.947406866	0.020121607
0.006399537	0.008951802	0.906476754	0.013640822
0.011918162	0.037723657	1	0.027576188
0.005388929	0.006687719	0.886935384	0.008557773
0.001189571	0.001598967	0.569188101	0.002123438
0.000999978	0.001978456	0.739308571	0.002819404
0.008032827	0.010553826	0.874359043	0.016139893
0.002712367	0.003952807	0.885955369	0.00623719
0.00328415	0.005864779	0.732970907	0.008505296
0.004755008	0.007857069	0.928985538	0.012207908
0.003830388	0.009353541	0.907002469	0.008852523
0.000335595	0.001182116	0.636987953	0.001935737
0.006404019	0.007055977	0.932515037	0.009836325
0.000995768	0.001496233	0.775689018	0.002648006
0.010132393	0.012277809	0.968512	0.015382054
0.001868103	0.004373998	0.693208763	0.007744287
0.000894839	0.001976463	0.819452992	0.003861785
0.002711934	0.003869008	0.795399039	0.005212777
0.000959678	0.002412191	0.931712803	0.00435593
0.008743676	0.009013498	1	0.011486129
0.001085061	0.003495707	0.9232	0.004634053
0.004940046	0.005807704	0.918277811	0.009324747
0.00434287	0.009724173	0.71349599	0.016224259
0.002275492	0.004040423	0.655198166	0.007889944
0.000984052	0.003574255	0.691915981	0.005749479
0.001484905	0.003702469	0.906958885	0.00733003
0.000865685	0.002126963	0.598716228	0.00302687
0.000904776	0.003665398	0.716773041	0.006317272
0.00363932	0.005624894	0.852423981	0.009992608
0.067418028	0.067641275	1	0.084365554
0.016459706	0.019590769	0.9753125	0.024413002
0.0022882	0.004467391	0.873362302	0.007704782
0.000445142	0.001420294	0.851750671	0.003383433
0.001138844	0.003192495	0.851533745	0.004970424
0.022034324	0.028178528	0.931903114	0.04346106
0.003162859	0.004567807	0.898596939	0.007807478
0.007016459	0.008829918	0.890225312	0.015728713
0.006308493	0.008516377	0.928304663	0.01327015
0.005429081	0.009012913	0.849823129	0.012212644
0.006683453	0.008892263	0.890991053	0.012701556
0.002269456	0.002826392	0.650620653	0.003818924
0.001086297	0.003984725	0.599573673	0.005739898
0.001106608	0.001625476	0.600951605	0.002023265
0.009570294	0.014609486	0.950445274	0.019805518
0.058962927	0.086380706	1	0.077577569
0.013402694	0.016549252	0.979594006	0.021294378
0.001654007	0.004142517	0.723977012	0.007246606
0.007289075	0.010774314	0.815753853	0.015887511
0.001781003	0.003452907	0.71270815	0.005964903
0.011356723	0.016238458	0.861884607	0.021747121
0.000308329	0.001242563	0.696879479	0.002219022
0.003850469	0.004536109	0.978144745	0.007290355
0.002051288	0.002361202	0.922283363	0.00286957
0.004057744	0.005470458	0.906268598	0.006966687
0.004270204	0.005857044	0.960542318	0.010412648
0.002678801	0.002951692	0.900277008	0.007330566
0.00170124	0.003287621	0.85875661	0.005554629
0.000650153	0.001726139	0.760618299	0.003075297
0.000591316	0.001795837	0.769040766	0.002748302
0.002781113	0.006071749	0.78125	0.012107483
0.043572781	0.050905057	1	0.076604705
0.001582069	0.002213068	0.777045412	0.003271304
0.014135168	0.015246284	0.954048443	0.01788056
0.001433749	0.003307097	0.752310609	0.006372612
0.013550887	0.016409142	0.943444245	0.019614107
0.001829678	0.003066472	0.761672791	0.004918656
0.001602935	0.003341167	0.800754333	0.005480159
0.005841168	0.010327303	0.937841932	0.016711729
0.000522693	0.0016795	0.620073528	0.002701144
0.000613579	0.002557722	0.689992425	0.004093805
0.00053526	0.001868017	0.643583705	0.003207962
0.004824709	0.006828614	0.8828125	0.009502128
0.004249333	0.005973043	0.888835479	0.008605582
0.00546492	0.006170783	0.815631451	0.008592453
0.002525297	0.003735705	0.848762536	0.005538997
0.001123903	0.0027059	0.810560908	0.00449048
0.011053032	0.016878867	1	0.022914354
0.001844799	0.002208901	0.73271139	0.002747532
0.000436975	0.000893654	0.660825002	0.001376191
0.045661551	0.047882096	1	0.064915627
0.001187498	0.002934849	0.746345906	0.005999477
0.001805287	0.003745204	0.834243107	0.005687127
0.004197055	0.011121896	0.954310728	0.012946753
0.007171606	0.008640927	0.899082687	0.011205514
0.02615978	0.031202244	1	0.048248688
0.041076513	0.156581196	0.702479339	0.094046815
0.000553275	0.001019164	0.675750222	0.001653371
0.006877686	0.010387021	0.93450158	0.013328003
0.005880989	0.008248799	0.946277586	0.014054526
0.000719364	0.00171936	0.748954006	0.002708341
0.000676428	0.002200248	0.754191877	0.004396251
0.024433119	0.025678352	1	0.031855622
0.002179915	0.002763855	0.760084379	0.004406812
0.048657129	0.055601573	1	0.054974443
0.008712698	0.009572058	1	0.012157711
0.010858379	0.014390165	1	0.021840401

wavelet.LHL_gldzm_LargeDistanceEmphasis	wavelet.LHL_gldzm_HighIntensitySmallDistanceEmphasis	wavelet.LHL_gldzm_LowIntensityLargeDistanceEmphasis	wavelet.LHL_gldzm_HighIntensityEmphasis
1.037974684	570.25	0.009241213	574.4367089
1.25	310.7110566	0.013805836	324.1176471
1.10619469	419.5862832	0.020226049	426.8141593
1.531325301	618.713755	0.007067267	673.253012
1.081081081	358.2331081	0.020543417	362.6216216
1.147540984	360	0.014598988	371.8770492
1	335.6923077	0.027576188	335.6923077
1.180451128	632.462406	0.009974505	643.5150376
2.219817768	1329.022202	0.0033358	1685.186788
1.546605293	720.7526211	0.003714436	806.1898734
1.202072539	389.6476684	0.017548211	401.8186528
1.195965418	682.5034422	0.006721674	709.3602305
1.528013582	666.2867384	0.017871453	698.0424448
1.110599078	280.1336406	0.012869894	286.0645161
1.146666667	508.0966667	0.009396628	524.2666667
2.338326446	864.976627	0.003419306	1045.998967
1.104895105	790.2062937	0.010084097	801.6293706
1.415711948	1428.8988	0.003251015	1520.01473
1.048	448.836	0.015687068	450.912
1.725244073	372.7391519	0.019344533	412.0264993
1.416938111	743.7355795	0.004851126	786.9568404
1.356275304	792.632816	0.006327479	839.3765182
1.120588235	526.6520425	0.004627625	540.0867647
1	1433.04902	0.011486129	1433.04902
1.12	1074.820714	0.00481741	1099.671429
1.12804878	645.3658537	0.010132169	657.4207317
1.69273743	242.3741562	0.02689726	276.1536313
1.845959596	454.2165053	0.010750636	538.956229
1.721120187	447.2121418	0.013380886	496.1516919
1.14673913	363.1915761	0.007790131	376.2961957
2.342657343	521.4784751	0.005490101	655.7280497
2.06779661	311.7714096	0.011076203	349.8386146
1.240671642	441.2458022	0.011482804	462.1436567
1	1270.333333	0.084365554	1270.333333
1.0375	365.115625	0.024634895	366.7
1.214953271	459.3509216	0.010403258	475.7757009
1.44200627	873.2085893	0.0039299	924.0407524
1.392712551	431.2991206	0.006059875	456.5937922
1.105882353	172.9897059	0.046352362	177.4588235
1.160714286	514.3805804	0.008228288	533.3214286
1.204268293	440.7386518	0.016159404	471.5731707
1.111702128	333.8045213	0.014532232	338.3882979
1.245327103	505.5560748	0.016479549	532.2336449
1.173553719	347.9504132	0.013424504	362.9628099
1.774240232	971.7403521	0.00507687	1124.904486
2.466063348	306.8546854	0.011633453	376.0776772
2.191950464	997.7004816	0.005642974	1273.954799
1.076271186	260.8898305	0.020478629	263.8389831
1	306.4444444	0.077577569	306.4444444
1.030927835	296.5979381	0.022153485	296.8762887
1.661322645	337.2559736	0.009709589	381.3567134
1.331632653	329.0038265	0.03428284	345.2193878
1.698536585	523.5310163	0.008309414	590.2653659
1.223880597	192.4496269	0.025294696	201.0970149
2.260153031	890.9994555	0.003857527	1009.490288
1.033149171	858.8301105	0.007326716	866.5911602
1.121495327	1572.52648	0.002974813	1612.610592
1.147887324	564.875	0.007353902	581.9190141
1.060402685	422.7818792	0.010517879	431.4899329
1.157894737	2550.315789	0.00738698	2663.333333
1.259493671	497.1678973	0.007123315	519.3080169
1.601053325	735.1928352	0.004064407	803.79921
1.48812095	731.2329764	0.003632198	797.6587473
1.375	607.7619048	0.015603123	632.2380952
1	169.9032258	0.076604705	169.9032258
1.414383562	877.693731	0.00373201	982.3715753
1.070588235	622.8	0.018478599	624.9529412
1.609988109	419.8682881	0.008608243	461.8347206
1.087378641	263.3131068	0.020279979	266.3203883
1.509352518	572.833783	0.005951278	637.2129496
1.377990431	557.6260383	0.007748153	585.5897129
1.096330275	269.7763761	0.017150029	275.8073394
2.660455487	626.3460912	0.005047651	770.0952381
2.291573034	438.9236362	0.007764292	498.5207865
3.340288925	578.3269379	0.007088787	689.0658106
1.1875	417.2833333	0.011724103	430.2208333
1.17721519	479.6265823	0.008947251	506.7594937
1.308219178	891.6643836	0.009169934	954.1917808
1.257990868	562.5450913	0.006336123	599.173516
1.388811189	538.936566	0.006271674	567.9482517
1	353.2125	0.022914354	353.2125
1.51980198	1257.389576	0.003281067	1435.174917
1.979064039	1365.898523	0.002035249	1627.964286
1	413.5909091	0.064915627	413.5909091
1.74904943	521.971758	0.010022437	564.6815589
1.273584906	522.0397013	0.006462554	552.7602201
1.070175439	364.5599415	0.013466354	368.5730994
1.159898477	609.6510152	0.012076093	628.6522843
1	141.6785714	0.048248688	141.6785714
1.545454545	324.9318182	0.096055849	362.0909091
1.875522139	1350.043497	0.002298971	1572.842105
1.101694915	499.6016949	0.013633946	510.220339
1.082872928	329.6864641	0.014743298	335.9226519
1.615606936	894.398237	0.004106119	958.0982659
2.247028862	595.7016942	0.006609093	660.4414261
1	884.7959184	0.031855622	884.7959184
1.443804035	1058.317643	0.004761083	1221.933718
1	396.6818182	0.054974443	396.6818182
1	743.075	0.012157711	743.075
1	336.7785235	0.021840401	336.7785235

wavelet.LHL_gldzm_DistanceZoneVariability	wavelet.LHL_gldzm_ZonePercentage	wavelet.LHL_gldzm_IntensityVariabilityNormalized	wavelet.LHL_gldzm_LowIntensitySmallDistanceEmphasis	wavelet.LHL_gldzm_IntensityVariability
154.0506329	0.43767313	0.043502644	0.009133575	6.873417722
353.7401961	0.290805417	0.044129662	0.010808216	18.00490196
105.2831858	0.638418079	0.04127183	0.018956324	4.663716814
616.0120482	0.429384377	0.030326608	0.00435829	25.17108434
70.10810811	0.732673267	0.047479912	0.020016154	3.513513514
110.5901639	0.5	0.044074174	0.013401281	5.37704918
52	0.742857143	0.043639053	0.027576188	2.269230769
235.924812	0.663341646	0.029283736	0.00820359	7.789473684
499.7471526	0.594850949	0.024688539	0.001901529	21.67653759
642.4591484	0.374730487	0.043192114	0.002627779	37.53394707
168.7512953	0.513297872	0.037826519	0.015787814	7.300518135
307.426513	0.458388375	0.030753515	0.006119877	10.67146974
431.7198642	0.431185944	0.031450964	0.006216247	18.524618
201.5898618	0.410984848	0.048355242	0.012042411	10.49308756
204.0755556	0.618131868	0.037353086	0.008716497	8.404444444
1233.208678	0.178795715	0.04896833	0.00173542	94.80268595
133.3496503	0.658986175	0.034965035	0.009774382	5
473.9459902	0.393685567	0.029039352	0.00250331	17.74304419
121.064	0.686813187	0.037696	0.0153058	4.712
497.0306834	0.258471521	0.042644367	0.004975394	30.57601116
1006.288274	0.241970443	0.040147641	0.00365154	49.30130293
392.9271255	0.543454345	0.028938353	0.004941885	14.29554656
633.5647059	0.232876712	0.049013841	0.004293799	33.32941176
102	0.766917293	0.031334102	0.011486129	3.196078431
323.12	0.364583333	0.034906122	0.004588213	12.21714286
150.597561	0.543046358	0.042162403	0.009122892	6.914634146
510.8631285	0.312800349	0.04510627	0.013766232	32.29608939
778.3754209	0.305006418	0.037681813	0.007290329	44.76599327
592.9719953	0.230624327	0.039846198	0.004007291	34.14819137
333.7608696	0.217365623	0.060609641	0.007215005	22.30434783
770.5477855	0.30635563	0.042666217	0.002619484	54.91142191
972.6610169	0.153628439	0.064518214	0.005541764	87.55121592
456.8992537	0.385889129	0.03892153	0.009620059	20.8619403
12	0.8	0.083333333	0.084365554	1
78.025	0.707964602	0.045	0.024357528	3.6
373.7990654	0.32083958	0.041062538	0.007036653	17.57476636
815.1253918	0.143027948	0.048618277	0.003296309	46.5276907
630.9865047	0.24439314	0.048548393	0.00478295	35.97435897
158.4235294	0.529595016	0.049480969	0.042738235	8.411764706
201.2857143	0.417132216	0.043287628	0.007702275	9.696428571
291.9939024	0.458741259	0.043054729	0.015629212	14.12195122
174.5212766	0.49343832	0.042949298	0.012954629	8.074468085
363.7242991	0.519417476	0.032120709	0.011145918	13.74766355
215.6198347	0.551252847	0.03910252	0.012520819	9.462809917
449.5788712	0.619730942	0.027450307	0.003536765	18.96816208
795.0346908	0.216595884	0.048238161	0.004855165	63.9638009
970.5368421	0.588128186	0.027728819	0.00126349	44.78204334
112.1525424	0.51754386	0.046969262	0.01963724	5.542372881
18	0.818181818	0.074074074	0.077577569	1.333333333
95.02061856	0.664383562	0.043256457	0.021079602	4.195876289
722.5290581	0.244607843	0.046021904	0.006745413	45.92985972
159.8877551	0.486352357	0.04092045	0.011328046	8.020408163
730.5258537	0.320813772	0.040628198	0.005469191	41.64390244
115.4925373	0.560669456	0.051793272	0.020860227	6.940298507
1183.998234	0.150274191	0.0504299	0.001988896	85.68040024
177.0441989	0.540298507	0.040383383	0.007281265	7.309392265
592.105919	0.736238532	0.023044225	0.002843259	14.79439252
257.3802817	0.620087336	0.035657608	0.006869883	10.12676056
143.1208054	0.420903955	0.052925544	0.01038634	7.88590604
153.9473684	0.36695279	0.033001607	0.007316463	5.643274854
407.0506329	0.325773196	0.043582759	0.005174519	20.65822785
1155.379197	0.224173554	0.041744226	0.00288933	63.40947992
712.1317495	0.227295042	0.045967467	0.002561402	42.56587473
393.75	0.282828283	0.031572814	0.011233574	15.91269841
31	0.58490566	0.065556712	0.076604705	2.032258065
453.7945205	0.506944444	0.036357666	0.003166642	21.23287671
81.09411765	0.80952381	0.035294118	0.01773105	3
632.6932224	0.24257283	0.045771058	0.005901724	38.49346017
97.17475728	0.72027972	0.044396267	0.019447639	4.572815534
529.3625899	0.387834821	0.040414057	0.00470155	28.08776978
669.430622	0.321662178	0.035269911	0.004932429	29.48564593
204.4495413	0.384479718	0.045787392	0.016602154	9.981651376
898.4865424	0.205328043	0.045942539	0.002428422	66.57073844
1228.186517	0.164297582	0.051666456	0.003536216	91.96629213
1603.810594	0.171637165	0.049010767	0.002891148	122.1348315
211.875	0.538116592	0.038854167	0.008946634	9.325
210.6540084	0.519736842	0.040858837	0.008520164	9.683544304
119.0821918	0.651785714	0.031525615	0.008448083	4.602739726
371.7579909	0.47973713	0.036415004	0.005347545	15.94977169
579.551049	0.264423077	0.041326226	0.00408344	29.54825175
80	0.536912752	0.0428125	0.022914354	3.425
444.0231023	0.700578035	0.0259016	0.002630734	15.69636964
1073.179803	0.329478596	0.037705659	0.001268732	61.23399015
22	0.709677419	0.061983471	0.064915627	1.363636364
785.1558935	0.212011286	0.042781087	0.005137691	45.00570342
1061.157233	0.362703165	0.041593736	0.00549327	52.9072327
163.1871345	0.468493151	0.041961629	0.012816853	7.175438596
354.2385787	0.66893039	0.029013889	0.010987869	11.43147208
28	0.56	0.079081633	0.048248688	2.214285714
7.727272727	0.846153846	0.090909091	0.093544557	1
808.8730159	0.349081365	0.037581422	0.001535477	44.98496241
165.4067797	0.582236842	0.031951227	0.013251518	5.655367232
171.2762431	0.435096154	0.045511431	0.013882333	8.237569061
777.4142582	0.28422782	0.034136345	0.002409664	35.43352601
888.4380306	0.163112711	0.056165525	0.004179002	66.16298812
49	0.777777778	0.029571012	0.031855622	1.448979592
263.7492795	0.510294118	0.026916593	0.004323982	9.340057637
22	0.916666667	0.05785124	0.054974443	1.272727273
120	0.736196319	0.03	0.012157711	3.6
149	0.532142857	0.047700554	0.021840401	7.10738255

wavelet.LHL_gldzm_HighIntensityLargeDistanceEmphasis	wavelet.LHL_gldzm_SmallDistanceEmphasis	wavelet.LHL_glcm_SumVariance	wavelet.LHL_glcm_Homogeneity1	wavelet.LHL_glcm_Homogeneity2
591.1835443	0.990506329	1809.816706	0.326000021	0.243365
386.5784314	0.945669935	813.2635756	0.430400744	0.363745606
455.7256637	0.973451327	1107.961155	0.22370572	0.136359234
932.5144578	0.885993976	1834.437658	0.333638737	0.260785709
380.1756757	0.97972973	1072.384169	0.239475905	0.14398083
419.3852459	0.963114754	968.0059128	0.281276971	0.192962911
335.6923077	1	1040.166376	0.188283895	0.105263571
687.7255639	0.954887218	1701.119461	0.200934741	0.116856326
3713.638952	0.781416097	5407.008519	0.200535305	0.11653208
1212.928654	0.88448568	2463.645501	0.324003183	0.236520754
450.5025907	0.949481865	906.1965507	0.277129596	0.186167895
827.5590778	0.954210695	2073.584016	0.299515411	0.214910407
840.7453311	0.88120166	1482.802231	0.316368709	0.236401097
309.7880184	0.97235023	765.2786018	0.357986312	0.274455546
588.9466667	0.963333333	1401.631891	0.230918213	0.143705409
2405.433368	0.826052562	3550.971343	0.516120791	0.466693545
847.3216783	0.973776224	2468.184428	0.225138591	0.140475113
1919.677578	0.903346063	4922.441231	0.351048489	0.276656602
459.216	0.988	1088.993444	0.203909956	0.11796078
627.2970711	0.859181389	1111.945369	0.432766503	0.367017769
1047.311889	0.923507962	2580.662302	0.498421968	0.447139448
1028.951417	0.913180387	2129.806065	0.258261762	0.173236975
601.4073529	0.973120915	1915.22262	0.531693742	0.487059755
1433.04902	1	4633.507281	0.171045164	0.094544354
1199.074286	0.97	3562.430713	0.410544905	0.348701127
705.6402439	0.967987805	2145.164252	0.272135167	0.186456366
460.8798883	0.870363904	689.3339693	0.355308901	0.271118796
996.6531987	0.837261504	1575.799401	0.399059779	0.33190095
739.4504084	0.858615325	1618.029617	0.505658549	0.454150692
428.7146739	0.963315217	1289.459076	0.524256279	0.475337364
1629.182595	0.802527195	2096.026091	0.33838958	0.252268073
691.9712601	0.870325761	1070.119157	0.559535746	0.516857063
545.7350746	0.93983209	1326.921476	0.373044844	0.301246122
1270.333333	1	4607.20526	0.168994696	0.09937493
373.0375	0.990625	950.3459477	0.223523031	0.132369875
545.6285047	0.948857736	1326.186281	0.404547933	0.336907987
1318.30094	0.937884883	3472.145166	0.610869708	0.583713422
620.7395412	0.938003824	1327.477379	0.498821497	0.445846759
195.3352941	0.973529412	452.7671634	0.277949941	0.184045766
609.0848214	0.959821429	1572.848347	0.350929775	0.270446144
617.8109756	0.955707995	1346.807077	0.289900264	0.200758217
356.7234043	0.972074468	934.5250567	0.305774147	0.218262194
638.9439252	0.938668224	1523.097215	0.289375085	0.210701282
423.0123967	0.95661157	910.1004927	0.264503665	0.17349778
1845.308249	0.833775527	3424.00358	0.200334473	0.114624959
892.7330317	0.802889015	1044.727116	0.442453203	0.376149417
3039.282353	0.803900069	4057.9596	0.209184615	0.123669386
275.6355932	0.980932203	534.2128665	0.287339505	0.197300852
306.4444444	1	877.5032162	0.16352902	0.081873895
297.9896907	0.992268041	740.1140701	0.228475063	0.138547399
621.8707415	0.876082164	1116.586898	0.484529784	0.430225441
413.3469388	0.922760771	773.1261512	0.282522982	0.191532886
963.6995122	0.869945799	1723.664596	0.396639684	0.327061135
235.6865672	0.944029851	465.3120806	0.268671303	0.175060952
2169.588582	0.859585321	3451.524533	0.581435943	0.548953599
897.6353591	0.991712707	2779.344281	0.276382177	0.187815206
1772.94704	0.969626168	4962.426396	0.182384165	0.100984793
650.0950704	0.963028169	1607.049321	0.223348805	0.134735661
466.3221477	0.984899329	1324.939868	0.392156231	0.317474576
3115.403509	0.960526316	9279.958945	0.380619789	0.313352078
624.6054852	0.942158931	1620.465964	0.416383868	0.349924757
1217.982883	0.894987748	2681.092367	0.512095471	0.463772028
1121.090713	0.899643029	2373.010972	0.470237698	0.411168354
730.1428571	0.90625	1814.463392	0.473823643	0.426798658
169.9032258	1	329.8196008	0.286359704	0.195775155
1425.047945	0.90401446	3291.795999	0.260390755	0.169663784
633.5647059	0.982352941	1750.427152	0.164554418	0.084569431
716.648038	0.890653983	1524.967799	0.48344335	0.42931981
278.3495146	0.97815534	637.6734211	0.229118147	0.136262886
955.1438849	0.895893285	1897.885774	0.30913151	0.221425657
719.0873206	0.915512028	1694.415133	0.418774834	0.356084474
299.9311927	0.975917431	678.2858104	0.355490417	0.275931263
2109.538302	0.81536849	2514.067256	0.450753345	0.385368548
1102.052809	0.855816769	1602.086999	0.54813435	0.505334086
2213.979133	0.827609125	2224.99261	0.498552793	0.444161233
481.9708333	0.953125	1108.452182	0.261418098	0.170528661
615.2911392	0.955696203	1365.352287	0.259164935	0.169242526
1204.30137	0.922945205	2754.936916	0.214968249	0.130287657
748.9748858	0.938039066	1799.222393	0.301146113	0.21747932
717.7818182	0.919995726	1732.272323	0.49469787	0.444288446
353.2125	1	746.3949755	0.296035008	0.209311417
2178.813531	0.881050605	4626.113572	0.179486563	0.097509463
3204.892241	0.840522886	5565.398321	0.355690602	0.277970616
413.5909091	1	1083.35944	0.19158117	0.108394248
893.7623574	0.887082013	1753.764348	0.507980923	0.458709788
675.6422956	0.931603774	1638.710458	0.344596481	0.26395242
384.625731	0.98245614	984.8930971	0.336395026	0.255500125
704.6573604	0.960025381	1741.316746	0.201897107	0.117135707
141.6785714	1	378.8141905	0.350887335	0.260968947
510.7272727	0.863636364	1191.377077	0.211697701	0.12985664
2910.233918	0.849233965	5348.900554	0.355720914	0.279060169
552.6949153	0.974576271	1238.213788	0.241588808	0.15658684
360.8674033	0.979281768	922.4381608	0.359688416	0.280900695
1362.263006	0.889141779	2845.856133	0.442260826	0.383074374
1404.441426	0.888199153	2058.810573	0.542020036	0.496991549
884.7959184	1	2670.172285	0.157948129	0.085514878
1911.236311	0.89545309	3977.466012	0.256538651	0.172947864
396.6818182	1	988.6318557	0.149188436	0.075783421
743.075	1	2411.309383	0.184415308	0.10241614
336.7785235	1	923.8233451	0.308130849	0.220262923

wavelet.LHL_glcm_ClusterShade	wavelet.LHL_glcm_MaximumProbability	wavelet.LHL_glcm_Idmn	wavelet.LHL_glcm_SumVariance2	wavelet.LHL_glcm_Contrast	wavelet.LHL_glcm_DifferenceEntropy
60.87514838	0.03489005	0.977194851	67.18020052	45.42911233	3.684428525
-36.584516	0.075488672	0.981709568	44.74721668	26.98633157	3.277865572
1885.40921	0.021322875	0.962231258	141.5875742	107.3178481	4.138401064
473.8149653	0.051494684	0.975406277	116.3863887	72.98854506	4.006148272
-151.2833804	0.029312586	0.95830702	94.57963465	51.85299794	3.575007071
241.6061042	0.020406135	0.960013466	63.91268148	53.34546012	3.782394305
-130.1923967	0.03946759	0.918979398	106.6798872	106.3589979	3.61242997
1489.126259	0.010796894	0.957191618	206.4142687	136.3601952	4.439809106
925.6197928	0.005636184	0.977250821	318.883245	144.8823218	4.580760223
275.0915761	0.021334532	0.989930961	57.54529134	37.24318501	3.629687061
704.2401316	0.021016435	0.96964241	84.5415517	55.9101329	3.823842196
1124.626219	0.030656366	0.982418162	145.8961955	63.44050092	3.91868686
8158.851958	0.033697301	0.986718041	226.8836088	79.68825142	3.96321935
168.5304203	0.033652749	0.974333347	48.33416665	28.27445506	3.390826459
754.0873808	0.014816087	0.958761002	117.9898575	92.76336446	4.174600872
7.488081853	0.114876248	0.993909885	25.38494532	20.36890876	2.838014947
319.6970095	0.022164187	0.953767685	139.2168751	115.5226833	4.20708206
562.1407369	0.067203106	0.988308484	160.1454116	77.99716653	3.881928754
752.3257042	0.019181455	0.940563583	126.7456853	107.1857845	4.11561546
28.31525065	0.067235688	0.983138401	39.27144939	32.61380074	3.352206425
-148.6779619	0.120714462	0.993176652	49.82046252	28.57071906	3.100544675
4253.031607	0.01566386	0.979628424	219.6636352	106.4904897	4.229861085
-186.7275359	0.149204047	0.99125338	28.54384154	20.56323742	2.8435678
-1836.087504	0.020736848	0.945759344	236.8178478	239.7794841	4.452087374
-628.1925627	0.077782731	0.986024795	89.74979819	72.60777902	3.61108289
-193.5640612	0.017358098	0.973635698	101.2911126	81.41153465	3.979388513
-5.386720536	0.02492207	0.983968663	40.84052385	28.23905573	3.423241304
-17.88191018	0.05883355	0.983744035	69.01733259	48.13769424	3.6114571
-291.8254006	0.137460945	0.990838338	73.9597596	28.35397915	3.119573958
-121.4368361	0.13735134	0.989824249	21.57825381	14.65065099	2.7774911
-209.6432067	0.020552898	0.987051399	57.51779024	31.95873677	3.530488498
-43.10192056	0.147122852	0.994514447	13.42255578	9.459952777	2.494254016
158.0283507	0.074082305	0.981306773	69.57988587	46.62902165	3.678495762
-2701.08602	0.168108664	0.900130806	257.4915492	342.8861159	2.14430833
-149.7425282	0.025637348	0.947585422	118.6561858	79.72728641	3.876166807
145.13132	0.071078391	0.98482728	62.55389562	43.52308635	3.510721815
-374.07794	0.207056059	0.996824423	29.82110006	17.82970209	2.386008879
44.57963806	0.118379916	0.988934627	25.52297691	19.60350088	2.97780246
0.12803421	0.019106672	0.950368186	55.83411621	42.64093081	3.707269209
134.4208563	0.0455041	0.984306215	51.00545057	39.67663802	3.597248856
601.4380888	0.015079709	0.978459814	85.67693896	68.29666531	3.882999761
19.40886604	0.023173539	0.966711761	53.79937426	44.69130856	3.668486925
-30.26728232	0.029147509	0.966405091	108.5610155	93.53860116	4.127342785
332.4508583	0.01880054	0.963672013	69.96607924	61.31165386	3.922927649
820.819827	0.007122707	0.974159072	199.465066	124.4270963	4.473062335
59.71306303	0.066029684	0.992319915	28.13346342	20.71865578	3.111919818
1155.698752	0.006114668	0.978614012	216.855914	132.3902089	4.521255186
469.3536279	0.018920843	0.96975483	63.47032717	47.56522207	3.701091659
102.7847848	0.091438119	0.877048271	174.8028357	136.2338066	2.676678796
308.7790345	0.019778968	0.941310862	92.81525705	71.7807641	3.926541493
-64.70448887	0.117832698	0.988147455	30.8157388	23.21804676	3.083791343
789.2264308	0.016363081	0.966377564	96.84537553	46.90798299	3.74334942
-5.106660997	0.082680716	0.98671777	61.00196521	46.17323976	3.478939987
88.88548126	0.017964738	0.956481225	49.11678919	41.69882494	3.67495085
-312.2909458	0.176115199	0.99558523	36.71162626	25.87711682	2.535548684
-294.7223735	0.021045952	0.979806914	83.54236786	60.16999574	3.887784972
-247.3895637	0.006913458	0.965529728	287.1852416	195.179401	4.682520508
395.4221675	0.012255184	0.970332398	131.3781334	98.56713411	4.210522201
-82.64749777	0.068340028	0.981571766	34.20523327	24.84997514	3.296492077
-1092.452984	0.068207032	0.980919708	163.1867346	159.0966992	3.683963155
-143.344482	0.072901593	0.984758989	46.53042081	34.71255619	3.391083486
29.86413317	0.139399062	0.9936142	40.91084386	23.06677473	2.996998053
426.2135206	0.094061611	0.994709889	41.69341261	22.34507616	3.057604544
-513.7488487	0.120293435	0.976893846	89.32484656	74.86384398	3.386075775
537.0306465	0.05418204	0.942124441	58.49411364	49.67704182	3.346058789
-85.14243404	0.013011564	0.981657249	94.01351491	67.07841993	4.035830222
215.475719	0.023002578	0.918083452	203.6702022	165.3990288	4.118700069
-227.0832051	0.122605418	0.988812134	40.85870825	25.2415048	3.103760801
96.24928082	0.019645591	0.93688825	77.1237815	64.21249475	3.807602497
361.3076836	0.017038408	0.986749298	65.36963233	52.69991155	3.779596581
96.77525933	0.077569598	0.984435666	76.7650362	50.61403264	3.486335335
10.01455949	0.038808153	0.973652183	45.55647789	35.59451988	3.500535528
-53.56531386	0.068804626	0.992799503	32.69269278	20.2900713	3.090943835
-140.8593024	0.135172242	0.99457491	28.16103984	15.55011266	2.645541784
61.78718276	0.100704483	0.995422603	25.1788593	18.38815975	2.817448474
295.7026396	0.017576146	0.962243379	78.06725835	64.27097543	3.954251638
380.0830233	0.013581191	0.974779113	84.33301055	64.68300965	3.983722398
2354.20902	0.01604701	0.961779047	209.7329552	139.6582623	4.343428995
-10.71121882	0.021325283	0.974751327	85.69289911	64.14273236	3.929687376
-7.021636611	0.133071692	0.988650214	45.69496434	30.54870991	3.138444172
999.161219	0.040553396	0.970141427	84.71714505	66.83632344	3.635078834
1524.28159	0.006020418	0.969817685	255.735852	182.9286845	4.693446153
184.9448401	0.035993467	0.991055302	58.46885037	50.38567237	3.693398343
1570.403739	0.068693497	0.940304441	204.2434874	116.3210057	3.15840978
-125.485933	0.115614728	0.989302887	44.27286112	32.1379292	2.959109033
-79.23786998	0.036025283	0.983164586	55.03746388	38.94773508	3.626523733
52.91179281	0.056994635	0.971321924	63.21295836	52.04984204	3.723173849
359.9134735	0.008393257	0.951930035	183.8373485	124.6037874	4.450144472
-26.6355066	0.073953481	0.959233115	26.33442393	17.38261709	2.696820725
-404.080049	0.077380952	0.912199258	199.1109851	102.3412698	2.602591499
-304.7223681	0.047862257	0.989435387	62.90881788	48.55068709	3.689535384
859.6488455	0.014108317	0.960966529	144.1786185	96.46955344	4.195417996
24.12701501	0.050649894	0.980030484	44.6888989	37.99760055	3.549869345
691.5640011	0.094984818	0.990902557	88.03702245	49.64796393	3.431757967
169.8429362	0.137008467	0.996387396	19.97941144	15.25394156	2.581005447
3065.608379	0.033850047	0.925743198	491.540841	328.7764126	4.193154222
692.3680093	0.017234256	0.98159496	203.1017855	102.3559459	4.249549941
455.7447155	0.077332976	0.857348786	206.1210835	236.1823802	2.922840278
73.88546754	0.016821667	0.947385217	280.285186	147.0335714	4.34048954
-227.8153528	0.035438326	0.969713852	67.22957406	44.95736656	3.676645385

wavelet.LHL_glcm_InverseVariance	wavelet.LHL_glcm_Entropy	wavelet.LHL_glcm_Dissimilarity	wavelet.LHL_glcm_DifferenceVariance	wavelet.LHL_glcm_Idn	wavelet.LHL_glcm_Idm	wavelet.LHL_glcm_Correlation
0.236929625	7.365258244	4.748165065	22.28960784	0.906583143	0.243365	0.197815523
0.320336071	7.039183096	3.292198373	15.76459795	0.924259378	0.363745606	0.256224104
0.135496951	7.299919049	7.692818493	46.85392932	0.875434667	0.136359234	0.139356956
0.230316446	8.406340729	5.731264853	38.9191464	0.908647735	0.260785709	0.233592414
0.173592707	6.615922056	5.725273649	17.59966047	0.862034945	0.14398083	0.299491806
0.187595313	7.379052018	5.507645917	21.47394858	0.872011119	0.192962911	0.099424951
0.109148482	5.737952734	8.581187997	28.54849213	0.808334301	0.105263571	-0.009083578
0.12234798	8.46353032	8.866949351	54.9990917	0.860063582	0.116856326	0.211679564
0.121122531	9.82486008	9.016669647	61.74091766	0.900944447	0.11653208	0.380801038
0.241698053	8.083658769	4.358201351	17.88254484	0.934988148	0.236520754	0.214046588
0.201125711	7.79047088	5.44659882	25.46541359	0.889833709	0.186167895	0.203274786
0.209807569	8.250828985	5.538182993	31.19205882	0.918043653	0.214910407	0.387501507
0.22801468	8.360008036	5.699004762	45.28968859	0.933459791	0.236401097	0.464096041
0.268824523	7.417938427	3.766335707	13.58398265	0.900583725	0.274455546	0.259295912
0.152193045	8.04688873	7.28626603	36.71520454	0.869496359	0.143705409	0.125030261
0.393412877	6.080144111	2.41360962	14.40021015	0.961427937	0.466693545	0.106755839
0.137144674	7.400039877	8.075453226	45.45396071	0.863751864	0.140475113	0.070608995
0.244134136	8.155000989	5.366300981	47.14646272	0.940447034	0.276656602	0.340368576
0.120101621	7.35043166	8.213569742	31.99310555	0.839719559	0.11796078	0.096026723
0.30734336	7.266218208	3.493769939	19.78823996	0.929311112	0.367017769	0.094287042
0.348374031	6.433750807	2.951101375	19.6126474	0.958349737	0.447139448	0.2726784
0.185242346	8.703848259	7.065223021	54.18796479	0.913625998	0.173236975	0.330642463
0.380418754	5.712493428	2.469437425	14.20864553	0.953889061	0.487059755	0.153975529
0.093304524	6.853409567	11.75445222	98.62076425	0.850622618	0.094544354	0.000630718
0.308255468	6.855460617	4.69342334	49.64793438	0.941026545	0.348701127	0.077975295
0.202261593	7.64359259	6.31493163	40.46164991	0.901107671	0.186456366	0.114862256
0.278681367	7.686829093	3.731868254	14.05059443	0.920601877	0.271118796	0.185755139
0.291765762	7.84179289	4.272865699	29.23782755	0.930217537	0.33190095	0.181725472
0.321156579	6.654174333	2.989253008	18.9519669	0.951537268	0.454150692	0.446073383
0.383786904	5.652131064	2.234969251	9.539243154	0.946740215	0.475337364	0.193633339
0.258531553	8.095186189	4.042579024	15.1712232	0.927061286	0.252268073	0.287106505
0.42050679	5.28218568	1.760912266	6.27117851	0.960796167	0.516857063	0.172081099
0.267066969	7.628931627	4.482787049	25.8414607	0.920720478	0.301246122	0.202542002
0.102996382	3.184061108	14.04130023	127.9239677	0.806057967	0.09937493	-0.185516339
0.125408999	6.696034308	7.051248853	27.60286179	0.848628797	0.132369875	0.215043198
0.295756134	7.322476756	4.041707175	26.26515619	0.932298181	0.336907987	0.187677356
0.424777039	4.59801883	1.896598499	14.12716811	0.976439651	0.583713422	0.247198554
0.361790303	6.165307153	2.612027627	12.60199031	0.944264216	0.445846759	0.135968382
0.176996382	7.634437543	5.088948873	16.16293848	0.853788199	0.184045766	0.135188813
0.253082637	7.303375695	4.327503051	20.31021855	0.923425447	0.270446144	0.126127141
0.202099087	8.0076471	5.568654386	36.4980705	0.911564558	0.200758217	0.116689801
0.224924865	7.546544195	4.85254415	20.27935334	0.885752293	0.218262194	0.099824543
0.207546028	8.242043577	6.571227373	47.45613447	0.891996153	0.210701282	0.081529962
0.182357216	7.876199546	5.807546201	26.51158459	0.878268122	0.17349778	0.066943001
0.119727994	9.376632674	8.553080527	48.8866432	0.892613551	0.114624959	0.235041239
0.348786374	6.901269376	2.865197143	12.36724369	0.949436727	0.376149417	0.152545655
0.130352094	9.763262007	8.525952546	57.59909083	0.904740942	0.123669386	0.249717395
0.212864001	7.315370963	5.078834038	21.23531273	0.88868524	0.197300852	0.14647431
0.086330244	4.034047974	9.824066683	25.97326068	0.766514528	0.081873895	0.132958918
0.149736327	7.062363912	6.7046819	25.33449592	0.840341035	0.138547399	0.133357336
0.353168703	6.419839784	2.855860842	14.80962491	0.942234252	0.430225441	0.144753213
0.204957751	7.886711257	5.143831452	19.22776734	0.881718111	0.191532886	0.352443833
0.320566905	7.241912978	4.010078463	29.25337117	0.938568153	0.327061135	0.137025846
0.18566645	7.369533038	5.128099048	14.86491193	0.860141482	0.175060952	0.076922042
0.430805146	5.042834508	2.19383305	20.87105345	0.973360127	0.548953599	0.16503641
0.196814198	7.689394006	5.656995537	27.43918277	0.908631635	0.187815206	0.167186142
0.11133979	9.271517867	10.39160026	78.96833388	0.881019708	0.100984793	0.200475122
0.129082705	8.312077441	7.483457789	41.24525869	0.886922644	0.134735661	0.139461002
0.300084265	6.578861585	3.457442351	12.62332959	0.917084302	0.317474576	0.161537134
0.305751652	6.763099514	6.101237032	118.5407372	0.939534373	0.313352078	-0.015653027
0.319836131	6.994170676	3.657133308	20.89815757	0.931846245	0.349924757	0.147621387
0.357828887	6.252553837	2.720093442	15.457961	0.958693472	0.463772028	0.27825366
0.361666468	6.681358507	2.790272506	14.36724741	0.960371523	0.411168354	0.297854739
0.347351392	6.432953759	4.401043565	53.72809218	0.932062902	0.426798658	0.076153588
0.194622388	5.48503585	5.223052858	21.54361859	0.849071023	0.195775155	0.091110409
0.169900743	8.613800294	6.056449811	29.61485663	0.911434124	0.169663784	0.167983716
0.095829138	6.676469702	10.36570248	48.23426618	0.810902508	0.084569431	0.11832284
0.350627857	6.465852636	2.937463425	16.26336231	0.944405259	0.42931981	0.23738002
0.157829252	7.08871034	6.358433051	21.21304229	0.834422275	0.136262886	0.102011117
0.228133696	8.124837958	4.93816003	27.74163554	0.929224292	0.221425657	0.111680724
0.321705634	7.194178905	4.114144096	32.35579633	0.93574911	0.356084474	0.199259168
0.277183154	7.359618048	4.086792291	18.28575112	0.902484085	0.275931263	0.130679821
0.344533904	6.964269409	2.805067941	12.23799545	0.951333892	0.385368548	0.234637624
0.412553507	5.693122415	2.073542856	11.12638967	0.964083398	0.505334086	0.288264825
0.397421801	6.010244388	2.378913752	12.53597034	0.965024756	0.444161233	0.153037506
0.175554271	7.975144724	5.945387541	27.95926947	0.875909141	0.170528661	0.101348673
0.166452058	8.049757199	6.050056262	27.21693368	0.895649366	0.169242526	0.134679842
0.126822568	7.722230111	8.712951158	59.84711304	0.87695402	0.130287657	0.204531265
0.219184095	8.170329843	5.541988825	32.44606362	0.903760203	0.21747932	0.150735679
0.344791405	6.349144291	3.098069032	20.56820932	0.94655174	0.444288446	0.199530704
0.225646863	6.587451478	5.499198771	35.57151554	0.897872632	0.209311417	0.088493188
0.102778622	9.346954332	10.19553337	75.867957	0.886225637	0.097509463	0.17235554
0.270325421	8.009046152	4.514048107	29.50630075	0.945121663	0.277970616	0.074497625
0.065973707	4.61466528	8.936176354	28.99591122	0.832530719	0.108394248	0.298738384
0.382381924	6.081984582	2.82741649	23.70212031	0.952729297	0.458709788	0.150906205
0.261783351	7.978621894	4.340756714	19.2414856	0.920158273	0.26395242	0.166560532
0.230180412	7.202542515	4.873179105	27.09324786	0.899770436	0.255500125	0.08548326
0.125007223	8.837919742	8.622839028	48.26539565	0.857390133	0.117135707	0.194545177
0.301364383	4.968011548	3.181838405	6.2418733	0.867544806	0.260968947	0.237869022
0.120244601	3.933084406	8.277777778	27.77412446	0.806651591	0.12985664	0.273626143
0.276021253	7.968262897	4.506127003	27.8351972	0.939874224	0.279060169	0.130235902
0.163220264	7.94639441	7.271973102	41.77742474	0.875205255	0.15658684	0.208024197
0.264244856	7.045513355	4.183062281	20.10876072	0.915187548	0.280900695	0.088432217
0.322649544	7.137119804	3.903960689	33.49853089	0.952095101	0.383074374	0.274905235
0.424266511	5.361920684	2.001733855	11.10589663	0.970894965	0.496991549	0.128533559
0.081979382	5.94348656	13.6077428	126.1275941	0.829657294	0.085514878	0.209612727
0.17246345	8.628119824	7.155188237	49.1649536	0.915109125	0.172947864	0.336612231
0.084514779	4.134041972	12.55031183	63.14706849	0.756200112	0.075783421	-0.064197589
0.095418439	7.232378482	9.652736258	49.51520475	0.847582135	0.10241614	0.324576698
0.215660753	7.157833022	4.867976728	20.88131252	0.890691629	0.220262923	0.197332485

wavelet.LHL_glcm_Autocorrelation	wavelet.LHL_glcm_SumEntropy	wavelet.LHL_glcm_AverageIntensity	wavelet.LHL_glcm_Energy	wavelet.LHL_glcm_SumSquares	wavelet.LHL_glcm_ClusterProminence	wavelet.LHL_glcm_SumAverage
547.7357404	4.829862307	23.51449771	0.009149004	27.82800236	18620.02848	46.57326786
263.54872	4.471850683	16.24933882	0.021377751	18.45542477	11229.68202	32.19353943
334.1624847	5.001508386	18.64720887	0.007265651	65.27180922	111079.8326	36.08410371
554.8426117	5.1980464	23.73547185	0.010288017	48.22415099	65333.08076	46.64671754
334.5274621	4.723556374	18.35307068	0.011280065	36.10038911	22758.24374	35.98441085
305.9427284	4.763805905	17.71930144	0.007296531	30.44766278	14906.7313	34.82929019
301.2702166	4.172174694	17.75206305	0.020946315	53.52866048	33078.85692	34.63781345
507.3708422	5.603279721	22.79661793	0.0033286	86.84436236	138998.02	44.26321321
1542.836491	6.111346225	39.50370641	0.001401982	116.0246678	328473.6945	77.44205805
731.5033156	4.852702445	27.19613419	0.006092244	23.85924057	19135.10965	53.9045363
289.6880963	4.951904649	17.13374434	0.006063582	35.4323781	31658.03174	33.61277167
627.4513832	5.363337069	25.05705311	0.005679003	53.0791833	116952.6033	49.26755141
452.7585058	5.350377202	20.77295153	0.006473825	78.64797771	615424.9823	40.7871094
252.4513886	4.684782359	15.84835305	0.008908864	19.64420664	9470.623963	31.45931966
427.4733971	5.216746381	21.01588016	0.004563282	53.87287264	49830.7178	41.04418407
1000.232502	3.836770796	31.737359	0.048746375	11.39254092	8806.373158	63.21321497
716.738917	5.065365765	27.28767077	0.007077177	65.18215484	75095.96088	53.31875697
1400.795319	5.294181992	37.55296714	0.011249516	59.38463961	182169.4771	74.30219491
329.9855898	5.040400276	18.65918057	0.006999703	58.51980069	50593.57525	36.05867115
347.805166	4.457905263	18.79625826	0.01812333	17.66628773	11061.02868	37.20943616
747.7945816	4.189693168	27.41372824	0.049999982	19.16880674	23172.402	54.49689847
635.7858307	5.587989666	25.19583714	0.003760443	84.03990967	282105.1846	49.28660943
558.0867826	3.727755323	23.69466935	0.069424742	11.88653246	8105.923961	47.16304471
1272.373311	5.053662665	36.81380151	0.009487155	115.5593725	225515.8169	71.35168361
1012.420738	4.573300672	32.10875351	0.025900778	39.00529311	77529.6013	63.50178503
636.7096714	5.059892508	25.62383105	0.006138671	45.663649	42314.17847	50.26784239
229.1603061	4.601876953	15.25064742	0.008629908	17.63705639	9102.202916	30.06715438
481.9204658	4.850070072	22.112502	0.014590343	29.38964325	28067.93559	43.66683329
490.0278671	4.4607237	22.03771708	0.04599989	25.49703997	33972.98738	43.75488134
387.7619909	3.688251553	19.75475292	0.061266676	8.794510144	4255.013789	39.29511496
631.479241	4.854125126	25.26381429	0.006307679	22.39837412	16622.58674	50.00352716
323.1623943	3.391731079	18.03923795	0.073926045	5.522303277	2038.083597	35.89825696
410.9866011	4.802699907	20.39488865	0.016256237	28.99153097	26161.70246	40.2613498
1137.086939	2.167643489	34.77148133	0.123377493	137.5519541	207730.4393	67.79354195
292.8450261	4.814038245	17.27659345	0.010654269	50.14941538	42968.44188	33.64925707
408.6980516	4.649328873	20.33946734	0.019881337	26.76293116	29955.04115	40.19613909
958.7592701	3.159561824	31.02679976	0.128997667	11.16063198	18661.95	61.83071615
401.4966552	3.918331989	20.11869665	0.049698484	11.11880475	5733.915451	40.00080794
156.0027367	4.791775149	12.39453401	0.006096813	25.01732607	8539.160685	24.71227612
479.3427208	4.648093924	22.07037513	0.011237737	23.05098506	14428.23337	43.65783792
414.8780726	5.011358058	20.63090157	0.005138832	40.77780521	52129.13093	40.5225316
298.2460843	4.731390969	17.46054327	0.007137205	25.04356664	10997.34187	34.40652875
460.4404628	5.130387906	21.8522774	0.006318448	50.46820017	60864.74167	42.73865608
288.8623889	4.878469201	17.32179865	0.005685431	33.36429482	21825.97507	33.86178442
996.3641845	5.747397669	32.01954453	0.001922412	81.19833337	139588.4563	62.53176757
327.509888	4.207970285	18.17414258	0.021831787	12.05180849	7114.62608	36.09182832
1170.482527	5.827865121	34.66146091	0.001646722	88.34859274	201458.178	67.80443038
178.0390262	4.689853623	13.43127454	0.007361779	29.65655086	20710.87789	26.38432248
225.9903571	2.923742953	14.95616787	0.065185479	78.44173106	58085.34947	29.37130767
234.6303076	4.847218701	15.43044018	0.008260936	42.68789711	22952.57209	30.28576722
343.989009	4.040460768	18.65806293	0.045650298	13.24965147	6764.648516	36.99123883
253.8458458	5.066034975	15.80056049	0.005381218	37.18501175	37262.11745	31.06940827
515.7790729	4.482240421	22.92984169	0.022368798	26.66613348	33325.02211	45.25760176
158.6144958	4.639482627	12.58144765	0.007065192	23.39030976	7210.245566	25.03925377
956.5293973	3.331730544	31.01791971	0.100113322	15.10922935	25303.8581	61.76779748
814.8423524	4.965232716	28.81480953	0.006285824	35.09984262	33062.99625	56.88467518
1402.364765	5.904824108	38.05980394	0.001947902	121.0922194	304267.36	74.27771754
487.0871337	5.351955224	22.4701559	0.003715308	58.40242089	56349.61135	43.76591806
406.8340418	4.297457141	20.3135878	0.020358713	14.04098821	6166.749427	40.22380278
2508.171743	4.661123302	50.63398758	0.020266738	77.19639305	335739.9887	100.1410051
488.5038798	4.397629511	22.20559356	0.02323886	19.74497708	12974.92536	44.06996968
772.9835007	4.061914066	27.87809046	0.059804739	15.74450332	14971.00924	55.44440216
694.7703257	4.24939508	26.41472907	0.031633616	16.28589533	24301.58768	52.53295164
528.5003844	4.285515898	23.21392058	0.048484031	39.26392399	62588.50406	45.81947024
106.1529469	3.92360119	10.24531651	0.024624753	27.89970718	19163.22608	20.37724399
960.3432066	5.2125677	31.31069545	0.003569114	40.43047213	36968.71366	61.76105675
499.8779294	4.960096075	22.94047677	0.010637396	90.7397789	102892.4389	44.2739875
459.0631649	4.145099031	21.47177687	0.04459614	16.17677717	11143.17022	42.66875282
205.5660604	4.773514233	14.44469954	0.008237267	37.06712939	16399.56346	28.44831657
571.4014953	4.867395748	24.19192477	0.005804839	29.80612687	32426.39767	47.67494814
507.9063964	4.563104504	22.68720898	0.024997381	31.81407624	43277.51846	44.78180953
223.7853886	4.598920805	15.03315631	0.010252404	21.40769159	9367.109906	29.75127518
735.5944279	4.315908274	27.2292043	0.021774204	12.85899493	9607.054728	54.12918536
473.4864597	3.70180954	21.76857215	0.066341637	10.87266031	9039.470481	43.3742738
642.433761	3.723597355	25.42152654	0.044606896	11.03861895	11219.62551	50.62550952
346.961683	4.968039988	18.94701471	0.00521341	35.68663399	25419.51965	37.06713001
421.2743442	5.018354034	20.78751082	0.004653084	37.95304525	34745.8373	40.80795465
796.290531	5.362020723	28.60459186	0.005411913	89.60407691	160387.9532	55.80900194
545.117613	5.069842004	23.57186637	0.005556682	38.01552714	34326.28656	46.46349444
514.4472713	4.127708972	22.75840424	0.053562121	19.31183886	16306.70363	45.1954028
233.3046737	4.530597753	15.47982323	0.013319911	39.36895043	59530.73005	30.24961824
1313.591802	5.874103569	36.91300751	0.001846012	111.9857406	220578.5953	71.98224232
1561.56852	4.773775963	39.81865044	0.008921497	27.62795676	26965.71389	78.98215444
297.0718844	3.526487014	17.20094138	0.044168793	81.39779352	124292.1781	33.1518356
515.2950859	3.920640494	22.78356873	0.050279072	18.80821378	24899.54918	45.26606686
500.7765785	4.781537191	22.48648633	0.008867254	23.88129553	15917.00403	44.57574206
309.6945914	4.677795631	17.81756299	0.012034946	28.92812908	22244.13225	35.0364622
523.1412515	5.627407052	23.21039339	0.002571415	77.00215631	101878.0769	45.09176805
127.2580018	3.588311669	11.17712037	0.037065981	10.92926026	1975.946334	22.35424073
320.5555556	2.933084406	17.20238095	0.066578483	75.36306374	86157.44857	34.4047619
1504.009202	4.765727094	39.09554036	0.010417034	27.02959051	31326.94794	77.47035803
380.2627688	5.308742219	19.68289547	0.004790702	61.42761696	82892.41422	38.38016773
292.6298866	4.488566969	17.3055378	0.013735762	20.20321825	11782.35293	34.11454817
825.3450857	4.607446721	28.86488291	0.032024272	35.04855282	84945.16137	57.12199664
591.4877694	3.439173673	24.37739886	0.067291513	8.915156343	10567.27343	48.59236419
699.7335216	4.677247097	26.87765001	0.017679282	213.8857208	629456.9384	51.2952724
1149.832396	5.636273044	34.16249206	0.003693963	75.45203796	189652.5449	67.07071309
232.881714	3.055241648	15.85054446	0.061715517	113.1482785	97843.81828	30.97233364
698.0102913	5.401317412	26.60712622	0.007307383	106.8118903	204872.3	51.56138474
294.5717091	4.732269271	17.26295818	0.009748807	29.22962804	19836.28523	33.99960345

wavelet.LHL_glcm_Imc2	wavelet.LHL_glcm_Imc1	wavelet.LHL_glcm_DifferenceAverage	wavelet.LHL_glcm_Id	wavelet.LHL_glcm_ClusterTendency	wavelet.LHL_firstorder_InterquartileRange	wavelet.LHL_firstorder_Skewness
0.929649791	-0.239885459	4.748165065	0.326000021	67.18020052	136.7597087	-0.059457564
0.819174568	-0.14699814	3.292198373	0.430400744	44.74721668	92.91669171	0.277742327
0.988176686	-0.409405581	7.692818493	0.22370572	141.5875742	191.3442214	1.069526515
0.866748869	-0.153378616	5.731264853	0.333638737	116.3863887	152.0889019	0.458457996
0.994679644	-0.513469652	5.725273649	0.239475905	94.57963465	239.9628205	-0.293849671
0.954170338	-0.28070391	5.507645917	0.281276971	63.91268148	179.1420909	0.540202425
0.998508957	-0.679630258	8.581187997	0.188283895	106.6798872	305.3270066	0.085098452
0.980297225	-0.321920524	8.866949351	0.200934741	206.4142687	323.4380057	0.503426038
0.930322505	-0.186002092	9.016669647	0.200535305	318.883245	330.2240208	0.229361602
0.702811452	-0.082378064	4.358201351	0.324003183	57.54529134	126.6803433	0.640859526
0.941907909	-0.245265382	5.44659882	0.277129596	84.5415517	194.0277268	0.694800811
0.937510182	-0.228332825	5.538182993	0.299515411	145.8961955	185.9232208	0.563734521
0.92095187	-0.205767663	5.699004762	0.316368709	226.8836088	169.538671	2.127003242
0.875088	-0.180958523	3.766335707	0.357986312	48.33416665	143.804431	0.226697682
0.966344391	-0.289771751	7.28626603	0.230918213	117.9898575	220.3520541	0.550792731
0.650856202	-0.09118461	2.41360962	0.516120791	25.38494532	42.62049587	-0.081677965
0.991751178	-0.432158399	8.075453226	0.225138591	139.2168751	246.5642554	-0.04367127
0.929434761	-0.220372295	5.366300981	0.351048489	160.1454116	154.4987253	0.546684854
0.991463385	-0.434637469	8.213569742	0.203909956	126.7456853	345.9562688	0.227525318
0.78408801	-0.126511371	3.493769939	0.432766503	39.27144939	83.58859854	0.30921581
0.784449387	-0.140615149	2.951101375	0.498421968	49.82046252	69.32989179	0.223201494
0.946783625	-0.232270199	7.065223021	0.258261762	219.6636352	227.7884752	1.26538307
0.765910882	-0.149951185	2.469437425	0.531693742	28.54384154	47.08375819	-0.588239494
0.998718498	-0.60800525	11.75445222	0.171045164	236.8178478	253.6010879	-0.434244426
0.910739992	-0.232415387	4.69342334	0.410544905	89.74979819	87.66021458	-0.137430016
0.971515864	-0.317338806	6.31493163	0.272135167	101.2911126	179.490923	-0.147648404
0.666252786	-0.074612202	3.731868254	0.355308901	40.84052385	107.223415	0.346689649
0.779819134	-0.116099483	4.272865699	0.399059779	69.01733259	89.98443721	0.332478903
0.842795428	-0.177524177	2.989253008	0.505658549	73.9597596	102.2559188	0.017806954
0.729578585	-0.12769624	2.234969251	0.524256279	21.57825381	50.36303042	-0.668727891
0.655619639	-0.070251892	4.042579024	0.33838958	57.51779024	129.1976376	-0.107359767
0.617519336	-0.090859773	1.760912266	0.559535746	13.42255578	35.48782437	-0.104621782
0.853756872	-0.160311939	4.482787049	0.373044844	69.57988587	126.4457726	0.36533116
0.996765254	-0.907267285	14.04130023	0.168994696	257.4915492	263.6682744	-1.300263805
0.995323651	-0.523241912	7.051248853	0.223523031	118.6561858	214.6678281	0.026585294
0.867585541	-0.17816236	4.041707175	0.404547933	62.55389562	104.2905129	0.679952208
0.720683214	-0.151775405	1.896598499	0.610869708	29.82110006	21.02672369	-0.972363336
0.710942483	-0.110324339	2.612027627	0.498821497	25.52297691	51.04718332	0.48074914
0.909657385	-0.206952952	5.088948873	0.277949941	55.83411621	178.2843367	0.060796943
0.887308952	-0.19267441	4.327503051	0.350929775	51.00545057	116.7588424	0.562934459
0.882843719	-0.1729044	5.568654386	0.289900264	85.67693896	148.7886745	1.05075373
0.916876163	-0.218953478	4.85254415	0.305774147	53.79937426	165.9603474	0.201295764
0.930325381	-0.219054473	6.571227373	0.289375085	108.5610155	174.2199503	0.258603787
0.924027814	-0.218256504	5.807546201	0.264503665	69.96607924	171.0557885	0.444762447
0.91266061	-0.175123873	8.553080527	0.200334473	199.465066	289.9638168	0.122896647
0.625739503	-0.07086383	2.865197143	0.442453203	28.13346342	65.19870711	0.819124416
0.841577335	-0.11920802	8.525952546	0.209184615	216.855914	261.6229874	0.406078042
0.949707865	-0.272504104	5.078834038	0.287339505	63.47032717	157.8137022	1.031705994
0.998611866	-0.852931068	9.824066683	0.16352902	174.8028357	429.3545065	-0.13960971
0.989863284	-0.434071978	6.7046819	0.228475063	92.81525705	230.5099146	0.140568551
0.743543477	-0.120462837	2.855860842	0.484529784	30.8157388	62.26434759	0.060752448
0.932947596	-0.22840789	5.143831452	0.282522982	96.84537553	179.5120405	0.673991258
0.786861363	-0.129518185	4.010078463	0.396639684	61.00196521	82.59453755	0.537256411
0.928313066	-0.236514159	5.128099048	0.268671303	49.11678919	164.794276	0.369796606
0.747106304	-0.15310974	2.19383305	0.581435943	36.71162626	28.51050422	-0.10221205
0.946864792	-0.258007854	5.656995537	0.276382177	83.54236786	168.6335586	-0.212665335
0.976432509	-0.286496224	10.39160026	0.182384165	287.1852416	381.4871235	-0.008335197
0.961174177	-0.269065257	7.483457789	0.223348805	131.3781334	251.4797655	0.331473793
0.876934244	-0.19949072	3.457442351	0.392156231	34.20523327	90.37194773	-0.425661055
0.961175107	-0.322829839	6.101237032	0.380619789	163.1867346	77.47580029	-1.148598986
0.816887436	-0.148608274	3.657133308	0.416383868	46.53042081	97.60836454	-0.021072048
0.757475895	-0.132603714	2.720093442	0.512095471	40.91084386	53.57673909	0.146920057
0.740408949	-0.115359269	2.790272506	0.470237698	41.69341261	57.14642671	1.489438305
0.911040942	-0.24704342	4.401043565	0.473823643	89.32484656	51.20551345	0.018915404
0.991171801	-0.543883418	5.223052858	0.286359704	58.49411364	121.3420577	1.07020462
0.848001643	-0.138198632	6.056449811	0.260390755	94.01351491	182.7715291	-0.222963378
0.998865956	-0.627223169	10.36570248	0.164554418	203.6702022	403.9348682	-0.089741546
0.751387495	-0.126308144	2.937463425	0.48344335	40.85870825	71.71890085	-0.310205421
0.984137154	-0.392495323	6.358433051	0.229118147	77.1237815	205.1337468	0.378629322
0.785594047	-0.112070848	4.93816003	0.30913151	65.36963233	137.054212	0.731885045
0.847925028	-0.167662996	4.114144096	0.418774834	76.7650362	86.70454336	0.476850817
0.873617029	-0.178523088	4.086792291	0.355490417	45.55647789	129.017443	0.484843541
0.663881476	-0.0822933	2.805067941	0.450753345	32.69269278	68.53711816	-0.092717752
0.726443384	-0.126033845	2.073542856	0.54813435	28.16103984	42.12697476	-0.147985062
0.641310457	-0.0900076	2.378913752	0.498552793	25.1788593	45.90804524	0.575586401
0.935019261	-0.231785344	5.945387541	0.261418098	78.06725835	166.8640167	0.33600147
0.926234499	-0.215895841	6.050056262	0.259164935	84.33301055	174.6746263	0.564949175
0.992546855	-0.428375687	8.712951158	0.214968249	209.7329552	267.5260567	0.582229994
0.886733526	-0.172441112	5.541988825	0.301146113	85.69289911	159.2054834	0.279734264
0.812847222	-0.160336552	3.098069032	0.49469787	45.69496434	63.31430014	0.399579967
0.982131858	-0.409293307	5.499198771	0.296035008	84.71714505	159.5625781	1.245986936
0.963002865	-0.246215508	10.19553337	0.179486563	255.735852	321.6881596	0.40390646
0.708164607	-0.085008726	4.514048107	0.355690602	58.46885037	110.491217	0.447097623
0.997308078	-0.732524031	8.936176354	0.19158117	204.2434874	368.3952257	0.49896178
0.772266767	-0.145370275	2.82741649	0.507980923	44.27286112	48.7201023	0.471184055
0.693434679	-0.085733256	4.340756714	0.344596481	55.03746388	114.0193998	0.002836322
0.938070076	-0.257926982	4.873179105	0.336395026	63.21295836	143.8682506	0.289460139
0.966042107	-0.266349212	8.622839028	0.201897107	183.8373485	321.8402478	0.124415972
0.97942428	-0.493329651	3.181838405	0.350887335	26.33442393	112.9603676	-0.484829231
0.996876421	-0.78886897	8.277777778	0.211697701	199.1109851	208.7511274	-0.346745951
0.744732854	-0.097892915	4.506127003	0.355720914	62.90881788	113.0452709	-0.412991461
0.983034412	-0.351401435	7.271973102	0.241588808	144.1786185	232.292125	0.482729612
0.899105837	-0.208989161	4.183062281	0.359688416	44.6888989	104.6779115	0.327996257
0.840732498	-0.161700489	3.903960689	0.442260826	88.03702245	80.50493425	1.056332714
0.660288848	-0.105723947	2.001733855	0.542020036	19.97941144	38.23280942	1.695417437
0.999690693	-0.766156835	13.6077428	0.157948129	491.540841	492.255138	0.550991103
0.962216167	-0.262818341	7.155188237	0.256538651	203.1017855	215.1090313	0.196735362
0.99920122	-0.884011941	12.55031183	0.149188436	206.1210835	370.1327593	0.409775141
0.998428824	-0.571719827	9.652736258	0.184415308	280.285186	383.2839423	0.051748396
0.952734627	-0.285781421	4.867976728	0.308130849	67.22957406	120.8850052	0.02388162

wavelet.LHL_firstorder_Uniformity	wavelet.LHL_firstorder_MeanAbsoluteDeviation	wavelet.LHL_firstorder_Energy	wavelet.LHL_firstorder_RobustMeanAbsoluteDeviation	wavelet.LHL_firstorder_Median
0.066428281	102.8265394	7602202.046	60.85304692	-21.28814516
0.101971186	76.9775087	19212973.51	42.5302787	-21.39548523
0.047655527	141.9356284	7055263.399	86.08568812	-19.12665145
0.061738975	126.0256827	63181985.82	73.05237144	-13.43456208
0.04950495	132.1310725	3353980.24	98.29237979	-75.19960379
0.056066917	114.2605173	5964018.178	76.12523428	-70.52123851
0.046122449	155.2054172	2749909.577	115.758546	57.27353688
0.033264719	191.8096574	25443523.28	133.9357358	-95.67539222
0.028054105	209.6185136	106731861	142.1081025	1.243766494
0.068487053	90.43448276	37992433.73	54.68108217	-19.72904917
0.051564622	125.3886625	10439042.44	80.43664337	-53.29752636
0.050046331	137.3886086	28621162.43	82.12285794	-17.97037248
0.052661478	148.9198098	76426205.93	76.75992053	-39.45857579
0.066101354	90.87315462	7585567.871	59.93892784	-20.76254202
0.04532967	141.5516987	12644998.43	90.4244902	-16.69255
0.175561341	48.06041581	83168450.06	20.40552694	-0.484731813
0.042961201	162.9288992	10522089.8	109.1279748	-79.46254681
0.063087868	133.4784122	65450560.82	73.17611186	-30.2779809
0.039548364	169.3663962	7779110.441	128.0364054	-57.0185183
0.099140021	72.18945412	33841511.34	38.66195855	-11.87038407
0.152039137	71.8607113	73923629.82	34.02078628	-8.440538118
0.042926075	170.2863792	51276739.87	100.5658393	-14.62441997
0.184448536	57.32877068	27467530.25	24.78464654	-5.021700376
0.038498502	198.7289693	9897609.988	120.7943173	-35.46190785
0.110737847	100.6742853	29161015.32	43.01433627	-16.22398439
0.051993334	125.8045825	9119503.242	75.75160725	2.391349432
0.079521964	78.10653669	27861748.67	46.73969595	-9.780862635
0.091174657	86.26652182	71727112.33	43.46772286	-9.712009222
0.141491105	92.12405228	73021633.83	51.46952177	-5.069221722
0.182198057	51.64437395	12262403.65	24.7957775	-6.571173883
0.067964854	88.55543194	61984424.92	55.50684678	-7.700449708
0.215722475	38.48878757	38754325.4	17.43603546	-4.470761572
0.080298198	99.11136801	29715101.36	56.89601131	-17.46595634
0.093333333	235.6726916	1425288.567	122.6129204	121.2572112
0.050199702	142.4286978	3724010.327	96.81686472	-26.46593517
0.087589264	91.02494136	26329984.21	49.91680515	-16.98019292
0.288400239	43.28388103	58295026.12	11.2202707	-1.355619323
0.159105208	55.22780723	25490505.13	25.24917606	-5.319561911
0.057481973	102.8232978	5045126.246	75.15931665	-5.893066296
0.075233468	91.99579103	9733509.324	52.27840793	-17.75139402
0.060714949	105.4977235	17075594.48	62.35605691	-21.70100166
0.058314561	105.0396012	8371360.087	69.05064073	-52.31960712
0.052152064	133.5959116	30410039.98	80.16296976	-34.0361996
0.051841782	119.1908846	12115712.14	77.45345114	-71.38571365
0.0328782	178.8805389	59781741.79	120.7619607	-28.18176438
0.123933626	56.88700991	49382758.75	29.62850327	-6.609636751
0.033908024	174.7663227	149279378.7	112.0214531	-3.489635276
0.059325946	108.0857772	6008154.658	68.63661884	-99.46881776
0.082644628	205.3108125	1279584.071	162.0873754	-38.57996653
0.046537812	132.7609744	3900534.602	96.36007037	-28.03364209
0.151560097	61.74401836	39479500.9	30.83745423	-7.095010027
0.051493452	125.8355079	13612434.92	83.51055335	-118.2971474
0.103806858	84.58863183	61485505.37	40.24897322	-9.708065759
0.060905796	99.85831787	3772160.322	71.19760818	-29.33219845
0.257842457	46.05493522	116912479.4	13.13311282	-2.07828626
0.052777902	117.7789703	9488389.927	73.492093	-43.47629761
0.025552879	229.2468811	76783889.04	158.4002145	-75.35583262
0.039663622	151.7797523	17399448.04	106.329083	-35.12714407
0.099221169	75.57453634	4464280.567	42.88729097	-15.85204297
0.096253385	124.700669	26183838.39	44.41501771	-5.581273476
0.102915648	81.71573073	22554135.77	45.36446079	-13.93018508
0.166910193	64.19777665	77628415.06	28.97706739	-3.607369447
0.134780614	60.9381883	43736819.89	27.85064226	-5.899500552
0.156121635	92.34975246	47914268.6	33.19345832	-5.206060564
0.0751157	99.46062549	1461940.874	55.91246492	-113.4003699
0.048695505	123.0169617	31606769.2	78.92723651	-18.17044356
0.035102041	211.2272825	6596782.491	164.4060518	-21.22307004
0.146879029	70.24111616	41895036.18	35.72192791	-5.822299584
0.053156634	126.0438449	3764259.102	89.79431285	-74.13700167
0.064101239	99.32081519	37710576.42	58.63254595	-18.54159442
0.106504248	92.43819076	57593364.51	43.86766134	-7.788291524
0.070493858	91.38953735	9596573.73	57.40511711	-33.06891698
0.116754722	61.37136179	65467886.53	32.066799	-4.041064745
0.19667848	50.22230214	84134856.93	21.90779788	-5.54215091
0.172057879	47.92185483	111711838.7	20.69030503	-1.587507814
0.051287981	117.1116143	11309395.29	75.70639439	-48.50567971
0.051371576	118.6095799	11583309.5	76.48632832	-37.51613892
0.038464605	186.4569799	13626554.33	122.564727	-100.7281967
0.055029638	117.00753	26611966.72	70.61469332	-47.60907096
0.152621801	71.06924888	36675809.91	33.63683231	-4.652119293
0.059231566	120.0355402	4856239.73	69.85985239	-59.28513891
0.028939156	205.9844852	60204390.98	138.8775224	-46.49443538
0.07626153	89.18946817	90729022.86	48.89086933	2.017540637
0.071800208	187.8877359	1732274.993	129.8143948	-28.52798731
0.169454805	62.426733	67714078.04	24.67009506	-5.977127892
0.075287691	88.00330694	57257064.51	51.58722908	-20.27602703
0.067622443	106.5400855	8621285.462	64.77940641	-36.18869369
0.031404844	186.8184744	32164642.37	133.9005246	-53.81851698
0.1136	68.47831956	406090.09	43.20325974	12.99612762
0.100591716	167.1164214	678051.5749	74.35089358	-41.42706288
0.074766962	92.61841847	65798543.25	50.95991063	-7.427115749
0.041248269	155.1062922	14247878.15	100.9571676	-84.94025802
0.086503791	83.98162216	6624220.091	47.6230318	-24.2631593
0.118414162	90.98956339	88635829.52	41.23935941	-7.937351897
0.205582944	42.45681933	49495458.46	17.40346999	-3.382181416
0.041068279	296.736054	8808513.275	207.6829342	-91.80236327
0.042011246	159.8528697	35268691.15	94.04298974	-34.23278833
0.0625	221.8378192	1761483.858	166.9309954	-148.1882837
0.031427604	211.1012868	10459174.8	153.6174733	-2.272742625
0.067091837	98.30376134	5280305.736	58.3561369	-13.19397652

wavelet.LHL_firstorder_TotalEnergy	wavelet.LHL_firstorder_Maximum	wavelet.LHL_firstorder_RootMeanSquared	wavelet.LHL_firstorder_90Percentile	wavelet.LHL_firstorder_Minimum	wavelet.LHL_firstorder_Entropy
205259455.2	441.7010483	145.1162687	136.2671062	-599.8659477	4.307958953
518750284.7	466.8095889	117.0222533	76.83301141	-420.7914213	3.911383354
190492111.8	757.5066188	199.6503073	223.3935688	-443.7112216	4.680885325
1705913617	672.1448412	180.7926243	217.3865129	-585.8940277	4.65001861
90557466.48	279.4343783	182.229869	117.6138349	-505.800017	4.507422964
161028490.8	362.2537481	156.341605	152.7476248	-451.6666737	4.438845866
74247558.57	498.3534169	198.2029831	307.6043253	-303.2484978	4.628445159
686975128.6	655.3428843	251.8931971	293.7947264	-605.0010264	5.145125065
2881760246	933.5539182	268.9080864	336.5441623	-926.0172539	5.421146927
1025795711	777.9129239	127.9965262	132.0369528	-657.2598276	4.272930408
281854145.9	531.5424035	166.62356	216.4746404	-449.3414953	4.608401001
772771385.5	778.0460352	194.4445139	202.3459037	-613.9488749	4.803020313
2063507560	1294.419915	236.5352114	233.7519139	-504.7838374	4.79239858
204810332.5	356.850845	119.8607768	127.5721739	-378.042217	4.224632738
341414957.5	575.0244854	186.3840301	278.7011516	-493.8366205	4.769193758
2245548152	597.5099329	87.64056737	71.46459887	-760.1623596	3.260337993
284096424.5	417.5450608	220.201937	226.3696621	-723.8630221	4.86520239
1767165142	940.7396508	205.3576225	133.8449252	-959.3721988	4.685981873
210035981.9	463.3938006	206.7422672	249.4380488	-487.8460855	4.840302006
913720806.1	531.8104541	110.4515072	95.48296118	-450.8643146	3.923231055
1995938005	831.1105251	120.6906478	38.66607551	-692.6047845	3.606807321
1384471976	1122.27787	237.5080101	355.7140728	-553.2554039	5.00962263
741623316.8	546.6083798	96.98808402	30.96006327	-591.5172121	3.27819709
267235469.7	536.2089512	272.7968479	260.7635293	-937.5491332	5.059693271
787347413.6	800.7459214	174.2872847	59.24659668	-815.6550302	4.070147982
246226587.5	669.0368706	173.7729283	145.6189726	-613.0548002	4.631991404
752267214.1	608.1344209	110.3268678	111.7445126	-371.8669021	4.070198723
1936632033	722.1514253	135.7025293	119.3835115	-538.4361578	4.149170028
1971584113	730.8050395	140.1806019	34.0266442	-553.4192308	3.765559292
331084898.7	376.4479394	85.10583264	27.94517675	-482.5561463	3.198455958
1673579473	562.5355406	121.4688577	118.4255886	-621.9752605	4.242218377
1046366786	525.0943765	66.2378116	29.3309465	-434.2708786	2.911462658
802307736.7	633.483756	146.2640131	124.2987396	-512.2739006	4.281682962
38482791.3	405.1793075	308.2519064	267.8690495	-811.2558552	3.506890596
100548278.8	426.5357246	181.5374441	190.8943603	-431.8352478	4.598032248
710909573.7	711.8107002	140.4906379	101.4331256	-506.2337162	4.165771084
1573965705	1016.811237	93.34052423	20.54004026	-755.718489	2.647201945
688243638.4	496.6249783	91.6905594	71.00069301	-487.265127	3.430604983
136218408.7	361.6961704	125.3670778	143.5985066	-292.6878614	4.302071398
262804751.8	639.7019901	134.6317782	127.2592872	-529.5296969	4.224705786
461041050.9	796.775107	154.5378605	134.5466905	-493.526379	4.417188832
226026722.4	367.6008699	148.2298004	117.7674481	-464.988518	4.405682246
821071079.5	601.3542606	192.1077519	154.0001054	-571.9099723	4.748732886
327124227.8	469.9883017	166.1277614	144.6791929	-469.5826839	4.576637786
1614107028	841.1678257	231.5511	297.3538093	-777.428097	5.186849239
1333334486	787.9242214	89.81337392	73.76484388	-448.9238056	3.599962551
4030543224	1028.774497	233.1576358	298.1239376	-806.4618787	5.209667327
162220175.8	520.4448016	162.331622	100.6008412	-377.8812964	4.353723909
34548769.91	287.7125649	241.1698827	212.6627045	-402.921981	3.754441846
105314434.3	389.968461	163.450269	202.2401389	-397.7884329	4.582154852
1065946524	560.2297167	98.36843121	46.87267354	-461.7482641	3.512609954
367535742.8	391.7820177	183.7872524	161.7278875	-460.4267474	4.566581163
1660108645	797.6191832	138.723789	87.99215604	-570.2155351	4.039165437
101848328.7	382.2092041	125.6307987	140.0363836	-321.2591133	4.244793685
3156636945	1005.122878	101.6894609	27.17340404	-756.0107292	2.825379512
256186528	532.636736	168.296025	131.2106032	-733.0780739	4.568176926
2073165004	727.8257387	296.7404904	304.8954605	-982.7182453	5.510168496
469785097.1	763.0285419	194.9103928	211.1663153	-568.6990416	4.874313905
120535575.3	346.0438052	112.2985399	81.96916298	-517.8965526	3.874299245
706963636.5	742.7330202	237.0411239	226.6791255	-1203.854167	4.257111994
608961665.7	528.7142909	124.5035109	57.77664443	-573.3720441	3.952590793
2095967207	725.3821918	107.0344695	54.07625403	-691.8837878	3.488599214
1180894137	915.4340215	103.6127233	94.69362679	-637.4538946	3.621050232
1293685252	647.36429	163.9753557	58.29532835	-592.0529598	3.747487813
39472403.59	299.6185151	166.0836839	80.14977283	-348.4725359	3.92415024
853382768.5	669.5552605	165.6394627	172.7735913	-751.3655706	4.683338563
178113127.3	390.1038234	250.6521492	290.3542932	-581.6857539	4.955745909
1131165977	569.0012301	109.926997	42.07919828	-532.1509405	3.597908596
101634995.7	321.0948031	162.2451547	175.8786795	-380.1908126	4.459106084
1018185563	888.069842	145.0649801	128.1099467	-586.5822378	4.391818332
1555020842	727.3407282	148.861738	100.66303	-570.259767	4.078129771
259107490.7	426.783209	130.0967875	91.418318	-382.9815238	4.206946462
1767632936	598.5043559	96.31725609	85.41854517	-652.8117261	3.704532977
2271641137	724.0937406	88.12387071	28.63389197	-548.0153872	3.145435431
3016219645	899.0099454	87.7164905	50.63146004	-623.7031807	3.246416119
305353672.9	437.9282224	159.2400344	165.1495238	-482.6859931	4.594683055
312749356.6	651.8864473	159.3800318	179.368215	-502.5023445	4.565774933
367916966.8	625.1240431	246.6431264	350.3723887	-746.1397922	4.984828637
718523101.4	533.4419379	170.7273491	108.5690538	-616.2062512	4.583835577
990246867.5	649.9664199	116.4626085	64.598701	-552.2672759	3.617229538
131118472.7	628.1862426	180.5331353	141.0023851	-403.0027176	4.433846271
1625518557	918.1107945	263.8189758	340.6484593	-881.3077882	5.371273222
2449683617	815.0453592	135.6730873	128.3025137	-961.9453504	4.245894128
46771424.81	575.2257361	236.3891674	184.495457	-420.3585439	3.977916875
1828280107	663.3395139	116.81836	34.40341624	-571.025801	3.405177189
1545940742	559.7040811	127.7752256	112.0482003	-568.029438	4.224804098
232774707.5	497.3152714	153.687866	86.92513965	-453.7617676	4.348565489
868445343.9	562.7362956	233.6854734	290.708531	-591.8647602	5.177337945
10964432.43	188.7294301	90.12103972	89.88361641	-270.3871701	3.501147632
18307392.52	294.1603229	228.3808518	247.4295508	-461.471273	3.39274741
1776560668	631.7046337	138.5238	129.3497048	-971.2968581	4.298250269
384692710	581.2194094	216.4902312	205.5575352	-525.1995682	4.918676428
178853942.5	553.0365255	126.1887712	81.05910544	-431.6418463	4.069881843
2393167397	1013.118975	155.7898906	97.43791349	-701.1547441	4.02022239
1336377378	931.6939489	82.78543471	35.04743784	-593.9750568	3.013119096
237829858.4	752.1343965	373.922012	507.123397	-660.4078432	4.980916432
952254661	917.4006186	227.7404713	260.2950919	-842.1785878	5.027318281
47560064.18	360.1933992	270.9154126	315.3749214	-465.3422322	4.136842188
282397719.5	563.1540463	253.311501	291.6421259	-632.3148698	5.165643379
142568254.9	453.2403954	137.3252882	135.7788859	-421.8677285	4.291389386

wavelet.LHL_firstorder_StandardDeviation	wavelet.LHL_firstorder_Range	wavelet.LHL_firstorder_Variance	wavelet.LHL_firstorder_10Percentile	wavelet.LHL_firstorder_Kurtosis	wavelet.LHL_firstorder_Mean
141.5927986	1041.566996	20048.52062	-214.7137767	4.324779908	-31.78381359
112.186769	887.6010102	12585.87115	-172.3907836	5.298140777	-33.29169007
199.4643686	1201.21784	39786.03435	-219.0976248	5.650029065	-8.614571909
179.5661691	1258.038869	32244.00908	-234.1479249	4.347198292	-21.02293796
159.5268457	785.2343954	25448.81451	-289.6521932	2.508183722	-88.0846788
146.8326597	813.9204218	21559.82995	-215.4392653	3.252821414	-53.69234113
186.5388627	801.6019147	34796.74731	-158.0368437	2.294369462	66.99011281
242.9285251	1260.343911	59014.2683	-368.1500506	2.944431122	-66.60266098
268.7816461	1859.571172	72243.5733	-331.7506947	3.447046872	8.245339136
127.4385332	1435.172752	16240.57975	-152.2437939	6.282331992	-11.93863303
164.4268461	980.8838988	27036.18773	-207.6509139	3.562191353	-26.96707288
192.9415977	1391.99491	37226.46011	-261.7247656	4.787386306	-24.129005
236.2155947	1799.203752	55797.8072	-229.0942716	9.661917507	-12.29223536
118.4396862	734.893062	14027.95927	-161.2821795	3.374147715	-18.40235174
186.1857751	1068.861106	34665.14286	-208.5675349	3.456823024	8.594405432
87.62877441	1357.672292	7678.802104	-64.11562888	14.44709651	1.437687249
211.0832165	1141.408083	44556.12429	-311.428275	3.072838597	-62.71179144
200.1549193	1900.11185	40061.99171	-271.2033322	6.619440483	-45.93213903
202.8129296	951.2398861	41133.08442	-282.2073358	2.213578677	-40.11584029
109.2819723	982.6747687	11942.54947	-133.2704577	6.536544489	-16.03078232
117.2638542	1523.71531	13750.81149	-164.3323445	10.11065893	-28.55557716
234.5313196	1675.533274	55004.93987	-198.2433133	5.596656238	37.48486349
93.8314935	1138.125592	8804.349172	-123.5846768	9.256374601	-24.54260112
265.8619727	1473.758084	70682.58854	-396.9820507	3.762312719	-61.118996
170.0112939	1616.400952	28903.84005	-201.0745605	8.746069397	-38.36948755
173.4260405	1282.091671	30076.59151	-220.7044744	4.803813216	-10.97447448
109.7508298	980.001323	12045.24464	-142.9538768	5.589333069	-11.25935737
134.7472471	1260.587583	18156.82061	-164.8596855	6.986613042	-16.07345189
133.7447848	1284.22427	17887.66746	-226.3109248	6.287703955	-41.98730376
81.99213485	859.0040857	6722.710177	-122.3993911	8.993646592	-22.80992267
120.7245408	1184.510801	14574.41475	-163.3856628	5.079761418	-13.42641592
65.12402923	959.3652551	4241.139183	-81.30201563	11.21650679	-12.09580517
142.6245982	1145.757657	20341.77602	-204.6702927	5.39330635	-32.42507531
308.0753854	1216.435163	94910.44306	-396.9201089	4.061350225	10.4304708
178.3083702	858.3709724	31793.87487	-257.0086874	2.843050514	-34.08766239
136.9859661	1218.044416	18765.15492	-182.1898582	7.249753979	-31.18436199
92.29525033	1772.529726	8518.413234	-75.77386583	23.14747878	-13.92983236
91.19229016	983.8901052	8316.033785	-103.0150833	7.899184751	-9.545936214
125.1167722	654.3840318	15654.20668	-169.8785958	2.61571776	-7.918176144
133.0940613	1169.231687	17714.02914	-164.8329424	5.854020491	-20.29006031
153.667292	1290.301486	23613.63662	-169.6137718	8.061310489	-16.38028391
137.0746511	832.5893879	18789.45997	-225.8107816	3.496491541	-56.41465898
185.7532734	1173.264233	34504.27856	-285.1512701	4.295401697	-49.00112003
155.654165	939.5709857	24228.21907	-240.6774296	3.465737718	-58.05354473
230.6362711	1618.595923	53193.08954	-304.3722024	3.217404411	-20.56264529
89.39765655	1236.848027	7991.940996	-103.9634091	10.89349286	-8.631404249
233.0938096	1835.236375	54332.72408	-275.3361391	4.122227979	5.455185081
143.1031927	898.3260979	20478.52377	-244.5655545	4.726058778	-76.63570806
230.3115088	690.6345459	53043.39108	-398.2913798	1.576859564	-71.55082972
161.3517922	787.7568939	26034.40085	-228.3519682	2.444583129	-26.10727066
95.78782927	1021.977981	9175.308236	-140.3442497	7.18840984	-22.38392332
161.8450143	852.2087651	26193.80865	-266.2139453	3.150765694	-87.08585126
137.3998264	1367.834718	18878.71231	-170.3602431	8.048080184	-19.12007646
123.6549797	703.4683175	15290.554	-171.1611203	2.91189618	-22.19332277
101.1436948	1761.133607	10230.04699	-62.60606025	18.78338917	-10.52138132
159.8920251	1265.71481	25565.45968	-250.0978326	4.708873525	-52.51754315
288.6693743	1710.543984	83330.00766	-424.2416296	2.926766328	-68.73798781
192.4280524	1331.727583	37028.55534	-280.7706361	3.561069305	-31.00815838
107.3531511	863.9403577	11524.69906	-182.0242112	5.370219366	-32.9585042
237.0269176	1946.587188	56181.75968	-125.5467361	10.74066903	2.595133214
119.5949277	1102.086335	14302.94673	-185.2202199	6.056885616	-34.61470082
105.4930378	1417.26598	11128.78102	-143.9139423	8.984264924	-18.09963113
103.5664136	1552.887916	10726.00202	-101.8747356	13.7829914	-3.097485289
162.0290023	1239.41725	26253.39758	-207.846683	7.114618459	-25.18967463
137.3498698	648.091051	18864.98674	-237.7731451	4.300529177	-93.37453256
164.017151	1420.920831	26901.62583	-219.7305531	4.390567955	-23.12586791
246.2607538	971.7895773	60644.35885	-368.3986663	2.002712256	-46.71339276
106.370552	1101.152171	11314.69433	-169.5092373	6.96476236	-27.7353626
153.6212734	701.2856156	23599.49563	-243.5676217	2.489144997	-52.1919016
144.086902	1474.65208	20761.03532	-167.8009717	7.6830867	-16.81704886
147.3265442	1297.600495	21705.11062	-203.905461	6.459058316	-21.32384658
123.4661265	809.7647328	15243.88439	-192.3571333	4.583710983	-41.00353325
95.87833358	1251.316082	9192.65485	-111.0483735	8.925060235	-9.184713932
86.12784187	1272.109128	7418.005146	-113.5557045	11.55572031	-18.64970358
87.60225211	1522.713126	7674.154575	-73.27491872	16.38108644	-4.475279877
153.7385837	920.6142155	23635.55213	-234.7051346	3.434448516	-41.49501673
157.423437	1154.388792	24782.13853	-214.6171786	4.325436518	-24.89690738
240.9322501	1371.263835	58048.34913	-333.5629479	3.077764048	-52.76819767
160.91961	1149.648189	25895.12088	-261.8360224	4.47137396	-57.03250696
114.6324382	1202.233696	13140.59588	-158.8698565	7.329610963	-20.56558507
173.892447	1031.18896	30238.58311	-248.1874739	6.054507588	-48.51422314
263.4355908	1799.418583	69398.31049	-333.55852	3.436218037	-14.21764817
135.6681426	1776.99071	18405.84492	-142.6575283	8.556988865	-1.158316816
235.9030423	995.58428	55650.24536	-275.7707158	2.957774644	-15.15233044
115.3562792	1234.365315	13307.07115	-125.2624348	11.44045872	-18.42438814
124.6238801	1127.733519	15531.1115	-181.7565454	5.099358511	-28.20277957
145.6995078	951.0770391	21228.34657	-235.9465567	4.389465518	-48.90412656
230.132033	1154.601056	52960.7526	-329.7583673	2.510962438	-40.59738727
89.89588387	459.1166002	8081.269936	-118.2892223	3.494926233	-6.366464041
221.9649058	755.6315959	49268.41941	-403.9149923	2.600072537	-53.75308401
137.7405456	1603.001492	18972.45789	-165.6931821	7.10638364	-14.71004033
203.3205636	1106.418978	41339.25158	-332.8185206	3.43488888	-74.35569004
121.4966143	984.6783718	14761.42728	-183.9375851	5.678177626	-34.09074225
154.752338	1714.273719	23948.28612	-183.0760488	9.537645404	-17.95003878
82.64622915	1525.669006	6830.399193	-70.631435	22.27458877	-4.798854672
368.2162487	1412.54224	135583.2058	-496.2577899	2.461370325	-65.07276872
225.0028824	1759.579206	50626.29709	-301.8272199	4.537161387	-35.20547102
256.5579564	825.5356314	65821.98497	-381.4662575	1.911713522	-87.02399555
252.4852351	1195.468916	63748.79395	-332.2526904	2.44448725	-20.44315535
136.9070441	875.1081239	18743.53873	-177.3193207	4.465845441	-10.70962369

wavelet.LHL_glrlm_ShortRunLowGrayLevelEmphasis	wavelet.LHL_glrlm_GrayLevelVariance	wavelet.LHL_glrlm_LowGrayLevelRunEmphasis	wavelet.LHL_glrlm_GrayLevelNonUniformityNormalized	wavelet.LHL_glrlm_RunVariance
0.005285244	33.92170006	0.005394657	0.062166352	0.105395245
0.006771236	22.77918272	0.007214687	0.085780985	0.283962914
0.013953952	64.95707524	0.014055896	0.046647636	0.041569597
0.003399008	56.86559426	0.003545098	0.050440985	0.226762324
0.016011215	41.46434779	0.016085105	0.049265606	0.024331008
0.009091647	35.86145882	0.00927303	0.054477074	0.069742776
0.036129461	56.92568763	0.037187407	0.045974871	0.021904247
0.006746325	95.83698823	0.00681431	0.032721689	0.034412189
0.001605954	117.6512764	0.001630118	0.027777911	0.041554973
0.001937004	27.59097743	0.002024521	0.065420977	0.103759783
0.010704196	44.8324513	0.010873974	0.049913889	0.059935784
0.004021295	63.11638371	0.004121928	0.045942968	0.100848199
0.005931179	96.63740843	0.006140323	0.04790426	0.146783342
0.007886865	23.77255974	0.008189793	0.062516522	0.126421582
0.009135131	56.52064343	0.009380334	0.044385769	0.040474112
0.001072322	15.6209794	0.0012341	0.139099576	0.582752886
0.007224553	72.25839372	0.007269641	0.04125514	0.041618462
0.001551086	71.09025964	0.001606988	0.051626529	0.224231934
0.012358976	65.84303204	0.012493682	0.039233524	0.034077628
0.004331931	22.39556033	0.004680429	0.083079659	0.376889654
0.00198791	27.57465986	0.002184054	0.109893562	0.587269951
0.003871849	91.22465407	0.003958219	0.041118163	0.061627365
0.002353723	18.03929795	0.002634282	0.136489222	0.628936083
0.009179347	114.9821465	0.009192993	0.037664834	0.022575538
0.003612034	52.8548777	0.003790085	0.090394797	0.247873731
0.005969015	49.31282977	0.006028679	0.050878959	0.053320697
0.009707492	20.68373433	0.010133415	0.075294249	0.126562675
0.004147527	33.15739858	0.004364767	0.076821848	0.27988501
0.003688549	35.48985237	0.00404952	0.095099867	0.688979024
0.0036734	13.41147786	0.004061371	0.14076037	0.532391657
0.002054403	24.751552	0.002173273	0.065423574	0.118009299
0.00332578	8.956400301	0.003904928	0.167544947	0.705332166
0.00588208	36.61465373	0.006097384	0.065545454	0.248320173
0.068734775	155.002599	0.068743975	0.092987964	0.015306122
0.019823926	52.00286809	0.020003738	0.049419182	0.033316347
0.004549951	34.19090259	0.004807907	0.071927173	0.273563358
0.001372899	19.87246571	0.001617651	0.218756889	1.05165153
0.003032596	16.67125605	0.003399165	0.119710733	0.546426994
0.028705188	25.74526999	0.029116167	0.056792327	0.056247768
0.004677707	31.10726488	0.004830851	0.067887236	0.141361518
0.009070453	39.62178744	0.009207052	0.059384712	0.068489052
0.00867962	31.4027174	0.008849566	0.056424632	0.074665662
0.009206472	58.82144682	0.009427093	0.047405351	0.104178035
0.008989898	39.68641227	0.009139875	0.05037342	0.054346283
0.002855137	86.6157122	0.002887542	0.032375846	0.039832536
0.003761454	14.86972873	0.004135266	0.108001534	0.288206867
0.001618322	88.93905805	0.001648915	0.033416807	0.043228927
0.014555845	33.79040524	0.014903296	0.058183621	0.057905688
0.084317472	82.53321678	0.085771258	0.081853147	0.013846154
0.016705561	41.89485887	0.016861397	0.046284381	0.032698531
0.004098136	18.15276762	0.004509881	0.113042195	0.525498454
0.010799559	43.31488387	0.011044484	0.05028367	0.060734714
0.003487455	34.39617681	0.003686668	0.08643422	0.238962056
0.016261309	24.87542711	0.016556338	0.060100721	0.046775846
0.001116907	22.87435078	0.001335567	0.197703313	0.82486058
0.004667866	42.98975021	0.00471737	0.050790552	0.060783974
0.002380445	134.8968917	0.002396983	0.025286458	0.026375839
0.005475772	60.03661826	0.005565216	0.039169983	0.042231705
0.006104328	20.51152583	0.006282432	0.086122881	0.168003953
0.003262125	100.8026364	0.003296532	0.081312568	0.1893859
0.003310926	26.1681364	0.003513282	0.08472923	0.26556694
0.00167327	22.96058584	0.001880953	0.117607415	0.700758418
0.001678982	20.64882969	0.00186986	0.109206042	0.38772669
0.006690981	51.48257153	0.007036595	0.115672979	0.435105462
0.052097893	30.39815433	0.052605173	0.073612978	0.046494922
0.002231679	44.57550395	0.002276824	0.047531402	0.06267159
0.015378265	96.7063227	0.015430728	0.035115504	0.016091849
0.003342382	22.03580728	0.003643684	0.108460621	0.537653818
0.016501086	38.22408889	0.016648723	0.052209844	0.031460666
0.003061638	35.18277723	0.003167781	0.061531759	0.094131198
0.00336678	40.12470866	0.003584285	0.084464978	0.305838708
0.010448414	25.97261977	0.010819413	0.06608816	0.123588043
0.001575024	17.31023492	0.001749289	0.097972471	0.373100439
0.002345982	15.29404403	0.002726216	0.149806599	0.655504942
0.001717937	15.27267028	0.001952804	0.141752412	0.463479628
0.006867873	38.94371083	0.006994331	0.050064709	0.056030296
0.006012632	40.94932003	0.006132301	0.050406475	0.059492578
0.006308644	95.48896613	0.006354334	0.037453915	0.03948922
0.003776857	43.82996685	0.003873909	0.052365145	0.09592395
0.002650802	26.18956057	0.002938185	0.10999147	0.545041942
0.016975592	50.58268087	0.017300687	0.057276463	0.053847175
0.002224708	112.4463833	0.002244359	0.028667521	0.028924723
0.00087266	32.56090181	0.00092548	0.069202315	0.174571974
0.049197739	92.31425073	0.049348565	0.069839594	0.031979226
0.002935825	26.81319554	0.003239731	0.130964621	0.537875454
0.003729876	26.73137566	0.003891479	0.069393151	0.129773121
0.011244375	36.63703204	0.01167901	0.05979899	0.14067813
0.008731449	85.61766652	0.008806924	0.031156459	0.032414521
0.0319874	13.61531543	0.03232004	0.11007316	0.056225434
0.123884409	74.18328525	0.140947601	0.09817106	0.037190083
0.001011442	33.37524295	0.001064586	0.066723449	0.180213223
0.010413327	68.15181096	0.010598726	0.040447475	0.048289069
0.008518712	25.66921713	0.008759105	0.077967052	0.139283659
0.001683015	46.12573543	0.001837993	0.087285922	0.436138735
0.002066331	14.22983354	0.002365541	0.163387257	0.59947115
0.026154765	220.4681692	0.026216816	0.039794304	0.0231542
0.002834195	85.03016451	0.002877738	0.039730265	0.076665041
0.05506204	107.184974	0.055532358	0.061600262	0.009597208
0.009728093	102.3488987	0.009771101	0.031255607	0.0288688
0.014769577	31.66673683	0.015003028	0.064509426	0.077153827

wavelet.LHL_glrlm_GrayLevelNonUniformity	wavelet.LHL_glrlm_LongRunEmphasis	wavelet.LHL_glrlm_ShortRunHighGrayLevelEmphasis	wavelet.LHL_glrlm_RunLengthNonUniformity	wavelet.LHL_glrlm_ShortRunEmphasis
20.66545062	1.287171772	538.1977814	286.2464472	0.942251885
101.9080724	1.683610091	251.2803326	904.8603637	0.89426088
7.967298928	1.116928023	387.6261026	159.6680922	0.974114304
86.36099112	1.505284457	572.7269713	1417.953808	0.926380659
4.854745259	1.075855345	358.1488354	93.79990089	0.981036164
12.50750736	1.20383481	323.7676994	204.7972974	0.954986258
3.147978946	1.069532751	314.5068871	65.55255404	0.982616812
12.70660482	1.101174125	605.3299526	364.6531933	0.976160211
39.41925448	1.123704183	1583.584982	1313.792239	0.970776878
139.4617742	1.288661608	715.5715133	1823.579305	0.940107751
17.82120142	1.169941936	340.8397129	323.8891806	0.962724574
32.19999141	1.269996815	630.1579543	610.4800496	0.946481375
59.45431174	1.361121846	515.0031886	1055.985603	0.93724397
30.02466944	1.338843553	254.8579771	405.6700746	0.934435779
15.56592252	1.118524351	481.017113	326.3247466	0.972473544
1157.787092	2.296245024	853.0417785	5526.08877	0.840340083
8.621899467	1.12209676	776.1603014	194.3865388	0.971991332
70.57825666	1.517803412	1358.151219	1114.063201	0.920278768
6.924075272	1.100980651	417.8719123	166.4029601	0.976641189
190.9029378	1.849088747	334.4529445	1705.79468	0.883483908
431.3870488	2.275991029	643.9930282	2660.816247	0.848956872
35.44331276	1.174809044	701.1555176	779.9186963	0.96159516
304.6206245	2.384877378	464.868994	1472.740431	0.838530936
4.902997852	1.067097045	1390.473364	124.7558704	0.983888298
74.92390116	1.598258992	963.1314974	650.368588	0.90571779
14.61220294	1.160965772	647.7293047	260.2792931	0.962187075
155.8852769	1.350411289	230.7706403	1722.247439	0.929205498
255.7964522	1.654349469	459.0666741	2578.912421	0.900751553
267.8233889	2.453917974	408.0160517	1872.690871	0.841341494
185.0811149	2.216137075	326.1561802	885.7638851	0.846218132
249.2132361	1.335929932	601.1637283	3171.537875	0.929596977
1088.642699	2.600840314	266.2530372	3995.122539	0.81037101
79.36021044	1.568834021	402.5227106	973.9053329	0.915304148
1.373626374	1.049450549	1284.517857	14.34065934	0.987637363
5.405903867	1.101593219	339.3940768	102.616526	0.975378696
81.97410789	1.653200217	396.7116962	883.1794171	0.900366579
995.505968	3.342367785	729.0344836	2479.512709	0.76469672
283.702184	2.201580675	358.8457077	1624.567288	0.853346026
17.31394346	1.165795412	167.6168223	275.833883	0.961807065
32.90944672	1.372278095	467.179334	403.8658479	0.92951943
39.90365995	1.201642634	416.7244178	596.103791	0.954273446
20.15064658	1.214684875	311.8614561	315.9270304	0.952912991
36.18691779	1.27315326	492.3146104	666.0873178	0.947235957
21.04228872	1.1600115	324.3633288	379.4342542	0.963226642
34.79877273	1.116383188	1063.743964	1001.369357	0.973026916
554.7337498	1.712639603	305.4190967	3820.806399	0.885349
88.19729155	1.126069334	1193.826971	2444.305705	0.970833635
12.59027177	1.16870266	210.2627041	195.7874351	0.961789397
1.776923077	1.046153846	297.3192308	21.13846154	0.988461538
6.551015138	1.097835299	275.3360669	133.2715409	0.976766568
363.069042	2.159155848	305.8292666	2221.339725	0.856716198
19.17819412	1.177884569	292.1920521	343.0479788	0.959585543
238.2793484	1.592340302	496.5224006	2148.250882	0.902870278
13.74999944	1.139097603	179.0386242	210.1326836	0.967498374
1585.668027	2.88535805	770.9663899	4656.265151	0.788023592
16.13170238	1.175166438	816.1117947	286.9214085	0.961022917
21.52236599	1.076849299	1539.186901	812.2412212	0.982082788
17.25031912	1.124300986	543.4083336	408.1964706	0.971063448
27.21629349	1.425684863	390.5968007	260.2193769	0.9248501
33.44788865	1.48154488	2359.557477	332.0659151	0.916735696
105.451097	1.641301924	455.4413563	962.0553579	0.899563382
601.6454799	2.482392809	654.018056	3361.732097	0.837421839
362.0475045	1.907002017	629.5729265	2383.413876	0.871492815
165.2760525	2.013164051	513.4489698	1002.721309	0.862170587
3.733454755	1.144297773	144.4863813	46.5491487	0.965744069
51.79754775	1.181487467	938.0068114	979.5564118	0.959267457
3.62753488	1.050056746	602.6732406	100.0424337	0.987485814
296.7907152	2.169943176	399.137767	1903.414938	0.85873175
7.265339837	1.088979587	242.4544778	132.1950339	0.980029805
101.8453507	1.267753841	568.8194135	1425.250589	0.942678615
185.9729804	1.70928376	491.9175431	1683.697525	0.895310684
34.05963169	1.336281386	230.2989513	433.7819021	0.933455852
565.4114932	1.873764411	656.2956386	4165.070904	0.873491011
1206.601369	2.486561973	392.3242407	5066.538545	0.820231572
1623.468185	2.092013533	557.3701087	7813.769773	0.849895638
21.26278013	1.159497425	382.1722338	387.2407437	0.964699872
21.79620443	1.172775635	447.1808051	390.8079416	0.96120774
[truncated: 1,182,173 more chars]
