# Supplementary material for: Peritumoral radiomics features predict distant metastasis in locally advanced NSCLC
Source: PLoS One. 2018 Nov 2;13(11):e0206108. doi: 10.1371/journal.pone.0206108 (PMC6214508; doi:10.1371/journal.pone.0206108)
Supplement: S8 File — (PDF) [file pone.0206108.s008.pdf]

X	general_info_BoundingBox
97	(6, 5, 6, 20, 16, 11)
98	(5, 6, 6, 9, 8, 6)
99	(6, 6, 6, 11, 14, 13)
100	(6, 6, 6, 8, 8, 7)
102	(6, 6, 5, 15, 16, 14)
103	(5, 6, 6, 9, 7, 8)
104	(6, 5, 6, 37, 36, 41)
105	(6, 6, 6, 22, 35, 33)
106	(6, 6, 6, 24, 31, 26)
109	(5, 6, 6, 16, 14, 13)
110	(6, 5, 6, 19, 36, 20)
111	(5, 6, 6, 19, 23, 27)
113	(6, 6, 6, 14, 14, 9)
115	(5, 5, 5, 13, 14, 10)
116	(6, 5, 6, 17, 14, 15)
118	(6, 6, 6, 28, 41, 30)
119	(6, 6, 5, 12, 12, 13)
120	(5, 6, 6, 9, 9, 10)
121	(6, 5, 6, 13, 17, 11)
122	(6, 6, 5, 35, 34, 41)
123	(5, 6, 6, 15, 17, 21)
124	(6, 6, 5, 10, 14, 8)
125	(6, 6, 5, 10, 10, 11)
127	(6, 6, 6, 26, 29, 18)
128	(6, 6, 6, 12, 20, 12)
129	(6, 5, 6, 17, 18, 23)
130	(5, 6, 6, 20, 24, 19)
131	(6, 6, 5, 19, 20, 22)
132	(5, 5, 6, 8, 6, 5)
133	(6, 6, 6, 17, 18, 12)
134	(6, 5, 6, 25, 25, 24)
137	(5, 6, 6, 10, 12, 14)
210	(6, 6, 5, 15, 26, 25)
310	(6, 5, 5, 22, 27, 19)
410	(6, 6, 6, 27, 26, 35)
510	(6, 6, 6, 17, 22, 19)
610	(6, 5, 6, 22, 24, 19)
710	(6, 5, 6, 24, 34, 42)
810	(5, 6, 6, 14, 10, 9)
910	(5, 5, 5, 31, 47, 41)
1010	(6, 6, 6, 15, 13, 15)
1110	(6, 5, 6, 33, 30, 29)
1210	(5, 5, 6, 27, 28, 12)
138	(5, 6, 6, 14, 19, 15)
142	(5, 6, 6, 16, 16, 16)
151	(5, 6, 5, 20, 28, 15)
161	(5, 5, 5, 31, 30, 28)
171	(6, 5, 6, 12, 9, 8)
181	(6, 6, 5, 12, 9, 6)
191	(6, 6, 6, 11, 8, 8)
201	(6, 6, 6, 18, 25, 25)
221	(6, 5, 5, 23, 23, 24)
231	(6, 5, 6, 33, 41, 27)
241	(6, 5, 6, 15, 19, 16)
251	(6, 6, 6, 9, 15, 10)
261	(6, 5, 6, 14, 15, 12)
271	(6, 6, 6, 27, 35, 23)
281	(6, 6, 5, 14, 17, 18)
291	(6, 5, 6, 13, 12, 13)
301	(6, 5, 5, 38, 42, 44)
311	(6, 6, 5, 13, 15, 15)
321	(6, 6, 5, 17, 23, 18)
331	(6, 6, 5, 31, 42, 40)
341	(6, 6, 6, 18, 18, 21)
351	(5, 6, 6, 10, 12, 13)
361	(6, 6, 6, 23, 22, 23)
371	(5, 6, 6, 9, 11, 8)
381	(6, 6, 6, 24, 33, 39)
391	(6, 6, 5, 31, 28, 32)
401	(5, 6, 6, 23, 19, 16)
411	(6, 5, 5, 13, 13, 7)
421	(6, 6, 5, 26, 26, 32)
431	(5, 6, 6, 14, 16, 14)
481	(6, 6, 6, 13, 15, 20)
491	(6, 6, 6, 23, 31, 32)
501	(6, 6, 6, 17, 8, 13)
541	(5, 6, 6, 18, 18, 19)
551	(5, 5, 6, 9, 9, 7)
561	(6, 6, 5, 21, 12, 14)
601	(5, 5, 5, 17, 17, 18)
611	(6, 5, 6, 20, 16, 11)
621	(6, 5, 6, 11, 12, 13)
641	(5, 6, 6, 15, 23, 27)
661	(5, 5, 6, 19, 19, 19)
681	(6, 6, 6, 15, 21, 24)
691	(5, 6, 5, 11, 10, 11)
701	(5, 6, 6, 17, 21, 10)
711	(6, 5, 5, 25, 27, 23)
721	(5, 6, 6, 19, 15, 14)
751	(5, 5, 6, 16, 15, 12)
771	(6, 6, 5, 11, 12, 14)
781	(5, 6, 5, 23, 24, 16)
791	(6, 6, 6, 30, 25, 29)
801	(6, 5, 6, 24, 28, 20)
811	(6, 5, 6, 28, 31, 23)
821	(6, 6, 5, 21, 19, 22)
841	(6, 6, 5, 20, 17, 19)
861	(5, 6, 5, 9, 8, 6)
871	(5, 5, 6, 8, 9, 6)
901	(6, 5, 6, 33, 40, 32)

general_info_GeneralSettings

{'distances': [1], 'verbose': True, 'additionalInfo': True, 'enableCExtensions': True, 'force2D': False, 'interpolator': 3, 'resampledPixelSpacing': [3, 3, 3], 'label': 1, 'normalizeScale': 1, 'normalize': False, 'force2Ddimension': 0, 'removeOutliers': None, 'minimumROISi
{'distances': [1], 'verbose': True, 'additionalInfo': True, 'enableCExtensions': True, 'force2D': False, 'interpolator': 3, 'resampledPixelSpacing': [3, 3, 3], 'label': 1, 'normalizeScale': 1, 'normalize': False, 'force2Ddimension': 0, 'removeOutliers': None, 'minimumROISi
{'distances': [1], 'verbose': True, 'additionalInfo': True, 'enableCExtensions': True, 'force2D': False, 'interpolator': 3, 'resampledPixelSpacing': [3, 3, 3], 'label': 1, 'normalizeScale': 1, 'normalize': False, 'force2Ddimension': 0, 'removeOutliers': None, 'minimumROISi
{'distances': [1], 'verbose': True, 'additionalInfo': True, 'enableCExtensions': True, 'force2D': False, 'interpolator': 3, 'resampledPixelSpacing': [3, 3, 3], 'label': 1, 'normalizeScale': 1, 'normalize': False, 'force2Ddimension': 0, 'removeOutliers': None, 'minimumROISi
{'distances': [1], 'verbose': True, 'additionalInfo': True, 'enableCExtensions': True, 'force2D': False, 'interpolator': 3, 'resampledPixelSpacing': [3, 3, 3], 'label': 1, 'normalizeScale': 1, 'normalize': False, 'force2Ddimension': 0, 'removeOutliers': None, 'minimumROISi
{'distances': [1], 'verbose': True, 'additionalInfo': True, 'enableCExtensions': True, 'force2D': False, 'interpolator': 3, 'resampledPixelSpacing': [3, 3, 3], 'label': 1, 'normalizeScale': 1, 'normalize': False, 'force2Ddimension': 0, 'removeOutliers': None, 'minimumROISi
{'distances': [1], 'verbose': True, 'additionalInfo': True, 'enableCExtensions': True, 'force2D': False, 'interpolator': 3, 'resampledPixelSpacing': [3, 3, 3], 'label': 1, 'normalizeScale': 1, 'normalize': False, 'force2Ddimension': 0, 'removeOutliers': None, 'minimumROISi
{'distances': [1], 'verbose': True, 'additionalInfo': True, 'enableCExtensions': True, 'force2D': False, 'interpolator': 3, 'resampledPixelSpacing': [3, 3, 3], 'label': 1, 'normalizeScale': 1, 'normalize': False, 'force2Ddimension': 0, 'removeOutliers': None, 'minimumROISi
{'distances': [1], 'verbose': True, 'additionalInfo': True, 'enableCExtensions': True, 'force2D': False, 'interpolator': 3, 'resampledPixelSpacing': [3, 3, 3], 'label': 1, 'normalizeScale': 1, 'normalize': False, 'force2Ddimension': 0, 'removeOutliers': None, 'minimumROISi
{'distances': [1], 'verbose': True, 'additionalInfo': True, 'enableCExtensions': True, 'force2D': False, 'interpolator': 3, 'resampledPixelSpacing': [3, 3, 3], 'label': 1, 'normalizeScale': 1, 'normalize': False, 'force2Ddimension': 0, 'removeOutliers': None, 'minimumROISi
{'distances': [1], 'verbose': True, 'additionalInfo': True, 'enableCExtensions': True, 'force2D': False, 'interpolator': 3, 'resampledPixelSpacing': [3, 3, 3], 'label': 1, 'normalizeScale': 1, 'normalize': False, 'force2Ddimension': 0, 'removeOutliers': None, 'minimumROISi
{'distances': [1], 'verbose': True, 'additionalInfo': True, 'enableCExtensions': True, 'force2D': False, 'interpolator': 3, 'resampledPixelSpacing': [3, 3, 3], 'label': 1, 'normalizeScale': 1, 'normalize': False, 'force2Ddimension': 0, 'removeOutliers': None, 'minimumROISi
{'distances': [1], 'verbose': True, 'additionalInfo': True, 'enableCExtensions': True, 'force2D': False, 'interpolator': 3, 'resampledPixelSpacing': [3, 3, 3], 'label': 1, 'normalizeScale': 1, 'normalize': False, 'force2Ddimension': 0, 'removeOutliers': None, 'minimumROISi
{'distances': [1], 'verbose': True, 'additionalInfo': True, 'enableCExtensions': True, 'force2D': False, 'interpolator': 3, 'resampledPixelSpacing': [3, 3, 3], 'label': 1, 'normalizeScale': 1, 'normalize': False, 'force2Ddimension': 0, 'removeOutliers': None, 'minimumROISi
{'distances': [1], 'verbose': True, 'additionalInfo': True, 'enableCExtensions': True, 'force2D': False, 'interpolator': 3, 'resampledPixelSpacing': [3, 3, 3], 'label': 1, 'normalizeScale': 1, 'normalize': False, 'force2Ddimension': 0, 'removeOutliers': None, 'minimumROISi
{'distances': [1], 'verbose': True, 'additionalInfo': True, 'enableCExtensions': True, 'force2D': False, 'interpolator': 3, 'resampledPixelSpacing': [3, 3, 3], 'label': 1, 'normalizeScale': 1, 'normalize': False, 'force2Ddimension': 0, 'removeOutliers': None, 'minimumROISi
{'distances': [1], 'verbose': True, 'additionalInfo': True, 'enableCExtensions': True, 'force2D': False, 'interpolator': 3, 'resampledPixelSpacing': [3, 3, 3], 'label': 1, 'normalizeScale': 1, 'normalize': False, 'force2Ddimension': 0, 'removeOutliers': None, 'minimumROISi
{'distances': [1], 'verbose': True, 'additionalInfo': True, 'enableCExtensions': True, 'force2D': False, 'interpolator': 3, 'resampledPixelSpacing': [3, 3, 3], 'label': 1, 'normalizeScale': 1, 'normalize': False, 'force2Ddimension': 0, 'removeOutliers': None, 'minimumROISi
{'distances': [1], 'verbose': True, 'additionalInfo': True, 'enableCExtensions': True, 'force2D': False, 'interpolator': 3, 'resampledPixelSpacing': [3, 3, 3], 'label': 1, 'normalizeScale': 1, 'normalize': False, 'force2Ddimension': 0, 'removeOutliers': None, 'minimumROISi
{'distances': [1], 'verbose': True, 'additionalInfo': True, 'enableCExtensions': True, 'force2D': False, 'interpolator': 3, 'resampledPixelSpacing': [3, 3, 3], 'label': 1, 'normalizeScale': 1, 'normalize': False, 'force2Ddimension': 0, 'removeOutliers': None, 'minimumROISi
{'distances': [1], 'verbose': True, 'additionalInfo': True, 'enableCExtensions': True, 'force2D': False, 'interpolator': 3, 'resampledPixelSpacing': [3, 3, 3], 'label': 1, 'normalizeScale': 1, 'normalize': False, 'force2Ddimension': 0, 'removeOutliers': None, 'minimumROISi
{'distances': [1], 'verbose': True, 'additionalInfo': True, 'enableCExtensions': True, 'force2D': False, 'interpolator': 3, 'resampledPixelSpacing': [3, 3, 3], 'label': 1, 'normalizeScale': 1, 'normalize': False, 'force2Ddimension': 0, 'removeOutliers': None, 'minimumROISi
{'distances': [1], 'verbose': True, 'additionalInfo': True, 'enableCExtensions': True, 'force2D': False, 'interpolator': 3, 'resampledPixelSpacing': [3, 3, 3], 'label': 1, 'normalizeScale': 1, 'normalize': False, 'force2Ddimension': 0, 'removeOutliers': None, 'minimumROISi
{'distances': [1], 'verbose': True, 'additionalInfo': True, 'enableCExtensions': True, 'force2D': False, 'interpolator': 3, 'resampledPixelSpacing': [3, 3, 3], 'label': 1, 'normalizeScale': 1, 'normalize': False, 'force2Ddimension': 0, 'removeOutliers': None, 'minimumROISi
{'distances': [1], 'verbose': True, 'additionalInfo': True, 'enableCExtensions': True, 'force2D': False, 'interpolator': 3, 'resampledPixelSpacing': [3, 3, 3], 'label': 1, 'normalizeScale': 1, 'normalize': False, 'force2Ddimension': 0, 'removeOutliers': None, 'minimumROISi
{'distances': [1], 'verbose': True, 'additionalInfo': True, 'enableCExtensions': True, 'force2D': False, 'interpolator': 3, 'resampledPixelSpacing': [3, 3, 3], 'label': 1, 'normalizeScale': 1, 'normalize': False, 'force2Ddimension': 0, 'removeOutliers': None, 'minimumROISi
{'distances': [1], 'verbose': True, 'additionalInfo': True, 'enableCExtensions': True, 'force2D': False, 'interpolator': 3, 'resampledPixelSpacing': [3, 3, 3], 'label': 1, 'normalizeScale': 1, 'normalize': False, 'force2Ddimension': 0, 'removeOutliers': None, 'minimumROISi
{'distances': [1], 'verbose': True, 'additionalInfo': True, 'enableCExtensions': True, 'force2D': False, 'interpolator': 3, 'resampledPixelSpacing': [3, 3, 3], 'label': 1, 'normalizeScale': 1, 'normalize': False, 'force2Ddimension': 0, 'removeOutliers': None, 'minimumROISi
{'distances': [1], 'verbose': True, 'additionalInfo': True, 'enableCExtensions': True, 'force2D': False, 'interpolator': 3, 'resampledPixelSpacing': [3, 3, 3], 'label': 1, 'normalizeScale': 1, 'normalize': False, 'force2Ddimension': 0, 'removeOutliers': None, 'minimumROISi
{'distances': [1], 'verbose': True, 'additionalInfo': True, 'enableCExtensions': True, 'force2D': False, 'interpolator': 3, 'resampledPixelSpacing': [3, 3, 3], 'label': 1, 'normalizeScale': 1, 'normalize': False, 'force2Ddimension': 0, 'removeOutliers': None, 'minimumROISi
{'distances': [1], 'verbose': True, 'additionalInfo': True, 'enableCExtensions': True, 'force2D': False, 'interpolator': 3, 'resampledPixelSpacing': [3, 3, 3], 'label': 1, 'normalizeScale': 1, 'normalize': False, 'force2Ddimension': 0, 'removeOutliers': None, 'minimumROISi
{'distances': [1], 'verbose': True, 'additionalInfo': True, 'enableCExtensions': True, 'force2D': False, 'interpolator': 3, 'resampledPixelSpacing': [3, 3, 3], 'label': 1, 'normalizeScale': 1, 'normalize': False, 'force2Ddimension': 0, 'removeOutliers': None, 'minimumROISi
{'distances': [1], 'verbose': True, 'additionalInfo': True, 'enableCExtensions': True, 'force2D': False, 'interpolator': 3, 'resampledPixelSpacing': [3, 3, 3], 'label': 1, 'normalizeScale': 1, 'normalize': False, 'force2Ddimension': 0, 'removeOutliers': None, 'minimumROISi
{'distances': [1], 'verbose': True, 'additionalInfo': True, 'enableCExtensions': True, 'force2D': False, 'interpolator': 3, 'resampledPixelSpacing': [3, 3, 3], 'label': 1, 'normalizeScale': 1, 'normalize': False, 'force2Ddimension': 0, 'removeOutliers': None, 'minimumROISi
{'distances': [1], 'verbose': True, 'additionalInfo': True, 'enableCExtensions': True, 'force2D': False, 'interpolator': 3, 'resampledPixelSpacing': [3, 3, 3], 'label': 1, 'normalizeScale': 1, 'normalize': False, 'force2Ddimension': 0, 'removeOutliers': None, 'minimumROISi
{'distances': [1], 'verbose': True, 'additionalInfo': True, 'enableCExtensions': True, 'force2D': False, 'interpolator': 3, 'resampledPixelSpacing': [3, 3, 3], 'label': 1, 'normalizeScale': 1, 'normalize': False, 'force2Ddimension': 0, 'removeOutliers': None, 'minimumROISi
{'distances': [1], 'verbose': True, 'additionalInfo': True, 'enableCExtensions': True, 'force2D': False, 'interpolator': 3, 'resampledPixelSpacing': [3, 3, 3], 'label': 1, 'normalizeScale': 1, 'normalize': False, 'force2Ddimension': 0, 'removeOutliers': None, 'minimumROISi
{'distances': [1], 'verbose': True, 'additionalInfo': True, 'enableCExtensions': True, 'force2D': False, 'interpolator': 3, 'resampledPixelSpacing': [3, 3, 3], 'label': 1, 'normalizeScale': 1, 'normalize': False, 'force2Ddimension': 0, 'removeOutliers': None, 'minimumROISi
{'distances': [1], 'verbose': True, 'additionalInfo': True, 'enableCExtensions': True, 'force2D': False, 'interpolator': 3, 'resampledPixelSpacing': [3, 3, 3], 'label': 1, 'normalizeScale': 1, 'normalize': False, 'force2Ddimension': 0, 'removeOutliers': None, 'minimumROISi
{'distances': [1], 'verbose': True, 'additionalInfo': True, 'enableCExtensions': True, 'force2D': False, 'interpolator': 3, 'resampledPixelSpacing': [3, 3, 3], 'label': 1, 'normalizeScale': 1, 'normalize': False, 'force2Ddimension': 0, 'removeOutliers': None, 'minimumROISi
{'distances': [1], 'verbose': True, 'additionalInfo': True, 'enableCExtensions': True, 'force2D': False, 'interpolator': 3, 'resampledPixelSpacing': [3, 3, 3], 'label': 1, 'normalizeScale': 1, 'normalize': False, 'force2Ddimension': 0, 'removeOutliers': None, 'minimumROISi
{'distances': [1], 'verbose': True, 'additionalInfo': True, 'enableCExtensions': True, 'force2D': False, 'interpolator': 3, 'resampledPixelSpacing': [3, 3, 3], 'label': 1, 'normalizeScale': 1, 'normalize': False, 'force2Ddimension': 0, 'removeOutliers': None, 'minimumROISi
{'distances': [1], 'verbose': True, 'additionalInfo': True, 'enableCExtensions': True, 'force2D': False, 'interpolator': 3, 'resampledPixelSpacing': [3, 3, 3], 'label': 1, 'normalizeScale': 1, 'normalize': False, 'force2Ddimension': 0, 'removeOutliers': None, 'minimumROISi
{'distances': [1], 'verbose': True, 'additionalInfo': True, 'enableCExtensions': True, 'force2D': False, 'interpolator': 3, 'resampledPixelSpacing': [3, 3, 3], 'label': 1, 'normalizeScale': 1, 'normalize': False, 'force2Ddimension': 0, 'removeOutliers': None, 'minimumROISi
{'distances': [1], 'verbose': True, 'additionalInfo': True, 'enableCExtensions': True, 'force2D': False, 'interpolator': 3, 'resampledPixelSpacing': [3, 3, 3], 'label': 1, 'normalizeScale': 1, 'normalize': False, 'force2Ddimension': 0, 'removeOutliers': None, 'minimumROISi
{'distances': [1], 'verbose': True, 'additionalInfo': True, 'enableCExtensions': True, 'force2D': False, 'interpolator': 3, 'resampledPixelSpacing': [3, 3, 3], 'label': 1, 'normalizeScale': 1, 'normalize': False, 'force2Ddimension': 0, 'removeOutliers': None, 'minimumROISi
{'distances': [1], 'verbose': True, 'additionalInfo': True, 'enableCExtensions': True, 'force2D': False, 'interpolator': 3, 'resampledPixelSpacing': [3, 3, 3], 'label': 1, 'normalizeScale': 1, 'normalize': False, 'force2Ddimension': 0, 'removeOutliers': None, 'minimumROISi
{'distances': [1], 'verbose': True, 'additionalInfo': True, 'enableCExtensions': True, 'force2D': False, 'interpolator': 3, 'resampledPixelSpacing': [3, 3, 3], 'label': 1, 'normalizeScale': 1, 'normalize': False, 'force2Ddimension': 0, 'removeOutliers': None, 'minimumROISi
{'distances': [1], 'verbose': True, 'additionalInfo': True, 'enableCExtensions': True, 'force2D': False, 'interpolator': 3, 'resampledPixelSpacing': [3, 3, 3], 'label': 1, 'normalizeScale': 1, 'normalize': False, 'force2Ddimension': 0, 'removeOutliers': None, 'minimumROISi
{'distances': [1], 'verbose': True, 'additionalInfo': True, 'enableCExtensions': True, 'force2D': False, 'interpolator': 3, 'resampledPixelSpacing': [3, 3, 3], 'label': 1, 'normalizeScale': 1, 'normalize': False, 'force2Ddimension': 0, 'removeOutliers': None, 'minimumROISi
{'distances': [1], 'verbose': True, 'additionalInfo': True, 'enableCExtensions': True, 'force2D': False, 'interpolator': 3, 'resampledPixelSpacing': [3, 3, 3], 'label': 1, 'normalizeScale': 1, 'normalize': False, 'force2Ddimension': 0, 'removeOutliers': None, 'minimumROISi
{'distances': [1], 'verbose': True, 'additionalInfo': True, 'enableCExtensions': True, 'force2D': False, 'interpolator': 3, 'resampledPixelSpacing': [3, 3, 3], 'label': 1, 'normalizeScale': 1, 'normalize': False, 'force2Ddimension': 0, 'removeOutliers': None, 'minimumROISi
{'distances': [1], 'verbose': True, 'additionalInfo': True, 'enableCExtensions': True, 'force2D': False, 'interpolator': 3, 'resampledPixelSpacing': [3, 3, 3], 'label': 1, 'normalizeScale': 1, 'normalize': False, 'force2Ddimension': 0, 'removeOutliers': None, 'minimumROISi
{'distances': [1], 'verbose': True, 'additionalInfo': True, 'enableCExtensions': True, 'force2D': False, 'interpolator': 3, 'resampledPixelSpacing': [3, 3, 3], 'label': 1, 'normalizeScale': 1, 'normalize': False, 'force2Ddimension': 0, 'removeOutliers': None, 'minimumROISi
{'distances': [1], 'verbose': True, 'additionalInfo': True, 'enableCExtensions': True, 'force2D': False, 'interpolator': 3, 'resampledPixelSpacing': [3, 3, 3], 'label': 1, 'normalizeScale': 1, 'normalize': False, 'force2Ddimension': 0, 'removeOutliers': None, 'minimumROISi
{'distances': [1], 'verbose': True, 'additionalInfo': True, 'enableCExtensions': True, 'force2D': False, 'interpolator': 3, 'resampledPixelSpacing': [3, 3, 3], 'label': 1, 'normalizeScale': 1, 'normalize': False, 'force2Ddimension': 0, 'removeOutliers': None, 'minimumROISi
{'distances': [1], 'verbose': True, 'additionalInfo': True, 'enableCExtensions': True, 'force2D': False, 'interpolator': 3, 'resampledPixelSpacing': [3, 3, 3], 'label': 1, 'normalizeScale': 1, 'normalize': False, 'force2Ddimension': 0, 'removeOutliers': None, 'minimumROISi
{'distances': [1], 'verbose': True, 'additionalInfo': True, 'enableCExtensions': True, 'force2D': False, 'interpolator': 3, 'resampledPixelSpacing': [3, 3, 3], 'label': 1, 'normalizeScale': 1, 'normalize': False, 'force2Ddimension': 0, 'removeOutliers': None, 'minimumROISi
{'distances': [1], 'verbose': True, 'additionalInfo': True, 'enableCExtensions': True, 'force2D': False, 'interpolator': 3, 'resampledPixelSpacing': [3, 3, 3], 'label': 1, 'normalizeScale': 1, 'normalize': False, 'force2Ddimension': 0, 'removeOutliers': None, 'minimumROISi
{'distances': [1], 'verbose': True, 'additionalInfo': True, 'enableCExtensions': True, 'force2D': False, 'interpolator': 3, 'resampledPixelSpacing': [3, 3, 3], 'label': 1, 'normalizeScale': 1, 'normalize': False, 'force2Ddimension': 0, 'removeOutliers': None, 'minimumROISi
{'distances': [1], 'verbose': True, 'additionalInfo': True, 'enableCExtensions': True, 'force2D': False, 'interpolator': 3, 'resampledPixelSpacing': [3, 3, 3], 'label': 1, 'normalizeScale': 1, 'normalize': False, 'force2Ddimension': 0, 'removeOutliers': None, 'minimumROISi
{'distances': [1], 'verbose': True, 'additionalInfo': True, 'enableCExtensions': True, 'force2D': False, 'interpolator': 3, 'resampledPixelSpacing': [3, 3, 3], 'label': 1, 'normalizeScale': 1, 'normalize': False, 'force2Ddimension': 0, 'removeOutliers': None, 'minimumROISi
{'distances': [1], 'verbose': True, 'additionalInfo': True, 'enableCExtensions': True, 'force2D': False, 'interpolator': 3, 'resampledPixelSpacing': [3, 3, 3], 'label': 1, 'normalizeScale': 1, 'normalize': False, 'force2Ddimension': 0, 'removeOutliers': None, 'minimumROISi
{'distances': [1], 'verbose': True, 'additionalInfo': True, 'enableCExtensions': True, 'force2D': False, 'interpolator': 3, 'resampledPixelSpacing': [3, 3, 3], 'label': 1, 'normalizeScale': 1, 'normalize': False, 'force2Ddimension': 0, 'removeOutliers': None, 'minimumROISi
{'distances': [1], 'verbose': True, 'additionalInfo': True, 'enableCExtensions': True, 'force2D': False, 'interpolator': 3, 'resampledPixelSpacing': [3, 3, 3], 'label': 1, 'normalizeScale': 1, 'normalize': False, 'force2Ddimension': 0, 'removeOutliers': None, 'minimumROISi
{'distances': [1], 'verbose': True, 'additionalInfo': True, 'enableCExtensions': True, 'force2D': False, 'interpolator': 3, 'resampledPixelSpacing': [3, 3, 3], 'label': 1, 'normalizeScale': 1, 'normalize': False, 'force2Ddimension': 0, 'removeOutliers': None, 'minimumROISi
{'distances': [1], 'verbose': True, 'additionalInfo': True, 'enableCExtensions': True, 'force2D': False, 'interpolator': 3, 'resampledPixelSpacing': [3, 3, 3], 'label': 1, 'normalizeScale': 1, 'normalize': False, 'force2Ddimension': 0, 'removeOutliers': None, 'minimumROISi
{'distances': [1], 'verbose': True, 'additionalInfo': True, 'enableCExtensions': True, 'force2D': False, 'interpolator': 3, 'resampledPixelSpacing': [3, 3, 3], 'label': 1, 'normalizeScale': 1, 'normalize': False, 'force2Ddimension': 0, 'removeOutliers': None, 'minimumROISi
{'distances': [1], 'verbose': True, 'additionalInfo': True, 'enableCExtensions': True, 'force2D': False, 'interpolator': 3, 'resampledPixelSpacing': [3, 3, 3], 'label': 1, 'normalizeScale': 1, 'normalize': False, 'force2Ddimension': 0, 'removeOutliers': None, 'minimumROISi
{'distances': [1], 'verbose': True, 'additionalInfo': True, 'enableCExtensions': True, 'force2D': False, 'interpolator': 3, 'resampledPixelSpacing': [3, 3, 3], 'label': 1, 'normalizeScale': 1, 'normalize': False, 'force2Ddimension': 0, 'removeOutliers': None, 'minimumROISi
{'distances': [1], 'verbose': True, 'additionalInfo': True, 'enableCExtensions': True, 'force2D': False, 'interpolator': 3, 'resampledPixelSpacing': [3, 3, 3], 'label': 1, 'normalizeScale': 1, 'normalize': False, 'force2Ddimension': 0, 'removeOutliers': None, 'minimumROISi
{'distances': [1], 'verbose': True, 'additionalInfo': True, 'enableCExtensions': True, 'force2D': False, 'interpolator': 3, 'resampledPixelSpacing': [3, 3, 3], 'label': 1, 'normalizeScale': 1, 'normalize': False, 'force2Ddimension': 0, 'removeOutliers': None, 'minimumROISi
{'distances': [1], 'verbose': True, 'additionalInfo': True, 'enableCExtensions': True, 'force2D': False, 'interpolator': 3, 'resampledPixelSpacing': [3, 3, 3], 'label': 1, 'normalizeScale': 1, 'normalize': False, 'force2Ddimension': 0, 'removeOutliers': None, 'minimumROISi
{'distances': [1], 'verbose': True, 'additionalInfo': True, 'enableCExtensions': True, 'force2D': False, 'interpolator': 3, 'resampledPixelSpacing': [3, 3, 3], 'label': 1, 'normalizeScale': 1, 'normalize': False, 'force2Ddimension': 0, 'removeOutliers': None, 'minimumROISi
{'distances': [1], 'verbose': True, 'additionalInfo': True, 'enableCExtensions': True, 'force2D': False, 'interpolator': 3, 'resampledPixelSpacing': [3, 3, 3], 'label': 1, 'normalizeScale': 1, 'normalize': False, 'force2Ddimension': 0, 'removeOutliers': None, 'minimumROISi
{'distances': [1], 'verbose': True, 'additionalInfo': True, 'enableCExtensions': True, 'force2D': False, 'interpolator': 3, 'resampledPixelSpacing': [3, 3, 3], 'label': 1, 'normalizeScale': 1, 'normalize': False, 'force2Ddimension': 0, 'removeOutliers': None, 'minimumROISi
{'distances': [1], 'verbose': True, 'additionalInfo': True, 'enableCExtensions': True, 'force2D': False, 'interpolator': 3, 'resampledPixelSpacing': [3, 3, 3], 'label': 1, 'normalizeScale': 1, 'normalize': False, 'force2Ddimension': 0, 'removeOutliers': None, 'minimumROISi
{'distances': [1], 'verbose': True, 'additionalInfo': True, 'enableCExtensions': True, 'force2D': False, 'interpolator': 3, 'resampledPixelSpacing': [3, 3, 3], 'label': 1, 'normalizeScale': 1, 'normalize': False, 'force2Ddimension': 0, 'removeOutliers': None, 'minimumROISi
{'distances': [1], 'verbose': True, 'additionalInfo': True, 'enableCExtensions': True, 'force2D': False, 'interpolator': 3, 'resampledPixelSpacing': [3, 3, 3], 'label': 1, 'normalizeScale': 1, 'normalize': False, 'force2Ddimension': 0, 'removeOutliers': None, 'minimumROISi
{'distances': [1], 'verbose': True, 'additionalInfo': True, 'enableCExtensions': True, 'force2D': False, 'interpolator': 3, 'resampledPixelSpacing': [3, 3, 3], 'label': 1, 'normalizeScale': 1, 'normalize': False, 'force2Ddimension': 0, 'removeOutliers': None, 'minimumROISi
{'distances': [1], 'verbose': True, 'additionalInfo': True, 'enableCExtensions': True, 'force2D': False, 'interpolator': 3, 'resampledPixelSpacing': [3, 3, 3], 'label': 1, 'normalizeScale': 1, 'normalize': False, 'force2Ddimension': 0, 'removeOutliers': None, 'minimumROISi
{'distances': [1], 'verbose': True, 'additionalInfo': True, 'enableCExtensions': True, 'force2D': False, 'interpolator': 3, 'resampledPixelSpacing': [3, 3, 3], 'label': 1, 'normalizeScale': 1, 'normalize': False, 'force2Ddimension': 0, 'removeOutliers': None, 'minimumROISi
{'distances': [1], 'verbose': True, 'additionalInfo': True, 'enableCExtensions': True, 'force2D': False, 'interpolator': 3, 'resampledPixelSpacing': [3, 3, 3], 'label': 1, 'normalizeScale': 1, 'normalize': False, 'force2Ddimension': 0, 'removeOutliers': None, 'minimumROISi
{'distances': [1], 'verbose': True, 'additionalInfo': True, 'enableCExtensions': True, 'force2D': False, 'interpolator': 3, 'resampledPixelSpacing': [3, 3, 3], 'label': 1, 'normalizeScale': 1, 'normalize': False, 'force2Ddimension': 0, 'removeOutliers': None, 'minimumROISi
{'distances': [1], 'verbose': True, 'additionalInfo': True, 'enableCExtensions': True, 'force2D': False, 'interpolator': 3, 'resampledPixelSpacing': [3, 3, 3], 'label': 1, 'normalizeScale': 1, 'normalize': False, 'force2Ddimension': 0, 'removeOutliers': None, 'minimumROISi
{'distances': [1], 'verbose': True, 'additionalInfo': True, 'enableCExtensions': True, 'force2D': False, 'interpolator': 3, 'resampledPixelSpacing': [3, 3, 3], 'label': 1, 'normalizeScale': 1, 'normalize': False, 'force2Ddimension': 0, 'removeOutliers': None, 'minimumROISi
{'distances': [1], 'verbose': True, 'additionalInfo': True, 'enableCExtensions': True, 'force2D': False, 'interpolator': 3, 'resampledPixelSpacing': [3, 3, 3], 'label': 1, 'normalizeScale': 1, 'normalize': False, 'force2Ddimension': 0, 'removeOutliers': None, 'minimumROISi
{'distances': [1], 'verbose': True, 'additionalInfo': True, 'enableCExtensions': True, 'force2D': False, 'interpolator': 3, 'resampledPixelSpacing': [3, 3, 3], 'label': 1, 'normalizeScale': 1, 'normalize': False, 'force2Ddimension': 0, 'removeOutliers': None, 'minimumROISi
{'distances': [1], 'verbose': True, 'additionalInfo': True, 'enableCExtensions': True, 'force2D': False, 'interpolator': 3, 'resampledPixelSpacing': [3, 3, 3], 'label': 1, 'normalizeScale': 1, 'normalize': False, 'force2Ddimension': 0, 'removeOutliers': None, 'minimumROISi
{'distances': [1], 'verbose': True, 'additionalInfo': True, 'enableCExtensions': True, 'force2D': False, 'interpolator': 3, 'resampledPixelSpacing': [3, 3, 3], 'label': 1, 'normalizeScale': 1, 'normalize': False, 'force2Ddimension': 0, 'removeOutliers': None, 'minimumROISi
{'distances': [1], 'verbose': True, 'additionalInfo': True, 'enableCExtensions': True, 'force2D': False, 'interpolator': 3, 'resampledPixelSpacing': [3, 3, 3], 'label': 1, 'normalizeScale': 1, 'normalize': False, 'force2Ddimension': 0, 'removeOutliers': None, 'minimumROISi
{'distances': [1], 'verbose': True, 'additionalInfo': True, 'enableCExtensions': True, 'force2D': False, 'interpolator': 3, 'resampledPixelSpacing': [3, 3, 3], 'label': 1, 'normalizeScale': 1, 'normalize': False, 'force2Ddimension': 0, 'removeOutliers': None, 'minimumROISi
{'distances': [1], 'verbose': True, 'additionalInfo': True, 'enableCExtensions': True, 'force2D': False, 'interpolator': 3, 'resampledPixelSpacing': [3, 3, 3], 'label': 1, 'normalizeScale': 1, 'normalize': False, 'force2Ddimension': 0, 'removeOutliers': None, 'minimumROISi
{'distances': [1], 'verbose': True, 'additionalInfo': True, 'enableCExtensions': True, 'force2D': False, 'interpolator': 3, 'resampledPixelSpacing': [3, 3, 3], 'label': 1, 'normalizeScale': 1, 'normalize': False, 'force2Ddimension': 0, 'removeOutliers': None, 'minimumROISi

general_info_ImageHash	general_info_ImageSpacing	general_info_InputImages	general_info_MaskHash	general_info_Version	general_info_VolumeNum
ed65c08c80974cb4fd975d41e064408072eb9fc5	(1.0, 1.0, 1.0)	{'LoG': {}, 'Original': {}, 'Wavelet': {}}	243fec6ba7126eb2e636d0ce84749a427561e549	1.1.1-beta1	1
65ad1ca0547df3f3064fed30066db1a82774efcf	(1.0, 1.0, 1.0)	{'LoG': {}, 'Original': {}, 'Wavelet': {}}	27d04867a6b490b0b38aa88de3b0bfafa72fac57	1.1.1-beta1	1
7da59d8ecc6ae1e315f9ebbade22cbdcbf329bff	(1.0, 1.0, 1.0)	{'LoG': {}, 'Original': {}, 'Wavelet': {}}	fa07ca4ab0633e99a1371ea4e9afb54e0bdc7cad	1.1.1-beta1	1
1ad8fc837aa7f23c48e4dbce3b8ae95447fca758	(1.0, 1.0, 1.0)	{'LoG': {}, 'Original': {}, 'Wavelet': {}}	a2dc8f36c124bdeb65c0c4dc581601bdbf74b290	1.1.1-beta1	1
314fd58bf855390c8419fda7f6ac787be3a080af	(1.0, 1.0, 1.0)	{'LoG': {}, 'Original': {}, 'Wavelet': {}}	6487ef64b57fcc0656e3d70b80b592bb65c43504	1.1.1-beta1	1
f7303295530dfe4c40be14bae21df46c9dac8f12	(1.0, 1.0, 1.0)	{'LoG': {}, 'Original': {}, 'Wavelet': {}}	8d9a0aa4623d3cf5ffc0c90dd071eb04441e5287	1.1.1-beta1	1
aa1d92a0b4bfd52bb01fba1ce0891d056f607af6	(1.0, 1.0, 1.0)	{'LoG': {}, 'Original': {}, 'Wavelet': {}}	a46f670eba28915ef7de0e2e6fc1fdf38b520bb8	1.1.1-beta1	1
ed7f9b7a46fb4f2c715cd36f3f6ca2aba60dbe72	(1.0, 1.0, 1.0)	{'LoG': {}, 'Original': {}, 'Wavelet': {}}	5eb2b658adc9c4d5b9e91627a0523c97bc96672f	1.1.1-beta1	1
a2885c5a291b9e749465e2a0ec811843c1356957	(1.0, 1.0, 1.0)	{'LoG': {}, 'Original': {}, 'Wavelet': {}}	345aee7609eb628d35fa6d38854412e60d7bc108	1.1.1-beta1	1
45186d25cd3d063f3db17aa6ad67e7f04c255dfd	(1.0, 1.0, 1.0)	{'LoG': {}, 'Original': {}, 'Wavelet': {}}	b90aca167688e33a9cdaf2b11e1c3657098d1b5d	1.1.1-beta1	1
73bb392ea6f27824996321b54d01efa59d755779	(1.0, 1.0, 1.0)	{'LoG': {}, 'Original': {}, 'Wavelet': {}}	b535cbd027dc61a849e577f4951a1bab31bc7983	1.1.1-beta1	1
df2a327f0fd4e4a0a788f198a38a51886ce4e7fe	(1.0, 1.0, 1.0)	{'LoG': {}, 'Original': {}, 'Wavelet': {}}	99370e0b8a35e570f9b1a9ca1abb0e20b35b3379	1.1.1-beta1	1
cb57eb7e4f3b9b6a9d2032f40065cf95023e077a	(1.0, 1.0, 1.0)	{'LoG': {}, 'Original': {}, 'Wavelet': {}}	ac118291bf8d966dfc2ec867778b55c97fa34bfc	1.1.1-beta1	1
8ef38ffe29df9cac94a667a31964ea2ca652e5bb	(1.0, 1.0, 1.0)	{'LoG': {}, 'Original': {}, 'Wavelet': {}}	2f0c19afa399d1773737d5e0b5b4f4e51f55ad42	1.1.1-beta1	1
89c8f005ffea423f2786070d8f173966baa33df8	(1.0, 1.0, 1.0)	{'LoG': {}, 'Original': {}, 'Wavelet': {}}	92a4b297f62da35f7be521853b7d20b67e652726	1.1.1-beta1	1
019cb604b3fbc1d0b89d44e8dcdeebed40cab556	(1.0, 1.0, 1.0)	{'LoG': {}, 'Original': {}, 'Wavelet': {}}	f3a5d31f8bb79db82cada99adbb39d8f6bb48909	1.1.1-beta1	1
e48cf6ca33cd38279eacb0fa088efc1e13829fcb	(1.0, 1.0, 1.0)	{'LoG': {}, 'Original': {}, 'Wavelet': {}}	c224d58c483a6882d95dd7130b4b2bd602c384e6	1.1.1-beta1	1
16dd0656efdc9e17c85f904f727385322aedba74	(1.0, 1.0, 1.0)	{'LoG': {}, 'Original': {}, 'Wavelet': {}}	12a782ec8c6c1fa8da71ea4b06bf837ccabb5bc8	1.1.1-beta1	1
16010807008a65eb9589d163a02dca4259c592eb	(1.0, 1.0, 1.0)	{'LoG': {}, 'Original': {}, 'Wavelet': {}}	d4635fdad1f14af654e25419d7282bb93442ca38	1.1.1-beta1	1
7f89bd9f0d38399c5078bdaf8c23dbb400da36d5	(1.0, 1.0, 1.0)	{'LoG': {}, 'Original': {}, 'Wavelet': {}}	feb5300687c209c9cd413f9d2bb4901e0dda7a8d	1.1.1-beta1	1
e4c2a9d419938e931e307ed2fef3aa78ae25b852	(1.0, 1.0, 1.0)	{'LoG': {}, 'Original': {}, 'Wavelet': {}}	2686fa3624edb49a258bb89e9a4dd8f751c5e9ef	1.1.1-beta1	1
b72f76ef35094b561f82869dbd9ce66688dfdc0d	(1.0, 1.0, 1.0)	{'LoG': {}, 'Original': {}, 'Wavelet': {}}	b5530652d580acaeb76493c51ffb0e13467d2e7a	1.1.1-beta1	1
598b8f00d15ac8a578c0f506c2fa81a09ca45dae	(1.0, 1.0, 1.0)	{'LoG': {}, 'Original': {}, 'Wavelet': {}}	3bf9a0f7da0e33d3d1fa373e8fe4b113dd7b43b4	1.1.1-beta1	1
585288372551a5a7b62426ba406e35cc42ba2307	(1.0, 1.0, 1.0)	{'LoG': {}, 'Original': {}, 'Wavelet': {}}	9b361628bbcc177c85c2170887806a1994bf9fae	1.1.1-beta1	1
88dfd708f1296121cc8bc50d5a588774b97fcaee	(1.0, 1.0, 1.0)	{'LoG': {}, 'Original': {}, 'Wavelet': {}}	5d6503911adbb54e37af55fc110e690317ce122a	1.1.1-beta1	1
0899aa8ed9ab2538553f22b4ca145c56d5b05c5d	(1.0, 1.0, 1.0)	{'LoG': {}, 'Original': {}, 'Wavelet': {}}	b36a32bf0bb41ca1d321cdee2df6c81d9288617f	1.1.1-beta1	1
75992e6c089e7148cd27cfcb442e09ecc06d0933	(1.0, 1.0, 1.0)	{'LoG': {}, 'Original': {}, 'Wavelet': {}}	981beb240773bf819116737f31d67f3443d857d0	1.1.1-beta1	1
4618b426281c2e2b2187e4f17e263612b29add3d	(1.0, 1.0, 1.0)	{'LoG': {}, 'Original': {}, 'Wavelet': {}}	cd5bddf7feb6863fac5b18bc99589a05903f2040	1.1.1-beta1	1
8fe9f25f5324cac50cf02b6ee52d869af931a01a	(1.0, 1.0, 1.0)	{'LoG': {}, 'Original': {}, 'Wavelet': {}}	b8c84b3004a99293c08cc27a9d573b6a16d2b829	1.1.1-beta1	1
c1d572ada6df0c9ad45d058195f67c5e18373f83	(1.0, 1.0, 1.0)	{'LoG': {}, 'Original': {}, 'Wavelet': {}}	95eed1e9e63d1e0f30bb600b0340367a6e43534d	1.1.1-beta1	1
10d556d912f25c09216bafa5a66952f7d81fe841	(1.0, 1.0, 1.0)	{'LoG': {}, 'Original': {}, 'Wavelet': {}}	3134aae3a9986e413bb39d375097893670b957d6	1.1.1-beta1	1
eb139341e5bdcb3228c5d66ca8cb59b543f7c121	(1.0, 1.0, 1.0)	{'LoG': {}, 'Original': {}, 'Wavelet': {}}	2443f6e46a58cceb45e84dee8c31b49f772ffc23	1.1.1-beta1	1
0f8bb6a679f462279fdc7030b67755129892e2a4	(1.0, 1.0, 1.0)	{'LoG': {}, 'Original': {}, 'Wavelet': {}}	42ebd28f04f48946728afbc8f18da7dcd806ece9	1.1.1-beta1	1
488004ba1d23d972f102431a393957dd51c2b9bb	(1.0, 1.0, 1.0)	{'LoG': {}, 'Original': {}, 'Wavelet': {}}	16ee9ec2d57c47172f2e64bc226a7a5595ed97c1	1.1.1-beta1	1
e8f2c5199d3012336ad8ba7b56d5e4f689a00ae8	(1.0, 1.0, 1.0)	{'LoG': {}, 'Original': {}, 'Wavelet': {}}	85abc8efb4773f16fb8845959f3f5bccffaeb981	1.1.1-beta1	1
a910d2fc98f00540b1f7953fcd1ae06a9ad06bc2	(1.0, 1.0, 1.0)	{'LoG': {}, 'Original': {}, 'Wavelet': {}}	22ef7c4410df45730ada00e7faa86152ffa78963	1.1.1-beta1	1
41230a7cf76b04c1c8be28f95e81055d7454f454	(1.0, 1.0, 1.0)	{'LoG': {}, 'Original': {}, 'Wavelet': {}}	db247351b88120bfcc0c8067a3d72ca72ec68a48	1.1.1-beta1	1
c9833ed94573172487d3a2392d8edbd9ce169831	(1.0, 1.0, 1.0)	{'LoG': {}, 'Original': {}, 'Wavelet': {}}	0434cbfb410cb6fbdf352d9743feccfc6fd92436	1.1.1-beta1	1
b3585b13296efcf884530d0066f687afb24148d1	(1.0, 1.0, 1.0)	{'LoG': {}, 'Original': {}, 'Wavelet': {}}	9341970133aec80ad86c17cf091fb6cb38d22a36	1.1.1-beta1	1
6baca2b585341fcdca50a5175b832a8667da594c	(1.0, 1.0, 1.0)	{'LoG': {}, 'Original': {}, 'Wavelet': {}}	ce434c1dac39fe89242d175a3209aeaf3b89e133	1.1.1-beta1	1
0040252eb47789872b18d46df6e5ebd5f36ed66a	(1.0, 1.0, 1.0)	{'LoG': {}, 'Original': {}, 'Wavelet': {}}	cb9df7cd6eac5389c82210a01dcc56fe2cef64ed	1.1.1-beta1	1
4c13260d78c556a515754d7d4d7105016427b20b	(1.0, 1.0, 1.0)	{'LoG': {}, 'Original': {}, 'Wavelet': {}}	96c9d6fafbbcb0ed3cf4ddde2dedd4684eb4ba19	1.1.1-beta1	3
0a3248d12a3ad904aff856fe2235bba407b54a68	(1.0, 1.0, 1.0)	{'LoG': {}, 'Original': {}, 'Wavelet': {}}	de579ac7e88b9de366fe951872be6443517e0276	1.1.1-beta1	1
300ec0022da2f22a510eeaf86521db72d2680cfe	(1.0, 1.0, 1.0)	{'LoG': {}, 'Original': {}, 'Wavelet': {}}	96a78ae2a49de36a379b19f8d3479bce1153b9b3	1.1.1-beta1	1
fa63779c19a9655e95a0cbbf52490f5fd7bb8592	(1.0, 1.0, 1.0)	{'LoG': {}, 'Original': {}, 'Wavelet': {}}	2ca948446f920b141fe1847b500a70bf040f5841	1.1.1-beta1	1
26420ffcbcae814458ecb352f9922a16cdcf7531	(1.0, 1.0, 1.0)	{'LoG': {}, 'Original': {}, 'Wavelet': {}}	021dd5bc402a5e3ab01086e45b31791aa697b1c1	1.1.1-beta1	1
afd835f657fc4009b364a8267baa496797ba8a7a	(1.0, 1.0, 1.0)	{'LoG': {}, 'Original': {}, 'Wavelet': {}}	ec5b4457a24076bebaa448e69c7a058e9d17938c	1.1.1-beta1	1
3ad47ff43e11e3e7c5d94798ba0a1dcc8d191316	(1.0, 1.0, 1.0)	{'LoG': {}, 'Original': {}, 'Wavelet': {}}	d8c4040915683d869b96a87f6d37a6e8a7292f7e	1.1.1-beta1	1
be465182b41363fb0a24c0f39e83be527145cef5	(1.0, 1.0, 1.0)	{'LoG': {}, 'Original': {}, 'Wavelet': {}}	90044d49612ec7c1f6d809405d57b0154f35fa95	1.1.1-beta1	1
5ccee5534ebee2035cdc08fb25c930f46d416e76	(1.0, 1.0, 1.0)	{'LoG': {}, 'Original': {}, 'Wavelet': {}}	899ca9d5008fbac0140269fa41ba645501dce3ed	1.1.1-beta1	1
6b086e9648536757c76fe9546dc229c2078bc31e	(1.0, 1.0, 1.0)	{'LoG': {}, 'Original': {}, 'Wavelet': {}}	f37f58045eba97417b5f45e8f5a13357a089f58f	1.1.1-beta1	1
deb3739483bf9882150472db24506ccdffc88835	(1.0, 1.0, 1.0)	{'LoG': {}, 'Original': {}, 'Wavelet': {}}	44eb9b6fd8152bc2fbf5e3272501e2b28875e8d6	1.1.1-beta1	2
ae2fb26d74deda60fcd9b5512d2d8c4cdc02f09b	(1.0, 1.0, 1.0)	{'LoG': {}, 'Original': {}, 'Wavelet': {}}	73b1f695458f08209cf374fb4d3923b7e5ab6448	1.1.1-beta1	1
e4c1f1b50a2622af66579a0cc705ed95224707ba	(1.0, 1.0, 1.0)	{'LoG': {}, 'Original': {}, 'Wavelet': {}}	bc3c8b4034149b483f7cc009872804211e843b5a	1.1.1-beta1	1
81301f5e7f57e66819369a50fb8580038cd4307f	(1.0, 1.0, 1.0)	{'LoG': {}, 'Original': {}, 'Wavelet': {}}	52774cd20c846489b5e9f70a27bcba245cf1525b	1.1.1-beta1	1
0988a7b4d40284e7aa00cdda8fdfd6e8170db508	(1.0, 1.0, 1.0)	{'LoG': {}, 'Original': {}, 'Wavelet': {}}	54f34b86dcf149c4c7079743f6af50593a683944	1.1.1-beta1	1
bba9fd993814f7c2d8700f76c15be963fa6a13f9	(1.0, 1.0, 1.0)	{'LoG': {}, 'Original': {}, 'Wavelet': {}}	22f208ec7015b71a07a1118f5f01d873b2f36289	1.1.1-beta1	1
d31fc0b625e1403fbcef8e23f7659a794f8d2a92	(1.0, 1.0, 1.0)	{'LoG': {}, 'Original': {}, 'Wavelet': {}}	b1b3a38ceec7a572d1f0f11bcc3049e7f0446771	1.1.1-beta1	1
ad39cbbd4fab07012ddc60e4825c63227300facc	(1.0, 1.0, 1.0)	{'LoG': {}, 'Original': {}, 'Wavelet': {}}	dea2ace08b2fb174f7d5213cc3f8592e418f377b	1.1.1-beta1	1
fbb72fe0721ecb66f8f14a631d06f617d8749d63	(1.0, 1.0, 1.0)	{'LoG': {}, 'Original': {}, 'Wavelet': {}}	73130206c6e42ec2fa68feea81c110a08dbfdcc6	1.1.1-beta1	1
1af4acb389de9db01f9f800373b5b5ee9f832914	(1.0, 1.0, 1.0)	{'LoG': {}, 'Original': {}, 'Wavelet': {}}	97721c344d170fe34c4b1cfca0e45345495d000e	1.1.1-beta1	1
934ce4d2a7a6080c14f4281770c6b6b8e7762379	(1.0, 1.0, 1.0)	{'LoG': {}, 'Original': {}, 'Wavelet': {}}	47e712a09010e36dc5400750acb03c7fd2d719c6	1.1.1-beta1	1
69b1efb62fc712b7b2c2e9f857250ca3efbaa6a1	(1.0, 1.0, 1.0)	{'LoG': {}, 'Original': {}, 'Wavelet': {}}	8c56ea5c0f9fbcaef95e096bb3dde5b7b45a26fe	1.1.1-beta1	1
6f2d3c3db98af76d85136544b1253c8130449a21	(1.0, 1.0, 1.0)	{'LoG': {}, 'Original': {}, 'Wavelet': {}}	48a1889b2f05b952b2b7f587b953498a71c59d76	1.1.1-beta1	1
cf2cdbe1128946803077cd3d274cbc70fc76011f	(1.0, 1.0, 1.0)	{'LoG': {}, 'Original': {}, 'Wavelet': {}}	2c5741bd03826c856af924e8dbfef335df6ae941	1.1.1-beta1	1
ef087ab4b8bf4d7fd7bef824258b7d926e5e1fe4	(1.0, 1.0, 1.0)	{'LoG': {}, 'Original': {}, 'Wavelet': {}}	cb83a715132017b6f1ec9ae43b34c437743c0334	1.1.1-beta1	1
054fba93d28e56e01c378e31bf15e0717dfbf45e	(1.0, 1.0, 1.0)	{'LoG': {}, 'Original': {}, 'Wavelet': {}}	5b2020da6c12d8e0b6006eeb953a6ebdfb72eefe	1.1.1-beta1	1
60e228850daf01015d767e6d5ac6f1d54dc80fbd	(1.0, 1.0, 1.0)	{'LoG': {}, 'Original': {}, 'Wavelet': {}}	b1b61d244aa172cc5ea41b2d3acc225c95620853	1.1.1-beta1	1
050ceb7940625f4865e6256707da469e33ecd8d7	(1.0, 1.0, 1.0)	{'LoG': {}, 'Original': {}, 'Wavelet': {}}	b3e6c5652b20101cca70c55993843d6b30162454	1.1.1-beta1	1
f218a4e109f512b25c86d77cc7e1ccf678b55635	(1.0, 1.0, 1.0)	{'LoG': {}, 'Original': {}, 'Wavelet': {}}	0e4293317d024c96be75d39210ae0e2b8a193fa5	1.1.1-beta1	1
8bf3c8a5059fea0ae370db32a372863938187979	(1.0, 1.0, 1.0)	{'LoG': {}, 'Original': {}, 'Wavelet': {}}	f6127cd81f5524b4e6832f7426dc79521f6dbd34	1.1.1-beta1	1
a4207feed93d67307a4d9c541fc4036fe9faf03f	(1.0, 1.0, 1.0)	{'LoG': {}, 'Original': {}, 'Wavelet': {}}	32217cfe7a2431db1bab76975be86bccb1d4082f	1.1.1-beta1	1
9a847ecddf2df6803b4253a70439f6719bd63f43	(1.0, 1.0, 1.0)	{'LoG': {}, 'Original': {}, 'Wavelet': {}}	58dea92e1a371493e9cd9fb33a76ba19abaf8591	1.1.1-beta1	1
2b60599e673e3162bc243e1fa284da7c7222285a	(1.0, 1.0, 1.0)	{'LoG': {}, 'Original': {}, 'Wavelet': {}}	418737f03cd7297fc1fd8c6f02fed8af7b842ef0	1.1.1-beta1	1
6871c8e2428a7f0df42ad9c0b267ce065b97fddd	(1.0, 1.0, 1.0)	{'LoG': {}, 'Original': {}, 'Wavelet': {}}	80393d04015b3b034149874852b4febf6d24aadd	1.1.1-beta1	1
78520834edfc54938eb50245deb0b7bda563bc0d	(1.0, 1.0, 1.0)	{'LoG': {}, 'Original': {}, 'Wavelet': {}}	d0e3defa24b66e349eced53cb423f8520bdc3fa5	1.1.1-beta1	1
19d2879a142774e357d38ac0e8fbb7492a584776	(1.0, 1.0, 1.0)	{'LoG': {}, 'Original': {}, 'Wavelet': {}}	b4a731f9b95f1038bb35f4540fa30598f88ff7bb	1.1.1-beta1	1
c5454410a0192ace0171bad6421cfd2e11cd6772	(1.0, 1.0, 1.0)	{'LoG': {}, 'Original': {}, 'Wavelet': {}}	5ee641890edf31b8cb20ebc1297620e28f7f8f13	1.1.1-beta1	1
e2e99cdda7f3e61d56e946a0b0bcc16d6fed45a6	(1.0, 1.0, 1.0)	{'LoG': {}, 'Original': {}, 'Wavelet': {}}	8c0f4016a383af7978b90ae653986bea577439c5	1.1.1-beta1	1
d6df8d2df93ef59b1bba950be2acb73a3f2f84c7	(1.0, 1.0, 1.0)	{'LoG': {}, 'Original': {}, 'Wavelet': {}}	bf65f36223f867da134b44a733713f0824b5f196	1.1.1-beta1	1
ed65c08c80974cb4fd975d41e064408072eb9fc5	(1.0, 1.0, 1.0)	{'LoG': {}, 'Original': {}, 'Wavelet': {}}	4f45b61838df1280d660eb029a5b9c47163f722e	1.1.1-beta1	1
b54fcec088350813b092acb7a2f155e1fbdb8574	(1.0, 1.0, 1.0)	{'LoG': {}, 'Original': {}, 'Wavelet': {}}	72b48726b31cac50a69e9c0a190d571cbc08a06f	1.1.1-beta1	1
f7bab373d06471ab6d846c8ae3aa0b8840feb195	(1.0, 1.0, 1.0)	{'LoG': {}, 'Original': {}, 'Wavelet': {}}	e961550f3450be0cba69f5a83edf4b868ef55150	1.1.1-beta1	1
8ea1a010ff3e496743303208f45e2b6a46e9638a	(1.0, 1.0, 1.0)	{'LoG': {}, 'Original': {}, 'Wavelet': {}}	eae9882a07414f296b9b4afea4ac6ec3de3664f1	1.1.1-beta1	2
6dff4408189ff68146aa5b8501b7c858b599c1a0	(1.0, 1.0, 1.0)	{'LoG': {}, 'Original': {}, 'Wavelet': {}}	d51ce09f6293f3ee34f54f1af5d81eec6e53dac7	1.1.1-beta1	1
c88b281b945d81f98496f84645bb20b68ffee8f9	(1.0, 1.0, 1.0)	{'LoG': {}, 'Original': {}, 'Wavelet': {}}	4e82526fe323a2a7a84e764cac7ca705347349e0	1.1.1-beta1	1
9e3af65703fe371a72314f30f8610cee46c07806	(1.0, 1.0, 1.0)	{'LoG': {}, 'Original': {}, 'Wavelet': {}}	212965589b47aeaeb4776915493d53d27fcf2153	1.1.1-beta1	1
6dc8bb58620b346a123fda450f59eb23c3f875b2	(1.0, 1.0, 1.0)	{'LoG': {}, 'Original': {}, 'Wavelet': {}}	3a124c3d03cb8851f8990d7e263ec5a4c4c289ab	1.1.1-beta1	1
8a6ee45d54882e971c721576805518b0223edd45	(1.0, 1.0, 1.0)	{'LoG': {}, 'Original': {}, 'Wavelet': {}}	fe3c59658f5810c410717836cbf81d5c4d8c29f6	1.1.1-beta1	1
62ec0bfda20ae2f050345ff4ab504a7f5d0ee9d6	(1.0, 1.0, 1.0)	{'LoG': {}, 'Original': {}, 'Wavelet': {}}	909949997bec2a21d8001b8002954e7dab65b117	1.1.1-beta1	1
0f7b3f928363b4c91b7257db151003acc51dc16d	(1.0, 1.0, 1.0)	{'LoG': {}, 'Original': {}, 'Wavelet': {}}	5f35405e39f131530047bd22021790382feff1db	1.1.1-beta1	1
21632cf06e539ea4939f3b3dd4814f74dfb8d133	(1.0, 1.0, 1.0)	{'LoG': {}, 'Original': {}, 'Wavelet': {}}	cf83d68ad49d08c76373b35480746bbcbf4a9cc4	1.1.1-beta1	1
285ebc6336a666cb2c34c76f74d2f91004ce6428	(1.0, 1.0, 1.0)	{'LoG': {}, 'Original': {}, 'Wavelet': {}}	168965e0d439305804a27b00301f4a050d8fb1c5	1.1.1-beta1	1
8652292d9b0a653fb4d837fd11c908a3093e7e67	(1.0, 1.0, 1.0)	{'LoG': {}, 'Original': {}, 'Wavelet': {}}	8ab7073489c0ed040f57254613e3bbf27d9154a2	1.1.1-beta1	1
38de3f3af0ee7c556478b9c3d7d7f92423576c6b	(1.0, 1.0, 1.0)	{'LoG': {}, 'Original': {}, 'Wavelet': {}}	47b8edc959d1af2196448740dc2f01201ded01a8	1.1.1-beta1	2
5a8ac6e079824e746b392f693cae4de3bf2660e8	(1.0, 1.0, 1.0)	{'LoG': {}, 'Original': {}, 'Wavelet': {}}	ed8e1d14175a406c0042ce15200e56588219a644	1.1.1-beta1	1
e7e5d7043754b07480d07b9ad918e2a543ea5b0d	(1.0, 1.0, 1.0)	{'LoG': {}, 'Original': {}, 'Wavelet': {}}	4e26309703d6efc75cd5cf344f0b9617a0acf24b	1.1.1-beta1	1
53184def7583c0f3c45375d7b7c7a6817f6be21a	(1.0, 1.0, 1.0)	{'LoG': {}, 'Original': {}, 'Wavelet': {}}	dcdd68f950b18d430f90b106a12606f5c934547a	1.1.1-beta1	1
d76dda1554fb0894e0ccc0d972f7c221c9e3eb53	(1.0, 1.0, 1.0)	{'LoG': {}, 'Original': {}, 'Wavelet': {}}	4e6595afe86030b2e11fafb0ad1cf39d3daf16bf	1.1.1-beta1	1
014c1dd75c7dc2418eda6e673725cd7aa7f1ef8e	(1.0, 1.0, 1.0)	{'LoG': {}, 'Original': {}, 'Wavelet': {}}	567c5407135f82c800e8fd371c3afb8f362e6d41	1.1.1-beta1	1

general_info_VoxelNum	original_shape_Maximum3DDiameter	original_shape_Compactness2	original_shape_Maximum2DDiameterSlice	original_shape_Sphericity	original_shape_Compactness1	original_shape_Elongation
1444	62.42595614	0.188028799	60.74537019	0.572894682	0.023004408	1.274639018
347	32.03123476	0.48302999	29.69848481	0.784617575	0.036871086	1.13152066
892	52.04805472	0.21526157	40.02499219	0.599315488	0.024613996	1.337818846
322	28.46049894	0.635955983	25.8069758	0.859954921	0.042307017	1.071285084
1411	52.13444159	0.221013514	49.92995093	0.604606683	0.02494068	1.048609197
242	27.82085549	0.60074919	24.73863375	0.84378357	0.041119277	1.07907888
10539	145.4303957	0.023021201	114.0394669	0.284474051	0.008049388	1.235655266
6068	111.5257818	0.048493423	97.67292358	0.364665166	0.011682621	1.095330676
4262	110.8377192	0.049583229	86.37708029	0.367376695	0.011813165	1.583869469
755	54.41507144	0.23555155	45	0.617582984	0.025747903	1.415600124
1556	117.3456433	0.065256094	72.24956747	0.402599926	0.013552188	2.964077023
3407	85.1704174	0.070044094	67.68308504	0.412215048	0.014040567	1.219211363
811	51.0881591	0.32015936	49.2036584	0.684103902	0.030018016	1.436221958
644	49.65883607	0.401838197	37.94733192	0.737933238	0.033629816	1.465084228
1575	54.5802162	0.171033557	48.46648326	0.555086216	0.021940147	1.041493484
5762	144.934468	0.024848551	110.063618	0.291810126	0.008362756	1.706871919
883	43.37049688	0.271949314	40.80441153	0.647882112	0.027665776	1.035025585
409	34.59768778	0.579004737	25.8069758	0.833477804	0.040368251	1.377841305
1094	55.39855594	0.227499894	51.26402247	0.61046448	0.025304017	1.474814297
9183	151.7300234	0.0282849	94.72592042	0.304685334	0.008922287	1.703061325
2078	65.7267069	0.136156247	50.28916384	0.514453182	0.019575716	1.317043087
496	40.91454509	0.383160576	40.80441153	0.726318221	0.032838952	1.33180789
558	36.74234614	0.307032823	36.12478374	0.674623712	0.029396206	1.09626075
3597	98.31581765	0.035515801	94.20191081	0.328705677	0.009997921	1.249688272
1228	60.9672043	0.172915181	57.70615219	0.557114388	0.022060504	1.566980814
2556	75.47847375	0.09988179	47.4341649	0.463975917	0.016766485	1.537017704
2004	96.23408959	0.158080922	61.84658438	0.540704296	0.021093012	2.161927775
2253	71.37226352	0.08779193	61.77378085	0.444445179	0.015719046	1.223436277
146	25.8069758	0.652490031	21.63330765	0.86734383	0.042853453	1.23928996
1610	57.39337941	0.129020787	53.16013544	0.505304574	0.019055867	1.035342049
4009	96.04686356	0.07256821	75.23961722	0.417108272	0.014291311	1.355498425
788	47.24404724	0.333521434	34.20526275	0.693491671	0.030638024	1.380398091
2335	82.1035931	0.111648683	75.95393341	0.481523923	0.01772661	1.239718373
2415	84.48076704	0.036054584	73.05477397	0.330359521	0.010073471	1.087114159
5133	123.7659081	0.058547978	74.09453421	0.388302899	0.012836742	1.948916678
1939	84.32081594	0.122208701	60.37383539	0.496250217	0.018545987	1.615596124
1512	90.09994451	0.205484101	64.76109943	0.590100626	0.0240485	1.742288113
5821	133.6936797	0.023807855	94.20191081	0.287678071	0.00818576	1.319914643
560	42	0.421462711	40.02499219	0.749755601	0.034441214	1.488714012
9447	158.006329	0.018848042	126.4634335	0.266126885	0.007283364	1.542010373
1340	51.8748494	0.24248504	45.69463864	0.623584029	0.026124101	1.165369609
5672	128.124939	0.029324349	100.8464179	0.308372845	0.009084752	1.405633616
2418	89.2972564	0.07227125	84.05355436	0.416538539	0.01426204	1.037587235
1420	67.14908786	0.19231789	56.92099788	0.57721804	0.023265303	1.791220059
1556	61.26173357	0.145365577	52.47856705	0.525799932	0.020226916	1.183153996
2401	85.01176389	0.083604768	80.04998438	0.437263959	0.015339613	1.414829681
5351	99.18165153	0.033118009	91.24143795	0.32113532	0.009654527	1.072018896
491	36.24913792	0.341260433	34.20526275	0.698814615	0.030991446	1.193726852
391	35.74912586	0.465625775	34.20526275	0.775078466	0.036200735	1.278526137
452	35.62302626	0.382531677	32.31098884	0.725920623	0.032811991	1.305217717
1287	95.67131231	0.083753354	68.41052551	0.437522847	0.015353238	1.207413776
2785	79.25906888	0.089207829	71.30918594	0.446821768	0.015845296	1.179953373
7976	126.1784451	0.032055362	112.0089282	0.317663193	0.009498373	1.200085789
1642	62.49799997	0.154903092	61.55485359	0.537056564	0.020879924	1.133214996
643	47.33920151	0.292185895	39	0.663569499	0.028676656	1.489295099
1176	49.47726751	0.204929774	44.59820624	0.589569516	0.024016041	1.030872462
4015	118.5284776	0.046183352	95.34149149	0.358780214	0.011400965	1.632933941
2067	58.86425061	0.138431978	52.39274759	0.517303568	0.019738634	1.031213411
763	49.02040392	0.340759225	39.11521443	0.698472332	0.03096868	1.417895741
10893	162.8312009	0.02059684	129.1394595	0.27411546	0.00761376	1.183244948
1329	54.66260147	0.160923413	53.41348144	0.543925907	0.021281807	1.019624141
2465	81	0.121865044	61.84658438	0.49578462	0.018519892	1.617290576
8234	169.5729931	0.044792788	100.623059	0.355142541	0.011228013	1.863013275
2096	73.11634564	0.163383323	57.62811814	0.546683427	0.021443849	1.418610335
677	43.47413024	0.279728347	33.54101966	0.654001623	0.028058672	1.312310756
3421	74.57881737	0.100983755	68.41052551	0.465675982	0.016858721	1.058438466
406	31.89043744	0.573807736	31.32091953	0.83097661	0.040186675	1.206005914
5085	118.8318139	0.050509633	97.16480844	0.36965059	0.011923012	1.407203451
7508	105.8536726	0.038657686	102	0.338126039	0.01043078	1.057509236
2124	71.56116265	0.110666913	71.30918594	0.480108357	0.017648499	1.256402581
492	48.37354649	0.299790912	46.86149806	0.669277391	0.029047456	1.420131173
4981	101.0247494	0.033191539	76.4852927	0.321372808	0.009665238	1.195124072
1335	51.35172831	0.200029512	47.4341649	0.584832311	0.023727169	1.184459574
950	65.03845017	0.089057855	43.26661531	0.446571233	0.015831971	1.480875065
4084	111.7676161	0.056902404	71.56116265	0.384630339	0.012655059	1.914745441
918	51.7880295	0.158903143	49.47726751	0.541640125	0.021147796	1.240309935
2209	64.89992296	0.130141542	64.20280368	0.506763487	0.019138454	1.089890765
323	29.69848481	0.646423207	26.83281573	0.86464728	0.042653763	1.182980032
1315	64.89992296	0.197273063	64.62197769	0.582133505	0.023563119	1.499156174
1800	59.16924877	0.110542947	48.46648326	0.479929022	0.017638612	1.162492167
1406	62.42595614	0.189094698	60.74537019	0.573975186	0.02306952	1.262423578
826	43.37049688	0.242394274	36.61966685	0.623506213	0.026119211	1.200549231
2282	86.74099377	0.055601465	70.22819946	0.381676491	0.012509559	1.344188118
1893	62.92853089	0.1492651	57.70615219	0.530460144	0.02049642	1.122928534
2715	78.97467949	0.105575408	59.09314681	0.472629606	0.017237737	1.213126276
531	36	0.381778963	31.32091953	0.725444175	0.032779692	1.081173951
1308	66.06814664	0.160885422	63.5688603	0.5438831	0.021279294	1.409926622
3836	80.94442538	0.062056996	79.42921377	0.395910406	0.013215824	1.068544171
1701	69.19537557	0.13498167	59.16924877	0.512969566	0.019491097	1.374584471
1367	57.78408085	0.189923583	53.41348144	0.574812625	0.023120026	1.11528916
804	49.02040392	0.382381623	36.12478374	0.725825693	0.032805555	1.296257018
2532	89.4483091	0.110418796	79.88116173	0.479749284	0.017628704	1.202462118
4501	98.81801455	0.050597957	89.49860334	0.369865929	0.011933432	1.445037353
3446	88.28363382	0.052434964	87.46427842	0.374288936	0.012148128	1.283311413
3307	107.0794098	0.083469346	76.4852927	0.43702774	0.015327184	1.442552764
2384	72.24956747	0.150122074	68.01470429	0.531473382	0.020555174	1.301479749
2452	68.60757976	0.124686287	57.31491952	0.499581365	0.018733038	1.130380894
313	31.32091953	0.62677186	30	0.855795168	0.042000419	1.256287272
268	28.46049894	0.628199826	25.8069758	0.856444591	0.042048236	1.15850577
8824	127.7732366	0.035861078	116.498927	0.329767445	0.010046402	1.285794048

original_shape_SurfaceVolumeRatio	original_shape_Volume	original_shape_SphericalDisproportion	original_shape_Flatness	original_shape_SurfaceArea	original_shape_Maximum2DDiameterColumn
0.248942482	38988	1.745521526	1.32131834	9705.769469	57.70615219
0.292366996	9369	1.274506245	1.26310296	2739.186389	26.83281573
0.279416737	24084	1.668570261	1.133980807	6729.472701	41.67733197
0.27348608	8694	1.162851652	1.100408429	2377.687981	25.8069758
0.237710075	38097	1.653967825	1.052779924	9056.040722	45.79301257
0.306567903	6534	1.185138032	1.251817147	2003.114677	27.65863337
0.258456273	284553	3.51525911	1.257381277	73544.50785	123.3288287
0.24235579	163836	2.742241633	1.50733222	39706.60316	98.27003612
0.270632605	115074	2.722001732	1.24148674	31142.77643	79.25906888
0.286649968	20385	1.619215596	1.758793889	5843.359594	43.68065934
0.345530176	42012	2.483855397	1.182684101	14516.41375	50.91168825
0.259885261	91989	2.425918233	1.171926938	23906.58532	83.57032966
0.252677857	21897	1.461766257	1.117120229	5532.887024	36.61966685
0.252959217	17388	1.355136141	1.460836537	4398.454867	36.49657518
0.249598655	42525	1.801521947	1.171970989	10614.18281	51.26402247
0.308132916	155574	3.426885881	1.270073914	47937.47028	83.57032966
0.259346254	23841	1.543490678	1.25442987	6183.074043	40.80441153
0.26055199	11043	1.199791998	1.116048744	2877.275627	32.44996148
0.256269367	29538	1.638096946	1.141003613	7569.684565	38.41874542
0.252648345	247941	3.282074613	1.17721487	62641.88337	135.8307771
0.245547301	56106	1.943811479	1.134659952	13776.67686	64.62197769
0.280376777	13392	1.376806985	1.299111368	3754.805791	28.3019434
0.290239512	15066	1.482307814	1.278214987	4372.748484	33.54101966
0.320067948	97119	3.042235258	1.51149395	31084.67906	84.05355436
0.270200225	33156	1.794963514	1.144517705	8958.758653	38.41874542
0.254105353	69012	2.155284278	1.068473667	17536.31863	71.11961755
0.236466965	54108	1.849439718	1.25053173	12794.75455	54.08326913
0.276667157	60831	2.249996283	1.223465879	16829.9398	66.40783086
0.352956045	3942	1.152945308	1.289852239	1391.352729	22.84731932
0.27218719	43470	1.97900445	1.421781259	11831.97715	53.66563146
0.243277558	108243	2.397459044	1.217621403	26333.09272	71.30918594
0.25165924	21276	1.441978385	1.424368985	5354.301993	42.95346319
0.252338029	63045	2.076740017	1.812764415	15908.65103	64.62197769
0.36369472	65205	3.027005233	1.343811122	23714.71424	68.41052551
0.240658838	138591	2.575309126	1.210229159	33353.149	109.2016483
0.260497099	52353	2.015112469	1.136831998	13637.8046	51.26402247
0.238005064	40824	1.694626232	2.674490328	9716.318733	64.76109943
0.311499183	157167	3.476107842	1.600708637	48957.39204	128.1600562
0.260843774	15120	1.333767962	1.221889564	3943.957863	40.80441153
0.28653399	255069	3.757606079	1.241249781	73085.93835	119.4738465
0.23447666	36180	1.603633117	1.125187575	8483.365576	49.92995093
0.293117221	153144	3.242827686	1.328047882	44889.1437	98.4073168
0.288329391	65286	2.400738244	2.626425359	18823.87264	80.77747211
0.248462124	38340	1.732447587	1.111208887	9526.037819	44.59820624
0.264569116	42012	1.901864072	1.269469178	11115.07771	57.62811814
0.275309882	64827	2.286948144	1.173896748	17847.51372	55.31726674
0.286987609	144477	3.113952086	1.073743921	41463.10872	91.24143795
0.292397548	13257	1.430994685	1.161053059	3876.314295	34.20526275
0.284418787	10557	1.290191954	1.520580678	3002.609133	34.20526275
0.289352734	12204	1.377561083	1.150941425	3531.260764	33.54101966
0.338716187	34749	2.285594928	1.95592319	11770.04877	65.7951366
0.256420936	75195	2.238028832	1.285185003	19281.57225	77.82673063
0.253980822	215352	3.147988255	1.313298895	54695.278	96.60745313
0.254420317	44334	1.862001263	1.649297111	11279.47035	53.41348144
0.28145316	17361	1.507001152	1.139818311	4886.308319	29.54657341
0.259035175	31752	1.696152824	1.19335891	8224.884881	44.59820624
0.282687021	108405	2.787221707	1.234972838	30644.68649	81.88406438
0.244626726	55809	1.933100912	1.221521085	13652.37296	55.15432893
0.252564409	20601	1.431695938	1.309090713	5203.079383	46.86149806
0.265285495	294111	3.648097771	1.456589618	78023.38214	133.1540461
0.269555459	35883	1.838485696	1.247840701	9672.458535	48.37354649
0.240693398	66555	2.017004883	1.093114314	16019.34911	55.15432893
0.224779423	222318	2.815770811	1.465810331	49972.51169	106.5316854
0.230407459	56592	1.82921221	1.059523994	13039.21895	63.5688603
0.280707804	18279	1.529048193	1.287184813	5131.057958	40.24922359
0.229735445	92367	2.147415884	1.196432573	21219.97386	71.30918594
0.261978344	10962	1.203403306	1.152530159	2871.806605	25.8069758
0.253595269	137295	2.705257413	1.363896662	34817.3624	109.4897255
0.243468529	202716	2.957477051	1.135491109	49354.96632	103.3150521
0.261199355	57348	2.08286314	1.16647844	14979.26061	66.2721661
0.305094947	13284	1.494148784	1.595150367	4052.881273	36.24913792
0.293707404	134487	3.111650935	1.081495633	39499.82762	96.04686356
0.250325114	36045	1.709891846	1.071876137	9022.96873	44.59820624
0.367195704	25650	2.239284411	1.393779454	9418.569805	57.70615219
0.262194808	110268	2.599898911	1.220040302	28911.69708	88.84255737
0.306223005	24786	1.846244314	1.785407744	7590.043393	51.26402247
0.244244981	59643	1.973307127	1.309312202	14567.50343	59.09314681
0.271720903	8721	1.156540965	1.145741282	2369.677995	28.3019434
0.252754185	35505	1.717819007	1.224322998	8974.037353	60.29925373
0.276118193	48600	2.083641445	1.11941916	13419.34416	55.80322571
0.250692483	37962	1.742235596	1.31219352	9516.788048	57.70615219
0.275546819	22302	1.603833257	1.043489467	6145.245163	40.24922359
0.320795976	61614	2.620019897	1.793850284	19765.52329	80.04998438
0.245655546	51111	1.885155769	1.244068553	12555.70061	55.31726674
0.244484878	73305	2.115821749	1.439215601	17921.96398	76.4852927
0.274406271	14337	1.37846582	1.042969389	3934.162701	33.9411255
0.271011711	35316	1.838630396	1.383309498	9571.049568	49.47726751
0.260099641	103572	2.525823987	1.144462745	26939.04007	78.23042886
0.263250881	45927	1.949433392	1.141741939	12090.3232	64.89992296
0.252685546	36909	1.739697348	1.1135441	9326.370802	48.46648326
0.238842633	21708	1.377741254	1.1717533	5184.795869	44.29446918
0.246524811	68364	2.084422078	1.837381731	16853.42215	68.41052551
0.263966535	121527	2.703682394	1.077919052	32079.06111	92.66067127
0.28513517	93042	2.671732724	1.256148118	26529.54648	69.26037828
0.247576338	89289	2.288184271	1.445319773	22105.84361	91.24143795
0.227045344	64368	1.881561775	1.169336941	14614.45473	64.76109943
0.239285556	66204	2.001675943	1.449681626	15841.66095	65.7951366
0.277424574	8451	1.16850391	1.197876656	2344.515072	25.8069758
0.291934441	7236	1.16761786	1.200441278	2112.437617	24.18677324
0.236555613	238248	3.032440031	1.241176171	56358.90172	114.0394669

original_shape_Maximum2DDiameterRow	log.sigma.5.0.mm.3D_gldm_GrayLevelVariance	log.sigma.5.0.mm.3D_gldm_HighGrayLevelEmphasis	log.sigma.5.0.mm.3D_gldm_GrayLevelNonUniformityNormalized
51.6139516	37.88614076	303.0526316	0.045254602
25.8069758	40.09241834	379.0979827	0.047396789
48.83646179	36.33177195	353.8665919	0.046140079
23.43074903	42.02361984	339.8540373	0.047201111
46.57252409	31.3831689	204.0885897	0.049803583
23.43074903	38.52272727	295.9793388	0.045522847
138.9748179	17.2872166	138.3206187	0.083364577
106.4048871	27.10866399	256.250824	0.057550681
102.6157883	22.09600607	158.7587987	0.068301696
53.07541804	25.0746476	188.1695364	0.05508004
116.7304588	10.61030359	95.80462725	0.093919549
82.54089895	17.68184272	148.9917816	0.075893835
37.10795063	39.29719136	335.298397	0.046902866
45.79301257	45.01377985	333.5388199	0.043439682
49.92995093	25.81329302	159.1974603	0.057378685
134.1640786	24.30438184	181.7280458	0.063243111
42.42640687	12.79587374	99.81540204	0.077804099
32.44996148	46.3279153	333.7506112	0.042963636
48.46648326	11.37627294	95.24771481	0.092831432
121.823643	24.37555322	719.8000653	0.060727469
62.64183905	29.14847071	207.033205	0.052120339
39.11521443	26.65322581	282.6532258	0.061532583
36.61966685	27.19681145	223.6989247	0.054842564
85.38149682	11.71587403	128.1061996	0.102021276
57.70615219	33.97288831	328.9283388	0.04998992
72.49827584	31.584952	242.6185446	0.05191167
64.89992296	31.375126	282.8602794	0.050803582
65.520989	17.79408587	152.3870395	0.066029631
17.49285568	10.73616063	124.0273973	0.090542316
53.66563146	21.50397786	177.1024845	0.061630338
89.49860334	26.51619227	190.1187329	0.055434079
45.79301257	32.33724619	312.8997462	0.050223531
77.12976079	28.40357909	235.640257	0.055619678
82.54089895	21.15027901	205.5925466	0.062224108
105.5130324	23.19527758	188.3826222	0.061356159
80.72174428	30.76574671	291.1578133	0.050767331
83.40863265	35.27884858	389.3756614	0.049589177
132.849539	26.27870171	248.0793678	0.071023554
29.54657341	18.20956314	175.3089286	0.069387755
155.2191998	17.59284268	332.9926961	0.075512599
45.79301257	32.25118011	225.5649254	0.048631098
112.6099463	9.138126087	173.942701	0.115255376
84.69356528	39.65525652	327.7841191	0.044370146
66.06814664	31.9596826	210.1633803	0.049104344
51	16.79912074	165.2442159	0.084112582
80.77747211	29.18107702	234.5618492	0.051194655
91.43850392	18.05502766	215.5176603	0.06739711
30	15.51847719	124.0855397	0.075397895
28.3019434	28.33631387	170.0741688	0.056952793
25.8069758	27.0099998	255.0066372	0.055172684
84.95881355	31.47654538	195.9417249	0.051916572
74.09453421	29.25433906	315.4775583	0.052337187
125.2836781	10.60142105	134.2582748	0.118383014
55.80322571	17.04147559	222.5499391	0.07264024
45.79301257	49.7408967	390.2954899	0.040718444
42.95346319	32.57579018	211.079932	0.047782463
107.3312629	22.55443296	185.4966376	0.061547962
56.60388679	28.06260379	230.2883406	0.051981643
38.18376618	24.56439281	264.4285714	0.057229086
159.0282994	14.51440484	196.4138438	0.092892516
50.91168825	23.75425437	207.2558315	0.059170294
78.23042886	36.59026517	367.5054767	0.046468161
138.0326048	30.55186831	389.639179	0.052198893
65.7951366	31.26858661	296.4642176	0.05226695
40.36087214	20.22742179	151.4771049	0.060792575
70.29224708	31.17516268	357.378252	0.05052045
31.32091953	31.59465772	298.1847291	0.056346915
118.6802427	25.99001958	256.0088496	0.058086937
98.68130522	17.64324561	186.4286095	0.069013865
60	24.7036044	257.6252354	0.056314082
31.32091953	28.34561108	175.7804878	0.053423888
93.77099765	26.83038123	221.7918089	0.060853246
47.4341649	36.39093493	212.693633	0.046766822
64.41273166	20.19825485	136.2705263	0.062255956
111.6064514	21.18562034	211.7526934	0.072564179
40.24922359	14.69914349	101.1405229	0.071646233
63.28506933	25.30871875	256.3377094	0.055236603
25.63201124	41.83590373	482.7058824	0.053091662
42.95346319	25.2917891	252.3726236	0.055334037
55.80322571	15.20222222	167.3133333	0.073364815
51.6139516	37.68899848	298.559744	0.045408926
41.67733197	26.50349272	285.027845	0.056144434
85.90692638	23.87143007	161.5788782	0.058190418
61.77378085	16.5868871	160.0295827	0.069780538
75.23961722	23.62192227	195.6906077	0.065466866
32.31098884	34.33189696	512.3841808	0.066197098
60.29925373	23.44292474	203.9220183	0.05717579
80.04998438	20.92263968	172.7145464	0.080019186
44.59820624	17.92267301	174.7095826	0.06906509
51.0881591	32.18676195	212.0841258	0.049488706
45.79301257	34.17024331	440.3631841	0.051601446
74.09453421	18.37615707	229.0616114	0.072721612
86.37708029	17.97550629	235.6445234	0.066777182
78.74642849	23.6587362	194.9677887	0.059223998
88.84255737	14.17859213	160.1155125	0.072893823
71.11961755	43.47002387	313.0687919	0.041783028
63.28506933	28.43668234	184.9416803	0.051431531
24.18677324	25.61355123	261.1821086	0.060743705
26.83281573	15.98144074	170.0932836	0.076603921
126.4634335	26.09250773	328.7081822	0.055206058

log.sigma.5.0.mm.3D_gldm_DependenceEntropy	log.sigma.5.0.mm.3D_gldm_DependenceNonUniformity	log.sigma.5.0.mm.3D_gldm_GrayLevelNonUniformity	log.sigma.5.0.mm.3D_gldm_SmallDependenceEmphasis
6.867788829	308.1204986	65.34764543	0.308164884
6.238583976	87.81268012	16.44668588	0.361809158
6.700300135	197.6188341	41.15695067	0.304520653
6.135573954	93.35403727	15.19875776	0.380543021
6.701157409	327.6888731	70.27285613	0.257206811
6.142387893	61.65289256	11.01652893	0.329066804
7.331958457	1050.372426	878.579277	0.148982107
7.188464403	965.2949901	349.2175346	0.236038343
7.064923935	595.2336931	291.1018301	0.193212459
6.580338774	147.392053	41.58543046	0.243613921
6.558365862	218.9935733	146.1388175	0.138859063
7.145659575	408.7681244	258.5702964	0.176240077
6.659869505	188.4229346	38.03822441	0.324379302
6.459293534	167.8726708	27.97515528	0.397122153
6.788185867	285.855873	90.37142857	0.279388371
7.32197462	745.0770566	364.4068032	0.205358947
6.42511379	145.7474519	68.70101925	0.162145655
6.30054406	121.2493888	17.57212714	0.369992529
6.664912243	136.9798903	101.5575868	0.165408082
7.12310833	1489.184689	557.6603506	0.205141945
6.717138719	449.0731473	108.3060635	0.250856113
6.397380692	103.7419355	30.52016129	0.27309595
6.35654054	129.4982079	30.60215054	0.2897737
6.922633227	377.5921601	366.970531	0.136250188
7.122589112	198.3843648	61.38762215	0.269253127
7.061899198	459.1048513	132.6862285	0.241781451
6.820026758	401.006986	101.8103792	0.296007535
6.799919192	361.6817577	148.7647581	0.199068684
5.758667261	21.4109589	13.21917808	0.189269741
6.554375613	325.2372671	99.22484472	0.257243354
6.986165606	719.2040409	222.2352208	0.231054595
6.500814176	188.8172589	39.57614213	0.309512358
7.037745266	394.3550321	129.8719486	0.23212009
6.927707331	380.7515528	150.2712215	0.202687437
7.053122116	781.9842198	314.941165	0.224514221
6.909037588	387.4610624	98.43785456	0.277346483
6.656385365	351.8809524	74.97883598	0.312323065
7.404142317	677.8002062	413.4281051	0.222206757
6.196739539	126.8607143	38.85714286	0.200449767
7.294350777	1085.225892	713.3675241	0.155427491
6.692558886	290.9402985	65.16567164	0.265846851
6.958664354	543.8882228	653.7284908	0.111320043
6.968440149	516.4681555	107.2870141	0.311200226
6.655228842	310.9295775	69.72816901	0.30603863
6.972627027	181.4807198	130.8791774	0.175874509
6.873192846	473.1682632	122.9183673	0.297547646
6.975612858	795.9871052	360.6419361	0.193057584
6.105671366	97.74949084	37.0203666	0.239230829
6.308085649	84.53452685	22.2685422	0.261083361
6.180543084	121.0530973	24.9380531	0.277131883
6.681065257	257.7583528	66.81662782	0.30291623
6.843035592	551.3798923	145.7590664	0.263018198
7.193843288	608.0230692	944.2229188	0.104436697
6.934814581	225.3398295	119.2752741	0.188207862
6.67110128	165.755832	26.18195956	0.384458877
6.593296086	278.7823129	56.19217687	0.283678565
7.113478596	601.1449564	247.1150685	0.210455221
6.644038553	453.2399613	107.4460571	0.250625044
6.529843626	158.1612058	43.66579292	0.263863188
7.397965881	981.477738	1011.878179	0.134765433
6.720695692	251.3205418	78.63732129	0.273063848
6.997027894	514.5667343	114.5440162	0.277212931
7.295544281	1317.465145	429.8056838	0.239370112
7.02458756	390.855916	109.5515267	0.241956913
6.371394677	141.0502216	41.15657312	0.262136754
6.941562727	689.0175387	172.8304589	0.254509703
6.10820452	114.9852217	22.87684729	0.330555556
6.953841703	878.2857424	295.3720747	0.223999277
7.051386166	1051.174214	518.1561002	0.160062565
6.692408007	433.8587571	119.6111111	0.233956934
6.323197074	112.9878049	26.28455285	0.28955477
7.111371625	785.9451917	303.1100181	0.23290636
6.588602593	326.694382	62.43370787	0.293574446
6.395692764	195.2021053	59.14315789	0.289822609
7.237164731	481.8682664	296.3521058	0.166410317
6.35261472	174.3572985	65.77124183	0.199334123
6.906720444	403.5875962	122.017655	0.221756047
6.144711142	94.93188854	17.14860681	0.371464568
6.693795723	261.695057	72.76425856	0.253256949
6.767581629	266.99	132.0566667	0.171155962
6.859819839	298.940256	63.84495021	0.308930246
6.495961013	175.7336562	46.37530266	0.267185
6.876272691	411.2340053	132.7905346	0.267565894
6.707894964	305.7321712	132.0945589	0.194524501
7.097563531	383.8581952	177.7425414	0.201204642
6.243135974	130.9623352	35.15065913	0.280642418
6.715761452	239.4174312	74.78593272	0.261223509
7.302313371	409.2445255	306.9535975	0.183207125
6.780838588	258.9952969	117.4797178	0.223695371
6.73609862	299.5135333	67.65106072	0.262029901
6.783140323	150.4701493	41.48756219	0.269752989
6.97513823	335.3309637	184.1311216	0.177008281
6.915070961	711.4825594	300.5640969	0.190768177
6.818035124	643.2112594	204.0858967	0.204396223
6.779148134	492.9770184	241.059873	0.162636525
6.953394036	538.7114094	99.61073826	0.321012143
6.860759535	469.5065253	126.1101142	0.245045669
6.175322839	71.56230032	19.01277955	0.287078565
5.788394572	67.73880597	20.52985075	0.211821589
7.159324382	1456.948323	487.1382593	0.223872387

log.sigma.5.0.mm.3D_gldm_DependenceNonUniformityNormalized	log.sigma.5.0.mm.3D_gldm_DependenceVariance	log.sigma.5.0.mm.3D_gldm_LargeDependenceEmphasis
0.213379847	2.338047974	10.15789474
0.253062479	1.332591418	7.178674352
0.221545778	1.703668885	9.076233184
0.28991937	1.056286409	6.111801242
0.232238748	1.7064876	9.746987952
0.254764019	1.241718462	7.47107438
0.099665284	22.67108755	56.94895151
0.159079596	7.393183451	21.30652604
0.139660651	10.70237985	29.49507274
0.195221262	2.283366519	11.91390728
0.140741371	6.383636442	25.99100257
0.119978904	10.33628257	32.31083064
0.232334075	2.016204439	9.024660912
0.260671849	1.252729447	6.534161491
0.181495792	4.855677904	15.56
0.129308757	14.2144203	35.35334953
0.165059402	3.233194261	17.79048698
0.296453273	1.031892444	6.124694377
0.125210137	7.759144946	28.28153565
0.162167558	5.523468557	19.46433627
0.216108348	2.029885477	10.77189605
0.209157128	2.192881764	10.78629032
0.232075641	1.603884842	8.946236559
0.10497419	12.67800098	41.34528774
0.161550786	8.285963246	21.39413681
0.179618486	4.768137691	16.32707355
0.200103286	2.939524145	11.70159681
0.160533403	4.645938965	18.54993342
0.146650403	5.040908238	21.15068493
0.202010725	2.288624667	11.46086957
0.179397366	4.664254741	16.51509105
0.239615811	1.587325105	8.474619289
0.168888665	4.538612401	16.83211991
0.157661098	5.592046946	20.0194617
0.152344481	6.085567125	20.18059614
0.1998252	4.043704307	13.58380609
0.232725498	1.605776364	8.555555556
0.11644051	22.49989303	49.33980416
0.22653699	1.886109694	11.69642857
0.114875187	10.66130131	34.48152853
0.217119626	2.012332368	10.37910448
0.095890025	16.25187184	51.59062059
0.213593116	2.351708752	10.13068652
0.218964491	1.846589962	9.297183099
0.116632853	10.40418382	32.82647815
0.19707133	3.461013485	12.50104123
0.148754832	6.193137191	21.79349654
0.199082466	2.335862221	11.95723014
0.216200836	1.704829246	10.01278772
0.267816587	1.234297909	8.026548673
0.20027844	2.817583522	11.35742036
0.197982008	2.374401207	11.50412926
0.076231578	25.05289011	75.37462387
0.137234975	5.933750321	22.75274056
0.25778512	1.393884131	6.916018663
0.23705979	1.585759984	8.943877551
0.149724771	7.324863021	22.74246575
0.219274292	2.036187337	10.68553459
0.207288605	2.413907302	11.3197903
0.090101693	30.30407463	72.92784357
0.189104998	3.329760774	12.97291196
0.208749182	3.353344223	12.36308316
0.160003054	6.435736019	19.83094486
0.186477059	3.628580065	14.36545802
0.20834597	2.561291125	11.54357459
0.201408225	2.37542626	11.57293189
0.283214832	1.044480575	6.729064039
0.172720893	3.780870685	15.62340216
0.140007221	5.835059119	23.71124134
0.204264952	2.306983945	11.92749529
0.22965001	1.549110979	8.922764228
0.157788635	6.771448267	20.78317607
0.244714893	1.535978903	8.547565543
0.2054759	2.097635457	10.30736842
0.117989291	10.24414715	32.86483839
0.189931698	2.409837622	13.66666667
0.182701492	3.130721865	14.32548665
0.293906776	1.02684776	6.120743034
0.199007648	2.213450534	11.51711027
0.148327778	4.7811	20.75444444
0.212617536	2.350952735	10.18776671
0.21275261	1.945077945	10.3874092
0.180207715	6.884683009	18.56178791
0.161506694	4.640813362	18.55414686
0.141384234	7.585381806	24.29134438
0.2466334	1.830238934	9.256120527
0.18304085	2.870645475	12.82110092
0.106685226	19.21345119	48.24296142
0.15226061	4.583136593	18.07936508
0.219102804	2.076996226	10.50694952
0.187151927	2.698887404	12.22885572
0.132437189	6.675342348	24.92969984
0.158072108	4.266692222	18.46323039
0.186654457	2.811975091	14.22170633
0.149070765	4.836344527	21.34593287
0.225969551	1.822175099	8.890100671
0.191479007	2.554634629	12.46737357
0.228633547	1.810103196	9.376996805
0.252756739	1.290487859	9.76119403
0.165112004	5.050835509	17.99093382

log.sigma.5.0.mm.3D_gldm_LargeDependenceLowGrayLevelEmphasis	log.sigma.5.0.mm.3D_gldm_SmallDependenceHighGrayLevelEmphasis	log.sigma.5.0.mm.3D_gldm_LargeDependenceHighGrayLevelEmphasis
0.078911297	104.2694586	2610.756233
0.053038445	139.8738881	2696.481268
0.047451769	115.1426659	3040.144619
0.068603579	137.5641303	1912.835404
0.192423734	54.51601209	1997.91708
0.100963199	101.3518365	2021.409091
0.584306272	26.76004395	7240.366164
0.136228369	74.08702762	4760.243902
0.36973969	43.25549382	3704.056312
0.1644822	54.31614645	1895.713907
0.454109744	16.52869534	2059.236504
0.421913607	34.2057352	3992.678309
0.073388564	104.5933084	3364.311961
0.190033733	144.6838833	1905.697205
0.3013902	60.31750767	1713.188571
0.345642929	44.2270032	5455.973794
0.453235059	19.07517432	1471.815402
0.100291026	128.1934821	2036.841076
0.631229899	21.85459924	2245.677331
0.030556185	153.7652524	13434.91953
0.180759372	53.86605087	2265.244947
0.107479061	78.74239812	2972.721774
0.125194084	69.58810814	1873.46595
0.474886565	23.54015214	4769.764248
0.099761928	99.22657409	6646.486971
0.156908699	70.38276709	3059.798513
0.090935537	101.9068954	2533.49501
0.254263805	37.64769766	2463.218819
0.248723722	23.42528018	2320.575342
0.171714303	53.14237155	1720.224224
0.221242749	53.36638181	2430.598653
0.059562505	97.07224177	2549.384518
0.158638822	65.319637	3106.222698
0.156299125	48.39472256	3875.354037
0.224665387	54.45209412	2857.436587
0.124300867	81.50841529	3686.487365
0.049152292	128.513544	3191.616402
0.266379012	67.64402216	11700.21474
0.185571955	36.46743771	2128.158929
0.126477627	61.18815571	10727.83106
0.160869364	67.75318274	1886.308209
0.366474277	24.58175331	8080.768865
0.080147397	112.6266159	2790.287014
0.186929239	74.57025314	1602.771831
0.30593981	37.09170792	4843.705656
0.133600003	80.68787838	2316.599334
0.160397462	49.26326271	4127.990095
0.415649351	32.6694081	1401.193483
0.461566076	57.95386658	1505.168798
0.070384126	70.54394174	2027.316372
0.209638768	76.58410824	1396.731158
0.061083598	91.92914604	3244.132496
0.676561991	18.72424138	9703.746991
0.144781454	49.97705304	4494.721681
0.127533825	147.3490034	2644.73717
0.143041909	65.03711519	1766.314626
0.252216988	48.62649445	3615.253051
0.109193403	65.15931986	2096.837929
0.084580362	75.16640399	2689.796855
0.448113826	31.7512486	13845.92325
0.123077314	64.91334218	2211.07374
0.054562198	100.5095562	4226.125355
0.077099208	103.6599294	6493.547607
0.105560814	77.51874407	4115.752385
0.207070458	45.10348581	1423.763663
0.052772275	95.02399009	3934.169249
0.062079639	103.2888663	1992.94335
0.115673711	70.46037412	3197.333137
0.209671409	34.4606129	4181.50293
0.070631819	57.00312775	3224.909605
0.213715396	58.86333316	1402.841463
0.192991453	62.36756771	4105.964465
0.197027319	69.76864645	1536.429963
0.220180503	47.84609426	1106.918947
0.249564858	47.83019582	6047.479922
0.537882906	22.95919963	1317.14488
0.112249844	57.47245953	3533.177909
0.025413176	167.7929033	3018.668731
0.078408247	66.61528466	2778.795437
0.21009578	33.96363235	3162.195556
0.082127722	103.2902978	2567.52845
0.05895492	67.91767198	3265.710654
0.43561963	53.35088098	2647.690184
0.216851976	39.02195923	2436.690967
0.231408272	49.0966051	3926.574586
0.024391301	131.7647868	5125.084746
0.160685702	59.15318469	2350.420489
0.482976988	43.41850306	6856.13634
0.179675591	45.57952175	2615.590241
0.18547001	61.12986796	2095.048281
0.042251977	117.4629854	5180.20398
0.165926465	51.27604284	5222.837283
0.120716232	50.87685352	3920.809598
0.161434264	47.84959058	2346.614916
0.250385699	30.1113295	2811.560327
0.093523524	107.5683645	2567.591443
0.225078534	50.63725006	2022.400082
0.065042342	77.62017681	2430.792332
0.123775759	34.41698444	1706.264925
0.076794203	75.27209961	5606.916478

log.sigma.5.0.mm.3D_gldm_SmallDependenceLowGrayLevelEmphasis	log.sigma.5.0.mm.3D_gldm_LowGrayLevelEmphasis	log.sigma.5.0.mm.3D_gldzm_DistanceZoneVariabilityNormalized
0.003057455	0.008125174	0.890635301
0.004929173	0.009640052	0.98726167
0.002995841	0.006774464	0.901144191
0.005284284	0.014847447	0.880013149
0.004864763	0.021164834	0.885291046
0.007098032	0.014030569	0.925346404
0.002366681	0.015492763	0.929076794
0.001628868	0.006998264	0.925527865
0.0023597	0.013536188	0.960514996
0.0043526	0.013699234	0.93052512
0.00312181	0.019483973	0.939971807
0.002571947	0.015857927	0.937298418
0.003793097	0.010155502	0.856776359
0.00350264	0.016512541	0.834710744
0.003132666	0.017958805	0.93956735
0.002445491	0.013231219	0.968624239
0.003591769	0.025187412	1
0.007901337	0.01644911	0.847942227
0.003754559	0.028786801	0.94035503
0.000444818	0.001707616	0.953793802
0.00433048	0.017840428	0.951011338
0.004124185	0.011474295	0.888038549
0.003981395	0.016450604	0.934654251
0.001636731	0.014198926	0.958193986
0.002309108	0.006949934	0.948884342
0.002548494	0.010666009	0.952968501
0.002304456	0.007859954	0.944719744
0.002805168	0.014432806	0.949450401
0.010259282	0.019271423	0.944489796
0.004156918	0.014066767	0.982907253
0.00246822	0.012919751	0.961412012
0.003810296	0.00868594	0.899653524
0.002239	0.00906201	0.889929596
0.001824267	0.008525853	0.969185633
0.002252807	0.010818046	0.941828186
0.002574443	0.010874258	0.828136836
0.002213259	0.006237856	0.700700178
0.001743911	0.007697787	0.94332011
0.003207679	0.017712826	0.876405709
0.000713809	0.00398143	0.955578512
0.003074281	0.012535392	0.891663364
0.000938076	0.007681786	0.947920046
0.002388534	0.007520569	0.944012075
0.004254496	0.015101503	0.908635762
0.002552063	0.012504913	0.887899654
0.002585034	0.009885855	0.937852141
0.001339736	0.007463978	0.964776432
0.006111467	0.032003765	0.985916201
0.004359234	0.032421562	0.879292404
0.004751837	0.011292673	0.963203125
0.003381296	0.013221945	0.987207353
0.002476943	0.006384719	0.918110194
0.001208545	0.011864385	0.928109372
0.001950448	0.007301698	0.933412604
0.004923532	0.016656633	0.898703594
0.004111814	0.014514243	0.948872472
0.001818825	0.011352227	0.965562759
0.002152712	0.009579882	0.947730035
0.003650779	0.008990935	0.871339811
0.001107761	0.008452994	0.942725074
0.004060341	0.010855085	0.945123875
0.001974104	0.005359373	0.908677375
0.001010273	0.003953881	0.887909878
0.002001679	0.008575067	0.866322119
0.005727842	0.020453136	0.963482964
0.001540034	0.005034059	0.897322449
0.00557258	0.012352189	0.868788927
0.001465165	0.007184315	0.890294813
0.001399741	0.009032814	0.96600708
0.002322535	0.007418334	0.944489796
0.007878303	0.022543294	0.977530992
0.002342289	0.011093697	0.943891358
0.004931808	0.018560998	0.948550796
0.005278044	0.019894279	0.987805337
0.001262204	0.009250108	0.813714664
0.005909796	0.035370534	0.973099174
0.002192548	0.009077995	0.939043474
0.005595719	0.007981047	0.771468144
0.003705559	0.009336079	0.917428947
0.002455909	0.011003246	0.96001666
0.003751844	0.009104196	0.894491036
0.003931013	0.008773863	0.977862553
0.003481106	0.02134862	0.975679363
0.002193944	0.012686384	0.956226331
0.002446587	0.011565182	0.909433621
0.003216407	0.005327593	0.91725347
0.002732874	0.012277302	0.886669529
0.001903388	0.012425991	0.975004006
0.002833311	0.011257982	0.968044961
0.005461415	0.020656977	0.942145021
0.0027611	0.005339194	0.788564389
0.001392322	0.007975434	0.939937504
0.001251094	0.006796872	0.947681813
0.002313606	0.011509864	0.961919125
0.001700716	0.010491612	0.957259693
0.002686824	0.009695207	0.841365838
0.005111122	0.017057886	0.882187755
0.006096546	0.011380085	0.91322314
0.00663823	0.017558167	0.971836735
0.001253629	0.004779316	0.923687558

log.sigma.5.0.mm.3D_gldzm_LowIntensityEmphasis	log.sigma.5.0.mm.3D_gldzm_LargeDistanceEmphasis	log.sigma.5.0.mm.3D_gldzm_HighIntensitySmallDistanceEmphasis
0.009725051	1.174157303	328.3515918
0.012987805	1.019230769	377.9166667
0.009198409	1.156441718	367.8519939
0.016154731	1.192307692	349.974359
0.02056509	1.183257919	207.9400452
0.019337936	1.116504854	299.0825243
0.017092653	1.110451306	185.2801366
0.006852971	1.116209774	311.3002086
0.011712137	1.060445387	232.0296925
0.016645665	1.108108108	222.0394144
0.022925811	1.092920354	126.3141593
0.014374678	1.097201767	196.660162
0.011247601	1.232919255	311.7639752
0.010742546	1.272727273	356.213474
0.012622931	1.093567251	218.3075049
0.012591881	1.047826087	215.2358696
0.024288343	1	131.2333333
0.018931564	1.248704663	332.6217617
0.022777328	1.092307692	139.3102564
0.002121481	1.070989115	744.6814955
0.018525547	1.075376884	211.8082077
0.015093114	1.178571429	281.5803571
0.015805728	1.101449275	239.5869565
0.012494727	1.06407767	187.2305825
0.008623472	1.078740157	367.996063
0.010711444	1.072289157	291.5686078
0.008208764	1.085348506	339.6891892
0.015023294	1.077844311	196.5249501
0.04576263	1.085714286	121.8285714
0.015482388	1.025862069	212.5721983
0.010848613	1.059044049	239.3626992
0.011635541	1.158940397	305.843543
0.009593188	1.17535545	275.3625592
0.008854107	1.046956522	235.5982609
0.010431266	1.089955022	245.8139055
0.009798975	1.284839204	285.0233538
0.007182906	1.549657534	383.3831336
0.007667876	1.087576375	306.3547183
0.020208574	1.198529412	178.0606618
0.004511094	1.068181818	404.458649
0.011211528	1.172413793	255.7534483
0.008878194	1.080267559	238.1024247
0.007857446	1.086474501	357.7427938
0.01364518	1.143953935	240.5076775
0.01439016	1.178807947	215.6456954
0.008955129	1.096313912	267.2901308
0.007186812	1.053800171	264.0730145
0.032736398	1.021276596	137.1276596
0.023634078	1.193548387	208.5947581
0.016050001	1.05625	256.6203125
0.010920717	1.019313305	255.0332618
0.008983139	1.128323699	346.0491329
0.012489687	1.11201867	188.876021
0.010026136	1.103448276	270.0610632
0.013269204	1.160535117	380.1580268
0.013902546	1.07875895	229.4749403
0.009467964	1.05257732	237.8690722
0.009265426	1.08056872	259.1986572
0.012661404	1.207317073	281.6676829
0.00951927	1.08852459	242.6740984
0.014239208	1.084705882	235.7094118
0.006846936	1.143884892	358.442446
0.00415602	1.178790535	425.6665206
0.009186599	1.216080402	314.5783082
0.022453655	1.055813953	171.5848837
0.006109696	1.162857143	365.6204762
0.01573203	1.211764706	301.3970588
0.007049734	1.17473525	316.1976172
0.008720227	1.051886792	225.9530267
0.009876781	1.085714286	240.094958
0.024588434	1.034090909	201.7982955
0.010693858	1.086666667	268.4114815
0.017000811	1.079268293	239.4522358
0.019286711	1.018404908	165.8228528
0.008617011	1.318059299	290.7118524
0.030927975	1.040909091	117.2420455
0.011053989	1.094405594	260.1966783
0.012894552	1.394736842	439.6743421
0.014011828	1.129441624	259.1510152
0.014541238	1.06122449	205.8199708
0.011790571	1.167630058	325.1339114
0.013965562	1.03358209	251.8152985
0.013437519	1.036935705	199.5222298
0.012838371	1.067164179	205.6996269
0.012492128	1.142630745	239.6473851
0.010074029	1.12972973	456.927027
0.011778434	1.180904523	222.7506281
0.009970848	1.037974684	246.6857595
0.01282512	1.048723898	208.8584687
0.020709095	1.089449541	233.3199541
0.009353961	1.360465116	408.6114341
0.009017544	1.092975207	295.7789256
0.00718159	1.080645161	273.141129
0.01224556	1.058252427	237.0855583
0.011399681	1.083484574	193.6069772
0.009073938	1.260586319	322.1881107
0.020010592	1.188571429	201.9864286
0.019256963	1.136363636	261.6318182
0.031209694	1.042857143	160.5285714
0.005615428	1.119205298	330.9178808

log.sigma.5.0.mm.3D_gldzm_LowIntensityLargeDistanceEmphasis	log.sigma.5.0.mm.3D_gldzm_HighIntensityEmphasis	log.sigma.5.0.mm.3D_gldzm_DistanceZoneVariability	log.sigma.5.0.mm.3D_gldzm_ZonePercentage
0.010728866	338.9569288	475.5992509	0.369806094
0.013027538	380.2435897	154.0128205	0.449567723
0.009775013	379.5736196	293.7730061	0.365470852
0.017275959	359.8205128	137.2820513	0.48447205
0.022230537	214.6696833	391.2986425	0.313253012
0.020069033	306.5242718	95.31067961	0.425619835
0.018096352	190.3159145	1564.565321	0.159787456
0.007656809	318.5101311	1553.035757	0.27653263
0.012363294	233.8144221	905.7656416	0.221257626
0.017663407	225.7792793	206.5765766	0.294039735
0.02392701	128.8628319	212.4336283	0.145244216
0.015090174	201.4064801	636.4256259	0.199295568
0.012556574	323.1677019	275.8819876	0.397040691
0.014270929	364.7727273	257.0909091	0.47826087
0.019664593	220.6920078	481.9980507	0.325714286
0.012886202	217.4326087	1336.701449	0.239500174
0.024288343	131.2333333	150	0.169875425
0.02130554	340.9533679	163.6528497	0.471882641
0.023702313	141.825641	183.3692308	0.178244973
0.002215753	758.6791292	2015.366304	0.230099096
0.019174566	214.3232831	567.7537688	0.287295476
0.016384124	289.25	149.1904762	0.338709677
0.01668482	243.0434783	193.47343	0.370967742
0.013165015	189.3029126	493.4699029	0.143174868
0.008995603	373.1102362	361.5249344	0.310260586
0.010997207	297.1659973	711.8674699	0.292253521
0.008903941	344.0953058	664.1379801	0.350798403
0.016679104	198.2015968	475.6746507	0.222370173
0.046199948	126.0285714	33.05714286	0.239726027
0.015713286	213.3771552	456.0689655	0.288198758
0.01128173	241.7160262	1025.826617	0.26615116
0.01268603	312.9139073	271.6953642	0.383248731
0.010890209	285.1800948	563.3254344	0.271092077
0.009075556	238.2434783	557.2817391	0.238095238
0.01143033	249.1034483	1256.398801	0.259887006
0.012792452	297.0796325	540.7733538	0.336771532
0.020836097	403.9297945	409.2089041	0.386243386
0.008141311	311.221317	1389.510523	0.253049304
0.025846997	181.4411765	119.1911765	0.242857143
0.004840323	410.5107323	1513.636364	0.167672277
0.012358189	264.1448276	387.8735632	0.324626866
0.009420951	242.2173913	566.8561873	0.105430183
0.008567182	361.827051	851.4988914	0.373035567
0.015462578	245.756238	473.3992322	0.366901408
0.016316754	221.1390728	268.1456954	0.194087404
0.009858334	271.4494649	788.7336504	0.350270721
0.007666142	266.2724167	1129.753202	0.2188376
0.033327414	137.3191489	139.0141844	0.287169043
0.026501885	215.0241935	109.0322581	0.31713555
0.016427967	259.4	154.1125	0.353982301
0.011102667	255.5965665	460.0386266	0.362082362
0.009624502	355.2	794.1653179	0.31059246
0.014318763	193.8424737	795.3897316	0.107447342
0.010521355	275.7614943	324.8275862	0.211936663
0.014707418	386.6923077	268.7123746	0.465007776
0.014597613	232.8400955	397.5775656	0.356292517
0.009970589	240	936.5958763	0.241594022
0.00977524	262.685624	599.9131122	0.306240929
0.014517449	289.4268293	214.3495935	0.322411533
0.010320915	246.9645902	1437.655738	0.139998164
0.01484064	240.1035294	401.6776471	0.319789315
0.007403289	370.7553957	757.8369305	0.338336714
0.004751647	442.2611744	2026.210342	0.277143551
0.010762613	325.1876047	517.1943049	0.284828244
0.023041827	173.1023256	207.1488372	0.317577548
0.007015147	375.8704762	942.1885714	0.306927799
0.017445739	309.0823529	147.6941176	0.418719212
0.008668735	326.0854766	1176.969743	0.259980334
0.009084544	228.2649371	1228.761006	0.169419286
0.010217758	246.0521008	561.9714286	0.280131827
0.025411124	202.4204545	172.0454545	0.357723577
0.011669568	271.3259259	1274.253333	0.271029914
0.018009596	241.6626016	466.6869919	0.368539326
0.019492346	166.2392638	322.0245399	0.343157895
0.020160098	303.3867925	603.7762803	0.181684623
0.031798124	117.75	214.0818182	0.239651416
0.01157421	265.4912587	537.1328671	0.258940697
0.015297112	463.6052632	117.2631579	0.470588235
0.014840766	265.4365482	361.4670051	0.299619772
0.017157207	207.9300292	329.2857143	0.190555556
0.012784807	335.1541426	464.2408478	0.36913229
0.014201024	253.4440299	262.0671642	0.324455206
0.013837668	200.6087551	713.2216142	0.320333041
0.013523787	207.641791	384.4029851	0.212361331
0.013703093	247.1901743	573.8526149	0.232412523
0.010454212	468.4486486	169.6918919	0.348399247
0.01370871	228.9522613	352.8944724	0.304281346
0.010325577	248.3481013	770.2531646	0.205943691
0.013259899	210.4176334	417.2273782	0.253380364
0.021271708	237.6100917	410.7752294	0.318946598
0.010343636	445.4806202	203.4496124	0.320895522
0.019210738	298.3140496	454.9297521	0.191153239
0.007603515	277.6612903	881.344086	0.206620751
0.012595396	240.243932	792.6213592	0.239117818
0.012501675	195.7477314	527.4500907	0.166616269
0.011761725	335.4136808	774.897937	0.386325503
0.021641667	209.63	617.5314286	0.28548124
0.020301782	267.1	100.4545455	0.3514377
0.031879337	161.2142857	68.02857143	0.26119403
0.006046653	340.7642384	2092.152318	0.25668631

log.sigma.5.0.mm.3D_gldzm_IntensityVariabilityNormalized	log.sigma.5.0.mm.3D_gldzm_LowIntensitySmallDistanceEmphasis	log.sigma.5.0.mm.3D_gldzm_IntensityVariability
0.042685407	0.009474097	22.79400749
0.047419461	0.012977872	7.397435897
0.044469118	0.009054258	14.49693252
0.042735043	0.015874424	6.666666667
0.048156262	0.020148728	21.28506787
0.046469978	0.019155162	4.786407767
0.05180658	0.016841729	87.24228029
0.046906542	0.006652012	78.70917759
0.050938489	0.011549348	48.0349947
0.05174093	0.01639123	11.48648649
0.070287415	0.022675511	15.88495575
0.054947391	0.014195804	37.30927835
0.044539177	0.010920358	14.34161491
0.044990724	0.00986045	13.85714286
0.05380193	0.010862516	27.60038986
0.048923545	0.0125183	67.51449275
0.069422222	0.024288343	10.41333333
0.042336707	0.018338069	8.170984456
0.0695595	0.022546082	13.56410256
0.044484496	0.002097913	93.99574065
0.051179965	0.018363292	30.55443886
0.054634354	0.014770362	9.178571429
0.051693155	0.015585955	10.70048309
0.064160618	0.012327155	33.04271845
0.043847865	0.008530439	16.70603675
0.047018955	0.010640003	35.1231593
0.046690773	0.00803497	32.82361309
0.060322469	0.014609341	30.22155689
0.067755102	0.045653301	2.371428571
0.054455262	0.015424663	25.26724138
0.053153785	0.010740334	56.71508903
0.051269681	0.011372919	15.48344371
0.049097929	0.009268933	31.07898894
0.0624	0.008798745	35.88
0.049989998	0.0101815	66.68665667
0.049804296	0.009050606	32.52220521
0.045857572	0.003769608	26.78082192
0.0479152	0.007549517	70.57909029
0.064770761	0.018798968	8.808823529
0.051546079	0.004428787	81.6489899
0.049153125	0.010924862	21.3816092
0.067985817	0.008742505	40.65551839
0.043987001	0.007680012	39.67627494
0.051852889	0.013190831	27.01535509
0.055567738	0.013908511	16.78145695
0.050818557	0.008729328	42.73840666
0.057354615	0.00706698	67.16225448
0.065439364	0.032588644	9.226950355
0.050988554	0.022917126	6.322580645
0.0528125	0.015955509	8.45
0.050139071	0.01087523	23.36480687
0.045736242	0.008822799	39.56184971
0.059112341	0.012032418	50.65927655
0.055687673	0.009902331	19.37931034
0.039831769	0.012909651	11.909699
0.050324389	0.013728779	21.08591885
0.053778297	0.009342308	52.16494845
0.05152375	0.009137973	32.61453397
0.054828475	0.012197392	13.48780488
0.054712604	0.009318858	83.43672131
0.050053979	0.01408885	21.27294118
0.042090069	0.006707848	35.10311751
0.04509936	0.004007114	102.9167397
0.044614474	0.008792596	26.63484087
0.057696052	0.022306612	12.40465116
0.045694331	0.005883333	47.97904762
0.052802768	0.015303603	8.976470588
0.049252611	0.006644983	65.11195159
0.058894278	0.008629148	74.91352201
0.050180072	0.009791536	29.85714286
0.054235537	0.024382761	9.545454545
0.047128669	0.010449931	63.6237037
0.0459961	0.016748614	22.6300813
0.058470398	0.019235302	19.06134969
0.051634324	0.006105603	38.31266846
0.06838843	0.030710438	15.04545455
0.048376449	0.010923934	27.67132867
0.045100416	0.012293912	6.855263158
0.049408642	0.013804594	19.46700508
0.059949511	0.013887246	20.56268222
0.043127996	0.011542012	22.38342967
0.052461573	0.013906697	14.05970149
0.054171244	0.013337481	39.59917921
0.06272122	0.012667017	25.21393035
0.049178599	0.012189387	31.03169572
0.044090577	0.009978984	8.156756757
0.056740991	0.011295865	22.58291457
0.050962987	0.009882165	40.26075949
0.057627812	0.012716425	24.83758701
0.045682182	0.020568442	19.91743119
0.04359714	0.009106543	11.24806202
0.052515197	0.006469245	25.41735537
0.057747716	0.007076109	53.70537634
0.049459775	0.012158101	40.75485437
0.067183573	0.01117934	37.01814882
0.040138357	0.008401992	36.96742671
0.049134694	0.019602823	34.39428571
0.054710744	0.018995758	6.018181818
0.064897959	0.031042283	4.542857143
0.047596938	0.005507621	107.807064

log.sigma.5.0.mm.3D_gldzm_HighIntensityLargeDistanceEmphasis	log.sigma.5.0.mm.3D_gldzm_SmallDistanceEmphasis	log.sigma.5.0.mm.3D_glcm_SumVariance	log.sigma.5.0.mm.3D_glcm_Homogeneity1
381.3782772	0.956460674	840.0306379	0.317882254
389.5512821	0.995192308	995.6755194	0.286227983
426.4601227	0.960889571	978.6755274	0.299343267
399.2051282	0.951923077	870.717205	0.275937275
241.5882353	0.95418552	510.0179452	0.334986314
336.2912621	0.970873786	801.8076448	0.308089153
210.4590261	0.972387173	356.8447657	0.494312434
347.3498212	0.970947557	700.0466923	0.37761199
240.9533404	0.984888653	393.3942316	0.420275582
240.7387387	0.972972973	467.9796926	0.3637751
139.0575221	0.976769912	229.3745135	0.466531677
220.3917526	0.975699558	371.1157731	0.450753603
368.7826087	0.941770186	898.68877	0.303158591
399.0097403	0.931818182	822.4886098	0.273008154
230.2300195	0.976608187	377.2232484	0.361721424
226.2195652	0.988043478	475.9474133	0.431662485
131.2333333	1	218.4158198	0.425839198
374.2797927	0.937823834	823.3390563	0.278095804
151.8871795	0.976923077	211.5701427	0.458903625
814.669664	0.982252721	2363.527953	0.390083437
224.3835846	0.981155779	516.2642202	0.34774626
319.9285714	0.955357143	730.9598294	0.341354568
256.8695652	0.974637681	555.3915847	0.335534463
197.592233	0.983980583	317.0657721	0.502548377
393.5669291	0.980314961	904.2780075	0.368013013
319.5555556	0.981927711	655.2617388	0.367840458
361.7197724	0.978662873	753.8882394	0.327690218
204.9081836	0.980538922	364.1079701	0.408411371
142.8285714	0.978571429	322.7677631	0.458068476
216.5969828	0.993534483	438.1013526	0.366496034
251.1293346	0.985238988	482.3226708	0.37357779
341.1953642	0.960264901	819.2821262	0.30036352
324.450237	0.956161137	629.2842512	0.380233175
248.8243478	0.98826087	557.1865635	0.412756363
262.2616192	0.977511244	484.7038322	0.395424667
345.3047473	0.928790199	772.3765925	0.336274974
486.1164384	0.862585616	1033.624509	0.304673458
330.6877122	0.978105906	679.6715723	0.451242177
194.9632353	0.950367647	408.2686081	0.367959513
434.7190657	0.982954545	979.6504932	0.46275096
297.7103448	0.956896552	570.0921243	0.33140598
258.6772575	0.97993311	474.8630771	0.535610938
378.1640798	0.978381375	891.9713709	0.307737043
266.7504798	0.964011516	528.4798814	0.327847446
243.1125828	0.955298013	415.842817	0.45570891
288.0868014	0.975921522	621.9535551	0.341186964
275.0700256	0.986549957	579.1120982	0.416996987
138.0851064	0.994680851	278.7668555	0.376721788
240.7419355	0.951612903	405.1227249	0.35053448
270.51875	0.9859375	654.7153146	0.323509187
257.8497854	0.995171674	444.9577709	0.348653515
391.8034682	0.967919075	897.5570613	0.348645525
213.7082847	0.971995333	348.8222179	0.558222433
298.5632184	0.974137931	613.349925	0.422841097
412.8294314	0.959866221	1045.767997	0.274887086
246.300716	0.980310263	526.4417129	0.325581099
248.5237113	0.98685567	474.6975084	0.407136092
276.6334913	0.97985782	595.3492588	0.33504955
320.4634146	0.948170732	683.6692782	0.344018717
264.1265574	0.977868852	537.320893	0.522860007
257.68	0.978823529	535.1107994	0.359160819
420.0071942	0.964028777	1048.206396	0.33168661
508.6397897	0.955302366	1121.697643	0.371377937
367.6247906	0.945979899	802.4848478	0.356577148
179.172093	0.986046512	357.8101348	0.361962442
416.8704762	0.959285714	1000.071428	0.337306774
339.8235294	0.947058824	749.2490966	0.29456657
365.6369138	0.956316188	701.0466309	0.375913506
237.5125786	0.987028302	501.4024998	0.433441177
269.8806723	0.978571429	712.3923173	0.354591498
204.9090909	0.991477273	397.9754096	0.329142805
282.9837037	0.978333333	592.0398428	0.381704107
250.504065	0.980182927	514.4463651	0.309860687
167.904908	0.995398773	313.9275318	0.373466586
354.1105121	0.92198263	558.6039554	0.442540273
119.7818182	0.989772727	227.5681329	0.39061106
286.6695804	0.976398601	706.8342543	0.372191229
559.3289474	0.901315789	1282.669132	0.269114577
290.5786802	0.967639594	683.5429766	0.358094353
216.3702624	0.984693878	417.839331	0.42928775
375.2350674	0.958092486	826.3382417	0.318977544
259.9589552	0.991604478	795.3037958	0.342027073
204.9548564	0.990766074	379.3115927	0.371828298
215.4104478	0.983208955	384.5361477	0.40551417
277.3613312	0.964342314	519.1737849	0.410751251
514.5351351	0.967567568	1428.686787	0.318342681
253.758794	0.954773869	508.4323579	0.365798052
254.9974684	0.990506329	444.966039	0.471295059
216.6542923	0.987819026	441.3553477	0.403373491
254.7706422	0.977637615	545.7852301	0.335814366
592.9573643	0.909883721	1278.093755	0.341413369
308.4545455	0.976756198	613.1726659	0.428075267
295.7419355	0.97983871	648.6333583	0.409538694
252.8774272	0.985436893	522.41531	0.380505153
205.8675136	0.983161928	401.3943379	0.436731135
388.3159609	0.93485342	836.4882949	0.300481803
240.2042857	0.952857143	465.1192113	0.354150924
288.9727273	0.965909091	649.6249711	0.335906461
163.9571429	0.989285714	392.5169046	0.357042292
380.1496689	0.970198675	963.9148244	0.374075594

log.sigma.5.0.mm.3D_glcm_Homogeneity2	log.sigma.5.0.mm.3D_glcm_ClusterShade	log.sigma.5.0.mm.3D_glcm_MaximumProbability	log.sigma.5.0.mm.3D_glcm_Idmn	log.sigma.5.0.mm.3D_glcm_SumVariance2
0.224144292	212.5089898	0.009329141	0.977842035	118.4355011
0.191514484	-220.5376038	0.015266779	0.970808667	124.2962344
0.207659744	-132.2678048	0.012535419	0.974521567	103.4165293
0.180251034	-77.26503853	0.01521097	0.968990336	128.9397141
0.241278288	61.83486393	0.010701892	0.977240142	101.7308356
0.211846812	-325.4937308	0.017027316	0.972260918	123.9487677
0.431080528	292.5188448	0.070702311	0.990865986	57.59536187
0.295519549	333.1040311	0.020907104	0.984500591	83.71751673
0.34535755	445.9696717	0.036853637	0.982102552	70.33276254
0.273326796	79.52071294	0.014742939	0.979593267	74.90268407
0.396854017	94.46896806	0.043046684	0.981893795	34.18923094
0.380817647	207.5540955	0.040448393	0.986747514	56.55070768
0.210407624	-385.1817199	0.013155017	0.973579099	116.7605789
0.176645667	-570.4602449	0.012724751	0.970895094	136.8326926
0.275806608	388.6380288	0.01786711	0.979598722	79.91919617
0.362114741	242.1440548	0.0531624	0.980412338	76.86081161
0.346010184	67.03900998	0.023230806	0.977922288	37.81960129
0.183227804	-222.4723145	0.014018541	0.971258614	144.9447281
0.388969907	72.52480901	0.048002511	0.981407755	34.10061523
0.306771489	316.1274448	0.021849763	0.993218745	73.88777562
0.257108345	-71.48020842	0.010800224	0.973192624	90.39643348
0.249667577	-410.7910321	0.018387995	0.973533962	89.94539009
0.244028576	48.29569049	0.017104827	0.978152389	84.95855387
0.441256698	89.1159395	0.0828085	0.989098835	39.22699988
0.286554136	101.3890581	0.032999556	0.98097481	102.3898637
0.282558348	407.3630491	0.016560008	0.985027701	101.0496909
0.235839472	176.2691621	0.013084769	0.978402129	91.37978321
0.326568363	85.87680172	0.020517552	0.983008089	55.50697883
0.383031716	24.40160235	0.050393554	0.983267349	31.40042874
0.2769415	120.8074754	0.014112897	0.982721591	66.01023827
0.289077351	363.8919571	0.014612431	0.981082297	82.00301624
0.204673318	-256.9177592	0.012382597	0.968102344	99.18795649
0.29662553	453.1262381	0.020062881	0.986760562	92.37712895
0.33542975	195.1941576	0.024314149	0.986428999	72.47549304
0.31435867	329.7413408	0.018080053	0.984536899	74.00688878
0.246886878	-227.9212998	0.015683658	0.974007936	95.82110176
0.211417995	-492.1655329	0.01210296	0.974552486	107.3292409
0.383511802	213.9848285	0.088489624	0.985891598	86.47989555
0.276792435	-178.7619421	0.018683552	0.972253106	60.77037981
0.394237411	234.1685949	0.040131781	0.993295632	57.9915118
0.238186701	132.1423476	0.01051775	0.978620138	102.1208906
0.480627505	91.12253376	0.087987745	0.993958336	32.03435032
0.216879187	155.7969635	0.010517713	0.976443274	120.7880667
0.233180246	312.1553215	0.009201441	0.980626326	104.6256083
0.38740392	146.0103169	0.043899425	0.987686948	53.41307788
0.25186997	118.5278475	0.013082886	0.97659958	93.22637631
0.338095394	117.1050115	0.022084884	0.988060718	58.54777652
0.289370214	-63.86222299	0.024059919	0.970784797	46.64631214
0.257121155	551.9962517	0.017825167	0.980600201	100.8858384
0.227684146	-102.5235	0.015309802	0.97273173	88.20649813
0.258700139	574.8458034	0.015565749	0.979867565	94.56118546
0.258337843	208.431699	0.010465279	0.983892753	91.57345631
0.507993187	141.4582075	0.103552451	0.992085098	34.83170791
0.346215713	152.246717	0.023378801	0.9867109	52.35306313
0.179372073	-505.1487706	0.010982701	0.970211955	151.4336501
0.230822257	269.9399444	0.010043898	0.97383856	103.7219602
0.328737514	285.3970246	0.02425904	0.986516986	74.14224347
0.242974296	86.45483409	0.010250358	0.975623666	81.47609759
0.252463671	-154.3711058	0.015074958	0.976377736	75.97584356
0.463738824	34.37288301	0.12269317	0.991823975	48.20942719
0.270474327	255.6980276	0.014795822	0.982069611	73.08794012
0.239856418	251.2282079	0.014788449	0.985014168	112.0994089
0.287426139	360.3558342	0.019997908	0.988052694	95.07693864
0.267163718	-18.55239328	0.012730585	0.984957072	104.312531
0.271449799	47.27614943	0.017107173	0.975889748	57.98553307
0.244426976	242.4681693	0.011564706	0.986517352	93.97466291
0.199007474	-453.0996819	0.013894766	0.964885929	98.57456019
0.289830648	359.1020567	0.015643416	0.987095947	83.54487645
0.357940316	140.8190986	0.021345361	0.990153249	59.42011438
0.263628134	30.59306682	0.014986474	0.979986137	77.5412216
0.235401186	41.29555368	0.015311854	0.968034285	84.35764724
0.301496927	181.8148313	0.031856505	0.981523221	86.55212741
0.216490839	221.1759342	0.009569774	0.971657634	107.7438344
0.285348479	182.3525102	0.016635717	0.981160757	59.99316799
0.369722736	346.2366092	0.038726105	0.99029999	76.30126536
0.303317221	-0.265704529	0.02303418	0.972035969	44.73778334
0.285315943	48.89902333	0.013622725	0.985011954	79.30338964
0.175696098	-785.0785238	0.015439007	0.967703755	131.7622723
0.267308078	5.7966684	0.013328648	0.980040799	83.2558497
0.35288519	42.73591275	0.025316429	0.987477749	47.95137013
0.225289281	229.1285869	0.009495367	0.978105972	119.3310928
0.248541427	-252.7545393	0.015086203	0.977144366	88.37757472
0.287366643	146.0092414	0.032319163	0.977793513	70.28354793
0.322752054	99.76099973	0.022709522	0.986180561	51.58177715
0.333844945	295.8054435	0.032080489	0.984979764	79.16830669
0.228467746	-480.4791296	0.023075026	0.980447177	101.0929903
0.278919092	61.55740556	0.012804968	0.980324577	77.32820869
0.406544894	301.2517958	0.081263647	0.98687806	68.24030561
0.32214952	112.7197625	0.025399445	0.984155489	54.44933367
0.242703609	147.9715497	0.011508793	0.976339126	103.1163189
0.252486951	-54.21898106	0.017790514	0.980086458	104.483839
0.352961149	19.99680529	0.030571791	0.986597447	59.93818659
0.327119485	114.7212081	0.01655726	0.989339717	59.38903315
0.294608672	275.7990664	0.014960326	0.985634239	78.7561679
0.35925681	61.22280518	0.018642641	0.986787001	45.12762861
0.20703031	189.9106857	0.008583146	0.977520324	129.4323893
0.264431642	127.1080604	0.010191675	0.980223652	88.74760307
0.243900416	-197.3915397	0.021805464	0.971044237	79.85104222
0.266702275	-159.3123924	0.025524537	0.967756345	50.11166614
0.288561911	110.8375681	0.016306353	0.985860603	80.16363441

log.sigma.5.0.mm.3D_glcm_Contrast	log.sigma.5.0.mm.3D_glcm_DifferenceEntropy	log.sigma.5.0.mm.3D_glcm_InverseVariance	log.sigma.5.0.mm.3D_glcm_Entropy	log.sigma.5.0.mm.3D_glcm_Dissimilarity
24.15288323	3.351590294	0.226522163	8.430392346	3.907251087
32.2178867	3.47356734	0.194948234	7.860591228	4.585541177
29.51934172	3.386035644	0.207470658	8.228723532	4.39722511
34.55554586	3.5053507	0.192426882	7.921987229	4.741299565
19.06301249	3.186481328	0.250917484	8.189248669	3.475431838
23.37232146	3.250985905	0.220216091	7.573473696	3.923341677
10.42787737	2.790199449	0.33301095	7.211628997	2.192847638
18.88446422	3.213157922	0.281948201	8.149218862	3.268486116
14.98924824	3.032089489	0.307635718	7.660495217	2.826479086
14.60200375	2.979898809	0.275472756	7.666179435	3.042032108
7.698950504	2.597406614	0.361299349	6.798816989	2.076993765
10.93728682	2.850142761	0.343357101	7.401986505	2.397228767
29.15415228	3.413794111	0.217592793	8.279701923	4.283458077
34.2053079	3.492713512	0.186741638	8.348789466	4.758385201
19.82945158	3.210014286	0.27726287	7.947431918	3.36275382
17.79015971	3.034667056	0.304605306	7.726909665	2.975264452
8.553357016	2.667979623	0.34799429	6.995815116	2.272986399
33.86688132	3.494136141	0.199401213	8.144053146	4.690016264
7.941404564	2.604504585	0.372752422	6.778401113	2.109289598
14.767858	3.034311015	0.303716047	7.931306514	2.891160733
18.15409722	3.146308415	0.266792918	8.107567527	3.348825483
19.24017027	3.171938075	0.249160008	7.695748686	3.482881263
19.55118873	3.169646645	0.260532535	7.819313127	3.509934017
7.749359501	2.624575504	0.365853071	6.853389681	1.949962897
23.49546893	3.269730924	0.26769822	8.081464189	3.59674077
18.21346438	3.168878973	0.280857892	8.153299707	3.253831359
23.66432929	3.284507512	0.239919575	8.215834935	3.799494235
11.21695348	2.869798743	0.318510541	7.535501432	2.579744073
6.36009955	2.41510505	0.364004461	6.054906577	1.967282377
14.29515332	2.974097432	0.29111357	7.722842843	2.975590532
16.9161784	3.094615455	0.288752044	8.00554828	3.146484208
27.20570031	3.381791097	0.204413143	8.171666225	4.208586711
16.01401406	3.077243151	0.297433396	8.029693508	3.043888735
14.59530884	3.016888124	0.306950548	7.70538091	2.822037218
14.72786357	3.046603259	0.309593293	7.87208251	2.879807797
23.62319523	3.3135741	0.239421515	8.295464251	3.785908129
30.16898278	3.329592005	0.225132909	8.357473487	4.2591693
17.26015764	3.079064059	0.287025591	7.694058689	2.886426413
13.20420664	2.827312696	0.282508481	7.328788159	2.910226207
9.98267404	2.794494608	0.353881996	7.409666706	2.254452748
20.44624153	3.220579135	0.24345219	8.205737752	3.598295198
5.232582888	2.39216956	0.397228143	6.46720541	1.607618101
30.9589866	3.347714455	0.216933654	8.507331569	4.356451897
19.64426124	3.182798851	0.246074272	8.200328684	3.55492489
10.92368315	2.819514227	0.351810943	7.171580808	2.362790366
21.07973483	3.25787847	0.253315483	8.260574796	3.597313372
11.98044321	2.918067157	0.320124269	7.622053358	2.599669452
12.67168305	2.902685305	0.304337134	7.194046246	2.828474749
16.13250226	3.041953546	0.263823104	7.601737593	3.187816501
19.82071423	3.168089359	0.234817833	7.726963102	3.607031932
19.25696251	3.202396671	0.25708138	7.945088655	3.432482116
18.48172114	3.182491985	0.269374425	8.21800921	3.350668374
6.471288923	2.484448237	0.365324752	6.431565837	1.676722874
11.77106764	2.829383022	0.335432513	7.320686737	2.543303012
35.03252862	3.538831429	0.183181771	8.457322074	4.808645666
20.51042584	3.21702092	0.238354743	8.172800878	3.63162325
14.49938141	3.001209041	0.311329502	7.787849222	2.827328822
20.45635937	3.231062184	0.24468159	8.085168152	3.590711228
18.40623458	3.143987403	0.255944846	7.860085785	3.405389939
8.724353953	2.681468444	0.328310141	6.983291828	1.989002148
17.06186706	3.121294186	0.276310596	7.89429172	3.219775741
21.42615603	3.271878049	0.244797637	8.415052303	3.661915034
18.97864364	3.198657869	0.282857069	8.269602643	3.276947858
18.2869164	3.180388582	0.266201672	8.257570672	3.306133506
14.89989179	3.00474157	0.280643831	7.553480702	3.05315494
19.20126072	3.206501507	0.251340736	8.283286019	3.478044017
28.15290292	3.358029914	0.210994206	7.83999718	4.274810671
15.58386281	3.083811216	0.289163915	8.040975984	3.033224152
9.808377968	2.785059177	0.34861833	7.493955573	2.35489496
16.64205588	3.108813933	0.265323281	7.995722244	3.224953088
19.98894046	3.122916082	0.238544249	7.655312366	3.60473533
20.16614785	3.240307487	0.276955787	8.137315747	3.343816823
25.7211562	3.35951141	0.222093421	8.300164858	4.062846144
13.48819889	2.948206532	0.300186487	7.520122834	2.887025383
12.3755941	2.924587178	0.321547482	7.65794084	2.532160215
10.86001629	2.771477849	0.303405576	7.172354791	2.639379734
15.10809373	3.054233354	0.291186062	7.965900963	3.020905172
38.2000002	3.573080729	0.188393161	7.878713651	5.012930683
15.46876651	3.040863521	0.27857139	7.960337109	3.107079253
10.28873996	2.810101018	0.344095145	7.318332947	2.404562077
23.84855389	3.34093459	0.22801409	8.421320253	3.881944911
17.7400226	3.104991837	0.251037592	7.889863339	3.367732147
18.68259398	3.163662639	0.268094002	7.892177254	3.301276899
11.35911266	2.858835078	0.319385864	7.468152315	2.587053816
14.26890205	2.992856511	0.31771133	7.769715774	2.799716303
26.81354819	3.397741845	0.238148461	7.785100996	4.060284302
16.41238762	3.079414152	0.288325622	7.895914643	3.139422997
12.50917354	2.905730788	0.330585217	7.401943427	2.447001166
12.17495339	2.914341712	0.321359015	7.513022021	2.666811607
19.88136709	3.21361954	0.245062402	8.216480841	3.534505189
21.74780956	3.259988691	0.260081359	8.025850415	3.606217881
11.89380838	2.87362616	0.336933608	7.486644713	2.532806404
10.61653434	2.836072365	0.322398156	7.593043708	2.519111151
14.45497031	3.024149449	0.292716761	7.94158578	2.941433236
7.948636136	2.611223542	0.35685058	7.152001906	2.180098323
29.42679153	3.479186888	0.215763343	8.708289228	4.301565073
17.66445168	3.133485044	0.267287321	8.107744044	3.292397962
19.66508834	3.152086254	0.25413644	7.480873	3.520133595
15.56021417	3.001020222	0.278102565	7.069123272	3.133331577
17.18631693	3.134005506	0.281708718	8.126714291	3.150452994

log.sigma.5.0.mm.3D_glcm_DifferenceVariance	log.sigma.5.0.mm.3D_glcm_Idn	log.sigma.5.0.mm.3D_glcm_Idm	log.sigma.5.0.mm.3D_glcm_Correlation	log.sigma.5.0.mm.3D_glcm_Autocorrelation
8.259079392	0.897017554	0.224144292	0.662959866	284.3129988
10.08969711	0.88167977	0.191514484	0.594478521	326.6682392
8.723379109	0.888662006	0.207659744	0.557529009	322.6306142
10.94017081	0.878337126	0.180251034	0.582959932	289.2573316
6.578726377	0.895507833	0.241278288	0.684206845	183.4349994
7.298309644	0.883675508	0.211846812	0.683069897	269.5473694
5.410527697	0.941569192	0.431080528	0.694586823	134.2579173
7.788664	0.917345898	0.295519549	0.634256723	241.5759409
6.4763146	0.914497223	0.34535755	0.647384542	145.6347077
4.835772907	0.900669788	0.273326796	0.671080211	169.8713289
3.115270888	0.911811777	0.396854017	0.628851384	92.00225905
4.989571218	0.925936611	0.380817647	0.675876168	139.2562596
9.694756689	0.888797222	0.210407624	0.604267429	299.4391896
10.19135263	0.880741276	0.176645667	0.606669549	275.6335268
7.954828722	0.905510183	0.275806608	0.605402285	140.0634594
7.852462007	0.91420402	0.362114741	0.628519747	171.823851
3.246367687	0.899402917	0.346010184	0.631275729	88.70668232
10.87690649	0.88249909	0.183227804	0.625258913	275.7810517
3.256866134	0.910600555	0.388969907	0.622040362	85.99420157
6.025079529	0.943232909	0.306771489	0.668960938	715.7788759
6.514912553	0.889015325	0.257108345	0.666024871	185.3399087
6.578570113	0.88873449	0.249667577	0.650043579	249.4048287
6.652962897	0.897827989	0.244028576	0.625358948	196.3515999
3.802381044	0.934544221	0.441256698	0.669558729	120.8945686
9.491739824	0.910610014	0.286554136	0.629801536	302.348368
7.123215931	0.917354709	0.282558348	0.691982032	228.7880568
8.008031857	0.89989259	0.235839472	0.595060662	257.1085358
4.327823831	0.911577225	0.326568363	0.663962393	137.36128
2.304013153	0.911044937	0.383031716	0.662905689	120.2301914
5.021432433	0.908765385	0.2769415	0.645156938	160.6798826
6.358293689	0.907857585	0.289077351	0.658479576	174.6150458
8.573117535	0.876921843	0.204673318	0.573270782	275.144672
6.30078377	0.922067201	0.29662553	0.705146223	220.9565176
6.229292465	0.923650079	0.33542975	0.666224262	197.7193153
6.150863727	0.917451304	0.31435867	0.669372148	175.4892167
8.287727037	0.89172128	0.246886878	0.608357956	262.8609357
9.160880294	0.892758617	0.211417995	0.578967457	338.9694944
8.33861526	0.927330496	0.383511802	0.670090733	234.570726
3.895021528	0.885202633	0.276792435	0.647567426	150.3901362
4.718460603	0.94665344	0.394237411	0.707893671	322.9236244
6.982685508	0.89853369	0.238186701	0.666681229	201.9566483
2.549587922	0.949972749	0.480627505	0.719189173	169.1470364
8.846546659	0.895787759	0.216879187	0.605210692	298.527191
6.446894664	0.902140241	0.233180246	0.685865606	189.1547859
5.125670911	0.929273575	0.38740392	0.661172693	152.3668569
7.489132249	0.896085741	0.25186997	0.6316896	217.7111602
4.980741861	0.926624603	0.338095394	0.661164721	204.4031771
4.391935063	0.883555269	0.289370214	0.573573607	107.9019298
5.52684212	0.902975926	0.257121155	0.726149274	148.9034466
6.201141882	0.884683059	0.227684146	0.634807376	226.4060339
7.053809886	0.902993614	0.258700139	0.656610498	161.6892302
6.852929162	0.9125012	0.258337843	0.666185697	301.5178072
3.553107537	0.947090099	0.507993187	0.687435543	129.4320872
4.617018687	0.923903618	0.346215713	0.635481855	213.4619464
10.86538692	0.879779141	0.179372073	0.628001207	343.0139897
6.780951174	0.887966568	0.230822257	0.668853371	187.8430664
5.929652149	0.92341448	0.328737514	0.674205437	172.5428337
7.079362932	0.892721293	0.242974296	0.597937244	208.6024014
6.292574468	0.894212454	0.252463671	0.611396101	235.4483725
4.552880652	0.945054435	0.463738824	0.695972215	189.7717252
6.210242394	0.9082379	0.270474327	0.623256594	190.5618075
7.434475725	0.914148946	0.239856418	0.68032952	346.6957954
7.632838106	0.926464681	0.287426139	0.668194269	367.2403343
6.923229491	0.915893796	0.267163718	0.703555778	274.0062278
5.146031164	0.893523007	0.271449799	0.590279817	134.0559173
6.628059048	0.917854448	0.244426976	0.663847492	331.8734278
8.955547136	0.871577253	0.199007474	0.559974236	252.7665192
6.025449242	0.922112437	0.289830648	0.686367598	242.666921
4.057517129	0.9327477	0.357940316	0.717058906	181.1570492
5.864045315	0.902155283	0.263628134	0.647745811	245.375207
5.989544477	0.877075032	0.235401186	0.615390493	145.8043975
8.520206725	0.911459183	0.301496927	0.625149715	208.2781857
8.465763623	0.88416924	0.216490839	0.615816771	182.8935447
4.80445818	0.90529778	0.285348479	0.630585434	120.2824937
5.61112838	0.93619947	0.369722736	0.724742207	199.1801222
3.457024415	0.885042823	0.303317221	0.610433595	91.38045263
5.642034146	0.915647438	0.285315943	0.680087886	244.2291975
11.9673951	0.875654452	0.175696098	0.558836539	408.2428775
5.39369948	0.90221362	0.267308078	0.687029006	237.2852676
4.31272594	0.925095808	0.35288519	0.646649578	153.7005489
8.146909434	0.897588534	0.225289281	0.668754843	280.3017425
5.779056831	0.894981096	0.248541427	0.668854925	270.063724
7.063722772	0.901196604	0.287366643	0.578985838	140.9565887
4.380473232	0.919700743	0.322752054	0.639934086	143.4685822
5.959393374	0.919733502	0.333844945	0.696094023	186.3583954
9.688555057	0.904136806	0.228467746	0.583467293	451.3804424
6.10926231	0.904886909	0.278919092	0.651050809	182.760536
6.299064691	0.929825506	0.406544894	0.690011514	162.2407904
4.810895188	0.915018956	0.32214952	0.634555491	161.2342416
6.976445173	0.89414177	0.242703609	0.677988082	194.5134073
7.991484236	0.904513991	0.252486951	0.657897564	411.488785
5.102826025	0.924350955	0.352961149	0.670396735	214.5121682
4.078614454	0.928174158	0.327119485	0.696738509	225.9711649
5.477160438	0.917681192	0.294608672	0.68955626	187.8007034
2.912104163	0.920761216	0.35925681	0.699733585	148.9285908
10.03329968	0.896490113	0.20703031	0.631366586	282.5732781
6.263083684	0.903585989	0.264431642	0.667854125	169.369265
6.555367368	0.883965854	0.243900416	0.608082759	223.4756275
5.220254546	0.878281382	0.266702275	0.531574787	142.9918224
6.755459721	0.919690643	0.288561911	0.649174625	320.5330327

log.sigma.5.0.mm.3D_glcm_SumEntropy	log.sigma.5.0.mm.3D_glcm_AverageIntensity	log.sigma.5.0.mm.3D_glcm_Energy	log.sigma.5.0.mm.3D_glcm_SumSquares	log.sigma.5.0.mm.3D_glcm_ClusterProminence
5.432602651	16.29587078	0.003603181	36.74803728	35781.01962
5.33236353	17.56559379	0.005177297	39.57867977	40357.00936
5.295089623	17.65494693	0.004157349	34.06147197	26303.5622
5.362736353	16.45552368	0.004930166	41.70651309	44026.8517
5.309905476	12.77652097	0.004120984	30.52604625	24973.25956
5.232040686	15.73433273	0.006044693	37.95243652	34969.05997
4.834084945	11.10470352	0.016453663	17.53162309	12853.81079
5.198474284	15.12879172	0.005314445	26.4659987	23557.24451
4.98658342	11.50111176	0.008415548	21.72301535	17355.94053
5.057261647	12.44328106	0.005944565	22.43061222	13209.67235
4.508749638	9.23945539	0.013663612	10.47204536	3424.141797
4.878469571	11.31284884	0.01067625	16.98877647	11922.23666
5.355696873	16.82315998	0.004118218	37.23167153	34205.04751
5.436270428	16.03314736	0.003761397	43.93388847	46447.97487
5.121610555	11.23168396	0.005513499	25.74474011	20721.4018
5.087073475	12.55887999	0.00952203	24.06852122	16496.54949
4.604401081	9.021137744	0.010080935	11.59323958	3982.112505
5.451270486	15.8695813	0.004206509	45.32238041	52061.20654
4.505197228	8.913243726	0.01484805	10.51050495	4032.056377
5.102625317	26.62763378	0.006241189	22.43621339	20130.25865
5.230596554	12.93261722	0.004412735	27.13763267	19173.56232
5.127109754	15.23024579	0.006232141	27.43202316	21480.79576
5.142996766	13.42951096	0.005538107	26.34851211	20324.18368
4.594532505	10.63176809	0.018452154	11.76000544	6101.388772
5.305114208	16.96570717	0.005826745	32.08565278	29872.66558
5.308013761	14.51582925	0.004871361	30.56714366	28971.78657
5.255811027	15.6535179	0.004505917	29.89586928	23288.14918
4.908479442	11.23750054	0.007123218	16.68098308	8563.871014
4.282990898	10.67549693	0.01866574	9.440132072	2613.955954
5.020819721	12.16822067	0.006150938	20.32322358	13496.80809
5.158651844	12.61675818	0.00524419	25.24529949	19395.92073
5.237202746	16.12830441	0.004185792	32.5654143	22828.17639
5.244547493	14.27562925	0.005527947	27.65602442	27245.81646
5.057805273	13.6186337	0.006654761	22.61144667	13495.34922
5.085363231	12.72225706	0.006345891	22.84957438	16088.85358
5.281947005	15.70474778	0.004114819	30.42738023	24183.27258
5.323257142	18.12500783	0.003986576	34.88495441	33357.72689
5.124203574	14.85552938	0.014382988	26.74060095	23728.66522
4.895383173	11.7666886	0.007648892	18.49364661	9528.650274
4.907079435	17.73046661	0.010459118	17.06115645	14903.41017
5.314503946	13.54385314	0.00407992	31.52883755	25287.7965
4.447472003	12.7496348	0.022985063	9.388018302	4802.894165
5.45975277	16.82892998	0.003623513	38.75920585	40270.67023
5.328242356	13.03768821	0.004080409	32.05641309	28976.9814
4.773674055	11.91374306	0.013115717	16.23592386	11682.71878
5.267049816	14.19504227	0.004175079	29.41170574	20969.81111
4.951870715	13.92745721	0.007145162	18.11425461	10180.48235
4.704801526	9.969360887	0.008838259	14.8294988	6151.702618
5.160009026	11.33651046	0.006297534	29.99378835	30803.25571
5.133819937	14.4739879	0.005694936	27.1395319	18690.72652
5.18540568	11.99489707	0.005281504	29.19074965	26038.44783
5.269838608	16.95580973	0.004450124	28.04988087	24672.45274
4.402025227	11.06472955	0.030694589	10.41277168	7220.838223
4.832369437	14.30597537	0.009029593	16.62224617	9306.320451
5.529987407	17.94874875	0.003440467	47.14972066	58604.59159
5.288578157	12.94267084	0.004109018	31.41955332	25250.36449
5.096299027	12.61005053	0.006625884	22.83151168	17247.14938
5.14084209	13.92851592	0.004310011	25.8151245	14727.22406
5.08427966	14.89431362	0.005356579	23.94685924	15556.11444
4.709591245	13.44401594	0.023669897	14.57909088	9454.723186
5.079914357	13.32776706	0.005559863	23.10323363	16808.3798
5.405454299	18.17363284	0.003931031	33.1382373	36725.73555
5.279864243	18.80665184	0.004961497	28.2058972	27390.68462
5.357511586	15.98416926	0.004487417	31.19433465	32143.6519
4.891292324	11.10216667	0.006548616	18.22135622	8276.34282
5.292254621	17.87255765	0.004260307	28.14889598	28935.80261
5.161304089	15.38083735	0.005264657	32.23602313	24821.0989
5.19546432	15.11299134	0.005411829	25.4113028	22616.3544
4.957708493	13.02317588	0.007930191	17.71926935	11217.82653
5.145076649	15.20418309	0.00502341	23.97429343	15450.62454
5.068481573	11.38628231	0.005938602	26.08664692	14897.57251
5.206600105	13.94532418	0.006140207	27.72976657	23671.30519
5.319203473	12.77288237	0.003755707	33.80727337	26866.78503
4.912932681	10.42289972	0.006779432	18.40136775	10397.67198
5.108745554	13.61772033	0.009065157	22.92302801	22439.32017
4.690135791	9.103434407	0.008537035	13.89944991	4462.357804
5.160708128	15.16306749	0.005135956	24.10173581	16896.70312
5.310231284	19.89579923	0.005142041	42.15371061	50707.50763
5.186727017	14.86579841	0.005110979	25.01110373	17738.57253
4.791214247	12.01990319	0.008725737	14.70982608	7153.273099
5.437343631	16.16011951	0.003638551	36.93362469	36912.49454
5.18632693	15.92393515	0.005226562	26.99447029	19987.56169
5.052954645	11.32430455	0.006114714	22.41066485	13327.30235
4.853596416	11.55592834	0.007788277	15.84340755	8253.881575
5.110625992	13.08170106	0.00793635	23.94458497	20663.84593
5.171796448	20.96110524	0.006445521	30.98881174	33841.44422
5.123538167	12.96034332	0.005283288	23.74085692	14781.91197
4.94712884	12.21162732	0.015483204	20.72035403	17262.48443
4.879319254	12.28073227	0.007993129	16.77110168	8955.78575
5.319773107	13.21829909	0.004193379	31.39474219	27444.2785
5.279570064	19.92302497	0.005245392	31.59065011	32753.52173
4.939614898	14.26961443	0.009401079	18.45977723	12751.30182
4.967856757	14.65273594	0.006872751	17.81826158	11424.34195
5.145401822	13.14174206	0.005496325	23.77436461	18072.55954
4.758045414	11.81636286	0.008908008	13.26906619	5250.483828
5.507210315	16.24969512	0.002993502	40.49969386	45388.07549
5.224633496	12.33071813	0.004443887	26.89878394	19155.18228
5.004244025	14.43423397	0.006878759	24.87903264	14773.01118
4.677950808	11.58840068	0.009162931	16.41797008	6777.796048
5.188523804	17.60219418	0.005012694	24.6448503	18141.494

log.sigma.5.0.mm.3D_glcm_SumAverage	log.sigma.5.0.mm.3D_glcm_Imc2	log.sigma.5.0.mm.3D_glcm_Imc1	log.sigma.5.0.mm.3D_glcm_DifferenceAverage	log.sigma.5.0.mm.3D_glcm_Id
32.29432704	0.866447379	-0.158950985	3.907251087	0.317882254
34.84257794	0.959906854	-0.281053967	4.585541177	0.286227983
34.87808045	0.883235749	-0.176816696	4.39722511	0.299343267
32.59315284	0.962480462	-0.283719574	4.741299565	0.275937275
25.51369898	0.863037493	-0.16150784	3.475431838	0.334986314
31.26359432	0.975503625	-0.334345757	3.923341677	0.308089153
22.1327617	0.855672907	-0.180115653	2.192847638	0.494312434
30.02430421	0.790641716	-0.122639547	3.268486116	0.37761199
22.96009227	0.806615936	-0.145783831	2.826479086	0.420275582
24.8812364	0.88002256	-0.185739657	3.042032108	0.3637751
18.47891078	0.791117533	-0.154279446	2.076993765	0.466531677
22.61367625	0.828345887	-0.156034455	2.397228767	0.450753603
33.31567939	0.895986069	-0.183566184	4.283458077	0.303158591
31.61501304	0.921317439	-0.207283088	4.758385201	0.273008154
22.36352508	0.824944728	-0.145800069	3.36275382	0.361721424
25.06328586	0.866839648	-0.181121796	2.975264452	0.431662485
18.04227549	0.808105968	-0.149709647	2.272986399	0.425839198
31.49054479	0.956164398	-0.263913988	4.690016264	0.278095804
17.82648745	0.808210363	-0.158890159	2.109289598	0.458903625
52.95253589	0.789480524	-0.129677368	2.891160733	0.390083437
25.86523445	0.826669144	-0.144850198	3.348825483	0.34774626
30.44115089	0.914515726	-0.214417628	3.482881263	0.341354568
26.82846464	0.911565942	-0.208591496	3.509934017	0.335534463
21.26214574	0.827832804	-0.168116611	1.949962897	0.502548377
33.62105724	0.89572035	-0.198043373	3.59674077	0.368013013
28.8485952	0.861728236	-0.163988792	3.253831359	0.367840458
30.99424557	0.82418166	-0.142437581	3.799494235	0.327690218
22.47500107	0.804432726	-0.140871109	2.579744073	0.408411371
21.35099385	0.944984956	-0.315715939	1.967282377	0.458068476
24.3095472	0.830991954	-0.150595615	2.975590532	0.366496034
25.16636996	0.810199473	-0.138179726	3.146484208	0.37357779
32.06781444	0.867533937	-0.163729057	4.208586711	0.30036352
28.41543744	0.854794907	-0.162757218	3.043888735	0.380233175
27.07256496	0.845296608	-0.162465896	2.822037218	0.412756363
25.35071552	0.800391739	-0.132715307	2.879807797	0.395424667
31.29174315	0.820350603	-0.135858885	3.785908129	0.336274974
35.75620761	0.849689613	-0.158650025	4.2591693	0.304673458
29.47927708	0.894450571	-0.197812121	2.886426413	0.451242177
23.5333772	0.882102332	-0.199084765	2.910226207	0.367959513
35.26581353	0.834546564	-0.161164077	2.254452748	0.46275096
26.94534776	0.862324115	-0.161785707	3.598295198	0.33140598
25.49080042	0.842190508	-0.18769604	1.607618101	0.535610938
33.22693816	0.855085575	-0.164143582	4.356451897	0.307737043
25.9138369	0.86930273	-0.165443374	3.55492489	0.327847446
23.81083848	0.855216334	-0.177652664	2.362790366	0.45570891
28.26012359	0.811742162	-0.132266152	3.597313372	0.341186964
27.76729598	0.798044095	-0.13524765	2.599669452	0.416996987
19.93872177	0.843521945	-0.165033682	2.828474749	0.376721788
22.59737169	0.947574722	-0.263918465	3.187816501	0.35053448
28.93051253	0.918526526	-0.219511027	3.607031932	0.323509187
23.89943928	0.865491285	-0.166916105	3.432482116	0.348653515
33.65945262	0.819279021	-0.136290681	3.350668374	0.348645525
22.12130541	0.845636096	-0.18875249	1.676722874	0.558222433
28.51754584	0.832088766	-0.166716173	2.543303012	0.422841097
35.43012883	0.930225006	-0.214741609	4.808645666	0.274887086
25.84504008	0.865970499	-0.165482565	3.63162325	0.325581099
25.10893211	0.8332575	-0.156631751	2.827328822	0.407136092
27.80904862	0.785407878	-0.122541638	3.590711228	0.33504955
29.7326785	0.859562093	-0.165307563	3.405389939	0.344018717
26.82513865	0.863882181	-0.19072236	1.989002148	0.522860007
26.57335269	0.827408002	-0.144682537	3.219775741	0.359160819
36.00058744	0.847782511	-0.149675042	3.661915034	0.33168661
37.32034721	0.798083388	-0.131077491	3.276947858	0.371377937
31.77916693	0.859217534	-0.159681402	3.306133506	0.356577148
22.20433333	0.838158714	-0.157871566	3.05315494	0.361962442
35.39327002	0.815263971	-0.133050127	3.478044017	0.337306774
30.66544202	0.925299469	-0.22338497	4.274810671	0.29456657
30.04469255	0.814806671	-0.138693927	3.033224152	0.375913506
25.98053136	0.831249515	-0.157538257	2.35489496	0.433441177
30.34098057	0.813841128	-0.136484092	3.224953088	0.354591498
22.77256462	0.910585589	-0.213178077	3.60473533	0.329142805
27.68931837	0.813307	-0.134903001	3.343816823	0.381704107
25.48464548	0.847944578	-0.148668929	4.062846144	0.309860687
20.843386	0.844760364	-0.161370355	2.887025383	0.373466586
27.06969419	0.868796233	-0.181582313	2.532160215	0.442540273
18.20686881	0.821728742	-0.15778749	2.639379734	0.39061106
30.21087302	0.835005251	-0.148906973	3.020905172	0.372191229
39.22952066	0.962075814	-0.282586478	5.012930683	0.269114577
29.6866367	0.853358547	-0.162338563	3.107079253	0.358094353
24.02278721	0.815397512	-0.149027588	2.404562077	0.42928775
32.02644962	0.871460556	-0.162629054	3.881944911	0.318977544
31.7735835	0.886999741	-0.186151699	3.367732147	0.342027073
22.63022589	0.798190089	-0.137968782	3.301276899	0.371828298
23.09994294	0.797065288	-0.139636126	2.587053816	0.40551417
26.08605412	0.856886326	-0.169243027	2.799716303	0.410751251
41.60368482	0.928188427	-0.227310306	4.060284302	0.318342681
25.88449387	0.843995652	-0.156700589	3.139422997	0.365798052
24.3553947	0.867038199	-0.180623456	2.447001166	0.471295059
24.548451	0.806897185	-0.140804732	2.666811607	0.403373491
26.35765896	0.864975692	-0.16274866	3.534505189	0.335814366
39.53628249	0.899936529	-0.195600699	3.606217881	0.341413369
28.45996124	0.839287307	-0.165808538	2.532806404	0.428075267
29.24203071	0.816687399	-0.144810553	2.519111151	0.409538694
26.20802985	0.824397668	-0.1448458	2.941433236	0.380505153
23.63272572	0.812934078	-0.158655225	2.180098323	0.436731135
32.09677353	0.82999002	-0.134971903	4.301565073	0.300481803
24.62450978	0.819035864	-0.141960647	3.292397962	0.354150924
28.86846793	0.934308315	-0.245855212	3.520133595	0.335906461
23.17680135	0.903547125	-0.218493057	3.133331577	0.357042292
34.91596498	0.779534899	-0.123083271	3.150452994	0.374075594

log.sigma.5.0.mm.3D_glcm_ClusterTendency	log.sigma.5.0.mm.3D_firstorder_InterquartileRange	log.sigma.5.0.mm.3D_firstorder_Skewness	log.sigma.5.0.mm.3D_firstorder_Uniformity
118.4355011	216.4847264	0.204572135	0.045254602
124.2962344	199.1555977	-0.363500776	0.047396789
103.4165293	236.7304878	-0.129744336	0.046140079
128.9397141	212.0634613	-0.189767594	0.047201111
101.7308356	202.4375916	-0.090175185	0.049803583
123.9487677	227.7712307	-0.295534852	0.045522847
57.59536187	105.5055456	0.726891255	0.083364577
83.71751673	161.9889612	0.534962968	0.057550681
70.33276254	132.7480354	0.740880224	0.068301696
74.90268407	189.2193413	0.057958336	0.05508004
34.18923094	106.3708019	0.528834626	0.093919549
56.55070768	115.8570004	0.508221647	0.075893835
116.7605789	238.8722343	-0.36617654	0.046902866
136.8326926	247.9908929	-0.396467977	0.043439682
79.91919617	171.2361979	0.562169303	0.057378685
76.86081161	160.4863682	0.387358093	0.063243111
37.81960129	125.863142	0.181002451	0.077804099
144.9447281	240.490695	-0.255461725	0.042963636
34.10061523	91.02700281	0.343672861	0.092831432
73.88777562	151.8408241	0.460238653	0.060727469
90.39643348	203.3690262	-0.189880641	0.052120339
89.94539009	167.4234695	-0.625687012	0.061532583
84.95855387	176.557023	-0.011897324	0.054842564
39.22699988	82.19750023	0.478720871	0.102021276
102.3898637	205.9682045	0.157366746	0.04998992
101.0496909	194.5390224	0.451882735	0.05191167
91.37978321	205.7594776	0.243931498	0.050803582
55.50697883	148.5138168	0.232939817	0.066029631
31.40042874	108.3445376	0.023487165	0.090542316
66.01023827	156.7001581	0.177691111	0.061630338
82.00301624	186.3470421	0.449229165	0.055434079
99.18795649	219.4408951	-0.385538199	0.050223531
92.37712895	172.1802845	0.502270346	0.055619678
72.47549304	165.9044018	0.408967455	0.062224108
74.00688878	165.9352074	0.537444033	0.061356159
95.82110176	200.2931671	-0.273716175	0.050767331
107.3292409	195.4429476	-0.473987735	0.049589177
86.47989555	138.1125488	0.388050825	0.071023554
60.77037981	151.6689711	-0.501965916	0.069387755
57.9915118	120.7639751	0.541618572	0.075512599
102.1208906	221.7681098	0.125603653	0.048631098
32.03435032	75.11422634	0.635604496	0.115255376
120.7880667	228.7605877	0.129716578	0.044370146
104.6256083	200.9077015	0.233087891	0.049104344
53.41307788	100.8527699	0.367743513	0.084112582
93.22637631	198.8041077	0.156867023	0.051194655
58.54777652	139.9998627	0.317626115	0.06739711
46.64631214	127.8188829	-0.233195398	0.075397895
100.8858384	158.8850746	0.393243557	0.056952793
88.20649813	186.2352715	-0.263826041	0.055172684
94.56118546	209.6551676	0.475622344	0.051916572
91.57345631	187.7901459	0.236100851	0.052337187
34.83170791	74.96979618	0.699926576	0.118383014
52.35306313	131.8540459	0.467582221	0.07264024
151.4336501	261.0159187	-0.368604019	0.040718444
103.7219602	214.8066788	0.118361624	0.047782463
74.14224347	157.6252499	0.507788715	0.061547962
81.47609759	217.5213642	0.0751303	0.051981643
75.97584356	180.3063116	-0.278186329	0.057229086
48.20942719	104.6441574	0.270561631	0.092892516
73.08794012	167.8670349	0.37174359	0.059170294
112.0994089	215.2093964	0.134303011	0.046468161
95.07693864	194.024292	0.321005795	0.052198893
104.312531	172.1362381	-0.003519842	0.05226695
57.98553307	171.015049	0.057539511	0.060792575
93.97466291	192.3163834	0.155040383	0.05052045
98.57456019	186.3505096	-0.649190678	0.056346915
83.54487645	168.4634743	0.535633584	0.058086937
59.42011438	142.8870831	0.378872056	0.069013865
77.5412216	177.5824337	-0.069000534	0.056314082
84.35764724	225.3272305	-0.060051	0.053423888
86.55212741	147.8131065	0.329863003	0.060853246
107.7438344	249.014698	0.090214585	0.046766822
59.99316799	160.13051	0.322391673	0.062255956
76.30126536	118.2615256	0.689472926	0.072564179
44.73778334	147.1485462	-0.022795915	0.071646233
79.30338964	183.0941162	0.021568577	0.055236603
131.7622723	204.1772308	-0.759440991	0.053091662
83.2558497	178.1452007	-0.087957981	0.055334037
47.95137013	130.7822533	0.11652393	0.073364815
119.3310928	215.378648	0.2039465	0.045408926
88.37757472	179.499301	-0.443070423	0.056144434
70.28354793	179.5335674	0.226292371	0.058190418
51.58177715	134.4963083	0.264281589	0.069780538
79.16830669	139.4776974	0.476693333	0.065466866
101.0929903	162.4469051	-0.767971757	0.066197098
77.32820869	186.5955896	0.030686758	0.05717579
68.24030561	112.1005964	0.555613036	0.080019186
54.44933367	138.3975029	0.259122109	0.06906509
103.1163189	199.8701859	0.047683849	0.049488706
104.483839	204.2665539	-0.137759816	0.051601446
59.93818659	121.2955103	0.1779608	0.072721612
59.38903315	146.8930054	0.265180183	0.066777182
78.7561679	168.5030785	0.42634568	0.059223998
45.12762861	135.3955135	0.175224213	0.072893823
129.4323893	245.0154076	0.123524726	0.041783028
88.74760307	207.3787384	0.115861654	0.051431531
79.85104222	193.0193367	-0.445646773	0.060743705
50.11166614	137.692276	-0.653860661	0.076603921
80.16363441	177.1414108	0.175627459	0.055206058

log.sigma.5.0.mm.3D_firstorder_MeanAbsoluteDeviation	log.sigma.5.0.mm.3D_firstorder_Energy	log.sigma.5.0.mm.3D_firstorder_RobustMeanAbsoluteDeviation	log.sigma.5.0.mm.3D_firstorder_Median
125.7919834	43435698.94	91.25007322	-88.62161255
127.4473055	9657861.891	89.74578661	-35.70673752
124.9547634	20569287.35	92.8590073	-10.9843812
130.3995061	9451789.791	91.02425823	-44.60678864
115.2510888	27689516.05	84.60434793	2.857310772
128.6442375	5845852.628	94.79589383	32.45644379
76.50453825	115109920.2	48.11483643	-16.47818565
101.0348331	109765618.9	67.94107161	-42.05788231
89.8584095	60361320.57	59.06276291	-34.73728371
104.1509866	12412131.61	77.94702347	35.89784622
64.59604741	10849195.13	44.56908437	5.450558662
78.86442683	37399683	50.2716886	-9.313735962
130.4650494	22678768.9	96.10173799	-31.54124832
138.5807688	26329250.07	101.3153086	-85.56402206
101.0145125	33775645.04	71.04515515	-88.03726959
96.08730879	87177098.43	66.49857431	-5.103800297
72.51160889	7098719.555	51.76072326	-6.851403713
137.7333156	17021561.85	97.99147471	-95.19457245
63.62179337	8920585.816	40.91412307	-36.5993576
95.28267824	143812914.2	63.72997491	12.75114918
112.1864742	37781904.57	83.26431368	11.592731
103.3223133	8372788.238	71.26772998	16.12412119
105.2477417	10336745.09	74.29090527	-44.0586834
61.61229711	27218896.53	37.28476907	-16.99254227
118.54528	30400855.95	85.29322245	-64.91657639
114.0889351	50310093.37	81.4364434	-22.24350452
114.7677242	47945500.12	83.51215542	-80.9569664
85.67310884	30146966.62	61.57111212	-49.39444733
65.96770585	1411329.357	46.00557315	45.04079056
92.57736041	22616201.76	65.09242514	-22.99939919
104.9336191	73408691.1	76.14655048	-58.36161041
118.9784796	26933690.14	89.95457697	-98.22337341
105.9276596	43010400.89	73.29428008	-44.72024155
93.18958106	33062995.17	67.69572691	-27.66505241
96.67455693	75251797.58	68.08974287	-34.73840714
113.4153995	39544322.98	82.49703817	-32.71643066
119.3200347	46040241.78	83.78087656	-72.89670181
96.39994788	96444504.42	63.23515465	-6.977231979
86.20763573	7661545.634	61.97436453	-32.14671803
78.3794821	106651457.3	50.38728863	-19.93476295
118.7744763	37606490.8	89.48752599	-92.29153061
53.97103647	32600165.04	32.6057127	3.488346457
129.2510256	60262291.71	94.03694793	-16.48976898
115.7039848	32316942.17	84.41900782	-59.58115578
74.20929053	16360752.59	44.38140358	-9.999939442
111.7211798	43650509.92	82.13652699	-16.41261673
84.08905856	60717965.63	58.50819589	-13.55196667
77.46375708	6078482.285	52.76918797	-42.19275665
103.7254907	6941717.837	69.40434604	0.517222106
104.6749426	7613892.625	75.00313124	-5.057620764
115.9823199	30872175.33	85.83768083	-85.79177856
109.0548909	54381659.12	77.57210072	26.3972435
56.36065452	53049327.96	32.90843973	-5.925783873
80.5153629	18766251.87	54.38800424	-40.47599411
144.0741636	19940066.45	104.623467	12.71061611
119.0014	24904935.86	88.98604668	-28.50274658
94.35911786	57725087.31	66.39445242	-23.84447861
112.9872027	42959613.03	87.63291182	-61.41459656
101.5721173	19331455.75	73.64646248	-88.87288666
69.54658578	98302334.18	43.65928526	4.0403409
97.19952607	20433077.89	67.8160148	-32.77810669
121.7530009	57190424.9	86.4770508	12.64208508
112.1287205	157854085.1	80.25099001	-8.344539642
109.6342823	40924179.26	74.0394155	-1.750467896
92.49858485	9000218.119	68.04820935	-29.41602516
112.0172992	66805491.71	79.51982791	-10.71682644
113.4491477	14761883	80.23358625	-102.4497604
100.9493143	87900498.11	69.96231098	-49.76289368
83.33693848	83573822.25	58.56979552	-11.48411465
101.4649143	33440065.77	73.02496267	27.70581722
113.9803667	10390793.39	88.66244354	-53.7456398
98.46633385	84202201.57	64.87292164	-14.85362053
129.2416285	34332436.75	100.9810829	-52.13392258
91.69882836	16262172.34	66.7035486	-73.26248169
84.77451449	55261729.54	52.87950278	-23.66396046
80.86983079	8393760.063	61.57841861	-3.360663891
102.8557463	35001473.31	74.81086998	-6.312240601
128.7398854	10908344.1	85.87465154	-46.64061737
101.6953174	22676217.65	72.50030162	40.89031982
77.34671358	22231912.11	54.21531448	-50.67543793
125.1188272	42859131.02	90.45255348	-92.24364853
104.8256635	14587483.4	75.66264184	-16.53439903
100.5913892	38399214.61	73.83946535	-50.26052666
81.13706863	26973721.03	56.85071413	-68.5474472
92.28431273	40170342.42	59.42665757	-20.87626648
112.6845204	12226970.89	72.29789032	-2.616868019
99.57801811	22521077.47	73.31826571	-53.99663734
83.18212577	49912505.9	51.13696458	-9.441860676
83.79452321	19861806.96	58.0740866	-36.45606613
115.4499195	27485908.65	83.06499002	5.887853146
115.1248371	17264540.05	80.36384827	11.81712532
80.99012137	30462659.84	52.31615505	-24.63309002
85.13147506	51960477.35	60.66139786	-23.2854023
97.78737035	51475486.61	69.24207761	0.932917267
77.12759847	29521967.79	56.36585357	-13.18666649
135.7193182	66428633.17	99.70238889	-23.7047596
111.6974917	43446590.61	84.50340121	-2.474427342
105.0934312	10105784.7	78.5912502	-114.8541183
80.00457195	2846287.734	56.09922847	-7.915333748
102.5028428	148189450.5	72.80613689	15.0891633

log.sigma.5.0.mm.3D_firstorder_TotalEnergy	log.sigma.5.0.mm.3D_firstorder_Maximum	log.sigma.5.0.mm.3D_firstorder_RootMeanSquared	log.sigma.5.0.mm.3D_firstorder_90Percentile	log.sigma.5.0.mm.3D_firstorder_Minimum
1172763871	312.032074	173.4362244	134.0296997	-459.5888672
260762271.1	277.1914673	166.8306177	143.4803284	-486.8921204
555370758.3	373.5210571	151.8543354	178.3531769	-439.3548584
255198324.4	310.91922	171.3282943	169.800293	-468.6414795
747616933.2	359.4605713	140.0858197	172.612381	-315.1899719
157838021	306.2545166	155.4233439	207.0106201	-370.8814392
3107967845	538.4494019	104.5097176	133.8959167	-267.137085
2963671710	429.935791	134.496313	137.9548401	-384.7572937
1629755655	394.3720398	119.0070373	155.459671	-287.9004517
335127553.4	353.1862793	128.2182102	200.8085754	-250.1890869
292928268.5	299.5086365	83.50143977	136.1261063	-183.0559845
1009791441	408.1882324	104.7726416	139.7555786	-266.3261414
612326760.2	302.6298218	167.2242707	120.6342926	-462.2403564
710889751.9	278.0654297	202.1977465	88.2465416	-523.0565186
911942416.1	376.8243408	146.4406159	101.479097	-337.4417725
2353781658	424.7654419	123.0026811	182.8904694	-283.3049011
191665428	238.2481537	89.66225513	115.947966	-213.1385651
459582170	287.7087402	204.0037025	114.6573822	-515.6225586
240855817	228.8666382	90.30005748	82.60814285	-240.1229248
3882948683	514.4251709	125.1430257	182.6525635	-617.6329956
1020111423	296.1750488	134.8401266	163.9666367	-318.309845
226065282.4	246.4473877	129.9254457	139.0194626	-377.0227661
279092117.5	329.9560242	136.1052269	129.7897354	-365.2116089
734910206.2	357.5086365	86.98914022	87.45931396	-260.5171814
820823110.8	369.1930542	157.341658	148.9229477	-461.9919128
1358372521	485.3682251	140.296597	200.1236115	-345.8586121
1294528503	330.3343506	154.6767606	121.73032	-436.5361328
813968098.6	296.6214294	115.6754544	90.08951111	-309.3175049
38105892.64	268.7146606	98.31906952	163.9380646	-175.0765991
610637447.5	362.5942078	118.5214339	120.9591522	-308.0752563
1982034660	373.7902527	135.3180442	138.3182922	-347.703186
727209633.8	169.9447021	184.877824	57.75551758	-511.0927734
1161280824	467.0109863	135.7198287	156.569989	-353.4013062
892700869.5	437.9380798	117.0071845	141.1823242	-325.5610962
2031798535	402.9729614	121.0801101	152.5940582	-304.696106
1067696720	296.4309082	142.8082074	141.1983826	-409.2975464
1243086528	270.2858582	174.4989838	85.6948349	-529.9584961
2604001619	473.7154846	128.7181956	165.7237549	-355.7312622
206861732.1	173.7042236	116.967224	74.55136032	-330.2492371
2879589347	485.2563171	106.251835	108.3168274	-434.4906921
1015375252	310.5802002	167.5247605	96.96193161	-402.7659607
880204456.1	424.9648132	75.81266934	100.8185699	-278.7063293
1627081876	438.3611145	157.8682059	185.461409	-406.3552856
872557438.6	386.7780457	150.8589075	140.3360718	-360.8354187
441740320	402.5710144	102.5408333	110.7459755	-282.9338989
1178563768	358.4256897	134.8337399	180.7187347	-326.48703
1639385072	402.7824402	106.5224461	128.6678772	-331.1473694
164119021.7	199.5023804	111.2645541	66.24622345	-288.9355164
187426381.6	418.9590454	133.2432139	191.3913727	-266.1334229
205575100.9	251.141861	129.7878854	160.5911377	-350.9295044
833548733.8	355.5379028	154.8796443	130.1278168	-354.679718
1468304796	426.2032166	139.7377116	221.8722076	-356.8908081
1432331855	410.83078	81.55439505	78.31203461	-253.6583099
506688800.4	342.0239563	106.906028	107.9578339	-353.2345886
538381794.3	368.9179688	176.0993748	224.256369	-449.9308472
672433268.2	324.6608887	145.5254842	168.5051727	-327.9355774
1558577357	463.8245239	119.905616	144.3291107	-314.1368713
1159909552	278.460907	144.1650351	123.6070801	-398.4609375
521949305.3	192.4259491	159.1732233	61.5877861	-456.9786072
2654163023	462.4416199	94.99662219	113.5749664	-321.8074036
551693103.1	398.3556519	123.9950662	137.4199646	-336.8207092
1544141472	490.7164307	152.3186913	224.132901	-412.4152527
4262060298	513.9681396	138.4594115	199.5203217	-445.5875549
1104952840	434.0725708	139.7315093	175.5614243	-388.8535767
243005889.2	278.5713501	115.3007635	128.4056946	-289.5634155
1803748276	464.4614563	139.7428336	168.211319	-426.0013733
398570841.1	129.3635406	190.681194	23.07150555	-520.5953369
2373313449	448.0667419	131.4771222	142.768573	-384.9571533
2256493201	427.8082581	105.50499	117.8178825	-317.5823975
902881775.7	336.1057434	125.4747402	178.9999863	-334.0100403
280551421.6	246.5954437	145.3254925	109.8106453	-330.9280396
2273459442	439.539093	130.0179914	156.6131897	-341.562439
926975792.3	348.3767395	160.3657728	138.6388733	-362.1427917
439078653.2	303.7845459	130.8360659	88.42691498	-316.6785889
1492066697	522.335144	116.3240113	133.6792801	-349.3266907
226631521.7	228.7175903	95.62180442	120.0277977	-223.2439423
945039779.3	379.634613	125.8766872	152.3628632	-356.6951599
294525290.7	219.22995	183.7714971	87.93317566	-575.6700439
612257876.6	341.9759521	131.3174474	202.4661194	-312.7051392
600261627	341.6942444	111.1353332	69.56253433	-349.7572937
1157196538	308.11203	174.5938816	129.6841202	-459.5888672
393862051.9	238.9956207	132.8924058	117.0621758	-400.213501
1036778795	352.302887	129.718928	124.0644615	-318.1836853
728290467.8	328.2798767	119.3699807	74.56728363	-326.7531433
1084599245	416.4957886	121.637602	153.6101776	-304.6375427
330128214	312.7130127	151.7442273	96.17681885	-555.7094116
608069091.6	306.4538879	131.2171831	101.7912476	-367.7503052
1347637659	435.8415222	114.06841	152.8822174	-276.6743469
536268787.8	341.4944153	108.0580737	123.0627289	-307.9526978
742119533.6	371.3859558	141.7982242	178.804715	-324.9685974
466142581.4	315.2445068	146.5377375	223.1434357	-463.0202637
822491815.6	328.3576355	109.6862174	107.6487312	-362.276001
1402932888	399.1308594	107.4439731	116.7870102	-358.9727783
1389838138	456.3809204	122.2200885	181.1914673	-295.1571655
797093130.3	281.1937561	94.48340882	117.7282516	-286.9425049
1793573096	431.7799377	166.9262099	187.4568359	-414.2307434
1173057946	403.5227966	133.1121254	175.6261337	-292.193512
272856186.9	114.1736832	179.6854253	14.63889542	-475.9481201
76849768.83	194.5317993	103.0556967	80.84061356	-316.4042664
4001115164	429.1925659	129.5913142	196.5262268	-385.1822815

log.sigma.5.0.mm.3D_firstorder_Entropy	log.sigma.5.0.mm.3D_firstorder_StandardDeviation	log.sigma.5.0.mm.3D_firstorder_Range	log.sigma.5.0.mm.3D_firstorder_Variance	log.sigma.5.0.mm.3D_firstorder_10Percentile
4.627004213	153.7074516	771.6209412	23625.98066	-272.3903748
4.596656193	158.3157714	764.0835876	25063.88346	-286.147699
4.57970671	150.8957123	812.8759155	22769.51599	-224.9277664
4.63162535	162.044141	779.5606995	26258.30362	-281.2143433
4.471079814	139.7956528	674.6505432	19542.82454	-211.8651581
4.578169888	154.8716529	677.1359558	23985.22888	-208.5595276
3.970198191	103.7732791	805.5864868	10768.89345	-137.0053619
4.375846181	130.0875836	814.6930847	16922.7794	-192.5435852
4.161338466	117.2660204	682.2724915	13751.31954	-159.6405869
4.300956335	124.3200358	603.3753662	15455.47131	-138.3637207
3.676949544	81.40869027	482.564621	6627.374851	-76.45198822
4.049594378	104.7658741	674.5143738	10975.88838	-133.6541412
4.602589795	157.1737227	764.8701782	24703.5791	-286.0708618
4.687547117	167.6625641	801.1219482	28110.73539	-359.5563629
4.322886516	126.5624009	714.2661133	16018.04132	-229.1431549
4.254819834	122.9966336	708.070343	15128.17188	-156.023407
3.851890615	89.54900493	451.3867188	8019.024284	-115.4338608
4.71508772	169.7654979	803.3312988	28820.32426	-359.5583923
3.742420185	83.924392	468.989563	7043.303572	-136.1891312
4.311622153	123.2485902	1132.058167	15190.21498	-128.3674286
4.397487635	134.7743363	614.4848938	18164.12174	-197.7574188
4.278527789	129.2992431	623.4701538	16718.29428	-203.3709946
4.395558016	130.8557019	695.1676331	17123.21472	-211.1912643
3.716355318	85.15667073	618.0258179	7251.65857	-122.4651245
4.534508294	146.1474325	831.184967	21359.07203	-243.0518097
4.482703584	140.2955499	831.2268372	19682.84132	-168.5464249
4.490641533	139.734774	766.8704834	19525.80707	-232.6344345
4.099993048	105.447176	605.9389343	11119.10694	-183.6606598
3.700823563	82.31355574	443.7912598	6775.521459	-46.85344315
4.228883099	115.7488785	670.6694641	13397.80287	-176.4726089
4.354175975	128.418722	721.4934387	16491.36816	-197.647699
4.448145967	141.7767366	681.0374756	20100.64304	-323.7685577
4.412242407	132.9109639	820.4122925	17665.32433	-186.6075775
4.185732849	114.7185781	763.499176	13160.35217	-163.8005096
4.252810827	119.9759337	707.6690674	14394.22468	-155.6341431
4.467497923	138.7438327	705.7284546	19249.85111	-223.4100281
4.546224696	148.2793461	800.2443542	21986.76447	-310.3920044
4.252396757	127.8267112	829.4467468	16339.6681	-180.5365143
4.041602228	106.1425508	503.9534607	11266.24109	-205.3866043
4.057454105	104.6276108	919.7470093	10946.93694	-143.447049
4.496873411	141.4206288	713.3461609	19999.79424	-273.3764771
3.53955224	75.35123078	703.6711426	5677.807981	-79.93086166
4.668150465	157.305015	844.7164001	24744.86773	-214.2463364
4.502028439	140.9414769	747.6134644	19864.4999	-238.607341
3.96438295	102.2346234	685.5049133	10451.91823	-139.4245605
4.44083496	134.7671601	684.9127197	18162.18744	-178.1065521
4.10737199	105.9362501	733.9298096	11222.48908	-148.6032562
3.949857492	98.28849065	488.4378967	9660.627393	-188.5276642
4.374045019	132.8182441	685.0924683	17640.68595	-152.717453
4.339271602	129.4234159	602.0713654	16750.42057	-188.5026459
4.434705255	139.7497588	710.2176208	19529.99509	-234.580835
4.455244966	135.1420762	783.0940247	18263.38076	-129.0871216
3.574024698	81.01266352	664.48909	6563.05165	-110.5553246
4.044146074	102.9575901	695.2585449	10600.26535	-148.7924438
4.766217668	176.0925968	818.8488159	31008.60264	-251.4230072
4.492025774	142.7104033	652.5964661	20366.2592	-221.6816254
4.242891123	118.4792827	777.9613953	14037.34043	-162.4434479
4.358255532	132.5168362	676.9218445	17560.71188	-236.314917
4.303879813	123.9414001	649.4045563	15361.47066	-267.6499451
3.877438627	94.99441534	784.2490234	9023.938947	-121.5947464
4.299471747	121.6678058	735.1763611	14803.05496	-169.5894714
4.617470391	151.3301358	903.1316833	22900.81	-179.5652069
4.478811845	137.9912679	959.5556946	19041.59001	-156.5707169
4.499204758	139.6240814	822.9261475	19494.8841	-190.2799911
4.170862496	112.3518047	568.1347656	12622.92802	-174.2362671
4.512925524	139.306123	890.4628296	19406.19592	-189.6881866
4.371105851	139.9276444	649.9588776	19579.74568	-339.9034729
4.349302546	127.1386078	833.0238953	16164.22559	-180.1886261
4.080187825	104.7394	745.3906555	10970.34191	-145.9207031
4.318085982	124.0533813	670.1157837	15389.2414	-147.5268539
4.334353726	132.9499462	577.5234833	17675.6882	-235.5560211
4.351644038	129.3169434	781.101532	16722.87185	-188.0460358
4.529781735	150.5904472	710.5195313	22677.48278	-255.4387878
4.16698547	111.8881118	620.4631348	12518.94957	-209.0465302
4.155464961	115.0076368	871.6618347	13226.75653	-154.7791214
3.926819619	95.49136306	451.9615326	9118.600419	-128.7291824
4.355411149	125.6444256	736.3297729	15786.52169	-170.4721161
4.538209125	161.7174222	794.8999939	26152.52466	-333.2759583
4.350524174	125.4619287	654.6810913	15740.69554	-128.8061859
3.989378384	97.15571892	691.4515381	9439.233719	-180.8846329
4.626104211	153.2449996	767.7008972	23484.0299	-275.4046631
4.338185955	127.9231981	639.2091217	16364.3446	-219.9150085
4.284738734	122.0027098	670.4865723	14884.6612	-198.0062073
4.055083741	101.448228	655.03302	10291.74296	-188.0325195
4.252744308	121.2582878	721.1333313	14703.57236	-155.7609558
4.370722295	146.1874435	868.4224243	21370.76864	-269.5550232
4.286546353	120.778416	674.2041931	14587.42577	-208.849147
4.092524079	113.9858514	712.5158691	12992.77432	-149.3010483
4.09466428	105.2122353	649.447113	11069.61445	-152.6164246
4.501714165	141.7816182	696.3545532	20102.02725	-199.5774902
4.490587961	145.6286228	778.2647705	21207.69578	-159.4764755
4.101964976	106.906437	690.6336365	11428.98627	-162.7770752
4.113246608	105.8181171	758.1036377	11197.4739	-151.4011536
4.292241109	121.3272682	751.5380859	14720.30602	-129.4805374
3.934009365	93.92251697	568.136261	8821.439195	-131.4053345
4.730223786	164.6799139	846.0106812	27119.47404	-245.2398773
4.40313827	133.1116881	695.7163086	17718.72152	-175.9039932
4.254987162	125.6891877	590.1218033	15797.7719	-303.8354248
3.926833422	99.45866373	510.9360657	9892.02579	-168.2715393
4.37777265	127.6509306	814.3748474	16294.76008	-144.061937

log.sigma.5.0.mm.3D_firstorder_Kurtosis	log.sigma.5.0.mm.3D_firstorder_Mean	log.sigma.5.0.mm.3D_glrlm_ShortRunLowGrayLevelEmphasis	log.sigma.5.0.mm.3D_glrlm_GrayLevelVariance
2.482806215	-80.33768266	0.007793795	38.13495286
2.665924254	-52.61721728	0.009480058	39.99326382
2.302628232	-17.03593798	0.006643783	36.59614502
2.595958718	-55.63345054	0.014529225	42.3117729
2.342879681	-9.01178928	0.020106489	31.50979486
2.284676175	13.08384246	0.013566561	38.26393853
4.038899627	-12.38497571	0.01460416	19.34085928
3.329114575	-34.15375275	0.006494941	28.11918069
3.37665771	-20.28189813	0.012234348	23.32339763
2.216633542	31.37575662	0.012957345	25.28364429
2.983841757	18.57728702	0.017948717	11.07993376
3.59908288	-1.190818321	0.01455846	18.79481551
2.460294481	-57.09971641	0.009846954	39.46082304
2.413831929	-113.0185529	0.014901519	44.61755005
3.132219499	-73.66690349	0.016320214	26.37251772
2.765860563	1.21970654	0.012396158	25.82910905
2.581047432	-4.505076037	0.023049231	13.03826843
2.497613771	-113.1246497	0.015915084	46.13809851
3.302898317	-33.32861846	0.026589708	12.12803821
3.490324585	21.69243905	0.001606133	25.32720428
2.236387366	-4.211652576	0.01695056	29.05977113
2.921399896	-12.74076803	0.010991809	26.81406553
2.78237523	-37.43551867	0.015742635	27.52153311
4.188362845	-17.76096693	0.012888754	12.79443682
2.591358568	-58.28657933	0.006729732	35.18285186
2.8101862	0.542031659	0.010042311	32.10147
2.612128904	-66.3256601	0.007423624	31.77130377
2.705146206	-47.5573739	0.013316718	18.12962981
2.829196365	53.76911727	0.019393433	11.21268366
2.892468305	-25.48582773	0.01321966	21.71889079
2.714737872	-42.65682742	0.011866595	26.87828598
2.310673793	-118.6556648	0.008481593	32.29293967
3.112650191	-27.4690294	0.008380922	28.86039271
2.787167393	-23.02887447	0.007935744	21.49141028
2.909563004	-16.31466778	0.009887668	23.89275314
2.526613719	-33.8279913	0.010284096	31.11514516
2.766616982	-91.99527644	0.005995013	35.48272773
3.24186065	-15.12302186	0.00722782	28.73054715
2.623419876	-49.14356926	0.016706583	18.28424858
4.332216658	-18.50717441	0.003611626	18.75671869
2.29777004	-89.80395946	0.01156713	32.1760453
5.173915202	8.351817257	0.006748507	10.09156176
2.577511168	-13.32301398	0.007127791	39.65991159
2.539903435	-53.79507485	0.013917759	31.75631465
4.075187973	-7.918602346	0.011716306	18.1543584
2.395787246	-4.236740757	0.009213877	29.33838204
3.029630505	-11.15985837	0.006832694	18.56049501
2.848896524	-52.14569584	0.029459559	15.66308554
3.043228096	10.63334869	0.028923559	28.62817128
2.485961548	-9.719805422	0.011066447	26.98131762
2.549635133	-66.76607766	0.011994032	31.54427085
2.786158145	35.54219036	0.006182144	29.66111565
5.663213218	-9.38443936	0.010722519	12.43530747
3.378725632	-28.7859944	0.006855402	17.73984382
2.473086495	-1.545050292	0.015717738	49.90194475
2.214965249	-28.48521271	0.013710801	32.42847425
3.14472428	-18.43953129	0.010266131	23.20997019
2.039936792	-56.77011063	0.008979416	28.09900977
2.548617676	-99.87314127	0.008707142	24.82637575
4.170373773	-0.647519241	0.007767347	16.60315974
3.073221966	-23.91069807	0.010406593	24.22658409
2.643601112	17.32552243	0.0051826	37.19025981
2.81388637	11.37623069	0.003672291	31.33919585
2.989603076	-5.478191672	0.00811334	31.82306092
2.327970038	-25.91019164	0.019542972	20.49437428
2.922030103	-11.0391861	0.004807373	31.46438367
2.738149655	-129.5359875	0.012140951	31.74889137
3.205242154	-33.49638878	0.006634608	26.52740371
3.249925099	-12.68704062	0.008205823	18.01681023
2.472687254	18.83265867	0.007166468	25.00959431
1.976424003	-58.68398896	0.021391284	28.3617698
3.280189581	-13.48355431	0.010323662	27.94019649
2.067492017	-55.13345907	0.017254066	36.19670129
2.594266477	-67.81686054	0.018722295	20.3488952
4.369503737	-17.4504746	0.008422743	22.71820382
2.175414106	-4.992901118	0.032192957	14.77553068
2.624733023	-7.643212307	0.008597742	25.70626906
3.131464178	-87.28939511	0.008074392	42.24359892
2.570600143	38.77597755	0.009138121	25.72413753
3.085282383	-53.96136179	0.010214523	15.63532498
2.506026842	-83.65998797	0.008810096	37.94805915
2.611568001	-36.00065178	0.008692418	26.6564274
2.510356882	-44.07197605	0.019010731	24.36640553
2.935258947	-62.90826132	0.011767587	17.06364855
3.414695229	-9.598638935	0.010850251	24.80620121
3.701438835	-40.68835073	0.00539844	35.26689478
2.474799215	-51.28862797	0.011450878	23.45743453
3.67916304	-4.339105895	0.011203635	23.00490261
2.898639189	-24.63600719	0.010606287	18.4790216
2.509236054	-2.17005641	0.0197566	32.48413473
2.850190121	16.29763003	0.005309773	34.66787896
3.548281041	-24.53731895	0.007422695	19.30768853
3.123619864	-18.62078054	0.006300571	18.30910933
2.919066011	14.74598294	0.010746902	24.07641694
2.502433857	-10.27985148	0.009434729	14.25764271
2.45250019	-27.29259072	0.009132148	43.60896081
2.274549656	0.341190543	0.016025697	28.49891711
2.445416096	-128.4098134	0.011372693	25.86905153
2.959827659	-26.98982828	0.017188409	16.16233883
2.66993139	22.34163455	0.004511239	26.7362716

log.sigma.5.0.mm.3D_glrlm_LowGrayLevelRunEmphasis	log.sigma.5.0.mm.3D_glrlm_GrayLevelNonUniformityNormalized	log.sigma.5.0.mm.3D_glrlm_RunVariance	log.sigma.5.0.mm.3D_glrlm_GrayLevelNonUniformity
0.008171392	0.045046267	0.093844933	60.55390624
0.009787839	0.04726291	0.064560375	15.50615012
0.006891528	0.045963389	0.101826344	38.29782017
0.014987498	0.046833344	0.05402553	14.35716851
0.021196099	0.049588994	0.094332283	65.03251276
0.014130381	0.04569767	0.070627752	10.42033172
0.016212005	0.073107258	0.508608755	627.6373157
0.007013424	0.055788037	0.169895725	303.0281932
0.013500498	0.064704871	0.232765901	240.5434641
0.013748093	0.054819163	0.114413729	38.0437993
0.019694933	0.090135206	0.220824131	121.8558885
0.016056757	0.071444451	0.25621278	209.0680244
0.010256671	0.046403585	0.08136694	35.2539805
0.015946752	0.043573624	0.056874124	26.66032349
0.017680077	0.056225174	0.123695027	80.82831179
0.013508326	0.057971951	0.293808295	288.0494593
0.025125843	0.076789499	0.150750794	60.46412654
0.016503136	0.042972967	0.055091899	16.7263865
0.029210786	0.087311013	0.224706232	82.61057968
0.001723359	0.058901246	0.167241189	484.0775475
0.017902604	0.052061595	0.108113776	100.0304825
0.011591727	0.060946501	0.087967394	27.98501683
0.016531355	0.054452851	0.08834273	28.38755129
0.014463259	0.092262727	0.347005564	276.7762321
0.007131017	0.048312169	0.164041367	53.37562164
0.010713477	0.050850507	0.142218763	117.9831437
0.007863458	0.050142843	0.108191821	92.92531023
0.014442774	0.065248358	0.155519576	131.5934348
0.020392111	0.087888957	0.15976782	11.35415217
0.014038938	0.061175853	0.106706045	90.8104532
0.012819422	0.054645581	0.144754397	198.4919423
0.008807664	0.050289199	0.079852916	37.15099866
0.009037346	0.054494841	0.143266526	114.988707
0.008566695	0.061498915	0.186894263	132.5645921
0.010756735	0.059725475	0.16406893	274.1480098
0.010915633	0.050191087	0.117228922	89.50079484
0.006272565	0.049251916	0.078580266	69.7892499
0.007937152	0.059756848	0.396815957	292.2337824
0.017803965	0.068970286	0.107712766	35.46308206
0.004015536	0.071963381	0.285034213	578.6301411
0.01236671	0.048638612	0.094797495	60.43008478
0.007758983	0.104535147	0.408721427	480.8404139
0.007503792	0.044334035	0.111878677	99.81639573
0.014845684	0.049223808	0.086930056	65.24487484
0.01284708	0.077858671	0.276484267	103.9089487
0.00981404	0.050778793	0.114700824	112.5164057
0.007444703	0.066129331	0.191027044	313.7099235
0.031734795	0.074359737	0.100246509	33.5630204
0.031492775	0.056318702	0.095560106	20.42828151
0.011475077	0.05516159	0.077158188	23.39293282
0.012918546	0.051388399	0.099913793	61.2554004
0.006483099	0.051851043	0.102316358	133.1830089
0.012425773	0.098801971	0.626502871	604.9622627
0.007419772	0.070783256	0.182904105	102.3785642
0.016510339	0.040623904	0.062673159	24.76824141
0.0144484	0.047859965	0.088648366	52.57310175
0.011263355	0.060125947	0.20328253	214.2783815
0.009533268	0.051852138	0.104995266	99.17340222
0.009128198	0.056881042	0.100079248	40.08715556
0.008820241	0.076211358	0.703630891	654.829551
0.010978816	0.05841226	0.109410327	71.34682655
0.005432185	0.045818716	0.117163408	104.2614386
0.003957818	0.05081572	0.164657542	375.6551397
0.008636164	0.051575023	0.124597054	98.64177017
0.020606665	0.060293345	0.104329134	37.68038738
0.005072788	0.050263046	0.105629745	158.5146024
0.012529135	0.056006945	0.059987844	21.5299216
0.0071538	0.057057243	0.136255524	262.917294
0.009019102	0.068087965	0.222071326	447.789769
0.007532607	0.055714967	0.115229799	108.7897447
0.022541695	0.053148653	0.086088097	24.42858944
0.011150743	0.057976727	0.182978956	258.4411852
0.018312256	0.046673246	0.083425954	58.35633528
0.019794803	0.061839468	0.100659779	54.5311559
0.009358622	0.068139101	0.260970676	238.258568
0.034934922	0.071365397	0.151361507	59.58078037
0.009155132	0.054717134	0.136818115	109.9679281
0.008208151	0.052621859	0.051720244	16.17602905
0.009550059	0.054757161	0.103765839	66.34363331
0.011097395	0.072052065	0.177876138	114.7736422
0.009203168	0.045200103	0.094545896	59.15489484
0.009015144	0.055792622	0.100864289	42.71144511
0.020810511	0.056671874	0.166725054	117.375698
0.012731996	0.068331903	0.15559111	115.7793173
0.011723398	0.061862823	0.226910738	148.1077819
0.005527641	0.06409355	0.081476964	31.78952535
0.012238086	0.057096576	0.115963303	68.49634647
0.012598155	0.068456176	0.385038292	218.6670358
0.011375604	0.067068971	0.159468697	102.3950064
0.020798046	0.049145786	0.102861063	62.27434319
0.00551314	0.050694079	0.104684289	37.48843987
0.008083231	0.069390476	0.228091747	153.6549906
0.006818182	0.066130341	0.161005045	265.97566
0.011510074	0.058503322	0.140551473	183.1911095
0.010403218	0.072625053	0.178770637	211.8480153
0.009642986	0.04170061	0.081630756	93.07438382
0.017053424	0.051305804	0.118217526	115.4061422
0.011720518	0.059899121	0.085951348	17.49113963
0.017938042	0.076104798	0.097285542	18.9017734
0.004823377	0.054132249	0.157095105	430.0135524

log.sigma.5.0.mm.3D_glrlm_LongRunEmphasis	log.sigma.5.0.mm.3D_glrlm_ShortRunHighGrayLevelEmphasis	log.sigma.5.0.mm.3D_glrlm_RunLengthNonUniformity	log.sigma.5.0.mm.3D_glrlm_ShortRunEmphasis
1.249866753	292.1271458	1181.506851	0.950117128
1.186608169	364.0417217	294.9378483	0.958470402
1.256286059	339.5552296	742.4429028	0.953702128
1.159760706	329.7084448	279.0385465	0.96354179
1.254105072	193.8310425	1148.893984	0.948808334
1.200073964	285.7620532	204.3496149	0.957141758
2.035288329	126.2143157	6320.061872	0.879933324
1.422150033	243.6486445	4496.479418	0.926461082
1.558459709	152.472453	2970.142805	0.911526286
1.302096605	180.666756	598.1116359	0.941875719
1.563830107	89.55289294	1062.108716	0.904095033
1.624434609	140.0352607	2265.354262	0.89975754
1.224145411	317.0896868	674.8285999	0.953797725
1.165660801	324.381957	554.7047123	0.96248196
1.328590313	155.9728103	1223.294398	0.937059491
1.657561759	170.5824046	3956.018961	0.908440903
1.410518117	94.34365824	640.0566078	0.920034211
1.160910367	322.3897417	354.0390619	0.963539551
1.57604151	89.55263182	738.298519	0.902103651
1.419473139	670.4295407	6791.607305	0.925891159
1.279442939	195.8923225	1675.259546	0.94689764
1.259633722	267.2038872	396.9908439	0.942902239
1.237462834	214.5897266	461.1009019	0.95212102
1.804345799	117.4720789	2229.34223	0.883469648
1.410356458	310.7742123	924.2296153	0.929099342
1.361571072	233.5522167	1963.749822	0.934745429
1.282082811	275.0612831	1614.106075	0.945968313
1.40929338	144.3393442	1657.382448	0.923580751
1.453544716	114.2782614	103.1463563	0.909907225
1.285562627	170.0844871	1283.371879	0.943646713
1.366740341	183.0782831	3065.742101	0.933761783
1.219864949	299.7948851	656.3816092	0.954243931
1.372784661	225.7648582	1767.697767	0.930837623
1.449927021	192.9810408	1785.489228	0.925739199
1.418863059	181.4718932	3782.341362	0.924808222
1.302703709	276.3951296	1540.297754	0.94295324
1.220509581	373.1031248	1256.78435	0.953299451
1.824556522	228.5445038	3769.386022	0.897870701
1.300049373	164.2045255	439.5149059	0.938494177
1.677492872	304.2638096	6157.577238	0.895277095
1.260362785	217.697626	1080.696537	0.946211025
1.949951689	156.177358	3266.323463	0.865706698
1.274128058	317.1504063	2003.197018	0.952710445
1.236152869	203.7632332	1169.333743	0.951595642
1.655267209	153.2078225	1032.091595	0.89894927
1.292474463	226.7118797	1926.716184	0.945457626
1.467514373	203.362779	3877.151932	0.921320747
1.28835737	117.5009128	385.0823029	0.938308276
1.261385673	163.7447566	316.4793529	0.94697661
1.215387178	243.6910793	376.498832	0.954114633
1.268846271	193.2239398	1038.014187	0.946255498
1.279901537	301.2139004	2215.25863	0.94296604
2.358204237	116.4619605	4083.655334	0.841207665
1.480113907	207.6463279	1158.611193	0.912931249
1.176965339	375.7813776	551.6596249	0.961523003
1.236513492	202.5883766	972.0219503	0.952620965
1.483832097	176.3951007	2929.647445	0.922328452
1.276230754	221.4903866	1665.89943	0.946408997
1.275020516	252.3671116	609.4246994	0.943712833
2.336692123	174.2541982	6134.068452	0.867476563
1.296692241	199.0822289	1048.035668	0.94056014
1.294038528	349.123179	1978.288639	0.94563474
1.409948086	369.5243193	6145.00125	0.927931255
1.328489858	279.4946249	1624.358607	0.936843872
1.280455092	146.0189624	541.6573988	0.944474499
1.284352999	339.0829536	2721.038171	0.943094168
1.177661944	286.6415012	345.9908139	0.95938609
1.357358823	245.5249705	3866.379095	0.932071922
1.534706129	172.7604164	5268.990084	0.913000354
1.302057723	241.2540754	1682.289793	0.942118562
1.238435257	169.1557968	406.29506	0.951180282
1.43707447	210.502628	3698.736041	0.927257183
1.22481711	205.9073354	1110.480318	0.954191061
1.264841797	132.2154	773.0573804	0.948580945
1.637737498	197.838369	2697.272651	0.898022854
1.373508808	95.45815539	711.9055636	0.935929978
1.347720141	241.0509543	1705.747511	0.936435972
1.156794926	463.5009319	278.8095979	0.962771961
1.286930597	238.6876906	1042.750471	0.941399461
1.46075838	156.4566481	1287.590722	0.91700912
1.250934574	287.9464404	1150.423877	0.950136347
1.269982812	266.6852247	667.5360457	0.946400101
1.394950655	155.120175	1770.062083	0.936784408
1.407645697	152.4870836	1394.494759	0.924336122
1.522841817	185.7672876	1944.419073	0.918616692
1.23075383	483.2511137	436.2274786	0.950660462
1.309583472	194.2006387	1027.798961	0.939733487
1.84271772	162.3974079	2394.968994	0.887585648
1.407250248	166.1098439	1264.540161	0.926636707
1.269352096	202.662019	1106.553372	0.947642622
1.28940083	415.7163252	634.6775936	0.940827394
1.547796839	213.6942532	1776.636612	0.912765755
1.417651287	221.0021327	3302.009872	0.923321142
1.355760883	186.0803825	2651.967992	0.935366708
1.468601188	150.310232	2335.623056	0.913467553
1.224653563	300.9113301	1978.347679	0.953347979
1.310793197	176.4199055	1930.776032	0.940341272
1.238370533	248.6185282	256.526299	0.94999371
1.266347914	160.5349489	215.7145727	0.94511403
1.395285777	307.3608324	6625.138491	0.92944342

log.sigma.5.0.mm.3D_glrlm_LongRunHighGrayLevelEmphasis	log.sigma.5.0.mm.3D_glrlm_RunPercentage	log.sigma.5.0.mm.3D_glrlm_LongRunLowGrayLevelEmphasis	log.sigma.5.0.mm.3D_glrlm_RunEntropy
371.8447521	0.930907735	0.010027141	4.982211119
451.3767511	0.945466637	0.011065947	4.84023024
441.9031504	0.934028976	0.008179102	4.898296893
392.007523	0.9519828	0.016867382	4.853281752
255.4674552	0.929400861	0.02637784	4.834024705
353.584532	0.942466624	0.016501146	4.794026055
270.9921879	0.813279613	0.028244297	4.836099849
354.5119578	0.894997718	0.009853495	4.912738504
233.9279476	0.871728694	0.020962551	4.789153867
239.9102069	0.919103413	0.017618427	4.687189905
144.3206292	0.868153055	0.029912365	4.314294853
231.5210212	0.858165316	0.024998863	4.737172273
415.2200926	0.936640425	0.012165392	4.9195436
385.0300658	0.950071667	0.020372506	4.930159293
201.202122	0.912625153	0.024330409	4.763798502
290.3303056	0.861626572	0.020752953	4.911766869
136.874503	0.891715306	0.035555339	4.37215436
387.9786146	0.951664472	0.018888544	4.933002452
144.1962038	0.864224441	0.044005509	4.395567459
1014.648666	0.894855879	0.002381337	4.858203112
265.4841509	0.924742726	0.022494355	4.779459549
355.7592094	0.9257134	0.014105247	4.632996867
274.9134594	0.934243176	0.019942607	4.704001869
223.5669523	0.832531383	0.024397086	4.522576872
456.9526917	0.899210724	0.009242103	5.006540759
319.3367635	0.907698327	0.014355539	4.945072518
352.7103511	0.924612314	0.009997137	4.875195917
209.122311	0.895045922	0.02018858	4.62093821
177.9227201	0.884088514	0.02533979	4.155639094
223.1390819	0.921978022	0.018114276	4.629726164
250.7191577	0.906057524	0.017762062	4.829220547
381.2108836	0.937524405	0.010278197	4.760079114
313.0311653	0.903607313	0.012437358	4.895975666
294.6441761	0.892371397	0.012136827	4.69792087
255.1206868	0.89406405	0.015432282	4.793153427
376.8039411	0.919665172	0.013904783	4.870838462
474.0406527	0.937067562	0.007498353	4.879692907
442.9974495	0.839202886	0.012922865	5.016912045
229.3610399	0.917994505	0.022537032	4.431214
548.3373542	0.850746269	0.006545418	4.786106483
278.424381	0.927210103	0.016127347	4.863374225
328.0617116	0.809821525	0.014610485	4.443886223
410.1866352	0.931189158	0.009589174	5.006599184
254.8742308	0.93347779	0.019151265	4.836630316
265.6416238	0.85633775	0.019344884	4.65737815
294.9553908	0.92282062	0.012887428	4.836159134
308.12649	0.886548884	0.010942105	4.670118628
158.9401792	0.919160269	0.041369768	4.343422196
211.2178539	0.92760181	0.042166355	4.704326747
310.7594478	0.938223281	0.013248964	4.630136254
237.2370752	0.926065388	0.017434081	4.806511438
398.255869	0.922248308	0.007932291	4.86831755
310.1127507	0.765623794	0.025402661	4.678322994
321.5051106	0.880727068	0.010498752	4.628614533
459.8976679	0.948079914	0.019742769	5.023005296
259.2996835	0.934131345	0.018094276	4.822576485
266.7712685	0.887441326	0.01677183	4.791320888
288.824188	0.925347028	0.012297468	4.744395714
332.7721237	0.923681823	0.011123665	4.679913609
453.1575294	0.787358148	0.017467196	4.854221009
262.5765344	0.919025294	0.013750979	4.717623589
472.037925	0.923014511	0.006723876	5.016316373
535.0825208	0.897694363	0.005549445	5.004321024
390.6146828	0.91243394	0.011157545	4.947263724
190.2141133	0.923190547	0.025714354	4.543233226
456.3037839	0.921817732	0.006355746	4.925421837
351.2104291	0.946760136	0.014126558	4.632102103
336.2062673	0.906103926	0.00977645	4.838101755
281.6372735	0.875845252	0.013791605	4.68393234
338.2642165	0.91916558	0.00933846	4.734119203
215.2080397	0.934021263	0.027723415	4.645655141
311.6310903	0.894491375	0.015487397	4.891045552
256.569392	0.936617689	0.023145716	4.854207859
167.209604	0.928259109	0.025599675	4.525106775
334.0485118	0.85553379	0.01457711	4.863163926
137.1435469	0.909418468	0.048791155	4.350694029
343.6992893	0.909774698	0.011922854	4.809901962
560.5914359	0.951655156	0.008755423	4.77137873
322.7680582	0.921146534	0.011488919	4.758104764
240.1406175	0.88474359	0.015716033	4.551260527
366.5665533	0.930791115	0.011128892	4.980605057
368.1467492	0.926708884	0.010514346	4.699024093
219.4558765	0.907031619	0.030453046	4.734744672
218.7950239	0.894997765	0.017609343	4.577718938
286.8710443	0.881257969	0.016964773	4.837460498
637.5447403	0.933652035	0.006099091	4.718191747
263.6783744	0.917137144	0.016098141	4.696141111
299.49716	0.831234459	0.02172276	4.898792277
238.6753729	0.897164564	0.015468208	4.609148958
266.7555851	0.926790839	0.025656843	4.873539558
566.0041501	0.919728282	0.006471214	4.890319258
347.0311237	0.87413416	0.01192159	4.713955134
328.3429568	0.893545024	0.009539672	4.649592487
257.5252573	0.908545024	0.015507467	4.760909733
228.1850934	0.882184643	0.015606024	4.504704957
380.3504467	0.936209344	0.011905738	5.070086226
238.3693477	0.917367298	0.022375702	4.825485902
324.4934885	0.932661588	0.013270252	4.561911064
217.4989389	0.92652124	0.021156253	4.256045554
454.9804947	0.900106353	0.006542332	4.892575653

log.sigma.5.0.mm.3D_glrlm_HighGrayLevelRunEmphasis	log.sigma.5.0.mm.3D_glrlm_RunLengthNonUniformityNormalized	log.sigma.5.0.mm.3D_glszm_GrayLevelVariance
305.7155421	0.878166206	42.45948533
379.2653388	0.897490301	40.83382643
355.0623756	0.888380563	40.26817908
341.0819875	0.909213014	46.80276134
204.2151266	0.875261498	32.60289511
297.2603432	0.894553618	39.2176454
141.3136133	0.735058783	28.8871198
260.098655	0.826780927	35.43652484
163.6493715	0.796823941	29.44268703
190.2447064	0.860437905	29.65313286
97.69662574	0.782116933	15.80760044
152.7816083	0.772356357	26.14631671
333.5478443	0.88711634	39.63230585
335.444514	0.90616571	40.77272727
163.3714677	0.849625822	27.6824626
185.2886407	0.792635708	31.18923073
101.3677753	0.812142739	16.45782222
334.0280005	0.908857905	46.46642863
97.51658306	0.777680565	17.30293228
722.3326092	0.825402603	39.40886886
206.8867002	0.871169874	29.19011024
282.9782275	0.862537017	30.57638889
224.6187787	0.883150565	31.60097085
130.8224332	0.741291433	19.39295315
332.0049875	0.83403649	40.83762168
246.8356434	0.844819731	35.17688783
287.282569	0.869556347	36.22510314
154.6023226	0.82029509	21.75200099
124.9103352	0.794312011	19.64408163
178.9290481	0.863691739	26.73680421
193.5787389	0.842661512	27.32824647
313.4836006	0.887585348	31.96302794
239.6594163	0.836225392	34.06024623
207.1449346	0.826314933	22.58786843
192.9423908	0.823059878	30.26753864
292.20736	0.862633613	31.53439069
390.360544	0.885692107	39.8650984
251.7217314	0.770066302	33.55667551
175.0058232	0.852806678	21.120891
336.8321756	0.763654803	30.67917775
228.1079826	0.868966907	32.3354525
177.5811743	0.707915232	19.91826713
330.7475938	0.886636203	40.61481999
212.3836186	0.881466425	30.32596402
168.1552607	0.771352317	28.64641902
237.6332571	0.86833344	29.14933103
218.5388483	0.816120573	23.62062832
124.6651179	0.851600943	19.5735627
171.6547281	0.871306044	33.86127732
255.1043973	0.88696366	29.119375
200.6805227	0.869750109	30.85400357
317.7660607	0.86177419	37.33026296
136.5318973	0.66512748	23.20023038
225.4013679	0.798729865	26.24860451
390.4444715	0.904240706	50.90813302
211.9153461	0.884080128	30.91531718
188.7687163	0.81939568	27.62196195
232.4183883	0.86996065	28.34901882
266.1581328	0.863659881	29.1161511
198.7887279	0.712410665	26.77791303
209.7695257	0.856837007	30.9779045
368.5969446	0.868455898	43.37974225
394.6970974	0.829959764	37.76129769
297.5892878	0.848500613	38.68803538
153.2047543	0.865652882	24.94247701
358.5830396	0.862140163	38.22110113
298.4197452	0.899167525	33.91280277
260.3156076	0.838142906	33.03355126
188.0429206	0.799047911	23.51853603
256.667716	0.860568047	29.64080503
176.9301542	0.881776003	28.66012397
224.7370417	0.82883644	35.1687155
214.4544456	0.887471121	34.93431489
138.1375979	0.875330482	22.68064285
216.5493892	0.769329362	32.2031317
101.6726999	0.84895745	16.38171488
256.9694576	0.847960499	32.71542313
481.8982567	0.906571971	50.33916205
253.0863803	0.859065604	31.51737999
169.1309338	0.80689958	23.03346395
301.2578262	0.878260702	42.21014178
283.0276882	0.870362155	29.22692972
163.8954311	0.851561947	25.72531678
162.7776403	0.821914189	21.6790426
199.5071286	0.810698895	32.43531637
509.879844	0.879101636	49.41668371
205.4236247	0.85526846	24.92190222
178.6697667	0.749163914	29.64518667
177.3596421	0.827085998	23.78780261
213.1088758	0.872652653	36.69897315
441.1668293	0.857280895	43.92873024
231.9104579	0.799640846	29.32694147
237.7909077	0.819739844	24.02997919
197.3525185	0.845846937	31.50455833
162.6703033	0.799027109	17.56641777
314.491769	0.885533877	46.59823329
186.4893059	0.856836712	31.02586735
261.3037877	0.877693052	30.7792562
169.7919403	0.867084396	21.63693878
329.9295911	0.832960384	33.3774238

log.sigma.5.0.mm.3D_glszm_SmallAreaHighGrayLevelEmphasis	log.sigma.5.0.mm.3D_glszm_GrayLevelNonUniformityNormalized	log.sigma.5.0.mm.3D_glszm_SizeZoneNonUniformityNormalized
206.5862079	0.042685407	0.325001052
235.1568494	0.047419461	0.342619987
226.3440623	0.044469118	0.312921073
218.9497755	0.042735043	0.338346483
107.5672118	0.048156262	0.241211277
170.2860542	0.046469978	0.295692337
108.1617664	0.05180658	0.265582597
190.9430096	0.046906542	0.294508901
133.4939678	0.050938489	0.253196799
127.8777007	0.05174093	0.264020778
59.64011855	0.070287415	0.185409977
113.1795868	0.054947391	0.261015832
187.4155927	0.044539177	0.320473747
241.4703583	0.044990724	0.380987519
143.206185	0.05380193	0.328887521
125.412629	0.048923545	0.282784079
59.55414528	0.069422222	0.218133333
202.3700255	0.042336707	0.326639641
81.99535065	0.0695595	0.258487837
399.2791166	0.044484496	0.260344712
119.2617532	0.051179965	0.265697555
157.018282	0.054634354	0.275226757
131.0994447	0.051693155	0.273448622
104.5658824	0.064160618	0.255077764
232.1872311	0.043847865	0.343418687
164.8027092	0.047018955	0.264427635
219.0764225	0.046690773	0.327936157
111.7598196	0.060322469	0.26417823
60.05097607	0.067755102	0.226122449
127.1144962	0.054455262	0.299290279
133.101269	0.053153785	0.270866912
170.0078835	0.051269681	0.291675804
163.9583971	0.049097929	0.277619301
131.9014419	0.0624	0.248514178
148.083698	0.049989998	0.292764038
159.9731493	0.049804296	0.288049736
236.5287588	0.045857572	0.297294286
199.4020877	0.0479152	0.346495346
80.4748606	0.064770761	0.180579585
225.7815383	0.051546079	0.260796889
140.6089357	0.049153125	0.264758885
131.9588444	0.067985817	0.251322692
219.8719526	0.043987001	0.326677352
149.5993436	0.051852889	0.317641771
130.1294568	0.055567738	0.286873383
169.8516816	0.050818557	0.327345143
142.2845134	0.057354615	0.261591507
73.18078459	0.065439364	0.255872441
136.699209	0.050988554	0.262617066
121.3546728	0.0528125	0.240546875
161.6454077	0.050139071	0.328095931
203.0134153	0.045736242	0.290940559
103.3072438	0.059112341	0.219594553
155.137069	0.055687673	0.268645131
236.3969015	0.039831769	0.366740864
125.3383573	0.050324389	0.263298796
136.3722887	0.053778297	0.273070464
136.1731778	0.05152375	0.239919239
154.2800304	0.054828475	0.266408884
137.5123355	0.054712604	0.263385972
143.7750342	0.050053979	0.308744637
191.9200031	0.042090069	0.283039755
254.8686753	0.04509936	0.304342332
178.4833514	0.044614474	0.265332806
95.08024471	0.057696052	0.264683613
198.6522124	0.045694331	0.268754649
175.4599407	0.052802768	0.29716263
182.58574	0.049252611	0.270641603
113.9578393	0.058894278	0.226889759
116.6262182	0.050180072	0.23614434
120.4566071	0.054235537	0.280798037
161.0257504	0.047128669	0.292228258
131.5449899	0.0459961	0.276563223
104.147476	0.058470398	0.320091084
176.8321879	0.051634324	0.271358098
58.30486344	0.06838843	0.210578512
131.7359633	0.048376449	0.24953543
249.9526082	0.045100416	0.325831025
145.513677	0.049408642	0.27634054
105.719418	0.059949511	0.226172768
205.4954023	0.043127996	0.327913098
127.1167704	0.052461573	0.272193139
123.5442972	0.054171244	0.299409949
115.7722915	0.06272122	0.256911958
142.3534453	0.049178599	0.266372146
230.258219	0.044090577	0.272169467
135.359301	0.056740991	0.305775107
151.1217055	0.050962987	0.30128505
123.1935607	0.057627812	0.302437002
125.994841	0.045682182	0.254744971
242.5958388	0.04359714	0.30394808
180.5375312	0.052515197	0.277124172
151.0098248	0.057747716	0.261995606
124.4181979	0.049459775	0.224903384
98.89704906	0.067183573	0.236023597
202.4959223	0.040138357	0.325729362
117.6070439	0.049134694	0.272253061
150.4407074	0.054710744	0.285950413
60.43181139	0.064897959	0.18
183.0066981	0.047596938	0.278736508

log.sigma.5.0.mm.3D_glszm_SizeZoneNonUniformity	log.sigma.5.0.mm.3D_glszm_GrayLevelNonUniformity	log.sigma.5.0.mm.3D_glszm_LargeAreaEmphasis	log.sigma.5.0.mm.3D_glszm_ZoneVariance
173.5505618	22.79400749	23.94007491	16.62781074
53.44871795	7.397435897	8.83974359	3.891970743
102.0122699	14.49693252	17.33742331	9.850653017
52.78205128	6.666666667	6.782051282	2.521531887
106.6153846	21.28506787	23.63574661	13.4449182
30.45631068	4.786407767	8.815533981	3.295315298
447.2410926	87.24228029	1993.157363	1953.990875
494.1859356	78.70917759	93.21454112	80.13756799
238.7645811	48.0349947	210.5153765	190.0884274
58.61261261	11.48648649	30.74324324	19.17711631
41.90265487	15.88495575	222.3274336	174.9248179
177.2297496	37.30927835	404.5346097	379.3575669
103.1925466	14.34161491	15.3136646	8.970149686
117.3441558	13.85714286	8.175324675	3.803423849
168.7192982	27.60038986	46.28654971	36.86057248
390.242029	67.51449275	388.126087	370.6924365
32.72	10.41333333	156.82	122.1671556
63.04145078	8.170984456	7.559585492	3.068699831
50.40512821	13.56410256	232.0205128	200.5455358
550.1083767	93.99574065	309.5575012	290.6701883
158.6214405	30.55443886	40.05360134	27.93807115
46.23809524	9.178571429	18.85714286	10.14058957
56.60386473	10.70048309	15.98067633	8.714135686
131.3650485	33.04271845	979.5203883	930.7376228
130.8425197	16.70603675	47.51706037	37.12870537
197.5274431	35.1231593	59.41633199	47.7083918
230.5391181	32.82361309	36.97581792	28.84966886
132.3532934	30.22155689	105.259481	85.03641818
7.914285714	2.371428571	36.34285714	18.94204082
138.8706897	25.26724138	45.70689655	33.66719307
289.0149953	56.71508903	92.12277413	78.00574621
88.08609272	15.48344371	14.0794702	7.271172317
175.7330174	31.07898894	102.6619273	89.0548031
142.8956522	35.88	107.5113043	89.87130435
390.5472264	66.68665667	125.464018	110.6582524
188.0964778	32.52220521	32.60796325	23.79079241
173.619863	26.78082192	13.54452055	6.841386752
510.3876443	70.57909029	570.7298031	555.1130874
24.55882353	8.808823529	46.82352941	29.86851211
413.1022727	81.6489899	690.2064394	654.6369627
115.1701149	21.3816092	23.30574713	13.81651473
150.2909699	40.65551839	2757.58194	2667.617666
294.6629712	39.67627494	24.94900222	17.76279861
165.4913628	27.01535509	22.0268714	14.59836944
86.63576159	16.78145695	382.2516556	355.7052761
275.2972652	42.73840666	31.24494649	23.09429491
306.323655	67.16225448	188.9385141	168.0572824
36.07801418	9.226950355	34.37588652	22.24968563
32.56451613	6.322580645	28.41129032	18.46845734
38.4875	8.45	15.2875	7.306875
152.8927039	23.36480687	26.5472103	18.91965684
251.6635838	39.56184971	37.64508671	27.27892011
188.1925321	50.65927655	4755.376896	4668.758797
93.48850575	19.37931034	181.1034483	158.8402365
109.6555184	11.909699	8.598662207	3.974004765
110.3221957	21.08591885	17.86634845	9.988881357
264.8783505	52.16494845	119.8381443	102.7053725
151.8688784	32.61453397	26.5892575	15.92640177
65.53658537	13.48780488	27.13414634	17.5140624
401.6636066	83.43672131	3146.257705	3095.235959
131.2164706	21.27294118	35.55058824	25.77209135
236.0551559	35.10311751	32.36330935	23.62752819
694.5092025	102.9167397	176.4382121	163.4188278
158.4036851	26.63484087	43.21273032	30.88639737
56.90697674	12.40465116	26.81395349	16.89877772
282.192381	47.97904762	32.76095238	22.1457678
50.51764706	8.976470588	10.24705882	4.543391003
357.7881997	65.11195159	119.6467474	104.8516099
288.6037736	74.91352201	434.2389937	399.3993018
140.5058824	29.85714286	38.83697479	26.09387473
49.42045455	9.545454545	16.5	8.685433884
394.5081481	63.6237037	176.7607407	163.1473739
136.0691057	22.6300813	16.70528455	9.342665576
104.3496933	19.06134969	24.36809816	15.87605856
201.3477089	38.31266846	488.9514825	458.6569917
46.32727273	15.04545455	55.99090909	38.5792562
142.7342657	27.67132867	56.9458042	42.03162441
49.52631579	6.855263158	7.480263158	2.964638158
108.8781726	19.46700508	30.05837563	18.91904584
77.57725948	20.56268222	175.9766764	148.4371308
170.1868979	22.38342967	24.06165703	16.72267329
72.94776119	14.05970149	24.09701493	14.59773892
218.8686731	39.59917921	54.39398085	44.64865138
103.278607	25.21393035	160.2910448	138.1167855
168.0808241	31.03169572	207.4659271	188.9527503
50.35135135	8.156756757	20.23243243	11.99398101
121.6984925	22.58291457	36.66834171	25.86770536
238.0151899	40.26075949	631.3620253	607.7842397
130.350348	24.83758701	139.1113689	123.5354353
111.0688073	19.91743119	25.93348624	16.10324783
78.41860465	11.24806202	34.86821705	25.15702181
134.1280992	25.41735537	366.5743802	339.2067823
243.655914	53.70537634	213.8247312	190.4012129
185.3203883	40.75485437	80.96601942	63.4765706
130.0490018	37.01814882	365.3629764	329.3411945
299.9967427	36.96742671	16.2432139	9.542929427
190.5771429	34.39428571	40.06571429	27.79570612
31.45454545	6.018181818	21.31818182	13.22157025
12.6	4.542857143	29.11428571	14.45632653
631.3381898	107.807064	179.0666667	163.8893643

log.sigma.5.0.mm.3D_glszm_ZonePercentage	log.sigma.5.0.mm.3D_glszm_LargeAreaLowGrayLevelEmphasis	log.sigma.5.0.mm.3D_glszm_LargeAreaHighGrayLevelEmphasis	log.sigma.5.0.mm.3D_glszm_HighGrayLevelZoneEmphasis
0.369806094	0.176384541	5403.955056	338.9569288
0.449567723	0.058171666	3407.480769	380.2435897
0.365470852	0.092888498	5720.322086	379.5736196
0.48447205	0.075998038	2098.262821	359.8205128
0.313253012	0.44458714	4580.255656	214.6696833
0.425619835	0.094135554	2502.067961	306.5242718
0.159787456	17.11616131	249657.788	190.3159145
0.27653263	0.554818827	19731.82241	318.5101311
0.221257626	2.391814917	24353.19194	233.8144221
0.294039735	0.421111133	4673.540541	225.7792793
0.145244216	4.130728806	15121.84956	128.8628319
0.199295568	4.117324465	48750.6377	201.4064801
0.397040691	0.11187733	5870.170807	323.1677019
0.47826087	0.205372922	2202.665584	364.7727273
0.325714286	0.983501705	4448.744639	220.6920078
0.239500174	2.886071058	60636.28768	217.4326087
0.169875425	3.601410138	11161.39333	131.2333333
0.471882641	0.116652449	2545.968912	340.9533679
0.178244973	4.288566593	17519.74872	141.825641
0.230099096	0.466648235	209963.0147	758.6791292
0.287295476	0.602718591	7746.21943	214.3232831
0.338709677	0.159025172	5109.22619	289.25
0.370967742	0.189561921	3171.864734	243.0434783
0.143174868	9.785635864	110628.5087	189.3029126
0.310260586	0.224812023	14031.29134	373.1102362
0.292253521	0.526153518	9593.358768	297.1659973
0.350798403	0.265149847	6926.395448	344.0953058
0.222370173	1.520056922	11719.37325	198.2015968
0.239726027	0.417039784	4238.342857	126.0285714
0.288198758	0.608324624	6595.5625	213.3771552
0.26615116	1.26102583	10311.63074	241.7160262
0.383248731	0.09380428	4394.043046	312.9139073
0.271092077	0.828442803	16009.14376	285.1800948
0.238095238	0.905647544	18655.80522	238.2434783
0.259887006	1.325049663	15019.18891	249.1034483
0.336771532	0.237900221	8500.050536	297.0796325
0.386243386	0.066978798	5327.996575	403.9297945
0.253049304	2.588769941	138205.7054	311.221317
0.242857143	0.51673788	8289.477941	181.4411765
0.167672277	2.435495439	210484.7967	410.5107323
0.324626866	0.333526584	3860.337931	264.1448276
0.105430183	18.55106912	428618.0318	242.2173913
0.373035567	0.176997906	6033.889135	361.827051
0.366901408	0.39536365	3193.523992	245.756238
0.194087404	3.045621793	54699.13907	221.1390728
0.350270721	0.29736096	5443.351962	271.4494649
0.2188376	1.325387353	34395.68659	266.2724167
0.287169043	0.872807242	4031.432624	137.3191489
0.31713555	0.676922888	3768.620968	215.0241935
0.353982301	0.122142408	3747.00625	259.4
0.362082362	0.510995864	2687.830472	255.5965665
0.31059246	0.194941782	9647.830058	355.2
0.107447342	38.82924727	607449.867	193.8424737
0.211936663	1.168079879	31245.73851	275.7614943
0.465007776	0.148138093	3276.695652	386.6923077
0.356292517	0.331698665	2906.02864	232.8400955
0.241594022	1.120644476	18238.32887	240
0.306240929	0.259254244	5103.096367	262.685624
0.322411533	0.190856549	6137.654472	289.4268293
0.139998164	16.74679501	613674.8472	246.9645902
0.319789315	0.315987254	5688.985882	240.1035294
0.338336714	0.137965423	10312.60072	370.7553957
0.277143551	0.674235764	50482.16608	442.2611744
0.284828244	0.282578547	11657.96817	325.1876047
0.317577548	0.441597457	3262.604651	173.1023256
0.306927799	0.138631628	10725.34	375.8704762
0.418719212	0.100247512	2964.052941	309.0823529
0.259980334	0.814899031	20788.62405	326.0854766
0.169419286	3.251316792	75097.2327	228.2649371
0.280131827	0.213301957	10072.09748	246.0521008
0.357723577	0.381731075	2526.034091	202.4204545
0.271029914	1.165490625	34786.15037	271.3259259
0.368539326	0.356991662	2579.780488	241.6626016
0.343157895	0.4644545	2443.279141	166.2392638
0.181684623	3.163705371	87203.18329	303.3867925
0.239651416	1.723020209	4633.695455	117.75
0.258940697	0.380499161	13562.39161	265.4912587
0.470588235	0.033545573	3639.684211	463.6052632
0.299619772	0.207717133	6972.385787	265.4365482
0.190555556	1.612950592	24643.99417	207.9300292
0.36913229	0.183184197	5330.460501	335.1541426
0.324455206	0.123739128	7964.63806	253.4440299
0.320333041	0.946896537	7405.790698	200.6087551
0.212361331	1.732066526	18370.70647	207.641791
0.232412523	1.636751684	31643.76228	247.1901743
0.348399247	0.051090145	11502.44324	468.4486486
0.304281346	0.413377404	6070.944724	228.9522613
0.205943691	5.570473245	87499.03924	248.3481013
0.253380364	1.223522679	18423.01856	210.4176334
0.318946598	0.443736358	5018.401376	237.6100917
0.320895522	0.113232536	14027.74419	445.4806202
0.191153239	1.962363884	75934.01653	298.3140496
0.206620751	1.385841352	38618.28172	277.6612903
0.239117818	0.825758392	11655.87985	240.243932
0.166616269	3.882930282	43730.75136	195.7477314
0.386325503	0.142389504	4415.187839	335.4136808
0.28548124	0.699813887	5761.567143	209.63
0.3514377	0.129073661	6236.818182	267.1
0.26119403	0.287529839	5227.842857	161.2142857
0.25668631	0.668561319	53326.99912	340.7642384

log.sigma.5.0.mm.3D_glszm_SmallAreaEmphasis	log.sigma.5.0.mm.3D_glszm_LowGrayLevelZoneEmphasis	log.sigma.5.0.mm.3D_glszm_ZoneEntropy	log.sigma.5.0.mm.3D_glszm_SmallAreaLowGrayLevelEmphasis
0.587515544	0.009725051	6.623105539	0.00656064
0.60343743	0.012987805	5.925331768	0.009531196
0.577190097	0.009198409	6.483559958	0.006743807
0.5900308	0.016154731	6.050514661	0.007664179
0.492721632	0.02056509	6.717753139	0.008198548
0.553263657	0.019337936	5.815154838	0.015067458
0.528751659	0.017092653	6.984776291	0.007870014
0.556932371	0.006852971	6.920428967	0.003890994
0.510543872	0.011712137	6.831997105	0.006152729
0.520798563	0.016645665	6.299921624	0.011194431
0.429425562	0.022925811	6.379681952	0.011012856
0.522042116	0.014374678	6.675546991	0.006990274
0.580962762	0.011247601	6.371044115	0.00714486
0.639116481	0.010742546	6.13363298	0.004530773
0.591297891	0.012622931	6.29885505	0.004801867
0.5440503	0.012591881	6.81846185	0.005853475
0.467440038	0.024288343	5.937738631	0.009329686
0.575034897	0.018931564	6.17216386	0.014316932
0.522465785	0.022777328	6.051711425	0.008068837
0.52021023	0.002121481	7.184187642	0.00134071
0.525950421	0.018525547	6.65210889	0.00834092
0.537489423	0.015093114	6.118487367	0.009774321
0.521601646	0.015805728	6.188234502	0.0053656
0.517077678	0.012494727	6.491142355	0.004877854
0.608322206	0.008623472	6.463773338	0.005522926
0.523128105	0.010711444	6.82883738	0.0053618
0.590626466	0.008208764	6.549711721	0.004675586
0.528176829	0.015023294	6.535557479	0.007579495
0.472247845	0.04576263	5.014997303	0.03884383
0.565656195	0.015482388	6.465109571	0.01043874
0.529600001	0.010848613	6.746710492	0.005135518
0.551785367	0.011635541	6.319931344	0.008130485
0.53822762	0.009593188	6.746842079	0.005578551
0.505700059	0.008854107	6.548316092	0.004820455
0.553658897	0.010431266	6.785363622	0.005484459
0.550028258	0.009798975	6.628331804	0.004167278
0.559157466	0.007182906	6.614804482	0.004289515
0.608057045	0.007667876	6.635091905	0.004786897
0.408150298	0.020208574	6.080038349	0.005504436
0.523622814	0.004511094	7.055388518	0.002719039
0.523523791	0.011211528	6.558855641	0.00622805
0.514331654	0.008878194	6.59128292	0.004758257
0.587815537	0.007857446	6.692301108	0.004675505
0.578783103	0.01364518	6.372639438	0.008390596
0.550443034	0.01439016	6.306287398	0.008538833
0.59156442	0.008955129	6.503388326	0.004818721
0.518393258	0.007186812	6.72210025	0.003529056
0.505839099	0.032736398	5.839916162	0.012199267
0.517401227	0.023634078	5.944853443	0.005096067
0.475812903	0.016050001	6.065372902	0.010527959
0.590535338	0.010920717	6.367402286	0.006593292
0.553271294	0.008983139	6.804828679	0.006421144
0.474768707	0.012489687	6.927850867	0.004707337
0.531195948	0.010026136	6.487084686	0.006822566
0.62637497	0.013269204	6.329408024	0.00690936
0.51697941	0.013902546	6.538552636	0.007661485
0.535211935	0.009467964	6.744467682	0.003961478
0.492796189	0.009265426	6.781442385	0.004012821
0.524005914	0.012661404	6.322113674	0.009245706
0.525023296	0.00951927	6.947935265	0.0041106
0.572884796	0.014239208	6.4558715	0.01017191
0.540663754	0.006846936	6.894488134	0.004514186
0.568431045	0.00415602	6.989143371	0.002545758
0.527092564	0.009186599	6.916270973	0.004198799
0.520914834	0.022453655	6.157869146	0.011067469
0.52260624	0.006109696	6.959430396	0.003603816
0.542532442	0.01573203	6.084415537	0.010693483
0.529610666	0.007049734	6.911074327	0.003343056
0.482801735	0.008720227	7.005992201	0.004329441
0.492388811	0.009876781	6.840726519	0.005881966
0.540043707	0.024588434	6.045794228	0.016813215
0.553031637	0.010693858	6.873678938	0.005354952
0.525141688	0.017000811	6.609224086	0.009148029
0.58193319	0.019286711	6.069730378	0.010214361
0.533702695	0.008617011	6.779197464	0.003416131
0.455585487	0.030927975	6.337854801	0.01295457
0.508130939	0.011053989	6.821324116	0.005503543
0.57815108	0.012894552	6.129419168	0.011084351
0.540434484	0.014011828	6.561170669	0.009865676
0.478877077	0.014541238	6.548198842	0.00877508
0.591170077	0.011790571	6.6134129	0.008337296
0.532857286	0.013965562	6.336666788	0.010005233
0.562418174	0.013437519	6.466487389	0.005586767
0.513523995	0.012838371	6.439476839	0.00503906
0.525904868	0.012492128	6.725496669	0.006436161
0.518596901	0.010074029	6.28968981	0.008271999
0.570611531	0.011778434	6.318343063	0.00541245
0.564249614	0.009970848	6.586478601	0.005300558
0.56740349	0.01282512	6.331449948	0.007442688
0.508774644	0.020709095	6.686279219	0.010115334
0.565904068	0.009353961	6.393933979	0.007618661
0.541335436	0.009017544	6.548851365	0.004053939
0.52501892	0.00718159	6.786224654	0.003232285
0.474804572	0.01224556	7.026555489	0.005610922
0.495761474	0.011399681	6.60701771	0.005529771
0.588818936	0.009073938	6.831478279	0.00449086
0.537315399	0.020010592	6.827471962	0.013246056
0.53948391	0.019256963	5.813673605	0.015125385
0.394695756	0.031209694	5.600432303	0.019948449
0.54182265	0.005615428	7.039542057	0.003361938

log.sigma.5.0.mm.3D_ngtdm_Coarseness	log.sigma.5.0.mm.3D_ngtdm_Complexity	log.sigma.5.0.mm.3D_ngtdm_Strength	log.sigma.5.0.mm.3D_ngtdm_Busyness	log.sigma.5.0.mm.3D_ngtdm_Contrast
0.009487064	0.502376804	2.848189951	0.253024278	0.000101397
0.027368798	2.601577963	8.826419517	0.069429565	0.000624732
0.012804889	1.063340656	4.269356056	0.139488053	0.000170684
0.033315131	2.759737517	10.77355008	0.058474669	0.000620604
0.009035411	0.372270356	2.216718195	0.339083042	0.000103745
0.057839459	2.123527548	13.81382112	0.045600136	0.000748918
0.001496508	0.047817943	0.64790372	1.639987436	4.00E-06
0.002165261	0.131795775	0.84987749	0.865662719	1.35E-05
0.003083841	0.111134828	0.828122451	1.053881012	2.07E-05
0.018108475	0.523253392	3.715597525	0.193658777	0.000155054
0.008956478	0.104740146	1.135423123	0.628078411	3.69E-05
0.004000265	0.125512555	1.14451879	0.713905579	1.82E-05
0.014661106	0.991166228	4.722168493	0.124050668	0.000204145
0.016285551	1.437039018	5.411455027	0.118962297	0.000340238
0.00775639	0.417764887	2.45412128	0.40568514	6.80E-05
0.002477161	0.091035624	0.651160263	1.158312769	1.61E-05
0.014795207	0.182900011	1.547146913	0.464490765	0.000101943
0.026434694	2.205803581	8.559519479	0.077956041	0.000554239
0.012238414	0.153797359	1.512961886	0.448590712	6.14E-05
0.001616369	0.093104308	0.85461073	0.54405086	5.68E-06
0.00569643	0.1943006	1.090516755	0.63135153	8.38E-05
0.021921486	0.946080741	5.166072239	0.10626422	0.000299146
0.023391887	0.987198828	5.817680799	0.11085388	0.000210544
0.003884473	0.074990151	0.958462516	0.790861431	1.12E-05
0.01144774	0.666662896	3.989268304	0.156797342	8.92E-05
0.005288985	0.286645193	1.979284626	0.439432511	3.95E-05
0.006266716	0.375640084	1.953299352	0.35449078	6.04E-05
0.006277932	0.141495027	1.217089648	0.580777164	3.44E-05
0.087594722	0.893691185	8.86203384	0.065651768	0.000517385
0.007173326	0.322613842	1.922431099	0.3965459	5.81E-05
0.003214967	0.137160517	0.884205355	1.00172896	2.70E-05
0.01195707	0.905810471	3.196371786	0.187703806	0.000247769
0.006300085	0.306653982	2.607184548	0.325625836	3.25E-05
0.00554013	0.231937215	1.893216312	0.43713084	2.87E-05
0.002585556	0.109135666	0.804827831	1.055113663	1.59E-05
0.006192033	0.321042153	1.568161776	0.373648463	7.61E-05
0.007274946	0.612262769	2.627367591	0.211048763	9.49E-05
0.002661856	0.118401541	1.042097295	0.665706697	1.17E-05
0.019552848	0.443619403	2.899704144	0.202193668	0.000229201
0.001629782	0.071967564	0.960953661	0.709590068	3.15E-06
0.009496304	0.465565948	2.731295955	0.292057389	9.95E-05
0.002491275	0.055639326	1.015551251	0.775253558	3.35E-06
0.004695453	0.439878312	1.828577283	0.395295935	6.23E-05
0.008492295	0.502546407	2.789081957	0.323762533	9.02E-05
0.008991865	0.254961484	2.556387015	0.279253073	3.56E-05
0.005096492	0.249792772	1.280404557	0.56260883	5.63E-05
0.002577627	0.095180862	0.803232686	0.826731297	1.04E-05
0.021009302	0.472686081	2.462007218	0.243251177	0.000268473
0.033518216	1.232938819	9.019192345	0.102103271	0.000305421
0.025190064	1.050152622	5.39932687	0.112769032	0.000336209
0.00866175	0.535587519	2.715786981	0.380784238	0.000109595
0.005066282	0.25276237	1.736056507	0.370519296	3.26E-05
0.001883328	0.039610783	0.674494646	1.261187031	3.19E-06
0.008209486	0.278618698	2.332120324	0.265800206	3.39E-05
0.017556434	1.499423619	6.076614144	0.104345208	0.000352713
0.010077683	0.439276603	2.227468786	0.380470969	0.000152359
0.003362407	0.161362135	1.316456981	0.689837317	1.67E-05
0.005188609	0.298967202	1.288896756	0.563437226	7.05E-05
0.014692949	0.681066352	3.432117783	0.167926761	0.000165391
0.001521473	0.042525427	0.61583607	1.150830798	2.79E-06
0.009739539	0.443029194	2.810667009	0.258026277	6.76E-05
0.00572672	0.402659335	2.519942339	0.26150952	4.02E-05
0.001813967	0.117437595	0.888113821	0.717159193	8.45E-06
0.007170527	0.313735864	2.482364419	0.255949541	4.43E-05
0.017796923	0.486218129	2.747607186	0.265037376	0.000194297
0.003967657	0.281330877	1.841112392	0.352012286	2.35E-05
0.022309743	1.581994499	5.784812125	0.099249165	0.000497921
0.002716474	0.141964524	1.126541121	0.690612439	1.38E-05
0.001779477	0.071582165	0.694943407	1.199429331	6.41E-06
0.005840916	0.222629557	1.30278977	0.454458849	5.59E-05
0.021558692	0.813087682	3.955657158	0.197219244	0.000393285
0.002819568	0.135300644	0.907822763	0.76297533	1.82E-05
0.007794206	0.52986286	2.178334453	0.408945441	0.000146438
0.01256936	0.369439623	2.389817592	0.377371008	0.000119652
0.004400703	0.139707717	2.203694187	0.388075139	9.55E-06
0.012746752	0.198850449	1.368915367	0.531485006	0.000127964
0.006047981	0.255971061	1.762716355	0.366590533	4.15E-05
0.025756812	3.206456963	11.25392788	0.054239425	0.000651439
0.011126611	0.311629329	2.368663128	0.24644082	8.27E-05
0.007514874	0.198718836	1.745978871	0.378052481	3.32E-05
0.009765678	0.511080635	2.944220844	0.246672947	0.000102823
0.013997889	0.593328629	3.452310544	0.166490505	0.000158379
0.005076579	0.250994883	1.303599653	0.653800521	5.04E-05
0.006809855	0.221910557	1.716596288	0.432106795	3.47E-05
0.005728167	0.176500745	1.697455223	0.435187896	2.71E-05
0.017129527	1.862866797	9.564620475	0.062625467	0.000218214
0.009288733	0.388478378	2.330652008	0.296471699	7.82E-05
0.003952214	0.123417178	1.28417723	0.62104814	1.62E-05
0.007540274	0.247479333	1.781004626	0.38246508	4.32E-05
0.009584639	0.374437549	2.242889017	0.336696709	0.000112199
0.019662525	0.82317926	6.672286886	0.083331907	0.000135729
0.005591657	0.173240037	1.534986265	0.376309273	2.36E-05
0.003100726	0.115654883	1.077639647	0.622263162	1.09E-05
0.003889084	0.175250098	1.345523015	0.643069275	2.21E-05
0.00419591	0.081569203	0.794735576	0.827056599	1.77E-05
0.005522637	0.410660623	2.063956918	0.365945307	6.45E-05
0.005400734	0.229551552	1.496327221	0.561991086	4.72E-05
0.031760597	1.446179749	7.264314703	0.083863591	0.000498634
0.033297472	1.08204812	5.093670201	0.108215811	0.000477441
0.001659849	0.082544681	0.616791039	0.96627211	8.06E-06

log.sigma.4.5.mm.3D_gldm_GrayLevelVariance	log.sigma.4.5.mm.3D_gldm_HighGrayLevelEmphasis	log.sigma.4.5.mm.3D_gldm_GrayLevelNonUniformityNormalized	log.sigma.4.5.mm.3D_gldm_DependenceEntropy
38.5536881	287.0090028	0.045587434	6.842870082
39.63311713	345.4293948	0.048542883	6.212453005
36.0524543	321.2533632	0.046338656	6.689068961
44.10868601	347.9720497	0.044153389	6.15991913
31.99550962	207.4500354	0.050114997	6.63304615
37.72290144	295.8429752	0.047537736	6.059050947
17.20848001	140.9669798	0.086702546	7.327842999
27.7967148	260.0125247	0.056528157	7.178684589
21.95374267	158.1342093	0.068878421	7.064967538
23.4531889	159.0291391	0.057336082	6.495204316
11.04825958	96.23071979	0.09315214	6.523665287
17.64152982	148.3668917	0.076714673	7.095972884
39.32036228	281.7348952	0.047717801	6.642209235
44.05150988	310.5419255	0.043695266	6.478075623
26.66348199	166.5034921	0.057125523	6.809798638
24.80499029	182.6775425	0.064045987	7.372678313
13.53329853	101.3363533	0.075159455	6.495348378
44.74217634	308.6259169	0.045785236	6.337800734
11.3362332	99.18829982	0.091509614	6.626982576
24.16483268	522.6702603	0.061416877	7.134692068
29.14120452	208.1573628	0.052122191	6.766737983
27.30504927	250.5544355	0.058622203	6.26912151
26.8043576	200.1182796	0.055568402	6.426106372
11.66684235	129.3805949	0.103749928	6.885074387
34.21951095	338.6197068	0.049902386	7.079256477
32.41469518	340.7496088	0.052219638	7.04764624
31.91757702	260.011976	0.051082466	6.803367866
18.33029177	157.0324012	0.065471318	6.780328876
10.53724901	100.3972603	0.09316945	5.683745302
21.15284325	180.3670807	0.061738359	6.608613752
26.15746399	193.3851334	0.055731986	7.052714548
34.4791978	333.356599	0.0489706	6.543858296
27.68677301	211.2051392	0.056377534	7.032652003
21.83371972	208.1929607	0.062838625	6.939915844
23.47442609	190.0820183	0.060988992	7.077501316
31.88631231	295.6848891	0.04965395	6.889142678
38.90210066	404.2883598	0.046864065	6.731711332
26.11358976	223.1394949	0.074183212	7.418048425
18.09617028	154.0267857	0.069885204	6.148882027
17.39935723	410.6995872	0.076721978	7.291966955
31.60884997	209.5126866	0.049345066	6.7497108
9.266510471	148.4499295	0.114844703	6.906661467
40.22003638	331.6153846	0.043653848	6.968880297
31.97748661	215.828169	0.049101369	6.657511322
16.71108769	164.937018	0.085069984	6.99238574
30.20377252	235.2265723	0.050300262	6.885282649
18.336067	246.2947113	0.067456971	7.026190775
16.07533568	130.5784114	0.074767402	6.211146665
28.81023803	167.8209719	0.056351018	6.297552977
29.93121525	288.9845133	0.053430182	6.019032542
30.56943229	173.2113442	0.05335345	6.626579238
30.33967478	312.5314183	0.051714977	6.895487313
10.52170929	113.6475677	0.119035265	7.156218429
17.28412367	259.406821	0.070148552	6.88746724
48.92062141	387.9844479	0.041086083	6.551438985
33.15130197	189.210034	0.047785356	6.600791243
22.56539186	185.5075965	0.061723146	7.119031756
28.81214299	267.6918239	0.051663328	6.710879292
24.82281949	221.2634338	0.056469857	6.4811003
14.65460275	223.0517764	0.092192737	7.359550271
24.3123696	212.2024078	0.057423081	6.758451552
35.67572366	366.4876268	0.046983942	6.941966645
31.12827393	390.5194316	0.051682453	7.276512329
31.493721	298.7132634	0.051665113	6.983167068
20.68368792	156.9556869	0.060447844	6.389584384
31.11495662	357.5609471	0.050471062	6.876884788
31.63455192	314.0566502	0.055267053	6.161136755
26.33823336	259.7457227	0.057781645	6.983937921
17.58220051	188.4573788	0.069048706	7.080709628
24.77130414	257.3338041	0.056091977	6.735055354
28.35876049	158.4207317	0.053225593	6.27396382
27.15108829	222.5332263	0.060097433	7.087535985
36.3550674	216.8397004	0.047382906	6.59984834
21.48554017	144.3452632	0.06014626	6.429784286
20.84810507	214.4422135	0.075461945	7.179813851
15.03969508	124.08061	0.070279237	6.274294038
25.30016122	257.9144409	0.055466126	6.840986453
44.75973123	495.0402477	0.050772077	6.247265999
25.83158004	222.7695817	0.054889907	6.62221732
15.30207284	149.1155556	0.073815432	6.788450435
38.11189041	282.9253201	0.046054402	6.841000884
26.00259279	264.0060533	0.055880611	6.482999197
24.62505406	163.7037686	0.057052834	6.884701869
16.20993685	141.8203909	0.0711111	6.602253073
23.08777578	197.0556169	0.066191305	7.072444162
31.18929923	473.4557439	0.07057359	6.16472021
24.56508992	211.67737	0.055753117	6.734789525
20.7296337	149.217414	0.078629302	7.273030921
19.0270011	203.3862434	0.068154051	6.774805208
31.56825882	240.7271397	0.050286056	6.726087801
33.08092003	438.1716418	0.052681246	6.740487751
18.19106511	229.7602686	0.072794923	6.942205866
18.01644148	210.4187958	0.066595831	6.847067726
23.54883285	194.4706907	0.058966817	6.86392094
14.50857566	187.8524342	0.072094829	6.718655134
44.1087393	352.2260906	0.041582798	6.964025267
28.43022758	213.3658238	0.051239924	6.761781359
25.53338301	281.942492	0.061988997	6.066839094
17.59649978	196.9365672	0.078107596	5.766110892
26.86188722	327.8041704	0.054627221	7.162016156

log.sigma.4.5.mm.3D_gldm_DependenceNonUniformity	log.sigma.4.5.mm.3D_gldm_GrayLevelNonUniformity	log.sigma.4.5.mm.3D_gldm_SmallDependenceEmphasis
312.2243767	65.82825485	0.323277332
95.11527378	16.8443804	0.369058173
199.6278027	41.33408072	0.330623707
99	14.2173913	0.382770876
332.4613749	70.71226081	0.267845509
64.78512397	11.50413223	0.389940312
1044.076193	913.7581364	0.153993804
973.9258405	343.0128543	0.248826603
577.9784139	293.5598311	0.201668201
150.8860927	43.28874172	0.253535567
220.3496144	144.9447301	0.164530324
423.1937188	261.3668917	0.183331984
186.9334155	38.69913687	0.309273797
172.4720497	28.13975155	0.396368387
284.4463492	89.97269841	0.288430121
719.1863936	369.0329747	0.206153671
144.997735	66.36579841	0.186184312
101.8997555	18.72616137	0.344104863
144.3053016	100.1115174	0.173032785
1459.348252	563.9911794	0.220509442
419.6236766	108.3099134	0.252059612
121.7943548	29.0766129	0.260055269
124.1290323	31.00716846	0.254297001
385.7111482	373.1884904	0.143218091
200.3355049	61.28013029	0.272636043
451.1494523	133.4733959	0.25747585
399.4600798	102.3692615	0.308219944
368.6493564	147.5068797	0.207738341
20.61643836	13.60273973	0.185412562
317.2720497	99.39875776	0.241178203
679.138688	223.4295335	0.227988333
187.3502538	38.58883249	0.343841433
388.5426124	131.6415418	0.240694053
365.6973085	151.7552795	0.220895841
787.2252094	313.0564972	0.227825866
394.9504899	96.2790098	0.295295031
353.5965608	70.85846561	0.334668698
656.7134513	431.8204776	0.219368626
125.0035714	39.13571429	0.202434237
1085.812745	724.7925267	0.162289772
281.3029851	66.12238806	0.276366841
566.3684767	651.3991537	0.123697981
529.4830438	105.5550041	0.338461312
315.7169014	69.72394366	0.294798459
183.5745501	132.3688946	0.163398721
485.4056643	120.7709288	0.292813586
764.7772379	360.96225	0.198609196
94.41344196	36.7107943	0.243584347
83.18414322	22.03324808	0.260561819
135.5132743	24.15044248	0.316142453
257.8142968	68.66588967	0.30584185
546.2007181	144.0262118	0.282276681
614.3252257	949.4252758	0.117907466
238.3520097	115.183922	0.215864863
175.8522551	26.41835148	0.409882266
276.3979592	56.19557823	0.296257619
590.4864259	247.8184309	0.215790836
422.7261732	106.7880987	0.274620265
159.9174312	43.08650066	0.292853876
1004.001928	1004.255485	0.143012273
257.4078254	76.31527464	0.293015938
530.3590264	115.8154158	0.302591242
1341.342968	425.5533155	0.246299551
397.8187023	108.2900763	0.262376198
143.2776957	40.92319055	0.278307102
712.1487869	172.6615025	0.283666353
110.4187192	22.43842365	0.341464149
877.9262537	293.8196657	0.25206247
1017.171018	518.4176878	0.169461744
428.2900188	119.1393597	0.240098208
111.8902439	26.18699187	0.3169924
799.5721743	299.3453122	0.236455768
314.2928839	63.25617978	0.299642018
204.64	57.13894737	0.302875269
484.2012733	308.1865818	0.183534703
184.1546841	64.51633987	0.225638442
415.9488456	122.5246718	0.242535594
89.90402477	16.3993808	0.388158298
277.5155894	72.18022814	0.267514763
262.4077778	132.8677778	0.191951142
302.197724	64.75248933	0.321488044
182.2784504	46.15738499	0.295379706
403.4750219	130.1945662	0.271156738
318.1621764	134.6133122	0.192498962
381.6069982	179.7093923	0.206407008
120.2429379	37.47457627	0.281620931
245.3073394	72.92507645	0.27358692
408.9077164	301.6220021	0.185744801
272.196943	115.9300412	0.235765035
292.9180688	68.74103877	0.258395947
149.1766169	42.35572139	0.282019186
331.7827804	184.3167457	0.19008836
732.0233281	299.7478338	0.205074087
617.1857226	203.1996518	0.226798642
514.9824614	238.417599	0.184084433
539.1090604	99.13338926	0.325225176
505.4763458	125.6402936	0.260865232
73.07667732	19.40255591	0.287152112
62.62686567	20.93283582	0.273110489
1490.014733	482.0305984	0.24385597

log.sigma.4.5.mm.3D_gldm_DependenceNonUniformityNormalized	log.sigma.4.5.mm.3D_gldm_DependenceVariance	log.sigma.4.5.mm.3D_gldm_LargeDependenceEmphasis
0.216221868	2.215889995	9.675900277
0.274107417	1.442417095	7.040345821
0.223797985	1.755338937	8.719730942
0.307453416	0.948072991	5.782608696
0.235621102	1.676568784	9.422395464
0.267707124	1.303189673	6.619834711
0.099067862	23.4392058	58.25021349
0.160501951	7.64142976	21.10118655
0.135612016	10.97509602	29.83294228
0.199849129	2.436338757	11.78940397
0.141612863	5.603604258	22.98971722
0.124213008	11.41471404	33.01115351
0.23049743	1.936082321	9.194821208
0.267813742	1.327823001	6.580745342
0.180600857	5.398624943	16.16952381
0.12481541	15.50083908	37.66365845
0.164210345	3.991458133	17.93318233
0.249143656	1.235944309	7.273838631
0.131906126	7.263815594	26.17915905
0.158918464	5.735056098	19.46368289
0.201936322	2.248758477	11.48123195
0.245553135	1.606594693	9.258064516
0.222453463	1.701699618	10.06810036
0.107231345	13.18559183	40.94328607
0.163139662	9.292435464	22.73289902
0.176506045	4.626085849	15.87323944
0.199331377	2.675645715	11.05788423
0.16362599	4.947677989	18.46382601
0.141208482	4.709326328	21.15068493
0.197063385	2.32123915	11.98136646
0.169403514	5.320307308	17.97430781
0.237754129	1.594250045	7.997461929
0.166399406	4.946944413	17.31263383
0.151427457	6.410734497	20.99585921
0.153365519	6.22000662	20.26826417
0.20368772	3.726658973	12.71428571
0.233860159	1.514975435	8.066137566
0.112817978	23.70133267	52.30664834
0.223220663	2.117295918	11.95
0.114937308	12.05777242	36.12247274
0.209927601	2.293203386	10.75522388
0.099853399	15.76595793	48.34767278
0.218975618	2.384781632	9.660049628
0.222335846	1.846290419	9.428169014
0.117978503	12.22985739	35.89845758
0.202168123	3.714918173	12.8000833
0.142922302	6.749092426	22.98467576
0.192288069	2.766406312	12.79633401
0.212747169	1.87135092	10.4168798
0.299808129	0.890594408	6.557522124
0.200321909	3.142507497	11.82828283
0.19612234	2.682892773	11.63985637
0.077021718	24.40541805	72.1948345
0.145159567	5.184877478	19.84043849
0.273487177	1.218568675	6.213063764
0.235032278	1.646536165	8.848639456
0.147070094	7.682981224	23.37957659
0.204511937	2.322592756	10.97194001
0.209590342	2.230091262	10.37090433
0.09216946	27.58714255	67.25759662
0.193685346	3.62324394	12.89616253
0.215155792	3.615418455	12.0831643
0.162902959	6.145272636	19.03157639
0.189798999	3.171339629	12.98473282
0.211636183	2.725465768	11.33973412
0.208169771	2.244554991	10.56679334
0.271967289	1.039554466	6.724137931
0.172650197	3.742805889	14.8159292
0.135478292	6.321796406	24.34017048
0.201643135	2.516089105	12.26553672
0.227419195	1.686727477	8.711382114
0.160524428	6.85065703	20.73940976
0.235425381	1.684482038	8.863670412
0.215410526	1.896970637	9.549473684
0.118560547	10.43288157	32.04946131
0.200604231	2.173480285	12.09803922
0.18829735	2.622957525	12.75373472
0.278340634	1.193704531	6.343653251
0.211038471	1.960689904	10.40380228
0.145782099	5.170369136	20.73777778
0.21493437	2.238850365	9.768136558
0.22067609	1.925467113	9.571428571
0.176807634	7.386276472	19.31025416
0.168072993	4.068455279	17.40464871
0.140555064	7.888621498	24.60662983
0.226446211	1.764371668	9.459510358
0.187543837	2.798737948	12.31039755
0.106597423	17.3580902	45.43482795
0.160021718	4.855786668	17.63609641
0.214278031	1.821116301	10.30212143
0.185543056	3.329038885	13.12189055
0.131035853	6.992409325	24.8507109
0.162635709	3.831307477	17.04754499
0.179102067	3.128459688	14.29251306
0.155724966	3.873971597	18.2827336
0.226136351	1.804439412	8.801174497
0.206148591	2.068101014	10.8588907
0.233471813	1.565883085	8.923322684
0.233682335	1.484127868	9.067164179
0.168859331	5.372964732	17.69605621

log.sigma.4.5.mm.3D_gldm_LargeDependenceLowGrayLevelEmphasis	log.sigma.4.5.mm.3D_gldm_SmallDependenceHighGrayLevelEmphasis	log.sigma.4.5.mm.3D_gldm_LargeDependenceHighGrayLevelEmphasis
0.08188392	108.2310732	2308.819252
0.067002455	124.0619052	2687.510086
0.056729749	109.5598536	2777.535874
0.072448548	151.7754848	1768.580745
0.161580554	57.7830701	1941.689582
0.067781872	120.3595742	1956.909091
0.575580534	27.54730215	7546.689155
0.126519591	79.08429216	4811.528675
0.363876435	45.92524891	3804.643829
0.240253551	47.47326071	1441.124503
0.422426301	19.1418051	1853.854113
0.406304691	35.08310991	4142.669797
0.122032236	82.16510842	2905.993835
0.195651695	131.8494111	1809.395963
0.271883478	64.72325206	1849.746667
0.370936391	44.04132614	5892.558834
0.447159155	23.61097375	1507.157418
0.128241758	117.4955318	2125.584352
0.48882558	23.13925836	2188.155393
0.043415335	119.1569226	9706.175868
0.184800873	54.50000952	2304.660731
0.332276926	67.35196298	2182.066532
0.241191845	56.05607919	1845.136201
0.463094925	24.3436324	4812.320267
0.097160983	103.3148043	7566.557818
0.076326658	102.2585126	4433.248044
0.107223345	99.67548505	2196.794411
0.236215277	41.34711506	2475.774523
0.378685446	21.77736582	1655.342466
0.160620228	49.46263641	1895.220497
0.205037622	53.14150246	2829.890995
0.048278235	118.0295668	2699.59264
0.181453229	60.74343758	2895.176017
0.157684979	52.52934076	4170.294824
0.218196353	54.53244592	2976.583479
0.100866695	88.62605523	3620.431666
0.052307717	142.4419531	3083.853175
0.322146303	57.90220786	11075.88954
0.491605685	32.51809315	1778.230357
0.100852157	77.6727436	14118.29353
0.20191033	68.69313335	1727.935075
0.422183374	23.07135588	6461.550423
0.078111321	119.7197863	2726.425972
0.155774897	74.14981261	1674.432394
0.329603387	33.94660466	5357.557841
0.144410573	82.14876935	2309.937943
0.133621153	59.1980518	5085.814801
0.424376255	35.57965567	1580.236253
0.569086382	54.43657018	1408.925831
0.055013599	90.7789104	1845.918142
0.334796391	69.55094136	1252.336441
0.068932889	97.33883264	3198.14219
0.820401297	18.16715505	7794.455491
0.10520245	62.65214164	4727.415347
0.067985672	163.2960865	2466.339036
0.230910502	62.26041032	1473.946429
0.237422067	50.12941924	3704.640349
0.083083313	81.3895076	2540.49492
0.145206796	74.42786914	1950.618611
0.346193329	38.29688598	14500.84449
0.120678496	71.6243892	2249.908954
0.051559539	113.3919137	4070.36998
0.072651719	105.9542024	6312.0436
0.08940528	84.57182633	3756.082538
0.206637319	49.92834802	1408.45938
0.054714585	108.5079142	3568.066647
0.054705964	109.497552	2075.800493
0.122006043	78.679211	3065.088102
0.208341353	37.30046113	4297.961375
0.074123583	59.72120428	3233.448682
0.353258861	59.82680317	1117.140244
0.182046082	62.91184988	4107.537041
0.156076102	76.26768206	1518.8397
0.193519375	51.54923624	1128.890526
0.233082154	52.84297203	6037.773262
0.261951328	30.37976652	1383.851852
0.118227315	63.11961182	3321.37483
0.027919214	183.0225897	3333.312693
0.095277037	63.29643472	2207.945247
0.246185762	35.66735042	2741.02
0.083826949	105.2608386	2305.175676
0.067774527	72.60697873	2726.986683
0.385614688	54.59577465	2775.410167
0.275002784	35.53998017	1970.824089
0.22352749	48.9249541	4025.436464
0.027940805	121.6013102	4856.397363
0.154972661	65.95177739	2328.284404
0.611682371	38.67197365	5494.58342
0.138333473	56.61922928	3042.360964
0.109441439	70.09572856	2266.08632
0.049877405	129.0808491	5611.014925
0.161779297	55.88767886	5188.446682
0.132786454	50.19874899	3179.119085
0.161947466	51.36700135	2329.972142
0.15786052	38.29751328	2974.774116
0.065283297	125.7984415	2911.303272
0.118640461	59.68023085	2120.550163
0.075193102	80.1510836	2484.977636
0.096998919	49.93945896	1883.966418
0.075188208	79.88361744	5593.614687

log.sigma.4.5.mm.3D_gldm_SmallDependenceLowGrayLevelEmphasis	log.sigma.4.5.mm.3D_gldm_LowGrayLevelEmphasis	log.sigma.4.5.mm.3D_gldzm_DistanceZoneVariabilityNormalized
0.003198759	0.009641512	0.888340407
0.004176661	0.013401322	0.916824197
0.003450714	0.008141539	0.8872
0.00482622	0.015372401	0.892565294
0.003938694	0.017935812	0.834710744
0.00707709	0.012827628	0.856866825
0.002428332	0.015110802	0.945008294
0.001730341	0.006930971	0.914163887
0.002371994	0.013530896	0.987792932
0.004675835	0.020399915	0.940481687
0.004914071	0.021585718	0.959062269
0.003409021	0.015302204	0.925934217
0.008612737	0.019021044	0.87099304
0.00543859	0.017952401	0.785486089
0.003601454	0.015559511	0.938689665
0.002775997	0.013123205	0.965478286
0.003886736	0.027385485	0.967222222
0.005482437	0.015450682	0.880918173
0.0044592	0.023160139	0.951952243
0.000661545	0.002456597	0.953297772
0.00340309	0.014577913	0.954422222
0.003381103	0.019673189	0.899163195
0.003773722	0.021765917	0.916390457
0.001848253	0.013373419	0.960589421
0.002242815	0.007328454	0.961180276
0.001533795	0.005180869	0.952822615
0.002867137	0.010265003	0.922894238
0.00296564	0.013848457	0.963112669
0.01069373	0.023106277	1
0.003133109	0.01244968	0.982341357
0.00227281	0.011177824	0.9608
0.004153103	0.00829059	0.898024311
0.002530841	0.011114955	0.873466964
0.002155774	0.008658771	0.974280894
0.002369956	0.010807832	0.936894199
0.003597189	0.010692407	0.7888
0.002351843	0.006615635	0.703046905
0.002188584	0.009431971	0.947059096
0.003475805	0.027918134	0.892963131
0.000562852	0.003055197	0.94375772
0.002766237	0.015305016	0.895498864
0.001397683	0.01028491	0.935208015
0.002436467	0.008261482	0.934598817
0.003024002	0.0133034	0.927028179
0.002388761	0.012263615	0.902221278
0.002606489	0.010168989	0.928995689
0.001243011	0.006043028	0.954010728
0.007307837	0.027679533	1
0.005829678	0.035736247	0.953152
0.005189793	0.010828775	0.936239585
0.003358359	0.019474391	0.970656406
0.002179479	0.00716557	0.901902497
0.001889606	0.016563546	0.944061875
0.001811397	0.00607358	0.94194066
0.004222655	0.01161878	0.90328208
0.005156611	0.01954643	0.93838843
0.002037373	0.010769839	0.958882
0.00221191	0.007305382	0.936268996
0.003196655	0.013416411	0.834168043
0.001083881	0.006643376	0.957368955
0.003007612	0.010686017	0.953710241
0.002422687	0.005614905	0.907586899
0.001156338	0.004046666	0.869763942
0.002238296	0.008317809	0.829687014
0.005824022	0.019466141	0.940227833
0.00150036	0.00553069	0.870920025
0.005271921	0.010275087	0.838240958
0.00171683	0.007883715	0.880613486
0.00149971	0.009015681	0.956267478
0.002375897	0.007493402	0.92697339
0.010084822	0.041075253	0.937887844
0.002468378	0.010956004	0.937216994
0.003923967	0.015782771	0.951642647
0.006918784	0.019739525	0.988372491
0.001379655	0.008909239	0.812825122
0.003930606	0.021117399	0.968262227
0.00256292	0.009626608	0.932286857
0.005894665	0.008312219	0.804703434
0.005509448	0.012949817	0.936861895
0.003374025	0.013769808	0.954339686
0.003227391	0.009862366	0.900980174
0.004052783	0.009553328	0.954273442
0.003343013	0.020389761	0.970751128
0.002916401	0.016374411	0.958448291
0.002233585	0.010546729	0.900277008
0.003400524	0.005779634	0.907436085
0.003552135	0.012908736	0.896936277
0.002734425	0.018714545	0.975731818
0.002678006	0.009390896	0.940733396
0.003125292	0.010816007	0.972608025
0.002583214	0.00538119	0.782661541
0.001396497	0.007780231	0.938356549
0.001612154	0.007905696	0.931449888
0.002589734	0.011404049	0.95083343
0.001771355	0.00819589	0.937003786
0.002136298	0.007614967	0.835198127
0.003334259	0.011506428	0.866707797
0.005424003	0.01183176	0.868431357
0.006699646	0.0152158	0.878071834
0.001405056	0.004922824	0.905322902

log.sigma.4.5.mm.3D_gldzm_LowIntensityEmphasis	log.sigma.4.5.mm.3D_gldzm_LargeDistanceEmphasis	log.sigma.4.5.mm.3D_gldzm_HighIntensitySmallDistanceEmphasis
0.010703812	1.178057554	321.2598921
0.014182547	1.130434783	328.7546584
0.010040254	1.18	320.3735714
0.015819044	1.170886076	378.528481
0.016958493	1.272727273	208.2008457
0.016606983	1.232758621	295.2543103
0.01741479	1.084889643	185.0271647
0.006979803	1.134812287	314.0489192
0.011206492	1.018423746	236.154043
0.021961373	1.092105263	188.9945175
0.028740368	1.06271777	120.3832753
0.018199265	1.115549215	192.6437233
0.026944635	1.207920792	256.9521452
0.015419051	1.366559486	320.6318328
0.012804032	1.094972067	223.6615456
0.013085415	1.052708638	215.715959
0.024073078	1.05	132.7138889
0.014862284	1.190751445	331.0245665
0.027691514	1.073891626	139.6921182
0.002959641	1.071770335	536.6780122
0.013503233	1.07	218.9683333
0.017371829	1.159763314	252.535503
0.018972106	1.131147541	216.8196721
0.012530833	1.060329068	181.8427788
0.009170325	1.059405941	371.5965347
0.005785585	1.072519084	395.8403308
0.010293572	1.120498615	315.3753463
0.014851923	1.056390977	204.1343985
0.048648116	1	116.6857143
0.012757787	1.026726058	212.6959911
0.009914966	1.06	237.3952381
0.011253714	1.161676647	333.616018
0.010937219	1.20361991	247.0542986
0.009586356	1.039087948	237.8831433
0.010791955	1.097850259	242.773536
0.012844857	1.36	285.3675
0.00732284	1.544117647	397.4505719
0.009785748	1.081632653	264.5215986
0.020253157	1.170212766	162.6507092
0.003414956	1.086879433	481.1422872
0.011184102	1.165929204	246.1753319
0.012739029	1.100558659	197.9752095
0.007436851	1.101538462	353.0553846
0.011516429	1.113772455	250.3178643
0.013941151	1.154639175	216.6632302
0.008955458	1.11058264	277.6495244
0.006121243	1.070647603	303.0264929
0.031000587	1	146.2649007
0.026066776	1.072	205.324
0.015314514	1.098901099	284.9656593
0.013462286	1.044680851	227.6202128
0.008431356	1.155172414	339.5689655
0.017652442	1.086395233	160.9270109
0.008059521	1.089775561	292.9326683
0.011486063	1.152866242	391.9482484
0.016887523	1.095454545	208.9585227
0.009502527	1.063	237.902
0.007787701	1.098853868	294.5272206
0.012617284	1.273722628	243.3512774
0.007834943	1.065371025	273.2446996
0.011266265	1.07112069	241.1196121
0.007560443	1.145695364	363.1272075
0.004556009	1.210062893	419.5010482
0.009442175	1.281972265	308.788906
0.022146766	1.092511013	176.3127753
0.005827609	1.208047945	368.7300942
0.014061007	1.266272189	311.1745562
0.007698532	1.191275168	306.6845638
0.009305914	1.067099567	225.6769481
0.009809235	1.113861386	246.390264
0.037133208	1.096256684	185.9318182
0.010962224	1.097332372	266.185292
0.01375626	1.074380165	256.4380165
0.022386274	1.01754386	170.4619883
0.008481057	1.319254658	289.3937198
0.020704251	1.048387097	138.2076613
0.011323645	1.105263158	259.2567783
0.012976441	1.329032258	459.6387097
0.01964203	1.097902098	232.2074592
0.018021322	1.07012987	189.0915584
0.010974054	1.156716418	317.9472948
0.012664795	1.070234114	242.5200669
0.012507494	1.044534413	202.224359
0.01611647	1.063679245	189.4563679
0.010614965	1.157894737	239.2174923
0.010807032	1.145945946	419.972973
0.014165625	1.163507109	236.4887441
0.014962617	1.036855037	216.6612408
0.011818274	1.091703057	236.231441
0.011850952	1.041666667	273.375
0.008602012	1.372180451	425.3909774
0.00807386	1.095505618	299.9382022
0.008379903	1.106614018	247.3526654
0.011846388	1.075657895	231.2916667
0.009503038	1.097674419	216.0775194
0.007515675	1.271834061	366.7259825
0.013140045	1.2154047	220.1204308
0.018054883	1.212389381	270.4668142
0.023469828	1.195652174	177.9673913
0.00577104	1.149461937	319.7364488

log.sigma.4.5.mm.3D_gldzm_LowIntensityLargeDistanceEmphasis	log.sigma.4.5.mm.3D_gldzm_HighIntensityEmphasis	log.sigma.4.5.mm.3D_gldzm_DistanceZoneVariability	log.sigma.4.5.mm.3D_gldzm_ZonePercentage
0.012157207	335.2158273	493.9172662	0.385041551
0.014647679	338.8819876	147.6086957	0.463976945
0.01075849	332.5942857	310.52	0.392376682
0.016694927	388.3734177	141.0253165	0.49068323
0.01978956	217.8794926	394.8181818	0.335223246
0.017894444	307.4482759	99.39655172	0.479338843
0.018142245	188.8947368	1669.829655	0.167662966
0.008111674	323.3441411	1607.100114	0.289716546
0.011406451	236.8433982	965.073695	0.229235101
0.023026223	191.5175439	214.4298246	0.301986755
0.029574674	122.087108	275.2508711	0.184447301
0.019223105	197.4550642	649.0798859	0.205752862
0.028798746	265.7491749	263.9108911	0.373612824
0.032507049	329.9742765	244.2861736	0.482919255
0.019658531	226.5344507	504.0763501	0.340952381
0.013468858	217.8967789	1318.843338	0.237070462
0.025013767	133.3888889	174.1	0.20385051
0.017522831	335.2427746	152.3988439	0.422982885
0.028349135	141.8719212	193.2463054	0.185557587
0.003089555	547.307525	2191.631579	0.250353915
0.014165979	221.3383333	572.6533333	0.288739172
0.019178409	257.7100592	151.9585799	0.340725806
0.020255282	220.7377049	167.6994536	0.327956989
0.013141062	183.8062157	525.4424132	0.15207117
0.009659166	375.1014851	388.3168317	0.328990228
0.005984747	404.0139949	748.9185751	0.307511737
0.01149244	321.9861496	666.3296399	0.360279441
0.015499952	205.7838346	512.3759398	0.236129605
0.048648116	116.6857143	35	0.239726027
0.012953447	213.674833	441.0712695	0.278881988
0.010382859	240.2638095	1008.84	0.261910701
0.012289792	341.3922156	299.9401198	0.423857868
0.012356935	257.3393665	579.1085973	0.283940043
0.009796852	239.7899023	598.2084691	0.254244306
0.012971447	246.0904374	1263.870274	0.262809273
0.020735604	299.5371429	552.16	0.36101083
0.020517613	419.0816993	430.2647059	0.404761905
0.010819935	268.314966	1392.176871	0.252533929
0.024011184	165.2624113	125.9078014	0.251785714
0.003674705	491.3031915	1596.838061	0.179104478
0.012641431	252.6283186	404.7654867	0.337313433
0.014203526	201.5167598	669.6089385	0.126234133
0.0083264	357.0369231	911.2338462	0.403225806
0.012452966	254.9061876	464.4411178	0.352816901
0.015669778	221.4570447	262.5463918	0.187017995
0.010136546	281.8953627	781.2853746	0.350270721
0.00661974	306.6534903	1134.318755	0.222201458
0.031000587	146.2649007	151	0.307535642
0.026539867	209.08	119.144	0.319693095
0.015804676	290.5494505	170.3956044	0.402654867
0.013956683	228.6893617	456.2085106	0.365190365
0.009279998	350.0204741	836.9655172	0.333213645
0.018954555	163.5888779	950.6703078	0.126253761
0.008397117	299.0897756	377.7182045	0.244214373
0.013443917	396.9808917	283.6305732	0.488335925
0.018053603	211.8477273	412.8909091	0.37414966
0.010688456	239.966	958.882	0.249066002
0.008380332	299.4484241	653.5157593	0.33768747
0.015886212	251.2372263	228.5620438	0.359108781
0.008368688	276.9310954	1625.612485	0.155879923
0.011742251	244.3362069	442.5215517	0.349134688
0.008075314	376.6412804	822.2737307	0.367545639
0.005355784	438.4343816	2074.387002	0.28965266
0.011437671	324.6209553	538.4668721	0.309637405
0.023397291	178.30837	213.4317181	0.335302806
0.006961339	383.0744863	1017.234589	0.341420637
0.017236837	320.1301775	141.6627219	0.416256158
0.009059928	318.3926174	1312.114094	0.293018682
0.009760365	229.1385281	1325.386724	0.18460309
0.010333406	253.5264026	561.7458746	0.285310734
0.039533172	187.6203209	175.3850267	0.380081301
0.012116286	269.9206921	1299.919971	0.278458141
0.014672251	258.446281	460.5950413	0.362546816
0.022611055	170.8216374	338.0233918	0.36
0.019886292	302.3763975	654.3242236	0.197110676
0.021361187	139.3508065	240.1290323	0.270152505
0.011822623	266.1419458	584.5438596	0.283838841
0.014497503	480.4064516	124.7290323	0.479876161
0.020405398	236.3752914	401.9137529	0.326235741
0.026342597	191.3766234	367.4207792	0.213888889
0.01241612	329.1455224	482.9253731	0.381223329
0.01310428	245.826087	285.3277592	0.361985472
0.012845071	203.8448043	719.3265857	0.324715162
0.017179882	190.754717	406.3820755	0.223983096
0.011846589	247.0309598	581.5789474	0.237937385
0.011315675	431.1621622	167.8756757	0.348399247
0.016427103	241.8009479	378.507109	0.322629969
0.015422111	217.8046683	794.2457002	0.212200209
0.012558501	239.6113537	430.8558952	0.26925338
0.012064191	276.0763889	420.1666667	0.316020483
0.009735918	462.5639098	208.1879699	0.330845771
0.017364635	303.6067416	501.082397	0.210900474
0.009131545	252.2813425	943.5587364	0.225061098
0.01244102	234.4627193	867.1600877	0.264654672
0.010677728	219.8356589	604.3674419	0.195040822
0.00959653	385.040393	765.0414847	0.384228188
0.01487139	229.5522193	663.8981723	0.312398042
0.019615582	280.0707965	98.13274336	0.361022364
0.025762494	182.6630435	80.7826087	0.343283582
0.006445486	331.2279793	2271.455161	0.284338169

log.sigma.4.5.mm.3D_gldzm_IntensityVariabilityNormalized	log.sigma.4.5.mm.3D_gldzm_LowIntensitySmallDistanceEmphasis	log.sigma.4.5.mm.3D_gldzm_IntensityVariability
0.042647896	0.010340464	23.71223022
0.047413294	0.014066263	7.633540373
0.045208163	0.009860695	15.82285714
0.045265182	0.015600073	7.151898734
0.049162156	0.016250726	23.25369979
0.050832342	0.016285118	5.896551724
0.050659949	0.017232926	89.51612903
0.046110678	0.006696836	81.0625711
0.052292806	0.011156502	51.09007165
0.053631887	0.02169516	12.22807018
0.067877478	0.028531792	19.48083624
0.054857438	0.017943305	38.45506419
0.042882506	0.026481107	12.99339934
0.042296916	0.011147051	13.15434084
0.054031467	0.011090407	29.01489758
0.049320563	0.012989554	67.37188873
0.069259259	0.023837906	12.46666667
0.044137793	0.014197148	7.63583815
0.068601519	0.027527109	13.92610837
0.045244817	0.002927163	104.0178338
0.051972222	0.013337546	31.18333333
0.052064003	0.016920185	8.798816568
0.050673355	0.018651312	9.273224044
0.065135073	0.012378276	35.62888483
0.042557102	0.009048114	17.19306931
0.046649703	0.005735795	36.66666667
0.046811335	0.009993855	33.79778393
0.05863107	0.014689916	31.19172932
0.071020408	0.048648116	2.485714286
0.057097931	0.012708873	25.63697105
0.05145034	0.009797993	54.02285714
0.048155187	0.010994695	16.08383234
0.049441612	0.01058229	32.77978884
0.059443602	0.009533732	36.49837134
0.05011482	0.010247082	67.60489251
0.04797551	0.01087217	33.58285714
0.044902602	0.004024147	27.48039216
0.047919848	0.009527201	70.44217687
0.068457321	0.01931365	9.65248227
0.052432809	0.003350019	88.71631206
0.0496515	0.01081977	22.44247788
0.064706158	0.012372904	46.32960894
0.044765286	0.007214463	43.64615385
0.052673097	0.011282295	26.38922156
0.056435328	0.013508994	16.42268041
0.048299049	0.008660186	40.61950059
0.056117862	0.005996619	66.72413793
0.064251568	0.031000587	9.701986755
0.050368	0.025948503	6.296
0.049993962	0.015191973	9.098901099
0.053463105	0.013338687	25.12765957
0.046844809	0.008219196	43.47198276
0.060120369	0.017326914	60.54121152
0.057555612	0.007975122	23.0798005
0.039514788	0.010996599	12.40764331
0.049607438	0.016596003	21.82727273
0.054054	0.009206045	54.054
0.050717974	0.007639543	35.40114613
0.05439821	0.011800053	14.90510949
0.053827617	0.007701507	91.39929329
0.050442182	0.011147268	23.40517241
0.041976716	0.007431725	38.03090508
0.043969955	0.004356066	104.8683438
0.045994668	0.008943301	29.85053929
0.05598789	0.021834134	12.7092511
0.045046854	0.005544177	52.61472603
0.050873569	0.013267049	8.597633136
0.049450025	0.007358183	73.68053691
0.058116727	0.009192301	80.54978355
0.050060452	0.009678192	30.33663366
0.054190855	0.036533218	10.13368984
0.048325449	0.010673709	67.02739726
0.046957175	0.013527262	22.72727273
0.055794945	0.022330079	19.08187135
0.050073685	0.005974814	40.30931677
0.071702653	0.020540017	17.78225806
0.048836387	0.0111989	30.62041467
0.04491155	0.012596176	6.961290323
0.047451383	0.019451188	20.35664336
0.06162928	0.015941003	23.72727273
0.04316106	0.010613537	23.13432836
0.053679489	0.012554924	16.05016722
0.055042881	0.0124231	40.78677463
0.065147739	0.015850617	27.62264151
0.050417429	0.010307059	32.56965944
0.043623083	0.010679871	8.07027027
0.051043328	0.013600256	21.54028436
0.050727744	0.014847744	41.29238329
0.05323125	0.011633218	24.37991266
0.047121485	0.011797642	20.35648148
0.045395443	0.008318535	12.07518797
0.057379119	0.005751166	30.64044944
0.057874483	0.008191992	58.62685094
0.051455736	0.01169773	46.92763158
0.066748393	0.009209365	43.05271318
0.039067714	0.006995461	35.7860262
0.049598129	0.012707209	37.9921671
0.054585324	0.017664708	6.168141593
0.068289225	0.022896662	6.282608696
0.047786359	0.005602428	119.8959745

log.sigma.4.5.mm.3D_gldzm_HighIntensityLargeDistanceEmphasis	log.sigma.4.5.mm.3D_gldzm_SmallDistanceEmphasis	log.sigma.4.5.mm.3D_glcm_SumVariance	log.sigma.4.5.mm.3D_glcm_Homogeneity1
391.0395683	0.955485612	788.6389228	0.308918054
379.3913043	0.967391304	889.0896337	0.28262514
381.4771429	0.955	876.649817	0.29473624
427.7531646	0.957278481	891.2807792	0.26875703
256.5940803	0.931818182	518.6886473	0.32757725
356.2241379	0.941810345	797.0852577	0.2987734
204.3650255	0.978777589	366.815675	0.489951751
360.5250284	0.966296928	709.6068433	0.368244434
239.6008188	0.995394063	389.5863407	0.417666666
201.6096491	0.976973684	376.0738239	0.360197715
128.902439	0.984320557	228.3214237	0.451994135
216.700428	0.971112696	366.9818027	0.443854623
300.9372937	0.948019802	741.6742825	0.306232491
367.3440514	0.908360129	758.969524	0.271561542
238.0260708	0.976256983	399.3023644	0.356570395
226.6200586	0.98682284	483.3281816	0.430714647
136.0888889	0.9875	220.4561633	0.416095693
352.1156069	0.952312139	747.7793192	0.282999249
150.591133	0.981527094	222.5846825	0.450354683
589.8255763	0.982057416	1640.475826	0.38763715
230.8183333	0.9825	521.6259732	0.348600772
278.408284	0.960059172	620.4364515	0.3296232
236.4098361	0.967213115	479.2266589	0.339130862
191.6599634	0.984917733	320.1048634	0.496514028
389.1212871	0.985148515	933.5132078	0.36598749
436.7086514	0.981870229	963.273342	0.357785036
348.4293629	0.969875346	680.6280376	0.321908358
212.3815789	0.985902256	376.5895342	0.400033289
116.6857143	1	245.5666141	0.453852495
217.5902004	0.993318486	447.7112026	0.366433621
251.7380952	0.985	496.6393129	0.373732153
372.497006	0.959580838	875.5433171	0.290072865
298.479638	0.949095023	553.1281134	0.375819768
247.4169381	0.990228013	564.5534695	0.409777367
259.358043	0.975537435	488.5127105	0.38997269
356.2157143	0.91	785.1186786	0.327207815
505.6062092	0.863970588	1070.601976	0.288887448
283.4884354	0.979591837	599.9612399	0.454098223
175.7092199	0.957446809	344.5061785	0.364588178
531.9468085	0.978280142	1240.046153	0.459660686
278.4402655	0.958517699	521.0796594	0.331662416
215.6829609	0.974860335	391.2904184	0.518352732
372.9630769	0.974615385	900.0356599	0.30074049
273.259481	0.971556886	544.3783389	0.325118026
240.6323024	0.961340206	415.0181976	0.457615542
298.8787158	0.97235434	625.5321509	0.335029347
321.1614802	0.982338099	677.0511833	0.414492027
146.2649007	1	296.2385085	0.373375395
224.104	0.982	390.862491	0.34095628
312.8846154	0.975274725	766.2612759	0.304334356
232.9659574	0.988829787	376.3070716	0.348186114
391.8265086	0.961206897	884.7114693	0.339618127
174.2363456	0.978401192	283.3007883	0.549400002
323.7182045	0.97755611	738.3103229	0.404008419
417.111465	0.961783439	1035.283507	0.270577747
223.4045455	0.976136364	457.2719844	0.322131441
248.222	0.98425	474.1788573	0.403076574
319.1332378	0.975286533	718.2882803	0.329412326
282.7810219	0.931569343	538.7676687	0.33491809
291.6766784	0.983657244	625.5048834	0.509815597
257.2025862	0.982219828	549.8286876	0.352103338
430.6975717	0.963576159	1046.554328	0.324411251
514.1677149	0.947484277	1123.025026	0.361861633
387.9491525	0.929506934	810.7208527	0.345253971
186.2907489	0.976872247	371.3991693	0.352145959
440.4520548	0.947988014	998.667887	0.323573453
355.9526627	0.933431953	803.5254853	0.292243423
365.2248322	0.952181208	714.4690724	0.362320578
242.9848485	0.983225108	507.1308855	0.429448265
282.0709571	0.971534653	711.5598239	0.35249903
194.3743316	0.975935829	345.5695865	0.322281864
284.8622927	0.975666907	591.9916311	0.377069188
266.4793388	0.981404959	530.4089032	0.307807323
172.2602339	0.995614035	335.9863211	0.362072403
354.3291925	0.921566598	565.098734	0.436303967
143.9233871	0.987903226	291.8683212	0.377751796
293.6826156	0.973684211	710.8615543	0.360747404
563.4774194	0.917741935	1312.871007	0.262782586
253.04662	0.975524476	583.551513	0.347684694
200.5168831	0.982467532	361.4030526	0.423689123
373.9384328	0.960820896	776.6278404	0.310789929
259.0501672	0.982441472	727.4982736	0.33575181
210.3265857	0.988866397	383.8511231	0.369013471
195.9481132	0.984080189	328.8462127	0.399413837
278.2848297	0.960526316	522.5225411	0.407938685
475.9189189	0.963513514	1321.958053	0.320122596
263.049763	0.959123223	533.8247884	0.35269851
222.3783784	0.990786241	367.2489236	0.467408857
253.1310044	0.977074236	530.6649363	0.392089442
286.8819444	0.989583333	638.8415389	0.33146689
611.2556391	0.906954887	1276.836937	0.342407408
318.2808989	0.976123596	616.4361263	0.423773946
271.9960513	0.973346496	564.8472091	0.399956193
247.1469298	0.981085526	518.8391022	0.374717359
234.8682171	0.975581395	487.463579	0.418930792
458.2980349	0.932041485	952.8417003	0.294050249
267.2793734	0.946148825	554.0589536	0.34255496
318.4867257	0.946902655	718.9788746	0.331797881
201.4456522	0.951086957	469.1300548	0.344082978
377.1941012	0.962634516	958.3622759	0.363360824

log.sigma.4.5.mm.3D_glcm_Homogeneity2	log.sigma.4.5.mm.3D_glcm_ClusterShade	log.sigma.4.5.mm.3D_glcm_MaximumProbability	log.sigma.4.5.mm.3D_glcm_Idmn	log.sigma.4.5.mm.3D_glcm_SumVariance2
0.216638952	318.2386311	0.010437021	0.975876794	117.4966544
0.188624943	-221.0909834	0.015983193	0.967648625	122.3023735
0.203419573	-153.1439304	0.012357188	0.973028278	103.1080991
0.173784837	-152.7291722	0.014622004	0.966164646	133.0257923
0.234192832	102.0404876	0.011409674	0.974792998	102.5582348
0.203024038	-222.8342097	0.019206767	0.969770699	121.2942292
0.426635654	268.8386332	0.076339072	0.990744866	56.27377734
0.285554496	343.189829	0.023470331	0.982338215	84.25526336
0.342717998	449.2603839	0.040307429	0.980830244	68.86603994
0.269828478	123.423036	0.015265493	0.974869151	68.69707886
0.380488942	93.02321062	0.042124301	0.981511003	35.69469524
0.373088722	198.6069364	0.040418734	0.984133419	54.72488577
0.21542842	-434.9274776	0.016943311	0.969215471	121.7249984
0.175712542	-452.8916273	0.011440785	0.96743073	135.0808923
0.271204232	431.927527	0.018117958	0.978494966	81.25141497
0.361714011	230.94135	0.058669453	0.979741959	78.89412854
0.335014875	59.64836069	0.024682206	0.974964285	39.51712212
0.189186605	-122.1080068	0.014458615	0.970470555	138.12392
0.379373002	66.69800236	0.03952358	0.980070172	33.69798291
0.305294897	321.6009054	0.023043354	0.991062256	77.93693303
0.259023348	-27.20181892	0.011553048	0.973858169	90.71646196
0.237171235	-321.7640905	0.015940257	0.970613366	89.56678205
0.248201522	33.54234908	0.017303627	0.975659928	83.01009589
0.434261603	90.47635395	0.087804495	0.986278189	38.50813817
0.284820313	75.32720833	0.035152338	0.979410399	101.8637157
0.272652706	498.3909894	0.016725466	0.985321602	97.21960469
0.230682699	230.3394347	0.0118837	0.977798166	92.09630832
0.317039284	105.163088	0.02127394	0.982330056	56.56352161
0.377703619	66.75510077	0.044173766	0.981720316	31.40874801
0.277279003	115.6141337	0.014504892	0.981782768	64.07900011
0.290071705	351.5951731	0.013766094	0.980926712	80.89324265
0.195399959	-258.1743726	0.011372333	0.966191664	102.6692244
0.29247714	443.9672469	0.017144327	0.986330559	88.91810066
0.332934723	189.8059097	0.029783872	0.984932522	74.13820472
0.30848201	331.6475409	0.017420066	0.982945089	73.29491624
0.238539646	-281.8106039	0.013179653	0.970678776	99.44559444
0.195560513	-564.1574962	0.010567524	0.969542887	116.0972018
0.387503193	209.5646095	0.099633148	0.984686983	84.94500576
0.273521489	-141.9056485	0.019185125	0.970630866	59.35751147
0.390464877	251.3056238	0.046959121	0.993235654	58.82378291
0.239542265	180.2966726	0.011687051	0.979043761	98.81600912
0.459295007	90.01217827	0.082857141	0.992733256	31.65369333
0.211013032	117.4464005	0.008924946	0.972764375	123.2020128
0.23192191	338.6222436	0.00939562	0.97877405	103.1595601
0.389501429	151.0594782	0.057964525	0.986155974	53.08564848
0.246391373	141.389383	0.013837492	0.975500365	96.04485643
0.336238621	118.3663863	0.025220534	0.987342423	58.23230801
0.284587376	-78.3087092	0.024233577	0.97177322	48.55398142
0.247344791	490.9230453	0.018570691	0.977492917	99.3784275
0.208211464	-183.1960187	0.013493318	0.969522252	98.21917104
0.258966565	566.513303	0.018550194	0.975759187	90.21304467
0.248679396	239.7728012	0.010744433	0.981680272	94.08118505
0.497893015	128.3970591	0.102899994	0.990762033	33.86967417
0.324527585	80.52181366	0.019010101	0.985563672	52.46357167
0.176348365	-476.3962199	0.010850614	0.968531591	147.1973608
0.228582433	349.5941505	0.009381218	0.973572125	104.1317339
0.324651147	282.8412388	0.02381358	0.984145935	72.93914457
0.237649269	49.80308883	0.010641786	0.974995967	82.91901653
0.243063141	-92.42061079	0.01435801	0.972272677	74.77900803
0.449357595	47.85211552	0.113863143	0.990657622	47.87631714
0.263675632	213.1868354	0.016441511	0.97999201	74.03544062
0.233273658	231.6717863	0.017232967	0.983416679	107.8683842
0.277712386	342.8428082	0.018800998	0.986908902	95.84686959
0.255099033	7.290347421	0.012075149	0.984378301	101.887594
0.260682149	30.4646069	0.017757099	0.975076001	58.01393415
0.230422697	169.4475159	0.010080409	0.984264633	91.44948807
0.196806932	-438.9094486	0.01586599	0.963469576	98.21711764
0.275169164	322.2710124	0.014162128	0.985886602	82.47334149
0.353093743	150.3286132	0.023174978	0.988583172	58.49382782
0.262231199	20.07279553	0.01331456	0.978612329	77.52377508
0.228347106	40.48673267	0.01582026	0.962178597	83.92626222
0.297355898	152.7020714	0.035583122	0.979820523	86.39727841
0.215206398	274.0626904	0.011478413	0.971593344	107.3366569
0.273459924	186.735235	0.015293906	0.979860601	62.6151449
0.363539214	365.8878417	0.044654767	0.9898731	73.21241493
0.288606696	-3.89698406	0.01792613	0.971749379	46.09551364
0.272136767	38.4927023	0.011529939	0.983610937	78.00635549
0.169831052	-903.6664356	0.014876481	0.964007786	140.1900145
0.25606223	48.12521738	0.012345299	0.977765086	84.04302455
0.346833506	79.84233063	0.025958066	0.986249016	47.80282401
0.218512882	337.8946255	0.010427146	0.976380699	117.4314029
0.242570689	-223.2017013	0.014824351	0.975866638	85.64497418
0.28482943	162.3306924	0.032685377	0.975687082	72.58200077
0.31613466	92.20691559	0.021723025	0.984108549	49.35819988
0.331545449	305.0338072	0.034057488	0.983730279	76.05463792
0.229855508	-587.7521392	0.022864788	0.978448019	89.87602835
0.264433227	80.79791689	0.013171761	0.977070735	80.00837077
0.402758218	303.7050103	0.070820617	0.985060928	65.74461718
0.310632732	88.2343507	0.024880438	0.983705538	56.96827521
0.238815011	137.4739139	0.011350046	0.977560829	99.02216768
0.254419765	-88.31485904	0.018953123	0.979224745	100.3714943
0.349138425	40.9685618	0.03167563	0.984504405	59.08526098
0.31664477	142.8651546	0.014841425	0.988784049	58.54349599
0.288762686	251.4383273	0.015388384	0.984315135	77.47216143
0.338219133	57.17567518	0.01717265	0.985819149	45.29286388
0.201188731	249.4305673	0.007341055	0.978775092	130.9149539
0.251957416	115.0381949	0.009076647	0.978174735	88.18354338
0.240298618	-291.3569717	0.020057005	0.97095296	79.84207921
0.252671741	-219.472788	0.028748276	0.966576691	55.63320162
0.277505024	82.31683409	0.017065426	0.983825976	81.25986345

log.sigma.4.5.mm.3D_glcm_Contrast	log.sigma.4.5.mm.3D_glcm_DifferenceEntropy	log.sigma.4.5.mm.3D_glcm_InverseVariance	log.sigma.4.5.mm.3D_glcm_Entropy	log.sigma.4.5.mm.3D_glcm_Dissimilarity
28.16033531	3.464308778	0.222390381	8.507321355	4.189388045
33.78263603	3.507841394	0.195221659	7.866640785	4.692996123
31.40983926	3.440791088	0.207071951	8.249217997	4.520896702
37.99627826	3.583517619	0.186299102	7.967541204	4.970891306
21.28020528	3.268260853	0.243317326	8.250105939	3.660089219
25.66379182	3.313994694	0.222832503	7.551709766	4.098740833
11.90943424	2.863163379	0.323772892	7.238252214	2.317352678
21.7018878	3.307809778	0.269353064	8.239702886	3.488224084
16.16839999	3.08491978	0.301543715	7.692116835	2.915145395
15.58504627	3.037707167	0.273646582	7.665260524	3.120541092
8.686034396	2.674223396	0.358400174	6.916379473	2.208818788
12.31551232	2.929589988	0.332579532	7.435508661	2.528971807
30.3001528	3.457685835	0.223075571	8.281840819	4.327259191
36.39286	3.546123731	0.183079544	8.388613584	4.877890723
22.48519284	3.29574111	0.269643571	8.009769761	3.545941751
19.78440421	3.104809035	0.295586109	7.767277936	3.104389178
9.811699495	2.763558873	0.335680303	7.123404023	2.419015875
34.91145549	3.541263705	0.192572657	8.084071131	4.733975674
8.570658518	2.651422693	0.372182195	6.836193244	2.186010406
16.36720594	3.1060601	0.299243097	8.036102667	3.0133045
19.15030032	3.190481169	0.264448429	8.129449968	3.413447339
21.56104959	3.250486866	0.241470598	7.822747097	3.6850688
20.48414147	3.219351939	0.250049131	7.837333052	3.565108764
8.433344698	2.676766098	0.360282878	6.877268153	2.025457101
25.62809133	3.332853667	0.260806403	8.111487633	3.724687248
21.14587795	3.266309171	0.273009216	8.197375657	3.485103415
25.95740073	3.352441744	0.237506865	8.262942631	3.960002071
12.65915854	2.953635865	0.305313235	7.613522365	2.727224006
6.986514451	2.497739519	0.349675974	6.000267885	2.04951546
15.14063799	3.026159715	0.284508932	7.751127681	3.04097087
18.29495002	3.152718458	0.282370823	8.025303592	3.241041042
31.07659326	3.47665767	0.197645583	8.261755941	4.495803861
17.5586192	3.144721031	0.290684487	8.053459895	3.171661286
16.32889017	3.088631702	0.300856375	7.760326723	2.949138733
16.36948441	3.119080537	0.29915352	7.934989369	3.016477779
26.94476642	3.406094434	0.234273491	8.407094424	4.027861036
36.6130435	3.464017404	0.203408197	8.53041463	4.707533849
18.8657845	3.128328734	0.278566745	7.683100409	2.97887066
14.04253181	2.888614797	0.276796371	7.364156854	2.99411931
11.17745309	2.865488781	0.339996021	7.478639966	2.359060372
21.42189237	3.2658438	0.245425706	8.214958593	3.653420268
6.32879956	2.514023767	0.378282536	6.592623266	1.768308071
34.12620059	3.41022884	0.21465507	8.590959999	4.571923111
21.68101206	3.265192778	0.24403997	8.260003647	3.700206638
11.5552434	2.858333758	0.343039922	7.17868139	2.405131232
23.72852488	3.343283756	0.245730213	8.350739648	3.79612531
13.60874402	3.0013944	0.311817494	7.689162806	2.728931331
13.48225134	2.954481949	0.28785301	7.236696448	2.911816188
18.88627329	3.156489257	0.242506147	7.648626519	3.44464677
24.15040553	3.290288162	0.224639919	7.862442131	3.9716711
20.48675822	3.25412763	0.253469842	7.954253736	3.514869736
21.19206977	3.280963063	0.254127639	8.317572171	3.571657436
7.072179406	2.538177176	0.363056815	6.470861834	1.754608526
13.72738201	2.929861705	0.32254462	7.471803692	2.760596626
37.25168608	3.591062093	0.190239703	8.454372715	4.934517407
22.37853915	3.283704849	0.238546209	8.204539562	3.761467911
16.16178851	3.078419148	0.300802417	7.840768727	2.962807782
22.56810271	3.304328256	0.237065019	8.149519742	3.756485072
20.26886221	3.216985629	0.249551965	7.919764196	3.572864968
10.03080741	2.768902265	0.326056483	7.0848375	2.129943703
19.20877137	3.203968691	0.269759892	7.993401587	3.393077148
23.84905032	3.350684242	0.242115242	8.476623961	3.844522175
21.98222584	3.298763165	0.272156472	8.375997839	3.506782904
20.15374002	3.245078113	0.260661352	8.31425714	3.478647968
16.74799214	3.086632184	0.266873809	7.614850994	3.235749633
22.56575712	3.316996167	0.2393355	8.372937648	3.763691394
29.44071355	3.403393299	0.207818294	7.86693955	4.358692912
18.13017009	3.186222391	0.276174851	8.143279325	3.270330842
10.72722011	2.84878699	0.336568028	7.543146646	2.446435974
17.88397069	3.167660821	0.261394606	8.053777071	3.321811897
22.15042516	3.197163324	0.231277307	7.726241339	3.770755089
22.16797018	3.303489594	0.272254828	8.196050753	3.4895181
27.65424931	3.419814454	0.218449231	8.326947099	4.186022309
15.62253521	3.054028689	0.290214433	7.647702709	3.092994555
13.71657749	2.987136444	0.319637234	7.667492429	2.646118618
12.20026708	2.857160548	0.294619528	7.306715893	2.791265768
16.59891075	3.119017467	0.280627978	8.02251675	3.173650494
43.10702905	3.659437561	0.176941657	7.948819417	5.29682091
17.39275753	3.125209793	0.269685196	8.038517413	3.283193841
11.38711596	2.874000557	0.338068492	7.374224021	2.506109727
27.52890496	3.447137005	0.224301404	8.478230744	4.14261571
18.81430237	3.151289181	0.253787536	7.953666351	3.462654838
20.65253612	3.234474201	0.260083352	7.943652799	3.437544732
12.23931791	2.913302477	0.314764978	7.479496836	2.678407401
15.55236743	3.054928284	0.314137445	7.792155249	2.898891634
26.54812064	3.392560286	0.236990258	7.675842574	4.028672975
19.3160818	3.191533067	0.267984045	8.047957403	3.403941603
13.43970271	2.946029176	0.330442805	7.42488481	2.520259929
14.49321736	3.02930518	0.311452713	7.642562839	2.884569718
21.59554656	3.27707601	0.239850074	8.238169555	3.667565655
22.81615414	3.294244679	0.261163086	8.007325952	3.657435191
12.93810422	2.928844781	0.335718933	7.517999273	2.626262457
11.93505346	2.915824648	0.314574571	7.65533073	2.659479357
15.86123898	3.089079671	0.286249517	7.989261583	3.067753814
9.286716696	2.71339524	0.342048843	7.271163552	2.360740606
32.63870305	3.554739529	0.207271919	8.7780955	4.521722482
19.63525924	3.206310445	0.261613532	8.195635323	3.46930133
21.44459289	3.22552672	0.253540487	7.476582865	3.639773781
17.78175224	3.074145312	0.268186613	7.094314581	3.343757587
19.79222102	3.232115806	0.27000089	8.237115425	3.376334056

log.sigma.4.5.mm.3D_glcm_DifferenceVariance	log.sigma.4.5.mm.3D_glcm_Idn	log.sigma.4.5.mm.3D_glcm_Idm	log.sigma.4.5.mm.3D_glcm_Correlation	log.sigma.4.5.mm.3D_glcm_Autocorrelation
9.957766972	0.893786892	0.216638952	0.616019525	267.6481684
10.57198426	0.876203778	0.188624943	0.573481703	294.743512
9.491455293	0.886143016	0.203419573	0.535087471	291.5983419
12.14568444	0.873508689	0.173784837	0.561692407	294.1170088
7.458927997	0.890944618	0.234192832	0.656091208	185.5423169
8.169490422	0.879463164	0.203024038	0.652468206	267.4137949
6.296602542	0.941902751	0.426635654	0.652871409	136.8795362
9.061841242	0.912683559	0.285554496	0.593483474	243.7313504
7.134211697	0.912383535	0.342717998	0.618207197	144.0556898
5.365099353	0.891572276	0.269828478	0.628000362	140.1167047
3.530927593	0.910797723	0.380488942	0.605589367	91.66794827
5.705596403	0.920068571	0.373088722	0.632479362	137.3788486
10.41729444	0.882017705	0.21542842	0.604182405	251.7267039
11.13137586	0.875361055	0.175712542	0.581629559	255.8675795
9.295386832	0.904132052	0.271204232	0.570212074	146.4534479
8.95110574	0.913863523	0.361714011	0.603487393	173.5427329
3.815649544	0.894172625	0.335014875	0.602496386	89.28783949
11.49604684	0.881813836	0.189186605	0.600341078	252.4427869
3.539657334	0.907875474	0.379373002	0.594103602	89.54001539
6.880826398	0.936182646	0.305294897	0.65499866	514.3592208
7.038967469	0.89111757	0.259023348	0.651520605	186.7294912
7.399736488	0.883377645	0.237171235	0.614684701	215.6454377
7.196232893	0.893595523	0.248201522	0.603739599	172.270843
4.184255135	0.92751967	0.434261603	0.64011972	121.5079674
10.64736422	0.908136829	0.284820313	0.601025274	310.3470609
8.411370375	0.918607533	0.272652706	0.639051186	319.9883457
8.991806918	0.899122585	0.230682699	0.567416663	234.3101486
4.969588891	0.910432556	0.317039284	0.634448472	141.0431288
2.619240289	0.908004055	0.377703619	0.63472306	95.26487807
5.469055857	0.907207731	0.277279003	0.618829313	163.306189
7.089276884	0.90843745	0.290071705	0.63180831	178.6494233
9.866401986	0.87377929	0.195399959	0.53961484	291.2013317
7.022014098	0.921331357	0.29247714	0.671449726	196.801008
7.20314739	0.920914464	0.332934723	0.640669538	199.5120655
6.964988432	0.914222182	0.30848201	0.636230067	176.2114694
9.600263553	0.88613427	0.238539646	0.578379975	265.9476651
10.8034076	0.883350764	0.195560513	0.539237432	348.7984894
9.361414417	0.925602291	0.387503193	0.639393943	209.4675993
4.235242854	0.882548597	0.273521489	0.621638477	129.8052115
5.416993634	0.947048536	0.390464877	0.682284233	397.8312395
7.560386306	0.90025734	0.239542265	0.644029764	186.206546
3.092339319	0.945483944	0.459295007	0.667083338	143.258326
9.663239243	0.888842933	0.211013032	0.579684776	300.2472327
7.408877463	0.898982651	0.23192191	0.65483815	193.554278
5.556770895	0.926130307	0.389501429	0.643539014	151.7679959
8.615862874	0.894499453	0.246391373	0.605209116	218.2756872
5.900375851	0.925793727	0.336238621	0.622734003	233.495001
4.709402472	0.885508443	0.284587376	0.566437318	113.4671413
6.446501535	0.896403442	0.247344791	0.68332198	143.8087727
7.651669488	0.87897829	0.208211464	0.607702149	259.523702
7.707765902	0.895286837	0.258966565	0.624639499	139.1025861
7.977484828	0.907651006	0.248679396	0.634895056	297.2063557
3.877407219	0.943106894	0.497893015	0.655553816	108.4788016
5.311641467	0.920551076	0.324527585	0.590150943	250.5705834
11.84188332	0.877349394	0.176348365	0.600324381	339.1877239
7.652297213	0.88834523	0.228582433	0.644901395	165.4161504
6.771285977	0.918173053	0.324651147	0.638659913	171.8525284
7.934267672	0.891939961	0.237649269	0.572122785	245.4414898
6.982813649	0.886326778	0.243063141	0.574544493	190.8282542
5.271736528	0.941634351	0.449357595	0.656394591	216.0878179
7.18807981	0.904188401	0.263675632	0.5902337	194.6976619
8.468288347	0.910546492	0.233273658	0.639762016	345.275905
8.995135343	0.923776175	0.277712386	0.628741031	366.9372333
7.578245775	0.914230477	0.255099033	0.672289251	275.8885547
5.788819066	0.89198196	0.260682149	0.551745196	137.9757824
7.845193139	0.912028423	0.230422697	0.608318897	330.4592941
9.475196793	0.869641843	0.196806932	0.543336113	268.5751913
7.02574565	0.918904441	0.275169164	0.641250488	246.0769402
4.527280089	0.928489734	0.353093743	0.69030096	182.5539836
6.473814429	0.89983161	0.262231199	0.626537839	244.7910694
6.83983168	0.868179546	0.228347106	0.58189222	128.2468384
9.500762064	0.908315928	0.297355898	0.595103113	207.7398512
9.35702513	0.884777627	0.215206398	0.591544693	187.263331
5.68799555	0.902856684	0.273459924	0.59835732	127.0395466
6.346053066	0.935397101	0.363539214	0.688192051	200.4149747
3.96741579	0.88463002	0.288606696	0.583530977	112.5293707
6.167114685	0.911899431	0.272136767	0.649416504	245.01419
13.69387756	0.870110255	0.169831052	0.539204628	416.525059
6.173801035	0.897589345	0.25606223	0.657484464	206.2877556
4.902026107	0.922493777	0.346833506	0.6155127	135.6420976
9.722536408	0.894785605	0.218512882	0.622944302	264.1765224
6.225762433	0.892522879	0.242570689	0.642658397	249.2652904
8.057769305	0.898065003	0.28482943	0.555998542	141.9155285
4.774246859	0.914632467	0.31613466	0.6037624	125.2377826
6.677311414	0.917461717	0.331545449	0.661966154	186.847043
9.669004424	0.900090662	0.229855508	0.548121126	419.3284681
7.184151762	0.898148937	0.264433227	0.612615552	190.139056
6.862607345	0.925998392	0.402758218	0.659849454	137.2102292
5.878359699	0.914685081	0.310632732	0.594450091	188.5076801
7.697591546	0.89709784	0.238815011	0.643952403	222.834924
8.632848503	0.903616655	0.254419765	0.632819787	410.247647
5.653440489	0.919644247	0.349138425	0.641908163	215.0579805
4.652234318	0.926793017	0.31664477	0.661298327	200.0664348
6.099227815	0.914707781	0.288762686	0.659786445	186.2569752
3.397643403	0.917984139	0.338219133	0.65893309	175.3019988
11.19837513	0.899350205	0.201188731	0.602984529	316.7600588
7.00275986	0.899191954	0.251957416	0.636165109	196.8129519
7.525162123	0.884844751	0.240298618	0.580222242	243.7176991
5.977765144	0.876471519	0.252671741	0.522425038	166.4591522
7.817460699	0.914754111	0.277505024	0.611062895	318.3558044

log.sigma.4.5.mm.3D_glcm_SumEntropy	log.sigma.4.5.mm.3D_glcm_AverageIntensity	log.sigma.4.5.mm.3D_glcm_Energy	log.sigma.4.5.mm.3D_glcm_SumSquares	log.sigma.4.5.mm.3D_glcm_ClusterProminence
5.418697713	15.85579844	0.003505765	37.72199464	36134.26966
5.331761715	16.63298767	0.005238698	39.63844963	38588.37814
5.273229508	16.75435756	0.004091342	34.70748693	25761.30244
5.34895309	16.62201288	0.004719898	43.66381342	47139.5876
5.308364743	12.87559868	0.004023621	31.33932318	25617.8677
5.214026808	15.71205759	0.006207914	37.89890872	32294.55734
4.808990689	11.27722895	0.017511224	17.73611285	12588.15377
5.198413839	15.23878256	0.005036498	27.38253949	23698.26043
4.97175249	11.46342491	0.008441096	21.68978706	17400.76682
4.992167646	11.26104133	0.00604154	21.07053128	11229.45226
4.550402502	9.214425452	0.012850763	11.09518241	3893.881836
4.848185628	11.26416872	0.010633094	16.86269451	11420.16091
5.358091778	15.23541134	0.004212387	39.0031369	33778.86393
5.432298846	15.38937558	0.003577449	44.18907504	42570.75058
5.123287296	11.54907149	0.005346231	26.97540104	21616.88795
5.089754747	12.6547285	0.009962009	25.36657915	17074.01719
4.643827892	9.047244822	0.00926232	12.3322054	4362.165154
5.418164393	15.17410987	0.004426033	44.00395441	47413.44291
4.505125653	9.124178145	0.013846795	10.56716036	3890.030209
5.146145186	22.45235797	0.005975747	23.53676618	21108.39739
5.228964922	12.99739035	0.004366581	27.55392353	19249.87704
5.147767465	14.09531322	0.00548689	27.84609906	20787.77047
5.125657554	12.52155216	0.00551886	26.01481346	19370.54165
4.57285444	10.67629329	0.018947781	11.73537072	5843.742581
5.295751403	17.23427782	0.005856822	32.42668861	29853.25852
5.267857798	17.50962097	0.004893872	29.82397932	29559.92797
5.254291788	14.93838256	0.004381684	30.81505456	24152.61656
4.919973644	11.40551179	0.006862486	17.33030874	9108.236222
4.251771362	9.442266662	0.01940203	9.598815616	2440.782222
4.995591057	12.30408583	0.006012919	20.041393	12594.7611
5.14419663	12.82614725	0.005154331	25.56296562	18603.01446
5.263041169	16.6602398	0.003962522	34.43890709	24456.43615
5.209638579	13.46102707	0.00546703	27.34078806	25642.54188
5.06207023	13.70067166	0.006857883	23.54175943	13756.89255
5.077313493	12.77869514	0.006080376	23.11919778	15848.26616
5.299086	15.81511462	0.00376023	32.25412275	25717.90574
5.37653	18.41713986	0.003486711	38.63336177	38547.69685
5.087013477	14.00631708	0.01628388	26.85795414	22525.26363
4.882538152	10.88268005	0.0074851	18.35001082	8807.23421
4.920758128	19.73239586	0.010459367	17.43865808	14872.0101
5.28560866	12.99372965	0.004104967	31.00751087	23914.38493
4.439158347	11.70544135	0.021413166	9.565462588	4738.131051
5.472634081	16.88066267	0.003341643	40.18291686	41393.85304
5.314602199	13.25102243	0.003948888	32.32967545	28328.62403
4.756511168	11.8988549	0.013595455	16.3254585	11787.60613
5.287401491	14.25378285	0.003945052	31.13399748	22376.23715
4.946074958	14.97322554	0.007080507	18.51914612	10315.01708
4.726045782	10.23121933	0.008531755	15.50905819	6362.851206
5.169979703	11.15411227	0.006059363	30.26917648	29650.42551
5.202336339	15.59000373	0.005189318	31.411757	23607.7485
5.145814965	11.05067208	0.005488782	28.13884221	24084.5048
5.287391484	16.84660786	0.004202983	29.44075268	26871.65845
4.383927342	10.09137767	0.03017583	10.30159596	6797.098964
4.852429572	15.60778899	0.007850181	17.31637001	8882.094577
5.50945293	17.90087481	0.003437176	46.66502434	53791.04522
5.289289819	12.05982478	0.004015683	32.02529069	26458.26297
5.081236663	12.61807077	0.006491973	23.03643137	16209.05539
5.148055303	15.23843897	0.004161752	26.98615741	14907.86366
5.082633881	13.31394427	0.005104913	23.82721287	14553.30917
4.71515744	14.42237232	0.021733195	14.91856965	9413.216945
5.093722255	13.50280726	0.005116092	23.93514612	16344.198
5.376847914	18.19982455	0.003851101	32.6419815	34414.6209
5.284802865	18.83861174	0.004675732	29.14363225	27496.26787
5.34196357	16.11493361	0.004277643	31.10503323	31180.5046
4.894435066	11.29766952	0.006277891	18.69048157	8548.503809
5.276903077	17.90274977	0.003962832	28.3498029	26967.61183
5.151677447	15.9270411	0.005200301	32.65772482	24237.35375
5.191343578	15.29632045	0.00503007	25.87438624	22432.29086
4.942662521	13.08803433	0.007727963	17.67337492	10826.67653
5.143504603	15.19565252	0.004806513	24.27959668	15549.04191
5.065985865	10.61792598	0.005632448	26.51917185	14713.66813
5.204280917	13.95803905	0.005979583	28.26564667	22705.00901
5.303199527	12.99253916	0.003786219	34.47979098	26902.75385
4.941404864	10.73730653	0.006258605	19.61186198	11155.49879
5.064113459	13.70443013	0.009599674	22.40226369	22149.19379
4.724315435	10.19933933	0.007712745	14.57394518	4866.011202
5.152533667	15.21192673	0.00492241	24.13112204	16796.05029
5.366465236	20.10958898	0.004892766	45.40615873	57108.30537
5.191102075	13.78465055	0.004875573	25.61052098	18798.72427
4.789016121	11.2585928	0.008581788	14.98003221	7577.840864
5.418678608	15.73685671	0.00358354	37.60016917	36721.43456
5.164732265	15.28077217	0.004998421	26.52923877	18801.58923
5.066637232	11.36637951	0.006031558	23.53655939	14313.46235
4.818615118	10.77003442	0.00780127	15.44310046	7670.475748
5.078555657	13.14556553	0.007922942	23.51691893	19286.95283
5.073058476	20.2639842	0.006955349	28.51832114	27541.71907
5.151632033	13.25251208	0.004834108	25.25048717	16254.17118
4.919415073	11.16236391	0.014680857	20.17576187	16572.67488
4.910397966	13.35715662	0.007594791	18.13552772	10124.55462
5.295116915	14.34121463	0.004114389	31.06203211	25454.49222
5.240572323	19.93749546	0.005411561	30.86094142	31612.91714
4.923804299	14.30772877	0.009211794	18.57983354	12115.06462
4.951528711	13.75114736	0.006564327	17.88997043	11239.47888
5.133332698	13.11243337	0.005292073	23.84507238	17128.66392
4.763380318	12.89547916	0.008192416	13.64489515	5255.85133
5.517497218	17.32210587	0.002848573	41.15260976	47710.82195
5.224333831	13.42996237	0.004191082	27.32584259	18732.45542
4.992138324	15.1361442	0.006891425	25.35897323	16682.16633
4.725147761	12.5269531	0.009256382	18.35373847	8373.304603
5.197301864	17.5648866	0.004710445	25.59069663	19105.79866

log.sigma.4.5.mm.3D_glcm_SumAverage	log.sigma.4.5.mm.3D_glcm_Imc2	log.sigma.4.5.mm.3D_glcm_Imc1	log.sigma.4.5.mm.3D_glcm_DifferenceAverage	log.sigma.4.5.mm.3D_glcm_Id
31.32436243	0.845726304	-0.143051584	4.189388045	0.308918054
33.01293819	0.957782332	-0.276238562	4.692996123	0.28262514
33.08402927	0.880520849	-0.172761979	4.520896702	0.29473624
32.87980614	0.967140802	-0.294122402	4.970891306	0.26875703
25.70530014	0.84583703	-0.148871457	3.660089219	0.32757725
31.20593422	0.97117032	-0.320672364	4.098740833	0.2987734
22.43098274	0.839127532	-0.167451788	2.317352678	0.489951751
30.2052916	0.767718252	-0.111084648	3.488224084	0.368244434
22.88006558	0.78892572	-0.135256063	2.915145395	0.417666666
22.52208265	0.855735116	-0.166542554	3.120541092	0.360197715
18.4288509	0.781364966	-0.145091812	2.208818788	0.451994135
22.5182602	0.803551019	-0.140467829	2.528971807	0.443854623
30.25235182	0.891017228	-0.178909096	4.327259191	0.306232491
30.40391039	0.912346488	-0.195592217	4.877890723	0.271561542
22.95674464	0.815589785	-0.138112224	3.545941751	0.356570395
25.19961131	0.864043542	-0.177697292	3.104389178	0.430714647
18.09448964	0.796344015	-0.139768303	2.419015875	0.416095693
30.10240664	0.949330421	-0.251980933	4.733975674	0.282999249
18.24835629	0.791672315	-0.148456055	2.186010406	0.450354683
44.67496346	0.787536685	-0.126403884	3.0133045	0.38763715
25.98523931	0.819885002	-0.140483746	3.413447339	0.348600772
28.18322983	0.906341482	-0.203230605	3.6850688	0.3296232
25.02665022	0.899716724	-0.197374903	3.565108764	0.339130862
21.35258658	0.812575508	-0.156792098	2.025457101	0.496514028
34.13238413	0.893629036	-0.193946033	3.724687248	0.36598749
34.69584113	0.835714196	-0.146202531	3.485103415	0.357785036
29.51320887	0.813357546	-0.135471492	3.960002071	0.321908358
22.80867176	0.790769156	-0.131807263	2.727224006	0.400033289
18.88453332	0.932135256	-0.294705918	2.04951546	0.453852495
24.58131797	0.814764145	-0.140716836	3.04097087	0.366433621
25.53383853	0.798757598	-0.131605901	3.241041042	0.373732153
33.06064172	0.862452687	-0.158155211	4.495803861	0.290072865
26.75483585	0.837806408	-0.150665404	3.171661286	0.375819768
27.2059074	0.83864662	-0.156415947	2.949138733	0.409777367
25.45388908	0.776542207	-0.119931018	3.016477779	0.38997269
31.48351713	0.804557129	-0.125365165	4.027861036	0.327207815
36.26909456	0.843388786	-0.150164982	4.707533849	0.288887448
27.7805343	0.887675305	-0.191322761	2.97887066	0.454098223
21.76536011	0.870367204	-0.186241774	2.99411931	0.364588178
39.28953697	0.823514312	-0.152648179	2.359060372	0.459660686
25.8328751	0.848608583	-0.151447148	3.653420268	0.331662416
23.40302692	0.805080143	-0.158609128	1.768308071	0.518352732
33.34133183	0.845630726	-0.156433513	4.571923111	0.30074049
26.31785701	0.852761066	-0.153000919	3.700206638	0.325118026
23.780676	0.850604287	-0.173117288	2.405131232	0.457615542
28.2970132	0.801561077	-0.125272569	3.79612531	0.335029347
29.82168544	0.779207941	-0.124333328	2.728931331	0.414492027
20.46243867	0.848733177	-0.167038573	2.911816188	0.373375395
22.23712197	0.94015266	-0.248413573	3.44464677	0.34095628
31.04349047	0.920153189	-0.216196885	3.9716711	0.304334356
22.05503707	0.84646621	-0.153781771	3.514869736	0.348186114
33.40534437	0.806635056	-0.127324184	3.571657436	0.339618127
20.17683669	0.826329972	-0.17369767	1.754608526	0.549400002
31.04070091	0.815182369	-0.150894691	2.760596626	0.404008419
35.30532968	0.925885697	-0.208876079	4.934517407	0.270577747
24.07691937	0.85805856	-0.158281218	3.761467911	0.322131441
25.11114973	0.808140519	-0.141614043	2.962807782	0.403076574
30.35418713	0.773208492	-0.114674642	3.756485072	0.329412326
26.61991051	0.845477196	-0.153099317	3.572864968	0.33491809
28.74881641	0.843479147	-0.172257818	2.129943703	0.509815597
26.90516508	0.813585732	-0.135465939	3.393077148	0.352103338
36.01417993	0.822582285	-0.133970094	3.844522175	0.324411251
37.33396601	0.774300128	-0.117848083	3.506782904	0.361861633
31.96468966	0.841025609	-0.146865651	3.478647968	0.345253971
22.59533905	0.834267304	-0.152376062	3.235749633	0.352145959
35.39662442	0.781714731	-0.115551397	3.763691394	0.323573453
31.7057384	0.925639675	-0.221612765	4.358692912	0.292243423
30.33057785	0.781309309	-0.120683022	3.270330842	0.362320578
26.12319249	0.810247513	-0.144238146	2.446435974	0.429448265
30.32319932	0.800776696	-0.128364049	3.321811897	0.35249903
21.23585197	0.899089553	-0.199122939	3.770755089	0.322281864
27.68944561	0.798527006	-0.125857381	3.4895181	0.377069188
25.87041688	0.834755267	-0.140580353	4.186022309	0.307807323
21.47045915	0.833787696	-0.150927896	3.092994555	0.362072403
27.24220508	0.85023451	-0.167361358	2.646118618	0.436303967
20.39867866	0.808003648	-0.145835032	2.791265768	0.377751796
30.30884994	0.816779791	-0.137249267	3.173650494	0.360747404
39.6049503	0.965128089	-0.2881362	5.29682091	0.262782586
27.5397328	0.844772373	-0.154443112	3.283193841	0.347684694
22.49687966	0.797508872	-0.138084915	2.506109727	0.423689123
31.09302592	0.850828252	-0.146596029	4.14261571	0.310789929
30.49874886	0.870110886	-0.17148231	3.462654838	0.33575181
22.70766218	0.801587399	-0.137937354	3.437544732	0.369013471
21.53587334	0.775022649	-0.12685028	2.678407401	0.399413837
26.20771355	0.837529302	-0.155621744	2.898891634	0.407938685
40.17050825	0.907703203	-0.205635665	4.028672975	0.320122596
26.45273449	0.825272716	-0.142723701	3.403941603	0.35269851
22.28202572	0.85528665	-0.170765257	2.520259929	0.467408857
26.67447215	0.792818903	-0.13053663	2.884569718	0.392089442
28.52737676	0.851730266	-0.151893732	3.667565655	0.33146689
39.53910454	0.889471455	-0.185614448	3.657435191	0.342407408
28.53147903	0.829838531	-0.157660027	2.626262457	0.423773946
27.4525666	0.788730523	-0.129184242	2.659479357	0.399956193
26.14183517	0.806817361	-0.133641188	3.067753814	0.374717359
25.79095832	0.779189628	-0.13805152	2.360740606	0.418930792
34.18561151	0.82157104	-0.12924582	4.521722482	0.294050249
26.80812706	0.798180707	-0.129184741	3.46930133	0.34255496
30.26766438	0.93461182	-0.244872645	3.639773781	0.331797881
25.05390621	0.912923766	-0.227686831	3.343757587	0.344082978
34.81284882	0.752219686	-0.109867382	3.376334056	0.363360824

log.sigma.4.5.mm.3D_glcm_ClusterTendency	log.sigma.4.5.mm.3D_firstorder_InterquartileRange	log.sigma.4.5.mm.3D_firstorder_Skewness	log.sigma.4.5.mm.3D_firstorder_Uniformity
117.4966544	215.8722219	0.286200923	0.045587434
122.3023735	201.1596947	-0.358670224	0.048542883
103.1080991	231.936697	-0.14712694	0.046338656
133.0257923	218.390274	-0.214660182	0.044153389
102.5582348	211.290844	-0.041513274	0.050114997
121.2942292	231.8377361	-0.247410521	0.047537736
56.27377734	101.6312819	0.707985462	0.086702546
84.25526336	166.4491291	0.541736471	0.056528157
68.86603994	133.9017658	0.755656299	0.068878421
68.69707886	186.8483696	0.142220156	0.057336082
35.69469524	105.9689445	0.485934752	0.09315214
54.72488577	111.2553444	0.510869658	0.076714673
121.7249984	244.9563599	-0.402588465	0.047717801
135.0808923	251.75105	-0.33910258	0.043695266
81.25141497	174.8320031	0.629920073	0.057125523
78.89412854	160.7867393	0.356983964	0.064045987
39.51712212	128.4275436	0.143640427	0.075159455
138.12392	247.4715977	-0.195284398	0.045785236
33.69798291	97.68639994	0.312772715	0.091509614
77.93693303	151.3755035	0.423609559	0.061416877
90.71646196	207.6552258	-0.141335094	0.052122191
89.56678205	172.843317	-0.529500334	0.058622203
83.01009589	173.6449661	-0.030875679	0.055568402
38.50813817	82.51060867	0.490894591	0.103749928
101.8637157	203.8037186	0.133932741	0.049902386
97.21960469	198.168642	0.486289951	0.052219638
92.09630832	206.5096693	0.312726227	0.051082466
56.56352161	151.0121975	0.265650671	0.065471318
31.40874801	113.455143	0.251498686	0.09316945
64.07900011	158.3023729	0.183482886	0.061738359
80.89324265	184.4765739	0.457402538	0.055731986
102.6692244	233.7609792	-0.361843488	0.0489706
88.91810066	168.0486546	0.52137899	0.056377534
74.13820472	168.3302212	0.390882089	0.062838625
73.29491624	167.9308243	0.547157897	0.060988992
99.44559444	206.8348274	-0.301102336	0.04965395
116.0972018	212.7615428	-0.493435354	0.046864065
84.94500576	138.9132233	0.368864905	0.074183212
59.35751147	151.1861639	-0.452988726	0.069885204
58.82378291	118.8156338	0.519066525	0.076721978
98.81600912	219.0284119	0.178868201	0.049345066
31.65369333	75.15871525	0.640936862	0.114844703
123.2020128	235.1526871	0.08489389	0.043653848
103.1595601	206.2682791	0.278185873	0.049101369
53.08564848	101.7474365	0.408266512	0.085069984
96.04485643	199.113266	0.202832127	0.050300262
58.23230801	140.612175	0.342470017	0.067456971
48.55398142	132.984477	-0.266494984	0.074767402
99.3784275	171.6097298	0.320772101	0.056351018
98.21917104	189.3289394	-0.336912963	0.053430182
90.21304467	207.9057102	0.489894149	0.05335345
94.08118505	191.8902054	0.267573694	0.051714977
33.86967417	73.4396348	0.683252462	0.119035265
52.46357167	137.0674362	0.319445111	0.070148552
147.1973608	261.7423134	-0.340920749	0.041086083
104.1317339	223.0864468	0.193426883	0.047785356
72.93914457	157.8235569	0.505515072	0.061723146
82.91901653	219.5047607	0.042429807	0.051663328
74.77900803	181.764254	-0.210601985	0.056469857
47.87631714	105.3938522	0.310049026	0.092192737
74.03544062	170.2033806	0.326539631	0.057423081
107.8683842	210.671875	0.126095697	0.046983942
95.84686959	194.5787544	0.28517668	0.051682453
101.887594	175.2893696	0.031091234	0.051665113
58.01393415	166.2095032	0.009350797	0.060447844
91.44948807	195.5234528	0.104367337	0.050471062
98.21711764	188.137759	-0.60519707	0.055267053
82.47334149	170.4155121	0.515179604	0.057781645
58.49382782	144.5851545	0.391667805	0.069048706
77.52377508	178.0791512	-0.088795761	0.056091977
83.92626222	219.7407894	-0.051969571	0.053225593
86.39727841	153.1845665	0.296052681	0.060097433
107.3366569	253.429882	0.15780451	0.047382906
62.6151449	169.0565591	0.319261285	0.06014626
73.21241493	112.790103	0.750914878	0.075461945
46.09551364	149.7757397	-0.052884947	0.070279237
78.00635549	185.0237198	-0.01370184	0.055466126
140.1900145	210.8889084	-0.793057455	0.050772077
84.04302455	175.0535107	-0.058202562	0.054889907
47.80282401	131.4986758	0.220058127	0.073815432
117.4314029	213.7245393	0.292287689	0.046054402
85.64497418	186.2025242	-0.390003687	0.055880611
72.58200077	184.2630348	0.229051422	0.057052834
49.35819988	132.8081183	0.262953582	0.0711111
76.05463792	140.586647	0.4921037	0.066191305
89.87602835	154.6829185	-0.939403851	0.07057359
80.00837077	183.7576685	0.069035224	0.055753117
65.74461718	111.9811001	0.59434281	0.078629302
56.96827521	141.4081116	0.191982946	0.068154051
99.02216768	198.0186272	0.09074765	0.050286056
100.3714943	190.4008541	-0.172086353	0.052681246
59.08526098	121.0516577	0.221530838	0.072794923
58.54349599	148.5270958	0.312565856	0.066595831
77.47216143	170.2910118	0.390851717	0.058966817
45.29286388	140.4253597	0.148972981	0.072094829
130.9149539	251.4870319	0.096030835	0.041582798
88.18354338	210.4916553	0.098699892	0.051239924
79.84207921	180.7094202	-0.539700619	0.061988997
55.63320162	138.2575579	-0.751452019	0.078107596
81.25986345	176.7824936	0.12735744	0.054627221

log.sigma.4.5.mm.3D_firstorder_MeanAbsoluteDeviation	log.sigma.4.5.mm.3D_firstorder_Energy	log.sigma.4.5.mm.3D_firstorder_RobustMeanAbsoluteDeviation	log.sigma.4.5.mm.3D_firstorder_Median
126.2875731	41629000.21	90.66038432	-82.15223312
127.1896045	9551874.19	89.79087701	-29.14000893
124.6066729	20332628.39	92.77899641	-3.850627422
133.6716062	9691246.711	93.09064908	-36.610466
116.7679978	28208821.61	86.14970756	6.102658749
128.3568833	5798871.224	95.41498205	24.47251034
75.31902084	113567746.1	46.64408873	-12.03291321
102.69245	111242730.7	69.63347207	-38.91337967
89.35051668	60108883.21	58.8390316	-33.02248383
100.9915931	11617695.93	75.62547023	27.36987305
64.77636834	11056452.58	43.96679072	5.362624407
78.71403082	37532971.19	50.16910368	-7.688241482
131.8049522	21890594.68	98.72223465	-14.5739603
137.7941136	24617710.77	101.9031687	-81.46355057
102.5276015	33294754.67	72.21029751	-81.49008942
96.62910339	89069746.66	66.4265066	-4.573322535
74.15841482	7385031.182	53.04071834	-4.353944778
136.3158982	16040497.06	97.85392458	-84.36707306
63.92283113	8539254.62	41.43160712	-31.73624802
94.33390379	142373477.3	62.67377237	14.27113342
112.2299112	37516105.95	83.81360219	13.69747925
104.4231107	8550835.604	72.13519041	7.749946117
103.9590617	9943047.819	73.324557	-34.69402122
61.48843305	26940141.06	37.36859093	-15.33845139
118.3176613	29536865.64	84.92251804	-55.42731094
115.2751581	51785076.89	81.87349802	-24.21371174
115.5260749	47004299.75	84.2137357	-74.68105698
86.47002556	29858958.42	61.92448808	-45.28836441
66.13609691	1332764.775	48.02453422	38.86591148
92.31233403	22116196.63	65.42892694	-22.17314339
104.1691759	71413458.56	75.57840935	-54.37331009
123.4139296	25575507.65	94.15597456	-86.32854843
103.9895595	41573614.83	71.55900322	-40.45474243
94.63003915	33981497.61	68.88994735	-21.85883141
97.10465911	76157080.34	68.19684034	-32.11759949
115.1176863	40298345.07	83.88899407	-25.37987518
125.9178752	47577824.5	88.95459494	-61.60402489
96.10543166	95519218.74	63.30655891	-2.882350445
86.36089453	7521072.991	62.36780956	-31.73837566
77.78651217	104839512	50.14637534	-16.65729904
118.1247119	34854940.53	88.90339948	-82.85037231
54.23303593	32975183.43	32.57009052	2.674381852
130.6969495	61114381.1	95.42800378	-14.4575367
115.760617	31478458.93	84.84754177	-51.9143486
73.70536049	16278481.96	44.21006248	-9.642529964
113.4943922	45282692.24	83.1476398	-17.81309891
84.68804859	61617375.35	58.82620021	-12.20396328
79.30729736	5930221.196	54.6752116	-32.88395691
105.3019595	7018331.298	71.82208978	1.273204327
109.9092234	8450732.458	78.32811654	-1.093531907
115.3137962	29796678.73	86.0337833	-85.51422119
110.7329298	55594336.22	78.48810668	20.3700695
56.13116812	52579626.9	32.72997815	-5.248245239
81.90267969	18585206.47	56.49201927	-29.2678833
144.1245366	19628415.77	105.925987	16.25004959
119.7156435	25019157.19	89.30358175	-25.98683548
94.59537392	57885046.38	66.64741751	-24.31864166
114.7881179	42387645.35	89.83029012	-56.14371109
102.7121503	17539371.26	75.32519575	-73.31077576
69.5441113	99207934.17	43.52142124	2.393574715
99.00565776	20680470.13	70.03121112	-30.34967041
119.7989725	55590311.38	84.98983396	14.76950455
112.8855581	160801764.1	80.3317466	-7.468591452
110.6147782	41308434.31	75.23756607	-2.992911935
93.01799071	8994290.872	67.79140092	-25.87529755
112.8059397	66826293.19	80.81343946	-8.565422058
114.3701517	13512436.41	82.05608396	-86.2492485
101.7011369	88165310.89	70.49146452	-46.94897461
83.76910985	83127047.17	59.41545228	-9.811391354
101.2934773	33478578.54	72.6472882	27.7800293
114.8381849	10126415.46	89.52782697	-48.70801353
100.0974403	85180642.54	67.25496476	-11.83949471
129.0874966	33719329.39	101.0561476	-53.37219238
95.02130595	16198757.18	69.36626597	-68.16340637
82.82595093	53700042.53	51.08342922	-19.89728832
81.00035124	8547788.776	60.92295893	0.73826474
102.5192127	34896770.97	74.48451388	-4.597083569
133.4962975	11168564.61	89.71795708	-36.67100143
102.4472504	23129547.08	72.3974223	41.94659042
77.39665673	21451209.21	53.94155392	-44.99217033
125.04263	40694786.05	89.2740762	-85.34717941
105.0357192	14036021.04	76.24503535	-8.901975632
102.2259794	38992666.62	75.52492436	-43.41969109
80.07018799	25336435	55.83759852	-63.57894516
91.36728183	39246472.45	59.17634493	-18.74855232
107.9488513	11050129.62	70.03430397	1.84559083
102.0839748	22743385.61	74.67014283	-50.78513145
82.96875837	49608381.16	51.31888144	-8.334357262
85.88513332	20892846.86	59.09148283	-34.5397377
114.2428469	26866198.78	82.43776678	3.771320343
112.6460685	16726512.91	77.67187388	15.55898142
80.6724102	30074091.91	52.39531145	-23.39423466
85.60032208	51624222.66	61.52167878	-21.3514843
97.49802352	51157003.1	69.13430651	2.432986856
78.21399439	30037210.86	57.35549228	-12.61081028
137.5402387	67063567.38	101.8702356	-19.32508659
112.1558647	43657833.41	84.90382064	-1.926961422
103.247057	8878141.039	75.7138324	-105.2157898
84.53462947	3159020.687	59.15093025	-4.108203173
103.2529395	151481307.9	72.83390731	18.08364487

log.sigma.4.5.mm.3D_firstorder_TotalEnergy	log.sigma.4.5.mm.3D_firstorder_Maximum	log.sigma.4.5.mm.3D_firstorder_RootMeanSquared	log.sigma.4.5.mm.3D_firstorder_90Percentile	log.sigma.4.5.mm.3D_firstorder_Minimum
1123983006	365.2684326	169.7908935	156.2331223	-446.6054993
257900603.1	285.3490601	165.9126727	134.7167603	-462.9414673
548980966.6	379.4460449	150.9782317	177.3081116	-424.7848511
261663661.2	319.4423218	173.4849834	172.4765549	-459.9526672
761638183.4	366.1048889	141.3933438	173.6889343	-309.5161438
156569523.1	307.5657349	154.797538	211.2646408	-361.1671143
3066329144	587.9163208	103.8072775	134.1006409	-269.3961487
3003553730	437.4927673	135.3982448	143.1502762	-381.9958191
1622939847	398.8898926	118.7579267	151.4506119	-288.1237488
313677790.2	334.0865784	124.0470747	199.8974396	-236.7127686
298524219.6	318.7491455	84.29525085	134.9668884	-199.7857056
1013390222	398.8483582	104.9591744	138.1597778	-269.7941589
591046056.3	305.4080811	164.2927325	131.4755707	-424.9298401
664678190.9	282.3500977	195.5153676	91.24611511	-492.0541077
898958376	419.7257996	145.3943836	113.8453903	-328.5666504
2404883160	435.1111755	124.3307294	185.5620789	-276.8880615
199395841.9	234.8946991	91.45254902	119.6630615	-208.3825378
433093420.5	308.5021973	198.0374187	112.7004257	-484.0950928
230559874.7	231.5220795	88.34893541	84.16169357	-231.7221985
3844083888	505.6256409	124.5151666	177.1589478	-519.8388672
1012934861	302.1151123	134.3649841	167.7163696	-304.9135132
230872561.3	253.6251373	131.2996112	146.0235901	-359.3850708
268462291.1	334.4221802	133.4881347	128.9358307	-346.1117249
727383808.6	318.2867432	86.54255521	91.41903687	-254.7512054
797495372.3	354.7940674	155.089723	158.4053482	-454.6213379
1398197076	479.6351013	142.3383372	205.7446823	-403.307251
1269116093	394.2582092	153.1510346	131.5734146	-420.7366028
806191877.3	309.1767883	115.1215773	96.44415436	-306.9285278
35984648.92	277.8617859	95.54331925	160.3169556	-162.7783966
597137309	367.1120911	117.2039594	122.9530411	-304.7332458
1928163381	386.0854492	133.4664181	143.0665924	-339.5946655
690538706.5	192.3185272	180.1561211	75.14476471	-501.0004883
1122487600	500.2604675	133.4336763	158.4018036	-349.1410217
917500435.4	435.7871704	118.6213029	148.4363342	-339.9953003
2056241169	407.1003113	121.8062326	156.340567	-322.4985657
1088055317	289.4875793	144.1632954	142.7469238	-418.0013123
1284601262	274.9222412	177.3888811	102.8662148	-535.5915527
2579018906	488.9932251	128.0992483	170.1578827	-337.7251892
203068970.8	187.712738	115.8899801	76.65620422	-319.1589355
2830666823	498.0964355	105.3453906	110.9777756	-478.901001
941083394.4	355.9929504	161.2797249	109.8020493	-385.6427917
890329952.6	425.6733398	76.24748074	101.5299278	-270.3477173
1650088290	416.3406982	158.980391	191.5162201	-406.6893005
849918391.2	391.3850708	148.8889815	142.9666901	-363.2185974
439519012.8	396.4577026	102.2826932	108.3019028	-279.4659119
1222632690	384.0218506	137.3314608	182.3181152	-333.2056274
1663669134	403.5235291	107.3084995	129.5246277	-352.7030945
160115972.3	212.2731628	109.8992428	70.85919189	-288.1540833
189494945	407.7485352	133.9764771	186.3307648	-271.2696533
228169776.4	272.4865723	136.7344534	165.1395187	-376.4799805
804510325.8	345.4647217	152.1579526	133.2076752	-345.7606812
1501047078	438.3114624	141.2871533	222.5639648	-366.1922302
1419649926	406.5428772	81.19254924	80.58870697	-240.8397064
501800574.8	342.3522339	106.389096	110.2597595	-379.0549316
529967225.9	353.4859619	174.7177941	222.3917572	-438.4941101
675517244.2	365.5485535	145.8588137	174.0997467	-315.2420044
1562896252	441.5848389	120.0716332	145.9194611	-313.2977295
1144466424	285.6445313	143.2021069	130.1380219	-417.366272
473563023.9	207.9799957	151.6158976	76.62192535	-417.509552
2678614223	445.4104309	95.43319227	111.440715	-332.9145813
558372693.4	399.1328125	124.7434391	140.8481079	-341.3734741
1500938407	498.3861389	150.1727354	218.8277679	-410.2076721
4341647630	537.0307617	139.746189	203.511824	-431.7410583
1115327726	463.5197144	140.3859768	179.1326447	-392.7488708
242845853.5	300.6739807	115.2627906	137.8556366	-295.9246826
1804309916	468.9857178	139.764588	165.095108	-443.7076111
364835783.1	142.4747314	182.4331743	44.00465393	-509.8379517
2380463394	458.9565735	131.67502	148.2902222	-398.5006104
2244430274	419.4129639	105.2226039	121.2527199	-319.174469
903921620.5	341.7781982	125.5469738	176.1982529	-347.7939453
273413217.4	243.6112366	143.4647875	110.469268	-313.6329041
2299877349	445.3247986	130.7712229	159.0355988	-348.3626404
910421893.4	365.125824	158.9274194	145.8855072	-355.6448669
437366443.9	325.8642883	130.5807158	100.5019028	-307.234375
1449901148	529.9769287	114.6685836	135.4204819	-346.795929
230790296.9	241.072464	96.49516438	124.1625282	-241.5798035
942212816.2	394.5853882	125.6882744	150.4221222	-370.3876038
301551244.5	212.4853058	185.9505295	98.75358276	-588.5628052
624497771.2	362.9626465	132.623562	205.0758087	-297.8050842
579182648.7	368.5841064	109.1665629	75.60513382	-320.7987976
1098759223	365.2684326	170.1283635	152.1841125	-446.6054993
378972568	258.8144531	130.3562869	128.8882141	-381.6014709
1052801999	353.8748779	130.7174744	126.5399521	-317.6835632
684083745	329.191803	115.690433	80.09520874	-320.7798157
1059654756	403.6074829	120.2307059	156.960144	-301.658905
298353499.9	287.0888367	144.2568437	87.77764893	-534.0634766
614071411.4	313.3414307	131.8632226	113.5973	-374.6351013
1339426291	431.711731	113.7203607	151.640213	-259.2255554
564106865.1	354.9901733	110.8272747	126.9618378	-326.9352112
725387367.1	390.4516907	140.1905871	179.5937012	-338.5934143
451615848.5	311.4137268	144.2363357	223.1367813	-460.9602966
812000481.5	324.5696716	108.9844183	107.699749	-353.3699036
1393854012	431.1026001	107.0957548	118.8392029	-334.9816895
1381239084	462.4424133	121.8414085	181.4253311	-298.5203857
811004693.2	278.3428955	95.30434572	122.0404892	-300.1594543
1810716319	479.2290955	167.7220648	191.1331055	-442.5575867
1178761502	389.5835876	133.435337	178.6788437	-304.612854
239709808	142.1698456	168.418134	28.9038681	-483.2492676
85293558.55	189.2019043	108.56975	85.55787354	-331.1756287
4089995313	426.0587463	131.022769	192.7611725	-376.0505981

log.sigma.4.5.mm.3D_firstorder_Entropy	log.sigma.4.5.mm.3D_firstorder_StandardDeviation	log.sigma.4.5.mm.3D_firstorder_Range	log.sigma.4.5.mm.3D_firstorder_Variance	log.sigma.4.5.mm.3D_firstorder_10Percentile
4.627928637	155.2804538	811.8739319	24112.01933	-261.5632477
4.574238296	157.9242965	748.2905273	24940.08343	-283.6489502
4.566714472	150.2009141	804.230896	22560.31461	-225.1717087
4.695807284	165.8024498	779.394989	27490.45236	-282.6743683
4.469993409	141.2596829	675.6210327	19954.29801	-211.6893311
4.527153793	154.1369466	668.7328491	23758.19829	-208.0798843
3.951988871	103.3856032	857.3124695	10688.58295	-134.1960175
4.39277538	131.6709221	819.4885864	17337.23173	-191.6187057
4.154389435	116.9409886	687.0136414	13675.19482	-159.3541412
4.241828783	120.7276118	570.7993469	14575.15624	-129.6558899
3.711724845	82.41517454	518.5348511	6792.260994	-82.55833817
4.037800639	104.9463411	668.6425171	11013.7345	-136.5623871
4.567731089	156.995305	730.3379211	24647.52581	-288.2608337
4.661253921	165.5088371	774.4042053	27393.17516	-347.6722107
4.333924234	128.8667468	748.29245	16606.63843	-224.8297485
4.254215409	124.3212832	711.9992371	15455.78146	-160.3010056
3.897311709	91.41010347	443.2772369	8355.807017	-124.6830643
4.653015476	167.2843003	792.59729	27984.03711	-356.3577332
3.745129065	83.73328743	463.244278	7011.263423	-131.8969055
4.309658607	122.8079588	1025.464508	15081.79474	-130.2471252
4.39135034	134.3297321	607.0286255	18044.47692	-196.9374573
4.32121012	130.6182006	613.0102081	17061.11432	-207.2071304
4.369296916	129.2756435	680.533905	16712.19201	-203.9678558
3.70402927	84.99317798	573.0379486	7223.840302	-120.8017029
4.536798294	146.3004572	809.4154053	21403.82378	-229.47229
4.489775229	142.3309918	882.9423523	20258.11123	-166.4746704
4.492993902	140.8605745	814.994812	19841.70145	-226.5312561
4.118212	106.702134	616.1053162	11385.3454	-176.8050232
3.637295167	81.18863007	440.6401825	6591.593653	-45.78141212
4.220551783	115.0642658	671.8453369	13239.78526	-173.4354889
4.34036702	127.6120102	725.6801147	16284.82515	-193.3121613
4.487287566	146.2913241	693.3190155	21401.15151	-309.1481781
4.389215386	131.2749986	849.4014893	17233.12526	-179.0937042
4.192455814	116.6888008	775.7824707	13616.27624	-165.5489655
4.258911869	120.9124263	729.598877	14619.81485	-155.4658783
4.486755483	140.6564075	707.4888916	19784.22496	-227.3592712
4.612020973	155.9566395	810.5137939	24322.47339	-315.6201569
4.222849752	127.565643	826.7184143	16272.99328	-178.2239075
4.034912008	106.2094618	506.8716736	11280.44977	-206.3466156
4.046443853	104.0246223	976.9974365	10821.12204	-139.8063141
4.478254492	140.568197	741.6357422	19759.41799	-262.1196564
3.546077049	75.88385984	696.0210571	5758.360185	-79.89699936
4.679442581	158.623522	823.0299988	25161.42174	-215.2481186
4.500767657	140.9262746	754.6036682	19860.21488	-229.4812698
3.951544626	101.9497862	675.9236145	10393.75892	-140.599144
4.466359784	137.2583894	717.227478	18839.86546	-181.8042755
4.11574756	106.8489444	756.2266235	11416.69691	-146.629425
3.97272122	99.92061944	500.4272461	9984.13019	-192.7015076
4.372570351	133.7889864	679.0181885	17899.49288	-167.8135834
4.398913632	136.3538227	648.9665527	18592.36495	-195.3432068
4.404006692	138.2151757	691.2254028	19103.43481	-225.7490051
4.478314599	137.4671789	804.5036926	18897.22528	-132.8981873
3.567130804	80.75082261	647.3825836	6520.695353	-110.3894348
4.075527881	103.7486738	721.4071655	10763.78732	-145.9070557
4.749302876	174.7030567	791.980072	30521.15801	-247.803479
4.493500595	143.6971841	680.7905579	20648.88071	-215.0059433
4.235537398	118.6395587	754.8825684	14075.34488	-164.2467224
4.368468137	133.7525892	703.0108032	17889.75513	-232.6683899
4.307428226	123.892763	625.4895477	15349.41672	-253.0135162
3.884914322	95.41369834	778.3250122	9103.773832	-122.0662277
4.322260181	123.1198454	740.5062866	15158.49633	-172.587915
4.604415786	149.1764198	908.593811	22253.60422	-179.5430054
4.495917578	139.2846169	968.7718201	19400.20451	-158.5747879
4.511413241	140.3346711	856.2685852	19693.8199	-184.0627441
4.191019648	113.3481736	596.5986633	12847.80847	-168.5279541
4.509358522	139.3560262	912.6933289	19420.10203	-190.6739807
4.380655913	140.2769267	652.3126831	19677.61615	-328.0942078
4.359350073	128.1019831	857.4571838	16410.11808	-177.2797638
4.075778027	104.6712231	738.5874329	10956.06494	-141.8417938
4.3244474	124.1435592	689.5721436	15411.62328	-150.1485916
4.326886672	133.3300884	557.2441406	17776.91248	-225.2884018
4.358466866	130.1255094	793.687439	16932.64819	-190.5637512
4.520337644	150.299994	720.7706909	22590.0882	-244.1907288
4.20945137	115.624491	633.0986633	13369.02293	-202.7893539
4.126478916	113.7457024	876.7728577	12938.08482	-147.172789
3.957982711	96.48737337	482.6522675	9309.813219	-128.2585205
4.356765044	125.5295658	764.9729919	15757.67189	-169.9942261
4.585357149	166.7634603	801.048111	27810.05169	-329.6659302
4.369405151	127.0127052	660.7677307	16132.22727	-133.6117889
3.990570788	97.86679186	689.3829041	9577.90895	-174.2483932
4.615895443	154.325245	811.8739319	23816.28126	-261.812088
4.335202855	127.575784	640.4159241	16275.58065	-207.1795959
4.302600946	123.6521849	671.5584412	15289.86284	-199.1520126
4.034273223	100.4219752	649.9716187	10084.57311	-182.2216827
4.230541255	119.9769951	705.2663879	14394.47935	-153.9075256
4.265804052	139.4114947	821.1523132	19435.56487	-262.0371399
4.330787821	123.7863424	687.976532	15323.05856	-203.8457703
4.091113248	113.6500489	690.9372864	12916.33363	-149.9910049
4.135410278	108.3845926	681.9253845	11747.21992	-156.8075409
4.486007721	140.1869152	729.045105	19652.3712	-197.1659851
4.457613832	143.3921372	772.3740234	20561.30502	-152.0482666
4.089816193	106.3087406	677.9395752	11301.54833	-159.8217804
4.108703418	105.9213314	766.0842896	11219.32844	-147.1070862
4.288449754	121.0069091	760.9627991	14642.67206	-133.6572495
3.951047112	94.95379436	578.5023499	9016.223064	-129.4995087
4.742365042	166.040122	921.7866821	27569.32211	-245.8263794
4.411359251	133.4146965	694.1964417	17799.48124	-173.4803818
4.238987241	125.2918961	625.4191132	15698.05923	-287.4943115
3.946492037	105.1050084	520.377533	11047.06279	-177.4812546
4.400062376	129.3227089	802.1093445	16724.36304	-150.7802383

log.sigma.4.5.mm.3D_firstorder_Kurtosis	log.sigma.4.5.mm.3D_firstorder_Mean	log.sigma.4.5.mm.3D_glrlm_ShortRunLowGrayLevelEmphasis	log.sigma.4.5.mm.3D_glrlm_GrayLevelVariance
2.548918425	-68.67989651	0.009265453	38.9647303
2.631735314	-50.86188673	0.013108313	39.55846092
2.290045427	-15.30071384	0.007963836	36.18905262
2.595405844	-51.05474627	0.014954363	44.23784931
2.336834738	-6.146517221	0.016983617	31.96147454
2.218097328	14.28563937	0.012625604	37.4421293
4.181061049	-9.347080057	0.014295289	19.36283809
3.294530352	-31.55079972	0.006489186	28.90488655
3.464621954	-20.69420985	0.012215737	23.20307648
2.21334901	28.5047451	0.019307316	23.5866239
3.105021625	17.70390698	0.020194823	11.50015537
3.620208981	-1.64127649	0.014173009	18.76920631
2.304714859	-48.42082341	0.018714142	39.51955216
2.286293703	-104.0821013	0.016449708	43.81837536
3.243792002	-67.32672828	0.014245449	27.26281986
2.740215703	1.532583693	0.012252699	26.36625883
2.527160257	-2.785983877	0.025314281	13.88388999
2.447332786	-105.994255	0.014619523	44.78525486
3.261410827	-28.18281328	0.021612403	11.97186644
3.587808184	20.54828401	0.002313632	25.18126651
2.208088695	-3.077668672	0.013587477	28.95939875
2.763657607	-13.3594009	0.017092809	27.2908785
2.744184039	-33.26995799	0.020124122	27.07260168
4.133499025	-16.30256297	0.012079174	12.67318923
2.596283993	-51.46842141	0.007172675	35.60213721
2.852818662	1.446026941	0.004900842	33.07348011
2.682727933	-60.11271033	0.009769911	32.2985436
2.742228526	-43.21610987	0.012857529	18.75870419
2.656195583	50.36796801	0.022565527	10.9278223
2.873813659	-22.29311173	0.011639535	21.31673378
2.698460125	-39.09551887	0.010263953	26.59370787
2.251310577	-105.1431237	0.008170516	34.50659616
3.190748033	-23.90440762	0.010336162	28.29095094
2.733101536	-21.32456945	0.008102868	22.30515814
2.924492536	-14.72900034	0.009914813	24.18602175
2.508085184	-31.6042841	0.010331683	32.25592091
2.715431369	-84.52420811	0.006312864	39.01339074
3.17222369	-11.68007423	0.008956944	28.7568665
2.565845777	-46.36849912	0.024494337	17.94557201
4.357966015	-16.62916926	0.002775159	18.67489507
2.340152554	-79.06789271	0.014006066	31.5968137
5.212620641	7.43761621	0.009224145	10.23422014
2.52914116	-10.64626623	0.007855132	40.20677106
2.546450513	-48.03867137	0.012316867	31.8276925
4.189353239	-8.245630019	0.011443095	18.03014971
2.427441501	-4.479360679	0.009408896	30.49141892
3.128497494	-9.920541799	0.005549274	18.96918211
2.772792816	-45.75711297	0.025427516	16.2115023
2.965963152	7.085445447	0.031487055	29.1224251
2.59168234	-10.19538112	0.010655146	30.02557296
2.52644424	-63.62867058	0.017218263	30.68003488
2.837967245	32.63180056	0.006892241	30.76213648
5.60007807	-8.457818842	0.015275066	12.31368662
3.310676213	-23.55530582	0.005732819	17.80690738
2.352836519	-2.269266709	0.011239741	49.09056242
2.271271364	-25.01824976	0.018084401	33.00969228
3.055773484	-18.48924631	0.009834221	23.27572585
2.002726546	-51.15748521	0.006900393	28.84845017
2.442600713	-87.39544438	0.012584315	25.00136244
4.231251145	-1.928822166	0.006092683	16.7174815
2.931733455	-20.06063984	0.010176194	24.82636179
2.671270962	17.26980723	0.005482372	36.40579757
2.786237415	11.34869281	0.003789378	32.02805426
2.963204703	-3.795072738	0.007948521	31.9716325
2.400904971	-20.92133887	0.018533393	20.9821641
2.881826823	-10.67885955	0.005244811	31.47671559
2.639612706	-116.637245	0.01010562	31.73487781
3.242251776	-30.46625702	0.007265563	26.82570913
3.220805593	-10.7578543	0.008241912	17.9820165
2.497883982	18.71949104	0.007225922	25.09518807
1.972217858	-52.96444808	0.038827762	28.37160942
3.139449672	-12.97938921	0.010306647	28.17314437
2.103040271	-51.65110296	0.014830767	36.2606414
2.549654055	-60.68196122	0.018749067	21.6421513
4.630988537	-14.5189272	0.008146375	22.36014608
2.27646214	-1.226185179	0.019648166	15.03757802
2.681031937	-6.314303417	0.00902362	25.54649168
3.13588495	-82.26510639	0.008400148	45.02837956
2.621700061	38.16781257	0.012727452	26.24828488
3.256339388	-48.36764932	0.012887606	15.81094531
2.579704682	-71.6057177	0.009480545	38.52049255
2.52418793	-26.78023332	0.00934884	26.07816949
2.490733592	-42.39334004	0.018388074	25.16292143
2.952710021	-57.44304284	0.015128318	16.61018136
3.37575201	-7.806618126	0.009800599	24.24166154
3.773903082	-37.07387341	0.00584615	31.97637434
2.503472735	-45.44063085	0.012153381	24.77631326
3.722279798	-3.998349876	0.017179977	22.80672898
2.955501216	-23.14011432	0.00892734	19.76190856
2.52879631	-1.014650631	0.010267049	31.77984093
2.97302414	15.58253863	0.005287711	33.42848551
3.51571062	-24.00114807	0.007233698	19.06542576
3.146309342	-15.81683467	0.007326022	18.31981169
2.854480225	14.23575628	0.010645882	23.9386026
2.460816109	-8.166715925	0.007509994	14.64829662
2.443308465	-23.69322479	0.007238728	44.38147478
2.255760341	2.346895034	0.01095557	28.56694652
2.759193933	-112.5460289	0.011634394	25.72815102
3.003060261	-27.20896602	0.014975952	17.72071946
2.716995026	21.03813077	0.004670364	27.54113417

log.sigma.4.5.mm.3D_glrlm_LowGrayLevelRunEmphasis	log.sigma.4.5.mm.3D_glrlm_GrayLevelNonUniformityNormalized	log.sigma.4.5.mm.3D_glrlm_RunVariance	log.sigma.4.5.mm.3D_glrlm_GrayLevelNonUniformity
0.00970335	0.045157567	0.089357346	60.86952993
0.013553639	0.048199995	0.064855382	15.84736563
0.008251997	0.046286412	0.102848383	38.68114821
0.015451894	0.044173796	0.052020058	13.56683871
0.017933142	0.050017292	0.086787694	65.73719519
0.012999143	0.047582797	0.061904468	10.93664708
0.015876576	0.074223944	0.501588494	635.926704
0.006973023	0.054723573	0.170237243	298.0185396
0.013499613	0.065155445	0.228000446	242.1177593
0.020452187	0.056979078	0.108053124	39.61598275
0.021933447	0.089336996	0.195748643	122.1570978
0.015565809	0.071996757	0.261136875	211.0704416
0.019397602	0.047159852	0.085096297	35.76156513
0.017479109	0.043711125	0.055344677	26.75133257
0.015365494	0.056041735	0.127381076	80.52053607
0.013394764	0.057991493	0.309536	286.7102093
0.027460578	0.073885917	0.145360366	58.3839986
0.015367313	0.045529863	0.067585477	17.57858131
0.023574522	0.0872969	0.210844287	83.24999624
0.002480226	0.059420481	0.165487311	488.9604472
0.014475838	0.052097464	0.111356738	99.76036297
0.018813562	0.058400866	0.082505777	26.99405632
0.021532983	0.055162022	0.096390833	28.54292561
0.013547629	0.093205863	0.344135672	280.7114156
0.007574413	0.047909989	0.165376166	52.82766253
0.00521281	0.050965885	0.141195867	118.499868
0.010287842	0.050401903	0.103535639	93.65388453
0.013898767	0.064483435	0.148586381	130.3532101
0.024049466	0.09012266	0.154424839	11.61785478
0.012407694	0.06143453	0.113173167	90.8969098
0.011103222	0.054974107	0.152161884	198.7036137
0.008435799	0.048938734	0.074273648	36.29190076
0.011121365	0.055065429	0.142826908	116.1440648
0.008732179	0.061393192	0.191057729	132.2371343
0.010773507	0.059413098	0.161504284	272.7675962
0.010844867	0.049230501	0.105642187	88.11785413
0.006618734	0.046780752	0.073652857	66.48961083
0.009822669	0.060473334	0.423951188	293.5606508
0.026954249	0.06963807	0.108018203	35.80527411
0.00308439	0.072275302	0.287818428	580.5330472
0.015036516	0.049217774	0.093028234	61.10795534
0.010484951	0.104325305	0.374976689	485.2944784
0.008249461	0.043661482	0.10085799	98.66149592
0.013119093	0.04923284	0.086095162	65.19114607
0.01259248	0.07821964	0.300334433	103.7904166
0.0100621	0.049831071	0.110786843	110.3801201
0.006049343	0.065891508	0.195495687	311.5405255
0.027432548	0.073719268	0.103434177	33.19011287
0.034496118	0.055637643	0.099579642	20.14517622
0.010987384	0.053261722	0.061684482	22.79662272
0.018798024	0.052403302	0.104943356	62.40151666
0.00724576	0.051228275	0.102730272	131.7390292
0.017535833	0.099398178	0.587455894	614.0466254
0.006163826	0.06907218	0.161978671	101.0895332
0.011637584	0.040912547	0.061984113	25.06063588
0.019229855	0.047850321	0.083724166	52.62829429
0.010744445	0.06011874	0.198508827	213.9289578
0.007297391	0.051462997	0.105955134	98.42867123
0.013362071	0.05620035	0.091094727	39.82687259
0.006891027	0.076422614	0.594791228	664.0518458
0.010759293	0.056631121	0.103798967	69.34238258
0.005715107	0.046191312	0.109430886	105.5176879
0.004067132	0.050240242	0.154243825	372.5076832
0.00840241	0.051141285	0.110779423	98.40323534
0.019530214	0.059967243	0.098611728	37.57463944
0.005540497	0.050129449	0.09466971	159.0683057
0.010424594	0.055091357	0.060849207	21.17706076
0.007831683	0.056875969	0.125206511	263.350053
0.009033834	0.067976026	0.225728073	446.6858528
0.007603923	0.055523356	0.114171576	108.3189708
0.040946541	0.053007816	0.080683988	24.4255239
0.011069763	0.057021475	0.177671615	254.3534319
0.015667217	0.047106891	0.084770744	58.8244321
0.019747508	0.059849278	0.085974739	52.99531621
0.009014964	0.070327316	0.251643839	247.0985508
0.021024173	0.070286742	0.118442745	59.18183866
0.009639923	0.055138031	0.115502899	111.5678453
0.008545891	0.050294301	0.053207327	15.45336141
0.01324669	0.054353152	0.092062412	66.23792228
0.013942497	0.072266829	0.172981649	115.3621279
0.009930058	0.045610139	0.090244312	59.83043134
0.009727014	0.055631706	0.086703218	42.83617825
0.019994256	0.055620122	0.175137309	115.0072635
0.016365402	0.069672715	0.1455399	118.4541424
0.010645348	0.062556277	0.218068788	149.7484769
0.00599718	0.068218723	0.084115203	33.78174485
0.012922632	0.055432682	0.10616707	66.6963765
0.019181766	0.068340358	0.364726272	219.2210062
0.009524345	0.065809359	0.148816134	100.8861656
0.010837537	0.050023171	0.099137684	63.35716004
0.005524491	0.051649287	0.109819864	38.13155217
0.007880285	0.069561864	0.211901686	154.3469913
0.007912823	0.066028128	0.146661287	267.0750255
0.011397091	0.058207472	0.133478258	182.5494355
0.008177332	0.071636772	0.156578941	211.4170827
0.007608974	0.041424141	0.081865483	92.5127363
0.011561178	0.051096602	0.101696999	115.821904
0.012072487	0.061417007	0.08448184	17.95878675
0.015547952	0.076785751	0.086032887	19.19941405
0.004977737	0.05345865	0.14949792	426.2549708

log.sigma.4.5.mm.3D_glrlm_LongRunEmphasis	log.sigma.4.5.mm.3D_glrlm_ShortRunHighGrayLevelEmphasis	log.sigma.4.5.mm.3D_glrlm_RunLengthNonUniformity	log.sigma.4.5.mm.3D_glrlm_ShortRunEmphasis
1.239228128	278.1148688	1190.001147	0.951810278
1.182274069	329.9014708	297.8885678	0.961221093
1.249955313	307.7453757	751.0809302	0.956934418
1.153201773	339.5592524	280.8228232	0.965117973
1.240924907	197.2014657	1151.751073	0.949283017
1.173190513	285.4354287	208.9923063	0.96298888
2.034220772	127.9139563	6254.639025	0.876908094
1.415731555	247.526391	4529.848153	0.928371775
1.553708655	151.6550263	2961.966374	0.910668142
1.291720574	153.5538706	599.6186658	0.942157114
1.506426197	90.22332751	1092.189649	0.910820259
1.624491842	139.4033214	2283.958498	0.902111019
1.232187307	265.5949129	670.7543451	0.952370254
1.163362042	301.6773583	554.5859791	0.962396169
1.333057777	163.300273	1221.407603	0.936830169
1.685184331	170.5765335	3908.09093	0.905968759
1.396082935	96.01283169	645.6250481	0.922223457
1.191371709	298.2788947	346.295634	0.958192383
1.540180548	93.34646741	753.7242711	0.907308151
1.414668085	487.4213062	6812.417081	0.92659331
1.290957669	197.3801116	1658.276863	0.944216
1.237260047	239.2251907	405.2284092	0.948701038
1.263168508	191.5019661	451.524035	0.94689975
1.787885248	118.6111574	2253.211034	0.886433457
1.417003462	317.4301414	919.742068	0.927891361
1.35470321	326.3169611	1973.073885	0.935990301
1.270447407	253.3411172	1625.590213	0.947815478
1.396186461	149.2614886	1663.775542	0.924387023
1.454948511	94.39293551	102.7185439	0.908542625
1.300435435	172.4207742	1272.111001	0.941387304
1.385803102	185.2784543	3026.898284	0.93076093
1.205491667	319.3057298	663.1464104	0.9567104
1.372732991	202.33287	1764.138117	0.930450244
1.455612702	195.2767898	1779.800882	0.925204362
1.415298077	182.5567239	3778.113942	0.924426281
1.281779456	280.1459018	1551.870587	0.944507588
1.208749805	388.5508512	1267.448786	0.955222455
1.874471586	204.3399482	3699.836461	0.893597063
1.299655985	145.5656768	439.2060819	0.938803262
1.682621057	373.7768614	6143.147995	0.894921846
1.25975202	203.019785	1078.029654	0.945561713
1.880390443	133.9620197	3347.293861	0.871325725
1.253591423	320.4972699	2015.527092	0.953892391
1.238089799	208.8812353	1164.95343	0.950398642
1.694521395	152.3065509	1021.351788	0.897201595
1.289354032	227.9478122	1921.898898	0.944599269
1.481331866	231.3704236	3830.92919	0.918029254
1.298901608	123.5344299	382.5262157	0.936513944
1.268360331	162.6288574	314.7324595	0.945911014
1.178127367	278.1449522	385.7365187	0.960173635
1.275960357	171.3239536	1036.741235	0.946280975
1.277257644	299.3056972	2225.171527	0.944214645
2.287292966	99.42585256	4156.872003	0.844662032
1.428130657	242.2302682	1193.6046	0.919959
1.165995456	375.0385104	561.5440154	0.966277279
1.228958259	182.412279	973.0658185	0.952677676
1.481620622	176.0712193	2908.495793	0.920370967
1.277003688	257.3299013	1667.301986	0.94671277
1.252619523	213.1338645	618.3281634	0.947356441
2.190235179	198.2295431	6223.265574	0.868960107
1.284178168	204.1734744	1054.490975	0.942265057
1.27671263	349.6566159	1994.142749	0.947549222
1.391648386	370.2139343	6175.894197	0.928871104
1.299816633	282.5128498	1647.755467	0.940082882
1.268687039	151.6786862	544.5733419	0.945521751
1.258809413	341.0410847	2764.253687	0.94687407
1.178683991	301.8735233	346.1091089	0.959507392
1.334965419	249.5065607	3912.766903	0.934686848
1.539993039	174.8901333	5266.715467	0.912999506
1.303118901	241.2953736	1676.184558	0.941092087
1.225132371	154.0651901	408.8187908	0.953073853
1.429718246	211.1722388	3703.538099	0.927522544
1.229008879	211.018231	1106.912573	0.953459684
1.240277676	139.7455193	778.0465449	0.949674002
1.614279818	200.6213738	2731.590671	0.901295335
1.31593343	117.6197617	722.6894532	0.939205652
1.309929302	242.1230887	1727.739041	0.938830392
1.160113848	474.1536415	278.6372105	0.962392505
1.259438384	211.7802523	1057.513894	0.945067373
1.450145413	140.312326	1293.605356	0.918051977
1.241447628	273.9670567	1157.1346	0.951451476
1.241461701	248.5724033	676.9975557	0.949743161
1.407529501	157.4818174	1767.722095	0.936712659
1.389657148	135.7740764	1402.614654	0.925285085
1.513807788	186.771957	1938.990724	0.917577464
1.239435025	445.1666509	433.338213	0.948580421
1.291779231	202.1257694	1032.734543	0.940705499
1.810182126	140.9828013	2407.252772	0.88825368
1.385318008	193.3749439	1276.435225	0.928842883
1.266293539	230.4808307	1103.300329	0.946705112
1.299741793	413.7533425	632.827309	0.940026679
1.524511438	214.6912822	1775.737371	0.912205087
1.38920868	198.3888357	3344.083521	0.926076077
1.345029306	185.7991303	2659.9465	0.935885334
1.416585593	176.5382566	2407.347302	0.920753242
1.223442633	338.7233669	1983.300588	0.954070781
1.275908641	203.2763075	1966.665844	0.944617272
1.234758976	268.4477274	258.1109132	0.951155649
1.239957603	186.1469845	219.7503575	0.949685373
1.378323705	306.2672919	6682.084539	0.931361739

log.sigma.4.5.mm.3D_glrlm_LongRunHighGrayLevelEmphasis	log.sigma.4.5.mm.3D_glrlm_RunPercentage	log.sigma.4.5.mm.3D_glrlm_LongRunLowGrayLevelEmphasis	log.sigma.4.5.mm.3D_glrlm_RunEntropy
348.5327911	0.933411464	0.011739293	4.974534728
413.0012253	0.94746176	0.01539933	4.807311518
400.2160297	0.936961021	0.009867952	4.860357127
397.0501317	0.953893932	0.017499998	4.892814846
257.1960946	0.93141798	0.022109761	4.823497362
347.5154914	0.949777495	0.014603687	4.735263275
276.7927927	0.811535177	0.027302939	4.838098284
358.2011469	0.897355611	0.009630378	4.92004813
232.7009774	0.871439916	0.020823335	4.786005117
199.1586666	0.920835456	0.0262398	4.625581521
140.1791138	0.878089777	0.031558064	4.310972739
231.0007418	0.859723194	0.023804729	4.71870646
351.7440375	0.934838281	0.022440115	4.894161371
358.3101354	0.950310559	0.021833837	4.906262537
210.8591172	0.912234432	0.021185873	4.774698602
296.6892749	0.857394601	0.020975135	4.929126354
137.6053112	0.894851468	0.037771716	4.408703975
367.0065923	0.943953357	0.018422307	4.9183369
147.3194951	0.871185487	0.034278104	4.367897147
733.7834369	0.895953225	0.003410116	4.853712424
267.8432214	0.921596209	0.019007137	4.786577572
309.5758666	0.93207196	0.025816182	4.643195608
250.6273667	0.927212572	0.027663498	4.710160908
223.925068	0.835824726	0.023053309	4.497033944
476.083575	0.897456778	0.009645901	5.018476127
447.5919896	0.909473938	0.006929203	4.952178617
320.9749403	0.927107324	0.012970687	4.870880794
213.2215801	0.89706033	0.019085326	4.635049877
141.2722502	0.882507903	0.031467989	4.130991292
230.7687734	0.91892021	0.016237793	4.633642255
259.6878768	0.901644377	0.015589848	4.834949446
402.1430379	0.941136275	0.009644067	4.786893219
280.4692567	0.903211991	0.01518495	4.876963554
299.7004473	0.891575092	0.012266713	4.714677562
257.9290616	0.894303826	0.015294854	4.800319019
377.6013144	0.923156266	0.013260394	4.880653363
486.7187138	0.940018315	0.007946293	4.925200741
409.4089623	0.832754087	0.015900446	5.021786086
199.7124693	0.917857143	0.03805297	4.424213855
681.6915543	0.849785443	0.005027384	4.783429857
257.042625	0.926578645	0.019824925	4.850455395
270.4150181	0.818921558	0.01845313	4.419817326
409.0202694	0.934720366	0.010383345	5.009203094
262.2405237	0.93255688	0.016819086	4.842528823
272.2310326	0.851344671	0.019371329	4.662733278
294.1555304	0.922532278	0.013282934	4.86445207
356.9656711	0.883486911	0.008884185	4.69540148
168.4546773	0.916653611	0.036202446	4.379560741
207.6511274	0.926027936	0.047185034	4.709844091
341.0274419	0.946902655	0.012369506	4.662237285
210.5331137	0.925109079	0.026087277	4.779135166
393.0765896	0.923353128	0.008936229	4.884601225
253.7150122	0.772577347	0.033497804	4.646292397
364.4951873	0.891220838	0.008437501	4.611516599
454.2241174	0.952506281	0.013431063	4.979156303
229.6697611	0.935243328	0.024726382	4.823601332
266.0533	0.886081042	0.015820799	4.795845866
336.2938346	0.925347028	0.009344285	4.754113296
272.5946843	0.928722653	0.016808836	4.660619128
481.9738583	0.796213518	0.013278021	4.838106224
266.7891781	0.921340511	0.013493398	4.729609085
463.5928299	0.926540802	0.006895295	4.992097691
530.191129	0.900394238	0.005564727	5.012911771
385.9904527	0.917975631	0.010561448	4.934976724
194.3952236	0.925576639	0.024660067	4.554609491
446.8144878	0.927506577	0.006906795	4.899827767
369.9857096	0.946760136	0.011758374	4.634781997
336.1006084	0.910475758	0.010575918	4.829369172
284.9299506	0.875199787	0.013735278	4.682655322
336.3864963	0.918368825	0.009465206	4.745180756
190.2909995	0.936522827	0.05021092	4.632833993
310.8284311	0.89512455	0.015153118	4.893945806
260.8985614	0.935407663	0.019576476	4.852808655
175.2484105	0.932064777	0.024431495	4.561729601
334.4182657	0.85963987	0.01383857	4.819445949
160.3872667	0.917295123	0.027893023	4.359028642
339.0590427	0.916042762	0.012433593	4.788747404
577.6062709	0.951178852	0.009145296	4.81263947
279.1348312	0.926703715	0.015599614	4.754074291
211.4266999	0.886709402	0.019376019	4.549442743
344.3847252	0.932924828	0.012015626	4.963842289
331.536043	0.932110263	0.011406366	4.675324348
223.3914723	0.905649565	0.029246023	4.750468514
190.3475791	0.898004795	0.022712679	4.544708435
287.5529246	0.881201303	0.015480704	4.81844372
593.9683794	0.931768796	0.006662362	4.619455837
269.7454737	0.91984239	0.016603291	4.73603451
253.4218285	0.834663512	0.031315985	4.884920124
274.4238257	0.900963234	0.012621418	4.63821065
301.3971977	0.92645321	0.013606426	4.861319958
567.355341	0.918101799	0.006628447	4.854158846
342.2797268	0.875926601	0.011491094	4.697150848
286.7059216	0.89863791	0.01093825	4.626194706
254.8943016	0.909951337	0.015287347	4.751597419
260.8598366	0.892465865	0.011621291	4.485310172
427.6381717	0.936725607	0.009268946	5.081522403
269.4229914	0.9244259	0.014538135	4.808107516
348.7472562	0.934136151	0.013972557	4.540531297
247.0459581	0.932548794	0.017983895	4.258390035
449.5673443	0.90344515	0.006625291	4.901656528

log.sigma.4.5.mm.3D_glrlm_HighGrayLevelRunEmphasis	log.sigma.4.5.mm.3D_glrlm_RunLengthNonUniformityNormalized	log.sigma.4.5.mm.3D_glszm_GrayLevelVariance
290.0078909	0.882079999	43.27312251
343.9480479	0.904544932	38.58107326
321.4795469	0.896075678	39.10844082
350.0974217	0.913204025	46.59161192
207.6018525	0.875770784	32.10201628
296.0407259	0.908177351	35.76694411
143.8247523	0.729024459	30.40843881
263.8127654	0.830811817	36.67956302
162.9438521	0.794985083	28.44447471
161.3757538	0.860842318	27.04816867
97.87802332	0.795532595	16.93530333
151.9122416	0.777315118	26.25967794
280.0061197	0.883548879	42.82357939
312.1167025	0.905818427	45.00513849
170.9679826	0.848835202	29.43794929
186.0596705	0.787290363	31.11233116
102.9000006	0.816487847	17.22839506
309.8450668	0.896294152	46.24277457
101.2751018	0.787813096	18.14739499
524.6625774	0.826890234	38.18591324
208.6461185	0.865127977	27.61833056
251.2788967	0.87505955	30.57483982
201.2327868	0.871341034	31.86299979
131.9029308	0.746753315	18.91620239
340.490214	0.8314977	43.1222613
345.5276168	0.847384298	36.6528838
264.142793	0.873601242	36.55401662
159.5306399	0.821727916	23.27998686
102.354571	0.792118905	18.95836735
181.9574888	0.85877776	24.7195004
196.6472591	0.836150928	28.90759637
333.4876753	0.893265219	36.39840977
214.8412984	0.835135505	32.8368288
209.7404013	0.824749773	24.5991602
194.3409016	0.82201078	30.13771726
296.2135678	0.865873679	34.12649796
405.5419895	0.890235677	41.63045196
226.2963007	0.761593516	33.68342866
154.5175808	0.852915426	18.18228459
414.521185	0.762898834	30.24686433
212.4740493	0.867487289	30.67105881
151.4205051	0.71784009	20.69690397
334.1753169	0.888869056	38.29541197
218.0530202	0.878810294	29.96606388
167.6524492	0.767986825	26.22604835
238.7806484	0.866360863	31.79674557
249.5179551	0.809005641	24.98403433
131.2580183	0.847984405	20.7061971
170.1314408	0.868373782	35.051136
289.2147295	0.900751733	33.84567081
177.6855366	0.869769944	28.13555455
315.0923533	0.864665538	35.76080257
115.8382775	0.670995248	22.4346575
261.6569849	0.81322803	25.31520326
387.844897	0.916170026	52.72477585
190.4927466	0.883924215	32.10495351
188.8375836	0.814962426	26.922784
270.0291395	0.870722742	29.36886602
223.3310531	0.871739287	26.78433854
225.7522548	0.714725468	27.54040886
214.7218211	0.860323445	30.31948573
368.0137804	0.872321184	43.55863656
395.2602525	0.831705164	39.40653649
299.7276162	0.855540434	37.92144368
158.9280663	0.867952911	25.50827689
359.0269475	0.870511473	37.99933076
314.3079517	0.899435313	34.14005112
263.9163492	0.843951827	32.73724652
190.2662806	0.799269297	23.56996683
256.8279621	0.858179976	30.6500806
160.2189046	0.885550239	28.48277045
225.4602802	0.829377556	33.73232649
219.3596021	0.885832434	34.80785124
145.9497298	0.877398978	25.06190794
219.0023061	0.775674717	33.53861965
124.8558293	0.855242398	15.7645519
257.7857321	0.852855617	32.25199871
493.6619254	0.906085787	49.10734651
223.4892248	0.866617961	33.59503589
151.1769284	0.808885154	22.84902007
285.8218324	0.881303681	42.67726665
262.6180279	0.87777005	27.10276171
166.2956016	0.851621935	25.23835281
144.5799339	0.823748934	20.64914115
200.8280717	0.808469003	31.31398748
470.9080183	0.87452323	45.09276844
213.4339849	0.857046365	29.69140406
154.6790241	0.749979214	29.60267342
206.1567258	0.831556434	27.5210999
242.2676285	0.87040174	35.32381151
439.2005671	0.855931448	41.23426989
232.8976044	0.797926034	24.82931448
212.599827	0.825431133	24.07155547
196.8957877	0.847015351	28.95540455
190.0315754	0.814138573	18.06354426
353.7811357	0.887312577	49.18275181
214.4487865	0.866141468	31.31611266
281.9800224	0.881091563	30.26298066
196.2225868	0.877589524	20.10054348
328.4605561	0.836997403	33.4901346

log.sigma.4.5.mm.3D_glszm_SmallAreaHighGrayLevelEmphasis	log.sigma.4.5.mm.3D_glszm_GrayLevelNonUniformityNormalized	log.sigma.4.5.mm.3D_glszm_SizeZoneNonUniformityNormalized
215.9577218	0.042647896	0.345284923
199.65513	0.047413294	0.34524131
206.2665176	0.045208163	0.348391837
245.5835774	0.045265182	0.343053998
112.8577944	0.049162156	0.252050472
196.2339324	0.050832342	0.36519025
107.7587817	0.050659949	0.271275017
200.8619208	0.046110678	0.317531687
144.4829562	0.052292806	0.282444012
107.5387492	0.053631887	0.275969529
61.12370955	0.067877478	0.215700081
112.8859224	0.054857438	0.268414187
149.1969022	0.042882506	0.30821597
213.9434065	0.042296916	0.370209158
148.9021609	0.054031467	0.341877941
128.2754523	0.049320563	0.299714463
72.84697515	0.069259259	0.234938272
212.2408987	0.044137793	0.33074944
83.76101134	0.068601519	0.272877284
299.5903661	0.045244817	0.285221311
120.9616213	0.051972222	0.281655556
120.2922403	0.052064003	0.227057876
113.9802938	0.050673355	0.243512795
100.5758846	0.065135073	0.250497144
234.1294111	0.042557102	0.322921772
237.1510591	0.046649703	0.300759474
214.5634949	0.046811335	0.349314385
118.1692771	0.05863107	0.261970716
61.26993578	0.071020408	0.239183673
117.6065726	0.057097931	0.271759565
136.1402545	0.05145034	0.278536054
207.3243739	0.048155187	0.335687906
149.536593	0.049441612	0.291574247
139.9464524	0.059443602	0.289026939
145.9477408	0.05011482	0.295966977
171.7663569	0.04797551	0.308297959
258.7762986	0.044902602	0.334379939
168.272928	0.047919848	0.337573233
65.62198675	0.068457321	0.183642674
273.5745236	0.052432809	0.260295285
146.2702208	0.0496515	0.294531678
108.3570487	0.064706158	0.249976592
221.9357178	0.044765286	0.356640105
152.910197	0.052673097	0.301389237
118.1119537	0.056435328	0.241482741
174.2807653	0.048299049	0.316283061
178.3305759	0.056117862	0.281715416
79.55694	0.064251568	0.267049691
120.0175412	0.050368	0.268224
148.9340626	0.049993962	0.288491728
147.2207909	0.053463105	0.337129923
207.7171123	0.046844809	0.314831674
92.7826082	0.060120369	0.246559091
172.2623555	0.057555612	0.297044173
263.1942265	0.039514788	0.391963163
116.5098743	0.049607438	0.272654959
141.2316526	0.054054	0.287
166.6192612	0.050717974	0.284037077
152.3665543	0.05439821	0.303026267
155.2549573	0.053827617	0.263544307
152.0673342	0.050442182	0.324037604
214.2873382	0.041976716	0.308731586
250.9230186	0.043969955	0.303359835
187.4030163	0.045994668	0.290125142
103.1355061	0.05598789	0.288400706
220.3284544	0.045046854	0.297927613
186.8346053	0.050873569	0.312559084
191.9013153	0.049450025	0.309410387
121.5391928	0.058116727	0.243947411
126.7752602	0.050060452	0.255579518
119.4845397	0.054190855	0.330492722
157.7038753	0.048325449	0.287052655
151.2644783	0.046957175	0.29478007
107.3612319	0.055794945	0.324219418
188.5234011	0.050073685	0.309156283
68.91007888	0.071702653	0.239268991
142.3762458	0.048836387	0.274553645
281.6063663	0.04491155	0.350426639
129.8550924	0.047451383	0.267348037
111.6069327	0.06162928	0.276532299
210.6982082	0.04316106	0.346534585
131.8133731	0.053679489	0.291283095
125.1980663	0.055042881	0.31453465
100.6164092	0.065147739	0.23257832
136.6789293	0.050417429	0.277789493
217.3166484	0.043623083	0.275500365
148.5186881	0.051043328	0.31864738
131.150146	0.050727744	0.299057042
146.2478648	0.05323125	0.310234359
149.9200863	0.047121485	0.259720079
275.262764	0.045395443	0.324975974
185.3743318	0.057379119	0.289743158
145.9144942	0.057874483	0.282281963
128.0305009	0.051455736	0.263905721
113.1361132	0.066748393	0.266154678
244.1160144	0.039067714	0.337793902
127.8490196	0.049598129	0.277795199
148.1569185	0.054585324	0.290625734
87.55533225	0.068289225	0.258034026
182.0162065	0.047786359	0.294649267

log.sigma.4.5.mm.3D_glszm_SizeZoneNonUniformity	log.sigma.4.5.mm.3D_glszm_GrayLevelNonUniformity	log.sigma.4.5.mm.3D_glszm_LargeAreaEmphasis	log.sigma.4.5.mm.3D_glszm_ZoneVariance
191.9784173	23.71223022	20.92086331	14.17581906
55.58385093	7.633540373	8.850931677	4.205701941
121.9371429	15.82285714	15.10285714	8.607640816
54.20253165	7.151898734	6.632911392	2.479570582
119.2198732	23.25369979	22.48837209	13.58956595
42.36206897	5.896551724	7.74137931	3.389120095
479.3429542	89.51612903	2111.022637	2075.44921
558.2207053	81.0625711	125.519909	113.606024
275.9477994	51.09007165	222.8577277	203.8277727
62.92105263	12.22807018	29.58333333	18.61794014
61.90592334	19.48083624	139.6655052	110.2717284
188.1583452	38.45506419	380.4693295	356.8477883
93.38943894	12.99339934	19.37623762	12.2122232
115.1350482	13.15434084	7.344051447	3.056089164
183.5884544	29.01489758	42.15456238	33.55230278
409.4099561	67.37188873	500.2489019	482.4560686
42.28888889	12.46666667	111.9388889	87.87441358
57.21965318	7.63583815	11.34682081	5.757559558
55.39408867	13.92610837	213.6650246	184.6219515
655.723793	104.0178338	305.7751196	289.8203247
168.9933333	31.18333333	41.25333333	29.25865556
38.37278107	8.798816568	16.47337278	7.859668779
44.56284153	9.273224044	22.21857923	12.92107856
137.0219378	35.62888483	930.8720293	887.6299844
130.460396	17.19306931	41.60891089	32.36971866
236.3969466	36.66666667	63.24681934	52.67191112
252.2049861	33.79778393	44.13573407	36.43164954
139.3684211	31.19172932	89.94172932	72.00682274
8.371428571	2.485714286	40.8	23.39918367
122.0200445	25.63697105	47.13585746	34.27828235
292.4628571	54.02285714	103.7857143	89.20786304
112.1197605	16.08383234	10.80838323	5.242174334
193.3137255	32.77978884	77.83559578	65.43202546
177.4625407	36.49837134	109.8583062	94.38804921
399.2594514	67.60489251	141.2031134	126.7247781
215.8085714	33.58285714	26.71285714	19.03995714
204.6405229	27.48039216	13.02287582	6.919069589
496.2326531	70.44217687	628.2578231	612.5773007
25.89361702	9.65248227	43.81560284	28.0417484
440.4196217	88.71631206	677.2913712	646.11776
133.1283186	22.44247788	27.89823009	19.10936643
178.9832402	46.32960894	1916.472067	1853.717347
347.7241026	43.64615385	18.69948718	12.54908718
150.996008	26.38922156	23.88822355	15.85478942
70.27147766	16.42268041	351.9243986	323.3331444
265.9940547	40.61950059	28.3293698	20.17871822
334.9596299	66.72413793	222.0058873	201.7521024
40.32450331	9.701986755	30.69536424	20.12209991
33.528	6.296	27.8	18.015616
52.50549451	9.098901099	11.74725275	5.579398623
158.4510638	25.12765957	36.58085106	29.08257583
292.1637931	43.47198276	34.08728448	25.0808178
248.285005	60.54121152	3991.831182	3929.095973
119.1147132	23.0798005	108.2693267	91.5022419
123.0764331	12.40764331	7.671974522	3.47860968
119.9681818	21.82727273	14.56818182	7.424710744
287	54.054	118.531	102.410775
198.2578797	35.40114613	24.95558739	16.18618279
83.02919708	14.90510949	19.15693431	11.40253876
447.4982332	91.39929329	2466.12662	2424.971901
150.3534483	23.40517241	27.36422414	19.16044423
279.7108168	38.03090508	25.79359823	18.39111467
723.5132075	104.8683438	170.8377358	158.9185948
188.2912173	29.85053929	32.44684129	22.01662864
65.46696035	12.7092511	21.63436123	12.7397776
347.9794521	52.61472603	23.80565068	15.22697079
52.82248521	8.597633136	10.68639053	4.915023984
461.0214765	73.68053691	74.37114094	62.72426692
338.1111111	80.54978355	379.6017316	350.2575456
154.8811881	30.33663366	34.88778878	22.60311081
61.80213904	10.13368984	17.92513369	11.00288827
398.1420332	67.02739726	178.3684211	165.4716746
142.6735537	22.72727273	29.94008264	22.3320598
110.8830409	19.08187135	21.18128655	13.46523717
248.8708075	40.30931677	454.757764	429.019473
59.33870968	17.78225806	37.5483871	23.84644901
172.1451356	30.62041467	44.15789474	31.74547795
54.31612903	6.961290323	7.141935484	2.799417274
114.6923077	20.35664336	21.78321678	12.38734847
106.4649351	23.72727273	155.412987	133.5543262
185.7425373	23.13432836	21.57835821	14.6975245
87.09364548	16.05016722	16.34782609	8.716188857
233.0701754	40.78677463	44.60728745	35.12321497
98.61320755	27.62264151	165.2287736	145.2959183
179.4520124	32.56965944	204.9241486	187.2607353
50.96756757	8.07027027	22.49189189	14.25344047
134.4691943	21.54028436	38.34597156	28.73890973
243.4324324	41.29238329	523.3808354	501.1728897
142.0873362	24.37991266	121.2991266	107.5055253
112.1990741	20.35648148	26.22916667	16.21604402
86.44360902	12.07518797	34.38345865	25.24761151
154.7228464	30.64044944	277.588015	255.1055001
285.9516288	58.62685094	143.7917078	124.0493447
240.6820175	46.92763158	73.05263158	58.77550304
171.6697674	43.05271318	216.296124	190.0086437
309.419214	35.7860262	17.19868996	10.42505864
212.7911227	37.9921671	32.25848564	22.01180048
32.84070796	6.168141593	18.7699115	11.09750176
23.73913043	6.282608696	18.06521739	9.579395085
739.27501	119.8959745	141.5464328	129.1775728

log.sigma.4.5.mm.3D_glszm_ZonePercentage	log.sigma.4.5.mm.3D_glszm_LargeAreaLowGrayLevelEmphasis	log.sigma.4.5.mm.3D_glszm_LargeAreaHighGrayLevelEmphasis	log.sigma.4.5.mm.3D_glszm_HighGrayLevelZoneEmphasis
0.385041551	0.171995772	4440.530576	335.2158273
0.463976945	0.091725678	3021.080745	338.8819876
0.392376682	0.101725269	4579.308571	332.5942857
0.49068323	0.078312469	1981.183544	388.3734177
0.335223246	0.325143459	4519.124736	217.8794926
0.479338843	0.075034667	2246.224138	307.4482759
0.167662966	17.83870086	265005.7765	188.8947368
0.289716546	0.712449782	27243.66325	323.3441411
0.229235101	2.485928188	27030.28454	236.8433982
0.301986755	0.597754172	3566.592105	191.5175439
0.184447301	2.587176489	10004.14983	122.087108
0.205752862	3.833103358	46680.43081	197.4550642
0.373612824	0.203992565	6324.214521	265.7491749
0.482919255	0.153269606	2039.430868	329.9742765
0.340952381	0.688596894	4553.364991	226.5344507
0.237070462	3.68770165	77874.89751	217.8967789
0.20385051	2.295181664	8880.366667	133.3888889
0.422982885	0.173334945	3248.289017	335.2427746
0.185557587	3.559627531	17342.64039	141.8719212
0.250353915	0.638229269	150565.06	547.307525
0.288739172	0.846626022	6966.851667	221.3383333
0.340725806	0.27729855	4104.846154	257.7100592
0.327956989	0.362533708	3947.661202	220.7377049
0.15207117	8.993903279	108692.2852	183.8062157
0.328990228	0.173971905	13760.39851	375.1014851
0.307511737	0.313476217	15553.88295	404.0139949
0.360279441	0.374333909	7116.567867	321.9861496
0.236129605	1.117487928	10602.97744	205.7838346
0.239726027	0.666684967	3535.085714	116.6857143
0.278881988	0.605055993	6667.64588	213.674833
0.261910701	1.216751147	13229.13048	240.2638095
0.423857868	0.067018687	3520.158683	341.3922156
0.283940043	0.722736282	11264.68175	257.3393665
0.254244306	0.828208014	20303.41694	239.7899023
0.262809273	1.468565934	16769.8169	246.0904374
0.36101083	0.17453162	7571.507143	299.5371429
0.404761905	0.070294564	5163.303922	419.0816993
0.252533929	3.129148509	137005.5367	268.314966
0.251785714	0.903971349	6905.723404	165.2624113
0.179104478	1.761321611	270898.8552	491.3031915
0.337313433	0.482615258	4012.904867	252.6283186
0.126234133	15.40308018	250211.8268	201.5167598
0.403225806	0.150408987	4699.277949	357.0369231
0.352816901	0.362978457	3581.477046	254.9061876
0.187017995	2.892083749	50863.42268	221.4570447
0.350270721	0.300159865	4734.667063	281.8953627
0.222201458	1.149706595	50500.20101	306.6534903
0.307535642	0.743135918	3851.152318	146.2649007
0.319693095	0.789947336	3856.944	209.08
0.402654867	0.083651485	3459.730769	290.5494505
0.365190365	0.876060585	2781.308511	228.6893617
0.333213645	0.206144334	7929.196121	350.0204741
0.126253761	38.30210958	433394.7974	163.5888779
0.244214373	0.570815524	23665.29676	299.0897756
0.488335925	0.076852248	2904.242038	396.9808917
0.37414966	0.410124858	2120.888636	211.8477273
0.249066002	1.0664864	18357.271	239.966
0.33768747	0.195826424	5382.095989	299.4484241
0.359108781	0.228064945	3660.875912	251.2372263
0.155879923	11.54422237	542096.1549	276.9310954
0.349134688	0.232323032	4852.769397	244.3362069
0.367545639	0.103477018	8654.309051	376.6412804
0.28965266	0.626375462	50201.81174	438.4343816
0.309637405	0.192548267	8995.48074	324.6209553
0.335302806	0.350919948	2847.440529	178.30837
0.341420637	0.112038939	7732.018836	383.0744863
0.416256158	0.08567783	3392.147929	320.1301775
0.293018682	0.495464722	14029.78322	318.3926174
0.18460309	2.7428627	66878.62482	229.1385281
0.285310734	0.199822317	8640.341584	253.5264026
0.380081301	0.604371627	2425.245989	187.6203209
0.278458141	1.145137125	36044.51766	269.9206921
0.362546816	0.567053639	3333.795455	258.446281
0.36	0.388408363	2320.649123	170.8216374
0.197110676	2.747067737	83745.7764	302.3763975
0.270152505	0.732743756	3787.112903	139.3508065
0.283838841	0.329403874	9983.570973	266.1419458
0.479876161	0.037331432	3627.070968	480.4064516
0.326235741	0.182881511	4657.407925	236.3752914
0.213888889	1.809965669	17975.64675	191.3766234
0.381223329	0.180990451	4498.878731	329.1455224
0.361985472	0.120757305	4546.277592	245.826087
0.324715162	0.955943266	5584.909582	203.8448043
0.223983096	2.089329183	16165.49528	190.754717
0.237937385	1.569864409	32136.13932	247.0309598
0.348399247	0.060328928	11840.93514	431.1621622
0.322629969	0.387537625	6376.92891	241.8009479
0.212200209	5.678530568	62009.01229	217.8046683
0.26925338	0.850149218	19731.05677	239.6113537
0.316020483	0.277124075	5361.898148	276.0763889
0.330845771	0.116017623	13609.15038	462.5639098
0.210900474	1.557853567	57222.88951	303.6067416
0.225061098	1.122789584	23035.74827	252.2813425
0.264654672	0.747741492	10654.50877	234.4627193
0.195040822	1.971885574	29281.00465	219.8356589
0.384228188	0.103263842	5794.658297	385.040393
0.312398042	0.333029079	5797.928198	229.5522193
0.361022364	0.123686191	5488.300885	280.0707965
0.343283582	0.156165708	3938.445652	182.6630435
0.284338169	0.52856152	43618.4719	331.2279793

log.sigma.4.5.mm.3D_glszm_SmallAreaEmphasis	log.sigma.4.5.mm.3D_glszm_LowGrayLevelZoneEmphasis	log.sigma.4.5.mm.3D_glszm_ZoneEntropy	log.sigma.4.5.mm.3D_glszm_SmallAreaLowGrayLevelEmphasis
0.606701502	0.010703812	6.550737312	0.006091213
0.59190628	0.014182547	5.93152365	0.005790491
0.61162613	0.010040254	6.395011235	0.007052438
0.582285406	0.015819044	5.911721078	0.0063675
0.499578073	0.016958493	6.622765106	0.005904792
0.620530855	0.016606983	5.745467419	0.013075017
0.533974708	0.01741479	6.980615092	0.007915656
0.579621911	0.006979803	6.818469084	0.004045079
0.545495124	0.011206492	6.707847638	0.006232948
0.536298861	0.021961373	6.231036406	0.008733326
0.471530305	0.028740368	6.417299448	0.01817379
0.530669561	0.018199265	6.697099747	0.011671724
0.570175129	0.026944635	6.438564701	0.01909264
0.629433041	0.015419051	6.254562704	0.008661414
0.600785881	0.012804032	6.262678706	0.006891067
0.561477801	0.013085415	6.747315494	0.007617507
0.493872949	0.024073078	5.963327521	0.006532769
0.591030195	0.014862284	6.148981427	0.009622653
0.530968421	0.027691514	5.933551881	0.014603518
0.547581351	0.002959641	7.06717555	0.00186407
0.546437134	0.013503233	6.588158037	0.007328425
0.455522567	0.017371829	6.32147328	0.00617972
0.489904393	0.018972106	6.246563585	0.005693442
0.511324926	0.012530833	6.535073628	0.006504376
0.587550954	0.009170325	6.56957379	0.004793806
0.560338922	0.005785585	6.698788033	0.003634793
0.611486067	0.010293572	6.508555722	0.005188234
0.524908064	0.014851923	6.558936321	0.007818132
0.488490548	0.048648116	4.843568731	0.040401123
0.531568849	0.012757787	6.518737023	0.007368473
0.538876231	0.009914966	6.742627091	0.005197161
0.594920713	0.011253714	6.320476531	0.008431809
0.552250395	0.010937219	6.621263333	0.005768559
0.554736476	0.009586356	6.501596811	0.005986183
0.558872555	0.010791955	6.79575249	0.005975175
0.57047773	0.012844857	6.570978195	0.007396507
0.597514844	0.00732284	6.591240517	0.004543108
0.601804593	0.009785748	6.692494959	0.006093809
0.394992	0.020253157	6.075350157	0.004583198
0.522391717	0.003414956	7.029642714	0.002009046
0.548972962	0.011184102	6.416628059	0.004240663
0.512862778	0.012739029	6.707784415	0.006305306
0.617430178	0.007436851	6.56516349	0.003926285
0.563253709	0.011516429	6.448915601	0.005072484
0.495855952	0.013941151	6.371929939	0.00816954
0.579192259	0.008955458	6.603967147	0.005019198
0.544888095	0.006121243	6.742268634	0.003601357
0.510725393	0.031000587	5.889525499	0.017088934
0.521016554	0.026066776	5.936437961	0.010613129
0.522984527	0.015314514	6.051543832	0.010788527
0.597710989	0.013462286	6.217871997	0.005298932
0.579778286	0.008431356	6.724752661	0.004559045
0.508698638	0.017652442	6.823340773	0.006815964
0.56234761	0.008059521	6.414052155	0.005621272
0.649421691	0.011486063	6.289837569	0.006169746
0.526984741	0.016887523	6.548598724	0.009317607
0.551433294	0.009502527	6.693188662	0.00490846
0.541905288	0.007787701	6.600800676	0.004800218
0.56506923	0.012617284	6.239195353	0.005406439
0.525164556	0.007834943	6.985029767	0.004244025
0.587700524	0.011266265	6.450766931	0.005670816
0.570100483	0.007560443	6.823781394	0.00541637
0.566757376	0.004556009	7.010206569	0.002939174
0.553953791	0.009442175	6.7983758	0.004637782
0.54384635	0.022146766	6.159320183	0.012126715
0.559002231	0.005827609	6.859337478	0.002966945
0.574415358	0.014061007	6.062589553	0.010839233
0.573805328	0.007698532	6.794398147	0.003766763
0.502919852	0.009305914	6.943965473	0.00449585
0.517624136	0.009809235	6.791008137	0.006024491
0.588936808	0.037133208	5.86842666	0.015545476
0.547972184	0.010962224	6.83201191	0.005562583
0.554408563	0.01375626	6.563397153	0.006278875
0.588218931	0.022386274	6.21012488	0.01486831
0.575106924	0.008481057	6.694089361	0.003769199
0.49226807	0.020704251	6.147733447	0.007889487
0.538816008	0.011323645	6.751121114	0.00642594
0.608860703	0.012976441	6.037451199	0.011455717
0.52750744	0.01964203	6.635590646	0.013660413
0.540555961	0.018021322	6.413863881	0.011419542
0.607993333	0.010974054	6.536533926	0.006156941
0.553824828	0.012664795	6.236407736	0.009010099
0.576331784	0.012507494	6.410118581	0.005633998
0.473507315	0.01611647	6.419698901	0.006604711
0.536679147	0.010614965	6.665239292	0.005890675
0.530887307	0.010807032	6.359975328	0.0087647
0.583197383	0.014165625	6.432514121	0.007799784
0.55999559	0.014962617	6.619924718	0.006545234
0.571045175	0.011818274	6.373213522	0.007240617
0.516562678	0.011850952	6.689582165	0.006906654
0.589836534	0.008602012	6.264289322	0.006853994
0.551693867	0.00807386	6.378826385	0.003702888
0.54827165	0.008379903	6.726630453	0.004311002
0.523651457	0.011846388	6.850327058	0.006248423
0.527678034	0.009503038	6.436412662	0.006102928
0.601839201	0.007515675	6.84328316	0.00360563
0.538180424	0.013140045	6.702393957	0.00735111
0.534010531	0.018054883	5.779923431	0.012876662
0.504936942	0.023469828	5.596559646	0.01605768
0.558631468	0.00577104	6.963772751	0.003515494

log.sigma.4.5.mm.3D_ngtdm_Coarseness	log.sigma.4.5.mm.3D_ngtdm_Complexity	log.sigma.4.5.mm.3D_ngtdm_Strength	log.sigma.4.5.mm.3D_ngtdm_Busyness	log.sigma.4.5.mm.3D_ngtdm_Contrast
0.008619482	0.601602863	2.803590178	0.272034357	0.000107789
0.025590932	2.716150492	8.306869487	0.07454857	0.000643734
0.012060469	1.044238737	3.788339743	0.163193585	0.000188946
0.034193749	2.829252921	10.4273685	0.059296807	0.000685545
0.008263971	0.401769484	2.053492145	0.373848651	0.000114571
0.049427293	2.13647301	11.70779773	0.055617053	0.000851542
0.001414853	0.060517524	0.7456964	1.533727318	3.77E-06
0.002042453	0.142414213	0.783116579	0.926166331	1.51E-05
0.002945021	0.116723626	0.786589367	1.102260156	2.17E-05
0.016612922	0.459353718	2.851291042	0.278761892	0.000180947
0.008541197	0.12725631	1.229872749	0.597117788	3.77E-05
0.003746052	0.122909367	0.937049701	0.81398158	2.12E-05
0.013322821	0.917283988	3.851401034	0.161855794	0.000244593
0.015509856	1.421774391	4.786524467	0.136738404	0.000372951
0.007227656	0.490467895	2.491645436	0.409170894	7.14E-05
0.002384517	0.106058961	0.687139579	1.103713536	1.62E-05
0.014263549	0.194352918	1.450021846	0.487910254	0.000116857
0.023760058	2.540518906	8.712602665	0.080551065	0.000521803
0.011651057	0.163475901	1.411001442	0.462137328	6.48E-05
0.001516443	0.098736134	0.7689993	0.678478847	6.08E-06
0.00542236	0.226414417	1.148292824	0.61227724	8.03E-05
0.020488667	1.005054492	4.534591422	0.125749325	0.000335354
0.021825762	0.967322059	5.120880211	0.129924981	0.000233702
0.003794438	0.069474822	0.769717706	0.861336592	1.27E-05
0.011033814	0.658283501	3.552325679	0.171095569	0.000100658
0.004917625	0.384063264	2.225794031	0.329060646	3.72E-05
0.005830153	0.430511725	2.041610654	0.37600341	6.14E-05
0.005880298	0.168665174	1.258701077	0.57389163	3.56E-05
0.079435232	0.857890612	7.509954478	0.091423367	0.000605716
0.006922957	0.335732605	1.829093186	0.406062978	5.95E-05
0.003062033	0.158933151	0.926681186	0.95407565	2.61E-05
0.011400802	1.0437368	3.301793077	0.183420401	0.000263585
0.005996133	0.310747046	2.346608886	0.378852228	3.59E-05
0.005385011	0.256673612	1.900167007	0.423946011	2.91E-05
0.002440697	0.116785756	0.750059254	1.116949138	1.72E-05
0.005708414	0.348914062	1.462284766	0.400969393	8.57E-05
0.006807698	0.678069841	2.423799964	0.23035944	0.000117632
0.002532211	0.110422017	0.853983428	0.820626971	1.40E-05
0.017994206	0.465025528	2.628049551	0.230444942	0.000242394
0.001533241	0.082604134	0.951518733	0.645522257	3.19E-06
0.009056511	0.455406371	2.475813342	0.346487458	0.00010941
0.002327402	0.058229617	0.867802358	0.950951161	4.08E-06
0.0044381	0.434347341	1.594661742	0.44536087	7.15E-05
0.008043863	0.530655563	2.594997014	0.342180117	9.61E-05
0.008408636	0.268829052	2.393819822	0.29608604	3.73E-05
0.004869363	0.288112739	1.326962	0.564115276	5.80E-05
0.002473208	0.116446694	0.881793659	0.71949338	9.89E-06
0.019881725	0.554061334	2.695672044	0.227772873	0.000261369
0.030342806	1.267759512	7.620646509	0.120453223	0.00037142
0.02374015	1.248750804	5.598176364	0.1064181	0.000382432
0.00815458	0.475209467	2.167646262	0.515119521	0.000129108
0.004664462	0.274000381	1.600224543	0.413361654	3.70E-05
0.001800681	0.039686022	0.601501311	1.518344119	3.64E-06
0.007600187	0.305461825	2.143349927	0.265612794	3.78E-05
0.016713147	1.58232002	5.793045048	0.107870627	0.000361487
0.009612382	0.520617915	2.402749798	0.391989293	0.000151394
0.003190458	0.158605211	1.109104319	0.772739399	1.90E-05
0.0048588	0.346337187	1.337173721	0.517494593	7.13E-05
0.013476191	0.663419934	2.825829414	0.219594041	0.000196369
0.001415572	0.045274247	0.541535655	1.163442172	3.07E-06
0.009184666	0.479376182	2.592142055	0.274774106	7.50E-05
0.005317589	0.409000175	2.204190005	0.294156867	4.45E-05
0.001696343	0.144342454	0.92133123	0.700203409	8.52E-06
0.006802655	0.390812295	2.708798357	0.243380614	4.24E-05
0.016409827	0.582073457	2.772301769	0.251574888	0.000199168
0.003642576	0.290987477	1.558116973	0.400691231	2.73E-05
0.021638887	1.683846492	5.711080149	0.101041065	0.000517872
0.002478716	0.168380467	1.113877993	0.713863152	1.46E-05
0.00166617	0.070371682	0.577655256	1.354147906	7.33E-06
0.005616731	0.232535488	1.243602751	0.473965109	5.88E-05
0.020284464	0.777742309	3.398036528	0.240072615	0.000455807
0.002689862	0.144652982	0.855460503	0.803717216	1.96E-05
0.007406421	0.55844719	2.111585153	0.439104797	0.000153121
0.011584853	0.450323449	2.422565247	0.380612075	0.000130278
0.003997716	0.163888309	2.211092755	0.400875539	9.68E-06
0.012199926	0.23924102	1.430446031	0.456813716	0.000126845
0.005580639	0.296010648	1.75118756	0.367687096	4.18E-05
0.025253146	3.703075438	11.74874892	0.052668689	0.000699445
0.010384417	0.331800533	2.169315458	0.28456383	9.16E-05
0.007265051	0.23013707	1.866269423	0.392616654	3.28E-05
0.008843804	0.615596949	2.907989796	0.262466739	0.000108239
0.013662056	0.613271889	3.208251245	0.178262242	0.000162118
0.004839853	0.265775876	1.230385625	0.688183373	5.53E-05
0.006463043	0.216191296	1.496581665	0.512536364	3.87E-05
0.005384831	0.190526908	1.574309021	0.456229776	2.84E-05
0.016002387	1.70116791	8.107578307	0.072110894	0.000225495
0.008676756	0.42523541	2.092360812	0.325915347	9.20E-05
0.003812374	0.122519282	1.146145035	0.741786114	1.83E-05
0.007183836	0.291562486	1.790898137	0.349288936	4.69E-05
0.009004107	0.473050651	2.513092369	0.2859234	9.97E-05
0.018686049	0.751400709	5.563643141	0.099110753	0.000156856
0.005254073	0.172210808	1.303236869	0.426112412	2.69E-05
0.002853675	0.138669553	1.129394966	0.680856187	1.12E-05
0.003702368	0.171727191	1.154700286	0.716852613	2.50E-05
0.003859378	0.09950693	0.797207935	0.771916674	1.85E-05
0.005135072	0.542126535	2.375660822	0.308691836	5.94E-05
0.005067708	0.240149089	1.336559102	0.563237386	5.07E-05
0.031256812	1.638670876	7.718964704	0.073112769	0.000475931
0.030357769	1.300886399	5.604969908	0.100280041	0.000511413
0.001538637	0.090309431	0.562204774	1.043081004	9.16E-06

log.sigma.4.0.mm.3D_gldm_GrayLevelVariance	log.sigma.4.0.mm.3D_gldm_HighGrayLevelEmphasis	log.sigma.4.0.mm.3D_gldm_GrayLevelNonUniformityNormalized	log.sigma.4.0.mm.3D_gldm_DependenceEntropy
39.42440397	302.1606648	0.045769676	6.852909224
37.98068251	282.1325648	0.050369989	6.269501578
36.02126903	292.2006726	0.046650345	6.63166423
44.01987771	323.6987578	0.044867096	6.244078429
32.42832237	211.1459957	0.049804588	6.653134085
37.04145892	266.7768595	0.048698859	6.027989583
17.06083259	166.8241769	0.090349599	7.318344813
28.46963202	264.1133817	0.055867929	7.114235823
21.64473755	157.9676208	0.068692896	7.038167933
22.08114381	179.4423841	0.059813166	6.486632541
11.17045015	114.5533419	0.09230956	6.467526988
17.82315006	172.0581156	0.076385063	7.064360834
39.28672796	326.0690506	0.048845939	6.646836763
42.0952606	324.4813665	0.043777246	6.457147706
27.53680383	173.7701587	0.056875586	6.856205086
25.18303163	182.9770913	0.065605474	7.362888436
14.03919255	103.3556059	0.073820459	6.425988619
42.00512909	257.2836186	0.046131958	6.385627144
11.26162732	102.8363803	0.08940072	6.569292279
23.89265624	358.0779702	0.061871865	7.172432998
28.04153509	182.0259865	0.05293922	6.736594448
27.50988147	223.1149194	0.058565297	6.326894161
26.0245115	179.7311828	0.05615935	6.411298885
11.52708034	109.706144	0.103655171	6.854464273
33.60713907	313.7605863	0.052076149	7.099756637
33.04156335	416.6404538	0.05318181	7.087001451
32.10310791	268.9466068	0.051058064	6.804121008
18.6711442	138.8814913	0.065236094	6.761739568
10.80657722	98.47260274	0.092137362	5.738539823
20.83043131	183.163354	0.061643455	6.659580279
25.6079863	197.329758	0.056152343	7.061842228
35.6074493	288.5304569	0.048664614	6.542198168
26.65201161	214.1601713	0.057624364	7.092464134
22.40888272	211.0815735	0.063712048	6.948755261
23.74548758	219.4946425	0.060717773	7.061309052
32.53623128	334.1021145	0.049334246	6.930379222
42.65745465	424.3015873	0.045256117	6.74877299
25.88476463	199.4849682	0.077311292	7.379235753
17.76823661	157.8946429	0.070408163	6.140870963
17.07343342	497.2060972	0.078423436	7.248545653
30.73801014	220.6634328	0.050744041	6.668547139
9.638359989	147.9661495	0.112765912	6.886664029
40.4034644	335.9789082	0.043574829	6.954869257
31.49950208	223.1464789	0.049788732	6.71842401
16.2791198	141.5147815	0.088767422	6.931628028
30.8810625	236.3931695	0.050130959	6.910033354
18.69831843	248.4040366	0.067338088	7.0293442
15.92827307	135.4541752	0.074418971	6.225049548
28.81429347	190.0588235	0.054794252	6.243698838
33.0543308	326.4336283	0.050933902	6.275512195
29.95935809	202.6969697	0.054096038	6.615496119
31.14683806	309.3716338	0.051259085	6.871283944
10.38964637	114.4623872	0.119349963	7.118144555
17.71453072	298.7771011	0.068831866	6.809754317
47.51737215	351.3499222	0.04118283	6.543442201
32.95767967	192.627551	0.04882225	6.559049548
22.50221235	185.7688667	0.061619797	7.092783427
29.22800316	309.0899855	0.051579068	6.632061982
24.93653561	181.5635649	0.056201893	6.367824326
14.72264618	251.7916093	0.092586155	7.301661506
24.91356049	245.6674191	0.056492291	6.79276177
34.71000926	329.7829615	0.047817683	6.91973449
31.44892684	390.7479961	0.051373243	7.257816895
31.226064	301.1822519	0.051373754	6.926611017
21.06657008	162.1742984	0.060111841	6.410043289
31.43372148	359.677872	0.050103471	6.848224174
30.68082215	301.226601	0.055279187	6.190000928
26.86042512	330.4285152	0.057167349	6.915492509
17.3361863	190.3736015	0.069777994	7.064232275
24.55626022	256.5447269	0.056318516	6.760829395
28.91719545	189.0528455	0.053680019	6.293383476
27.30412984	283.7861875	0.059830206	7.071154406
35.9806837	195.2696629	0.047921559	6.687989513
22.97041551	152.6905263	0.058504155	6.407118673
20.05911222	217.1851126	0.078864775	7.122891486
15.32898553	149.3638344	0.069835438	6.330569399
25.05831303	259.651426	0.055886645	6.753276887
45.82319393	469.1888545	0.050810417	6.279412568
26.37883792	250.626616	0.055039107	6.627308716
15.56918395	155.0977778	0.073817284	6.759542327
38.66731484	298.2759602	0.046505628	6.845555995
25.69240161	244.3813559	0.056320316	6.482547117
25.11916373	166.8773006	0.057978033	6.91110954
15.76456983	146.844691	0.072081115	6.572105731
22.48341816	198.6051565	0.06628247	7.041140048
28.06320023	399.2241055	0.077255365	6.110655687
25.66670174	219.4892966	0.05500145	6.669029613
20.20677136	148.9606361	0.080444197	7.224264611
20.02893481	207.031746	0.065746504	6.831074328
30.49432945	240.7125091	0.051086617	6.665380817
30.89407905	395.7325871	0.05430868	6.785358354
17.63560813	176.0951817	0.074016569	6.955320758
18.19459676	186.7847145	0.066045261	6.820251469
22.99563079	192.9930354	0.058992586	6.859596995
14.91310832	190.5657696	0.070747566	6.649746339
44.36936681	395.4286913	0.041750301	6.905684987
28.5851913	215.6231648	0.051045656	6.747735285
25.26648225	303.399361	0.062560606	6.117777004
19.08559813	201.738806	0.077940521	5.845565618
27.36856653	361.9259973	0.054343646	7.165011424

log.sigma.4.0.mm.3D_gldm_DependenceNonUniformity	log.sigma.4.0.mm.3D_gldm_GrayLevelNonUniformity	log.sigma.4.0.mm.3D_gldm_SmallDependenceEmphasis
313.4889197	66.09141274	0.333293085
85.78386167	17.47838617	0.362225424
209.5784753	41.61210762	0.327363312
88.69565217	14.44720497	0.418528295
316.794472	70.27427356	0.310536738
63.52066116	11.78512397	0.364535124
1012.367872	952.1944207	0.165334511
1002.906065	339.006592	0.262121849
575.6081652	292.7691225	0.217343421
148.4543046	45.1589404	0.269937987
237.4447301	143.6336761	0.171662453
428.5837981	260.2439096	0.197992214
175.8557337	39.61405672	0.312923428
173.1708075	28.19254658	0.409148309
269.16	89.57904762	0.307287351
693.8514405	378.0187435	0.212825402
153.4779162	65.18346546	0.198019028
101.2885086	18.86797066	0.357649735
150.2888483	97.80438757	0.174580491
1396.297615	568.1693346	0.228090182
421.9345525	110.0076997	0.237417161
113.1008065	29.0483871	0.32287757
112.4982079	31.33691756	0.294263524
387.8345844	372.8476508	0.155723151
190.2947883	63.9495114	0.286572473
425.9209703	135.9327074	0.270534282
408.6786427	102.3203593	0.304339776
382.6591212	146.9769197	0.222769276
23.82191781	13.45205479	0.199499593
299.7614907	99.24596273	0.249703536
658.4203043	225.1147418	0.231539034
181.4517766	38.34771574	0.358723871
364.7319058	134.5528908	0.242961744
358.6447205	153.8645963	0.227504824
815.6362751	311.6643289	0.234276733
385.7797834	95.65910263	0.314453356
362.1626984	68.42724868	0.353209303
633.5349596	450.0290328	0.2187973
120.8428571	39.42857143	0.242984846
1089.107547	740.8662009	0.179573316
284.8895522	67.99701493	0.278145809
594.2863188	639.6082511	0.140260658
537.1894127	105.3639371	0.336404238
300.128169	70.7	0.30121833
181.1696658	138.122108	0.17300038
473.0824656	120.3644315	0.298644807
753.1468884	360.3261073	0.204804548
92.11201629	36.53971487	0.235462017
95.0971867	21.42455243	0.3116329
113.9115044	23.02212389	0.343587758
256.1406371	69.62160062	0.324468775
565.081149	142.756553	0.293897204
624.3219659	951.9353059	0.125233832
261.6138855	113.0219245	0.222636999
173.2581649	26.48055988	0.387874088
270.2619048	57.41496599	0.287314439
604.9556663	247.4034869	0.224959264
453.6976294	106.6139332	0.291676574
170.378768	42.88204456	0.303988112
1032.286881	1008.54099	0.152709746
251.8036117	75.07825433	0.292701465
531.9809331	117.8705882	0.299025386
1371.969152	423.0072869	0.26700808
411.7290076	107.6793893	0.27100842
139.5317578	40.6957164	0.290447143
737.7407191	171.4039754	0.305065354
104.6748768	22.44334975	0.344945265
936.1815143	290.6959685	0.268152635
1011.39984	523.8931806	0.181233097
422.720339	119.6205273	0.251484246
116.6260163	26.41056911	0.330812109
781.2087934	298.0142542	0.245084869
290.7138577	63.9752809	0.320731403
204.8357895	55.57894737	0.322525413
484.3266405	322.0837414	0.194267846
187.4858388	64.10893246	0.21758312
425.432775	123.4535989	0.246863406
88.18266254	16.41176471	0.382908497
280.3201521	72.37642586	0.28236417
271.8377778	132.8711111	0.199925094
303.4139403	65.38691323	0.326559936
180.9491525	46.52058111	0.301709589
387.2559159	132.305872	0.26787213
327.4384575	136.449551	0.213916185
381.1178637	179.9569061	0.207292361
113.8888889	41.02259887	0.269711794
260.6880734	71.94189602	0.301605699
406.6506778	308.5839416	0.186195237
269.6078777	111.8348031	0.251648154
294.9517191	69.835406	0.303248931
141.539801	43.6641791	0.257941692
321.4186414	187.4099526	0.195994892
764.7053988	297.2697178	0.213367371
610.2315728	203.2884504	0.230335059
555.1548231	233.9622014	0.204155593
555.4790268	99.53271812	0.356665544
523.3972268	125.1639478	0.273221842
66.65495208	19.58146965	0.333273873
60.82835821	20.8880597	0.249342319
1489.946056	479.5283318	0.261848255

log.sigma.4.0.mm.3D_gldm_DependenceNonUniformityNormalized	log.sigma.4.0.mm.3D_gldm_DependenceVariance	log.sigma.4.0.mm.3D_gldm_LargeDependenceEmphasis
0.21709759	2.52741308	9.972299169
0.24721574	1.327741282	7.201729107
0.234953448	1.715156146	8.479820628
0.275452336	1.109563674	5.944099379
0.224517698	1.772697159	9.014174344
0.262482071	1.115702479	6.702479339
0.096059196	24.40532348	60.04981497
0.165277862	6.877532005	19.41529334
0.135055881	11.45132864	29.92397935
0.196628218	2.314887944	11.42649007
0.152599441	4.541791952	20.1940874
0.125795068	10.98241065	31.48547109
0.216838143	2.252058244	9.914919852
0.268898769	1.448449134	6.630434783
0.170895238	4.825890249	15.52190476
0.120418508	15.90723529	38.56959389
0.173814175	3.139608228	15.55266138
0.247649165	1.450170671	7.4400978
0.137375547	5.872868129	23.4606947
0.152052446	6.249075743	20.45137755
0.203048389	2.21046742	11.75553417
0.228025819	1.68496358	8.596774194
0.201609692	2.059338909	10.40501792
0.10782168	13.9359507	41.18626633
0.154963183	11.76663413	26.49022801
0.166635747	4.497195221	15.84428795
0.203931458	2.426452484	10.58483034
0.169844262	4.643076874	17.12250333
0.163163821	3.807656221	17.5890411
0.186187261	2.782176614	12.85590062
0.164235546	5.420099986	18.35245697
0.230268752	1.617240846	8.007614213
0.156202101	5.311833976	18.3490364
0.148507131	7.264795168	22.27039337
0.158900502	6.7511017	20.39918177
0.198958114	3.501208201	12.19443012
0.239525594	1.600527351	7.857142857
0.108836104	23.31123357	52.54475176
0.215790816	1.874885204	10.81071429
0.115286075	12.05925979	35.16576691
0.212604143	2.215491201	10.54776119
0.104775444	15.61459852	45.12447109
0.222162702	2.340107725	9.557485525
0.211357866	2.386929181	10.42535211
0.116432947	14.36828332	38.70437018
0.197035596	3.790070464	12.96584756
0.140748811	6.711723918	22.79405719
0.187600848	3.003430382	13.58248473
0.243215311	1.444705359	8.130434783
0.252016603	1.238331114	7.225663717
0.199021474	3.092821708	11.48795649
0.202901669	2.310675619	10.63447038
0.078275071	22.87230322	67.85105316
0.159326361	4.38581926	17.35809988
0.269452822	1.21713198	6.465007776
0.229814545	1.567515271	8.962585034
0.150673889	8.088189836	23.33026152
0.219495708	2.252251088	10.17755201
0.223301138	2.18561964	9.796854522
0.094766077	25.61188941	62.57110071
0.189468481	3.387744934	12.70654628
0.215813766	3.138151402	11.48356998
0.166622438	6.273913521	18.44328394
0.196435595	2.840951139	12.08301527
0.20610304	2.453722108	10.86115214
0.215650605	2.098262866	9.782227419
0.257819894	1.189982771	7.064039409
0.184106492	3.540261184	13.62104228
0.134709622	6.737127636	24.43580181
0.199020875	2.556038778	12.03107345
0.237044748	1.594817899	8.195121951
0.156837742	6.230868467	19.74804256
0.217763189	2.488683528	10.08314607
0.21561662	2.073178947	9.471578947
0.118591244	11.0406776	32.49363369
0.20423294	2.094161315	11.9912854
0.192590663	2.376844306	12.10819375
0.273011339	1.23179557	6.523219814
0.213171218	1.988101028	10.08136882
0.151020988	5.40881358	20.26555556
0.215799389	2.552527372	10.13655761
0.219066771	1.925666446	9.518159806
0.169700226	9.70189979	23.03943909
0.1729733	3.82527671	16.08082409
0.140374904	7.580551944	24.11233886
0.214480017	1.947808385	10.23917137
0.199302808	2.769842606	11.33486239
0.10600904	16.9696759	44.87539103
0.158499634	4.845203275	17.30805409
0.215765705	1.934273707	9.57644477
0.176044529	3.392063315	13.99502488
0.126942591	7.414739985	25.77725118
0.169896778	3.742995718	16.14196845
0.177084032	3.078609061	14.18456181
0.167872641	3.41316936	16.11279105
0.233002948	1.807170144	8.255872483
0.21345727	1.990338479	10.26182708
0.212955118	1.986485521	9.274760383
0.226971486	1.588382713	9.843283582
0.168851548	5.372073731	17.25362647

log.sigma.4.0.mm.3D_gldm_LargeDependenceLowGrayLevelEmphasis	log.sigma.4.0.mm.3D_gldm_SmallDependenceHighGrayLevelEmphasis	log.sigma.4.0.mm.3D_gldm_LargeDependenceHighGrayLevelEmphasis
0.067932141	117.2071062	2546.509695
0.206295542	103.3698263	2116.259366
0.092227686	96.10820634	2540.272422
0.059513267	154.017657	1740.773292
0.153082124	71.0060454	1806.299787
0.090124292	104.937314	1595.231405
0.454211273	33.7534025	9324.00854
0.113879628	84.72439003	4478.281147
0.35753108	47.25556909	3887.058189
0.139258427	56.94285565	1746.549669
0.277857254	22.75012314	1995.827121
0.292140547	44.22856229	4652.757264
0.069088832	92.89502747	3726.554871
0.074046654	145.5233268	1876.63354
0.246305917	74.41424227	1785.452698
0.372701706	45.30218497	6116.816383
0.436161262	24.0340822	1376.284258
0.218855802	105.3634716	1606.026895
0.420801689	23.25117624	2157.607861
0.070971034	87.09820722	6923.199608
0.292509702	46.66265799	1977.000962
0.172080003	73.92693996	1841.5625
0.360440752	64.99828521	1600.362007
0.575636344	23.17393321	4048.651932
0.126376692	101.1553801	8309.356678
0.057533693	133.7707597	5489.614632
0.086220566	100.4911871	2240.412675
0.300009971	37.77783951	2097.636041
0.326461658	25.71090433	1315.308219
0.163190135	52.80027814	1963.550932
0.199755897	54.8678482	2965.548266
0.146047051	109.0807056	2144.606599
0.170862187	60.59802394	3245.035546
0.162260476	54.30470245	4511.988406
0.15805987	64.09952012	3633.4549
0.069695551	107.5609741	3902.757091
0.046580903	155.5280371	3238.374339
0.391122496	51.71948387	9783.036248
0.281496628	40.14495685	1718.5875
0.079599862	102.902969	16606.98592
0.144495343	77.32986796	1815.033582
0.386581035	26.14397538	6019.586037
0.071141164	122.2684162	2832.343672
0.135829771	80.87396412	1759.426761
0.453573612	32.36537224	4866.232005
0.148372087	85.25922247	2326.198251
0.135496134	62.4200926	4965.605681
0.282536786	38.30259785	1825.173116
0.130339467	66.76652186	1328.104859
0.050051937	106.2142761	2417.730088
0.150053394	84.56055645	1510.139083
0.065595013	99.1159424	2930.94219
0.761124726	18.85894817	7344.475426
0.075488974	71.34153711	4911.17905
0.080192366	134.2322233	2349.813375
0.178795279	62.15418743	1466.880952
0.225050597	52.20745591	3725.640847
0.058031657	98.45659822	2811.436381
0.707128709	65.59530478	1362.418087
0.279202231	46.08631746	15214.59965
0.090024791	82.05074604	2720.620015
0.05701766	100.4610086	3628.711156
0.068055565	112.8408522	6242.66274
0.075269238	87.39006135	3545.228053
0.162216988	51.8372925	1521.60709
0.053101483	116.3522548	3274.405145
0.048499489	104.594426	2104.896552
0.068030054	104.9346941	3669.293019
0.2019107	40.43182599	4289.288093
0.073176159	62.41659297	3249.84322
0.142821544	72.77583733	1372.036585
0.107976759	81.34351394	5108.082112
0.272921063	76.34404992	1378.173034
0.182582855	57.25658967	1191.212632
0.217667553	56.12449914	6186.826151
0.154673724	34.01409802	1725.305011
0.139759527	63.95499198	3132.779538
0.032543835	185.1114284	3109.869969
0.073878051	73.68264415	2440.660076
0.236586567	38.83276986	2736.497778
0.068972028	112.5897781	2571.315789
0.087256402	70.0914383	2491.173123
0.348922678	54.8252405	3437.713409
0.222590047	39.47882619	1942.032224
0.208828927	50.73940957	4048.263352
0.035857936	94.22267616	4578.120527
0.123068078	75.65254849	2066.600917
0.551505533	39.05256158	5471.338113
0.143316142	59.4692216	3098.864785
0.098554739	82.73930245	2064.926116
0.062098795	103.519844	5286.54602
0.324179137	46.36431998	3962.406398
0.147507458	44.33181008	2685.113975
0.161645214	52.32093185	2354.387115
0.133833993	43.12110023	2697.130027
0.048059746	152.19905	3015.385906
0.113341653	62.95478848	2033.00571
0.054541862	100.7128665	2887.591054
0.097698635	45.64014001	2195.067164
0.063475984	92.74731576	6135.841002

log.sigma.4.0.mm.3D_gldm_SmallDependenceLowGrayLevelEmphasis	log.sigma.4.0.mm.3D_gldm_LowGrayLevelEmphasis	log.sigma.4.0.mm.3D_gldzm_DistanceZoneVariabilityNormalized
0.004323442	0.008961708	0.902221278
0.005450147	0.022322357	0.949719043
0.003269774	0.011415029	0.899488156
0.010770624	0.01693598	0.886134068
0.004229419	0.015412429	0.854320278
0.007944489	0.015550926	0.883781032
0.002122629	0.010703455	0.952142926
0.001975661	0.00700137	0.895644474
0.002353136	0.013623164	0.963044535
0.004386471	0.013181022	0.952029024
0.00334756	0.015348178	0.949560646
0.002556144	0.010918409	0.947472591
0.005184052	0.010526177	0.863028711
0.004436862	0.00985037	0.820498636
0.003550771	0.01409595	0.924772989
0.002736895	0.013326747	0.969238281
0.004552012	0.027667722	0.97030378
0.00696639	0.023282439	0.832289698
0.003702544	0.020354031	0.971434573
0.001176941	0.004087811	0.95970443
0.003345793	0.020282546	0.945342398
0.004271026	0.018890339	0.87331511
0.004221595	0.02833473	0.939756772
0.002817522	0.019426382	0.967646295
0.002154289	0.008278058	0.958255655
0.001356226	0.003932547	0.929090293
0.002415126	0.0078458	0.933145848
0.003710616	0.019560081	0.936203945
0.00997107	0.0229193	0.947406866
0.003303446	0.011757178	0.983194484
0.002081751	0.010266357	0.950940174
0.003967723	0.016741449	0.884417919
0.002629043	0.010114505	0.858963211
0.001909386	0.008998047	0.969379537
0.002016313	0.008219983	0.942707305
0.002629527	0.00706568	0.79961903
0.002635309	0.006708697	0.677543195
0.00303619	0.012726552	0.941503682
0.003647876	0.022243493	0.84120983
0.000506849	0.002444233	0.939078405
0.002462385	0.012383535	0.891573342
0.00155608	0.01044973	0.90878124
0.002431258	0.00792302	0.92446223
0.003257241	0.01115955	0.932413814
0.003184604	0.017868171	0.950032873
0.00237749	0.01060334	0.917563943
0.00123034	0.006037847	0.952671894
0.004548591	0.022714259	1
0.007178995	0.017686093	0.925577523
0.005511561	0.010585605	0.881078357
0.003544112	0.011619371	0.976565795
0.002323205	0.007421008	0.903202
0.002177374	0.016088787	0.939182651
0.001770678	0.005213991	0.93048493
0.004121574	0.012305286	0.890613944
0.004734822	0.016695489	0.957163408
0.002264001	0.010652881	0.944489796
0.001887084	0.005787291	0.926326959
0.00348939	0.02437038	0.859340765
0.000920763	0.00552735	0.9393566
0.002933221	0.007944073	0.946210955
0.00294394	0.006854895	0.907876376
0.001246292	0.004085791	0.843338067
0.002286474	0.007389952	0.806086074
0.008365412	0.020561543	0.975004006
0.001601141	0.005598059	0.867150102
0.005580333	0.010200949	0.822780581
0.00132282	0.004867807	0.866292102
0.001501253	0.008567555	0.95019648
0.002446475	0.007517137	0.908764503
0.006380098	0.017964854	0.940649729
0.001563725	0.006259191	0.922689043
0.004919686	0.02159914	0.944694288
0.006190946	0.018155242	0.994609204
0.001408628	0.008270915	0.827836257
0.003218786	0.014966496	0.9608
0.00249578	0.010616543	0.925374214
0.006506967	0.009500119	0.866547055
0.003652369	0.009269767	0.924486566
0.002559056	0.013581222	0.949108841
0.003695187	0.008415656	0.920960676
0.004076269	0.010286482	0.974280894
0.003305473	0.017506969	0.967922968
0.002773944	0.014691292	0.953416368
0.002069033	0.010007768	0.889433815
0.005689976	0.008414867	0.863184401
0.003962571	0.011719156	0.916824197
0.002416986	0.015693497	0.973011188
0.003582648	0.012040726	0.932887534
0.003246196	0.009887596	0.931384649
0.002069873	0.006987907	0.794631635
0.001827069	0.013714483	0.944296707
0.002370002	0.009835663	0.928358273
0.002611571	0.011387626	0.942080642
0.001887692	0.008149042	0.900530569
0.001911459	0.005991843	0.808948911
0.003597786	0.012336609	0.885354098
0.005627651	0.01063631	0.839488
0.007710418	0.016768776	0.931903114
0.001329535	0.004280274	0.895353556

log.sigma.4.0.mm.3D_gldzm_LowIntensityEmphasis	log.sigma.4.0.mm.3D_gldzm_LargeDistanceEmphasis	log.sigma.4.0.mm.3D_gldzm_HighIntensitySmallDistanceEmphasis
0.012069045	1.154639175	338.8556701
0.018991794	1.077419355	277.3129032
0.011171756	1.159217877	288.400838
0.023006974	1.181818182	348.9166667
0.014123426	1.237288136	219.757533
0.019374665	1.185840708	275.8318584
0.01318989	1.073590814	207.6417015
0.007477805	1.165683646	316.7919571
0.011120406	1.056497175	226.9981168
0.015420699	1.073770492	210.7366803
0.019165369	1.077669903	136.8511327
0.012655984	1.080976864	224.0215296
0.015145319	1.221864952	285.642283
0.010253658	1.299065421	345.4719626
0.011341019	1.117437722	238.9430605
0.012796753	1.046875	211.0644531
0.026333342	1.045226131	127.8555276
0.021612433	1.277173913	283.4442935
0.02123937	1.043478261	137.9673913
0.00514496	1.061712846	381.0126994
0.015100846	1.084358524	196.4112478
0.016805888	1.203883495	220.9381068
0.016194215	1.093264249	217.5595855
0.019126375	1.049342105	159.4239309
0.009290918	1.063981043	344.5503555
0.00471995	1.110429448	483.1883436
0.007957978	1.103878116	325.2624654
0.019115962	1.098958333	174.718316
0.042388928	1.081081081	129.5945946
0.01276726	1.025423729	213.8908898
0.008855716	1.075489282	241.7993942
0.013938304	1.184750733	296.1341642
0.010925918	1.229041916	245.7020958
0.009172101	1.046656299	240.2391135
0.008534968	1.088552916	275.9868611
0.008180909	1.338842975	326.3973829
0.007583934	1.612307692	405.385812
0.014177709	1.090472927	237.9705278
0.020909808	1.260869565	161.0481366
0.002752748	1.09434968	572.2869136
0.010360954	1.172566372	270.5049779
0.012574	1.143712575	193.4314371
0.0078161	1.117943548	357.6658266
0.010453717	1.105058366	265.8273346
0.0195011	1.076923077	188.9102564
0.008762173	1.129220023	281.6702561
0.00601804	1.072756669	306.9553355
0.021805089	1	161.5602837
0.020968391	1.116129032	215.3725806
0.015136098	1.19047619	302.6653439
0.0107407	1.035573123	261.7282609
0.00863708	1.153	330.17175
0.019282218	1.094182825	155.9272853
0.0077257	1.108173077	316.2313702
0.01200683	1.174193548	344.041129
0.015129932	1.065693431	223.8321168
0.010080526	1.085714286	235.4185714
0.00616499	1.114911081	334.8026676
0.015236425	1.228373702	208.3892734
0.006205697	1.093904448	307.1052993
0.009590371	1.082978723	274.3079787
0.009224056	1.145214521	328.0225523
0.004577185	1.257011141	408.0822128
0.008397388	1.326378539	307.4944113
0.028395176	1.037974684	179.4409283
0.005837298	1.214630225	366.9933682
0.014788115	1.294797688	296.566474
0.004981548	1.216133005	380.348984
0.008580755	1.076664425	227.9583053
0.009505779	1.14374034	243.8593509
0.018323695	1.091836735	218.8418367
0.006301483	1.120833333	330.8418403
0.017254812	1.085388994	232.3164137
0.019252536	1.008108108	176.4601351
0.00778082	1.301754386	293.0390838
0.017421408	1.06	156.722
0.011761978	1.116459627	256.384705
0.014661599	1.215686275	471.8431373
0.012413789	1.11790393	253.3629913
0.014354696	1.078384798	196.6425178
0.010631333	1.123655914	336.7719534
0.012806767	1.039087948	230.8697068
0.013284698	1.048913043	204.3250679
0.015223462	1.071583514	188.5759219
0.009915485	1.176197836	245.4656105
0.018384398	1.221590909	325.7627841
0.012897061	1.130434783	245.7789855
0.01239712	1.041044776	220.4306592
0.016410865	1.104294479	234.4125767
0.010602765	1.106719368	265.7653162
0.009853029	1.348547718	382.1275934
0.009962487	1.086021505	240.561828
0.011538733	1.111615245	209.7883394
0.011821534	1.089552239	226.3403518
0.009106876	1.157469717	211.4017497
0.006021234	1.320902846	395.2944063
0.013620772	1.183150183	226.0418193
0.016412323	1.264	290.812
0.029302298	1.105882353	181.9058824
0.005027613	1.166174298	346.5362814

log.sigma.4.0.mm.3D_gldzm_LowIntensityLargeDistanceEmphasis	log.sigma.4.0.mm.3D_gldzm_HighIntensityEmphasis	log.sigma.4.0.mm.3D_gldzm_DistanceZoneVariability	log.sigma.4.0.mm.3D_gldzm_ZonePercentage
0.014537719	351.814433	525.0927835	0.403047091
0.019673865	280.3612903	147.2064516	0.446685879
0.01189441	298	322.0167598	0.401345291
0.02408657	358.3939394	146.2121212	0.51242236
0.01614422	228.8775895	453.6440678	0.376328845
0.020596685	285.0707965	99.86725664	0.466942149
0.013733834	211.2719207	1824.305846	0.18180093
0.00972475	328.0841823	1670.376944	0.307350033
0.011744581	228.6873823	1022.753296	0.249178789
0.015941117	214.1147541	232.295082	0.323178808
0.019791495	139.4724919	293.4142395	0.198586118
0.013267019	227.9344473	737.1336761	0.22835339
0.016405261	297.4951768	268.4019293	0.383477189
0.026838283	353.7943925	263.3800623	0.498447205
0.013952625	242.8932384	519.7224199	0.356825397
0.013097629	213.196733	1364.6875	0.244359597
0.027027309	128.6582915	193.0904523	0.225368063
0.027613315	289.8967391	153.1413043	0.449877751
0.021862365	139.057971	201.0869565	0.189213894
0.005304799	387.9047019	2286.015953	0.259392355
0.016060341	198.5254833	537.8998243	0.273820982
0.019348852	227.0582524	179.9029126	0.415322581
0.017429103	219.5025907	181.373057	0.345878136
0.025141196	160.1529605	588.3289474	0.169029747
0.009935137	348.014218	404.3838863	0.343648208
0.00503385	495.8932515	757.208589	0.31885759
0.008698159	330.9238227	673.7313019	0.360279441
0.021805905	176.9878472	539.2534722	0.255659121
0.047456496	129.9189189	35.05405405	0.253424658
0.012950176	214.815678	464.0677966	0.293167702
0.00967853	244.9804287	1020.358807	0.267647792
0.015485675	302.941349	301.5865103	0.432741117
0.012976228	256.8577844	573.7874251	0.28608137
0.009390266	243.0419907	623.311042	0.266252588
0.009567779	279.737941	1309.420446	0.270601987
0.017140532	341.362259	580.523416	0.374419804
0.016643818	432.4076923	440.4030769	0.42989418
0.015027447	241.5935572	1373.653873	0.250644219
0.045634927	165.1242236	135.4347826	0.2875
0.002987289	584.8981876	1761.711087	0.19858156
0.012647478	277.5154867	402.9911504	0.337313433
0.014350758	198.9760479	758.8323353	0.147214386
0.010425177	361.46875	917.0665323	0.41025641
0.012746151	271.4202335	479.2607004	0.361971831
0.020958879	190.3717949	296.4102564	0.200514139
0.010019606	287.145518	788.1874272	0.357767597
0.006479815	311.1139854	1178.455133	0.231171744
0.021805089	161.5602837	141	0.287169043
0.02322227	217.8064516	143.4645161	0.396419437
0.016368866	311.9153439	166.5238095	0.418141593
0.01314797	262.3241107	494.1422925	0.393162393
0.00964175	340.896	903.202	0.359066427
0.021579585	158.6565097	1017.134811	0.135782347
0.0081814	323.3798077	387.0817308	0.253349574
0.013554819	352.1483871	276.0903226	0.482115086
0.015991602	225.5328467	393.3941606	0.349489796
0.016054077	238.2514286	991.7142857	0.261519303
0.006662751	342.3406293	677.1450068	0.353652637
0.020478689	213.3460208	248.349481	0.378768021
0.006821565	313.0801757	1710.568369	0.167171578
0.010247787	278.2893617	444.7191489	0.35364936
0.009889838	339.7832783	825.259626	0.368762677
0.005714341	430.1552055	2195.20899	0.316128249
0.011460023	326.5350224	540.8837556	0.320133588
0.028743969	180.535865	231.0759494	0.350073855
0.007059535	382.5836013	1078.734727	0.363636364
0.018120775	304.6473988	142.3410405	0.426108374
0.006164239	396.8799261	1406.858374	0.319370698
0.009206898	231.8298588	1412.942165	0.198055408
0.010213102	252.3632148	587.9706337	0.304613936
0.01950943	221.5204082	184.3673469	0.398373984
0.009729685	336.6569444	1328.672222	0.289098575
0.018479225	234.5806452	497.8538899	0.394756554
0.019352636	176.6243243	368.0054054	0.389473684
0.018206771	304.9017544	707.8	0.209353575
0.017992063	158.36	240.2	0.272331155
0.012434344	262.7934783	595.9409938	0.291534631
0.015967878	482.6470588	132.5816993	0.473684211
0.013256444	259.2860262	423.4148472	0.348288973
0.014974582	199.4608076	399.5748219	0.233888889
0.012890477	346.7706093	513.8960573	0.396870555
0.013206818	232.6775244	299.1042345	0.371670702
0.013608753	206.4375	712.3913043	0.322524102
0.016304061	190.2255965	439.5249458	0.24352879
0.011065762	254.9258114	575.4636785	0.238305709
0.019282498	340.7159091	151.9204545	0.331450094
0.014228893	250.8364389	442.826087	0.369266055
0.012778408	222.1032338	782.300995	0.209593326
0.017161904	238.5582822	456.1820041	0.287477954
0.011370676	270.7233202	471.2806324	0.370153621
0.011371421	407.6431535	191.5062241	0.299751244
0.016463104	243.2473118	526.9175627	0.220379147
0.012489126	214.2059891	1023.050817	0.244834481
0.012668631	230.0863539	883.6716418	0.272199652
0.010583823	219.1480485	669.0942127	0.224674932
0.007541357	419.9656526	824.3189401	0.427432886
0.015244186	233.4529915	725.1050061	0.334013051
0.018609612	302.152	104.936	0.399361022
0.030253279	184.9411765	79.21176471	0.317164179
0.005767295	359.6166913	2424.61743	0.306890299

log.sigma.4.0.mm.3D_gldzm_IntensityVariabilityNormalized	log.sigma.4.0.mm.3D_gldzm_LowIntensitySmallDistanceEmphasis	log.sigma.4.0.mm.3D_gldzm_IntensityVariability
0.039814126	0.011451876	23.17182131
0.042913632	0.018821276	6.651612903
0.046658968	0.010991093	16.70391061
0.040440771	0.022737075	6.672727273
0.049088349	0.013618227	26.06591337
0.051296108	0.019069159	5.796460177
0.050414377	0.013053904	96.59394572
0.045245779	0.006916069	84.38337802
0.054353262	0.010964362	57.72316384
0.052405267	0.015290595	12.78688525
0.068086845	0.019008838	21.03883495
0.052233332	0.012503225	40.63753213
0.047238966	0.014830333	14.69131833
0.045253831	0.006107502	14.52647975
0.051880042	0.010688117	29.15658363
0.049311362	0.012721534	69.43039773
0.066690235	0.02615985	13.27135678
0.045545841	0.020112213	8.380434783
0.069056454	0.021083621	14.29468599
0.044631898	0.005105	106.3131822
0.051942637	0.014860972	29.55536028
0.052785371	0.016170147	10.87378641
0.054954495	0.015885493	10.60621762
0.06760193	0.017622669	41.10197368
0.042990948	0.009129863	18.14218009
0.045344575	0.004641475	36.95582822
0.045652658	0.007772932	32.96121884
0.057159047	0.018443476	32.92361111
0.07669832	0.041122036	2.837837838
0.057454754	0.012721531	27.11864407
0.05319331	0.008650012	57.07642125
0.048597793	0.013551461	16.57184751
0.050364839	0.010413341	33.64371257
0.057025171	0.00911756	36.66718507
0.049510164	0.008276765	68.76961843
0.049157996	0.005941003	35.68870523
0.043081657	0.005425801	28.00307692
0.048803461	0.013965275	71.20424949
0.064696578	0.014728528	10.41614907
0.052656494	0.002694113	98.78358209
0.048838985	0.009789323	22.07522124
0.064772491	0.012129811	54.08502994
0.044692215	0.007163831	44.33467742
0.050848612	0.009880608	26.13618677
0.059377055	0.019136655	18.52564103
0.048082279	0.008447815	41.30267753
0.054785452	0.005902597	67.76960388
0.065238167	0.021805089	9.19858156
0.050655567	0.020404922	7.851612903
0.046723216	0.014827906	8.830687831
0.051711478	0.010138883	26.16600791
0.04662	0.008385912	46.62
0.062190881	0.018707876	67.35272392
0.057345599	0.007611775	23.85576923
0.041789802	0.011619833	12.95483871
0.04947283	0.014914514	20.33333333
0.053745125	0.008587138	56.43238095
0.051929314	0.006040549	37.96032832
0.055471079	0.013925859	16.03114187
0.053965859	0.006051729	98.27182867
0.049425079	0.009426017	23.22978723
0.043192328	0.00905761	39.26182618
0.044363392	0.004292896	115.4779101
0.046635024	0.007631729	31.29210134
0.054033364	0.028307978	12.80590717
0.045097497	0.005531738	56.10128617
0.050285676	0.01395495	8.699421965
0.048677928	0.004685876	79.05295567
0.058456409	0.00842422	86.92468056
0.051100191	0.009328948	33.0618238
0.052374011	0.018027262	10.26530612
0.047660108	0.005444433	68.63055556
0.046509367	0.016948709	24.51043643
0.054740687	0.019227511	20.25405405
0.052284122	0.005577722	44.70292398
0.064032	0.017278745	16.008
0.050837159	0.011593886	32.73913043
0.046776881	0.014335029	7.156862745
0.047853779	0.012203126	21.91703057
0.059156741	0.014199724	24.90498812
0.040576303	0.010066548	22.64157706
0.052976689	0.012706754	16.26384365
0.054314597	0.013203684	39.97554348
0.06386663	0.014953312	29.44251627
0.052084403	0.009627916	33.69860896
0.047262397	0.018159873	8.318181818
0.052222779	0.012564103	25.22360248
0.052675058	0.012301798	42.35074627
0.051409119	0.016223105	25.1390593
0.047571435	0.010410788	24.07114625
0.048019146	0.009473431	11.57261411
0.058214823	0.008337333	32.48387097
0.056653305	0.011301135	62.43194192
0.051481854	0.01160976	48.28997868
0.065198923	0.008737639	48.44279946
0.039669248	0.005641203	40.42296369
0.049694302	0.013214919	40.6996337
0.054208	0.015863001	6.776
0.063252595	0.029064553	5.376470588
0.047100718	0.004842693	127.5487445

log.sigma.4.0.mm.3D_gldzm_HighIntensityLargeDistanceEmphasis	log.sigma.4.0.mm.3D_gldzm_SmallDistanceEmphasis	log.sigma.4.0.mm.3D_glcm_SumVariance	log.sigma.4.0.mm.3D_glcm_Homogeneity1
403.6494845	0.961340206	830.8493183	0.302079452
292.5548387	0.980645161	696.253258	0.285048611
336.396648	0.960195531	787.0614255	0.291432606
396.3030303	0.954545455	819.6534406	0.267098707
265.3578154	0.940677966	527.8517417	0.319364052
322.0265487	0.953539823	692.1576053	0.298470571
225.7927975	0.981602296	449.8641197	0.485054249
373.2530831	0.958579088	720.2359376	0.358628161
235.4444444	0.985875706	387.2151154	0.413694132
227.6270492	0.981557377	436.9302459	0.355747509
149.9579288	0.980582524	282.7626226	0.438710193
243.5861183	0.979755784	442.2796358	0.433022676
344.9067524	0.944533762	884.9354782	0.30335361
387.0841121	0.925233645	807.5410341	0.268168119
258.6939502	0.970640569	418.6740849	0.349324578
221.7258523	0.98828125	482.8075786	0.430252046
131.8693467	0.988693467	224.9015605	0.401892881
315.7065217	0.930706522	594.3683649	0.284204927
143.4202899	0.989130435	232.9786583	0.439156157
415.472712	0.984571788	1055.367251	0.379959638
206.9824253	0.978910369	439.345119	0.351361229
251.538835	0.949029126	529.1432227	0.317636072
227.2746114	0.976683938	418.1480155	0.340142875
163.0690789	0.987664474	259.5800056	0.490682169
361.8696682	0.984004739	856.2393411	0.370225923
546.7128834	0.972392638	1207.529078	0.355413594
353.5692521	0.974030471	705.4493862	0.316880577
186.0659722	0.975260417	320.0368335	0.390075956
131.2162162	0.97972973	235.5234553	0.43178062
218.5148305	0.993644068	455.3278654	0.365531896
257.7045666	0.981127679	509.0418299	0.373996803
330.170088	0.953812317	738.7147473	0.28783019
301.4805389	0.942739521	561.4931564	0.374780209
254.2534992	0.988335925	573.5977606	0.408589199
294.7422606	0.977861771	581.9672829	0.381603784
401.2217631	0.915289256	911.4021788	0.316166929
540.6046154	0.848632479	1124.685557	0.275853977
256.0856751	0.977381768	527.4289984	0.454448449
181.4285714	0.934782609	355.6081964	0.355741723
635.3432836	0.97641258	1553.772897	0.454026392
305.5575221	0.956858407	555.0020812	0.329764703
221.154491	0.964071856	389.2182315	0.498157232
376.6804435	0.970514113	910.7360687	0.294808136
293.7918288	0.973735409	565.6843344	0.321571168
196.2179487	0.980769231	340.8889133	0.459099431
309.0465658	0.967694994	626.4719279	0.330468768
327.7485853	0.981810833	680.4834826	0.410126001
161.5602837	1	310.0944583	0.37670162
227.5419355	0.970967742	450.8143133	0.321432926
348.9153439	0.952380952	868.9607501	0.294156272
264.7075099	0.991106719	465.5556572	0.342884757
383.793	0.96175	867.2108741	0.327240778
169.5734072	0.976454294	285.548856	0.539779329
351.9735577	0.972956731	872.5064446	0.386753302
384.5774194	0.956451613	919.9175593	0.270402416
232.3357664	0.983576642	462.5462726	0.32178687
249.5828571	0.978571429	472.0241939	0.397490319
372.4924761	0.97127223	860.9323675	0.319167488
233.1730104	0.942906574	417.0866333	0.323506127
336.9796815	0.976523888	725.4849632	0.493733425
294.2148936	0.979255319	661.0451739	0.344236845
386.8261826	0.96369637	935.0353728	0.318763964
518.4471763	0.935747215	1124.37227	0.353281767
402.6974665	0.918405365	815.6900609	0.336367198
184.9156118	0.990506329	385.3100191	0.34609881
444.9445338	0.946342444	1004.448952	0.311122357
336.9710983	0.926300578	758.9147297	0.29629067
463.0036946	0.945966749	937.2676392	0.345280908
247.3160726	0.980833894	514.0346302	0.423387067
286.3786708	0.964064915	709.3416051	0.349405397
232.2346939	0.977040816	430.1285019	0.314069496
359.9173611	0.969791667	784.870399	0.364958602
243.6375712	0.978652751	462.1240896	0.308843461
177.2810811	0.997972973	358.1920349	0.349710917
352.7578947	0.928460039	575.9880117	0.431084418
164.912	0.985	364.7660495	0.372231417
288.4285714	0.970885093	716.3618486	0.351015707
525.8627451	0.946078431	1238.521407	0.259875181
282.9781659	0.970524017	671.4757029	0.336966295
210.7339667	0.9804038	379.9549631	0.41532031
386.765233	0.969086022	819.517121	0.304732694
239.9087948	0.990228013	657.491187	0.33271735
214.8872283	0.987771739	390.9320543	0.372186759
196.824295	0.982104121	344.3346495	0.391547409
292.7666151	0.955950541	523.2745013	0.403505058
400.5284091	0.944602273	1116.264683	0.325560139
271.0662526	0.967391304	560.3918351	0.338870849
228.7935323	0.989738806	365.5776159	0.465198972
255.1411043	0.97392638	544.8165564	0.381728251
290.555336	0.973320158	638.9368996	0.325100708
509.7053942	0.912863071	1149.777261	0.346956447
253.9892473	0.978494624	440.5437635	0.425304509
231.876588	0.972096189	488.248126	0.38940984
245.0703625	0.97761194	512.5476037	0.371085211
250.1332436	0.960632571	494.6823707	0.401955473
518.6506379	0.919774289	1088.263986	0.285160783
263.0976801	0.954212454	559.6835135	0.333828621
347.512	0.934	801.2694087	0.324249456
197.0823529	0.973529412	479.953956	0.339974112
411.9383309	0.958456425	1065.475299	0.352831777

log.sigma.4.0.mm.3D_glcm_Homogeneity2	log.sigma.4.0.mm.3D_glcm_ClusterShade	log.sigma.4.0.mm.3D_glcm_MaximumProbability	log.sigma.4.0.mm.3D_glcm_Idmn	log.sigma.4.0.mm.3D_glcm_SumVariance2
0.21135972	420.1993334	0.010854172	0.974776363	115.1533723
0.191806199	-120.367978	0.016473738	0.962920018	120.1931222
0.200174292	-178.2307983	0.013129573	0.967807723	104.6963268
0.173545853	-175.6662416	0.014766936	0.962499754	132.5016283
0.226509317	143.379654	0.01017518	0.973763182	102.3352341
0.202676981	-69.20175782	0.01742079	0.962644082	120.0244684
0.421635275	241.6553238	0.081831218	0.990403617	53.7937925
0.276269632	357.4878596	0.023861498	0.979839737	84.68113999
0.338822883	432.8435365	0.042745956	0.979487229	66.659548
0.264993582	133.5854942	0.014954573	0.973052497	64.04058604
0.364900749	87.42201075	0.039107975	0.981295738	35.80993233
0.361329602	200.2921003	0.039645264	0.984960075	54.37557854
0.213416341	-527.2542151	0.014488551	0.968382446	117.8309168
0.1728055	-250.4805452	0.011020309	0.967454417	123.9233342
0.264253436	452.4236234	0.015838325	0.979217536	80.75475857
0.362313731	213.626254	0.062918971	0.977866696	79.33200812
0.318320363	48.37666208	0.020866962	0.972153413	40.50312914
0.19045429	23.41377427	0.014204206	0.96858461	129.5039525
0.365333318	51.36875261	0.032506843	0.978376314	32.90044231
0.297367303	310.2112065	0.020164868	0.988356451	69.01843357
0.263046903	6.263763471	0.011875633	0.971360849	85.74056446
0.223766263	-167.0725908	0.015799179	0.965366567	87.11944309
0.25059805	57.75964309	0.015306475	0.972821899	80.27841591
0.427355384	96.28304875	0.08663548	0.98384163	37.34845578
0.291305923	52.92416913	0.044184206	0.976618089	99.11600983
0.271227028	622.6711949	0.018513427	0.984572805	97.43394235
0.226265899	299.0077881	0.011444435	0.978326131	89.83972344
0.306482373	114.9822746	0.018843158	0.978678595	56.47704394
0.35309616	96.91819867	0.043414156	0.97742421	32.36399963
0.277076239	106.509625	0.014866544	0.98050684	62.14756398
0.291724412	337.6503143	0.013817453	0.979696827	78.53650031
0.193831064	-214.595088	0.010820317	0.961708523	107.3529627
0.292254708	414.0099664	0.017713183	0.985035201	83.65457027
0.332808094	191.7134159	0.033500844	0.982309294	75.75671612
0.299860323	306.2273455	0.018738763	0.982911149	71.53673636
0.22762851	-323.9457608	0.012772855	0.970482987	97.9865034
0.183383768	-579.8736385	0.010269327	0.9641073	124.957339
0.389102383	197.9214438	0.103693529	0.982494569	83.61764896
0.264027919	-114.770583	0.018547505	0.971084679	57.02279612
0.38457863	217.2492618	0.048871155	0.993083752	58.53713827
0.238573262	199.6801662	0.01281704	0.978956527	93.74734971
0.435171364	101.0002711	0.074728283	0.990927807	31.9821731
0.205231196	62.86659855	0.009558841	0.970327309	122.6734065
0.228434999	330.7980468	0.01066169	0.976545335	99.08867696
0.391505698	157.1338163	0.074012035	0.985636706	50.35780075
0.242516258	173.3542983	0.012915935	0.974697681	96.15145692
0.332698845	135.6880301	0.024002685	0.985648484	58.43970276
0.288700112	-73.40922092	0.025851148	0.973762332	48.13646257
0.226677061	417.2687695	0.015148163	0.974180635	95.62805529
0.199136572	-316.1976614	0.013981807	0.96774023	104.3150513
0.255184382	524.7685117	0.017013973	0.97632879	87.17717506
0.23571657	271.9196204	0.010843834	0.981056232	93.84234377
0.487185524	115.2464401	0.099339465	0.989826974	32.76230282
0.304646738	22.63488711	0.016448768	0.984388453	51.74627625
0.176578922	-370.825687	0.010991173	0.967091433	141.7280737
0.229537832	406.4331101	0.009778442	0.9734286	100.6290735
0.319213493	282.704811	0.023807981	0.981378304	71.24709379
0.228151863	5.730560674	0.01003723	0.975567462	81.11155638
0.230033118	-33.04152367	0.013773453	0.967050863	73.73170559
0.431419605	59.8662114	0.104142707	0.989693362	46.75154287
0.255938672	146.4004076	0.015136939	0.978643051	74.29840277
0.227900056	152.4668839	0.014106318	0.980483281	104.6319847
0.269233747	300.0646644	0.017998078	0.985667764	96.72198311
0.245495446	59.81189871	0.011203492	0.98361742	99.67681321
0.255760363	12.83740681	0.016606292	0.972442727	57.64708216
0.218110237	124.2375943	0.009214916	0.981550618	90.45705942
0.202273419	-335.092488	0.014442676	0.96075797	94.2950421
0.256740044	338.087942	0.012511171	0.984967418	80.06427271
0.346588637	151.4295622	0.025823837	0.987321718	56.446931
0.260118487	0.831687609	0.012866658	0.977030819	76.32031143
0.220483321	63.66020775	0.013931733	0.963996062	84.83435213
0.285478773	107.207489	0.033280476	0.980503326	81.25949346
0.217671644	339.4237292	0.013736807	0.969819183	105.0524706
0.259910813	190.300059	0.016021152	0.976314236	65.12986142
0.357691594	364.5940172	0.050567977	0.988859808	68.55632509
0.282539879	-14.96073658	0.017123957	0.971621712	46.84217922
0.260858594	26.47259738	0.011261076	0.982970676	75.80821792
0.166605488	-906.4166787	0.014478703	0.959426018	142.6764503
0.244577559	50.31960618	0.012967133	0.977749495	83.9068457
0.337602499	105.2330719	0.026167685	0.985521801	47.88851871
0.213991151	441.8639813	0.011101794	0.975489992	113.7147489
0.240635183	-179.5611985	0.012946077	0.971966822	81.91500187
0.289259987	175.8700909	0.043846193	0.971678334	73.44142716
0.307816385	85.3647473	0.02058308	0.981739491	47.06214737
0.326522626	283.1543804	0.033641083	0.981318211	72.10358471
0.235457263	-658.7635978	0.025275099	0.975419839	79.80185466
0.250008532	125.6231461	0.014048947	0.97347587	81.99361582
0.400893824	309.9988737	0.068936369	0.983008746	62.99657347
0.299438115	53.463312	0.02304894	0.982088856	59.92150772
0.232659996	150.4644709	0.010809239	0.97631452	94.28724097
0.260179715	-135.2162563	0.017644249	0.977431514	94.18842567
0.351389478	74.80077046	0.034418968	0.982677491	55.2357362
0.305280221	154.3818764	0.013903374	0.987140803	57.81306687
0.285686341	213.8749896	0.015070279	0.9830069	74.85777138
0.318737526	54.83768163	0.016466126	0.98335464	45.62764011
0.192961009	285.7819186	0.007287916	0.978587403	132.0984129
0.242763787	94.21947161	0.009219498	0.975742673	87.8943749
0.230822761	-356.3722592	0.017846639	0.9706812	79.10419019
0.249029737	-262.7273138	0.026245367	0.962418103	60.19997492
0.267148902	66.43216867	0.017846446	0.982581979	79.27411578

log.sigma.4.0.mm.3D_glcm_Contrast	log.sigma.4.0.mm.3D_glcm_DifferenceEntropy	log.sigma.4.0.mm.3D_glcm_InverseVariance	log.sigma.4.0.mm.3D_glcm_Entropy	log.sigma.4.0.mm.3D_glcm_Dissimilarity
33.33274024	3.58626466	0.215028463	8.585223699	4.505658896
34.35255908	3.52142271	0.195006379	7.841816107	4.711867277
33.60723879	3.513614645	0.202703481	8.310255625	4.649855831
39.90527808	3.622624845	0.187643022	7.988358179	5.086031607
23.86008965	3.348155297	0.238645021	8.302185161	3.860385762
28.05361655	3.392063163	0.211437611	7.53721769	4.23096184
13.8412219	2.944664466	0.312327823	7.26529368	2.470275123
25.04042056	3.399574612	0.265186767	8.332196727	3.720886544
17.4292305	3.135806011	0.298798089	7.71710972	3.009693387
16.87767726	3.10597696	0.267244471	7.676031611	3.223051685
9.654479745	2.747599277	0.350462131	7.006033269	2.335265343
14.39589546	3.027228818	0.323370588	7.517020309	2.714616344
33.38167069	3.538815776	0.215585138	8.288686006	4.513353038
38.71061242	3.59906004	0.181437078	8.365771382	5.01176923
26.15869983	3.398534693	0.259372174	8.077737461	3.786062054
21.84194889	3.164720948	0.292256027	7.784991718	3.223119585
11.01254605	2.845780898	0.324011163	7.222836746	2.570448101
35.16855112	3.560900483	0.193274721	8.065378506	4.731987059
9.35237608	2.712674453	0.358854464	6.892152614	2.292727261
18.56617256	3.194814454	0.287693877	8.021566968	3.198706004
19.61289369	3.212963133	0.267646919	8.103709056	3.426215935
23.83920247	3.310664412	0.229471091	7.871682696	3.886268663
21.5325587	3.261290658	0.254741555	7.83529081	3.614310386
9.240377438	2.733000555	0.351980203	6.904891645	2.110413437
27.79834077	3.388322701	0.260170324	8.07539359	3.822099463
23.58638212	3.337902457	0.271012555	8.205487361	3.627281399
28.52294672	3.425563075	0.234042896	8.296440426	4.125527623
14.33136506	3.035352841	0.299839342	7.692608781	2.884417027
8.766952264	2.642164469	0.34042676	6.184503068	2.281661247
16.29240812	3.087122209	0.278744405	7.779774766	3.130195871
19.61006298	3.202795805	0.283459949	8.048790283	3.319051517
33.2795148	3.541913029	0.194697732	8.318849286	4.62546851
19.3465216	3.216482502	0.283011766	8.067815865	3.295138246
18.22522456	3.157108188	0.296352265	7.793985461	3.073555611
18.70443762	3.205625766	0.290916222	8.002085655	3.201215537
31.03671874	3.5057921	0.224759176	8.472357028	4.314599518
43.79052718	3.59148985	0.187496938	8.650505709	5.156666603
20.56158756	3.173257122	0.278023808	7.677758884	3.07118835
15.17663142	2.963589685	0.273565651	7.431157167	3.111717034
12.62267991	2.937327116	0.332948932	7.543077121	2.482215159
22.995162	3.322942293	0.246784124	8.212043624	3.748497345
7.968800319	2.65847163	0.358842073	6.7461366	1.980857291
37.46675379	3.474841811	0.205435482	8.632700799	4.782402052
24.16073315	3.353755339	0.229835767	8.27765449	3.877524256
12.06452235	2.881231964	0.34029051	7.123236861	2.432762484
26.27300617	3.418210981	0.239227259	8.399129932	3.972054737
15.59614333	3.083731859	0.308587409	7.761823488	2.874516178
13.70937375	2.972026233	0.286787707	7.218234571	2.914572032
21.89848853	3.230277329	0.238296618	7.739095385	3.718933457
29.70243877	3.455207759	0.204263729	7.990198702	4.370667388
22.97946555	3.337300086	0.255479303	7.98799748	3.68759286
24.73089065	3.388781155	0.24170643	8.416668195	3.843516607
7.828582748	2.596614067	0.362504212	6.527614005	1.844479454
15.91643951	3.028929247	0.30959141	7.567039676	2.97916281
39.20932137	3.638554211	0.185530238	8.457425375	5.032264104
24.26153215	3.34778155	0.237222276	8.211712833	3.871393429
18.00534028	3.150614431	0.295730817	7.89966434	3.103242677
25.16477811	3.379056846	0.238004923	8.193212638	3.961869108
22.6077445	3.300811244	0.233673036	7.965625409	3.78009281
11.83059597	2.871759948	0.321179567	7.174556411	2.312997153
22.01957892	3.302631508	0.259246885	8.067783951	3.609824953
26.86534498	3.442325278	0.231661001	8.540068053	4.050761811
25.41794783	3.395710753	0.26222568	8.488077619	3.747769528
22.42691422	3.319280016	0.249012839	8.374685619	3.665740804
18.68235192	3.167704364	0.264128961	7.681085556	3.399851118
26.69847486	3.435306305	0.227061433	8.48873407	4.076797062
29.59868003	3.424163017	0.213652803	7.890931854	4.343376676
21.63733704	3.307190274	0.259712641	8.242011106	3.578073935
11.98630305	2.92428161	0.32799941	7.589106066	2.567044108
19.34771072	3.228435734	0.262193056	8.10127386	3.426760202
24.88128721	3.296671941	0.226453232	7.78449414	3.973559808
25.50590041	3.391333897	0.265292225	8.236695341	3.753875634
29.62802119	3.484456174	0.216901723	8.346852131	4.290788787
18.58231668	3.18493282	0.269094653	7.76569241	3.361278224
15.18297171	3.052144219	0.310249056	7.660445228	2.764718197
13.5704091	2.951268266	0.283503032	7.414956328	2.919644281
18.41777938	3.19326377	0.264641854	8.060390613	3.347054831
46.3665114	3.708465799	0.166399742	7.965780928	5.467622416
20.11660221	3.230950836	0.254813897	8.137813073	3.515058486
12.94640982	2.955586157	0.329851958	7.437674368	2.646930838
32.30717906	3.56296423	0.217211091	8.546974513	4.437053999
20.51922466	3.228735325	0.250440221	7.991601488	3.593710525
22.73102299	3.303071539	0.248148635	7.955334247	3.56147637
13.15184265	2.960210884	0.313743796	7.500728849	2.775253955
16.86289673	3.115476468	0.305297471	7.826401369	3.004916729
25.46733899	3.377098496	0.242025985	7.531925919	3.925067701
22.6118466	3.29467852	0.256868832	8.131902398	3.676831337
14.42013522	2.98448795	0.329227482	7.415184164	2.589656525
17.18446732	3.147057276	0.295825137	7.788142566	3.113614629
22.8952846	3.31739214	0.242677616	8.241752508	3.769533602
23.48015452	3.317607416	0.261312454	7.990333543	3.670224423
13.5910304	2.964061364	0.333204179	7.494915073	2.663842144
13.77504942	3.015890043	0.303488929	7.74409845	2.841537656
17.27568586	3.150086455	0.28205463	8.018937352	3.18230768
10.99427578	2.828312923	0.327121708	7.401451833	2.566633626
36.5101475	3.632761548	0.203186775	8.84526808	4.773770138
22.03398543	3.290026684	0.252392681	8.28092449	3.658802685
23.41107216	3.303390897	0.236452034	7.527475597	3.794561015
20.26410974	3.171849276	0.251295066	7.159907728	3.546171031
22.68047927	3.325330022	0.26311298	8.301116122	3.607022816

log.sigma.4.0.mm.3D_glcm_DifferenceVariance	log.sigma.4.0.mm.3D_glcm_Idn	log.sigma.4.0.mm.3D_glcm_Idm	log.sigma.4.0.mm.3D_glcm_Correlation	log.sigma.4.0.mm.3D_glcm_Autocorrelation
12.33748583	0.892898125	0.21135972	0.554565488	278.9163456
10.96058604	0.8690204	0.191806199	0.561324061	236.0797998
10.54933575	0.87744737	0.200174292	0.515979857	264.4083542
12.96225437	0.867820551	0.173545853	0.543006437	272.1522602
8.506978721	0.889393706	0.226509317	0.621696934	187.6721054
9.396453241	0.869024286	0.202676981	0.622070414	235.0853579
7.442153374	0.941628454	0.421635275	0.594514377	162.1618376
10.65571177	0.907912224	0.276269632	0.547503371	246.0872758
7.82541777	0.91012087	0.338822883	0.583753086	142.8703481
6.040390192	0.888810117	0.264993582	0.58173866	158.883426
3.918903944	0.910013643	0.364900749	0.572648135	109.285506
6.789723439	0.922545284	0.361329602	0.58174026	160.4796143
11.77067224	0.881352714	0.213416341	0.562889023	293.6156634
12.08783571	0.875891953	0.1728055	0.531594739	269.7268937
11.15599227	0.906547555	0.264253436	0.515747182	151.6011605
10.16875741	0.911448692	0.362313731	0.571753183	172.6359886
4.244505202	0.888552273	0.318320363	0.573113487	90.54136458
11.84461288	0.878953354	0.19045429	0.575370795	205.004933
3.823527943	0.903959898	0.365333318	0.557219285	92.80199121
7.875115973	0.928204525	0.297367303	0.580556289	344.7487579
7.404059126	0.88742684	0.263046903	0.627686079	160.4308615
8.072117503	0.874019994	0.223766263	0.572908503	187.0589063
7.91387768	0.889396299	0.25059805	0.575936159	152.4615314
4.633277459	0.922199463	0.427355384	0.602678586	101.6507738
12.08023715	0.904169054	0.291305923	0.566025979	286.5622772
9.796017481	0.917951357	0.271227028	0.605089161	389.9015926
10.16678054	0.900803258	0.226265899	0.525945806	240.9491089
5.734347984	0.902988117	0.306482373	0.595704194	122.3561928
3.35128115	0.899349817	0.35309616	0.574327445	91.42295218
6.050373939	0.905077006	0.277076239	0.585607823	165.2101139
7.877732958	0.906845179	0.291724412	0.600830582	182.0328001
10.92990529	0.867320209	0.193831064	0.529959667	249.8083641
7.984567607	0.918879785	0.292254708	0.626018866	198.6791399
8.335778732	0.916100007	0.332808094	0.61325421	201.8254458
8.108219716	0.914752196	0.299860323	0.587828711	204.4183736
11.14644226	0.886004321	0.22762851	0.526299854	302.4498976
12.73520484	0.874271993	0.183383768	0.500495439	363.3259362
10.46551368	0.9219585	0.389102383	0.607770734	186.3831225
4.688382295	0.883311772	0.264027919	0.58355968	132.9694625
6.252389622	0.947047443	0.38457863	0.646858031	486.55251
8.419713067	0.901024023	0.238573262	0.606657807	196.2575436
3.917313561	0.939691725	0.435171364	0.601956256	142.2048478
10.59904767	0.884673443	0.205231196	0.546935576	302.5135573
8.534978221	0.895106435	0.228434999	0.610168499	199.3800133
5.932463951	0.925574234	0.391505698	0.614253964	127.8222527
9.75704842	0.89356478	0.242516258	0.572997261	217.8784279
7.048978377	0.922649063	0.332698845	0.580652266	233.9913988
4.907553868	0.890023127	0.288700112	0.557657911	117.6817795
7.323274644	0.889374064	0.226677061	0.63128771	162.4085842
9.720875886	0.876835828	0.199136572	0.560735857	289.4104161
8.940626882	0.897405315	0.255184382	0.578390152	166.9660119
9.440691129	0.906582063	0.23571657	0.586228104	291.100329
4.303975716	0.940563527	0.487185524	0.615379454	108.9025305
6.175805246	0.917453345	0.304646738	0.537054559	289.3854882
12.82904118	0.875576372	0.176578922	0.571248659	304.1030218
8.67842712	0.889295554	0.229537832	0.610106847	166.483745
7.714852727	0.912608628	0.319213493	0.597299654	170.5176287
8.904064231	0.893192499	0.228151863	0.528508816	287.2191905
7.788797411	0.876882543	0.230033118	0.531714759	152.0389457
6.242693309	0.938915314	0.431419605	0.600267267	245.178994
8.451421418	0.901958701	0.255938672	0.54600042	228.0201759
9.826190574	0.904374158	0.227900056	0.593591283	311.5059596
10.59841391	0.921071203	0.269233747	0.586252973	366.6274816
8.471076658	0.912431173	0.245495446	0.634919162	276.6466642
6.578142671	0.887596983	0.255760363	0.510980046	141.8011502
9.441423329	0.905833538	0.218110237	0.549391588	331.0545231
9.832211003	0.866264365	0.202273419	0.525786512	255.2532326
8.364843539	0.916411145	0.256740044	0.576996119	311.1034239
5.172360074	0.925525817	0.346588637	0.649895484	184.1085478
7.234225491	0.897386651	0.260118487	0.597147538	243.6948645
7.913080981	0.871677522	0.220483321	0.546228548	155.0335596
10.84750184	0.909543534	0.285478773	0.529618195	264.9597452
10.44950721	0.882744696	0.217671644	0.561041223	165.0111079
6.892073711	0.895866442	0.259910813	0.553958715	133.5806119
7.151734797	0.932952692	0.357691594	0.641562036	202.8269531
4.601203512	0.885260056	0.282539879	0.553702604	135.7898141
6.823361989	0.910219732	0.260858594	0.609631644	246.0086174
14.99267568	0.863559605	0.166605488	0.519246387	394.0966225
7.292336651	0.897986158	0.244577559	0.61454814	232.5293163
5.724859456	0.921325456	0.337602499	0.575320993	141.0969853
11.94265355	0.894264593	0.213991151	0.561043584	275.6761196
7.037246013	0.885776398	0.240635183	0.601596094	227.5187146
9.222328315	0.892241667	0.289259987	0.526350726	143.5319503
5.148964695	0.909074869	0.307816385	0.564735248	129.7834975
7.365079001	0.912481987	0.326522626	0.62206539	186.5111543
9.438563097	0.894475514	0.235457263	0.52129231	358.1375429
8.438777304	0.891388981	0.250008532	0.569710439	197.587714
7.479328301	0.922122353	0.400893824	0.626021095	136.1468551
7.160740828	0.911705356	0.299438115	0.554801879	192.4704841
8.220247498	0.894730139	0.232659996	0.61117458	222.2912056
9.171467655	0.900999824	0.260179715	0.605554964	372.9978937
6.119609495	0.916298501	0.351389478	0.605385152	160.463569
5.473575081	0.922487718	0.305280221	0.615105546	175.9566974
6.777043739	0.91209998	0.285686341	0.624577494	183.8153619
4.057862879	0.911815859	0.318737526	0.61082754	177.1110757
12.62192872	0.899148467	0.192961009	0.569885044	355.9931635
8.017990967	0.894677595	0.242763787	0.599999519	197.9726221
8.426986239	0.884523021	0.230822761	0.547596789	267.8545435
6.918904677	0.87071908	0.249029737	0.504401115	169.1969754
9.042377129	0.912038533	0.267148902	0.55984953	348.6348945

log.sigma.4.0.mm.3D_glcm_SumEntropy	log.sigma.4.0.mm.3D_glcm_AverageIntensity	log.sigma.4.0.mm.3D_glcm_Energy	log.sigma.4.0.mm.3D_glcm_SumSquares	log.sigma.4.0.mm.3D_glcm_ClusterProminence
5.40096487	16.31642515	0.003420094	38.18890714	37478.93636
5.299312651	14.69404977	0.00529787	39.33716905	35971.10775
5.286838776	15.88321225	0.003908159	35.95370996	25792.57269
5.346480886	15.96750639	0.004650385	44.45295236	45211.62012
5.298797403	12.98942534	0.003866586	31.97225712	25662.22586
5.207473302	14.60088587	0.006226024	37.63984192	30937.87738
4.770391779	12.43659181	0.018567774	17.68197876	12245.44764
5.198773923	15.36096507	0.004800785	28.41150366	23953.89237
4.948479598	11.44959114	0.008470982	21.45485898	16795.94689
4.945531874	12.12700715	0.006134343	20.22956582	10311.41115
4.55764967	10.13597898	0.012042734	11.36610302	4019.902182
4.839062642	12.29074395	0.010204974	17.54613825	11257.23516
5.31845435	16.69168607	0.004162677	38.76843858	32012.71167
5.37631573	16.02453466	0.003646866	41.90886297	35868.57364
5.107825064	11.85456703	0.005116966	27.99919128	21860.08724
5.073691996	12.63961184	0.010739394	26.00642606	17252.83775
4.659743749	9.11916244	0.008559793	12.8789188	4497.944808
5.37804384	13.53789009	0.00444029	41.88609982	40372.42893
4.500262233	9.322424155	0.012618216	10.5632046	3599.826124
5.042908385	18.42330916	0.006142823	21.50163323	19340.62346
5.186932839	11.99471115	0.00447862	26.33836454	17403.23161
5.147382766	13.08292854	0.005319365	27.73966139	19093.63436
5.093603307	11.74324458	0.005450913	25.61200271	17940.84254
4.548303374	9.727132497	0.018790862	11.6472083	5568.765821
5.270051491	16.579394	0.006678376	32.4940752	27686.80384
5.227065923	19.43098263	0.005214575	30.18929066	31425.99781
5.229339173	15.23410243	0.004256772	30.79798758	24243.41065
4.914304779	10.57409184	0.00654446	17.70210225	9129.950202
4.243075195	9.247726637	0.016958108	10.28273797	2658.786243
4.969959421	12.4126716	0.005845554	19.84421093	11757.1641
5.120194107	13.00009086	0.005104791	25.33873108	17514.09055
5.289402149	15.30019362	0.003771925	36.20720779	25658.15636
5.166428554	13.60906443	0.005500675	26.50750873	23231.10602
5.069632791	13.79653866	0.007251887	24.53500119	14221.27768
5.063012321	13.91773409	0.005821031	23.44021932	15039.40636
5.28522991	17.09150867	0.003654275	33.06049356	25770.45204
5.422419295	18.83753483	0.003215775	42.50684304	43337.02076
5.057247041	13.17891488	0.017353116	27.05700494	21254.77016
4.856610819	11.06646134	0.007201687	18.04985689	8136.317286
4.92411966	21.86333869	0.01050104	18.0330794	13835.95068
5.249002336	13.45659512	0.004224011	30.15013953	21928.7442
4.440458299	11.67579385	0.019538774	10.08012302	4883.739048
5.465077087	16.99298269	0.003251683	40.91596141	40492.93997
5.279991898	13.54595754	0.003960002	32.03963021	25740.94445
4.703401407	10.8788411	0.015406524	15.71090267	11232.06964
5.284280204	14.29048275	0.003849524	31.9680731	22780.55729
4.944444177	15.01597251	0.00691204	19.141881	10842.79975
4.702281024	10.44281374	0.008626924	15.46145908	6007.610957
5.151738343	12.04240037	0.005591499	30.21190564	26537.79855
5.257370439	16.55683783	0.00477119	34.36223837	28250.52668
5.116861195	12.3438252	0.005444648	28.42069711	21997.62638
5.285545363	16.72941397	0.003938967	30.17224224	28032.01373
4.366012791	10.13482166	0.029134214	10.20158093	6183.983769
4.843250539	16.88964706	0.007254172	17.71892768	8633.321628
5.479155538	16.94318032	0.00340563	46.02563779	47647.42982
5.256641398	12.16270943	0.004081248	31.62820368	26221.4469
5.056790994	12.58801305	0.006384005	22.9784264	15353.05218
5.134015633	16.70067468	0.004050748	27.49582947	14446.22257
5.071370648	11.79896718	0.004869048	24.08486252	13757.43537
4.701320167	15.47036931	0.020237079	15.23200032	9204.494643
5.099285369	14.75507805	0.004833392	24.93086433	15437.93785
5.362875102	17.26027789	0.003644003	33.0308778	31863.18603
5.295072159	18.84320661	0.00430703	30.16310563	27291.04649
5.324759732	16.17211665	0.004019266	31.01057783	29658.0925
4.881697047	11.49091817	0.006102328	19.08235852	8697.788126
5.270514329	17.9842293	0.003654288	29.14081554	26130.91787
5.143460533	15.49450342	0.005045774	31.37795558	21634.22057
5.160003834	17.4106343	0.004763663	25.65838954	22688.90245
4.914051193	13.18245917	0.00763632	17.50454448	9973.613972
5.135302696	15.18154704	0.004677339	24.33911584	15255.06024
5.085190059	11.83151734	0.005411043	27.42890983	15255.14153
5.161479376	16.09012639	0.005659584	27.61607407	20396.1739
5.282211107	12.14122523	0.003833123	34.37582036	26376.09355
4.966998257	11.04282829	0.005778749	20.98191667	11749.77229
5.004268017	13.85983165	0.01013243	21.66111267	20456.91477
4.749972065	11.28934945	0.007222313	15.10314708	5303.486085
5.131517776	15.27951486	0.004771134	23.99404905	16000.97213
5.368003347	19.53369553	0.004810141	47.34582574	57782.30445
5.193524749	14.74855541	0.004640884	26.41006165	19434.98723
4.786914546	11.51985027	0.008361262	15.45351316	7967.239334
5.390755732	16.21508175	0.003519789	37.63275203	37252.9878
5.140781013	14.57469846	0.004826709	25.74615526	16854.61336
5.059646843	11.45083682	0.006567365	24.26599808	14543.984
4.785949652	11.01499209	0.007644818	15.08324019	6910.234055
5.042147911	13.17341685	0.007756073	22.7306323	17252.71187
4.93008978	18.73547321	0.008015849	26.5792259	22543.38819
5.163637871	13.5555032	0.00464644	26.72813394	17374.61888
4.876644373	11.15295158	0.015012857	19.70177871	15631.89707
4.945154569	13.52729279	0.006863785	19.78637366	11401.92725
5.258867111	14.37796856	0.004091839	30.22336617	23045.44712
5.209987729	19.02790682	0.005438252	29.9033318	28404.08188
4.869806519	12.2672012	0.009523031	17.50339122	10882.601
4.939317506	12.86429062	0.006187505	18.15435423	10995.67834
5.111094056	13.06090175	0.005154607	23.57710509	15783.14243
4.766675066	12.97867108	0.00748569	14.15547897	5287.528749
5.524563242	18.49198598	0.002707998	42.04249934	47967.73683
5.224032222	13.49932353	0.003963048	27.8506035	18463.50107
4.997157394	15.96731637	0.006593036	25.99793398	17879.3423
4.748477665	12.6147941	0.008809694	20.11602116	9790.547652
5.174071234	18.51304456	0.004681242	25.43315848	19047.99857

log.sigma.4.0.mm.3D_glcm_SumAverage	log.sigma.4.0.mm.3D_glcm_Imc2	log.sigma.4.0.mm.3D_glcm_Imc1	log.sigma.4.0.mm.3D_glcm_DifferenceAverage	log.sigma.4.0.mm.3D_glcm_Id
32.15299846	0.831406554	-0.132483662	4.505658896	0.302079452
29.28922714	0.959025735	-0.278573295	4.711867277	0.285048611
31.40752964	0.86755501	-0.161457853	4.649855831	0.291432606
31.55428815	0.963576542	-0.283489144	5.086031607	0.267098707
25.92484235	0.834402925	-0.14003236	3.860385762	0.319364052
29.12436771	0.967420589	-0.310117534	4.23096184	0.298470571
24.67165041	0.818819626	-0.153338721	2.470275123	0.485054249
30.40883428	0.740470523	-0.099287949	3.720886544	0.358628161
22.85224885	0.775653233	-0.127376515	3.009693387	0.413694132
24.2540143	0.834358737	-0.151452631	3.223051685	0.355747509
20.27195796	0.764973869	-0.133059967	2.335265343	0.438710193
24.53387369	0.78067592	-0.126714484	2.714616344	0.433022676
33.01209314	0.874988465	-0.16628205	4.513353038	0.30335361
31.51732772	0.908677441	-0.190972865	5.01176923	0.268168119
23.49000346	0.799738408	-0.127591944	3.786062054	0.349324578
25.15991328	0.858036236	-0.172368563	3.223119585	0.430252046
18.23832488	0.780049977	-0.128181396	2.570448101	0.401892881
26.93025032	0.947306779	-0.248063563	4.731987059	0.284204927
18.64484831	0.774466093	-0.136678799	2.292727261	0.439156157
36.44901209	0.737741298	-0.104428364	3.198706004	0.379959638
23.9894223	0.805209586	-0.133557237	3.426215935	0.351361229
26.16585707	0.896195036	-0.191263061	3.886268663	0.317636072
23.47076751	0.887770331	-0.185774626	3.614310386	0.340142875
19.45426499	0.796113637	-0.145579177	2.110413437	0.490682169
32.78397466	0.891175327	-0.19092714	3.822099463	0.370225923
38.54409915	0.820387271	-0.13750454	3.627281399	0.355413594
30.04033563	0.798074729	-0.125600974	4.125527623	0.316880577
21.14818369	0.764592458	-0.118268238	2.884417027	0.390075956
18.49545327	0.92177634	-0.27065297	2.281661247	0.43178062
24.79802229	0.799695493	-0.131612135	3.130195871	0.365531896
25.86859481	0.775435186	-0.120979994	3.319051517	0.373996803
30.41272911	0.855938038	-0.151714385	4.62546851	0.28783019
27.02568047	0.815389772	-0.136670115	3.295138246	0.374780209
27.3803042	0.838600241	-0.153882845	3.073555611	0.408589199
27.65545549	0.747978819	-0.106829762	3.201215537	0.381603784
33.80500628	0.789077776	-0.11627482	4.314599518	0.316166929
37.03801864	0.840841083	-0.144980755	5.156666603	0.275853977
26.12353645	0.879516988	-0.184245818	3.07118835	0.454448449
22.13292269	0.847414088	-0.167325336	3.111717034	0.355741723
43.59227538	0.806056283	-0.141091528	2.482215159	0.454026392
26.72444384	0.831408201	-0.140863335	3.748497345	0.329764703
23.34092693	0.763615712	-0.132206729	1.980857291	0.498157232
33.53458745	0.841699604	-0.151312957	4.782402052	0.294808136
26.87912814	0.83483939	-0.140684241	3.877524256	0.321571168
21.74797544	0.843612312	-0.169102663	2.432762484	0.459099431
28.31213684	0.788517964	-0.118249599	3.972054737	0.330468768
29.88467343	0.760383545	-0.114313904	2.874516178	0.410126001
20.88562748	0.857441994	-0.173791106	2.914572032	0.37670162
23.99249058	0.935233933	-0.237767394	3.718933457	0.321432926
32.90431322	0.925911036	-0.220075317	4.370667388	0.294156272
24.56447754	0.830465607	-0.14228109	3.68759286	0.342884757
33.0948784	0.783386703	-0.114540534	3.843516607	0.327240778
20.26489413	0.806238172	-0.158889759	1.844479454	0.539779329
33.49175569	0.795643606	-0.135863831	2.97916281	0.386753302
33.37023924	0.915308507	-0.196989807	5.032264104	0.270402416
24.27704331	0.844780093	-0.149132734	3.871393429	0.32178687
25.07521505	0.78496773	-0.129005786	3.103242677	0.397490319
33.05897213	0.75391462	-0.105174896	3.961869108	0.319167488
23.59793437	0.825378619	-0.139803473	3.78009281	0.323506127
30.75362207	0.820968612	-0.153942397	2.312997153	0.493733425
29.32143231	0.80464346	-0.127648604	3.609824953	0.344236845
34.17906986	0.799526622	-0.120379191	4.050761811	0.318763964
37.3515815	0.750432393	-0.106106917	3.747769528	0.353281767
32.08211467	0.822762795	-0.134483908	3.665740804	0.336367198
22.98183635	0.827156053	-0.146350838	3.399851118	0.34609881
35.50253808	0.743133606	-0.098125248	4.076797062	0.311122357
30.92015417	0.916101152	-0.209998613	4.343376676	0.29629067
34.4378507	0.736174389	-0.100500438	3.578073935	0.345280908
26.30489156	0.778420812	-0.127005674	2.567044108	0.423387067
30.29479644	0.784034015	-0.118622098	3.426760202	0.349405397
23.66303468	0.895288299	-0.193126794	3.973559808	0.314069496
31.68698419	0.766560991	-0.109506336	3.753875634	0.364958602
24.17715588	0.81897478	-0.131088619	4.290788787	0.308843461
22.08137257	0.819331452	-0.138698664	3.361278224	0.349710917
27.53016069	0.826685455	-0.151334368	2.764718197	0.431084418
22.5786989	0.79215323	-0.134026683	2.919644281	0.372231417
30.440438	0.791777565	-0.12391763	3.347054831	0.351015707
38.46609303	0.962495767	-0.281140275	5.467622416	0.259875181
29.43240358	0.828248142	-0.140729409	3.515058486	0.336966295
23.00888569	0.789804633	-0.130745332	2.646930838	0.41532031
31.95741899	0.834435474	-0.134676532	4.437053999	0.304732694
29.13100374	0.844993673	-0.152604911	3.593710525	0.33271735
22.87642327	0.795386041	-0.133668995	3.56147637	0.372186759
22.02706385	0.747549256	-0.113790661	2.775253955	0.391547409
26.28251792	0.815682179	-0.141488374	3.004916729	0.403505058
37.1212138	0.883678206	-0.185870814	3.925067701	0.325560139
27.03454231	0.8138148	-0.134513995	3.676831337	0.338870849
22.27016052	0.840010506	-0.160309027	2.589656525	0.465198972
26.96512302	0.781936195	-0.122055817	3.113614629	0.381728251
28.59499111	0.832625006	-0.140046537	3.769533602	0.325100708
37.69883406	0.886069111	-0.182696513	3.670224423	0.346956447
24.49841255	0.813130385	-0.146944276	2.663842144	0.425304509
25.68613101	0.753321671	-0.111500225	2.841537656	0.38940984
26.0320202	0.785943215	-0.123028342	3.18230768	0.371085211
25.95734216	0.740280255	-0.117550624	2.566633626	0.401955473
36.44513875	0.805951724	-0.120287575	4.773770138	0.285160783
26.94443383	0.773780079	-0.116005206	3.658802685	0.333828621
31.86599472	0.929032315	-0.234192544	3.794561015	0.324249456
25.2295882	0.917145008	-0.229793401	3.546171031	0.339974112
36.57780224	0.719060232	-0.096200982	3.607022816	0.352831777

log.sigma.4.0.mm.3D_glcm_ClusterTendency	log.sigma.4.0.mm.3D_firstorder_InterquartileRange	log.sigma.4.0.mm.3D_firstorder_Skewness	log.sigma.4.0.mm.3D_firstorder_Uniformity
115.1533723	211.7391624	0.353454744	0.045769676
120.1931222	209.7981071	-0.336187365	0.050369989
104.6963268	225.0226803	-0.153011987	0.046650345
132.5016283	222.2261524	-0.210876582	0.044867096
102.3352341	217.8192863	0.01583027	0.049804588
120.0244684	228.0665283	-0.237239657	0.048698859
53.7937925	98.56971741	0.692324181	0.090349599
84.68113999	170.1745577	0.553150486	0.055867929
66.659548	135.922431	0.757681611	0.068692896
64.04058604	179.3789406	0.173420903	0.059813166
35.80993233	104.9674082	0.433036257	0.09230956
54.37557854	109.8364162	0.529091347	0.076385063
117.8309168	245.8794632	-0.457880441	0.048845939
123.9233342	242.9623966	-0.229074249	0.043777246
80.75475857	176.2861052	0.683997267	0.056875586
79.33200812	158.9812584	0.323315448	0.065605474
40.50312914	132.5114861	0.110018788	0.073820459
129.5039525	232.8814373	-0.114037467	0.046131958
32.90044231	99.03547096	0.292471283	0.08940072
69.01843357	147.6985798	0.373983385	0.061871865
85.74056446	206.1588669	-0.090824927	0.05293922
87.11944309	172.3822975	-0.367665363	0.058565297
80.27841591	176.934206	-0.026392071	0.05615935
37.34845578	82.5219574	0.51488133	0.103655171
99.11600983	203.0811892	0.129922914	0.052076149
97.43394235	197.9487877	0.529701912	0.05318181
89.83972344	207.5400753	0.406560477	0.051058064
56.47704394	150.9004745	0.316271172	0.065236094
32.36399963	131.3914728	0.466342796	0.092137362
62.14756398	163.0438919	0.182017887	0.061643455
78.53650031	179.6629791	0.457329385	0.056152343
107.3529627	242.4239931	-0.324437115	0.048664614
83.65457027	163.6153564	0.538498432	0.057624364
75.75671612	170.2596016	0.3712264	0.063712048
71.53673636	166.3305588	0.55643809	0.060717773
97.9865034	209.9522209	-0.314974809	0.049334246
124.957339	228.4315519	-0.503449943	0.045256117
83.61764896	137.2924194	0.343250145	0.077311292
57.02279612	155.69522	-0.397224791	0.070408163
58.53713827	115.5400696	0.517113897	0.078423436
93.74734971	211.9669333	0.223416179	0.050744041
31.9821731	75.47616196	0.658557763	0.112765912
122.6734065	242.204464	0.033067175	0.043574829
99.08867696	211.234416	0.30450179	0.049788732
50.35780075	101.7668161	0.473532174	0.088767422
96.15145692	204.0144653	0.260479875	0.050130959
58.43970276	140.9276409	0.385906728	0.067338088
48.13646257	134.6524582	-0.260665772	0.074418971
95.62805529	175.2865448	0.278964973	0.054794252
104.3150513	190.3673878	-0.418889176	0.050933902
87.17717506	206.5166225	0.504665412	0.054096038
93.84234377	193.6595459	0.307158222	0.051259085
32.76230282	71.95311737	0.663215213	0.119349963
51.74627625	143.0841236	0.181462513	0.068831866
141.7280737	262.5405884	-0.28581176	0.04118283
100.6290735	215.3577862	0.261014898	0.04882225
71.24709379	154.7359943	0.493787669	0.061619797
81.11155638	226.6470146	0.029208074	0.051579068
73.73170559	181.7206564	-0.111228244	0.056201893
46.75154287	103.5994186	0.38155124	0.092586155
74.29840277	178.4845352	0.273173401	0.056492291
104.6319847	205.0227127	0.113955966	0.047817683
96.72198311	194.035677	0.242263086	0.051373243
99.67681321	179.6898785	0.054701681	0.051373754
57.64708216	168.5650406	-0.015171575	0.060111841
90.45705942	198.7907257	0.071914724	0.050103471
94.2950421	194.5868744	-0.535070398	0.055279187
80.06427271	171.244072	0.482509521	0.057167349
56.446931	143.4276457	0.413430742	0.069777994
76.32031143	173.400219	-0.097087697	0.056318516
84.83435213	217.8057327	-0.048338701	0.053680019
81.25949346	159.3252869	0.263048288	0.059830206
105.0524706	251.6216774	0.219807607	0.047921559
65.12986142	183.3742018	0.300708509	0.058504155
68.55632509	110.9077587	0.816887118	0.078864775
46.84217922	144.7583332	-0.091616651	0.069835438
75.80821792	183.447403	-0.046271479	0.055886645
142.6764503	218.9370365	-0.770833648	0.050810417
83.9068457	170.0372543	-0.03764449	0.055039107
47.88851871	129.3958626	0.32123329	0.073817284
113.7147489	208.4129791	0.373590919	0.046505628
81.91500187	187.4762726	-0.325519716	0.056320316
73.44142716	191.223382	0.241588158	0.057978033
47.06214737	131.5185919	0.274966895	0.072081115
72.10358471	138.39674	0.486577822	0.06628247
79.80185466	151.2225418	-1.082658298	0.077255365
81.99361582	188.2165232	0.108792022	0.05500145
62.99657347	110.4098492	0.627132486	0.080444197
59.92150772	144.5872002	0.114718673	0.065746504
94.28724097	203.5774155	0.104267799	0.051086617
94.18842567	176.5250072	-0.172397572	0.05430868
55.2357362	121.027071	0.319058681	0.074016569
57.81306687	151.5788612	0.325352022	0.066045261
74.85777138	169.8689556	0.347994599	0.058992586
45.62764011	144.0855064	0.128081795	0.070747566
132.0984129	255.3036079	0.068692483	0.041750301
87.8943749	209.4644222	0.080240179	0.051045656
79.10419019	181.0638156	-0.619571775	0.062560606
60.19997492	142.8048754	-0.803707144	0.077940521
79.27411578	175.5550804	0.066215157	0.054343646

log.sigma.4.0.mm.3D_firstorder_MeanAbsoluteDeviation	log.sigma.4.0.mm.3D_firstorder_Energy	log.sigma.4.0.mm.3D_firstorder_RobustMeanAbsoluteDeviation	log.sigma.4.0.mm.3D_firstorder_Median
126.2691393	40287174.01	89.17963873	-70.54283524
124.8529101	9034188.572	88.49629375	-24.22523689
124.7871685	20221048.38	93.15176135	-1.46702835
134.1437206	9459338.446	93.84774923	-29.12835598
118.1885181	28608672.82	87.95627473	7.519221306
127.5207428	5716223.215	94.66862727	22.73516178
74.12432004	112185136.1	45.22022495	-8.418064117
104.0706152	112458540.7	71.13353829	-34.93615341
89.11285264	59308129.9	59.41400903	-31.15486813
97.57082682	10937184.94	72.57264581	22.3481636
65.07962161	11248648.13	43.73417168	5.614125967
78.91716595	37772346.81	50.48050971	-8.296096802
132.0308448	21013322.21	100.08556	-2.262992859
136.3873649	22497362.45	102.7168306	-68.95158768
104.6006556	32800063.74	73.78851449	-78.46102905
96.82659755	90277808.2	66.33293335	-4.300155401
75.796338	7673167.701	54.41149143	-3.094323874
133.0512212	14463702.54	97.05547993	-83.68445587
64.66733145	8229627.958	42.64622794	-26.50182438
93.35322517	139898293.9	61.84962208	14.53067493
110.8622581	36259212.04	83.4079437	14.9090414
105.8337304	8630996.728	74.3896758	2.801261067
102.661261	9481296.288	72.70717708	-26.22268963
61.37004278	26510683.16	37.58395624	-13.63275528
117.020166	28149184.54	84.00185368	-45.09950829
115.5547923	52714696.8	81.28771486	-23.65532017
115.7140936	45587138.11	84.43097567	-71.16275787
86.96873122	29435599.13	62.00917449	-40.5153389
67.536369	1323771.626	48.60884999	34.84669113
92.07601349	21568905.44	66.06634392	-16.05925274
103.158589	68821121.24	74.82574605	-49.42379761
126.4461508	23931382.91	97.16456794	-70.18219757
101.6408502	39824555.69	69.79092431	-36.40133286
95.07169682	34489432.21	69.01720706	-17.61371422
97.18717558	76501997.38	68.16667528	-29.1986351
116.5743384	40919515.68	85.08172101	-20.14752197
132.4148837	48514961.82	94.51766047	-49.24699402
95.45550184	94198244.16	63.14513739	0.039333027
85.23918461	7098976.985	61.74387992	-26.25557899
76.84467799	102320450.7	49.52076547	-12.82324982
115.9979415	31718664.29	87.07904171	-72.80480957
55.2441619	34234054.28	33.10442079	1.83191818
131.4510087	61077857.63	96.77079115	-8.402295113
115.6741169	30397668.36	85.42799551	-48.97872734
72.26459608	15809175.65	43.42295576	-8.370322227
114.8043924	46431758.2	84.10033581	-18.63955688
85.26827357	62530566.51	59.28307547	-9.810009003
80.38500945	5651615.551	56.32950155	-24.32400513
107.8434885	7073198.88	75.53669747	5.137577057
114.5775813	9242742.861	80.81186527	2.74335146
114.3772379	28497707.35	85.48346247	-85.5966568
111.9510425	56378209.49	79.20232293	17.48047447
55.83610837	51924264.34	32.50369446	-4.732750177
83.61384893	18561851.17	58.65889092	-22.26714134
143.1515653	19040233.75	106.385764	17.58605766
119.3548532	24654169.9	88.85435958	-23.23920441
94.42159216	57615626.57	66.39558904	-24.71305847
116.3673808	41710114.72	91.74730137	-48.20438766
103.7771573	15992339.2	77.03762143	-61.04524612
69.16804062	99645188.27	43.01631371	0.641360581
100.7958669	21014669.62	71.98592436	-25.50425911
117.8705582	54068625.6	83.37608454	15.37764072
113.0852957	162519332.2	80.09327627	-6.160000086
110.6288565	40828896.66	76.12514581	0.396014899
93.56182002	9032090.453	67.43179598	-16.72692299
113.8099008	67432826.45	81.92869389	-4.811909676
114.416898	11974105.25	83.66160514	-73.55862427
102.5969174	88546608.57	71.35209663	-42.77553558
83.34552946	81452576.25	59.34369941	-9.362093449
101.0304887	33284264.97	72.52205536	26.53838253
115.1985652	9850034.434	89.70208422	-42.9766655
101.5162314	85484521.9	69.70099228	-7.706257343
128.1366645	32727651.53	100.1780293	-53.84558105
99.23785574	16223751.24	73.47569404	-61.05987358
80.64145812	51504029.47	49.43183767	-16.41531277
80.90990717	8729663.359	59.90890126	5.64717555
101.9104175	34347831.97	74.30765829	-1.767778993
136.745728	10975981.47	93.21395914	-28.97210503
102.9977021	23404916.76	72.18505074	41.73491287
77.31091413	20709368.06	53.34541138	-41.72647858
124.5793861	38963447.78	87.58719895	-74.12563705
104.9042488	13510281.83	76.71658778	-0.682727396
103.7950473	39375873.66	77.32051114	-40.12779808
78.92960981	23606637.54	54.92479328	-56.08880234
90.19027183	38037559.08	58.86555006	-15.55037117
102.8462284	9730540.349	67.30236259	9.048331261
104.5411117	23024172.04	76.14776381	-46.22221756
82.05945369	48427387.03	51.02009585	-7.885185242
88.42020553	22007439.64	60.7999397	-30.04524994
112.8258563	25910977.98	82.20517955	7.226892471
108.8675619	15680040.55	74.39577655	17.30229664
79.60601353	29100842.97	52.06960925	-22.73117828
86.17871301	51724251.74	62.24810167	-17.83931351
96.68676788	50070675.5	68.91752161	2.50009799
79.51045685	30734155.92	58.52101188	-8.789875031
138.5353886	66845060.69	103.5877759	-11.64147758
111.9887357	43712831.81	84.62511211	-0.430930451
102.0278744	7709600.919	73.07626701	-81.69120789
87.65662342	3315912.836	61.2803729	2.989732504
103.8942316	153735319.3	72.92696784	21.51839542

log.sigma.4.0.mm.3D_firstorder_TotalEnergy	log.sigma.4.0.mm.3D_firstorder_Maximum	log.sigma.4.0.mm.3D_firstorder_RootMeanSquared	log.sigma.4.0.mm.3D_firstorder_90Percentile	log.sigma.4.0.mm.3D_firstorder_Minimum
1087753698	424.0751648	167.0320478	171.5050461	-434.9532776
243923091.4	281.1963196	161.354038	136.5735443	-422.7549744
545968306.3	369.1703491	150.5633978	181.3778275	-394.4076538
255402138	317.5029602	171.3966962	179.6745117	-432.9391479
772434166.2	393.3486938	142.3919209	175.8167267	-300.213623
154338026.8	299.4476013	153.6904587	210.7684891	-349.7799072
3028998675	614.6448975	103.173451	135.574118	-289.027771
3036380600	440.7656555	136.1361411	147.7946991	-395.0310669
1601319507	389.0384827	117.964245	146.9007233	-291.2433777
295303993.2	305.9439087	120.3592026	193.9697174	-252.1726837
303713499.6	324.8802795	85.02475118	133.6639328	-208.4039764
1019853364	443.2417603	105.2933437	138.2622253	-286.2835083
567359699.8	305.741394	160.9670307	141.4339752	-432.323822
607428786.3	306.2570496	186.9058389	107.4431046	-479.7702637
885601721.1	484.4734497	144.310212	120.9867325	-325.7639465
2437500821	433.5355225	125.1710445	188.2398132	-280.8027039
207175527.9	233.2618561	93.21954809	122.3572998	-204.4493561
390519968.5	327.962738	188.0520563	104.1160034	-429.7589722
222199954.9	233.6549683	86.73241519	85.76090012	-225.2739258
3777253935	530.2390747	123.428064	171.1732239	-403.267334
978998725.1	321.8068848	132.0950092	166.5154404	-283.2482605
233036911.6	263.7513123	131.9136198	144.5009155	-344.3067627
255994999.8	330.6473694	130.3517181	129.952034	-322.0176392
715788445.2	320.6708374	85.84998838	93.03680267	-244.1469421
760027982.6	357.6663513	151.4027368	164.0606201	-444.6971436
1423296814	458.2610168	143.6102479	216.1515274	-457.1113586
1230852729	447.4501648	150.8246429	136.1496521	-402.2316895
794761176.5	316.8258667	114.3025302	105.385495	-297.883606
35741833.9	281.0547485	95.22042294	163.4387665	-150.9983978
582360446.8	363.4380188	115.7447004	123.0717819	-307.6620789
1858170274	392.8161926	131.0215835	146.8299072	-333.8278809
646147338.5	211.5415955	174.2692608	90.84296112	-469.4194946
1075263004	514.6450806	130.5966471	158.1271057	-329.7631836
931214669.7	419.5223999	119.5045538	154.2543549	-342.9489136
2065553929	437.7695923	122.0817525	159.1435699	-334.8326721
1104826923	317.4692078	145.2701343	148.1116425	-442.041748
1309903969	268.3015137	179.1273708	117.8965088	-548.0222778
2543352592	487.3226929	127.2103959	176.9825897	-313.7612305
191672378.6	201.6334686	112.5910503	80.38534546	-314.5253296
2762652170	495.8433533	104.0720872	113.9235367	-525.2261963
856403935.8	391.8666382	153.852673	117.4343811	-388.7819214
924319465.6	430.131897	77.68927281	102.1771126	-270.8396606
1649102156	403.8842773	158.9328786	196.5080521	-409.6533203
820737045.6	385.601532	146.3106582	148.5146454	-356.1316528
426847742.6	400.9717102	100.7975128	104.6062965	-265.3331299
1253657471	400.1410217	139.0629649	191.1786194	-338.9881287
1688325296	419.257782	108.1007498	130.0346375	-366.390625
152593619.9	230.746933	107.2866191	74.13272095	-292.4429932
190976369.8	385.4519958	134.4991551	179.7395172	-285.2260742
249554057.2	299.4099731	142.9984131	165.6198959	-410.3894958
769438098.5	358.5541687	148.8043708	139.0371277	-352.5092163
1522211656	495.4399414	142.2797324	221.6429321	-366.8484497
1401955137	406.6934814	80.68496287	83.72771835	-244.0231781
501169981.6	336.8690491	106.3222275	113.661676	-406.6059265
514086311.2	387.9840393	172.0801007	221.5625763	-410.3495178
665662587.4	399.4164429	144.7909879	169.2540436	-315.0031738
1555621917	407.8511658	119.791877	148.9715149	-311.8688965
1126173097	317.2944031	142.0530144	133.9416656	-428.2269897
431793158.4	240.3786926	144.7750513	83.52312775	-374.7554016
2690420083	425.7626953	95.6432696	111.6534729	-350.1290894
567396079.8	391.3566589	125.7473359	144.7386475	-353.5632019
1459852891	488.4878845	148.1031185	213.1228485	-394.2730713
4388021969	554.4678345	140.4905405	205.887439	-440.5011902
1102380210	484.3884888	139.568748	181.2188492	-383.5427246
243866442.2	317.1088867	115.5047394	136.2653564	-287.5032959
1820686314	472.8664246	140.3974257	163.0159149	-441.2908936
323300841.7	149.615097	171.7348831	56.59980583	-479.4120789
2390758431	464.0297852	131.9594471	153.5841492	-429.6397095
2199219559	409.2507324	104.157436	126.6561722	-319.2648926
898675154.1	343.0302734	125.1820989	173.1085419	-347.2637939
265950929.7	256.6573181	141.4934458	128.8678726	-333.0393066
2308082091	454.6965942	131.0042764	162.953949	-375.6911621
883646591.2	386.7478943	156.5729695	150.8372223	-347.765625
438041283.6	335.4458618	130.6814175	108.9028366	-307.6215515
1390608796	538.3688965	112.2994779	133.6717941	-332.0502319
235700910.7	249.0591278	97.5163433	127.1969009	-264.7533264
927391463.2	416.1325378	124.6957946	147.7555481	-372.1721802
296351499.8	214.534729	184.3403558	105.0092072	-574.3199463
631932752.4	386.8916931	133.4107034	205.6484497	-304.5531616
559152937.6	376.7010803	107.2623162	83.88232956	-321.6419983
1052013090	424.0751648	166.4700191	169.9408722	-431.5130615
364777609.5	264.9568176	127.8916471	137.7919159	-351.2741394
1063148589	345.6013184	131.358228	130.4262192	-321.905426
637379213.6	320.5197449	111.6713481	86.82641144	-310.2997131
1027014095	380.2282104	118.3644823	155.4384277	-303.4093933
262724589.4	250.0029602	135.3696227	88.74176788	-485.02948
621652645.2	317.4883423	132.6747075	127.9169403	-372.0228882
1307539450	411.2489929	112.3585741	149.824707	-256.9203186
594200870.3	391.5532227	113.7450754	130.5646515	-341.4146118
699596405.6	395.6189575	137.6758116	175.7433777	-345.2795715
423361095	324.595459	139.6514877	215.475322	-445.3809204
785722760.3	335.8049011	107.2064547	111.5715668	-324.6665039
1396554797	458.5746765	107.199461	124.2121964	-312.0631409
1351908238	450.357666	120.5408053	178.9434357	-292.561554
829822209.8	291.6941223	96.40366561	125.4046158	-302.9229126
1804816639	520.9736938	167.4486056	191.3164322	-463.7131958
1180246459	378.8035278	133.5193588	180.3897141	-308.7012024
208159224.8	162.7716827	156.9436614	46.27994461	-476.3115234
89529646.56	178.353363	111.2331295	86.69478302	-329.2181396
4150853622	425.9917908	131.9939658	190.5977615	-408.8885498

log.sigma.4.0.mm.3D_firstorder_Entropy	log.sigma.4.0.mm.3D_firstorder_StandardDeviation	log.sigma.4.0.mm.3D_firstorder_Range	log.sigma.4.0.mm.3D_firstorder_Variance	log.sigma.4.0.mm.3D_firstorder_10Percentile
4.647613207	157.0106001	859.0284424	24652.32855	-248.6927933
4.53763855	154.4589268	703.9512939	23857.56008	-273.9466125
4.555985779	150.0590655	763.5780029	22517.72315	-224.0689026
4.67474249	165.5670619	750.4421082	27412.45199	-279.9311615
4.475564697	142.3508635	693.5623169	20263.76834	-205.816391
4.466139759	152.8090046	649.2275085	23350.5919	-192.8320587
3.929780776	102.9692419	903.6726685	10602.66478	-131.5880737
4.407380356	133.0908167	835.7967224	17713.16548	-189.5464432
4.149106229	116.192701	680.2818604	13500.74378	-156.4773483
4.210238251	117.5142564	558.1165924	13809.60046	-121.5189377
3.73142506	83.4124486	533.284256	6957.636582	-86.92258072
4.04427603	105.2732306	729.5252686	11082.45309	-137.0095245
4.53059653	156.1724023	738.0652161	24389.81924	-278.4882507
4.651295946	162.2285482	786.0273132	26318.10184	-335.3105835
4.345422635	131.151613	810.2373962	17200.74559	-214.5503754
4.24170744	125.1605188	714.3382263	15665.15547	-164.2486755
3.918466342	93.21092693	437.7112122	8688.276899	-129.9381744
4.626399278	161.8744116	757.7217102	26203.32512	-336.430127
3.747316315	83.62023602	458.928894	6992.343872	-129.66577
4.304799433	121.9003996	933.5064087	14859.70742	-132.7194672
4.366313408	132.0814148	605.0551453	17445.50014	-192.9800461
4.323334773	131.2574715	608.058075	17228.52383	-211.227356
4.336808196	127.2894384	652.6650085	16202.60113	-197.8628937
3.696394226	84.59765249	564.8177795	7156.762807	-120.2349289
4.500055638	144.8379132	802.3634949	20978.0211	-221.156189
4.483293223	143.5895867	915.3723755	20617.96942	-160.39991
4.490052035	141.2594351	849.6818542	19954.22801	-220.4160934
4.124596483	107.734536	614.7094727	11606.73024	-174.589447
3.628879542	82.99531017	432.0531464	6888.22151	-47.00176239
4.209806612	114.179985	671.1000977	13037.06897	-170.1938812
4.320963187	126.330304	726.6440735	15959.34572	-189.8854401
4.492486964	149.0839239	680.9610901	22226.01637	-299.9802765
4.360893002	129.0272879	844.4082642	16648.04103	-170.9011627
4.193849382	117.9384553	762.4713135	13909.47923	-164.5681396
4.267460867	121.4304995	772.6022644	14745.36622	-154.3807068
4.501692323	142.5376382	759.5109558	20316.97829	-228.026651
4.656127243	163.0449678	816.3237915	26583.66151	-312.7703247
4.190733061	126.9418874	801.0839233	16114.24278	-176.8843994
4.030761765	104.4830123	516.1587982	10916.69985	-193.7532471
4.025802228	103.0488867	1021.06955	10619.07304	-136.9141724
4.457957959	138.0725677	780.6485596	19064.03395	-241.2820129
3.570534083	77.41798191	700.9715576	5993.543923	-81.06867752
4.68001719	158.7521324	813.5375977	25202.23954	-212.3502747
4.479798055	140.2014981	741.7331848	19656.46006	-221.1707199
3.918324246	100.470201	666.3048401	10094.26129	-140.5387497
4.473968888	139.001855	739.1291504	19321.51569	-181.1162262
4.125354939	107.7690472	785.648407	11614.16753	-146.0289154
3.975204432	99.9711135	523.1899261	9994.223535	-183.6386414
4.395745048	134.4219287	670.6780701	18069.25492	-174.7398071
4.475448435	142.6838022	709.799469	20358.66741	-204.8487442
4.391564283	136.7591388	711.063385	18703.06204	-217.5810303
4.499181361	139.2028593	862.2883911	19377.43604	-137.4630096
3.563347733	80.34298676	650.7166595	6454.995522	-109.982811
4.097560415	104.7270124	743.4749756	10967.74712	-143.0901169
4.716447697	172.0692104	798.3335571	29607.81316	-249.1883575
4.479553162	143.1881528	714.4196167	20502.84709	-207.1662216
4.235029192	118.4238317	719.7200623	14024.20392	-164.1294708
4.373402762	134.9460778	745.5213928	18210.4439	-224.297525
4.301261596	124.0849558	615.1340942	15397.07626	-241.8962433
3.883681707	95.6004595	775.8917847	9139.447856	-122.0563232
4.338342137	124.7013569	744.9198608	15550.4284	-175.9829193
4.582773574	147.1177819	882.7609558	21643.64176	-179.7519623
4.505581799	140.0220005	994.9690247	19606.16063	-159.6084473
4.507389672	139.5584492	867.9312134	19476.56073	-181.9848557
4.209095533	114.426347	604.6121826	13093.38888	-165.0639954
4.518041387	140.0619638	914.1573181	19617.3537	-192.1325073
4.369569746	138.6121387	629.0271759	19213.32501	-307.3003845
4.37761624	129.1830753	893.6694946	16688.26696	-175.8630341
4.062610893	103.7941463	728.515625	10773.2248	-135.8900177
4.320788209	123.8170186	690.2940674	15330.65409	-155.7779572
4.343671514	133.8538281	589.6966248	17916.84729	-228.8431854
4.354349613	130.4560831	830.3877563	17018.78961	-189.6647186
4.510829815	149.3340102	734.5135193	22300.6466	-232.4970245
4.23838588	119.6056109	643.0674133	14305.50216	-198.6762573
4.082443888	111.7063217	870.4191284	12478.30232	-138.6716553
3.984998392	97.49182751	513.8124542	9504.656432	-130.4322632
4.340986976	124.6193379	788.304718	15529.97937	-167.9347778
4.572635847	169.1346992	788.8546753	28606.54647	-316.8282166
4.386812222	128.2713264	691.4448547	16453.53319	-140.9210236
4.004359916	98.53302682	698.3430786	9708.757374	-165.5860336
4.630781825	155.4789926	855.5882263	24173.71714	-247.1667938
4.314655943	126.6328052	616.230957	16035.86735	-198.6372452
4.291049121	125.2258588	667.5067444	15681.51572	-197.571553
4.011517639	99.28834319	630.819458	9858.175094	-176.0654358
4.218653152	118.2221614	683.6376038	13976.47945	-151.8836823
4.137883087	131.8253483	735.0324402	17377.92246	-238.7417145
4.355961277	126.7128408	689.5112305	16056.14403	-197.2545731
4.06129861	112.2994189	668.1693115	12611.15948	-148.2762604
4.180781915	111.8198064	732.9678345	12503.66911	-161.9363251
4.455244948	137.6754353	740.8985291	18954.52547	-188.198703
4.428612031	138.826746	769.9763794	19272.86541	-139.9123184
4.064467863	104.7780159	660.471405	10978.43262	-153.1530319
4.113748747	106.4119385	770.6378174	11323.50066	-146.0971069
4.274309737	119.7702222	742.91922	14344.90613	-139.5966797
3.970704762	96.22486004	594.6170349	9259.22369	-127.9521973
4.740088646	166.3329429	984.6868896	27666.6479	-239.2543839
4.41707896	133.4517183	687.5047302	17809.36113	-171.037178
4.229312531	124.9273899	639.0832062	15606.85274	-268.3893616
3.969700527	108.4837221	507.5715027	11768.71796	-178.9765137
4.413999647	130.5071912	834.8803406	17032.12696	-157.3191666

log.sigma.4.0.mm.3D_firstorder_Kurtosis	log.sigma.4.0.mm.3D_firstorder_Mean	log.sigma.4.0.mm.3D_glrlm_ShortRunLowGrayLevelEmphasis	log.sigma.4.0.mm.3D_glrlm_GrayLevelVariance
2.682771267	-56.98575645	0.008785175	40.11163068
2.567524238	-46.66439206	0.02061955	38.1611963
2.262480448	-12.31314767	0.010850898	36.02446067
2.550384144	-44.32127571	0.016969922	44.63065612
2.313056233	-3.41917868	0.014469189	32.37009407
2.22331196	16.43670258	0.015219812	36.82555382
4.36399164	-6.488159671	0.010062694	19.30315623
3.287288455	-28.63360644	0.006612988	29.59641931
3.502204408	-20.36711355	0.012337317	22.8287374
2.301713222	26.01417303	0.012602479	22.33665129
3.159566617	16.47943358	0.0143081	11.60419548
3.687803993	-2.057946632	0.010074346	19.035399
2.227815222	-38.99443208	0.010404753	39.30814637
2.205863897	-92.82074545	0.009439041	41.79513841
3.316998501	-60.20541263	0.012894597	28.21272858
2.704825135	1.623243739	0.012468132	26.84683024
2.458140616	-1.267772387	0.02544844	14.2423425
2.371595527	-95.70919898	0.021797398	41.91155137
3.190053257	-23.02537671	0.018765064	11.7573199
3.694669016	19.35922397	0.003878386	25.02425578
2.193943105	-1.895076479	0.018510008	27.92367268
2.556324818	-13.14074754	0.017627708	27.64011649
2.673568048	-28.08859701	0.025658638	26.19630575
4.126151601	-14.61019159	0.018071378	12.60203455
2.568145819	-44.09951942	0.008024416	35.19475851
2.951059863	2.435952903	0.003727003	33.81459801
2.805603194	-52.85683404	0.007402721	32.49823626
2.819831623	-38.18819418	0.01812709	19.09261557
2.679187184	46.67662622	0.022208125	11.22202275
2.829362952	-18.96751719	0.010995873	20.99698989
2.669618986	-34.74636122	0.009349835	26.00571846
2.185486856	-90.24277748	0.015661218	35.49718838
3.254378375	-20.18522247	0.009454096	27.33631381
2.682768678	-19.28365014	0.0084609	22.99202075
2.94640673	-12.59317562	0.007622928	24.5415573
2.499144383	-28.04342372	0.006808618	32.83889927
2.64015928	-74.18189435	0.006477565	42.79698802
3.088976579	-8.26087407	0.012261924	28.57375255
2.53214906	-41.95288747	0.020247962	17.75295916
4.346604724	-14.55768846	0.002224351	18.32933716
2.396697313	-67.8720196	0.011504368	30.88449796
5.225040908	6.486847222	0.009556053	10.62252371
2.452115089	-7.57762193	0.007573464	40.44435523
2.517887442	-41.83716814	0.010398497	31.57244026
4.33359573	-8.116483235	0.0169787	17.71308589
2.443772149	-4.122197702	0.009792389	31.18249922
3.2799933	-8.461948437	0.005522847	19.35239185
2.693338377	-38.93835003	0.021228076	16.06921408
2.80748457	4.557169813	0.01708527	29.07954046
2.716624175	-9.480439723	0.010516175	33.40777177
2.503175133	-58.64877422	0.010841784	30.25200291
2.90919622	29.42934288	0.007147494	31.52346825
5.450662644	-7.420762149	0.014861591	12.07110139
3.283750557	-18.34854059	0.004984038	18.20047664
2.225268655	-1.935945303	0.011819288	47.34169322
2.3647252	-21.48448504	0.015584389	32.88486621
2.958891526	-18.05242012	0.009795717	23.27946545
1.98811595	-44.36907689	0.005516481	29.25895233
2.342089155	-74.5837731	0.01946227	24.87440045
4.353563398	-2.8613219	0.005042112	16.73443171
2.798589466	-16.18530469	0.007595242	25.39699626
2.695036769	17.05555478	0.00671484	35.32297063
2.765457887	11.46435058	0.003856087	32.36620681
2.910176066	-1.695492624	0.007070207	31.61458426
2.467947337	-15.74661737	0.020151188	21.49285987
2.837861238	-9.699661753	0.005308781	31.73552723
2.490070391	-101.3880913	0.010142701	30.92156265
3.244743909	-26.92635756	0.004549852	27.38778449
3.201829382	-8.691758973	0.007805111	17.79192404
2.515556894	18.43648029	0.007243549	24.76910156
1.989417794	-45.86445135	0.017207165	29.00271548
2.98656878	-11.97208548	0.005872249	28.2709519
2.140989012	-47.05792365	0.019896866	35.90880827
2.453923879	-52.65102784	0.017160743	23.11992532
4.823256871	-11.52694339	0.007629242	21.58863238
2.382828306	2.186499208	0.014245413	15.4359276
2.69687312	-4.365983429	0.009863203	25.20949953
3.04170546	-73.31316609	0.009595552	46.22253975
2.695248823	36.672641	0.009014761	26.7674684
3.457544797	-42.38451491	0.012610428	16.19420602
2.719449137	-59.48571371	0.008188103	39.3231927
2.420801099	-17.89988976	0.009907385	25.66633219
2.46720457	-39.66696804	0.016006841	25.81274139
2.925751124	-51.11080993	0.013675656	16.11570913
3.296968137	-5.802691489	0.009290722	23.55010258
3.764751151	-30.77356461	0.008645808	28.74685497
2.520273806	-39.32472489	0.011148062	25.92517415
3.750901888	-3.645503341	0.014222472	22.19634969
2.999289136	-20.8392195	0.011684258	20.84329793
2.500705176	0.321918135	0.009372627	30.63260092
3.056887806	15.15495274	0.006853509	31.46924535
3.507041582	-22.68901283	0.012339638	18.49660552
3.15340005	-12.97011134	0.009240799	18.53222809
2.796870459	13.60807177	0.010621546	23.37876302
2.42542556	-5.868820374	0.007558463	15.07251808
2.441465541	-19.29734765	0.005723352	44.52503052
2.257125369	4.249475746	0.011810064	28.75042212
2.985975545	-94.99715836	0.01065964	25.67022554
2.993552592	-24.57826529	0.016677991	19.3915565
2.754937378	19.75550659	0.004071268	28.07275354

log.sigma.4.0.mm.3D_glrlm_LowGrayLevelRunEmphasis	log.sigma.4.0.mm.3D_glrlm_GrayLevelNonUniformityNormalized	log.sigma.4.0.mm.3D_glrlm_RunVariance	log.sigma.4.0.mm.3D_glrlm_GrayLevelNonUniformity
0.00912003	0.045115291	0.080911406	60.81821381
0.021910437	0.04978116	0.073214378	16.33196985
0.011394273	0.046595877	0.090309157	39.00297163
0.01730376	0.044383467	0.052585347	13.63448888
0.015305284	0.049650196	0.082663111	65.50173892
0.015718645	0.048864066	0.059831259	11.20234342
0.011212057	0.075788505	0.490925011	647.2753071
0.007065654	0.05406985	0.158014936	296.0721845
0.013617685	0.065041547	0.224365067	242.1584862
0.013293036	0.059282829	0.099845862	41.2846117
0.015506123	0.089091314	0.177163046	122.9187211
0.011066295	0.071514279	0.237033782	210.7807281
0.010777898	0.048238072	0.087121471	36.46642164
0.009827059	0.043859717	0.05578701	26.85788533
0.013902546	0.055666736	0.129750029	80.03267679
0.013644443	0.058492929	0.306063379	288.5938361
0.027565932	0.073071212	0.131775948	58.26511796
0.023016449	0.046019112	0.067776992	17.77498625
0.020522405	0.086600665	0.182457202	83.13987322
0.004150035	0.059780814	0.168208756	490.5981328
0.01993186	0.052855798	0.116388057	101.0016719
0.018707414	0.057927786	0.078735344	26.93428837
0.02772314	0.055740426	0.09330317	28.84615587
0.020038679	0.092813655	0.323757718	280.1544576
0.00853998	0.048793827	0.182540817	53.40691118
0.003965054	0.051835753	0.137793662	120.4623152
0.007831367	0.050490643	0.094216982	93.96653101
0.019568255	0.064230331	0.140799212	130.6348537
0.023585234	0.089745575	0.14965267	11.73764309
0.011734349	0.061385829	0.115902489	90.56598945
0.010163386	0.055525884	0.148707777	200.360692
0.016564135	0.048694088	0.07108565	36.11578414
0.010166117	0.05623723	0.14931406	118.1444148
0.009109572	0.061402523	0.194789749	131.9901438
0.00823375	0.059047175	0.158559131	271.7266752
0.007147056	0.04893353	0.101858199	87.7734921
0.006749399	0.045014779	0.068884302	64.13493075
0.013404962	0.062177406	0.403632617	301.0682435
0.02186867	0.069841597	0.099666559	36.12377318
0.002465419	0.073344343	0.279635853	591.7864121
0.0122627	0.050396527	0.092234485	62.62709672
0.010707162	0.102867347	0.342724388	484.6363936
0.007932487	0.043499899	0.098046301	98.33879605
0.011054046	0.049564864	0.092496995	65.41520778
0.018543054	0.079693839	0.321840948	105.4464579
0.010487728	0.049606386	0.109133523	109.8086151
0.006030156	0.065871942	0.193465065	311.7202061
0.022723629	0.073434884	0.112886621	32.93513512
0.017814508	0.054289207	0.072685109	19.9334505
0.010809699	0.050588207	0.066848731	21.59334474
0.011529129	0.052956683	0.095658905	63.18981696
0.007499635	0.05084564	0.091242375	131.3362078
0.016988811	0.10032734	0.535917044	626.0995008
0.005311935	0.06795097	0.137335653	100.4165391
0.012293252	0.041163927	0.059541379	25.15425168
0.016533524	0.048755222	0.08172267	53.54708572
0.010659381	0.059902972	0.19279193	213.6962324
0.005798101	0.051415412	0.097553409	98.85591045
0.022302808	0.056057631	0.081448173	39.87643581
0.005694806	0.077308879	0.52176496	678.7413328
0.008014508	0.055812119	0.104872928	68.31856811
0.006988416	0.047118588	0.108179532	107.7339887
0.004118069	0.049877371	0.144396475	371.4306723
0.007459881	0.050914044	0.100705596	98.34394409
0.020961532	0.05946377	0.095877517	37.3138957
0.005598466	0.049827913	0.084637192	158.8463064
0.010413495	0.05485408	0.065459935	21.0510904
0.004858332	0.056241887	0.112217215	262.0720874
0.008564053	0.068416034	0.21741079	450.348889
0.00762268	0.055885182	0.104270541	109.2173122
0.017995368	0.053407983	0.081827709	24.69254029
0.00630303	0.056818455	0.173471685	254.0161659
0.021218407	0.047476268	0.088330181	59.10091008
0.018101258	0.058135327	0.081814186	51.5765696
0.008402424	0.072647259	0.248618881	255.4423324
0.015093761	0.069615934	0.12040408	58.63161682
0.010556576	0.055656376	0.110558337	112.9205886
0.009768535	0.050389798	0.056399097	15.46317743
0.009411305	0.054392209	0.08822478	66.45911566
0.013676646	0.071938618	0.168571598	115.3007896
0.008532107	0.045845028	0.082167711	60.11264107
0.01038074	0.056153762	0.081067524	43.24838457
0.017361802	0.055619534	0.189316599	114.0353903
0.014721626	0.070855066	0.13164045	121.2464299
0.010097789	0.062675835	0.201640482	150.209402
0.00882403	0.074569274	0.09486829	36.75951484
0.011769842	0.054517189	0.094630155	66.02672119
0.015949301	0.069821279	0.356566125	224.1499852
0.012357187	0.063589341	0.140699939	97.65757726
0.009879316	0.05090828	0.089121422	64.87431287
0.007185197	0.053461883	0.115556161	39.25554809
0.01364774	0.070726668	0.219621539	156.5375958
0.009904372	0.065468784	0.134936101	266.0992836
0.011375865	0.05838185	0.129836443	183.160926
0.008151501	0.070257861	0.137402221	209.4299369
0.005993219	0.041609568	0.072783447	93.3225757
0.012418655	0.05086539	0.09512626	115.7261705
0.010964837	0.061641019	0.082327495	18.03671255
0.017271848	0.076514021	0.094521649	19.03811284
0.004329044	0.053080151	0.144351044	424.3483063

log.sigma.4.0.mm.3D_glrlm_LongRunEmphasis	log.sigma.4.0.mm.3D_glrlm_ShortRunHighGrayLevelEmphasis	log.sigma.4.0.mm.3D_glrlm_RunLengthNonUniformity	log.sigma.4.0.mm.3D_glrlm_ShortRunEmphasis
1.229927544	292.0952286	1184.221449	0.95026049
1.198941323	270.356841	296.7645897	0.959841805
1.232084543	279.0417447	749.8169273	0.956151727
1.152977078	315.3780751	280.5714758	0.96518471
1.227954111	202.3872728	1165.327704	0.952323017
1.176100028	258.5895234	206.8664391	0.96006751
2.03358932	149.9067946	6175.737918	0.873407816
1.39000797	252.213037	4588.033146	0.931238526
1.544391571	151.1487987	2970.38387	0.911106317
1.278403833	171.9309225	600.5038431	0.942392881
1.462263504	107.4992398	1118.162079	0.916615032
1.583888103	161.5270674	2303.462919	0.903881576
1.241037788	305.1232296	663.3552645	0.94941192
1.162402978	315.8534838	555.7680645	0.963013747
1.334665842	171.1006824	1222.500737	0.936945133
1.687966753	169.944852	3870.053045	0.903160405
1.359875556	97.91298489	661.0750161	0.928021635
1.190770441	250.596635	346.4472587	0.958316152
1.492209881	95.88890589	762.8171045	0.909097674
1.424310939	333.9254941	6759.853088	0.924629862
1.300819865	173.0666262	1648.506772	0.942886907
1.220225819	213.6626025	412.0896675	0.953020812
1.258349244	173.4682458	450.5746596	0.946495851
1.761201377	101.0673059	2254.272179	0.885795977
1.453059433	292.1546186	904.5082459	0.923964214
1.354055461	398.9593239	1965.10973	0.934545113
1.257253739	261.2894461	1627.704026	0.947813209
1.372417161	131.9775379	1693.053941	0.928833993
1.407667517	94.32959391	107.4925784	0.922054363
1.309794644	175.4919114	1261.693719	0.939211566
1.386359135	188.7909696	3002.920817	0.928486019
1.201958255	277.8563436	661.2829291	0.955815299
1.389890507	203.8900099	1742.392785	0.927166961
1.465119998	197.3153099	1767.027784	0.923459299
1.406648101	210.1580586	3806.245327	0.926302515
1.272722145	317.3880326	1558.993586	0.94569888
1.199255049	407.2325548	1275.299694	0.956438692
1.863147853	182.2367349	3648.64504	0.889197358
1.276024551	149.0299706	447.0270929	0.94324392
1.661110589	453.5448545	6208.171578	0.897436954
1.256366628	214.2261376	1081.185753	0.946409857
1.808504633	134.4318848	3461.445335	0.879439272
1.250740405	323.8180571	2017.005367	0.953702484
1.252119825	217.2907511	1154.952518	0.948456185
1.72724567	131.1920333	1021.027907	0.897551427
1.289572691	229.2637436	1915.985169	0.943519797
1.476756365	234.1391053	3838.151229	0.918463455
1.316709137	127.6405214	379.1662196	0.934664267
1.208963913	184.0862201	326.0019962	0.954140638
1.190071981	311.9091765	383.0583188	0.958279019
1.26156132	199.617636	1038.546587	0.946396409
1.255278575	296.1937132	2244.817185	0.946035088
2.200474241	100.4331884	4234.477572	0.847990843
1.378151595	278.9676909	1221.066964	0.925222152
1.168329829	337.5632263	555.613747	0.963335217
1.230394863	186.0191619	967.008426	0.950913485
1.469048033	176.4375537	2925.575491	0.921693069
1.255278047	296.6924974	1688.48901	0.949925585
1.233717384	176.370486	623.3783465	0.94910954
2.082498277	224.2951815	6327.815297	0.871583966
1.287281972	235.1279506	1053.994487	0.941891407
1.273614362	313.7639419	1999.326566	0.948169298
1.370724972	370.3443279	6243.610621	0.931493446
1.280235749	285.3463459	1661.004419	0.941826392
1.263346436	155.6668764	547.0015254	0.946451585
1.237541763	344.0022819	2794.562928	0.949463062
1.185577986	289.2519876	345.1527273	0.959367264
1.305853007	317.4106027	3971.811106	0.938280692
1.525896478	177.3945877	5275.314411	0.913328363
1.288093573	239.925175	1676.792604	0.940873547
1.217970898	183.1320965	413.5384451	0.956230073
1.420365802	268.3526655	3721.999079	0.928548396
1.240448062	191.2194152	1095.439693	0.950667999
1.23098835	147.776709	780.7719642	0.950528977
1.607910875	203.1458922	2732.327436	0.901254245
1.317699212	140.8501781	723.223314	0.939365288
1.298538945	244.292332	1739.658518	0.94048691
1.165971264	451.4862408	278.0853679	0.962042153
1.248925902	238.297296	1065.138612	0.947061755
1.435014078	146.6989214	1306.137501	0.920522342
1.23372099	287.9807507	1149.613915	0.94946811
1.233808443	230.2362473	674.5942617	0.948829305
1.442872638	159.8162751	1727.195819	0.931085424
1.359141161	140.3840822	1425.314885	0.929156474
1.494817163	187.8228683	1937.25452	0.916791829
1.260191358	372.9893738	429.7861619	0.946963289
1.264116998	211.3836653	1050.132003	0.944802358
1.800541581	140.5167422	2404.561134	0.887441153
1.371477076	196.2615839	1278.746995	0.929009525
1.242088857	231.5315425	1119.744556	0.950167045
1.317645394	372.5400274	622.2627213	0.935901694
1.540492668	165.8465498	1767.269674	0.911056449
1.364324045	176.1418233	3381.153853	0.928715777
1.340092717	183.986945	2655.352197	0.935331428
1.371967016	179.5936803	2472.967519	0.927388331
1.204517194	381.0348813	2002.131608	0.956122186
1.260221652	205.9802756	1985.321223	0.946996524
1.230190353	287.9984788	257.9026894	0.951114017
1.260777901	189.1809287	217.2730408	0.946897242
1.365914817	338.0664047	6731.237425	0.933241434

log.sigma.4.0.mm.3D_glrlm_LongRunHighGrayLevelEmphasis	log.sigma.4.0.mm.3D_glrlm_RunPercentage	log.sigma.4.0.mm.3D_glrlm_LongRunLowGrayLevelEmphasis	log.sigma.4.0.mm.3D_glrlm_RunEntropy
364.5975103	0.933518006	0.010672679	5.004237341
340.4477562	0.945244957	0.027239477	4.782073019
361.133485	0.938427044	0.013971729	4.852823148
369.445164	0.953893932	0.018715647	4.889190286
257.4800551	0.934961566	0.019060374	4.81763441
310.8610298	0.947552448	0.017945026	4.6837594
329.9477631	0.808834585	0.019658432	4.839823778
357.2556138	0.902274225	0.009513202	4.914767925
232.1375087	0.873154532	0.020707796	4.770337647
225.1441883	0.922363729	0.016565172	4.596532436
162.9182383	0.886296223	0.02202564	4.2965774
262.6709085	0.864306518	0.01671797	4.713263652
412.5034718	0.931992791	0.012431493	4.875051638
373.8205571	0.950907788	0.011472064	4.897993631
218.4149734	0.912673993	0.019319244	4.790080569
298.3487847	0.855365391	0.021175464	4.931432492
137.3340894	0.902953219	0.037654428	4.39105535
302.8281202	0.944329509	0.028040307	4.881570105
149.7148775	0.877162143	0.0298853	4.341009484
504.9137188	0.89351561	0.005651492	4.861096549
234.6799363	0.919634264	0.027044631	4.770117591
271.5301691	0.937344913	0.023181416	4.629697176
221.7412644	0.927350427	0.036537179	4.683792373
186.6558377	0.837685251	0.031809116	4.484975709
453.404646	0.890879479	0.011155401	5.008889874
548.8029911	0.908902131	0.005236288	4.950736694
329.6422513	0.92860433	0.009859856	4.868401797
186.1867321	0.902591417	0.026579007	4.618046017
133.1807601	0.895679663	0.030610939	4.064100962
235.0162352	0.91638796	0.015423196	4.632217484
264.9502647	0.900147744	0.014430352	4.822166638
344.2397154	0.941233893	0.020364502	4.787399512
288.6130336	0.899588206	0.013876755	4.868149441
305.7126249	0.889472846	0.012732227	4.729600169
297.9276423	0.896371892	0.01146699	4.800549062
423.3799117	0.925060499	0.008763195	4.887439864
508.2586821	0.942256817	0.00792834	4.962983901
362.1844626	0.830507579	0.020913773	5.011266153
202.0080472	0.923489011	0.028818935	4.40060069
814.0957623	0.853579891	0.003981695	4.751820283
270.1446435	0.927439724	0.015778605	4.830411026
259.6690881	0.829526961	0.017923143	4.401175722
414.686093	0.935133931	0.009927036	5.009854238
271.5080371	0.929414951	0.014174256	4.839973787
236.4987756	0.84872454	0.028121366	4.637770423
295.1721804	0.921955595	0.013856791	4.875432428
357.6284932	0.88422006	0.008880657	4.702066374
177.203767	0.913363622	0.029806828	4.38621818
226.4023153	0.939012394	0.021065787	4.690750452
390.5946236	0.944349898	0.012071935	4.747921344
244.5083782	0.927021696	0.014846588	4.768279944
383.3536341	0.927496202	0.009119607	4.889606184
245.931337	0.780514621	0.031571048	4.613279287
408.1127151	0.899934414	0.006945575	4.601724505
411.5973718	0.950352913	0.01435112	4.960565372
233.3157899	0.933869702	0.020830638	4.820414382
263.987036	0.888303477	0.015466471	4.788971132
383.1836635	0.930184958	0.007243943	4.740061203
218.7063894	0.932352052	0.034023528	4.633809337
516.8923836	0.804638123	0.010726996	4.810626056
310.8682776	0.921051108	0.010043237	4.745430841
417.384958	0.927352161	0.008365736	4.965046647
524.5096653	0.904289905	0.005507753	5.007823218
383.4907984	0.921535526	0.009272167	4.920125422
201.4449223	0.92694012	0.025155028	4.573324358
441.7587075	0.931846289	0.006902031	4.891522305
356.9096262	0.945244411	0.011611633	4.634242431
420.0603781	0.916345208	0.006373334	4.82577201
284.1751753	0.876654645	0.013007853	4.668501172
332.4455603	0.920070984	0.009415159	4.737046909
227.5691134	0.939649781	0.021694476	4.631323539
395.3009839	0.897054963	0.008714478	4.88348475
234.6397558	0.932469029	0.027660159	4.856558339
183.8717889	0.933846154	0.022454599	4.580736758
337.0953629	0.860317939	0.01276333	4.779859023
195.0271473	0.917462712	0.01930144	4.387701978
337.8037838	0.918480343	0.013822527	4.762529581
547.6414149	0.949988092	0.010491779	4.80087346
311.710968	0.92904358	0.011246028	4.762620493
216.7673773	0.890213675	0.019024569	4.552248451
361.2416796	0.932541854	0.010123257	4.990853081
304.1774089	0.932482771	0.012441413	4.657381657
233.3001081	0.897997708	0.025259045	4.771574737
194.023665	0.903815677	0.019794715	4.502347886
287.1558103	0.882079615	0.014565394	4.805820137
512.0330514	0.927712589	0.009638978	4.512236228
271.6198944	0.925899788	0.014671233	4.739652554
252.6156157	0.835285153	0.026740116	4.853770277
278.078207	0.90268168	0.015640313	4.678828121
295.2595017	0.932136627	0.012264804	4.807714207
519.213277	0.91322235	0.008691597	4.848451354
262.8968822	0.873648074	0.021038669	4.678668538
250.6464223	0.903030096	0.013222627	4.617199426
252.6875883	0.910286173	0.015247979	4.737710692
256.8478328	0.901397967	0.011161025	4.473026725
472.9357152	0.940791172	0.00718432	5.062113163
269.4875103	0.927845401	0.015314246	4.799177497
375.3967704	0.934627673	0.012295031	4.536470992
258.6681392	0.927956372	0.019834264	4.300408137
493.4063501	0.905886045	0.005708584	4.904764182

log.sigma.4.0.mm.3D_glrlm_HighGrayLevelRunEmphasis	log.sigma.4.0.mm.3D_glrlm_RunLengthNonUniformityNormalized	log.sigma.4.0.mm.3D_glszm_GrayLevelVariance
305.1560856	0.877871947	48.74342533
281.6944377	0.902193127	43.00253902
291.9141839	0.893498973	36.06657096
325.426543	0.912761074	51.82567493
211.9156629	0.882818389	31.92728782
268.1788954	0.90098674	33.86232281
169.5825869	0.722225876	31.19961423
267.9489518	0.836846606	38.09889354
162.460002	0.795707249	26.62999138
181.182872	0.861004563	27.63558183
116.1305634	0.807307342	16.61427928
175.8141337	0.780271369	28.57311444
323.2426494	0.876506662	37.46658947
326.3548432	0.907213305	39.36747508
178.8972979	0.849058645	31.04055166
186.1046203	0.781378853	31.2342615
104.5898231	0.828602748	18.24994318
259.4766374	0.896362103	40.63442226
104.3256555	0.791696837	17.4194497
359.9016109	0.822704521	39.69531439
182.8878991	0.86197287	27.23305154
223.6587275	0.88496653	29.15072109
181.7117518	0.869842721	27.83076056
112.1387899	0.74538084	18.237467
315.031291	0.823593643	41.84715078
422.55888	0.844165455	38.87803982
272.7327065	0.873345055	37.52772961
140.6898985	0.831251798	23.28811909
100.6226999	0.81857822	19.38495252
185.2524056	0.854005626	24.76141464
200.7616484	0.830907543	27.27356646
289.7126164	0.890730188	34.50051169
217.5072221	0.827960482	32.35152524
212.5230774	0.820878762	24.75630126
223.7235802	0.826180409	30.60273951
334.9313966	0.868262682	33.18084868
424.9850888	0.893320276	44.56011598
202.6343466	0.752781313	33.2968472
157.8937686	0.862936289	19.17981559
501.7750577	0.767716466	29.51208481
223.8611345	0.869364616	32.50988233
150.7283296	0.733042051	20.8894202
338.0868337	0.888742779	39.24453592
226.5335733	0.874335444	30.50635135
144.2199783	0.769519783	26.66305062
240.2272287	0.864031735	32.48485596
252.0787791	0.809909439	26.35859176
136.0465979	0.843765165	19.25738142
191.6552512	0.88700465	32.49531738
325.8005186	0.896582225	39.7526385
207.4131036	0.869576012	29.79174804
311.4851524	0.868392099	37.369916
116.609082	0.676604564	21.50926985
300.3028215	0.824106964	26.9772328
350.8223446	0.908580011	45.33361082
194.3117371	0.879693633	31.30013438
189.0291914	0.817730817	26.92290612
310.996759	0.877572951	28.74546608
183.9517249	0.875448203	25.87401971
254.7740656	0.719231318	27.43119581
247.9334469	0.859783463	31.86616569
330.529863	0.873781456	40.97649347
394.7691775	0.837321217	38.86637821
302.1622906	0.859146716	36.72372352
163.526686	0.8702676	28.00815396
361.1601955	0.87610387	37.89034956
301.3660844	0.898956337	34.77657122
334.9606994	0.851428801	33.79441043
192.6018041	0.799569028	23.69054016
255.6874143	0.857109898	28.65964181
190.4148338	0.892978888	28.36713869
286.7885738	0.831580478	34.13856289
198.5892691	0.879220721	34.84383698
154.3182657	0.879008367	25.86574872
221.7990377	0.775448681	31.66665436
149.7777353	0.855673617	18.1744
259.628755	0.856493353	30.13624812
469.1650764	0.905480075	51.47404844
251.1880095	0.87096368	32.90837799
157.4901814	0.813757996	23.5991898
301.1551038	0.876106387	47.31936897
243.346282	0.874773822	27.45090134
169.6483027	0.839179226	26.09011697
149.2184759	0.831878163	19.69938971
202.202464	0.80684339	28.71863242
395.9778873	0.871210961	39.22004132
221.9162516	0.865965497	28.25227936
154.2915298	0.748379211	28.08328228
209.417408	0.831603715	29.58140021
242.3869885	0.877991004	34.07434892
396.9469865	0.846387858	39.51285274
179.4205021	0.795864001	23.91015018
188.4398221	0.830790162	24.53160645
195.1778651	0.845393347	29.00911639
192.3545919	0.828218499	18.18021951
397.1133762	0.891809479	47.99443161
216.6841036	0.871326837	30.48617615
302.9410881	0.880364119	30.825216
200.4304444	0.871721499	24.69536332
362.1665072	0.841058744	34.44576821

log.sigma.4.0.mm.3D_glszm_SmallAreaHighGrayLevelEmphasis	log.sigma.4.0.mm.3D_glszm_GrayLevelNonUniformityNormalized	log.sigma.4.0.mm.3D_glszm_SizeZoneNonUniformityNormalized
227.9808076	0.039814126	0.350420992
175.6890499	0.042913632	0.346097815
174.0595396	0.046658968	0.328438563
252.0100665	0.040440771	0.39261708
137.2192872	0.049088349	0.312245311
172.591097	0.051296108	0.332132508
128.1588321	0.050414377	0.302384927
206.0567173	0.045245779	0.324855063
139.3028297	0.054353262	0.308493019
130.660482	0.052405267	0.301027949
66.68741675	0.068086845	0.217184571
137.3529684	0.052233332	0.288978397
167.433264	0.047238966	0.315598474
237.8149777	0.045253831	0.391019109
172.3145215	0.051880042	0.386779549
132.0510514	0.049311362	0.318833532
65.85428258	0.066690235	0.233807227
184.4346563	0.045545841	0.336956522
82.10682625	0.069056454	0.263506733
226.3731839	0.044631898	0.309636858
107.6730275	0.051942637	0.263320783
128.8529889	0.052785371	0.310632482
145.9159084	0.054954495	0.341995758
91.99490666	0.06760193	0.271262552
230.6815827	0.042990948	0.360751106
313.9512656	0.045344575	0.334144304
214.7132689	0.045652658	0.335924371
100.1408205	0.057159047	0.269042969
70.68649246	0.07669832	0.237399562
124.6486106	0.057454754	0.285119219
138.7191553	0.05319331	0.284826492
193.1087818	0.048597793	0.365158538
150.1435467	0.050364839	0.306980351
141.7442436	0.057025171	0.297794891
168.1005673	0.049510164	0.299416738
209.6925762	0.049157996	0.34646237
272.5440064	0.043081657	0.349528994
152.946896	0.048803461	0.349397115
88.01693621	0.064696578	0.246093901
348.2056174	0.052656494	0.298198317
170.781069	0.048838985	0.296088182
112.0711707	0.064772491	0.264848507
226.587279	0.044692215	0.344351587
166.7208425	0.050848612	0.321912519
113.1036378	0.059377055	0.257581361
180.871094	0.048082279	0.327986491
186.003279	0.054785452	0.291703073
93.74745632	0.065238167	0.248729943
123.6518218	0.050655567	0.300062435
177.149913	0.046723216	0.328070323
173.1140197	0.051711478	0.359012014
199.6423972	0.04662	0.308574
90.37314023	0.062190881	0.259162632
182.4862171	0.057345599	0.291524131
214.4554219	0.041789802	0.362226847
123.1752951	0.04947283	0.277674179
141.2285865	0.053745125	0.291348753
198.1413139	0.051929314	0.299136726
129.7566912	0.055471079	0.304701812
180.0398723	0.053965859	0.282597531
174.386705	0.049425079	0.326002716
189.9888232	0.043192328	0.30076572
254.8979595	0.044363392	0.325812337
187.9855366	0.046635024	0.296718868
105.6543221	0.054033364	0.306592604
228.2074997	0.045097497	0.319404524
177.9150493	0.050285676	0.324735207
239.3639427	0.048677928	0.309469715
127.8604269	0.058456409	0.266584327
128.8774001	0.051100191	0.260369462
141.1884821	0.052374011	0.327467722
202.6626784	0.047660108	0.305601852
148.4550467	0.046509367	0.321288018
112.7070515	0.054740687	0.335368882
195.1809415	0.052284122	0.338123867
72.67472812	0.064032	0.211424
139.5214021	0.050837159	0.275413757
299.913406	0.046776881	0.347515913
144.4794119	0.047853779	0.283766519
111.7004359	0.059156741	0.255702687
220.2569037	0.040576303	0.34343084
129.7580406	0.052976689	0.306305637
125.7247507	0.054314597	0.31209756
108.3060937	0.06386663	0.264213889
145.0446722	0.052084403	0.278969157
168.8018599	0.047262397	0.274405992
154.0823238	0.052222779	0.324168735
136.0351811	0.052675058	0.310366699
148.3257953	0.051409119	0.332446753
165.8444744	0.047571435	0.308503492
227.6914183	0.048019146	0.309722629
153.0106478	0.058214823	0.297812207
115.7288862	0.056653305	0.265463552
129.9090261	0.051481854	0.265067444
118.4987166	0.065198923	0.269575708
275.828755	0.039669248	0.366260867
127.5371077	0.049694302	0.276454654
187.8799157	0.054208	0.357056
83.04874971	0.063252595	0.235986159
202.5476139	0.047100718	0.319289473

log.sigma.4.0.mm.3D_glszm_SizeZoneNonUniformity	log.sigma.4.0.mm.3D_glszm_GrayLevelNonUniformity	log.sigma.4.0.mm.3D_glszm_LargeAreaEmphasis	log.sigma.4.0.mm.3D_glszm_ZoneVariance
203.9450172	23.17182131	19.86941581	13.7135603
53.64516129	6.651612903	9.15483871	4.14301769
117.5810056	16.70391061	15.2849162	9.07674542
64.78181818	6.672727273	6.509090909	2.700679522
165.8022599	26.06591337	17.61016949	10.54918943
37.53097345	5.796460177	7.079646018	2.493225781
579.3695198	96.59394572	2243.927453	2213.671712
605.8546917	84.38337802	93.33726542	82.75122685
327.6195857	57.72316384	217.8907721	201.785137
73.45081967	12.78688525	30.5942623	21.01980314
67.11003236	21.03883495	113.2750809	87.91782658
224.8251928	40.63753213	289.5745501	270.3973556
98.1511254	14.69131833	17.1221865	10.32199833
125.517134	14.52647975	7.794392523	3.769431585
217.3701068	29.15658363	40.82384342	32.96988703
448.9176136	69.43039773	511.90625	495.1590889
46.52763819	13.27135678	84.80904523	65.12042625
62	8.380434783	8.972826087	4.031870274
54.54589372	14.29468599	218.9468599	191.0153796
737.5549958	106.3131822	296.663728	281.8014406
149.8295255	29.55536028	50.88224956	37.54499152
63.99029126	10.87378641	12.18446602	6.38712414
66.00518135	10.60621762	24.16580311	15.80681361
164.9276316	41.10197368	811.2944079	776.2939507
152.2369668	18.14218009	45.46919431	37.00137014
272.3276074	36.95582822	56.72392638	46.88819903
242.5373961	32.96121884	24.76731302	17.06322849
154.96875	32.92361111	72.28645833	56.98695204
8.783783784	2.837837838	38.37837838	22.80788897
134.5762712	27.11864407	43.80932203	32.17428541
305.6188257	57.07642125	101.2199441	87.2603539
124.5190616	16.57184751	10.81524927	5.475219511
205.0628743	33.64371257	87.55239521	75.33381217
191.4821151	36.66718507	96.12597201	82.01969771
415.8898488	68.76961843	118.7566595	105.1002026
251.5316804	35.68870523	26.71212121	19.57895446
227.1938462	28.00307692	11.8	6.389008284
509.7703907	71.20424949	747.878684	731.9608264
39.62111801	10.41614907	31.36645963	19.26816095
559.4200426	98.78358209	664.8630064	639.504588
133.8318584	22.07522124	34.5619469	25.77308325
221.148503	54.08502994	1442.452695	1396.310368
341.5967742	44.33467742	18.35282258	12.41141633
165.463035	26.13618677	25.6536965	18.02148405
80.36538462	18.52564103	324.1538462	299.2818869
281.7403958	41.30267753	31.26309662	23.45045339
360.8367017	67.76960388	184.7962813	166.0838378
35.07092199	9.19858156	30.30496454	18.17876364
46.50967742	7.851612903	13.92903226	7.565619147
62.00529101	8.830687831	11.14285714	5.423420397
181.6600791	26.16600791	28.91501976	22.4457381
308.574	46.62	22.953	15.196775
280.6731302	67.35272392	3664.915974	3610.676761
121.2740385	23.85576923	83.91346154	68.33374168
112.2903226	12.95483871	7.474193548	3.171914672
114.1240876	20.33333333	18.67153285	10.48441579
305.9161905	56.43238095	130.4828571	115.8613379
218.6689466	37.96032832	25.94664843	17.9511379
88.05882353	16.03114187	18.44636678	11.47603597
514.6101043	98.27182867	2527.55464	2491.771775
153.2212766	23.22978723	27.60638298	19.61072431
273.3960396	39.26182618	28.60176018	21.24805726
848.0895121	115.4779101	143.88398	133.877683
199.0983607	31.29210134	30.24739195	20.4899154
72.66244726	12.80590717	22.35021097	14.19038972
397.3392283	56.10128617	21.88987138	14.32737138
56.17919075	8.699421965	10.45086705	4.943299141
502.5788177	79.05295567	55.03140394	45.2272558
396.4108944	86.92468056	415.872226	390.3788946
168.4590417	33.0618238	29.70942813	18.93236409
64.18367347	10.26530612	12.95918367	6.658059142
440.0666667	68.63055556	201.0881944	189.1233213
169.3187856	24.51043643	15.11385199	8.696715143
124.0864865	20.25405405	17.77297297	11.18056976
289.0959064	44.70292398	496.0678363	473.2518505
52.856	16.008	32.968	19.484416
177.3664596	32.73913043	36.54192547	24.77617328
53.16993464	7.156862745	7.549019608	3.092229484
129.9650655	21.91703057	22.00655022	13.76288114
107.6508314	24.90498812	105.3539192	87.07372448
191.6344086	22.64157706	20.62365591	14.27470099
94.03583062	16.26384365	17.99348534	10.75440588
229.7038043	39.97554348	67.48913043	57.87576057
121.802603	29.44251627	128.3015184	111.4398954
180.4930448	33.69860896	209.8438949	192.2350403
48.29545455	8.318181818	29.01704545	19.91448218
156.573499	25.22360248	22.28985507	14.95618739
249.5348259	42.35074627	624.0298507	601.266033
162.5664622	25.1390593	111.7770961	99.6769418
156.1027668	24.07114625	17.37351779	10.07497774
74.64315353	11.57261411	38.37344398	27.24388354
166.1792115	32.48387097	270.3620072	249.7718811
292.5408348	62.43194192	87.71597096	71.03371283
248.6332623	48.28997868	66.84434968	53.34773437
200.294751	48.44279946	142.4427995	122.6325127
373.2198234	40.42296369	12.80863592	7.335148373
226.4163614	40.6996337	26.21245421	17.24904698
44.632	6.776	15.992	9.721984
20.05882353	5.376470588	23.27058824	13.32955017
864.6358936	127.5487445	135.1070901	124.4893112

log.sigma.4.0.mm.3D_glszm_ZonePercentage	log.sigma.4.0.mm.3D_glszm_LargeAreaLowGrayLevelEmphasis	log.sigma.4.0.mm.3D_glszm_LargeAreaHighGrayLevelEmphasis	log.sigma.4.0.mm.3D_glszm_HighGrayLevelZoneEmphasis
0.403047091	0.13520052	4483.666667	351.814433
0.446685879	0.184565366	2685.819355	280.3612903
0.401345291	0.153153066	4223.879888	298
0.51242236	0.073814102	1869.909091	358.3939394
0.376328845	0.346656794	2977.109228	228.8775895
0.466942149	0.109276148	1692.690265	285.0707965
0.18180093	15.09815894	344882.4186	211.2719207
0.307350033	0.478575037	21770.76139	328.0841823
0.249178789	2.384925964	26242.72128	228.6873823
0.323178808	0.317330907	4735.512295	214.1147541
0.198586118	1.496074738	10759.65372	139.4724919
0.22835339	2.364514254	42107.76093	227.9344473
0.383477189	0.105984757	6764.758842	297.4951768
0.498447205	0.09244834	2045.183801	353.7943925
0.356825397	0.630959387	4268.010676	242.8932384
0.244359597	3.795531925	79256.61932	213.196733
0.225368063	1.700004373	6929.623116	128.6582915
0.449877751	0.229699784	1927.581522	289.8967391
0.189213894	3.189133874	19027.10628	139.057971
0.259392355	0.899567384	102504.0672	387.9047019
0.273820982	1.130330402	8228.776801	198.5254833
0.415322581	0.174585248	2773.082524	227.0582524
0.345878136	0.695549644	3612.953368	219.5025907
0.169029747	9.478715619	79266.58717	160.1529605
0.343648208	0.204826842	13868.4455	348.014218
0.31885759	0.213752889	17933.84663	495.8932515
0.360279441	0.186343629	4915.860111	330.9238227
0.255659121	1.051510598	8073.96875	176.9878472
0.253424658	0.679032079	2901.216216	129.9189189
0.293167702	0.544163879	6276.358051	214.815678
0.267647792	1.167016707	13174.3383	244.9804287
0.432741117	0.155130325	2919.777126	302.941349
0.28608137	0.709575879	13621.98802	256.8577844
0.266252588	0.689416918	18581.48989	243.0419907
0.270601987	0.972568911	17637.49604	279.737941
0.374419804	0.139539641	8455.264463	341.362259
0.42989418	0.060117328	5208.027692	432.4076923
0.250644219	4.379331483	142308.9938	241.5935572
0.2875	0.491533651	5094.068323	165.1242236
0.19858156	1.435504657	317626.6322	584.8981876
0.337313433	0.420173451	4844.621681	277.5154867
0.147214386	11.90521655	184579.6599	198.9760479
0.41025641	0.13122354	4761.757056	361.46875
0.361971831	0.332757121	3770.754864	271.4202335
0.200514139	3.151925212	39779.41346	190.3717949
0.357767597	0.328227392	5139.797439	287.145518
0.231171744	0.996621304	41486.52708	311.1139854
0.287169043	0.617936934	3910.865248	161.5602837
0.396419437	0.207656639	2358.96129	217.8064516
0.418141593	0.071869836	3753.724868	311.9153439
0.393162393	0.416268746	2964.934783	262.3241107
0.359066427	0.131912267	5906.176	340.896
0.135782347	35.18517094	397790.0166	158.6565097
0.253349574	0.346238528	23505.60096	323.3798077
0.482115086	0.086562637	2553.780645	352.1483871
0.349489796	0.448449194	2486.379562	225.5328467
0.261519303	1.204437305	19712.49333	238.2514286
0.353652637	0.173497748	6064.630643	342.3406293
0.378768021	0.495859016	2545.989619	213.3460208
0.167171578	10.59261801	619008.5327	313.0801757
0.35364936	0.184009338	5693.344681	278.2893617
0.368762677	0.129291704	8614.480748	339.7832783
0.316128249	0.519613737	42766.22628	430.1552055
0.320133588	0.185868584	8507.591654	326.5350224
0.350073855	0.324348005	2873.240506	180.535865
0.363636364	0.099822497	7368.803859	382.5836013
0.426108374	0.071905379	3088.104046	304.6473988
0.319370698	0.260286671	13619.63116	396.8799261
0.198055408	2.713024094	77070.13248	231.8298588
0.304613936	0.173377388	7867.958269	252.3632148
0.398373984	0.241865189	2007.05102	221.5204082
0.289098575	0.889629758	54140.39653	336.6569444
0.394756554	0.35961433	2098.184061	234.5806452
0.389473684	0.30400357	2100.637838	176.6243243
0.209353575	2.862426983	93750.10994	304.9017544
0.272331155	0.398472771	4520.616	158.36
0.291534631	0.296433263	8723.313665	262.7934783
0.473684211	0.044030225	3477.372549	482.6470588
0.348288973	0.141081877	5380.633188	259.2860262
0.233888889	1.014860346	14274.4228	199.4608076
0.396870555	0.140334331	4608.247312	346.7706093
0.371670702	0.130173584	4918.739414	232.6775244
0.322524102	0.944001202	9521.350543	206.4375
0.24352879	1.324115785	15525.41215	190.2255965
0.238305709	1.546107404	33918.03555	254.9258114
0.331450094	0.088192142	13560.71591	340.7159091
0.369266055	0.226615106	3799.395445	250.8364389
0.209593326	5.781042007	77957.84204	222.1032338
0.287477954	0.763205853	18840.27607	238.5582822
0.370153621	0.155314096	3872.924901	270.7233202
0.299751244	0.153667948	13817.09129	407.6431535
0.220379147	2.219463706	41758.91577	243.2473118
0.244834481	0.782066998	13277.57713	214.2059891
0.272199652	0.634919938	10344.20469	230.0863539
0.224674932	1.197428764	20079.9825	219.1480485
0.427432886	0.064162747	4904.361138	419.9656526
0.334013051	0.28602277	4520.483516	233.4529915
0.399361022	0.088244962	5048.512	302.152
0.317164179	0.189536605	5203.929412	184.9411765
0.306890299	0.431137881	48259.1562	359.6166913

log.sigma.4.0.mm.3D_glszm_SmallAreaEmphasis	log.sigma.4.0.mm.3D_glszm_LowGrayLevelZoneEmphasis	log.sigma.4.0.mm.3D_glszm_ZoneEntropy	log.sigma.4.0.mm.3D_glszm_SmallAreaLowGrayLevelEmphasis
0.609913647	0.012069045	6.572886645	0.009196262
0.606653159	0.018991794	6.086590235	0.008193124
0.587488026	0.011171756	6.323126638	0.005708111
0.648002525	0.023006974	6.027982745	0.018883532
0.57530593	0.014123426	6.475044945	0.007618411
0.586378844	0.019374665	5.710585352	0.014802931
0.568154442	0.01318989	6.876292688	0.007793975
0.587606793	0.007477805	6.803888644	0.004625679
0.571771005	0.011120406	6.540328188	0.005468397
0.56466853	0.015420699	6.184779105	0.010005666
0.469446999	0.019165369	6.422530207	0.011161594
0.549980297	0.012655984	6.672317514	0.008042599
0.578303453	0.015145319	6.29610428	0.01154602
0.645456206	0.010253658	6.093780794	0.007438675
0.645194469	0.011341019	6.168990086	0.006955597
0.582841455	0.012796753	6.679358232	0.007055954
0.488503462	0.026333342	6.078562042	0.009089222
0.594452241	0.021612433	6.100754112	0.010968525
0.524239549	0.02123937	5.954610085	0.011508062
0.572888842	0.00514496	6.996934113	0.003327215
0.522916793	0.015100846	6.600260036	0.005740729
0.566008512	0.016805888	6.035863631	0.006572842
0.605360322	0.016194215	5.978713727	0.005152933
0.534245353	0.019126375	6.390623096	0.007988086
0.623045001	0.009290918	6.445825768	0.004238815
0.596602163	0.00471995	6.633741059	0.003408937
0.599186738	0.007957978	6.613989198	0.004875475
0.53123409	0.019115962	6.58580223	0.00837753
0.479066512	0.042388928	4.864726676	0.034663283
0.548716919	0.01276726	6.475167278	0.007940279
0.545971243	0.008855716	6.685772445	0.004774619
0.626671114	0.013938304	6.22410345	0.005606538
0.569916275	0.010925918	6.568831074	0.006447749
0.56051209	0.009172101	6.437094011	0.004323482
0.561096581	0.008534968	6.764091961	0.005097327
0.609903168	0.008180909	6.460069324	0.005494896
0.609436404	0.007583934	6.547037469	0.004809385
0.612959106	0.014177709	6.626654259	0.008657549
0.505339289	0.020909808	6.05984673	0.006644096
0.564215288	0.002752748	6.874313648	0.001754517
0.55231368	0.010360954	6.481012617	0.003864628
0.525778676	0.012574	6.632495607	0.005523855
0.604713896	0.0078161	6.588082863	0.004106316
0.583147553	0.010453717	6.402944089	0.006562428
0.515912439	0.0195011	6.405214118	0.008241712
0.590207299	0.008762173	6.536924399	0.004152588
0.555949138	0.00601804	6.744222929	0.003438384
0.504847406	0.021805089	5.922779101	0.008686358
0.546984318	0.020968391	6.004944931	0.014533949
0.58947172	0.015136098	6.097040658	0.01115489
0.616929608	0.0107407	6.193491276	0.006689285
0.571272349	0.00863708	6.769551307	0.004573314
0.520790999	0.019282218	6.729109855	0.009044289
0.552725531	0.0077257	6.44246897	0.005544223
0.617654234	0.01200683	6.219856154	0.006076935
0.536916864	0.015129932	6.496592774	0.009374354
0.554069953	0.010080526	6.644418848	0.005648431
0.560112932	0.00616499	6.494589585	0.003922062
0.557890812	0.015236425	6.122607297	0.005156333
0.543982325	0.006205697	6.858627224	0.003355417
0.588692129	0.009590371	6.425508261	0.006664251
0.560925177	0.009224056	6.816769212	0.006535687
0.589306108	0.004577185	6.893222033	0.00294588
0.561656934	0.008397388	6.754893409	0.005090597
0.570165993	0.028395176	6.151729036	0.019171804
0.582027404	0.005837298	6.798167163	0.003036485
0.584612548	0.014788115	6.007851518	0.011433133
0.571286149	0.004981548	6.783983281	0.002917265
0.529984085	0.008580755	6.870432381	0.004422434
0.52071679	0.009505779	6.751515119	0.005866459
0.590089518	0.018323695	6.023209441	0.011965485
0.568724919	0.006301483	6.796155699	0.003651458
0.582437225	0.017254812	6.402474912	0.007481917
0.598165225	0.019252536	6.149506104	0.012297014
0.602427363	0.00778082	6.516326511	0.003542222
0.448882422	0.017421408	6.337985601	0.006476817
0.539183133	0.011761978	6.768906708	0.005667615
0.605520796	0.014661599	6.04709681	0.012635905
0.543361331	0.012413789	6.623267323	0.008390782
0.513589335	0.014354696	6.555434999	0.006359963
0.602074907	0.010631333	6.550407892	0.007741764
0.566878341	0.012806767	6.242343743	0.008817602
0.574169055	0.013284698	6.442687535	0.006127803
0.522442979	0.015223462	6.338834279	0.005989644
0.541078609	0.009915485	6.627629466	0.005417147
0.525448126	0.018384398	6.268023434	0.016015853
0.587228469	0.012897061	6.351025573	0.008357749
0.572705703	0.01239712	6.538014688	0.00656189
0.596656526	0.016410865	6.402830727	0.008855022
0.572012325	0.010602765	6.525541944	0.00656134
0.573356817	0.009853029	6.277269766	0.004650601
0.563858728	0.009962487	6.357792708	0.003600985
0.52659014	0.011538733	6.777766141	0.006290355
0.525843559	0.011821534	6.849135437	0.006195877
0.533663249	0.009106876	6.520392004	0.005593668
0.626441687	0.006021234	6.691165515	0.003173982
0.535264726	0.013620772	6.740797591	0.007180496
0.617050861	0.016412323	5.635745538	0.012217293
0.477623452	0.029302298	5.74745373	0.020542803
0.581715432	0.005027613	6.823522339	0.003230872

log.sigma.4.0.mm.3D_ngtdm_Coarseness	log.sigma.4.0.mm.3D_ngtdm_Complexity	log.sigma.4.0.mm.3D_ngtdm_Strength	log.sigma.4.0.mm.3D_ngtdm_Busyness	log.sigma.4.0.mm.3D_ngtdm_Contrast
0.008014695	0.763575536	2.96246847	0.253926607	0.000108598
0.024063553	2.34471699	6.750556398	0.097254694	0.000723406
0.011263593	0.946060547	3.0555868	0.20783981	0.00022921
0.03171783	2.772336951	9.146105417	0.069435453	0.000770834
0.007537731	0.481772799	2.075392539	0.385825274	0.000117824
0.044102578	2.228379753	9.92558492	0.070251544	0.000979176
0.001318449	0.073522173	0.779755983	1.352279258	3.59E-06
0.001901252	0.154841047	0.715284106	1.002005801	1.69E-05
0.002813557	0.12313565	0.741820452	1.144329416	2.26E-05
0.015338947	0.490610759	2.586441399	0.279452625	0.000182328
0.007955895	0.152745024	1.236385753	0.532768477	3.78E-05
0.003514732	0.160160258	1.035258689	0.705440334	2.03E-05
0.012178886	1.078111998	3.98002221	0.149267578	0.000240446
0.014738449	1.623152646	4.781313656	0.137035803	0.000351756
0.006641842	0.636180734	2.792134212	0.391210497	7.14E-05
0.002241881	0.113768175	0.646120079	1.154338637	1.76E-05
0.013326045	0.209202024	1.348108608	0.519777364	0.00013161
0.022716298	2.278057627	7.341291137	0.105992268	0.000574063
0.010776125	0.177634668	1.296788201	0.489610417	6.93E-05
0.001421863	0.117700766	0.782745311	0.80062727	5.85E-06
0.0052201	0.211203277	1.015179814	0.739727007	8.57E-05
0.018596225	0.98177975	3.767605077	0.163881738	0.000398113
0.020016413	0.949850614	4.465554309	0.159021603	0.000257024
0.003662731	0.066864353	0.667118662	1.048360885	1.45E-05
0.010210356	0.652035057	3.081335532	0.19690061	0.000111895
0.004554996	0.444656958	2.171440403	0.299409312	3.88E-05
0.00546083	0.538571236	2.260036604	0.352984854	5.86E-05
0.005523815	0.166212349	1.073483551	0.715873369	4.27E-05
0.071261782	0.892375483	6.318867873	0.133401747	0.000799088
0.006513384	0.356995247	1.711548407	0.425742155	6.20E-05
0.002911817	0.167995306	0.87107111	0.98263514	2.68E-05
0.010725717	0.997506231	2.806720631	0.227557429	0.000311239
0.005526239	0.335942057	2.141373841	0.397351851	3.73E-05
0.00513063	0.248319055	1.583388843	0.471307623	3.40E-05
0.002296054	0.14663943	0.812764162	0.963922309	1.65E-05
0.005338835	0.445057745	1.599725889	0.352992003	8.25E-05
0.006281692	0.753919842	2.269876255	0.249615745	0.000143603
0.002372311	0.130704491	0.905239309	0.864689435	1.38E-05
0.016797948	0.556884057	2.714198042	0.224503382	0.000231145
0.001418388	0.082179139	0.798377316	0.681422171	3.81E-06
0.008354995	0.574465308	2.723828519	0.313716374	9.88E-05
0.002145155	0.072265975	0.849154135	0.993060212	4.54E-06
0.004129873	0.466223234	1.492255447	0.469176545	7.68E-05
0.007388869	0.575997901	2.35567442	0.365749648	0.000102699
0.008171326	0.281439021	2.467148252	0.326491011	3.73E-05
0.004604578	0.333816914	1.37273554	0.564661309	5.91E-05
0.002330809	0.117298047	0.775331809	0.808306838	1.17E-05
0.019008746	0.640875338	2.9516493	0.211868951	0.000240896
0.02833865	1.4700717	7.149523305	0.113036089	0.000380044
0.022214947	1.623300117	6.032355662	0.094966028	0.000412206
0.007559227	0.609150063	2.335290826	0.423409523	0.000117975
0.004263221	0.349886379	1.70419469	0.408426518	3.74E-05
0.001695043	0.042761471	0.55358646	1.605485119	3.88E-06
0.007005399	0.363940795	2.123519267	0.252694847	4.00E-05
0.015712447	1.462063134	4.838854459	0.138447852	0.000416264
0.0090156	0.60158453	2.504799921	0.383269955	0.000147531
0.003014904	0.157065945	0.920171331	0.874137729	2.19E-05
0.004548558	0.398379459	1.387286823	0.475045739	7.16E-05
0.012424214	0.648267417	2.361695721	0.289932474	0.000233276
0.001299273	0.052932297	0.526587109	1.139961674	3.18E-06
0.008441431	0.565789025	2.533172046	0.261543624	7.89E-05
0.004852631	0.445882832	1.99957529	0.332191413	4.71E-05
0.00157745	0.158817984	0.844672228	0.753095882	9.47E-06
0.006276157	0.426856097	2.516467472	0.265007384	4.55E-05
0.015615792	0.61902332	2.561519533	0.259148973	0.000219762
0.003337364	0.344970149	1.496310107	0.414113701	2.92E-05
0.020886642	1.610103154	5.080979516	0.116243639	0.000560468
0.002264026	0.211722361	1.10936201	0.621666619	1.49E-05
0.001530827	0.076427811	0.527821446	1.463804243	7.86E-06
0.005274326	0.246056941	1.173460759	0.498558329	6.16E-05
0.019136579	1.027877329	3.821429026	0.192866861	0.000422211
0.002532799	0.192230623	0.987782877	0.63602973	1.75E-05
0.006995388	0.648314915	2.219448451	0.458875795	0.000149537
0.010399622	0.509744735	2.169719811	0.425593892	0.000158266
0.003553878	0.169504628	1.823503685	0.461292107	1.09E-05
0.011731545	0.284625545	1.525737335	0.392351412	0.000124773
0.005072768	0.353196155	1.754022867	0.374129237	4.22E-05
0.024679826	3.363263205	9.981638363	0.061876453	0.000845544
0.009655034	0.431204075	2.369181096	0.250052106	8.87E-05
0.006933671	0.289853683	2.118628126	0.361374442	3.10E-05
0.00818963	0.780072499	3.072436447	0.246964056	0.000108026
0.012554969	0.609781967	2.692587775	0.217580487	0.000187072
0.004456677	0.258402785	1.028751543	0.79302551	6.50E-05
0.006087529	0.206264232	1.207853886	0.569096865	4.33E-05
0.005113091	0.18734496	1.312654582	0.507700691	3.17E-05
0.014218762	1.437043087	6.146338257	0.095139767	0.000249738
0.007939295	0.471197382	1.88383404	0.367057604	0.000107658
0.003558552	0.119514707	0.951631574	0.844159592	2.04E-05
0.00670732	0.348386438	1.802482728	0.354222588	5.10E-05
0.008256027	0.509945729	2.349895727	0.30689958	0.000101927
0.017580803	0.750899006	5.033901792	0.106399806	0.000151769
0.005012563	0.165236731	1.144842396	0.546756474	2.97E-05
0.002611961	0.145192016	0.969557582	0.841570217	1.34E-05
0.003489558	0.200012617	1.190120994	0.701015489	2.42E-05
0.003529533	0.111092621	0.716730636	0.852595288	2.12E-05
0.004661984	0.666684324	2.482004453	0.286410072	5.83E-05
0.004700813	0.259459304	1.231675172	0.602882952	5.53E-05
0.029688339	1.804815696	7.552073498	0.073549363	0.000504695
0.027406691	1.405604835	5.298907377	0.109483563	0.000605549
0.001424514	0.092757913	0.484825194	1.127571949	1.09E-05

log.sigma.3.5.mm.3D_gldm_GrayLevelVariance	log.sigma.3.5.mm.3D_gldm_HighGrayLevelEmphasis	log.sigma.3.5.mm.3D_gldm_GrayLevelNonUniformityNormalized	log.sigma.3.5.mm.3D_gldm_DependenceEntropy
40.07578355	352.2929363	0.046337505	6.819454942
35.06234584	256.0172911	0.054024201	6.160425148
35.93258964	265.4618834	0.046748376	6.598917095
41.61556267	268.8571429	0.046101616	6.223657621
32.33261993	213.2551382	0.050506776	6.560585255
36.03749744	300.5702479	0.047469435	6.10064995
16.76344875	194.5108644	0.092988265	7.269583768
28.68304141	370.6708965	0.056028328	7.131516885
21.16429147	183.5682778	0.069665114	7.021928218
21.14438139	175.8913907	0.061139424	6.411841076
11.40291623	113.1715938	0.090200633	6.491127743
17.86970247	197.5256824	0.076309079	7.062552703
38.09944034	338.1023428	0.051616111	6.644339223
39.61234134	273.8354037	0.045026234	6.418572859
28.43632794	182.464127	0.05678448	6.800391798
25.00920223	158.5997917	0.068407649	7.296208731
14.33828103	104.7281993	0.07312018	6.412351349
37.42336547	211.7408313	0.049527442	6.232142067
11.26059377	88.42413163	0.087915136	6.515455094
23.22186192	355.1376456	0.063069434	7.170401683
26.03772437	181.7107796	0.055534353	6.795308434
27.50584921	196.8729839	0.057565362	6.210212401
24.93135045	210.3888889	0.058413946	6.523308282
11.27642618	110.99861	0.104444295	6.876321732
32.20579197	289.5887622	0.054174315	7.105368683
32.79608736	458.6952269	0.054659814	7.067979737
31.79570873	220.2000998	0.05190567	6.796972274
18.73150501	144.26498	0.066360599	6.692355372
12.29705386	79.17808219	0.092794145	5.469341406
20.40068516	161.552795	0.06179777	6.631149829
24.79377338	176.2451983	0.057010103	7.02405829
35.95804292	308.501269	0.048516452	6.469056468
25.34017873	190.2783726	0.059717822	7.108823025
22.34468252	213.6542443	0.065548397	6.96267744
23.52021006	222.4494448	0.061138986	7.056345404
33.14712608	378.1217122	0.04912572	6.889793253
45.41619775	531.2804233	0.043703284	6.708621699
25.2423458	229.8979557	0.080500817	7.332264998
16.49355867	161.7821429	0.071192602	6.184457474
16.56928107	545.5498042	0.080859024	7.163123764
28.52848908	231.3783582	0.053128759	6.624666472
10.28766541	172.1854725	0.109385164	6.851932399
39.35152745	339.738627	0.044110856	6.973349977
30.80070373	202.3922535	0.050440389	6.64878238
15.30020412	140.938946	0.091324899	6.867206302
30.90622105	237.4106622	0.050395322	6.924285312
18.84575692	250.7774248	0.06734626	7.009026148
15.51839423	141.6802444	0.077770542	6.247074985
29.1213035	188.8107417	0.054231723	6.182158569
34.91441675	366.8473451	0.05272535	6.372137432
29.40153673	184.2657343	0.055394058	6.59497181
31.41447366	304.7378815	0.051158005	6.822876124
10.19803159	115.2605316	0.1204244	7.06090697
17.74342955	340.7356882	0.068367503	6.775502609
45.35648169	284.7153966	0.043112935	6.514520375
31.61805772	195.5059524	0.051340009	6.612570948
22.09280416	213.2346202	0.062134306	7.039013594
29.45054604	286.6129657	0.051965728	6.764418792
25.00931516	195.3066841	0.055428922	6.442510714
14.64468642	282.7556229	0.094822677	7.205653889
25.50635955	250.6869827	0.056031425	6.7361111
33.66013866	363.336714	0.048838053	6.89113207
31.18893839	429.6456157	0.051605726	7.220440382
30.08719421	271.0920802	0.05170654	6.802094877
21.15539274	167.183161	0.060330025	6.51575821
31.79659925	326.7503654	0.049576267	6.845487728
29.63435779	229.4458128	0.055133587	6.224148918
27.21785783	372.519764	0.056654919	6.880676296
16.71297537	191.8733351	0.071604176	7.01111346
24.23154865	255.7612994	0.056958232	6.686817532
28.35535643	196.8800813	0.054200542	6.409190845
27.04180532	318.806063	0.060049066	7.085897521
34.93714093	199.6411985	0.048994375	6.61661015
24.48195125	162.6557895	0.057480332	6.468421141
19.0614569	191.9865328	0.083192118	7.043316187
15.32005852	152.2712418	0.070794234	6.301783029
24.22518521	293.1317338	0.057328543	6.693314509
45.42301757	448.0278638	0.049966932	6.157307259
26.69709032	247.3155894	0.055077275	6.70386063
15.81521944	186.5094444	0.074958025	6.747307266
39.02575277	348.4203414	0.047315002	6.823172928
25.20414612	196.9854722	0.057868077	6.422238176
25.6685396	171.2927257	0.057627387	6.954250761
15.23127467	153.3465399	0.073484233	6.527437591
21.53823062	199.9156538	0.067887088	7.045066965
24.8100482	370.5178908	0.088533521	6.034240809
26.4622904	257.3256881	0.054425133	6.696553447
19.34226508	148.5461418	0.081527182	7.155755984
21.3134702	240.2351558	0.063209006	6.851454377
28.79020747	241.1989759	0.052523989	6.582474518
27.81829039	354.960199	0.058064776	6.767042944
16.76453106	177.5793839	0.07693186	6.939533539
18.3540833	217.0444346	0.06571168	6.82278492
22.12972252	191.5676146	0.0595024	6.847473914
15.21995101	167.9482915	0.070179181	6.653368262
43.56102695	401.602349	0.042473453	6.912653521
28.24481314	216.6349918	0.051720272	6.759277695
25.37386316	296.7284345	0.062336045	5.978278502
19.14819559	181.4253731	0.079388505	5.753163733
27.26987161	397.1651179	0.054635543	7.141813633

log.sigma.3.5.mm.3D_gldm_DependenceNonUniformity	log.sigma.3.5.mm.3D_gldm_GrayLevelNonUniformity	log.sigma.3.5.mm.3D_gldm_SmallDependenceEmphasis
315.1468144	66.91135734	0.356179227
88.60230548	18.74639769	0.343435374
220.6928251	41.69955157	0.342328762
84.67701863	14.8447205	0.381780538
327.8703047	71.26506024	0.296473426
61.04958678	11.48760331	0.396266609
1016.527659	980.003321	0.171641015
971.0698748	339.9798945	0.266765207
578.5598311	296.912717	0.219102728
154.9496689	46.1602649	0.288139779
239.2056555	140.3521851	0.187692094
427.6715586	259.9850308	0.216083651
162.3440197	41.86066584	0.309863202
165.9409938	28.99689441	0.436204131
278.7752381	89.43555556	0.304026685
685.0857341	394.1648733	0.215428906
155.9830125	64.56511891	0.198891175
102.7408313	20.25672372	0.411131933
152.4753199	96.17915905	0.18340744
1360.342916	579.1666122	0.244638126
388.4177093	115.400385	0.236714646
114.7137097	28.55241935	0.319391504
103.1469534	32.59498208	0.258877884
386.9099249	375.6861273	0.161407939
180.2296417	66.52605863	0.270871516
417.0446009	139.7104851	0.270797941
384.1546906	104.0189621	0.310961764
388.8375499	149.5104305	0.238837186
26.94520548	13.54794521	0.279122869
299.0757764	99.49440994	0.26454654
642.1224744	228.5535046	0.243449936
189.819797	38.23096447	0.363883812
356.7601713	139.4411135	0.253810176
343.9780538	158.2993789	0.24229329
805.7703098	313.8264173	0.24831142
400.8793192	95.2547705	0.324481348
381.7566138	66.07936508	0.374511864
619.8706408	468.5952585	0.21377642
116.0821429	39.86785714	0.23197324
1103.254472	763.8751985	0.191890837
271.9925373	71.19253731	0.300266495
623.280677	620.4326516	0.168469535
511.0132341	106.6600496	0.345680026
308.1169014	71.62535211	0.322503265
180.3791774	142.1015424	0.18853786
454.0845481	120.999167	0.318949288
768.5402728	360.3698374	0.218112136
80.14867617	38.18533605	0.248483456
99.62915601	21.20460358	0.338562092
113.7256637	23.83185841	0.327990087
252.0753691	71.29215229	0.347522801
590.8398564	142.4750449	0.311325585
633.8658475	960.505015	0.136556849
262.5761267	112.2594397	0.243879778
166.7542768	27.72161742	0.385029778
251.9727891	60.37585034	0.293658902
622.1088418	249.4692403	0.241065147
408.6516691	107.4131592	0.278935543
165.1179554	42.29226737	0.335154378
1052.561002	1032.903424	0.168262204
258.8254327	74.46576373	0.300656677
541.8275862	120.3858012	0.316254583
1379.97231	424.9215448	0.274730483
451.0639313	108.3769084	0.271398103
132.3441654	40.84342688	0.292776738
734.7211342	169.6004092	0.317852631
96.80788177	22.38423645	0.295024589
964.4941986	288.0902655	0.287788237
1037.664624	537.6041556	0.188940627
429.5998117	120.9792844	0.273082933
106.1544715	26.66666667	0.33009757
755.6974503	299.1043967	0.245515435
294.5925094	65.40749064	0.323537757
204.4526316	54.60631579	0.332645511
488.7301665	339.7566112	0.203312787
183.7647059	64.98910675	0.228268891
437.6871888	126.6387506	0.262226372
92.12074303	16.13931889	0.431889405
270.3505703	72.42661597	0.292901479
264.6155556	134.9244444	0.219266404
301.5675676	66.52489331	0.356143333
184.5859564	47.79903148	0.307699515
371.0438212	131.5056968	0.266598483
324.8235605	139.1056524	0.214141784
380.2883978	184.3134438	0.21559445
102.0508475	47.01129944	0.287237496
266.3577982	71.18807339	0.308092105
399.8373306	312.738269	0.19551346
280.3697825	107.5185185	0.26151428
308.4264813	71.80029261	0.300314755
138.5273632	46.6840796	0.246164835
304.2669826	194.7914692	0.211956696
759.6114197	295.7682737	0.239184115
602.7701683	205.0452699	0.235383797
555.0127003	232.0825522	0.224172976
549.3800336	101.2567114	0.354937721
505.8083197	126.8181077	0.269730699
73.5686901	19.51118211	0.342779498
62.94776119	21.2761194	0.266572283
1478.132593	482.1040345	0.270177147

log.sigma.3.5.mm.3D_gldm_DependenceNonUniformityNormalized	log.sigma.3.5.mm.3D_gldm_DependenceVariance	log.sigma.3.5.mm.3D_gldm_LargeDependenceEmphasis
0.218245716	2.352402913	9.394736842
0.255338056	1.314021377	7.328530259
0.247413481	1.816414969	8.213004484
0.262972108	1.394544964	6.906832298
0.232367332	1.657884969	8.899362155
0.25227102	1.520661157	7.107438017
0.096453901	23.57926919	58.10200209
0.160031291	7.00230284	19.80520765
0.135748435	11.60282271	30.01501642
0.20523135	2.19894566	10.52847682
0.153731141	4.730597538	19.55012853
0.125527314	11.18550518	30.9823892
0.200177583	2.690520753	11.03699137
0.257672351	1.588200687	6.770186335
0.177000151	3.82194971	13.70349206
0.118897212	15.65920818	38.33148212
0.176651203	3.035954079	15.27406569
0.251200077	1.767038695	7.356968215
0.13937415	5.332516736	21.83363803
0.148137092	6.659593694	20.88652946
0.186919013	3.049399687	13.54475457
0.23127764	1.5316077	8.358870968
0.184851171	2.752598245	12.65591398
0.107564616	14.52910292	41.80845149
0.146766809	12.94460684	29.50977199
0.163162989	4.638558267	16.20813772
0.191693957	2.584283529	11.10578842
0.172586573	4.527932082	16.42476698
0.184556202	3.108087821	12.8630137
0.185761352	2.843885653	12.61242236
0.160170236	5.038034589	17.61761038
0.240888067	1.576631709	7.687817259
0.152788082	7.392901797	21.4385439
0.142433977	7.51028021	22.69606625
0.156978436	7.52170363	21.26495227
0.206745394	3.618869592	12.03455389
0.252484533	1.496771857	7.260582011
0.106488686	22.97408051	52.79007043
0.207289541	2.150395408	11.65
0.116783579	11.77885592	33.98253414
0.202979505	2.263642237	10.46716418
0.109887284	14.74828955	40.54795487
0.211337152	2.502299749	9.925558313
0.216983733	2.120650665	9.509859155
0.115924921	14.01782634	37.47429306
0.189123094	4.00799646	13.21407747
0.143625542	6.420064386	21.58942254
0.163235593	3.252384053	14.75152749
0.254806026	1.271237106	7.31202046
0.251605451	1.360541154	7.654867257
0.195862758	3.034926396	11.17094017
0.212150756	2.074791538	9.728904847
0.079471646	22.2407785	64.94182548
0.159912379	4.229290206	16.46650426
0.259337911	1.735067687	7.404354588
0.214262576	2.010687098	9.894557823
0.154946162	8.815052892	23.63611457
0.197702791	2.534771652	11.43057571
0.216406233	2.554780485	9.99344692
0.096627284	23.13391775	57.20508583
0.194752019	3.194656901	12.10458992
0.219808351	3.705620842	11.92494929
0.167594402	6.311821415	18.26524168
0.215202257	2.310041664	10.79103053
0.195486212	3.060421662	11.98966027
0.214767943	2.023556552	9.482315113
0.238443059	1.372151714	8.448275862
0.189674375	3.111460569	12.38249754
0.138207862	6.546282864	23.47575919
0.202259798	2.341185483	11.10546139
0.215761121	1.956656091	9.264227642
0.151716011	6.639991736	20.71491668
0.220668546	1.981363745	9.200749064
0.215213296	2.325810526	9.724210526
0.119669482	11.91104029	33.25073457
0.200179418	2.294283775	12.1503268
0.198138157	2.351834065	11.46627433
0.285203539	1.113209175	5.743034056
0.205589787	2.003779728	10.10570342
0.147008642	5.82251358	20.44888889
0.214486179	2.400290161	9.551920341
0.223469681	2.290093745	9.869249395
0.16259589	9.938289291	23.81244522
0.17159195	3.551085002	15.63708399
0.140069391	8.103027651	24.6228361
0.192186153	3.012487543	12.17137476
0.20363746	2.825461287	11.16819572
0.104232881	16.47924742	43.94056309
0.164826445	4.908320001	16.81305115
0.225622883	1.780448218	9.158010241
0.172297715	3.216745873	14.17910448
0.120168635	8.438412335	27.58767773
0.168765034	3.7117772	15.32259498
0.174918795	3.417728934	14.66337783
0.167829664	3.564626043	15.62171152
0.230444645	1.825976307	8.347315436
0.20628398	2.054144301	10.65252855
0.235043738	1.628045606	8.111821086
0.234879706	1.546892404	9.253731343
0.16751276	5.133271402	16.76767906

log.sigma.3.5.mm.3D_gldm_LargeDependenceLowGrayLevelEmphasis	log.sigma.3.5.mm.3D_gldm_SmallDependenceHighGrayLevelEmphasis	log.sigma.3.5.mm.3D_gldm_LargeDependenceHighGrayLevelEmphasis
0.04944897	148.615086	2717.24169
0.099395708	85.39335536	1974.340058
0.086908371	94.2187321	2143.107623
0.230327209	124.142795	1555.447205
0.14455159	70.6741558	1679.803685
0.051636929	117.8860135	2022.355372
0.357904908	39.82814484	10564.06281
0.071517159	116.1492743	6605.567073
0.254115968	52.89368993	4697.774754
0.151166519	58.75400582	1638.291391
0.269231449	24.46840116	1946.765424
0.225044723	53.68751413	5326.227766
0.057566139	89.66933606	4473.628853
0.106836511	137.1262293	1435.282609
0.188690498	74.75468282	1760.385397
0.529058621	39.03418864	5234.453662
0.416017863	24.82698189	1385.613817
0.238503963	98.41530374	1221.031785
0.547084832	21.32545106	1732.577697
0.073452603	92.31682362	6962.735381
0.240149777	46.85974976	2165.551973
0.205405507	70.69818561	1397.824597
0.15812685	66.96437101	2309.012545
0.554760094	23.79398871	4157.463998
0.14270859	87.14990097	8380.766287
0.053399178	147.9017795	6260.75313
0.124328791	87.97458752	1899.076347
0.24931232	43.63631447	2022.281846
0.377996655	30.84496934	668.6575342
0.205007389	53.01317566	1669.771429
0.220936589	52.06080224	2570.629833
0.065987205	114.658489	2146.643401
0.21604158	57.66278082	3346.747752
0.157493063	57.74729484	4660.628157
0.153596393	70.03554051	3854.159556
0.050840442	124.5689867	4490.298092
0.02607358	197.327468	3918.085979
0.302861266	57.3216486	11301.45405
0.151090359	43.04921077	1857.221429
0.068580559	118.2612934	17724.24103
0.105318575	84.79238005	1899.557463
0.285253262	35.4952746	6360.868124
0.070496056	120.1971669	3120.82713
0.14550644	78.23937474	1472.259859
0.398886691	36.61821793	4615.640746
0.139111628	91.59557592	2309.303207
0.123229968	65.95501062	4747.175855
0.247748158	41.48702978	2011.415479
0.120544104	74.81863313	1169.859335
0.037787702	113.4627377	2946.236726
0.190738353	84.65191587	1357.381507
0.063212991	104.3455154	2647.922083
0.707985736	20.26838244	7074.335507
0.060510396	87.74614252	5424.704019
0.151737506	116.1809469	2057.060653
0.165828187	64.99906014	1608.941327
0.168852967	62.51656079	4460.639851
0.075383753	85.06203375	2935.167392
0.167968875	75.28445746	1471.568807
0.224889205	57.40556885	15510.52336
0.081199141	85.30178922	2719.296464
0.049005003	120.6752777	4091.079513
0.057754111	128.39103	6976.576391
0.090287445	79.49267835	2799.379294
0.159326405	51.77658566	1723.564254
0.05445467	110.1028542	2970.246419
0.245430212	66.94355688	1931.844828
0.050384355	123.2237006	3937.527827
0.183906223	42.41578384	4091.31553
0.065159721	68.23440172	2999.101224
0.135424027	73.34001666	1643.392276
0.094210454	90.12886194	6032.291708
0.180325483	76.77594391	1384.992509
0.154873435	63.19617623	1249.036842
0.255752144	51.17288067	5506.514447
0.147801207	35.87803955	1783.454248
0.062332666	75.20953079	3417.472159
0.037877841	192.8864792	2629.043344
0.078961774	75.60725597	2451.565019
0.160880119	51.50087175	3373.311667
0.049376283	145.5767852	2750.265292
0.15898774	60.18212872	2019.871671
0.285556626	54.79465301	3646.40929
0.189305582	41.58360291	1953.799789
0.193268093	52.09870032	4219.28582
0.041640721	87.94041756	5283.290019
0.083161579	91.45731347	2382.293578
0.494887791	39.72020516	5439.149374
0.108223895	67.94257965	3648.032922
0.078179409	79.38306023	2100.84199
0.06146504	90.89166829	4799.174129
0.257560597	50.52340084	4251.642575
0.109922823	57.57165825	3026.603644
0.153883277	51.92320154	2486.691817
0.158534086	42.47679887	2265.232235
0.044224565	145.7807836	3255.586409
0.115092043	61.971087	2177.064845
0.058135759	101.6080014	2384.217252
0.18045775	49.55659532	1774.238806
0.053163815	104.9311082	6644.327403

log.sigma.3.5.mm.3D_gldm_SmallDependenceLowGrayLevelEmphasis	log.sigma.3.5.mm.3D_gldm_LowGrayLevelEmphasis	log.sigma.3.5.mm.3D_gldzm_DistanceZoneVariabilityNormalized
0.002727478	0.006214895	0.891996432
0.008091455	0.016519126	0.934715121
0.005463628	0.013781798	0.884856889
0.007768994	0.02790182	0.887899654
0.003921272	0.013697005	0.889273356
0.009833665	0.013786449	0.847222222
0.001696696	0.00816519	0.947406866
0.001133991	0.004015911	0.889550222
0.001758191	0.009861885	0.958614363
0.004265506	0.015914294	0.939223376
0.003640936	0.016432536	0.956066153
0.002096515	0.008427738	0.929319001
0.005272214	0.00950726	0.830405827
0.005320624	0.012672743	0.814344736
0.003733567	0.013283797	0.940718648
0.004570117	0.021595733	0.958467457
0.004402977	0.025724016	0.960991065
0.010510631	0.02908834	0.835458701
0.005503538	0.031454896	0.9561624
0.001344186	0.004216603	0.95179057
0.003398627	0.01525898	0.931475949
0.005403141	0.018223262	0.885047897
0.004385423	0.012304399	0.975907198
0.002862145	0.018214104	0.957466919
0.002841085	0.008021698	0.950281133
0.000939117	0.003760599	0.931626342
0.003876681	0.011498816	0.930939751
0.00396797	0.017021046	0.95011072
0.020208989	0.04008165	0.82
0.003944134	0.016205746	0.980546624
0.002732242	0.012583463	0.953474712
0.004280858	0.009152617	0.876881142
0.003768044	0.012458243	0.877410773
0.002321377	0.008464857	0.960187141
0.002195324	0.008017631	0.938971664
0.00221666	0.00548502	0.789200139
0.00193207	0.004158015	0.68069431
0.001793855	0.008353454	0.942906574
0.003811125	0.016438625	0.883492149
0.000491356	0.002194671	0.933538885
0.002853467	0.009662569	0.866830458
0.00149376	0.008220955	0.890346272
0.002394281	0.007090866	0.937867766
0.004046467	0.014202786	0.917110871
0.00387061	0.015390468	0.953106259
0.002492405	0.009352287	0.925485247
0.001329254	0.00596646	0.948603063
0.004536452	0.018794458	0.986302021
0.007117801	0.016957918	0.898024311
0.004673987	0.008381185	0.95791421
0.004509302	0.015635771	0.973836375
0.002027884	0.00691702	0.907071608
0.002421961	0.015488098	0.926624478
0.001650559	0.004503432	0.95791421
0.005977789	0.019492092	0.925346404
0.004427859	0.014606264	0.925864422
0.001897404	0.008124054	0.947848913
0.002151194	0.00655851	0.924275135
0.004617269	0.01348264	0.863834478
0.000875868	0.004666987	0.925506562
0.002943866	0.00788755	0.956109954
0.002139562	0.005195228	0.896491445
0.001096167	0.003534102	0.855749757
0.003283063	0.010179769	0.824363847
0.010204707	0.019655389	0.933134686
0.002663909	0.006536991	0.843585052
0.005153092	0.022062693	0.863249403
0.001281906	0.004105665	0.852101455
0.00172219	0.008346914	0.948344081
0.00266864	0.007442411	0.924237255
0.005649823	0.015622469	0.930438941
0.00136051	0.005101523	0.930339239
0.005005576	0.017261661	0.965983728
0.007425727	0.017585902	0.974558933
0.002054785	0.009832879	0.820173748
0.003456179	0.014670231	0.970266999
0.002163915	0.006832564	0.911669784
0.005769652	0.009722362	0.807666105
0.003531542	0.010223601	0.905583363
0.002381235	0.009757075	0.973219222
0.002671536	0.005995341	0.903320929
0.004253968	0.017217042	0.96846236
0.004311787	0.016245405	0.973014498
0.002657184	0.011776038	0.970345705
0.002171142	0.009463442	0.890358654
0.003867247	0.006843853	0.875555556
0.002905848	0.008052145	0.91087481
0.00227881	0.014651752	0.970018483
0.003195773	0.009264466	0.930493395
0.003149804	0.009161042	0.946195066
0.002751242	0.006389619	0.790095066
0.002030376	0.010160475	0.941516628
0.001927907	0.007639793	0.916635482
0.002537904	0.010640384	0.929173875
0.002463082	0.010049894	0.894356576
0.001958346	0.005740702	0.784492509
0.003316384	0.012124191	0.87010031
0.005464097	0.010025086	0.89956382
0.005363882	0.023289066	0.895061728
0.001220025	0.003757966	0.882891022

log.sigma.3.5.mm.3D_gldzm_LowIntensityEmphasis	log.sigma.3.5.mm.3D_gldzm_LargeDistanceEmphasis	log.sigma.3.5.mm.3D_gldzm_HighIntensitySmallDistanceEmphasis
0.007476859	1.171849427	396.8686579
0.022417966	1.101351351	234.625
0.015950581	1.184	268.858
0.022804243	1.178807947	313.4519868
0.013065468	1.176470588	233.7828431
0.021517321	1.25	284.1520833
0.009930668	1.081081081	230.9755528
0.004171181	1.176	425.9881333
0.00821759	1.063419118	248.6091452
0.017559528	1.094117647	200.4264706
0.019680379	1.06741573	133.0351124
0.009405146	1.110059172	247.0005917
0.015292465	1.280645161	280.1153226
0.012090922	1.310650888	303.7995562
0.012242187	1.091726619	244.948741
0.02201116	1.063649222	181.4729491
0.024023487	1.059701493	130.4029851
0.027291329	1.271356784	235.0753769
0.031524967	1.067264574	117.0627803
0.005441045	1.074146724	374.1240683
0.014234527	1.106571936	198.1771758
0.016918776	1.183673469	216.0127551
0.01616084	1.036585366	259.4969512
0.020323199	1.065217391	154.5636646
0.010388761	1.076530612	317.8450255
0.00396753	1.106329114	543.6132911
0.0123201	1.107438017	276.1866391
0.017867204	1.0768	185.7184
0.065601933	1.3	108.91
0.016479765	1.029469548	197
0.011189291	1.071491615	218.2266108
0.011183952	1.197707736	311.9899713
0.014566075	1.196793003	224.4435131
0.009407815	1.06095791	237.0870827
0.008769769	1.094520548	281.0664384
0.006711328	1.359210526	366.6611842
0.004943394	1.598265896	491.003974
0.00860352	1.088235294	266.7862395
0.019266106	1.186335404	184.9440994
0.002476971	1.103244838	614.5773107
0.009247157	1.215189873	278.1344937
0.009570916	1.174647887	209.5816901
0.006859805	1.096288867	350.0549147
0.013333667	1.129963899	238.8131769
0.020490737	1.072072072	195.5938438
0.007989838	1.11627907	284.5542636
0.006124937	1.07918552	302.515083
0.020849522	1.020689655	164.4896552
0.019188341	1.161676647	218.1616766
0.012795645	1.064516129	348.8534946
0.014192743	1.039772727	240.0359848
0.007138611	1.146551724	328.8235153
0.018807531	1.114427861	151.990257
0.006479783	1.064516129	357.7225806
0.017619636	1.116504854	296.3624595
0.014482367	1.115662651	224.2662651
0.007822703	1.080380294	259.4991357
0.007450964	1.118248175	303.0547445
0.013400337	1.220447284	219.2907348
0.005235828	1.116244411	340.5730253
0.009374708	1.067346939	275.2826531
0.006353921	1.164256198	366.5147211
0.003947118	1.234743542	448.1758705
0.01234405	1.291845494	282.9531474
0.032373099	1.103896104	179.9989177
0.008031619	1.256564141	330.685859
0.021407516	1.22147651	221.2399329
0.004350898	1.243916242	410.0198076
0.009208537	1.079595704	227.6577701
0.009362425	1.118309859	245.2901408
0.016464552	1.108247423	221.6159794
0.00533644	1.108408617	368.5955525
0.01607238	1.051923077	239.6134615
0.021033462	1.038659794	187.820232
0.010642184	1.299674267	249.4660695
0.017213239	1.045283019	159.7160377
0.008790474	1.138929088	281.8180174
0.012642443	1.323353293	427.5404192
0.012528481	1.149028078	253.3693305
0.011977488	1.040723982	240.9366516
0.007228851	1.152801358	393.5878608
0.016020683	1.048076923	194.6009615
0.015891106	1.041039672	208.3142955
0.012202407	1.04516129	198.2107527
0.009934466	1.174626866	242.4820896
0.012541928	1.2	290.8388889
0.009077138	1.140243902	293.6920732
0.012229478	1.045667447	210.8665105
0.012831886	1.108159393	257.8178368
0.01001515	1.083003953	261.9288538
0.010490045	1.357446809	348.5638298
0.009558386	1.090452261	239.2943886
0.008408388	1.130744849	239.8474643
0.010655356	1.110294118	221.1622899
0.011051726	1.167857143	187.6720238
0.005904038	1.372930867	385.4223466
0.012817545	1.209476309	223.6165835
0.014700647	1.159090909	294.6212121
0.027307857	1.166666667	174.4333333
0.004485802	1.187365398	375.2431802

log.sigma.3.5.mm.3D_gldzm_LowIntensityLargeDistanceEmphasis	log.sigma.3.5.mm.3D_gldzm_HighIntensityEmphasis	log.sigma.3.5.mm.3D_gldzm_DistanceZoneVariability	log.sigma.3.5.mm.3D_gldzm_ZonePercentage
0.008358153	416.2324059	545.00982	0.423130194
0.023144718	239.2972973	138.3378378	0.426512968
0.01711001	277.912	331.8213333	0.420403587
0.024307372	320.7483444	134.0728477	0.468944099
0.014591906	240.2254902	453.5294118	0.361445783
0.023260961	295.0083333	101.6666667	0.495867769
0.010397203	236.0206388	1927.972973	0.193092324
0.005114431	442.8133333	1667.906667	0.308998022
0.008837948	251.1727941	1042.972426	0.255279212
0.018500579	204.3764706	239.5019608	0.337748344
0.020475527	134.9353933	340.3595506	0.228791774
0.010275378	252.4662722	785.2745562	0.248018785
0.017261823	294.2709677	257.4258065	0.382244143
0.033349607	309.5976331	275.2485207	0.52484472
0.013763466	247.7517986	523.0395683	0.353015873
0.022603207	183.5707214	1355.272984	0.245400902
0.024969797	131.5223881	193.159204	0.227633069
0.053744968	238.919598	166.2562814	0.486552567
0.032395669	118.67713	213.2242152	0.203839122
0.005626111	382.751275	2426.114162	0.277578134
0.015136856	201.714032	524.4209591	0.27093359
0.019730806	220	173.4693878	0.39516129
0.016494916	260.6402439	160.0487805	0.29390681
0.0260527	155.8726708	616.6086957	0.179038087
0.011115172	322.125	372.5102041	0.319218241
0.004210675	556.9139241	735.9848101	0.309076682
0.01339242	281.2465565	675.862259	0.362275449
0.020301312	187.3024	593.8192	0.277407901
0.152685266	109.9	41	0.342465753
0.016720433	197.9312377	499.0982318	0.316149068
0.012569549	221.0326567	1080.286849	0.282614118
0.013331165	318.9484241	306.0315186	0.442893401
0.016843623	232.6049563	601.9037901	0.29379015
0.009722784	240.605225	661.5689405	0.285300207
0.010516209	284.7527397	1370.89863	0.284434054
0.015709361	382.2039474	599.7921053	0.391954616
0.008979745	526.2312139	471.0404624	0.457671958
0.009185371	271.3445378	1346.470588	0.245318674
0.050311208	186.6770186	142.242236	0.2875
0.002717221	628.9936087	1898.818092	0.215306446
0.011945049	286.0759494	410.8776371	0.353731343
0.012221243	217.2676056	948.2187793	0.187764457
0.008813871	352.8284855	935.0541625	0.412324235
0.021190718	244.2743682	508.0794224	0.390140845
0.022096694	196.8258258	317.3843844	0.214010283
0.012451163	289.6256921	835.7131783	0.376093294
0.006807656	307.5806938	1257.847662	0.247804149
0.021172797	164.8206897	143.0137931	0.295315682
0.023138607	220.011976	149.9700599	0.427109974
0.013192198	351.7688172	178.172043	0.411504425
0.021843679	240.4393939	514.1856061	0.41025641
0.007935731	338.3285441	946.9827586	0.37486535
0.023501746	155.615257	1117.509121	0.151203611
0.006679185	363.2645161	445.4301075	0.28319123
0.019919247	299.1197411	285.9320388	0.480559876
0.016345591	227.3277108	384.2337349	0.352891156
0.012455089	262.7389801	1096.661193	0.288169365
0.00804173	310.3532847	633.1284672	0.331398162
0.026512869	224.658147	270.3801917	0.410222805
0.005988576	349.9880775	1863.044709	0.184797576
0.009838835	278.9836735	468.4938776	0.368698269
0.007034625	381.518595	867.803719	0.392697769
0.004883799	471.0928491	2285.7076	0.324386689
0.019897534	297.5765379	576.230329	0.333492366
0.033714697	182.3593074	215.5541126	0.341211226
0.012247983	346.3390848	1124.498875	0.389652148
0.025661912	225.0604027	128.6241611	0.366995074
0.005769804	430.4352009	1505.663271	0.347492625
0.009736877	232.5590651	1501.22868	0.210841769
0.010009953	251.556338	656.2084507	0.334274953
0.018011209	224.3608247	180.5051546	0.394308943
0.0083405	374.2515636	1338.758165	0.288897812
0.018026976	240.8076923	502.3115385	0.389513109
0.021405947	189.0515464	378.128866	0.408421053
0.019996516	261.1411509	755.3800217	0.225514202
0.017905232	161.0150943	257.1207547	0.288671024
0.009540064	290.1895803	629.9638205	0.312811227
0.015095089	444.1976048	134.8802395	0.517027864
0.014122942	260.6846652	419.2850972	0.352091255
0.012258081	242.8371041	430.1628959	0.245555556
0.008063951	409.5670628	532.0560272	0.418918919
0.016662492	196.2115385	302.1602564	0.377723971
0.017180473	209.8399453	711.2735978	0.320333041
0.014491627	198.8623656	451.2107527	0.245641838
0.011245473	250.9044776	596.5402985	0.246777164
0.013553881	301.5722222	157.6	0.338983051
0.017035608	299.2987805	448.1504065	0.376146789
0.01280266	212.1030445	828.3957845	0.222627737
0.013706929	262.2296015	490.370019	0.309817754
0.010580233	266.2924901	478.7747036	0.370153621
0.012068415	374.4212766	185.6723404	0.292288557
0.017262324	241.8157454	562.0854271	0.235781991
0.009558229	245.5752773	1156.793978	0.280382137
0.011644688	226.0892857	884.5735294	0.276262333
0.014057767	193.6595238	751.2595238	0.254006653
0.007815416	414.2259007	805.6738072	0.430788591
0.015018458	231.4102244	697.8204489	0.327079935
0.017287394	299.5984848	118.7424242	0.42172524
0.02941999	178.0333333	80.55555556	0.335820896
0.00532424	391.269921	2459.734386	0.315729828

log.sigma.3.5.mm.3D_gldzm_IntensityVariabilityNormalized	log.sigma.3.5.mm.3D_gldzm_LowIntensitySmallDistanceEmphasis	log.sigma.3.5.mm.3D_gldzm_IntensityVariability
0.038677171	0.007256535	23.63175123
0.046749452	0.022236279	6.918918919
0.045319111	0.015660724	16.99466667
0.046620762	0.022428461	7.039735099
0.049803922	0.012683859	25.4
0.045972222	0.021081411	5.516666667
0.051456996	0.009814034	104.7149877
0.04557056	0.003935369	85.4448
0.054817596	0.0080625	59.64154412
0.054117647	0.017324265	13.8
0.067731347	0.019481593	24.11235955
0.052982739	0.009187588	44.7704142
0.044703434	0.014800125	13.85806452
0.047512342	0.006776251	16.0591716
0.048192381	0.011861867	26.79496403
0.050974394	0.021863148	72.07779349
0.068636915	0.023786909	13.7960199
0.048811899	0.02067792	9.713567839
0.070079833	0.031307291	15.62780269
0.045340745	0.005394778	115.5735583
0.054879184	0.014008945	30.89698046
0.052998751	0.016215769	10.3877551
0.052498513	0.016077321	8.609756098
0.06628313	0.018890824	42.6863354
0.044916181	0.010207158	17.60714286
0.044031405	0.003906744	34.78481013
0.045587354	0.012052021	33.09641873
0.05619456	0.017258677	35.1216
0.0656	0.0438311	3.28
0.056928142	0.016419598	28.97642436
0.053658635	0.010844227	60.79523389
0.046559552	0.010647148	16.24928367
0.050110923	0.013996687	34.37609329
0.05554631	0.009329072	38.27140784
0.048509101	0.008333159	70.82328767
0.046464681	0.00446182	35.31315789
0.043732667	0.003934306	30.26300578
0.048454676	0.008458058	69.19327731
0.069480344	0.01150483	11.1863354
0.054289952	0.002416908	110.425762
0.051647706	0.008572685	24.48101266
0.06271507	0.008908334	66.7915493
0.046489519	0.006371288	46.35005015
0.050912953	0.011369405	28.20577617
0.056029002	0.020089248	18.65765766
0.048919009	0.006874506	44.17386489
0.055245024	0.005954258	73.25490196
0.066539834	0.020768703	9.648275862
0.050449998	0.018200775	8.425149701
0.042952943	0.012696507	7.989247312
0.050454832	0.012280009	26.64015152
0.046461444	0.006939331	48.50574713
0.063154377	0.017633978	76.1641791
0.054475662	0.006429932	25.3311828
0.042783381	0.017044734	13.22006472
0.048221803	0.01401656	20.01204819
0.054004448	0.006664607	62.48314607
0.049927007	0.007303272	34.2
0.057048658	0.010122204	17.85623003
0.054081407	0.005047641	108.8658718
0.050020825	0.009258676	24.51020408
0.043401236	0.006183745	42.01239669
0.043806903	0.003712948	117.0082366
0.049105507	0.010455679	34.32474964
0.051067259	0.032037699	11.7965368
0.045023071	0.006977528	60.01575394
0.052385028	0.020343917	7.805369128
0.048618498	0.004000538	85.90888512
0.058860149	0.009076451	93.17561592
0.051081135	0.009200543	36.26760563
0.052928048	0.016077887	10.26804124
0.047816678	0.004585425	68.80820014
0.048128698	0.015583731	25.02692308
0.052117653	0.020940341	20.22164948
0.053120516	0.0083036	48.92399566
0.06494838	0.017040241	17.21132075
0.052146578	0.008603077	36.03328509
0.045716949	0.012029282	7.634730539
0.048080646	0.012129866	22.26133909
0.055250711	0.01190734	24.42081448
0.039135711	0.007020076	23.05093379
0.054487179	0.01586023	17
0.052692842	0.015568764	38.51846785
0.064076772	0.011630101	29.79569892
0.051267543	0.009606714	34.34925373
0.054135802	0.012288939	9.744444444
0.049722387	0.007087521	24.46341463
0.052454629	0.012086182	44.79625293
0.049332263	0.012613125	25.99810247
0.049305566	0.009873879	24.9486166
0.046808511	0.010095453	11
0.058632077	0.007632402	35.00335008
0.056335251	0.008120928	71.09508716
0.054028229	0.010408023	51.43487395
0.063157596	0.010300216	53.05238095
0.041289279	0.005428072	42.40408958
0.049119719	0.012267317	39.39401496
0.055211203	0.014053961	7.287878788
0.065679012	0.026779824	5.911111111
0.046702335	0.004276192	130.1127064

log.sigma.3.5.mm.3D_gldzm_HighIntensityLargeDistanceEmphasis	log.sigma.3.5.mm.3D_gldzm_SmallDistanceEmphasis	log.sigma.3.5.mm.3D_glcm_SumVariance	log.sigma.3.5.mm.3D_glcm_Homogeneity1
493.6873977	0.957037643	979.3414266	0.294940684
257.9864865	0.974662162	619.4872856	0.287095971
314.128	0.954	694.454489	0.290468122
349.9337748	0.955298013	651.4189062	0.272169476
265.9960784	0.955882353	536.8460286	0.313845355
338.4333333	0.9375	807.0786518	0.292552961
256.2009828	0.97972973	540.6978257	0.476583311
510.1141333	0.956	1058.017991	0.351769048
261.4273897	0.984145221	468.9981975	0.407878407
220.1764706	0.976470588	423.9729544	0.347456237
142.5365169	0.983146067	276.4307595	0.427463581
274.3289941	0.972485207	525.3188938	0.422251348
350.8935484	0.92983871	936.4161683	0.306325328
332.7899408	0.922337278	663.5254812	0.273398062
258.9640288	0.977068345	442.497972	0.338668222
191.9618105	0.984087694	397.8328926	0.433265296
136	0.985074627	228.0548964	0.396326174
254.2964824	0.932160804	465.3732531	0.28843329
125.1345291	0.983183857	190.7814042	0.429583862
417.260102	0.981463319	1046.458462	0.376782035
215.8614565	0.973357016	441.7918558	0.358157443
235.9489796	0.954081633	447.1680286	0.313505005
265.2134146	0.990853659	512.9039851	0.346881289
161.1086957	0.983695652	263.0594914	0.486860877
339.244898	0.980867347	781.4486271	0.379674612
610.1164557	0.973417722	1350.946676	0.355334969
301.4862259	0.973140496	555.8276342	0.318687689
193.6384	0.9808	336.6884976	0.382707593
113.86	0.925	171.9157717	0.387587974
201.6561886	0.992632613	387.2776696	0.361472305
232.2568402	0.982127096	436.5156478	0.373180987
346.782235	0.950573066	805.4977333	0.282771397
265.2507289	0.950801749	487.3286035	0.378186772
254.6777939	0.984760522	582.3794733	0.407134861
299.4979452	0.976369863	593.1919534	0.375755719
444.375	0.910197368	1044.258469	0.308771832
667.1401734	0.850433526	1446.501909	0.265298767
289.5777311	0.977941176	625.6794167	0.454355714
193.6086957	0.953416149	369.9374764	0.357448321
686.6588004	0.974188791	1736.459756	0.446114398
317.8417722	0.946202532	594.5324384	0.326808208
248.0112676	0.956338028	469.403772	0.473474114
363.9227683	0.975927783	925.2510487	0.292801175
266.1191336	0.967509025	494.7570512	0.316133735
201.7537538	0.981981982	340.1277165	0.45632968
309.9114064	0.970930233	627.284917	0.327997043
327.8431373	0.98020362	687.7711914	0.399021295
166.1448276	0.994827586	328.8052379	0.380725775
227.4131737	0.959580838	441.6416726	0.308772742
363.4301075	0.983870968	987.0815892	0.2893633
242.0530303	0.990056818	406.6785181	0.339833831
376.348659	0.963362069	848.5355635	0.31462498
170.115257	0.971393035	288.178479	0.531355544
385.4322581	0.983870968	1008.56225	0.375054736
310.1488673	0.970873786	724.5465063	0.276761245
239.573494	0.971084337	473.4759796	0.323491812
275.6983578	0.979904927	556.8772626	0.391534885
339.5474453	0.970437956	786.1841783	0.321961499
246.1277955	0.944888179	459.3771836	0.31675961
387.6482861	0.970938897	828.4184579	0.480551556
293.7877551	0.983163265	679.0310916	0.334725196
441.5340909	0.95893595	1042.199272	0.310439815
562.7607638	0.941314115	1257.898565	0.346265566
356.0701001	0.927038627	728.3155469	0.327381643
191.8008658	0.974025974	399.262701	0.347207591
408.951988	0.935858965	902.7962891	0.301750247
240.3422819	0.944630872	534.7037352	0.301868893
512.45897	0.939649752	1068.129906	0.332126028
252.1642451	0.980101074	520.5046935	0.41596106
276.6211268	0.970422535	709.7432403	0.340736812
235.3402062	0.972938144	453.7976822	0.317184954
396.8756081	0.972897846	891.6712212	0.364666035
245.5846154	0.987019231	475.7477972	0.30577093
193.9768041	0.990335052	386.6684869	0.339067862
307.8414767	0.925081433	502.1606569	0.426903936
166.2113208	0.988679245	370.6301686	0.368097468
323.6758321	0.965267728	831.7570596	0.338332204
510.8263473	0.919161677	1176.164628	0.253509962
289.9460043	0.962742981	662.4561386	0.329681826
250.438914	0.989819005	480.422646	0.406454396
473.483871	0.96179966	967.7729946	0.297878056
202.6538462	0.987980769	503.8484053	0.330264286
215.9425445	0.989740082	403.1363379	0.373593492
201.4688172	0.988709677	365.7327029	0.387428932
284.5940299	0.956343284	527.307101	0.400177681
344.5055556	0.95	1048.043619	0.339064095
321.7256098	0.964939024	683.2253742	0.328559041
217.0491803	0.988583138	363.5509826	0.461450991
279.8766603	0.972960152	653.6888245	0.366539639
283.7470356	0.979249012	642.2860349	0.321680082
477.8510638	0.910638298	1023.71662	0.349367583
251.9011725	0.977386935	447.6364977	0.427256283
268.4865293	0.967313788	588.8305455	0.374427519
245.7972689	0.972426471	501.3895368	0.370735256
217.6095238	0.958035714	421.9251266	0.388417764
531.932814	0.907849183	1108.817158	0.281585081
262.584788	0.947630923	564.986145	0.331043275
319.5075758	0.960227273	783.0155207	0.311928305
192.4333333	0.958333333	418.2879774	0.334491488
455.3768844	0.95315865	1172.605897	0.346863449

log.sigma.3.5.mm.3D_glcm_Homogeneity2	log.sigma.3.5.mm.3D_glcm_ClusterShade	log.sigma.3.5.mm.3D_glcm_MaximumProbability	log.sigma.3.5.mm.3D_glcm_Idmn	log.sigma.3.5.mm.3D_glcm_SumVariance2
0.20546956	639.6596941	0.012124528	0.976373079	113.4481962
0.194475555	-82.58823763	0.016606901	0.959993633	108.8884614
0.200000713	-122.824145	0.01234678	0.961021688	105.5145316
0.178499422	-132.4438675	0.013645874	0.957364895	127.091068
0.221340946	186.5877011	0.009870399	0.972872753	100.5711337
0.198749267	-149.3283925	0.017336149	0.96296012	113.9695388
0.41312501	222.0522941	0.085480427	0.989062606	51.07546514
0.269533557	436.5896229	0.025050023	0.981573604	81.18970335
0.33246182	383.71852	0.0447537	0.980080797	64.09112583
0.256378351	95.34954486	0.015495369	0.972649962	60.69696239
0.351418955	73.50647192	0.036990076	0.978837107	36.07942682
0.34973292	187.4650174	0.041721509	0.985317511	52.67638355
0.217627792	-576.088601	0.017007122	0.964776609	114.0501687
0.180214805	-37.36301203	0.01029593	0.965176748	117.5012915
0.254144809	459.2134734	0.013992556	0.978418497	79.93716668
0.367121285	193.7323867	0.070862552	0.975036192	76.67226362
0.31325741	43.41936951	0.021080702	0.969037653	40.58770068
0.195380097	176.6195767	0.0135138	0.965788954	113.6443497
0.35394839	48.57183846	0.029557011	0.974035623	32.14956127
0.295481818	255.1602663	0.020054165	0.987012555	65.03907756
0.271735518	35.42241123	0.013543389	0.973142644	79.06637686
0.221340804	-14.21093839	0.014865256	0.962199563	84.04463718
0.258550605	56.53419344	0.019217953	0.971449905	75.94798257
0.423056687	98.37492538	0.085284631	0.983701101	35.83051811
0.303424579	60.96366563	0.052256219	0.975426603	93.85954303
0.273047386	676.0128783	0.016603113	0.984460141	97.08586867
0.229496099	378.1816812	0.011325831	0.97668912	89.23320335
0.299392195	148.0068036	0.019313519	0.977702126	55.78971071
0.301055356	135.6668344	0.043631552	0.965394538	37.29388662
0.273676344	99.89192951	0.013709416	0.977563888	59.61598175
0.291661089	323.7817802	0.014252154	0.977267609	74.31020584
0.190584151	-200.9968904	0.011283002	0.962837183	104.0068348
0.297282869	407.5103593	0.023168334	0.98195443	79.10821568
0.332518514	175.0104218	0.039564572	0.98045068	74.68298497
0.293971953	286.8123548	0.021254505	0.981971514	68.50743462
0.221752042	-284.0268866	0.012367462	0.969777007	97.07920758
0.174811098	-469.3682711	0.008431954	0.964161827	133.699207
0.39083655	170.8446288	0.104354087	0.980659489	78.92136435
0.265433465	-75.32726595	0.0188354	0.970511623	51.49812837
0.3764383	203.9493146	0.051112373	0.992081244	54.98688322
0.236094628	186.4241108	0.014967636	0.979055743	84.25590046
0.407099915	113.1164856	0.068688491	0.990191036	33.48668221
0.20410318	-11.65762507	0.009151134	0.966581382	117.2487471
0.22413138	365.0958386	0.010539523	0.970857557	94.30385194
0.388852513	158.6366577	0.075067477	0.985122499	46.20303113
0.241169494	231.130548	0.012864537	0.972507695	94.37952941
0.321203388	134.3404754	0.02132891	0.98614934	56.21722866
0.293470699	-51.43343733	0.02556729	0.973354048	46.45969473
0.213952709	384.7665661	0.013830026	0.970310983	91.92054634
0.197522411	-359.0657023	0.0158321	0.967080137	104.1222169
0.253496073	511.1206691	0.019532216	0.972180361	82.20556213
0.222938812	328.2681299	0.009454696	0.980298862	92.18389349
0.477973189	103.5966615	0.098169812	0.98867224	31.33257499
0.291610617	-0.049598093	0.019223796	0.984284501	48.63029798
0.184495002	-256.4419805	0.01294358	0.964207199	137.8864246
0.232370075	406.0302911	0.01177842	0.973698514	94.96445892
0.313182128	256.1194202	0.026858624	0.978115897	67.67803753
0.232923969	22.64184163	0.010656673	0.974902122	81.6988983
0.224384416	45.96861549	0.014575018	0.96522668	72.56827689
0.417503932	90.99132504	0.092869993	0.988652756	44.48870002
0.246373138	103.6598882	0.016431923	0.975536592	74.94787468
0.220162812	172.556296	0.014373011	0.978918559	98.11371467
0.262642609	273.4169246	0.020586021	0.984531304	96.68830658
0.236285985	31.62906773	0.010473635	0.98110452	93.95190177
0.258537451	10.48203584	0.018012374	0.970038979	56.56186045
0.209681043	50.44076864	0.008854462	0.977065277	89.47966796
0.207939736	-237.4814924	0.01375604	0.953764684	88.25654644
0.243258359	365.0712085	0.013661349	0.98475899	80.16495647
0.338806246	153.479427	0.025091527	0.985842716	52.89280549
0.251286731	-18.40910694	0.012708648	0.977829994	74.01904343
0.225740129	69.46805214	0.01580989	0.961665317	82.21408543
0.286556373	117.1715788	0.034929967	0.979719347	78.54279089
0.215625441	351.9109276	0.013012005	0.96778035	100.5464396
0.249028587	187.8028474	0.016462821	0.972084947	67.50462368
0.353754841	352.8257254	0.058363774	0.986971417	63.70016901
0.278521718	-17.85226979	0.016879791	0.971788926	46.40390419
0.247101249	-4.200901443	0.013799195	0.982008004	69.37606562
0.160928301	-780.2427704	0.016124014	0.957437773	138.7058665
0.23776632	28.93870505	0.013227891	0.976040748	83.46096758
0.327521449	128.6475059	0.02628597	0.983284517	47.60603805
0.208380918	665.3361894	0.012466686	0.977191536	110.5074805
0.238880602	-144.0085186	0.015685167	0.967673955	77.11196577
0.293524733	199.3750154	0.043209065	0.970577955	74.52173282
0.303436156	94.36815205	0.02051156	0.980500275	44.48786917
0.323305781	246.4414877	0.039119283	0.979798263	67.50453758
0.251931791	-631.8535312	0.037846872	0.97114191	74.35641751
0.239795228	129.5451625	0.014102513	0.970949796	81.33208422
0.396873442	288.769039	0.074220485	0.98180028	58.72255769
0.282716098	0.522775276	0.021633051	0.980869513	61.69624601
0.22971745	107.8721118	0.01241502	0.975002694	87.38040591
0.26351359	-101.3841031	0.017221225	0.976981088	85.3321077
0.354826575	117.6153484	0.039655441	0.982693042	51.03295653
0.288798196	121.9807516	0.013266226	0.986382015	55.88984993
0.28662464	177.5487159	0.015979178	0.979355247	70.18955323
0.303083971	52.36977344	0.017387268	0.978970564	45.3132134
0.190692628	269.0481017	0.007377818	0.978476406	129.3074513
0.240909874	84.80212328	0.010026229	0.971568526	86.31922208
0.217343055	-355.9363964	0.017994896	0.965001677	77.6269017
0.244851062	-250.6356893	0.024617709	0.956611627	59.54837676
0.262329362	65.50162466	0.017310011	0.982308265	78.40628848

log.sigma.3.5.mm.3D_glcm_Contrast	log.sigma.3.5.mm.3D_glcm_DifferenceEntropy	log.sigma.3.5.mm.3D_glcm_InverseVariance	log.sigma.3.5.mm.3D_glcm_Entropy	log.sigma.3.5.mm.3D_glcm_Dissimilarity
38.79078322	3.689393596	0.214854517	8.612483868	4.791241929
34.89476665	3.541587386	0.195887725	7.791381134	4.725066367
36.49476224	3.590549213	0.204236337	8.39121521	4.77793122
40.34395073	3.657852712	0.178742378	7.948516986	5.084814785
26.49859237	3.427153027	0.229761789	8.324502837	4.048908978
32.44858512	3.474979741	0.21027828	7.623285917	4.495726559
15.89834809	3.023135926	0.308986566	7.304459857	2.632139188
28.46500712	3.485606598	0.256329547	8.363972218	3.940801385
19.41806758	3.208868735	0.288911386	7.753054913	3.157947473
18.68323993	3.179941267	0.263354688	7.743530238	3.370801584
11.030376	2.845441672	0.334618283	7.12366325	2.489088043
17.02386369	3.130641953	0.312643393	7.59921919	2.926827703
35.345278	3.584361624	0.213831753	8.241244528	4.593473128
39.33011536	3.626624036	0.193906592	8.365811701	4.995591479
30.55518561	3.503965069	0.254983282	8.15197241	4.068808994
23.42023794	3.203169096	0.296711594	7.751550501	3.282281662
12.41447836	2.931798401	0.318981994	7.262831035	2.702591996
34.02807648	3.554058008	0.20710758	7.969810622	4.620985287
10.30080091	2.782255925	0.348864469	6.966621018	2.404810061
20.84718481	3.27018196	0.285966991	8.051728185	3.349805993
19.85678477	3.231309276	0.271504342	8.039219215	3.407704464
26.3209305	3.390124159	0.227740587	7.883181092	4.062686826
22.81664079	3.311074146	0.24377628	7.784428956	3.67032636
10.17401504	2.793464407	0.343456014	6.937698599	2.19778441
29.53872889	3.425641401	0.261897315	8.041729442	3.856151285
26.43885112	3.40434463	0.272753265	8.219885971	3.771905455
31.02952463	3.495723355	0.233575467	8.314433905	4.23311777
16.35072521	3.121956523	0.295930705	7.734147541	3.046626544
12.56461339	2.847557138	0.305577804	6.27458948	2.749009808
17.62280103	3.148255664	0.27805758	7.813036759	3.236307361
20.76613149	3.247303981	0.285372507	8.060657388	3.38177653
37.0175198	3.614378076	0.198006219	8.341344244	4.843926099
21.05295262	3.278972272	0.278628178	8.055889075	3.383079628
20.37564065	3.21642913	0.295040106	7.810550435	3.200142667
21.10741964	3.286963019	0.281348973	8.040891782	3.374702177
36.17326903	3.61112916	0.221150344	8.563441402	4.617398728
51.97169159	3.724406503	0.180056272	8.777797533	5.607430249
22.95453684	3.221274565	0.279963383	7.653053729	3.1985577
15.55714266	3.015088096	0.266182768	7.430519139	3.13026096
14.53564192	3.018769111	0.326835679	7.563505047	2.640053271
24.36820899	3.368252279	0.247030408	8.16466456	3.837797314
10.53050467	2.828066121	0.343316107	6.955753142	2.26830789
40.2309439	3.536839728	0.202038426	8.656697627	4.933494766
26.81467217	3.434462964	0.231386901	8.298737399	4.047927575
12.56110481	2.902506448	0.343939902	7.094124174	2.466681146
28.83343759	3.486755191	0.23709511	8.426422062	4.123585118
18.01418347	3.172126552	0.304774412	7.826811462	3.066942559
13.98062818	2.99048687	0.28336448	7.151465657	2.920115312
25.56581659	3.336822142	0.22855279	7.758705241	4.00017457
34.77546315	3.568359944	0.202243534	7.997357684	4.685308596
25.67889786	3.408593592	0.256209431	7.999533482	3.84777394
28.84501877	3.496896263	0.230696704	8.48985352	4.140731174
8.790134124	2.659525457	0.361198305	6.565804196	1.941617687
18.18776295	3.122449112	0.298167008	7.60297779	3.175827566
40.64031431	3.682552149	0.189796182	8.424897095	5.047352964
25.78758725	3.394723895	0.235357749	8.183219474	3.94641301
20.07122442	3.22480978	0.289123968	7.936806156	3.254402016
27.67496148	3.458823783	0.232230617	8.256304795	4.100790118
26.05524129	3.413213449	0.227815865	8.033544456	4.018049881
13.92484152	2.967532578	0.31936444	7.220079541	2.496653873
25.55898778	3.410780973	0.24878467	8.149837943	3.860775968
30.86330057	3.542813563	0.223847102	8.567596652	4.314137017
28.93986268	3.481758036	0.256034839	8.568502172	3.970471811
24.69842751	3.382603495	0.245789587	8.406106292	3.835243542
20.57452305	3.238269804	0.261464664	7.728121699	3.513376318
31.90123754	3.566934753	0.214421285	8.601952567	4.420700872
30.34229524	3.464506463	0.20006635	7.860220553	4.372564724
25.68661995	3.424399168	0.247753062	8.353752111	3.879925853
13.49893724	2.999508322	0.322595038	7.613349309	2.700344461
21.41417317	3.297853712	0.258121549	8.148704752	3.592943976
26.78604455	3.364274614	0.22457154	7.781119925	4.075618441
28.17782489	3.456482608	0.260897007	8.250190504	3.906132587
31.93545106	3.54189155	0.220591573	8.360483451	4.41561262
22.22120685	3.317269752	0.24970294	7.862274576	3.65565316
16.9844271	3.119165643	0.305461422	7.640298103	2.890477448
14.83025396	3.025770392	0.276038541	7.476938855	3.033772564
20.7548993	3.272809666	0.25343886	8.056581454	3.560977355
48.90565977	3.747097973	0.170459023	7.990880346	5.61725136
23.3520965	3.338470291	0.243147682	8.216289902	3.754537037
15.1413975	3.061527161	0.314087812	7.50127568	2.833161822
37.33037663	3.662497895	0.218188023	8.559909819	4.702140911
22.22028633	3.296910162	0.247370446	7.989505179	3.709219216
25.61934727	3.381087258	0.251208866	8.004904882	3.715833503
14.12998835	3.013721879	0.308445685	7.50408537	2.861421112
18.3841915	3.175918455	0.299770547	7.834898867	3.114102742
24.88196681	3.36417377	0.255482097	7.392763848	3.807294291
26.87039192	3.412232793	0.243381971	8.226786651	3.976881497
15.58193336	3.029064303	0.325861598	7.397786913	2.671896395
21.00296878	3.282982425	0.274969722	7.925534862	3.427865663
24.27190054	3.362386661	0.241858795	8.228455527	3.867368374
24.00500784	3.331078801	0.261253016	7.937009366	3.69162316
14.67250359	3.011506044	0.332459408	7.445321251	2.725430738
16.51753037	3.140491479	0.286452909	7.846596092	3.101920956
18.62769769	3.205419548	0.280753556	8.024093384	3.273115855
12.99650569	2.951070709	0.306428835	7.514663362	2.782984427
40.49149669	3.709248822	0.198825605	8.875291121	4.997336286
24.48088067	3.370925951	0.24511863	8.31495256	3.822313236
26.36983854	3.384850864	0.224020159	7.55052731	4.030741974
21.67824754	3.214246691	0.254988171	7.170105367	3.665843128
25.77319994	3.412486349	0.258500899	8.377182238	3.820629576

log.sigma.3.5.mm.3D_glcm_DifferenceVariance	log.sigma.3.5.mm.3D_glcm_Idn	log.sigma.3.5.mm.3D_glcm_Idm	log.sigma.3.5.mm.3D_glcm_Correlation	log.sigma.3.5.mm.3D_glcm_Autocorrelation
15.12307483	0.897430255	0.20546956	0.494082859	321.2113425
11.41958692	0.86516918	0.194475555	0.520146395	211.9990542
12.29818835	0.868286518	0.200000713	0.487198175	235.7506225
13.51381386	0.860817714	0.178499422	0.522642476	220.7417109
9.640215343	0.888276727	0.221340946	0.582753248	189.7169948
11.41793547	0.870938074	0.198749267	0.560436535	268.9058951
8.630694816	0.938378386	0.41312501	0.53022537	189.0991774
12.3399513	0.912191002	0.269533557	0.485421306	344.4788147
8.871962072	0.911841965	0.33246182	0.534111447	168.0117487
6.876918032	0.888605679	0.256378351	0.529187089	154.418469
4.551489126	0.905066438	0.351418955	0.529515501	107.0465308
8.183363746	0.924031347	0.34973292	0.512988823	185.3119852
12.99102642	0.876815742	0.217627792	0.531073703	307.7830152
12.93332307	0.873522904	0.180214805	0.503886317	225.6914934
13.30806521	0.905531745	0.254144809	0.45242428	157.9986665
11.40264692	0.908042952	0.367121285	0.532958603	144.9324307
4.924338681	0.884189141	0.31325741	0.53213594	91.19364125
11.9019253	0.875106701	0.195380097	0.539605015	164.0027766
4.247150851	0.895650625	0.35394839	0.514316774	78.40827367
9.14597927	0.925448889	0.295481818	0.520345063	341.0054929
7.774205592	0.891913281	0.271735518	0.59809692	160.8296099
9.130381509	0.869603694	0.221340804	0.525324447	160.551731
8.805470048	0.888450167	0.258550605	0.53719178	181.672708
5.181432029	0.922492324	0.423056687	0.557181697	102.4251675
13.62739047	0.904106006	0.303424579	0.525136672	263.2930777
11.53261883	0.919178262	0.273047386	0.566709925	429.957768
11.7632207	0.899076238	0.229496099	0.490720958	194.1625394
6.767202396	0.901993814	0.299392195	0.547476062	127.1248442
4.641399776	0.876776266	0.301055356	0.500495539	69.43896784
6.699567907	0.899449786	0.273676344	0.544462148	143.2416147
8.666716875	0.902876295	0.291661089	0.561737887	158.7141858
12.53006957	0.870089259	0.190584151	0.479844408	268.8051878
9.111958447	0.913069036	0.297282869	0.581490322	174.7747835
9.664387326	0.913544804	0.332518514	0.572089317	203.6856167
9.34206438	0.913304908	0.293971953	0.532056905	206.9916825
13.39982189	0.885832551	0.221752042	0.466787851	340.311429
15.33464314	0.874710691	0.174811098	0.458497137	455.1834684
12.00502564	0.919602795	0.39083655	0.552516166	215.4187987
5.070422881	0.882945063	0.265433465	0.53911805	137.0760126
7.33877342	0.944138009	0.3764383	0.584062989	536.3432465
9.119516897	0.901774031	0.236094628	0.553124556	207.6247789
5.228762476	0.937729956	0.407099915	0.523548731	166.4004972
11.65602008	0.878934263	0.20410318	0.506545812	305.7721488
9.855442696	0.885320285	0.22413138	0.558433053	176.1822722
6.270565252	0.924787261	0.388852513	0.573068886	126.9897131
11.07881897	0.890485527	0.241169494	0.534499343	217.2639648
8.300924268	0.924420767	0.321203388	0.518220111	235.2160999
5.141083413	0.890104583	0.293470699	0.537937587	123.2985595
8.645482887	0.882561917	0.213952709	0.568995103	158.3897765
11.80795234	0.876941901	0.197522411	0.504889841	322.7626682
10.4060973	0.891071774	0.253496073	0.518686033	147.305496
11.1461828	0.905114374	0.222938812	0.527757227	284.2192912
4.896236814	0.937929276	0.477973189	0.562982424	109.3072492
7.17592097	0.917402295	0.291610617	0.465354405	327.5114423
14.17351219	0.872714764	0.184495002	0.54846285	244.2681768
9.631933829	0.891007793	0.232370075	0.571333439	169.3731358
8.789881137	0.9065455	0.313182128	0.543343461	195.988197
10.26771079	0.893374629	0.232923969	0.495998584	264.418615
9.370518771	0.875055066	0.224384416	0.472364152	164.3984537
7.44680473	0.936445861	0.417503932	0.528116326	274.2296851
10.10646973	0.896444596	0.246373138	0.495184247	232.5983765
11.58734809	0.901593124	0.220162812	0.52574515	341.5030806
12.35934416	0.918886923	0.262642609	0.5429105	404.4784329
9.455468149	0.906779613	0.236285985	0.587101447	249.4562691
7.656320914	0.885036818	0.258537451	0.46769627	145.7080035
11.63857841	0.896946912	0.209681043	0.479938852	299.6984553
10.33473893	0.856959239	0.207939736	0.491431886	186.4868797
10.1112848	0.916284125	0.243258359	0.517635973	348.1971385
5.974716331	0.922316285	0.338806246	0.593541957	185.2452141
8.126709805	0.899332997	0.251286731	0.553278372	243.0800534
8.967997815	0.869531404	0.225740129	0.509000295	161.8233215
12.32334105	0.90892799	0.286556373	0.479916049	295.5268589
11.69798276	0.880264795	0.215625441	0.51885051	168.664371
8.435577342	0.888475856	0.249028587	0.502409687	141.8648478
8.239499103	0.928744999	0.353754841	0.583499884	179.0592309
5.19127634	0.886244536	0.278521718	0.518904234	137.3764068
7.645713165	0.907718104	0.247101249	0.541446429	279.1255384
15.80526809	0.86053908	0.160928301	0.488942078	375.2178271
8.760803366	0.895452575	0.23776632	0.56446227	228.9371038
6.883795202	0.916774623	0.327521449	0.518701974	171.6900836
14.53324961	0.899032507	0.208380918	0.498749867	317.876099
7.930777229	0.879181456	0.238880602	0.554578077	179.5158338
10.95505515	0.892331147	0.293524733	0.487443361	146.6415132
5.639441679	0.906789155	0.303436156	0.51950415	136.0173844
8.229054452	0.909975731	0.323305781	0.572618673	187.0370516
9.767816255	0.88864853	0.251931791	0.503399134	336.9674571
10.24772186	0.887667128	0.239795228	0.506547968	233.9415527
8.202695298	0.920188393	0.396873442	0.578954346	134.8608473
8.859986752	0.909384576	0.282716098	0.494541179	224.8290671
8.839874535	0.892550746	0.22971745	0.56697278	222.6015842
9.544090972	0.900675686	0.26351359	0.566358372	335.362795
6.890175702	0.917596264	0.354826575	0.552822716	161.8787957
6.639930466	0.920686804	0.288798196	0.544672454	205.8581519
7.52533467	0.904923772	0.28662464	0.579692269	179.7547514
4.883847991	0.902188186	0.303083971	0.553318286	154.0912271
14.32767368	0.899587331	0.190692628	0.527212091	360.7574127
9.215076123	0.887804966	0.240909874	0.559290408	198.9088051
9.581147405	0.874768275	0.217343055	0.496525536	261.2347293
7.415559014	0.862192438	0.244851062	0.475161684	149.6918365
10.50717995	0.912053478	0.262329362	0.511395091	378.8853898

log.sigma.3.5.mm.3D_glcm_SumEntropy	log.sigma.3.5.mm.3D_glcm_AverageIntensity	log.sigma.3.5.mm.3D_glcm_Energy	log.sigma.3.5.mm.3D_glcm_SumSquares	log.sigma.3.5.mm.3D_glcm_ClusterProminence
5.361566141	17.68522246	0.003542849	38.35344586	41653.45789
5.225919823	13.94403944	0.005518655	36.57450552	28905.90626
5.294634302	14.88846903	0.003665069	36.61248002	25424.15105
5.319790835	14.21693071	0.004708983	43.16646022	39654.22923
5.281156933	13.1269944	0.003806674	32.37909499	24508.52606
5.202282683	15.90535684	0.005868825	37.96960433	29506.1717
4.728311303	13.55698156	0.018889651	17.48684966	11837.49936
5.148794754	18.43160414	0.004853241	27.53344467	24181.36623
4.924942704	12.57688523	0.008469803	21.58855239	15052.26797
4.932605535	11.99526921	0.005845038	19.84505058	9788.401502
4.574693413	10.03878051	0.011061247	11.7774507	4034.074227
4.822594773	13.34697839	0.009780865	18.04305378	10634.04141
5.270124964	17.16749663	0.004458011	38.29749759	29984.54704
5.348060041	14.53533061	0.003609662	40.53644724	31110.40873
5.096115417	12.21967367	0.004936579	29.10701129	21791.3602
5.02379618	11.48881401	0.011944382	25.2672799	16492.77901
4.654550251	9.17277858	0.008295539	13.25054476	4430.865336
5.253777397	12.03256992	0.004760028	37.42435702	30675.31692
4.486285429	8.540439031	0.011797579	10.61259055	3362.327857
5.001769715	18.3842816	0.006147294	21.07061048	17694.76432
5.122581887	12.08562541	0.004781277	24.7802458	14980.15467
5.120178599	12.08471084	0.005163956	27.59139192	17396.38555
5.048978246	12.99272362	0.005832604	24.97207578	15509.53837
4.523796008	9.798163235	0.018513115	11.50113329	5223.000819
5.223943291	15.91202745	0.007629051	31.68297908	24259.7881
5.199880959	20.47519514	0.005320324	31.01751061	31545.67765
5.202556584	13.59571025	0.004207359	31.54904539	24520.93073
4.897571409	10.83645606	0.006544368	18.18286595	9397.469347
4.304708532	7.953083932	0.01671124	12.464625	3795.9312
4.941264025	11.52786386	0.005666486	19.44163618	10684.83838
5.07845996	12.08346878	0.005138095	24.19558552	16077.03029
5.266717314	16.06230255	0.003766755	36.54082833	24024.93905
5.114203248	12.73439398	0.005892847	25.84507229	21142.66822
5.04391604	13.90566662	0.007765104	24.83609168	13638.91297
5.032587416	14.09781598	0.005728941	23.41976869	13960.32499
5.28339791	18.28940304	0.003465955	33.54788645	25742.5037
5.466880474	21.15310759	0.002843602	45.23896279	46073.59029
5.001993076	14.33988334	0.01813324	26.41942145	18935.85466
4.78980533	11.31608967	0.007169455	16.76381776	6648.468831
4.87369499	23.01561884	0.010898248	17.86032275	12130.64728
5.170411856	14.02120733	0.004451293	28.27597889	17915.98409
4.471866968	12.70155144	0.017501165	11.31999976	5153.715777
5.426880063	17.18556939	0.003190565	40.33081344	36172.17593
5.231863737	12.68837679	0.003953682	31.23062948	23579.5512
4.634423203	10.89511725	0.015927281	14.81364314	9914.86088
5.261276893	14.32276299	0.003825971	32.27120763	22056.06467
4.913739419	15.15551872	0.006636803	19.28050269	10619.16332
4.660776533	10.73092997	0.009228107	15.11008073	5323.614337
5.115267154	11.95154242	0.00550345	30.17099506	23484.88876
5.238282162	17.66319759	0.00493907	35.3743005	30162.77641
5.066087147	11.57727734	0.005655757	27.57893847	20054.16015
5.262910907	16.6090525	0.003748017	30.74162771	28846.21732
4.337548816	10.18450368	0.028924928	10.08635173	5458.506133
4.78888786	18.08870391	0.007085604	17.13225754	7982.196629
5.440902159	15.02274676	0.003517258	46.05627933	41860.51966
5.208280703	12.36763796	0.004275143	30.73738301	24630.13778
5.017624256	13.61996969	0.006406082	22.57071531	13708.52661
5.138108813	16.00929146	0.003978514	28.27403501	14589.19519
5.052062235	12.36962403	0.004663466	24.83541398	13143.80228
4.656415395	16.44632145	0.01953313	15.10358014	8985.977179
5.103355893	14.95564797	0.004564728	26.09475081	15104.25719
5.312103858	18.26400298	0.003645509	31.90612426	28761.54716
5.295198442	19.89072631	0.004153649	31.06177617	26474.63381
5.285837979	15.39454508	0.003883264	30.46649752	25376.32213
4.872118465	11.69165445	0.006062324	19.28409588	8687.956836
5.263053014	17.12615298	0.003362726	30.60028668	24616.37327
5.100510896	13.11306985	0.005108511	29.64971042	18001.6787
5.150834495	18.50660536	0.004486267	26.31275596	22539.78598
4.863093401	13.2785004	0.00768699	17.0263054	8825.720936
5.113129212	15.21673347	0.004535592	24.37757309	14463.52431
5.050940392	12.16190218	0.005530827	27.2500325	14483.31743
5.125831686	17.08565755	0.005775902	27.17359427	18651.98294
5.247477615	12.37148164	0.0038747	33.9253602	24149.04732
4.986480923	11.42944177	0.005477312	22.5504757	12022.17695
4.93552087	13.03910831	0.010966718	21.08514268	17593.21717
4.751513598	11.37825018	0.006972583	15.30853954	5364.726017
5.067016119	16.47805032	0.004845326	22.97404785	13696.57558
5.354690554	19.11085556	0.004765845	47.05586823	52675.41961
5.188879048	14.67075678	0.004432852	27.21984113	19697.63382
4.774853466	12.81776975	0.008271609	16.08698998	8251.623109
5.337640495	17.5958584	0.003673116	37.28253006	40388.9836
5.094317597	12.87553488	0.004978469	24.83306303	14617.94136
5.059368122	11.6094822	0.006478574	25.32246106	15056.75585
4.741692857	11.33381587	0.007647212	14.68147563	6161.516596
4.995891327	13.25261336	0.008074479	21.94586505	14802.4048
4.829356293	18.08158183	0.009607618	25.21611645	18679.9661
5.152736101	14.92900942	0.004426518	27.93740268	17384.0571
4.81868797	11.15831437	0.015553037	18.93696326	13749.54886
4.971244087	14.75475531	0.006158278	21.44588055	12033.32328
5.206170258	14.45926987	0.004132711	28.7685135	19216.27376
5.145691481	18.0565166	0.005669476	27.98248437	23451.4948
4.806457054	12.38616884	0.010174116	16.81072711	9248.15978
4.915509266	14.06364098	0.005772018	18.55477328	10122.51645
5.069807335	12.93732212	0.0051064	22.49038707	13788.21682
4.760301709	12.08328062	0.006963192	14.57742977	5212.130987
5.501957196	18.69674664	0.00265681	42.13132996	44836.16697
5.209982438	13.57251164	0.003907014	28.11012663	17951.03074
4.963276947	15.7786254	0.006486249	26.20788765	16944.68504
4.743790867	11.83820551	0.008679316	20.30665608	9261.967043
5.169187605	19.37057418	0.004468029	25.37881491	18621.42117

log.sigma.3.5.mm.3D_glcm_SumAverage	log.sigma.3.5.mm.3D_glcm_Imc2	log.sigma.3.5.mm.3D_glcm_Imc1	log.sigma.3.5.mm.3D_glcm_DifferenceAverage	log.sigma.3.5.mm.3D_glcm_Id
34.78744279	0.82243388	-0.126892871	4.791241929	0.294940684
27.80987966	0.948821778	-0.25946058	4.725066367	0.287095971
29.56090115	0.852237286	-0.149721727	4.77793122	0.290468122
28.21092741	0.956541883	-0.268828112	5.084814785	0.272169476
26.16634543	0.814114442	-0.128641971	4.048908978	0.313845355
31.52696685	0.972101052	-0.319736919	4.495726559	0.292552961
26.85546638	0.796017791	-0.138750472	2.632139188	0.476583311
36.40290398	0.706599612	-0.08716012	3.940801385	0.351769048
25.04698587	0.75700237	-0.116473878	3.157947473	0.407878407
23.99053842	0.816767138	-0.139133192	3.370801584	0.347456237
20.07756102	0.738434186	-0.117657565	2.489088043	0.427463581
26.56260878	0.752415606	-0.111922973	2.926827703	0.422251348
33.94436549	0.863716061	-0.158298685	4.593473128	0.306325328
28.71009238	0.893460234	-0.177270238	4.995591479	0.273398062
24.136785	0.780188315	-0.115756902	4.068808994	0.338668222
22.9445373	0.847923495	-0.165939238	3.282281662	0.433265296
18.34555716	0.766697483	-0.121091323	2.702591996	0.396326174
24.00035058	0.928447772	-0.221911967	4.620985287	0.28843329
17.08087806	0.752316431	-0.12324154	2.404810061	0.429583862
36.32932048	0.706980975	-0.091823059	3.349805993	0.376782035
24.16635953	0.786315301	-0.124169438	3.407704464	0.358157443
24.16942167	0.880952246	-0.17694282	4.062686826	0.313505005
25.94913717	0.887850928	-0.185932144	3.67032636	0.346881289
19.59632647	0.772342332	-0.132239557	2.19778441	0.486860877
31.44368425	0.884267687	-0.18435156	3.856151285	0.379674612
40.60867743	0.809068644	-0.130793008	3.771905455	0.355334969
26.80272133	0.784390178	-0.117853935	4.23311777	0.318687689
21.65703524	0.741916331	-0.106865632	3.046626544	0.382707593
15.90616786	0.943978501	-0.303457067	2.749009808	0.387587974
23.04196114	0.771590017	-0.117495827	3.236307361	0.361472305
24.10996923	0.749129961	-0.109526491	3.38177653	0.373180987
31.74950767	0.847295776	-0.14540327	4.843926099	0.282771397
25.31840415	0.800571285	-0.12766102	3.383079628	0.378186772
27.57424987	0.830118277	-0.147003246	3.200142667	0.407134861
27.93834617	0.718047127	-0.094643479	3.374702177	0.375755719
36.05891283	0.766816333	-0.105314836	4.617398728	0.308771832
41.6949174	0.841748736	-0.140972268	5.607430249	0.265298767
28.384438	0.865303892	-0.173159644	3.1985577	0.454355714
22.63217933	0.833186724	-0.154735896	3.13026096	0.357448321
45.87933116	0.774088399	-0.124008824	2.640053271	0.446114398
27.75866445	0.803934561	-0.126179316	3.837797314	0.326808208
25.35033968	0.724885612	-0.110463163	2.26830789	0.473474114
33.84940484	0.822951137	-0.139178529	4.933494766	0.292801175
25.24178562	0.804162821	-0.12384125	4.047927575	0.316133735
21.7783397	0.824333986	-0.15692615	2.466681146	0.45632968
28.34529528	0.76936241	-0.109628465	4.123585118	0.327997043
30.04385218	0.726868271	-0.099450301	3.066942559	0.399021295
21.46185994	0.853552182	-0.17147805	2.920115312	0.380725775
23.81015859	0.926042095	-0.22509998	4.00017457	0.308772742
34.94843308	0.928659238	-0.222155108	4.685308596	0.2893633
23.07506505	0.80232543	-0.125844185	3.84777394	0.339833831
32.76464235	0.755606565	-0.101252317	4.140731174	0.31462498
20.36365011	0.786144685	-0.145327961	1.941617687	0.531355544
35.77141991	0.764813054	-0.119856328	3.175827566	0.375054736
29.65697286	0.90665761	-0.187452899	5.047352964	0.276761245
24.65979785	0.833564147	-0.14215647	3.94641301	0.323491812
27.13481998	0.760069455	-0.115849915	3.254402016	0.391534885
31.67981847	0.737340265	-0.097731649	4.100790118	0.321961499
24.71683725	0.818832046	-0.133723895	4.018049881	0.31675961
32.65498787	0.788327088	-0.133509966	2.496653873	0.480551556
29.68094655	0.786116727	-0.116284989	3.860775968	0.334725196
36.03758007	0.76590111	-0.104342001	4.314137017	0.310439815
39.3713034	0.729059888	-0.095768796	3.970471811	0.346265566
30.47132429	0.797579841	-0.120646784	3.835243542	0.327381643
23.38330889	0.81882241	-0.139467893	3.513376318	0.347207591
33.78158078	0.703575711	-0.083075095	4.420700872	0.301750247
26.22613971	0.905491544	-0.198826329	4.372564724	0.301868893
36.58255637	0.694531512	-0.084861492	3.879925853	0.332126028
26.48706069	0.737183025	-0.10840906	2.700344461	0.41596106
30.32632455	0.758679375	-0.106489638	3.592943976	0.340736812
24.32380436	0.892238254	-0.190214413	4.075618441	0.317184954
33.64113654	0.753127074	-0.103824954	3.906132587	0.364666035
24.61641474	0.801717183	-0.121178953	4.41561262	0.30577093
22.84699705	0.810211717	-0.131396019	3.65565316	0.339067862
25.87466069	0.802765783	-0.137074951	2.890477448	0.426903936
22.75650036	0.774350944	-0.123175683	3.033772564	0.368097468
32.67808014	0.753252912	-0.106643897	3.560977355	0.338332204
37.55901402	0.962949406	-0.281595949	5.61725136	0.253509962
29.25017277	0.811460101	-0.129248278	3.754537037	0.329681826
25.57862011	0.772044992	-0.120236265	2.833161822	0.406454396
34.61655198	0.820793724	-0.126747195	4.702140911	0.297878056
25.75106977	0.825660785	-0.140018186	3.709219216	0.330264286
23.18581984	0.789269705	-0.12865412	3.715833503	0.373593492
22.66465717	0.718520442	-0.102046093	2.861421112	0.387428932
26.43861879	0.793000105	-0.128853719	3.114102742	0.400177681
36.03013122	0.856378235	-0.16876727	3.807294291	0.339064095
29.68538074	0.794453754	-0.123413031	3.976881497	0.328559041
22.2768421	0.825509274	-0.150652678	2.671896395	0.461450991
29.30191799	0.769884443	-0.1128911	3.427865663	0.366539639
28.76114675	0.807378916	-0.12612928	3.867368374	0.321680082
35.77778699	0.87934158	-0.176445139	3.69162316	0.349367583
24.72074634	0.788631163	-0.13423012	2.725430738	0.427256283
28.00095554	0.704524559	-0.091186076	3.101920956	0.374427519
25.83499418	0.751696684	-0.108474935	3.273115855	0.370735256
24.16656123	0.6960559	-0.097699549	2.782984427	0.388417764
36.79781529	0.789132751	-0.11105994	4.997336286	0.281585081
27.08816055	0.755989238	-0.107336686	3.822313236	0.331043275
31.51868387	0.927108502	-0.230718828	4.030741974	0.311928305
23.67641103	0.906765325	-0.216661398	3.665843128	0.334491488
38.24779711	0.694319727	-0.085895795	3.820629576	0.346863449

log.sigma.3.5.mm.3D_glcm_ClusterTendency	log.sigma.3.5.mm.3D_firstorder_InterquartileRange	log.sigma.3.5.mm.3D_firstorder_Skewness	log.sigma.3.5.mm.3D_firstorder_Uniformity
113.4481962	207.0096331	0.410281899	0.046337505
108.8884614	206.9379044	-0.311270958	0.054024201
105.5145316	227.1638145	-0.150331853	0.046748376
127.091068	223.3964481	-0.197060169	0.046101616
100.5711337	229.5125008	0.073894517	0.050506776
113.9695388	222.8029938	-0.280242146	0.047469435
51.07546514	97.30997849	0.670736254	0.092988265
81.18970335	175.681345	0.563225393	0.056028328
64.09112583	137.9048986	0.745362235	0.069665114
60.69696239	167.4220028	0.139245426	0.061139424
36.07942682	104.3728542	0.376655882	0.090200633
52.67638355	115.1868534	0.557605684	0.076309079
114.0501687	242.5707817	-0.521044144	0.051616111
117.5012915	243.1836872	-0.100924526	0.045026234
79.93716668	183.417469	0.710054896	0.05678448
76.67226362	159.2619572	0.289797981	0.068407649
40.58770068	132.9809875	0.090718723	0.07312018
113.6443497	231.9689217	-0.043880647	0.049527442
32.14956127	101.7218676	0.285492581	0.087915136
65.03907756	143.2679825	0.310936188	0.063069434
79.06637686	202.382864	-0.039787473	0.055534353
84.04463718	192.0089512	-0.171721857	0.057565362
75.94798257	175.8500023	-0.001062425	0.058413946
35.83051811	82.71870995	0.55242974	0.104444295
93.85954303	199.4877329	0.135559028	0.054174315
97.08586867	190.6229496	0.598231008	0.054659814
89.23320335	211.4542561	0.508741506	0.05190567
55.78971071	146.1576691	0.378642789	0.066360599
37.29388662	120.762394	0.609002646	0.092794145
59.61598175	163.8355598	0.174428577	0.06179777
74.31020584	179.580555	0.44874502	0.057010103
104.0068348	250.2879934	-0.276806825	0.048516452
79.10821568	162.3759499	0.546407922	0.059717822
74.68298497	164.8654976	0.352725218	0.065548397
68.50743462	163.9578857	0.562277639	0.061138986
97.07920758	212.353138	-0.314304739	0.04912572
133.699207	238.0595226	-0.51416895	0.043703284
78.92136435	139.3340378	0.31337883	0.080500817
51.49812837	143.7699385	-0.339499566	0.071192602
54.98688322	113.1362343	0.542599067	0.080859024
84.25590046	205.8963118	0.253659075	0.053128759
33.48668221	78.98153305	0.69914791	0.109385164
117.2487471	247.0112934	-0.016716193	0.044110856
94.30385194	212.5146008	0.310856982	0.050440389
46.20303113	97.73148632	0.54845646	0.091324899
94.37952941	207.871582	0.319756809	0.050395322
56.21722866	143.4560795	0.449061228	0.06734626
46.45969473	137.1125183	-0.231947936	0.077770542
91.92054634	190.8912048	0.266251151	0.054231723
104.1222169	195.4229755	-0.488169436	0.05272535
82.20556213	201.4525909	0.517362445	0.055394058
92.18389349	196.2531586	0.356543183	0.051158005
31.33257499	71.41852474	0.643136682	0.1204244
48.63029798	147.6813459	0.057309967	0.068367503
137.8864246	263.7229729	-0.21866682	0.043112935
94.96445892	209.356638	0.314581051	0.051340009
67.67803753	151.967205	0.479100054	0.062134306
81.6988983	230.4364052	0.041306884	0.051965728
72.56827689	190.2484913	0.00516093	0.055428922
44.48870002	100.3364906	0.488812754	0.094822677
74.94787468	180.0456924	0.225645017	0.056031425
98.11371467	198.8995514	0.103458935	0.048838053
96.68830658	191.6727543	0.200131106	0.051605726
93.95190177	183.1286297	0.061588421	0.05170654
56.56186045	163.5560989	-0.027371592	0.060330025
89.47966796	202.8261719	0.060595528	0.049576267
88.25654644	199.9442286	-0.45997657	0.055133587
80.16495647	174.1363449	0.450056054	0.056654919
52.89280549	139.8845015	0.450223679	0.071604176
74.01904343	176.2936821	-0.09182478	0.056958232
82.21408543	213.194334	-0.038498664	0.054200542
78.54279089	167.7409477	0.233932897	0.060049066
100.5464396	247.8709793	0.264270189	0.048994375
67.50462368	196.2766075	0.279920124	0.057480332
63.70016901	105.8401823	0.875712231	0.083192118
46.40390419	138.8943529	-0.118623031	0.070794234
69.37606562	180.2644882	-0.070671177	0.057328543
138.7058665	221.2758312	-0.705223211	0.049966932
83.46096758	172.2426529	-0.032263835	0.055077275
47.60603805	125.5053363	0.425127175	0.074958025
110.5074805	201.3831739	0.450153334	0.047315002
77.11196577	184.8926888	-0.251153417	0.057868077
74.52173282	196.1673212	0.260175905	0.057627387
44.48786917	130.0184898	0.305689712	0.073484233
67.50453758	135.6025429	0.463574611	0.067887088
74.35641751	141.1110077	-1.179208716	0.088533521
81.33208422	186.9105949	0.14544136	0.054425133
58.72255769	114.4343967	0.650664704	0.081527182
61.69624601	144.6902542	0.043981328	0.063209006
87.38040591	205.3089218	0.071096057	0.052523989
85.3321077	161.6568451	-0.142426221	0.058064776
51.03295653	120.7845912	0.454459269	0.07693186
55.88984993	150.7268639	0.293180602	0.06571168
70.18955323	170.4798965	0.299526195	0.0595024
45.3132134	146.7041359	0.122435657	0.070179181
129.3074513	256.3619976	0.042418231	0.042473453
86.31922208	209.6989326	0.074669091	0.051720272
77.6269017	176.4427299	-0.618422811	0.062336045
59.54837676	147.2707224	-0.791779563	0.079388505
78.40628848	173.4562454	-0.007346899	0.054635543

log.sigma.3.5.mm.3D_firstorder_MeanAbsoluteDeviation	log.sigma.3.5.mm.3D_firstorder_Energy	log.sigma.3.5.mm.3D_firstorder_RobustMeanAbsoluteDeviation	log.sigma.3.5.mm.3D_firstorder_Median
125.4746717	39179473.52	86.82295725	-60.0077877
120.64181	8183359.429	85.93973669	-13.53582954
125.26366	20122661.49	93.76496437	4.562054634
131.4058258	8788913.322	93.25780542	-22.98870564
119.0478702	28525407.53	90.02604067	10.74506187
125.2549758	5570943.005	91.73108562	26.62275505
72.80352245	110080287.5	43.80892411	-5.636076927
104.7382972	112347071.4	72.01916443	-32.57392311
88.5105352	57638540.92	59.81796113	-27.86474991
94.20558248	10361388.11	68.74014299	18.45251846
65.55627611	11363092.44	44.06592074	5.602751255
78.99812288	37968686.71	50.6873692	-7.001959801
130.2127779	19927208.27	98.91456882	9.895960808
133.0905589	20052798.87	101.5658593	-67.02474976
106.2402219	32194515.44	74.77440306	-70.51444244
96.30795749	89732966.86	66.12280667	-4.556544065
76.99930529	7866338.61	55.72247117	-0.254358917
128.4508609	12437518.58	96.98060319	-66.52571106
65.27246959	7955842.418	43.7731689	-21.03092384
91.75041832	135590795.1	60.70612229	14.79699039
107.578442	33794666.29	81.62305894	13.79689598
107.0946163	8587794.478	77.07706076	-1.383321553
101.1240198	8944618.706	72.41718685	-22.94885731
60.77671448	25836086.03	37.3193098	-12.43942261
113.7749476	26126001.37	81.88443233	-33.80582619
114.2405349	52461313.36	79.36538029	-24.91924572
114.884277	43527390.31	83.8457645	-65.11919403
86.63138861	28683049.46	61.30605772	-35.76835632
69.57488613	1391930.271	47.68997154	32.8273983
91.55251318	20868520.4	66.38858984	-15.48600149
101.5093859	65185769.81	73.99966803	-44.24580765
127.7187975	21959064.31	98.9661797	-51.14849472
98.91566335	37599440.9	67.74811972	-32.22949982
94.41082132	34219571.72	68.30757212	-13.58131313
96.725221	75816548.17	67.96034667	-25.47935104
117.5133842	41079832.96	86.01087858	-15.41820908
137.7873043	48456903.42	99.58681307	-34.02672195
94.09912438	91796200.74	62.50979487	1.867460489
82.7884842	6465061.93	59.98463447	-24.56615162
75.17083716	98653653.38	48.11227027	-11.08237553
112.4460265	28208027.22	84.47412613	-68.24890137
56.95611831	36379371.8	34.03363155	0.495187491
131.0084538	59569101.47	97.71139426	-5.142157555
115.0955123	29055586.07	85.69555677	-40.8470993
69.98092273	14879759.98	42.15670922	-7.897403717
114.8201246	46429664.14	84.37021259	-18.64176178
85.35224899	63005749.87	59.25151559	-9.31832695
79.90263743	5211094.422	57.55689833	-14.36674023
108.726687	7075966.653	77.53600653	4.423104286
118.6212236	9862140.481	82.92090759	16.18105888
113.1915552	26984865.56	84.08791803	-78.92079163
112.9620524	56507881.73	80.27299409	12.29921055
55.44164097	50843989.19	32.25619156	-4.614058495
84.42399178	18451933.22	59.79778777	-14.68018866
141.2049217	18157489.81	106.7372124	20.57526779
117.3871686	23580714.94	87.51724155	-19.89853668
93.40641257	56452587.46	65.46017775	-24.31816292
117.1736157	40697455.64	92.84129629	-36.53765488
104.9241058	14785418.68	78.55658022	-54.23906708
68.34063161	99310966.3	42.10321452	-1.142561197
102.3541793	21331385.3	73.77556662	-20.65362358
115.8476735	52565576.01	81.74439708	15.36587048
112.2092508	161448481	79.12143836	-4.6504848
109.5215246	39341075.3	76.51901782	1.498742223
93.3128551	9022838.752	66.46736465	-12.40772533
114.9125722	68273641.13	83.00000142	-3.351587296
113.6123855	10323680.09	84.79554329	-58.8134861
103.5631697	88773115.47	72.33369835	-37.96728134
81.87934832	78385118.21	58.23709368	-8.957266331
100.4223893	32659194.8	72.55233283	24.95919323
114.6586434	9473587.906	89.23288986	-44.76767349
102.1278198	84485766.98	71.52871487	-4.226017952
126.3032666	31198960.06	98.4578983	-53.58705902
103.7526001	16299869.55	78.31145793	-54.3490696
78.1616742	48777015.15	47.78302076	-13.25799179
80.60435475	8831311.849	59.05696352	9.363640308
101.1663969	33375307.36	74.17325829	2.533991575
137.797676	10324265.15	96.82653388	-14.99584579
102.9570731	23254087.41	71.79606379	41.76436996
76.8737897	20019852.4	52.13796984	-38.46079445
123.5014562	37490229.66	85.26115547	-61.98815727
103.9771009	13017737.88	76.50309591	7.886797428
104.9915501	39337490.32	79.00750002	-33.18221092
77.50127919	21654667.95	53.83458063	-50.97580338
88.50579608	36412628.01	58.24183041	-12.29813766
97.75649269	8491783.217	64.42778334	19.09057617
105.8712298	23121578.94	76.73900898	-40.38877869
80.25559584	46159609.87	50.1853207	-7.28896451
90.64638873	23074928.42	62.17128532	-22.6022644
111.2219921	24575042.09	82.47901121	9.925151825
103.2749254	14190554.25	69.90283746	19.71371269
77.78216312	27527026.29	51.19801282	-21.49589443
86.63187978	52110476.52	62.44688446	-14.50043488
95.03499196	47999482.16	68.30237574	6.569545507
80.66148538	31391430.38	59.65456117	-6.637613773
138.2968773	65331963.56	104.2422393	-6.253500462
111.0131696	43163632.77	83.76310449	-0.760005832
103.3125315	6772816.72	74.22407459	-52.26595688
88.451206	3296224.445	62.63753039	9.997353077
103.8589258	153174811.5	72.80149707	23.73817539

log.sigma.3.5.mm.3D_firstorder_TotalEnergy	log.sigma.3.5.mm.3D_firstorder_Maximum	log.sigma.3.5.mm.3D_firstorder_RootMeanSquared	log.sigma.3.5.mm.3D_firstorder_90Percentile	log.sigma.3.5.mm.3D_firstorder_Minimum
1057845785	478.9492188	164.7197602	180.5228455	-455.2085876
220950704.6	286.2527161	153.5681229	130.927478	-385.1043091
543311860.3	337.1661987	150.1966629	180.7070007	-369.1929016
237300659.7	302.447052	165.2112644	173.5115067	-392.6557007
770186003.3	401.1671448	142.1845546	174.9210815	-308.9163513
150415461.1	300.4783325	151.7248353	212.5320297	-351.7454834
2972167762	598.3810425	102.2009835	137.2709961	-314.0350952
3033370927	454.7615356	136.068655	153.0363373	-456.9634399
1556240605	410.933197	116.2919804	144.2520477	-314.1509399
279757479.1	327.5464172	117.1481663	179.1884521	-260.4783325
306803496	309.3952026	85.45617965	132.2143021	-203.5031586
1025154541	483.5266113	105.5666454	139.2333191	-313.6583862
538034623.2	288.0982361	156.7518961	143.2936249	-433.4658203
541425569.4	344.69104	176.4593053	118.4512138	-441.75354
869251916.9	534.2099609	142.971893	134.3757141	-336.70224
2422790105	429.1560364	124.792759	188.5592163	-272.7251587
212391142.5	241.356781	94.38564905	124.6303085	-220.8900909
335813001.6	348.0813293	174.3834306	98.06470032	-389.4121399
214807745.3	239.6832581	85.27749316	87.81276016	-215.940033
3660951468	549.7582397	121.5130191	167.0422394	-417.0053711
912455989.8	339.4850769	127.5267552	155.0522675	-277.7923889
231870450.9	281.1105957	131.5830603	146.4957047	-314.3707886
241504705	321.8539124	126.6087778	133.1459564	-325.882782
697574322.8	349.8340759	84.75067036	93.85331879	-247.2037659
705402036.9	375.5764771	145.8603627	166.1622559	-409.2463379
1416455461	483.6218872	143.2646868	218.2793655	-495.8490601
1175239538	478.8580322	147.3779313	144.7646484	-367.1344299
774442335.4	343.3305054	112.8319425	109.7428772	-286.6471252
37582117.32	288.5819702	97.64102099	165.0195465	-142.3574829
563450050.8	366.7657471	113.8499623	127.7118263	-298.9885864
1760015785	395.290863	127.5141473	148.0562225	-311.480835
592894736.5	253.4428558	166.9336182	106.7961182	-451.5813599
1015184904	491.6695557	126.8958009	153.917334	-311.2303467
923928436.6	406.10672	119.0361076	157.1149841	-328.2933655
2047046801	466.6491089	121.5336026	164.8025787	-342.453949
1109155490	349.6062317	145.5544307	154.7889832	-462.7019958
1308336392	283.0792542	179.0201568	141.6406418	-597.7460327
2478497420	467.5494995	125.5779976	179.1620178	-325.8493042
174556672.1	211.2290955	107.4465144	83.09056244	-312.011261
2663648641	471.8371277	102.1902893	117.3737946	-574.1071167
761616735	408.2597961	145.0888231	126.3146217	-375.3555298
982243038.5	477.2757568	80.08653179	104.58825	-282.8883362
1608365740	393.5349731	156.9576098	195.7942154	-405.0612488
784500823.8	366.0162659	143.0443299	149.0969574	-339.5325928
401753519.4	404.5476685	97.78971188	98.48695374	-252.1848907
1253600932	402.9790649	139.059829	193.6208954	-337.2556458
1701155247	491.1434326	108.5107129	131.3966217	-359.3352051
140699549.4	238.9586487	103.020517	75.91371155	-299.0227356
191051099.6	388.5411377	134.5254675	184.7622833	-277.2364197
266277793	310.5022888	147.7122028	176.0394684	-427.1882629
728591370.2	362.8755493	144.8007649	145.0497711	-343.0355835
1525712807	548.1263428	142.4432631	219.3212769	-354.1603088
1372787708	402.171875	79.84123319	87.88396732	-243.5806732
498202196.8	356.7323303	106.0069552	117.6170288	-432.2080688
490252225	401.9849243	168.0437707	216.3581635	-373.0667114
636679303.2	417.1227112	141.6037724	167.0930023	-315.6006775
1524219861	367.1705017	118.5766431	149.3367859	-342.5990906
1098831302	367.8819275	140.3180016	140.7239563	-421.7946167
399206304.3	269.8624268	139.204907	100.3258942	-359.9692688
2681396090	449.0080872	95.48273535	113.660083	-379.6986084
575947403	385.7913208	126.6913721	148.9638336	-352.7173767
1419270552	494.0980225	146.0300561	205.6482178	-405.8930664
4359108987	551.0843506	140.0269245	202.9634415	-456.4082947
1062209033	488.1392517	137.0021788	180.7737885	-374.4393921
243616646.3	322.5020447	115.4455677	136.9207703	-292.114502
1843388311	473.5267639	141.2700163	165.7745972	-416.393158
278739362.5	167.6781769	159.4609152	76.16137695	-423.2909546
2396874118	502.802063	132.1281189	158.0211151	-450.5843811
2116398192	416.7040405	102.1773599	128.6046448	-304.9897461
881798259.6	375.2849121	124.0010836	166.3768616	-326.6332703
255786873.5	274.089447	138.7633236	139.5519836	-341.9475708
2281115708	458.8828125	130.2367364	166.1310577	-416.3809509
842371921.5	389.6273499	152.8725205	156.2921906	-349.3070679
440096477.9	326.5084534	130.9876232	124.5003326	-308.1118164
1316979409	533.3250732	109.2860536	132.9674408	-322.1991272
238445419.9	265.9413452	98.08244192	129.0985794	-271.2840881
901133298.7	417.7484436	122.9178001	145.6171295	-392.9076233
278755159	242.0232849	178.7838616	127.4484726	-528.0695801
627860360	413.2790833	132.9801363	194.5044128	-317.0590515
540536014.8	363.894928	105.461558	89.00203476	-348.5601196
1012236201	478.9492188	163.2925571	176.5491562	-455.2085876
351478922.9	282.8000793	125.538732	145.3611603	-313.3320007
1062112239	361.5075378	131.2941889	135.7692474	-315.9490967
584676034.5	302.3250427	106.9548437	91.67058868	-313.4918518
983140956.3	379.6047363	115.8086757	156.9589783	-300.7570496
229278146.9	201.9605408	126.4597121	90.08052063	-450.9888
624282631.4	318.3130493	132.9550605	143.4336227	-380.5328979
1246309467	406.7005005	109.696246	147.5197372	-272.1052246
623023067.4	412.8624573	116.4710591	140.8713837	-365.361145
663526136.4	382.5666504	134.0796532	168.2721924	-338.131897
383144964.9	329.6266479	132.8530884	203.6788788	-420.6661072
743229709.9	352.7694397	104.2672209	112.622789	-305.7895813
1406982866	491.3318176	107.5989457	127.9878159	-346.4553833
1295986018	417.6781921	118.0213667	170.5399704	-276.1153564
847568620.4	294.2980347	97.42904714	130.3340912	-288.3412781
1763963016	556.0657349	165.542584	192.488942	-468.3565063
1165418085	368.8693542	132.6779527	180.8722366	-317.6687622
182866051.4	172.7457581	147.0999391	68.73665771	-468.6528015
88998060.03	190.6412506	110.9024118	88.69646301	-306.677124
4135719910	456.0169373	131.7531258	186.3066513	-440.6280518

log.sigma.3.5.mm.3D_firstorder_Entropy	log.sigma.3.5.mm.3D_firstorder_StandardDeviation	log.sigma.3.5.mm.3D_firstorder_Range	log.sigma.3.5.mm.3D_firstorder_Variance	log.sigma.3.5.mm.3D_firstorder_10Percentile
4.65551252	158.3000419	934.1578064	25058.90326	-238.0992386
4.453859086	148.153519	671.3570251	21949.46519	-271.5598022
4.55051011	149.9585139	706.3591003	22487.5559	-221.8980911
4.60474596	161.2611216	695.1027527	26005.14935	-273.0986877
4.454040313	142.1815233	710.0834961	20215.58557	-196.2773132
4.539542413	150.5067791	652.2238159	22652.29056	-184.4327881
3.911165347	102.1255597	912.4161377	10429.62993	-130.3139038
4.408973844	133.7141469	911.7249756	17879.47307	-185.4424164
4.13255737	114.712039	725.084137	13158.85189	-152.9130127
4.200891676	114.709562	588.0247498	13158.2836	-119.718396
3.756457123	84.15969721	512.8983612	7082.854634	-89.24571228
4.04664889	105.5420756	797.1849976	11139.12973	-136.1479279
4.47388535	153.9730092	721.5640564	23707.68756	-264.0572815
4.603295121	157.4656903	786.4445801	24795.44362	-300.1275421
4.359854975	133.1428524	870.9122009	17727.01915	-206.2276764
4.206379642	124.7843017	701.8811951	15571.12195	-166.7899734
3.921451184	94.38561437	462.2468719	8908.6442	-130.6051849
4.502202482	153.6730038	737.4934692	23615.39209	-304.4579956
3.755085644	83.37482762	455.623291	6951.36188	-127.1474907
4.285001378	120.1770472	966.7636108	14442.52267	-131.8779114
4.304405203	127.5249138	617.2774658	16262.60365	-183.8853973
4.314012436	131.0523925	595.4813843	17174.72957	-196.0966415
4.297137854	124.6539815	647.7366943	15538.6151	-195.4046692
3.682074616	83.80183956	597.0378418	7022.748313	-116.6463547
4.45459588	141.2591793	784.8228149	19954.15572	-211.7654221
4.463313172	143.2233283	979.4709473	20512.92176	-155.0773621
4.466234273	140.4651801	845.9924622	19730.46681	-208.0010941
4.116139277	108.0449151	629.9776306	11673.70368	-167.3166382
3.71318596	87.7867209	430.9394531	7706.508367	-46.31147575
4.191894575	112.7922227	665.7543335	12722.08551	-163.2628998
4.29699008	124.0246201	706.771698	15382.1064	-181.5780792
4.50847064	149.4608076	705.0242157	22338.533	-280.4617035
4.320278623	125.8343058	802.8999023	15834.27252	-167.1699707
4.178745868	117.8510082	734.4000854	13888.86014	-165.2143372
4.258033292	121.126335	809.1030579	14671.58904	-151.7212097
4.511718249	143.687565	812.3082275	20646.11632	-226.6606232
4.689157924	168.1980867	880.8252869	28290.59637	-310.1014069
4.150486696	125.4778474	793.3988037	15744.69018	-172.9008179
4.004877701	101.2320449	523.2403564	10247.92691	-180.0242798
3.993953474	101.4453703	1045.944244	10291.16316	-132.6994904
4.401578457	133.7379675	783.6153259	17885.84395	-221.8031067
3.619293927	79.8972442	760.164093	6383.569631	-84.73924866
4.652020711	156.9006225	798.5962219	24617.80533	-206.7857193
4.449323245	138.6578944	705.5488586	19226.01169	-210.7288101
3.878170622	97.50362975	656.7325592	9506.957814	-135.2776413
4.465193706	139.0230162	740.2347107	19327.39902	-176.0372314
4.130851008	108.2962633	850.4786377	11728.08064	-141.1792908
3.929477754	98.00327068	537.9813843	9604.641064	-173.7414398
4.387550172	134.4893545	665.7775574	18087.38647	-175.0658112
4.491611175	147.527048	737.6905518	21764.22989	-220.1022079
4.369317119	135.3065422	705.9111328	18307.86035	-208.4527496
4.501110075	140.0751574	902.2866516	19621.04971	-142.9418427
3.551544026	79.59415957	645.7525482	6335.230237	-107.0962448
4.095321896	105.1654421	788.9403992	11059.77022	-140.2487167
4.655824905	168.0423462	775.0516357	28238.23011	-248.6116943
4.438059969	140.4750155	732.7233887	19733.22999	-197.4549026
4.221866511	117.3575802	709.7695923	13772.80163	-161.2349854
4.377775908	135.4882136	789.6765442	18357.05603	-212.1366333
4.316115292	124.9188183	629.8316956	15604.71117	-230.1849823
3.863056933	95.42364578	828.7066956	9105.672175	-119.7415802
4.342511289	126.0808536	738.5086975	15896.38163	-177.2061279
4.556115082	145.0948164	899.9910889	21052.50576	-181.9519562
4.500718744	139.5537804	1007.492645	19475.25763	-161.88564
4.489004397	137.0008195	862.5786438	18769.22456	-176.543251
4.215425328	114.9502987	614.6165466	13213.57118	-165.4391724
4.524133468	141.0338349	889.9199219	19890.5426	-193.6062622
4.335737269	135.4130313	590.9691315	18336.68903	-282.6182556
4.389513918	130.1332277	953.3864441	16934.65696	-172.5038666
4.033122492	101.9635522	721.6937866	10396.56598	-129.0561691
4.310614183	122.7233488	701.9181824	15061.02033	-155.9576889
4.329127115	133.5815927	616.0370178	17844.04191	-218.6343109
4.339217234	129.8200692	875.2637634	16853.25036	-185.3972626
4.487489396	147.2066539	738.9344177	21669.79896	-223.7404755
4.27633977	123.4618261	634.6202698	15242.82252	-195.6143799
4.025346014	108.9452142	855.5242004	11869.0597	-131.6092224
3.983007441	97.94997299	537.2254333	9594.197209	-129.4824097
4.31026899	122.8994252	810.6560669	15104.26872	-169.2636841
4.594254422	168.0688986	770.092865	28247.15466	-306.8365906
4.393145156	128.5742399	730.3381348	16531.33517	-139.746048
4.003905934	99.13345795	712.4550476	9827.442485	-156.0386276
4.629269899	156.2001451	934.1578064	24398.48531	-237.7975464
4.289576361	125.1613452	596.1320801	15665.36234	-188.3417435
4.295839649	126.3813283	677.4566345	15972.24014	-198.0273087
3.982490995	97.52293539	615.8168945	9510.722927	-165.1279327
4.1883175	115.7506242	680.3617859	13398.207	-149.7940704
3.966623199	124.4008855	652.9493408	15475.58031	-223.330658
4.379397431	128.8285738	698.8459473	16596.80144	-190.3100418
4.031985858	109.6510037	678.8057251	12023.34262	-143.8689041
4.229561206	115.0934642	778.2236023	13246.50551	-165.7891693
4.40875344	134.0695985	720.6985474	17974.65724	-180.8423431
4.364166299	132.0351651	750.2927551	17433.28483	-135.2093781
4.009611104	102.2319546	658.559021	10451.37253	-147.157074
4.124111844	107.122653	837.7872009	11475.26278	-143.0514069
4.243988106	117.3202786	693.7935486	13764.04777	-139.591629
3.98165964	97.36830107	582.6393127	9480.586053	-127.9806503
4.716077349	164.9183627	1024.422241	27198.06635	-230.6488144
4.402637049	132.5501074	686.5381165	17569.53097	-167.7004272
4.231632755	125.6795901	641.3985596	15795.35937	-254.789798
3.937496224	109.1536682	497.3183746	11914.52329	-184.2300461
4.412594768	130.4699085	896.644989	17022.39702	-159.6999893

log.sigma.3.5.mm.3D_firstorder_Kurtosis	log.sigma.3.5.mm.3D_firstorder_Mean	log.sigma.3.5.mm.3D_glrlm_ShortRunLowGrayLevelEmphasis	log.sigma.3.5.mm.3D_glrlm_GrayLevelVariance
2.882167092	-45.53785383	0.006034963	40.90967321
2.489530141	-40.41909434	0.016202626	35.18178912
2.2131296	-8.454681789	0.013460657	36.08196454
2.451235794	-35.91117535	0.026176162	41.79932411
2.262319909	-0.928441118	0.012846261	32.14000446
2.393357674	19.18684639	0.013963669	36.30298096
4.518561424	-3.925697683	0.007621314	19.02093987
3.306675276	-25.20328931	0.003764328	29.80876323
3.475565419	-19.10426197	0.009019901	22.42451101
2.425129014	23.77833601	0.015124591	21.37167842
3.124880059	14.82916065	0.015474499	11.87759947
3.799590212	-2.277472845	0.007778085	19.09899341
2.243932429	-29.38484923	0.009547443	38.24738826
2.162095329	-79.63945516	0.011972063	39.30100091
3.346336324	-52.09551834	0.01235356	29.20309936
2.655801802	1.452843362	0.020497179	26.58442562
2.446404739	0.080911451	0.023619238	14.50823902
2.305511197	-82.42686928	0.027672032	37.16978061
3.102662847	-17.91337375	0.029409178	11.69867366
3.798162316	17.96917212	0.004009506	24.39928384
2.200698655	-0.685300818	0.014049134	25.97537488
2.391536325	-11.80560023	0.016843008	27.46364277
2.612250514	-22.16229969	0.011634971	25.20659417
4.184614888	-12.6462569	0.016962102	12.34365621
2.503258739	-36.34679751	0.007804475	33.88193198
3.117301487	3.442197716	0.003553191	33.76211672
2.990261353	-44.60703795	0.010904125	32.30433345
2.958075652	-32.51681988	0.015876068	19.21821348
3.045861387	42.74646901	0.039465086	12.78170676
2.759818126	-15.48316526	0.015188137	20.64425387
2.628750975	-29.62686912	0.011577564	25.21042966
2.107729412	-74.35253787	0.0088746	35.87254011
3.288225947	-16.37900364	0.011796883	26.38229143
2.635692326	-16.7551418	0.007972839	23.00589204
2.981245231	-9.941202876	0.007497144	24.48802316
2.49171244	-23.23738298	0.005325124	33.57257161
2.55678581	-61.2994305	0.004048682	45.35076694
3.001978879	-5.014310197	0.007879939	27.98866671
2.522829605	-36.01147781	0.015788927	16.63949877
4.335891951	-12.31633341	0.00200603	17.8600693
2.447527197	-56.25764514	0.009096652	28.71645202
5.244203255	5.50299395	0.00751937	11.31772056
2.356929833	-4.229175959	0.006726543	39.15384462
2.455177191	-35.15207875	0.013364422	30.92177661
4.475735405	-7.474619491	0.014639268	16.77437509
2.444821665	-3.19953413	0.008626402	31.19822296
3.502866629	-6.818664371	0.005502115	19.5768288
2.641030758	-31.75824088	0.017687632	15.58115219
2.63581386	3.116880133	0.016339054	29.28714234
2.787654316	-7.393576691	0.008382181	35.4455978
2.489589492	-51.56938196	0.014577204	29.6774309
2.987599998	25.86568189	0.006586678	31.76806319
5.245232083	-6.276326911	0.014389669	11.8078462
3.317653296	-13.33057897	0.004325822	18.22302023
2.11076286	-0.691938892	0.018447869	45.26318053
2.468280679	-17.84372036	0.013692228	31.69225039
2.886269846	-16.95932375	0.007532484	22.92716073
2.002873784	-36.49774719	0.006217031	29.42865723
2.310084648	-61.42715171	0.012530482	24.97568156
4.584739205	-3.358656678	0.004235083	16.61510561
2.707165297	-12.42264563	0.00758328	25.98861476
2.717752788	16.50065229	0.005041009	34.3690605
2.762345353	11.50138976	0.003339283	32.18322357
2.867899331	0.610276244	0.00979142	30.34006468
2.540359028	-10.68213111	0.019440509	21.65510772
2.782193992	-8.165471313	0.006304382	32.10666602
2.322816808	-84.20863642	0.020173927	29.56678494
3.220736044	-22.87319085	0.003871554	27.76192795
3.22465365	-6.606579416	0.007699634	17.21998402
2.521251211	17.75523597	0.007209998	24.51272195
2.008662101	-37.56618237	0.014933521	28.39705195
2.845399927	-10.40947369	0.004774015	28.01654981
2.160882144	-41.23358556	0.016203442	34.89554821
2.327255792	-43.75996924	0.016961742	24.75087788
4.89710548	-8.624489337	0.009144351	20.57613756
2.46073443	5.09590061	0.014028829	15.50098487
2.676513085	-2.125291715	0.006604019	24.4033974
2.836721151	-60.9632227	0.009667687	45.50712195
2.786159307	33.94674495	0.009933231	27.07497618
3.74058142	-35.98190847	0.009241929	16.60875003
2.922617461	-47.60224684	0.005814023	39.85176733
2.357028457	-9.726812791	0.01634415	25.24434804
2.444336303	-35.57982417	0.015327919	26.53139049
2.885299148	-43.91600688	0.010896354	15.57755237
3.198420218	-3.666382194	0.008850418	22.69666061
3.715642134	-22.72616298	0.006918155	25.40119464
2.538366888	-32.8671064	0.00769167	26.84943911
3.764816586	-3.150201174	0.013394893	21.27653614
3.02216748	-17.86062946	0.009014119	22.1814
2.390948349	1.641997679	0.008781605	28.94705052
3.093046469	14.71931623	0.00628019	28.52323084
3.54403031	-20.5007517	0.009221562	17.67882997
3.151202161	-10.11287945	0.007178797	18.73624608
2.74004974	12.84504653	0.009925092	22.49425157
2.40489344	-3.439938085	0.009337851	15.43417705
2.420117917	-14.36247819	0.00551493	43.64065793
2.285266669	5.823071846	0.011579127	28.3999757
2.905794828	-76.43973253	0.009899764	25.68609539
2.89450832	-19.61687179	0.022325253	19.26057636
2.790484437	18.34364033	0.003586719	28.03139566

log.sigma.3.5.mm.3D_glrlm_LowGrayLevelRunEmphasis	log.sigma.3.5.mm.3D_glrlm_GrayLevelNonUniformityNormalized	log.sigma.3.5.mm.3D_glrlm_RunVariance	log.sigma.3.5.mm.3D_glrlm_GrayLevelNonUniformity
0.006282173	0.045566872	0.079864342	61.62062498
0.016750292	0.053420026	0.066272267	17.50879835
0.013947695	0.04645954	0.082541409	39.00882892
0.027520725	0.045789313	0.061880089	13.9814929
0.013576647	0.050413313	0.079973955	66.50781765
0.014211236	0.047432854	0.066098201	10.87596731
0.00849765	0.077436138	0.469172659	664.4348493
0.004036973	0.054051238	0.157149923	295.5090227
0.009901061	0.065444609	0.22260682	243.7132695
0.015976495	0.060609012	0.08898655	42.44017616
0.016674571	0.086971447	0.162503706	120.5472373
0.00853102	0.07124418	0.229105255	210.6649659
0.00983887	0.050634437	0.094025561	38.08925623
0.012541504	0.045142515	0.055510635	27.64237028
0.013212552	0.055494651	0.111602581	80.2034087
0.022316897	0.060275414	0.295220102	297.3468866
0.025569603	0.072446009	0.126535591	57.8195653
0.028939301	0.049181039	0.063124609	19.05967055
0.031924221	0.085683245	0.172419513	82.71804923
0.004288958	0.060801917	0.162494545	498.9267819
0.015104222	0.055330386	0.123375944	105.0728546
0.017927838	0.057322203	0.071504619	26.66677023
0.012361323	0.057655346	0.111877469	29.52057622
0.018802113	0.093472808	0.328577506	282.0941116
0.008318178	0.049737077	0.204912209	53.89588776
0.003788534	0.053163798	0.140205835	123.3750807
0.01152125	0.051168613	0.095328072	94.97292006
0.017069105	0.065007036	0.129348386	132.6817133
0.041206607	0.088916888	0.112022798	11.93582007
0.016195187	0.061422895	0.108580326	90.80403501
0.012521245	0.056269719	0.14101445	203.476691
0.009217447	0.048390176	0.068228688	35.97372818
0.012638713	0.05758084	0.167823072	120.2679857
0.008583554	0.062443403	0.187129215	134.0841175
0.00806562	0.059193745	0.162407703	272.2488664
0.005565918	0.048526203	0.100049606	87.21572307
0.004194054	0.04365183	0.064686884	62.4426714
0.008689432	0.064360418	0.402980676	311.0080518
0.016654444	0.070683489	0.105396732	36.42068112
0.002215887	0.074907076	0.265429825	607.0304072
0.009635693	0.052548134	0.088183716	65.36419033
0.008367252	0.099610993	0.304458195	476.9735732
0.00707973	0.044145714	0.097913806	99.62647523
0.014125024	0.050197256	0.081845527	66.56785643
0.015960289	0.082001358	0.302909855	108.9181366
0.009235963	0.049803296	0.110975	110.2216796
0.005978475	0.065780369	0.175597678	312.8534539
0.018910749	0.076776683	0.126514331	34.24147672
0.017018733	0.053949691	0.062170624	19.91297982
0.008601453	0.051983969	0.066941034	22.13674297
0.015500326	0.054162264	0.091979192	64.75138235
0.006933474	0.050799689	0.08092136	131.8628802
0.016342519	0.101269449	0.504400236	637.290604
0.004592619	0.067409322	0.126681324	100.0535221
0.01934925	0.042850727	0.063506822	26.08948456
0.014509702	0.050998951	0.085810133	55.80165455
0.008157413	0.06017043	0.186026224	215.1360545
0.006571647	0.051701286	0.103965378	98.71676352
0.013320214	0.055484024	0.082556466	39.51826756
0.004776015	0.079910777	0.448236035	709.64877
0.007973447	0.055236427	0.10045814	67.80795399
0.005267186	0.047928318	0.100034486	109.6716136
0.003562736	0.049955555	0.141076522	372.4975019
0.010288834	0.051498121	0.09120129	99.99948579
0.020193659	0.059556441	0.098358374	37.2376562
0.006594506	0.049327738	0.080927146	157.5118292
0.021623202	0.055203324	0.073306063	20.97674847
0.004110053	0.055780411	0.101685159	261.3484447
0.008386296	0.069993405	0.202895959	462.5946704
0.007553857	0.05644042	0.096010574	110.8459916
0.015673187	0.053812151	0.08366975	24.7420916
0.005136802	0.056757105	0.174949945	252.9142509
0.017122489	0.048549159	0.082459339	60.60690611
0.017737638	0.056770399	0.081405687	50.36675227
0.010034314	0.075664884	0.250895264	266.2287591
0.014835201	0.070295962	0.107725739	59.22385681
0.006936451	0.056971798	0.098581999	116.0840348
0.009884124	0.04952776	0.049718902	15.29149113
0.010379467	0.054569458	0.086968743	66.66707332
0.009921729	0.072610974	0.16511729	116.5272274
0.006057424	0.046527011	0.080983146	61.21155062
0.017222267	0.057493961	0.082019006	44.29223724
0.016431086	0.055176908	0.183519612	112.7656051
0.011747493	0.072172712	0.134052431	123.6223184
0.009589733	0.063703345	0.199084794	152.6894865
0.007127783	0.083608367	0.109748991	40.9788805
0.008096332	0.053713274	0.094923989	65.15064364
0.014959277	0.07047683	0.349398164	226.7154889
0.009510733	0.061217065	0.131065549	94.33226436
0.009204048	0.052185874	0.084439904	66.63492212
0.006584008	0.056786855	0.111139479	41.59819971
0.010185295	0.07302224	0.221966313	160.9976502
0.007679871	0.065072187	0.124701851	265.7737257
0.010632876	0.058875141	0.127334151	184.536584
0.010052181	0.06954131	0.127585431	208.1009415
0.005759563	0.042354087	0.074459787	94.93926745
0.012198039	0.051503961	0.092910951	116.9033529
0.010222286	0.061575845	0.068695166	18.12858483
0.023465899	0.078174061	0.086750478	19.52545541
0.003806616	0.053341541	0.14100527	427.0765819

log.sigma.3.5.mm.3D_glrlm_LongRunEmphasis	log.sigma.3.5.mm.3D_glrlm_ShortRunHighGrayLevelEmphasis	log.sigma.3.5.mm.3D_glrlm_RunLengthNonUniformity	log.sigma.3.5.mm.3D_glrlm_ShortRunEmphasis
1.222239731	342.3417482	1197.918294	0.953188376
1.192700707	244.0949598	294.3853095	0.957764081
1.214718348	255.1794669	753.962931	0.95796503
1.176069235	262.806924	276.2230673	0.961305938
1.224726331	205.3168905	1163.366953	0.951881337
1.18259156	289.7156668	207.5551297	0.961170326
1.99824038	174.4161331	6229.963769	0.874954807
1.393229107	351.850298	4565.021179	0.929859363
1.541299457	174.4583902	2972.516171	0.911445812
1.25367468	168.416514	607.5035997	0.945237795
1.433442737	106.1276839	1128.409059	0.919021834
1.566566426	185.1343074	2321.863496	0.905593097
1.260027495	312.6941488	654.8027289	0.946156201
1.162328618	268.5138157	555.8611565	0.96294932
1.302341147	178.7387409	1234.717014	0.939118841
1.675236795	147.183126	3857.379038	0.902421239
1.352682907	99.19850542	662.6681653	0.928338284
1.177608751	207.2927124	350.2293456	0.961308472
1.466630957	82.68593913	773.1988727	0.91244153
1.418732298	331.2672735	6734.357647	0.923345617
1.323358424	172.7239002	1619.262826	0.938181485
1.20990588	190.3482177	411.4331105	0.952827891
1.30262389	201.7275701	437.8622688	0.939487484
1.765753764	102.2433999	2258.193859	0.886534829
1.499874646	268.5530544	881.4743014	0.918102057
1.358833977	438.8254713	1957.433122	0.933754012
1.263917901	214.187483	1612.47669	0.945378579
1.351440607	137.8787652	1704.657597	0.930382677
1.308361943	77.95967544	115.3019747	0.939053956
1.297649424	155.4615222	1266.04476	0.939754344
1.372957076	168.4284077	3009.956994	0.928765547
1.19400854	297.4257821	666.5024936	0.95780414
1.422474296	181.5496037	1723.421446	0.925207317
1.460081408	199.1290611	1756.157694	0.921508363
1.411382433	213.1048548	3804.345552	0.926367354
1.266361867	358.5258645	1567.421688	0.947024805
1.185175735	509.2281794	1290.456148	0.959623411
1.870724575	209.2429299	3620.154286	0.886990521
1.291409768	153.1798794	442.6209786	0.940785526
1.634032089	497.9696588	6266.368204	0.899658313
1.25037064	224.3957135	1082.311512	0.946380297
1.721916083	157.5078581	3606.497258	0.889255032
1.253391155	325.6961987	2006.750396	0.952491029
1.230241873	197.1981674	1167.139446	0.95078964
1.695476221	131.6822607	1028.919767	0.899069978
1.291264603	231.1298703	1916.157645	0.943873666
1.445984454	236.5563379	3874.343021	0.920277839
1.3477382	133.391541	374.4529545	0.931254
1.18636241	183.4343994	330.0556497	0.956975339
1.196318581	349.1288982	380.019656	0.955979352
1.253519859	181.5558497	1044.391889	0.947695928
1.232976184	292.5471956	2270.945919	0.948868537
2.137977794	101.3751834	4311.440416	0.851957633
1.355393124	317.7775932	1232.83902	0.92764277
1.180619488	274.4480897	549.563862	0.960486204
1.242456405	188.6923978	956.7235809	0.948371767
1.455135004	202.0383864	2940.259846	0.923003077
1.278119298	273.6435797	1653.053276	0.944410536
1.232028056	190.1669369	626.2856214	0.950390666
1.970872785	253.3669358	6456.351387	0.875357468
1.275761208	239.7337926	1061.790578	0.943793181
1.262938147	346.6416758	2000.628927	0.948159198
1.363950163	406.4694949	6259.917186	0.932120397
1.258001881	257.8148806	1683.132051	0.944974057
1.275114635	159.9134151	540.6477544	0.943270569
1.229910941	312.1704817	2804.734751	0.950239102
1.216658064	218.3974197	335.0504408	0.951232438
1.282301129	357.1609254	4025.755426	0.941413537
1.500126519	179.1728434	5325.186103	0.915429078
1.267960618	240.028004	1697.97949	0.943897012
1.232790463	189.747106	406.2417616	0.951502336
1.429948035	300.7841126	3685.765113	0.926046513
1.228193504	194.7807305	1102.788663	0.952098425
1.23063152	157.8602314	780.794886	0.95047849
1.608011834	180.1749591	2738.844849	0.90204737
1.301972376	143.3176544	719.2347146	0.937821928
1.276426364	275.6667836	1754.312481	0.942213944
1.145745584	432.393568	282.9170779	0.966531582
1.247547862	234.7583405	1063.227819	0.946556138
1.42811108	176.1866804	1309.21648	0.921001986
1.225309115	338.198959	1163.649618	0.95259795
1.234420326	186.2220525	675.866771	0.949380389
1.443774728	163.0825544	1704.40682	0.927373691
1.359805681	146.8285222	1431.609034	0.93014087
1.491851654	188.6450653	1933.735135	0.916149494
1.290658513	342.6491825	424.3523703	0.943938178
1.260267034	248.2840562	1057.339538	0.946705948
1.78852852	139.3056555	2416.329992	0.888245981
1.353233938	226.907779	1288.388124	0.930681348
1.232662991	231.0661107	1125.546094	0.951345359
1.318415856	332.9676547	615.3206277	0.93267549
1.552033158	167.3107609	1743.251124	0.907607273
1.342166283	204.7670354	3420.363427	0.9313378
1.340284106	181.8386437	2643.452196	0.93381603
1.352517949	158.854621	2493.56254	0.929131384
1.207545469	384.9295964	1998.936983	0.95580193
1.262962547	205.7790633	1964.847584	0.944109026
1.201347864	283.8081836	261.9499944	0.954867472
1.242281151	171.5531328	219.1118363	0.948994748
1.35864683	370.7248787	6753.507447	0.934020755

log.sigma.3.5.mm.3D_glrlm_LongRunHighGrayLevelEmphasis	log.sigma.3.5.mm.3D_glrlm_RunPercentage	log.sigma.3.5.mm.3D_glrlm_LongRunLowGrayLevelEmphasis	log.sigma.3.5.mm.3D_glrlm_RunEntropy
421.2608885	0.93639463	0.007437317	4.998941991
307.3317239	0.944136555	0.019157702	4.716100337
323.0810377	0.941186616	0.016241036	4.846349525
310.6713994	0.948160535	0.033724255	4.832836513
257.4257161	0.934961566	0.01709968	4.796441529
355.1844206	0.947552448	0.015318622	4.745475363
379.0660002	0.812476735	0.015031597	4.814030817
505.2813863	0.900841742	0.005520627	4.92419366
271.4210659	0.873425261	0.014924466	4.758190023
217.2041861	0.927457972	0.019718269	4.570607614
158.6552793	0.890399446	0.022862842	4.307055141
299.1782597	0.867331964	0.012822499	4.705787594
436.5271102	0.927345158	0.011200154	4.839455932
312.3331195	0.950907788	0.014984015	4.847686375
226.7870335	0.917557998	0.017449215	4.790161273
256.7251426	0.855325341	0.033415248	4.899165765
138.5663372	0.903911491	0.035168745	4.391386215
245.191645	0.947526801	0.034427813	4.751522499
126.5147678	0.882224722	0.044693243	4.324556103
498.2061734	0.89338996	0.005791111	4.845068575
237.0411567	0.91385948	0.020503089	4.736053883
234.5330686	0.937965261	0.022620075	4.62145088
268.704241	0.917424869	0.015814679	4.688778875
189.4589175	0.837578324	0.029798269	4.471747494
430.6381351	0.881921824	0.011090473	5.002416668
605.6938034	0.907638137	0.005020127	4.938106565
270.3477499	0.926186089	0.0144521	4.858432001
189.7092324	0.905800813	0.022731692	4.602064688
98.57255984	0.91833509	0.049626546	4.067086931
204.4690584	0.918251314	0.020966977	4.612401396
234.1490143	0.902047317	0.017394192	4.795522665
366.1262146	0.943381492	0.010776815	4.799671207
261.5940946	0.894317246	0.017142908	4.849182949
309.135845	0.888580984	0.011935556	4.726287914
302.2416682	0.895877355	0.011162882	4.795410748
478.4628765	0.926885389	0.006738346	4.89408863
631.5581499	0.946123321	0.004838425	4.972093165
418.9947631	0.828446077	0.014269472	4.98574213
208.0001338	0.919917582	0.020589079	4.394343125
880.0381583	0.857227773	0.003513914	4.711289829
281.7432467	0.928300804	0.01213845	4.774624637
288.7797881	0.843102419	0.013608015	4.399977508
423.2765313	0.933670548	0.008879193	4.98356897
242.3906554	0.933911159	0.017619467	4.796466923
230.3494262	0.85218509	0.023925615	4.591093176
295.4593143	0.921763368	0.012313634	4.869760453
353.8521112	0.888662076	0.008560115	4.695925941
188.9764273	0.908036973	0.025020038	4.36526148
220.2223398	0.94393075	0.020010738	4.661783095
441.4037045	0.941967325	0.009533295	4.782469459
220.8085453	0.928755006	0.019860206	4.740201034
370.9009089	0.932053584	0.008476231	4.875344455
240.5084721	0.787130623	0.029706376	4.576274539
459.0261343	0.903916425	0.005896294	4.585765282
335.1260504	0.946883599	0.023203472	4.917173438
238.2074669	0.930468341	0.018374201	4.793291423
302.0857807	0.8903918	0.011622027	4.766932864
361.3412346	0.92374679	0.008359131	4.773371017
234.6293912	0.933561851	0.016941067	4.643826791
547.9808331	0.813959565	0.008729578	4.764699893
315.2891772	0.923655727	0.009845322	4.741884477
455.6191807	0.928194726	0.006369094	4.942174702
575.318768	0.905485697	0.004739665	4.999168517
338.5418046	0.926453318	0.012514205	4.879247892
209.9708535	0.923531417	0.023829268	4.602179466
399.4764743	0.933420277	0.007895881	4.891010631
280.1579943	0.936150057	0.027571368	4.633803567
468.0355129	0.921352394	0.005251747	4.817972901
281.3506199	0.880209827	0.012400501	4.630994774
325.9869133	0.924598001	0.009172925	4.711790588
239.7116713	0.934490306	0.01908088	4.642422685
447.4137808	0.894167066	0.00716309	4.88415618
238.6964416	0.935119562	0.021534192	4.826255588
195.872961	0.933846154	0.021261524	4.62312744
297.2323048	0.860788819	0.01502084	4.723541368
196.7050567	0.917714094	0.018698727	4.39486532
375.6993942	0.922345649	0.008454618	4.718938108
514.7287983	0.955703739	0.010772054	4.801680284
307.5244965	0.928985083	0.012376168	4.768825344
259.5613945	0.891367521	0.013451062	4.554737398
417.9113357	0.935605646	0.007201247	4.97662172
244.2305905	0.932575899	0.021210622	4.634301258
239.7908785	0.895199892	0.022748793	4.798673388
202.4167295	0.904750295	0.016053671	4.466854673
289.0804112	0.88213628	0.01365864	4.779413172
490.5867752	0.922062871	0.008112467	4.372439417
317.0485441	0.927370031	0.010026161	4.759230258
251.6634956	0.836909441	0.024650685	4.817401353
320.6955907	0.905756795	0.01188198	4.718008391
295.3239909	0.933993585	0.011165713	4.757023941
465.794861	0.911117489	0.00796423	4.810225707
266.5164334	0.870154332	0.015498978	4.64238178
287.1703742	0.907405192	0.010146029	4.610689353
251.8278354	0.909482566	0.014275015	4.71245937
222.7359921	0.90491033	0.013568571	4.471813739
483.638771	0.940242643	0.006873662	5.039443397
272.0021218	0.925680763	0.015159325	4.796748471
356.3129058	0.940525928	0.011587072	4.513258289
226.9936949	0.931687715	0.028236231	4.259439069
540.0319406	0.907272125	0.004969023	4.900280583

log.sigma.3.5.mm.3D_glrlm_HighGrayLevelRunEmphasis	log.sigma.3.5.mm.3D_glrlm_RunLengthNonUniformityNormalized	log.sigma.3.5.mm.3D_glszm_GrayLevelVariance
356.2508569	0.885107045	51.80368101
255.2992175	0.896561703	38.73046019
265.8239574	0.896818069	37.12436622
271.395914	0.90387161	41.12793299
214.6976823	0.881523267	30.99777393
300.9953763	0.90392522	41.93326389
197.2852553	0.72514025	30.97512693
375.0763486	0.833900501	38.21351822
188.0001689	0.79625465	26.28307472
177.2141545	0.866665875	26.38948097
114.5032833	0.81160781	16.82245771
201.4258969	0.783708168	28.14032282
333.7529615	0.869338842	39.30218522
276.6474709	0.907233862	36.2726095
186.9295284	0.853307508	34.78082139
161.3189418	0.779358477	29.43515992
105.9488471	0.829486306	16.89393827
213.8784263	0.903446485	34.8174541
89.66592856	0.798081274	17.68814977
357.2529293	0.819730408	38.69171012
183.088119	0.851760354	25.33864826
198.5172508	0.883636842	28.82705123
212.7720265	0.854365855	31.41325848
113.4095604	0.746895739	18.21127657
291.1625321	0.811095359	38.56345663
465.2667518	0.842309927	40.87656465
223.7915377	0.867564768	38.77104061
146.4725079	0.834222758	24.44396544
81.33572952	0.856105621	20.4084
163.7509069	0.855127145	24.30272386
179.2008088	0.83120041	26.66033128
309.6126074	0.895670227	36.92018949
193.7177708	0.824018347	33.20256228
214.9958436	0.816560197	25.67856067
226.8514537	0.82632985	32.22581113
378.3511763	0.871221179	36.09708968
530.8110466	0.900532634	44.51752481
233.4141889	0.748538682	32.65502083
162.3200485	0.85758809	19.28806759
549.9869145	0.771880912	27.49025103
234.6253273	0.869308361	30.21095266
174.9676061	0.75173088	21.68803721
340.9808555	0.885790811	35.22389234
205.2433236	0.879309348	30.18570554
144.0727755	0.772443037	27.98194591
241.7710298	0.864593763	31.2814796
254.2928124	0.813458971	27.08135014
142.5473882	0.837227602	18.32722949
190.462862	0.893391099	32.96131091
365.8556068	0.891455264	44.01159094
188.4792179	0.872768464	30.51881026
306.7902821	0.874446414	37.0533857
117.299723	0.683436989	20.12256847
341.8021016	0.828793521	27.07454735
285.2796722	0.901874985	43.53381301
197.4952911	0.873717349	34.08421542
216.4544573	0.820207956	26.39818504
288.4696969	0.864989779	30.85346262
197.9875543	0.878380313	25.24404659
286.3908197	0.725841414	28.55822104
252.5886104	0.863865619	31.53151187
364.6305163	0.873630825	40.87208439
433.4642684	0.838483445	39.69481687
272.1054682	0.866027972	35.03455376
168.5314871	0.863101822	30.03455707
327.762228	0.877791576	38.28786942
229.3900948	0.880453626	30.18629791
376.4568113	0.858291166	34.38690909
194.1282027	0.803924199	23.63514186
254.9575255	0.863762434	28.79020234
198.2787522	0.881918546	27.49388883
322.2065971	0.826149383	33.32446621
202.4545622	0.882495156	32.89347633
164.5922183	0.878984703	28.25868849
196.348849	0.777007695	30.70149875
152.6497585	0.851319698	18.37038092
292.7283847	0.860094664	28.92984223
447.579884	0.915903999	47.78235146
247.7185925	0.869552978	33.34600619
189.2229726	0.814655784	27.10614033
352.2338664	0.883757727	51.01451339
196.5004663	0.876325753	25.54076266
173.9754575	0.830787284	28.1619991
155.8826139	0.834375257	19.53282923
203.3355064	0.805352665	29.29459568
365.9909088	0.865087768	30.96972222
260.2122105	0.870599683	31.12487193
153.2679894	0.750263381	28.1100209
242.2071366	0.835080979	31.96193412
242.1410899	0.880758273	31.58524582
356.0530897	0.838941674	38.51817112
181.3157725	0.788482938	23.4495313
218.6750595	0.836366028	25.11631476
193.4209735	0.842112684	26.69234054
169.6952032	0.831833256	19.04217687
402.231465	0.890951924	45.1520438
217.3817932	0.864427112	31.09243879
296.9550953	0.888825029	32.57317493
180.9674433	0.876000508	22.33839506
397.0307545	0.842615863	35.08220365

log.sigma.3.5.mm.3D_glszm_SmallAreaHighGrayLevelEmphasis	log.sigma.3.5.mm.3D_glszm_GrayLevelNonUniformityNormalized	log.sigma.3.5.mm.3D_glszm_SizeZoneNonUniformityNormalized
286.1370583	0.038677171	0.390888806
141.8345958	0.046749452	0.321128561
164.8014032	0.045319111	0.323406222
219.6109772	0.046620762	0.365554142
138.1139718	0.049803922	0.286705113
184.8118795	0.045972222	0.375833333
143.1682833	0.051456996	0.302780216
281.4403002	0.04557056	0.34454272
149.8066817	0.054817596	0.302347467
128.8116066	0.054117647	0.319492503
66.24951385	0.067731347	0.222888524
157.8863301	0.052982739	0.319463604
160.9255639	0.044703434	0.319646202
223.2378467	0.047512342	0.433545744
171.1670935	0.048192381	0.379062937
113.6620167	0.050974394	0.33394185
68.40751881	0.068636915	0.239771293
166.2179749	0.048811899	0.419863135
72.16176757	0.070079833	0.269963201
232.6099985	0.045340745	0.338985542
110.7524962	0.054879184	0.272143333
132.63309	0.052998751	0.309766764
167.6039055	0.052498513	0.316255205
88.90616876	0.06628313	0.263362717
208.6816364	0.044916181	0.355880362
357.7726904	0.044031405	0.354321423
195.2646529	0.045587354	0.367502979
113.55409	0.05619456	0.29211392
70.84505076	0.0656	0.3184
124.7307885	0.056928142	0.301623816
132.450859	0.053658635	0.315879469
196.9202658	0.046559552	0.36402821
144.3183845	0.050110923	0.333559996
147.5723273	0.05554631	0.329159654
182.4836075	0.048509101	0.326169075
234.854088	0.046464681	0.343320637
326.4404106	0.043732667	0.359154165
171.8813068	0.048454676	0.344737895
99.70087623	0.069480344	0.239766984
376.1049572	0.054289952	0.311173183
183.3327869	0.051647706	0.334419342
127.5626119	0.06271507	0.288462166
221.2394526	0.046489519	0.373834643
154.5366834	0.050912953	0.340040923
125.2804834	0.056029002	0.29477225
191.5250681	0.048919009	0.369467347
187.4285903	0.055245024	0.310100348
104.8826562	0.066539834	0.31529132
136.0203658	0.050449998	0.315931012
185.2734224	0.042952943	0.295583304
173.5441863	0.050454832	0.401644284
205.6280991	0.046461444	0.32741005
91.03780707	0.063154377	0.28033272
215.0052859	0.054475662	0.316739507
190.4717321	0.042783381	0.356542139
131.0094284	0.048221803	0.302737698
156.9246637	0.054004448	0.297297499
177.6398956	0.049927007	0.310582343
141.3861422	0.057048658	0.343220815
213.0544885	0.054081407	0.308349183
174.8771002	0.050020825	0.320982924
223.2812373	0.043401236	0.31760766
289.4576708	0.043806903	0.339761861
163.8432081	0.049105507	0.274360879
107.8511643	0.051067259	0.338093364
209.4684461	0.045023071	0.325983784
119.3696821	0.052385028	0.275528129
263.7874959	0.048618498	0.322132576
128.4369062	0.058860149	0.26782152
135.2540258	0.051081135	0.278885142
143.9343712	0.052928048	0.356786056
224.0448415	0.047816678	0.31551957
151.1071533	0.048128698	0.338949704
122.0291907	0.052117653	0.345360825
167.2379092	0.053120516	0.338796403
75.22814193	0.06494838	0.22511926
154.8117101	0.052146578	0.28164262
297.5948648	0.045716949	0.410520277
154.1653288	0.048080646	0.317942426
150.1769751	0.055250711	0.308030139
282.0460893	0.039135711	0.396732974
112.9152946	0.054487179	0.304692636
127.0784841	0.052692842	0.317656042
113.28916	0.064076772	0.262582958
147.4113205	0.051267543	0.30345734
162.4961336	0.054135802	0.32617284
183.9035539	0.049722387	0.328656884
134.4280703	0.052454629	0.331398187
153.4987786	0.049332263	0.316866442
154.5407124	0.049305566	0.29695824
212.6193347	0.046808511	0.302851969
162.0910174	0.058632077	0.349045619
140.6971519	0.056335251	0.291750071
128.0053092	0.054028229	0.27758677
111.1981672	0.063157596	0.290042517
257.3335859	0.041289279	0.360595945
130.1553494	0.049119719	0.28201939
176.8188213	0.055211203	0.344467401
95.84061808	0.065679012	0.252839506
229.2679529	0.046702335	0.336668214

log.sigma.3.5.mm.3D_glszm_SizeZoneNonUniformity	log.sigma.3.5.mm.3D_glszm_GrayLevelNonUniformity	log.sigma.3.5.mm.3D_glszm_LargeAreaEmphasis	log.sigma.3.5.mm.3D_glszm_ZoneVariance
238.8330606	23.63175123	19.6792144	14.09384417
47.52702703	6.918918919	10.34459459	4.847470782
121.2773333	16.99466667	11.744	6.085944889
55.1986755	7.039735099	8.344370861	3.797026446
146.2196078	25.4	18.80196078	11.14751634
45.1	5.516666667	7.133333333	3.066388889
616.1577396	104.7149877	1799.504177	1772.683485
646.0176	85.4448	96.86506667	86.39164473
328.9540441	59.64154412	217.5827206	202.2376426
81.47058824	13.8	28.51372549	19.74748174
79.34831461	24.11235955	84.80337079	65.69959601
269.9467456	44.7704142	297.7715976	281.5149554
99.09032258	13.85806452	17.17096774	10.32683663
146.5384615	16.0591716	7.923076923	4.292811876
210.7589928	26.79496403	40.31834532	32.29396447
472.1937765	72.07779349	614.4526167	597.8472799
48.19402985	13.7960199	82.34328358	63.0445286
83.55276382	9.713567839	10.27638191	6.052220904
60.20179372	15.62780269	175.8116592	151.7444952
864.0741467	115.5735583	250.9992154	238.0205661
153.2166963	30.89698046	55.61278863	41.98974032
60.71428571	10.3877551	13.06122449	6.657226156
51.86585366	8.609756098	45.12195122	33.5453599
169.6055901	42.6863354	704.9177019	673.7209671
139.505102	17.60714286	62.04081633	52.22730112
279.9139241	34.78481013	89.59493671	79.12684506
266.8071625	33.09641873	30.55647383	22.93704893
182.5712	35.1216	58.0464	45.05181696
15.92	3.28	24.32	15.7936
153.5265226	28.97642436	36.23968566	26.23470652
357.8914387	60.79523389	95.58781995	83.06759036
127.0458453	16.24928367	11.92550143	6.827480891
228.8221574	34.37609329	118.1239067	106.5381197
226.7910015	38.27140784	96.87808418	84.59249959
476.2068493	70.82328767	121.3006849	108.9401628
260.9236842	35.31315789	25.61710526	19.10789301
248.5346821	30.26300578	9.297687861	4.523589161
492.2857143	69.19327731	807.3312325	790.7147619
38.60248447	11.1863354	28.54658385	16.44828517
632.9262537	110.425762	789.8254671	768.2536895
158.5147679	24.48101266	34.27004219	26.27808934
307.2122066	66.7915493	1079.844131	1051.479756
372.7131394	46.35005015	17.82146439	11.93950155
188.3826715	28.20577617	19.74729242	13.17741662
98.15915916	18.65765766	279.4894895	257.6556196
333.6290144	44.17386489	32.58582503	25.51599749
411.1930618	73.25490196	190.3898944	174.1050785
45.71724138	9.648275862	41.73103448	30.26463734
52.76047904	8.425149701	10.29341317	4.811646169
54.97849462	7.989247312	10.75268817	4.84726558
212.0681818	26.64015152	28.19128788	22.24988163
341.816092	48.50574713	21.02586207	13.90964148
338.0812604	76.1641791	3454.40796	3410.668273
147.283871	25.3311828	58.15913978	45.68988785
110.171521	13.22006472	7.770226537	3.440056137
125.6361446	20.01204819	20.64578313	12.61572942
343.9732066	62.48314607	119.653414	107.6112543
212.7489051	34.2	30.11532847	21.00991209
107.428115	17.85623003	15.4600639	9.517684165
620.7069051	108.8658718	2190.115748	2160.833294
157.2816327	24.51020408	24.54897959	17.19270721
307.4442149	42.01239669	23.93285124	17.44825213
907.5039311	117.0082366	131.5949083	122.091619
191.7782546	34.32474964	23.53361946	14.5422011
78.0995671	11.7965368	27.94372294	19.35450985
434.5363841	60.01575394	16.13728432	9.550918487
41.05369128	7.805369128	13.27516779	5.850457187
569.2082626	85.90888512	34.89473684	26.61324048
423.9614656	93.17561592	408.5887555	386.0937192
198.0084507	36.26760563	22.06478873	13.11542154
69.21649485	10.26804124	16.93814433	10.50643001
454.0326616	68.80820014	204.666435	192.6849267
176.2538462	25.02692308	22.32884615	15.73777737
134	20.22164948	17.09793814	11.10301307
312.0314875	48.92399566	573.4527687	553.7896588
59.65660377	17.21132075	28.42264151	16.42229975
194.6150507	36.03328509	29.70332851	19.48369464
68.55688623	7.634730539	7.778443114	4.03757754
147.2073434	22.26133909	31.62634989	23.55976844
136.1493213	24.42081448	94.7239819	78.13955488
233.6757216	23.05093379	20.36332767	14.66509666
95.06410256	17	18.23717949	11.22826266
232.2065663	38.51846785	50.07387141	40.32854194
122.1010753	29.79569892	103.9978495	87.42507111
203.3164179	34.34925373	225.1656716	208.7450323
58.71111111	9.744444444	57.48333333	48.78083333
161.699187	24.46341463	29.04878049	21.98096371
283.0140515	44.79625293	638.3981265	618.2218298
166.9886148	25.99810247	77.06072106	66.64264805
150.2608696	24.9486166	19.27075099	11.97221094
71.17021277	11	49.62553191	37.92039837
208.3802345	35.00335008	287.8358459	269.8480173
368.1885895	71.09508716	62.63787639	49.91751892
264.262605	51.43487395	70.19327731	57.09070246
243.6357143	53.05238095	101.1964286	85.69720947
370.3320351	42.40408958	12.5842259	7.195679649
226.1795511	39.39401496	25.46134663	16.1139172
45.46969697	7.287878788	12.08333333	6.46068641
22.75555556	5.911111111	20.66666667	11.79950617
937.9576454	130.1127064	156.6547021	146.6231352

log.sigma.3.5.mm.3D_glszm_ZonePercentage	log.sigma.3.5.mm.3D_glszm_LargeAreaLowGrayLevelEmphasis	log.sigma.3.5.mm.3D_glszm_LargeAreaHighGrayLevelEmphasis	log.sigma.3.5.mm.3D_glszm_HighGrayLevelZoneEmphasis
0.423130194	0.094833614	5424.708674	416.2324059
0.426512968	0.114240677	3036.243243	239.2972973
0.420403587	0.124907314	3046.192	277.912
0.468944099	0.253006018	1793.92053	320.7483444
0.361445783	0.36825011	2920.37451	240.2254902
0.495867769	0.055284268	2139.55	295.0083333
0.193092324	10.12159599	328673.4486	236.0206388
0.308998022	0.329794901	31889.36373	442.8133333
0.255279212	1.800999341	32396.2739	251.1727941
0.337748344	0.31651375	4139.152941	204.3764706
0.228791774	1.155413913	7743.463483	134.9353933
0.248018785	1.883689606	52050.59408	252.4662722
0.382244143	0.087364599	6962.158065	294.2709677
0.52484472	0.129141264	1456.08284	309.5976331
0.353015873	0.554443225	4619.332734	247.7517986
0.245400902	5.855971399	79956.25177	183.5707214
0.227633069	1.95095785	6646.542289	131.5223881
0.486552567	0.397197797	1465.748744	238.919598
0.203839122	3.4535321	12344.64574	118.67713
0.277578134	0.779119247	85421.00863	382.751275
0.27093359	1.20679752	7675.0746	201.714032
0.39516129	0.254730536	2277.882653	220
0.29390681	0.390280627	9452.384146	260.6402439
0.179038087	8.082459989	69065.24534	155.8726708
0.319218241	0.292882736	17133.14541	322.125
0.309076682	0.287066749	31039.90506	556.9139241
0.362275449	0.320610083	4691.676309	281.2465565
0.277407901	0.752091562	6844.5904	187.3024
0.342465753	0.708372281	1229.24	109.9
0.316149068	0.525292307	4697.850688	197.9312377
0.282614118	1.395829369	10487.46602	221.0326567
0.442893401	0.098555839	2989.578797	318.9484241
0.29379015	1.050911528	16255.96501	232.6049563
0.285300207	0.657998336	18955.61248	240.605225
0.284434054	0.884578017	19491.75411	284.7527397
0.391954616	0.095131956	9599.673684	382.2039474
0.457671958	0.031354814	5153.069364	526.2312139
0.245318674	3.903905903	177339.0462	271.3445378
0.2875	0.364857445	4244.21118	186.6770186
0.215306446	1.541570203	413545.9366	628.9936087
0.353731343	0.343611111	4918.21308	286.0759494
0.187764457	7.232748509	165819.2864	217.2676056
0.412324235	0.130952039	5015.971916	352.8284855
0.390140845	0.299304239	2608.871841	244.2743682
0.214010283	2.697545089	34079.48649	196.8258258
0.376093294	0.330384807	5169.591362	289.6256921
0.247804149	1.003797858	42660.62896	307.5806938
0.295315682	0.560996324	5676.227586	164.8206897
0.427109974	0.177376042	1582.08982	220.011976
0.411504425	0.053724744	4148.403226	351.7688172
0.41025641	0.434218595	2772.738636	240.4393939
0.37486535	0.124023641	5272.953065	338.3285441
0.151203611	32.98154786	374539.2919	155.615257
0.28319123	0.209353482	18870.18495	363.2645161
0.480559876	0.152788031	2044.200647	299.1197411
0.352891156	0.349694931	2846.228916	227.3277108
0.288169365	0.836393167	21442.03198	262.7389801
0.331398162	0.194185888	7709.764964	310.3532847
0.410222805	0.223189317	2228.482428	224.658147
0.184797576	8.206745667	596656.7258	349.9880775
0.368698269	0.159554137	5270.871429	278.9836735
0.392697769	0.093148661	8060.905992	381.518595
0.324386689	0.414699932	44519.25197	471.0928491
0.333492366	0.175734677	5914.573677	297.5765379
0.341211226	0.321075002	3954.255411	182.3593074
0.389652148	0.083700634	5006.054764	346.3390848
0.366995074	0.258147556	3017.966443	225.0604027
0.347492625	0.141679712	10210.35597	430.4352009
0.210841769	3.421945594	61419.14656	232.5590651
0.334274953	0.127333973	5861.987324	251.556338
0.394308943	0.289178994	2491.438144	224.3608247
0.288897812	0.753568465	63154.14524	374.2515636
0.389513109	0.430049628	2713.871154	240.8076923
0.408421053	0.270530947	1953.786082	189.0515464
0.225514202	3.79422665	93863.53637	261.1411509
0.288671024	0.367863211	3708.298113	161.0150943
0.312811227	0.146367633	8883.386397	290.1895803
0.517027864	0.045331926	3676.676647	444.1976048
0.352091255	0.195007333	7696.116631	260.6846652
0.245555556	0.75271373	14265.94796	242.8371041
0.418918919	0.09741015	5581.186757	409.5670628
0.377723971	0.233072174	3903.916667	196.2115385
0.320333041	0.789235464	6827.228454	209.8399453
0.245641838	1.049623364	12505.11613	198.8623656
0.246777164	1.530347816	37581.73284	250.9044776
0.338983051	0.146532228	26806.36111	301.5722222
0.376146789	0.21221889	5297.317073	299.2987805
0.222627737	6.180322432	78507.9192	212.1030445
0.309817754	0.449711948	15247.28653	262.2296015
0.370153621	0.168215623	4207.685771	266.2924901
0.292288557	0.192401921	17054.69787	374.4212766
0.235781991	2.327042583	43417.93802	241.8157454
0.280382137	0.456419291	10961.00872	245.5752773
0.276262333	0.657224442	11293.55252	226.0892857
0.254006653	1.018458073	12622.45595	193.6595238
0.430788591	0.064953121	4706.948393	414.2259007
0.327079935	0.255842446	4933.295511	231.4102244
0.42172524	0.074655504	3528.318182	299.5984848
0.335820896	0.273610801	4086.866667	178.0333333
0.315729828	0.430860294	63130.28033	391.269921

log.sigma.3.5.mm.3D_glszm_SmallAreaEmphasis	log.sigma.3.5.mm.3D_glszm_LowGrayLevelZoneEmphasis	log.sigma.3.5.mm.3D_glszm_ZoneEntropy	log.sigma.3.5.mm.3D_glszm_SmallAreaLowGrayLevelEmphasis
0.645591437	0.007476859	6.474222333	0.005369808
0.580177928	0.022417966	6.030267515	0.016134052
0.583551115	0.015950581	6.379516573	0.009879716
0.620626512	0.022804243	5.880017279	0.011490242
0.546247625	0.013065468	6.540316035	0.007493026
0.632030472	0.021517321	5.828556509	0.01875549
0.567944592	0.009930668	6.863628176	0.00602865
0.607801813	0.004171181	6.76225976	0.002683755
0.563779963	0.00821759	6.553307946	0.003898498
0.583728127	0.017559528	6.140273361	0.008611412
0.469326349	0.019680379	6.36342847	0.010075516
0.583264315	0.009405146	6.562983239	0.006220071
0.582110863	0.015292465	6.3679205	0.012223957
0.683705997	0.012090922	5.973442199	0.008629435
0.637436326	0.012242187	6.306843877	0.00776087
0.597062467	0.02201116	6.557054302	0.011817516
0.495234242	0.024023487	6.073029485	0.009907739
0.671543538	0.027291329	5.796794282	0.016505836
0.52949315	0.031524967	5.994645672	0.013649421
0.603041859	0.005441045	6.868656832	0.003747986
0.533421121	0.014234527	6.574376237	0.00776928
0.570463341	0.016918776	5.983817031	0.010087457
0.580531611	0.01616084	6.062212658	0.011912103
0.525634506	0.020323199	6.474009226	0.008334102
0.619431984	0.010388761	6.363386235	0.007155003
0.616778281	0.00396753	6.569750818	0.001968462
0.62918608	0.0123201	6.510036791	0.008274285
0.557202101	0.017867204	6.47039631	0.009614917
0.570765636	0.065601933	4.97366069	0.052862947
0.564450313	0.016479765	6.397759889	0.008095167
0.577359531	0.011189291	6.509406743	0.006247158
0.623454564	0.011183952	6.223093402	0.008278304
0.595941152	0.014566075	6.516258264	0.009682922
0.5922629	0.009407815	6.361760294	0.00603635
0.589871585	0.008769769	6.69803572	0.00560277
0.605274299	0.006711328	6.507613802	0.004552461
0.619533283	0.004943394	6.51250082	0.003524179
0.609005943	0.00860352	6.650657837	0.004914913
0.485618774	0.019266106	5.912113955	0.007443177
0.576911802	0.002476971	6.773946183	0.001624308
0.598580568	0.009247157	6.348926099	0.005679742
0.550981212	0.009570916	6.551563106	0.004956103
0.633527338	0.006859805	6.431015493	0.004373284
0.599070276	0.013333667	6.333742571	0.007146868
0.557057557	0.020490737	6.310288846	0.013201961
0.629902139	0.007989838	6.364932324	0.004711544
0.573560634	0.006124937	6.650970711	0.003591435
0.580978979	0.020849522	5.729384032	0.010218411
0.571946292	0.019188341	5.973307215	0.01361038
0.553442861	0.012795645	6.261544954	0.009839399
0.657323944	0.014192743	6.111174345	0.008167206
0.59060599	0.007138611	6.689486488	0.003691928
0.543247965	0.018807531	6.616814407	0.009654799
0.580060021	0.006479783	6.428738152	0.004759144
0.613765528	0.017619636	6.195749101	0.009014776
0.566531552	0.014482367	6.474058454	0.009112679
0.557464144	0.007822703	6.57808439	0.004373533
0.573515398	0.007450964	6.558917994	0.004967544
0.604802036	0.013400337	6.073274117	0.00896642
0.573401056	0.005235828	6.786410255	0.003148905
0.584423422	0.009374708	6.459051874	0.00632442
0.574192111	0.006353921	6.723763417	0.00441901
0.602694494	0.003947118	6.835615985	0.00255688
0.533524536	0.01234405	6.738986781	0.007128173
0.602050042	0.032373099	6.162261763	0.026612149
0.588480111	0.008031619	6.764752464	0.005614562
0.534767688	0.021407516	5.969824668	0.009770169
0.583383812	0.004350898	6.74933497	0.002763243
0.527934333	0.009208537	6.806499027	0.005243694
0.541538077	0.009362425	6.682009956	0.006062635
0.619085631	0.016464552	5.993743191	0.011238212
0.577931059	0.00533644	6.744916212	0.003391924
0.599145618	0.01607238	6.338931169	0.009012419
0.604877701	0.021033462	6.158841002	0.014973933
0.601062083	0.010642184	6.513383673	0.005830994
0.468517363	0.017213239	6.277220616	0.00699974
0.544288506	0.008790474	6.668990888	0.004924341
0.663309851	0.012642443	5.86194793	0.009873998
0.579941705	0.012528481	6.498829507	0.00734728
0.572779743	0.011977488	6.407863358	0.006414361
0.652648095	0.007228851	6.459933844	0.005356479
0.565343687	0.016020683	6.169270619	0.00645558
0.581104433	0.015891106	6.492103599	0.009427965
0.515713005	0.012202407	6.367337501	0.006925829
0.566158324	0.009934466	6.59806039	0.005820239
0.589152187	0.012541928	5.986103634	0.010240731
0.58744425	0.009077138	6.361256118	0.006074786
0.594848269	0.012229478	6.484318593	0.005648536
0.579692414	0.012831886	6.477788076	0.007857177
0.555459216	0.01001515	6.508446204	0.006338271
0.560846492	0.010490045	6.312010138	0.007943593
0.613035074	0.009558386	6.226196571	0.00582638
0.555813067	0.008408388	6.696963295	0.004601148
0.540010853	0.010655356	6.739952907	0.006038331
0.555258338	0.011051726	6.472987134	0.006661707
0.620804564	0.005904038	6.667526545	0.003332934
0.545013823	0.012817545	6.764828543	0.006417068
0.601599532	0.014700647	5.583907995	0.011641583
0.499084969	0.027307857	5.471314525	0.008883689
0.598495113	0.004485802	6.764483837	0.002942618

log.sigma.3.5.mm.3D_ngtdm_Coarseness	log.sigma.3.5.mm.3D_ngtdm_Complexity	log.sigma.3.5.mm.3D_ngtdm_Strength	log.sigma.3.5.mm.3D_ngtdm_Busyness	log.sigma.3.5.mm.3D_ngtdm_Contrast
0.00734124	1.046064113	3.293467845	0.214175915	0.000104611
0.02155035	2.32238987	5.835954452	0.114262182	0.000742499
0.010452118	0.926623346	2.602225872	0.252782971	0.000262687
0.028469732	2.566062703	7.321076337	0.096035198	0.000874213
0.006761103	0.530935424	1.921295497	0.432953076	0.000127804
0.046216351	2.663635324	10.72291059	0.057059563	0.000887588
0.001216536	0.084884382	0.740096713	1.279760269	3.67E-06
0.001745031	0.208206535	0.799981234	0.775528449	1.58E-05
0.002577076	0.148312081	0.767903077	1.053877795	2.22E-05
0.013961171	0.593855301	2.570469012	0.265533534	0.000176676
0.007239386	0.169304388	1.122860778	0.592118164	4.29E-05
0.003327752	0.205687685	1.130981835	0.616053382	1.94E-05
0.011062896	0.97645308	3.280565164	0.175311156	0.000279832
0.013579379	1.584193059	4.195458746	0.17370721	0.000367714
0.006120007	0.700535921	2.52589701	0.419171154	8.18E-05
0.002074215	0.114538621	0.577000978	1.375452477	1.99E-05
0.012095539	0.230891573	1.237516536	0.569705977	0.000147218
0.020062921	2.115032713	6.009307677	0.142650271	0.000600074
0.010148568	0.167862644	1.072150944	0.635604142	8.33E-05
0.001311067	0.127936397	0.71678952	0.85822781	6.19E-06
0.004849603	0.246783381	1.072218038	0.704350357	7.50E-05
0.016941095	1.083334886	3.443181344	0.202312936	0.00043028
0.018083922	1.008559186	4.067546507	0.156179842	0.000259677
0.003469109	0.080648321	0.71145633	1.018111942	1.40E-05
0.009607596	0.649207507	2.697141431	0.22728094	0.00012064
0.004249688	0.504204484	2.159321035	0.286847691	3.91E-05
0.005042597	0.554894821	2.105070494	0.453863024	6.60E-05
0.00504225	0.20166193	1.091100532	0.712590751	4.34E-05
0.063106631	1.231061334	6.63754571	0.137123311	0.000837667
0.0061364	0.351949742	1.493281571	0.515836355	6.94E-05
0.00267299	0.167608985	0.756455696	1.203177675	2.97E-05
0.010019313	1.248630249	3.062065575	0.208605715	0.000291872
0.005126923	0.341023939	1.845723031	0.472337213	4.07E-05
0.004735074	0.268761548	1.45576564	0.498612443	3.67E-05
0.002117611	0.172693994	0.819751573	0.972744072	1.67E-05
0.004897842	0.567582575	1.717155438	0.322712883	8.11E-05
0.005748763	0.910067233	2.261959219	0.236686905	0.000160202
0.00217977	0.138671641	0.79200252	0.854285376	1.45E-05
0.016608133	0.575143211	2.598059154	0.225758729	0.000219981
0.001285815	0.091226486	0.714217745	0.709505829	4.10E-06
0.007515501	0.669767068	2.688132869	0.308646253	9.15E-05
0.001988726	0.097470042	0.878338874	0.913764334	4.96E-06
0.003808418	0.466175203	1.30167174	0.523619257	8.41E-05
0.006693468	0.536964524	1.813252031	0.495712583	0.000125656
0.007526851	0.300712469	2.298150677	0.350553903	3.72E-05
0.004300908	0.358187389	1.282840142	0.620323746	6.33E-05
0.002178525	0.148531588	0.877936409	0.775775801	1.12E-05
0.017187674	0.667742195	2.831327185	0.224798816	0.000241814
0.025435755	1.523364956	5.850639235	0.141053975	0.000464559
0.019875714	1.885861602	6.005012124	0.091864086	0.000447098
0.00692948	0.614360877	1.986011186	0.522606874	0.000135258
0.003816504	0.452176954	1.811881692	0.414666715	3.76E-05
0.00159684	0.046464189	0.504883267	1.694056974	4.15E-06
0.006326602	0.440574728	2.130197942	0.244872151	4.15E-05
0.013876776	1.469753649	4.149534624	0.17760764	0.000449543
0.008174919	0.636714367	2.358567235	0.401945369	0.000149002
0.002833384	0.154258589	0.733933956	0.916998163	2.49E-05
0.004226439	0.424145607	1.313050645	0.525018482	7.72E-05
0.011331663	0.79778012	2.318814004	0.295009038	0.000241469
0.001160566	0.06270545	0.50724641	1.14904438	3.31E-06
0.007696256	0.584621538	2.138157288	0.302071095	9.56E-05
0.004403334	0.524351366	1.923073513	0.324174717	4.75E-05
0.001448413	0.166667892	0.727583183	0.807908121	1.09E-05
0.005668585	0.439957717	2.145946245	0.324086264	5.08E-05
0.015070175	0.65104824	2.390725553	0.263698616	0.00023598
0.00298577	0.367776417	1.240330334	0.511760033	3.54E-05
0.019610685	1.357897343	3.748060914	0.162864424	0.000669242
0.002042447	0.287284718	1.213884981	0.565343475	1.46E-05
0.001384256	0.083745214	0.476433176	1.597497017	8.31E-06
0.004814616	0.322969063	1.292569144	0.465889832	5.69E-05
0.017948752	1.102664789	3.580981478	0.204941812	0.000438095
0.002353386	0.221613103	0.975794316	0.611520508	1.75E-05
0.006466495	0.633926341	1.864199787	0.534459232	0.000166415
0.009096545	0.69333835	2.279024711	0.403143984	0.000165156
0.003087593	0.181428773	1.521598884	0.584362638	1.23E-05
0.010951055	0.339568773	1.593273551	0.369867635	0.000120861
0.004571966	0.38572899	1.632114206	0.380879136	4.41E-05
0.023413531	3.700432053	9.740771511	0.062953214	0.000826935
0.008582986	0.526279229	2.321575218	0.262170052	9.36E-05
0.006284581	0.309686344	1.782249716	0.364142142	3.50E-05
0.007460706	1.008265653	3.258320679	0.220923613	0.000108999
0.011684392	0.589088503	2.228442252	0.280041653	0.000213336
0.004066201	0.311085774	1.050941556	0.793649528	6.73E-05
0.005664834	0.24286894	1.23483401	0.549166821	4.10E-05
0.00468377	0.206138968	1.192670105	0.541705115	3.30E-05
0.012177036	1.226981603	4.853782523	0.125917292	0.000277933
0.007337066	0.569382029	1.854923491	0.353777842	0.000114952
0.003363768	0.12953052	0.879588024	0.879561137	2.09E-05
0.006306826	0.421364744	1.791022664	0.332942963	5.67E-05
0.007367174	0.520137356	2.025500246	0.355046227	0.000110536
0.015619179	0.84850001	4.759949606	0.111394984	0.000135222
0.004664516	0.197302941	1.186339337	0.540042529	2.79E-05
0.002380702	0.200679119	1.100440595	0.729457709	1.26E-05
0.003227412	0.181411623	0.894273527	0.844484178	2.86E-05
0.00317708	0.113124362	0.573455356	1.101662892	2.67E-05
0.004170599	0.783232542	2.441867107	0.296534726	5.93E-05
0.004289387	0.257701237	1.022901465	0.700466124	6.40E-05
0.026432952	1.795613748	5.983555753	0.090812939	0.000610945
0.024924668	1.367433349	4.383930038	0.135093981	0.000715624
0.00129977	0.117469689	0.511130766	1.041048193	1.06E-05

log.sigma.3.0.mm.3D_gldm_GrayLevelVariance	log.sigma.3.0.mm.3D_gldm_HighGrayLevelEmphasis	log.sigma.3.0.mm.3D_gldm_GrayLevelNonUniformityNormalized	log.sigma.3.0.mm.3D_gldm_DependenceEntropy
40.22489612	368.5020776	0.047918217	6.854121678
31.32701044	205.6887608	0.058226545	6.156095085
35.39332683	240.3452915	0.047007279	6.655518135
37.88508352	276.0217391	0.047008217	6.244136679
31.21635062	243.1034727	0.052088958	6.602307458
34.51125265	335.8636364	0.051567516	6.144200133
16.2516935	196.336939	0.095369256	7.215845148
28.33286592	414.6807844	0.056451732	7.139051242
20.11973062	184.9366495	0.070775183	7.000203719
20.32196132	172.7708609	0.062135871	6.307300667
11.45550642	111.6998715	0.088697207	6.476843775
17.84722005	225.978867	0.076992077	7.032670599
35.81112356	314.2194821	0.055113034	6.513375881
36.87545571	229.5419255	0.046140195	6.433520503
29.05488093	192.6349206	0.057040867	6.837259816
24.01985471	181.2084346	0.073534116	7.211566626
14.37493924	147.6296716	0.073422865	6.425915934
32.67601222	197.9462103	0.054226123	6.183198369
11.08054988	91.57129799	0.087485671	6.477220056
22.08332493	388.6538168	0.064720544	7.13561864
23.23253091	155.759384	0.059902957	6.805434652
26.90459157	150.9395161	0.056232115	6.287361595
23.65848653	188.6290323	0.058664457	6.410275871
10.92227259	133.8565471	0.106571449	6.846809542
29.47873916	208.2060261	0.059715488	7.034116281
31.55077255	501.4021909	0.057326221	7.030207168
30.47176381	201.6192615	0.053800582	6.834716459
18.42331643	173.4385264	0.06744531	6.654292559
14.27378495	95.52054795	0.089041096	5.646404525
19.58454728	189.473913	0.062955904	6.646163368
23.18044938	156.5717136	0.059036226	6.98577465
34.45517792	265.2791878	0.049366771	6.449430226
23.67280441	167.6522484	0.061820083	7.003030598
21.61847082	164.5966874	0.068432717	6.95204487
22.94037492	286.3052796	0.061975644	7.04665648
32.87434147	349.8024755	0.049849178	6.931387314
46.10126352	604.8670635	0.043902746	6.682667711
24.10530857	204.2901563	0.084218556	7.295439881
15.31070153	144.2142857	0.073278061	6.199603569
15.73544539	697.1294591	0.085058773	7.087908051
26.05526565	214.2977612	0.055370907	6.637065337
11.06245263	171.8942172	0.108391803	6.800994487
37.18758744	308.7965261	0.045675493	6.885469199
29.75787542	208.6014085	0.051266614	6.621448537
14.030356	119.0269923	0.095341195	6.837670438
29.84247678	238.182424	0.051880195	6.813235081
18.6723828	223.0592413	0.068050058	7.011735444
14.33769563	171.6843177	0.081611575	6.142726
28.60533356	163.4859335	0.053302896	6.217003802
36.02621094	336.8030973	0.056239721	6.329085853
28.92812411	248.3224553	0.057105029	6.517008704
31.37617797	267.1888689	0.051275073	6.717480925
9.856648116	137.5160481	0.12160796	7.004839651
17.60410309	346.7326431	0.069273976	6.738578747
42.56640601	284.7729393	0.045376818	6.539620497
28.99795224	171.5323129	0.054822354	6.553741207
21.18539189	214.3780822	0.064230183	7.000117814
29.15685915	266.1112724	0.053389254	6.708715348
25.6869294	210.2621232	0.05537739	6.464982743
14.4720296	282.2974387	0.097423289	7.14747074
25.92364575	255.096313	0.055278413	6.7369717
32.69716477	360.5789047	0.049391357	6.953606321
30.23467351	389.1148895	0.052332288	7.204554357
28.0725662	338.2256679	0.053327221	6.811753604
20.93765396	196.6469719	0.06263841	6.52266797
32.32236318	329.8319205	0.049304889	6.81013265
27.69417482	219.5098522	0.057438909	6.145762565
27.40511188	342.7486726	0.056383893	6.857306647
15.85675792	281.8794619	0.074343715	6.925318978
23.23567621	196.2250471	0.058239881	6.643418355
27.71518276	205.9268293	0.053688281	6.345027958
26.20476598	356.5669544	0.06054128	7.086583009
33.05678351	232.9985019	0.050729285	6.55237795
25.84062161	172.8115789	0.056170637	6.437322657
17.96029344	193.7889324	0.087913489	7.000690266
15.23192528	154.3845316	0.071572662	6.299656283
23.24410346	295.3820733	0.058549934	6.678191758
42.49552857	390.0928793	0.0512705	6.27743421
26.07751681	242.0669202	0.056516069	6.561990847
15.96128858	221.3261111	0.077028395	6.684159275
38.89353694	364.7396871	0.04908045	6.829710994
24.51735661	178.1452785	0.058891123	6.429280202
25.92418047	202.8071867	0.060215565	7.042536616
14.42861779	159.8520866	0.075814948	6.515938662
20.31691205	175.028361	0.069347361	7.016544235
22.48290366	276.2580038	0.094307369	6.054064676
26.66018339	265.8685015	0.054601652	6.724608168
17.92183132	171.7309698	0.085095946	7.091978055
22.26693984	244.9800118	0.061495451	6.811662352
26.78569842	240.1550841	0.054923532	6.611277883
24.44937471	281.9241294	0.062244746	6.668117564
15.71287879	155.0778041	0.081096561	6.915412693
18.58702204	249.8553655	0.065317387	6.771017316
20.64666596	164.131747	0.061129022	6.857231562
15.45369239	146.776837	0.069667854	6.607726731
41.74905339	407.5788591	0.043970429	6.911588836
26.9975956	190.1117455	0.053037571	6.772593986
26.6026192	288.4632588	0.059886291	5.966056301
18.43979728	161.9029851	0.076854533	5.844761883
26.5422364	432.9594288	0.055827021	7.141133013

log.sigma.3.0.mm.3D_gldm_DependenceNonUniformity	log.sigma.3.0.mm.3D_gldm_GrayLevelNonUniformity	log.sigma.3.0.mm.3D_gldm_SmallDependenceEmphasis
302.9293629	69.19390582	0.351598717
81.31123919	20.20461095	0.365914185
201.6188341	41.93049327	0.355041721
82.95652174	15.13664596	0.447818323
304.4372785	73.49751949	0.326078488
57.08264463	12.47933884	0.362836857
1023.103995	1005.096594	0.179487314
963.8806856	342.5491101	0.271202374
567.8385734	301.6438292	0.232118347
168.7986755	46.91258278	0.295956978
238.6401028	138.0128535	0.207381911
427.5471089	262.3120047	0.233380375
179.6855734	44.69667078	0.336685103
161.3198758	29.71428571	0.388477103
272.5365079	89.83936508	0.319437865
663.7733426	423.7035751	0.212893173
153.419026	64.83238958	0.209710357
98.34474328	22.17848411	0.347326093
154.0968921	95.70932358	0.184678249
1341.967004	594.3287597	0.241845813
339.6217517	124.4783446	0.228996147
110.0282258	27.89112903	0.34084172
110.1577061	32.73476703	0.297134512
383.6649986	383.3375035	0.168870634
172.495114	73.33061889	0.256805169
400.7237872	146.5258216	0.271002691
376.1027944	107.8163673	0.309389645
383.4749223	151.9542832	0.257357077
29.65753425	13	0.338283702
293.8310559	101.3590062	0.265236771
638.588426	236.6762285	0.24712723
182.0532995	38.90101523	0.346196347
354.9914347	144.3498929	0.257770873
329.842236	165.2650104	0.236164118
775.2969024	318.1209819	0.260801323
379.7447138	96.65755544	0.326892311
371.7301587	66.38095238	0.395454932
591.6318502	490.2362137	0.207996231
113.7642857	41.03571429	0.251541472
1079.92495	803.5502276	0.202002951
256.5238806	74.19701493	0.292167231
652.0141044	614.7983075	0.186501743
513.0934657	110.4433416	0.357117075
310.6957746	72.79859155	0.348684895
174.9254499	148.3508997	0.181282117
463.1249479	124.5643482	0.318178591
756.0655952	364.1358625	0.228199294
87.40733198	40.0712831	0.219355985
92.20204604	20.84143223	0.370839727
111.659292	25.42035398	0.321124177
258.7933178	73.49417249	0.345272139
620.5935368	142.8010772	0.352183469
643.719659	969.9450853	0.14505156
257.9585871	113.7478685	0.254643586
155.251944	29.17729393	0.381237777
240.7772109	64.47108844	0.30870344
610.7036115	257.8841843	0.252102253
401.3009192	110.3555878	0.289919248
166.051114	42.25294889	0.360204009
1059.373084	1061.231892	0.176560575
254.2069225	73.46501129	0.314967058
507.4868154	121.7496957	0.333872926
1391.25504	430.9040564	0.287463571
429.4723282	111.773855	0.291701
126.3914328	42.40620384	0.294867012
754.3645133	168.6720257	0.352151915
99.44334975	23.32019704	0.360246476
995.8715831	286.7120944	0.300929228
1045.724827	558.1726159	0.20136606
430.3973635	123.7015066	0.308548223
108.7560976	26.41463415	0.328486781
725.2971291	301.5561132	0.266201612
296.282397	67.72359551	0.346543725
208.2315789	53.36210526	0.380409172
479.6371205	359.0386876	0.209354077
186.7167756	65.7037037	0.247202199
441.2453599	129.336804	0.273704259
83.31578947	16.56037152	0.423913829
294.1604563	74.31863118	0.307386699
264.3022222	138.6511111	0.229785215
290.5618777	69.00711238	0.343190541
173.5326877	48.6440678	0.321208711
335.5311131	137.4119194	0.288849322
308.9820391	143.5176968	0.223492372
374.9690608	188.2780847	0.229833937
97.51977401	50.07721281	0.27114718
263.5122324	71.41896024	0.317524562
399.6079249	326.4280501	0.195807362
300.3004115	104.6037625	0.29015519
291.0307242	75.08046818	0.305243794
137.5920398	50.04477612	0.264557946
304.1461295	205.3364929	0.213656513
785.3630304	293.993557	0.262202644
589.7190946	210.6506094	0.244353266
570.8355005	230.3915936	0.247179547
526.0083893	104.8255034	0.357577538
490.1590538	130.048124	0.278822037
77.42172524	18.74440895	0.385504915
61.44029851	20.59701493	0.2974233
1435.365367	492.6176337	0.273083756

log.sigma.3.0.mm.3D_gldm_DependenceNonUniformityNormalized	log.sigma.3.0.mm.3D_gldm_DependenceVariance	log.sigma.3.0.mm.3D_gldm_LargeDependenceEmphasis
0.209784877	2.294963974	9.559556787
0.234326338	1.667865359	7.910662824
0.226030083	1.867843713	8.493273543
0.25762895	1.599475329	6.739130435
0.215759942	2.261094237	9.694542877
0.235878697	1.860187146	8.297520661
0.097077901	21.89438404	55.01556125
0.15884652	7.510348874	20.55735003
0.133232889	11.23870438	29.31816049
0.223574405	1.838610587	9.362913907
0.153367675	4.328939803	18.16580977
0.125490786	11.10371566	30.24273554
0.22156051	2.344878756	9.577065351
0.250496702	1.632624127	7.425465839
0.173039053	5.012266667	15.33777778
0.115198428	15.82838381	39.31100312
0.173747481	3.239691723	15.41449604
0.240451695	1.570172345	7.875305623
0.140856391	4.497137452	20.4095064
0.146136013	6.443296681	20.78547316
0.163436839	3.455827168	15.47449471
0.2218311	2.117650884	9.157258065
0.197415244	2.844593466	11.63082437
0.106662496	13.32976736	39.62496525
0.140468334	14.83364545	33.67263844
0.156777695	5.336804867	17.63615023
0.187676045	2.963712296	11.8502994
0.170206357	4.012867983	15.23968043
0.203133796	2.368737099	10.17808219
0.182503761	3.307927935	13.42111801
0.159288707	4.901320993	17.39610876
0.231032106	2.020368987	8.748730964
0.152030593	7.188814475	21.00428266
0.136580636	7.749461655	23.78592133
0.151041672	7.839335587	21.79719462
0.195845649	3.59772281	12.26663228
0.24585328	1.600380378	7.313492063
0.101637494	22.64460558	53.45559182
0.20315051	2.198456633	11.43571429
0.114314063	11.94982366	34.19741717
0.191435732	2.606892404	11.4641791
0.114953121	13.34908359	36.77045134
0.212197463	2.418992927	9.631927213
0.218799841	2.25193414	9.338028169
0.112419955	13.58084635	37.77763496
0.192888358	3.773327822	12.71345273
0.141294262	6.23914965	21.21117548
0.178019006	3.414528727	15.02443992
0.235810859	1.568671058	7.659846547
0.247033832	1.937837732	8.730088496
0.201082609	2.554258139	10.27894328
0.222834304	1.943411131	8.703411131
0.080707079	21.10056542	61.75877633
0.157100236	4.081021778	16.12302071
0.241449369	2.119095705	8.287713841
0.204742526	2.005966843	9.870748299
0.152105507	8.927882582	23.5882939
0.19414655	2.497700521	11.26124819
0.217629245	2.867936974	10.12188729
0.097252647	19.99872182	51.64334894
0.191276842	3.234609546	12.05492852
0.205877004	3.455106748	11.59553753
0.168964664	6.385926196	18.03230508
0.20490092	2.519153167	10.94465649
0.186693401	3.182115904	12.4859675
0.220509943	2.046183104	8.91055247
0.244934359	1.494552161	7.536945813
0.195844952	3.027262206	11.81612586
0.13928141	6.169501103	22.27836974
0.202635294	2.332374867	10.43502825
0.221048979	2.093082821	9.31300813
0.145612754	7.641869056	22.11423409
0.22193438	2.091097364	9.031460674
0.219191136	2.512137396	9.36
0.117442978	13.20251218	35.39666993
0.20339518	2.086462472	11.24400871
0.199748918	2.359701382	11.20823902
0.257943621	1.413240806	6.591331269
0.223696164	1.808263239	9.141444867
0.146834568	5.693569136	19.94
0.206658519	2.325354253	9.792318634
0.210088	1.889294069	9.401937046
0.147033792	12.33467498	28.0832603
0.163223475	3.863976298	16.25303751
0.138110151	8.513165722	24.88360958
0.183653058	3.692730555	13.93032015
0.201461951	2.822952613	11.06880734
0.104173077	17.14580354	45.02398332
0.176543452	5.338428375	16.20399765
0.212897384	1.983686959	9.674469642
0.171134378	3.374427613	14.04228856
0.120120904	10.25094088	30.27409163
0.174486343	3.64628631	14.32281715
0.171131484	3.796167653	15.18630296
0.172614303	3.465203106	14.59177502
0.220641103	2.068273952	8.884228188
0.199901735	2.917575691	11.87846656
0.247353755	1.432024416	7.127795527
0.229254845	1.563098686	8.820895522
0.162666066	4.625090261	16.16999093

log.sigma.3.0.mm.3D_gldm_LargeDependenceLowGrayLevelEmphasis	log.sigma.3.0.mm.3D_gldm_SmallDependenceHighGrayLevelEmphasis	log.sigma.3.0.mm.3D_gldm_LargeDependenceHighGrayLevelEmphasis
0.050048919	147.6541141	3001.189058
0.172118896	75.49238793	1706.74928
0.118501752	94.78055214	1986.197309
0.093064797	139.5622248	1519.338509
0.105154609	86.25583371	1906.226081
0.044095925	107.3888916	2831.227273
0.336243416	41.7563161	10004.57339
0.06389025	132.7995092	7715.727587
0.236403973	56.80834873	4636.57954
0.113159243	57.00720305	1478.765563
0.273933072	26.90400365	1771.265424
0.174792059	64.98525832	6071.471089
0.05672794	92.71084227	3531.482121
0.135441333	106.2518853	1276.513975
0.165820648	79.98192857	2277.765079
0.360494093	43.57664113	6106.71555
0.192846084	34.99823601	2018.866365
0.185362187	76.55991867	1241.652812
0.450145276	20.47581517	1680.984461
0.064836546	98.01088383	7680.085593
0.353365672	41.05184593	2070.088547
0.566239518	61.21892744	1008.350806
0.156251509	68.48527428	1931.747312
0.37933102	29.21084142	4834.043369
0.277805575	62.40332328	6649.57899
0.048263062	166.4208923	7430.553991
0.147977466	78.42328282	1831.955589
0.150676235	56.14754931	2230.533955
0.373772662	42.50354798	719.3013699
0.130942566	58.11015128	2142.963354
0.254420692	46.93833105	2282.532801
0.123622645	99.60501914	1866.586294
0.251184117	53.82041488	2892.08394
0.25426957	44.46103893	3648.106418
0.105690152	90.67461608	5378.109682
0.066078266	117.4318153	4212.155235
0.018948687	223.91584	4771.693783
0.358813262	48.98647125	10044.51555
0.211013789	44.02557028	1470.2
0.05280986	158.3118216	22927.35493
0.132874841	76.36176692	1923.706716
0.261439077	39.35256052	5659.112835
0.094659436	112.0123376	2737.93962
0.124738963	86.96468576	1440.797183
0.522763043	29.18849923	3840.722365
0.118429889	90.5207288	2312.852145
0.142553714	63.79605036	4066.814801
0.154331554	42.47380146	2319.081466
0.244715957	71.32177465	992.4475703
0.058516875	107.8071334	3165.723451
0.082425321	107.4798514	1879.3108
0.065468966	109.4324809	1963.566607
0.527188121	25.4305872	8013.750752
0.055270304	91.41979942	5680.437881
0.107867841	116.5577907	2086.11042
0.20006825	62.02142017	1433.396259
0.163208997	64.43001295	4433.888418
0.083957982	84.59033077	2597.778423
0.114534152	89.9997385	1678.466579
0.206606415	60.1463334	13803.50151
0.076512164	89.36863843	2826.050414
0.048146692	123.3643852	3989.148479
0.063478052	119.6097384	6298.859121
0.048292185	101.7829292	3702.869752
0.135360036	65.53673447	2099.456425
0.047207991	125.8280443	2812.293774
0.10221958	79.44422209	1638.864532
0.053897947	117.5503202	3441.771288
0.10111265	63.834904	5759.913026
0.111541017	60.72381957	2117.863465
0.118760183	75.07819159	1622.504065
0.082175169	108.2706514	7304.698655
0.100670096	97.18042969	1609.443446
0.145104389	73.9242029	1275.268421
0.250373537	52.8936762	5891.071499
0.116964797	39.51623339	1707.948802
0.064326592	81.29440336	3409.509733
0.04221239	159.2047549	2761.356037
0.076302504	79.23277731	2095.648669
0.12424727	64.71637304	3849.816111
0.04549027	143.6136511	3043.173542
0.21631966	58.38556297	1598.03632
0.241327576	69.55624273	4972.721297
0.174573305	46.22130123	2126.431062
0.224548267	48.55189035	3743.793738
0.085195544	63.68846355	4605.719397
0.074152089	97.00426272	2455.714067
0.376356571	46.14190063	6569.525547
0.100817201	77.6939988	3691.83833
0.079265635	75.60989702	2261.673738
0.090751034	76.54465656	3770.991294
0.294263698	45.0042607	4094.245261
0.079952238	71.15562028	3311.790935
0.207787372	45.72667469	2154.277423
0.190104304	42.34987075	1837.035077
0.038296102	151.0623392	3576.10151
0.177559597	57.4883494	1979.584013
0.070587096	115.4579878	2036.642173
0.256323544	51.68805037	1378.761194
0.0461757	115.5678197	7021.172031

log.sigma.3.0.mm.3D_gldm_SmallDependenceLowGrayLevelEmphasis	log.sigma.3.0.mm.3D_gldm_LowGrayLevelEmphasis	log.sigma.3.0.mm.3D_gldzm_DistanceZoneVariabilityNormalized
0.002383249	0.006540079	0.860394802
0.01138359	0.024491843	0.926035503
0.006789065	0.017136216	0.880488909
0.007412481	0.013508879	0.867346939
0.003482832	0.009291648	0.840826484
0.009082877	0.012227136	0.897735573
0.001684886	0.008113299	0.932717257
0.001004693	0.003442301	0.886049801
0.001963195	0.009375588	0.961919125
0.005703762	0.016052007	0.951079765
0.004121588	0.01707949	0.943924332
0.001976092	0.006881567	0.909771682
0.005910765	0.010633782	0.821919036
0.006669277	0.018405809	0.859282488
0.005729481	0.014773179	0.907503858
0.002890005	0.01280646	0.963648834
0.003105979	0.013646765	0.95518054
0.01161323	0.023383811	0.843265306
0.007612984	0.026508136	0.957102185
0.001186933	0.00371646	0.943480702
0.003964872	0.018370159	0.944296707
0.009027626	0.038562193	0.907198096
0.004214956	0.015495632	0.912380258
0.002224362	0.011848797	0.956731146
0.003804471	0.017423278	0.942004371
0.001045781	0.003016096	0.953514739
0.00474486	0.013140061	0.934026845
0.003439185	0.01094465	0.932095527
0.009854361	0.040477519	0.871581451
0.00310082	0.010203832	0.988281657
0.003528598	0.015321462	0.942472269
0.00449056	0.012172751	0.920591016
0.00437954	0.015764871	0.884381081
0.003500374	0.014797261	0.96056226
0.001434811	0.005227928	0.931515003
0.002088725	0.006674508	0.773410665
0.001848099	0.003394523	0.649177318
0.002499242	0.011023409	0.945875308
0.004240887	0.019574853	0.816156191
0.000404175	0.001677142	0.921663695
0.00293066	0.010414927	0.884020681
0.001797036	0.008223321	0.869173775
0.002858622	0.008406793	0.943497457
0.004347817	0.012537531	0.924951214
0.004055305	0.019574548	0.951981065
0.002780466	0.008721019	0.910334739
0.001640248	0.007038516	0.94744419
0.004578453	0.011727901	1
0.01062495	0.025679766	0.902432653
0.005266661	0.010911063	0.916824197
0.002909923	0.00780931	0.973589638
0.003296762	0.008280696	0.879821789
0.001898895	0.010917863	0.922793788
0.001800982	0.004542565	0.979383681
0.004552367	0.011763255	0.880985671
0.006986483	0.018957644	0.933116076
0.001854912	0.008068207	0.952564871
0.002458049	0.007383086	0.946349584
0.004141991	0.011085066	0.869221633
0.000872246	0.004713759	0.932166143
0.003183079	0.007754155	0.961104812
0.002246367	0.005187148	0.900562174
0.001388666	0.004109259	0.842788425
0.002211607	0.005491304	0.801999646
0.00402311	0.013130614	0.959531453
0.002343909	0.005870329	0.832564185
0.006485431	0.015207816	0.872933884
0.001708312	0.004836949	0.850099638
0.001024322	0.004667295	0.946706699
0.005188816	0.013099071	0.931122449
0.005339163	0.013636336	0.95172042
0.00123534	0.004248487	0.894225721
0.003532568	0.010077154	0.957542126
0.006629883	0.017748717	0.96280407
0.002078759	0.008912672	0.841060968
0.004746883	0.013370303	0.964550561
0.001876452	0.00674786	0.906958885
0.006496687	0.010368659	0.789164187
0.003977122	0.01100017	0.920290168
0.002165793	0.007386943	0.974472354
0.002453302	0.005595125	0.88397283
0.007243517	0.019014911	0.968262227
0.003477359	0.01086081	0.969238281
0.002292932	0.010401973	0.97233746
0.003414017	0.011896195	0.903768062
0.005918132	0.014171573	0.863184401
0.003021175	0.007625499	0.904638635
0.001893977	0.010109848	0.967450777
0.003485564	0.009419029	0.930668277
0.003224411	0.008816254	0.956799084
0.003193031	0.008176964	0.74863891
0.002255274	0.0117451	0.93079791
0.001955552	0.006318236	0.888970993
0.003855216	0.014061602	0.936728021
0.003705065	0.013229392	0.860292534
0.002139689	0.004996524	0.768510405
0.005201671	0.017147206	0.893521875
0.003744783	0.012549271	0.873995617
0.006098439	0.027791134	0.926035503
0.001148916	0.003354243	0.875183688

log.sigma.3.0.mm.3D_gldzm_LowIntensityEmphasis	log.sigma.3.0.mm.3D_gldzm_LargeDistanceEmphasis	log.sigma.3.0.mm.3D_gldzm_HighIntensitySmallDistanceEmphasis
0.007453694	1.226510067	399.9907718
0.02839913	1.115384615	203.3269231
0.019109274	1.191489362	254.2859043
0.015057674	1.214285714	300.9970238
0.009946979	1.261565836	257.5369217
0.021077171	1.162162162	308.7905405
0.009816268	1.104569006	229.4878709
0.003609079	1.181962025	473.8264768
0.008443807	1.058252427	249.978376
0.020565183	1.075268817	186.9829749
0.020225624	1.086614173	130.7755906
0.008153354	1.142070485	275.9446586
0.015966873	1.296407186	266.9086826
0.018347124	1.228476821	269.772351
0.018067863	1.145833333	248.7209201
0.013615948	1.055555556	202.7977208
0.015889792	1.068807339	170.7637615
0.029789187	1.257142857	218.2514286
0.039261155	1.065789474	115.2785088
0.004865668	1.087320574	402.1259968
0.016707122	1.086021505	179.5344982
0.029542923	1.146341463	177.7353659
0.015638109	1.137755102	228.7933673
0.013212619	1.066371681	177.5058997
0.017674996	1.089673913	236.453125
0.003654896	1.071428571	613.2139724
0.015005021	1.102459016	248.3893443
0.012844484	1.105571848	216.2532991
0.039307288	1.206896552	120.9439655
0.011521564	1.017681729	218.1281925
0.014452497	1.088927637	192.4553182
0.013083796	1.124260355	284.4837278
0.01767512	1.184813754	205.3595989
0.016120091	1.060371517	189.1652477
0.00538267	1.106508876	346.700526
0.007063467	1.390789474	340.7125
0.004382888	1.680672269	518.7839636
0.012957006	1.083511777	236.8149536
0.021116252	1.307228916	170.0225904
0.001947538	1.122507123	771.4241453
0.010061908	1.185430464	258.7036424
0.00970892	1.211092715	205.901697
0.008216166	1.087293889	312.9561106
0.012733528	1.117147708	247.9499151
0.023192644	1.073846154	163.6761538
0.008540274	1.141137856	281.8632385
0.007190479	1.081021898	280.2443431
0.019830535	1	190.752
0.026593697	1.154285714	191.6057143
0.015810058	1.130434783	323.7866848
0.00810185	1.040152964	310.6878585
0.009280427	1.192636986	297.4437072
0.013121888	1.120662461	179.5711751
0.00673008	1.03125	361.4020833
0.011407698	1.190635452	299.7006689
0.021076312	1.103926097	202.4382217
0.007757113	1.072925398	258.1213328
0.008168715	1.082758621	290.1275862
0.011433021	1.211009174	244.1353211
0.005129394	1.105457909	341.5366559
0.009690344	1.05952381	275.9310516
0.00636502	1.157416751	359.9482846
0.004766285	1.258006477	399.5236596
0.00730326	1.33423913	328.0896739
0.015302387	1.061983471	219.7117769
0.006529839	1.276668961	334.3463524
0.017556349	1.204545455	218.1747159
0.005516509	1.244831338	373.1592764
0.005001742	1.082191781	319.5081894
0.0162957	1.107142857	195.484375
0.015722792	1.074257426	230.345297
0.004455112	1.168078176	402.321987
0.009878705	1.065099458	280.6518987
0.018686817	1.056872038	194.7606635
0.010402242	1.261139896	251.511399
0.01827126	1.054151625	162.2987365
0.007605386	1.14673913	289.5512908
0.014060895	1.359281437	360.2215569
0.013671974	1.124752475	248.5940594
0.009484325	1.038793103	285.5371767
0.006925549	1.185512367	406.1775618
0.022776166	1.048387097	181.8491935
0.011504338	1.046875	243.2926432
0.010138592	1.042084168	208.751002
0.014283136	1.152054795	211.1873288
0.023194472	1.221590909	223.8948864
0.009059725	1.15060241	301.1204819
0.009467311	1.04964539	240.3900709
0.012446436	1.107876712	262.817637
0.010106448	1.06626506	246.7038153
0.012129664	1.442231076	267.5747012
0.01146449	1.10766721	213.8613377
0.007472303	1.176984705	264.6640568
0.01547482	1.098116947	188.9167493
0.014901873	1.231656184	167.3077685
0.0058066	1.400774443	387.7253146
0.020000962	1.169267707	202.3565426
0.012418254	1.202702703	288.0371622
0.024312193	1.115384615	165.8509615
0.004129411	1.200643777	408.9214056

log.sigma.3.0.mm.3D_gldzm_LowIntensityLargeDistanceEmphasis	log.sigma.3.0.mm.3D_gldzm_HighIntensityEmphasis	log.sigma.3.0.mm.3D_gldzm_DistanceZoneVariability	log.sigma.3.0.mm.3D_gldzm_ZonePercentage
0.00861414	423.6208054	512.795302	0.412742382
0.030646915	206.1346154	144.4615385	0.449567723
0.020203279	264.3829787	331.0638298	0.421524664
0.016758659	308.7559524	145.7142857	0.52173913
0.014670997	268.1637011	472.544484	0.398299079
0.022634577	313.7567568	99.64864865	0.458677686
0.010810509	235.2359868	1980.158738	0.201442262
0.004482821	495.3818565	1679.950422	0.3124588
0.009193435	251.9602824	1089.854369	0.265837635
0.023761478	189.1577061	265.3512545	0.369536424
0.021828504	132.9370079	359.6351706	0.244858612
0.009509953	283.2059471	826.0726872	0.266510126
0.021320082	279.9191617	274.5209581	0.411837238
0.043173736	272.4470199	259.5033113	0.468944099
0.022162626	253.9461806	522.7222222	0.365714286
0.01401152	205.1438746	1352.962963	0.243665394
0.016575204	172.6697248	208.2293578	0.246885617
0.092823685	221.9542857	147.5714286	0.427872861
0.040304446	116.7587719	218.2192982	0.208409506
0.005096422	412.5637959	2366.249601	0.273113362
0.017602978	181.8261649	526.9175627	0.26852743
0.034626696	179.3121951	185.9756098	0.413306452
0.017376584	231.877551	178.8265306	0.35125448
0.019929786	179.20059	648.6637168	0.188490409
0.019037057	241.2119565	346.6576087	0.299674267
0.00381352	622.1115288	760.9047619	0.312206573
0.017193487	252.1885246	683.7076503	0.365269461
0.01518077	219.3533724	635.6891496	0.302707501
0.102080277	121.6034483	50.55172414	0.397260274
0.011723708	218.6247544	503.0353635	0.316149068
0.017989005	195.9006103	1081.015693	0.286106261
0.015597953	288.8017751	311.1597633	0.42893401
0.024015203	212.4040115	617.2979943	0.298929336
0.016503906	192.0108359	620.5232198	0.267494824
0.006435124	352.9638396	1416.83432	0.296317943
0.016891479	357.7828947	587.7921053	0.391954616
0.007827764	568.1064426	463.512605	0.472222222
0.013913974	240.0992148	1325.171306	0.240680295
0.059169158	172.8373494	135.4819277	0.296428571
0.002159898	793.3618234	1941.023742	0.222927914
0.012393947	265.9403974	400.4613687	0.338059701
0.012230767	216.6456954	1049.961921	0.212976023
0.010022004	314.4888458	972.7458778	0.426385443
0.022224647	251.008489	544.7962649	0.414788732
0.026668705	164.8184615	309.3938462	0.208868895
0.015392013	287.2658643	832.0459519	0.380674719
0.008370774	284.2029197	1297.99854	0.256026911
0.019830535	190.752	125	0.254582485
0.03420122	193.5771429	157.9257143	0.447570332
0.016542941	330.2228261	168.6956522	0.407079646
0.009146741	311.5181644	509.1873805	0.406371406
0.014067405	308.8715753	1027.631849	0.419389587
0.018466368	183.6388013	1170.102524	0.158976931
0.006839464	363.8770833	470.1041667	0.292326431
0.02379105	305.0535117	263.4147157	0.465007776
0.024003827	204.8891455	404.039261	0.368197279
0.011506571	261.2464376	1136.409891	0.297135741
0.008658987	294.6896552	686.1034483	0.350749879
0.026376787	248.8623853	284.235474	0.428571429
0.005874092	349.6614246	2015.343201	0.198476086
0.011607558	278.5634921	484.3968254	0.379232506
0.007133141	373.975782	892.457114	0.402028398
0.006407538	420.8445484	2342.109032	0.337503036
0.01538013	349.2078804	590.2717391	0.351145038
0.015735132	222.0454545	232.2066116	0.35745938
0.010987272	353.732278	1209.71576	0.424729611
0.041232671	221.9886364	153.6363636	0.433497537
0.007216229	392.6376496	1562.483134	0.361455261
0.005335233	326.0875521	1589.520548	0.22362813
0.017129832	199.692602	730	0.369114878
0.017335965	232.0643564	192.2475248	0.410569106
0.007552628	413.8143322	1372.636482	0.30817105
0.016136118	282.4014467	529.5207957	0.41423221
0.019216759	196.6587678	406.3033175	0.444210526
0.019782413	261.1937824	811.6238342	0.236287953
0.01932813	163.4223827	267.1805054	0.301742919
0.008619556	298.4320652	667.5217391	0.333182435
0.017720631	375.2754491	131.7904192	0.517027864
0.020835591	253.5841584	464.7465347	0.384030418
0.009789856	286.8383621	452.1551724	0.257777778
0.007949175	423.5017668	500.3286219	0.402560455
0.023704847	182.6258065	300.1612903	0.375302663
0.011879056	245.3111979	744.375	0.336546889
0.011243508	209.8016032	485.1963928	0.263602747
0.017489025	216.2369863	659.7506849	0.268876611
0.024569516	234.0795455	151.9204545	0.331450094
0.018414562	307	450.5100402	0.380733945
0.009890473	242.1737589	818.463357	0.220542231
0.013299712	267.4486301	543.510274	0.343327454
0.010621449	249.935743	476.4859438	0.36430139
0.015123864	291.5179283	187.9083665	0.312189055
0.019647869	216.2446982	570.5791191	0.242101106
0.00877792	274.3932993	1220.557174	0.305043324
0.016562326	192.4876115	945.1585728	0.29280325
0.022122647	175.5136268	820.7190776	0.288478984
0.008769613	420.7754114	793.8712488	0.433305369
0.022011122	208.4861945	744.3037215	0.339722675
0.01369612	297.777027	129.3513514	0.47284345
0.025833939	168.2307692	96.30769231	0.388059701
0.004970511	427.555794	2447.013591	0.316863101

log.sigma.3.0.mm.3D_gldzm_IntensityVariabilityNormalized	log.sigma.3.0.mm.3D_gldzm_LowIntensitySmallDistanceEmphasis	log.sigma.3.0.mm.3D_gldzm_IntensityVariability
0.038843971	0.007163582	23.15100671
0.051117686	0.027837184	7.974358974
0.047688434	0.018835773	17.93085106
0.047548186	0.014632428	7.988095238
0.050056357	0.008765974	28.1316726
0.047479912	0.020687819	5.27027027
0.051325134	0.009567707	108.9632595
0.04514946	0.003390643	85.60337553
0.053688238	0.0082564	60.82877317
0.055356432	0.01976611	15.44444444
0.066746578	0.019824904	25.43044619
0.051662656	0.007814205	46.90969163
0.046380293	0.01462857	15.49101796
0.048221569	0.012140471	14.56291391
0.045175058	0.017044173	26.02083333
0.051778192	0.013517056	72.6965812
0.067418567	0.015718439	14.69724771
0.053126531	0.014030562	9.297142857
0.069213604	0.039000332	15.78070175
0.046912082	0.00480798	117.6555024
0.056801685	0.016483158	31.6953405
0.057037478	0.028271979	11.69268293
0.054768846	0.01520349	10.73469388
0.064957667	0.011533328	44.04129794
0.046860232	0.017334481	17.24456522
0.042933147	0.00361524	34.26065163
0.04669817	0.014457905	34.18306011
0.056797757	0.012260413	38.73607038
0.063614744	0.023614041	3.689655172
0.057468514	0.011471028	29.25147348
0.054623372	0.01356837	62.65300785
0.049998249	0.012455257	16.89940828
0.051284472	0.016090099	35.7965616
0.05507577	0.016024137	35.57894737
0.049353755	0.005119557	75.06706114
0.047434211	0.004606464	36.05
0.041742187	0.003521669	29.80392157
0.049573543	0.012717765	69.4525339
0.069821454	0.011603025	11.59036145
0.053465024	0.001894448	112.5973409
0.052829067	0.009478899	23.93156733
0.059831532	0.009078458	72.27649007
0.047968834	0.007764707	49.45586809
0.049143753	0.010360748	28.94567063
0.059218935	0.022323629	19.24615385
0.048934876	0.00682734	44.72647702
0.056271512	0.006895405	77.0919708
0.064064	0.019830535	8.008
0.049991837	0.024691816	8.748571429
0.044482514	0.015626837	8.184782609
0.050111688	0.007840628	26.208413
0.045017534	0.008083682	52.58047945
0.063127556	0.011785769	80.04574132
0.057265625	0.006702733	27.4875
0.04506661	0.008311859	13.47491639
0.049992266	0.020344433	21.64665127
0.053030074	0.006819748	63.26487846
0.050969798	0.008046147	36.95310345
0.053951688	0.00769708	17.64220183
0.054556416	0.00494322	117.9509713
0.048910305	0.00921104	24.65079365
0.044903628	0.00617299	44.49949546
0.043743358	0.004355972	121.5627924
0.049149338	0.005284043	36.17391304
0.053343351	0.015194201	12.90909091
0.044371258	0.005415481	64.4714384
0.056495351	0.011637269	9.943181818
0.047654343	0.005091579	87.58868335
0.058940966	0.004918369	98.96188207
0.053236282	0.016087167	41.7372449
0.05327909	0.015319498	10.76237624
0.049270761	0.003680733	75.63061889
0.048307931	0.008314351	26.71428571
0.052705465	0.018554332	22.24170616
0.054157696	0.0080572	52.26217617
0.061085118	0.018007042	16.92057762
0.053827239	0.007351844	39.61684783
0.044784682	0.01314596	7.479041916
0.050108813	0.011881069	25.3049505
0.056656882	0.009407942	26.2887931
0.0392813	0.006669643	22.23321555
0.052528616	0.022543995	16.28387097
0.053429498	0.011410659	41.03385417
0.067184469	0.009862363	33.5250501
0.05211109	0.013481664	38.04109589
0.058819731	0.022850711	10.35227273
0.048305672	0.006721016	24.0562249
0.053591089	0.009361521	45.33806147
0.049458153	0.012233117	28.88356164
0.053813648	0.009977698	26.79919679
0.049602387	0.011381114	12.4501992
0.057213341	0.009418645	35.07177814
0.05453579	0.007145898	74.8776402
0.054676396	0.015202943	55.16848365
0.064734605	0.013114878	61.75681342
0.040775418	0.005065847	42.12100678
0.052891745	0.019498423	44.05882353
0.057341125	0.012098788	8.486486486
0.068232249	0.023931757	7.096153846
0.046676634	0.003919136	130.5078684

log.sigma.3.0.mm.3D_gldzm_HighIntensityLargeDistanceEmphasis	log.sigma.3.0.mm.3D_gldzm_SmallDistanceEmphasis	log.sigma.3.0.mm.3D_glcm_SumVariance	log.sigma.3.0.mm.3D_glcm_Homogeneity1
518.1409396	0.943372483	1033.659836	0.291254503
217.3653846	0.971153846	468.3345391	0.29297059
304.7712766	0.95212766	613.0669763	0.29421806
339.7916667	0.946428571	683.0235244	0.274807328
310.6708185	0.934608541	633.6956697	0.310723802
333.6216216	0.959459459	924.637282	0.295076282
258.2284503	0.973857748	546.5625484	0.471274382
581.6033755	0.954509494	1200.59286	0.350756517
259.8879082	0.985436893	474.0476857	0.404603748
197.8566308	0.981182796	414.1008596	0.336145098
141.5826772	0.978346457	271.4430627	0.415759771
312.2511013	0.964482379	616.2902156	0.414242806
331.9610778	0.925898204	864.8094019	0.303628408
283.1456954	0.942880795	543.1817398	0.279906005
274.8472222	0.963541667	470.4166137	0.3344954
214.52849	0.986111111	476.5123487	0.434908752
180.293578	0.982798165	356.3733931	0.390354427
236.7657143	0.935714286	430.4352521	0.297453826
122.6798246	0.983552632	200.4047876	0.420917054
454.314992	0.978169856	1163.823789	0.376428168
190.9928315	0.978494624	365.8156843	0.368370836
185.6195122	0.963414634	314.3820892	0.310903699
244.2142857	0.965561224	447.8370614	0.341405973
185.979351	0.98340708	333.8351149	0.479368216
260.2472826	0.977581522	526.7406555	0.401326459
657.7017544	0.982142857	1507.543312	0.360866415
267.3852459	0.974385246	498.6181948	0.323322999
231.7536657	0.973607038	428.6033102	0.375212535
124.2413793	0.948275862	211.3964303	0.357455578
220.611002	0.995579568	475.051929	0.359768332
209.6817786	0.977768091	376.2515747	0.372292652
306.0739645	0.968934911	670.8006926	0.291116956
240.5816619	0.953796562	414.077538	0.379194266
203.3931889	0.984907121	413.7715305	0.417817176
378.017094	0.973372781	794.6970991	0.368913498
426.0644737	0.902302632	957.1366946	0.30743783
765.3963585	0.829831933	1692.300298	0.258647917
253.2362598	0.979122056	546.5334961	0.462628403
184.0963855	0.923192771	322.162361	0.357275144
881.1125356	0.969373219	2297.515179	0.435905165
294.8874172	0.953642384	544.4538986	0.330964645
259.6216887	0.947226821	468.903816	0.452811273
320.6197866	0.978176528	835.6493291	0.29211947
263.2427844	0.970713073	513.8342234	0.309125171
169.3876923	0.981538462	274.7342479	0.458150615
308.8763676	0.964715536	628.4364262	0.324673674
300.0372263	0.979744526	596.805067	0.39311024
190.752	1	422.1053726	0.38570411
201.4628571	0.961428571	359.4710609	0.304665301
355.9673913	0.967391304	892.8431627	0.292184735
314.8393881	0.989961759	607.0140443	0.328882563
354.5830479	0.951840753	730.4350321	0.300238246
199.909306	0.969834385	359.5180496	0.522801304
373.7770833	0.9921875	1030.151962	0.369211284
326.4648829	0.952341137	726.8791205	0.280509908
214.6928406	0.974018476	400.6025854	0.326475496
273.7468567	0.98176865	563.3064785	0.388526334
312.937931	0.979310345	720.8817383	0.318945509
267.7706422	0.947247706	510.4298112	0.310014712
382.1604995	0.973635523	828.5585762	0.463696947
289.093254	0.985119048	695.1138134	0.327059715
430.0857719	0.960645812	1036.739289	0.304292719
506.1281036	0.935498381	1119.321779	0.339879157
433.6807065	0.916440217	941.7198553	0.320187673
231.3801653	0.984504132	491.3598861	0.348807194
431.2759807	0.93083276	911.1478984	0.288147453
237.2443182	0.948863636	511.9953917	0.298417046
470.5511425	0.938792165	973.0372238	0.320069098
352.405003	0.979452055	830.2664449	0.402705987
216.5255102	0.973214286	513.2573478	0.334651313
238.9405941	0.981435644	481.3506457	0.31449882
459.7837134	0.957980456	1013.72258	0.363664647
289.3996383	0.983725136	584.0413311	0.30159715
204.2511848	0.985781991	415.6445688	0.328045467
299.9233161	0.934715026	511.5918455	0.425749249
167.9169675	0.986462094	375.1965782	0.361301351
333.955163	0.963315217	844.7024413	0.333570889
435.491018	0.910179641	1015.223939	0.261112149
273.5445545	0.968811881	646.1351006	0.319568044
292.0431034	0.990301724	599.6761155	0.396276079
492.7985866	0.953621908	1021.221545	0.295064341
185.7322581	0.987903226	447.0762336	0.326019867
253.3854167	0.98828125	502.8505294	0.38059931
214.004008	0.989478958	388.2916195	0.38607308
236.4356164	0.961986301	447.3418501	0.398054528
274.8181818	0.944602273	737.1523716	0.346679061
330.5180723	0.962349398	717.7074767	0.322357411
249.3085106	0.987588652	436.3221058	0.461005763
285.9726027	0.973030822	668.1020299	0.352690661
262.8634538	0.983433735	640.7717765	0.322886328
387.2908367	0.889442231	790.1095009	0.351181131
225.7781403	0.973083197	377.4017089	0.430282363
313.3102695	0.955753824	698.6084663	0.356221273
206.7710605	0.975470763	413.556186	0.370010912
208.6352201	0.943250641	355.4670227	0.374159942
552.9757986	0.899806389	1132.490866	0.28140831
233.0048019	0.957683073	478.7936273	0.331149291
336.7364865	0.949324324	764.0960895	0.292052023
177.75	0.971153846	362.1018992	0.330314687
502.0933476	0.949839056	1294.248816	0.344527827

log.sigma.3.0.mm.3D_glcm_Homogeneity2	log.sigma.3.0.mm.3D_glcm_ClusterShade	log.sigma.3.0.mm.3D_glcm_MaximumProbability	log.sigma.3.0.mm.3D_glcm_Idmn	log.sigma.3.0.mm.3D_glcm_SumVariance2
0.202688945	693.6461034	0.012901909	0.973944737	110.3610885
0.201997362	-27.64951843	0.015909366	0.954242525	93.33350257
0.205126265	-82.51852878	0.010024129	0.952696778	102.964276
0.182807931	-96.59906054	0.011920262	0.954568436	113.4566229
0.219455233	185.1872948	0.013464712	0.970170985	94.41144229
0.203581233	-236.9581339	0.020003694	0.959332039	104.8452449
0.408609867	191.7652291	0.086382111	0.987120113	48.07343214
0.270666745	456.1427571	0.025755952	0.980082587	79.65113737
0.329730861	320.0750671	0.043231893	0.979827104	58.90708165
0.244515354	63.54570334	0.014402421	0.969712738	57.06538412
0.338622804	63.23900561	0.036052244	0.973476623	35.20998265
0.342231219	180.7028828	0.045269904	0.983702305	49.82640889
0.215517219	-550.5826762	0.017959573	0.96106915	108.1590103
0.188050122	117.8628508	0.010760275	0.962702741	108.189722
0.250711754	442.0137379	0.016716104	0.974987415	77.39177272
0.370450587	157.4191352	0.082080747	0.9745055	74.00655068
0.306823675	42.93932984	0.0202994	0.977552424	39.82361795
0.205932527	179.4240726	0.013727199	0.966380338	95.67006371
0.343961181	39.10022038	0.027677283	0.971160916	30.75818675
0.296494867	193.4081524	0.021130964	0.986796838	61.64548405
0.284323928	45.92103369	0.015881651	0.971252884	68.6359151
0.218909017	132.4106477	0.015138262	0.956256357	78.13762148
0.253454602	75.74675507	0.018248086	0.969967131	70.45968993
0.414712283	97.8158448	0.083345558	0.98441305	33.63602004
0.329216681	105.2183364	0.068388882	0.969808895	86.91673523
0.280873663	664.8880953	0.017118636	0.984615099	92.64294488
0.236032425	425.382612	0.013385241	0.973970275	83.90433697
0.292285936	165.0814372	0.01899023	0.979358053	53.42874122
0.268285206	175.5250337	0.036993263	0.967472373	41.98369383
0.272669656	90.88757778	0.015498783	0.977222494	55.8737695
0.291210104	281.9308495	0.014281548	0.974550653	68.12435914
0.201249423	-89.58410987	0.011995031	0.956875925	99.95037836
0.300651983	370.8261771	0.025980487	0.976891721	71.74875626
0.345921615	198.2263478	0.045605542	0.975624971	69.89026497
0.287523563	260.766578	0.020581387	0.983494073	61.22947206
0.22255263	-257.9065293	0.013707816	0.966252566	94.89122255
0.169501133	-414.2052511	0.008478438	0.961776692	135.6475208
0.402088709	156.8796783	0.108238651	0.97718797	74.80016873
0.26735242	-45.71348446	0.019049419	0.966016785	45.93696947
0.36605746	168.0621263	0.055238474	0.991813128	47.14723072
0.240969371	193.71677	0.013801475	0.975918879	75.88410839
0.384344413	129.3726599	0.061449657	0.98787923	34.44733959
0.204087106	-73.86809319	0.010024759	0.961441059	110.1550706
0.21784772	320.6780728	0.011828264	0.965190554	87.61643689
0.391163678	142.9095254	0.079387259	0.983385321	40.23989787
0.238241814	242.0476171	0.014244346	0.970509432	88.50998294
0.315137883	156.3846783	0.022426942	0.985853682	54.03199209
0.301106479	-29.71059785	0.029076017	0.975298316	41.46040983
0.21081892	336.3882497	0.015133156	0.965083394	83.96401125
0.204662554	-419.5264758	0.021002483	0.960727139	107.9866894
0.242586403	428.7542052	0.01841716	0.971466074	77.1005398
0.208254999	331.2716526	0.009603643	0.976859742	89.88590935
0.468660699	88.00974551	0.092971752	0.987224265	29.29266011
0.285888933	-24.06264271	0.019323421	0.982355023	46.33595871
0.190779769	-140.638078	0.012647594	0.961634879	125.8425845
0.23702311	376.4076494	0.012779041	0.971326764	84.62508786
0.310916456	237.3846474	0.03114801	0.974803791	63.41275714
0.231845624	44.0988842	0.012201384	0.973684657	79.4091481
0.220013915	120.8794734	0.013154234	0.964319037	72.7662718
0.399202906	129.9588286	0.077922621	0.98854789	41.30572478
0.239787274	75.92260734	0.014913328	0.971574034	73.77999687
0.215591796	146.1793755	0.01408482	0.975509542	92.39139495
0.257034553	200.225586	0.020384336	0.979857583	87.1566776
0.228894505	52.96640027	0.011363312	0.980018172	83.15169576
0.262390701	10.66044118	0.019133103	0.969559759	54.41004676
0.196836133	51.42437412	0.008579842	0.973916463	86.48043525
0.206221206	-168.7592885	0.014775627	0.948805126	80.01744299
0.231535124	326.9026619	0.012422219	0.981688557	77.28753407
0.324358266	122.2521105	0.022622727	0.985210226	46.91954637
0.2450786	-8.896483871	0.012371176	0.974501522	69.45250919
0.224343985	86.04280529	0.016597886	0.958500678	78.5951202
0.287083853	122.5359225	0.036786624	0.977602191	74.19575936
0.212147256	301.9714993	0.012651331	0.965311812	92.75166021
0.240271234	185.2532907	0.015621299	0.966226478	68.6504774
0.353400801	327.5635978	0.068865957	0.983552582	57.41688094
0.273139022	-11.05223788	0.016924974	0.970961021	44.82974108
0.242786002	-14.47202288	0.013023527	0.978928259	65.67009484
0.170382956	-643.9393396	0.015194442	0.95525306	127.2024057
0.227876347	19.88387468	0.012396962	0.97288174	79.1564203
0.31810049	142.9532275	0.02650006	0.983331987	46.19314969
0.20657076	718.6892775	0.013204193	0.974930767	106.1833528
0.235597156	-86.83081686	0.01360959	0.966786171	72.36261905
0.305004645	198.5451597	0.052344403	0.970250373	73.7720425
0.302067394	91.75061171	0.021631392	0.977844259	40.71778855
0.321580512	221.9120486	0.04037954	0.975323937	61.69574348
0.261481009	-534.8701442	0.036291974	0.956635964	67.94430731
0.235090738	135.6417099	0.013462728	0.96902818	78.33129582
0.39671842	239.5382304	0.076990715	0.98184384	52.50519364
0.268800883	-20.50837656	0.022333844	0.97743325	63.00952433
0.23228564	52.31866287	0.010841428	0.970031398	79.23410345
0.266100203	-60.30852874	0.020551713	0.973666771	76.71553637
0.359137681	144.7063288	0.041850045	0.980424879	44.72972358
0.269106554	81.10255173	0.013198712	0.984998658	52.68964167
0.286845342	139.3907648	0.015559617	0.97484249	63.82295183
0.287418045	55.94568059	0.016537356	0.97701105	44.01468596
0.192896817	191.0200832	0.007842378	0.974875951	120.0055158
0.242598765	82.92907488	0.011110808	0.970931205	80.06275911
0.197837194	-294.0946709	0.017527739	0.957528911	77.49691008
0.240112653	-193.4832388	0.024072594	0.95572744	55.99730958
0.261232772	29.63544262	0.015328457	0.981244351	75.74676477

log.sigma.3.0.mm.3D_glcm_Contrast	log.sigma.3.0.mm.3D_glcm_DifferenceEntropy	log.sigma.3.0.mm.3D_glcm_InverseVariance	log.sigma.3.0.mm.3D_glcm_Entropy	log.sigma.3.0.mm.3D_glcm_Dissimilarity
45.7265954	3.799439567	0.206932092	8.63038826	5.100864372
35.11135049	3.564404561	0.204159797	7.749386713	4.691091435
39.77626548	3.666367127	0.207989813	8.432473978	4.879282066
40.52692441	3.667552421	0.194431176	7.973723229	5.053148224
29.42752512	3.509981244	0.225030209	8.311325184	4.233313929
36.26844143	3.571111389	0.206208275	7.537056991	4.67982816
17.93987066	3.087103568	0.309902733	7.32237124	2.769718515
32.71394419	3.566739331	0.257099952	8.406545076	4.150011039
21.07224627	3.258109885	0.288872383	7.756634116	3.262176152
20.89683125	3.258643531	0.256627184	7.768196138	3.564807115
12.88026278	2.950708323	0.324825297	7.21354438	2.671456022
20.2624098	3.230634918	0.307088556	7.658252738	3.150017615
37.13060856	3.622268401	0.223366479	8.236030973	4.661125282
39.99219674	3.672670503	0.196816083	8.343985563	4.980496671
35.92839034	3.612013536	0.242606932	8.211748529	4.357085537
25.77520516	3.24709494	0.298696886	7.697061496	3.386502307
13.98980872	3.011099495	0.307714325	7.312214539	2.844066963
33.44577619	3.558863254	0.213882195	7.871103976	4.535439858
11.57074993	2.868405423	0.336611008	7.028955535	2.540419353
23.50804803	3.346228086	0.281234663	8.087850986	3.499921291
19.84643678	3.240552964	0.275154644	7.91634686	3.362080199
28.62803561	3.456595013	0.218358766	7.872249358	4.210646847
24.24096824	3.352743124	0.253004765	7.826957204	3.750088383
11.40301592	2.861736406	0.337132254	6.965175571	2.312147533
29.24984967	3.400928952	0.277269109	7.863637711	3.688747193
28.9942057	3.45389233	0.275186763	8.196421421	3.858106269
33.28868683	3.549263313	0.238653159	8.29563253	4.295213066
18.82885541	3.208160013	0.293777338	7.774011947	3.223570779
17.66450144	3.046634289	0.280095183	6.430285833	3.232369405
19.29274953	3.22102012	0.271620363	7.828085956	3.355498886
21.97169245	3.286606071	0.285289593	8.028193185	3.445940503
38.25113795	3.657056444	0.20412674	8.327663832	4.849109897
22.8009522	3.323432092	0.28692629	8.010567369	3.468168394
21.3293659	3.233135126	0.298890987	7.72738847	3.192523014
24.16311695	3.376574574	0.272970225	8.044586809	3.587466334
41.00733899	3.70037353	0.22048804	8.587655395	4.853214954
58.8862127	3.834732115	0.172882828	8.815459518	5.960362064
24.46839335	3.228832253	0.290204333	7.558332741	3.214777747
16.67143477	3.088370286	0.276224388	7.411471805	3.192054636
17.2622225	3.123455542	0.317172671	7.542601862	2.862242653
25.0320068	3.400676695	0.243183742	8.115300297	3.856699565
14.02290742	2.99992354	0.326711037	7.125226371	2.589771927
41.72677822	3.579872711	0.2058856	8.613976605	4.993428738
30.51128193	3.531652178	0.225270247	8.343886024	4.284355104
13.18190459	2.934756444	0.339534753	7.022928283	2.501764436
31.24841462	3.546415521	0.236193484	8.417126248	4.253986606
20.68639534	3.256454332	0.296462995	7.869499592	3.241223467
14.15366344	3.00829212	0.297227089	7.104625759	2.899516946
28.51114114	3.424106545	0.220805241	7.783412533	4.194554131
39.66167994	3.662866574	0.206811592	8.01578525	4.92945733
30.21822757	3.514716972	0.24739127	8.024473308	4.145915257
34.3388487	3.616780523	0.218823552	8.573569399	4.499721964
10.01431349	2.728557168	0.358430051	6.604683783	2.055795278
20.62952479	3.209478504	0.290792488	7.630044199	3.347374512
44.07154784	3.751599159	0.193482472	8.365431424	5.183427026
26.61742781	3.418141474	0.244428202	8.109839018	3.958819641
21.88049674	3.283540777	0.287697622	7.932250562	3.371232753
31.06759797	3.5396896	0.235432489	8.28999332	4.291281574
31.29070433	3.547394924	0.226049912	8.097541491	4.336205533
16.70510098	3.076443583	0.316424631	7.265899005	2.722639127
30.20006032	3.527335996	0.239501454	8.223281452	4.156777514
36.35432164	3.658475297	0.21779358	8.627335326	4.626949366
33.11309127	3.569961914	0.251792963	8.580874856	4.210319721
27.80683769	3.470704849	0.233492303	8.393157861	4.048749165
22.85702889	3.313493599	0.264050076	7.722806701	3.630232556
38.77890501	3.705044115	0.204056558	8.695009289	4.843540326
31.33932418	3.491170392	0.217481857	7.857555887	4.423891516
31.2693594	3.559289652	0.23458649	8.448730616	4.243587781
16.12833688	3.111273732	0.311646737	7.641167194	2.926527849
23.31148677	3.356755182	0.2547047	8.155565424	3.728418299
29.37032863	3.446881553	0.223828943	7.838848243	4.230980302
31.41443198	3.525535592	0.259219766	8.256051961	4.077021536
34.72847918	3.604159013	0.220774017	8.343263913	4.571927269
27.48281058	3.458027829	0.245632312	7.977823341	4.019963324
19.43725517	3.199470796	0.295702259	7.604892352	3.043624649
16.78782365	3.117234236	0.278848022	7.535514421	3.194751698
23.14236587	3.350025488	0.247713274	8.103182867	3.72494705
48.65853366	3.765431657	0.178242981	7.977848481	5.557235842
26.75270561	3.433962065	0.238023468	8.236232805	3.994756858
18.45254951	3.182258964	0.308124136	7.542537935	3.083616666
43.82219588	3.770440098	0.210653541	8.5628591	4.992509517
24.88570155	3.388177731	0.243371621	7.976203214	3.884640696
29.94330358	3.468251354	0.252988821	7.989632028	3.914760276
15.00640652	3.059803013	0.301725283	7.481870459	2.930327255
19.88041955	3.227179325	0.297581578	7.828376021	3.206791452
26.66505694	3.415191078	0.249862036	7.334927059	3.872800032
30.96155422	3.508389246	0.240749538	8.267407918	4.226403333
16.72703538	3.065793184	0.322037279	7.34298905	2.739054179
25.14388789	3.400777953	0.265574215	8.073656518	3.733703549
25.77089495	3.413923598	0.240370857	8.168100428	3.952800276
24.40127144	3.348796438	0.265341291	7.932489155	3.693496024
15.64674569	3.048743685	0.330673141	7.358082373	2.773248105
20.50930703	3.288632421	0.267618226	7.955677481	3.444244196
20.0391847	3.257352414	0.27937199	8.01184222	3.361517932
15.54024478	3.082371775	0.289521244	7.611851103	3.030347479
45.61723381	3.798259675	0.198083742	8.883915464	5.240231569
27.02835058	3.446447207	0.243071189	8.328934334	3.961645399
32.59494394	3.541326073	0.205547202	7.66496911	4.511849949
22.23546945	3.243840657	0.254106112	7.2314307	3.712390555
29.00222139	3.491646776	0.256289859	8.420320554	3.99830236

log.sigma.3.0.mm.3D_glcm_DifferenceVariance	log.sigma.3.0.mm.3D_glcm_Idn	log.sigma.3.0.mm.3D_glcm_Idm	log.sigma.3.0.mm.3D_glcm_Correlation	log.sigma.3.0.mm.3D_glcm_Autocorrelation
18.9804965	0.894761869	0.202688945	0.418988491	335.0526865
12.06971918	0.858278773	0.201997362	0.457961449	164.6201555
14.69102819	0.859291333	0.205126265	0.442977725	209.8218331
14.17519235	0.858012703	0.182807931	0.47744171	230.1475446
11.03348134	0.884360223	0.219455233	0.524963423	218.5258099
13.54291307	0.8674049	0.203581233	0.490624109	302.1543946
9.924942304	0.934203506	0.408609867	0.461453768	189.8170878
14.86467593	0.91070159	0.270666745	0.423170595	384.4023622
9.874351956	0.912074577	0.329730861	0.472209866	168.8262172
7.746695377	0.883299311	0.244515354	0.464252663	150.5577995
5.455671367	0.895459748	0.338622804	0.461957753	104.8392293
10.0425366	0.921410248	0.342231219	0.424175952	211.6009943
14.23362556	0.87229631	0.215517219	0.491777077	285.4524824
13.96409221	0.871021842	0.188050122	0.463131633	187.915713
16.23316223	0.900363914	0.250711754	0.371765895	165.3793077
13.02155511	0.908764224	0.370450587	0.483607016	168.5925158
5.68607553	0.900799776	0.306823675	0.480803671	132.202577
12.20306252	0.877438972	0.205932527	0.481579088	152.8450556
4.847437408	0.890911926	0.343961181	0.453017727	81.23958406
10.76996099	0.926139674	0.296494867	0.45348932	373.7815564
8.101253668	0.890054283	0.284323928	0.550674243	136.0505045
10.2286402	0.861628224	0.218909017	0.465443341	116.6098111
9.649271448	0.886724828	0.253454602	0.487316386	160.7892101
5.890649916	0.924532172	0.414712283	0.493130658	124.4217879
14.86366781	0.898486189	0.329216681	0.495936052	184.657378
13.40701197	0.921456629	0.280873663	0.517822469	473.4101933
13.57501629	0.895947719	0.236032425	0.437185926	175.2978318
8.122315373	0.906614152	0.292285936	0.48053832	155.4125329
6.702123653	0.880861418	0.268285206	0.413598614	81.73830641
7.604724675	0.89973908	0.272669656	0.48745662	170.0961646
9.475881959	0.898674098	0.291210104	0.508674207	138.8282611
13.81361928	0.863310913	0.201249423	0.448650807	227.452087
10.31185667	0.904186531	0.300651983	0.518818857	150.790027
10.73239133	0.906760982	0.345921615	0.528840318	150.360717
10.89873302	0.917326551	0.287523563	0.438625275	265.8172116
15.94748527	0.881600528	0.22255263	0.406750779	313.223179
18.06288691	0.87135425	0.169501133	0.411387329	523.6037542
13.49529019	0.915685684	0.402088709	0.506307503	190.2180622
5.947389747	0.876957065	0.26735242	0.469433788	121.1053501
8.812680274	0.943617044	0.36605746	0.469163336	688.5380739
9.694289478	0.896263272	0.240969371	0.504447973	191.3539514
7.128037618	0.932076001	0.384344413	0.42506103	165.5255494
12.62228187	0.871635922	0.204087106	0.466109385	277.9101643
11.60429635	0.876617232	0.21784772	0.48366727	181.023474
6.731880057	0.921673631	0.391163678	0.506572897	105.2834355
12.43865864	0.887930235	0.238241814	0.480899333	216.5874455
9.864241874	0.924682133	0.315137883	0.450592821	206.8529152
5.463672389	0.895136601	0.301106479	0.491246695	152.028977
9.935535629	0.87444838	0.21081892	0.495951715	131.1417851
14.24600148	0.869013737	0.204662554	0.467961774	293.6564147
12.54534184	0.890591759	0.242586403	0.433730603	208.4025928
13.49915769	0.898277758	0.208254999	0.452605159	247.2798911
5.668349029	0.934906225	0.468660699	0.491614243	131.0680584
8.51515124	0.913807006	0.285888933	0.394307076	332.4341241
16.25819906	0.870582167	0.190779769	0.485238673	243.7059419
10.4015761	0.888012711	0.23702311	0.519606862	145.6010777
9.855342238	0.901123871	0.310916456	0.485794588	196.9614762
12.0381458	0.892470345	0.231845624	0.440554075	243.7920216
11.92269199	0.875477382	0.220013915	0.399182579	178.899767
9.047256336	0.936376378	0.399202906	0.430824465	272.5077573
12.3597386	0.890193511	0.239787274	0.42340239	236.1430631
14.2740776	0.895960269	0.215591796	0.440904427	337.9714707
14.5718409	0.909353139	0.257034553	0.454321402	362.2776048
10.87645918	0.904799856	0.228894505	0.502648543	311.4879661
9.117326132	0.886343628	0.262390701	0.409816846	173.5717758
14.53816422	0.891446285	0.196836133	0.388935155	300.0979818
10.92113088	0.850956199	0.206221206	0.439574986	178.8296589
12.67936455	0.909754082	0.231535124	0.428679752	318.760689
7.311751824	0.921392833	0.324358266	0.490534823	276.1319193
9.033681178	0.893295401	0.2450786	0.499075851	182.2158195
10.318338	0.865951654	0.224343985	0.45648169	169.6339603
14.20024473	0.905919248	0.287083853	0.414303109	329.9279562
13.11476452	0.87709514	0.212147256	0.456990815	201.528281
10.87276902	0.879851415	0.240271234	0.4262477	149.7396158
9.782312045	0.921954275	0.353400801	0.498514944	180.4947962
6.157011699	0.885910651	0.273139022	0.458753626	138.1924846
8.828858708	0.90178099	0.242786002	0.481100968	281.7882536
16.38049897	0.85862626	0.170382956	0.455426465	327.6433912
10.29258278	0.890067142	0.227876347	0.496946398	222.7264019
8.691801296	0.918052018	0.31810049	0.432203843	206.7273544
18.20007347	0.89664433	0.20657076	0.420934229	331.4670936
9.301757059	0.878877195	0.235597156	0.489282117	160.8440142
13.71589926	0.894778202	0.305004645	0.421723335	176.6102842
6.130232828	0.901840738	0.302067394	0.463062782	142.3697011
9.175522434	0.902311978	0.321580512	0.511931101	161.3297205
10.98700409	0.868409451	0.261481009	0.441720129	244.7650616
12.18266796	0.885511151	0.235090738	0.437362388	243.0379735
8.993647401	0.921116216	0.39671842	0.515343368	156.9387586
10.76065272	0.902694205	0.268800883	0.432670285	228.3164941
9.698347686	0.884408326	0.23228564	0.509660607	221.049073
10.03880742	0.895157652	0.266100203	0.52175938	265.7272963
7.628203072	0.914103414	0.359137681	0.48077339	138.7877117
8.356796622	0.917402299	0.269106554	0.442264416	237.4421813
8.350569282	0.897034407	0.286845342	0.520966642	151.4051427
5.98961564	0.898562722	0.287418045	0.476917228	132.3907282
16.91952948	0.893731343	0.192896817	0.454168858	365.7551413
10.67445939	0.888220339	0.242598765	0.496796542	171.1219687
11.69966867	0.862517526	0.197837194	0.410641384	254.1490015
7.655742334	0.860751073	0.240112653	0.43998942	131.9032294
12.35860679	0.910960235	0.261232772	0.452783987	412.7369093

log.sigma.3.0.mm.3D_glcm_SumEntropy	log.sigma.3.0.mm.3D_glcm_AverageIntensity	log.sigma.3.0.mm.3D_glcm_Energy	log.sigma.3.0.mm.3D_glcm_SumSquares	log.sigma.3.0.mm.3D_glcm_ClusterProminence
5.329239791	18.19855936	0.003667204	39.56879002	42914.58066
5.136293145	12.26322314	0.005617358	32.43498722	21203.45648
5.27259723	13.99311191	0.003521167	36.48883214	24051.86855
5.249198299	14.65839016	0.00459792	39.73216817	30161.34486
5.222225312	14.2880965	0.003922278	31.6831718	21093.86318
5.132308307	17.04754358	0.006431299	36.60863627	27621.2884
4.675866292	13.62035266	0.019261155	17.20835804	10671.74853
5.128663702	19.55705343	0.004912554	28.14647283	23178.68333
4.873019859	12.68908418	0.008462881	20.73872494	12503.35346
4.896053287	11.89484132	0.005756512	19.49055384	8898.501072
4.555396001	9.962470156	0.010282514	12.02256136	3741.743064
4.779831797	14.38537232	0.009756229	18.18061868	9950.255904
5.215134341	16.48658956	0.004564444	37.15771438	26938.21272
5.285963215	13.1911082	0.003676498	38.31636437	26328.14329
5.075792455	12.63927506	0.005002639	29.92186554	20650.02122
4.959941183	12.56723393	0.013610346	25.52353261	14980.8572
4.63497524	11.21307284	0.007992254	13.45335667	4369.719426
5.137098475	11.73998545	0.005107139	32.6664654	21973.68542
4.460997662	8.742752584	0.01111762	10.58223417	2980.685216
4.971364267	19.2790286	0.006066166	20.72461466	15436.01583
5.018502524	11.12808224	0.005328422	22.12058797	11519.36515
5.048813906	10.20565957	0.005158554	26.69141427	15407.61257
5.005473095	12.2274043	0.005575903	23.89975098	13331.73029
4.479403787	10.90234533	0.018225898	11.26479588	4764.85855
5.123080049	13.1022462	0.010171164	29.66994522	19991.298
5.162903079	21.53235078	0.005540457	31.01697692	29652.10868
5.141470918	12.91262691	0.004357651	30.75767	22946.405
4.859527032	12.14016728	0.006430444	18.44290144	8935.846983
4.380588604	8.697652971	0.014350375	14.91204882	5727.18887
4.899281981	12.69816208	0.005678758	18.98523089	9066.338964
5.010669725	11.29658804	0.005301024	22.77594041	13503.41185
5.2297391	14.64723505	0.003793994	35.49495922	21403.50916
5.03934137	11.80980292	0.006309266	24.19498554	17951.27872
4.969415675	11.76765389	0.009085619	22.99433375	12577.70644
4.951740378	16.22390695	0.005720307	21.92895046	12180.99241
5.262057646	17.59362323	0.003449869	34.26558753	24353.66811
5.4617846	22.7971862	0.002751556	46.93144005	44615.47655
4.936288796	13.42954861	0.019826136	25.7158739	16604.96422
4.713326482	10.66578784	0.007334298	15.65210106	5347.807813
4.756537641	26.25662956	0.011539323	16.74366153	9325.121162
5.084694276	13.45436541	0.004640437	26.14617532	14686.27712
4.487772043	12.71824392	0.016441649	12.64940421	5426.673785
5.362144279	16.38362497	0.003296703	39.26374581	30282.40998
5.18053929	12.97816687	0.003872348	30.46940911	20031.20935
4.537925788	9.928533824	0.016941006	13.42265083	7831.295192
5.207904967	14.38795823	0.003896177	31.42323204	19435.90897
4.881877173	14.2429985	0.006530897	19.4935379	10472.20098
4.589536036	12.0489002	0.009700941	13.90351832	4146.744693
5.059737866	10.84189839	0.005463029	28.41822143	19587.0196
5.246090274	16.77359651	0.005171842	37.84827914	31374.14324
5.027892856	14.1538015	0.005611379	28.03637792	17273.83053
5.245211797	15.53175698	0.003502645	31.9420047	27407.22756
4.300042051	11.24011046	0.028328015	9.900819155	4498.880044
4.745616822	18.28000954	0.00710105	17.11095476	7381.081477
5.367810507	15.15876595	0.003684853	43.85918857	34157.77124
5.123267766	11.47000646	0.004599024	28.17283521	20754.03714
4.960125923	13.70325874	0.006679449	21.88658665	12089.19121
5.116320048	15.43229373	0.004028775	28.6308826	14063.52331
5.04290265	13.0263978	0.004496198	26.61336615	13459.95734
4.582976224	16.50020447	0.018452592	14.92559937	8723.251658
5.089703034	15.14792827	0.004360307	27.05601157	14571.27662
5.267355904	18.28347457	0.003477249	31.85058839	25955.12401
5.222818983	18.92004413	0.004240905	29.62708439	22768.97994
5.203667897	17.45101936	0.003916714	27.9337616	20580.50507
4.839740208	12.87435997	0.0062953	19.36728962	8238.033824
5.234100344	17.29193811	0.003158091	31.43923391	23009.74723
5.034711928	12.90791678	0.005152332	27.83919179	14153.92952
5.12821574	17.79094205	0.004203026	27.22501943	21056.30452
4.779555747	16.49986011	0.007637151	16.43524851	6987.502338
5.062138742	13.08601705	0.004529146	23.47681117	12816.67477
5.016210265	12.54110337	0.005317674	26.99136221	13467.3863
5.082340293	18.16774974	0.005920495	26.58659033	16250.31742
5.185851174	13.78320905	0.003976196	32.95615593	20105.27343
4.990171211	11.81666544	0.005078848	24.20171773	11959.79944
4.841825707	13.17710223	0.012188368	20.15879584	14373.90983
4.73060057	11.45275578	0.006806984	15.40439118	5153.630853
5.022279932	16.588066	0.004681867	22.67102947	11872.46934
5.300609859	17.86635452	0.004831287	44.61856341	42007.51576
5.145434521	14.523535	0.004371917	26.98339479	18375.15414
4.743271822	14.21900431	0.008311278	16.94021745	8240.841552
5.296110876	18.10749483	0.003823036	38.01876273	40481.37336
5.053132831	12.21150427	0.004960892	24.42201897	12871.03884
5.026503885	12.93196692	0.007441886	26.60291438	14528.16923
4.675318009	11.6592149	0.007896839	13.93104877	5150.123989
4.928117706	12.30184994	0.008288118	20.70411341	12519.44734
4.75420038	15.30994143	0.010094179	23.65234106	14958.56019
5.123723468	15.34580814	0.004357742	28.47733342	16599.40005
4.739491588	12.19080498	0.016543157	17.70048779	10913.6917
4.988353869	14.91609176	0.005625046	22.88715604	12653.8185
5.125318805	14.45633033	0.004261651	26.83313801	15036.1401
5.080423047	15.99247684	0.005903004	26.0642504	18288.65327
4.696670997	11.47921198	0.011353871	15.27943657	7483.790061
4.876751964	15.28848389	0.005413534	18.80676515	9144.968879
5.002055163	11.86056741	0.005201262	21.12578115	11314.48705
4.736697472	11.19225431	0.006515646	14.88873268	4973.344684
5.444188399	18.93926196	0.002701213	40.9691201	37929.27584
5.160257262	12.58306105	0.003934937	27.0474935	15934.83411
4.9685454	15.61010129	0.00597157	27.79625035	15758.20167
4.727383585	11.10790924	0.008196707	19.55819476	7873.751696
5.14533957	20.26749691	0.004376668	25.25873395	17230.44539

log.sigma.3.0.mm.3D_glcm_SumAverage	log.sigma.3.0.mm.3D_glcm_Imc2	log.sigma.3.0.mm.3D_glcm_Imc1	log.sigma.3.0.mm.3D_glcm_DifferenceAverage	log.sigma.3.0.mm.3D_glcm_Id
35.71495253	0.814179283	-0.121748308	5.100864372	0.291254503
24.48993541	0.921089903	-0.219170792	4.691091435	0.29297059
27.85574592	0.834288957	-0.138353404	4.879282066	0.29421806
29.10903391	0.949649446	-0.254387946	5.053148224	0.274807328
28.44342444	0.786547037	-0.114552344	4.233313929	0.310723802
33.76262546	0.968586829	-0.311745286	4.67982816	0.295076282
27.00244015	0.778450843	-0.127626422	2.769718515	0.471274382
38.60899982	0.692351426	-0.081402808	4.150011039	0.350756517
25.24779891	0.734495888	-0.105753208	3.262176152	0.404603748
23.78968263	0.799598463	-0.127762386	3.564807115	0.336145098
19.92494031	0.714168958	-0.103958066	2.671456022	0.415759771
28.58011872	0.726681754	-0.099433145	3.150017615	0.414242806
32.7199742	0.852830557	-0.149950604	4.661125282	0.303628408
26.13718972	0.87670244	-0.163943653	4.980496671	0.279906005
24.9003188	0.770025526	-0.109240581	4.357085537	0.3344954
25.02230434	0.844063932	-0.162463362	3.386502307	0.434908752
22.42614568	0.752115023	-0.113131323	2.844066963	0.390354427
23.42767255	0.906161152	-0.198003823	4.535439858	0.297453826
17.48550517	0.719359357	-0.106664401	2.540419353	0.420917054
38.17037611	0.686490052	-0.083278174	3.499921291	0.376428168
22.25616447	0.746972939	-0.108667054	3.362080199	0.368370836
20.41131915	0.861892988	-0.16200984	4.210646847	0.310903699
24.42853133	0.869904562	-0.168928238	3.750088383	0.341405973
21.80427091	0.750256429	-0.119814516	2.312147533	0.479368216
26.09286816	0.884681671	-0.186947686	3.688747193	0.401326459
42.77722774	0.797541826	-0.124260342	3.858106269	0.360866415
25.5054672	0.761169293	-0.107673003	4.295213066	0.323322999
24.22857801	0.719461219	-0.096162666	3.223570779	0.375212535
17.39530594	0.949376123	-0.308985386	3.232369405	0.357455578
25.372543	0.738646273	-0.10298787	3.355498886	0.359768332
22.56403891	0.719240441	-0.097667709	3.445940503	0.372292652
29.11874951	0.834627767	-0.137266385	4.849109897	0.291116956
23.54117815	0.782115909	-0.117782943	3.468168394	0.379194266
23.51144074	0.829916434	-0.147576602	3.192523014	0.417817176
32.03421443	0.672229883	-0.078551954	3.587466334	0.368913498
34.62533532	0.756873811	-0.099725862	4.853214954	0.30743783
44.91138969	0.827631164	-0.131275152	5.960362064	0.258647917
26.65544608	0.862094624	-0.171736091	3.214777747	0.462628403
21.33157568	0.805673838	-0.136760183	3.192054636	0.357275144
52.19446297	0.720414247	-0.099901731	2.862242653	0.435905165
26.73005164	0.771341412	-0.110834952	3.856699565	0.330964645
25.33125827	0.680342224	-0.090360726	2.589771927	0.452811273
32.29464071	0.801809231	-0.128370065	4.993428738	0.29211947
25.82432142	0.770097485	-0.106490069	4.284355104	0.309125171
19.85092342	0.797703815	-0.141313937	2.501764436	0.458150615
28.44360454	0.742888611	-0.098287493	4.253986606	0.324673674
28.17869328	0.701186904	-0.088825166	3.241223467	0.39311024
24.0978004	0.82152791	-0.151481462	2.899516946	0.38570411
21.65318137	0.911340674	-0.206879934	4.194554131	0.304665301
33.25459989	0.934142282	-0.228373327	4.92945733	0.292184735
28.04471833	0.785599139	-0.115898797	4.145915257	0.328882563
30.55419902	0.722227332	-0.08731367	4.499721964	0.300238246
22.47192662	0.762163734	-0.130547323	2.055795278	0.522801304
36.11122428	0.748520796	-0.110580243	3.347374512	0.369211284
29.87969813	0.889951941	-0.173399439	5.183427026	0.280509908
22.89672587	0.808518771	-0.129161847	3.958819641	0.326475496
27.31793909	0.731530747	-0.103002524	3.371232753	0.388526334
30.44315853	0.707468882	-0.086694976	4.291281574	0.318945509
25.96104484	0.811001718	-0.127140809	4.336205533	0.310014712
32.64086069	0.750337052	-0.112791282	2.722639127	0.463696947
30.01588066	0.778869819	-0.111043591	4.156777514	0.327059715
35.99720519	0.732838336	-0.090344249	4.626949366	0.304292719
37.34984407	0.689694268	-0.081040892	4.210319721	0.339879157
34.50425683	0.758405159	-0.103291972	4.048749165	0.320187673
25.74248043	0.808323273	-0.133205591	3.630232556	0.348807194
33.95099341	0.659108656	-0.068618702	4.843540326	0.288147453
25.81583356	0.877388944	-0.173300519	4.423891516	0.298417046
35.05719025	0.651299023	-0.070417502	4.243587781	0.320069098
32.76774104	0.666560032	-0.083004808	2.926527849	0.402705987
26.12830817	0.723048324	-0.092915116	3.728418299	0.334651313
25.08220675	0.882530507	-0.178205214	4.230980302	0.31449882
35.73395417	0.733365996	-0.09528103	4.077021536	0.363664647
27.3500652	0.776918839	-0.109582724	4.571927269	0.30159715
23.61384625	0.792861383	-0.119821057	4.019963324	0.328045467
26.15301719	0.769807407	-0.11956965	3.043624649	0.425749249
22.90551156	0.751222089	-0.110786974	3.194751698	0.361301351
32.93314322	0.721142405	-0.092766164	3.72494705	0.333570889
35.09572026	0.956080753	-0.266706473	5.557235842	0.261112149
28.95581011	0.789114101	-0.116245609	3.994756858	0.319568044
28.26918209	0.752517975	-0.109466857	3.083616666	0.396276079
35.54559041	0.810647658	-0.120954129	4.992509517	0.295064341
24.40996717	0.810621426	-0.129117137	3.884640696	0.326019867
25.73955154	0.788284833	-0.126325003	3.914760276	0.38059931
23.31842979	0.678548934	-0.087441639	2.930327255	0.38607308
24.56574056	0.760157597	-0.11347817	3.206791452	0.398054528
30.61988287	0.837102411	-0.156672514	3.872800032	0.346679061
30.40893937	0.786771617	-0.117476004	4.226403333	0.322357411
24.32980906	0.801360696	-0.136928307	2.739054179	0.461005763
29.58669341	0.747973447	-0.101603989	3.733703549	0.352690661
28.82107343	0.776554388	-0.112104412	3.952800276	0.322886328
31.78872486	0.857939319	-0.159580626	3.693496024	0.351181131
22.93554282	0.755403807	-0.118936943	2.773248105	0.430282363
30.29162922	0.638343834	-0.069772601	3.444244196	0.356221273
23.70285119	0.712422094	-0.093576094	3.361517932	0.370010912
22.38450862	0.64474329	-0.078524681	3.030347479	0.374159942
37.26292492	0.756546711	-0.097467975	5.240231569	0.28140831
25.12825055	0.716044236	-0.09243206	3.961645399	0.331149291
31.16850347	0.915407512	-0.213153331	4.511849949	0.292052023
22.21581848	0.900692859	-0.20783597	3.712390555	0.330314687
40.05226097	0.670178752	-0.076462853	3.99830236	0.344527827

log.sigma.3.0.mm.3D_glcm_ClusterTendency	log.sigma.3.0.mm.3D_firstorder_InterquartileRange	log.sigma.3.0.mm.3D_firstorder_Skewness	log.sigma.3.0.mm.3D_firstorder_Uniformity
110.3610885	199.3038101	0.46449047	0.047918217
93.33350257	209.030117	-0.29694702	0.058226545
102.964276	236.7480431	-0.144261714	0.047007279
113.4566229	219.0089169	-0.203415203	0.047008217
94.41144229	229.7735672	0.11328892	0.052088958
104.8452449	200.9022598	-0.363331248	0.051567516
48.07343214	94.4040451	0.630790808	0.095369256
79.65113737	172.787324	0.562938142	0.056451732
58.90708165	136.6143112	0.716157894	0.070775183
57.06538412	161.6821232	0.065715121	0.062135871
35.20998265	106.5134115	0.327475199	0.088697207
49.82640889	117.990715	0.569585319	0.076992077
108.1590103	228.1087532	-0.568940875	0.055113034
108.189722	249.347167	-0.004810618	0.046140195
77.39177272	182.3349094	0.696352893	0.057040867
74.00655068	158.928421	0.259656125	0.073534116
39.82361795	138.2022972	0.101016943	0.073422865
95.67006371	227.1710892	-0.032191159	0.054226123
30.75818675	105.7741299	0.300854714	0.087485671
61.64548405	139.9735756	0.227565418	0.064720544
68.6359151	190.7565174	0.013602491	0.059902957
78.13762148	194.1148033	0.015396324	0.056232115
70.45968993	178.8914852	0.045345583	0.058664457
33.63602004	83.08046913	0.602280214	0.106571449
86.91673523	197.0050144	0.138571871	0.059715488
92.64294488	181.6486549	0.696394391	0.057326221
83.90433697	204.9851303	0.59271954	0.053800582
53.42874122	143.8562851	0.448344664	0.06744531
41.98369383	99.89670753	0.661935699	0.089041096
55.8737695	166.4442272	0.159790535	0.062955904
68.12435914	178.5873871	0.431909276	0.059036226
99.95037836	254.5688477	-0.238039383	0.049366771
71.74875626	158.9759769	0.537944065	0.061820083
69.89026497	160.0136261	0.33637448	0.068432717
61.22947206	162.715416	0.561833705	0.061975644
94.89122255	219.1564026	-0.302553988	0.049849178
135.6475208	250.5295334	-0.531691498	0.043902746
74.80016873	138.4947624	0.279431601	0.084218556
45.93696947	135.3597155	-0.275613026	0.073278061
47.14723072	107.3469715	0.589775697	0.085058773
75.88410839	196.0356741	0.25982636	0.055370907
34.44733959	78.78389454	0.773461802	0.108391803
110.1550706	244.602869	-0.053395243	0.045675493
87.61643689	207.5173244	0.302428761	0.051266614
40.23989787	94.68015194	0.603475036	0.095341195
88.50998294	206.6569748	0.366677058	0.051880195
54.03199209	143.8353062	0.52818106	0.068050058
41.46040983	140.3411255	-0.210809383	0.081611575
83.96401125	194.1318207	0.248367713	0.053302896
107.9866894	197.3505287	-0.524328035	0.056239721
77.1005398	197.123848	0.510500145	0.057105029
89.88590935	197.9297256	0.413700241	0.051275073
29.29266011	70.65790272	0.622676725	0.12160796
46.33595871	143.2779722	-0.051443309	0.069273976
125.8425845	263.7104759	-0.162663287	0.045376818
84.62508786	207.4067039	0.349606452	0.054822354
63.41275714	149.9568195	0.470866987	0.064230183
79.4091481	230.8028107	0.077791667	0.053389254
72.7662718	192.2457485	0.118486801	0.05537739
41.30572478	96.06177902	0.624396946	0.097423289
73.77999687	186.2746506	0.206674356	0.055278413
92.39139495	196.1506271	0.09976275	0.049391357
87.1566776	187.5518703	0.162340258	0.052332288
83.15169576	182.0349808	0.046030446	0.053327221
54.41004676	149.2725296	-0.06344249	0.06263841
86.48043525	209.1367798	0.055071068	0.049304889
80.01744299	201.8005619	-0.412405589	0.057438909
77.28753407	175.8555145	0.429668442	0.056383893
46.91954637	133.6944914	0.507525984	0.074343715
69.45250919	176.7488213	-0.083247976	0.058239881
78.5951202	219.3732491	-0.01373267	0.053688281
74.19575936	173.8676376	0.209047089	0.06054128
92.75166021	238.202755	0.283823309	0.050729285
68.6504774	206.581831	0.271361454	0.056170637
57.41688094	102.8022351	0.911496511	0.087913489
44.82974108	135.2276258	-0.098324895	0.071572662
65.67009484	177.6808319	-0.086227465	0.058549934
127.2024057	229.7344437	-0.618764782	0.0512705
79.1564203	176.0561352	-0.042987061	0.056516069
46.19314969	118.6925497	0.547785041	0.077028395
106.1833528	196.4636326	0.523071852	0.04908045
72.36261905	182.0372181	-0.167268284	0.058891123
73.7720425	204.0941162	0.274496478	0.060215565
40.71778855	123.584362	0.346919813	0.075814948
61.69574348	133.9717922	0.437834536	0.069347361
67.94430731	125.9104671	-1.229834386	0.094307369
78.33129582	183.6255035	0.181873398	0.054601652
52.50519364	108.5705032	0.662745922	0.085095946
63.00952433	147.4372749	0.002387977	0.061495451
79.23410345	207.6618118	-0.017474591	0.054923532
76.71553637	149.6660366	-0.114411093	0.062244746
44.72972358	115.0639625	0.589056962	0.081096561
52.68964167	152.588623	0.223187674	0.065317387
63.82295183	164.1100407	0.255729849	0.061129022
44.01468596	148.6838379	0.14365875	0.069667854
120.0055158	258.6355457	0.019517465	0.043970429
80.06275911	207.1366043	0.09795197	0.053037571
77.49691008	192.4651146	-0.5139819	0.059886291
55.99730958	144.626545	-0.707200205	0.076854533
75.74676477	170.2750111	-0.092659949	0.055827021

log.sigma.3.0.mm.3D_firstorder_MeanAbsoluteDeviation	log.sigma.3.0.mm.3D_firstorder_Energy	log.sigma.3.0.mm.3D_firstorder_RobustMeanAbsoluteDeviation	log.sigma.3.0.mm.3D_firstorder_Median
123.7903745	37937855.26	84.16469845	-52.76396179
114.5608761	7137239.282	82.67055464	-7.775464058
125.3073029	19772649.15	94.7309725	14.06828737
127.1434944	7821795.924	92.6177925	-7.107287407
118.173853	27484915.41	91.13078497	8.442392349
119.9445334	5327715.734	85.28161139	28.01685429
71.13750007	106224448.8	42.32407109	-3.834082365
104.2741896	109785853	71.96695956	-30.02239227
86.99060207	54762526.34	59.43146866	-24.6799078
91.9892164	9853776.804	66.77023085	19.10024643
65.82354792	11292176.68	44.52142311	3.983475208
78.67763009	37946374.02	50.37351112	-6.074318409
126.0099133	18428092.26	95.13489413	21.4829464
128.2708269	17414245.39	98.5050058	-59.66781044
106.8112321	31381430.11	74.61117985	-61.58728027
94.33477445	86139071.07	65.1553704	-5.043613434
77.34857011	7878278.711	56.5473466	3.125073433
121.7693452	10182459.59	94.17573135	-46.35409927
65.50133759	7696345.709	44.64749389	-16.75851345
89.36183948	128899345.7	59.06025936	14.55076694
101.7471799	30076532.36	77.70587734	15.40237999
107.1472521	8360060.288	78.95125659	0.558910772
98.92302684	8333536.324	71.78315244	-20.53822136
59.55456499	24794054.42	36.31359518	-12.58741665
108.8171412	23567878.81	78.68934602	-19.39120483
110.5197397	50220129.3	75.76199995	-22.96063709
112.5550572	40649870.86	81.92672466	-57.28652
85.30307074	27423732.78	59.82746078	-31.62035751
71.26942022	1508407.126	45.52346644	21.9473629
90.16207885	19897575.94	65.90060721	-13.85849905
98.63296953	60057340.06	72.58265949	-37.08595276
126.7427654	19658787.28	99.25052451	-34.02852058
95.14750065	34716296.67	65.03220396	-26.21103859
92.04700094	32884773.42	65.90539898	-10.34063339
95.48496327	73623764.55	67.34078164	-22.18962288
117.5837752	40411258	86.60457867	-10.54720116
140.0144126	46828540.63	103.0072503	-17.82886028
91.63864542	87551084.54	61.1104963	2.657375097
79.67728494	5736511.956	57.60815402	-14.25229549
72.69971448	93557070.84	45.93514772	-10.74439049
107.3409413	24408189.26	81.09210746	-57.88094139
58.79520981	39181777.15	34.80373371	-1.738405228
128.3363984	55940678.44	97.33233268	1.100718319
114.2387011	27518644.23	85.77651584	-40.74789619
67.18924383	13579214.04	40.9156484	-7.380651474
112.6239399	44619666.65	83.13834938	-17.16169739
84.53893346	62369041.78	58.41436786	-8.36641407
77.22701568	4652218.752	56.48672553	-8.208753586
108.791971	6954713.555	78.96750149	1.06557548
120.7125635	10095534.26	83.55584182	36.99918365
111.6490487	25390089.72	82.34692132	-73.79059601
113.1905626	55666523.59	81.21070347	7.860368252
54.76216115	49111916.12	32.00074463	-4.482076168
84.0402168	18079752.53	59.76313643	-4.528052807
137.9117152	16947803.13	106.731029	23.59497261
112.7494769	21594327.9	84.5103343	-16.78911877
91.03779152	53864631.46	63.53938001	-23.43216705
116.4418829	39009306.27	92.4142658	-30.05809593
106.351357	13978526.42	80.14616947	-45.70529175
67.15868858	98008292.37	40.80979118	-2.716148376
103.4453882	21467742.22	75.1259492	-14.68237114
114.467551	50950418.19	80.95553905	17.41522408
109.9816923	156359269.8	77.32517146	-2.267643809
106.7239112	36784064.77	75.79013959	4.490544796
91.7734553	8871704.404	64.41571639	-4.196543694
116.3811484	69046927.85	84.75445974	-0.05704689
111.6082274	8747725.875	85.22356793	-33.91064453
104.1445695	88231896.22	72.87928315	-34.37677002
79.21751134	74047290.36	55.87119483	-9.10602951
98.92290276	31318676.42	72.30418177	26.08765984
112.3712226	8898905.207	87.23420953	-33.78638458
101.3181822	81543976.16	72.05900834	-2.133594275
123.2557756	28947529.59	96.35447516	-48.91659546
107.8076519	16359883.35	82.72629488	-50.31559181
75.53958918	45720862.1	45.83418582	-11.55039787
79.90596355	8795262.298	58.09413319	14.02712774
99.36012784	31959260.42	73.25531727	8.255544662
135.8276591	9251969.801	98.59933825	2.576978445
101.8979102	22397783.27	71.37119463	38.42235184
75.86040006	19381948.02	50.28790765	-35.54528809
121.582719	35974705.59	82.6131045	-55.27204895
101.7753374	12579517.87	74.86974747	15.78041506
105.4859596	38739660.98	80.20568047	-24.46349812
75.26346454	19372740.26	52.31505136	-44.05838394
86.07592913	34219447.44	57.11720852	-8.941986084
92.94026095	7459994.82	61.47760976	29.6035614
105.5088517	22660968.19	76.14792896	-34.46063614
77.22280104	42646816.84	48.48924121	-6.818733692
92.02220785	23843694.79	62.54750837	-15.01935291
109.2263174	22917724.47	82.97303158	12.89465523
96.08629812	12407168.89	64.43430305	18.83132648
74.81537958	25463337.74	49.0080271	-20.4046936
86.61365322	52286369.8	62.08974974	-10.49406815
92.19991432	44803500.31	66.75568159	10.09999371
81.27825873	31810887.75	60.21966656	-6.900758266
136.2782696	62221884.19	103.7664861	-0.748387486
108.9928291	41500171.48	82.35940379	1.639333189
107.1246022	6260186.25	78.44168458	-29.55004501
87.02125681	3104309.484	62.35525288	14.68672037
102.2195749	148090689.4	71.69489344	25.34686089

log.sigma.3.0.mm.3D_firstorder_TotalEnergy	log.sigma.3.0.mm.3D_firstorder_Maximum	log.sigma.3.0.mm.3D_firstorder_RootMeanSquared	log.sigma.3.0.mm.3D_firstorder_90Percentile	log.sigma.3.0.mm.3D_firstorder_Minimum
1024322092	519.3672485	162.0887194	188.9118851	-466.5336914
192705460.6	288.7602234	143.4169199	120.3690231	-339.1442566
533861527.1	312.1351318	148.884677	179.3945419	-334.5285339
211188489.9	286.4030151	155.8566383	167.0324127	-383.4868469
742092716	388.4704895	139.5673059	173.1798096	-326.5643616
143848324.8	295.1630859	148.37572	219.2014221	-377.5888672
2868060119	553.4954834	100.3951056	136.3185211	-316.4351196
2964218031	460.0154114	134.5087089	158.8010971	-475.0149841
1478588211	446.0078125	113.353521	144.4038971	-321.2486877
266051973.7	334.3353882	114.242549	171.2719116	-255.1529846
304888770.3	281.5892639	85.18910115	130.8759918	-210.5840149
1024552098	489.1643982	105.5356222	137.5913666	-339.2250061
497558491.2	298.3910828	150.7404342	143.2572021	-408.4406738
470184625.7	350.4594116	164.4407301	130.9865417	-386.3984985
847298613.1	549.132019	141.1549416	147.9385742	-340.4560852
2325754919	430.6154785	122.2681849	182.2885849	-282.0615845
212713525.2	301.3699036	94.4572546	125.1257431	-256.0116272
274926409	364.9348755	157.7846292	95.79302979	-367.3748169
207801334.1	249.5227051	83.87521041	92.8747139	-207.9787292
3480282333	569.9041138	118.4767314	160.4688812	-426.9324036
812066373.7	341.7145691	120.3070589	140.9911087	-256.598053
225721627.8	307.3649292	129.8266547	155.1348038	-268.1448059
225005480.7	340.1821289	122.2074176	134.6313843	-321.4710693
669439469.3	369.9726868	83.02398141	95.78060913	-255.699585
636332728	355.4933472	138.5354963	164.1826309	-346.4102478
1355943491	511.6389465	140.1711023	209.1873856	-501.5238953
1097546513	498.2145081	142.4231958	144.9443268	-341.6858826
740440785	385.0107117	110.3272234	119.5716141	-315.9401245
40726992.39	363.6553955	101.6442603	180.2412643	-172.4731293
537234550.3	353.5385437	111.1698827	131.6770081	-315.506134
1621548182	383.821167	122.3953782	147.8014954	-297.4903564
530787256.6	266.9799194	157.948408	120.0822281	-411.4066467
937340010	425.6700439	121.9335598	152.5647095	-295.9295349
887888882.4	379.7564392	116.6914024	158.6711731	-291.5168762
1987841643	502.4709167	119.7631962	168.1941772	-375.7274475
1091103966	365.4648132	144.3651214	157.0167084	-449.5092163
1264370597	295.1434021	175.9865251	155.8641205	-620.7609253
2363879283	443.5126343	122.6399502	179.139679	-324.2359314
154885822.8	203.8529358	101.2115179	86.72611542	-296.8381958
2526040913	457.7689514	99.51564229	120.2946625	-632.0452881
659021109.9	391.2377625	134.9632047	131.6560532	-356.559845
1057907983	514.1298218	83.11395599	107.9361771	-275.4735107
1510398318	372.0310974	152.1022773	193.457843	-381.7730408
743003394.3	347.0731506	139.209651	154.8884079	-331.5773926
366638779	405.3267212	93.41842061	99.1562233	-237.7936554
1204730999	406.9502258	136.3223552	191.615387	-339.9108582
1683964128	566.3738403	107.9610402	130.9143372	-336.9050598
125609906.3	226.988327	97.33954631	80.04598236	-318.1426086
187777266	378.3847656	133.3678791	179.6402283	-264.5013733
272579424.9	312.6574402	149.4498336	176.9096008	-400.95224
685532422.4	351.8525391	140.4568241	156.5696045	-376.6888733
1502996137	569.9093018	141.3788518	211.0554169	-347.0612793
1326021735	386.6386108	78.46949662	90.8024826	-257.9275513
488153318.4	368.9972229	104.9324141	114.7292992	-447.9263916
457590684.5	413.9359741	162.3495984	209.6140778	-373.839386
583046853.4	404.1596985	135.5083899	152.2899857	-293.1612549
1454345049	345.2247314	115.8268036	154.1468872	-341.7435303
1053251269	401.4995422	137.3769516	147.1087402	-383.1683044
377420213.3	321.0904846	135.3531685	114.5006256	-371.6260986
2646223894	503.0067444	94.85443891	114.6333694	-384.9418335
579629040	390.7051697	127.0956526	151.5817352	-364.1618652
1375661291	491.141449	143.7690539	199.3888641	-417.1319885
4221700283	512.8549805	137.8022743	199.8381393	-432.4773254
993169748.9	465.5987549	132.4750891	172.2472	-411.5543518
239536018.9	309.372406	114.4746165	139.8837616	-323.2953796
1864267052	493.2775879	142.067796	168.217804	-402.7621765
236188598.6	168.7003174	146.7859763	86.2122879	-390.1206665
2382261198	525.1934814	131.7247333	157.6507019	-448.8283691
1999276840	393.7878418	99.30987916	128.5967117	-389.4060059
845604263.4	409.6420288	121.4295662	163.361264	-297.5691223
240270440.6	272.5224609	134.4886824	143.6618423	-328.2986145
2201687356	431.8902893	127.9492284	163.7575378	-432.8624268
781583298.8	366.049469	147.2533285	160.7580078	-357.5756836
441716850.4	338.8762817	131.2285405	137.7495407	-320.4442139
1234463277	494.6437378	105.8069849	135.389183	-310.1142578
237472082.1	298.5479126	97.88205019	133.7348297	-252.8383942
862900031.3	389.9524536	120.2819617	146.4142639	-390.2079773
249803184.6	261.6185608	169.244998	138.4047729	-475.1358643
604740148.2	424.7561035	130.5087537	179.4568909	-310.1366882
523312596.6	402.1906433	103.7677643	101.1216522	-360.3675842
971317051	519.3672485	159.9579994	186.4763489	-466.5336914
339646982.6	330.9922485	123.4076194	150.2989349	-279.1264038
1045970847	381.2444458	130.2927012	141.9324768	-339.0440369
523063987	276.4034729	101.1626555	96.46377411	-311.8257141
923925080.9	374.3856812	112.2668608	155.242157	-279.6028748
201419860.1	156.7643738	118.5282855	89.95472717	-397.5597229
611846141.1	328.9143372	131.624083	155.6970825	-392.704071
1151464055	414.65802	105.4396635	142.1893234	-276.4964294
643779759.3	406.361145	118.395343	142.3372345	-366.5784607
618778560.8	335.0852966	129.4796419	165.7477112	-333.714447
334993560.1	343.7062378	124.2248047	184.0057327	-368.3515625
687510119	363.0527649	100.2826529	119.8608604	-280.7803345
1411731985	510.4329529	107.780387	132.7498779	-353.7810669
1209694508	397.9708557	114.0245418	163.2676086	-270.6842957
858893969.1	334.3323975	98.07781842	132.5721649	-270.8603821
1679990873	547.1856689	161.5542746	188.1789093	-451.5601501
1120504630	409.6445313	130.0962304	174.3220276	-294.2025757
169025028.7	181.2002106	141.42346	93.10486603	-432.2878723
83816356.08	207.8989258	107.6254819	100.8489479	-294.755249
3998448614	456.395752	129.5481238	178.2768356	-452.4659424

log.sigma.3.0.mm.3D_firstorder_Entropy	log.sigma.3.0.mm.3D_firstorder_StandardDeviation	log.sigma.3.0.mm.3D_firstorder_Range	log.sigma.3.0.mm.3D_firstorder_Variance	log.sigma.3.0.mm.3D_firstorder_10Percentile
4.647780625	158.3543511	985.9009399	25076.10051	-223.2717743
4.340370781	139.6770838	627.90448	19509.68775	-239.0188904
4.527919548	148.8202668	646.6636658	22147.47182	-217.6040924
4.561035377	153.5967796	669.8898621	23591.9707	-257.2867523
4.409775064	139.5620453	715.0348511	19477.56449	-182.5618286
4.450611861	146.7271229	672.7519531	21528.8486	-172.063385
3.888965858	100.3788932	869.930603	10075.9222	-126.9098389
4.399093962	132.8518306	935.0303955	17649.60891	-178.2014191
4.103429512	112.115373	767.2565002	12569.85685	-146.1147995
4.178567764	112.1817541	589.4883728	12584.74595	-119.2360901
3.765044228	84.2396873	492.1732788	7096.324916	-92.58572006
4.045182768	105.5143393	828.3894043	11133.2758	-131.562619
4.419559718	149.3648618	706.8317566	22309.86195	-251.7839966
4.558693906	151.0288777	736.8579102	22809.72191	-272.4151337
4.375863654	134.5049461	889.5881042	18091.58053	-195.4046631
4.145785518	122.2636074	712.677063	14948.3897	-166.9128204
3.927953223	94.44722989	557.3815308	8920.279234	-127.3125229
4.383230443	143.0633159	732.3096924	20467.11235	-267.2515503
3.747305293	82.86819017	457.5014343	6867.136942	-121.5962753
4.25071238	117.368895	996.8365173	13775.45752	-131.4481689
4.206473569	120.3058644	598.3126221	14473.50102	-170.1374252
4.308051803	129.5060884	575.5097351	16771.82693	-189.8520355
4.279950685	121.1772633	661.6531982	14683.92915	-181.9049622
3.660357415	82.37198746	625.6722717	6785.144318	-111.1741776
4.352762006	135.5904936	701.903595	18384.78195	-202.3876572
4.411594264	140.1017359	1013.162842	19628.49641	-145.3617401
4.422322057	137.9218837	839.9003906	19022.446	-196.487915
4.104257394	107.1573871	700.9508362	11482.70562	-156.814035
3.798055029	94.03850692	536.1285248	8843.240784	-58.04512215
4.160174545	110.5439101	669.0446777	12219.95606	-156.1495239
4.243904736	120.0582103	681.3115234	14413.97386	-170.7324158
4.466454587	146.9274002	678.3865662	21587.66093	-260.8421997
4.267690002	121.2850955	721.5995789	14710.07439	-158.4066132
4.135946062	115.9055398	671.2733154	13434.09415	-159.4870453
4.237210116	119.566392	878.1983643	14296.12209	-147.1673248
4.498221653	143.3045907	814.9740295	20536.20571	-218.2229797
4.668691878	169.6793961	915.9043274	28791.09747	-298.8147247
4.091799728	122.6204937	767.7485657	15035.78549	-168.4784393
3.961082436	97.0251659	500.6911316	9413.882818	-167.4706772
3.940395763	99.02055307	1089.81424	9805.069931	-128.3904266
4.334379697	127.4667247	747.7976074	16247.76592	-200.5520782
3.653046957	82.99365888	789.6033325	6887.947415	-90.16797943
4.590058321	152.1001794	753.8041382	23134.46458	-193.5992828
4.428409714	136.3702586	678.6505432	18596.84742	-197.6311935
3.815149572	93.20300061	643.1203766	8686.799323	-126.7275734
4.430287186	136.310727	746.861084	18580.61428	-166.8701477
4.121664326	107.8451424	903.2789001	11630.57473	-134.7964935
3.867772797	94.2639375	545.1309357	8885.689913	-159.8548737
4.37487178	133.3370551	642.8861389	17778.77026	-172.3551636
4.4765669	149.3987268	713.6096802	22319.97957	-225.3137314
4.348320162	133.9798331	728.5414124	17950.59567	-191.9502625
4.492540827	139.678732	916.9705811	19510.14816	-143.7427307
3.534334332	78.30743738	644.5661621	6132.054749	-103.79702
4.084065703	104.5665345	816.9236145	10934.16014	-138.6534439
4.585595336	162.3446643	787.7753601	26355.79002	-228.3312469
4.365068509	134.7773959	697.3209534	18164.94644	-182.3804016
4.183513101	114.844127	686.9682617	13189.17351	-154.663681
4.361498337	134.5321119	784.6678467	18098.88913	-199.1177216
4.322728012	126.5991361	692.7165833	16027.34125	-211.7185791
3.836994537	94.79473411	887.9485779	8986.041616	-115.9185593
4.358727263	126.778693	754.8670349	16072.83699	-174.6631104
4.535948666	142.9432219	908.2734375	20432.76467	-182.2439789
4.481561217	137.3480481	945.3323059	18864.48633	-159.5369949
4.440523787	132.4459343	877.1531067	17541.92551	-170.4248352
4.188436872	114.3139	632.6677856	13067.66774	-162.089502
4.526346987	141.9291752	896.0397644	20143.89077	-193.9275513
4.27343892	131.2206884	558.8209839	17218.86907	-264.9736023
4.39557094	130.4389528	974.0218506	17014.32041	-167.8872955
3.991060731	99.20018052	783.1938477	9840.675815	-121.7133667
4.274126966	120.3185828	707.2111511	14476.56136	-154.1638062
4.318956246	131.4532539	600.8210754	17279.95795	-204.9662827
4.314891573	127.6818309	864.7527161	16302.64995	-178.6088867
4.447199752	143.2380523	723.6251526	20517.13964	-207.7642242
4.299738576	126.6998361	659.3204956	16052.84846	-189.2357864
3.966904865	105.6386305	804.7579956	11159.52024	-124.0836754
3.981708677	97.60239131	551.3863068	9526.22679	-127.4446808
4.281786688	120.2819353	780.1604309	14467.74397	-162.1709961
4.557519764	162.8342658	736.754425	26514.99812	-293.9834106
4.363137941	127.0720225	734.8927917	16147.29891	-143.4553375
3.990149888	99.5858785	762.5582275	9917.347197	-144.6527878
4.610962159	155.7920662	985.9009399	24271.1679	-221.9318542
4.264776111	123.378411	610.1186523	15222.23229	-180.0686417
4.265849129	126.8471856	720.2884827	16090.2085	-190.2885376
3.939023962	94.58000901	588.229187	8945.378105	-153.5962616
4.148954365	112.256373	653.9885559	12601.49328	-143.3633179
3.853595988	117.6680679	554.3240967	13845.77421	-212.3392029
4.384853781	129.0111895	721.6184082	16643.88703	-183.5178375
3.972325339	105.4128602	691.1544495	11111.8711	-135.3763809
4.267214569	117.517556	772.9396057	13810.37598	-163.3986206
4.338413655	129.4537164	668.7997437	16758.26468	-172.6177765
4.284094651	123.430772	712.0578003	15235.15547	-135.258139
3.944211096	98.76242399	643.8330994	9754.016393	-137.6876419
4.13900817	107.5307693	864.2140198	11562.86635	-140.7185059
4.199053252	113.4077384	668.6551514	12861.31512	-135.2004395
3.991576119	98.07298402	605.1927795	9618.310194	-126.4470444
4.675470273	161.283968	998.7458191	26012.51832	-216.5773636
4.370656944	129.9194872	703.8471069	16879.07315	-159.9132446
4.259412203	129.0883744	613.4880829	16663.80841	-237.7414276
3.958608494	106.8159303	502.6541748	11409.64297	-178.8863403
4.391146238	128.482331	908.8616943	16507.70937	-159.6559937

log.sigma.3.0.mm.3D_firstorder_Kurtosis	log.sigma.3.0.mm.3D_firstorder_Mean	log.sigma.3.0.mm.3D_glrlm_ShortRunLowGrayLevelEmphasis	log.sigma.3.0.mm.3D_glrlm_GrayLevelVariance
3.14384936	-34.59266452	0.006336025	41.10973047
2.415051438	-32.53805726	0.023745581	31.26973943
2.14299229	-4.378950875	0.016627047	35.44896314
2.288612352	-26.44467789	0.013052711	37.80188669
2.189472022	1.211774704	0.008764616	31.06735821
2.704259168	22.05687362	0.012518419	34.94798062
4.553743421	-1.804170509	0.007600073	18.44430409
3.314675662	-21.04718206	0.003225621	29.55120899
3.389663823	-16.70819779	0.008601464	21.38889095
2.502196811	21.60125114	0.015661335	20.58118631
3.026980964	12.68298225	0.016022242	11.87239164
3.896278847	-2.119372561	0.006431883	19.21030207
2.315186349	-20.31788737	0.010646745	36.06725326
2.120854355	-65.046382	0.017652384	36.85267612
3.318655712	-42.81514923	0.014300517	30.1329717
2.600262367	1.057988445	0.012045821	25.71940665
2.572477435	1.376122089	0.012841951	14.60133945
2.27081065	-66.54980748	0.022599684	32.50226021
3.053049957	-12.95816266	0.02495852	11.46076953
3.891919085	16.16410731	0.003532757	23.17272918
2.237185142	0.536111525	0.016599469	23.18050013
2.316609549	-9.117748201	0.034686684	26.69595171
2.571016202	-15.83425904	0.014775657	23.96271505
4.30325449	-10.38446781	0.010908908	12.00020131
2.430998579	-28.41305636	0.017172798	31.44123194
3.327018937	4.409251587	0.002854124	32.7775244
3.19707208	-35.52352318	0.012488597	30.97708397
3.179021239	-26.25624876	0.010348705	18.97420243
3.6781842	38.57868416	0.038148502	14.73368258
2.689429664	-11.78077919	0.009648034	19.88840869
2.578771531	-23.80451082	0.014194554	23.57445185
2.024863855	-57.96584055	0.011461722	34.14167786
3.277775846	-12.55860732	0.014962123	24.67443975
2.599258317	-13.51995774	0.014096804	22.40748594
3.033817765	-6.863023041	0.004857802	23.93252392
2.466957329	-17.46661325	0.006405917	33.16101867
2.483797813	-46.69217884	0.00334426	46.01837328
2.920419249	-2.18446747	0.010665159	26.77708534
2.531859107	-28.80778595	0.018404536	15.48772965
4.372560985	-9.914289178	0.00153496	17.06612193
2.445569099	-44.35426354	0.009685855	26.25580365
5.344353944	4.47015281	0.007571998	12.17660847
2.251746899	-0.798855303	0.007876816	36.78369628
2.395370566	-27.9728351	0.011882424	29.96251156
4.536367833	-6.340503662	0.018411823	15.32157321
2.450521111	-1.780517624	0.008138154	30.19397697
3.810359653	-5.00114731	0.006496456	19.41385516
2.695589152	-24.27544775	0.011306695	14.47493969
2.519480088	2.867216512	0.024181303	28.66777017
2.737440101	-3.908095481	0.010821825	36.48934035
2.503709971	-42.16068987	0.007403636	29.33236808
3.057952861	21.85935915	0.007983572	31.81805937
5.014863281	-5.040550672	0.009972693	11.39993391
3.41830341	-8.755077719	0.004404484	18.10197381
2.023120007	1.265736181	0.011196659	42.27281814
2.549584669	-14.05621872	0.017919785	29.14495828
2.855114688	-15.05572738	0.007518144	22.07709709
2.04951512	-27.81254585	0.006992616	29.18260361
2.359058034	-47.88673078	0.010494636	25.87891248
4.952310653	-3.364961303	0.004277671	16.30672642
2.69848212	-8.970391258	0.00749322	26.46095455
2.758353187	15.38753303	0.005034203	33.24043377
2.769944962	11.17946725	0.003909336	31.25012397
2.875214328	2.779156498	0.005328401	28.32147606
2.661307551	-6.063833589	0.012579258	21.40601472
2.695757254	-6.274383041	0.005653526	32.67174067
2.163429893	-65.78186512	0.014723555	27.52617133
3.1862005	-18.35987322	0.00460648	27.96290843
3.358418995	-4.666506619	0.004293718	16.42367177
2.496487462	16.38835563	0.012666736	23.42451417
2.007014757	-28.41210582	0.013134599	27.72339311
2.739950647	-8.267714425	0.003983455	27.26633208
2.143248999	-34.15264442	0.009531819	33.13035685
2.233091582	-34.17720553	0.01709926	26.10406289
4.830592916	-5.966389307	0.008312445	19.55420525
2.526371787	7.393846061	0.012974972	15.51046784
2.658493483	-0.079607709	0.006436158	23.47536491
2.549722039	-46.13969261	0.010353069	42.60288287
2.853721513	29.75291397	0.010723126	26.43942823
4.179876684	-29.16164783	0.006987964	16.82087656
3.190116254	-36.26835654	0.005433151	39.79418029
2.39698187	-2.684815383	0.017854656	24.52944697
2.40801586	-29.76540759	0.010218093	26.94235399
2.857804866	-35.89574846	0.009580849	14.77385146
3.110535956	-1.534522887	0.0113022	21.41609257
3.709562622	-14.25413115	0.014347684	23.05057195
2.574743666	-26.09621047	0.007331606	27.18852813
3.756284157	-2.37729663	0.009182123	19.82161161
3.035709603	-14.39031895	0.00920696	23.16383564
2.224612882	2.590943882	0.008462425	26.73614325
3.111734772	14.02307522	0.007911146	24.96842717
3.633116537	-17.39523181	0.010842747	16.82263035
3.135664418	-7.331130367	0.006011513	19.07404083
2.68428811	11.84402887	0.013147574	21.01082022
2.417173891	-0.973792396	0.012386043	15.65178622
2.360893236	-9.341590434	0.004840223	42.0028561
2.339917652	6.779086032	0.016378027	27.14624982
2.573778381	-57.76492565	0.01214811	26.60431846
2.712629841	-13.17578738	0.026119859	18.46837745
2.859487523	16.58333563	0.003203436	27.23182107

log.sigma.3.0.mm.3D_glrlm_LowGrayLevelRunEmphasis	log.sigma.3.0.mm.3D_glrlm_GrayLevelNonUniformityNormalized	log.sigma.3.0.mm.3D_glrlm_RunVariance	log.sigma.3.0.mm.3D_glrlm_GrayLevelNonUniformity
0.006611457	0.047026544	0.07799083	63.48080818
0.024707862	0.057490787	0.066518316	18.80396318
0.017303354	0.04691541	0.075856225	39.31371057
0.013543627	0.04700859	0.052620263	14.39779019
0.009245098	0.051654222	0.083533054	68.04321523
0.012723787	0.051000068	0.071010776	11.61737472
0.008446827	0.07958983	0.441625242	686.7380574
0.00346205	0.054395605	0.154931305	296.9557566
0.009425221	0.066428043	0.213683762	247.8016396
0.016304475	0.06157568	0.079873391	43.37404248
0.017254987	0.085966752	0.145460047	119.8258924
0.006999204	0.071348637	0.223042964	211.6604925
0.010947431	0.053767444	0.078394934	40.77948127
0.018399831	0.046073385	0.064102786	28.06457172
0.015050663	0.055282686	0.116249844	79.66518633
0.013170311	0.063216049	0.298987435	310.7922968
0.013757394	0.072766199	0.119936294	58.10726824
0.023518336	0.054032265	0.071643944	20.81587397
0.026986628	0.085590226	0.159786882	82.88993332
0.003781036	0.06249209	0.161541315	512.4208734
0.018074457	0.059538075	0.127754307	111.9848441
0.037363607	0.056253454	0.076926428	26.12906099
0.015594931	0.05810213	0.095387561	29.97732536
0.012130148	0.09558221	0.295179028	289.6420438
0.018284271	0.052554123	0.23369279	56.2814596
0.003045408	0.055374828	0.143416451	127.9273143
0.013202952	0.052992676	0.097992282	98.1074648
0.011030053	0.066043911	0.120908285	135.3615828
0.04041407	0.086334933	0.086028574	11.74253131
0.010248132	0.06237304	0.114249461	92.00909779
0.01531084	0.058247864	0.136561875	210.7306259
0.012090734	0.04938779	0.07050782	36.530769
0.016023061	0.059489432	0.159596548	124.418788
0.015129335	0.064334421	0.185290943	137.5224171
0.005244962	0.059985857	0.159929136	275.53739
0.006736308	0.049293079	0.102709459	88.4359969
0.003445829	0.043726884	0.06440611	62.58363039
0.011664645	0.0670005	0.406056049	322.3992112
0.019587493	0.072843085	0.106844753	37.5998745
0.001694714	0.077936175	0.263975508	631.513495
0.010334672	0.054801357	0.09903293	67.85112753
0.008361549	0.098254284	0.285331803	475.666524
0.008329732	0.045823031	0.092179778	103.5875285
0.012502022	0.05084391	0.078302342	67.58204621
0.020184607	0.085569972	0.300380265	113.2284149
0.008668813	0.051190527	0.101173523	113.5085481
0.007052923	0.066555923	0.170249038	316.8503889
0.01195083	0.080359417	0.128844362	35.81701401
0.025445621	0.053096084	0.062525722	19.58756297
0.011164898	0.054589295	0.07439467	23.15472828
0.007803953	0.055696575	0.086951449	66.78317296
0.008333048	0.050801358	0.071584612	132.7773875
0.011356684	0.102802775	0.474065554	651.7985623
0.004653655	0.068204038	0.127197003	101.355012
0.011717405	0.045101953	0.067336261	27.34619894
0.018931246	0.05428134	0.080921938	59.40498535
0.008117802	0.061709062	0.187297461	220.8509426
0.007393922	0.052913525	0.097297692	101.1285006
0.011063899	0.055126171	0.077025797	39.31970419
0.004813364	0.083298415	0.390886108	747.0582115
0.007862343	0.054575044	0.096041518	67.03870652
0.005258963	0.04874059	0.094258633	111.589818
0.004156222	0.050633678	0.137903882	378.2813663
0.005574807	0.053111661	0.089722564	103.1741459
0.013270699	0.061656413	0.100891225	38.44622691
0.005906119	0.048983969	0.074143721	157.134784
0.015311872	0.057271761	0.064351058	21.94769939
0.004861579	0.055430595	0.096147521	260.571504
0.00467721	0.072349498	0.18718589	480.306295
0.013260324	0.057824664	0.087512261	114.087488
0.013727813	0.053470555	0.08646962	24.60227012
0.004285827	0.056925384	0.177321668	253.074655
0.010028653	0.050102563	0.080652234	62.68340826
0.017856591	0.055452964	0.075152358	49.40397814
0.009114441	0.078379152	0.261635709	274.615439
0.013605792	0.070807114	0.1016627	59.94599459
0.006798271	0.058080521	0.092704084	118.5548477
0.010588406	0.050713556	0.057558582	15.57826739
0.01116326	0.056048029	0.078127051	68.86383664
0.007501353	0.074474332	0.159059951	119.7783161
0.005665417	0.048168382	0.080128825	63.21490076
0.018913492	0.058722485	0.082736334	45.25559616
0.011007031	0.056523628	0.206131202	114.3320891
0.010365147	0.074370618	0.128522304	127.1641992
0.012163973	0.064934878	0.1903168	155.7541595
0.014828356	0.089119006	0.117658499	43.36757235
0.0076968	0.053757147	0.090632231	65.25670711
0.010281304	0.073178122	0.34598282	234.9829687
0.009664001	0.05950483	0.129555547	92.29579014
0.008870334	0.054863535	0.085128165	69.88376425
0.008344545	0.060675527	0.107330355	44.53237073
0.011898047	0.075797735	0.234738125	166.4089052
0.00638075	0.064406153	0.114386559	264.6202484
0.014093629	0.060486137	0.125364651	189.4223189
0.013261737	0.069071838	0.115232907	207.8801419
0.005042431	0.043637063	0.074900521	97.58950845
0.017283467	0.052800732	0.098112743	119.5401961
0.012621366	0.059382838	0.056747209	17.59755212
0.027635142	0.076078552	0.079190652	19.06647805
0.003399479	0.05464446	0.133275875	437.7501873

log.sigma.3.0.mm.3D_glrlm_LongRunEmphasis	log.sigma.3.0.mm.3D_glrlm_ShortRunHighGrayLevelEmphasis	log.sigma.3.0.mm.3D_glrlm_RunLengthNonUniformity	log.sigma.3.0.mm.3D_glrlm_ShortRunEmphasis
1.224268114	356.189695	1188.213468	0.95085881
1.195757903	195.9815683	292.0595113	0.955970838
1.212408194	230.8925883	746.6738398	0.955015455
1.158699638	270.0734908	277.7452108	0.962583303
1.231829799	235.0472328	1159.953298	0.95115949
1.205953591	318.9088486	203.0941804	0.954887863
1.951591146	176.4189191	6284.297872	0.876469347
1.393595853	392.8672418	4537.561217	0.928436415
1.526828949	175.6873953	2977.818555	0.911699172
1.230713476	165.4965414	617.4288893	0.949185956
1.400502799	105.1233832	1142.831437	0.921789036
1.549790884	211.3727364	2342.686136	0.908095035
1.224575875	293.7304671	668.5037457	0.95118456
1.182998215	224.9830518	548.6970335	0.959801667
1.313986459	187.5083922	1226.747751	0.937653654
1.688418613	168.192233	3808.443212	0.899302333
1.345836273	139.1932159	661.6103446	0.927379088
1.200271065	193.023321	344.8051453	0.957296289
1.444445318	85.54720432	776.2154715	0.913010171
1.41925545	361.2688176	6709.626785	0.922290644
1.351629726	147.7440141	1569.099356	0.92969481
1.219294577	147.3783225	410.6488364	0.952385869
1.268513547	181.2779532	445.5393454	0.943218314
1.718887464	122.4625913	2265.822163	0.886250188
1.558251105	193.0053508	854.2707254	0.91126839
1.373048574	479.338278	1931.602859	0.930419761
1.27268199	195.9201187	1598.964993	0.943166045
1.33231635	166.1364647	1723.044342	0.933044254
1.245130656	93.46162811	119.2360436	0.948303099
1.308739301	181.4300083	1259.663865	0.938678258
1.367526858	149.3696939	3008.867086	0.928427532
1.207413004	256.8211292	655.1810619	0.95325927
1.410548034	160.3547668	1722.425276	0.924645466
1.4687587	153.591457	1727.137182	0.917348595
1.411701314	272.1182824	3780.752376	0.924618158
1.27389881	331.5444953	1559.548124	0.945619723
1.184278078	576.4215443	1292.490581	0.959935076
1.886676337	185.6521774	3575.622541	0.883795224
1.289044289	137.9975532	444.8193029	0.942027028
1.632350567	635.1964255	6254.91235	0.899229693
1.271956182	207.5340972	1070.202025	0.943959831
1.671604041	158.7530895	3708.65889	0.896006217
1.244196374	295.6455639	2009.730802	0.95241349
1.220842987	203.7895165	1174.051611	0.952338932
1.699429318	111.3921346	1016.073707	0.896144266
1.277059678	231.2547274	1919.064171	0.943784466
1.437468136	211.1101792	3879.874042	0.920514923
1.348831632	162.0982336	374.5279709	0.931983151
1.187083955	159.5842739	329.4570569	0.956638516
1.212693544	319.5178651	375.9842587	0.953453115
1.241050571	242.9630811	1051.694445	0.949460655
1.208303837	258.8369223	2314.396605	0.95341078
2.079933358	121.4151295	4382.817082	0.855742407
1.353067026	322.1603175	1239.259888	0.929118887
1.193170595	275.2091273	542.5233521	0.957367818
1.23695139	165.4669703	953.2889486	0.947167582
1.453016181	203.4079699	2949.70264	0.924143639
1.269513393	254.7946194	1654.445549	0.94422767
1.222595883	204.5684921	627.5414913	0.950895703
1.88234782	254.1352536	6557.318576	0.877777973
1.269425517	243.035636	1060.458669	0.943330842
1.255179782	343.1150371	1997.089935	0.947540245
1.355989346	367.9716107	6290.763152	0.933317381
1.255311791	319.9348307	1683.322585	0.945026553
1.283263015	188.2603094	535.8380738	0.940971138
1.212605688	316.2364468	2838.838437	0.953177302
1.188008317	210.4372756	343.2130312	0.957636398
1.268675722	329.2051102	4064.393161	0.943822971
1.472134599	262.8354925	5363.233293	0.916796979
1.248177743	185.3912267	1718.215626	0.94674316
1.233972095	198.7763899	407.4868456	0.952414049
1.437525841	335.2123188	3657.545182	0.924140784
1.221135271	227.6498462	1110.598007	0.954025206
1.213234093	168.0859579	789.5267345	0.953719044
1.629214443	181.8401918	2713.049764	0.900133785
1.282759006	145.6736285	728.3721277	0.94144892
1.266176967	277.9223054	1759.923979	0.942710367
1.16485763	374.1300092	279.2765692	0.963351873
1.225362186	231.3359554	1080.002861	0.95044391
1.417016248	209.4768854	1314.171939	0.92154171
1.229998344	352.3060413	1152.032664	0.949787209
1.233452114	169.9855223	676.7352166	0.949871413
1.491636414	192.7631162	1658.937183	0.921611382
1.359130719	152.7481122	1418.141712	0.927207188
1.480604457	164.8455446	1931.950772	0.915588878
1.317733923	252.5593349	414.9331729	0.937698861
1.254964148	256.5912198	1057.622013	0.946488471
1.790341178	160.3896172	2401.446997	0.886588923
1.3359748	232.0933214	1317.495425	0.936637439
1.238464755	228.5530033	1116.877658	0.949404263
1.310493888	264.6296834	617.830339	0.933426425
1.57687417	145.9896662	1724.967412	0.905199374
1.317097516	235.9992591	3473.428796	0.935120847
1.340742808	155.9643552	2629.640258	0.932212567
1.325280735	139.4879418	2527.267725	0.93236802
1.212801577	389.5393862	1983.444759	0.953821294
1.274846483	181.5365593	1953.017836	0.942561581
1.174433608	276.6922852	265.9965816	0.958454982
1.226733139	154.8754805	220.9768983	0.951084917
1.349232603	403.5171475	6738.363141	0.933155174

log.sigma.3.0.mm.3D_glrlm_LongRunHighGrayLevelEmphasis	log.sigma.3.0.mm.3D_glrlm_RunPercentage	log.sigma.3.0.mm.3D_glrlm_LongRunLowGrayLevelEmphasis	log.sigma.3.0.mm.3D_glrlm_RunEntropy
443.1182779	0.934796505	0.007836388	5.001318589
247.5847294	0.942363112	0.028685894	4.609798319
290.6264645	0.939461883	0.020313461	4.832958869
314.9019612	0.951266125	0.015545536	4.784214324
293.7255764	0.933598648	0.011572954	4.76005191
406.1636875	0.940877305	0.013652254	4.70398307
373.1408257	0.817111534	0.014592911	4.780787475
565.9031391	0.899536028	0.004733918	4.921555054
271.0801202	0.874923294	0.014056931	4.727597304
210.2130104	0.932959755	0.019191058	4.52715415
152.9494915	0.895392525	0.023445207	4.294794071
339.6818631	0.870199363	0.010313844	4.698700855
391.4557325	0.935027981	0.01233211	4.761379781
264.6939086	0.945891065	0.021776636	4.827571292
243.1741938	0.914871795	0.018705622	4.819058072
294.99287	0.852081275	0.020323086	4.865916724
195.2775709	0.904259953	0.018118854	4.404992236
233.3567036	0.941884521	0.02771884	4.659264731
129.4027242	0.885037266	0.037005225	4.309882366
546.3232012	0.892803592	0.005112472	4.81287463
206.4401422	0.905123269	0.025545577	4.681323676
178.9578248	0.936414392	0.04965471	4.609615912
234.8371684	0.924455473	0.019386763	4.645555738
223.8456743	0.841235217	0.019372342	4.444790468
319.597483	0.871523428	0.024342463	4.955642159
668.0399496	0.903575298	0.004066952	4.910426286
248.9401879	0.923806234	0.016537088	4.825040525
224.870786	0.909590631	0.014356798	4.578872036
115.0316486	0.930979979	0.050091366	4.108569188
242.5920413	0.916149068	0.013238286	4.590868756
208.0629504	0.902507819	0.020932432	4.742845932
314.3901779	0.938695822	0.014866693	4.773155793
228.2896086	0.895503212	0.021395507	4.796643777
237.9007381	0.884440197	0.020769585	4.70648602
392.4488769	0.894768392	0.007318476	4.780504832
444.1134781	0.925219185	0.008320295	4.88603181
722.7498383	0.946530322	0.003902957	4.955067574
374.8034836	0.824970597	0.018402856	4.946171932
182.8619601	0.921565934	0.024948862	4.343429383
1124.834136	0.857048636	0.002681923	4.662235137
264.7159911	0.923995408	0.013394911	4.725809109
279.0590699	0.852324509	0.013252474	4.40183973
380.3516483	0.935165744	0.010653047	4.913929811
247.2467537	0.936078007	0.015396214	4.768574961
193.7423898	0.849268341	0.031014459	4.539535951
294.5529003	0.923461378	0.011235002	4.832213779
311.6480042	0.889639607	0.010050548	4.683289709
228.3026885	0.907410309	0.015318348	4.310797172
190.0297562	0.943537281	0.031034277	4.645745863
411.5224618	0.938223281	0.012622978	4.780535767
297.9625143	0.931564162	0.009699379	4.71311943
317.0225354	0.938461538	0.009890518	4.840774091
279.1837863	0.793216187	0.021053817	4.537354468
469.3207464	0.90499391	0.005884518	4.573484349
335.9224701	0.942935758	0.014182758	4.873232661
208.1580412	0.930599163	0.023542861	4.728420065
302.6290601	0.891196475	0.011492159	4.728854822
332.1260105	0.924602732	0.009363546	4.756435939
251.2961828	0.934872467	0.013559778	4.656438251
520.5941598	0.822101703	0.008479207	4.713799295
320.187057	0.924234531	0.009582005	4.755151635
449.8776364	0.92872523	0.006334711	4.919102538
519.0774737	0.907204649	0.005441455	4.973607517
423.9669071	0.926820317	0.006701333	4.829612317
247.7375095	0.921145324	0.016491452	4.581776114
397.3714179	0.937692533	0.007029383	4.87637712
261.1567507	0.943918151	0.017784721	4.543775309
426.0938728	0.924438393	0.006080922	4.812673363
407.3871105	0.884092865	0.006838735	4.581482808
245.4636816	0.928980154	0.015955288	4.654942735
249.4837281	0.935115697	0.016588089	4.621109976
503.4081639	0.892143993	0.005991205	4.869441654
277.0938139	0.937136272	0.012406567	4.778828662
205.3976411	0.937813765	0.021261554	4.631665927
303.5891575	0.857266632	0.013795602	4.688341355
197.2113718	0.922071393	0.016577171	4.38620146
375.4868863	0.924051955	0.008393865	4.688033072
457.8779788	0.9509407	0.011565224	4.785290551
295.0971174	0.934308277	0.0130889	4.713763587
305.674811	0.893290598	0.010140965	4.534365809
440.4208047	0.933362512	0.006727163	4.971269211
218.9390203	0.933041535	0.02353273	4.60666941
292.3525517	0.885828895	0.015670882	4.812637587
210.618947	0.903084237	0.014211162	4.434306887
251.3465353	0.882844596	0.016783379	4.739645775
374.8012256	0.915399102	0.016989903	4.291856922
326.9479497	0.928016937	0.009388892	4.764338608
292.9131651	0.835385418	0.017274283	4.767427141
323.7778346	0.911680912	0.011901351	4.723703404
296.471825	0.931798998	0.010796613	4.694687554
367.499202	0.912839648	0.010321696	4.725493355
235.5084406	0.866356787	0.017915773	4.60220254
325.2646458	0.912788611	0.008173669	4.606417858
215.3238592	0.908656636	0.01870663	4.673229809
190.4861184	0.910167244	0.017451698	4.46064611
493.3843558	0.938048529	0.00594717	5.017186052
238.6455334	0.923327896	0.021497318	4.774626379
338.4249669	0.946669943	0.014574562	4.515627438
198.2133189	0.934845006	0.034251621	4.261290797
584.5625385	0.907777739	0.00441085	4.880477492

log.sigma.3.0.mm.3D_glrlm_HighGrayLevelRunEmphasis	log.sigma.3.0.mm.3D_glrlm_RunLengthNonUniformityNormalized	log.sigma.3.0.mm.3D_glszm_GrayLevelVariance
371.9779765	0.879407643	52.08871222
205.2131558	0.891682856	31.58986686
241.0521671	0.889620348	35.69779878
278.5138833	0.906227142	36.70234552
245.4441332	0.880104578	30.82829498
334.9221169	0.889824057	41.65538511
199.2216115	0.727576644	30.65032796
419.4943696	0.830426547	38.82938098
189.309754	0.796520946	26.80138414
173.7038163	0.875620325	25.15250318
113.0501016	0.817526611	17.41682683
229.8612576	0.788576471	29.98251834
311.1850511	0.880478298	36.68992255
232.3963227	0.900162014	35.8781742
196.8000032	0.850302944	38.58036446
184.6578882	0.772664162	28.65529906
149.0331318	0.827522519	18.13517381
199.984598	0.894454305	31.35653878
92.75174611	0.798946263	16.84647199
390.4724274	0.817304428	37.0263762
157.5326523	0.833030943	23.68877905
153.1210667	0.883222445	24.70419988
190.6115679	0.862467389	27.2124115
136.2342899	0.746148948	19.05492469
210.1993148	0.796085784	35.50776819
509.3471817	0.834943673	43.40158196
205.0331535	0.862466672	37.15755173
176.0510581	0.839864469	24.86949072
97.24110187	0.87504699	20.56629013
191.6627544	0.852801579	23.61368066
159.0124842	0.83037631	25.60607445
267.7033073	0.884844182	32.89644095
170.9244091	0.822472275	30.8110114
166.170011	0.807230005	25.29716809
290.8867672	0.822297365	31.04333242
350.2950951	0.868182635	34.49303151
602.2610478	0.901426479	45.52940392
207.6496892	0.742303598	31.32662455
145.5020707	0.860238133	18.98769778
702.2596051	0.770814524	27.81227876
217.4909187	0.863751026	28.45392746
174.8863685	0.764840173	23.18707239
309.8224127	0.885504654	33.31677644
211.7085363	0.882675485	32.43058794
122.0227862	0.76605192	24.92415621
242.1367877	0.86422382	31.79075911
226.6165259	0.813817792	27.17415099
172.9819112	0.83867523	21.543936
165.3528393	0.892361547	31.19673469
335.7501788	0.885562469	43.25186082
252.6451021	0.876427499	31.73977165
269.6641438	0.884935382	38.98969084
139.9067783	0.689858867	20.37167501
346.7262678	0.832127972	26.31301649
286.3521551	0.894282089	38.13004329
173.3176113	0.870510795	32.02434276
217.6848692	0.822418849	26.64059627
268.3049118	0.864713963	30.41418121
212.9718246	0.879183854	27.42614258
286.2802862	0.72998388	27.78927929
256.5633887	0.862393251	33.74034706
361.5302328	0.871783338	38.17293075
392.1712792	0.841074523	39.29531166
338.4225679	0.865920927	34.38650276
198.5873724	0.857779694	28.23763746
330.9411257	0.88443263	38.51705824
219.5515334	0.894939343	25.03292872
346.2604146	0.863694818	35.18493158
284.644215	0.806341721	23.40216336
195.9978961	0.870003795	26.72313457
207.6049289	0.884043822	28.64898049
360.0962293	0.821848463	31.77101762
236.2299848	0.886973138	32.95569457
174.7465066	0.885739038	28.64311224
198.4081092	0.773085667	29.96472818
154.6131416	0.858745898	20.80532784
294.9806877	0.861168624	27.43605269
388.9507727	0.908537857	43.68661479
242.8034307	0.878319126	32.07545927
224.6637185	0.815795616	28.00296801
368.2709209	0.876988214	51.27087365
178.571604	0.877209731	27.90430801
206.4849803	0.817910593	28.32448832
162.610576	0.827887574	17.98742977
177.9168918	0.803986439	27.87076187
272.1824039	0.851369177	27.76549587
268.8242376	0.870048601	32.82492218
176.8801062	0.746951908	27.09731933
246.5452599	0.848409885	31.74201304
240.5142628	0.876144611	27.21907711
282.8016939	0.840611035	32.32075681
158.5664494	0.783590915	24.06278857
251.3221272	0.844464501	26.11853619
165.9645986	0.838379235	25.31219422
148.3989513	0.83847077	18.66032022
408.0211366	0.886259159	45.96131344
191.5595494	0.861195263	28.57855939
288.5996391	0.896798995	25.74995435
162.3412196	0.880566602	20.04696746
432.6798406	0.840348786	35.52712438

log.sigma.3.0.mm.3D_glszm_SmallAreaHighGrayLevelEmphasis	log.sigma.3.0.mm.3D_glszm_GrayLevelNonUniformityNormalized	log.sigma.3.0.mm.3D_glszm_SizeZoneNonUniformityNormalized
286.8342133	0.038843971	0.401749921
131.4218631	0.051117686	0.373109796
178.8357736	0.047688434	0.370826732
227.7029566	0.047548186	0.464923469
161.474328	0.050056357	0.336761186
166.3540369	0.047479912	0.348916484
146.9508954	0.051325134	0.317948965
323.5410465	0.04514946	0.357666707
161.2070911	0.053688238	0.337992302
112.1662316	0.055356432	0.299752059
72.74912052	0.066746578	0.268122981
184.8262171	0.051662656	0.35546731
156.9633942	0.046380293	0.338897056
188.2481845	0.048221569	0.391759133
180.0914283	0.045175058	0.413230613
126.8190142	0.051778192	0.338905528
90.48209981	0.067418567	0.25052605
134.1306486	0.053126531	0.336555102
63.49189501	0.069213604	0.263465682
248.4783767	0.046912082	0.341415286
103.834267	0.056801685	0.281516168
116.1323126	0.057037478	0.347769185
152.0982137	0.054768846	0.33819242
107.0379546	0.064957667	0.283211946
159.0843317	0.046860232	0.353305175
411.1167175	0.042933147	0.361056149
170.7317042	0.04669817	0.363059512
138.3657177	0.056797757	0.311912522
88.99783731	0.063614744	0.395362663
131.070779	0.057468514	0.306232414
119.3166228	0.054623372	0.320782999
177.0718318	0.049998249	0.343580407
135.8905207	0.051284472	0.343552188
120.7990649	0.05507577	0.343590948
231.2476029	0.049353755	0.359351719
227.123555	0.047434211	0.362977839
362.9805724	0.041742187	0.400058062
150.1976428	0.049573543	0.34617111
104.7669617	0.069821454	0.275947162
501.6307225	0.053465024	0.344359128
171.9969757	0.052829067	0.346324966
128.9868832	0.059831532	0.297663754
202.7424909	0.047968834	0.384412034
166.6899443	0.049143753	0.374514659
100.4120819	0.059218935	0.290433136
186.1102718	0.048934876	0.362060628
184.66501	0.056271512	0.336739304
107.3982281	0.064064	0.25888
129.5029487	0.049991837	0.376979592
186.2688237	0.044482514	0.297849716
217.83228	0.050111688	0.402820176
208.7052248	0.045017534	0.374069068
111.0644412	0.063127556	0.305297097
224.1907646	0.057265625	0.348463542
198.287358	0.04506661	0.375130032
128.9122817	0.049992266	0.336494408
160.4034333	0.053030074	0.329907838
175.3040826	0.050969798	0.319986683
171.3860884	0.053951688	0.391353141
213.507977	0.054556416	0.315670299
184.5402281	0.048910305	0.357386936
231.1684688	0.044903628	0.359179131
262.5395036	0.043743358	0.355798468
203.7540436	0.049149338	0.310561643
138.2881385	0.053343351	0.334949799
232.6703342	0.044371258	0.366295805
137.1668268	0.056495351	0.356146694
244.5113901	0.047654343	0.333862208
186.5753601	0.058940966	0.297875412
119.0442221	0.053236282	0.334967852
141.5563311	0.05327909	0.332712479
266.9334242	0.049270761	0.365833908
186.5944429	0.048307931	0.364596856
136.2985615	0.052705465	0.425405988
166.9168372	0.054157696	0.338804263
83.4573603	0.061085118	0.25071355
166.163599	0.053827239	0.292326323
244.2357963	0.044784682	0.407436624
149.428363	0.050108813	0.299080482
187.1921515	0.056656882	0.332286712
285.0921318	0.0392813	0.396864738
114.4628274	0.052528616	0.363225806
163.1641329	0.053429498	0.392100016
126.2230055	0.067184469	0.284934599
132.2448939	0.05211109	0.320217677
127.2035506	0.058819731	0.315082645
194.5529222	0.048305672	0.346018613
158.2272223	0.053591089	0.339467051
167.9903994	0.049458153	0.345169122
147.9605529	0.053813648	0.320131933
170.5501275	0.049602387	0.323185346
142.1110437	0.057213341	0.339338796
165.0762707	0.05453579	0.321157332
108.34567	0.054676396	0.287628391
105.8660211	0.064734605	0.310050587
273.0674423	0.040775418	0.37520488
118.3505141	0.052891745	0.297982819
196.4423969	0.057341125	0.380934989
94.1298202	0.068232249	0.270340237
257.3331644	0.046676634	0.352266983

log.sigma.3.0.mm.3D_glszm_SizeZoneNonUniformity	log.sigma.3.0.mm.3D_glszm_GrayLevelNonUniformity	log.sigma.3.0.mm.3D_glszm_LargeAreaEmphasis	log.sigma.3.0.mm.3D_glszm_ZoneVariance
239.442953	23.15100671	29.88255034	24.01249944
58.20512821	7.974358974	10.32692308	5.37915023
139.4308511	17.93085106	12.18617021	6.558171118
78.10714286	7.988095238	7.476190476	3.802579365
189.2597865	28.1316726	24.03024911	17.72675435
38.72972973	5.27027027	9.711711712	4.958526094
675.0056524	108.9632595	2001.277909	1976.634611
678.1360759	85.60337553	112.7310127	102.4883121
382.945278	60.82877317	222.5631068	208.4127604
83.63082437	15.44444444	21.37275986	14.0498195
102.1548556	25.43044619	77.42782152	60.74885128
322.7643172	46.90969163	326.6795154	312.6004907
113.1916168	15.49101796	14.66167665	8.765794758
118.3112583	14.56291391	9.145695364	4.59835095
238.0208333	26.02083333	43.55729167	36.08048503
475.8233618	72.6965812	798.8105413	781.9678188
54.6146789	14.69724771	64.15137615	47.74516034
58.89714286	9.297142857	12.41714286	6.954906122
60.07017544	15.78070175	131.9122807	108.8891197
856.2695375	117.6555024	277.7515949	264.3451353
157.0860215	31.6953405	59.2688172	45.40053442
71.29268293	11.69268293	14.05853659	8.204497323
66.28571429	10.73469388	22.78571429	14.68065389
192.0176991	44.04129794	714.0722714	685.92596
130.0163043	17.24456522	100.6086957	89.47341682
288.122807	34.26065163	88.86716792	78.60791075
265.7595628	34.18306011	30.07377049	22.57874227
212.7243402	38.73607038	60.47067449	49.5574363
22.93103448	3.689655172	15.37931034	9.042806183
155.8722986	29.25147348	40.82121807	30.81623894
367.9380994	62.65300785	86.6233653	74.40690813
116.1301775	16.89940828	13.70414201	8.268898148
239.7994269	35.7965616	115.5229226	104.3320765
221.9597523	35.57894737	104.2337461	90.25818564
546.5739645	75.06706114	106.0282709	94.63931001
275.8631579	36.05	26.57236842	20.06315616
285.6414566	29.80392157	9.254901961	4.770472895
484.9857245	69.4525339	862.8686652	845.6055596
45.80722892	11.59036145	32.3373494	20.95688779
725.2203229	112.5973409	1022.67189	1002.549892
156.8852097	23.93156733	47.31567329	38.56556974
359.5778146	72.27649007	954.3758278	932.3293825
396.328807	49.45586809	15.3385063	9.838093778
220.5891341	28.94567063	22.19694397	16.38466971
94.39076923	19.24615385	312.9107692	289.9887716
330.9234136	44.72647702	32.53063457	25.62995394
461.3328467	77.0919708	151.5525547	136.2969732
32.36	8.008	54.84	39.410816
65.97142857	8.748571429	10.34857143	5.356538776
54.80434783	8.184782609	13.31521739	7.280718336
210.6749522	26.208413	37.91778203	31.86223033
436.9126712	52.58047945	16.36900685	10.68355857
387.1167192	80.04574132	3301.323344	3261.756466
167.2625	27.4875	75.4875	63.78539931
112.1638796	13.47491639	10.04347826	5.418820819
145.7020785	21.64665127	19.90762125	12.5313165
393.5800503	63.26487846	122.4015088	111.0751527
231.9903448	36.95310345	29.81103448	21.68263686
127.9724771	17.64220183	15.86238532	10.41794088
682.4791859	117.9509713	2111.558279	2086.172902
180.1230159	24.65079365	22.08928571	15.13601899
355.9465187	44.49949546	25.03834511	18.85125361
988.7639439	121.5627924	140.027348	131.2483564
228.5733696	36.17391304	22.88586957	14.77575614
81.05785124	12.90909091	28.21900826	20.39288641
532.2278045	64.4714384	13.3964212	7.853037762
62.68181818	9.943181818	10.51136364	5.189953512
613.6387378	87.58868335	38.71001088	31.05596783
500.1328172	98.96188207	443.6581298	423.6619453
262.6147959	41.7372449	19.10969388	11.7700177
67.20792079	10.76237624	13.02970297	7.097343398
561.5550489	75.63061889	206.523127	195.9934191
201.6220615	26.71428571	18.14647378	12.3185714
179.521327	22.24170616	17.50236967	12.43453651
326.946114	52.26217617	557.7139896	539.8031131
69.44765343	16.92057762	28.80866426	17.82554184
215.1521739	39.61684783	26.10190217	17.09374815
68.04191617	7.479041916	6.113772455	2.372906881
151.0356436	25.3049505	14.50891089	7.728301147
154.1810345	26.2887931	102.5818966	87.5328478
224.6254417	22.23321555	31.75618375	25.5854362
112.6	16.28387097	17.49677419	10.39712799
301.1328125	41.03385417	65.2734375	56.44449192
142.1823647	33.5250501	114.1663327	99.77502902
233.7589041	38.04109589	188.5164384	174.6841527
55.45454545	10.35227273	42.90340909	33.80084582
172.3172691	24.0562249	25.35341365	18.45487976
287.1891253	45.33806147	692.106383	671.5466973
201.5787671	28.88356164	52.75513699	44.27148328
159.4257028	26.79919679	20.52811245	12.99319769
81.11952191	12.4501992	44.33466135	34.07425279
208.0146819	35.07177814	310.2479608	293.1868834
440.9490168	74.8776402	50.5600874	39.81334222
290.2170466	55.16848365	54.33696729	42.67294842
295.78826	61.75681342	72.53563941	60.51931512
387.5866409	42.12100678	13.0571152	7.730984014
248.2196879	44.05882353	35.45738295	26.79273486
56.37837838	8.486486486	8.560810811	4.088157414
28.11538462	7.096153846	12.11538462	5.474852071
984.9384835	130.5078684	149.2947067	139.334768

log.sigma.3.0.mm.3D_glszm_ZonePercentage	log.sigma.3.0.mm.3D_glszm_LargeAreaLowGrayLevelEmphasis	log.sigma.3.0.mm.3D_glszm_LargeAreaHighGrayLevelEmphasis	log.sigma.3.0.mm.3D_glszm_HighGrayLevelZoneEmphasis
0.412742382	0.133568801	8473.062081	423.6208054
0.449567723	0.280184348	2033.679487	206.1346154
0.421524664	0.186691546	2717.111702	264.3829787
0.52173913	0.110149799	1709.613095	308.7559524
0.398299079	0.30404225	3479.033808	268.1637011
0.458677686	0.061105615	2996.441441	313.7567568
0.201442262	11.19752452	366174.3217	235.2359868
0.3124588	0.335769472	41337.10918	495.3818565
0.265837635	1.708869368	33967.32921	251.9602824
0.369536424	0.223855834	3202.344086	189.1577061
0.244858612	1.094836108	7116.548556	132.9370079
0.266510126	1.7537986	65558.78855	283.2059471
0.411837238	0.083382901	5558.191617	279.9191617
0.468944099	0.162054445	1500.407285	272.4470199
0.365714286	0.502609695	5405.819444	253.9461806
0.243665394	5.824671765	121138.0848	205.1438746
0.246885617	0.904642693	7303.004587	172.6697248
0.427872861	0.318465007	1717.611429	221.9542857
0.208409506	2.416394561	10356.40789	116.7587719
0.273113362	0.78057379	104962.6539	412.5637959
0.26852743	1.717326941	6766.752688	181.8261649
0.413306452	0.747944972	1360.712195	179.3121951
0.35125448	0.293240182	3601.056122	231.877551
0.188490409	6.283372464	86259.06785	179.20059
0.299674267	0.741009681	19397.23641	241.2119565
0.312206573	0.248642387	34297.09273	622.1115288
0.365269461	0.339605605	4256.088798	252.1885246
0.302707501	0.52546991	8890.268328	219.3533724
0.397260274	0.445718428	1103.724138	121.6034483
0.316149068	0.432151704	5762.548134	218.6247544
0.286106261	1.371067873	9165.693112	195.9006103
0.42893401	0.163320967	2668.727811	288.8017751
0.298929336	1.168932446	14536.36963	212.4040115
0.267494824	1.052926142	15536.55573	192.0108359
0.296317943	0.522371023	24171.72847	352.9638396
0.391954616	0.118100755	9256.411842	357.7828947
0.472222222	0.023605128	6145.530812	568.1064426
0.240680295	4.886595631	164950.5632	240.0992148
0.296428571	0.483657527	3870.301205	172.8373494
0.222927914	1.510076904	699561.3124	793.3618234
0.338059701	0.47568379	6553.838852	265.9403974
0.212976023	6.420084142	144908.1573	216.6456954
0.426385443	0.133886366	4075.85063	314.4888458
0.414788732	0.28570246	2744.20034	251.008489
0.208868895	3.525147844	32149.28	164.8184615
0.380674719	0.311779522	5083.085339	287.2658643
0.256026911	1.104482574	25271.72263	284.2029197
0.254582485	0.487216899	9086.088	190.752
0.447570332	0.284111835	1282.531429	193.5771429
0.407079646	0.077663977	4927.853261	330.2228261
0.406371406	0.318013454	5439.099426	311.5181644
0.419389587	0.117292842	3446.023116	308.8715753
0.158976931	25.97048252	430081.1562	183.6388013
0.292326431	0.246380548	26231.72708	363.8770833
0.465007776	0.139501468	2400.351171	305.0535117
0.368197279	0.429425123	2339.676674	204.8891455
0.297135741	0.875680948	20857.78206	261.2464376
0.350749879	0.241425243	6102.791724	294.6896552
0.428571429	0.170504113	2456.122324	248.8623853
0.198476086	7.896786261	572316.6633	349.6614246
0.379232506	0.139834041	5126.085317	278.5634921
0.402028398	0.099683671	8165.377397	373.975782
0.337503036	0.474415472	43981.84095	420.8445484
0.351145038	0.097383076	7427.237772	349.2078804
0.35745938	0.246994663	4787.140496	222.0454545
0.424729611	0.067285423	4218.292498	353.732278
0.433497537	0.142127545	2274.232955	221.9886364
0.361455261	0.172419315	10069.98205	392.6376496
0.22362813	1.947965147	111843.648	326.0875521
0.369114878	0.1886461	3738.905612	199.692602
0.410569106	0.161701872	2146.326733	232.0643564
0.30817105	0.674047552	70089.91661	413.8143322
0.41423221	0.208134709	2725.640145	282.4014467
0.444210526	0.264795827	2206.609005	196.6587678
0.236287953	3.499996377	93352.74197	261.1937824
0.301742919	0.307234605	3996.285199	163.4223827
0.333182435	0.133512264	7720.710598	298.4320652
0.517027864	0.041914104	2596.754491	375.2754491
0.384030418	0.112960593	3436.938614	253.5841584
0.257777778	0.610545439	19123.56681	286.8383621
0.402560455	0.137186371	8899.462898	423.5017668
0.375302663	0.267670138	3138.754839	182.6258065
0.336546889	0.666967579	10086.38542	245.3111979
0.263602747	1.081054869	13799.55511	209.8016032
0.268876611	1.460928353	27428.3589	216.2369863
0.331450094	0.20447699	14345.71023	234.0795455
0.380733945	0.165598822	5135.883534	307
0.220542231	5.04863424	101960.3073	242.1737589
0.343327454	0.284464595	11921.67979	267.4486301
0.36430139	0.180431977	4404.606426	249.935743
0.312189055	0.204932263	12735.73307	291.5179283
0.242101106	2.702369932	41557.38499	216.2446982
0.305043324	0.283472942	10815.55645	274.3932993
0.29280325	0.67113977	7205.062438	192.4876115
0.288478984	0.892760739	7649.372117	175.5136268
0.433305369	0.05614487	5079.194579	420.7754114
0.339722675	0.503357671	4690.393758	208.4861945
0.47284345	0.080221905	2491.574324	297.777027
0.388059701	0.270771079	2044.807692	168.2307692
0.316863101	0.376514011	65701.29828	427.555794

log.sigma.3.0.mm.3D_glszm_SmallAreaEmphasis	log.sigma.3.0.mm.3D_glszm_LowGrayLevelZoneEmphasis	log.sigma.3.0.mm.3D_glszm_ZoneEntropy	log.sigma.3.0.mm.3D_glszm_SmallAreaLowGrayLevelEmphasis
0.656483637	0.007453694	6.477726147	0.004417569
0.627805084	0.02839913	5.80591388	0.021480491
0.632273726	0.019109274	6.215127978	0.012520513
0.70829762	0.015057674	5.645078453	0.012768445
0.598181528	0.009946979	6.37998598	0.007111566
0.604795164	0.021077171	5.725684421	0.018844999
0.583205327	0.009816268	6.778208858	0.005555651
0.620556306	0.003609079	6.732227441	0.002398929
0.60007058	0.008443807	6.455507285	0.004845407
0.54980159	0.020565183	6.109384214	0.011426665
0.523957451	0.020225624	6.259338259	0.011473745
0.617989049	0.008153354	6.4446032	0.005772364
0.599752238	0.015966873	6.296167047	0.012666164
0.647415548	0.018347124	6.035956098	0.010787943
0.667567676	0.018067863	6.270952934	0.012725493
0.602044718	0.013615948	6.510252901	0.00826297
0.508672449	0.015889792	6.193025525	0.00786457
0.5957509	0.029789187	5.977328738	0.024032449
0.526474316	0.039261155	6.111005676	0.028815974
0.604874568	0.004865668	6.829209514	0.003372637
0.544061237	0.016707122	6.488089238	0.009853844
0.609359508	0.029542923	5.904149934	0.014931038
0.601474964	0.015638109	5.984657833	0.007743645
0.544840556	0.013212619	6.422502092	0.00736774
0.61480454	0.017674996	6.230193058	0.006291896
0.622428288	0.003654896	6.603844082	0.002709916
0.623583182	0.015005021	6.461472644	0.010396051
0.575824677	0.012844484	6.422040784	0.008684672
0.650080894	0.039307288	5.116264616	0.016998587
0.56633953	0.011521564	6.356524116	0.007283856
0.581875844	0.014452497	6.503926318	0.0081122
0.60129062	0.013083796	6.178865155	0.008534378
0.604340477	0.01767512	6.388631778	0.010546331
0.607321354	0.016120091	6.322629315	0.008285791
0.621942425	0.00538267	6.565562975	0.003571623
0.624385971	0.007063467	6.407165357	0.003806687
0.656829208	0.004382888	6.421737101	0.003452576
0.609600131	0.012957006	6.608272855	0.00695849
0.538138182	0.021116252	5.864796568	0.008489448
0.60840692	0.001947538	6.60995609	0.001343354
0.608598956	0.010061908	6.186181741	0.006249892
0.559103978	0.00970892	6.550833325	0.005836846
0.643938967	0.008216166	6.39739279	0.005231033
0.633203076	0.012733528	6.248144453	0.008051246
0.552402135	0.023192644	6.271137962	0.013097589
0.621330293	0.008540274	6.402611207	0.005669614
0.601729501	0.007190479	6.601275198	0.004433197
0.514350378	0.019830535	5.890555261	0.014956651
0.635272435	0.026593697	5.857217024	0.020278125
0.550185163	0.015810058	6.243914114	0.010950642
0.657242733	0.00810185	6.099033723	0.005840149
0.63314071	0.009280427	6.544545919	0.006239311
0.570207011	0.013121888	6.523832285	0.007968904
0.60974491	0.00673008	6.260864515	0.005172532
0.633505047	0.011407698	6.138040178	0.007695213
0.600976058	0.021076312	6.331491661	0.015411476
0.590663592	0.007757113	6.47038993	0.004078282
0.582735877	0.008168715	6.475525787	0.00543153
0.64781354	0.011433021	5.996878501	0.007777075
0.579482402	0.005129394	6.73289974	0.002862071
0.619405889	0.009690344	6.405164215	0.006966105
0.62054244	0.00636502	6.583385826	0.004652826
0.617852193	0.004766285	6.756947552	0.003206465
0.572538596	0.00730326	6.656909591	0.005101032
0.594082265	0.015302387	6.05405881	0.008083311
0.627320657	0.006529839	6.617083255	0.004416748
0.617128167	0.017556349	5.751300311	0.012107945
0.596150958	0.005516509	6.714671476	0.003710611
0.561057769	0.005001742	6.667111387	0.003083416
0.599682366	0.0162957	6.462784056	0.011353363
0.588585965	0.015722792	5.946153348	0.010314119
0.627579487	0.004455112	6.516632642	0.003044228
0.624089974	0.009878705	6.247498675	0.0065521
0.676849052	0.018686817	5.925003127	0.011463772
0.601211538	0.010402242	6.50709337	0.006133476
0.506155769	0.01827126	6.304337596	0.011894404
0.554002718	0.007605386	6.631079296	0.003936212
0.66033215	0.014060895	5.912561985	0.011480438
0.560177007	0.013671974	6.527751341	0.007749703
0.596138701	0.009484325	6.311494751	0.006342455
0.651916646	0.006925549	6.462967227	0.005120801
0.625685109	0.022776166	6.115225016	0.015974118
0.650123413	0.011504338	6.214975963	0.008220001
0.540732815	0.010138592	6.170072182	0.005626365
0.584487252	0.014283136	6.523676608	0.009510352
0.567190481	0.023194472	5.893325666	0.014036145
0.607795922	0.009059725	6.379960392	0.006433832
0.602976278	0.009467311	6.422562031	0.005690153
0.607588063	0.012446436	6.424108861	0.007938729
0.584604496	0.010106448	6.35516598	0.006807567
0.5849989	0.012129664	6.135821353	0.008476648
0.601949475	0.01146449	6.256641702	0.005700299
0.5857526	0.007472303	6.635717613	0.004745507
0.547863491	0.01547482	6.623967805	0.009498977
0.57534473	0.014901873	6.343674243	0.009442436
0.633939053	0.0058066	6.630994288	0.004074551
0.557290822	0.020000962	6.556921908	0.010943003
0.638820796	0.012418254	5.628302288	0.004999465
0.524391833	0.024312193	5.602237801	0.007644339
0.614780872	0.004129411	6.743823982	0.002855485

log.sigma.3.0.mm.3D_ngtdm_Coarseness	log.sigma.3.0.mm.3D_ngtdm_Complexity	log.sigma.3.0.mm.3D_ngtdm_Strength	log.sigma.3.0.mm.3D_ngtdm_Busyness	log.sigma.3.0.mm.3D_ngtdm_Contrast
0.006531972	1.226186632	3.107818002	0.218623127	0.000112077
0.018825556	2.124815401	4.580935757	0.158571837	0.000796615
0.009439222	0.84265351	2.027099113	0.342647035	0.000324637
0.025190332	2.606086163	6.148533649	0.1112292	0.000915646
0.005943604	0.586896053	1.70242217	0.457640085	0.000134787
0.039129976	2.903899404	9.354135412	0.062492592	0.001017658
0.001102827	0.092872806	0.633204992	1.386775771	3.96E-06
0.001595827	0.217666861	0.691838057	0.835686897	1.82E-05
0.002361812	0.188254276	0.816625019	0.984022073	1.99E-05
0.012158523	0.669113718	2.286379189	0.294005712	0.000189058
0.006526972	0.169114979	0.865697232	0.725461559	5.37E-05
0.003084373	0.244450432	1.098131444	0.584989419	2.02E-05
0.009817075	1.055828311	2.994879794	0.195657201	0.000282187
0.012409396	1.586573187	3.650172022	0.222407673	0.000388135
0.005524356	0.836253517	2.395620583	0.425348835	8.90E-05
0.001896354	0.131521452	0.566901857	1.273991744	1.90E-05
0.010769435	0.459488495	2.008038911	0.324921348	0.000100926
0.017607919	1.938430017	4.802087132	0.175079781	0.000626279
0.009118792	0.186934075	0.955716667	0.690780705	9.07E-05
0.00116825	0.159761314	0.735602985	0.832736155	5.89E-06
0.00439546	0.23943393	0.93320587	0.855297461	7.44E-05
0.015637764	1.084354765	2.926756959	0.287218287	0.000498292
0.016986216	1.086477419	3.795670681	0.176666981	0.000263816
0.003191789	0.096547882	0.700841077	0.924894354	1.36E-05
0.008498833	0.552829189	1.930112487	0.344544904	0.000145679
0.003883488	0.524204787	2.040273108	0.305224983	4.24E-05
0.004601756	0.566893557	1.851545326	0.544572486	7.23E-05
0.00458479	0.291868285	1.296489149	0.569359561	3.80E-05
0.058120985	1.929404848	7.694126787	0.106549095	0.000955145
0.005610768	0.414796281	1.456515814	0.476792857	6.68E-05
0.002385843	0.170158068	0.645035327	1.483110352	3.22E-05
0.009169958	1.145608296	2.382418444	0.278488949	0.000345688
0.004530381	0.324344546	1.392558997	0.62463247	4.77E-05
0.004342591	0.232844914	1.033265792	0.746553444	4.84E-05
0.001914798	0.219370955	0.86962396	0.826253454	1.59E-05
0.004399133	0.540756507	1.37058687	0.415275765	0.000100335
0.005076642	1.121254008	2.293133767	0.234779469	0.000169474
0.001936855	0.132468898	0.614421701	1.095908142	1.71E-05
0.015433892	0.554348148	2.104038654	0.279200526	0.000241798
0.001161293	0.097273159	0.624604317	0.709988898	4.57E-06
0.006670027	0.636465293	2.057728818	0.392581239	0.000103404
0.001822905	0.113423257	0.765031298	1.016426665	6.27E-06
0.003429675	0.434278201	1.044580794	0.661312915	9.53E-05
0.005987429	0.546293166	1.444755209	0.588039976	0.000145697
0.006884886	0.310401616	1.989115147	0.433619115	3.98E-05
0.003890401	0.389755708	1.177447265	0.670934032	6.58E-05
0.001982314	0.188762525	0.961556442	0.823846072	1.09E-05
0.016079575	0.781893476	3.013526309	0.194817108	0.000210389
0.023163454	1.427302351	4.449411959	0.204240136	0.000594541
0.017265456	2.049560868	5.494975075	0.107330623	0.000506096
0.006169614	0.742045167	1.880926664	0.42713778	0.000137782
0.003321805	0.530559084	1.657126701	0.517546021	4.30E-05
0.001470911	0.049784438	0.418505661	1.664933561	4.43E-06
0.005670418	0.523910547	2.067829498	0.251614878	4.29E-05
0.012118681	1.664479606	3.784021668	0.19947884	0.000458913
0.00730917	0.690319989	2.259019185	0.444561097	0.000143694
0.002566863	0.143141446	0.561431042	1.145436011	3.02E-05
0.003800158	0.462584581	1.231881392	0.601692042	8.29E-05
0.010170528	0.999228533	2.326290643	0.303099206	0.000260428
0.00101528	0.087476841	0.584210876	1.15956244	3.14E-06
0.006839137	0.705182225	2.023108512	0.315029461	0.000103179
0.00390569	0.592339676	1.697468523	0.360705377	5.20E-05
0.001282101	0.164475149	0.551769457	1.039202126	1.32E-05
0.004852571	0.565722593	2.112052676	0.29473825	4.73E-05
0.013930319	0.784022596	2.453639125	0.230318422	0.000230967
0.002586089	0.432050686	1.095108301	0.588007704	4.17E-05
0.017240859	1.34653625	3.140119052	0.20074258	0.000730733
0.001824363	0.311230143	1.018338633	0.693775029	1.77E-05
0.001240457	0.092685456	0.418842658	1.425843712	8.72E-06
0.004276788	0.300856961	1.00072789	0.669066121	6.87E-05
0.016819083	1.108535775	3.049935483	0.237254136	0.000504559
0.002136469	0.227929763	0.826212785	0.662217113	1.95E-05
0.005865563	0.742692495	1.776071619	0.489644767	0.000158195
0.007984621	0.724578531	1.776753708	0.504818791	0.000219401
0.002670931	0.193018176	1.167780507	0.690558837	1.41E-05
0.010073177	0.414939886	1.616393907	0.365415294	0.000120509
0.004134578	0.418621795	1.440219158	0.41207657	4.63E-05
0.021458905	3.549309889	8.18108327	0.076519646	0.000859586
0.007459267	0.596918354	2.061456394	0.296971821	0.000102557
0.00562101	0.434674335	2.068168819	0.315806497	3.26E-05
0.00664795	1.18686329	3.081940297	0.223420883	0.000115351
0.01041399	0.743971419	2.2060453	0.303680088	0.000213686
0.003559847	0.408727341	1.108124278	0.700965806	6.55E-05
0.005225194	0.217178659	0.934148155	0.667001541	4.90E-05
0.004161157	0.192708022	0.890704381	0.735822721	3.97E-05
0.011293456	0.925209321	3.248276331	0.190967047	0.000365673
0.006880926	0.674282571	1.839636896	0.344631924	0.00011868
0.003028188	0.152753761	0.850342495	0.818181322	1.94E-05
0.00595528	0.461850017	1.617662082	0.352996949	6.60E-05
0.006231982	0.529374193	1.613422872	0.420714982	0.00012115
0.013581611	0.784608675	3.482425843	0.154138176	0.000148244
0.004261972	0.199674558	1.005388957	0.665180306	3.05E-05
0.002170474	0.224003627	0.935385878	0.743652135	1.46E-05
0.002941411	0.166921943	0.685286336	1.112905245	3.33E-05
0.002776169	0.145412113	0.570905933	1.252718358	2.88E-05
0.003636246	0.821349379	2.025419726	0.340537824	6.62E-05
0.00382003	0.318617075	1.046519638	0.75121108	6.25E-05
0.022405129	1.94546077	4.449762249	0.123190202	0.000845975
0.024695584	1.397932516	4.016167364	0.147431567	0.000716762
0.001161203	0.129340011	0.462035726	1.087393339	1.14E-05

log.sigma.2.5.mm.3D_gldm_GrayLevelVariance	log.sigma.2.5.mm.3D_gldm_HighGrayLevelEmphasis	log.sigma.2.5.mm.3D_gldm_GrayLevelNonUniformityNormalized	log.sigma.2.5.mm.3D_gldm_DependenceEntropy
39.55397969	420.8580332	0.049632254	6.814570005
27.4341619	185.092219	0.062080077	6.120612681
34.17428839	243.2230942	0.047962457	6.652690307
33.70272173	225.121118	0.051849851	6.175711492
28.80276214	213.9440113	0.053962463	6.635942177
32.36460966	337.4586777	0.053377502	6.124765321
15.43783591	225.0320714	0.098377922	7.128766726
27.51359097	311.8816744	0.057726235	7.123512714
19.05818958	214.3165181	0.072115038	6.954677135
19.95379852	146.7324503	0.062160432	6.283785871
11.26601727	109.3727506	0.088983023	6.494388442
18.07163725	288.3927209	0.077106139	6.997938045
32.39576963	257.2108508	0.059199874	6.552237821
33.66901354	216.3385093	0.047876239	6.395730621
29.64365674	231.4279365	0.057892265	6.74638676
22.11523792	232.9696286	0.078981841	7.094637811
14.16587896	199.0396376	0.074692602	6.448625889
27.52549303	184.4792176	0.060060617	6.241262102
10.96622762	135.6946984	0.087186549	6.515609713
20.97774203	463.859523	0.066610415	7.023205469
19.84072473	131.7237729	0.065009388	6.665082809
25.93910965	152.9556452	0.057443418	6.157075592
22.23057258	168.9964158	0.060430878	6.458751647
10.50651668	159.1946066	0.1091897	6.782279399
26.82066123	241.8680782	0.068366773	6.973720811
28.76347454	379.3290297	0.061413692	6.964814455
28.79061936	267.5578842	0.056017207	6.778425183
17.94370814	206.2654239	0.06997722	6.708508587
15.79301933	134.0958904	0.08969788	5.462229718
18.62642838	280.3223602	0.064409552	6.62625373
21.12374521	184.8603143	0.061612992	6.898631499
32.55907779	283.3071066	0.053238295	6.484934149
21.67558566	221.7738758	0.065262347	6.97125195
20.48024828	192.9755694	0.072575218	6.892097241
22.10116682	323.390415	0.063854746	6.963672013
32.30175111	358.2913873	0.050268358	6.920811629
44.86586628	631.8062169	0.045741651	6.652846513
22.64958645	262.9342037	0.092097132	7.181778016
14.10141582	149.9071429	0.074068878	6.214422762
14.95914052	867.4297661	0.091512745	6.949532582
23.07864279	197.4171642	0.059472043	6.569357586
12.09354936	198.2149154	0.109198105	6.7203342
33.32698602	276.792804	0.048678884	6.807368844
28.90623091	216.056338	0.052632414	6.58451997
12.94167036	140.6491003	0.09506777	6.796766332
27.46949079	299.6851312	0.054654272	6.749219768
18.27228224	254.6823024	0.069978589	6.902352425
13.18592506	233.3849287	0.085402831	6.080492765
27.43758871	163.6879795	0.055121303	6.132845857
34.47996613	275.0730088	0.059479991	6.383206482
28.967695	401.5446775	0.056416777	6.499850902
30.75640611	293.1382406	0.052024406	6.701222554
9.577539477	138.4182548	0.122250779	6.889156591
17.18572906	351.9732034	0.073061579	6.769796433
38.72994251	283.8009331	0.048109924	6.465677109
25.55542829	172.2619048	0.057872299	6.594570643
19.80686957	189.0724782	0.067587332	6.952628117
28.12703883	245.4862119	0.055008919	6.727603669
27.06116265	200.0026212	0.054467002	6.527479524
14.41091369	316.6338015	0.09935372	7.00179881
26.01765784	228.8389767	0.055181031	6.690087903
32.24517459	322.7229209	0.049768894	6.908140508
28.96138639	349.5818557	0.053183483	7.159188985
25.88193956	453.7361641	0.055251551	6.668231005
20.65689494	289.6750369	0.065383164	6.51432821
33.25521178	299.695703	0.048611919	6.769270756
26.08743236	212.1477833	0.062292218	6.09464799
27.53451208	315.1054081	0.056234689	6.860856712
15.06839224	390.5803143	0.077637379	6.802123485
21.9161001	219.6600753	0.06060058	6.590020798
26.15471528	163.8353659	0.054960672	6.24371754
24.89082275	323.3180084	0.061608495	7.040716143
30.40137805	239.5558052	0.05375135	6.540557334
27.10353019	184.2231579	0.055401662	6.464656065
17.12832045	223.2659158	0.091889733	6.90120661
15.25784955	155.9825708	0.072607402	6.353571548
22.05627089	366.5291987	0.060881644	6.593381778
38.18658283	272.380805	0.055238716	6.092086578
24.52471136	264.0326996	0.058299527	6.496592895
16.23034444	230.0722222	0.081417284	6.626082589
38.07979013	417.1379801	0.050767995	6.800787298
24.05854669	208.9987893	0.059081662	6.454586738
26.14310006	270.0749343	0.061996066	6.909092192
13.25518907	143.4537771	0.080052207	6.509793074
18.92249714	175.3705341	0.071397766	6.940468767
20.04854572	251.7645951	0.101393455	6.04833162
25.75597067	242.7775229	0.05655622	6.684893996
16.03123821	170.9350886	0.08979581	7.015440139
22.67255455	220.4708995	0.063429507	6.820572864
25.07198255	300.9429407	0.057960956	6.512807078
21.04415732	246.7910448	0.06661345	6.548797654
14.62496397	157.8582148	0.085808445	6.842964418
18.63378051	253.0277716	0.065746332	6.729578361
18.97545827	160.9973883	0.063791946	6.797579741
15.8458177	199.629876	0.069539839	6.541786264
39.37539958	305.0079698	0.046218709	6.903462835
25.44660549	215.3287113	0.055066743	6.783635958
29.97248109	282.1022364	0.056619951	5.973981846
16.58536144	165.7350746	0.073902874	5.890815848
25.03238135	428.057117	0.058145761	7.080729991

log.sigma.2.5.mm.3D_gldm_DependenceNonUniformity	log.sigma.2.5.mm.3D_gldm_GrayLevelNonUniformity	log.sigma.2.5.mm.3D_gldm_SmallDependenceEmphasis
305.901662	71.66897507	0.385155137
82.24495677	21.54178674	0.335302169
194.8116592	42.78251121	0.348725712
79.86335404	16.69565217	0.370009894
286.4472006	76.14103473	0.337260001
64.04132231	12.91735537	0.411652284
1029.056931	1036.804915	0.189250202
935.7643375	350.282795	0.268659866
574.8122947	307.3542938	0.244964944
172.4887417	46.93112583	0.337571768
230.6311054	138.4575835	0.225749032
436.5039624	262.7006164	0.241029727
162.4278668	48.01109741	0.32789039
158.6677019	30.83229814	0.401782319
283.1269841	91.18031746	0.324400733
655.9597362	455.0933704	0.205610207
145.8878822	65.95356738	0.242817328
89.53300733	24.56479218	0.34261069
151.4003656	95.3820841	0.216450756
1375.657737	611.6834368	0.257575049
349.280077	135.0895091	0.23742861
120.0040323	28.49193548	0.34350705
105.8960573	33.72043011	0.300511561
382.1637476	392.7553517	0.192773913
159.1970684	83.95439739	0.253174105
389.2190923	156.9733959	0.269269635
366.8512974	112.258483	0.33447791
364.8428762	157.6586773	0.274827077
33.31506849	13.09589041	0.330977813
282.4248447	103.6993789	0.274026623
642.2696433	247.0064854	0.252797876
173.1091371	41.95177665	0.379586442
343.4599572	152.3875803	0.269002991
318.7871636	175.2691511	0.233841023
793.3183324	327.7664134	0.263515276
370.8751934	97.47034554	0.307916384
368.0780423	69.16137566	0.401667892
586.0599553	536.0974059	0.21158934
110.0642857	41.47857143	0.257287891
1073.168307	864.5209061	0.212582404
262.7358209	79.69253731	0.319080254
673.3409732	619.3716502	0.220733978
504.7411084	117.7055418	0.3472676
307.1295775	74.73802817	0.361008024
177.4524422	147.9254499	0.208740775
456.3186172	131.2249063	0.32504882
795.8843207	374.4554289	0.24190241
90.56008147	41.93279022	0.242393677
96.10485934	21.55242967	0.418551174
99.77433628	26.88495575	0.35167168
282.2494172	72.60839161	0.380499625
614.5705566	144.8879713	0.359526584
664.9543631	975.0722166	0.15832178
240.2436054	119.9671133	0.270272847
154.8382582	30.93468118	0.4021414
241.0663265	68.05782313	0.279066431
591.0493151	271.3631382	0.26104684
394.8964683	113.7034349	0.306615372
157.9646134	41.55832241	0.354895565
1120.634811	1082.260075	0.201749308
266.8419865	73.33559067	0.329048778
506.4231237	122.6803245	0.342860106
1384.30289	437.9128006	0.302746613
447.8482824	115.8072519	0.312368639
118.4534712	44.26440177	0.276343559
770.8965215	166.3013739	0.373220768
97.84236453	25.29064039	0.351122253
982.679056	285.9533923	0.327412132
1071.020778	582.9014385	0.220906699
422.2900188	128.7156309	0.292931219
112.1219512	27.04065041	0.344823091
715.0714716	306.8719133	0.261361049
290.764794	71.75805243	0.355064635
212.2189474	52.63157895	0.393106903
485.7428991	375.277669	0.217206436
176.0305011	66.65359477	0.277860791
439.2851969	134.4875509	0.29830267
80.75851393	17.84210526	0.393998968
292.9726236	76.66387833	0.331573125
263.6466667	146.5511111	0.257223953
292.4352774	71.37980085	0.379771403
172.7215496	48.80145278	0.343894353
347.4250657	141.4750219	0.287781013
299.528262	151.5388273	0.249888826
373.5053407	193.8449355	0.230658412
94.15630885	53.83992467	0.267730221
247.2262997	73.97553517	0.329421139
391.8675704	344.4567258	0.206071951
300.4920635	107.893592	0.288236636
288.4952451	79.23262619	0.305088945
143.2164179	53.55721393	0.282604102
298.1082148	217.2669826	0.215775088
802.8600311	295.9242391	0.288302267
586.6366802	219.8270459	0.25704321
602.9159359	229.9682492	0.263440199
503.8154362	110.1854027	0.344695618
463.0562806	135.0236542	0.301569122
84.25878594	17.72204473	0.415336989
60.2238806	19.80597015	0.316274876
1428.56709	513.0781958	0.280678268

log.sigma.2.5.mm.3D_gldm_DependenceNonUniformityNormalized	log.sigma.2.5.mm.3D_gldm_DependenceVariance	log.sigma.2.5.mm.3D_gldm_LargeDependenceEmphasis
0.211843256	2.804549919	9.927977839
0.237017166	1.555813934	8.060518732
0.218398721	1.896227553	8.778026906
0.248022839	1.536823425	7.434782609
0.203010064	2.723761515	10.5804394
0.264633563	1.371900826	6.537190083
0.097642749	19.14367309	49.91621596
0.154212976	7.839496908	21.32102835
0.134869145	9.625485421	26.43125293
0.22846191	1.768476821	8.528476821
0.148220505	4.817383245	18.7596401
0.128119742	10.76648751	29.26885823
0.20028097	2.66567131	10.70160296
0.246378419	1.699529339	7.388198758
0.179763165	4.782559234	14.59238095
0.11384237	14.00632542	37.2440125
0.16521844	4.043006891	15.90373726
0.218907108	2.215398043	9.356968215
0.138391559	5.309743357	20.58683729
0.149804828	5.397546771	18.48818469
0.168084734	3.177614887	14.62271415
0.241943613	1.724489464	8.157258065
0.189777881	3.332703845	12.48387097
0.106245134	12.40070058	36.8587712
0.129639307	19.06819966	41.63843648
0.15227664	5.898606733	18.80907668
0.18305953	3.237126545	12.11776447
0.161936474	4.444761623	15.91078562
0.228185401	1.770688685	8.616438356
0.175419158	3.816996258	14.29689441
0.160206945	4.541809863	16.63083063
0.219681646	2.542876652	9.403553299
0.147092059	7.156909335	21.18329764
0.132002966	6.912597508	23.08198758
0.154552568	7.126401333	20.47671927
0.191271374	3.58935038	12.65755544
0.243437859	1.659611993	7.366402116
0.100680288	21.84602515	52.36780622
0.196543367	2.351071429	11.65357143
0.113598847	11.40633387	33.20091034
0.196071508	2.802517264	11.18656716
0.118713148	12.42925285	33.43511989
0.208743221	2.266138097	9.59057072
0.216288435	2.29010117	9.301408451
0.114043986	12.94415514	35.4151671
0.190053568	4.094416442	13.23490212
0.148735623	5.776292982	19.51653896
0.184440084	2.659147755	13.15885947
0.245792479	1.839456832	7.460358056
0.220739682	2.036612107	8.898230088
0.219308017	2.35300226	9.124320124
0.220671654	2.077956222	8.867863555
0.083369404	19.28305227	56.79714142
0.146311575	5.534371055	18.79780755
0.240806	1.781936829	7.600311042
0.204988373	2.699150238	11.46598639
0.14721029	10.02153543	25.26749689
0.191048122	2.661247811	11.31059507
0.207030948	2.52091403	9.988204456
0.102876601	16.04877197	43.06719912
0.200784038	3.577493672	11.98269375
0.205445486	3.330545528	11.3464503
0.168120341	6.01539869	17.32766578
0.213668074	2.001431298	9.563931298
0.1749682	3.030181376	13.10635155
0.22534245	1.912833548	8.363928676
0.240991046	1.71768303	8.054187192
0.193250552	3.09927674	11.57384464
0.14265061	5.23277419	19.8857219
0.198818276	2.446662836	10.97834275
0.227890145	1.899795096	8.62601626
0.143559822	6.677610374	20.87633005
0.217801344	2.445678856	9.576779026
0.223388366	2.245234349	8.755789474
0.118938026	12.55132209	34.00881489
0.191754358	2.594296591	11.7254902
0.198861565	2.181017161	10.49388864
0.250026359	1.457025372	7.037151703
0.22279287	2.020329338	9.100380228
0.14647037	5.889288889	19.53
0.207990951	2.874890987	10.19487909
0.209105992	2.371022871	9.857142857
0.152245866	9.999945464	24.36809816
0.158229404	4.166275005	16.30375066
0.137571028	9.483797605	26.37090239
0.177318849	3.388936768	13.72316384
0.189010932	3.166524049	11.83027523
0.102155258	18.22855017	46.51564129
0.176656122	6.090398807	17.32333921
0.211042608	2.031074192	9.803218727
0.178129873	2.937879013	12.6840796
0.117736262	10.91403994	31.69352291
0.178373702	3.848110971	13.88691402
0.170236994	3.699286799	14.73186303
0.18231507	3.251075392	13.36105231
0.211331978	2.242347614	9.526006711
0.188848402	3.0530073	12.0725938
0.269197399	1.529769621	6.71884984
0.224715972	1.514089998	8.611940299
0.161895636	4.582858739	16.00793291

log.sigma.2.5.mm.3D_gldm_LargeDependenceLowGrayLevelEmphasis	log.sigma.2.5.mm.3D_gldm_SmallDependenceHighGrayLevelEmphasis	log.sigma.2.5.mm.3D_gldm_LargeDependenceHighGrayLevelEmphasis
0.03710855	181.5447571	3636.500693
0.151728457	64.59988165	1447.657061
0.098887598	90.64594139	2068.528027
0.130792328	85.20097661	1439.723602
0.129398107	76.47975518	1818.30404
0.040602654	134.184964	2158.020661
0.26012398	49.07021236	10412.11415
0.094771982	101.3226252	5841.561635
0.169004085	65.29277046	4962.485453
0.155089006	54.55953518	1124.064901
0.277466699	27.99960819	1790.8509
0.124057189	82.51142152	7645.090696
0.107795718	77.20419827	3112.799014
0.128694797	89.35436349	1365.81677
0.110618786	97.82782686	2753.576508
0.223309487	52.49873043	7718.941513
0.123323014	54.46582262	2651.336353
0.186450603	67.60568326	1410.371638
0.209539398	36.3928444	2559.745887
0.046485506	123.1220979	8244.957639
0.33516748	36.35374854	1758.102984
0.227864879	57.85077997	1027.322581
0.187195388	59.88083785	1719.236559
0.283519412	39.05164306	5274.756464
0.223674052	68.39999147	9559.946254
0.069199679	124.6423102	6022.156103
0.077550062	112.0819458	2603.908184
0.118458174	69.83021352	2797.873058
0.109488101	49.44517302	982.0410959
0.073494408	83.2515497	3487.199379
0.161443972	54.8305115	2690.462709
0.081872062	115.3679118	2263.936548
0.134400148	69.46856745	4215.072377
0.177711515	51.83277362	4145.361491
0.083141733	100.0891111	5811.19287
0.061324698	114.1420252	4521.659103
0.016095699	232.0898317	5169.581349
0.238845703	63.09126722	13000.57963
0.130495658	42.79696278	1622.757143
0.04062225	202.3916872	27775.24918
0.125429001	72.19114581	1748.879851
0.201114205	53.82884659	5960.02768
0.081422223	100.6575487	2501.67866
0.108561376	92.783997	1567.890141
0.348397901	38.00742427	4320.047558
0.076172569	117.3984623	3096.8788
0.106377714	73.78886002	4275.189124
0.094112956	63.7268067	2810.672098
0.220705355	84.60548437	854.8030691
0.108020236	102.1467788	2525.736726
0.034371387	175.203104	3052.714841
0.055574119	118.5040819	2180.728187
0.484249508	27.12271658	7308.274072
0.060385799	94.4150932	6810.343484
0.07638012	119.2255519	1884.37325
0.176625353	55.84738574	1506.772109
0.192564274	59.86429592	4196.266999
0.089889731	82.70957569	2439.784228
0.127305718	81.98698479	1507.887287
0.152050366	76.90740838	12833.98421
0.088321104	84.96901414	2525.039127
0.055569412	116.991352	3505.083164
0.068804399	113.7826915	5502.831795
0.027549627	142.7215059	4265.649332
0.072210183	84.44682902	3584.573117
0.052862761	120.3560614	2389.32739
0.117166179	77.02037017	1583.192118
0.056517425	117.3124098	3168.315241
0.060261029	95.41159549	7166.226292
0.077708221	62.71563019	2495.655367
0.223820029	68.5646085	1123.432927
0.085640371	93.74879149	6288.127886
0.09027428	98.78504567	1735.794007
0.130977763	83.22633425	1263.063158
0.189646803	59.64936437	6768.021548
0.135121793	49.85000107	1673.749455
0.036987993	110.9657749	3868.507469
0.135598584	102.7727563	2106.04644
0.056212825	91.75298834	2319.977947
0.115198436	72.56153321	3907.796667
0.036755795	178.4923037	3698.248933
0.094451002	73.11480401	2124.245763
0.127971198	87.96002505	6036.128396
0.185017649	45.13813234	1925.347068
0.226684899	49.37698353	3943.919337
0.092319786	57.75038177	3993.233522
0.103729141	93.96030698	2395.965596
0.365909488	47.25439411	6814.885036
0.130442129	71.52237656	3522.59318
0.053052081	93.60930278	2832.742502
0.086137683	70.55387821	3114.034826
0.27343689	45.42075059	4416.887441
0.076852354	79.48258564	3246.680293
0.184892279	46.29751863	2057.719965
0.10401882	59.5306193	2269.29241
0.07357025	109.6474581	2764.205117
0.116062236	73.27263601	2196.411093
0.047746857	115.8564253	1882.84345
0.120450329	56.77162313	1290.600746
0.044888878	116.4422596	6918.233454

log.sigma.2.5.mm.3D_gldm_SmallDependenceLowGrayLevelEmphasis	log.sigma.2.5.mm.3D_gldm_LowGrayLevelEmphasis	log.sigma.2.5.mm.3D_gldzm_DistanceZoneVariabilityNormalized
0.002517319	0.005059031	0.871672502
0.01171652	0.024330422	0.921699292
0.00507238	0.01307534	0.88456919
0.011617975	0.0198842	0.882048022
0.004852932	0.011391407	0.852680437
0.008587001	0.012579937	0.890991053
0.001442135	0.006421674	0.920479676
0.001699068	0.005410776	0.88034937
0.001904212	0.007326205	0.954948838
0.009099606	0.024269559	0.924644368
0.006260389	0.019078821	0.915261229
0.001475244	0.004999085	0.909205778
0.007512323	0.016770732	0.813990744
0.009171214	0.017541261	0.836620644
0.003921611	0.01041196	0.910454484
0.001619289	0.007208352	0.96753652
0.003120946	0.008466041	0.968999938
0.006832851	0.020417556	0.810657439
0.0032872	0.011950142	0.948753463
0.001133934	0.003050973	0.932272983
0.004313054	0.021082717	0.934332007
0.008428795	0.023158805	0.881751343
0.005274671	0.016100599	0.932122926
0.001973867	0.00893112	0.934992277
0.002594399	0.009503185	0.951900919
0.001389214	0.004107748	0.962700053
0.002498267	0.006736679	0.922043255
0.002246597	0.008463557	0.918404571
0.013403331	0.021431207	0.787752675
0.002238697	0.005785828	0.992218018
0.002493816	0.009857459	0.955954573
0.003661124	0.010058515	0.903217264
0.002440749	0.007785715	0.877173456
0.002675249	0.009494218	0.96541952
0.001339796	0.00443331	0.921166335
0.00220042	0.00590382	0.797003297
0.001831756	0.003144119	0.654148369
0.001729922	0.006404813	0.949578294
0.005803406	0.014262455	0.837372449
0.000355663	0.001339379	0.917739307
0.003796763	0.010832756	0.878071834
0.001739512	0.006874215	0.843265306
0.003583669	0.009578791	0.937443118
0.00505781	0.012949568	0.895383505
0.003221268	0.012506648	0.942906574
0.001919044	0.005611077	0.921905502
0.001568064	0.005750082	0.926577705
0.002473074	0.009738159	0.985715025
0.011383308	0.026057914	0.940057392
0.006110248	0.014774575	0.912803731
0.001985406	0.004189201	0.972179452
0.002583823	0.006355697	0.880273121
0.002116749	0.011000545	0.915752742
0.002625369	0.005072536	0.969119569
0.00503213	0.010235418	0.913756504
0.004454336	0.014531644	0.942358592
0.002692864	0.00943664	0.928043013
0.00293921	0.008215454	0.916085474
0.005316854	0.013161449	0.86125
0.000929946	0.004003434	0.933124835
0.003714805	0.009314051	0.930110425
0.002857139	0.006861664	0.887751272
0.001790975	0.004902049	0.815669348
0.001567346	0.003492753	0.800727997
0.002551625	0.008168385	0.954775377
0.004128918	0.008189786	0.842921165
0.005350069	0.015907247	0.861004377
0.001927612	0.005279215	0.82220326
0.000796589	0.003137935	0.931592978
0.003837526	0.009269352	0.928396198
0.005448233	0.021961175	0.971297955
0.001710716	0.005040797	0.89874808
0.003428537	0.008860963	0.948753463
0.006135259	0.016223644	0.972978045
0.001668682	0.006612308	0.77655515
0.004226709	0.012799056	0.937952005
0.001671803	0.004230221	0.908739161
0.006445148	0.020341482	0.79471384
0.003489928	0.00776605	0.929671266
0.002376445	0.007157273	0.916522941
0.002387494	0.004690153	0.88579328
0.006034393	0.012624921	0.934404054
0.00193094	0.006899009	0.968919668
0.003008385	0.011600764	0.942599622
0.002945595	0.010747666	0.893430928
0.006317612	0.014652614	0.820936356
0.002957175	0.009869721	0.904638635
0.002004532	0.009536515	0.977301517
0.004284193	0.01237197	0.91322314
0.002490096	0.005990753	0.932413814
0.00370317	0.009296696	0.752779424
0.002888943	0.010750636	0.943176681
0.002084965	0.006509553	0.889751558
0.004191697	0.013755158	0.935077973
0.002291644	0.007731645	0.869262695
0.003499114	0.009276117	0.768691195
0.00340515	0.009869405	0.872799916
0.007455207	0.011286018	0.90328208
0.008531842	0.016814335	0.942906574
0.001387023	0.003573464	0.859417581

log.sigma.2.5.mm.3D_gldzm_LowIntensityEmphasis	log.sigma.2.5.mm.3D_gldzm_LargeDistanceEmphasis	log.sigma.2.5.mm.3D_gldzm_HighIntensitySmallDistanceEmphasis
0.006387446	1.206738132	442.5470904
0.032670919	1.12244898	189.3758503
0.014630573	1.184491979	251.7580214
0.027229891	1.188811189	234.5786713
0.013525441	1.240213523	225.2989324
0.018962155	1.173553719	326.9152893
0.007675033	1.124442462	255.3458965
0.006255182	1.191729323	365.3617078
0.007630587	1.069171648	272.4880444
0.029543939	1.117647059	156.9199346
0.02751883	1.133004926	123.0344828
0.005926779	1.143008475	337.596928
0.023661375	1.311320755	219.6383648
0.021027259	1.269230769	225.1530449
0.012412987	1.140939597	294.272651
0.007607432	1.049512378	257.7271193
0.011771101	1.047244094	229.5994094
0.022150692	1.317647059	191.3352941
0.014860765	1.078947368	169.1907895
0.004250325	1.107129385	470.3708139
0.017624708	1.1019678	156.6618962
0.022339177	1.189320388	171.2330097
0.017210565	1.105527638	196.1168342
0.010154793	1.100905563	204.0268435
0.011505554	1.073972603	266.8938356
0.004874973	1.057034221	469.2937262
0.007352611	1.121887287	328.1448231
0.008995741	1.127840909	252.7638494
0.036326946	1.362068966	148.9310345
0.007613437	1.01171875	311.3925781
0.009651562	1.067590988	219.1605286
0.010740531	1.152974504	299.0169972
0.008913401	1.197202797	252.079021
0.011270083	1.0528	222.408
0.004873898	1.126444159	378.2603409
0.006819772	1.343922652	355.3874309
0.004238649	1.667132867	534.2601399
0.007938387	1.077641984	298.4590223
0.020777865	1.267857143	168.391369
0.001611671	1.128932211	935.2712672
0.011562237	1.195652174	225.4313241
0.007863358	1.257142857	230.3167857
0.010488852	1.096969697	289.5977273
0.014081053	1.166123779	252.4743485
0.01569555	1.088235294	180.4525401
0.005615497	1.122112211	361.1185369
0.006345787	1.114503817	306.5924705
0.013038733	1.021582734	262.1816547
0.026833502	1.092783505	195.9510309
0.01749221	1.137055838	274.1586294
0.004914447	1.042328042	462.6776896
0.006952252	1.191860465	314.5957226
0.013729329	1.132196162	175.2260128
0.008944592	1.047058824	349.4867647
0.011439513	1.135483871	300.0532258
0.014998982	1.089108911	202.3434406
0.01018379	1.112126246	229.4892027
0.00931026	1.131648936	265.6894947
0.014717811	1.225	231.646875
0.004513993	1.10391198	377.726467
0.010995661	1.108778626	248.6932252
0.008319104	1.179060665	328.9567025
0.005826266	1.308147632	354.154422
0.004619384	1.336696091	433.5419294
0.011039779	1.069444444	306.2986111
0.010441331	1.257766028	302.8623596
0.017265551	1.225433526	216.8973988
0.005848297	1.29825462	334.4595882
0.003507323	1.106382979	432.0324604
0.012389624	1.111553785	211.9624834
0.018485895	1.04368932	194.9987864
0.006266045	1.160460393	357.838524
0.009251107	1.078947368	279.879386
0.016046784	1.04109589	213.05879
0.007669079	1.389060888	267.32846
0.015130257	1.096153846	175.6602564
0.005409614	1.143782383	357.9705311
0.019603155	1.348387097	249.0145161
0.009952283	1.109489051	269.067062
0.009176652	1.130929791	280.6190702
0.005987153	1.1824	447.7716
0.016994149	1.101851852	210.0501543
0.007153021	1.047368421	306.2526316
0.011926799	1.088724584	182.9898336
0.012438798	1.169421488	213.5581956
0.024947314	1.298245614	205.1681287
0.010077245	1.15060241	284.0090361
0.009548252	1.034443169	234.4649828
0.015626655	1.136363636	239.7739899
0.007502053	1.105058366	303.4421206
0.013330419	1.433460076	232.7737643
0.012880981	1.087804878	216.350813
0.007552147	1.175657895	266.6121711
0.016156297	1.100767754	182.365643
0.008394101	1.2109375	220.8161621
0.010481543	1.400404449	294.6370071
0.010981323	1.204778157	234.7059158
0.016300108	1.152866242	270.8869427
0.024549458	1.088235294	176.8676471
0.004821155	1.228237792	395.7498231

log.sigma.2.5.mm.3D_gldzm_LowIntensityLargeDistanceEmphasis	log.sigma.2.5.mm.3D_gldzm_HighIntensityEmphasis	log.sigma.2.5.mm.3D_gldzm_DistanceZoneVariability	log.sigma.2.5.mm.3D_gldzm_ZonePercentage
0.007407992	468.0689127	569.202144	0.452216066
0.039020802	192.2176871	135.4897959	0.423631124
0.015678382	261.6764706	330.828877	0.419282511
0.030448951	239.1888112	126.1328671	0.444099379
0.022183656	231.2455516	479.2064057	0.398299079
0.020518287	332.9834711	107.8099174	0.5
0.009093021	263.0472792	2063.715433	0.212733656
0.007831966	383.5961332	1639.210526	0.306855636
0.011473183	274.9385141	1118.24509	0.274753637
0.040888748	160.8267974	282.9411765	0.405298013
0.029376897	126.7364532	371.5960591	0.26092545
0.007284225	347.8379237	858.2902542	0.277076607
0.037244164	232.8176101	258.8490566	0.392108508
0.049519951	228.1987179	261.025641	0.48447205
0.015443006	300.7936242	542.6308725	0.378412698
0.007864866	260.7096774	1289.726182	0.231343284
0.012253391	230.8661417	246.1259843	0.287655719
0.057927013	195.6764706	137.8117647	0.415647922
0.015784991	171.7030075	252.3684211	0.243144424
0.004520508	484.4326669	2471.455677	0.288685615
0.018998468	158.9749553	522.2915921	0.269008662
0.048516113	172.6456311	181.6407767	0.415322581
0.018282706	199.0603015	185.4924623	0.356630824
0.012195012	207.7360931	722.7490298	0.214901307
0.013894027	270.5123288	347.4438356	0.29723127
0.005073136	474.148289	759.5703422	0.308685446
0.00888201	333.8243775	703.5190039	0.380738523
0.010928813	257.4417614	646.5568182	0.312472259
0.1174189	150.4827586	45.68965517	0.397260274
0.00766622	312.0546875	508.015625	0.318012422
0.010617443	222.0103986	1103.171577	0.287852332
0.013045867	304.9065156	318.8356941	0.447969543
0.010683639	262.9440559	627.179021	0.30620985
0.011628647	224.6976	603.3872	0.258799172
0.005728431	387.2689345	1435.17715	0.303526203
0.016976834	371.2140884	577.0303867	0.373388345
0.007518825	583.3216783	467.7160839	0.472883598
0.008624957	302.023005	1320.863408	0.238962378
0.058413998	170.1904762	140.6785714	0.3
0.001776905	964.2507754	2071.337616	0.238911824
0.015577596	230.5909091	444.3043478	0.37761194
0.010060203	247.0128571	1180.571429	0.246826516
0.021843855	290.969697	928.0686869	0.40942928
0.02172212	258.6905537	549.7654723	0.432394366
0.019558163	182.6122995	352.6470588	0.240359897
0.010857206	366.6622662	838.0121012	0.378592253
0.007526193	313.5058987	1335.198473	0.269295459
0.013134657	263.3956835	137.0143885	0.283095723
0.043338816	197.7680412	182.371134	0.496163683
0.019556526	278.3350254	179.822335	0.435840708
0.005315965	463.9805996	551.2257496	0.440559441
0.0085257	329.8828904	1059.848837	0.432315978
0.017954291	179.7526652	1288.464108	0.176404213
0.009154949	352.8235294	494.2509804	0.310596833
0.023627396	303.2225806	283.2645161	0.482115086
0.018370194	204.5915842	380.7128713	0.343537415
0.016042639	233.7923588	1117.363787	0.299875467
0.010386429	272.3736702	688.8962766	0.363812288
0.034998813	235.6125	275.6	0.419397117
0.005098913	387.5036675	2289.888346	0.225282291
0.011726062	254.7290076	487.3778626	0.394281415
0.010100213	343.1252446	907.2818004	0.414604462
0.0094612	377.7851671	2342.602368	0.348797668
0.010803807	463.5523329	634.9773014	0.378339695
0.011938843	309.0694444	206.2314815	0.319054653
0.017007245	320.3681428	1275.339722	0.442268343
0.028175505	220.0751445	148.9537572	0.426108374
0.008110663	358.448152	1601.651951	0.383087512
0.003793603	444.3016912	1707.609929	0.244139584
0.01330708	216.5152722	699.0823373	0.354519774
0.019485982	195.7087379	200.0873786	0.418699187
0.008336345	368.3182126	1327.450914	0.296526802
0.016378272	281.5701754	540.7894737	0.426966292
0.016859284	214.1324201	426.1643836	0.461052632
0.016215603	284.7254902	752.4819401	0.237267385
0.016429312	177.9775641	292.6410256	0.339869281
0.006013123	371.6541451	701.5466321	0.349479402
0.029495921	256.083871	123.1806452	0.479876161
0.016743345	273.7846715	509.459854	0.416730038
0.010269069	286.9392789	483.0075901	0.292777778
0.006985754	467.088	553.6208	0.444523471
0.017802052	213.7932099	302.7469136	0.392251816
0.007725918	307.9855263	736.3789474	0.333041192
0.015010204	184.5397412	509.9463956	0.285789752
0.015788265	220.4022039	648.630854	0.267403315
0.029421054	215.1111111	140.380117	0.322033898
0.018760173	288.8433735	450.5100402	0.380733945
0.009848921	235.5809414	851.2296211	0.227059437
0.017128131	245.0151515	542.4545455	0.349206349
0.008239212	309.8871595	479.2607004	0.376005852
0.0281916	252.6501901	197.9809886	0.327114428
0.020603956	218.1837398	580.0536585	0.242890995
0.008977504	276.9532895	1352.422368	0.337702733
0.017190241	186.1285988	974.3512476	0.302379571
0.010782266	231.0078125	890.125	0.309646205
0.018801661	319.4014156	760.2355915	0.414848993
0.013403129	244.8065984	767.1911263	0.358482871
0.017656685	276.910828	141.8152866	0.501597444
0.025569792	178.7941176	96.17647059	0.380597015
0.005904201	417.636235	2428.714084	0.320262919

log.sigma.2.5.mm.3D_gldzm_IntensityVariabilityNormalized	log.sigma.2.5.mm.3D_gldzm_LowIntensitySmallDistanceEmphasis	log.sigma.2.5.mm.3D_gldzm_IntensityVariability
0.040024953	0.00613231	26.13629403
0.051783979	0.031083448	7.612244898
0.047013069	0.014368621	17.5828877
0.050026896	0.026425127	7.153846154
0.049967706	0.011360887	28.08185053
0.047332832	0.018573122	5.727272727
0.05155291	0.007320536	115.5816236
0.043956177	0.005860986	81.84640172
0.053050485	0.006669938	62.12211785
0.056153616	0.026707737	17.18300654
0.068759252	0.027054313	27.91625616
0.051000072	0.005587417	48.1440678
0.046161149	0.020265678	14.67924528
0.050562952	0.013904085	15.77564103
0.043590379	0.011655482	25.97986577
0.05447217	0.007543073	72.61140285
0.065751132	0.011650528	16.7007874
0.055224913	0.013206612	9.388235294
0.066114534	0.014629709	17.58646617
0.047805708	0.004183216	126.732931
0.062470998	0.017281268	34.92128801
0.056555755	0.015794942	11.65048544
0.057094518	0.01694253	11.36180905
0.063436252	0.009644738	49.03622251
0.047220867	0.010908436	17.23561644
0.045243454	0.004825432	35.69708492
0.044906204	0.006970261	34.26343381
0.056733439	0.008512473	39.94034091
0.061831153	0.016053958	3.586206897
0.059906006	0.007600242	30.671875
0.056758348	0.009410092	65.49913345
0.051561284	0.010164197	18.20113314
0.050594161	0.008470841	36.17482517
0.05350656	0.011180442	33.4416
0.04880191	0.004662241	76.03337612
0.048228076	0.004280506	34.91712707
0.042441195	0.003418605	30.34545455
0.04836008	0.007766744	67.26887132
0.069515306	0.011368831	11.67857143
0.053996929	0.001570362	121.8710678
0.055874955	0.010558397	28.27272727
0.057546939	0.007314146	80.56571429
0.049658198	0.007650102	49.16161616
0.049618564	0.012170786	30.46579805
0.05899511	0.014729897	22.06417112
0.050894308	0.00430507	46.26292629
0.054596695	0.006050685	78.67383761
0.073753946	0.013014753	10.25179856
0.055425656	0.022707174	10.75257732
0.04697364	0.016976131	9.253807107
0.048689069	0.004814067	27.60670194
0.046270185	0.00655889	55.70930233
0.061618499	0.012673088	86.69722814
0.055401769	0.008892003	28.25490196
0.046097815	0.008392542	14.29032258
0.053034016	0.014156179	21.42574257
0.054210218	0.008719077	65.26910299
0.051090708	0.009041218	38.42021277
0.050722656	0.009647561	16.23125
0.052764842	0.004367764	129.4849226
0.048576715	0.010813061	25.45419847
0.043782384	0.007873827	44.74559687
0.043992132	0.004917533	126.3454039
0.050741911	0.003073279	40.23833544
0.051483196	0.010815013	11.12037037
0.043874	0.008799852	66.38136153
0.056433559	0.014538063	9.76300578
0.046727755	0.005285615	91.02566735
0.056251561	0.003435753	103.1091107
0.05338716	0.01216026	40.20053121
0.055471769	0.018235873	11.42718447
0.048316112	0.00574847	71.36289777
0.049547553	0.007469315	28.24210526
0.049654928	0.015843659	21.74885845
0.051547402	0.005604114	49.9494324
0.06235618	0.014805493	19.45512821
0.054108835	0.005258737	41.77202073
0.051404787	0.017129964	7.967741935
0.049996004	0.008254518	27.39781022
0.054365947	0.008903548	28.65085389
0.04052736	0.005737503	25.3296
0.053193111	0.016792173	17.2345679
0.051520083	0.007009796	39.15526316
0.067435194	0.011155948	36.48243993
0.052629981	0.011601431	38.20936639
0.062412366	0.023828879	10.67251462
0.049402429	0.007906512	24.60240964
0.055461015	0.009473085	48.3065442
0.046582548	0.015251287	27.67003367
0.054421717	0.007317763	27.97276265
0.056311353	0.009615124	14.80988593
0.057449931	0.010950237	35.33170732
0.053828774	0.007195808	81.81973684
0.057054756	0.015897811	59.45105566
0.060375214	0.00779706	61.82421875
0.042042856	0.008401514	41.58038423
0.051681182	0.010375872	45.42775882
0.049535478	0.015960964	7.777070064
0.070934256	0.024294374	7.235294118
0.04737377	0.004550394	133.8782732

log.sigma.2.5.mm.3D_gldzm_HighIntensityLargeDistanceEmphasis	log.sigma.2.5.mm.3D_gldzm_SmallDistanceEmphasis	log.sigma.2.5.mm.3D_glcm_SumVariance	log.sigma.2.5.mm.3D_glcm_Homogeneity1
570.1562021	0.948315467	1204.559869	0.287967531
203.585034	0.969387755	411.0656376	0.296687692
301.3502674	0.953877005	628.5262003	0.30039691
257.6293706	0.952797203	523.3257421	0.286439351
255.0320285	0.939946619	541.6482706	0.312192183
357.2561983	0.95661157	933.2055433	0.280323606
293.85281	0.968889384	645.8692584	0.457971239
456.5338346	0.952067669	868.5672158	0.349321319
284.7403928	0.982707088	569.2854856	0.396774776
176.4542484	0.970588235	334.662942	0.321068152
141.544335	0.966748768	264.2835557	0.410713422
388.8019068	0.964247881	823.2604947	0.402510568
285.5345912	0.922169811	684.3129323	0.312135412
240.3814103	0.932692308	505.9191343	0.278582731
326.8775168	0.964765101	587.0730352	0.325985052
272.63991	0.987621905	657.9985378	0.425351749
235.9330709	0.988188976	522.8983393	0.382357773
213.0411765	0.920588235	399.8870702	0.307431356
181.7518797	0.980263158	335.9771116	0.411703549
542.2912109	0.973636783	1438.020523	0.37017779
168.2271914	0.97450805	299.0074748	0.367904831
178.2961165	0.952669903	319.8309421	0.305230228
210.8341709	0.97361809	391.9210776	0.342617057
222.5730918	0.974773609	416.6527963	0.469320894
284.9863014	0.981506849	646.1053625	0.414766871
493.5665399	0.985741445	1085.562695	0.359777138
356.542595	0.969528178	709.9474134	0.315950778
276.1534091	0.968039773	540.825874	0.368616014
156.6896552	0.909482759	320.9814102	0.33515636
314.703125	0.997070313	778.2895111	0.355616423
233.4098787	0.983102253	467.0962777	0.366758426
328.4645892	0.961756374	731.300704	0.293817222
306.4041958	0.950699301	592.5455004	0.374984855
233.856	0.9868	504.5097212	0.41679395
424.3331194	0.969102125	911.2483029	0.361301977
434.5207182	0.914019337	990.2633445	0.307470789
779.5678322	0.833216783	1822.358288	0.253800476
316.278936	0.980589504	741.5792312	0.456942808
177.3869048	0.933035714	345.4625687	0.35686419
1080.168808	0.967766947	2903.513787	0.427519654
251.229249	0.951086957	489.7941915	0.329317355
313.7971429	0.935714286	557.4212895	0.429128807
296.4575758	0.975757576	742.6226512	0.295625248
283.5553746	0.958469055	540.2426601	0.300435644
191.2513369	0.977941176	342.049452	0.448162704
388.8371837	0.969471947	828.0338216	0.324361815
341.1596114	0.971374046	696.9529401	0.379598947
268.2517986	0.994604317	623.7081215	0.378713092
205.0360825	0.976804124	354.4533565	0.29600878
295.0406091	0.965736041	695.412441	0.293903866
469.1922399	0.989417989	1082.870543	0.308832995
391.0315615	0.952034884	808.8462219	0.288596882
197.8592751	0.966950959	362.8921235	0.510980471
366.1705882	0.988235294	1051.61659	0.370025503
315.9	0.966129032	729.7923928	0.276128895
213.5841584	0.977722772	408.3065089	0.332935258
251.0049834	0.971968439	482.4031645	0.388412345
299.1103723	0.967087766	657.7560168	0.318116088
251.475	0.94375	478.6661044	0.305498237
426.6124694	0.974022005	940.3883231	0.442949988
278.8721374	0.972805344	607.003042	0.31659742
399.7994129	0.955234834	917.5252089	0.295035517
472.3081476	0.922963092	1003.820684	0.332206208
583.593947	0.915825977	1323.475599	0.309937612
320.1527778	0.982638889	809.8670609	0.345591258
390.3912756	0.935558493	816.9077144	0.276054812
232.7861272	0.943641618	499.6091427	0.298222824
454.8495893	0.926006731	882.5985108	0.310736995
493.3786143	0.973404255	1187.853997	0.387607448
234.7264276	0.972111554	595.5598171	0.331141476
198.5485437	0.98907767	360.5705016	0.310429618
410.2369668	0.959884902	917.2331897	0.361498963
288.3333333	0.980263158	609.776078	0.300491749
218.4269406	0.989726027	449.8255348	0.313189035
354.3869969	0.903881436	610.104962	0.41425749
187.2467949	0.975961538	379.2443338	0.355290732
426.388601	0.964054404	1084.841455	0.317457005
284.3612903	0.912903226	665.8322112	0.274565398
292.6551095	0.972627737	725.8945467	0.309690037
312.2201139	0.967267552	633.2467295	0.380972072
544.3536	0.9544	1191.581984	0.29208089
228.7654321	0.974537037	548.8118885	0.322043999
314.9171053	0.988157895	716.929352	0.364403629
190.7393715	0.977818854	342.4598953	0.383586504
247.7782369	0.957644628	448.8260534	0.397220852
254.8830409	0.925438596	675.7014442	0.351559554
308.1807229	0.962349398	641.5483443	0.326568148
240.0447761	0.991389208	436.9310573	0.461464608
265.979798	0.965909091	583.9414588	0.350990712
335.6673152	0.973735409	849.141142	0.316999037
332.1558935	0.891634981	679.6786561	0.343993466
225.5154472	0.97804878	390.4563585	0.430255261
318.3177632	0.956085526	713.88154	0.342140283
201.1804223	0.974808061	404.3808626	0.365639599
271.7744141	0.947265625	527.7006153	0.356767749
418.4590495	0.899898888	814.2358937	0.28070443
285.2093288	0.948805461	570.0787941	0.325222691
301.0063694	0.961783439	755.0046838	0.269130366
186.5	0.977941176	378.9197946	0.330188337
505.1818825	0.942940552	1281.760602	0.340891115

log.sigma.2.5.mm.3D_glcm_Homogeneity2	log.sigma.2.5.mm.3D_glcm_ClusterShade	log.sigma.2.5.mm.3D_glcm_MaximumProbability	log.sigma.2.5.mm.3D_glcm_Idmn	log.sigma.2.5.mm.3D_glcm_SumVariance2
0.201552623	692.9345717	0.013418428	0.972275069	104.861281
0.207312153	-35.65115771	0.017707284	0.949096743	75.42709787
0.214976181	-84.01108545	0.010500692	0.951237093	96.41709597
0.197317663	-72.61829778	0.015354225	0.946636733	94.7811025
0.222867284	178.2026157	0.012890539	0.963539959	83.90159026
0.188916079	-226.5733283	0.016049019	0.954535617	92.16447042
0.395162764	139.7475139	0.086969203	0.984230126	43.07906058
0.271594472	324.2944823	0.028778351	0.972723029	71.60086915
0.323067406	242.2078522	0.041813003	0.978221895	52.53503238
0.229304386	43.51603839	0.014469375	0.9613067	53.15454991
0.334360685	52.03139787	0.03798434	0.969438664	32.99656853
0.330721538	156.0849277	0.045023803	0.979798559	46.3244457
0.226210747	-451.6643529	0.019367858	0.959726891	95.25178719
0.186083998	161.0256205	0.010887526	0.953154018	93.33912379
0.243801542	391.3911664	0.019186289	0.970122063	72.26715455
0.361156479	83.89352896	0.080804745	0.973624837	65.67454585
0.29916937	56.50453805	0.020197613	0.980485897	38.00811901
0.218208007	121.7816212	0.015909362	0.962156582	76.47760561
0.333983986	37.74575733	0.029200471	0.978153388	28.62014111
0.290698726	104.0805194	0.019938889	0.9868114	57.33363724
0.284681893	51.74757604	0.015763743	0.966272482	56.45023833
0.215105875	204.5274969	0.017148739	0.955234856	70.44016184
0.257575903	84.41903751	0.018891527	0.96688678	62.60433454
0.405043777	101.02478	0.082478359	0.983309643	30.94970617
0.345953675	81.7750169	0.093344834	0.965187162	75.18448532
0.280236442	632.7787684	0.022007656	0.98250772	76.93401332
0.228959349	376.087975	0.014252896	0.97484638	71.94865229
0.287088398	150.5235622	0.020379758	0.979254234	49.2881122
0.245636949	190.9983046	0.03686904	0.968415861	44.56325196
0.269441204	60.62667266	0.017722697	0.975656385	50.74577447
0.285587641	213.2289843	0.015004365	0.973853738	59.81001652
0.206731932	-32.32004842	0.013613516	0.949973258	89.75575014
0.29721145	245.1562297	0.029662508	0.976761999	61.33910789
0.346936204	170.0035981	0.048339896	0.970141849	63.35014218
0.280547525	250.8215807	0.025345886	0.983908529	54.63167807
0.225186442	-213.4293849	0.015001678	0.959053366	89.87522228
0.166555726	-441.1436452	0.008965616	0.964407125	129.1801252
0.39810759	102.5280229	0.116873573	0.974110923	65.19722726
0.269224347	-16.46674575	0.017447258	0.962658641	39.47111124
0.357786676	191.6062102	0.063708606	0.991212233	41.64848952
0.240195501	172.2418279	0.015971915	0.971525507	63.34238324
0.358779645	135.3773257	0.062414832	0.98532699	34.41768606
0.208257239	-72.31955821	0.011101354	0.95841504	96.92057085
0.210790229	242.8543502	0.011886701	0.963223081	78.48610479
0.381177958	102.5023805	0.072357029	0.979702438	34.13785056
0.239420041	218.3732716	0.016294316	0.969967324	75.48700077
0.301144144	170.503525	0.023647599	0.985633571	48.49403195
0.292825141	-9.778734379	0.026170582	0.977935212	35.09781404
0.202846163	223.3199924	0.014356003	0.954782286	72.53721583
0.207527565	-328.9917039	0.020468628	0.955704373	101.1433357
0.221127946	376.2776974	0.015846883	0.973572405	67.23427082
0.197292872	346.3752406	0.009719894	0.976167641	81.38817336
0.456376842	68.62072397	0.09580791	0.984911659	26.63418272
0.288668529	-38.90066905	0.024950318	0.979970261	42.22826567
0.1873951	-86.29647393	0.013790254	0.95800933	109.2625172
0.245080874	300.6225813	0.014306441	0.968622629	72.1443524
0.311369583	209.1954147	0.040747461	0.972580366	56.00770656
0.231723822	86.64979606	0.012314965	0.967554331	76.21402992
0.218322223	179.4738296	0.013860722	0.957002363	71.24160643
0.377080878	174.0420126	0.065919949	0.987247256	38.5470468
0.230660522	79.81129443	0.013583102	0.970868099	69.34638641
0.208530954	106.7909749	0.013397448	0.970060309	85.56549504
0.25098291	77.64229815	0.01934832	0.970901304	77.74774881
0.218549496	76.30541702	0.010597777	0.979559557	73.61874549
0.259758875	-12.21588865	0.018529452	0.969318392	52.03582532
0.186559206	-0.919802245	0.007948349	0.965995181	83.99289342
0.208462968	-127.2119146	0.016367027	0.944543639	71.24599643
0.224357662	278.0321814	0.011673256	0.978339187	73.37290727
0.307690646	154.6004578	0.021223768	0.987408265	39.7673537
0.242406568	-24.65699074	0.013735187	0.973131471	62.95184775
0.221066403	105.0044687	0.014336363	0.951899229	71.37159117
0.287246919	76.29056012	0.032777577	0.970160367	68.65041918
0.211848399	251.0281967	0.014897147	0.962590509	81.85308523
0.225816666	186.680323	0.016144492	0.957665455	67.89752015
0.34264547	264.6354145	0.067289118	0.982785699	49.06748415
0.268309745	14.13061538	0.018487394	0.968712057	42.35781058
0.225842661	-13.40055157	0.012988992	0.97665822	56.43493154
0.185698995	-374.7311119	0.017019015	0.945840003	118.4224484
0.218139033	-23.35560743	0.011405105	0.970873628	70.12527993
0.301359893	157.5258685	0.029926231	0.981604514	43.25494961
0.20564935	703.9802468	0.013732527	0.973456725	100.0983595
0.233107769	-47.56132025	0.014841109	0.968956736	66.63783589
0.290111725	148.7915116	0.04875755	0.966505877	67.74025134
0.300406079	85.14866103	0.022893812	0.972490382	35.21768265
0.32215735	176.1083609	0.043211503	0.97259056	54.02408589
0.269262361	-450.7224559	0.039703042	0.950986545	59.52258377
0.24176832	163.4559368	0.015775584	0.964571788	71.47621211
0.397634921	195.3548683	0.087814436	0.979525925	44.91458804
0.268202729	-5.567081446	0.03036119	0.969111878	62.39090809
0.228266189	-49.61149774	0.01278398	0.967367008	68.9559238
0.257681843	-48.78334382	0.020505489	0.970491719	63.78535901
0.360294497	152.8637329	0.043841017	0.978199391	38.66088974
0.255407431	39.58062567	0.016372286	0.980667264	49.22605692
0.283589546	103.4529995	0.014291775	0.971977156	56.02593877
0.269744401	64.80664278	0.015122335	0.980725829	42.91099042
0.194085376	48.79039379	0.009160219	0.965248751	104.25127
0.237877846	67.96140755	0.011096823	0.972133834	72.60100096
0.176704763	-233.1339485	0.017864794	0.953194986	79.97399266
0.238978779	-110.1478877	0.020146408	0.956760883	48.48092164
0.259102922	-25.91701492	0.015602696	0.977615117	67.36654832

log.sigma.2.5.mm.3D_glcm_Contrast	log.sigma.2.5.mm.3D_glcm_DifferenceEntropy	log.sigma.2.5.mm.3D_glcm_InverseVariance	log.sigma.2.5.mm.3D_glcm_Entropy	log.sigma.2.5.mm.3D_glcm_Dissimilarity
54.4918014	3.910960786	0.208141627	8.625493159	5.445451249
36.8635815	3.613698587	0.208041587	7.690203303	4.746946637
45.1022326	3.749120163	0.222934446	8.426170634	5.033962933
42.40390213	3.710319017	0.203455994	7.832905251	5.05346878
32.04233123	3.577767845	0.223516898	8.285466163	4.371344355
41.28625229	3.643068165	0.203499732	7.588733207	4.99589058
21.04230739	3.181748762	0.309830117	7.359125276	2.999066141
39.42462202	3.671554162	0.252455393	8.429921833	4.467614259
24.49250008	3.338609472	0.293933296	7.764023039	3.468666729
25.23888894	3.392765172	0.242187929	7.855486748	3.910572902
15.13512952	3.063311112	0.315588641	7.256826746	2.853911241
25.65227866	3.365479541	0.297302999	7.729523787	3.489147843
38.88540932	3.663068395	0.227853352	8.149146855	4.680919487
42.37986589	3.732485708	0.190174962	8.32794143	5.08245375
43.82757464	3.729219258	0.240174677	8.262105842	4.747038356
30.44518153	3.32797474	0.296905706	7.608147641	3.652420495
16.43859564	3.117661851	0.299248917	7.355849633	3.046273798
33.2704011	3.558277897	0.221536401	7.734228016	4.472162496
13.7039109	2.985525118	0.325237884	7.088340243	2.726252046
27.18241399	3.426296984	0.281472603	8.120725293	3.694380148
20.09569937	3.250268776	0.284180311	7.790614065	3.365335371
32.05936713	3.544940312	0.224080853	7.832309191	4.412956198
27.1226083	3.438337451	0.256049426	7.75474404	3.906403448
13.26305405	2.943006054	0.339916233	6.990981188	2.471014745
32.73184575	3.44245605	0.273823497	7.647509512	3.768503461
31.78934818	3.515244685	0.268919319	8.066670598	3.995857727
38.03942801	3.64959676	0.231164518	8.292258733	4.558622095
23.1819929	3.337135496	0.286374338	7.817047457	3.502632547
23.89821827	3.243577993	0.256629216	6.55791498	3.735993634
22.32172002	3.324840337	0.263240248	7.836314264	3.559305217
24.35383844	3.357707021	0.280340152	7.993094379	3.598737402
42.41174056	3.72732805	0.212020658	8.258895287	5.013724348
26.15760354	3.405706295	0.281738275	7.973358771	3.667190976
25.09457266	3.303936703	0.300944889	7.687872028	3.380577046
27.5546689	3.457268417	0.272775882	8.022454732	3.793295257
48.2186447	3.805871058	0.219734203	8.61432871	5.16338533
66.68424361	3.959306545	0.169521667	8.845883508	6.320773949
30.12727463	3.314799853	0.29148215	7.48281871	3.501741676
18.6620831	3.183935692	0.279196621	7.417810825	3.317881216
20.29659463	3.213038628	0.312579744	7.488732394	3.067453773
26.34001089	3.439136326	0.246631047	8.024571411	3.929425036
19.38300502	3.19642376	0.30942102	7.280576323	3.011985841
42.75896234	3.633590867	0.209380359	8.53160539	4.999376234
37.4357805	3.676404448	0.216465618	8.384669943	4.680452949
15.15676285	3.01680333	0.338553803	7.054680355	2.670666152
34.07360267	3.606266319	0.237667908	8.336762816	4.390527086
24.63995881	3.364130384	0.290941489	7.883337788	3.509760043
14.97187843	3.038172871	0.298024829	7.03582436	2.956875725
32.82672302	3.537193322	0.214952579	7.782766303	4.466522383
42.59127177	3.714239152	0.209136178	7.965396166	5.040611385
37.47992367	3.658056182	0.228519034	8.105242859	4.617235903
41.46074485	3.757448535	0.200826953	8.605691531	4.930180275
12.01472801	2.818289884	0.358928951	6.646907493	2.224531278
23.75291634	3.31005311	0.284350496	7.60048271	3.524075313
48.88513198	3.823782102	0.195426006	8.316731235	5.40795287
27.52220762	3.459161757	0.245079631	8.042577374	3.971286442
24.08511276	3.352563963	0.278642946	7.883539927	3.499481253
34.76532689	3.621230825	0.23345089	8.310371231	4.453326274
38.99911855	3.69152461	0.221955464	8.158292482	4.732457233
20.88856403	3.20418453	0.316219698	7.329723661	3.023225038
37.41811114	3.665460285	0.229840559	8.296160321	4.574073758
45.3161929	3.813168634	0.209214622	8.70168683	5.107337742
39.89924373	3.69024908	0.245918728	8.633462336	4.575228911
31.8790811	3.565346498	0.228586257	8.377607516	4.298648971
27.16654285	3.423653023	0.252610718	7.708742805	3.865332639
49.25723976	3.874883285	0.191444682	8.821148877	5.399275535
34.46697073	3.57707374	0.217516155	7.757674787	4.600567907
39.42136482	3.71621055	0.225617461	8.558544732	4.697264188
19.28295081	3.218812613	0.303797593	7.629912618	3.16498897
26.4486337	3.453534996	0.244234036	8.131279428	3.935011304
32.14892463	3.520079349	0.225168447	7.863700399	4.386046882
36.28900777	3.60290528	0.264841313	8.264339193	4.306130186
37.97051152	3.675337364	0.217395202	8.305517267	4.726741272
35.57119708	3.630371459	0.226616088	8.084964266	4.524053153
24.28657775	3.33109326	0.287372521	7.602519763	3.369565043
20.06268485	3.245241622	0.271766011	7.573891121	3.424580044
27.44382751	3.474550096	0.230509635	8.110552051	4.067622873
46.14117959	3.753435155	0.192616424	7.878031417	5.340152395
30.86978254	3.542320515	0.226535099	8.245514314	4.27891041
23.24599142	3.330604632	0.287432906	7.578191124	3.429289847
51.92724119	3.877148735	0.212248058	8.561841285	5.312587009
29.02178928	3.496569192	0.240421072	7.998853279	4.116218787
38.894081	3.599413444	0.253331218	7.981662351	4.411390499
16.13127409	3.111510297	0.302735056	7.419939845	3.01209853
22.41398384	3.303739522	0.293315063	7.786829317	3.357469747
28.56157013	3.436142957	0.264803284	7.240817135	3.915095327
33.81184819	3.571614643	0.244719871	8.220150654	4.33669205
17.85626446	3.100897876	0.318888323	7.270714052	2.798596529
29.38232312	3.505786686	0.255010414	8.117913034	3.963029495
30.48777131	3.536837871	0.235512704	8.137817331	4.242705999
25.84513334	3.401613918	0.259880271	7.872271829	3.79820587
17.66417558	3.119653168	0.325349198	7.292653496	2.897300377
25.42104098	3.435368969	0.255668552	8.052072006	3.802251819
22.67608236	3.3388639	0.278723526	7.997761656	3.528347221
19.87066162	3.255634705	0.274633235	7.743960305	3.391490498
53.24529576	3.907906022	0.194784152	8.855826848	5.557387858
31.50971151	3.552442536	0.237720713	8.326686791	4.226429066
45.17140086	3.753970028	0.182013013	7.762131689	5.299109101
21.73433492	3.233935072	0.253299007	7.229630516	3.670825508
33.37653558	3.580882704	0.25587792	8.413045891	4.224662828

log.sigma.2.5.mm.3D_glcm_DifferenceVariance	log.sigma.2.5.mm.3D_glcm_Idn	log.sigma.2.5.mm.3D_glcm_Idm	log.sigma.2.5.mm.3D_glcm_Correlation	log.sigma.2.5.mm.3D_glcm_Autocorrelation
24.15048219	0.894012546	0.201552623	0.3217775	381.9827604
13.43571606	0.853151702	0.207312153	0.346527211	145.3510156
18.57308747	0.861522688	0.214976181	0.36366962	212.963335
16.1779586	0.85080536	0.197317663	0.383261547	179.5450338
12.50374337	0.874852162	0.222867284	0.44685197	188.8672902
15.52287733	0.8602062	0.188916079	0.386447815	302.6727654
11.69217612	0.92786796	0.395162764	0.350520604	217.9565021
18.79311937	0.899113344	0.271594472	0.297336652	285.1634544
11.91946173	0.91022559	0.323067406	0.364407361	196.5286147
9.512844076	0.869969105	0.229304386	0.356767803	123.8561315
6.710676238	0.890135982	0.334360685	0.368889541	101.6351936
13.14718594	0.91467711	0.330721538	0.29149522	270.8295451
15.99800735	0.872735204	0.226210747	0.42134236	230.2751883
15.58403919	0.858175855	0.186083998	0.375175275	175.3175069
20.56409714	0.893573861	0.243801542	0.251495986	199.2783056
15.61648784	0.908213225	0.361156479	0.374959502	221.6608454
6.933350951	0.908039321	0.29916937	0.39671625	182.3969568
12.71952496	0.872382368	0.218208007	0.392793346	142.662494
6.011753568	0.905159675	0.333983986	0.352080193	124.097483
13.09645817	0.927506029	0.290698726	0.360936162	450.2908202
8.38433818	0.882375498	0.284681893	0.47360652	113.6711428
12.00353584	0.861423753	0.215105875	0.375320913	117.1829196
11.36656175	0.883487859	0.257575903	0.394999664	141.8280766
6.994176349	0.922852477	0.405043777	0.399527462	149.1013619
17.83394329	0.8951092	0.345953675	0.391949912	218.6550015
15.19571432	0.91760778	0.280236442	0.409584355	349.0902864
16.13861352	0.898324999	0.228959349	0.31553144	237.9361234
10.58760058	0.908370332	0.287088398	0.363621709	188.4084689
9.352839352	0.883282739	0.245636949	0.307461849	115.7428367
9.252570856	0.89812634	0.269441204	0.38986917	259.923583
10.82674959	0.898220326	0.285587641	0.417961408	166.3439671
16.39539132	0.856582269	0.206731932	0.359396251	244.1398946
12.26095991	0.905271993	0.29721145	0.404137962	204.5460871
13.27148758	0.900159577	0.346936204	0.427358235	177.1386
12.80265862	0.919091724	0.280547525	0.334196296	297.8204529
20.06230948	0.873320812	0.225186442	0.311870861	320.6780581
21.97730042	0.876055939	0.166555726	0.335620448	557.3473464
17.14822534	0.912498232	0.39810759	0.370867963	246.1288121
7.278436766	0.873770301	0.269224347	0.358557187	127.4061279
10.63343527	0.942456563	0.357786676	0.350262473	850.0537725
10.49422191	0.888824348	0.240195501	0.411895273	173.1665778
10.09189353	0.92662812	0.358779645	0.285489392	190.7890275
14.10715124	0.86859897	0.208257239	0.399930085	248.4895903
14.97697322	0.875248237	0.210790229	0.355294903	187.1101872
7.830401722	0.914675759	0.381177958	0.384518626	125.6968374
14.1550461	0.888379381	0.239420041	0.381887288	274.8277931
12.01787797	0.924714086	0.301144144	0.33166038	234.8946724
5.985025167	0.900753945	0.292825141	0.40098579	211.703352
12.01047981	0.859836646	0.202846163	0.380604823	128.3751294
16.11709492	0.863677376	0.207527565	0.41072109	233.1191546
15.61823488	0.89431515	0.221127946	0.282553115	346.1104102
16.5716856	0.897213123	0.197292872	0.331769223	268.1750572
6.956315859	0.93057702	0.456376842	0.379177518	131.0855362
10.50868462	0.910425986	0.288668529	0.290973345	336.5426156
18.79111818	0.866563165	0.1873951	0.386549311	242.8088494
11.2936481	0.884936276	0.245080874	0.4450618	147.3005044
11.26260819	0.898389869	0.311369583	0.395551903	170.7427212
14.37308213	0.883931878	0.231723822	0.374501683	223.4100079
16.03745511	0.867471296	0.218322223	0.292484771	166.8770713
11.50605033	0.93352812	0.377080878	0.304261969	302.752393
15.8906868	0.890318985	0.230660522	0.304300922	207.1928389
18.57124598	0.887487363	0.208530954	0.314841332	299.9724939
18.17385466	0.894039646	0.25098291	0.328748808	326.0532965
12.92176793	0.904519268	0.218549496	0.399794222	419.7820822
11.66524434	0.888169479	0.259758875	0.318084384	267.6532405
19.29769049	0.878960967	0.186559206	0.268646049	269.226483
12.5298968	0.846880156	0.208462968	0.349927326	173.4607284
16.74475127	0.90405042	0.224357662	0.306838745	289.7527825
9.037824637	0.927694055	0.307690646	0.349365547	376.1811431
10.60107649	0.891816426	0.242406568	0.40995812	206.0282484
11.92086124	0.857986053	0.221066403	0.379500369	130.3895553
17.18922762	0.894834951	0.287246919	0.318467006	299.8760664
14.98564636	0.874200619	0.211848399	0.368277008	207.9216218
14.58050065	0.868356722	0.225816666	0.310285323	158.2899575
12.50364221	0.921211532	0.34264547	0.343791437	208.0489823
7.942800134	0.884012591	0.268309745	0.361088241	138.3681162
10.45551473	0.896822518	0.225842661	0.351189106	349.2597864
16.4661562	0.848026544	0.185698995	0.441574421	223.0094595
12.07559188	0.886856266	0.218139033	0.391591644	244.8286508
11.22638175	0.914998801	0.301359893	0.305377719	214.8170599
23.04108624	0.896182107	0.20564935	0.322959322	378.3355495
11.6317152	0.884537663	0.233107769	0.392776533	190.8330887
18.32389871	0.89048584	0.290111725	0.275103044	238.7893006
6.808285726	0.892561115	0.300406079	0.372923422	126.8399604
10.76983272	0.89900183	0.32215735	0.412008863	160.4395113
12.52980095	0.863747955	0.269262361	0.357895208	225.0785591
14.11583155	0.880559393	0.24176832	0.359280401	218.5865545
9.830893124	0.9175495	0.397634921	0.428941125	155.8681882
13.2501519	0.88968035	0.268202729	0.361577364	201.6019937
12.03013555	0.881480265	0.228266189	0.388055172	281.0176344
10.86368205	0.889533986	0.257681843	0.425384555	231.0600555
8.979810162	0.91134944	0.360294497	0.37060258	141.3278022
10.67726208	0.908056348	0.255407431	0.323030949	240.2729345
9.859303383	0.89323732	0.283589546	0.42293736	147.2643987
8.028316207	0.907261259	0.269744401	0.365677725	184.4491335
21.19232137	0.879317724	0.194085376	0.331124448	268.5789686
13.00316383	0.891722334	0.237877846	0.397150901	197.7793917
16.45706003	0.856923147	0.176704763	0.281558172	248.4521557
7.599185571	0.861915291	0.238978779	0.387984728	137.0918893
14.92485174	0.905000272	0.259102922	0.345522678	406.6184903

log.sigma.2.5.mm.3D_glcm_SumEntropy	log.sigma.2.5.mm.3D_glcm_AverageIntensity	log.sigma.2.5.mm.3D_glcm_Energy	log.sigma.2.5.mm.3D_glcm_SumSquares	log.sigma.2.5.mm.3D_glcm_ClusterProminence
5.277208299	19.59572764	0.003773935	40.37604515	41227.78276
4.978560106	11.64479407	0.005948193	28.07266984	13978.64865
5.225821762	14.22811242	0.003592887	36.21869261	20827.44673
5.101325339	12.91654947	0.005182741	34.56301195	20223.78589
5.13027592	13.28973077	0.004084972	29.41236442	16373.77619
5.055160905	17.23116727	0.006075975	34.64378124	23189.97116
4.59960027	14.73920454	0.019285543	16.72688775	8416.337927
5.063285795	16.93273552	0.005057012	28.46072695	18669.69138
4.800910513	13.86615877	0.008456978	20.02246223	9653.924283
4.843048643	10.80968361	0.005331591	19.59835971	7778.648028
4.506661906	9.857246255	0.010031384	12.03292451	3160.045272
4.724629734	16.46769238	0.009746726	18.54864508	8977.428705
5.135220736	14.77421079	0.004995572	34.09855457	21423.11135
5.188071136	12.78632595	0.003762074	34.4555631	19880.98462
5.035405456	14.11795558	0.005119564	30.50589724	18800.86973
4.841319445	14.81213139	0.014230066	24.97041373	11625.86159
4.588056459	13.31445104	0.007880659	13.75859718	4320.57086
4.982073024	11.49062693	0.005722631	27.61484914	13961.3428
4.410516207	10.97094798	0.010629033	10.581013	2574.487291
4.925809114	21.19910972	0.006053416	20.82058181	12795.18414
4.878338998	10.22583133	0.005791164	19.13648442	8011.799033
4.952591084	10.36910937	0.00544542	25.62488224	13106.88111
4.91355622	11.54147578	0.0059371	22.63954241	10383.58034
4.4178864	12.0327089	0.018141317	11.12868441	4277.249116
4.9530496	14.50230337	0.014774441	27.67411015	15264.90957
4.999588393	18.58490001	0.006212055	27.5162118	24107.12415
5.036981916	15.43913362	0.004476716	28.71037337	19044.2608
4.801914519	13.58512252	0.006542805	18.98366306	7955.072853
4.404950863	10.52728907	0.013060513	17.36545379	7519.743968
4.827357667	15.95823628	0.005691709	18.75560955	7183.003819
4.916685089	12.57991013	0.005445552	21.42126244	10162.23913
5.153924744	15.32590134	0.004116184	33.83828041	17137.83638
4.93404539	14.08628301	0.006797562	22.65088016	12720.49848
4.886222894	12.96263793	0.009953233	22.35032159	10670.45586
4.852361501	17.31436029	0.006164707	20.67956592	10502.05027
5.221379033	17.913367	0.003475503	34.7207023	21817.94977
5.399651646	23.67834588	0.002767506	47.7433184	38996.64477
4.805639408	15.60079642	0.022349261	24.55101352	13012.21246
4.615624581	11.05359661	0.007276207	14.53329858	4031.608927
4.631620676	29.25857067	0.013019633	15.62295616	7736.481673
4.954981611	12.83229435	0.005048402	22.77643366	10522.72721
4.482550519	13.79541563	0.015649399	14.26195039	5481.041929
5.24335263	15.532939	0.003521085	36.19345299	22314.10185
5.107735619	13.42687357	0.003874163	30.23999291	15965.24076
4.44796965	10.99922925	0.01613682	12.35674759	5477.489605
5.092515736	16.48708438	0.004221453	28.61268456	14851.10177
4.796355518	15.35076295	0.006698209	18.88252831	9623.462494
4.490407914	14.37520064	0.009971575	12.51742312	3052.997988
4.975307741	10.88120427	0.00540375	26.34098471	14159.0237
5.184859473	14.86557478	0.005358203	36.76167433	26210.83267
4.936052126	18.66006466	0.005240315	26.41818602	14954.49656
5.16523695	16.39558065	0.00341573	31.18298256	23405.44919
4.239782841	11.29118129	0.02821687	9.707152858	3525.666735
4.666713221	18.46835184	0.007872554	16.76380192	6492.59704
5.26972087	15.35052417	0.003900647	40.71895508	25382.04805
5.000777684	11.68597854	0.004901193	25.24909612	15578.11252
4.866058289	12.78292734	0.007402941	20.37110177	9806.6631
5.076871384	14.74964134	0.004072632	28.86379461	13120.40984
5.019109267	12.67353552	0.004449377	28.43120889	13711.49586
4.514291814	17.48532787	0.017669288	15.06109161	8446.077266
5.040692969	14.29419451	0.004133761	27.72480207	13404.23209
5.209483708	17.374433	0.003319254	32.75156614	22930.2561
5.154203183	18.08686785	0.004057791	29.54836875	18467.14836
5.111120121	20.44890654	0.003914785	25.67719307	16219.21724
4.808689904	16.26750991	0.006421377	20.50830033	7745.431163
5.210118258	16.47547717	0.002863979	33.78114425	20720.92542
4.935732005	12.81530561	0.005648745	26.42824179	10927.20989
5.094883814	17.06207182	0.003915019	28.41309266	18584.22556
4.642361119	19.43546358	0.007990636	14.52647361	6120.29503
4.985917599	14.07626813	0.004622374	22.77635778	10384.5809
4.955293904	10.97917698	0.005186284	25.88012895	11280.2952
5.032890726	17.41660792	0.005774596	26.9977961	13464.25553
5.090983478	14.1594908	0.004182576	31.10603627	15491.04327
4.969194339	12.27778879	0.004793484	26.19861369	11641.83003
4.728682611	14.36941566	0.012576731	19.27504398	10818.53646
4.692439946	11.52297082	0.006704698	15.60512386	4867.482379
4.91809184	18.74077686	0.004772032	20.98448494	9320.641204
5.234425589	14.36649524	0.005092139	41.84314668	30920.77781
5.052250415	15.4200977	0.004304812	25.89487383	14613.5596
4.68016513	14.63026702	0.008569827	17.65792183	8029.818879
5.239823758	19.50377536	0.003939821	38.44425153	38087.97904
4.980116443	13.50136641	0.004935512	24.3167579	11397.06228
4.955755505	15.45529756	0.007441238	27.56696176	12517.15644
4.568483122	11.0483242	0.008376482	12.83723919	3950.009112
4.831250548	12.36629453	0.008793377	19.35440608	9569.273333
4.661818772	14.74101257	0.011019512	22.02103848	11810.01181
5.048549818	14.56195025	0.004658502	27.27752853	14274.71168
4.622304022	12.23301901	0.018064524	16.01444431	8101.118132
4.972293984	13.97331067	0.005960841	23.62284664	12671.98067
5.016378715	16.57458414	0.004396584	25.50744289	10823.42041
4.953322229	14.93657373	0.006013708	22.93274161	12779.90949
4.574070907	11.6780545	0.012357725	14.28242571	6002.923944
4.834187987	15.50382811	0.005193578	19.27746817	7740.797004
4.909451191	11.79371549	0.005300022	19.79445297	8705.836153
4.716705225	13.40988321	0.006022322	16.07581984	4891.277743
5.343080557	16.36778295	0.002814455	39.74215264	28427.86364
5.082153168	13.78484923	0.003965645	26.74033732	13148.72099
4.986208544	15.63096853	0.005529422	32.2194524	15859.4579
4.660820481	11.41670838	0.008043655	17.55381414	5752.539671
5.057700707	20.24006127	0.004502697	24.21081224	13947.06577

log.sigma.2.5.mm.3D_glcm_SumAverage	log.sigma.2.5.mm.3D_glcm_Imc2	log.sigma.2.5.mm.3D_glcm_Imc1	log.sigma.2.5.mm.3D_glcm_DifferenceAverage	log.sigma.2.5.mm.3D_glcm_Id
38.43880589	0.817972407	-0.122588592	5.445451249	0.287967531
23.28958814	0.902540919	-0.199038598	4.746946637	0.296687692
28.29145269	0.822875875	-0.131459965	5.033962933	0.30039691
25.79684089	0.930716843	-0.228300425	5.05346878	0.286439351
26.52418932	0.748450453	-0.097861386	4.371344355	0.312192183
34.05409285	0.961028708	-0.289692289	4.99589058	0.280323606
29.1510997	0.755442045	-0.113585325	2.999066141	0.457971239
33.29370263	0.681301816	-0.075385905	4.467614259	0.349321319
27.53284202	0.711735508	-0.094230389	3.468666729	0.396774776
21.61936722	0.766492014	-0.109770946	3.910572902	0.321068152
19.71449251	0.678140592	-0.089165442	2.853911241	0.410713422
32.59805243	0.719406983	-0.093782916	3.489147843	0.402510568
29.4037095	0.824853952	-0.134629734	4.680919487	0.312135412
25.49584123	0.848977268	-0.144536087	5.08245375	0.278582731
27.7245412	0.767587294	-0.105116095	4.747038356	0.325985052
29.17869898	0.837210729	-0.155140863	3.652420495	0.425351749
26.60778812	0.736888729	-0.104688517	3.046273798	0.382357773
22.96219037	0.882511346	-0.179348837	4.472162496	0.307431356
21.94189596	0.695330967	-0.094418797	2.726252046	0.411703549
42.08331741	0.661330012	-0.073529542	3.694380148	0.37017779
20.45166266	0.68951655	-0.088851437	3.365335371	0.367904831
20.73821875	0.848877148	-0.153086375	4.412956198	0.305230228
23.05829354	0.852150036	-0.156558731	3.906403448	0.342617057
24.05607598	0.716627712	-0.103809636	2.471014745	0.469320894
28.84526975	0.889701729	-0.19375387	3.768503461	0.414766871
36.75804298	0.758326639	-0.106336006	3.995857727	0.359777138
30.29522932	0.730377028	-0.091847618	4.558622095	0.315950778
26.97232822	0.687305303	-0.083281366	3.502632547	0.368616014
21.02920847	0.952021714	-0.308164716	3.735993634	0.33515636
31.8000081	0.714802936	-0.09127379	3.559305217	0.355616423
25.09786941	0.681775293	-0.082430048	3.598737402	0.366758426
30.47999187	0.800958961	-0.119476899	5.013724348	0.293817222
27.98183774	0.746044101	-0.10190003	3.667190976	0.374984855
25.88864068	0.819725861	-0.140158007	3.380577046	0.41679395
34.12026416	0.635703396	-0.066720514	3.793295257	0.361301977
35.22719624	0.750086814	-0.095466614	5.16338533	0.307470789
46.54382658	0.801906181	-0.115281043	6.320773949	0.253800476
30.81279671	0.84885771	-0.161380964	3.501741676	0.456942808
22.10719322	0.778768128	-0.121276391	3.317881216	0.35686419
58.12795235	0.67390463	-0.083351497	3.067453773	0.427519654
25.60502971	0.721848644	-0.09202994	3.929425036	0.329317355
27.35172718	0.635969827	-0.072844343	3.011985841	0.429128807
30.65196563	0.770026515	-0.11276382	4.999376234	0.295625248
26.59542492	0.728370969	-0.089073003	4.680452949	0.300435644
21.99525575	0.756267848	-0.11938273	2.670666152	0.448162704
32.52477517	0.696318649	-0.081768846	4.390527086	0.324361815
30.2605466	0.66320143	-0.075078096	3.509760043	0.379598947
28.75040128	0.785962732	-0.131759769	2.956875725	0.378713092
21.76240853	0.890934723	-0.184866466	4.466522383	0.29600878
29.55519234	0.92087637	-0.212109789	5.040611385	0.293903866
36.80357586	0.750109756	-0.098263092	4.617235903	0.308832995
32.13660159	0.676403934	-0.071725102	4.930180275	0.288596882
22.57693859	0.742348494	-0.118130142	2.224531278	0.510980471
36.43742154	0.73568154	-0.10259053	3.524075313	0.370025503
30.17649318	0.86523852	-0.154883313	5.40795287	0.276128895
23.33388205	0.779219636	-0.114122164	3.971286442	0.332935258
25.51512078	0.704973889	-0.090870057	3.499481253	0.388412345
29.19133447	0.692808466	-0.079986387	4.453326274	0.318116088
25.20206565	0.809165124	-0.124339278	4.732457233	0.305498237
34.54485894	0.714076252	-0.09665521	3.023225038	0.442949988
28.2278097	0.754095089	-0.099551309	4.574073758	0.31659742
34.05290954	0.708001559	-0.079555769	5.107337742	0.295035517
35.58552354	0.647367369	-0.06648932	4.575228911	0.332206208
40.46382958	0.717061188	-0.086830371	4.298648971	0.309937612
32.33698368	0.818935788	-0.137167763	3.865332639	0.345591258
32.28241021	0.615807423	-0.055829586	5.399275535	0.276054812
25.63061122	0.854972357	-0.156970126	4.600567907	0.298222824
33.54172047	0.604315607	-0.056634699	4.697264188	0.310736995
38.5257023	0.581861949	-0.05931106	3.16498897	0.387607448
28.06388237	0.685517745	-0.079326698	3.935011304	0.331141476
21.95835396	0.858106902	-0.158759494	4.386046882	0.310429618
34.16329748	0.714159171	-0.086252766	4.306130186	0.361498963
28.06689953	0.738504095	-0.093585942	4.726741272	0.300491749
24.50884881	0.789564878	-0.115786628	4.524053153	0.313189035
28.41482603	0.718423558	-0.09633862	3.369565043	0.41425749
23.04594164	0.731342569	-0.100269832	3.424580044	0.355290732
36.98665272	0.65232207	-0.07056619	4.067622873	0.317457005
28.62680046	0.941802053	-0.243798811	5.340152395	0.274565398
30.65960698	0.753707052	-0.100049625	4.27891041	0.309690037
28.96970905	0.714197955	-0.092405092	3.429289847	0.380972072
38.27726371	0.81429057	-0.121495665	5.312587009	0.29208089
26.93798991	0.800410384	-0.122740713	4.116218787	0.322043999
30.43387494	0.774989806	-0.116358115	4.411390499	0.364403629
22.09664841	0.617165219	-0.068884082	3.01209853	0.383586504
24.70070899	0.733915571	-0.100898677	3.357469747	0.397220852
29.48202513	0.808115885	-0.141328838	3.915095327	0.351559554
28.92426264	0.763998799	-0.107761236	4.33669205	0.326568148
24.42099288	0.77888579	-0.125046654	2.798596529	0.461464608
27.80933527	0.73765258	-0.095926963	3.963029495	0.350990712
32.94761634	0.716758606	-0.088162578	4.242705999	0.316999037
29.76973465	0.832026053	-0.142489417	3.79820587	0.343993466
23.3300524	0.720672272	-0.103024494	2.897300377	0.430255261
30.61505187	0.582660442	-0.054256569	3.802251819	0.342140283
23.57315476	0.661080319	-0.076150094	3.528347221	0.365639599
26.73450698	0.594273544	-0.060773288	3.391490498	0.356767749
31.98815593	0.708700469	-0.08003095	5.557387858	0.28070443
27.38619808	0.684338009	-0.080123132	4.226429066	0.325222691
30.96514418	0.92941164	-0.2273765	5.299109101	0.269130366
22.83341677	0.885184143	-0.193218518	3.670825508	0.330188337
39.90574028	0.630695706	-0.063781378	4.224662828	0.340891115

log.sigma.2.5.mm.3D_glcm_ClusterTendency	log.sigma.2.5.mm.3D_firstorder_InterquartileRange	log.sigma.2.5.mm.3D_firstorder_Skewness	log.sigma.2.5.mm.3D_firstorder_Uniformity
104.861281	193.2370939	0.497079898	0.049632254
75.42709787	200.8851357	-0.296092834	0.062080077
96.41709597	239.7479515	-0.139425836	0.047962457
94.7811025	231.2108002	-0.257820946	0.051849851
83.90159026	223.4649849	0.098975795	0.053962463
92.16447042	190.1344795	-0.450967147	0.053377502
43.07906058	91.12353516	0.561589597	0.098377922
71.60086915	171.1717119	0.532323656	0.057726235
52.53503238	134.0927649	0.64891914	0.072115038
53.15454991	159.8557167	0.002608695	0.062160432
32.99656853	107.0180073	0.289614268	0.088983023
46.3244457	116.9926548	0.521908201	0.077106139
95.25178719	206.695858	-0.578134414	0.059199874
93.33912379	236.8892365	-0.002299705	0.047876239
72.26715455	181.0159588	0.646302936	0.057892265
65.67454585	153.5723076	0.235768948	0.078981841
38.00811901	135.1910324	0.146457327	0.074692602
76.47760561	218.8647461	-0.118672677	0.060060617
28.62014111	107.4203463	0.329468981	0.087186549
57.33363724	135.6031132	0.096735601	0.066610415
56.45023833	178.3565788	0.076878363	0.065009388
70.44016184	193.2589569	0.143448955	0.057443418
62.60433454	177.5135746	0.122259598	0.060430878
30.94970617	79.06284523	0.655645076	0.1091897
75.18448532	190.3366146	0.13515454	0.068366773
76.93401332	175.237093	0.803138297	0.061413692
71.94865229	193.1241055	0.621242477	0.056017207
49.2881122	139.3472824	0.526129035	0.06997722
44.56325196	95.92265606	0.628185894	0.08969788
50.74577447	156.710598	0.109212963	0.064409552
59.81001652	173.2525635	0.399934348	0.061612992
89.75575014	245.2980309	-0.234605448	0.053238295
61.33910789	154.5644951	0.503359802	0.065262347
63.35014218	146.6291275	0.329274423	0.072575218
54.63167807	160.7666397	0.553136039	0.063854746
89.87522228	225.3190079	-0.283862501	0.050268358
129.1801252	252.0562496	-0.555358054	0.045741651
65.19722726	131.1756058	0.23346139	0.092097132
39.47111124	134.283555	-0.180166663	0.074068878
41.64848952	98.15432644	0.632645527	0.091512745
63.34238324	189.0553532	0.228411058	0.059472043
34.41768606	76.91722679	0.897687035	0.109198105
96.92057085	238.7691364	-0.068008184	0.048678884
78.48610479	209.4424095	0.285971825	0.052632414
34.13785056	94.44303417	0.573070389	0.09506777
75.48700077	199.2999878	0.384853017	0.054654272
48.49403195	136.6840858	0.605182523	0.069978589
35.09781404	129.8165436	-0.28153315	0.085402831
72.53721583	198.0558128	0.179187395	0.055121303
101.1433357	190.9245682	-0.509795051	0.059479991
67.23427082	201.2114525	0.43730937	0.056416777
81.38817336	198.7163086	0.461878138	0.052024406
26.63418272	69.30251837	0.596674321	0.122250779
42.22826567	139.476675	-0.161396496	0.073061579
109.2625172	268.4500504	-0.143825377	0.048109924
72.1443524	196.9613857	0.357872305	0.057872299
56.00770656	143.6454601	0.477471004	0.067587332
76.21402992	223.0657463	0.133804764	0.055008919
71.24160643	199.0523643	0.209644416	0.054467002
38.5470468	89.36870956	0.761423977	0.09935372
69.34638641	187.9111252	0.232933881	0.055181031
85.56549504	195.5719528	0.097576301	0.049768894
77.74774881	184.5487175	0.116640377	0.053183483
73.61874549	182.0373116	-0.011779872	0.055251551
52.03582532	135.8954468	-0.190314612	0.065383164
83.99289342	215.4730988	0.027327434	0.048611919
71.24599643	210.9481649	-0.41259615	0.062292218
73.37290727	181.2859268	0.415943067	0.056234689
39.7673537	123.5512409	0.582987429	0.077637379
62.95184775	177.990387	-0.095292367	0.06060058
71.37159117	215.7523842	0.017910157	0.054960672
68.65041918	179.0521393	0.18588952	0.061608495
81.85308523	236.1344757	0.281344985	0.05375135
67.89752015	208.9596748	0.305281844	0.055401662
49.06748415	98.70777416	0.888261136	0.091889733
42.35781058	135.6987705	0.032027352	0.072607402
56.43493154	173.4495392	-0.106641389	0.060881644
118.4224484	247.5292511	-0.525782318	0.055238716
70.12527993	171.2434864	-0.072770535	0.058299527
43.25494961	108.8924274	0.701224675	0.081417284
100.0983595	187.5728846	0.559195734	0.050767995
66.63783589	182.3286591	-0.099269592	0.059081662
67.74025134	206.8847523	0.253856621	0.061996066
35.21768265	119.6143513	0.383020763	0.080052207
54.02408589	136.0423164	0.425530778	0.071397766
59.52258377	114.706852	-1.256573545	0.101393455
71.47621211	183.2156734	0.23519965	0.05655622
44.91458804	104.4389906	0.657899911	0.08979581
62.39090809	148.3329849	-0.000690132	0.063429507
68.9559238	211.4327698	-0.15921606	0.057960956
63.78535901	143.1409645	-0.170662165	0.06661345
38.66088974	105.1777139	0.678502267	0.085808445
49.22605692	151.2461777	0.143880722	0.065746332
56.02593877	158.7893009	0.234416117	0.063791946
42.91099042	149.2414589	0.212531913	0.069539839
104.25127	249.7458611	0.010713205	0.046218709
72.60100096	197.8272858	0.155425311	0.055066743
79.97399266	206.9917717	-0.388439494	0.056619951
48.48092164	146.4005566	-0.553484631	0.073902874
67.36654832	163.9597569	-0.196672145	0.058145761

log.sigma.2.5.mm.3D_firstorder_MeanAbsoluteDeviation	log.sigma.2.5.mm.3D_firstorder_Energy	log.sigma.2.5.mm.3D_firstorder_RobustMeanAbsoluteDeviation	log.sigma.2.5.mm.3D_firstorder_Median
122.0359128	36572925.93	81.90211693	-44.23847198
108.8804102	6197809.422	80.2334916	-0.153331012
124.1294491	19071997.95	95.17169376	16.69987106
122.4816065	6843568.434	93.24576965	5.632953405
115.258684	25409277.5	90.1679453	10.7506752
112.2281552	5032384.856	77.00954167	25.56489754
69.12728911	100935325.7	40.87839834	-2.920827627
102.5519671	105624251.8	70.66264559	-27.25176525
84.54889862	51175030.97	57.90054073	-21.51014423
91.68746438	9637436.661	67.00074739	22.46835136
65.75549523	11064068.44	44.99782078	1.505053699
78.8065847	38286123.48	50.21734778	-5.215919018
119.0951668	16538639.98	88.5992288	30.28409958
123.5609415	15019881.56	95.23977306	-46.36895943
107.2614523	30749525.1	74.08126086	-51.08846283
90.38101332	79257361.03	62.48043918	-5.691233635
76.57201476	7800409.611	55.97014493	5.425635815
112.05253	8025195.061	87.33201183	-27.93809319
65.33688794	7599267.447	44.65407276	-11.74583864
86.97820255	121793694.9	57.61251632	14.18562889
93.57483201	25576695.05	71.44712923	11.20212507
106.0858376	7968372.427	79.75329323	5.946998358
97.77307991	7785825.174	72.63652136	-13.03264952
57.96615114	23590759.17	34.96035254	-11.02164459
102.4311039	21101505.47	73.36167805	-12.4481287
104.9704325	45837090.56	71.38650157	-22.67240047
109.023791	37369012.05	78.5641803	-50.65889549
82.86322009	25991046.83	57.02844466	-27.87781334
73.75797053	1626063.169	45.00422204	18.97387314
88.25867174	18793841.25	64.7236179	-12.76826668
94.51071748	53768950.71	70.09440978	-28.85303879
124.1910042	17344840.9	98.43625426	-21.0206871
90.73981784	31598069.95	62.04581135	-20.96787643
88.29825713	30949120.63	62.07851998	-9.350196838
93.24349426	70661536.05	65.50917086	-16.97916985
117.5750205	39220101.24	87.75947635	-4.849358559
138.9389212	43661066.51	103.59313	0.630978048
88.19547156	82062555.62	58.56253298	3.079864979
76.60834066	5134807.64	55.48494099	-15.62680244
69.82354512	88285812.46	43.20683284	-10.06983852
101.4597599	20661129.36	77.26517384	-45.68122673
60.71620675	42894609.43	34.95143791	-4.072368622
122.7311292	50199715.46	94.71623485	4.399989367
112.8508934	26300156.63	84.850445	-37.56027603
65.09211471	12454686.98	40.16091571	-7.368881226
108.4256188	41104786.14	80.03915845	-17.27012634
82.67937633	60822571.74	56.36345821	-9.26834774
73.06648026	4161835.789	52.98073156	-3.141365051
108.3917294	6706904.638	79.87786734	4.223293781
120.0223994	9800557.152	83.74500181	39.38054276
110.8247781	24425500.69	81.15819574	-58.64375305
112.7460357	54328249.36	81.29466929	2.390286922
53.97882056	47471836.86	31.45253293	-4.601719379
82.45355788	17652131.38	57.91677366	6.008216381
134.0339271	15525843.73	106.0166097	23.83160973
105.4821945	18808617.33	79.42654741	-12.44539928
87.51620767	50095125.85	60.70844674	-21.90449905
114.1994785	36819646.96	90.19188675	-24.62770844
108.2836082	13719526.3	80.43297437	-45.19758987
66.33535506	97464604.68	39.63784781	-4.531127453
104.2523596	21600446.24	76.19426099	-9.438174248
113.951221	50096967.06	81.07219106	15.66049004
107.3253128	149521986.6	75.26914278	-0.204608046
102.939113	33753451.09	74.12990694	6.140152931
89.21069724	8626851.333	61.32595602	1.464674234
119.0914136	71008505.47	87.86827678	5.627802372
109.6985733	7477674.553	86.03838252	-13.51016092
104.7703722	87806674.49	73.5944393	-31.736063
75.79391584	70446803.07	52.03111611	-10.9517808
96.88013139	29328827.18	71.96957551	26.52768993
109.3818531	8165634.542	85.605586	-20.36293221
99.33988246	77516683.3	71.29811491	-0.585435569
118.7294357	26148017.78	92.99222373	-44.04240036
110.0509939	16629236.35	83.66988424	-44.88981628
73.70250077	43492963.99	44.53858591	-10.48462534
79.06716532	8811684.353	56.72419464	14.59827232
96.87134211	30410370.07	71.21374167	12.94906998
130.7269586	7937646.548	98.82307563	17.83978271
99.87963871	20928240.58	70.82082595	33.353302
74.62537813	19043011.95	47.55040603	-31.70048237
119.6430706	34424389.21	80.26467687	-45.50813866
100.7288192	12346973.98	74.58025804	18.12045193
105.501321	38316789.98	80.11119289	-16.88609505
72.35886366	17015364.35	50.27879219	-37.08747864
83.59655368	31840684.47	56.02354349	-8.35593605
87.43110534	6637317.997	56.97611606	33.46289063
103.1136076	21513227.6	74.08907079	-31.34316826
73.25453794	38221635.35	46.01725104	-6.635032892
91.52876637	24165377.81	60.98754483	-8.447523117
107.0867177	21460176.54	83.015651	19.70372391
89.0640922	10713824.12	59.02916266	23.20961857
71.50400324	23483339.01	45.99276758	-18.74412346
86.06281906	52246110.51	61.00215917	-8.200744629
88.58122747	41081536.23	64.19020915	9.239818573
81.90011487	32445158.14	60.40720587	-7.493485928
133.5778474	58688039.09	102.3271643	8.175157547
105.758211	38914199.79	80.17263173	-1.66593045
115.4849683	6395923.12	86.07738033	-8.821956635
83.33240352	2776791.403	60.91449466	15.15543747
98.95603645	139357434.3	69.15058366	24.34867477

log.sigma.2.5.mm.3D_firstorder_TotalEnergy	log.sigma.2.5.mm.3D_firstorder_Maximum	log.sigma.2.5.mm.3D_firstorder_RootMeanSquared	log.sigma.2.5.mm.3D_firstorder_90Percentile	log.sigma.2.5.mm.3D_firstorder_Minimum
987469000.1	540.31073	159.1461939	199.9667328	-497.1176758
167340854.4	299.8975525	133.6455097	118.0534424	-324.6645813
514943944.7	336.6878662	146.2229928	183.526091	-334.6622314
184776347.7	283.439209	145.785178	158.3120758	-335.6244507
686050492.4	368.2736511	134.1938618	172.3574219	-309.7700806
135874391.1	295.7994385	144.2046419	202.1676605	-388.6081543
2725253793	518.0369873	97.86375873	132.6764069	-330.7472839
2851854798	471.0429077	131.9347009	166.3476563	-423.6062317
1381725836	438.6266479	109.5777388	145.8217255	-334.9649658
260210789.8	339.1399841	112.9814886	162.8158783	-247.2906342
298729847.8	282.40448	84.32427781	126.6752777	-210.3824463
1033725334	437.8989563	106.0070212	139.2233215	-376.8724365
446543279.3	331.9633789	142.8037031	140.232254	-370.2122192
405536802.1	307.6470947	152.7180325	135.2853424	-357.5965576
830237177.7	503.8623657	139.7265472	160.6537872	-369.8922119
2139948748	433.6469421	117.2824898	172.7160324	-341.1359863
210611059.5	363.8449402	93.98928646	123.4794617	-302.9759827
216680266.7	341.9093018	140.0767783	101.4850601	-336.690918
205180221.1	319.6582947	83.34454954	97.76496964	-271.2780762
3288429763	589.6609497	115.1648937	152.5148834	-496.1234131
690570766.5	322.234436	110.9428814	130.386261	-225.3896484
215146055.5	342.5088806	126.7488343	158.0170898	-261.7281189
210217279.7	365.8043213	118.1232056	141.5425095	-284.3113098
636950497.7	364.6121826	80.98428266	99.19760284	-277.2101135
569740647.7	321.3891602	131.0863699	163.1536423	-373.4764404
1237601445	562.9950562	133.914652	196.8509369	-443.5852966
1008963325	504.0837097	136.5547934	150.5770645	-390.7501221
701758264.5	434.6479797	107.4066794	123.9556747	-334.3681335
43903705.56	421.765564	105.5339707	175.6143341	-222.6649933
507433713.8	324.8695679	108.0425533	130.9429184	-394.5531616
1451761669	377.7887878	115.8104509	142.1313995	-301.2145996
468310704.3	243.2124176	148.3617856	130.8011337	-407.6433411
853147888.7	429.5539856	116.3286895	150.0342529	-346.73172
835626257	343.1064453	113.2049949	159.9090698	-304.6530151
1907861473	555.288147	117.3291447	164.582486	-414.2946167
1058942733	345.8440247	142.2215641	160.6110352	-441.0027161
1178848796	382.0549011	169.9304762	158.422168	-605.2342529
2215689002	419.4975281	118.7336228	174.966507	-360.12677
138639806.3	210.4633484	95.75645857	92.36145554	-294.7677612
2383716936	445.7388916	96.67150979	119.8029755	-722.2014771
557850492.7	360.5555115	124.1722725	132.4833328	-337.1735535
1158154455	526.1002808	86.96274658	114.1510719	-303.308197
1355392317	359.4801636	144.0862304	186.4194031	-353.3366699
710104229.1	376.1082458	136.0927517	162.4541061	-347.6654663
336276548.5	365.506134	89.46673988	101.0477371	-266.1504211
1109829226	396.7337036	130.8428865	185.4401245	-383.9531555
1642209437	608.3082886	106.6141661	130.9979706	-363.9738464
112369566.3	242.585144	92.06651926	86.33249664	-368.319397
181086425.2	337.4398193	130.9702592	170.4380646	-263.396759
264615043.1	325.5952148	147.2502922	165.5592712	-364.3292847
659488518.6	381.6558228	137.7629595	172.0655609	-496.085907
1466862733	601.8947144	139.6690746	201.7159943	-354.6177368
1281739595	397.351532	77.14813745	94.46175385	-270.3049316
476607547.3	365.8157959	103.6840611	118.2687195	-445.6898804
419197780.8	422.7173462	155.3896609	199.6069824	-355.3597717
507832667.8	390.0384216	126.4662889	145.8812027	-295.4942932
1352568398	351.2909851	111.7004599	153.0477295	-314.943512
994130467.9	395.0186462	133.4656702	150.9200531	-353.3315735
370427210.1	345.3329468	134.093365	129.224704	-334.9737854
2631544326	537.6364136	94.59097696	115.2690094	-411.0905762
583212048.5	483.905426	127.4878717	151.5379608	-343.6841736
1352618111	501.4241943	142.5598583	193.7777008	-393.4122925
4037093638	436.8510742	134.7556856	191.7676865	-413.6876526
911343179.3	429.5827332	126.9005349	165.4240112	-477.8769226
232924986	288.9725037	112.8838525	147.2429749	-385.1905212
1917229648	483.6075439	144.0716894	170.9992981	-394.7763062
201897212.9	192.1238251	135.7126288	100.0329628	-361.750885
2370780211	589.1763916	131.4069351	160.6059021	-405.9393311
1902063683	456.6435852	96.86536353	125.1175629	-453.8070679
791878333.8	404.1607971	117.508719	155.8092148	-310.3559875
220472132.6	291.1138306	128.8286388	147.880336	-290.8497925
2092950449	392.8912659	124.7496455	162.6709442	-419.4128418
705996480	359.6708984	139.9518852	156.4726288	-365.8230591
448989381.5	343.1248474	132.3044191	154.1296432	-315.6094971
1174310028	526.9066162	103.1968963	140.1570374	-330.3479614
237915477.5	323.6175232	97.9733877	130.1469025	-262.2016296
821079992	355.0950928	117.3310601	143.6781158	-445.8873291
214316456.8	253.1669006	156.7633794	136.8332489	-388.3331909
565062495.6	405.1456299	126.1547139	169.6417999	-327.3566895
514161322.6	456.8293762	102.8564586	110.0721611	-360.4901123
929458508.8	540.31073	156.4733791	195.7104492	-497.1176758
333368297.5	390.8172913	122.2616473	152.0737152	-312.7663269
1034553329	390.1535339	129.5796311	154.3858459	-393.2624817
459414837.4	253.4152985	94.80807301	105.2044235	-289.9747925
859698480.8	370.0076599	108.2944701	152.3074188	-299.1766663
179207585.9	165.8122559	111.801866	93.65538788	-371.5653076
580857145.3	347.916748	128.2475018	158.6254883	-371.027771
1031984155	380.5645142	99.81948691	135.3970871	-289.8032227
652465200.8	358.4796753	119.1913223	140.8101807	-335.8670654
579424766.5	308.1999817	125.2946098	154.1398529	-393.8475342
289273251.2	341.9179077	115.4367874	166.8310745	-343.0881958
634050153.4	352.0846558	96.30483095	125.2161415	-289.2841187
1410644984	493.7812805	107.7388848	135.631073	-363.4515686
1109201478	390.8553162	109.1856845	152.7844696	-266.2371216
876019269.9	431.3106384	99.05076868	134.4736328	-321.6662903
1584577055	505.1541748	156.8995413	185.5076645	-392.4454346
1050683394	472.2739258	125.9777418	167.9555664	-304.9459839
172689924.2	290.3845825	142.9484493	119.9353607	-408.0979004
74973367.89	207.7748413	101.789793	112.9319771	-293.2141418
3762650725	439.6902771	125.6702043	169.6424149	-452.9008179

log.sigma.2.5.mm.3D_firstorder_Entropy	log.sigma.2.5.mm.3D_firstorder_StandardDeviation	log.sigma.2.5.mm.3D_firstorder_Range	log.sigma.2.5.mm.3D_firstorder_Variance	log.sigma.2.5.mm.3D_firstorder_10Percentile
4.624446976	157.2675175	1037.428406	24733.07206	-204.7384781
4.25028524	131.57756	624.5621338	17312.65428	-219.163385
4.499607773	146.2199114	671.3500977	21380.2625	-202.1999741
4.416836462	144.8263381	619.0636597	20974.66821	-233.2958786
4.357917028	134.1626886	678.0437317	17999.62702	-169.1387787
4.431505947	142.0961064	684.4075928	20191.30344	-148.850351
3.857194904	97.86340718	848.7842712	9577.246465	-122.7782104
4.378804496	130.9556429	894.6491394	17149.3804	-169.0730606
4.071971065	108.7965349	773.5916138	11836.68601	-139.2801727
4.171136452	111.3256455	586.4306183	12393.39935	-126.5258652
3.750311302	83.72624651	492.7869263	7010.084355	-95.99095535
4.056700115	105.9973728	814.7713928	11235.44303	-129.5595917
4.332768526	142.2469337	702.1755981	20234.19016	-239.9354248
4.495664551	144.3907876	665.2436523	20848.69955	-235.7890549
4.389957039	135.9293326	873.7545776	18476.78345	-189.8054565
4.064771812	117.2810853	774.7829285	13754.85297	-160.9701797
3.918322011	93.94560684	666.8209229	8825.777045	-120.6138916
4.254538584	131.2657748	678.6002197	17230.70363	-229.7070648
3.748817295	82.92508953	590.9363708	6876.570474	-112.3849564
4.211315258	114.345808	1085.784363	13074.96381	-131.4602386
4.09012091	110.9292692	547.6240845	12305.30276	-149.8038071
4.281251946	126.6469649	604.2369995	16039.45373	-177.8743439
4.22024919	117.7342265	650.1156311	13861.34808	-163.7797607
3.630799859	80.60294359	641.8222961	6496.834516	-102.9265793
4.244110803	129.4635754	694.8656006	16760.81737	-189.5586319
4.325135863	133.8074463	1006.580353	17904.43269	-136.2625656
4.388240805	134.0676003	894.8338318	17974.12145	-180.3910873
4.081522043	105.6227513	769.0161133	11156.1656	-142.2993683
3.838080764	99.88128663	644.4305573	9976.271418	-70.5848732
4.125096386	107.7598632	719.4227295	11612.18812	-146.0551987
4.178927105	114.4874986	679.0033875	13107.38733	-158.7068207
4.38497855	142.3824686	650.8557587	20272.76735	-234.8773529
4.20329117	115.99419	776.2857056	13454.65211	-149.8862549
4.082218984	112.8188723	647.7594604	12728.09794	-153.7950806
4.209376507	117.2764192	969.5827637	13753.75851	-138.2564697
4.486510059	141.7759973	786.8467407	20100.43341	-203.2201752
4.632095031	166.937935	987.2891541	27868.27414	-286.3412354
4.015718596	118.7335985	779.6242981	14097.66741	-162.0558472
3.932391157	93.47611659	505.2311096	8737.784373	-148.8712662
3.874985144	96.3894486	1167.940369	9290.925802	-120.4219971
4.236686908	119.8548626	697.7290649	14365.18808	-180.1227386
3.680030489	86.89732666	829.4084778	7551.14538	-91.95536804
4.496144888	144.0659937	712.8168335	20755.01055	-177.9069366
4.408373933	134.5438293	723.7737122	18102.04199	-186.2291443
3.788579158	89.33706952	631.6565552	7981.11199	-113.6426392
4.35741876	130.842883	780.6868591	17119.86002	-157.5934753
4.101327777	106.5712607	972.282135	11357.43361	-128.4319305
3.793989851	90.54808575	610.904541	8198.955832	-139.2195129
4.328992266	130.9130116	600.8365784	17138.21661	-164.571701
4.395422681	147.2488721	689.9244995	21682.23035	-234.404512
4.362403162	134.3772502	877.7417297	18057.24537	-181.4657684
4.470054313	138.5817714	956.5124512	19204.90735	-146.6088745
3.523089168	77.05747402	667.6564636	5937.854303	-98.49753189
4.045969936	103.5637153	811.5056763	10725.44313	-133.4648178
4.512303494	155.3472546	778.0771179	24132.76952	-215.3561005
4.286726327	126.059551	685.5327148	15891.01039	-165.2811432
4.130710514	111.0228423	666.2344971	12326.07152	-146.1816498
4.337110996	132.1384558	748.3502197	17460.57151	-185.4609039
4.352490265	129.6803992	680.3067322	16817.00593	-196.3128662
3.818182608	94.54716236	948.7269897	8939.165911	-111.4923813
4.355726089	127.3418769	827.5895996	16215.95362	-171.8167847
4.528722386	141.9133573	894.8364868	20139.40098	-179.3158539
4.456880164	134.3678257	850.5387268	18054.71259	-159.5023636
4.375419802	126.8246832	907.4596558	16084.50027	-154.1826172
4.176835431	112.8606081	674.1630249	12737.51685	-151.8807983
4.541410994	144.0053817	878.3838501	20737.54997	-193.0622406
4.179509518	127.2436317	553.8747101	16190.9418	-243.5568314
4.398327056	130.7077967	995.1157227	17084.52813	-165.6247742
3.954623689	96.81679177	910.4506531	9373.491169	-114.2499039
4.217585236	116.6634654	714.5167847	13610.36415	-150.6432938
4.286114195	127.4165802	581.963623	16234.98491	-186.2452667
4.28060738	124.6243061	812.3041077	15531.21767	-168.1703339
4.378448216	137.5239068	725.4939575	18912.82493	-192.2023376
4.338482314	130.0580589	658.7343445	16915.09869	-180.7178131
3.915100377	103.1299158	857.2545776	10635.77954	-119.1349403
3.979366378	97.55874223	585.8191528	9517.708185	-126.0591286
4.24063742	117.3248653	800.9824219	13765.12401	-153.9237854
4.412790178	153.8391611	641.5000916	23666.4875	-265.5705383
4.322556762	123.8373061	732.5023193	15335.67839	-144.0233551
3.961652509	100.4665915	817.3194885	10093.53601	-133.9046265
4.59308477	154.3301221	1037.428406	23817.7866	-203.8777084
4.263934249	122.2303321	703.5836182	14940.25408	-170.9072952
4.24078441	127.7067781	783.4160156	16309.02118	-180.4494263
3.866676823	90.82802983	543.3900909	8249.731002	-137.5797363
4.097191488	108.2937157	669.1843262	11727.52887	-133.8056793
3.759479856	111.5888632	537.3775635	12452.07439	-190.8917084
4.345756964	126.8001789	718.944519	16078.28537	-170.9041077
3.898232654	99.81171349	670.3677368	9962.378149	-127.6383209
4.262786158	118.7104889	694.3467407	14092.18018	-161.1341705
4.261067184	125.2652483	702.0475159	15691.38243	-168.8115784
4.190856232	114.710993	685.0061035	13158.61192	-138.0455643
3.879653616	95.35875742	641.3687744	9093.292616	-123.3757545
4.142965308	107.632239	857.2328491	11584.69888	-138.7010956
4.14258111	108.6886536	657.0924377	11813.22342	-129.828064
4.006411538	99.0410889	752.9769287	9809.137291	-122.3198273
4.611694365	156.8255154	897.5996094	24594.24228	-207.7850906
4.327186414	125.7914414	777.2199097	15823.48674	-155.1604965
4.350213693	137.1688675	698.4824829	18815.2982	-236.4775879
3.939854437	101.5905889	500.9889832	10320.64776	-164.1771149
4.344490574	124.8578384	892.591095	15589.47981	-156.8919754

log.sigma.2.5.mm.3D_firstorder_Kurtosis	log.sigma.2.5.mm.3D_firstorder_Mean	log.sigma.2.5.mm.3D_glrlm_ShortRunLowGrayLevelEmphasis	log.sigma.2.5.mm.3D_glrlm_GrayLevelVariance
3.414170876	-24.38111913	0.004961168	40.5756621
2.379537895	-23.41939301	0.023856169	27.62454709
2.086891993	-0.949277712	0.012646813	34.2601752
2.067006635	-16.69281017	0.019516559	33.58806056
2.110884051	2.892324018	0.010829306	28.75978999
3.001850122	24.56980461	0.012721104	32.91975813
4.46914833	-0.262313303	0.005945228	17.43150954
3.268279581	-16.04321947	0.005128265	28.87426658
3.254959801	-13.06119596	0.006800613	20.19714852
2.497156575	19.27219279	0.023707213	20.20771714
2.954640102	10.0249425	0.018309864	11.68419467
3.927148444	-1.430215322	0.004680352	19.49578462
2.395160114	-12.59791533	0.016600013	32.74880081
2.074133252	-49.74030458	0.017000859	33.4435659
3.230083569	-32.35312263	0.010093856	30.83506321
2.587322444	0.573963549	0.00665753	23.56600962
2.927564187	2.865122139	0.008049415	14.4952665
2.242524046	-48.89580947	0.019511712	27.41928204
3.215710695	-8.351255176	0.011252181	11.50489512
4.084857372	13.71090523	0.00292479	21.96224752
2.331163977	1.737864202	0.019363258	19.90215857
2.338437546	-5.080675456	0.021798473	25.80888043
2.536079751	-9.578289603	0.015273105	22.51487203
4.446014685	-7.849810333	0.008197858	11.59142249
2.47493837	-20.56256333	0.009321132	29.17405418
3.49549607	5.357362158	0.003878762	29.98852845
3.305884	-25.94398092	0.006403504	29.60070264
3.531637661	-19.49433708	0.007988804	18.61575073
4.312054238	34.07561515	0.021532638	16.31174036
2.740360017	-7.810582839	0.005537834	18.99627794
2.536800857	-17.45489076	0.00914934	21.48085263
1.98212061	-41.69474862	0.009698294	32.56392601
3.259028873	-8.815435265	0.007373175	22.73698602
2.627970838	-9.341997827	0.008964814	21.36102154
3.150266833	-3.517057207	0.004151841	23.10062819
2.427268993	-11.24899541	0.005676301	32.60394627
2.450917635	-31.75047402	0.003118106	44.96959509
2.901229931	-0.075994737	0.006080122	25.28118849
2.583621052	-20.77293874	0.013877046	14.35554476
4.541448686	-7.379363279	0.001231208	16.32626912
2.332721261	-32.4586687	0.010220536	23.2511178
5.593942171	3.37252315	0.006353667	13.31928501
2.155684673	2.414797138	0.009225906	33.23078883
2.409577796	-20.47425394	0.012461287	29.16004344
4.375223076	-4.81513816	0.011710113	14.11469425
2.47754241	0.030412077	0.005271254	27.92361872
4.16936111	-3.02436669	0.005354779	19.06823248
3.238168087	-16.65197094	0.009425245	13.3687817
2.402530686	3.871974468	0.024825292	27.54535566
2.578665271	0.646700014	0.014339075	34.79919614
2.584260363	-30.35436772	0.00405828	29.38313089
3.084265622	17.39376459	0.006090893	31.1614974
4.811872915	-3.739092055	0.010147677	11.0545724
3.677416245	-4.994135345	0.005041422	17.91040611
1.954724727	3.630039762	0.009895505	38.54309072
2.604445551	-10.13468408	0.013686754	25.75848794
2.870842686	-12.28499971	0.008893992	20.81303209
2.151749306	-18.77534604	0.00782161	28.30284477
2.449949538	-34.11780491	0.012584535	27.37546294
5.380889375	-2.878716946	0.00364482	16.15559438
2.839165399	-6.099493956	0.009016623	26.64234003
2.824847898	13.56142436	0.006705954	33.01640948
2.784168965	10.21676115	0.004698974	30.00764024
2.938145098	4.386967172	0.003400286	26.11575145
3.045735102	-2.290699189	0.007978087	21.24928207
2.586564097	-4.370552615	0.008029129	33.65771826
2.036971457	-47.19084463	0.015325299	26.00600659
3.135220942	-13.5371514	0.005040173	28.12124424
3.795977922	-3.06716195	0.002910697	15.75757107
2.438602428	14.06893367	0.00903151	22.21452915
1.975403201	-19.02191535	0.020483332	26.2034677
2.720753177	-5.59074122	0.004802954	25.94482497
2.117383621	-25.95583248	0.008416399	30.53458524
2.315175086	-24.27675082	0.015623933	27.43168707
4.685833316	-3.717508733	0.006140664	18.6344846
2.767917398	9.004249706	0.012227094	15.52209184
2.72022062	1.205678972	0.004092104	22.31298953
2.222350845	-30.13751176	0.019497966	37.88476821
2.836900589	24.06934687	0.007577345	24.91895168
4.776128747	-22.04348131	0.006801558	17.19798788
3.47467404	-25.8095285	0.004590085	39.10643421
2.552958719	2.767003362	0.012345584	24.2231786
2.368814972	-21.95130097	0.006561597	27.19850822
2.830375889	-27.18160602	0.010794403	13.66017299
3.059988864	0.404214654	0.010103647	20.0719067
3.796144079	-6.898032834	0.01475482	20.60105172
2.634060184	-19.21292132	0.009350858	26.33993133
3.730539189	-1.245719903	0.008715522	17.86264236
3.112059617	-10.69537896	0.012108348	23.75585965
2.135228631	2.712342697	0.005784803	25.07917501
3.166714222	12.92439425	0.00906998	21.37253301
3.791658169	-13.46580291	0.010047791	15.81145848
3.076964753	-4.792537779	0.006219478	19.18073713
2.665662257	10.4062618	0.013029047	19.33240311
2.567285265	1.384732967	0.007255736	16.14313158
2.308096176	-4.819106256	0.008960121	39.66044427
2.416415739	6.848700187	0.009344225	25.68785071
2.395898243	-40.23631407	0.011292823	30.20524488
2.505345463	-6.365076061	0.016427651	16.64895845
3.064536808	14.2660594	0.003454143	25.80998282

log.sigma.2.5.mm.3D_glrlm_LowGrayLevelRunEmphasis	log.sigma.2.5.mm.3D_glrlm_GrayLevelNonUniformityNormalized	log.sigma.2.5.mm.3D_glrlm_RunVariance	log.sigma.2.5.mm.3D_glrlm_GrayLevelNonUniformity
0.005145496	0.048379959	0.077839912	65.38040273
0.024715188	0.061278063	0.068967027	19.9979421
0.013181622	0.047765762	0.075703336	39.94674544
0.020138697	0.05169936	0.063430294	15.7327889
0.01140355	0.053422514	0.08471791	70.1515658
0.012937956	0.052768929	0.053534959	12.14482718
0.006618386	0.08244155	0.397906811	718.2724384
0.005484471	0.055279996	0.156957243	301.0214749
0.007392555	0.068067236	0.193867984	255.5972634
0.02459132	0.061698057	0.076006454	43.71609698
0.019532418	0.08617366	0.1486293	120.043309
0.005083555	0.071177885	0.215167314	211.8185584
0.017204756	0.057642317	0.084992001	43.46334564
0.01763462	0.047916641	0.061737294	29.21216845
0.010598885	0.055823979	0.113674618	80.71776148
0.007331365	0.068020695	0.287684533	334.7837634
0.008569938	0.073822749	0.122584318	59.06107709
0.02046977	0.05969359	0.076654856	22.84588403
0.012130917	0.084753329	0.157417169	82.38063831
0.00311029	0.064489792	0.14620516	532.6203581
0.020892906	0.064529082	0.122595476	121.8069232
0.02296162	0.057113654	0.069386264	26.65294982
0.016166218	0.059839477	0.097842954	30.790573
0.009091435	0.098313965	0.272974071	300.3840386
0.010009576	0.056546956	0.286547274	59.50859982
0.004149191	0.059120787	0.145903974	136.0736438
0.006766516	0.054789033	0.09612834	101.4356401
0.008530445	0.068153101	0.12043313	139.4768155
0.022094734	0.087206138	0.067571736	11.943347
0.005855213	0.063706451	0.116343114	93.74330949
0.00984459	0.060909593	0.133444426	220.9056945
0.010122878	0.052794287	0.076700131	39.0135325
0.007906403	0.062359103	0.15517978	130.2691519
0.009665256	0.067883683	0.183237698	145.0180443
0.004461541	0.061542995	0.149765766	283.6775017
0.00596908	0.049737168	0.10708827	88.98296559
0.003204167	0.045343915	0.063123382	64.90542379
0.006692264	0.071895801	0.389800282	346.3766187
0.014534964	0.07348319	0.09669593	37.90901077
0.001356357	0.082458615	0.247973453	669.6198728
0.010820873	0.058712879	0.089277359	72.94078922
0.006974661	0.098217605	0.247800321	480.9268321
0.009641059	0.048640066	0.093870432	109.8690377
0.013027305	0.052036514	0.075982636	69.20867958
0.012821853	0.085823452	0.265524924	114.4437279
0.00559858	0.053689648	0.102635287	118.8832817
0.005775982	0.068141554	0.153795727	326.684981
0.009932939	0.084517916	0.116669647	37.94347366
0.025945199	0.054711716	0.058807097	20.26557367
0.014920926	0.058123683	0.077541434	24.63595825
0.004224212	0.055238082	0.075593526	66.71478683
0.006374133	0.05154867	0.071358225	134.6927293
0.011457788	0.103403646	0.429328802	663.3766514
0.005285532	0.070783124	0.142302407	104.439598
0.010278737	0.047706506	0.063750619	29.01209032
0.014508368	0.057196315	0.095041899	62.18933533
0.009566561	0.064224009	0.191628227	229.1085228
0.008250923	0.054480156	0.094711518	104.2084321
0.013210431	0.054056653	0.083114036	38.49798509
0.004067557	0.086367231	0.314643715	789.8904358
0.00944085	0.054459916	0.095188228	67.0987489
0.006984507	0.048961401	0.088363511	112.1975664
0.004976983	0.051444114	0.130232955	385.1404138
0.003542578	0.055074664	0.079510526	107.6616981
0.008366831	0.064037553	0.109247661	39.735202
0.0083108	0.048239157	0.068925386	155.253704
0.015983164	0.061666476	0.065372609	23.57609721
0.005308591	0.055223789	0.093292678	260.1703947
0.003150724	0.075369879	0.164169412	504.4076722
0.009433428	0.05983241	0.090930303	117.6960612
0.021696564	0.054775019	0.073456248	25.29771218
0.005130704	0.058433071	0.16683828	260.1407076
0.008847048	0.05281944	0.078751762	65.98715987
0.016264064	0.054649361	0.073279878	48.81960639
0.006730373	0.081625564	0.255680093	287.0253534
0.012901469	0.071858532	0.104281822	60.83674088
0.004285906	0.060298105	0.086887162	123.5523249
0.020338531	0.054672291	0.058290305	16.73497542
0.007878004	0.057620554	0.073982721	70.93343046
0.007276128	0.077854725	0.151166299	125.671019
0.004767184	0.049468431	0.080091252	64.99542958
0.012837716	0.058574322	0.080230805	45.15279686
0.007012657	0.058070019	0.196490738	118.38379
0.011614173	0.077969059	0.12819061	133.5090758
0.010932533	0.066484842	0.193646066	159.0238227
0.015293413	0.096078632	0.1029671	46.70780896
0.009893148	0.055495889	0.097312847	67.16159958
0.009729452	0.076413258	0.342470257	244.8465103
0.012721464	0.060366559	0.134021091	93.42515343
0.006050666	0.0576842	0.082228225	73.43326145
0.009506442	0.065519336	0.099385635	48.3727128
0.01098192	0.079724281	0.230837445	174.3491744
0.006579965	0.064536408	0.107679171	266.2648804
0.013867101	0.063113899	0.121049457	198.090761
0.00773703	0.068709094	0.105675761	208.1695293
0.009362139	0.045731814	0.077409382	101.9037817
0.009898336	0.054689365	0.096564824	123.7722284
0.011536643	0.055804946	0.056888827	16.61116458
0.017081831	0.073732902	0.071088269	18.49855281
0.003645445	0.056734286	0.129747166	454.8535567

log.sigma.2.5.mm.3D_glrlm_LongRunEmphasis	log.sigma.2.5.mm.3D_glrlm_ShortRunHighGrayLevelEmphasis	log.sigma.2.5.mm.3D_glrlm_RunLengthNonUniformity	log.sigma.2.5.mm.3D_glrlm_ShortRunEmphasis
1.220968896	406.6887183	1193.028898	0.952161176
1.202368879	177.3599492	290.0677258	0.954418523
1.215295886	232.9252463	740.9250897	0.953369933
1.184643295	218.0843777	273.2684221	0.958570219
1.240215146	206.2881014	1147.031635	0.948208391
1.160521767	324.9821104	208.7492811	0.96252906
1.877760754	202.6192839	6400.455394	0.880021075
1.402377218	296.346978	4505.269338	0.926512278
1.488440517	202.2588844	3019.271546	0.914899369
1.21299521	141.4236688	629.085229	0.954283523
1.40396364	102.7728521	1139.404399	0.921381563
1.532218108	268.9999404	2361.389478	0.910081884
1.244838375	239.9949905	656.6105034	0.946730143
1.178848097	209.5983786	549.4778499	0.95999511
1.303010358	224.4325615	1240.435334	0.940619565
1.673604777	214.3614732	3802.63953	0.898337495
1.343192251	189.2711005	666.9252296	0.929740255
1.220285549	178.3077067	337.6415056	0.951893718
1.432597092	126.8255045	785.0280522	0.916183388
1.385246728	432.3301159	6832.901227	0.926703512
1.33823679	124.5473722	1584.385258	0.931980503
1.20031605	148.5005052	416.0878074	0.955731281
1.275732498	162.8064954	442.6996752	0.942064066
1.672320459	146.3946441	2309.936315	0.890739588
1.660792304	221.1691983	818.4785464	0.901839774
1.383408182	361.7711458	1908.442855	0.927484463
1.270159597	259.1263625	1599.02756	0.94327151
1.33532356	196.998445	1714.100787	0.93158693
1.207182138	129.4510221	120.7548313	0.9514577
1.316532086	266.8790499	1250.56551	0.936831179
1.357698834	175.6427169	3032.28926	0.930580707
1.215829054	273.9041042	655.9992236	0.954009096
1.408959493	208.9313071	1712.026094	0.92287518
1.469225464	179.572872	1720.559107	0.915902679
1.39227403	306.4947598	3810.980831	0.926390527
1.284813297	338.6167224	1547.889484	0.943710634
1.181779873	600.3765394	1289.897707	0.959608314
1.864633767	237.1428935	3572.775112	0.883525011
1.277594893	142.2086106	442.1400106	0.940547232
1.609832026	788.9539117	6267.437505	0.899237133
1.254289221	190.8500399	1077.829984	0.945415665
1.600762708	183.9938279	3809.786761	0.902288856
1.245930621	265.0837263	2004.835795	0.952419365
1.217330171	210.5478648	1175.54535	0.952513778
1.640495744	131.3610119	1031.907274	0.899254399
1.280716251	290.5885033	1910.815601	0.942994681
1.402418876	241.3787388	3942.771917	0.924345258
1.320448198	221.1700437	382.0233081	0.936664671
1.174513956	161.3211459	333.4974724	0.959776109
1.218129938	262.2191418	376.4572648	0.953740249
1.212353171	389.9276133	1073.038476	0.954590762
1.208209589	283.7739337	2311.823685	0.953226039
1.993473905	123.2046321	4499.082924	0.861617384
1.386479118	324.5677789	1216.477675	0.924767485
1.183285191	274.9166469	547.3531382	0.959432877
1.266498336	166.488389	939.9057652	0.943931331
1.46434464	179.1179293	2922.757997	0.922094986
1.264439932	234.9728067	1656.854556	0.944578643
1.232872253	194.8587298	626.3869063	0.950477227
1.745744255	287.5871281	6840.310914	0.886798487
1.261453371	218.6531838	1071.433113	0.94606233
1.247007163	307.376024	1999.317046	0.947670439
1.342617341	330.4336976	6311.074568	0.934019965
1.229968993	431.3445223	1712.036775	0.949268111
1.304300473	273.3136731	528.014245	0.937324624
1.199612655	288.0532464	2863.719444	0.955401228
1.193908378	203.8194123	340.3208233	0.955570125
1.260119657	302.5933384	4089.029948	0.945461557
1.42716621	364.8603953	5457.149192	0.920843722
1.258578439	206.7442834	1703.602782	0.944620651
1.210046542	159.7326175	410.1327389	0.954186797
1.422409075	302.9945489	3658.022764	0.923936376
1.222266118	233.5255117	1103.080723	0.952187992
1.205776054	179.6354632	796.3392521	0.955702088
1.612106476	207.6227567	2735.310018	0.902340639
1.285080007	148.7489146	729.5192244	0.941873998
1.250813086	346.4556383	1776.847409	0.945260589
1.173395718	259.2370701	275.7686823	0.959926252
1.2163741	252.2353634	1084.905108	0.951568555
1.400267854	218.205735	1326.68314	0.923804859
1.226710353	402.8660637	1156.972678	0.951167968
1.229612662	198.4413346	676.0081991	0.949663583
1.462560641	254.9055708	1696.165442	0.926918979
1.353977176	137.1408728	1424.757041	0.928799602
1.49044672	165.5574944	1919.070338	0.91421322
1.303355258	230.3340579	410.9549155	0.934404453
1.267991856	234.165561	1050.18122	0.944960461
1.788861163	159.016626	2387.507332	0.885519285
1.345449449	208.8632905	1307.731096	0.934898851
1.237234668	286.1870726	1112.611002	0.948094951
1.287311142	231.4440395	629.0867305	0.938184532
1.581828643	147.7636945	1702.963566	0.901707284
1.299923884	239.7111704	3511.25147	0.937829358
1.33012036	153.0769731	2647.073052	0.933992519
1.298616275	190.5966975	2572.815445	0.937162091
1.223806852	291.473403	1960.393708	0.950736926
1.273714472	206.462869	1948.638774	0.94203891
1.164190144	271.7025848	270.5497375	0.963359351
1.214945274	159.1765971	220.6488773	0.950456123
1.343787583	398.6408098	6748.147596	0.933439885

log.sigma.2.5.mm.3D_glrlm_LongRunHighGrayLevelEmphasis	log.sigma.2.5.mm.3D_glrlm_RunPercentage	log.sigma.2.5.mm.3D_glrlm_LongRunLowGrayLevelEmphasis	log.sigma.2.5.mm.3D_glrlm_RunEntropy
505.8319251	0.935808651	0.005978298	4.977171233
222.0440496	0.940367989	0.028331634	4.542248132
294.2550826	0.937564677	0.015657537	4.821422989
263.6987406	0.945054945	0.023101393	4.672255666
259.9499646	0.930654746	0.014127631	4.725082774
391.75826	0.951048951	0.013837487	4.659782334
412.2246017	0.825103827	0.011367938	4.722701928
425.9146199	0.897241519	0.007378203	4.914345104
309.0686485	0.880789084	0.010681665	4.67510176
175.8586473	0.938461538	0.028726095	4.492929865
150.2802097	0.894848725	0.025606026	4.28416989
431.0904261	0.873021607	0.007409276	4.700894771
324.5849285	0.929431851	0.019876858	4.701683502
252.2513869	0.946727186	0.020486317	4.762940528
291.5282401	0.917997558	0.013083133	4.823613775
378.7933227	0.85305583	0.011648784	4.787198013
260.8582696	0.906002265	0.011151417	4.388468075
221.4272541	0.935678014	0.024768594	4.564844291
190.3016993	0.888131065	0.01670877	4.302579158
638.2633702	0.899303898	0.004081316	4.748134783
173.3320269	0.908343822	0.028769772	4.557801526
180.5700302	0.940911911	0.028229779	4.567043126
210.2303445	0.922111938	0.020373911	4.592194095
258.5330272	0.84824961	0.01441913	4.392619837
394.8571604	0.85573791	0.014092503	4.914062412
508.3384569	0.900264837	0.005583808	4.840023082
330.7763787	0.923844618	0.00848248	4.801029734
268.5513046	0.908224931	0.011119279	4.566944094
159.4522298	0.937829294	0.024480754	4.138205995
362.231517	0.913951266	0.007459578	4.567605746
245.497164	0.90473358	0.013403535	4.667564031
339.1566514	0.937719641	0.012064617	4.701788013
305.9432203	0.894416076	0.010621332	4.745006626
279.2268248	0.883803153	0.013447994	4.663897367
439.2998617	0.897930435	0.006096686	4.741526857
459.6700731	0.922640536	0.007409128	4.883597975
755.7135041	0.946581197	0.003578896	4.923903604
480.4797523	0.825974918	0.010919194	4.879003157
189.4233198	0.921153846	0.017626322	4.326000782
1381.880249	0.858905147	0.002107728	4.599850131
241.3427247	0.927095293	0.013611703	4.622576982
308.8166167	0.862184008	0.010672048	4.39687314
341.4418326	0.934370427	0.011805494	4.833673263
256.3534561	0.936619718	0.01557518	4.749455169
222.290913	0.856140004	0.019446235	4.492229881
371.74387	0.922179861	0.007216928	4.768764703
347.8953082	0.895892932	0.00798686	4.642485565
304.8114956	0.913833621	0.012333205	4.213131522
186.9710752	0.947275231	0.030866963	4.590847487
335.9936347	0.93771273	0.017659817	4.698790828
477.5207763	0.938377861	0.005002168	4.698138529
348.1911291	0.938240575	0.007643141	4.817941768
268.3673779	0.802889438	0.020316285	4.488707117
490.2285954	0.898388457	0.006517668	4.568299131
332.1666313	0.945687283	0.012022743	4.78849361
211.8674348	0.924581371	0.018498114	4.679515272
268.7825138	0.888284318	0.013373388	4.692582204
305.6887518	0.925347028	0.010321365	4.734477344
239.6449346	0.933360218	0.016085826	4.691374573
541.6255192	0.838541336	0.006767722	4.635879198
285.3709347	0.926954911	0.011430019	4.739582575
399.9143874	0.929567795	0.008265399	4.913698321
462.6981099	0.909101101	0.006383263	4.943103812
557.0729272	0.932692308	0.004187617	4.740868246
375.0261543	0.916373139	0.010155662	4.601593888
357.192674	0.940773053	0.009556947	4.879938172
251.8487383	0.941644562	0.01881363	4.473455356
390.0585114	0.926495726	0.006584212	4.80732385
548.5746987	0.891233966	0.004447537	4.523880689
277.264197	0.926119079	0.011294574	4.619395078
193.8645731	0.938711695	0.027080132	4.584922041
452.269621	0.89353389	0.006939236	4.832682258
285.1033302	0.935753385	0.010872881	4.723233915
216.5833163	0.940323887	0.019642965	4.653134168
349.4804426	0.860299103	0.010256895	4.629271783
197.5331002	0.922238981	0.016212669	4.377726686
458.7639846	0.927569036	0.005165181	4.633220774
322.6785511	0.947606573	0.023790494	4.656033709
319.9921795	0.936121673	0.009192654	4.673633973
313.6725811	0.896410256	0.009700338	4.502261924
503.4369645	0.934402013	0.005578472	4.952570162
257.4552032	0.933227789	0.015032318	4.612804663
386.4304958	0.892671745	0.009706666	4.767024002
188.5161924	0.904465846	0.015660879	4.362819871
252.9214008	0.88040799	0.015424046	4.696755228
334.9487079	0.914819644	0.017612809	4.206934626
300.5399669	0.925252882	0.012404373	4.737659943
291.3650392	0.8339015	0.016301078	4.705486846
292.3643336	0.909555465	0.015635127	4.736475063
370.7023968	0.931236284	0.007294768	4.625460372
317.3938425	0.918388825	0.011482624	4.595615823
241.1182414	0.863136469	0.016297917	4.557493062
325.2061453	0.91659973	0.008287644	4.595816032
209.8309119	0.9107103	0.018005457	4.608943582
253.9580865	0.9161685	0.010032182	4.455336067
371.3269148	0.934660558	0.01114517	4.97087303
268.6306318	0.922951437	0.012499623	4.737811848
329.0533302	0.950847874	0.012578057	4.587263262
199.6966307	0.935993111	0.01982231	4.241097811
576.0475512	0.908457703	0.004627851	4.834833144

log.sigma.2.5.mm.3D_glrlm_HighGrayLevelRunEmphasis	log.sigma.2.5.mm.3D_glrlm_RunLengthNonUniformityNormalized	log.sigma.2.5.mm.3D_glszm_GrayLevelVariance
424.4041739	0.882307843	51.79643957
185.4679667	0.887748766	31.08315979
243.8293274	0.885181678	35.14982556
226.4655444	0.897260888	33.27634603
216.0728183	0.873086648	30.231684
337.51313	0.906301945	40.03619971
228.0589355	0.733951334	29.9198737
316.4302599	0.826452247	39.65500524
218.1539648	0.802595306	27.15166335
147.5959882	0.887206123	24.63942287
110.6218073	0.816069328	16.4864532
292.4707843	0.79253069	30.326297
255.1621234	0.870119739	36.64146988
217.499542	0.900556038	32.01918968
235.5441672	0.856971172	41.39990034
236.348013	0.770583252	26.31886066
201.6439408	0.832737121	19.58732717
186.0943427	0.881713245	28.05013841
137.2590203	0.805375678	18.25983662
465.4241753	0.82647842	35.8910783
132.8469555	0.838036792	20.20739821
154.3231573	0.890860123	25.01133472
171.2009265	0.859647426	25.58889927
162.0681561	0.754569669	20.04963776
243.9230147	0.776981449	35.54122725
385.5505267	0.828268133	39.19585363
271.5855175	0.86265259	40.12654057
209.1910856	0.836730792	26.13395653
135.1994862	0.880701338	25.65992866
282.9915891	0.848691379	23.32025146
187.0773802	0.835019805	24.06451826
285.5186951	0.886974696	32.70325578
224.395748	0.818526114	30.66940388
194.7088903	0.804428656	27.53106944
327.6736434	0.826030631	31.92949595
358.577711	0.864014611	34.51186434
628.1893006	0.900021469	46.31397917
266.0991463	0.741315715	32.45901428
150.6871236	0.85618071	19.25170068
872.9893599	0.770724947	27.24531193
199.9943308	0.866949885	25.46737568
201.4257777	0.777059224	25.21566327
277.7432117	0.88487258	31.40179574
218.9168634	0.883195199	31.3391654
143.7280329	0.771994448	23.27230547
304.5907516	0.862116814	29.62275545
258.353674	0.82152348	28.6202272
234.9037839	0.849194837	17.87609337
166.1548804	0.899772918	28.90203529
274.9100499	0.886803576	39.26377902
405.5345485	0.888017936	33.2900037
295.7463119	0.884336393	37.28538523
140.9592591	0.700133613	20.49436441
351.3255078	0.822818811	28.39986159
285.3825276	0.899444241	36.69062435
174.6918431	0.863751104	28.37253088
192.0643314	0.817891654	26.13564144
247.3755162	0.86543274	31.25719889
202.886377	0.878756579	31.1225
320.8725586	0.746857562	29.47065118
230.1529582	0.868770054	33.71879552
323.6929199	0.871992733	40.66481823
352.0461592	0.84224094	38.684227
454.1314161	0.875345816	31.92943934
290.7723347	0.849573381	31.13732425
300.7245426	0.889419481	39.71153375
212.7984054	0.889793831	26.49537238
317.9595544	0.86721462	35.94334288
393.84658	0.814288145	25.47084026
219.2381982	0.865294519	26.71250721
165.8340284	0.887217172	25.69752097
326.0147157	0.820913076	33.30677979
242.8309339	0.882381297	31.43097876
186.4241351	0.89072282	31.32720335
227.1298961	0.777108989	30.07767309
157.1112272	0.85988147	20.20141971
366.4866075	0.866475931	27.34016182
271.0497585	0.900237562	35.3217898
264.6047807	0.880729841	31.26656988
233.5354773	0.820652893	28.68767035
420.7701469	0.880068811	50.928
208.9374379	0.87640205	28.44683547
272.9913741	0.829604613	29.22735284
145.8282899	0.830925706	17.69548416
178.6322964	0.801070691	27.47546084
248.8119322	0.843780457	26.24958107
245.7054501	0.866662554	32.45768617
175.8428465	0.744551204	24.9478739
222.1476761	0.844305652	34.66749708
301.50699	0.873156826	27.0716135
246.7921579	0.851009616	26.73671732
161.2255823	0.776618304	24.22716637
254.5111637	0.850273694	26.75165815
162.6846243	0.842258525	23.30903953
201.7822693	0.848526118	20.92770004
305.8902796	0.879032371	43.14945492
217.556034	0.859802802	29.12780192
281.9935742	0.908500305	34.78137044
166.6501696	0.878477182	19.09390619
427.5479314	0.840949884	34.91194694

log.sigma.2.5.mm.3D_glszm_SmallAreaHighGrayLevelEmphasis	log.sigma.2.5.mm.3D_glszm_GrayLevelNonUniformityNormalized	log.sigma.2.5.mm.3D_glszm_SizeZoneNonUniformityNormalized
334.1085031	0.040024953	0.437066291
114.6869952	0.051783979	0.333055671
169.4253649	0.047013069	0.367067974
142.4956184	0.050026896	0.364663309
145.3166018	0.049967706	0.377920746
207.0644781	0.047332832	0.390888601
166.3137633	0.05155291	0.341404586
253.1646615	0.043956177	0.367731705
181.6088418	0.053050485	0.372418123
102.6413089	0.056153616	0.348904268
75.19770808	0.068759252	0.307663375
225.3537652	0.051000072	0.361352252
143.5182874	0.046161149	0.366342312
146.7974907	0.050562952	0.402942143
216.4592047	0.043590379	0.403196928
158.2475687	0.05447217	0.343486284
129.6328223	0.065751132	0.297135594
120.6552927	0.055224913	0.34615917
108.9963388	0.066114534	0.325795692
304.473144	0.047805708	0.370115915
95.45858715	0.062470998	0.310428474
103.8435869	0.056555755	0.338863229
127.2914682	0.057094518	0.337036944
134.9377931	0.063436252	0.340607736
177.608871	0.047220867	0.364924001
311.9724344	0.045243454	0.371870186
245.838757	0.044906204	0.429670766
172.7361036	0.056733439	0.36639253
92.60966105	0.061831153	0.338287753
187.0288815	0.059906006	0.340835571
139.0769433	0.056758348	0.340488513
204.9978292	0.051561284	0.408742547
175.1207493	0.050594161	0.382561494
148.778183	0.05350656	0.36922112
247.6024487	0.04880191	0.35507736
228.3145387	0.048228076	0.342636824
375.7226738	0.042441195	0.41672649
197.5352973	0.04836008	0.374128952
97.3919861	0.069515306	0.28125
615.2862195	0.053996929	0.362699495
146.5193739	0.055874955	0.360753956
165.5547932	0.057546939	0.365654082
189.684347	0.049658198	0.385827977
175.7666778	0.049618564	0.391547921
119.1773379	0.05899511	0.335668735
246.7834071	0.050894308	0.387409368
200.7084223	0.054596695	0.352694294
157.9028208	0.073753946	0.302727602
147.7171383	0.055425656	0.436550112
184.8290532	0.04697364	0.363034348
328.517437	0.048689069	0.429199755
219.1001388	0.046270185	0.379230086
109.1365693	0.061618499	0.326198735
223.894966	0.055401769	0.380638216
198.6929803	0.046097815	0.409552549
115.6716196	0.053034016	0.285633761
153.3094506	0.054210218	0.365422291
171.9421937	0.051090708	0.354296344
157.4022897	0.050722656	0.401601563
254.216013	0.052764842	0.358724741
170.8637764	0.048576715	0.362704679
220.9724469	0.043782384	0.370196193
249.3125943	0.043992132	0.387447044
270.8249889	0.050741911	0.327297968
191.4319649	0.051483196	0.343792867
217.8609859	0.043874	0.39897928
135.3049277	0.056433559	0.347722944
240.9460339	0.046727755	0.38248675
267.8573343	0.056251561	0.333584532
123.5633585	0.05338716	0.317636933
130.4514036	0.055471769	0.351446885
236.8359423	0.048316112	0.379096954
184.9905746	0.049547553	0.378861188
149.9775388	0.049654928	0.429943079
186.5604271	0.051547402	0.367513667
108.1444415	0.06235618	0.2984262
228.1104629	0.054108835	0.339942012
166.6572117	0.051404787	0.384391259
166.3992358	0.049996004	0.330225372
190.5946656	0.054365947	0.368996396
334.2017543	0.04052736	0.43709184
143.0283468	0.053193111	0.412056089
205.5961715	0.051520083	0.390654432
118.8893535	0.067435194	0.338720313
134.8491247	0.052629981	0.326506993
115.5326516	0.062412366	0.310351903
197.6156613	0.049402429	0.402783826
159.5329319	0.055461015	0.373255598
154.5863065	0.046582548	0.334432994
178.5495226	0.054421717	0.316250057
155.5122582	0.056311353	0.347973803
143.425896	0.057449931	0.352004759
175.0932323	0.053828774	0.349124827
108.5337607	0.057054756	0.315083204
136.0141629	0.060375214	0.308769226
203.7947176	0.042042856	0.369378635
154.5687442	0.051681182	0.345784135
181.5411854	0.049535478	0.402004138
109.9563419	0.070934256	0.334102268
260.4446477	0.04737377	0.376860504

log.sigma.2.5.mm.3D_glszm_SizeZoneNonUniformity	log.sigma.2.5.mm.3D_glszm_GrayLevelNonUniformity	log.sigma.2.5.mm.3D_glszm_LargeAreaEmphasis	log.sigma.2.5.mm.3D_glszm_ZoneVariance
285.4042879	26.13629403	19.05359877	14.16360818
48.95918367	7.612244898	11.44897959	5.876810588
137.2834225	17.5828877	13.30481283	7.616460293
52.14685315	7.153846154	10.68531469	5.614944496
212.3914591	28.08185053	21.80960854	15.50611378
47.29752066	5.727272727	7.090909091	3.090909091
765.4290812	115.5816236	2482.227921	2460.131213
684.7164339	81.84640172	138.1740064	127.5538286
436.1016225	62.12211785	260.4355252	247.1886605
106.7647059	17.18300654	14.36928105	8.281611773
124.91133	27.91625616	67.09359606	52.40544541
341.1165254	48.1440678	287.6324153	274.606739
116.4968553	14.67924528	19.61320755	13.10910367
125.7179487	15.77564103	10.88461538	6.624095989
240.3053691	25.97986577	47.2533557	40.26992872
457.8672168	72.61140285	1016.886722	998.2020182
75.47244094	16.7007874	48.13779528	36.05259161
58.84705882	9.388235294	13.07647059	7.288200692
86.66165414	17.58646617	129.7593985	112.8444231
981.1772916	126.732931	244.5277254	232.5285966
173.529517	34.92128801	58.18962433	44.37091535
69.80582524	11.65048544	15.33980583	9.542463946
67.07035176	11.36180905	23.87939698	16.01686826
263.2897801	49.03622251	723.6235446	701.9703546
133.1972603	17.23561644	142.3123288	130.993252
293.4055767	35.69708492	89.28770596	78.7930624
327.8387942	34.26343381	44.11533421	37.21696621
257.9403409	39.94034091	71.57244318	61.33062492
19.62068966	3.586206897	15.96551724	9.62901308
174.5078125	30.671875	52.27734375	42.38926697
392.9237435	65.49913345	111.1793761	99.110676
144.286119	18.20113314	13.90368272	8.92054346
273.5314685	36.17482517	118.4405594	107.7755391
230.7632	33.4416	264.0656	249.135104
553.2105263	76.03337612	141.7041078	130.8496628
248.0690608	34.91712707	29.46546961	22.29283866
297.9594406	30.34545455	10.05874126	5.586847279
520.4133717	67.26887132	1132.561467	1115.049257
47.25	11.67857143	32.20238095	21.09126984
818.6127603	121.8710678	1360.525033	1343.005412
182.541502	28.27272727	29.7826087	22.76953241
511.9157143	80.56571429	781.5928571	765.1787837
381.969697	49.16161616	20.81010101	14.84466483
240.4104235	30.46579805	20.26384365	14.91524579
125.540107	22.06417112	301.8395722	284.5304127
352.1551155	46.26292629	39.59185919	32.61505468
508.2324774	78.67383761	213.2387231	199.449432
42.07913669	10.25179856	43.10071942	30.62305264
84.69072165	10.75257732	11.57216495	7.510070146
71.5177665	9.253807107	16.67005076	11.40570486
243.356261	27.60670194	29.22222222	24.07004283
456.5930233	55.70930233	15.20348837	9.852950712
458.9616205	86.69722814	3272.331201	3240.195921
194.1254902	28.25490196	92.48235294	82.11647828
126.9612903	14.29032258	9.693548387	5.391269511
115.3960396	21.42574257	25.7029703	17.22968336
439.9684385	65.26910299	144.7018272	133.5814857
266.4308511	38.42021277	31.99069149	24.43550355
128.5125	16.23125	20.171875	14.48663086
880.3105134	129.4849226	1785.041157	1765.337543
190.0572519	25.45419847	18.46374046	12.03112799
378.3405088	44.74559687	23.09491194	17.27747002
1112.747911	126.3454039	151.3259053	143.1062642
259.5472888	40.23833544	20.5851198	13.59899753
74.25925926	11.12037037	41.86574074	32.04215964
603.655651	66.38136153	13.01189689	7.899456091
60.15606936	9.76300578	13.26011561	7.752547696
745.0841889	91.02566735	43.11755647	36.30352697
611.4604474	103.1091107	333.8396072	317.0622482
239.1806109	40.20053121	22.36122178	14.40477664
72.39805825	11.42718447	12.50485437	6.800640965
559.9262018	71.36289777	244.6411645	233.2682417
215.9508772	28.24210526	20.35614035	14.87068329
188.3150685	21.74885845	15.74429224	11.03994913
356.120743	49.9494324	683.5810114	665.8177006
93.10897436	19.45512821	21.90384615	13.2466716
262.4352332	41.77202073	25.16450777	16.97690374
59.58064516	7.967741935	8.264516129	3.921997919
180.9635036	27.39781022	14.70620438	8.947956071
194.4611006	28.65085389	89.78368121	78.11763266
273.1824	25.3296	20.0384	14.97769984
133.5061728	17.2345679	22.36419753	15.8648453
296.8973684	39.15526316	102.0710526	93.05525623
183.2476895	36.48243993	129.025878	116.7823467
237.0440771	38.20936639	206.4393939	192.4542666
53.07017544	10.67251462	35.87719298	26.2345337
200.5863454	24.60240964	40.41767068	33.51913679
325.1056257	48.3065442	699.1021814	679.7057923
198.6531987	27.67003367	61.33838384	53.13797062
162.5525292	27.97276265	18.99805447	11.9249383
91.51711027	14.80988593	32.49429658	23.14883835
216.4829268	35.33170732	336.3154472	319.3651557
530.6697368	81.81973684	50.70197368	41.93336176
328.3166987	59.45105566	55.16314779	44.22622596
316.1796875	61.82421875	49.09277344	38.66315365
365.3154702	41.58038423	17.7330637	11.92247789
303.9442548	45.42775882	28.12286689	20.34136941
63.11464968	7.777070064	7.751592357	3.777029494
34.07843137	7.235294118	14.33333333	7.429834679
1065.007785	133.8782732	190.606511	180.8569135

log.sigma.2.5.mm.3D_glszm_ZonePercentage	log.sigma.2.5.mm.3D_glszm_LargeAreaLowGrayLevelEmphasis	log.sigma.2.5.mm.3D_glszm_LargeAreaHighGrayLevelEmphasis	log.sigma.2.5.mm.3D_glszm_HighGrayLevelZoneEmphasis
0.452216066	0.066457165	6788.243492	468.0689127
0.423631124	0.188839014	2145.217687	192.2176871
0.419282511	0.155128957	2833.526738	261.6764706
0.444099379	0.224688305	1919.839161	239.1888112
0.398299079	0.267358918	3447.151246	231.2455516
0.5	0.04947951	2302.305785	332.9834711
0.212733656	12.10992475	516304.6044	263.0472792
0.306855636	0.553424132	37849.83673	383.5961332
0.274753637	1.628431483	46839.90692	274.9385141
0.405298013	0.230658421	1874.343137	160.8267974
0.26092545	0.991220959	5990.689655	126.7364532
0.277076607	1.128852989	76415.86441	347.8379237
0.392108508	0.155105193	5885	232.8176101
0.48447205	0.190690311	1575.557692	228.1987179
0.378412698	0.349981487	8011.461409	300.7936242
0.231343284	5.375349082	206402.3068	260.7096774
0.287655719	0.381729217	7634.125984	230.8661417
0.415647922	0.279858636	1847.670588	195.6764706
0.243144424	1.289971832	15296.4812	171.7030075
0.288685615	0.572655463	110215.3157	484.4326669
0.269008662	1.588256091	5508.275492	158.9749553
0.415322581	0.506564527	1455.048544	172.6456311
0.356630824	0.34481657	3269.788945	199.0603015
0.214901307	5.283846992	102537.2432	207.7360931
0.29723127	0.727638724	32306.49863	270.5123288
0.308685446	0.332040536	26430.50063	474.148289
0.380738523	0.279104486	8355.347313	333.8243775
0.312472259	0.50714791	11741.07386	257.4417614
0.397260274	0.209828311	1686.896552	150.4827586
0.318012422	0.284103495	11425.08398	312.0546875
0.287852332	1.241443517	13891.67504	222.0103986
0.447969543	0.113840185	3276.560907	304.9065156
0.30620985	0.698787174	22273.11608	262.9440559
0.258799172	1.656554751	47826.352	224.6976
0.303526203	0.553001334	38858.87484	387.2689345
0.373388345	0.126567507	10176.97099	371.2140884
0.472883598	0.022457733	7151.316084	583.3216783
0.238962378	4.717676096	283514.3436	302.023005
0.3	0.36803847	4053.214286	170.1904762
0.238911824	1.612065654	1152746.385	964.2507754
0.37761194	0.340577353	3713.373518	230.5909091
0.246826516	4.489688442	138266.2164	247.0128571
0.40942928	0.179382529	4578.685859	290.969697
0.432394366	0.240084281	2688.094463	258.6905537
0.240359897	2.588424625	37576.41176	182.6122995
0.378592253	0.24422767	8147.636964	366.6622662
0.269295459	1.223950623	40264.48092	313.5058987
0.283095723	0.309822013	8187.165468	263.3956835
0.496163683	0.248388177	1302.391753	197.7680412
0.435840708	0.129004688	5061.192893	278.3350254
0.440559441	0.11362638	8406.723104	463.9805996
0.432315978	0.092832391	3462.495017	329.8828904
0.176404213	26.10016758	421160.7647	179.7526652
0.310596833	0.268315406	35319.07843	352.8235294
0.482115086	0.115161773	2064.193548	303.2225806
0.343537415	0.430439397	2828.277228	204.5915842
0.299875467	1.096202077	21864.62791	233.7923588
0.363812288	0.267160965	5665.654255	272.3736702
0.419397117	0.248714029	2591.88125	235.6125
0.225282291	5.983201397	538341.3203	387.5036675
0.394281415	0.136666879	3811.318702	254.7290076
0.414604462	0.111559257	6610.78865	343.1252446
0.348797668	0.556419567	43642.38405	377.7851671
0.378339695	0.062988727	8375.718789	463.5523329
0.319054653	0.19575778	11276.71296	309.0694444
0.442268343	0.08017016	3646.202908	320.3681428
0.426108374	0.199023846	2428.739884	220.0751445
0.383087512	0.208395309	10165.72433	358.448152
0.244139584	1.01326586	116052.2668	444.3016912
0.354519774	0.148738366	5159.580345	216.5152722
0.418699187	0.281108684	1577.305825	195.7087379
0.296526802	0.876183757	74532.3541	368.3182126
0.426966292	0.194440756	3077.050877	281.5701754
0.461052632	0.224478798	1879.474886	214.1324201
0.237267385	3.614375735	134141.4696	284.7254902
0.339869281	0.235751814	3095.910256	177.9775641
0.349479402	0.08481526	9509.843264	371.6541451
0.479876161	0.133830904	2498.754839	256.083871
0.416730038	0.082737636	3870.118613	273.7846715
0.292777778	0.518891059	16961.87097	286.9392789
0.444523471	0.068551131	7068.7808	467.088
0.392251816	0.187965762	4713.478395	213.7932099
0.333041192	0.497489418	24961.45921	307.9855263
0.285789752	1.298180225	14172.34011	184.5397412
0.267403315	1.562255785	30315.04821	220.4022039
0.322033898	0.191491749	10676.62573	215.1111111
0.380733945	0.300493672	6926.540161	288.8433735
0.227059437	5.052612828	102565.8037	235.5809414
0.349206349	0.373506503	12337.65152	245.0151515
0.376005852	0.110004452	5045.538911	309.8871595
0.327114428	0.189390275	7887.718631	252.6501901
0.242890995	2.636224888	46696.56748	218.1837398
0.337702733	0.28322752	10588.45526	276.9532895
0.302379571	0.640585163	7413.866603	186.1285988
0.309646205	0.36980388	7748.009766	231.0078125
0.414848993	0.121801862	5034.698686	319.4014156
0.358482871	0.264725756	4672.532423	244.8065984
0.501597444	0.053503564	2227.859873	276.910828
0.380597015	0.187926587	2154.264706	178.7941176
0.320262919	0.475767033	81939.30432	417.636235

log.sigma.2.5.mm.3D_glszm_SmallAreaEmphasis	log.sigma.2.5.mm.3D_glszm_LowGrayLevelZoneEmphasis	log.sigma.2.5.mm.3D_glszm_ZoneEntropy	log.sigma.2.5.mm.3D_glszm_SmallAreaLowGrayLevelEmphasis
0.687017335	0.006387446	6.393406368	0.004858508
0.582773531	0.032670919	5.827912205	0.023786086
0.628323618	0.014630573	6.252174842	0.009482465
0.623031216	0.027229891	5.843584541	0.023792815
0.637543221	0.013525441	6.226876423	0.010392525
0.646335713	0.018962155	5.82784449	0.015884564
0.604678632	0.007675033	6.662638957	0.004818446
0.628689096	0.006255182	6.706809363	0.004274717
0.632512525	0.007630587	6.358278646	0.005066339
0.607161594	0.029543939	6.018112229	0.016907104
0.574501191	0.02751883	6.207676857	0.018831312
0.621731286	0.005926779	6.424226123	0.004152239
0.62613568	0.023661375	6.206326781	0.016003509
0.658186946	0.021027259	5.967537882	0.016846631
0.658830921	0.012412987	6.344010045	0.008276137
0.605205182	0.007607432	6.4333651	0.00493977
0.559886852	0.011771101	6.051040153	0.009009116
0.608735745	0.022150692	5.879465628	0.012174089
0.586543205	0.014860765	5.904199347	0.010075875
0.631803752	0.004250325	6.717308275	0.003244154
0.576711643	0.017624708	6.313380541	0.00980006
0.598205376	0.022339177	5.856294993	0.015985172
0.599952195	0.017210565	5.894366781	0.011009353
0.604046312	0.010154793	6.253121582	0.006510498
0.627667502	0.011505554	6.317881736	0.006081124
0.631621855	0.004874973	6.500017298	0.003632829
0.681654115	0.007352611	6.310273437	0.005329554
0.628056381	0.008995741	6.256177791	0.004904397
0.5979016	0.036326946	5.314709012	0.030931686
0.604321134	0.007613437	6.200788931	0.005849162
0.602690918	0.009651562	6.389740169	0.006093846
0.664192044	0.010740531	5.973099308	0.006214966
0.642864665	0.008913401	6.371862673	0.006168144
0.631280358	0.011270083	6.254735687	0.007726971
0.615822904	0.004873898	6.545988576	0.0033884
0.603696859	0.006819772	6.457886067	0.004692931
0.67104229	0.004238649	6.387289014	0.003468161
0.634944302	0.007938387	6.519732062	0.005559906
0.545594038	0.020777865	5.888897075	0.015625951
0.623466405	0.001611671	6.483863907	0.001157419
0.62256733	0.011562237	6.175267099	0.007990848
0.627676279	0.007863358	6.374736452	0.005214708
0.644859956	0.010488852	6.324511998	0.006823025
0.646779508	0.014081053	6.156807432	0.00931435
0.598379323	0.01569555	6.113598402	0.010077347
0.645521843	0.005615497	6.248427141	0.003991828
0.614942493	0.006345787	6.504121956	0.00430633
0.566381422	0.013038733	5.711884239	0.00540036
0.685481754	0.026833502	5.629472801	0.019235321
0.618867398	0.01749221	6.053194747	0.011491907
0.680443406	0.004914447	6.082305937	0.003861829
0.638353585	0.006952252	6.503962532	0.004868055
0.587661113	0.013729329	6.357487578	0.008065333
0.640577908	0.008944592	6.214505619	0.007655967
0.664234354	0.011439513	6.059589696	0.009045187
0.542728496	0.014998982	6.405214188	0.009328621
0.626419438	0.01018379	6.37280585	0.006722137
0.61599996	0.00931026	6.433167205	0.006411397
0.657980523	0.014717811	5.98678955	0.010520141
0.621264787	0.004513993	6.592908554	0.003056656
0.624246013	0.010995661	6.352373922	0.007569893
0.629781447	0.008319104	6.558781495	0.005572075
0.646967978	0.005826266	6.675032972	0.004093528
0.589803376	0.004619384	6.537198858	0.003481901
0.60761191	0.011039779	6.117133802	0.005569769
0.655755702	0.010441331	6.524178609	0.008108733
0.606616944	0.017265551	5.874868186	0.008969283
0.641752428	0.005848297	6.555374656	0.003978999
0.597445609	0.003507323	6.549938815	0.002362859
0.581024927	0.012389624	6.483975291	0.008838776
0.611034328	0.018485895	5.824192988	0.008123979
0.638903668	0.006266045	6.512601457	0.004652355
0.635722492	0.009251107	6.187338934	0.006501191
0.681387128	0.016046784	5.989030404	0.010146053
0.628004533	0.007669079	6.385543903	0.005174514
0.561691221	0.015130257	6.199889686	0.009388218
0.604609049	0.005409614	6.468811827	0.003849994
0.641855677	0.019603155	5.656138921	0.009164452
0.58766644	0.009952283	6.372958491	0.007028946
0.630268252	0.009176652	6.266123331	0.006549498
0.687107086	0.005987153	6.371644143	0.004748393
0.667284802	0.016994149	5.955216693	0.013285736
0.649383354	0.007153021	6.256424584	0.004172018
0.601731309	0.011926799	6.046387554	0.0075081
0.590374517	0.012438798	6.447046789	0.008274726
0.573458807	0.024947314	5.884651645	0.015991785
0.659239657	0.010077245	6.168371556	0.005816097
0.63463826	0.009548252	6.320714849	0.006181763
0.593839628	0.015626655	6.470565191	0.00913212
0.577459261	0.007502053	6.346811136	0.005467273
0.61180135	0.013330419	6.097114791	0.009189077
0.612383973	0.012880981	6.237799456	0.009068399
0.612210073	0.007552147	6.493296109	0.00462071
0.577500619	0.016156297	6.49048871	0.010330816
0.573101417	0.008394101	6.45903009	0.005445171
0.62988215	0.010481543	6.595867991	0.006577664
0.609689443	0.010981323	6.491268059	0.007472851
0.654545324	0.016300108	5.770967068	0.013812736
0.5967113	0.024549458	5.482228377	0.019835725
0.637848905	0.004821155	6.667083563	0.003565557

log.sigma.2.5.mm.3D_ngtdm_Coarseness	log.sigma.2.5.mm.3D_ngtdm_Complexity	log.sigma.2.5.mm.3D_ngtdm_Strength	log.sigma.2.5.mm.3D_ngtdm_Busyness	log.sigma.2.5.mm.3D_ngtdm_Contrast
0.00567112	1.670134012	3.247695341	0.199674897	0.000108478
0.016315528	2.143822272	3.680469799	0.195910854	0.000821601
0.008379446	1.036472786	1.975425963	0.340314163	0.000320262
0.020203804	2.572410337	4.449303137	0.153615352	0.001052782
0.005172989	0.606323822	1.362772288	0.573931025	0.000149174
0.035279638	3.527059164	8.624250087	0.062793906	0.001010246
0.000967555	0.095727482	0.467020271	1.508733672	4.55E-06
0.001433367	0.241636462	0.589405575	1.069055814	2.00E-05
0.002087784	0.228152714	0.752597671	0.942511565	1.99E-05
0.010336906	0.72228739	1.769298847	0.394188904	0.000238824
0.005718709	0.194897076	0.758872312	0.82805631	6.03E-05
0.002848472	0.27073884	0.911134509	0.556037966	2.35E-05
0.008551843	1.094811531	2.405618196	0.249961414	0.000299855
0.011035588	1.48988663	2.668439306	0.285798642	0.000450206
0.004936523	0.999635411	2.090823169	0.399977025	9.90E-05
0.001667281	0.154502915	0.52554841	1.150710979	1.78E-05
0.009627586	0.592267875	2.141296523	0.287102961	0.000103403
0.015061625	2.098080047	4.191030819	0.188085998	0.000552487
0.008122125	0.354927739	1.412339808	0.437715508	6.98E-05
0.001003195	0.214474195	0.76694401	0.781994427	5.56E-06
0.00386101	0.214575183	0.685095511	1.156188579	8.03E-05
0.013642824	1.385262963	2.844529206	0.310513044	0.000499974
0.013902116	1.194739741	3.066311312	0.242882407	0.000307488
0.00279619	0.130313834	0.732841749	0.841158077	1.27E-05
0.007450532	0.622079521	1.641420524	0.337191265	0.000147842
0.003344973	0.58166804	1.794040717	0.393054113	4.24E-05
0.00409178	0.769984964	1.972235005	0.42514396	6.39E-05
0.004030987	0.395840929	1.37748922	0.51481303	3.70E-05
0.048836811	2.90197161	8.147994942	0.085308418	0.00106005
0.005068556	0.429561141	1.218136953	0.456092457	7.57E-05
0.002082057	0.205474114	0.59806182	1.346755468	3.01E-05
0.0076791	1.214921452	2.005057475	0.326333411	0.00038862
0.003987931	0.394747172	1.282333172	0.537593368	4.57E-05
0.003764373	0.262539998	0.849464295	0.750976303	5.21E-05
0.001688923	0.278613165	0.860458914	0.77875463	1.54E-05
0.003792396	0.658851534	1.262673262	0.443950533	0.00010533
0.004390719	1.27245214	2.074954211	0.255717688	0.000184509
0.00167229	0.158404726	0.55590366	1.0084558	1.69E-05
0.014256736	0.621120843	1.846888918	0.298732043	0.00024929
0.001008149	0.105042303	0.504115351	0.747494302	5.29E-06
0.0055914	0.660619833	1.636525711	0.481155141	0.000108051
0.001614938	0.146077267	0.725232263	1.033330122	7.73E-06
0.00301951	0.440243903	0.881055754	0.784319866	9.66E-05
0.005220444	0.692844407	1.366153618	0.5827966	0.000152512
0.006148072	0.318475486	1.37796034	0.463499642	4.59E-05
0.003432868	0.439069714	1.056732201	0.640037634	6.59E-05
0.00173715	0.243479562	0.967205531	0.796472756	1.09E-05
0.014995412	0.800305789	2.69660358	0.189180434	0.000227504
0.019894511	1.64539494	3.734060327	0.215756185	0.000650449
0.014610592	1.960831331	4.198711367	0.151927845	0.000605488
0.005537043	0.876497596	1.702106307	0.363606175	0.000159463
0.002838275	0.662957983	1.519320167	0.54325521	4.67E-05
0.001320516	0.057653502	0.358296429	1.8393268	4.99E-06
0.005072268	0.581951462	1.878366939	0.270369582	4.67E-05
0.010105486	1.894472349	3.233647082	0.225916061	0.000466058
0.006694616	0.673758283	1.835283631	0.487239713	0.000143953
0.002249618	0.176302081	0.542043634	1.236341398	2.93E-05
0.003369553	0.553145767	1.144199318	0.647640236	8.42E-05
0.008999478	1.148947441	1.981936639	0.368095463	0.00032298
0.000878644	0.11553827	0.570058935	1.168371911	3.32E-06
0.005848179	0.918938252	1.98374833	0.363215613	0.000113744
0.003293051	0.708306647	1.449875414	0.448087239	6.10E-05
0.001103525	0.165970733	0.398462618	1.381450498	1.65E-05
0.004016127	0.643259071	1.739849734	0.313511994	5.38E-05
0.012316503	1.036830125	2.704713497	0.178644924	0.00021635
0.002202524	0.481140194	0.859854018	0.769573704	5.45E-05
0.014067585	1.538864193	2.770437385	0.235328409	0.00075591
0.001572564	0.370956661	0.88623813	0.841033543	2.10E-05
0.001093205	0.134956779	0.504711543	1.137137741	7.83E-06
0.003677316	0.40681387	1.037965845	0.617519066	6.28E-05
0.014833488	1.20801517	2.673877577	0.294112069	0.000524674
0.001843304	0.223083219	0.617745142	0.871530799	2.35E-05
0.005133018	0.770183769	1.492941167	0.556794401	0.000170547
0.007055349	0.913882256	1.635752552	0.519193085	0.000253975
0.002296461	0.234226453	1.011644362	0.727695519	1.59E-05
0.009051642	0.516036085	1.59319479	0.375166686	0.00012566
0.003608818	0.478370185	1.284383462	0.412679419	4.97E-05
0.017604519	2.890866276	5.163804049	0.133301085	0.001096513
0.006254823	0.751351265	1.909591528	0.307453264	0.000103779
0.004926017	0.544103753	2.035942123	0.333155969	3.59E-05
0.005818828	1.697923751	3.377324062	0.192581275	0.000105236
0.00918401	1.107225386	2.524520181	0.257586564	0.000191601
0.003091642	0.544433155	1.096801928	0.596071554	6.62E-05
0.004563998	0.19653454	0.661995448	0.921059759	5.92E-05
0.003565858	0.224609459	0.766307741	0.824362354	4.22E-05
0.010311526	0.90041512	2.644240528	0.226827651	0.000379809
0.006129988	0.684186215	1.522674123	0.428502779	0.000135347
0.002754137	0.153312014	0.683805578	0.913947386	2.02E-05
0.00542111	0.463196246	1.287906798	0.431391384	8.13E-05
0.005123322	0.63414658	1.409056622	0.422949023	0.000130671
0.011000224	0.848319543	2.654239463	0.200956325	0.000157305
0.003675277	0.22665058	0.872049235	0.738755284	3.20E-05
0.001914737	0.241432984	0.724989703	0.874553151	1.81E-05
0.00256455	0.188951801	0.602638515	1.22351278	3.43E-05
0.002403325	0.211866368	0.632655261	1.089926455	3.00E-05
0.003103017	0.745192637	1.376927373	0.53703884	8.81E-05
0.003272685	0.465276827	1.177498645	0.663854863	5.48E-05
0.019335388	3.124783131	4.910287135	0.121062062	0.000927403
0.025900369	1.317792059	3.627930041	0.159853902	0.000718202
0.001024964	0.163688307	0.465602533	1.088059623	1.08E-05

log.sigma.2.0.mm.3D_gldm_GrayLevelVariance	log.sigma.2.0.mm.3D_gldm_HighGrayLevelEmphasis	log.sigma.2.0.mm.3D_gldm_GrayLevelNonUniformityNormalized	log.sigma.2.0.mm.3D_gldm_DependenceEntropy
41.40376263	564.8753463	0.048534964	6.786698405
27.68401033	251.389049	0.061050254	6.182379077
34.01536327	307.5246637	0.048988015	6.672281129
32.91586937	263.257764	0.055186914	6.238274143
27.75117948	305.135365	0.054666659	6.517315909
33.3400724	454.7768595	0.056587665	5.910050621
15.49406658	288.4474808	0.102295	6.933175025
28.37983267	392.2788398	0.059631037	6.985214174
19.06312598	249.0387142	0.073839818	6.786979964
23.48641551	317.8701987	0.058104469	6.312235181
11.88592917	201.7268638	0.086836923	6.342509557
20.36904527	400.1899031	0.076262041	6.915404235
30.53269699	294.1245376	0.05971681	6.4985952
33.95309546	327.0326087	0.048315073	6.382268853
32.63911071	343.9879365	0.056402318	6.749811627
20.52210578	292.9267615	0.082116118	6.892760118
15.39531788	261.0645527	0.074890116	6.40967173
24.57915723	228.2518337	0.062571362	6.128451738
12.63665615	312.8382084	0.084407555	6.461407022
21.97685341	693.8663835	0.066237488	6.876870711
17.1293935	151.680462	0.069634588	6.507970716
26.04031852	182.3407258	0.057736082	6.120847561
23.1360851	151.8243728	0.058966354	6.424899506
10.91090241	162.1523492	0.111215452	6.642586219
26.80039244	314.3900651	0.079917294	6.849629666
26.60747978	244.2738654	0.063438753	6.809868133
29.48053071	348.1671657	0.056032147	6.725216338
18.75716217	274.9844652	0.071974459	6.559704865
19.05629574	155.2739726	0.075811597	5.469914611
19.18681725	519.1397516	0.064630223	6.482590764
19.74788218	217.3295086	0.062829885	6.781996508
32.04085876	336.2068528	0.054439692	6.301139973
21.1972635	254.8222698	0.067645961	6.848826895
20.96017729	255.500207	0.077235189	6.70973055
22.90068658	443.058835	0.064437112	6.868345694
33.3791917	367.5956679	0.048750692	6.824195404
44.36643711	562.7116402	0.047842131	6.552630744
22.46410067	404.7435149	0.094685194	7.022161953
14.91366071	183.2642857	0.07408801	6.134089394
15.45378419	1333.57341	0.098528761	6.785652402
21.37044943	181.5440299	0.060965694	6.430270224
14.67363348	324.190409	0.106295916	6.623738171
29.92919608	308.7142266	0.051463349	6.733778076
31.24916634	319.2683099	0.051251736	6.522261738
13.72407333	220.0784062	0.09275398	6.685023208
25.99157161	300.8109121	0.057005784	6.649486217
19.03411714	359.6714633	0.072981886	6.756480298
13.64698172	459.7942974	0.083013593	6.029396509
28.02278897	190.3734015	0.05339447	6.262575907
33.61825515	280.3584071	0.058970945	6.218861007
32.69838182	806.5928516	0.053827982	6.555897527
32.62327692	516.9709156	0.0509757	6.608465278
10.29818818	217.6242477	0.126467762	6.741885984
18.45317904	476.4183922	0.072693649	6.613486566
36.93143773	316.8040435	0.049208004	6.316740854
22.42791198	225.0544218	0.061259198	6.441241754
19.41449055	192.2371108	0.071664943	6.803902963
28.30557083	255.6337687	0.054285687	6.589980285
30.62086439	308.1074705	0.052467582	6.457878188
16.02643704	474.7088956	0.100728518	6.790372526
28.52288108	263.8878856	0.054206079	6.676632892
36.13843957	394.7529412	0.047185712	6.815301003
30.38348114	507.4085499	0.05299761	7.073926238
25.7203395	686.1073473	0.056310002	6.532209865
21.01726489	442.4490399	0.067421001	6.39898396
38.84122014	633.7106109	0.045055981	6.737227539
26.87189692	230.8128079	0.062789682	6.070912108
30.24778165	324.2707965	0.05378663	6.825021507
16.53982447	356.0945658	0.078603246	6.757678939
21.46570869	275.5258945	0.062250187	6.476813552
25.59840373	171.6544715	0.056356996	6.270227418
25.6298569	527.5211805	0.058745175	6.930942207
29.22275246	279.7393258	0.053816437	6.521874197
31.53757784	317.1831579	0.053289751	6.401183686
18.5191039	287.8117042	0.094800449	6.798581001
17.10965868	238.788671	0.071378055	6.351582047
23.02956771	625.8220914	0.059837723	6.471225379
34.96479406	256.9102167	0.05583299	6.103588463
24.63493415	286.8532319	0.057366161	6.471033755
18.25865154	303.7372222	0.083244444	6.481142782
39.61895878	560.5362731	0.049744137	6.769764228
25.52483746	305.7021792	0.056944697	6.402183403
28.98115208	513.0775635	0.058714276	6.855800674
13.27095879	151.9704173	0.082314273	6.414572091
18.90192933	293.692081	0.07385733	6.825387071
18.66762425	288.7777778	0.100010285	5.987448765
25.4584327	249.9541284	0.057325422	6.720100416
15.00181659	225.3349844	0.096208359	6.820263982
24.29031026	287.2410347	0.06371222	6.746330987
26.29778952	491.5566935	0.057231032	6.483654561
20.24810494	274.8569652	0.071112101	6.384524257
14.91866182	244.3597946	0.089056288	6.722830699
20.13333264	288.551433	0.063794409	6.634167858
18.81670035	210.0841555	0.064662015	6.690090823
17.81026913	294.6044754	0.067697343	6.559080423
41.10613243	459.1405201	0.044785074	6.796516786
25.3440829	242.7622349	0.055448959	6.668633992
38.78643244	380.3865815	0.051169247	6.082779812
14.92164179	147.1716418	0.07426487	5.981173099
25.01002899	647.3779465	0.059117545	6.919975276

log.sigma.2.0.mm.3D_gldm_DependenceNonUniformity	log.sigma.2.0.mm.3D_gldm_GrayLevelNonUniformity	log.sigma.2.0.mm.3D_gldm_SmallDependenceEmphasis
320.666205	70.08448753	0.40574944
81.85878963	21.18443804	0.386438896
195.5	43.69730942	0.370683763
74.66459627	17.77018634	0.367303071
312.8724309	77.13465627	0.351736863
66.2231405	13.69421488	0.422693663
1121.349369	1078.08701	0.224110516
1027.847726	361.8411338	0.307797416
628.3758799	314.7053027	0.27590707
175.9033113	43.86887417	0.394579194
250.1092545	135.1182519	0.262612321
462.8597006	259.8247725	0.279601394
161.2219482	48.43033292	0.337186402
156.5341615	31.11490683	0.397227709
303.7834921	88.83365079	0.363525894
744.7368969	473.1530719	0.23860424
155.4507361	66.12797282	0.302259161
93.24449878	25.59168704	0.357714934
171.714808	92.34186472	0.260567104
1540.046717	608.2588479	0.298286795
372.425409	144.7006737	0.254404115
122.8467742	28.63709677	0.427444225
108.9641577	32.90322581	0.343639155
411.6249652	400.0419794	0.217889734
171.2947883	98.13843648	0.283314714
419.2629108	162.1494523	0.302175376
394.9091816	112.2884232	0.349063352
392.7745229	162.1584554	0.311241182
38.79452055	11.06849315	0.454423516
319.3925466	104.0546584	0.304741048
680.8987279	251.8850087	0.281494997
192.5304569	42.89847716	0.382853285
363.4179872	157.9533191	0.30083961
369.0298137	186.5229814	0.278906516
838.5675044	330.7556984	0.296694099
413.2588963	94.52759154	0.359359197
403.8743386	72.33730159	0.439934202
677.3157533	551.162515	0.243146099
116.45	41.48928571	0.333844614
1146.962104	930.8012067	0.246680638
281.8134328	81.69402985	0.33087845
733.7263752	602.9104372	0.263347574
506.1364764	124.4383788	0.352590446
335.771831	72.77746479	0.403380747
193.9203085	144.3251928	0.249612157
476.2811329	136.8708871	0.343380266
827.7482714	390.5260699	0.271385514
91.94093686	40.75967413	0.290245078
94.18158568	20.87723785	0.410780969
106.960177	26.65486726	0.384197742
288.8181818	69.27661228	0.404947529
664.897307	141.967325	0.410266116
738.221665	1008.706871	0.196338635
272.1924482	119.362972	0.290221345
166.8942457	31.6407465	0.416947874
238.0680272	72.04081633	0.315786142
619.1305106	287.7347447	0.286551946
438.1349782	112.2085148	0.334417499
176.6618611	40.0327654	0.400868014
1313.920775	1097.235748	0.248518572
277.7990971	72.03987961	0.369734839
570.810142	116.3127789	0.41654354
1481.101287	436.3823172	0.338846047
479.360687	118.0257634	0.358351691
125.5051699	45.64401773	0.338466495
866.7474423	154.1365098	0.426525086
100.1921182	25.49261084	0.359948644
1056.482006	273.5050147	0.36160277
1120.258125	590.15317	0.262205115
444.8691149	132.2193974	0.320861256
110.2601626	27.72764228	0.368779765
815.0871311	292.6097169	0.299183579
293.1153558	71.84494382	0.356136818
237.0547368	50.62526316	0.433848194
516.2889324	387.1650343	0.260819059
175.4422658	65.52505447	0.322771
494.0203712	132.1815301	0.330315482
78.82043344	18.03405573	0.417159443
298.3885932	75.4365019	0.367660499
284.5911111	149.84	0.296620116
306.3100996	69.94025605	0.397620284
190.9975787	47.03631961	0.380542284
400.3593339	133.9859772	0.324542005
314.076598	155.8209192	0.251990053
389.9510129	200.5226519	0.263638645
94.46516008	53.10546139	0.293827896
249.3180428	74.98165138	0.352888239
431.5312826	369.0552659	0.223188348
309.2504409	108.3744856	0.341198291
297.6217996	78.23482078	0.36125592
157.6890547	57.17412935	0.322279268
316.3120063	225.4905213	0.255612566
877.6918463	287.1386359	0.335161581
625.540917	222.8253047	0.297881635
616.8627155	223.8751134	0.313947575
547.114094	106.7676174	0.39539106
511.3368679	135.9608483	0.347207149
92.73801917	16.01597444	0.473519647
56.47014925	19.90298507	0.325948029
1566.855621	521.6532185	0.302511445

log.sigma.2.0.mm.3D_gldm_DependenceNonUniformityNormalized	log.sigma.2.0.mm.3D_gldm_DependenceVariance	log.sigma.2.0.mm.3D_gldm_LargeDependenceEmphasis
0.222068009	2.716310111	9.403047091
0.235904293	1.688661147	7.703170029
0.219170404	2.098051439	8.816143498
0.231877628	1.932062806	8.354037267
0.22173808	1.995159024	8.815733522
0.273649341	1.56246158	6.652892562
0.106399978	15.76495031	41.45279438
0.169388221	7.028951601	18.58536585
0.147436856	7.785426458	21.95635852
0.232984518	1.674937064	7.659602649
0.160738595	4.23173089	16.03598972
0.135855504	9.226577751	25.2571177
0.198794018	2.456762062	10.31196054
0.243065468	1.538038656	7.226708075
0.192878408	5.453652205	14.56063492
0.129249722	10.64848658	29.14057619
0.176048399	3.821554492	13.78369196
0.22798166	1.642386165	8.070904645
0.156960519	4.657657357	16.87568556
0.167706274	4.816838291	15.6516389
0.179223007	2.679237365	12.88354187
0.247674948	1.774908949	7.282258065
0.195276268	4.106704693	12.93548387
0.114435631	10.63556949	31.73116486
0.13949087	18.69615593	39.85016287
0.164030873	5.998649347	17.57355243
0.19706047	2.89833407	10.89221557
0.174334009	3.930974512	13.84243231
0.265715894	1.29198724	6.095890411
0.198380464	3.634683847	12.57515528
0.169842536	4.293624453	15.15614867
0.244327991	1.735493056	7.659898477
0.155639395	7.759292766	20.83982869
0.152807376	5.139212565	17.4952381
0.163367914	6.341458942	18.17436197
0.21312991	3.400259753	10.99896854
0.267112658	1.479380127	6.447089947
0.116357285	19.05688108	44.47088129
0.207946429	2.570395408	10.31071429
0.121410194	10.49450392	29.67068911
0.210308532	2.307262196	9.849253731
0.129359375	9.616963716	26.84414669
0.209320296	2.416675732	9.741108354
0.236459036	1.8671097	7.804225352
0.124627448	13.38815663	33.40745501
0.198367819	3.24808159	11.4423157
0.154690389	5.835708205	18.48196599
0.187252417	2.532260941	11.75356415
0.240873621	2.262033869	8.227621483
0.23663756	2.298496358	8.615044248
0.224411952	2.420498089	8.895881896
0.238742301	1.796075539	7.588868941
0.092555374	16.64698333	47.44558676
0.165768848	4.235172638	15.09866017
0.259555592	1.379734865	6.570762053
0.202438799	2.22690777	10.13945578
0.154204361	8.718227196	22.37783313
0.211966608	2.058259249	9.358974359
0.23153586	2.634204157	9.05242464
0.120620653	11.55936513	31.52327183
0.209028666	3.352942209	10.90820166
0.231565981	2.828021345	9.258823529
0.17987628	5.857370067	16.04396405
0.228702618	1.874693163	8.409351145
0.185384298	3.399588505	12.24076809
0.253360843	1.696409055	7.060216311
0.246778616	1.399500109	7.369458128
0.207764406	2.946616893	10.46941986
0.149208594	4.902025799	17.72163026
0.209448736	2.18281429	9.825800377
0.224106022	1.945468967	8.504065041
0.163639255	5.268410403	16.66191528
0.219562064	2.14998443	9.05093633
0.249531302	1.68993241	7.082105263
0.126417466	11.93095101	30.99559256
0.191113579	3.156074824	11.83224401
0.223639824	1.892522073	8.89859665
0.24402611	1.616271602	7.167182663
0.226911478	1.968376585	8.508745247
0.158106173	4.951980247	16.54222222
0.217859246	2.762946446	9.635846373
0.231231935	1.77298923	8.004842615
0.175442302	8.145348922	19.87642419
0.165914737	3.850472548	15.32012678
0.143628366	8.450061421	23.63278085
0.17790049	3.123843368	12.83804143
0.190610124	3.737267252	12.3470948
0.112495121	16.00201782	40.54014599
0.18180508	6.037687828	16.36743092
0.217718946	2.014191768	8.822970007
0.196130665	2.377472092	10.49004975
0.124925753	8.955786782	26.56793049
0.194999299	3.582440121	12.10375472
0.181526674	3.42771671	12.99709808
0.186532421	3.393851402	12.4702147
0.229494167	1.989875878	8.202181208
0.20853869	2.886951824	10.52365416
0.296287601	1.224632282	5.523961661
0.210709512	2.252784585	9.835820896
0.1775675	4.052251329	13.95648232

log.sigma.2.0.mm.3D_gldm_LargeDependenceLowGrayLevelEmphasis	log.sigma.2.0.mm.3D_gldm_SmallDependenceHighGrayLevelEmphasis	log.sigma.2.0.mm.3D_gldm_LargeDependenceHighGrayLevelEmphasis
0.02187288	246.4486002	4858.203601
0.059490525	100.2793796	1858.391931
0.051548467	122.1296787	2584.695067
0.075969627	99.55027225	2098.65528
0.048325263	112.5849833	2408.97803
0.023282065	185.610697	3124.198347
0.162309448	71.79869624	11175.87171
0.058885037	136.3963383	6659.997363
0.112538454	81.20340115	4902.332004
0.035269603	133.9486523	2284.137748
0.101016542	57.43517769	2930.992931
0.072483687	122.7315975	9398.155269
0.057155011	92.41669319	3289.75709
0.045403839	140.5349785	2083.007764
0.058060893	149.4054654	4388.884444
0.120541131	75.19281776	7885.453315
0.068481136	86.65389025	3245.392978
0.068508189	82.20962855	1761.757946
0.063405005	86.84923449	4961.081353
0.024801154	209.1460785	10650.8715
0.154221164	43.19942471	1803.250241
0.109456058	90.95069057	975.0100806
0.253836063	64.9930201	1413.78853
0.236535608	43.27391075	4656.028913
0.148813945	97.03684951	11777.76954
0.106916307	94.50908333	3516.388889
0.046435025	137.4835923	3233.917665
0.065132976	97.47840035	3388.281403
0.072633126	82.24554985	850.4246575
0.029086565	172.3112491	6076.526087
0.100861929	68.59625875	3032.424046
0.047585788	133.278225	2396.699239
0.103475393	85.45792477	4851.647109
0.090596621	79.20874933	4131.798344
0.048831705	148.7398347	7395.468342
0.053022593	138.3940661	3723.448169
0.017906125	237.0531917	3802.851852
0.120151966	105.8663414	17344.85037
0.094045476	72.79487741	1538.967857
0.023254325	351.8043757	38454.05314
0.105023638	65.08100829	1571.454478
0.095261349	102.0917579	7923.076516
0.056232938	117.215188	2710.570306
0.040251096	145.9657284	2121.85
0.179382214	66.45690265	6654.028278
0.05969178	118.0558761	2922.316951
0.064888115	113.4352554	5913.449449
0.032489838	142.1773071	4959.753564
0.114245515	88.64397426	1303.150895
0.097366867	113.0455271	2071.495575
0.014009377	354.3034111	6540.255633
0.020124209	231.6646512	3493.252424
0.24100725	48.87417817	9736.007648
0.034405156	134.2834794	7357.456151
0.044831517	137.5738388	1949.186625
0.078914758	84.21390115	1920.146259
0.161977825	65.3528719	3759.205978
0.068225012	95.97108121	2129.584422
0.049460456	138.7149066	2291.653997
0.071916856	132.6502078	14264.10631
0.063398931	105.6252244	2668.367946
0.034361497	170.0737215	3364.060852
0.038459883	179.4262691	7565.156667
0.014702825	250.8928081	5619.460401
0.04366218	156.3218491	5195.932053
0.014788514	281.2362593	4335.614148
0.068064475	85.6102427	1734.581281
0.049136591	130.6395203	2962.623206
0.058818981	102.9143031	5846.769845
0.049172047	87.14423932	2725.892185
0.146896628	71.22036475	1219.215447
0.037166983	173.0252634	8340.060831
0.05417572	110.5859073	2213.033708
0.037549525	155.4801573	1881.254737
0.125358137	85.31504094	8185.000735
0.06793847	83.78225586	2552.834423
0.016788154	210.9609116	5440.975555
0.081160732	109.2107422	1827.380805
0.043953861	108.1299549	2364.95057
0.066891884	107.2751985	4484.190556
0.022366172	241.5468979	4944.667141
0.038345634	117.9935339	2463.569007
0.048020519	180.7078478	9678.692813
0.150176725	46.69776621	1979.41046
0.098245335	88.35703173	6275.20663
0.052310332	69.82130886	4214.774011
0.082052128	102.9218387	2594.648318
0.21629702	63.06055446	8137.658238
0.073516683	107.2213324	4400.780129
0.023098159	177.8406363	4262.168252
0.055954818	87.72854833	2953.949005
0.130236657	75.71407133	5911.263428
0.054269972	102.8036318	3233.33126
0.096292868	70.48897111	2384.635519
0.057261871	103.6321497	3213.670094
0.026695848	192.2757502	3573.505453
0.070751936	92.7630689	2225.202692
0.046918646	177.810784	2180.335463
0.138776215	51.901471	1406.985075
0.024237205	194.653351	8927.312897

log.sigma.2.0.mm.3D_gldm_SmallDependenceLowGrayLevelEmphasis	log.sigma.2.0.mm.3D_gldm_LowGrayLevelEmphasis	log.sigma.2.0.mm.3D_gldzm_DistanceZoneVariabilityNormalized
0.001956152	0.003337457	0.886838264
0.00773508	0.012175748	0.907731864
0.004148267	0.007962747	0.878166594
0.007030471	0.01294369	0.944489796
0.003466986	0.006776522	0.866297527
0.00622651	0.007918153	0.892656488
0.001318699	0.004703393	0.902976589
0.001630072	0.004092582	0.852640776
0.001935838	0.006204492	0.948983977
0.003264589	0.005964555	0.908811744
0.002708439	0.007336746	0.925577523
0.001424452	0.003596135	0.892481439
0.006360123	0.010726733	0.832887952
0.004552869	0.007721507	0.86694819
0.002275852	0.005217109	0.900277008
0.001449028	0.005018613	0.97473566
0.003244905	0.006638733	0.942542417
0.006786589	0.01182786	0.85654321
0.001973876	0.004718617	0.933613281
0.000991008	0.002107366	0.933038044
0.003946495	0.013094326	0.941516628
0.009519112	0.017282214	0.892244898
0.0084218	0.025255619	0.948971225
0.002734856	0.00928809	0.922521149
0.002776011	0.006312006	0.95654321
0.002572096	0.007185769	0.946289599
0.002198658	0.004886864	0.878166594
0.002166135	0.005502508	0.906252547
0.020528906	0.025918766	0.793557092
0.001454437	0.003035315	0.989726301
0.00250019	0.007536423	0.951855298
0.002952755	0.007800174	0.920770439
0.002768443	0.006619708	0.878872658
0.002257211	0.006391261	0.960953001
0.00116461	0.003131759	0.906054501
0.003020245	0.006411795	0.802055291
0.002411807	0.00390985	0.665882115
0.001151827	0.003535758	0.940567507
0.004926562	0.010893396	0.904174733
0.000293693	0.000890084	0.903393205
0.004774646	0.011923499	0.875769182
0.001145681	0.003891545	0.825150627
0.002711628	0.006546989	0.920290168
0.003004658	0.006068979	0.908651371
0.002584657	0.007063002	0.954048443
0.002122444	0.005996961	0.931537955
0.00108239	0.003913601	0.910925972
0.002992117	0.004722731	0.987654797
0.010309648	0.019857222	0.89037261
0.006180123	0.011555829	0.96280407
0.001362379	0.002247489	0.970642735
0.001410715	0.00291425	0.86524036
0.001497417	0.005919726	0.894364048
0.00179133	0.003446956	0.955163803
0.004159854	0.008033739	0.876646493
0.003371803	0.008440069	0.925874507
0.003154069	0.009157136	0.937016953
0.004081729	0.009392799	0.921246704
0.003920036	0.007222942	0.867009691
0.000733731	0.002540769	0.920392711
0.004940682	0.00897075	0.935989268
0.002968856	0.005202477	0.876700541
0.001139977	0.002831961	0.81850642
0.001144418	0.002229392	0.803638098
0.00224573	0.006198808	0.941658658
0.001260898	0.002455896	0.837748228
0.005782496	0.012120278	0.911303407
0.002549503	0.005628613	0.829221212
0.001219464	0.003704277	0.931183922
0.003166196	0.006650595	0.936013192
0.010629605	0.023491814	0.938116386
0.001123944	0.002787249	0.899443966
0.003001976	0.006758964	0.945248959
0.003593143	0.006426174	0.955034051
0.001765317	0.005042492	0.782160402
0.0031361	0.007002856	0.944180209
0.001152509	0.002375098	0.911255526
0.007997068	0.01426408	0.839405087
0.00360249	0.007024867	0.902695595
0.001934043	0.004914703	0.904244051
0.001921541	0.003313289	0.898254133
0.003773622	0.0068219	0.936743164
0.0010457	0.003389497	0.94835098
0.003158228	0.010798919	0.938579027
0.001657875	0.004957665	0.899482132
0.006793781	0.010045747	0.863184401
0.003395676	0.008089624	0.890055717
0.001757872	0.006385979	0.981074283
0.004271784	0.007603204	0.930625392
0.00184772	0.003475379	0.952489981
0.004074397	0.007899569	0.767881815
0.002245042	0.00618399	0.915003633
0.002435961	0.005549895	0.874048683
0.003357585	0.008706759	0.926331166
0.001768372	0.004832617	0.841970801
0.002022313	0.004020878	0.788745129
0.003365562	0.007909568	0.894172451
0.005429686	0.012178587	0.842449465
0.007654711	0.017075906	0.91322314
0.000848033	0.002101909	0.860913667

log.sigma.2.0.mm.3D_gldzm_LowIntensityEmphasis	log.sigma.2.0.mm.3D_gldzm_LargeDistanceEmphasis	log.sigma.2.0.mm.3D_gldzm_HighIntensitySmallDistanceEmphasis
0.004489133	1.18061674	586.3645374
0.017633078	1.145454545	254.9469697
0.010358911	1.195488722	309.0588972
0.017925829	1.085714286	266.0392857
0.009124147	1.216123499	316.3722127
0.01285823	1.170731707	429.4085366
0.005865782	1.153376574	312.1458413
0.005154441	1.24028436	419.8529621
0.007015915	1.078582435	299.0859014
0.007764868	1.143661972	323.534507
0.009862755	1.116129032	213.7344086
0.00484713	1.171028037	433.1331776
0.017438643	1.27607362	264.1587423
0.010382298	1.214983713	347.0032573
0.006203323	1.157894737	397.7666409
0.006017502	1.038387716	313.0089571
0.009970117	1.088815789	288.3618421
0.016636893	1.233333333	235.3541667
0.007131642	1.103125	332.071875
0.003139137	1.104051864	686.6074554
0.015407911	1.090452261	170.1587102
0.021270469	1.171428571	208.5336735
0.028307888	1.07860262	182.2456332
0.012378774	1.121107266	199.1744521
0.009386154	1.066666667	338.3302469
0.008221799	1.082853855	312.8895282
0.006029563	1.195488722	383.3997494
0.006754731	1.147914033	310.7664349
0.04034449	1.350649351	175.7564935
0.004365279	1.015490534	566.0938038
0.008647349	1.074044586	245.0694666
0.008615898	1.123966942	342.4710744
0.008762129	1.194271482	277.8228518
0.007969056	1.059760956	283.3290173
0.003790379	1.151087596	492.3718401
0.007971571	1.334131737	371.7649701
0.00511212	1.636015326	498.9907407
0.004627345	1.091968118	427.31729
0.013928393	1.151376147	217.6605505
0.0011484	1.152680653	1378.616744
0.013806883	1.199630314	194.0702403
0.004309104	1.290381125	357.9432849
0.007480064	1.124752475	330.5638614
0.007212705	1.143928036	350.0925787
0.010141734	1.070588235	266.1335294
0.006443928	1.106471816	344.3593424
0.0042153	1.143217666	417.5667368
0.009376142	1.01863354	492.0124224
0.02473646	1.174603175	209.6746032
0.014979277	1.056872038	288.0936019
0.003097303	1.044701987	868.8046358
0.003299446	1.217977528	531.2035581
0.007518431	1.167844523	245.288722
0.005860851	1.068833652	460.8542065
0.009587761	1.198113208	322.9638365
0.01006744	1.115646259	263.2108844
0.010791247	1.097653293	226.8705526
0.012090635	1.123188406	278.1497585
0.00919555	1.214876033	341.2865014
0.002888125	1.124584718	523.019186
0.012645383	1.099303136	278.385453
0.006668095	1.198019802	389.1485149
0.003296767	1.302803738	496.5767913
0.002962164	1.331081081	659.5166104
0.007955369	1.090225564	461.4605263
0.002835805	1.267171129	614.9727881
0.015263625	1.139534884	236.4491279
0.006795606	1.282833021	338.9695122
0.004567349	1.107043559	388.3919138
0.009157244	1.099264706	269.8051471
0.029036571	1.095890411	192.0273973
0.003609107	1.159292035	560.1671091
0.007943088	1.084507042	310.7433979
0.007817472	1.069037657	353.8964435
0.006602468	1.392611684	313.8699885
0.00915057	1.086206897	256.8369253
0.00319573	1.139614075	617.5831442
0.017996493	1.264150943	257.5141509
0.009233816	1.153846154	283.6769231
0.006350948	1.151260504	353.8966387
0.004486413	1.161290323	590.5199693
0.009317524	1.098092643	304.739782
0.003702596	1.079584775	549.3422722
0.012627904	1.095149254	188.0876866
0.006069596	1.159227986	326.3525332
0.021565596	1.221590909	230.2940341
0.009693669	1.175141243	283.5188324
0.00765022	1.02866242	288.0504246
0.011677236	1.107946027	306.3568216
0.004771565	1.073043478	491.2291304
0.01202988	1.402061856	250.402921
0.008494433	1.133428981	300.3547346
0.006979902	1.20261067	294.8481839
0.010846838	1.114904246	230.4875104
0.005449148	1.263422819	313.567953
0.004890342	1.360108303	448.8348375
0.009340838	1.168168168	260.1881882
0.013240909	1.25862069	361.5876437
0.020985209	1.136363636	157.6090909
0.002727286	1.225593668	611.5216854

log.sigma.2.0.mm.3D_gldzm_LowIntensityLargeDistanceEmphasis	log.sigma.2.0.mm.3D_gldzm_HighIntensityEmphasis	log.sigma.2.0.mm.3D_gldzm_DistanceZoneVariability	log.sigma.2.0.mm.3D_gldzm_ZonePercentage
0.005156568	609.6784141	603.9368576	0.471606648
0.024347812	258.0242424	149.7757576	0.475504323
0.011636654	326.8671679	350.3884712	0.447309417
0.019124716	269.5857143	132.2285714	0.434782609
0.017158253	325.1406518	505.051458	0.41318214
0.013920422	437.1219512	109.796748	0.508264463
0.006969917	323.9252194	2366.701641	0.248695322
0.006578728	448.2758294	1799.072038	0.347725775
0.010132266	301.751926	1231.781202	0.304551854
0.009111661	332.8788732	322.628169	0.470198675
0.010944168	219.7505376	430.3935484	0.298843188
0.006374822	447.2878505	954.9551402	0.31405929
0.027440626	275.9815951	271.5214724	0.401972873
0.015864391	352.52443	266.1530945	0.476708075
0.007512546	408.5789474	581.5789474	0.41015873
0.006306297	316.0492642	1523.511836	0.271259979
0.010935181	291.2828947	286.5328947	0.344280861
0.028705537	239.3666667	154.1777778	0.4400978
0.007608013	339.05625	298.75625	0.29250457
0.003323068	706.313128	2878.422366	0.335946858
0.016736451	172.9664992	562.0854271	0.287295476
0.046295129	209.4979592	218.6	0.493951613
0.033500257	184.1484716	217.3144105	0.410394265
0.018546224	203.5801615	799.8258362	0.241034195
0.017454733	343.6098765	387.4	0.32980456
0.009502846	316.7871116	822.3256617	0.339984351
0.007275593	395.2907268	700.7769424	0.398203593
0.008356062	317.94311	716.8457649	0.351087439
0.138815479	177.7532468	61.1038961	0.52739726
0.004426281	567.2065404	575.0309811	0.360869565
0.009707578	248.5406051	1195.530255	0.313295086
0.010279195	348.3471074	334.2396694	0.460659898
0.010911846	288.3462017	705.7347447	0.343897216
0.008400892	287.0451527	723.5976096	0.311801242
0.004392162	506.5714286	1541.198707	0.331385155
0.021343929	387.3245509	669.7161677	0.430634348
0.009429468	542.4418902	521.385696	0.517857143
0.005151183	435.685469	1534.065604	0.280192407
0.02111482	218.8990826	197.1100917	0.389285714
0.00126713	1433.929681	2325.33411	0.27246745
0.025129006	199.792976	473.7911275	0.403731343
0.005392702	388.8058076	1363.973987	0.291431594
0.015357609	333.580198	929.4930693	0.417700579
0.009003556	358.4662669	606.0704648	0.46971831
0.011392319	268.0235294	405.4705882	0.273136247
0.010813588	349.3361169	892.4133612	0.399000416
0.005120984	428.5949527	1443.817666	0.296206317
0.009433653	493.5217391	159.0124224	0.32790224
0.049376978	212.6190476	168.2804233	0.483375959
0.015615686	289.6966825	203.1516588	0.466814159
0.003219347	873.2698675	586.2682119	0.469308469
0.003952118	561.4906367	1155.09588	0.47935368
0.009457074	255.3297998	1518.630153	0.212888666
0.006055786	468.1032505	499.5506692	0.318514007
0.014435191	328.4685535	278.7735849	0.494556765
0.013156338	265.9795918	408.3106576	0.375
0.013026556	231.3739591	1237.799394	0.329016189
0.017411358	284.1243961	762.7922705	0.400580552
0.0134368	347.3526171	314.7245179	0.475753604
0.003244643	540.2156146	2770.38206	0.276324245
0.014852225	282.4216028	537.2578397	0.431903687
0.008513328	411.5321782	1062.561056	0.49168357
0.005483113	533.147352	2627.405607	0.389846976
0.007328004	704.8434685	713.6306306	0.423664122
0.008612455	466.0451128	250.481203	0.392909897
0.003578124	655.9150175	1439.251455	0.502192341
0.018348488	239.4709302	156.744186	0.42364532
0.009481908	363.3606942	1767.899625	0.41927237
0.004915054	399.8577386	2009.494903	0.287426745
0.009818111	275.7647059	763.7867647	0.384180791
0.031166291	194.3835616	205.4474886	0.445121951
0.00418979	580.0896755	1524.557522	0.340293114
0.009785692	312.9260563	536.9014085	0.425468165
0.008398283	357.2949791	456.5062762	0.503157895
0.013826128	335.1116838	910.4347079	0.285014691
0.010240926	260.3304598	328.5747126	0.379084967
0.003502273	641.8513053	802.816118	0.398822997
0.028230298	262.1509434	133.4654088	0.492260062
0.016024744	293.6512821	528.0769231	0.44486692
0.007387875	363.6857143	538.0252101	0.330555556
0.005095323	608.7388633	584.7634409	0.463015647
0.009792946	311.2752044	343.7847411	0.444309927
0.004048514	556.8777393	822.2202999	0.379929886
0.015947701	189.6100746	503.0783582	0.283148442
0.007230992	337.0036188	745.6706876	0.3053407
0.027021935	237.3977273	151.9204545	0.331450094
0.019163505	290.7645951	472.6195857	0.405963303
0.00841657	288.7335456	924.1719745	0.2455683
0.01265795	313.1754123	620.7271364	0.392122281
0.00509999	496.9721739	547.6817391	0.420629115
0.01911046	275.6219931	223.4536082	0.361940299
0.010093148	304.7675753	637.7575323	0.275276461
0.008662117	308.5822928	1540.07378	0.391468563
0.011889452	238.4796003	1112.52373	0.348520023
0.006923282	333.2911074	1003.629195	0.360447536
0.010099748	482.400722	873.9296029	0.464765101
0.011659006	269.1971972	893.2782783	0.407422512
0.015857959	376.4885057	146.5862069	0.555910543
0.025282558	159.9272727	100.4545455	0.410447761
0.003273729	646.7971636	2610.290237	0.343608341

log.sigma.2.0.mm.3D_gldzm_IntensityVariabilityNormalized	log.sigma.2.0.mm.3D_gldzm_LowIntensitySmallDistanceEmphasis	log.sigma.2.0.mm.3D_gldzm_IntensityVariability
0.03639159	0.004322274	24.78267254
0.051753903	0.015954394	8.539393939
0.043146714	0.010039475	17.21553885
0.048265306	0.017626108	6.757142857
0.050372327	0.007115621	29.3670669
0.046731443	0.012592682	5.74796748
0.050297679	0.005589749	131.8302175
0.041361155	0.00479837	87.27203791
0.049760091	0.006236828	64.58859784
0.051140647	0.007428169	18.15492958
0.065565961	0.009592401	30.48817204
0.046370862	0.004465207	49.61682243
0.047687154	0.014938148	15.54601227
0.046122505	0.009011775	14.15960912
0.041134296	0.005876017	26.57275542
0.050670721	0.005945303	79.19833653
0.060876904	0.009728851	18.50657895
0.057962963	0.013619732	10.43333333
0.059902344	0.007012549	19.16875
0.047339745	0.003093154	146.0431118
0.058368335	0.015075776	34.84589615
0.053527697	0.015014304	13.11428571
0.05173433	0.027009795	11.84716157
0.05985055	0.010836911	51.89042676
0.043097089	0.007369009	17.45432099
0.04387806	0.007901537	38.13003452
0.043671208	0.005718055	34.84962406
0.051615759	0.006354398	40.82806574
0.05987519	0.015726742	4.61038961
0.055483305	0.004350028	32.23580034
0.053526512	0.008382291	67.22929936
0.048941709	0.008200074	17.76584022
0.048298023	0.0082247	38.78331258
0.049023913	0.007861098	36.91500664
0.045523244	0.003644469	77.43503821
0.044873606	0.004628482	37.46946108
0.042311964	0.004032783	33.1302682
0.044982616	0.004496386	73.36664623
0.068891507	0.012131786	15.01834862
0.051159796	0.001118718	131.6853147
0.054499609	0.010976352	29.48428835
0.053580924	0.004038205	88.569268
0.048528576	0.005510677	49.01386139
0.043788451	0.006764992	29.20689655
0.053032526	0.009829088	22.53882353
0.048868772	0.005351513	46.81628392
0.050612107	0.003993027	80.22018927
0.069403187	0.009361764	11.17391304
0.050250553	0.018576331	9.497354497
0.047505671	0.014820174	10.02369668
0.04233367	0.003066792	25.56953642
0.043192638	0.003136278	57.66217228
0.056092458	0.00703377	95.24499411
0.05186653	0.005812117	27.12619503
0.046794035	0.008375903	14.88050314
0.054313789	0.009295215	23.95238095
0.050423457	0.01023242	66.60938683
0.04636631	0.010760455	38.39130435
0.045830203	0.008135237	16.63636364
0.050733215	0.002798996	152.7069767
0.045484345	0.012093672	26.10801394
0.041563191	0.006206786	50.37458746
0.041645947	0.00275018	133.6834891
0.049194465	0.001870704	43.68468468
0.050794279	0.007791097	13.5112782
0.040203637	0.002650225	69.06984866
0.05509735	0.01449241	9.476744186
0.042914368	0.006124031	91.4934334
0.05115178	0.004480422	110.3855422
0.053206699	0.008992027	43.41666667
0.053897959	0.028504142	11.80365297
0.045328878	0.003463936	76.83244838
0.046853303	0.007482436	26.61267606
0.044458255	0.00767227	21.25104603
0.047546085	0.004859741	55.34364261
0.059320914	0.008877981	20.64367816
0.049939381	0.003119094	43.99659478
0.051066018	0.015438042	8.119496855
0.049894076	0.007536084	29.18803419
0.048767742	0.006091717	29.01680672
0.03703861	0.004334185	24.11213518
0.047836126	0.009198668	17.55585831
0.044109998	0.003616117	38.24336794
0.062430385	0.011797954	33.46268657
0.049259285	0.005779248	40.83594692
0.058238636	0.020201512	10.25
0.048506708	0.00732621	25.75706215
0.052702161	0.007458632	49.64543524
0.04556867	0.011432058	30.39430285
0.050368242	0.004689459	28.96173913
0.053790106	0.010259735	15.65292096
0.05274295	0.008094755	36.76183644
0.050347157	0.006559349	88.71169126
0.053856729	0.010586184	64.68193172
0.056181309	0.005087088	66.96812081
0.03852357	0.003587991	42.68411552
0.048622196	0.008761296	48.57357357
0.041551064	0.012586647	7.229885057
0.075206612	0.019910872	8.272727273
0.04584672	0.002590676	139.0072559

log.sigma.2.0.mm.3D_gldzm_HighIntensityLargeDistanceEmphasis	log.sigma.2.0.mm.3D_gldzm_SmallDistanceEmphasis	log.sigma.2.0.mm.3D_glcm_SumVariance	log.sigma.2.0.mm.3D_glcm_Homogeneity1
702.9339207	0.954845815	1712.65736	0.275207306
270.3333333	0.963636364	624.3578671	0.278830349
398.1002506	0.95112782	842.5486187	0.282756524
283.7714286	0.978571429	655.7084519	0.282656771
360.2144082	0.945969125	849.1598325	0.286990127
467.9756098	0.957317073	1290.93996	0.258264116
371.0427318	0.961655857	858.6074299	0.428778908
561.9672986	0.93992891	1123.126921	0.329199659
312.4160247	0.980354391	685.0304965	0.370104793
370.256338	0.964084507	885.4209615	0.280395796
243.8150538	0.970967742	561.2706678	0.386150153
503.9065421	0.957242991	1184.206173	0.376446936
323.2730061	0.930981595	822.7330199	0.300819746
374.6091205	0.946254072	876.2191052	0.257215542
451.8281734	0.960526316	937.0199409	0.305709201
328.2104926	0.990403071	855.7808233	0.396317238
302.9671053	0.977796053	736.6815746	0.34857406
255.4166667	0.941666667	543.034463	0.294492059
366.99375	0.97421875	938.9169849	0.36661939
785.1358185	0.973987034	2256.417931	0.335644486
184.1976549	0.977386935	363.8791502	0.35428294
213.355102	0.957142857	412.2147734	0.285962253
191.7598253	0.980349345	341.5202122	0.326769312
221.2029988	0.969723183	427.1228265	0.445816669
364.7283951	0.983333333	888.4207101	0.392371553
332.3774453	0.979286536	648.0508683	0.347346081
442.8546366	0.95112782	964.5625728	0.298196212
346.6498104	0.963021492	765.7814776	0.338716673
185.7402597	0.912337662	374.425982	0.291432068
571.6574871	0.996127367	1571.942466	0.331772853
262.4251592	0.981488854	579.3955247	0.348597933
371.8512397	0.969008264	913.5153903	0.270529226
330.4396015	0.95143213	701.1685679	0.352114556
301.9096946	0.985059761	718.8799144	0.3729398
563.7460317	0.962881312	1313.247095	0.346165886
449.5628743	0.916467066	1020.889913	0.28465903
716.2464879	0.840996169	1616.756684	0.239573174
469.1581852	0.977007971	1210.344159	0.426920597
223.853211	0.962155963	452.4784034	0.331830781
1655.18143	0.961829837	4641.219183	0.405439595
222.6839187	0.950092421	442.9490485	0.313805476
512.2558984	0.927404719	976.7253348	0.394839738
345.6455446	0.968811881	852.0163449	0.290048168
391.9610195	0.964017991	861.9473798	0.270514637
275.5835294	0.982352941	602.021822	0.420640698
369.243215	0.973382046	833.0203796	0.310485547
473.1817035	0.9648966	1036.514794	0.361334373
499.5590062	0.995341615	1395.291926	0.348526422
224.3968254	0.956349206	433.9882707	0.286339628
296.1090047	0.985781991	721.3601793	0.279829899
891.1307947	0.988824503	2477.002254	0.286556935
682.6389513	0.945505618	1577.369375	0.267642586
295.4941107	0.958038869	631.5789478	0.47543144
497.0994264	0.982791587	1458.227702	0.349744037
350.4874214	0.950471698	840.2183789	0.258493988
277.0544218	0.971088435	584.4602334	0.320077448
249.3875852	0.975586677	495.4278761	0.371413093
308.0229469	0.969202899	680.4354907	0.291442013
371.6170799	0.946280992	819.9380429	0.276988852
609.0013289	0.968853821	1498.848128	0.413704889
298.5662021	0.975174216	717.4020495	0.295418117
501.0668317	0.95049505	1141.83716	0.264245278
679.429595	0.924299065	1536.881139	0.314003649
886.1509009	0.91722973	2193.107424	0.286681887
484.3834586	0.977443609	1306.492994	0.32611219
819.6839348	0.933207218	1960.399429	0.247379502
251.5581395	0.965116279	564.4240098	0.284881005
460.9254221	0.929291745	908.5876314	0.288066219
445.721038	0.97323911	1064.930687	0.362166816
299.6029412	0.975183824	791.1123092	0.311854312
203.8082192	0.976027397	386.4025861	0.302469185
659.779941	0.960176991	1612.920803	0.341626074
321.6566901	0.978873239	739.2333036	0.285847223
370.8891213	0.982740586	878.5234162	0.272488189
423.709622	0.906619893	819.5026768	0.384910567
274.3045977	0.978448276	654.1800308	0.328951215
738.9239501	0.965096481	1999.563462	0.303507518
280.6981132	0.933962264	621.097267	0.272655232
333.5487179	0.961538462	808.7842185	0.286575585
402.8420168	0.962184874	868.48526	0.351417894
681.6144393	0.959677419	1700.26044	0.279115162
337.4168937	0.975476839	869.7275377	0.292745631
587.0196078	0.980103806	1485.456632	0.332383876
195.6996269	0.976212687	371.1102008	0.371189
379.6079614	0.960193004	844.0041954	0.370608643
265.8125	0.944602273	812.6770711	0.344528797
319.747646	0.956214689	669.3179655	0.315043218
291.4660297	0.992834395	621.8312696	0.43805741
340.4497751	0.973013493	805.3374843	0.32877799
519.9443478	0.98173913	1477.179126	0.28913722
376.4982818	0.899484536	777.9352543	0.31934274
322.4189383	0.966642755	684.6202394	0.400471021
363.5187287	0.949347333	824.9843138	0.315714735
270.44796	0.971273938	570.8971013	0.340217107
412.7206376	0.935076435	834.107224	0.321635741
616.6642599	0.909972924	1310.201467	0.262201666
305.2332332	0.957957958	663.4458782	0.299799159
436.091954	0.935344828	1055.986511	0.235369043
169.2	0.965909091	332.5090384	0.332496074
787.8990765	0.943601583	2093.959803	0.324364764

log.sigma.2.0.mm.3D_glcm_Homogeneity2	log.sigma.2.0.mm.3D_glcm_ClusterShade	log.sigma.2.0.mm.3D_glcm_MaximumProbability	log.sigma.2.0.mm.3D_glcm_Idmn	log.sigma.2.0.mm.3D_glcm_SumVariance2
0.191212501	489.5786294	0.012249419	0.968763812	101.2021805
0.190382075	-44.99821846	0.017009969	0.953498358	68.38237928
0.197675032	-116.218465	0.013785792	0.950959977	84.67306807
0.196188158	-81.18028162	0.015291448	0.945847601	83.77431681
0.197337288	49.31218259	0.011801515	0.960909693	70.06504967
0.167479389	-126.0441279	0.022123725	0.957934288	79.74777077
0.363744079	93.88602964	0.081696179	0.982033689	37.4901746
0.250290607	331.257044	0.03157461	0.971795898	67.75332701
0.294146733	161.354408	0.041137658	0.973421926	47.05264647
0.189171091	1.886570754	0.013264707	0.966361586	52.91080807
0.307981365	41.23650135	0.040506126	0.970749373	31.43611393
0.303125561	170.7458161	0.04213974	0.976430339	45.94599118
0.21530297	-379.1028227	0.016695519	0.954945938	80.48531755
0.166862312	21.02712533	0.011710051	0.948021627	79.4408746
0.22352865	394.8356894	0.026476879	0.968218119	69.12583027
0.328853631	63.08221995	0.071914197	0.969793256	55.65947116
0.262873944	42.02248079	0.023102199	0.979325821	37.01506747
0.203440398	9.777764176	0.014450005	0.957445611	63.02750983
0.283960825	23.66823289	0.024312345	0.980921901	28.628976
0.252889629	47.14102036	0.020007477	0.986832015	50.99566181
0.268908798	51.97241535	0.016116533	0.967641275	44.77905798
0.195097335	219.9372091	0.015576704	0.96079336	64.78748858
0.241919647	108.5370293	0.021454307	0.960446278	57.8966703
0.379632481	96.72397145	0.074165136	0.978254562	29.04598828
0.324449304	24.68315061	0.100948118	0.960889096	63.99878156
0.267372152	475.0971242	0.025558661	0.973874943	64.98393874
0.211050534	339.2363375	0.015142539	0.97174852	64.91246363
0.254342061	128.1085261	0.020207293	0.979588624	42.88013238
0.197675916	173.6165749	0.027615235	0.961613262	49.16230736
0.24469352	65.45272659	0.017314278	0.97877683	46.29961164
0.266046033	145.0213961	0.014412725	0.970311373	52.17546358
0.182789213	-50.5169813	0.01316442	0.945089865	77.76149254
0.272373703	154.7650563	0.03646934	0.977039618	51.07334467
0.299538794	125.9725046	0.039693151	0.965804275	58.29888976
0.265424801	247.3663781	0.028063138	0.982075572	55.61245714
0.201910026	-148.022313	0.013408442	0.951323936	83.08102334
0.153481032	-390.6052541	0.009785731	0.961276405	116.977778
0.365331252	111.4369118	0.105701758	0.977534434	58.62822561
0.242279551	6.948418442	0.019537187	0.964281936	36.70138378
0.334760256	177.344417	0.0685552	0.991603222	38.71341807
0.223229855	113.8774871	0.012408027	0.959969437	52.74566226
0.322937587	177.6285018	0.06784746	0.982033731	34.02741377
0.20312819	-70.97247862	0.010662527	0.956720064	78.22084185
0.18212367	199.6478338	0.01062597	0.96286783	69.04364351
0.350369224	66.12765172	0.077365114	0.977722256	31.74197724
0.22518919	174.6379646	0.014739369	0.964722691	64.78120904
0.281484126	199.0573098	0.033891057	0.982065199	46.44898621
0.258918092	-6.414381923	0.022153966	0.981254568	29.00001362
0.195223818	153.0733829	0.01574587	0.947733179	66.27656633
0.191451733	-254.7221552	0.017235157	0.951627273	91.42798334
0.199821779	280.5018227	0.015660636	0.977847387	67.82444402
0.178177172	282.1612481	0.009151674	0.975557837	83.14083289
0.416944776	63.79678361	0.097929374	0.982926308	26.37731783
0.267561392	-33.57309633	0.022864302	0.97912011	42.32947812
0.169655834	-91.70255925	0.012648588	0.946309904	94.13351157
0.230907044	174.7659385	0.014949901	0.970678346	58.43604218
0.293953474	177.8782615	0.04284833	0.968319252	49.56293935
0.204206254	106.2137698	0.009792879	0.967539143	67.60596613
0.189094279	153.1342593	0.013841083	0.958363358	66.68734299
0.346164808	193.9211831	0.058924605	0.986208007	41.76692728
0.211603624	104.5762929	0.014591905	0.966095906	65.29773745
0.178958515	145.6554785	0.010293115	0.962819285	87.4591793
0.233313927	80.88965323	0.021242895	0.971017306	78.19958986
0.195626463	-89.22961182	0.009484995	0.977415202	67.19428194
0.239685846	-27.05388606	0.020132398	0.974627618	44.63129682
0.159685236	-58.07967597	0.0075272	0.970112806	89.90171872
0.195562802	-114.1325741	0.017243821	0.941399642	65.83805455
0.203036831	252.069051	0.012259304	0.969046934	71.17950715
0.280990699	163.8151371	0.02091141	0.984717677	39.53958191
0.222852493	-56.29468792	0.013364831	0.966527909	55.95882298
0.214518296	77.25178762	0.016942447	0.943639532	63.76882511
0.265654014	94.17328169	0.02573156	0.97752311	69.06180111
0.197882908	175.2863854	0.011414899	0.963346807	68.53881641
0.185387406	216.5316265	0.013405795	0.962216986	67.01047329
0.312240472	210.7795069	0.068556859	0.980851892	44.59552444
0.240894899	58.63024358	0.016196087	0.975848797	42.94243464
0.21306861	-36.4978645	0.010969525	0.978123805	59.17763638
0.184708156	-175.0892823	0.016456186	0.941213416	97.00502997
0.195791126	-62.38655632	0.012786589	0.965842924	61.7538966
0.269372184	207.1839133	0.033322027	0.982078779	41.12046611
0.19494757	475.4692049	0.0126515	0.970135103	96.08997986
0.202802586	-42.241968	0.013732841	0.968647039	58.06295028
0.256053236	196.8984132	0.045322649	0.967131277	68.64260375
0.287880308	78.05939106	0.030098278	0.969116114	31.33277708
0.294693588	110.7942613	0.045421025	0.975198818	46.33965451
0.262165401	-368.7818976	0.035793765	0.953399994	52.19541408
0.231075028	139.0070299	0.014747653	0.967896243	61.57085208
0.372331498	135.7571901	0.090456406	0.979941973	38.60531359
0.246606782	-11.23857222	0.028948078	0.969046836	58.75189251
0.201335704	-67.82873687	0.011375272	0.964962673	61.3240968
0.232186553	-102.0164778	0.020110588	0.961725608	54.98144724
0.327697005	129.1715288	0.040387043	0.976792562	35.57566224
0.228516733	28.82801554	0.014979111	0.974848614	46.57874536
0.256450746	79.83856311	0.014749259	0.970874228	51.53997112
0.232597207	69.72754591	0.015506059	0.981596645	38.74807244
0.176847282	149.8843799	0.008429203	0.963578399	101.7240719
0.212461293	56.97727391	0.011343252	0.968966377	63.32019589
0.146546757	-98.4692887	0.015060603	0.950893296	85.95487542
0.240746504	-45.79934143	0.020099981	0.947574972	40.23734705
0.241610167	-126.021442	0.014871098	0.979691699	63.47288967

log.sigma.2.0.mm.3D_glcm_Contrast	log.sigma.2.0.mm.3D_glcm_DifferenceEntropy	log.sigma.2.0.mm.3D_glcm_InverseVariance	log.sigma.2.0.mm.3D_glcm_Entropy	log.sigma.2.0.mm.3D_glcm_Dissimilarity
71.70133424	4.09059805	0.196187005	8.767613941	6.176193283
48.06685229	3.797668687	0.189629306	7.79351081	5.387846209
59.72077291	3.939632827	0.201774528	8.453785978	5.741098397
54.5802276	3.87359419	0.194468158	7.826423142	5.583470701
42.39807543	3.774915244	0.201914483	8.349736173	5.03759773
55.88517095	3.848697995	0.17021347	7.555174581	5.811837734
27.01213849	3.345362031	0.306732263	7.42983858	3.428764045
50.89191784	3.831203489	0.241525947	8.50455621	5.016365182
32.40066366	3.5305813	0.274726399	7.89886635	4.00008658
43.22227584	3.76956215	0.195539774	8.110700376	5.103996909
20.53576946	3.262898911	0.296803985	7.412199144	3.296869551
36.28330358	3.573499328	0.281799337	7.883029611	4.094043975
47.60849946	3.802574112	0.216789676	8.163111736	5.122632268
58.61924155	3.966431907	0.164644848	8.372438625	5.964841972
58.14628353	3.922983826	0.221958406	8.327630263	5.437239138
37.57963825	3.494519684	0.288105139	7.744276135	4.108895249
24.3682975	3.375812379	0.268880868	7.558213277	3.685641597
38.13293294	3.660025084	0.206402778	7.781989758	4.767946252
22.47039129	3.308143062	0.286459292	7.370618282	3.469059967
37.94944766	3.659239515	0.251331781	8.255388375	4.392214892
22.70835596	3.34638317	0.270037569	7.773086639	3.581045308
39.7889679	3.698686152	0.205555095	7.887000628	4.912631713
35.79635279	3.631626928	0.233491595	7.855170037	4.44144646
17.74871735	3.120010095	0.327889228	7.099175149	2.841983082
45.79995921	3.651300243	0.259993074	7.656300846	4.460976499
38.08089863	3.635536477	0.258798361	8.109169591	4.34388001
48.11964924	3.820710301	0.214985546	8.367616375	5.102797845
32.16378637	3.56075808	0.255780614	7.933287548	4.122809013
34.29673894	3.498752014	0.211240409	6.793453479	4.555406376
29.95929416	3.520739329	0.247086602	7.945482835	4.074097657
29.97481326	3.503976007	0.262579554	8.064026311	3.993034575
54.4325864	3.905692389	0.189080158	8.288289565	5.701513263
34.53043286	3.604948103	0.253124138	8.058466105	4.216835807
38.41673797	3.573611571	0.279073917	7.879514683	4.220032227
35.91721459	3.623768295	0.25931629	8.183884851	4.261071223
62.67770134	3.998739448	0.200959595	8.761854028	5.888096628
80.60564745	4.137462745	0.162404462	8.892078774	6.934435524
40.27727396	3.513977725	0.283754783	7.606012506	4.058105538
25.31084554	3.406109778	0.253241169	7.517438993	3.832898963
27.49154423	3.395902924	0.299470673	7.564987001	3.542091764
30.94818759	3.559942264	0.227821374	8.043755778	4.265604049
28.58463158	3.423646756	0.303121077	7.416688753	3.620572768
47.8913106	3.761939123	0.20185666	8.485372741	5.261660487
54.30539468	3.936747617	0.190403865	8.539064235	5.651259494
22.3257707	3.262876839	0.305858533	7.253243597	3.215930748
40.87431276	3.736246773	0.22623301	8.356848934	4.782737292
32.93139557	3.557059652	0.264815266	7.978588108	4.015374927
20.99748619	3.221011057	0.26654265	7.117654372	3.453634525
41.95610665	3.723471158	0.194360991	7.863342902	5.003221942
50.72848016	3.847395887	0.187483392	7.964181913	5.466850921
52.90425466	3.89747644	0.200225668	8.281707777	5.454512705
56.41715828	3.973831782	0.183973832	8.823894797	5.717733763
18.13181909	3.055957084	0.345198167	6.857045043	2.720763773
31.42497406	3.490291551	0.271654906	7.757809016	3.99612435
60.68061606	3.980365984	0.175311711	8.364802133	6.032389282
31.32799634	3.556124542	0.233586772	8.017467847	4.232090337
30.37397043	3.506625663	0.266558356	7.916436957	3.910367535
46.97744735	3.838494556	0.206882146	8.438596703	5.184345785
55.6270356	3.932634587	0.191723939	8.294884677	5.628806519
30.38901668	3.412540992	0.31037874	7.577163061	3.583376002
52.5503941	3.887233669	0.216944023	8.400541234	5.369717881
67.25299311	4.090770798	0.181169275	8.969191557	6.229391231
54.64629057	3.901956174	0.224949663	8.821940951	5.300872599
43.41898135	3.785546209	0.203181209	8.493407069	5.002165536
34.90623163	3.568634992	0.242867075	7.735153197	4.313939646
72.89636635	4.150749167	0.165278821	9.095427725	6.547775065
44.00137388	3.760176789	0.200539792	7.841943885	5.123193085
55.47542256	3.949971744	0.206004423	8.756829596	5.535249895
29.13751824	3.477433638	0.275632922	7.853915472	3.802936145
33.73255888	3.621620573	0.226964341	8.190826077	4.426179393
38.92578336	3.66357032	0.219380926	7.87448521	4.75768833
47.73502013	3.778590905	0.252383034	8.475490967	4.851746176
47.82334352	3.837933818	0.200297555	8.336691597	5.273231321
58.74285729	3.96800671	0.188510386	8.293817689	5.811967688
35.2354624	3.576441772	0.263918171	7.747985873	4.069726014
30.90110979	3.525089064	0.23421217	7.744858409	4.146002273
36.17352073	3.661228989	0.220768862	8.316925894	4.556045667
51.09188918	3.842330344	0.190394867	7.871447218	5.553628763
41.55087445	3.758142071	0.201961282	8.340883468	4.974093374
32.97094254	3.555585054	0.260619435	7.716733371	4.06055453
68.18196773	4.056249615	0.199354871	8.701231756	6.022185193
40.51801542	3.715044682	0.216683418	8.103494462	4.838753093
53.83131957	3.83757127	0.230752263	8.236545429	5.208057416
20.06252107	3.261253907	0.288351664	7.454316657	3.317125454
31.7741709	3.525963092	0.273181883	7.885152941	3.982065957
32.47682417	3.51214026	0.260155528	7.27394415	4.122467139
41.65245122	3.717569705	0.231171203	8.24109664	4.781688439
22.77920106	3.251780195	0.312294205	7.318651316	3.162657797
40.4383289	3.716040092	0.237158523	8.228652614	4.624752416
45.7911654	3.812163767	0.209046143	8.279972132	5.134793855
34.64950221	3.605239654	0.238495744	7.923548	4.386302056
24.91622012	3.338832946	0.303929464	7.422360347	3.433175109
35.74591874	3.66865771	0.229202722	8.262783797	4.490090124
31.37515707	3.557068267	0.253615911	8.155297177	4.113765979
29.97927285	3.545838913	0.234594846	7.9411947	4.163670469
73.03628557	4.124152649	0.182441287	9.029178909	6.397032111
42.39050596	3.762622022	0.2182346	8.446491062	4.90548106
73.59315566	4.074939421	0.152261756	7.973417352	6.735681319
22.32633101	3.278389821	0.246283316	7.202917776	3.686103921
42.76065992	3.743227745	0.240065916	8.517518727	4.714193678

log.sigma.2.0.mm.3D_glcm_DifferenceVariance	log.sigma.2.0.mm.3D_glcm_Idn	log.sigma.2.0.mm.3D_glcm_Idm	log.sigma.2.0.mm.3D_glcm_Correlation	log.sigma.2.0.mm.3D_glcm_Autocorrelation
32.93181687	0.889361067	0.191212501	0.176047967	523.1778616
18.18861586	0.859747194	0.190382075	0.175182587	207.9328559
25.53840271	0.862189067	0.197675032	0.179086228	273.1601399
22.8734456	0.853423805	0.196188158	0.212778984	216.7230174
16.64255705	0.870654153	0.197337288	0.24958449	278.0377472
21.33689647	0.864860353	0.167479389	0.18301042	400.9321903
14.92361017	0.922725795	0.363744079	0.172044727	277.2162558
25.12729804	0.89858125	0.250290607	0.149429366	355.5339781
15.91126375	0.901414744	0.294146733	0.187506481	228.5549011
16.67525525	0.878514116	0.189171091	0.108793494	286.1164586
9.40006264	0.893068164	0.307981365	0.206808968	191.5550839
19.203111	0.909475419	0.303125561	0.122349412	371.766114
20.58240745	0.867450933	0.21530297	0.260371773	268.7745225
22.23805765	0.851640954	0.166862312	0.155453578	282.6267756
27.88708303	0.891104455	0.22352865	0.092724139	299.2106438
19.35249977	0.901049646	0.328853631	0.205409923	277.0926777
10.53817009	0.906111579	0.262873944	0.210229921	243.9094764
14.91961663	0.865775242	0.203440398	0.244512618	185.4979061
10.18019902	0.911310436	0.283960825	0.128102698	300.537594
18.26034989	0.926999449	0.252889629	0.15485587	673.8600106
9.589610936	0.884317727	0.268908798	0.325065439	133.1300951
15.24815818	0.869520899	0.195097335	0.238014817	144.5309859
15.68496036	0.874963998	0.241919647	0.234770665	123.1845903
9.533228248	0.91335321	0.379632481	0.240893295	150.6255376
25.07990079	0.889482675	0.324449304	0.167092845	285.6611512
18.67312042	0.901626724	0.267372152	0.25893421	217.8188165
21.26731691	0.893278332	0.211050534	0.153740676	309.547732
14.86115949	0.909052045	0.254342061	0.150169519	252.0950108
13.15540101	0.870032296	0.197675916	0.180798638	130.5272363
13.05080944	0.905102263	0.24469352	0.214578346	484.1338638
13.50075686	0.892169763	0.266046033	0.268479904	198.6190269
21.08295448	0.849809243	0.182789213	0.180446959	294.5173737
16.36445403	0.905677141	0.272373703	0.197435267	233.8125697
20.13773999	0.89288474	0.299538794	0.205161014	238.1753234
17.46861361	0.916343885	0.265424801	0.217699761	411.4721086
26.69826242	0.862818688	0.201910026	0.150279861	325.4036355
28.98929641	0.871558666	0.153481032	0.198453824	494.1572065
23.15611169	0.917628071	0.365331252	0.188703007	378.1760618
10.36701688	0.877095685	0.242279551	0.183185361	158.582803
14.70707374	0.944133643	0.334760256	0.175710595	1313.07586
12.44498173	0.869951336	0.223229855	0.259219742	156.5609627
15.27107493	0.920162583	0.322937587	0.095035263	309.6373733
17.32398829	0.866938089	0.20312819	0.253267063	278.1471205
21.86123403	0.874557488	0.18212367	0.125161514	279.1021391
11.78991271	0.911825228	0.350369224	0.173593321	202.61275
17.493604	0.880505857	0.22518919	0.230776957	273.4010397
16.54361652	0.917504737	0.281484126	0.175070992	331.1603005
8.850660369	0.90837686	0.258918092	0.165404162	429.3338988
16.2866182	0.852014373	0.195223818	0.227420059	151.2280383
20.0339769	0.858736628	0.191451733	0.28902336	238.2826119
22.57122795	0.902963381	0.199821779	0.125239007	733.7022598
23.17306365	0.896666594	0.178177172	0.197676372	487.1152437
10.62151158	0.9268504	0.416944776	0.187800392	209.9761074
14.7676936	0.909909448	0.267561392	0.154150437	449.8672836
23.56982514	0.85081407	0.169655834	0.222005301	272.4292193
13.07149882	0.888519702	0.230907044	0.301269926	200.2497482
14.61640338	0.892089885	0.293953474	0.237154422	172.2957522
19.56660322	0.883609156	0.204206254	0.183490762	226.7879814
23.40726597	0.869793311	0.189094279	0.093785141	265.8215601
17.33979706	0.932264223	0.346164808	0.161504925	459.5826473
23.10728034	0.883701488	0.211603624	0.116499926	236.2313964
27.84253257	0.875822928	0.178958515	0.137858593	360.3636381
25.81390947	0.895357576	0.233313927	0.182604709	475.6461084
17.99733625	0.900341462	0.195626463	0.220254264	659.9590332
15.73221693	0.898543024	0.239685846	0.133613809	406.9199744
29.27023498	0.886182943	0.159685236	0.113373571	592.3128676
17.11099725	0.844796846	0.195562802	0.200436414	190.7146934
24.25689405	0.887978899	0.203036831	0.131792597	293.1547181
14.47182807	0.922678357	0.280990699	0.154738468	338.3979915
13.81428675	0.880947365	0.222852493	0.250453005	261.7380905
15.51415813	0.849553782	0.214518296	0.241965814	136.8319489
23.6988257	0.908927126	0.265654014	0.18848852	496.7990835
19.4469525	0.876039139	0.197882908	0.18282027	243.7614424
24.28294862	0.874999384	0.185387406	0.072266885	281.8730767
18.23691413	0.917165128	0.312240472	0.123907169	265.9259953
13.29437541	0.898837553	0.240894899	0.170854313	219.1876879
15.0503324	0.902018688	0.21306861	0.242587936	605.9399483
19.48115165	0.844074381	0.184708156	0.309860295	207.3909972
16.37191308	0.878337495	0.195791126	0.201435749	265.7134802
16.26482991	0.916422564	0.269372184	0.114810465	280.3152962
31.30575195	0.891628047	0.19494757	0.175623141	519.842278
16.67210083	0.884424432	0.202802586	0.181705813	282.8058561
25.55817628	0.890790906	0.256053236	0.126249711	457.4140002
8.869324108	0.888129952	0.287880308	0.219199813	134.0247903
15.52057015	0.904027379	0.294693588	0.189842335	275.4930579
14.78287954	0.867996783	0.262165401	0.240013613	263.9036794
17.98871661	0.886654844	0.231075028	0.196921094	223.9785086
12.61643593	0.918307705	0.372331498	0.258127051	209.5735741
18.62575523	0.890335913	0.246606782	0.189571099	264.509679
18.9634943	0.878809417	0.201335704	0.151230134	456.9738094
14.98087778	0.87591257	0.232186553	0.229407542	256.8100698
12.85224158	0.909071204	0.327697005	0.177743311	227.5808659
15.32197601	0.89681792	0.228516733	0.136920843	269.9757309
14.13000162	0.892144298	0.256450746	0.244415276	196.0890861
12.36804811	0.90918069	0.232597207	0.132777731	271.9593743
31.11702366	0.878617641	0.176847282	0.171755749	408.7880944
17.7365593	0.88628007	0.212461293	0.20435299	222.6314635
27.45281405	0.854472121	0.146546757	0.083724133	330.4617959
8.297745036	0.850526853	0.240746504	0.289630272	121.7406935
20.04271495	0.910294693	0.241610167	0.201900257	631.749702

log.sigma.2.0.mm.3D_glcm_SumEntropy	log.sigma.2.0.mm.3D_glcm_AverageIntensity	log.sigma.2.0.mm.3D_glcm_Energy	log.sigma.2.0.mm.3D_glcm_SumSquares	log.sigma.2.0.mm.3D_glcm_ClusterProminence
5.27954373	23.14294834	0.003366456	44.39033227	39811.34423
4.904956974	14.30263297	0.005493389	29.66809288	12178.29407
5.145480638	16.66749579	0.003669132	36.81358618	17604.96426
5.027317102	14.59978121	0.005240032	35.46697656	15907.1385
5.018995621	16.71145677	0.003898123	28.9712889	11525.75909
4.944736263	20.27222027	0.006464161	33.19223964	19214.87451
4.487015651	16.82510226	0.019046133	16.43410886	6758.283369
5.000491341	19.10833934	0.005378428	29.80336881	17818.16092
4.734622934	15.18965496	0.008163202	20.73198816	7734.841507
4.83269753	17.17324101	0.004471072	24.47973941	7905.459669
4.464981239	13.74145016	0.00930059	12.99297085	2953.951405
4.698798997	19.49015222	0.009318807	20.3861695	9855.875935
5.03879034	16.36107422	0.004880726	32.75940893	16737.47789
5.083742977	17.0110234	0.003725194	35.53624491	15271.87589
4.976033737	17.61667474	0.005358459	32.642287	19512.20909
4.7335668	16.8442857	0.013003861	23.78630828	9383.600785
4.580582157	15.67839302	0.007141436	16.01934406	4464.370756
4.868877556	13.40117949	0.005486639	25.46662891	9405.046796
4.411968161	17.52561726	0.008758318	13.407928	2859.881916
4.829908495	26.24845405	0.005454256	21.65762921	11835.95719
4.729192342	11.29610657	0.005985194	16.87185348	5527.95757
4.87815673	11.78827273	0.005277126	26.5872818	11619.12111
4.852133987	10.85770545	0.005805898	23.60873254	9198.616823
4.363379239	12.16539321	0.017321639	11.81513773	3957.645771
4.819596859	17.02211321	0.017755303	28.22269206	13242.4915
4.911124276	14.73975983	0.006121968	26.75628368	17893.54629
4.9541907	17.84508213	0.004490515	28.75136589	16823.49462
4.698939335	16.05149591	0.006331605	19.47226931	7374.733613
4.486364666	11.28107046	0.010545941	21.27590689	9446.911604
4.760413629	22.0726034	0.005383915	18.33470936	6737.844108
4.828413016	13.98352039	0.005152168	21.22641527	7673.708978
5.071629021	17.26871108	0.00401524	33.98971093	13932.07102
4.813031568	15.37939176	0.00675443	22.08525736	9376.074086
4.840171096	15.52254984	0.008840915	25.35503009	10030.00114
4.862882871	20.4047199	0.005904017	22.92687442	10991.62464
5.169408178	18.30030029	0.003084859	36.36236829	19765.80917
5.319198027	22.5386891	0.002821083	47.8409201	32999.92806
4.719923163	19.61268584	0.021488216	24.48090307	11963.10841
4.56738647	12.47875341	0.00704955	15.50305733	3847.158574
4.553558463	36.44839105	0.013851928	16.85514431	6868.503601
4.831618931	12.29352153	0.004866988	20.93827366	7248.060905
4.412261191	17.80541349	0.015969376	16.14018278	6633.359492
5.078415457	16.74389423	0.003659509	32.54774878	15073.39286
5.032459878	16.99119111	0.003544883	31.6126896	14213.12156
4.421864967	14.19847995	0.015312537	13.89980715	4349.739855
4.988858179	16.61788215	0.004258208	27.49160524	11665.35103
4.744221284	18.33554016	0.007109801	19.9191521	9471.337448
4.380290283	20.89070225	0.009035122	12.09464626	2865.514459
4.91860025	12.05771626	0.005174036	27.21168292	11934.04536
5.108037862	15.24778877	0.005144564	36.49800208	21070.42586
4.952024189	27.38192298	0.004709365	30.54241117	16410.80692
5.182368799	22.27275184	0.002928038	35.61004566	21653.43738
4.237726502	14.4975651	0.026401147	11.6702563	3286.954736
4.663004835	21.38799187	0.007148557	17.66532226	7015.810433
5.185264669	16.60334903	0.003744203	39.68642386	19713.00936
4.883138695	13.98167053	0.005010944	23.07768803	10189.69514
4.768713029	12.99725287	0.007870149	20.51525282	8023.398178
5.018790502	15.19719002	0.003715545	29.56384289	12089.14459
4.992084837	16.55552208	0.004176095	31.56408236	13753.10732
4.571163414	21.53945541	0.016168807	18.68627778	9491.833596
4.994944833	15.64405672	0.003964685	30.13241209	13804.65533
5.227995861	19.30170355	0.002694591	38.10561631	24510.28419
5.154827502	21.97061606	0.00384296	32.76011999	18576.30356
5.039720284	25.83999682	0.0035809	28.03927375	14110.3875
4.700705206	20.4039464	0.006403138	19.03740597	7326.720731
5.250677799	24.73990376	0.002369005	40.9368313	23531.80958
4.892048624	13.60989735	0.005475243	27.45985711	9625.712745
5.07534076	17.41505053	0.003506882	32.08260307	17981.29718
4.627821435	18.55340666	0.007356609	17.03979099	7189.798509
4.89751638	16.13683306	0.004486915	23.07863281	8119.70813
4.894755011	11.42746982	0.005252978	25.67365212	9308.285087
5.046035338	22.43735071	0.005000781	29.68118221	14044.97404
4.994203321	15.74283835	0.003984507	30.30125458	11966.30113
4.967949452	17.15983481	0.004166107	32.47031509	13441.98795
4.633428197	16.50763272	0.013012411	20.44189668	9549.081944
4.682439006	14.86569189	0.006061988	19.3634629	5847.799847
4.94751529	24.63019359	0.004120467	23.66535424	10509.20803
5.100335624	14.02427516	0.005134474	37.38455872	19640.55566
4.958063237	16.4073989	0.004015706	26.51154696	11291.05492
4.599383291	16.93320447	0.008544609	18.94761505	9240.54688
5.240892146	23.06201016	0.003527928	42.18001428	36136.76286
4.881756248	16.9589125	0.004625599	25.05709222	9859.437173
4.959969187	21.63920703	0.006832148	29.95693208	14581.62017
4.47607088	11.45448785	0.008711968	12.84882454	3234.600833
4.732824498	16.70080068	0.008797771	20.09445984	7246.168008
4.608322981	16.09174066	0.010660667	21.16805956	9580.347625
4.944053203	15.03300958	0.004585819	26.90919704	11185.90587
4.528671959	14.44199827	0.018555755	16.01382245	5950.599392
4.920851709	16.3616221	0.005870307	25.58627121	12126.59437
4.943717789	21.62762351	0.004041986	25.81954718	9718.867076
4.843713135	15.95139802	0.005892362	22.99038759	9778.099555
4.517924206	15.14467394	0.011462247	16.13253772	5173.252544
4.796699534	16.61699616	0.004643135	20.98579112	7115.450558
4.854845256	13.92276084	0.004898065	21.54969638	7483.473481
4.647022793	16.71641761	0.005501791	17.36785385	4865.141337
5.317033467	20.50032257	0.002545899	43.22544107	27372.80058
4.991274966	15.01479732	0.003751477	27.33993785	10913.0104
5.040744977	18.55125886	0.004742976	39.59446687	21100.65275
4.561184613	10.82659702	0.008018011	15.64091952	3933.904165
5.001893651	25.31591276	0.004287282	26.56652774	13348.08965

log.sigma.2.0.mm.3D_glcm_SumAverage	log.sigma.2.0.mm.3D_glcm_Imc2	log.sigma.2.0.mm.3D_glcm_Imc1	log.sigma.2.0.mm.3D_glcm_DifferenceAverage	log.sigma.2.0.mm.3D_glcm_Id
45.42239031	0.831476096	-0.126094845	6.176193283	0.275207306
28.47860328	0.913190714	-0.2071573	5.387846209	0.278830349
32.67425759	0.835202278	-0.134285876	5.741098397	0.282756524
28.93829845	0.931762673	-0.229165673	5.583470701	0.282656771
32.93093476	0.690585002	-0.075983817	5.03759773	0.286990127
39.74498462	0.959933545	-0.288431218	5.811837734	0.258264116
33.14186922	0.714893488	-0.093423072	3.428764045	0.428778908
37.48688286	0.68063057	-0.072644077	5.016365182	0.329199659
29.99258266	0.689463409	-0.081000148	4.00008658	0.370104793
33.68528375	0.769523897	-0.105530488	5.103996909	0.280395796
27.48290032	0.67423878	-0.081867475	3.296869551	0.386150153
38.43684046	0.721255947	-0.091116762	4.094043975	0.376446936
32.28155997	0.819154827	-0.128277838	5.122632268	0.300819746
33.30984289	0.855029399	-0.146167206	5.964841972	0.257215542
34.43594649	0.78744892	-0.111046093	5.437239138	0.305709201
33.01946195	0.814397968	-0.135134806	4.108895249	0.396317238
31.03148843	0.732992512	-0.097489336	3.685641597	0.34857406
26.77600957	0.865056362	-0.163567942	4.767946252	0.294492059
34.58286498	0.676474046	-0.080356278	3.469059967	0.36661939
51.79173061	0.59456004	-0.052983303	4.392214892	0.335644486
22.59221315	0.617691027	-0.065023724	3.581045308	0.35428294
23.51217545	0.851512965	-0.152019625	4.912631713	0.285962253
21.6915126	0.840930023	-0.145631333	4.44144646	0.326769312
24.31436531	0.67660414	-0.085507167	2.841983082	0.445816669
33.53164954	0.880386001	-0.183103211	4.460976499	0.392371553
29.05754969	0.732155046	-0.093205422	4.34388001	0.347346081
34.94811226	0.714977707	-0.082685154	5.102797845	0.298196212
31.58563925	0.645662727	-0.067200925	4.122809013	0.338716673
22.51911058	0.960628658	-0.319168882	4.555406376	0.291432068
43.81971039	0.672164154	-0.074645175	4.074097657	0.331772853
27.78956303	0.641634211	-0.066410002	3.993034575	0.348597933
33.97958218	0.785858393	-0.110836296	5.701513263	0.270529226
30.30992001	0.698496179	-0.081190553	4.216835807	0.352114556
30.54122196	0.802302491	-0.124042985	4.220032227	0.3729398
40.32594228	0.643561067	-0.065367135	4.261071223	0.346165886
35.79262361	0.735789556	-0.086529111	5.888096628	0.28465903
44.04178192	0.796916035	-0.109223898	6.934435524	0.239573174
38.65669341	0.842203509	-0.151982724	4.058105538	0.426920597
24.95750683	0.766366826	-0.112004822	3.832898963	0.331830781
72.39527818	0.634248039	-0.068319885	3.542091764	0.405439595
24.58478494	0.652984815	-0.068511534	4.265604049	0.313805476
35.1155805	0.626637261	-0.065779313	3.620572768	0.394839738
32.89446628	0.722909323	-0.090492155	5.261660487	0.290048168
33.19080622	0.709112859	-0.079094679	5.651259494	0.270514637
28.30232738	0.749979566	-0.109837422	3.215930748	0.420640698
32.70577348	0.662461793	-0.068944571	4.782737292	0.310485547
36.20922305	0.642491922	-0.066493482	4.015374927	0.361334373
41.34292819	0.74483818	-0.108358844	3.453634525	0.348526422
24.0923177	0.898356123	-0.190226096	5.003221942	0.286339628
30.19993423	0.909098639	-0.197915294	5.466850921	0.279829899
54.03459967	0.781482538	-0.107970746	5.454512705	0.286556935
43.83756022	0.65652853	-0.063316216	5.717733763	0.267642586
28.83844485	0.693020723	-0.09235659	2.720763773	0.47543144
42.29124443	0.753904796	-0.104573529	3.99612435	0.349744037
32.49727843	0.841205624	-0.137448161	6.032389282	0.258493988
27.81769019	0.745419258	-0.098057559	4.232090337	0.320077448
25.88396882	0.668719457	-0.074976998	3.910367535	0.371413093
29.77383314	0.659796306	-0.067333629	5.184345785	0.291442013
32.43655323	0.826349214	-0.129687311	5.628806519	0.276988852
42.74282005	0.684030018	-0.08158308	3.583376002	0.413704889
30.53108126	0.764704852	-0.100722072	5.369717881	0.295418117
37.69901837	0.699293581	-0.073098591	6.229391231	0.264245278
43.34743548	0.639731362	-0.0603718	5.300872599	0.314003649
51.14704304	0.670429369	-0.069193166	5.002165536	0.286681887
40.22312277	0.809929145	-0.130276029	4.313939646	0.32611219
48.49980978	0.632333881	-0.055708585	6.547775065	0.247379502
27.2197947	0.857189842	-0.156720841	5.123193085	0.284881005
34.01330968	0.584361912	-0.048469885	5.535249895	0.288066219
36.64947389	0.551596778	-0.047656947	3.802936145	0.362166816
32.01104561	0.618961175	-0.059073133	4.426179393	0.311854312
22.85493964	0.849978357	-0.151516815	4.75768833	0.302469185
44.33799555	0.73131208	-0.088525135	4.851746176	0.341626074
30.89172204	0.718713054	-0.084230031	5.273231321	0.285847223
33.45360735	0.805567056	-0.118465311	5.811967688	0.272488189
32.47050895	0.700170932	-0.085083716	4.069726014	0.384910567
29.40522456	0.736899747	-0.09738926	4.146002273	0.328951215
48.99706335	0.66355289	-0.069148086	4.556045667	0.303507518
27.98997263	0.919837871	-0.212724024	5.553628763	0.272655232
32.28947725	0.710477741	-0.081736254	4.974093374	0.286575585
33.36319757	0.679274332	-0.077829987	4.06055453	0.351417894
45.29288823	0.82742543	-0.124873265	6.022185193	0.279115162
33.37112483	0.78937327	-0.11460424	4.838753093	0.292745631
42.59946065	0.802229913	-0.1217069	5.208057416	0.332383876
22.9089757	0.565872705	-0.053278422	3.317125454	0.371189
32.97569165	0.700287533	-0.084659618	3.982065957	0.370608643
32.18348132	0.806587751	-0.138904015	4.122467139	0.344528797
29.59628193	0.74939743	-0.097668845	4.781688439	0.315043218
28.67843055	0.746849648	-0.107074427	3.162657797	0.43805741
32.24438507	0.737331833	-0.092133044	4.624752416	0.32877799
42.57138254	0.697653063	-0.078432833	5.134793855	0.28913722
31.7310672	0.792643907	-0.118598119	4.386302056	0.31934274
29.9941914	0.683851839	-0.083850303	3.433175109	0.400471021
32.69657598	0.525065681	-0.039549391	4.490090124	0.315714735
27.64380113	0.620405426	-0.060080105	4.113765979	0.340217107
32.84904673	0.527467417	-0.041449715	4.163670469	0.321635741
40.07984493	0.720754098	-0.079471071	6.397032111	0.262201666
29.48863675	0.638107473	-0.063020952	4.90548106	0.299799159
36.18451402	0.950494496	-0.256605676	6.735681319	0.235369043
21.65319403	0.856415926	-0.168975118	3.686103921	0.332496074
50.06276688	0.609860719	-0.055341	4.714193678	0.324364764

log.sigma.2.0.mm.3D_glcm_ClusterTendency	log.sigma.2.0.mm.3D_firstorder_InterquartileRange	log.sigma.2.0.mm.3D_firstorder_Skewness	log.sigma.2.0.mm.3D_firstorder_Uniformity
101.2021805	197.1269855	0.41094792	0.048534964
68.38237928	202.5859756	-0.280451491	0.061050254
84.67306807	229.2081223	-0.127749811	0.048988015
83.77431681	222.487896	-0.359798449	0.055186914
70.06504967	210.5405464	-0.0041385	0.054666659
79.74777077	185.7545118	-0.463012308	0.056587665
37.4901746	88.11381912	0.454598004	0.102295
67.75332701	166.4490814	0.445325176	0.059631037
47.05264647	131.6562986	0.494204738	0.073839818
52.91080807	180.5545273	-0.069886371	0.058104469
31.43611393	110.4024525	0.221001919	0.086836923
45.94599118	116.7759113	0.396267634	0.076262041
80.48531755	195.6825523	-0.53347399	0.05971681
79.4408746	234.9556198	-0.121489872	0.048315073
69.12583027	186.0641136	0.576592099	0.056402318
55.65947116	133.0878487	0.213836294	0.082116118
37.01506747	130.6988487	0.151046102	0.074890116
63.02750983	199.6321487	-0.297626147	0.062571362
28.628976	107.6958351	0.27399154	0.084407555
50.99566181	139.7927303	-0.116385463	0.066237488
44.77905798	159.4316635	0.151626266	0.069634588
64.78748858	192.3663158	0.179832354	0.057736082
57.8966703	182.0152073	0.201732878	0.058966354
29.04598828	74.93892097	0.675692043	0.111215452
63.99878156	165.1208	0.108820141	0.079917294
64.98393874	163.1030121	0.85974779	0.063438753
64.91246363	185.0432262	0.548968751	0.056032147
42.88013238	127.6246414	0.559065748	0.071974459
49.16230736	137.7774353	0.485108403	0.075811597
46.29961164	157.6881905	-0.057610613	0.064630223
52.17546358	161.217308	0.320837641	0.062829885
77.76149254	237.2076321	-0.234634151	0.054439692
51.07334467	142.530241	0.440200174	0.067645961
58.29888976	120.0891819	0.354745232	0.077235189
55.61245714	155.5010986	0.527520687	0.064437112
83.08102334	217.1057281	-0.249038137	0.048750692
116.977778	252.9505234	-0.568814919	0.047842131
58.62822561	112.0858765	0.159730231	0.094685194
36.70138378	137.8918648	-0.063335889	0.07408801
38.71341807	88.9377985	0.636761091	0.098528761
52.74566226	182.2546692	0.139526227	0.060965694
34.02741377	78.03965425	1.028679937	0.106295916
78.22084185	228.3626804	-0.055604534	0.051463349
69.04364351	209.6727657	0.256112194	0.051251736
31.74197724	96.2352705	0.363285607	0.09275398
64.78120904	191.021637	0.364262935	0.057005784
46.44898621	133.7050304	0.621753693	0.072981886
29.00001362	121.5434532	-0.573112478	0.083013593
66.27656633	197.1920395	0.09831589	0.05339447
91.42798334	201.0557976	-0.392514604	0.058970945
67.82444402	211.2767715	0.240646254	0.053827982
83.14083289	200.6886063	0.44908191	0.0509757
26.37731783	65.12744379	0.554498061	0.126467762
42.32947812	132.8105373	-0.366372296	0.072693649
94.13351157	256.634285	-0.177300836	0.049208004
58.43604218	177.2306538	0.306478701	0.061259198
49.56293935	131.8777981	0.499518479	0.071664943
67.60596613	213.7281609	0.193316316	0.054285687
66.68734299	201.0763779	0.240651334	0.052467582
41.76692728	84.00526619	0.837993996	0.100728518
65.29773745	189.6405258	0.260508892	0.054206079
87.4591793	215.0031967	0.054801983	0.047185712
78.19958986	180.6283531	0.040394309	0.05299761
67.19428194	182.9950981	-0.131890828	0.056310002
44.63129682	141.7932816	-0.453715137	0.067421001
89.90171872	228.0761719	-0.006395993	0.045055981
65.83805455	202.8968525	-0.423595892	0.062789682
71.17950715	184.6712494	0.384018585	0.05378663
39.53958191	117.8432884	0.577134293	0.078603246
55.95882298	177.8201885	-0.149956126	0.062250187
63.76882511	213.023798	-0.012287995	0.056356996
69.06180111	169.2923889	0.147421216	0.058745175
68.53881641	222.0442772	0.285643331	0.053816437
67.01047329	202.7619629	0.439341941	0.053289751
44.59552444	92.77298546	0.741798728	0.094800449
42.94243464	141.893692	0.299897307	0.071378055
59.17763638	170.2926941	-0.18706733	0.059837723
97.00502997	255.17173	-0.402519355	0.05583299
61.7538966	177.1220131	-0.099736395	0.057366161
41.12046611	105.7046909	0.826093614	0.083244444
96.08997986	190.9457569	0.445738019	0.049744137
58.06295028	181.5806999	-0.09258799	0.056944697
68.64260375	194.6301594	0.140213668	0.058714276
31.33277708	117.3629837	0.401493744	0.082314273
46.33965451	132.0596409	0.402107209	0.07385733
52.19541408	112.727417	-1.27367521	0.100010285
61.57085208	177.1389751	0.327542495	0.057325422
38.60531359	97.63119221	0.624779445	0.096208359
58.75189251	142.8036194	-0.007897211	0.06371222
61.3240968	204.8599243	-0.322357542	0.057231032
54.98144724	138.1496878	-0.368426572	0.071112101
35.57566224	96.48380375	0.681690616	0.089056288
46.57874536	148.4339371	0.112588034	0.063794409
51.53997112	149.913887	0.243757204	0.064662015
38.74807244	151.5533981	0.343297909	0.067697343
101.7240719	244.4088631	0.055891336	0.044785074
63.32019589	190.0488701	0.231384652	0.055448959
85.95487542	221.9141159	-0.355598971	0.051169247
40.23734705	149.8020897	-0.334591948	0.07426487
63.47288967	162.02075	-0.322748965	0.059117545

log.sigma.2.0.mm.3D_firstorder_MeanAbsoluteDeviation	log.sigma.2.0.mm.3D_firstorder_Energy	log.sigma.2.0.mm.3D_firstorder_RobustMeanAbsoluteDeviation	log.sigma.2.0.mm.3D_firstorder_Median
123.4392259	37570555.93	81.79468142	-32.08492088
109.1345241	6030024.365	81.31034336	14.68978024
122.7316284	18961073.92	92.68897826	15.8843956
121.7637926	6556481.05	93.31453319	26.26322365
113.5018005	24335510.24	88.62257186	12.49250984
110.3273737	5172828.779	72.83782961	30.79449844
68.66069901	101565174.7	39.80954174	-2.702620506
102.3016168	108341935.3	68.15518544	-23.44810104
83.45029943	50930445.37	55.53963532	-16.62078953
99.27995809	11199997.9	72.49826133	24.5636158
67.1734218	11539794.76	45.97942692	-1.094243705
81.99805502	43141198.24	50.72319337	-5.981599808
113.3379175	15478545.12	82.53210676	26.22511101
123.0261389	14376483.8	93.41191433	-27.95520782
111.1102509	32908738.21	75.42445493	-38.76916504
85.40978215	73735676.27	56.49204896	-5.883657217
77.2594885	8487122.32	53.92235503	4.140498161
104.0727402	6586074.185	79.7301641	-9.527280807
68.06058841	8586174.13	45.33177784	-7.706543684
87.91799349	126737475.4	57.79278343	11.20355034
86.30671611	22110662.02	64.86174044	9.822251797
106.7976212	7987995.619	81.12318584	5.585412264
98.7244994	8043461.092	71.8840387	-9.650437832
58.1660017	24351008.2	34.08361052	-10.36371231
97.2694998	20651278.29	64.68015755	-8.411814213
99.87357228	42451246.56	66.70693074	-17.51112175
108.4547083	37174143.17	76.13317888	-40.48438263
82.40931338	26634884.38	54.46178615	-23.09540939
83.44380253	1842550.753	54.51186317	15.23104
87.96755029	19150554.83	63.50308611	-10.63104725
90.98602915	49953352.51	66.69638134	-19.69469643
123.8319908	16334000.19	98.48388712	-12.60013723
88.1998064	30980868.39	58.62029324	-13.94355774
85.57499467	31447531.47	56.06395455	-6.661393642
92.67642821	73134868.08	63.08078057	-12.32384396
119.5643374	40502448.08	88.56652531	-0.191049784
138.3279055	42380445.08	102.1515529	23.38770676
85.12002414	81569420.08	52.89792208	2.634006023
77.37961308	5276862.625	54.75780728	-15.63209724
69.04930773	90831615.59	40.55789926	-10.22163582
97.33188056	18366838.45	74.14830311	-31.48840714
65.81794686	51818327.84	36.9169169	-7.449956656
116.3699423	45051322.53	90.18223702	4.147949696
115.3637909	28004529.99	84.49810993	-32.86960983
67.35230246	13271471.21	41.27745691	-7.781461477
104.7014349	38966913.45	76.36127867	-17.35937691
82.16075239	63304888.36	53.86772812	-10.7876749
71.65139068	4241739.864	49.92795187	-0.630555868
110.1540989	6875536.111	81.55792934	1.197095275
119.624344	9515958.394	86.2891358	42.26897049
115.1305122	26659016.63	82.63535411	-38.94148254
115.7603452	56899937.62	83.1209785	-7.751588821
54.87188538	51053241.01	30.45159828	-5.397175789
81.75782645	18812247.04	54.5914663	7.906589031
132.0556224	14840920.05	104.6337953	25.51877975
98.2798057	16458639.5	73.28794135	-12.11217022
84.96889325	48853765.25	57.23423504	-18.94124222
112.9387208	36654690	86.83220804	-22.26378822
113.1818261	14975609.54	81.26476777	-36.11824417
68.60549717	108516256.5	39.21801336	-6.806200981
107.9922849	23745162.58	77.53638742	-6.641019344
120.7441252	55981248.46	85.91484108	14.96948147
108.2446538	156545240.9	74.49640255	1.239823043
102.61921	33748931.9	73.88434136	7.651390076
87.14759427	8862383.341	57.92217684	5.387043476
128.0496843	82926516.59	93.69884864	6.345829964
110.3704793	7148969.974	84.63104895	5.379456282
109.5015363	96478417.45	76.10396028	-25.75938797
76.34998629	77180865.69	49.80390254	-12.52958822
95.93866945	28662796.35	71.19943651	23.87658119
107.5090753	7900638.78	83.04329481	-8.19093585
99.09797385	79606474.77	69.07868154	-3.202980995
114.8964896	24689273.37	88.66527909	-36.81420135
115.0373682	18849591.8	83.72914633	-41.21191788
75.42126582	47089306.98	44.20552378	-8.649067402
81.86832504	9842042.708	57.53898894	13.91213894
97.12294571	31678243.55	69.97255639	7.632684708
128.182209	7082199.082	101.2301131	22.13393974
101.0065576	20610747.1	72.96802893	28.75512695
76.94492357	20825158.24	46.6649512	-26.63299179
120.5528299	35062617.33	79.83649806	-34.41554451
102.8585725	13177611.18	74.76856243	19.40073204
106.5068426	41484646.6	76.06656704	-12.93519592
71.68208309	16101203.67	49.37147663	-31.34010887
82.86432341	31950931.15	54.62317658	-6.450710773
82.8518382	6152455.346	52.84954744	34.53995514
101.4005732	20905518.61	71.98467183	-25.39970875
70.03546329	35732981.08	42.73001379	-7.039115667
93.12837186	25755377.14	60.69569249	-6.068511486
108.2545636	22375732.91	82.72605364	22.62646103
87.33319833	10330334.52	57.92645644	27.12389755
70.44827624	23694805.52	43.32931168	-17.27138233
88.53125676	56363997.77	61.49526376	-5.643055916
87.25603549	40681346.48	62.04719692	3.448819757
85.4798362	36619726.36	61.70384519	-7.688063622
134.1344404	61164669.91	100.2814142	11.18460846
103.8912585	38611797.01	76.85042198	-4.686014175
128.0558541	7762577.764	93.01327368	-3.703729391
79.4336496	2474878.189	59.08819064	9.006129265
97.39509141	138545198	66.87018036	19.63025475

log.sigma.2.0.mm.3D_firstorder_TotalEnergy	log.sigma.2.0.mm.3D_firstorder_Maximum	log.sigma.2.0.mm.3D_firstorder_RootMeanSquared	log.sigma.2.0.mm.3D_firstorder_90Percentile	log.sigma.2.0.mm.3D_firstorder_Minimum
1014405010	545.5999146	161.3021715	204.0288208	-573.2005615
1628810657.9	370.5771179	131.8240943	126.4123566	-364.5021973
511948995.9	391.6779175	145.7971513	182.1610306	-394.0130005
177024988.4	335.3166199	142.6945773	162.4479233	-371.2202454
657058776.6	357.8622437	131.3278158	171.9575958	-388.2380981
139666377	357.3494873	146.2030285	198.2583633	-451.3300171
2742259716	514.2249146	98.16862489	131.1296661	-385.4432068
2925232253	520.0740356	133.6212433	174.5160217	-471.8108521
1375122025	436.5942993	109.3155676	148.9089478	-356.2031555
302399943.3	441.5582581	121.7966925	163.8750183	-387.4719543
311574458.6	286.6324463	86.11806165	124.4210052	-309.8744812
1164812353	432.1756287	112.5278536	147.3714966	-471.0185242
417920718.2	338.1746521	138.1511936	143.5458679	-389.2127991
388165062.5	305.5406799	149.4112867	150.7323013	-444.8761597
888535931.7	546.2224121	144.5490815	183.1221771	-437.6911011
1990863259	410.6968994	113.1233368	165.8168747	-385.2404785
229152302.6	449.1854553	98.03922713	131.2683197	-353.1376038
177824003	316.9475098	126.8970877	109.3011353	-363.5951233
231826701.5	388.5715942	88.5913225	112.1119293	-415.9173889
3421911835	656.6977539	117.478998	148.157251	-623.5596313
596987874.4	337.2879639	103.1521065	124.7961525	-254.4185486
215675881.7	432.7124939	126.9048064	158.0003357	-298.3540039
217173449.5	410.3111267	120.0616709	145.129361	-260.903656
657477221.4	352.9813538	82.27885788	103.2873276	-289.099823
557584513.9	347.5653076	129.6803835	165.6768417	-401.8007202
1146183657	544.8531494	128.8738489	185.9830399	-334.6131287
1003701866	524.937439	136.1982806	162.5272247	-443.3910217
719141878.3	538.2858887	108.7288544	133.696991	-377.2817078
49748870.33	442.4202576	112.3397109	157.9142151	-248.4385071
517064980.3	326.7007446	109.063076	138.6073288	-534.6704102
1348740518	385.2026367	111.6257248	143.0869904	-329.4125061
441018005.1	266.8411255	143.9737012	144.9370895	-439.2941895
836483446.6	531.6846924	115.1869691	147.7730072	-371.7156677
849083349.6	387.5237427	114.1128922	164.0954895	-364.3425598
1974641438	552.1087036	119.3648919	163.1638855	-487.1204224
1093566098	370.0876465	144.5279133	172.6315125	-432.9807739
1144272017	482.5213013	167.4198163	164.3889923	-558.8303223
2202374342	501.1498413	118.3763344	173.8599854	-469.281311
142475290.9	276.3640747	97.07197831	114.3378792	-316.4803772
2452453621	480.427002	98.05541041	128.9436432	-891.4644775
495904638.1	310.9621582	117.0751718	132.4593582	-302.3399048
1399094852	515.531311	95.58144372	124.1055443	-423.1250916
1216385708	353.1683044	136.4977913	182.2819031	-375.8929749
756122309.8	455.7204895	140.4332582	175.5852539	-408.2692261
358329722.7	375.5430298	92.35379334	116.3787041	-331.8054199
1052106663	391.8266907	127.3948651	179.9264526	-398.4610901
1709231986	563.9628906	108.7680013	138.7670441	-445.3214722
114526976.3	271.4783325	92.94612091	97.15570831	-509.0142517
185639475	331.4841003	132.6065282	182.2998657	-285.1836548
256930876.6	352.5649719	145.0965378	160.4330338	-366.2515259
719793449.1	472.9263916	143.9238571	192.6181274	-689.5767822
1536298316	606.7729492	142.93655	202.5025452	-508.8204346
1378437507	402.7177429	80.00536044	100.4290314	-346.3016052
507930670.1	379.2640991	107.0369586	119.3518219	-518.0689087
400704841.5	363.8092041	151.9234892	190.4810272	-388.2339478
444383266.5	412.9626465	118.302332	142.0516205	-325.1042175
1319051662	382.2340698	110.3078058	152.9208649	-318.1847229
989676629.9	515.050415	133.1663626	159.7819733	-371.7287903
404341457.5	405.2081604	140.0973708	161.1872345	-410.9971008
2929938925	575.7545166	99.80990675	123.4608292	-501.20047
641119389.5	549.2401123	133.6672716	157.3549805	-369.3627319
1511493709	547.9959106	150.6998528	196.2077576	-426.7508545
4226721505	482.5871887	137.8841999	191.2522369	-515.0167236
911221161.3	424.6585388	126.8920393	163.734787	-622.439209
239284350.2	351.51828	114.4144643	142.8447968	-490.2373657
2239015948	558.8729858	155.6933898	191.7047272	-586.4830322
193022189.3	235.4867706	132.6962713	116.0618553	-374.8371582
2604917271	562.8271484	137.743022	172.6073181	-416.1877136
2083883374	594.675293	101.3894322	127.408197	-441.9614563
773895501.5	368.7166443	116.1667991	151.0222656	-361.0347595
213317247.1	282.4521484	126.7209884	157.2926559	-285.3809814
2149374819	528.7920532	126.4200406	164.311615	-544.2445679
666610381.1	441.4034119	135.9920503	159.9274689	-392.9262695
508938978.7	491.2376404	140.8604827	186.7043747	-401.2574158
1271411289	618.7843628	107.3787349	149.9272293	-386.8760376
265735153.1	494.9014893	103.5431283	129.822406	-326.8404541
855312575.9	368.1360779	119.7519761	146.3597229	-591.7672729
191219375.2	286.2709351	148.0753624	153.0416351	-359.1786194
556490171.8	438.8244934	125.194137	172.0710724	-357.104248
562279272.6	579.4671631	107.5617607	128.6820862	-404.8309326
946690667.8	545.5999146	157.9172257	198.3739471	-573.2005615
355795501.9	431.0697021	126.307262	157.5423355	-396.6838379
1120085458	391.153717	134.829796	175.2570633	-544.4668579
434732499.2	286.4511108	92.22610447	114.7115173	-288.9115601
862675140.9	438.9404297	108.48179	152.0380066	-381.3379211
166116294.3	191.6143494	107.6408143	96.98383331	-376.6915894
564449002.5	472.6063843	126.4231482	160.1412598	-364.475708
964790489.1	425.4172058	96.51511343	136.1857834	-347.8658752
695395182.6	446.1471558	123.0500516	145.4245453	-389.6320496
604144788.4	309.856842	127.9394199	156.8427948	-514.0950928
278919032	313.4004822	113.351995	148.0199799	-363.0718689
639759749.1	376.2067261	96.73746969	127.1960297	-363.7564392
1521827940	483.0945435	111.9042003	144.7564697	-379.218689
1098396355	449.0032043	108.6525749	150.857811	-322.3371887
988732611.7	561.0523682	105.2302131	145.7725769	-389.078125
1651446087	529.8233032	160.1759073	194.1386154	-488.8381958
1042518519	522.621582	125.4872998	174.2327713	-348.3817749
209589599.6	404.5044556	157.4819607	153.6212891	-473.2068176
66821711.1	189.9126282	96.0969308	121.419548	-256.9656982
3740720346	494.3762512	125.3034386	162.7722305	-586.5256348

log.sigma.2.0.mm.3D_firstorder_Entropy	log.sigma.2.0.mm.3D_firstorder_StandardDeviation	log.sigma.2.0.mm.3D_firstorder_Range	log.sigma.2.0.mm.3D_firstorder_Variance	log.sigma.2.0.mm.3D_firstorder_10Percentile
4.669083272	160.5790429	1118.800476	25785.629	-200.2430069
4.295297992	131.1019936	735.0793152	17187.73272	-200.8957306
4.516229129	145.792689	785.690918	21255.50815	-197.4079544
4.378374144	142.4941778	706.5368652	20304.5907	-216.2110947
4.346444784	131.2630359	746.1003418	17229.9846	-164.3999634
4.425764396	143.8240174	808.6795044	20685.34797	-169.0437469
3.84816072	98.16651824	899.6681213	9636.665304	-120.842305
4.393727531	133.2128989	991.8848877	17745.67642	-167.6369202
4.085178597	108.991767	792.7974548	11879.20527	-137.264856
4.279313084	120.6509311	829.0302124	14556.64716	-142.5018463
3.798920319	85.82961216	596.5069275	7366.722324	-98.48327255
4.125769966	112.5276601	903.1941528	12662.47428	-132.2349609
4.333156852	137.9831473	727.3874512	19039.34895	-219.2999573
4.516303969	145.2754655	750.4168396	21104.96087	-223.3741867
4.453764994	142.9786224	983.9135132	20442.88646	-185.7668884
4.055813255	113.1231891	795.9373779	12796.85592	-155.2276016
3.981552166	97.92194959	802.3230591	9588.708211	-117.6088394
4.193536255	123.044095	680.5426331	15139.84932	-204.921579
3.837386194	88.48394704	804.4889832	7829.408885	-112.4497536
4.231007916	117.0094305	1280.257385	13691.20682	-132.6506409
4.025348922	103.1144267	591.7065125	10632.58498	-133.4209824
4.298193273	126.9045296	731.0664978	16104.75963	-161.4692307
4.254025708	119.9936477	671.2147827	14398.4755	-150.9582825
3.63452045	82.11456219	642.0811768	6742.801323	-98.59975281
4.198107	129.0114664	749.3660278	16643.95847	-184.6156693
4.283968479	128.7153518	879.4662781	16567.64178	-135.9835281
4.414392306	135.2021922	968.3284607	18279.63278	-167.6548477
4.100850182	108.0081	915.5675964	11665.74967	-135.7998871
3.994666921	108.5360439	690.8587646	11780.07283	-91.26829529
4.139505702	108.9983348	861.3711548	11880.637	-137.1858383
4.157479217	111.0800621	714.6151428	12338.7802	-149.8815491
4.371619987	141.530113	706.1353149	20030.77288	-221.0707703
4.190529704	115.0641397	903.4003601	13239.75624	-144.7536591
4.092453613	114.0352016	751.8663025	13004.02721	-156.3515381
4.240490397	119.3648103	1039.229126	14247.95793	-135.6640198
4.532004575	144.4307051	803.0684204	20860.22859	-199.9361603
4.630523186	166.4082206	1041.351624	27691.69587	-271.3889069
4.031755864	118.371284	970.4311523	14011.76088	-158.3540649
3.963896265	96.24372983	592.8444519	9262.855532	-131.9835495
3.849210023	97.93529296	1371.891479	9591.321608	-117.5480545
4.185330283	115.1373377	613.302063	13256.60652	-163.1697723
3.771044087	95.554185	938.6564026	9130.602272	-102.8635864
4.420456336	136.4047527	729.0612793	18606.25655	-164.9188766
4.476325111	139.8157587	863.9897156	19548.44638	-180.4872116
3.85256869	92.30186334	707.3484497	8519.633976	-109.7408714
4.330521781	127.3783857	790.2877808	16225.25314	-150.1253357
4.102144755	108.7631596	1009.284363	11829.42488	-124.949707
3.81083604	92.48158359	780.4925842	8552.843303	-124.843132
4.380692118	132.4785699	616.6677551	17550.57148	-171.7146912
4.380505338	144.9976757	718.8164978	21024.32596	-228.3581085
4.460228055	142.9604566	1162.503174	20437.69216	-183.3620605
4.513701062	142.3709442	1115.593384	20269.48576	-155.6248291
3.538603816	79.9680454	749.0193481	6394.888285	-96.65417862
4.095060445	107.0097443	897.3330078	11451.08538	-135.6011871
4.483706186	151.8113321	752.0431519	23046.68056	-203.6709045
4.223544997	118.1351285	738.066864	13955.9086	-148.9316177
4.100742261	109.9535269	700.4187927	12089.77808	-142.2692902
4.37427603	132.7798094	886.7792053	17630.47778	-173.7840027
4.459268761	138.5269356	816.2052612	19189.71189	-188.2023254
3.852515907	99.79017629	1076.954987	9958.079285	-112.9190292
4.417831757	133.605253	918.6028442	17850.36363	-170.5766449
4.615855652	150.288965	974.7467651	22586.77299	-181.6375549
4.489100131	137.6209308	997.6039124	18939.52059	-167.0518326
4.360989882	126.7884247	1047.097748	16075.30463	-149.2425156
4.167721311	114.4140122	841.7556458	13090.56618	-139.249884
4.659587194	155.6670986	1145.356018	24232.24559	-207.0516357
4.217646826	129.2576203	610.3239288	16707.5324	-228.6150436
4.469530027	137.4668555	979.0148621	18897.13637	-172.4261749
4.002863819	101.3705036	1036.636749	10275.97901	-112.0424156
4.19679836	115.6651569	729.7514038	13378.42851	-145.9747238
4.277229691	126.2973678	567.8331299	15951.02511	-172.9240448
4.345203752	126.3938118	1073.036621	15975.39566	-162.3339691
4.376930576	134.9036202	834.3296814	18198.98673	-182.2780762
4.444283061	140.0826369	892.4950562	19623.14517	-179.5394943
3.947509627	107.3593506	1005.6604	11526.03017	-125.9762054
4.034381729	103.0840859	821.7419434	10626.32877	-123.5888046
4.279128422	119.7448672	959.9033508	14338.83322	-155.63302
4.360973601	147.3550354	645.4495544	21713.50646	-228.3601654
4.327705896	123.9960004	795.9287415	15375.00811	-147.3284302
4.004780752	106.5040704	984.2980957	11343.117	-130.9973419
4.634013507	157.0356484	1118.800476	24660.19487	-196.108963
4.330690764	126.1502341	827.75354	15913.88157	-163.9332581
4.365917562	134.2458885	935.620575	18021.95859	-187.5551056
3.856888684	90.41395462	575.3626709	8174.68319	-125.4122849
4.092775406	108.4639797	820.2783508	11764.43489	-132.6379852
3.775709097	107.6253991	568.3059387	11583.22653	-171.4761505
4.34097357	125.7899313	837.0820923	15823.10682	-158.8218094
3.852558244	96.51489939	773.2830811	9315.125804	-115.1237831
4.298813509	122.8371397	835.7792053	15088.96288	-170.7235718
4.307626936	127.9270888	823.9519348	16365.34004	-165.3360504
4.133121776	112.7766191	676.4723511	12718.56582	-147.6011917
3.874546604	96.31126997	739.9631653	9275.860723	-117.933165
4.203769869	111.8712523	862.3132324	12515.1771	-141.2124176
4.149356205	108.3260496	771.3403931	11734.53301	-126.3369446
4.089105372	105.1748422	950.1304932	11061.74744	-121.6880585
4.673061678	160.1708439	1018.661499	25654.69925	-210.6345139
4.343886783	125.3456348	871.0033569	15711.52817	-150.6064743
4.547078687	155.4203696	877.7112732	24155.49129	-242.8232178
3.893028599	96.09604384	446.8783264	9234.449643	-132.6886841
4.340366315	124.7877196	1080.901886	15571.97496	-152.09263

log.sigma.2.0.mm.3D_firstorder_Kurtosis	log.sigma.2.0.mm.3D_firstorder_Mean	log.sigma.2.0.mm.3D_glrlm_ShortRunLowGrayLevelEmphasis	log.sigma.2.0.mm.3D_glrlm_GrayLevelVariance
3.572731967	-15.25652424	0.003304985	42.78661101
2.560129791	-13.77893729	0.012174585	28.03146457
2.252142905	1.140691177	0.007840015	34.50911858
2.177258082	-7.559874042	0.012804016	33.10061193
2.150181128	4.124392134	0.006637845	28.01270721
3.339569408	26.26742391	0.008075268	33.95919805
4.600668913	0.643124087	0.004387493	17.3925643
3.404832209	-10.43840226	0.003954718	29.90369457
3.246042595	-8.407619054	0.00587916	20.25166394
2.678878186	16.66694753	0.00586894	23.86130619
3.12906163	7.042600252	0.007020368	12.3823162
4.227973164	-0.208686384	0.003444492	22.02590209
2.550263543	-6.812000508	0.010827315	31.08178894
2.243477145	-34.91091138	0.007606477	34.17634665
3.254287354	-21.24971765	0.005058624	34.09548142
2.88424279	0.182757381	0.004730833	22.01451283
3.690496757	4.793938496	0.006495695	15.99576197
2.362550735	-31.03258857	0.011711672	24.77565424
3.939126281	-4.360451529	0.004562396	13.24289766
4.826349934	10.49324317	0.002059658	23.06215163
2.597683304	2.78784825	0.012423028	17.42441857
2.479092972	-0.265048876	0.016904594	26.22387614
2.644780148	-4.040956396	0.024303812	23.6995954
4.733018261	-5.197030922	0.008750514	12.00527449
2.908119644	-13.15459608	0.006286451	29.63537877
3.683837501	6.389612273	0.006839499	27.9798326
3.34192572	-16.44198483	0.004724392	30.25814305
4.108519653	-12.49856388	0.005277926	19.58056866
4.195999563	28.98513078	0.026402997	19.57075051
3.479745288	-3.757333097	0.002970146	19.79289888
2.64513185	-11.02371293	0.00715776	20.29817878
2.074159838	-26.41313601	0.007592284	32.24472553
3.495391343	-5.318046226	0.006405428	22.44702695
3.091574278	-4.210101099	0.006122996	21.95797843
3.630443495	-0.139577971	0.002987248	24.10320119
2.50930041	-5.2999192	0.006264558	33.96310507
2.601577807	-18.37658893	0.00388161	44.58295705
3.347951694	1.093465728	0.003368601	25.16615664
2.891561386	-12.65359397	0.010582215	15.25423121
5.204300424	-4.851999952	0.00083312	16.91263508
2.268098043	-21.21295208	0.01148982	21.58927203
5.90844704	2.282566833	0.003609169	16.03732231
2.185616528	5.038897635	0.00631671	30.16840143
2.600905743	-13.15498436	0.00591782	31.83257921
4.219882476	-3.096638112	0.006772715	15.15081035
2.622423481	2.049030379	0.005750225	26.62744353
4.476656052	-1.026271045	0.003685842	20.05651094
5.484091558	-9.281060771	0.004760629	14.06655121
2.373580128	5.824075046	0.019604847	28.32429225
2.48249054	5.35530879	0.011245818	33.64009143
2.931482582	-16.62481509	0.002230544	33.5244168
3.098293354	12.70321016	0.002827036	33.20896317
4.955765774	-2.443238566	0.00542495	11.81293584
4.499456962	-2.413528139	0.003414286	19.33780193
1.963478866	5.83661077	0.007859237	36.86695128
2.751582252	-6.287539633	0.0081129	22.79538124
3.051215254	-8.833682071	0.008732672	20.5288265
2.482174267	-10.13914874	0.009181333	28.76555102
2.755136273	-20.91796885	0.007098972	31.2715527
5.83482815	-1.98449013	0.002352465	17.69981775
3.27786787	-4.071345181	0.008903135	29.3780141
2.975815737	11.12081983	0.005138335	37.01914759
2.968295664	8.51657142	0.002717774	31.69190658
3.221668065	5.126891538	0.002187793	26.15829331
4.316474128	0.321644299	0.006118461	21.94923001
2.718644189	-2.861124623	0.002398473	39.3498246
2.154436113	-30.01279775	0.011905511	27.00862317
3.194810558	-8.718012333	0.005461801	31.0761879
4.812665747	-1.959067311	0.003504058	17.44398572
2.557161844	10.78409493	0.006522852	21.80616869
2.050397951	-10.35296074	0.023051236	25.80662957
3.110382234	-2.575071536	0.002689984	26.80010471
2.384793635	-17.17122602	0.006544507	29.64812778
2.814569174	-14.78277414	0.00630693	32.07431759
4.798545062	-2.040228539	0.004792571	20.31525219
3.63556731	9.739129763	0.00679401	17.59797391
3.209276363	1.304825901	0.002324945	23.50985232
1.966100162	-14.58788855	0.014024387	34.85534345
2.806016238	17.27899975	0.006914827	25.08770167
5.310885349	-15.04710447	0.004717328	19.4238554
3.63710183	-16.66299171	0.003278967	40.9821708
2.866383698	6.296257449	0.006750982	26.00683539
2.622420489	-12.534564	0.003278035	30.46587485
2.910794611	-18.19261264	0.010194607	13.74825533
3.232598061	1.965675141	0.004698928	20.18914257
4.113288053	-1.821640756	0.010355074	19.36752428
2.870575629	-12.63746695	0.00782494	26.14047964
3.965585414	0.203265289	0.005941503	16.72758756
3.420431173	-7.235489659	0.007598441	25.58038435
2.597490198	1.77627054	0.003420923	26.64998118
3.296283317	11.40653113	0.007814951	20.65566322
4.201320876	-9.070684563	0.005889327	16.19849366
3.10276913	-2.715317068	0.005384288	20.8316678
2.919062193	8.417185796	0.008373835	19.33078777
3.118729844	3.413255353	0.004595187	18.29949302
2.569229089	-1.273586361	0.003932925	41.79993656
2.631019478	5.96105944	0.007655843	25.90088785
2.730408274	-25.39835922	0.011975381	39.36046998
2.341121556	-0.412875081	0.0165709	14.96288507
3.669379175	11.35679377	0.002029537	25.91717347

log.sigma.2.0.mm.3D_glrlm_LowGrayLevelRunEmphasis	log.sigma.2.0.mm.3D_glrlm_GrayLevelNonUniformityNormalized	log.sigma.2.0.mm.3D_glrlm_RunVariance	log.sigma.2.0.mm.3D_glrlm_GrayLevelNonUniformity
0.003407836	0.047216651	0.072469528	64.0214238
0.012480585	0.060343611	0.06370528	19.77142144
0.008105423	0.048446519	0.074020767	40.57168949
0.013196231	0.054693497	0.066416159	16.57174949
0.00687917	0.054093088	0.073923398	71.59603425
0.008179844	0.055305869	0.057720104	12.74085901
0.00482823	0.085834783	0.325886644	764.3976321
0.004171775	0.056159257	0.139738728	309.367677
0.006296538	0.068943138	0.16297784	262.6545459
0.0060562	0.057633464	0.064982372	41.09363081
0.007449198	0.083670661	0.130446405	118.0296856
0.003685416	0.069662509	0.185675001	210.0037018
0.011115997	0.058347906	0.085453662	44.04776495
0.007836567	0.047993468	0.059490704	29.26138234
0.005299551	0.053619154	0.105219816	77.90568767
0.005120348	0.071394106	0.219537928	359.4503316
0.006800207	0.073043383	0.105083234	59.16095565
0.012071935	0.061984413	0.065328683	23.85467503
0.004826197	0.081947629	0.132691445	81.07339018
0.0021601	0.064050002	0.122754523	536.3830118
0.013188256	0.068749275	0.108119014	130.8073569
0.017456332	0.057084849	0.05885321	26.84827894
0.025563699	0.057841892	0.095465959	29.83044086
0.009534747	0.100607211	0.234126847	312.1943676
0.006678005	0.062284623	0.269090721	65.99696834
0.007268757	0.060829381	0.130629257	141.1450863
0.004940984	0.054735307	0.088037185	101.9755518
0.005571393	0.069475405	0.105803858	143.6140071
0.026717177	0.074543203	0.058090438	10.38535048
0.003099006	0.063538335	0.099188385	94.46910484
0.007602617	0.06192234	0.119878845	226.3098019
0.007870251	0.053889415	0.064120556	40.12270024
0.006784465	0.063511903	0.145725108	133.4147263
0.006499942	0.072312384	0.141918379	157.850004
0.00317294	0.061548922	0.135229977	286.3031942
0.00651382	0.048187147	0.087028139	87.12342819
0.003975357	0.047404302	0.053586145	68.29299424
0.003666742	0.074412314	0.326684077	366.3673521
0.011039903	0.073094703	0.082152542	38.12956245
0.000905726	0.087336235	0.219118116	718.3356135
0.012021324	0.060332048	0.080838519	75.41797869
0.003928978	0.095408057	0.195877446	476.0307867
0.00660043	0.051132507	0.082781793	115.5267044
0.00612875	0.050488084	0.063798669	67.7340017
0.007296704	0.082128297	0.241735756	110.7687334
0.006041082	0.055834643	0.090071288	124.4597509
0.003944894	0.069496701	0.144628464	335.367581
0.004911018	0.082085344	0.093793208	37.15119497
0.020199371	0.05295025	0.065787735	19.55510248
0.011669796	0.057798403	0.070129328	24.61036465
0.002295455	0.052461407	0.074092032	63.50871216
0.002932991	0.050265671	0.060835322	132.415398
0.006071061	0.10691434	0.351096147	704.3653821
0.003563367	0.070159911	0.116219732	105.0399538
0.008107501	0.048807798	0.054060523	29.83963631
0.008504431	0.060409214	0.084028489	66.09082604
0.009314777	0.067155791	0.164871851	241.7340185
0.009539594	0.05361362	0.079529704	103.5673052
0.007338196	0.051517473	0.070344294	36.98732386
0.002573665	0.088939908	0.225740272	840.169395
0.009196663	0.053345925	0.087573193	66.13283576
0.005299533	0.046529475	0.072165336	107.9282401
0.002867337	0.050801484	0.120550828	383.0730903
0.002264156	0.055773004	0.068768755	109.9018803
0.006366784	0.065379416	0.090198593	40.909251
0.002477092	0.044689474	0.057878117	145.1471212
0.012293638	0.06177447	0.060529623	23.69024052
0.005695117	0.052583481	0.084745449	249.4728173
0.003746093	0.075767848	0.140249552	512.477723
0.006775578	0.061411785	0.081716155	121.592215
0.023864942	0.055860386	0.074033616	25.83583269
0.002839429	0.056142034	0.133072456	254.1450576
0.006817503	0.053131507	0.075879967	66.49340657
0.006497988	0.052472489	0.059452012	47.31271327
0.005172156	0.082167148	0.233013601	292.3842708
0.007112215	0.070447573	0.096137789	59.83717283
0.002412431	0.059146221	0.073355029	122.3774754
0.014457778	0.055282172	0.05807402	16.92752717
0.007148806	0.056731507	0.069079871	70.13432773
0.00499816	0.078445931	0.127623025	128.1838264
0.003383974	0.048394757	0.074276466	63.79770774
0.006953861	0.056340931	0.066090051	43.86009899
0.00345937	0.054093258	0.153452709	111.9967426
0.010883098	0.079503682	0.119960062	136.6899018
0.005042528	0.067896357	0.179097013	163.8897652
0.010599263	0.095072346	0.103675843	46.40501563
0.008196932	0.056207308	0.09474469	68.04290343
0.006532092	0.081378438	0.300293841	265.0501934
0.007884761	0.060222869	0.123420059	93.75629758
0.003538977	0.056626935	0.071538369	72.61789791
0.008097543	0.069462348	0.084701837	51.87739177
0.006341855	0.082527514	0.191470326	183.3558308
0.005632534	0.062271953	0.093273814	259.6065842
0.008818711	0.063655822	0.104330526	201.7139357
0.004857026	0.066358903	0.096755684	202.4612421
0.004070918	0.044225014	0.068555609	99.38586802
0.007993091	0.054688624	0.085503406	125.0046296
0.012284753	0.050145725	0.047416197	15.05016291
0.01730874	0.073876841	0.077576602	18.46671764
0.002134748	0.057689441	0.11128602	467.0654487

log.sigma.2.0.mm.3D_glrlm_LongRunEmphasis	log.sigma.2.0.mm.3D_glrlm_ShortRunHighGrayLevelEmphasis	log.sigma.2.0.mm.3D_glrlm_RunLengthNonUniformity	log.sigma.2.0.mm.3D_glrlm_ShortRunEmphasis
1.207467177	544.3112988	1204.109505	0.954531587
1.187836968	241.6751303	293.6552179	0.957582505
1.211647556	295.2023002	744.341666	0.954147373
1.196407417	252.5126595	269.3744482	0.955165587
1.21119871	293.6123303	1172.105532	0.953542075
1.164086512	437.0859634	209.8052391	0.963800276
1.739081304	261.9351962	6744.320181	0.892132159
1.355407381	372.326191	4655.038057	0.934865343
1.41804942	235.2825286	3132.184392	0.924113355
1.187820977	306.5660021	639.640934	0.958170596
1.352068876	190.2092881	1181.260847	0.930974024
1.468058133	372.7927724	2439.748721	0.917758528
1.242168302	276.1680715	660.0392463	0.948203045
1.176257092	316.8804672	548.0825369	0.959302691
1.282198988	331.3193573	1256.094991	0.943841017
1.538167982	270.5855856	4007.231053	0.910718039
1.296348184	248.1666085	688.730734	0.937608385
1.197308704	218.6089914	341.8634288	0.954279802
1.361080044	293.4777603	824.8252319	0.929032974
1.327271656	650.7335524	7090.430733	0.935818986
1.302767913	143.8180032	1615.512282	0.936984813
1.172541348	179.1086814	424.6965348	0.960744546
1.268717732	148.0557224	445.4490134	0.943325896
1.587330678	149.9758996	2403.036097	0.90042306
1.623741133	289.0911371	835.9890753	0.906973932
1.347778754	235.2934861	1950.878624	0.932842424
1.246183008	335.4902299	1626.690945	0.947849441
1.295768085	262.3188972	1760.795226	0.938336769
1.157839006	151.615448	127.725764	0.966560703
1.273969109	494.3998733	1284.261733	0.943425633
1.324592717	205.9196956	3090.226041	0.935276601
1.18579919	324.0962082	668.7189128	0.958631314
1.384600549	239.859499	1738.6565	0.926964761
1.370996611	240.8594799	1823.375669	0.930289496
1.354458913	419.150117	3904.068892	0.932489043
1.238855357	352.1476946	1590.312786	0.950505054
1.157080707	540.3874638	1313.542512	0.964273659
1.734698353	366.9381621	3774.661373	0.896500826
1.236005943	177.517819	455.8246696	0.948330882
1.544500805	1220.163978	6484.292019	0.907768467
1.231725942	174.3343479	1096.105181	0.949458956
1.495530761	302.5839144	3979.051621	0.912481381
1.233253466	296.4970775	1994.71007	0.950891923
1.184919441	310.4626528	1203.543995	0.958525276
1.578857384	204.7177269	1062.92727	0.907316692
1.251739257	289.9911651	1941.417331	0.946931048
1.376470029	340.7800801	4017.914799	0.929307707
1.273405738	436.7776008	387.963224	0.940477969
1.187732649	184.944948	331.3678789	0.958488448
1.199425998	270.7696041	380.1034448	0.956357317
1.205348047	778.589421	1080.660164	0.956398938
1.179136696	500.8305789	2365.060415	0.958933118
1.827687197	194.9713579	4808.323249	0.877503834
1.322686103	443.7972995	1263.753696	0.934200075
1.161482474	306.4528422	554.3324224	0.962400227
1.240615738	217.082034	954.2225864	0.947642029
1.413024587	182.7240405	2981.54068	0.926873075
1.225824906	246.0468697	1700.531234	0.951057022
1.20074499	299.53591	639.7966302	0.955787066
1.561271389	436.0032233	7402.478654	0.905532669
1.238787045	252.808206	1090.611321	0.950651144
1.202711787	380.1993765	2069.66719	0.956330364
1.314953966	480.6981489	6442.176294	0.939232932
1.200644318	656.6372711	1749.498275	0.954715652
1.263457426	419.1561596	538.3638792	0.942077719
1.167926003	611.7736652	2939.891575	0.962014394
1.182703455	220.6985044	342.9343989	0.957254851
1.235151239	312.4876818	4171.238121	0.950485374
1.375480746	334.310151	5602.057288	0.927215877
1.23421235	261.1449797	1733.699565	0.94894566
1.209172926	166.5449829	412.2484371	0.955093512
1.346103175	497.4465655	3819.428308	0.934440618
1.215129367	270.0987064	1108.485405	0.953383676
1.170363109	309.5826003	817.15	0.962250712
1.556874887	267.4114652	2825.046873	0.910358142
1.267520371	227.8947494	734.4398936	0.943872793
1.21418335	597.180082	1825.174214	0.952038744
1.172266202	247.0020414	275.9033178	0.960214088
1.201735961	274.569796	1097.367086	0.954434156
1.344348933	288.6965216	1369.354927	0.931877216
1.212596001	539.728914	1167.531708	0.953495841
1.192989887	292.5197695	694.6770647	0.956436282
1.375847647	484.6550008	1757.653376	0.935705378
1.3344024	144.8320656	1437.768003	0.931173833
1.450484024	275.4757906	1967.311839	0.920488095
1.295109234	265.6533878	417.3372616	0.938623116
1.264325989	240.9155219	1050.633665	0.945065468
1.696771066	208.7055785	2490.716766	0.895957736
1.320804865	272.4163096	1328.622153	0.938798535
1.208714325	469.0239044	1134.042076	0.953099734
1.2443024	259.6616933	649.4416267	0.946585101
1.496291509	228.3184969	1769.422142	0.911432487
1.260195275	275.0849544	3614.219294	0.945213235
1.289008952	200.708381	2714.374695	0.940387701
1.272461809	281.9108468	2625.050799	0.942379343
1.195485141	441.8224535	2010.651329	0.957197293
1.237506223	233.6469841	2005.036066	0.949788916
1.136223532	367.9531451	277.0629897	0.969413052
1.231298253	140.1916482	218.8438738	0.947986149
1.30095508	608.5374301	6918.733555	0.939563639

log.sigma.2.0.mm.3D_glrlm_LongRunHighGrayLevelEmphasis	log.sigma.2.0.mm.3D_glrlm_RunPercentage	log.sigma.2.0.mm.3D_glrlm_LongRunLowGrayLevelEmphasis	log.sigma.2.0.mm.3D_glrlm_RunEntropy
675.1355722	0.939004901	0.003876172	5.012195926
297.9035914	0.944136555	0.013793692	4.568731961
370.0554461	0.93877199	0.009335653	4.839080286
313.7789865	0.940993789	0.014976598	4.658079032
365.591747	0.938014501	0.007988089	4.685087139
531.5539787	0.951684679	0.008637113	4.652211442
491.71955	0.843526243	0.007783378	4.650168783
522.6923814	0.907712591	0.005345865	4.891778423
344.9316786	0.893675775	0.008546136	4.644199918
375.3795344	0.944370861	0.006878216	4.578545905
268.357349	0.906317975	0.00966879	4.287013423
577.9780818	0.884468628	0.005045702	4.731220764
369.0666295	0.930664896	0.012451123	4.695680261
380.3423432	0.946727186	0.008845727	4.791566307
431.339675	0.922393162	0.006486476	4.869884478
442.8556333	0.873067578	0.007432401	4.711553418
333.7154031	0.917065947	0.008239029	4.412925262
271.9988128	0.940944141	0.013662024	4.494322328
421.2839001	0.904021938	0.006167081	4.334391534
918.1428542	0.911860545	0.002672524	4.716691591
195.4335615	0.915599319	0.016828285	4.470428716
208.7144536	0.948200993	0.019899714	4.565898082
185.7816732	0.924179763	0.031199537	4.6262035
250.7571015	0.861807917	0.01403999	4.347775408
501.9671059	0.861563518	0.009142829	4.886307438
317.825909	0.907608041	0.009480182	4.771886856
425.7354986	0.929717488	0.005975691	4.801381721
350.2209841	0.917375124	0.006968835	4.553389267
177.1767075	0.954162276	0.028217504	4.210794375
654.789784	0.92345915	0.003721326	4.547770992
283.9214751	0.911698678	0.00982313	4.623739557
396.2261375	0.944845763	0.009082491	4.667606466
346.7905438	0.899357602	0.008754293	4.71728935
344.8488465	0.903264851	0.008493876	4.604894424
591.0090515	0.906157743	0.004137636	4.745464644
450.8535591	0.932439402	0.007708076	4.892720652
654.3863114	0.952737078	0.004381834	4.896456409
694.0527317	0.844568076	0.005715637	4.831738575
221.291445	0.931456044	0.013154589	4.315576022
2043.671247	0.870036072	0.001333263	4.539989094
220.1171531	0.932835821	0.014481382	4.546119339
474.5088169	0.878824455	0.005708768	4.425188503
376.0311938	0.934370427	0.007962786	4.77233335
372.6671906	0.944745395	0.007058217	4.786542095
338.7884823	0.866373344	0.01047055	4.521993248
369.0835996	0.928363182	0.007396901	4.723555278
484.8575244	0.901686241	0.005298939	4.627554441
579.7202377	0.921666928	0.005601572	4.207978249
222.3447375	0.944520952	0.022797393	4.644842958
332.5572835	0.941797141	0.013623404	4.67022727
961.9829754	0.940589325	0.00260194	4.792333583
602.8904334	0.945891451	0.003397679	4.829645704
390.0694236	0.825013502	0.010345192	4.428227861
632.0321445	0.911693057	0.004289475	4.567577166
365.9463259	0.950831439	0.009178977	4.746802424
273.2799487	0.930272109	0.010349333	4.600674597
264.3224454	0.896311907	0.012481782	4.643405161
309.0888762	0.934539094	0.011187589	4.736974267
362.8370815	0.94102228	0.008417398	4.773126071
731.1754366	0.866611585	0.003873844	4.563454007
323.8161983	0.932742953	0.010578386	4.778635829
470.9678772	0.940926822	0.006037784	4.94926698
660.1028346	0.915705985	0.003635275	4.949769828
821.5547308	0.940142396	0.002602013	4.701349395
555.8264584	0.924099534	0.007441581	4.567388903
737.659134	0.94938502	0.002821472	4.957534033
272.937658	0.944486548	0.013987763	4.498035282
394.1283241	0.932970275	0.00680205	4.852031253
482.8898046	0.900752018	0.004996645	4.541112786
340.1715321	0.932130958	0.007934201	4.571941616
203.7572025	0.939962477	0.0275008	4.575645091
702.7894197	0.908637438	0.003622211	4.835278643
334.8335232	0.937424373	0.008091678	4.719252213
365.0919915	0.949149798	0.007369965	4.727270482
438.603392	0.870526633	0.007471104	4.631662845
298.9016918	0.925171778	0.008624445	4.424141547
757.9358591	0.936657729	0.002803722	4.636982605
301.1955996	0.947844725	0.016292472	4.611677882
344.0509183	0.940099444	0.008167075	4.660724672
400.1074796	0.907521368	0.006402921	4.510630206
672.6256152	0.937629938	0.003863199	4.98373957
364.6976148	0.942447383	0.00785869	4.642216756
698.2017765	0.906728241	0.004453832	4.843979832
197.8211587	0.908204315	0.014213267	4.34338282
417.0149875	0.888596118	0.006959075	4.668838034
381.4717001	0.918586122	0.011750545	4.206766492
308.9110304	0.92560574	0.009985519	4.736056584
369.667643	0.847938558	0.010360773	4.615456421
375.1739484	0.91484647	0.009339238	4.754601265
593.3508855	0.938101401	0.004063822	4.650467826
343.0102325	0.928913127	0.009401156	4.508802055
357.1808119	0.877050674	0.008839079	4.508577896
359.9775692	0.926187343	0.006804973	4.618489562
266.0063282	0.919483013	0.010961758	4.584365837
368.5852191	0.922588449	0.006094451	4.515255173
546.0215084	0.94259809	0.004694211	4.997955313
295.5343893	0.932174677	0.009607445	4.71932701
433.2378721	0.958712214	0.013571296	4.754151449
180.4277085	0.932548794	0.020475499	4.209774707
840.908493	0.917419276	0.00265783	4.795417032

log.sigma.2.0.mm.3D_glrlm_HighGrayLevelRunEmphasis	log.sigma.2.0.mm.3D_glrlm_RunLengthNonUniformityNormalized	log.sigma.2.0.mm.3D_glszm_GrayLevelVariance
567.9298009	0.887638518	59.39362301
251.882452	0.895232733	33.93241506
308.6040096	0.887613634	40.67235759
263.8591123	0.888722635	36.80530612
306.7643862	0.885213155	32.27877925
453.9926636	0.909902001	43.59957697
291.4653174	0.756981586	31.16411061
395.7607022	0.844473523	44.78007322
252.2327539	0.821379231	30.34026272
318.9976496	0.896358276	30.21689347
203.2712319	0.836101321	19.26929818
403.6908268	0.808361977	37.58485108
292.5032692	0.873552893	37.53494674
328.9117677	0.898441105	39.56873813
348.0290452	0.86397069	47.46129072
295.4362254	0.794584237	30.69222164
263.0943377	0.849628073	24.43347472
228.757368	0.8872078	28.14888889
314.6180756	0.832193308	22.43871094
694.785339	0.846039059	38.6477323
152.6762101	0.848347657	23.07836222
184.6638096	0.902295499	29.2810329
154.7393821	0.862735906	29.91159589
164.6383151	0.773274577	22.65128797
317.0497432	0.788124956	41.79432404
248.842328	0.839905374	40.89845212
351.4969427	0.872518922	41.20963436
277.5764251	0.851223613	30.40270361
156.2376722	0.916262408	28.40580199
521.9160747	0.862915477	28.02375274
218.9524293	0.844804858	26.99065315
337.2907916	0.897444807	35.16437098
257.1189842	0.82707695	33.85828362
257.5032227	0.834676895	32.0216434
446.738706	0.838847613	37.61088836
369.3175511	0.878884011	38.8204898
561.3537291	0.910907488	49.40907438
407.408718	0.766448127	38.20753857
185.4999027	0.87333049	19.54540864
1339.582395	0.787696893	30.24261517
182.8144557	0.876136745	25.12328439
328.3291897	0.796949098	29.80303754
310.5365844	0.880783218	32.67793354
321.8718991	0.896780657	40.00164086
223.2492959	0.787230355	28.7572263
303.9358466	0.870420511	33.03100579
363.7051319	0.832088092	32.44683338
462.3842996	0.856226441	23.35928398
191.8967729	0.89678735	32.15038773
281.9368451	0.892073134	37.92551829
810.9398665	0.892250329	43.27666276
519.9871813	0.897482754	41.71894907
220.206898	0.729076466	24.74789991
475.6470343	0.842828088	33.26513094
317.7198715	0.906022809	36.14458487
227.4198894	0.871767291	28.56809663
195.0521294	0.82743756	29.11431651
257.4141064	0.879741029	36.06189614
311.1500983	0.890549005	38.54219126
478.5406151	0.783087566	33.0682073
265.0861874	0.879087352	40.06071459
396.3736228	0.891888886	46.35016924
510.0082066	0.853703579	43.41458846
687.0618922	0.887596222	34.16179566
443.8397096	0.859570836	34.15546385
634.9376256	0.904893748	47.10678822
230.7135797	0.893714698	28.37706193
326.8825725	0.878593364	41.93903385
358.6882206	0.82743809	31.51306409
275.3677554	0.874960821	27.16147035
173.2379646	0.889925429	27.15969225
530.3367106	0.843239289	38.17522507
281.8155767	0.885163854	35.77390399
319.824325	0.905814033	39.66503125
291.2288836	0.793417317	35.50056683
240.3184951	0.863636908	24.42557636
626.6509229	0.881659077	32.52925875
257.0449718	0.900663307	35.0706855
287.2845382	0.887144009	31.59883118
307.1680718	0.837371063	34.88825365
563.6688027	0.885237397	57.75158152
305.7580678	0.891870181	34.64670463
515.9460129	0.847606912	39.59072169
153.9911293	0.835493578	20.46452091
296.9469434	0.814004827	31.49201517
285.531345	0.85335969	27.4771436
252.8415632	0.866796442	33.43707818
229.6986045	0.764548092	28.33262675
288.9261172	0.852877979	38.5205748
491.8784547	0.883949279	32.8881845
274.5127749	0.869264229	28.12746661
247.7985621	0.79530241	28.97889088
290.0007103	0.866467136	29.98413473
212.0182539	0.855931276	26.25705196
297.2157778	0.860011192	25.1884818
460.5042337	0.89408533	51.72585007
244.6705314	0.876747327	32.54871288
379.9145915	0.922913517	48.54554763
147.5571504	0.873842322	15.49057851
647.6373936	0.854071831	38.45576374

log.sigma.2.0.mm.3D_glszm_SmallAreaHighGrayLevelEmphasis	log.sigma.2.0.mm.3D_glszm_GrayLevelNonUniformityNormalized	log.sigma.2.0.mm.3D_glszm_SizeZoneNonUniformityNormalized
435.4314563	0.03639159	0.456465723
169.0743448	0.051753903	0.383067034
219.2099648	0.043146714	0.38953901
175.0699943	0.048265306	0.379693878
205.9807283	0.050372327	0.378467676
284.9658672	0.046731443	0.411593628
217.6731514	0.050297679	0.395610629
308.0626354	0.041361155	0.410349273
207.1749105	0.049760091	0.408452259
231.7060671	0.051140647	0.413608411
139.0305511	0.065565961	0.356614638
305.9557051	0.046370862	0.425212682
172.1196803	0.047687154	0.379220144
239.001229	0.046122505	0.404089168
305.4053741	0.041134296	0.456162716
199.9043616	0.050670721	0.357539944
190.1270182	0.060876904	0.38214162
144.1151226	0.057962963	0.366296296
212.0250195	0.059902344	0.362851563
464.4350018	0.047339745	0.403256306
105.9436575	0.058368335	0.324512007
155.3039523	0.053527697	0.454127447
130.7628083	0.05173433	0.392193131
135.9161623	0.05985055	0.37535071
231.3149725	0.043097089	0.394397196
226.1909274	0.04387806	0.410602507
277.6848007	0.043671208	0.424067688
218.4081133	0.051615759	0.411597923
137.6819797	0.05987519	0.477483555
353.9175695	0.055483305	0.342850626
164.398267	0.053526512	0.382879376
225.6898819	0.048941709	0.386335177
195.9851302	0.048298023	0.408376744
194.913449	0.049023913	0.407191068
350.8272517	0.045523244	0.412071814
251.6282059	0.044873606	0.389221557
365.9306072	0.042311964	0.442720388
284.909537	0.044982616	0.377384301
147.313184	0.068891507	0.373411329
975.3430948	0.051159796	0.411122461
123.0941049	0.054499609	0.34579286
278.2408939	0.053580924	0.420152693
221.0309157	0.048528576	0.390738163
258.2244847	0.043788451	0.434227414
192.8373736	0.053032526	0.423468512
237.772496	0.048868772	0.406392057
291.6886528	0.050612107	0.403902915
317.8313444	0.069403187	0.37880483
154.8543159	0.050250553	0.432098765
190.2895249	0.047505671	0.385548393
631.0183826	0.04233367	0.456854963
398.9400938	0.043192638	0.435633548
171.1695274	0.056092458	0.401531074
304.4050352	0.05186653	0.413283418
222.911843	0.046794035	0.415707448
174.2844146	0.054313789	0.349777099
156.211058	0.050423457	0.390186248
186.9611788	0.04636631	0.363692852
242.5805433	0.045830203	0.427862396
370.4494816	0.050733215	0.409048465
198.9698864	0.045484345	0.420346247
284.9523737	0.041563191	0.44483384
361.6082491	0.041645947	0.422183985
450.2007694	0.049194465	0.378208445
313.989388	0.050794279	0.415682062
459.5149513	0.040203637	0.437067088
153.9943137	0.05509735	0.367563548
251.4519515	0.042914368	0.416748801
267.5312205	0.05115178	0.40123952
164.9850994	0.053206699	0.346086842
128.3574794	0.053897959	0.380580055
394.1312417	0.045328878	0.399330671
206.5700928	0.046853303	0.380201101
263.4233627	0.044458255	0.464531433
232.0163808	0.047546085	0.428948052
171.2463513	0.059320914	0.379161712
387.2940704	0.049939381	0.338490339
180.7239304	0.051066018	0.430085835
190.9623867	0.049894076	0.383025787
259.8931725	0.048767742	0.422283737
434.0181244	0.03703861	0.449911633
209.0540851	0.047836126	0.412498422
376.2122058	0.044109998	0.397415687
121.7688947	0.062430385	0.3388909
219.4229532	0.049259285	0.364141546
142.7769679	0.058238636	0.393401343
209.6397188	0.048506708	0.434709056
197.0218902	0.052702161	0.383756835
220.3452905	0.04556867	0.425209434
323.3775417	0.050368242	0.400372023
179.7052798	0.053790106	0.399676433
218.3206746	0.05274295	0.443793754
203.7214784	0.050347157	0.392084245
153.939707	0.053856729	0.359323794
221.4806854	0.056181309	0.384494223
336.9863418	0.03852357	0.424876839
177.9205037	0.048622196	0.386553721
261.3330984	0.041551064	0.450389748
97.64625627	0.075206612	0.321157025
415.208961	0.04584672	0.389282569

log.sigma.2.0.mm.3D_glszm_SizeZoneNonUniformity	log.sigma.2.0.mm.3D_glszm_GrayLevelNonUniformity	log.sigma.2.0.mm.3D_glszm_LargeAreaEmphasis	log.sigma.2.0.mm.3D_glszm_ZoneVariance
310.8531571	24.78267254	16.16446402	11.66832054
63.20606061	8.539393939	8.466666667	4.043930211
155.4260652	17.21553885	11.96491228	6.967054227
53.15714286	6.757142857	12.48571429	7.195714286
220.6466552	29.3670669	25.60034305	19.74278073
50.62601626	5.74796748	8.991869919	5.120893648
1036.89546	131.8302175	2441.498283	2425.329968
865.8369668	87.27203791	207.7004739	199.4300793
530.1710324	64.58859784	256.4021572	245.6206989
146.8309859	18.15492958	9.670422535	5.147312041
165.8258065	30.48817204	59.2688172	48.07151809
454.9775701	49.61682243	303.028972	292.8904018
123.6257669	15.54601227	18.85582822	12.66702736
124.0553746	14.15960912	10.33876221	5.938333563
294.6811146	26.57275542	42.08204334	36.13780684
558.8349328	79.19833653	829.3410109	815.7507263
116.1710526	18.50657895	43.04934211	34.61261037
65.93333333	10.43333333	11.21666667	6.05367284
116.1125	19.16875	87.3125	75.62464844
1244.045705	146.0431118	169.8589951	160.9984829
193.7336683	34.84589615	53.16247906	41.04694887
111.2612245	13.11428571	9.559183673	5.46062474
89.81222707	11.84716157	18.84716157	12.90974619
325.4290657	51.89042676	715.9250288	698.7125793
159.7308642	17.45432099	182.4345679	173.240945
356.8135788	38.13003452	90.12197929	81.47066387
338.406015	34.84962406	28.04010025	21.73358208
325.573957	40.82806574	95.27054362	87.1577689
36.76623377	4.61038961	7.74025974	4.145049755
199.1962134	32.23580034	32.49913941	24.82023101
480.8964968	67.22929936	72.17117834	61.98308692
140.2396694	17.76584022	11.7630854	7.050717544
327.9265255	38.78331258	118.0660025	109.6104366
306.6148738	36.91500664	170.2350598	159.9491119
700.9341564	77.43503821	157.3691946	148.2630634
325	37.46946108	21.20598802	15.81358098
346.6500639	33.1302682	8.114942529	4.386048355
615.5137952	73.36664623	1090.326793	1077.589203
81.40366972	15.01834862	23.70642202	17.10765087
1058.229215	131.6853147	1468.793706	1455.323609
187.0739372	29.48428835	15.00184843	8.866841373
694.5124017	88.569268	671.4797338	659.7056605
394.6455446	49.01386139	17.81584158	12.08432114
289.6296852	29.20689655	14.62068966	10.08832315
179.9741176	22.53882353	274.8235294	261.4193163
389.3235908	46.81628392	38.84864301	32.56728854
640.1861199	80.22018927	155.9249211	144.5273747
60.98757764	11.17391304	31.79503106	22.49442537
81.66666667	9.497354497	9.91005291	5.630189524
81.3507109	10.02369668	9.744075829	5.155140271
275.9403974	25.56953642	19.09768212	14.55739606
581.5707865	57.66217228	11.22771536	6.875725568
681.7997644	95.24499411	2815.954064	2793.889522
216.1472275	27.12619503	108.3059273	98.44896885
132.1949686	14.88050314	9.669811321	5.581276453
154.2517007	23.95238095	32.39002268	25.27891156
515.4360333	66.60938683	132.2172597	122.9795254
301.1376812	38.39130435	19.02777778	12.79588059
155.3140496	16.63636364	17.3030303	12.88492741
1231.23588	152.7069767	1298.84485	1285.748146
241.2787456	26.10801394	20.27351916	14.91276148
539.1386139	50.37458746	15.70379538	11.56733749
1355.210592	133.6834891	129.8093458	123.2295614
335.8490991	43.68468468	24.54279279	18.97149176
110.5714286	13.5112782	25.07894737	18.6013483
750.8812573	69.06984866	9.228754366	5.263602464
63.22093023	9.476744186	12.97674419	7.404948621
888.5084428	91.4934334	34.88602251	29.19739479
865.8748842	110.3855422	211.9675626	199.8630962
282.4068627	43.41666667	20.52696078	13.75165802
83.34703196	11.80365297	10.98630137	5.9392006
676.8654867	76.83244838	165.9781711	157.342548
215.9542254	26.61267606	14.57570423	9.051549172
222.0460251	21.25104603	10.17991632	6.229967963
499.2955326	55.34364261	699.3556701	687.0454588
131.9482759	20.64367816	19.98850575	13.0298256
298.2099886	43.99659478	17.31101022	11.02406588
68.3836478	8.119496855	8.132075472	4.005300423
224.0700855	29.18803419	12.54188034	7.488991161
251.2588235	29.01680672	122.0268908	112.8749947
292.8924731	24.11213518	16.90015361	12.2356153
151.386921	17.55585831	12.0599455	6.994379645
344.5594002	38.24336794	99.0911188	92.16335479
181.6455224	33.46268657	193.2033582	180.7303374
301.8733414	40.83594692	224.1435464	213.4177239
69.23863636	10.25	70.53977273	61.43720945
230.8305085	25.75706215	30.92278719	24.85505442
361.4989384	49.64543524	764.9171975	748.3344918
283.6146927	30.39430285	59.23388306	52.73023608
230.213913	28.96173913	16.1373913	10.48540189
116.3058419	15.65292096	35.67697595	28.04343359
309.3242468	36.76183644	359.3055954	346.1090017
690.8524404	88.71169126	36.52270148	29.99731531
431.5478768	64.68193172	52.2764363	44.04369381
458.3171141	66.96812081	62.05788591	54.36098529
470.7635379	42.68411552	12.25270758	7.623219383
386.1671672	48.57357357	23.02502503	17.00067836
78.36781609	7.229885057	5.304597701	2.068734311
35.32727273	8.272727273	11.49090909	5.555041322
1180.304749	139.0072559	129.9564644	121.4866751

log.sigma.2.0.mm.3D_glszm_ZonePercentage	log.sigma.2.0.mm.3D_glszm_LargeAreaLowGrayLevelEmphasis	log.sigma.2.0.mm.3D_glszm_LargeAreaHighGrayLevelEmphasis	log.sigma.2.0.mm.3D_glszm_HighGrayLevelZoneEmphasis
0.471606648	0.038229845	7986.948605	609.6784141
0.475504323	0.066274543	2192.060606	258.0242424
0.447309417	0.068097053	3668.721805	326.8671679
0.434782609	0.099502886	3440.778571	269.5857143
0.41318214	0.154487306	5496.850772	325.1406518
0.508264463	0.032603778	4240.146341	437.1219512
0.248695322	9.150349914	656576.2877	323.9252194
0.347725775	0.616272634	71994.0673	448.2758294
0.304551854	1.199306711	56815.93143	301.751926
0.470198675	0.04319155	2958.495775	332.8788732
0.298843188	0.368277149	10625.09462	219.7505376
0.31405929	0.824795703	113387.0813	447.2878505
0.401972873	0.101863802	5973.745399	275.9815951
0.476708075	0.066180882	2688.04886	352.52443
0.41015873	0.154313851	12746.23684	408.5789474
0.271259979	3.226645172	216928.8548	316.0492642
0.344280861	0.210051223	10075.44079	291.2828947
0.4400978	0.100517709	2220.761111	239.3666667
0.29250457	0.323123354	25098.17813	339.05625
0.335946858	0.258494144	114417.4784	706.313128
0.287295476	0.5767875	7526.80402	172.9664992
0.493951613	0.139198292	1364.077551	209.4979592
0.410394265	0.330771597	2079.458515	184.1484716
0.241034195	5.119303365	102140.083	203.5801615
0.32980456	0.648485788	53673.20741	343.6098765
0.339984351	0.517181909	17491.92635	316.7871116
0.398203593	0.121018134	7704.853383	395.2907268
0.351087439	0.445642877	21430.92288	317.94311
0.52739726	0.106818157	971.2987013	177.7532468
0.360869565	0.076844076	15013.08606	567.2065404
0.313295086	0.458983488	13600.29299	248.5406051
0.460659898	0.067865514	3562.460055	348.3471074
0.343897216	0.558589683	26489.94022	288.3462017
0.311801242	0.800021112	39252.85392	287.0451527
0.331385155	0.416656902	61498.01705	506.5714286
0.430634348	0.092160088	6956.366467	387.3245509
0.517857143	0.022845079	4828.462324	542.4418902
0.280192407	2.81250486	426113.4237	435.685469
0.389285714	0.221670493	3192.743119	218.8990826
0.27246745	1.128347746	1915046.729	1433.929681
0.403731343	0.166151441	2211.462107	199.792976
0.291431594	2.309845203	196647.1349	388.8058076
0.417700579	0.110605761	4442.135644	333.580198
0.46971831	0.072687831	3790.955022	358.4662669
0.273136247	1.443444201	53948.58353	268.0235294
0.399000416	0.205746591	8586.836117	349.3361169
0.296206317	0.53582019	47906.00757	428.5949527
0.32790224	0.086959336	13542.70186	493.5217391
0.483375959	0.13220069	1584.656085	212.6190476
0.466814159	0.10191188	2525.526066	289.6966825
0.469308469	0.030221634	13546.28974	873.2698675
0.47935368	0.030583174	4943.916105	561.4906367
0.212888666	13.8773933	576517.0501	255.3297998
0.318514007	0.232096239	53206.11281	468.1032505
0.494556765	0.071005426	2512.022013	328.4685535
0.375	0.284787876	4669.401361	265.9795918
0.329016189	0.876651883	21567.75019	231.3739591
0.400580552	0.143475069	3932.873188	284.1243961
0.475753604	0.094342344	3952.506887	347.3526171
0.276324245	2.851480107	594663.8252	540.2156146
0.431903687	0.115972206	4882.074913	282.4216028
0.49168357	0.055771209	5561.448845	411.5321782
0.389846976	0.293181745	59184.85358	533.147352
0.423664122	0.04792916	13906.75113	704.8434685
0.392909897	0.07966384	10608.59398	466.0451128
0.502192341	0.019304586	5596.798603	655.9150175
0.42364532	0.12066722	2991.197674	239.4709302
0.41927237	0.153494883	9057.619606	363.3606942
0.287426745	0.698957001	66912.75996	399.8577386
0.384180791	0.099879969	5759.209559	275.7647059
0.445121951	0.197889318	1465.424658	194.3835616
0.340293114	0.338072315	84330.04425	580.0896755
0.425468165	0.09367611	3229.15493	312.9260563
0.503157895	0.054976734	2540.112971	357.2949791
0.285014691	2.701360622	183245.9734	335.1116838
0.379084967	0.118375476	4239.45977	260.3304598
0.398822997	0.03427883	10008.45062	641.8513053
0.492260062	0.094103999	2094.616352	262.1509434
0.44486692	0.062968353	3514.078632	293.6512821
0.330555556	0.479828122	32068.2437	363.6857143
0.463015647	0.039874088	8307.883257	608.7388633
0.444309927	0.056826032	3736.495913	311.2752044
0.379929886	0.213196572	48611.98039	556.8777393
0.283148442	1.745371293	22459.3806	189.6100746
0.3053407	0.896706422	57560.13028	337.0036188
0.331450094	0.234441769	24285.5625	237.3977273
0.405963303	0.209195132	5757.774011	290.7645951
0.2455683	3.898350974	152877.2495	288.7335456
0.392122281	0.250944964	15378.09145	313.1754123
0.420629115	0.043042978	7572.045217	496.9721739
0.361940299	0.156304757	10390.1134	275.6219931
0.275276461	1.715156565	78049.87231	304.7675753
0.391468563	0.16046128	9320.934166	308.5822928
0.348520023	0.347078351	9358.827644	238.4796003
0.360447536	0.293291213	14126.30285	333.2911074
0.464765101	0.039821632	5185.674188	482.400722
0.407422512	0.165117262	4275.43043	269.1971972
0.555910543	0.045255704	2077.902299	376.4885057
0.410447761	0.182525146	1516.436364	159.9272727
0.343608341	0.211583371	83279.77408	646.7971636

log.sigma.2.0.mm.3D_glszm_SmallAreaEmphasis	log.sigma.2.0.mm.3D_glszm_LowGrayLevelZoneEmphasis	log.sigma.2.0.mm.3D_glszm_ZoneEntropy	log.sigma.2.0.mm.3D_glszm_SmallAreaLowGrayLevelEmphasis
0.702084003	0.004489133	6.435001857	0.003773347
0.64164634	0.017633078	5.90889947	0.014945514
0.647971707	0.010358911	6.338281504	0.008115511
0.638817011	0.017925829	5.817161996	0.014173666
0.637721057	0.009124147	6.24754094	0.007337022
0.662237145	0.01285823	5.663463184	0.011781127
0.653821512	0.005865782	6.471651223	0.004120869
0.665561005	0.005154441	6.605161473	0.003862394
0.664213352	0.007015915	6.30812393	0.004827473
0.668228397	0.007764868	5.988479049	0.006195467
0.620272656	0.009862755	6.080235188	0.007446119
0.6780263	0.00484713	6.368606572	0.003877318
0.638791206	0.017438643	6.147373783	0.014296411
0.659535436	0.010382298	6.070637673	0.0087067
0.702003522	0.006203323	6.278161421	0.004666166
0.620032784	0.006017502	6.516490224	0.004038797
0.641400661	0.009970117	5.926700322	0.008279887
0.623281103	0.016636893	5.75834551	0.013887993
0.624398296	0.007131642	5.995620736	0.005774113
0.660372369	0.003139137	6.617045687	0.002582265
0.588538735	0.015407911	6.318836359	0.010065626
0.700636704	0.021270469	5.728305452	0.017123784
0.649337492	0.028307888	5.91914092	0.015443544
0.636429794	0.012378774	6.215500212	0.008952027
0.65200416	0.009386154	6.247189321	0.007237418
0.666439756	0.008221799	6.417461425	0.006076976
0.677368853	0.006029563	6.392722868	0.004706457
0.666832281	0.006754731	6.206175611	0.005094841
0.717523346	0.04034449	5.193680171	0.037422715
0.604298411	0.004365279	6.244409197	0.003492993
0.643477952	0.008647349	6.37960738	0.006196741
0.643161016	0.008615898	6.054118341	0.004836899
0.664056006	0.008762129	6.278855353	0.006831806
0.662269624	0.007969056	6.240421495	0.005846222
0.667387581	0.003790379	6.466544546	0.002869654
0.645897857	0.007971571	6.3839162	0.005981462
0.691027492	0.00511212	6.303514192	0.004236595
0.63664998	0.004627345	6.562575422	0.003301168
0.632291313	0.013928393	5.669045492	0.010585098
0.66665977	0.0011484	6.385038306	0.000888611
0.606408544	0.013806883	6.23559995	0.009544684
0.673994038	0.004309104	6.259897426	0.003059548
0.649134032	0.007480064	6.336978304	0.005304718
0.684451447	0.007212705	6.21654377	0.005495124
0.675839586	0.010141734	5.985598902	0.007949186
0.661898108	0.006443928	6.247729491	0.004088936
0.660631596	0.0042153	6.411375688	0.002612834
0.637871897	0.009376142	5.541958721	0.008547648
0.682338323	0.02473646	5.760854356	0.018260113
0.642740575	0.014979277	5.964292447	0.011816934
0.702483658	0.003097303	6.180210126	0.002663938
0.686522124	0.003299446	6.401772915	0.002518007
0.657568899	0.007518431	6.214989317	0.00549149
0.668426984	0.005860851	6.187333401	0.005046827
0.669622716	0.009587761	5.986560043	0.00730366
0.61155032	0.01006744	6.223588809	0.007354732
0.648607759	0.010791247	6.343638464	0.007637448
0.62382249	0.012090635	6.443542912	0.00844157
0.678793722	0.00919555	6.047981627	0.007352479
0.664576494	0.002888125	6.465018641	0.002074771
0.673925297	0.012645383	6.278852239	0.010213337
0.693382104	0.006668095	6.395321252	0.005437807
0.67570932	0.003296767	6.594289126	0.002411563
0.637544347	0.002962164	6.41781728	0.002349478
0.669596565	0.007955369	5.975571967	0.004261611
0.68725498	0.002835805	6.482787606	0.00217308
0.628229827	0.015263625	5.753397697	0.01127067
0.670852304	0.006795606	6.515899999	0.005147686
0.658117579	0.004567349	6.444800075	0.003380475
0.608696546	0.009157244	6.384479528	0.007040932
0.639127805	0.029036571	5.848317326	0.019805281
0.656520408	0.003609107	6.50494394	0.00275529
0.639351888	0.007943088	6.314492715	0.005906072
0.708654239	0.007817472	6.099558683	0.006396775
0.681019092	0.006602468	6.306627586	0.00510285
0.638626379	0.00915057	5.987441534	0.007049637
0.60037697	0.00319573	6.511338753	0.002472517
0.679979033	0.017996493	5.719250708	0.014454089
0.641980605	0.009233816	6.281573186	0.007059334
0.675834283	0.006350948	6.190054185	0.004895004
0.696965344	0.004486413	6.426656511	0.003770271
0.667909461	0.009317524	6.098900904	0.007530864
0.654874153	0.003702596	6.385622838	0.001947914
0.601765279	0.012627904	6.131341053	0.008222373
0.624988831	0.006069596	6.380173297	0.004336844
0.651465262	0.021565596	5.76740491	0.019233093
0.684997779	0.009693669	6.097413661	0.006954336
0.643462903	0.00765022	6.328636165	0.005526636
0.67792149	0.011677236	6.266055543	0.009862191
0.657190791	0.004771565	6.204932945	0.003874428
0.65706311	0.01202988	5.974167727	0.009849406
0.692762006	0.008494433	6.035417439	0.006872059
0.650103356	0.006979902	6.37881923	0.005231947
0.621086635	0.010846838	6.412264853	0.00780664
0.643972973	0.005449148	6.240737021	0.003913162
0.677823704	0.004890342	6.520642815	0.003766958
0.645593197	0.009340838	6.376399098	0.006800975
0.697555289	0.013240909	5.956160074	0.007749346
0.583553591	0.020985209	5.479982983	0.015660509
0.647742763	0.002727286	6.653492643	0.00203856

log.sigma.2.0.mm.3D_ngtdm_Coarseness	log.sigma.2.0.mm.3D_ngtdm_Complexity	log.sigma.2.0.mm.3D_ngtdm_Strength	log.sigma.2.0.mm.3D_ngtdm_Busyness	log.sigma.2.0.mm.3D_ngtdm_Contrast
0.005149665	2.063901836	2.891029322	0.181509383	0.000129911
0.014298476	3.640305236	4.35896452	0.145923951	0.00074363
0.0075692	1.522922891	2.168199566	0.274274787	0.000303041
0.016266092	3.71200664	4.445992117	0.140131253	0.001053356
0.004416699	0.804640957	1.232924935	0.498376584	0.000161798
0.02679858	5.627810645	8.813283707	0.056051402	0.001068501
0.000834019	0.118204803	0.398419641	1.461780895	5.18E-06
0.001280887	0.364117612	0.645218655	0.86746775	2.01E-05
0.001885235	0.265253974	0.608881603	0.944220407	2.38E-05
0.0083611	1.412361875	2.126560932	0.254426785	0.000276233
0.005069666	0.325328715	0.887702903	0.537924501	5.95E-05
0.002514965	0.42806782	1.011484272	0.448413725	2.60E-05
0.007829352	1.456994291	2.355278688	0.230962911	0.000288144
0.009417678	2.301379629	2.784661786	0.218118954	0.000451597
0.004437095	1.271461445	1.824393699	0.344089433	0.000125957
0.001520636	0.185375015	0.482046242	1.046464981	1.78E-05
0.008529988	0.992098097	2.519144465	0.219593273	0.000104066
0.013623964	2.160686604	3.345115307	0.184744623	0.000592079
0.006899554	0.61644014	1.641209892	0.277753209	8.18E-05
0.000856236	0.347939123	0.8838365	0.621817244	5.59E-06
0.003337693	0.301758637	0.695187961	1.000639127	6.76E-05
0.011723899	1.992163532	2.924636428	0.278546412	0.000515072
0.012667236	1.298933935	2.378158983	0.326940481	0.000424492
0.002395887	0.156158557	0.595774422	0.974452371	1.62E-05
0.006379946	0.934577693	1.768299414	0.277339607	0.000144351
0.003035127	0.623731372	1.451745258	0.567809948	4.76E-05
0.003537946	0.971719107	1.635918247	0.400013559	7.58E-05
0.003500077	0.553735362	1.338053657	0.453582119	4.22E-05
0.041811156	4.228475883	7.502942234	0.094273514	0.001519047
0.004455352	0.692214279	1.543318814	0.311608582	7.54E-05
0.001832262	0.256792115	0.532346615	1.251596759	3.08E-05
0.006471557	1.579008658	1.859368735	0.327207135	0.000420155
0.003482617	0.510688268	1.16308043	0.524815452	5.09E-05
0.003241816	0.403721515	0.926039472	0.604623458	5.30E-05
0.001501775	0.393318454	0.873803728	0.649165516	1.65E-05
0.0034262	0.832031067	1.125526842	0.478449616	0.000123928
0.00377727	1.94287964	2.277307725	0.244168062	0.000163696
0.00154747	0.290614601	0.780721233	0.63804688	1.34E-05
0.012147246	0.962469423	1.908603777	0.27984766	0.000275962
0.000871017	0.157062231	0.564439199	0.611146754	5.62E-06
0.0048565	0.538882635	0.937957656	0.746711604	0.000157587
0.001422039	0.22017811	0.694593731	0.827894022	1.01E-05
0.002647955	0.544118905	0.834624405	0.758418904	9.03E-05
0.004613516	1.155519321	1.544487447	0.415644326	0.000158159
0.005531311	0.540015174	1.515910546	0.327879674	4.64E-05
0.002969836	0.504444975	0.893090054	0.692318731	7.27E-05
0.001493504	0.324457697	0.858912011	0.706831861	1.26E-05
0.013818912	1.237593834	3.593497535	0.137395471	0.000256555
0.017176973	2.12940329	3.318259711	0.215665223	0.000749908
0.01289173	2.698894173	4.147986286	0.152405703	0.000594108
0.005048309	1.549317889	2.161955564	0.201365573	0.000163549
0.002426833	0.929581243	1.424817173	0.434822847	5.60E-05
0.00110583	0.082229162	0.335787925	1.568285182	5.84E-06
0.004668106	0.950076166	2.325200733	0.2022846	4.52E-05
0.008467019	1.926184928	2.307050161	0.288363654	0.000611902
0.005857366	0.954937641	1.974967182	0.365829076	0.000115555
0.00195148	0.22652012	0.502105248	1.270927627	3.18E-05
0.003052014	0.78109886	1.228765473	0.637872957	8.94E-05
0.008384823	2.178779616	2.71094788	0.226452674	0.000296912
0.000747812	0.17231825	0.579917913	1.015593519	4.04E-06
0.005025943	1.466840925	2.228059621	0.323513901	0.000122255
0.002764918	1.062858598	1.375721528	0.434306585	7.62E-05
0.000948431	0.250450058	0.406337968	1.141105352	1.84E-05
0.003204591	0.931194833	1.611685849	0.285057755	6.08E-05
0.011382031	1.562265018	3.44588431	0.125118258	0.000203367
0.001901228	0.984817101	1.138494133	0.44939426	5.48E-05
0.013039047	1.919015874	2.577372915	0.229700233	0.000841112
0.001377798	0.484230962	0.756527121	0.929952757	2.77E-05
0.000949919	0.275177032	0.743676938	0.963498422	6.66E-06
0.003138189	0.438160357	0.810101963	0.67512899	7.81E-05
0.012711689	1.413279964	2.207054294	0.325007838	0.000614034
0.001651308	0.405088722	0.828439229	0.57650592	2.12E-05
0.004627141	1.055596343	1.54892632	0.473584184	0.000166469
0.006366362	1.732274838	2.18256736	0.326639071	0.000264528
0.001994567	0.358528755	0.981115326	0.645172412	1.86E-05
0.007891314	0.884991299	2.057888226	0.290213733	0.000138332
0.003179875	0.730937774	1.46833895	0.312425279	5.28E-05
0.015121057	3.16510963	4.053315709	0.170157883	0.00124436
0.005244851	1.002639819	1.693157758	0.334935601	0.000120508
0.004310495	0.797945589	2.161002523	0.304918206	4.50E-05
0.005288204	1.992127508	2.876604026	0.182042154	0.000131268
0.008218311	1.572994095	2.587580737	0.220084413	0.000218421
0.003100882	0.648452388	0.975089198	0.464401663	8.99E-05
0.003888597	0.287186386	0.668404958	0.874302256	5.95E-05
0.00313225	0.379101921	0.922961117	0.54578875	3.85E-05
0.009970604	1.080639759	2.495180764	0.208199311	0.000365518
0.005527096	1.016046016	1.773242577	0.380506642	0.000125922
0.00233929	0.224312638	0.746642677	0.756709605	1.79E-05
0.004848177	0.801945079	1.640216804	0.319806142	7.58E-05
0.004543336	0.995909204	1.595955281	0.308933511	0.000138374
0.008471411	1.080241465	2.142940204	0.233790985	0.00018974
0.003180547	0.29527101	0.8132518	0.62786936	3.64E-05
0.001689492	0.318487323	0.640671592	0.898720816	2.31E-05
0.00223299	0.312636256	0.699922058	0.938989889	3.13E-05
0.002074563	0.365346732	0.773288763	0.834563875	3.23E-05
0.002819778	1.070171995	1.35377294	0.430607638	9.74E-05
0.002864228	0.596017978	1.039672487	0.695789579	6.26E-05
0.017414909	6.089661991	6.519697921	0.089664677	0.001047202
0.024639143	1.087762575	2.46711365	0.231701634	0.000870807
0.000876037	0.264768423	0.561736972	0.837935288	1.00E-05

log.sigma.1.5.mm.3D_gldm_GrayLevelVariance	log.sigma.1.5.mm.3D_gldm_HighGrayLevelEmphasis	log.sigma.1.5.mm.3D_gldm_GrayLevelNonUniformityNormalized	log.sigma.1.5.mm.3D_gldm_DependenceEntropy
38.84297235	529.6925208	0.052813821	6.650331531
24.5653398	257.3256484	0.062013637	5.949083518
28.22992997	336.7993274	0.052796155	6.488173432
29.96397708	333.3944099	0.057096563	6.103083847
25.11946481	270.9489724	0.05616044	6.379390124
32.29717915	408.5289256	0.055802199	5.922963682
14.34434892	357.8259797	0.113917292	6.708037343
26.48950944	616.2476928	0.069000026	6.773910618
17.41722656	317.8887846	0.084506363	6.563073785
27.17083286	556.4145695	0.055515109	6.270403683
11.40234171	257.1825193	0.094107064	6.182737238
21.51759327	725.8614617	0.085028674	6.658655703
27.40282886	328.9025894	0.060768928	6.463183025
33.52799853	418.3944099	0.048937155	6.228672786
32.06554558	357.575873	0.062805543	6.657952783
17.10838142	257.390663	0.097091068	6.669344115
15.85239756	405.7248018	0.077206425	6.314893352
20.11411936	303.2420538	0.065404917	6.01307421
13.8963701	431.4149909	0.086807215	6.321928374
22.03026743	968.7031471	0.070168898	6.621901855
13.1907698	147.8758422	0.078206908	6.308866877
24.562939	209.983871	0.057272698	6.098419337
22.80516694	204.5232975	0.062531314	6.274232809
10.80186984	217.6833472	0.123810663	6.361281207
24.76544579	321.4576547	0.09671588	6.701149059
23.02850132	304.0105634	0.072479128	6.64675582
28.65219162	476.7644711	0.057890208	6.530854377
17.74305049	250.0044385	0.080766405	6.423463277
19.969225	171.630137	0.07149559	5.37607623
18.60093553	719.5347826	0.070547433	6.339083576
17.59747341	221.2629085	0.069695331	6.580137231
28.37129757	349.6814721	0.05275516	6.267260566
19.68981911	288.4672377	0.076587632	6.61200517
19.79034142	260.2136646	0.090747699	6.470660778
22.23493802	532.5300994	0.069838801	6.688234962
29.99883768	334.4734399	0.051901458	6.64145955
40.75007305	487.5747354	0.049636418	6.476743648
20.12967739	528.6201684	0.114456685	6.713951202
15.34910395	279.4696429	0.074789541	6.021703074
14.77597502	1646.492961	0.110578873	6.512049233
18.6661233	244.1813433	0.064748274	6.305321149
15.88550194	480.6558533	0.103999915	6.481114887
23.42335024	337.6129032	0.05685406	6.537633064
31.74146548	327.8964789	0.05196588	6.537390265
13.98261766	284.1793059	0.100333893	6.502645931
22.8894562	369.5997501	0.062695139	6.524584367
18.39935032	439.1590357	0.085031534	6.53593601
13.29418743	655.8289206	0.085112473	5.98989013
27.07395948	244.8439898	0.056782726	6.074641267
29.12774591	380.8561947	0.056562769	6.262733552
33.91700159	1078.105672	0.055290216	6.411633214
30.58021061	598.2685817	0.053247166	6.51016849
10.54503793	249.2932548	0.136277187	6.51604676
18.20041066	771.9403167	0.079166549	6.49276966
33.36332172	346.9782271	0.049802999	6.307415507
18.34736611	254.4243197	0.067168194	6.225474357
17.44779332	221.8273973	0.082117402	6.591982552
25.28043489	325.791969	0.056368782	6.45999902
29.89381434	432.4141547	0.055215925	6.361359573
16.75279806	521.3331497	0.105466965	6.544024522
28.08962197	403.6734387	0.057739006	6.644126081
38.7126405	654.3363083	0.04710079	6.698075177
30.28291901	642.4336896	0.05719453	6.861764259
25.05432899	1028.00334	0.057540535	6.447119621
17.9931883	482.5007386	0.078517833	6.241856195
42.05093273	849.4346682	0.044409493	6.681535614
25.99820428	217.4729064	0.060617826	6.104126046
30.94320307	443.6202557	0.054404562	6.699389921
17.82709565	665.6407832	0.08270592	6.512549653
19.03939086	371.6266478	0.0656558	6.327401731
23.92594934	259.4735772	0.05883568	6.343235834
24.75496701	576.4248143	0.06703002	6.667790691
25.64855728	285.4981273	0.056812692	6.467984952
32.80166205	364.8084211	0.054313573	6.239114276
18.90570258	359.5088149	0.101665226	6.629269252
16.90034697	236.379085	0.073122398	6.215204945
24.1909465	955.3110005	0.061724322	6.476640201
31.28898005	368.3467492	0.051922284	6.042716347
23.48463387	461.0737643	0.058863942	6.374387165
18.77777747	279.2594444	0.085640123	6.398791539
36.87286755	525.3897582	0.054219976	6.61161538
24.44203959	454.9794189	0.059999179	6.39337629
26.85444829	719.1761613	0.072049212	6.678328521
12.22465005	183.6782884	0.08788321	6.281573822
17.34902055	327.0441989	0.086695902	6.602625397
14.52832839	321.4708098	0.101819046	5.886974581
21.52604064	223.5657492	0.063947806	6.520578097
13.42373986	395.4040667	0.112001471	6.616759354
23.96824153	615.9077014	0.069364392	6.622771022
25.85479125	782.849305	0.058079755	6.333516688
19.89832554	303.5945274	0.072464172	6.288881222
14.00800746	348.8459716	0.099196322	6.461421277
19.88618234	258.3179293	0.067341178	6.531006018
18.04996052	235.1889147	0.070718479	6.488935709
18.60080673	404.8593892	0.06915415	6.484295144
40.17159119	547.3091443	0.045751388	6.716473536
23.13982992	334.8955954	0.060613036	6.509463362
42.52420664	481.2460064	0.04888281	6.154027347
12.05040098	123.8731343	0.081727556	5.816867998
23.68109346	797.2194016	0.064240879	6.689663104

log.sigma.1.5.mm.3D_gldm_DependenceNonUniformity	log.sigma.1.5.mm.3D_gldm_GrayLevelNonUniformity	log.sigma.1.5.mm.3D_gldm_SmallDependenceEmphasis
343.232687	76.26315789	0.42403375
91.6685879	21.51873199	0.439552465
207.8609865	47.0941704	0.400163541
85.05590062	18.38509317	0.437280883
334.4727144	79.24238129	0.377770168
67.85123967	13.50413223	0.477681898
1177.444255	1200.574343	0.24169873
1064.801252	418.6921556	0.346155484
678.470671	360.1661192	0.309098659
195.1298013	41.91390728	0.428425328
276.5732648	146.4305913	0.303245529
514.737012	289.6926915	0.320634497
175.7644883	49.28360049	0.384411677
185.6118012	31.51552795	0.474061853
309.5219048	98.91873016	0.384947273
810.9468934	559.4387365	0.270773832
164.9524349	68.17327293	0.339337744
101.5232274	26.75061125	0.386738828
183.4241316	94.96709324	0.296540746
1752.065883	644.3609931	0.341172241
386.2791145	162.5139557	0.265441816
129.4637097	28.40725806	0.400668946
119.7096774	34.89247312	0.399339923
455.8551571	445.3469558	0.251926978
177.737785	118.767101	0.315018838
437.4319249	185.256651	0.319132419
444.6097804	116.011976	0.396284895
393.6302708	181.9667111	0.32310167
42.60273973	10.43835616	0.481082572
327.5975155	113.5813665	0.343350619
715.7014218	279.4085807	0.315778917
207.6954315	41.57106599	0.420269693
392.9820128	178.8321199	0.324890203
395.7031056	219.1556936	0.307154397
900.6314046	358.4825638	0.337679797
450.6926251	100.6369263	0.409571192
413.5	75.05026455	0.461685137
735.1535819	666.2523621	0.274872573
124.0285714	41.88214286	0.36046746
1237.025405	1044.638615	0.277604835
296.1328358	86.76268657	0.358783197
815.3737659	589.8875176	0.296460599
523.2894955	137.4731183	0.376439101
340.4661972	73.7915493	0.424172253
206.6658098	156.1195373	0.2864231
493.9287797	150.5310287	0.367912776
848.5066343	455.0037376	0.312922662
95.99796334	41.79022403	0.326808577
101.6189258	22.20204604	0.458717588
112.9557522	25.56637168	0.43555454
303.4801865	71.15850816	0.450679514
699.2247756	148.2933573	0.426764508
816.3718656	1086.946841	0.221802101
271.1205847	129.9914738	0.309985089
181.5381026	32.02332815	0.442750994
252.0102041	78.98979592	0.346648015
652.3853051	329.7013699	0.318471799
482.7397194	116.5142719	0.372748356
195.5111402	42.12975098	0.448453623
1512.408978	1148.851648	0.283717079
272.6704289	76.7351392	0.386721705
642.7241379	116.1034483	0.468706144
1639.071168	470.939762	0.380349777
503.7853053	120.6049618	0.406402277
128.0812408	53.15657312	0.339821247
950.3154049	151.9248758	0.473707812
100.7142857	24.61083744	0.444462362
1133.515241	276.6471976	0.407404468
1264.836175	620.9560469	0.307128341
481.9020716	139.452919	0.356582326
104.2439024	28.94715447	0.365455578
924.4306364	333.8765308	0.340846936
295.3565543	75.84494382	0.385346703
262.2442105	51.59789474	0.479080245
545.7272282	415.2007835	0.294732332
183.8801743	67.12636166	0.346067088
478.483024	136.3490267	0.36517048
94.49845201	16.77089783	0.463376333
314.9726236	77.40608365	0.396505525
312.5444444	154.1522222	0.320494745
331.6756757	76.23328592	0.41914198
183.2227603	49.55932203	0.374706986
410.3549518	164.4163015	0.353047638
312.2477549	166.362916	0.288934957
403.7388582	235.3793738	0.286752826
97.6252354	54.06591337	0.287324813
259.3042813	83.64373089	0.363415706
443.4770594	429.6376434	0.245027965
316.5002939	117.9888301	0.359423859
327.6891002	79.3950256	0.399993269
166.0671642	58.26119403	0.357232989
350.2259084	251.1650869	0.270492617
897.1919573	303.1026439	0.356741746
667.9001741	243.6958793	0.327386297
648.1000907	228.6927729	0.35616838
590.2374161	109.0713087	0.431083341
538.7650897	148.6231648	0.36558528
91.97763578	15.30031949	0.496380014
55.05970149	21.90298507	0.292091907
1714.822303	566.8615141	0.340858584

log.sigma.1.5.mm.3D_gldm_DependenceNonUniformityNormalized	log.sigma.1.5.mm.3D_gldm_DependenceVariance	log.sigma.1.5.mm.3D_gldm_LargeDependenceEmphasis
0.237695767	2.769267808	8.96398892
0.264174605	1.435424262	6.423631124
0.233028012	2.072462748	8.221973094
0.26414876	1.381968288	6.354037267
0.237046573	1.604263554	7.620836286
0.280377023	1.29239806	5.768595041
0.111722578	13.44392693	36.47281526
0.175478123	6.677577979	17.4218853
0.159190678	6.529680846	18.77803848
0.258450068	1.6772317	6.933774834
0.177746314	3.827773409	13.67994859
0.151082187	7.941820777	21.46962137
0.216725633	2.36712223	9.155363748
0.288217083	1.151865283	5.48136646
0.196521844	6.066481633	15.34285714
0.140740523	9.119002752	25.0607428
0.1868091	3.743926104	12.67610419
0.24822305	1.438286476	7.097799511
0.16766374	4.29438954	14.96709324
0.190794499	4.182988041	13.10345203
0.185889853	2.574312125	12.23869105
0.261015544	1.445434443	6.802419355
0.214533472	3.575211007	10.91756272
0.126732043	8.740575445	26.13761468
0.14473761	15.82694777	35.0781759
0.171139251	5.934597412	16.81220657
0.221861168	2.279031757	8.801397206
0.174713835	4.222036643	14.11673324
0.291799587	1.18708951	5.465753425
0.203476718	3.822084025	12.04223602
0.178523677	4.298678684	14.11249688
0.263572883	1.477009199	6.637055838
0.168300648	6.666380423	18.22055675
0.163852218	5.048876544	16.2679089
0.17545907	5.659466604	16.07149815
0.232435598	3.025684087	9.503352243
0.273478836	1.447026959	6.175925926
0.126293349	15.52057832	37.34701941
0.221479592	2.417844388	9.421428571
0.130943729	9.002763189	25.67788716
0.220994654	2.345787481	9.27761194
0.14375419	7.946427257	22.2612835
0.216414183	2.244138769	9.045492142
0.239764928	2.172384448	8.047887324
0.132818644	10.47738087	27.51156812
0.205717942	3.454342656	11.2482299
0.158569732	5.796776476	17.55279387
0.1955152	3.326599774	11.89205703
0.259894951	2.070473113	7.404092072
0.249902107	1.851515389	7.309734513
0.235804341	2.633200694	8.757575758
0.251068142	1.819641127	7.288689408
0.102353544	13.63497237	39.22517553
0.165116069	4.497242749	15.23264312
0.282329864	1.20024477	5.839813375
0.214294391	2.004431024	9.06122449
0.162487	7.366744323	19.45080946
0.233546066	2.062813035	8.41848089
0.256240027	2.543965756	8.190039318
0.138842282	8.572173551	23.99219682
0.205169623	4.203656239	12.15274643
0.260740015	2.699161733	8.176470588
0.199061352	5.284052409	14.1085742
0.240355585	1.938829322	7.812977099
0.189189425	5.033078858	14.40915805
0.277788777	1.752942283	6.517100263
0.248064743	1.861389502	7.359605911
0.222913518	2.721131521	9.363028515
0.168465127	4.431143594	14.95817794
0.226884214	2.082420086	8.77306968
0.21187785	2.250528786	9.296747967
0.185591375	4.095384263	13.24051395
0.221240865	2.800519575	9.662172285
0.276046537	1.470289197	6.090526316
0.133625668	10.49237247	27.59598433
0.200305201	3.257232498	11.31590414
0.216606167	2.061289252	8.895880489
0.292564867	1.1292354	5.470588235
0.239522908	1.94833755	7.903422053
0.173635802	5.15094321	15.57666667
0.235900196	2.801879367	9.083926031
0.221819322	1.989529164	8.552058111
0.179822503	7.903296845	19.25328659
0.164948629	3.973565132	14.76650819
0.148706762	8.545322385	23.10313076
0.183851667	3.026340522	12.48399247
0.198244863	3.856220015	12.12844037
0.115609244	14.81822556	37.72627737
0.186067192	5.795458009	15.55144033
0.239714046	1.618519722	7.386247257
0.206551199	2.355591446	9.694029851
0.138319869	8.169881754	23.67456556
0.199331695	3.331036412	11.37413908
0.193818971	3.211041493	11.73012188
0.195978255	3.578060265	11.85999395
0.247582809	1.758180234	7.224832215
0.219724751	2.768798118	9.865415987
0.293858261	1.132460268	5.300319489
0.205446647	2.336489196	10.63432836
0.194336163	3.733602168	12.31165005

log.sigma.1.5.mm.3D_gldm_LargeDependenceLowGrayLevelEmphasis	log.sigma.1.5.mm.3D_gldm_SmallDependenceHighGrayLevelEmphasis	log.sigma.1.5.mm.3D_gldm_LargeDependenceHighGrayLevelEmphasis
0.023483113	234.8683339	4474.710526
0.044055304	124.1908958	1499.56196
0.034976143	145.5233511	2609.84417
0.031225538	146.8584791	2060.263975
0.045036438	109.1389068	1920.764706
0.02411659	200.4547377	2241.752066
0.110887213	92.65756103	12399.68327
0.031783256	231.5751927	10081.17419
0.067963933	107.6500976	5601.651807
0.016456195	249.0736125	3616.25298
0.062609349	83.14555497	3292.706941
0.032086577	248.8862498	14876.74141
0.040369317	125.3373747	3061.011097
0.019359185	205.264717	2203.350932
0.054664869	156.5847738	4959.815873
0.11504311	74.54618713	5995.467025
0.037596636	148.0954062	4733.894677
0.034293106	124.1821687	2043.339853
0.03868545	135.715713	6251.351005
0.014424607	332.9284111	12448.37537
0.130889454	44.37531674	1640.285852
0.061240719	92.14969699	1237.443548
0.085489574	94.3862804	1888.268817
0.136620155	62.80909103	5253.665555
0.13027327	107.7846698	10539.53257
0.068536572	114.2227385	4567.534038
0.024019788	208.902546	3799.887226
0.070138319	91.07427647	3208.709277
0.076057884	98.8964003	747.4794521
0.019460311	266.2814668	8147.375776
0.083078887	75.42679631	2887.370167
0.028508367	147.4688815	2209.980964
0.075359203	105.1257101	4832.820985
0.076350552	90.44214335	3957.901449
0.034132705	194.1942623	8044.027859
0.045912677	142.8583257	2934.236204
0.018769477	218.2458688	3141.946429
0.07475091	153.9878626	19166.81945
0.043204076	107.3294487	2460.380357
0.016106157	478.3243493	41476.49296
0.051616061	92.94608242	2093.247015
0.050794694	162.8983551	10057.57228
0.037226165	132.9305396	2798.533499
0.036968184	154.3241717	2285.600704
0.11071158	92.64528322	7191.981362
0.039023944	152.4920794	3697.484798
0.046712508	155.8601881	7139.036442
0.022377052	227.843849	7248.105906
0.054059422	125.2031684	1490.173913
0.028824893	175.681596	2698.577434
0.009856263	516.8434664	8889.034965
0.01540633	271.9254264	3991.840575
0.171282604	62.16615664	9269.306294
0.020946291	240.3465231	11806.79659
0.027459312	154.2448168	2034.832037
0.057002121	99.72419867	1996.211735
0.108377476	82.20188246	3892.974595
0.035249262	128.8930615	2556.644412
0.025737534	212.5758536	3155.365662
0.049574206	163.5365424	11922.83834
0.038228979	162.6729257	4659.838977
0.015403502	315.8721913	5075.685598
0.025126349	255.3288486	8558.225528
0.008843008	426.9888039	7771.308683
0.036313128	169.3676697	6593.753323
0.009655717	420.4095993	5352.679918
0.06917947	96.11509649	1577.812808
0.027498941	197.3823897	3736.587807
0.024598164	214.8300795	9502.914092
0.028212436	133.7504697	3222.15678
0.055353499	100.1760111	2224.010163
0.026228127	205.436476	7301.762297
0.047193017	124.8759274	2513.289888
0.025883225	200.1585486	1944.090526
0.085733983	117.2990629	9302.950539
0.065136613	93.02890438	2341.026144
0.010683893	358.6012924	8243.150747
0.02734973	183.1166546	1783.126935
0.021521656	189.3927558	3484.028897
0.068526116	103.1103512	3912.821667
0.023737808	232.0524261	4497.354196
0.023179734	173.0366731	3891.717918
0.029386471	265.5654328	13326.79667
0.104314521	61.99032525	2410.898574
0.081586148	104.6845618	6939.26814
0.040787244	76.20819661	4467.975518
0.077456235	94.23280873	2359.119266
0.104788961	111.5172866	13927.89885
0.027979286	228.3642638	9260.417989
0.011224352	310.5695282	5727.156547
0.039583316	104.1073726	2995.863184
0.074802914	106.9027887	7813.799368
0.056993132	98.63516113	2724.31082
0.067465251	86.21909835	2490.274811
0.035746255	156.7930216	4325.265195
0.01804212	250.5905586	3791.433305
0.039211595	137.052708	2932.424144
0.019879253	242.6044524	2563.750799
0.141377535	36.46224387	1227.462687
0.016742661	274.1457619	9662.480734

log.sigma.1.5.mm.3D_gldm_SmallDependenceLowGrayLevelEmphasis	log.sigma.1.5.mm.3D_gldm_LowGrayLevelEmphasis	log.sigma.1.5.mm.3D_gldzm_DistanceZoneVariabilityNormalized
0.003644576	0.005149247	0.854443288
0.006806088	0.010424329	0.93450158
0.003331705	0.005964674	0.87087063
0.007398068	0.009722099	0.918751148
0.004853899	0.008402525	0.852909633
0.007304379	0.009072346	0.929671266
0.001157849	0.003591114	0.896848028
0.000925987	0.002172857	0.853799433
0.001710143	0.00440058	0.944305933
0.002389093	0.003616776	0.899022222
0.002350833	0.005545849	0.903696702
0.000840483	0.001878739	0.905891579
0.004135937	0.007237965	0.85356405
0.003707338	0.005452303	0.81117284
0.002583168	0.005108024	0.891872837
0.002363851	0.00611374	0.968052502
0.002184619	0.004085156	0.965527855
0.004568218	0.007318003	0.898254133
0.001743321	0.003596554	0.945546846
0.000597633	0.00132012	0.925691019
0.003953367	0.011697328	0.952931628
0.006639822	0.011682855	0.919819331
0.00748637	0.012821098	0.910975185
0.002125422	0.006239761	0.920678641
0.002417673	0.006572195	0.931563758
0.002107848	0.005083517	0.953168611
0.002178052	0.003785907	0.87710304
0.003101619	0.006640992	0.899463496
0.011366843	0.017946879	0.864366449
0.001383197	0.002404204	0.990491501
0.003361312	0.00763079	0.953323403
0.003569818	0.005888123	0.913844762
0.002609177	0.005616306	0.885635308
0.00273873	0.006433528	0.96687331
0.001111903	0.00256079	0.897804535
0.004260368	0.007400812	0.816707752
0.003755507	0.005307551	0.676751502
0.000939914	0.00250581	0.924565549
0.004176776	0.006988622	0.865785423
0.000279244	0.000734565	0.893223952
0.003355357	0.006926803	0.87658801
0.000878462	0.002551231	0.831605505
0.002391333	0.004882777	0.916974012
0.004016167	0.006554586	0.891075545
0.002337997	0.005345355	0.930842636
0.00186853	0.004089503	0.932966821
0.001000291	0.003125245	0.910706234
0.002591206	0.003703971	0.96757638
0.007326929	0.011016332	0.907625601
0.00394371	0.006159686	0.94786352
0.001258983	0.001837752	0.960681489
0.001296207	0.002466772	0.866111122
0.001620231	0.005240062	0.905047848
0.001134469	0.002087715	0.943709305
0.004348316	0.006936979	0.906047188
0.002459378	0.007363079	0.949613555
0.002878901	0.007156756	0.92881291
0.002782125	0.005396752	0.919886818
0.003050757	0.004730551	0.856299545
0.000770188	0.00230862	0.91863005
0.002527819	0.004729032	0.950198654
0.001658051	0.002645761	0.864491981
0.000994448	0.002140308	0.810572055
0.000930865	0.001562904	0.806441564
0.004262466	0.005796909	0.955413552
0.001339657	0.002091173	0.830213267
0.015138315	0.019572719	0.825433499
0.001795436	0.003560285	0.817248479
0.000717624	0.001870001	0.938499953
0.001957204	0.004036418	0.949149725
0.005516068	0.009252071	0.926712018
0.001211758	0.002561413	0.894198955
0.003348453	0.006533121	0.947406866
0.00336977	0.005518211	0.977145912
0.001523711	0.003915634	0.734347547
0.003766613	0.007448314	0.937074493
0.000922846	0.001656271	0.898971376
0.005097153	0.007374423	0.91322314
0.001947488	0.00355764	0.875555556
0.003077225	0.006182282	0.888464057
0.003570947	0.00509951	0.867164474
0.002485903	0.004154155	0.961867284
0.001103427	0.002130285	0.957376318
0.003682128	0.008762913	0.935242937
0.001748594	0.004402859	0.892527699
0.004136741	0.006554136	0.84162518
0.004078347	0.008551415	0.900277008
0.001007944	0.003197959	0.975004006
0.001394759	0.002573182	0.924801512
0.001409174	0.002258278	0.898947431
0.004282126	0.006886085	0.73964497
0.001500937	0.003916073	0.89400612
0.003523798	0.006900526	0.863100189
0.004251086	0.008217228	0.913286765
0.001411123	0.00333184	0.836471767
0.002381524	0.00377116	0.78077391
0.002121704	0.00468094	0.865833907
0.006988342	0.008459206	0.885246914
0.008595555	0.018277222	0.916824197
0.000776797	0.001681563	0.862587091

log.sigma.1.5.mm.3D_gldzm_LowIntensityEmphasis	log.sigma.1.5.mm.3D_gldzm_LargeDistanceEmphasis	log.sigma.1.5.mm.3D_gldzm_HighIntensitySmallDistanceEmphasis
0.007974473	1.237068966	529.0132902
0.014435835	1.101694915	273.8333333
0.007902605	1.208133971	334.937201
0.015152909	1.127272727	327.7166667
0.011970479	1.239804241	282.632137
0.013508	1.109489051	416.2627737
0.004695415	1.163655685	371.3748672
0.002612604	1.238216561	625.8167728
0.005441766	1.086006826	347.8675768
0.005113418	1.16	560.7993333
0.007309442	1.152173913	263.0557065
0.002520563	1.148514851	756.1159241
0.01057294	1.238636364	310.7535511
0.007168076	1.316666667	418.8118056
0.00652013	1.172058824	394.4724265
0.008465163	1.048712206	273.8390258
0.005989727	1.052631579	436.4466374
0.010720694	1.161290323	316.8629032
0.005638372	1.084033613	449.0371148
0.001683711	1.117329545	952.4557607
0.014642407	1.072347267	166.1953376
0.01509475	1.125523013	230.6820084
0.017834263	1.140077821	230.1459144
0.008263861	1.124117053	246.9296165
0.008586713	1.106430155	329.3564302
0.006378205	1.071973828	353.9852781
0.00518725	1.197324415	509.7460981
0.009280385	1.159259259	274.9401235
0.022329536	1.219512195	197.0335366
0.003714031	1.01433121	778.7014331
0.010283498	1.071729958	238.2489451
0.007725211	1.135338346	350.0889724
0.007696904	1.182669789	310.4028103
0.008804896	1.050541516	288.3354392
0.003182178	1.164524422	557.1027135
0.009936931	1.306188925	335.4231813
0.007510003	1.60815822	437.0361557
0.0033068	1.117775354	545.1245911
0.010540742	1.217021277	292.637234
0.000975599	1.169771529	1654.120562
0.008809926	1.198214286	254.6517857
0.002917763	1.278433476	503.3678916
0.005996925	1.130188679	354.2127358
0.008825385	1.173410405	353.2463873
0.007930367	1.107594937	324.035865
0.004868236	1.104166667	407.8392857
0.003441665	1.140522876	483.7580338
0.007161589	1.049450549	698.1978022
0.014853686	1.145631068	269.3859223
0.008467489	1.080357143	399.7064732
0.002607811	1.060185185	1134.831019
0.002891295	1.216450216	598.202381
0.007234947	1.149920255	276.098219
0.003503107	1.086956522	760.4927536
0.009027439	1.148255814	342.8473837
0.008045756	1.077586207	287.6373922
0.008951632	1.110878661	251.2548815
0.006973098	1.125412541	337.9290429
0.006397237	1.233766234	461.6402597
0.002682895	1.12747123	557.5660962
0.006394207	1.076660988	414.4889267
0.003334514	1.219291635	631.1633384
0.002546186	1.319101124	616.3158786
0.002104486	1.325697211	973.3336653
0.011274397	1.068441065	499.0570342
0.00264181	1.281000532	815.4873603
0.030918622	1.289855072	212.6449275
0.004244161	1.30517024	447.7357083
0.002294635	1.095275903	687.7227074
0.005211934	1.078320091	370.2548241
0.013977591	1.114285714	272.6238095
0.003395462	1.168123393	583.1348329
0.0083246	1.081081081	318.2804054
0.006657482	1.034682081	408.0920039
0.00512245	1.482784431	360.2295201
0.010153061	1.097560976	262.901084
0.002345147	1.160085379	937.8988794
0.009983665	1.136363636	386.6860795
0.004607297	1.2	449.0654472
0.009165386	1.177847114	312.2265991
0.007909917	1.214605067	531.266766
0.00611304	1.058333333	457.1590278
0.002951648	1.065359477	743.836329
0.012322226	1.100502513	215.4932998
0.005808245	1.170949721	356.4879888
0.013305217	1.260115607	256.7109827
0.011005062	1.157894737	254.5780075
0.004007054	1.037974684	456.8108569
0.003668968	1.117391304	620.3764493
0.00323665	1.160125589	750.1236264
0.011231192	1.461538462	263.5184615
0.005386504	1.168449198	389.8739973
0.009465694	1.22173913	262.7199728
0.012175299	1.136258661	252.8966513
0.003837934	1.272999252	416.5177221
0.005140336	1.375952583	529.4002964
0.00557025	1.216936251	353.7963844
0.012621805	1.183333333	469.4263889
0.027856599	1.130434783	125.1847826
0.002183159	1.222642608	760.8848952

log.sigma.1.5.mm.3D_gldzm_LowIntensityLargeDistanceEmphasis	log.sigma.1.5.mm.3D_gldzm_HighIntensityEmphasis	log.sigma.1.5.mm.3D_gldzm_DistanceZoneVariability	log.sigma.1.5.mm.3D_gldzm_ZonePercentage
0.008770759	556.3850575	594.6925287	0.48199446
0.016418198	277.4011299	165.4067797	0.510086455
0.009191644	356.6244019	364.0239234	0.468609865
0.020520581	333.630303	151.5939394	0.51242236
0.021840817	292.6280587	522.8336052	0.434443657
0.015700112	418.1678832	127.3649635	0.566115702
0.005591659	386.5451647	2531.801984	0.267862226
0.003221626	667.4798301	2010.697665	0.388101516
0.006283146	351.2730375	1383.408191	0.343735336
0.005789512	579.5573333	337.1333333	0.496688742
0.008217263	274.8804348	498.8405797	0.354755784
0.002897735	779.0231023	1097.940594	0.355738186
0.015362942	324.5880682	300.4545455	0.434032059
0.009839454	433.4638889	292.0222222	0.559006211
0.008240204	406.0852941	606.4735294	0.431746032
0.009369981	276.5996641	1728.941769	0.309961819
0.006279447	439.997076	330.2105263	0.387315968
0.013713981	321.016129	167.0752688	0.454767726
0.005884553	457.3585434	337.5602241	0.326325411
0.001835745	981.2963068	3258.432386	0.383316999
0.016359168	168.1414791	592.7234727	0.299326275
0.019668388	231.7112971	219.8368201	0.481854839
0.033071724	234.5175097	234.1206226	0.460573477
0.010774853	253.2633703	912.3925328	0.275507367
0.009161612	339.9512195	420.135255	0.367263844
0.0072557	358.612868	874.0556161	0.358763693
0.00601528	525.6332219	786.761427	0.44760479
0.014777349	282.6641975	728.5654321	0.359520639
0.06751553	199.4756098	70.87804878	0.561643836
0.003748183	780.5286624	622.0286624	0.390062112
0.011147491	241.5253165	1355.625879	0.354701921
0.008937695	356.5137845	364.6240602	0.506345178
0.009479321	323.5269321	756.3325527	0.365738758
0.009305015	292.2623345	803.4717208	0.344099379
0.003660439	577.0971722	1746.22982	0.378920709
0.016828797	348.3474484	752.1878393	0.474987107
0.014155228	473.5488257	547.4919654	0.53505291
0.003801604	558.540349	1695.653217	0.31506614
0.01322476	298.8382979	203.4595745	0.419642857
0.001086073	1726.759578	2541.222144	0.301153805
0.017022131	260.775	490.8892857	0.417910448
0.00353045	547.7977468	1550.112661	0.328631876
0.012504159	357.3188679	971.9924528	0.438378825
0.01190493	362.4436416	616.6242775	0.487323944
0.009053539	329.3206751	441.2194093	0.304627249
0.005842215	416.5029762	940.4305556	0.419825073
0.004099586	498.2265795	1672.056645	0.343113437
0.007294882	702.8296703	176.0989011	0.370672098
0.018858091	272.3932039	186.9708738	0.52685422
0.009011019	404.15625	212.3214286	0.495575221
0.002702997	1145.083333	622.5216049	0.503496503
0.003404293	635.5021645	1200.430014	0.497666068
0.010255579	286.2030835	1702.395003	0.235832497
0.003642292	775.9601449	520.9275362	0.336175396
0.013432092	347.5	311.6802326	0.534992224
0.009138677	289.9439655	440.6206897	0.394557823
0.010411283	257.1136681	1331.917713	0.357160648
0.011085841	346.7656766	836.1771177	0.439767779
0.008295192	473.238961	329.6753247	0.504587156
0.003017576	577.7202715	3113.237238	0.311117231
0.00713487	419.1396934	557.7666099	0.441685478
0.003951278	673.250942	1147.180859	0.538336714
0.004440178	669.9662921	2885.636517	0.432353656
0.005540975	1049.938247	809.6673307	0.479007634
0.011660136	503.0038023	251.2737643	0.388478582
0.003138419	879.749867	1559.970729	0.549254604
0.045583474	216.2753623	170.8647343	0.509852217
0.005644969	483.2517865	1944.234132	0.467846608
0.00244072	707.1691147	2364.081382	0.335508791
0.005672291	376.2905789	836.2009081	0.414783427
0.016107088	275.4952381	194.6095238	0.426829268
0.003992437	605.151671	1739.216967	0.390483839
0.009730101	320.2871622	560.8648649	0.443445693
0.006903322	410.7957611	507.1387283	0.546315789
0.008620397	397.4109281	981.0883234	0.327130264
0.013809139	266.8401084	345.7804878	0.401960784
0.002533256	982.4482391	842.3361793	0.424173834
0.011642588	391.0625	160.7272727	0.544891641
0.00547401	473.803252	538.4666667	0.467680608
0.010517634	323.0577223	569.5054602	0.356111111
0.008650017	553.7198212	581.8673621	0.477240398
0.006261821	463.3777778	346.2722222	0.435835351
0.00315657	751.956427	878.8714597	0.402278703
0.019367464	217.6264657	558.3400335	0.315372425
0.00707148	367.803352	798.8122905	0.329650092
0.017033969	266.9942197	145.6011561	0.325800377
0.019842453	259.7913534	478.9473684	0.406727829
0.004214677	458.872444	1001.329114	0.267726799
0.003925533	637.8623188	638.1130435	0.405643739
0.003536959	780.0141287	572.6295133	0.465983906
0.019971958	292.0369231	240.3846154	0.404228856
0.006531515	398.2152406	668.7165775	0.295418641
0.012360356	276.4396739	1588.104348	0.408798045
0.015780549	263.4526559	1186.359507	0.376958793
0.004836455	442.9887809	1118.362752	0.404293922
0.009430762	578.6443692	922.0939881	0.495385906
0.00701368	372.7611798	909.9914367	0.42862969
0.014073886	483.5888889	159.3444444	0.575079872
0.035908128	126.6521739	84.34782609	0.343283582
0.002613125	804.2229336	2963.849243	0.389392566

log.sigma.1.5.mm.3D_gldzm_IntensityVariabilityNormalized	log.sigma.1.5.mm.3D_gldzm_LowIntensitySmallDistanceEmphasis	log.sigma.1.5.mm.3D_gldzm_IntensityVariability
0.037699003	0.007775401	26.23850575
0.054486259	0.013940245	9.644067797
0.044069504	0.007580345	18.42105263
0.046391185	0.013810991	7.654545455
0.048473929	0.009502895	29.71451876
0.046725984	0.012959972	6.401459854
0.049861789	0.004471353	140.75983
0.041932195	0.002460349	98.75031847
0.049916015	0.005231421	73.12696246
0.047239111	0.004944394	17.71466667
0.06477762	0.007082487	35.75724638
0.045182117	0.00242627	54.76072607
0.046745868	0.009375439	16.45454545
0.042916667	0.006500232	15.45
0.039814014	0.006090112	27.07352941
0.052013358	0.008238959	92.89585666
0.058513731	0.005917297	20.01169591
0.059428836	0.009972372	11.05376344
0.058023209	0.005576827	20.71428571
0.046906315	0.001645899	165.1102273
0.061212146	0.014213216	38.07395498
0.05103202	0.01395134	12.19665272
0.0474042	0.014024897	12.18287938
0.058617365	0.007636112	58.08980827
0.041538636	0.008442988	18.73392461
0.044596849	0.006158831	40.8953108
0.042740014	0.004980242	38.33779264
0.049169334	0.007906144	39.82716049
0.062760262	0.011033037	5.146341463
0.057061138	0.003705493	35.8343949
0.052139278	0.0100675	74.14205345
0.048171808	0.00742209	19.22055138
0.047131833	0.0072513	40.25058548
0.047829229	0.008679866	39.74608905
0.045335413	0.003065528	88.17737789
0.044460242	0.008213965	40.94788274
0.040244102	0.005848697	32.55747837
0.044156839	0.003183098	80.98364231
0.060787687	0.009869737	14.28510638
0.050718771	0.00094798	144.2949033
0.053839286	0.006756874	30.15
0.051473365	0.002764591	95.94635193
0.051082236	0.004370117	54.14716981
0.04061696	0.008055499	28.10693642
0.051621001	0.007649574	24.46835443
0.04778636	0.004624741	48.16865079
0.047895278	0.003277184	87.93572985
0.072515397	0.007128266	13.1978022
0.050146102	0.013852585	10.33009709
0.048628827	0.008331607	10.89285714
0.040442577	0.002584014	26.20679012
0.04336084	0.002763045	60.0981241
0.053087748	0.006479789	99.85805423
0.05060649	0.003468311	27.93478261
0.044652515	0.007926276	15.36046512
0.058031733	0.007772526	26.92672414
0.050146959	0.008586719	71.91073919
0.047423334	0.005944912	43.10781078
0.044277281	0.005922749	17.04675325
0.048593766	0.002599224	164.6842726
0.044156613	0.006209041	25.91993186
0.038223574	0.003180323	50.72268274
0.040114411	0.002073333	142.8073034
0.048902002	0.001245363	49.09760956
0.055212595	0.011177962	14.52091255
0.03755437	0.002517658	70.56466205
0.048892623	0.027252409	10.12077295
0.041129641	0.00389396	97.84741488
0.050028832	0.002258114	126.022628
0.053572648	0.005096844	47.19750284
0.049251701	0.013445217	10.34285714
0.044059185	0.003246219	85.69511568
0.046361395	0.007973225	27.44594595
0.044330842	0.006596021	23.00770713
0.045174621	0.004274475	60.35329341
0.056528668	0.009239041	20.85907859
0.047394612	0.00229812	44.40875133
0.049715909	0.009568934	8.75
0.049782537	0.004390618	30.61626016
0.04948148	0.008827324	31.71762871
0.038683727	0.007724892	25.95678092
0.048564815	0.006075845	17.48333333
0.042210736	0.002900417	38.74945534
0.062605041	0.010560916	37.37520938
0.047762554	0.005492436	42.74748603
0.062314143	0.012373028	10.78034682
0.048794449	0.008795714	25.95864662
0.052243752	0.003955148	53.65433301
0.044238605	0.003604826	30.52463768
0.04766998	0.003161572	30.36577708
0.05172071	0.009046	16.80923077
0.050516171	0.005100251	37.78609626
0.048338256	0.008742029	88.9423913
0.051985865	0.011273986	67.52963818
0.052804116	0.003590382	70.59910247
0.037783534	0.004067729	44.62235394
0.047632584	0.005209392	50.06184586
0.042222222	0.012258785	7.6
0.073487713	0.025843716	6.760869565
0.045960663	0.002075667	157.9208382

log.sigma.1.5.mm.3D_gldzm_HighIntensityLargeDistanceEmphasis	log.sigma.1.5.mm.3D_gldzm_SmallDistanceEmphasis	log.sigma.1.5.mm.3D_glcm_SumVariance	log.sigma.1.5.mm.3D_glcm_Homogeneity1
665.8721264	0.940732759	1591.93847	0.26590262
291.6723164	0.974576271	668.7097528	0.267119277
443.3732057	0.947966507	960.2693921	0.274321942
357.2848485	0.968181818	915.3102266	0.258117983
332.6117455	0.94004894	741.983976	0.278729656
425.7883212	0.972627737	1155.534147	0.245001198
447.2263549	0.959086079	1091.865285	0.415072438
834.1320594	0.94044586	1943.86555	0.317640719
364.8948805	0.978498294	921.012085	0.350828749
654.5893333	0.96	1685.343824	0.264633074
322.1793478	0.961956522	755.6641764	0.365710262
870.6518152	0.962871287	2352.363741	0.354710379
379.9261364	0.940340909	946.2102816	0.279817668
492.0722222	0.920833333	1179.423963	0.236293311
452.5367647	0.956985294	986.9493309	0.297429887
287.6422172	0.987821948	744.3355391	0.37887028
454.1988304	0.986842105	1206.360825	0.323277862
337.6290323	0.959677419	830.8803156	0.284511314
490.6442577	0.978991597	1333.048732	0.348404043
1098.678693	0.97098327	3288.917166	0.312932669
175.926045	0.981913183	365.3124138	0.349435698
235.8284519	0.968619247	516.7495239	0.274295715
252.0038911	0.964980545	513.6922456	0.30634779
278.5983855	0.968970737	622.5599851	0.417955373
382.3303769	0.973392461	923.957438	0.379714687
377.1232279	0.982006543	851.4150258	0.335356949
589.1817168	0.950668896	1416.265496	0.279057018
313.5604938	0.960185185	693.4571327	0.330997642
209.2439024	0.945121951	424.7612086	0.272508235
787.8375796	0.996417197	2348.644563	0.316733158
254.6308017	0.982067511	602.0236876	0.328198989
382.2130326	0.966165414	974.3161401	0.255346035
376.0234192	0.954332553	821.2881145	0.338451769
307.9699158	0.987364621	732.6709409	0.351439399
657.5228792	0.95944016	1659.505313	0.331144987
400.0445168	0.923452769	934.463132	0.269586727
619.5995056	0.847960445	1384.004467	0.231474353
612.2033806	0.970556161	1683.804132	0.40966178
323.6425532	0.945744681	785.3892604	0.312187496
2017.315641	0.957557118	5842.979124	0.389492861
285.2678571	0.950446429	665.751536	0.303681755
725.5171674	0.930391631	1528.896947	0.378353862
369.7433962	0.96745283	963.8888591	0.287881435
399.232659	0.956647399	899.9007688	0.258047327
350.4599156	0.973101266	831.875542	0.392423446
451.1577381	0.973958333	1063.324427	0.300659891
556.1007625	0.964869281	1321.498251	0.350594099
721.3571429	0.987637363	2116.051396	0.34698412
284.4223301	0.963592233	630.7157165	0.270424747
421.9553571	0.979910714	1055.552108	0.26174109
1186.092593	0.984953704	3459.873322	0.273940359
784.7012987	0.945887446	1887.970531	0.257023445
326.6225412	0.962519936	743.2635809	0.447283419
837.8297101	0.97826087	2570.266848	0.339601938
366.1104651	0.962936047	956.7238183	0.244996638
299.1702586	0.980603448	702.1865607	0.308965369
280.5488145	0.972280335	608.06327	0.355366453
382.1122112	0.968646865	910.829961	0.274502548
519.6337662	0.941558442	1230.133896	0.2658348
658.3369726	0.968132192	1677.206999	0.379420663
437.7427598	0.980834753	1173.649235	0.292362415
841.6013564	0.945177091	2069.264743	0.243239085
885.172191	0.920536829	2038.570063	0.292157888
1356.356574	0.918575697	3426.037445	0.267807482
518.7908745	0.982889734	1469.788305	0.332968479
1136.799894	0.929749867	2717.661101	0.228323257
230.7971014	0.927536232	543.8022312	0.270378601
625.3160992	0.92370744	1323.793311	0.270054811
784.9547439	0.976181024	2190.813481	0.33456759
400.4335982	0.980419977	1113.19364	0.291949143
286.9809524	0.971428571	686.7321025	0.296433335
693.2190231	0.957969152	1806.773739	0.312393349
328.3141892	0.97972973	771.6020035	0.282417314
421.61079	0.99132948	1025.086652	0.255824873
549.6661677	0.881798902	1057.912164	0.367582218
282.596206	0.975609756	654.0564751	0.316270126
1160.645678	0.959978655	3180.897628	0.287364218
408.5681818	0.965909091	1010.763809	0.247994366
572.7544715	0.95	1378.566873	0.272475458
366.3822153	0.955538222	796.4187277	0.334860843
643.5320417	0.946348733	1578.948311	0.270462291
488.2527778	0.985416667	1365.223812	0.288434628
784.4368192	0.983660131	2272.689414	0.325756075
226.159129	0.974874372	484.5439765	0.362975284
413.0648045	0.95726257	967.548314	0.362440592
308.1271676	0.934971098	952.0415186	0.348685049
280.6447368	0.960526316	598.2437606	0.312716429
467.1187926	0.990506329	1204.685407	0.419394561
707.8057971	0.970652174	1965.296246	0.316473827
899.5761381	0.959968603	2577.852001	0.272291001
406.1107692	0.884615385	878.7521245	0.294593107
431.5802139	0.957887701	1054.334558	0.381230432
331.3184783	0.944565217	730.3316708	0.303865156
305.6766744	0.965935335	658.8954099	0.317182077
550.2026926	0.932581235	1201.92666	0.312381209
775.6206605	0.906011854	1649.869049	0.246049407
448.6203616	0.945765937	970.4011618	0.28618338
540.2388889	0.954166667	1392.571123	0.22699206
132.5217391	0.967391304	279.1329711	0.3431394
977.5750873	0.944339348	2619.043965	0.302741487

log.sigma.1.5.mm.3D_glcm_Homogeneity2	log.sigma.1.5.mm.3D_glcm_ClusterShade	log.sigma.1.5.mm.3D_glcm_MaximumProbability	log.sigma.1.5.mm.3D_glcm_Idmn	log.sigma.1.5.mm.3D_glcm_SumVariance2
0.181451722	225.8928502	0.013717621	0.967971848	84.81123326
0.177428086	-17.10974195	0.0159954	0.956794345	52.60285591
0.187638807	-58.34800068	0.012583951	0.951741445	64.56800002
0.168936108	-98.61163117	0.018461995	0.947226495	64.8453649
0.188033604	34.20672086	0.010643949	0.950304505	59.05719036
0.155273819	-21.10454588	0.01722411	0.955808178	70.54644514
0.348554821	71.67850271	0.078705626	0.983006036	32.48215591
0.238640379	177.6087766	0.041772363	0.978327446	58.76264956
0.272325957	86.79356737	0.043393784	0.975086858	37.53009793
0.175980105	25.91271002	0.011681429	0.968752941	60.47647556
0.284942582	31.42427767	0.041273425	0.970846477	27.90880741
0.279474823	132.3266198	0.048098181	0.983937774	43.91021743
0.191964619	-216.8728397	0.018436515	0.957535285	61.2378539
0.146812938	34.98335722	0.011500241	0.948221133	67.6645798
0.216061496	307.5373812	0.035833029	0.965002155	62.68410582
0.308177016	33.24002244	0.066344215	0.972395088	43.57474576
0.233247668	70.87878943	0.020410523	0.982375509	33.53842287
0.191640011	-57.57472973	0.016199178	0.96425199	46.21872271
0.264076252	42.34329438	0.031681357	0.979883596	28.44512495
0.227905479	-46.48757847	0.020715341	0.987296644	47.56928152
0.261658138	31.78681925	0.016109631	0.966154492	31.18849088
0.182904357	133.7981074	0.012682242	0.963118156	53.8807508
0.220361444	76.57431149	0.026953843	0.950814571	50.96823664
0.348127213	72.21787237	0.075745269	0.979101817	26.51938859
0.312552382	3.920267131	0.090483591	0.959308646	52.59868436
0.253390345	334.2790849	0.033885386	0.976524607	48.8792031
0.190666854	224.481836	0.013291439	0.972713526	62.41119792
0.246506646	85.81767241	0.030842027	0.978309414	36.75405408
0.179715716	86.0900461	0.026171679	0.947813712	46.02888066
0.228866413	2.518460759	0.023989645	0.9822839	39.63588602
0.243053402	61.84719441	0.021598793	0.975958687	40.62977438
0.166339909	-9.43399259	0.010835707	0.951227725	59.97555111
0.257813386	98.63719172	0.037250377	0.975883389	41.54382356
0.27583086	109.560571	0.043974929	0.970966463	48.00619336
0.249749616	144.9197515	0.02858906	0.980867865	49.25771675
0.184619378	-88.33042147	0.015729944	0.94970896	67.53813002
0.144902334	-254.8505167	0.009109827	0.960103814	96.97230335
0.347621111	31.91704821	0.090637293	0.981767067	51.03967456
0.221078137	7.966325561	0.018462931	0.966706843	34.45449811
0.317605077	132.4775541	0.067874971	0.99352374	34.49037999
0.212763694	50.95677522	0.014260269	0.963976881	42.66552682
0.305001499	187.0605474	0.054035083	0.982977513	37.57502707
0.199534315	-29.36458938	0.010803131	0.963212044	54.28852437
0.169909767	166.2458254	0.011461011	0.956965173	62.98112003
0.319116165	27.0567948	0.069634793	0.973706945	28.62069558
0.214606231	144.517162	0.019808299	0.969576379	49.69705839
0.271564131	161.5639338	0.044430384	0.979396643	42.85101066
0.255993716	-16.99302052	0.025999043	0.982985542	26.40822055
0.179759854	94.15843425	0.016523939	0.955112488	57.91169767
0.172108586	-100.3882819	0.017516517	0.957337088	67.05917427
0.189273121	151.5123247	0.023209084	0.976428521	70.11560074
0.167927584	138.8503991	0.009917435	0.973904514	68.43737462
0.385172045	46.55843177	0.08985143	0.982132535	23.73709335
0.25677249	-102.8475823	0.028240524	0.982840767	36.60168041
0.156536307	-101.5917426	0.011650652	0.941651764	75.63199906
0.217861048	81.97076793	0.016066109	0.969601675	43.32580595
0.276889026	115.9063298	0.04459785	0.970312569	40.22777623
0.18466347	114.7568537	0.011652195	0.970232883	53.78550433
0.176662582	189.9351125	0.016772621	0.967104735	59.95703528
0.30685287	159.9253503	0.054127885	0.984897687	38.72586677
0.209226359	158.5389576	0.020380104	0.970120269	60.19255434
0.158042412	-11.33104696	0.010290828	0.964680831	85.33469625
0.210725461	-21.77491328	0.023301075	0.97450883	66.74254904
0.176839888	-86.42595858	0.008855003	0.980713216	58.98438947
0.246939509	-43.91702417	0.033119903	0.976451889	36.57398098
0.141439769	49.02497066	0.006714559	0.969417111	89.3804226
0.180359737	-86.98010737	0.017069699	0.948205602	58.06834587
0.185046803	179.2043909	0.013844798	0.974571082	71.35474765
0.250430341	89.94490974	0.029570364	0.98611851	36.58805409
0.200486326	-23.19230339	0.013078182	0.972056088	41.68594335
0.210352063	5.612399068	0.015306296	0.950613273	55.31226579
0.232915587	39.8127269	0.027189304	0.975930472	59.82460562
0.194469224	130.1614833	0.013470156	0.968914215	53.7715216
0.168111273	298.0538531	0.015158603	0.965477262	65.51554711
0.294999715	153.698762	0.069276431	0.978301977	42.31097025
0.227534453	79.26750289	0.020013898	0.975066203	38.01514449
0.1963923	-52.8792508	0.011219516	0.980759117	53.31431817
0.157910266	-88.91637135	0.014675695	0.946982227	70.85026701
0.18111351	29.28660868	0.010304429	0.969711097	54.09371962
0.252031409	174.7865007	0.0353719	0.978765093	38.49203821
0.185891565	221.9582356	0.014402133	0.966887127	80.09382252
0.198751176	26.16689279	0.012918983	0.968714608	50.00799825
0.25155322	23.25539456	0.053580113	0.969957065	60.6357051
0.278254141	53.12492319	0.028798578	0.977497138	26.03546795
0.286548378	64.86754803	0.051101491	0.974520449	37.10647943
0.265532902	-211.3465065	0.035252977	0.962965644	37.60294675
0.226697941	117.13309	0.017899603	0.968855522	47.79966341
0.35073407	92.98484608	0.090535831	0.981788535	29.50145938
0.234305931	-27.70978445	0.03379521	0.978806811	55.17059878
0.182109434	-141.0151311	0.011790463	0.972875053	55.53649935
0.205806858	-102.8918699	0.017792104	0.966051338	45.9117827
0.305183409	78.74606405	0.050956648	0.977362985	28.23332673
0.216466043	19.72993232	0.018777958	0.971090761	42.26206015
0.230825221	58.88154259	0.0188786	0.973153299	42.85949832
0.224043359	93.96429784	0.017123077	0.980194192	38.24200202
0.159905181	57.74829499	0.007757197	0.965984698	91.34138321
0.198030251	84.32066899	0.013046778	0.967312527	50.55010296
0.140299123	-17.92734057	0.012895622	0.949615386	90.42051906
0.252199548	-9.302022295	0.022944209	0.951382909	29.14703628
0.217290591	-66.63544315	0.017476426	0.981259415	52.37527894

log.sigma.1.5.mm.3D_glcm_Contrast	log.sigma.1.5.mm.3D_glcm_DifferenceEntropy	log.sigma.1.5.mm.3D_glcm_InverseVariance	log.sigma.1.5.mm.3D_glcm_Entropy	log.sigma.1.5.mm.3D_glcm_Dissimilarity
77.07416227	4.157765324	0.186911602	8.713095147	6.436811796
50.60863672	3.846147007	0.188336861	7.709811108	5.539660835
58.20095133	3.957207386	0.193640362	8.41163335	5.766043801
60.40589495	3.952196256	0.180291034	7.819957853	5.970052288
45.63587179	3.836283827	0.192385252	8.39216578	5.224227567
66.03409468	3.97758057	0.16096214	7.605607616	6.327441454
29.69322215	3.429378018	0.300407622	7.394154837	3.626942451
56.98130892	3.936251269	0.227776793	8.487192062	5.358532861
35.72974763	3.627619681	0.260384793	7.850198265	4.276649853
58.48456008	3.978310589	0.182956676	8.322458418	5.842785393
23.81313916	3.371002121	0.285045668	7.430876942	3.577850059
43.6835631	3.705618127	0.270697597	7.889431003	4.510111835
53.44708067	3.907252243	0.194779756	8.198790751	5.527263714
70.11246534	4.094909792	0.157021847	8.441433281	6.570119704
64.70261266	4.023114899	0.208271911	8.3147756	5.78080351
38.0411795	3.578915731	0.277279608	7.742547741	4.237385834
30.26853411	3.537805314	0.231236977	7.609687977	4.14145682
38.59068976	3.668224704	0.205860435	7.704616297	4.821807059
28.56290796	3.477309432	0.264849555	7.449097943	3.873747597
45.46154109	3.800836861	0.229125636	8.334615207	4.857347342
21.9421429	3.343951788	0.262215194	7.58353752	3.562660834
44.97597683	3.800142886	0.189210117	7.936528974	5.239902204
42.74314489	3.759421792	0.217788835	7.937359878	4.889429047
22.35880477	3.275386332	0.314645911	7.122885785	3.214285438
50.48292216	3.742327616	0.258100443	7.572138193	4.758417939
39.78474892	3.690566054	0.250900977	7.975532637	4.477850047
56.2467989	3.947815487	0.196503452	8.514758395	5.566416373
36.1944883	3.654906612	0.242496601	7.884574578	4.373787724
41.36963786	3.63572076	0.192616332	6.795802077	5.077055669
35.42924464	3.639495777	0.232233255	7.924653819	4.420319527
34.04141769	3.620194701	0.239451027	8.049348134	4.337069603
57.37336841	3.960058364	0.175734043	8.319122588	5.958964451
38.57138875	3.688033859	0.248264123	7.973013871	4.464663615
45.10502782	3.734841223	0.254362235	7.931989642	4.667313026
42.22913803	3.745126389	0.245907941	8.226771649	4.632815009
64.7324903	4.05255861	0.187542427	8.730099038	6.085537135
87.52625504	4.241469057	0.14807115	8.945855976	7.241914704
44.21726408	3.60810382	0.286764845	7.56971553	4.309606365
31.65920734	3.555325004	0.228998739	7.583159153	4.277628058
31.89299414	3.506542528	0.289845461	7.543803131	3.836546006
34.23271071	3.639239731	0.220423172	8.025175958	4.488790429
36.18265745	3.577145947	0.283826135	7.652881762	4.049769548
45.1640489	3.771727232	0.204329451	8.334470213	5.141623139
64.29231465	4.068463971	0.169466779	8.613413251	6.157104971
28.63305347	3.441295545	0.288101356	7.309926407	3.676265755
43.85687325	3.80615562	0.215638547	8.281558303	4.987624168
38.21672012	3.669577122	0.255843462	7.98059775	4.339656226
23.07712478	3.274851081	0.255348455	7.10720953	3.53568146
50.15895324	3.840634117	0.185841469	7.864800871	5.460868448
56.11501488	3.92908555	0.173581053	7.991691006	5.833803747
64.11829884	4.042329969	0.18892051	8.402383381	6.011388717
63.05944068	4.064481338	0.172165249	8.792787402	6.084326971
22.82923643	3.216861892	0.333752291	6.918318402	3.092154937
35.96812921	3.605201704	0.255086847	7.716933025	4.279273421
66.81775205	4.074162453	0.16519542	8.386941906	6.3919287
32.54158521	3.600195196	0.224521966	7.927083456	4.357905781
34.27852749	3.5989698	0.261168632	7.856708273	4.185386907
50.09571037	3.901820668	0.189582847	8.4450867	5.431332003
59.35081952	3.980233215	0.184957995	8.231592568	5.82463417
37.98049081	3.591830626	0.28788933	7.710404855	4.094726927
59.21785046	3.976421584	0.206788526	8.381749227	5.642587654
84.14236902	4.258521877	0.160540481	9.098827348	6.994613394
64.82602997	4.049917533	0.20789203	8.847022913	5.857622406
51.82458291	3.921243578	0.182071244	8.543504621	5.530084805
34.2782454	3.570040606	0.242688788	7.557156675	4.231735717
91.56430769	4.32378275	0.144108986	9.274063605	7.363247704
51.56993059	3.871316069	0.18357062	7.940880892	5.540694835
66.82774927	4.096642295	0.185788827	8.912936748	6.132156696
37.37249471	3.649379466	0.253461585	7.912977542	4.320068219
37.56032461	3.708784146	0.208870123	8.152837988	4.739238642
45.55045137	3.789890003	0.206703485	7.882722173	5.125039291
56.19878842	3.931747701	0.228131465	8.547588891	5.378925367
50.14413335	3.88509043	0.196896449	8.308803891	5.375923805
68.84113123	4.084098146	0.177303829	8.299956682	6.279992436
42.23276261	3.707668163	0.25758624	7.828325635	4.483611103
36.22339768	3.632659308	0.222938549	7.731991112	4.453156304
46.48708691	3.822864639	0.199912646	8.349697991	5.074567562
59.62747219	3.94877649	0.172708387	7.92016766	6.067151299
48.28021146	3.875551055	0.184963382	8.38471818	5.374235929
39.50659519	3.689012297	0.245188195	7.754564303	4.466023752
73.21462789	4.119964171	0.191128413	8.636659238	6.266483151
45.49382855	3.806672958	0.203255289	8.080887504	5.093065175
59.25301945	3.931031404	0.225906042	8.216371077	5.515959753
22.38142971	3.329999114	0.279297888	7.382506479	3.465191423
34.81659993	3.592581129	0.272402783	7.767827861	4.15755126
29.2552207	3.452081371	0.268182904	7.153256507	3.92194585
40.47677418	3.726085285	0.224302599	8.082395648	4.725528187
25.89200731	3.365238233	0.296840894	7.227694794	3.418645695
46.75032121	3.825975096	0.225428805	8.220366256	4.980938487
53.31620094	3.916328981	0.192890482	8.351898815	5.501944022
44.61395296	3.786453193	0.209815639	7.975835832	5.004643776
27.39245478	3.423886342	0.292947576	7.357946843	3.650491794
41.62463009	3.784349495	0.213647833	8.299092628	4.849152771
38.8105932	3.715725074	0.227337253	8.175710414	4.596669702
35.93405966	3.677142126	0.224493151	8.053148381	4.508702929
85.25405767	4.247648674	0.163922906	9.114018207	6.922273372
47.47501004	3.862126893	0.201402538	8.407755023	5.235590838
89.845713	4.211680834	0.145294087	8.051144449	7.351839038
20.43695516	3.254775741	0.251743055	7.030096701	3.521656058
48.67191237	3.861385562	0.218827641	8.508789343	5.101205518

log.sigma.1.5.mm.3D_glcm_DifferenceVariance	log.sigma.1.5.mm.3D_glcm_Idn	log.sigma.1.5.mm.3D_glcm_Idm	log.sigma.1.5.mm.3D_glcm_Correlation	log.sigma.1.5.mm.3D_glcm_Autocorrelation
35.34014851	0.887370425	0.181451722	0.053465787	485.5479149
19.50624284	0.863543753	0.177428086	0.020058722	219.1377506
24.26561417	0.860745455	0.187638807	0.058757667	306.2291798
24.47805404	0.852267152	0.168936108	0.041646417	290.7158003
18.17798083	0.855566837	0.188033604	0.128679712	244.5226751
25.60419242	0.86178969	0.155273819	0.039622515	358.9668255
16.36059892	0.923922529	0.348554821	0.052034854	341.6554006
27.95800166	0.909193444	0.238640379	0.021167049	584.31654
17.1857463	0.902469825	0.272325957	0.02810761	294.4464224
24.06148399	0.884140907	0.175980105	0.020517944	512.1406476
10.87575396	0.892349468	0.284942582	0.076469353	246.878439
23.20062704	0.923646687	0.279474823	0.007145147	693.7644235
22.4771818	0.868523043	0.191964619	0.075350095	301.9404455
26.63639166	0.850672938	0.146812938	-0.012760705	366.8510366
30.89615757	0.885257745	0.216061496	-0.009363816	311.0823145
19.27282541	0.902368101	0.308177016	0.076256355	242.8231036
13.00232664	0.911737802	0.233247668	0.055417532	376.1209997
15.12537168	0.874878166	0.191640011	0.092162585	269.1448945
13.44420596	0.909714711	0.264076252	0.002676862	410.2391295
21.65518656	0.927338965	0.227905479	0.029585326	952.6217593
9.125621328	0.880390616	0.261658138	0.172257089	131.9338008
17.40833384	0.872562179	0.182904357	0.090499075	175.3190745
18.66778229	0.860729061	0.220361444	0.086967799	174.8688755
11.95995688	0.914215491	0.348127213	0.085727898	206.861294
27.28668365	0.885974436	0.312552382	0.022204173	292.5738768
19.49350477	0.905283555	0.253390345	0.103881979	274.8827009
24.85924272	0.893883944	0.190666854	0.055494886	437.261939
16.92279711	0.906478533	0.246506646	0.014073004	228.1747545
15.44359306	0.849046331	0.179715716	0.053726569	144.1010936
15.77555103	0.912560401	0.228866413	0.057374434	695.3272769
14.88802532	0.90011393	0.243053402	0.091543782	202.9480098
21.41756719	0.855010803	0.166339909	0.028623391	309.7033481
18.46657275	0.903147799	0.257813386	0.041336664	266.129788
23.06342731	0.898539104	0.27583086	0.031420964	239.0025079
20.6417925	0.913508118	0.249749616	0.07917305	506.245346
26.9756424	0.858221925	0.184619378	0.029659918	297.9529113
33.21528576	0.869261943	0.144902334	0.060054698	424.8679788
25.30149648	0.923895927	0.347621111	0.072960348	508.3270156
13.23999136	0.880914862	0.221078137	0.042244864	255.545858
17.06191783	0.950154869	0.317605077	0.044490288	1627.62551
13.95475312	0.875869577	0.212763694	0.109520671	222.1579682
19.66449807	0.922600139	0.305001499	0.022797129	465.3502494
17.14906931	0.875435847	0.199534315	0.102062071	308.7228014
26.18267403	0.865507462	0.169909767	-0.00498228	287.0906771
15.01132809	0.903828387	0.319116165	0.001499069	267.2257402
18.77885319	0.887386161	0.214606231	0.067374595	337.1477396
19.2614162	0.911840376	0.271564131	0.059752086	409.8566762
10.49327731	0.913841391	0.255993716	0.067886917	626.1682768
20.04474051	0.862011909	0.179759854	0.076009964	208.9010781
21.72759609	0.864565129	0.172108586	0.095520943	333.4665072
27.65427551	0.899992542	0.189273121	0.047945369	1000.809436
25.78564779	0.892899007	0.167927584	0.047454	570.4817322
13.22064192	0.924342363	0.385172045	0.022578275	240.187558
17.27216739	0.917551243	0.25677249	0.014727836	752.9230756
25.65796175	0.843260174	0.156536307	0.067730052	304.0421562
13.41479552	0.88538729	0.217861048	0.14240708	233.1929158
16.50338852	0.894462536	0.276889026	0.079697354	203.9143976
20.37561077	0.886157378	0.18466347	0.040652614	292.4793765
25.22737165	0.882428465	0.176662582	0.007777514	382.2760333
21.11928807	0.927708283	0.30685287	0.014255681	506.3491436
27.0275352	0.891125584	0.209226359	0.014599851	366.9535773
34.9178302	0.877976137	0.158042412	0.014785857	619.2080202
30.13893405	0.899732363	0.210725461	0.021407852	611.579207
21.07407187	0.906103224	0.176839888	0.069611821	993.0771244
16.0924427	0.902729707	0.246939509	0.038716724	450.1236733
36.99216676	0.884490449	0.141439769	-0.002929959	798.2449088
20.59817929	0.852937283	0.180359737	0.060288354	182.2329988
28.92401444	0.89660988	0.185046803	0.039271278	410.8573669
18.63061612	0.925653031	0.250430341	-0.004585368	649.159038
14.96354695	0.888501375	0.200486326	0.05867377	351.5148146
18.88118538	0.858835285	0.210352063	0.097786636	226.694034
27.00225527	0.903647029	0.232915587	0.03838304	547.3295584
20.9713266	0.885064901	0.194469224	0.040174494	251.3003071
29.05211682	0.879862804	0.168111273	-0.018899572	321.5027001
21.90361127	0.911803554	0.294999715	0.006531234	332.4628012
16.17706166	0.897776964	0.227534453	0.030528824	216.693899
20.57034596	0.908690578	0.1963923	0.071746996	925.2862178
22.47265903	0.849176385	0.157910266	0.092236256	319.6775603
19.21717386	0.884423148	0.18111351	0.061818297	427.2918983
19.48009162	0.909313103	0.252031409	-0.008303157	256.9598178
33.63701982	0.885792103	0.185891565	0.049861445	482.05353
19.31732333	0.885218363	0.198751176	0.051948782	422.5615517
28.03895362	0.894181745	0.25155322	0.01829939	674.1853125
10.29536611	0.903841821	0.278254141	0.074931161	166.7007008
17.31521863	0.90294449	0.286548378	0.036407168	307.991738
13.4173684	0.880738547	0.265532902	0.131430855	302.760136
17.79248242	0.887558561	0.226697941	0.084850782	201.0213409
14.14295391	0.92065811	0.35073407	0.06801327	373.4408602
21.7154689	0.907514343	0.234305931	0.087327706	591.9161176
22.83544353	0.89226199	0.182109434	0.026966496	759.5173134
19.32712534	0.881741437	0.205806858	0.021120225	282.0700271
13.94846118	0.908735477	0.305183409	0.018382467	331.0339187
18.00911601	0.889987478	0.216466043	0.012148191	239.747637
17.52987131	0.895292938	0.230825221	0.053977363	219.3149501
15.49141423	0.907130148	0.224043359	0.033184227	375.8230981
36.84274797	0.881693383	0.159905181	0.041605953	501.7717444
19.75543055	0.882422226	0.198030251	0.037460611	309.9886079
35.3571534	0.854225711	0.140299123	0.008121075	423.8843664
7.864170308	0.855911773	0.252199548	0.175419648	103.8160741
22.40692617	0.912053039	0.217290591	0.044719701	771.7609857

log.sigma.1.5.mm.3D_glcm_SumEntropy	log.sigma.1.5.mm.3D_glcm_AverageIntensity	log.sigma.1.5.mm.3D_glcm_Energy	log.sigma.1.5.mm.3D_glcm_SumSquares	log.sigma.1.5.mm.3D_glcm_ClusterProminence
5.160648607	22.45485131	0.003696575	40.89081386	28722.6321
4.750374387	14.96400425	0.005723648	26.46256017	8101.918713
4.979219087	17.81895909	0.003783054	31.25290194	11388.97218
4.872907812	17.38413865	0.005455303	31.96563702	11844.88067
4.926154543	15.6360883	0.003766172	26.93921071	8764.12813
4.893994044	19.36433955	0.00606364	33.4715432	16181.7224
4.382102537	18.72097104	0.020661625	15.29918423	5389.029714
4.909123882	24.54434794	0.006448479	29.83967335	14198.11368
4.56928898	17.40261244	0.009319033	18.68666578	5553.879496
4.929225193	22.88959637	0.003910694	29.27991112	10652.7647
4.382612991	15.70618903	0.00962528	13.15858732	2551.259619
4.630276531	26.67597907	0.010751434	21.86062378	10442.17144
4.891963025	17.70750528	0.004826907	28.928593	11021.50796
4.994987062	19.64933652	0.003551392	33.94169785	12848.40579
4.901682781	18.09225438	0.006316799	32.31743396	16910.88409
4.605166692	15.8590646	0.013466278	20.94263985	6657.671533
4.498833843	19.62042295	0.007073021	15.35320453	4543.490953
4.682540348	16.56444274	0.005808989	21.91318634	5726.19505
4.390822705	20.48391695	0.009053112	13.89371953	3496.439206
4.779265274	31.22541297	0.005413452	23.01983461	11009.50905
4.490951044	11.38493617	0.006866988	13.28265844	2940.854709
4.797972787	13.31108909	0.004906867	25.67099787	8227.355976
4.780077467	13.17772216	0.005781298	23.75310471	7760.821528
4.279255645	14.43320496	0.018560188	12.84637546	3345.245688
4.659891642	17.36536539	0.019496398	26.44210683	10970.30869
4.692545534	16.80640047	0.007720223	22.67602449	12409.54112
4.952938216	21.22135806	0.004073381	30.03833775	14597.91836
4.575383357	15.39262196	0.007398694	18.95612241	6139.110847
4.45029437	11.97869755	0.010430518	22.23949845	7033.294918
4.646054888	26.60459739	0.005976827	19.11051433	6089.783712
4.682395489	14.40330534	0.005652857	19.42438013	5139.551545
4.921349847	17.98367505	0.003826523	29.7996087	9483.019377
4.657385444	16.55504665	0.007859542	20.48979491	7091.175237
4.706375941	15.77628938	0.010004707	24.06904419	8900.356313
4.793709983	22.70378679	0.00647601	23.52653422	9560.947511
5.037630838	17.66123845	0.003214807	33.36469191	13847.87931
5.233136589	21.0463589	0.002694549	44.20260032	25642.0497
4.609131473	22.8031021	0.023928501	24.23034677	10339.43773
4.524298295	16.05004382	0.006649813	17.05055959	3757.812233
4.458141446	40.600557	0.015541367	17.04081312	5682.781304
4.706356253	14.91145537	0.004983827	19.78370803	5102.452479
4.510180932	21.73415586	0.013263919	19.29913043	7442.028173
4.850041008	17.8444166	0.004031847	25.34630847	8211.874731
4.977053209	17.38505608	0.00347974	32.56549991	13127.16833
4.352419098	16.51241992	0.014891161	15.00424001	3505.026323
4.80592122	18.64613158	0.004841828	23.55109863	8346.653053
4.674424497	20.41608211	0.008704318	20.24832511	8113.614239
4.300648824	25.04651581	0.009259169	11.87394983	3047.472091
4.838640473	14.58676794	0.005273193	28.18683743	9933.257475
4.931594655	18.58561369	0.004953842	30.48386393	12385.03527
5.001834612	32.02918192	0.004572158	33.61206134	17077.89122
5.057231147	24.27162039	0.003052264	33.81910554	15515.45508
4.157376558	15.63654012	0.026200496	12.25811856	2865.434715
4.537593696	27.73315249	0.008072313	18.06796051	6771.053551
5.063716391	17.7876471	0.003667839	36.31913909	14725.01434
4.695725257	15.3159749	0.005358872	19.74783704	5813.741311
4.626137284	14.39971687	0.008932024	19.62248737	5659.73615
4.874505	17.46769681	0.003797318	26.01880318	9264.765533
4.887772599	19.94416671	0.004523521	29.52508323	12916.62712
4.517984998	22.75446229	0.014053192	20.11865687	8775.66106
4.930689833	19.5428647	0.004318997	29.38784594	13372.22021
5.214424536	25.42301589	0.002547882	42.53448688	24248.57381
5.035577251	25.15611621	0.00419392	32.8766903	16031.00423
4.943099686	31.80807907	0.003402931	27.30004461	12390.69381
4.547064158	21.42596482	0.007825655	16.65573776	5616.147474
5.25898499	28.86467231	0.002131013	45.02555249	24962.16758
4.838416234	13.50247933	0.00506333	27.93379256	8853.364186
5.093497481	20.6682316	0.003162601	35.2130481	16610.65093
4.551280787	25.82213679	0.007901847	18.7025575	7904.105819
4.708483152	19.04791969	0.004663966	19.50775683	5315.258744
4.821513962	15.08711291	0.005309659	26.06045112	7952.253303
4.954830786	23.76687595	0.0052	29.66037266	12667.82376
4.855148495	16.17337887	0.00414553	26.81884152	9519.974493
4.930379495	18.43863151	0.004335633	34.08680454	14940.72182
4.59752959	18.54571663	0.013648859	20.96563065	8409.866886
4.590330374	14.93493147	0.006251224	19.44714146	5713.113821
4.856074196	30.71962088	0.004230067	24.09958003	12639.09307
4.943824949	18.21260854	0.004829439	33.36604498	11879.32352
4.878307576	20.93611498	0.003836317	24.63017607	8821.846845
4.545268814	16.34103077	0.008666808	20.22177921	8069.554121
5.117956423	22.31852466	0.003932155	38.68733686	25646.5143
4.791513169	20.84865061	0.004764862	23.08240593	8125.317332
4.884194882	26.30501586	0.008875605	30.50463505	12855.13274
4.3388419	12.90655817	0.009500282	12.3615517	2482.887527
4.563588811	17.8051331	0.010786806	18.29309823	5300.719688
4.440131562	17.3493305	0.01098852	16.75718991	5235.071442
4.765004276	14.31821769	0.005297315	23.1769922	7459.86433
4.321670235	19.5020708	0.020378323	13.76164225	4197.774671
4.866962841	24.59162685	0.00650581	25.90600248	11273.95255
4.875843241	27.93589366	0.004006669	27.3566704	10426.47591
4.711478871	17.0923674	0.005648021	22.84353249	7500.431203
4.344308599	18.42374114	0.012870624	14.53818956	3826.279077
4.726185694	15.76402798	0.004869171	21.6451634	6078.655174
4.730288052	15.02647158	0.005189654	21.31491703	6231.248028
4.6296696	19.58730148	0.005461475	18.17598668	5250.472204
5.254129478	22.88005183	0.002390643	44.47945723	25853.81148
4.84026708	17.91619199	0.004083481	24.78258873	8336.034471
5.084196988	21.09134651	0.004393536	43.94142892	25131.488
4.351729401	10.08023246	0.009226172	12.39599786	2139.166016
4.86552274	28.15695192	0.004554875	24.46459511	11083.31683

log.sigma.1.5.mm.3D_glcm_SumAverage	log.sigma.1.5.mm.3D_glcm_Imc2	log.sigma.1.5.mm.3D_glcm_Imc1	log.sigma.1.5.mm.3D_glcm_DifferenceAverage	log.sigma.1.5.mm.3D_glcm_Id
43.98228992	0.815967993	-0.118538703	6.436811796	0.26590262
29.56897727	0.898685966	-0.193481709	5.539660835	0.267119277
34.90706815	0.808330816	-0.119232985	5.766043801	0.274321942
34.03301729	0.930917978	-0.228834344	5.970052288	0.258117983
31.05869029	0.665746679	-0.067512141	5.224227567	0.278729656
37.83096917	0.963413906	-0.294882437	6.327441454	0.245001198
36.93006963	0.667972801	-0.077325663	3.626942451	0.415072438
48.32681368	0.634240815	-0.059236836	5.358532861	0.317640719
34.29252982	0.622850689	-0.060933545	4.276649853	0.350828749
45.23839401	0.830271873	-0.13138168	5.842785393	0.264633074
31.35939674	0.633188549	-0.067267751	3.577850059	0.365710262
52.67660562	0.693650343	-0.080400121	4.510111835	0.354710379
34.63974714	0.807477867	-0.121157278	5.527263714	0.279817668
38.33735958	0.856915898	-0.145628288	6.570119704	0.236293311
35.30330676	0.77819715	-0.106879551	5.78080351	0.297429887
31.07660651	0.742000614	-0.099992389	4.237385834	0.37887028
38.74509872	0.717042177	-0.090395539	4.14145682	0.323277862
32.69332409	0.840512915	-0.147264367	4.821807059	0.284511314
40.50996871	0.672226803	-0.077745866	3.873747597	0.348404043
61.71200948	0.53812563	-0.040619462	4.857347342	0.312932669
22.76987234	0.538041129	-0.045866985	3.562660834	0.349435698
26.30989592	0.832053183	-0.138111783	5.239902204	0.274295715
26.29005508	0.831970623	-0.138269801	4.889429047	0.30634779
28.69281795	0.616981417	-0.065263487	3.214285438	0.417955373
34.17804355	0.854587116	-0.160867588	4.758417939	0.379714687
33.02151464	0.683702586	-0.076575896	4.477850047	0.335356949
41.74750834	0.704430703	-0.077693915	5.566416373	0.279057018
30.20154133	0.625111317	-0.060805271	4.373787724	0.330997642
23.90896332	0.958421883	-0.315460125	5.077055669	0.272508235
52.69806312	0.64027951	-0.064389734	4.420319527	0.316733158
28.37598962	0.573745745	-0.048849695	4.337069603	0.328198989
35.15874943	0.771321168	-0.103319132	5.958964451	0.255346035
32.5812139	0.662885704	-0.070030373	4.464663615	0.338451769
30.87216517	0.751201717	-0.099610803	4.667313026	0.351439399
44.92157491	0.608498866	-0.055194387	4.632815009	0.331144987
34.48080834	0.700612344	-0.07488275	6.085537135	0.269586727
41.10520316	0.787200572	-0.103331222	7.241914704	0.231474353
45.01643787	0.794250464	-0.124296418	4.309606365	0.40966178
31.92713839	0.771244155	-0.112059654	4.277628058	0.312187496
80.67155686	0.586412933	-0.055116757	3.836546006	0.389492861
29.66782365	0.637972143	-0.063262016	4.488790429	0.303681755
43.12779674	0.618729506	-0.061452479	4.049769548	0.378353862
35.00941024	0.668377957	-0.07152569	5.141623139	0.287881435
33.90648666	0.722376945	-0.081747585	6.157104971	0.258047327
32.69410874	0.718259469	-0.094593238	3.676265755	0.392423446
36.64334144	0.630492857	-0.059493852	4.987624168	0.300659891
40.43248224	0.606101017	-0.056340952	4.339656226	0.350594099
50.01286654	0.726689928	-0.100836007	3.53568146	0.34698412
28.77090453	0.895086264	-0.185879496	5.460868448	0.270424747
36.36898663	0.890161031	-0.178720685	5.833803747	0.26174109
63.2225417	0.805489387	-0.117098003	6.011388717	0.273940359
47.71312664	0.62836153	-0.055546369	6.084326971	0.257023445
30.98129453	0.622208191	-0.068107327	3.092154937	0.447283419
54.87303635	0.718442842	-0.090144043	4.279273421	0.339601938
34.74502886	0.847969792	-0.140907283	6.3919287	0.244996638
30.36380687	0.692079877	-0.078933896	4.357905781	0.308965369
28.45526543	0.613888162	-0.05909376	4.185386907	0.355366453
34.14966441	0.632752606	-0.05895071	5.431332003	0.274502548
39.09510435	0.830754487	-0.133261981	5.82463417	0.2658348
44.99609092	0.595766172	-0.05532437	4.094726927	0.379420663
38.29907427	0.782483286	-0.107341235	5.642587654	0.292362415
49.75566641	0.710591306	-0.074368488	6.994613394	0.243239085
49.44078126	0.588446028	-0.047325685	5.857622406	0.292157888
62.96923421	0.635996512	-0.058933974	5.530084805	0.267807482
42.40468427	0.794495417	-0.123579745	4.231735717	0.332968479
56.52564433	0.636915066	-0.054680555	7.363247704	0.228323257
26.87690594	0.872628072	-0.165615705	5.540694835	0.270378601
40.48329237	0.578245417	-0.045283732	6.132156696	0.270054811
50.96486613	0.51666375	-0.038702884	4.320068219	0.33456759
37.44226039	0.56268489	-0.045743506	4.739238642	0.291949143
29.94854133	0.87162889	-0.165682788	5.125039291	0.296433335
46.75133968	0.667568344	-0.067401509	5.378925367	0.312393349
31.64737693	0.707910176	-0.08036888	5.375923805	0.282417314
35.90665777	0.826490953	-0.129158644	6.279992436	0.255824873
36.46590971	0.678609645	-0.076454861	4.483611103	0.367582218
29.41016536	0.748587509	-0.100439471	4.453156304	0.316270126
60.78067641	0.658970573	-0.066084139	5.074567562	0.287364218
35.60023085	0.919242776	-0.211080874	6.067151299	0.247994366
41.27140014	0.692677444	-0.074830703	5.374235929	0.272475458
32.07565622	0.684387162	-0.077863621	4.466023752	0.334860843
43.83288469	0.812475423	-0.117946	6.266483151	0.270462291
41.05710022	0.787093283	-0.112834571	5.093065175	0.288434628
51.91628833	0.784676824	-0.112100931	5.515959753	0.325756075
25.75156252	0.559957085	-0.050340079	3.465191423	0.362975284
35.06666191	0.660765115	-0.071676776	4.15755126	0.362440592
34.67892936	0.766564496	-0.120400999	3.92194585	0.348685049
28.2262982	0.719016481	-0.086463629	4.725528187	0.312716429
38.60242618	0.674732817	-0.080802793	3.418645695	0.419394561
48.57180055	0.731245395	-0.089008815	4.980938487	0.316473827
55.09831125	0.715454796	-0.082485133	5.501944022	0.272291001
33.57014483	0.78165235	-0.111869305	5.004643776	0.294593107
36.37704632	0.625002706	-0.064937643	3.650491794	0.381230432
30.95722809	0.499535669	-0.033915412	4.849152771	0.303865156
29.55003697	0.583568387	-0.049597576	4.596669702	0.317182077
38.74245956	0.533847172	-0.04081107	4.508702929	0.312381209
44.73211415	0.729835051	-0.080438475	6.922273372	0.246049407
35.16920116	0.611223003	-0.054295998	5.235590838	0.28618338
41.16833002	0.960278806	-0.274794224	7.351839038	0.22699206
20.16046492	0.810312021	-0.141977348	3.521656058	0.3431394
55.52775682	0.531091407	-0.038590267	5.101205518	0.302741487

log.sigma.1.5.mm.3D_glcm_ClusterTendency	log.sigma.1.5.mm.3D_firstorder_InterquartileRange	log.sigma.1.5.mm.3D_firstorder_Skewness	log.sigma.1.5.mm.3D_firstorder_Uniformity
84.81123326	165.4990711	0.195172622	0.052813821
52.60285591	174.287468	-0.179785774	0.062013637
64.56800002	186.7374287	-0.076823237	0.052796155
64.8453649	197.5290928	-0.45165627	0.057096563
59.05719036	193.3186646	-0.105045661	0.05616044
70.54644514	169.0454798	-0.310982844	0.055802199
32.48215591	73.6879406	0.338582883	0.113917292
58.76264956	140.491375	0.366750757	0.069000026
37.53009793	106.9069262	0.317473642	0.084506363
60.47647556	179.755394	-0.160835587	0.055515109
27.90880741	99.45549488	0.152553608	0.094107064
43.91021743	104.0829315	0.299826342	0.085028674
61.2378539	173.1990662	-0.409160994	0.060768928
67.6645798	204.575449	-0.168508675	0.048937155
62.68410582	159.7480431	0.493409104	0.062805543
43.57474576	88.05569172	0.18630227	0.097091068
33.53842287	117.11199	0.096789638	0.077206425
46.21872271	165.8154297	-0.386570459	0.065404917
28.44512495	106.2202024	0.187220325	0.086807215
47.56928152	127.7480602	-0.329347301	0.070168898
31.18849088	127.0302773	0.154933355	0.078206908
53.8807508	187.8874245	0.172638242	0.057272698
50.96823664	152.4266472	0.140406872	0.062531314
26.51938859	65.63730621	0.630351832	0.123810663
52.59868436	94.55430889	0.06169542	0.09671588
48.8792031	126.0634394	0.838932425	0.072479128
62.41119792	162.2157059	0.402769289	0.057890208
36.75405408	110.1161041	0.44986808	0.080766405
46.02888066	158.1011887	0.3337365	0.07149559
39.63588602	137.5981312	-0.237597357	0.070547433
40.62977438	133.7719002	0.218797272	0.069695331
59.97555111	209.6186886	-0.148049388	0.05275516
41.54382356	120.3215199	0.418396016	0.076587632
48.00619336	93.35556221	0.396708168	0.090747699
49.25771675	133.2557983	0.443826461	0.069838801
67.53813002	187.5695839	-0.17070847	0.051901458
96.97230335	213.9126034	-0.558744188	0.049636418
51.03967456	71.35542679	0.105542963	0.114456685
34.45449811	127.8834839	-0.095974025	0.074789541
34.49037999	74.02033234	0.6437076	0.110578873
42.66552682	155.3581944	0.053556978	0.064748274
37.57502707	79.1208663	1.013467737	0.103999915
54.28852437	183.2878857	-0.028345722	0.05685406
62.98112003	187.1779099	0.220115478	0.05196588
28.62069558	83.19452572	0.164086449	0.100333893
49.69705839	161.5986938	0.335665944	0.062695139
42.85101066	107.5441494	0.5843383	0.085031534
26.40822055	114.498703	-0.795595752	0.085112473
57.91169767	178.0869675	0.119659463	0.056782726
67.05917427	185.0544434	-0.237231275	0.056562769
70.11560074	190.6898689	0.066952501	0.055290216
68.43737462	182.5774078	0.358652404	0.053247166
23.73709335	57.17342901	0.489860706	0.136277187
36.60168041	110.2061071	-0.659659418	0.079166549
75.63199906	229.8535233	-0.211346695	0.049802999
43.32580595	152.262104	0.200220227	0.067168194
40.22777623	110.3051319	0.490829731	0.082117402
53.78550433	183.7338905	0.184690906	0.056368782
59.95703528	178.0757866	0.209427694	0.055215925
38.72586677	76.16234779	0.81865701	0.105466965
60.19255434	168.0426483	0.223194645	0.057739006
85.33469625	201.6718521	-0.008901166	0.04710079
66.74254904	162.9718876	-0.016053002	0.05719453
58.98438947	169.1821518	-0.240738813	0.057540535
36.57398098	118.1910019	-0.651044832	0.078517833
89.3804226	218.2587891	0.014435348	0.044409493
58.06834587	174.0032959	-0.377290996	0.060617826
71.35474765	177.2051468	0.351722043	0.054404562
36.58805409	108.7592697	0.364217099	0.08270592
41.68594335	154.5081501	-0.175299976	0.0656558
55.31226579	180.9541416	-0.144801486	0.05883568
59.82460562	141.1828079	0.108966245	0.06703002
53.7715216	184.3660049	0.306759923	0.056812692
65.51554711	189.7964725	0.541046921	0.054313573
42.31097025	86.0177927	0.536258525	0.101665226
38.01514449	134.0248737	0.415566046	0.073122398
53.31431817	154.1708832	-0.363147593	0.061724322
70.85026701	217.3416328	-0.264573234	0.051922284
54.09371962	168.8301659	-0.061511653	0.058863942
38.49203821	101.876205	0.771917863	0.085640123
80.09382252	158.8575802	0.19902218	0.054219976
50.00799825	164.5491219	-0.144709392	0.059999179
60.6357051	160.2778072	-0.003405526	0.072049212
26.03546795	106.4016418	0.365107316	0.08788321
37.10647943	100.9980927	0.291814136	0.086695902
37.60294675	100.0255356	-1.243524644	0.101819046
47.79966341	148.082242	0.424262238	0.063947806
29.50145938	77.7382946	0.569355454	0.112001471
55.17059878	130.7964554	-0.114291973	0.069364392
55.53649935	177.589119	-0.407569013	0.058079755
45.9117827	128.8188372	-0.373635093	0.072464172
28.23332673	84.73830652	0.587695754	0.099196322
42.26206015	135.8327637	0.135151344	0.067341178
42.85949832	131.5628672	0.284706207	0.070718479
38.24200202	142.423996	0.391135962	0.06915415
91.34138321	209.6966724	0.119960727	0.045751388
50.55010296	160.5030193	0.276066613	0.060613036
90.42051906	223.5672073	-0.349026768	0.04888281
29.14703628	128.0108232	-0.170093619	0.081727556
52.37527894	141.7933493	-0.388045372	0.064240879

log.sigma.1.5.mm.3D_firstorder_MeanAbsoluteDeviation	log.sigma.1.5.mm.3D_firstorder_Energy	log.sigma.1.5.mm.3D_firstorder_RobustMeanAbsoluteDeviation	log.sigma.1.5.mm.3D_firstorder_Median
115.7071706	35027845.26	72.95449529	-14.89337254
101.2157843	5355038.022	73.56762507	8.130699158
108.0041734	15696662.68	77.62048261	11.15215302
112.7738567	6016148.759	81.90574505	26.85040951
104.9067423	22112130.87	78.56754181	2.946782827
108.4718103	5007591.7	70.43822395	29.70170689
64.00844726	93999773.1	35.3522547	-2.022999287
94.34585664	100233032.6	58.9712609	-12.90393448
76.77126153	46387666.4	47.97154727	-10.22986078
104.3277513	12842189.72	74.067181	10.33950901
64.11024322	11038420.18	42.13654294	-2.554917693
80.8030175	45688247.64	46.99878343	-5.26137352
103.3953656	13921237.29	71.24528858	15.96823215
117.5399534	13662057.95	84.24130632	-11.57928896
105.323308	31698059.17	67.3761665	-17.46670151
73.89297909	61441266.09	43.91807826	-4.522493839
75.95740179	8805881.378	50.39486557	1.873006225
90.26768742	5206288.847	65.39803574	-6.795936108
69.1944012	9511028.799	44.69216441	-4.91472435
85.48155077	126485731.7	54.04046443	5.269351959
73.53929702	17053956.88	52.77954549	6.026717663
102.6124854	7629662.566	75.73196039	-1.086315691
93.4022834	7903516.872	63.62207324	-9.933445454
55.73422022	24029631.94	30.72091588	-7.382033825
86.60777335	18960964.37	49.88784928	-5.682983398
88.1928271	36571172.56	55.02201096	-9.023310184
102.5982248	35974674.86	67.60717701	-21.01679516
77.06326126	24999431.89	47.66710846	-14.12742901
89.68305595	1901036.28	63.04373499	7.675280094
82.47134662	18544353.3	56.24355182	-7.219209909
81.72772694	43870159.67	56.06365547	-9.932735443
113.2162744	14200045.36	86.67809055	-9.129918575
81.37739233	28564281.77	50.91884834	-9.665901184
78.36801759	29861450.13	45.47539586	-3.810948372
87.5059618	71128763.7	56.08645683	-6.341662407
109.3181924	36320745.89	77.00484212	-1.109832287
127.9668446	38620218.87	89.20563896	24.07515335
74.78656581	72647035.07	39.98367884	0.62068516
76.8365491	5354838.023	53.50479708	-5.451764822
65.13183499	86846109.55	35.94708151	-8.141720772
87.20452788	15637420.09	63.077395	-14.2492795
67.71587604	55974454.18	37.50673896	-7.859449148
100.1584322	35333207.4	74.4984836	4.245599985
110.8446231	28028399.78	76.51184426	-17.61495399
65.67549192	13547995.28	38.23730674	-5.568698645
94.16715781	34222247.79	65.07158469	-10.3400631
76.64497283	61312527.2	46.03050624	-7.668366909
68.20538539	4045749.965	46.58269537	-4.551373959
104.5794525	6576437.459	74.03265442	7.524903297
109.5749604	8197988.478	78.71234255	31.52674961
112.7139486	27256388.56	76.90106275	-18.08969498
109.9945654	53314158.84	76.60243365	-9.884284973
53.55278911	52108971.73	27.86847597	-4.647448063
77.23286978	18555058.28	48.39988464	6.705311775
122.6253603	13434541.08	93.08168718	25.36512375
86.3502998	13380302.39	61.96254287	-13.51171064
77.58194222	43786705.12	49.18840966	-12.58511734
101.420307	32568201.17	73.06148678	-11.69555569
106.6531499	14345917.64	72.22256107	-24.07471848
67.88695293	113306954.5	36.34525905	-5.947703362
101.7767652	23153293.52	69.07307603	-6.275305748
121.3615775	59587603.56	82.77814642	3.086875916
104.1770885	156048409.6	68.03514072	2.117035389
99.84924425	32713366.44	70.98155957	7.969847918
77.5481372	7572797.395	49.49649949	4.159261227
128.9244855	89985834.05	90.05351199	4.134140015
103.9702293	6656015.603	75.00328644	11.22097874
107.4630234	98079265.45	72.1462117	-15.91502953
75.62619889	83502145.87	46.37033539	-10.0119276
88.05082206	25382132.37	62.75135071	15.90158367
100.1106643	7335237.068	72.80882332	-4.661228418
92.25354935	76735898.89	59.24143722	-3.996406555
102.2449615	21573446.83	73.670907	-16.45744514
112.4801294	19409533.37	77.25579624	-26.72694492
73.82436717	47940063	40.95237895	-5.50305438
79.61227189	9741545.807	54.3512121	5.556734085
94.22450439	33356110.27	63.70483825	0.324691355
117.9758342	6309368.781	89.35236981	17.23803329
97.49016778	19415117.87	69.00058877	15.7987175
76.90777317	21214473.97	45.52960074	-19.89539051
112.6357298	32395869.07	70.96984902	-15.84728527
97.41824277	12662853.56	67.18098271	14.38983297
96.47180213	38256619.22	62.7138779	-6.188660145
67.03119798	14481729.24	44.70815351	-18.93992805
75.73133763	29315886.38	46.22657509	-4.539249897
72.16008983	4777958.184	45.48713259	25.89038849
90.93210705	17646641.86	62.23644352	-18.03095627
63.40284631	32056519.22	35.88667792	-5.215508461
89.93159919	25419108.05	56.40273193	-5.148171902
101.7378053	21991444.88	72.65387251	14.36000061
84.81085667	10065347.01	54.91550128	23.01612663
65.88330739	22086966.47	38.22448074	-11.58618736
85.6668881	55925586.13	56.99084872	-4.404631615
81.48116653	38789199.24	54.50508653	0.670353115
84.76163547	38321162.54	58.54601781	-8.361621857
125.355551	59777619.14	87.25730158	3.501947999
95.74427332	35545895.73	66.78636159	-8.378259182
129.4344019	8373087.942	89.93623996	3.300762177
70.74191848	1978120.348	51.45761713	8.507395744
91.26617188	130731478.8	59.67762642	10.14524317

log.sigma.1.5.mm.3D_firstorder_TotalEnergy	log.sigma.1.5.mm.3D_firstorder_Maximum	log.sigma.1.5.mm.3D_firstorder_RootMeanSquared	log.sigma.1.5.mm.3D_firstorder_90Percentile	log.sigma.1.5.mm.3D_firstorder_Minimum
945751822.1	587.6693115	155.7482281	199.3410599	-538.2664185
144586026.6	405.8955078	124.2271558	130.2914368	-353.9539795
423809892.2	365.8826599	132.6542704	166.2153503	-409.8631287
162436016.5	336.54245	136.6883016	153.2412155	-408.4519958
597027533.4	324.1528015	125.1848551	162.3842773	-374.9009705
135204975.9	428.4788818	143.8489753	198.8815445	-447.02948
2537993874	528.0172729	94.44167921	121.3584885	-440.4091492
2706291880	619.1951294	128.5235311	171.5477264	-575.0892944
1252466993	467.6722107	104.3264765	145.0428299	-410.6760559
346739122.4	463.5272217	130.4205613	171.5733795	-534.7626343
298037344.8	290.1026917	84.22648261	119.9017258	-357.7702332
1233582686	595.7967529	115.802033	147.6055908	-629.6640625
375873406.8	387.9664917	131.0172534	147.3526917	-424.6177368
368875564.7	337.7005005	145.6515558	160.0629089	-497.3175049
855847597.5	540.8491821	141.8652595	182.2401184	-445.749115
1658914184	497.1834717	103.262694	146.2226273	-372.4168396
237758797.2	507.6724854	99.86333457	134.0181702	-454.8577576
140569798.9	330.7967529	112.8242553	111.1288788	-400.9917297
256797777.6	385.0445557	93.24060597	112.5074448	-491.1888428
3415114755	675.416748	117.3622634	144.9085846	-734.0783691
460456835.9	324.7512512	90.59199187	110.6421364	-272.0113831
206000889.3	481.664032	124.0257401	170.2604446	-307.9771118
213394955.5	346.756958	119.0126411	165.4885483	-319.1108093
648800062.3	421.8455505	81.73411047	101.3480652	-330.9771118
511946037.9	359.3904724	124.259908	157.5718582	-424.3149719
987421659.1	551.8752441	119.6159291	164.8512344	-387.8126526
971316221.1	544.2067261	133.9829637	167.6922165	-509.5943298
674984661.1	590.1654663	105.3378558	133.7757446	-350.0316467
51327979.56	374.1416016	114.1087045	165.1413345	-265.6412964
500697539.1	401.5098267	107.3230258	137.2643784	-629.5054932
1184494311	540.5509033	104.6084048	133.8211731	-345.6689148
383401224.8	334.9980164	134.2399424	151.9639526	-441.6339417
771235607.8	534.414978	110.6033323	145.8263	-395.9159546
806259153.6	545.5079346	111.1979753	152.5530853	-355.8911133
1920476620	557.7457886	117.716407	157.1405457	-539.1708984
980660139.1	396.3413696	136.8637625	173.1476166	-424.3653564
1042745909	552.5957031	159.8201264	164.8918945	-513.0869141
1961469947	600.1633301	111.7146547	154.1898346	-535.8204956
144580626.6	306.5601807	97.78655713	117.1708237	-389.7402649
2344844958	710.866333	95.88004438	122.4980942	-975.3607788
422210342.4	327.3530884	108.026462	127.2800369	-351.6542358
1511310263	554.3046875	99.34061004	126.4672897	-519.1929321
953996599.9	386.5455322	120.8824861	166.115213	-413.1477356
756766794.1	454.3097229	140.4930949	179.8152618	-423.0765076
365795872.5	364.78479	93.31097385	116.8446045	-399.65448
924000690.3	443.2987366	119.3873152	167.4885864	-430.1982422
1655438234	519.2166748	107.0427193	140.5340881	-483.7328796
109235249.1	234.1988983	90.7734357	108.9654007	-604.1706543
177563811.4	404.2420959	129.6901428	167.9809723	-325.9648132
221345688.9	375.5904846	134.6742093	169.437735	-425.0924988
735922491.2	435.7727051	145.5274376	201.1184387	-776.8497925
1439482289	583.2359619	138.3594035	195.4033447	-562.9511108
1406942237	453.1119385	80.8283436	99.72266769	-372.3085632
500986573.6	417.5357056	106.3027709	119.3614349	-666.4331055
362732609.1	330.7764282	144.5459512	182.9856537	-401.6888428
361268164.5	394.5870667	106.6668091	132.3717117	-372.5253906
1182241038	429.5100098	104.4307407	146.7886993	-336.1956177
879341431.7	538.6271362	125.5239646	160.6312103	-415.0302429
387339776.2	499.1443787	137.1203451	165.6105713	-488.9719238
3059287772	641.7009277	101.9892813	121.0795563	-541.7261963
625138925.1	553.2860107	131.9908721	153.6465942	-462.7819519
1608865296	535.7236938	155.4781978	206.302179	-576.4471436
4213307059	591.4620361	137.6652228	186.6383362	-598.8540649
883260893.7	473.6910095	124.9300691	160.9270859	-761.7989502
204465529.7	350.8988953	105.7630179	123.7166885	-508.295105
2429617519	578.3529663	162.1849443	199.9571075	-693.6226807
179712421.3	351.4292603	128.0395529	125.9292412	-344.2732239
2648140167	702.5320435	138.8810923	181.7820923	-492.489624
2254557938	619.6088257	105.4597375	127.2768021	-620.0002441
685317574.1	409.7781677	109.3167713	140.7748123	-441.7339783
198051400.8	324.704071	122.1024915	148.1747391	-351.7420044
2071869270	574.763916	124.119787	162.574234	-568.852478
582483064.5	527.9073486	127.1215374	151.5560638	-398.9577026
524057400.9	587.4760742	142.9373561	183.8722275	-438.9112549
1294381701	597.6796265	108.3443912	141.3190125	-425.0083008
263021736.8	545.0387573	103.0131335	124.2343788	-349.0053711
900614977.3	422.8044434	122.8824446	146.1961548	-738.2035522
170352957.1	317.9619751	139.762833	168.418988	-442.1019287
524208182.5	447.3302307	121.5086342	164.7841339	-491.0145874
572790797.2	615.7662964	108.5625114	135.6268814	-388.0861816
874688464.8	529.9468384	151.7931443	193.2660294	-538.2664185
341897046.1	394.4266968	123.8157146	146.0112915	-485.2907104
1032928719	394.7739868	129.4778485	172.1335495	-625.5123291
391006689.4	409.6388245	87.46512168	111.2977875	-309.6222229
791528932.2	426.1820679	103.9122139	149.7964691	-416.51474
129004871	203.2319794	94.85798866	93.33600616	-405.0350952
476459330.2	490.4629822	116.1521261	149.6397736	-340.2556763
865526018.8	402.4967957	91.41529869	122.5879631	-451.0883789
686315917.5	508.5470276	122.2441252	147.7545013	-582.406189
593769011.7	354.8549194	126.8360256	146.6705719	-650.8979492
271764369.4	433.0534668	111.8887319	137.672406	-380.4950562
596348094.7	373.2137451	93.39770082	113.9794632	-433.0514526
1509990825	501.128418	111.4681429	141.6039886	-363.3389587
1047308379	526.6922607	106.0956971	144.0996399	-345.5814209
1034671389	541.8444824	107.6470767	145.0923523	-457.8477173
1613995717	616.0355225	158.3493122	193.375676	-534.1970215
959739184.6	455.340271	120.402221	165.3990585	-407.5941467
226073374.4	446.6956787	163.557573	168.2096283	-505.433197
53409249.39	205.6000061	85.91301457	113.8783936	-225.3208313
3529749927	525.138916	121.7187089	156.3565689	-663.1251831

log.sigma.1.5.mm.3D_firstorder_Entropy	log.sigma.1.5.mm.3D_firstorder_StandardDeviation	log.sigma.1.5.mm.3D_firstorder_Range	log.sigma.1.5.mm.3D_firstorder_Variance	log.sigma.1.5.mm.3D_firstorder_10Percentile
4.604597408	155.5297373	1125.93573	24189.49918	-184.9064056
4.243406286	124.0633346	759.8494873	15391.71099	-166.1837341
4.431720774	132.6430942	775.7457886	17594.19044	-179.6427277
4.382470374	136.6768945	744.9944458	18680.57348	-189.9213806
4.326393608	125.1102813	699.053772	15652.58248	-155.4123688
4.434684948	142.0021726	875.5083618	20164.61701	-159.4077225
3.754409269	94.43801617	968.4264221	8918.538898	-112.8703064
4.304799824	128.4109205	1194.284424	16489.36449	-157.1250351
3.996244339	104.246314	878.3482666	10867.29398	-131.7564346
4.39365255	129.7908177	998.289856	16845.65636	-156.2567993
3.743393627	84.11572321	647.8729248	7075.454892	-101.4024162
4.095055562	115.7987796	1225.460815	13409.35736	-132.2857391
4.332607165	130.9764083	812.5842285	17154.81953	-195.1026917
4.545192688	144.0123685	835.0180054	20739.5623	-216.0076523
4.416006421	141.3964151	986.5982971	19992.9462	-180.1263672
3.912502284	103.2626934	869.6003113	10663.18384	-138.0280609
3.985884534	99.69531885	962.5302429	9939.156601	-111.3311584
4.125231863	111.6278094	731.7884827	12460.76784	-169.2646667
3.866439653	93.22605415	876.2333984	8691.097172	-111.2009453
4.201943393	117.1741948	1409.495117	13729.79192	-131.9304504
3.880168059	90.55013762	596.7626343	8199.327422	-115.4843407
4.289734663	123.9969029	789.6411438	15375.23192	-156.5767441
4.252530357	119.0100106	665.8677673	14163.38262	-150.2354507
3.568313427	81.68694571	752.8226624	6672.757099	-90.35579987
4.073442177	124.0645633	783.7054443	15392.01588	-176.2525818
4.17910522	119.4422549	939.6878967	14266.45225	-126.8472519
4.417261207	133.7217747	1053.801056	17881.51302	-162.1036835
4.030678746	105.1404041	940.197113	11054.50457	-128.9539246
4.010722653	112.1402664	639.7828979	12575.43935	-111.0832176
4.087694733	107.3195627	1031.01532	11517.48853	-127.5859047
4.092729222	104.4579176	886.2198181	10911.45654	-139.6842804
4.380123556	133.4838776	776.631958	17817.94557	-191.2504898
4.106868889	110.5736817	930.3309326	12226.53909	-133.7391724
4.013236276	111.1979624	901.3990479	12364.98684	-138.1081207
4.208818799	117.697482	1096.916687	13852.69728	-134.0827545
4.478153516	136.8584365	820.7067261	18730.23163	-183.5399048
4.61641022	159.5650604	1065.682617	25461.00849	-238.9811142
3.876750416	111.7086841	1135.983826	12478.83011	-146.6985626
3.974398084	97.58790341	696.3004456	9523.398892	-127.5891998
3.761950779	95.84199824	1686.227112	9185.688626	-112.0893066
4.130698501	107.3628934	679.0073242	11526.79088	-148.9411194
3.817572965	99.33020918	1073.49762	9866.490457	-106.8470825
4.293601602	120.7545706	799.6932678	14581.66632	-146.827536
4.510755923	140.3137518	877.3862305	19687.94894	-176.7967972
3.811133999	93.29679957	764.43927	8704.29281	-109.5955238
4.260074608	119.3440817	873.4969788	14243.00984	-135.2591553
4.019187955	107.0424717	1002.949554	11458.09074	-122.7063446
3.774903214	90.70814811	838.3695526	8227.968133	-108.1168594
4.361243793	129.5399743	730.2069092	16780.60493	-164.7844696
4.366817795	134.5139232	800.6829834	18093.99553	-189.1805679
4.489997988	145.4600202	1212.622498	21158.61747	-173.6431488
4.475169195	138.1147254	1146.187073	19075.67737	-153.5625824
3.495897716	80.81701929	825.4205017	6531.390608	-91.5786972
4.034714816	106.2966938	1083.968811	11298.98711	-136.5066101
4.491606632	144.431909	732.465271	20860.57633	-188.5825043
4.116953259	106.6183093	767.1124573	11367.46389	-128.4290695
4.004734372	104.3002992	765.7056274	10878.55241	-126.5396835
4.350567092	125.458911	953.6573792	15739.93834	-158.7257385
4.442044853	136.6930013	988.1163025	18684.97661	-171.2806122
3.840846819	101.9845709	1183.427124	10400.85271	-112.5218307
4.400518757	131.9616464	1016.067963	17413.87613	-165.8191162
4.666234823	155.2812243	1112.170837	24112.25862	-186.3274841
4.46728772	137.5287891	1190.316101	18914.16783	-168.265239
4.338005473	124.8459679	1235.48996	15586.51571	-148.1255722
4.017638964	105.7521273	859.1940002	11183.51244	-122.3065842
4.726082443	162.1727166	1271.975647	26299.99001	-211.2630005
4.277559156	126.9806568	695.7024841	16124.0872	-194.0754013
4.488158008	138.8033961	1195.021667	19266.38276	-169.6027313
4.013440421	105.4525172	1239.60907	11120.23339	-112.4435524
4.149052777	109.102266	851.512146	11903.30444	-138.0566864
4.284004219	122.0321954	676.4460754	14891.85671	-156.5512009
4.288821374	124.1195372	1143.616394	15405.65951	-156.6894073
4.340013916	126.7798819	926.8650513	16073.13846	-167.278302
4.463451979	142.745143	1026.387329	20376.17586	-174.4949677
3.933503653	108.3394389	1022.687927	11737.43402	-127.7204582
4.026002399	102.7175442	894.0441284	10550.89388	-118.0254868
4.29594592	122.8818462	1161.007996	15099.94812	-144.7313538
4.416432233	139.7123208	760.0639038	19519.5326	-196.1018768
4.303821331	121.0720404	938.3448181	14658.43897	-144.6369873
4.002412151	108.1900296	1003.852478	11705.08251	-124.6568604
4.565872499	151.4925061	1068.213257	22949.9794	-183.4478302
4.308334919	123.6423722	879.7174072	15287.43621	-152.2056961
4.268893924	129.3877251	1020.286316	16741.18341	-176.9112671
3.804593912	86.87990336	719.2610474	7548.117607	-108.5238358
3.986143234	103.8852277	842.6968079	10792.14054	-121.3630249
3.690394452	94.85769478	608.2670746	8997.982259	-141.1951447
4.219714388	115.9302993	830.7186584	13439.83429	-146.2277191
3.72573545	91.40627542	853.5851746	8355.107186	-105.2414742
4.252331013	122.1625988	1090.953217	14923.70054	-161.820816
4.333176145	126.8349904	1005.752869	16087.1148	-157.8784912
4.126603727	111.5583577	813.5485229	12445.26718	-143.5323792
3.80415527	93.26502598	806.2651978	8698.36507	-109.4420135
4.182467551	111.4593898	864.4673767	12423.19558	-139.568634
4.107844978	105.9323053	872.2736816	11221.6533	-121.0225945
4.117585455	107.5756407	999.6921997	11572.51847	-121.7716629
4.689775653	158.3480183	1150.232544	25074.0949	-199.9592361
4.288158436	120.3314501	862.9344177	14479.65789	-140.3361786
4.62167358	162.9113182	952.1288757	26540.09761	-225.4258789
3.778029658	85.88094482	430.9208374	7375.536684	-108.5969025
4.283571124	121.4730531	1188.264099	14755.70262	-143.8865143

log.sigma.1.5.mm.3D_firstorder_Kurtosis	log.sigma.1.5.mm.3D_firstorder_Mean	log.sigma.1.5.mm.3D_glrlm_ShortRunLowGrayLevelEmphasis	log.sigma.1.5.mm.3D_glrlm_GrayLevelVariance
3.853238978	-8.246901575	0.005211469	40.15215327
2.935074125	-6.377714387	0.010414112	25.00538845
2.642027313	1.721926024	0.005897719	28.93066519
2.812697805	-1.765876468	0.009860502	30.57676393
2.483498091	4.32035587	0.008335635	25.54400032
3.64143185	22.9763067	0.009228919	32.95915804
5.23365796	0.831789517	0.003364494	16.12710883
4.029152596	-5.378992653	0.002088854	28.165437
3.655921455	-4.088975526	0.004218076	18.57659162
3.286120965	12.80103312	0.003612507	27.79284183
3.562585611	4.318041249	0.005361132	11.90367383
5.384383998	0.868032385	0.001812967	23.32758951
2.927672636	-3.27126464	0.007219252	28.11398161
2.722842329	-21.79021349	0.005450404	34.17144982
3.584239716	-11.52413348	0.005015047	33.83191262
3.655083809	0.011657154	0.005908214	18.45272671
4.518361821	5.790422351	0.004022512	16.54135305
2.923215931	-16.38733481	0.007303599	20.42765343
4.912139586	-1.647249368	0.003531045	14.65039467
5.749116121	6.641456405	0.001278952	23.15493502
2.98035733	2.753464933	0.011153926	13.50411817
2.686344335	2.674375693	0.011523877	24.93462055
2.89011368	-0.791270079	0.012782845	23.71058987
5.394625719	-2.776277247	0.005901663	11.8631277
3.62014809	-6.964829743	0.006537551	27.56146678
4.433166565	6.443464322	0.004901297	24.4360739
3.707691248	-8.361909751	0.003743858	29.63537146
4.571531644	-6.446649739	0.006484171	18.7586603
3.226293191	21.10348511	0.017747209	20.30647957
5.050938966	-0.862166811	0.002388348	19.33327548
3.219233791	-5.609082696	0.007421568	18.3458831
2.305294213	-14.22731753	0.005839195	28.70243273
4.210878597	-2.560864711	0.005478893	21.02325097
4.149540742	0.053543458	0.006275957	21.02480492
4.410568256	2.110735667	0.002467887	23.55102245
2.796564109	-1.207413419	0.007337704	30.75835279
3.120068004	-9.025758001	0.005338576	41.48087673
4.428860492	1.154971985	0.00238628	22.64541412
3.568605946	-6.229916696	0.006964614	15.84857252
6.471861389	-2.700793178	0.000695989	16.23738551
2.714248072	-11.95514946	0.006769172	19.1757949
6.198476935	1.437478947	0.002400577	17.28509776
2.521156509	5.559596648	0.004755271	23.8679043
3.023462308	-7.096532211	0.006503112	32.69639623
4.682516541	-1.626354806	0.005176256	15.38066339
3.153227379	3.21266013	0.003959569	23.71663843
4.803593893	0.230237524	0.002975393	19.67194779
7.685191311	-3.44216444	0.003773162	13.79396876
2.89564296	6.239247638	0.010986558	27.68287211
2.607797337	6.568646629	0.006145324	29.58305059
3.488019103	-4.429178211	0.001842519	35.10190479
3.226959799	8.224789811	0.002410384	31.27532228
5.612411749	-1.352967748	0.0048965	12.02326275
5.858585341	-1.136659513	0.002073235	19.30342886
2.2935975	5.740703086	0.006906189	33.71035943
3.128313632	-3.216250094	0.007096975	18.79124995
3.560567139	-5.217968189	0.006903603	18.58313902
3.007538859	-4.040710849	0.005299288	25.89216492
3.409433623	-10.81722829	0.004710837	30.84929882
6.441302917	-0.980201743	0.002166901	18.33892547
3.996122368	-2.777441535	0.004677549	29.27418639
3.403887704	7.823770375	0.002622245	39.88940342
3.481753938	6.127457967	0.002071706	31.78167874
3.860774071	4.583279163	0.001547877	25.64387244
5.534663432	1.51773147	0.005968569	19.01371172
3.214373408	-1.991521001	0.002076648	42.96638757
2.648513126	-16.4328909	0.019897766	26.5155564
3.533333611	-4.644894358	0.003471407	31.99209056
5.981913332	-1.234041028	0.001788091	18.91309458
3.080040795	6.844855607	0.003949688	19.51100969
2.595756262	-4.142670464	0.00922839	24.53264618
3.950385672	-0.249010497	0.002499684	25.94310034
3.228199909	-9.313796892	0.006416374	26.46521579
3.3348854	-7.410256276	0.005451942	33.47657866
5.085529482	-1.035894076	0.003750675	20.84516855
4.414302415	7.798191369	0.007295995	17.55645965
4.948895831	0.383489403	0.001635846	24.97054199
2.321294294	-3.757243811	0.00737114	31.53003473
3.091017153	10.29122072	0.00350295	24.00013741
5.403393063	-8.985341271	0.00607291	19.96411777
3.854805376	-9.548782645	0.005158535	38.15688245
3.39799726	6.549425293	0.004136996	25.05280284
3.341847282	-4.830096692	0.002093973	28.63351123
3.604210474	-10.10098526	0.008473192	12.81320911
3.78184883	2.368050808	0.004227011	18.78625033
4.580039447	0.2361212	0.006666741	15.14190473
3.477450723	-7.17510423	0.008362212	22.41871492
4.807569434	1.284386273	0.002979079	15.09381392
4.066424554	-4.463809609	0.002540633	25.38162898
3.87938296	0.512423606	0.002249747	26.44945182
3.833174709	8.591923383	0.006907363	20.55074883
4.806713252	-4.976489493	0.003745094	15.2937534
3.436381119	-1.396890738	0.006785287	20.71109669
3.784550292	5.885884541	0.008113164	18.77816694
3.644280579	3.921052546	0.003199061	19.30095494
3.317313012	0.640136415	0.003758656	41.16021902
3.163966254	4.127583496	0.004541571	23.88744434
3.237492121	-14.5252223	0.008613781	43.25687016
2.45878138	2.347208847	0.017977857	12.26469962
4.583868304	7.729261636	0.001634915	24.73284801

log.sigma.1.5.mm.3D_glrlm_LowGrayLevelRunEmphasis	log.sigma.1.5.mm.3D_glrlm_GrayLevelNonUniformityNormalized	log.sigma.1.5.mm.3D_glrlm_RunVariance	log.sigma.1.5.mm.3D_glrlm_GrayLevelNonUniformity
0.00531565	0.051103884	0.067931419	69.57218061
0.010634783	0.061346823	0.053422693	20.27729195
0.006074234	0.052039516	0.071379577	43.77810919
0.010006021	0.056427003	0.051223093	17.31177093
0.008568856	0.0555443	0.065631981	73.99438963
0.009335197	0.055093805	0.046747933	12.7603477
0.003680011	0.096279454	0.273135587	867.4461222
0.002204237	0.062899874	0.127200748	348.3086327
0.004475373	0.077706418	0.139317983	299.395966
0.003693227	0.054799885	0.056454142	39.31812595
0.005637216	0.089893623	0.109807832	128.4279519
0.001922927	0.076031357	0.156898594	232.4285605
0.007425117	0.059110404	0.074706472	44.98175433
0.005551433	0.048458884	0.045238957	29.91134866
0.005226008	0.057535897	0.110481544	83.49899991
0.006295839	0.08561658	0.183895197	436.7497734
0.004185754	0.074810261	0.096239257	61.00710568
0.007479269	0.065055477	0.060875746	25.1962891
0.003696648	0.082880021	0.114184033	82.78385934
0.001339441	0.067258863	0.101907333	570.4899916
0.011795375	0.077155113	0.101150882	147.3208079
0.011853654	0.056706752	0.054987527	26.70608425
0.013168575	0.060105763	0.083517934	31.33847699
0.006370583	0.111111422	0.196567596	351.1898902
0.006930078	0.077493727	0.236791842	82.82057604
0.005171726	0.068205782	0.124934591	158.9644359
0.003860244	0.0562872	0.071225595	106.0570237
0.006790617	0.076303577	0.107170944	157.7570339
0.018157315	0.070428176	0.046587566	9.859779298
0.00247014	0.068223378	0.092487325	101.9592538
0.007778578	0.067351518	0.111381322	247.8677422
0.005986524	0.052328701	0.054112379	39.21955005
0.005760187	0.070924575	0.131700011	150.3624271
0.006593065	0.083235787	0.127059537	182.9310164
0.002600623	0.065669601	0.117858725	308.2318466
0.007558182	0.050850545	0.073096123	92.74263167
0.005431794	0.048924739	0.050981729	70.63477422
0.002582954	0.093211016	0.268828654	466.3065199
0.00716861	0.073388641	0.076003268	38.49560289
0.000749255	0.097668976	0.185542601	813.5762165
0.007024006	0.063721997	0.076520851	80.02789975
0.002579916	0.094124392	0.16401221	477.0032763
0.004940605	0.056304462	0.077453339	127.7333589
0.006681838	0.05075567	0.063542648	68.12868888
0.005521055	0.088936848	0.200407659	121.7906173
0.004135773	0.060692358	0.08767832	135.6975022
0.0031625	0.078382119	0.136364136	380.3594757
0.003866588	0.083484573	0.0913187	37.95697102
0.011244305	0.055767663	0.056921103	20.70777358
0.006291319	0.055602012	0.058416779	23.84351835
0.001885717	0.052859893	0.070411221	64.18449495
0.00248949	0.052297552	0.058732381	138.1499022
0.005389525	0.116399891	0.281398824	783.9213135
0.002159639	0.075184545	0.117542295	112.6552818
0.007055315	0.049387552	0.048822997	30.34703311
0.007416912	0.066039576	0.073483194	72.71514009
0.007300778	0.075918921	0.142562321	275.8317282
0.005477961	0.055549624	0.068847053	108.1081463
0.004828715	0.053696942	0.061485453	38.80241597
0.002336481	0.094533234	0.17097096	914.0685836
0.004844351	0.055808836	0.090457506	68.98879884
0.002691349	0.046070677	0.0611612	107.7134686
0.002169214	0.054047969	0.104921845	411.3419522
0.001591803	0.05681352	0.061951945	112.5632846
0.00610091	0.074835034	0.108277235	46.65957833
0.002124201	0.043804852	0.051614864	143.0420617
0.02021255	0.059519055	0.058413866	22.9162314
0.003602403	0.052822051	0.073994181	252.3362556
0.001891794	0.078432217	0.116435376	538.1140127
0.004093583	0.064688676	0.072366591	129.0232775
0.009499501	0.057813537	0.07544506	26.6378339
0.002611015	0.063300303	0.106021692	290.7881575
0.006642408	0.055660597	0.076837325	69.68168639
0.005585758	0.05335631	0.050574632	48.44895371
0.004019375	0.087248621	0.205288259	313.5930337
0.007588719	0.071688858	0.095646595	61.1702897
0.001690078	0.060691227	0.070800899	125.7453528
0.007505842	0.051757006	0.048612436	16.02063991
0.003612705	0.058091107	0.063779371	72.16230199
0.006349739	0.080189484	0.117681034	131.9985399
0.00526434	0.052434761	0.068901599	69.45689429
0.004255131	0.059117345	0.068798561	45.8981582
0.00219127	0.06377138	0.143658144	132.3546923
0.008930047	0.084365534	0.116289868	145.6753754
0.004503701	0.077781735	0.171894665	188.4181208
0.006855808	0.097211966	0.099046847	47.52025663
0.008708489	0.062054815	0.094938532	75.31451237
0.003263672	0.094244822	0.267742948	309.1353472
0.002644033	0.064514442	0.118641038	100.8019105
0.00230695	0.057324435	0.061479589	74.13920958
0.0071004	0.070629615	0.077537699	53.05898274
0.004007763	0.090282565	0.168317009	202.8195772
0.007046479	0.065131667	0.088910792	272.4691921
0.008421184	0.068688388	0.095783857	219.2620063
0.003358554	0.067113719	0.090723079	205.916898
0.003848082	0.045073249	0.061259062	101.9261862
0.004730155	0.05925162	0.078970907	135.9920013
0.008698794	0.048334183	0.043067852	14.52320251
0.018702044	0.080890211	0.082907282	20.11043968
0.001708598	0.061961082	0.095135019	506.2254495

log.sigma.1.5.mm.3D_glrlm_LongRunEmphasis	log.sigma.1.5.mm.3D_glrlm_ShortRunHighGrayLevelEmphasis	log.sigma.1.5.mm.3D_glrlm_RunLengthNonUniformity	log.sigma.1.5.mm.3D_glrlm_ShortRunEmphasis
1.193818697	510.0869635	1217.621634	0.957343175
1.156583304	250.0936743	301.0122031	0.964149225
1.197408376	324.9511433	755.0908702	0.95829124
1.153848559	321.7866126	279.0019483	0.963804907
1.188067341	261.3462907	1194.251228	0.958280538
1.138968237	396.7545189	212.7023735	0.967451062
1.649416665	325.3453973	6921.216982	0.897908633
1.329627407	583.8635946	4711.524496	0.937787999
1.365122475	299.2811048	3221.266859	0.930911437
1.164313928	538.483871	650.5595862	0.962696126
1.299839752	243.639859	1220.976276	0.939351033
1.401613403	678.7475107	2528.434609	0.926710448
1.21144706	313.3072778	674.5711561	0.95396793
1.134311808	406.4090539	567.7898535	0.96831202
1.28951561	342.9017686	1255.092843	0.94408037
1.465047754	239.2933242	4134.078458	0.918320188
1.270221093	386.5671731	703.4311757	0.943097792
1.177129433	292.5648719	349.0005978	0.960096461
1.31626798	405.6214129	844.1562356	0.935264502
1.275142293	916.5103844	7336.904432	0.944354133
1.286538231	140.6375457	1628.634877	0.939066733
1.164815333	204.2159077	425.3879625	0.961350986
1.229992314	198.2102531	459.8044913	0.951854992
1.49707228	202.2354718	2518.313807	0.912165155
1.563347942	296.9990062	851.6967292	0.911227383
1.330187181	289.8879195	1972.935437	0.935822365
1.203200308	460.3225141	1677.585311	0.955513579
1.296265271	238.0500794	1763.212929	0.938954236
1.135132644	169.1757586	129.2518057	0.969331862
1.254329096	686.9365164	1302.977737	0.947334875
1.299220493	210.3566506	3154.393502	0.940616921
1.160839101	337.4558915	680.0213759	0.962680859
1.347030352	273.0888908	1784.207502	0.933555461
1.337379007	246.0690631	1855.571731	0.934779764
1.314628266	504.4076893	3994.359986	0.938056431
1.204244941	322.0339155	1626.498338	0.9561656
1.148935051	469.4036066	1320.95794	0.965930478
1.627368108	482.1450427	3907.730751	0.904275516
1.216503231	268.0797768	463.590237	0.95278773
1.474745398	1513.77087	6681.526291	0.914958759
1.215809042	234.4856844	1111.773297	0.953374606
1.420672093	449.9254371	4147.95304	0.922884777
1.217701144	324.5946666	2016.86727	0.953859828
1.183172205	318.5433929	1205.507184	0.959076975
1.495448235	265.0921554	1101.93973	0.915770601
1.242326322	355.7057811	1959.246375	0.949353316
1.354280045	415.3689554	4079.037489	0.933193048
1.259686344	624.4425076	393.7351768	0.944538796
1.16671837	239.1278681	336.1164927	0.961958069
1.170739288	367.7668635	387.6635385	0.961334315
1.195122042	1040.362899	1090.067236	0.958656077
1.170654809	579.3092344	2386.512927	0.961336874
1.688309245	225.5113468	5062.293138	0.889657196
1.320651129	721.9584686	1268.823985	0.935798396
1.14447488	335.3486971	562.4863468	0.966349937
1.214569286	245.6148609	969.629432	0.95166877
1.365950967	210.50593	3047.554176	0.932143884
1.197647989	313.6521635	1735.823302	0.956337375
1.176896882	419.7855994	651.6596554	0.960525669
1.442105725	484.6074683	7838.865667	0.919215532
1.248070456	384.9606341	1081.209394	0.9485515
1.173210486	631.9697163	2115.594687	0.961887828
1.276512395	611.4913432	6606.534031	0.945645439
1.181557318	988.0845333	1777.129109	0.958641159
1.28977747	457.3942456	536.8139342	0.942423949
1.149442753	823.3901882	2985.799026	0.965993751
1.171244424	209.2228213	347.3950262	0.960799705
1.208258122	428.2312368	4247.788357	0.95505703
1.315522314	627.7612009	5819.249285	0.936798464
1.207365755	355.0362695	1770.165563	0.95438974
1.217818031	248.9006511	406.9901784	0.952157694
1.282847474	547.1380453	3968.391622	0.943802069
1.214923623	275.4633855	1110.798825	0.954176822
1.145959717	357.0270383	833.0534182	0.967029169
1.501164006	333.8266911	2891.115964	0.91583312
1.25626361	227.5649203	746.1824142	0.948282828
1.20817946	913.2374013	1830.531713	0.952813083
1.137864603	359.9315921	285.5933072	0.969308219
1.185209184	443.6161738	1113.65952	0.958320426
1.315313688	266.2219831	1399.389892	0.937587182
1.196399768	505.8762944	1183.241373	0.956860397
1.201478607	434.6208202	688.9297222	0.954434204
1.357927175	677.6231879	1763.123294	0.936547008
1.319341034	174.7878693	1458.560146	0.935202569
1.432889175	306.7238784	1985.817435	0.923104047
1.283471575	297.1871757	418.5607467	0.939967784
1.257864531	215.6351776	1059.573323	0.947669813
1.640376195	362.5660804	2528.40359	0.899362519
1.305634795	582.7620654	1343.989245	0.942058253
1.179155317	751.2564654	1162.146619	0.959306794
1.223817472	287.6807859	659.2310394	0.950265524
1.441509395	324.7669923	1819.176664	0.918483388
1.247271257	247.0035258	3650.501531	0.947902664
1.26263824	225.2620087	2772.398197	0.945895516
1.253050333	387.7644353	2668.716423	0.946642569
1.17322031	529.0251876	2047.646266	0.962055264
1.221271641	323.0529439	2026.728898	0.952434277
1.129080671	466.7185563	277.3730452	0.96946007
1.246284382	117.6842928	215.1780356	0.944445911
1.26263632	754.6542494	7081.916696	0.945285607

log.sigma.1.5.mm.3D_glrlm_LongRunHighGrayLevelEmphasis	log.sigma.1.5.mm.3D_glrlm_RunPercentage	log.sigma.1.5.mm.3D_glrlm_LongRunLowGrayLevelEmphasis	log.sigma.1.5.mm.3D_glrlm_RunEntropy
628.4837437	0.942733859	0.005781182	4.934511703
294.8053802	0.952560408	0.011622122	4.484232776
400.6555301	0.943083822	0.006887401	4.736817123
383.9331227	0.952699474	0.010628491	4.628862798
319.327124	0.944120373	0.009623661	4.6426798
463.8072852	0.957088366	0.009780834	4.636585529
582.1105595	0.853890677	0.00567033	4.529453905
810.4742159	0.912390345	0.002811082	4.79874095
428.6546643	0.903855178	0.00582002	4.527442892
644.1332321	0.950280183	0.004047609	4.66917238
331.1569567	0.917737789	0.006993659	4.191901302
1007.776964	0.89699939	0.002521949	4.664691846
399.1029508	0.938252869	0.008343833	4.671375707
473.1983597	0.958432871	0.005975568	4.77827765
453.0453579	0.921318681	0.006297918	4.849447877
371.5841685	0.884895736	0.008487688	4.533461528
509.4288069	0.923512501	0.004966101	4.395644109
354.565773	0.946962573	0.00827615	4.400147708
564.6418426	0.912811138	0.004504074	4.335001193
1232.139902	0.923587901	0.001638752	4.641264993
187.7733674	0.918893907	0.014845258	4.315086412
241.8726423	0.949441687	0.013246004	4.556719558
246.4918085	0.934243176	0.01496407	4.599185324
319.9445759	0.878039392	0.008991537	4.227815505
494.5020782	0.86970684	0.009211531	4.759219262
396.5565447	0.91161069	0.006540256	4.663787237
567.144717	0.940234915	0.004393787	4.763395042
319.5582022	0.917477551	0.008258064	4.493659878
191.3467042	0.95890411	0.01989383	4.19528278
894.5481246	0.928189202	0.002866321	4.485749382
284.0955124	0.917973022	0.009561982	4.541775358
404.5118493	0.951093323	0.006633533	4.64828292
382.7846365	0.907725251	0.007212351	4.611469697
343.6393637	0.909635292	0.008216626	4.519741145
692.8159537	0.914355078	0.003270952	4.687751929
399.8497687	0.940572063	0.008544314	4.812201624
562.8775776	0.954822955	0.005829624	4.881355946
852.8388687	0.858773935	0.003853554	4.643816937
337.4619813	0.936675824	0.008108919	4.312063623
2416.564589	0.881403132	0.001046171	4.417298848
294.2330601	0.937198622	0.008210057	4.478663105
673.4731386	0.892942389	0.003548398	4.412540165
407.344378	0.938156137	0.005817177	4.634023653
382.5347956	0.945232936	0.007480211	4.827385794
417.0222837	0.879721179	0.007525886	4.440858969
452.3309803	0.931086406	0.004973368	4.647930918
586.3220084	0.906588273	0.004131902	4.542473494
819.2612894	0.925896914	0.004304868	4.155645065
281.0283389	0.949636042	0.012363269	4.623824648
443.9341293	0.948604493	0.006926675	4.632582336
1279.103196	0.943278943	0.002089089	4.816102755
694.6485648	0.948515398	0.002839722	4.782438196
414.3901106	0.843897076	0.008424656	4.320758872
1020.083816	0.912442612	0.002589324	4.513319315
397.2614702	0.955616701	0.00768689	4.731165751
304.2160408	0.936289901	0.008818926	4.470118308
297.118351	0.904760992	0.009402727	4.526927026
387.2028886	0.94149827	0.006287357	4.686265833
503.1358883	0.947071277	0.005354637	4.73910185
743.7787132	0.887429471	0.003256803	4.46999891
500.5301721	0.930022573	0.005631964	4.785746389
763.9673	0.948447496	0.003004078	4.971557488
813.606877	0.92420732	0.002662552	4.898884629
1210.720026	0.945243688	0.001785596	4.657883915
618.2139102	0.920690831	0.006762432	4.438807564
972.4591029	0.954511726	0.002333427	5.004451754
254.7258951	0.948275862	0.02154417	4.552141827
529.5819338	0.93933893	0.004207898	4.845534535
869.1711504	0.913671571	0.002409083	4.504391704
447.8882582	0.938975808	0.004749743	4.499472783
313.2951237	0.936366479	0.010725331	4.612319795
734.526883	0.922150325	0.003168502	4.732485875
342.8840931	0.937712475	0.007687619	4.685286752
412.7647484	0.955789474	0.00617949	4.717234179
531.628839	0.879398026	0.005593392	4.59400681
292.1305658	0.929277694	0.009019059	4.403081184
1150.473589	0.937911342	0.001928514	4.654992374
415.2288378	0.95832341	0.008104308	4.616002452
544.4414429	0.944603685	0.004096559	4.618561427
361.1305948	0.914273504	0.007729522	4.480858912
624.5686349	0.942061495	0.005737737	4.898863136
546.6033151	0.939932948	0.004772788	4.636926047
969.3734969	0.908885593	0.002737024	4.768312411
237.9075609	0.912105327	0.011172787	4.282864675
460.5670229	0.891712707	0.006044939	4.567545505
419.5345006	0.920179632	0.007732402	4.11782272
275.8391577	0.927840508	0.010381632	4.610556695
636.1263283	0.854375551	0.005059112	4.484054744
799.723205	0.918328585	0.003186569	4.700588826
922.4667977	0.946091948	0.002558949	4.643798344
372.6809014	0.934270953	0.007955097	4.491340497
496.5518703	0.887015433	0.005413072	4.410371456
319.1800211	0.929383214	0.008272821	4.589006112
292.9214973	0.926202063	0.009904039	4.522307238
500.7658282	0.927775581	0.004115208	4.526303492
638.8398648	0.948535106	0.004256319	4.990565787
403.3087938	0.936002008	0.005606431	4.652522781
543.5879827	0.959941017	0.009051138	4.823214298
153.7635031	0.927669346	0.021832821	4.126396189
1004.711833	0.925814213	0.002061641	4.710039249

log.sigma.1.5.mm.3D_glrlm_HighGrayLevelRunEmphasis	log.sigma.1.5.mm.3D_glrlm_RunLengthNonUniformityNormalized	log.sigma.1.5.mm.3D_glszm_GrayLevelVariance
531.4581886	0.894103187	57.86395164
258.523823	0.910135769	32.84100993
338.0546869	0.89687658	39.22204734
333.6865146	0.909048691	40.90931129
271.9951159	0.896202798	33.74888296
409.3672311	0.918000672	42.03441846
360.4522826	0.767955257	32.32423081
619.8801756	0.850602356	46.16863862
319.9670538	0.835587608	31.41263101
558.0894929	0.906506182	38.09545956
258.4409698	0.853986225	18.76447963
729.6319063	0.826714928	42.87696195
328.6286971	0.88617567	38.95886331
419.1191734	0.919657602	43.9498071
360.8341422	0.864574743	50.4300692
259.2820615	0.809542971	29.70865002
408.1246429	0.861952227	25.95787593
304.1480762	0.900583747	26.32168459
432.7203228	0.844568538	25.95284388
969.8007237	0.864692462	39.73056875
148.9381643	0.852482306	20.33530464
211.3301665	0.903051238	30.87852103
206.6635877	0.881683352	34.24545413
219.9622587	0.796291535	24.58549549
324.118323	0.796093449	43.59232255
307.2759632	0.846056977	40.94152736
479.6130153	0.88993801	44.42517049
251.9799725	0.852535294	33.18022253
173.2697849	0.922737041	25.68604997
722.7833192	0.871436518	29.69885188
222.6354772	0.856658973	28.41048987
350.2112712	0.906692366	34.04580373
290.7960842	0.841208286	35.87897701
261.9580993	0.844006219	36.33934439
535.5061871	0.850789089	39.00029897
335.7937881	0.891475913	38.95424531
486.5772962	0.914435194	53.37484205
531.2611438	0.781092583	40.29537222
280.5560648	0.883499048	23.27829787
1651.011163	0.801816098	32.59083015
245.2291582	0.884922321	26.90352041
484.5032811	0.818275702	32.98988717
339.1683971	0.8873419	29.98201851
330.3169502	0.897951373	46.04591492
287.1982652	0.804165043	30.62894123
372.5117385	0.875949086	33.73393704
442.5592651	0.84040605	35.41431714
658.8297868	0.865262041	22.53716339
246.9655188	0.904798578	36.03989537
381.8016469	0.903616414	35.48196349
1082.204219	0.897542928	49.35073731
600.7668937	0.903216958	41.49343153
251.6478559	0.751442925	27.74746203
771.6912602	0.846090178	36.15956469
346.9818828	0.915108891	38.80327204
256.5151449	0.880446238	25.60121879
224.1328832	0.838323388	30.2899036
327.0580157	0.891666901	35.31501511
435.0402371	0.90153191	43.51144544
524.6230748	0.810506495	37.02398479
404.9484203	0.874178809	43.49322197
655.8979411	0.904715569	53.92751894
644.9162563	0.867809051	47.60395278
1029.609282	0.896780049	34.46226647
484.1457784	0.860619533	32.62522228
851.1391606	0.914253823	55.73296917
217.5912706	0.901949167	34.74204766
446.3633586	0.888893016	45.44145821
668.2282517	0.847871926	35.76469172
371.8794679	0.887204282	27.56356993
260.5948411	0.882581521	34.21079365
578.354719	0.863616422	40.69225025
287.2994426	0.886968749	37.91092951
367.2908864	0.917086563	42.88049124
362.4054461	0.804198556	39.39127826
238.4271195	0.873772721	27.21977659
957.0597939	0.883233031	38.50950774
370.0674653	0.922474544	35.86851111
462.0848689	0.896218572	31.57312711
281.9020067	0.849825963	36.22835809
527.2419768	0.892977912	55.31918684
455.1474048	0.887000195	35.11774691
721.7614691	0.848860597	43.86316754
185.5086705	0.844328345	22.23570673
330.0684286	0.819247985	33.34259979
318.4712085	0.855373008	23.05676768
225.8436683	0.872522482	33.60558469
399.8285882	0.770863962	29.20857396
617.5266929	0.85996552	41.29939298
783.04251	0.898349132	36.87400467
303.1067416	0.87735097	31.28895621
351.4864424	0.809264462	31.14447618
259.6100142	0.872405761	32.41792031
236.8843885	0.868167752	29.59109897
407.5957949	0.869555007	28.3775541
548.7841197	0.905260043	56.32256566
337.3437538	0.882703741	33.93501907
481.289125	0.922722701	54.17839506
124.2495087	0.864779501	15.94517958
797.893252	0.866541967	39.52982459

log.sigma.1.5.mm.3D_glszm_SmallAreaHighGrayLevelEmphasis	log.sigma.1.5.mm.3D_glszm_GrayLevelNonUniformityNormalized	log.sigma.1.5.mm.3D_glszm_SizeZoneNonUniformityNormalized
403.7841086	0.037699003	0.477568866
204.0714291	0.054486259	0.448625874
254.730266	0.044069504	0.428950802
232.4231021	0.046391185	0.444481175
197.4483633	0.048473929	0.408333311
299.5757745	0.046725984	0.46752624
262.8081595	0.049861789	0.416477347
485.589529	0.041932195	0.461603581
247.5226437	0.049916015	0.438548148
407.8576562	0.047239111	0.443896889
176.9834662	0.06477762	0.36970962
567.3875313	0.045182117	0.47391051
225.1644744	0.046745868	0.44594202
311.3864001	0.042916667	0.465478395
306.4432589	0.039814014	0.486682526
181.0837776	0.052013358	0.383839594
301.6231394	0.058513731	0.421907595
214.8870273	0.059428836	0.395594866
313.5092797	0.058023209	0.412580719
665.4742038	0.046906315	0.428499161
106.162206	0.061212146	0.332290816
154.1339617	0.05103202	0.390346808
174.1572099	0.0474042	0.460097806
175.7579512	0.058617365	0.420418479
238.6055211	0.041538636	0.437554388
257.5099474	0.044596849	0.425182158
387.0436999	0.042740014	0.465566505
201.4130964	0.049169334	0.433811919
156.4136065	0.062760262	0.481261154
534.9709255	0.057061138	0.413023855
163.234419	0.052139278	0.410719832
234.3832287	0.048171808	0.414149409
231.5671235	0.047131833	0.436921718
208.7920147	0.047829229	0.41976451
410.4356556	0.045335413	0.446858004
245.3767423	0.044460242	0.448600103
333.4692919	0.040244102	0.469323021
383.5204157	0.044156839	0.419607106
201.5522587	0.060787687	0.405595292
1235.068262	0.050718771	0.458294236
172.4516623	0.053839286	0.393246173
401.8806512	0.051473365	0.44886395
240.8953058	0.051082236	0.422716269
266.1241732	0.04061696	0.464858498
246.1764372	0.051621001	0.493590771
296.2369087	0.04778636	0.435330845
366.7250925	0.047895278	0.457236296
470.4136834	0.072515397	0.402306485
204.5584604	0.050146102	0.487793383
296.0347886	0.048628827	0.472496811
889.2114652	0.040442577	0.538180155
451.9137398	0.04336084	0.445760428
201.3351311	0.053087748	0.439442128
543.7430895	0.05060649	0.449649496
234.1406517	0.044652515	0.419770822
197.6610664	0.058031733	0.403119426
188.0278108	0.050146959	0.442531352
229.4224703	0.047423334	0.388781782
359.8061553	0.044277281	0.505495024
414.5426174	0.048593766	0.443884241
301.4691574	0.044156613	0.463532703
500.2927908	0.038223574	0.50189701
481.5696542	0.040114411	0.46291693
717.9525846	0.048902002	0.426566642
338.9190876	0.055212595	0.416617271
649.8244377	0.03755437	0.481059672
155.2895295	0.048892623	0.486055684
352.4226229	0.041129641	0.462946148
487.6800047	0.050028832	0.434347707
242.098365	0.053572648	0.383037798
186.4283207	0.049251701	0.414058957
414.7229243	0.044059185	0.424847047
233.6174146	0.046361395	0.434760774
321.9092136	0.044330842	0.501108178
291.3626004	0.045174621	0.464224246
185.8971851	0.056528668	0.40153201
660.4763471	0.047394612	0.41086986
279.3097226	0.049715909	0.446862087
322.4042567	0.049782537	0.412138277
231.7548171	0.04948148	0.435155191
402.2881439	0.038683727	0.471656291
311.5795505	0.048564815	0.414814815
533.3154292	0.042210736	0.451018364
152.3687076	0.062605041	0.415587148
247.9360232	0.047762554	0.397139914
145.6616681	0.062314143	0.357479368
191.1323609	0.048794449	0.457749166
322.7008827	0.052243752	0.419543788
452.3185934	0.044238605	0.45915984
522.0588832	0.04766998	0.423282705
193.4029934	0.05172071	0.42504142
281.1481399	0.050516171	0.428372273
192.0845486	0.048338256	0.427743974
178.205673	0.051985865	0.388105139
310.8488631	0.052804116	0.437061171
423.1042936	0.037783534	0.465697707
256.4727776	0.047632584	0.402636789
359.7101365	0.042222222	0.497469136
72.40875852	0.073487713	0.318761815
538.4854976	0.045960663	0.411978524

log.sigma.1.5.mm.3D_glszm_SizeZoneNonUniformity	log.sigma.1.5.mm.3D_glszm_GrayLevelNonUniformity	log.sigma.1.5.mm.3D_glszm_LargeAreaEmphasis	log.sigma.1.5.mm.3D_glszm_ZoneVariance
332.387931	26.23850575	17.52011494	13.21568239
79.40677966	9.644067797	7.644067797	3.800695841
179.3014354	18.42105263	13.35885167	8.8050182
73.33939394	7.654545455	7.975757576	4.167346189
250.3083197	29.71451876	16.05057096	10.75231326
64.05109489	6.401459854	5.357664234	2.237412755
1175.715551	140.75983	2559.149486	2545.212238
1087.076433	98.75031847	346.2454352	339.6063335
642.4730375	73.12696246	398.6552901	390.1917581
166.4613333	17.71466667	10.312	6.258488889
204.0797101	35.75724638	46.94565217	38.99978996
574.379538	54.76072607	365.1674917	357.2654553
156.9715909	16.45454545	18.83238636	13.52407509
167.5722222	15.45	6.072222222	2.872098765
330.9441176	27.07352941	89.38088235	84.01620891
685.537514	92.89585666	807.5285554	797.1201644
144.2923977	20.01169591	34.34795322	27.68189357
73.58064516	11.05376344	10.87634409	6.041074113
147.2913165	20.71428571	95.08123249	85.69052719
1508.317045	165.1102273	163.1247159	156.3188429
206.6848875	38.07395498	57.32475884	46.16357358
93.29288703	12.19665272	8.050209205	3.743281805
118.2451362	12.18287938	18.14007782	13.42594135
416.6347124	58.08980827	807.3794147	794.2049322
197.3370288	18.73392461	194.713969	187.3001214
389.8920393	40.8953108	134.7328244	126.9635041
417.613155	38.33779264	33.17948718	28.1882231
351.3876543	39.82716049	114.1666667	106.4300274
39.46341463	5.146341463	6.024390244	2.854253421
259.3789809	35.8343949	52.60191083	46.02938253
584.0436006	74.14205345	150.9894515	143.0411758
165.245614	19.22055138	9.493734336	5.593356826
373.1311475	40.25058548	154.1651054	146.6892993
348.8243081	39.74608905	228.0204573	219.5748239
869.1388175	88.17737789	189.6930591	182.7283446
413.1606949	40.94788274	20.68078176	16.24840818
379.6823239	32.55747837	7.423980222	3.930916253
769.5594329	80.98364231	1163.055071	1152.981196
95.31489362	14.28510638	18.34042553	12.66183794
1303.8471	144.2949033	1785.153954	1774.12782
220.2178571	30.15	17.94642857	12.22066327
836.6824034	95.94635193	527.9388412	518.6794885
448.0792453	54.14716981	17.37169811	12.16813457
321.6820809	28.10693642	13.42774566	9.216946774
233.9620253	24.46835443	248.3206751	237.544553
438.8134921	48.16865079	46.81448413	41.14082479
839.4858388	87.93572985	510.2325708	501.7383304
73.21978022	13.1978022	28.99450549	21.7163688
100.4854369	10.33009709	10.54854369	6.94591856
105.8392857	10.89285714	10.92857143	6.85682398
348.7407407	26.20679012	35.17746914	31.23283179
617.8239538	60.0981241	11.73953824	7.701932227
826.5906433	99.85805423	2867.870282	2849.890157
248.2065217	27.93478261	123.8949275	115.0464582
144.4011628	15.36046512	6.444767442	2.950910965
187.0474138	26.92672414	40.18103448	33.75743163
634.5899582	71.91073919	163.4525802	155.6133612
353.4026403	43.10781078	13.37183718	8.201091396
194.6155844	17.04675325	20.88051948	16.95291617
1504.323694	164.6842726	1034.412806	1024.08158
272.0936968	25.91993186	23.68824532	18.56230247
666.0173323	50.72268274	16.5403165	13.08973729
1647.98427	142.8073034	203.0865169	197.7369117
428.2729084	49.09760956	12.21314741	7.854867383
109.5703422	14.52091255	42.4904943	35.86427446
903.9111229	70.56466205	7.271420969	3.956657162
100.6135266	10.12077295	8.115942029	4.269037784
1101.348886	97.84741488	28.44766709	23.87896299
1094.121874	126.022628	245.4267566	236.5430912
337.4562997	47.19750284	15.03291714	9.220494202
86.95238095	10.34285714	14.71428571	9.225306122
826.3275064	85.69511568	221.7239075	215.1655683
257.3783784	27.44594595	23.16722973	18.0819
260.0751445	23.00770713	6.986512524	3.635990362
620.2035928	60.35329341	708.4550898	699.1105364
148.1653117	20.85907859	22.65582656	16.46665345
384.9850587	44.40875133	17.38633938	11.82841991
78.64772727	8.75	6.676136364	3.308077221
253.4650407	30.61626016	10.51219512	5.940247207
278.9344774	31.71762871	119.3572543	111.4717595
316.4813711	25.95678092	18.03576751	13.64515004
149.3333333	17.48333333	14.77777778	9.513302469
414.0348584	38.74945534	177.3834423	171.2040478
248.1055276	37.37520938	210.681742	200.6274252
355.4402235	42.74748603	292.0927374	282.8904966
61.84393064	10.78034682	53.67052023	44.24952387
243.5225564	25.95864662	38.59022556	32.54528238
430.8714703	53.65433301	904.06037	890.1090176
316.8202899	30.52463768	85.8884058	79.81110901
269.6310832	30.36577708	11.05023548	6.444932954
138.1384615	16.80923077	21.41538462	15.29546982
320.4224599	37.78609626	330.4224599	318.9640539
787.048913	88.9423913	68.68532609	62.70145292
504.1485758	67.52963818	55.00538876	47.96798864
584.3507853	70.59910247	54.59760658	48.47966148
549.9889924	44.62235394	11.05842506	6.983564926
423.1712655	50.06184586	26.51189343	21.06892896
89.54444444	7.6	5.472222222	2.448487654
29.32608696	6.760869565	21.7173913	13.231569
1415.558207	157.9208382	129.7299185	123.1347683

log.sigma.1.5.mm.3D_glszm_ZonePercentage	log.sigma.1.5.mm.3D_glszm_LargeAreaLowGrayLevelEmphasis	log.sigma.1.5.mm.3D_glszm_LargeAreaHighGrayLevelEmphasis	log.sigma.1.5.mm.3D_glszm_HighGrayLevelZoneEmphasis
0.48199446	0.044121845	8678.683908	556.3850575
0.510086455	0.054346887	1781.627119	277.4011299
0.468609865	0.05219058	4464.997608	356.6244019
0.51242236	0.04121992	2633.933333	333.630303
0.434443657	0.098917805	3566.504078	292.6280587
0.566115702	0.026902771	2113.664234	418.1678832
0.267862226	7.570547942	868688.0213	386.5451647
0.388101516	0.604451443	199404.1023	667.4798301
0.343735336	1.364128256	117700.0177	351.2730375
0.496688742	0.025098813	5284.317333	579.5573333
0.354755784	0.213158887	10914.93478	274.8804348
0.355738186	0.532058007	251980.6997	779.0231023
0.434032059	0.074521988	6282.153409	324.5880682
0.559006211	0.023190353	2370.805556	433.4638889
0.431746032	0.290698695	28577.42353	406.0852941
0.309961819	3.523845426	186672.8975	276.5996641
0.387315968	0.102183505	12413.24269	439.997076
0.454767726	0.054277723	3019.795699	321.016129
0.326325411	0.24232037	38527.39496	457.3585434
0.383316999	0.176055825	152457.0929	981.2963068
0.299326275	0.544672451	7443.414791	168.1414791
0.481854839	0.079705572	1418.761506	231.7112971
0.460573477	0.138644463	3058.964981	234.5175097
0.275507367	4.089092051	160594.1221	253.2633703
0.367263844	0.672992001	58159.04656	339.9512195
0.358763693	0.523750805	35681.28135	358.612868
0.44760479	0.085299429	13957.42029	525.6332219
0.359520639	0.526997466	25552.30864	282.6641975
0.561643836	0.079855878	817.0731707	199.4756098
0.390062112	0.084180067	34349.63217	780.5286624
0.354701921	0.798107177	29763.6308	241.5253165
0.506345178	0.04376966	2837.441103	356.5137845
0.365738758	0.600265128	40598.03864	323.5269321
0.344099379	0.995479657	53291.22744	292.2623345
0.378920709	0.384330759	94788.39846	577.0971722
0.474987107	0.087120211	6317.724213	348.3474484
0.53505291	0.023687891	3765.637824	473.5488257
0.31506614	2.284116619	594360.9716	558.540349
0.419642857	0.08630522	4628.191489	298.8382979
0.301153805	1.104867712	2887163.594	1726.759578
0.417910448	0.104924093	3776.398214	260.775
0.328631876	1.183449289	236610.7425	547.7977468
0.438378825	0.075175986	4918.026415	357.3188679
0.487323944	0.06060702	3768.859827	362.4436416
0.304627249	0.969760512	64750.89662	329.3206751
0.419825073	0.15324817	15086.16964	416.5029762
0.343113437	1.26419426	207359.2963	498.2265795
0.370672098	0.055135868	17504.96703	702.8296703
0.52685422	0.083121627	1902.009709	272.3932039
0.495575221	0.04166872	4078.209821	404.15625
0.503496503	0.036763017	35738.98148	1145.083333
0.497666068	0.025156201	6189.822511	635.5021645
0.235832497	12.27310519	673712.0005	286.2030835
0.336175396	0.168003031	93377.7029	775.9601449
0.534992224	0.031272846	2273.944767	347.5
0.394557823	0.239102925	7658.079741	289.9439655
0.357160648	0.834619314	33018.51813	257.1136681
0.439767779	0.056544166	3958.607261	346.7656766
0.504587156	0.063433702	7743.090909	473.238961
0.311117231	2.083222136	515448.6497	577.7202715
0.441685478	0.073864117	8795.584327	419.1396934
0.538336714	0.030533837	9955.730972	673.250942
0.432353656	0.346812295	120128.2542	669.9662921
0.479007634	0.014227427	11947.25	1049.938247
0.388478582	0.104676037	19296.47529	503.0038023
0.549254604	0.01126733	5932.161788	879.749867
0.509852217	0.080643815	1815.004831	216.2753623
0.467846608	0.079029287	11065.05633	483.2517865
0.335508791	0.399256917	152130.4141	707.1691147
0.414783427	0.049086981	5465.681044	376.2905789
0.426829268	0.091188968	3358.061905	275.4952381
0.390483839	0.409415718	121472.1347	605.151671
0.443445693	0.108967463	5750.442568	320.2871622
0.546315789	0.031676123	2158.296724	410.7957611
0.327130264	2.129118022	237156.0958	397.4109281
0.401960784	0.127942765	4726.948509	266.8401084
0.424173834	0.021814964	15359.83565	982.4482391
0.544891641	0.034633678	2120.761364	391.0625
0.467680608	0.029276599	4615.938211	473.803252
0.356111111	0.499498384	29446.04836	323.0577223
0.477240398	0.045145995	8917.248882	553.7198212
0.435835351	0.040045752	6685.275	463.3777778
0.402278703	0.260805451	122062.5795	751.956427
0.315372425	1.429032958	32091.75042	217.6264657
0.329650092	0.992586445	87036.10391	367.803352
0.325800377	0.164381998	19291.48555	266.9942197
0.406727829	0.242858491	7080.755639	259.7913534
0.267726799	2.463504942	333599.889	458.872444
0.405643739	0.148826071	50837.96377	637.8623188
0.465983906	0.017595865	8365.66248	780.0141287
0.404228856	0.080975061	6889.766154	292.0369231
0.295418641	1.013205134	109175.2112	398.2152406
0.408798045	0.319434061	15625.53207	276.4396739
0.376958793	0.293029427	11339.93303	263.4526559
0.404293922	0.172193654	18043.49289	442.9887809
0.495385906	0.027392976	5822.879763	578.6443692
0.42862969	0.103915515	7506.125595	372.7611798
0.575079872	0.024289871	2657.688889	483.5888889
0.343283582	0.268441073	2645.108696	126.6521739
0.389392566	0.170745255	99895.12864	804.2229336

log.sigma.1.5.mm.3D_glszm_SmallAreaEmphasis	log.sigma.1.5.mm.3D_glszm_LowGrayLevelZoneEmphasis	log.sigma.1.5.mm.3D_glszm_ZoneEntropy	log.sigma.1.5.mm.3D_glszm_SmallAreaLowGrayLevelEmphasis
0.718359384	0.007974473	6.348308765	0.00712537
0.696544507	0.014435835	5.606894274	0.012264401
0.680562796	0.007902605	6.155369429	0.00633112
0.692505903	0.015152909	5.833006046	0.01377917
0.664249375	0.011970479	6.206344423	0.009989575
0.709379562	0.013508	5.665346455	0.012446783
0.671052114	0.004695415	6.403378928	0.003535974
0.706549057	0.002612604	6.407837783	0.002023252
0.68796076	0.005441766	6.151451435	0.004117702
0.692765481	0.005113418	6.033787498	0.004453322
0.630257689	0.007309442	5.981296786	0.005586176
0.715849416	0.002520563	6.276838615	0.00207331
0.694065125	0.01057294	6.046966141	0.008505948
0.708772785	0.007168076	6.022723831	0.006151744
0.724997028	0.00652013	6.191868058	0.005258193
0.643051705	0.008465163	6.376909862	0.006324828
0.674355432	0.005989727	5.857239251	0.005059332
0.653455667	0.010720694	5.686396884	0.009159272
0.667809941	0.005638372	5.982097622	0.004691792
0.680892186	0.001683711	6.479455803	0.001328043
0.597588172	0.014642407	6.212164322	0.010194615
0.646495647	0.01509475	5.930015244	0.01222271
0.704240309	0.017834263	5.840671302	0.014682834
0.673729695	0.008263861	6.05655421	0.006346711
0.686689677	0.008586713	6.156118007	0.005180986
0.678065845	0.006378205	6.360604758	0.004911765
0.709196191	0.00518725	6.250053986	0.004389597
0.684527834	0.009280385	6.217022903	0.007453415
0.718496478	0.022329536	5.063174377	0.018701857
0.668053537	0.003714031	6.039189531	0.003215799
0.666305606	0.010283498	6.235165353	0.008038904
0.666552583	0.007725211	6.014366389	0.006388055
0.687046326	0.007696904	6.248852498	0.006190871
0.673444777	0.008804896	6.2804755	0.006665739
0.695218536	0.003182178	6.34474218	0.002497938
0.696283324	0.009936931	6.253858854	0.008090391
0.712461241	0.007510003	6.327435126	0.006575285
0.671785465	0.0033068	6.384166056	0.002520347
0.659613166	0.010540742	5.691931412	0.009050694
0.704117429	0.000975599	6.214010953	0.000788807
0.651010413	0.008809926	6.045951091	0.006864583
0.696810677	0.002917763	6.199380945	0.0021725
0.676117587	0.005996925	6.210282939	0.004735238
0.709012008	0.008825385	6.270575195	0.007527808
0.729412627	0.007930367	5.900231831	0.006748052
0.686081129	0.004868236	6.236323315	0.003774927
0.70329376	0.003441665	6.224198378	0.002202348
0.657676106	0.007161589	5.496092984	0.006623693
0.726018181	0.014853686	5.687655516	0.01292774
0.714384877	0.008467489	5.812774172	0.007281
0.761333239	0.002607811	6.044560305	0.002357751
0.694123695	0.002891295	6.326866978	0.002271313
0.689297538	0.007234947	6.170521395	0.005623191
0.697348708	0.003503107	6.11894098	0.003053476
0.670806477	0.009027439	6.104339121	0.007377454
0.659388976	0.008045756	5.991626199	0.004499468
0.691768654	0.008951632	6.160046603	0.006717485
0.64767925	0.006973098	6.341803513	0.005479875
0.736941569	0.006397237	5.914201581	0.005576625
0.692829528	0.002682895	6.369681599	0.001992372
0.707668589	0.006394207	6.172183131	0.005078262
0.736297772	0.003334514	6.311313537	0.002826235
0.707665251	0.002546186	6.46290107	0.001972811
0.678356092	0.002104486	6.211667666	0.001761892
0.67098078	0.011274397	5.902257926	0.010487169
0.721177331	0.00264181	6.42272858	0.00223567
0.72297855	0.030918622	5.693976566	0.028545182
0.707662678	0.004244161	6.425777785	0.003344563
0.685297154	0.002294635	6.377092804	0.001751719
0.642477679	0.005211934	6.292361371	0.004007601
0.66852448	0.013977591	5.882219386	0.01179971
0.677473883	0.003395462	6.431071549	0.002690068
0.685440249	0.0083246	6.159920686	0.00655957
0.735626094	0.006657482	6.020979567	0.005576099
0.70841639	0.00512245	6.240602729	0.003950104
0.658799623	0.010153061	5.983537276	0.008230425
0.666559471	0.002345147	6.402209348	0.00195151
0.693632199	0.009983665	5.71188425	0.008709389
0.666792467	0.004607297	6.191373623	0.003678654
0.685459522	0.009165386	6.131867542	0.00763514
0.713941537	0.007909917	6.317939978	0.007034835
0.66951949	0.00611304	6.083218469	0.00518396
0.698453102	0.002951648	6.269535942	0.002449396
0.669817639	0.012322226	5.86117234	0.009908536
0.653530537	0.005808245	6.287774404	0.004466927
0.618123926	0.013305217	5.776379579	0.011743082
0.703030487	0.011005062	6.024115965	0.008622766
0.673627319	0.004007054	6.175668642	0.003044709
0.704650405	0.003668968	6.222811351	0.003095586
0.676581568	0.00323665	6.195096014	0.002767354
0.677665504	0.011231192	6.04032274	0.009726239
0.678788818	0.005386504	6.090337331	0.004277007
0.679995567	0.009465694	6.280753392	0.0075784
0.647228684	0.012175299	6.366046594	0.009938611
0.687597104	0.003837934	6.14637975	0.002916675
0.709695449	0.005140336	6.485096308	0.004399244
0.657585033	0.00557025	6.301938559	0.004158653
0.731965964	0.012621805	5.820008257	0.011759633
0.582535753	0.027856599	5.387199593	0.021080222
0.667288726	0.002183159	6.542380551	0.001704167

log.sigma.1.5.mm.3D_ngtdm_Coarseness	log.sigma.1.5.mm.3D_ngtdm_Complexity	log.sigma.1.5.mm.3D_ngtdm_Strength	log.sigma.1.5.mm.3D_ngtdm_Busyness	log.sigma.1.5.mm.3D_ngtdm_Contrast
0.005010882	2.191104178	2.689841541	0.18069979	0.000130355
0.014510696	3.502234997	3.880316766	0.150823672	0.000733148
0.007474682	1.568270575	1.999403114	0.250205848	0.000259285
0.016893535	4.438423541	4.932734364	0.10567939	0.000884348
0.004256879	0.783589341	0.990621079	0.56388415	0.000173139
0.026081196	6.318634671	7.733813721	0.062607727	0.001181195
0.000744649	0.160259554	0.477517297	1.223761274	4.20E-06
0.001200352	0.544963677	0.892187235	0.556484677	1.51E-05
0.001712663	0.310042297	0.607570917	0.812438536	2.22E-05
0.007998243	2.646152598	3.155265023	0.140134513	0.000241253
0.004601821	0.404435062	0.916421304	0.466315225	5.83E-05
0.002292623	0.696381906	1.478509428	0.282739091	2.28E-05
0.008209566	1.678170012	2.344623534	0.202156047	0.00026335
0.009518828	2.618907704	2.748424881	0.178615819	0.000463905
0.004317202	1.509368676	1.882300948	0.300261065	0.000119052
0.001304516	0.19744716	0.464760142	1.20266679	1.57E-05
0.007714764	1.252544107	2.533051058	0.181805828	0.00011641
0.014278745	2.05796811	3.057940491	0.165756067	0.000535538
0.006415096	0.886448566	1.923415406	0.21154124	8.33E-05
0.000786683	0.416412413	0.870177159	0.549103243	6.10E-06
0.003127581	0.28445642	0.564422971	1.0585933	5.83E-05
0.011467449	2.181394141	2.700817712	0.262635191	0.000526975
0.012389571	1.656171191	2.340697986	0.23468963	0.000403134
0.002168252	0.220820027	0.695296658	0.76852524	1.46E-05
0.005724388	0.961168251	1.609637705	0.284998625	0.00014397
0.002934598	0.70128349	1.442977841	0.450636418	4.14E-05
0.003488821	1.188880716	1.668615728	0.299606145	7.37E-05
0.003238684	0.664796038	1.389391722	0.465982943	4.13E-05
0.035284853	4.33601439	5.682375152	0.135273727	0.002280908
0.004234745	0.960232396	2.02432956	0.2342615	7.05E-05
0.001747373	0.305574525	0.572871902	1.107535957	2.78E-05
0.006780924	1.793772554	1.861755434	0.302254397	0.000365603
0.00321131	0.623234301	1.204194944	0.451966696	4.54E-05
0.002928785	0.626312067	1.301967003	0.48882075	4.03E-05
0.001439122	0.524245902	0.986046672	0.514734861	1.47E-05
0.003402545	0.871942597	1.017303741	0.494173985	0.0001185
0.003808922	2.020579597	2.064291884	0.258053864	0.000161407
0.001265253	0.329321046	0.805271095	0.623250761	1.23E-05
0.011150968	1.513056949	2.487846789	0.193640377	0.000251978
0.000805713	0.191795277	0.62255058	0.539182054	5.26E-06
0.004793873	0.745996018	1.133577146	0.477248428	0.000115636
0.001390115	0.299569306	0.796081096	0.620473997	1.05E-05
0.00259494	0.564528606	0.801663636	0.6887262	7.13E-05
0.004649089	1.401506089	1.596007247	0.352637427	0.000158275
0.004907889	0.599927507	1.335626604	0.31677006	5.36E-05
0.002838892	0.706767071	1.101651905	0.473320873	5.34E-05
0.001386967	0.332405507	0.699865737	0.687575668	1.42E-05
0.012747268	1.33150802	3.104218631	0.124178088	0.000278022
0.016369887	3.296238654	4.248453009	0.138661764	0.000595602
0.013905466	2.737869585	3.660627023	0.134619417	0.000586982
0.004915258	2.012802954	2.391611618	0.151631427	0.000159992
0.002323992	0.995546936	1.250013607	0.401312613	5.71E-05
0.000990724	0.115298865	0.397367518	1.40435476	5.55E-06
0.004331123	1.078630527	2.260003819	0.166205955	4.93E-05
0.008455331	2.353821766	2.355434587	0.23819228	0.000523681
0.005642411	0.927759269	1.649506742	0.350075316	0.000105917
0.001828397	0.303191288	0.601884979	0.983260113	2.50E-05
0.003069855	1.043164587	1.424241308	0.442031413	6.76E-05
0.008414974	2.880675838	3.307369003	0.152581231	0.000258851
0.00069314	0.211424525	0.567501544	0.977723409	4.37E-06
0.005028884	2.025161548	2.741550471	0.201517559	9.97E-05
0.002724047	1.536162288	1.648295264	0.28201529	7.35E-05
0.000908351	0.387464251	0.538489583	0.80241912	1.52E-05
0.002896467	1.080174435	1.499184847	0.252668025	6.75E-05
0.010469093	1.848199845	3.791135727	0.108163711	0.00015512
0.001890596	1.332841202	1.298829671	0.341465168	5.64E-05
0.014118731	2.428559632	2.849634913	0.192642456	0.000765238
0.001331264	0.674786529	0.880910003	0.688938357	2.64E-05
0.000923579	0.389575631	0.872394837	0.609322395	6.53E-06
0.003070393	0.591735658	0.968605974	0.481528816	6.03E-05
0.012419313	2.185093138	2.85246108	0.186871883	0.000461474
0.001510222	0.511674528	0.87691705	0.514501224	1.96E-05
0.004741954	1.351733634	1.861483299	0.356478344	0.000125983
0.005946249	2.703391589	2.816986124	0.242303996	0.000230706
0.001938678	0.438773994	0.974263046	0.537451371	1.91E-05
0.007390824	1.232509369	2.447411906	0.258973989	0.000122161
0.003128816	1.136080923	2.03566714	0.198177756	4.52E-05
0.017068013	3.520002964	3.957743971	0.134919066	0.00112658
0.004895276	1.208389337	1.68832635	0.267639266	0.000120165
0.00391626	0.938529237	1.951248876	0.335850024	4.98E-05
0.005118289	2.094347315	2.590412362	0.181546678	0.000130627
0.007891525	1.635055971	2.371380484	0.183875422	0.000226741
0.002821642	0.845067791	1.216241004	0.335535311	7.30E-05
0.003715753	0.406761798	0.895404469	0.63258064	4.58E-05
0.002903136	0.403036177	0.843007459	0.517581757	3.82E-05
0.010212757	1.270596566	2.697201455	0.17801096	0.000261327
0.005307687	1.071889015	1.797336128	0.378520504	0.000108055
0.002036518	0.262190986	0.743364857	0.580358018	1.63E-05
0.004549698	1.104745562	1.964457117	0.195482361	6.97E-05
0.004592657	1.546189836	2.282163237	0.187121353	0.00011088
0.00808415	1.659161674	2.652436268	0.18308078	0.000160657
0.002996292	0.430986134	1.025404123	0.428401574	2.80E-05
0.001580056	0.351784495	0.582103437	0.994949136	2.55E-05
0.002094095	0.425912819	0.827830656	0.77044786	2.77E-05
0.001972167	0.459117047	0.78955926	0.661393301	3.38E-05
0.002848421	1.604000957	1.770982457	0.301641856	7.87E-05
0.0026864	0.718778436	1.049387818	0.531287633	5.52E-05
0.017173387	8.028110985	7.411492135	0.071732819	0.001077315
0.023143033	1.1072909	2.207290302	0.251545722	0.00071505
0.000822938	0.392872863	0.736095091	0.643994284	8.00E-06

log.sigma.1.0.mm.3D_gldm_GrayLevelVariance	log.sigma.1.0.mm.3D_gldm_HighGrayLevelEmphasis	log.sigma.1.0.mm.3D_gldm_GrayLevelNonUniformityNormalized	log.sigma.1.0.mm.3D_gldm_DependenceEntropy
14.27671816	281.6807479	0.091515374	6.347684939
8.876811534	116.7492795	0.098588976	5.753382691
9.587196354	142.6894619	0.092546603	6.132404574
11.18784962	167.5931677	0.088653987	5.781739132
9.526302651	144.3231751	0.09073238	6.046848472
12.44600779	156.0495868	0.087698928	5.879498247
5.465256294	162.0938419	0.172175829	6.256226746
9.912296842	313.4286421	0.115432862	6.331434062
6.561793174	137.4446269	0.137143343	6.175346686
11.63406868	260.4794702	0.086955835	6.039368356
4.426865323	96.11118252	0.150901065	5.83832063
8.824580585	390.1112416	0.135830348	6.334075706
10.67881974	191.5166461	0.096778117	6.106484528
13.3745901	159.8447205	0.079414374	6.008356596
12.24764082	140.1314286	0.102144822	6.340767545
6.006333853	116.2381118	0.161564517	6.224867806
6.540053791	192.8640997	0.122344935	6.097389511
7.192950783	111.4425428	0.109713596	5.734601997
5.93180018	187.9104205	0.135420392	6.000932401
8.849526208	433.9644996	0.114749925	6.261559586
4.450979647	61.53416747	0.136125652	5.971186672
9.892023283	121.9274194	0.090433143	5.827156361
9.002367005	98.91218638	0.101668786	6.004144295
4.347501304	113.4242424	0.191451747	5.91399721
9.274355696	162.0423453	0.157404853	6.219597043
8.47824919	144.2335681	0.1252097	6.220989443
11.4057683	247.5279441	0.094679902	6.207201097
6.720843876	114.6759876	0.134751337	6.061463767
8.255582661	71.16438356	0.103021205	5.219427152
7.324458547	313.2701863	0.117532503	6.135403547
6.636526035	115.006236	0.120122404	6.217857044
10.39813413	136.8464467	0.085086578	6.01308346
7.499066161	138.8689507	0.129274012	6.282671302
7.62779831	118.5701863	0.151495356	6.059050579
8.746887744	220.6923826	0.115225172	6.348542661
10.65843875	166.7988654	0.091950706	6.238933699
15.40807753	250.9656085	0.079177865	6.186924031
7.219693779	218.3549218	0.178762297	6.306342438
6.170277423	136.3339286	0.119445153	5.813986925
5.686202027	653.2532021	0.171044704	6.110374634
6.938547561	93.23432836	0.109888617	6.04438623
6.472602375	217.6183004	0.158626507	6.151110632
7.893510964	120.7204301	0.099623995	6.21768195
12.3736957	165.6450704	0.085992859	6.220672589
5.531526936	136.9697943	0.159678762	6.114072002
8.543177119	142.6822157	0.105747796	6.146451898
7.28336954	189.7781723	0.140112906	6.158176289
5.104126829	276.1792261	0.135734463	5.621349294
10.72232652	123.8644501	0.090796109	5.803855212
10.38924348	145.9159292	0.089699663	5.88737098
13.64196142	393.0893551	0.089003718	6.189215044
11.32693804	226.1572711	0.089027459	6.179951027
4.262692148	135.9509779	0.202719587	6.110999629
7.079446577	425.8824604	0.132438531	6.207720919
13.06464401	148.7527216	0.080017124	5.931115467
6.553519367	95.83673469	0.113747455	5.84356275
6.507895764	95.05529265	0.135279315	6.225942401
9.176834861	140.1669086	0.097673979	6.090240444
11.03391627	188.3460026	0.093386972	6.064176343
6.76052466	216.753695	0.164215624	6.177215005
10.32218593	191.0820166	0.097718828	6.285804201
15.69132414	293.2482759	0.077366292	6.356413132
12.15683686	256.3344668	0.095625265	6.493335054
9.763886638	476.1054389	0.092992924	6.062602827
6.071328674	189.3397341	0.136895112	6.009544785
17.32960314	477.9909383	0.071471057	6.309790461
9.872042515	114.3546798	0.097818438	5.821948923
12.25515785	249.9120944	0.089453257	6.282388237
7.472988269	386.8456313	0.132786438	6.157783183
7.030331101	166.4863465	0.108924195	6.04232022
9.237639632	118.1260163	0.095594554	5.93986595
9.676573187	220.1274844	0.114893088	6.287870832
9.488059027	116.4516854	0.097378838	6.142197287
12.75167202	142.0884211	0.086003324	6.027727011
7.50801748	163.1709109	0.150529263	6.280387095
6.451061083	99.18082789	0.120729919	5.913291862
10.38761837	430.6097782	0.100493249	6.161955001
12.07622042	167.8235294	0.081118385	5.797284099
9.265981003	255.2190114	0.094772514	6.048289637
7.513110802	136.0961111	0.136919753	6.026183098
13.51952969	280.0263158	0.094194966	6.328434632
9.183188328	195.1125908	0.099777803	6.050058341
9.559786186	279.9763365	0.118941363	6.313473078
4.601839178	111.0850502	0.144511335	5.891902488
6.417293733	139.9027624	0.144964643	6.258022768
4.5691851	115.472693	0.165806619	5.583790974
7.611823617	95.16590214	0.109645185	6.121833501
5.214137497	162.2275808	0.176169237	6.22245308
9.408335035	348.3492063	0.113852522	6.27833337
10.02321521	352.1514265	0.094952665	6.030898168
8.072478095	122.0360697	0.11564689	5.893259405
5.286538937	159.5876777	0.159094709	6.129280861
7.830422142	117.5840924	0.111112735	6.144367395
7.052409718	119.7658154	0.120666959	6.07448158
7.584002339	165.9008164	0.109896455	6.141233839
15.38445463	288.4517617	0.078304116	6.364288624
8.806738149	167.0101958	0.102259633	6.240230174
16.7898621	191.485623	0.075095183	5.817368142
4.209957674	47.14179104	0.137836935	5.620415481
9.316652063	395.4587489	0.108706916	6.334748827

log.sigma.1.0.mm.3D_gldm_DependenceNonUniformity	log.sigma.1.0.mm.3D_gldm_GrayLevelNonUniformity	log.sigma.1.0.mm.3D_gldm_SmallDependenceEmphasis
229.2077562	132.1481994	0.318923364
65.3054755	34.21037464	0.298821348
148.5807175	82.55156951	0.243417242
67.97515528	28.54658385	0.28118744
246.6754075	128.0233877	0.268514145
47.33057851	21.2231405	0.345574159
928.4944492	1814.561059	0.161489589
780.2452208	700.4466051	0.239350849
488.5321445	584.5049273	0.207030096
143.6649007	65.65165563	0.322281173
198.2120823	234.8020566	0.203336624
372.8720282	462.7739947	0.239126899
135.4562269	78.48705302	0.268340818
138.3913043	51.14285714	0.354218772
213.9955556	160.8780952	0.292837099
603.9107949	930.9347449	0.177196553
119.3352208	108.0305776	0.22054926
67.86552567	44.87286064	0.259042495
133.8244973	148.1499086	0.211635385
1274.919634	1053.748557	0.24152805
262.226179	282.8691049	0.167253523
92.89112903	44.85483871	0.283119547
90.27956989	56.7311828	0.278262292
346.294412	688.6519322	0.171438153
127.1026059	193.2931596	0.208564115
316.8607199	320.0359937	0.232670962
327.6976048	189.738523	0.274366986
282.7328007	303.5947625	0.226816025
33.31506849	15.04109589	0.342998361
225.684472	189.2273292	0.232773194
522.4272886	481.5707159	0.212006259
149.180203	67.04822335	0.304208364
275.1670236	301.854818	0.227931229
291.2492754	365.8612836	0.220138403
640.6801091	591.4508085	0.24035238
330.9432697	178.2924188	0.287556581
307.8571429	119.7169312	0.337494046
537.9984539	1040.575331	0.188009611
86.64285714	66.88928571	0.219249364
912.8700116	1615.85932	0.185012523
203.5402985	147.2507463	0.251008248
597.5987306	899.7295487	0.209849533
373.261373	240.8908189	0.239671968
248.9802817	122.1098592	0.322246758
151.8688946	248.4601542	0.194550405
366.2378176	253.9004581	0.249098236
617.0452252	749.74416	0.216297946
72.9185336	66.64562118	0.220307923
77.23017903	35.50127877	0.350899332
92.69026549	40.54424779	0.280375063
213.048174	114.5477855	0.32631027
510.5159785	247.9414722	0.310179296
634.5644433	1616.891424	0.160286672
187.09257	217.4640682	0.210711555
134.5832037	51.45101089	0.304570768
187.7585034	133.7670068	0.236173397
465.1594022	543.1464508	0.215313048
348.329463	201.8921142	0.265868195
122.8086501	71.2542595	0.30603624
1110.341963	1788.800789	0.209305535
192.9021821	129.868322	0.26678548
476.9553753	190.7079108	0.34394235
1207.073111	787.3784309	0.278852516
383.1832061	194.9131679	0.290289049
84.30576071	92.67799114	0.211101642
719.1444022	244.5024847	0.370072966
72.71921182	39.71428571	0.290453686
871.0047198	454.8698132	0.302903523
900.8558871	996.9605754	0.215035474
339.0122411	231.3549906	0.244522534
84.74796748	47.03252033	0.276793647
673.3814495	572.2824734	0.246601802
213.6202247	130.0007491	0.257123683
186.1852632	81.70315789	0.36655642
415.1748286	614.7615083	0.215683487
135.5729847	110.8300654	0.24663853
357.4382073	221.989588	0.262726181
68.15789474	26.20123839	0.357123648
237.1536122	124.6258555	0.289533142
224.9955556	246.4555556	0.241962208
220.6514936	132.4381223	0.315629081
140.0823245	82.41646489	0.269260713
297.0096407	271.4241893	0.26255318
226.0575806	273.5599577	0.190934923
277.9053407	393.5790055	0.199734681
63.5386064	88.0433145	0.171252265
192.411315	143.4159021	0.24949385
341.0776851	675.7851929	0.171308131
230.1040564	193.6631393	0.261575872
242.6298464	129.8002926	0.27311819
124.8532338	92.9800995	0.253564029
243.8483412	402.8278041	0.192569795
668.6534104	500.1184181	0.261334298
487.9640163	415.8183401	0.231389698
477.9900212	363.4275779	0.257460167
440.8431208	186.6770134	0.324344541
368.3083197	250.7406199	0.261393948
75	23.50479233	0.382551695
39.55970149	36.94029851	0.190373504
1238.517679	959.2298277	0.234817096

log.sigma.1.0.mm.3D_gldm_DependenceNonUniformityNormalized	log.sigma.1.0.mm.3D_gldm_DependenceVariance	log.sigma.1.0.mm.3D_gldm_LargeDependenceEmphasis
0.158731133	6.680316296	18.78531856
0.188200218	2.668455016	11.59942363
0.166570311	4.174194132	16.12780269
0.211102967	2.402376452	10.88819876
0.17482311	2.928572254	13.24946846
0.195580903	3.25735947	11.52892562
0.088100811	13.79113963	46.3143562
0.128583589	8.94715353	26.47989453
0.114625093	9.479810559	30.25903332
0.190284637	3.093881847	11.77880795
0.127385657	6.348737122	24.00385604
0.10944292	12.43759283	35.23422366
0.167023708	3.850365124	14.87669544
0.21489333	2.362611396	9.51242236
0.135870194	8.582494331	24.20380952
0.104809232	11.4176672	36.66122874
0.135147475	6.695923631	22.88674972
0.165930381	3.225674165	14.46210269
0.122325866	8.137241861	26.98903108
0.138834764	6.606436509	21.66840902
0.126191616	5.225149858	24.25120308
0.187280502	3.015462409	12.43145161
0.161791344	6.382703203	19.05017921
0.09627312	10.82334033	38.18988046
0.103503751	15.59742278	43.4771987
0.123967418	8.576550067	26.58920188
0.163521759	4.036140294	15.22055888
0.1254917	7.686053748	25.29027963
0.228185401	1.842184275	8.616438356
0.140176691	6.707304502	21.99006211
0.130313617	6.72583345	23.76178598
0.189314978	2.709397305	11.49746193
0.11784455	10.68935636	30.99914347
0.120600114	7.015806832	25.16728778
0.124815918	9.008042717	27.050263
0.170677292	4.502614423	15.30531202
0.203609221	2.281990986	9.895502646
0.092423717	15.68457534	46.57189486
0.154719388	4.016313776	17.26071429
0.096630678	11.42197192	38.22536255
0.151895745	4.65890176	17.45820896
0.105359438	10.06856734	32.93018336
0.154367813	3.97106619	16.58560794
0.175338227	4.091955961	13.91267606
0.097602117	13.77715254	41.33290488
0.152535534	5.006725471	17.98292378
0.115314002	8.070490128	27.7153803
0.148510252	4.804468208	19.01629328
0.197519639	3.313570686	11.56265985
0.205066959	2.487313024	11.11946903
0.165538597	5.169914433	16.13131313
0.183309148	3.428473774	12.70197487
0.079559233	16.04298201	54.10581745
0.113941882	9.147992481	29.92204629
0.209305138	1.985850734	9.796267496
0.159658591	3.780600676	16.00680272
0.115855393	9.390173772	29.57633873
0.168519334	3.731970287	14.65360426
0.16095498	5.310681263	16.91349934
0.101931696	11.56084795	36.11668044
0.145148369	5.725526698	19.23626787
0.193491024	4.482663825	13.42434077
0.1465962	8.265805644	22.77677921
0.182816415	2.742574005	12.05438931
0.12452845	7.833743883	26.50073855
0.210214675	2.557775577	9.741303712
0.179111359	2.791817321	12.27093596
0.17128903	4.49057569	14.95201573
0.119986133	7.530154658	26.15050613
0.159610283	3.781504357	15.87382298
0.172251966	5.43623174	16.90243902
0.135190012	6.558520853	21.93354748
0.16001515	4.25658152	16.16554307
0.195984488	3.116183934	11.12210526
0.101658871	12.69614659	38.04505387
0.14768299	4.556277025	17.98474946
0.161809963	3.616137202	14.96287913
0.211015154	2.274036941	9.445820433
0.180344952	3.237334644	12.88745247
0.124997531	8.584961728	26.32777778
0.156935628	6.787581772	19.11237553
0.169591192	3.570297065	14.30266344
0.130153217	9.782365669	27.11305872
0.119417634	6.810394678	26.69360803
0.102359242	13.00652063	38.71123389
0.119658392	5.997623785	26.20527307
0.147103452	5.966898596	19.81804281
0.088914934	15.70656864	48.2346194
0.135275753	8.126872148	23.9159318
0.177490744	2.805381741	12.73226042
0.155290092	4.102342764	16.56218905
0.096306612	13.75823207	41.80805687
0.148556634	6.002718397	19.39724506
0.141603023	5.26425349	19.684852
0.144538863	5.838981537	19.65255519
0.184917416	3.051940228	11.8716443
0.150207308	5.365825813	18.37520392
0.239616613	1.542406271	7.453674121
0.147610826	4.520438851	19.75373134
0.140357851	6.210408603	21.18653672

log.sigma.1.0.mm.3D_gldm_LargeDependenceLowGrayLevelEmphasis	log.sigma.1.0.mm.3D_gldm_SmallDependenceHighGrayLevelEmphasis	log.sigma.1.0.mm.3D_gldm_LargeDependenceHighGrayLevelEmphasis
0.075078641	96.28059994	5048.918975
0.13677711	37.00366782	1229.596542
0.135807555	37.96629167	2241.438341
0.081384639	44.63610724	1812.779503
0.115015813	39.98218985	1831.93905
0.098329419	56.8867044	1717.396694
0.308052775	28.12845957	7176.534301
0.091629874	82.02945188	7896.079927
0.249198538	31.99238785	3874.331534
0.055186039	92.75557689	2828.037086
0.296460227	20.70935266	2129.402956
0.095900497	99.97459222	13176.32375
0.088611083	51.82482372	2876.789149
0.08211777	61.00554618	1430.360248
0.215748614	49.19984663	3037.358095
0.359288618	22.4069245	3991.706005
0.13353727	45.63088276	4151.711212
0.170193785	31.29415364	1487.628362
0.15638378	43.1568211	4843.711152
0.052760054	105.328946	9161.001851
0.510937603	11.62699061	1383.684312
0.150748064	39.89390301	1296.951613
0.267805591	32.41634709	1625.657706
0.377045007	22.70450088	4000.882124
0.295795209	37.4394469	6676.723127
0.213791759	39.67503811	3515.827465
0.071369712	74.58394657	3519.121756
0.262527259	30.81989717	2628.857079
0.200268124	27.55439859	535.4657534
0.081470945	78.16661416	6518.712422
0.241979655	26.61108729	2577.905712
0.114581428	42.54143542	1449.189086
0.253996291	36.86950221	4008.063812
0.248337845	29.96097629	2819.884472
0.135565933	58.164674	5642.575882
0.11411112	50.25830278	2384.098504
0.047664623	82.49726542	2534.69709
0.224435793	44.38673301	9922.964439
0.154023548	33.63676665	2182.3625
0.060221164	128.2679806	24460.90685
0.240405093	26.07060381	1506.131343
0.165119724	53.3496914	6734.974436
0.185219875	31.79213584	1838.221671
0.107996048	61.23532325	2056.685915
0.337090234	30.49377638	5252.449229
0.155274795	40.13350332	2308.379842
0.16416667	47.55493523	4881.426836
0.077421899	66.20176105	4931.596741
0.130092402	47.61461993	1253.88491
0.10127647	43.80453292	1568.20354
0.0468701	135.8416787	5901.894328
0.068464264	75.84568726	2612.389946
0.425320201	24.2189026	7045.33488
0.072242222	89.2779726	12714.47442
0.094826479	47.532212	1415.604977
0.22696466	25.81024183	1355.25
0.374769994	23.75175466	2553.353674
0.131061291	41.15099926	1899.854378
0.109233424	65.17456221	2876.306684
0.177854302	51.05881664	7501.650234
0.115646417	55.88912408	3526.862302
0.054572153	106.5974126	3712.00284
0.099629681	73.95925793	5524.376731
0.02736492	139.7029599	5649.77624
0.155049289	41.60943424	4768.308715
0.022650357	184.9710793	4559.975153
0.156286789	36.66840328	1339.53202
0.070614877	83.13076794	3428.056441
0.071750172	87.26242365	9764.213239
0.107046481	41.50449062	2604.387476
0.184403376	35.25941837	1800.012195
0.111335008	58.65691377	4590.384059
0.181811958	33.51811888	1717.008989
0.112137437	62.98190819	1362.050526
0.258878018	39.14631952	5803.414789
0.228179715	28.20200166	1626.616558
0.037859142	118.2380276	6289.156632
0.084515302	65.81091948	1398.944272
0.05796467	77.69349422	3140.274525
0.223975319	39.03092362	3251.652778
0.076376378	94.99074305	5116.872688
0.083787508	54.13903018	2763.403148
0.105497465	78.09859478	7245.773883
0.278867437	24.04410747	2716.489171
0.310781351	31.20556209	5024.497238
0.225837959	16.07232471	3346.301318
0.262071237	27.51195753	1713.652141
0.327426608	31.95486393	7286.459072
0.073483059	96.2614971	8063.706055
0.039656299	96.47128001	4426.451353
0.161933745	30.34508173	2003.165423
0.286837734	35.8150031	6252.99842
0.198833878	34.38099808	2104.59098
0.20058752	32.14943214	2150.792513
0.140169997	46.99260549	2955.38857
0.048355025	100.6499774	3316.549077
0.132424231	47.35824446	2791.313622
0.081272197	75.07819223	1381.632588
0.631150124	10.5931384	829.0597015
0.056744287	93.64210325	8190.19311

log.sigma.1.0.mm.3D_gldm_SmallDependenceLowGrayLevelEmphasis	log.sigma.1.0.mm.3D_gldm_LowGrayLevelEmphasis	log.sigma.1.0.mm.3D_gldzm_DistanceZoneVariabilityNormalized
0.002608425	0.005487626	0.912730377
0.008157008	0.016005784	0.966111772
0.004096579	0.011075421	0.84091183
0.007305417	0.012573806	0.889273356
0.004492229	0.010922491	0.884163999
0.013615965	0.018920298	0.880488909
0.001673248	0.007516227	0.909695613
0.001158734	0.003870332	0.855990793
0.002916492	0.009816651	0.945649208
0.003027717	0.006142785	0.903365289
0.003474237	0.014164539	0.915318058
0.001043622	0.003137977	0.929917355
0.003718696	0.008388904	0.867346939
0.008171235	0.013915213	0.856979749
0.005092021	0.012497578	0.899723183
0.002860876	0.011654574	0.976302406
0.002592635	0.007161793	0.954362826
0.007709341	0.016131656	0.848497601
0.002453602	0.007096695	0.941475922
0.001002978	0.002840586	0.930575896
0.00619911	0.024907331	0.935930469
0.00750171	0.015925208	0.940532199
0.009285654	0.021430176	0.90980708
0.002531133	0.010944852	0.920504465
0.00330537	0.009406144	0.96001666
0.002973506	0.009521017	0.956610517
0.002104139	0.00556393	0.898543527
0.004227119	0.012840769	0.918298505
0.017569902	0.032620175	0.82
0.001220312	0.004801071	0.98984798
0.00358092	0.012132575	0.947406866
0.00548075	0.012345932	0.929423446
0.003314601	0.010109976	0.894348942
0.003795712	0.012982395	0.961687105
0.00190413	0.005887847	0.911360416
0.004247689	0.009729776	0.827410838
0.00362979	0.006876991	0.712372176
0.001475322	0.005673194	0.92675668
0.005835939	0.01259922	0.884497041
0.000398115	0.001689191	0.906476754
0.006663053	0.017894211	0.882187755
0.001303288	0.005365997	0.849070374
0.003868528	0.01289356	0.940956813
0.004365198	0.009896261	0.908953826
0.003639358	0.010386733	0.942173589
0.003359903	0.010043402	0.92262965
0.00190394	0.006700767	0.919041879
0.002900888	0.005937036	0.981819712
0.008163573	0.015450275	0.868137357
0.005550345	0.012280924	0.973860796
0.001843645	0.003754886	0.982943693
0.002336108	0.006019947	0.86863973
0.001833571	0.008707758	0.911601705
0.001228293	0.003160252	0.951772129
0.007110997	0.013965099	0.884733764
0.004828005	0.016821247	0.944681552
0.00513712	0.016178265	0.942049593
0.004413257	0.011117476	0.926272222
0.004042725	0.008589685	0.857485726
0.001379557	0.005431149	0.914749094
0.003183984	0.007928843	0.932724306
0.003248583	0.005981178	0.876629594
0.002149167	0.005445855	0.822074946
0.001191619	0.002777406	0.79337083
0.003517205	0.008100763	0.945987654
0.001299303	0.002788484	0.830648947
0.010726321	0.019987198	0.884497041
0.001986181	0.005326666	0.8321749
0.00082131	0.003025977	0.932903778
0.002770878	0.008008356	0.942185186
0.007591694	0.015969979	0.936776454
0.002092697	0.006093558	0.889114526
0.006984618	0.015695332	0.948489684
0.005497684	0.012507845	0.974751604
0.002746545	0.008338992	0.741973482
0.006241187	0.016180891	0.966254674
0.001296476	0.003197389	0.901802453
0.006832861	0.011980792	0.87112426
0.00219554	0.005376968	0.868788927
0.004075076	0.010762207	0.924168975
0.002538825	0.005438769	0.920613488
0.004466498	0.008867136	0.94404647
0.001782407	0.004743818	0.9559048
0.003081289	0.011533841	0.908567142
0.002845741	0.009553472	0.89260521
0.005059698	0.013199648	0.830078125
0.006030499	0.016787657	0.907071608
0.001727388	0.007473426	0.96784485
0.001539246	0.003859954	0.938711745
0.001859517	0.004085015	0.889273356
0.007494086	0.015553153	0.730687077
0.002044395	0.007755077	0.942469028
0.005041164	0.013201788	0.869619845
0.004066794	0.012427989	0.891394733
0.002522258	0.007790212	0.868448428
0.002587248	0.005428429	0.774955582
0.002875204	0.008227374	0.878562995
0.00899902	0.01829725	0.89367189
0.010653781	0.038972976	0.961553249
0.00097333	0.003037504	0.8719382

log.sigma.1.0.mm.3D_gldzm_LowIntensityEmphasis	log.sigma.1.0.mm.3D_gldzm_LargeDistanceEmphasis	log.sigma.1.0.mm.3D_gldzm_HighIntensitySmallDistanceEmphasis
0.007925864	1.137176938	295.9746521
0.025818645	1.051724138	125.1918103
0.016465556	1.261410788	144.0010373
0.024229657	1.176470588	158.1911765
0.016393083	1.185185185	149.2685185
0.035940308	1.191489362	164.7952128
0.010590846	1.142196532	172.6229769
0.004787498	1.234314981	322.0201665
0.014004878	1.083870968	156.8768817
0.008895036	1.152727273	275.6527273
0.018918966	1.132911392	101.2310127
0.00423981	1.109090909	413.059375
0.013518539	1.214285714	183.7415966
0.021876812	1.23255814	168.128876
0.017136807	1.158823529	163.020098
0.016457604	1.03597786	127.4107472
0.011271744	1.070093458	209.0922897
0.028948588	1.247706422	124.3394495
0.011670298	1.090517241	201.8286638
0.004101435	1.10802603	429.0280911
0.039080573	1.09939759	72.3501506
0.024059592	1.09202454	143.5322086
0.03385666	1.142011834	116.2766272
0.014876379	1.124401914	133.5865231
0.01527763	1.06122449	177.4991497
0.012656442	1.066561014	172.4255151
0.007496878	1.16080402	267.6494975
0.018727603	1.128014842	134.5449907
0.047298583	1.3	77.69583333
0.006382098	1.015306122	342.4547194
0.017375042	1.081081081	126.3739718
0.017147285	1.10989011	140.040293
0.014531165	1.167870036	157.3222022
0.018245456	1.058614565	136.6283304
0.007881391	1.146945089	239.9005757
0.014385526	1.286184211	170.9473684
0.0101955	1.522413793	226.3021552
0.00777694	1.114213198	232.1588409
0.025744224	1.184615385	151.3096154
0.002138396	1.147540984	675.7535331
0.026760369	1.188571429	103.4721429
0.006195412	1.246677741	241.0006229
0.016962587	1.091346154	133.4507212
0.01320376	1.143426295	186.4800797
0.018721011	1.089403974	159.990894
0.01320238	1.120930233	161.8821705
0.008807679	1.126796281	219.8755283
0.01303928	1.027522936	307.6146789
0.022119661	1.212903226	133.383871
0.017974722	1.039735099	157.3923841
0.005342074	1.025806452	421.6994624
0.007345429	1.212025316	233.8702532
0.011562375	1.139041634	151.6559309
0.005706137	1.074175824	416.8516484
0.021735611	1.184210526	153.6513158
0.023016975	1.085409253	112.5516014
0.024307702	1.08960177	112.0459071
0.016057676	1.115	152.3908333
0.012642207	1.231660232	208.7007722
0.00659172	1.133848134	239.7110682
0.012093098	1.104557641	204.9121984
0.008860173	1.198142415	293.1963364
0.007545891	1.299801193	248.6771482
0.004010556	1.351014041	449.74883
0.016684327	1.083333333	198.5659722
0.003403151	1.280196629	461.5935744
0.035318501	1.184615385	121.1480769
0.006435264	1.277385159	257.9477326
0.003898955	1.104268293	400.3588415
0.011389641	1.089385475	167.2146182
0.025731814	1.098039216	129.9281046
0.008410008	1.176740627	230.5577659
0.026088271	1.079365079	132.0992063
0.014916108	1.038363171	168.7557545
0.012629282	1.465811966	167.8337488
0.025518048	1.051502146	116.1223176
0.004882898	1.155339806	431.4478155
0.017923707	1.207692308	179.6038462
0.007294661	1.211764706	254.8252941
0.016602521	1.118421053	160.6962719
0.007816463	1.124223602	296.4632505
0.015813642	1.086419753	201.8497942
0.006543891	1.067669173	295.4860902
0.016325371	1.144067797	128.305791
0.014219237	1.170818505	156.4088078
0.029297415	1.28125	89.20052083
0.024036427	1.146551724	110.5984195
0.01000276	1.049034175	191.8276374
0.005769906	1.094936709	359.9905063
0.006515763	1.176470588	340.3743873
0.030062063	1.481132075	106.5589623
0.010500571	1.088932806	188.6245059
0.019492511	1.210314263	126.3716761
0.01785167	1.172869148	134.9105642
0.009727404	1.212359551	175.7092697
0.007683093	1.38766007	283.9452852
0.010767675	1.194805195	178.1713564
0.024624157	1.169014085	191.1144366
0.059784058	1.058823529	58.05882353
0.004106319	1.206275958	382.0913706

log.sigma.1.0.mm.3D_gldzm_LowIntensityLargeDistanceEmphasis	log.sigma.1.0.mm.3D_gldzm_HighIntensityEmphasis	log.sigma.1.0.mm.3D_gldzm_DistanceZoneVariability	log.sigma.1.0.mm.3D_gldzm_ZonePercentage
0.008785809	304.8449304	459.1033797	0.34833795
0.027571519	125.5862069	112.0689655	0.334293948
0.021767706	156.2655602	202.659751	0.270179372
0.034353749	160.4117647	90.70588235	0.316770186
0.022145449	152.4148148	358.0864198	0.287030475
0.051886691	166.4787234	82.76595745	0.388429752
0.012605257	178.5843931	1573.77341	0.164152197
0.005811489	345.3066581	1337.057618	0.257415953
0.015797743	158.7172043	879.4537634	0.218207414
0.010206252	285.2254545	248.4254545	0.364238411
0.021383739	104.7721519	289.2405063	0.203084833
0.004733632	422.9977273	818.3272727	0.258291752
0.018270698	191.197479	206.4285714	0.293464858
0.027725727	171.6666667	221.1007752	0.400621118
0.02085968	168.7921569	458.8588235	0.323809524
0.018136675	128.1116236	1058.311808	0.188129122
0.012073589	211.0373832	204.2336449	0.242355606
0.046870988	126.0183486	92.48623853	0.266503667
0.012340004	206.7974138	218.4224138	0.212065814
0.004444546	440.8065076	2144.97744	0.251007296
0.044568154	73.46385542	310.7289157	0.159769009
0.027929735	144.1165644	153.3067485	0.328629032
0.058087738	118.9171598	153.7573964	0.302867384
0.01783222	137.5135566	577.1562998	0.174311927
0.016351815	180.2619048	282.244898	0.239413681
0.014234553	174.2345483	603.6212361	0.24687011
0.008753423	275.1809045	536.4304858	0.297904192
0.026408751	138.2115028	494.9628942	0.239236573
0.131999546	78.73333333	49.2	0.410958904
0.006464479	343.4515306	388.0204082	0.243478261
0.019104285	128.2150411	806.2432432	0.212272387
0.018607017	143.040293	253.7326007	0.346446701
0.01835365	164.3537906	495.4693141	0.237259101
0.019168302	139.4511545	541.4298401	0.233126294
0.009031087	247.12529	1178.389018	0.251899474
0.02183145	177.4407895	503.0657895	0.313563693
0.017157357	245.5137931	413.1758621	0.383597884
0.009441524	237.7267343	1095.426396	0.203057894
0.030227057	153.7846154	114.9846154	0.232142857
0.002374104	702.5500283	1603.557377	0.187255213
0.042500062	106.2857143	308.7657143	0.26119403
0.00738735	260.744186	1022.280731	0.212270804
0.0320426	134.0288462	587.1570513	0.258064516
0.015686723	192.3545817	456.2948207	0.353521127
0.020227916	162.4470199	284.5364238	0.194087404
0.015551545	166.8728682	595.096124	0.268638067
0.010208465	225.6365173	1087.226543	0.221080172
0.013230411	308.6055046	107.0183486	0.221995927
0.029325318	137.0322581	134.5612903	0.396419437
0.019461722	157.8741722	147.0529801	0.334070796
0.005464877	423.2494624	457.0688172	0.361305361
0.008690978	248.3386076	823.4704641	0.340394973
0.016034839	156.6732129	1160.468971	0.159603811
0.005943917	423.3791209	346.4450549	0.221680877
0.03352646	155.6842105	201.7192982	0.354587869
0.027023499	113.0427046	265.455516	0.238945578
0.028532382	114.4037611	851.6128319	0.225155666
0.022240115	157.5483333	555.7633333	0.290275762
0.017025945	213.2702703	222.0888031	0.339449541
0.007492873	249.1389961	2132.280137	0.213990636
0.014271191	207.9383378	347.9061662	0.280662152
0.010077166	310.494324	849.4540764	0.393103448
0.013213905	268.3033797	2067.518489	0.305440855
0.009825308	486.0624025	508.550702	0.305820611
0.017932715	200.1180556	136.2222222	0.212703102
0.004265311	498.2514045	1182.844101	0.416252558
0.048083676	124.1769231	114.9846154	0.320197044
0.008425606	276.6195524	1413.03298	0.333923304
0.004197	411.8402439	1529.962195	0.218433671
0.012643303	170.7243948	505.9534451	0.252824859
0.029814698	131.4379085	143.3267974	0.31097561
0.010419967	240.2853864	1162.072686	0.262397109
0.02955271	133.015873	358.5291005	0.283146067
0.015414593	170.0946292	381.1278772	0.411578947
0.021833426	183.5512821	694.4871795	0.229187071
0.029580411	117.0042918	225.1373391	0.253812636
0.005307139	451.1553398	557.3139159	0.279764599
0.022898399	183.7461538	113.2461538	0.40247678
0.008627465	270.84	369.2352941	0.323193916
0.018719133	164.8903509	421.4210526	0.253333333
0.008537791	303.9399586	444.6563147	0.343527738
0.016618902	204.473251	229.4032922	0.294188862
0.007094581	299.7413534	635.6766917	0.291411043
0.021484125	130.5536723	321.6327684	0.187004754
0.017580482	160.8807829	501.6441281	0.206998158
0.039478693	92.27083333	79.6875	0.18079096
0.037588895	113.4971264	315.6609195	0.266055046
0.010750308	192.8172363	651.359584	0.17544317
0.00611521	369.0126582	444.9493671	0.278659612
0.007299569	355.7990196	362.8235294	0.298463789
0.062629746	119.6981132	154.9056604	0.263681592
0.011930796	190.8418972	476.8893281	0.199842022
0.026137632	132.7784045	1079.198227	0.275716507
0.025474932	142.270108	742.5318127	0.241729541
0.011605425	186.011236	772.9191011	0.269126096
0.010851345	309.2444703	665.6868452	0.360318792
0.01362488	186.2005772	608.8441558	0.282626427
0.027401169	198.0492958	126.9014085	0.453674121
0.074489941	58.11764706	49.03921569	0.190298507
0.004918971	402.2648823	1889.490078	0.245580236

log.sigma.1.0.mm.3D_gldzm_IntensityVariabilityNormalized	log.sigma.1.0.mm.3D_gldzm_LowIntensitySmallDistanceEmphasis	log.sigma.1.0.mm.3D_gldzm_IntensityVariability
0.05631025	0.007710878	28.32405567
0.075356718	0.025380427	8.74137931
0.064892133	0.015140018	15.63900415
0.065743945	0.021698634	6.705882353
0.068934614	0.014954992	27.91851852
0.065640561	0.031953712	6.170212766
0.068954526	0.010087244	119.2913295
0.060240737	0.0045315	94.09603073
0.071989825	0.013556662	66.95053763
0.067345455	0.008567233	18.52
0.088026759	0.018302772	27.8164557
0.062541322	0.004116354	55.03636364
0.064578773	0.012330499	15.3697479
0.061264347	0.020414583	15.80620155
0.060438293	0.016206089	30.82352941
0.07722866	0.016037837	83.71586716
0.081753865	0.011071283	17.4953271
0.086608871	0.024467987	9.440366972
0.077809156	0.011502871	18.05172414
0.063859477	0.004015657	147.1960954
0.088837277	0.037708678	29.4939759
0.077646882	0.023092056	12.65644172
0.074682259	0.027798891	12.62130178
0.083837621	0.014137419	52.5661882
0.06106252	0.015009084	17.95238095
0.065021436	0.012261915	41.02852615
0.060057406	0.007182741	35.85427136
0.072844992	0.016807316	39.26345083
0.084444444	0.026123342	5.066666667
0.079198771	0.006361503	31.04591837
0.074279102	0.016942732	63.21151586
0.07802332	0.016782352	21.3003663
0.067790536	0.013575544	37.55595668
0.067410378	0.018014744	37.95204263
0.063725253	0.007607932	82.39675174
0.062462128	0.012524044	37.97697368
0.058222354	0.008455036	33.76896552
0.064813145	0.007360794	76.60913706
0.08035503	0.024623516	10.44615385
0.072227205	0.00207947	127.7699265
0.078546939	0.022825446	27.49142857
0.070283441	0.005897428	84.62126246
0.075787927	0.013192583	47.29166667
0.060165712	0.012583019	30.20318725
0.074908995	0.018344284	22.62251656
0.070055886	0.012615088	45.18604651
0.069626128	0.008457483	82.36770921
0.113205959	0.012991497	12.33944954
0.069719043	0.020318247	10.80645161
0.075215999	0.017602972	11.35761589
0.056797318	0.005311374	26.41075269
0.063520358	0.007009042	60.21729958
0.078776128	0.010444259	100.282011
0.074809806	0.005646691	27.23076923
0.063404124	0.018787898	14.45614035
0.081825205	0.022015344	22.99288256
0.073782207	0.023251533	66.69911504
0.06885	0.014512066	41.31
0.064981142	0.011546273	16.83011583
0.068087392	0.006366432	158.7117117
0.060009056	0.011548575	22.38337802
0.05436115	0.008555925	52.67595459
0.056298867	0.006136956	141.5916501
0.067413679	0.002556868	43.21216849
0.077739198	0.01637223	11.19444444
0.051780867	0.003187611	73.73595506
0.073491124	0.032127207	9.553846154
0.058630607	0.005937679	99.55477032
0.070974123	0.003824444	116.397561
0.075171048	0.011076226	40.36685289
0.070784741	0.024711094	10.83006536
0.060586601	0.007907519	79.18668707
0.065465692	0.025222162	24.74603175
0.06444882	0.014791487	25.19948849
0.063840036	0.010406546	59.7542735
0.074619168	0.024502457	17.38626609
0.06377185	0.004776838	39.41100324
0.071715976	0.016680034	9.323076923
0.068334948	0.00696146	29.04235294
0.066895583	0.016073368	30.50438596
0.056997972	0.007636131	27.5300207
0.067384714	0.015612326	16.3744856
0.064078241	0.006406218	42.61203008
0.096619745	0.015035682	34.20338983
0.069781284	0.013378925	39.21708185
0.113498264	0.026752096	10.89583333
0.073754789	0.02064831	25.66666667
0.075272283	0.009815872	50.65824666
0.064430558	0.00568358	30.54008439
0.064121972	0.006319811	26.16176471
0.071288715	0.021920142	15.11320755
0.080004374	0.010143015	40.48221344
0.068612625	0.01783123	85.14826753
0.072387659	0.015945854	60.29891957
0.072074233	0.009257899	64.14606742
0.053584521	0.00689103	46.02910361
0.068245847	0.010053374	47.29437229
0.065760762	0.023929904	9.338028169
0.124183007	0.056107588	6.333333333
0.062683552	0.003903156	135.8352561

log.sigma.1.0.mm.3D_gldzm_HighIntensityLargeDistanceEmphasis	log.sigma.1.0.mm.3D_gldzm_SmallDistanceEmphasis	log.sigma.1.0.mm.3D_glcm_SumVariance	log.sigma.1.0.mm.3D_glcm_Homogeneity1
340.3260437	0.965705765	830.5002308	0.356106434
127.1637931	0.987068966	278.4506339	0.358776272
205.3236515	0.934647303	367.9973344	0.37359263
169.2941176	0.955882353	433.4237358	0.343112601
165	0.953703704	372.1388866	0.360456674
173.212766	0.95212766	397.8163162	0.336562986
202.4300578	0.964450867	467.9888853	0.501288612
438.4526248	0.941421255	956.9310368	0.39444961
166.0784946	0.979032258	365.9956937	0.445910698
323.5163636	0.961818182	749.8075662	0.341339946
118.9367089	0.966772152	248.8635495	0.453107376
462.7511364	0.972727273	1223.314056	0.433426666
221.0210084	0.946428571	528.9204698	0.3617961
185.8178295	0.941860465	405.4363197	0.318739048
191.8803922	0.960294118	348.4425457	0.389468833
130.9151292	0.991005535	306.7898136	0.473946748
218.817757	0.982476636	551.7782618	0.418910039
132.733945	0.938073394	265.4412507	0.383677408
226.6724138	0.97737069	546.3401507	0.441859639
487.9201735	0.972993492	1386.161389	0.394941788
77.9186747	0.975150602	135.7241258	0.449820277
146.4539877	0.976993865	290.2393428	0.358452234
129.4792899	0.964497041	230.2002241	0.385118544
153.2216906	0.968899522	305.9488975	0.500808665
191.3129252	0.984693878	441.9666105	0.463532124
181.4706815	0.983359746	379.2507974	0.429075461
305.3065327	0.959798995	717.1102777	0.360029875
152.877551	0.967996289	291.1656791	0.430999238
82.88333333	0.925	150.2389148	0.344688603
347.4387755	0.996173469	967.8238477	0.411732019
135.5793184	0.97972973	292.4139664	0.420226996
155.040293	0.972527473	339.4197174	0.344857127
192.4801444	0.958032491	369.4852607	0.434942799
150.7424512	0.985346359	303.9382978	0.444965452
277.1825213	0.964982384	637.3972118	0.417646943
203.4144737	0.928453947	438.8457967	0.359659944
322.3603448	0.869396552	702.4718228	0.31018467
259.998308	0.971446701	650.2709863	0.491800341
163.6846154	0.953846154	360.2500874	0.401881573
809.736009	0.963114754	2242.825283	0.470097667
117.54	0.952857143	221.3443708	0.393729786
339.7184385	0.938330565	651.3634701	0.460168451
136.3413462	0.977163462	301.4126193	0.391333075
215.8525896	0.964143426	429.0211641	0.34695676
172.2715232	0.977649007	375.3043203	0.48247267
186.8356589	0.969767442	369.4128812	0.392145329
248.6804734	0.96830093	537.7991651	0.437808558
312.5688073	0.993119266	851.9951186	0.435329289
151.6258065	0.946774194	295.392182	0.346803164
159.8013245	0.990066225	365.478698	0.35238209
429.4494624	0.993548387	1182.348857	0.353695753
306.2120253	0.946993671	646.5334292	0.345971519
176.7423409	0.965239592	387.3117051	0.528878629
449.489011	0.981456044	1386.036129	0.428789608
163.8157895	0.953947368	367.0761059	0.327140825
115.0071174	0.978647687	229.4734541	0.399446217
123.835177	0.977599558	231.8078408	0.4468872
178.1783333	0.97125	361.2103199	0.367600667
231.5482625	0.942084942	508.1439817	0.367135553
286.8507079	0.966537967	648.5679099	0.464719209
220.0428954	0.97386059	533.6080838	0.38556125
379.6862745	0.950464396	876.585064	0.315692123
347.2500994	0.925933289	756.0692581	0.367627374
631.3166927	0.91224649	1515.186992	0.341613886
206.3263889	0.979166667	544.6692787	0.444447507
644.8827247	0.929950843	1474.971625	0.296802914
136.2923077	0.953846154	272.1852367	0.350210634
351.3068316	0.93065371	730.8628975	0.342309184
457.7658537	0.973932927	1238.07231	0.414933352
184.7635009	0.977653631	465.7294065	0.389898588
137.4771242	0.975490196	283.5446453	0.377159272
279.1958684	0.955814843	629.2370863	0.399379264
136.6825397	0.98015873	280.0715197	0.377436745
175.4501279	0.990409207	358.6620669	0.344379305
247.8696581	0.885921178	453.2906958	0.454346927
120.5321888	0.987124464	243.2133821	0.410685804
529.9854369	0.961165049	1368.755725	0.363244484
200.3153846	0.948076923	423.1348246	0.331357924
334.8988235	0.947058824	753.3758797	0.356678236
181.6666667	0.970394737	362.4335106	0.430248502
333.8467909	0.968944099	827.7089787	0.362219691
214.9670782	0.978395062	550.7143622	0.380721226
316.762406	0.983082707	826.1987797	0.411686908
139.5451977	0.963983051	291.0902374	0.457104064
178.7686833	0.957295374	379.8644922	0.463066287
104.5520833	0.9296875	309.3415873	0.455631357
125.091954	0.963362069	226.6483652	0.404831384
196.7756315	0.987741456	461.0350627	0.50431777
405.1012658	0.976265823	1090.647141	0.392960116
417.497549	0.955882353	1100.045968	0.354291428
172.254717	0.879716981	312.2070539	0.382600239
199.7114625	0.977766798	452.2685309	0.484138278
158.4053183	0.947421434	300.048054	0.395135975
171.7082833	0.956782713	309.2481797	0.409364371
227.2191011	0.946910112	455.9921939	0.399090652
410.4412107	0.903084983	849.6447344	0.317900067
218.3174603	0.951298701	455.8919297	0.381835203
225.7887324	0.957746479	512.5593941	0.30281262
58.35294118	0.985294118	91.1435149	0.441176511
482.9589294	0.948431011	1246.540119	0.379232222

log.sigma.1.0.mm.3D_glcm_Homogeneity2	log.sigma.1.0.mm.3D_glcm_ClusterShade	log.sigma.1.0.mm.3D_glcm_MaximumProbability	log.sigma.1.0.mm.3D_glcm_Idmn	log.sigma.1.0.mm.3D_glcm_SumVariance2
0.274588159	22.04859089	0.03850036	0.974615017	29.15423887
0.270211306	3.629207379	0.023941131	0.960938899	18.21656929
0.289868313	3.237141293	0.029115615	0.958546629	22.01567186
0.254551814	-17.35180613	0.025037067	0.952471452	24.16053681
0.272538009	6.218400674	0.022103332	0.960358924	20.57965943
0.247301717	-3.013693978	0.031319109	0.959281787	27.05777307
0.444755606	10.00774342	0.109339627	0.985385637	11.99293352
0.318958143	33.3119662	0.068715123	0.982210654	20.99806392
0.376943718	17.51545511	0.083340513	0.977964284	14.07166589
0.252906668	0.059609696	0.024739933	0.970094527	24.5709308
0.382269154	6.735995758	0.078372839	0.973277839	10.2273652
0.364258623	31.54770812	0.083157384	0.988206453	17.38357695
0.276426882	-34.51341936	0.040631245	0.969855645	24.10302183
0.227111511	4.756736185	0.019805476	0.952382909	27.74336927
0.312712935	54.45157247	0.067074792	0.967199204	24.14361464
0.41189134	9.559654371	0.104683302	0.980151056	15.00561009
0.339199318	16.29102446	0.052089339	0.984497785	13.4700924
0.297647686	-8.669305197	0.032356463	0.965581996	15.83864493
0.369545559	10.73889573	0.067725891	0.981853016	11.93951439
0.315997358	-8.05857628	0.047591563	0.987917438	18.70430322
0.376376777	5.152004547	0.041685415	0.967864256	10.06877643
0.270210341	30.20841952	0.029342559	0.966131311	20.07031436
0.303786644	10.3115347	0.059618126	0.953460006	18.98134389
0.44201729	14.43858505	0.126378339	0.981674624	9.758992638
0.402295021	2.479356191	0.116417316	0.968454326	19.17804337
0.35713125	70.32585643	0.060168846	0.980218186	18.13313914
0.275164664	39.34281885	0.032217554	0.976945543	23.73690172
0.357914387	18.57048751	0.077824428	0.980800627	13.89884959
0.253810672	11.85240256	0.033156744	0.941413081	17.769103
0.333304771	9.331874129	0.053893944	0.983416273	15.38340559
0.343364747	13.00781418	0.052106043	0.982544494	14.64017364
0.255759008	8.888809324	0.020115169	0.959610588	22.19386801
0.363709888	23.15177608	0.074115012	0.98048754	15.74432928
0.37811302	27.1935581	0.082756076	0.976718578	18.29789039
0.343218765	34.254804	0.057105537	0.983106904	18.14185465
0.275574449	-5.291224514	0.035422882	0.963203643	24.03923935
0.219815483	-65.82845837	0.018690935	0.967773277	35.10419565
0.436033259	10.53337974	0.114647864	0.984252275	17.30904012
0.318467841	0.516394754	0.0349648	0.969075671	12.92705201
0.406913365	23.83421668	0.103293463	0.994145438	12.45157919
0.310349451	7.387133407	0.036931783	0.964199115	14.88635197
0.395447959	44.39826393	0.091236683	0.984402393	14.34785795
0.309204581	1.388313285	0.026122575	0.969825193	18.07274494
0.259738489	42.99768338	0.028877646	0.966331581	24.84783616
0.420450504	5.989240435	0.11383994	0.97791887	10.75223735
0.311058758	30.10087674	0.045128875	0.972724804	18.51604516
0.367154691	33.36758489	0.081591394	0.981304835	16.06701199
0.359155654	0.723403635	0.053153695	0.984718318	9.482830608
0.256689236	26.29382529	0.030319938	0.960441169	21.74817733
0.26376405	-11.85263974	0.025170233	0.964073104	23.94772877
0.27084269	26.36858922	0.048595284	0.97602069	25.55610393
0.257673081	37.34875315	0.027082138	0.973551953	24.82121381
0.478249401	10.08062835	0.129007552	0.986192741	9.015040601
0.356852779	-22.697427	0.079875437	0.987063304	12.92900489
0.237067118	-14.85625386	0.020575841	0.950042587	29.35420947
0.316261205	14.98547761	0.035973906	0.970103135	14.38004508
0.377496277	23.28942362	0.080133849	0.974748977	13.93358207
0.281438439	19.76907334	0.030289068	0.971894843	20.08901707
0.282350194	26.5767019	0.046069977	0.973968952	21.61438258
0.401918876	41.92636749	0.089495108	0.984907763	15.09664561
0.305957751	25.74348668	0.040105923	0.975928635	21.8148194
0.228567193	-4.16008471	0.024971221	0.969171718	33.63772521
0.288344315	-5.444962112	0.045535194	0.978607295	26.27176163
0.250958831	-19.10295148	0.019617665	0.982851423	20.9074507
0.371756726	-10.72863597	0.075755799	0.977861158	11.91810362
0.206852064	17.65783446	0.014522274	0.974003284	36.20921797
0.260839621	-16.17157454	0.033255502	0.959918517	21.0863258
0.257210822	31.12068255	0.035846028	0.978179821	26.49445381
0.339065649	18.52318006	0.063464499	0.987724452	14.65102441
0.305319699	-5.336038572	0.028002941	0.976263466	15.75765913
0.294454353	-2.849222861	0.043156891	0.960459054	19.94245357
0.324573203	20.14539257	0.05617233	0.978350735	22.88871296
0.293867571	30.64503414	0.035222912	0.972224875	19.82857424
0.25705322	61.92163933	0.033072223	0.968752909	25.61487661
0.388718259	23.48853016	0.102712343	0.978766146	16.28899095
0.330690526	19.72199423	0.040402181	0.975521381	14.03879287
0.27637905	-26.42212252	0.024267549	0.983285228	21.21247951
0.240759786	-4.470755514	0.021843611	0.955049897	26.99283756
0.268859702	1.371623906	0.022324651	0.974146767	20.78966654
0.358092972	37.27198716	0.080643436	0.980030664	15.36141995
0.281177722	24.7528269	0.039869867	0.973161528	27.8940744
0.296974649	2.404690823	0.03111823	0.970009013	18.32160203
0.339292181	8.332435048	0.077201294	0.976422423	21.43946049
0.386910356	8.034410861	0.06723762	0.982167891	9.217513835
0.397437567	12.89726928	0.094108777	0.974929489	13.7072948
0.386202458	-30.83454398	0.071081571	0.963278574	11.35089084
0.325158185	27.23357649	0.039024513	0.974129013	15.88444355
0.447217928	18.96180016	0.127491203	0.983138349	11.26602986
0.314520775	-6.516090642	0.060436883	0.982628183	19.57604472
0.266277738	-28.33018788	0.021077464	0.976892301	21.80945243
0.299795847	-17.69990176	0.047016736	0.972742798	18.50533365
0.421911796	16.98763948	0.106453062	0.981577772	10.50520926
0.314639746	7.98323806	0.045252711	0.973257042	16.45829516
0.330799662	18.82674459	0.048589089	0.978608905	16.13208367
0.318320846	19.50005883	0.044091953	0.97738709	14.90839822
0.230249492	7.961201088	0.018696605	0.971835055	32.49325187
0.298695103	21.23768212	0.037749393	0.972720083	19.25267833
0.212315734	-15.06487301	0.020111006	0.95871669	35.39937236
0.364543955	-0.447611344	0.047498254	0.954382431	9.51826829
0.297871867	-12.73976646	0.038098154	0.984530502	19.57238119

log.sigma.1.0.mm.3D_glcm_Contrast	log.sigma.1.0.mm.3D_glcm_DifferenceEntropy	log.sigma.1.0.mm.3D_glcm_InverseVariance	log.sigma.1.0.mm.3D_glcm_Entropy	log.sigma.1.0.mm.3D_glcm_Dissimilarity
28.91983948	3.502880809	0.265042606	7.471540792	3.893708255
17.74561606	3.175814202	0.273907715	6.771431347	3.260138686
19.11167755	3.248406096	0.274772655	7.187562981	3.297717481
22.29021261	3.323080595	0.258253711	7.036351284	3.625536799
18.01228786	3.218736842	0.273241771	7.188132024	3.275530866
24.70727658	3.374887026	0.241477119	6.962240448	3.814667616
10.72653343	2.792064981	0.37190346	6.071915231	2.167620007
22.19893167	3.329040673	0.293897602	7.157526477	3.357634613
12.83180664	2.967782981	0.342497086	6.500787713	2.539649128
24.35267615	3.400491209	0.253234275	7.317040659	3.734934836
9.579791318	2.804041299	0.351818183	6.193593072	2.273778372
18.1984744	3.153962038	0.324140807	6.717241098	2.90397947
20.94685257	3.305530392	0.26922817	7.221957522	3.451062588
26.88120813	3.463387941	0.235426487	7.488949558	4.020491971
23.61914443	3.363383379	0.283968036	7.162921642	3.456489041
12.53118433	2.903867855	0.34992738	6.331664636	2.422237048
12.08175009	2.940854346	0.317190092	6.479219402	2.57705822
13.95465764	3.022348068	0.298225256	6.610811337	2.873694421
11.41848974	2.882700674	0.348995824	6.299670928	2.418354613
18.56226255	3.208169938	0.305435856	7.062086037	3.085917473
8.096272781	2.699572435	0.361999951	6.174079479	2.149434735
18.28869454	3.215678214	0.269947444	6.934678639	3.305941636
17.7452405	3.196159705	0.287242837	6.888867106	3.156610934
8.90066026	2.710081559	0.376979593	5.853175042	2.030927802
17.80997757	3.094247774	0.324714869	6.339028836	2.818481176
14.75033426	3.040017409	0.339265693	6.687649837	2.699554844
22.84667252	3.348608342	0.275112584	7.319500518	3.523003617
13.07849526	2.987736811	0.336872182	6.547025105	2.599769804
17.74873771	3.10118867	0.268840433	6.282898664	3.343660066
14.04515299	3.036074727	0.318004292	6.693336593	2.740015937
12.63710226	2.977941731	0.325082741	6.694022872	2.628037234
20.10441137	3.275309426	0.262740542	7.203257589	3.501319502
14.42338574	3.045134505	0.328565188	6.636238308	2.689244207
16.23379256	3.085500179	0.330019571	6.611604239	2.771664151
16.48449625	3.132890343	0.318420473	6.904088051	2.877000743
22.08996132	3.34768861	0.270877423	7.410958863	3.539960034
32.34611526	3.58336648	0.22353577	7.813128077	4.366121244
15.47297037	2.972914331	0.345356304	6.237638498	2.54050873
12.56923006	2.960861325	0.310617022	6.423302916	2.680120633
12.78695859	2.933348136	0.35195987	6.264209716	2.430048728
13.11704086	3.008655604	0.312063839	6.760141674	2.768629256
14.34429476	2.989479956	0.349658489	6.404350017	2.545990891
14.78877629	3.050504232	0.305535915	6.920264578	2.906915259
23.7311185	3.399824812	0.256064983	7.429836011	3.685053636
10.90247069	2.8327231	0.360803261	6.065896045	2.248305748
15.97598275	3.139412185	0.301891063	6.993289103	2.982323611
14.95069105	3.063387091	0.327027026	6.658492842	2.70175659
8.743557461	2.682374393	0.367615178	5.940618043	2.214234125
19.90264576	3.248892568	0.262294335	6.993476338	3.450615852
19.51383136	3.244291374	0.267720192	7.057677133	3.408054096
26.87240523	3.471685684	0.254863096	7.340189322	3.849558166
22.82288591	3.378806651	0.258574776	7.481950174	3.624209776
8.622741067	2.634010255	0.394476175	5.643178326	1.909062511
14.11541004	3.003461821	0.342678104	6.396570531	2.665339499
25.8693831	3.448204312	0.23776179	7.44729351	3.93641746
12.00512168	2.9442025	0.321158367	6.622681226	2.649180008
12.41256713	2.949518978	0.34251043	6.496228914	2.50532469
17.87337272	3.214547284	0.281051448	7.166193881	3.220057729
21.04783682	3.30257009	0.270869353	7.10591691	3.409827236
14.93671118	2.985039811	0.358858599	6.39241392	2.541202129
20.98081305	3.289665885	0.28943681	7.149557288	3.303927768
35.21227656	3.666949389	0.227551486	7.941189428	4.482412625
26.97949857	3.471347268	0.271293728	7.572791813	3.758204476
21.17143002	3.315908193	0.258741871	7.297653281	3.536776568
10.85088637	2.84697732	0.345973093	6.217688687	2.37408506
39.00910497	3.737609053	0.214352344	8.133090624	4.759681059
20.15262393	3.26883066	0.25679616	6.976053475	3.467670907
27.29237655	3.498706461	0.253210506	7.651162046	3.92009661
16.15372262	3.094995422	0.327289016	6.674480682	2.817449778
13.65264715	3.038975592	0.303297039	6.860406933	2.824000699
18.09563368	3.198280856	0.283555586	6.935824796	3.218579692
21.46734187	3.301670318	0.298757637	7.235424246	3.284108218
17.87464648	3.19805366	0.29134466	7.061515264	3.156916529
25.68754095	3.433668883	0.251834856	7.302494187	3.789394357
15.87653536	3.07713607	0.325268119	6.590438585	2.721356315
13.2822532	2.982644071	0.320558424	6.553324961	2.67433342
21.18750491	3.277984137	0.277587905	7.171971409	3.356707199
22.9057944	3.335999166	0.254711142	7.150111415	3.716908687
19.11915819	3.255116416	0.274473266	7.211797605	3.347410641
14.91014449	3.049738342	0.333933827	6.539667644	2.708709558
27.0525449	3.455928991	0.271675201	7.386240607	3.761246183
16.32545137	3.127332134	0.304281291	6.910899075	3.024254508
20.34613529	3.258676165	0.294839376	6.951204328	3.209004014
8.544283676	2.707968852	0.375578785	6.031297691	2.134485381
12.42510207	2.934967707	0.344633511	6.401490556	2.44579583
9.56110793	2.762568264	0.361252646	5.907991581	2.252844602
13.87570934	3.031999665	0.318428828	6.742513678	2.76659318
9.790673499	2.749303342	0.373330813	5.948086394	2.08740793
19.18699978	3.239259276	0.295800832	7.01219389	3.176955536
21.4682639	3.308481938	0.269720361	7.252030393	3.459598752
17.55322826	3.174390778	0.296822932	6.877010219	3.101445012
9.76955818	2.773457387	0.370046811	6.015924038	2.154115272
15.67175358	3.13009418	0.306272237	6.971890473	2.943150821
14.73883027	3.070296813	0.316109391	6.841275548	2.792217748
14.22523118	3.062654202	0.313107764	6.805620743	2.821193978
34.19571458	3.6341202	0.236112647	7.895431483	4.373488873
17.43852948	3.197644546	0.288967736	7.099624464	3.12257226
34.73052388	3.595991473	0.226847219	7.378771158	4.501506523
7.572647618	2.626811535	0.360593809	5.887723726	2.133806936
20.26396743	3.285663741	0.288737174	7.211985303	3.292528613

log.sigma.1.0.mm.3D_glcm_DifferenceVariance	log.sigma.1.0.mm.3D_glcm_Idn	log.sigma.1.0.mm.3D_glcm_Idm	log.sigma.1.0.mm.3D_glcm_Correlation	log.sigma.1.0.mm.3D_glcm_Autocorrelation
13.69726622	0.900036481	0.274588159	0.006885824	267.5096725
7.054840217	0.870216685	0.270211306	0.011464116	101.9289437
8.117104443	0.870312909	0.289868313	0.071351096	131.0711222
9.102832414	0.859129843	0.254551814	0.040676386	150.2471303
7.253339979	0.869896861	0.272538009	0.066594136	132.2543523
10.07566669	0.868778709	0.247301717	0.04377613	139.0808133
6.002561527	0.929241323	0.444755606	0.056536485	157.0009721
10.87319403	0.916578916	0.318958143	-0.023966385	301.7706554
6.333910297	0.90846407	0.376943718	0.045223652	128.3000512
10.32397873	0.887232247	0.252906668	0.005303977	244.0011
4.387309346	0.89651219	0.382269154	0.030091932	91.60444715
9.744748013	0.933849666	0.364258623	-0.020287096	374.2797752
8.935119174	0.887663722	0.276426882	0.070883923	179.5693996
10.66432738	0.857693585	0.227111511	0.01470048	142.0619792
11.60580635	0.889694622	0.312712935	0.011008064	123.8217421
6.51837268	0.916074916	0.41189134	0.085782059	110.1638656
5.42309536	0.918039866	0.339199318	0.053866701	183.319831
5.663641311	0.878088011	0.297647686	0.06247919	98.19543863
5.547995592	0.914907049	0.369545559	0.023005788	180.814892
8.994301834	0.929353635	0.315997358	0.006539032	422.1316202
3.459537627	0.884114671	0.376376777	0.107573441	56.01655102
7.348906126	0.878582959	0.270210341	0.046039035	106.2329372
7.745496147	0.864174765	0.303786644	0.034001454	87.10570669
4.764406869	0.919052861	0.44201729	0.045203172	108.0708535
9.750162738	0.898747768	0.402295021	0.035359121	150.2809955
7.42373414	0.91318505	0.35713125	0.100019289	133.126509
10.33561506	0.902363891	0.275164664	0.01965842	234.3819208
6.299341261	0.912554032	0.357914387	0.030639444	105.5374369
6.516854169	0.840681554	0.253810672	0.000740076	59.51079034
6.521054373	0.916538718	0.333304771	0.043877601	303.5206404
5.658957982	0.914027938	0.343364747	0.072377863	106.7382076
7.772952713	0.867531413	0.255759008	0.049112302	122.0043999
7.167721485	0.913355808	0.363709888	0.043680288	129.8181479
8.512970654	0.909027851	0.37811302	0.053481174	109.6082439
8.191758362	0.918692633	0.343218765	0.04776549	209.941936
9.443737885	0.877346294	0.275574449	0.042418532	152.758958
12.89654811	0.881945056	0.219815483	0.041519465	231.3510686
8.967602244	0.928848307	0.436033259	0.051297424	210.9134634
5.361628487	0.885408288	0.318467841	0.013735564	126.3765564
6.8631047	0.952397941	0.406913365	-0.009450102	649.2697894
5.435089752	0.876681771	0.310349451	0.062664577	84.49829667
7.841892389	0.925664014	0.395447959	0.001275937	211.1237437
6.018247523	0.887319205	0.309204581	0.098063832	110.2218883
10.12484748	0.881287562	0.259738489	0.02269016	149.3872521
5.831391961	0.911845968	0.420450504	-0.007649499	129.2206537
7.057655531	0.893656614	0.311058758	0.073446349	131.0399932
7.632189654	0.916035418	0.367154691	0.035622481	179.8019442
3.826755386	0.917104142	0.359155654	0.040720367	266.1931694
7.956767951	0.869353809	0.256689236	0.045414293	107.8795404
7.853086545	0.875634284	0.26376405	0.101997684	130.3097441
11.99067595	0.900265556	0.27084269	-0.021615413	366.62575
9.660668953	0.893216521	0.257673081	0.042424824	214.6211567
4.973487988	0.93236479	0.478249401	0.022238133	131.2766357
6.921559752	0.927978408	0.356852779	-0.037618126	416.6388964
10.32382707	0.854981812	0.237067118	0.063042282	130.8116305
4.971079732	0.886137747	0.316261205	0.088559054	87.01092661
6.09618005	0.902278188	0.377496277	0.055153517	87.10184355
7.47714805	0.889874796	0.281438439	0.057939909	128.8339361
9.399997242	0.896016688	0.282350194	0.011891333	172.3872228
8.46782799	0.928306434	0.401918876	0.005954571	210.0955703
10.01353915	0.90263514	0.305957751	0.019802006	180.315555
15.05561189	0.886219179	0.228567193	-0.018717138	281.6171652
12.78211279	0.907823963	0.288344315	-0.010160638	245.9193529
8.63710786	0.910895632	0.250958831	-0.003320052	459.251296
5.176218061	0.906018233	0.371756726	0.045889517	180.4757718
16.28610809	0.893628215	0.206852064	-0.032876816	451.8360517
8.078374847	0.868955931	0.260839621	0.023128853	100.3051845
11.87733095	0.903462096	0.257210822	-0.011808373	238.9137573
8.20490633	0.930207812	0.339065649	-0.043414334	377.098791
5.66304671	0.89761927	0.305319699	0.071772128	159.6772644
7.650933807	0.872670835	0.294454353	0.048248149	104.253798
10.63682775	0.909102508	0.324573203	0.03300372	208.7422617
7.861507024	0.89226329	0.293867571	0.052226614	103.3508714
11.26706113	0.8863319	0.25705322	-0.002065572	127.0936872
8.434973846	0.913501417	0.388718259	0.012581791	154.1994359
6.098117689	0.899522412	0.330690526	0.029061424	90.75724208
9.881885144	0.915812332	0.27637905	0.003101444	417.8797727
9.024647569	0.861182592	0.240759786	0.080965637	147.71366
7.890919719	0.89341507	0.268859702	0.042158268	244.8833899
7.563155814	0.912990117	0.358092972	0.014996639	127.0367971
12.84404962	0.897562237	0.281177722	0.017340549	266.650012
7.142335117	0.888175336	0.296974649	0.056962199	185.0648906
9.912466052	0.90555386	0.339292181	0.026706149	264.6336528
3.977307633	0.913800359	0.386910356	0.037989637	104.1269935
6.409521272	0.904955185	0.397437567	0.048069032	132.3599019
4.366748323	0.881095715	0.386202458	0.090703732	109.8183875
6.168198928	0.896417314	0.325158185	0.066473881	86.04660164
5.428314359	0.923750804	0.447217928	0.069295805	154.6828314
9.046130797	0.915742471	0.314520775	0.014082354	339.7075998
9.466999078	0.900604617	0.266277738	0.009923645	343.4907855
7.895245544	0.894115887	0.299795847	0.026572949	112.7666027
5.114860273	0.917920998	0.421911796	0.034530905	152.032952
7.001545587	0.894917887	0.314639746	0.024387759	109.2174967
6.92295365	0.90673331	0.330799662	0.044896205	112.0499091
6.245926196	0.901903023	0.318320846	0.023355454	155.9665514
14.97696257	0.891555199	0.230249492	-0.021023078	273.693114
7.641665702	0.893245283	0.298695103	0.049847997	157.280259
14.38870247	0.86838871	0.212315734	0.007993457	173.2538731
2.99052271	0.860901405	0.364543955	0.112752533	40.42200915
9.372097067	0.919357796	0.297871867	-0.010575011	383.634074

log.sigma.1.0.mm.3D_glcm_SumEntropy	log.sigma.1.0.mm.3D_glcm_AverageIntensity	log.sigma.1.0.mm.3D_glcm_Energy	log.sigma.1.0.mm.3D_glcm_SumSquares	log.sigma.1.0.mm.3D_glcm_ClusterProminence
4.399826899	16.53878363	0.010502752	15.13480733	3545.890706
4.048042244	10.08956939	0.011298565	8.990546338	1033.010305
4.233127961	11.41674047	0.009432792	10.28183735	1389.957246
4.246721682	12.23801685	0.009506172	11.61268735	1736.161462
4.194647244	11.47218924	0.008961641	9.647986821	1177.307249
4.281932338	11.7677039	0.010252132	12.94126241	2403.394737
3.680801719	12.51793957	0.041344807	5.686562993	758.3321329
4.167387873	17.57884712	0.016196562	11.25712669	1905.796267
3.866918742	11.31323323	0.024173269	6.725868134	816.7811606
4.307455915	15.64279211	0.008485601	12.42610908	1961.640623
3.677455492	9.562541563	0.023039424	4.951789129	359.0994927
3.976644035	19.51391481	0.02393626	8.535679611	1732.696987
4.273645061	13.37075212	0.010107912	11.2624686	1764.937734
4.385313188	11.90963362	0.007286856	13.65614435	2209.06964
4.234902419	11.12155349	0.015691999	11.94068977	2465.164991
3.851584545	10.46637831	0.032420965	6.884198606	875.0941438
3.851941621	13.53075696	0.017246628	6.439780305	768.8522147
3.972154974	9.885290781	0.013149738	7.448325642	747.126802
3.766706002	13.44184723	0.02197671	5.839501032	650.8686988
4.110845107	20.67797381	0.013898319	8.729456256	1697.947821
3.69306877	7.451315478	0.019396824	4.541262302	319.6207939
4.133659801	10.28493375	0.010431785	9.589752224	1239.205664
4.099465527	9.316211783	0.013386852	9.181646097	1137.863121
3.560845512	10.38536877	0.041207189	4.664913225	500.3843167
3.928321957	12.24484032	0.039801181	9.247005235	1620.161462
3.999516249	11.50612715	0.020063052	8.288876225	1774.769433
4.272519558	15.39888483	0.010279203	12.19956559	2219.306599
3.874960415	10.26312316	0.02106936	6.744336212	888.0900016
3.919105154	7.713498518	0.015311263	8.879460178	929.3669021
3.96287042	17.45378517	0.015727676	7.57648677	985.2474008
3.94790138	10.30788237	0.015857143	6.831551942	764.6683325
4.23284166	11.02168557	0.008422805	10.57456985	1346.734375
3.950546222	11.38205223	0.021774569	7.580320341	1074.952444
3.98887158	10.44466486	0.026659113	8.632920737	1445.989972
4.065288395	14.50802235	0.016979354	8.912615253	1479.184983
4.312579251	12.33965404	0.008825573	11.53230017	1786.623108
4.540977878	15.24810658	0.006263309	17.20869034	3749.342534
3.855478356	14.5353993	0.045417485	8.384957652	1400.98874
3.838803597	11.23762528	0.015568265	6.374070518	591.2124184
3.738015083	25.62313409	0.033270019	6.677610554	784.955183
3.96780514	9.168142602	0.013136672	7.000848206	692.4932145
3.820990256	14.55929997	0.029139485	7.422657243	1159.66085
4.085581293	10.459099	0.011408146	8.215380309	968.4639136
4.317848843	12.21089643	0.008864618	12.14473867	2121.75989
3.645207466	11.36914632	0.035100451	5.41367701	542.8590718
4.106711458	11.41943931	0.012677178	8.623006976	1217.599009
3.955847173	13.40957672	0.022622073	7.861709354	1220.934574
3.593442962	16.30966991	0.021679433	4.556597017	387.051809
4.186325198	10.36406422	0.010410678	10.41270577	1538.121753
4.252896533	11.36606223	0.009445606	10.86539003	1580.363399
4.300308179	19.37856405	0.010938526	13.4004622	2526.933384
4.331627608	14.65845502	0.008299732	12.12989257	2094.735865
3.456843833	11.45331628	0.053595669	4.409445417	450.5127847
3.782533494	20.61348552	0.023576053	6.562942328	943.7761003
4.421015483	11.39878151	0.007379354	13.80589814	2452.582276
3.926056909	9.296014689	0.014229601	6.596291691	686.7191243
3.864524233	9.312418264	0.022773272	6.5865373	773.6850564
4.18262162	11.32600221	0.010044696	9.490597446	1286.134199
4.190910926	13.13171243	0.011508616	10.75949825	1786.45589
3.817749056	14.53819645	0.032560444	7.846574128	1398.844013
4.217911136	13.44491908	0.011098327	10.93680438	1915.269462
4.552828643	17.03455057	0.006245822	17.9361946	3877.222174
4.360182869	15.86559568	0.010991023	13.82641242	2571.595468
4.207526278	21.59939597	0.008497045	10.03088228	1722.53682
3.766866936	13.42409759	0.022344687	5.692247497	604.3451918
4.614814769	21.53487794	0.005048354	18.10411374	4421.05766
4.161066108	10.00334335	0.010489296	10.30973743	1262.277255
4.386629113	15.64325402	0.008201472	14.1635016	2397.947272
3.879990521	19.62965486	0.019361138	7.548719647	1415.11235
4.018502106	12.61547943	0.011885579	7.352576572	780.9755366
4.139626901	10.18743583	0.011295277	9.509521812	1170.018172
4.247100894	14.48178348	0.014600609	11.42062145	2070.320642
4.152138673	10.1420502	0.010981813	9.425805179	1487.962714
4.299333211	11.28075019	0.009378767	12.91628516	2349.303401
3.922615216	12.41556233	0.029016465	8.06486067	1236.66114
3.894991089	9.51662976	0.015350292	6.830261518	834.5499674
4.175115079	20.62615979	0.010406242	10.15583661	2667.594329
4.319921986	12.1113137	0.008578098	12.47465799	1859.18966
4.204497777	15.64570267	0.009114298	10.06352733	1378.80041
3.902281596	11.2684724	0.022367526	7.600739234	1220.232531
4.365003019	16.46504806	0.011190832	14.3740258	3240.889945
4.097340734	13.58541854	0.011659175	8.66176335	1148.306712
4.149922388	16.30052297	0.019685642	10.73285561	1767.749418
3.603001112	10.19599782	0.02464554	4.440449378	343.7620573
3.846475536	11.49081284	0.027841846	6.533099219	770.1824396
3.653845471	10.45788577	0.027018112	5.227999691	466.4875945
3.980246715	9.248914179	0.014285501	7.440038222	928.1661064
3.637000508	12.42230113	0.042361261	5.264175841	653.6362772
4.129833458	18.60614744	0.015111194	10.01299787	1579.79354
4.225956236	18.6479561	0.008986457	11.16243405	1773.178104
4.078251087	10.60786299	0.013416669	9.014640478	1257.86931
3.627265974	12.32267584	0.033625904	5.06869186	555.5431905
4.042497519	10.44129165	0.013390998	8.032512186	976.695433
4.017356093	10.56887953	0.014689334	7.717728485	1033.356588
3.961680883	12.48179906	0.013818286	7.283407351	840.1864919
4.527163701	16.81787302	0.006157226	17.36407065	3682.116056
4.150245901	12.52302065	0.01120194	9.172801954	1280.658066
4.468543504	13.17887973	0.007494674	17.78905824	4076.817153
3.603918187	6.318927739	0.021538649	4.272728977	239.5418836
4.153908979	19.82953834	0.011953058	9.565962734	1663.320062

log.sigma.1.0.mm.3D_glcm_SumAverage	log.sigma.1.0.mm.3D_glcm_Imc2	log.sigma.1.0.mm.3D_glcm_Imc1	log.sigma.1.0.mm.3D_glcm_DifferenceAverage	log.sigma.1.0.mm.3D_glcm_Id
32.70781037	0.6571215	-0.072651324	3.893708255	0.356106434
20.17913878	0.724909103	-0.104644341	3.260138686	0.358776272
22.83348093	0.599122769	-0.061606562	3.297717481	0.37359263
24.4760337	0.788373288	-0.130189435	3.625536799	0.343112601
22.94437848	0.5003127	-0.039583452	3.275530866	0.360456674
23.53540779	0.863685437	-0.179988564	3.814667616	0.336562986
25.03470967	0.558098915	-0.060559988	2.167620007	0.501288612
34.76034963	0.507433267	-0.040866824	3.357634613	0.39444961
22.62646646	0.517362313	-0.048034174	2.539649128	0.445910698
31.23732319	0.67892969	-0.081265534	3.734934836	0.341339946
19.12508313	0.480395433	-0.041949992	2.273778372	0.453107376
38.70312251	0.576140132	-0.058251806	2.90397947	0.433426666
26.74150424	0.636703903	-0.070327298	3.451062588	0.3617961
23.81926723	0.68971505	-0.082844938	4.020491971	0.318739048
22.24310698	0.632843808	-0.069772677	3.456489041	0.389468833
20.93275663	0.609301054	-0.074357662	2.422237048	0.473946748
27.0533383	0.544194845	-0.05305984	2.57705822	0.418910039
19.77058156	0.648283969	-0.080168677	2.873694421	0.383677408
26.88369446	0.549259161	-0.0558197	2.418354613	0.441859639
41.0899084	0.426426544	-0.028223882	3.085917473	0.394941788
14.90263096	0.385041438	-0.026723676	2.149434735	0.449820277
20.5698675	0.642801977	-0.074229775	3.305941636	0.358452234
18.63242357	0.663268737	-0.081041114	3.156610934	0.385118544
20.77073754	0.474383553	-0.043115803	2.030927802	0.500808665
24.48968064	0.69580124	-0.101595756	2.818481176	0.463532124
23.00257549	0.550932147	-0.053513949	2.699554844	0.429075461
30.60441365	0.552727811	-0.048615425	3.523003617	0.360029875
20.52624631	0.487337349	-0.041081461	2.599769804	0.430999238
15.42699704	0.876894248	-0.213611038	3.343660066	0.344688603
34.82441687	0.513767739	-0.044832087	2.740015937	0.411732019
20.61425415	0.436120613	-0.032079054	2.628037234	0.420226996
22.04337115	0.581363214	-0.056007212	3.501319502	0.344857127
22.75847755	0.543306015	-0.0520038	2.689244207	0.434942799
20.88932971	0.616925122	-0.070996244	2.771664151	0.444965452
28.95010492	0.483688338	-0.038201013	2.877000743	0.417646943
24.67930807	0.522824897	-0.042634217	3.539960034	0.359659944
30.37333193	0.631031762	-0.063588529	4.366121244	0.31018467
29.01405498	0.663748353	-0.089803381	2.54050873	0.491800341
22.47525056	0.599554745	-0.06753154	2.680120633	0.401881573
50.96482873	0.487782523	-0.042460316	2.430048728	0.470097667
18.3362852	0.456855893	-0.034302253	2.768629256	0.393729786
29.06012681	0.509320595	-0.046344049	2.545990891	0.460168451
20.918198	0.53709598	-0.051004244	2.906915259	0.391333075
24.42179286	0.553527352	-0.048175958	3.685053636	0.34695676
22.73829263	0.575534021	-0.064996988	2.248305748	0.48247267
22.83887862	0.483721913	-0.037724715	2.982323611	0.392145329
26.79718494	0.47956173	-0.039051472	2.70175659	0.437808558
32.61933981	0.556056247	-0.060898608	2.214234125	0.435329289
20.72812843	0.734499513	-0.105727825	3.450615852	0.346803164
22.73212446	0.701590664	-0.092127366	3.408054096	0.35238209
38.31161771	0.656707586	-0.073747996	3.849558166	0.353695753
29.26573696	0.463753937	-0.031894387	3.624209776	0.345971519
22.90663256	0.521044993	-0.054894814	1.909062511	0.528878629
40.83793286	0.583391496	-0.063347452	2.665339499	0.428789608
22.79756302	0.697104324	-0.08591949	3.93641746	0.327140825
18.59202938	0.500701097	-0.042944538	2.649180008	0.399446217
18.62483653	0.490709731	-0.042388313	2.50532469	0.4468872
22.65200443	0.474271148	-0.035330251	3.220057729	0.367600667
26.24808273	0.658609118	-0.077370424	3.409827236	0.367135553
28.98657989	0.489810301	-0.042391328	2.541202129	0.464719209
26.84070197	0.627108943	-0.068012189	3.303927768	0.38556125
33.58630081	0.553221138	-0.044831792	4.482412625	0.315692123
31.37487745	0.485824162	-0.035123135	3.758204476	0.367627374
42.8633183	0.480428078	-0.035143839	3.536776568	0.341613886
26.84819519	0.601873423	-0.070812191	2.37408506	0.444447507
42.54572862	0.499198895	-0.034584898	4.759681059	0.296802914
20.0066867	0.710801539	-0.096421666	3.467670907	0.350210634
30.92655371	0.434826896	-0.02735042	3.92009661	0.342309184
38.85739076	0.427311198	-0.029960584	2.817449778	0.414933352
25.23095886	0.414702418	-0.027496549	2.824000699	0.389898588
20.37487166	0.716993424	-0.099359647	3.218579692	0.377159272
28.87119579	0.546471599	-0.048378336	3.284108218	0.399379264
20.28410041	0.570687781	-0.054812567	3.156916529	0.377436745
22.54832398	0.674106471	-0.07980043	3.789394357	0.344379305
24.82707347	0.568409494	-0.058404185	2.721356315	0.454346927
19.03325952	0.580023033	-0.060915082	2.67433342	0.410685804
40.88382895	0.549053305	-0.048722427	3.356707199	0.363244484
24.22262739	0.783282482	-0.125501975	3.716908687	0.331357924
31.27067959	0.52277187	-0.043368319	3.347410641	0.356678236
22.53205144	0.533121563	-0.050399434	2.708709558	0.430248502
32.64590001	0.650069392	-0.071489406	3.761246183	0.362219691
27.17083708	0.601290847	-0.06314685	3.024254508	0.380721226
32.51808214	0.63946692	-0.075025092	3.209004014	0.411686908
20.39199564	0.436457332	-0.034842173	2.134485381	0.457104064
22.98162567	0.548825878	-0.055324347	2.44579583	0.463066287
20.91577153	0.564652266	-0.067617023	2.252844602	0.455631357
18.49782836	0.537900688	-0.049869783	2.76659318	0.404831384
24.84460227	0.544854156	-0.057916259	2.08740793	0.50431777
36.85696088	0.565673854	-0.053559398	3.176955536	0.392960116
37.06232552	0.579787474	-0.054759651	3.459598752	0.354291428
21.21572598	0.613030204	-0.066531772	3.101445012	0.382600239
24.64535169	0.49961928	-0.047339525	2.154115272	0.484138278
20.88258331	0.375644458	-0.021701353	2.943150821	0.395135975
21.13775905	0.436158738	-0.030708297	2.792217748	0.409364371
24.96359812	0.400113996	-0.025545753	2.821193978	0.399090652
33.11292075	0.565586859	-0.047570753	4.373488873	0.317900067
25.0460413	0.461095953	-0.033647633	3.12257226	0.381835203
26.31179901	0.868204971	-0.174814429	4.501506523	0.30281262
12.63785548	0.612668279	-0.077391496	2.133806936	0.441176511
39.18194598	0.410715249	-0.025507203	3.292528613	0.379232222

log.sigma.1.0.mm.3D_glcm_ClusterTendency	log.sigma.1.0.mm.3D_firstorder_InterquartileRange	log.sigma.1.0.mm.3D_firstorder_Skewness	log.sigma.1.0.mm.3D_firstorder_Uniformity
29.15423887	90.99412346	0.051899139	0.091515374
18.21656929	102.4360867	-0.123083412	0.098588976
22.01567186	98.91063213	-0.028763365	0.092546603
24.16053681	112.4937258	-0.498420359	0.088653987
20.57965943	108.5772839	-0.127673466	0.09073238
27.05777307	107.2741976	-0.203877004	0.087698928
11.99293352	40.73439789	0.250829062	0.172175829
20.99806392	76.65166092	0.307509514	0.115432862
14.07166589	60.18944883	0.246729657	0.137143343
24.5709308	107.3958931	-0.173686896	0.086955835
10.2273652	59.39945173	0.15454734	0.150901065
17.38357695	60.44308567	0.267344396	0.135830348
24.10302183	95.95055199	-0.302716955	0.096778117
27.74336927	117.8062897	-0.124563128	0.079414374
24.14361464	88.01048088	0.415536502	0.102144822
15.00561009	45.58182001	0.177931893	0.161564517
13.4700924	71.7556839	0.123937796	0.122344935
15.83864493	92.66801071	-0.345833757	0.109713596
11.93951439	64.40148449	0.18614887	0.135420392
18.70430322	75.38456345	-0.406784607	0.114749925
10.06877643	70.66233635	0.111617415	0.136125652
20.07031436	110.7855501	0.144208432	0.090433143
18.98134389	89.8019886	0.062150516	0.101668786
9.758992638	38.30013561	0.561345522	0.191451747
19.17804337	42.6636343	0.044201991	0.157404853
18.13313914	66.179142	0.808709625	0.1252097
23.73690172	93.468009	0.286435373	0.094679902
13.89884959	62.18253326	0.342048921	0.134751337
17.769103	104.748023	0.286521309	0.103021205
15.38340559	75.14587116	-0.246536688	0.117532503
14.64017364	74.54393768	0.175779747	0.120122404
22.19386801	119.5792303	-0.070009756	0.085086578
15.74432928	68.30997753	0.407181034	0.129274012
18.29789039	50.2287674	0.384976933	0.151495356
18.14185465	74.818573	0.365396106	0.115225172
24.03923935	104.889452	-0.099158665	0.091950706
35.10419565	121.9081221	-0.521118693	0.079177865
17.30904012	33.09444809	0.091420393	0.178762297
12.92705201	79.94373417	-0.140200973	0.119445153
12.45157919	43.34276581	0.627943456	0.171044704
14.88635197	84.23589325	0.023114158	0.109888617
14.34785795	49.26298237	0.927461306	0.158626507
18.07274494	98.92201805	-0.003438199	0.099623995
24.84783616	106.6391001	0.197889152	0.085992859
10.75223735	49.66019821	0.09552256	0.159678762
18.51604516	88.42170334	0.329259092	0.105747796
16.06701199	57.56080055	0.552466485	0.140112906
9.482830608	69.76793289	-0.708306407	0.135734463
21.74817733	99.11705399	0.127058888	0.090796109
23.94772877	116.1917725	-0.176036239	0.089699663
25.55610393	111.1721725	0.054585412	0.089003718
24.82121381	106.01717	0.293870168	0.089027459
9.015040601	34.20778513	0.432800549	0.202719587
12.92900489	64.92709923	-0.806901738	0.132438531
29.35420947	132.6513062	-0.217399238	0.080017124
14.38004508	85.59141827	0.149356858	0.113747455
13.93358207	63.06382561	0.438384887	0.135279315
20.08901707	98.58103752	0.143480863	0.097673979
21.61438258	100.8818378	0.173929171	0.093386972
15.09664561	46.55091667	0.748262571	0.164215624
21.8148194	93.15677261	0.16638411	0.097718828
33.63772521	117.6711159	-0.035979778	0.077366292
26.27176163	93.15035248	-0.024447786	0.095625265
20.9074507	102.0807352	-0.293518469	0.092992924
11.91810362	64.28373718	-0.668509733	0.136895112
36.20921797	128.9220581	0.012916587	0.071471057
21.0863258	105.1934814	-0.323006515	0.097818438
26.49445381	103.9878693	0.313826688	0.089453257
14.65102441	65.41240788	0.200231134	0.132786438
15.75765913	85.87813854	-0.14775763	0.108924195
19.94245357	98.95590973	-0.170586818	0.095594554
22.88871296	75.90084839	0.11539636	0.114893088
19.82857424	100.5747643	0.288496358	0.097378838
25.61487661	111.8963394	0.538180056	0.086003324
16.28899095	50.22670841	0.400739379	0.150529263
14.03879287	77.87888145	0.367079406	0.120729919
21.21247951	90.03972626	-0.485714736	0.100493249
26.99283756	121.9061394	-0.191692428	0.081118385
20.78966654	100.5044918	-0.033149373	0.094772514
15.36141995	60.5859437	0.649725205	0.136919753
27.8940744	87.81135654	0.046065605	0.094194966
18.32160203	92.43257332	-0.181373505	0.099777803
21.43946049	78.10068607	-0.078522287	0.118941363
9.217513835	63.42399788	0.293333843	0.144511335
13.7072948	56.70551586	0.197724206	0.144964643
11.35089084	55.68988323	-1.150797282	0.165806619
15.88444355	84.58353996	0.469026623	0.109645185
11.26602986	40.93097353	0.520761165	0.176169237
19.57604472	75.28398514	-0.201687827	0.113852522
21.80945243	99.56093979	-0.39554515	0.094952665
18.50533365	77.59634829	-0.230286817	0.11564689
10.50520926	49.89897966	0.503089954	0.159094709
16.45829516	80.39464951	0.139592588	0.111112735
16.13208367	74.9416647	0.321282132	0.120666959
14.90839822	85.81666756	0.351191634	0.109896455
32.49325187	118.6682081	0.105936391	0.078304116
19.25267833	90.1914854	0.265446713	0.102259633
35.39937236	131.3106766	-0.280383246	0.075095183
9.51826829	73.25444126	-0.114863015	0.137836935
19.57238119	80.1256628	-0.370693905	0.108706916

log.sigma.1.0.mm.3D_firstorder_MeanAbsoluteDeviation	log.sigma.1.0.mm.3D_firstorder_Energy	log.sigma.1.0.mm.3D_firstorder_RobustMeanAbsoluteDeviation	log.sigma.1.0.mm.3D_firstorder_Median
68.67320526	12884181.02	41.96294212	-5.718544006
59.31822625	1913418.433	41.65972157	3.027422905
60.50555019	5251673.939	41.65658547	1.265231848
66.51447499	2224056.207	46.33425961	10.01734591
62.17840246	8342458.884	44.47496945	1.519762278
67.72862106	1910438.4	44.92857775	13.24318123
38.38996175	35352784.72	20.47274904	-1.006545305
55.8071664	37311680.13	33.48845131	-4.248829365
45.68141294	17152154.18	27.51277934	-3.966849208
66.77257517	5480583.318	46.0452166	5.86350584
39.1033797	4240232.534	25.10475685	-1.33566314
50.14695274	18765682.69	27.7873503	-1.978546858
61.69701891	5293417.573	40.7990232	6.052425861
72.32351327	5409127.324	49.68659417	-3.319126725
62.93954278	11938476.77	38.21809754	-7.012942314
42.06980458	21295438.05	23.48785686	-1.927897632
47.45452634	3557705.196	30.58621654	0.610860288
52.58398824	1819552.519	37.08904121	-2.400640726
44.12272296	4029202.075	27.6057999	-2.589696407
52.83186627	50344461.86	32.48433249	1.540524602
41.30274134	5674862.361	28.5508493	1.42165333
63.32996438	3053512.435	45.32501538	-1.544438303
57.19426858	3096544.282	37.64503731	-4.279285669
34.2900682	9551130.298	18.17344087	-3.627890587
50.51815788	7057732.094	26.57538155	-2.082142115
51.29576067	13383823.47	30.24982293	-2.76540494
62.87480771	14204324	39.80748263	-7.869100094
46.26688414	9365091.081	27.76288101	-5.731249809
59.03242194	766648.1153	42.89575249	1.152755082
49.80363009	7311600.528	32.15374156	-2.780446649
48.11935704	16428179.2	31.15013601	-2.924561024
65.79177561	5087856.461	48.16429029	-3.938171506
48.66555328	10740359.66	29.47577332	-4.054333687
46.6342698	11387514.42	25.15634315	-0.055372555
53.26297044	27784911.8	32.75242093	-2.421774864
63.04499614	12853742.69	42.54926244	0.623713791
76.0609081	14440999.64	51.05074581	10.79997253
42.70260339	25840951.45	20.82394459	0.341322958
47.96724611	2118832.83	33.07473047	-2.295158148
39.38117787	33044312.18	21.09383562	-3.785253048
51.40505611	5782820.027	35.39116685	-6.593677759
42.55925989	22560928.36	23.20192985	-3.892554998
56.323621	11819146.51	40.14976064	2.736107469
67.16820707	10933513.79	44.47716793	-7.038567066
40.26326665	5296517.038	22.76641409	-2.793160677
55.32343637	12629982.91	36.52414544	-4.226624012
46.27991351	23978959.86	26.31770381	-3.083236217
42.0127091	1541042.704	28.45948021	-2.224421501
63.53500747	2560518.426	43.51003891	1.426737428
64.91850844	2979844.977	45.98408837	7.284533501
69.16106152	10931105.53	44.99854694	-5.354504108
65.42828245	19701208.56	44.22473108	-3.91284585
33.24391575	20785254.13	16.81436967	-2.099058032
46.89082217	7189817.028	28.45841215	3.287656188
73.49643387	5200147.66	52.945284	9.698767662
50.63308336	4805510.332	35.1501896	-6.304404497
46.13128668	16170998.26	28.13038532	-6.033608437
58.74983962	11764420.91	40.13981407	-4.530095577
63.2077843	5249111.915	41.67917127	-9.866363525
42.42177586	45448278.41	22.16297192	-3.074004173
59.81582734	8542079.757	38.83460062	-0.86370331
75.11314203	24073612.7	49.46294814	0.89477706
63.93924252	62172464.9	40.0462195	0.90015921
61.15509216	12754899.63	42.40326502	3.748855352
44.18555027	2522273.08	27.52357018	-0.053877302
80.00494039	36734926.91	53.71804611	1.161364913
62.96264697	2516703.838	44.44675575	7.51198411
65.89139878	38755531.78	42.76769699	-5.993932724
47.38669988	34665799.85	27.99884976	-5.17079711
51.70504905	9226906.054	35.63144191	5.500459671
59.58563097	2838719.245	40.66586539	-0.01948119
55.26387589	29745060.55	33.32572203	-1.279211044
59.21738991	7891455.852	40.14937621	-4.605704308
68.59329984	7521149.728	45.94531181	-10.5530405
45.61566398	18870555.69	24.60629905	-2.481255412
47.78519909	3640156.013	31.53165826	0.450876281
58.90275644	14160339.61	38.08335374	-0.843600571
70.6129862	2447919.821	50.56899102	2.393562317
59.8115298	7549017.555	41.34919026	2.513601542
47.78004351	8405026.876	27.76434917	-8.490349293
66.80346099	11880944.82	40.75439541	-6.182931185
58.40214198	4757106.501	38.91067135	7.475433111
54.9926185	13473928.41	33.49782919	-1.791591167
40.21699554	5363877.21	26.37342223	-7.575514793
44.43779377	10774793.19	25.85814316	-1.700743198
40.02290728	1491415.869	25.37611087	11.98884296
52.60544067	6148979.701	35.05479781	-8.135350227
37.92102914	12300436.2	20.23779834	-2.37322998
54.80805261	9903338.941	33.18370164	-2.499107361
60.5512676	8538112.801	40.80349942	3.656088829
52.49738136	4021509.254	33.21248538	9.669095993
39.63071014	8278881.418	22.46133851	-4.621096373
52.36859577	21704089.23	33.86343173	-2.451417446
49.16681392	15140733.62	31.36920185	-0.307338029
52.94683795	15567585.63	35.54508224	-3.89423728
74.3909099	22841213.06	49.04378479	1.335747361
56.67981787	13357015.36	37.81885604	-4.013462782
79.97574065	3274821.574	54.11439869	3.487994671
41.38778211	694205.8257	29.54568884	5.759557962
55.25868282	51041973.33	34.54021792	3.684756517

log.sigma.1.0.mm.3D_firstorder_TotalEnergy	log.sigma.1.0.mm.3D_firstorder_Maximum	log.sigma.1.0.mm.3D_firstorder_RootMeanSquared	log.sigma.1.0.mm.3D_firstorder_90Percentile	log.sigma.1.0.mm.3D_firstorder_Minimum
347872887.6	378.5635986	94.45931947	121.9089622	-376.069519
51662297.69	240.9088898	74.25748554	83.26446686	-226.4657135
141795196.4	216.7897949	76.73022115	99.9317688	-253.1793823
60049517.58	185.1084595	83.10840423	95.18113174	-285.6713562
225246389.9	209.1028595	76.8924202	99.99069977	-250.9291687
51581836.8	288.8214417	88.8502873	119.6718018	-257.8325806
954525187.5	343.1422729	57.91780736	71.3128891	-284.5823975
1007415364	406.2414551	78.41508485	99.46800613	-404.2095947
463108163	279.3725586	63.43845731	86.00234222	-269.2929993
147975749.6	275.7363281	85.2000631	115.0065475	-367.5427551
114486278.4	207.3050385	52.20234838	73.06109238	-215.22995
506673432.7	474.7500305	74.2157563	91.72066345	-457.7991638
142922274.5	275.125885	80.79000784	96.9511261	-304.7511597
146046437.7	241.3022919	91.64750968	101.4087997	-292.134613
322338872.7	329.2265625	87.06311072	108.8008087	-259.9203491
574976827.4	330.9566345	60.79343207	83.35308533	-238.5953369
96058040.28	332.8282776	63.47528028	83.82409668	-305.3337097
49127918.01	208.8096008	66.69920288	71.97247162	-240.0183563
108788456	250.917984	60.68772585	72.62099304	-303.135498
1359300470	428.0661926	74.04292372	91.66637726	-497.0871277
153221283.8	191.2230835	52.25825391	64.55308456	-165.243042
82444835.73	299.93396	78.46193389	103.9355927	-237.6585999
83606695.61	215.4037476	74.49404347	99.98119354	-215.1373749
257880518	265.713623	51.52964684	61.43939209	-230.7844238
190558766.5	238.8903503	75.81120523	97.98038254	-276.3608093
361363233.6	361.211853	72.36185226	95.12311935	-260.0905457
383516748	354.9360962	84.19017774	106.2633186	-374.5545044
252857459.2	362.8723145	64.47263302	82.70444489	-238.8330231
20699499.11	215.0432892	72.46388403	106.4226151	-150.8139038
197413214.3	273.4078979	67.38966381	84.64690323	-424.8871765
443560838.5	383.4008484	64.01425385	81.42527008	-233.3686371
137372124.4	226.4182434	80.35341085	98.67190094	-274.6787109
289989710.8	363.9093018	67.8212781	87.26158142	-252.23909
307462889.2	363.5706787	68.66823778	91.53765564	-229.6319733
750192618.6	376.9551086	73.57307014	97.1731308	-332.8459473
347051052.5	256.2391357	81.4190248	106.6857346	-291.6473083
389906990.3	367.5771484	97.72883755	105.198317	-372.6120605
697705689.1	390.6174622	66.62779646	85.63345337	-328.8513794
57208486.41	189.593811	61.51121892	76.65796051	-257.550354
892196428.9	519.5818481	59.1427345	72.44528656	-603.9848633
156136140.7	217.4914398	65.69274947	80.14879456	-223.6814117
609145065.6	368.4029541	63.0681907	79.22134323	-328.9936218
319116955.8	259.3389893	69.9141223	93.50431595	-246.5545197
295204872.3	311.9914551	87.74769284	110.4641418	-282.4235535
143005960	247.398941	58.34321947	71.62893295	-254.9285431
341009538.6	296.6433716	72.52793315	99.01245117	-263.168335
647431916.1	344.1807556	66.94184761	86.14470673	-311.0241089
41608153	150.0272522	56.02302959	66.83608246	-383.2335205
69133997.51	271.7538147	80.92367068	106.269722	-248.1631927
80455814.39	254.1652527	81.19468815	101.0926613	-271.7284851
295139849.3	314.7595215	92.16006451	125.4935043	-459.75354
531932631.1	347.7097473	84.10732769	118.0104767	-340.0836182
561201861.4	309.8388977	51.04874817	58.64011765	-251.9780579
194125059.8	283.4214172	66.17170771	73.51335373	-486.7410583
140403986.8	229.0256348	89.92953575	111.8702866	-253.1250153
129748779	233.381424	63.92431704	78.57555008	-224.5290527
436616952.9	298.9759827	63.46373683	84.70226898	-224.332016
317639364.5	314.1717529	75.44232061	98.23870392	-272.8842468
141726021.7	326.2698975	82.94317336	98.41974182	-322.2857056
1227103517	396.7739563	64.59292725	74.48107605	-329.5369873
230636153.4	350.8166199	80.17137287	92.50868225	-303.009613
649987542.8	379.0881958	98.82394184	128.3136749	-393.8576355
1678656552	460.2585144	86.89476471	115.6549706	-366.517395
344382290.1	309.2497253	78.00867182	99.49201202	-520.0335083
68101373.17	190.1060181	61.03820002	71.71974335	-316.85495
991843026.5	394.0896606	103.6246443	125.593483	-506.447937
67951003.63	251.8085785	78.73231818	81.66866302	-240.4444885
1046399358	461.3330383	87.30143283	112.9413895	-359.5181885
935976596.1	382.462677	67.9498466	80.20094299	-457.0647583
249126463.5	268.7092285	65.90992132	83.84435959	-291.7625732
76645419.61	240.6417084	75.95890048	93.21977463	-227.7784119
803116634.9	385.4848633	77.27680498	100.1129532	-334.1961365
213069308	335.933197	76.88434747	92.03314514	-237.2803192
203071042.7	377.9238586	88.97752364	121.980677	-257.8494873
509505003.7	342.3821411	67.97504095	84.408358	-292.5636902
98284212.35	306.9004822	62.97071987	75.34582596	-216.5404816
382329169.4	334.3684998	80.0643121	92.62189484	-494.6673279
66093835.18	219.4716492	87.05572432	110.1771164	-294.2213135
203823474	269.0997314	75.76739246	103.117807	-351.4590759
226935725.6	361.592041	68.33344258	82.71997299	-274.1381531
320785510.1	340.6847534	91.92482866	117.7436218	-376.069519
128441875.5	221.3606415	75.88945144	87.79878616	-307.8907166
363796067	290.7486267	76.84034351	100.3642311	-388.3951721
144824684.7	265.4313049	53.23093649	67.32459564	-229.9672852
290919416	245.4405518	62.99695449	86.79546356	-252.7921143
40268228.46	112.2001495	52.99710167	54.83575439	-234.9232178
166022451.9	307.4196167	68.56423862	83.73920975	-201.6394806
332111777.4	272.1608276	56.62666139	73.02317047	-299.7366943
267390151.4	329.8318787	76.30248193	93.28028107	-425.7826538
230529045.6	268.9262695	79.03085617	90.79937592	-433.2331543
108580749.8	327.9466553	70.72395055	82.13122864	-239.8294983
223529798.3	227.9448853	57.18129416	68.61860046	-282.9904175
586010409.1	304.7079773	69.44104961	87.93519592	-239.6143799
408799807.8	355.326355	66.28508581	88.80973053	-231.2309418
420324812	297.1731567	68.61096603	92.83082581	-285.3250122
616712752.7	415.8943176	97.88281641	118.975058	-388.7364197
360639414.8	282.4336548	73.8064777	100.0687576	-286.2273865
88420182.51	335.6208496	102.2872853	107.3059753	-312.5441284
18743557.29	132.2735748	50.89518882	65.77510529	-147.3970337
1378133280	378.0944214	76.05556175	96.97534256	-455.8091736

log.sigma.1.0.mm.3D_firstorder_Entropy	log.sigma.1.0.mm.3D_firstorder_StandardDeviation	log.sigma.1.0.mm.3D_firstorder_Range	log.sigma.1.0.mm.3D_firstorder_Variance	log.sigma.1.0.mm.3D_firstorder_10Percentile
3.86724287	94.38797515	754.6331177	8909.089854	-117.0258827
3.562524504	74.21277156	467.3746033	5507.535462	-97.8331131
3.659986498	76.72345507	469.9691772	5886.488557	-100.6778915
3.731040635	83.10752232	470.7798157	6906.860266	-113.3916679
3.659657636	76.85111011	460.0320282	5906.093125	-95.88706207
3.789241105	88.00505049	546.6540222	7744.888911	-95.72501373
3.086258298	57.91605801	627.7246704	3354.269775	-67.2014267
3.586654869	78.38599986	810.4510498	6144.364974	-96.07588196
3.30397247	63.42005533	548.6655579	4022.103419	-78.69731216
3.778055751	84.93397412	643.2790833	7213.779959	-101.2903732
3.070589893	52.16145103	422.5349884	2720.816973	-63.16022301
3.431943739	74.21162833	932.5491943	5507.365779	-82.43320007
3.685897462	80.77698103	579.8770447	6524.920664	-116.4090881
3.890916846	91.06238953	533.4369049	8292.358787	-132.3029175
3.739641654	86.92315925	589.1469116	7555.635613	-113.8392822
3.159915525	60.79342729	569.5519714	3695.840802	-79.53042068
3.341635526	63.37109952	638.1619873	4015.896254	-73.34017334
3.41422653	66.32060603	448.8279572	4398.422784	-93.73144989
3.252348271	60.68517674	554.0534821	3682.690675	-69.6894455
3.526285696	73.98226929	925.1533203	5473.37617	-81.57005005
3.114510578	52.23929923	356.4661255	2728.944384	-64.47996902
3.637678906	78.43796677	537.5925598	6152.514631	-102.2144661
3.582966365	74.49383368	430.5411224	5549.331256	-93.85030746
2.91371153	51.51798294	496.4980469	2654.102566	-55.50499039
3.33638627	75.75702656	515.2511597	5739.127074	-104.9471725
3.450593741	72.26277085	621.3023987	5221.908051	-77.41374588
3.747657616	84.12397788	729.4906006	7076.843655	-103.6888481
3.323486017	64.41949034	601.7053375	4149.870735	-79.25876007
3.473840659	71.73179125	365.857193	5145.449875	-73.13986206
3.41261269	67.38963995	698.2950745	4541.363572	-77.04763641
3.377325183	63.9748212	616.7694855	4092.777747	-84.46334534
3.703890956	80.10872976	501.0969543	6417.408584	-110.9198288
3.393802507	67.81425506	616.1483917	4598.773189	-79.55479736
3.296431777	68.66257772	593.202652	4714.549579	-81.08993835
3.524992705	73.55442704	709.8010559	5410.253737	-83.97794952
3.724727658	81.41901634	547.8864441	6629.056222	-107.3289841
3.951492772	97.65122344	740.189209	9535.76144	-140.5899429
3.150098578	66.6256857	719.4688416	4438.981995	-85.14344788
3.30760673	61.45652916	447.144165	3776.904976	-76.30093842
3.089793427	59.13114299	1123.566711	3496.492071	-66.50644989
3.433362879	65.47411342	441.1728516	4286.859528	-89.41835938
3.188181313	63.063773	697.3965759	3977.039465	-68.85214157
3.526226865	69.84644032	505.8935089	4878.525226	-87.33939438
3.832976544	87.69316006	594.4150085	7690.090321	-110.3550819
3.141226136	58.33927521	502.3274841	3403.471032	-66.11373138
3.554250605	72.49644888	559.8117065	5255.735101	-82.85915375
3.330838073	66.94138523	655.2048645	4481.149057	-77.72507477
3.11821048	56.01691872	533.2607727	3137.895182	-65.54801941
3.710316754	80.84543542	519.9170074	6535.984428	-100.8586426
3.674086726	81.1207849	525.8937378	6580.581742	-112.7351654
3.850411688	92.15992651	774.5130615	8493.452055	-107.7609009
3.766901577	84.01227345	687.7933655	7058.062091	-96.34905243
2.865320045	51.04547436	561.8169556	2605.640452	-56.22707939
3.328880929	66.16887241	770.1624756	4378.319676	-83.02646103
3.854773241	89.87992392	482.15065	8078.400723	-115.7998672
3.384273397	63.91068856	457.9104767	4084.576112	-77.28724289
3.303106125	63.42311569	523.3079987	4022.491604	-77.06605682
3.623828546	75.42853194	587.0559998	5689.463431	-92.86990967
3.731608824	82.80661886	648.555603	6856.936127	-103.0810501
3.1851469	64.59201007	726.3109436	4172.127765	-71.05477905
3.677247305	80.15727644	653.8262329	6425.188966	-98.32985992
3.995746763	98.75196685	772.9458313	9751.950956	-119.5813843
3.782945858	86.83971387	826.7759094	7541.135904	-104.6858498
3.66704523	77.96904603	829.2832336	6079.172138	-94.80099487
3.242566471	61.02983758	506.960968	3724.641075	-68.0265152
4.083880422	103.6181033	900.5375977	10736.71132	-131.8702698
3.622183608	78.39821427	492.253067	6146.280001	-118.0225182
3.812682506	87.27926115	820.8512268	7617.669426	-107.1279694
3.363932186	67.9470052	839.5274353	4616.795516	-72.11769104
3.444249445	65.83746781	560.4718018	4334.572168	-81.90749283
3.629154383	75.94636261	468.4201202	5767.849993	-100.4929893
3.580594346	77.27635852	719.6809998	5971.635586	-96.28151703
3.634704662	76.78487272	573.2135162	5895.916678	-98.29664459
3.819691996	88.92415042	635.7733459	7907.504529	-105.7351364
3.304306007	67.97333987	634.9458313	4620.374933	-80.19969254
3.344883562	62.86785821	523.4409637	3952.367595	-70.62301788
3.649370817	80.06410536	829.0358276	6410.260967	-88.78535919
3.79483022	87.05388345	513.6929626	7578.378623	-114.9044525
3.634825872	75.63049681	620.5588074	5719.972048	-88.49129181
3.347104389	68.20572005	635.7301941	4652.020248	-78.63455658
3.824165316	91.82038375	716.7542725	8430.982872	-114.3698654
3.603523707	75.80880821	529.251358	5746.975403	-93.80825043
3.542689566	76.82516269	679.1437988	5902.105623	-101.4914223
3.100965694	53.05428406	495.3985901	2814.757057	-64.46042328
3.262791724	62.98413275	498.232666	3967.000979	-71.5388504
2.95550665	52.99697121	347.1233673	2808.678958	-74.56428528
3.477367692	68.48856373	509.0590973	4690.683361	-84.63019028
3.043597373	56.61807918	571.897522	3205.60689	-64.98139572
3.568539149	76.27270972	755.6145325	5817.526248	-91.76207733
3.667620578	79.03085608	702.1594238	6245.876213	-97.3143158
3.468385468	70.59676492	567.7761536	4983.903217	-89.18160248
3.110329995	57.14438362	510.9353027	3265.480579	-64.5205925
3.499357056	69.43778831	544.3223572	4821.606446	-85.97930145
3.401613174	66.22003281	586.5572968	4385.092745	-72.8851738
3.476237269	68.57527961	582.4981689	4702.568973	-78.18383179
3.984138796	97.88088866	804.6307373	9580.668365	-119.8584709
3.582095021	73.78260316	568.6610413	5443.872529	-83.70635452
4.001694034	102.0604967	648.164978	10416.34499	-140.5961761
3.05840118	50.87364608	279.6706085	2588.127866	-64.52061691
3.585136107	75.96589095	833.903595	5770.816587	-87.5808815

log.sigma.1.0.mm.3D_firstorder_Kurtosis	log.sigma.1.0.mm.3D_firstorder_Mean	log.sigma.1.0.mm.3D_glrlm_ShortRunLowGrayLevelEmphasis	log.sigma.1.0.mm.3D_glrlm_GrayLevelVariance
4.18745642	-3.670583202	0.005364618	15.29028857
3.170633029	-2.576566788	0.015759624	9.219651871
2.942814183	1.018960289	0.010725067	10.06540623
3.167051581	-0.382867314	0.012624604	11.58459247
2.776675959	2.520150622	0.010587763	9.899079197
3.657896937	12.22639124	0.019361851	13.04403937
5.732153694	0.450149022	0.006821143	6.313297437
4.578500928	-2.135546465	0.0036174	10.85936036
4.063841545	-1.527889847	0.009216668	7.248042541
3.611572802	6.728357407	0.00601744	12.10799584
3.781048402	2.065962915	0.013247338	4.752360839
6.418783322	0.782753798	0.002926675	9.976346452
3.298027612	-1.450759492	0.008218737	11.18399445
3.016950951	-10.33959591	0.013899362	13.83208774
3.852588648	-4.934534873	0.012128133	13.34016594
4.223903568	0.024112468	0.010837056	6.673604304
4.783819653	3.635237622	0.006836989	7.030136935
3.285009317	-7.096540036	0.015809432	7.500439953
5.20296822	-0.556231287	0.006742648	6.518138533
6.232143266	2.996395098	0.002698353	9.573493698
3.208109828	1.407379724	0.023389343	4.733191954
2.909191792	1.939184993	0.015545904	10.22402553
3.086483116	-0.176793771	0.021093592	9.550665089
5.864394788	-1.096328789	0.00995925	4.943503345
4.100381613	-2.865617761	0.009066107	10.67123669
5.153840733	3.785447362	0.008963734	9.311022865
4.017200677	-3.338019156	0.005337553	12.06161849
4.754251093	-2.61718813	0.012214799	7.345127304
2.756932477	10.27446414	0.032273992	8.469782811
5.85430595	-0.056704222	0.00458812	7.848258558
3.750156947	-2.246541444	0.011466341	7.127228234
2.517618918	-6.265943744	0.012015028	10.69916096
4.673150396	-0.975999061	0.009562094	8.306823845
4.847762992	0.881646871	0.012413199	8.355769264
4.826020115	1.656173885	0.005547186	9.597362705
3.103261893	-0.037110172	0.009466115	11.16799611
3.485570955	-3.894130129	0.006792313	15.88473678
5.15641493	0.53034608	0.005230314	8.32927868
3.947016362	-2.593275353	0.012381792	6.50245919
7.222406511	-1.170885808	0.001527512	6.471924876
3.132905611	-5.355166202	0.017276461	7.317911027
6.341152777	0.74646669	0.004882735	7.272187189
2.877588833	3.075592689	0.012247127	8.184730613
3.373221842	-3.09310153	0.009605578	13.04036595
5.175609912	-0.678399849	0.009869857	6.326083201
3.65660074	2.13681684	0.00955673	9.065359576
5.152709117	0.248806765	0.006240772	8.024738887
7.813857495	-0.827443044	0.00586465	5.437406063
3.258137739	3.557533989	0.015203666	11.18649864
2.84001319	3.463472433	0.012018797	10.69059242
3.782250371	0.159486869	0.003657482	14.47648386
3.40823649	3.997559228	0.005765261	11.81088394
6.095945812	-0.578132662	0.007760755	5.049281942
7.006919332	-0.612556122	0.003038119	7.835387032
2.677802145	2.986750135	0.013805724	13.4306261
3.328375182	-1.319923219	0.016038052	6.868259029
4.031264192	-2.270305703	0.015389293	7.146127704
3.312349274	-1.442327174	0.010744671	9.613520102
3.862897385	-4.757507794	0.008366597	11.75863647
6.803921681	-0.344217878	0.004964947	7.682701466
4.458226927	-1.503350044	0.007674592	11.04398588
3.807267412	3.771010958	0.005919872	16.45917247
3.909843898	3.092608926	0.005232403	13.11594306
4.323324426	2.486109487	0.002693673	10.15307942
5.647278567	1.010339923	0.007899336	6.639398139
3.639659119	-1.164297312	0.00271029	17.99543166
3.044627786	-7.245545224	0.019877377	10.22176352
3.824443673	-1.967421557	0.005083643	12.93286048
6.856329463	-0.621399984	0.002808411	8.222927664
3.498994158	3.089588974	0.007660167	7.368531833
3.082534688	-1.38006154	0.015738953	9.781546736
4.457447532	0.262681509	0.00579907	10.45614366
3.870121564	-3.909757988	0.015367796	10.01490465
3.570147554	-3.081425802	0.01213997	13.2839289
5.186999524	-0.480894403	0.00786217	8.524716315
4.507989909	3.597772305	0.015627037	6.859171155
6.578955716	-0.181949835	0.003093158	10.98749752
2.80811075	-0.566139987	0.011835327	12.35922707
3.377302933	4.552550091	0.005192233	9.652180733
5.407752937	-4.176018123	0.010306919	8.304282662
4.166996521	-4.380782183	0.005307702	14.503877
3.798244595	3.497632973	0.00878797	9.638464844
3.983585109	-1.527339989	0.004522736	10.45469723
4.141755432	-4.333075416	0.01066591	5.005654547
4.266038595	1.270942984	0.008913819	7.251788551
4.648098618	0.117590062	0.012972539	4.869762945
3.940236998	-3.220474498	0.016112203	8.128649446
5.517701679	0.985845236	0.006740138	6.027598608
4.766828451	-2.131314213	0.003708218	10.23751427
4.690935988	-0.003704205	0.003990097	10.48885786
4.38520983	4.23957125	0.015443922	8.562210765
5.17565757	-2.054220696	0.007104826	6.055747021
3.707258	-0.672997055	0.012739468	8.380541873
4.497844198	2.935959061	0.011863747	7.549772538
3.83394236	2.212619896	0.007321594	8.0841296
3.861002332	0.614314883	0.005315034	16.05681643
3.64334908	1.877131202	0.007813967	9.341748062
3.477362309	-6.80762407	0.018206621	17.19261976
2.676077723	1.480668594	0.037009966	4.391901066
5.148748818	3.69213837	0.002867234	10.02374264

log.sigma.1.0.mm.3D_glrlm_LowGrayLevelRunEmphasis	log.sigma.1.0.mm.3D_glrlm_GrayLevelNonUniformityNormalized	log.sigma.1.0.mm.3D_glrlm_RunVariance	log.sigma.1.0.mm.3D_glrlm_GrayLevelNonUniformity
0.005642688	0.084835032	0.135850262	110.841986
0.016405862	0.097006085	0.091327818	31.08849505
0.011332836	0.089641097	0.125327535	72.40428186
0.013013429	0.087202453	0.091278146	26.01469531
0.011137387	0.08870191	0.107080702	114.5156723
0.019790472	0.084965137	0.083477436	19.08202203
0.007694802	0.148518121	0.348987355	1283.087376
0.003924342	0.103185524	0.189702906	549.4798228
0.010050186	0.12289584	0.218103283	452.2270007
0.006271512	0.084689526	0.094063814	59.15952382
0.014395421	0.141413945	0.179331802	193.042339
0.003208281	0.117961354	0.247177333	343.6515187
0.008622181	0.092178055	0.117825831	68.10600218
0.014301897	0.077680876	0.076869905	46.80777916
0.01290474	0.091004822	0.167535743	127.1002123
0.01195453	0.142270852	0.274332062	693.0865748
0.007339456	0.115580354	0.165038247	90.19279553
0.01659862	0.107371692	0.107825229	39.94380252
0.007290721	0.125163057	0.193150136	119.371518
0.002893994	0.107110671	0.161961001	874.7688451
0.02544162	0.131285545	0.18507651	237.5646983
0.016278865	0.088460422	0.091856685	40.38747403
0.022179167	0.095211782	0.130662385	47.90283022
0.011145398	0.169012158	0.273642963	509.4260703
0.009887682	0.129382008	0.297973419	132.8265477
0.00970746	0.113966102	0.196922036	255.0277613
0.00565264	0.090339094	0.117770428	164.7417963
0.013163323	0.12249517	0.188707631	242.164221
0.033313052	0.101129275	0.07137212	13.85752263
0.004914462	0.110924674	0.159556541	158.6257173
0.012374413	0.112961263	0.179979157	398.3904217
0.012559842	0.083926654	0.095435207	61.14262191
0.010382858	0.115878782	0.218137559	234.1689031
0.013365862	0.13685668	0.189171634	289.2154657
0.006004679	0.105070339	0.194515515	472.0194078
0.009950097	0.088253066	0.120221857	156.0801166
0.007036395	0.077558715	0.080481396	109.3502861
0.005837366	0.15093078	0.33665124	724.8545911
0.013045331	0.116328556	0.130547073	58.53439259
0.001710863	0.149905454	0.278217448	1189.028013
0.018321745	0.105870461	0.129753568	127.8061468
0.005418899	0.141315628	0.240033784	685.4793451
0.013071417	0.097581605	0.137789301	212.8401795
0.010083105	0.082282448	0.104447777	107.2587024
0.010820028	0.138356481	0.299658613	180.1985675
0.010195846	0.100338974	0.13920001	216.842991
0.006812534	0.126242161	0.207578955	586.4954091
0.006176033	0.130924346	0.145043071	57.44744066
0.015801321	0.087945771	0.084302924	31.91389154
0.012540882	0.087969448	0.085495379	36.79989722
0.003842939	0.082917381	0.122428626	97.25677655
0.006078737	0.08605273	0.097033067	220.8134655
0.008898678	0.172808601	0.390509908	1104.881239
0.003280907	0.120574913	0.209006	170.9398373
0.014301321	0.078618025	0.082795249	47.06391464
0.017082275	0.110115371	0.122878867	117.0627899
0.016669857	0.122748268	0.213586756	426.7114624
0.011329464	0.094360406	0.117988863	177.7734761
0.008811793	0.087955771	0.118537344	60.90370738
0.00551256	0.144205552	0.255570797	1332.318397
0.008141937	0.093038658	0.13929207	110.9397174
0.006145594	0.074286814	0.099616259	169.1156772
0.005573296	0.087684995	0.163534206	644.0825503
0.002824633	0.091016944	0.099013285	175.7204963
0.00844675	0.12695937	0.188368909	75.0230279
0.002821742	0.069659378	0.077409736	222.9145876
0.020643465	0.095105056	0.096246527	35.52892986
0.005382809	0.084952909	0.11530753	394.9090841
0.003066437	0.12150514	0.18973899	796.0982398
0.00814121	0.105951144	0.123336552	203.6048117
0.016481234	0.091716701	0.126286193	40.98896395
0.006216578	0.105408761	0.170162392	466.1756906
0.016160285	0.093697576	0.122965189	113.3027571
0.012674369	0.083358502	0.090193196	73.63071185
0.00861294	0.129303155	0.282808849	446.4477941
0.016584317	0.116319955	0.152920546	95.88177825
0.003266593	0.097110011	0.111634569	194.9843872
0.012235999	0.080215963	0.075406166	24.23865222
0.005459824	0.092444272	0.102782853	111.7219299
0.011076326	0.123379235	0.189066399	194.7067473
0.005590076	0.087283469	0.137345449	110.8920339
0.009163664	0.09658472	0.114261272	72.79766734
0.004859644	0.10574767	0.199128673	212.0555087
0.011703259	0.135288495	0.194306941	222.0650845
0.009824194	0.126484191	0.277376328	289.7994538
0.013880264	0.155083269	0.199052648	71.33582738
0.017170202	0.104450575	0.143285951	122.3326642
0.007616814	0.150406649	0.348623364	472.8193852
0.003962453	0.103747689	0.171144962	156.3276107
0.004179345	0.092651	0.101088015	116.1796403
0.016173364	0.109818353	0.121646495	79.71989045
0.00792223	0.13922266	0.299471058	294.4371701
0.013522061	0.104779084	0.143558704	423.4052056
0.012682691	0.114234905	0.152417997	351.3867184
0.007871455	0.104447373	0.142063945	309.3370776
0.005542365	0.075927862	0.095585584	167.3067386
0.008343964	0.097647325	0.140259295	215.4441349
0.018696971	0.074179773	0.062672055	21.94084218
0.039823838	0.134220402	0.142350996	31.96506064
0.003082963	0.101952453	0.160047518	800.4396846

log.sigma.1.0.mm.3D_glrlm_LongRunEmphasis	log.sigma.1.0.mm.3D_glrlm_ShortRunHighGrayLevelEmphasis	log.sigma.1.0.mm.3D_glrlm_RunLengthNonUniformity	log.sigma.1.0.mm.3D_glrlm_ShortRunEmphasis
1.358872591	264.5289101	1093.32837	0.931471972
1.265041395	111.0807865	275.6607737	0.942212495
1.347418401	133.4441543	673.586877	0.929830713
1.257438758	158.234196	258.5750903	0.945226671
1.302505726	135.8953916	1093.241901	0.936388346
1.246092275	148.3338562	194.3507847	0.944868532
1.844044398	143.0296625	6175.813465	0.870228774
1.490185481	288.9910748	4240.349618	0.912122125
1.563652751	125.8726386	2846.394793	0.9005722
1.264061644	248.451518	604.4371253	0.944328521
1.483010442	88.52973025	1077.849636	0.908845875
1.618057235	353.9316196	2231.157311	0.896874387
1.325574431	178.7240275	621.7259346	0.933557057
1.219798019	153.2246437	531.251536	0.951909022
1.441963461	131.7381035	1128.955686	0.917945239
1.680345841	104.9581327	3665.004827	0.889039849
1.44773187	178.2953663	624.0631203	0.913765658
1.317258482	104.8799455	310.0527303	0.930535572
1.512498833	171.8425291	746.2859417	0.905457859
1.428070742	400.1378469	6613.330443	0.918881578
1.506321375	56.39387006	1408.462678	0.903363308
1.273250206	116.7332979	388.8295244	0.938720557
1.361731478	93.66276497	415.241917	0.926408073
1.703875447	102.1757982	2211.832291	0.880591111
1.7357959	145.4271137	760.3364506	0.883448582
1.504956198	134.131762	1774.233777	0.910339321
1.327899627	233.2667357	1531.728203	0.93276962
1.491260024	106.5911953	1567.765835	0.910520091
1.209252876	68.70263836	121.5203149	0.953314274
1.429065288	290.3838303	1155.188325	0.917805651
1.47449092	106.1466816	2814.13022	0.912613802
1.266887355	130.1634353	629.8725685	0.944092652
1.556540769	127.7477974	1571.034328	0.902869501
1.498953311	109.2481632	1667.296686	0.908556184
1.501425175	203.4463959	3557.646742	0.910154103
1.323485576	157.36026	1495.21773	0.935517234
1.233416526	237.5821192	1234.187314	0.948601005
1.810619777	193.2193768	3496.38702	0.876889337
1.370631059	127.3577922	412.0244661	0.923217997
1.699810469	581.2239812	5869.247355	0.883793478
1.362867191	87.37744292	994.3386576	0.925681948
1.612814033	198.1883069	3687.637431	0.894301037
1.37582333	113.2420254	1813.864104	0.926988607
1.291831113	158.066627	1111.64771	0.939080586
1.732046346	123.7308989	965.3917809	0.884297185
1.375261369	134.3854804	1784.639944	0.926475044
1.53660788	174.670732	3625.744728	0.904379629
1.400489059	257.1130289	357.5207522	0.920959849
1.246397916	118.8164934	314.6981501	0.945509428
1.253519105	138.3347856	359.7916698	0.942702913
1.328845703	371.4393648	991.0279699	0.935129947
1.275530711	215.2016809	2198.652859	0.941067719
1.951425906	118.6002507	4417.711404	0.857266776
1.555384933	382.9371188	1093.211136	0.899189356
1.237489047	141.60788	522.6589478	0.948050028
1.347200947	90.47408981	880.7212591	0.9279616
1.551027969	87.7137143	2702.69281	0.902590196
1.323220062	132.4765034	1588.564957	0.934555165
1.333562471	178.1478236	577.8262149	0.930783937
1.648383198	195.6623626	6953.245475	0.890432616
1.383691233	177.8451519	977.5568209	0.92354909
1.273052109	278.6895892	1965.825221	0.943788584
1.421557343	238.4692594	6010.048805	0.922761968
1.278050535	448.8136039	1654.465592	0.9411157
1.507255418	172.8502739	462.5611272	0.905006476
1.220661333	456.3384855	2819.575995	0.951788201
1.278732783	108.3132982	319.0619781	0.939539131
1.313122304	236.9846362	3943.294963	0.936917581
1.504734416	353.2151446	5134.809353	0.906281488
1.345846245	154.9037824	1595.43971	0.928733874
1.340880748	111.6266202	375.5914862	0.932831983
1.441035975	204.2427715	3582.691743	0.918827619
1.343761607	109.7557553	1007.279214	0.929589007
1.248742678	137.3344361	772.1074569	0.948313437
1.68742902	147.9813079	2605.019081	0.890945432
1.398522647	93.16232408	678.7374056	0.924903815
1.322795451	402.4238628	1676.024109	0.930934578
1.218744884	161.4530924	266.0721967	0.951412122
1.287611717	241.2218267	1031.996655	0.939643952
1.492401238	126.5651159	1250.011699	0.910311504
1.363711905	262.7393102	1060.597772	0.930524323
1.316481133	182.9384977	636.7672317	0.9353037
1.500280137	258.1802223	1606.747633	0.913694686
1.526568933	101.9679469	1268.083788	0.900626008
1.686933461	126.7007741	1715.410782	0.887983941
1.543895209	101.3916187	355.5783328	0.899219516
1.393143901	89.34908865	957.9379252	0.922381282
1.841441296	144.7707574	2255.913558	0.87183149
1.447262481	321.5471777	1215.128068	0.917314054
1.290089769	330.6454808	1065.185665	0.937692755
1.349965267	113.0618265	598.4066323	0.926048037
1.739933097	143.8128687	1562.606054	0.882420443
1.385167797	110.2367544	3325.8404	0.925271244
1.409524477	112.1143427	2504.470391	0.921013023
1.38994644	155.4404381	2420.067996	0.922522626
1.267204428	273.7313391	1904.350735	0.943976976
1.377213415	156.8911206	1821.635379	0.926248406
1.183457106	184.0533151	265.1318992	0.958231602
1.412460385	43.96126226	190.7056081	0.914234161
1.424813402	364.7123399	6363.006871	0.919261358

log.sigma.1.0.mm.3D_glrlm_LongRunHighGrayLevelEmphasis	log.sigma.1.0.mm.3D_glrlm_RunPercentage	log.sigma.1.0.mm.3D_glrlm_LongRunLowGrayLevelEmphasis	log.sigma.1.0.mm.3D_glrlm_RunEntropy
379.5441865	0.904645216	0.007076079	4.38427838
146.1852912	0.923520284	0.01937776	3.954143847
191.2019086	0.90548465	0.014317451	4.148291978
210.8511129	0.926421405	0.014826038	4.099618036
186.8688287	0.914899417	0.0137381	4.116778472
193.1048788	0.927844882	0.021704893	4.157014771
294.7185001	0.819118731	0.013350385	4.005696787
462.0853977	0.877414938	0.005629636	4.241384538
211.276299	0.863137566	0.014744318	4.01658361
325.7387263	0.92511462	0.007475182	4.181645318
140.3861722	0.876853866	0.020429289	3.703050154
624.2806604	0.854823779	0.004890129	4.194574738
254.1538323	0.910746467	0.010570554	4.157748129
193.5903493	0.935618729	0.016117137	4.242452274
197.4209996	0.886446886	0.01698351	4.33810217
191.9844815	0.845219342	0.018761006	3.954463929
275.9710936	0.883700671	0.009941634	3.932098741
145.0414752	0.909535452	0.020254227	3.874490657
281.4439464	0.871466742	0.010252315	3.908090417
617.0753119	0.889193242	0.003927627	4.127005682
91.24097072	0.870696676	0.036259405	3.766700909
152.5749879	0.920440447	0.019492088	4.039401647
131.633175	0.901571547	0.027405366	4.080754103
189.1896089	0.837257544	0.018116337	3.749918471
276.9256467	0.835379604	0.014983146	4.18819114
212.6867242	0.875225713	0.013827144	4.110005957
325.3564465	0.909834178	0.007181394	4.239577731
167.3574101	0.877086961	0.018325679	3.971547556
84.87349925	0.938356164	0.037943059	3.774453662
443.2421427	0.888103201	0.006568589	3.99432243
167.4553131	0.879712954	0.017290786	4.003594579
171.7127795	0.924443577	0.015161167	4.101782496
212.4553244	0.865129303	0.01499996	4.094683721
175.262675	0.874597866	0.018473629	3.97019263
327.1110919	0.875091789	0.00853859	4.185062332
218.6702752	0.912048241	0.012345751	4.201348664
310.6553807	0.932336182	0.008124251	4.329242704
392.0474237	0.824706302	0.009795798	4.063101093
184.6202121	0.898489011	0.016267923	3.824182078
1103.818552	0.839338496	0.002813075	3.926571853
125.4811183	0.900861079	0.023396639	3.954840402
345.0957427	0.854562222	0.008509684	3.955001391
163.6888494	0.901857861	0.017318687	4.034512504
210.4204052	0.917930661	0.012355161	4.2845249
232.3046442	0.83656318	0.01679058	3.964130237
192.7063712	0.899913498	0.013445877	4.091030559
286.4581238	0.867990167	0.010009959	4.032513845
382.5800677	0.893467022	0.007758479	3.650647896
152.1101592	0.927995278	0.018492773	4.081165218
182.0476977	0.925459496	0.014826608	4.061378668
516.54393	0.911123065	0.004781825	4.330027473
284.9475224	0.921336832	0.007551105	4.208583153
261.3228896	0.801172749	0.016431367	3.867177046
662.2067267	0.863159374	0.004615098	4.03037163
183.4734089	0.930972604	0.016526562	4.225840332
126.9351372	0.903976975	0.022036656	3.890406114
144.1582749	0.86565763	0.02379677	4.001900829
183.0770844	0.911354248	0.014224528	4.104924964
247.1727959	0.907450348	0.010947175	4.221374163
352.9291768	0.847869839	0.008730204	3.992238905
262.306709	0.897088615	0.010465411	4.22178153
370.3833623	0.923451396	0.007236088	4.42539442
360.8962859	0.891948955	0.0074209	4.368640308
607.1143518	0.921095126	0.003444394	4.104404461
282.6855626	0.87228724	0.01136762	3.8854759
581.6001471	0.935376521	0.003330264	4.461475834
145.3090643	0.920045472	0.024074961	4.030022166
323.8905244	0.914060964	0.006860965	4.293698628
577.7013813	0.87249498	0.004453123	4.044596079
223.4807362	0.904715341	0.010475854	3.956301744
156.0134677	0.90822389	0.020187922	4.09442357
313.8278201	0.887649993	0.008471214	4.190307775
154.1742581	0.905733218	0.020028991	4.134426802
173.6645338	0.929635628	0.015236109	4.203131137
270.2962613	0.844816545	0.013310425	4.114014342
136.3323362	0.897519692	0.021618934	3.876878009
567.3581093	0.908904133	0.004075584	4.150713456
201.9184298	0.935460824	0.014058691	4.118138635
326.3621058	0.918982159	0.006739131	4.076710217
198.844452	0.876452991	0.015266035	4.002211522
378.6815085	0.903435824	0.007045264	4.347214054
256.2180085	0.912460421	0.010990254	4.065530922
415.0507653	0.878345581	0.006826179	4.184104684
166.4769161	0.866959243	0.017198685	3.779038744
231.2754899	0.843462247	0.015375897	4.060461741
182.3683905	0.865565696	0.018666591	3.610033122
130.234678	0.895318749	0.022462955	4.026301768
292.1859945	0.81910243	0.013351515	3.95639524
500.827696	0.885632886	0.005332666	4.17488618
453.4402831	0.917280963	0.005062135	4.121350478
164.5964747	0.902698048	0.019598234	3.979047624
272.4218691	0.83476121	0.013022349	3.937302561
160.5892005	0.897697947	0.017504142	4.053970665
165.9406388	0.892405911	0.016892704	3.978261692
226.8938461	0.895513014	0.010637529	4.03465964
363.5884085	0.924238513	0.00662086	4.40894685
226.4469477	0.899736479	0.011052931	4.118478861
225.8785689	0.944949619	0.020759255	4.277053338
65.45833196	0.888346728	0.052957055	3.587372043
561.216808	0.88961922	0.004218409	4.181667746

log.sigma.1.0.mm.3D_glrlm_HighGrayLevelRunEmphasis	log.sigma.1.0.mm.3D_glrlm_RunLengthNonUniformityNormalized	log.sigma.1.0.mm.3D_glszm_GrayLevelVariance
282.9954405	0.836603245	27.88386974
117.3982207	0.859657664	16.41973841
143.1509064	0.833065693	18.37365059
167.4951942	0.866394154	19.68704344
144.7849268	0.846569357	17.51089163
156.5971282	0.865140829	22.22329108
163.3897759	0.714490406	17.29439306
315.3610735	0.795969092	23.26272376
138.7707036	0.772787285	16.18441554
261.9764711	0.864704252	19.95020165
96.86828042	0.789055655	10.38723762
392.6716304	0.765495719	22.81588843
191.4510478	0.840915371	19.45535273
160.4765754	0.881417751	21.65164353
142.0306167	0.808044869	24.22689735
117.3034037	0.75086615	14.01217559
194.1414262	0.799021614	14.51366932
112.1638847	0.833254621	14.31866005
188.9721284	0.782066006	16.66659854
434.8625169	0.809451643	22.04044476
62.05653577	0.777733119	10.80614022
123.2374715	0.851281793	14.88290865
100.1543185	0.825064619	15.93669689
114.8872976	0.733567881	13.54823989
163.5224238	0.740033588	22.819161
145.9735405	0.792389105	20.64743157
248.9612966	0.839228823	23.3978996
116.0413909	0.792555561	16.35284196
71.68187489	0.885827324	12.03888889
315.0351769	0.807380157	16.22927296
115.7671056	0.797112234	15.54592164
137.4604372	0.86411322	15.59025346
140.3304905	0.777110737	18.70287636
119.5219681	0.788623243	18.60740956
222.3089205	0.791701684	21.01022281
167.6122439	0.84504223	19.21087258
250.6368414	0.874381292	24.53019025
219.4831599	0.72747	19.00215371
137.243226	0.818359823	14.18130178
655.6941794	0.739676358	17.25885189
93.8863909	0.823363221	13.68633469
219.8458585	0.760011974	18.27479829
121.5791074	0.828754621	13.66262944
167.1642925	0.852589894	22.83625657
138.6529603	0.741004489	16.93312793
144.0489926	0.825504412	17.12357671
191.6933176	0.78008585	18.5004298
277.8807906	0.814079373	12.38666779
124.8793929	0.866890826	18.15192508
146.3566536	0.859923209	15.61720977
395.4465275	0.844475894	25.39710949
227.6076091	0.85669283	19.68465145
137.2837169	0.690830447	14.60853585
425.8968116	0.770042455	18.75042265
149.08194	0.872679448	21.00207756
96.76731533	0.828220904	13.6822482
96.2635775	0.776725722	15.39685836
141.0896413	0.842840347	17.48110833
190.0581618	0.834298646	21.89135523
218.4152079	0.752443413	19.81988092
191.9214194	0.819297412	23.10957457
294.4308404	0.863272964	26.73351928
257.5773598	0.817988257	24.33512737
476.5902958	0.856821381	18.53141421
190.2961353	0.781787226	16.92934992
478.8191868	0.880997902	28.90839028
114.8429447	0.853656435	17.13402367
251.6484137	0.848026147	23.13864853
388.4972651	0.783462737	20.86196312
166.6772156	0.829946814	14.54421939
119.0236071	0.83972405	17.38109274
221.3741013	0.809730047	21.57591194
117.3772553	0.832315294	19.23809524
143.6624905	0.873686786	20.20439427
164.8623755	0.754184916	19.89721218
100.0672756	0.822559904	14.76008031
431.5563853	0.834483436	23.24126266
168.9126491	0.880276622	18.5247929
256.0903771	0.853700385	16.71889273
137.7562372	0.791931242	19.6244229
281.3460603	0.834580261	26.64506256
195.3846528	0.844454021	17.92037122
281.5498595	0.800167231	19.92140652
112.2787284	0.772095147	12.51833764
141.5034189	0.748175823	17.43540799
113.9349073	0.771020276	8.624565972
96.12589611	0.817149726	16.18003534
164.3971031	0.717521338	15.32991264
349.6028636	0.806314367	21.51272054
352.4464206	0.849260031	20.58600058
122.0778496	0.824058485	17.42114632
161.4796961	0.738028527	15.20083113
118.4771787	0.822869619	17.2288977
120.8901348	0.813937304	16.25398587
167.3772636	0.816855107	15.9579144
289.3022238	0.863836948	27.40330487
168.3799396	0.825104083	17.7254216
191.8183763	0.895978344	23.69772863
47.65028999	0.799918191	7.735486351
396.2249478	0.810190971	22.09586555

log.sigma.1.0.mm.3D_glszm_SmallAreaHighGrayLevelEmphasis	log.sigma.1.0.mm.3D_glszm_GrayLevelNonUniformityNormalized	log.sigma.1.0.mm.3D_glszm_SizeZoneNonUniformityNormalized
221.1418634	0.05631025	0.471220391
81.05216299	0.075356718	0.383026159
98.38684796	0.064892133	0.334997676
87.83268005	0.065743945	0.314878893
97.42649086	0.068934614	0.392848651
112.3237322	0.065640561	0.418741512
114.204019	0.068954526	0.378996959
234.2003953	0.060240737	0.399873926
104.3288839	0.071989825	0.38626662
196.6919041	0.067345455	0.39932562
67.79817961	0.088026759	0.399214869
295.5458558	0.062541322	0.443070764
121.0953468	0.064578773	0.36371019
119.2022692	0.061264347	0.420016826
125.772139	0.060438293	0.463414072
77.97937076	0.07722866	0.331749295
129.1973297	0.081753865	0.348633068
84.0786917	0.086608871	0.425469237
141.9898673	0.077809156	0.412752675
290.7938894	0.063859477	0.414378438
46.17868917	0.088837277	0.353951952
89.04145593	0.077646882	0.330799052
81.87807852	0.074682259	0.395399321
91.80399206	0.083837621	0.39367485
115.1286323	0.06106252	0.354528206
121.0453897	0.065021436	0.412714957
185.6275621	0.060057406	0.39656406
96.00488748	0.072844992	0.400181054
52.13300188	0.084444444	0.363888889
225.4846911	0.079198771	0.393729175
86.32754668	0.074279102	0.402660311
88.03187438	0.07802332	0.374310671
116.5626341	0.067790536	0.423568664
95.98921269	0.067410378	0.407254337
170.8724559	0.063725253	0.429982014
117.1689558	0.062462128	0.404123745
159.0939086	0.058222354	0.40098692
156.6441205	0.064813145	0.378063794
98.73289611	0.08035503	0.340473373
482.3152648	0.072227205	0.423120567
72.32546494	0.078546939	0.403346939
190.0565865	0.070283441	0.45605043
86.72451896	0.075787927	0.365245932
138.3065472	0.060165712	0.440985064
114.0798077	0.074908995	0.441932371
107.1646536	0.070055886	0.373326122
160.5866654	0.069626128	0.437386074
201.8812686	0.113205959	0.391128693
95.05688948	0.069719043	0.429760666
90.25674562	0.075215999	0.293978334
297.5388401	0.056797318	0.461884611
167.6506577	0.063520358	0.411492549
107.4331496	0.078776128	0.437660171
279.3314614	0.074809806	0.404782031
96.68980315	0.063404124	0.339912281
74.43926537	0.081825205	0.380782918
77.31765948	0.073782207	0.407109014
102.6794916	0.06885	0.374822222
153.4121975	0.064981142	0.442077488
178.7123981	0.068087392	0.44984293
150.3950417	0.060009056	0.441870494
213.1008539	0.05436115	0.419554167
180.2485345	0.056298867	0.418965175
322.5489988	0.067413679	0.412545238
136.3919933	0.077739198	0.433256173
354.1210672	0.051780867	0.445151733
85.22234902	0.073491124	0.387100592
190.1643122	0.058630607	0.414706694
279.8106124	0.070974123	0.425259518
112.573934	0.075171048	0.398368063
81.9056626	0.070784741	0.360075185
162.6574666	0.060586601	0.406892087
86.43758485	0.065465692	0.376501218
129.8287792	0.06444882	0.466591663
127.9829906	0.063840036	0.428297812
83.30316107	0.074619168	0.433476395
301.9556032	0.06377185	0.396811931
128.3524588	0.071715976	0.419408284
174.0007623	0.068334948	0.374449827
118.2115218	0.066895583	0.432556171
220.6280166	0.056997972	0.471068074
129.5749491	0.067384714	0.375908144
203.4389684	0.064078241	0.415930804
90.25445749	0.096619745	0.43911392
108.2211692	0.069781284	0.411570269
47.94674721	0.113498264	0.316189236
76.20935759	0.073754789	0.386329106
128.8954316	0.075272283	0.400524144
258.8153872	0.064430558	0.434679271
223.9136403	0.064121972	0.37468762
79.79635341	0.071288715	0.424394802
131.6447411	0.080004374	0.409614273
92.69334674	0.068612625	0.416954043
95.94156787	0.072387659	0.383878401
128.3181749	0.072074233	0.429438202
217.1956404	0.053584521	0.423782426
122.6579969	0.068245847	0.403488607
130.6538105	0.065760762	0.403094624
35.61716344	0.124183007	0.314109958
259.2709134	0.062683552	0.39205718

log.sigma.1.0.mm.3D_glszm_SizeZoneNonUniformity	log.sigma.1.0.mm.3D_glszm_GrayLevelNonUniformity	log.sigma.1.0.mm.3D_glszm_LargeAreaEmphasis	log.sigma.1.0.mm.3D_glszm_ZoneVariance
237.0238569	28.32405567	178.7077535	170.4664024
44.43103448	8.74137931	30.07758621	21.12923603
80.73443983	15.63900415	92.44813278	78.74891961
32.11764706	6.705882353	29.80392157	19.83813918
159.1037037	27.91851852	107.6765432	95.53863131
39.36170213	6.170212766	18.08510638	11.45722046
655.6647399	119.2913295	7551.201734	7514.090397
624.603073	94.09603073	1625.99872	1610.907335
359.227957	66.95053763	1558.184946	1537.18293
109.8145455	18.52	40.94181818	33.40429752
126.1518987	27.8164557	341.1898734	316.9435988
389.9022727	55.03636364	1103.180682	1088.191466
86.56302521	15.3697479	54.34033613	42.72885036
108.3643411	15.80620155	26.31007752	20.07944234
236.3411765	30.82352941	270.7901961	261.2529988
359.6162362	83.71586716	3084.302583	3056.048062
74.60747664	17.4953271	223.9205607	206.8952965
46.37614679	9.440366972	75.88073394	61.80102685
95.75862069	18.05172414	421.2327586	398.9966558
955.1422993	147.1960954	1582.677223	1566.805382
117.5120482	29.4939759	791.6746988	752.4991653
53.9202454	12.65644172	37.80368098	28.54416802
66.82248521	12.62130178	88.69822485	77.79650572
246.8341308	52.5661882	2746.23126	2713.319903
104.2312925	17.95238095	644.8163265	627.3700773
260.4231379	41.02852615	751.4675119	735.0592348
236.7487437	35.85427136	172.1273032	160.8593049
215.6975881	39.26345083	696.0500928	678.578003
21.83333333	5.066666667	13	7.078888889
154.3418367	31.04591837	335.1581633	318.2895408
342.6639248	63.21151586	1097.453584	1075.260738
102.1868132	21.3003663	48.68864469	40.35706906
234.6570397	37.55595668	786.5902527	768.8257015
229.2841918	37.95204263	1092.904085	1074.5041
555.966744	82.39675174	1024.027069	1008.267458
245.7072368	37.97697368	135.5279605	125.3573164
232.5724138	33.76896552	25.44137931	18.64546968
446.8714044	76.60913706	3328.398477	3304.145769
44.26153846	10.44615385	122.9692308	104.4130178
748.5002826	127.7699265	6488.273036	6459.754175
141.1714286	27.49142857	209.9314286	195.2734694
549.0847176	84.62126246	2659.692691	2637.499514
227.9134615	47.29166667	199.3557692	184.3401442
221.374502	30.20318725	100.7011952	92.69973492
133.4635762	22.62251656	996.0397351	969.4933556
240.7953488	45.18604651	344.7705426	330.9136807
517.4277261	82.36770921	2302.528318	2282.068564
42.63302752	12.33944954	170.2477064	149.956401
66.61290323	10.80645161	25.7483871	19.38497399
44.39072848	11.35761589	23.66887417	14.70856541
214.7763441	26.41075269	119.344086	111.6836906
390.0949367	60.21729958	176.8512658	168.2208102
557.1413983	100.282011	8310.743126	8271.486454
147.3406593	27.23076923	647.7032967	627.3542748
77.5	14.45614035	27.96052632	20.00713681
107	22.99288256	253.2597865	235.7451147
368.0265487	66.69911504	1317.797566	1298.071784
224.8933333	41.31	161.705	149.836975
114.4980695	16.83011583	84.99227799	76.31368048
1048.58387	158.7117117	5878.079365	5856.241486
164.8176944	22.38337802	155.1179625	142.4229744
406.5479876	52.67595459	55.35500516	48.88378325
1053.697416	141.5916501	667.4290258	656.7102362
264.4414977	43.21216849	126.9235569	116.2313711
62.38888889	11.19444444	276.2986111	254.1955536
633.8960674	73.73595506	30.1369382	24.36547183
50.32307692	9.553846154	40.58461538	30.83100592
704.171967	99.55477032	333.3080094	324.3397834
697.4256098	116.397561	2451.284146	2430.325616
213.9236499	40.36685289	140.7523277	125.1078722
55.09150327	10.83006536	59.9869281	49.64628989
531.8079572	79.18668707	923.6472839	909.1234287
142.3174603	24.74603175	153.9920635	141.5188335
182.4373402	25.19948849	47.73401535	41.83073109
400.8867521	59.7542735	1843.895299	1824.857367
101	17.38626609	119.2360515	103.7131279
245.2297735	39.41100324	175.3932039	162.6166279
54.52307692	9.323076923	20.00769231	13.8343787
159.1411765	29.04235294	63.67764706	54.10408304
197.245614	30.50438596	577.9561404	562.3744229
227.5258799	27.5300207	181.552795	173.0790307
91.34567901	16.3744856	98.05761317	86.5032092
276.593985	42.61203008	426.4902256	414.7144915
155.4463277	34.20338983	960.240113	931.6448099
231.3024911	39.21708185	1180.841637	1157.503454
30.35416667	10.89583333	335.53125	304.9365234
134.4425287	25.66666667	214.4712644	200.3440349
269.5527489	50.65824666	2808.805349	2776.317043
206.0379747	30.54008439	299.3945148	286.5164103
152.872549	26.16176471	71.31617647	60.09039192
89.97169811	15.11320755	173.3867925	159.004094
207.2648221	40.48221344	1542.747036	1517.707494
517.4399678	85.14826753	494.4165995	481.262096
319.7707083	60.29891957	637.4765906	620.3630235
382.2	64.14606742	501.2955056	487.4888537
364.0291036	46.02910361	45.26891735	37.56651547
279.6176046	47.29437229	309.3939394	296.8748004
57.23943662	9.338028169	10.86619718	6.00758778
16.01960784	6.333333333	127.0588235	99.44482891
849.5879096	135.8352561	1371.463775	1354.882681

log.sigma.1.0.mm.3D_glszm_ZonePercentage	log.sigma.1.0.mm.3D_glszm_LargeAreaLowGrayLevelEmphasis	log.sigma.1.0.mm.3D_glszm_LargeAreaHighGrayLevelEmphasis	log.sigma.1.0.mm.3D_glszm_HighGrayLevelZoneEmphasis
0.34833795	0.68444382	47669.42942	304.8449304
0.334293948	0.360041979	3063.275862	125.5862069
0.270179372	0.720727791	12987.19917	156.2655602
0.316770186	0.212465546	5216.892157	160.4117647
0.287030475	0.972341874	12973.93086	152.4148148
0.388429752	0.176116999	2538.510638	166.4787234
0.164152197	49.48748498	1160982.803	178.5843931
0.257415953	5.442271625	487935.4923	345.3066581
0.218207414	12.56647963	195412.0204	158.7172043
0.364238411	0.187604391	9641.592727	285.2254545
0.203084833	4.020259661	29964.61076	104.7721519
0.258291752	2.977768177	410502.758	422.9977273
0.293464858	0.307517542	10660.63866	191.197479
0.400621118	0.221768674	3766.434109	171.6666667
0.323809524	2.221936779	33861.49216	168.7921569
0.188129122	29.21255423	329363.4668	128.1116236
0.242355606	1.292044181	39701.99533	211.0373832
0.266503667	0.938380355	7155.59633	126.0183486
0.212065814	2.446828603	73806.99138	206.7974138
0.251007296	3.845438936	653966.1267	440.8065076
0.159769009	15.59146782	43927.37952	73.46385542
0.328629032	0.464077847	3702.883436	144.1165644
0.302867384	1.191809053	7257.177515	118.9171598
0.174311927	27.21633127	279858.0654	137.5135566
0.239413681	4.248230057	99135.72789	180.2619048
0.24687011	5.913969546	96818.97781	174.2345483
0.297904192	0.790790829	38320.0871	275.1809045
0.239236573	6.932340472	70977.84601	138.2115028
0.410958904	0.307783659	819.4333333	78.73333333
0.243478261	1.162108623	98422.73724	343.4515306
0.212272387	10.75357603	114434.2139	128.2150411
0.346446701	0.504319731	5307.307692	143.040293
0.237259101	6.225614952	100908.139	164.3537906
0.233126294	9.988814388	121408.2504	139.4511545
0.251899474	5.064791743	208662.9946	247.12529
0.313563693	0.953521151	20233.61349	177.4407895
0.383597884	0.112477083	6881.303448	245.5137931
0.203057894	15.9432444	698920.0575	237.7267343
0.232142857	1.058815431	15624.91538	153.7846154
0.187255213	10.13670177	4160366.566	702.5500283
0.26119403	2.701085079	17396.69714	106.2857143
0.212270804	13.3526797	532691.1669	260.744186
0.258064516	2.248678159	19638.62179	134.0288462
0.353521127	0.735229766	14463.67729	192.3545817
0.194087404	7.940904368	126834.5	162.4470199
0.268638067	2.902798565	41973.71783	166.8728682
0.221080172	13.15533445	406245.9552	225.6365173
0.221995927	0.68020168	43921.02752	308.6055046
0.396419437	0.298876516	2606.909677	137.0322581
0.334070796	0.229996213	3204.688742	157.8741722
0.361305361	0.335796199	43171.33333	423.2494624
0.340394973	0.916199518	34840.59072	248.3386076
0.159603811	64.8387577	1074625.988	156.6732129
0.221680877	1.534427798	275442.0082	423.3791209
0.354587869	0.241928497	4278.631579	155.6842105
0.238945578	3.699144978	18572.21352	113.0427046
0.225155666	16.41495925	107632.6184	114.4037611
0.290275762	1.390716887	19611.38167	157.5483333
0.339449541	0.52559565	14335.78378	213.2702703
0.213990636	28.58626786	1215139.955	249.1389961
0.280662152	0.923903908	27252.44504	207.9383378
0.393103448	0.217328596	14943.34056	310.494324
0.305440855	2.832620311	158716.5113	268.3033797
0.305820611	0.291711745	56435.49922	486.0624025
0.212703102	1.588943642	49277.99306	200.1180556
0.416252558	0.069455299	13769.58919	498.2514045
0.320197044	0.466821248	4704.569231	124.1769231
0.333923304	1.479748695	75950.39164	276.6195524
0.218433671	6.746150274	893689.3957	411.8402439
0.252824859	0.933349453	22721.05587	170.7243948
0.31097561	0.62835244	6481.294118	131.4379085
0.262397109	4.521213616	190416.4705	240.2853864
0.283146067	1.655722426	15338.37302	133.015873
0.411578947	0.432833831	5746.480818	170.0946292
0.229187071	12.22425065	280373.203	183.5512821
0.253812636	1.475040905	10484.00858	117.0042918
0.279764599	0.446905764	70287.53883	451.1553398
0.40247678	0.17570981	2896.623077	183.7461538
0.323193916	0.30080297	14169.82824	270.84
0.253333333	4.851466267	69838.08114	164.8903509
0.343527738	0.695666673	48383.00414	303.9399586
0.294188862	0.554729848	18371.43621	204.473251
0.291411043	1.61200583	113887.9338	299.7413534
0.187004754	10.25989217	92198.22599	130.5536723
0.206998158	9.444800434	149510.9288	160.8807829
0.18079096	2.689064052	43216.8125	92.27083333
0.266055046	2.862718473	16986.07184	113.4971264
0.17544317	18.87576271	421255.11	192.8172363
0.278659612	0.904434212	100161.0464	369.0126582
0.298463789	0.227169705	23374.72304	355.7990196
0.263681592	1.526465222	20752.71226	119.6981132
0.199842022	10.5751019	227806.4585	190.8418972
0.275716507	4.798801622	52149.88558	132.7784045
0.241729541	6.25168156	66350.64706	142.270108
0.269126096	3.582689173	71452.14382	186.011236
0.360318792	0.182197115	12205.30501	309.2444703
0.282626427	2.221173021	44086.8961	186.2005772
0.453674121	0.115300828	1940.5	198.0492958
0.190298507	3.782812896	5412.196078	58.11764706
0.245580236	3.550155228	532200.4079	402.2648823

log.sigma.1.0.mm.3D_glszm_SmallAreaEmphasis	log.sigma.1.0.mm.3D_glszm_LowGrayLevelZoneEmphasis	log.sigma.1.0.mm.3D_glszm_ZoneEntropy	log.sigma.1.0.mm.3D_glszm_SmallAreaLowGrayLevelEmphasis
0.71373291	0.007925864	5.825945917	0.006587616
0.641063424	0.025818645	5.343491639	0.021552849
0.597628498	0.016465556	5.84488654	0.01215516
0.575144681	0.024229657	5.608665602	0.020685193
0.651840674	0.016393083	5.761403946	0.01314181
0.671043755	0.035940308	5.383789783	0.033242037
0.639858379	0.010590846	6.094590312	0.007630293
0.657202378	0.004787498	6.123226085	0.00350258
0.644666426	0.014004878	5.831928447	0.010571506
0.656340873	0.008895036	5.691189773	0.007261718
0.656717119	0.018918966	5.447582596	0.012051051
0.69229682	0.00423981	5.889988678	0.003390105
0.626802369	0.013518539	5.828800406	0.010779529
0.670463582	0.021876812	5.674105465	0.018413461
0.707833384	0.017136807	5.743395202	0.013366415
0.596770856	0.016457604	6.083235481	0.011184477
0.608635457	0.011271744	5.614153932	0.008927108
0.67666828	0.028948588	5.109467069	0.025593051
0.667238337	0.011670298	5.544237714	0.009566861
0.66939853	0.004101435	6.119579966	0.003281309
0.617178308	0.039080573	5.525700454	0.028142432
0.594196566	0.024059592	5.535266999	0.019702546
0.652866961	0.03385666	5.406958679	0.025594909
0.650532867	0.014876379	5.600643374	0.010973221
0.613104067	0.01527763	5.878099864	0.011488987
0.667828941	0.012656442	5.920264515	0.009623788
0.654444433	0.007496878	6.03140167	0.005761538
0.657140171	0.018727603	5.741069794	0.014167497
0.617638233	0.047298583	4.90291008	0.037719234
0.652394345	0.006382098	5.697174345	0.003331084
0.659784552	0.017375042	5.80747451	0.012954635
0.634159964	0.017147285	5.58149733	0.013398878
0.676882756	0.014531165	5.794747701	0.011321821
0.663670723	0.018245456	5.850832591	0.012308323
0.681993237	0.007881391	6.002563542	0.006075284
0.66106223	0.014385526	5.905474345	0.01152833
0.658519153	0.0101955	6.079963186	0.008341361
0.639024366	0.00777694	6.133247612	0.005646139
0.601320937	0.025744224	5.427326756	0.022156555
0.676556895	0.002138396	5.815372671	0.00162829
0.660457727	0.026760369	5.557193634	0.020591476
0.702289272	0.006195412	5.729768146	0.00463563
0.627821259	0.016962587	5.873592869	0.011244235
0.690666257	0.01320376	5.794116673	0.010448477
0.690677141	0.018721011	5.492641852	0.016026354
0.632432583	0.01320238	5.894234547	0.009797976
0.687949988	0.008807679	5.825448809	0.006765529
0.649702091	0.01303928	4.936422775	0.011770501
0.680452159	0.022119661	5.338014229	0.018238404
0.555159116	0.017974722	5.698281521	0.013901621
0.70518343	0.005342074	5.745468796	0.004548633
0.666844235	0.007345429	5.945748805	0.005553453
0.687783635	0.011562375	5.632601915	0.008760891
0.661306136	0.005706137	5.673520842	0.004813563
0.602516625	0.021735611	5.774030673	0.017385801
0.639887621	0.023016975	5.507826208	0.014311217
0.66352971	0.024307702	5.779924023	0.01827747
0.635157515	0.016057676	5.894564541	0.012585475
0.691336721	0.012642207	5.61457713	0.010498984
0.697582544	0.00659172	5.872818217	0.004938673
0.689975207	0.012093098	5.830512195	0.009576704
0.673591993	0.008860173	6.153845258	0.007401959
0.673262572	0.007545891	6.208966202	0.005889434
0.667880383	0.004010556	5.897405725	0.003265602
0.683030093	0.016684327	5.334681794	0.014699104
0.693946101	0.003403151	6.161931179	0.002649292
0.646010033	0.035318501	5.407348559	0.029990956
0.669719816	0.006435264	6.104648334	0.004803598
0.678109048	0.003898955	5.928915721	0.002874605
0.65632215	0.011389641	5.767787758	0.008735787
0.620908573	0.025731814	5.428651612	0.021024524
0.663531522	0.008410008	6.116084836	0.006448882
0.63487399	0.026088271	5.862809231	0.021349354
0.710182073	0.014916108	5.617493152	0.011264223
0.680658869	0.012629282	5.89177891	0.01000544
0.682184329	0.025518048	5.520094315	0.020519522
0.654646039	0.004882898	6.044512764	0.003877644
0.672900049	0.017923707	5.33757551	0.015334738
0.635105355	0.007294661	5.859456187	0.005622213
0.683566043	0.016602521	5.653507212	0.013559925
0.713560875	0.007816463	5.793276689	0.006472459
0.634552149	0.015813642	5.772285676	0.013427182
0.670741087	0.006543891	5.858917893	0.00516742
0.688969139	0.016325371	5.306547352	0.013046814
0.666926382	0.014219237	5.753536582	0.01113387
0.578588658	0.029297415	5.186910424	0.023719296
0.645738299	0.024036427	5.707840065	0.018215497
0.658084466	0.01000276	5.794608601	0.007610196
0.685630631	0.005769906	5.777985998	0.004702873
0.636066091	0.006515763	5.93719381	0.005372867
0.677115465	0.030062063	5.539146519	0.024967794
0.665236685	0.010500571	5.593455918	0.008049013
0.671647367	0.019492511	5.859954434	0.015035835
0.64325476	0.01785167	5.92343257	0.013194208
0.681604516	0.009727404	5.726067236	0.007422506
0.677101309	0.007683093	6.156854511	0.006211629
0.660261893	0.010767675	5.863794035	0.008234735
0.658623722	0.024624157	5.547064726	0.016678332
0.574696931	0.059784058	4.733713519	0.038881755
0.651226056	0.004106319	6.221577804	0.00313085

log.sigma.1.0.mm.3D_ngtdm_Coarseness	log.sigma.1.0.mm.3D_ngtdm_Complexity	log.sigma.1.0.mm.3D_ngtdm_Strength	log.sigma.1.0.mm.3D_ngtdm_Busyness	log.sigma.1.0.mm.3D_ngtdm_Contrast
0.005114026	0.689490204	1.401560327	0.31050553	6.15E-05
0.016194947	0.994854889	1.850620998	0.283103911	0.000355775
0.0075545	0.358816856	0.760744956	0.598104095	0.00014407
0.018249861	1.27389234	2.318445344	0.201364612	0.000474845
0.004473371	0.268766202	0.529539758	0.908327268	8.37E-05
0.025494168	2.106560536	3.758774303	0.134719939	0.000597976
0.000688365	0.04084117	0.176603319	3.134801371	2.73E-06
0.001193775	0.179661315	0.49034259	1.006509266	6.99E-06
0.001690983	0.088176573	0.269279437	1.777444585	1.22E-05
0.008290612	0.837198087	1.531551479	0.277119027	0.000148059
0.004496348	0.122531235	0.413300087	1.088905845	3.27E-05
0.002246474	0.27374359	0.952744559	0.477963111	1.06E-05
0.008616307	0.582525889	1.253636051	0.335031859	0.000135264
0.009821584	0.813094759	1.283862151	0.373073301	0.0002583
0.004601341	0.40564975	0.827673445	0.649189249	6.67E-05
0.001176755	0.061169715	0.227854645	2.666855974	7.49E-06
0.007715621	0.354741841	1.116763408	0.416538623	8.14E-05
0.014845003	0.595418438	1.414512809	0.353349395	0.000262116
0.006428718	0.338259921	1.11506109	0.387029771	4.16E-05
0.000772302	0.131329328	0.431239316	1.179605111	3.45E-06
0.003181801	0.067380292	0.218757896	2.4507636	3.14E-05
0.01180506	0.586694056	1.098894492	0.548768094	0.000362097
0.012198013	0.496898258	1.012566161	0.470832636	0.000241326
0.002018928	0.074016959	0.340678018	1.558292064	8.15E-06
0.005332413	0.329597451	0.851481923	0.542895492	6.92E-05
0.002884735	0.220022922	0.712431693	0.851736979	1.99E-05
0.003531056	0.409171584	0.886238112	0.515497183	3.74E-05
0.003275896	0.197458364	0.652794858	0.945808972	2.20E-05
0.033389037	1.632271575	2.699915216	0.249925281	0.001141027
0.004289471	0.325756307	0.999608818	0.415528379	3.24E-05
0.001726256	0.102822621	0.314276794	1.950035058	1.35E-05
0.007335999	0.534751572	0.897377923	0.599557395	0.00017729
0.003138772	0.227080076	0.718523781	0.814531557	2.07E-05
0.002764793	0.196600515	0.644654911	1.04375503	2.18E-05
0.001415237	0.160852824	0.470573037	1.138886857	8.23E-06
0.003406228	0.274657738	0.523336612	0.87790537	5.41E-05
0.004078984	0.686584481	1.121781114	0.431551823	7.42E-05
0.001119264	0.097542643	0.374161925	1.543586666	6.24E-06
0.010737088	0.4725964	1.153640041	0.387664957	0.000140512
0.000778432	0.057482467	0.308175206	1.297606411	2.98E-06
0.004964112	0.189633745	0.459122102	1.081705781	6.71E-05
0.001328203	0.100397994	0.385098818	1.303229196	5.72E-06
0.002639186	0.143048652	0.337195375	1.587479597	3.55E-05
0.004804294	0.472221444	0.849020998	0.586954083	7.80E-05
0.004797051	0.195005664	0.680112287	0.628621113	2.85E-05
0.002861916	0.194417497	0.474159374	1.074924576	3.00E-05
0.00137322	0.103726564	0.340723596	1.474479142	7.92E-06
0.012505637	0.438987821	1.618085533	0.260249594	0.000146494
0.01675116	0.871659048	1.729961861	0.303138532	0.000394488
0.013987746	0.787220631	1.629986693	0.315258529	0.000316125
0.005242503	0.604005923	1.147723291	0.325031118	8.95E-05
0.002366538	0.286786503	0.569395777	0.873080133	2.95E-05
0.000905841	0.04481423	0.228279568	2.622504297	2.72E-06
0.004330747	0.339479199	1.216752214	0.317379646	2.73E-05
0.008787501	0.74348209	1.150030904	0.456321803	0.000275603
0.005550379	0.235812865	0.635050517	0.868606339	6.11E-05
0.001818499	0.083574463	0.264239412	2.166909813	1.40E-05
0.003112656	0.271277152	0.588132518	0.954215222	3.70E-05
0.008872284	0.794853306	1.586670698	0.301396775	0.000133278
0.000664241	0.064272476	0.257191842	2.266470708	2.53E-06
0.005169064	0.548766609	1.249634976	0.408603711	5.27E-05
0.002756193	0.450597571	0.76191948	0.595319345	4.39E-05
0.000896713	0.111254143	0.243412832	1.886362542	9.26E-06
0.002970573	0.354811376	0.804057518	0.475168258	3.46E-05
0.01031164	0.451006987	1.590276275	0.270129767	8.20E-05
0.001945903	0.437465432	0.68564706	0.586583807	3.10E-05
0.014154674	0.970215241	1.765069831	0.298595731	0.00031593
0.001350951	0.209189944	0.435538733	1.238777987	1.47E-05
0.000949399	0.127689737	0.463033654	1.066914769	3.67E-06
0.003079639	0.196028155	0.516328597	0.921187344	2.84E-05
0.013508625	0.725761653	1.511307541	0.319282342	0.000225886
0.001484599	0.171278582	0.469730321	1.099776938	9.80E-06
0.004942206	0.422085037	0.971923793	0.666037839	5.91E-05
0.006304883	0.70068869	1.17097424	0.54264334	0.00013739
0.001889354	0.132650297	0.434331312	1.153522005	1.07E-05
0.007448005	0.382649462	1.163656147	0.522840731	6.20E-05
0.003138541	0.495772739	1.440651821	0.326692455	2.00E-05
0.018661283	1.160019327	2.013686236	0.244900158	0.000580674
0.00485352	0.363388066	0.778011709	0.523475616	7.11E-05
0.004090985	0.261719976	0.810025943	0.665196976	2.94E-05
0.005202191	0.645163832	1.318911262	0.317506849	6.27E-05
0.008095185	0.429876785	0.994390109	0.407521147	0.000131223
0.002912562	0.293123705	0.716073645	0.607622716	2.91E-05
0.003700641	0.146351198	0.537112823	0.971727583	2.00E-05
0.00277603	0.116835028	0.381797099	1.130695524	1.98E-05
0.011253666	0.290372393	1.091704288	0.429701143	0.000125715
0.005226972	0.321507702	0.872340411	0.758676962	5.10E-05
0.001899303	0.091352235	0.388358702	1.265136013	8.21E-06
0.004474159	0.401296447	1.164131909	0.330952068	3.36E-05
0.004868344	0.559779902	1.374264295	0.314580902	4.89E-05
0.008467374	0.479778882	1.226563916	0.414105352	0.0001001
0.002904311	0.133727556	0.500877111	0.89771564	1.45E-05
0.001568184	0.104253867	0.264272379	2.073584896	1.44E-05
0.00206003	0.154742807	0.48979547	1.337860259	1.31E-05
0.002001143	0.143806965	0.356046492	1.371460785	1.90E-05
0.002907602	0.492309783	0.884608567	0.538027652	4.06E-05
0.002715213	0.197843065	0.457464236	1.056828489	3.14E-05
0.01855179	2.271186791	3.232650902	0.156995581	0.000625978
0.024173875	0.306291609	0.959242109	0.536194057	0.000358397
0.000815779	0.130694194	0.395396905	1.23284249	4.10E-06

log.sigma.0.5.mm.3D_gldm_GrayLevelVariance	log.sigma.0.5.mm.3D_gldm_HighGrayLevelEmphasis	log.sigma.0.5.mm.3D_gldm_GrayLevelNonUniformityNormalized	log.sigma.0.5.mm.3D_gldm_DependenceEntropy
1.016142352	20.81371191	0.298895612	5.453089224
0.577780731	12.63688761	0.376890432	4.989255598
0.680412838	12.99327354	0.338947093	5.376710901
0.734462405	13.00621118	0.339126577	5.201664714
0.677738594	13.05457123	0.341069786	5.32105443
0.879260296	14.01239669	0.307048699	5.049865635
0.473499301	12.67340355	0.414127876	5.200337324
0.729471505	30.56756757	0.348988382	5.27410135
0.534995126	12.49601126	0.392818929	5.106596281
0.821044691	21.37615894	0.314675672	5.315615826
0.384941201	12.60347044	0.441566273	5.138547195
0.6855178	30.85324332	0.366858526	5.281210958
0.792539694	21.04808878	0.328788346	5.298632099
0.889934802	12.60248447	0.298271672	5.303291565
0.860450088	12.66349206	0.322964172	5.295231293
0.519090413	12.65636932	0.390004363	5.192898743
0.5209	21.0419026	0.397503364	5.070401061
0.520812286	12.38875306	0.395807055	5.09879975
0.481653293	20.52650823	0.416439679	5.041055648
0.665003935	43.15103997	0.368407709	5.281345408
0.361501073	6.671799808	0.447351186	5.026244814
0.741898901	12.94959677	0.322913957	5.118418044
0.654195732	12.70430108	0.34732339	5.201982364
0.42092687	12.28384765	0.440995257	5.050036951
0.710534727	12.6490228	0.337397479	5.28246289
0.635680476	13.09194053	0.378091012	5.227807172
0.813113294	20.68762475	0.330050478	5.331877627
0.532588102	12.38659565	0.398538891	5.186866848
0.584537437	7.287671233	0.370519797	4.514822066
0.544223217	30.64409938	0.395710814	5.036045236
0.524400418	12.55624844	0.387979068	5.244234346
0.718523732	12.55456853	0.32467018	5.373722413
0.564856366	12.68479657	0.388189959	5.245627641
0.617572196	12.94741201	0.362701199	5.076726745
0.660554859	21.00974089	0.364512766	5.33688792
0.76571852	12.99226405	0.320902269	5.428382041
1.042803953	21.03174603	0.297540312	5.448241429
0.614675032	20.99553341	0.365562744	5.324765657
0.438364158	12.47678571	0.422984694	4.93165057
0.488879786	56.17741082	0.420305373	5.154987443
0.500712854	12.28507463	0.393780352	5.165768323
0.532858811	20.63081805	0.408724109	5.213441507
0.561310573	12.97105045	0.37320626	5.383249693
0.873566753	12.82957746	0.309976195	5.275317358
0.476742736	12.5340617	0.414938277	5.068513989
0.635645532	12.92211579	0.366410046	5.299601085
0.578337644	20.70155111	0.387335422	5.19414215
0.38017098	20.51120163	0.453279188	4.702551296
0.755371825	13.23017903	0.323918603	5.181989894
0.752466912	13.31415929	0.328667084	5.117757096
0.953244114	30.90909091	0.306852519	5.249689327
0.802126808	21.31310592	0.324615003	5.461530734
0.42141218	12.5	0.431710377	5.084649537
0.56789906	30.84470158	0.39268457	5.338990779
0.861054205	13.40124417	0.307537326	5.335440867
0.474974981	12.48214286	0.408066373	5.073153659
0.520069416	12.46600249	0.404512034	5.196399435
0.651870608	12.72472182	0.352145787	5.259384055
0.754643411	20.44823067	0.341651651	5.262714892
0.569171768	20.663729	0.397367105	5.190172438
0.722249115	20.82016554	0.348735535	5.476328354
1.087852079	21.63651116	0.2806323	5.456578857
0.872758919	21.3705368	0.311579463	5.535663946
0.701650681	43.28101145	0.3472463	5.393041303
0.495185773	20.71196455	0.406710027	5.107725894
1.190513465	43.2829582	0.269114427	5.540425613
0.728002378	12.5	0.348807299	5.037191206
0.858262014	20.77600787	0.317869493	5.468500955
0.577293153	30.5	0.399978258	5.233282607
0.509803661	12.98493409	0.396290178	5.198288115
0.647675821	12.71341463	0.35196477	5.277844678
0.734590843	20.92049789	0.340322295	5.308458883
0.682122628	12.59625468	0.353043527	5.274463966
0.864979501	12.81473684	0.317697507	5.259337267
0.596791269	12.63687561	0.380872874	5.311931806
0.493206554	12.9422658	0.406740522	5.156819042
0.732522064	30.90538705	0.359645026	5.351155961
0.850329247	13.21981424	0.301613166	5.228625184
0.658734404	21.30418251	0.348908326	5.235347074
0.571041667	12.30166667	0.407566667	5.024825924
0.963689957	20.70483642	0.305861488	5.422143061
0.648196624	21.22639225	0.364998915	5.408813989
0.713344	20.55915863	0.337667575	5.295677536
0.37637427	12.05810882	0.45389986	5.034322005
0.523233791	12.71786372	0.393354293	5.218750079
0.401339192	13.0433145	0.483176751	4.831010739
0.552877961	12.54204893	0.388316079	5.235522873
0.454528799	20.53128259	0.428566671	5.047517264
0.695879348	30.61375661	0.352992551	5.249070691
0.703419349	30.82882224	0.353495418	5.308762143
0.615967117	13.34950249	0.384204599	5.128360792
0.458978628	12.40402844	0.429142677	5.102630242
0.594136305	12.73783604	0.371591719	5.256947483
0.547237218	12.95414974	0.389721157	5.197357047
0.568058207	12.86997279	0.382142451	5.324401891
1.042770983	21.32676174	0.286610471	5.592373633
0.629208869	12.93923328	0.367034668	5.365509857
1.178189019	20.81469649	0.282038196	5.242215822
0.339051014	6.701492537	0.459512141	4.906044197
0.688603002	31.40537171	0.355764234	5.390406178

log.sigma.0.5.mm.3D_gldm_DependenceNonUniformity	log.sigma.0.5.mm.3D_gldm_GrayLevelNonUniformity	log.sigma.0.5.mm.3D_gldm_SmallDependenceEmphasis
98.54293629	431.6052632	0.076473416
24.26801153	130.7809798	0.072646344
59.81390135	302.3408072	0.047953246
23.45962733	109.1987578	0.062939499
86.35931963	481.2494685	0.065704598
23.25619835	74.30578512	0.078181548
729.2463232	4364.49369	0.040249119
385.0481213	2117.661503	0.054018412
266.2820272	1674.194275	0.049324615
54.70066225	237.5801325	0.073478409
101.0501285	687.0771208	0.054526051
218.5256824	1249.886997	0.055733204
56.58816276	266.647349	0.059190565
47.11180124	192.0869565	0.084558082
99.97142857	508.6685714	0.067609006
415.4866366	2247.205137	0.038370357
56.92298981	350.99547	0.046312884
24.28117359	161.8850856	0.062840151
73.68190128	455.5850091	0.054772091
637.0185125	3383.087989	0.057070504
147.2502406	929.5957652	0.036683891
36.06048387	160.1653226	0.054472807
35.77777778	193.8064516	0.050757701
232.4795663	1586.259939	0.044352644
91.23452769	414.3241042	0.043700865
163.3974961	966.400626	0.054849272
126.0129741	661.4211577	0.063978647
136.0856636	897.9081225	0.048248143
13.78082192	54.09589041	0.056594458
102.5142857	637.0944099	0.05291206
248.7640309	1555.408082	0.046887476
48.91624365	255.8401015	0.060833487
145.6569593	906.4235546	0.052351246
212.7167702	875.9233954	0.046949256
314.5308786	1871.044029	0.056756767
134.8504384	622.2294997	0.058129797
110.7777778	449.8809524	0.080264099
423.3538911	2127.940732	0.046078216
37.02857143	236.8714286	0.044511038
602.4576056	3970.624854	0.04463104
81.53283582	527.6656716	0.058780836
368.4894217	2318.283145	0.052861404
153.7799835	902.4127378	0.04796475
94.09577465	440.1661972	0.077311915
98.7377892	645.6439589	0.052220945
145.3840067	879.7505206	0.059845516
337.0754999	2072.631845	0.051365458
33.37474542	222.5600815	0.048587519
27.0971867	126.6521739	0.080023498
36.61504425	148.5575221	0.052979465
91.33566434	394.9191919	0.097324816
182.5439856	904.0527828	0.066992493
553.8355065	3443.321966	0.037859337
99.3361754	644.7880633	0.050990124
48.90357698	197.7465008	0.07344358
71.43707483	479.8860544	0.050063375
238.5521793	1624.115816	0.052735125
132.2162554	727.8853411	0.055683526
46.07470511	260.6802097	0.082043757
746.9451024	4328.519875	0.049466774
81.17456734	463.469526	0.068486442
191.3870183	691.7586207	0.075832003
559.5462716	2565.5453	0.05677321
136.2041985	727.8282443	0.062737267
44.49778434	275.3426883	0.054666194
257.4086524	920.640456	0.090586202
31.77339901	141.6157635	0.068852981
335.2296952	1616.366372	0.07216763
484.8353756	3003.036761	0.050606932
146.106403	841.720339	0.056160459
31.45121951	173.1666667	0.058523155
353.3814495	1695.145352	0.054578634
80.49662921	471.3131086	0.056905086
72.18526316	301.8126316	0.08867776
264.596474	1555.484819	0.051854521
60.66884532	373.3877996	0.056274937
147.6591218	794.4558624	0.061367923
25.05572755	97.42105263	0.079991319
90.05551331	458.8144487	0.057486461
105.1866667	733.62	0.062080541
95.49075391	430.0412518	0.076068247
50.65133172	301.4891041	0.064217388
157.5486415	770.5574058	0.06516635
108.6682515	859.2324353	0.046870885
169.8802947	1067.956906	0.04508642
36.40489642	256.566855	0.033152573
77.51681957	507.9174312	0.05859823
239.8378519	1643.981752	0.037543532
116.9494415	600.4403292	0.055182035
89.80175567	483.228237	0.065206634
55.2761194	308.9004975	0.054589251
144.5473934	1086.589258	0.044621942
303.6047545	1672.534326	0.058335321
233.2205456	1342.979106	0.051429133
194.2766858	1263.745086	0.062502536
162.8464765	683.2793624	0.073611701
147.7463295	899.9690049	0.054163595
27.07028754	88.27795527	0.081604435
18.34328358	123.1492537	0.033346714
589.1949229	3139.263599	0.054006103

log.sigma.0.5.mm.3D_gldm_DependenceNonUniformityNormalized	log.sigma.0.5.mm.3D_gldm_DependenceVariance	log.sigma.0.5.mm.3D_gldm_LargeDependenceEmphasis
0.068243031	16.39249622	77.41412742
0.069936633	16.64063318	88.2795389
0.067055943	18.51666533	95.88565022
0.072855985	15.42984453	81.13043478
0.061204337	20.62142094	104.9461375
0.096099993	10.43439656	56.02479339
0.069195021	16.10604538	113.0049341
0.063455524	18.49962543	96.61865524
0.062478186	18.76614284	105.6030033
0.072451208	15.57338011	73.63576159
0.064942242	17.53549739	103.0385604
0.064140206	17.97032164	99.66921045
0.069775786	15.49844387	82.99753391
0.073154971	15.30788743	68.43478261
0.063473923	20.38671706	89.61968254
0.072108059	14.5005215	94.39222492
0.064465447	17.70965603	107.2831257
0.059367173	25.57888822	118.2371638
0.067350915	17.63408855	114.226691
0.069369325	16.07510426	97.18098661
0.070861521	17.55290448	148.9961501
0.072702588	17.09027055	79.80645161
0.064117881	20.01583998	93.82795699
0.064631517	17.96455314	108.7408952
0.074295218	13.88491125	83.96416938
0.063927033	18.58799817	108.7339593
0.062880726	19.9864393	93.32634731
0.060401981	20.60097766	108.8508655
0.094389191	10.07299681	60.42465753
0.063673469	18.40490876	106.3217391
0.062051392	19.27042889	114.943128
0.062076451	21.4334626	94.80964467
0.062379854	19.10908005	105.2449679
0.088081478	10.11073647	67.43726708
0.061276228	19.99482846	106.9859731
0.069546384	15.83786297	83.80041258
0.073265726	14.29396573	70.06878307
0.072728722	14.10158281	87.05566054
0.066122449	17.96651786	110.6071429
0.063772373	18.54399572	107.7881867
0.0608454	20.76087325	117.1522388
0.0649664	17.93335087	100.887165
0.063598008	19.35703823	106.4218362
0.06626463	19.24372347	82.05492958
0.063456163	18.6510068	113.9768638
0.06055144	21.04779679	102.2278217
0.062992992	18.5794699	99.85554102
0.067973005	19.85243134	122.9307536
0.069302268	18.08468024	76.83631714
0.081006735	11.60198528	74.12389381
0.070967882	21.69511142	77.78787879
0.065545417	18.0004675	84.77019749
0.069437752	16.24050254	117.584002
0.060497062	20.2892198	113.5140073
0.07605533	13.75732436	68.72006221
0.060745812	20.72115785	122.5663265
0.059415238	20.95718925	111.4463263
0.06396529	18.7501337	98.41461055
0.060386245	24.31720342	97.09174312
0.06857111	15.78359656	98.87569999
0.061079434	21.25206922	102.2520692
0.077641792	12.67186946	64.31926978
0.067955583	16.54263456	84.94923488
0.064982919	17.96097255	92.63167939
0.065727894	18.7859507	115.6499261
0.075243687	13.87284394	64.25752704
0.078259603	13.04904268	77.28571429
0.06592521	18.51583958	83.22576205
0.064575836	18.14458348	112.4062334
0.068788325	17.32523381	108.759887
0.063925243	20.97103245	97.56910569
0.070945884	14.7927401	82.04195945
0.060297101	21.64392038	105.3355805
0.075984488	16.713241	66.00842105
0.064788559	17.73209827	99.53623898
0.066088067	17.98893588	104.8562092
0.066844329	17.39563713	99.98415573
0.077571912	15.0420305	70.94427245
0.06848328	15.7076702	87.12623574
0.058437037	21.96234444	108.0188889
0.06791661	16.51096803	78.83783784
0.061321225	21.0946655	104.0677966
0.06903972	16.35524061	79.33567046
0.05740531	23.57843072	145.3000528
0.062571011	19.15748793	116.0077348
0.068559127	18.9623033	132.8606403
0.059263624	20.94007706	109.4266055
0.062522902	18.8663591	123.7205422
0.068753346	16.06131953	91.10111699
0.065692579	18.46722167	101.9348939
0.068751392	16.59459543	95.41542289
0.057088228	22.83592761	129.9731438
0.067452734	16.5704996	93.74561209
0.067678626	16.39070154	100.8641904
0.058747108	21.96877558	112.1871787
0.068308086	16.58020402	78.83389262
0.060255436	20.72956324	107.6631321
0.086486542	10.56262695	54.93610224
0.068445088	19.10731789	129.9850746
0.066771863	16.90430985	99.51971895

log.sigma.0.5.mm.3D_gldm_LargeDependenceLowGrayLevelEmphasis	log.sigma.0.5.mm.3D_gldm_SmallDependenceHighGrayLevelEmphasis	log.sigma.0.5.mm.3D_gldm_LargeDependenceHighGrayLevelEmphasis
4.299374339	1.823932783	1503.050554
8.217092539	1.143181067	1043.357349
8.624309604	0.757391099	1194.11435
7.197399931	0.79109715	1038.639752
9.78248307	0.979397068	1244.881644
4.898025712	1.016364712	734.6983471
10.27909406	0.594035922	1358.131227
3.577253721	1.881912864	2702.755768
10.47744897	0.728007991	1146.857813
3.965208647	1.674002849	1467.476821
9.39205959	0.744077249	1226.890103
3.584935047	1.862048726	2872.773408
4.129169274	1.309634255	1810.293465
6.792656573	1.206116751	794.7888199
9.436718382	1.192439673	930.2304762
8.811273741	0.555375539	1114.594932
5.786816635	0.961894017	2093.691959
11.55866001	1.053719393	1307.914425
6.23223769	1.326863376	2197.127971
2.335741893	2.448319492	4157.899161
28.03214563	0.246031791	933.2098171
7.786447395	0.846528549	909.0705645
9.338636499	0.751487116	1031.865591
10.80382502	0.700585535	1170.354462
8.204094055	0.707310177	975.0692182
10.44861283	0.951584954	1231.102113
5.349887821	1.594170152	1719.237525
10.94300596	0.749391058	1162.762983
12.55126332	0.693268252	349.9178082
3.882533128	1.746038451	3005.876398
10.987774	0.667316105	1311.261661
9.378503948	0.88809319	1064.151015
10.15160385	0.816152783	1184.710493
6.263472273	0.723801026	815.5718427
5.877715822	1.316983609	2053.923437
8.01300957	0.869871887	1004.62919
3.594568121	1.58243859	1523.493386
4.552105948	1.001177511	1778.862223
10.4698879	0.569413531	1264.773214
2.061835498	2.794582696	5723.138139
11.2821733	0.826312007	1320.876119
5.664002961	1.375071405	1882.232722
9.769360008	0.734033837	1281.019851
8.418189059	1.225760246	880.0450704
11.01842045	0.823579305	1271.387532
10.06534135	0.877855371	1125.697626
5.673202445	1.314719477	1841.45057
6.975457683	1.068244199	2251.741344
7.376469167	1.284568915	882.8056266
6.465579523	0.860236846	988.2522124
2.994972106	3.26012001	2086.884227
4.715534357	1.697874812	1625.190305
11.03785337	0.558364607	1357.442076
3.775710496	1.436528657	3540.160171
6.096248056	1.124815018	896.7760498
11.86940169	0.804588736	1364.806973
11.19134693	0.796193491	1188.784807
9.563898225	0.826578856	1111.227866
5.685180622	1.962087727	1732.091743
5.454970018	1.255603652	1887.444047
5.443637393	1.573087451	2039.610233
3.438130394	1.81043135	1319.032049
4.536683124	1.259246341	1719.772529
2.244446929	2.795400938	3937.679866
6.219838339	1.183520019	2261.595273
1.585807261	4.511712695	2726.555685
6.984068829	1.051908545	993.5541872
4.763762746	1.821652839	1549.231465
4.052701804	1.629920316	3224.10975
9.09636561	0.752673525	1423.516008
9.158421409	0.917605921	1145.636179
4.49297117	1.200509748	1600.848223
10.39106591	0.883353871	1164.046442
6.833626161	1.605631071	702.6105263
9.568396604	0.816584694	1132.695642
9.662103749	0.838827899	1237.088235
3.478636682	2.055386815	2995.786329
6.914605263	1.389794185	848.7058824
4.572635697	1.273319774	1765.257034
11.17024035	1.024507364	1103.489444
4.379119979	1.810475227	1525.824324
5.152966589	1.399609011	2225.112591
4.49663392	1.529276123	1488.086766
14.58951165	0.666484371	1535.712625
10.99508645	0.633930762	1330.822468
9.770638209	0.263525539	1935.224105
10.67100354	0.864523498	1212.500765
7.00040874	0.888391604	2281.211679
3.272422397	1.657518311	2638.51264
3.442308396	1.983446998	3144.051939
7.5437626	0.719891736	1324.767413
13.0271191	0.720463354	1380.063586
8.767835942	0.912672866	1101.925572
9.292197289	0.801395337	1198.283227
10.73896549	0.971788255	1270.576656
4.186879777	1.764570227	1619.567534
10.34292142	0.803252546	1223.696166
2.869471562	1.738082802	1179.824281
25.1620284	0.2500176	787.619403
3.301824182	1.633579903	3127.984474

log.sigma.0.5.mm.3D_gldm_SmallDependenceLowGrayLevelEmphasis	log.sigma.0.5.mm.3D_gldm_LowGrayLevelEmphasis	log.sigma.0.5.mm.3D_gldzm_DistanceZoneVariabilityNormalized
0.009453321	0.064438166	0.978949751
0.010780797	0.100307397	1
0.00681851	0.099830904	0.808673469
0.014600399	0.110831608	1
0.011071645	0.101097527	0.92866941
0.021719122	0.108510101	0.802469136
0.0059163	0.094733277	0.896651193
0.002495873	0.036403816	0.822740887
0.007063444	0.097996441	0.955330612
0.006731598	0.057270892	0.809868719
0.006739596	0.091218759	0.84120983
0.002876114	0.035987595	0.935967721
0.005867766	0.058178979	0.900277008
0.020700449	0.122057885	1
0.012763096	0.111473088	0.867346939
0.004483745	0.095177671	0.959613136
0.004638737	0.053884711	0.950032873
0.009229704	0.102641266	0.9232
0.004260669	0.054618953	0.962976148
0.002007239	0.02506427	0.946108838
0.016754525	0.197208186	0.905
0.009157366	0.103277593	1
0.008142913	0.101650239	0.916824197
0.004897894	0.094300729	0.907908429
0.005707812	0.105582021	1
0.007428009	0.094452467	0.966676242
0.005461524	0.058423495	0.94786352
0.006153159	0.098758931	0.955079931
0.010560065	0.204611872	1
0.002437448	0.036360433	1
0.006676397	0.096722892	0.959476813
0.010182083	0.106543993	1
0.009237506	0.097870561	0.904091419
0.006327946	0.097438686	1
0.004836284	0.055580528	0.959229352
0.011679966	0.10643115	0.809422222
0.010742734	0.065083161	0.724519077
0.003672164	0.054832561	0.921185964
0.009909247	0.097209325	0.827664399
0.000913416	0.018515759	0.90443416
0.008599538	0.099100332	0.78125
0.003165334	0.053883496	0.870364149
0.006020424	0.095270311	0.914152254
0.016049798	0.112968402	0.957010023
0.008923618	0.096324264	0.971434573
0.009904294	0.098570326	0.936069941
0.003302986	0.054563002	0.952634556
0.005076356	0.054317153	0.91322314
0.012998145	0.101585678	1
0.008779888	0.102134341	1
0.005228603	0.037832464	1
0.004781329	0.055923015	0.875555556
0.004937265	0.092832751	0.906211667
0.003304216	0.035999737	1
0.015307584	0.111157335	0.854570637
0.005852787	0.09580428	0.926035503
0.006774649	0.096417633	0.976334756
0.009426766	0.101041427	0.954570038
0.007530921	0.05872991	0.932286857
0.00337973	0.054452	0.908424932
0.005787873	0.057072989	0.975907198
0.010121921	0.062270528	0.793702382
0.006017872	0.058148788	0.792387543
0.002298398	0.025251451	0.827664399
0.005279773	0.055101756	1
0.003001949	0.02644528	0.755932315
0.01740811	0.114018199	0.847222222
0.005896977	0.05849503	0.810460862
0.002432482	0.035963664	0.933412604
0.009253745	0.094661279	0.943971893
0.00882229	0.103705962	1
0.00515267	0.057054001	0.857988166
0.010506982	0.1046833	0.875555556
0.011152344	0.108367681	0.970594787
0.008286088	0.099745716	0.75878251
0.008596444	0.092558239	0.957466919
0.004324461	0.038139025	0.865123967
0.015032561	0.10869023	1
0.004816299	0.054477768	0.87359954
0.009953281	0.10105856	0.905891579
0.008863937	0.063674286	1
0.006899205	0.056152077	0.958352196
0.004584905	0.057683826	0.943971893
0.006702098	0.095273522	0.8828125
0.007517213	0.095957131	0.922456892
0.008208343	0.092795564	0.867346939
0.008010521	0.097078973	0.939453125
0.002520149	0.053522075	0.980584391
0.003438676	0.036718883	1
0.004263246	0.036892679	0.881429258
0.011739133	0.098446127	0.728268809
0.006004408	0.095088863	0.9232
0.009496696	0.099126643	0.870756173
0.008491927	0.095386106	0.864670467
0.008883548	0.095423176	0.870628271
0.008391009	0.061137329	0.735687235
0.008506289	0.098173826	0.913968022
0.011514304	0.07129091	0.84120983
0.011937732	0.195092247	0.802469136
0.003199596	0.035882006	0.862465306

log.sigma.0.5.mm.3D_gldzm_LowIntensityEmphasis	log.sigma.0.5.mm.3D_gldzm_LargeDistanceEmphasis	log.sigma.0.5.mm.3D_gldzm_HighIntensitySmallDistanceEmphasis
0.146996837	1.031914894	24.27659574
0.157789855	1	16.52173913
0.152748016	1.321428571	17.41071429
0.282795139	1	12.6875
0.196227709	1.111111111	13.93209877
0.30779321	1.333333333	13.86111111
0.181047167	1.163987138	15.38906752
0.050388973	1.294871795	34.28418803
0.170752381	1.068571429	15.28
0.101677401	1.319148936	21.9893617
0.140954106	1.260869565	13.61956522
0.058133744	1.099337748	32.86092715
0.109074621	1.157894737	24.10526316
0.313022487	1	14.97619048
0.211944242	1.214285714	19.26785714
0.14572013	1.06185567	15.20103093
0.108965238	1.076923077	20.26282051
0.1733	1.12	16.72
0.082999722	1.056603774	25.47641509
0.03916565	1.083140878	41.40127021
0.561805556	1.15	6.875
0.174756519	1	18.6
0.169565217	1.130434783	16.75
0.129090502	1.14516129	16.42540323
0.172547619	1	19.17142857
0.150573956	1.050847458	18.1779661
0.091747462	1.080357143	26.49776786
0.154618357	1.068965517	16.10632184
0.190401235	1	13.22222222
0.059084221	1	32.73913043
0.164261436	1.062068966	14.56724138
0.181184211	1	16
0.200583442	1.151515152	16.85353535
0.182092309	1	17.11666667
0.097886399	1.082987552	23.6120332
0.259537037	1.32	15.96666667
0.165049589	1.494845361	18.04639175
0.099034582	1.123287671	22.87671233
0.251600529	1.285714286	13.0952381
0.021540859	1.150943396	62.28066038
0.158209877	1.375	14.00694444
0.063162632	1.209016393	26.54405738
0.145689763	1.134831461	16.22191011
0.241225487	1.065934066	16.70604396
0.184967794	1.043478261	16.89855072
0.190705853	1.099173554	14.85330579
0.069945416	1.072815534	26.99029126
0.108510101	1.136363636	22.96590909
0.171268519	1	16.83333333
0.191351852	1	19.13333333
0.05690691	1	34.23423423
0.079399452	1.2	24.94166667
0.161668497	1.147982063	14.53699552
0.072947481	1	27.72857143
0.255277778	1.236842105	16.20394737
0.148637821	1.115384615	15.48076923
0.151679091	1.035928144	15.40718563
0.193515728	1.069767442	15.59593023
0.096090872	1.105263158	24.74122807
0.072860835	1.144303797	27.11518987
0.091108588	1.036585366	23.40243902
0.165219154	1.350364964	24.33394161
0.138517009	1.352941176	20.90686275
0.039917232	1.285714286	42.91428571
0.10504085	1	21.85294118
0.035185159	1.426829268	46.88821138
0.269131944	1.25	16.4375
0.091811867	1.318021201	25.17756184
0.054566778	1.103448276	31.49451411
0.180683761	1.086538462	14.04326923
0.166884921	1	17.03571429
0.120675467	1.230769231	21.97337278
0.211479025	1.2	17.1625
0.13685518	1.044776119	20.13432836
0.200908644	1.450331126	16.12490802
0.170757665	1.065217391	15.32608696
0.085227533	1.218181818	32.88863636
0.217324561	1	18.84210526
0.092442638	1.203389831	21.8220339
0.17648919	1.148514851	17.1509901
0.137634341	1	24.42857143
0.115513461	1.063829787	22.49468085
0.082365635	1.086538462	24.26201923
0.158017361	1.1875	14.428125
0.201781706	1.121212121	13.91919192
0.298988095	1.214285714	6.785714286
0.153092315	1.09375	14.91015625
0.077971411	1.029411765	24.71813725
0.066419083	1	32.12658228
0.068477461	1.189873418	30.88607595
0.24142995	1.486486486	13.56756757
0.157461111	1.12	16.6125
0.184087538	1.208333333	16.14236111
0.200163034	1.218978102	15.69160584
0.156167558	1.20855615	15.38636364
0.133124599	1.470149254	23.36567164
0.184974975	1.135135135	15.51576577
0.183540927	1.260869565	19.91304348
0.406635802	1.333333333	8.027777778
0.071734509	1.222857143	28.06

log.sigma.0.5.mm.3D_gldzm_LowIntensityLargeDistanceEmphasis	log.sigma.0.5.mm.3D_gldzm_HighIntensityEmphasis	log.sigma.0.5.mm.3D_gldzm_DistanceZoneVariability	log.sigma.0.5.mm.3D_gldzm_ZonePercentage
0.15497556	24.30851064	92.0212766	0.065096953
0.157789855	16.52173913	23	0.066282421
0.267152778	19.07142857	22.64285714	0.031390135
0.282795139	12.6875	16	0.049689441
0.208449931	14.43209877	75.22222222	0.057406095
0.641126543	13.94444444	14.44444444	0.074380165
0.233619514	15.78456592	278.8585209	0.029509441
0.061720742	37.79059829	192.5213675	0.038562953
0.187895238	15.34857143	167.1828571	0.041060535
0.143760192	23.61702128	38.06382979	0.062251656
0.178780193	14.56521739	58.04347826	0.044344473
0.063349743	34.07284768	141.3311258	0.044320517
0.148548305	24.26315789	34.21052632	0.046855734
0.313022487	14.97619048	42	0.065217391
0.268928369	20.21428571	72.85714286	0.053333333
0.177885078	15.40206186	93.08247423	0.016834432
0.117512246	20.43589744	37.05128205	0.04416761
0.2033	16.84	23.08	0.061124694
0.08928903	25.60377358	51.03773585	0.048446069
0.041693346	42.45265589	409.665127	0.047152347
0.664930556	7.1	54.3	0.028873917
0.174756519	18.6	20	0.040322581
0.3	16.7826087	21.08695652	0.041218638
0.144762545	17.14516129	112.5806452	0.034473172
0.172547619	19.17142857	35	0.028501629
0.157636102	18.43220339	114.0677966	0.046165884
0.09824649	26.94642857	106.1607143	0.055888224
0.164618357	16.35632184	83.09195402	0.03861518
0.190401235	13.22222222	9	0.061643836
0.059084221	32.73913043	69	0.042857143
0.175433849	14.73793103	139.1241379	0.036168621
0.181184211	16	38	0.04822335
0.283916775	16.95959596	89.50505051	0.042398287
0.182092309	17.11666667	60	0.02484472
0.103903467	24.04979253	231.1742739	0.046951101
0.481137037	16.34666667	60.70666667	0.038679732
0.274559372	19.2371134	70.27835052	0.064153439
0.132978297	23.47260274	134.4931507	0.025081601
0.323029101	13.38095238	17.38095238	0.0375
0.023999513	65.0754717	287.6100629	0.03366148
0.274459877	14.56944444	56.25	0.053731343
0.076381675	28.12704918	212.3688525	0.043018336
0.255240325	16.28089888	81.35955056	0.036807279
0.243459919	17.20879121	87.08791209	0.064084507
0.186175523	17.28985507	67.02898551	0.044344473
0.22858464	15.1322314	113.2644628	0.050395668
0.074775018	27.7184466	196.2427184	0.038497477
0.123661616	23.27272727	20.09090909	0.044806517
0.171268519	16.83333333	30	0.076726343
0.191351852	19.13333333	15	0.033185841
0.05690691	34.23423423	111	0.086247086
0.093861612	26.44666667	131.3333333	0.053859964
0.197124401	15.03139013	202.0852018	0.027958877
0.072947481	27.72857143	70	0.042630938
0.492119883	16.26315789	32.47368421	0.059097978
0.177483974	15.59615385	48.15384615	0.044217687
0.156669111	15.58682635	163.0479042	0.041594022
0.229368441	15.91860465	82.09302326	0.041606193
0.11509672	24.9122807	53.14035088	0.074705111
0.081474834	28.47088608	358.8278481	0.03626182
0.092124848	23.73170732	80.02439024	0.061700527
0.209605202	26.69343066	108.7372263	0.055578093
0.223562138	22.96078431	242.4705882	0.037162983
0.080404445	45.51428571	86.9047619	0.05009542
0.10504085	21.85294118	34	0.050221566
0.047193879	53.58943089	185.9593496	0.071908799
0.399131944	17.25	20.33333333	0.0591133
0.124247026	27.23674912	229.360424	0.055653884
0.05896309	32.36677116	297.7586207	0.042488013
0.190202991	14.43269231	98.17307692	0.048964218
0.166884921	17.03571429	28	0.056910569
0.155524318	23.22485207	145	0.03392893
0.325979025	17.55	52.53333333	0.04494382
0.138098961	20.53731343	65.02985075	0.070526316
0.406140432	17.13907285	114.5761589	0.036973555
0.187062013	15.39130435	44.04347826	0.050108932
0.096532226	34.87272727	95.16363636	0.049796288
0.217324561	18.84210526	19	0.058823529
0.119941773	23.16949153	51.54237288	0.04486692
0.200779619	17.69306931	91.4950495	0.056111111
0.137634341	24.42857143	91	0.064722617
0.122605659	22.63829787	45.04255319	0.056900726
0.090967153	24.90384615	98.17307692	0.045574058
0.233017361	14.5875	70.625	0.042260961
0.232084736	14.04040404	91.32323232	0.036464088
0.352559524	7	12.14285714	0.026365348
0.201269398	15.34375	60.125	0.048929664
0.081239385	24.78431373	100.0196078	0.026590198
0.066419083	32.12658228	79	0.046443269
0.080839684	32.13924051	69.63291139	0.057790783
0.45061914	14.78378378	26.94594595	0.0460199
0.209961111	16.71	92.32	0.039494471
0.249581365	16.83333333	188.0833333	0.047989336
0.289579092	16.38686131	118.459854	0.039756239
0.187896613	16.14438503	162.8074866	0.056546719
0.223008407	26.02985075	98.58208955	0.056208054
0.213083083	15.79279279	101.4504505	0.045269168
0.219772811	21.2173913	19.34782609	0.073482428
0.739969136	8.111111111	7.222222222	0.03358209
0.086835335	29.55142857	301.8628571	0.039664551

log.sigma.0.5.mm.3D_gldzm_IntensityVariabilityNormalized	log.sigma.0.5.mm.3D_gldzm_LowIntensitySmallDistanceEmphasis	log.sigma.0.5.mm.3D_gldzm_IntensityVariability
0.172928927	0.145002156	16.25531915
0.323251418	0.157789855	7.434782609
0.285714286	0.124146825	8
0.2578125	0.282795139	4.125
0.299192196	0.193172154	24.2345679
0.203703704	0.224459877	3.666666667
0.3077615	0.16790408	95.71382637
0.219519322	0.04755603	51.36752137
0.339559184	0.166466667	59.42285714
0.231326392	0.091156704	10.87234043
0.31779038	0.131497585	21.92753623
0.233630104	0.056829745	35.2781457
0.254847645	0.0992062	9.684210526
0.252834467	0.313022487	10.61904762
0.231575964	0.19769821	19.45238095
0.208417473	0.137678893	20.21649485
0.333333333	0.106828486	13
0.344	0.1658	8.6
0.349234603	0.081427395	18.50943396
0.253054846	0.038533726	109.5727483
0.408333333	0.536024306	24.5
0.35	0.174756519	7
0.266540643	0.136956522	6.130434783
0.31594693	0.125172491	39.17741935
0.263673469	0.172547619	9.228571429
0.289141051	0.148808419	34.11864407
0.228954082	0.090122705	25.64285714
0.334654512	0.152118357	29.11494253
0.358024691	0.190401235	3.222222222
0.352657005	0.059084221	24.33333333
0.377502973	0.161468332	54.73793103
0.346260388	0.181184211	13.15789474
0.303744516	0.179750109	30.07070707
0.234444444	0.182092309	14.06666667
0.26953737	0.096894401	64.95850622
0.2288	0.204137037	17.16
0.223084281	0.137672143	21.63917526
0.164946519	0.090548654	24.08219178
0.319727891	0.233743386	6.714285714
0.3220996	0.020926196	102.427673
0.376929012	0.129147377	27.13888889
0.283693899	0.059857872	69.22131148
0.32685267	0.118302122	29.08988764
0.245260234	0.240666878	22.31868132
0.342995169	0.184665862	23.66666667
0.300730824	0.181236156	36.38842975
0.294184183	0.068738015	60.60194175
0.338842975	0.104722222	7.454545455
0.297777778	0.171268519	8.933333333
0.28	0.191351852	4.2
0.174417661	0.05690691	19.36036036
0.241333333	0.075783912	36.2
0.343843632	0.152804521	76.67713004
0.287755102	0.072947481	20.14285714
0.213296399	0.196067251	8.105263158
0.412721893	0.141426282	21.46153846
0.32489512	0.150431586	54.25748503
0.274202271	0.18455255	23.58139535
0.254539858	0.09133941	14.50877193
0.252606954	0.070707335	99.77974684
0.219512195	0.090854523	18
0.199211466	0.154122642	27.2919708
0.211393054	0.117255727	64.68627451
0.278730159	0.029795429	29.26666667
0.316608997	0.10504085	10.76470588
0.201368233	0.032182979	49.53658537
0.319444444	0.236631944	7.666666667
0.216546592	0.083703077	61.28268551
0.298729376	0.0534677	95.29467085
0.317492604	0.178303953	33.01923077
0.298469388	0.166884921	8.357142857
0.192535275	0.111963254	32.53846154
0.273333333	0.182854025	16.4
0.203831588	0.136544235	13.65671642
0.248190869	0.156959049	37.47682119
0.306238185	0.166681578	14.08695652
0.239834711	0.08240136	26.38181818
0.329639889	0.217324561	6.263157895
0.30767021	0.085567854	18.15254237
0.310851877	0.170416582	31.3960396
0.184881053	0.137634341	16.82417582
0.232231779	0.113740411	10.91489362
0.221153846	0.080215256	23
0.39875	0.139267361	31.9
0.326191205	0.194205948	32.29292929
0.326530612	0.285595238	4.571428571
0.326660156	0.141048044	20.90625
0.298539023	0.077154417	30.45098039
0.246595097	0.066419083	19.48101266
0.273834321	0.065386905	21.63291139
0.292914536	0.189132653	10.83783784
0.3434	0.144336111	34.34
0.320558985	0.167714081	69.24074074
0.282966594	0.177809019	38.76642336
0.323114759	0.148235294	60.42245989
0.182445979	0.110653647	24.44776119
0.302491681	0.177947948	33.57657658
0.179584121	0.174482956	4.130434783
0.283950617	0.323302469	2.555555556
0.215657143	0.067959302	75.48

log.sigma.0.5.mm.3D_gldzm_HighIntensityLargeDistanceEmphasis	log.sigma.0.5.mm.3D_gldzm_SmallDistanceEmphasis	log.sigma.0.5.mm.3D_glcm_SumVariance	log.sigma.0.5.mm.3D_glcm_Homogeneity1
24.43617021	0.992021277	41.89067934	0.628717789
16.52173913	1	22.83862261	0.676678184
25.71428571	0.919642857	22.87358151	0.672939188
12.6875	1	22.06258177	0.651423044
16.43209877	0.972222222	23.27162198	0.673901694
14.27777778	0.916666667	23.56462554	0.613395906
17.36655949	0.959003215	25.55760405	0.710613728
51.81623932	0.926282051	74.37452311	0.663510235
15.62285714	0.982857143	23.63207418	0.694452631
30.12765957	0.920212766	44.80722212	0.633459422
18.34782609	0.934782609	26.66287077	0.724284862
38.9205298	0.975165563	77.26589752	0.679053749
24.89473684	0.960526316	44.50032306	0.646336988
14.97619048	1	20.25033138	0.613799456
24	0.946428571	21.0172028	0.646757002
16.20618557	0.984536082	24.10385953	0.694055857
21.12820513	0.980769231	49.14561882	0.700238097
17.32	0.97	22.49399463	0.702440818
26.11320755	0.985849057	48.92148195	0.710293654
46.65819861	0.979214781	118.2287384	0.676232119
8	0.9625	10.42795073	0.756219826
18.6	1	21.85007574	0.650200885
16.91304348	0.967391304	22.39469174	0.673169954
20.02419355	0.963709677	25.19583932	0.7226784
19.17142857	1	22.22329984	0.661346116
19.44915254	0.987288136	24.31706205	0.69388243
28.74107143	0.979910714	43.53078858	0.652267995
17.35632184	0.982758621	23.53167928	0.698402312
13.22222222	1	9.677531288	0.652221498
32.73913043	1	78.9409772	0.699742888
15.42068966	0.984482759	23.78497874	0.700370245
16	1	21.17879353	0.657845241
17.38383838	0.962121212	24.02268854	0.688252527
17.11666667	1	23.4300974	0.667521343
25.96680498	0.983863532	46.70842249	0.682784988
17.86666667	0.92	21.96468891	0.64572672
24	0.87628866	40.97967525	0.604817055
25.85616438	0.969178082	46.62848686	0.679937979
14.52380952	0.928571429	25.04058443	0.707765643
76.25471698	0.962264151	166.7917381	0.707523985
16.81944444	0.90625	23.27597967	0.701206706
34.45901639	0.947745902	48.43997236	0.695223081
16.51685393	0.966292135	24.13614159	0.694188955
19.21978022	0.983516484	21.12129247	0.634447328
18.85507246	0.989130435	25.17560407	0.71021843
16.24793388	0.975206612	23.342618	0.682783106
30.63106796	0.981796117	46.72954181	0.6895005
24.5	0.965909091	51.86833049	0.739185084
16.83333333	1	22.67933915	0.644309815
19.13333333	1	22.20022924	0.65028369
34.23423423	1	70.74029399	0.641912268
32.46666667	0.95	45.43190505	0.644429198
17.00896861	0.963004484	25.94256326	0.71802159
27.72857143	1	80.37931026	0.698708339
16.5	0.940789474	21.70202805	0.625150422
16.05769231	0.971153846	24.16272709	0.718194277
16.30538922	0.991017964	24.09282559	0.705174948
17.20930233	0.98255814	22.67809998	0.67093373
25.59649123	0.973684211	43.55357777	0.658721971
33.89367089	0.963924051	47.69234218	0.687780457
25.04878049	0.990853659	45.3570335	0.676320669
36.13138686	0.912408759	43.51314051	0.598665421
31.17647059	0.911764706	44.74042417	0.63649528
55.91428571	0.928571429	115.7193652	0.657340638
21.85294118	1	49.04521182	0.717000413
80.39430894	0.893292683	107.7695967	0.590615921
20.5	0.9375	20.98089372	0.645136542
35.47349823	0.9204947	43.16991659	0.636140078
35.85579937	0.974137931	79.1425247	0.697955522
15.99038462	0.978365385	25.42213999	0.701016851
17.03571429	1	22.24629416	0.682046465
28.23076923	0.942307692	44.27010238	0.656409337
19.1	0.95	21.91164823	0.677103334
22.14925373	0.98880597	21.4954031	0.63504718
21.22516556	0.89477557	23.49114558	0.679713019
15.65217391	0.983695652	25.59429313	0.709498661
42.80909091	0.945454545	76.92263782	0.675468614
18.84210526	1	21.24944491	0.628530968
28.55932203	0.949152542	46.96484462	0.658039519
19.86138614	0.962871287	23.41272651	0.69431464
24.42857143	1	42.13267703	0.633271894
23.21276596	0.984042553	47.97965222	0.687174863
27.47115385	0.978365385	43.17654875	0.657604815
15.225	0.953125	25.26826473	0.743850663
14.52525253	0.96969697	24.65178369	0.703295292
7.857142857	0.946428571	27.78022532	0.739865328
17.078125	0.9765625	23.74014597	0.697482945
25.04901961	0.992647059	48.97715692	0.723679086
32.12658228	1	75.57186468	0.669199551
37.15189873	0.952531646	76.14513064	0.676090644
19.64864865	0.878378378	24.77195459	0.666912238
17.1	0.97	24.97961326	0.720683188
19.59722222	0.947916667	23.88614662	0.675382719
19.16788321	0.945255474	24.71616216	0.694792078
19.17647059	0.947860963	24.65160618	0.692281324
36.68656716	0.882462687	42.3970097	0.618638991
16.9009009	0.966216216	23.83148669	0.680552758
26.43478261	0.934782609	40.61800086	0.591239615
8.444444444	0.916666667	10.56702196	0.750703027
35.51714286	0.944285714	78.4301612	0.671521855

log.sigma.0.5.mm.3D_glcm_Homogeneity2	log.sigma.0.5.mm.3D_glcm_ClusterShade	log.sigma.0.5.mm.3D_glcm_MaximumProbability	log.sigma.0.5.mm.3D_glcm_Idmn	log.sigma.0.5.mm.3D_glcm_SumVariance2
0.598111123	0.231993342	0.192785734	0.973594913	2.047242356
0.65709433	0.038463006	0.214532048	0.970925999	1.173732952
0.648960859	0.071020083	0.19583327	0.967360928	1.550216063
0.624626376	-0.218259658	0.194582641	0.950799546	1.552863103
0.650583755	0.152254217	0.21351616	0.968522702	1.464298223
0.580969123	-0.180834866	0.161757298	0.957454521	1.899610813
0.698092016	0.188691311	0.247948177	0.982455814	1.008284398
0.6395982	0.676851437	0.249419632	0.986321254	1.545243286
0.678134624	0.368723132	0.283301102	0.973764327	1.132078707
0.603007516	0.040578581	0.173393215	0.969186718	1.696363615
0.714370668	0.149883498	0.2654371	0.979815245	0.844739226
0.659076206	0.537867549	0.244367871	0.987703006	1.358476086
0.618908052	-0.67414394	0.218662257	0.971891453	1.744261641
0.579690306	0.009494528	0.158064389	0.956467393	1.80853016
0.619202958	0.997552747	0.251748425	0.969963762	1.691565059
0.678035142	0.254684678	0.240013542	0.980292324	1.233111804
0.686835679	0.216284361	0.240796046	0.985847077	1.060272668
0.686473908	-0.081828886	0.264798433	0.974710692	1.120254956
0.698239442	0.198216029	0.264823816	0.982552657	0.971184517
0.656425835	-0.118623093	0.201457996	0.98991254	1.3558487
0.749797932	0.060095819	0.274495737	0.965222521	0.824602288
0.624454949	0.418249781	0.203773687	0.97394301	1.523393856
0.650568218	0.276049501	0.247920356	0.968739001	1.405694338
0.712011296	0.402533843	0.320427411	0.978789344	0.900475266
0.638898361	0.189426927	0.205449175	0.966249926	1.456949241
0.677017463	1.105572393	0.267286699	0.979257413	1.342532194
0.626620414	0.597145271	0.237270696	0.978258147	1.622816674
0.682289189	0.42046104	0.296378223	0.980525356	1.103925935
0.630364519	0.370695226	0.214669744	0.956559689	1.244109718
0.68520068	0.131911216	0.274780641	0.985253931	1.096290818
0.684984444	0.268390492	0.261019195	0.981247996	1.151637946
0.631117775	0.252824703	0.217392306	0.965878268	1.54491305
0.67123545	0.386434772	0.25890978	0.979528692	1.178085968
0.646679738	0.290480103	0.199724561	0.976289987	1.413833984
0.663596006	0.481782085	0.245821931	0.982434159	1.33540236
0.61653858	0.001590542	0.178123632	0.961854083	1.703229159
0.566233682	-1.052949848	0.188654116	0.970050901	2.317871218
0.660843118	0.04804328	0.196809189	0.982086628	1.402989099
0.696469333	-0.039673835	0.257140905	0.968641173	0.896162957
0.694231412	0.527104539	0.29758302	0.994489594	1.029059893
0.68672681	0.095617762	0.262446622	0.975803553	1.083793623
0.679254657	0.675249207	0.280625887	0.984073013	1.114634591
0.676784352	0.021903102	0.231643481	0.973349906	1.267440623
0.604722265	0.725038712	0.224613225	0.969469723	1.746532075
0.697386597	0.242542717	0.284354264	0.976542355	0.934705086
0.662513342	0.56382079	0.267306218	0.977789668	1.368379394
0.671609435	0.598639898	0.277163035	0.983386564	1.234117768
0.733813307	0.155580929	0.321057279	0.982653584	0.698994843
0.619142702	0.428177024	0.196360779	0.965363925	1.522189717
0.623791845	-0.252086474	0.20732075	0.965015734	1.731767232
0.611895566	0.733618101	0.260412245	0.98048695	1.872663207
0.616126011	0.555539181	0.211611357	0.977295418	1.717765375
0.707259834	0.233457889	0.277292415	0.98392653	0.880416699
0.682637177	-0.447572215	0.242409444	0.984486313	1.125693533
0.592778759	-0.367122674	0.175441026	0.957926509	1.957843732
0.705711413	0.270437221	0.29207266	0.97781982	1.031310526
0.689969931	0.530444369	0.310419098	0.981398735	1.098412854
0.649037126	0.36726687	0.237848206	0.976465958	1.415182894
0.634390662	0.52420159	0.257036232	0.979318701	1.456555341
0.671702236	0.645743415	0.253703893	0.983228642	1.187959271
0.65316481	0.284964765	0.214614594	0.979908005	1.507496608
0.559906048	0.203818971	0.139700846	0.968330318	2.337562135
0.606947726	0.024912244	0.168825572	0.97991352	1.865152051
0.633791648	-0.483849053	0.184723551	0.986863576	1.50471392
0.704538738	-0.040004736	0.258019611	0.977403776	0.984016413
0.549814843	-0.02250344	0.13759477	0.981050116	2.471299866
0.617844214	-0.475865571	0.206974099	0.963719652	1.555843327
0.60525806	0.66316069	0.214825506	0.980123386	1.822043468
0.682080228	0.384257335	0.271167867	0.98692065	1.181096429
0.686037234	-0.127762499	0.244138679	0.975321562	1.139042874
0.659941039	-0.000678626	0.222453451	0.969525029	1.411744093
0.630570677	0.215652199	0.206254967	0.978191565	1.655866545
0.655492707	0.519042733	0.252944088	0.976222589	1.432161246
0.604078061	0.869267289	0.233027326	0.968491947	1.754959703
0.659727908	0.434232718	0.249785418	0.977081746	1.272296221
0.695603998	0.355819672	0.253476725	0.981561785	1.057067117
0.654921977	-0.395030668	0.207316727	0.984500688	1.520531503
0.597900605	-0.026013216	0.159472189	0.960039004	1.840970002
0.634366373	0.078176812	0.187028997	0.975008971	1.459488189
0.676994449	0.648537768	0.322523083	0.979080792	1.155120657
0.603908053	0.173382832	0.197545695	0.974734325	1.924341838
0.667884983	-0.105069942	0.238214907	0.978022458	1.31206216
0.632492671	0.168449938	0.228889205	0.97326877	1.540036766
0.736602013	0.208281569	0.35330866	0.981967119	0.762533536
0.688405228	0.282031017	0.264468616	0.975015758	1.106410486
0.728205172	-0.73863411	0.383621257	0.971724718	0.952249282
0.681587149	0.458919172	0.276691906	0.980539872	1.157814049
0.712785639	0.45962282	0.315042487	0.983876999	0.965670827
0.647809672	0.127426364	0.22275617	0.984751305	1.45867068
0.65338126	-0.470887181	0.214597815	0.980181655	1.513581175
0.644358807	-0.355504955	0.255699661	0.975491395	1.37480815
0.708899613	0.407563752	0.327963502	0.978263102	0.931120888
0.655851292	0.197929765	0.221838211	0.977714177	1.242057563
0.677853017	0.306701604	0.237902123	0.979373162	1.224000293
0.675154029	0.390545729	0.260623521	0.973269192	1.132753673
0.583455776	0.248192958	0.156204076	0.976261617	2.23748465
0.659847429	0.432023815	0.251986577	0.969830681	1.382641975
0.551328365	-0.509487844	0.161288837	0.966839339	2.452406012
0.745208263	-0.055127853	0.276091489	0.965661013	0.7313786
0.649732631	-0.315764945	0.212686958	0.984494044	1.491942647

log.sigma.0.5.mm.3D_glcm_Contrast	log.sigma.0.5.mm.3D_glcm_DifferenceEntropy	log.sigma.0.5.mm.3D_glcm_InverseVariance	log.sigma.0.5.mm.3D_glcm_Entropy	log.sigma.0.5.mm.3D_glcm_Dissimilarity
1.876682422	1.810942032	0.475986043	3.977956535	0.974804549
1.156112491	1.538438023	0.48321815	3.228193217	0.763463444
1.315315032	1.626819346	0.452554919	3.551902068	0.803665472
1.442704943	1.665700285	0.471642345	3.60018641	0.865392678
1.259003364	1.602952989	0.457541927	3.494261922	0.791644374
1.748260188	1.745134592	0.49785215	3.874281642	0.987686364
0.920783042	1.420926573	0.47369091	2.988987136	0.65608575
1.445979874	1.675077364	0.46830746	3.589025257	0.838355744
1.037620179	1.489180633	0.474822927	3.15567297	0.708922793
1.679825935	1.750658643	0.473619571	3.801516187	0.938289574
0.77722776	1.330708133	0.469853656	2.755918615	0.60550487
1.301130683	1.608353328	0.470691768	3.396990546	0.781273683
1.51459804	1.69366695	0.474991654	3.710452395	0.886084975
1.790810029	1.775464281	0.482958292	3.900877642	0.996961225
1.652921479	1.747704792	0.468621039	3.71973062	0.905765206
1.036321772	1.474811612	0.477835004	3.213513812	0.709090112
0.956097976	1.432276837	0.48746853	3.034774707	0.680465739
0.995347657	1.468035777	0.462374893	3.113276333	0.688205438
0.916531125	1.410346168	0.477980066	2.925606724	0.655018324
1.277252102	1.595339218	0.478665582	3.41016265	0.781926978
0.624071632	1.226847215	0.435077218	2.590378851	0.521015385
1.388239211	1.642955936	0.484041131	3.56189699	0.856398279
1.248916693	1.595266987	0.464661047	3.438978087	0.790264746
0.82628626	1.367548956	0.465821499	2.804779565	0.61744477
1.377400597	1.627375074	0.485794349	3.526511594	0.829728458
1.105222931	1.522511277	0.469607097	3.252806591	0.721175627
1.512480894	1.69019857	0.476347837	3.657432693	0.8710495
1.025442529	1.486684699	0.468629287	3.119292843	0.699826371
1.238160171	1.536092891	0.514069204	3.279125341	0.821982999
1.004536878	1.461152319	0.478541823	3.077937029	0.690254422
0.980809654	1.456458629	0.471506579	3.140464565	0.688270865
1.364243534	1.640026902	0.461688702	3.573274496	0.841995168
1.082132817	1.509825895	0.480964495	3.212794343	0.727382473
1.265497662	1.588025134	0.488909917	3.464553721	0.798711381
1.207195353	1.573913785	0.471817927	3.365037931	0.759945341
1.557627184	1.714422484	0.461649226	3.754825789	0.897359799
2.118198646	1.868986539	0.457323781	4.10708817	1.069580784
1.232839377	1.575335012	0.477406311	3.434079201	0.768962756
0.875303683	1.378199984	0.490276119	2.865580387	0.651549106
0.953139653	1.440139052	0.473291318	2.985575182	0.66759539
0.943805564	1.436602421	0.477799898	3.092590881	0.679406794
1.089991553	1.512402264	0.474062774	3.109580297	0.714706987
1.052067709	1.473446425	0.466760392	3.229505774	0.713825896
1.661109499	1.746377574	0.477461245	3.794519504	0.932967579
0.922243151	1.423552	0.472109757	2.928438368	0.657771805
1.178434105	1.567119492	0.464669236	3.36927461	0.758107046
1.135232599	1.542052533	0.469987133	3.251360654	0.735284118
0.667627298	1.218880339	0.476670282	2.433374605	0.554291105
1.391060607	1.629854646	0.500384718	3.588337129	0.865837512
1.405807978	1.649033192	0.477613164	3.64493637	0.860809629
1.70255306	1.767847744	0.458840665	3.795941344	0.925807138
1.574146912	1.725180582	0.470512929	3.756442719	0.899605338
0.834845679	1.369585531	0.474449266	2.82784498	0.626862383
1.057811097	1.495482335	0.467249453	3.123730763	0.703936022
1.733386088	1.768640321	0.474397966	3.921757871	0.965481444
0.863240721	1.395494383	0.460319852	2.968534628	0.634249615
0.97511083	1.460681105	0.463106408	3.054134345	0.678882424
1.251179349	1.594079635	0.474534451	3.462324742	0.79264466
1.432319339	1.663339447	0.475546409	3.536058175	0.845394651
1.157521203	1.531401861	0.486382071	3.203731711	0.737528504
1.397095193	1.660897539	0.449032507	3.550438605	0.8080256
2.264844349	1.926473375	0.464007325	4.251582699	1.101420734
1.756980136	1.781598562	0.471517487	3.904801701	0.942768097
1.384904363	1.639290553	0.48622296	3.562276509	0.837683395
0.888268106	1.396579976	0.462168387	2.925216882	0.639687508
2.455361765	1.975187934	0.459431087	4.348364762	1.146135638
1.470380725	1.672267186	0.48016586	3.584905514	0.880620469
1.728700531	1.780361936	0.463149454	3.862236072	0.942263205
1.130079103	1.525611431	0.468040946	3.162092423	0.71481375
0.967225546	1.452756165	0.473662223	3.141073735	0.684279712
1.22032154	1.58323371	0.451058954	3.423165578	0.769460317
1.519518596	1.705176036	0.466108171	3.71307572	0.865510835
1.277601318	1.605247861	0.463118717	3.459843869	0.7850928
1.723567904	1.772821026	0.464860067	3.774595116	0.943754442
1.224785009	1.582407881	0.471456415	3.349885943	0.770120525
0.979003165	1.452466404	0.463970857	3.032608784	0.668771609
1.360243134	1.621437124	0.472597661	3.483833153	0.794891749
1.630172133	1.723899754	0.483937316	3.814993054	0.940595027
1.33046132	1.622362487	0.48517964	3.543825517	0.830435483
1.114962969	1.533663465	0.46498035	3.1377281	0.722896743
1.785816939	1.78219523	0.478784637	3.903592865	0.951362469
1.172197833	1.559728947	0.463127508	3.337832133	0.747556042
1.44055592	1.667348268	0.471476125	3.61287487	0.851151717
0.695125726	1.274053794	0.452016268	2.586000383	0.554631414
0.98615831	1.463844489	0.468911021	3.109549134	0.683327937
0.781711819	1.343084971	0.424462882	2.5915865	0.583276684
1.024645823	1.480968952	0.472159564	3.149072727	0.700865589
0.842115762	1.378347198	0.461521283	2.852829162	0.618606165
1.313134744	1.612167728	0.479952148	3.498140907	0.803300981
1.382518526	1.65174246	0.453681737	3.534578328	0.804030193
1.313551188	1.61656112	0.475878539	3.427860231	0.809997182
0.845743626	1.384746131	0.460971292	2.837864137	0.626004905
1.182021253	1.559263225	0.48534614	3.345578934	0.769723575
1.099186341	1.523120485	0.467460821	3.248324515	0.71860535
1.058741745	1.503992349	0.472605424	3.173688317	0.717230451
2.107437991	1.890760731	0.455028689	4.156167408	1.035354412
1.212766903	1.581486757	0.464866892	3.401280636	0.767936316
2.381092129	1.942015986	0.46678818	4.22827804	1.13390309
0.608489223	1.192361611	0.45394899	2.469676904	0.526067765
1.339022219	1.629806594	0.471302571	3.533956233	0.804016207

log.sigma.0.5.mm.3D_glcm_DifferenceVariance	log.sigma.0.5.mm.3D_glcm_Idn	log.sigma.0.5.mm.3D_glcm_Idm	log.sigma.0.5.mm.3D_glcm_Correlation	log.sigma.0.5.mm.3D_glcm_Autocorrelation
0.9227929	0.900775785	0.598111123	0.043054213	19.60374454
0.56966819	0.897595743	0.65709433	0.00601566	11.52644867
0.663012963	0.893814577	0.648960859	0.082608282	12.16296899
0.691285839	0.868630807	0.624626376	0.036884732	11.80503689
0.630547304	0.894853322	0.650583755	0.07531926	12.19327958
0.767763463	0.871509792	0.580969123	0.040760558	12.73972788
0.488880189	0.921474652	0.698092016	0.045815882	12.22100061
0.740695383	0.928101291	0.6395982	0.032984921	29.5351885
0.532216646	0.904305804	0.678134624	0.043083946	11.76757815
0.793801262	0.892259305	0.603007516	0.005715199	20.15333608
0.409026907	0.916488736	0.714370668	0.04085541	12.20514061
0.689522999	0.932641395	0.659076206	0.021314682	29.93708833
0.724172634	0.897432878	0.618908052	0.070246776	20.1006069
0.793572561	0.870758175	0.579690306	0.004028736	11.39860364
0.828545497	0.896255765	0.619202958	0.011734175	11.50337046
0.525466696	0.915707014	0.678035142	0.084951979	12.15056316
0.491941887	0.927312071	0.686835679	0.051270221	20.26869017
0.519293839	0.906943701	0.686473908	0.058545731	11.32971757
0.485784882	0.92155153	0.698239442	0.029148016	19.92717482
0.663509339	0.937819122	0.656425835	0.029877152	42.61102991
0.350464356	0.899180871	0.749797932	0.138066829	6.231205126
0.654156059	0.899981056	0.624454949	0.046274464	11.72128116
0.622887162	0.894902094	0.650568218	0.059136518	11.76653221
0.444519763	0.915355513	0.712011296	0.042921655	11.78064596
0.680479799	0.890493988	0.638898361	0.027787992	11.75249998
0.582297349	0.91484771	0.677017463	0.095046742	12.26390737
0.74749031	0.909836042	0.626620414	0.034513713	19.56496121
0.534089258	0.916835863	0.682289189	0.036888263	11.6587935
0.556287911	0.872460768	0.630364519	0.003116828	6.388859935
0.526800113	0.926589141	0.68520068	0.042713322	29.77008697
0.502461608	0.917883887	0.684984444	0.079039838	11.89062946
0.651845284	0.888646739	0.631117775	0.062028522	11.49454148
0.551702396	0.913749718	0.67123545	0.042540439	12.0051363
0.625536338	0.906262707	0.646679738	0.051688149	12.17840276
0.628800493	0.920220022	0.663596006	0.050259544	20.12729213
0.74383862	0.882823383	0.61653858	0.044882305	11.99681725
0.947714132	0.891872062	0.566233682	0.045838494	19.51536462
0.638435109	0.919378357	0.660843118	0.062064629	20.28772486
0.448776015	0.896462038	0.696469333	0.01209421	11.77951961
0.50682088	0.95362448	0.694231412	0.038299859	55.55556401
0.480918762	0.907512664	0.68672681	0.068767777	11.61003754
0.578271386	0.924496539	0.679254657	0.011570717	20.07638592
0.520428837	0.90380959	0.676784352	0.092723853	12.16460412
0.789151876	0.892769032	0.604722265	0.024897117	11.68324301
0.488687537	0.910512544	0.697386597	0.006138074	11.89626016
0.60194572	0.910788049	0.662513342	0.074468737	12.04610342
0.593503748	0.922499361	0.671609435	0.041488307	19.87140985
0.358740864	0.922703924	0.733813307	0.023015757	19.77262959
0.638506085	0.88518148	0.619142702	0.045500713	12.0267004
0.66201273	0.886266009	0.623791845	0.103771228	12.10147928
0.842625617	0.913979709	0.611895566	0.046313486	28.95438225
0.76303956	0.907065363	0.616126011	0.043924867	20.42428877
0.441477774	0.924421058	0.707259834	0.026596595	12.05210032
0.557794595	0.92549822	0.682637177	0.029693197	30.23970832
0.797726192	0.874786634	0.592778759	0.060861034	12.14493946
0.459647514	0.913344389	0.705711413	0.087616554	11.77880958
0.511410136	0.919089918	0.689969931	0.058167326	11.8253618
0.621093221	0.906969197	0.649037126	0.061063477	11.91497467
0.715860977	0.912155168	0.634390662	0.007380484	19.24520956
0.613064387	0.922359705	0.671702236	0.012991107	20.02180938
0.741335619	0.916294388	0.65316481	0.038001606	20.00755388
1.048142135	0.889339818	0.559906048	0.015853312	20.55765442
0.863972339	0.912565518	0.606947726	0.030092111	20.42743871
0.681829996	0.927748874	0.633791648	0.04149266	42.32305096
0.473438852	0.912819376	0.704538738	0.050141004	19.97545804
1.137706061	0.912186541	0.549814843	0.003456816	41.93954436
0.691915034	0.884087128	0.617844214	0.028694586	11.3868006
0.838493221	0.912451941	0.60525806	0.026563298	19.79458558
0.618432546	0.932004774	0.682080228	0.021891349	29.95310945
0.497778991	0.907103876	0.686037234	0.081768301	12.49398282
0.622077853	0.897715771	0.659941039	0.072677794	11.69897256
0.768098352	0.910572047	0.630570677	0.043377122	19.95579462
0.657657476	0.908255622	0.655492707	0.057494285	11.60389197
0.829382711	0.892038853	0.604078061	0.008206426	11.76469215
0.629306367	0.909664931	0.659727908	0.019053384	11.94742652
0.529217802	0.920422259	0.695603998	0.039133796	12.28822904
0.726392179	0.925178752	0.654921977	0.053184517	30.20611852
0.741771752	0.877150822	0.597900605	0.06014121	11.83164102
0.639597213	0.902793281	0.634366373	0.046484474	20.5356887
0.591787684	0.914743187	0.676994449	0.017794436	11.62906149
0.877043236	0.902749261	0.603908053	0.037185406	19.53446438
0.610529679	0.912104556	0.667884983	0.056230526	20.51279146
0.709864436	0.901267293	0.632492671	0.033717617	19.36193874
0.386693595	0.923164213	0.736602013	0.046279039	11.47856147
0.517053192	0.907519609	0.688405228	0.05686174	12.10786019
0.433165954	0.907399805	0.728205172	0.101502985	12.46150441
0.529678048	0.916677766	0.681587149	0.060658682	11.84091582
0.459151621	0.925629055	0.712785639	0.068019767	19.85711176
0.665505755	0.924020365	0.647809672	0.051707026	29.77367337
0.733751653	0.916591194	0.65338126	0.045180115	30.036078
0.655237707	0.905316094	0.644358807	0.022870473	12.56545793
0.452482991	0.914352332	0.708899613	0.047477924	11.77554426
0.588957654	0.909214933	0.655851292	0.024772291	12.0996994
0.58155826	0.915123722	0.677853017	0.053793056	12.35013212
0.542904092	0.903336791	0.675154029	0.033851369	12.13403537
1.030633076	0.905332093	0.583455776	0.02991768	20.06157508
0.62028272	0.897756627	0.659847429	0.065881139	12.26998944
1.090741771	0.886521839	0.551328365	0.01357755	19.4864248
0.329334263	0.897533829	0.745208263	0.091355156	6.066681171
0.690086794	0.924146286	0.649732631	0.054334775	30.78716208

log.sigma.0.5.mm.3D_glcm_SumEntropy	log.sigma.0.5.mm.3D_glcm_AverageIntensity	log.sigma.0.5.mm.3D_glcm_Energy	log.sigma.0.5.mm.3D_glcm_SumSquares	log.sigma.0.5.mm.3D_glcm_ClusterProminence
2.533656869	4.422784629	0.099289272	0.980981194	16.17507087
2.134698005	3.394310694	0.142902398	0.582461361	4.274702776
2.340771868	3.4790843	0.116782749	0.716382774	6.699426748
2.335084232	3.431741932	0.110874355	0.748892012	7.011268321
2.299453564	3.484517922	0.121631914	0.680825397	6.06860417
2.474150079	3.563803767	0.092257095	0.91196775	10.9166193
2.030855723	3.492722318	0.174949533	0.48226686	3.799829608
2.330828998	5.432342935	0.128659697	0.74780579	9.239766134
2.110780385	3.426939795	0.16252836	0.542424721	4.375631912
2.412099312	4.48876302	0.100252088	0.844047387	8.786798525
1.901444513	3.491162394	0.191771897	0.405491746	2.094502375
2.22793815	5.47016627	0.145232068	0.664901692	8.208398272
2.415334472	4.47693278	0.108275252	0.81471492	8.929174721
2.456790715	3.375459212	0.08908964	0.899835047	9.378736558
2.384610861	3.390222313	0.120563448	0.836121634	10.90511007
2.176376159	3.478698731	0.148301088	0.567358394	4.705429342
2.064074212	4.499167062	0.166076518	0.504092661	3.982349357
2.099746585	3.361272349	0.159521687	0.528900653	3.921085286
2.000393809	4.462445146	0.181557871	0.47192891	3.498250119
2.241746068	6.526204177	0.139219293	0.6582752	7.922543864
1.873518	2.486157475	0.208946712	0.36216848	1.794779722
2.328968225	3.418621822	0.11117986	0.727908267	7.052371377
2.267739582	3.424472185	0.128082953	0.663652758	5.709316039
1.930304066	3.42958777	0.199881616	0.431690382	3.036377116
2.294553373	3.425264487	0.122130692	0.708587459	7.340291301
2.194071081	3.49349613	0.156876226	0.611938781	7.947023955
2.366951904	4.42009817	0.121233321	0.783824392	9.734624863
2.087525144	3.411615591	0.168312604	0.532342116	4.575328127
2.151806183	2.527222496	0.136750895	0.620567472	4.923582823
2.085338605	5.454090297	0.166730407	0.525206924	4.521278849
2.127434536	3.442074799	0.156721941	0.5331119	4.128258723
2.336459857	3.383645557	0.111350447	0.727289146	6.549451624
2.143283982	3.461369766	0.155346367	0.565054696	4.930132369
2.277703926	3.484427859	0.127360202	0.669832911	6.742075921
2.229665028	4.482768458	0.143706554	0.635649428	6.822584399
2.415773579	3.458344171	0.10125091	0.815214086	8.478261891
2.606013832	4.411793948	0.08525701	1.109017466	16.19959054
2.274549652	4.499461667	0.132913849	0.658957119	6.900082664
1.949294735	3.431348922	0.183886207	0.44286666	2.632708555
2.029737133	7.452286593	0.181234363	0.495549887	4.143901749
2.09368116	3.402197764	0.159939419	0.506899797	3.458891624
2.080701789	4.479979589	0.169942746	0.551156536	5.158823198
2.179226531	3.479978367	0.148932532	0.579877083	4.783358202
2.428326774	3.414932418	0.107277355	0.851910394	10.06294094
1.974072399	3.448633982	0.182924572	0.464237059	2.987871806
2.240194766	3.463883864	0.143112717	0.636703375	6.362076127
2.165032772	4.454956986	0.156483128	0.592337592	5.685747685
1.73830094	4.445736985	0.22756881	0.341655535	1.585427775
2.327101876	3.463180446	0.112483582	0.728312581	6.906704674
2.410001037	3.466860826	0.106528404	0.784393803	8.18702516
2.455251903	5.376900301	0.116357777	0.893804067	12.67509503
2.41910199	4.51534437	0.108004171	0.822978072	9.681748084
1.933816485	3.469970531	0.189115063	0.428815595	2.642878059
2.092925056	5.49751467	0.162419378	0.545876157	5.201983691
2.510609977	3.476818647	0.090310054	0.922807455	10.99471797
2.042541914	3.425885528	0.176563955	0.473637812	3.305428854
2.073826327	3.434313438	0.175606789	0.518380921	4.164872856
2.280695217	3.445848769	0.128284252	0.666590561	6.182074101
2.284355914	4.386206494	0.130612114	0.72221867	7.669407549
2.128093704	4.473721151	0.162150296	0.586370119	6.436701936
2.317943159	4.469878494	0.128032029	0.72614795	8.070105056
2.647572384	4.53204223	0.076704863	1.150601621	18.21634965
2.485710333	4.516673597	0.099306347	0.905533047	12.06401557
2.319609825	6.503306219	0.120261352	0.722404571	9.227867292
2.00097102	4.466692256	0.178341035	0.46807113	3.282698834
2.690194826	6.475763198	0.072054059	1.231665408	20.51604014
2.335278605	3.371223847	0.113607286	0.756556013	6.807816305
2.462799179	4.446483156	0.103205933	0.887686	11.09713196
2.114063619	5.471776049	0.167742576	0.577793883	6.94032872
2.129453033	3.528593232	0.156134673	0.526567105	3.848677276
2.26252379	3.413280763	0.128488578	0.658016408	5.586721003
2.398891395	4.463371383	0.112731966	0.793846285	9.463178423
2.276270935	3.400760135	0.134666612	0.677440641	7.322512467
2.415093784	3.428754657	0.108513284	0.869631902	9.895616018
2.196179981	3.454783559	0.145308275	0.624270308	5.953922037
2.051957033	3.502656278	0.170758433	0.50901757	4.120910847
2.301683186	5.49234947	0.134399855	0.720193659	12.11989628
2.459129518	3.431986165	0.090906773	0.867785534	8.94694767
2.310474146	4.528058482	0.117996143	0.697487377	6.488073804
2.099664799	3.408663971	0.177336231	0.567520906	5.407001718
2.490976106	4.415854078	0.103553903	0.927539694	14.2762127
2.219319306	4.525226966	0.142231568	0.621064998	5.76752842
2.342354532	4.397368963	0.118149041	0.745148172	7.746149297
1.820838037	3.385508902	0.223808343	0.364414815	1.853489427
2.098516308	3.475303769	0.16276886	0.523142199	3.96999261
1.868598899	3.523994937	0.227663391	0.433490275	2.899653718
2.120725598	3.436204667	0.161585058	0.545614968	4.504464566
1.976378442	4.452661537	0.194867746	0.451946647	3.47165448
2.297700369	5.453179285	0.129929449	0.692951356	7.930194193
2.316263558	5.477517284	0.126803705	0.724024925	8.300499586
2.248470916	3.542596809	0.138322365	0.672089834	6.483299378
1.953072068	3.428429845	0.198325824	0.444216128	3.075514505
2.194091662	3.476301011	0.139870536	0.606019704	5.121649856
2.173037503	3.509829466	0.151461689	0.580796658	5.177716753
2.111986656	3.480731914	0.157335781	0.547873854	4.347882316
2.613809865	4.475364972	0.083157283	1.08623066	16.67336892
2.2557409	3.496779802	0.138565665	0.648852219	6.193225338
2.647015557	4.412259155	0.076769321	1.208374535	19.49524511
1.777492307	2.456723456	0.217088716	0.334966956	1.353532828
2.319030395	5.545170116	0.127178326	0.707741216	8.35548644

log.sigma.0.5.mm.3D_glcm_SumAverage	log.sigma.0.5.mm.3D_glcm_Imc2	log.sigma.0.5.mm.3D_glcm_Imc1	log.sigma.0.5.mm.3D_glcm_DifferenceAverage	log.sigma.0.5.mm.3D_glcm_Id
8.845569257	0.299885225	-0.02506125	0.974804549	0.628717789
6.788621387	0.309121864	-0.03200109	0.763463444	0.676678184
6.9581686	0.295220074	-0.029621556	0.803665472	0.672939188
6.863483865	0.281426207	-0.024040261	0.865392678	0.651423044
6.969035844	0.273786088	-0.023346212	0.791644374	0.673901694
7.127607534	0.348538	-0.033711041	0.987686364	0.613395906
6.985444636	0.205651811	-0.015064892	0.65608575	0.710613728
10.86468587	0.229702019	-0.016085832	0.838355744	0.663510235
6.85387959	0.201396527	-0.014734394	0.708922793	0.694452631
8.97752604	0.274986529	-0.021815813	0.938289574	0.633459422
6.982324788	0.187404545	-0.014084918	0.60550487	0.724284862
10.94033254	0.251291341	-0.020100347	0.781273683	0.679053749
8.953865559	0.27679284	-0.023403562	0.886084975	0.646336988
6.750918423	0.25920052	-0.018058813	0.996961225	0.613799456
6.780444626	0.265777092	-0.02115524	0.905765206	0.646757002
6.957397462	0.292591919	-0.031222879	0.709090112	0.694055857
8.998334123	0.242187753	-0.021046661	0.680465739	0.700238097
6.722544698	0.281650511	-0.028753359	0.688205438	0.702440818
8.924890291	0.21508988	-0.017198754	0.655018324	0.710293654
13.05240835	0.211207998	-0.014387124	0.781926978	0.676232119
4.97231495	0.225511472	-0.024634424	0.521015385	0.756219826
6.837243643	0.241551041	-0.017452377	0.856398279	0.650200885
6.84894437	0.276246615	-0.023092168	0.790264746	0.673169954
6.859175541	0.187066197	-0.013316481	0.61744477	0.7226784
6.850528975	0.322055761	-0.035049304	0.829728458	0.661346116
6.98699226	0.25557794	-0.023100989	0.721175627	0.69388243
8.84019634	0.267958764	-0.021772965	0.8710495	0.652267995
6.823231183	0.204008832	-0.014875858	0.699826371	0.698402312
5.054444993	0.377487495	-0.047742087	0.821982999	0.652221498
10.90818059	0.221403461	-0.017124384	0.690254422	0.699742888
6.884149598	0.242815395	-0.021249315	0.688270865	0.700370245
6.767291113	0.255657923	-0.020803641	0.841995168	0.657845241
6.922739533	0.18795159	-0.011917991	0.727382473	0.688252527
6.968855718	0.260735249	-0.021486523	0.798711381	0.667521343
8.965536917	0.214930899	-0.014738516	0.759945341	0.682784988
6.916688341	0.263614882	-0.021098072	0.897359799	0.64572672
8.823587897	0.335865588	-0.031941231	1.069580784	0.604817055
8.998923333	0.300204806	-0.028724678	0.768962756	0.679937979
6.862697845	0.216558806	-0.01683417	0.651549106	0.707765643
14.90457319	0.178729184	-0.011604525	0.66759539	0.707523985
6.804395527	0.18426557	-0.012301902	0.679406794	0.701206706
8.959959179	0.203828289	-0.014056114	0.714706987	0.695223081
6.959956734	0.346976694	-0.046735198	0.713825896	0.694188955
6.829864836	0.217473221	-0.013659714	0.932967579	0.634447328
6.897267965	0.19866032	-0.014963	0.657771805	0.71021843
6.927767729	0.23311489	-0.017457706	0.758107046	0.682783106
8.909913971	0.204104641	-0.013572136	0.735284118	0.6895005
8.891473969	0.226305488	-0.023129648	0.554291105	0.739185084
6.926360893	0.259210288	-0.019965207	0.865837512	0.644309815
6.933721651	0.3094597	-0.028237873	0.860809629	0.65028369
10.7538006	0.292452632	-0.024403174	0.925807138	0.641912268
9.03068874	0.209478608	-0.012664084	0.899605338	0.644429198
6.939941063	0.170323323	-0.010652573	0.626862383	0.71802159
10.99502934	0.263977074	-0.024687095	0.703936022	0.698708339
6.953637295	0.274808923	-0.0203876	0.965481444	0.625150422
6.851771055	0.243077744	-0.021441011	0.634249615	0.718194277
6.868626876	0.213770004	-0.016400414	0.678882424	0.705174948
6.891697538	0.22072495	-0.015817857	0.79264466	0.67093373
8.772412987	0.275867709	-0.023322271	0.845394651	0.658721971
8.947442303	0.2251395	-0.016505553	0.737528504	0.687780457
8.939756987	0.299590313	-0.027852597	0.8080256	0.676320669
9.064084461	0.239230131	-0.014545067	1.101420734	0.598665421
9.033347194	0.248628007	-0.017179236	0.942768097	0.63649528
13.00661244	0.214507844	-0.013513046	0.837683395	0.657340638
8.933384513	0.270906653	-0.029261835	0.639687508	0.717000413
12.9515264	0.236376876	-0.014021817	1.146135638	0.590615921
6.742447694	0.263408884	-0.020468417	0.880620469	0.645136542
8.892966313	0.191825339	-0.011065762	0.942263205	0.636140078
10.9435521	0.23367728	-0.018446469	0.71481375	0.697955522
7.057186465	0.189463278	-0.012766731	0.684279712	0.701016851
6.826561526	0.336298485	-0.036732384	0.769460317	0.682046465
8.926742767	0.24206656	-0.017374124	0.865510835	0.656409337
6.801520269	0.273455202	-0.024232129	0.7850928	0.677103334
6.857509313	0.271237459	-0.021153356	0.943754442	0.63504718
6.909567118	0.216077815	-0.015547732	0.770120525	0.679713019
7.005312557	0.268456816	-0.025442969	0.668771609	0.709498661
10.98469894	0.285785183	-0.025590924	0.794891749	0.675468614
6.86397233	0.316441119	-0.028313977	0.940595027	0.628530968
9.056116965	0.209260789	-0.013135596	0.830435483	0.658039519
6.817327942	0.200841213	-0.014011028	0.722896743	0.69431464
8.831708156	0.292624737	-0.024205732	0.951362469	0.633271894
9.050453931	0.264823158	-0.02278165	0.747556042	0.687174863
8.794737926	0.261219964	-0.022505241	0.851151717	0.657604815
6.771017805	0.198785009	-0.016652324	0.554631414	0.743850663
6.950607539	0.216527953	-0.017497799	0.683327937	0.703295292
7.047989873	0.234583258	-0.033168985	0.583276684	0.739865328
6.872409333	0.24061475	-0.020527266	0.700865589	0.697482945
8.905323073	0.211855971	-0.016449552	0.618606165	0.723679086
10.90635857	0.265793466	-0.022174308	0.803300981	0.669199551
10.95503457	0.298164655	-0.02743189	0.804030193	0.676090644
7.085193619	0.237105011	-0.017736309	0.809997182	0.666912238
6.856859689	0.172569891	-0.011568852	0.626004905	0.720683188
6.952602023	0.157575136	-0.007827511	0.769723575	0.675382719
7.019658932	0.213644358	-0.015171396	0.71860535	0.694792078
6.961463829	0.181041459	-0.011230322	0.717230451	0.692281324
8.950729944	0.284035645	-0.021380797	1.035354412	0.618638991
6.993559603	0.215418524	-0.015469462	0.767936316	0.680552758
8.82451831	0.413315242	-0.043995669	1.13390309	0.591239615
4.913446912	0.24132475	-0.025950413	0.526067765	0.750703027
11.09034023	0.229311668	-0.016530412	0.804016207	0.671521855

log.sigma.0.5.mm.3D_glcm_ClusterTendency	log.sigma.0.5.mm.3D_firstorder_InterquartileRange	log.sigma.0.5.mm.3D_firstorder_Skewness	log.sigma.0.5.mm.3D_firstorder_Uniformity
2.047242356	22.60274887	0.046250022	0.298895612
1.173732952	25.4666872	-0.119778135	0.376890432
1.550216063	24.64581823	-0.023528184	0.338947093
1.552863103	28.51613879	-0.499567755	0.339126577
1.464298223	27.04688883	-0.126751989	0.341069786
1.899610813	26.51212692	-0.200070648	0.307048699
1.008284398	10.12256694	0.244874633	0.414127876
1.545243286	19.0354495	0.304552329	0.348988382
1.132078707	14.97043705	0.245819292	0.392818929
1.696363615	26.89122343	-0.170767963	0.314675672
0.844739226	14.80272341	0.15826089	0.441566273
1.358476086	15.05080128	0.266812542	0.366858526
1.744261641	23.80792999	-0.29922929	0.328788346
1.80853016	29.28995347	-0.120479265	0.298271672
1.691565059	22.11430311	0.412111267	0.322964172
1.233111804	11.35415554	0.179302494	0.390004363
1.060272668	17.96272731	0.13192218	0.397503364
1.120254956	23.43714619	-0.338315171	0.395807055
0.971184517	16.14848077	0.188283034	0.416439679
1.3558487	18.84676218	-0.409713041	0.368407709
0.824602288	17.53788185	0.10741439	0.447351186
1.523393856	27.46664	0.144643433	0.322913957
1.405694338	22.30605483	0.058518971	0.34732339
0.900475266	9.703754187	0.556130784	0.440995257
1.456949241	10.57094431	0.044812446	0.337397479
1.342532194	16.52397072	0.810078773	0.378091012
1.622816674	23.38881135	0.280380244	0.330050478
1.103925935	15.6657033	0.337542145	0.398538891
1.244109718	26.37461376	0.282420053	0.370519797
1.096290818	18.66529655	-0.242788378	0.395710814
1.151637946	18.70558834	0.175338679	0.387979068
1.54491305	29.779037	-0.06516655	0.32467018
1.178085968	16.96984172	0.406002348	0.388189959
1.413833984	12.53934455	0.383187118	0.362701199
1.33540236	18.65825176	0.361168014	0.364512766
1.703229159	26.16900015	-0.09331563	0.320902269
2.317871218	30.40431929	-0.517911043	0.297540312
1.402989099	8.244603157	0.092598999	0.365562744
0.896162957	20.23184061	-0.139072203	0.422984694
1.029059893	10.7734133	0.626935262	0.420305373
1.083793623	21.07422996	0.022924474	0.393780352
1.114634591	12.31627965	0.921821538	0.408724109
1.267440623	24.63151097	-0.000846924	0.37320626
1.746532075	26.66054368	0.198950652	0.309976195
0.934705086	12.39367175	0.094910396	0.414938277
1.368379394	22.09700108	0.331910145	0.366410046
1.234117768	14.36099458	0.551336928	0.387335422
0.698994843	17.4060111	-0.685531509	0.453279188
1.522189717	24.82977009	0.124833123	0.323918603
1.731767232	29.00382876	-0.17450458	0.328667084
1.872663207	27.48916864	0.059163272	0.306852519
1.717765375	26.58724213	0.291272875	0.324615003
0.880416699	8.561159849	0.431428382	0.431710377
1.125693533	16.17113805	-0.816408204	0.39268457
1.957843732	33.29306889	-0.219052248	0.307537326
1.031310526	21.35649633	0.150684814	0.408066373
1.098412854	15.65646577	0.434904684	0.404512034
1.415182894	24.46952009	0.140441204	0.352145787
1.456555341	25.04026508	0.169468024	0.341651651
1.187959271	11.65755892	0.744454751	0.397367105
1.507496608	23.46340275	0.161924647	0.348735535
2.337562135	29.31831646	-0.037257232	0.2806323
1.865152051	23.354285	-0.02433285	0.311579463
1.50471392	25.62553072	-0.299148372	0.3472463
0.984016413	16.03820705	-0.662301939	0.406710027
2.471299866	32.16341114	0.00957618	0.269114427
1.555843327	26.27192521	-0.320826928	0.348807299
1.822043468	26.11984825	0.309479295	0.317869493
1.181096429	16.4161787	0.191070334	0.399978258
1.139042874	21.55567575	-0.1444271	0.396290178
1.411744093	24.83942151	-0.16698252	0.35196477
1.655866545	18.83152008	0.117648603	0.340322295
1.432161246	24.86629963	0.286343925	0.353043527
1.754959703	27.80186057	0.536308966	0.317697507
1.272296221	12.57236934	0.392086788	0.380872874
1.057067117	19.38597178	0.360701905	0.406740522
1.520531503	22.5872488	-0.495324038	0.359645026
1.840970002	30.85902309	-0.189271667	0.301613166
1.459488189	25.08590174	-0.032042519	0.348908326
1.155120657	15.11324751	0.641823327	0.407566667
1.924341838	22.03346992	0.040345687	0.305861488
1.31206216	23.11782932	-0.184518508	0.364998915
1.540036766	19.05096722	-0.081461989	0.337667575
0.762533536	15.8531599	0.28850353	0.45389986
1.106410486	13.96066451	0.192759051	0.393354293
0.952249282	13.9900279	-1.141115549	0.483176751
1.157814049	21.01244307	0.472390989	0.388316079
0.965670827	10.35417664	0.520270288	0.428566671
1.45867068	19.08290005	-0.206866606	0.352992551
1.513581175	24.78730011	-0.392113531	0.353495418
1.37480815	19.50484157	-0.219922429	0.384204599
0.931120888	12.44693112	0.499582435	0.429142677
1.242057563	20.17763424	0.13901194	0.371591719
1.224000293	18.78253818	0.323318526	0.389721157
1.132753673	21.37642145	0.347599382	0.382142451
2.23748465	29.42389441	0.102352808	0.286610471
1.382641975	22.77118611	0.263758515	0.367034668
2.452406012	32.81038952	-0.273518059	0.282038196
0.7313786	18.29784346	-0.112959676	0.459512141
1.491942647	19.98754191	-0.368983453	0.355764234

log.sigma.0.5.mm.3D_firstorder_MeanAbsoluteDeviation	log.sigma.0.5.mm.3D_firstorder_Energy	log.sigma.0.5.mm.3D_firstorder_RobustMeanAbsoluteDeviation	log.sigma.0.5.mm.3D_firstorder_Median
17.1615499	805914.997	10.47141632	-1.416698158
14.79849186	119434.8887	10.36756607	0.802561939
15.06612629	326490.0053	10.35224842	0.422078073
16.63666014	139234.5209	11.58415622	2.312417507
15.55984153	523965.758	11.10755455	0.27688691
16.97553109	119806.4149	11.27507391	3.289371133
9.608110638	2219081.155	5.11597533	-0.247105256
13.95143004	2338202.483	8.356972526	-1.042491794
11.42460598	1074811.717	6.870696224	-0.992257148
16.75674904	345792.1004	11.53701914	1.077572465
9.793975784	266466.7877	6.278880363	-0.307809293
12.56720512	1183199.922	6.939897703	-0.507450938
15.4597435	332984.6159	10.20815712	1.501202345
18.14936473	340915.157	12.46112323	-1.030069679
15.73663797	748120.7022	9.535000998	-1.722862244
10.50541949	1329959.327	5.856688264	-0.47202456
11.89775367	223949.504	7.661929722	0.090966076
13.14554929	113803.4291	9.267039521	-0.660416842
11.08284739	254557.6467	6.924036208	-0.671892107
13.24714392	3170516.038	8.135308105	0.35411492
10.30435178	353576.3149	7.116140705	0.238323405
15.90048387	192787.7137	11.36483859	-0.386502624
14.33606275	194782.5255	9.422517852	-1.124367356
8.598094964	601662.2014	4.547650477	-0.890116096
12.63016508	442398.0467	6.619802785	-0.528590947
12.82943571	839456.5212	7.550018479	-0.728114009
15.76570249	894428.7333	9.970797742	-1.840326548
11.57840513	587103.5951	6.944106502	-1.405902624
14.84543403	48335.81606	10.80865449	0.370674983
12.4706918	460102.5409	8.028644606	-0.610797018
12.0484478	1033428.552	7.776588466	-0.792035997
16.42732708	318346.5559	11.999177	-0.974738359
12.18469857	674150.9469	7.371882924	-1.048910618
11.66467825	715236.9885	6.268162894	-0.025762511
13.34669579	1748345.725	8.190812635	-0.618191242
15.72965006	802300.6436	10.59549861	0.163695499
19.0301333	906125.7715	12.76294336	2.571509838
10.6577696	1613222.68	5.183405289	0.080569141
12.02244596	133069.7255	8.291504882	-0.613125741
9.855953498	2072728.058	5.273638263	-0.948575497
12.87179503	363556.4751	8.842221907	-1.713215113
10.67077039	1419560.517	5.815748406	-0.94575277
14.04921363	737226.6977	9.99622661	0.625036687
16.80764983	686686.6446	11.10890346	-1.64863193
10.07000215	331955.8778	5.685694254	-0.665468395
13.8378671	792446.9666	9.116749421	-1.082214236
11.59296929	1509026.682	6.578479402	-0.750914216
10.5255292	96601.03758	7.122148322	-0.616002738
15.92029712	161039.3855	10.88737412	0.403192043
16.25509705	186756.6299	11.51194199	1.80699414
17.32951226	688656.5493	11.24042821	-1.260266781
16.35464963	1233052.019	11.04105389	-0.994434595
8.334620931	1307607.368	4.212122306	-0.533591419
11.73852621	451782.3698	7.111202422	0.819048971
18.42171466	327958.7	13.23021144	2.335279465
12.68262681	301875.4367	8.7920527	-1.634017467
11.54257299	1014046.105	7.027878332	-1.505457878
14.67094032	736070.5176	9.999171826	-1.078948975
15.78091699	327894.2619	10.400358	-2.540031433
10.63613822	2858294.625	5.553964179	-0.746865153
14.93413044	533592.6603	9.677839254	-0.305766761
18.81392147	1513828.583	12.36973975	0.304243743
16.02752577	3916287.936	10.01924666	0.245992623
15.30501304	800874.1978	10.59639315	0.94227019
11.0076849	156467.8383	6.848694714	-0.000703282
20.04110832	2311930.291	13.42680656	0.370992988
15.765826	157877.8875	11.12929629	1.859569788
16.49994566	2436555.373	10.69001587	-1.474339008
11.87377572	2183759.423	7.00184693	-1.260330558
12.92624014	578003.9401	8.894655415	1.383925617
14.91795963	178717.4885	10.15156248	-0.03067033
13.83317232	1869916.759	8.318370043	-0.274886459
14.80005931	495084.793	10.00260772	-1.164684296
17.16259017	471731.7419	11.47524891	-2.670646667
11.42734369	1185257.02	6.157827454	-0.61230123
11.93036438	227369.9001	7.854635009	0.176034823
14.78406782	895363.3196	9.54319074	-0.192441523
17.68629411	154294.6905	12.62874618	0.71231389
14.99677349	475218.2013	10.35749874	0.612408519
11.9664422	528548.7705	6.939361686	-2.110541701
16.69232669	743039.3519	10.16819165	-1.506004274
14.61894143	298469.5432	9.73178184	1.858498871
13.68608759	838498.1028	8.302298373	-0.463598877
10.06695257	335798.8346	6.606092292	-1.872435093
11.10793769	674432.3772	6.453337985	-0.420089036
9.959861313	92235.97952	6.318420846	3.02596736
13.13993139	384299.1986	8.743497533	-1.970540106
9.500209189	774311.5851	5.058272119	-0.610064834
13.71913604	621739.2947	8.294899012	-0.640839159
15.14588043	536259.4405	10.17855156	0.928395689
13.16570358	253406.8767	8.32409749	2.290732741
9.918612528	519040.1776	5.61954728	-1.150121152
13.11449128	1363058.021	8.470751139	-0.618353605
12.31736658	952582.0045	7.845035728	-0.090843022
13.28433209	981425.9475	8.909730992	-1.054808259
18.59931554	1432155.997	12.23459954	0.345354021
14.17472967	837785.7741	9.440893526	-0.973167628
20.01601241	205383.7812	13.54278832	0.886279941
10.35674143	43535.34421	7.387846632	1.505455315
13.85024631	3215716.033	8.639215505	0.920158148

log.sigma.0.5.mm.3D_firstorder_TotalEnergy	log.sigma.0.5.mm.3D_firstorder_Maximum	log.sigma.0.5.mm.3D_firstorder_RootMeanSquared	log.sigma.0.5.mm.3D_firstorder_90Percentile	log.sigma.0.5.mm.3D_firstorder_Minimum
21759704.92	94.70183563	23.62441277	30.68381062	-94.50418854
3224741.995	59.91615295	18.5524328	20.89319229	-57.29773331
8815230.142	54.10358047	19.13165401	24.79041767	-63.21552658
3759332.065	46.63504028	20.79435848	23.95570526	-71.95840454
14147075.47	52.74554825	19.2702765	25.20632744	-63.36029434
3234773.203	72.53591156	22.25011978	30.05953751	-64.24664307
59915191.19	86.0532074	14.51065078	17.85761223	-71.9930954
63131467.04	101.7739868	19.62990838	24.8341383	-102.2825012
29019916.37	69.6341629	15.88032819	21.52446194	-67.47471619
9336386.71	69.33596039	21.40099956	28.70111275	-92.271492
7194603.268	52.26457977	13.08629623	18.10408497	-53.46176529
31946397.89	120.3382797	18.63558367	22.99510689	-115.4561234
8990584.63	69.39421844	20.26290263	24.3621273	-76.78535461
9204709.239	60.59555817	23.00807167	25.48995762	-73.35352325
20199258.96	82.18744659	21.79443212	27.0610878	-66.45133972
35908901.84	83.05810547	15.19261572	20.79681187	-59.38868713
6046636.608	83.71735382	15.92555956	20.90290833	-76.62635803
3072692.586	52.57818985	16.68076716	18.22848625	-59.69537735
6873056.462	63.2084465	15.25402356	18.21726341	-75.47196198
85603933.02	107.1272278	18.5811541	23.0595356	-125.3843231
9546560.503	47.55777359	13.04424091	16.08993969	-41.26625443
5205268.27	75.0248642	19.71509337	25.74720955	-60.07752228
5259128.187	54.01549149	18.68348539	24.79794769	-54.03317261
16244879.44	66.29079437	12.93320452	15.52478924	-58.19195175
11944747.26	60.34669113	18.98048987	24.54819736	-69.49440765
22665326.07	90.71054077	18.1225238	23.93498135	-65.34146118
24149575.8	89.23529816	21.12632773	26.5960062	-94.17276001
15851797.07	90.58837891	16.14272224	20.66051483	-60.38187408
1305067.034	53.98145676	18.19525304	26.59502697	-37.46021271
12422768.6	68.83269501	16.90496896	20.94378109	-106.5677719
27902570.91	96.60604095	16.05543956	20.34692001	-58.96482849
8595357.01	57.11478424	20.09957938	24.71218109	-69.04097748
18202075.57	91.50338745	16.9916335	21.9065567	-63.36093521
19311398.69	90.9141922	17.2094272	22.71857338	-57.96119308
47205334.58	94.63259888	18.45559392	24.42840271	-84.55981445
21662117.38	64.18099213	20.341345	26.65677795	-73.4654007
24465395.83	92.27498627	24.48039075	26.25903168	-94.21153259
43557012.35	98.08522034	16.64747456	21.38955116	-82.73415375
3592882.59	47.42783356	15.41507411	19.18650246	-64.52837372
55963657.56	130.3555145	14.81235819	18.13961029	-150.676239
9816024.829	54.94510269	16.47151488	19.90493622	-56.04413605
38328133.96	92.64977264	15.82008617	19.90656586	-82.16801453
19905120.84	65.28276062	17.46113133	23.23887138	-61.55477905
18540539.4	78.5819931	21.99050122	27.53661156	-70.74180603
8962808.7	62.85979462	14.60613751	17.74074554	-63.64617538
21396068.1	75.13191986	18.16724293	24.81826019	-65.54750061
40743720.42	86.51483154	16.79310425	21.45569801	-78.51861572
2608228.015	37.78222275	14.02652692	16.83627319	-95.57606506
4348063.409	68.01107788	20.29446812	26.64712524	-62.95898438
5042429.006	63.86701965	20.32678983	25.38089314	-68.07666779
18593726.83	80.67814636	23.13194056	31.47423744	-114.1103363
33292404.5	87.1714325	21.04156798	29.52641182	-84.81663513
35305398.93	77.91589355	12.80401301	14.64666557	-63.17291641
12198123.98	70.97736359	16.58739028	18.28951244	-123.4610672
8854884.901	58.05886078	22.58416779	27.89452896	-64.66975403
8150636.79	58.15773392	16.0217602	19.62929249	-55.77552414
27379244.84	75.10037231	15.89227516	21.12709351	-56.45471573
19873903.97	78.01078796	18.87076356	24.40613899	-68.6184082
8853145.072	81.70671844	20.73025476	24.75405846	-80.9850235
77173954.88	99.03471375	16.19868442	18.6042942	-82.15599823
14407001.83	87.60350037	20.03744914	23.13430252	-76.02488708
40873371.75	95.72117615	24.78163116	32.10331192	-99.86207581
105739774.3	116.3924255	21.80880534	29.11195736	-91.88887024
21623603.34	77.16647339	19.54728801	24.84748554	-131.023468
4224631.633	46.77918625	15.20261183	17.94955559	-79.21020508
62422117.87	100.2580414	25.99625752	31.39915276	-128.440567
4262702.964	63.46259689	19.7195789	20.34216022	-60.72721863
65786995.08	115.4824371	21.88984388	28.30810738	-90.97620392
58961504.42	96.42149353	17.05454998	20.09679737	-115.3256683
15606106.38	67.44889069	16.49636067	20.93710346	-73.29929352
4825372.189	60.66031265	19.05903796	23.35032158	-56.91573334
50487752.49	96.94837189	19.37549766	25.12209892	-83.41521454
13367289.41	84.0149765	19.25746692	23.08522034	-59.24807739
12736757.03	94.57662201	22.28362018	30.56249008	-63.97800064
32001939.53	85.29210663	17.03583407	21.17293339	-73.37625885
6138987.302	75.48858643	15.73784069	18.7864069	-54.1991806
24174809.63	84.7786026	20.1326893	23.21269379	-124.1488266
4165956.643	54.8727417	21.85617853	27.70905457	-74.02263641
12830891.43	67.2983551	19.0100674	26.11695251	-88.68835449
14270816.8	89.9009552	17.13587482	20.78467731	-69.13052368
20062062.5	85.37745667	22.98863829	29.36466503	-94.50418854
8058677.666	55.91159058	19.00903134	21.89352417	-77.49937439
22639448.78	73.23734283	19.16872479	24.94349136	-96.86349487
9066568.536	65.91126251	13.31877509	16.79085693	-57.9830246
18209674.19	61.72421646	15.76101886	21.67237358	-63.1554985
2490371.447	27.81188774	13.179621	13.68864632	-58.21278763
10376078.36	77.09790802	17.14079131	20.86086655	-50.31368256
20906412.8	68.29853821	14.20752992	18.23981667	-75.14370728
16786960.96	82.8755188	19.11842014	23.26322174	-107.4044418
14479004.89	67.72375488	19.80629385	22.92032928	-108.7183914
6841985.67	82.53102112	17.7533851	20.56561642	-60.00958633
14014084.8	57.09532166	14.31754767	17.06795654	-71.04335785
36802566.56	75.97262573	17.40214027	21.98923302	-59.84584427
25719714.12	88.96992493	16.62622103	22.26459312	-58.1257019
26498500.58	73.99133301	17.22708	23.36152153	-71.13514709
38668211.91	104.2291031	24.50992811	29.76403885	-98.20574188
22620215.9	70.79436493	18.4844383	25.18311176	-72.21094513
5545362.092	85.13973999	25.61597577	26.95522766	-78.41326904
1175454.294	33.04092026	12.74540365	16.37148476	-37.10702515
86824332.9	95.72816467	19.09000736	24.38484535	-115.0189438

log.sigma.0.5.mm.3D_firstorder_Entropy	log.sigma.0.5.mm.3D_firstorder_StandardDeviation	log.sigma.0.5.mm.3D_firstorder_Range	log.sigma.0.5.mm.3D_firstorder_Variance	log.sigma.0.5.mm.3D_firstorder_10Percentile
2.037421613	23.60637758	189.2060242	557.2610625	-29.26355343
1.633594615	18.54080666	117.2138863	343.7615115	-24.51971092
1.767279205	19.12998187	117.3191071	365.9562062	-24.99589291
1.791713486	20.79404262	118.5934448	432.3922084	-28.30260906
1.765618878	19.26008639	116.1058426	370.9509278	-23.99276161
1.93910577	22.04282939	136.7825546	485.8863275	-24.02025394
1.49204018	14.51023314	158.0463028	210.5468659	-16.81460381
1.796259596	19.62269959	204.056488	385.0503392	-23.95451183
1.582887697	15.87575901	137.1088791	252.0397242	-19.56850281
1.90075784	21.3349042	161.6074524	455.1781372	-25.27175484
1.349826979	13.07601579	105.7263451	170.982189	-15.89822626
1.73369942	18.63454193	235.7944031	347.2461529	-20.48548584
1.854149985	20.25952787	146.1795731	410.4484695	-29.28491592
1.958653074	22.86265029	133.9490814	522.7007783	-33.27210503
1.91075056	21.75934079	148.6387863	473.4689117	-28.82225113
1.568536789	15.19261379	142.4467926	230.8155137	-19.80246201
1.553960417	15.89964874	160.3437119	252.79883	-18.45017509
1.562591536	16.584941	112.2735672	275.060268	-23.24137917
1.492694809	15.25336199	138.6804085	232.665052	-17.33704739
1.714819146	18.56621797	232.5115509	344.7044496	-20.45609665
1.310264836	13.03971432	88.82402802	170.0341495	-16.17135792
1.813615274	19.70928568	135.1023865	388.4559421	-25.41727448
1.73379973	18.68340394	108.0486641	349.0695827	-23.41072292
1.396290669	12.93036905	124.4827461	167.1944439	-14.03090496
1.797631893	18.9670007	129.8410988	359.7471157	-26.33108196
1.670699622	18.09787304	156.052002	327.5330085	-19.25931263
1.878173084	21.10982625	183.4080582	445.6247645	-26.05424442
1.572401334	16.12940644	150.970253	260.1577521	-19.73841286
1.635615534	18.01581097	91.44166946	324.5694449	-18.39951801
1.568078453	16.90495927	175.4004669	285.7776479	-19.30999146
1.577872034	16.04564556	155.5708694	257.4627415	-21.22279015
1.801813171	20.03774761	126.1557617	401.5113294	-27.82139797
1.614115135	16.98988553	154.8643227	288.6562104	-20.16655083
1.693861071	17.20805157	148.8753853	296.1170387	-20.16410637
1.720957396	18.45098341	179.1924133	340.4387887	-21.10469284
1.853763635	20.34134019	137.6463928	413.7701206	-26.80193405
2.03064741	24.46065224	186.4865189	598.3235081	-35.22460175
1.690914724	16.64698348	180.8193741	277.122059	-21.23573685
1.43871651	15.40121313	111.9562073	237.1973659	-18.95709457
1.489290027	14.80948389	281.0317535	219.3208131	-16.54932175
1.547977958	16.41672469	110.9892387	269.5088495	-22.26396427
1.544578702	15.81895411	174.8177872	250.239309	-17.29755135
1.630426985	17.44467467	126.8375397	304.3166742	-21.83049221
1.938763665	21.97679479	149.3237991	482.9795091	-27.79044876
1.494717125	14.60514754	126.50597	213.3103346	-16.39691639
1.700261693	18.15936136	140.6794205	329.7624051	-20.76685905
1.623250045	16.7930055	165.0334473	282.0050336	-19.56194496
1.302967083	14.0250558	133.3582878	196.7021901	-16.5938015
1.843452946	20.27535949	130.9700623	411.0902023	-25.00210381
1.820155464	20.30937074	131.9436874	412.4705397	-27.61895657
1.982421649	23.13188625	194.7884827	535.0841167	-27.21001167
1.872136171	21.01776602	171.9880676	441.7464886	-24.10944176
1.409705123	12.80320438	141.08881	163.9220423	-14.14750147
1.600141616	16.58664055	194.4384308	275.1166447	-20.69610081
1.927204237	22.57227118	122.7286148	509.5074264	-29.14355698
1.504723032	16.01835047	113.9332581	256.5875518	-19.38384056
1.547764307	15.88228442	131.555088	252.2469585	-19.33001213
1.732795503	18.86713859	146.6291962	355.9689185	-23.18267403
1.823475379	20.69546492	162.6917419	428.3022682	-25.95533867
1.59574524	16.19846023	181.190712	262.3901137	-17.78859634
1.793753934	20.03385643	163.6283875	401.3554034	-24.47260017
2.097943645	24.76400921	195.583252	613.2561521	-30.09582291
1.941372357	21.79511739	208.2812958	475.0271421	-26.23912697
1.772910809	19.53749559	208.1899414	381.7137338	-24.02428055
1.514628102	15.20056794	125.9893913	231.0572658	-16.84373245
2.162081722	25.99456898	228.6986084	675.7176167	-33.04892731
1.766683932	19.63591434	124.1898155	385.5691319	-29.51208591
1.920230246	21.88429613	206.4586411	478.9224172	-27.05261803
1.596940573	17.05383886	211.7471619	290.8334199	-18.07378941
1.555888121	16.47851532	140.7481842	271.541467	-20.43733196
1.730903826	19.05585771	117.576046	363.1257133	-25.3012373
1.813885469	19.37539478	180.3635864	375.4059228	-24.03601456
1.757615488	19.23267883	143.2630539	369.895935	-24.60826607
1.915067655	22.2701898	158.5546227	495.9613536	-26.55734768
1.652421778	17.0354039	158.6683655	290.204986	-20.07902184
1.519652727	15.71348556	129.687767	246.9136285	-17.71735554
1.765339269	20.13262333	208.9274292	405.3225221	-22.25295601
1.920855907	21.85560894	128.8953781	477.6676423	-28.97154274
1.742564628	18.97622716	155.9867096	360.0971974	-22.2184761
1.590322543	17.10402096	159.0314789	292.5475331	-19.58980713
2.000209884	22.96236317	179.8816452	527.2701223	-28.65921879
1.713058826	18.98956164	133.410965	360.6034512	-23.57082939
1.798680581	19.16471711	170.1008377	367.2863819	-25.24779644
1.330620164	13.2747195	123.8942871	176.2181779	-16.0616188
1.569620546	15.75797777	124.879715	248.3138636	-17.80789108
1.263493457	13.17961107	86.02467537	173.7021478	-18.81534195
1.601975973	17.12179887	127.4115906	293.1559967	-21.18238754
1.443325501	14.20539138	143.4422455	201.7931443	-16.31131935
1.767358117	19.11101718	190.2799606	365.2309776	-22.93497086
1.765055323	19.80629367	176.4421463	392.2892689	-24.45179024
1.662073315	17.72234109	142.5406075	314.0813737	-22.06502419
1.452311237	14.30846369	128.1386795	204.7321333	-16.17835121
1.665287461	17.40129042	135.81847	302.8049083	-21.53423691
1.595445359	16.61003168	147.0956268	275.8931523	-18.31522083
1.62335397	17.21841244	145.1264801	296.4737269	-19.68676453
2.068733248	24.50947379	202.434845	600.7143054	-29.95517426
1.693100906	18.47864013	143.0053101	341.4601411	-21.00201702
2.125363709	25.55880429	163.553009	653.2524767	-35.373526
1.259466675	12.74045442	70.1479454	162.3191788	-16.33812733
1.754248345	19.0678985	210.7471085	363.5847533	-21.93647537

log.sigma.0.5.mm.3D_firstorder_Kurtosis	log.sigma.0.5.mm.3D_firstorder_Mean	log.sigma.0.5.mm.3D_glrlm_ShortRunLowGrayLevelEmphasis	log.sigma.0.5.mm.3D_glrlm_GrayLevelVariance
4.202056738	-0.922938928	0.056658397	1.208933255
3.18076161	-0.656697269	0.083972646	0.684473966
2.958950045	0.252940873	0.082581245	0.803523396
3.175000764	-0.114612582	0.099569477	0.85072973
2.787324079	0.626600815	0.083596932	0.820441948
3.662688234	3.030099464	0.098771395	0.993885354
5.760894304	0.110091948	0.072896015	0.563587998
4.613445103	-0.531943514	0.028302813	0.880464105
4.086066012	-0.380918852	0.075487726	0.640869399
3.619231345	1.680667938	0.048098511	0.953062256
3.782775141	0.51861342	0.069813991	0.439837793
6.478679151	0.19704231	0.027931387	0.838266974
3.31654079	-0.369802052	0.049421127	0.920290178
3.026813605	-2.582747275	0.110059058	1.024402895
3.867242584	-1.236268411	0.092472248	1.032448161
4.25026393	0.007653706	0.074390839	0.595061457
4.791775648	0.90808445	0.041370638	0.624061336
3.294020029	-1.785420103	0.082842307	0.638737444
5.200668286	-0.142065792	0.040926408	0.587810829
6.252690051	0.744874486	0.019815668	0.811594817
3.211832091	0.343615147	0.148259387	0.425182654
2.918870934	0.478502444	0.086538301	0.859065851
3.097662555	-0.05516824	0.081794611	0.768974099
5.887569675	-0.27080475	0.069031044	0.493268021
4.126146677	-0.715457894	0.086591826	0.81872794
5.195652514	0.944912978	0.071794965	0.773519396
4.026017445	-0.834840547	0.047230547	0.984232337
4.759856672	-0.655537267	0.075644817	0.640686839
2.738776717	2.549075997	0.165188898	0.649733716
5.870636459	-0.018104236	0.028054049	0.657555855
3.77561553	-0.560712026	0.074436214	0.629066215
2.532083591	-1.575360871	0.088856098	0.8496128
4.687026086	-0.24371818	0.076919695	0.680687392
4.886061257	0.217590923	0.081354678	0.690668665
4.844505667	0.412502518	0.043393906	0.809996962
3.123233801	-0.013993185	0.09015444	0.884505217
3.497183007	-0.982864875	0.058614334	1.200203717
5.185332704	0.127867643	0.044263291	0.707826863
3.952709789	-0.653562522	0.076807063	0.520498393
7.232287252	-0.29179109	0.013726361	0.582888086
3.154054834	-1.342368363	0.077144089	0.609982649
6.33963912	0.189254929	0.040294904	0.633313361
2.89484783	0.757913723	0.074850758	0.667418007
3.392084722	-0.776295693	0.097597772	1.036011203
5.210294612	-0.170054275	0.073575652	0.576175273
3.685536915	0.535080085	0.078582931	0.762130162
5.17257892	0.057589967	0.041462769	0.686577706
7.722071212	-0.203143434	0.039445541	0.445800478
3.268079285	0.880473626	0.085905141	0.88358269
2.850707058	0.841335179	0.086623591	0.853442778
3.792951113	0.050122349	0.031284988	1.115894223
3.419205035	1.000547034	0.045045513	0.939223171
6.116125825	-0.143898934	0.068223866	0.49653635
7.104286201	-0.157707309	0.028663215	0.69752545
2.701475882	0.732944858	0.099691793	0.984965903
3.325522618	-0.330527039	0.07196658	0.577512585
4.055027727	-0.563428124	0.072027706	0.622623466
3.322541047	-0.36986313	0.081791617	0.780628358
3.881922226	-1.200497555	0.047646457	0.923655506
6.819165664	-0.085223889	0.041457609	0.680649452
4.479373871	-0.379426968	0.046272004	0.887152944
3.829390864	0.934393178	0.055223525	1.25519808
3.931417953	0.772559475	0.048775338	1.02743028
4.348829042	0.61865552	0.020271451	0.842929774
5.608604605	0.249280509	0.04207925	0.606392131
3.665046141	-0.296291358	0.022443174	1.380026796
3.063499644	-1.814568853	0.100814896	0.835797181
3.839690159	-0.492796085	0.047882163	1.014954051
6.921420831	-0.155740042	0.027177011	0.711661177
3.517907351	0.767103848	0.077699344	0.613321968
3.102669057	-0.348158778	0.086690146	0.778379323
4.478551277	0.063140083	0.046897991	0.859061625
3.906155271	-0.976778991	0.085521584	0.83879413
3.577445606	-0.773546856	0.092606318	0.995507707
5.183733279	-0.121064051	0.079902731	0.71316143
4.486829292	0.875214946	0.071687027	0.589997751
6.671937895	-0.051541565	0.031291259	0.905281275
2.830792141	-0.157790629	0.092986126	0.964755609
3.390480792	1.133783566	0.043901611	0.775519711
5.406966162	-1.044352792	0.077961234	0.706433439
4.182618987	-1.098803125	0.055544161	1.148654223
3.811999427	0.860128737	0.046723673	0.793314936
4.028186231	-0.39195424	0.047167391	0.827177269
4.143887583	-1.082401091	0.065659246	0.459177065
4.28938609	0.309599448	0.073470222	0.631360097
4.616803147	0.016180376	0.078088013	0.468382358
3.958250124	-0.806678391	0.075228564	0.672355993
5.552466131	0.246499855	0.037949831	0.541334653
4.805525328	-0.531987823	0.029186663	0.834251412
4.711035847	-0.002689213	0.030159142	0.865563504
4.401098016	1.04943256	0.085470336	0.742317524
5.195678875	-0.509939191	0.068166972	0.561377582
3.719353789	-0.171981293	0.080500491	0.705799899
4.526250349	0.733535015	0.07566951	0.653081467
3.836610883	0.546404934	0.07362072	0.690292077
3.886208762	0.14923251	0.052817759	1.232312719
3.67017732	0.462945031	0.077620285	0.760077134
3.495475385	-1.71047888	0.066265548	1.335207867
2.683620702	0.355155514	0.14579462	0.384426439
5.179012027	0.918492029	0.029025945	0.833132652

log.sigma.0.5.mm.3D_glrlm_LowGrayLevelRunEmphasis	log.sigma.0.5.mm.3D_glrlm_GrayLevelNonUniformityNormalized	log.sigma.0.5.mm.3D_glrlm_RunVariance	log.sigma.0.5.mm.3D_glrlm_GrayLevelNonUniformity
0.067573572	0.266052609	0.564509223	283.6810437
0.103715488	0.350311974	0.685780935	86.83923792
0.103303854	0.308030443	0.7883468	192.6347864
0.118549021	0.313054101	0.547456414	73.35312074
0.105241339	0.306635867	0.797976012	296.7612912
0.114228295	0.286527726	0.378413167	54.03552295
0.096834768	0.383477342	0.954943904	2668.688276
0.036407024	0.309441502	0.77276992	1312.399956
0.09866915	0.352688971	0.857963023	1023.268195
0.058569033	0.288588776	0.509462674	162.5117997
0.091753095	0.418559826	0.796578764	445.1246242
0.036220822	0.329056948	0.772035023	775.0949563
0.060681423	0.295770601	0.583355715	173.5264815
0.128852353	0.275056076	0.457939279	134.3876096
0.114228034	0.280453886	0.681961315	317.5102478
0.096084541	0.36295999	0.796633584	1453.62331
0.054465313	0.366542195	0.797443308	218.3645912
0.106420972	0.365800336	0.822245352	100.0669189
0.055168218	0.381633019	0.850610942	276.0224351
0.025509249	0.333792307	0.751938081	2123.092816
0.205971076	0.420820935	1.320237393	523.1629864
0.105784638	0.297194356	0.563945354	108.2283711
0.103200368	0.313847593	0.667838308	124.0412787
0.09324295	0.405066154	0.850856062	979.8882867
0.108289174	0.310775526	0.623087528	273.3462482
0.094826505	0.336366383	0.887453296	579.4654401
0.059285128	0.289486901	0.726146315	411.9575207
0.099121492	0.356360161	0.862928371	544.0219593
0.204671477	0.347527509	0.361019492	38.85139293
0.036635627	0.360232939	0.878650155	393.6537328
0.098143601	0.352285069	0.978328061	935.9852156
0.109962185	0.29595321	0.727872567	165.5806623
0.099582472	0.350062146	0.79869719	557.3783431
0.099020608	0.342595257	0.515723645	618.5332227
0.056305255	0.324038559	0.833377667	1131.316692
0.110405186	0.295738108	0.66189602	414.0392014
0.069029034	0.267615942	0.530553708	304.7631506
0.055795719	0.340415281	0.684692345	1407.036776
0.099997165	0.395451036	0.826262217	148.1714952
0.018362439	0.381616325	0.861558246	2435.14221
0.101394715	0.35660396	0.912772164	316.1069107
0.053383901	0.371030424	0.749954222	1449.115929
0.097202236	0.341491865	1.033952046	564.0016366
0.117271311	0.27450734	0.620981223	286.1774369
0.097431854	0.37839179	0.901733432	390.5477212
0.099963793	0.32656183	0.826501432	543.1805546
0.054171528	0.349557736	0.795959577	1294.642748
0.054193159	0.431297497	0.896508009	137.3349027
0.104141269	0.295891661	0.509399049	86.1459671
0.106112231	0.302657844	0.51172429	100.6176564
0.03794712	0.269977664	0.583039875	260.9039955
0.056362263	0.291952324	0.613535905	589.1783882
0.093121663	0.40023445	0.93491509	2079.892752
0.037304721	0.355723766	0.873707422	390.1087368
0.117337597	0.283300561	0.480663236	137.3528105
0.096684423	0.369571486	0.973970546	282.9253354
0.095924335	0.359137381	0.919849919	970.9748722
0.10334477	0.315813203	0.799857267	454.3080535
0.059447631	0.300247395	0.722473352	162.8323841
0.054476453	0.362917048	0.759266993	2720.378353
0.058782587	0.311320017	0.761680051	286.8839506
0.064884431	0.257022216	0.483082299	483.0685448
0.060010884	0.282799879	0.641840677	1678.048061
0.025687755	0.314185947	0.667507051	465.2634079
0.056304337	0.372969977	0.848715317	166.9979213
0.026891903	0.245063259	0.449214834	642.0084919
0.121272279	0.319907714	0.51593987	94.94041742
0.059209348	0.28377512	0.629825579	1052.760962
0.03617377	0.359099947	0.979450274	1794.204057
0.099362867	0.36110976	0.858713241	515.0810933
0.108094064	0.320665062	0.702920519	110.9323461
0.058117852	0.311701158	0.667811614	1123.399474
0.108235166	0.313800717	0.850884894	288.2332117
0.109963478	0.28516066	0.491770427	208.3959938
0.101865305	0.344718048	0.782872886	972.6982516
0.093751937	0.37576436	0.825518955	235.0484889
0.039647933	0.323209986	0.72287265	492.1579509
0.112194288	0.281513634	0.456059952	68.34197742
0.05539093	0.318535668	0.61505129	299.0221514
0.101415909	0.350848758	0.844159514	430.9370428
0.066600473	0.272374084	0.573001133	281.5488798
0.058807325	0.326308049	0.753283932	185.6674546
0.058263783	0.308546598	0.64355247	516.7025437
0.094210457	0.411522012	1.160450798	478.7293995
0.097636669	0.355585572	0.897684653	637.5904277
0.102225711	0.429220176	1.032820969	143.4798272
0.098102842	0.347452931	0.913938304	308.139386
0.052700379	0.389728926	1.04705833	964.2038365
0.037241007	0.319793281	0.656727566	383.8658987
0.038249066	0.315873115	0.749682769	297.0033973
0.105440564	0.341410269	0.682331405	191.4783335
0.094115892	0.381249717	1.09740089	618.8563586
0.10179928	0.338914928	0.685440583	1069.206719
0.097465397	0.357569382	0.801341795	844.8074635
0.096434145	0.341716826	0.859517231	761.3852095
0.063660288	0.258273501	0.610183668	452.9052809
0.100133576	0.32641044	0.846250935	544.7639436
0.075871164	0.258505287	0.361342056	63.36953392
0.200512971	0.445528797	0.885883957	75.7755959
0.036973534	0.321477852	0.797228389	1955.656115

log.sigma.0.5.mm.3D_glrlm_LongRunEmphasis	log.sigma.0.5.mm.3D_glrlm_ShortRunHighGrayLevelEmphasis	log.sigma.0.5.mm.3D_glrlm_RunLengthNonUniformity	log.sigma.0.5.mm.3D_glrlm_ShortRunEmphasis
2.413939962	17.16003247	644.068358	0.804220856
2.686805577	10.21814204	144.3554478	0.787954208
2.87530852	10.31954895	352.6782968	0.775059173
2.457955456	10.24338999	136.9825775	0.790748641
2.94520817	10.39039442	525.7590727	0.763019605
2.042817532	11.86986808	121.604478	0.827823002
3.278045	9.647389609	3525.206594	0.734968598
2.839999155	24.70948247	2375.036853	0.77483729
3.053837047	10.13795873	1553.784468	0.756298416
2.327703525	17.65327843	341.77022	0.804419845
2.981062243	9.813234584	567.0283088	0.753905649
2.88686316	24.43597811	1283.975117	0.764416283
2.52496562	16.3276411	339.7358655	0.785462578
2.211431013	10.57083559	304.7070268	0.815703674
2.638642538	10.93103584	661.5054336	0.790754822
2.917703396	10.0380978	2217.32097	0.76526112
3.015589829	16.32510019	312.4109274	0.748103591
3.085011725	9.725315912	142.9556815	0.747964147
3.175560701	15.5794894	363.3970134	0.731418083
2.85940489	33.07519099	3463.685414	0.763460135
4.209479785	4.726462625	555.9928426	0.682167123
2.43037989	10.84404134	217.3578963	0.799962724
2.676129988	10.53812368	224.6457743	0.779744501
3.087856627	9.818268211	1254.116012	0.744187182
2.602579797	10.25665267	503.9646649	0.78048124
3.127054954	10.6646397	908.493459	0.750479743
2.746938991	17.0614609	820.1319668	0.783744333
3.069160009	10.09529454	815.4392476	0.756169065
2.086502041	6.425634031	68.80659098	0.807024568
3.087695408	24.17130351	585.7911532	0.757546547
3.297113933	9.821056948	1382.367528	0.743931847
2.743460761	10.28858123	321.8681436	0.785162148
2.972060441	10.19449914	850.025929	0.756344264
2.318613519	10.73019192	1103.72098	0.80816663
3.012345251	16.71120199	1868.909435	0.758060718
2.613169821	10.61517778	820.5189413	0.790083916
2.35029223	16.81398853	705.7063469	0.809016912
2.688864679	16.55493075	2349.063501	0.77920395
3.098034528	9.58165852	193.1164261	0.742120252
3.071175207	43.55390737	3360.053886	0.751405877
3.231379458	9.491593118	454.4873209	0.740526656
2.879383611	16.52553662	2098.168307	0.758761806
3.370076856	10.18257193	907.5979821	0.754168968
2.492015265	11.03115323	630.7950443	0.804613707
3.20070035	9.934163408	531.0272654	0.74218209
2.941488576	10.71940237	925.0698998	0.771844606
2.901317498	16.81596426	2034.393625	0.767250832
3.330943674	15.79726359	157.3017525	0.723685032
2.336503646	11.22202001	176.876499	0.805095098
2.385593066	10.62348254	194.7412268	0.791642297
2.375914605	26.66495443	610.3684805	0.820432681
2.53234904	17.61743774	1177.93249	0.79120487
3.309774701	9.554740576	2570.123507	0.727098838
3.164765291	22.63133108	567.4951363	0.742473479
2.256644294	11.03068467	299.9052801	0.812754676
3.371375384	9.713357036	384.4257987	0.731987949
3.168585891	10.14084139	1423.252014	0.749532245
2.893680565	10.39889116	802.4650986	0.772552927
2.724613555	17.11274808	315.928828	0.789960567
2.885613899	16.37641626	4019.629431	0.758487197
2.88047855	16.30897893	505.3766714	0.765449674
2.215123257	18.12128053	1185.860169	0.821107061
2.582494943	17.2086514	3445.62484	0.789028824
2.685594057	33.83998129	830.4738237	0.775782197
3.225509415	15.54023992	225.5058406	0.727830371
2.163318244	36.01309917	1658.883309	0.822786864
2.404090947	9.764893226	172.116887	0.788100084
2.526037231	17.37004065	2208.476429	0.798717187
3.257049765	23.50627564	2581.114575	0.744485934
3.111897704	9.665929717	744.8936456	0.74714279
2.782299189	10.13979054	194.2541208	0.771378007
2.596235983	17.01903973	2113.593246	0.791844293
3.012636848	10.26387734	503.2409457	0.764413635
2.203548436	11.32324774	473.6108404	0.829934779
2.898029687	10.21422098	1546.847136	0.766637223
3.054624588	10.23296615	333.0590942	0.752034401
2.844589129	23.88305918	819.7081988	0.759733972
2.240270785	11.08043434	147.8651082	0.806476335
2.590946376	16.94695042	529.6606936	0.778282452
3.013328717	10.31808162	662.4974709	0.761119436
2.439304809	17.00228248	619.8920783	0.801342107
2.892225562	16.17721892	309.6982661	0.76236275
2.541035554	17.07814834	1010.17396	0.801224575
3.847167759	9.181762626	532.789374	0.697689274
3.228964172	9.88908769	909.423766	0.735465681
3.742008628	8.197546046	159.4445089	0.700248722
3.150858918	10.07418493	474.9669941	0.755570803
3.471780465	15.9190467	1222.217559	0.726618736
2.681560607	24.21368586	666.6258849	0.771791539
2.89162761	23.37726058	509.4772378	0.761336262
2.757137583	10.02238109	305.5108435	0.76378614
3.580066387	9.902653302	800.1770128	0.725337555
2.734539619	10.19663648	1741.481482	0.769687174
2.957212042	10.29500179	1270.952029	0.758243565
3.088997655	10.37007735	1175.307669	0.751702073
2.475947258	17.47380053	1054.635559	0.802291203
3.04045387	10.45437505	893.6837486	0.75701345
2.010137044	17.05800293	158.6080807	0.828941072
3.451517953	4.92505956	79.93685388	0.700268884
2.923333364	23.95564111	3308.1689	0.763396202

log.sigma.0.5.mm.3D_glrlm_LongRunHighGrayLevelEmphasis	log.sigma.0.5.mm.3D_glrlm_RunPercentage	log.sigma.0.5.mm.3D_glrlm_LongRunLowGrayLevelEmphasis	log.sigma.0.5.mm.3D_glrlm_RunEntropy
48.95225778	0.738014063	0.146462756	3.218304863
33.45862333	0.712923964	0.257360757	2.857304521
36.63634392	0.700155226	0.274972655	3.058218115
31.95650933	0.726708075	0.24993878	2.962462103
36.85273643	0.685275037	0.286404779	3.111954977
28.10852264	0.778766688	0.206088627	2.916520018
40.48148653	0.659856796	0.304135034	2.970895105
83.58049459	0.698519345	0.104058827	3.12561445
35.92945894	0.680052702	0.299775934	2.968427148
48.65934381	0.74538971	0.129671133	3.030074508
36.48017697	0.682815899	0.27237541	2.717997619
86.45516158	0.690817548	0.103695782	3.105901031
53.78922252	0.722469885	0.138628656	3.065968816
27.19836705	0.758122312	0.25039178	3.024359989
30.90817213	0.718437118	0.286462098	3.144253476
35.84014485	0.694684004	0.275914588	2.897251505
61.43772055	0.674448994	0.161988597	2.954451245
36.56032108	0.668233967	0.307118881	2.936278319
63.31152733	0.660455632	0.172826128	2.96079159
122.9218821	0.692081522	0.070276225	3.089178755
27.16837639	0.597504997	0.805727312	2.990184994
30.16960546	0.733870968	0.244280435	2.950642425
32.15535039	0.70802316	0.268653998	2.983353968
35.73670146	0.672013002	0.298101341	2.813533228
31.87648332	0.716487096	0.267335389	3.035194103
38.35903866	0.673287589	0.296858503	3.096220466
54.19023149	0.709081836	0.158757531	3.15699026
35.58953805	0.677148417	0.305080507	2.969111432
14.32301809	0.765542677	0.42627001	2.645277207
91.50359339	0.677830865	0.111932087	2.950451619
39.54579532	0.662259915	0.317103466	3.022868354
32.99906147	0.70900039	0.281805388	3.032402547
35.87168874	0.681502224	0.287410072	3.009515881
29.35026182	0.747252747	0.222303773	2.823894983
60.72647856	0.679734448	0.166301331	3.12849313
32.88797606	0.721386916	0.265810615	3.074252332
50.08700675	0.751221001	0.140197706	3.146230135
55.69477089	0.709949387	0.145104618	2.973705084
37.30286178	0.668269231	0.295365769	2.843443917
168.092081	0.675118678	0.057716992	2.910028052
38.09630418	0.660849598	0.315494877	2.989512589
56.87116923	0.688157752	0.157857803	2.922339381
42.02829055	0.679582618	0.315889258	3.007393058
29.92890072	0.733640303	0.269364425	3.105719114
38.01302427	0.662942456	0.307789564	2.946467572
35.50147645	0.691923237	0.28835359	3.040948728
57.17003282	0.691718299	0.160920352	2.974301364
65.15476122	0.647971173	0.183403284	2.751494283
29.49427077	0.743655322	0.231006286	2.952484161
31.78202665	0.734343091	0.230021453	2.973576479
69.97065608	0.750403443	0.089986809	3.075530278
51.79204166	0.724181743	0.141097274	3.100182187
39.81752887	0.651271121	0.308656323	2.91394938
98.13190265	0.667103907	0.109591233	3.051385111
29.95617216	0.753319775	0.232130377	3.022625567
39.8490433	0.650313972	0.3240758	2.986668329
36.77748468	0.67259316	0.311101934	2.962716578
34.98877648	0.695173235	0.286803851	3.05118196
52.79043404	0.710353866	0.158894011	3.071666578
57.57484178	0.687865884	0.157948961	2.980899127
58.95019284	0.692307692	0.158859852	3.149613935
47.01276066	0.762053362	0.13035686	3.186098377
54.05270246	0.720352759	0.144754038	3.19491658
115.3106423	0.70610687	0.066578935	3.070587585
65.22698881	0.659925009	0.174790017	2.964162037
92.83997541	0.765453196	0.055890765	3.242526165
30.34798811	0.730200834	0.252084182	2.953508293
50.30874091	0.729067393	0.146036669	3.13515296
96.17439705	0.665044465	0.117403295	3.068984449
40.37972625	0.670686658	0.278970413	2.985688449
34.23951407	0.701844903	0.275615313	2.99378224
52.7320092	0.723055302	0.145586783	3.050954219
35.91377659	0.686603284	0.305054122	3.102209558
26.48187097	0.768421053	0.235101777	2.958581358
34.94835973	0.690593686	0.284000971	3.018573416
37.99863566	0.679989945	0.280707962	2.903498694
86.65262167	0.688929902	0.104017748	3.149100461
28.72164819	0.75089307	0.23313026	2.986846858
54.0235632	0.713424978	0.139010818	3.002673042
34.1314995	0.681666667	0.30680972	2.981912126
49.2333401	0.734817814	0.146882442	3.193201223
61.5914273	0.688116968	0.153311828	3.076973136
50.44658199	0.73322996	0.145363328	2.976846288
43.41266076	0.614124914	0.37601576	2.937079642
39.18026947	0.659951835	0.307056059	3.038786263
51.83782805	0.627987831	0.309031272	2.814517474
37.31695177	0.676664314	0.305775856	2.995011582
67.65877389	0.644621802	0.190903327	2.961338163
80.411459	0.705286483	0.097176768	3.072418479
89.00764316	0.687074447	0.102568156	3.136484282
37.36590874	0.69699579	0.248087716	2.992539734
41.14128503	0.640357273	0.349064207	2.985190509
33.66431847	0.700579358	0.263887983	2.990224497
36.87876083	0.684963614	0.276835149	2.978902051
37.50758514	0.67314089	0.294477791	3.040811527
52.03961187	0.734996128	0.142853094	3.259846782
37.10630618	0.679853181	0.294970088	3.08803325
42.22877284	0.782256083	0.130610638	3.108483862
22.23712545	0.633467279	0.663485845	2.73890468
91.72849187	0.688873004	0.101317741	3.129965883

log.sigma.0.5.mm.3D_glrlm_HighGrayLevelRunEmphasis	log.sigma.0.5.mm.3D_glrlm_RunLengthNonUniformityNormalized	log.sigma.0.5.mm.3D_glszm_GrayLevelVariance
21.12344088	0.602581081	4.439678588
12.8097001	0.579385295	2.540642722
13.17467635	0.560363117	2.494897959
12.99183952	0.583021536	2.52734375
13.38732631	0.541699768	2.567901235
14.18487771	0.64257248	3.200617284
12.9175072	0.504429665	2.792134076
31.31861836	0.558275339	3.862462561
13.00387075	0.532721373	2.552620408
21.69171407	0.604256389	3.271163422
12.83549836	0.52990802	1.957571939
31.47158309	0.543512763	4.222183238
20.89719206	0.576037699	3.296398892
12.80207182	0.621771753	3.383786848
13.31128503	0.582301941	3.443310658
12.91245214	0.547913685	2.233393559
21.48575525	0.522146651	2.967784352
12.75263494	0.520938648	2.4
20.94869382	0.499942603	2.816660733
43.25330705	0.542612984	3.80044696
6.824267288	0.441913651	1.962222222
13.29426193	0.595847648	2.6
13.16517818	0.566520591	2.143667297
12.78181647	0.516609352	2.285379813
12.92136372	0.568893816	2.942040816
13.69972751	0.524935475	3.16927607
21.3108623	0.572945151	3.527742347
12.93094387	0.53198659	2.572598758
7.629782078	0.611767303	1.358024691
31.36249681	0.533818821	2.88636841
12.92513939	0.516523376	2.512057075
12.88004504	0.573542123	2.426592798
13.12626961	0.532095846	3.217222732
13.13181099	0.609518512	3.303055556
21.59435047	0.534056339	3.518741069
13.24805728	0.582462366	3.386666667
20.8849157	0.61380458	3.890105218
21.14398477	0.565759321	3.099127416
12.74198212	0.513230372	2.585034014
57.16912354	0.525243094	2.873976504
12.59799347	0.510646981	2.222029321
21.26075628	0.535683611	2.839340903
13.27791494	0.536292086	2.28153011
13.33983433	0.603600266	3.412872841
13.00718914	0.512651586	2.761604705
13.48212916	0.554149386	2.795300867
21.38313765	0.547750993	2.815439721
21.23572833	0.489797175	2.611570248
13.63136192	0.605411902	2.645555556
13.35431351	0.584229731	2.595555556
31.82672059	0.629391203	3.436734031
21.85986456	0.582544553	3.085555556
12.84619255	0.493278739	2.544832995
30.66460038	0.513929809	3.155306122
13.49146638	0.616835271	3.074792244
12.93481878	0.499707106	2.385724852
13.05863635	0.523408439	2.462476245
13.14333975	0.555729526	2.841130341
21.15453349	0.580835615	3.460757156
21.16777855	0.535097832	3.394879026
21.08830651	0.545871719	3.151100535
21.88832681	0.629664275	5.34498375
21.63044179	0.579184523	4.521337947
43.48655018	0.559627774	3.449795918
21.07116674	0.497796656	2.647923875
43.54400643	0.632176366	4.986995175
12.43123247	0.578707815	3.1875
21.33014214	0.593912788	3.869944687
31.10323984	0.515706783	3.463507631
12.96638516	0.519958896	2.383783284
12.96640143	0.556634309	2.432397959
21.2587526	0.58457337	3.89608207
13.06177809	0.544804813	3.236388889
13.29630396	0.64568593	2.56983738
13.02535071	0.546405593	3.286873383
13.30929285	0.527975723	2.680529301
31.20368078	0.536824965	4.321983471
13.50293219	0.607642598	2.842105263
21.58178759	0.563349982	2.995690893
13.00139922	0.538359809	3.011273405
21.00851718	0.598117523	4.227991788
21.21361855	0.541661648	3.052965143
20.99036238	0.599376712	2.705251479
12.65909961	0.455777598	2.24984375
13.12759282	0.504582583	2.658300173
12.35122874	0.46845457	1.102040816
12.9939403	0.532191759	2.65234375
21.28949782	0.492717476	2.823144944
31.03781579	0.55408583	3.861881109
30.79380572	0.539695912	3.739785291
13.21356354	0.543322615	3.002191381
13.08887054	0.490314192	2.6475
13.02368084	0.550896464	2.909379287
13.29654961	0.535646216	3.120251479
13.38376042	0.525652617	2.489976837
21.56908435	0.599902969	4.759857429
13.43318568	0.533203502	2.871682493
20.68758968	0.644539784	4.514177694
6.888245828	0.464663535	1.580246914
31.39755091	0.542155162	3.715991837

log.sigma.0.5.mm.3D_glszm_SmallAreaHighGrayLevelEmphasis	log.sigma.0.5.mm.3D_glszm_GrayLevelNonUniformityNormalized	log.sigma.0.5.mm.3D_glszm_SizeZoneNonUniformityNormalized
16.41240427	0.172928927	0.356269805
11.8684875	0.323251418	0.413988658
8.705080213	0.285714286	0.18622449
7.60851823	0.2578125	0.34375
9.930372097	0.299192196	0.363816491
5.432580027	0.203703704	0.290123457
9.841784919	0.3077615	0.352860289
22.37227471	0.219519322	0.333808167
8.200369623	0.339559184	0.268702041
13.55935612	0.231326392	0.329108194
9.160028909	0.31779038	0.429531611
20.5386345	0.233630104	0.354501996
12.56815069	0.254847645	0.289473684
9.926907063	0.252834467	0.404761905
12.35907645	0.231575964	0.340136054
11.16906417	0.208417473	0.461579339
8.749714909	0.333333333	0.255752794
12.59491979	0.344	0.3568
17.90221743	0.349234603	0.445354219
26.73679867	0.253054846	0.375227347
4.728106326	0.408333333	0.492777778
7.928529184	0.35	0.185
5.938011884	0.266540643	0.168241966
10.43955177	0.31594693	0.330905307
10.34253984	0.263673469	0.252244898
11.91707625	0.289141051	0.361246768
15.34434055	0.228954082	0.283163265
9.628758027	0.334654512	0.292905272
5.296829571	0.358024691	0.160493827
20.37720095	0.352657005	0.349716446
9.345892908	0.377502973	0.350107015
8.877137614	0.346260388	0.272853186
9.993781345	0.303744516	0.323742475
11.35401991	0.234444444	0.323888889
13.54871264	0.26953737	0.332139598
10.9394233	0.2288	0.409422222
12.1093246	0.223084281	0.413115103
14.9275329	0.164946519	0.423343967
7.252913592	0.319727891	0.306122449
37.8858466	0.3220996	0.306791662
9.569691998	0.376929012	0.391203704
17.91609025	0.283693899	0.355919108
8.78253543	0.32685267	0.276858982
10.94581853	0.245260234	0.379302017
11.27589051	0.342995169	0.407687461
8.121133442	0.300730824	0.302506659
17.20383264	0.294184183	0.3388161
14.9659448	0.338842975	0.483471074
8.878950451	0.297777778	0.284444444
11.55321343	0.28	0.324444444
20.96010512	0.174417661	0.376349322
16.94991438	0.241333333	0.342755556
9.267047077	0.343843632	0.331979328
16.50648804	0.287755102	0.368571429
10.14325292	0.213296399	0.332409972
11.39421005	0.412721893	0.357988166
10.13906239	0.32489512	0.379898885
9.216213198	0.274202271	0.32747431
16.20100385	0.254539858	0.383194829
18.58346137	0.252606954	0.399493671
14.01504205	0.219512195	0.335217133
16.87019597	0.199211466	0.413607544
14.53045785	0.211393054	0.383848093
27.60114782	0.278730159	0.35600907
13.00957207	0.316608997	0.35467128
38.99227556	0.201368233	0.477824047
11.64709051	0.319444444	0.427083333
18.65874448	0.216546592	0.423066838
20.20247755	0.298729376	0.3858944
8.869865932	0.317492604	0.378143491
8.492186382	0.298469388	0.204081633
14.12672251	0.192535275	0.389587199
9.61735889	0.273333333	0.268333333
13.43399122	0.203831588	0.316997104
11.77306341	0.248190869	0.410903031
9.03228251	0.306238185	0.345935728
23.89635289	0.239834711	0.440330579
14.72292388	0.329639889	0.556786704
12.56876369	0.30767021	0.333524849
10.58090514	0.310851877	0.330457798
16.76619605	0.184881053	0.366743147
12.93490681	0.232231779	0.328202807
16.33076983	0.221153846	0.39589497
10.33751013	0.39875	0.4728125
8.183238555	0.326191205	0.312723192
1.525691132	0.326530612	0.173469388
10.35343837	0.326660156	0.388183594
14.95642131	0.298539023	0.35005767
15.01310833	0.246595097	0.299471239
19.82682821	0.273834321	0.399455216
7.047754727	0.292914536	0.310445581
9.731216158	0.3434	0.2964
11.10491897	0.320558985	0.378386488
10.63305136	0.282966594	0.378869412
9.749581035	0.323114759	0.344733907
16.68125308	0.182445979	0.427378035
8.299558192	0.302491681	0.297134973
11.59852481	0.179584121	0.258979206
3.812585451	0.283950617	0.259259259
19.57726214	0.215657143	0.429061224

log.sigma.0.5.mm.3D_glszm_SizeZoneNonUniformity	log.sigma.0.5.mm.3D_glszm_GrayLevelNonUniformity	log.sigma.0.5.mm.3D_glszm_LargeAreaEmphasis	log.sigma.0.5.mm.3D_glszm_ZoneVariance
33.4893617	16.25531915	6128.744681	5892.762789
9.52173913	7.434782609	1928.913043	1701.296786
5.214285714	8	9150.071429	8135.193878
5.5	4.125	2144.25	1739.234375
29.4691358	24.2345679	7893.888889	7590.441091
5.222222222	3.666666667	931.5555556	750.8024691
109.7395498	95.71382637	145704.8521	144556.4922
78.11111111	51.36752137	52563.37607	51890.92695
47.02285714	59.42285714	39606.19429	39013.06305
15.46808511	10.87234043	3549.851064	3291.804436
29.63768116	21.92753623	15079.15942	14570.62424
53.52980132	35.2781457	27515.17219	27006.0871
11	9.684210526	5423.710526	4968.225069
17	10.61904762	2727.857143	2492.746032
28.57142857	19.45238095	8868.678571	8517.116071
44.77319588	20.21649485	129451.7113	125923.1064
9.974358974	13	7838.384615	7325.768573
8.92	8.6	2589.8	2322.1504
23.60377358	18.50943396	9334.415094	8908.343183
162.4734411	109.5727483	69712.73672	69262.96373
29.56666667	24.5	31931.3	30731.83222
3.7	7	3736.3	3121.26
3.869565217	6.130434783	4435.73913	3847.149338
41.03225806	39.17741935	45206.71774	44365.24993
8.828571429	9.228571429	14052	12820.99265
42.62711864	34.11864407	20434.72881	19965.52916
31.71428571	25.64285714	11038.19643	10718.04209
25.48275862	29.11494253	22737.57471	22066.94332
1.444444444	3.222222222	856	592.8395062
24.13043478	24.33333333	14589.47826	14045.03382
50.76551724	54.73793103	41993.68966	41229.2625
10.36842105	13.15789474	4942.105263	4512.088643
32.05050505	30.07070707	20981.16162	20424.86889
19.43333333	14.06666667	34432.45	32812.3875
80.04564315	64.95850622	38404.53527	37950.8983
30.70666667	17.16	15116.92	14448.52516
40.07216495	21.63917526	6542.329897	6299.355723
61.80821918	24.08219178	79437.03425	77847.42827
6.428571429	6.714285714	6280	5568.888889
97.55974843	102.427673	116149.4308	115266.8925
28.16666667	27.13888889	9574.833333	9228.459877
86.8442623	69.22131148	52563.94262	52023.57068
24.64044944	29.08988764	23581.79775	22843.66822
34.51648352	22.31868132	6379.472527	6135.975365
28.13043478	23.66666667	14408.34783	13899.81264
36.60330579	36.38842975	16712.73554	16318.99187
69.7961165	60.60194175	52402.02427	51727.28582
10.63636364	7.454545455	4932.5	4434.39876
8.533333333	8.933333333	1553.433333	1383.565556
4.866666667	4.2	4402.933333	3494.915556
41.77477477	19.36036036	3762.351351	3627.916728
51.41333333	36.2	15564.7	15219.97889
74.03139013	76.67713004	121631.9013	120352.6362
25.8	20.14285714	14866.22857	14315.99102
12.63157895	8.105263158	3043.184211	2756.862188
18.61538462	21.46153846	10734.11538	10222.65976
63.44311377	54.25748503	38045.88623	37467.87249
28.1627907	23.58139535	16665.05814	16087.3825
21.84210526	14.50877193	3323.877193	3144.693136
157.8	99.77974684	117182.9494	116422.4466
27.48780488	18	7020.47561	6757.798483
56.66423358	27.2919708	11722.9562	11399.21892
117.4575163	64.68627451	65613.04575	64888.97856
37.38095238	29.26666667	13914	13515.52236
12.05882353	10.76470588	5393.147059	4996.668685
117.5447154	49.53658537	11743.93089	11550.54004
10.25	7.666666667	2365.166667	2078.993056
119.7279152	61.28268551	27044.85866	26722.00252
123.1003135	95.29467085	69660.01254	69106.06688
39.32692308	33.01923077	16696.5	16279.39793
5.714285714	8.357142857	2877.285714	2568.530612
65.84023669	32.53846154	47728.88166	46860.20195
16.1	16.4	10132.78333	9637.720833
21.23880597	13.65671642	3566.029851	3364.982847
62.04635762	37.47682119	41046.21192	40314.70646
15.91304348	14.08695652	7305.043478	6906.780718
48.43636364	26.38181818	15549.40909	15146.12967
10.57894737	6.263157895	1605.105263	1316.105263
19.6779661	18.15254237	9828.728814	9331.967825
33.37623762	31.3960396	12843.36634	12525.75042
33.37362637	16.82417582	6150.637363	5911.917884
15.42553191	10.91489362	5074.638298	4765.776369
41.17307692	23	15480.55769	14999.09283
37.825	31.9	20017.1125	19457.19859
30.95959596	32.29292929	28668.33333	27916.24426
2.428571429	4.571428571	9382.214286	7943.637755
24.84375	20.90625	10130.75	9713.058594
35.70588235	30.45098039	61098.37255	59684.02268
23.65822785	19.48101266	12515.43038	12051.81862
31.55696203	21.63291139	8127.481013	7828.059606
11.48648649	10.83783784	6584.378378	6112.197224
29.64	34.34	27184.62	26543.5176
81.73148148	69.24074074	33853.83796	33419.61726
51.90510949	38.76642336	33040.93431	32408.24658
64.46524064	60.42245989	21341.80214	21029.06088
57.26865672	24.44776119	11292.8209	10976.29962
32.98198198	33.57657658	19207.74775	18719.77567
5.956521739	4.130434783	1149.695652	964.4990548
2.333333333	2.555555556	3632	2745.283951
150.1714286	75.48	76002.26286	75366.64673

log.sigma.0.5.mm.3D_glszm_ZonePercentage	log.sigma.0.5.mm.3D_glszm_LargeAreaLowGrayLevelEmphasis	log.sigma.0.5.mm.3D_glszm_LargeAreaHighGrayLevelEmphasis	log.sigma.0.5.mm.3D_glszm_HighGrayLevelZoneEmphasis
0.065096953	327.3380316	121407.2872	24.30851064
0.066282421	161.5446377	25126.73913	16.52173913
0.031390135	798.1382242	115856.7143	19.07142857
0.049689441	178.1281944	29053.625	12.6875
0.057406095	680.2678292	99840.22222	14.43209877
0.074380165	78.22111111	12381.72222	13.94444444
0.029509441	13049.3898	1763968.486	15.78456592
0.038562953	1868.470265	1526666.222	37.79059829
0.041060535	3712.716037	455829.6114	15.34857143
0.062251656	181.5592153	73450.91489	23.61702128
0.044344473	1350.643635	182525.7246	14.56521739
0.044320517	961.9415648	813612.5497	34.07284768
0.046855734	268.9502644	117315.0526	24.26315789
0.065217391	244.997123	34168.42857	14.97619048
0.053333333	865.3235759	98462.46429	20.21428571
0.016834432	11828.48827	1545550.742	15.40206186
0.04416761	403.5846063	160075.7436	20.43589744
0.061124694	225.6543667	32441.24	16.84
0.048446069	492.6198593	185776.2642	25.60377358
0.047152347	1671.939844	2977050.166	42.45265589
0.028873917	5719.079514	209337.8	7.1
0.040322581	336.207701	46037.75	18.6
0.041218638	412.5853986	52182.04348	16.7826087
0.034473172	4300.414626	511053.3145	17.14516129
0.028501629	1295.379944	169144.8286	19.17142857
0.046165884	1890.230856	239068.1102	18.43220339
0.055888224	606.9668463	210896.6964	26.94642857
0.03861518	2153.964486	258736.6322	16.35632184
0.061643836	165.5461111	5302	13.22222222
0.042857143	509.0946358	432007.058	32.73913043
0.036168621	3802.992397	503460.6069	14.73793103
0.04822335	441.3078216	60966.18421	16
0.042398287	1940.484961	245393.7273	16.95959596
0.02484472	3021.120055	430777	17.11666667
0.046951101	2049.748306	755193.805	24.04979253
0.038679732	1316.839104	191575.0667	16.34666667
0.064153439	323.140805	143877.3196	19.2371134
0.025081601	4093.048845	1632138.432	23.47260274
0.0375	553.8144974	77381.66667	13.38095238
0.03366148	2182.543671	6280694.604	65.0754717
0.053731343	866.105108	114989.8611	14.56944444
0.043018336	2889.162367	999661.1885	28.12704918
0.036807279	2028.581064	298560.2921	16.28089888
0.064084507	607.6220649	73463.75824	17.20879121
0.044344473	1323.228301	169845.8986	17.28985507
0.050395668	1552.238771	194593.719	15.1322314
0.038497477	2841.957848	1012269.466	27.7184466
0.044806517	259.2602146	98559.95455	23.27272727
0.076726343	136.7120741	19430.56667	16.83333333
0.033185841	374.554463	59713.86667	19.13333333
0.086247086	138.8095399	105014.1622	34.23423423
0.053859964	838.1652679	304161.94	26.44666667
0.027958877	11188.76653	1429828.036	15.03139013
0.042630938	487.1253031	468698.1286	27.72857143
0.059097978	253.5772003	40902.60526	16.26315789
0.044217687	979.4550481	127494.0577	15.59615385
0.041594022	3652.897433	425424.0299	15.58682635
0.041606193	1534.50879	197071.6744	15.91860465
0.074705111	180.7793872	64199.47368	24.9122807
0.03626182	6309.807986	2281562.765	28.47088608
0.061700527	363.6159308	142822.8049	23.73170732
0.055578093	620.9393736	239355.3285	26.69343066
0.037162983	3379.785991	1353162.114	22.96078431
0.05009542	331.8778746	598303.6857	45.51428571
0.050221566	278.0035294	110024.5882	21.85294118
0.071908799	289.6626995	494969.7846	53.58943089
0.0591133	203.6557523	31600.45833	17.25
0.055653884	1476.468948	521102.0353	27.23674912
0.042488013	2473.261156	2023763.787	32.36677116
0.048964218	1383.075155	218459.0962	14.43269231
0.056910569	250.8327381	36263.53571	17.03571429
0.03392893	2511.624797	958859.7811	23.22485207
0.04494382	925.1945133	120852.9333	17.55
0.070526316	356.6966976	38859.8209	20.53731343
0.036973555	3818.548669	477937.0795	17.13907285
0.050108932	641.4948338	90341.8913	15.39130435
0.049796288	531.4536022	471130.3818	34.87272727
0.058823529	145.5015351	20742.10526	18.84210526
0.04486692	502.5090943	202563.1695	23.16949153
0.056111111	1264.929547	139063.3564	17.69306931
0.064722617	330.2245205	120810.978	24.42857143
0.056900726	248.9525396	108765.8511	22.63829787
0.045574058	834.5416055	302755.4231	24.90384615
0.042260961	1904.908632	226166.475	14.5875
0.036464088	2612.221277	340909.5253	14.04040404
0.026365348	671.6284921	138141.4286	7
0.048929664	939.7209178	118257.0781	15.34375
0.026590198	3333.098102	1172239.078	24.78431373
0.046443269	435.1301043	372913.1392	32.12658228
0.057790783	270.41059	253256.1899	32.13924051
0.0460199	514.0858444	91442.78378	14.78378378
0.039494471	2597.22335	305712.21	16.71
0.047989336	3028.109972	410660.0972	16.83333333
0.039756239	2927.747913	404827.4745	16.38686131
0.056546719	1959.046777	251585.3102	16.14438503
0.056208054	589.8104596	232522.903	26.02985075
0.045269168	1780.315501	224467.973	15.79279279
0.073482428	59.11505108	24595.95652	21.2173913
0.03358209	624.1149691	24809.11111	8.111111111
0.039664551	2514.303446	2376423.494	29.55142857

log.sigma.0.5.mm.3D_glszm_SmallAreaEmphasis	log.sigma.0.5.mm.3D_glszm_LowGrayLevelZoneEmphasis	log.sigma.0.5.mm.3D_glszm_ZoneEntropy	log.sigma.0.5.mm.3D_glszm_SmallAreaLowGrayLevelEmphasis
0.613555327	0.146996837	4.40501501	0.10235334
0.63015732	0.157789855	2.940676219	0.103867188
0.428147812	0.152748016	4.137537511	0.09651678
0.589195385	0.282795139	3.25	0.184225227
0.624921782	0.196227709	4.016910684	0.127324824
0.535851444	0.30779321	3.530493057	0.232921827
0.616423732	0.181047167	4.250417872	0.115761567
0.600102867	0.050388973	4.676273841	0.035219906
0.531286012	0.170752381	4.308503732	0.098024074
0.586415021	0.101677401	4.057130545	0.07148651
0.678723889	0.140954106	3.366126279	0.099579473
0.615663935	0.058133744	4.3051514	0.041553881
0.550466656	0.109074621	3.914479238	0.080792409
0.655887184	0.313022487	3.621352175	0.227128621
0.602982484	0.211944242	4.283658482	0.16318014
0.69739524	0.14572013	4.051556756	0.105605039
0.50466637	0.108965238	3.687869846	0.077486961
0.596224706	0.1733	3.044889452	0.085977994
0.692022073	0.082999722	3.213556194	0.062240736
0.635040857	0.03916565	4.42155318	0.027175245
0.725629279	0.561805556	2.931687774	0.434386484
0.397732652	0.174756519	3.646439345	0.114632927
0.3674309	0.169565217	4.001822826	0.103566613
0.591281549	0.129090502	3.993839191	0.074892987
0.480531246	0.172547619	3.921721231	0.073318415
0.624192909	0.150573956	4.060768851	0.103804387
0.549274458	0.091747462	4.543381098	0.060036477
0.551507381	0.154618357	4.032882836	0.078486299
0.271479459	0.190401235	2.947702779	0.01736743
0.611316379	0.059084221	3.838578349	0.030634907
0.613614332	0.164261436	3.786215323	0.108104709
0.529146899	0.181184211	3.750720856	0.121735274
0.587047846	0.200583442	4.042087217	0.150583666
0.581782363	0.182092309	4.149791896	0.108910454
0.596070514	0.097886399	4.344244404	0.067263056
0.652678208	0.259537037	4.010001915	0.199103971
0.66724502	0.165049589	4.105107556	0.124211001
0.675638101	0.099034582	4.468651756	0.078154513
0.563145887	0.251600529	3.386630971	0.193640201
0.570838544	0.021540859	4.243193986	0.013522294
0.648380569	0.158209877	3.378601595	0.108409828
0.61858358	0.063162632	4.167739299	0.041213796
0.531572353	0.145689763	4.159789781	0.084084216
0.636372613	0.241225487	4.036885356	0.17696578
0.661126019	0.184967794	3.466772923	0.149100486
0.568772126	0.190705853	4.242525051	0.12975704
0.603016692	0.069945416	4.301888616	0.045789306
0.704547153	0.108510101	2.699019984	0.095328368
0.507118362	0.171268519	3.577747163	0.110644963
0.574149798	0.191351852	3.189898095	0.154082009
0.635145639	0.05690691	4.477434767	0.043290462
0.605675212	0.079399452	4.371694397	0.050598456
0.59338108	0.161668497	4.05340306	0.100607142
0.62897294	0.072947481	3.961731227	0.055281235
0.594378962	0.255277778	3.998350054	0.176750517
0.598031496	0.148637821	2.99180318	0.066709245
0.639789142	0.151679091	3.995617411	0.098520265
0.592624305	0.193515728	4.155446082	0.144533032
0.640001365	0.096090872	3.971282923	0.07340173
0.656920333	0.072860835	4.312552208	0.053838954
0.597390583	0.091108588	4.316139605	0.062537226
0.668031737	0.165219154	4.177457864	0.13657188
0.642831859	0.138517009	4.370368856	0.103497692
0.618043641	0.039917232	4.261187606	0.029562124
0.613705708	0.10504085	3.550266885	0.077393256
0.717315373	0.035185159	4.249500548	0.026728647
0.672981419	0.269131944	3.000961983	0.236841556
0.675545469	0.091811867	4.351886992	0.069095676
0.644021647	0.054566778	4.023626835	0.036003129
0.638205554	0.180683761	3.913126475	0.128830869
0.423369401	0.166884921	3.937193209	0.073656832
0.648684552	0.120675467	4.375440153	0.093473627
0.52860834	0.211479025	4.215922064	0.142166559
0.582733207	0.13685518	4.37616185	0.076633471
0.664194869	0.200908644	3.973157354	0.139971118
0.601301781	0.170757665	3.671061798	0.121631479
0.688721624	0.085227533	4.059309269	0.063193181
0.73842102	0.217324561	2.629249224	0.190888843
0.59306481	0.092442638	3.940487432	0.068635439
0.594016579	0.17648919	3.87200525	0.124490904
0.624018857	0.137634341	4.291997313	0.094545847
0.588571262	0.115513461	4.073532671	0.088657609
0.647730365	0.082365635	4.230450592	0.052096681
0.71352328	0.158017361	2.92367593	0.120573394
0.577631455	0.201781706	4.083966416	0.133250881
0.389843316	0.298988095	3.324862958	0.200301303
0.646169143	0.153092315	3.708918899	0.100536385
0.610477706	0.077971411	3.975793371	0.051860732
0.563342935	0.066419083	4.167693163	0.051433748
0.653300836	0.068477461	3.713626662	0.055396297
0.572425472	0.24142995	3.662986313	0.181948238
0.555674358	0.157461111	3.808925574	0.094558401
0.63867675	0.184087538	4.048435026	0.133391065
0.638368352	0.200163034	4.07886237	0.145200517
0.609187956	0.156167558	4.069246888	0.106663404
0.676037354	0.133124599	4.293227858	0.109194882
0.557331838	0.184974975	4.162744478	0.114355055
0.500035195	0.183540927	3.936180434	0.099543921
0.423624965	0.406635802	2.947702779	0.168598308
0.680599137	0.071734509	4.38299434	0.053976565

log.sigma.0.5.mm.3D_ngtdm_Coarseness	log.sigma.0.5.mm.3D_ngtdm_Complexity	log.sigma.0.5.mm.3D_ngtdm_Strength	log.sigma.0.5.mm.3D_ngtdm_Busyness	log.sigma.0.5.mm.3D_ngtdm_Contrast
0.0043509	0.013868596	0.090121756	5.322599611	1.94E-05
0.015057459	0.027192345	0.191349778	2.619351804	7.01E-05
0.007397686	0.009895757	0.07976525	5.698405163	3.09E-05
0.017562838	0.019918277	0.134997784	3.218779289	0.000156765
0.004492823	0.006377806	0.049237857	9.311445531	1.99E-05
0.02406556	0.042221043	0.251558304	1.819736525	0.000181975
0.000482474	0.00119261	0.011062791	63.251839	1.22E-06
0.000973741	0.005011131	0.044333924	13.0269811	1.78E-06
0.001302211	0.002065784	0.016582533	31.79174225	4.91E-06
0.007770904	0.02010175	0.129138117	3.416219935	3.80E-05
0.003111208	0.00543585	0.047925211	11.96076001	8.76E-06
0.001621229	0.008728928	0.079978629	7.693543998	2.93E-06
0.007614909	0.017220133	0.129218853	3.359248212	3.14E-05
0.009221254	0.016261199	0.092691431	5.078436651	7.06E-05
0.00393224	0.009294241	0.063107882	9.276797694	1.90E-05
0.001010003	0.002122815	0.021052432	31.10785301	2.37E-06
0.005952507	0.019109952	0.179339849	3.291781856	1.23E-05
0.013569001	0.020734045	0.178546042	2.881115822	4.80E-05
0.004600765	0.011606351	0.104646261	5.219653107	1.21E-05
0.000600311	0.003961029	0.04068243	15.15407913	8.48E-07
0.002636819	0.001348488	0.013529608	37.70673841	1.33E-05
0.011928071	0.022904072	0.164568274	3.683133917	6.83E-05
0.010991793	0.01655549	0.122662918	3.940781481	4.89E-05
0.001386828	0.002354865	0.02056218	28.23953586	4.23E-06
0.004769795	0.007858202	0.052299059	9.248272286	2.62E-05
0.002226814	0.004947413	0.044403327	14.73387904	7.10E-06
0.003058886	0.009504203	0.069374955	7.223796621	1.01E-05
0.002427544	0.005700873	0.051124687	13.25324943	6.63E-06
0.033595365	0.045130019	0.282645069	2.372182115	0.00026714
0.003364418	0.01126021	0.114725028	4.88772341	7.08E-06
0.001439185	0.003104957	0.029828327	21.91940097	3.51E-06
0.007989029	0.012143953	0.085196479	5.543175507	3.95E-05
0.002303119	0.005588987	0.047847735	13.95170474	6.98E-06
0.002312555	0.005595223	0.044835833	13.806179	7.65E-06
0.001112934	0.003444029	0.029128347	18.72065876	2.91E-06
0.003172025	0.004965764	0.033448119	13.82114028	1.79E-05
0.003965678	0.013863745	0.085061082	5.510341205	1.93E-05
0.001006434	0.002959088	0.02661528	20.08551925	2.28E-06
0.008600952	0.010840273	0.082319995	5.947119857	4.52E-05
0.000539506	0.002791948	0.039436624	18.632839	8.25E-07
0.004238509	0.006380706	0.05493158	9.462135796	1.39E-05
0.000880842	0.003113239	0.026763089	22.87493758	2.05E-06
0.00253453	0.003468799	0.030936439	15.60799609	8.64E-06
0.004366757	0.010522511	0.069203847	8.368955136	2.16E-05
0.003241479	0.005692112	0.044815781	12.18530131	1.19E-05
0.002453719	0.005385442	0.046681229	13.41239232	7.69E-06
0.001022831	0.003253652	0.028680126	20.05781822	2.35E-06
0.009494234	0.019585309	0.195641784	3.041020683	2.75E-05
0.015380442	0.024747364	0.165515761	2.846796941	8.77E-05
0.013464799	0.020948937	0.149427305	3.080856549	7.24E-05
0.004889871	0.020714321	0.147563329	3.14783887	1.53E-05
0.002129559	0.006990166	0.049550464	10.28502719	7.22E-06
0.000609256	0.00157669	0.014786197	50.03052033	1.40E-06
0.003180878	0.01124841	0.108640984	5.030596603	7.56E-06
0.009850006	0.015171378	0.099573889	4.430510754	6.32E-05
0.004837729	0.006936472	0.065088675	8.179865651	1.43E-05
0.001370999	0.003131794	0.029334142	23.22748845	3.53E-06
0.002974214	0.006409497	0.053973105	11.15670719	9.29E-06
0.007844095	0.024785321	0.182423205	2.775671201	2.44E-05
0.000465691	0.001635861	0.013670633	43.95491849	1.17E-06
0.00459039	0.013420287	0.110505379	4.576634352	1.26E-05
0.002514092	0.008634317	0.050078778	9.203211016	1.29E-05
0.000736361	0.003207473	0.023158175	24.69176379	2.16E-06
0.002748953	0.012268307	0.113904025	4.388479711	6.14E-06
0.008043156	0.014065975	0.14035301	3.722401599	2.71E-05
0.001820513	0.013685201	0.085156631	5.560047577	5.46E-06
0.012954397	0.024577465	0.156368971	3.154893418	8.25E-05
0.001185741	0.005173185	0.037915468	15.79472214	3.42E-06
0.000693513	0.003031162	0.029180807	20.25013301	1.31E-06
0.002693489	0.003926031	0.035112341	13.98666507	8.82E-06
0.013232925	0.017427483	0.146389366	3.119650098	5.21E-05
0.001236438	0.003632742	0.02930767	17.12436448	3.45E-06
0.004729189	0.009697894	0.084219808	7.091274204	1.51E-05
0.00625279	0.015948233	0.102310773	5.851647517	3.22E-05
0.001329239	0.003280649	0.026607428	24.56532363	4.37E-06
0.005880125	0.013332092	0.128454728	5.14633197	1.43E-05
0.002635349	0.010424836	0.097018303	5.42986575	5.91E-06
0.018933461	0.031386425	0.194629482	2.364155283	0.000126209
0.004412529	0.010585709	0.081906809	5.697312189	1.54E-05
0.002987158	0.007232978	0.062454426	10.81985762	9.22E-06
0.004414794	0.014182578	0.093322756	5.178955045	1.86E-05
0.007045863	0.015916137	0.134051972	3.448809089	2.33E-05
0.002705925	0.006086993	0.046103934	9.810777594	9.75E-06
0.002754402	0.004212005	0.041497989	14.01320523	6.68E-06
0.002049342	0.003183742	0.026504253	19.61236378	7.37E-06
0.009219656	0.010354432	0.102331782	4.632749261	3.77E-05
0.0043598	0.009699648	0.088948955	7.412871759	1.17E-05
0.001343338	0.003203878	0.031503393	17.80149601	3.06E-06
0.003421152	0.013979518	0.123821614	4.24276332	7.36E-06
0.00452408	0.01345839	0.129069312	3.713966505	1.16E-05
0.006760217	0.016904858	0.131847717	4.400292064	2.31E-05
0.002044676	0.003355599	0.029016346	19.37263873	6.67E-06
0.001210007	0.003027404	0.023945611	26.45669904	3.97E-06
0.001620754	0.003624293	0.033482313	19.13332424	4.38E-06
0.001630127	0.002762245	0.020582132	25.23292004	7.04E-06
0.002788445	0.011273041	0.077920631	6.812840172	9.37E-06
0.002375888	0.003609781	0.028453147	17.94032427	1.04E-05
0.018308362	0.070520471	0.381854457	1.252455154	0.00011577
0.018131149	0.011474253	0.097139924	5.16824915	0.000102994
0.000667683	0.002651358	0.024473852	21.00528172	1.38E-06

original_gldm_GrayLevelVariance	original_gldm_HighGrayLevelEmphasis	original_gldm_GrayLevelNonUniformityNormalized	original_gldm_DependenceEntropy	original_gldm_DependenceNonUniformity
198.5582422	1482.768006	0.030103552	7.115418283	404.1038781
104.5193133	350.204611	0.036268053	6.203714969	126.129683
192.4719882	736.4282511	0.033848459	6.989697586	210.1479821
132.4263339	414.7080745	0.037884341	6.311830343	94.1863354
129.8025878	500.8632176	0.03805927	6.838327403	349.897236
236.8178403	784.2231405	0.031725975	6.444080996	69.30578512
139.4641727	1396.963659	0.10062941	7.285065581	1268.698833
162.6906969	1237.791365	0.072109533	7.087019785	968.5702044
115.5668103	1070.118724	0.073138235	6.947852625	682.4448616
146.8786211	665.3907285	0.028881189	6.791040554	227.392053
92.3564554	549.9479434	0.059829766	6.736294852	295.8817481
111.8933104	1262.889052	0.069153969	7.043282943	529.6786029
152.5188461	411.3279901	0.046249093	6.777407451	186.2848335
159.5950388	636.515528	0.029180008	6.750060682	210.9782609
149.2756398	1128.230476	0.064154397	6.86879759	278.0755556
194.6563406	1105.057619	0.095412728	6.8511289	753.9951406
75.93381977	799.0747452	0.044826848	6.731032016	192.8686297
125.3096646	482.8801956	0.038229088	6.356201333	123.9046455
98.35184186	740.9789762	0.057428085	6.873372194	198.3400366
167.9109022	1185.439617	0.040682902	7.596015768	1785.833497
121.3454792	482.6015399	0.035288799	7.002441028	507.8700674
92.18076467	435.4012097	0.037851197	6.564165777	134.4475806
150.2555498	665.2526882	0.042573965	6.592960607	137.781362
80.22205574	1076.478176	0.098644354	6.816311638	509.8940784
214.372685	1210.782573	0.100194697	6.826227167	159.8778502
121.1378075	1226.856416	0.052834963	7.093735218	462.3943662
152.563188	1223.263473	0.035883323	7.097758781	505.9341317
110.5000539	1087.63826	0.043210522	7.018762108	488.8180204
70.82083881	435.2123288	0.038937887	5.998636867	47.42465753
117.1779441	717.5496894	0.045556884	6.884727886	340.7006211
153.0436768	908.9575954	0.052875853	7.222988212	735.8421053
146.3065075	519.8553299	0.035616738	6.72185291	213.3680203
147.6806185	917.2381156	0.044517055	7.147458731	446.9263383
151.4379305	1046.500621	0.106317057	6.61313333	329.7701863
128.5825703	1219.087473	0.053186183	7.237585908	916.4689266
195.7076751	706.7148014	0.04072616	6.9706738	443.4878804
182.8333176	537.3783069	0.030608746	6.988813596	474.5251323
199.1043301	1207.235011	0.106526108	7.181064009	615.0267995
76.51884885	563.4303571	0.036084184	6.652329917	144.3285714
89.18645775	1294.930666	0.108462225	6.856339722	1216.892664
134.0167582	663.9440299	0.034739363	6.942516473	331.0477612
62.59083842	1175.92842	0.071735852	6.944912899	956.9400564
182.2762367	858.0181969	0.028322185	7.203292739	666.0554177
134.7854091	913.706338	0.033731402	6.901526685	403.1647887
94.79373773	905.0302057	0.079313182	6.76455308	228.0989717
148.0592752	874.9466889	0.042786039	6.920829354	577.0982924
113.3122395	1107.276023	0.085640338	7.012193944	779.8331153
88.1793671	558.389002	0.051513807	6.484616381	111.6435845
82.4844029	520.8030691	0.032149188	6.473205226	122.8414322
116.1347159	326.8340708	0.048085206	6.294822885	130.6061947
169.5937693	984.3923854	0.04662548	6.784104798	313.4428904
136.1458235	1023.586715	0.026076216	7.131709295	858.0850987
72.14856982	1140.764669	0.108064131	6.985467953	914.8871615
160.7534646	1469.941535	0.060955491	7.147625912	274.8331303
166.4928008	568.7978227	0.031503281	6.804583012	177.6189736
116.6734687	589.7729592	0.033886344	6.81138909	314.5646259
110.9021322	930.7865504	0.068193155	6.9516994	714.0052304
168.9608469	922.0875665	0.036222211	7.063966378	507.7460087
141.5594613	1121.175623	0.029745658	6.883464769	226.5465269
85.32903413	1338.71523	0.081341973	7.053734701	1621.379693
156.0790322	979.1083521	0.040519386	6.953880925	294.4778029
195.7599174	887.6953347	0.031329485	7.198822296	660.2170385
188.736841	1025.557931	0.034339745	7.519234172	1619.836774
117.6451527	983.4813931	0.026864602	7.160754245	629.9933206
137.7562755	813.6794682	0.044913152	6.769051635	157.3249631
175.7886837	766.2318036	0.028312243	7.140742741	1023.151418
125.9356512	496.4507389	0.034070227	6.442218878	127.9162562
140.2845656	1074.190954	0.04212575	7.174062081	1115.565978
93.67608516	1198.893181	0.051126357	7.227559129	1459.018114
119.9708834	512.0630885	0.033363657	6.929360136	570.5725047
146.4739573	544.8495935	0.042922533	6.569989663	115.0853659
185.236709	1154.156997	0.066474043	7.011822346	816.8968079
158.4735575	995.6546816	0.045325927	6.843993491	329.540824
129.1835391	818.0526316	0.037429363	6.773776153	261.8421053
123.9714898	1206.097943	0.08110471	7.12489631	581.3805093
97.23602033	838.2854031	0.041683873	6.768383019	217.4008715
122.2822348	769.7071073	0.027683982	7.173750333	609.4762336
144.1193149	437.5572755	0.03436245	6.337728525	103.7678019
118.4120054	561.4904943	0.031285113	6.858336953	365.9475285
84.26198889	1175.683333	0.064074691	6.804851874	336.7411111
196.0748449	1294.200569	0.030713726	7.11948042	385.4978663
116.7379961	607.8486683	0.033042346	6.721832729	250.3099274
181.3566265	981.2151621	0.099380435	6.854161943	335.5390009
77.03600191	720.6619123	0.043341764	6.870957591	416.9704173
127.4603863	903.293186	0.052775353	7.157944635	466.3627993
93.05627374	259.2768362	0.067558989	6.189544964	121.8060264
141.9574408	775.3570336	0.035232491	7.030295655	315.0351682
114.1526776	932.8793014	0.102852239	6.916577199	477.24244
135.5066733	953.0094062	0.040811142	7.218891725	360.5390947
128.9675029	502.1609364	0.032868498	6.808492935	379.2955377
155.9662307	428.6679104	0.045203089	6.801596197	176.0074627
77.00427888	964.2535545	0.059757692	7.025311178	390.3649289
116.3507816	1006.022884	0.033366551	7.169334294	1136.007332
130.4388519	902.0240859	0.045725629	7.062974502	732.01509
75.29509816	861.4980345	0.037121594	6.924764772	831.4366495
196.6381608	876.8544463	0.031781719	7.183854574	581.965604
135.2565898	824.6402936	0.038711948	7.081652498	575.7128874
141.8446039	548.5175719	0.030632139	6.281181939	118.0798722
70.41928882	312.1753731	0.03920695	6.149643531	76.52238806
204.0487234	899.2231414	0.043506326	7.412766475	1643.050091

original_gldm_GrayLevelNonUniformity	original_gldm_SmallDependenceEmphasis	original_gldm_DependenceNonUniformityNormalized	original_gldm_DependenceVariance	original_gldm_LargeDependenceEmphasis
43.46952909	0.494762566	0.279850331	1.686890447	6.325484765
12.58501441	0.591539385	0.363486118	0.870582764	3.991354467
30.19282511	0.444674021	0.235591908	2.826228157	9.087443946
12.19875776	0.518301645	0.292504147	2.601365688	7.49068323
53.70163005	0.472463823	0.247978197	3.931263973	10.3579022
7.67768595	0.527381667	0.286387542	2.421897411	7.438016529
1060.533352	0.300522398	0.12038133	18.28027547	42.3739444
437.560646	0.372836177	0.159619348	9.134092785	23.04251813
311.7151572	0.361545833	0.160123149	7.81053964	20.72125763
21.80529801	0.518164037	0.301181527	1.430509188	5.574834437
93.09511568	0.392504318	0.190155365	4.457259733	13.3496144
235.6075726	0.349998624	0.155467744	7.853550017	21.16201937
37.5080148	0.438883657	0.229697699	2.860440825	9.281134402
18.79192547	0.572088142	0.327605995	2.698805987	7.093167702
101.0431746	0.392921297	0.176555908	7.16936256	18.6431746
549.7681361	0.294831344	0.130856498	8.143266378	24.31030892
39.58210646	0.413963043	0.218424269	3.817232255	11.14949037
15.63569682	0.548822117	0.302945343	3.442650391	8.535452323
62.82632541	0.371329695	0.181298022	6.31883065	16.63619744
373.5910922	0.395181007	0.194471686	5.253524767	14.31242513
73.33012512	0.433193123	0.244403305	4.543167757	11.35129933
18.77419355	0.492784988	0.271063671	3.23073296	8.681451613
23.7562724	0.469093508	0.246920004	4.010739841	10.52329749
354.823742	0.317640038	0.141755373	13.27806719	30.52237976
123.0390879	0.334426208	0.130193689	21.85189233	46.46254072
135.0461659	0.3763099	0.180905464	6.168927143	16.55164319
71.91017964	0.477118851	0.252462142	2.900969518	8.719560878
97.3533067	0.417578446	0.216963169	3.076239621	10.08122503
5.684931507	0.529680365	0.324826421	0.837305311	4.410958904
73.34658385	0.415176542	0.211615293	4.098783226	11.83478261
211.9792966	0.400787482	0.183547544	7.962841526	19.21351958
28.06598985	0.509399406	0.270771599	4.335650236	10.45939086
103.9473233	0.400499571	0.191403143	5.411596917	14.76445396
256.7556936	0.292075833	0.136550802	6.457044096	20.97267081
273.0046756	0.384716085	0.178544502	6.929503684	17.80420807
78.96802476	0.447724529	0.228719897	3.687814069	10.57607014
46.28042328	0.537848842	0.313839373	1.390036673	5.337301587
620.0884728	0.273193275	0.105656554	16.33944115	42.57052053
20.20714286	0.4686468	0.257729592	2.67244898	8.228571429
1024.642638	0.304583832	0.128812603	11.61951587	30.33862602
46.55074627	0.460938134	0.247050568	3.706535977	10.0238806
406.8857546	0.34574451	0.168712986	6.822835829	18.2122708
68.48304384	0.510496927	0.275457162	2.687800553	7.910669975
47.89859155	0.521423145	0.283918865	2.866415394	8.008450704
123.4113111	0.338823964	0.146593169	9.074930115	23.92416452
102.7292795	0.464935386	0.240357473	4.320483222	11.16826322
458.2614465	0.346332714	0.145735959	12.44507221	30.02672398
25.29327902	0.437752385	0.227380009	4.584384501	11.93279022
12.57033248	0.498721561	0.314172461	1.281009413	5.352941176
21.73451327	0.506343566	0.288951758	1.456770303	5.818584071
60.00699301	0.489998649	0.243545369	4.751982197	12.06759907
72.62226212	0.518459344	0.308109551	1.325298583	5.341113106
861.9195085	0.275117828	0.11470501	14.51129989	36.87888666
100.088916	0.376019988	0.167377059	6.585673572	18.4591961
20.25660964	0.517554819	0.276234796	2.436363373	7.556765163
39.85034014	0.481004261	0.267486927	1.995276852	7.027210884
273.7955168	0.382998276	0.177834428	7.820970613	19.06475716
74.87131108	0.466191925	0.245643933	3.099534253	9.194484761
22.69593709	0.542205199	0.2969155	2.645242189	7.487549148
886.0581107	0.330567475	0.14884602	8.887183947	23.12944093
53.85026336	0.429239554	0.221578482	5.917283089	14.01128668
77.22718053	0.501636609	0.267836527	3.223484976	8.865314402
282.7534613	0.402013104	0.196725379	5.734349698	14.81175613
56.30820611	0.518452022	0.300569332	1.660989125	5.920801527
30.40620384	0.455454963	0.23238547	4.003857491	10.95568685
96.8561824	0.527919909	0.299079631	2.864845217	7.657117802
13.83251232	0.531199179	0.31506467	1.334587105	5.256157635
214.2094395	0.434853124	0.219383673	4.960508175	12.81042281
383.8566862	0.386035807	0.194328465	5.008427097	13.94938732
70.86440678	0.462588193	0.268631123	1.64343739	6.535781544
21.11788618	0.457355877	0.233913345	3.923094719	10.77642276
331.1072074	0.363065093	0.164002571	6.931140905	19.07107007
60.51011236	0.481478792	0.246847059	6.967889015	14.91310861
35.55789474	0.513050449	0.275623269	2.758621607	8.063157895
331.2316357	0.33684105	0.142355658	13.03575214	31.33839373
38.26579521	0.449185862	0.236820121	4.474774659	11.51633987
61.1539158	0.467964817	0.275905945	1.357175192	5.975101856
11.09907121	0.560758918	0.321262544	1.401316988	5.26625387
41.13992395	0.485394332	0.278287094	2.119230291	6.972623574
115.3344444	0.395742993	0.187078395	5.757011111	15.51222222
43.18349929	0.484206613	0.274180559	1.729144957	6.509246088
27.29297821	0.510959449	0.303038653	1.803539916	6.159806295
226.7861525	0.356995933	0.147037248	16.31089785	35.62927257
82.0459588	0.418885241	0.220269634	3.883093076	11.20707871
143.2850829	0.355226178	0.17177267	6.905711059	18.22651934
35.87382298	0.420793595	0.22938988	2.727490681	9.124293785
46.08409786	0.463818625	0.240852575	3.149073217	9.383792049
394.5411887	0.314124773	0.124411481	17.20219212	40.36548488
69.41975309	0.428337603	0.21195714	6.367011002	15.17754262
44.93123628	0.483711697	0.277465646	1.638091732	6.310899781
36.34328358	0.41431003	0.218914755	3.083401401	9.957711443
151.3064771	0.328699145	0.154172563	6.062720714	18.37361769
150.1828483	0.456229629	0.252389987	2.607022824	8.297489447
157.5705165	0.425904668	0.212424576	4.702182651	12.72721997
122.7611128	0.445017405	0.251417191	2.475125795	8.177502268
75.76761745	0.462141931	0.244113089	2.679478824	8.578020134
94.92169657	0.447552538	0.234793184	4.823364221	11.98531811
9.587859425	0.571713703	0.377251988	0.900876808	4.022364217
10.50746269	0.482291667	0.285531299	1.317442638	5.746268657
383.8998187	0.39138798	0.186202413	5.980089484	15.84156845

original_gldm_LargeDependenceLowGrayLevelEmphasis	original_gldm_SmallDependenceHighGrayLevelEmphasis	original_gldm_LargeDependenceHighGrayLevelEmphasis	original_gldm_SmallDependenceLowGrayLevelEmphasis
0.009300665	604.3912341	11823.78047	0.002061518
0.09959906	205.7550768	1368.354467	0.014624187
0.273390499	277.9828779	9669.132287	0.007391944
0.149048984	182.1243556	5183.534161	0.020854435
0.10483713	195.9345805	9080.120482	0.007102567
0.123025099	364.9407953	8451.983471	0.020424356
0.064542753	321.7517968	75529.62311	0.001292972
0.029097503	332.66485	38746.31444	0.001601107
0.022944996	273.0102914	29933.19005	0.001582156
0.074310309	300.7834232	4948.410596	0.005502219
0.063623698	166.5662808	10447.17802	0.005163131
0.016864926	337.4430339	33837.67684	0.00118441
0.312422722	199.6086762	2750.669544	0.007296173
0.07107532	311.5012531	7447.257764	0.010977173
0.024162095	298.4377247	29564.98603	0.003236613
0.105498097	236.2060357	35420.38077	0.002838219
0.015343242	268.8368641	12283.49604	0.002323117
0.068530166	202.0697218	7945.93154	0.011160238
0.032518814	209.9047908	16756.08135	0.003519774
0.023599006	448.3581508	20764.6353	0.001362151
0.106359165	185.7352525	9905.662175	0.006305487
0.045779651	159.7691662	6560.171371	0.010322219
0.044517977	215.0082573	11820.48566	0.008286359
0.036696621	257.0058304	42369.37225	0.001658084
0.07856556	255.0180697	82033.4487	0.004657855
0.014746032	353.7985317	26958.60994	0.001279418
0.015997645	481.2041782	14227.93413	0.00193844
0.012945621	355.4747827	14233.25832	0.00154022
0.030440505	218.2156107	1998.458904	0.011281341
0.028578109	226.9388392	12402.09627	0.003508386
0.063241551	273.1630676	25028.56448	0.002863214
0.129746389	220.9208863	10085.47716	0.011796765
0.03019513	260.4599788	19898.85182	0.003175951
0.075213783	230.0435623	27326.48986	0.003927165
0.016631916	366.297572	28897.30918	0.001081701
0.087216689	250.6257013	11902.03713	0.005498058
0.113142245	319.7902633	2626.142857	0.009074362
0.154922989	210.4899251	67423.30304	0.004137944
0.020718518	207.3150909	6752.983929	0.005504247
0.02303329	291.1355844	48872.69694	0.000957269
0.063765601	227.918231	10697.89627	0.005299684
0.015199495	325.3205126	26129.08145	0.00115212
0.060825448	353.3198971	9911.099256	0.004735611
0.014992671	387.3061196	10566.07394	0.002968672
0.029422308	211.1325921	29092.18959	0.003152956
0.022912826	305.0927063	15198.15285	0.002671554
0.030018159	270.2200287	45640.51878	0.001437892
0.033970229	215.8515866	9283.847251	0.005689716
0.061852659	241.1001262	3502.235294	0.005533852
0.1846607	163.7146468	2028.440265	0.012149962
0.038852837	339.6308264	17925.93395	0.004814026
0.016498977	460.5842322	6902.396768	0.002647708
0.030019581	234.3887491	51545.39957	0.000905024
0.026431834	546.5861091	28284.93179	0.002327777
0.163382024	264.8565147	6294.785381	0.007455673
0.035888867	237.1528987	5747.118197	0.004762677
0.040324588	258.5175302	24196.79253	0.003783036
0.022977461	349.7413849	12219.9821	0.002520175
0.010162613	510.7450344	11992.42857	0.003447424
0.017614401	340.6731478	37789.92224	0.001159166
0.022648318	351.7383899	21750.9082	0.002108345
0.03367551	373.6173093	11870.00446	0.00410302
0.035302194	344.9176419	22311.93199	0.001524338
0.01794247	508.3709753	6609.327767	0.001915461
0.024157425	328.8963856	11712.71935	0.004842961
0.038130777	364.8593194	9210.043555	0.003370791
0.04967096	242.0801655	3515.519704	0.007095043
0.018102854	373.0345038	20031.76814	0.001451713
0.013063436	409.4673967	20260.15024	0.000988292
0.057571039	231.7360381	4092.547081	0.003917955
0.095920952	182.858153	9647.394309	0.00910072
0.025871426	289.1106248	31783.96065	0.001895634
0.018747872	366.5376173	24372.58202	0.002579426
0.023240302	328.5857335	9173.865263	0.004939502
0.032181312	304.0444299	50051.1523	0.001453206
0.016201173	314.7139263	14953.69281	0.002194978
0.023274468	378.319766	4493.426437	0.001927453
0.115604805	241.2232069	2993.006192	0.02241115
0.039738758	249.0561396	5875.51635	0.003909939
0.01354409	349.921147	23424.24556	0.001564315
0.017416458	510.2323015	10719.0128	0.004127738
0.025280506	302.6483463	4745.531477	0.003410266
0.061467252	228.7747692	51973.58414	0.004065513
0.019492875	224.3191618	11431.02747	0.003920489
0.028165063	223.2514752	23287.84788	0.002670201
0.266806303	157.4507192	973.1563089	0.007618923
0.063044973	287.0821616	10516.02217	0.004940595
0.048620193	187.2274531	52762.38816	0.002798384
0.024378347	347.9375376	20911.46326	0.003002355
0.089830691	225.1542307	4016.359912	0.005747384
0.242310139	174.5037339	5835.620647	0.008358009
0.021065858	228.4709105	22380.36019	0.001896556
0.01302416	391.3929031	11214.69162	0.001268575
0.027004506	307.5449624	17012.79251	0.002127458
0.011502226	328.4469698	9174.996069	0.001356176
0.04310321	362.1277853	9237.059983	0.002274661
0.028765359	301.3797126	15554.34829	0.003443872
0.047229269	301.0267643	2244.881789	0.008722169
0.154755221	155.54037	2000.570896	0.007428673
0.078419614	331.4135097	19214.50102	0.002483007

original_gldm_LowGrayLevelEmphasis	original_gldzm_DistanceZoneVariabilityNormalized	original_gldzm_LowIntensityEmphasis	original_gldzm_LargeDistanceEmphasis	original_gldzm_HighIntensitySmallDistanceEmphasis
0.002926723	0.84876144	0.004007508	1.247232472	1154.608549
0.024315377	0.878071834	0.024786019	1.195652174	321.4467391
0.023789092	0.810014069	0.019906413	1.318872017	570.7223427
0.033052573	0.821668837	0.038417161	1.296875	322.3359375
0.014929483	0.822108726	0.015556817	1.296052632	344.1434211
0.031140458	0.84625165	0.038047903	1.251748252	624.2202797
0.004049843	0.867946214	0.004708598	1.21323741	984.7603597
0.003246445	0.843768564	0.004564872	1.256232121	789.7798324
0.002839772	0.926837492	0.004469289	1.114081996	691.3817588
0.014066878	0.914881375	0.011807403	1.13363029	546.669265
0.010221662	0.900414513	0.013653145	1.157664234	395.2036496
0.002018169	0.901501096	0.003396417	1.155844156	890.9100458
0.023105498	0.774280011	0.01729676	1.389027431	402.117207
0.018514783	0.817293881	0.019942158	1.305084746	445.7935835
0.005025814	0.91636138	0.008307774	1.131195335	709.4271137
0.008709966	0.948478634	0.010177945	1.07938258	746.8127067
0.003382085	0.949459327	0.005234342	1.077830189	622.7323113
0.018847512	0.847771407	0.021413432	1.249011858	307.5731225
0.00557717	0.900483124	0.009104714	1.157549234	524.7527352
0.002996446	0.903086815	0.003518532	1.154372252	1071.481253
0.015456938	0.906111873	0.015147976	1.148148148	401.1944444
0.014106489	0.911454082	0.019557331	1.139285714	300.6258929
0.012566125	0.879862126	0.017263351	1.192567568	428.9510135
0.003287301	0.920853107	0.005195732	1.123831776	758.232866
0.008634981	0.934572915	0.014071695	1.101580135	727.4012415
0.002199428	0.92211435	0.003396323	1.121771218	886.8892989
0.003674773	0.917567928	0.004429866	1.129213483	955.073736
0.002599665	0.895892126	0.003698999	1.165266106	799.0816993
0.013573069	0.857988166	0.018866327	1.230769231	378.260989
0.00600261	0.956094505	0.008446188	1.067371202	516.8536988
0.006300954	0.924359849	0.007089758	1.118110236	640.3818898
0.022388195	0.846303037	0.022932399	1.251655629	373.0871965
0.005311621	0.842858433	0.007838788	1.257879656	588.889446
0.009915567	0.936616343	0.014805969	1.098296199	735.5419397
0.001999613	0.901220042	0.002805232	1.158519872	883.0572179
0.012063201	0.763658768	0.012765218	1.410750507	452.7378296
0.017767083	0.638802174	0.016593202	1.709677419	375.5045699
0.011004023	0.89582318	0.016051623	1.165382336	691.0828394
0.007089424	0.868174763	0.010997348	1.212837838	393.8023649
0.001949275	0.879417284	0.00337317	1.193333333	874.8307143
0.01156197	0.823858805	0.012410128	1.292786421	424.1541726
0.001877385	0.81796872	0.003225204	1.303814714	862.8444596
0.009290312	0.932122926	0.009571205	1.105527638	665.8438622
0.004287715	0.894942311	0.00558675	1.166868198	686.3440145
0.004605277	0.902221278	0.009058995	1.154639175	573.6898625
0.004616301	0.893253758	0.005803493	1.169721116	600.8189243
0.003177655	0.891900673	0.00465135	1.172011662	714.6977648
0.00870511	0.959367091	0.012918638	1.062240664	469.3672199
0.011492966	0.856299545	0.011534465	1.233766234	431.6774892
0.028079429	0.858487104	0.025062917	1.229885057	286.1254789
0.007538182	0.965822306	0.010014945	1.052173913	661.3391304
0.004125346	0.830471426	0.004843035	1.280524122	833.3257892
0.001780744	0.900074018	0.0033174	1.160269928	784.1448873
0.00470275	0.914090931	0.007177935	1.134932534	1399.865442
0.018195009	0.86966093	0.015013602	1.210242588	443.5256065
0.007914029	0.917314434	0.009578222	1.12962963	461.8765432
0.006847165	0.925451636	0.010190969	1.116333725	630.7962691
0.004442667	0.877969011	0.005366586	1.195829556	693.3723935
0.004109401	0.819316605	0.005861639	1.301282051	829.5395299
0.001909143	0.922484671	0.003409718	1.121166791	984.0982299
0.003591546	0.904589145	0.004620391	1.150684932	775.4813546
0.006986158	0.849111052	0.008134596	1.246604718	697.5010722
0.00371815	0.779075753	0.003856722	1.379356931	752.76709
0.003608101	0.751712018	0.003720986	1.435714286	831.65
0.006919436	0.955079931	0.010297548	1.068965517	689.0991379
0.006373656	0.787248377	0.006473126	1.363066538	610.1997348
0.011966441	0.857459184	0.013169019	1.231707317	400.1117886
0.002777939	0.807647421	0.003407782	1.323389021	760.5179992
0.001773701	0.918882973	0.002503447	1.127056673	1011.078458
0.008795268	0.895322366	0.008633462	1.166226913	467.6244503
0.016994478	0.924644368	0.020447498	1.117647059	362.15
0.00371649	0.885281101	0.005391783	1.183274912	714.880696
0.003809654	0.915620452	0.005187739	1.132413793	700.3486207
0.006837833	0.957617068	0.009133394	1.064981949	645.5379061
0.003274275	0.713812086	0.004822652	1.52734375	705.8177445
0.003309265	0.908213898	0.004590714	1.144654088	659.2106918
0.003906189	0.880761683	0.004084867	1.191020408	764.7955102
0.032639654	0.772221699	0.038414296	1.393203883	342.4235437
0.007620354	0.865207617	0.008165208	1.218034993	474.7442799
0.002295025	0.905687314	0.003855054	1.148854962	832.7961196
0.005827472	0.864205893	0.008205623	1.219794344	1003.908419
0.005579451	0.89970176	0.006354168	1.15885947	562.7479633
0.007638226	0.947750101	0.011583741	1.080536913	595.4633669
0.005334284	0.914040011	0.008742628	1.135016465	505.6352909
0.004661648	0.859521304	0.007477948	1.228054299	574.1520362
0.021077908	0.830078125	0.017968673	1.28125	336.1953125
0.010887102	0.857988166	0.011534552	1.230769231	559.6669086
0.005110283	0.954403079	0.009114572	1.07003012	555.8840361
0.004862668	0.916919974	0.006940186	1.130277443	763.4942702
0.013527068	0.903832012	0.012636756	1.151948052	437.2737013
0.023260448	0.717592719	0.020016462	1.510471204	362.5229058
0.00315405	0.929731083	0.005892223	1.109392265	637.3110497
0.002580621	0.854683423	0.002979904	1.236641221	786.980704
0.004276994	0.898114814	0.005203795	1.161524501	665.3498185
0.002312863	0.81320346	0.002975123	1.318235294	662.6399183
0.00544338	0.720430837	0.005293968	1.504038772	652.0961228
0.006002429	0.815588433	0.007652592	1.308300395	586.2393281
0.012420913	0.851845197	0.014328323	1.241706161	478.5485782
0.025441527	0.867346939	0.018406524	1.214285714	295.3603896
0.006869573	0.823515078	0.006486287	1.29342723	774.2161581

original_gldzm_LowIntensityLargeDistanceEmphasis	original_gldzm_HighIntensityEmphasis	original_gldzm_DistanceZoneVariability	original_gldzm_ZonePercentage	original_gldzm_IntensityVariabilityNormalized
0.004575813	1232.349323	690.0430504	0.563019391	0.02119464
0.02529858	346.0956522	201.9565217	0.662824207	0.03436673
0.021339429	611.2125813	373.4164859	0.516816143	0.02756904
0.039253601	360.625	157.7604167	0.596273292	0.032986111
0.016087941	394.7960526	624.8026316	0.538625089	0.031547784
0.038488451	677.1398601	121.013986	0.590909091	0.027531909
0.005111947	1037.594245	3016.113094	0.329727678	0.023635961
0.005129279	856.9926441	2064.701676	0.403263019	0.025656964
0.00459771	721.8104575	1559.867499	0.394885031	0.029501615
0.012077092	568.0935412	410.7817372	0.594701987	0.027018715
0.014506115	408.1649635	616.7839416	0.440231362	0.034100911
0.003617778	930.9786096	1180.064935	0.384208982	0.026194046
0.018512035	459.2817955	310.4862843	0.494451295	0.029757278
0.020220123	538.2106538	337.5423729	0.641304348	0.027044774
0.008522671	742.4052478	628.6239067	0.435555556	0.026115819
0.010357602	762.6400221	1720.540243	0.314821243	0.028991708
0.005341466	639.1367925	402.5707547	0.4801812	0.034487362
0.021752596	360.5296443	214.486166	0.618581907	0.035198175
0.009481621	546.6323851	411.5207877	0.41773309	0.030993684
0.003878588	1098.865901	3697.237421	0.445823805	0.021256577
0.015604782	417.4700855	954.1358025	0.506737247	0.030707723
0.019750539	327.2535714	255.2071429	0.564516129	0.035790816
0.017678092	456.8175676	260.4391892	0.53046595	0.030017348
0.005350481	787.1557632	1182.375389	0.356964137	0.03374021
0.014275492	743.6049661	414.0158014	0.360749186	0.028071481
0.00354451	917.6586716	999.5719557	0.424100156	0.027351888
0.004533892	1002.41573	979.9625468	0.532934132	0.023985117
0.003878373	843.4164332	959.5004669	0.475366178	0.027719149
0.019250646	418.1098901	78.07692308	0.623287671	0.039487985
0.008547042	529.651255	723.7635403	0.470186335	0.030309799
0.007270494	665.6861642	1643.511811	0.44350212	0.026067256
0.023397074	417.8587196	383.3752759	0.574873096	0.030295942
0.008376043	634.5835721	882.4727794	0.448394004	0.026519578
0.015018949	753.249017	714.63827	0.315942029	0.032215731
0.002999641	930.3586112	1972.770672	0.426456263	0.026365684
0.013526867	535.6227181	752.9675456	0.508509541	0.028212418
0.01748194	577.544086	594.0860215	0.615079365	0.026000694
0.016639589	727.2566686	1511.253705	0.289812747	0.025767322
0.011298736	435.3006757	256.9797297	0.528571429	0.034445763
0.003654398	922.6571429	2770.164444	0.333439187	0.031089141
0.01284802	482.2362093	582.4681754	0.52761194	0.02867466
0.00373229	931.475931	1801.167121	0.388222849	0.034610184
0.009671169	696.6460876	1298.447236	0.576095947	0.024374256
0.005826538	732.47763	740.1172914	0.582394366	0.026164997
0.009316257	603.7955326	525.0927835	0.37403599	0.032085119
0.006087373	636.9545817	1121.033466	0.522698875	0.027499881
0.004923963	753.2735666	1835.531584	0.384601009	0.028726693
0.013020109	478.9460581	231.2074689	0.490835031	0.033935366
0.011862822	480.9177489	197.8051948	0.590792839	0.034838178
0.025763888	313.8467433	224.0651341	0.577433628	0.036772801
0.010056518	678.6304348	666.4173913	0.536130536	0.025503046
0.005412373	900.0315664	1394.361525	0.602872531	0.024654838
0.003555988	817.4609869	2134.075496	0.2972668	0.031600204
0.007319365	1438.448276	609.6986507	0.406211937	0.018011234
0.01530383	498.9811321	322.6442049	0.576982893	0.027760624
0.009803188	485.8024691	594.4197531	0.551020408	0.030564129
0.010362519	655.2849589	1575.118684	0.423910336	0.028818657
0.005610728	741.9673617	968.3998187	0.533623609	0.025973012
0.006089068	944.6068376	383.4401709	0.613368283	0.025997151
0.003639286	1016.837946	3700.086013	0.368218122	0.029585245
0.00483798	811.7701674	594.3150685	0.494356659	0.02815482
0.009408653	735.4567548	1187.906362	0.567545639	0.024039943
0.005562388	832.5682248	2883.359362	0.449477775	0.024529252
0.004375279	966.9142857	947.1571429	0.601145038	0.026455026
0.010385056	703.9655172	332.3678161	0.514032496	0.026885982
0.007489216	681.0298939	1632.753134	0.606255481	0.024924003
0.013495967	450.0569106	210.9349593	0.60591133	0.031661048
0.004138701	841.2402546	2030.425617	0.49439528	0.026896318
0.002679346	1043.449116	3015.773918	0.437133724	0.026495786
0.009254448	486.9832894	1017.98153	0.535310734	0.030109788
0.020653708	383.0117647	235.7843137	0.518292683	0.033417916
0.005702668	759.6309464	1767.90636	0.400923509	0.025511728
0.00530628	743.0924138	663.8248276	0.543071161	0.027900119
0.009314284	659.833935	530.5198556	0.583157895	0.027642743
0.005531701	875.3157552	1096.415365	0.376101861	0.025828891
0.004793119	688.1949686	433.2180294	0.519607843	0.033266089
0.004632026	792.7836735	1078.933061	0.55454957	0.027029404
0.039042355	426.0048544	159.0776699	0.637770898	0.030398718
0.009049055	501.7967699	642.8492598	0.565019011	0.029930314
0.004006188	875.9020356	711.870229	0.436666667	0.03029479
0.008639902	1063.915167	672.3521851	0.553342817	0.021431262
0.006701424	590.5315682	441.7535642	0.594430993	0.030541602
0.011811067	611.7829978	847.2885906	0.391761613	0.028307033
0.008919821	533.1646542	832.6904501	0.481246698	0.03311761
0.007969748	612.7665158	949.7710407	0.406998158	0.0279724
0.018966419	366.7695313	212.5	0.482109228	0.036010742
0.012240394	609.2409289	591.1538462	0.52675841	0.026240676
0.009226275	568.7402108	1267.447289	0.346193952	0.029978725
0.007255516	783.3027744	760.1266586	0.487360376	0.025535438
0.013177894	456.6402597	695.9506494	0.563277249	0.029080789
0.023526782	416.6701571	274.1204188	0.475124378	0.032194841
0.006023851	664.9657459	841.4066298	0.357424961	0.03227374
0.003301444	842.2574215	2015.343511	0.523883581	0.027563216
0.00566883	694.7640653	1484.583787	0.479686593	0.027834413
0.003491877	729.5094118	1382.445882	0.514061083	0.033103806
0.006329913	776.9232633	891.8933764	0.519295302	0.024564087
0.008289895	649.798419	1031.719368	0.515905383	0.027120561
0.014922139	523.2322275	179.7393365	0.674121406	0.030210462
0.018916067	322.0194805	133.5714286	0.574626866	0.038033395
0.00746544	814.9741784	3157.356808	0.434496827	0.023315262

original_gldzm_LowIntensitySmallDistanceEmphasis	original_gldzm_IntensityVariability	original_gldzm_HighIntensityLargeDistanceEmphasis	original_gldzm_SmallDistanceEmphasis	original_glcm_SumVariance
0.003865431	17.23124231	1543.312423	0.938191882	4980.295322
0.024657879	7.904347826	444.6913043	0.951086957	1158.173421
0.019548159	12.70932755	773.1735358	0.920281996	2343.021649
0.038208052	6.333333333	513.78125	0.92578125	1412.495584
0.015424036	23.97631579	597.4065789	0.925986842	1587.509264
0.037937766	3.937062937	888.8181818	0.937062937	2526.793245
0.00460776	82.13496403	1248.929784	0.946690647	4610.572276
0.004423771	62.78259093	1125.84389	0.93594197	4110.172303
0.004437184	49.65121806	843.5252525	0.971479501	3472.718764
0.011739981	12.13140312	653.7906459	0.966592428	2097.349212
0.013439903	23.35912409	460.010219	0.960583942	1549.807816
0.003341077	34.28800611	1091.252865	0.961038961	4164.476585
0.016992942	11.93266833	687.9401496	0.902743142	1300.568697
0.019872667	11.16949153	907.8789346	0.923728814	2157.295908
0.008254049	17.9154519	874.3177843	0.967201166	3837.452189
0.010133031	52.59095921	825.9492834	0.980154355	3467.789139
0.005207561	14.62264151	704.754717	0.980542453	2637.087838
0.021328641	8.90513834	572.3557312	0.937747036	1740.685121
0.009010487	14.16411379	634.1509847	0.960612691	2398.585987
0.003430402	87.02442599	1208.560821	0.961678337	3710.947963
0.015033774	32.33523267	482.5726496	0.962962963	1490.963807
0.019509028	10.02142857	433.7642857	0.965178571	1440.470406
0.017159666	8.885135135	568.2837838	0.951858108	2121.299679
0.005157045	43.32242991	902.847352	0.969042056	3467.334552
0.014020746	12.43566591	808.4198646	0.974604966	4044.105587
0.003359277	29.64944649	1040.736162	0.969557196	4073.369245
0.00440386	25.61610487	1191.783708	0.967696629	4143.241924
0.003654156	29.68720822	1020.755369	0.958683473	3613.298779
0.018770247	3.593406593	577.5054945	0.942307692	1311.044495
0.008420974	22.94451783	580.8414795	0.983157199	2298.553226
0.007044574	46.34758155	766.9032621	0.970472441	2892.886852
0.02281623	13.72406181	596.9448124	0.937086093	1716.535426
0.007704475	27.76599809	817.3600764	0.935530086	2876.91523
0.014752724	24.58060288	824.0773263	0.97542595	3309.986709
0.002756804	57.7144815	1125.484696	0.96087762	4063.184416
0.012574805	27.81744422	867.1622718	0.897312373	2154.585769
0.016371018	24.18064516	1385.702151	0.822580645	1817.249476
0.015904631	43.46947244	871.9519858	0.958654416	3864.341717
0.010922001	10.19594595	601.2939189	0.946790541	1868.74352
0.003302864	97.93079365	1113.962857	0.951666667	4246.734977
0.012300655	20.27298444	714.5643564	0.926803395	2167.176646
0.003098433	76.21162579	1206.001817	0.924046322	3825.167487
0.009546214	33.95333812	819.8549892	0.97361809	2739.97988
0.005526803	21.63845224	917.0120919	0.95828295	3032.59067
0.008994679	18.67353952	724.2182131	0.961340206	2947.549956
0.005732523	34.5123506	781.4972112	0.957569721	2822.622245
0.004583196	59.11953353	907.5767736	0.956997085	3540.595359
0.01289327	8.178423237	517.2614108	0.984439834	1785.580087
0.011452375	8.047619048	677.8787879	0.941558442	1757.948683
0.024887674	9.597701149	424.7318008	0.942528736	1018.050523
0.010004552	17.59710145	747.7956522	0.986956522	3358.52639
0.004700701	41.3954735	1166.854675	0.92986897	3243.391919
0.003258032	74.92408267	953.8764235	0.960401143	3766.007858
0.007142578	12.01349325	1592.77961	0.966266867	4939.697702
0.014941045	10.29919137	720.8032345	0.947439353	1833.722083
0.009521981	19.80555556	581.5061728	0.967592593	1936.247228
0.010148082	49.0493537	753.239718	0.970916569	2986.466235
0.00530555	28.64823209	936.3472348	0.951042611	2969.596498
0.005804781	12.16666667	1404.876068	0.924679487	3855.231751
0.003352325	118.6664174	1147.796809	0.969708302	4450.52418
0.004565994	18.49771689	956.9254186	0.962328767	3203.682672
0.007816082	33.63187991	887.2794853	0.938348821	2695.097646
0.003430305	90.78276142	1151.772764	0.905160767	3228.346883
0.003557412	33.33333333	1507.971429	0.891071429	3250.849319
0.010275671	9.356321839	763.4310345	0.982758621	2557.327437
0.006219103	51.69238187	964.3505304	0.909233365	2347.150692
0.013087282	7.788617886	649.8373984	0.942073171	1679.902373
0.003225052	67.61734288	1164.129276	0.919152745	3456.523927
0.002459472	86.95917124	1172.931749	0.968235832	3932.452379
0.008478215	34.2348285	564.4186456	0.958443272	1492.387231
0.020395945	8.521568627	466.4588235	0.970588235	1781.14856
0.005314061	50.94692038	938.6319479	0.954181272	3578.786876
0.005158104	20.22758621	914.0675862	0.966896552	3348.104853
0.009088172	15.31407942	717.0180505	0.983754513	2514.217413
0.004646702	39.67317708	1572.931641	0.870334201	3935.948136
0.004540113	15.86792453	804.1320755	0.963836478	2634.791803
0.003948077	33.11102041	904.7363265	0.952244898	2392.288642
0.038257281	6.262135922	760.3300971	0.901699029	1519.226561
0.007944246	22.23822342	610.0067295	0.945491252	1692.932825
0.003817271	23.81170483	1048.3257	0.96278626	3951.225231
0.008097053	16.67352185	1303.942159	0.945051414	4298.31639
0.006267354	14.99592668	701.6659878	0.960285132	1868.308075
0.011526909	25.3064877	677.0615213	0.979865772	3164.873602
0.008698329	30.1701427	643.2821076	0.966245884	2411.391214
0.007354998	30.90950226	767.2244344	0.942986425	2835.515907
0.017719237	9.21875	489.0664063	0.9296875	849.5354611
0.011358092	18.07982583	807.5370102	0.942307692	2457.15571
0.009086646	39.81174699	620.1649096	0.98249247	3034.932705
0.006861354	21.16887817	862.5367913	0.967430639	3042.130833
0.012501471	22.39220779	534.1064935	0.962012987	1519.95663
0.019138883	12.29842932	633.2591623	0.872382199	1265.630902
0.005859316	29.20773481	775.5845304	0.972651934	3206.81377
0.00289952	64.99406277	1063.364292	0.940839695	3177.067214
0.005087536	46.01028433	812.4210526	0.959618875	2749.39451
0.002847501	56.27647059	1001.601765	0.921748366	2805.844431
0.005034982	30.41033926	1276.231826	0.873990307	2821.626933
0.007493267	34.30750988	904.0347826	0.922924901	2570.343362
0.01417987	6.374407583	701.9668246	0.93957346	1784.799684
0.018279139	5.857142857	428.6558442	0.946428571	1025.783856
0.006241499	89.39071466	978.0062598	0.926643192	2757.024544

original_glcm_Homogeneity1	original_glcm_Homogeneity2	original_glcm_ClusterShade	original_glcm_MaximumProbability	original_glcm_Idmn	original_glcm_SumVariance2	original_glcm_Contrast	original_glcm_DifferenceEntropy
0.219497384	0.142634694	-7694.934845	0.011588352	0.951133449	527.1308623	191.7589891	4.718512937
0.191426995	0.113591513	1331.561655	0.011218481	0.907180498	272.7240911	154.0051478	4.515806326
0.226264132	0.155854495	145.008141	0.01782055	0.925360341	530.5322298	200.7312449	4.70379222
0.217333184	0.145106582	501.4251859	0.020525112	0.908536711	358.8077243	172.2922698	4.563803292
0.228217619	0.154108204	402.9424189	0.021672514	0.919181942	352.9201645	140.186039	4.550916624
0.218137172	0.143807635	2472.970692	0.017085022	0.96882795	678.0419874	179.2508378	4.544944225
0.388042811	0.330082227	-9929.938639	0.107685521	0.982977977	446.6142834	89.01115616	3.972509582
0.312557516	0.251513366	-7464.136724	0.046258064	0.974823835	439.8534049	157.05231	4.39638798
0.316903571	0.252315742	-4750.310424	0.044444079	0.948594246	322.3591141	116.6548291	4.2510161
0.218116578	0.138212011	-827.5342539	0.01106708	0.955874767	421.5695895	126.3015046	4.467960361
0.302124757	0.227344116	-1281.427707	0.037969301	0.9642558	277.8719737	71.57988922	4.036858216
0.325265186	0.259177746	-4895.014273	0.041680343	0.979913574	311.5729485	104.3732069	4.188146142
0.235437612	0.164166625	4134.549645	0.015598814	0.919314569	437.8590066	176.6179937	4.619094333
0.195005891	0.120855442	-732.0270288	0.013934948	0.920179348	419.5754805	195.2945491	4.74245871
0.294054987	0.230574048	-5929.492106	0.045161059	0.940252829	367.170234	167.7536447	4.463903548
0.362208094	0.305865983	-11732.33986	0.062249384	0.96181087	618.94569	149.43115	4.094135408
0.275119532	0.19293243	-1176.975003	0.025513898	0.981562986	211.3336412	70.67374537	4.086828667
0.217691803	0.142191837	14.87475787	0.019776038	0.919385428	347.426445	147.4526976	4.518742641
0.311596372	0.237705008	-3023.31579	0.039985723	0.980433512	282.2108355	73.68804901	4.018608356
0.283875983	0.208172212	-2710.698238	0.027642198	0.985693071	518.9263203	108.1108013	4.323336089
0.246944876	0.168367428	487.7516465	0.021818543	0.93430792	341.8476717	105.0356457	4.371433607
0.224591359	0.147263725	-107.1080522	0.021618458	0.920281632	239.3993454	121.8839968	4.434294326
0.248479627	0.176686536	-1657.85866	0.030324137	0.927230646	414.9099459	140.8863256	4.471981467
0.362897332	0.296993474	-4151.505545	0.096113708	0.972414575	242.779758	69.20923651	3.941693921
0.382819752	0.33053182	-11794.48262	0.114656442	0.949016009	628.0843592	171.2827106	4.14093263
0.290891382	0.219305674	-4219.231666	0.030351515	0.959514596	334.372735	114.6308191	4.334562659
0.232896241	0.156582523	-4671.014216	0.013519845	0.961416336	404.6227565	154.2405613	4.572335191
0.26694343	0.188246715	-3356.897952	0.018746637	0.962446077	304.2201258	99.83348051	4.291960738
0.220984007	0.131711661	755.8285918	0.020771939	0.963629525	194.461755	78.46986842	4.035586796
0.267994652	0.193302189	-1403.080779	0.022252965	0.982865553	317.9881891	108.0889293	4.310942117
0.284247421	0.215963795	-4720.325475	0.036623242	0.966301261	430.5693776	128.2840603	4.407921008
0.218959669	0.148491424	-782.5840296	0.016845781	0.92222064	379.0878332	179.8593249	4.671739127
0.282507131	0.211406917	-3583.866768	0.02187763	0.946911141	426.1918351	119.4446203	4.354226731
0.374333935	0.317185107	-10130.66927	0.057634058	0.954767232	482.9178025	126.3735074	3.977350358
0.295149843	0.224566648	-4474.372382	0.027396231	0.981002956	365.0066782	112.3735316	4.318725184
0.23432944	0.166330609	708.8344994	0.020676845	0.924188185	525.6513907	195.1215471	4.702300681
0.183823898	0.109840984	6296.867373	0.006073817	0.948743305	524.3050682	222.2589798	4.813354262
0.409975125	0.362769475	-14494.58948	0.085623557	0.950808341	628.6025087	152.9917719	3.977999807
0.247432015	0.164598187	-776.3438329	0.014666414	0.965907668	206.8362249	84.06996207	4.187002185
0.371450656	0.309471327	-5234.264018	0.079298078	0.992819915	272.4935397	80.73029599	3.966997136
0.239257132	0.164101941	-1999.177816	0.016936323	0.935407646	357.0405653	127.3855428	4.479809084
0.327401536	0.253786713	-2695.598284	0.035765929	0.971200654	188.7871641	69.98168603	4.014947386
0.211971201	0.13864101	-1670.825031	0.010417919	0.937945566	498.1087982	179.0914321	4.593602224
0.218966634	0.142995563	-2590.678741	0.013005078	0.95531801	342.8860164	155.8673031	4.633700734
0.340091748	0.273107672	-3941.696929	0.062621048	0.979026612	268.8722661	79.81045196	4.015413344
0.243136372	0.170987122	-2307.490479	0.028169682	0.94789079	409.288595	141.6984892	4.525906352
0.341367295	0.279196236	-4343.251378	0.07445016	0.98208624	323.8675082	106.1625914	4.205957659
0.277750298	0.196317089	-915.0160953	0.038234792	0.984226882	221.0043328	73.12451717	4.049366829
0.217875065	0.133656417	-1128.235964	0.011254232	0.937518257	222.778688	102.9535639	4.309419153
0.223255847	0.149673369	2418.10676	0.013177084	0.914364659	318.7672082	151.389795	4.487647776
0.251905677	0.183926358	-5534.424767	0.031577039	0.965223575	409.5179233	180.7084208	4.636120836
0.201751223	0.121144365	-2962.89961	0.004914004	0.950054529	369.9352385	147.1810631	4.637409628
0.392991092	0.330926591	-3941.468437	0.094263469	0.982837162	221.7671746	60.90486144	3.7715051
0.315732005	0.247160393	-7865.632596	0.034716856	0.982080407	473.7685898	109.5706735	4.190803943
0.211216443	0.14186167	1782.861437	0.012804029	0.917001	451.5629151	204.4192362	4.741117172
0.221577209	0.142103441	-742.3347006	0.012196253	0.932138107	312.3795895	120.0560139	4.458411927
0.307533765	0.239669525	-5007.32957	0.03984671	0.955488555	303.0483326	107.7908434	4.254797335
0.225952613	0.152782406	-2149.109109	0.021813162	0.942956757	451.7674983	170.8288526	4.669787704
0.211258426	0.135712195	-1651.423983	0.011199672	0.946825487	357.1103356	162.2330025	4.637730583
0.345891536	0.278445833	-4841.690811	0.057583416	0.973927482	253.2687444	84.40085502	4.039968915
0.25098419	0.178721037	-1339.550318	0.027184369	0.942584753	441.207063	147.2628662	4.554436446
0.22079436	0.147819237	623.1707829	0.012907893	0.969143324	548.9877127	181.7161283	4.730874988
0.267872112	0.196840074	-2665.138844	0.015014192	0.965331411	572.1899919	152.0335261	4.529758649
0.209109406	0.126033525	-1336.693648	0.005290796	0.975398386	327.7726103	123.9459129	4.509686377
0.262610928	0.186565656	-2333.493977	0.025478186	0.974243948	376.0884416	112.7813906	4.316981635
0.201157786	0.125852764	2583.922904	0.013217558	0.972711036	473.5403183	185.4472918	4.787760386
0.193613986	0.11651747	1380.774278	0.010576566	0.93104741	337.2388696	162.0651935	4.608640377
0.253795429	0.182891384	-2586.200594	0.027027945	0.961646257	379.2726235	147.3666735	4.569131151
0.281435853	0.203715702	-2541.906368	0.028781617	0.984159634	267.7089332	85.21125351	4.226103572
0.225132265	0.145223962	1923.160051	0.007291781	0.954725328	326.0524579	119.8133779	4.461524361
0.242473251	0.171429365	-746.1139702	0.025939932	0.917205905	386.9765684	154.0165375	4.482939225
0.307797516	0.246490848	-5921.354187	0.042017051	0.954701866	541.9285201	157.4583616	4.42912038
0.237982639	0.169291978	-2287.803197	0.041476172	0.936246256	415.353826	171.2395724	4.64173451
0.237301975	0.165382794	-2339.215226	0.019505686	0.935166205	328.6252999	161.1135914	4.584336154
0.344694269	0.281271998	-6260.004001	0.071820234	0.976350227	394.9276005	101.2342621	4.17250443
0.253231487	0.175780852	-36.42855141	0.032594481	0.952948943	262.6922864	95.85119708	4.279619274
0.22422089	0.138536638	2004.021762	0.004631418	0.985223002	337.6625904	100.2910574	4.354340033
0.18980752	0.116064331	2119.931517	0.009568462	0.911633888	402.7486448	189.986702	4.668737659
0.221495953	0.141769228	1740.561726	0.011586813	0.940577817	331.6273063	119.8580339	4.472654849
0.292788687	0.217680783	-3151.838697	0.040440688	0.966024739	217.5004875	94.54441417	4.236071238
0.222415575	0.145477738	-7824.089897	0.009417487	0.947767229	527.3219974	186.762166	4.700023403
0.212513169	0.132740836	2305.69009	0.00923653	0.943111872	314.0380446	130.4790054	4.501267843
0.345975932	0.294200177	-6665.87408	0.107370187	0.959644742	493.7816898	187.0922477	4.347703824
0.270720088	0.188540576	-1999.964343	0.020213525	0.955122207	198.4728542	76.45368901	4.149512199
0.300296411	0.230298594	-4261.466925	0.028872412	0.960171915	376.9103278	107.2243998	4.267149639
0.249414109	0.172493358	4044.279262	0.027517965	0.936506141	284.3918607	119.8175933	4.37342921
0.245408309	0.169922892	-2938.517914	0.015746766	0.943841181	399.7640033	131.1636601	4.460308922
0.376691884	0.318557568	-5640.774687	0.100406934	0.961480673	331.1213619	88.76329064	4.005479436
0.270036788	0.19548126	-2697.86487	0.027086506	0.978583204	394.2146906	107.1972674	4.355716201
0.215866001	0.137744526	333.1500523	0.009185383	0.934424238	345.2814274	142.0052917	4.570481164
0.25937828	0.183719602	5738.946511	0.016179946	0.955941795	462.7226264	119.7259015	4.384731735
0.323779645	0.252124306	-3458.800332	0.027874026	0.961971599	205.3589789	79.23990837	4.045715875
0.243698845	0.163049654	-867.3635789	0.012304805	0.970591168	334.9622378	98.0979967	4.351581216
0.26621335	0.191932731	-2206.977565	0.032135849	0.96387159	377.8880212	111.3825511	4.387232227
0.250672814	0.166426219	-1054.361772	0.011239899	0.962507994	203.5669617	78.61464283	4.190473625
0.218419804	0.147244144	647.8117607	0.009542416	0.95434943	531.9756307	209.4489298	4.79737308
0.242859639	0.168665805	-2074.460069	0.021801725	0.942368746	372.9692493	135.1582056	4.524052106
0.179398652	0.102103894	1169.12404	0.009763093	0.928106255	367.6917212	186.2375185	4.671272522
0.23271077	0.145819801	44.49184417	0.014124061	0.940541476	204.1194471	76.66617719	4.049411381
0.284570947	0.213943358	356.1863974	0.031288145	0.981697271	635.6956705	126.1968331	4.393802202

original_glcm_InverseVariance	original_glcm_Entropy	original_glcm_Dissimilarity	original_glcm_DifferenceVariance	original_glcm_Idn	original_glcm_Idm	original_glcm_Correlation	original_glcm_Autocorrelation
0.151104252	9.549550829	9.962782403	90.3550607	0.866737708	0.142634694	0.470041852	1414.699962
0.124720455	8.368798507	9.840790087	53.08224221	0.805714235	0.113591513	0.287810312	344.2865405
0.149297929	9.081154381	10.49206605	85.04436662	0.835492955	0.155854495	0.458803038	676.1760345
0.148627564	8.193392031	10.01654225	69.28981669	0.814618818	0.145106582	0.35952632	412.5518465
0.144052052	9.2219575	9.091922606	55.24996898	0.823091537	0.154108204	0.437967499	475.4398741
0.13698944	7.879807915	10.0068808	74.98056414	0.887517835	0.143807635	0.585915588	723.0471609
0.260893254	8.476358746	5.902762895	53.27768613	0.929004585	0.330082227	0.66818045	1320.063639
0.237157588	8.836347973	8.354249853	85.11530127	0.909951807	0.251513366	0.478677758	1170.911669
0.229855487	8.665324127	7.374914093	59.31229954	0.872094575	0.252315742	0.475558176	1003.979156
0.147802344	9.087734383	8.618329635	49.85965571	0.864960375	0.138212011	0.54280506	626.6237902
0.218131837	8.544385869	6.133302951	32.20743902	0.884239393	0.227344116	0.592542279	477.97102
0.241522509	8.675629407	6.848870126	56.48106707	0.917887144	0.259177746	0.503378778	1199.303952
0.158862942	8.868354985	9.804517867	75.76347484	0.829986967	0.164166625	0.435055	377.8748839
0.11544669	9.038251085	10.81882894	73.76707084	0.822111315	0.120855442	0.374220994	626.3931616
0.222963723	8.484621184	8.79670071	87.9884552	0.863789297	0.230574048	0.378536461	1091.454797
0.273183366	8.26529859	7.543885502	86.18922095	0.899890501	0.305865983	0.611825515	986.7377254
0.189897535	8.57563597	6.276085279	30.57080755	0.911314666	0.19293243	0.50339411	787.039369
0.141935471	8.349071527	9.338250617	57.47240167	0.822890375	0.142191837	0.412771469	515.3228989
0.235840118	8.323114499	6.021175861	36.102812	0.914713559	0.237705008	0.588482284	717.0331316
0.200532642	9.854031368	7.18777238	54.96521894	0.926813492	0.208172212	0.657209903	1089.414456
0.156026925	9.406411218	7.763170657	42.69772977	0.840209028	0.168367428	0.532804712	456.9554521
0.136076656	8.496245882	8.627336244	45.62573505	0.821169845	0.147263725	0.331739785	436.1495394
0.17323	8.482670459	8.768700037	60.97104661	0.837277751	0.176686536	0.497792813	624.4574385
0.242997724	8.189776611	5.55743443	37.47531938	0.905197916	0.296993474	0.558742546	1008.333558
0.252439662	7.61840801	7.951561685	103.9437417	0.887643276	0.33053182	0.57402648	1134.318328
0.204865324	9.00377084	7.456028281	57.37892497	0.882155172	0.219305674	0.494085067	1177.176564
0.158853252	9.491592664	9.079839561	66.70365569	0.878782774	0.156582523	0.45394648	1191.319816
0.190134203	9.192292309	7.210595116	46.19859915	0.881623202	0.188246715	0.513209272	1054.204531
0.136009156	7.228975815	7.048922534	27.91005187	0.870761722	0.131711661	0.430500376	401.9990675
0.193196243	8.906322213	7.546892938	48.36080137	0.916786999	0.193302189	0.498975855	685.0132253
0.193936489	9.260878126	8.046886435	59.97607256	0.889792024	0.215963795	0.543740143	849.8134264
0.138501388	8.974334777	10.26718928	70.53716413	0.827203575	0.148491424	0.364806621	502.8855984
0.203356883	9.147664466	7.742065017	57.53678382	0.864948161	0.211406917	0.568583234	846.0548318
0.288588985	7.854768535	6.820409406	77.84532608	0.89335929	0.317185107	0.584525496	946.6989977
0.212063587	9.197320797	7.366507979	56.80950121	0.916742908	0.224566648	0.533648985	1175.549786
0.159158639	9.424986922	10.34502417	82.75560693	0.834657764	0.166330609	0.465253858	623.2083277
0.110730554	9.810851395	11.50897014	76.9040347	0.854646574	0.109840984	0.420807543	521.545232
0.285175826	8.033202641	7.29663868	96.88251287	0.893097635	0.362769475	0.610763966	1093.656661
0.169357043	8.580936586	6.997733246	33.15210612	0.880085963	0.164598187	0.426736996	569.3018676
0.266068195	8.204928575	5.738710846	47.00393623	0.951147194	0.309471327	0.5473886	1216.773348
0.164211409	9.254948877	8.481054732	53.40289406	0.842868426	0.164101941	0.478581321	645.8062788
0.238179993	8.704018549	5.705111151	36.82008718	0.900514729	0.253786713	0.464994779	1110.20536
0.135029899	9.766772468	10.21842452	59.94572552	0.84389941	0.13864101	0.479324184	797.3928243
0.144431193	9.406367324	9.426013807	64.83531574	0.866405865	0.142995563	0.3804581	883.6149038
0.248749254	8.065827776	6.038138591	42.14799299	0.915545502	0.273107672	0.547784049	864.4144781
0.168647373	9.328495754	8.87428321	59.44562351	0.8591361	0.170987122	0.49285739	825.6276405
0.224920979	8.621705277	6.897028624	56.84798631	0.922148814	0.279196236	0.511562424	1025.614858
0.193878579	7.990444474	6.292590286	32.4797282	0.918350912	0.196317089	0.504122215	543.6841779
0.136697824	8.451170172	7.939499121	37.91827843	0.838393869	0.133656417	0.373763425	532.8309929
0.163579827	8.39610631	9.348721716	60.20426987	0.82047667	0.149673369	0.36357672	300.8049725
0.174730403	8.859922183	9.708240951	83.52351204	0.886628824	0.183926358	0.394487442	963.4792597
0.123335291	10.08570512	9.316694	58.79219068	0.856077161	0.121144365	0.437570841	949.0651316
0.280699132	7.969315249	4.974135978	35.6428912	0.927399536	0.330926591	0.575051	1088.777092
0.235249731	8.606032452	6.983461618	57.44670899	0.922232902	0.247160393	0.61841422	1416.998003
0.141325301	8.977998713	10.9144867	81.37168206	0.822570433	0.14186167	0.386850085	530.1462551
0.141293051	9.212101764	8.472554935	46.41263907	0.83461882	0.142103441	0.450500822	582.2699965
0.220183593	8.771417067	7.18506482	53.6107466	0.87806081	0.239669525	0.475238447	872.9529787
0.148282594	9.620738694	9.773152493	72.30403626	0.852750304	0.152782406	0.458206398	863.4066017
0.136388013	9.112544428	9.753580481	64.82800314	0.8536699	0.135712195	0.383447711	1110.481348
0.256623994	8.684312541	5.955042911	48.00794524	0.909848425	0.278445833	0.504132059	1276.697654
0.160906617	9.056841659	8.914482766	65.4349257	0.854836817	0.178721037	0.505270166	930.4478668
0.147095608	9.872372588	10.04447712	78.61627096	0.888610747	0.147819237	0.50789145	784.1447693
0.187775647	9.835851256	8.669272414	74.43295159	0.889495846	0.196840074	0.585232087	939.5787861
0.130225115	9.937432155	8.552474836	49.19901828	0.895354467	0.126033525	0.460080698	957.1558436
0.185610164	8.495738638	7.687489897	51.08239937	0.899963635	0.186565656	0.543572692	756.8362697
0.123907635	10.13674834	10.38839731	75.16986797	0.891795913	0.125852764	0.444317806	686.0855553
0.116336997	8.578126554	10.014886	58.54027932	0.830779092	0.11651747	0.361582919	496.1948762
0.175962083	9.644344624	8.842214115	67.28219202	0.880004126	0.182891384	0.445748466	1003.948653
0.194729749	9.409390269	6.664414051	39.84942411	0.919849565	0.203715702	0.521790366	1148.667774
0.148368533	9.558063742	8.379319237	47.65542378	0.863708351	0.145223962	0.470064002	454.5856503
0.160929236	8.30285782	9.284238224	63.13057622	0.82632252	0.171429365	0.438827845	524.4801028
0.220647804	9.056237916	8.529207237	82.35595328	0.880288866	0.246490848	0.551730769	1028.03706
0.154217317	9.082555123	9.811086645	71.52765694	0.845184255	0.169291978	0.425922348	964.8145424
0.164674462	8.928811453	9.419411317	70.33555365	0.845557786	0.165382794	0.34920762	735.4161102
0.227613406	8.75975464	6.687604907	55.28394899	0.912256388	0.281271998	0.595458433	1138.987752
0.160360416	8.850544504	7.491851679	37.52923508	0.861440522	0.175780852	0.46753403	783.8561545
0.143777673	9.761053086	7.61988115	41.17968166	0.91764874	0.138536638	0.548658059	720.7348606
0.117167311	8.384027077	10.82086547	69.10831456	0.811461084	0.116064331	0.370412826	440.1716751
0.142061738	9.473621086	8.442103903	46.98247867	0.844455512	0.141769228	0.473779834	513.1729964
0.205018398	8.626970565	6.803960488	47.32529326	0.890040224	0.217680783	0.400987134	1140.08996
0.152068477	9.525000555	9.814156463	88.34562158	0.862735383	0.145477738	0.48018084	1229.08682
0.134863889	9.113202581	8.849827779	49.97423153	0.84698681	0.132740836	0.421768256	559.816589
0.225542757	8.125223414	9.022747864	100.8049821	0.890729894	0.294200177	0.455917953	898.0204158
0.186915374	8.872808918	6.466161129	33.43309113	0.867978149	0.188540576	0.448046028	722.5969903
0.215394383	8.979166999	7.21424104	53.39616766	0.883392215	0.230298594	0.562359332	834.7050788
0.167098803	8.198159393	8.093928899	51.54331599	0.846466051	0.172493358	0.417637404	257.0863855
0.168710309	9.22937498	8.463091421	56.40108192	0.855211188	0.169922892	0.511406803	727.7907512
0.248489087	8.034175643	6.142078622	50.02103583	0.893026549	0.318557568	0.580005622	883.5774389
0.186636207	9.18911524	7.51430006	49.43028272	0.907857204	0.19548126	0.580820002	896.869276
0.140884546	9.445875934	9.095379371	57.37957311	0.839126786	0.137744526	0.424374475	456.8611704
0.184775798	8.715200906	7.894980534	55.66596858	0.872864521	0.183719602	0.589858265	379.0042849
0.23971939	8.518031313	6.074651914	40.96058393	0.887629233	0.252124306	0.448594207	938.8121987
0.166481375	9.782780954	7.4903724	40.99898146	0.888880566	0.163049654	0.552859576	941.3270697
0.180178204	9.404614204	7.699205688	50.0520481	0.882585499	0.191932731	0.549347425	814.574624
0.171072582	9.338509814	6.754733093	31.61275766	0.874777634	0.166426219	0.447457775	836.3040169
0.145302706	9.839944066	10.75064234	90.14426452	0.867876306	0.147244144	0.442574937	813.3495091
0.158175677	9.528720965	8.666605385	56.68332782	0.851927275	0.168665805	0.474454387	759.3525408
0.107572923	8.378004322	10.7459086	68.70855493	0.827316996	0.102103894	0.337509175	519.7077047
0.147626541	7.960075793	6.935970035	26.04781125	0.840155384	0.145819801	0.461719334	324.3317502
0.210158905	9.60591322	7.795085938	63.75448414	0.91826584	0.213943358	0.671900345	811.0193553

original_glcm_SumEntropy	original_glcm_AverageIntensity	original_glcm_Energy	original_glcm_SumSquares	original_glcm_ClusterProminence	original_glcm_SumAverage	original_glcm_Imc2	original_glcm_Imc1
6.230577553	37.27751469	0.002129236	171.3804168	658541.874	72.95814526	0.94349457	-0.20681174
5.726445934	18.3711946	0.003473435	107.5325265	161501.3545	35.44579064	0.981718379	-0.330992539
6.160910635	25.35691412	0.002831262	183.0691305	515795.2024	48.72528358	0.95882112	-0.245959349
5.803150953	19.93165244	0.00426871	133.3258003	238399.1582	38.23950989	0.983045176	-0.342579271
5.961877187	21.26474943	0.002910158	122.9078908	232234.117	41.09440003	0.862532654	-0.13886772
5.926743842	25.12647778	0.004989182	212.6488769	898585.6293	48.91195229	0.996324505	-0.475742343
5.63414425	35.48211394	0.020734	129.7334664	650305.3104	70.15699693	0.861485269	-0.152369702
5.755875646	33.91464054	0.009484146	141.0661955	506121.4827	66.33739648	0.770040848	-0.100606442
5.599178671	31.51525834	0.009074872	102.2451802	267552.4613	61.72546685	0.777999465	-0.106502277
6.088805143	24.09440473	0.002356907	134.9612462	335240.0332	47.01740449	0.967352177	-0.262721444
5.638259942	21.00267598	0.006654071	85.44077111	137494.0827	41.28176674	0.881170827	-0.166427197
5.678119785	34.47174318	0.008364054	97.80577958	282701.0845	67.74874032	0.83889753	-0.13331872
5.989218953	18.5003775	0.003243057	159.5852892	384330.0558	35.3463534	0.941607432	-0.219713161
6.080032719	24.73081567	0.002505538	151.1923856	338653.906	47.75090394	0.969328844	-0.26854398
5.638866341	33.1155288	0.008554567	124.3357985	355196.8873	64.54578223	0.881159706	-0.162883165
5.596617118	29.85004571	0.013784654	187.5318613	802926.2611	58.96095269	0.825113493	-0.141413821
5.589301134	27.86463803	0.004375099	68.43525524	103275.3637	54.83878991	0.906090097	-0.182067473
5.818557099	22.33836898	0.004122492	123.7229461	223361.3478	43.1342734	0.974450448	-0.302331984
5.567503617	26.14515905	0.007773219	86.58646333	188043.5854	51.57049279	0.918273855	-0.201419294
6.326415611	31.90921376	0.003527528	154.6761594	710032.4045	62.81967137	0.878161414	-0.143967877
5.989558843	20.38235173	0.002505097	111.5580074	222319.6375	39.88447527	0.835545068	-0.124297421
5.683645897	20.69896772	0.003726657	89.55797544	116266.2379	40.32708312	0.953268783	-0.24673283
5.849814978	24.18718229	0.004771987	136.467814	303523.2511	47.1502757	0.96769745	-0.279192681
5.343320335	31.50410604	0.016480145	73.05008173	205399.1506	62.12306736	0.828364086	-0.135222645
5.433686826	32.63229612	0.023354319	190.137145	815870.2197	63.86907517	0.936235074	-0.245624223
5.852676907	34.17433185	0.005301331	105.9746884	270649.3256	66.99847893	0.855309001	-0.138141404
6.049266586	34.39761242	0.002554169	133.6635341	374958.393	67.1913121	0.889847337	-0.154368026
5.819514427	32.28576862	0.003552437	97.02801552	214352.0325	63.34203527	0.850859898	-0.133178772
5.212038039	19.71808013	0.007368736	66.81278552	80769.45733	38.62383947	0.994828012	-0.482020324
5.797751865	25.79792344	0.004412413	102.9352874	252115.2043	50.2974353	0.875551733	-0.153064325
6.029306579	28.53001903	0.005353194	134.6055815	407381.1713	55.64749243	0.840187564	-0.129012823
6.003039835	22.16412789	0.002890307	138.4406121	274588.7126	42.56000869	0.934685449	-0.207662791
5.970518987	28.46836852	0.004071683	129.9587891	340061.8984	55.47284308	0.8858257	-0.157327948
5.403386825	29.69245644	0.015893609	147.3702321	586958.4737	58.54767813	0.878353956	-0.175982766
5.892825077	33.99738494	0.005093329	114.3944531	328614.6651	66.70287974	0.808880481	-0.112143107
6.141069137	24.19160391	0.002804815	177.0749623	475351.6538	46.49728199	0.877768127	-0.147687671
6.288613108	22.17095137	0.001474981	191.3861863	644115.6536	42.21558917	0.93595655	-0.192606352
5.560147258	31.79736227	0.01920109	186.8283528	946651.6277	62.43500329	0.859236222	-0.158658444
5.65141017	23.68907939	0.003392992	71.3768673	95271.67967	46.41156066	0.943275106	-0.227907752
5.335042536	34.65301369	0.017513917	82.83884942	269612.4956	68.3756613	0.748227346	-0.098637491
5.969045454	24.94898935	0.002804364	118.3835324	248829.377	48.50890844	0.896229863	-0.162982593
5.439815918	33.28276254	0.007272523	59.81556236	126695.597	65.74135107	0.757693152	-0.095608993
6.233143899	27.80035381	0.001746734	165.1850663	462077.5465	53.56458289	0.904819508	-0.165602915
5.996286029	29.73452178	0.002321346	120.3783346	254035.8457	57.8522836	0.900108307	-0.16196471
5.41607586	29.09763696	0.011863266	81.59288565	199415.0073	57.16980338	0.886057543	-0.174469094
5.965183286	28.37281576	0.003981817	133.2952973	308564.3711	55.08653564	0.852606743	-0.132717804
5.612278095	31.74858599	0.013139175	100.984087	273043.0983	62.32491962	0.804771493	-0.117669908
5.466811522	22.94638282	0.006905983	71.96772633	135274.0668	45.01646082	0.94933674	-0.252143912
5.671345325	22.88292979	0.00333029	81.10171119	126829.8927	44.84250899	0.976105715	-0.306222771
5.744143262	16.74631834	0.003782146	119.4930368	211702.2694	32.16772454	0.966270625	-0.27777731
5.906672794	31.07361359	0.004851347	139.2308092	412920.9337	60.19894211	0.927120412	-0.197681807
6.174585417	30.70746885	0.001179725	125.3952815	329513.4312	59.77794847	0.836952752	-0.113930314
5.229863309	32.81389343	0.018656171	65.89183797	174201.2862	64.76170433	0.7665944	-0.107700387
6.000716478	36.85632797	0.007243461	145.6567595	818734.9017	72.82100894	0.951893343	-0.250752736
6.10921795	22.61508099	0.002549982	164.6307419	399259.8931	43.26887881	0.970441091	-0.273595248
5.928891531	23.76070078	0.002337104	108.0272813	185504.2178	46.22104358	0.891489343	-0.159069001
5.614999084	29.19671468	0.008101938	98.30021126	271396.5956	57.41247158	0.785835661	-0.108165421
6.149290655	29.04161563	0.002426825	151.9801756	375867.7051	56.32322903	0.873486988	-0.140806306
6.02587629	33.49646384	0.002371553	124.3333022	272289.0624	65.16425885	0.962163343	-0.248813452
5.484263151	35.53601872	0.011030336	79.12426372	249293.8045	70.26953007	0.753407294	-0.095572182
5.989028208	30.02596185	0.003771855	145.5106692	336768.0486	58.54454205	0.910566021	-0.178641439
6.29853283	27.12509451	0.001858491	179.5909841	579617.442	52.62061328	0.880320452	-0.142754631
6.239116988	29.67262451	0.002389062	176.791137	552878.93	57.77500986	0.804964792	-0.105582023
6.141265935	30.85200768	0.001275481	112.4213418	291212.512	60.20500428	0.884446242	-0.143379438
5.871004282	26.92237953	0.004791778	118.1789594	344745.9446	52.57058185	0.968488124	-0.281257255
6.275755547	25.67573773	0.001360589	163.2257661	468928.4927	49.55895222	0.812919843	-0.103348751
5.899572132	22.10179727	0.003060795	125.6174643	230469.868	42.52982641	0.979407839	-0.314192858
6.04058933	31.50916804	0.003346305	127.3888717	296824.2434	61.51229818	0.765958423	-0.091671234
5.887153677	33.73839584	0.004246513	85.31590659	246088.635	66.42367782	0.786014029	-0.100288644
6.000033254	20.76272178	0.001759299	112.3170961	214615.8933	40.14853019	0.83448278	-0.11926863
5.848951504	22.49456119	0.004739755	133.1285947	261799.098	43.17488974	0.969061379	-0.286965356
5.947200535	31.03908754	0.00743249	169.3561437	542758.4125	61.05406809	0.828323126	-0.124380517
5.971728855	31.01544435	0.004534242	141.6009815	323246.0642	60.12284496	0.906758794	-0.17390879
5.921336557	27.10050819	0.003264956	117.3375784	233620.3837	52.65981413	0.942108222	-0.21701906
5.780339845	33.16926412	0.012599352	118.0042785	423019.9905	65.28234964	0.877683849	-0.15910626
5.781788772	27.72716392	0.003891582	88.48691883	135878.9289	54.47767275	0.922004898	-0.195059134
6.107788893	26.38463884	0.001404691	109.5069502	412766.8712	51.43173045	0.87837759	-0.141909554
5.934111312	20.58469638	0.003389714	148.9959702	336910.161	39.32760581	0.989803397	-0.376927438
6.011039866	22.00186395	0.001883673	113.6374765	225620.3347	42.90205674	0.890971973	-0.155249253
5.510023154	33.89421796	0.006800417	72.57246895	159863.8965	66.61246771	0.858944737	-0.143444701
6.237089472	34.57352082	0.002083349	170.7501008	663540.3385	67.64047906	0.945474349	-0.210746725
5.916497532	23.41268056	0.002241858	111.8725211	211597.9556	45.33706409	0.941106291	-0.21231839
5.636447083	29.43251748	0.019008163	161.3990444	512263.9797	57.31002008	0.861899124	-0.159748401
5.574109408	26.75865339	0.003909926	66.11360519	108195.5926	52.61423148	0.807210875	-0.114499628
5.815763288	28.34452716	0.005284148	114.2727	290949.2031	55.39801799	0.856378486	-0.141339062
5.620140268	15.2752458	0.005560559	107.3436823	224590.045	29.37425956	0.956120211	-0.26057363
6.048683987	26.39202359	0.002872088	127.0155687	323148.6224	51.40097018	0.928419408	-0.195324553
5.378011897	29.18635453	0.02098433	99.38045926	293554.9936	57.37279453	0.823407521	-0.134177115
5.991834283	29.48897656	0.004182634	121.1794566	342419.0979	57.44908217	0.926922437	-0.193018697
6.028230188	20.8754484	0.001861304	123.2766224	225007.0329	40.29812716	0.877672721	-0.146160269
5.914665305	17.56020503	0.003517494	147.1981035	432726.1115	34.24397215	0.948029532	-0.233054845
5.457076883	30.64134498	0.006291315	65.93541425	158177.6889	60.24123211	0.822527162	-0.126718198
6.089530173	30.3422724	0.001901709	105.0831753	228421.2922	59.399262	0.82402271	-0.112008654
6.000350504	27.9353076	0.003876614	118.9561241	280204.8031	54.69089865	0.835814684	-0.123009571
5.735538487	28.8796994	0.002291141	67.95664357	95496.91574	56.74579296	0.753649303	-0.08909307
6.288102911	28.06806627	0.001694476	184.9905557	506274.6468	54.13275399	0.868929649	-0.13585265
6.035390486	27.18757516	0.002952625	123.6653539	286319.2414	52.91010067	0.838162164	-0.123599557
5.918046066	22.74816467	0.00337648	140.0202191	270017.8164	43.54640191	0.991660155	-0.392642407
5.543755233	17.51414606	0.004640388	70.9773844	88828.89556	34.19008144	0.98296728	-0.351818444
6.236090175	26.7430502	0.004437295	189.2028625	794085.8395	52.28728677	0.85920787	-0.135035211

original_glcm_DifferenceAverage	original_glcm_Id	original_glcm_ClusterTendency	original_firstorder_InterquartileRange	original_firstorder_Skewness	original_firstorder_Uniformity	original_firstorder_MeanAbsoluteDeviation
9.962782403	0.219497384	527.1308623	579.25	-0.630764584	0.030103552	307.7611149
9.840790087	0.191426995	272.7240911	434.5	0.525753051	0.036268053	223.1329552
10.49206605	0.226264132	530.5322298	707.25	0.116916431	0.033848459	316.1502845
10.01654225	0.217333184	358.8077243	555	0.355075033	0.037884341	260.1108754
9.091922606	0.228217619	352.9201645	576.5	0.17474643	0.03805927	253.7938211
10.0068808	0.218137172	678.0419874	723.5	0.315907101	0.031725975	345.5326822
5.902762895	0.388042811	446.6142834	340	-1.176643247	0.10062941	240.2324604
8.354249853	0.312557516	439.8534049	498	-0.835796531	0.072109533	277.0259265
7.374914093	0.316903571	322.3591141	424	-0.921651745	0.073138235	233.2551083
8.618329635	0.218116578	421.5695895	583.5	-0.082675131	0.028881189	268.0505557
6.133302951	0.302124757	277.8719737	444.25	-0.50545849	0.059829766	215.7109539
6.848870126	0.325265186	311.5729485	347.5	-0.968633586	0.069153969	219.5195844
9.804517867	0.235437612	437.8590066	529	0.711361963	0.046249093	270.5392165
10.81882894	0.195005891	419.5754805	608.75	0.088625478	0.029180008	281.7504967
8.79670071	0.294054987	367.170234	510	-0.849086405	0.064154397	268.2216272
7.543885502	0.362208094	618.94569	576.75	-0.928043514	0.095412728	307.5042918
6.276085279	0.275119532	211.3336412	382	-0.310716078	0.044826848	191.3584801
9.338250617	0.217691803	347.426445	529	0.329554691	0.038229088	248.3353758
6.021175861	0.311596372	282.2108355	404.5	-0.590803581	0.057428085	215.9855185
7.18777238	0.283875983	518.9263203	456	-0.209609867	0.040682902	267.9959962
7.763170657	0.246944876	341.8476717	526	0.168739626	0.035288799	244.7947247
8.627336244	0.224591359	239.3993454	461	0.122715437	0.037851197	212.3145405
8.768700037	0.248479627	414.9099459	626	-0.164803937	0.042573965	279.4566488
5.55743443	0.362897332	242.779758	278	-1.31776731	0.098644354	182.872679
7.951561685	0.382819752	628.0843592	670	-0.805188308	0.100194697	330.7221456
7.456028281	0.290891382	334.372735	459	-0.739254069	0.052834963	241.7975257
9.079839561	0.232896241	404.6227565	552	-0.521727942	0.035883323	273.2331385
7.210595116	0.26694343	304.2201258	445	-0.659411433	0.043210522	230.4027515
7.048922534	0.220984007	194.461755	359.75	0.304207993	0.038937887	176.2010696
7.546892938	0.267994652	317.9881891	491	-0.103641638	0.045556884	239.1330612
8.046886435	0.284247421	430.5693776	551	-0.531795318	0.052875853	275.4784293
10.26718928	0.218959669	379.0878332	611.5	0.126969276	0.035616738	273.8151105
7.742065017	0.282507131	426.1918351	563.5	-0.50881787	0.044517055	275.0893134
6.820409406	0.374333935	482.9178025	376.5	-1.153546336	0.106317057	260.9884907
7.366507979	0.295149843	365.0066782	463	-0.630018575	0.053186183	246.6882925
10.34502417	0.23432944	525.6513907	753.5	0.062513993	0.04072616	323.669385
11.50897014	0.183823898	524.3050682	540.5	0.753365653	0.030608746	288.8155987
7.29663868	0.409975125	628.6025087	498	-1.088874613	0.106526108	303.9732465
6.997733246	0.247432015	206.8362249	389	-0.193189284	0.036084184	189.4959503
5.738710846	0.371450656	272.4935397	254	-1.327978512	0.108462225	187.8389401
8.481054732	0.239257132	357.0405653	568.25	-0.203896537	0.034739363	258.5497939
5.705111151	0.327401536	188.7871641	232	-1.398044049	0.071735852	156.5010778
10.21842452	0.211971201	498.1087982	664.75	-0.13961352	0.028322185	305.6194187
9.426013807	0.218966634	342.8860164	546.25	-0.329741156	0.033731402	258.9324479
6.038138591	0.340091748	268.8722661	352	-0.993372623	0.079313182	206.0165336
8.87428321	0.243136372	409.288595	600	-0.291212205	0.042786039	276.6185341
6.897028624	0.341367295	323.8675082	408	-0.890980228	0.085640338	228.7623584
6.292590286	0.277750298	221.0043328	409	0.176935099	0.051513807	198.3036656
7.939499121	0.217875065	222.778688	367.5	-0.102959522	0.032149188	194.0330322
9.348721716	0.223255847	318.7672082	442.5	0.654379384	0.048085206	238.5239251
9.708240951	0.251905677	409.5179233	597	-0.532485937	0.04662548	290.942313
9.316694	0.201751223	369.9352385	487	-0.39290724	0.026076216	248.8679782
4.974135978	0.392991092	221.7671746	204	-1.372118249	0.108064131	167.9105053
6.983461618	0.315732005	473.7685898	144.75	-0.736600862	0.060955491	223.6972662
10.9144867	0.211216443	451.5629151	621	0.330743942	0.031503281	287.7393729
8.472554935	0.221577209	312.3795895	527.25	0.016802283	0.033886344	242.3395113
7.18506482	0.307533765	303.0483326	373	-1.014018475	0.068193155	223.4852353
9.773152493	0.225952613	451.7674983	646	-0.161284387	0.036222211	297.9326645
9.753580481	0.211258426	357.1103356	546	-0.21013999	0.029745658	264.7397405
5.955042911	0.345891536	253.2687444	263	-1.400259792	0.081341973	184.7515523
8.914482766	0.25098419	441.207063	642	-0.131660446	0.040519386	285.3998452
10.04447712	0.22079436	548.9877127	688	0.006744448	0.031329485	317.5803282
8.669272414	0.267872112	572.1899919	686	-0.247444705	0.034339745	315.153761
8.552474836	0.209109406	327.7726103	437.25	-0.125905234	0.026864602	226.2495302
7.687489897	0.262610928	376.0884416	491	-0.172406371	0.044913152	251.382976
10.38839731	0.201157786	473.5403183	631	0.250557603	0.028312243	294.9128803
10.014886	0.193613986	337.2388696	489.75	0.456018652	0.034070227	245.0700696
8.842214115	0.253795429	379.2726235	556	-0.395227725	0.04212575	265.3673488
6.664414051	0.281435853	267.7089332	320	-0.640166752	0.051126357	195.6701441
8.379319237	0.225132265	326.0524579	511	0.359024729	0.033363657	242.5855606
9.284238224	0.242473251	386.9765684	639.75	0.042194502	0.042922533	279.416047
8.529207237	0.307797516	541.9285201	642	-0.631882359	0.066474043	308.9509846
9.811086645	0.237982639	415.353826	647.5	-0.19801009	0.045325927	286.601612
9.419411317	0.237301975	328.6252999	511.75	-0.449689033	0.037429363	254.2935047
6.687604907	0.344694269	394.9276005	368.75	-1.011878912	0.08110471	232.5735528
7.491851679	0.253231487	262.6922864	472.5	-0.05090533	0.041683873	217.4247939
7.61988115	0.22422089	337.6625904	430	0.509159147	0.027683982	227.7598282
10.82086547	0.18980752	402.7486448	506.5	0.528532258	0.03436245	264.0531204
8.442103903	0.221495953	331.6273063	489	0.284285043	0.031285113	238.2875693
6.803960488	0.292788687	217.5004875	326.25	-1.084805863	0.064074691	189.861842
9.814156463	0.222415575	527.3219974	564.75	-0.657077286	0.030713726	304.8278218
8.849827779	0.212513169	314.0380446	474	0.45559346	0.033042346	235.0464563
9.022747864	0.345975932	493.7816898	628	-0.667256078	0.099380435	304.18823
6.466161129	0.270720088	198.4728542	352	-0.674097807	0.043341764	185.3222189
7.21424104	0.300296411	376.9103278	474	-0.736536095	0.052775353	249.2578824
8.093928899	0.249414109	284.3918607	262.5	1.268019223	0.067558989	193.5339568
8.463091421	0.245408309	399.7640033	543.25	-0.392570341	0.035232491	265.0148814
6.142078622	0.376691884	331.1213619	389	-1.038151447	0.102852239	228.3849888
7.51430006	0.270036788	394.2146906	504	-0.345733277	0.040811142	254.3230247
9.095379371	0.215866001	345.2814274	555	0.189359231	0.032868498	253.8368685
7.894980534	0.25937828	462.7226264	581	0.624221222	0.045203089	275.4984221
6.074651914	0.323779645	205.3589789	273	-1.23825086	0.059757692	176.7412039
7.4903724	0.243698845	334.9622378	497	-0.257484961	0.033366551	239.2186883
7.699205688	0.26621335	377.8880212	540	-0.397108476	0.045725629	254.3719578
6.754733093	0.250672814	203.5669617	368	-0.391455782	0.037121594	187.7473028
10.75064234	0.218419804	531.9756307	728.25	0.150439821	0.031781719	320.8104097
8.666605385	0.242859639	372.9692493	554.25	-0.288323359	0.038711948	259.3017831
10.7459086	0.179398652	367.6917212	550	0.401309428	0.030632139	262.8165848
6.935970035	0.23271077	204.1194471	359.5	0.281281224	0.039262642	180.3537536
7.795085938	0.284570947	635.6956705	683	0.008618425	0.043506326	320.9874671

original_firstorder_Energy	original_firstorder_RobustMeanAbsoluteDeviation	original_firstorder_Median	original_firstorder_TotalEnergy	original_firstorder_Maximum	original_firstorder_RootMeanSquared
231894229	243.7754015	-36.5	6261144183	337	400.7387819
99687622	178.5924642	-558	2691565794	43	535.9890048
272255632	275.8094473	-505	7350902064	129	552.4665767
70341667	221.4099532	-459	1899225009	165	467.388898
319633540	217.3981204	-421	8630105580	62	475.9514623
70606836	289.6666667	-450.5	1906384572	840	540.1516316
1086696154	163.4786455	32	29340796158	723	321.1103935
788502505	218.6197017	17	21289567635	882	360.4779899
424695013	182.6522031	-14	11466765351	149	315.6689444
145904188	224.6992255	-317	3939413076	351	439.6027526
153773141	182.2585768	-109.5	4151874807	301	314.3659111
282975935	157.3650011	19	7640350245	750	288.1964886
310366810	221.179697	-670	8379903870	99	618.624633
150835121	238.4591742	-393	4072548267	177	483.9587032
213354860	214.0398604	-25	5760581220	188	368.053533
952411037	250.4762616	5	25715097999	462	406.5608754
66191820	155.7454671	-123	1787179140	663	273.7926653
94245493	210.3214047	-464	2544628311	84	480.0302889
102521234	171.0306847	-60.5	2768073318	662	306.1246147
1215546808	198.6375906	-57	32819763816	1143	363.825849
470203218	206.9608468	-417.5	12695486886	49	475.6856244
80824969	176.9364096	-355.5	2182274163	79	403.6750754
109675763	245.6773358	-297	2961245601	91	443.3413432
235552467	127.5803262	-6	6359916609	271	255.9019585
240891316	279.1281727	-14	6504065532	230	442.9058216
256693614	192.0630207	-10.5	6930727578	230	316.9035524
287724657	223.1650778	-110	7768565739	449	378.9131538
229590787	184.4237512	-78	6198951249	246	319.2248066
25595619	134.4369798	-386	691081713	267	418.7033065
199786742	200.5353313	-164.5	5394242034	1078	352.2657294
587309468	228.2335569	-91	15857355636	542	382.7502411
200970157	237.6764641	-451.5	5426194239	185	505.0131387
303937195	233.4433917	-59	8206304265	190	360.7850084
275736191	193.5940098	30	7444877157	294	337.9000986
547006573	196.2967942	-17	14769177471	851	326.4454655
544158588	291.1026196	-440	14692281876	173	529.7535056
659142046	224.1112746	-691	17796835242	482	660.2579092
890813878	233.9801912	38	24051974706	270	391.1962541
51947959	152.3748394	-189	1402594893	408	304.5721795
661763618	123.6425935	2	17867617686	1624	264.6698589
264703535	220.4958924	-315.5	7146995445	95	444.4546675
282513127	104.7976429	-16	7627854429	220	223.1779094
512044273	262.1355579	-302.5	13825195371	263	460.1777415
219188700	216.088141	-205.5	5918094900	402	392.8845116
127822957	153.2023146	-25	3451219839	611	286.615474
423014258	239.6820533	-226	11421384966	247	419.741029
542242042	177.8083409	-25	14640535134	892	318.3311352
41343744	159.7917385	-124	1116281088	948	290.177781
53120479	151.4907298	-309	1434252933	115	368.5892057
124481442	195.4645988	-573.5	3360998934	112	524.7870238
216536381	243.0352342	-107	5846482287	745	410.1815894
476553293	194.7640418	-256	12866938911	205	413.6596286
432614906	107.7450937	7	11680602462	517	232.8939286
170222007	118.0090756	13.5	4595994189	902	321.9743514
189348427	243.6929927	-521	5112407529	156	542.6569435
205621078	205.8447261	-336	5551769106	96	418.1481262
365276439	168.5008536	-4	9862463853	258	301.6255004
439101763	258.7573514	-283	11855747601	226	460.9059898
117485456	219.2070341	-240	3172107312	247	392.4007099
695920973	122.4430131	12	18789866271	345	252.7587569
288635692	250.8996346	-339	7793163684	84	466.0285533
612806878	273.9393707	-349	16545785706	814	498.6012394
1662368186	275.3172127	-204.5	44883941022	532	449.3225119
378207362	172.103084	-331.5	10211598774	636	424.7851736
89318730	198.4422535	-94	2411605710	723	363.226006
1085437430	249.4658633	-508	29306810610	919	563.2819823
107603531	198.7630769	-505.5	2905295337	177	514.8138766
827047728	224.4289584	-210	22330288656	423	403.2921864
524732831	137.1549308	-19	14167786437	834	264.3668442
487106471	201.5963876	-471	13151874717	323	478.8888146
101828815	248.5613716	-371.5	2749378005	111	454.938607
796052314	263.9387061	0	21493412478	373	399.772148
264342635	251.0503978	-284	7137251145	129	444.9825439
108254400	209.7896814	-82	2922868800	279	337.567771
428152646	169.9841657	-14	11560121442	572	323.7847943
116268946	183.1706105	-264.5	3139261542	124	355.8856913
338481860	175.4121121	-284	9139010220	1090	391.444181
121617366	213.7583309	-633	3283668882	65	613.6158024
347475320	195.8040186	-481	9381833640	63	514.0425927
117357568	137.1291956	-7	3168654336	292	255.340261
219571270	239.5496448	-27.5	5928424290	337	395.1801271
216039304	189.3819284	-513	5833061208	110	511.418414
380032138	258.8022271	-10	10260867726	646	408.0866075
169765501	142.488607	-156	4583668527	105	299.467304
343733910	202.7159252	-66	9280815570	284	355.8166546
232004035	132.8550111	-723	6264108945	44	660.9985527
187499501	220.4112249	-169.5	5062486527	229	378.6135792
387852801	174.1033448	-19	10472025627	207	317.9758719
210368989	206.2246331	-115	5679962703	791	351.6727383
307790500	216.1417402	-416	8310343500	176	474.5077918
300160461	229.7525548	-653.5	8104332447	259	611.0105619
157450430	119.333447	-32	4251161610	186	249.3676283
591382619	198.2667245	-213	15967330713	379	362.4764298
529324191	214.7073	-213	14291753157	340	391.9252415
271265561	149.6455215	-159	7324170147	184	286.4047608
648157059	280.8998202	-450.5	17500240593	532	521.4191797
437773344	217.9083628	-273.5	11819880288	134	422.5366882
93947203	214.8552765	-540	2536574481	176	547.8602087
49345680	142.0030287	-394.5	1332333360	50	429.0986735
1938847253	274.8060823	-229	52348875831	1028	468.7475903

original_firstorder_90Percentile	original_firstorder_Minimum	original_firstorder_Entropy	original_firstorder_StandardDeviation	original_firstorder_Range	original_firstorder_Variance	original_firstorder_10Percentile
176.7	-1066	5.398074783	351.7647987	1403	123738.4736	-755
-74	-834	4.962051568	255.6222957	877	65342.75808	-754
28	-998	5.173451383	346.8095939	1127	120276.8944	-844
54.8	-774	4.936377296	287.9599488	939	82920.93211	-696
18	-849	4.950410545	284.376348	911	80869.90732	-732
51.6	-936	5.2308477	385.0461924	1776	148260.5702	-859.8
79	-999	4.483797645	295.1772986	1722	87129.63762	-641
92	-964	4.64852159	318.8144291	1846	101642.6402	-715
64	-910	4.526675925	268.6483097	1059	72171.9143	-625
63	-870	5.255931353	302.7641251	1221	91666.11542	-733.6
42	-704	4.578263743	239.9651221	1005	57583.25984	-573
92	-928	4.650987736	264.2971819	1678	69853.00034	-569
-19	-901	4.856877093	309.08349	1000	95532.60378	-835
55.7	-896	5.260544953	315.6289105	1073	99621.60917	-768.7
60	-951	4.712542802	304.9447433	1139	92991.29645	-705
63	-926	4.264200104	348.8600512	1388	121703.3353	-816
69	-804	4.781936509	217.9784452	1467	47514.60257	-471.6
19	-834	4.949556537	279.2897975	918	78002.79097	-707.2
44	-786	4.715657557	248.0453116	1448	61526.47662	-573.7
115	-934	5.295879038	323.9969784	2077	104974.042	-678
15	-847	4.99265068	275.4386932	896	75866.47369	-729.3
11.5	-771	4.89714667	239.9938435	850	57597.0449	-626.5
37	-859	4.920744597	306.108799	950	93702.59681	-737
50	-898	4.254409097	223.7524787	1169	50065.1717	-490
43	-1006	4.304729606	366.2682078	1236	134152.4001	-849
98	-966	4.862730339	275.3549831	1196	75820.36671	-591
93	-1019	5.200594126	308.8163557	1468	95367.54152	-697.1
79	-947	4.943916901	262.4778148	1193	68894.60326	-592.4
-74	-802	4.814658914	211.0240485	1069	44531.14904	-613.5
53	-809	4.87840056	270.6424519	1887	73247.33675	-613
62	-894	4.932939041	309.1265194	1436	95559.20497	-712.2
1	-875	5.020058519	302.3150782	1060	91394.40651	-783
112	-865	4.940615102	303.7707325	1055	92276.65792	-657
80	-873	4.145761569	307.5164847	1167	94566.38835	-711
90	-951	4.873187352	283.2141055	1802	80210.22958	-618
42	-933	4.989634304	349.4529238	1106	122117.346	-826
-4.1	-1009	5.332192176	337.9429827	1491	114205.4596	-917
85	-941	4.177693379	352.6680741	1211	124374.7705	-818
63.1	-735	4.92307187	218.9774024	1143	47951.10278	-512
49	-963	4.197435219	235.9291841	2587	55662.57993	-521
8	-899	5.080700292	289.1313303	994	83596.92614	-741
61	-906	4.43969979	197.5252873	1126	39016.23912	-414
93	-940	5.335802189	337.2634771	1203	113746.653	-770
58	-931	5.166461322	289.9968113	1333	84098.15055	-685
48	-832	4.432778736	243.4142097	1443	59250.47749	-555
49	-928	4.990510214	304.2631252	1175	92576.04936	-723
50	-943	4.472047675	266.0359945	1835	70775.15039	-621
50	-698	4.711780998	234.2094823	1646	54854.08161	-488
19	-784	5.052274051	227.4940711	899	51753.55237	-592
-0.2	-780	4.750246302	269.943625	892	72869.56069	-719.9
70	-948	5.001383623	325.7311921	1693	106100.8095	-756.4
68	-1002	5.375645272	291.6718051	1207	85072.44191	-721
56	-897	4.134534067	212.2992965	1414	45070.99128	-475
252	-943	4.971741086	316.6296693	1845	100254.3475	-613.9
39	-923	5.177278833	322.4765821	1079	103991.146	-808
41.5	-829	5.031084559	269.8876013	925	72839.31733	-673.5
77.6	-846	4.604315507	263.2746638	1104	69313.54861	-595
38	-978	5.161990018	324.8031786	1204	105497.1048	-767
96.6	-1005	5.271837715	297.5976961	1252	88564.38871	-648.8
76	-961	4.47251775	230.8503097	1306	53291.86547	-475.8
27	-1033	4.965711424	312.3581366	1117	97567.60548	-770
55	-977	5.303802626	349.806623	1791	122364.6735	-812
89	-982	5.178670263	343.298034	1514	117853.5401	-771
26.5	-1030	5.374339893	270.7822465	1666	73323.02503	-674
35	-834	4.998545463	293.7966993	1557	86316.50054	-648
2	-1034	5.337783083	331.3067031	1953	109764.1315	-858
7	-900	5.068486104	280.1116309	1077	78462.52577	-739
44	-1013	5.043485182	296.0429379	1436	87641.42107	-710
95	-912	4.94601351	241.755105	1746	58445.53082	-471.3
17.7	-851	5.066847633	273.6933856	1174	74908.06933	-709
40	-810	4.880113689	303.2512205	921	91961.30271	-715
97	-969	4.678987122	340.2229572	1342	115751.6606	-748
46	-1011	4.982250637	314.5804831	1140	98960.88032	-738
134.1	-814	5.06414324	284.0745783	1093	80698.36605	-609.1
50	-973	4.614713033	278.1924521	1545	77391.04043	-641
65	-901	4.873517509	246.1798234	1025	60604.50544	-588.6
53	-891	5.302643841	276.5043372	1981	76454.64848	-641
-56.2	-948	5.097796476	300.0295065	1013	90017.70474	-860.8
-18	-946	5.128114076	271.739972	1009	73842.61236	-760
85.1	-916	4.664281291	229.3889312	1208	52619.28177	-474.2
182	-985	5.379891575	349.5197817	1322	122164.0778	-753.5
-2	-956	5.08317902	270.2151188	1066	73016.21045	-738.5
51	-920	4.355969517	336.4701154	1566	113212.1386	-770.9
35.8	-822	4.812307018	219.5001726	927	48180.32576	-541.8
44	-877	4.769499522	282.0883928	1161	79573.86134	-683.6
-188	-918	4.500157255	241.1661889	962	58161.13065	-815
89	-841	5.139470003	297.6907613	1070	88619.78935	-687
40	-856	4.254247082	267.0417374	1063	71311.28951	-647
89	-895	5.100530533	290.5545998	1686	84421.97548	-625
19	-831	5.077231113	283.693066	1007	80481.75569	-729.4
-30.3	-902	4.846303547	311.9126976	1161	97289.53089	-837.7
72	-829	4.589588277	219.5193067	1015	48188.72601	-480.9
68	-973	5.164107368	269.4596462	1352	72608.50094	-618
41	-931	4.95592501	285.3537939	1271	81426.78771	-687
68	-856	4.927867798	216.827383	1040	47014.11402	-505
65	-1006	5.246208223	350.4366594	1538	122805.8523	-807.7
31	-936	5.07107671	290.7507413	1070	84535.99359	-723.9
-13.4	-932	5.200005852	297.5995708	1108	88565.50454	-801.2
-60.4	-745	4.83456136	210.2233222	795	44193.84518	-624
43.7	-942	5.125693929	356.8925653	1970	127372.3032	-787

original_firstorder_Kurtosis	original_firstorder_Mean	original_glrlm_ShortRunLowGrayLevelEmphasis	original_glrlm_GrayLevelVariance	original_glrlm_LowGrayLevelRunEmphasis	original_glrlm_GrayLevelNonUniformityNormalized
2.044820416	-191.9716066	0.002952372	199.6920978	0.002998235	0.029078349
1.939158767	-471.1066282	0.023757928	103.8110079	0.024293568	0.036057688
1.470423686	-430.0493274	0.02191118	187.5608369	0.023305055	0.032380862
1.583797076	-368.1459627	0.032892893	129.1525779	0.033610734	0.036874433
1.548577178	-381.654146	0.014580463	125.4515721	0.015106648	0.035734236
2.187637458	-378.8181818	0.031114312	236.5678158	0.031787847	0.030801303
3.394535895	-126.4209128	0.004074161	148.6727455	0.004355701	0.074821788
2.331915351	-168.2312129	0.003303604	166.9690031	0.003435283	0.061170471
2.373316038	-165.7557485	0.00290942	118.3238983	0.003009262	0.063148151
1.638044937	-318.7231788	0.013549918	145.3941144	0.014016678	0.028463586
1.752758101	-203.0829049	0.010251201	91.88565705	0.010590247	0.054141556
2.930005726	-114.9095979	0.002078962	117.0077415	0.002147162	0.059705119
1.969472835	-535.8766954	0.021156386	151.0790434	0.022537104	0.043995328
1.574349809	-366.871118	0.018382814	156.6146515	0.018785945	0.028228726
2.208620819	-206.087619	0.005256697	152.713685	0.005372425	0.055329776
2.121100405	-208.7783756	0.008572354	198.0588707	0.00909561	0.082480428
2.272524904	-165.6738392	0.003449546	76.02844413	0.003520975	0.042125584
1.636422945	-390.4180929	0.018890501	120.8776028	0.019281062	0.036727916
2.299399042	-179.4040219	0.005737042	100.3091685	0.005885498	0.05069181
2.920697006	-165.5149733	0.002961641	171.8892794	0.003079519	0.036164378
1.607673376	-387.827719	0.015050408	117.0816893	0.015645919	0.033559416
1.638186274	-324.5866935	0.014344977	89.91555926	0.014568196	0.036509426
1.452051108	-320.7007168	0.012810323	147.8311589	0.013043611	0.038951908
3.689850596	-124.1798721	0.003374829	83.84278219	0.003523619	0.078929398
1.89663191	-249.02443	0.009336612	223.1089208	0.009666375	0.073513935
2.17379	-156.8677621	0.002245911	123.3525232	0.002310725	0.046449506
1.896957153	-219.5623752	0.003667202	152.9456892	0.003759848	0.033763129
2.099810972	-181.6861962	0.002616731	111.2261153	0.002683406	0.040686977
2.34022721	-361.6369863	0.013752537	70.80256715	0.013883077	0.038996934
2.770686356	-225.4857143	0.006082479	117.010645	0.006235315	0.041952334
1.869952974	-225.6956847	0.006206236	153.4486113	0.006503399	0.045333099
1.487414771	-404.5291878	0.02209711	141.5780539	0.022767026	0.034070887
1.705841775	-194.6513919	0.005409303	147.5899866	0.00555808	0.040787765
2.642748716	-140.0360248	0.010084874	156.6663523	0.01051886	0.093978699
2.306738184	-162.3465809	0.00201684	130.9303979	0.002088329	0.047176927
1.351722303	-398.1474987	0.011758926	191.0808126	0.012220102	0.038063069
2.34610023	-567.2169312	0.017061518	181.7493704	0.01764287	0.030092714
2.454638004	-169.2918742	0.011353547	210.2167098	0.012047062	0.087579013
2.011314675	-211.6910714	0.007263429	76.27552752	0.007353486	0.035342034
4.327649759	-119.9481317	0.002020073	94.96974185	0.002105612	0.088763832
1.588155435	-337.5544776	0.01138767	131.9121688	0.011765825	0.032753666
4.339552449	-103.8852257	0.001919023	65.06269824	0.001978664	0.064537943
1.532341091	-313.0765095	0.009026345	179.5746336	0.009336942	0.027392657
1.743904552	-265.0661972	0.004330576	134.3154118	0.004405457	0.032059414
2.823344053	-151.3206941	0.004887817	98.36587092	0.005002465	0.066435004
1.557557144	-289.1478551	0.004636559	146.3884802	0.004756407	0.038728651
2.572980326	-174.8129322	0.003310258	116.9076213	0.003436366	0.06793063
3.854829913	-171.3156823	0.008918446	89.40287978	0.009085748	0.0464241
2.022245471	-290.0076726	0.011091829	81.4917523	0.011465087	0.032187004
1.926423817	-450.0353982	0.02687561	114.5284589	0.027885616	0.04694168
1.853001352	-249.2952603	0.007607224	169.5790256	0.007803107	0.041440936
2.076993547	-293.3289048	0.004082293	135.4709522	0.004170339	0.025841688
3.808017025	-95.75275827	0.00183471	77.51438631	0.001928799	0.086767779
3.841348117	-58.42204629	0.004852548	172.6689164	0.004994661	0.053067359
1.628120733	-436.4463453	0.017144629	162.3053083	0.01794658	0.030660486
1.560458725	-319.3877551	0.007857969	114.7516033	0.008046298	0.033000132
2.668332445	-147.1882939	0.007032374	113.5401916	0.007239192	0.059187163
1.479478131	-327.0125786	0.004417779	167.2874834	0.004539063	0.033623243
1.789626959	-255.7614679	0.004214331	140.706221	0.004254208	0.028876701
4.125003365	-102.9326173	0.00197279	89.88622097	0.002038699	0.069674184
1.452640499	-345.8540256	0.003606805	152.069786	0.003713471	0.036755536
1.692588912	-355.3006085	0.006954078	193.8291105	0.007129654	0.029717914
1.488202081	-289.8916687	0.00361298	185.8146045	0.003784245	0.032316843
2.33157664	-327.2910305	0.003524833	116.9089786	0.003624878	0.026724292
2.553961402	-213.5805022	0.007109588	140.873544	0.007222415	0.041174381
1.802621832	-455.5463315	0.006216049	172.3651075	0.006407497	0.027427315
1.856775819	-431.9384236	0.011793583	123.8383756	0.012057915	0.033737124
1.730257391	-273.86706	0.002767391	138.7708387	0.002857767	0.037992301
3.346912435	-106.9780234	0.001778819	96.48202179	0.001836439	0.046002248
1.705825208	-392.9712806	0.008462357	118.1744708	0.008781127	0.032933933
1.379635755	-339.1280488	0.01687063	142.9879811	0.017402518	0.039968938
1.733869537	-209.9192933	0.003796402	187.2293387	0.003924633	0.057564534
1.497938954	-314.7198502	0.003900124	155.5309742	0.003980846	0.03875411
1.773726508	-182.3557895	0.006930671	129.394232	0.007044616	0.035396446
2.911112597	-165.6669931	0.003398784	130.1043838	0.00354128	0.063410459
1.662633804	-257.0021786	0.003356069	94.5386792	0.003430011	0.038317319
3.89309527	-277.0810321	0.003782189	122.4060403	0.003909725	0.027597275
1.902182231	-535.2631579	0.032485683	141.677519	0.033088503	0.033977245
1.750327368	-436.3452471	0.007461907	115.6684626	0.007682917	0.030751218
3.269799577	-112.1577778	0.002363198	86.69047793	0.002418591	0.056293824
2.084917915	-184.3997155	0.00589827	197.2352688	0.005984922	0.029675468
1.864906627	-434.2033898	0.005513484	114.8765018	0.005641705	0.03269295
1.808488863	-230.9167397	0.008097956	186.259541	0.008379387	0.074917366
2.392970903	-203.7163233	0.005501458	77.35544361	0.005588124	0.040710427
2.013060652	-216.8677716	0.004801692	129.2822251	0.004937293	0.047292749
3.400102221	-615.433145	0.019533422	94.46924291	0.020678257	0.063396013
1.738159593	-233.9411315	0.010685244	140.9299253	0.011047565	0.033262858
2.586657218	-172.6191345	0.005516526	119.3259378	0.00570441	0.077650799
2.199733728	-198.1205173	0.004977128	136.224355	0.005095938	0.036386066
1.564243445	-380.3628383	0.012959536	126.7017674	0.01346005	0.032232789
1.830779565	-525.3992537	0.021882437	152.3658878	0.023086601	0.04358019
3.539197102	-118.3025276	0.003263478	79.95680439	0.003357136	0.054734914
1.781138967	-242.4472339	0.00255044	115.5474535	0.002624282	0.032106864
1.745953317	-268.6607661	0.004262604	128.936252	0.004401918	0.041009368
2.033520509	-187.1191412	0.002301595	74.96503942	0.002360777	0.03607663
1.470266277	-386.0985738	0.005234819	193.1939811	0.005449695	0.030635574
1.702731291	-306.596248	0.006050606	133.1100503	0.006203807	0.035299771
1.780159416	-459.9840256	0.012269002	141.0949634	0.01249346	0.030525993
1.941308424	-374.0746269	0.024177485	69.56669284	0.025197481	0.038823771
2.065908846	-303.8947189	0.006581236	203.7345772	0.006949214	0.038627839

original_glrlm_RunVariance	original_glrlm_GrayLevelNonUniformity	original_glrlm_LongRunEmphasis	original_glrlm_ShortRunHighGrayLevelEmphasis	original_glrlm_RunLengthNonUniformity	original_glrlm_ShortRunEmphasis
0.052672556	40.12875822	1.148383481	1404.396321	1267.304132	0.967381202
0.03405971	12.14391703	1.096499439	342.6176435	318.4682892	0.978446481
0.075749798	27.23000623	1.20560334	680.9043191	755.5022084	0.958186824
0.054059924	11.32148075	1.154677776	384.0816699	280.3954096	0.965425527
0.079390316	47.45447038	1.209770454	452.5432027	1191.07298	0.958141807
0.053658894	7.100313272	1.158541989	731.4036073	209.8428157	0.963639
0.306841046	671.5695215	1.697489851	1166.599247	6900.734869	0.898319423
0.164588321	332.4987078	1.415632019	1074.219677	4493.801205	0.926353401
0.144262784	242.4689261	1.380655429	933.3497499	3184.7966	0.927819309
0.047462684	20.63544854	1.132918782	632.8469438	671.7506279	0.970776852
0.107426857	77.91772807	1.2825684	494.4622102	1248.600733	0.945052484
0.149711548	182.8295458	1.391677808	1115.150881	2532.670888	0.926742924
0.073646276	33.58967168	1.204407709	402.0204865	681.7261617	0.956553484
0.050685012	17.41424743	1.141309247	593.0555388	568.7357107	0.968971544
0.13213474	79.20930364	1.347453157	986.4077864	1208.831878	0.934524809
0.180585715	420.5684783	1.466009042	953.5248193	4110.72734	0.916273223
0.079289167	34.76121398	1.225907723	730.9477316	725.5615398	0.950851224
0.057007883	14.29716672	1.162521962	435.1998353	354.4416241	0.963981295
0.125551821	50.7725267	1.322882232	662.2678503	855.3483686	0.93912005
0.107362447	306.6085322	1.283325931	1094.347963	7337.87677	0.944495959
0.090916966	65.43230288	1.228305661	437.6671818	1745.243388	0.957168066
0.061041006	17.18251778	1.173475181	396.4333652	426.3466099	0.961972696
0.079900259	20.45098749	1.212174668	597.0458615	469.883535	0.957417315
0.228238738	249.9937912	1.528022774	931.2934973	2563.555543	0.917938804
0.304748939	76.61304126	1.700313981	957.4963456	801.8201897	0.898097083
0.123022025	108.6455647	1.320907224	1099.964791	1998.529615	0.939174202
0.067412971	64.00284175	1.186473453	1139.991515	1708.665675	0.960214423
0.079845172	85.89108202	1.221335609	1003.619239	1869.054019	0.95322066
0.036401981	5.498421981	1.109435676	422.5859325	131.6955267	0.973879843
0.087218523	62.94927534	1.242060265	648.3213138	1317.197564	0.949603306
0.139281313	165.4329933	1.350449671	799.6401344	3098.229865	0.936730657
0.072141898	25.33071593	1.197245533	465.5248386	668.3311777	0.958978602
0.109845293	87.7406744	1.291040908	814.3303207	1856.551194	0.943228917
0.154713688	202.5557506	1.414690751	921.6832933	1757.315037	0.921021747
0.125799055	220.83374	1.3303391	1088.458774	3974.055667	0.936926912
0.079896668	69.22050643	1.219286402	639.8033766	1615.185539	0.95435222
0.042373095	43.78214994	1.123942795	526.4794668	1350.74723	0.971500054
0.295917359	429.6326173	1.715019444	978.4143181	3685.620505	0.888737783
0.066118764	18.76000642	1.179971163	522.5192564	480.6447409	0.962058968
0.215026166	732.076136	1.533234505	1113.270751	6532.880862	0.909934361
0.07230958	41.34151582	1.200549941	601.9735425	1129.257373	0.957360078
0.132727518	332.8922628	1.346370355	1061.433735	4346.456379	0.934098696
0.061805181	62.9793151	1.171164345	797.9173763	2095.959617	0.963875304
0.058226995	43.31304632	1.164013653	850.2489445	1229.868306	0.964110856
0.172255349	92.11152353	1.437059804	781.5207964	1134.731721	0.92245522
0.082217177	87.25049507	1.220763106	791.6530751	2004.984499	0.955179005
0.212348081	319.2892151	1.51534316	934.2608432	3778.123782	0.91536627
0.087443373	21.30317516	1.234458965	512.5905461	405.6602949	0.952653514
0.044492125	12.09210949	1.128856182	498.5542826	348.1666172	0.970708781
0.047177396	20.33363341	1.137485949	315.3141202	398.8463613	0.968462018
0.085271959	49.86136825	1.231815617	884.1592894	1063.265994	0.95247239
0.043333169	69.19120536	1.125632258	977.1961915	2481.89805	0.971149043
0.264421705	593.4698895	1.631308313	969.8609721	5291.344927	0.900580295
0.130200476	79.00128898	1.350958543	1361.458671	1246.041529	0.93143646
0.059597701	18.76015319	1.165608675	532.4650327	558.1431548	0.964566662
0.056233459	36.95696752	1.159869149	553.2448768	1021.008981	0.964653437
0.135396252	216.2559107	1.346478002	823.1451832	3093.435669	0.935786144
0.072940386	65.59466372	1.197562424	851.6062407	1753.54685	0.959040058
0.053487902	21.01811872	1.153482053	1048.078276	665.2967495	0.965608979
0.163287307	678.446506	1.418557461	1185.684009	7991.823998	0.92382274
0.099983471	45.40113477	1.260414616	879.8078268	1082.966776	0.949280742
0.063567253	69.39037965	1.179020161	820.0451264	2108.567621	0.961026583
0.106611441	245.5440236	1.282782716	921.710058	6572.263366	0.944205415
0.047219953	53.72289688	1.134904341	948.6891458	1856.69685	0.969659182
0.081604383	26.13117582	1.221685435	753.155987	563.0919718	0.954202157
0.057048243	89.54776481	1.155908972	717.5938874	2994.40708	0.966857877
0.042779843	13.18153355	1.123186911	473.3773899	363.163736	0.972066585
0.095628959	179.9147088	1.251765962	973.859337	4162.5528	0.950434621
0.107748942	319.0790424	1.281821669	1105.810288	6017.871269	0.945338941
0.054574912	66.69433837	1.155348562	488.8171983	1850.044685	0.965465298
0.077735229	18.45002749	1.216573605	489.5588823	409.7589044	0.954157253
0.14225739	259.5115117	1.366664488	1001.535706	3777.544245	0.931882331
0.107866916	48.14368624	1.265431588	886.2778088	1099.129814	0.952527214
0.059970512	31.95118592	1.169552956	760.925466	819.0593235	0.962730725
0.219828049	226.7246328	1.533388658	1023.861177	2852.986145	0.912643768
0.092763345	32.97693733	1.237078114	762.8321312	766.1952121	0.954921163
0.049417416	58.26878684	1.144348869	746.2206407	1934.778286	0.96680254
0.039111379	10.56901071	1.11890632	419.055837	288.6289267	0.971423968
0.05269391	38.56916806	1.152561468	527.3659929	1145.148156	0.965373228
0.115110839	93.08983219	1.302143147	1062.542168	1421.032536	0.941651699
0.054156215	39.82299808	1.152833892	1222.879416	1229.262593	0.966398113
0.047241008	25.87653641	1.137116931	580.8825391	729.241163	0.968745386
0.260213714	149.0048142	1.589895863	802.5407338	1596.305172	0.914223112
0.085848633	72.03473765	1.232864241	657.0254807	1564.021254	0.952410831
0.133964934	116.8262409	1.347457831	793.7179118	2089.429862	0.935162813
0.077847424	31.71825057	1.210012321	262.7308753	447.8014447	0.957287465
0.073858188	41.0257779	1.201996158	713.9547432	1107.103558	0.958179268
0.286789032	254.536585	1.663477484	754.4166087	2539.725757	0.901098489
0.108321526	57.23840317	1.280566814	858.5988775	1367.623469	0.946101386
0.051792961	42.09649135	1.148382316	477.7445136	1197.670738	0.966809934
0.074665922	32.86200913	1.213284014	399.657861	667.2175864	0.953258928
0.136428379	125.297826	1.364625665	856.5057009	1909.016349	0.929670579
0.063707074	136.8217037	1.180075111	940.2029631	3843.860205	0.960604337
0.096297851	131.4207402	1.254954717	814.2766785	2811.145896	0.949619176
0.064398828	112.9449005	1.181030309	807.5316678	2823.471269	0.960528111
0.068159319	69.03175751	1.188811729	826.5744955	2029.282946	0.959815073
0.087227545	81.02061075	1.231130534	746.6910367	2035.726924	0.953943266
0.030976924	9.273936379	1.093197971	534.6774813	286.38976	0.977549788
0.046014504	9.962554852	1.137671084	300.2796563	235.6589017	0.967458308
0.115853172	312.9582808	1.30475688	816.4205222	6952.283035	0.941111521

original_glrlm_LongRunHighGrayLevelEmphasis	original_glrlm_RunPercentage	original_glrlm_LongRunLowGrayLevelEmphasis	original_glrlm_RunEntropy	original_glrlm_HighGrayLevelRunEmphasis
1742.966837	0.9556254	0.003203131	5.648038958	1464.920769
383.6291511	0.970516515	0.02672112	5.094423902	350.2360505
926.339964	0.942221456	0.029485297	5.459641586	721.2090369
500.3777947	0.953416149	0.037055295	5.143721974	405.0662505
651.6054487	0.940958404	0.017565594	5.267826246	483.6314125
940.1683924	0.952320407	0.034677074	5.432772525	769.9069699
2556.29972	0.849671915	0.00574437	5.348596689	1338.85291
1874.498485	0.895023072	0.004075146	5.257184017	1192.17678
1570.961183	0.900263509	0.003492753	5.099600907	1033.30997
774.2034147	0.960163016	0.015965543	5.45742971	657.3008523
747.3403695	0.923769033	0.012134743	5.000720924	534.1465509
1845.937067	0.898150866	0.002481694	5.235318698	1228.550484
482.2253938	0.941003509	0.029166269	5.176772483	416.5852935
760.3428204	0.957835643	0.020454917	5.466405772	622.2614814
1625.689996	0.908180708	0.00590169	5.229178084	1086.217561
1722.803086	0.883814381	0.01163033	4.927690947	1067.636051
1020.446208	0.934314836	0.003833801	5.131178669	781.6627539
601.650778	0.951664472	0.020906778	5.176392635	464.8942301
1035.604131	0.914920546	0.006581153	5.174917219	720.0531952
1564.546362	0.922699972	0.003621296	5.752166723	1169.437446
643.4780669	0.938106167	0.018265205	5.31812378	466.4188787
542.834298	0.948666253	0.015514347	5.139875726	421.8254394
868.4022235	0.940308795	0.014056597	5.232054868	640.6133873
1757.215425	0.878745108	0.004272174	4.936608973	1039.564481
2322.710694	0.847657229	0.011608319	5.127175727	1125.361794
1704.249203	0.914529915	0.002624799	5.342264739	1194.325837
1501.206188	0.945685552	0.004150462	5.514705751	1202.316863
1375.630053	0.936665642	0.002976393	5.303248764	1067.668104
485.7691628	0.965753425	0.014410565	4.931349152	434.3002251
939.8719612	0.931485905	0.006899081	5.256402508	697.3772555
1316.921814	0.909453729	0.007890655	5.442817911	876.1376552
670.7984856	0.943283873	0.025938467	5.302647912	499.690125
1263.312743	0.920836765	0.006237963	5.370822517	885.849041
1548.749333	0.891925466	0.012419314	4.769743451	1020.138203
1706.430168	0.911627628	0.002435565	5.377989018	1185.765291
915.7545583	0.937517356	0.014279812	5.340547775	685.2763061
598.4533608	0.962047212	0.020285761	5.547549322	539.9579525
2251.721352	0.841475824	0.015582199	5.050410191	1146.048385
693.9669641	0.947802198	0.007758687	5.17797503	551.5521428
2100.082279	0.872055435	0.00255649	4.942440424	1253.621557
846.3263137	0.941791045	0.013333305	5.391553398	643.4350945
1642.1895	0.908660627	0.002274536	4.965073028	1153.039258
1046.870066	0.950563085	0.010751532	5.605300227	840.3543447
1106.509341	0.951245937	0.004722168	5.441471997	895.2287115
1390.562734	0.890251137	0.005590577	5.040668369	871.4692233
1135.578177	0.937814372	0.005285147	5.346440432	847.8357785
1821.96304	0.87719046	0.004071307	5.17220724	1056.477961
719.4755268	0.934200219	0.009827499	5.051133555	546.4270573
598.9411679	0.960849892	0.012974445	5.214892536	516.4372939
374.3930239	0.958134786	0.032414217	4.971452571	325.9930763
1290.511384	0.934433088	0.008620025	5.374406117	951.8740975
1174.470549	0.961386549	0.004543313	5.601786614	1013.599085
1971.045311	0.856560065	0.002476774	4.938777157	1103.603222
1998.748341	0.905930854	0.005622046	5.517822299	1465.073738
686.2177604	0.951429597	0.021789925	5.412051062	558.9667726
706.4416839	0.95218472	0.008886906	5.290996424	580.2627316
1330.757747	0.909493246	0.008174259	5.129145038	901.0216579
1154.661021	0.943507871	0.005079347	5.481827152	901.9724006
1339.612686	0.953825991	0.004425119	5.507067181	1100.602152
1982.291761	0.893311866	0.00237435	5.094026912	1306.90072
1318.24931	0.929038606	0.004213566	5.343006992	947.8976043
1094.18333	0.947105633	0.007908623	5.607449051	867.6366801
1393.22893	0.92258179	0.004579793	5.606168797	996.3079988
1125.28134	0.959079565	0.004049274	5.603086782	980.2695335
1028.501159	0.937052608	0.007752798	5.338060837	799.5247675
922.9311196	0.954264385	0.007278052	5.598998498	751.745109
568.5102665	0.962296324	0.013261941	5.241271288	491.1408039
1421.152873	0.930701157	0.003265611	5.441283317	1044.821762
1580.871204	0.923456006	0.002116671	5.399611835	1181.77575
600.7773937	0.953389831	0.010171033	5.330944025	508.5756923
709.1072658	0.937773609	0.019638287	5.186534592	526.1728564
1698.742325	0.904452303	0.004530403	5.234347652	1107.958973
1369.427152	0.930048977	0.00436036	5.364548151	956.0422627
993.4969379	0.949878543	0.007527745	5.336550862	802.1248125
1990.547691	0.873916974	0.004232452	5.333955703	1156.775417
1103.946471	0.936400201	0.00378227	5.196892229	814.4013829
878.3060502	0.955810147	0.004462512	5.553321579	770.8996641
496.4413076	0.962848297	0.035600939	5.267992135	434.1298269
668.7796873	0.953729161	0.008641558	5.374442516	552.5248833
1599.215751	0.918333333	0.002685133	5.130374595	1148.288717
1529.126893	0.954371375	0.006370342	5.635100984	1277.662579
700.2882362	0.958185882	0.006219399	5.299607495	603.285613
1753.879295	0.869412796	0.009786269	5.094219513	918.9393317
933.4167322	0.934373603	0.005980356	5.173842682	702.5884608
1300.965	0.909052274	0.005565798	5.270199743	871.3837747
291.4947933	0.941184992	0.026534015	4.818906421	268.2053869
975.6118962	0.942425312	0.01265689	5.448238544	757.5737631
1723.179262	0.853352852	0.006699579	5.068530196	876.6281833
1292.834956	0.924297924	0.005626362	5.514977923	925.9580776
588.0439144	0.955320466	0.015668009	5.322709787	497.4139958
532.6706971	0.937619594	0.028636272	5.176179393	423.2258997
1376.856877	0.90351197	0.003802601	5.112749215	939.7196834
1225.006508	0.946712696	0.002940125	5.472704733	990.119328
1200.380416	0.929505782	0.00504526	5.358961277	875.6226647
1046.498465	0.946616734	0.002620956	5.233426379	849.3751056
1064.269876	0.945050336	0.006456749	5.554311702	867.6966228
1080.78784	0.935531434	0.006876092	5.433151581	799.5832306
601.1830436	0.970508725	0.013433367	5.32460614	547.6714615
355.9870723	0.957520092	0.029399962	5.024716027	311.314168
1226.446903	0.917680801	0.008701172	5.603805922	880.1179933

original_glrlm_RunLengthNonUniformityNormalized	original_glszm_GrayLevelVariance	original_glszm_SmallAreaHighGrayLevelEmphasis	original_glszm_GrayLevelNonUniformityNormalized
0.91804595	201.4101781	861.0262698	0.02119464
0.945253332	93.42389414	274.4807822	0.03436673
0.897492501	139.4466429	446.2697619	0.02756904
0.91311759	103.9578993	261.3531991	0.032986111
0.896726395	91.44049688	312.6068456	0.031547784
0.909430137	234.3758619	539.8119949	0.027531909
0.769435063	160.7185877	772.8317091	0.023635961
0.826530608	153.8435007	672.9462479	0.025656964
0.829102975	104.0383987	540.6447923	0.029501615
0.926272871	123.8135228	423.9461758	0.027018715
0.867318414	76.60605466	300.9413024	0.034100911
0.826844669	132.4952176	694.4384568	0.026194046
0.892653082	124.9665612	346.1821021	0.029757278
0.921716633	132.6710246	428.6292331	0.027044774
0.844081146	138.0769152	543.3008181	0.026115819
0.805464431	165.5732217	564.4043668	0.028991708
0.879034519	72.83150142	443.9275209	0.034487362
0.909983899	86.25415176	281.227825	0.035198175
0.853669254	100.655622	389.373741	0.030993684
0.865440086	201.6922085	846.2577255	0.021256577
0.894818477	91.89552754	305.5662523	0.030707723
0.905455833	69.07753827	227.0962787	0.035790816
0.895017693	111.0586194	323.2885084	0.030017348
0.809634239	86.72078833	542.8522986	0.03374021
0.769402779	191.7262355	562.536933	0.028071481
0.854130412	115.2642019	648.0558622	0.027351888
0.901154915	142.0811451	741.9028165	0.023985117
0.884958034	105.2110849	579.5093622	0.027719149
0.933545301	71.57734573	293.4826007	0.039487985
0.877262947	100.4992557	382.7028486	0.030309799
0.848940283	127.6444812	501.6438513	0.026067256
0.898586294	106.1374794	340.5596315	0.030295942
0.862760068	120.58928	452.7362206	0.026519578
0.814761362	162.1155128	532.7983523	0.032215731
0.84878816	125.888658	686.7070773	0.026365684
0.887782013	141.2015067	408.1259631	0.028212418
0.928011103	170.1988438	467.7489956	0.026000694
0.750723928	187.2108649	543.9544852	0.025767322
0.905302422	70.82613906	309.9054508	0.034445763
0.792070376	111.2573964	664.6122453	0.031089141
0.894438937	101.9586275	346.7395253	0.02867466
0.842493702	78.74045146	627.7217235	0.034610184
0.910659049	147.8154015	514.591592	0.024374256
0.910177025	119.750518	560.7016007	0.026164997
0.818174281	101.2500709	432.788555	0.032085119
0.889789914	113.985105	474.3733558	0.027499881
0.803875784	105.054049	564.4409699	0.028726693
0.88390838	101.8451129	366.5841618	0.033935366
0.926135342	70.46067353	341.2826085	0.034838178
0.920465333	92.39585444	242.5457427	0.036772801
0.883535738	143.2781453	525.6289405	0.025503046
0.926769627	126.3700975	630.0247999	0.024654838
0.773503042	92.57747053	588.0006334	0.031600204
0.836767839	284.3844959	1150.23069	0.018011234
0.911721109	122.1399002	400.7433837	0.027760624
0.911465251	91.21612559	350.1514197	0.030564129
0.84640396	106.6190191	473.8958724	0.028818657
0.898589808	139.9168295	542.3769252	0.025973012
0.913736233	128.210534	725.4809288	0.025997151
0.820563963	108.3362873	702.8933606	0.029585245
0.876379436	114.9548175	590.5449031	0.02815482
0.902868255	172.6922028	562.8223503	0.024039943
0.86465492	147.333985	628.9080652	0.024529252
0.923348149	111.4910658	736.0084977	0.026455026
0.887161574	167.0047562	545.0854568	0.026885982
0.916908108	148.0300762	524.7963138	0.024924003
0.929268829	101.9159231	340.1929566	0.031661048
0.878920323	111.9395076	616.4498176	0.026896318
0.867398807	119.7484865	750.0223281	0.026495786
0.913299101	99.47869728	362.3104348	0.030109788
0.887322921	102.3507266	283.139033	0.033417916
0.837823465	152.8240789	563.6833878	0.025511728
0.884646805	119.9530958	571.5348292	0.027900119
0.907057327	119.816875	468.9440484	0.027642743
0.798160973	139.7267147	648.9500268	0.025828891
0.889941737	74.25669695	504.3688642	0.033266089
0.916183418	129.1219565	581.024963	0.027029404
0.927500252	122.1288764	339.8976686	0.030398718
0.912853635	93.62626144	373.4631701	0.029930314
0.859183583	90.54776172	624.6159467	0.03029479
0.915713566	199.9481301	730.7496325	0.021431262
0.921060835	100.8694837	438.7344601	0.030541602
0.80288577	157.2444134	473.9087741	0.028307033
0.883519331	71.83461317	355.9812268	0.03311761
0.845412321	115.0122446	404.6492339	0.0279724
0.894879674	99.01255798	291.4385955	0.036010742
0.897114414	117.7403949	452.7077978	0.026240676
0.775010068	103.1491049	423.9537917	0.029978725
0.869192208	135.9716868	600.5926564	0.025535438
0.916663229	101.4891027	335.4370121	0.029080789
0.884593559	123.2027631	307.1592204	0.032194841
0.833239155	85.45987485	466.886467	0.03227374
0.901778395	101.0464053	606.22881	0.027563216
0.877121861	104.1829184	526.3559414	0.027834413
0.901608477	68.53555848	508.6364852	0.033103806
0.900247278	150.9131625	579.427594	0.024564087
0.886932598	106.1529785	486.7910967	0.027120561
0.942477742	133.4036971	385.6183862	0.030210462
0.91782884	59.9580452	236.2881336	0.038033395
0.857994171	195.5303715	648.735433	0.023315262

original_glszm_SizeZoneNonUniformityNormalized	original_glszm_SizeZoneNonUniformity	original_glszm_GrayLevelNonUniformity	original_glszm_LargeAreaEmphasis	original_glszm_ZoneVariance	original_glszm_ZonePercentage
0.524119891	426.1094711	17.23124231	8.516605166	5.361939819	0.563019391
0.581625709	133.773913	7.904347826	3.647826087	1.371663516	0.662824207
0.492313701	226.9566161	12.70932755	19.44902386	15.70509267	0.516816143
0.536241319	102.9583333	6.333333333	6.40625	3.593641493	0.596273292
0.531592798	404.0105263	23.97631579	34.35394737	30.90706198	0.538625089
0.586679055	83.8951049	3.937062937	6.363636364	3.499731038	0.590909091
0.51987512	1806.566043	82.13496403	2155.191079	2145.993168	0.329727678
0.589602474	1442.757254	62.78259093	646.6465059	640.497241	0.403263019
0.552060396	929.1176471	49.65121806	491.8633393	485.4503774	0.394885031
0.526748379	236.5100223	12.13140312	7.516703786	4.689212851	0.594701987
0.512681549	351.1868613	23.35912409	60.66569343	55.50583196	0.440231362
0.536028704	701.6615737	34.28800611	339.1512605	332.3769519	0.384208982
0.514493069	206.3117207	11.93266833	14.47630923	10.38602994	0.494451295
0.600666006	248.0750605	11.16949153	8.532687651	6.101202446	0.641304348
0.560697498	384.638484	17.9154519	124.6399417	119.3687005	0.435555556
0.528837329	959.3109151	52.59095921	1099.973539	1089.883986	0.314821243
0.472921858	200.5188679	14.62264151	28.62028302	24.2832803	0.4801812
0.588307894	148.8418972	8.90513834	9.300395257	6.686997141	0.618581907
0.496947555	227.1050328	14.16411379	88.92778993	83.19716159	0.41773309
0.502741987	2058.225696	87.02442599	288.9127992	283.8815771	0.445823805
0.462448447	486.9582146	32.33523267	31.5631529	27.6688086	0.506737247
0.522780612	146.3785714	10.02142857	10.09285714	6.954897959	0.564516129
0.536545836	158.8175676	8.885135135	21.89189189	18.33815741	0.53046595
0.476710727	612.0965732	43.32242991	608.4571651	600.6093127	0.356964137
0.583080678	258.3047404	12.43566591	272.2799097	264.5958756	0.360749186
0.499918302	541.9114391	29.64944649	147.5350554	141.9752046	0.424100156
0.549827112	587.2153558	25.61610487	20.11048689	16.58959306	0.532934132
0.496734642	532.0028011	29.68720822	39.8627451	35.43743772	0.475366178
0.498611279	45.37362637	3.593406593	3.56043956	0.986354305	0.623287671
0.506464543	383.3936592	22.94451783	61.58520476	57.06185684	0.470186335
0.553170168	983.5365579	46.34758155	230.6169854	225.5329496	0.44350212
0.569901905	258.1655629	13.72406181	12.6799117	9.65400153	0.574873096
0.523851747	548.4727794	27.76599809	68.27411652	63.30040713	0.448394004
0.491766137	375.2175623	24.58060288	477.8047182	467.7866221	0.315942029
0.531673484	1163.833257	57.7144815	119.0182732	113.5196874	0.426456263
0.526210764	518.8438134	27.81744422	47.26673428	43.39948837	0.508509541
0.540924962	503.0602151	24.18064516	5.578494624	2.935248006	0.615079365
0.560531404	945.616479	43.46947244	1157.036752	1145.130775	0.289812747
0.519699598	153.8310811	10.19594595	12.25	8.670745069	0.528571429
0.514367952	1620.259048	97.93079365	2233.803492	2224.809205	0.333439187
0.51400523	363.4016973	20.27298444	17.55021216	13.95792729	0.52761194
0.471346336	1037.904632	76.21162579	195.5776567	188.9427042	0.388222849
0.561336577	781.9418521	33.95333812	12.34170854	9.328630642	0.576095947
0.579529454	479.2708585	21.63845224	16.45465538	13.50639613	0.582394366
0.528170428	307.395189	18.67353952	216.7697595	209.6219459	0.37403599
0.545322773	684.3800797	34.5123506	71.42868526	67.76855225	0.522698875
0.53764399	1106.471331	59.11953353	792.9936832	786.2331778	0.384601009
0.516399511	124.4522822	8.178423237	26.25311203	22.10233984	0.490835031
0.476546541	110.0822511	8.047619048	5.034632035	2.16959952	0.590792839
0.524258305	136.8314176	9.597701149	5.762452107	2.763318213	0.577433628
0.620163831	427.9130435	17.59710145	60.00724638	56.52821046	0.536130536
0.508149048	853.1822513	41.3954735	5.069088743	2.317718685	0.602872531
0.505962761	1199.637706	74.92408267	1917.694644	1906.378272	0.2972668
0.580848257	387.4257871	12.01349325	137.0614693	131.0011621	0.406211937
0.584193663	216.7358491	10.29919137	8.283018868	5.279197332	0.576982893
0.511397843	331.3858025	19.80555556	13.70061728	10.40706447	0.551020408
0.531333842	904.3301998	49.0493537	248.166275	242.6014439	0.423910336
0.523611941	577.543971	28.64823209	45.34270172	41.83090048	0.533623609
0.583789904	273.2136752	12.16666667	7.455128205	4.797113558	0.613368283
0.495198103	1986.239591	118.6664174	860.9640987	853.588629	0.368218122
0.497174788	326.6438356	18.49771689	43.73363775	39.64179229	0.494356659
0.556382814	778.3795568	33.63187991	15.83345247	12.72890419	0.567545639
0.517461451	1915.124831	90.78276142	105.3936774	100.4439241	0.449477775
0.518804485	653.6936508	33.33333333	6.815873016	4.048667171	0.601145038
0.53776919	187.1436782	9.356321839	29.03735632	25.25276622	0.514032496
0.543663617	1127.558341	51.69238187	16.2270974	13.50634736	0.606255481
0.532255932	130.9349593	7.788617886	5.138211382	2.414369753	0.60591133
0.51925858	1305.41607	67.61734288	107.7696897	103.6784835	0.49439528
0.485583099	1593.683729	86.95917124	282.6197441	277.3864954	0.437133724
0.480651222	546.5004398	34.2348285	11.66051011	8.170810098	0.535310734
0.532610534	135.8156863	8.521568627	20.01568627	16.29305652	0.518292683
0.530558231	1059.524787	50.94692038	394.6664997	388.4452598	0.400923509
0.563411177	408.4731034	20.22758621	57.31724138	53.92656361	0.543071161
0.559658017	310.0505415	15.31407942	12.15162455	9.211080556	0.583157895
0.5204383	799.3932292	39.67317708	637.9791667	630.9096612	0.376101861
0.499931877	238.4675052	15.86792453	29.0230608	25.31925161	0.519607843
0.460690046	564.3453061	33.11102041	5.852244898	2.600479134	0.55454957
0.570883212	117.6019417	6.262135922	4.004854369	1.546352154	0.637770898
0.496740326	369.0780619	22.23822342	11.74831763	8.615941701	0.565019011
0.539941987	424.394402	23.81170483	127.216285	121.9718354	0.436666667
0.512731214	398.9048843	16.67352185	8.871465296	5.605500889	0.553342817
0.502640191	246.796334	14.99592668	5.433808554	2.603738992	0.594430993
0.566656157	506.590604	25.3064877	421.6196868	415.1040594	0.391761613
0.487009004	443.6652031	30.1701427	52.01646542	47.69864602	0.481246698
0.466805348	515.8199095	30.90950226	65.20452489	59.16760918	0.406998158
0.479705811	122.8046875	9.21875	12.40234375	8.099960327	0.482109228
0.537104952	370.065312	18.07982583	21.64296081	18.03902503	0.52675841
0.537585961	713.9141566	39.81174699	940.6114458	932.2677003	0.346193952
0.51806135	429.4728589	21.16887817	66.32931242	62.11914307	0.487360376
0.492926294	379.5532468	22.39220779	8.77012987	5.618352167	0.563277249
0.477645898	182.460733	12.29842932	14.28272251	9.852909734	0.475124378
0.510881841	462.3480663	29.20773481	167.361326	159.5336968	0.357424961
0.497240729	1172.493639	64.99406277	28.68066158	25.03706383	0.523883581
0.523736747	865.7368421	46.01028433	111.9600726	107.6141215	0.479686593
0.476945329	810.8070588	56.27647059	18.73941176	14.95524256	0.514061083
0.53455858	661.7835218	30.41033926	22.03069467	18.32242582	0.519295302
0.507646737	642.1731225	34.30750988	36.55019763	32.79303598	0.515905383
0.517396285	109.1706161	6.374407583	3.056872038	0.85635992	0.674121406
0.471496036	72.61038961	5.857142857	5.441558442	2.413054478	0.574626866
0.525443762	2014.551382	89.39071466	343.0213876	337.7244265	0.434496827

original_glszm_LargeAreaLowGrayLevelEmphasis	original_glszm_LargeAreaHighGrayLevelEmphasis	original_glszm_HighGrayLevelZoneEmphasis	original_glszm_SmallAreaEmphasis	original_glszm_LowGrayLevelZoneEmphasis
0.010781063	16487.88807	1232.349323	0.752007521	0.004007508
0.081450878	1298.813043	346.0956522	0.790335108	0.024786019
0.191956169	27565.54881	611.2125813	0.729357218	0.019906413
0.131954911	4206.791667	360.625	0.759851738	0.038417161
0.110503483	38918.17895	394.7960526	0.757437473	0.015556817
0.103989599	7146.699301	677.1398601	0.789535153	0.038047903
1.240984702	3960457.068	1037.594245	0.749189851	0.004708598
0.3853303	1151721.827	856.9926441	0.795730737	0.004564872
0.331274673	758588.4242	721.8104575	0.771157914	0.004469289
0.071679126	8089.96882	568.0935412	0.753937351	0.011807403
0.125742942	54251.74745	408.1649635	0.743738952	0.013653145
0.211050869	561185.22	930.9786096	0.760226765	0.003396417
0.490621961	3231.0798	459.2817955	0.744848202	0.01729676
0.060662533	10108.41162	538.2106538	0.803246021	0.019942158
0.091849361	205536.7945	742.4052478	0.776858383	0.008307774
0.957303517	1772729.222	762.6400221	0.754710316	0.010177945
0.030889096	34317.77594	639.1367925	0.714659767	0.005234342
0.059590505	9325.731225	360.5296443	0.79491621	0.021413432
0.10544987	96176.21663	546.6323851	0.731748528	0.009104714
0.199437969	463486.1553	1098.865901	0.736989463	0.003518532
0.136449499	34277.20038	417.4700855	0.706412336	0.015147976
0.050711937	8067.996429	327.2535714	0.750384656	0.019557331
0.058354934	26877.21284	456.8175676	0.760656538	0.017263351
0.44322852	873694.324	787.1557632	0.717709339	0.005195732
0.230325592	491045.0677	743.6049661	0.789823536	0.014071695
0.09197676	254686.9013	917.6586716	0.734736316	0.003396323
0.022799161	35409.51685	1002.41573	0.769578126	0.004429866
0.032569608	61342.51634	843.4164332	0.73256947	0.003698999
0.031109419	1606.472527	418.1098901	0.729242979	0.018866327
0.073661962	72500.43461	529.651255	0.73927849	0.008446188
0.273278068	329117.5202	665.6861642	0.772111471	0.007089758
0.181017221	12250.90508	417.8587196	0.783062519	0.022932399
0.07150533	98562.78032	634.5835721	0.751709773	0.007838788
0.448972438	665503.9043	753.249017	0.728672583	0.014805969
0.078648252	204946.7492	930.3586112	0.757343112	0.002805232
0.110601973	70472.62069	535.6227181	0.753464907	0.012765218
0.113304509	3529.507527	577.544086	0.763888291	0.016593202
1.159820558	1914819.995	727.2566686	0.775409234	0.016051623
0.028180401	10937.50338	435.3006757	0.748228818	0.010997348
1.359962915	3690181.072	922.6571429	0.745315325	0.00337317
0.064872237	20379.90806	482.2362093	0.744870768	0.012410128
0.134415248	293780.5622	931.475931	0.713742905	0.003225204
0.073442267	17359.87796	696.6460876	0.777338732	0.009571205
0.02138617	24073.39903	732.47763	0.78946317	0.00558675
0.186309036	275073.0808	603.7955326	0.754897741	0.009058995
0.060943247	111456.3355	636.9545817	0.766968584	0.005803493
0.51380362	1259494.583	753.2735666	0.761727708	0.00465135
0.05622786	21836.26971	478.9460581	0.746036928	0.012918638
0.047292639	3304.354978	480.9177489	0.714209057	0.011534465
0.172578768	2217.835249	313.8467433	0.751900562	0.025062917
0.062057211	97382.01884	678.6304348	0.814915955	0.010014945
0.015297333	6671.30137	900.0315664	0.740961349	0.004843035
1.340584856	2759023.192	817.4609869	0.73932807	0.0033174
0.106328851	215987.1454	1438.448276	0.789689259	0.007177935
0.133507017	7704.110512	498.9811321	0.790713274	0.015013602
0.041931882	14911.71759	485.8024691	0.743288419	0.009578222
0.217947019	332234.2609	655.2849589	0.757242796	0.010190969
0.044704375	72919.98821	741.9673617	0.751891234	0.005366586
0.011159157	12039.07906	944.6068376	0.791936163	0.005861639
0.517961279	1447774.565	1016.837946	0.731557927	0.003409718
0.042526089	77149.0761	811.7701674	0.732812216	0.004620391
0.036611009	24630.9757	735.4567548	0.774254973	0.008134596
0.1042839	189394.0389	832.5682248	0.747399549	0.003856722
0.016635416	8785.735714	966.9142857	0.748527219	0.003720986
0.042799673	33141.46264	703.9655172	0.761350209	0.010297548
0.035462937	25667.94262	681.0298939	0.76570896	0.006473126
0.048289278	3489.394309	450.0569106	0.757354728	0.013169019
0.070686587	193933.5827	841.2402546	0.748777906	0.003407782
0.184593469	445508.3571	1043.449116	0.724528261	0.002503447
0.065659843	10204.6051	486.9832894	0.72061411	0.008633462
0.095772922	20670.03137	383.0117647	0.757833407	0.020447498
0.238466925	716708.9855	759.6309464	0.756613295	0.005391783
0.042220294	102589.0607	743.0924138	0.779077351	0.005187739
0.027085711	14389.68953	659.833935	0.776112794	0.009133394
0.402787554	1053075.042	875.3157552	0.749588972	0.004822652
0.028331646	42760.54507	688.1949686	0.735034215	0.004590714
0.021514131	4526.890612	792.7836735	0.705569514	0.004084867
0.101706525	1989.373786	426.0048544	0.782211319	0.038414296
0.041899437	13039.15478	501.7967699	0.732581785	0.008165208
0.085059585	201578.9059	875.9020356	0.762937141	0.003855054
0.019808643	15082.05656	1063.915167	0.743943288	0.008205623
0.024749078	3811.366599	590.5315682	0.736896704	0.006354168
0.33991015	638352.3658	611.7829978	0.780912965	0.011583741
0.057302509	59812.79363	533.1646542	0.724985479	0.008742628
0.066814054	88315.98281	612.7665158	0.709997657	0.007477948
0.34736749	1383.710938	366.7695313	0.719821357	0.017968673
0.071615008	27056.98258	609.2409289	0.760793745	0.011534552
0.733427196	1261156.675	568.7402108	0.761469189	0.009114572
0.059156231	100242.6043	783.3027744	0.747779736	0.006940186
0.080365062	7208.594805	456.6402597	0.729540221	0.012636756
0.376135912	8226.15445	416.6701571	0.718264083	0.020016462
0.140651348	216038.8221	664.9657459	0.742312247	0.005892223
0.025084199	45992.66794	842.2574215	0.73294	0.002979904
0.091328349	174445.9413	694.7640653	0.751946266	0.005203795
0.019340015	24057.27882	729.5094118	0.717994248	0.002975123
0.067340773	31841.07835	776.9232633	0.759191193	0.005293968
0.045795563	53951.38656	649.798419	0.74000942	0.007652592
0.032245164	1879.630332	523.2322275	0.745563085	0.014328323
0.146592536	1551.292208	322.0194805	0.712721171	0.018406524
0.372940344	523454.1771	814.9741784	0.752944856	0.006486287

original_glszm_ZoneEntropy	original_glszm_SmallAreaLowGrayLevelEmphasis	original_ngtdm_Coarseness	original_ngtdm_Complexity	original_ngtdm_Strength	original_ngtdm_Busyness	original_ngtdm_Contrast
6.854578434	0.00342821	0.004261289	5.309152025	5.73196488	0.109618387	0.000599395
5.835683785	0.019858811	0.012142902	9.879107424	7.139291909	0.255808488	0.003062851
6.483942451	0.011125999	0.004888584	6.45211919	5.67621915	0.197404706	0.001403359
5.878357281	0.031845793	0.012040282	13.07057553	9.372878417	0.152214964	0.003829603
6.284583636	0.011165549	0.003126562	2.399064981	2.236506725	0.51721925	0.000789911
5.992962096	0.032181543	0.024394583	31.00570984	42.7143004	0.034435695	0.004590678
6.996200968	0.00305505	0.000484344	0.709171817	1.902143376	0.551452826	2.09E-05
6.583446574	0.003472225	0.000574143	2.11200274	2.288906801	0.514171095	6.74E-05
6.422746498	0.003602059	0.001009105	0.895702198	1.57121867	0.546419245	0.000134473
6.399030027	0.006754971	0.008738678	6.427041858	7.813063535	0.108417547	0.000968552
6.258741546	0.010161876	0.003630592	1.233118745	2.69317523	0.299527197	0.000354434
6.665095089	0.00280263	0.001440188	2.357974803	3.849689237	0.217574309	7.34E-05
6.319463361	0.011217865	0.004813506	5.688040205	5.509866274	0.421294887	0.001332667
6.216680649	0.01529565	0.00750687	8.370322102	6.449225668	0.167679292	0.001783277
6.420079204	0.006833191	0.002281562	3.61226015	3.708639603	0.224980923	0.000557951
6.649258479	0.006943723	0.000574919	0.996924962	1.632075502	0.755033579	0.000128824
6.285620964	0.004529648	0.00732347	3.695782635	6.907024252	0.100929219	0.000367843
5.838496414	0.016127056	0.010488467	8.819768991	7.859955388	0.152101646	0.002533666
6.364388506	0.007746786	0.005305006	3.548904686	6.978294808	0.119497858	0.00032107
7.220306008	0.002555256	0.000731979	1.518175038	3.140439826	0.400101244	2.58E-05
6.56362909	0.00983105	0.002785864	1.210916447	1.707433776	0.663112311	0.000431717
5.990768502	0.017251383	0.009355677	5.527978375	4.900330701	0.197267143	0.001749273
6.134147012	0.01430275	0.008577005	6.362837562	7.336302528	0.121929491	0.001967664
6.542096465	0.004125149	0.001493845	0.78230182	2.527493612	0.324732113	7.45E-05
6.297531908	0.011498155	0.002699559	5.015631896	7.189440188	0.156879955	0.000756309
6.605220163	0.002718973	0.002006127	1.701872068	2.905209167	0.260246277	0.000201396
6.666060541	0.003139941	0.002682402	3.676725623	4.002617314	0.17856823	0.000296857
6.630459849	0.002928092	0.002915254	1.738676958	3.1084111	0.202357804	0.00020897
5.53923458	0.017532633	0.044217893	12.96230555	19.28923026	0.052360146	0.003952771
6.3928476	0.006645503	0.003133766	3.495457517	5.596371617	0.232688019	0.000338862
6.608880512	0.005548992	0.001146106	1.85538786	2.46131706	0.416292891	0.000118007
6.158612155	0.017873819	0.005006118	6.094322688	4.331881449	0.271984815	0.001591402
6.583646774	0.006442935	0.002534825	1.507475217	2.703951005	0.282166355	0.000319255
6.609452293	0.009713659	0.001440246	1.702556478	3.328974073	0.336741292	0.000261926
6.722393807	0.002256915	0.001003133	2.066240168	3.064501282	0.306698778	5.20E-05
6.493427264	0.00912077	0.002038332	3.078575742	2.692386537	0.448501366	0.000640701
6.635515959	0.012825692	0.003193598	7.439801305	6.458683471	0.427532025	0.000513961
6.670065069	0.011756082	0.000579985	0.941933412	1.695630771	0.703875504	0.000128417
6.066809668	0.009944353	0.012203613	4.631647366	6.91692548	0.100824052	0.000845726
6.658408129	0.002494042	0.000458303	0.802839802	2.156199134	0.588119204	1.58E-05
6.408778416	0.008195702	0.004306465	2.505789576	3.132038099	0.256920569	0.000668748
6.626693965	0.002734238	0.001090725	0.580012839	1.602120747	0.416951324	3.63E-05
6.619673043	0.007192642	0.002414853	2.483172186	2.502761942	0.329363024	0.000397848
6.412667615	0.004753731	0.00368694	4.399375418	3.96031312	0.182060515	0.000496544
6.343864032	0.007919033	0.003226718	2.638110414	5.409921578	0.153219292	0.000203472
6.454895323	0.004545471	0.002049374	1.895088018	2.178311109	0.370997117	0.000356575
6.524777386	0.003095056	0.000800417	1.165023166	2.198475079	0.491689993	6.20E-05
6.072271026	0.010596784	0.011530029	6.716987563	12.99159176	0.085066487	0.000946316
5.984763179	0.008006236	0.016200544	6.579886819	7.373672425	0.103054671	0.001627897
6.013900349	0.0170567	0.007937368	8.181399485	7.142702192	0.329906542	0.002212822
6.286708453	0.008331244	0.003235341	5.873199463	5.08891354	0.164809843	0.000668073
6.763485146	0.004035819	0.002507822	2.026431489	2.253730772	0.27584946	0.000230665
6.625534841	0.002728727	0.000626865	0.505746952	1.473103369	0.573734261	1.88E-05
6.885899211	0.004863086	0.003504874	5.448530498	11.62780182	0.081130014	0.000172765
6.170607724	0.010701751	0.00695637	8.382006004	6.33236419	0.211415221	0.001970505
6.350447094	0.007777513	0.004665384	2.621342747	2.995993871	0.300677366	0.000736668
6.533341394	0.007920778	0.001169796	0.895409868	1.488004438	0.504161994	0.00013665
6.629655431	0.004221335	0.002304315	3.041355404	2.879101884	0.316452582	0.000427087
6.336530477	0.005468102	0.007281253	8.374620285	7.953591359	0.091631551	0.000951491
6.793604066	0.002896702	0.000508997	0.381071702	1.008321081	0.746607838	2.23E-05
6.474019576	0.003860929	0.003429847	3.66311233	3.707968267	0.232185167	0.000758282
6.696597411	0.006478875	0.002066752	5.660495624	5.52406725	0.22610894	0.000237072
6.884728926	0.002769248	0.000685154	1.072978108	1.294286923	0.70964042	7.30E-05
6.684930481	0.002759318	0.003677334	3.274339279	4.266856056	0.165385366	0.00018308
6.353066841	0.00877094	0.009301533	9.004872925	14.45633888	0.064266805	0.000698583
6.688272968	0.004864383	0.001505267	3.831433212	3.367146102	0.414747529	0.00018439
6.033947813	0.010456271	0.011495482	10.9261119	8.954511107	0.148139454	0.002360722
6.670007702	0.00256783	0.000909083	1.405933654	1.441315931	0.561183208	0.000114714
6.97287096	0.002021497	0.000809585	1.228623419	2.219420812	0.374197314	2.21E-05
6.58048132	0.006062186	0.002751089	1.986636321	2.386479893	0.484201854	0.00030511
5.984567441	0.015522828	0.008108632	7.507841371	7.278192114	0.162417896	0.002399222
6.692521755	0.004094184	0.000762924	1.372752942	1.68981398	0.581387521	0.000148057
6.334950559	0.004423205	0.002949652	4.621540628	4.118556772	0.224417734	0.000688657
6.313389576	0.007986962	0.00498269	5.177943209	4.45916902	0.158371316	0.00089313
6.771119412	0.00323559	0.001372838	1.56341814	3.393336918	0.243891378	6.44E-05
6.212880099	0.003926132	0.006440082	2.71785807	4.18283207	0.177913215	0.00069891
6.842144685	0.002988859	0.00365308	4.2222028	7.919898096	0.16206934	0.000138835
5.909663639	0.032822746	0.013096464	14.8918318	11.51260293	0.169264184	0.003588841
6.429117012	0.005975198	0.004767729	2.581733386	3.144833125	0.343720887	0.000562683
6.373140008	0.003370706	0.003096221	2.155232444	4.135495846	0.15544553	0.00018311
6.868094197	0.006989499	0.004417142	5.094109578	5.70178908	0.113173965	0.000618074
6.34830952	0.005171821	0.007041914	4.693010971	5.041982538	0.224640142	0.000885189
6.414870312	0.009176122	0.001105943	3.590657247	3.404728269	0.4191823	0.000363397
6.382109874	0.007712046	0.003546184	1.260488594	2.296315703	0.256614114	0.000234542
6.684100099	0.005876182	0.002117108	1.403173843	2.532295728	0.287464934	0.000200453
6.148500031	0.012810917	0.007354111	5.980871822	8.78733268	0.416106759	0.001068369
6.493985455	0.007811335	0.004512742	3.094816261	3.940110704	0.188984048	0.00060791
6.420798582	0.00719451	0.001071513	0.829845563	1.900356696	0.509088991	0.000121777
6.709718036	0.005602413	0.004168687	4.352145924	7.223473921	0.107942123	0.00020763
6.457010706	0.008380631	0.003723669	2.961417767	2.881430075	0.405273155	0.000653587
6.411562587	0.013886276	0.006139108	5.453615154	8.442839092	0.246152841	0.000959451
6.414209423	0.004834791	0.002416384	1.121537211	2.572265466	0.245472973	0.000133293
6.709332378	0.002039658	0.001606596	1.173913438	1.861317749	0.360089941	8.47E-05
6.590573897	0.003805137	0.001569288	1.452149514	2.214509576	0.367456086	0.000140344
6.507332985	0.002363832	0.002060771	0.854859648	1.319471417	0.436941432	0.000125918
6.696167313	0.003572699	0.001842488	3.873615977	3.055517378	0.396509736	0.000397829
6.579267834	0.005937566	0.002061057	1.738028826	2.129389597	0.392828846	0.000305265
6.083685431	0.012187858	0.015525754	17.20860144	12.68897451	0.105096607	0.003310157
5.732306308	0.007576781	0.023448142	6.237881154	9.886003822	0.124732377	0.002023076
7.028816728	0.004545107	0.000601481	1.841651553	3.284746677	0.587930527	3.73E-05

wavelet.LHL_gldm_GrayLevelVariance	wavelet.LHL_gldm_HighGrayLevelEmphasis	wavelet.LHL_gldm_GrayLevelNonUniformityNormalized	wavelet.LHL_gldm_DependenceEntropy	wavelet.LHL_gldm_DependenceNonUniformity
97.28928329	1447.546399	0.033209345	6.7838493	509.3891967
78.23908512	711.3342939	0.034756538	6.411608407	111.5014409
69.06623273	898.4674888	0.036618472	6.707796065	282.9147982
64.77957062	927.3509317	0.039060993	6.351487619	98.42857143
51.67118535	620.5832743	0.039306934	6.716199101	415.8348689
95.69976436	713.0785124	0.032921249	6.185349538	91.14876033
42.01998154	1068.304583	0.073242377	7.15676018	1675.78034
60.76514988	1227.00791	0.039418995	6.9945053	1645.175676
31.48280544	1060.609808	0.074290914	6.790414688	746.4016893
52.01395377	510.3708609	0.040340336	6.443203688	244.2211921
11.93310413	292.9910026	0.097070136	6.222580311	267.8329049
51.81229515	1237.156149	0.061011235	6.911100316	693.2254183
68.20925894	1286.1418	0.042527151	6.698350341	224.2996301
46.92984453	451.1645963	0.042156938	6.508863494	192.1583851
119.1805275	1622.798095	0.033342807	7.142631149	455.0380952
41.19174785	951.8024991	0.118979258	6.766197538	697.9843804
29.20949507	486.7236693	0.057231794	6.450287666	206.5605889
58.19380563	677.8264059	0.040536582	6.338777695	129.1271394
32.04554425	640.1142596	0.058542691	6.43040451	258.8738574
91.40842123	2529.012741	0.041101461	7.238767922	2285.813895
26.32650245	352.0091434	0.057101715	6.68240443	419.4340712
40.23233855	457.4294355	0.046728668	6.42448053	142.5725806
73.91441849	613.7688172	0.035322003	6.587849919	178.702509
23.73016079	629.7631359	0.083668788	6.617945797	637.647762
43.84983926	1223.447883	0.081350996	6.79868403	216.9478827
32.52058981	613.6776213	0.056009549	6.77449689	556.4507042
71.06135708	2422.875749	0.03964775	6.845867532	574.1606786
49.55397754	1147.242344	0.049073002	6.819841158	540.2436751
57.05310565	635.0273973	0.043066241	5.861827494	50.35616438
33.11856024	1035.765217	0.060280853	6.503089159	362.5639752
31.17298724	532.5240708	0.055260237	6.799944056	843.2519332
61.08367099	843.0241117	0.036972738	6.469545402	263.2918782
49.44107956	875.8496788	0.052152837	6.943247788	498.8226981
34.42777671	532.1888199	0.079155897	6.643583806	438.2248447
38.68393748	640.0370154	0.049024224	6.799169824	1263.578999
80.77838701	1476.623517	0.036092306	6.979569315	555.915936
96.89950589	1376.191799	0.031266622	6.819091578	538.494709
49.2620366	2228.825288	0.055145046	7.050763925	1232.43721
43.78387755	454.1357143	0.044553571	6.467097911	159.55
23.78071454	1505.113899	0.089470453	6.752959756	1436.841219
38.20300958	459.5552239	0.046950323	6.642014483	336.9462687
30.87443102	971.5964386	0.060683863	6.745860905	1216.405148
33.00916137	665.7667494	0.057925772	6.68927898	526.1480562
66.32792055	1670.217606	0.036066257	6.696894437	465.7422535
33.57586356	974.6773779	0.05830321	6.685277749	338.3046272
39.54387532	630.69596	0.046846196	6.693147282	623.1399417
62.26778822	3116.343113	0.054378167	6.933564029	1201.447767
69.86481722	2144.472505	0.04953107	6.446487946	131.5784114
39.79853612	735.2199488	0.049705326	6.233881646	111.8337596
57.11024258	606.9712389	0.04229971	6.352731437	142.3495575
71.3675757	1024.291375	0.034119813	6.681089443	450.7871018
86.84112	1591.863196	0.031678168	6.966226453	918.3450628
22.52931833	787.3402708	0.112594145	6.730212013	1003.648696
21.54980966	600.5060901	0.073595671	6.432735822	313.4360536
39.6077388	524.4292379	0.049009672	6.501284591	166.5396579
66.20309825	830.5187075	0.036422902	6.821421818	339.3843537
46.54744993	1905.155915	0.047900138	6.805148472	1025.400498
56.70731417	951.7793904	0.039320171	6.812610055	594.5713595
53.39532335	703.0104849	0.039976364	6.604956165	219.9331586
25.4797589	1697.848986	0.075338085	6.688024035	1956.153034
61.30958233	618.3619263	0.037587736	6.694669663	404.9277652
70.1714344	1802.419473	0.03756691	6.823825538	776.1610548
67.38220861	1405.943162	0.03571213	7.031098775	2356.319893
35.69915957	620.0882634	0.050842481	6.663385589	507.0982824
40.58347606	476.7577548	0.051441214	6.405827953	184.5952733
70.64561859	1691.243496	0.03539182	6.763712832	1176.223034
65.53915407	608.91133	0.036606081	6.332389437	141.9359606
47.17162302	849.1695182	0.044159147	6.836381494	1363.230875
38.86074004	859.0941662	0.057130749	6.898800729	1534.173415
45.625174	1169.421375	0.043439075	6.782516627	544.4934087
42.88192792	501.7296748	0.046153083	6.385411237	134.2276423
46.53186167	865.9325437	0.047352999	6.897006382	1241.33688
49.65111251	883.9737828	0.046393132	6.798258451	320.1550562
46.5800831	616.7842105	0.045185596	6.478574842	286.9873684
49.14127005	1337.343046	0.04715051	6.789961114	1052.80999
49.35648207	560.372549	0.042459928	6.531444593	273.2418301
67.44952467	1576.854685	0.036556693	6.853409622	660.3915799
79.56704272	670.9752322	0.034074898	6.239082637	119.7244582
53.09210427	623.6661597	0.039895907	6.688695885	385.8030418
23.93123333	520.1811111	0.067995679	6.582968321	346.8022222
92.64894205	1432.958748	0.033905696	6.749513447	496.2830725
36.9336339	1095.782082	0.053092883	6.444576254	208.0774818
35.7349535	516.1936897	0.069778267	6.896122869	407.4680105
15.35812621	265.6428949	0.078311984	6.438809525	329.7860539
37.41560188	513.9090239	0.050919488	6.853935591	626.0143646
63.2920794	1030.841808	0.046371661	6.6043397	140.1412429
37.72165409	374.1131498	0.057183973	6.62943065	288.1987768
21.6307331	953.7262774	0.081445224	6.772805505	601.9327424
69.40722561	1068.762493	0.040001023	6.869882711	482.1992945
43.32387251	551.3035845	0.043880496	6.718088408	347.8310168
55.80182421	714.579602	0.042932106	6.541585188	241.0696517
15.24338748	317.6488942	0.093204143	6.563452836	367.4470774
47.90907666	945.1917352	0.042725947	6.711237067	1295.255943
20.95964948	489.4698201	0.07186055	6.572044907	652.8520023
27.40323158	407.3054128	0.057436421	6.610275842	749.0822498
95.00371165	987.0432047	0.030487084	7.025004289	761.8271812
86.38601159	1141.708401	0.032025659	6.940716145	799.6133768
97.5633925	901.3674121	0.03218365	6.299889755	124.6102236
28.53697928	382.2014925	0.060369793	6.08587174	64.52238806
39.68789129	797.3783998	0.052984284	6.867324719	1966.101088

wavelet.LHL_gldm_GrayLevelNonUniformity	wavelet.LHL_gldm_SmallDependenceEmphasis	wavelet.LHL_gldm_DependenceNonUniformityNormalized	wavelet.LHL_gldm_DependenceVariance
47.95429363	0.586349887	0.352762602	1.016800823
12.06051873	0.539275112	0.321329801	1.209593967
32.66367713	0.536304808	0.317169056	1.204267128
12.57763975	0.535425293	0.305678793	1.372516492
55.46208363	0.496825501	0.294709333	1.355868967
7.966942149	0.618841757	0.37664777	1.340823714
771.9014138	0.341693185	0.159007528	12.18691698
239.1944628	0.472600979	0.271123216	1.845146025
316.6278742	0.355381195	0.175129444	6.669521076
30.45695364	0.545787884	0.323471778	1.08265427
151.0411311	0.295251842	0.172129116	3.905292392
207.8652774	0.412772265	0.203470918	6.039302877
34.48951911	0.477044687	0.276571677	1.855780795
27.14906832	0.49093533	0.298382586	1.255294935
52.51492063	0.527438502	0.288913076	2.882887982
685.5584866	0.281260295	0.121135783	10.30861871
50.53567384	0.408969442	0.233930452	2.178743063
16.5794621	0.533819614	0.315714277	1.104417119
64.04570384	0.424980354	0.236630583	2.093309359
377.4347163	0.452315884	0.248917989	3.057828106
118.6573628	0.336480195	0.201845078	2.454415431
23.17741935	0.496215086	0.287444719	1.579994797
19.70967742	0.522663017	0.320255392	1.027620406
300.9566305	0.349816859	0.177272105	7.460707263
99.8990228	0.385107958	0.176667657	13.93458551
143.1604069	0.396486088	0.217703718	4.548268397
79.45409182	0.495459747	0.286507325	1.608411919
110.5614736	0.437591558	0.239788582	2.421479552
6.287671233	0.569849696	0.344905236	0.986676675
97.05217391	0.40054427	0.225195016	2.226858532
221.5382889	0.381834998	0.210339719	2.983385163
29.13451777	0.558730425	0.334126749	1.005424
121.7768737	0.399166008	0.213628564	3.931752633
191.1614907	0.367289417	0.181459563	4.279498819
251.6413403	0.434631791	0.246167738	2.938595791
69.98298092	0.499208336	0.286702391	1.678452204
47.27513228	0.570491071	0.356147294	0.836718947
320.9993128	0.413635368	0.211722592	4.893595011
24.95	0.493689717	0.284910714	1.445663265
845.2273738	0.313425139	0.152094974	8.138766315
62.91343284	0.427139211	0.251452439	1.803642237
344.1988717	0.396323723	0.214457889	3.870466931
140.0645161	0.384604421	0.217596384	2.628420976
51.21408451	0.539374493	0.327987502	1.039295775
90.71979434	0.417018395	0.217419426	3.999479583
112.4777176	0.440144493	0.259533503	1.559043929
290.9775743	0.419335511	0.224527708	2.958603406
24.3197556	0.47365211	0.267980471	1.48958234
19.43478261	0.499977122	0.286019846	1.879003931
19.11946903	0.499938507	0.314932649	1.216814159
43.91219891	0.570552089	0.350261928	0.97555436
88.22369838	0.549037356	0.329746881	1.182182569
898.0509027	0.286859946	0.125833588	13.05898482
120.8440926	0.338787866	0.190886756	3.088183063
31.51321928	0.444560539	0.259004134	1.923903553
42.83333333	0.498339202	0.288592138	1.457552409
192.3190535	0.454148572	0.255392403	1.936070371
81.27479439	0.487697516	0.287649424	1.409128496
30.50196592	0.490742398	0.288247914	1.367125354
820.6577619	0.343914209	0.179578907	4.408602632
49.95410083	0.512869659	0.304686054	1.281287208
92.60243408	0.543704317	0.314872639	1.748349674
294.0536799	0.482661838	0.286169528	1.521747154
106.5658397	0.420709709	0.241936203	1.942227289
34.82570162	0.446008055	0.272666578	1.755852237
121.0754165	0.563722583	0.34382433	1.068998066
14.86206897	0.569025725	0.349595962	0.910771919
224.5492625	0.469491494	0.268088668	2.090915634
428.9376665	0.372525458	0.204338494	3.003496253
92.2645951	0.426469013	0.256352829	1.577142761
22.70731707	0.44371556	0.272820411	1.186297839
235.8652881	0.446636101	0.249214391	2.335212535
61.93483146	0.448462181	0.239816521	2.525368009
42.92631579	0.528832021	0.302091967	1.630688089
192.5626836	0.450097758	0.25778893	1.898526871
38.97821351	0.503548248	0.297649052	1.322321424
80.75373472	0.489239216	0.298954993	1.132964225
11.00619195	0.589718782	0.370663957	0.682590651
52.46311787	0.507349032	0.293386344	1.488397403
122.3922222	0.357563473	0.192667901	4.721865432
47.67140825	0.587567419	0.352975158	1.025547896
43.85472155	0.449022419	0.251909784	1.772801623
159.2340053	0.368966098	0.17855741	7.743700845
148.2445853	0.292620671	0.174213446	3.32392173
138.2464088	0.400477054	0.230576193	3.248171912
24.62335217	0.454353457	0.263919478	2.127599207
74.79663609	0.403067343	0.220335456	2.701827848
312.423879	0.318779888	0.156916773	9.079085031
68.04174015	0.506901618	0.283479891	2.429386041
59.98463789	0.440351225	0.25444844	1.908639693
34.51741294	0.517002574	0.299837875	1.337417391
235.992891	0.285410293	0.145121279	7.084180624
192.3094868	0.480861493	0.287770705	1.31029146
247.6314568	0.335532226	0.189452119	3.307880915
189.9422437	0.387893334	0.226514137	2.340056815
72.68120805	0.536769956	0.319558381	1.069337389
78.5269168	0.533083945	0.326106597	1.48404738
10.07348243	0.628989173	0.39811573	0.836060386
16.17910448	0.413429749	0.240755179	2.442637558
467.5333182	0.393896703	0.222812907	2.707163723

wavelet.LHL_gldm_LargeDependenceEmphasis	wavelet.LHL_gldm_LargeDependenceLowGrayLevelEmphasis	wavelet.LHL_gldm_SmallDependenceHighGrayLevelEmphasis	wavelet.LHL_gldm_LargeDependenceHighGrayLevelEmphasis
4.308864266	0.004571358	897.7267977	5739.549169
4.971181556	0.017875189	381.6869049	3855.247839
5.02690583	0.009042864	505.8363693	4183.545964
5.422360248	0.010725001	499.0557497	5101.295031
5.618001417	0.013592606	317.7248487	3401.890149
4.58677686	0.020533101	405.6725115	3660.252066
27.20723029	0.028277546	389.5011369	27095.26767
6.746539222	0.00668587	597.6356832	8083.182268
17.53073674	0.017482195	382.8617001	18372.11427
4.745695364	0.02640076	292.8689018	2247.210596
14.15681234	0.059212024	90.34791207	3963.533419
15.00763135	0.013566567	528.2242747	17908.77928
6.689272503	0.007652401	599.5397489	8926.686806
5.456521739	0.024888589	231.2775192	2288.708075
7.874920635	0.007034	876.3003418	12380.42857
29.36619229	0.034879672	292.9480498	25681.09441
8.268403171	0.021831687	208.6223552	3813.242356
4.882640587	0.013473402	366.6630277	3125.621027
7.996343693	0.015201112	287.4130524	4949.033821
8.991506044	0.003997838	1179.493794	22035.58532
10.21270452	0.043557105	125.1779573	3342.901347
6.044354839	0.021691409	232.8188299	2636.784274
4.759856631	0.0191808	330.6008603	2842.790323
18.75979983	0.032717632	228.7154226	11275.55518
28.9218241	0.025753593	497.1017849	34279.03583
12.17762128	0.022958984	252.0526861	7197.457746
6.101796407	0.00324715	1221.104019	14384.76697
8.348424323	0.008719563	515.4207748	9231.502441
4.356164384	0.01690748	368.9995852	2531.452055
8.617391304	0.009948519	436.6903917	8443.513043
10.28510851	0.023656812	215.0745409	5283.439012
4.494923858	0.009077048	471.2040572	3666.483503
11.43254818	0.015796045	363.3926978	9523.606424
13.55486542	0.032739686	213.6010498	6732.828986
8.917397234	0.01673195	287.5568237	5492.480031
6.177926766	0.005705153	752.195025	8844.417225
4.03968254	0.00480181	786.9362888	5535.427249
12.85741282	0.006284892	944.3211898	27766.2144
5.885714286	0.03350213	233.1364318	2571.807143
21.72954377	0.015024997	486.6595966	32064.04499
7.259701493	0.029040416	211.7875816	3128.698507
11.32475317	0.012975662	402.4075628	10512.38769
9.52936311	0.020249585	268.302014	6031.211745
4.649295775	0.00395245	916.2105773	7604.366901
11.32005141	0.01346812	422.8939701	10491.3072
6.671803415	0.017045109	281.1364916	4158.729279
9.602877967	0.003454324	1348.534909	28988.7393
6.283095723	0.005322329	1055.404151	12873.88595
6.483375959	0.011949068	360.8528076	5143.951407
5.216814159	0.014605128	309.8532236	3052.705752
4.28982129	0.00626641	589.2815731	4250.142191
4.829802513	0.004156405	895.477425	7323.81939
32.8891675	0.044813097	239.4133862	24778.97969
11.52009744	0.020873403	202.2406825	6982.229598
7.214618974	0.019625846	239.3686526	3851.539658
5.833333333	0.010987154	439.5452929	4439.437075
7.238107098	0.006197911	884.4802892	13525.87522
5.815674891	0.011811478	472.4901003	5431.492017
5.726081258	0.012714287	347.317881	4109.770642
13.89984394	0.008431171	593.1342595	23615.30644
5.326561324	0.014483746	342.5231486	2957.693755
5.84137931	0.003974837	983.1733041	10379.75254
6.02331795	0.005248706	688.4744147	8290.537527
7.678435115	0.015448356	266.6443433	4697.877385
6.76366322	0.021114397	228.4764992	3015.189069
4.502484654	0.003393655	971.5271559	7464.195265
4.197044335	0.017563751	358.1061008	2399.591133
7.158505408	0.010277889	403.8950533	5912.082793
10.56526372	0.01422346	328.919907	8833.092834
6.834274953	0.007043747	503.8998325	8101.356403
5.898373984	0.019253343	229.3505036	2949.28252
7.969283276	0.011163407	398.2228404	6669.620157
8.492134831	0.012381699	406.5462373	7016.208989
5.852631579	0.013832807	343.114814	3392.034737
7.14446621	0.006287583	620.7489254	9172.137855
5.516339869	0.015744282	296.5058249	2879.392157
5.262562245	0.004450606	776.9425732	8230.464464
3.656346749	0.013571433	388.6018877	2477.303406
5.801520913	0.014844392	323.0176728	3453.363498
13.60888889	0.029890378	197.6389659	6789.566667
4.320056899	0.004591107	891.0572023	5684.870555
7.075060533	0.008220943	497.8051137	7682.244552
18.94653812	0.044677592	202.8886699	8949.437336
13.25990491	0.068506219	80.62313933	3370.833597
9.996685083	0.026016579	218.2984155	4710.761326
7.380414313	0.023315394	463.792933	7875.99435
9.418960245	0.037980118	166.8106061	3070.66208
22.57455683	0.025245701	317.7072472	20781.47602
7.323339212	0.00887986	546.5177046	7782.519106
7.269202634	0.024136869	244.3055696	4189.893197
5.457711443	0.011213719	366.0747129	4017.206468
20.93127962	0.074851839	99.89519986	6258.371643
5.693179293	0.007243495	462.5072368	5349.719618
11.94254208	0.027793601	171.088474	5609.022925
8.870577563	0.028645743	165.8854643	3439.220139
4.782718121	0.008883187	545.4150875	4548.157299
5.303425775	0.006973071	615.095862	5806.781811
3.670926518	0.015968428	564.3040584	3358.670927
8.507462687	0.034437112	172.577473	3035.276119
9.423390752	0.013944348	323.0148041	7283.608908

wavelet.LHL_gldm_SmallDependenceLowGrayLevelEmphasis	wavelet.LHL_gldm_LowGrayLevelEmphasis	wavelet.LHL_gldzm_DistanceZoneVariabilityNormalized	wavelet.LHL_gldzm_LowIntensityEmphasis
0.001299808	0.001673958	0.87326246	0.00207376
0.005725932	0.007203658	0.95518054	0.009679457
0.002150106	0.002884391	0.879292404	0.003665771
0.004061557	0.004798675	0.859310985	0.006880888
0.00240546	0.003596973	0.891188598	0.004539695
0.009052616	0.010569653	0.899163195	0.013573251
0.00052478	0.001245484	0.91715258	0.001500707
0.000768813	0.001292274	0.885510556	0.001549858
0.000680928	0.001344858	0.948548536	0.001835284
0.00387259	0.007306143	0.900672002	0.007662565
0.00172968	0.005452025	0.948280518	0.00662825
0.000826404	0.001380064	0.89956382	0.001900775
0.002534583	0.003004264	0.846610646	0.004676262
0.004881112	0.007373687	0.805309206	0.009563549
0.001293937	0.001750993	0.874679697	0.002330882
0.000807156	0.001694919	0.958306521	0.002764408
0.002531988	0.004038841	0.953406508	0.00571484
0.005060916	0.005973531	0.872776246	0.008566673
0.001969434	0.003021002	0.913066334	0.004326848
0.000334591	0.000580026	0.943983255	0.000704363
0.00195442	0.00543001	0.958955241	0.006371904
0.005128765	0.006776616	0.944871988	0.009509313
0.004478579	0.006167342	0.954570038	0.007826877
0.00112367	0.002291612	0.937647773	0.003043558
0.001342782	0.001900262	0.942599622	0.003173068
0.001372014	0.002565349	0.952134528	0.003280476
0.000758291	0.000983937	0.855151504	0.001386563
0.00096936	0.001549588	0.915916919	0.002076177
0.008406234	0.009303858	0.88166205	0.013189598
0.001133202	0.001780314	0.983762928	0.002619586
0.001501741	0.003015443	0.941165311	0.003820392
0.00254952	0.003238552	0.8698	0.004207872
0.001341018	0.002200818	0.84955387	0.003148314
0.002955663	0.00465363	0.964438543	0.007680321
0.001372456	0.002461881	0.884799102	0.003009334
0.001152281	0.001567323	0.824056094	0.002129106
0.001468707	0.001903285	0.667042	0.002372187
0.000386171	0.000668783	0.932298657	0.000877981
0.003214661	0.007644975	0.864630889	0.007806121
0.000338981	0.000819937	0.910356119	0.001043377
0.003023089	0.005976387	0.877621882	0.007296222
0.000688041	0.001382982	0.851064443	0.001667721
0.001261101	0.003002628	0.949309711	0.003583184
0.001096678	0.001425439	0.893615534	0.001855598
0.001186543	0.001857614	0.937074493	0.002646734
0.001940662	0.003677762	0.935757272	0.004475829
0.000343676	0.000541989	0.933513094	0.000762256
0.002386806	0.002651384	1	0.004469405
0.003617341	0.00438421	0.892466913	0.006428315
0.00463243	0.005782289	0.927112029	0.008309457
0.001677091	0.002213329	0.958110002	0.002731977
0.000841305	0.001195156	0.863510808	0.001440087
0.000719914	0.001760829	0.905628638	0.002458722
0.001452795	0.002663475	0.923434801	0.004048186
0.003545862	0.005094351	0.931709609	0.007268803
0.001851733	0.002722053	0.928872055	0.003466535
0.000490715	0.001178694	0.929323181	0.001256541
0.001079299	0.002550204	0.929659778	0.002563478
0.00314117	0.004197085	0.867346939	0.005861701
0.000319427	0.00072279	0.919830267	0.000899908
0.002665708	0.003896493	0.938936056	0.004843978
0.00080157	0.001081766	0.883242667	0.001371723
0.000609222	0.001056934	0.807485016	0.001213565
0.001854479	0.002996251	0.815038835	0.004097639
0.003416908	0.005149753	0.94509879	0.007082166
0.000725383	0.001030639	0.82920396	0.001216075
0.008281335	0.009496501	0.875126104	0.012939164
0.001147407	0.001908487	0.831644461	0.002320215
0.000993214	0.001851493	0.933352164	0.002534849
0.00095204	0.001524134	0.933532071	0.002049032
0.00426605	0.00588323	0.962976148	0.008489083
0.001117149	0.00189513	0.924630333	0.002368324
0.001605409	0.002345627	0.943068945	0.003309196
0.002465823	0.003524467	0.969132805	0.004377507
0.000712587	0.00117697	0.803897726	0.001485011
0.003500881	0.004776458	0.949583335	0.006337563
0.000876963	0.001265602	0.897242234	0.001643449
0.006613256	0.007542587	0.80715851	0.009957906
0.003267776	0.00434513	0.856299545	0.005868773
0.00150555	0.003005242	0.924790696	0.004034441
0.001310429	0.001681514	0.896442041	0.002086572
0.001741377	0.002295048	0.967676167	0.003517689
0.002050076	0.003705676	0.961397084	0.005281729
0.002276722	0.006822004	0.944232046	0.008161926
0.002270472	0.003871924	0.917192416	0.005248303
0.001907515	0.005375476	0.864800347	0.00546892
0.004835912	0.007153497	0.890821674	0.01102204
0.000655088	0.001422559	0.958737197	0.001957426
0.001639033	0.002215518	0.932953619	0.003006995
0.002868685	0.005469952	0.907198096	0.006814615
0.002917086	0.003804303	0.750868056	0.005163152
0.001952455	0.004632711	0.936807151	0.006630027
0.000956006	0.001614184	0.875235652	0.001899417
0.001239879	0.002810567	0.944931854	0.003548536
0.002376864	0.004438324	0.82672123	0.005847276
0.002400904	0.003135996	0.803957488	0.004089533
0.001429157	0.002027673	0.855766367	0.002503083
0.011103494	0.011789221	0.836714995	0.015832654
0.00624875	0.008639958	0.923784329	0.013963493
0.000906591	0.001852494	0.890505784	0.002233554

wavelet.LHL_gldzm_LargeDistanceEmphasis	wavelet.LHL_gldzm_HighIntensitySmallDistanceEmphasis	wavelet.LHL_gldzm_LowIntensityLargeDistanceEmphasis	wavelet.LHL_gldzm_HighIntensityEmphasis
1.203974895	1422.631276	0.002266614	1509.770921
1.068807339	679.6490826	0.009799688	699.8715596
1.193548387	877.6590502	0.004307609	937.9265233
1.228426396	882.8032995	0.007530502	931.4010152
1.173218673	605.7632064	0.00517874	634.6953317
1.159763314	640.9201183	0.014446838	665.6035503
1.129895403	1096.988142	0.001678426	1137.830212
1.182882883	1194.775	0.001820094	1256.612613
1.079271873	1054.319289	0.001920581	1078.544334
1.157232704	501.5078616	0.016492197	529.2578616
1.079696395	301.5683112	0.007097923	306.740038
1.159090909	1225.375473	0.002566388	1277.863636
1.251101322	1185.104075	0.006637769	1260.482379
1.327868852	437.3709016	0.017981736	468.6311475
1.201516793	1557.01273	0.002510739	1656.366197
1.063901345	1025.597814	0.002821893	1052.329596
1.071599045	497.4146778	0.005843737	511.9761337
1.204819277	647.8524096	0.021269252	688.8433735
1.136622391	646.5981973	0.004724026	671.345351
1.086520376	2552.430512	0.000753536	2600.182863
1.062885327	366.25709	0.006945604	371.8446363
1.085106383	464.6374113	0.01015887	470.1879433
1.069767442	617.8168605	0.008059854	624.6540698
1.096641535	633.5221042	0.003285543	654.5051405
1.088724584	1257.735675	0.003298501	1279.589649
1.073604061	618.6146362	0.003423935	635.2351946
1.23580786	2327.044541	0.001502833	2466.671616
1.131926121	1129.644239	0.002200734	1175.92788
1.189473684	613.3763158	0.013875678	639.4526316
1.024556617	1083.535812	0.002647558	1091.204638
1.091013165	545.2939325	0.004056379	559.8723526
1.21	796.49	0.004615885	845.012
1.245810056	856.066108	0.003766355	910.7029795
1.054325956	561.6576962	0.007741942	576.1257545
1.184098804	631.8502509	0.003493631	660.7896565
1.292418773	1387.269179	0.002807442	1504.391697
1.633	1175.776	0.003558732	1375.69
1.105244123	2209.17387	0.000927619	2277.155877
1.219047619	434.0238095	0.008405708	468.0380952
1.141102457	1489.519398	0.00113397	1551.81456
1.196428571	468.59375	0.008236324	492.4017857
1.244937695	950.2677938	0.001976035	1013.58528
1.078066914	674.9372677	0.003945395	694.9098513
1.169109357	1615.438839	0.001964843	1694.6708
1.097560976	980.5067751	0.00273403	1013.588076
1.099675851	623.2795786	0.005545988	640.4529984
1.103286385	3121.152973	0.000796391	3205.789124
1	2214.992481	0.004469405	2214.992481
1.171052632	697.4638158	0.006723677	727.3684211
1.113636364	586.467803	0.008470075	617.9393939
1.064209275	1014.376635	0.002808053	1033.567182
1.221016562	1531.5998	0.003355521	1627.084523
1.148952676	800.5338441	0.002729624	835.3611327
1.119617225	581.9110845	0.004382323	599.1323764
1.10619469	515.0442478	0.007495655	534.4955752
1.110782866	843.062777	0.003679958	871.2171344
1.110052406	1884.528585	0.00138417	1937.757504
1.10950764	937.1256367	0.002756132	963.3820034
1.214285714	665.3870968	0.006313655	708.9700461
1.125505111	1666.273057	0.000995828	1717.78203
1.094577554	649.1778058	0.005162915	660.3114754
1.186762779	1728.660059	0.001554096	1811.557667
1.325671321	1300.320193	0.002215182	1421.425994
1.309338521	582.422179	0.005964613	629.8881323
1.084745763	492.9152542	0.016697621	503.5
1.282864914	1589.468948	0.001449256	1716.947869
1.200743494	585.5910781	0.024515634	624.267658
1.278361725	797.7662196	0.0030231	862.793041
1.103545654	859.7902416	0.002744411	884.2764355
1.103255814	1147.10814	0.002193972	1174.92093
1.056603774	508.4726415	0.00865914	514.0226415
1.117669903	864.5317476	0.003846085	891.0042718
1.08797654	894.2851906	0.00347	912.4809384
1.047038328	635.5030488	0.004468641	644.6271777
1.343500704	1259.313742	0.001799142	1375.699672
1.077634011	568.4269871	0.006516437	584.3807763
1.162992126	1524.727559	0.001808026	1584.514961
1.324324324	612.4087838	0.01205392	660.2972973
1.233766234	606.8714286	0.011600619	639.4545455
1.117408907	529.6815115	0.004339339	548.1551957
1.16433942	1429.144737	0.002249681	1495.559613
1.049295775	1091.127347	0.003582425	1102.523474
1.059067358	541.5901554	0.005810797	550.6601036
1.086124402	274.9011164	0.00890896	279.2838915
1.129829985	518.7386012	0.006397078	541.6669243
1.21875	954.4722222	0.006250679	1019.190972
1.17384106	389.4850993	0.012004065	413.5099338
1.063226744	975.5703125	0.002021892	995.75
1.104187947	1056.265832	0.004400217	1076.972421
1.146341463	539.0900287	0.008011378	553.8350072
1.4375	630.9744792	0.014202825	710.2979167
1.09798995	341.8702889	0.006979319	351.4497487
1.200553797	910.1514043	0.002281352	956.4363133
1.085011186	497.0415735	0.003794836	510.2602535
1.306245803	399.1723379	0.007893702	427.9267965
1.333558406	930.4513842	0.00492271	1014.469953
1.234714004	1093.527449	0.003338741	1156.995398
1.269058296	840.2242152	0.017311595	897.8834081
1.119047619	405.0535714	0.020567774	413.047619
1.176762661	784.6323582	0.002644205	820.3624628

wavelet.LHL_gldzm_DistanceZoneVariability	wavelet.LHL_gldzm_ZonePercentage	wavelet.LHL_gldzm_IntensityVariabilityNormalized	wavelet.LHL_gldzm_LowIntensitySmallDistanceEmphasis	wavelet.LHL_gldzm_IntensityVariability
834.8389121	0.662049861	0.027732795	0.002025546	26.5125523
208.2293578	0.628242075	0.03219426	0.009649399	7.018348624
490.6451613	0.625560538	0.032200254	0.003505312	17.96774194
169.284264	0.611801242	0.034656909	0.006718485	6.827411168
725.4275184	0.576895819	0.035747273	0.004379934	29.0982801
151.9585799	0.698347107	0.029515773	0.013354855	4.98816568
3770.414254	0.39007496	0.034953241	0.001456278	143.6927755
2948.75015	0.548780488	0.032406821	0.0014823	107.9147147
1615.378156	0.399577663	0.038150699	0.00181396	64.97064005
429.6205451	0.631788079	0.035999807	0.005455157	17.17190776
499.743833	0.338688946	0.061905671	0.006510832	32.62428843
1424.909091	0.464925154	0.032659295	0.001734371	51.73232323
384.3612335	0.559802713	0.031972287	0.004185885	14.5154185
294.7431694	0.568322981	0.03681806	0.007459003	13.47540984
807.3293608	0.586031746	0.02346557	0.002285918	21.65872156
1709.618834	0.309614717	0.03367062	0.002750037	60.06838565
399.477327	0.474518686	0.043956232	0.005682615	18.4176611
217.3212851	0.608801956	0.035531685	0.005391028	8.847389558
481.1859583	0.481718464	0.041713325	0.004227554	21.9829222
4516.959875	0.521071545	0.028701107	0.00069207	137.3347962
777.7127004	0.390279115	0.045689586	0.006228479	37.05425401
266.4539007	0.568548387	0.040918465	0.009346924	11.53900709
328.372093	0.616487455	0.03174013	0.007768633	10.91860465
1368.028101	0.405615791	0.046257283	0.002983062	67.48937629
509.9463956	0.440553746	0.035051814	0.003141709	18.96303142
1125.423012	0.462441315	0.041344934	0.003244611	48.86971235
979.1484716	0.571357285	0.03069583	0.001357496	35.14672489
1041.397537	0.504660453	0.034775741	0.002045038	39.54001759
83.75789474	0.650684932	0.038448753	0.013018078	3.652631579
721.0982265	0.455279503	0.039652775	0.002612593	29.06548431
1644.215799	0.435769519	0.041289852	0.003761395	72.13337149
434.9	0.634517766	0.034936	0.004105869	17.468
912.4208566	0.459957173	0.033982502	0.002993804	36.4972067
958.6519115	0.411594203	0.039035015	0.007664916	38.80080483
2292.514473	0.504773037	0.038086146	0.002888259	98.68120417
913.0541516	0.571428571	0.028442636	0.001959522	31.51444043
667.042	0.661375661	0.027826	0.002075551	27.826
2577.805787	0.475004295	0.035752905	0.000865572	98.85678119
272.3587302	0.5625	0.039778282	0.007656224	12.53015873
3038.768724	0.353339685	0.044062399	0.001020729	147.0802876
589.7619048	0.501492537	0.039461097	0.007061196	26.51785714
2185.533489	0.452750353	0.041789797	0.001591016	107.3161994
1021.457249	0.444995864	0.041465707	0.003492631	44.61710037
792.6369786	0.624647887	0.033513013	0.001828286	29.72604284
691.5609756	0.474293059	0.041807125	0.00262491	30.85365854
1154.724473	0.51395252	0.04042276	0.004208289	49.88168558
2386.059468	0.477667726	0.035072284	0.000753722	89.64475743
266	0.541751527	0.03513483	0.004469405	9.345864662
203.4824561	0.583120205	0.042282241	0.006354475	9.640350877
244.7575758	0.584070796	0.035009183	0.008269303	9.242424242
805.7705113	0.653457653	0.031109276	0.002712959	26.16290131
1512.007424	0.628725314	0.027929867	0.000961228	48.90519703
2334.710628	0.323219659	0.04389773	0.002390997	113.1683476
578.9936204	0.381851401	0.047752773	0.003964652	29.94098884
315.8495575	0.527216174	0.040801942	0.00721209	13.83185841
628.8463811	0.575680272	0.03246358	0.00341318	21.97784343
1950.649357	0.522789539	0.038472232	0.001224634	80.75321582
1095.139219	0.569908079	0.035454758	0.002515314	41.76570458
376.4285714	0.568807339	0.035496613	0.005748713	15.40552995
3869.725933	0.386211328	0.044638326	0.000875928	187.7934395
744.5762926	0.59668924	0.032355939	0.004764244	25.65825977
1347.828309	0.619066937	0.030103801	0.001326129	45.93840105
3758.84275	0.565338839	0.030969875	0.000963388	144.1647691
837.8599222	0.490458015	0.040428318	0.003630896	41.56031128
334.5649718	0.522895126	0.043043187	0.004678302	15.23728814
1829.223935	0.64484069	0.031094624	0.00115778	68.59474161
235.4089219	0.662561576	0.0330703	0.010045047	8.895910781
2294.507068	0.542576205	0.036003689	0.002144494	99.33417905
2974.593348	0.424480554	0.03733348	0.002482458	118.9818011
1003.546977	0.506120527	0.038068578	0.002012797	40.92372093
255.1886792	0.538617886	0.041082236	0.008446568	10.88679245
2380.923107	0.516964465	0.036900443	0.001998883	95.01864078
643.1730205	0.510861423	0.034829422	0.003268995	23.75366569
556.28223	0.604210526	0.03717418	0.004354724	21.33797909
1713.106053	0.52179236	0.035235263	0.001408978	75.08634444
513.7245841	0.589324619	0.036773825	0.006292844	19.89463956
1139.497638	0.574920779	0.033245706	0.001602305	42.22204724
179.1891892	0.687306502	0.031734437	0.009433902	7.045045045
659.3506494	0.585551331	0.034734357	0.004435811	26.74545455
685.2699055	0.411666667	0.045390389	0.003958217	33.634278
834.5875403	0.662162162	0.02819347	0.002045794	26.2481203
412.2300469	0.515738499	0.040611431	0.003501504	17.30046948
927.7481865	0.422874671	0.038649091	0.005149462	37.29637306
592.0334928	0.331220285	0.054040786	0.007975168	33.88357257
1186.846986	0.476611418	0.039630061	0.004961109	51.2812983
249.0625	0.542372881	0.034288194	0.00527348	9.875
538.0562914	0.4617737	0.038430332	0.010776534	23.21192053
1319.222384	0.358706986	0.045420455	0.001941309	62.49854651
913.3615935	0.575543798	0.030857403	0.00265869	30.20939734
632.3170732	0.50987564	0.037971713	0.006515424	26.46628407
360.4166667	0.597014925	0.035842014	0.002903234	17.20416667
745.6984925	0.314375987	0.052397667	0.006542704	41.70854271
2212.595728	0.561652966	0.036191178	0.001803934	91.49129747
1267.153617	0.389146837	0.047996392	0.003486961	64.36316182
1230.987911	0.450257031	0.044448183	0.005376847	66.18334453
1190.66104	0.621224832	0.027361205	0.003882541	40.52194463
1301.620644	0.620309951	0.028371158	0.002294168	43.15253123
186.5874439	0.712460064	0.027971606	0.015462919	6.237668161
116.3968254	0.470149254	0.04925674	0.012312422	6.206349206
3586.957299	0.456482321	0.037568821	0.002131321	151.3272095

wavelet.LHL_gldzm_HighIntensityLargeDistanceEmphasis	wavelet.LHL_gldzm_SmallDistanceEmphasis	wavelet.LHL_glcm_SumVariance	wavelet.LHL_glcm_Homogeneity1	wavelet.LHL_glcm_Homogeneity2
1858.329498	0.949006276	4786.288163	0.194991044	0.110210525
780.7614679	0.982798165	2195.49094	0.218668426	0.131186471
1178.996416	0.951612903	2844.291333	0.212580954	0.127795623
1125.791878	0.942893401	2870.75403	0.219260152	0.134069327
750.4238329	0.956695332	1889.300115	0.232502128	0.143439487
764.3372781	0.960059172	2194.217504	0.180947246	0.100393435
1301.198492	0.967526149	3583.654542	0.34237272	0.269090837
1503.963063	0.954279279	4073.409075	0.237046516	0.150188478
1175.44451	0.980182032	3571.179146	0.325992686	0.247674799
640.2578616	0.960691824	1502.172309	0.208895604	0.12339526
327.426945	0.980075901	897.8433684	0.368596639	0.284659593
1487.816288	0.960227273	4162.282161	0.2915468	0.214071209
1561.995595	0.93722467	4259.224792	0.234945018	0.150436851
593.6721311	0.918032787	1259.368565	0.233929122	0.14422087
2053.780065	0.949620802	5409.791566	0.215451444	0.137253088
1159.256726	0.984024664	3290.436878	0.403626543	0.342009576
570.221957	0.982100239	1475.787116	0.275549926	0.185238563
852.8072289	0.948795181	2069.833578	0.215668379	0.129543507
770.3339658	0.965844402	2038.362555	0.272077898	0.181871692
2791.192268	0.978369906	8935.413197	0.250723131	0.166295996
394.1948212	0.984278668	1011.672303	0.308976553	0.220971978
492.3900709	0.978723404	1298.943296	0.243058595	0.152637355
652.002907	0.98255814	1831.641782	0.215019293	0.128743528
738.4372858	0.975839616	2057.103871	0.339319697	0.261705381
1367.005545	0.977818854	4121.607354	0.336157342	0.265537309
701.7174281	0.981598985	1943.654757	0.283927682	0.197151438
3025.179913	0.941048035	8436.875863	0.226076145	0.138527104
1361.062445	0.96701847	3833.638451	0.256424345	0.171216412
743.7578947	0.952631579	1962.061356	0.217315197	0.130236777
1121.879945	0.993860846	3442.308791	0.288438346	0.200921984
618.1860332	0.977246709	1639.083075	0.291340942	0.204206319
1039.1	0.9475	2669.234272	0.210815672	0.122890397
1129.250466	0.938547486	2833.400347	0.282896114	0.200005472
633.9979879	0.986418511	1706.131699	0.334575377	0.258301658
776.547279	0.953975299	2026.511014	0.262062947	0.174179675
1972.881769	0.926895307	4947.950902	0.217518849	0.1342687
2175.346	0.84175	4439.872699	0.183649046	0.10241337
2549.083906	0.973688969	7864.864603	0.292185882	0.211118986
604.0952381	0.945238095	1319.171685	0.247556723	0.157140658
1800.995207	0.964724386	5271.1246	0.356963217	0.280232155
587.6339286	0.950892857	1336.54146	0.260550783	0.17049648
1268.855919	0.939198252	3219.855241	0.291609834	0.206250923
774.8001859	0.980483271	2116.314144	0.292975787	0.205523518
2011.598647	0.957722661	5689.324282	0.205108825	0.117544342
1145.913279	0.975609756	3273.974527	0.285454564	0.20100444
709.1466775	0.975081037	1970.707532	0.260788081	0.169839908
3544.333725	0.974178404	11239.09578	0.275091359	0.188987038
2214.992481	1	7569.59299	0.246701734	0.159619044
846.9868421	0.957236842	2398.67492	0.245506327	0.155736539
743.8257576	0.971590909	1876.682629	0.221263329	0.1345316
1110.32937	0.983947681	3344.62427	0.207990504	0.121155931
2009.023415	0.94474586	5370.444609	0.202109236	0.116839884
974.670287	0.962761831	2639.483788	0.391820063	0.323157628
668.0175439	0.970095694	1921.925063	0.325479522	0.23907257
612.300885	0.973451327	1593.850747	0.25387152	0.165066764
983.8345643	0.972304284	2595.977264	0.231795234	0.143300202
2150.673178	0.972486899	6603.61207	0.258369255	0.169181731
1068.40747	0.97262309	3089.6418	0.230043604	0.141724775
883.3018433	0.946428571	2170.6491	0.233214342	0.145369131
1923.817923	0.968623722	5915.142154	0.322272318	0.237753794
704.8461538	0.976355612	1851.49401	0.214925797	0.129888681
2143.1481	0.953309305	6170.717307	0.208484536	0.12493718
1910.016756	0.919059554	4712.442076	0.222976877	0.136655143
819.7519455	0.92266537	1940.461369	0.260641856	0.170387191
545.8389831	0.978813559	1414.980069	0.259319782	0.169875037
2226.863554	0.929283772	5778.758122	0.19440272	0.110015364
778.9739777	0.949814126	1830.747936	0.190218764	0.104986407
1122.900326	0.930409569	2738.671456	0.240047988	0.153055006
982.2212112	0.974113586	2805.325189	0.292344022	0.205105546
1286.172093	0.974186047	3876.182378	0.251846896	0.161238519
536.2226415	0.985849057	1488.788643	0.252069178	0.15996874
996.8943689	0.970582524	2795.810719	0.255348993	0.169893502
985.2639296	0.978005865	2824.5411	0.264706941	0.177615431
681.1236934	0.988240418	1896.463879	0.245136958	0.157780754
1858.049273	0.917253246	4549.530542	0.24761113	0.160081875
648.1959335	0.980591497	1709.328249	0.232697841	0.144629496
1823.664567	0.959251969	5300.713831	0.223911973	0.134325932
851.8513514	0.918918919	1961.085399	0.188234448	0.10424391
769.787013	0.941558442	1902.635074	0.226700411	0.139765386
622.0499325	0.970647773	1620.768542	0.313749333	0.230163244
1761.219119	0.958915145	4768.128931	0.19663412	0.111516516
1148.107981	0.987676056	3660.743039	0.257945171	0.169406056
586.9398964	0.985233161	1606.66695	0.338945738	0.263253035
296.814992	0.9784689	776.2021921	0.348287412	0.260735014
633.3802164	0.967542504	1577.85012	0.27531799	0.188855036
1278.065972	0.9453125	3307.287113	0.233713941	0.151057824
509.6092715	0.956539735	1071.368805	0.283914088	0.198107318
1076.46875	0.984193314	3240.676046	0.357650705	0.280355905
1159.798774	0.973953013	3481.067451	0.231367156	0.147143119
612.8149211	0.963414634	1647.980522	0.25234874	0.163042924
1027.591667	0.890625	2239.221938	0.21777205	0.132210492
389.7675879	0.975502513	956.0523236	0.37284704	0.292788481
1141.575949	0.949861551	3059.6783	0.233188062	0.144156086
563.1349739	0.978747204	1539.743788	0.325900159	0.239950899
546.2518469	0.927915827	1203.730096	0.286336794	0.19603323
1352.272789	0.917360642	3094.182744	0.196875387	0.11316705
1410.867193	0.941321499	3638.136518	0.198591262	0.116225205
1128.520179	0.932735426	2748.046766	0.169556808	0.093230191
445.0238095	0.970238095	1101.75488	0.278449261	0.187600657
966.1516882	0.956361028	2590.618454	0.27321583	0.186887379

wavelet.LHL_glcm_ClusterShade	wavelet.LHL_glcm_MaximumProbability	wavelet.LHL_glcm_Idmn	wavelet.LHL_glcm_SumVariance2	wavelet.LHL_glcm_Contrast	wavelet.LHL_glcm_DifferenceEntropy
2447.908801	0.007172327	0.981759773	239.0476281	150.6294949	4.589849868
-941.6641859	0.012262328	0.96046886	238.8172189	100.8252061	4.250225074
445.0434599	0.007204993	0.966533091	182.3480413	129.0893855	4.465100256
-595.0676105	0.013541127	0.953722512	177.1757427	110.7215932	4.288434923
31.62522801	0.006146029	0.963495693	142.255751	80.56978294	4.174843242
-679.0132917	0.015036175	0.935594979	213.8212295	174.3422931	4.554265626
816.7762721	0.050155491	0.98844311	122.0275313	62.27580147	3.935273915
-48.99343272	0.006510619	0.980531225	166.6378037	95.5530227	4.30738999
-32.1618713	0.037563218	0.982772789	79.61560462	59.64386868	3.888303532
-69.05683357	0.008390846	0.945947381	118.0068007	114.4406279	4.372870553
16.1426599	0.035416003	0.978650213	32.59585144	20.93366353	3.268649471
307.393321	0.032691172	0.981398092	134.7830512	99.95634653	4.21595364
-1495.476182	0.011609748	0.969339147	174.6733683	111.2174087	4.317838044
140.727617	0.009130426	0.959553932	118.238699	78.45797759	4.11997541
953.9660895	0.0133909	0.975011108	285.2804643	196.9314249	4.698654636
1038.535056	0.077666215	0.980153367	123.3400059	80.86485364	3.721474409
118.96719	0.012887106	0.969575969	67.31166883	55.04817374	3.910391212
-208.9991493	0.011478968	0.953458622	140.3380956	112.1309196	4.305586149
-16.01252172	0.015018233	0.97710954	69.43160698	61.46599264	3.946125952
4950.095893	0.011923614	0.990259478	287.6183746	112.6337806	4.345319563
101.2754402	0.012440463	0.970696087	67.7649698	43.77163896	3.702732628
166.7014341	0.013162295	0.959242207	97.4673052	67.80792302	4.013955273
2.750921939	0.010055739	0.961184911	196.4595537	107.576852	4.31921775
86.21271797	0.045376435	0.979485363	62.04051804	48.14452455	3.793030445
137.7616968	0.060073722	0.984471187	100.889848	79.60226632	4.022949566
115.3729114	0.019417587	0.98215517	76.12768091	58.77682872	3.971621294
309.4500785	0.008087912	0.984842871	155.7756076	116.066224	4.373124817
390.2631342	0.011883753	0.98231977	120.7440345	95.99936846	4.22434979
313.0042714	0.021638684	0.954027071	135.2395368	94.82848622	4.059289597
112.5756833	0.016742793	0.981625653	83.87160428	57.18509902	3.89029276
79.54067921	0.013491908	0.975331943	82.60723845	50.48811221	3.880264689
-166.557364	0.007661604	0.965672505	171.7137195	95.90828084	4.299288831
484.5980991	0.019688951	0.979080766	128.5366862	82.12400409	4.112860976
303.5641741	0.036838143	0.976447049	97.93456906	65.90319157	3.891833635
202.9807309	0.011446598	0.976769576	92.34019987	66.29383169	4.077011111
-861.179643	0.009002448	0.973376864	214.107904	144.1698475	4.541457409
-1348.351991	0.006347409	0.965007608	257.9530545	179.4513454	4.671214224
500.6718467	0.01820336	0.989080597	130.9672315	86.84628153	4.142678288
29.07203795	0.01079675	0.962402594	118.9123723	61.52382938	3.972613781
152.452323	0.048497809	0.992321992	62.05981815	42.90617829	3.688970626
287.2919565	0.009209726	0.965228355	97.58944295	64.17657749	3.997949449
86.72696282	0.018837202	0.985923393	73.88703146	55.90691143	3.937810574
240.8111687	0.014039377	0.982112045	83.05149758	60.95042882	3.883971155
133.3725534	0.006536728	0.978667545	166.9778738	106.4860772	4.388010495
225.2031961	0.018251975	0.981820672	75.2820166	70.49028145	4.01797697
-77.22841693	0.009426132	0.981604251	107.3307542	60.19991212	3.99202316
2548.79999	0.017686393	0.99341447	166.7765853	89.57152628	4.138302241
-257.3296825	0.013504154	0.982578903	134.6146918	123.6508295	4.180190925
219.7331645	0.014290461	0.967443396	86.92713887	72.14668439	4.032344106
-154.6547016	0.010960242	0.956640945	145.0264519	102.8167259	4.310138643
428.342266	0.006416448	0.972833374	181.2392911	112.2770249	4.403674303
557.2284575	0.005504281	0.978205319	240.0877693	126.6883486	4.497895683
140.9943166	0.071049658	0.990806567	52.91702	45.80395299	3.613192949
-50.14957877	0.020489284	0.981221161	54.49636844	38.51686643	3.622902397
-40.42759793	0.011957609	0.969481532	100.8459449	73.87004254	4.076505725
1019.915107	0.00767023	0.976908089	188.6136634	88.09224674	4.24163763
-636.1471793	0.010455454	0.986729064	124.9100785	74.13858765	4.102370755
232.7031724	0.005824663	0.974488623	150.6193958	96.23171271	4.297226073
-101.1239604	0.009042629	0.963290928	129.2811931	90.33206665	4.2279181
-74.48730745	0.023235324	0.992787567	61.1025342	48.53950619	3.773701367
428.653717	0.006703392	0.961832734	144.6586341	109.0009215	4.406893739
-379.0583355	0.008106877	0.977087375	167.6709784	137.7376365	4.539792141
-359.2173562	0.005789131	0.980724895	185.5301296	108.3780308	4.404920794
-245.3475698	0.010082673	0.972969571	85.63281117	64.00310485	4.015504616
517.8836509	0.013316572	0.967273854	104.6245433	70.15669578	4.017296875
-47.66254036	0.004863027	0.97571623	169.4995031	133.5898076	4.560893332
-30.05084682	0.009076289	0.948996813	147.5187569	132.4491425	4.481343139
135.6948434	0.008848815	0.978358223	123.3461808	84.85499752	4.231402364
132.452605	0.013539751	0.986716499	105.1777215	58.59048605	3.933610945
-318.0059524	0.008323918	0.980883163	126.1274999	67.11135387	4.071860801
442.2274811	0.012629503	0.966111658	118.4435719	61.50440734	3.948759986
423.9603595	0.009607442	0.976708887	125.8255375	86.74626885	4.238817873
491.5951287	0.012275304	0.979401636	128.3739868	70.76476617	4.060433189
182.6032441	0.011150418	0.967645435	104.9119736	77.22140692	4.144484142
390.1126122	0.011374262	0.984138312	126.6337787	86.86079161	4.219960832
213.9244773	0.009938211	0.97172202	121.031848	85.50626508	4.186332579
-231.2840861	0.006049327	0.982087383	193.5093271	89.19864598	4.255757015
106.203562	0.01174621	0.953353257	185.701458	135.9191104	4.41311546
-53.98277394	0.007256648	0.966263801	140.3817961	95.11172509	4.27898334
14.64310464	0.028943056	0.9764272	59.02738227	46.01649567	3.78803101
2396.479362	0.007363278	0.98219396	229.2872192	146.9899219	4.569129256
-115.1199061	0.012952709	0.977852164	79.91667353	68.01853119	4.021041894
391.9381511	0.042782519	0.973022066	99.46216514	64.17558402	3.896357907
-8.391955117	0.020921902	0.976380495	39.78487912	25.47408326	3.373152218
215.0451663	0.013436585	0.971925822	99.12572487	64.00489875	4.034669779
-1129.666955	0.014073855	0.956513006	163.1132778	138.1872907	4.369964115
567.702201	0.016362585	0.975565427	94.44430804	69.30945625	3.995232028
163.7220055	0.042039175	0.988403405	52.11505514	42.03717051	3.669464681
19.65664415	0.010928177	0.980931242	193.481055	118.0847912	4.41728737
-119.1716146	0.008327174	0.964571058	113.0444218	72.44684103	4.116599453
97.38619218	0.010796476	0.956457304	137.3841636	113.4267545	4.356781694
76.4286962	0.04543023	0.978647141	34.45349134	27.59637509	3.412529603
-19.43340691	0.006765761	0.978928554	120.2732935	82.3870805	4.222554176
128.0326041	0.021960273	0.981353274	58.22845425	37.69928597	3.64872162
-17.67031127	0.012645952	0.981568935	67.68449636	47.82466486	3.819571257
1468.500273	0.005231733	0.974039218	251.9850629	145.3645558	4.606326015
-57.0722524	0.006079496	0.965748145	219.0202368	156.2475819	4.595351108
-551.0099093	0.010147492	0.931972179	222.5240293	208.7160252	4.702472119
-82.34547173	0.021136573	0.964731393	72.6504794	49.80531379	3.746318871
146.3918993	0.013701072	0.979110237	107.473856	69.63635769	4.068213876

wavelet.LHL_glcm_InverseVariance	wavelet.LHL_glcm_Entropy	wavelet.LHL_glcm_Dissimilarity	wavelet.LHL_glcm_DifferenceVariance	wavelet.LHL_glcm_Idn	wavelet.LHL_glcm_Idm	wavelet.LHL_glcm_Correlation
0.11909446	9.535054634	9.136290662	65.47835192	0.91078874	0.110210525	0.235352265
0.1366469	8.368007934	7.674620947	39.71499184	0.870410465	0.131186471	0.412089205
0.135447692	9.144921254	8.465349913	55.48128716	0.882645398	0.127795623	0.179904537
0.13983223	8.236055858	7.976719143	44.07237699	0.862136465	0.134069327	0.244290079
0.152630547	9.204135305	6.887331267	31.20724076	0.87460376	0.143439487	0.284313601
0.096820725	7.929867894	10.29404234	62.38803659	0.835838285	0.100393435	0.109509098
0.236478869	8.71962285	5.321023005	33.1167235	0.935120114	0.269090837	0.331321773
0.156543865	9.690876146	7.209061077	42.31167591	0.909211578	0.150188478	0.278272572
0.237787422	8.440372671	5.254202219	30.59762479	0.921021232	0.247674799	0.14462133
0.127375538	8.874215382	8.255438364	42.62034705	0.849488052	0.12339526	0.03397176
0.279066317	7.400733439	3.386332878	9.04521514	0.905485114	0.284659593	0.223950014
0.206048784	8.929579369	6.704108467	53.17105786	0.918891657	0.214071209	0.152556125
0.158261406	8.91835914	7.650638532	49.45704274	0.889684086	0.150436851	0.229552873
0.153004511	8.661916593	6.797020324	29.98602774	0.868667227	0.14422087	0.208204017
0.137268261	9.57984685	9.878607243	93.15543246	0.90247035	0.137253088	0.194208279
0.305880031	7.802629728	5.038902601	53.89024111	0.930217227	0.342009576	0.210483366
0.187256388	8.299230148	5.538024675	23.75156769	0.887563	0.185238563	0.105102508
0.13899384	8.406152312	8.004350407	44.58997357	0.86181874	0.129543507	0.122884897
0.189930967	8.326279987	5.707000199	28.03079852	0.903229108	0.181871692	0.070758638
0.169381628	9.818597456	7.344140751	56.8018627	0.937927813	0.166295996	0.431315241
0.228898196	8.399000351	4.754310268	19.95326773	0.893051778	0.220971978	0.221661747
0.15883865	8.304637627	6.363523262	25.82894137	0.86727383	0.152637355	0.185661887
0.137268042	8.781109818	7.914217278	42.054087	0.87160411	0.128743528	0.304559633
0.242562887	8.183562247	4.828270959	24.14047587	0.912666086	0.261705381	0.129067438
0.228191118	8.058621751	5.888595867	43.90112355	0.926837345	0.265537309	0.12299773
0.194624513	8.661228582	5.606271007	26.76937219	0.913517965	0.197151438	0.13539489
0.14508706	9.29209311	7.769224252	53.07029781	0.92066863	0.138527104	0.151428392
0.172909527	9.066109192	6.915019887	45.70500787	0.916509042	0.171216412	0.122659883
0.136945119	7.153709948	7.567702707	35.98637583	0.85899347	0.130236777	0.1825506
0.210735524	8.452279952	5.403899427	27.02292755	0.913947187	0.200921984	0.191335199
0.207961413	8.783809729	5.256775467	22.25181255	0.898904868	0.204206319	0.245548806
0.133326196	9.047411176	7.675681719	35.64613154	0.875762718	0.122890397	0.290027157
0.200651923	8.94846225	6.267086738	41.10174998	0.911782641	0.200005472	0.226268412
0.251857278	8.340012723	5.303838205	36.78612782	0.911988632	0.258301658	0.199322815
0.179512487	9.075539777	6.099416218	28.38295575	0.900366702	0.174179675	0.172207232
0.135048134	9.622838534	8.697176039	65.71176293	0.896804819	0.1342687	0.203270639
0.109972633	9.76177221	10.15345815	68.0824556	0.878322109	0.10241337	0.195329363
0.209051155	9.103111307	6.279727363	46.54567082	0.936502899	0.211118986	0.208939012
0.170429911	8.500726387	6.097583982	23.1396891	0.871557271	0.157140658	0.320653063
0.259204752	8.14145647	4.43966749	22.48721066	0.945957504	0.280232155	0.190299825
0.179180684	8.802282274	5.980420981	26.86902207	0.880216837	0.17049648	0.212644465
0.207809009	8.701622497	5.447796629	25.38679777	0.922946798	0.206250923	0.147374331
0.214539329	8.519449116	5.401388222	30.16621309	0.917290126	0.205523518	0.164557061
0.123371358	9.371007102	8.021870547	40.72473057	0.900451599	0.117544342	0.229936441
0.202751975	8.505094967	5.89497908	34.34892665	0.91582194	0.20100444	0.04246828
0.180470963	8.986054507	5.829200849	25.6001528	0.909940846	0.169839908	0.28585809
0.192943973	9.089640171	6.326631204	48.53336275	0.949846776	0.188987038	0.301975649
0.169472295	8.182618014	7.331401006	67.17754582	0.921157249	0.159619044	0.053434698
0.162962587	8.035166595	6.416633515	30.03178253	0.88208512	0.155736539	0.099671223
0.138609459	8.441742575	7.720687207	41.8809438	0.865391201	0.1345316	0.177893421
0.128721124	9.329585448	8.103067465	45.26560019	0.8904726	0.121155931	0.241536194
0.122057175	9.898008208	8.588461914	49.95979739	0.901153208	0.116839884	0.318225487
0.288828936	7.650186411	4.254862374	26.99436012	0.945448435	0.323157628	0.077484953
0.247544707	7.985996644	4.395081986	18.5902488	0.914001485	0.23907257	0.179475989
0.16594354	8.454823635	6.389290518	31.84478773	0.887297406	0.165066764	0.154412245
0.150848886	9.209214763	7.091011382	36.54977228	0.899668432	0.143300202	0.367924084
0.177257206	9.116046827	6.23967161	34.28794109	0.924925509	0.169181731	0.262997849
0.148262024	9.391296576	7.290426559	41.90113175	0.896233375	0.141724775	0.226797163
0.154978082	8.811248372	7.121711201	38.2809355	0.87659122	0.145369131	0.185351891
0.23909886	8.308073344	4.775677899	25.14220312	0.946681694	0.237753794	0.118250285
0.134127373	9.267453552	8.030720118	43.10311748	0.871906514	0.129888681	0.147535684
0.129517315	9.649320035	8.780283792	57.49666208	0.901041205	0.12493718	0.108756
0.14343139	9.940343688	7.776207706	46.19698234	0.908642446	0.136655143	0.271078215
0.17498278	8.817874408	5.982885456	26.99349927	0.893112906	0.170387191	0.151683591
0.179457936	8.381176017	6.150603829	31.17128717	0.885009184	0.169875037	0.206994066
0.117253548	9.842850397	8.929582345	52.02846929	0.895110696	0.110015364	0.129474444
0.105675615	8.562480825	9.02719639	49.45209083	0.851268551	0.104986407	0.061616577
0.15648037	9.425662048	6.952039178	34.66148966	0.902777213	0.153055006	0.193186511
0.209541629	8.91417538	5.414636061	28.55528251	0.926947939	0.205105546	0.287956353
0.163742932	9.110556255	6.201079218	27.79945468	0.907743973	0.161238519	0.312048473
0.161486014	8.308916589	6.017955962	23.72955767	0.878527784	0.15996874	0.319938256
0.171691356	9.373383776	6.802410403	39.41749438	0.9027076	0.169893502	0.191968621
0.183377066	8.853418492	6.183648254	31.00315124	0.907324857	0.177615431	0.29269094
0.165053657	8.671429322	6.661727987	31.4797893	0.882805208	0.157780754	0.157026665
0.165767566	9.296032681	6.791865141	39.56562828	0.918124254	0.160081875	0.1944869
0.15289	8.809652079	7.022876765	34.10440742	0.889199799	0.144629496	0.185986867
0.142966089	9.506678466	7.189118347	36.09416833	0.909812866	0.134325932	0.378048065
0.113285452	8.324021016	9.121338029	48.02628229	0.8572699	0.10424391	0.162665074
0.147001451	9.208760771	7.430735414	37.39350294	0.880136904	0.139765386	0.203314828
0.223176255	8.244176039	4.939097845	20.72803318	0.902628315	0.230163244	0.129420831
0.121249144	9.484941942	9.008788655	64.26978275	0.911889138	0.111516516	0.226538047
0.175170686	8.417966591	6.187926603	27.7034575	0.902311074	0.169406056	0.093805298
0.243219149	8.413661806	5.286609313	35.07459746	0.905637953	0.263253035	0.21931752
0.263335935	7.734400239	3.683196182	11.44313432	0.901531948	0.260735014	0.224413172
0.18997746	8.965601333	5.8918528	28.47868512	0.893102937	0.188855036	0.221182426
0.147325068	8.514598176	8.381346749	61.10322529	0.872955394	0.151057824	0.100101073
0.202969907	8.524550175	5.870668994	33.06709263	0.903552767	0.198107318	0.160985925
0.263689872	8.015618705	4.36814167	22.59420081	0.934786433	0.280355905	0.108378634
0.150704144	9.384278612	7.879427648	54.55855357	0.91141906	0.147143119	0.248254464
0.164643837	8.95722791	6.366075515	31.21071835	0.878762505	0.163042924	0.223902042
0.13945953	8.903812999	8.036132775	45.39610056	0.866171874	0.132210492	0.108086126
0.279186452	7.517154178	3.666944371	13.66567585	0.910487511	0.292788481	0.119275016
0.151467769	9.390245752	6.91330741	33.45135796	0.902959638	0.144156086	0.194363814
0.240147579	8.251885492	4.423632373	17.527297	0.91320848	0.239950899	0.219496808
0.202422257	8.598472646	5.150374558	20.24423525	0.91081798	0.19603323	0.179178716
0.11745286	9.967118721	9.127449287	60.35454399	0.893737997	0.11316705	0.277658109
0.120627304	9.875803302	9.362722526	62.804929	0.880666597	0.116225205	0.179570371
0.099164415	8.362419398	11.34501583	73.46071166	0.830976895	0.093230191	0.044466295
0.188706813	7.563706222	5.318531923	20.39333028	0.878220775	0.187600657	0.193468853
0.1907239	9.17000821	6.046234331	31.60551623	0.908042165	0.186887379	0.220656475

wavelet.LHL_glcm_Autocorrelation	wavelet.LHL_glcm_SumEntropy	wavelet.LHL_glcm_AverageIntensity	wavelet.LHL_glcm_Energy	wavelet.LHL_glcm_SumSquares	wavelet.LHL_glcm_ClusterProminence	wavelet.LHL_glcm_SumAverage
1364.155237	5.834897521	37.47706251	0.001800605	98.59133541	289264.0453	73.26789957
657.3914199	5.681408533	25.50501947	0.003495778	82.47505769	130421.6053	49.91346264
833.3692789	5.679291632	29.35524808	0.002171226	78.13065009	104770.4541	57.27243467
840.4022188	5.503094767	29.31498057	0.003845197	69.33912123	83397.38572	57.40124522
575.6789819	5.54185913	24.13192004	0.002104922	55.61018116	54930.66715	47.3393072
634.3090156	5.480950958	25.91126292	0.004684659	94.00755019	132709.9744	49.97053328
1042.202209	5.266265158	32.48581267	0.009433845	46.64827058	89321.37509	64.10168293
1180.649867	5.697865469	34.69586022	0.001819723	65.59244644	101570.7096	68.20187378
1032.114461	5.008643198	32.43396429	0.007722138	34.38278806	32964.253	64.097399
453.8454718	5.360660615	21.89491019	0.002574682	57.17584454	40535.88278	42.56470456
290.1699279	4.482132529	17.06309119	0.009884347	13.93386825	4257.077946	33.89699082
1193.063855	5.366574576	34.93629613	0.00575531	58.64377744	102515.1462	68.82892329
1223.147129	5.581821228	35.38334943	0.002785212	70.18331812	122276.5047	69.49122694
392.9616917	5.360411423	20.01597742	0.002983013	48.14377594	41352.66248	39.14065129
1524.949048	5.948011346	39.6484176	0.002209745	120.9639016	356008.606	77.5331208
945.6440144	4.879274676	30.95313963	0.020465244	52.51730705	101235.1426	61.15595505
454.8830673	4.982992927	21.58335947	0.004222737	30.15154953	16780.67034	42.51148837
615.1869937	5.395317101	25.27715382	0.00350863	61.96620782	63792.51188	49.32021044
610.3401077	4.957333621	25.09516327	0.00449089	33.14915598	24647.10397	49.32927757
2489.173652	5.90876983	50.13671052	0.002257251	100.7574323	569196.3609	98.90040535
325.4965476	5.026053041	18.16864759	0.004202447	27.9686844	14886.03987	35.74884793
404.4417418	5.188310065	20.30678315	0.003917004	40.25612797	29953.28287	39.84957731
553.7388634	5.673359868	23.72301954	0.002709364	75.88728881	112797.5969	46.1072005
616.4708364	4.851844685	25.07560851	0.009349422	27.82177102	18395.29646	49.51745137
1177.679277	5.070373701	34.72210277	0.012971748	45.06125629	57693.14435	68.47913736
587.4465028	5.080632299	24.58575304	0.004138111	33.82403676	23233.54325	48.29435507
2339.839243	5.538144204	48.97742015	0.002311838	68.44894984	135557.1177	96.53810463
1106.33738	5.403602032	33.73968577	0.00290551	54.92128907	68322.31767	66.33675971
581.342237	5.059891704	24.38846627	0.007857946	55.95531853	50596.20688	47.79844696
1000.93079	5.112359434	31.89403183	0.004599196	34.82714079	33162.01328	63.06341224
506.3761431	5.195287172	22.64326274	0.003547076	33.28194996	22607.67094	44.64709925
791.9548779	5.630809751	28.38445807	0.002260752	65.90257178	77540.28117	55.60554052
836.2199447	5.423928412	29.1768955	0.00373799	52.37923486	78458.43607	57.4319705
519.5876813	5.133623972	22.97893079	0.008009501	41.62836438	46509.48689	45.23478168
612.718251	5.263569646	25.08669361	0.002916822	40.00109467	31118.77714	49.24237581
1409.213151	5.808784882	38.04686978	0.001832641	88.09049466	176628.5747	74.61140636
1265.028371	5.91211701	36.25967689	0.001489519	106.2167969	201959.8329	70.57899927
2190.792801	5.433244037	47.2424988	0.003925196	54.72228215	88127.27355	93.37561274
414.4318339	5.359345386	20.37106806	0.003379374	44.53165748	42169.89116	40.0029676
1488.143978	4.854860464	38.84747821	0.010592928	26.38873645	22547.97762	77.02864238
417.6599471	5.263712932	20.63667068	0.002972171	39.92644987	30128.52368	40.46223195
939.5089168	5.067100659	31.01206058	0.00441004	32.68025991	24056.23825	61.15591776
634.9312978	5.084218159	25.50880205	0.004446167	35.85443471	37510.33895	50.17556987
1613.56803	5.648837602	40.67739753	0.001890083	68.21128107	94640.82037	79.9607973
949.809808	5.042028165	31.24765834	0.004375778	36.91991473	26929.89316	61.59847997
600.6622602	5.367032113	24.70385149	0.002684774	41.83212216	39274.91737	48.53360368
3082.500742	5.467673253	55.93254322	0.003607327	64.73674032	286375.8964	110.6918775
2093.411987	5.222479263	46.37931033	0.004415825	63.10577035	127758.3605	91.44637622
709.9177392	5.06931362	26.96702882	0.004624882	39.26481754	26887.82152	53.14955826
564.1424787	5.444436171	24.11994061	0.00344282	61.21441167	62724.50327	47.05604697
976.4152032	5.697437347	31.62838678	0.001928448	72.98164812	102125.7436	61.93866413
1532.126997	5.930452171	39.55363742	0.001392737	91.55324992	201334.9625	77.5566586
769.7888031	4.567988841	28.05051113	0.018620647	24.96758961	23505.29035	55.4259848
580.905951	4.824804812	24.2422267	0.006448855	23.34896746	13566.42457	48.0374769
487.8229583	5.227796222	22.37710712	0.003601979	43.20386738	36777.26822	43.86683388
775.2863285	5.712983264	27.95176273	0.002136218	69.09120177	123132.65	54.77687956
1857.947555	5.422623701	43.47593116	0.002771464	48.84176438	96637.55639	85.91274226
908.1007158	5.603867627	30.48565007	0.001943168	61.42210017	72975.9057	59.81635894
650.3753201	5.44026179	25.82481202	0.002753558	54.65573442	51674.60798	50.62109191
1657.701192	4.841386911	41.05766598	0.006665141	27.10748559	25779.67141	81.35246446
558.0468944	5.553895969	24.07709463	0.00204818	63.87106138	66601.60597	46.86671217
1735.293993	5.654650596	42.36055366	0.001773942	75.34326739	109448.0818	83.13369627
1353.761466	5.778710349	37.22148762	0.001481517	72.87986395	113589.6109	73.06071132
587.3530615	5.179875551	24.54136286	0.00312322	37.42348892	26601.57008	48.24680692
437.9278345	5.241368166	21.14686306	0.003929894	43.74913711	41712.50442	41.43867728
1632.693237	5.696222258	41.11346944	0.001466261	74.87854274	99981.51451	80.59046124
543.6555227	5.444170772	23.94744661	0.00303974	69.85725131	65831.95622	46.4701608
810.8813411	5.472884844	28.85374049	0.00212849	51.8268645	54277.19431	56.61294102
831.3424092	5.298516346	29.06197912	0.003995651	41.03146574	51282.12268	57.26011354
1127.503053	5.47802295	33.83121479	0.002393702	47.45499244	50591.46709	66.71555263
462.1127758	5.308430715	21.53128358	0.003870926	44.6549753	44195.52083	42.32577769
826.1934218	5.474840742	29.12934578	0.00239627	53.49565682	59878.31366	57.14551913
836.6926254	5.426302783	29.12426401	0.003161933	49.83788484	60016.10302	57.35050876
573.5580684	5.28198269	24.28211553	0.003161979	46.21373805	39543.36242	47.60795515
1304.548079	5.456445756	36.56108786	0.002583076	53.4975055	68284.65221	71.96112523
520.7730827	5.398484023	23.209112	0.002807005	52.24215127	48276.80572	45.24822562
1517.869029	5.78336651	39.2736017	0.001828951	69.05461161	130265.0885	77.24610124
580.1499615	5.518473116	24.60356271	0.003587578	78.63032696	98650.22403	47.65238821
575.7139706	5.534687026	24.3506448	0.002163935	58.08861447	58768.7827	47.51356859
496.4829749	4.898888292	22.45901055	0.005880257	26.17816028	12660.46851	44.41743072
1359.062466	5.799863364	37.42533695	0.001861776	95.22261244	274955.3129	73.17053933
1056.952401	5.090157054	32.91543302	0.003891588	36.70056958	23665.64452	64.92986612
492.2006215	5.148840598	22.3084058	0.008386837	40.97704305	40971.69246	43.97147848
256.317567	4.658962363	16.02931849	0.007113563	17.06519535	5550.556006	31.79554878
487.5291319	5.306427104	22.26090002	0.003097843	40.78764483	33099.40208	43.76046812
953.0571847	5.468070958	31.48600714	0.003632213	73.47324962	109311.4979	61.54039284
338.3970509	5.191679299	18.65221001	0.004219483	41.58654812	39954.2048	36.44719374
939.7387565	4.760792797	30.90379991	0.009246386	23.83580679	14581.97993	61.2279901
1013.275926	5.731424615	32.22003509	0.002209002	77.98450778	164328.2324	63.06873254
506.6768118	5.387387478	22.70827789	0.002546306	45.96927195	36606.98396	44.56546357
663.5189319	5.439285269	26.30294224	0.002764577	61.3539206	70627.18617	51.28433483
304.99636	4.472548855	17.60395805	0.011024441	15.92117579	5901.17736	34.82995095
899.8854181	5.463446051	30.3944808	0.002077475	50.25068399	49586.87885	59.67946904
476.20391	4.918028402	21.91732863	0.005629708	24.13596543	12864.14498	43.40824941
380.3000924	5.042471394	19.68662594	0.003754325	28.6519169	15670.25592	38.74702447
905.0388012	5.962841271	30.47087308	0.001304344	99.31094076	211633.6562	59.2744034
1050.340014	5.85834958	32.99718578	0.001442856	92.93070881	160879.0511	64.33124577
783.6573906	5.609636831	28.92667313	0.003417721	106.0010289	134155.3848	55.86352559
347.5296222	4.896635574	18.83643381	0.006514756	30.35158056	17730.12673	36.97572813
770.9613054	5.359464137	28.06613894	0.003124081	43.9650885	47437.60013	55.19019179

wavelet.LHL_glcm_Imc2	wavelet.LHL_glcm_Imc1	wavelet.LHL_glcm_DifferenceAverage	wavelet.LHL_glcm_Id	wavelet.LHL_glcm_ClusterTendency	wavelet.LHL_firstorder_InterquartileRange	wavelet.LHL_firstorder_Skewness
0.917744263	-0.176707456	9.136290662	0.194991044	239.0476281	276.4111668	0.563559941
0.98570805	-0.352702223	7.674620947	0.218668426	238.8172189	327.2166409	-0.41757066
0.94155945	-0.21353671	8.465349913	0.212580954	182.3480413	254.2774077	0.147389484
0.983156915	-0.342466121	7.976719143	0.219260152	177.1757427	272.1608961	-0.527633835
0.842075876	-0.127547274	6.887331267	0.232502128	142.255751	251.5005358	-0.043419288
0.993796058	-0.433099735	10.29404234	0.180947246	213.8212295	330.2266795	-0.336463289
0.750487854	-0.093831139	5.321023005	0.34237272	122.0275313	122.8345828	0.520925574
0.718426752	-0.074270743	7.209061077	0.237046516	166.6378037	225.4033408	-0.00675101
0.753119386	-0.099127492	5.254202219	0.325992686	79.61560462	114.7682025	-0.164672122
0.922414749	-0.194763716	8.255438364	0.208895604	118.0068007	237.1419585	-0.063166719
0.670488887	-0.081310594	3.386332878	0.368596639	32.59585144	89.68012973	0.027222348
0.835419462	-0.128266057	6.704108467	0.2915468	134.7830512	139.9841149	0.151376339
0.941675045	-0.218171664	7.650638532	0.234945018	174.6733683	224.9740771	-0.768893086
0.920241437	-0.19733773	6.797020324	0.233929122	118.238699	227.634287	-0.007226117
0.943676421	-0.208970687	9.878607243	0.215451444	285.2804643	269.9446265	0.111694968
0.862430391	-0.165106771	5.038902601	0.403626543	123.3400059	66.78172696	0.556936715
0.840311103	-0.137496621	5.538024675	0.275549926	67.31166883	158.1259284	0.099447328
0.970854816	-0.291203486	8.004350407	0.215668379	140.3380956	243.6817043	-0.336561182
0.835760402	-0.134652845	5.707000199	0.272077898	69.43160698	148.7068229	0.11493732
0.808205707	-0.107441055	7.344140751	0.250723131	287.6183746	207.3668638	0.774929855
0.743860625	-0.097395941	4.754310268	0.308976553	67.7649698	162.0998031	0.0926621
0.928180011	-0.214195224	6.363523262	0.243058595	97.4673052	199.7766601	-0.141427531
0.96644328	-0.267753869	7.914217278	0.215019293	196.4595537	271.3518632	0.047258305
0.728618294	-0.089414509	4.828270959	0.339319697	62.04051804	101.075415	0.142619434
0.905429052	-0.193540059	5.888595867	0.336157342	100.889848	119.617199	-0.005271974
0.719087802	-0.08185162	5.606271007	0.283927682	76.12768091	159.5896874	0.208466148
0.862801706	-0.138112156	7.769224252	0.226076145	155.7756076	223.1117564	0.09915226
0.825721443	-0.122211309	6.915019887	0.256424345	120.7440345	177.4004603	0.300185241
0.994634831	-0.483644731	7.567702707	0.217315197	135.2395368	274.6287859	0.008798599
0.813187362	-0.123421781	5.403899427	0.288438346	83.87160428	144.0899688	-0.020744594
0.660611005	-0.066058469	5.256775467	0.291340942	82.60723845	162.8315446	0.108094762
0.921354513	-0.189311595	7.675681719	0.210815672	171.7137195	267.9986277	-0.18582588
0.844867207	-0.134092323	6.267086738	0.282896114	128.5366862	171.4364966	0.235750783
0.849860243	-0.143919268	5.303838205	0.334575377	97.93456906	103.7380114	0.301860735
0.625964717	-0.054933415	6.099416218	0.262062947	92.34019987	188.5477046	0.201159257
0.896193063	-0.156448143	8.697176039	0.217518849	214.107904	254.3212915	-0.165885242
0.921753656	-0.178988229	10.15345815	0.183649046	257.9530545	307.1211871	-0.380883277
0.762123029	-0.093430582	6.279727363	0.292185882	130.9672315	151.1100733	0.251850786
0.922730803	-0.202060594	6.097583982	0.247556723	118.9123723	208.6461534	-0.067333486
0.662064673	-0.072293508	4.43966749	0.356963217	62.05981815	95.02863479	0.22909899
0.817029827	-0.119697293	5.980420981	0.260550783	97.58944295	189.359716	0.011419365
0.642837757	-0.060826338	5.447796629	0.291609834	73.88703146	143.0663633	0.204165459
0.803372127	-0.118528877	5.401388222	0.292975787	83.05149758	152.9806302	0.04096055
0.863139815	-0.136536115	8.021870547	0.205108825	166.9778738	264.8084073	-0.012935484
0.815021423	-0.121865952	5.89497908	0.285454564	75.2820166	154.9020443	0.235024586
0.756820718	-0.091724568	5.829200849	0.260788081	107.3307542	201.952386	0.034450254
0.794038877	-0.105675628	6.326631204	0.275091359	166.7765853	154.6229932	0.631046272
0.963099008	-0.277505421	7.331401006	0.246701734	134.6146918	174.6120999	-0.668009326
0.943513427	-0.241965364	6.416633515	0.245506327	86.92713887	215.6278987	-0.088148653
0.967979038	-0.281233396	7.720687207	0.221263329	145.0264519	223.3916002	-0.086286223
0.905581942	-0.169083632	8.103067465	0.207990504	181.2392911	278.2297969	0.145984994
0.845538551	-0.121147492	8.588461914	0.202109236	240.0877693	298.9451352	0.114237004
0.713855947	-0.092161004	4.254862374	0.391820063	52.91702	69.59119959	0.325935079
0.7526801	-0.102047202	4.395081986	0.325479522	54.49636844	116.3642329	-0.123830441
0.917718265	-0.198109513	6.389290518	0.25387152	100.8459449	191.6531685	-0.007945724
0.909713959	-0.174956512	7.091011382	0.231795234	188.6136634	269.0196547	0.244582738
0.745468466	-0.086718055	6.23967161	0.258369255	124.9100785	190.2275928	-0.374623028
0.822996334	-0.114914099	7.290426559	0.230043604	150.6193958	235.5600348	0.06218576
0.912846295	-0.185324453	7.121711201	0.233214342	129.2811931	244.4854065	-0.084029616
0.611729401	-0.056628002	4.775677899	0.322272318	61.1025342	112.8670199	-0.031331129
0.862198003	-0.136976843	8.030720118	0.214925797	144.6586341	252.895965	0.23227606
0.831038104	-0.116590406	8.780283792	0.208484536	167.6709784	243.1009202	-0.169332243
0.674624733	-0.061057621	7.776207706	0.222976877	185.5301296	259.2462288	-0.111905422
0.746432868	-0.090634734	5.982885456	0.260641856	85.63281117	183.8658082	-0.210459715
0.914900439	-0.195568485	6.150603829	0.259319782	104.6245433	176.9079209	0.383357185
0.756670054	-0.083758681	8.929582345	0.19440272	169.4995031	258.2363117	-0.076328997
0.972145015	-0.290284842	9.02719639	0.190218764	147.5187569	278.4641488	-0.040010301
0.672742451	-0.06538515	6.952039178	0.240047988	123.3461808	207.1416201	0.060321931
0.683581711	-0.070972586	5.414636061	0.292344022	105.1777215	148.4955957	0.065653208
0.785854213	-0.102181949	6.201079218	0.251846896	126.1274999	228.1302308	-0.294212133
0.945977751	-0.239765702	6.017955962	0.252069178	118.4435719	199.2034318	0.003466351
0.705811776	-0.072974142	6.802410403	0.255348993	125.8255375	186.2800049	0.163544471
0.864590836	-0.147873586	6.183648254	0.264706941	128.3739868	198.8130984	0.124912846
0.889840532	-0.166410448	6.661727987	0.245136958	104.9119736	202.7888906	0.216038254
0.739876112	-0.083598969	6.791865141	0.24761113	126.6337787	191.63242	0.192398274
0.899244013	-0.171878793	7.022876765	0.232697841	121.031848	225.5154326	0.088525083
0.846996048	-0.126032036	7.189118347	0.223911973	193.5093271	257.4798894	-0.234527842
0.986479106	-0.357468473	9.121338029	0.188234448	185.701458	339.4205656	-0.19728723
0.857773797	-0.137510889	7.430735414	0.226700411	140.3817961	240.4882657	-0.091537113
0.748124396	-0.096899638	4.939097845	0.313749333	59.02738227	130.9600475	0.017796384
0.917675841	-0.177423413	9.008788655	0.19663412	229.2872192	271.1749904	0.55301244
0.868150652	-0.15604409	6.187926603	0.257945171	79.91667353	178.3872869	-0.319804215
0.860166946	-0.150812912	5.286609313	0.338945738	99.46216514	129.9675652	0.236626652
0.659644668	-0.076623	3.683196182	0.348287412	39.78487912	114.8014177	0.024711912
0.738832986	-0.085907853	5.8918528	0.27531799	99.12572487	177.3389133	0.210849678
0.970653167	-0.289923701	8.381346749	0.233713941	163.1132778	201.2685909	-0.641245534
0.851815033	-0.144244149	5.870668994	0.283914088	94.44430804	150.510908	0.635119983
0.70672622	-0.083674355	4.36814167	0.357650705	52.11505514	104.6333992	0.305746332
0.89439867	-0.159209964	7.879427648	0.231367156	193.481055	239.8716305	0.140134456
0.831048745	-0.123742949	6.366075515	0.25234874	113.0444218	227.2245944	-0.14858471
0.927456584	-0.19956691	8.036132775	0.21777205	137.3841636	206.0638444	-0.138642424
0.657668018	-0.075281447	3.666944371	0.37284704	34.45349134	90.27258484	0.304847684
0.678464368	-0.065357071	6.91330741	0.233188062	120.2732935	215.5410182	0.01033549
0.661817002	-0.071301816	4.423632373	0.325900159	58.22845425	121.5385366	0.304200734
0.654987642	-0.066269972	5.150374558	0.286336794	67.68449636	157.9175903	-0.03282895
0.85857998	-0.126164102	9.127449287	0.196875387	251.9850629	304.9105187	0.265505324
0.857147832	-0.13051082	9.362722526	0.198591262	219.0202368	296.1190267	-0.031096335
0.99226681	-0.400016599	11.34501583	0.169556808	222.5240293	327.8797057	-0.310301961
0.950886808	-0.269974878	5.318531923	0.278449261	72.6504794	171.3565622	-0.106797649
0.640384257	-0.060830939	6.046234331	0.27321583	107.473856	165.2420555	0.055392227

wavelet.LHL_firstorder_Uniformity	wavelet.LHL_firstorder_MeanAbsoluteDeviation	wavelet.LHL_firstorder_Energy	wavelet.LHL_firstorder_RobustMeanAbsoluteDeviation	wavelet.LHL_firstorder_Median
0.033209345	182.5275164	90244541.82	116.018337	-60.58688356
0.034756538	181.4481489	17333557.52	131.1673903	-3.514694294
0.036618472	161.2558479	38660541.6	107.7213206	-18.07156464
0.039060993	160.9382475	12960223.69	111.3395327	23.40582636
0.039306934	144.6844279	45814868.79	102.6626056	-16.25580344
0.032921249	197.5606274	14836470.15	138.0065069	-11.75011514
0.073242377	107.9691686	277814891.2	57.61661712	1.073844597
0.039418995	147.8631764	230821690.5	96.31320914	-7.630755245
0.074290914	95.85529667	84067795.75	53.01761338	-6.534143877
0.040340336	143.5295528	24970008.99	98.81499236	24.66342526
0.097070136	63.41030344	11604274.6	39.49241633	2.550549545
0.061011235	121.2448651	110334826.2	66.31517717	-5.145798176
0.042527151	153.3434586	34759244.4	95.44691378	12.74064185
0.042156938	136.1330112	18970848.9	94.13422624	-6.218751795
0.033342807	197.675311	117624013.7	121.3148162	-15.5116983
0.118979258	92.89181777	149890394.2	35.32802027	0.767767565
0.057231794	103.0540676	16103658.18	67.31874584	-6.333800275
0.040536582	149.2100261	15530347.26	100.7737971	-26.82070286
0.058542691	103.7859992	22337143.61	64.76376883	-20.12266582
0.041101461	162.1805081	524086841.6	91.3236095	-11.38543337
0.057101715	100.3722838	34364544.17	68.24289413	-13.91059582
0.046728668	124.6739402	12415645.53	85.38820444	-3.566850108
0.035322003	168.4158188	27479311.3	114.3358612	-48.58460894
0.083668788	83.62596734	53158598.82	46.73111472	-2.016777708
0.081350996	107.7869695	33542944.24	57.04196004	-3.355512918
0.056009549	106.2466051	52344181.85	68.06330071	11.87528572
0.03964775	153.5501888	90029177.61	95.34652822	-31.71074545
0.049073002	126.3780355	69624749.6	76.67249878	-11.66533052
0.043066241	154.3820063	5250581.416	110.1566326	1.944049527
0.060280853	104.0184704	33256387.07	63.663217	-3.678867017
0.055260237	105.8042169	77915056.37	69.10197936	-4.094880821
0.036972738	156.2252169	30135007.62	110.5624242	-8.257996127
0.052152837	124.5732199	72181257.26	73.70963068	-1.817301803
0.079155897	96.40814361	51953012.74	49.48450094	-6.253491141
0.049024224	118.814197	123817710	78.28750497	-6.055501871
0.036092306	167.6036243	97698224.53	107.2556832	-8.073160257
0.031266622	192.6828452	91919587.52	129.8424522	11.58695957
0.055145046	120.4326847	178900744.2	68.19873916	-2.537723843
0.044553571	130.0935955	15388286.1	88.6787864	-1.474275537
0.089470453	81.3204677	139987436.2	44.07215003	-3.887442712
0.046950323	121.087408	32683436.45	82.54999669	-25.98509725
0.060683863	101.1065292	109343044.4	62.59073386	-1.777380504
0.057925772	104.6425081	50182206.07	64.84059	-8.867795139
0.036066257	160.6083622	59038517.18	111.7562263	-11.37181125
0.05830321	105.5892505	32622090.27	65.65964056	-2.919192619
0.046846196	122.4058176	59455583.71	83.43908315	-5.509524772
0.054378167	127.1291174	208396211.7	68.84094027	-12.11909063
0.04953107	138.0524243	21728434	75.82569529	-25.65030022
0.049705326	122.7958501	9914012.511	84.19013084	21.0511283
0.04229971	144.6205878	16141901.36	93.37001631	1.357956682
0.034119813	167.6727536	57492727.91	116.6425664	-0.388325835
0.031678168	182.928051	151266760.3	124.3823459	-3.178025359
0.112594145	73.18759294	112043711.9	35.26840334	-1.14819258
0.073595671	83.90464846	22238490.12	51.18912047	-4.712375133
0.049009672	121.02448	15998876.15	80.3826295	-11.26654163
0.036422902	160.9332901	49064424.26	111.8192887	-33.68522542
0.047900138	124.9308858	116961959.4	79.58733959	-6.763098998
0.039320171	145.7978979	73601670.22	98.51094336	-13.70871679
0.039976364	145.0416096	25220485.03	101.5305116	-6.0760603
0.075338085	86.48092593	175253175.2	49.61302138	-8.375259238
0.037587736	154.2529015	51427878.4	106.6665433	-29.64721649
0.03756691	157.7666247	108184222.9	101.4797322	10.51252775
0.03571213	159.5964984	346127945.2	107.9982787	6.123002034
0.050842481	115.2842546	46779593.24	77.20249924	1.37465771
0.051441214	118.5574586	18265296.78	74.5642335	-44.17447248
0.03539182	162.4171771	150742106.3	109.1366287	-4.140271776
0.036606081	160.8239396	16666148.23	111.6434698	-8.174516815
0.044159147	131.1554568	149844757	86.60807059	-7.604792016
0.057130749	110.9195811	185764525.1	65.52837427	-21.32519075
0.043439075	133.1664782	60322292.9	92.41365303	11.17417201
0.046153083	127.4581926	13579247.3	84.53108668	-23.73620371
0.047352999	126.8677382	146980695.4	80.21355767	-21.35898744
0.046393132	133.1134779	41639010.47	86.19164208	-8.114055325
0.045185596	130.7172727	27781336.87	85.86714009	-19.90804993
0.04715051	129.5942587	125731480.2	81.48196225	-2.268296296
0.042459928	137.5710007	28671014.56	94.92201597	-26.87792877
0.036556693	159.3628033	93623864.04	107.0419109	-12.63674727
0.034074898	184.6289438	16277092.07	136.1549954	-6.685892309
0.039895907	144.3140139	43739336.02	99.18293105	11.99682559
0.067995679	89.96998847	26810355.46	56.50440722	-5.803713803
0.033905696	177.9480345	84199496.37	113.3422737	-61.23872742
0.053092883	114.9954444	19079255.96	74.65411228	11.384392
0.069778267	104.67519	51194290.83	59.3213193	0.529049652
0.078311984	74.11755041	18599313.91	48.07359007	-18.54931038
0.050919488	116.2236894	63656076.93	75.57363409	-0.954816699
0.046371661	144.9356572	20986755.51	87.85159847	12.50324501
0.057183973	110.6628849	30782573.09	66.40070968	-16.1919685
0.081445224	80.59443513	51648476.57	46.7245565	-4.425738478
0.040001023	153.4962893	73685179.38	97.88923674	-3.879691559
0.043880496	130.0236836	36782937.04	90.26274987	3.010960071
0.042932106	140.7316521	27944210.13	90.44112143	12.05660509
0.093204143	68.46930613	24031621.99	39.78734914	-6.170545069
0.042725947	134.1295765	135191068.1	90.49811107	-14.091607
0.07186055	84.16255739	44954605.15	52.51563283	-3.695430421
0.057436421	100.1413911	56375403.02	66.66720122	-0.313907479
0.030487084	190.0158478	141757723.5	128.702414	-24.32831646
0.032025659	180.9497918	132275553.7	121.9671966	-8.072312368
0.03218365	199.9679363	19433835.69	141.6775362	3.371184795
0.060369793	101.9805688	4760487.881	68.13217646	8.796363368
0.052984284	115.1149092	218010921.5	71.09703459	1.285058088

wavelet.LHL_firstorder_TotalEnergy	wavelet.LHL_firstorder_Maximum	wavelet.LHL_firstorder_RootMeanSquared	wavelet.LHL_firstorder_90Percentile	wavelet.LHL_firstorder_Minimum	wavelet.LHL_firstorder_Entropy
2436602629	1215.984872	249.9924401	268.735298	-930.5414487	5.24957396
468006053	501.1962239	223.5008173	229.320666	-642.3850044	5.050333213
1043834623	711.7711889	208.186009	236.9035382	-703.9734647	5.04772596
349926039.7	393.5832606	200.6218879	245.2653464	-706.2832761	4.911234899
1237001457	478.5147575	180.1937472	207.3806481	-592.7967423	4.872744724
400584694	514.304262	247.6039739	256.7599559	-630.4379293	5.136701589
7501002062	980.9862213	162.3596319	205.4996388	-769.2832955	4.471736146
6232185644	811.5625243	195.0363329	227.0457263	-842.5063584	4.979545536
2269830485	593.7182655	140.4455902	154.9545215	-776.2064158	4.342224863
674190242.7	527.1651485	181.8594501	246.1688416	-487.6393952	4.854391491
313315414.3	325.463136	86.35832345	115.7141205	-392.7285122	3.758673685
2979040309	850.3270582	179.95762	215.4259619	-836.9207289	4.66259511
938499598.9	505.9530487	207.0259263	211.8276206	-869.6965605	4.948917022
512212920.3	513.9224115	171.6328637	211.6093506	-480.0630113	4.787519645
3175848369	1085.894736	273.2799178	334.2394366	-971.4290408	5.353006638
4047040644	715.9813636	161.287338	177.2666114	-702.5910553	4.018115429
434798770.7	452.3443347	135.0460606	162.8172922	-502.4191432	4.431601771
419319376.1	469.3491256	194.8627958	186.4457703	-649.6822605	4.88645276
603102877.4	579.1387512	142.8910926	141.0794904	-612.5747513	4.458495473
14150344724	1357.789355	238.8962518	257.7008945	-1201.356659	5.11344569
927842692.5	427.2021873	128.5974989	156.7357465	-449.8459954	4.384041368
335222429.2	439.9030863	158.2136006	202.6254664	-482.8615926	4.647669386
741941405.1	591.3594771	221.9145728	203.3255011	-608.9732422	5.070410614
1435282168	537.8054568	121.5672363	157.1386057	-583.3937981	4.149819432
905659494.6	829.4827416	165.2728083	191.8088447	-833.7042574	4.404578183
1413292910	777.4708705	143.1046623	193.3018003	-560.5633429	4.49874128
2430787795	872.8909057	211.954569	228.1977936	-1201.385463	5.026450698
1879868239	917.4164164	175.7928582	206.8327366	-806.8134245	4.755176078
141765698.2	451.8696209	189.6388315	257.6168603	-558.6474415	4.754028715
897922450.9	528.600803	143.7224444	189.3582876	-763.9176229	4.460847414
2103706522	506.9753192	139.4095953	177.1424673	-543.426042	4.494276826
813645205.6	541.6643955	195.5566298	234.0896571	-698.5617748	4.949677961
1948893946	770.9460576	175.8202036	218.5209348	-692.0213869	4.733004256
1402731344	660.150029	146.6718605	161.4708265	-548.868974	4.321250973
3343078169	672.6998658	155.3122644	205.7396763	-599.5107738	4.65499535
2637852062	788.8595201	224.467997	269.7272285	-902.1115943	5.14663673
2481828863	764.457177	246.5631319	276.098943	-896.1995127	5.280531298
4830320094	977.914995	175.3102358	211.8630324	-1125.300857	4.68871398
415483724.8	446.1009659	165.7681584	205.130727	-489.9079811	4.721579269
3779660778	861.216231	121.729986	147.1193795	-935.367018	4.105748258
882452784.1	450.8098924	156.1749795	181.5181545	-522.5000283	4.653025033
2952262198	767.3355558	138.8441128	177.0213443	-741.637734	4.443431033
1354919564	736.1738169	144.0611	158.7069633	-615.397048	4.487130067
1594039964	695.1672263	203.9029685	247.0190654	-992.5142092	5.018094128
880796437.2	714.5062402	144.7941768	184.6699112	-729.2095836	4.473596449
1605300760	755.8494132	157.3621366	182.9136801	-592.8193018	4.672655782
5626697716	1427.242645	197.3455939	196.9408379	-1366.572632	4.752881513
586667718	790.459857	210.3649917	190.0126469	-1127.12305	4.790783423
267678337.8	467.869665	159.2342008	212.8656726	-613.6103492	4.591728941
435831336.7	529.3839297	188.9766418	221.6071703	-567.1116624	4.866281413
1552303654	756.3079081	211.3572669	282.1448668	-731.3564864	5.093869517
4084202528	873.2084615	233.0553908	301.4658825	-944.4407469	5.24147793
3025180223	1018.422845	118.5226004	134.5589107	-657.2795865	3.906166888
600439233.1	452.6419651	116.3767092	116.5747146	-580.9136646	4.155631241
431969656.1	593.6837571	157.7390612	181.2317313	-529.3427293	4.630442354
1324739455	759.7351028	204.2582904	233.3621438	-682.5401881	5.0267674
3157972905	716.9915046	170.6787851	189.1047962	-1059.567465	4.72962951
1987245096	684.2494171	188.7007371	221.4401142	-738.6246159	4.930742201
680953095.7	506.1152419	181.8086107	235.0518676	-618.9853856	4.850751358
4731835730	960.8755173	126.8408652	113.5418247	-1000.664292	4.219756007
1388552717	639.904638	196.7146995	248.9109429	-595.401248	4.968668716
2920974019	813.9427703	209.4949233	255.3190983	-1001.55712	5.06127998
9345454521	876.7272536	205.0278704	257.692297	-899.1992104	5.069776283
1263049018	527.6034732	149.3938025	169.6706339	-590.3427774	4.590139221
493163013.1	516.9836047	164.255164	147.402059	-531.7107261	4.613058632
4070036870	769.8319173	209.913685	261.4277711	-986.0148525	5.092496858
449986002.1	578.6110782	202.6070733	260.6505846	-551.3603325	4.984602669
4045808439	781.2072891	171.6624463	215.0790802	-685.2222999	4.801385658
5015642177	863.3211833	157.2965723	164.8505779	-711.0740718	4.577876648
1628701908	588.093903	168.5239634	201.2415984	-822.4418693	4.767694496
366639677.2	428.5504013	166.1327666	178.4108759	-525.1911315	4.705049105
3968478776	721.8540487	171.7797151	191.595394	-707.9144537	4.760754788
1124253283	682.939671	176.6076726	255.5138707	-699.5724028	4.776771144
750096095.5	547.9957593	171.0073463	204.1854916	-578.945126	4.757350902
3394749965	891.7556831	175.4604131	232.9095623	-866.0651259	4.786131516
774117393.2	710.553104	176.7258951	199.0141151	-565.0580285	4.808183843
2527844329	709.6613969	205.8711179	231.593362	-962.5937279	5.047382185
439481485.9	600.4077285	224.484908	232.1088666	-605.2314591	5.077198856
1180962072	669.9703724	182.3783224	245.0870489	-565.5242958	4.881183131
723879597.5	489.1844521	122.0436067	154.2410351	-536.9180839	4.261665617
2273386402	1215.984872	244.715843	253.1874156	-930.5414487	5.220335702
515139911	503.6541468	151.9814885	168.4097369	-776.648046	4.557648726
1382245853	578.4757904	149.7796963	217.5015469	-504.2143846	4.431511225
502181475.7	367.7829149	99.12270729	104.1391403	-390.9814752	3.985356629
1718714077	583.6509828	153.1210921	213.1294258	-502.3009021	4.619104055
566642398.9	483.3725046	198.8041249	220.7374394	-761.4833041	4.844890422
831129473.5	779.2156393	153.4082048	188.7856226	-446.5503259	4.534700081
1394508867	683.2663433	116.0351208	141.2393187	-725.6579494	4.115198144
1989499843	1080.0844	208.1315487	251.0658849	-762.7630361	5.017567016
993139300.1	499.521876	164.0359147	209.9875865	-540.3912587	4.737275536
754493673.5	543.3101384	186.4308994	240.7984376	-620.5843321	4.868103019
648853793.7	414.7273457	97.42259452	110.8967878	-408.3711299	3.882521792
3650158838	742.1725186	173.3083522	201.8158271	-726.3735541	4.820851014
1213774339	534.9380755	114.2166603	149.3405599	-506.1854374	4.176262318
1522135881	729.7115169	130.5652884	165.5601775	-456.12939	4.409393261
3827458535	1021.668971	243.8485849	299.5708264	-742.9426231	5.29548054
3571439949	769.1924613	232.2627486	304.2052435	-788.156673	5.231356403
524713563.5	508.900346	249.1765075	285.470696	-722.7977534	5.184866481
128533172.8	402.2911212	133.2779595	176.5127834	-426.5230219	4.332994409
5886294880	690.769879	157.1832903	190.6780796	-665.0562343	4.637442135

wavelet.LHL_firstorder_StandardDeviation	wavelet.LHL_firstorder_Range	wavelet.LHL_firstorder_Variance	wavelet.LHL_firstorder_10Percentile	wavelet.LHL_firstorder_Kurtosis	wavelet.LHL_firstorder_Mean
246.1673527	2146.52632	60598.36553	-325.8208694	4.788607351	-43.56437271
220.9281419	1143.581228	48809.24388	-349.5515847	2.633235159	-33.81377594
207.4576386	1415.744654	43038.67179	-281.3703676	3.346246405	-17.39949886
200.5952515	1099.866537	40238.45492	-279.1151589	3.053877942	-3.269094992
179.4770905	1071.3115	32212.02602	-246.5772687	2.807458642	-16.05492163
244.0985786	1144.742191	59584.11606	-406.4998382	2.588999936	-41.51640433
161.8119963	1750.269517	26183.12214	-164.8935963	6.016103775	13.32396097
194.8413534	1654.068883	37963.15299	-255.0223557	3.801408517	-8.718839129
140.0528739	1369.924681	19614.80749	-178.9150149	5.087819793	-10.49553825
180.4593655	1014.804544	32565.58259	-217.010496	2.936403397	22.52281072
86.07640434	718.1916482	7409.147384	-97.10647516	4.334226311	6.97227684
179.9472692	1687.247787	32381.01968	-206.6617536	5.608932307	-1.930110506
206.486093	1375.649609	42636.50659	-292.3755841	4.341648272	-14.94080218
171.3432827	993.9854228	29358.52053	-227.7474564	2.945756666	-9.965911003
272.68139	2057.323777	74355.14046	-364.419255	4.216106448	-18.07686329
160.3900825	1418.572419	25724.97858	-101.8510862	7.683389609	16.98902023
135.0200202	954.7634779	18230.40586	-156.0587527	3.860581697	-2.65190758
190.6529595	1119.031386	36348.55095	-283.2634104	3.272549098	-40.28595597
141.2858606	1191.713502	19961.69439	-175.8303468	4.882615901	-21.35813582
238.8745557	2559.146014	57061.05338	-263.7870202	6.65164664	-3.219584521
128.0843775	877.0481827	16405.60775	-170.0177397	3.266230754	-11.47645331
158.2062335	922.7646789	25029.2123	-199.5495176	3.11407069	-1.526793671
214.5935027	1200.332719	46050.37138	-357.0746662	3.00657632	-56.53057773
121.5337502	1121.199255	14770.45243	-135.4079978	5.05493745	2.853158585
165.2334046	1663.186999	27302.07799	-191.422991	6.433717324	-3.608764847
142.3060446	1338.034213	20251.01034	-152.3746051	4.327635193	15.09748471
210.4803911	2074.276369	44301.99502	-258.4133747	5.114578166	-24.95484529
175.5417107	1724.229841	30814.89221	-218.1572143	5.112961414	-9.393442949
189.1414666	1010.517062	35774.49438	-189.8475559	2.902033129	13.72559741
143.6603988	1292.518426	20638.31018	-162.5442651	4.921608776	4.222660198
139.385046	1050.401361	19428.19105	-176.5828	3.619900065	-2.616145162
195.0706582	1240.22617	38052.56168	-277.7686197	2.976053641	-13.77801763
175.7179157	1462.967445	30876.78591	-191.7771757	4.876787346	5.996507211
146.599051	1209.019003	21491.28176	-172.9037974	5.539141545	-4.620921291
155.3117008	1272.21064	24121.7244	-185.6835437	3.577684205	0.418404352
224.4416653	1690.971114	50374.06112	-278.0181235	3.981211306	-3.438101046
245.8794335	1660.65669	60456.6958	-361.7033689	3.190914571	-18.34890174
175.248009	2103.215852	30711.86466	-186.5541043	6.074233408	4.670559961
165.6658424	936.0089471	27445.17133	-231.8283359	2.967931151	-5.823314678
121.7294824	1796.583249	14818.06688	-135.5850735	6.638984394	-0.350172753
154.2394298	973.3099207	23789.80169	-222.4587746	3.100500064	-24.51168131
138.7754075	1508.97329	19258.61373	-156.615868	4.775057275	4.367370016
143.8024788	1351.570865	20679.1529	-171.5815394	5.126371416	-8.628303413
203.5798777	1687.681436	41444.76662	-266.8377889	3.496437806	-11.47405431
144.7326798	1443.715824	20947.54859	-164.7332566	5.140081235	4.219603204
157.2893498	1348.668715	24739.93956	-201.1403752	3.794023438	-4.78565221
197.2553521	2793.815278	38909.67392	-196.410457	9.990796287	-5.967371435
209.0229771	1917.582907	43690.60498	-218.9584414	8.012627626	-23.72392799
157.7283386	1081.480014	24878.22881	-161.9190634	3.75005207	21.84724027
188.9739865	1096.495592	35711.16758	-256.4283859	3.396887886	-1.001781187
211.1499315	1487.664395	44584.29358	-256.63576	3.135993365	9.359523663
232.9362451	1817.649208	54259.29428	-275.2520255	3.402673127	7.451235383
[truncated: 1,205,139 more chars]
