# Supplementary material for: Peritumoral radiomics features predict distant metastasis in locally advanced NSCLC
Source: PLoS One. 2018 Nov 2;13(11):e0206108. doi: 10.1371/journal.pone.0206108 (PMC6214508; doi:10.1371/journal.pone.0206108)
Supplement: S9 File — (PDF) [file pone.0206108.s009.pdf]

X	general_info_BoundingBox
1	(6, 6, 5, 15, 16, 17)
2	(6, 5, 6, 19, 25, 21)
3	(6, 5, 5, 12, 17, 11)
4	(6, 6, 5, 18, 23, 25)
5	(6, 6, 6, 13, 13, 11)
6	(6, 6, 6, 15, 15, 13)
8	(5, 6, 6, 16, 14, 9)
9	(6, 6, 6, 19, 18, 15)
10	(6, 6, 6, 23, 22, 19)
11	(5, 6, 6, 25, 22, 28)
12	(5, 6, 5, 15, 17, 14)
13	(6, 5, 6, 17, 20, 23)
14	(6, 5, 6, 23, 22, 20)
15	(5, 5, 6, 18, 20, 16)
16	(5, 6, 6, 19, 16, 17)
17	(6, 6, 6, 32, 26, 40)
18	(6, 6, 6, 18, 13, 14)
19	(6, 6, 5, 25, 20, 25)
20	(6, 6, 6, 13, 15, 15)
21	(6, 5, 6, 25, 28, 28)
22	(6, 6, 6, 28, 28, 38)
23	(6, 6, 6, 19, 14, 25)
24	(5, 5, 6, 24, 32, 35)
25	(5, 6, 6, 15, 13, 13)
26	(6, 6, 5, 21, 26, 20)
27	(6, 6, 6, 18, 22, 14)
29	(6, 6, 5, 22, 31, 24)
30	(5, 5, 6, 20, 36, 34)
31	(5, 5, 5, 17, 29, 32)
32	(6, 6, 6, 22, 24, 24)
33	(5, 6, 6, 19, 32, 31)
34	(6, 6, 6, 37, 34, 36)
35	(6, 5, 6, 19, 21, 22)
36	(6, 5, 6, 9, 9, 9)
37	(6, 6, 5, 12, 13, 14)
38	(6, 6, 6, 22, 21, 19)
39	(5, 5, 6, 26, 36, 34)
40	(5, 6, 6, 26, 29, 22)
41	(6, 6, 5, 15, 18, 14)
42	(6, 5, 6, 17, 17, 17)
43	(6, 5, 6, 12, 23, 20)
44	(6, 6, 6, 20, 17, 16)
45	(5, 6, 6, 29, 18, 18)
46	(5, 5, 6, 18, 18, 19)
48	(5, 6, 5, 24, 21, 18)
49	(7, 6, 4, 26, 38, 30)
50	(6, 5, 0, 24, 22, 31)
51	(6, 5, 5, 14, 14, 13)
52	(6, 6, 5, 9, 10, 10)
53	(6, 5, 6, 12, 16, 13)
54	(6, 6, 5, 30, 29, 29)
55	(5, 5, 6, 16, 16, 15)
56	(6, 6, 6, 22, 26, 34)
57	(6, 6, 5, 15, 15, 15)
58	(6, 6, 6, 29, 42, 36)
59	(6, 6, 5, 16, 17, 15)
60	(6, 6, 6, 27, 24, 25)
61	(6, 6, 5, 21, 17, 19)
62	(6, 6, 5, 16, 17, 16)
63	(6, 6, 6, 20, 13, 13)
64	(6, 5, 5, 21, 23, 20)
65	(5, 6, 6, 26, 31, 37)
66	(5, 6, 5, 24, 23, 38)
67	(5, 6, 6, 26, 30, 23)
68	(6, 6, 6, 12, 11, 10)
69	(5, 5, 5, 26, 20, 17)
70	(6, 6, 6, 14, 12, 12)
71	(6, 6, 6, 27, 25, 25)
72	(6, 6, 6, 14, 13, 13)
73	(6, 6, 6, 20, 26, 24)
74	(6, 6, 5, 27, 29, 26)
75	(5, 6, 6, 18, 18, 16)
76	(6, 6, 6, 27, 35, 33)
77	(7, 5, 6, 35, 43, 41)
78	(6, 6, 5, 35, 43, 26)
79	(5, 6, 6, 19, 21, 16)
80	(6, 6, 6, 19, 21, 14)
81	(6, 5, 6, 14, 14, 15)
82	(6, 5, 5, 25, 23, 16)
83	(5, 5, 5, 22, 33, 26)
84	(6, 6, 6, 16, 15, 10)
85	(6, 6, 6, 22, 27, 15)
86	(5, 6, 5, 18, 28, 28)
87	(6, 5, 6, 10, 11, 9)
88	(6, 5, 6, 26, 31, 30)
89	(6, 6, 6, 33, 27, 26)
90	(6, 6, 6, 16, 15, 15)
91	(5, 6, 6, 20, 30, 17)
92	(6, 6, 6, 10, 13, 9)
93	(6, 6, 6, 11, 10, 7)
94	(6, 6, 5, 27, 35, 25)
95	(6, 6, 6, 15, 16, 18)
96	(6, 5, 6, 16, 21, 15)
107	(6, 6, 6, 28, 24, 27)
108	(5, 6, 5, 30, 32, 32)
112	(6, 6, 6, 11, 11, 11)
114	(6, 6, 6, 20, 16, 17)
126	(6, 6, 5, 9, 10, 9)
135	(6, 6, 6, 13, 13, 11)
139	(6, 5, 5, 13, 19, 16)

general_info_GeneralSettings
{'distances': [1], 'verbose': True, 'additionalInfo': True, 'enableCExtensions': True, 'force2D': False, 'interpolator': 3, 'resampledPixelSpacing': [3, 3, 3], 'label': 1, 'normalizeScale': 1, 'normalize': False, 'force2Ddimension': 0, 'removeOutliers': None, 'minimumROISi
{'distances': [1], 'verbose': True, 'additionalInfo': True, 'enableCExtensions': True, 'force2D': False, 'interpolator': 3, 'resampledPixelSpacing': [3, 3, 3], 'label': 1, 'normalizeScale': 1, 'normalize': False, 'force2Ddimension': 0, 'removeOutliers': None, 'minimumROISi
{'distances': [1], 'verbose': True, 'additionalInfo': True, 'enableCExtensions': True, 'force2D': False, 'interpolator': 3, 'resampledPixelSpacing': [3, 3, 3], 'label': 1, 'normalizeScale': 1, 'normalize': False, 'force2Ddimension': 0, 'removeOutliers': None, 'minimumROISi
{'distances': [1], 'verbose': True, 'additionalInfo': True, 'enableCExtensions': True, 'force2D': False, 'interpolator': 3, 'resampledPixelSpacing': [3, 3, 3], 'label': 1, 'normalizeScale': 1, 'normalize': False, 'force2Ddimension': 0, 'removeOutliers': None, 'minimumROISi
{'distances': [1], 'verbose': True, 'additionalInfo': True, 'enableCExtensions': True, 'force2D': False, 'interpolator': 3, 'resampledPixelSpacing': [3, 3, 3], 'label': 1, 'normalizeScale': 1, 'normalize': False, 'force2Ddimension': 0, 'removeOutliers': None, 'minimumROISi
{'distances': [1], 'verbose': True, 'additionalInfo': True, 'enableCExtensions': True, 'force2D': False, 'interpolator': 3, 'resampledPixelSpacing': [3, 3, 3], 'label': 1, 'normalizeScale': 1, 'normalize': False, 'force2Ddimension': 0, 'removeOutliers': None, 'minimumROISi
{'distances': [1], 'verbose': True, 'additionalInfo': True, 'enableCExtensions': True, 'force2D': False, 'interpolator': 3, 'resampledPixelSpacing': [3, 3, 3], 'label': 1, 'normalizeScale': 1, 'normalize': False, 'force2Ddimension': 0, 'removeOutliers': None, 'minimumROISi
{'distances': [1], 'verbose': True, 'additionalInfo': True, 'enableCExtensions': True, 'force2D': False, 'interpolator': 3, 'resampledPixelSpacing': [3, 3, 3], 'label': 1, 'normalizeScale': 1, 'normalize': False, 'force2Ddimension': 0, 'removeOutliers': None, 'minimumROISi
{'distances': [1], 'verbose': True, 'additionalInfo': True, 'enableCExtensions': True, 'force2D': False, 'interpolator': 3, 'resampledPixelSpacing': [3, 3, 3], 'label': 1, 'normalizeScale': 1, 'normalize': False, 'force2Ddimension': 0, 'removeOutliers': None, 'minimumROISi
{'distances': [1], 'verbose': True, 'additionalInfo': True, 'enableCExtensions': True, 'force2D': False, 'interpolator': 3, 'resampledPixelSpacing': [3, 3, 3], 'label': 1, 'normalizeScale': 1, 'normalize': False, 'force2Ddimension': 0, 'removeOutliers': None, 'minimumROISi
{'distances': [1], 'verbose': True, 'additionalInfo': True, 'enableCExtensions': True, 'force2D': False, 'interpolator': 3, 'resampledPixelSpacing': [3, 3, 3], 'label': 1, 'normalizeScale': 1, 'normalize': False, 'force2Ddimension': 0, 'removeOutliers': None, 'minimumROISi
{'distances': [1], 'verbose': True, 'additionalInfo': True, 'enableCExtensions': True, 'force2D': False, 'interpolator': 3, 'resampledPixelSpacing': [3, 3, 3], 'label': 1, 'normalizeScale': 1, 'normalize': False, 'force2Ddimension': 0, 'removeOutliers': None, 'minimumROISi
{'distances': [1], 'verbose': True, 'additionalInfo': True, 'enableCExtensions': True, 'force2D': False, 'interpolator': 3, 'resampledPixelSpacing': [3, 3, 3], 'label': 1, 'normalizeScale': 1, 'normalize': False, 'force2Ddimension': 0, 'removeOutliers': None, 'minimumROISi
{'distances': [1], 'verbose': True, 'additionalInfo': True, 'enableCExtensions': True, 'force2D': False, 'interpolator': 3, 'resampledPixelSpacing': [3, 3, 3], 'label': 1, 'normalizeScale': 1, 'normalize': False, 'force2Ddimension': 0, 'removeOutliers': None, 'minimumROISi
{'distances': [1], 'verbose': True, 'additionalInfo': True, 'enableCExtensions': True, 'force2D': False, 'interpolator': 3, 'resampledPixelSpacing': [3, 3, 3], 'label': 1, 'normalizeScale': 1, 'normalize': False, 'force2Ddimension': 0, 'removeOutliers': None, 'minimumROISi
{'distances': [1], 'verbose': True, 'additionalInfo': True, 'enableCExtensions': True, 'force2D': False, 'interpolator': 3, 'resampledPixelSpacing': [3, 3, 3], 'label': 1, 'normalizeScale': 1, 'normalize': False, 'force2Ddimension': 0, 'removeOutliers': None, 'minimumROISi
{'distances': [1], 'verbose': True, 'additionalInfo': True, 'enableCExtensions': True, 'force2D': False, 'interpolator': 3, 'resampledPixelSpacing': [3, 3, 3], 'label': 1, 'normalizeScale': 1, 'normalize': False, 'force2Ddimension': 0, 'removeOutliers': None, 'minimumROISi
{'distances': [1], 'verbose': True, 'additionalInfo': True, 'enableCExtensions': True, 'force2D': False, 'interpolator': 3, 'resampledPixelSpacing': [3, 3, 3], 'label': 1, 'normalizeScale': 1, 'normalize': False, 'force2Ddimension': 0, 'removeOutliers': None, 'minimumROISi
{'distances': [1], 'verbose': True, 'additionalInfo': True, 'enableCExtensions': True, 'force2D': False, 'interpolator': 3, 'resampledPixelSpacing': [3, 3, 3], 'label': 1, 'normalizeScale': 1, 'normalize': False, 'force2Ddimension': 0, 'removeOutliers': None, 'minimumROISi
{'distances': [1], 'verbose': True, 'additionalInfo': True, 'enableCExtensions': True, 'force2D': False, 'interpolator': 3, 'resampledPixelSpacing': [3, 3, 3], 'label': 1, 'normalizeScale': 1, 'normalize': False, 'force2Ddimension': 0, 'removeOutliers': None, 'minimumROISi
{'distances': [1], 'verbose': True, 'additionalInfo': True, 'enableCExtensions': True, 'force2D': False, 'interpolator': 3, 'resampledPixelSpacing': [3, 3, 3], 'label': 1, 'normalizeScale': 1, 'normalize': False, 'force2Ddimension': 0, 'removeOutliers': None, 'minimumROISi
{'distances': [1], 'verbose': True, 'additionalInfo': True, 'enableCExtensions': True, 'force2D': False, 'interpolator': 3, 'resampledPixelSpacing': [3, 3, 3], 'label': 1, 'normalizeScale': 1, 'normalize': False, 'force2Ddimension': 0, 'removeOutliers': None, 'minimumROISi
{'distances': [1], 'verbose': True, 'additionalInfo': True, 'enableCExtensions': True, 'force2D': False, 'interpolator': 3, 'resampledPixelSpacing': [3, 3, 3], 'label': 1, 'normalizeScale': 1, 'normalize': False, 'force2Ddimension': 0, 'removeOutliers': None, 'minimumROISi
{'distances': [1], 'verbose': True, 'additionalInfo': True, 'enableCExtensions': True, 'force2D': False, 'interpolator': 3, 'resampledPixelSpacing': [3, 3, 3], 'label': 1, 'normalizeScale': 1, 'normalize': False, 'force2Ddimension': 0, 'removeOutliers': None, 'minimumROISi
{'distances': [1], 'verbose': True, 'additionalInfo': True, 'enableCExtensions': True, 'force2D': False, 'interpolator': 3, 'resampledPixelSpacing': [3, 3, 3], 'label': 1, 'normalizeScale': 1, 'normalize': False, 'force2Ddimension': 0, 'removeOutliers': None, 'minimumROISi
{'distances': [1], 'verbose': True, 'additionalInfo': True, 'enableCExtensions': True, 'force2D': False, 'interpolator': 3, 'resampledPixelSpacing': [3, 3, 3], 'label': 1, 'normalizeScale': 1, 'normalize': False, 'force2Ddimension': 0, 'removeOutliers': None, 'minimumROISi
{'distances': [1], 'verbose': True, 'additionalInfo': True, 'enableCExtensions': True, 'force2D': False, 'interpolator': 3, 'resampledPixelSpacing': [3, 3, 3], 'label': 1, 'normalizeScale': 1, 'normalize': False, 'force2Ddimension': 0, 'removeOutliers': None, 'minimumROISi
{'distances': [1], 'verbose': True, 'additionalInfo': True, 'enableCExtensions': True, 'force2D': False, 'interpolator': 3, 'resampledPixelSpacing': [3, 3, 3], 'label': 1, 'normalizeScale': 1, 'normalize': False, 'force2Ddimension': 0, 'removeOutliers': None, 'minimumROISi
{'distances': [1], 'verbose': True, 'additionalInfo': True, 'enableCExtensions': True, 'force2D': False, 'interpolator': 3, 'resampledPixelSpacing': [3, 3, 3], 'label': 1, 'normalizeScale': 1, 'normalize': False, 'force2Ddimension': 0, 'removeOutliers': None, 'minimumROISi
{'distances': [1], 'verbose': True, 'additionalInfo': True, 'enableCExtensions': True, 'force2D': False, 'interpolator': 3, 'resampledPixelSpacing': [3, 3, 3], 'label': 1, 'normalizeScale': 1, 'normalize': False, 'force2Ddimension': 0, 'removeOutliers': None, 'minimumROISi
{'distances': [1], 'verbose': True, 'additionalInfo': True, 'enableCExtensions': True, 'force2D': False, 'interpolator': 3, 'resampledPixelSpacing': [3, 3, 3], 'label': 1, 'normalizeScale': 1, 'normalize': False, 'force2Ddimension': 0, 'removeOutliers': None, 'minimumROISi
{'distances': [1], 'verbose': True, 'additionalInfo': True, 'enableCExtensions': True, 'force2D': False, 'interpolator': 3, 'resampledPixelSpacing': [3, 3, 3], 'label': 1, 'normalizeScale': 1, 'normalize': False, 'force2Ddimension': 0, 'removeOutliers': None, 'minimumROISi
{'distances': [1], 'verbose': True, 'additionalInfo': True, 'enableCExtensions': True, 'force2D': False, 'interpolator': 3, 'resampledPixelSpacing': [3, 3, 3], 'label': 1, 'normalizeScale': 1, 'normalize': False, 'force2Ddimension': 0, 'removeOutliers': None, 'minimumROISi
{'distances': [1], 'verbose': True, 'additionalInfo': True, 'enableCExtensions': True, 'force2D': False, 'interpolator': 3, 'resampledPixelSpacing': [3, 3, 3], 'label': 1, 'normalizeScale': 1, 'normalize': False, 'force2Ddimension': 0, 'removeOutliers': None, 'minimumROISi
{'distances': [1], 'verbose': True, 'additionalInfo': True, 'enableCExtensions': True, 'force2D': False, 'interpolator': 3, 'resampledPixelSpacing': [3, 3, 3], 'label': 1, 'normalizeScale': 1, 'normalize': False, 'force2Ddimension': 0, 'removeOutliers': None, 'minimumROISi
{'distances': [1], 'verbose': True, 'additionalInfo': True, 'enableCExtensions': True, 'force2D': False, 'interpolator': 3, 'resampledPixelSpacing': [3, 3, 3], 'label': 1, 'normalizeScale': 1, 'normalize': False, 'force2Ddimension': 0, 'removeOutliers': None, 'minimumROISi
{'distances': [1], 'verbose': True, 'additionalInfo': True, 'enableCExtensions': True, 'force2D': False, 'interpolator': 3, 'resampledPixelSpacing': [3, 3, 3], 'label': 1, 'normalizeScale': 1, 'normalize': False, 'force2Ddimension': 0, 'removeOutliers': None, 'minimumROISi
{'distances': [1], 'verbose': True, 'additionalInfo': True, 'enableCExtensions': True, 'force2D': False, 'interpolator': 3, 'resampledPixelSpacing': [3, 3, 3], 'label': 1, 'normalizeScale': 1, 'normalize': False, 'force2Ddimension': 0, 'removeOutliers': None, 'minimumROISi
{'distances': [1], 'verbose': True, 'additionalInfo': True, 'enableCExtensions': True, 'force2D': False, 'interpolator': 3, 'resampledPixelSpacing': [3, 3, 3], 'label': 1, 'normalizeScale': 1, 'normalize': False, 'force2Ddimension': 0, 'removeOutliers': None, 'minimumROISi
{'distances': [1], 'verbose': True, 'additionalInfo': True, 'enableCExtensions': True, 'force2D': False, 'interpolator': 3, 'resampledPixelSpacing': [3, 3, 3], 'label': 1, 'normalizeScale': 1, 'normalize': False, 'force2Ddimension': 0, 'removeOutliers': None, 'minimumROISi
{'distances': [1], 'verbose': True, 'additionalInfo': True, 'enableCExtensions': True, 'force2D': False, 'interpolator': 3, 'resampledPixelSpacing': [3, 3, 3], 'label': 1, 'normalizeScale': 1, 'normalize': False, 'force2Ddimension': 0, 'removeOutliers': None, 'minimumROISi
{'distances': [1], 'verbose': True, 'additionalInfo': True, 'enableCExtensions': True, 'force2D': False, 'interpolator': 3, 'resampledPixelSpacing': [3, 3, 3], 'label': 1, 'normalizeScale': 1, 'normalize': False, 'force2Ddimension': 0, 'removeOutliers': None, 'minimumROISi
{'distances': [1], 'verbose': True, 'additionalInfo': True, 'enableCExtensions': True, 'force2D': False, 'interpolator': 3, 'resampledPixelSpacing': [3, 3, 3], 'label': 1, 'normalizeScale': 1, 'normalize': False, 'force2Ddimension': 0, 'removeOutliers': None, 'minimumROISi
{'distances': [1], 'verbose': True, 'additionalInfo': True, 'enableCExtensions': True, 'force2D': False, 'interpolator': 3, 'resampledPixelSpacing': [3, 3, 3], 'label': 1, 'normalizeScale': 1, 'normalize': False, 'force2Ddimension': 0, 'removeOutliers': None, 'minimumROISi
{'distances': [1], 'verbose': True, 'additionalInfo': True, 'enableCExtensions': True, 'force2D': False, 'interpolator': 3, 'resampledPixelSpacing': [3, 3, 3], 'label': 1, 'normalizeScale': 1, 'normalize': False, 'force2Ddimension': 0, 'removeOutliers': None, 'minimumROISi
{'distances': [1], 'verbose': True, 'additionalInfo': True, 'enableCExtensions': True, 'force2D': False, 'interpolator': 3, 'resampledPixelSpacing': [3, 3, 3], 'label': 1, 'normalizeScale': 1, 'normalize': False, 'force2Ddimension': 0, 'removeOutliers': None, 'minimumROISi
{'distances': [1], 'verbose': True, 'additionalInfo': True, 'enableCExtensions': True, 'force2D': False, 'interpolator': 3, 'resampledPixelSpacing': [3, 3, 3], 'label': 1, 'normalizeScale': 1, 'normalize': False, 'force2Ddimension': 0, 'removeOutliers': None, 'minimumROISi
{'distances': [1], 'verbose': True, 'additionalInfo': True, 'enableCExtensions': True, 'force2D': False, 'interpolator': 3, 'resampledPixelSpacing': [3, 3, 3], 'label': 1, 'normalizeScale': 1, 'normalize': False, 'force2Ddimension': 0, 'removeOutliers': None, 'minimumROISi
{'distances': [1], 'verbose': True, 'additionalInfo': True, 'enableCExtensions': True, 'force2D': False, 'interpolator': 3, 'resampledPixelSpacing': [3, 3, 3], 'label': 1, 'normalizeScale': 1, 'normalize': False, 'force2Ddimension': 0, 'removeOutliers': None, 'minimumROISi
{'distances': [1], 'verbose': True, 'additionalInfo': True, 'enableCExtensions': True, 'force2D': False, 'interpolator': 3, 'resampledPixelSpacing': [3, 3, 3], 'label': 1, 'normalizeScale': 1, 'normalize': False, 'force2Ddimension': 0, 'removeOutliers': None, 'minimumROISi
{'distances': [1], 'verbose': True, 'additionalInfo': True, 'enableCExtensions': True, 'force2D': False, 'interpolator': 3, 'resampledPixelSpacing': [3, 3, 3], 'label': 1, 'normalizeScale': 1, 'normalize': False, 'force2Ddimension': 0, 'removeOutliers': None, 'minimumROISi
{'distances': [1], 'verbose': True, 'additionalInfo': True, 'enableCExtensions': True, 'force2D': False, 'interpolator': 3, 'resampledPixelSpacing': [3, 3, 3], 'label': 1, 'normalizeScale': 1, 'normalize': False, 'force2Ddimension': 0, 'removeOutliers': None, 'minimumROISi
{'distances': [1], 'verbose': True, 'additionalInfo': True, 'enableCExtensions': True, 'force2D': False, 'interpolator': 3, 'resampledPixelSpacing': [3, 3, 3], 'label': 1, 'normalizeScale': 1, 'normalize': False, 'force2Ddimension': 0, 'removeOutliers': None, 'minimumROISi
{'distances': [1], 'verbose': True, 'additionalInfo': True, 'enableCExtensions': True, 'force2D': False, 'interpolator': 3, 'resampledPixelSpacing': [3, 3, 3], 'label': 1, 'normalizeScale': 1, 'normalize': False, 'force2Ddimension': 0, 'removeOutliers': None, 'minimumROISi
{'distances': [1], 'verbose': True, 'additionalInfo': True, 'enableCExtensions': True, 'force2D': False, 'interpolator': 3, 'resampledPixelSpacing': [3, 3, 3], 'label': 1, 'normalizeScale': 1, 'normalize': False, 'force2Ddimension': 0, 'removeOutliers': None, 'minimumROISi
{'distances': [1], 'verbose': True, 'additionalInfo': True, 'enableCExtensions': True, 'force2D': False, 'interpolator': 3, 'resampledPixelSpacing': [3, 3, 3], 'label': 1, 'normalizeScale': 1, 'normalize': False, 'force2Ddimension': 0, 'removeOutliers': None, 'minimumROISi
{'distances': [1], 'verbose': True, 'additionalInfo': True, 'enableCExtensions': True, 'force2D': False, 'interpolator': 3, 'resampledPixelSpacing': [3, 3, 3], 'label': 1, 'normalizeScale': 1, 'normalize': False, 'force2Ddimension': 0, 'removeOutliers': None, 'minimumROISi
{'distances': [1], 'verbose': True, 'additionalInfo': True, 'enableCExtensions': True, 'force2D': False, 'interpolator': 3, 'resampledPixelSpacing': [3, 3, 3], 'label': 1, 'normalizeScale': 1, 'normalize': False, 'force2Ddimension': 0, 'removeOutliers': None, 'minimumROISi
{'distances': [1], 'verbose': True, 'additionalInfo': True, 'enableCExtensions': True, 'force2D': False, 'interpolator': 3, 'resampledPixelSpacing': [3, 3, 3], 'label': 1, 'normalizeScale': 1, 'normalize': False, 'force2Ddimension': 0, 'removeOutliers': None, 'minimumROISi
{'distances': [1], 'verbose': True, 'additionalInfo': True, 'enableCExtensions': True, 'force2D': False, 'interpolator': 3, 'resampledPixelSpacing': [3, 3, 3], 'label': 1, 'normalizeScale': 1, 'normalize': False, 'force2Ddimension': 0, 'removeOutliers': None, 'minimumROISi
{'distances': [1], 'verbose': True, 'additionalInfo': True, 'enableCExtensions': True, 'force2D': False, 'interpolator': 3, 'resampledPixelSpacing': [3, 3, 3], 'label': 1, 'normalizeScale': 1, 'normalize': False, 'force2Ddimension': 0, 'removeOutliers': None, 'minimumROISi
{'distances': [1], 'verbose': True, 'additionalInfo': True, 'enableCExtensions': True, 'force2D': False, 'interpolator': 3, 'resampledPixelSpacing': [3, 3, 3], 'label': 1, 'normalizeScale': 1, 'normalize': False, 'force2Ddimension': 0, 'removeOutliers': None, 'minimumROISi
{'distances': [1], 'verbose': True, 'additionalInfo': True, 'enableCExtensions': True, 'force2D': False, 'interpolator': 3, 'resampledPixelSpacing': [3, 3, 3], 'label': 1, 'normalizeScale': 1, 'normalize': False, 'force2Ddimension': 0, 'removeOutliers': None, 'minimumROISi
{'distances': [1], 'verbose': True, 'additionalInfo': True, 'enableCExtensions': True, 'force2D': False, 'interpolator': 3, 'resampledPixelSpacing': [3, 3, 3], 'label': 1, 'normalizeScale': 1, 'normalize': False, 'force2Ddimension': 0, 'removeOutliers': None, 'minimumROISi
{'distances': [1], 'verbose': True, 'additionalInfo': True, 'enableCExtensions': True, 'force2D': False, 'interpolator': 3, 'resampledPixelSpacing': [3, 3, 3], 'label': 1, 'normalizeScale': 1, 'normalize': False, 'force2Ddimension': 0, 'removeOutliers': None, 'minimumROISi
{'distances': [1], 'verbose': True, 'additionalInfo': True, 'enableCExtensions': True, 'force2D': False, 'interpolator': 3, 'resampledPixelSpacing': [3, 3, 3], 'label': 1, 'normalizeScale': 1, 'normalize': False, 'force2Ddimension': 0, 'removeOutliers': None, 'minimumROISi
{'distances': [1], 'verbose': True, 'additionalInfo': True, 'enableCExtensions': True, 'force2D': False, 'interpolator': 3, 'resampledPixelSpacing': [3, 3, 3], 'label': 1, 'normalizeScale': 1, 'normalize': False, 'force2Ddimension': 0, 'removeOutliers': None, 'minimumROISi
{'distances': [1], 'verbose': True, 'additionalInfo': True, 'enableCExtensions': True, 'force2D': False, 'interpolator': 3, 'resampledPixelSpacing': [3, 3, 3], 'label': 1, 'normalizeScale': 1, 'normalize': False, 'force2Ddimension': 0, 'removeOutliers': None, 'minimumROISi
{'distances': [1], 'verbose': True, 'additionalInfo': True, 'enableCExtensions': True, 'force2D': False, 'interpolator': 3, 'resampledPixelSpacing': [3, 3, 3], 'label': 1, 'normalizeScale': 1, 'normalize': False, 'force2Ddimension': 0, 'removeOutliers': None, 'minimumROISi
{'distances': [1], 'verbose': True, 'additionalInfo': True, 'enableCExtensions': True, 'force2D': False, 'interpolator': 3, 'resampledPixelSpacing': [3, 3, 3], 'label': 1, 'normalizeScale': 1, 'normalize': False, 'force2Ddimension': 0, 'removeOutliers': None, 'minimumROISi
{'distances': [1], 'verbose': True, 'additionalInfo': True, 'enableCExtensions': True, 'force2D': False, 'interpolator': 3, 'resampledPixelSpacing': [3, 3, 3], 'label': 1, 'normalizeScale': 1, 'normalize': False, 'force2Ddimension': 0, 'removeOutliers': None, 'minimumROISi
{'distances': [1], 'verbose': True, 'additionalInfo': True, 'enableCExtensions': True, 'force2D': False, 'interpolator': 3, 'resampledPixelSpacing': [3, 3, 3], 'label': 1, 'normalizeScale': 1, 'normalize': False, 'force2Ddimension': 0, 'removeOutliers': None, 'minimumROISi
{'distances': [1], 'verbose': True, 'additionalInfo': True, 'enableCExtensions': True, 'force2D': False, 'interpolator': 3, 'resampledPixelSpacing': [3, 3, 3], 'label': 1, 'normalizeScale': 1, 'normalize': False, 'force2Ddimension': 0, 'removeOutliers': None, 'minimumROISi
{'distances': [1], 'verbose': True, 'additionalInfo': True, 'enableCExtensions': True, 'force2D': False, 'interpolator': 3, 'resampledPixelSpacing': [3, 3, 3], 'label': 1, 'normalizeScale': 1, 'normalize': False, 'force2Ddimension': 0, 'removeOutliers': None, 'minimumROISi
{'distances': [1], 'verbose': True, 'additionalInfo': True, 'enableCExtensions': True, 'force2D': False, 'interpolator': 3, 'resampledPixelSpacing': [3, 3, 3], 'label': 1, 'normalizeScale': 1, 'normalize': False, 'force2Ddimension': 0, 'removeOutliers': None, 'minimumROISi
{'distances': [1], 'verbose': True, 'additionalInfo': True, 'enableCExtensions': True, 'force2D': False, 'interpolator': 3, 'resampledPixelSpacing': [3, 3, 3], 'label': 1, 'normalizeScale': 1, 'normalize': False, 'force2Ddimension': 0, 'removeOutliers': None, 'minimumROISi
{'distances': [1], 'verbose': True, 'additionalInfo': True, 'enableCExtensions': True, 'force2D': False, 'interpolator': 3, 'resampledPixelSpacing': [3, 3, 3], 'label': 1, 'normalizeScale': 1, 'normalize': False, 'force2Ddimension': 0, 'removeOutliers': None, 'minimumROISi
{'distances': [1], 'verbose': True, 'additionalInfo': True, 'enableCExtensions': True, 'force2D': False, 'interpolator': 3, 'resampledPixelSpacing': [3, 3, 3], 'label': 1, 'normalizeScale': 1, 'normalize': False, 'force2Ddimension': 0, 'removeOutliers': None, 'minimumROISi
{'distances': [1], 'verbose': True, 'additionalInfo': True, 'enableCExtensions': True, 'force2D': False, 'interpolator': 3, 'resampledPixelSpacing': [3, 3, 3], 'label': 1, 'normalizeScale': 1, 'normalize': False, 'force2Ddimension': 0, 'removeOutliers': None, 'minimumROISi
{'distances': [1], 'verbose': True, 'additionalInfo': True, 'enableCExtensions': True, 'force2D': False, 'interpolator': 3, 'resampledPixelSpacing': [3, 3, 3], 'label': 1, 'normalizeScale': 1, 'normalize': False, 'force2Ddimension': 0, 'removeOutliers': None, 'minimumROISi
{'distances': [1], 'verbose': True, 'additionalInfo': True, 'enableCExtensions': True, 'force2D': False, 'interpolator': 3, 'resampledPixelSpacing': [3, 3, 3], 'label': 1, 'normalizeScale': 1, 'normalize': False, 'force2Ddimension': 0, 'removeOutliers': None, 'minimumROISi
{'distances': [1], 'verbose': True, 'additionalInfo': True, 'enableCExtensions': True, 'force2D': False, 'interpolator': 3, 'resampledPixelSpacing': [3, 3, 3], 'label': 1, 'normalizeScale': 1, 'normalize': False, 'force2Ddimension': 0, 'removeOutliers': None, 'minimumROISi
{'distances': [1], 'verbose': True, 'additionalInfo': True, 'enableCExtensions': True, 'force2D': False, 'interpolator': 3, 'resampledPixelSpacing': [3, 3, 3], 'label': 1, 'normalizeScale': 1, 'normalize': False, 'force2Ddimension': 0, 'removeOutliers': None, 'minimumROISi
{'distances': [1], 'verbose': True, 'additionalInfo': True, 'enableCExtensions': True, 'force2D': False, 'interpolator': 3, 'resampledPixelSpacing': [3, 3, 3], 'label': 1, 'normalizeScale': 1, 'normalize': False, 'force2Ddimension': 0, 'removeOutliers': None, 'minimumROISi
{'distances': [1], 'verbose': True, 'additionalInfo': True, 'enableCExtensions': True, 'force2D': False, 'interpolator': 3, 'resampledPixelSpacing': [3, 3, 3], 'label': 1, 'normalizeScale': 1, 'normalize': False, 'force2Ddimension': 0, 'removeOutliers': None, 'minimumROISi
{'distances': [1], 'verbose': True, 'additionalInfo': True, 'enableCExtensions': True, 'force2D': False, 'interpolator': 3, 'resampledPixelSpacing': [3, 3, 3], 'label': 1, 'normalizeScale': 1, 'normalize': False, 'force2Ddimension': 0, 'removeOutliers': None, 'minimumROISi
{'distances': [1], 'verbose': True, 'additionalInfo': True, 'enableCExtensions': True, 'force2D': False, 'interpolator': 3, 'resampledPixelSpacing': [3, 3, 3], 'label': 1, 'normalizeScale': 1, 'normalize': False, 'force2Ddimension': 0, 'removeOutliers': None, 'minimumROISi
{'distances': [1], 'verbose': True, 'additionalInfo': True, 'enableCExtensions': True, 'force2D': False, 'interpolator': 3, 'resampledPixelSpacing': [3, 3, 3], 'label': 1, 'normalizeScale': 1, 'normalize': False, 'force2Ddimension': 0, 'removeOutliers': None, 'minimumROISi
{'distances': [1], 'verbose': True, 'additionalInfo': True, 'enableCExtensions': True, 'force2D': False, 'interpolator': 3, 'resampledPixelSpacing': [3, 3, 3], 'label': 1, 'normalizeScale': 1, 'normalize': False, 'force2Ddimension': 0, 'removeOutliers': None, 'minimumROISi
{'distances': [1], 'verbose': True, 'additionalInfo': True, 'enableCExtensions': True, 'force2D': False, 'interpolator': 3, 'resampledPixelSpacing': [3, 3, 3], 'label': 1, 'normalizeScale': 1, 'normalize': False, 'force2Ddimension': 0, 'removeOutliers': None, 'minimumROISi
{'distances': [1], 'verbose': True, 'additionalInfo': True, 'enableCExtensions': True, 'force2D': False, 'interpolator': 3, 'resampledPixelSpacing': [3, 3, 3], 'label': 1, 'normalizeScale': 1, 'normalize': False, 'force2Ddimension': 0, 'removeOutliers': None, 'minimumROISi
{'distances': [1], 'verbose': True, 'additionalInfo': True, 'enableCExtensions': True, 'force2D': False, 'interpolator': 3, 'resampledPixelSpacing': [3, 3, 3], 'label': 1, 'normalizeScale': 1, 'normalize': False, 'force2Ddimension': 0, 'removeOutliers': None, 'minimumROISi
{'distances': [1], 'verbose': True, 'additionalInfo': True, 'enableCExtensions': True, 'force2D': False, 'interpolator': 3, 'resampledPixelSpacing': [3, 3, 3], 'label': 1, 'normalizeScale': 1, 'normalize': False, 'force2Ddimension': 0, 'removeOutliers': None, 'minimumROISi
{'distances': [1], 'verbose': True, 'additionalInfo': True, 'enableCExtensions': True, 'force2D': False, 'interpolator': 3, 'resampledPixelSpacing': [3, 3, 3], 'label': 1, 'normalizeScale': 1, 'normalize': False, 'force2Ddimension': 0, 'removeOutliers': None, 'minimumROISi
{'distances': [1], 'verbose': True, 'additionalInfo': True, 'enableCExtensions': True, 'force2D': False, 'interpolator': 3, 'resampledPixelSpacing': [3, 3, 3], 'label': 1, 'normalizeScale': 1, 'normalize': False, 'force2Ddimension': 0, 'removeOutliers': None, 'minimumROISi
{'distances': [1], 'verbose': True, 'additionalInfo': True, 'enableCExtensions': True, 'force2D': False, 'interpolator': 3, 'resampledPixelSpacing': [3, 3, 3], 'label': 1, 'normalizeScale': 1, 'normalize': False, 'force2Ddimension': 0, 'removeOutliers': None, 'minimumROISi
{'distances': [1], 'verbose': True, 'additionalInfo': True, 'enableCExtensions': True, 'force2D': False, 'interpolator': 3, 'resampledPixelSpacing': [3, 3, 3], 'label': 1, 'normalizeScale': 1, 'normalize': False, 'force2Ddimension': 0, 'removeOutliers': None, 'minimumROISi

general_info_ImageHash	general_info_ImageSpacing	general_info_InputImages	general_info_MaskHash	general_info_Version	general_info_VolumeNum
7d71e808fe6671534a3019b7be487ba8a0f98390	(1.0, 1.0, 1.0)	{'LoG': {}, 'Original': {}, 'Wavelet': {}}	12ff1c2f813d0146b95c2508180070919a18ad76	1.1.1-beta1	1
acd8228c36628cbfaefc90db997bb9ba7b57d5b7	(1.0, 1.0, 1.0)	{'LoG': {}, 'Original': {}, 'Wavelet': {}}	c6668afdb4b3dbc86c9ae7da3be3604d9460a0c0	1.1.1-beta1	1
a7529fcb4f8c9ad8d0d680fb4c32473100b7d25e	(1.0, 1.0, 1.0)	{'LoG': {}, 'Original': {}, 'Wavelet': {}}	c83c2193b77440caa3c814da7b8b8c466892715f	1.1.1-beta1	1
dbc259ac4a070f69d98a1a383291a01e3a986bc5	(1.0, 1.0, 1.0)	{'LoG': {}, 'Original': {}, 'Wavelet': {}}	cd50c211b8c0eaa52bbf83d26b094784d4b3adac	1.1.1-beta1	2
89de885ea7aa7e2148a38702fb9ea9a34295bb7a	(1.0, 1.0, 1.0)	{'LoG': {}, 'Original': {}, 'Wavelet': {}}	fc4080b27a133711cfac0c565ebf435d72fbf2f9	1.1.1-beta1	1
60fced09f191524b644aded04cb0252ee2b6fc2b	(1.0, 1.0, 1.0)	{'LoG': {}, 'Original': {}, 'Wavelet': {}}	b59c8440c60020148cab10dfa0329b57a477a402	1.1.1-beta1	1
4995f6ecbf9ab9416d2b5b690525de6bf6f58dd9	(1.0, 1.0, 1.0)	{'LoG': {}, 'Original': {}, 'Wavelet': {}}	b8862c79b7eea511a83ac1c8dc265d9865b05486	1.1.1-beta1	1
cd5f1a7045e07876b5e7fb747ce134ffc613ac6b	(1.0, 1.0, 1.0)	{'LoG': {}, 'Original': {}, 'Wavelet': {}}	9c24d7beafafc8aa59000ea90c3de249721bcc47	1.1.1-beta1	1
de70f76ee5c3c953e9b5710409ec3282e7258cde	(1.0, 1.0, 1.0)	{'LoG': {}, 'Original': {}, 'Wavelet': {}}	1b35455759de0f9ca4c01edfaa0b9488745d96ca	1.1.1-beta1	1
be3af75292a035c2daeed04f83ce4a3e84e6f871	(1.0, 1.0, 1.0)	{'LoG': {}, 'Original': {}, 'Wavelet': {}}	8d0866b7982a7c3f99eeb4dd3e98a2e333115339	1.1.1-beta1	1
778f4b4a8ea6a18c8da5046951d7aed66d34a6b2	(1.0, 1.0, 1.0)	{'LoG': {}, 'Original': {}, 'Wavelet': {}}	9f6fe293abd4771e3889a2aef0aec4f5621f6bd4	1.1.1-beta1	1
43820772bb86565ffd947ea76a7e037d208aa784	(1.0, 1.0, 1.0)	{'LoG': {}, 'Original': {}, 'Wavelet': {}}	480787f6f57065c056a54f76f7d576bae10ae85f	1.1.1-beta1	1
5ae037077e6ef4bfb56a0af8bd4e0f4ba3c34c57	(1.0, 1.0, 1.0)	{'LoG': {}, 'Original': {}, 'Wavelet': {}}	3926f7355380a7c1f8732135d36946b0753dc80e	1.1.1-beta1	1
4824fd3555f87f78f06c42bcaece5153075edba7	(1.0, 1.0, 1.0)	{'LoG': {}, 'Original': {}, 'Wavelet': {}}	66f56e5fcd663fe708d69cf8e3db85120eb54431	1.1.1-beta1	1
069fd1be988755e457288f8d74b75c3c15fefbc4	(1.0, 1.0, 1.0)	{'LoG': {}, 'Original': {}, 'Wavelet': {}}	2d38d6b649b67f248f66d8f2e87ec205af5320d4	1.1.1-beta1	1
cc30fa586f37ea761ac29cb87ac70d41ad928a62	(1.0, 1.0, 0.9999999999999999)	{'LoG': {}, 'Original': {}, 'Wavelet': {}}	23af1f3d93a11b5838d2f66eee5ca743ea69576e	1.1.1-beta1	1
24235a029862f96ddac62459ec91f1189d5dc2d9	(1.0, 1.0, 1.0)	{'LoG': {}, 'Original': {}, 'Wavelet': {}}	6b766ef17082cea8ce6a928a64d738e177a2fdd6	1.1.1-beta1	1
95880cb500e05e8a3cd7fd96ee5264dcb1e450d9	(1.0, 1.0, 0.9999999999999999)	{'LoG': {}, 'Original': {}, 'Wavelet': {}}	b2c3fb1f2838afe5f65e21cac4205a2689dd39f3	1.1.1-beta1	2
d02f4eeadf32f7bc50c30c0d58b7691f5080827b	(1.0, 1.0, 1.0)	{'LoG': {}, 'Original': {}, 'Wavelet': {}}	19e565657d204a20db360478f1e450d95ba3af99	1.1.1-beta1	1
1ba33fcb87a615e09a14f5560918e3602925af8c	(1.0, 1.0, 1.0)	{'LoG': {}, 'Original': {}, 'Wavelet': {}}	888396bc47783d33b1d0e77f964a4b1182d26d08	1.1.1-beta1	1
56955aa6d8ad23566e6500c3745a0238a7b67410	(1.0, 1.0, 1.0)	{'LoG': {}, 'Original': {}, 'Wavelet': {}}	c690578303006cb2d5b5620b39cf9199393d6ad1	1.1.1-beta1	1
78c291ea9c332118a69a05e48f1c08b14d5e7c7f	(1.0, 1.0, 0.9999999999999999)	{'LoG': {}, 'Original': {}, 'Wavelet': {}}	8099876420bf5e6b4e29e6547fac4937b354de83	1.1.1-beta1	2
03fd1116f7ebbc9b0236bacd710d54e70f43222b	(1.0, 1.0, 1.0)	{'LoG': {}, 'Original': {}, 'Wavelet': {}}	5a054124271644672192c0e80897eb9c061aa64e	1.1.1-beta1	1
e83cabcb67c6acdba6290c6523c55038a9d3bec9	(1.0, 1.0, 1.0)	{'LoG': {}, 'Original': {}, 'Wavelet': {}}	4019df79f82537bf535f3f2999cff28951da6b19	1.1.1-beta1	2
bab90b53bb641677fc25b517e30253e88e6c1551	(1.0, 1.0, 0.9999999999999999)	{'LoG': {}, 'Original': {}, 'Wavelet': {}}	7967d9a2d4251211726864bc5a6c101663a20682	1.1.1-beta1	1
331a674ae99f13aad88c4d4d11ba8a24a766d352	(1.0, 1.0, 1.0)	{'LoG': {}, 'Original': {}, 'Wavelet': {}}	c8d4d94cf86353b2dbd212baffd8d8318e3d873d	1.1.1-beta1	1
df35a3918b83254f64f08cd4d9c7ea699dc12827	(1.0, 1.0, 1.0)	{'LoG': {}, 'Original': {}, 'Wavelet': {}}	c4aab37a766e0b2c42a15c787ab8cd6535d54bc5	1.1.1-beta1	4
90a2432541c8208da8e51d3eed094103df5926b0	(1.0, 1.0, 1.0)	{'LoG': {}, 'Original': {}, 'Wavelet': {}}	a2103eb557846f6b96a0f9315c7496e012f4eee6	1.1.1-beta1	1
1b368f9f9427ad60615923d9ece22534d031e675	(1.0, 1.0, 1.0)	{'LoG': {}, 'Original': {}, 'Wavelet': {}}	fc2fc18097e98720fabc417a06d25cef418b7939	1.1.1-beta1	1
4b5e7d8d777c8fcef9804e75c9fc5e48ab1a0412	(1.0, 1.0, 1.0)	{'LoG': {}, 'Original': {}, 'Wavelet': {}}	83e1357223456100ac336d9aa238d8f7163067c8	1.1.1-beta1	1
86c04aea820ab661dfd5c877cbf21e4cf8defd7d	(1.0, 1.0, 1.0)	{'LoG': {}, 'Original': {}, 'Wavelet': {}}	cdf5fa460976e71d326c51c379f257340741c20d	1.1.1-beta1	1
3bc54655740cee2499184a8461d0ed43db11a9ac	(1.0, 1.0, 1.0)	{'LoG': {}, 'Original': {}, 'Wavelet': {}}	82c762f5f92bc8fa457e0c3293eda29f43d39ffa	1.1.1-beta1	1
bd7e8746c1d829d9a88e7d61e8e554f2b32f7740	(1.0, 1.0, 1.0)	{'LoG': {}, 'Original': {}, 'Wavelet': {}}	7d3118d9567403a8230e857830a85a7550f158d0	1.1.1-beta1	1
f3c7c875021eddbd98b64055ac1af6012a53427c	(1.0, 1.0, 1.0)	{'LoG': {}, 'Original': {}, 'Wavelet': {}}	4dd4812aa1a3ead0af46d4497b8e0ec9d7b4bedf	1.1.1-beta1	1
b843096e4fc13a50b9dd6fd33ac8111282f33a52	(1.0, 1.0, 1.0)	{'LoG': {}, 'Original': {}, 'Wavelet': {}}	60095bf8fd10b2ca0ebd90f6c98bc199cc2138b2	1.1.1-beta1	1
62f24f52dfe1b55bef588eae84ed26a62bec8c90	(1.0, 1.0, 1.0)	{'LoG': {}, 'Original': {}, 'Wavelet': {}}	a1faa28862fea752814bfe7420c0df3374a2b7ff	1.1.1-beta1	1
001216bee1af2b3881edcf5e01759d697a0150ff	(1.0, 1.0, 1.0)	{'LoG': {}, 'Original': {}, 'Wavelet': {}}	f5dbc4bbbd33f8831ef2dfa4782bdef105ec6c7c	1.1.1-beta1	2
425d9499496351afcb3b43d88e01590e34770cc2	(1.0, 1.0, 1.0)	{'LoG': {}, 'Original': {}, 'Wavelet': {}}	a18627c1df195d20d624d9464f3fcea216bbf2a9	1.1.1-beta1	1
e5b1250a787ca1ed95ef710f82c70cfe8b39a0b0	(1.0, 1.0, 1.0)	{'LoG': {}, 'Original': {}, 'Wavelet': {}}	efc648895704bcc714cc974b261393284575e332	1.1.1-beta1	1
92005832804dbdf44d0453ea2aaf4b3be7cf95ee	(1.0, 1.0, 1.0)	{'LoG': {}, 'Original': {}, 'Wavelet': {}}	e4914c860cd5e606ed31b52ced47d0abff719618	1.1.1-beta1	1
2c3510223208746f011c6cc80d234d225f19eb6c	(1.0, 1.0, 1.0)	{'LoG': {}, 'Original': {}, 'Wavelet': {}}	4095180ddc4a2b708ef49c7d8b50c58f5d35fbd7	1.1.1-beta1	1
7b9e96024ec8928dc40f07539fda7467232b1db0	(1.0, 1.0, 1.0)	{'LoG': {}, 'Original': {}, 'Wavelet': {}}	b8ddfb676af2eee3326b2fe0743e32459fc0ed73	1.1.1-beta1	1
f1a89c80faf8e38162c556eca91776d271727649	(1.0, 1.0, 1.0)	{'LoG': {}, 'Original': {}, 'Wavelet': {}}	ac87ed2c0fcf6a6dfe60c9cf2a2edf9f965157ae	1.1.1-beta1	1
ecf3abdee7a7aa29590029351da7e80de30b5748	(1.0, 1.0, 1.0)	{'LoG': {}, 'Original': {}, 'Wavelet': {}}	ccc1ecd57722aa063fb267a9f9387e175464be51	1.1.1-beta1	1
7e56a6fa7582b1efd41526d493b9dd57779f00b0	(1.0, 1.0, 1.0)	{'LoG': {}, 'Original': {}, 'Wavelet': {}}	92defeee0494993e08a684baf3a7175eb916ad87	1.1.1-beta1	1
124810c6a78ec91138f06ece964dbe2c6aaa17fe	(1.0, 1.0, 1.0)	{'LoG': {}, 'Original': {}, 'Wavelet': {}}	16832dcc4591c0a042f76708decd52f5bfc4a95f	1.1.1-beta1	1
f60bb3e1c364756a8ccbe1d8f9fc0855f7ccd1f6	(1.0, 1.0, 1.0)	{'LoG': {}, 'Original': {}, 'Wavelet': {}}	47c6c39cf64531ad53b523ba0f8d6200e8a0a420	1.1.1-beta1	2
6a36618ca2f75a85a5128fc656f10e6180d8cc57	(1.0, 1.0, 1.0)	{'LoG': {}, 'Original': {}, 'Wavelet': {}}	6fd8a6c3eb1ae8ec9a6b84e001fe389b9b205d22	1.1.1-beta1	1
08940100339b89405caae24ed3babf29aa4c22bb	(1.0, 1.0, 1.0)	{'LoG': {}, 'Original': {}, 'Wavelet': {}}	83c7cd2673ebf4f0187a18f9b23543d4411a4c5f	1.1.1-beta1	1
39ccd562aa9d39222569d219fb2eb8003df0d30c	(1.0, 1.0, 1.0)	{'LoG': {}, 'Original': {}, 'Wavelet': {}}	9914e62463994ccaef7a6056d4a23f2329b6af8f	1.1.1-beta1	1
2c2efec1dbcd2cd1429b1960dadedb922e5aa603	(1.0, 1.0, 1.0)	{'LoG': {}, 'Original': {}, 'Wavelet': {}}	956c5db47b17f2df7ab041f72c2408978d20e9c7	1.1.1-beta1	1
ff839a7865e4a66e9ed3f151a45992caa8c43eb4	(1.0, 1.0, 1.0)	{'LoG': {}, 'Original': {}, 'Wavelet': {}}	16acb6b34a25af2fa7cc80cf022e91c3fd780293	1.1.1-beta1	1
40b3697fef4a5dc9de3a9bc4bb29356337cf24da	(1.0, 1.0, 1.0)	{'LoG': {}, 'Original': {}, 'Wavelet': {}}	69fca78ea25bb3144e03bd8ca2b6a43824696389	1.1.1-beta1	1
8b1df46b0cfc61193922dedcc4296bb21c2e773c	(1.0, 1.0, 1.0)	{'LoG': {}, 'Original': {}, 'Wavelet': {}}	741657d554d33961e93a392d3dc9a7c221b6d2fd	1.1.1-beta1	1
303aa75ad2b720c7d7329d77466a4c9967dcdd29	(1.0, 1.0, 1.0)	{'LoG': {}, 'Original': {}, 'Wavelet': {}}	d288fd0018ca808c4f33495544f5d77192df2340	1.1.1-beta1	1
dcd6cede20943bb052f20e3f05664f6ce47536a3	(1.0, 1.0, 1.0)	{'LoG': {}, 'Original': {}, 'Wavelet': {}}	cf4fbc77fff9f7952b62a1040d256a3a1a5c724a	1.1.1-beta1	1
882c441365cfbede7ef6456bba09d3eb7759da54	(1.0, 1.0, 1.0)	{'LoG': {}, 'Original': {}, 'Wavelet': {}}	92995f17de6527b6a22dc1e6e3d639525ee460d0	1.1.1-beta1	2
082856f2a12425cee867d722afa3b93a8d1c8f30	(1.0, 1.0, 1.0)	{'LoG': {}, 'Original': {}, 'Wavelet': {}}	c6f7bbc56bcb2be72af60864ec99e95db286f09a	1.1.1-beta1	1
0f850fec7bb5bc5714a52d63844862f8e428496d	(1.0, 1.0, 1.0)	{'LoG': {}, 'Original': {}, 'Wavelet': {}}	b6ac922f9e77988096e7c23d26670e348d4b581d	1.1.1-beta1	1
425351daad40d6a5866008f88407c9c301ab9d74	(1.0, 1.0, 1.0)	{'LoG': {}, 'Original': {}, 'Wavelet': {}}	6303b73b1d3ed2c3c02ca7cb0e9547bbab7dec4a	1.1.1-beta1	1
286477e11a09101dd019b73125ded81c1fb2f575	(1.0, 1.0, 1.0)	{'LoG': {}, 'Original': {}, 'Wavelet': {}}	46d64b28854bdc47a713d69130e189e62ecb33bd	1.1.1-beta1	1
05028e3b7ffbbe3f7d7095782ca680d0b92bd767	(1.0, 1.0, 1.0)	{'LoG': {}, 'Original': {}, 'Wavelet': {}}	b3e302c1f53ca500fe9a6bdf3662858fcd7e9fc4	1.1.1-beta1	2
a1ef95f7dc3da464757d168b8b06989cff9fa3d9	(1.0, 1.0, 1.0)	{'LoG': {}, 'Original': {}, 'Wavelet': {}}	e185020dc5627c6079b826591b5bdd5a22b82798	1.1.1-beta1	1
53e9cc2d3a433a1c0d3f6efa685675ffdc80dd24	(1.0, 1.0, 1.0)	{'LoG': {}, 'Original': {}, 'Wavelet': {}}	7574188be7392e03b663aea316fd8324edbde594	1.1.1-beta1	1
99adb35d859ae1c7f7c50bfcdd3d64a7cbf59bf0	(1.0, 1.0, 1.0)	{'LoG': {}, 'Original': {}, 'Wavelet': {}}	dcca8442960e08e37e0da678a5a578fbc1aa70b5	1.1.1-beta1	1
bec1de8d9329b8187163106b831361859cd7bf20	(1.0, 1.0, 1.0)	{'LoG': {}, 'Original': {}, 'Wavelet': {}}	b5eab4a8666ad66853708e1f2d53ffd9f042c301	1.1.1-beta1	1
2ca04a708c5b413bac22cde7644c886624834bf3	(1.0, 1.0, 1.0)	{'LoG': {}, 'Original': {}, 'Wavelet': {}}	ef2ae8342ae588bcf32a11abcbb22422bd741f83	1.1.1-beta1	1
68faced8e8d9f36b5bf826127fc1ef66dc61dcfc	(1.0, 1.0, 1.0)	{'LoG': {}, 'Original': {}, 'Wavelet': {}}	e3b4094c1418437d7c0d5d0016e45a0e95336993	1.1.1-beta1	1
5627858df0e21252bee88b75a293758b47022e9a	(1.0, 1.0, 1.0)	{'LoG': {}, 'Original': {}, 'Wavelet': {}}	f026bae7627ecfb2ccfc28279161900a6fafe3c8	1.1.1-beta1	1
8d140ccfe8a2e02737dd8387970765181737d54f	(1.0, 1.0, 1.0)	{'LoG': {}, 'Original': {}, 'Wavelet': {}}	2a0b38ed3fed93531ac4d4c3e4a49cc7584c6746	1.1.1-beta1	1
f8fcbac275778cd107756269b7306710466567cc	(1.0, 1.0, 1.0)	{'LoG': {}, 'Original': {}, 'Wavelet': {}}	d23a21d7904298a7db7b1f17bf84ec9be47f3631	1.1.1-beta1	1
ceb6b308f0598622f4c495c05a54895ecfa308f4	(1.0, 1.0, 1.0)	{'LoG': {}, 'Original': {}, 'Wavelet': {}}	2aed3127c80f5502f1c54d0d0854788e0f6c06cc	1.1.1-beta1	1
e567a92e50fcdc68a599ba598cdd80985307759c	(1.0, 1.0, 1.0)	{'LoG': {}, 'Original': {}, 'Wavelet': {}}	bd2f4a5f27d888d3f8fed27da2dad4c87a3f1abc	1.1.1-beta1	3
bb83d60db485faac72a5af79b593e920ddc3806a	(1.0, 1.0, 1.0)	{'LoG': {}, 'Original': {}, 'Wavelet': {}}	b896d43d03ac83ad9e3c57d681d4600a6dd00f2d	1.1.1-beta1	2
a73d9a89b71cea5258e59ad0bfee7eb241a94258	(1.0, 1.0, 1.0)	{'LoG': {}, 'Original': {}, 'Wavelet': {}}	a9e991edd3a62c9d34ae6a5fcb09d77190d461be	1.1.1-beta1	1
6f7dbc63238a8f35b442abd4d0df881a5c90a25c	(1.0, 1.0, 1.0)	{'LoG': {}, 'Original': {}, 'Wavelet': {}}	259c87451aac0c06fc5002925527ee01798b065c	1.1.1-beta1	1
ab6ef8e1c396418632c92d0f36915895773e5e7c	(1.0, 1.0, 1.0)	{'LoG': {}, 'Original': {}, 'Wavelet': {}}	42c1637bdc9a2f5c3f860d3278986af8bc161e7d	1.1.1-beta1	1
d9f46cc9c5c2019d299615d06d6993a53e2bd258	(1.0, 1.0, 1.0)	{'LoG': {}, 'Original': {}, 'Wavelet': {}}	ba065be739e7acf8efb96bbf3f9ef42802b79c36	1.1.1-beta1	1
5b2de6388b14d2c1e149157e5ff79b32e4fcc5e4	(1.0, 1.0, 1.0)	{'LoG': {}, 'Original': {}, 'Wavelet': {}}	d2fc85893a3d6f4948021226ccfd031cf2a1d29c	1.1.1-beta1	1
341adba7f1fdbadb81a40490335321ac03811a01	(1.0, 1.0, 1.0)	{'LoG': {}, 'Original': {}, 'Wavelet': {}}	a18e1e7570dd60c21791c64f5d4557419dae0a11	1.1.1-beta1	1
7505a548e249926abe434b898d8fc2e5763f238d	(1.0, 1.0, 1.0)	{'LoG': {}, 'Original': {}, 'Wavelet': {}}	2fc26b392eb46cb3f186a093dfc060211b057b14	1.1.1-beta1	1
eb16fbadd3bea123c4b0fae2635d350cb7791af5	(1.0, 1.0, 1.0)	{'LoG': {}, 'Original': {}, 'Wavelet': {}}	7bfdc672d279c9961df5b1d2b06515196f9796d1	1.1.1-beta1	1
2f247bc2a882d7a7b134d0035e403c3a80a8d389	(1.0, 1.0, 1.0)	{'LoG': {}, 'Original': {}, 'Wavelet': {}}	0571220c784655f108430a1a731b306c76330cee	1.1.1-beta1	1
88011fc8e4004e2da019b1e69eeb737f6b1b8a5c	(1.0, 1.0, 1.0)	{'LoG': {}, 'Original': {}, 'Wavelet': {}}	3cd381d0041b6fae670491dabf87b4d5ce04c32f	1.1.1-beta1	1
27878584982c4556d58290555364c0bf9271a6b2	(1.0, 1.0, 1.0)	{'LoG': {}, 'Original': {}, 'Wavelet': {}}	b07a492374c4fc5a67b446167ff0b709e6d88266	1.1.1-beta1	1
bb975f93f3220af49485a0f8e3cbc6248da84fe9	(1.0, 1.0, 1.0)	{'LoG': {}, 'Original': {}, 'Wavelet': {}}	12f73b1a168834cb492c4d90bbf9dc8d5294b780	1.1.1-beta1	1
c038c6ada1b41149a97d5d58676ae2c625c43d0e	(1.0, 1.0, 1.0)	{'LoG': {}, 'Original': {}, 'Wavelet': {}}	12a70e62daa0b8d8a6e91b7ed0317fdd77c44de1	1.1.1-beta1	1
7b65b3b77f26f606f1ad35358001d7167203c041	(1.0, 1.0, 1.0)	{'LoG': {}, 'Original': {}, 'Wavelet': {}}	6b734380fd8b9c717c355aea94d3d8c93a57cbc6	1.1.1-beta1	1
1e75d9bc15f8092472c67d8c924d8786f94c1e78	(1.0, 1.0, 1.0)	{'LoG': {}, 'Original': {}, 'Wavelet': {}}	93bde50cd1915246616ae6784d50e5d3a5abd851	1.1.1-beta1	1
38e36f9da7fef61903791c86f3239e06c671c416	(1.0, 1.0, 1.0)	{'LoG': {}, 'Original': {}, 'Wavelet': {}}	2ad557894a3cee1e51e0be824d805587d4c113fb	1.1.1-beta1	1
fd24cf5e6b7434ab0bfc65f6c8a8ca90b14ee927	(1.0, 1.0, 1.0)	{'LoG': {}, 'Original': {}, 'Wavelet': {}}	fa7956576630561c50ba10504bf775d395590da8	1.1.1-beta1	1
a2f8c625d6f5fe6dfeda8762d8a180e51e1e0860	(1.0, 1.0, 1.0)	{'LoG': {}, 'Original': {}, 'Wavelet': {}}	026a13c06ba0d34091170422363c68e19c59aabe	1.1.1-beta1	1
98c354e74e35b0436885e9fa927228ab2a4fab7d	(1.0, 1.0, 1.0)	{'LoG': {}, 'Original': {}, 'Wavelet': {}}	bc3f9e086373d5aa2e9528230b09e3b277b59e02	1.1.1-beta1	1
c907b4bccf40f0ddf63e2ab0ea5b2631e2a3fa9e	(1.0, 1.0, 1.0)	{'LoG': {}, 'Original': {}, 'Wavelet': {}}	eee4a37cac2b16166775954c88daf722cdb6fc8e	1.1.1-beta1	1
bab82adc990efd6447998cba8f0e2c5158114246	(1.0, 1.0, 1.0)	{'LoG': {}, 'Original': {}, 'Wavelet': {}}	15c249626ad147dceb97c054b0d2e079caf3056b	1.1.1-beta1	1
a82808907bd5f4f4342a69e2ffa3a4b2a00a0435	(1.0, 1.0, 1.0)	{'LoG': {}, 'Original': {}, 'Wavelet': {}}	8116118057b7ee9940dd019fc2bc8f9b16e92ff0	1.1.1-beta1	1
a3ef48a21870bef679038cdf1da0a554774524e5	(1.0, 1.0, 1.0)	{'LoG': {}, 'Original': {}, 'Wavelet': {}}	5e2b92cb38bdca874d12f3b9c4c1c998b1fa39a5	1.1.1-beta1	1
060480befcbe7178b54f76651aef987231588545	(1.0, 1.0, 1.0)	{'LoG': {}, 'Original': {}, 'Wavelet': {}}	977980c1ae0faf105446724df8640748171320c6	1.1.1-beta1	1
d271553e8e5eca7e73252b2e6e52b09a3019aca8	(1.0, 1.0, 1.0)	{'LoG': {}, 'Original': {}, 'Wavelet': {}}	cc3e940df7031b112708efd3849dee178c2b3dd9	1.1.1-beta1	1
1751d2ed951684b0961cc9a1157a921e2543d279	(1.0, 1.0, 1.0)	{'LoG': {}, 'Original': {}, 'Wavelet': {}}	b675f062529109be6da148399282776d8448534e	1.1.1-beta1	1

general_info_VoxelNum	original_shape_Maximum3DDiameter	original_shape_Compactness2	original_shape_Maximum2DDiameterSlice	original_shape_Sphericity	original_shape_Compactness1	original_shape_Elongation
1203	56.36488268	0.114244269	48.46648326	0.485226831	0.017931478	1.119311266
2000	79.65550828	0.073445473	77.82673063	0.418782325	0.014377434	1.451876183
1118	59.01694672	0.10798821	55.07267925	0.476202986	0.017433599	1.365876018
2453	87.82368701	0.054725634	70.80254233	0.379661827	0.012410643	1.195226281
949	51.35172831	0.133646433	45	0.511272527	0.019394454	1.187674799
1638	56.92099788	0.111813158	49.92995093	0.481760259	0.017739662	1.017220325
1057	62.42595614	0.125164961	57.62811814	0.500219851	0.018768962	1.637885747
1805	67.08203932	0.078051653	61.77378085	0.427360161	0.014821424	1.174373826
2027	78.2879301	0.053325869	67.68308504	0.376396849	0.012250896	1.228987343
3003	101.9117265	0.041904284	71.30918594	0.347338408	0.010859957	1.555484733
1807	67.94850992	0.085432418	58.24946352	0.440427302	0.015506373	1.184888204
1685	84.26743143	0.079608261	64.41273166	0.430182474	0.014968488	1.191996789
2929	80.27452896	0.050003275	67.68308504	0.368411194	0.011863098	1.044547667
1858	66.13622306	0.082082964	60.74537019	0.434594617	0.015199363	1.167848869
1548	71.8748913	0.07680865	61.77378085	0.425079385	0.014702932	1.3461619
4109	141.5733026	0.029608975	87	0.309367337	0.009128734	1.771549852
1605	66.47555942	0.100263706	58.24946352	0.46456653	0.01679851	1.150836472
2558	97.71898485	0.05486686	86.53323061	0.379988133	0.012426646	1.124318122
1136	62.92853089	0.094590293	51.6139516	0.45563337	0.016316318	1.19018416
2901	102.7472627	0.054765285	74.09453421	0.379753498	0.012415138	1.40702559
6950	125.0679815	0.02703392	89.19641248	0.300125576	0.008722749	1.387927897
2121	89.09545443	0.062962136	59.09314681	0.39782599	0.013311856	1.400631502
4121	115.2562363	0.032485081	97.9489663	0.319076376	0.009561826	1.260347156
1030	61.26173357	0.124753703	51.6139516	0.499671387	0.018738102	1.123991834
2604	89.49860334	0.057313389	83.19254774	0.385554132	0.012700678	1.459179277
1352	72.56031973	0.091143049	69.0651866	0.450029708	0.016016243	1.542104414
2211	98.81801455	0.018026754	98.08669635	0.262203917	0.007122913	1.213843318
3355	126.9251748	0.034256791	112.0089282	0.324774726	0.009819112	1.386344059
3399	106.573918	0.047964877	87.46427842	0.363335455	0.01161878	1.05974054
3159	88.43641784	0.034419638	73.23933369	0.325288544	0.009842423	1.102019733
4142	124.1652125	0.036879647	88.84255737	0.332860492	0.010188078	1.493036397
5000	141.0638153	0.022410372	104.9571341	0.281935451	0.007941882	1.300690992
2531	79.7684148	0.067339453	68.41052551	0.406839577	0.013766821	1.077064044
481	38.41874542	0.225839066	31.89043744	0.608975316	0.025211484	1.10070798
888	58.94064811	0.133508763	41.67733197	0.511096913	0.019384463	1.253393422
3217	81.49846624	0.052992807	70.80254233	0.375611582	0.012212578	1.119809205
3569	121.1197754	0.045297785	105.5130324	0.35647219	0.011291129	1.479956638
3391	102.7472627	0.050767975	92.61209424	0.370279738	0.011953465	1.050724961
1484	60.44832504	0.091858429	56.36488268	0.451204065	0.016078976	1.239480385
1636	64.20280368	0.097689015	58.24946352	0.460555434	0.016581421	1.001990304
1316	68.73863542	0.096378466	66.61080993	0.458486624	0.016469821	1.158462933
1932	67.74953874	0.077588065	63.07138812	0.426512379	0.014777342	1.06662025
3027	87.20665112	0.057382361	84.85281374	0.385708732	0.012708318	1.367294743
1884	76.13146524	0.076473692	55.07267925	0.424460569	0.014670837	1.284046543
3494	85.1704174	0.057857018	72.49827584	0.386769319	0.012760771	1.110252568
3575	137.5099996	0.042323194	118.6802427	0.348492	0.010914104	1.38467322
3686	102.9660138	0.036879112	76.4852927	0.332858883	0.010188004	1.333481365
1516	57.31491952	0.117233999	51.0881591	0.489423171	0.018164593	1.034230864
564	40.36087214	0.20505767	34.20526275	0.58969214	0.024023533	1.054183838
1090	52.82045058	0.121375117	49.65883607	0.495119336	0.018482627	1.149954351
3984	106.8690788	0.037444956	90.24965374	0.334552625	0.010265865	1.331684262
1956	62.92853089	0.099808584	51.6139516	0.463862536	0.01676034	1.058157693
3797	104.0096149	0.039800017	81.9390017	0.341426754	0.010583887	1.306934722
1171	56.44466317	0.111899112	51.6139516	0.481883674	0.017746479	1.051262662
3937	159.3674998	0.03890104	128.1600562	0.338834069	0.01046356	2.124135405
1888	64.96922348	0.084362572	56.60388679	0.438581125	0.015408976	1.116471538
3279	121.2682976	0.041803698	89.19641248	0.347060272	0.010846915	1.498912357
2432	83.89278872	0.067624665	68.01470429	0.407413151	0.013795945	1.337235515
1372	64.41273166	0.099637141	58.24946352	0.463596789	0.016745939	1.125562495
1405	63.63961031	0.108068978	58.94064811	0.476321679	0.017440117	1.352938049
2941	77.59510294	0.047894066	69.26037828	0.363156567	0.011610201	1.090908909
5487	122.1556384	0.029676927	90.44888059	0.30960382	0.009139203	1.384528949
2271	129.3483668	0.059831234	76.4852927	0.391119366	0.012976658	1.333525748
1868	98.27003612	0.070547952	90.24965374	0.4132011	0.014090976	1.767038979
590	44.39594576	0.191565791	40.36087214	0.576464612	0.023219767	1.050276549
1543	87.25823743	0.085616811	70.22819946	0.440743939	0.015523098	1.407042732
1058	51.35172831	0.123743851	47.4341649	0.498319493	0.018662108	1.1681128
3566	95.1052049	0.045948757	87.6184912	0.358171689	0.011371971	1.149888588
1293	52.13444159	0.139972555	45	0.519215478	0.019848163	1.056512683
2507	81.44323176	0.060838832	77.88452991	0.393302725	0.01308547	1.098999426
3808	106.0660172	0.035871572	84.53401682	0.329799608	0.010047872	1.428764893
2061	72.86974681	0.077730339	66.06814664	0.426772919	0.014790885	1.287708026
4679	123.073149	0.028587729	105.5130324	0.305768836	0.008969923	1.279397995
6511	140.2640367	0.024858404	131.041978	0.291848688	0.008364414	1.142464085
4262	131.487642	0.032399079	121.4907404	0.318794552	0.009549161	1.293120225
2331	82.15838363	0.0716882	76.4852927	0.415415368	0.014204394	1.479097242
2355	75.95393341	0.071884998	69.97142274	0.415795153	0.014223878	1.196569773
1359	58.24946352	0.100159426	51.0881591	0.464405415	0.016789772	1.086695359
3276	84.53401682	0.056637899	75.89466384	0.384033442	0.012625612	1.028106857
4453	109.8590005	0.038514693	98.68130522	0.337708621	0.010411471	1.164257503
1362	63.14269554	0.107356214	59.54829972	0.475272183	0.017382509	1.075983448
2657	91.04394543	0.057368883	84.69356528	0.385678532	0.012706826	1.570859144
2474	91.68424074	0.061555683	84.69356528	0.394841432	0.013162335	1.137367162
523	41.78516483	0.215203164	36.61966685	0.59926128	0.024610657	1.208974472
3862	98.49873096	0.033343069	92.0271699	0.321861125	0.009687276	1.017752753
3474	103.0970417	0.042922107	93.77099765	0.350128137	0.010991055	1.20154139
1428	60.9672043	0.08345518	53.07541804	0.437003014	0.015325884	1.148719828
3043	105.6834897	0.055241387	95.34149149	0.380850788	0.012468987	2.007862405
411	46.86149806	0.220398607	43.26661531	0.604045447	0.024905961	1.334818317
578	40.80441153	0.21468611	36.61966685	0.59878096	0.024581074	1.118174365
3211	126.3566381	0.043983907	96.93296653	0.352991786	0.011126172	1.700341144
1105	68.54195795	0.105054757	51.6139516	0.471851392	0.01719518	1.441033175
1562	68.93475176	0.084896367	65.7951366	0.439504206	0.015457649	1.432605855
4353	100.1748471	0.039866473	89.09545443	0.341614218	0.010592605	1.069106329
4706	111.5257818	0.032485646	97.34988444	0.319078226	0.00956191	1.172234218
884	48.74423043	0.159520283	40.36087214	0.542340416	0.021188823	1.076949356
1763	73.60706488	0.088051425	62.42595614	0.444882645	0.01574226	1.223981244
428	38.18376618	0.225909009	34.20526275	0.609038177	0.025215388	1.062312257
888	51	0.133943973	45	0.511651665	0.019416031	1.207840322
1154	62.49799997	0.094763779	56.36488268	0.455911756	0.016331274	1.233488062

original_shape_SurfaceVolumeRatio	original_shape_Volume	original_shape_SphericalDisproportion	original_shape_Flatness	original_shape_SurfaceArea	original_shape_Maximum2DDiameterColumn
0.31236535	32481	2.060891805	1.316449712	10145.93894	52.47856705
0.305514055	54000	2.387875374	1.154243487	16497.75897	68.41052551
0.326154585	30186	2.099944835	1.290930102	9845.302299	44.59820624
0.314823218	66231	2.633922949	1.276231409	20851.05653	79.25906888
0.320839878	25623	1.955904036	1.143894277	8220.880181	46.86149806
0.28385329	44226	2.075721236	1.115789907	12553.69561	51.6139516
0.316356638	28539	1.999120981	1.149950029	9028.502083	47.4341649
0.309796447	48735	2.339946702	1.272344676	15097.92986	59.09314681
0.338401522	54729	2.656770379	1.258186305	18520.37689	68.41052551
0.321680659	81081	2.879036628	1.098052789	26082.18948	99.04544412
0.300494079	48789	2.270522276	1.035862187	14660.80564	53.07541804
0.314903042	45495	2.324594937	1.635485024	14326.51388	78.23042886
0.305813694	79083	2.714358348	1.186748021	24184.66434	73.23933369
0.301714807	50166	2.300994906	1.310846732	15135.82499	59.16924877
0.327820298	41796	2.352501756	1.141701679	13701.57717	68.01470429
0.325319201	110943	3.2324033	1.26119815	36091.88816	138.9748179
0.296362516	43335	2.152544221	1.142280315	12842.86963	55.07267925
0.310188711	69066	2.631661128	1.218211925	21423.49355	70.29224708
0.339068857	30672	2.194747063	1.449923058	10399.91999	48.37354649
0.297631238	78327	2.633287131	1.482518405	23312.56195	90.69729875
0.281448062	187650	3.33193863	1.225435127	52813.72876	120.9338662
0.315371779	57267	2.513661817	1.646587613	18060.39569	82.37718131
0.315113745	111267	3.134045874	1.460053757	35061.76108	103.4456379
0.319447368	27810	2.001315315	1.651843169	8883.831291	50.91168825
0.30389987	70308	2.593669516	1.27617935	21366.59205	69.0651866
0.323938666	36504	2.222075527	1.028294946	11825.05705	45.79301257
0.471911738	59697	3.813825554	1.442625562	28171.71503	77.4209274
0.331550589	90585	3.079057332	2.117257397	30033.51011	107.3312629
0.295078819	91773	2.752277509	2.14969698	27080.26842	99.72462083
0.337736159	85293	3.074193726	1.215281294	28806.53019	76.4852927
0.301553186	111834	3.004261621	1.609692527	33723.899	99.31767214
0.334366825	135000	3.546911174	1.204631895	45139.52132	125.6065285
0.290742794	68337	2.457971287	1.480807884	19868.49034	74.09453421
0.337842919	12987	1.642102681	1.186881482	4387.565993	31.89043744
0.328137066	23976	1.956576092	1.511998353	7867.414288	51.0881591
0.290719084	86859	2.662324724	1.047518002	25251.56891	68.47627326
0.29590686	96363	2.805267922	1.615108924	28514.4727	101.2027668
0.289772383	91557	2.700660874	1.777007438	26530.69008	87.6184912
0.313216933	40068	2.216292089	1.220981134	12549.97606	51.0881591
0.297043367	44172	2.171291287	1.510694094	13120.99962	58.24946352
0.320836845	35532	2.181088711	1.858167702	11399.97477	59.77457654
0.303455856	52164	2.344597836	1.391274096	15829.47125	61.84658438
0.288912229	81729	2.59262992	1.131477699	23612.50756	85.90692638
0.307490629	50868	2.355931439	1.272252869	15641.43331	66.06814664
0.274664936	94338	2.58552049	1.191332204	25911.3407	81.60882305
0.302513464	96525	2.869506334	2.556323001	29200.11212	107.4150827
0.31350967	99522	3.00427614	1.38118191	31201.10942	96.93296653
0.286711608	40932	2.04322161	1.064329096	11735.67952	49.2036584
0.330860176	15228	1.695800117	1.355249904	5038.338755	36.12478374
0.316357025	29430	2.0197151	1.246331043	9310.387247	45
0.303942887	107568	2.989066366	1.208377859	32694.52847	89.19641248
0.277875733	52812	2.155811093	1.042721041	14675.17323	55.15432893
0.302634514	102519	2.928885887	1.291817326	31025.78772	101.8675611
0.317371817	31617	2.075189622	1.497872943	10034.34474	49.2036584
0.301291811	106299	2.951297087	1.06544945	32027.01818	109.2016483
0.29738035	50976	2.280079881	1.080670571	15159.26075	55.31726674
0.312639726	88533	2.88134391	1.164023018	27678.9329	91.83136719
0.294220753	65664	2.454510853	1.007172491	19319.71154	76.4852927
0.312923188	37044	2.157046864	1.47088072	11591.92659	55.80322571
0.302160075	37935	2.099421555	1.278389599	11462.44246	61.84658438
0.309816084	79407	2.753633258	1.091980925	24601.56577	72.62231062
0.295196751	148149	3.22993431	1.336591824	43733.10342	112.0892502
0.313555559	61317	2.556764218	2.313738546	19226.28621	122.4499898
0.316768832	50436	2.420129082	1.138147263	15976.5528	71.30918594
0.333405593	15930	1.73471186	1.601984542	5311.151089	38.41874542
0.316510323	41661	2.268891097	2.481255942	13186.13656	78
0.317462992	28566	2.006744696	1.073773392	9068.647816	46.66904756
0.294585366	96282	2.791957129	1.53851541	28363.26821	88.58893836
0.284980885	34911	1.925982647	1.061231067	9948.967685	45
0.301706363	67689	2.542570739	1.508464886	20422.20202	78.23042886
0.313001982	102816	3.032144294	1.147614648	32181.6118	84.69356528
0.296806462	55647	2.343166482	1.12190772	16516.38919	61.84658438
0.315199341	126333	3.270444474	1.513904586	39820.07839	110.1453585
0.295793908	175797	3.426433079	1.725419301	51999.68166	131.5902732
0.311875192	115074	3.13681647	2.335765075	35888.72589	108.1665383
0.292661922	62937	2.407229192	1.082071495	18419.26337	62.42595614
0.29139794	63585	2.40503044	1.223880814	18528.538	60
0.31337122	36693	2.153290997	1.137915563	11498.53019	53.41348144
0.28262627	88452	2.603939894	1.438477185	24998.85886	76.89603371
0.290137028	120231	2.961132583	1.467424694	34883.465	83.19254774
0.305981219	36774	2.104057499	1.453715305	11252.15336	51
0.301768256	71739	2.592832938	1.193518216	21648.55294	66.40783086
0.301860939	66798	2.532662273	2.304776798	20163.70703	84.69356528
0.333871557	14121	1.668721197	1.268177771	4714.60026	34.20526275
0.319220119	104274	3.10693005	1.662699886	33286.35872	95.34149149
0.303989969	93798	2.856097226	1.198356657	28513.65107	99.72462083
0.327568678	38556	2.288313735	1.090441828	12629.73795	51
0.292083726	82161	2.625700224	1.091509746	23997.89102	57.31491952
0.358932387	11097	1.655504574	2.01722217	3983.072702	31.89043744
0.323185803	15606	1.670059784	1.324868033	5043.637645	34.98571137
0.30954099	86697	2.832927107	1.072917568	26836.27525	80.49844719
0.330448311	29835	2.119311329	1.46983131	9858.925367	57.70615219
0.316110915	42174	2.275291082	1.257002587	13331.66171	55.15432893
0.28899969	117531	2.927278634	1.203419782	33966.42257	99.04544412
0.30147298	127062	3.134027706	1.598649176	38305.75977	106.0660172
0.309699244	23868	1.843860369	1.081802405	7391.901545	42.42640687
0.299939251	47601	2.247783797	1.412982822	14277.40826	66.06814664
0.351212876	11556	1.641933194	1.459727254	4058.615998	31.89043744
0.327781287	23976	1.954454697	1.118762093	7858.884137	43.26661531
0.33709073	31158	2.19340692	1.558675406	10503.07296	52.47856705

original_shape_Maximum2DDiameterRow	log.sigma.5.0.mm.3D_gldm_GrayLevelVariance	log.sigma.5.0.mm.3D_gldm_HighGrayLevelEmphasis	log.sigma.5.0.mm.3D_gldm_GrayLevelNonUniformityNormalized
51.26402247	26.68777903	248.2842893	0.054324565
72.99315036	17.85774375	362.7955	0.0731035
52.39274759	18.61256284	232.1958855	0.070738701
80.72174428	16.76234779	140.2865879	0.072723916
45	17.31917242	138.5511064	0.082223982
49.92995093	15.08537467	131.035409	0.091193436
45.79301257	13.33534243	64.20719016	0.106591279
57.70615219	23.39030732	300.6819945	0.06183777
66.61080993	20.56648272	230.3660582	0.065223333
85.38149682	23.2016515	578.9080919	0.060297899
60	10.64249373	91.59269507	0.093884724
68.41052551	15.6004142	268.9863501	0.074569997
69.0651866	12.0646683	140.7620348	0.089546023
61.55485359	13.74770492	136.5780409	0.075542182
53.41348144	25.70823902	267.2416021	0.061376186
124.3100961	23.9547283	368.4641032	0.062122743
49.2036584	18.90742908	353.6492212	0.07189643
90	18.69981679	249.0320563	0.06856351
57.31491952	12.92971385	250.0246479	0.079959953
99.90495483	17.95081348	240.4860393	0.074110949
119.7747887	17.81846532	374.0392806	0.070866932
76.89603371	19.28018573	260.1956624	0.067282764
112.7297654	20.81642402	346.3557389	0.066357936
48.83646179	20.91870676	171.231068	0.065640494
81.9390017	22.49996048	231.2380952	0.061436854
66.40783086	23.08363623	269.9593195	0.064582166
90.7964757	20.84238853	231.3876074	0.062250525
125.1758763	18.36962302	292.2992548	0.06700918
104.4844486	17.3771328	405.1391586	0.077485279
80.77747211	19.6142187	472.9711934	0.068952162
119.0965994	21.31771584	336.7274264	0.065275548
112.4499889	20.15580096	397.8828	0.06689576
69.97142274	23.50904041	614.0892928	0.071755198
33.9411255	18.47416807	177.5696466	0.067414128
50.91168825	14.35128921	117.8727477	0.077517551
76.83749085	12.77279053	198.5436742	0.083271387
118.7939392	28.06271071	458.27935	0.057755912
85.90692638	13.17678964	259.3733412	0.081999196
57.70615219	10.51935243	164.5033693	0.099345399
55.80322571	16.01310781	133.3698044	0.080982748
64.41273166	12.48095627	156.3168693	0.081238856
59.54829972	12.13455842	164.3478261	0.082631414
66.06814664	13.82124561	257.9012223	0.08048923
72.24956747	16.9759859	161.8052017	0.079411042
71.56116265	26.4041302	397.9645106	0.06237498
108.3743512	18.19314361	165.7255944	0.064649812
96.93296653	21.09524475	454.3358654	0.064917659
50.91168825	15.26349197	294.5237467	0.089962128
36.12478374	13.8346254	98.61170213	0.08392435
49.65883607	18.63668378	209.1779817	0.068529585
99.85990186	17.03790608	358.2763554	0.078781491
57.31491952	10.21672396	218.101227	0.11123448
101.2027668	18.45167647	213.545694	0.070368752
48.37354649	17.57793269	404.1989752	0.070843127
143.1223253	19.90239722	284.2600965	0.062556835
58.24946352	26.40174227	296.871822	0.056390944
87.20665112	21.50684557	511.1726136	0.066427973
59.54829972	16.24190008	169.1587171	0.096636405
57.31491952	14.6085363	210.3841108	0.081690027
46.86149806	7.684302884	149.3366548	0.103713479
76.66159404	20.52683207	202.1026862	0.062191362
115.7626883	18.75438797	381.4322945	0.073149787
92.4175308	16.29042581	319.0902686	0.073930269
89.49860334	18.34771355	176.8811563	0.064081636
36.61966685	15.42706406	207.9779661	0.072536627
60.74537019	20.35326726	133.5832793	0.066472506
40.80441153	24.35530176	157.1493384	0.063323459
81.88406438	23.31120055	191.823051	0.060664392
45	13.67590614	202.2761021	0.077135555
75.89466384	22.37282382	337.5309134	0.064398066
101.9117265	34.15148387	399.5254727	0.051009236
63.78087488	13.70828216	174.6817079	0.090451091
110.3086579	20.78855879	424.4644155	0.068487562
131.7269904	16.31323256	341.7634772	0.074458566
128.26535	25.2706183	344.2475364	0.057529011
68.47627326	22.0819389	342.0789361	0.062489958
63.28506933	12.9498576	231.1987261	0.085297668
48.37354649	16.62271256	220.0051508	0.075184595
69.26037828	15.86035376	275.6446886	0.078033569
104.7854952	18.06413034	345.1434988	0.078592797
48.37354649	21.7992743	613.0888399	0.080076807
81.60882305	23.61041349	397.4949191	0.062198135
85.38149682	21.33645173	390.7405012	0.067163496
36.61966685	8.368831093	90.19694073	0.111224038
95.34149149	21.08220727	368.3322113	0.065258943
87	19.03460835	147.6030512	0.066083968
53.41348144	24.76799102	313.2682073	0.059529694
96.60745313	13.23864504	144.0752547	0.079275267
38.41874542	6.714168161	81.23600973	0.115799693
32.44996148	15.68848553	129.9342561	0.073957448
103.4456379	17.30240189	291.2765494	0.069972313
64.20280368	18.46154174	189.680543	0.067670195
64.62197769	11.90756991	188.5320102	0.08796464
93.1933474	16.27082706	314.0374454	0.080429281
106.0660172	13.67302766	191.9460263	0.078581493
42.42640687	45.6859145	550.7149321	0.046789583
61.84658438	11.31432622	171.1366988	0.096404346
32.44996148	15.1137381	103.4649533	0.073543541
41.67733197	11.21387139	96.13175676	0.093471005
59.09314681	34.84064545	349.1499133	0.052418083

log.sigma.5.0.mm.3D_gldm_DependenceEntropy	log.sigma.5.0.mm.3D_gldm_DependenceNonUniformity	log.sigma.5.0.mm.3D_gldm_GrayLevelNonUniformity	log.sigma.5.0.mm.3D_gldm_SmallDependenceEmphasis
7.001440053	174.1970075	65.3524522	0.231235572
6.930574137	256.888	146.207	0.156636365
6.773858495	166.3703041	79.08586762	0.182682425
7.047320075	304.3102324	178.3917652	0.16674492
6.800706237	100.8651212	78.03055848	0.168839821
6.86422049	181.1489621	149.3748474	0.121435944
6.743063885	83.41248817	112.666982	0.113332842
7.153770068	228.567867	111.6171745	0.168717013
7.054858499	262.6171682	132.2076961	0.206300011
7.106436947	446.5930736	181.0745921	0.198952747
6.890978013	182.5307139	169.6496956	0.114835396
6.871707171	229.8498516	125.6504451	0.196911888
7.147067183	283.3523387	262.2803004	0.130814301
6.763876586	259.2863294	140.3573735	0.169730308
7.213496135	200.4095607	95.01033592	0.21712718
7.380211126	508.7517644	255.2623509	0.175328279
7.050819503	206.9252336	115.3937695	0.164974402
7.238128107	271.3479281	175.3854574	0.156721481
6.696637329	154.7007042	90.83450704	0.193097911
7.179526279	329.7528438	214.9958635	0.148428166
7.244756478	858.5772662	492.5251799	0.155431941
7.133146323	275.8137671	142.7067421	0.178374896
7.131864503	570.0419801	273.4610531	0.173579792
6.912352977	139.7728155	67.60970874	0.189031466
6.993385832	399.4961598	159.9815668	0.197917711
6.932415648	205.8964497	87.31508876	0.226660991
7.020721142	310.7033017	137.6359114	0.202946409
6.849229344	536.1958271	224.8157973	0.211013339
7.012254858	420.6187114	263.3724625	0.164217948
7.068652304	404.9702437	217.8198797	0.204848208
7.293636267	518.1825205	270.3713182	0.16644346
7.222658207	639.91	334.4788	0.200243838
7.220901439	281.6527064	181.6124062	0.1594122
6.633998769	63.46985447	32.42619543	0.240717245
6.853868597	108.1981982	68.83558559	0.161495263
6.949370895	384.850171	267.8840535	0.14057764
7.220323411	526.6295881	206.130849	0.205575303
6.967522741	412.5128281	278.0592746	0.142922206
6.959710589	138.3530997	147.4285714	0.107342361
7.0807825	167.6381418	132.4877751	0.154411063
6.7424056	168.2537994	106.9103343	0.179729615
6.936951711	224.6563147	159.6438923	0.153135118
7.040077911	330.872481	243.6408986	0.124376188
7.058445713	195.2643312	149.6104034	0.166726262
7.467268716	404.6164854	217.9381797	0.180179914
7.138674132	470.3046154	231.1230769	0.154644261
7.230359785	487.5029843	239.2864894	0.191513851
6.937765892	158.8007916	136.3825858	0.138762084
6.377069941	62.69858156	47.33333333	0.179552154
6.993672578	128.5706422	74.69724771	0.160451705
7.186949365	404.9643574	313.8654618	0.131875779
6.894967307	173.9570552	217.5746421	0.096766661
7.178821749	422.7498025	267.1901501	0.140460155
6.895888188	156.9146029	82.95730145	0.178297377
7.207547122	533.8945898	246.2862586	0.183929303
7.246564765	266.6493644	106.4661017	0.194133575
7.135300633	434.9957304	217.8173224	0.204032497
7.169971664	198.8824013	235.0197368	0.123729312
6.749058118	184.1997085	112.0787172	0.165133287
6.5394005	166.8633452	145.7174377	0.104304673
7.332198773	322.2784767	182.9047943	0.171914403
7.222996312	636.8899216	401.3728814	0.155165268
7.081910874	275.4297666	167.8956407	0.173164572
6.718655325	306.6905782	119.7044968	0.210040172
6.422598684	95.11186441	42.79661017	0.244429897
6.863153096	211.8412184	102.5670771	0.202655407
7.090061614	122.6786389	66.99621928	0.15644522
7.201069365	456.7178912	216.3292204	0.169150641
6.79798889	163.8321732	99.73627224	0.146901347
7.189699214	325.1930594	161.4459513	0.172705815
7.275215437	603.4957983	194.2431723	0.229372755
6.86787504	223.7501213	186.4196992	0.147093384
7.229442199	587.7941868	320.453302	0.184260053
7.056562801	848.3524804	484.7997235	0.160605409
7.36149541	561.8456124	245.1886438	0.193728755
7.122285022	311.7353067	145.6640927	0.183672887
7.052366483	239.8985138	200.8760085	0.13736288
6.85443921	181.6799117	102.1758646	0.163508041
6.979404379	421.3431013	255.6379731	0.16278756
7.164308322	518.2113182	349.9737256	0.14788351
6.964458717	167.0763583	109.0646109	0.192543437
7.034511026	408.7372977	165.2604441	0.195959934
7.0378337	344.4850445	166.1624899	0.184281037
6.41793598	47.84512428	58.17017208	0.130756892
7.149602611	539.701709	252.0300363	0.178699467
7.368297515	372.8796776	229.5757052	0.148293933
7.027368308	215.5182073	85.00840336	0.210749361
6.951346966	356.497864	241.2346369	0.143536567
5.872222793	50.44038929	47.59367397	0.185811349
6.604815737	78.84429066	42.74740484	0.180920765
7.064516638	390.5316101	224.6810962	0.184685273
6.825780035	157.4886878	74.77556561	0.196721581
6.708924791	210.1126761	137.4007682	0.16125049
7.168942109	481.997473	350.1086607	0.128203734
7.107406491	555.1840204	369.8045049	0.157052425
6.841819471	180.2058824	41.36199095	0.321635434
6.815390015	183.332388	169.9608622	0.131259202
6.356187026	56.60747664	31.47663551	0.193771089
6.819575682	87.48873874	83.00225225	0.129894027
6.82377544	234.202773	60.49046794	0.294609394

log.sigma.5.0.mm.3D_gldm_DependenceNonUniformityNormalized	log.sigma.5.0.mm.3D_gldm_DependenceVariance	log.sigma.5.0.mm.3D_gldm_LargeDependenceEmphasis
0.144802167	5.08036227	19.16625104
0.128444	5.304311	24.515
0.148810648	5.108579402	20.72450805
0.124056352	6.397058503	26.32001631
0.106285691	9.992074182	34.59325606
0.110591552	8.657362309	35.42124542
0.078914369	19.23921167	62.90917692
0.126630397	5.841356036	24.84709141
0.12955953	7.826434403	25.62259497
0.148715642	5.024126944	19.97369297
0.101013123	9.647780613	39.77919203
0.136409407	5.374538474	21.56023739
0.0967403	13.09810059	44.39160123
0.139551308	4.599048017	21.61356297
0.129463541	7.455574919	24.67700258
0.123814009	14.1403143	36.62715989
0.128925379	6.786993915	26.2623053
0.106078158	9.976192063	35.16419077
0.136180197	5.288670403	21.77112676
0.113668681	7.855634657	31.17511203
0.123536297	7.057550313	27.76201439
0.130039494	5.79540399	23.87694484
0.13832613	5.625525735	22.91021597
0.135701763	7.024786502	24.84466019
0.153416344	4.873632908	19.29262673
0.152290273	5.294860124	19.08136095
0.140526143	4.787349794	20.15332429
0.159819919	4.036321526	17.23129657
0.123747782	6.012482428	25.90791409
0.128195709	6.03183055	23.23108579
0.125104423	6.438252514	26.09512313
0.127982	6.78610224	24.49
0.111281196	8.589903373	32.21849072
0.131953959	9.264085131	26.6049896
0.121844818	8.594346035	30.22522523
0.119630143	6.730148325	29.22256761
0.147556623	5.367043235	20.25077052
0.121649315	6.896746388	29.29076968
0.093229852	10.53581237	44.47978437
0.102468302	12.32588429	39.97188264
0.127852431	7.040432923	25.95896657
0.116281736	8.793153556	31.9047619
0.109307063	8.457599695	35.38982491
0.103643488	9.889953841	35.15605096
0.11580323	12.1922917	35.09730967
0.131553739	5.472303115	24.60895105
0.132257999	6.369932248	23.78459034
0.104749863	9.005282266	35.38258575
0.111167698	7.904808611	30.08156028
0.117954718	8.0021345	29.87706422
0.10164768	9.703452364	37.8062249
0.0889351	12.38150978	50.32106339
0.111337846	7.681624647	32.32473005
0.134000515	5.437601414	23.05465414
0.135609497	6.457016011	23.97688595
0.141233774	5.513730789	21.46292373
0.132661095	6.508379728	23.51174138
0.081777303	16.92338718	56.62582237
0.134256347	5.625139185	23.74344023
0.118763947	6.360412862	32.83345196
0.109581257	9.884364623	33.36450187
0.116072521	7.134777501	29.26608347
0.121281271	7.03696814	27.11889036
0.164181252	3.635056101	16.30835118
0.16120655	4.912887101	17.43728814
0.137291781	4.900220048	20.61633182
0.115953345	8.187252761	30.71833648
0.128075685	5.446500869	24.22265844
0.126707017	5.549260729	25.85846868
0.129714025	7.409359259	26.51814918
0.158481039	4.315350865	17.06670168
0.108563863	7.79192042	32.30907327
0.125623891	7.372487541	26.39431502
0.130295267	5.611898025	24.51589618
0.131826751	5.952226314	23.13327076
0.13373458	5.530103739	22.84298584
0.101867734	9.486215803	36.96518047
0.133686469	6.027260013	24.57615894
0.12861511	6.038951281	25.13553114
0.116373528	7.597612693	30.21401302
0.122669867	7.196308012	26.24229075
0.153834135	4.013378002	18.05683101
0.139242136	4.875616353	21.18755053
0.091482073	15.79233646	51.13001912
0.139746688	5.596636792	22.41895391
0.107334392	10.47718872	36.28382268
0.150923114	4.775614167	19.00140056
0.117153422	6.722391045	29.66973382
0.122726008	6.313827174	25.34549878
0.136408807	5.914129381	23.06920415
0.121623049	6.448181426	25.84272812
0.142523699	5.039328433	20.65791855
0.134515158	6.379425898	25.36491677
0.110727653	8.360549315	34.0172295
0.117973655	7.31445457	29.00637484
0.203852808	2.57372392	10.55882353
0.103988876	9.435607266	37.44809983
0.132260459	5.6511049	22.71495327
0.098523354	12.23382335	42.70720721
0.202948677	2.92066777	11.59098787

log.sigma.5.0.mm.3D_gldm_LargeDependenceLowGrayLevelEmphasis	log.sigma.5.0.mm.3D_gldm_SmallDependenceHighGrayLevelEmphasis	log.sigma.5.0.mm.3D_gldm_LargeDependenceHighGrayLevelEmphasis
0.154759161	58.66210611	3987.115544
0.088220975	56.38773506	8143.8035
0.114996332	38.34173469	5405.170841
0.367866724	24.87135243	3094.586629
0.5749287	26.92338172	3426.119073
0.442040266	18.12270976	3868.39072
3.519780213	9.518296407	2183.964995
0.136427917	50.45864091	6783.754571
0.18111182	46.30763008	4840.26739
0.041295441	110.3864497	11768.5005
0.834046029	10.95058648	2965.121195
0.104973433	49.30976701	5764.456973
0.465093742	19.56417832	5197.088426
0.284418117	21.02056407	3044.911733
0.149512072	57.97226254	5571.095607
0.164591669	66.09778181	10884.46118
0.086192953	54.24536431	9465.322741
0.231219129	40.499872	7059.08835
0.107428218	44.54543194	5503.066901
0.227417396	35.91077856	6730.547397
0.090705523	55.27941008	10292.90777
0.126795735	47.48702366	6070.330504
0.086994589	62.85683294	7373.448192
0.330912184	34.23341617	3630.316505
0.207836783	42.42051604	4750.850998
0.10857786	61.50085311	4458.475592
0.12416542	44.29264497	4679.160561
0.079088613	54.56904932	5473.410432
0.076287123	60.57936154	10573.11974
0.053906118	90.64927978	11096.62805
0.102777821	52.58699722	8942.496137
0.108319856	76.33778437	9162.7456
0.068031317	97.63758739	18484.69696
0.31559329	38.55862183	3439.790021
0.468352938	18.26457908	3477.875
0.195721957	27.97318273	5445.693192
0.057964098	90.53723603	9278.568507
0.137605802	35.17121116	7480.847832
0.362415833	18.9988359	6425.705526
0.579999386	23.06871574	3888.168093
0.235194478	26.43176228	3662.588906
0.30095185	26.24825584	4316.186335
0.187685081	31.2933369	8055.847043
0.390977854	30.54706076	4290.806263
0.111005197	63.44724421	13699.61305
0.330266408	23.73829484	4317.313287
0.068480183	86.08043457	10390.33587
0.128732742	34.78110759	11363.14908
0.750166743	19.63435245	2214.427305
0.24360293	36.94293679	4848.699083
0.138398538	47.14375842	12147.25477
0.292684383	20.67487871	9943.740286
0.244806008	32.3656196	5704.946273
0.068653762	66.12396388	9109.850555
0.117390767	48.40751479	6656.134112
0.118209611	59.87212399	5844.327331
0.066636296	98.38430533	11078.6688
0.560252843	25.25742108	6876.599507
0.158378743	34.41846426	4690.182945
0.256687637	13.06457943	4970.062633
0.313377164	34.26218504	5281.378443
0.094683836	57.50661752	10818.2156
0.103441912	52.76273021	8533.889916
0.146234467	32.37576729	3359.255889
0.116594315	44.27873528	3521.29661
0.392410715	31.82816716	2192.690214
0.559414804	32.26324372	3211.746692
0.243877445	34.40634357	4034.281828
0.170164122	26.6098542	5243.180974
0.112617028	59.38284744	7989.462306
0.091754209	84.16900254	6991.182511
0.266443248	26.61964434	4780.868025
0.088570614	77.56239772	10272.79526
0.085005317	50.53884374	8798.121333
0.100051674	71.06730394	7126.156969
0.090743432	60.52217627	7493.241527
0.199168034	28.93368936	8184.740127
0.180848198	35.00652392	5081.104489
0.109233424	41.79509824	7131.667277
0.10449578	47.43997306	10522.38491
0.047737598	108.7210535	15978.53818
0.057331387	73.61212508	7381.631163
0.070182796	74.39682422	7712.676637
1.104190112	11.63903256	3139.382409
0.093113906	61.42375559	8411.086225
0.58465309	21.85665981	4065.520725
0.088029342	64.09803353	5606.822829
0.334463851	20.54610982	3803.022346
0.408254499	11.23444942	2434.705596
0.390940723	21.30382828	2994.221453
0.123022576	55.04931375	6589.434756
0.16693351	33.71854374	3757.628054
0.167610856	26.70679128	5002.848271
0.135808328	39.86609481	10259.57569
0.23553303	28.24686032	5288.59201
0.044219318	156.2799847	6423.547511
0.306419576	22.99970623	5470.983551
0.654477338	20.37882335	1967.455607
0.831789251	12.36696928	3116.809685
0.109339239	89.7090218	4458.208839

log.sigma.5.0.mm.3D_gldm_SmallDependenceLowGrayLevelEmphasis	log.sigma.5.0.mm.3D_gldm_LowGrayLevelEmphasis	log.sigma.5.0.mm.3D_gldzm_DistanceZoneVariabilityNormalized
0.003036404	0.009218212	1
0.001334783	0.004429805	1
0.002601414	0.008192108	1
0.003537886	0.015062675	1
0.002564926	0.018674469	1
0.002341393	0.013853279	1
0.003753819	0.043856264	1
0.001385251	0.007171829	1
0.002251988	0.007294923	1
0.000837513	0.002716958	1
0.002861332	0.022426615	1
0.001920261	0.006327161	1
0.00228167	0.011504673	1
0.003158921	0.015179315	1
0.002464045	0.006831935	1
0.000830538	0.005079153	1
0.001646698	0.005051981	1
0.00140843	0.006221541	1
0.002114812	0.006194213	1
0.001628032	0.009014832	1
0.000844375	0.003725909	1
0.001772003	0.006718397	1
0.00099247	0.00432389	1
0.003413283	0.017184238	1
0.001963984	0.010760489	1
0.003334616	0.007937475	1
0.002244194	0.007405347	1
0.001595572	0.005706657	1
0.001131503	0.003797817	1
0.001011795	0.003022238	1
0.001294913	0.005035935	0.997445726
0.000860464	0.004789345	0.99823009
0.000912452	0.003003732	1
0.004398668	0.014040272	1
0.004938267	0.023870598	1
0.001341465	0.007800779	1
0.000994543	0.003996393	0.997578696
0.001098485	0.005865089	1
0.001931173	0.009118812	1
0.002969143	0.014373158	1
0.002885204	0.010470496	1
0.001869997	0.009090119	1
0.001577953	0.005864331	1
0.002610121	0.011470177	1
0.001411418	0.004609461	1
0.002262704	0.014111624	1
0.000795806	0.00372186	0.997555016
0.001873551	0.006106709	1
0.005105812	0.02315082	1
0.002249633	0.008193499	1
0.000693661	0.004348772	1
0.001082029	0.007858945	1
0.001271757	0.007394124	1
0.001576078	0.00397772	1
0.001427687	0.005756715	1
0.00183209	0.006758297	1
0.000854138	0.003546821	0.997297302
0.001593911	0.009875059	1
0.001884679	0.008707356	1
0.002068382	0.009780906	1
0.001946435	0.008744065	1
0.00085215	0.004060649	1
0.001392992	0.004793221	1
0.003553504	0.012906076	1
0.00436434	0.009229364	1
0.00391817	0.018732932	1
0.002145357	0.018388129	1
0.002275994	0.010954309	1
0.002019767	0.007860885	1
0.001390432	0.004982372	1
0.001584746	0.006073252	1
0.002373933	0.009768981	1
0.000865734	0.003819459	1
0.000930457	0.004150422	0.992914173
0.001189192	0.00506771	1
0.001434303	0.005092852	1
0.001627874	0.006853711	1
0.001812791	0.010081728	1
0.001441834	0.005623288	1
0.001157836	0.004723317	1
0.001276287	0.002903565	1
0.001107975	0.004422498	1
0.001151407	0.004023594	1
0.003457736	0.020391639	1
0.001207039	0.005484688	0.99746515
0.002640747	0.014827556	1
0.002134238	0.006376315	1
0.002368817	0.011912715	1
0.011619746	0.025006577	1
0.004482423	0.024333136	1
0.001218638	0.004774411	1
0.00373115	0.010575171	1
0.001960536	0.009568385	1
0.000886201	0.004773823	1
0.001582757	0.008924682	1
0.002710379	0.005406882	1
0.001913921	0.008634243	1
0.005906262	0.02524147	1
0.003931487	0.019450353	1
0.002494374	0.009754398	0.99530519

log.sigma.5.0.mm.3D_gldzm_LowIntensityEmphasis	log.sigma.5.0.mm.3D_gldzm_LargeDistanceEmphasis	log.sigma.5.0.mm.3D_gldzm_HighIntensitySmallDistanceEmphasis
0.01299379	1	263.191358
0.008201041	1	377.0674157
0.013315676	1	215.8015267
0.022000644	1	157.7404063
0.019166875	1	168.1620112
0.020020989	1	154.5668203
0.033392667	1	85.90298507
0.009983244	1	307.3105413
0.010373859	1	231.4123711
0.004147864	1	560.8323944
0.032281539	1	94.93087558
0.009317197	1	260.9143577
0.017738055	1	159.8968825
0.020769585	1	127.3068493
0.010518243	1	276.7005076
0.005947281	1	375.0176056
0.009603069	1	339.164557
0.008708572	1	266.875
0.010454606	1	234.036
0.013197725	1	243.4244306
0.005398523	1	354.8033708
0.010164689	1	269.1617312
0.005827028	1	373.4575243
0.020704238	1	188.7627119
0.01098705	1	214.0031397
0.014024588	1	285.3697479
0.010886403	1	222.1111111
0.007698982	1	258.4444444
0.007080732	1	373.3902848
0.004931009	1	440.0998613
0.007974996	1.003836317	315.476023
0.005210275	1.002657219	383.9904783
0.005985802	1	622.881549
0.020540865	1	170.6865672
0.034450156	1	115.0166667
0.011341959	1	201.2515856
0.005696289	1.003636364	441.7818182
0.008966872	1	245.6785714
0.017636943	1	180.3426966
0.018617373	1	160.972028
0.016293667	1	150.0538462
0.012119075	1	179.4523077
0.013132457	1	252.237467
0.016330025	1	188.9505814
0.007860336	1	354.380109
0.016783741	1	155.096828
0.004952812	1.003671971	456.6058752
0.013943537	1	244.2025316
0.026276928	1	114.5203252
0.013252818	1	238.2713568
0.006556691	1	364.0566372
0.016593804	1	214.1308901
0.009456167	1	241.479927
0.008443292	1	374.9521739
0.00795391	1	265.491206
0.009634375	1	313.4730679
0.005095857	1.00405954	488.1948579
0.012865693	1	214.7163743
0.013464802	1	214.5313653
0.021715402	1	124.2551724
0.011274723	1	204.7814208
0.006365007	1	367.6739368
0.00781016	1	312.8454746
0.018830352	1	150.8017241
0.015837362	1	188.2234637
0.019514658	1	166.810585
0.016780373	1	214.1485149
0.014598425	1	213.5987749
0.01404447	1	182.902439
0.007827699	1	353.3917323
0.007146244	1	365.2892483
0.017436091	1	189.7009346
0.004650846	1	429.2971014
0.006018985	1.010666667	312.0851111
0.006622129	1	374.462845
0.008039205	1	329.8360324
0.013145812	1	203.2063037
0.013273645	1	221.6830189
0.008930164	1	256.8267327
0.008078501	1	315.6315087
0.006207321	1	580.7359736
0.006516753	1	374.2252836
0.006437025	1	412.1565558
0.031847941	1	89.08860759
0.007778871	1.003807107	340.2449239
0.019000873	1	155.6892857
0.010206298	1	310.1675978
0.016914306	1	148.6861472
0.055934038	1	61.57777778
0.030176927	1	114.6451613
0.006382761	1	308.8542636
0.018091682	1	180.1377953
0.014464163	1	165.6228956
0.007567826	1	312.5784148
0.010898658	1	184.7568238
0.007903661	1	494.7748538
0.01412614	1	178.8918919
0.029568621	1	108.9484536
0.027655987	1	102.3285714
0.010110096	1.007058824	309.9864706

log.sigma.5.0.mm.3D_gldzm_LowIntensityLargeDistanceEmphasis	log.sigma.5.0.mm.3D_gldzm_HighIntensityEmphasis	log.sigma.5.0.mm.3D_gldzm_DistanceZoneVariability	log.sigma.5.0.mm.3D_gldzm_ZonePercentage
0.01299379	263.191358	324	0.269326683
0.008201041	377.0674157	356	0.178
0.013315676	215.8015267	262	0.234347048
0.022000644	157.7404063	443	0.18059519
0.019166875	168.1620112	179	0.1886196
0.020020989	154.5668203	217	0.132478632
0.033392667	85.90298507	134	0.126773888
0.009983244	307.3105413	351	0.194459834
0.010373859	231.4123711	485	0.239269857
0.004147864	560.8323944	710	0.236430236
0.032281539	94.93087558	217	0.120088545
0.009317197	260.9143577	397	0.235608309
0.017738055	159.8968825	417	0.142369409
0.020769585	127.3068493	365	0.196447793
0.010518243	276.7005076	394	0.254521964
0.005947281	375.0176056	852	0.20734972
0.009603069	339.164557	316	0.196884735
0.008708572	266.875	440	0.172009382
0.010454606	234.036	250	0.220070423
0.013197725	243.4244306	483	0.166494312
0.005398523	354.8033708	1246	0.179280576
0.010164689	269.1617312	439	0.206977841
0.005827028	373.4575243	824	0.199951468
0.020704238	188.7627119	236	0.229126214
0.01098705	214.0031397	637	0.244623656
0.014024588	285.3697479	357	0.264053254
0.010886403	222.1111111	513	0.23202171
0.007698982	258.4444444	828	0.246795827
0.007080732	373.3902848	597	0.175639894
0.004931009	440.0998613	721	0.228236784
0.007982248	315.983376	780.0025575	0.188797682
0.0052163	384.2834367	1127.001771	0.2258
0.005985802	622.881549	439	0.17344923
0.020540865	170.6865672	134	0.278586279
0.034450156	115.0166667	180	0.202702703
0.011341959	201.2515856	473	0.147031396
0.00569984	442.7127273	823.0024242	0.231157187
0.008966872	245.6785714	504	0.148628723
0.017636943	180.3426966	178	0.119946092
0.018617373	160.972028	286	0.174816626
0.016293667	150.0538462	260	0.197568389
0.012119075	179.4523077	325	0.168219462
0.013132457	252.237467	379	0.125206475
0.016330025	188.9505814	344	0.182590234
0.007860336	354.380109	734	0.210074413
0.016783741	155.096828	599	0.167552448
0.004964145	456.9033048	815.002448	0.221649485
0.013943537	244.2025316	237	0.156332454
0.026276928	114.5203252	123	0.218085106
0.013252818	238.2713568	199	0.182568807
0.006556691	364.0566372	565	0.141817269
0.016593804	214.1308901	191	0.097648262
0.009456167	241.479927	548	0.144324467
0.008443292	374.9521739	230	0.196413322
0.00795391	265.491206	796	0.202184404
0.009634375	313.4730679	427	0.226165254
0.005106006	488.6008119	737.0027064	0.22537359
0.012865693	214.7163743	342	0.140625
0.013464802	214.5313653	271	0.197521866
0.021715402	124.2551724	145	0.103202847
0.011274723	204.7814208	549	0.1866712
0.006365007	367.6739368	917	0.167122289
0.00781016	312.8454746	453	0.199471598
0.018830352	150.8017241	464	0.248394004
0.015837362	188.2234637	179	0.303389831
0.019514658	166.810585	359	0.232663642
0.016780373	214.1485149	202	0.190926276
0.014598425	213.5987749	653	0.18311834
0.01404447	182.902439	205	0.158546017
0.007827699	353.3917323	508	0.202632629
0.007146244	365.2892483	1051	0.275997899
0.017436091	189.7009346	321	0.155749636
0.004650846	429.2971014	966	0.206454371
0.006063681	312.7564444	1117.028444	0.172784519
0.006622129	374.462845	942	0.221022994
0.008039205	329.8360324	494	0.211926212
0.013145812	203.2063037	349	0.148195329
0.013273645	221.6830189	265	0.194996321
0.008930164	256.8267327	606	0.184981685
0.008078501	315.6315087	749	0.168201213
0.006207321	580.7359736	303	0.22246696
0.006516753	374.2252836	617	0.232216786
0.006437025	412.1565558	511	0.2065481
0.031847941	89.08860759	79	0.151051625
0.007816942	340.3401015	786.0025381	0.204039358
0.019000873	155.6892857	560	0.161197467
0.010206298	310.1675978	358	0.25070028
0.016914306	148.6861472	462	0.151823858
0.055934038	61.57777778	90	0.218978102
0.030176927	114.6451613	124	0.214532872
0.006382761	308.8542636	645	0.200872002
0.018091682	180.1377953	254	0.229864253
0.014464163	165.6228956	297	0.190140845
0.007567826	312.5784148	593	0.136227889
0.010898658	184.7568238	806	0.171270718
0.007903661	494.7748538	342	0.386877828
0.01412614	178.8918919	259	0.146908678
0.029568621	108.9484536	97	0.226635514
0.027655987	102.3285714	140	0.157657658
0.010197242	310.1294118	423.0047059	0.368284229

log.sigma.5.0.mm.3D_gldzm_IntensityVariabilityNormalized	log.sigma.5.0.mm.3D_gldzm_LowIntensitySmallDistanceEmphasis	log.sigma.5.0.mm.3D_gldzm_IntensityVariability
0.053974242	0.01299379	17.48765432
0.060740437	0.008201041	21.62359551
0.06010722	0.013315676	15.7480916
0.061019419	0.022000644	27.03160271
0.069941637	0.019166875	12.51955307
0.062753509	0.020020989	13.61751152
0.104143462	0.033392667	13.95522388
0.054260923	0.009983244	19.04558405
0.057549155	0.010373859	27.91134021
0.052612577	0.004147864	37.35492958
0.077618977	0.032281539	16.84331797
0.05822637	0.009317197	23.11586902
0.061734785	0.017738055	25.74340528
0.06889848	0.020769585	25.14794521
0.050194543	0.010518243	19.77664975
0.052064736	0.005947281	44.35915493
0.052014901	0.009603069	16.43670886
0.060743802	0.008708572	26.72727273
0.07248	0.010454606	18.12
0.059175529	0.013197725	28.58178054
0.057131778	0.005398523	71.18619583
0.057341961	0.010164689	25.17312073
0.052582124	0.005827028	43.3276699
0.064062051	0.020704238	15.11864407
0.058858612	0.01098705	37.49293564
0.047069808	0.014024588	16.80392157
0.056461817	0.010886403	28.96491228
0.062341011	0.007698982	51.61835749
0.051258526	0.007080732	30.60134003
0.049013064	0.004931009	35.33841886
0.05352202	0.007973183	41.85421995
0.055839252	0.005208768	63.0425155
0.049050181	0.005985802	21.53302961
0.063934061	0.020540865	8.567164179
0.068641975	0.034450156	12.35555556
0.065986081	0.011341959	31.21141649
0.04441359	0.005695401	36.64121212
0.064633724	0.008966872	32.57539683
0.069372554	0.017636943	12.34831461
0.065577779	0.018617373	18.75524476
0.070236686	0.016293667	18.26153846
0.070655621	0.012119075	22.96307692
0.06522511	0.013132457	24.72031662
0.058443753	0.016330025	20.10465116
0.044873746	0.007860336	32.9373297
0.066357117	0.016783741	39.74791319
0.053212862	0.004949978	43.4749082
0.053819723	0.013943537	12.75527426
0.088373323	0.026276928	10.8699187
0.057296533	0.013252818	11.40201005
0.057128984	0.006556691	32.27787611
0.059949015	0.016593804	11.45026178
0.061231286	0.009456167	33.55474453
0.059508507	0.008443292	13.68695652
0.056415873	0.00795391	44.90703518
0.047436228	0.009634375	20.25526932
0.050283728	0.00509332	37.15967524
0.056581512	0.012865693	19.35087719
0.059571629	0.013464802	16.14391144
0.080808561	0.021715402	11.71724138
0.062053543	0.011274723	34.06739526
0.051028138	0.006365007	46.79280262
0.058706002	0.00781016	26.59381898
0.063113109	0.018830352	29.28448276
0.065697076	0.015837362	11.75977654
0.063772007	0.019514658	22.89415042
0.057200274	0.016780373	11.55445545
0.054874545	0.014598425	35.8330781
0.071314694	0.01404447	14.6195122
0.050677351	0.007827699	25.74409449
0.047266841	0.007146244	49.67745005
0.063207849	0.017436091	20.28971963
0.05739662	0.004650846	55.44513458
0.061140543	0.006007811	68.78311111
0.048228236	0.006622129	45.43099788
0.05160714	0.008039205	25.49392713
0.058644839	0.013145812	20.46704871
0.064293343	0.013273645	17.03773585
0.058213247	0.008930164	35.27722772
0.053185288	0.008078501	39.83578104
0.044734176	0.006207321	13.55445545
0.047069918	0.006516753	29.04213938
0.053205219	0.006437025	27.18786693
0.0985419	0.031847941	7.784810127
0.05736105	0.007769353	45.20050761
0.061913265	0.019000873	34.67142857
0.048172654	0.010206298	17.24581006
0.073490002	0.016914306	33.95238095
0.11382716	0.055934038	10.24444444
0.073491155	0.030176927	9.112903226
0.05877291	0.006382761	37.90852713
0.059520119	0.018091682	15.11811024
0.070752418	0.014464163	21.01346801
0.054619806	0.007567826	32.38954469
0.066751227	0.010898658	53.80148883
0.037960398	0.007903661	12.98245614
0.076251099	0.01412614	19.74903475
0.087682007	0.029568621	8.505154639
0.078265306	0.027655987	10.95714286
0.046687889	0.010088309	19.84235294

log.sigma.5.0.mm.3D_gldzm_HighIntensityLargeDistanceEmphasis	log.sigma.5.0.mm.3D_gldzm_SmallDistanceEmphasis	log.sigma.5.0.mm.3D_glcm_SumVariance	log.sigma.5.0.mm.3D_glcm_Homogeneity1
263.191358	1	728.5413701	0.42661616
377.0674157	1	1131.104236	0.464814179
215.8015267	1	661.7887271	0.4415556
157.7404063	1	363.3521952	0.482310857
168.1620112	1	362.2538924	0.502927285
154.5668203	1	345.6985271	0.512415323
85.90298507	1	143.4726339	0.5792656
307.3105413	1	905.9949519	0.463224872
231.4123711	1	679.7882922	0.461382268
560.8323944	1	1849.112604	0.434021516
94.93087558	1	223.2145571	0.533471272
260.9143577	1	808.1566699	0.445627239
159.8968825	1	360.0809873	0.533704391
127.3068493	1	356.4737138	0.459138083
276.7005076	1	806.5699855	0.436599223
375.0176056	1	1138.465361	0.468578816
339.164557	1	1079.594951	0.455332221
266.875	1	735.9710932	0.495836989
234.036	1	744.4006885	0.457168956
243.4244306	1	699.3468836	0.489534155
354.8033708	1	1139.879202	0.463297207
269.1617312	1	759.9173944	0.461130255
373.4575243	1	1048.546912	0.453424482
188.7627119	1	448.676894	0.454576658
214.0031397	1	650.0602505	0.43033339
285.3697479	1	801.3551684	0.4228861
222.1111111	1	737.5820074	0.481641343
258.4444444	1	884.8332361	0.423047752
373.3902848	1	1268.197022	0.460755952
440.0998613	1	1530.77142	0.457448072
318.0127877	0.999040921	1015.585152	0.468833963
385.4552702	0.999335695	1241.395652	0.457436739
622.881549	1	1977.254332	0.488891484
170.6865672	1	490.694122	0.453199073
115.0166667	1	293.1674492	0.484490978
201.2515856	1	558.147174	0.492334084
446.4363636	0.999090909	1420.68627	0.424994267
245.6785714	1	754.7581267	0.489512869
180.3426966	1	453.8038536	0.553362188
160.972028	1	349.6082323	0.510535186
150.0538462	1	432.9782495	0.472370362
179.4523077	1	449.6938785	0.492136616
252.237467	1	771.6609927	0.50994957
188.9505814	1	442.7091154	0.49822192
354.380109	1	1221.354464	0.450994016
155.096828	1	444.1546976	0.471702157
458.0930233	0.999082007	1392.90397	0.44992041
244.2025316	1	893.6563908	0.501519454
114.5203252	1	243.8465342	0.489818812
238.2713568	1	590.5171527	0.486069012
364.0566372	1	1117.442961	0.514948471
214.1308901	1	632.2829501	0.556378221
241.479927	1	603.9118954	0.503677944
374.9521739	1	1295.195928	0.452488709
265.491206	1	856.2888124	0.445671908
313.4730679	1	873.7903391	0.432321718
490.2246279	0.998985115	1618.030592	0.445636764
214.7163743	1	466.9333798	0.552093988
214.5313653	1	590.2966873	0.465749266
124.2551724	1	409.1661094	0.52532681
204.7814208	1	571.4040284	0.49338791
367.6739368	1	1163.900136	0.479374478
312.8454746	1	964.6307386	0.469401679
150.8017241	1	492.5299229	0.41532731
188.2234637	1	618.5900642	0.415323228
166.810585	1	336.5291823	0.444007688
214.1485149	1	413.4077698	0.490630696
213.5987749	1	522.9178835	0.462403412
182.902439	1	572.3298533	0.479543933
353.3917323	1	1023.427406	0.459045776
365.2892483	1	1234.313103	0.399601397
189.7009346	1	488.0824052	0.500751869
429.2971014	1	1318.964349	0.463039961
315.4417778	0.997333333	1064.41236	0.462531676
374.462845	1	1014.661174	0.447183624
329.8360324	1	1026.679276	0.441429507
203.2063037	1	676.8212115	0.514919621
221.6830189	1	632.7690391	0.460311775
256.8267327	1	816.0651354	0.458488605
315.6315087	1	1054.011562	0.478193603
580.7359736	1	2010.247522	0.451357601
374.2252836	1	1192.403413	0.419655362
412.1565558	1	1185.155779	0.442038258
89.08860759	1	228.2170796	0.553522875
340.7208122	0.999048223	1114.814128	0.452267465
155.6892857	1	392.0070242	0.503850583
310.1675978	1	935.5074384	0.428745358
148.6861472	1	381.0218675	0.49320155
61.57777778	1	211.3452227	0.478258791
114.6451613	1	329.0873532	0.463573705
308.8542636	1	882.6255637	0.46954079
180.1377953	1	531.5762897	0.446150798
165.6228956	1	525.4454141	0.475978546
312.5784148	1	944.3205449	0.503626152
184.7568238	1	534.7138233	0.487523376
494.7748538	1	1686.318809	0.328755681
178.8918919	1	471.2761388	0.523283524
108.9484536	1	258.0987801	0.46505285
102.3285714	1	239.9982516	0.539721929
310.7011765	0.998235294	1033.58961	0.362344147

log.sigma.5.0.mm.3D_glcm_Homogeneity2	log.sigma.5.0.mm.3D_glcm_ClusterShade	log.sigma.5.0.mm.3D_glcm_MaximumProbability	log.sigma.5.0.mm.3D_glcm_Idmn	log.sigma.5.0.mm.3D_glcm_SumVariance2
0.351749252	-41.14717845	0.020846947	0.984962822	98.45140966
0.395694079	38.93824725	0.029549651	0.991998333	62.64630722
0.367510495	-116.3237763	0.030083846	0.989778746	65.27846112
0.416784827	238.2748477	0.029599935	0.989787008	61.34840678
0.444359686	211.4291364	0.057492564	0.985262059	63.63818418
0.454465615	317.3087124	0.064057709	0.990660614	56.1014979
0.53463674	185.709708	0.094777503	0.985853244	48.97999852
0.394747594	70.08850157	0.030194969	0.991661168	83.92357094
0.392699201	200.0340696	0.037520031	0.989044315	74.00848954
0.357548832	-197.0975433	0.020992647	0.993511931	83.88017318
0.480520557	113.9550882	0.054150076	0.986680815	37.91468333
0.371391237	-73.17134995	0.022695775	0.989763646	53.74586202
0.479327245	145.5688412	0.065278502	0.990692359	41.33395774
0.387174003	-0.94256131	0.025001361	0.985514463	47.79442618
0.36539767	526.7030781	0.02694599	0.985355085	88.38763138
0.39941938	-103.2698086	0.056821567	0.991460416	86.51425216
0.383487232	-121.1433532	0.03316972	0.991722285	62.86874592
0.435287176	188.8622551	0.035158713	0.992618152	69.95512989
0.38583211	-105.5779751	0.02668804	0.986656491	41.92938659
0.426898395	-92.75768332	0.034342154	0.991656037	66.49003186
0.394318609	-158.0981973	0.026227894	0.99376561	60.77509465
0.391235196	65.6986242	0.02161053	0.993236678	70.75653541
0.38116551	260.7187456	0.025938234	0.993891021	72.59472574
0.382757565	-6.938593142	0.03682356	0.984109529	76.13954609
0.352840426	-349.9196237	0.022481054	0.986390391	81.3983014
0.34504579	201.5816957	0.024380489	0.988429824	81.99161686
0.415284889	99.7737221	0.023487799	0.991205973	71.72514097
0.343493256	-247.4595756	0.018706119	0.988785887	66.29019977
0.391154008	-255.2092899	0.03011441	0.991842731	54.87851088
0.388768307	-76.47916618	0.028369998	0.994110286	61.56941361
0.400828907	-162.0157893	0.023528158	0.992132243	77.84825252
0.387131384	-112.9181008	0.024460995	0.992739401	68.80286179
0.425687623	-405.8492348	0.04153394	0.994765359	87.33448692
0.382131873	56.15642421	0.042289405	0.981526231	60.84834808
0.420483449	61.25930041	0.043129551	0.983403393	51.09637231
0.429756297	86.53164554	0.035912348	0.990159802	44.12102493
0.347059248	65.84208209	0.022420692	0.993322079	100.0901791
0.425097425	-26.98226809	0.035537454	0.991839358	46.83477659
0.505391658	141.5279337	0.052122494	0.992936272	36.27438958
0.452236989	224.8669964	0.053630961	0.987658892	58.6732046
0.404546967	43.63824561	0.038522821	0.985306022	40.81061953
0.428453146	100.6050546	0.041216244	0.98893078	41.39489391
0.45105269	136.7274206	0.039207678	0.992084248	49.87501753
0.438661054	230.3136521	0.05076961	0.986748452	59.21773832
0.383511648	-398.7119406	0.035266953	0.987845014	87.05946898
0.402247369	-28.72640695	0.018662023	0.989723938	68.6547897
0.377419263	-127.1190389	0.022061827	0.993357028	75.30316802
0.443022344	-357.9121763	0.04421118	0.990550159	53.71814743
0.427171302	80.517106	0.053949984	0.978564608	50.41453225
0.423330908	253.3151657	0.03703387	0.990083997	66.30706147
0.458069582	168.5272995	0.048254646	0.994078119	58.72393743
0.510294418	48.89238635	0.06925724	0.992224706	35.24448457
0.444533579	257.4615696	0.035748771	0.992559533	67.60355813
0.381618126	62.08030165	0.027149612	0.989279059	57.03517513
0.370850452	-64.88785517	0.019458346	0.988470013	69.68709795
0.356171806	-45.99154276	0.018596282	0.989893849	93.62027164
0.374079388	-203.9616731	0.025265275	0.992277958	75.55704194
0.503872685	416.9371762	0.084289875	0.992285857	60.40357957
0.396525056	84.67118815	0.034656205	0.989393344	50.32490829
0.470208403	-20.03156905	0.041944162	0.98958444	26.64523096
0.432294478	141.905078	0.028295333	0.988870853	76.02839427
0.414067458	-48.85385361	0.033410174	0.993778825	64.45212146
0.401633632	-104.3414517	0.027269425	0.991917209	57.06519366
0.333053694	-117.1450639	0.020422322	0.981616414	62.31061817
0.336373075	-53.37335468	0.027142151	0.979805611	45.85828996
0.371486815	304.7028302	0.029384171	0.985984561	72.81648052
0.429123487	521.8664692	0.032780365	0.989550083	92.29714461
0.393561348	276.3452594	0.021858662	0.986821719	84.32125683
0.414120262	0.785647583	0.030916963	0.98754947	48.48345459
0.388244341	-1.148904915	0.029114906	0.992435318	78.56372287
0.318967522	-910.8241347	0.015092296	0.987950191	117.685192
0.441914687	219.1473933	0.045251814	0.987696839	47.37563634
0.393726361	-189.8447295	0.03134299	0.993898996	75.37604512
0.392322367	-226.6093959	0.026272713	0.992319697	54.9611417
0.374135211	157.7199378	0.019912884	0.994196951	90.65963422
0.36844918	-117.546451	0.024703752	0.992195447	73.31123049
0.458875445	-65.83385605	0.038821852	0.991820871	44.79916087
0.390521726	-50.99334168	0.036390427	0.986988457	59.31196187
0.388437588	-130.7838192	0.029686379	0.990528472	55.70238096
0.412551259	-274.1337196	0.034411234	0.99280805	63.73770218
0.384860181	-385.6191027	0.041447147	0.991872168	75.9948172
0.341316781	-138.3085704	0.0221974	0.991641368	81.60663032
0.367485104	229.4317074	0.029476009	0.993188822	71.17116353
0.504074052	43.42942335	0.091205332	0.979628372	27.67395369
0.379495382	-307.5227585	0.022781392	0.992699639	74.08675654
0.443538854	253.5193226	0.029483839	0.990124216	70.5600483
0.352602717	-85.64924371	0.023659362	0.989529787	85.74012939
0.430249741	75.0519531	0.033672783	0.987735583	47.08249495
0.411392333	-25.94114841	0.066025609	0.967438476	18.13210792
0.392481033	-15.98833932	0.038016417	0.9825013	55.59648688
0.402840939	183.5059554	0.027602831	0.991038294	60.97833455
0.37185421	98.63273182	0.030327737	0.986040218	64.19194119
0.408121367	-81.11258684	0.038036433	0.988027239	42.64559731
0.443118909	13.76300407	0.04036271	0.994153118	58.10406097
0.423454448	-31.44662306	0.030565881	0.989761754	47.92146647
0.239155365	-958.1475475	0.015611862	0.983049827	158.2597922
0.468462032	116.1878992	0.052338131	0.990354558	40.04410914
0.394734326	32.92931777	0.038588154	0.979879012	53.04712688
0.487223263	109.4985873	0.075924839	0.986509609	39.17381888
0.272663785	-48.24727314	0.018409192	0.986661081	123.2369244

log.sigma.5.0.mm.3D_glcm_Contrast	log.sigma.5.0.mm.3D_glcm_DifferenceEntropy	log.sigma.5.0.mm.3D_glcm_InverseVariance	log.sigma.5.0.mm.3D_glcm_Entropy	log.sigma.5.0.mm.3D_glcm_Dissimilarity
11.76088222	2.862650534	0.353677676	7.655807187	2.489428451
7.463546751	2.585004228	0.386476617	7.141353704	2.014913493
8.91210065	2.717620004	0.35776657	7.262267749	2.252150378
6.169957494	2.472136308	0.40485079	7.074762322	1.835248781
7.017435407	2.494406627	0.405663986	6.664689372	1.848859209
5.163936341	2.356376609	0.423057243	6.648298598	1.650213653
3.859403517	2.13417965	0.416127284	5.788723412	1.352303755
8.371025442	2.647645658	0.384683528	7.38992729	2.084833035
9.088912344	2.708101039	0.375904308	7.323528369	2.15385706
9.105796003	2.736632355	0.353694859	7.642284803	2.282912554
4.582328362	2.291527306	0.430937344	6.447441637	1.534854075
8.363167714	2.663322042	0.365359532	7.221550008	2.168132736
4.713001468	2.322934241	0.417697177	6.592800252	1.549680548
6.741196208	2.531195815	0.383873499	7.03762445	1.977988003
14.25422957	2.915561	0.343000085	7.600193897	2.62044989
9.084459487	2.729593409	0.349960516	7.566343129	2.167246818
8.256552801	2.647728594	0.367050745	7.223215538	2.127746669
7.371488548	2.555203593	0.399040177	7.154865869	1.901014738
8.183501751	2.60790121	0.37425743	6.914361174	2.099114831
6.798059635	2.537669582	0.401064324	7.139597025	1.877045551
7.844392906	2.636064289	0.382309674	7.361880903	2.060028191
7.577339973	2.596940774	0.384248519	7.323345474	2.041039393
7.669811228	2.623690843	0.376940743	7.422441481	2.08008104
8.148486505	2.614179058	0.362858253	7.184960477	2.143574051
8.904515002	2.714043401	0.35252058	7.579104347	2.286905684
10.92136011	2.836155055	0.341160926	7.573910642	2.461683811
6.692464515	2.504961357	0.390023544	7.176376482	1.891466948
9.122714164	2.735320873	0.344870444	7.518363603	2.335546556
8.142454717	2.618796033	0.381888474	7.138072085	2.082006156
9.228332356	2.707485587	0.381726925	7.356755405	2.174817881
7.336256096	2.590089752	0.389541833	7.445336371	1.990702396
8.686478486	2.685191855	0.373256919	7.437869736	2.143572782
7.394959286	2.547954468	0.396480134	7.166454472	1.90878571
10.75826623	2.730441279	0.350247628	6.967615279	2.319868343
7.093891886	2.543729451	0.388542162	6.894782703	1.940771782
5.926396881	2.449691865	0.412437495	6.871289492	1.782216419
10.46928713	2.8109959	0.345422486	7.799039366	2.408165067
5.703998333	2.440910816	0.405181983	6.913398872	1.779247959
3.864676241	2.161161485	0.446074801	6.24393771	1.399965753
6.360629222	2.460398383	0.402862979	6.772717755	1.772893495
6.897864652	2.539119246	0.390322702	6.827335616	1.944343793
6.151304259	2.47309616	0.398098857	6.789206115	1.814451759
5.574636258	2.411931487	0.415251867	6.804009317	1.692476973
8.340462236	2.574645446	0.396908999	6.924874796	1.94748817
14.13601358	2.926053742	0.347700522	7.709967056	2.563353217
6.144465993	2.495014062	0.389694096	7.310247285	1.884812552
8.867396547	2.711348207	0.365354519	7.560479421	2.191107448
7.245514098	2.546875943	0.406495776	6.841967296	1.869176764
6.799001964	2.406397326	0.397749826	6.354656195	1.884472998
7.633189658	2.571509048	0.399048959	7.00636427	1.944635512
5.886435205	2.428672626	0.417255628	6.900950788	1.701515741
4.703101193	2.266916834	0.442368645	6.228652821	1.463415291
6.078466264	2.451044115	0.418451133	7.033829365	1.749989687
10.45657596	2.676212647	0.372215109	7.137219268	2.239682266
8.823280642	2.706112535	0.352498895	7.512825233	2.21433586
10.12989272	2.794167739	0.348845154	7.740742097	2.363084527
10.4341812	2.779072862	0.366550853	7.538519085	2.306530699
6.412228652	2.414760602	0.40981531	6.581968314	1.627204324
6.887947645	2.552051654	0.386776501	6.981209214	1.979083259
3.921102904	2.198907059	0.445680121	6.266006724	1.481286184
7.390526291	2.579363739	0.400072763	7.277023922	1.906141012
7.429312852	2.574763657	0.393756149	7.232312323	1.952876817
7.539736823	2.58969367	0.387920222	7.163695546	2.005842611
9.441967533	2.742661808	0.3320138	7.426416169	2.39452499
11.5424205	2.806122325	0.334246565	7.051094065	2.54884053
9.325317428	2.713866756	0.370353125	7.290892406	2.242518934
7.472816193	2.560039227	0.400025461	7.137900699	1.923197886
8.837721092	2.679516	0.380091432	7.484388663	2.118716116
6.373744829	2.444702325	0.406640826	6.844510599	1.863205853
7.992567207	2.65039731	0.372245252	7.430760657	2.093745515
13.71635637	2.991810836	0.320480013	8.100666779	2.779047202
7.105526561	2.473342022	0.418206651	6.678945856	1.822649443
8.137467112	2.646057808	0.378758192	7.412297031	2.078356669
7.135804506	2.577010363	0.386015841	7.219803324	1.997946687
9.069120609	2.719737175	0.364467429	7.706379587	2.212500065
9.894094725	2.766008794	0.361825261	7.51600952	2.305219352
5.773041943	2.416274148	0.423899064	6.761751767	1.693894799
8.613260952	2.65262294	0.371360593	7.137008273	2.134865565
8.301899505	2.654142123	0.376850526	7.241498827	2.113330218
7.13066467	2.57454578	0.391889074	7.197068177	1.948638598
13.68824378	2.897421019	0.369246305	7.326342941	2.489899557
10.58249403	2.818885782	0.346381284	7.691928367	2.447151395
8.617694186	2.676430606	0.36972986	7.375233233	2.185875958
5.160159164	2.222186492	0.410415232	5.68534065	1.534801813
7.693357587	2.615625406	0.374038705	7.457347336	2.08874345
6.515224903	2.509038574	0.400422442	7.176915915	1.798885152
10.53907608	2.798075062	0.352707414	7.631655974	2.396872157
5.702997909	2.426863837	0.410637353	6.856487976	1.763191104
6.271753321	2.225019872	0.397388551	5.552323815	1.867755948
6.744207668	2.500215027	0.381509412	6.805328891	1.967470928
7.882982112	2.61894493	0.393656681	7.197845752	2.023170877
8.503214926	2.680413241	0.361358985	7.239160589	2.181148359
6.045837153	2.475187819	0.39454898	6.86528128	1.864266416
5.399268026	2.404378011	0.414801815	6.978173288	1.703728005
6.182601421	2.484977742	0.405199262	7.043070245	1.823137938
25.86463073	3.393344995	0.241523173	8.296586645	3.92191942
4.902704099	2.29472104	0.433500203	6.38405265	1.5845655
7.053384621	2.462640085	0.378054047	6.649135724	1.994960152
4.669701333	2.256081178	0.421526917	6.280965352	1.526746923
16.12426293	3.070605364	0.279368081	8.038589041	3.120962078

log.sigma.5.0.mm.3D_glcm_DifferenceVariance	log.sigma.5.0.mm.3D_glcm_Idn	log.sigma.5.0.mm.3D_glcm_Idm	log.sigma.5.0.mm.3D_glcm_Correlation	log.sigma.5.0.mm.3D_glcm_Autocorrelation
5.359100871	0.920853427	0.351749252	0.786424887	251.7385136
3.238282822	0.939950684	0.395694079	0.787254397	367.5306322
3.665285674	0.93137484	0.367510495	0.760301964	229.6710475
2.699148738	0.932542871	0.416784827	0.817529385	138.0425548
3.335234623	0.924729906	0.444359686	0.801820714	135.741542
2.337871466	0.936506061	0.454465615	0.828057204	130.9101477
1.823185065	0.927742999	0.53463674	0.850324547	60.51059499
3.889478684	0.940129276	0.394747594	0.81790951	304.9495731
4.321749078	0.932675728	0.392699201	0.781139106	235.5956732
3.744679756	0.94413325	0.357548832	0.80400834	575.9123498
2.137031465	0.926339741	0.480520557	0.782892778	90.64246691
3.475777385	0.931602319	0.371391237	0.731617088	272.5898186
2.231606883	0.937775383	0.479327245	0.793992725	135.6289314
2.706065411	0.918468364	0.387174003	0.752431536	135.2502267
6.851662816	0.925329032	0.36539767	0.725284754	273.57504
4.174154503	0.939831946	0.39941938	0.810803027	373.3062135
3.538332478	0.938723135	0.383487232	0.765946144	352.8118835
3.593548825	0.945219142	0.435287176	0.810089355	253.0991032
3.476232089	0.924189427	0.38583211	0.675121237	251.3836868
3.163275615	0.940350026	0.426898395	0.814322184	241.7913661
3.454520534	0.946735126	0.394318609	0.770787329	370.8169928
3.248385273	0.944204236	0.391235196	0.807434946	261.0923415
3.210326702	0.946067696	0.38116551	0.808752858	345.9189448
3.182277709	0.916385446	0.382757565	0.806405703	164.7147502
3.470680242	0.920441329	0.352840426	0.802160546	228.0756971
4.633598342	0.928102684	0.34504579	0.766583683	273.1099803
2.904676399	0.937737549	0.415284889	0.826231629	254.0974721
3.499261872	0.926492822	0.343493256	0.758553813	296.9571449
3.590250399	0.940072877	0.391154008	0.74258338	404.9705757
4.325017813	0.949507136	0.388768307	0.739442445	481.7477638
3.258202131	0.940640473	0.400828907	0.826980937	337.3365797
3.918520258	0.943419544	0.387131384	0.77497889	400.448877
3.579578325	0.953104204	0.425687623	0.844505064	608.8696875
4.845016778	0.915083575	0.382131873	0.698934514	175.7665371
3.110578575	0.917327589	0.420483449	0.758305115	114.3628776
2.657541667	0.934420283	0.429756297	0.760328014	197.0781677
4.459206163	0.944232672	0.347059248	0.811201506	455.9316075
2.452154174	0.938832654	0.425097425	0.782377746	256.4461942
1.774384223	0.94542575	0.505391658	0.805894922	163.4186031
2.974950089	0.930176133	0.452236989	0.8041513	132.4021793
2.983684373	0.920213684	0.404546967	0.709820879	157.688569
2.70258294	0.930839217	0.428453146	0.740137442	163.2433902
2.634988292	0.941926948	0.45105269	0.798260459	261.0436488
4.247738046	0.930362846	0.438661054	0.754375103	161.6072355
7.116659137	0.932880631	0.383511648	0.717703752	395.1835581
2.507347704	0.930558848	0.402247369	0.835511892	165.0019747
3.899039781	0.945079495	0.377419263	0.790197027	445.565498
3.626209437	0.939066623	0.443022344	0.761844695	295.9888797
2.735407426	0.907578439	0.427171302	0.764263334	96.76809558
3.714894652	0.936680306	0.423330908	0.792718298	208.304584
2.899760565	0.950403798	0.458069582	0.817462275	362.5525164
2.499040537	0.945878617	0.510294418	0.763785315	215.8545606
2.932895392	0.944103635	0.444533579	0.834114004	212.7012387
4.893705275	0.934742864	0.381618126	0.698927175	412.939334
3.746258966	0.928122475	0.370850452	0.775291855	289.4172365
4.337048772	0.932684859	0.356171806	0.805390189	296.6003603
4.896197617	0.94277299	0.374079388	0.757853019	508.3603002
3.655162918	0.948654461	0.503872685	0.808570956	168.4206991
2.821758014	0.930199876	0.396525056	0.759023415	207.1692205
1.664266956	0.931241703	0.470208403	0.744341907	148.9473748
3.674186655	0.933487154	0.432294478	0.822312253	203.5341193
3.475969866	0.94812231	0.414067458	0.792247091	377.6277193
3.351427007	0.940311704	0.401633632	0.767550299	319.1661334
3.519294681	0.907117037	0.333053694	0.737223044	178.2574699
4.420618287	0.906596756	0.336373075	0.600078201	213.0939193
4.079965544	0.922510538	0.371486815	0.772970561	128.3455426
3.607912535	0.935193888	0.429123487	0.848912656	154.0356539
4.23333861	0.926728509	0.393561348	0.810090601	188.683835
2.627870798	0.92621499	0.414120262	0.763477763	201.4532348
3.455476963	0.941315535	0.388244341	0.81423541	339.3638367
5.712251686	0.926355486	0.318967522	0.791397343	402.0036875
3.59477689	0.931488698	0.441914687	0.737698047	174.0764142
3.657938246	0.947736584	0.393726361	0.804462373	423.3577091
3.033831594	0.940253362	0.392322367	0.769034398	347.7948779
4.035917844	0.948485102	0.374135211	0.81754574	338.3930803
4.38514319	0.941074987	0.36844918	0.760087484	339.2556668
2.802210241	0.942081574	0.458875445	0.770677851	232.5004506
3.769922265	0.925955034	0.390521726	0.746694143	220.3675045
3.636832295	0.935489451	0.388437588	0.742059393	275.1377543
3.205150742	0.94355412	0.412551259	0.79862397	345.4411629
7.238550664	0.944893091	0.384860181	0.696178964	614.7676319
4.361293693	0.937516427	0.341316781	0.771280613	388.3402553
3.705211677	0.943642829	0.367485104	0.782783648	384.1279817
2.365593384	0.915237074	0.504074052	0.68895187	89.34797035
3.149554309	0.941257467	0.379495382	0.811395094	365.4883814
3.203780433	0.936723177	0.443538854	0.831016904	147.5293692
4.554078717	0.931897048	0.352602717	0.779856551	313.850006
2.473728392	0.926788814	0.430249741	0.78226372	142.8213892
1.989053963	0.884215996	0.411392333	0.498637463	82.15129369
2.652224356	0.91163766	0.392481033	0.782101151	125.9451006
3.679455329	0.938146806	0.402840939	0.771968322	294.9033662
3.584954977	0.921304076	0.37185421	0.765099551	190.1666531
2.464425481	0.925790253	0.408121367	0.751897951	186.9135347
2.406355976	0.948468394	0.443118909	0.829786429	313.7018594
2.765529158	0.933059254	0.423454448	0.771400305	190.9372077
9.897270595	0.911515715	0.239155365	0.721187618	531.1738925
2.238546224	0.936471379	0.468462032	0.781488238	168.6144229
2.684754716	0.906563844	0.394734326	0.761630835	102.2006613
2.119882315	0.92692714	0.487223263	0.787084067	95.88870664
5.870093018	0.919910483	0.272663785	0.768086495	343.2593721

log.sigma.5.0.mm.3D_glcm_SumEntropy	log.sigma.5.0.mm.3D_glcm_AverageIntensity	log.sigma.5.0.mm.3D_glcm_Energy	log.sigma.5.0.mm.3D_glcm_SumSquares	log.sigma.5.0.mm.3D_glcm_ClusterProminence
5.235443572	15.20685548	0.006951928	28.07121714	21083.93874
4.928681065	18.87694284	0.010681923	17.878565	12740.06559
4.941991459	14.69601095	0.00954092	18.73912679	13199.97345
4.915281033	11.14623479	0.011049475	16.87959107	10181.36366
4.773260605	11.02584747	0.017667521	17.6639049	8993.548313
4.724210538	10.8701494	0.01822479	15.31635856	9533.497975
4.312158924	7.015247427	0.032867147	13.20985051	4399.325669
5.155871767	16.98773442	0.009250406	23.55239345	21560.1477
5.009943937	14.84421689	0.009868519	21.18804312	12130.70232
5.196371204	23.6554286	0.007080352	22.8439312	23473.9619
4.532365985	9.071428223	0.018139261	10.62425292	3668.770185
4.860043286	16.19142596	0.009675099	15.82784854	10207.48096
4.638541345	11.24553598	0.017931046	11.5117398	5233.044355
4.790436639	11.17953502	0.010268713	13.6339056	5777.271451
5.141446803	16.04652545	0.007908062	26.39486747	23531.99141
5.19352619	18.88418477	0.009930593	24.04529603	23912.93704
4.947033351	18.47183178	0.01071158	17.9586251	16258.14878
5.012037263	15.45744735	0.011250449	19.7619796	13487.27093
4.66980501	15.58635372	0.011263281	12.52822209	5137.831959
4.967817247	15.08097325	0.011981873	18.55822875	14814.28992
4.970243107	18.98749525	0.009572907	17.07972354	13411.35973
5.072575335	15.72364158	0.00905995	19.9953837	15461.42181
5.074469428	18.22187858	0.008992655	20.1270606	18190.93786
5.007192437	12.15294388	0.010347545	21.07200815	13028.72329
5.1331472	14.48934826	0.00724867	22.5757041	18014.26093
5.138148744	16.03195331	0.008037738	23.78021414	21512.79042
5.041497306	15.4428302	0.009249023	19.87451638	11803.55384
5.015600858	16.84235218	0.007426908	19.15919157	13027.75525
4.830314276	19.91469962	0.011794503	15.82747153	14616.90074
4.967243209	21.69495354	0.010216114	17.42223901	14657.99434
5.138480199	17.91605956	0.008917796	21.52949039	20291.67844
5.021217139	19.68296044	0.008827411	19.33369747	20629.3104
5.060685692	24.2933019	0.012456164	23.54994238	39769.24546
4.822348241	12.7754982	0.011399516	17.90165358	7718.557433
4.775083172	10.16593993	0.013063909	14.54756605	7234.666236
4.716168025	13.69373181	0.013076306	12.51185545	5863.74518
5.302771838	20.88516627	0.00699143	27.33460382	34457.03911
4.772524799	15.6904203	0.012645046	13.1497181	7118.483654
4.491689909	12.46245881	0.021595326	10.03476646	4350.294601
4.79061504	10.92279913	0.015363685	16.25845846	8076.643031
4.627402894	12.21481407	0.012443207	11.92712104	3819.946991
4.647794137	12.42676	0.013110012	11.88654954	4265.55186
4.75465746	15.81592057	0.013662967	13.94482155	6518.128062
4.821549871	12.20118075	0.014862891	16.88955014	8775.634578
5.152077432	19.53698399	0.009117187	25.18038904	27883.96491
5.066777849	12.21893975	0.0083708	18.69981392	11948.66889
5.123766058	20.76052866	0.008164744	20.79403341	21276.25931
4.745408799	16.87529063	0.016047399	15.381645	12169.15444
4.627765266	9.262436165	0.017896816	14.30338355	4893.855871
4.935482697	13.9333859	0.011764327	18.78321718	11418.76312
4.843601157	18.73318308	0.015093967	16.32704702	10454.15768
4.425407429	14.429501	0.025459649	9.98689644	5884.940514
4.935815324	14.06087828	0.012176281	18.63117688	10896.84875
4.878001394	20.12861141	0.009925591	16.98361474	9298.475631
5.071902977	16.59571298	0.007587083	20.01032646	13440.24844
5.278757654	16.66917174	0.006794206	26.42239689	25989.44524
5.091325539	22.22789735	0.008492449	21.12847471	23848.69463
4.73104923	12.46095485	0.024678144	16.94892705	13213.36222
4.784918066	14.01056179	0.012299723	14.30321398	8387.850436
4.380962475	11.96901537	0.018121941	7.641583466	2066.630795
5.046932934	13.65153671	0.009448127	20.85473014	11809.39779
4.966719991	19.11018201	0.011446438	17.97339111	15897.77092
4.904856023	17.56406691	0.010666876	16.46023592	11159.02841
4.952284406	12.84616312	0.007373973	17.93814642	9088.406806
4.670893134	14.30000521	0.009856297	14.35017761	4923.314482
4.971952214	10.60384145	0.009581931	20.53544949	12773.37341
5.131404088	11.52876023	0.011356681	25.03289295	22640.64197
5.120510956	13.02928293	0.008396651	23.28974448	16008.38779
4.747187202	13.81661109	0.011885682	13.71429985	5797.977241
5.134873885	17.98754069	0.009068626	21.89408907	19972.16705
5.366505443	19.4912814	0.005275021	32.88939162	46047.91276
4.620266842	12.80650779	0.016948711	13.62029072	6604.074412
5.061539501	20.2140884	0.009483335	20.71749783	25273.75508
4.880166851	18.375408	0.01017346	15.78097567	11457.419
5.267965821	17.8837524	0.007289795	24.93817751	26961.93885
5.090231935	18.06179884	0.008161071	20.91885741	17658.35017
4.70918913	14.92484794	0.015196631	12.65497914	7351.13857
4.876533524	14.41126881	0.011140279	16.9813057	11249.2352
4.877709411	16.24619092	0.011018571	16.19793835	11090.91524
4.934515078	18.25674705	0.012260639	17.94236893	17978.80013
4.976832072	24.56632049	0.012047682	22.23004539	30828.39802
5.172513996	19.33354066	0.007507651	22.89274824	24084.92883
5.015834893	19.27051094	0.009570656	19.88919563	18002.60536
4.139415337	9.148910626	0.031201625	8.208528214	1455.32684
5.096953023	18.73577133	0.008484367	20.61664223	20918.44068
5.008160606	11.46674972	0.010403986	19.2688183	12183.72205
5.203329171	17.24159425	0.007553353	24.49594149	22315.61583
4.745664941	11.50940601	0.01256291	13.19637321	5209.202668
3.898772665	8.898294705	0.028346864	6.100965311	692.5848362
4.792349121	10.66329245	0.012316213	15.58517364	7403.689772
4.899348763	16.83108532	0.010184472	17.6334724	8719.554264
4.932737785	13.27495567	0.009431268	18.17378903	10021.95355
4.693327346	13.33201587	0.013061165	12.17285862	6365.136876
4.902211882	17.36358709	0.013910219	16.10680226	12620.27627
4.80713461	13.43467496	0.011452494	13.52601697	7362.172304
5.545331094	22.45178223	0.004369963	45.39071159	79857.48903
4.518911478	12.64194187	0.019477241	11.23670331	4312.137807
4.730713395	9.520563686	0.012926459	15.02512787	5739.536666
4.512362002	9.340798975	0.020757125	10.96088005	3527.210947
5.407596022	17.86917265	0.005437603	34.968257	48395.7003

log.sigma.5.0.mm.3D_glcm_SumAverage	log.sigma.5.0.mm.3D_glcm_Imc2	log.sigma.5.0.mm.3D_glcm_Imc1	log.sigma.5.0.mm.3D_glcm_DifferenceAverage	log.sigma.5.0.mm.3D_glcm_Id
30.33203793	0.935000454	-0.245161733	2.489428451	0.42661616
37.61434426	0.904206744	-0.223168914	2.014913493	0.464814179
29.3639192	0.900816291	-0.213681798	2.252150378	0.4415556
22.29246957	0.911763749	-0.232028542	1.835248781	0.482310857
22.05169495	0.938941698	-0.287045112	1.848859209	0.502927285
21.7402988	0.920817248	-0.25872445	1.650213653	0.512415323
14.03049485	0.939142332	-0.323582535	1.352303755	0.5792656
33.8239352	0.939396561	-0.260796514	2.084833035	0.463224872
29.61957487	0.90945059	-0.219642291	2.15385706	0.461382268
47.21050308	0.909795158	-0.214737294	2.282912554	0.434021516
18.14285645	0.888447786	-0.228980379	1.534854075	0.533471272
32.32490096	0.871621411	-0.189525941	2.168132736	0.445627239
22.49107196	0.897104281	-0.23201044	1.549680548	0.533704391
22.35907004	0.871571373	-0.194512953	1.977988003	0.459138083
31.93954509	0.907593434	-0.219318906	2.62044989	0.436599223
37.62598299	0.922594285	-0.234955911	2.167246818	0.468578816
36.83052144	0.904806359	-0.224332123	2.127746669	0.455332221
30.81712037	0.928891381	-0.254317271	1.901014738	0.495836989
31.17270744	0.852903929	-0.189193719	2.099114831	0.457168956
30.12363436	0.918165255	-0.238104119	1.877045551	0.489534155
37.81944769	0.870600756	-0.188622762	2.060028191	0.463297207
31.32182202	0.920627746	-0.237334218	2.041039393	0.461130255
36.31402792	0.903424892	-0.214180953	2.08008104	0.453424482
24.30588775	0.929290009	-0.25767753	2.143574051	0.454576658
28.97869652	0.896560803	-0.2054795	2.286905684	0.43033339
31.95748776	0.911618558	-0.217610873	2.461683811	0.4228861
30.84046378	0.930417387	-0.260564678	1.891466948	0.481641343
33.62397086	0.863536166	-0.175753327	2.335546556	0.423047752
39.66251091	0.861675665	-0.190428479	2.082006156	0.460755952
43.29668553	0.881352827	-0.195282908	2.174817881	0.457448072
35.75855928	0.919082101	-0.231922368	1.990702396	0.468833963
39.26308654	0.884080152	-0.197448532	2.143572782	0.457436739
48.53335788	0.941929261	-0.273894173	1.90878571	0.488891484
25.5509964	0.928071338	-0.262932534	2.319868343	0.453199073
20.33187985	0.90890305	-0.237718123	1.940771782	0.484490978
27.38746362	0.874448168	-0.201517229	1.782216419	0.492334084
41.64141449	0.92015167	-0.222229065	2.408165067	0.424994267
31.37857356	0.887748039	-0.211046688	1.779247959	0.489512869
24.92491761	0.908362335	-0.265776019	1.399965753	0.553362188
21.84559825	0.921175456	-0.256857181	1.772893495	0.510535186
24.42962813	0.857806765	-0.187279115	1.944343793	0.472370362
24.85352001	0.861737558	-0.19563673	1.814451759	0.492136616
31.62015447	0.905422675	-0.2315342	1.692476973	0.50994957
24.4023615	0.911260175	-0.241041261	1.94748817	0.49822192
38.82969358	0.889310995	-0.202115462	2.563353217	0.450994016
24.4378795	0.919375673	-0.234009878	1.884812552	0.471702157
41.42223669	0.899525389	-0.207384882	2.191107448	0.44992041
33.72567326	0.898656295	-0.225165088	1.869176764	0.501519454
18.52487233	0.940842213	-0.306838543	1.884472998	0.489818812
27.82851784	0.934694903	-0.266343524	1.944635512	0.486069012
37.38042034	0.915810828	-0.243371243	1.701515741	0.514948471
28.859002	0.899990096	-0.244239834	1.463415291	0.556378221
28.09220506	0.926250012	-0.25141994	1.749989687	0.503677944
40.06285174	0.896181952	-0.222584735	2.239682266	0.452488709
33.11688681	0.887852093	-0.196747788	2.21433586	0.445671908
33.20962428	0.923802161	-0.229066624	2.363084527	0.432321718
44.36536452	0.893900581	-0.202749711	2.306530699	0.445636764
24.89281982	0.932171342	-0.277703462	1.627204324	0.552093988
28.02112358	0.886924886	-0.208950427	1.979083259	0.465749266
23.93803075	0.86315866	-0.20641946	1.481286184	0.52532681
27.30307341	0.924817137	-0.240881829	1.906141012	0.49338791
38.12442199	0.899163785	-0.217290735	1.952876817	0.479374478
35.029484	0.89672073	-0.214578204	2.005842611	0.469401679
25.69232624	0.864796271	-0.177614213	2.39452499	0.41532731
28.60001043	0.861900574	-0.19006986	2.54884053	0.415323228
21.2076829	0.902352384	-0.216297687	2.242518934	0.444007688
23.04865099	0.957806024	-0.3050813	1.923197886	0.490630696
26.05856585	0.911244787	-0.220856443	2.118716116	0.462403412
27.63322217	0.893345478	-0.230426429	1.863205853	0.479543933
35.87237177	0.921185852	-0.234487046	2.093745515	0.459045776
38.7810577	0.894744718	-0.190434952	2.779047202	0.399601397
25.61301557	0.887625589	-0.222529236	1.822649443	0.500751869
40.32479041	0.902916303	-0.216311253	2.078356669	0.463039961
36.65144719	0.86724213	-0.18591258	1.997946687	0.462531676
35.66141383	0.920585837	-0.226895475	2.212500065	0.447183624
35.96393795	0.898494937	-0.208695195	2.305219352	0.441429507
29.8482174	0.895761903	-0.225353285	1.693894799	0.514919621
28.82253763	0.894501841	-0.218041449	2.134865565	0.460311775
32.45148202	0.865708018	-0.18686446	2.113330218	0.458488605
36.40192474	0.892405702	-0.210346613	1.948638598	0.478193603
48.95600787	0.913055371	-0.224865386	2.489899557	0.451357601
38.50081477	0.891501612	-0.198194327	2.447151395	0.419655362
38.39154085	0.900206233	-0.211428762	2.185875958	0.442038258
18.29782125	0.908802911	-0.289578624	1.534801813	0.553522875
37.35602505	0.905810488	-0.218458352	2.08874345	0.452267465
22.93349943	0.924409663	-0.244300827	1.798885152	0.503850583
34.35230906	0.919010735	-0.227517812	2.396872157	0.428745358
23.01881201	0.886574945	-0.214224495	1.763191104	0.49320155
17.79658941	0.860925115	-0.238417666	1.867755948	0.478258791
21.32658491	0.925116368	-0.262161562	1.967470928	0.463573705
33.56283258	0.89102307	-0.204722844	2.023170877	0.46954079
26.54991135	0.905725199	-0.219422305	2.181148359	0.446150798
26.66403173	0.870648536	-0.197174307	1.864266416	0.475978546
34.67034817	0.917494688	-0.243680209	1.703728005	0.503626152
26.86934993	0.876171869	-0.197147992	1.823137938	0.487523376
44.63186729	0.943074432	-0.236899604	3.92191942	0.328755681
25.28388374	0.901231799	-0.246167366	1.5845655	0.523283524
19.04112737	0.935727058	-0.284622943	1.994960152	0.46505285
18.68159795	0.912160598	-0.266521968	1.526746923	0.539721929
35.5761659	0.928809878	-0.228128584	3.120962078	0.362344147

log.sigma.5.0.mm.3D_glcm_ClusterTendency	log.sigma.5.0.mm.3D_firstorder_InterquartileRange	log.sigma.5.0.mm.3D_firstorder_Skewness	log.sigma.5.0.mm.3D_firstorder_Uniformity
98.45140966	203.0938549	0.030913366	0.054324565
62.64630722	137.9914188	0.075222382	0.0731035
65.27846112	148.1486702	-0.212733134	0.070738701
61.34840678	134.9378052	0.475806556	0.072723916
63.63818418	155.8632765	0.424725319	0.082223982
56.1014979	105.3696136	0.751395728	0.091193436
48.97999852	161.170969	0.536282337	0.106591279
83.92357094	166.9980927	0.13552899	0.06183777
74.00848954	174.284565	0.33255214	0.065223333
83.88017318	160.3629074	-0.323039554	0.060297899
37.91468333	113.5700779	0.477547026	0.093884724
53.74586202	127.2066765	-0.1509038	0.074569997
41.33395774	105.2757225	0.486578096	0.089546023
47.79442618	128.1571822	-0.017884391	0.075542182
88.38763138	159.0594158	0.612181417	0.061376186
86.51425216	161.1335559	-0.032255424	0.062122743
62.86874592	121.8075371	-0.23238664	0.07189643
69.95512989	160.3854065	0.407727112	0.06856351
41.92938659	130.2984428	-0.329519249	0.079959953
66.49003186	131.4989738	-0.135741443	0.074110949
60.77509465	127.7437485	-0.340775127	0.070866932
70.75653541	144.0057697	0.180409889	0.067282764
72.59472574	142.9197559	0.372713887	0.066357936
76.13954609	176.0086515	0.009477203	0.065640494
81.3983014	160.2694826	-0.446265552	0.061436854
81.99161686	142.2129831	0.256433601	0.064582166
71.72514097	170.2461529	0.25873306	0.062250525
66.29019977	150.2112974	-0.411483244	0.06700918
54.87851088	117.4461002	-0.645754551	0.077485279
61.56941361	132.0889921	-0.241775625	0.068952162
77.84825252	140.480031	-0.233820853	0.065275548
68.80286179	146.5702381	-0.143649778	0.06689576
87.33448692	140.341383	-0.561730963	0.071755198
60.84834808	173.9573059	0.058214874	0.067414128
51.09637231	118.6033539	0.158994369	0.077517551
44.12102493	113.7327194	0.264224982	0.083271387
100.0901791	153.1815834	-0.050613519	0.057755912
46.83477659	111.0782242	-0.147165569	0.081999196
36.27438958	94.09337521	0.502426593	0.099345399
58.6732046	151.1575527	0.52823321	0.080982748
40.81061953	128.1933022	0.139519028	0.081238856
41.39489391	128.770195	0.348418536	0.082631414
49.87501753	135.4437504	0.300184079	0.08048923
59.21773832	142.9286661	0.517800037	0.079411042
87.05946898	140.2349603	-0.469898488	0.06237498
68.6547897	149.6800537	-0.028103508	0.064649812
75.30316802	141.43402	-0.241195081	0.064917659
53.71814743	101.4213839	-0.951645128	0.089962128
50.41453225	152.4350133	0.329202659	0.08392435
66.30706147	160.744132	0.448026838	0.068529585
58.72393743	130.2365236	0.247414222	0.078781491
35.24448457	84.08697796	0.126958842	0.11123448
67.60355813	161.2881699	0.430695899	0.070368752
57.03517513	141.1248178	0.013599264	0.070843127
69.68709795	156.433815	-0.06703321	0.062556835
93.62027164	162.0892941	-0.004968649	0.056390944
75.55704194	143.3188362	-0.431080746	0.066427973
60.40357957	120.0975575	0.879371313	0.096636405
50.32490829	111.5105147	0.177486659	0.081690027
26.64523096	94.51195526	-0.240856703	0.103713479
76.02839427	179.4976196	0.238606735	0.062191362
64.45212146	126.1007423	-0.109022899	0.073149787
57.06519366	128.0786724	-0.168491908	0.073930269
62.31061817	165.6830873	-0.193973797	0.064081636
45.85828996	151.4445343	-0.246763367	0.072536627
72.81648052	174.7812233	0.482678744	0.066472506
92.29714461	172.9010868	0.605742579	0.063323459
84.32125683	182.8606739	0.36547285	0.060664392
48.48345459	133.8834152	0.006356558	0.077135555
78.56372287	147.8976822	0.025947371	0.064398066
117.685192	187.809691	-0.582316801	0.051009236
47.37563634	116.8276062	0.586229411	0.090451091
75.37604512	142.8272552	-0.241403895	0.068487562
54.9611417	122.8555031	-0.463810201	0.074458566
90.65963422	166.6827726	0.1412019	0.057529011
73.31123049	159.4119701	-0.12363842	0.062489958
44.79916087	108.8950644	-0.232845382	0.085297668
59.31196187	138.2765045	-0.018172324	0.075184595
55.70238096	111.150383	-0.271501926	0.078033569
63.73770218	108.5296192	-0.465713048	0.078592797
75.9948172	106.2476192	-0.702472828	0.080076807
81.60663032	146.7447218	-0.224750233	0.062198135
71.17116353	146.6790504	0.230790936	0.067163496
27.67395369	120.9501638	0.278217114	0.111224038
74.08675654	142.2892094	-0.462337088	0.065258943
70.5600483	170.0450449	0.421476643	0.066083968
85.74012939	153.4243975	-0.086733699	0.059529694
47.08249495	135.8015804	0.255712392	0.079275267
18.13210792	112.8551407	-0.346798886	0.115799693
55.59648688	145.4667072	-0.013020228	0.073957448
60.97833455	153.023304	0.37679419	0.069972313
64.19194119	151.367939	0.151827489	0.067670195
42.64559731	101.3542316	-0.304804117	0.08796464
58.10406097	109.2582054	0.015222176	0.080429281
47.92146647	119.5623569	-0.016602455	0.078581493
158.2597922	209.0638275	-0.615902481	0.046789583
40.04410914	115.5754929	0.425205814	0.096404346
53.04712688	161.9957151	0.161071257	0.073543541
39.17381888	123.3518176	0.464285429	0.093471005
123.2369244	179.4090738	-0.165777263	0.052418083

log.sigma.5.0.mm.3D_firstorder_MeanAbsoluteDeviation	log.sigma.5.0.mm.3D_firstorder_Energy	log.sigma.5.0.mm.3D_firstorder_RobustMeanAbsoluteDeviation	log.sigma.5.0.mm.3D_firstorder_Median
109.3993244	50671261.98	82.93410344	156.6721649
83.5151758	54568153.51	58.16786436	110.6672516
86.04895338	13760773.62	60.76593003	44.67402267
81.84159911	73689324.53	57.39658456	125.312355
87.77467918	17651495.34	65.68703292	55.38141632
75.02373208	26248238.74	48.74758518	60.67072105
81.2709831	17481082.06	66.36187038	50.94300079
97.15488413	69115462.41	68.39385597	138.1669769
94.88632802	71464852.96	71.0084105	129.5261536
95.12468074	91626921.87	66.54503086	124.2381973
66.06665746	34721194.02	47.07876303	96.32828522
77.33692775	37084127.83	52.68774081	110.8255539
68.34872791	48865087.62	46.42464492	81.17990875
74.64355999	30794024.01	53.19006062	86.44659424
100.2536211	49499527.76	67.99899183	106.89505
96.98687639	85444615.21	66.52528641	72.97403717
81.30053289	26674753.97	52.15795742	70.35092926
88.90713082	101011313.1	65.58620779	154.7067184
73.3776852	26237018.06	53.67467604	131.368454
82.05831014	67912873.06	55.25725784	101.4562149
81.7187935	101593699.6	55.02122646	65.84662247
86.3551773	46971935.65	59.75538517	93.29627228
88.42182174	85620565.52	59.86767136	76.5307312
95.42057716	22519398.49	71.43340072	74.45458603
95.8214995	37914697.37	67.6525702	40.18155861
92.84745945	42431094.26	61.5033012	119.207325
94.52463964	50819613.4	70.5877432	88.05103302
86.03776999	57824772.84	60.91944652	86.39180756
77.37568284	74040104.62	49.15912673	107.8182755
84.2571768	66978054.33	55.28273295	96.88465881
88.67557723	101867095.5	59.00137909	103.5304794
87.16371027	172743986.3	59.95765474	142.5322647
90.10232108	73569731.59	59.41982679	105.0075455
91.54088256	13417130.93	70.42652379	122.936348
74.97046661	11985660.65	50.74072021	59.9706459
70.39416944	59864219.68	48.3385639	92.79167175
101.4796718	123709240.5	67.74843454	127.2690964
70.32619982	64941525.35	47.41143217	100.3943863
62.28305375	17584543.64	40.71491372	60.0169754
82.9704338	45424316.88	60.79399054	108.9875412
73.01264462	26676787.09	53.95312084	98.92610168
71.99069102	42859522.11	53.1446246	109.0534744
75.42001132	75596447.19	54.93933506	113.7783737
84.18748863	44086024.91	60.25623067	87.5332756
96.63575425	74257832.2	61.76108645	73.88216782
86.3887251	70087742.73	61.99529797	94.18724823
88.3845304	91374216.45	59.40505334	106.4651985
72.55924509	15702018.18	45.26337496	47.97138786
80.33573109	9745252.032	63.06423867	71.26777267
89.11868706	31820546.68	65.61476358	118.6166153
81.24888752	104043084.1	55.90455168	104.7933159
58.28812909	31065986	35.88272119	87.7308197
89.09658342	142755417.9	66.22683804	141.0965576
83.79730323	25465224.87	59.49344958	94.05534363
90.32231973	130386660.1	64.7264522	142.4955597
100.2852151	41058242.74	68.28461272	72.45593262
88.54947742	87675735.36	59.67049134	109.9283066
78.7883866	47314415.01	52.21180871	66.24386978
73.50438159	22996708.23	48.33987671	79.18572998
55.07611095	23750673.88	38.24515386	110.3157043
95.68426846	126670728.3	73.5556931	162.9573364
82.53731605	134884307.9	54.45291074	105.3055191
78.69773394	56991343.07	53.58361523	122.317749
89.71226199	51618461.96	67.47936044	130.719223
82.07103767	16333204.13	61.75040245	140.9452667
94.43250037	55743771.37	70.98811145	131.8939972
101.289486	32736474.17	72.99416031	96.11288452
99.94952991	145232635.1	73.89718917	139.327652
75.27611615	25396230.03	55.19904243	97.55041504
92.2122813	63274968.74	62.554715	95.48274994
115.901026	132081095.2	80.09858513	135.9212723
72.91856396	52018528.85	49.80712698	107.6170883
87.24226156	152359395.7	59.02470286	132.0315399
77.76965869	150400904.1	51.62142259	120.5127869
99.31120058	143526904.7	68.9307769	126.4821968
93.38460558	59960252.54	65.67134212	104.0917435
68.76276767	34646736.82	45.29698407	78.80161285
81.41154204	33462937.84	57.28216145	106.8556519
75.75418632	59337427.84	48.73619159	94.24589539
77.90574978	68778801.66	47.9411928	66.70449066
80.52603402	25215343.28	46.72448671	70.79949951
93.33318294	52525439.68	61.75369624	73.97309113
89.5464894	83427632.1	60.22723115	128.4991302
61.88697881	6779850.896	48.87323945	73.09944153
88.42508253	114272449.2	59.14384774	134.1064987
90.68656005	91985608.3	67.72599787	103.162117
96.38371033	39895521.55	64.61853068	107.4864349
75.0663722	71290625.35	55.34854977	111.3437424
55.52445921	8500171.419	43.85555762	133.9368591
81.39912926	11954928.42	59.42335034	93.94072342
85.06636674	108198046.9	62.15176321	136.7431793
85.84983504	27183454.72	60.56241321	107.1634827
65.62715522	14729089.62	42.71587847	47.71681213
75.04731666	82297842.79	47.62449007	88.55639648
72.71192276	140599453.5	49.92851256	140.8203964
133.2175301	25648928.21	89.72857582	52.80794907
68.00241239	31359965.96	48.44843777	84.49852753
82.58444426	13277656.88	63.75991063	145.5060272
69.57317758	13901295.19	51.1909658	72.13737869
114.6813326	28629284.74	76.35026027	71.5796051

log.sigma.5.0.mm.3D_firstorder_TotalEnergy	log.sigma.5.0.mm.3D_firstorder_Maximum	log.sigma.5.0.mm.3D_firstorder_RootMeanSquared	log.sigma.5.0.mm.3D_firstorder_90Percentile	log.sigma.5.0.mm.3D_firstorder_Minimum
1368124073	467.6130371	205.2334032	330.1539429	-177.8731842
1473340145	414.8034363	165.1789235	279.1588165	-307.8885803
371540887.7	386.7808838	110.9431579	150.0309021	-303.506073
1989611762	473.5629883	173.3219289	295.7374451	-118.7165833
476590374.2	327.7531128	136.3821853	248.0746246	-173.0295563
708702445.9	388.3225098	126.5881739	234.714975	-155.7884369
471989215.5	306.066803	128.6016859	229.2711121	-57.93478394
1866117485	515.6990356	195.6811562	317.8147034	-231.8709259
1929551030	479.2218018	187.7670477	311.4239319	-181.7367249
2473926890	472.9743958	174.6762591	280.6538574	-444.5741272
937472238.5	335.0647583	138.6175599	237.3002197	-97.58974457
1001271451	413.8696289	148.3522311	237.4495483	-263.5618591
1319357366	361.928833	129.1634555	221.6285645	-163.5466766
831438648.1	332.035675	128.7390699	215.6127533	-164.3271637
1336487250	488.9830017	178.8195714	308.9738434	-236.8022766
2307004611	424.9164429	144.2029943	239.124762	-372.5736389
720218357.1	393.5294189	128.9177428	203.1484619	-357.8866882
2727305454	564.3227539	198.7168702	308.5832916	-177.3131409
708399487.7	339.0438538	151.9735721	231.0418854	-245.7256927
1833647573	445.9039001	153.0037896	255.5253296	-230.3931885
2743029890	450.2581787	120.9040881	185.3329193	-376.0184631
1268242263	545.4836426	148.8157489	246.3537292	-272.8634949
2311755269	512.6994629	144.141069	246.8258667	-326.7945251
608023759.3	330.4571838	147.863091	251.84021	-186.6587067
1023696829	290.5362244	120.6655528	165.7128754	-301.9927979
1145639545	491.3103027	177.1551452	287.0331604	-242.34729
1372129562	402.5353394	151.6077167	266.3613892	-244.4653625
1561268867	367.9674377	131.283669	205.4104401	-315.0016785
1999082825	390.2709961	147.5903388	226.2349426	-364.7140808
1808407467	530.5113525	145.6100834	230.3931671	-414.1296692
2750411578	408.7776794	156.8237788	254.6116592	-310.5089417
4664087631	520.942627	185.8730676	295.4500183	-319.8171997
1986382753	437.1296692	170.4918068	281.0908203	-458.9022827
362262535.1	377.9332275	167.0156972	268.0210571	-160.5352631
323612837.4	308.6714172	116.1781631	198.7426208	-165.9504547
1616333931	355.7694702	136.4137452	233.6903534	-202.1735535
3340149494	577.3346558	186.1777581	314.3272034	-359.144104
1753421185	354.395813	138.3876542	228.7753906	-265.312561
474782678.3	345.4055481	108.8550547	195.0489471	-209.1839905
1226456556	415.9264221	166.6297538	282.6904755	-105.0551376
720273251.5	329.2774353	142.3766653	236.82444	-154.842453
1157207097	392.0244141	148.9430014	244.9787796	-152.6175079
2041104074	396.7602844	158.0317983	262.1313049	-246.6510925
1190322673	403.1963501	152.9713226	269.369693	-158.5724335
2004961469	400.9769897	145.7839399	232.2408432	-382.27948
1892369054	395.9755859	140.0177239	228.3772186	-189.3388977
2467103844	479.8208923	157.4469193	254.6964111	-390.0973206
423954490.9	296.8333435	101.7719597	128.1198349	-353.7850952
263121804.9	288.1822815	131.4489089	234.3249237	-102.1182251
859154760.5	468.1905212	170.8600658	280.6797028	-193.4210968
2809163270	429.7339172	161.602079	278.1003784	-304.1123962
838781621.9	345.0541382	126.0254178	206.5127335	-249.7216949
3854396284	512.444519	193.8991929	319.3966248	-156.4782562
687561071.5	370.7293701	147.4671579	253.9862823	-362.6174316
3520439822	421.9385986	181.9842793	292.9811829	-235.0833588
1108572554	442.015625	147.4684733	232.9985199	-319.0941162
2367244855	456.8626709	163.5192928	261.5060669	-410.7597656
1277489205	477.740387	139.4809678	248.8893555	-195.5917816
620911122.1	363.7752075	129.4660154	219.7626846	-248.1342316
641268194.8	291.7472229	130.0168998	196.5618134	-152.0744781
3420109665	465.7007141	207.5346522	334.9185181	-135.077179
3641876314	487.4434509	156.7881493	263.1807434	-347.0692749
1538766263	433.0770264	158.4148457	242.9178619	-278.0527649
1393698473	364.7963257	166.2317948	263.0240234	-161.0091858
440996511.6	360.3218689	166.3832829	252.2648987	-191.7619934
1505081827	513.4564819	190.0707161	310.3728821	-92.87191772
883884802.7	493.0117188	175.9029478	300.9907349	-147.5634918
3921281148	463.965271	201.8094223	343.846756	-139.7599487
685698210.8	314.2058716	140.1475028	235.7676758	-200.9791718
1708424156	458.3327026	158.8688686	269.3768127	-317.7370605
3566189571	474.3698425	186.2395274	287.3528625	-349.1570435
1404500279	393.6040649	158.8693201	275.5969849	-171.222641
4113703685	541.8868408	180.4505035	287.9021057	-333.2347717
4060824411	422.1365051	151.9852268	237.7764587	-305.7270203
3875226428	657.3631592	183.5100852	296.3969025	-280.2168274
1618926819	537.3075562	160.3838335	259.6723938	-313.9035645
935461894.2	359.9733582	121.292993	198.8793823	-258.6355591
903499321.7	378.1682129	156.9178341	261.0553284	-211.6801147
1602110552	402.5404663	134.5836839	210.3207932	-299.8149109
1857027645	388.5843506	124.2799171	191.6312042	-353.3007507
680814268.5	450.3031921	136.0642051	202.7618881	-523.1647949
1418186871	454.6391907	140.6012142	216.4087189	-379.4892273
2252546067	543.439209	183.6348527	306.0807068	-324.2994385
183055974.2	248.1055603	113.8568664	188.3587921	-109.759491
3085356129	454.1156616	172.014332	266.5792511	-308.5063171
2483611424	474.2394104	162.7215361	275.8534454	-146.1125183
1077179082	472.5598755	167.1467628	270.9423309	-289.4262695
1924846884	370.6831055	153.061243	254.4854889	-126.5528412
229504628.3	227.6400909	143.8112732	209.4817657	-53.23660278
322783067.4	301.2162476	143.8167833	238.9856354	-139.1432495
2921347267	457.497467	183.5648644	302.3753662	-225.9435577
733953277.4	378.303894	156.8451833	274.3626343	-181.2636566
397685419.8	272.593689	97.10630691	152.4136642	-272.1708679
2222041755	407.3033447	137.4991116	229.792099	-321.677063
3796185245	420.0178528	172.8485955	268.4817352	-157.9635773
692521061.8	402.4629822	170.3367978	210.7663559	-502.5805969
846719080.8	340.2141113	133.3710699	235.1139038	-185.2118225
358496735.7	373.3604431	176.132231	280.6879333	-67.38301086
375334970.1	305.0176697	125.1183907	220.7835678	-104.140419
772990688.1	468.6372986	157.5079012	221.6099915	-374.6587219

log.sigma.5.0.mm.3D_firstorder_Entropy	log.sigma.5.0.mm.3D_firstorder_StandardDeviation	log.sigma.5.0.mm.3D_firstorder_Range	log.sigma.5.0.mm.3D_firstorder_Variance	log.sigma.5.0.mm.3D_firstorder_10Percentile
4.354477602	129.2343579	645.4862213	16701.51926	-0.03651547
4.039184754	105.5489941	722.6920166	11140.59015	13.90281763
4.075204686	107.3890557	690.2869568	11532.40929	-121.5624954
4.007191628	102.0224235	592.2795715	10408.57489	22.92245483
3.898565964	104.1962948	500.7826691	10856.86786	-23.73282051
3.824427145	96.69726499	544.1109467	9350.361056	-23.42842884
3.50024526	91.20721343	364.0015869	8318.755782	-5.943104935
4.273548961	120.9389354	747.5699615	14626.2261	15.80474415
4.136185229	113.4040356	660.9585266	12860.47529	20.19939613
4.280251777	120.1768363	917.5485229	14442.47199	-24.38287048
3.647367479	81.39093079	432.6545029	6624.483616	19.06330872
4.000579717	98.72372258	677.431488	9746.373401	-10.67129803
3.773574836	86.62247558	525.4755096	7503.453275	-2.853321075
3.907434375	92.52143578	496.3628387	8560.216079	-30.47539215
4.287347604	126.6432452	725.7852783	16038.51156	-14.40722656
4.287690382	122.240357	797.4900818	14942.70489	-63.54470901
4.121246875	108.232302	751.4161072	11714.23119	-60.99386292
4.087689961	107.9098431	741.6358948	11644.53425	43.34753456
3.84190183	89.45009803	584.7695465	8001.320038	12.02904558
4.054637023	105.8501921	676.2970886	11204.26316	-4.409684658
4.097896487	105.3778145	826.2766418	11104.48378	-80.06858902
4.149002284	109.633076	818.3471375	12019.41136	-27.36564636
4.192285669	113.7240172	839.493988	12933.15209	-44.47069931
4.119855187	114.1658357	517.1158905	13033.83805	-53.60966835
4.226093037	118.2593448	592.5290222	13985.27264	-148.7334518
4.253375404	119.9577562	733.6575928	14389.86328	-3.246427035
4.171598288	113.7463682	647.0007019	12938.23628	-35.2815094
4.110881818	106.8741152	682.9691162	11422.07649	-67.28707275
4.014982835	103.9274444	754.9850769	10800.91371	-9.52615509
4.159619088	110.4627908	944.6410217	12202.02815	-43.46726837
4.214273893	115.1435313	719.2866211	13258.03281	-28.48638115
4.167968067	111.9630045	840.7598267	12535.71437	23.85098343
4.160066099	121.0555341	896.0319519	14654.44233	7.034891605
4.049836491	107.2720531	538.4684906	11507.29338	-1.209183216
3.918291733	94.98347984	474.6218719	9021.861443	-56.23178749
3.840679841	89.0003346	557.9430237	7921.059559	-0.235088757
4.406091911	132.1137259	936.4787598	17454.03657	-25.92233696
3.869060664	90.45122479	619.708374	8181.424067	-3.890602827
3.652210452	80.93695314	554.5895386	6550.790384	-15.87795496
3.888789268	100.018667	520.9815598	10003.73374	23.24343586
3.810348242	88.03313907	484.1198883	7749.833575	3.719556928
3.785771701	86.80646461	544.641922	7535.362299	18.47202721
3.853749648	92.65779916	643.411377	8585.467745	24.79972916
3.947312242	102.691056	561.7687836	10545.45297	3.633896208
4.327068648	128.3289041	783.2564697	16468.30763	-103.5255676
4.122919993	106.1958134	585.3144836	11277.55078	-50.25080719
4.220455022	114.5244043	869.9182129	13115.83918	-30.61098862
3.855461854	97.16051795	650.6184387	9440.166249	-100.8899193
3.744985279	93.1761974	390.3005066	8681.803761	-15.49402142
4.072380855	107.9142831	661.611618	11645.49249	9.416876602
3.987398654	103.066489	733.8463135	10622.70115	16.42807369
3.576725368	79.60137997	594.7758331	6336.379694	21.39945602
4.047036631	106.8935445	668.9227753	11426.22986	42.06848297
4.044124394	104.4795884	733.3468018	10915.98439	-12.07932663
4.184552097	111.2136645	657.0219574	12368.47917	2.962689734
4.389704786	127.794118	761.1097412	16331.33658	-97.14306412
4.194381125	115.7015124	867.6224365	13386.83997	-9.985748672
3.829842307	100.4471038	673.3321686	10089.62067	4.408094978
3.920530938	95.55639498	611.9094391	9131.024622	-22.15379772
3.494802534	69.15494447	443.821701	4782.406344	21.65741501
4.138822377	112.7727049	600.7778931	12717.68297	37.80895233
4.099332841	108.160497	834.5127258	11698.6931	-6.434247208
4.029550051	100.5842148	711.1297913	10117.18427	-2.057870865
4.091008205	106.9418794	525.8055115	11436.56557	-17.10298729
3.955507767	97.97870513	552.0838623	9599.826659	9.861934471
4.108030136	112.3851429	606.3283997	12630.42033	23.68930321
4.221606242	123.1288594	640.5752106	15160.71602	-8.539797592
4.219433793	120.4213804	603.7252197	14501.30885	21.13987064
3.871490576	91.88788526	515.1850433	8443.383457	-7.069479275
4.23924048	118.2169587	776.0697632	13975.24933	-32.96044769
4.514480887	146.0219821	823.526886	21322.41926	-86.01108856
3.784541625	91.92565826	564.8267059	8450.326647	33.46629333
4.162963761	113.7028567	875.1216125	12928.33962	19.063311
4.025680363	100.8654229	727.8635254	10173.83353	-16.71190643
4.362656721	125.3409198	937.5799866	15710.34617	-14.61962786
4.245519688	117.2004898	851.2111206	13735.9548	-31.00496483
3.838061402	89.49909604	618.6089172	8010.088193	-16.31679802
3.994714784	101.8549651	589.8483276	10374.43391	10.39028091
3.996927135	99.69785976	702.3553772	9939.663241	-40.09703636
4.047965506	106.1827417	741.8851013	11274.77463	-64.16998596
4.10224745	116.3214024	973.4679871	13530.66866	-50.4788578
4.296405698	121.4530494	834.128418	14750.84321	-87.43919373
4.195356762	115.3989302	867.7386475	13316.9131	16.82218685
3.370893474	71.17225201	357.8650513	5065.489456	11.08354549
4.210888294	114.6594632	762.6219788	13146.79251	-17.5847271
4.092853217	108.974493	620.3519287	11875.44013	-7.798955727
4.33405902	124.0042211	761.986145	15377.04685	-45.40071449
3.845164509	90.64383633	497.2359467	8216.305064	15.89558487
3.241192563	64.75494422	280.8766937	4193.202801	40.80340195
3.928805703	98.68554752	440.3594971	9738.837289	-19.09504662
4.033078728	103.8153699	683.4410248	10777.63102	32.02458954
4.090721824	107.2455104	559.5675507	11501.5995	-18.80991936
3.799030442	86.00483575	544.7645569	7396.831773	-64.08424873
3.995275543	100.4119918	728.9804077	10082.56811	-21.77381287
3.916414114	92.10460411	577.9814301	8483.258098	35.06903076
4.69535871	168.6208981	905.0435791	28433.00729	-206.8632767
3.659461867	84.02897266	525.4259338	7060.868246	12.77452888
3.892576807	96.71699699	440.743454	9354.177507	25.40673904
3.656743924	83.60059566	409.1580887	6989.059595	-4.373005867
4.544846555	147.2801521	843.2960205	21691.44321	-143.8250534

log.sigma.5.0.mm.3D_firstorder_Kurtosis	log.sigma.5.0.mm.3D_firstorder_Mean	log.sigma.5.0.mm.3D_glrlm_ShortRunLowGrayLevelEmphasis	log.sigma.5.0.mm.3D_glrlm_GrayLevelVariance
2.268858841	159.4340946	0.008716054	26.99951789
3.456623513	127.0570211	0.004219893	18.28301224
3.096748592	27.85632748	0.007948432	18.93690846
2.792137914	140.1139399	0.013877705	17.0951292
2.338014468	87.99563972	0.016752216	17.86947416
3.161187354	81.695806	0.012510432	15.8123207
1.868804974	90.66221836	0.033170043	13.472879
3.077835267	153.8339651	0.006762471	24.00003233
2.424549011	149.6528948	0.006800133	20.89063555
3.55114313	126.7648354	0.002607958	23.74922075
2.609992514	112.2067034	0.020020475	10.88863464
3.467983131	110.7339653	0.00605022	16.15987437
3.125425925	95.81098558	0.010439344	12.85557404
2.564326494	89.51833354	0.014222244	13.84805938
3.228665411	126.2455051	0.006471097	26.36003748
3.179890285	76.4970502	0.004640092	24.72709429
3.965499317	70.03965459	0.004949874	19.97884751
2.934740266	166.8647963	0.005572886	18.99034204
2.946631629	122.8602725	0.00593394	13.15134266
3.387021804	110.480299	0.008313195	18.56985009
3.484375972	59.27322101	0.003460222	18.35851561
3.392003707	100.6315844	0.00636267	19.79460264
3.434373446	88.56351219	0.004034765	21.4854131
2.266993929	93.96624731	0.016065843	21.31675923
2.705798138	23.97713494	0.009885969	22.43392801
3.406055701	130.3613523	0.007794718	23.91505474
2.416529603	100.2330459	0.007059582	21.00020041
2.983740868	76.24516542	0.005433914	18.51787262
4.64307955	104.7950113	0.003634922	18.18195311
3.763142543	94.86974359	0.002909199	20.67167089
3.422257349	106.4690791	0.004756375	21.96018912
3.938764253	148.3680656	0.004343243	20.74590296
5.352211036	120.0542121	0.002894597	24.95416133
2.240979079	128.0115218	0.013387722	18.53095888
2.817937149	66.89920888	0.022774889	14.88483855
3.066779616	103.3810927	0.00723476	13.19073882
3.521206013	131.179728	0.00385665	29.00719572
3.364030477	104.7364252	0.005498753	13.69875846
3.431448248	72.79170664	0.008240821	11.16440173
2.475145887	133.2731823	0.012831719	16.52506211
2.428798981	111.898531	0.009848693	12.84305147
2.577951271	121.0316296	0.008151085	12.38675035
2.920899059	128.0178954	0.00544022	14.06500835
2.678027273	113.3788894	0.010378159	17.63571332
3.474603235	69.17116087	0.004467056	28.07551138
2.607881495	91.25465587	0.012845699	18.18112038
3.869332803	108.0448667	0.003530096	21.86385727
4.398349374	30.28804275	0.006037998	16.41918152
1.972064118	92.7200728	0.020420611	13.73479665
2.79965398	132.4676172	0.00748736	19.10063104
3.3336522	124.4689953	0.003996698	17.96327035
4.641861932	97.70376777	0.007357329	11.22766184
2.530894516	161.7735057	0.006604933	18.77630563
3.26913164	104.0700642	0.003868205	17.92210895
2.741314743	144.0479043	0.00541859	20.26435348
3.015983099	73.59085555	0.006408867	27.04501215
4.476660868	115.5496393	0.003358572	22.36011448
3.755498817	96.77458197	0.008493907	17.60300405
3.355363838	87.35230119	0.008284593	15.10250234
3.116250408	110.0998995	0.009174834	7.982691761
2.179604954	174.2209772	0.007775882	20.62952063
3.790094935	113.5069631	0.003830673	19.57366253
3.539596858	122.3849626	0.004553666	16.91554486
2.290074854	127.2652507	0.01251452	18.32319311
2.524754372	134.4751657	0.009080916	15.63081899
2.493627071	153.28554	0.017097093	20.48876644
2.773273075	125.6229717	0.016145577	24.98469643
2.32895566	161.9436139	0.01012913	23.63512253
2.516162516	105.820315	0.007379268	13.75706727
3.196242103	106.132314	0.004688796	23.0973103
3.005928154	115.5973283	0.005730629	34.37812797
3.085229311	129.5728915	0.009142591	14.38435997
4.260898384	140.1215351	0.003554294	21.3928773
3.524018777	113.6911414	0.003892872	16.65958624
3.307462948	134.0358355	0.004760289	25.99455554
3.31314281	109.4852468	0.004860844	22.79397932
3.641613301	81.865145	0.00647708	13.74757592
3.093345075	119.3682233	0.009638027	16.9654782
3.549854181	90.40522507	0.005338467	16.52752238
4.208489315	64.5811363	0.00451218	19.05856114
5.990438774	70.58894557	0.002887392	23.5439727
3.537483427	70.83684222	0.004265976	24.34408016
3.504629437	142.8455321	0.003832872	21.97300238
2.100461403	88.87011072	0.017667608	8.415998264
3.698017559	128.2269	0.005193081	21.50366923
2.513553364	120.8422864	0.01300551	19.15754488
3.116006035	112.0758379	0.006171935	25.636531
2.406137002	123.3346628	0.010824946	13.44306463
2.208398301	128.4074744	0.024939344	6.64508434
2.405173394	104.6156292	0.022996281	15.84044025
2.588325681	151.3883365	0.004375631	17.65827137
2.585936573	114.4500415	0.010190614	18.81459435
3.557586666	45.0866174	0.009179284	12.30959254
3.785823068	93.93315489	0.004396182	17.27904148
3.090460068	146.264756	0.008145252	14.01723525
3.268741003	24.11674496	0.005275068	46.27218787
2.792565622	103.5711062	0.007785514	11.77005338
2.077113564	147.2018522	0.022495981	14.80276407
2.402474058	93.08894727	0.017046064	11.64366293
3.175969024	55.83274792	0.009077793	35.17655441

log.sigma.5.0.mm.3D_glrlm_LowGrayLevelRunEmphasis	log.sigma.5.0.mm.3D_glrlm_GrayLevelNonUniformityNormalized	log.sigma.5.0.mm.3D_glrlm_RunVariance	log.sigma.5.0.mm.3D_glrlm_GrayLevelNonUniformity
0.009350173	0.053511106	0.155102996	57.56495016
0.004562943	0.071134938	0.201940352	123.8012136
0.00847689	0.069053057	0.161451466	68.45209186
0.015253457	0.070992098	0.223053731	151.0625878
0.018807805	0.075469993	0.256138382	60.77981001
0.014069003	0.084408534	0.262483197	116.1632065
0.041394189	0.092916125	0.439863446	77.19489376
0.007350725	0.059994975	0.212364017	94.3231696
0.007379132	0.062914928	0.2101039	111.7639479
0.002786522	0.059349335	0.161339956	158.5794192
0.022712055	0.089975497	0.322509561	134.5490758
0.006497969	0.072954326	0.174794679	108.6810461
0.011747897	0.083500794	0.357238268	201.5409577
0.015431448	0.074852342	0.185653671	122.3540887
0.006984066	0.05986416	0.187982673	81.45683309
0.005138184	0.059133075	0.284888872	208.1390707
0.005299009	0.068985578	0.210363546	96.30280126
0.006242055	0.066696245	0.286187995	144.3184462
0.006367108	0.07910903	0.176693044	79.28352315
0.009213055	0.071218067	0.236565764	176.2470659
0.003796911	0.069565388	0.209243275	417.4847379
0.006892605	0.066339638	0.195965552	123.1241188
0.004386244	0.064575546	0.184262458	233.8675276
0.017511405	0.063018185	0.215757065	56.93085348
0.010736378	0.060797276	0.160825069	141.2573376
0.008214505	0.062706568	0.156787716	75.94986085
0.007561448	0.061816657	0.171201776	121.3362786
0.005806558	0.066579062	0.147411491	200.7692773
0.003925665	0.075250096	0.240664702	221.9632258
0.003116083	0.066282689	0.195060132	184.1354816
0.005176355	0.063864858	0.199100393	229.6195984
0.004794579	0.0655329	0.203149179	287.3296453
0.003142715	0.06787375	0.260767373	146.422293
0.014414079	0.065646652	0.177354104	27.73871286
0.024790622	0.074366454	0.221602399	56.76201614
0.007962159	0.080900497	0.242694818	222.8663717
0.004110466	0.056248594	0.169268811	178.7280233
0.006028415	0.079391325	0.23560987	230.6507591
0.009366018	0.094001897	0.363674445	113.805174
0.014460878	0.076002972	0.297731146	104.0326069
0.010695006	0.078766366	0.199139642	90.31761649
0.00910267	0.080434855	0.245357341	132.6419563
0.006024244	0.07834025	0.29396292	199.0654777
0.011587703	0.074325745	0.288388301	118.4743483
0.004828038	0.058551105	0.261460449	174.9318905
0.014189524	0.064599506	0.201980756	200.9949072
0.003813687	0.063501178	0.186454232	205.5447469
0.006525248	0.084045245	0.263982452	107.2761707
0.023034122	0.079834488	0.237923877	38.68592463
0.008276431	0.066181293	0.243015952	61.95296331
0.004457704	0.073721636	0.313110021	245.3246705
0.008283177	0.101891321	0.397791814	160.0072128
0.007404038	0.067700063	0.261624522	217.9561952
0.004133946	0.069895937	0.184122663	71.78571184
0.005862293	0.061820923	0.210579094	213.5767214
0.006903784	0.05552893	0.169880088	92.78860168
0.003626869	0.064944458	0.198352298	187.4464518
0.009922284	0.083939053	0.446622815	163.0779447
0.008960169	0.079055214	0.186226184	94.92304931
0.010137471	0.101314728	0.259055567	119.7261347
0.00871761	0.061089672	0.249528609	153.0964666
0.004184277	0.07046631	0.244762845	331.71118
0.004938228	0.07195563	0.208544098	141.5878071
0.013247153	0.063915602	0.146067569	107.6577037
0.009547665	0.071424559	0.127711794	38.02690536
0.018709256	0.064861653	0.168232775	88.69772776
0.018285918	0.060379994	0.219324831	54.69840588
0.011086723	0.059352598	0.200100138	184.552496
0.008061863	0.076241662	0.206771448	85.27098206
0.005095115	0.062450588	0.199953482	136.3096914
0.006127927	0.050495633	0.140878094	173.2692712
0.010051793	0.085501935	0.276375633	149.6982464
0.003876716	0.066638833	0.217229493	271.562106
0.004234967	0.073044567	0.205814932	414.3818758
0.005158327	0.056515984	0.187024687	211.7722039
0.005230944	0.061119848	0.189850431	125.1731755
0.007132985	0.081880798	0.296876142	161.5372309
0.010378345	0.072993319	0.204193109	86.60682242
0.005787573	0.075326942	0.203062668	214.9394658
0.004910497	0.074797058	0.243701116	285.1376047
0.00306401	0.075081537	0.216041851	89.12786957
0.004543521	0.060936866	0.150359125	144.8238745
0.004119006	0.065464095	0.180485337	143.1054261
0.020534224	0.102696974	0.357512756	43.55966303
0.005613726	0.0644328	0.171670057	219.1483475
0.014748656	0.064736877	0.271331198	189.6569564
0.006565616	0.057755492	0.145044467	73.70606877
0.012035637	0.077407536	0.250296808	201.2865595
0.026526275	0.113541269	0.241601683	40.70736174
0.024948486	0.072006294	0.185415515	36.60140436
0.004803592	0.068264877	0.21372217	190.583455
0.01089101	0.066286902	0.167877773	64.92271422
0.009915896	0.085371473	0.18298283	116.1505477
0.004889301	0.075955398	0.264138423	279.085312
0.009025188	0.077160363	0.23793411	312.1415974
0.00550344	0.045811451	0.095630943	37.6650843
0.008766979	0.090783231	0.295779696	133.6927399
0.024935799	0.072838081	0.19203761	27.42948957
0.019524387	0.086947725	0.303401781	63.85437398
0.009696518	0.051570537	0.09244802	55.0710637

log.sigma.5.0.mm.3D_glrlm_LongRunEmphasis	log.sigma.5.0.mm.3D_glrlm_ShortRunHighGrayLevelEmphasis	log.sigma.5.0.mm.3D_glrlm_RunLengthNonUniformity	log.sigma.5.0.mm.3D_glrlm_ShortRunEmphasis
1.409039716	233.8069301	881.8264823	0.923119907
1.53323414	331.6602622	1360.02937	0.903432011
1.443505686	208.2467906	798.243919	0.914541275
1.566681599	130.159471	1663.918192	0.903164446
1.66226139	130.2805004	606.0982746	0.887926441
1.696819521	119.6505339	1012.588681	0.879180069
2.100665109	62.19517363	557.6425824	0.841393822
1.539439359	276.1009598	1239.095853	0.906596041
1.51895973	214.5940782	1419.378938	0.912309907
1.429723842	529.8676862	2169.92327	0.918895534
1.798313648	83.1133693	1085.327631	0.873976811
1.464277519	245.2909052	1194.619276	0.913599525
1.849366204	127.6742934	1754.993748	0.874516539
1.485468388	123.3596481	1304.732268	0.911663513
1.494941619	248.0829296	1085.685657	0.910598347
1.661577667	340.1013533	2730.342263	0.89999641
1.554723372	317.0497051	1089.564093	0.901413697
1.699962749	228.48881	1632.548384	0.889079803
1.471901555	227.410663	804.8309996	0.913176263
1.622382985	217.9807665	1870.737949	0.890548847
1.558125454	335.2907879	4624.157316	0.898269176
1.512992923	236.7153225	1461.526589	0.906245323
1.485850543	318.8584205	2875.432547	0.910305906
1.537197511	158.6974279	719.1962334	0.908905376
1.425503741	210.7522429	1901.04556	0.920875605
1.408053603	252.5960774	995.3194339	0.923759054
1.448509563	211.6971797	1597.301692	0.918737105
1.391167312	266.6362618	2489.825177	0.925285969
1.591817344	364.6693766	2322.527765	0.904667842
1.496993648	429.5920081	2222.298595	0.912785411
1.534839824	302.0152218	2791.749267	0.901185576
1.512571768	364.3996995	3496.261526	0.911345469
1.650793321	554.1666419	1637.350571	0.892290358
1.481090412	166.5023325	335.7793119	0.909505959
1.591309787	105.5745793	586.2602975	0.894747109
1.61535866	179.2992621	2104.721776	0.895045942
1.437753575	420.9551099	2590.948367	0.920317472
1.608223368	231.7028582	2214.985427	0.894242197
1.909364748	146.1309932	854.5749795	0.860184938
1.738797471	123.7292022	1010.347524	0.88093627
1.525856126	142.900119	901.6084954	0.905297451
1.627817318	150.9126841	1255.200945	0.892666546
1.729084081	232.1940844	1888.016443	0.883355752
1.70343531	151.1531944	1203.120303	0.888956527
1.64343302	354.0175487	2288.746076	0.895397684
1.529428141	148.0303606	2431.215694	0.903881906
1.491264973	414.8940128	2573.498075	0.910209123
1.692308156	252.7291856	941.5830562	0.880748376
1.639462711	92.21597991	372.9583341	0.891481319
1.614028741	195.7629976	722.2063655	0.897836734
1.761432163	322.4196383	2465.647382	0.882201029
1.975137931	190.6549577	1087.003082	0.855361848
1.662880786	195.9716141	2427.775679	0.889658433
1.495663405	366.3192665	812.2563217	0.907579371
1.515298929	259.1888354	2767.29965	0.913183967
1.454181301	274.7480567	1342.944143	0.914694659
1.499076672	470.3345309	2314.865145	0.913448396
2.053756413	156.872354	1339.256202	0.853389585
1.502729499	191.7489024	942.8579796	0.905088003
1.687958745	130.0002523	868.9450902	0.879533158
1.635170525	186.7026735	1902.256526	0.892286816
1.617226833	342.9339551	3620.663281	0.896616384
1.553851484	287.2733892	1531.243328	0.900837349
1.381774722	160.438985	1401.680507	0.928372489
1.3619414	191.4640266	440.4673532	0.925726371
1.450627411	126.2064093	1104.218251	0.915776854
1.595680568	150.1351228	687.0503621	0.891401769
1.523055417	177.3795726	2442.7713	0.905906331
1.554936415	181.2131358	866.4451726	0.898749864
1.528535752	309.4586889	1707.740501	0.904032612
1.378506986	367.98412	2847.929303	0.927093505
1.685838684	160.1382502	1323.280627	0.889431008
1.545670771	388.8926911	3214.08964	0.906986186
1.530096622	306.1945972	4459.103392	0.906056068
1.489318097	318.4889299	2984.552438	0.911055466
1.492916527	312.8481497	1633.402193	0.911588135
1.739647774	204.2234638	1463.040452	0.882152747
1.528974075	200.4287883	936.8007869	0.906799501
1.53394447	247.5268933	2233.187239	0.903825685
1.622688124	306.3074705	2903.412751	0.893168743
1.546168125	553.5745015	932.3222882	0.905424993
1.406701484	363.9242178	1940.858478	0.921007609
1.471211829	360.7082633	1755.135663	0.914219178
1.945912123	82.76545832	299.7260396	0.852710063
1.467995068	333.3202463	2706.05638	0.910575682
1.69041657	136.1279068	2182.752724	0.884814126
1.403307596	289.8544075	1037.992311	0.919610194
1.635321895	131.0263339	1983.28977	0.893204336
1.642667588	70.16283357	288.3867755	0.899731749
1.492666839	117.5191907	404.6986852	0.909794112
1.545594568	268.5932272	2190.85081	0.90520312
1.450040936	173.8754537	790.4509584	0.915532581
1.507803517	167.5763986	1057.291828	0.901893185
1.679636247	279.6077728	2734.914201	0.884644
1.60398798	172.8476505	3110.311372	0.897018653
1.255942858	515.932141	719.362573	0.948248207
1.752695032	155.0133224	1084.305493	0.878192605
1.504704986	96.07827766	301.3002972	0.910505928
1.788358312	88.20974751	527.9873656	0.869554538
1.263143036	325.061807	921.7595761	0.943319822

log.sigma.5.0.mm.3D_glrlm_LongRunHighGrayLevelEmphasis	log.sigma.5.0.mm.3D_glrlm_RunPercentage	log.sigma.5.0.mm.3D_glrlm_LongRunLowGrayLevelEmphasis	log.sigma.5.0.mm.3D_glrlm_RunEntropy
339.9581929	0.894110877	0.012625419	4.860809279
547.0821996	0.869884615	0.006460972	4.665254837
341.511762	0.886473098	0.011084316	4.620784146
212.6800077	0.866787921	0.022919715	4.641347563
215.0532136	0.847693929	0.03043545	4.591073673
213.717259	0.839485301	0.022854371	4.583114571
116.8071983	0.784295175	0.099768738	4.424204975
454.9400746	0.870786277	0.010449896	4.883038871
337.987018	0.876209632	0.010909761	4.730349952
829.3962802	0.889751274	0.003703879	4.829601828
156.6877885	0.827338129	0.039447026	4.429000234
393.4603933	0.883725177	0.008799957	4.575822985
248.5808657	0.823305407	0.020559104	4.595072371
204.0533314	0.879812867	0.021770836	4.478113637
389.848213	0.878851123	0.009845307	4.866109735
587.4353587	0.856075781	0.008158358	4.955962642
550.8611804	0.868727534	0.007196266	4.751796768
405.3193476	0.845432128	0.010562019	4.795713801
370.332076	0.882313109	0.008661308	4.404293047
381.7250943	0.852729828	0.013959668	4.756596829
582.7998084	0.863453237	0.005620781	4.765144997
393.4636532	0.874913865	0.009647504	4.751117142
507.5228339	0.878558229	0.006252605	4.798539166
255.174993	0.876101568	0.025069216	4.68666836
332.8003458	0.892414038	0.015467074	4.746911681
371.0181746	0.895653163	0.010532122	4.774725278
335.130547	0.887694395	0.010345378	4.707809975
412.3644573	0.89875043	0.007644841	4.617575537
645.9299525	0.866906556	0.005673633	4.662140096
710.5359217	0.878953905	0.004275964	4.765989313
520.1783821	0.867938194	0.007327169	4.859095207
595.658313	0.876630769	0.00709591	4.775050389
999.7939886	0.851502902	0.004545862	4.871245796
252.2647334	0.878298417	0.019926773	4.577860906
187.2889682	0.859580735	0.035296591	4.558116262
316.3712207	0.856053179	0.012139184	4.525625407
659.8540012	0.8900791	0.005425336	4.958902775
416.4922847	0.856452601	0.00896189	4.559526636
301.9929146	0.814379017	0.016734753	4.497493452
215.5672558	0.836232838	0.02495704	4.642605236
234.4268402	0.871171382	0.015428727	4.42527012
257.1252513	0.853559484	0.014850701	4.468510463
434.5572571	0.838860512	0.009736795	4.594051011
259.1431271	0.845133105	0.019188902	4.670379421
654.4478966	0.854387742	0.006930256	5.022971555
255.7933102	0.87020979	0.021235092	4.732212447
672.8142894	0.877958179	0.005290434	4.827558903
510.527804	0.840927542	0.009168888	4.619907438
152.701305	0.857337698	0.038145545	4.351246671
323.4528183	0.858574453	0.012980682	4.719076186
615.3261714	0.834569045	0.007299732	4.773991081
419.2922635	0.801557338	0.014163391	4.497978548
340.3434549	0.847531452	0.012329585	4.765341491
603.4742403	0.877028181	0.005571175	4.624991135
429.9531971	0.877474063	0.008394585	4.772141448
427.2078082	0.884859844	0.009411613	4.947447526
756.9616041	0.879864874	0.005059323	4.786932661
315.9967198	0.796115891	0.020122647	4.7808153
312.7007862	0.874747701	0.012408245	4.528691369
253.3157136	0.840569395	0.015560668	4.235494086
315.2670137	0.852091125	0.01446465	4.821745108
614.740753	0.857523377	0.00619865	4.794341659
494.5517745	0.866104393	0.007085927	4.67396888
250.357405	0.901540109	0.016863677	4.568049858
283.5662217	0.902346806	0.011854441	4.423805721
186.3658369	0.88598634	0.027136642	4.65660014
233.0502773	0.855896466	0.029301917	4.88005418
284.4372078	0.871802062	0.016303841	4.83990684
316.4923324	0.865131775	0.011658993	4.487676445
505.3527626	0.8703323	0.007345483	4.864966999
554.5800519	0.901119101	0.008273894	5.013686737
282.3956582	0.848020005	0.015721665	4.508756709
645.4871149	0.870715307	0.005739695	4.792844
528.5265925	0.87123566	0.006111554	4.655023706
502.3692646	0.879038371	0.007299805	4.959543905
507.0817021	0.878427878	0.007227407	4.834685136
400.1211879	0.836844684	0.011099452	4.598190583
332.582216	0.87281372	0.014663272	4.594624242
426.0134767	0.870386024	0.008157325	4.637481294
562.9859555	0.855551141	0.007126162	4.757956226
946.4535616	0.870608833	0.004062648	4.74760029
563.3759697	0.894328479	0.005894321	4.830837912
567.7237203	0.88312294	0.005675003	4.774806711
161.2035082	0.809824974	0.039541886	4.156219977
543.7538266	0.880711469	0.007678297	4.795905953
237.3342785	0.843076037	0.025316583	4.813320279
435.3943609	0.89339582	0.00848227	4.865382518
230.2070413	0.854217751	0.019089065	4.527191808
137.6257346	0.870671907	0.037094701	3.773654875
194.7098186	0.879158903	0.03415377	4.468308628
438.1617832	0.869079846	0.007336083	4.666548247
274.4603685	0.886460146	0.014521785	4.631864536
287.3887962	0.870875603	0.013499299	4.426486564
523.2160533	0.843644525	0.007659078	4.754105416
304.6500154	0.859328517	0.013931613	4.589935224
701.3746345	0.929777236	0.006500574	5.051613681
291.2448736	0.834896811	0.014727384	4.42334173
150.289872	0.879583034	0.038747233	4.397469481
161.4117003	0.826143451	0.034595086	4.434820242
446.4496276	0.925209972	0.012342468	4.922353736

log.sigma.5.0.mm.3D_glrlm_HighGrayLevelRunEmphasis	log.sigma.5.0.mm.3D_glrlm_RunLengthNonUniformityNormalized	log.sigma.5.0.mm.3D_glszm_GrayLevelVariance
251.4101069	0.818910173	30.7229843
365.5028756	0.77911316	24.13420023
229.6727806	0.802412384	23.41827399
142.5797555	0.779368476	20.43279711
143.4017318	0.749885237	17.2428451
133.9389785	0.732850001	21.73322857
69.7244065	0.666506562	9.013644464
303.0650814	0.78601549	29.15276662
233.5393266	0.797374314	23.75535764
577.7390564	0.810456993	29.69220591
93.72527139	0.72287346	13.18261165
268.793275	0.799737362	23.50603075
143.9353344	0.724329501	20.29444071
135.9363847	0.795911707	16.3364984
270.3617731	0.794593414	33.53052127
374.5348779	0.773330411	30.11341858
352.7304666	0.776678205	31.36196122
254.0148987	0.751653058	23.58590393
249.4270503	0.799798267	17.004176
243.0687893	0.753652105	23.91842736
373.7012233	0.768482265	25.49448057
260.7812237	0.784828739	27.39110943
348.7155465	0.792484237	29.45407054
173.3476294	0.792126813	20.86261132
229.9345623	0.815314896	22.37409955
272.3029844	0.82049394	36.22518811
230.9997919	0.811248149	25.07270233
289.9699595	0.823899805	20.88253518
404.1645532	0.783671808	32.90433743
471.7569106	0.798272664	33.07902993
335.7554645	0.774338652	28.76323578
399.3126017	0.795413224	26.33982745
618.2994282	0.757233409	45.02127947
180.9673136	0.792276302	20.53263533
117.9574164	0.763715966	17.77515432
199.6908028	0.762258295	18.63036084
457.8347477	0.813699598	40.86818145
259.3213706	0.760526506	19.60586735
167.7352819	0.700016081	17.85181795
137.7624932	0.735949748	18.81226466
157.3741535	0.783684563	16.20674556
167.2955857	0.758425426	16.08458225
260.9777625	0.740525816	19.97665012
166.739759	0.751943695	23.03521329
396.8481444	0.763845607	38.13154935
164.6853415	0.77955132	17.86675065
455.42029	0.792953026	30.53388146
290.284355	0.735322294	28.59605832
101.4789823	0.761603092	11.24185339
214.3018113	0.768623336	25.31673443
362.7118614	0.738601659	26.798039
221.0680821	0.689439689	23.22820098
217.7319383	0.752200378	21.7588144
404.0709485	0.787773708	24.61143667
284.1544154	0.799201286	24.18854795
298.8262791	0.801780692	34.26227314
513.639242	0.79983708	34.6052395
177.8188634	0.686752932	25.29185903
211.3200306	0.782721868	22.84682943
148.6005632	0.732759796	12.479239
206.4880175	0.756970403	20.48472301
382.06437	0.766371596	31.42952756
319.1489859	0.774957235	24.29563031
174.5785807	0.830596567	18.84897072
207.4715522	0.825038365	18.49642645
136.1245197	0.804900952	20.73070507
163.5507145	0.755711165	26.10567591
194.2486092	0.783859257	25.7450945
201.8230219	0.770950576	16.42950625
340.5439733	0.780338025	32.00847464
398.0863997	0.827946398	35.26723224
177.9972716	0.752922171	20.78192176
427.2856052	0.786545004	25.60331284
339.6328333	0.78416857	21.51567328
347.5052991	0.79440554	34.99591149
342.9016436	0.795697187	33.00638021
231.536536	0.738805702	22.38472591
220.8079937	0.786546644	19.93583482
274.5833439	0.7802291	24.49066268
343.9879475	0.759068133	28.67062269
612.5055852	0.783435298	45.03501835
396.1486431	0.814805275	35.53405536
393.0467639	0.800987866	31.79206575
94.25467109	0.694823783	8.770709822
367.0982603	0.793376518	25.77296503
151.3521588	0.742641691	20.38253827
314.4996635	0.811774132	34.49630161
145.6755743	0.759604322	14.63786754
79.44226052	0.786748878	7.473580247
129.5049123	0.792865818	15.289282
294.7321558	0.782878712	22.693766
189.8539601	0.804115361	23.29668609
187.0838227	0.775372345	16.07552517
315.2633215	0.74227804	27.80567555
192.5534867	0.766392266	17.43008085
546.8897804	0.873913169	56.10510071
174.1851338	0.731930644	15.46676406
104.8758268	0.795355238	11.81422043
99.27837628	0.715515152	12.89102041
346.6228214	0.862286251	37.37447197

log.sigma.5.0.mm.3D_glszm_SmallAreaHighGrayLevelEmphasis	log.sigma.5.0.mm.3D_glszm_GrayLevelNonUniformityNormalized	log.sigma.5.0.mm.3D_glszm_SizeZoneNonUniformityNormalized
142.3011461	0.053974242	0.32123533
169.1515171	0.060740437	0.235860371
90.28042429	0.06010722	0.203601189
81.02132035	0.061019419	0.288719943
90.41565436	0.069941637	0.309634531
67.67752853	0.062753509	0.19705239
39.69422219	0.104143462	0.212296725
149.4303718	0.054260923	0.258496278
118.3634488	0.057549155	0.295610586
279.5977442	0.052612577	0.254858163
42.23379409	0.077618977	0.218798445
124.0159735	0.05822637	0.263646112
75.8230597	0.061734785	0.257261816
57.98041182	0.06889848	0.233071871
150.2537915	0.050194543	0.320144812
186.5867802	0.052064736	0.243999207
146.8568102	0.052014901	0.219355872
137.6773943	0.060743802	0.278595041
118.205617	0.07248	0.282848
117.3204818	0.059175529	0.246445396
164.8721865	0.057131778	0.226713283
139.8290784	0.057341961	0.257942829
181.8168787	0.052582124	0.238638774
94.94056663	0.064062051	0.256104568
100.5490471	0.058858612	0.23399274
151.3557587	0.047069808	0.300331897
116.2907479	0.056461817	0.287955648
126.9653396	0.062341011	0.261222549
191.9984449	0.051258526	0.29552284
256.1500558	0.049013064	0.335862312
156.2810358	0.05352202	0.25540453
211.0927753	0.055839252	0.309579717
335.4589262	0.049050181	0.298498866
88.83876689	0.063934061	0.356426821
48.14532153	0.068641975	0.205864198
99.08575201	0.065986081	0.250825776
234.6946537	0.04441359	0.28368191
123.5942025	0.064633724	0.256574389
76.52065128	0.069372554	0.198144174
81.09480318	0.065577779	0.280111497
75.25748091	0.070236686	0.287810651
86.63420544	0.070655621	0.252052071
118.6303208	0.06522511	0.250868485
106.2267805	0.058443753	0.31941252
176.6616412	0.044873746	0.27462896
73.5767346	0.066357117	0.238731219
241.1054121	0.053212862	0.277983607
116.5622192	0.053819723	0.236055475
57.39840281	0.088373323	0.27992597
115.5129012	0.057296533	0.255675362
173.7045058	0.057128984	0.255603415
91.05210629	0.059949015	0.212740879
126.9572399	0.061231286	0.2857238
187.2632582	0.059508507	0.278676749
133.1328398	0.056415873	0.274879422
161.2701021	0.047436228	0.258713644
268.9567844	0.050283728	0.325651275
105.9700462	0.056581512	0.259874833
95.61462677	0.059571629	0.219863564
43.60212008	0.080808561	0.183638526
105.6792972	0.062053543	0.304561033
196.3448938	0.051028138	0.280530486
157.2537072	0.058706002	0.26763446
73.93842475	0.063113109	0.251263377
91.37441374	0.065697076	0.279922599
92.85068531	0.063772007	0.298639831
103.7161955	0.057200274	0.219390256
112.0390295	0.054874545	0.288560983
77.77066472	0.071314694	0.23302796
171.2380207	0.050677351	0.242645235
187.6435266	0.047266841	0.278946878
94.31469485	0.063207849	0.2878757
224.687502	0.05739662	0.284346883
157.2788817	0.061140543	0.255798519
208.6571854	0.048228236	0.291977137
167.574343	0.05160714	0.267411366
102.9974412	0.058644839	0.266689108
97.0385033	0.064293343	0.214866501
121.3025908	0.058213247	0.236371162
149.7143734	0.053185288	0.232268748
304.7535005	0.044734176	0.305754338
182.1928542	0.047069918	0.247674611
216.9664453	0.053205219	0.272559465
39.14979349	0.0985419	0.248517866
167.0621959	0.05736105	0.245271715
71.3356169	0.061913265	0.269566327
152.1098186	0.048172654	0.263927468
71.74392831	0.073490002	0.269260321
28.68408677	0.11382716	0.307654321
53.38583775	0.073491155	0.233350676
172.9462016	0.05877291	0.332943934
82.00792578	0.059520119	0.268057536
74.21858891	0.070752418	0.224682289
143.9087761	0.054619806	0.22012575
88.94924613	0.066751227	0.261681926
279.2796554	0.037960398	0.349697343
82.93226739	0.076251099	0.243869352
53.56425443	0.087682007	0.271548517
36.41118864	0.078265306	0.204795918
159.2569476	0.046687889	0.302388927

log.sigma.5.0.mm.3D_glszm_SizeZoneNonUniformity	log.sigma.5.0.mm.3D_glszm_GrayLevelNonUniformity	log.sigma.5.0.mm.3D_glszm_LargeAreaEmphasis	log.sigma.5.0.mm.3D_glszm_ZoneVariance
104.0802469	17.48765432	77.87962963	64.09353567
83.96629213	21.62359551	287.1516854	255.5900139
53.34351145	15.7480916	62.38931298	44.18052561
127.9029345	27.03160271	400.1963883	369.5352944
55.42458101	12.51955307	241.4692737	213.3615056
42.76036866	13.61751152	476.0921659	419.1140181
28.44776119	13.95522388	679.619403	617.3979171
90.73219373	19.04558405	211.7863248	185.3415313
143.371134	27.91134021	171.5195876	154.0523584
180.9492958	37.35492958	82.35352113	64.4641956
47.47926267	16.84331797	681.0460829	611.7040073
104.6675063	23.11586902	108.0881612	90.07380289
107.2781775	25.74340528	729.1726619	679.8363554
85.07123288	25.14794521	152.0986301	126.1863464
126.1370558	19.77664975	95.9035533	80.46703084
207.8873239	44.35915493	220.3603286	197.1012186
69.3164557	16.43670886	162.2879747	136.4905764
122.5818182	26.72727273	362.7454545	328.9470868
70.712	18.12	101.352	80.704064
119.0331263	28.58178054	507.3064182	471.2318455
282.4847512	71.18619583	287.9390048	256.826604
113.2369021	25.17312073	118.7312073	95.38844236
196.6383495	43.3276699	170.5279126	145.5157752
60.44067797	15.11864407	103.0932203	84.0451738
149.0533752	37.49293564	74.44897959	57.73795435
107.2184874	16.80392157	84.76190476	70.41966591
147.7212476	28.96491228	84.50097466	65.92537875
216.2922705	51.61835749	75.0615942	58.64343829
176.4271357	30.60134003	378.9631491	346.5475788
242.1567268	35.33841886	248.4008322	229.2040374
199.7263427	41.85421995	189.9897698	161.9350017
349.5155004	63.0425155	216.5314438	196.9180781
131.0410023	21.53302961	504.3166287	471.07707
47.76119403	8.567164179	45.08208955	32.19720428
37.05555556	12.35555556	129.0222222	104.6844444
118.640592	31.21141649	472.9830867	426.7258335
234.0375758	36.64121212	116.3793939	97.66459357
129.3134921	32.57539683	305.0218254	259.7534919
35.26966292	12.34831461	620.6966292	551.1897488
80.11188811	18.75524476	334.7132867	301.9916866
74.83076923	18.26153846	191.4923077	165.8731361
81.91692308	22.96307692	291.9876923	256.6492402
95.07915567	24.72031662	779.944591	716.1554988
109.877907	20.10465116	425.3372093	395.3424824
201.5776567	32.9373297	258.7493188	236.0896435
143	39.74791319	217.4207012	181.8003294
227.1126071	43.4749082	181.0893513	160.7345649
55.94514768	12.75527426	368.8185654	327.9017608
34.43089431	10.8699187	118.9430894	97.91750942
50.87939698	11.40201005	193.678392	163.6766243
144.4159292	32.27787611	1173.79115	1124.069932
40.63350785	11.45026178	1607.277487	1502.40273
156.5766423	33.55474453	856.2755474	808.2668329
64.09565217	13.68695652	121.6565217	95.73514178
218.8040201	44.90703518	200.4660804	176.0033632
110.470726	20.25526932	103.1381733	83.58810721
240.6562923	37.15967524	131.7577808	112.0701273
88.87719298	19.35087719	1027.789474	977.2215724
59.58302583	16.14391144	190.597786	164.9665446
26.62758621	11.71724138	577.6758621	483.7864447
167.2040073	34.06739526	262.1621129	233.4645273
257.2464558	46.79280262	703.8091603	668.005185
121.2384106	26.59381898	185.5916115	160.4589857
116.5862069	29.28448276	61.88793103	45.68036564
50.10614525	11.75977654	33.24022346	22.37601823
107.2116992	22.89415042	173.1169916	154.6437566
44.31683168	11.55445545	145.039604	117.6069013
188.4303216	35.8330781	349.3843798	319.5624107
47.77073171	14.6195122	185.6634146	145.881166
123.2637795	25.74409449	141.8562992	117.5016856
293.1731684	49.67745005	63.34538535	50.21769127
92.40809969	20.28971963	541.8037383	500.580138
274.679089	55.44513458	250.7215321	227.2602448
287.7733333	68.78311111	389.2702222	355.7744229
275.0424628	45.43099788	137.9532909	117.4829495
132.1012146	25.49392713	146.0222672	123.7568596
93.07449857	20.46704871	560.3810888	514.8475957
56.93962264	17.03773585	168.9471698	142.6476896
143.2409241	35.27722772	222.7524752	193.5282815
173.9692924	39.83578104	657.7022697	622.3561491
92.64356436	13.55445545	210.3168317	190.1113616
152.815235	29.04213938	103.0940032	84.54960348
139.2778865	27.18786693	171.2837573	147.8437583
19.63291139	7.784810127	248.6708861	204.8431341
193.2741117	45.20050761	115.8172589	91.79730861
150.9571429	34.67142857	394.8714286	356.3871301
94.48603352	17.24581006	76.79329609	60.88255672
124.3982684	33.95238095	692.5822511	649.1992138
27.68888889	10.24444444	89.78888889	68.93444444
28.93548387	9.112903226	86.27419355	64.54656608
214.7488372	37.90852713	357.7131783	332.9297614
68.08661417	15.11811024	86.87007874	67.94415339
66.73063973	21.01346801	181.4680135	153.8082055
130.53457	32.38954469	906.6627319	852.7777244
210.9156328	53.80148883	308.0521092	273.9615785
119.5964912	12.98245614	21.3625731	14.68140624
63.16216216	19.74903475	480.8146718	434.4801061
26.34020619	8.505154639	69.73195876	50.26293974
28.67142857	10.95714286	263.7857143	223.5538776
128.5152941	19.84235294	18.20705882	10.83423668

log.sigma.5.0.mm.3D_glszm_ZonePercentage	log.sigma.5.0.mm.3D_glszm_LargeAreaLowGrayLevelEmphasis	log.sigma.5.0.mm.3D_glszm_LargeAreaHighGrayLevelEmphasis	log.sigma.5.0.mm.3D_glszm_HighGrayLevelZoneEmphasis
0.269326683	0.603369481	13928.87037	263.191358
0.178	1.061526641	82899.16011	377.0674157
0.234347048	0.322746673	16250.14885	215.8015267
0.18059519	4.980055064	37777.7991	157.7404063
0.1886196	3.394003077	21670.57542	168.1620112
0.132478632	5.679009356	46662.3871	154.5668203
0.126773888	39.13032036	18622.12687	85.90298507
0.194459834	1.061921959	49877.55271	307.3105413
0.239269857	1.298398669	27434.6433	231.4123711
0.236430236	0.163844787	46505.52817	560.8323944
0.120088545	12.28823215	46438.31336	94.93087558
0.235608309	0.501339451	26610.20655	260.9143577
0.142369409	7.599241334	76174.43645	159.8968825
0.196447793	1.584377785	18679.32603	127.3068493
0.254521964	0.594784863	18981.56345	276.7005076
0.20734972	0.802078534	71710.95892	375.0176056
0.196884735	0.532131619	53724.25633	339.164557
0.172009382	2.403744732	64590.70455	266.875
0.220070423	0.485889683	24741.696	234.036
0.166494312	3.015008074	94356.48447	243.4244306
0.179280576	0.86601065	104266.2528	354.8033708
0.206977841	0.605748595	28274.60364	269.1617312
0.199951468	0.621650462	51071.32767	373.4575243
0.229126214	1.133243857	13750.5678	188.7627119
0.244623656	0.543279629	19129.56044	214.0031397
0.264053254	0.452413806	18647.95238	285.3697479
0.23202171	0.512040558	17660.24172	222.1111111
0.246795827	0.28313272	24813.66425	258.4444444
0.175639894	1.044724269	145038.6466	373.3902848
0.228236784	0.538432789	119424.5992	440.0998613
0.188797682	0.654171528	64081.11637	315.983376
0.2258	0.731766965	73114.76882	384.2834367
0.17344923	0.993797567	266181.4169	622.881549
0.278586279	0.501084729	6407.052239	170.6865672
0.202702703	1.725343989	13211.91667	115.0166667
0.147031396	2.983083836	81391.74419	201.2515856
0.231157187	0.298865817	52055.88727	442.7127273
0.148628723	1.380600058	73728.64881	245.6785714
0.119946092	4.819167888	86021.24157	180.3426966
0.174816626	4.679671294	28279.68182	160.972028
0.197568389	1.743575438	23936.26538	150.0538462
0.168219462	2.802256996	34558.48615	179.4523077
0.125206475	4.340715859	155485.4644	252.237467
0.182590234	4.678020279	43666.57849	188.9505814
0.210074413	0.744333408	98262.85831	354.380109
0.167552448	1.974937435	38700.9399	155.096828
0.221649485	0.470861902	75598.47491	456.9033048
0.156332454	1.242372199	116483.7553	244.2025316
0.218085106	3.007907724	7119.130081	114.5203252
0.182568807	1.558603952	29018.1608	238.2713568
0.141817269	4.12566151	346708.4938	364.0566372
0.097648262	8.712484978	307814.2618	214.1308901
0.144324467	7.026402735	118691.4325	241.479927
0.196413322	0.380225762	43463.5	374.9521739
0.202184404	0.84031545	56730.90452	265.491206
0.226165254	0.514197582	26764.57143	313.4730679
0.22537359	0.314170348	62501.26522	488.6008119
0.140625	9.711288957	114494.4386	214.7163743
0.197521866	1.144380658	34977.369	214.5313653
0.103202847	4.514356999	80797.4069	124.2551724
0.1866712	2.670011945	36996.36794	204.7814208
0.167122289	2.194813759	238227.7481	367.6739368
0.199471598	0.732428488	51941.5011	312.8454746
0.248394004	0.485384209	12637.72845	150.8017241
0.303389831	0.230530404	6436.843575	188.2234637
0.232663642	3.383247005	11892.71031	166.810585
0.190926276	2.365684469	13777.08911	214.1485149
0.18311834	3.50407472	43089.82389	213.5987749
0.158546017	1.162910683	36448.37561	182.902439
0.202632629	0.564770794	40588.98425	353.3917323
0.275997899	0.266995984	26643.62988	365.2892483
0.155749636	4.50758103	69912.28349	189.7009346
0.206454371	0.781840416	89004.34679	429.2971014
0.172784519	1.21715989	133523.6942	312.7564444
0.221022994	0.571444053	39274.46391	374.462845
0.211926212	0.561961577	44400.17409	329.8360324
0.148195329	2.727259094	123435.0745	203.2063037
0.194996321	1.160659691	29097.60377	221.6830189
0.184981685	0.881723515	61292.33333	256.8267327
0.168201213	1.992460537	227008.7664	315.6315087
0.22246696	0.368031865	124704.637	580.7359736
0.232216786	0.287445903	41876.18801	374.2252836
0.2065481	0.573612719	55756.49706	412.1565558
0.151051625	5.038030282	15870.03797	89.08860759
0.204039358	0.383472786	43921.10787	340.3401015
0.161197467	5.770735813	39665.09821	155.6892857
0.25070028	0.323286182	21529.65642	310.1675978
0.151823858	7.689471717	73504.53463	148.6861472
0.218978102	1.414463595	8419.466667	61.57777778
0.214532872	1.169348535	11352.51613	114.6451613
0.200872002	1.905852781	72938.9845	308.8542636
0.229864253	0.68956883	13803.41339	180.1377953
0.190140845	1.051911628	34854.57576	165.6228956
0.136227889	3.233169862	263694.9258	312.5784148
0.171270718	2.123246399	52457.52854	184.7568238
0.386877828	0.061557506	13200.05263	494.7748538
0.146908678	3.832857781	65436.54826	178.8918919
0.226635514	1.893647635	5162.628866	108.9484536
0.157657658	5.098629103	17138.05	102.3285714
0.368284229	0.114460946	6938.614118	310.1294118

log.sigma.5.0.mm.3D_glszm_SmallAreaEmphasis	log.sigma.5.0.mm.3D_glszm_LowGrayLevelZoneEmphasis	log.sigma.5.0.mm.3D_glszm_ZoneEntropy	log.sigma.5.0.mm.3D_glszm_SmallAreaLowGrayLevelEmphasis
0.580886299	0.01299379	6.253621947	0.009266618
0.493988931	0.008201041	6.494324926	0.005976412
0.447056356	0.013315676	6.489397452	0.008652736
0.554963405	0.022000644	6.412806756	0.0145671
0.575748667	0.019166875	5.695892449	0.007777795
0.449897054	0.020020989	6.490386171	0.011555742
0.465512198	0.033392667	5.591171867	0.016065015
0.51871756	0.009983244	6.50053037	0.004622267
0.559097876	0.010373859	6.364025086	0.007550923
0.517264542	0.004147864	6.870758323	0.002750831
0.47193007	0.032281539	6.132715806	0.012555674
0.52323836	0.009317197	6.438025226	0.00625772
0.518117227	0.017738055	6.431121927	0.011468509
0.494862854	0.020769585	6.417689311	0.010052398
0.585181648	0.010518243	6.405393804	0.008178238
0.501389045	0.005947281	6.896020733	0.002295453
0.475864291	0.009603069	6.750389868	0.006479363
0.543074116	0.008708572	6.426610866	0.006138497
0.549688223	0.010454606	6.089500784	0.007934651
0.508317828	0.013197725	6.62204383	0.006530548
0.481742672	0.005398523	7.05854169	0.00331301
0.522850327	0.010164689	6.612292469	0.006412862
0.498954047	0.005827028	6.998465564	0.003481925
0.5192462	0.020704238	6.161063731	0.009887641
0.486331741	0.01098705	6.653118233	0.004454525
0.565055444	0.014024588	6.471682819	0.010693557
0.555752269	0.010886403	6.552329332	0.007496808
0.525097651	0.007698982	6.668300601	0.004693828
0.560517861	0.007080732	6.63705765	0.005137054
0.600186091	0.004931009	6.544573957	0.003604431
0.519900855	0.007974996	6.903292093	0.005084177
0.575632515	0.005210275	6.68691051	0.002417569
0.565927931	0.005985802	6.687851816	0.004304179
0.619453157	0.020540865	5.489847609	0.012135359
0.452923003	0.034450156	6.167082316	0.016174504
0.51431803	0.011341959	6.478034322	0.005322485
0.54955993	0.005696289	6.996681044	0.002765505
0.524414166	0.008966872	6.56937618	0.004451419
0.451288293	0.017636943	6.192622145	0.01248257
0.547218106	0.018617373	6.237920052	0.012559094
0.554058907	0.016293667	6.007696148	0.01111906
0.516969338	0.012119075	6.300556147	0.007974505
0.515638451	0.013132457	6.432148226	0.010276295
0.58568274	0.016330025	6.203562315	0.010993457
0.53793076	0.007860336	6.89455563	0.005224025
0.50196631	0.016783741	6.635741625	0.007402134
0.542413911	0.004952812	6.757535958	0.002252128
0.494032953	0.013943537	6.411361368	0.009263803
0.538196555	0.026276928	5.412481351	0.018276842
0.516311034	0.013252818	6.208513676	0.010056061
0.513712297	0.006556691	6.640027337	0.003012562
0.465074925	0.016593804	6.261432297	0.005893507
0.552514524	0.009456167	6.451090303	0.005851176
0.545884593	0.008443292	6.252413389	0.006927776
0.540091266	0.00795391	6.646721006	0.004965789
0.521033142	0.009634375	6.756050309	0.005909379
0.591974166	0.005095857	6.670028841	0.002600926
0.524826533	0.012865693	6.466902668	0.008176233
0.47140463	0.013464802	6.483825103	0.005994167
0.434845056	0.021715402	6.053412063	0.014206767
0.571625978	0.011274723	6.351185794	0.007804775
0.545199945	0.006365007	6.864649219	0.003336873
0.531370389	0.00781016	6.553666396	0.00547339
0.508430528	0.018830352	6.422750316	0.00980543
0.542953432	0.015837362	5.921107067	0.01255707
0.564090668	0.019514658	6.222090807	0.011491
0.466440727	0.016780373	6.328346929	0.005371134
0.554395692	0.014598425	6.58325566	0.008344883
0.49292885	0.01404447	6.113957521	0.009683168
0.505797799	0.007827699	6.867110886	0.005298892
0.539393204	0.007146244	6.8945891	0.004018858
0.553927398	0.017436091	6.227252615	0.01142813
0.550647665	0.004650846	6.715471729	0.002863294
0.51913402	0.006018985	6.81792355	0.003675319
0.557855181	0.006622129	6.889480504	0.003665837
0.531186414	0.008039205	6.728277134	0.005158075
0.527628244	0.013145812	6.371402699	0.008063623
0.465664885	0.013273645	6.363268135	0.004304952
0.495060362	0.008930164	6.763354529	0.005619504
0.487360959	0.008078501	6.912960203	0.004885694
0.570222128	0.006207321	6.493294314	0.005071489
0.502007966	0.006516753	6.899826308	0.003104896
0.536240243	0.006437025	6.657859647	0.004240951
0.510600745	0.031847941	5.24955021	0.015127816
0.507481294	0.007778871	6.865725323	0.00396203
0.532177148	0.019000873	6.464757905	0.011446046
0.527646772	0.010206298	6.642581369	0.006690603
0.534062788	0.016914306	6.255051812	0.011192779
0.555161208	0.055934038	4.921027998	0.048114361
0.48538272	0.030176927	5.708539178	0.011204564
0.599174071	0.006382761	6.383290675	0.004749701
0.533703202	0.018091682	6.245738817	0.013512346
0.481363105	0.014464163	6.327706585	0.005998414
0.475411572	0.007567826	6.951291205	0.004337871
0.527888928	0.010898658	6.592420848	0.005704955
0.611711795	0.007903661	6.570263191	0.006263964
0.506117806	0.01412614	6.141528208	0.010170259
0.536302203	0.029568621	5.328444647	0.020512739
0.451183803	0.027655987	5.9093961	0.019086037
0.563230728	0.010110096	6.526557408	0.004911153

log.sigma.5.0.mm.3D_ngtdm_Coarseness	log.sigma.5.0.mm.3D_ngtdm_Complexity	log.sigma.5.0.mm.3D_ngtdm_Strength	log.sigma.5.0.mm.3D_ngtdm_Busyness	log.sigma.5.0.mm.3D_ngtdm_Contrast
0.012324817	0.321433805	2.829199767	0.239962083	8.84E-05
0.006911703	0.223580165	2.785695208	0.227123047	2.26E-05
0.013292989	0.330153917	3.914978613	0.156861598	4.93E-05
0.005945523	0.098837737	1.244199588	0.696240108	2.59E-05
0.014726944	0.18929055	2.250021753	0.351062936	9.23E-05
0.008872375	0.121691389	1.807972237	0.467894291	3.43E-05
0.014551583	0.074588385	1.387027356	0.791247985	8.37E-05
0.008797591	0.247253443	3.105245225	0.212036388	3.20E-05
0.006619213	0.20519042	1.837280584	0.390212959	3.46E-05
0.005026379	0.212270885	2.575349687	0.216423862	1.69E-05
0.007372357	0.064788768	0.810908438	0.99568096	3.58E-05
0.007976774	0.213731343	2.120892313	0.261022602	3.16E-05
0.00490148	0.061260007	0.844350889	0.809033207	1.66E-05
0.007552037	0.098448527	1.060258774	0.61462333	3.79E-05
0.00847375	0.33934225	2.61580735	0.272019573	5.69E-05
0.004373335	0.10944793	1.669194244	0.354235341	1.24E-05
0.010542852	0.258617805	3.864928718	0.140062711	2.64E-05
0.00532502	0.18992944	2.04826076	0.363638553	1.85E-05
0.010091289	0.226385954	1.800918083	0.280639447	6.28E-05
0.005293787	0.112492952	1.580535213	0.402391506	1.67E-05
0.002361015	0.07830458	1.191559913	0.491536888	4.39E-06
0.00716386	0.222993919	2.998069653	0.244108093	1.98E-05
0.003820143	0.140108571	1.902203646	0.349403105	9.31E-06
0.015385414	0.193084512	2.4163176	0.288876386	9.60E-05
0.006248943	0.102905193	1.306041941	0.445696803	3.24E-05
0.009474754	0.364406553	3.110627705	0.216479413	5.49E-05
0.007528684	0.139609979	1.932755989	0.381874569	2.78E-05
0.003942936	0.12359597	1.157626639	0.483371675	1.83E-05
0.004079469	0.128853965	1.629635679	0.335935526	1.32E-05
0.004933179	0.207445544	2.624616411	0.206391536	1.14E-05
0.004213737	0.090792447	1.447102563	0.404741492	1.20E-05
0.002724247	0.123820111	1.322767183	0.462685993	8.88E-06
0.006609692	0.258230331	4.774690122	0.130281737	1.41E-05
0.028915675	0.483372698	4.236257372	0.150951707	0.000214512
0.01573343	0.177647219	2.001747765	0.357050061	9.13E-05
0.004314349	0.075153064	0.913203078	0.662788241	1.46E-05
0.004198405	0.235192355	2.601502946	0.245195163	1.38E-05
0.004185986	0.085724519	1.139934039	0.503567498	1.19E-05
0.01020528	0.118825584	2.13625361	0.301403051	2.22E-05
0.008439017	0.119027574	1.506761254	0.568125403	4.29E-05
0.009322136	0.137784108	1.236972031	0.505074587	5.77E-05
0.006941032	0.097586018	1.068278934	0.610437326	3.12E-05
0.00482996	0.096414919	1.304271307	0.469609215	1.38E-05
0.007402395	0.136011001	1.466799416	0.50734208	3.69E-05
0.004791049	0.162057464	1.772452374	0.282883302	1.86E-05
0.004349562	0.065859755	0.844394606	0.841393434	1.94E-05
0.004086281	0.178247473	2.259259885	0.263592063	1.06E-05
0.010553357	0.180629486	3.259935153	0.162212639	2.61E-05
0.02230252	0.208528443	2.154424071	0.404721568	0.000191162
0.014286217	0.274511245	3.285033841	0.212425571	5.83E-05
0.004068171	0.10179306	1.679425839	0.35647978	8.79E-06
0.007108913	0.104722527	1.795375497	0.325058322	1.60E-05
0.004010938	0.093011298	1.150626127	0.6372566	1.38E-05
0.011870817	0.301660283	3.282963126	0.164130314	5.63E-05
0.003675075	0.093610869	0.956837411	0.63447057	1.77E-05
0.009425614	0.230220517	3.006303688	0.205769679	3.50E-05
0.004308722	0.205568626	2.309956032	0.243906493	1.38E-05
0.005879964	0.137172055	1.934428384	0.427583071	1.81E-05
0.010220623	0.19573935	2.305147327	0.259437975	3.79E-05
0.009556793	0.090237503	1.340299837	0.41557604	2.67E-05
0.004918717	0.101801334	1.028440371	0.741144858	2.80E-05
0.002762008	0.098623952	1.333892876	0.445304402	6.64E-06
0.006000024	0.193498337	2.190260731	0.266716535	1.89E-05
0.006917294	0.129789649	1.061395636	0.589124884	5.64E-05
0.020124504	0.460206786	3.094199849	0.167664582	0.000162955
0.008412184	0.214532975	1.934994035	0.531695836	5.74E-05
0.016982848	0.248335672	4.025060405	0.255332834	7.31E-05
0.004174884	0.083006306	0.853950089	0.975856574	2.69E-05
0.01102426	0.143336156	1.732702319	0.34703518	5.15E-05
0.006256732	0.189003495	2.514665766	0.251672263	1.99E-05
0.004093068	0.161234939	1.585683284	0.360564074	2.40E-05
0.006285257	0.11159625	1.197925537	0.538415658	2.85E-05
0.003067268	0.138992804	1.764369817	0.358583003	8.07E-06
0.002180328	0.06562344	0.824103323	0.68761281	6.29E-06
0.003793688	0.176361696	2.236868361	0.329679334	1.02E-05
0.006611982	0.267414287	3.105818143	0.203041058	1.91E-05
0.006154997	0.117211023	1.606838105	0.354866149	1.65E-05
0.008372736	0.24749023	1.968219108	0.327713426	5.35E-05
0.004085612	0.124824889	1.32549165	0.429666385	1.39E-05
0.003776592	0.087431363	1.472067888	0.376495421	8.45E-06
0.010116208	0.542752468	6.275473289	0.081717478	3.07E-05
0.006233425	0.208840406	2.652944825	0.207577095	1.86E-05
0.005954548	0.248706907	3.008222461	0.208776723	1.70E-05
0.022556692	0.136223888	1.462874335	0.470476527	0.000167285
0.003779684	0.129913556	1.599883664	0.374777327	1.26E-05
0.004545823	0.076857833	1.007629113	0.913297404	1.93E-05
0.010312658	0.353397147	3.462614699	0.17449293	4.95E-05
0.004472543	0.059264313	0.677315733	1.05055572	2.11E-05
0.019861964	0.19687686	1.247082199	0.447160893	0.000248387
0.02333469	0.259630457	2.704940596	0.27074576	0.000178093
0.004437521	0.110355839	1.167158831	0.508444634	1.78E-05
0.011694034	0.258020395	2.256963098	0.297988107	7.77E-05
0.008874468	0.121604851	1.595897029	0.34961714	3.22E-05
0.003610494	0.082912774	1.380730391	0.439615667	8.09E-06
0.002889683	0.052881639	0.59573048	1.018458664	1.12E-05
0.015185951	1.126561425	7.350303416	0.073860717	0.000147529
0.007816371	0.106177592	1.446141796	0.429574205	2.53E-05
0.031527115	0.302136839	3.185619998	0.264024576	0.000256194
0.016256211	0.119925898	1.846747267	0.449450185	6.92E-05
0.011978756	0.601330369	4.943430902	0.13184561	8.41E-05

log.sigma.4.5.mm.3D_gldm_GrayLevelVariance	log.sigma.4.5.mm.3D_gldm_HighGrayLevelEmphasis	log.sigma.4.5.mm.3D_gldm_GrayLevelNonUniformityNormalized	log.sigma.4.5.mm.3D_gldm_DependenceEntropy
26.38617919	233.286783	0.056029917	6.993985409
17.40672775	310.1075	0.0741125	6.858730978
18.83263622	233.2271914	0.070337076	6.728358178
16.20348504	145.2523441	0.073812793	6.916469103
16.40053253	132.8767123	0.088810694	6.787027007
13.91328506	122.5665446	0.092294424	6.779106472
12.69903486	73.54493851	0.111363716	6.749926093
22.08584771	311.2554017	0.064370593	7.110858553
19.94076027	217.0335471	0.066443662	7.001758531
22.52831041	602.5954046	0.06135423	7.02673233
10.2395609	120.9490869	0.09669921	6.793595018
16.43300549	257.6498516	0.072384189	6.839687506
11.61969502	178.7695459	0.094353786	7.048664496
13.96583476	129.1410118	0.076279111	6.724111754
25.05247206	252.6763566	0.062143201	7.1608901
22.99374226	353.5449014	0.065205328	7.363402335
18.77855378	380.3943925	0.073637096	6.940394627
17.53990959	260.1223612	0.071608732	7.13193082
13.6942044	271.443662	0.07715483	6.623300497
17.39374493	225.634264	0.075271861	7.037923579
17.94328857	365.1461871	0.070834512	7.161132026
18.73716707	313.601603	0.06912821	7.04705729
19.86199614	300.1502063	0.068142699	7.0549003
20.81357809	187.6747573	0.0663908	6.817893854
21.92514706	201.9423963	0.062065981	6.935305643
23.41235207	327.6464497	0.064088705	6.902716718
19.24882352	217.47128	0.06594203	6.851935834
18.72288166	254.7275708	0.066858327	6.80710374
17.22792116	349.5954693	0.078036122	6.930405187
18.67499713	416.980057	0.071491423	6.99920584
20.52114843	395.5446644	0.06676306	7.158498068
19.40346684	339.291	0.06795448	7.155165014
22.1227132	583.7621493	0.071948768	7.113740739
18.77197972	169.3825364	0.070413769	6.658375315
15.76442532	159.4132883	0.075255154	6.793487547
13.10301883	242.8464408	0.082686408	6.854705947
27.07562656	476.1616699	0.058855634	7.072139134
13.37019176	276.9943969	0.083481601	6.921585261
10.80265101	181.9292453	0.099795846	6.901629601
15.7894273	123.1448655	0.082434646	7.04221245
12.50959144	146.6010638	0.082048392	6.702407854
12.57973158	155.0434783	0.082759474	6.887407028
13.36560145	271.7922035	0.082377095	6.982947071
16.32226093	153.2892781	0.082826777	7.007753245
26.18054599	426.9135661	0.063054205	7.353209203
17.89548352	184.4967832	0.065953191	7.035207124
21.16963984	396.7048291	0.066308441	7.208230944
16.06023002	293.2519789	0.091121268	6.78929348
13.42727667	77.34219858	0.089080026	6.373231655
17.99950257	193.233945	0.075241141	6.979798444
16.05456156	337.9525602	0.081050734	7.073859401
9.725491488	261.7167689	0.115036948	6.729788674
17.71077398	220.8014222	0.071870291	7.083766604
16.93581945	430.9940222	0.071235472	6.834126424
19.28753445	296.2981966	0.064190774	7.070640602
24.68668755	318.0974576	0.059266487	7.04103168
22.25470801	496.3226593	0.065705678	7.097717315
15.97924534	185.3429276	0.101124602	7.096689233
15.20206504	261.271137	0.079338753	6.742917731
7.18339636	139.9003559	0.10887856	6.523150649
19.49179332	239.83407	0.064291834	7.330917982
18.43447114	440.0193184	0.074501757	7.130782098
16.6965784	302.9335095	0.073252413	7.059363658
17.88685697	217.5310493	0.065458941	6.645956497
15.59254237	200.5525424	0.073059466	6.378251127
21.5103377	166.6636423	0.066159173	6.789827755
23.25150443	167.9555766	0.069884327	6.958884716
22.53874184	227.857263	0.062232294	7.126849205
13.43693001	219.058778	0.079023285	6.844518468
21.75548035	393.320702	0.066117384	7.067243928
32.69096786	382.041229	0.051370732	7.237846604
13.80883396	217.4352256	0.09122327	6.777157015
20.21686087	366.7065612	0.069342994	7.181253422
15.94506255	360.7713101	0.074901374	6.968486656
25.26028564	358.8925387	0.058855324	7.243772384
22.62112232	326.2715573	0.064673793	7.074065917
12.58264433	250.9605096	0.088082005	6.963859212
15.98998853	242.5378955	0.077415383	6.760511919
16.54493208	332.6920024	0.076988115	6.936579058
18.03749116	372.5726477	0.079045765	7.062440852
22.63591214	713.9552129	0.078075776	6.921938897
24.25414295	427.0289801	0.063389695	7.000425283
20.90003457	400.0945837	0.066859282	6.973296008
8.477733622	86.44359465	0.114602108	6.486537479
20.88484379	349.5624029	0.064793238	7.090093148
17.48083095	134.9542314	0.069179917	7.225953607
24.6641637	337.6666667	0.060692905	7.007794738
13.45429521	157.1232337	0.079523219	6.903100565
7.165089006	74.756691	0.108672101	5.870639805
15.35565008	143.2647059	0.076986626	6.524432694
17.12013622	304.1118032	0.070882645	6.942268345
18.40242419	209.8678733	0.067458897	6.709608295
12.72104979	214.371959	0.086022713	6.634368963
15.78397338	371.5132093	0.081514216	7.077521631
13.61253829	177.3829154	0.079107988	7.048846933
48.71458406	553.2352941	0.046454311	6.80167459
10.93709834	211.3289847	0.099158379	6.726511658
14.91466504	94.07476636	0.076109267	6.397302527
10.32371053	87.91441441	0.099350195	6.776167992
36.02957087	351.1074523	0.051174575	6.756382847

log.sigma.4.5.mm.3D_gldm_DependenceNonUniformity	log.sigma.4.5.mm.3D_gldm_GrayLevelNonUniformity	log.sigma.4.5.mm.3D_gldm_SmallDependenceEmphasis
170.3516209	67.40399002	0.230528821
272.843	148.225	0.175555425
177.2576029	78.63685152	0.219415355
329.2095393	181.0627803	0.17645613
98.91148577	84.28134879	0.174500414
196.6630037	151.1782662	0.137083089
83.14947966	117.7114475	0.118480327
226.2044321	116.1889197	0.185805743
274.8677849	134.6813024	0.220255049
463.1425241	184.2467532	0.214683522
193.2955174	174.7354732	0.118304755
250.8041543	121.9673591	0.220795815
285.142028	276.3622397	0.136109737
275.3713671	141.7265877	0.178002756
208.5943152	96.19767442	0.224509126
504.0523242	267.9286931	0.195302455
207.7464174	118.1875389	0.186581346
282.4222048	183.1751368	0.158194392
166.0158451	87.64788732	0.220759078
350.7235436	218.3636677	0.160854965
907.1766906	492.2998561	0.181708006
283.3507779	146.6209335	0.189685566
583.9512254	280.8160641	0.189733426
143.1359223	68.38252427	0.193617595
409.7296467	161.6198157	0.215436735
208.5547337	86.64792899	0.247892642
338.1334238	145.797829	0.221927931
558.9266766	224.309687	0.235272604
438.3203883	265.2447779	0.183598231
424.9455524	225.8414055	0.210522552
549.9531627	276.532593	0.183553318
669.782	339.7724	0.197607774
311.9877519	182.1023311	0.171076102
65.25363825	33.86902287	0.212940247
116.1599099	66.82657658	0.195592391
411.0447622	266.0021759	0.147197109
557.1143177	210.0557579	0.217493658
422.597169	283.0861103	0.161040532
146.4797844	148.097035	0.112517418
173.4633252	134.8630807	0.163100686
177.8693009	107.9756839	0.199909938
230.7070393	159.8913043	0.160192175
350.2362075	249.3554675	0.12879249
198.4819533	156.0456476	0.175073892
444.1333715	220.311391	0.19865303
502.1793007	235.7826573	0.175047259
484.0846446	244.4129137	0.208521261
171.5897098	138.1398417	0.153606482
62.67021277	50.24113475	0.178595708
119.7137615	82.01284404	0.19285566
429.8669679	322.9061245	0.14336987
187.8813906	225.0122699	0.098156085
450.9926258	272.8914933	0.148138495
164.8872758	83.41673783	0.201261323
573.3972568	252.7190754	0.20323372
291.4597458	111.8951271	0.21332637
447.8429399	215.4489174	0.224959247
207.4662829	245.9350329	0.133224803
190.3746356	108.8527697	0.183394783
164.1985765	152.9743772	0.108966
321.482829	189.0822849	0.179963187
665.3196647	408.7911427	0.169440446
282.0444738	166.3562307	0.191334181
320.4057816	122.2773019	0.220667334
92.25084746	43.10508475	0.271922197
226.3959819	102.0836034	0.211850391
120.7561437	73.93761815	0.179144296
476.9024117	221.9203589	0.18282188
158.9535963	102.1771075	0.151242347
348.484643	165.7562824	0.191343956
622.2988445	195.6197479	0.251223712
240.0606502	188.0111596	0.14729736
596.2472751	324.4558666	0.190446516
897.8732914	487.6828444	0.17312722
598.6133271	250.841389	0.212191065
313.9412269	150.7546118	0.202176496
247.9299363	207.433121	0.144501409
188.714496	105.2075055	0.173521743
445.957265	252.2130647	0.178330147
539.0107792	351.9907927	0.170814905
173.0499266	106.339207	0.200073997
405.8724125	168.4264208	0.230433922
365.6313662	165.4098626	0.207456715
45.24474187	59.93690249	0.132218249
556.814086	250.2314863	0.203011025
402.337939	240.3310305	0.161959144
218.4313725	86.66946779	0.230985751
378.2783437	241.9891554	0.148316575
56.17274939	44.66423358	0.201169585
83.61937716	44.4982699	0.191743773
427.4254126	227.6041732	0.18577411
173.3674208	74.54208145	0.213229033
223.2650448	134.3674776	0.16783349
513.7296118	354.8313807	0.146880893
577.358691	372.2821929	0.16575849
186.760181	41.06561086	0.36260041
191.9642655	174.8162223	0.139900101
58.39252336	32.57476636	0.201963996
88.15315315	88.22297297	0.123846329
253.6897747	59.05545927	0.328629564

log.sigma.4.5.mm.3D_gldm_DependenceNonUniformityNormalized	log.sigma.4.5.mm.3D_gldm_DependenceVariance	log.sigma.4.5.mm.3D_gldm_LargeDependenceEmphasis
0.14160567	5.541766255	20.22028263
0.1364215	5.277756	22.6
0.15854884	4.375350821	17.57602862
0.134206906	5.877829073	23.50550347
0.104227066	11.34242356	36.80821918
0.120062884	6.517140979	29.50793651
0.078665544	18.11362552	60.51750237
0.125321015	5.672481335	23.97950139
0.135603249	6.948520577	23.22200296
0.154226615	4.681801271	18.39127539
0.106970403	8.128006164	35.49474267
0.148845195	4.964867525	19.0462908
0.097351324	12.6275494	42.95834756
0.148208486	4.301698297	19.9590958
0.13475085	6.627891954	22.65891473
0.122670315	15.81083176	38.3253833
0.12943702	6.09983754	23.89719626
0.11040743	8.770448962	32.45895231
0.146140709	4.268485915	18.33098592
0.120897464	6.879285525	27.61771803
0.13052902	6.414153802	24.48460432
0.133593012	6.364408718	23.73927393
0.141701341	5.245558214	21.27469061
0.138966915	5.600279008	21.92815534
0.157346254	4.469449105	17.84639017
0.15425646	5.570636095	18.78106509
0.152932349	4.20382852	17.65490728
0.166595135	3.609731677	15.41013413
0.128955689	5.430061504	23.33598117
0.13451901	6.032128166	22.2073441
0.132774786	5.4149958	22.72718493
0.1339564	6.03386224	22.7816
0.123266595	6.342023312	26.40576847
0.13566245	10.36922385	28.97920998
0.130810709	6.764771528	24.38963964
0.127772696	5.966427576	26.29623873
0.156098156	4.046161174	17.26281872
0.12462317	6.289506779	26.51164848
0.098706054	10.69916486	42.57142857
0.106028927	11.21922544	36.67359413
0.135159043	6.456197282	23.28115502
0.119413581	8.080159159	29.71325052
0.115704066	8.299715511	33.14139412
0.105351355	9.622809805	33.74946921
0.127113157	10.46734102	30.06582713
0.140469734	4.53219197	21.0441958
0.131330614	6.889332987	23.8898535
0.113185824	7.218640569	29.91292876
0.111117399	8.202039636	30.54609929
0.109829139	10.41725781	33.1412844
0.107898335	7.906051667	32.82078313
0.096053881	10.06138001	44.59202454
0.11877604	6.790998868	29.09217804
0.140808946	5.381878167	21.17079419
0.145643195	5.009591955	20.01955804
0.154374865	4.326000745	17.86122881
0.136579122	6.260718885	21.86550778
0.08530686	15.80920444	52.15542763
0.138757023	5.00881648	21.53790087
0.116867314	6.670212383	32.6911032
0.10931072	10.41752979	33.96361782
0.121253812	6.806823053	26.95443776
0.124193956	6.797299281	25.57507706
0.171523438	3.07093549	14.80192719
0.156357369	4.751462223	16.89491525
0.146724551	4.719324913	19.16720674
0.114136242	8.111795627	29.49149338
0.133735954	4.880983829	22.07347168
0.122933949	5.970636104	26.67208043
0.139004644	5.90138979	22.46469884
0.163418814	4.177937811	15.97268908
0.116477754	6.211111794	28.43037361
0.127430493	7.029419257	25.3787134
0.137900982	5.073940746	22.1138074
0.14045362	5.201625531	20.37541061
0.134680921	5.818839247	22.2020592
0.105278105	8.276070519	33.85690021
0.138862764	4.98277366	21.78734364
0.136128591	5.275836326	22.32295482
0.121044415	7.430592415	27.80305412
0.127055746	6.425298807	24.09985316
0.152755895	4.518734288	17.70681219
0.147789558	4.403779583	18.8585287
0.086510023	15.88070004	52.50286807
0.14417765	4.847183823	19.88037286
0.11581403	8.159339765	30.417962
0.152963146	4.851881929	18.21428571
0.12431099	5.94994562	26.74630299
0.136673356	5.31010354	21.60340633
0.144670203	5.371978305	21.04152249
0.133112866	5.251218052	22.43070694
0.156893593	4.003952417	17.25882353
0.142935368	4.533825933	21.18565941
0.11801737	7.277916917	29.51045256
0.122685655	6.462279709	26.58053549
0.211267173	2.56003665	9.79638009
0.108885006	7.64912397	33.16222348
0.136431129	7.683247445	24.90186916
0.099271569	13.40746997	44.58108108
0.21983516	2.613584278	10.14038128

log.sigma.4.5.mm.3D_gldm_LargeDependenceLowGrayLevelEmphasis	log.sigma.4.5.mm.3D_gldm_SmallDependenceHighGrayLevelEmphasis	log.sigma.4.5.mm.3D_gldm_LargeDependenceHighGrayLevelEmphasis
0.172470905	58.6160064	3879.070657
0.101746575	55.62713103	6246.5925
0.102716952	47.36908086	4409.805009
0.283300953	27.09568115	2945.436608
0.532433174	27.54343016	3467.829294
0.389669894	18.82258646	3145.349206
2.166742596	11.762244	2615.641438
0.111421989	59.22742382	6705.561219
0.176404338	47.70227339	4056.442526
0.035101839	125.8532034	11197.72494
0.442157572	14.73396049	3691.256779
0.100856498	55.74424647	4803.956083
0.319318678	26.42438388	6588.061454
0.303715023	22.2295141	2571.430571
0.135512965	55.52515842	5007.812016
0.188524009	71.40297639	10851.46167
0.069676349	65.21661585	9390.835514
0.192133344	44.20216289	6932.3319
0.08518555	59.78177758	4832.640845
0.229976808	36.56190284	5566.18304
0.080806838	62.31693329	8891.08259
0.096059724	59.33333926	7258.430457
0.099470445	58.51135913	6034.691822
0.229044761	40.14589179	3517.230097
0.250938898	40.91704559	3804.110599
0.082964217	83.79005327	5291.053254
0.113951404	46.95148018	3851.911805
0.099164706	53.14656546	4217.204471
0.084336267	59.30770628	7995.928508
0.058811993	82.96103061	9314.232352
0.069181421	68.26052273	9251.93578
0.112016639	65.76398566	7347.2234
0.059688377	101.017182	14487.50217
0.365335002	36.99698371	3292.276507
0.223076011	28.53052901	3997.850225
0.135990816	35.45635493	5996.276344
0.046719999	97.49693401	8174.257495
0.113759207	42.3175686	7294.056031
0.307432446	21.76622666	6739.231132
0.60497132	23.63938033	3242.21577
0.238579184	28.09759519	3059.508359
0.306145796	26.88756007	3682.704969
0.161671666	34.91060509	7949.243145
0.396446802	31.37974336	3875.896497
0.088473212	77.89119127	12408.21294
0.218443555	31.10938163	4161.955245
0.091865224	83.0943535	8928.118285
0.128409799	39.56567688	9485.856201
1.572969967	15.65264833	1581.455674
0.284766225	43.74146135	4759.905505
0.127644179	49.45749681	9922.323042
0.210851916	25.64041892	10907.35992
0.20205402	36.42873569	5365.167501
0.058027308	79.52520493	9013.140905
0.091787594	58.90589428	5699.114046
0.082881573	71.23913618	5272.572034
0.059069684	107.0066026	10047.72736
0.442605557	29.48322822	7098.149671
0.109272252	44.9938536	5398.120991
0.280446799	13.81472529	4578.566548
0.234290064	42.68260753	6504.50799
0.071389408	71.59023625	11533.98323
0.105356801	57.25395084	7608.691325
0.094383864	41.27744551	3576.061028
0.118514598	46.83023084	3285.711864
0.25295833	41.87171822	2507.942968
0.377020528	39.99266375	3522.44707
0.157766014	42.86505132	4627.783791
0.156322154	30.18690517	5822.458623
0.075111367	76.99354393	7990.619465
0.08139841	91.76930069	6173.561712
0.173750142	33.58269969	5418.014556
0.106715095	70.76618996	8339.003633
0.071380308	58.02269667	8209.885732
0.080589473	81.55466151	6656.679493
0.104144386	64.34306648	6817.909481
0.159605069	33.68085007	8263.790234
0.126956665	40.34327264	4962.988227
0.076888209	56.10592275	7610.765873
0.086644119	59.18514711	10358.13294
0.037051138	136.9001543	16963.55874
0.052955468	92.35136329	7641.981935
0.059581927	85.6781886	7071.315279
1.17865336	11.71247928	3029.770554
0.085687061	67.44644699	7027.182289
0.489752878	22.80338787	3312.916811
0.074061193	79.62213152	5842.952381
0.26458202	23.7705135	3771.781137
0.452541582	12.34749478	1833.510949
0.297815933	26.38518006	3030.614187
0.101709555	59.30066835	5955.535036
0.116771966	42.24359118	3601.230769
0.120277636	32.16109151	4810.457746
0.093812526	54.31356308	10662.14725
0.243668721	29.25602062	4416.225244
0.0389021	172.8565482	6153.975113
0.198144261	30.47862912	6340.177538
0.915529743	19.11606171	1527.64486
0.919948141	12.01855279	2940.932432
0.073572108	105.6542704	4003.195841

log.sigma.4.5.mm.3D_gldm_SmallDependenceLowGrayLevelEmphasis	log.sigma.4.5.mm.3D_gldm_LowGrayLevelEmphasis	log.sigma.4.5.mm.3D_gldzm_DistanceZoneVariabilityNormalized
0.002462488	0.01075233	1
0.001410219	0.005256233	1
0.00286161	0.008430709	1
0.003049273	0.013556267	1
0.002796637	0.017051178	1
0.002823587	0.015599919	1
0.002976603	0.031140486	1
0.001727601	0.005754498	1
0.002496749	0.007961555	1
0.000827659	0.002453883	1
0.002331396	0.013166177	1
0.001887348	0.007041926	1
0.001621187	0.008054949	1
0.00447616	0.02001941	1
0.002646753	0.007275564	1
0.001055522	0.005550359	0.997870077
0.001857902	0.004584752	1
0.001301717	0.005746097	1
0.002050027	0.005715361	1
0.00157468	0.010598445	1
0.001018225	0.003917344	0.99724708
0.001427964	0.005014852	1
0.001182848	0.00557272	1
0.003526879	0.014168709	1
0.003158544	0.014593966	0.997118162
0.002186797	0.005605392	1
0.002736013	0.008025606	1
0.002095094	0.007908487	1
0.00190864	0.005018021	0.997063149
0.001290276	0.003517968	1
0.001068698	0.003866278	0.997674422
0.000983124	0.005543437	1
0.00095252	0.003101172	1
0.004383764	0.015238649	1
0.003998518	0.015764892	1
0.001167431	0.006059516	1
0.001177117	0.003485661	0.997755334
0.001332604	0.005603727	1
0.002012205	0.008344645	1
0.003733683	0.017001873	1
0.004260618	0.012952048	1
0.002278726	0.010090049	1
0.00152286	0.00544464	1
0.003048583	0.013235108	1
0.001301878	0.004222232	1
0.002104849	0.012376577	1
0.001000963	0.004481914	0.988876877
0.002119321	0.008099482	1
0.005379921	0.044312053	1
0.002516641	0.008892217	1
0.000860396	0.004607619	1
0.001193108	0.005747912	1
0.00126625	0.006920513	1
0.001535952	0.003673907	1
0.001406059	0.005270507	1
0.001802545	0.005675002	1
0.001445898	0.003701814	0.992907891
0.00151262	0.008544164	1
0.001762127	0.007142217	1
0.001744128	0.011035631	1
0.001629909	0.006729938	1
0.000901807	0.003283141	1
0.001516553	0.00539614	1
0.003019198	0.008423876	1
0.004531383	0.009422069	1
0.002262422	0.012872638	1
0.002821633	0.013070059	1
0.002025171	0.00800488	1
0.002125098	0.007279867	1
0.00116968	0.00401757	1
0.002052046	0.006002022	1
0.001729795	0.006785766	1
0.000901476	0.004761774	1
0.000893112	0.003819402	1
0.001309401	0.00470507	1
0.001425884	0.006268516	1
0.001642626	0.006141076	1
0.002158132	0.007474405	1
0.001240258	0.004424335	1
0.001060438	0.004025124	1
0.001215379	0.002510268	1
0.001226196	0.004272776	1
0.001277494	0.003825029	1
0.00360139	0.021166696	1
0.001495016	0.005503098	0.997780247
0.003038297	0.015784547	1
0.001945553	0.005533758	1
0.002341028	0.010837864	1
0.008588547	0.02502815	1
0.00452711	0.021226409	1
0.001142373	0.004529494	1
0.005963554	0.011532351	1
0.00192631	0.008506757	1
0.000801408	0.00372476	1
0.002203094	0.010527573	1
0.004228427	0.007319958	1
0.0016729	0.006726826	1
0.009277204	0.030776534	1
0.004033091	0.021174592	1
0.004415275	0.009870712	1

log.sigma.4.5.mm.3D_gldzm_LowIntensityEmphasis	log.sigma.4.5.mm.3D_gldzm_LargeDistanceEmphasis	log.sigma.4.5.mm.3D_gldzm_HighIntensitySmallDistanceEmphasis
0.01315124	1	260.1487342
0.008205934	1	324.8303797
0.012784987	1	215.2508039
0.018284301	1	160.0785124
0.019413109	1	167.6030151
0.023048228	1	143.1890756
0.029027725	1	102.3897059
0.009347828	1	328.1917098
0.01116534	1	220.4772277
0.003699563	1	591.0091027
0.021971473	1	124.7042254
0.00958809	1	255.3456221
0.012082962	1	200.7103448
0.028350768	1	126.9573333
0.010954052	1	257.7559809
0.006493279	1.003198294	367.3763326
0.009347412	1	352.3887324
0.008140818	1	285.0693512
0.008990629	1	271.8339223
0.013299463	1	233.1401152
0.005717102	1.004135079	339.3895589
0.007443967	1	320.6277056
0.007019113	1	312.5264293
0.020068918	1	209.6224066
0.015214666	1.004329004	189.8860029
0.008403036	1	347.535
0.012058529	1	215.8435252
0.009871673	1	228.4984227
0.010326665	1.004411765	328.9444853
0.005867908	1	395.6284224
0.005976442	1.003492433	367.0279395
0.005674952	1	339.0744681
0.005791155	1	600.6773504
0.022644469	1	180.1102362
0.022207394	1	149.6495327
0.00925921	1	239.916996
0.005420143	1.003370787	455.5598315
0.009397076	1	262.2852234
0.017901081	1	194.0110497
0.022698558	1	155.4115646
0.021658849	1	143.5748299
0.01366774	1	175.0655271
0.012297729	1	272.308094
0.018985022	1	189.6747967
0.006647716	1	389.0325407
0.014656617	1	177.8909884
0.005584489	1.016778523	402.8548658
0.016639425	1	251.6641509
0.034507632	1	90.95762712
0.012451777	1	239.2833333
0.007182711	1	352.6176471
0.012915508	1	265.7355769
0.008776037	1	260.5927835
0.007157743	1	406.0378788
0.007037426	1	292.8026608
0.008345764	1	338.4894515
0.006403593	1.010676157	481.8650652
0.011630851	1	234.7247956
0.011274464	1	253.6
0.020619763	1	122.1768293
0.009084028	1	244.7487179
0.005572739	1	421.8670635
0.008128679	1	305.6966068
0.013448258	1	185.8574423
0.015645889	1	176.6630435
0.011968611	1	210.2624672
0.015351662	1	230.9910714
0.01129062	1	241.3225806
0.013843472	1	197.0542986
0.006056	1	409.6189624
0.008039434	1	365.1089799
0.011520546	1	242.8266254
0.005164681	1	382.1743389
0.00543981	1	329.0751819
0.006320235	1	391.3731778
0.00851644	1	322.6593807
0.011942113	1	227.184492
0.01214306	1	239.5944056
0.006923387	1	311.6443452
0.006323864	1	344.4597049
0.005563927	1	699.9213836
0.00607169	1	400.9299451
0.006019902	1	417.9468268
0.030661376	1	92.79761905
0.008000284	1.003333333	334.7058333
0.020659834	1	143.7601958
0.008187318	1	345.3274559
0.016354248	1	168.7452632
0.041232992	1	61.41304348
0.026395958	1	134.9264706
0.005957524	1	329.0877458
0.024964212	1	206.7900356
0.013436066	1	190.5590062
0.005685525	1	373.9157143
0.01427549	1	180.9420804
0.010807359	1	484.0798969
0.01173882	1	223.1685393
0.040764204	1	107.3047619
0.030532859	1	102.8175182
0.013148787	1	322.6485356

log.sigma.4.5.mm.3D_gldzm_LowIntensityLargeDistanceEmphasis	log.sigma.4.5.mm.3D_gldzm_HighIntensityEmphasis	log.sigma.4.5.mm.3D_gldzm_DistanceZoneVariability	log.sigma.4.5.mm.3D_gldzm_ZonePercentage
0.01315124	260.1487342	316	0.262676642
0.008205934	324.8303797	395	0.1975
0.012784987	215.2508039	311	0.278175313
0.018284301	160.0785124	484	0.197309417
0.019413109	167.6030151	199	0.209694415
0.023048228	143.1890756	238	0.145299145
0.029027725	102.3897059	136	0.128666036
0.009347828	328.1917098	386	0.213850416
0.01116534	220.4772277	505	0.249136655
0.003699563	591.0091027	769	0.256077256
0.021971473	124.7042254	213	0.117874931
0.00958809	255.3456221	434	0.257566766
0.012082962	200.7103448	435	0.148514851
0.028350768	126.9573333	375	0.201829925
0.010954052	257.7559809	418	0.27002584
0.006499887	367.7633262	936.0021322	0.228279387
0.009347412	352.3887324	355	0.221183801
0.008140818	285.0693512	447	0.174745895
0.008990629	271.8339223	283	0.249119718
0.013299463	233.1401152	521	0.179593244
0.005724682	339.9627843	1447.005513	0.208776978
0.007443967	320.6277056	462	0.217821782
0.007019113	312.5264293	927	0.224945402
0.020068918	209.6224066	241	0.233980583
0.015236752	190.0981241	691.002886	0.266129032
0.008403036	347.535	400	0.295857988
0.012058529	215.8435252	556	0.251469923
0.009871673	228.4984227	951	0.283457526
0.010381131	329.0338235	678.0029412	0.200058841
0.005867908	395.6284224	767	0.242798354
0.005981608	367.6181607	857.0023283	0.207387735
0.005674952	339.0744681	1128	0.2256
0.005791155	600.6773504	468	0.184907151
0.022644469	180.1102362	127	0.264033264
0.022207394	149.6495327	214	0.240990991
0.00925921	239.916996	506	0.1572894
0.005424767	456.1741573	888.0022472	0.249369571
0.009397076	262.2852234	582	0.171630787
0.017901081	194.0110497	181	0.121967655
0.022698558	155.4115646	294	0.179706601
0.021658849	143.5748299	294	0.223404255
0.01366774	175.0655271	351	0.181677019
0.012297729	272.308094	383	0.126527915
0.018985022	189.6747967	369	0.195859873
0.006647716	389.0325407	799	0.228677733
0.014656617	177.8909884	688	0.192447552
0.006015842	403.9295302	884.0559284	0.242539338
0.016639425	251.6641509	265	0.174802111
0.034507632	90.95762712	118	0.209219858
0.012451777	239.2833333	240	0.220183486
0.007182711	352.6176471	612	0.153614458
0.012915508	265.7355769	208	0.106339468
0.008776037	260.5927835	582	0.153278904
0.007157743	406.0378788	264	0.225448335
0.007037426	292.8026608	902	0.229108458
0.008345764	338.4894515	474	0.251059322
0.006423555	483.3855279	837.0213523	0.257090576
0.011630851	234.7247956	367	0.150904605
0.011274464	253.6	305	0.222303207
0.020619763	122.1768293	164	0.116725979
0.009084028	244.7487179	585	0.198911935
0.005572739	421.8670635	1008	0.183706944
0.008128679	305.6966068	501	0.220607662
0.013448258	185.8574423	477	0.255353319
0.015645889	176.6630435	184	0.311864407
0.011968611	210.2624672	381	0.246921581
0.015351662	230.9910714	224	0.211720227
0.01129062	241.3225806	713	0.199943915
0.013843472	197.0542986	221	0.17092034
0.006056	409.6189624	559	0.222975668
0.008039434	365.1089799	1147	0.301207983
0.011520546	242.8266254	323	0.156720039
0.005164681	382.1743389	1021	0.218209019
0.00543981	329.0751819	1237	0.189986177
0.006320235	391.3731778	1029	0.241435946
0.00851644	322.6593807	549	0.235521236
0.011942113	227.184492	374	0.15881104
0.01214306	239.5944056	286	0.210448859
0.006923387	311.6443452	672	0.205128205
0.006323864	344.4597049	881	0.19784415
0.005563927	699.9213836	318	0.233480176
0.00607169	400.9299451	728	0.273993225
0.006019902	417.9468268	583	0.235650768
0.030661376	92.79761905	84	0.160611855
0.008041436	334.7733333	898.0022222	0.233039876
0.020659834	143.7601958	613	0.176453656
0.008187318	345.3274559	397	0.278011204
0.016354248	168.7452632	475	0.156095958
0.041232992	61.41304348	92	0.223844282
0.026395958	134.9264706	136	0.235294118
0.005957524	329.0877458	661	0.205854874
0.024964212	206.7900356	281	0.254298643
0.013436066	190.5590062	322	0.206145967
0.005685525	373.9157143	700	0.160808638
0.01427549	180.9420804	846	0.179770506
0.010807359	484.0798969	388	0.438914027
0.01173882	223.1685393	267	0.151446398
0.040764204	107.3047619	105	0.245327103
0.030532859	102.8175182	137	0.154279279
0.013148787	322.6485356	478	0.414211438

log.sigma.4.5.mm.3D_gldzm_IntensityVariabilityNormalized	log.sigma.4.5.mm.3D_gldzm_LowIntensitySmallDistanceEmphasis	log.sigma.4.5.mm.3D_gldzm_IntensityVariability
0.054057843	0.01315124	17.08227848
0.060612081	0.008205934	23.94177215
0.058942732	0.012784987	18.33118971
0.060105184	0.018284301	29.09090909
0.066993258	0.019413109	13.33165829
0.063801991	0.023048228	15.18487395
0.098724048	0.029027725	13.42647059
0.052873903	0.009347828	20.40932642
0.058374669	0.01116534	29.47920792
0.052348735	0.003699563	40.25617685
0.074918998	0.021971473	15.95774648
0.057911614	0.00958809	25.13364055
0.059912802	0.012082962	26.06206897
0.066339556	0.028350768	24.87733333
0.050777226	0.010954052	21.22488038
0.049752001	0.006491627	46.6673774
0.050648681	0.009347412	17.98028169
0.060883143	0.008140818	27.2147651
0.069160559	0.008990629	19.57243816
0.057968398	0.013299463	30.20153551
0.056383083	0.005715207	81.81185389
0.055555556	0.007443967	25.66666667
0.053042548	0.007019113	49.17044229
0.060725538	0.020068918	14.63485477
0.057384815	0.015209144	39.76767677
0.0480875	0.008403036	19.235
0.057372807	0.012058529	31.89928058
0.059543278	0.009871673	56.6256572
0.050860727	0.010313048	34.58529412
0.048777047	0.005867908	37.41199478
0.052042267	0.00597515	44.70430733
0.056352485	0.005674952	63.56560284
0.049127036	0.005791155	22.99145299
0.061194122	0.022644469	7.771653543
0.06157743	0.022207394	13.17757009
0.060233717	0.00925921	30.47826087
0.044842823	0.005418987	39.91011236
0.060544868	0.009397076	35.2371134
0.061506059	0.017901081	11.13259669
0.063654033	0.022698558	18.71428571
0.068142903	0.021658849	20.03401361
0.068936129	0.01366774	24.1965812
0.064142506	0.012297729	24.56657963
0.05563267	0.018985022	20.52845528
0.045164403	0.006647716	36.08635795
0.061963392	0.014656617	42.63081395
0.052287434	0.005476651	46.74496644
0.054353863	0.016639425	14.40377358
0.090922149	0.034507632	10.72881356
0.0575	0.012451777	13.8
0.053184673	0.007182711	32.54901961
0.059356509	0.012915508	12.34615385
0.060025271	0.008776037	34.9347079
0.062213039	0.007157743	16.42424242
0.054881736	0.007037426	49.50332594
0.047819972	0.008345764	22.66666667
0.049795328	0.006398603	41.97746145
0.060264758	0.011630851	22.11716621
0.061306101	0.011274464	18.69836066
0.080160619	0.020619763	13.14634146
0.060594638	0.009084028	35.44786325
0.050906242	0.005572739	51.31349206
0.059326457	0.008128679	29.72255489
0.061798716	0.013448258	29.47798742
0.066280718	0.015645889	12.19565217
0.060326121	0.011968611	22.98425197
0.056760204	0.015351662	12.71428571
0.053510344	0.01129062	38.15287518
0.071517782	0.013843472	15.80542986
0.050572675	0.006056	28.27012522
0.046180134	0.008039434	52.96861378
0.06041465	0.011520546	19.51393189
0.056307263	0.005164681	57.48971596
0.061137691	0.00543981	75.62732417
0.048395368	0.006320235	49.79883382
0.050998504	0.00851644	27.99817851
0.059981698	0.011942113	22.43315508
0.063523889	0.01214306	18.16783217
0.056888641	0.006923387	38.22916667
0.050256326	0.006323864	44.27582293
0.044638266	0.005563927	14.19496855
0.047888238	0.00607169	34.86263736
0.051755132	0.006019902	30.17324185
0.094671202	0.030661376	7.952380952
0.05522716	0.007989996	49.70444444
0.061950294	0.020659834	37.97553018
0.049051767	0.008187318	19.47355164
0.065945706	0.016354248	31.32421053
0.113185255	0.041232992	10.41304348
0.067041522	0.026395958	9.117647059
0.059930743	0.005957524	39.61422088
0.05563506	0.024964212	15.63345196
0.065911809	0.013436066	21.22360248
0.052130612	0.005685525	36.49142857
0.063589468	0.01427549	53.79669031
0.038354235	0.010807359	14.8814433
0.07012302	0.01173882	18.72284644
0.079002268	0.040764204	8.295238095
0.071767276	0.030532859	9.832116788
0.044677089	0.013148787	21.35564854

log.sigma.4.5.mm.3D_gldzm_HighIntensityLargeDistanceEmphasis	log.sigma.4.5.mm.3D_gldzm_SmallDistanceEmphasis	log.sigma.4.5.mm.3D_glcm_SumVariance	log.sigma.4.5.mm.3D_glcm_Homogeneity1
260.1487342	1	672.8770317	0.425482167
324.8303797	1	943.1083614	0.450680909
215.2508039	1	663.8812722	0.420498879
160.0785124	1	373.5800154	0.464293929
167.6030151	1	342.8079292	0.506025594
143.1890756	1	318.2517083	0.493386114
102.3897059	1	167.6789523	0.575684471
328.1917098	1	933.7974348	0.460184317
220.4772277	1	626.3679806	0.449311462
591.0091027	1	1930.755955	0.421705322
124.7042254	1	313.2424399	0.522270544
255.3456221	1	759.5958462	0.425163085
200.7103448	1	480.400143	0.528356197
126.9573333	1	328.4752403	0.446343459
257.7559809	1	753.2869844	0.427277675
369.3113006	0.999200426	1080.674645	0.459711633
352.3887324	1	1169.732803	0.445260225
285.0693512	1	768.9088671	0.487937142
271.8339223	1	810.4156239	0.437848036
233.1401152	1	641.5673438	0.475404025
342.2556857	0.99896623	1106.693233	0.445161377
320.6277056	1	928.5428466	0.451628554
312.5264293	1	893.127062	0.439578481
209.6224066	1	496.6655795	0.445938374
190.9466089	0.998917749	553.3561052	0.417761702
347.535	1	990.6052834	0.409270289
215.8435252	1	676.5057255	0.46559983
228.4984227	1	749.7128536	0.406010906
329.3911765	0.998897059	1073.751865	0.446076038
395.6284224	1	1323.279134	0.444631112
369.9790454	0.999126892	1211.09569	0.450744349
339.0744681	1	1034.915886	0.446545101
600.6773504	1	1857.831012	0.468792255
180.1102362	1	462.2701627	0.456103491
149.6495327	1	417.5715668	0.454847674
239.916996	1	703.1420944	0.475337263
458.6314607	0.999157303	1473.466704	0.408386777
262.2852234	1	814.4442339	0.472055744
194.0110497	1	507.1384056	0.540582029
155.4115646	1	314.1488964	0.49837521
143.5748299	1	395.579663	0.454343508
175.0655271	1	415.7183239	0.480654556
272.308094	1	819.2139341	0.497795433
189.6747967	1	412.259373	0.491278533
389.0325407	1	1311.505508	0.432186095
177.8909884	1	502.2136659	0.453019334
408.2281879	0.995805369	1189.08882	0.440406158
251.6641509	1	890.3192231	0.478248746
90.95762712	1	179.8961449	0.491368918
239.2833333	1	534.9781819	0.487665456
352.6176471	1	1042.527579	0.502191628
265.7355769	1	784.9199754	0.542196419
260.5927835	1	621.1387647	0.49127428
406.0378788	1	1377.0921	0.435779737
292.8026608	1	886.6477517	0.4282229
338.4894515	1	942.8703649	0.413187632
489.4673784	0.997330961	1552.559526	0.430142213
234.7247956	1	517.8007914	0.537729336
253.6	1	761.286478	0.446825552
122.1768293	1	379.0684349	0.519928065
244.7487179	1	697.5064595	0.488566198
421.8670635	1	1349.75869	0.464386125
305.6966068	1	900.4487313	0.455881229
185.8574423	1	623.6857917	0.402250859
176.6630435	1	590.6332861	0.410827921
210.2624672	1	437.1683767	0.43067941
230.9910714	1	449.5097847	0.480658148
241.3225806	1	648.1354106	0.446397521
197.0542986	1	627.2292165	0.476563997
409.6189624	1	1204.078997	0.441126685
365.1089799	1	1165.114765	0.388866716
242.8266254	1	629.3463024	0.488517057
382.1743389	1	1116.734537	0.453878489
329.0751819	1	1122.195772	0.446485964
391.3731778	1	1056.24763	0.431831667
322.6593807	1	968.7074505	0.432494722
227.184492	1	748.8195201	0.501222333
239.5944056	1	706.2805208	0.449520235
311.6443452	1	1015.204524	0.436115365
344.4597049	1	1143.41889	0.460137111
699.9213836	1	2378.506111	0.43706279
400.9299451	1	1286.04981	0.405982794
417.9468268	1	1207.471487	0.425798465
92.79761905	1	213.9113373	0.557987244
335.0433333	0.999166667	1044.853311	0.43625972
143.7601958	1	349.1524245	0.487374478
345.3274559	1	1011.388331	0.422643375
168.7452632	1	418.4669292	0.478500379
61.41304348	1	185.7859477	0.460672724
134.9264706	1	368.7874989	0.453351529
329.0877458	1	918.4142521	0.450702437
206.7900356	1	593.5864711	0.426402827
190.5590062	1	608.144506	0.454924268
373.9157143	1	1135.401841	0.480280668
180.9420804	1	481.9881332	0.477358425
484.0798969	1	1687.94219	0.3106493
223.1685393	1	600.1000427	0.51077995
107.3047619	1	227.8198065	0.466274346
102.8175182	1	214.2727196	0.539918425
322.6485356	1	1035.079584	0.343266707

log.sigma.4.5.mm.3D_glcm_Homogeneity2	log.sigma.4.5.mm.3D_glcm_ClusterShade	log.sigma.4.5.mm.3D_glcm_MaximumProbability	log.sigma.4.5.mm.3D_glcm_Idmn	log.sigma.4.5.mm.3D_glcm_SumVariance2
0.35087838	86.33946848	0.026804241	0.984761927	95.19140018
0.37902807	67.90981496	0.03084376	0.990938856	60.4263782
0.343024353	-126.3052351	0.025756116	0.987765429	64.56830277
0.394466749	212.0249015	0.029797875	0.989234162	57.5561921
0.448107356	246.1952256	0.062152825	0.985037185	59.26720126
0.430708126	271.046608	0.047711531	0.988581026	50.244941
0.530539136	203.6690885	0.098856841	0.987069568	45.64312659
0.390988468	125.9674191	0.027020249	0.991976688	77.84043351
0.377901821	225.1152716	0.033219357	0.988093238	71.25802403
0.342708289	-171.0251637	0.021039829	0.993155822	79.82865879
0.467649983	107.3543113	0.048149108	0.988280563	36.18225857
0.346138629	-57.04154961	0.022425376	0.988735993	55.18935915
0.473115036	138.1790403	0.059532921	0.992210023	38.78860821
0.371594151	-11.77549092	0.028810218	0.983837782	47.69087515
0.354033595	517.7674831	0.025390414	0.985327613	84.08200524
0.388677686	-101.4812406	0.064226612	0.990689574	80.41048436
0.372240778	-168.1986413	0.032207757	0.991434279	60.31288991
0.425598113	178.092559	0.033371166	0.992337867	63.95884467
0.363286993	-61.85014747	0.022502771	0.986806561	44.13510161
0.409359133	-56.72622339	0.036269593	0.990179552	63.02375101
0.372452144	-148.2703349	0.022487563	0.992956855	60.6759015
0.379176629	66.4624917	0.025097601	0.993598667	66.46934636
0.363877977	173.4384306	0.026209741	0.992116092	68.83831045
0.372938778	22.49588997	0.038080712	0.984676573	74.77836344
0.338510624	-293.4420149	0.02145637	0.98442412	77.67317279
0.329292795	163.001763	0.022678676	0.987874669	79.21929766
0.396059726	140.0166375	0.021941158	0.990349904	66.43345849
0.323253304	-221.5217218	0.018530921	0.986639419	65.67301959
0.373259976	-229.8501842	0.028014629	0.99079392	54.44658858
0.372983299	-42.57584692	0.028395211	0.993319609	56.87817232
0.379357176	-223.8719223	0.02488922	0.991347306	70.76319044
0.373711497	-70.66324629	0.022648191	0.991645974	63.95220491
0.401426679	-348.3611603	0.032604414	0.994097828	79.76134209
0.386833858	111.6781782	0.051398876	0.979870366	60.9630272
0.385003123	-3.761829415	0.041694987	0.982034328	54.56100833
0.408814473	77.54485794	0.035047468	0.990111086	44.45318973
0.327825733	-0.057285328	0.0177541	0.991875898	93.17797274
0.4038425	-49.82628265	0.03367229	0.990968718	46.62967161
0.489153015	116.9458475	0.060273026	0.992622417	35.9339809
0.436486549	254.1582712	0.047250596	0.987985311	56.96499274
0.382142348	45.6655552	0.035746634	0.983237245	39.65711369
0.414730261	114.8436355	0.040996504	0.987505455	41.89577179
0.436500334	128.7459988	0.039262876	0.991879291	47.77933485
0.429655614	253.3603885	0.049971418	0.986082805	55.30903348
0.361180416	-340.614706	0.037011826	0.987684513	84.81762355
0.379843432	-48.24786541	0.018606973	0.989884818	66.49408409
0.366621212	-117.366186	0.025760311	0.992108267	71.52410911
0.414866404	-387.1535425	0.04102436	0.989176661	54.83545937
0.429493735	107.3831581	0.060126444	0.976234259	48.8078761
0.425513398	318.957324	0.050713593	0.989661475	63.23062935
0.443203217	145.5909164	0.042590947	0.993640192	54.28382637
0.492981772	25.62937638	0.061601743	0.992486367	33.27211973
0.429295755	261.9454129	0.036559576	0.992544555	63.2020243
0.360174911	66.09574571	0.022785745	0.989041646	53.21807142
0.350175135	-40.88152113	0.019563224	0.988807364	65.41484679
0.334227376	-3.343396371	0.019537912	0.988954182	84.18920848
0.355241889	-189.5587723	0.025045701	0.99102393	76.34608149
0.486533595	416.8140195	0.079541119	0.991661051	58.45240102
0.373168824	18.61488149	0.029717295	0.988005208	52.2058591
0.462934961	-21.66108332	0.051028266	0.987747586	24.30641075
0.425618877	132.6351178	0.034747611	0.989803005	72.22482166
0.396385195	-94.20027985	0.029857061	0.994177464	62.19890758
0.385109656	-94.87405395	0.028474373	0.990489213	57.71713516
0.317588618	-130.2191078	0.018154221	0.980993818	59.52808468
0.330722976	-39.62427982	0.029983897	0.978794392	46.87312915
0.355487604	404.1096094	0.026122073	0.986180405	77.08899059
0.417118953	577.0224338	0.042242369	0.989235605	87.29291064
0.374343919	248.1419371	0.020898051	0.987921346	81.75981371
0.410040251	-7.470747264	0.029677702	0.987500134	47.08510642
0.366957272	26.55715805	0.028348993	0.992239949	73.5502438
0.306209196	-700.5677812	0.0138436	0.987370651	111.9732342
0.426550551	237.2700279	0.04325393	0.989554442	47.41162287
0.382558906	-129.5483389	0.027569724	0.992432806	71.33269489
0.373137939	-192.2333929	0.026539275	0.992195403	51.43415294
0.356414954	170.6973487	0.021574679	0.9936482	88.82912133
0.358500703	-80.55428733	0.027581259	0.99157379	74.32935188
0.44196452	-61.10535322	0.036831475	0.99189498	43.42796443
0.377736209	-41.91706229	0.030132739	0.986182241	55.98258902
0.36149114	-149.4165637	0.025676393	0.990004664	55.47043733
0.390525432	-254.5296044	0.033807466	0.991917431	61.36483902
0.367371469	-387.5140124	0.034705272	0.991650791	76.65478099
0.32584122	-199.5442803	0.021254529	0.990236772	83.11840364
0.348043609	208.9973777	0.026046905	0.992350872	67.77890919
0.509825474	65.41000389	0.095348931	0.981329255	28.09728183
0.360100337	-263.3363234	0.019683127	0.991068377	71.55983987
0.423609339	235.2423382	0.026970208	0.989493246	63.28489596
0.346542417	-47.78239599	0.024822229	0.989245721	83.32988721
0.411805837	83.00161549	0.032002873	0.988440753	46.9325962
0.389728788	-19.01158756	0.0587017	0.965021311	20.59626754
0.380742883	-7.716810989	0.039972464	0.982647078	53.31553166
0.379719474	181.1208278	0.025590448	0.991080963	58.07319636
0.347877377	59.66467729	0.026994593	0.985238778	61.88805675
0.382352918	-117.8882429	0.030931607	0.987640853	44.43772397
0.414277164	-21.70030287	0.034783831	0.993372075	52.8841313
0.410921964	-0.81378093	0.027999456	0.989804639	46.93486469
0.22062498	-1049.90191	0.016547308	0.980076604	165.1343291
0.452299637	92.29767873	0.048186758	0.99131657	37.87942461
0.396322979	65.63325983	0.050800757	0.980521995	52.45617401
0.486505283	116.4483228	0.076208977	0.986200396	35.45182049
0.25163481	-222.3330465	0.0173529	0.984061072	124.3464542

log.sigma.4.5.mm.3D_glcm_Contrast	log.sigma.4.5.mm.3D_glcm_DifferenceEntropy	log.sigma.4.5.mm.3D_glcm_InverseVariance	log.sigma.4.5.mm.3D_glcm_Entropy	log.sigma.4.5.mm.3D_glcm_Dissimilarity
12.88711981	2.915248979	0.346351399	7.632729986	2.572468077
8.475163995	2.678104913	0.374418969	7.208055094	2.157499853
10.7540954	2.837067857	0.343814016	7.385563575	2.474617412
7.05247855	2.562616892	0.387637179	7.162083166	1.982398174
7.170942744	2.506426003	0.404268035	6.582973336	1.847980248
5.830136267	2.433856785	0.411789382	6.723318928	1.768873124
3.96854717	2.154460098	0.417910539	5.824351455	1.36990439
8.578719781	2.662367575	0.382957556	7.35805425	2.108550884
9.922813074	2.765269551	0.365555965	7.405658006	2.261879622
10.14889901	2.808692138	0.340955239	7.698504768	2.414178486
4.939496083	2.335603401	0.432592135	6.497293484	1.600967639
9.90555232	2.773879461	0.341592217	7.367447565	2.373752144
5.058089364	2.369990347	0.414283622	6.595691319	1.600447964
7.55256136	2.611674136	0.370647347	7.129338047	2.099662013
15.25905013	2.969117111	0.33510555	7.647589717	2.718324765
10.58111617	2.8291622	0.331658803	7.595688155	2.32073961
9.129798732	2.70365303	0.36534683	7.214697823	2.227224203
7.658696987	2.585780423	0.395557975	7.113431493	1.950543478
9.476781429	2.707870138	0.36467306	7.051541629	2.27306197
7.488192514	2.60017971	0.390731144	7.193079188	1.982119358
9.410016695	2.75293592	0.363417194	7.486431035	2.255117737
8.524125905	2.674836081	0.366631673	7.360459799	2.159967917
8.860080918	2.720930526	0.356196506	7.506133708	2.235967068
8.56955071	2.645121134	0.363480513	7.210987943	2.205327588
10.26044157	2.811036955	0.339718624	7.651340952	2.450891099
13.05907436	2.955702466	0.322948299	7.643365971	2.675659305
7.357279232	2.577703091	0.38274517	7.218854075	2.005712382
10.96053772	2.858043462	0.323229158	7.642534023	2.553430956
9.232740056	2.702005844	0.367602768	7.219255456	2.22461538
10.51006521	2.792404726	0.365408434	7.383119186	2.321515941
8.673718101	2.68260645	0.38011894	7.464410581	2.152971595
9.463433507	2.742293513	0.362938058	7.471432504	2.244027702
8.362368479	2.630644485	0.387088511	7.261473623	2.05462147
11.91938761	2.778648109	0.34650721	6.917027243	2.391958499
9.370515154	2.714379824	0.365670699	7.09910082	2.228461632
6.989521692	2.555621918	0.39488162	6.993738017	1.937534573
12.15255638	2.91140477	0.331148157	7.868740586	2.604282355
6.83952302	2.556389298	0.388659471	7.016417733	1.941555131
4.397691826	2.24979616	0.432622036	6.341271139	1.49590952
6.746950911	2.514094847	0.39178163	6.827525332	1.847166543
7.916044299	2.635649934	0.371796745	6.925179877	2.100128302
6.996909089	2.556434424	0.387519672	6.870925321	1.929610889
6.175692867	2.473580635	0.409288366	6.839498507	1.785024969
8.834719871	2.614275085	0.389603645	6.9143411	2.002430591
16.15261323	3.013997482	0.337788789	7.803211553	2.752987758
7.093956299	2.581359379	0.378550106	7.39377825	2.034021458
10.01760746	2.788986052	0.356818251	7.572696377	2.317249844
8.988948482	2.678361357	0.38878777	6.942232867	2.085453241
6.745256682	2.410631582	0.401426432	6.274561835	1.874293743
7.997907946	2.59626213	0.395548276	6.91464024	1.969725049
6.34470167	2.467467806	0.419157722	6.91208653	1.774243033
5.351848123	2.32780827	0.437775478	6.238637783	1.554833393
6.530613925	2.498118392	0.412490165	7.067420876	1.828786302
11.43525741	2.750686154	0.353280077	7.19196836	2.372544026
9.8618424	2.773201225	0.343875635	7.55761321	2.359080842
11.83603912	2.889072908	0.338214852	7.767724487	2.56544269
12.22704375	2.878952021	0.347506317	7.645608924	2.494454562
6.918585812	2.479920359	0.404089707	6.599074085	1.721229022
8.482990185	2.690945532	0.361220099	7.135988245	2.188269689
4.170901534	2.241942311	0.436052367	6.233373867	1.526284155
7.880378786	2.620247788	0.388529376	7.293968734	1.962327032
8.676243197	2.667642482	0.383730798	7.297808758	2.102009172
8.941035793	2.697200988	0.368881282	7.283481016	2.171758701
10.68047857	2.824755062	0.316197971	7.482940651	2.55341374
12.19131321	2.846441006	0.326706588	7.100108257	2.611948298
10.73007342	2.808542337	0.354999615	7.389750382	2.401972069
8.321466265	2.630838318	0.390946262	7.089036529	2.025587485
10.10359417	2.764976648	0.367618643	7.559584502	2.273095471
7.024837033	2.519094122	0.39393938	6.899426842	1.931664241
9.267928367	2.742488912	0.359830839	7.487617271	2.261633655
15.30107589	3.067674042	0.305970451	8.177841724	2.935829039
7.625306371	2.519390163	0.41082134	6.736306644	1.900051773
9.066765164	2.721087929	0.366166659	7.465464241	2.189502216
8.260290853	2.665822116	0.373597139	7.270416983	2.149168074
10.47735476	2.807741369	0.355790135	7.767619984	2.373828032
11.34976228	2.856434258	0.35226343	7.563797175	2.447816476
6.636485965	2.498383838	0.411659309	6.806184496	1.812936073
9.174137892	2.692845844	0.368273922	7.13105171	2.214613599
10.02951543	2.775112221	0.35712783	7.3609527	2.334377894
8.575154416	2.692491475	0.372808083	7.281734748	2.133732887
15.52828133	2.973557195	0.35477244	7.393611134	2.65676552
13.17823964	2.963439166	0.325312322	7.813066415	2.695946535
9.690027671	2.761440496	0.355731336	7.460356316	2.340116021
5.371358055	2.243043572	0.409029102	5.708346334	1.538369094
8.894534328	2.711971301	0.358862215	7.550313318	2.246448862
6.945553449	2.550311279	0.398124802	7.178910008	1.885215411
11.58440808	2.854753593	0.353071018	7.645900938	2.489543366
6.430773492	2.503960798	0.396558547	6.950425025	1.878771092
6.719852059	2.236125101	0.38894501	5.690114811	1.969801153
7.41880978	2.561068818	0.378633811	6.841344027	2.057226222
8.94562415	2.70619207	0.378259315	7.282029887	2.179277988
9.789966503	2.767955072	0.346416815	7.333707645	2.354316522
7.450022106	2.608022542	0.372905696	7.002452078	2.064539423
6.558433515	2.521234478	0.396231912	7.056316866	1.885590386
6.676069013	2.534506157	0.397317754	7.08609441	1.901615317
32.33299912	3.545662434	0.221961265	8.385756862	4.379453989
5.214144747	2.355596392	0.422894457	6.441862387	1.655350884
6.794929185	2.430757838	0.382550758	6.600996396	1.967704957
4.786989288	2.288193333	0.411650086	6.253214305	1.541305732
19.4363319	3.197284587	0.260539012	8.166972634	3.432822263

log.sigma.4.5.mm.3D_glcm_DifferenceVariance	log.sigma.4.5.mm.3D_glcm_Idn	log.sigma.4.5.mm.3D_glcm_Idm	log.sigma.4.5.mm.3D_glcm_Correlation	log.sigma.4.5.mm.3D_glcm_Autocorrelation
6.047872212	0.921341248	0.35087838	0.761223371	234.293249
3.664545038	0.936108789	0.37902807	0.753820791	312.4437867
4.439695954	0.925404573	0.343024353	0.715209053	229.8818076
3.002650534	0.930195573	0.394466749	0.781809013	141.0443922
3.522048616	0.924958034	0.448107356	0.783557366	129.0035002
2.601473762	0.929624947	0.430708126	0.788417122	121.7458578
1.904190633	0.930657168	0.530539136	0.834539053	69.23324079
4.003028055	0.941218846	0.390988468	0.800332799	312.439378
4.681529041	0.929628981	0.377901821	0.755384979	219.2491753
4.161107533	0.942610795	0.342708289	0.773874299	597.9243027
2.2940049	0.930211308	0.467649983	0.758611477	119.6707719
4.060578697	0.928059837	0.346138629	0.696574627	258.3174663
2.422329133	0.942897427	0.473115036	0.767267167	172.3359751
3.025070701	0.914082862	0.371594151	0.725847445	126.1336428
7.332927008	0.925015456	0.354033595	0.694924761	257.0796626
4.960204115	0.937881601	0.388677686	0.768787315	355.3021291
3.969030292	0.937998792	0.372240778	0.734792096	377.6103873
3.697444442	0.943865304	0.425598113	0.786609536	262.0663585
4.003332765	0.924104035	0.363286993	0.647761803	271.023212
3.442969593	0.935235506	0.409359133	0.787131999	224.0890404
4.156570769	0.943646135	0.372452144	0.73088204	360.8653808
3.680370863	0.945741205	0.379176629	0.772886188	310.0340505
3.711944175	0.939272756	0.363877977	0.772161485	299.9858305
3.340059476	0.917461577	0.372938778	0.793781054	179.5380418
4.03455397	0.915532303	0.338510624	0.765984037	197.8903679
5.645424047	0.927003286	0.329292795	0.718354655	327.9498368
3.155752061	0.934247662	0.396059726	0.797559418	235.1434022
4.243972021	0.920523587	0.323253304	0.71437003	256.5734167
4.039799476	0.936369902	0.373259976	0.71110202	349.1562624
4.919982017	0.946425782	0.372983299	0.688753173	421.505526
3.909043316	0.938208095	0.379357176	0.780751205	392.4487434
4.247746918	0.939402383	0.373711497	0.741451051	340.2969926
3.957506085	0.94974562	0.401426679	0.810716888	574.6742354
5.607147406	0.913349932	0.386833858	0.671533764	166.2250108
4.128013532	0.914215551	0.385003123	0.70944361	153.8181167
3.135012155	0.93419963	0.408814473	0.725007433	240.223491
5.142061563	0.938693089	0.327825733	0.769666164	469.8131644
2.976666466	0.936094317	0.4038425	0.743298672	273.5756114
2.025707409	0.944233747	0.489153015	0.77916262	179.5273743
3.134166228	0.930186929	0.436486549	0.786702935	120.8032969
3.362537288	0.914544785	0.382142348	0.665671931	145.8220756
3.123821745	0.92703782	0.414730261	0.711142657	152.4848107
2.907945912	0.941086255	0.436500334	0.7708222	274.3472667
4.562740975	0.928650644	0.429655614	0.724228684	151.5117542
8.091577805	0.932040982	0.361180416	0.677627933	420.4334034
2.861880229	0.930724898	0.379843432	0.806849365	182.8571997
4.466442341	0.940804143	0.366621212	0.755351781	386.0003522
4.490904238	0.934974646	0.414866404	0.717614447	294.3711523
2.768371598	0.903244193	0.429493735	0.758562492	74.73582601
3.983929873	0.936103366	0.425513398	0.774445343	190.6643092
3.102530476	0.948435247	0.443203217	0.790048544	340.1710321
2.857082303	0.946824741	0.492981772	0.722847946	259.5919858
3.103077264	0.943559979	0.429295755	0.811839628	217.4872873
5.320739834	0.933087627	0.360174911	0.654600407	435.2328211
4.113818267	0.928506682	0.350175135	0.738198527	297.5933299
5.012148121	0.929543352	0.334227376	0.754355387	315.7599013
5.745071907	0.938623248	0.355241889	0.724317277	489.4981651
3.837778786	0.945837995	0.486533595	0.788632299	183.3304188
3.534230344	0.926369282	0.373168824	0.719859004	258.0060176
1.774200679	0.925970268	0.462934961	0.707983565	138.9049478
3.938718422	0.936264276	0.425618877	0.802901394	241.6262759
4.107426716	0.949895155	0.396385195	0.754479978	430.0556479
4.030163193	0.935992423	0.385109656	0.732818927	300.2172364
3.938510766	0.905464351	0.317588618	0.696369298	217.8529138
4.727261239	0.904699159	0.330722976	0.590161256	204.712849
4.728258927	0.923084182	0.355487604	0.755577172	160.2596755
4.075202465	0.934405473	0.417118953	0.824206354	164.5386638
4.801188751	0.929366908	0.374343919	0.780278033	226.7746188
3.069348308	0.926951224	0.410040251	0.735418312	217.7644281
3.982754317	0.940394985	0.366957272	0.774446478	390.487835
6.375169714	0.92470976	0.306209196	0.759570512	381.5408752
3.841534964	0.936076602	0.426550551	0.719779174	216.4551824
4.114067949	0.94233149	0.382558906	0.773775604	364.8073444
3.508580364	0.939794326	0.373137939	0.722180078	363.3642482
4.693621871	0.94634126	0.356414954	0.788237349	349.815642
5.151802216	0.939466613	0.358500703	0.732205904	321.7848054
3.235509093	0.942415918	0.44196452	0.734009633	253.1144425
3.985988299	0.923447211	0.377736209	0.718123539	241.7496532
4.329010156	0.933507948	0.36149114	0.696113346	332.5980246
3.881925005	0.940493487	0.390525432	0.754504341	370.2859933
8.200556396	0.944072236	0.367371469	0.664529309	716.4081115
5.635122584	0.933733086	0.32584122	0.727618903	414.6499439
4.074121724	0.939965346	0.348043609	0.748506193	389.9286332
2.586872219	0.919957394	0.509825474	0.680846338	84.75842621
3.664662934	0.935461542	0.360100337	0.778252147	344.7292408
3.314724775	0.933819467	0.423609339	0.802254807	133.3735386
5.167001934	0.931648064	0.346542417	0.754317835	335.485829
2.780079975	0.928469574	0.411805837	0.75677303	154.4395445
1.938115021	0.878369994	0.389728788	0.516439684	74.32785024
2.975284817	0.912193565	0.380742883	0.752338682	138.4234404
4.085181164	0.93758443	0.379719474	0.734148482	304.9337883
4.070736707	0.918692865	0.347877377	0.726002512	208.7692865
3.059776709	0.924826596	0.382352918	0.712936906	211.7928759
2.886193514	0.945108669	0.414277164	0.778930421	367.4905942
2.963314092	0.93292435	0.410921964	0.75048828	174.590522
12.45872152	0.904910778	0.22062498	0.675183461	530.4425283
2.351358614	0.938714139	0.452299637	0.756674103	207.2136207
2.503022077	0.907540453	0.396322979	0.766515773	91.87568701
2.225833166	0.92637952	0.486505283	0.761569789	87.03683379
7.109002516	0.912929381	0.25163481	0.729499643	343.1143952

log.sigma.4.5.mm.3D_glcm_SumEntropy	log.sigma.4.5.mm.3D_glcm_AverageIntensity	log.sigma.4.5.mm.3D_glcm_Energy	log.sigma.4.5.mm.3D_glcm_SumSquares	log.sigma.4.5.mm.3D_glcm_ClusterProminence
5.204060399	14.66502946	0.007603877	27.63782151	20515.27088
4.899847279	17.3717708	0.010380219	17.71144684	11352.24205
4.942435638	14.72803437	0.00871742	19.03046916	13045.71208
4.887472295	11.33187764	0.010617948	16.15216766	9477.260424
4.700398713	10.76882729	0.019815745	16.609536	8342.45103
4.666462137	10.5179961	0.016423753	14.01876932	8298.29757
4.291124967	7.667679161	0.03340303	12.40291844	4330.803169
5.102288759	17.24529441	0.009484405	21.98606317	19194.17624
4.999401077	14.31058169	0.009228763	20.70562436	12064.97453
5.164984401	24.13841184	0.006882113	22.23838144	21919.54027
4.507574136	10.5754792	0.017817414	10.28043866	3643.68049
4.891995283	15.74560969	0.00874314	16.57169005	10387.3106
4.590265438	12.80198502	0.018661204	10.96167439	5228.51922
4.793514209	10.7745585	0.009954155	13.81085912	6133.21569
5.106997465	15.56564514	0.007708428	25.53162638	22430.83985
5.134386416	18.47704193	0.010584085	22.91627824	22150.80314
4.892864189	19.16305222	0.01101049	17.44269387	16180.55922
4.944758419	15.80393519	0.011493344	18.3680902	11697.60991
4.713415048	16.20756383	0.010100013	13.52792717	5848.273344
4.944077158	14.5076229	0.011671885	17.76250693	13439.92193
4.97009128	18.72682504	0.008752247	17.4273244	13690.53461
5.022212243	17.24994409	0.009096672	18.94238664	15579.52473
5.053037054	16.94939555	0.008511481	19.773179	15692.42726
4.993710817	12.76588802	0.010415548	20.83697854	13123.64345
5.100188726	13.45462939	0.006891193	21.98340359	15796.15372
5.104597467	17.72022788	0.007592668	23.41697889	21560.90467
4.991616386	14.86376398	0.009170361	18.70799343	10708.5182
5.016457942	15.60429029	0.006975881	19.39263315	12830.24507
4.835133813	18.4617417	0.011226868	16.19828459	13854.06816
4.906255884	20.30708451	0.010083621	16.50871574	13322.58169
5.060463099	19.48089245	0.008848605	19.94917141	18444.66263
4.988168868	18.14487058	0.00858222	18.53088313	16756.38483
5.02708402	23.62711117	0.011102977	21.82592692	34436.41768
4.785351334	12.40719311	0.012950095	18.2206037	7835.076499
4.823646093	11.93745285	0.011453297	15.98288087	8265.510918
4.722996912	15.19748193	0.012340341	12.91642218	6291.079794
5.253407955	21.27631056	0.00666305	25.94492961	31142.29848
4.763858035	16.24799559	0.012193697	13.49865082	7653.873605
4.495368895	13.10117685	0.021350622	10.08291818	4445.417331
4.77178574	10.4037382	0.014887096	15.92798591	8303.836815
4.619303869	11.74216769	0.011668304	11.8932895	3884.912321
4.645412028	11.98951125	0.012886788	12.22317022	4510.716267
4.718119773	16.26290687	0.01349793	13.69048783	6149.149567
4.762566894	11.82712522	0.015221426	16.03593834	8599.747789
5.139584964	20.21465964	0.008525979	24.79955813	26975.01584
5.050192904	12.96078431	0.008062435	18.42850517	11945.34716
5.073152895	19.33667181	0.008548788	20.26007899	20435.01483
4.735608288	16.83926758	0.015180386	16.14651571	13557.15595
4.581590306	8.010791161	0.020161373	13.8882832	4792.831148
4.87809216	13.31699062	0.014247595	18.11690678	11665.68894
4.795597747	18.15872256	0.014874735	15.37800296	9687.82838
4.371449077	15.89838124	0.024902919	9.722974092	6060.571528
4.897375729	14.27868384	0.011951114	17.69168748	10167.30507
4.836353622	20.72272234	0.00931927	16.09168595	8335.943581
5.030090811	16.89247258	0.007466556	19.2265864	12287.29931
5.203202295	17.32512697	0.006778251	24.35967445	21941.30187
5.097043037	21.81805832	0.008022042	21.73412699	24541.44937
4.678623085	13.07368702	0.02489048	16.64384991	12878.62176
4.808752906	15.72502929	0.010767539	15.26857086	9336.806509
4.305720754	11.56995981	0.018998122	7.119328072	1803.88508
5.030477475	15.03280405	0.009710655	20.23655369	11256.90517
4.942621164	20.47116132	0.010980765	17.36450801	15814.26957
4.912840671	17.0173464	0.009901576	16.99095368	11654.29846
4.928689663	14.33966775	0.007165061	17.55214081	8857.878763
4.685399011	14.00047071	0.009861032	14.76611059	4985.450563
4.996913818	11.99874902	0.009004913	22.21646146	15534.60976
5.034415901	12.05239602	0.012646322	24.25864989	21358.07466
5.105906986	14.49861184	0.008085133	23.54801033	15685.23777
4.740434316	14.41241389	0.011960555	13.52748586	6095.135217
5.076575442	19.39604829	0.008968187	20.71792877	18592.85732
5.356880114	18.99371122	0.004954709	31.83819248	39321.43876
4.617482065	14.37579185	0.016312171	13.83516943	7217.617489
5.043309666	18.75679863	0.00929354	20.17277522	21564.61582
4.831389606	18.84457853	0.010065003	15.05070588	10232.6785
5.241363112	18.23250689	0.007240669	24.81553591	27273.96806
5.081913426	17.56528183	0.008357752	21.54219449	19952.21883
4.676438433	15.63139558	0.014922942	12.67311537	7424.003078
4.833926397	15.16710289	0.010818881	16.28918173	10107.15031
4.866698058	17.99626055	0.010104416	16.6573272	11852.76966
4.899314232	18.96371019	0.011802969	17.61736293	16774.46341
4.980198086	26.60418411	0.011097485	22.87940188	33468.78551
5.174653041	20.01288257	0.007100091	23.75337734	26346.3686
4.991496946	19.45820078	0.008746591	19.27351184	16215.63047
4.15428302	8.891821096	0.032342408	8.367159971	1623.647006
5.082564217	18.20606445	0.007822879	20.39847433	18450.92506
4.936990379	10.92083829	0.010246189	17.55761235	10124.48867
5.175828022	17.88467087	0.007712636	24.00149148	22055.78709
4.750861431	12.01244544	0.012052366	13.34084242	5610.000261
3.984411843	8.417258969	0.025131978	6.829029899	829.3737013
4.773477299	11.26565536	0.012532506	15.18358536	7760.743538
4.882604914	17.18253371	0.009573752	17.21324384	8454.069944
4.923568401	13.99026242	0.008789677	17.91950581	9906.539482
4.721571066	14.23120592	0.011662613	12.97193652	7213.45065
4.829632177	18.90848264	0.013004047	14.97476598	11576.49697
4.795796954	12.82624585	0.011159003	13.40273342	7056.425825
5.575254312	22.47796815	0.004162644	48.63273938	85350.27687
4.505859195	14.10815897	0.018770444	10.77339234	4339.218175
4.699458862	8.967094609	0.01420973	14.8127758	5533.825789
4.446177322	8.908476641	0.022167509	10.05970245	3225.976866
5.424251463	17.89444701	0.004890287	36.0634826	48604.13912

log.sigma.4.5.mm.3D_glcm_SumAverage	log.sigma.4.5.mm.3D_glcm_Imc2	log.sigma.4.5.mm.3D_glcm_Imc1	log.sigma.4.5.mm.3D_glcm_DifferenceAverage	log.sigma.4.5.mm.3D_glcm_Id
29.23549352	0.933282195	-0.2430079	2.572468077	0.425482167
34.60834641	0.884545789	-0.200912525	2.157499853	0.450680909
29.42183777	0.882613714	-0.192347417	2.474617412	0.420498879
22.66375527	0.886608264	-0.203909847	1.982398174	0.464293929
21.53765458	0.932005198	-0.277418545	1.847980248	0.506025594
21.03599221	0.897354535	-0.226226149	1.768873124	0.493386114
15.33535832	0.929963867	-0.306248799	1.36990439	0.575684471
34.35641699	0.930443569	-0.248007952	2.108550884	0.460184317
28.55761632	0.897213639	-0.203646891	2.261879622	0.449311462
48.18694641	0.890205686	-0.193782066	2.414178486	0.421705322
21.1509584	0.874497971	-0.211406103	1.600967639	0.522270544
31.43134704	0.85680887	-0.174054938	2.373752144	0.425163085
25.60397004	0.88002435	-0.21375583	1.600447964	0.528356197
21.54911699	0.852828883	-0.176388594	2.099662013	0.446343459
30.97538044	0.895514058	-0.204983021	2.718324765	0.427277675
36.76018373	0.902077078	-0.209991081	2.32073961	0.459711633
38.19853268	0.892996828	-0.211230409	2.227224203	0.445260225
31.49379757	0.914833255	-0.236149337	1.950543478	0.487937142
32.39421302	0.851037566	-0.181222519	2.27306197	0.437848036
28.99647219	0.901415263	-0.217085699	1.982119358	0.475404025
37.31179873	0.84437178	-0.16563235	2.255117737	0.445161377
34.38173859	0.901459594	-0.215059	2.159967917	0.451628554
33.76285996	0.879459411	-0.189054416	2.235967068	0.439578481
25.53177605	0.92842701	-0.25409114	2.205327588	0.445938374
26.90925879	0.870155462	-0.179983187	2.450891099	0.417761702
35.29216961	0.896170534	-0.198392828	2.675659305	0.409270289
29.68720008	0.913324518	-0.232567346	2.005712382	0.46559983
31.16915183	0.835165755	-0.153770913	2.553430956	0.406010906
36.76115023	0.845965964	-0.17603646	2.22461538	0.446076038
40.49226477	0.850657588	-0.169838701	2.321515941	0.444631112
38.82772608	0.891205288	-0.201374557	2.152971595	0.450744349
36.14712683	0.862760223	-0.178421863	2.244027702	0.446545101
47.19369567	0.923080573	-0.242120411	2.05462147	0.468792255
24.81438621	0.931470062	-0.267494459	2.391958499	0.456103491
23.87490571	0.895060911	-0.21675365	2.228461632	0.454847674
30.38717654	0.855681625	-0.181994578	1.937534573	0.475337263
42.40460863	0.894352745	-0.193135022	2.604282355	0.408386777
32.47276541	0.864246465	-0.186978195	1.941555131	0.472055744
26.2023537	0.896723436	-0.247651662	1.49590952	0.540582029
20.8074764	0.909167078	-0.238406508	1.847166543	0.49837521
23.48433538	0.828455669	-0.163348449	2.100128302	0.454343508
23.97902251	0.847692484	-0.181670534	1.929610889	0.480654556
32.49217548	0.888972578	-0.212470217	1.785024969	0.497795433
23.65425044	0.898802612	-0.225193849	2.002430591	0.491278533
40.16248072	0.872530688	-0.183222363	2.752987758	0.432186095
25.91827762	0.902611717	-0.211976516	2.034021458	0.453019334
38.5029578	0.878669404	-0.187936636	2.317249844	0.440406158
33.63898353	0.878456277	-0.201190298	2.085453241	0.478248746
16.02158232	0.940757555	-0.309845385	1.874293743	0.491368918
26.59591401	0.93096138	-0.262249034	1.969725049	0.487665456
36.23095547	0.900753488	-0.224699475	1.774243033	0.502191628
31.78717027	0.879598964	-0.221674988	1.554833393	0.542196419
28.51620703	0.911533221	-0.231483201	1.828786302	0.49127428
41.21952392	0.881578066	-0.201351646	2.372544026	0.435779737
33.68623911	0.864120146	-0.175324273	2.359080842	0.4282229
34.5059539	0.89741581	-0.198813341	2.56544269	0.413187632
43.51830515	0.879672871	-0.187292879	2.494454562	0.430142213
26.11041813	0.916034047	-0.253190688	1.721229022	0.537729336
31.43638118	0.871153191	-0.189366321	2.188269689	0.446825552
23.13991962	0.838042357	-0.187070351	1.526284155	0.519928065
30.0353569	0.919653314	-0.233074326	1.962327032	0.488566198
40.82498431	0.878740691	-0.194215828	2.102009172	0.464386125
33.94121421	0.881627501	-0.196413593	2.171758701	0.455881229
28.67933551	0.841000409	-0.158827889	2.55341374	0.402250859
28.00094142	0.859744195	-0.185035597	2.611948298	0.410827921
23.96970185	0.894671702	-0.204307097	2.401972069	0.43067941
24.06508666	0.947032318	-0.283316981	2.025587485	0.480658148
28.900853	0.899341592	-0.204977092	2.273095471	0.446397521
28.82482777	0.884920644	-0.216888503	1.931664241	0.476563997
38.6992887	0.895110834	-0.204678575	2.261633655	0.441126685
37.80773856	0.877188507	-0.173855397	2.935829039	0.388866716
28.74063885	0.881133323	-0.212585239	1.900051773	0.488517057
37.37458142	0.88672272	-0.197877962	2.189502216	0.453878489
37.55351149	0.833161357	-0.160939326	2.149168074	0.446485964
36.34090533	0.9049439	-0.207072952	2.373828032	0.431831667
34.98504797	0.884430742	-0.193937465	2.447816476	0.432494722
31.23487837	0.877474886	-0.20520166	1.812936073	0.501222333
30.33420578	0.87849807	-0.202054849	2.214613599	0.449520235
35.84566649	0.837349946	-0.163755953	2.334377894	0.436115365
37.79304602	0.865187996	-0.183475127	2.133732887	0.460137111
52.9567883	0.904874288	-0.213488383	2.65676552	0.43706279
39.85777422	0.875018047	-0.178963797	2.695946535	0.405982794
38.75014681	0.876526137	-0.186798103	2.340116021	0.425798465
17.78364219	0.906988921	-0.287896745	1.538369094	0.557987244
36.27909436	0.883881147	-0.193950571	2.246448862	0.43625972
21.84167658	0.904171441	-0.21856837	1.885215411	0.487374478
35.63806603	0.911769071	-0.217214265	2.489543366	0.422643375
24.02489088	0.872550053	-0.197904826	1.878771092	0.478500379
16.83451794	0.870903518	-0.242845713	1.969801153	0.460672724
22.53131072	0.917689247	-0.249809121	2.057226222	0.453351529
34.21366869	0.866512459	-0.180278193	2.179277988	0.450702437
27.98052483	0.890598129	-0.200460829	2.354316522	0.426402827
28.46241184	0.850730328	-0.177544121	2.064539423	0.454924268
37.73032748	0.880454703	-0.202411256	1.885590386	0.480280668
25.65249169	0.861452029	-0.183920956	1.901615317	0.477358425
44.59500258	0.93813322	-0.227063199	4.379453989	0.3106493
28.21631794	0.883137892	-0.223192048	1.655350884	0.51077995
17.93418922	0.936277728	-0.287950651	1.967704957	0.466274346
17.81695328	0.896789004	-0.247909702	1.541305732	0.539918425
35.59924876	0.919543845	-0.211630962	3.432822263	0.343266707

log.sigma.4.5.mm.3D_glcm_ClusterTendency	log.sigma.4.5.mm.3D_firstorder_InterquartileRange	log.sigma.4.5.mm.3D_firstorder_Skewness	log.sigma.4.5.mm.3D_firstorder_Uniformity
95.19140018	192.7025127	0.146363823	0.056029917
60.4263782	138.5392962	0.134439895	0.0741125
64.56830277	145.9514275	-0.234098076	0.070337076
57.5561921	131.307518	0.442026799	0.073812793
59.26720126	145.1376657	0.541529933	0.088810694
50.244941	98.92381096	0.728460289	0.092294424
45.64312659	151.0042496	0.63447603	0.111363716
77.84043351	155.1583405	0.185649663	0.064370593
71.25802403	166.2583733	0.380137459	0.066443662
79.82865879	157.1531925	-0.297276153	0.06135423
36.18225857	102.902071	0.471527968	0.09669921
55.18935915	129.2297096	-0.112405133	0.072384189
38.78860821	98.46950912	0.527986481	0.094353786
47.69087515	121.8843322	-0.055547806	0.076279111
84.08200524	156.7161584	0.635099333	0.062143201
80.41048436	153.253767	-0.040137022	0.065205328
60.31288991	119.7628818	-0.345037192	0.073637096
63.95884467	153.2738228	0.441217597	0.071608732
44.13510161	135.1064262	-0.162282591	0.07715483
63.02375101	127.2613068	-0.10118885	0.075271861
60.6759015	129.0991299	-0.333900049	0.070834512
66.46934636	139.0273533	0.144954446	0.06912821
68.83831045	136.3161345	0.295589397	0.068142699
74.77836344	169.7073538	0.038518941	0.0663908
77.67317279	159.446496	-0.398232561	0.062065981
79.21929766	146.9607439	0.219041848	0.064088705
66.43345849	157.2379072	0.329749303	0.06594203
65.67301959	146.9910285	-0.392423263	0.066858327
54.44658858	116.2699585	-0.546863866	0.078036122
56.87817232	125.3918085	-0.193167327	0.071491423
70.76319044	138.9714766	-0.385996436	0.06676306
63.95220491	141.3252907	-0.063334247	0.06795448
79.76134209	133.1565895	-0.553076137	0.071948768
60.9630272	175.9122314	0.164528503	0.070413769
54.56100833	127.157707	-0.02353912	0.075255154
44.45318973	111.7616386	0.195041218	0.082686408
93.17797274	150.5563622	-0.124949733	0.058855634
46.62967161	107.2521744	-0.244660933	0.083481601
35.9339809	92.51977444	0.368017155	0.099795846
56.96499274	143.4924765	0.610008172	0.082434646
39.65711369	123.7922888	0.139655655	0.082048392
41.89577179	129.2660809	0.389350723	0.082759474
47.77933485	130.5757027	0.286599807	0.082377095
55.30903348	135.0994148	0.617481267	0.082826777
84.81762355	134.7385589	-0.479656606	0.063054205
66.49408409	143.9789014	-0.087785006	0.065953191
71.52410911	136.3805385	-0.207958632	0.066308441
54.83545937	96.17874718	-0.983375143	0.091121268
48.8078761	143.722167	0.426451534	0.089080026
63.23062935	151.8205624	0.618010189	0.075241141
54.28382637	120.9671955	0.234565023	0.081050734
33.27211973	76.9119072	-0.003438344	0.115036948
63.2020243	152.1167908	0.480708127	0.071870291
53.21807142	139.8569622	0.014679986	0.071235472
65.41484679	153.3284302	-0.03425601	0.064190774
84.18920848	154.7513618	0.037289317	0.059266487
76.34608149	145.0519371	-0.419825366	0.065705678
58.45240102	111.0908113	0.936614116	0.101124602
52.2058591	113.5919132	-0.036788092	0.079338753
24.30641075	88.22724533	-0.291618746	0.10887856
72.22482166	170.6579666	0.220237701	0.064291834
62.19890758	123.3333855	-0.236174457	0.074501757
57.71713516	129.6450672	-0.153803288	0.073252413
59.52808468	160.4594564	-0.224606618	0.065458941
46.87312915	152.6459723	-0.140002177	0.073059466
77.08899059	177.2810631	0.560443887	0.066159173
87.29291064	160.6712132	0.729640297	0.069884327
81.75981371	170.9220114	0.362901766	0.062232294
47.08510642	125.6080856	-0.019201352	0.079023285
73.5502438	142.7516365	0.018822908	0.066117384
111.9732342	187.6637979	-0.485670565	0.051370732
47.41162287	111.5562248	0.634834865	0.09122327
71.33269489	138.896513	-0.134273085	0.069342994
51.43415294	120.9129028	-0.423253616	0.074901374
88.82912133	161.9018879	0.135277599	0.058855324
74.32935188	151.6151638	-0.074843135	0.064673793
43.42796443	105.6883354	-0.239004667	0.088082005
55.98258902	132.3885307	-0.019456154	0.077415383
55.47043733	113.0744395	-0.270603412	0.076988115
61.36483902	107.6802988	-0.473461393	0.079045765
76.65478099	108.802598	-0.638367698	0.078075776
83.11840364	142.2160246	-0.331331025	0.063389695
67.77890919	144.3161526	0.192128468	0.066859282
28.09728183	120.0952377	0.422716129	0.114602108
71.55983987	142.5574322	-0.419497594	0.064793238
63.28489596	156.8437805	0.450668645	0.069179917
83.32988721	149.2560382	-0.063656817	0.060692905
46.9325962	131.7283154	0.26527783	0.079523219
20.59626754	116.210556	-0.153879155	0.108672101
53.31553166	124.1710148	-0.031985243	0.076986626
58.07319636	145.3423138	0.407930736	0.070882645
61.88805675	143.9515533	0.049119938	0.067458897
44.43772397	104.5884367	-0.439697346	0.086022713
52.8841313	106.1177692	-0.056674804	0.081514216
46.93486469	117.9061909	0.04414702	0.079107988
165.1343291	226.4868298	-0.609713082	0.046454311
37.87942461	107.3441048	0.364198388	0.099158379
52.45617401	158.9608898	0.244869553	0.076109267
35.45182049	109.0291748	0.566227577	0.099350195
124.3464542	184.3689117	-0.273697579	0.051174575

log.sigma.4.5.mm.3D_firstorder_MeanAbsoluteDeviation	log.sigma.4.5.mm.3D_firstorder_Energy	log.sigma.4.5.mm.3D_firstorder_RobustMeanAbsoluteDeviation	log.sigma.4.5.mm.3D_firstorder_Median
107.2099193	45703051.89	79.72874204	129.2425232
82.49876016	48073986.68	57.63198118	99.29100037
85.72902749	13977621.6	59.6719334	41.94304085
80.17488883	61035698.66	55.77304034	109.4135437
83.54500956	16010525.41	60.49228219	51.39489365
70.95330874	22972843.23	45.00609659	55.75324249
78.18006386	15539168.1	61.81843724	43.64720917
92.76270836	59044335.71	64.08011826	122.7004395
92.3722289	63914560.05	68.02055632	120.6454544
93.07212302	81557182.39	64.3714865	111.9323502
63.33210202	29769023.09	43.84887935	85.63670349
79.1155955	34394959.72	53.66564	101.500824
65.71098294	42681653.47	43.1660514	71.38282013
74.01158463	28125980.8	51.69048534	77.84116364
98.79789172	44453702.44	66.8602834	93.56149673
93.93392774	77256003.17	63.67239906	61.7368927
80.4861618	25038724.71	50.96350979	66.29743958
86.06273677	86965759.6	63.14650984	134.0727615
74.84826183	24315809.64	54.32771125	113.8395729
80.23959904	59211130.22	53.79790669	88.99223328
81.49696686	97245591.19	54.57828997	59.49960709
83.73564346	42521157.55	56.87377176	84.25402832
86.01177184	78085072.73	57.52529656	70.92806244
94.07696574	20883214.47	69.19374952	64.3091774
94.90365347	36966569.77	67.18865624	39.723629
93.03198699	40335370.87	61.53453546	111.4441833
89.80985668	44324647.6	65.72881548	76.82337952
86.46031876	56072454.27	60.59694189	84.50457764
76.99244557	65766111.38	48.96466429	93.80889893
81.74996563	60138393.37	53.07347274	87.57181549
86.81632399	91493830.81	57.66904103	96.30727005
85.39551978	150898314.3	58.30985	128.4715271
86.8351327	62869728.2	56.34996884	95.19835663
92.31505707	12526588.21	70.96363626	112.5131836
78.23882557	12075662.25	53.3345239	55.88537598
70.63304071	53039435.98	47.88954974	82.41602325
99.22669534	108942199.9	65.56022166	116.1307755
69.63749918	57623803.73	45.87096001	91.15193176
62.52665697	16137442.64	40.66166549	53.02594948
80.92541936	40220701.99	57.9236216	98.7611351
71.79383993	23972047.99	51.7552785	91.92298889
72.71441656	38853540.9	52.92587222	96.43879318
74.06225806	64851442.32	53.69345112	102.216713
81.22400448	40035398.69	56.26934039	80.0257988
95.65849337	70668873.58	60.69855314	67.8696022
85.19067705	65772724.24	60.42664276	89.35555267
87.54542388	83410086.07	57.69146975	95.69247437
73.36753599	16409116.56	44.17753996	46.5976696
77.93274261	9063537.057	59.67531591	61.60245705
86.07768698	27600690.56	61.43168184	96.46959686
77.69149248	88708977.95	52.45191819	93.4735527
55.72799594	25625126.87	33.53892864	76.66300583
86.05219831	120358204.1	62.4372915	122.8970718
81.64418673	23107892.13	57.32212042	88.22219849
88.62352136	112249413.4	63.20125105	125.560791
96.83245963	37289585.28	65.38623654	65.14424133
89.88294568	82943492.55	60.1009237	103.8666229
77.31046749	43017656.34	50.38631259	58.1045742
74.5121429	21736383.23	49.171158	76.72043991
52.35433466	20333640.31	35.98291161	98.98912811
92.5408024	109691835.1	69.870436	146.1634674
80.96523998	118599633	52.94625091	94.22639465
79.6344244	51319869.43	54.15928387	107.3405685
88.00605622	45888045.53	65.63548189	117.3427429
82.90903334	15176602.01	62.7651047	131.1544724
96.48855156	50287445.54	71.76013644	112.6553574
98.03707221	28932682.23	69.32898038	80.53637314
96.38614701	125729352.4	69.48567302	127.003212
73.43999762	22643609.17	52.22593235	87.30974579
90.41088731	56428552.42	60.60574179	83.04223633
113.3244649	119185549.9	78.40121479	120.9270172
72.13754274	46868413.18	48.03345062	99.29243469
86.220425	135327635.1	57.99669914	120.2932434
77.19338707	131895822.7	51.59665082	109.0177612
98.56753291	127923617	67.42004769	111.1119804
93.34156081	55307406.87	64.42260009	88.72575378
67.02054673	31087936.22	43.5991887	69.78274536
79.53074068	31088090.45	55.81040179	99.78246307
76.57076426	55814142.25	48.96467042	84.90942383
77.49328066	65019942.11	47.50253007	60.60166931
82.41738701	25944830.49	47.90567205	68.6530571
93.09382572	51205102.31	60.31892854	70.0451889
88.68361217	70368549.25	60.02174154	112.6185341
62.41009516	6389050.058	48.24252272	63.19939423
88.45706818	101802401.5	59.62033375	119.3274765
86.01453697	78540454.72	63.40383663	93.28811646
95.62635129	37620815.04	63.4171251	100.7456398
74.33756315	63599652.73	53.47284902	101.4026718
57.82339353	7564681.732	46.49311572	120.2557831
78.32739616	10782156.22	54.84264605	83.59020233
83.94384178	93579510.13	60.752804	121.9459229
85.20709924	25687598.45	59.65477512	102.8530121
67.68413011	15159332.92	43.98222432	48.36479187
73.83020652	73504425.75	46.37373546	78.95328522
72.12626239	122438485.4	49.06744293	127.8292465
139.2039295	27366585.84	95.27433186	62.7989521
65.4202522	26797836.77	45.24314892	75.27788544
83.01011936	11839536.57	64.7021644	128.5342789
65.08248417	11720238.46	45.6786971	61.40489197
116.7525663	29490510.4	77.94123663	74.08115768

log.sigma.4.5.mm.3D_firstorder_TotalEnergy	log.sigma.4.5.mm.3D_firstorder_Maximum	log.sigma.4.5.mm.3D_firstorder_RootMeanSquared	log.sigma.4.5.mm.3D_firstorder_90Percentile	log.sigma.4.5.mm.3D_firstorder_Minimum
1233982401	479.459198	194.9125428	320.5663574	-189.1743011
1297997640	425.2293396	155.0386834	265.4071167	-278.2629395
377395783.3	376.753418	111.8138851	153.1987869	-300.0209351
1647963864	466.6896973	157.7404902	269.0125305	-146.3869019
432284186	330.4551697	129.8881963	238.9299957	-158.1827545
620266767.3	373.3007507	118.4269176	220.9579636	-174.6829376
419557538.8	313.2479858	121.248504	218.7496857	-80.18283081
1594197064	513.1928101	180.8633278	300.2508057	-259.6239929
1725693121	484.1246338	177.5714036	294.3362793	-181.5777283
2202043925	465.9420471	164.7985707	264.7732971	-461.6306152
803763623.4	329.6294556	128.3521699	223.5848969	-129.5970459
928663912.5	428.8523865	142.8721072	232.1460846	-262.6592102
1152404644	381.4482422	120.7149147	205.4799744	-200.1195374
759401481.7	338.5384827	123.0356543	206.3528275	-167.7284851
1200249966	508.7695923	169.4605029	286.571283	-243.0021515
2085912086	427.21875	137.1191295	224.282019	-372.4768982
676045567.1	392.4682922	124.9017675	195.2026306	-375.7919312
2348075509	545.2633057	184.3842738	289.0713348	-218.0897522
656526860.4	364.5314636	146.3036637	233.1867294	-256.1541443
1598700516	411.2175293	142.8656466	241.9408875	-247.6917725
2625630962	481.6154175	118.2885091	180.419017	-385.6148376
1148071254	560.5811768	141.5898772	232.4201813	-313.8063354
2108296964	476.6466064	137.6520559	234.3078613	-314.5095825
563846790.7	348.5715332	142.3901875	245.604007	-213.8170471
998097383.7	300.7848816	119.1472674	165.1375534	-284.6443481
1089055014	475.2451477	172.7247932	284.4107635	-279.6371765
1196765485	400.1138	141.5885957	249.6465759	-237.0319214
1513956265	381.1372681	129.2791597	198.2710938	-297.8055725
1775685007	420.6773071	139.0994778	221.2110992	-343.3890076
1623736621	551.1885986	137.9752208	217.5617371	-393.4112549
2470333432	394.3282471	148.6246547	240.8267715	-359.4608459
4074254485	520.0289307	173.7229485	276.7375122	-299.9740601
1697482661	431.45401	157.6067156	261.2070007	-466.2998352
338217881.6	390.5213013	161.3778266	268.680481	-164.7341309
326042880.6	309.8855896	116.6135444	193.8623215	-219.0653992
1432064772	369.2380981	128.4026297	218.4876465	-258.0314331
2941439398	534.8813477	174.7128292	294.7128601	-375.5905762
1555842701	356.3572388	130.3578132	212.5817108	-280.6392822
435710951.3	335.0706787	104.2798518	184.580954	-240.1103821
1085958954	429.4807129	156.795348	268.1877747	-114.8531113
647245295.7	343.2867737	134.966077	227.6754761	-159.7125397
1049045604	379.8718567	141.8115948	239.4182785	-161.3981628
1750988943	378.6881714	146.3705178	246.2042236	-262.6759949
1080955765	424.3314819	145.7745232	258.3564148	-163.8084869
1908059587	426.9166565	142.2173671	221.7371536	-424.7333069
1775863555	401.3068237	135.6390977	220.9336334	-214.8862
2252072324	490.526886	150.4290176	242.7597275	-365.6493225
443046147.1	318.0700378	104.0382406	126.7014008	-372.4957275
244715500.5	299.5419922	126.7679027	232.4403305	-99.53375244
745218645.1	470.922821	159.1280442	268.3417358	-190.1766815
2395142405	445.2599487	149.2189992	257.8487335	-312.4914856
691878425.6	334.9941406	114.4586415	185.9896698	-285.13974
3249671511	511.7883911	178.0399858	300.0721069	-179.3765259
623913087.4	361.9417114	140.475866	242.2378235	-376.902832
3030734161	429.7220764	168.8532144	275.3454773	-260.4873352
1006818803	449.6275024	140.5376806	222.1180328	-347.7894287
2239474299	468.3968506	159.0451576	251.84776	-413.6183472
1161476721	467.0596924	132.9969212	241.0464935	-217.6643982
586882347.1	331.2854309	125.8683634	216.2524948	-299.6166992
549008288.3	271.2592773	120.3010468	183.6112549	-165.9358826
2961679547	454.6721802	193.1255078	315.1946716	-183.1508636
3202190091	536.1015625	147.0192465	244.5658905	-377.2096558
1385636474	434.1564941	150.3260355	234.4789124	-297.4569702
1238977229	349.8392029	156.733324	248.5916992	-200.2140198
409768254.3	360.9482117	160.3840835	247.9003876	-176.7023773
1357761030	508.7153931	180.5289369	300.7292969	-141.0582428
781182420.2	492.2850952	165.3680155	292.6151917	-155.6507416
3394692515	478.0354309	187.7706328	319.8089752	-188.2088623
611377447.5	319.6350708	132.3346468	228.324176	-244.5092621
1523570915	455.8796082	150.0279889	255.1203918	-370.3500977
3218009846	479.0710449	176.9144605	277.5491394	-343.656311
1265447156	401.0619202	150.7999262	261.986145	-208.3134308
3653846149	517.7772827	170.0656971	275.0879028	-312.1151123
3561187214	443.1529541	142.3284347	223.052475	-336.5160828
3453937659	663.446228	173.2481647	281.865036	-307.7500305
1493299986	568.0654907	154.0353897	247.429184	-316.3577271
839374278	385.640625	114.8948309	185.3227051	-287.0529785
839378442.2	359.7559509	151.2471832	257.0130554	-228.6284485
1506981841	402.9491882	130.5269472	202.9831619	-341.7380066
1755538437	382.2661743	120.8361673	183.5297638	-384.2154236
700510423.3	466.7802734	138.0183608	206.7615387	-556.1010742
1382537762	458.9469604	138.8228135	208.7992889	-414.9472961
1899950830	516.2463379	168.651206	284.5004822	-339.9477539
172504351.6	256.4765015	110.5267248	190.6082153	-115.5737
2748664839	445.6204224	162.3576949	257.4197235	-303.7266541
2120592277	452.831665	150.3598205	257.8631897	-138.9308929
1015762006	455.2728577	162.3117635	266.8612335	-303.4648438
1717190624	376.6848145	144.5694058	245.4972839	-173.0685272
204246406.8	232.7720337	135.6670618	206.717392	-55.2414093
291118218	301.7399902	136.5805605	229.6249023	-170.6187897
2526646774	483.315094	170.7144294	286.7289124	-263.1173096
693565158.1	376.3678284	152.4686703	261.841571	-205.6701355
409301988.9	275.6375427	98.51435782	148.1334564	-296.4422302
1984619495	392.5576172	129.945849	213.190509	-355.0616455
3305839105	437.4032898	161.2994969	254.4451218	-167.9047089
738897817.8	440.4220886	175.9479363	213.8666962	-505.7550659
723541592.7	326.81073	123.2888247	216.2856262	-234.6962738
319667487.5	368.3713379	166.3203795	272.2542114	-73.28664398
316446438.3	305.6477966	114.88458	205.4844696	-112.3780441
796243780.8	450.4569397	159.8594226	227.4742661	-363.9634705

log.sigma.4.5.mm.3D_firstorder_Entropy	log.sigma.4.5.mm.3D_firstorder_StandardDeviation	log.sigma.4.5.mm.3D_firstorder_Range	log.sigma.4.5.mm.3D_firstorder_Variance	log.sigma.4.5.mm.3D_firstorder_10Percentile
4.346269094	127.9305534	668.6334991	16366.2265	-4.171365499
4.026943577	103.906205	703.4922791	10796.49944	3.419018459
4.090634466	108.1320345	676.774353	11692.5369	-126.4264885
4.000093061	100.3897801	613.0765991	10078.10796	5.44810524
3.83757572	100.7018198	488.6379242	10140.85651	-20.86977615
3.798633749	93.32072808	547.9836884	8708.758289	-30.50253525
3.491592337	89.0812708	393.4308167	7935.472807	-8.769769859
4.230394967	117.1802803	772.816803	13731.21809	9.218829918
4.128924977	111.5572592	665.7023621	12445.02208	12.29649868
4.262611552	118.3274769	927.5726624	14001.39179	-34.00903625
3.635095641	79.58224128	459.2265015	6333.333128	11.65178738
4.048220572	101.1070183	691.5115967	10222.62915	-23.16182747
3.742530548	85.14435061	581.5677795	7249.560441	-8.899749947
3.920047419	92.87683622	506.2669678	8626.106706	-41.64564514
4.280870222	125.0626019	751.7717438	15640.65439	-24.65931835
4.250620614	119.5951271	799.6956482	14302.99442	-67.9405304
4.103764877	107.8948094	768.2602234	11641.28989	-70.41678619
4.03203458	104.7905546	763.3530579	10981.06033	33.49044342
3.894130991	92.14722042	620.6856079	8491.110231	2.028494954
4.041349336	103.7648886	658.9093018	10767.15211	-17.50611687
4.101763028	105.622566	867.2302551	11156.12646	-83.00171051
4.127962585	107.8708336	874.3875122	11636.11674	-37.94635773
4.163051355	111.3144948	791.156189	12390.91675	-52.24151993
4.12937874	114.0358575	562.3885803	13004.17679	-54.60335922
4.207440933	116.9496384	585.4292297	13677.21791	-151.9078247
4.262237604	120.5739394	754.8823242	14538.07486	-10.76197844
4.114271527	109.5417497	637.1457214	11999.39493	-38.76651382
4.12841981	107.8809881	678.9428406	11638.3076	-73.27537842
4.015791274	103.3488758	764.0663147	10680.99014	-14.65491447
4.122212179	107.71479	944.5998535	11602.47599	-48.28620453
4.180198411	113.0395331	753.789093	12777.93604	-38.7901783
4.149923819	109.7846374	820.0029907	12052.6666	10.55399113
4.142940331	117.5771806	897.7538452	13824.3934	-10.29020119
4.038401785	108.2768081	555.2554321	11723.86718	-3.10487175
3.981072026	98.73528305	528.9509888	9748.65612	-67.03862381
3.866056192	90.21890251	627.2695313	8139.45037	-13.71364326
4.384299059	129.7028356	910.4719238	16822.82556	-37.40752563
3.874933437	90.98172242	636.996521	8277.673814	-15.76907539
3.677101657	81.73880788	575.1810608	6681.232713	-24.10300388
3.884809662	98.96415427	544.3338242	9793.903831	17.19692135
3.815462624	88.15035486	502.9993134	7770.485061	-2.227361441
3.810907352	88.28434122	541.2700195	7794.124905	9.090358829
3.831555502	91.28278286	641.3641663	8332.546446	13.80722294
3.917870631	101.044556	588.1399689	10210.0023	-0.075228157
4.325412352	127.522942	851.6499634	16262.10074	-102.595343
4.118671595	105.6082828	616.1930237	11153.10939	-56.08203278
4.215543083	114.7730558	856.1762085	13172.85433	-37.0487442
3.863851313	99.98913608	690.5657654	9997.827334	-106.4958801
3.712422687	91.66820976	399.0757446	8403.060681	-14.56298532
4.018873447	106.121724	661.0995026	11261.82031	5.090171623
3.952086032	99.92589819	757.7514343	9985.185128	8.817609406
3.532060043	77.75472885	620.1338806	6045.797858	9.985962391
4.031240145	104.8573054	691.164917	10995.05449	26.87390366
4.033270528	102.496955	738.8445435	10505.62579	-16.32401657
4.165320055	109.4766223	690.2094116	11985.13082	-5.448956299
4.336751445	124.1493385	797.4169312	15413.05826	-95.74315109
4.221881228	117.7783078	882.0151978	13871.72979	-20.98381042
3.791518474	99.75093748	684.7240906	9950.249529	-0.473054272
3.949695581	97.29845234	630.9021301	9466.988827	-31.05258751
3.440515269	66.50532646	437.1951599	4422.958447	17.58620033
4.122592926	110.1521885	637.8230438	12133.50462	26.35171509
4.08186445	107.0727558	913.3112183	11464.57503	-15.23499985
4.047354742	101.8930136	731.6134644	10382.18621	-11.35969925
4.076979163	105.5963848	550.0532227	11150.59648	-24.53813782
3.958088042	98.18684621	537.650589	9640.656768	0.0683855
4.132548141	115.7598394	649.7736359	13400.34041	7.602234364
4.142894393	120.363838	647.9358368	14487.45349	-14.42678814
4.215516309	118.2198384	666.2442932	13975.93018	9.920064449
3.872396085	91.32511192	564.1443329	8340.276068	-14.6098259
4.220514938	116.5724247	826.2297058	13589.13021	-41.02689819
4.504121492	142.7633832	822.727356	20381.38358	-95.21902847
3.794379418	92.36194796	609.375351	8530.729431	23.90832901
4.154943641	112.1831634	829.892395	12585.06215	7.077593327
4.015501156	99.73981217	779.6690369	9948.030132	-29.52472878
4.354918402	125.6941032	971.1962585	15799.00758	-28.45188065
4.234388703	118.6625481	884.4232178	14080.80032	-35.40486526
3.811049703	88.28631874	672.6936035	7794.474076	-22.61790314
3.957424312	99.96054337	588.3843994	9992.110231	9.637925911
4.026497318	101.4728682	744.6871948	10296.74298	-47.10151291
4.045842056	105.9020584	766.4815979	11215.24597	-70.50503235
4.126798428	118.9767692	1022.881348	14155.47162	-48.28255119
4.30260015	122.9068494	873.8942566	15106.09363	-94.92221069
4.189711364	113.9703106	856.1940918	12989.23169	-2.438681221
3.36932335	72.58254024	372.0502014	5268.225148	7.290597534
4.208850643	114.1231516	749.3470764	13024.09374	-28.97489567
4.038207414	104.158232	591.762558	10848.93729	-12.90442085
4.324360911	123.9088113	758.7377014	15353.39352	-51.35581779
3.868718099	91.41866534	549.7533417	8357.372373	6.37355032
3.291481558	66.66692583	288.013443	4444.479	27.8412056
3.930990296	97.78747903	472.3587799	9562.391056	-27.53673916
4.027850514	103.1093245	746.4324036	10631.5328	16.0631237
4.10534041	106.8995993	582.0379639	11427.52432	-22.60813522
3.835178501	88.84025205	572.0797729	7892.590385	-69.74468689
3.975103191	99.30921759	747.6192627	9862.320698	-27.18882256
3.915255467	92.01149726	605.3079987	8466.115629	22.41637039
4.722747393	174.2765858	946.1771545	30372.32837	-207.455719
3.644747156	82.62767205	561.5070038	6827.332188	4.405003071
3.867833582	96.72647665	441.6579819	9356.011286	18.5790266
3.599662551	80.30665595	418.0258408	6449.15899	-7.529152489
4.572651552	149.6760009	814.4204102	22402.90525	-150.3348251

log.sigma.4.5.mm.3D_firstorder_Kurtosis	log.sigma.4.5.mm.3D_firstorder_Mean	log.sigma.4.5.mm.3D_glrlm_ShortRunLowGrayLevelEmphasis	log.sigma.4.5.mm.3D_glrlm_GrayLevelVariance
2.444267315	147.0532992	0.01021201	26.87311361
3.326191563	115.067345	0.004951932	17.84677326
3.117614068	28.45712582	0.008203983	19.18702434
2.901235499	121.6715015	0.012617312	16.63847354
2.514078076	82.03710758	0.015567782	17.22196835
3.399770774	72.91211511	0.014382945	14.56222187
2.092834343	82.25403888	0.024750922	13.12685216
3.240044288	137.7691012	0.00546383	22.80014513
2.596632801	138.1541939	0.007447538	20.37255594
3.619014657	114.7047388	0.002360108	23.13054231
2.84473475	100.7022661	0.011860429	10.59000376
3.390696771	100.944588	0.00675497	16.99096751
3.556641534	85.57178388	0.007287968	12.53366355
2.710844554	80.69489156	0.01902379	14.20751339
3.335838758	114.3512468	0.006957159	25.90784935
3.343221083	67.07206019	0.005018912	24.05837357
4.203263949	62.92186935	0.00453754	19.85896285
3.10489208	151.7118984	0.005171877	17.90803871
3.038426554	113.6382496	0.00548905	13.95719598
3.379821181	98.20102275	0.009783098	18.08576623
3.60825165	53.25452973	0.003684671	18.55225126
3.731795745	91.71464762	0.004764244	19.35766329
3.378507821	80.97636544	0.005216554	20.47562875
2.410623419	85.26891998	0.013595749	21.35285733
2.612885418	22.77835428	0.013588861	21.85498217
3.505738197	123.6761066	0.005433936	24.26182534
2.57608559	89.71028656	0.007702522	19.47573477
2.997091726	71.23758506	0.007505855	18.99854137
4.487253062	93.10034684	0.00488803	18.04706157
3.937699305	86.22462276	0.003406052	19.71860495
3.624943789	96.49534671	0.003674079	21.15125299
3.762234265	134.6365339	0.005104989	19.89026118
5.428624956	104.9546731	0.002976311	23.28607328
2.279303148	119.6617556	0.014485775	19.06399556
2.842644187	62.04887288	0.015475209	16.31759921
3.263292225	91.36621332	0.005672918	13.62190177
3.59279057	117.0544623	0.003358214	27.95014872
3.689932328	93.3567654	0.005332587	13.97896379
3.591302151	64.75380128	0.00767421	11.59074839
2.709170005	121.6177509	0.015352631	16.37159989
2.64088377	102.2025287	0.012393097	12.86200587
2.656544342	110.9792932	0.009127878	12.90454659
3.033150029	114.4193255	0.005078423	13.71064473
2.993479396	105.0724004	0.012183556	17.13066379
3.663398008	62.95775378	0.00408888	27.79164494
2.72684498	85.11671654	0.011516911	17.9838028
3.917496689	97.24214621	0.004172513	21.98983135
4.628034136	28.7424456	0.008041734	17.21566148
2.137289513	87.5616382	0.037416886	13.50597591
3.113428952	118.5745089	0.008109206	18.82777061
3.592211579	110.8202355	0.004260964	16.97542929
5.385062738	83.99394473	0.005273541	10.63068271
2.705066011	143.8860036	0.006245985	18.15717997
3.373280804	96.06166327	0.003583266	17.18243656
2.836661485	128.5545689	0.004980628	19.67987924
3.121332612	65.86183573	0.005425323	25.35049929
4.442499226	106.881394	0.003596493	23.25562718
3.974522404	87.96551331	0.007461164	17.35639848
3.576812015	79.84895798	0.0068938	15.69941242
3.295221662	100.2466129	0.0103505	7.488521672
2.342380703	158.6315138	0.006071235	19.80352107
4.168981431	100.7476243	0.003116977	19.32416505
3.610263389	110.524797	0.005153542	17.3162766
2.414719601	115.8220116	0.00816333	17.93304771
2.437847216	126.8163928	0.009228711	15.84271415
2.650756519	138.5292627	0.01170441	21.77038787
2.976222518	113.3980911	0.011763571	24.08890339
2.528276641	145.8831052	0.007477717	22.89408899
2.815154681	95.77151291	0.006890309	13.62190551
3.346094613	94.44187231	0.00381226	22.55446319
2.899132395	104.4860888	0.005721355	32.91527391
3.33498793	119.2052361	0.006292022	14.4774597
4.00528796	127.8173666	0.004410582	20.81870404
3.511848247	101.534985	0.003596795	16.34350852
3.474145005	119.2305288	0.004449735	26.0543548
3.644617639	98.21456592	0.005963991	23.41492334
3.97992532	73.52787293	0.005841767	13.448061
3.18527899	113.5059478	0.00717205	16.37953462
3.727122849	82.10079768	0.004223998	17.25255795
4.196894639	58.19049192	0.003826091	19.12273796
6.18649097	69.95424424	0.002514558	24.36926203
3.794781567	64.54207862	0.004140901	25.14146936
3.505768739	124.3141086	0.003657507	21.48439666
2.214249716	83.35425455	0.018153161	8.694580547
3.545000673	115.4812857	0.005239057	21.30178451
2.592617036	108.4395607	0.0141821	17.65740304
3.205858849	104.8413804	0.005353433	25.48361695
2.620787856	111.995271	0.009962169	13.77888429
1.915327324	118.1569831	0.023814838	6.9560375
2.755252436	95.35123734	0.020318836	15.65097098
2.732023144	136.0583831	0.004170493	17.43898753
2.752420752	108.7160113	0.011573444	18.82517186
3.696627431	42.57332864	0.008238661	13.16397055
4.012063957	83.8069387	0.003454987	16.68363056
3.154036607	132.4817424	0.009705931	14.00513138
3.209977162	24.19396454	0.00733111	49.44291862
3.134157096	91.50301695	0.006190919	11.47815363
2.073018692	135.3013575	0.027458292	14.72993308
2.695890842	82.15417055	0.018558301	10.92582335
3.077007334	56.14383103	0.009625116	36.36864738

log.sigma.4.5.mm.3D_glrlm_LowGrayLevelRunEmphasis	log.sigma.4.5.mm.3D_glrlm_GrayLevelNonUniformityNormalized	log.sigma.4.5.mm.3D_glrlm_RunVariance	log.sigma.4.5.mm.3D_glrlm_GrayLevelNonUniformity
0.010956609	0.054614535	0.162388965	58.55884001
0.005360616	0.071805912	0.184239003	126.1677132
0.008688723	0.06870552	0.138415915	69.05035239
0.013745504	0.071782417	0.19019181	154.5323921
0.017376604	0.079835362	0.25138985	64.04051863
0.015923955	0.087326229	0.228046406	122.2004682
0.030194588	0.096535372	0.409570537	80.57703414
0.005906631	0.062349114	0.198029458	98.37855703
0.008054037	0.06420755	0.186485021	115.0097519
0.002512464	0.060248936	0.145722515	162.132568
0.013348305	0.092600945	0.275948426	140.1450782
0.007209448	0.070862589	0.154480977	106.7948057
0.008208435	0.087692941	0.327072576	212.5113548
0.020476911	0.075049142	0.16316731	123.5987643
0.007461253	0.060471757	0.178098929	82.80335003
0.005585275	0.060860016	0.294548164	214.1874325
0.004814594	0.070481769	0.196632508	99.23726091
0.005766479	0.06950868	0.273060316	151.4002793
0.005849009	0.076513104	0.152356096	77.72926026
0.010791426	0.071943071	0.215761754	180.2539166
0.004000368	0.069462958	0.184778977	422.4425432
0.005141372	0.06775213	0.194978683	126.2114966
0.005653402	0.066326695	0.165367056	241.819305
0.014597503	0.063843001	0.196938729	58.16481168
0.014624683	0.061458415	0.145356496	143.6484191
0.005749032	0.062230119	0.158896957	75.63347125
0.008198471	0.065433827	0.149934786	129.8339556
0.008010286	0.066079494	0.129929089	200.9500733
0.005220467	0.075611098	0.222219289	225.2873258
0.003631562	0.068796174	0.182885513	192.1575472
0.003966434	0.065380047	0.175550853	237.8954179
0.005578493	0.066726693	0.183381841	294.0213038
0.003217136	0.068958029	0.211484105	151.2871216
0.015663877	0.0667522	0.192613722	28.02412059
0.016509651	0.072420091	0.175287821	56.4081538
0.006188841	0.080028604	0.229227142	222.8037935
0.003566911	0.057409187	0.150509359	184.155381
0.005783248	0.080535383	0.206710027	236.4392167
0.008617748	0.093391202	0.354654672	114.1086719
0.017173267	0.077388382	0.263911434	106.952053
0.013306272	0.079731207	0.184850895	92.43008949
0.010127568	0.079876135	0.235970607	132.6755909
0.005591381	0.079831908	0.264779869	204.7919574
0.01349069	0.077134606	0.268219391	123.5737321
0.004392606	0.059307682	0.228890611	180.0746649
0.012537664	0.065748389	0.173083684	207.3862497
0.004544413	0.064542486	0.179857557	209.372923
0.008622021	0.085460523	0.243434424	110.9204686
0.043119237	0.082911493	0.237408763	40.1419095
0.008996871	0.070203216	0.236320788	65.49932524
0.004716327	0.076446395	0.27159992	258.0187586
0.005957724	0.10666208	0.380592415	169.8301517
0.006941651	0.069156825	0.243376086	225.0655004
0.003807979	0.070614427	0.167232241	73.23095714
0.005351221	0.063380167	0.181222146	221.9645896
0.005788836	0.058057688	0.149368485	98.33550687
0.003827929	0.063932419	0.177223657	185.9154155
0.008612096	0.088219433	0.41665033	173.6285588
0.007384583	0.077250011	0.16708347	93.5256689
0.011440181	0.10566801	0.25304281	125.0871412
0.006763133	0.06263391	0.250326377	156.9346312
0.003379719	0.071674498	0.227643895	340.6973702
0.005566608	0.071445427	0.197778061	141.5242075
0.008621078	0.065222208	0.135359996	110.4866621
0.009712136	0.071543944	0.13271775	38.18844969
0.01279703	0.064391913	0.152903283	88.67697091
0.013112359	0.065659942	0.209433893	59.81869037
0.008120179	0.0609367	0.186691418	191.0143958
0.007511766	0.077985478	0.198449331	87.03123039
0.004107809	0.063805859	0.173618959	141.1371136
0.006085957	0.050902646	0.131720107	175.6878339
0.006917738	0.08678037	0.247341084	153.5286946
0.004816381	0.067359586	0.20970672	275.4887539
0.00389046	0.073447311	0.186730903	420.7025914
0.004786708	0.057546774	0.166532298	217.9904103
0.006423138	0.062689316	0.175335562	129.036393
0.006395824	0.084276117	0.273939895	167.6565905
0.007690782	0.07528374	0.181364001	90.13527174
0.004545719	0.074492925	0.189580955	214.8456521
0.004152885	0.074805814	0.222786368	288.2449594
0.002651744	0.07367987	0.196594618	88.08112351
0.004391828	0.06167182	0.142249851	147.3097629
0.003909566	0.065349105	0.154868487	144.2927889
0.021227892	0.104076656	0.367145426	43.95532215
0.005623973	0.064022559	0.157631849	219.8958953
0.015837063	0.067864118	0.225605183	202.0862174
0.005683754	0.058833871	0.136700011	75.4448875
0.010984414	0.077499394	0.224018722	203.6195661
0.025671267	0.107961074	0.194653941	39.20318948
0.021843996	0.074502163	0.161920536	38.17518775
0.00455353	0.069403529	0.184991555	195.9751652
0.012087443	0.066173768	0.142556667	65.69854367
0.008820292	0.083920463	0.159580387	115.5634034
0.003803993	0.077686552	0.232719465	289.9703301
0.010677317	0.077557965	0.21509985	316.1621535
0.007542642	0.045404386	0.084440879	37.54485632
0.006880729	0.093849215	0.260764139	139.760468
0.030413753	0.074035838	0.191984266	27.8624694
0.021323843	0.091515772	0.296931619	67.01510397
0.010030746	0.050420991	0.076508188	54.28506362

log.sigma.4.5.mm.3D_glrlm_LongRunEmphasis	log.sigma.4.5.mm.3D_glrlm_ShortRunHighGrayLevelEmphasis	log.sigma.4.5.mm.3D_glrlm_RunLengthNonUniformity	log.sigma.4.5.mm.3D_glrlm_ShortRunEmphasis
1.426070642	220.0725108	873.7946038	0.920701438
1.485673224	286.6172426	1393.690203	0.910138156
1.383320821	212.7867799	827.1376325	0.923701789
1.500147331	134.9673537	1710.426828	0.909730037
1.668184705	125.3797996	597.3998666	0.884091955
1.61011094	112.2754551	1057.605444	0.890306243
2.051584814	70.3786975	559.0951596	0.840710043
1.514610704	287.146646	1245.876822	0.907622774
1.473809317	203.737521	1443.687324	0.916032243
1.395703671	554.6958541	2207.021146	0.922918606
1.714037912	109.176872	1107.825561	0.878385296
1.411846467	237.941093	1233.928224	0.922020769
1.804109923	161.4703969	1760.115099	0.874911695
1.44285425	117.8050842	1325.449277	0.915268254
1.466916136	234.6447741	1103.390554	0.914799028
1.669943075	327.7878949	2740.227931	0.90167597
1.51460809	342.9156916	1118.626277	0.90887817
1.6639413	239.9667986	1660.246302	0.89352223
1.409663401	251.0530783	833.1796127	0.922127658
1.565158951	206.4252385	1932.04057	0.898665638
1.497171174	330.1006168	4782.645798	0.90651573
1.502373344	285.57109	1480.102404	0.909670332
1.449151332	276.3078124	2921.740092	0.913910736
1.499647031	175.2932531	735.4707811	0.914224707
1.393655114	184.7043746	1925.525467	0.923760757
1.401888628	308.5604187	1006.342035	0.926790626
1.397213511	200.7841569	1636.654455	0.924752739
1.351166627	234.2591098	2542.768152	0.930574198
1.544339296	318.7372155	2386.270538	0.911557959
1.471145353	380.350813	2251.928159	0.915692975
1.477969385	357.3034488	2882.152355	0.909171348
1.479083154	311.2312239	3530.110979	0.913346722
1.552239926	531.2136245	1701.360998	0.900994799
1.514909963	160.6775317	330.526313	0.905356373
1.48681108	143.0073416	614.5983183	0.906327056
1.572251706	220.9298365	2168.814782	0.902781462
1.392970828	440.5408608	2653.843821	0.926395189
1.549329426	248.8771196	2272.871326	0.900311467
1.870215114	163.1805417	879.3902884	0.868203271
1.674955552	115.4290644	1031.990661	0.885909868
1.482894224	135.4286457	930.5990985	0.913428185
1.597160588	143.9121502	1276.872086	0.897379268
1.671168611	246.0651781	1931.022788	0.888829511
1.666799477	143.8905567	1214.956419	0.891312747
1.565839845	386.0705574	2385.539353	0.905510204
1.463993172	166.3867188	2518.542548	0.912346548
1.477333004	363.8219815	2584.842012	0.911569066
1.622531304	256.4442356	986.2230466	0.892747163
1.641973665	73.34459466	372.8029127	0.890648627
1.619102506	182.0512289	712.8117404	0.893569325
1.677824278	306.463569	2542.320153	0.889024475
1.918714045	229.5602295	1128.183112	0.863934091
1.612991351	203.9613547	2497.44814	0.896740282
1.449815625	393.1104368	834.5210678	0.914873196
1.450241506	273.8785642	2864.157572	0.921460309
1.39768311	296.9668337	1391.728765	0.923739878
1.457382459	458.5410079	2354.802658	0.917386289
1.981035412	171.9557617	1380.806446	0.860380113
1.459174563	238.4266887	961.2838482	0.910189737
1.677043544	122.3608674	872.869625	0.880054569
1.635927951	220.9595047	1901.415024	0.892431859
1.573884616	397.9697566	3716.008011	0.902883845
1.525495743	274.2720811	1557.388108	0.90489778
1.356470765	199.6030964	1423.000821	0.932084098
1.361446961	185.5923535	445.0420168	0.92862949
1.414357063	158.3722469	1121.988382	0.920120162
1.568717422	159.9286167	696.9969542	0.895286156
1.487311134	210.2030669	2498.070622	0.91150829
1.551170125	195.6427995	856.9071055	0.895535355
1.46603135	363.3430465	1764.894809	0.912131913
1.354464648	354.8638085	2893.579222	0.931143202
1.624192732	199.2413297	1354.213919	0.894893174
1.528482028	338.1531024	3237.051508	0.90858277
1.484564312	326.7041585	4573.462486	0.912344176
1.438660399	334.0760865	3069.869064	0.918221798
1.465653416	299.9696785	1650.763048	0.913711771
1.691246441	222.6632072	1488.727598	0.886068492
1.482725994	222.3168301	958.2992117	0.911840497
1.493640322	301.5048664	2301.695694	0.911183891
1.569276448	334.1815449	2987.313017	0.900644715
1.507278945	650.0504698	950.6307828	0.910269663
1.386001361	393.3712782	1969.773439	0.924852673
1.416962154	371.2508999	1797.239321	0.919871915
1.959018963	80.19189693	295.4619528	0.851022472
1.427827489	319.552701	2777.583735	0.917227701
1.597220491	124.3877479	2269.312854	0.893603488
1.380528132	313.3279951	1051.072027	0.923139879
1.578641095	143.771744	2031.940602	0.899036249
1.54132523	66.19636637	298.9528181	0.909536593
1.445652343	130.2751622	411.0782344	0.913338937
1.486068989	282.2019149	2249.748848	0.911270833
1.387957191	193.7120379	818.3406367	0.924295155
1.452169914	192.4952343	1089.965813	0.90916547
1.60335643	333.6102573	2851.642518	0.894702551
1.558170854	161.2665981	3162.061656	0.900885376
1.231557716	518.6931254	730.7162515	0.952033837
1.678797223	190.5193859	1108.441284	0.883726
1.509437124	89.47691563	301.8603421	0.910091577
1.787929292	81.06241484	521.9029988	0.866686052
1.226851653	328.7435652	941.2328401	0.948555564

log.sigma.4.5.mm.3D_glrlm_LongRunHighGrayLevelEmphasis	log.sigma.4.5.mm.3D_glrlm_RunPercentage	log.sigma.4.5.mm.3D_glrlm_LongRunLowGrayLevelEmphasis	log.sigma.4.5.mm.3D_glrlm_RunEntropy
322.9162466	0.891105569	0.01473076	4.861681321
452.3292426	0.878384615	0.007535107	4.624549531
326.8522894	0.898720242	0.011000301	4.591722343
212.3312354	0.876979523	0.019751888	4.606114526
206.3891901	0.844370593	0.027489285	4.565826063
192.1314736	0.854043393	0.024145614	4.49945858
132.2706752	0.788006695	0.066813411	4.428986973
461.462736	0.873897294	0.00830676	4.839360871
309.1026479	0.883306136	0.011533676	4.701195437
842.3345876	0.896052665	0.003279194	4.791256729
200.4119549	0.837256822	0.022137184	4.391853832
363.0008792	0.894133759	0.009474733	4.581153989
311.0543831	0.826640754	0.014101629	4.56599062
186.668745	0.886271425	0.027546617	4.477211621
363.617247	0.884466309	0.010200944	4.840422278
564.1732387	0.855963457	0.008970233	4.927739092
580.2546555	0.87620417	0.006348205	4.710317371
414.8590087	0.851266013	0.009540741	4.722647075
380.9505172	0.894230769	0.007730078	4.410441159
345.9752793	0.863309734	0.015845801	4.706205006
547.2791911	0.874964029	0.005652827	4.728257233
471.2448427	0.87814166	0.007172997	4.717635187
430.5328469	0.884475389	0.007844017	4.74742707
274.2122924	0.88304705	0.019862371	4.674887421
283.798582	0.897790382	0.020527347	4.709897806
448.1848997	0.898668639	0.007515128	4.7664496
303.9769407	0.897401106	0.010777444	4.620432625
348.7161019	0.906339562	0.010343904	4.612622965
538.7135438	0.87571005	0.007152558	4.6301319
614.3528328	0.883775294	0.004885991	4.712945743
588.8929821	0.878431081	0.005447253	4.786203293
497.5290854	0.881061538	0.008038962	4.741009436
896.1461615	0.866182415	0.004488461	4.799946815
242.2495503	0.87254118	0.022021877	4.591447532
239.0289417	0.876992377	0.021475462	4.560638356
377.5040647	0.865043877	0.009159845	4.522041995
663.3955215	0.898635688	0.004636212	4.907020466
429.0697331	0.865503709	0.008212412	4.538273741
327.3885434	0.821324902	0.014834867	4.49494802
192.8295437	0.844414143	0.028089961	4.616571549
213.6865346	0.880699088	0.018268368	4.393902539
236.8933276	0.859571588	0.016073786	4.475076867
443.9042931	0.846763742	0.008728654	4.550369403
239.8255199	0.849542708	0.021296035	4.633774752
666.7995902	0.868191625	0.006114473	4.973677489
272.8519287	0.882173211	0.017556868	4.688705855
580.7448663	0.879878125	0.00640411	4.816381516
484.6741429	0.855236452	0.011602686	4.572149599
118.2013847	0.856655756	0.074953573	4.330623086
296.1442147	0.855116443	0.014105957	4.697981241
552.6639618	0.846482082	0.007399692	4.698100038
492.7226138	0.812450842	0.010322686	4.411811553
342.4468706	0.856830291	0.011146684	4.71784601
626.1103186	0.885633581	0.005004552	4.576763049
427.8916052	0.889451164	0.007396924	4.712825265
440.0538054	0.896960561	0.007629435	4.852542968
716.4395729	0.886527318	0.005053421	4.793834628
336.2972645	0.806585273	0.016768287	4.712008712
379.1148272	0.882092397	0.009801882	4.529177169
235.1187088	0.84226663	0.017387467	4.172950674
375.9689604	0.851829571	0.010986657	4.812848696
690.0048551	0.865822714	0.004899673	4.745670039
460.9825471	0.871794872	0.007773007	4.669766042
300.4235762	0.90672871	0.010884483	4.539967566
273.2313533	0.904432855	0.012126757	4.416256598
226.6195413	0.89226781	0.018326918	4.662893856
246.6717088	0.860622364	0.020384301	4.795828186
333.7932564	0.878985288	0.011590593	4.810595198
341.555068	0.863466	0.010685326	4.500690198
567.4424115	0.881930594	0.005670278	4.809367076
519.1933815	0.906371202	0.007895642	4.981542561
341.6805536	0.857164185	0.01071976	4.487193305
549.7314812	0.8737074	0.007109847	4.774332806
539.2886468	0.879694718	0.00547458	4.615588676
507.8838964	0.888640219	0.006549698	4.919260864
474.1435679	0.882783883	0.008735222	4.812769055
422.7864782	0.843932713	0.009663244	4.556226353
356.5437427	0.880794702	0.010542315	4.53239696
499.9295148	0.879660937	0.006274061	4.629010185
586.9818248	0.864862064	0.005951931	4.724063811
1072.826567	0.876764938	0.003422106	4.749162123
594.6184243	0.898786949	0.005605319	4.825914022
561.1793861	0.89223307	0.005213811	4.7356889
153.8989005	0.805706722	0.041460126	4.187263213
500.9948127	0.889335936	0.007523568	4.760512388
207.7633046	0.857003676	0.025046163	4.715795042
462.5291878	0.897866839	0.007271322	4.839054352
243.5116608	0.863065295	0.016627542	4.5252704
117.401886	0.883211679	0.037067992	3.765903412
207.5011134	0.886212404	0.028818876	4.45000185
441.5591072	0.879045588	0.006686219	4.623167999
291.4507577	0.898433693	0.01465713	4.603921451
315.4456988	0.881512853	0.011550789	4.423040715
592.418109	0.857110039	0.005742882	4.68234301
272.6261419	0.865948544	0.015910806	4.568986589
692.5408289	0.93499826	0.008457726	5.052181527
346.9575216	0.84419041	0.0108883	4.386189326
133.0814316	0.878864127	0.04754118	4.383589465
147.58531	0.823717949	0.037301949	4.398198951
436.0917066	0.932942274	0.01172688	4.927308327

log.sigma.4.5.mm.3D_glrlm_HighGrayLevelRunEmphasis	log.sigma.4.5.mm.3D_glrlm_RunLengthNonUniformityNormalized	log.sigma.4.5.mm.3D_glszm_GrayLevelVariance
236.6935432	0.813887433	30.85630308
312.9602294	0.791860508	23.91767986
231.593444	0.820897729	23.2813143
147.1167797	0.79235302	21.67592719
138.0531393	0.742164261	19.10613368
124.5300643	0.7532771	22.28149495
79.31369883	0.665292797	10.59639922
314.336132	0.787750744	30.13742651
220.3253511	0.804676865	24.19990197
601.8013383	0.818660293	30.13859554
122.9290128	0.72996958	14.89744098
257.9178554	0.81705134	23.94123893
182.1852917	0.724245035	21.51980975
128.9279022	0.80290847	17.85234489
254.9630529	0.802856265	32.18145189
359.9755946	0.77665365	32.14717609
378.6835909	0.791482603	32.83534219
265.2510046	0.75995058	22.87573633
271.9587499	0.817891304	18.65297357
228.0385797	0.769335016	24.47349516
364.6398668	0.784699776	25.95754538
313.8972826	0.791884412	28.23251064
301.5161495	0.799866443	28.23845582
190.0586909	0.803343648	23.8262771
200.8659864	0.8211303	22.93150845
330.9274666	0.82688525	34.5989
217.3168085	0.822995896	24.29991654
252.9984919	0.834852786	22.48084865
349.7247115	0.797492498	32.73835424
416.2160784	0.804357842	33.43839507
394.0659119	0.790225943	30.23952643
340.3448141	0.799233922	25.33239022
587.1718196	0.773972761	41.88028253
174.1942959	0.784593137	22.18252837
158.5382456	0.78570993	20.6041794
244.0276877	0.777583217	21.62513084
475.8940123	0.826116583	39.96831334
276.7628024	0.77245195	22.7271643
185.4774583	0.714801173	21.40081194
127.3728164	0.74487937	20.17206951
147.6706486	0.800302221	16.74433107
158.2749591	0.766857586	16.92236264
274.8758483	0.750717751	21.46204555
158.1248664	0.755930599	25.08467182
426.8227351	0.783816546	38.41980511
183.3381722	0.79672508	20.36659808
398.2442309	0.795245263	31.05947805
289.9229456	0.757932972	29.14782485
80.25078766	0.761273535	11.02190463
199.25089	0.760953261	25.39270833
342.085065	0.751202745	29.67560554
264.1231899	0.705353686	25.63459227
224.8526584	0.765763587	22.78670835
430.3902325	0.802371236	22.53093434
296.8781227	0.816279299	25.32425111
320.0112971	0.820224566	34.22987769
498.4751799	0.807757998	35.93506499
193.8832823	0.698911733	24.90917595
261.7577228	0.792200722	23.15764579
139.5055309	0.734194847	12.05484087
244.6117964	0.757105004	21.43784352
440.570702	0.779005561	33.6832483
303.1228796	0.783054224	24.31439715
215.5629011	0.83852228	20.27396068
200.2399208	0.831607602	19.05139414
169.8982875	0.813083228	23.26940432
174.0382365	0.76302169	27.67402742
229.5104482	0.795229335	27.12929781
218.642205	0.764176683	16.81456154
396.3365194	0.79630434	31.70258032
381.4936782	0.836546523	36.32071231
220.6269765	0.762918099	22.63042874
370.0400012	0.78958816	25.81165169
359.2912014	0.796844219	21.79157073
361.8035146	0.808872654	36.40414189
327.5376208	0.800042539	34.0927336
251.2762745	0.745790408	23.39741628
243.4123746	0.797090626	20.63719497
331.6387254	0.795495682	25.72241488
371.7425873	0.773354321	30.71548815
714.3856281	0.793389691	47.42416241
426.0389075	0.822955103	35.47949938
402.1385984	0.812338492	31.21402576
91.00042563	0.690522072	9.857568027
348.9024591	0.806829699	27.51138765
137.4766269	0.75969524	20.162387
338.7694641	0.818600843	34.34267079
158.7554491	0.770695991	18.20527867
73.69185197	0.805674305	6.459829868
142.9321547	0.799441711	18.7307526
307.5045514	0.794944095	22.86422031
209.7571391	0.822098754	26.21765175
212.8537391	0.789828936	18.96527912
372.3801136	0.761882214	28.96469184
178.4173771	0.773635896	19.08714211
548.4413087	0.882730772	55.44050244
213.7993914	0.741307609	18.04606601
96.7477121	0.796063801	13.86031746
91.09385382	0.709900201	15.25163834
348.5968087	0.873615867	40.15542445

log.sigma.4.5.mm.3D_glszm_SmallAreaHighGrayLevelEmphasis	log.sigma.4.5.mm.3D_glszm_GrayLevelNonUniformityNormalized	log.sigma.4.5.mm.3D_glszm_SizeZoneNonUniformityNormalized
153.7917263	0.054057843	0.33610399
161.6723064	0.060612081	0.262413075
106.6933584	0.058942732	0.251744709
80.31715491	0.060105184	0.272274776
85.51004534	0.066993258	0.28400798
71.21400932	0.063801991	0.249346798
52.89499835	0.098724048	0.255730969
173.0938828	0.052873903	0.294531397
120.3432386	0.058374669	0.318451132
306.8174163	0.052348735	0.266492041
55.8122622	0.074918998	0.216469395
138.3636653	0.057911614	0.295037057
103.2717868	0.059912802	0.267728894
63.11448656	0.066339556	0.244814222
134.1041786	0.050777226	0.303083721
200.4012406	0.049752001	0.289335382
176.7777552	0.050648681	0.266764531
148.0660613	0.060883143	0.273185893
158.0218275	0.069160559	0.324651325
111.605599	0.057968398	0.251586901
172.9595756	0.056383083	0.265193661
169.3102637	0.055555556	0.277178089
159.8480261	0.053042548	0.251516008
108.1783365	0.060725538	0.24305711
96.09847668	0.057384815	0.255223853
193.4698914	0.0480875	0.3148125
119.8903148	0.057372807	0.310931111
114.9703257	0.059543278	0.277121542
173.8253991	0.050860727	0.313715398
218.0828959	0.048777047	0.309574036
194.3439706	0.052042267	0.277780564
181.2993004	0.056352485	0.29073613
336.1510356	0.049127036	0.312258017
89.24712447	0.061194122	0.274722549
72.62139051	0.06157743	0.258406848
118.0301	0.060233717	0.245184271
235.098959	0.044842823	0.289791693
138.5348106	0.060544868	0.281125636
84.53432177	0.061506059	0.2040536
82.17122597	0.063654033	0.289300754
74.40015581	0.068142903	0.301471609
83.52464025	0.068936129	0.239665262
130.4770947	0.064142506	0.240502014
105.0083994	0.05563267	0.313900456
203.7199022	0.045164403	0.2884253
94.28170045	0.061963392	0.263305334
225.3239926	0.052287434	0.300704673
127.3946563	0.054353863	0.258184407
46.3208612	0.090922149	0.285119219
136.05616	0.0575	0.322847222
177.1905975	0.053184673	0.269522406
107.1394473	0.059356509	0.194156805
138.0482856	0.060025271	0.287715072
207.1939212	0.062213039	0.2899736
158.1090174	0.054881736	0.292424816
177.7167171	0.047819972	0.266695152
271.180605	0.049795328	0.329373573
116.0014419	0.060264758	0.270319031
115.5179223	0.061306101	0.243020693
48.50264347	0.080160619	0.179803688
130.1447749	0.060594638	0.320937979
224.8775471	0.050906242	0.289351852
169.7935397	0.059326457	0.299333469
89.93638299	0.061798716	0.270827015
97.67590797	0.066280718	0.371632798
115.3701896	0.060326121	0.293625698
130.8963632	0.056760204	0.281529018
131.3977118	0.053510344	0.302644339
87.60416844	0.071517782	0.224258308
210.5073161	0.050572675	0.262867182
201.5664286	0.046180134	0.305468418
117.1615913	0.06041465	0.269963289
201.6582268	0.056307263	0.290125772
166.4638722	0.061137691	0.255487466
225.0602816	0.048395368	0.308543965
169.6285438	0.050998504	0.29024124
116.8904437	0.059981698	0.270096371
102.8474666	0.063523889	0.213580126
156.4132475	0.056888641	0.252661742
171.4831852	0.050256326	0.258370364
370.1036607	0.044638266	0.297812587
217.5679671	0.047888238	0.30079776
232.2081268	0.051755132	0.295211084
36.9057219	0.094671202	0.235260771
177.2807802	0.05522716	0.284903704
74.78595168	0.061950294	0.282617778
190.6700339	0.049051767	0.286817377
79.58080597	0.065945706	0.256571745
32.33793553	0.113185255	0.327032136
65.32253509	0.067041522	0.247945502
179.6303803	0.059930743	0.298834343
99.11476517	0.05563506	0.269614113
82.84029178	0.065911809	0.212588249
179.9803451	0.052130612	0.234510204
93.54434028	0.063589468	0.27844564
289.0359288	0.038354235	0.386863641
114.5054082	0.07012302	0.280057232
46.59287019	0.079002268	0.266938776
36.67857328	0.071767276	0.180457137
181.2640462	0.044677089	0.325396964

log.sigma.4.5.mm.3D_glszm_SizeZoneNonUniformity	log.sigma.4.5.mm.3D_glszm_GrayLevelNonUniformity	log.sigma.4.5.mm.3D_glszm_LargeAreaEmphasis	log.sigma.4.5.mm.3D_glszm_ZoneVariance
106.2088608	17.08227848	102.4272152	87.93425533
103.6531646	23.94177215	292.1518987	266.5149816
78.2926045	18.33118971	46.23794212	33.31495745
131.7809917	29.09090909	293.7541322	268.0676653
56.51758794	13.33165829	195.1507538	172.4088786
59.34453782	15.18487395	450.1092437	402.7424617
34.77941176	13.42647059	724.1544118	663.7495134
113.6891192	20.40932642	208.5880829	186.7215563
160.8178218	29.47920792	144.1207921	128.0097089
204.9323797	40.25617685	79.84005202	64.59046843
46.10798122	15.95774648	792.0798122	720.1088849
128.0460829	25.13364055	90.21889401	75.14517722
116.462069	26.06206897	836.0206897	790.6829119
91.80533333	24.87733333	157.2213333	132.6726116
126.6889952	21.22488038	68.37799043	54.66319452
271.3965885	46.6673774	223.2761194	204.0864892
94.70140845	17.98028169	140.0366197	119.5960325
122.114094	27.2147651	394.0357942	361.2876998
91.87632509	19.57243816	77.76678445	61.65351047
131.0767754	30.20153551	455.2034549	424.1992919
384.7960028	81.81185389	228.4231564	205.4809711
128.0562771	25.66666667	114.1450216	93.06857536
233.1553398	49.17044229	128.5361381	108.7734616
58.57676349	14.63485477	85.36929461	67.10342453
176.8701299	39.76767677	54.83982684	40.72045127
125.925	19.235	63.57	52.1456
172.8776978	31.89928058	74.88309353	59.0695972
263.5425868	56.6256572	59.20399579	46.75816148
213.3264706	34.58529412	304.8191176	279.8338214
237.4432855	37.41199478	168.1003911	151.1371622
238.6135041	44.70430733	153.5227008	130.272117
327.9503546	63.56560284	228.8865248	209.2383683
146.1367521	22.99145299	439.715812	410.4680537
34.88976378	7.771653543	59.58267717	45.23826648
55.29906542	13.17757009	100.7570093	83.53838763
124.0632411	30.47826087	430.8754941	390.4550493
257.9146067	39.91011236	102.5629213	86.48192021
163.6151203	35.2371134	380.242268	346.2946263
36.93370166	11.13259669	599.9337017	532.711822
85.05442177	18.71428571	332.2108844	301.2458235
88.63265306	20.03401361	147.8231293	127.7868481
84.12250712	24.1965812	264.8091168	234.51209
92.11227154	24.56657963	764.6083551	702.1447143
115.8292683	20.52845528	363.1111111	337.0430299
230.4518148	36.08635795	192.5607009	173.4378674
181.1540698	42.63081395	164.1758721	137.1751601
268.8299776	46.74496644	173.163311	156.1638315
68.41886792	14.40377358	319.9849057	287.2578711
33.6440678	10.72881356	147.2033898	124.3582304
77.48333333	13.8	203.3166667	182.6899306
164.9477124	32.54901961	1061.071895	1018.694348
40.38461538	12.34615385	1401.865385	1313.433062
167.4501718	34.9347079	777.2319588	734.6686653
76.5530303	16.42424242	74.92045455	55.24585342
263.767184	49.50332594	153.0898004	134.0388014
126.4135021	22.66666667	81.75949367	65.89422991
277.6619217	41.97746145	96.58244365	81.45283537
99.20708447	22.11716621	900.2179837	856.304791
74.12131148	18.69836066	125.6655738	105.4303252
29.48780488	13.14634146	471.9817073	398.5869646
187.7487179	35.44786325	265.4102564	240.1360041
291.6666667	51.31349206	793.8541667	764.2229928
149.9660679	29.72255489	108.6646707	88.11717882
129.1844864	29.47798742	62.15932914	46.82315661
68.38043478	12.19565217	39.5	29.21821834
111.8713911	22.98425197	137.8818898	121.4804527
63.0625	12.71428571	161.3303571	139.021604
215.7854137	38.15287518	249.3969144	224.3828872
49.56108597	15.80542986	144.1674208	109.9369792
146.9427549	28.27012522	126.8604651	106.7470854
350.3722755	52.96861378	47.67741935	36.65525091
87.19814241	19.51393189	670.1826625	629.4679907
296.2184133	57.48971596	186.8393732	165.8376656
316.0379951	75.62732417	302.7978981	275.0930361
317.4917396	49.79883382	123.9844509	106.8292369
159.3424408	27.99817851	170.3515483	152.3238675
101.0160428	22.43315508	417.8850267	378.2354443
61.08391608	18.16783217	123.6678322	101.0887207
169.7886905	38.22916667	183.2440476	159.4784226
227.6242906	44.27582293	514.6935301	489.1457262
94.70440252	14.19496855	172.7358491	154.3915984
218.9807692	34.86263736	76.58379121	63.26329663
172.1080617	30.17324185	98.22984563	80.22197835
19.76190476	7.952380952	244.0833333	205.3178855
256.4133333	49.70444444	86.76666667	68.3530321
173.2446982	37.97553018	288.4665579	256.3492837
113.8664987	19.47355164	54.44836272	41.51011681
121.8715789	31.32421053	596.2926316	555.2517496
30.08695652	10.41304348	80.72826087	60.7706758
33.72058824	9.117647059	73.38235294	55.31985294
197.5295008	39.61422088	265.9258699	242.3277343
75.76156584	15.63345196	73.98220641	58.5185598
68.45341615	21.22360248	151.3043478	127.7728097
164.1571429	36.49142857	576.9614286	538.290798
235.5650118	53.79669031	282.643026	251.699976
150.1030928	14.8814433	14.80927835	9.618397279
74.7752809	18.72284644	642.588015	598.9884554
28.02857143	8.295238095	69.18095238	52.56562358
24.72262774	9.832116788	294.4379562	252.4248495
155.539749	21.35564854	13.66945607	7.840969171

log.sigma.4.5.mm.3D_glszm_ZonePercentage	log.sigma.4.5.mm.3D_glszm_LargeAreaLowGrayLevelEmphasis	log.sigma.4.5.mm.3D_glszm_LargeAreaHighGrayLevelEmphasis	log.sigma.4.5.mm.3D_glszm_HighGrayLevelZoneEmphasis
0.262676642	0.852068152	16104.8481	260.1487342
0.1975	1.324224325	68858.59494	324.8303797
0.278175313	0.236176034	12029.44695	215.2508039
0.197309417	3.336821722	29878.70661	160.0785124
0.209694415	2.83995367	15583.92965	167.6030151
0.145299145	5.551837246	41108.60504	143.1890756
0.128666036	27.01873058	25155.40441	102.3897059
0.213850416	0.954532459	49998.6114	328.1917098
0.249136655	1.154239615	21298.42178	220.4772277
0.256077256	0.150499735	45823.25358	591.0091027
0.117874931	9.401149691	74926.84038	124.7042254
0.257566766	0.412717827	22947.01843	255.3456221
0.148514851	6.17544571	117852.6161	200.7103448
0.201829925	1.834096568	18088.26133	126.9573333
0.27002584	0.437306487	13246.86842	257.7559809
0.228279387	0.873258662	65335.04691	367.7633262
0.221183801	0.399508248	52613.56338	352.3887324
0.174745895	2.344672158	74107.59955	285.0693512
0.249119718	0.365685291	19290.61484	271.8339223
0.179593244	2.807500081	81664.06718	233.1401152
0.208776978	0.713330958	78864.4397	339.9627843
0.217821782	0.448266203	33502.05844	320.6277056
0.224945402	0.533665832	34507.45092	312.5264293
0.233980583	0.799234419	12520.77593	209.6224066
0.266129032	0.535949011	12210.75036	190.0981241
0.295857988	0.277473288	16677.4	347.535
0.251469923	0.465852492	15114.28597	215.8435252
0.283457526	0.271530722	16791.1346	228.4984227
0.200058841	0.985456758	99569.28971	329.0338235
0.242798354	0.425830631	69273.94524	395.6284224
0.207387735	0.43158532	61158.54482	367.6181607
0.2256	0.942901697	62998.55053	339.0744681
0.184907151	0.899723912	224215.6838	600.6773504
0.264033264	0.71798652	6852.133858	180.1102362
0.240990991	0.877629901	14376.47196	149.6495327
0.1572894	2.211479141	89858.3419	239.916996
0.249369571	0.250731913	46373.96292	456.1741573
0.171630787	1.537829548	98959.96907	262.2852234
0.121967655	4.227050429	90673.62983	194.0110497
0.179706601	5.504559596	24113.85374	155.4115646
0.223404255	1.41257171	17983.44558	143.5748299
0.181677019	2.664000557	29196.83761	175.0655271
0.126527915	3.896245495	162621.2037	272.308094
0.195859873	3.928008495	36879.60163	189.6747967
0.228677733	0.506527197	80613.1164	389.0325407
0.192447552	1.200513614	32615.21802	177.8909884
0.242539338	0.538178064	60930.87919	403.9295302
0.174802111	1.136804066	98363.0717	251.6641509
0.209219858	5.507152917	6504.70339	90.95762712
0.220183486	1.784827652	25688.9875	239.2833333
0.153614458	4.022467572	288559.5441	352.6176471
0.106339468	5.969574062	338606.9904	265.7355769
0.153278904	5.886906671	111746.4828	260.5927835
0.225448335	0.208908274	30305.27273	406.0378788
0.229108458	0.6609533	40625.90022	292.8026608
0.251059322	0.336212875	24145.94937	338.4894515
0.257090576	0.237541481	43456.78292	483.3855279
0.150904605	7.49215595	113381.951	234.7247956
0.222303207	0.57568214	30456.51148	253.6
0.116725979	3.87335336	62622.43902	122.1768293
0.198911935	1.898467289	45797.2547	244.7487179
0.183706944	2.121321119	305114.5119	421.8670635
0.220607662	0.447818927	30166.6507	305.6966068
0.255353319	0.365982098	14456.38994	185.8574423
0.311864407	0.274126439	7498.304348	176.6630435
0.246921581	1.853191154	12854.69291	210.2624672
0.211720227	2.0153395	16278.51339	230.9910714
0.199943915	1.820414297	40730.82328	241.3225806
0.17092034	0.755387128	33353.86878	197.0542986
0.222975668	0.40637715	43189.13596	409.6189624
0.301207983	0.197927308	18585.74106	365.1089799
0.156720039	4.170493709	112485.7647	242.8266254
0.218209019	0.718967724	56070.6856	382.1743389
0.189986177	0.864780069	112131.6653	329.0751819
0.241435946	0.476690988	36737.12536	391.3731778
0.235521236	0.709268121	46596.87978	322.6593807
0.15881104	1.890306034	98301.82353	227.184492
0.210448859	0.722579268	24382.51399	239.5944056
0.205128205	0.575830159	62258.37946	311.6443452
0.19784415	1.500553191	181978.9489	344.4597049
0.233480176	0.261274315	118119.1918	699.9213836
0.273993225	0.202787392	32565.39835	400.9299451
0.235650768	0.311833123	34092.80274	417.9468268
0.160611855	5.323572388	13616.16667	92.79761905
0.233039876	0.315551288	29885.96889	334.7733333
0.176453656	4.363118097	27202.92822	143.7601958
0.278011204	0.207625447	16875.7733	345.3274559
0.156095958	5.838258937	67864.59368	168.7452632
0.223844282	1.773395027	6095.293478	61.41304348
0.235294118	0.886393434	9756.375	134.9264706
0.205854874	1.312607371	57999.27534	329.0877458
0.254298643	0.489838912	13583.80071	206.7900356
0.206145967	0.753210835	33457.11491	190.5590062
0.160808638	1.747542117	195906.3471	373.9157143
0.179770506	2.079976722	44874.48227	180.9420804
0.438914027	0.049818444	9412.458763	484.0798969
0.151446398	3.875097758	110872.161	223.1685393
0.245327103	2.322969578	3667.390476	107.3047619
0.154279279	5.878203413	17656.21898	102.8175182
0.414211438	0.078413541	5305.937238	322.6485356

log.sigma.4.5.mm.3D_glszm_SmallAreaEmphasis	log.sigma.4.5.mm.3D_glszm_LowGrayLevelZoneEmphasis	log.sigma.4.5.mm.3D_glszm_ZoneEntropy	log.sigma.4.5.mm.3D_glszm_SmallAreaLowGrayLevelEmphasis
0.600802858	0.01315124	6.248117083	0.00592851
0.525403373	0.008205934	6.422742064	0.005442553
0.507853682	0.012784987	6.431690669	0.008059677
0.529565027	0.018284301	6.407820773	0.010593965
0.543800148	0.019413109	5.849007144	0.008057234
0.512993217	0.023048228	6.2672115	0.012445903
0.521092389	0.029027725	5.472968387	0.012452524
0.560651836	0.009347828	6.433148424	0.006523706
0.58489908	0.01116534	6.349894701	0.007990368
0.529725127	0.003699563	6.788617674	0.002567964
0.475945452	0.021971473	6.188365255	0.013161559
0.559696646	0.00958809	6.423792117	0.0050704
0.528027782	0.012082962	6.461431828	0.007845756
0.507527251	0.028350768	6.413920345	0.014484487
0.567520402	0.010954052	6.48144499	0.00818873
0.553120706	0.006493279	6.800759705	0.003137644
0.527861424	0.009347412	6.602795342	0.007184256
0.536042962	0.008140818	6.495078375	0.005651995
0.590831385	0.008990629	6.049549479	0.006823339
0.51546787	0.013299463	6.648721672	0.004800191
0.529196251	0.005717102	6.918888267	0.003594331
0.543276946	0.007443967	6.623792257	0.005006875
0.514428685	0.007019113	6.954242394	0.003480958
0.503004198	0.020068918	6.340136125	0.010114527
0.513532202	0.015214666	6.654185796	0.007393553
0.574745658	0.008403036	6.474548599	0.006078694
0.577701793	0.012058529	6.431020284	0.008753707
0.541026083	0.009871673	6.647631298	0.005185562
0.580090229	0.010326665	6.646997528	0.008220324
0.573560317	0.005867908	6.675760659	0.004426759
0.543597452	0.005976442	6.829658812	0.00387078
0.555702925	0.005674952	6.697380677	0.002586346
0.579776285	0.005791155	6.70148972	0.004194605
0.535947139	0.022644469	5.835774132	0.012252493
0.517835044	0.022207394	6.1483882	0.010043763
0.501405697	0.00925921	6.587541865	0.004521221
0.555015281	0.005420143	6.911036296	0.003769431
0.549050543	0.009397076	6.609652064	0.005370247
0.457684958	0.017901081	6.408933164	0.013079735
0.556183877	0.022698558	6.162397445	0.015228156
0.568068158	0.021658849	6.008906703	0.015600155
0.501929183	0.01366774	6.451467648	0.009190516
0.503839831	0.012297729	6.585060532	0.009732808
0.579978047	0.018985022	6.304171989	0.011733588
0.552946015	0.006647716	6.88321923	0.004312926
0.526648291	0.014656617	6.623988929	0.005872286
0.566765444	0.005584489	6.744605429	0.002731977
0.518431433	0.016639425	6.419558399	0.008796297
0.549845535	0.034507632	5.341235916	0.016196555
0.587759652	0.012451777	6.070215177	0.009347964
0.534834626	0.007182711	6.764277807	0.003797269
0.437049843	0.012915508	6.405741084	0.008832755
0.554437257	0.008776037	6.512629175	0.00560937
0.556722225	0.007157743	6.212372275	0.005880301
0.557773021	0.007037426	6.661960811	0.004441112
0.52793324	0.008345764	6.730194761	0.005565501
0.594399324	0.006403593	6.701204974	0.004788064
0.534754228	0.011630851	6.415221992	0.007304573
0.500536431	0.011274464	6.348858632	0.005213928
0.420597817	0.020619763	6.152649642	0.008048602
0.586559149	0.009084028	6.335164185	0.006283875
0.554416739	0.005572739	6.801807238	0.00374121
0.566643269	0.008128679	6.459226734	0.005160336
0.534172115	0.013448258	6.43676992	0.009519331
0.632743939	0.015645889	5.633497329	0.012924107
0.557178912	0.011968611	6.271672631	0.00564005
0.545111173	0.015351662	6.181678986	0.009975829
0.569878334	0.01129062	6.596578228	0.007573175
0.486580836	0.013843472	6.309561298	0.009945449
0.527016094	0.006056	6.801929657	0.003988257
0.568774294	0.008039434	6.805007232	0.005477774
0.533574853	0.011520546	6.33318852	0.008648707
0.555907917	0.005164681	6.685954739	0.002497726
0.517722957	0.00543981	6.825024291	0.003175995
0.574249709	0.006320235	6.829204678	0.00406178
0.549433765	0.00851644	6.624413388	0.004055057
0.533684577	0.011942113	6.440904473	0.008011436
0.465858993	0.01214306	6.42211686	0.007568027
0.512744943	0.006923387	6.732528728	0.004495927
0.521060515	0.006323864	6.913999421	0.00397167
0.560849148	0.005563927	6.614548677	0.004685387
0.56405884	0.00607169	6.692460538	0.003168385
0.561992312	0.006019902	6.666302416	0.004366846
0.496588752	0.030661376	5.344815269	0.015470418
0.55022403	0.008000284	6.77159084	0.004671325
0.549952287	0.020659834	6.498991175	0.01196634
0.550250936	0.008187318	6.52723111	0.005544095
0.521256896	0.016354248	6.482729579	0.010869771
0.590208143	0.041232992	4.866667519	0.032400154
0.489495616	0.026395958	5.797468794	0.010848163
0.564795786	0.005957524	6.539290494	0.004205111
0.527235455	0.024964212	6.33593949	0.02117842
0.460774443	0.013436066	6.509547993	0.005638109
0.493746006	0.005685525	6.921671815	0.003492404
0.545683557	0.01427549	6.622304973	0.00862604
0.645291979	0.010807359	6.427365346	0.008675519
0.545592121	0.01173882	6.072407363	0.00882515
0.527755593	0.040764204	5.528497961	0.03219084
0.407875214	0.030532859	6.072882652	0.019308723
0.582427585	0.013148787	6.502620699	0.009053134

log.sigma.4.5.mm.3D_ngtdm_Coarseness	log.sigma.4.5.mm.3D_ngtdm_Complexity	log.sigma.4.5.mm.3D_ngtdm_Strength	log.sigma.4.5.mm.3D_ngtdm_Busyness	log.sigma.4.5.mm.3D_ngtdm_Contrast
0.011749571	0.366593921	2.862536046	0.244946652	8.65E-05
0.006299126	0.238948572	2.327661777	0.269254496	2.45E-05
0.011763682	0.401037399	3.595942136	0.167421119	5.32E-05
0.00550276	0.120275678	1.249816798	0.655800221	2.55E-05
0.013920054	0.196556935	2.14336276	0.368816666	9.02E-05
0.008248599	0.11683048	1.471633857	0.547261138	3.71E-05
0.013998001	0.08678233	1.51587041	0.652011731	7.15E-05
0.008261921	0.278934248	3.191584989	0.203746234	2.95E-05
0.006256431	0.215085864	1.724979171	0.422988833	3.56E-05
0.004657517	0.24746024	2.583729377	0.215707138	1.67E-05
0.007070988	0.086101369	1.003035675	0.675736235	2.86E-05
0.007510338	0.272573182	2.256581222	0.255491623	3.18E-05
0.004659341	0.085284771	1.129421617	0.570649455	1.27E-05
0.006973089	0.105341903	0.983915419	0.670328562	4.17E-05
0.00796531	0.39380207	2.65773776	0.274619497	5.55E-05
0.003983915	0.126378494	1.594369546	0.366265945	1.23E-05
0.009498161	0.301258609	3.900754868	0.139933063	2.66E-05
0.004968749	0.18838398	1.855561048	0.391205395	1.93E-05
0.009561413	0.296064981	2.011567321	0.258324984	6.09E-05
0.004956548	0.110378068	1.328689698	0.469815166	1.87E-05
0.002117565	0.09437724	1.126457107	0.53301007	4.74E-06
0.006529937	0.261728152	2.973469103	0.225352091	1.98E-05
0.003417745	0.134736181	1.468841752	0.453919269	1.10E-05
0.014550582	0.223755839	2.558762733	0.267741144	9.15E-05
0.005648314	0.113904485	1.139524983	0.523430052	3.50E-05
0.008684833	0.475953061	3.324444787	0.189279976	5.45E-05
0.006581063	0.141195892	1.579130338	0.458247254	3.06E-05
0.003562687	0.136268606	0.987667701	0.575610744	2.11E-05
0.003792921	0.148571948	1.506302212	0.371008028	1.35E-05
0.004513374	0.237428658	2.517992377	0.227381655	1.12E-05
0.003819841	0.101611481	1.364921872	0.399259776	1.27E-05
0.002531504	0.122453472	1.106156295	0.562336535	9.72E-06
0.005921082	0.28261158	4.0917053	0.14813796	1.48E-05
0.026288871	0.579480265	4.256576862	0.155695526	0.000217809
0.014222513	0.257857291	2.202882346	0.279499262	9.72E-05
0.004066972	0.090096664	0.943894864	0.588159475	1.51E-05
0.003837611	0.247637234	2.206240083	0.270466665	1.56E-05
0.0038032	0.102300755	1.13428677	0.497183726	1.25E-05
0.009924081	0.139062239	2.236221713	0.272378503	2.25E-05
0.007963607	0.140553242	1.635301386	0.574545694	4.10E-05
0.008389907	0.16972623	1.226802891	0.518769482	5.83E-05
0.006587398	0.126259337	1.197784589	0.555872611	2.89E-05
0.004505596	0.111770069	1.334343799	0.449325264	1.32E-05
0.006887169	0.142857895	1.374910111	0.54156088	3.77E-05
0.004375305	0.191900909	1.755625149	0.283746965	1.92E-05
0.003949371	0.087766881	0.93916285	0.730569245	1.78E-05
0.00379014	0.176734241	1.884586969	0.31682829	1.22E-05
0.009251385	0.224821221	2.98271319	0.176113599	2.98E-05
0.021591542	0.177703928	1.803868859	0.558410792	0.000217984
0.013095535	0.282544045	3.187573407	0.227477508	5.84E-05
0.003815041	0.105823754	1.54682996	0.384665232	8.70E-06
0.006842244	0.134508042	2.145033032	0.265561994	1.36E-05
0.003848583	0.104748315	1.206318134	0.592777087	1.28E-05
0.011035756	0.352982429	3.190082353	0.157069144	5.45E-05
0.003390811	0.117615312	1.027492759	0.577377807	1.59E-05
0.008166722	0.282493436	2.865994537	0.208616486	3.44E-05
0.004086593	0.234489217	2.260487045	0.251736798	1.47E-05
0.005337476	0.144539841	1.767202201	0.438509163	1.89E-05
0.009539186	0.243222567	2.42638894	0.230607074	4.06E-05
0.008717936	0.085620553	1.109306208	0.497561033	2.95E-05
0.004770841	0.1258136	1.200147943	0.561758111	2.34E-05
0.002536626	0.13094615	1.531935341	0.39032613	6.03E-06
0.005514116	0.211325618	1.951438177	0.299395482	2.14E-05
0.006255585	0.162661935	1.124814322	0.527782811	5.50E-05
0.018695077	0.479013742	2.843620183	0.184971575	0.000174482
0.008057508	0.250417025	2.030383003	0.463991118	6.02E-05
0.014613832	0.305647131	3.967035241	0.240269905	7.07E-05
0.003952469	0.114852543	1.054402541	0.689264309	2.19E-05
0.01023938	0.189890603	2.026592818	0.289700972	4.54E-05
0.00563584	0.228220705	2.464173245	0.241428301	2.04E-05
0.003751211	0.187291851	1.511161173	0.383695845	2.36E-05
0.005979261	0.156424438	1.560521695	0.397869022	2.35E-05
0.002885212	0.129380551	1.396695708	0.44867955	9.43E-06
0.002048129	0.076192773	0.807538441	0.673904042	6.24E-06
0.003471489	0.215818314	2.317951498	0.321054544	1.01E-05
0.006056154	0.310680396	3.102618197	0.214765369	2.02E-05
0.005634779	0.150244504	1.768487385	0.324222828	1.50E-05
0.007673425	0.271096396	1.91646799	0.330293609	5.44E-05
0.003782291	0.148895075	1.336550504	0.404875679	1.50E-05
0.00342474	0.09795015	1.307481768	0.398802048	9.51E-06
0.009931125	0.657816389	7.095152034	0.072103188	3.06E-05
0.005424553	0.270687656	2.710026646	0.207660972	1.95E-05
0.005515697	0.274514087	2.737041712	0.221919784	1.84E-05
0.021686647	0.164079127	1.635699845	0.429542289	0.000152403
0.003553735	0.13073231	1.353921554	0.434020463	1.45E-05
0.004127188	0.084301978	0.947396308	1.008762149	1.89E-05
0.00981044	0.370620081	3.302058493	0.175839489	5.19E-05
0.004218195	0.078202001	0.784665992	0.87232895	1.93E-05
0.018970033	0.21063675	1.138103658	0.538841779	0.000281473
0.021887338	0.305834968	2.858589711	0.234291768	0.000166753
0.004079425	0.131182101	1.204833627	0.495621157	1.77E-05
0.01090694	0.311933464	2.261624148	0.27725015	7.90E-05
0.008135744	0.166316106	1.765801842	0.309566094	3.26E-05
0.003232215	0.095007903	1.261375612	0.443765968	8.87E-06
0.002777725	0.060687414	0.623553731	1.024892361	1.08E-05
0.013428921	1.449994127	7.348712101	0.076146889	0.000162014
0.007203125	0.139951311	1.716875371	0.348030018	2.20E-05
0.031748764	0.298069191	3.248050758	0.285224264	0.00024887
0.015342751	0.124948076	1.764786732	0.477237819	6.58E-05
0.010958679	0.678712527	4.395930278	0.145236593	9.87E-05

log.sigma.4.0.mm.3D_gldm_GrayLevelVariance	log.sigma.4.0.mm.3D_gldm_HighGrayLevelEmphasis	log.sigma.4.0.mm.3D_gldm_GrayLevelNonUniformityNormalized	log.sigma.4.0.mm.3D_gldm_DependenceEntropy
25.44284343	215.2776392	0.059931219	6.913864788
17.10544975	258.9235	0.075141	6.862783008
19.17144082	204.2093023	0.069468224	6.692849971
15.75694978	174.6628618	0.075438463	6.864750755
15.14143111	105.2813488	0.095610598	6.618241309
13.24689457	134.6636142	0.092262371	6.717154926
11.77887472	82.75591296	0.11819299	6.65825149
20.38515235	319.4930748	0.06989664	6.955146524
19.06497751	200.1114948	0.06884197	6.97647341
21.42923566	575.5511156	0.063858109	6.91190591
9.807371505	153.6480354	0.099603124	6.761174045
17.03704919	244.3038576	0.071568474	6.731329912
11.26309456	193.112325	0.098371018	6.928320948
13.90444515	142.0522067	0.077303975	6.70436351
23.74967283	234.5245478	0.063005362	7.033788096
21.71067185	374.1338525	0.069149563	7.296372307
17.93986316	367.2454829	0.077817956	6.853019441
16.54006548	300.7353401	0.075419922	7.014945722
14.4166584	257.9524648	0.076237354	6.689196857
16.49638507	238.2609445	0.076211284	6.954710262
18.08330285	355.2046043	0.070928751	7.06379059
17.90720442	370.2347949	0.072192247	6.934269353
19.0122859	287.340694	0.069652947	6.968841615
20.19822415	203.4194175	0.070040532	6.744941178
21.44453927	199.3759601	0.062345594	6.923686765
23.42354084	350.7337278	0.064291123	6.833263059
17.78922828	203.813659	0.069187593	6.800385853
19.26687032	278.4816692	0.066828832	6.717234371
16.72465129	329.9282142	0.080546595	6.899791326
17.9318971	361.9116809	0.074246131	6.89903127
19.72084902	415.8865282	0.068593539	7.068765766
18.472551	316.2766	0.06944792	6.996703238
20.69799582	506.9083366	0.073812032	6.907357089
18.62267193	160.5841996	0.078193818	6.514138891
16.93174255	234.1621622	0.072650353	6.767006609
13.35673159	289.6375505	0.083882262	6.829070963
26.18537339	448.8800785	0.059823465	7.036579814
13.59635829	364.6254792	0.085486666	6.829663774
10.74172848	199.0822102	0.099357205	6.812137083
15.36127086	132.0171149	0.085001435	6.839131209
12.76594821	160.2887538	0.081442106	6.631480729
12.98644278	168.615942	0.082617483	6.816888686
13.11903954	252.6987116	0.084117843	6.957693392
15.63222871	167.037155	0.086442542	6.867806765
25.33045545	497.416142	0.064751939	7.265750875
17.64254925	202.3591608	0.067367832	6.943713954
20.85566753	341.6698318	0.068133622	7.131237632
16.79009257	360.0903694	0.090057853	6.786989051
12.7437849	88.93439716	0.092764448	6.409273218
17.11206885	176.033945	0.083296019	6.860273035
14.88248753	315.5532129	0.085409157	7.015140579
9.521604909	274.4821063	0.118763618	6.695667576
16.56004363	197.8548854	0.075129035	7.025192172
16.32531991	414.6532878	0.072914243	6.657887181
18.533479	305.9380239	0.066491165	6.918283681
23.51608032	340.028072	0.061150038	6.991948412
22.75154794	614.936871	0.066400815	7.032444121
15.46269027	200.5546875	0.105163743	6.9881885
15.98573511	352.2215743	0.077024667	6.693647814
6.668638948	128.6099644	0.112323299	6.404684577
18.49999162	246.7143829	0.067271898	7.176341845
18.16731729	457.6759614	0.077098415	7.047765606
17.09416356	318.898723	0.072753716	7.008426947
17.23581514	230.6804069	0.067354956	6.586323748
15.24245906	163.3389831	0.073323758	6.401948319
22.25821461	201.6584576	0.067312543	6.764681395
21.90015491	178.4338374	0.074017031	6.965076577
21.33396014	264.8199663	0.065284426	7.034064491
13.19176552	265.2227378	0.080882305	6.75898415
21.23929917	452.2281611	0.06740202	7.060031751
30.72611729	437.2652311	0.05244722	7.166432603
13.70264478	231.5264435	0.091849959	6.73598204
19.44025136	310.0192349	0.0706234	7.083022978
15.69707651	377.6782368	0.075523739	6.924201993
25.1575073	372.1726889	0.060327194	7.120517135
22.62181026	343.4174174	0.067668514	7.037242047
12.22311584	270.1562633	0.091433594	6.804995266
15.55458202	231.7895511	0.077340662	6.710530506
16.97816137	355.3315018	0.076433335	6.83665945
17.78307375	399.369863	0.08020446	6.970333791
23.0269778	706.7995595	0.077446141	6.867554029
24.67669257	456.8690252	0.065213016	6.966959425
20.25216104	369.8544867	0.067856557	6.897419865
8.635449989	82.04397706	0.118382329	6.429117645
20.62590523	364.7850854	0.065094813	7.015357667
15.77120382	121.3802533	0.07324946	7.051594171
24.10307456	323.7226891	0.062659377	7.013008204
13.6260857	222.3397963	0.081243765	6.823293307
7.771727612	67.90024331	0.104457113	5.961240977
14.77396104	180.9965398	0.084194394	6.517673871
16.77549621	313.8788539	0.072080063	6.830605952
18.70428861	258.8561086	0.067273807	6.717895373
13.50059799	240.7919334	0.084569341	6.595546155
15.87246969	392.1504709	0.082563369	6.958032014
13.48023872	186.9290268	0.080078619	6.986475246
51.21791118	598.9049774	0.045829836	6.813992943
10.50895752	254.9177538	0.103905225	6.662845317
14.52849594	101.7850467	0.07988689	6.434046453
9.596522198	78.74099099	0.107922957	6.659827205
36.59906241	385.8743501	0.049815426	6.803956267

log.sigma.4.0.mm.3D_gldm_DependenceNonUniformity	log.sigma.4.0.mm.3D_gldm_GrayLevelNonUniformity	log.sigma.4.0.mm.3D_gldm_SmallDependenceEmphasis
174.8004988	72.09725686	0.242360261
273.185	150.282	0.193365474
182.842576	77.66547406	0.242312549
334.4651447	185.0505503	0.194041625
102.1928346	90.73445732	0.170589047
211.2246642	151.1257631	0.147818028
93.24030274	124.9299905	0.103797024
236.2686981	126.1634349	0.192907084
285.2427232	139.5426739	0.231209181
480.8141858	191.7659008	0.234390948
197.1527393	179.9828445	0.121930972
272.6605341	120.5928783	0.24470782
300.0720382	288.1287129	0.146922132
279.3498385	143.6307858	0.192071358
218.9354005	97.53229974	0.244329845
499.1932344	284.1355561	0.211473886
207.5121495	124.8978193	0.188957278
304.6364347	192.9241595	0.163370454
163.0880282	86.6056338	0.244579334
381.5425715	221.0889349	0.17663341
967.5571223	492.9548201	0.202171631
293.4507308	153.1197548	0.205244325
614.1671924	287.0397962	0.213805274
140.4446602	72.14174757	0.203170522
415.9815668	162.3479263	0.227879982
216.8402367	86.92159763	0.258707529
344.1279964	152.9737675	0.234683074
588.7991058	224.2107303	0.26612284
437.2471315	273.7778758	0.193126013
432.3111744	234.5435264	0.211649028
571.8536939	284.1144375	0.196457414
731.8788	347.2396	0.211499941
342.7226393	186.8182537	0.183483026
66.24324324	37.61122661	0.246829811
130.9369369	64.51351351	0.20595143
412.4964252	269.8492384	0.16764139
566.502942	213.5099468	0.240057204
432.9551755	289.8852846	0.170505752
157.7398922	147.4460916	0.120768401
190.0378973	139.0623472	0.16477264
187.6474164	107.1778116	0.228875216
242.0590062	159.6169772	0.181021507
348.3861909	254.6247109	0.152457969
213.6857749	162.8577495	0.176775829
471.2593017	226.2432742	0.213245627
538.2095105	240.84	0.19098317
509.7021161	251.1405317	0.217895541
183.969657	136.5277045	0.179297837
62.76595745	52.31914894	0.175763844
118.9247706	90.79266055	0.205374765
433.2464859	340.2700803	0.147132034
189.7801636	232.301636	0.116605416
454.7318936	285.264946	0.160235025
182.4859095	85.38257899	0.230996626
607.014732	261.7757176	0.207448783
298.5603814	115.4512712	0.237678054
477.9929857	217.7282708	0.230801509
219.5559211	255.7582237	0.150463954
200.0830904	105.6778426	0.211355756
171.6192171	157.8142349	0.120786712
340.6817409	197.8466508	0.182483915
673.2861309	423.0390013	0.18909988
304.9321885	165.22369	0.204471457
331.987152	125.8190578	0.236307463
96.65084746	43.26101695	0.265409767
229.4977317	103.8632534	0.237968799
126.6086957	78.3100189	0.190089392
492.1228267	232.8042625	0.196780963
164.4508894	104.5808198	0.179664208
346.597128	168.9768648	0.219753342
638.7888655	199.7190126	0.2758917
250.2799612	189.3027656	0.149652216
620.7862791	330.4468904	0.200764713
924.4950084	491.7350637	0.184033894
649.8836227	257.1145002	0.209973608
316.4886315	157.7353067	0.221098154
273.9927813	215.3261146	0.149577054
195.1942605	105.1059603	0.204078154
476.2557998	250.3956044	0.201985973
560.4358859	357.1504604	0.191894968
174.9309838	105.4816446	0.224376218
414.6785849	173.2709823	0.253625917
379.9943411	167.8771221	0.225895597
47.44359465	61.91395793	0.150273379
589.0409114	251.3961678	0.216070247
427.3851468	254.4686241	0.175523846
212.0168067	89.47759104	0.239924272
387.1873151	247.2247782	0.166868577
55.16058394	42.93187348	0.21160957
75.87197232	48.66435986	0.190685691
459.7075677	231.4490813	0.190770475
177.2244344	74.33755656	0.249910766
232.346991	132.0973111	0.192429075
545.3482656	359.398346	0.178693816
592.401615	376.8499788	0.173617269
190.3914027	40.51357466	0.37057773
196.3556438	183.1849121	0.13784625
55.67757009	34.19158879	0.175891395
91.06756757	95.83558559	0.152351894
247.9202773	57.48700173	0.352721742

log.sigma.4.0.mm.3D_gldm_DependenceNonUniformityNormalized	log.sigma.4.0.mm.3D_gldm_DependenceVariance	log.sigma.4.0.mm.3D_gldm_LargeDependenceEmphasis
0.145303823	5.221505671	19.04655029
0.1365925	6.6286	23.603
0.163544343	4.008666127	15.97853309
0.136349427	5.464999803	22.06074195
0.107684757	8.863996376	32.43097998
0.128952786	6.859021321	27.45665446
0.088212207	15.41084933	53.89309366
0.130896786	5.432412581	22.63767313
0.14072162	7.376681694	22.86383818
0.160111284	4.14893487	16.63902764
0.109105002	8.485988664	35.20807969
0.161816341	4.11720311	16.14183976
0.10244863	9.895370124	36.7234551
0.150349752	4.536791415	19.32077503
0.141431137	4.785559762	18.68863049
0.121487767	16.52555251	38.81893405
0.129291059	5.884610592	23.52461059
0.119091648	7.861108139	29.2634871
0.143563405	4.855720591	18.70774648
0.131521052	6.625135742	24.97518097
0.139216852	5.935794214	21.88978417
0.138354894	5.348009766	21.11126827
0.149033534	4.635994013	18.7408396
0.136354039	5.91528325	22.1961165
0.159747145	4.679882185	17.52841782
0.16038479	5.112406341	17.39349112
0.155643599	4.501222762	17.40072365
0.175498988	3.238757554	13.58777943
0.128639933	6.037783987	23.73021477
0.136850641	5.08235914	20.55745489
0.138062215	5.167467739	21.2409464
0.14637576	5.27349056	19.9332
0.135409972	5.330960023	22.40734887
0.13771984	10.1772641	27.65696466
0.147451506	5.839663988	20.98648649
0.128223943	6.181992444	25.15604601
0.158728759	3.886868658	16.15158308
0.127677728	5.996599495	24.89383663
0.106293728	9.317474444	37.2277628
0.116160084	7.376758269	28.95721271
0.142589222	4.853985551	19.16261398
0.125289341	7.557471634	26.85921325
0.115092894	8.57981132	31.84043608
0.113421324	8.071720286	29.75053079
0.134876732	10.07438805	27.96336577
0.150548115	4.041417693	18.63916084
0.138280552	7.322824348	23.35756918
0.121352016	6.946582104	26.54881266
0.111287159	8.925821136	31.60638298
0.109105294	10.23167747	32.57247706
0.108746608	7.953754526	32.44929719
0.097024624	10.51257418	42.40388548
0.119760836	6.681426898	27.55359494
0.155837668	3.818209928	16.63962425
0.15418205	4.262734719	18.05461011
0.158135795	4.582410541	17.24364407
0.145774012	5.901667073	20.28331808
0.090277928	15.49411087	48.42434211
0.145833156	4.370364389	18.6909621
0.122148909	5.748032573	29.69181495
0.115838742	8.42508539	29.48010881
0.122705692	6.724915983	25.62292692
0.13427221	6.139568441	22.67591369
0.177723315	2.907071196	13.74625268
0.163814996	4.21897156	15.7559322
0.148734758	4.78144729	18.213221
0.119667954	7.93673193	27.8563327
0.138004158	4.786860638	20.75546831
0.127185529	5.411097533	23.78886311
0.138251746	6.341906642	21.95811727
0.167749177	4.12962731	15.1092437
0.121436177	6.096341544	27.25812712
0.13267499	5.990822289	22.75721308
0.141989711	4.710950109	20.70480725
0.152483253	4.608064766	18.56780854
0.135773759	6.065960058	21.76876877
0.11634513	7.008029354	29.27600849
0.143630802	4.759724097	19.74613687
0.145377228	4.517127561	19.33821734
0.125855802	6.918570479	25.32966539
0.128436846	5.833327943	22.21145374
0.156070224	4.450102548	16.77794505
0.153595126	4.637646561	18.10185934
0.09071433	13.06900548	45.25239006
0.152522245	4.276579003	17.872087
0.123023934	6.631229474	26.21070812
0.148471153	5.715960894	19.5952381
0.127238684	5.727082159	24.80414065
0.134210667	5.169836788	20.99513382
0.131266388	6.590115061	24.29411765
0.14316648	5.068129535	20.91155403
0.160384104	3.898421408	15.81809955
0.148749674	4.658707688	19.66069142
0.125281017	6.512475085	25.38594073
0.125882196	6.107389198	25.07224819
0.21537489	2.654040663	9.805429864
0.111375861	7.529206423	32.50652297
0.130087781	6.421761726	24.94392523
0.102553567	11.66597679	39.34684685
0.214835596	2.377888379	9.547660312

log.sigma.4.0.mm.3D_gldm_LargeDependenceLowGrayLevelEmphasis	log.sigma.4.0.mm.3D_gldm_SmallDependenceHighGrayLevelEmphasis	log.sigma.4.0.mm.3D_gldm_LargeDependenceHighGrayLevelEmphasis
0.271648252	58.52922492	3185.85453
0.138107402	52.78888432	5216.8635
0.143745754	46.65365219	3323.9839
0.194142742	35.20995454	3353.947004
0.614914119	22.78286553	2413.804004
0.301260339	21.3943044	3278.826007
1.384367586	10.91978452	2783.081362
0.098292792	62.10399462	6536.67867
0.19651528	47.23236661	3681.050814
0.03314283	133.7346943	9530.374292
0.311905096	18.18580828	4864.401771
0.089132795	58.38376517	3889.190504
0.244243536	31.84355229	6278.561967
0.226504842	25.99616207	2710.017761
0.117332326	56.78933108	4007.881137
0.145361937	81.25966918	11943.8311
0.074183598	65.34240117	8941.391277
0.138639501	53.59564029	7282.989445
0.097285479	64.77367579	4421.353873
0.176446406	43.20855493	5319.917615
0.075626802	69.11738634	7642.603741
0.067070306	76.32385424	7725.845356
0.088792861	62.26799169	5249.259646
0.218012681	44.45956724	3957.561165
0.196586535	44.23866678	3560.306836
0.070428517	91.6271079	5306.244083
0.113814032	46.76785481	3511.797377
0.066205753	66.47185273	4047.791952
0.090692392	62.59143791	7420.077081
0.068211716	74.55510822	7289.207028
0.058236836	75.28187986	9214.914051
0.090266988	67.54388263	5765.7346
0.060729466	93.81441962	10666.37179
0.354494404	43.62370814	3015.902287
0.115749543	45.41369998	4882.128378
0.105883909	47.25359399	6841.857631
0.047569897	104.8715259	7026.38246
0.075850657	59.0205703	9035.795636
0.244344374	25.90417438	6506.649596
0.411271756	26.72355028	2822.949878
0.187063243	36.9025169	2891.762918
0.246218627	33.49496885	3642.977226
0.168749763	39.33570464	7074.841427
0.284902852	33.33795349	3837.235669
0.066084153	100.07926	13584.22038
0.170620853	36.88000489	3920.582378
0.096315287	75.56571417	7434.836408
0.080237701	54.8442514	10094.75264
0.843974336	19.23023058	1870.278369
0.292667419	42.02331579	4368.514679
0.131861637	48.24749429	9102.836847
0.207193147	32.95023017	10944.3001
0.213811274	36.11388436	4559.557282
0.047090511	90.36164107	6820.552519
0.079082007	63.00899441	5178.527813
0.067751734	83.13999273	5612.398835
0.039218625	140.5099246	11702.07929
0.362269937	38.46346815	7253.454359
0.063090184	69.76157245	6606.784257
0.282937559	13.90620926	3939.415658
0.19161975	47.06114105	5995.592996
0.065580638	83.59954102	11235.62238
0.086802863	64.0871542	7063.55218
0.079263517	48.35976254	3518.199679
0.154513419	40.02748662	2366.644068
0.17408727	56.83088291	2822.624109
0.269920034	44.45791585	3825.191871
0.116065233	55.25206852	4971.964666
0.106158302	42.82338989	6252.113689
0.059964393	102.4043626	9146.866374
0.056852181	115.4683691	6398.978992
0.15456995	37.6148059	5497.350801
0.123232899	64.79854322	6290.639453
0.061879272	65.12633256	8001.452772
0.069911834	81.82686093	6352.809479
0.088411468	74.34636154	6945.800944
0.124588209	37.08284447	7723.530361
0.129488283	48.29879753	4273.533481
0.062870589	69.17010524	6952.910867
0.072212728	72.78613746	9988.640467
0.035233464	154.8378252	15410.13142
0.04321272	108.5951581	7880.509974
0.065728077	87.66479776	6145.092158
1.005300766	13.697053	2614.544933
0.062689048	74.52444608	6616.830658
0.452584458	22.16890861	2648.253022
0.082802714	81.39422073	5886.904762
0.149878134	38.18213709	4944.028919
0.658876253	13.2075376	1308.172749
0.201903939	30.70204314	4220.681661
0.090041084	63.52176592	5728.580193
0.083126581	64.41623101	3892.921267
0.097686924	41.40481495	5028.202945
0.073786903	68.97740196	9746.173444
0.192456333	33.24118857	4392.042924
0.054613752	199.7421537	6413.079186
0.153606032	37.58256308	7781.857062
0.619031382	18.77156065	1810.626168
0.861856779	13.62385204	2375.959459
0.057673978	127.2715451	3972.070191

log.sigma.4.0.mm.3D_gldm_SmallDependenceLowGrayLevelEmphasis	log.sigma.4.0.mm.3D_gldm_LowGrayLevelEmphasis	log.sigma.4.0.mm.3D_gldzm_DistanceZoneVariabilityNormalized
0.003262472	0.01521203	1
0.001602339	0.007009207	1
0.003928592	0.013102412	1
0.002982997	0.010236749	1
0.004382959	0.024730336	1
0.002990995	0.013244304	1
0.002984758	0.023458835	1
0.002092762	0.005842487	1
0.004067897	0.011141961	1
0.000879345	0.002521496	1
0.001589969	0.009892825	1
0.002621364	0.007354845	1
0.00145504	0.007612407	1
0.004038691	0.016888669	1
0.003040892	0.007902125	1
0.001211589	0.004277472	0.998019804
0.001710697	0.005325063	1
0.001174637	0.004735231	1
0.002495096	0.00628762	1
0.001420801	0.008401635	1
0.001083062	0.004090972	0.995221055
0.001280641	0.003984905	0.996070742
0.001335691	0.005733196	0.998086126
0.003268767	0.013543192	1
0.003508093	0.012746491	1
0.003798064	0.006626643	1
0.002641022	0.008115337	1
0.00230639	0.006284627	1
0.001714776	0.005053052	0.997237574
0.001353324	0.004509033	1
0.001162673	0.003625371	0.997892521
0.001505803	0.00506944	1
0.001043834	0.003590472	1
0.006338544	0.017376945	1
0.002908928	0.008293396	1
0.001080291	0.005000372	1
0.001319538	0.003805064	0.997993984
0.001008096	0.00374967	1
0.001569818	0.008082372	1
0.00341472	0.014852761	1
0.003342147	0.012366561	1
0.002230939	0.009121197	1
0.001597607	0.006252366	1
0.002930297	0.010471261	1
0.001068638	0.003308225	1
0.002083176	0.011001848	1
0.001254141	0.005436932	0.991498561
0.001872886	0.004821917	1
0.003809499	0.027800792	1
0.003140148	0.009908483	1
0.000957515	0.004784814	1
0.000865907	0.006666769	1
0.001643556	0.008003002	1
0.001644861	0.003781412	1
0.001373152	0.004954965	1
0.001783273	0.005013644	1
0.000863028	0.002364659	0.995423365
0.001491127	0.007623642	1
0.001862886	0.004724929	1
0.0020392	0.012473913	1
0.001379297	0.007097653	1
0.000927617	0.003161607	1
0.00166998	0.005072691	1
0.002492238	0.008115436	1
0.005329917	0.012213614	1
0.002170642	0.009441112	1
0.002827316	0.010843249	1
0.001860214	0.006371169	1
0.001951331	0.00568644	1
0.001136392	0.003316092	1
0.001382741	0.00426253	1
0.002004892	0.006652649	1
0.001091461	0.005972184	1
0.000939321	0.003567357	0.998498499
0.001236946	0.004750881	1
0.001535325	0.00577535	1
0.001597662	0.005481707	1
0.002828713	0.00919018	1
0.001221384	0.004134836	1
0.001028568	0.003595332	1
0.001278821	0.002635063	1
0.001540971	0.003723171	1
0.001313857	0.004647023	1
0.005207811	0.021073811	1
0.001394995	0.00443272	0.995955527
0.00392675	0.018724925	0.997080298
0.002358998	0.006030841	1
0.001565708	0.0065158	1
0.009275333	0.029621625	1
0.003415095	0.013525942	1
0.001117713	0.004341569	1
0.003864044	0.008175489	1
0.002179116	0.007677351	1
0.000935061	0.003513762	1
0.001875899	0.008663031	1
0.00224884	0.007128472	1
0.00099229	0.005895776	1
0.005540583	0.023037047	1
0.005075957	0.025013178	1
0.002608752	0.008176962	1

log.sigma.4.0.mm.3D_gldzm_LowIntensityEmphasis	log.sigma.4.0.mm.3D_gldzm_LargeDistanceEmphasis	log.sigma.4.0.mm.3D_gldzm_HighIntensitySmallDistanceEmphasis
0.017181862	1	252.652819
0.0092462	1	281.0393873
0.018317341	1	193.9630769
0.015312994	1	189.1881919
0.030138764	1	141.2108108
0.020990056	1	143.3709091
0.028397589	1	108.0671642
0.010966844	1	324.3376289
0.017219507	1	209.4055046
0.003619362	1	572.7372781
0.015061364	1	148.2490272
0.010521336	1	241.98778
0.01091899	1	222.0705394
0.024087403	1	136.955711
0.011816286	1	239.7636364
0.005853582	1.002973241	385.9199703
0.009635311	1	347.1517615
0.006953922	1	338.1295117
0.009544275	1	273.921875
0.010006457	1	248.4466667
0.00545078	1.007185629	339.9967066
0.006029584	1.005905512	376.5688976
0.007017425	1.002873563	293.4233716
0.018183955	1	220.2879377
0.015410542	1	194.3748309
0.012909492	1	363.8419811
0.0110637	1	200.5960265
0.008608754	1	251.6225536
0.009020066	1.004149378	327.3872752
0.006924833	1	352.5229358
0.005847523	1.003164557	381.378692
0.007103488	1	325.5102208
0.005899976	1	518.3436293
0.026832283	1	179.4125874
0.013557234	1	222.7402597
0.007393006	1	282.391231
0.005509383	1.003012048	444.8125
0.005936384	1	349.6324655
0.015003163	1	218.6439024
0.020251291	1	169.016129
0.016483073	1	164.2332362
0.01196816	1	190.7167488
0.011570962	1	260.5424431
0.016378644	1	200.3881402
0.005087164	1	466.2370787
0.013064348	1	192.4309677
0.006566966	1.01280683	351.3241729
0.009949593	1	308.6435331
0.026977532	1	112.5932203
0.014306575	1	213.7878788
0.007625452	1	334.9170579
0.011214929	1	283.3647541
0.010683806	1	236.1872075
0.006492579	1	400.3738318
0.006728079	1	310.24946
0.007155136	1	351.7753623
0.003581279	1.006880734	616.4762041
0.009956397	1	266.7535885
0.008184228	1	336.2253521
0.021071189	1	115.2786885
0.008821921	1	264.6839465
0.005053686	1	441.8353448
0.00794259	1	323.6466302
0.011457762	1	205.458891
0.018908428	1	152.4408602
0.009879531	1	247.6052632
0.013839739	1	248.1880342
0.009533	1	288.7390728
0.010655417	1	237.0196078
0.004969815	1	470.6615147
0.005296714	1	420.851461
0.012727756	1	266.4188791
0.006155759	1	331.1397059
0.005194428	1.002253944	349.5905334
0.006409858	1	398.4966249
0.008337755	1	340.336163
0.010747396	1	250.8941799
0.015109453	1	241.1323077
0.006201913	1	335.1044177
0.00546285	1	375.4496439
0.005393075	1	695.2096317
0.005853625	1	429.8246914
0.006529519	1	392.7476341
0.031235992	1	95.72631579
0.00639119	1.006079027	348.1469098
0.024939038	1.004385965	130.5789474
0.009596696	1	342.5311721
0.009445618	1	234.0217786
0.041289326	1	63.92929293
0.022014358	1	163.3769231
0.005589088	1	345.7945402
0.014607293	1	259.2676923
0.012529419	1	214.4553073
0.005320366	1	388.593786
0.011209981	1	196.3593407
0.007294815	1	551.3120393
0.009011563	1	276.1845018
0.030529015	1	114.8021978
0.033988431	1	93.41509434
0.008628182	1	363.2738095

log.sigma.4.0.mm.3D_gldzm_LowIntensityLargeDistanceEmphasis	log.sigma.4.0.mm.3D_gldzm_HighIntensityEmphasis	log.sigma.4.0.mm.3D_gldzm_DistanceZoneVariability	log.sigma.4.0.mm.3D_gldzm_ZonePercentage
0.017181862	252.652819	337	0.280133001
0.0092462	281.0393873	457	0.2285
0.018317341	193.9630769	325	0.290697674
0.015312994	189.1881919	542	0.220953934
0.030138764	141.2108108	185	0.194942044
0.020990056	143.3709091	275	0.167887668
0.028397589	108.0671642	134	0.126773888
0.010966844	324.3376289	388	0.214958449
0.017219507	209.4055046	545	0.268870252
0.003619362	572.7372781	845	0.281385281
0.015061364	148.2490272	257	0.142224682
0.010521336	241.98778	491	0.291394659
0.01091899	222.0705394	482	0.164561284
0.024087403	136.955711	429	0.230893434
0.011816286	239.7636364	440	0.284237726
0.005871175	386.0455897	1007.001982	0.24555853
0.009635311	347.1517615	369	0.229906542
0.006953922	338.1295117	471	0.184128225
0.009544275	273.921875	320	0.281690141
0.010006457	248.4466667	600	0.206825233
0.005470889	340.7431138	1662.019162	0.24028777
0.006037116	377.726378	506.003937	0.239509665
0.00703738	293.5268199	1042.001916	0.253336569
0.018183955	220.2879377	257	0.249514563
0.015410542	194.3748309	739	0.283794163
0.012909492	363.8419811	424	0.313609467
0.0110637	200.5960265	604	0.273179557
0.008608754	251.6225536	1073	0.319821162
0.0090849	327.4536653	721.0027663	0.21270962
0.006924833	352.5229358	763	0.24153213
0.005851559	381.9989451	946.0021097	0.22887494
0.007103488	325.5102208	1223	0.2446
0.005899976	518.3436293	518	0.204662189
0.026832283	179.4125874	143	0.297297297
0.013557234	222.7402597	231	0.260135135
0.007393006	282.391231	593	0.18433323
0.005516213	445.1445783	994.002008	0.279069767
0.005936384	349.6324655	653	0.192568564
0.015003163	218.6439024	205	0.138140162
0.020251291	169.016129	310	0.189486553
0.016483073	164.2332362	343	0.260638298
0.01196816	190.7167488	406	0.210144928
0.011570962	260.5424431	483	0.159563925
0.016378644	200.3881402	371	0.196921444
0.005087164	466.2370787	890	0.254722381
0.013064348	192.4309677	775	0.216783217
0.006626767	352.3383138	929.0341515	0.2542051
0.009949593	308.6435331	317	0.209102902
0.026977532	112.5932203	118	0.209219858
0.014306575	213.7878788	264	0.242201835
0.007625452	334.9170579	639	0.160391566
0.011214929	283.3647541	244	0.124744376
0.010683806	236.1872075	641	0.168817487
0.006492579	400.3738318	321	0.27412468
0.006728079	310.24946	926	0.23520447
0.007155136	351.7753623	552	0.292372881
0.003594891	617.3474771	868.0091743	0.265934736
0.009956397	266.7535885	418	0.171875
0.008184228	336.2253521	355	0.258746356
0.021071189	115.2786885	183	0.13024911
0.008821921	264.6839465	598	0.2033322
0.005053686	441.8353448	1160	0.211408784
0.00794259	323.6466302	549	0.241743725
0.011457762	205.458891	523	0.279978587
0.018908428	152.4408602	186	0.315254237
0.009879531	247.6052632	418	0.270900843
0.013839739	248.1880342	234	0.221172023
0.009533	288.7390728	755	0.211721817
0.010655417	237.0196078	255	0.197215777
0.004969815	470.6615147	647	0.258077383
0.005296714	420.851461	1232	0.323529412
0.012727756	266.4188791	339	0.164483261
0.006155759	331.1397059	1088	0.232528318
0.005205928	349.7009767	1329.001503	0.204423284
0.006409858	398.4966249	1037	0.243312999
0.008337755	340.336163	589	0.252681253
0.010747396	250.8941799	378	0.160509554
0.015109453	241.1323077	325	0.239146431
0.006201913	335.1044177	747	0.228021978
0.00546285	375.4496439	983	0.220750056
0.005393075	695.2096317	353	0.25917768
0.005853625	429.8246914	810	0.3048551
0.006529519	392.7476341	634	0.256265158
0.031235992	95.72631579	95	0.181644359
0.006429184	348.526849	983.0081054	0.255567064
0.025007568	130.6491228	682.002924	0.196891192
0.009596696	342.5311721	401	0.280812325
0.009445618	234.0217786	551	0.181071311
0.041289326	63.92929293	99	0.240875912
0.022014358	163.3769231	130	0.224913495
0.005589088	345.7945402	696	0.216754905
0.014607293	259.2676923	325	0.294117647
0.012529419	214.4553073	358	0.229193342
0.005320366	388.593786	869	0.199632437
0.011209981	196.3593407	910	0.193370166
0.007294815	551.3120393	407	0.46040724
0.009011563	276.1845018	271	0.153715258
0.030529015	114.8021978	91	0.212616822
0.033988431	93.41509434	159	0.179054054
0.008628182	363.2738095	504	0.436741768

log.sigma.4.0.mm.3D_gldzm_IntensityVariabilityNormalized	log.sigma.4.0.mm.3D_gldzm_LowIntensitySmallDistanceEmphasis	log.sigma.4.0.mm.3D_gldzm_IntensityVariability
0.050726871	0.017181862	17.09495549
0.060086474	0.0092462	27.4595186
0.055015385	0.018317341	17.88
0.058380196	0.015312994	31.64206642
0.064368152	0.030138764	11.90810811
0.063986777	0.020990056	17.59636364
0.08398307	0.028397589	11.25373134
0.054349559	0.010966844	21.08762887
0.056409393	0.017219507	30.74311927
0.05271104	0.003619362	44.5408284
0.075625672	0.015061364	19.43579767
0.058142284	0.010521336	28.54786151
0.059184587	0.01091899	28.52697095
0.065642982	0.024087403	28.16083916
0.051508264	0.011816286	22.66363636
0.050358469	0.005849183	50.81169475
0.051284876	0.009635311	18.92411924
0.057748568	0.006953922	27.19957537
0.067636719	0.009544275	21.64375
0.05665	0.010006457	33.99
0.055418265	0.005445753	92.54850299
0.05488561	0.006027701	27.88188976
0.055995948	0.007012436	58.45977011
0.056336962	0.018183955	14.47859922
0.057234569	0.015410542	42.29634641
0.049005429	0.012909492	20.77830189
0.06059493	0.0110637	36.59933775
0.058071151	0.008608754	62.31034483
0.052009741	0.009003858	37.60304288
0.050265473	0.006924833	38.3525557
0.051888052	0.005846513	49.18987342
0.054276543	0.007103488	66.38021259
0.050327216	0.005899976	26.06949807
0.064208519	0.026832283	9.181818182
0.057588876	0.013557234	13.3030303
0.058492986	0.007393006	34.68634064
0.045235157	0.005507676	45.05421687
0.057261925	0.005936384	37.39203675
0.059036288	0.015003163	12.10243902
0.060957336	0.020251291	18.89677419
0.064301439	0.016483073	22.05539359
0.064197141	0.01196816	26.06403941
0.065382423	0.011570962	31.57971014
0.055426799	0.016378644	20.56334232
0.046446156	0.005087164	41.33707865
0.062126951	0.013064348	48.1483871
0.051743285	0.006552016	48.48345784
0.055160266	0.009949593	17.48580442
0.082160299	0.026977532	9.694915254
0.058482553	0.014306575	15.43939394
0.054606547	0.007625452	34.89358372
0.061979307	0.011214929	15.12295082
0.056237694	0.010683806	36.04836193
0.061344513	0.006492579	19.69158879
0.055112913	0.006728079	51.03455724
0.049602237	0.007155136	27.38043478
0.048312432	0.003577876	42.12844037
0.054783544	0.009956397	22.89952153
0.05915493	0.008184228	21
0.082026934	0.021071189	15.01092896
0.056190647	0.008821921	33.60200669
0.050870987	0.005053686	59.01034483
0.054946732	0.00794259	30.16575592
0.062183534	0.011457762	32.52198853
0.063359926	0.018908428	11.78494624
0.058274765	0.009879531	24.35885167
0.052523924	0.013839739	12.29059829
0.051785448	0.009533	39.09801325
0.06638985	0.010655417	16.92941176
0.050459976	0.004969815	32.64760433
0.04858139	0.005296714	59.85227273
0.058327025	0.012727756	19.77286136
0.054505028	0.006155759	59.30147059
0.058815361	0.005191553	78.28324568
0.047052686	0.006409858	48.79363549
0.050486998	0.008337755	29.73684211
0.059054898	0.010747396	22.32275132
0.05923787	0.015109453	19.25230769
0.055101262	0.006201913	41.16064257
0.051169992	0.00546285	50.30010173
0.044659696	0.005393075	15.76487252
0.046252096	0.005853625	37.46419753
0.050672213	0.006529519	32.12618297
0.093185596	0.031235992	8.852631579
0.054313985	0.006381692	53.60790274
0.063104887	0.024921905	43.16374269
0.046995976	0.009596696	18.84538653
0.063790962	0.009445618	35.14882033
0.1184573	0.041289326	11.72727273
0.067692308	0.022014358	8.8
0.060126007	0.005589088	41.84770115
0.054011834	0.014607293	17.55384615
0.062139134	0.012529419	22.24581006
0.052278918	0.005320366	45.43037975
0.063004468	0.011209981	57.33406593
0.036915405	0.007294815	15.02457002
0.071635735	0.009011563	19.41328413
0.073300326	0.030529015	6.67032967
0.075985918	0.033988431	12.08176101
0.045934114	0.008628182	23.15079365

log.sigma.4.0.mm.3D_gldzm_HighIntensityLargeDistanceEmphasis	log.sigma.4.0.mm.3D_gldzm_SmallDistanceEmphasis	log.sigma.4.0.mm.3D_glcm_SumVariance	log.sigma.4.0.mm.3D_glcm_Homogeneity1
252.652819	1	603.0047101	0.418406101
281.0393873	1	755.8099575	0.442079319
193.9630769	1	564.9382535	0.401117323
189.1881919	1	462.202527	0.450053038
141.2108108	1	255.657153	0.498189904
143.3709091	1	353.9156846	0.474112858
108.0671642	1	192.006949	0.564205486
324.3376289	1	955.610474	0.45137534
209.4055046	1	561.7032455	0.439153748
572.7372781	1	1819.607274	0.408067558
148.2490272	1	417.0591863	0.514271201
241.98778	1	706.3441114	0.406456246
222.0705394	1	527.1415825	0.511704195
136.955711	1	364.5880535	0.436017073
239.7636364	1	688.5438743	0.413380662
386.5480674	0.99925669	1138.487785	0.453860243
347.1517615	1	1126.780506	0.440182224
338.1295117	1	897.8310925	0.473694831
273.921875	1	755.0607515	0.425805433
248.4466667	1	678.5844186	0.459157557
343.7287425	0.998203593	1070.079831	0.424046127
382.3562992	0.998523622	1105.062049	0.435237435
293.940613	0.999281609	847.5061765	0.421479597
220.2879377	1	543.5369213	0.443128824
194.3748309	1	543.2514851	0.405500853
363.8419811	1	1062.36178	0.396599323
200.5960265	1	616.9711128	0.454355855
251.6225536	1	833.1521404	0.38360041
327.7192254	0.998962656	999.9651485	0.439455614
352.5229358	1	1125.684348	0.43250049
384.4799578	0.999208861	1269.841361	0.434301711
325.5102208	1	946.1686732	0.432427372
518.3436293	1	1574.298218	0.449847258
179.4125874	1	432.3220571	0.453524947
222.7402597	1	654.645477	0.430001577
282.391231	1	861.5313726	0.458605793
446.4728916	0.999246988	1368.338531	0.392736919
349.6324655	1	1114.690791	0.455514177
218.6439024	1	560.665728	0.521719657
169.016129	1	337.3358279	0.480718865
164.2332362	1	433.7925854	0.431610448
190.7167488	1	454.8629841	0.465671426
260.5424431	1	742.1829591	0.48802119
200.3881402	1	454.7157757	0.479614049
466.2370787	1	1553.428206	0.419948706
192.4309677	1	556.7421786	0.433905795
356.3948773	0.996798292	1003.069886	0.429459621
308.6435331	1	1129.605329	0.451445901
112.5932203	1	211.5033579	0.492988294
213.7878788	1	475.992313	0.483745802
334.9170579	1	961.2074386	0.49472273
283.3647541	1	828.185405	0.526953173
236.1872075	1	539.6044186	0.480746811
400.3738318	1	1315.783382	0.414668306
310.24946	1	914.0835338	0.416472923
351.7753623	1	1016.315286	0.397116048
620.8325688	0.998279817	1978.764945	0.414605826
266.7535885	1	565.8749737	0.521636778
336.2253521	1	1076.623298	0.421427008
115.2786885	1	342.7484623	0.508771097
264.6839465	1	711.6651373	0.47528572
441.8353448	1	1410.688098	0.453305315
323.6466302	1	950.3815923	0.435057389
205.458891	1	662.5728032	0.391152948
152.4408602	1	461.9736599	0.406655522
247.6052632	1	542.8915636	0.418476189
248.1880342	1	482.763069	0.465382547
288.7390728	1	766.3044515	0.433504649
237.0196078	1	782.3766161	0.461961477
470.6615147	1	1395.120535	0.427624187
420.851461	1	1338.090966	0.378375631
266.4188791	1	673.4776139	0.479417769
331.1397059	1	918.322895	0.441168921
350.1427498	0.999436514	1166.529895	0.430798673
398.4966249	1	1090.952967	0.416959057
340.336163	1	1021.726688	0.420550171
250.8941799	1	812.0594911	0.481838243
241.1323077	1	665.8905152	0.431881485
335.1044177	1	1090.713865	0.415392421
375.4496439	1	1231.423382	0.443925555
695.2096317	1	2351.196943	0.425333751
429.8246914	1	1377.682447	0.39150181
392.7476341	1	1101.876227	0.413111705
95.72631579	1	197.8696247	0.543410386
350.0466059	0.998480243	1087.922716	0.419631307
130.9298246	0.998903509	304.2476981	0.472058418
342.5311721	1	959.7756648	0.420101878
234.0217786	1	629.836855	0.46424864
63.92929293	1	159.4872284	0.455872731
163.3769231	1	488.7737249	0.459092697
345.7945402	1	940.6505681	0.43731916
259.2676923	1	760.8138057	0.405836585
214.4553073	1	692.6151222	0.43734899
388.593786	1	1199.142946	0.453863886
196.3593407	1	508.510336	0.467151073
551.3120393	1	1838.173648	0.299095941
276.1845018	1	743.2070481	0.505110247
114.8021978	1	248.5331426	0.476352469
93.41509434	1	185.8107311	0.525973271
363.2738095	1	1143.850064	0.328187182

log.sigma.4.0.mm.3D_glcm_Homogeneity2	log.sigma.4.0.mm.3D_glcm_ClusterShade	log.sigma.4.0.mm.3D_glcm_MaximumProbability	log.sigma.4.0.mm.3D_glcm_Idmn	log.sigma.4.0.mm.3D_glcm_SumVariance2
0.342389527	210.1611494	0.031798148	0.984764036	88.98999399
0.368200662	103.2685197	0.036062166	0.98900029	57.83949885
0.319398053	-130.2362846	0.027079928	0.984125414	64.49218095
0.376574908	180.4862951	0.031351069	0.988418018	53.95894662
0.438905739	263.6749815	0.068948859	0.983568886	53.39724855
0.407117982	219.5062452	0.050638298	0.987384769	45.88038703
0.516933644	208.9044858	0.101874519	0.987789769	41.06222166
0.380488317	118.1722494	0.031039897	0.991894918	69.4060688
0.36525108	217.1198247	0.037022058	0.98698951	67.01708916
0.326589167	-115.0276132	0.024383128	0.991893063	72.77504034
0.456957819	85.10879182	0.048326454	0.988307284	33.96141374
0.324537674	-57.30447069	0.020954864	0.986896542	55.46876745
0.453119591	130.8533825	0.057154806	0.991852942	36.63905873
0.359814979	-22.45005346	0.026144022	0.984750369	46.20349665
0.337814049	455.5932458	0.0216659	0.984507178	77.78987894
0.382751351	-63.17363481	0.069898382	0.990490441	73.16590396
0.36649075	-187.0766184	0.035456858	0.990572176	55.91562632
0.408091051	165.8921383	0.036479332	0.992279665	57.24634691
0.347395333	-11.70843113	0.024049795	0.986002543	46.0118726
0.389107212	-35.90828381	0.03792596	0.988312635	58.01364596
0.347239153	-140.5642464	0.022431135	0.991409486	58.90022137
0.359458061	85.21285702	0.027093693	0.99389102	61.48813104
0.342586835	95.00324938	0.027235918	0.989577609	64.75055054
0.370170185	42.33759636	0.040760635	0.986051296	70.76528591
0.324345105	-234.9388683	0.019221131	0.981718083	73.46630012
0.315166892	115.560199	0.022832567	0.985850623	76.44550681
0.381543548	155.1303278	0.029989795	0.989295593	60.62399423
0.297558317	-230.5969932	0.017281233	0.985525746	64.48850117
0.365246511	-173.6256443	0.033557655	0.990302573	52.106224
0.358582961	-12.95349738	0.032792337	0.991618763	51.69792864
0.359384717	-244.8194564	0.026265651	0.990759658	64.57096869
0.35678495	-8.607746253	0.021056564	0.991352618	59.73541756
0.378852052	-267.2537831	0.032159121	0.99307492	71.06695234
0.38571448	180.4200737	0.06664781	0.978706841	59.28139093
0.354896678	-74.35680501	0.035121664	0.982203922	56.58034397
0.388469577	48.31564167	0.03729177	0.990280946	44.0929934
0.310206538	-50.39715892	0.017544669	0.989804956	85.66158385
0.384038207	-89.66868588	0.034697309	0.991120044	44.59831912
0.465247117	85.48785743	0.056175867	0.991738891	34.28325869
0.415662323	263.8371405	0.048131376	0.987117213	53.83935728
0.354604448	46.41257603	0.027432631	0.983416351	39.16649397
0.397110065	119.6774387	0.037877774	0.986989683	42.03095921
0.424472585	121.2540218	0.043106755	0.990245633	44.60811263
0.41555882	279.6366532	0.044769959	0.987371919	51.62963599
0.34592559	-306.6729146	0.033560113	0.987722376	82.68190197
0.356685565	-75.73540994	0.017778083	0.988749146	63.80207309
0.353956147	-117.8150275	0.025921651	0.990979029	67.96482428
0.382769255	-416.3742824	0.039653303	0.988321399	53.66699349
0.431014285	128.7411398	0.061345603	0.980897707	45.94293299
0.420537013	357.4992244	0.048182415	0.989984149	58.6879224
0.43299907	115.9588174	0.042280402	0.993071268	49.0428767
0.473373627	-12.66268219	0.067601705	0.991164754	31.16041371
0.415469613	261.6596594	0.033964121	0.991572524	57.35589283
0.33582128	62.77657435	0.023202056	0.98785904	49.9103079
0.336691688	-23.92014264	0.022315885	0.988491001	60.88916153
0.316035047	17.84900053	0.021057827	0.98762683	75.52812975
0.33723363	-205.0353489	0.026713966	0.991329773	79.13444312
0.466601275	396.142503	0.078807298	0.991422484	54.96136155
0.343055644	-83.95036654	0.026025903	0.986837616	52.79215411
0.449208777	-20.57411916	0.044235715	0.985190473	21.82645469
0.409740544	118.4888619	0.032823098	0.988965707	66.50128249
0.383644612	-162.2555353	0.034780646	0.993911535	60.33888018
0.360281565	-98.9426252	0.027574596	0.989347125	56.20380541
0.304858139	-133.8099282	0.018282284	0.980668748	56.02813803
0.326349238	-3.811463219	0.027027213	0.976012804	45.72335987
0.340945754	458.1761228	0.025382388	0.986369177	77.63302196
0.397209267	587.0741199	0.044628524	0.98875703	80.42737882
0.359120973	201.8803001	0.022832186	0.988097421	74.31319472
0.393025616	-10.46570019	0.02997178	0.987894795	45.18822644
0.351452484	17.23654544	0.026484287	0.991604044	69.20553694
0.294580126	-470.2707202	0.013890825	0.986778028	102.1324177
0.414830092	240.1956873	0.043101544	0.988915872	46.24424254
0.367584497	-71.29568292	0.02819494	0.991580945	67.18090463
0.353736618	-154.5547882	0.02514154	0.991682612	48.98811703
0.338677511	149.9526198	0.022151482	0.992508493	85.79958442
0.344595229	-48.6565969	0.032323381	0.990810743	71.2492766
0.418105554	-52.99175185	0.038047086	0.991202059	40.34642129
0.355521967	-37.33178973	0.028467836	0.985871402	53.34212603
0.336754075	-144.7451871	0.025970987	0.988339718	54.71623029
0.371825732	-237.6101117	0.036540864	0.990348011	57.71898803
0.35325533	-377.4448126	0.031969524	0.990642613	76.23768237
0.310455841	-218.0138575	0.019656311	0.988630127	82.61999596
0.332716068	146.6225453	0.02092567	0.990589488	64.35593879
0.493805791	88.23875186	0.088434169	0.980276364	28.25340834
0.34019617	-210.9405262	0.019120034	0.989528182	67.69313482
0.404789888	204.472983	0.028012005	0.98781601	55.37210265
0.343053537	-15.88803896	0.025609644	0.988265919	80.03497812
0.394561086	95.14178524	0.034377233	0.990173243	46.73589213
0.384484513	-9.061016294	0.045613085	0.962968556	23.18164341
0.387907292	-9.26366888	0.050539539	0.984167241	50.15995791
0.362774019	182.7293838	0.028126055	0.990769931	54.94520207
0.323448994	12.98011293	0.022665963	0.984565381	62.01622366
0.362189545	-168.1696914	0.030657258	0.985938243	45.61620627
0.382752823	-49.12891222	0.03431139	0.991959361	50.05339494
0.398059129	24.13488065	0.028024277	0.989673658	45.39461819
0.209947725	-1176.388904	0.015323125	0.977882633	169.6977252
0.444885771	65.57731324	0.055513172	0.991509205	35.48302457
0.409608182	113.7522411	0.050337417	0.982831454	50.16662276
0.470760685	111.6784339	0.070980464	0.985238662	31.92416608
0.235600112	-390.0865274	0.014734373	0.980812123	121.0482855

log.sigma.4.0.mm.3D_glcm_Contrast	log.sigma.4.0.mm.3D_glcm_DifferenceEntropy	log.sigma.4.0.mm.3D_glcm_InverseVariance	log.sigma.4.0.mm.3D_glcm_Entropy	log.sigma.4.0.mm.3D_glcm_Dissimilarity
13.85315388	2.951905136	0.340173886	7.611852756	2.658547845
9.682125509	2.780747931	0.352394346	7.272659952	2.300959693
13.18391504	2.972590052	0.313732703	7.544015476	2.741054439
8.228760181	2.66671735	0.365581697	7.234535232	2.140283748
7.186956267	2.518802913	0.407973119	6.532343836	1.871685086
7.059816828	2.56575868	0.39100078	6.851195614	1.952362545
4.189341142	2.200500716	0.420551419	5.869557065	1.419665399
9.226355975	2.713406779	0.376240689	7.312783673	2.192315558
10.87099117	2.835505788	0.35037236	7.453368584	2.379946091
11.44599199	2.887060622	0.327396551	7.702081531	2.570899069
5.434323278	2.40080635	0.417508759	6.51944718	1.677513334
11.60566214	2.877485563	0.328746511	7.485091469	2.577206
5.732527055	2.444008299	0.408854078	6.632562226	1.711432041
8.512271606	2.696323208	0.360302889	7.185733076	2.226762618
16.13111546	3.014813409	0.331262804	7.693161828	2.830579272
12.20204946	2.914808	0.324374162	7.58932076	2.457145774
10.10115125	2.771489636	0.357203603	7.204314185	2.321350452
8.207517408	2.642853985	0.387095236	7.10209424	2.047575166
10.84776372	2.809499555	0.334462682	7.151171064	2.442505897
8.322224833	2.671789787	0.37654151	7.252040748	2.109916759
11.54977204	2.88870866	0.34050243	7.604730986	2.503363277
9.530391208	2.748955281	0.354882645	7.363790489	2.302776968
10.42832571	2.828281011	0.339567736	7.595162449	2.435253357
9.175052776	2.702516903	0.358969195	7.190109225	2.269750872
12.16660283	2.930946966	0.319710201	7.733878479	2.655216308
15.38345261	3.06285116	0.309500678	7.708031684	2.887073975
8.184972457	2.664253051	0.362760331	7.256717958	2.125498706
13.69481289	3.005169321	0.30125556	7.771767137	2.852372935
10.40040443	2.786758474	0.352185291	7.254580775	2.345741616
11.98739138	2.874673367	0.353208718	7.371624373	2.471351121
10.55294429	2.805146878	0.359028011	7.510647099	2.356783154
10.40236932	2.807103572	0.353077082	7.511932226	2.370322056
9.332881582	2.699327291	0.379704342	7.294959268	2.195851433
12.8002188	2.802091291	0.358072453	6.778707894	2.441942021
12.03467246	2.875705561	0.338392093	7.287974601	2.51781227
8.547201851	2.687248691	0.373268577	7.103278332	2.131650039
14.57532243	3.036492555	0.313051901	7.955283675	2.848653305
8.316231319	2.681430184	0.370811848	7.095269426	2.12594815
4.937186814	2.328514136	0.419735319	6.418446788	1.60642062
7.242071663	2.568130462	0.392324871	6.893676975	1.943684513
9.355034766	2.748973178	0.351294465	7.07818705	2.3094596
7.954753505	2.638507642	0.379917181	6.966586813	2.060541405
6.944303541	2.546349067	0.397966442	6.880958195	1.884964212
9.344822562	2.651327647	0.387716887	6.912818338	2.072291292
18.02427973	3.098680839	0.319688729	7.889476867	2.917035685
8.548096569	2.703610167	0.357493378	7.494407371	2.232550512
11.51647704	2.877791321	0.344399687	7.612387208	2.472079059
11.18546994	2.810051339	0.371232685	7.07421877	2.339148861
6.722951329	2.430583031	0.396738801	6.236458948	1.871959159
8.314517569	2.633144806	0.38843363	6.818258377	2.014518446
6.941300498	2.532130041	0.402708352	6.895509031	1.853623133
6.373817797	2.43029292	0.419144762	6.293636095	1.687577652
6.89757272	2.545641256	0.398812628	7.066786649	1.903133933
12.68826241	2.833597198	0.3478077	7.260214202	2.544617352
10.86027625	2.838959559	0.337498412	7.568038352	2.480706366
14.17011566	3.010616514	0.320170622	7.80514623	2.80639821
14.57890409	2.991966317	0.331349375	7.751398792	2.713618222
7.606423403	2.557909156	0.392544284	6.615743563	1.837065297
10.86469582	2.848838653	0.337287329	7.290608497	2.472216835
4.53812657	2.292559375	0.429764993	6.225791011	1.598150621
8.564491169	2.670734536	0.383608607	7.312180229	2.059444169
10.59651034	2.791298689	0.367732892	7.372659285	2.287170237
10.76042503	2.814590114	0.351809846	7.387772585	2.386582097
11.83748094	2.891448383	0.306111185	7.520390331	2.690894977
12.77840224	2.887309911	0.326612362	7.114181959	2.666756638
12.20827961	2.898359277	0.341190489	7.435040937	2.557271541
9.358115948	2.722154721	0.36656463	7.117711413	2.171646333
11.40543585	2.848058387	0.355502879	7.591781526	2.421062744
8.030733191	2.610808169	0.387892591	6.955050974	2.061607272
11.30626914	2.869483614	0.342187899	7.567993956	2.469896219
17.02920063	3.141251345	0.297790611	8.207098073	3.094222418
8.099524384	2.573400305	0.398609529	6.778984699	1.976591185
10.12100408	2.795164092	0.356234678	7.515923131	2.320842906
9.959215998	2.793021272	0.347016661	7.36199651	2.353987145
12.44188662	2.918500154	0.337486513	7.832451774	2.572995187
13.13392054	2.951836327	0.338517742	7.594739174	2.620923991
7.763853749	2.592288038	0.397689379	6.848933281	1.967774942
10.12449913	2.771898645	0.348429575	7.204534584	2.364016773
11.76996781	2.877737021	0.339016453	7.455506557	2.548705853
10.33052433	2.80504713	0.360795461	7.33639429	2.32563057
17.53189488	3.054954673	0.341149896	7.446094558	2.820732776
16.34833366	3.10225227	0.311351469	7.9025339	2.970308118
11.31957496	2.875452046	0.332927356	7.559352795	2.528555614
5.70921714	2.280082433	0.421577933	5.743397666	1.601623583
10.50154715	2.825168734	0.340300196	7.63125382	2.439010393
7.464957371	2.601256646	0.390320896	7.171914116	1.982251165
12.73373014	2.923495121	0.337710327	7.647177396	2.585013911
7.492637916	2.601174351	0.382542658	7.029302845	2.020377596
7.136373041	2.293206487	0.38448087	5.858889181	2.031700139
8.165818053	2.63753589	0.365601728	6.782869915	2.113941408
9.857499985	2.78138382	0.359687039	7.339085103	2.310000369
11.96979827	2.894947721	0.322489988	7.47405846	2.607530014
9.285238779	2.747250656	0.357021476	7.150172748	2.281753163
8.541279524	2.683451722	0.374838079	7.170452083	2.14460178
7.315471729	2.595703412	0.384756828	7.127707718	1.995039285
39.99929003	3.70006275	0.203766292	8.509425498	4.839679495
5.522935134	2.407558865	0.41351154	6.440155238	1.707161812
6.643354338	2.413636337	0.391773413	6.481364632	1.918365112
5.145668497	2.338856926	0.418568824	6.24059803	1.611133738
23.65952271	3.347824054	0.238814615	8.27834965	3.777626691

log.sigma.4.0.mm.3D_glcm_DifferenceVariance	log.sigma.4.0.mm.3D_glcm_Idn	log.sigma.4.0.mm.3D_glcm_Idm	log.sigma.4.0.mm.3D_glcm_Correlation	log.sigma.4.0.mm.3D_glcm_Autocorrelation
6.555846735	0.921536354	0.342389527	0.730182418	212.3324787
4.239438374	0.930330198	0.368200662	0.711749292	256.6354795
5.455498401	0.915903046	0.319398053	0.661282022	199.4824403
3.506994663	0.927863202	0.376574908	0.735126044	168.3432529
3.480750993	0.920630521	0.438905739	0.760454974	100.2369427
3.142174509	0.926155544	0.407117982	0.729808705	132.8377314
2.016549193	0.931771356	0.516933644	0.806629275	77.70310298
4.299564467	0.94075238	0.380488317	0.763938296	317.4280461
5.082267662	0.92634856	0.36525108	0.720532711	199.1504787
4.655411465	0.937747029	0.326589167	0.727622747	565.1049751
2.5319537	0.930355049	0.456957819	0.723328525	151.6283061
4.740657602	0.922603811	0.324537674	0.654448482	242.1841079
2.725549539	0.94139574	0.453119591	0.726061191	185.7831815
3.426494802	0.916478949	0.359814979	0.687742776	137.2588235
7.598365808	0.922097412	0.337814049	0.657863293	237.0903629
5.912898653	0.938157799	0.382751351	0.716083989	370.472326
4.5114693	0.935783143	0.36649075	0.691476102	363.9796741
3.853660167	0.942859732	0.408091051	0.748953854	298.6970029
4.552003256	0.921749658	0.347395333	0.619513321	254.6916199
3.751493888	0.929087738	0.389107212	0.7482578	234.5763273
5.09015157	0.938076101	0.347239153	0.671879699	349.3576859
4.04057643	0.946447017	0.359458061	0.732136365	360.0639754
4.332100994	0.930643416	0.342586835	0.722931487	285.7355256
3.686814498	0.921390704	0.370170185	0.769375212	193.5003095
4.884651489	0.909618398	0.324345105	0.715058221	194.0508762
6.792114497	0.922093042	0.315166892	0.66620617	347.9081705
3.515810291	0.930715923	0.381543548	0.758667916	216.4364598
5.316564136	0.917573475	0.297558317	0.650615638	280.3642129
4.63708141	0.93524107	0.365246511	0.668374407	327.0487927
5.661948958	0.940610006	0.358582961	0.625157845	363.0231862
4.848274068	0.936733408	0.359384717	0.718371494	407.573376
4.598712396	0.937944004	0.35678495	0.701739492	313.723301
4.313810208	0.945167928	0.378852052	0.768159852	493.5157108
6.244801701	0.912172474	0.38571448	0.642543091	155.9327893
5.393786163	0.914659274	0.354896678	0.652037533	225.7890433
3.889618458	0.93506417	0.388469577	0.672476763	286.2405369
6.205586049	0.932002627	0.310206538	0.709271974	438.2204714
3.691506768	0.93700157	0.384038207	0.684837231	359.5677592
2.222352046	0.940393968	0.465247117	0.74428746	195.2276713
3.3043176	0.926735481	0.415662323	0.759630666	128.0134866
3.871122472	0.913999276	0.354604448	0.612399562	157.3988931
3.563210149	0.925513288	0.397110065	0.677365904	164.3047897
3.299790738	0.936089899	0.424472585	0.730100572	250.9867495
4.829183045	0.931292302	0.41555882	0.690311799	164.0918401
9.029450503	0.931835613	0.34592559	0.639831738	489.0865591
3.455130979	0.927233067	0.356685565	0.763040823	199.0836054
5.206398109	0.937339069	0.353956147	0.711337959	331.1334072
5.540955265	0.932207439	0.382769255	0.65445823	362.1568491
2.825522062	0.912475562	0.431014285	0.745031534	85.41650656
4.136738716	0.936841894	0.420537013	0.750629675	171.4543342
3.406229476	0.946364039	0.43299907	0.751036459	315.5110857
3.429563412	0.942844506	0.473373627	0.661032777	270.9563945
3.186986527	0.939496937	0.415469613	0.784100604	192.0859564
5.756026835	0.928562185	0.33582128	0.602356426	416.842801
4.526183098	0.927432039	0.336691688	0.697517711	304.6427717
6.012907739	0.925795035	0.316035047	0.685559982	335.4370978
6.920567462	0.939809031	0.33723363	0.689947495	609.3112406
4.105370451	0.944178113	0.466601275	0.75727341	197.0025186
4.572419017	0.923220992	0.343055644	0.658785227	349.4222276
1.911659913	0.918918186	0.449208777	0.656668617	126.956252
4.220948877	0.933384152	0.409740544	0.771320426	245.3408839
5.199754388	0.949645407	0.383644612	0.700406432	446.3630845
4.840111031	0.932367731	0.360281565	0.680215373	314.029046
4.34666936	0.904631586	0.304858139	0.65192359	228.9171695
5.1208102	0.899379777	0.326349238	0.566544825	165.384578
5.443829757	0.923700784	0.340945754	0.728072994	192.5492571
4.507089903	0.932291348	0.397209267	0.78945881	174.0493135
5.400640922	0.929698437	0.359120973	0.734477434	261.6803704
3.612745604	0.928129825	0.393025616	0.693289637	263.1689732
5.011149295	0.938840225	0.351452484	0.717291952	443.7821203
7.138928192	0.923149238	0.294580126	0.714684535	430.8614349
4.036800401	0.933687415	0.414830092	0.697285472	229.2761734
4.573336607	0.939199899	0.367584497	0.737366249	306.4728322
4.25093127	0.938158285	0.353736618	0.661261637	375.1297362
5.653842098	0.94233702	0.338677511	0.745770569	358.8717769
6.057323974	0.937270243	0.344595229	0.685848995	335.9166438
3.762498083	0.939900175	0.418105554	0.676916001	270.5239134
4.249268252	0.921391328	0.355521967	0.680106955	229.1931135
4.965366282	0.9280131	0.336754075	0.648761402	353.6813935
4.768512708	0.935823968	0.371825732	0.696193944	394.070097
9.305184855	0.941022244	0.35325533	0.627752821	708.0598867
7.221437102	0.92969195	0.310455841	0.672225079	439.7818742
4.770132955	0.933966336	0.332716068	0.699136418	358.8886681
2.752734977	0.91691153	0.493805791	0.663421171	79.37582074
4.375226249	0.930560936	0.34019617	0.730297656	356.3629782
3.459056224	0.928072936	0.404789888	0.762326783	118.3595917
5.847284409	0.92948785	0.343053537	0.723713849	319.5544201
3.281951474	0.933848844	0.394561086	0.720653195	218.4216361
2.09483966	0.875303808	0.384484513	0.533673851	66.01008456
3.481816267	0.917658504	0.387907292	0.715062467	175.0973046
4.408772979	0.935970868	0.362774019	0.696322966	310.7399676
4.965613812	0.916858549	0.323448994	0.675992044	258.6412879
3.928985662	0.920999685	0.362189545	0.661835176	236.5361683
3.790135444	0.9401229	0.382752823	0.707935134	384.2736265
3.231531113	0.932369632	0.398059129	0.721533143	182.3339908
15.73839313	0.900980755	0.209947725	0.62182396	571.4626078
2.507372337	0.939239105	0.444885771	0.728412623	248.7684465
2.554016315	0.913935713	0.409608182	0.761382813	98.33412615
2.400978185	0.923320825	0.470760685	0.720905121	77.01130491
8.833467519	0.905507392	0.235600112	0.673564724	373.8501505

log.sigma.4.0.mm.3D_glcm_SumEntropy	log.sigma.4.0.mm.3D_glcm_AverageIntensity	log.sigma.4.0.mm.3D_glcm_Energy	log.sigma.4.0.mm.3D_glcm_SumSquares	log.sigma.4.0.mm.3D_glcm_ClusterProminence
5.153209838	13.95532288	0.008328657	26.30372141	19747.95717
4.860162176	15.67766995	0.010304346	17.27510277	10424.56024
4.954099633	13.67528414	0.007862961	19.61733296	12969.68467
4.847852912	12.52792753	0.010407295	15.57900943	8924.678957
4.612815377	9.416750402	0.020930897	15.14605121	7425.467788
4.64228724	11.09596207	0.014935053	13.23505096	7460.658842
4.265090122	8.274050983	0.033415676	11.3128907	4140.006184
5.009621855	17.45884713	0.010320941	19.98193239	16148.36576
4.970045881	13.63158754	0.009103065	19.83621063	11731.80782
5.099371011	23.50745044	0.007053385	20.57165818	18585.48308
4.468350614	12.01993337	0.01763017	9.848934254	3506.860284
4.899826787	15.23229016	0.00810263	17.04850626	10297.75099
4.540045132	13.34518318	0.018197151	10.62741362	5340.273339
4.769911951	11.3059612	0.00977985	13.67894206	6130.547843
5.064422787	14.96282081	0.007316613	24.17516751	19741.70774
5.056028543	18.95664172	0.011459406	21.21129581	19667.52412
4.827846076	18.84433091	0.01145347	16.63931418	14833.94327
4.856026465	16.98810247	0.011709016	16.73311942	10134.88364
4.7344381	15.69490457	0.009581839	14.3862324	6319.582266
4.898438313	14.91022521	0.010982986	16.66733157	11412.27913
4.944544714	18.46181805	0.008140648	17.52991778	13238.931
4.955596451	18.71910118	0.009289132	17.54134919	14464.62219
5.016551994	16.56240708	0.008086871	19.26540034	13553.02685
4.94787742	13.34500075	0.011078148	19.98508467	12844.54481
5.063512385	13.36850232	0.006481593	21.40822574	13822.17073
5.077794587	18.33299471	0.007227649	23.29553698	20885.58976
4.932429274	14.27324511	0.009375225	17.41795046	9735.414308
4.996649988	16.41649399	0.006519847	19.99338741	12646.62305
4.800550717	17.87041948	0.011363045	15.8893655	12604.80261
4.810077781	18.88761604	0.010576787	15.69114315	12016.46772
4.985358822	19.94567005	0.008691053	18.63364369	16559.08227
4.948204938	17.42284914	0.008326804	17.72154899	13785.23885
4.958603969	21.90684287	0.010768985	19.81178019	27736.54563
4.712873086	12.01217276	0.016309913	18.02040243	7772.151989
4.84670501	14.6503408	0.009800318	17.15375411	9551.520698
4.716940877	16.68113386	0.011964305	13.41104481	6564.634223
5.195317078	20.60365211	0.006346341	24.60347091	27104.0817
4.730902856	18.76431456	0.011947029	13.44132906	7900.094175
4.47170693	13.7072597	0.019737881	9.805111375	4113.501163
4.737117933	10.7868291	0.014406502	15.27035724	8250.093255
4.62536649	12.24488593	0.010507551	12.13038218	4218.698806
4.644273083	12.48075211	0.012346087	12.49642818	4696.753885
4.673475806	15.54537971	0.013607925	12.93365987	5725.776353
4.703831621	12.39387888	0.015167153	15.30971121	8681.669512
5.14369555	21.86138569	0.007893786	24.48394994	25867.62304
5.020852571	13.61340387	0.007713914	18.16197815	11649.34161
5.030674976	17.90152017	0.008565662	19.96689906	19002.01895
4.69767637	18.83033523	0.013857846	16.49094156	13861.38371
4.525994834	8.69401567	0.020901643	13.16647108	4559.225353
4.780154041	12.62149093	0.016041242	17.06472602	11432.26845
4.725710664	17.50799794	0.015136094	14.26258393	8677.887404
4.311325761	16.27667779	0.024685576	9.452881503	6095.383272
4.83327131	13.4077633	0.01199089	16.24285256	9280.961817
4.796711258	20.3102827	0.008998286	15.6712317	7595.539393
4.974507828	17.15953517	0.007663498	18.42073471	11161.77745
5.110419776	18.00219838	0.00683937	22.7151906	18926.43666
5.12545922	24.44210708	0.00772259	23.38770684	25666.9153
4.611541185	13.63156023	0.024660822	16.01088665	11955.08223
4.823423738	18.46615361	0.009475087	16.30486644	10342.23318
4.233942081	11.07380615	0.018850108	6.591145314	1490.855538
4.987914613	15.20863923	0.009967517	18.96891504	10528.70333
4.914700343	20.88096026	0.011067206	17.3291685	16012.30996
4.892087907	17.48100281	0.009391509	17.1376277	11588.50267
4.892947977	14.75994541	0.007123353	16.96640474	8501.527189
4.670791694	12.53466248	0.009561624	14.62544053	4792.579593
4.977626037	13.29715445	0.008814068	22.85340742	16582.40756
4.944161532	12.53136151	0.012503223	22.94753659	19628.38049
5.060379994	15.75955332	0.008207568	22.14793877	14325.97512
4.715524364	15.93250211	0.01177153	13.30473991	6048.493453
5.02644654	20.77244515	0.00853552	19.87412032	17254.1769
5.320516312	20.3480231	0.004847119	29.42497268	31605.43332
4.60256711	14.83446753	0.015843001	13.73026653	7365.087381
5.013842123	17.16490541	0.009062607	19.58004868	17652.71857
4.799892751	19.19953854	0.009475356	14.6617742	9349.151583
5.203097538	18.53119012	0.00713606	24.41536927	26473.25823
5.024711864	18.02949235	0.008681761	21.10648332	20452.05644
4.616483826	16.21720678	0.014623424	12.21569024	6980.235911
4.806282527	14.78105221	0.010076437	15.90876286	9215.202938
4.85063921	18.61551309	0.009543562	16.89446856	12102.83896
4.841928285	19.63702304	0.011789918	17.04429593	15149.45327
4.968059179	26.4630113	0.010617788	23.2103989	34190.81333
5.157286644	20.6926856	0.006915133	24.1260924	27163.0126
4.971702019	18.65676178	0.00797875	18.9844704	14632.74338
4.150773021	8.586647607	0.03214015	8.490656369	1813.804105
5.048943176	18.57053546	0.007361289	19.82170402	16051.43991
4.853247415	10.31337191	0.010321472	15.709265	8154.160968
5.137848166	17.4669695	0.008002942	23.54798907	20935.87791
4.739079385	14.44792	0.011982793	13.63174817	6108.696859
4.074135303	7.873312164	0.021522571	7.579504114	1014.358337
4.71643076	12.8280106	0.014506169	14.58144399	8027.879488
4.849479091	17.37773203	0.009379477	16.58046145	8057.713099
4.942667829	15.71714355	0.007908633	18.85614871	10713.35261
4.727251048	15.0809745	0.010837381	13.72536126	7921.599631
4.774643051	19.39978627	0.01235399	14.69830804	11205.62168
4.771765888	13.14666454	0.010996669	13.19992046	6691.876889
5.587839542	23.42251346	0.00395368	51.42871732	87842.18058
4.463303594	15.53297945	0.019285595	10.25148993	4353.803129
4.620029252	9.349444553	0.016132648	14.20249427	5239.087657
4.366513943	8.385049691	0.023415572	9.267458645	2971.845977
5.408620003	18.82146485	0.00437786	36.14642225	45573.68714

log.sigma.4.0.mm.3D_glcm_SumAverage	log.sigma.4.0.mm.3D_glcm_Imc2	log.sigma.4.0.mm.3D_glcm_Imc1	log.sigma.4.0.mm.3D_glcm_DifferenceAverage	log.sigma.4.0.mm.3D_glcm_Id
27.82250903	0.921756664	-0.227883399	2.658547845	0.418406101
31.27793864	0.861543916	-0.178295341	2.300959693	0.442079319
27.32324001	0.859060743	-0.168415458	2.741054439	0.401117323
25.05236865	0.860657537	-0.179721021	2.140283748	0.450053038
18.8335008	0.915387485	-0.254430706	1.871685086	0.498189904
22.19192413	0.862398076	-0.189027765	1.952362545	0.474112858
16.54810197	0.91162046	-0.27819499	1.419665399	0.564205486
34.77705178	0.908687525	-0.22043325	2.192315558	0.45137534
27.20954427	0.87834203	-0.184599898	2.379946091	0.439153748
46.89416846	0.861841253	-0.169953248	2.570899069	0.408067558
24.03986674	0.852940251	-0.191470179	1.677513334	0.514271201
30.41106405	0.833891582	-0.155208571	2.577206	0.406456246
26.6864693	0.851941864	-0.188164288	1.711432041	0.511704195
22.6119224	0.833270477	-0.160993815	2.226762618	0.436017073
29.77724364	0.875620265	-0.185250059	2.830579272	0.413380662
37.69456611	0.878572689	-0.1879084	2.457145774	0.453860243
37.54985263	0.869392373	-0.189149073	2.321350452	0.440182224
33.84751924	0.888049866	-0.207781345	2.047575166	0.473694831
31.36141537	0.845869776	-0.173037263	2.442505897	0.425805433
29.80936269	0.872895062	-0.188627063	2.109916759	0.459157557
36.74312743	0.801235569	-0.137195658	2.503363277	0.424046127
37.25900469	0.880158333	-0.192901957	2.302776968	0.435237435
32.99377779	0.844999164	-0.160699779	2.435253357	0.421479597
26.6900015	0.922356556	-0.245229718	2.269750872	0.443128824
26.73700463	0.838523105	-0.155113487	2.655216308	0.405500853
36.47525009	0.87340462	-0.1762874	2.887073975	0.396599323
28.51629965	0.892021261	-0.206639875	2.125498706	0.454355855
32.71993251	0.791064952	-0.126733575	2.852372935	0.38360041
35.58738266	0.823843355	-0.159549803	2.345741616	0.439455614
37.58142275	0.812868112	-0.14545403	2.471351121	0.43250049
39.70122109	0.856201153	-0.170639476	2.356783154	0.434301711
34.72013214	0.834906714	-0.157915469	2.370322056	0.432427372
43.72971428	0.89874876	-0.212195606	2.195851433	0.449847258
24.02434553	0.930251309	-0.269459621	2.441942021	0.453524947
29.30068161	0.881121282	-0.195727396	2.51781227	0.430001577
33.30729418	0.830588477	-0.160476831	2.131650039	0.458605793
41.00905162	0.855697353	-0.16064011	2.848653305	0.392736919
37.44284321	0.826184736	-0.157292459	2.12594815	0.455514177
27.41451941	0.878842485	-0.224437998	1.60642062	0.521719657
21.5736582	0.891462313	-0.215484544	1.943684513	0.480718865
24.48977185	0.800122195	-0.141842094	2.3094596	0.431610448
24.96150422	0.826938859	-0.164206542	2.060541405	0.465671426
31.08438564	0.863568433	-0.18874556	1.884964212	0.48802119
24.77991921	0.880721975	-0.20614081	2.072291292	0.479614049
43.49320218	0.855981846	-0.167486423	2.917035685	0.419948706
27.21834379	0.872117646	-0.181286643	2.232550512	0.433905795
35.61001314	0.851890682	-0.165814073	2.472079059	0.429459621
37.49800967	0.845928453	-0.169999684	2.339148861	0.451445901
17.38803134	0.941376886	-0.308981104	1.871959159	0.492988294
25.20695055	0.916714774	-0.244595534	2.014518446	0.483745802
34.92696218	0.877767234	-0.201443503	1.853623133	0.49472273
32.54268028	0.846051421	-0.190202953	1.687577652	0.526953173
26.79136521	0.888631021	-0.207314935	1.903133933	0.480746811
40.37425681	0.854229487	-0.175287903	2.544617352	0.414668306
34.18333762	0.83857988	-0.155896878	2.480706366	0.416472923
35.7822682	0.862090537	-0.168315957	2.80639821	0.397116048
48.71003576	0.869064906	-0.176174886	2.713618222	0.414605826
27.21445554	0.896763547	-0.2282799	1.837065297	0.521636778
36.82000156	0.846336283	-0.165489104	2.472216835	0.421427008
22.14761229	0.795538124	-0.158782191	1.598150621	0.508771097
30.38731595	0.903560367	-0.21404428	2.059444169	0.47528572
41.66163768	0.849159804	-0.168933894	2.287170237	0.453305315
34.79356694	0.855387997	-0.172519341	2.386582097	0.435057389
29.51989082	0.818998204	-0.144188623	2.690894977	0.391152948
25.06932497	0.862793701	-0.181764328	2.666756638	0.406655522
26.54528491	0.883687755	-0.19150848	2.557271541	0.418476189
25.00152243	0.93079917	-0.25332547	2.171646333	0.465382547
31.3634546	0.877582728	-0.183250875	2.421062744	0.433504649
31.86500422	0.870051055	-0.196658949	2.061607272	0.461961477
41.43923332	0.866454327	-0.177472964	2.469896219	0.427624187
40.47573897	0.853188047	-0.155027638	3.094222418	0.378375631
29.64690345	0.867076593	-0.197383364	1.976591185	0.479417769
34.18665636	0.865263133	-0.177484668	2.320842906	0.441168921
38.22928664	0.790366218	-0.134726475	2.353987145	0.430798673
36.90376747	0.881613535	-0.182896908	2.572995187	0.416959057
35.85193167	0.861235431	-0.172882886	2.620923991	0.420550171
32.39565522	0.845050031	-0.176570946	1.967774942	0.481838243
29.55554361	0.862152673	-0.184327901	2.364016773	0.431881485
37.03716247	0.804878319	-0.142312757	2.548705853	0.415392421
39.10069559	0.830784771	-0.157273077	2.32563057	0.443925555
52.66361485	0.898108766	-0.203798869	2.820732776	0.425333751
41.14412246	0.850804909	-0.157651313	2.970308118	0.39150181
37.18161245	0.84870939	-0.163032768	2.528555614	0.413111705
17.17329521	0.899878691	-0.275202268	1.601623583	0.543410386
36.98898137	0.851019226	-0.165233295	2.439010393	0.419631307
20.62674382	0.873280265	-0.188498533	1.982251165	0.472058418
34.79631494	0.90112309	-0.204685818	2.585013911	0.420101878
28.88520983	0.852078779	-0.178917672	2.020377596	0.46424864
15.74662433	0.870430444	-0.239207233	2.031700139	0.455872731
25.65602121	0.911540589	-0.244730637	2.113941408	0.459092697
34.60992207	0.838287606	-0.158874817	2.310000369	0.43731916
31.37576081	0.876466996	-0.183122635	2.607530014	0.405836585
30.16194901	0.822392606	-0.15487564	2.281753163	0.43734899
38.6721027	0.833600549	-0.163856085	2.14460178	0.453863886
26.29071518	0.839348019	-0.166792213	1.995039285	0.467151073
46.4322079	0.933035968	-0.216827739	4.839679495	0.299095941
31.0659589	0.861726789	-0.202188919	1.707161812	0.505110247
18.69888911	0.934060095	-0.290259763	1.918365112	0.476352469
16.77009938	0.87598044	-0.222414721	1.611133738	0.525973271
37.38732307	0.903382239	-0.190160108	3.777626691	0.328187182

log.sigma.4.0.mm.3D_glcm_ClusterTendency	log.sigma.4.0.mm.3D_firstorder_InterquartileRange	log.sigma.4.0.mm.3D_firstorder_Skewness	log.sigma.4.0.mm.3D_firstorder_Uniformity
88.98999399	180.5195675	0.265666925	0.059931219
57.83949885	137.0218058	0.192842013	0.075141
64.49218095	141.6388931	-0.265721254	0.069468224
53.95894662	123.300663	0.376875193	0.075438463
53.39724855	123.0043411	0.670552998	0.095610598
45.88038703	96.8779633	0.629232326	0.092262371
41.06222166	134.639472	0.730495793	0.11819299
69.4060688	143.6617546	0.190978301	0.06989664
67.01708916	155.5563221	0.366557921	0.06884197
72.77504034	147.9437408	-0.264914594	0.063858109
33.96141374	97.64597702	0.383096589	0.099603124
55.46876745	125.4713211	-0.101582598	0.071568474
36.63905873	91.72904587	0.523694257	0.098371018
46.20349665	116.7877653	-0.10849859	0.077303975
77.78987894	154.9183216	0.618001126	0.063005362
73.16590396	141.9928665	-0.050353023	0.069149563
55.91562632	113.4626627	-0.422975356	0.077817956
57.24634691	147.9901085	0.450477859	0.075419922
46.0118726	135.4285879	-0.012404184	0.076237354
58.01364596	122.3147488	-0.075025824	0.076211284
58.90022137	129.1875443	-0.332655042	0.070928751
61.48813104	131.7557621	0.08065965	0.072192247
64.75055054	128.3145523	0.190270226	0.069652947
70.76528591	156.6198635	0.059438766	0.070040532
73.46630012	156.4807072	-0.33531882	0.062345594
76.44550681	145.944376	0.13803672	0.064291123
60.62399423	146.8988048	0.380746136	0.069187593
64.48850117	146.288697	-0.388090059	0.066828832
52.106224	111.6764736	-0.428909407	0.080546595
51.69792864	120.8684502	-0.129522911	0.074246131
64.57096869	134.0911036	-0.516868712	0.068593539
59.73541756	136.9509077	0.027472347	0.06944792
71.06695234	123.7654066	-0.498139776	0.073812032
59.28139093	165.541605	0.302842864	0.078193818
56.58034397	126.5713443	-0.197370311	0.072650353
44.0929934	107.8599148	0.097375612	0.083882262
85.66158385	148.0304279	-0.176094584	0.059823465
44.59831912	102.2927999	-0.348859267	0.085486666
34.28325869	93.4124707	0.247967355	0.099357205
53.83935728	130.6536741	0.676349355	0.085001435
39.16649397	122.6876187	0.095209949	0.081442106
42.03095921	127.2146826	0.401914562	0.082617483
44.60811263	125.2008686	0.261151605	0.084117843
51.62963599	123.9729023	0.733818242	0.086442542
82.68190197	129.8574533	-0.49193496	0.064751939
63.80207309	141.2217062	-0.162573082	0.067367832
67.96482428	132.3073163	-0.186256468	0.068133622
53.66699349	96.48866105	-1.004661685	0.090057853
45.94293299	131.2830319	0.507293784	0.092764448
58.6879224	139.0712686	0.765223745	0.083296019
49.0428767	114.4219885	0.228472487	0.085409157
31.16041371	74.46293736	-0.162221584	0.118763618
57.35589283	138.4738541	0.531025919	0.075129035
49.9103079	134.2098684	0.030917711	0.072914243
60.88916153	147.6648636	0.002674145	0.066491165
75.52812975	149.8394423	0.049873069	0.061150038
79.13444312	142.9455023	-0.406823834	0.066400815
54.96136155	103.0481009	0.975535363	0.105163743
52.79215411	114.7542195	-0.271891096	0.077024667
21.82645469	82.960392	-0.321090354	0.112323299
66.50128249	156.030056	0.186981732	0.067271898
60.33888018	120.7303171	-0.346861686	0.077098415
56.20380541	128.3591404	-0.140831377	0.072753716
56.02813803	155.0722108	-0.256400001	0.067354956
45.72335987	149.832572	-0.033949955	0.073323758
77.63302196	174.6575298	0.626352848	0.067312543
80.42737882	153.7293916	0.830500998	0.074017031
74.31319472	157.3304644	0.3415532	0.065284426
45.18822644	120.4086342	-0.091846683	0.080882305
69.20553694	137.3626108	-0.021972927	0.06740202
102.1324177	183.783619	-0.399335092	0.05244722
46.24424254	104.5649796	0.667167245	0.091849959
67.18090463	133.9178848	-0.045033427	0.0706234
48.98811703	119.8689804	-0.404727547	0.075523739
85.79958442	157.1894755	0.076568933	0.060327194
71.2492766	146.812623	-0.063484584	0.067668514
40.34642129	99.20548344	-0.241015055	0.091433594
53.34212603	127.0383663	-0.033002441	0.077340662
54.71623029	114.051908	-0.294597485	0.076433335
57.71898803	105.8839965	-0.493953385	0.08020446
76.23768237	111.7029538	-0.623198674	0.077446141
82.61999596	134.185123	-0.428543797	0.065213016
64.35593879	135.1485977	0.129391631	0.067856557
28.25340834	113.43015	0.594219242	0.118382329
67.69313482	142.5494661	-0.386479064	0.065094813
55.37210265	142.283041	0.469525239	0.07324946
80.03497812	142.2399845	-0.043195794	0.062659377
46.73589213	122.7800732	0.256018409	0.081243765
23.18164341	122.2653103	0.000988141	0.104457113
50.15995791	107.2635574	-0.059842012	0.084194394
54.94520207	143.0409317	0.431708045	0.072080063
62.01622366	141.438942	-0.081343941	0.067273807
45.61620627	106.6097685	-0.590457281	0.084569341
50.05339494	103.7681427	-0.150785093	0.082563369
45.39461819	115.4510765	0.097851011	0.080078619
169.6977252	235.9721298	-0.614158005	0.045829836
35.48302457	91.03699017	0.256986505	0.103905225
50.16662276	154.6460991	0.355047287	0.07988689
31.92416608	96.73387003	0.637493112	0.107922957
121.0482855	186.5674133	-0.385603757	0.049815426

log.sigma.4.0.mm.3D_firstorder_MeanAbsoluteDeviation	log.sigma.4.0.mm.3D_firstorder_Energy	log.sigma.4.0.mm.3D_firstorder_RobustMeanAbsoluteDeviation	log.sigma.4.0.mm.3D_firstorder_Median
103.1646392	39914702.36	74.31129659	110.4634399
82.0194923	41751894.76	57.23453885	85.86351013
85.81964718	14150147.24	59.08398397	40.76102066
78.3829759	49841800.02	53.67216797	89.75480652
78.50309945	14188955.98	54.47371085	45.87165833
67.90931383	19832536.06	42.48264296	49.89552689
72.97277352	13295290.64	55.20160216	37.74140167
87.85412095	48720769.09	59.34863034	104.1186829
88.96246906	55118928.97	64.17320236	107.1015015
90.32975032	70758668.23	61.73454466	97.29753876
61.3536345	24848847.11	41.73748138	74.99407959
80.28900423	31351557.95	53.87949543	90.58778381
63.1830853	36880230.7	40.21444713	63.17995834
73.22341985	25284112.69	50.20321932	70.30644226
95.8965351	38618744.53	64.85744861	82.38533401
89.88616278	68731717.09	59.90407361	50.14094925
78.14617567	22834745.44	48.63419144	61.03133774
82.71116361	72226437.55	60.21943972	116.5418472
76.25672699	22213837.59	54.69210094	99.29914474
78.17484229	50612673.74	52.24387748	75.46367645
81.39223951	93127270.09	54.05876358	52.53112221
80.87317463	37709347.42	53.92453691	76.56671906
83.62992854	70089591.5	55.45848561	65.03215027
90.80458431	18921579.93	65.13920764	57.05234718
93.61553163	35914085.91	66.53964635	35.42722511
93.15119234	37510382.76	61.91233384	104.4871101
84.61251164	38189534.17	60.03112885	67.84184265
86.89842301	54298455.98	60.24636218	77.42855835
75.76392099	57078677.19	48.05139474	77.44163513
79.29827416	53555885.78	50.7650429	75.44591522
84.66532605	80698085.02	55.93576366	89.50206375
83.35626029	128143786.6	56.53110981	114.0666809
83.41237255	52418080.38	53.30833825	81.29885864
92.08104378	11454571.89	70.30102881	84.7095108
80.27585942	12221809.84	54.15771557	51.97660446
70.34117507	46232310.98	46.76108433	69.80947876
97.1909645	94728423.91	63.54630936	101.1873703
68.95861921	50847877.41	44.26633171	80.49082947
62.03476711	14469458.53	40.42019284	45.39864349
78.33950843	34574917.35	54.66222908	83.52592468
70.79841984	21311651.95	49.73656241	84.06551361
73.28257164	34627675.78	52.53436267	83.12303162
72.67972539	54372427.97	51.90328951	86.94786835
77.79539603	35481459.13	52.24509156	72.1868515
93.73018368	65885809.55	58.9543743	62.13419724
83.91653242	60649871.92	58.94709654	82.44489288
86.18517874	74817305.23	55.78177746	84.05160522
74.11059193	16862895.1	43.66016101	42.28700256
74.88540908	8204634.595	55.57177118	54.53925133
82.10106033	23208388.42	56.30852841	77.02233505
73.95028304	73423588.19	49.20530542	80.26910019
54.44007874	21000654.67	32.37508124	62.18640518
82.07768974	97672146.18	58.07084451	104.6702881
79.6626379	20672543.83	55.07085473	81.49378967
86.47675998	94170872	61.24938173	106.0024185
93.94618316	33876584.52	62.86665545	56.21142387
90.25352611	76567713.51	59.64891996	91.43475342
74.70960439	38023647.89	47.37775331	48.49665642
75.96962503	20598273.45	49.96617389	70.19846344
50.19607072	16798078.47	34.23702439	89.29424286
88.84308772	91795236.89	65.41682451	124.9402542
79.31537984	102932263.3	51.13206027	80.20233917
79.87973577	45553560.27	53.54735571	94.75169373
85.50774215	39720030.92	62.92692508	103.7581902
82.20387412	13590558.1	62.20696827	116.0394211
97.25705852	44477304.85	70.8315443	91.33415985
93.84823959	25094891.58	64.18336452	66.60555267
92.27472845	105425248.3	64.67777476	112.355484
71.28637404	19764890.77	49.22963653	79.86642456
88.09507772	49824361.27	58.00326934	72.39105988
109.9679108	104818087.9	76.50985137	104.1530457
70.77280785	40960538.26	46.00591648	87.99728394
84.72568172	116977437	56.59907861	104.9243164
76.39424272	113975839.8	51.23869466	94.26741028
97.10172955	112483033.8	65.30362031	94.16007614
91.70030016	49766216.42	61.91540936	73.62129211
65.26104411	27495260.7	41.81623811	61.14022446
77.05433997	28125580.1	53.24103404	96.31303406
77.24703512	51767728.6	49.17460969	75.97558212
76.67117911	60900791.9	46.71161455	54.64432907
83.25896706	25669806.89	48.49024928	65.53549576
92.6702348	49697397.08	58.49391675	64.30639648
86.78632148	58353265.87	58.29192179	95.81608963
62.16665928	5914916.959	46.38165147	53.24175644
88.10396887	89301097.03	59.34510029	105.1760712
80.81458979	65109771.39	58.4673434	79.6776619
93.83837497	34522161.06	61.35882098	89.78142929
73.59495266	55283401.72	51.54151482	86.30812073
60.57857508	6563441.646	49.11129669	104.9044342
74.36308114	9539260.871	49.21829838	72.81263351
82.72166124	78632393.22	59.20564289	102.8961639
84.61109824	23921242.21	58.02094369	97.3546524
69.73322774	15489424.38	45.24501644	47.90473557
72.98878867	65666252	45.18603947	70.14796448
71.49135837	103577493.3	48.2750667	112.8311653
143.5250045	28823421.53	98.65736661	63.25636101
61.79195543	22229471.89	40.94504989	64.67415619
81.33382436	10163293.13	63.14081931	108.5610542
60.49920018	9645692.752	40.36275803	53.52959442
118.6256635	29921742.48	80.07061866	68.401474

log.sigma.4.0.mm.3D_firstorder_TotalEnergy	log.sigma.4.0.mm.3D_firstorder_Maximum	log.sigma.4.0.mm.3D_firstorder_RootMeanSquared	log.sigma.4.0.mm.3D_firstorder_90Percentile	log.sigma.4.0.mm.3D_firstorder_Minimum
1077696964	508.9642334	182.1518699	306.5983643	-198.3729095
1127301159	427.9973755	144.4851113	250.7515503	-257.9555359
382053975.5	376.4356995	112.5018276	151.1998947	-291.9514465
1345728601	438.5185852	142.5437189	244.6836121	-194.9060822
383101811.5	330.6635742	122.2762509	230.8338654	-139.6258392
535478473.7	355.3322754	110.0353369	200.0479416	-196.6223145
358972847.4	305.6370544	112.1531366	206.4350006	-117.353508
1315460765	504.7410889	164.2927748	278.9687317	-283.7278442
1488211082	476.3285828	164.9010841	277.3747925	-199.0179138
1910484042	443.6617432	153.5013358	250.8547333	-454.5973206
670918872	314.737854	117.2665223	207.1984222	-185.6530609
846492064.6	425.0263977	136.4047867	219.4013275	-258.1670532
995766229	399.2998047	112.2114382	188.646286	-246.8540039
682671042.6	352.4287415	116.6543676	194.1882477	-191.5140228
1042706102	509.1394653	157.9478059	258.3695923	-242.8032532
1855756361	460.2111816	129.3333498	212.0380249	-378.8318481
616538126.9	395.4154968	119.2780605	177.9262207	-381.5922546
1950113814	511.9669495	168.0342559	272.0957611	-260.0113525
599773615	391.2640991	139.8371714	230.7146835	-255.4546967
1366542191	363.2749329	132.085692	225.8798676	-260.4897156
2514436292	496.7239685	115.7566724	175.3194962	-377.3588867
1018152380	592.1253052	133.3380729	213.7807465	-352.3625488
1892418971	435.018158	130.4143728	220.3036194	-312.3344727
510882658	365.12146	135.537692	239.7291687	-248.3393402
969680319.5	301.2964478	117.4388848	165.1320282	-280.0671387
1012780334	464.8789368	166.566401	279.2906647	-314.0967407
1031117423	405.6692505	131.4249466	235.3072968	-243.306778
1466058311	407.2314758	127.2176819	193.2326965	-310.7629395
1541124284	434.542572	129.5869796	206.5204803	-343.0074463
1446008916	546.7841797	130.2053383	206.4121002	-359.099884
2178848296	416.6679688	139.5810841	221.7363373	-397.0520935
3459882239	533.6499023	160.0898414	257.4404816	-292.1000977
1415288170	435.9195251	143.9111632	235.705719	-446.9565735
309273441	387.4577332	154.318109	264.8760986	-169.4091797
329988865.6	304.9941406	117.317089	191.9774887	-276.6963196
1248272396	375.8885803	119.8801481	202.5616028	-306.6171265
2557667446	517.6179199	162.9171791	270.312384	-380.760437
1372892690	350.9841309	122.4538712	197.1958008	-350.1433105
390675380.2	320.2022095	98.74365286	170.1190521	-256.7692566
933522768.5	422.0855103	145.3747315	252.9933853	-140.2893677
575414602.6	354.7653809	127.2566871	214.8035507	-195.3883209
934947246.1	394.1714478	133.8776592	230.1230728	-178.5074158
1468055555	364.3085327	134.0241782	228.1251068	-265.4551086
957999396.5	441.8468323	137.2335464	242.3341949	-177.0245972
1778916858	436.0883789	137.3202208	210.9801498	-461.0301514
1637546542	415.1477356	130.2497568	208.7185181	-244.758316
2020067241	523.9443359	142.4699807	225.5401001	-340.2112122
455298167.7	324.6190796	105.4669698	126.9751091	-417.1572266
221525134.1	301.097168	120.6118753	223.2731064	-116.9840469
626626487.5	486.8777771	145.9181259	255.7681091	-180.6888275
1982436881	449.6086731	135.75572	234.099823	-305.0076599
567017676	323.4584961	103.6172332	165.3859329	-323.17453
2637147947	492.0683289	160.3854869	274.4827087	-188.3345947
558158683.5	373.5534668	132.8674234	224.9581909	-383.0102539
2542613544	443.1139221	154.6591392	253.7550659	-276.175354
914667782.1	438.3588257	133.9518802	209.888591	-368.0892944
2067328265	486.9122925	152.8101249	242.8789917	-487.5776367
1026638493	454.2242737	125.0388885	227.7682007	-238.2750854
556153383.1	317.5547791	122.528849	202.9084106	-351.7255249
453548118.8	249.5477753	109.343165	169.60867	-173.7041931
2478471396	443.5423889	176.6698992	292.5136719	-224.8750153
2779171109	578.43573	136.9645834	223.725943	-404.6947632
1229946127	429.1343079	141.6291326	220.9362793	-315.6098022
1072440835	349.0704651	145.8197512	230.6470657	-238.5870361
366945068.7	355.3141479	151.7723435	233.5597885	-158.2566223
1200887231	517.2658691	169.779898	289.8205688	-195.8855591
677562072.6	478.4535522	154.0103217	274.7375977	-178.649353
2846481703	480.3119202	171.9418704	296.2207031	-243.2488403
533652050.7	315.5758667	123.6368543	213.1482086	-287.1577148
1345257754	466.5295715	140.9755193	236.2754028	-422.1524658
2830088373	462.9862061	165.9088858	265.5483307	-382.9991455
1105934533	393.7525024	140.975561	246.8370361	-246.0521088
3158390798	525.2277832	158.11553	258.9031372	-293.3361816
3077347674	452.48703	132.3069179	207.9685364	-362.978363
3037041913	626.309082	162.4563861	266.6131104	-349.0558777
1343687843	568.8529663	146.1154619	234.8300171	-345.8927917
742372038.8	388.6207581	108.052163	173.3213165	-310.6419067
759390662.8	378.4158325	143.860318	245.7254822	-248.0049591
1397728672	391.0390625	125.7064635	194.8024826	-374.2521362
1644321381	374.5063782	116.9459328	174.3470123	-400.8699036
693084786	456.5274353	137.2848922	200.6227631	-573.0613403
1341829721	457.7301941	136.7637638	199.3309967	-443.7295532
1575538179	477.1224365	153.5793185	259.822879	-340.4105835
159702757.9	273.841095	106.3465686	190.3183502	-114.7945328
2411129620	419.7356262	152.0625476	243.6506927	-341.6448975
1757963828	428.8503418	136.901491	237.987886	-144.7710266
932098348.7	458.6351013	155.483706	259.6887512	-313.9842224
1492651847	401.053894	134.7865021	231.3877625	-246.9373627
177212924.4	233.6637573	126.370267	202.3735199	-57.42510986
257560043.5	300.7825623	128.4675505	222.3043823	-209.4291687
2123074617	482.599823	156.4878393	268.3776855	-291.3833008
645873539.6	386.5813904	147.1332151	243.4579346	-254.3426819
418214458.2	286.5895996	99.58114707	147.0591675	-316.6665955
1772988804	393.8118591	122.8221742	196.0806915	-379.9271545
2796592318	439.7510681	148.3565539	236.8793182	-199.0449829
778232381.4	467.2836609	180.5704308	222.7253098	-543.5610962
600195741	318.6906738	112.2893108	200.1270905	-288.6992798
274408914.5	356.0550842	154.0974107	259.8827332	-80.16239166
260433704.3	304.7786865	104.2221983	184.2693817	-115.0721283
807887047	438.9031372	161.0239723	232.5254379	-390.8805237

log.sigma.4.0.mm.3D_firstorder_Entropy	log.sigma.4.0.mm.3D_firstorder_StandardDeviation	log.sigma.4.0.mm.3D_firstorder_Range	log.sigma.4.0.mm.3D_firstorder_Variance	log.sigma.4.0.mm.3D_firstorder_10Percentile
4.307702255	125.6557305	707.3371429	15789.36262	-12.45160522
4.016292677	102.9759244	685.9529114	10604.041	-8.491569614
4.113297008	109.0205744	668.387146	11885.48564	-126.9604172
3.992906744	99.05076241	633.4246674	9811.053534	-11.85199089
3.771314473	96.727953	470.2894135	9356.296893	-23.35476341
3.796170746	90.36021687	551.9545898	8164.968793	-37.89697647
3.467270909	85.4067494	422.9905624	7294.312843	-10.30204468
4.153963556	112.6092288	788.4689331	12680.8384	-0.504858887
4.102838836	108.8527792	675.3464966	11848.92753	1.721822071
4.224041558	115.5940471	898.2590637	13361.98373	-46.48553696
3.607159418	78.03785426	500.3909149	6089.906698	2.353256226
4.074826549	103.0542927	683.1934509	10620.18725	-39.07909088
3.711031692	83.77566458	646.1538086	7018.361977	-15.10427494
3.923306392	92.80353706	543.9427643	8612.496491	-52.65713577
4.258533891	121.6476819	751.9427185	14798.15851	-35.99494324
4.20008741	116.1055546	839.0430298	13480.49982	-73.0569397
4.054649896	105.9351291	777.0077515	11222.25158	-79.30882416
3.980667967	101.3928301	771.978302	10280.506	23.87456512
3.931955305	94.68497693	646.7187958	8965.244856	-6.055978537
4.012976486	101.2439105	623.7646484	10250.32942	-28.73882675
4.10526074	105.9870372	874.0828552	11233.25205	-86.34967728
4.088534929	105.6565741	944.487854	11163.31164	-44.65112305
4.140322345	108.7737916	747.3526306	11831.73773	-60.17802048
4.104803581	112.3301511	613.4608002	12618.06284	-54.91736755
4.198140203	115.5489016	581.3635864	13351.54866	-153.1262253
4.250616784	120.8463095	778.9756775	14603.83052	-20.28062668
4.07204939	105.3148465	648.9760284	11091.21689	-43.04907227
4.14654719	109.3980567	717.9944153	11967.93481	-83.04196014
3.991572783	101.9849559	777.5500183	10400.93123	-26.76660042
4.084445014	105.4865134	905.8840637	11127.40451	-50.35536423
4.149807374	110.7822923	813.7200623	12272.71628	-48.71127739
4.123218784	107.1565094	825.75	11482.51752	-1.742196107
4.110860392	113.2954182	882.8760986	12835.85179	-27.09883118
3.973385884	108.1014127	556.8669128	11685.91543	-4.439368725
4.042543539	102.9928165	581.6904602	10607.52025	-78.53093567
3.878761109	91.08099496	682.5057068	8295.747644	-25.78700981
4.366777523	127.5444856	898.3783569	16267.5958	-52.189077
3.872303089	91.89268072	701.1274414	8444.264771	-26.83583832
3.681371062	81.57764455	576.9714661	6654.912091	-32.92888794
3.875690629	97.46570446	562.3748779	9499.563545	7.140196562
3.849903333	88.8043968	550.1537018	7886.220891	-13.02785587
3.833055677	89.84481319	572.6788635	8072.090457	-3.379171777
3.820426577	90.11494899	629.7636414	8120.704032	2.523724174
3.884036349	98.86768003	618.8714294	9774.818154	-6.394656992
4.304185794	125.5668637	897.1185303	15767.03725	-106.5676949
4.106617962	104.7661065	659.9060516	10975.93708	-64.94404144
4.196303074	114.1248685	864.1555481	13024.48561	-45.58563042
3.895910769	102.3067492	741.7763062	10466.67094	-114.794735
3.694008563	89.40271879	418.0812149	7992.846127	-14.03309526
3.936359375	103.2237888	667.5666046	10655.15058	-0.698740113
3.894421259	96.14654855	754.616333	9244.158798	-1.296383286
3.501964759	76.99969978	646.6330261	5928.953766	-1.914124489
3.986116118	101.7094055	680.4029236	10344.80317	11.465098
4.011961277	101.0207646	756.5637207	10205.19488	-27.19605255
4.135251118	107.3435422	719.2892761	11522.63605	-16.36723595
4.304769989	121.1463894	806.4481201	14676.44767	-101.1883636
4.229759501	118.9351865	974.4899292	14145.57858	-34.22902374
3.752808423	98.05130439	692.4993591	9614.058292	-7.724707127
3.982914875	99.79854002	669.280304	9959.74859	-45.42933922
3.38742018	64.23166791	423.2519684	4125.707162	7.693253994
4.095377329	107.4316273	668.4174042	11541.55454	13.48608112
4.053145584	106.1213229	983.1304932	11261.73518	-28.49757385
4.066732967	103.2201746	744.7441101	10654.40445	-25.66179466
4.061957266	103.7580241	587.6575012	10765.72756	-33.14086533
3.947592769	97.3146235	513.5707703	9470.135947	-8.459269905
4.13795811	117.8109842	713.1514282	13879.428	-10.4826683
4.091116901	117.222132	657.1029053	13741.02824	-22.79799576
4.187414171	115.2937299	723.5607605	13292.64416	-2.577205062
3.85808468	90.48080399	602.7335815	8186.775891	-19.99770432
4.203957678	115.0597944	888.6820374	13238.75629	-49.30955811
4.473716467	138.3074548	845.9853516	19128.95205	-103.2174271
3.790212611	92.22675245	639.8046112	8505.773868	11.45674801
4.135693034	109.9834273	818.5639648	12096.35428	-6.010187626
4.003358387	98.71728491	815.4653931	9745.102341	-43.62390137
4.341263835	125.2959773	975.3649597	15699.08194	-40.37573242
4.212745138	118.6048839	914.7457581	14067.11847	-44.55111694
3.780459724	87.04352626	699.2626648	7576.575464	-30.13273506
3.95616897	98.18384051	626.4207916	9640.066536	0.041246772
4.04250568	102.8006078	765.2911987	10567.96496	-59.47485924
4.031972082	105.2666215	775.3762817	11081.06161	-77.87787781
4.134643764	120.1879605	1029.588776	14445.14586	-53.42317085
4.292341293	124.2208675	901.4597473	15430.82392	-100.8642776
4.17099874	112.2030969	817.53302	12589.53496	-21.30046902
3.373176523	73.62603827	388.6356277	5420.793511	0.908777046
4.198948632	113.4912226	761.3805237	12880.25761	-41.3631958
3.971606537	98.91658755	573.6213684	9784.491293	-20.19570408
4.299194077	122.7416236	772.6193237	15065.50616	-56.54820938
3.874328598	92.03219064	647.9912567	8469.924114	-3.748160219
3.35264275	69.06717442	291.0888672	4770.274582	17.31737518
3.892864108	96.40174078	510.211731	9293.295625	-25.08991051
4.01974572	102.0483806	773.9831238	10413.87198	3.293146849
4.127798268	107.5167552	640.9240723	11559.85265	-34.22112122
3.867508903	91.493641	603.2561951	8371.086344	-76.08623581
3.972886807	99.15346046	773.7390137	9831.408721	-40.91761246
3.906443666	91.5578227	638.796051	8382.834897	8.06710577
4.759240132	179.1371403	1010.844757	32090.11502	-236.2891983
3.606876297	80.43757868	607.3899536	6470.204064	-6.542617702
3.826742576	95.09832648	436.2174759	9043.691699	12.88261347
3.546212814	76.87126774	419.8508148	5909.191805	-16.23858089
4.583474179	151.3369063	829.7836609	22902.8592	-159.0326126

log.sigma.4.0.mm.3D_firstorder_Kurtosis	log.sigma.4.0.mm.3D_firstorder_Mean	log.sigma.4.0.mm.3D_glrlm_ShortRunLowGrayLevelEmphasis	log.sigma.4.0.mm.3D_glrlm_GrayLevelVariance
2.710681352	131.8709259	0.013936377	26.17642784
3.239570645	101.3504138	0.006609505	17.56099785
3.076278232	27.7700482	0.012670297	19.66743868
3.085793352	102.5068693	0.009654963	16.3014082
2.758368112	74.80096688	0.023313973	16.03529767
3.599195727	62.7917716	0.012350032	13.96196168
2.409668151	72.69121828	0.019382983	12.44229725
3.443262674	119.6297515	0.005654708	21.11285422
2.799293566	123.8686401	0.010819883	19.58396874
3.582011148	100.9983978	0.002427839	22.05632316
3.083690053	87.53016938	0.009022262	10.14999471
3.340851489	89.36486218	0.007124578	17.56799114
4.037118523	74.65282906	0.006976963	12.13234481
2.88098905	70.68058431	0.016267037	14.28591556
3.408971406	100.7439868	0.0076041	24.56710023
3.544907635	56.97908005	0.004000793	22.95515002
4.378260464	54.81791807	0.00526473	19.04444635
3.262565493	133.9962879	0.004323283	17.02492551
3.150881134	102.9037883	0.006036539	14.74268254
3.386558416	84.83101215	0.007732942	17.19754903
3.679912702	46.5441205	0.00385815	18.74057574
4.058758857	81.33713818	0.003809823	18.61183097
3.336391121	71.94561076	0.005414195	19.53404794
2.659582053	75.84459841	0.012846843	20.85726986
2.55807424	20.98435152	0.012054895	21.50966505
3.594481199	114.6321745	0.006654124	24.24401574
2.799214207	78.62124203	0.007784752	18.17153932
3.048918443	64.93384156	0.006081564	19.58014322
4.443136704	79.94907167	0.004872018	17.62130081
4.11109183	76.32840635	0.004357872	18.87919132
3.861877318	84.91267734	0.003480349	20.42685448
3.627025843	118.9379662	0.004785124	19.00410659
5.286666414	88.73878022	0.003429662	21.72508125
2.369490279	110.1279408	0.016893477	19.0960859
3.109551294	56.17632166	0.008109873	17.61116555
3.535039234	77.94550834	0.004699181	13.99115488
3.617892944	101.3627716	0.003670768	27.06719892
4.111152871	80.9363071	0.003542908	14.34796456
3.727786491	55.63629112	0.007530103	11.5263794
3.012469291	107.862176	0.013535703	16.02140982
2.907605207	91.14846959	0.011746305	13.12241359
2.756196718	99.25289504	0.008319362	13.38594516
3.162744017	99.20572717	0.005881607	13.57425463
3.399120733	95.17472403	0.009713426	16.45094223
3.898638518	55.58602146	0.003204218	26.85283786
2.882448833	77.3890306	0.010309467	17.83117389
4.005904602	85.28311547	0.005171811	21.78760073
4.749968526	25.62441783	0.004761572	17.91478612
2.367095232	80.95911519	0.024745109	13.10946433
3.498997061	103.135585	0.009140349	18.13741553
3.857274844	95.84078835	0.004452216	15.7543491
6.178378969	69.33669483	0.006164117	10.49574836
2.925709016	124.0108916	0.007295499	17.11181492
3.445171486	86.30502491	0.003689347	16.628579
2.963338317	111.3409775	0.004692933	18.92932035
3.20569769	57.15468958	0.004828668	24.22608743
4.469779125	95.94454492	0.002268507	23.78861352
4.224679684	77.59294649	0.006700814	16.87500858
3.926126374	71.08846778	0.004606051	16.51354459
3.388413426	88.48853359	0.011686504	6.924177297
2.615896025	140.2522682	0.00650989	19.0134969
4.619590302	86.58846306	0.00300624	19.22186616
3.720856428	96.97632067	0.004877133	17.85611293
2.603045009	102.458149	0.007894292	17.38765189
2.405674951	116.4676277	0.011844246	15.49305616
2.832615606	122.2529581	0.008721029	22.60017311
3.232871613	99.89069499	0.010026221	22.99037053
2.813321763	127.5592515	0.00599782	21.88714073
3.132031005	84.2573193	0.005442268	13.57716813
3.606384781	81.45760092	0.00316749	22.14284613
2.8525823	91.6340893	0.004068067	30.87831532
3.603010552	106.6223942	0.00627652	14.45904779
3.790798823	113.5965076	0.005492982	20.04749416
3.557187399	88.09096544	0.003377533	16.1701725
3.604898193	103.406941	0.004539731	26.04131091
4.048343428	85.33820787	0.005585212	23.58422433
4.347014693	64.02104709	0.005229457	13.008381
3.31863281	105.1462057	0.008891754	15.98463537
3.871429844	72.34742564	0.003958836	17.67838744
4.226250768	50.94398486	0.00342018	18.9170337
6.354864021	66.34753774	0.002637593	24.78105668
4.034713238	57.21453634	0.003646115	25.70050006
3.555418778	104.8669257	0.00445599	20.98253919
2.421543811	76.73851148	0.018299217	8.960374072
3.474021262	101.2065254	0.004236877	21.13713382
2.709842546	94.64421247	0.017165339	16.05512242
3.331743699	95.44462612	0.005876835	25.15939007
2.921077379	98.47576881	0.006024101	14.06639337
1.816138564	105.8261301	0.027187396	7.587052035
3.247904684	84.91534559	0.013244923	15.38063556
2.89048596	118.6363009	0.004017113	17.10016712
3.037048995	100.4406807	0.008109167	19.3393887
3.844985833	39.31053939	0.007491831	14.04585531
4.236245988	72.48363779	0.00331182	16.82378958
3.221355576	116.7340232	0.008013167	13.94527817
3.119971795	22.70606634	0.006814662	52.11417647
3.664127622	78.34976239	0.005501373	11.1170403
2.151911686	121.2531248	0.020784767	14.62548481
3.065585781	70.37808471	0.022661447	10.27590845
3.02598424	55.00782178	0.00791736	36.85011043

log.sigma.4.0.mm.3D_glrlm_LowGrayLevelRunEmphasis	log.sigma.4.0.mm.3D_glrlm_GrayLevelNonUniformityNormalized	log.sigma.4.0.mm.3D_glrlm_RunVariance	log.sigma.4.0.mm.3D_glrlm_GrayLevelNonUniformity
0.01519684	0.057373224	0.152764615	61.82946234
0.007151614	0.07225083	0.173332513	127.186365
0.013419922	0.067612094	0.131023869	68.46242649
0.010433442	0.0729968	0.180045749	158.0113914
0.025501623	0.085981326	0.243568215	69.58506347
0.013548544	0.08702368	0.202972287	123.1969052
0.023114177	0.102731976	0.36193348	87.03299989
0.006047907	0.06714685	0.19084613	106.55029
0.011484086	0.066418539	0.177590458	119.4603394
0.002576658	0.062320504	0.130769366	168.9241279
0.010102751	0.095769671	0.256116656	145.3393586
0.007532673	0.070074946	0.130216662	106.8873262
0.007786687	0.092085965	0.275327793	226.507751
0.017371981	0.075705067	0.153831233	125.2911318
0.008101147	0.061596387	0.147012908	85.34012708
0.004369023	0.063549969	0.297794737	223.8968746
0.005593052	0.074044983	0.188579137	104.314995
0.004776327	0.072741528	0.230185029	160.1953286
0.006432527	0.075248321	0.143661486	76.52745117
0.008493124	0.072913142	0.19235106	184.8756105
0.004165422	0.069317826	0.16346454	426.336514
0.004086068	0.070367591	0.171841375	132.2260807
0.005820501	0.067929769	0.145675769	250.31257
0.013869381	0.066554473	0.193647378	60.61628098
0.012894312	0.061767082	0.136154395	144.8314231
0.006926812	0.062421138	0.133788426	76.28080323
0.008270913	0.068016498	0.14988839	135.4271652
0.0064044	0.065940509	0.117567154	202.3899349
0.005223428	0.077320205	0.217033783	230.6108236
0.004646261	0.071493489	0.17118121	200.4616693
0.003731293	0.067159747	0.162656123	246.0020057
0.005143363	0.068133038	0.161111582	303.6522971
0.003693576	0.070738238	0.178176947	157.5557707
0.01802869	0.071226511	0.19809887	30.0861172
0.008608277	0.070031624	0.156625159	55.27107442
0.00510873	0.080581714	0.201892777	225.867855
0.003889165	0.058271655	0.134551705	187.991032
0.003845849	0.081788456	0.187774785	241.7146215
0.008362595	0.093329305	0.297981556	115.8767389
0.014999214	0.079882255	0.221414576	112.4281406
0.012601339	0.079643122	0.153085486	93.61006214
0.009164631	0.079500391	0.200071006	133.5881553
0.006439039	0.080696713	0.249897847	208.513295
0.010670778	0.081076161	0.228893732	131.3707407
0.003429085	0.061107969	0.210193128	187.1220944
0.011142731	0.066957132	0.152756849	213.4431846
0.005563365	0.065914952	0.174046434	214.932989
0.005080027	0.084888718	0.202310541	111.8130312
0.027897172	0.086000359	0.227810741	41.5272854
0.010092558	0.07671305	0.222834465	71.68379904
0.004912035	0.080827257	0.252566969	273.3424779
0.006927676	0.109659299	0.353518376	176.4684577
0.008058691	0.072213663	0.222151838	236.676244
0.003896216	0.07195409	0.130448705	75.8928494
0.005023713	0.065409648	0.16229876	230.6874033
0.005125413	0.059692054	0.133213679	101.6250725
0.002419934	0.064131368	0.159084615	187.7962183
0.007682621	0.091277429	0.359916929	181.9862252
0.004885018	0.075227068	0.145915609	92.17090809
0.01288681	0.109493942	0.221856612	130.8365423
0.007181479	0.065048168	0.224597553	164.9382891
0.003249773	0.073369448	0.212933008	350.9856163
0.005227401	0.070771949	0.172861733	141.8314077
0.0083046	0.066928722	0.127522963	114.0168807
0.012499385	0.071999392	0.124280859	38.56733194
0.009441063	0.065333477	0.144657115	90.5094265
0.011000101	0.069355552	0.20233451	63.63783813
0.006475903	0.063493627	0.170830887	200.3701547
0.005870759	0.079160657	0.180853845	89.41053226
0.003395499	0.064907946	0.167377212	144.3142003
0.004315211	0.052089668	0.125583527	180.7250135
0.006832051	0.087122376	0.239415456	154.9067879
0.00600391	0.068453488	0.188044938	282.2789442
0.003636493	0.074023068	0.174904342	426.3982577
0.004848526	0.058762512	0.151183041	224.1069793
0.005960578	0.064852814	0.168796385	133.9985285
0.005683353	0.087559745	0.239665571	176.8561805
0.009478538	0.075457162	0.163171064	91.2119542
0.004234547	0.073946315	0.164880853	215.8636974
0.003695533	0.075680508	0.199412992	294.4759923
0.002775719	0.073388861	0.18052511	88.30561145
0.003839908	0.063119849	0.130711996	151.5400793
0.004749822	0.065996021	0.13944328	146.5638378
0.021112059	0.106682418	0.334320208	45.89715784
0.004526121	0.06417317	0.136239366	222.2170465
0.018923734	0.071576616	0.197189432	215.9478454
0.006225934	0.060216612	0.140943383	76.99237742
0.006597526	0.07855423	0.203098851	208.1798744
0.029725005	0.103843728	0.184767724	37.80109799
0.01414097	0.079392985	0.182051233	40.25098635
0.004365224	0.070242729	0.169719541	199.7431851
0.008482306	0.065724228	0.133858557	65.78875495
0.00796033	0.082015811	0.150113565	113.9732758
0.00359622	0.078444663	0.20893327	297.6813104
0.008780004	0.078188207	0.20063297	320.6028659
0.007157036	0.044585437	0.08212443	36.89364963
0.006079207	0.097839924	0.247685097	146.0918727
0.022983465	0.076788814	0.201618593	28.71311626
0.025506405	0.098777315	0.275995264	73.42965106
0.008266338	0.049302879	0.075566377	53.22643632

log.sigma.4.0.mm.3D_glrlm_LongRunEmphasis	log.sigma.4.0.mm.3D_glrlm_ShortRunHighGrayLevelEmphasis	log.sigma.4.0.mm.3D_glrlm_RunLengthNonUniformity	log.sigma.4.0.mm.3D_glrlm_ShortRunEmphasis
1.403859812	205.0498133	883.7416965	0.923247304
1.469350711	240.8197798	1397.84729	0.910627441
1.355126945	188.9844647	844.2847128	0.929458743
1.475517595	162.4313321	1732.663535	0.912765605
1.635630257	99.90843743	612.5606814	0.890337022
1.552152115	123.4341176	1087.01207	0.896817403
1.946835908	78.73292064	578.0334616	0.84913711
1.491872645	295.3110107	1264.67164	0.911520996
1.452864891	188.0376701	1461.685359	0.91934063
1.362156083	533.5182517	2244.352635	0.92691786
1.687807669	137.0847392	1110.771701	0.877641783
1.356045112	227.1328452	1273.405205	0.929538321
1.707015604	174.5841124	1811.925485	0.880502008
1.420514939	130.2080184	1341.09282	0.918027297
1.403875797	218.1487806	1133.633876	0.920885495
1.668653193	346.2222695	2748.208708	0.902644923
1.503946332	330.963844	1118.959698	0.908380958
1.589955291	278.19247	1697.847048	0.898188442
1.396869328	240.3155209	832.9958872	0.921897111
1.508713348	219.6652558	1994.758445	0.906632126
1.445825053	324.4855226	4926.056487	0.913729519
1.455017792	338.962013	1511.777976	0.914850703
1.401618767	265.3695651	3002.535476	0.920458161
1.492444255	189.470295	734.3130319	0.914137358
1.375535719	183.6847945	1939.940779	0.925676139
1.361210224	330.4709593	1016.911911	0.929068035
1.388285239	189.0995801	1655.243142	0.928036335
1.317067613	258.223001	2611.362367	0.937151271
1.534713758	302.7651826	2389.562944	0.911854309
1.447489027	332.4029626	2270.334314	0.91777473
1.447209135	376.1309426	2926.331173	0.912691192
1.426067732	293.9824905	3633.115016	0.92034994
1.477520973	465.8256218	1769.87034	0.910589804
1.503949275	153.6053053	337.2727345	0.911777004
1.433189927	213.917585	639.6287957	0.916406292
1.525808005	264.3338868	2198.288013	0.905623312
1.363185932	418.0086101	2687.914739	0.929051363
1.511636841	328.9312577	2304.818873	0.90328709
1.754927466	179.9586878	913.4477775	0.878028576
1.581653645	124.5262544	1078.615073	0.895979461
1.413996789	148.5374002	960.9492758	0.920985309
1.529637851	157.2575282	1311.386745	0.903320711
1.633631953	230.0550427	1962.602158	0.892742974
1.592671786	156.4103971	1244.242763	0.896776735
1.521055301	452.259279	2436.371371	0.910911536
1.415255984	184.7556981	2589.628084	0.919324063
1.457126035	314.9799068	2623.609359	0.915544994
1.539420582	319.5679426	1021.289591	0.900439167
1.621224259	84.68426287	367.9416578	0.88886401
1.596488348	165.5881535	711.1099348	0.892586041
1.654062664	286.4706351	2545.294592	0.888462345
1.855719739	242.6187855	1161.469433	0.871643418
1.572626031	183.7179524	2537.342574	0.900314916
1.368135853	383.0306498	870.0302745	0.924900771
1.413480118	284.9303242	2911.943324	0.925315006
1.368727263	316.727502	1409.850953	0.926494737
1.421292983	569.9095605	2392.399349	0.920968162
1.875482773	186.8868565	1421.247964	0.867118231
1.40565461	322.4775151	994.459146	0.918781125
1.617103374	112.4336079	890.6823393	0.884562711
1.577175216	228.2246315	1957.205718	0.899135148
1.539967819	416.8319683	3771.577754	0.906677291
1.467365893	291.6650304	1602.678553	0.912143678
1.334704328	213.0556352	1446.753331	0.936295296
1.340957395	152.764429	448.2821049	0.931147165
1.390713296	192.7815106	1139.251824	0.924117401
1.539827026	168.5440985	710.4659298	0.900764721
1.453483862	245.8096037	2536.886615	0.915171499
1.499794166	239.2690391	884.795854	0.904018862
1.445324032	418.4984807	1791.49545	0.916126891
1.334642223	409.0791513	2933.555972	0.934867118
1.60062827	213.3477621	1375.15297	0.899294324
1.484950258	287.5545262	3303.17429	0.913172004
1.458630218	343.6886421	4638.760754	0.915528056
1.403951003	347.1974887	3123.757025	0.922657063
1.449210051	317.0085167	1668.347626	0.916445589
1.612351322	242.2798405	1548.125355	0.895593121
1.437815735	214.6485441	983.2114267	0.917958435
1.437274951	325.4506959	2377.182107	0.918865175
1.517415225	361.9410065	3065.914256	0.907163225
1.469980954	647.4134662	964.3183052	0.914035114
1.360297029	421.6117319	1995.094814	0.928093786
1.386059856	345.2816004	1821.391422	0.923150557
1.861272552	76.25295414	308.2178059	0.863192535
1.383691541	334.9870881	2827.409213	0.921561196
1.53071747	112.2904276	2342.397715	0.901432109
1.392246485	301.607333	1044.496325	0.921779596
1.532577149	204.4178447	2076.185048	0.904342591
1.50277391	62.36104696	298.3437398	0.912400923
1.493131477	163.9692144	400.5514449	0.90728338
1.450519917	292.5994432	2290.445372	0.915883524
1.358523521	242.045115	837.6368788	0.930348572
1.41921731	217.9979989	1120.288959	0.916353319
1.534317786	356.1557858	2981.354424	0.90575463
1.527821152	170.8129197	3209.747668	0.904304528
1.227940525	564.0494127	730.8084354	0.951928616
1.658311065	228.639307	1112.742573	0.884463774
1.534945446	96.15224976	295.9984365	0.905347974
1.721983673	72.95106087	542.8866453	0.876873603
1.21959851	363.7210389	949.9850884	0.951019721

log.sigma.4.0.mm.3D_glrlm_LongRunHighGrayLevelEmphasis	log.sigma.4.0.mm.3D_glrlm_RunPercentage	log.sigma.4.0.mm.3D_glrlm_LongRunLowGrayLevelEmphasis	log.sigma.4.0.mm.3D_glrlm_RunEntropy
290.5617668	0.895453674	0.021037713	4.823887977
371.4601606	0.88	0.009924867	4.608996222
278.0201997	0.905394248	0.016834722	4.593640911
251.6412346	0.881777415	0.014580084	4.583325754
159.9798009	0.851746778	0.038624682	4.475774787
204.9105845	0.863905325	0.019692882	4.464829711
144.301288	0.799869005	0.046666761	4.380981921
468.5045533	0.87892606	0.008173491	4.748308263
281.7516847	0.887101059	0.015380388	4.665269532
783.9237712	0.902533364	0.003300676	4.73043111
254.7470515	0.839640713	0.016147219	4.359852784
331.1697028	0.905090162	0.00952506	4.562507517
320.5622958	0.839246789	0.012512298	4.494318259
202.0175471	0.89057713	0.022675108	4.468705044
325.2345269	0.895050686	0.010608891	4.783197471
598.6108716	0.856862048	0.006857017	4.882286825
556.353913	0.876923077	0.007298436	4.662804318
462.7131419	0.860528057	0.007434751	4.649244324
355.7314375	0.895314193	0.008445119	4.449712688
352.7465097	0.873704028	0.012299541	4.64075464
513.0945669	0.884836746	0.005726921	4.695130363
538.5878305	0.885757807	0.005533469	4.657884094
400.8713991	0.894013776	0.007797753	4.68601872
297.1906867	0.883271098	0.019028938	4.657759257
275.3310664	0.900596715	0.017246955	4.695748142
468.8683212	0.903675467	0.008308035	4.741766656
282.2466568	0.900323557	0.010875398	4.570035213
370.9930031	0.91473117	0.007959307	4.597068512
501.6858576	0.876683187	0.007255544	4.608625918
522.047091	0.887160007	0.00616546	4.664235619
607.5340522	0.884262526	0.004977309	4.736751831
445.1604322	0.8912	0.007040625	4.67805156
741.5605011	0.879524663	0.005015778	4.717693398
226.775422	0.877658724	0.024455809	4.526485377
336.7183128	0.888773389	0.011093733	4.58500061
438.1004852	0.87092609	0.007330252	4.519666788
609.537711	0.90376533	0.004971815	4.871581705
551.2884407	0.871265567	0.005425443	4.521469252
338.7471606	0.835268505	0.013362454	4.458222502
197.2294709	0.859789355	0.023077748	4.556015802
224.7550614	0.892974047	0.016804808	4.386407863
248.0046149	0.869485587	0.013889632	4.466916196
402.9307174	0.852964347	0.009726827	4.523301909
253.1390745	0.859382656	0.016172923	4.572410406
755.7085956	0.875787064	0.004692053	4.927866523
288.5293159	0.891511565	0.01507609	4.646919149
492.7244248	0.884448433	0.007554794	4.783531193
561.6889387	0.86817536	0.006759673	4.553137826
134.1994812	0.855291871	0.044309868	4.339054506
266.7316788	0.856669019	0.015340493	4.625670676
508.8400937	0.848103954	0.007572176	4.638741046
501.8584147	0.821260028	0.011354231	4.354477657
299.9816512	0.862745893	0.012446868	4.657279215
567.1090886	0.900742298	0.004909801	4.505361195
428.7463369	0.89562533	0.006810832	4.661660288
462.5995501	0.90160528	0.006589209	4.806464371
866.3160518	0.89260328	0.003224963	4.782293468
348.7488451	0.817750506	0.014121947	4.633924479
495.8465132	0.892913209	0.006257967	4.518692026
209.3636286	0.85026006	0.019055366	4.095355335
375.1253769	0.861977873	0.010953047	4.755750894
700.196066	0.871262144	0.004627519	4.704675452
466.141998	0.882058056	0.007050078	4.656240034
313.2234018	0.911834953	0.010303234	4.506324229
217.3481913	0.907822686	0.015697762	4.391428387
270.3059278	0.897502368	0.013113103	4.651079939
260.3276944	0.866802385	0.0163006	4.732575824
378.3599599	0.884766383	0.008984497	4.767611808
398.9051124	0.87357963	0.008072175	4.462831317
645.7207715	0.886471725	0.00459517	4.781123065
582.9437767	0.911017292	0.005540883	4.928294444
358.4266215	0.861530997	0.010204853	4.469339855
451.2690695	0.88097391	0.008719444	4.731799615
553.7171227	0.88464492	0.005025164	4.587646427
515.8223611	0.89475869	0.006416081	4.885987013
492.7735243	0.886050886	0.007873234	4.786757044
434.9639239	0.85696554	0.008284856	4.476192295
330.2904276	0.889568121	0.012429034	4.494219821
512.4228826	0.89039166	0.005677676	4.603093167
607.0006206	0.873430185	0.005179078	4.680041163
1035.360815	0.882808088	0.003516927	4.733511467
625.1572081	0.903419125	0.004778195	4.804726981
506.2257183	0.897332255	0.006182873	4.701772874
140.3287413	0.820267686	0.039346185	4.159719168
507.1587778	0.896645819	0.00589854	4.730997871
180.4706052	0.868274213	0.027983936	4.610640955
445.7405452	0.895173454	0.007918856	4.828915729
334.2032632	0.870471953	0.009771132	4.508857746
100.9183008	0.885457608	0.044189971	3.83856822
269.7141125	0.87663029	0.018538521	4.459876768
445.8674247	0.885370002	0.006246035	4.592095582
349.2110362	0.905673512	0.010348873	4.602342594
346.0899678	0.889490791	0.01017703	4.426472113
600.6993705	0.871370761	0.005153681	4.625292704
282.1718021	0.870966687	0.012880381	4.545538749
743.4673212	0.935607379	0.008604339	5.090952118
416.9792665	0.84624111	0.009266293	4.345772303
147.2893698	0.872933142	0.035576608	4.381621393
127.9587372	0.836105336	0.041459258	4.319882825
474.5542391	0.93547527	0.009735426	4.921913175

log.sigma.4.0.mm.3D_glrlm_HighGrayLevelRunEmphasis	log.sigma.4.0.mm.3D_glrlm_RunLengthNonUniformityNormalized	log.sigma.4.0.mm.3D_glszm_GrayLevelVariance
219.3290148	0.819140924	33.40196709
262.1448793	0.792833093	23.28584767
203.7186085	0.832524414	25.84193136
176.6310382	0.798340947	22.95902834
109.3344437	0.754282423	20.32970051
136.2953924	0.765718128	21.56958678
88.32130641	0.679159039	12.64051014
322.1299514	0.795313871	28.55082235
202.8891773	0.811534789	25.04391886
575.5690053	0.826760247	29.70939953
155.1434664	0.729068795	13.9777438
244.3447428	0.833145004	24.07967447
196.0659797	0.73441404	23.03815361
141.9466602	0.808565583	18.65839677
235.9163577	0.81535113	32.92694215
380.2594167	0.778451773	32.05385819
365.6137601	0.790829141	32.93050139
306.0095214	0.768903329	24.8169635
259.5709629	0.816992255	19.41967773
240.5987632	0.785168592	24.24595556
355.2006352	0.799398347	26.05250995
370.4469357	0.802255644	29.40440743
287.9321835	0.813346749	25.99755582
205.6692022	0.802697951	24.94108919
198.8683207	0.825021851	23.32807565
353.7290473	0.831379865	34.64203787
203.848221	0.829956507	22.34700232
276.7989704	0.84938549	23.59285139
331.2284376	0.797933588	31.45187277
362.2162877	0.808306356	31.77032786
413.7370167	0.797201771	31.06907169
318.1932804	0.813542244	27.2067654
509.592512	0.793160549	37.56604702
165.3231254	0.796606494	22.37488386
233.747316	0.806920674	23.94246735
290.968704	0.783038849	23.03459984
449.5701039	0.831821975	39.09780084
364.4101511	0.778236235	24.52892411
202.4617714	0.731766424	23.18986318
136.026351	0.764548297	21.35443288
160.8992303	0.815492774	18.8304193
171.8714328	0.778687574	18.56425174
255.6870191	0.757881335	20.32453309
171.3617245	0.76597827	25.80640943
497.064746	0.794289178	37.65403989
201.5625207	0.811039919	20.43197336
343.1760894	0.803325961	30.75428404
357.2171128	0.773152157	28.47718656
92.5082341	0.755591249	12.59322034
181.3700712	0.758483703	25.76561065
319.647819	0.750309215	28.58489277
276.7190202	0.719086671	24.33947528
201.5276716	0.772750063	24.96338356
414.4629223	0.822988606	22.24702788
307.1546776	0.824283588	25.7480151
341.1155445	0.826430476	32.58347774
617.3454074	0.815132486	37.21163912
209.1026643	0.710609028	26.94835855
351.6320194	0.809903213	24.08106328
127.860595	0.742448702	11.30556302
251.0024724	0.770227898	24.53761423
458.9578526	0.786262257	33.27276382
319.4412974	0.797223568	27.48071174
228.8167493	0.847826833	20.2803944
163.7992807	0.83578384	19.32385247
205.6528336	0.821134053	24.82464344
183.4886883	0.773032567	30.05785667
267.0463943	0.80251869	30.44521556
264.9056742	0.780598318	18.44367551
454.9300319	0.804498918	32.44844234
437.4958245	0.844246723	33.95661091
235.0369887	0.771396428	24.93277991
312.9616761	0.798994198	26.81681174
376.4360425	0.803524263	22.99059869
374.5863282	0.817947455	37.86297355
344.9514187	0.805707097	34.76914341
270.390651	0.764143716	23.5069287
232.9644335	0.809863293	22.82031716
354.6788201	0.811615494	26.95388784
399.1359637	0.786331955	31.33393426
707.7752213	0.800230021	48.20627724
455.3918503	0.829633974	38.27342783
372.2696157	0.818940468	31.50784663
85.71812475	0.709547795	10.69850416
364.0349163	0.815226462	28.30905931
123.2526041	0.774596641	19.10587873
325.7052195	0.815937263	36.49933769
224.5335016	0.781169737	20.15106011
68.19977979	0.806910884	6.69013366
180.9419395	0.787532944	20.66633136
317.2056277	0.804069665	22.98310337
259.6663104	0.83498845	27.29136095
239.1305419	0.804650804	20.71690178
392.7187636	0.78374366	29.20992477
188.0904256	0.780707889	19.58624441
595.3234319	0.882249892	60.58613092
256.9241116	0.742470559	18.5286965
104.5158465	0.786126286	15.02185726
81.28184826	0.728001148	14.21628891
383.9825804	0.879388009	38.27303792

log.sigma.4.0.mm.3D_glszm_SmallAreaHighGrayLevelEmphasis	log.sigma.4.0.mm.3D_glszm_GrayLevelNonUniformityNormalized	log.sigma.4.0.mm.3D_glszm_SizeZoneNonUniformityNormalized
145.4506404	0.050726871	0.331807095
143.0228028	0.060086474	0.26306566
104.3132683	0.055015385	0.292364497
98.95339332	0.058380196	0.288102014
77.6818998	0.064368152	0.295193572
66.97841902	0.063986777	0.209652893
37.20218213	0.08398307	0.157384718
179.4522739	0.054349559	0.312360506
117.2690167	0.056409393	0.321929131
314.7688437	0.05271104	0.289657925
59.25146235	0.075625672	0.194264864
132.7918169	0.058142284	0.301595729
120.0896509	0.059184587	0.275434307
64.90887226	0.065642982	0.24047359
137.8203448	0.051508264	0.346342975
221.0924617	0.050358469	0.317877458
177.62373	0.051284876	0.264209282
176.130113	0.057748568	0.268989952
162.2603981	0.067636719	0.348710938
121.2897287	0.05665	0.250566667
178.1018425	0.055418265	0.272097243
207.5819457	0.05488561	0.289602579
157.936774	0.055995948	0.277579601
116.3995416	0.056336962	0.260851792
102.373645	0.057234569	0.268345293
200.3766391	0.049005429	0.312511125
111.877111	0.06059493	0.306450375
136.4027027	0.058071151	0.311286348
183.1360431	0.052009741	0.319092685
200.778863	0.050265473	0.317809777
201.1759664	0.051888052	0.283336894
173.3981305	0.054276543	0.286809308
276.509879	0.050327216	0.288405063
102.7173866	0.064208519	0.331605457
107.0697919	0.057588876	0.255186372
140.2807702	0.058492986	0.264840793
245.9377159	0.045235157	0.315089676
173.7959555	0.057261925	0.260095354
90.74100859	0.059036288	0.192004759
89.9091183	0.060957336	0.269594173
95.6333624	0.064301439	0.332506014
98.5206123	0.064197141	0.271724623
138.1086882	0.065382423	0.286884508
107.9148376	0.055426799	0.31007476
251.5939932	0.046446156	0.292599419
99.03692378	0.062126951	0.257719875
197.807116	0.051743285	0.301068717
144.6989942	0.055160266	0.267820358
59.74542844	0.082160299	0.276070095
121.9486991	0.058482553	0.334538567
171.7286592	0.054606547	0.274707889
142.1202464	0.061979307	0.246439129
126.8384002	0.056237694	0.28387538
212.0901384	0.061344513	0.298978077
162.2017656	0.055112913	0.285146173
192.6678804	0.049602237	0.285129175
350.742586	0.048312432	0.323783772
149.988523	0.054783544	0.290561114
168.1296712	0.05915493	0.267716723
50.2550921	0.082026934	0.209292604
144.7238727	0.056190647	0.305376897
245.8878739	0.050870987	0.312012485
170.0213614	0.054946732	0.289079996
103.3835131	0.062183534	0.27353224
83.65290804	0.063359926	0.330442826
150.5290369	0.058274765	0.336874156
144.9168173	0.052523924	0.296332822
167.9286867	0.051785448	0.325233104
122.5789153	0.06638985	0.298946559
267.4701914	0.050459976	0.308533739
245.6078598	0.04858139	0.340100038
127.3482852	0.058327025	0.252599612
178.1244421	0.054505028	0.2890625
181.2260811	0.058815361	0.268495976
211.682002	0.047052686	0.275469165
193.8117539	0.050486998	0.324601278
119.7951269	0.059054898	0.268581507
127.5614063	0.05923787	0.281325444
184.5391059	0.055101262	0.284599352
207.1318587	0.051169992	0.299509774
411.7761727	0.044659696	0.350062997
242.1952105	0.046252096	0.319158665
226.1843195	0.050672213	0.309237827
45.32248571	0.093185596	0.27534626
179.3626454	0.054313985	0.275308494
67.73696938	0.063104887	0.285818713
200.0749578	0.046995976	0.314755505
121.2686211	0.063790962	0.275740198
33.40621674	0.1184573	0.320885624
78.04745347	0.067692308	0.266627219
181.2346077	0.060126007	0.274726681
147.2156548	0.054011834	0.317017751
101.8278423	0.062139134	0.247214506
206.0882621	0.052278918	0.28007405
102.4687202	0.063004468	0.27313368
330.305427	0.036915405	0.383032798
143.3233211	0.071635735	0.251045057
49.57700799	0.073300326	0.23970535
45.34638541	0.075985918	0.259206519
218.6488017	0.045934114	0.357197972

log.sigma.4.0.mm.3D_glszm_SizeZoneNonUniformity	log.sigma.4.0.mm.3D_glszm_GrayLevelNonUniformity	log.sigma.4.0.mm.3D_glszm_LargeAreaEmphasis	log.sigma.4.0.mm.3D_glszm_ZoneVariance
111.8189911	17.09495549	109.3976261	96.65463287
120.2210066	27.4595186	178.8665208	159.7139273
95.01846154	17.88	46.46153846	34.62793846
156.1512915	31.64206642	204.3228782	183.8397387
54.61081081	11.90810811	317.4	291.0858729
57.65454545	17.59636364	227.8690909	192.3908231
21.08955224	11.25373134	657.0820896	594.8606037
121.1958763	21.08762887	231.5695876	209.9279081
175.4513761	30.74311927	135.2458716	121.4129316
244.7609467	44.5408284	66.75857988	54.1287574
49.92607004	19.43579767	553.5525292	504.1157625
148.0835031	28.54786151	51.33401222	39.55695389
132.7593361	28.52697095	641.7738589	604.8468045
103.1631702	28.16083916	117.6270396	98.86945844
152.3909091	22.66363636	84.13636364	71.75876033
320.7383548	50.81169475	225.6858276	209.1018023
97.49322493	18.92411924	142.5718157	123.6528521
126.6942675	27.19957537	331.5923567	302.0966187
111.5875	21.64375	52.125	39.5225
150.34	33.99	275.5616667	252.1844417
454.4023952	92.54850299	140.1113772	122.7918247
147.1181102	27.88188976	94.26574803	76.83347929
289.7931034	58.45977011	155.1503831	139.5690637
67.03891051	14.47859922	87.3463035	71.28398613
198.3071719	42.29634641	51.0473613	38.63103598
132.504717	20.77830189	48.93396226	38.76628693
185.0960265	36.59933775	75.26655629	61.86659302
334.0102516	62.31034483	45.04100652	35.26445699
230.7040111	37.60304288	256.8506224	234.7489196
242.4888598	38.3525557	200.4416776	183.3001242
268.6033755	49.18987342	119.3797468	100.2898529
350.7677841	66.38021259	199.3753066	182.6610489
149.3938224	26.06949807	334.0752896	310.2013126
47.41958042	9.181818182	70.27272727	58.95867769
58.94805195	13.3030303	67.65367965	52.8761455
157.0505902	34.68634064	381.3035413	351.8733737
313.8293173	45.05421687	65.67570281	52.83542503
169.8422665	37.39203675	198.3139357	171.3471479
39.36097561	12.10243902	457.4634146	405.0599405
83.57419355	18.89677419	341.6967742	313.8456191
114.0495627	22.05539359	101.1020408	86.38150771
110.320197	26.06403941	172.5960591	149.9515882
138.5652174	31.57971014	559.4513458	520.1750447
115.0377358	20.56334232	360.0916442	334.3038629
260.4134831	41.33707865	146.2269663	130.8147254
199.7329032	48.1483871	119.56	98.28112383
282.1013874	48.48345784	127.0394877	111.5644584
84.89905363	17.48580442	208.2397476	185.3690255
32.57627119	9.694915254	163.2542373	140.4090779
88.31818182	15.43939394	210.1666667	193.1197773
175.5383412	34.89358372	957.9687011	919.096696
60.13114754	15.12295082	1343.54918	1279.286617
181.9641186	36.04836193	573.5709828	538.482456
95.97196262	19.69158879	50.94392523	37.63620306
264.0453564	51.03455724	166.0917927	148.0155211
157.3913043	27.38043478	61.86956522	50.17118252
282.3394495	42.12844037	113.3107798	99.17076503
121.4545455	22.89952153	919.8851675	886.0339278
95.03943662	21	59.74647887	44.80988693
38.30054645	15.01092896	508.3879781	449.4425035
182.6153846	33.60200669	266.5033445	242.3160283
361.9344828	59.01034483	581.4508621	559.076331
158.704918	30.16575592	118.9307832	101.8192242
143.0573614	32.52198853	43.95028681	31.19323362
61.46236559	11.78494624	35.65591398	25.59405712
140.8133971	24.35885167	103.5909091	89.96456697
69.34188034	12.29059829	154.1196581	133.6768939
245.5509934	39.09801325	287.3324503	265.0240323
76.23137255	16.92941176	125.7843137	100.0734487
199.6213292	32.64760433	137.0370943	122.0229665
419.0032468	59.85227273	38.88149351	29.3277745
85.63126844	19.77286136	502.4867257	465.52463
314.5	59.30147059	174.8023897	156.3076468
357.3681443	78.28324568	276.8166792	252.8868676
285.6615236	48.79363549	135.2092575	118.3177123
191.1901528	29.73684211	194.4125637	178.7503207
101.5238095	22.32275132	469.7698413	430.9549635
91.43076923	19.25230769	97.64	80.15473609
212.5957162	41.16064257	130.4712182	111.2382381
294.4181078	50.30010173	430.5279756	410.0069834
123.572238	15.76487252	159.7620397	144.8751214
258.5185185	37.46419753	51.60123457	40.84121475
196.0567823	32.12618297	75.36908517	60.14185632
26.15789474	8.852631579	237.5894737	207.2815512
271.7294833	53.60790274	59.20364742	43.89311711
195.5	43.16374269	282.0643275	256.2686211
126.2169576	18.84538653	58.06982544	45.38841176
151.9328494	35.14882033	310.9274047	280.4273438
31.76767677	11.72727273	75.82828283	58.59320478
34.66153846	8.8	100.9384615	81.17017751
191.2097701	41.84770115	220.4956897	199.2112544
103.0307692	17.55384615	46.97538462	35.41538462
88.5027933	22.24581006	97.6424581	78.60556787
243.3843498	45.43037975	407.2278481	382.1357035
248.5516484	57.33406593	236.3032967	209.5596232
155.8943489	15.02457002	15.04176904	10.32422773
68.03321033	19.41328413	650.8302583	608.5082719
21.81318681	6.67032967	78.65934066	56.53834078
41.21383648	12.08176101	309.1069182	277.915747
180.0277778	23.15079365	11.73015873	6.487512598

log.sigma.4.0.mm.3D_glszm_ZonePercentage	log.sigma.4.0.mm.3D_glszm_LargeAreaLowGrayLevelEmphasis	log.sigma.4.0.mm.3D_glszm_LargeAreaHighGrayLevelEmphasis	log.sigma.4.0.mm.3D_glszm_HighGrayLevelZoneEmphasis
0.280133001	1.003309024	15092.40059	252.652819
0.2285	1.049190084	33523.80525	281.0393873
0.290697674	0.3040997	10328.97846	193.9630769
0.220953934	1.757689468	26204.70111	189.1881919
0.194942044	5.817264761	19705.92432	141.2108108
0.167887668	2.201860976	27143.34182	143.3709091
0.126773888	17.9033775	28059.85821	108.0671642
0.214958449	0.965873564	59594.75	324.3376289
0.268870252	1.150847614	18802.75229	209.4055046
0.281385281	0.130222202	36778.17515	572.7372781
0.142224682	4.844458784	68863.50195	148.2490272
0.291394659	0.261427962	12135.63747	241.98778
0.164561284	4.182270413	102530.1743	222.0705394
0.230893434	1.198862602	14783.95571	136.955711
0.284237726	0.535376986	15354.55909	239.7636364
0.24555853	0.779469943	70524.89594	386.0455897
0.229906542	0.417946857	52495.53117	347.1517615
0.184128225	1.635582746	72663.46709	338.1295117
0.281690141	0.278106483	11394.025	273.921875
0.206825233	1.526054748	54213.955	248.4466667
0.24028777	0.444165125	48113.9509	340.7431138
0.239509665	0.303851237	32237.22835	377.726378
0.253336569	0.634335799	40750.04885	293.5268199
0.249514563	0.690455209	14834.69261	220.2879377
0.283794163	0.458508327	10440.67524	194.3748309
0.313609467	0.197371671	14126.03302	363.8419811
0.273179557	0.458877395	14713.02318	200.5960265
0.319821162	0.17677655	13839.51538	251.6225536
0.21270962	0.901404782	77173.72614	327.4536653
0.24153213	0.600741012	70476.87156	352.5229358
0.22887494	0.306775216	51150.55274	381.9989451
0.2446	0.833521908	51548.06132	325.5102208
0.204662189	0.801566843	146352.5849	518.3436293
0.297297297	0.893820689	7076.468531	179.4125874
0.260135135	0.357195193	15197.76623	222.7402597
0.18433323	1.562048908	97023.32884	282.391231
0.279069767	0.179032516	27496.59337	445.1445783
0.192568564	0.593446894	69286.98162	349.6324655
0.138140162	2.884439257	76725.13171	218.6439024
0.189486553	4.718715732	27855.07097	169.016129
0.260638298	0.83387573	14245.00875	164.2332362
0.210144928	1.522724624	21823.38916	190.7167488
0.159563925	3.02466604	111987.588	260.5424431
0.196921444	3.093439789	44949.52561	200.3881402
0.254722381	0.327197446	69563.31236	466.2370787
0.216783217	0.748000927	26347.31871	192.4309677
0.2542051	0.466593427	38828.24013	352.3383138
0.209102902	0.58178233	78614.61514	308.6435331
0.209219858	3.966022497	8659.533898	112.5932203
0.242201835	1.966833535	24287.41288	213.7878788
0.160391566	3.860266573	244349.759	334.9170579
0.124744376	5.508264109	337759.5615	283.3647541
0.168817487	4.702590429	75525.63651	236.1872075
0.27412468	0.144919817	20201.79128	400.3738318
0.23520447	0.698265065	43516.92657	310.24946
0.292372881	0.224777913	19446.00543	351.7753623
0.265934736	0.218419297	61837.3922	617.3474771
0.171875	6.824302215	127324.6244	266.7535885
0.258746356	0.197194898	20183.1493	336.2253521
0.13024911	4.564807637	60654.48634	115.2786885
0.2033322	1.730839662	47220.89799	264.6839465
0.211408784	1.442133342	239519.9948	441.8353448
0.241743725	0.438408525	35351.06193	323.6466302
0.279978587	0.218466571	11635.96367	205.458891
0.315254237	0.328589572	5301.172043	152.4408602
0.270900843	1.013970648	12907.75837	247.6052632
0.221172023	1.510377044	18146.38889	248.1880342
0.211721817	1.606746709	57017.97881	288.7390728
0.197215777	0.525082523	34448.75686	237.0196078
0.258077383	0.357724877	55234.20711	470.6615147
0.323529412	0.125074692	16541.87338	420.851461
0.164483261	2.910423423	90463.43363	266.4188791
0.232528318	0.806169709	43624.95956	331.1397059
0.204423284	0.760280329	105236.402	349.7009767
0.243312999	0.496412581	40811.24783	398.4966249
0.252681253	0.741732257	55664.62139	340.336163
0.160509554	1.961147888	118010.672	250.8941799
0.239146431	0.597905076	18977.32308	241.1323077
0.228021978	0.389690709	46908.78715	335.1044177
0.220750056	1.097031921	173666.0814	375.4496439
0.25917768	0.24406019	108485.4249	695.2096317
0.3048551	0.122970839	24074.76667	429.8246914
0.256265158	0.261316059	24436.86435	392.7476341
0.181644359	5.348247313	12369.12632	95.72631579
0.255567064	0.196835654	21161.49341	348.526849
0.196891192	4.534439588	22682.00439	130.6491228
0.280812325	0.232681586	16880.23441	342.5311721
0.181071311	1.812804213	57687.2559	234.0217786
0.240875912	2.432463932	4080.353535	63.92929293
0.224913495	0.766514312	16203.32308	163.3769231
0.216754905	0.999755082	51741.24282	345.7945402
0.294117647	0.227186205	11664.33846	259.2676923
0.229193342	0.435823023	24757.25419	214.4553073
0.199632437	1.137571438	149194.3303	388.593786
0.193370166	1.649831483	38908.16703	196.3593407
0.46040724	0.054183908	9933.552826	551.3120393
0.153715258	3.084662174	141262.2435	276.1845018
0.212616822	1.872300537	5275.296703	114.8021978
0.179054054	6.378348995	16946.33962	93.41509434
0.436741768	0.064235213	4834.323413	363.2738095

log.sigma.4.0.mm.3D_glszm_SmallAreaEmphasis	log.sigma.4.0.mm.3D_glszm_LowGrayLevelZoneEmphasis	log.sigma.4.0.mm.3D_glszm_ZoneEntropy	log.sigma.4.0.mm.3D_glszm_SmallAreaLowGrayLevelEmphasis
0.596125237	0.017181862	6.313636194	0.008043022
0.525935861	0.0092462	6.541234161	0.004531406
0.556603844	0.018317341	6.313558261	0.010040859
0.555156111	0.015312994	6.48917542	0.010600881
0.560791978	0.030138764	5.884092175	0.011502036
0.4620134	0.020990056	6.466424447	0.012537584
0.370828412	0.028397589	5.897006304	0.015019219
0.578420706	0.010966844	6.329793578	0.008198108
0.585286776	0.017219507	6.355917016	0.012044289
0.554007664	0.003619362	6.704044379	0.002486056
0.433113486	0.015061364	6.268162355	0.00641934
0.564319236	0.010521336	6.406659144	0.007057269
0.535127153	0.01091899	6.495411574	0.005901817
0.494370875	0.024087403	6.44822744	0.011382155
0.611094917	0.011816286	6.382869832	0.009020458
0.58242486	0.005853582	6.688970652	0.003840736
0.524562759	0.009635311	6.610710791	0.005661217
0.531633719	0.006953922	6.582179965	0.004946367
0.613215625	0.009544275	6.065251086	0.007600688
0.509675365	0.010006457	6.68876559	0.004170552
0.534907323	0.00545078	6.899136962	0.003259138
0.554925471	0.006029584	6.605218702	0.004242445
0.541882159	0.007017425	6.804549267	0.003443288
0.522097418	0.018183955	6.334961012	0.009493448
0.524916715	0.015410542	6.573785725	0.008810695
0.576296389	0.012909492	6.478991253	0.011163769
0.572771856	0.0110637	6.429933255	0.007521666
0.575552144	0.008608754	6.507521422	0.005730747
0.585305734	0.009020066	6.629108937	0.006662803
0.581969331	0.006924833	6.614983966	0.004197909
0.548271206	0.005847523	6.875056054	0.004063663
0.54989256	0.007103488	6.798619379	0.004735372
0.553370384	0.005899976	6.686958977	0.003979922
0.588134851	0.026832283	5.655448128	0.017232136
0.505885231	0.013557234	6.271593991	0.008918123
0.526798861	0.007393006	6.598671962	0.003814061
0.581026618	0.005509383	6.830152527	0.003771168
0.525739291	0.005936384	6.747323882	0.003964385
0.43551936	0.015003163	6.495392047	0.007493343
0.531731854	0.020251291	6.309617584	0.013702962
0.597338551	0.016483073	6.054564318	0.009625554
0.53217536	0.01196816	6.33882961	0.008036225
0.552446412	0.011570962	6.333336506	0.00753092
0.57515003	0.016378644	6.301711617	0.011864861
0.554578044	0.005087164	6.829331755	0.003269989
0.52051584	0.013064348	6.661064921	0.005440328
0.566763827	0.006566966	6.753609172	0.003106016
0.529631263	0.009949593	6.416421117	0.007559478
0.540393861	0.026977532	5.483373876	0.009843327
0.598379878	0.014306575	6.084234742	0.011029032
0.537160656	0.007625452	6.731379352	0.003914988
0.505812336	0.011214929	6.314913181	0.0039298
0.549117236	0.010683806	6.632105895	0.006817553
0.564785831	0.006492579	6.188884651	0.005213886
0.54838385	0.006728079	6.705380162	0.004282887
0.54523552	0.007155136	6.655722153	0.004922358
0.588202774	0.003581279	6.727158689	0.002685209
0.556575356	0.009956397	6.515357986	0.006504725
0.530262867	0.008184228	6.403640692	0.006127645
0.465806758	0.021071189	6.022459158	0.009389118
0.571428207	0.008821921	6.488219752	0.004464232
0.576777536	0.005053686	6.748713976	0.003421311
0.551828638	0.00794259	6.58138147	0.00554816
0.53679948	0.011457762	6.440689216	0.006309911
0.595004951	0.018908428	5.826429197	0.014597966
0.601446609	0.009879531	6.197740285	0.005351743
0.562204414	0.013839739	6.26588399	0.009898112
0.591190886	0.009533	6.591838832	0.006910845
0.566915093	0.010655417	6.070692124	0.008278711
0.571799397	0.004969815	6.619765135	0.003584199
0.604299128	0.005296714	6.653798411	0.003240044
0.512263072	0.012727756	6.389746788	0.010066985
0.554777399	0.006155759	6.759811048	0.002895421
0.533298007	0.005194428	6.832074515	0.00333219
0.538410238	0.006409858	6.973205909	0.003434943
0.589730819	0.008337755	6.52603501	0.004115076
0.527706713	0.010747396	6.355664888	0.00788626
0.542814067	0.015109453	6.272137161	0.009047202
0.550685883	0.006201913	6.7316889	0.003994862
0.563434518	0.00546285	6.734351161	0.003576869
0.61283175	0.005393075	6.474172112	0.004436166
0.583742381	0.005853625	6.760419515	0.004265954
0.57632905	0.006529519	6.665967848	0.003651459
0.536550397	0.031235992	5.355558277	0.024452981
0.540192972	0.00639119	6.856017736	0.004241792
0.551495714	0.024939038	6.448549735	0.014056152
0.580846056	0.009596696	6.564749369	0.006938494
0.541959541	0.009445618	6.469580405	0.006434186
0.584938639	0.041289326	4.874201623	0.032346806
0.529123932	0.022014358	5.81238719	0.010065686
0.539675007	0.005589088	6.605019041	0.003858436
0.582865581	0.014607293	6.298479215	0.011486479
0.505914067	0.012529419	6.464954552	0.00668099
0.544203875	0.005320366	6.806243185	0.003511059
0.538914086	0.011209981	6.653027439	0.006664542
0.63880709	0.007294815	6.450787955	0.003805389
0.516088044	0.009011563	6.257152427	0.003668367
0.49858664	0.030529015	5.576276443	0.019989431
0.522822301	0.033988431	5.845076861	0.020635818
0.618346008	0.008628182	6.391020124	0.004162417

log.sigma.4.0.mm.3D_ngtdm_Coarseness	log.sigma.4.0.mm.3D_ngtdm_Complexity	log.sigma.4.0.mm.3D_ngtdm_Strength	log.sigma.4.0.mm.3D_ngtdm_Busyness	log.sigma.4.0.mm.3D_ngtdm_Contrast
0.01098852	0.420767905	2.971195017	0.247342825	8.19E-05
0.005784896	0.241047048	1.855479599	0.340326227	2.88E-05
0.010649405	0.426246105	2.874498279	0.213160486	6.74E-05
0.00503707	0.143676937	1.198707826	0.583402519	2.54E-05
0.012703728	0.181609979	1.820104476	0.492500515	9.61E-05
0.007644623	0.144698949	1.441317074	0.49348734	3.63E-05
0.01279534	0.104608037	1.580947026	0.564394714	6.25E-05
0.007216792	0.325973698	3.149217923	0.206566158	2.76E-05
0.005718858	0.232786058	1.594040476	0.463268779	3.68E-05
0.004203958	0.249580699	2.121957743	0.251055034	1.83E-05
0.006709982	0.090911612	0.981537636	0.602456033	2.86E-05
0.006873343	0.280402198	1.86312343	0.30635935	3.99E-05
0.004228769	0.100098171	1.15208652	0.55152723	1.23E-05
0.00645471	0.144202592	1.134971791	0.568850148	3.77E-05
0.007270874	0.432408688	2.461156378	0.304030811	5.69E-05
0.003610808	0.159068301	1.659417029	0.3439238	1.13E-05
0.008536751	0.32928242	3.480710424	0.154369514	2.79E-05
0.004572242	0.211257862	1.8582495	0.358613565	1.79E-05
0.008878985	0.415320842	2.428242048	0.239057369	5.50E-05
0.004462674	0.113420833	1.111979591	0.535317675	2.11E-05
0.00187913	0.102059015	0.913621891	0.636415362	5.84E-06
0.00586281	0.330588522	3.226686174	0.203467136	1.84E-05
0.003010837	0.131369115	1.096613829	0.571934869	1.35E-05
0.013312329	0.28777813	2.882884206	0.225758304	7.98E-05
0.005083016	0.129055018	0.999385816	0.589426669	3.88E-05
0.008099362	0.486175456	2.867262433	0.207058871	6.36E-05
0.005916486	0.16812777	1.539002092	0.460436769	2.86E-05
0.00322667	0.168316218	0.965387167	0.575432294	2.32E-05
0.00342535	0.173254544	1.430873573	0.399700405	1.35E-05
0.004116318	0.215202756	1.860049844	0.301537722	1.39E-05
0.003405682	0.130212863	1.386981319	0.380666222	1.20E-05
0.002331685	0.141407989	1.094929137	0.595302688	9.39E-06
0.005429509	0.282699425	3.240633023	0.178382387	1.61E-05
0.024185061	0.612812626	4.014187413	0.168231354	0.000228336
0.012799626	0.411422686	2.727191555	0.203623675	9.44E-05
0.003776382	0.128453692	1.135777821	0.470825217	1.36E-05
0.003477495	0.260466349	1.817506522	0.316682127	1.79E-05
0.003486497	0.121778339	1.165918195	0.439542291	1.30E-05
0.009214059	0.152825594	2.08760308	0.277634914	2.44E-05
0.007376167	0.149501255	1.462391341	0.588516545	4.34E-05
0.007736278	0.232373763	1.371824604	0.446261212	5.55E-05
0.006300224	0.149728245	1.237592936	0.512408152	2.98E-05
0.004198604	0.111238527	1.0991548	0.526247621	1.53E-05
0.006204991	0.187125407	1.564445935	0.474250525	3.30E-05
0.003933181	0.257194695	1.973827492	0.251721568	1.71E-05
0.003515764	0.107650168	0.913989944	0.711823685	1.82E-05
0.003433081	0.193604704	1.671626596	0.373032026	1.33E-05
0.008296999	0.309679619	3.21751378	0.161743973	3.16E-05
0.020939071	0.245415968	2.284157281	0.377318975	0.00016613
0.011449314	0.361422428	3.627141997	0.223936694	5.06E-05
0.003413348	0.112522283	1.395825492	0.431444799	8.66E-06
0.006293778	0.149908819	1.989763523	0.27714297	1.47E-05
0.003517888	0.102928484	1.028928058	0.707418303	1.37E-05
0.009966927	0.358048812	2.633814441	0.186329483	6.25E-05
0.003125675	0.136696896	1.025630075	0.571123772	1.54E-05
0.007347489	0.344826813	2.717781134	0.207415311	3.48E-05
0.003786064	0.274552504	2.262712403	0.233208982	1.57E-05
0.005006057	0.167944615	1.792867632	0.414199681	1.82E-05
0.008718975	0.34172901	2.886244777	0.190711015	4.18E-05
0.007902533	0.082843073	0.884701638	0.615526362	3.33E-05
0.004404066	0.132660176	1.125789968	0.567892802	2.35E-05
0.002328735	0.164940292	1.631802996	0.379293201	5.90E-06
0.005182256	0.249486257	1.924062172	0.29657403	2.27E-05
0.005757082	0.193584059	1.140729749	0.499041716	5.24E-05
0.017677912	0.452357157	2.334652528	0.243052393	0.000198636
0.007505735	0.318722129	2.233956186	0.379066544	5.80E-05
0.01311253	0.367771543	3.928108689	0.228885293	6.72E-05
0.00359346	0.145244354	1.145270551	0.573844419	1.94E-05
0.009465512	0.228675835	2.093657581	0.257478632	4.45E-05
0.005103548	0.293008948	2.539135136	0.221601583	2.00E-05
0.003338183	0.226293422	1.487502645	0.381034816	2.30E-05
0.005728636	0.165115084	1.506196384	0.399114596	2.45E-05
0.002660538	0.130926981	1.175514084	0.552726272	1.05E-05
0.001868168	0.092290779	0.812197907	0.685101392	6.42E-06
0.003113046	0.225296814	1.91811946	0.361014641	1.18E-05
0.00544021	0.363276314	3.016400385	0.219848595	2.11E-05
0.005154881	0.155460353	1.549133738	0.355961827	1.73E-05
0.007404773	0.307710689	1.892287329	0.323611204	5.22E-05
0.003433018	0.166315775	1.225486666	0.434080758	1.72E-05
0.003091197	0.111029278	1.192262595	0.423423455	1.06E-05
0.009497972	0.743362677	6.877320361	0.073275301	3.21E-05
0.004719748	0.330989023	2.544764084	0.218236023	2.17E-05
0.005027395	0.282675345	2.25989246	0.262132104	2.09E-05
0.02043711	0.201614358	1.741898005	0.404110516	0.000144462
0.003248346	0.134389049	1.128882721	0.49243549	1.70E-05
0.003668115	0.084315191	0.775597012	1.21725522	2.01E-05
0.009277129	0.420761553	3.340444299	0.176758471	5.02E-05
0.003894896	0.105570602	0.932136393	0.661714869	1.81E-05
0.018837902	0.211048089	1.086060417	0.651041896	0.000315467
0.020795957	0.402831147	3.475710715	0.168541631	0.00013805
0.003727047	0.144492365	1.120871604	0.526759915	1.90E-05
0.010111665	0.415347611	2.518496757	0.227745804	7.64E-05
0.007489104	0.206431762	1.776870597	0.296454066	3.62E-05
0.002851935	0.117818601	1.180713013	0.460820704	9.72E-06
0.0026361	0.070708717	0.648636335	0.975061765	1.05E-05
0.012066337	1.867912172	7.273298019	0.075854373	0.000177514
0.006536611	0.136332949	1.506725335	0.361235766	2.43E-05
0.030700858	0.349285787	3.637747267	0.250520743	0.000217841
0.013308555	0.13758669	1.612109655	0.536297577	6.73E-05
0.009933819	0.773909976	3.915675248	0.152046651	0.000114223

log.sigma.3.5.mm.3D_gldm_GrayLevelVariance	log.sigma.3.5.mm.3D_gldm_HighGrayLevelEmphasis	log.sigma.3.5.mm.3D_gldm_GrayLevelNonUniformityNormalized	log.sigma.3.5.mm.3D_gldm_DependenceEntropy
23.90296633	221.4655029	0.064328649	6.8820456
16.782279	211.078	0.077179	6.792118232
19.41437479	202.0509839	0.068844186	6.564855847
15.4463812	206.580106	0.079770372	6.762880106
13.79768621	116.4920969	0.103025646	6.57275892
12.60661584	146.2362637	0.092143103	6.684025299
10.48886148	91.19962157	0.126993177	6.566018417
18.61675279	324.7795014	0.075702611	6.777746494
17.70223298	266.6941293	0.071283115	6.854994976
19.9621147	458.3366633	0.066588201	6.807004134
9.585490111	217.6026563	0.100776694	6.638376376
17.54024214	229.1952522	0.071390256	6.669754052
10.95852415	236.994196	0.10209288	6.809941181
13.79322419	155.0678149	0.078321308	6.691297191
21.75775528	215.629199	0.064800626	6.877455338
19.98783739	394.5125335	0.075227076	7.206968309
16.86230374	352.9277259	0.082740268	6.817321289
15.22289452	306.5402658	0.08020859	6.836638985
14.86718595	275.2235915	0.076899115	6.594934732
15.43651567	250.5687694	0.078964201	6.856809678
18.1683677	309.4378417	0.071134952	6.939230246
16.99324663	389.0957096	0.07502199	6.788186053
18.09881702	272.7107498	0.070567764	6.828868915
19.10612122	248.3786408	0.076474691	6.766629422
20.90195988	223.844086	0.062115533	6.835802933
23.37739181	448.066568	0.063738577	6.772345969
16.38057318	190.1886024	0.073880628	6.667240954
19.88029486	302.0602086	0.066636401	6.629073304
15.84995639	309.308326	0.086057448	6.841070923
17.23116385	345.5302311	0.077713114	6.829227581
18.82368069	434.0799131	0.071534056	6.93181179
17.33580316	291.8278	0.07179288	6.92297438
18.86234524	394.4654287	0.079604762	6.818593453
18.22851734	172.7422037	0.088290593	6.469348544
18.51776946	290.2477477	0.072290196	6.593634962
13.37501842	304.1321107	0.087651097	6.7821183
25.18373127	420.3121322	0.06189463	7.038464329
13.95861458	463.846358	0.087165612	6.756548655
10.24519357	187.921159	0.102808211	6.720956454
14.68236493	163.3404645	0.088340128	6.739062332
12.95521741	199.4354103	0.082040308	6.476232161
13.32759448	180.6495859	0.083414777	6.783681954
12.77346847	295.395441	0.087339154	6.8803715
14.80403842	155.0705945	0.092372465	6.714720377
23.96061578	525.1104751	0.067442462	7.151753641
17.34936625	249.0360839	0.069178855	6.853816276
20.37953797	358.2270754	0.070267343	7.012636434
17.28714295	391.7453826	0.089060575	6.715316933
12.14019353	101.2819149	0.103258136	6.31974162
15.71165727	158.612844	0.0936773	6.597014605
13.51216305	292.5715361	0.09161067	6.867331542
9.59705625	322.2684049	0.123079424	6.592369327
15.24418173	228.5949434	0.081263509	6.898662121
16.04674014	396.146883	0.073707685	6.539058531
17.63320198	280.5803912	0.070044721	6.83433077
22.58286838	325.4682203	0.063773094	6.872104284
22.49036049	688.8670326	0.068861416	6.92414497
14.51030125	214.5838816	0.108249654	6.88101647
17.03585878	376.3206997	0.075038887	6.569662585
6.341047352	116.7480427	0.115639366	6.222159725
17.56863701	316.0204012	0.072191037	7.041790289
17.80096906	474.211591	0.081509457	6.957482839
17.40804519	333.5931308	0.07386512	6.894527733
16.35095305	271.8715203	0.069837887	6.475346252
14.51726803	127.5610169	0.073720195	6.419636079
22.16510581	237.7200259	0.07000486	6.736067601
20.53514049	186.915879	0.076670324	6.763497948
19.92967553	336.6918116	0.069121846	6.980824052
12.76062731	280.3727765	0.08442449	6.69327446
20.74801549	513.651775	0.070219818	6.924237505
28.22076416	494.8511029	0.053953061	7.110207256
13.29760264	274.9961184	0.09513784	6.57411573
18.41527159	287.6725796	0.072938748	7.002956207
15.24928901	393.3609277	0.077190519	6.818696228
24.66097062	423.6105115	0.062908362	7.028503071
21.76117136	395.7048477	0.07051637	6.909591604
11.83737434	256.2318471	0.09540076	6.729579649
15.1491872	248.615894	0.07664327	6.595168915
16.99448118	376.4996947	0.077287588	6.833167978
17.45080068	349.0783741	0.082752217	6.912741064
22.98144734	692.8105727	0.07851458	6.769641424
25.31378713	486.5366955	0.066317036	6.879096813
19.56940851	339.8694422	0.069864832	6.840988175
8.864917431	77.34608031	0.121482548	6.233826654
20.27380067	458.2881926	0.066100777	6.898281699
14.11293907	150.7820956	0.078164829	6.892697562
23.37841166	307.7542017	0.066622728	6.854350378
13.55981744	296.9559645	0.084173187	6.734360356
8.172838191	59.97080292	0.103853281	6.065867655
14.36961962	221.9688581	0.09608362	6.392121836
16.08477089	321.3491124	0.075609273	6.867330844
19.17190066	311.2570136	0.069172212	6.56920511
14.2567119	267.4577465	0.084475073	6.576267168
15.98270142	373.5688031	0.084850925	6.813817647
13.14531354	222.4842754	0.082154077	6.921087635
52.64223066	642.35181	0.045149055	6.718407257
9.671126634	301.6874645	0.113553993	6.577049049
13.49276684	107.4649533	0.085717093	6.302198809
8.814518911	86.96171171	0.119001603	6.544932207
36.9298439	421.0762565	0.049022464	6.705895342

log.sigma.3.5.mm.3D_gldm_DependenceNonUniformity	log.sigma.3.5.mm.3D_gldm_GrayLevelNonUniformity	log.sigma.3.5.mm.3D_gldm_SmallDependenceEmphasis
173.7082294	77.38736492	0.232840399
279.479	154.358	0.20542532
204.8318426	76.96779964	0.281660704
343.9359967	195.6767224	0.212944883
102.5279241	97.77133825	0.172372322
208.4188034	150.9304029	0.181592862
94.54020814	134.2317881	0.120377929
239.8631579	136.6432133	0.2137912
290.4385792	144.4908732	0.236964896
511.8984349	199.964369	0.248201574
212.5882678	182.1034864	0.154819309
285.2207715	120.2925816	0.269604726
316.4810516	299.0300444	0.165086405
282.4413348	145.5209903	0.217668463
233.7609819	100.3113695	0.249382481
509.9218788	309.1080555	0.222977385
207.6330218	132.7981308	0.199391156
317.1383894	205.1735731	0.17089664
175.5774648	87.35739437	0.254216713
394.9351948	229.0751465	0.193532248
1055.103309	494.3879137	0.226344942
316.1466289	159.1216407	0.217076282
665.955108	290.8097549	0.235238929
135.4019417	78.76893204	0.194387898
448.5599078	161.7488479	0.253923716
234.2781065	86.17455621	0.283851697
360.8787879	163.3500678	0.264836186
638.2274218	223.5651267	0.286818801
427.8708444	292.5092674	0.20226749
444.0174106	245.4957265	0.237022539
610.7416707	296.2940608	0.210525079
757.6528	358.9644	0.226899685
345.4140656	201.4796523	0.192856599
61.83575884	42.46777547	0.230454033
145.7972973	64.19369369	0.250209449
412.5760025	281.9735779	0.190765055
559.3054077	220.9019333	0.254283325
460.1436155	295.5785904	0.200289398
166.6239892	152.5673854	0.136294363
204.3447433	144.5244499	0.167078186
206.9057751	107.9650456	0.247567973
247.742236	161.1573499	0.193640287
350.0981169	264.3756194	0.171110001
229.4087049	174.029724	0.188229798
484.485976	235.6439611	0.225819776
555.1706294	247.3144056	0.215845278
543.2354856	259.0054259	0.243379428
202.182058	135.0158311	0.19907153
61.96453901	58.23758865	0.195168382
128.8385321	102.1082569	0.204600479
439.7334337	364.9769076	0.156664881
199.4059305	240.7433538	0.137856033
464.4943376	308.5575454	0.181443434
194.0111016	86.3116994	0.244281611
599.7818136	275.7660655	0.216881068
306.6302966	120.4036017	0.255121956
502.4656908	225.7965843	0.245137128
227.6907895	263.2631579	0.164299718
220.8061224	102.9533528	0.252691167
187.6918149	162.4733096	0.142251675
365.8092486	212.3138388	0.199400345
697.6382358	447.2423911	0.201475563
324.3535887	167.7476882	0.227815765
356.6584582	130.4571734	0.23926456
94.79322034	43.49491525	0.262140619
231.808814	108.0174984	0.262187101
143.6994329	81.11720227	0.218817524
509.3213685	246.4885025	0.207285844
167.8538283	109.1608662	0.184854375
371.3278819	176.041085	0.226693051
637.4164916	205.4532563	0.284310063
261.5046094	196.0790878	0.167899708
629.4005129	341.2804018	0.212693229
974.2440485	502.5874674	0.203366553
651.4645706	268.1154388	0.23594753
342.007293	164.3736594	0.228424697
280.6186837	224.6687898	0.165217333
214.2715232	104.1582046	0.232669466
467.490232	253.1941392	0.213406205
574.9389176	368.4956209	0.201349261
192.195301	106.9368576	0.229762498
432.1215657	176.2043658	0.271962211
391.9805982	172.8455942	0.246231635
51.73422562	63.53537285	0.169196665
637.0994303	255.2812015	0.237199012
454.3126079	271.5446172	0.186742487
226.245098	95.1372549	0.256460604
401.4134078	256.1390076	0.181879982
52.29440389	42.6836983	0.220309836
75.01384083	55.53633218	0.192357567
447.7355964	242.7813765	0.21292426
183.8669683	76.43529412	0.272709464
224.503201	131.950064	0.22471295
588.7574087	369.3560763	0.198035251
600.2277943	386.6170846	0.187767043
208.6651584	39.91176471	0.398477333
192.7231991	200.1956892	0.145986477
57.73831776	36.68691589	0.187492629
93.34009009	105.6734234	0.150519062
258.2772964	56.57192374	0.374925186

log.sigma.3.5.mm.3D_gldm_DependenceNonUniformityNormalized	log.sigma.3.5.mm.3D_gldm_DependenceVariance	log.sigma.3.5.mm.3D_gldm_LargeDependenceEmphasis
0.144395868	4.973117221	19.07148795
0.1397395	6.078711	21.943
0.183212739	4.030337845	14.26118068
0.140210353	5.495792485	20.85976355
0.108037855	9.520269242	33.18967334
0.127239807	6.557138832	25.08180708
0.089442013	14.03855542	49.28192999
0.132888176	5.746652036	21.84875346
0.143284943	5.371332108	19.55155402
0.170462349	3.985092497	15.40692641
0.117647077	7.30816486	29.63862756
0.169270488	3.724524826	14.53827893
0.108050888	8.73356381	32.35336292
0.152013636	4.537875953	18.31324004
0.151008386	4.262622772	16.96899225
0.124098778	16.8295306	38.62472621
0.129366369	6.281966984	23.60934579
0.123979042	6.842127688	26.61297889
0.154557627	4.427293072	16.83802817
0.136137606	6.132572764	22.96208204
0.151813426	4.69228806	18.17122302
0.149055459	4.417311806	18.32201792
0.161600366	3.983559683	16.23610774
0.131458196	6.466419078	23.94368932
0.172258029	3.977101871	15.21658986
0.173282623	4.491918613	15.17011834
0.163219714	4.702507364	16.45273632
0.19023172	2.836391177	11.98002981
0.12588139	7.326517733	25.5425125
0.140556319	5.663574159	20.25989237
0.14745091	4.6697299	19.08112023
0.15153056	5.22431616	19.0092
0.136473357	5.401390986	21.85657843
0.128556671	10.2795026	29.03326403
0.164186146	4.309892663	16.16891892
0.12824868	6.995222478	25.2026733
0.156712078	4.616326684	16.9557299
0.135695552	5.743782025	22.13063993
0.112280316	9.103375811	34.52291105
0.1249051	7.792785792	28.05378973
0.157223233	4.220734287	16.60942249
0.128230971	6.904858352	25.11283644
0.115658446	8.350200033	30.03865213
0.121766828	7.708966106	27.48407643
0.138662271	8.645438791	24.94447624
0.155292484	3.82713005	17.08895105
0.147378048	6.393725404	20.49593055
0.133365474	5.612923887	22.29551451
0.109866204	9.45071928	31.92907801
0.118200488	8.897180372	28.9412844
0.110374858	8.05150925	31.54869478
0.101945772	10.02759691	37.71370143
0.12233193	7.279004556	26.86726363
0.165679848	3.256332038	14.66353544
0.152344885	4.706219606	18.498095
0.162410115	4.11070813	15.83262712
0.153237478	5.830708434	19.19030192
0.093622858	12.95315746	42.66282895
0.160937407	4.25002125	16.2361516
0.13358848	4.491500361	24.34946619
0.124382607	7.671461444	26.43352601
0.127143837	7.156820291	25.10679789
0.142824125	5.668170707	20.36767944
0.190930652	2.553750992	12.63811563
0.160666475	4.214179833	15.95932203
0.150232543	4.724046758	17.43421905
0.13582177	6.391290054	22.3610586
0.14282708	5.343337352	20.59842961
0.129817346	5.598366838	23.27996906
0.148116427	5.581682498	19.78739529
0.167388785	4.142555919	14.94537815
0.126882392	5.364663545	24.20135856
0.134516032	5.892887425	21.94250908
0.149630479	4.63258782	19.12071878
0.152854193	4.442177672	17.48474894
0.146721275	5.629786801	19.96954097
0.119158677	6.781091683	27.45944798
0.157668523	3.958227953	16.51140545
0.142701536	4.927571818	19.69230769
0.129112714	6.792987859	24.32450034
0.141112556	6.074359422	21.10279001
0.16263514	4.087954798	15.39141889
0.158440015	5.218659508	17.99191593
0.098918213	10.28143999	36.99235182
0.164966191	3.936412122	15.98964267
0.130775074	5.908946101	23.50834773
0.158434943	5.508107164	18.19327731
0.131913706	5.439115908	22.92047322
0.127236992	6.362915209	23.33090024
0.129781732	7.677542175	26.17647059
0.139438056	6.756991814	22.67985051
0.166395446	3.24212854	13.94479638
0.143728042	4.612935909	18.76440461
0.135253253	5.848754175	22.35814381
0.127545218	6.131560672	24.19677008
0.236046559	2.111253865	8.226244344
0.109315484	7.983296919	32.43051617
0.134902612	6.806249454	24.53271028
0.105112714	13.34390472	41.02477477
0.223810482	1.932466081	8.414211438

log.sigma.3.5.mm.3D_gldm_LargeDependenceLowGrayLevelEmphasis	log.sigma.3.5.mm.3D_gldm_SmallDependenceHighGrayLevelEmphasis	log.sigma.3.5.mm.3D_gldm_LargeDependenceHighGrayLevelEmphasis
0.168143879	56.97295396	3474.965087
0.18172036	46.98070576	3813.781
0.105020749	52.60466993	2987.984794
0.142850463	46.90463178	3815.154912
0.468711896	24.92891672	2883.286617
0.228476717	27.22497317	3519.602564
0.961689183	13.86394293	3072.406812
0.094157038	71.12673925	6363.227147
0.101951489	63.2091099	4556.978293
0.03914418	110.4095679	6972.66367
0.1623236	32.49474876	6104.143885
0.096893557	61.03240195	3272.845104
0.164219589	43.12261106	6932.204165
0.180145989	33.22517549	2812.579117
0.112878031	52.29614209	3441.268734
0.127174653	89.52093561	12863.24897
0.073580583	63.75131101	8726.472897
0.116893967	57.94654304	6907.643471
0.081908626	74.27168914	4081.507042
0.131380108	49.51671259	5222.496381
0.073784982	66.54521674	5585.116403
0.053221318	83.51890969	7175.680339
0.08144341	64.11890603	4389.275661
0.146686319	51.47321636	5143.75534
0.119888569	55.03452749	3309.466974
0.044050312	126.9332639	6143.902367
0.113568543	48.52872724	2992.755767
0.052647183	79.53573307	3827.120715
0.101966141	63.35539276	7275.198882
0.068936531	80.72280992	6680.031022
0.048727493	83.50683194	8631.624577
0.088775194	66.20618695	5038.6262
0.090804234	78.98078891	8025.856974
0.298817623	42.40270543	3512.862786
0.078960565	70.55722597	4571.371622
0.099504302	58.23940942	7147.649052
0.053756136	106.6635818	6733.218268
0.051074003	87.25924054	10277.47007
0.234258629	27.45534905	5772.302561
0.263697116	32.88734258	3624.452934
0.123028386	48.42527512	3135.985562
0.201462286	37.6469899	3779.403209
0.125953125	51.96848769	7920.326396
0.285956394	33.71372719	3272.798832
0.055065655	111.5636018	12907.74184
0.094945361	50.93959269	4431.246993
0.074791605	87.41083609	6913.135377
0.062371151	70.97967163	9200.279683
0.625976593	24.90713631	2205.757092
0.284495662	38.85502324	3486.878899
0.139835513	48.91627024	8092.644327
0.135232076	45.0848896	11422.77352
0.163979939	47.83507493	5134.829866
0.043904561	93.17954854	5634.283518
0.090093336	62.41793191	4677.602489
0.067543576	83.37002463	4879.189619
0.031973951	165.6637887	12460.4419
0.279775874	42.94007624	7215.976151
0.058214877	89.29548623	6135.930029
0.262762828	15.01186499	2886.154448
0.115160938	64.60205219	7054.513431
0.061250717	94.11634493	11241.33607
0.074292562	77.09332928	6528.366358
0.056699931	60.18414828	3632.342612
0.21858513	30.67326087	1865.452542
0.128223881	74.29291925	3215.351912
0.183197827	50.94665094	3436.830813
0.079901896	73.03019701	6265.235838
0.09408074	46.27015052	6590.776489
0.044612839	116.7828112	9633.024731
0.042105383	138.2385451	7024.738708
0.10996591	52.09574888	5905.398836
0.110346296	64.06622958	5650.6433
0.054802742	75.57357967	7542.252649
0.053874644	102.7001358	6944.189582
0.065157955	87.21301706	7519.941656
0.129092084	41.31004473	6768.841614
0.086319016	58.09475678	3958.699779
0.058558171	75.70669866	7526.341575
0.080990345	66.13268247	8417.410734
0.033641438	152.3142627	14370.56975
0.036630078	124.2881385	7697.775687
0.071940162	86.84474697	5795.657639
0.900834007	16.49539249	1894.114723
0.040997798	105.7350487	7373.115225
0.243061171	29.97742587	3052.486183
0.087188966	81.61148151	5121.895658
0.095400569	56.06499161	6209.942491
0.970491509	12.73906511	1071.221411
0.159270708	39.56784786	5415.453287
0.093372263	72.39986684	6258.46839
0.055846889	81.0136093	4330.467873
0.08263938	54.05222399	5310.739437
0.067600819	73.66466051	8231.426832
0.140722049	43.23831582	4992.808117
0.021513581	236.8162183	5774.946833
0.125079938	44.93959769	9296.782189
0.478167662	22.65642027	1818.030374
0.681449513	14.79259014	2931.677928
0.037079681	140.9671379	3906.958406

log.sigma.3.5.mm.3D_gldm_SmallDependenceLowGrayLevelEmphasis	log.sigma.3.5.mm.3D_gldm_LowGrayLevelEmphasis	log.sigma.3.5.mm.3D_gldzm_DistanceZoneVariabilityNormalized
0.003151438	0.012876699	1
0.002179362	0.010106861	1
0.005367728	0.012405763	1
0.002608046	0.008712593	1
0.003875128	0.015878204	1
0.003815172	0.013260209	1
0.003537723	0.020640129	1
0.001680105	0.005275956	1
0.002107506	0.005949982	1
0.001199129	0.003227831	0.9978022
0.001734555	0.006333828	1
0.003050949	0.009863603	1
0.001445706	0.005691723	1
0.004393101	0.014101248	1
0.003178673	0.008576876	1
0.001133216	0.003717798	0.994392568
0.001762426	0.004877155	1
0.001235844	0.004580687	1
0.002276976	0.005823681	1
0.001519554	0.007109625	1
0.001706375	0.005274403	0.995746962
0.001324783	0.003751499	1
0.001621289	0.006231408	0.998280311
0.003269437	0.008838829	1
0.002970626	0.008932622	1
0.001987144	0.004032904	1
0.003414849	0.008864059	1
0.002250732	0.005633152	1
0.00210687	0.005509142	1
0.001707713	0.00443661	1
0.001193637	0.003409545	1
0.001616076	0.005150138	1
0.001201433	0.004839314	1
0.006713171	0.013605438	1
0.002287321	0.007907795	1
0.00120285	0.004852016	1
0.001442319	0.004076311	1
0.000931432	0.002870613	1
0.001712225	0.008542046	1
0.002169014	0.01013114	1
0.002909441	0.009701104	1
0.00241505	0.008542972	1
0.001210622	0.004580802	1
0.003226655	0.012279507	1
0.00120462	0.003092018	1
0.001891349	0.006898029	1
0.001450972	0.004894365	0.988636735
0.001614452	0.004241627	1
0.003551315	0.022596857	1
0.004365489	0.01205094	1
0.000984267	0.005064523	1
0.000980844	0.005006782	1
0.001447229	0.006289963	0.997333338
0.001715983	0.003910604	1
0.001868495	0.005847977	0.9979798
0.001951859	0.005438432	1
0.000804615	0.002060648	0.997874604
0.001338217	0.007181921	1
0.001528495	0.005462452	1
0.002767274	0.013833924	1
0.001437636	0.004684097	1
0.000946742	0.002979124	1
0.001695568	0.004830136	1
0.002204976	0.005938981	1
0.008473601	0.018974485	1
0.001821053	0.007588188	1
0.002949594	0.009778364	1
0.001301671	0.004378868	1
0.001942994	0.005379837	1
0.001102524	0.002860411	1
0.001129133	0.003142449	1
0.001399753	0.005086406	1
0.001554126	0.005826561	1
0.000962718	0.003363414	0.998664887
0.001312255	0.00389401	1
0.001436353	0.00465685	1
0.002297704	0.006966913	1
0.002707853	0.007212309	1
0.001289422	0.003884426	1
0.001352314	0.004380815	1
0.001540858	0.002788648	1
0.001523462	0.003546658	1
0.001854499	0.005587501	1
0.005376872	0.022665389	1
0.001018055	0.003088992	0.998142991
0.002484928	0.010880809	0.997260279
0.002756561	0.007606685	1
0.001237552	0.004585054	1
0.01216625	0.038757904	1
0.00301134	0.011011009	1
0.001179028	0.004198629	1
0.002815594	0.005912027	1
0.002716074	0.007451112	1
0.001084692	0.003859086	1
0.001638673	0.006437536	0.997952919
0.002829456	0.004769034	1
0.000911641	0.004985152	1
0.005749097	0.019344773	1
0.00504029	0.020752741	1
0.003391296	0.006587647	1

log.sigma.3.5.mm.3D_gldzm_LowIntensityEmphasis	log.sigma.3.5.mm.3D_gldzm_LargeDistanceEmphasis	log.sigma.3.5.mm.3D_gldzm_HighIntensitySmallDistanceEmphasis
0.017162767	1	252.3333333
0.011378498	1	237.614
0.018476216	1	189.2282051
0.012828384	1	224.0562914
0.021300731	1	151.0461538
0.022136449	1	151.5367232
0.031095491	1	122.56
0.007983796	1	340.295045
0.00871568	1	273.400369
0.004583679	1.00330033	449.5976348
0.011014264	1	211.0512821
0.012965982	1	227.2342342
0.008424904	1	266.6972973
0.020634767	1	153.791498
0.012428868	1	212.0845987
0.005041127	1.008434864	402.43791
0.0087027	1	325.1105263
0.00696829	1	345.5376782
0.008469189	1	297.4117647
0.009208422	1	261.4153846
0.007533354	1.006393181	294.2815663
0.005765417	1	387.4181818
0.007570373	1.002581756	273.685241
0.01587976	1	269.9268293
0.0113765	1	219.0779874
0.006399967	1	454.4365591
0.012439394	1	188.548105
0.007691433	1	279.3794872
0.010377822	1	315.3626667
0.006875801	1	343.6214367
0.005437022	1	401.9525194
0.006961122	1	298.208622
0.006518837	1	413.433213
0.025992354	1	186.9022556
0.010933745	1	282.4836364
0.007006395	1	304.4900285
0.005670791	1	423.0709617
0.004582724	1	437.4511873
0.014825335	1	212.1764706
0.013438663	1	201.5587302
0.012871663	1	201.0104167
0.012057571	1	200.8837209
0.007146389	1	306.4082734
0.017977913	1	188.1489899
0.005110041	1	494.627409
0.009246142	1	232.0885936
0.006484232	1.017142857	360.1840476
0.007930419	1	352.4444444
0.022730792	1	136.778626
0.019553146	1	195.701107
0.007232498	1	316.6205882
0.009248986	1	328.0440678
0.008101339	1.00400534	270.0186916
0.006363061	1	388.6397695
0.008608652	1.003033367	292.117543
0.007447235	1	333.4941176
0.003097642	1.003191489	686.7542553
0.009071528	1	270.9262473
0.007413728	1	356.2711864
0.023089813	1	105.8779343
0.006977901	1	333.6820276
0.004652109	1	467.4496374
0.007413217	1	340.1459732
0.0088106	1	254.6405959
0.030661179	1	119.4456522
0.007830616	1	292.3390558
0.012822062	1	239.5107143
0.006223686	1	363.1818182
0.01011956	1	244.6457565
0.004549685	1	517.5468053
0.003957806	1	489.695962
0.00805627	1	323.8453333
0.007524013	1	306.2340792
0.00472234	1.002004008	371.1731797
0.005372841	1	440.9370805
0.007048981	1	378.9712
0.014866966	1	253.4052133
0.011530078	1	251.8870968
0.005992124	1	353.4136646
0.006674533	1	327.5148042
0.005972276	1	672.3342246
0.005507253	1	458.3969555
0.008113837	1	355.7793696
0.028866266	1	104.6857143
0.004181617	1.002788104	447.0966543
0.013688419	1.004115226	164.7301097
0.011480169	1	321.4908257
0.006711347	1	311.0210016
0.052469104	1	59.11428571
0.018587889	1	210.0592593
0.00530483	1	347.1047382
0.009696086	1	300.6685237
0.01278886	1	240.2971014
0.005669834	1	373.4246714
0.008671902	1.00307377	236.1270492
0.00670869	1	601.0188679
0.007819551	1	310.814433
0.029559618	1	128.4565217
0.033308354	1	100.1089744
0.008868162	1	380.925144

log.sigma.3.5.mm.3D_gldzm_LowIntensityLargeDistanceEmphasis	log.sigma.3.5.mm.3D_gldzm_HighIntensityEmphasis	log.sigma.3.5.mm.3D_gldzm_DistanceZoneVariability	log.sigma.3.5.mm.3D_gldzm_ZonePercentage
0.017162767	252.3333333	333	0.27680798
0.011378498	237.614	500	0.25
0.018476216	189.2282051	390	0.348837209
0.012828384	224.0562914	604	0.246229107
0.021300731	151.0461538	195	0.205479452
0.022136449	151.5367232	354	0.216117216
0.031095491	122.56	150	0.141911069
0.007983796	340.295045	444	0.24598338
0.00871568	273.400369	542	0.267390232
0.004592821	449.8954895	907.0022002	0.302697303
0.011014264	211.0512821	312	0.172661871
0.012965982	227.2342342	555	0.329376855
0.008424904	266.6972973	555	0.189484466
0.020634767	153.791498	494	0.265877287
0.012428868	212.0845987	461	0.297803618
0.005076134	403.158388	1061.01687	0.259673887
0.0087027	325.1105263	380	0.236760125
0.00696829	345.5376782	491	0.191946833
0.008469189	297.4117647	340	0.299295775
0.009208422	261.4153846	650	0.224060669
0.007554769	294.8129995	1869.017048	0.270071942
0.005765417	387.4181818	550	0.259311645
0.00759171	273.7633391	1160.001721	0.281970396
0.01587976	269.9268293	246	0.238834951
0.0113765	219.0779874	795	0.305299539
0.006399967	454.4365591	465	0.343934911
0.012439394	188.548105	686	0.310266848
0.007691433	279.3794872	1170	0.348733234
0.010377822	315.3626667	750	0.220653133
0.006875801	343.6214367	877	0.2776195
0.005437022	401.9525194	1032	0.249154998
0.006961122	298.208622	1299	0.2598
0.006518837	413.433213	554	0.218885816
0.025992354	186.9022556	133	0.276507277
0.010933745	282.4836364	275	0.309684685
0.007006395	304.4900285	702	0.218215729
0.005670791	423.0709617	1071	0.300084057
0.004582724	437.4511873	758	0.223532881
0.014825335	212.1764706	221	0.148921833
0.013438663	201.5587302	315	0.192542787
0.012871663	201.0104167	384	0.291793313
0.012057571	200.8837209	430	0.222567288
0.007146389	306.4082734	556	0.183680211
0.017977913	188.1489899	396	0.210191083
0.005110041	494.627409	934	0.267315398
0.009246142	232.0885936	903	0.252587413
0.006540008	361.7047619	1038.068571	0.284861639
0.007930419	352.4444444	342	0.225593668
0.022730792	136.778626	131	0.232269504
0.019553146	195.701107	271	0.248623853
0.007232498	316.6205882	680	0.170682731
0.009248986	328.0440678	295	0.150817996
0.008121775	270.2149533	747.0026702	0.197260996
0.006363061	388.6397695	347	0.296327925
0.008619148	292.3367037	987.0020222	0.251206502
0.007447235	333.4941176	595	0.315148305
0.003104236	687.1404255	938.0021277	0.286672766
0.009071528	270.9262473	461	0.189555921
0.007413728	356.2711864	413	0.301020408
0.023089813	105.8779343	213	0.151601423
0.006977901	333.6820276	651	0.221353281
0.004652109	467.4496374	1241	0.22617095
0.007413217	340.1459732	596	0.262439454
0.0088106	254.6405959	537	0.287473233
0.030661179	119.4456522	184	0.311864407
0.007830616	292.3390558	466	0.302009073
0.012822062	239.5107143	280	0.264650284
0.006223686	363.1818182	814	0.228266966
0.01011956	244.6457565	271	0.209590101
0.004549685	517.5468053	673	0.268448345
0.003957806	489.695962	1263	0.331670168
0.00805627	323.8453333	375	0.181950509
0.007524013	306.2340792	1162	0.248343663
0.004727891	371.3540414	1495.001336	0.229918599
0.005372841	440.9370805	1192	0.279680901
0.007048981	378.9712	625	0.268125268
0.014866966	253.4052133	422	0.179193206
0.011530078	251.8870968	372	0.273730684
0.005992124	353.4136646	805	0.245726496
0.006674533	327.5148042	1047	0.235122389
0.005972276	672.3342246	374	0.274596182
0.005507253	458.3969555	854	0.32141513
0.008113837	355.7793696	698	0.282134196
0.028866266	104.6857143	105	0.200764818
0.004200979	447.197026	1074.001859	0.278612118
0.013739224	164.8134431	727.0027435	0.20984456
0.011480169	321.4908257	436	0.305322129
0.006711347	311.0210016	619	0.20341768
0.052469104	59.11428571	105	0.255474453
0.018587889	210.0592593	135	0.233564014
0.00530483	347.1047382	802	0.249766428
0.009696086	300.6685237	359	0.324886878
0.01278886	240.2971014	414	0.265044814
0.005669834	373.4246714	989	0.227199632
0.008702639	236.2038934	974.0020492	0.207394815
0.00670869	601.0188679	424	0.479638009
0.007819551	310.814433	291	0.165059558
0.029559618	128.4565217	92	0.214953271
0.033308354	100.1089744	156	0.175675676
0.008868162	380.925144	521	0.451473137

log.sigma.3.5.mm.3D_gldzm_IntensityVariabilityNormalized	log.sigma.3.5.mm.3D_gldzm_LowIntensitySmallDistanceEmphasis	log.sigma.3.5.mm.3D_gldzm_IntensityVariability
0.04881458	0.017162767	16.25525526
0.058768	0.011378498	29.384
0.056725838	0.018476216	22.12307692
0.056390071	0.012828384	34.05960265
0.066719264	0.021300731	13.01025641
0.059832743	0.022136449	21.18079096
0.072711111	0.031095491	10.90666667
0.055616427	0.007983796	24.69369369
0.056637301	0.00871568	30.69741697
0.05395138	0.004581394	49.04180418
0.074375411	0.011014264	23.20512821
0.056479182	0.012965982	31.34594595
0.057492087	0.008424904	31.90810811
0.063466046	0.020634767	31.35222672
0.054691066	0.012428868	25.21258134
0.05160612	0.005032375	55.06373008
0.053933518	0.0087027	20.49473684
0.057536679	0.00696829	28.25050916
0.062785467	0.008469189	21.34705882
0.057448521	0.009208422	37.34153846
0.05464262	0.007528001	102.5641982
0.055960331	0.005765417	30.77818182
0.05601358	0.007565039	65.08777969
0.054068346	0.01587976	13.30081301
0.056144931	0.0113765	44.63522013
0.050378078	0.006399967	23.42580645
0.059741264	0.012439394	40.98250729
0.055783476	0.007691433	65.26666667
0.053066667	0.010377822	39.8
0.051636331	0.006875801	45.28506271
0.052565952	0.005437022	54.24806202
0.056008619	0.006961122	72.7551963
0.052620261	0.006518837	29.15162455
0.064277234	0.025992354	8.54887218
0.053963636	0.010933745	14.84
0.056927298	0.007006395	39.96296296
0.046203231	0.005670791	49.48366013
0.057044994	0.004582724	43.24010554
0.06189472	0.014825335	13.67873303
0.058886369	0.013438663	18.54920635
0.060858832	0.012871663	23.36979167
0.061103299	0.012057571	26.2744186
0.059714818	0.007146389	33.20143885
0.054280175	0.017977913	21.49494949
0.044997684	0.005110041	42.02783726
0.061226943	0.009246142	55.28792913
0.051735147	0.006470288	54.32190476
0.052939366	0.007930419	18.10526316
0.084901812	0.022730792	11.1221374
0.059571629	0.019553146	16.14391144
0.056012111	0.007232498	38.08823529
0.061465096	0.009248986	18.13220339
0.058393835	0.00809623	43.73698264
0.058691626	0.006363061	20.36599424
0.055634221	0.008606028	55.02224469
0.049705529	0.007447235	29.57478992
0.047716161	0.003095994	44.85319149
0.056225032	0.009071528	25.9197397
0.057155755	0.007413728	23.60532688
0.089642708	0.023089813	19.09389671
0.055839887	0.006977901	36.35176651
0.051022641	0.004652109	63.3190975
0.054141705	0.007413217	32.26845638
0.060862992	0.0088106	32.68342644
0.066398866	0.030661179	12.2173913
0.056512369	0.007830616	26.33476395
0.050765306	0.012822062	14.21428571
0.049028367	0.006223686	39.90909091
0.068749064	0.01011956	18.63099631
0.048557279	0.004549685	32.67904903
0.048443143	0.003957806	61.18368963
0.059697778	0.00805627	22.38666667
0.0539147	0.007524013	62.64888124
0.057006018	0.004720952	85.33800935
0.047251531	0.005372841	56.3238255
0.0507264	0.007048981	31.704
0.056770962	0.014866966	23.95734597
0.059197595	0.011530078	22.02150538
0.05231434	0.005992124	42.11304348
0.051029503	0.006674533	53.42788921
0.045397352	0.005972276	16.97860963
0.045354826	0.005507253	38.73302108
0.051957701	0.008113837	36.26647564
0.087709751	0.028866266	9.20952381
0.05316227	0.004176777	57.20260223
0.061747212	0.013675717	45.01371742
0.048301911	0.011480169	21.05963303
0.060885633	0.006711347	37.68820679
0.104761905	0.052469104	11
0.061947874	0.018587889	8.362962963
0.059903234	0.00530483	48.04239401
0.051310899	0.009696086	18.42061281
0.057585941	0.01278886	23.84057971
0.052313569	0.005669834	51.73811931
0.059006316	0.008664217	57.59016393
0.035599858	0.00670869	15.09433962
0.070629775	0.007819551	20.5532646
0.073251418	0.029559618	6.739130435
0.071581197	0.033308354	11.16666667
0.043571163	0.008868162	22.70057582

log.sigma.3.5.mm.3D_gldzm_HighIntensityLargeDistanceEmphasis	log.sigma.3.5.mm.3D_gldzm_SmallDistanceEmphasis	log.sigma.3.5.mm.3D_glcm_SumVariance	log.sigma.3.5.mm.3D_glcm_Homogeneity1
252.3333333	1	621.1509051	0.413377884
237.614	1	584.422134	0.431903333
189.2282051	1	554.6533177	0.3816147
224.0562914	1	562.3604225	0.435478785
151.0461538	1	287.7246514	0.495963954
151.5367232	1	388.0557307	0.452824762
122.56	1	216.2712243	0.554617779
340.295045	1	968.3155506	0.442284789
273.400369	1	777.593045	0.427771786
451.0869087	0.999174917	1401.281142	0.392635784
211.0512821	1	626.440691	0.489785038
227.2342342	1	647.721589	0.391234957
266.6972973	1	676.6858285	0.491802974
153.791498	1	401.1052663	0.421134292
212.0845987	1	621.0771047	0.40268741
406.0402999	0.997891284	1200.458214	0.449198341
325.1105263	1	1080.284712	0.438017958
345.5376782	1	910.900877	0.460422797
297.4117647	1	811.8278657	0.411869183
261.4153846	1	718.7738301	0.44463128
296.938732	0.998401705	918.6531179	0.398535908
387.4181818	1	1163.153473	0.417348155
274.0757315	0.999354561	794.1397384	0.403407705
269.9268293	1	692.4724616	0.443758917
219.0779874	1	624.108532	0.386114209
454.4365591	1	1375.547121	0.377052836
188.548105	1	559.2415903	0.439634566
279.3794872	1	911.9197875	0.362176264
315.3626667	1	928.8304302	0.437758202
343.6214367	1	1067.405509	0.420546822
401.9525194	1	1321.471358	0.416899006
298.208622	1	861.9798265	0.420827078
413.433213	1	1186.390198	0.435332786
186.9022556	1	471.0890542	0.455391644
282.4836364	1	850.1533552	0.397579775
304.4900285	1	910.6225511	0.4471749
423.0709617	1	1265.237507	0.382501071
437.4511873	1	1437.638796	0.430289101
212.1764706	1	521.1580845	0.50794294
201.5587302	1	432.1863962	0.467967226
201.0104167	1	556.500952	0.412149758
200.8837209	1	488.8252807	0.455759343
306.4082734	1	887.5506347	0.472158469
188.1489899	1	413.7795813	0.467487445
494.627409	1	1653.311397	0.408017903
232.0885936	1	707.4359874	0.415436488
367.787619	0.995714286	1046.798654	0.41146379
352.4444444	1	1232.354195	0.42538154
136.778626	1	246.3099906	0.491915046
195.701107	1	418.8744634	0.471583757
316.6205882	1	880.5880515	0.487942178
328.0440678	1	993.7478115	0.501804564
271	0.998998665	636.9472474	0.468252484
388.6397695	1	1241.673774	0.398649585
293.2133468	0.999241658	817.4947168	0.411113749
333.4941176	1	967.1269901	0.380965755
688.6851064	0.999202128	2232.896797	0.402184365
270.9262473	1	612.3840114	0.507376303
356.2711864	1	1147.173719	0.399748751
105.8779343	1	304.9162699	0.485813521
333.6820276	1	944.5429762	0.451279084
467.4496374	1	1468.509477	0.440126677
340.1459732	1	993.0029502	0.418277235
254.6405959	1	798.4695017	0.382440055
119.4456522	1	340.2938043	0.405859172
292.3390558	1	659.1889639	0.405503534
239.5107143	1	507.0846984	0.438863801
363.1818182	1	995.5274432	0.425105852
244.6457565	1	834.4153017	0.450639124
517.5468053	1	1608.627652	0.413647495
489.695962	1	1526.165383	0.372485084
323.8453333	1	824.8487747	0.461912986
306.2340792	1	841.9576701	0.430372169
372.0774883	0.999498998	1213.202236	0.41599728
440.9370805	1	1254.676397	0.403104787
378.9712	1	1190.848866	0.408552944
253.4052133	1	761.0671883	0.467693069
251.8870968	1	718.9277802	0.411782879
353.4136646	1	1157.609031	0.407327799
327.5148042	1	1058.318242	0.433728227
672.3342246	1	2291.533802	0.413602523
458.3969555	1	1471.919387	0.375204821
355.7793696	1	999.7217557	0.400839908
104.6857143	1	180.8561265	0.522422146
447.598513	0.999302974	1385.025991	0.400751395
165.1467764	0.998971193	393.6312064	0.455992507
321.4908257	1	903.4915784	0.405809668
311.0210016	1	889.9355028	0.444279773
59.11428571	1	132.0299175	0.459141728
210.0592593	1	625.1430293	0.464120868
347.1047382	1	960.5473109	0.428578889
300.6685237	1	931.0055475	0.387261938
240.2971014	1	779.2796426	0.42055531
373.4246714	1	1129.464185	0.430709935
236.5112705	0.999231557	624.7996558	0.45666559
601.0188679	1	1993.343224	0.285576752
310.814433	1	906.4835591	0.499257695
128.4565217	1	261.9542235	0.476909427
100.1089744	1	210.7359053	0.522395701
380.925144	1	1250.161435	0.313419919

log.sigma.3.5.mm.3D_glcm_Homogeneity2	log.sigma.3.5.mm.3D_glcm_ClusterShade	log.sigma.3.5.mm.3D_glcm_MaximumProbability	log.sigma.3.5.mm.3D_glcm_Idmn	log.sigma.3.5.mm.3D_glcm_SumVariance2
0.336745787	264.1686149	0.029051238	0.984532415	81.08740024
0.35663353	130.2555512	0.03464161	0.986588034	54.3157391
0.298256028	-132.7064095	0.024127646	0.979338721	62.33337737
0.359697954	136.7175755	0.039322368	0.988697111	50.25593974
0.434801642	265.7964211	0.068947596	0.984743616	46.62483921
0.381096111	141.2047319	0.040313097	0.985494579	40.90856924
0.506287644	184.3766098	0.114372386	0.988575928	35.11668211
0.369530217	102.225483	0.034377668	0.991558589	60.75446884
0.352832077	147.9975355	0.028972688	0.986475042	59.65912271
0.308994798	-72.52320002	0.024004172	0.989676987	61.51857473
0.426617793	54.36368473	0.046043124	0.98823122	31.7648866
0.307588928	-64.7030979	0.020282296	0.982472904	54.59128361
0.429414004	110.7115997	0.057257203	0.992079624	35.00184837
0.341765002	-36.97026275	0.023448022	0.983611544	44.05090827
0.324286305	368.888882	0.021426327	0.982776424	69.0963886
0.378824759	-36.15303185	0.074473025	0.990287486	65.2741441
0.365273644	-179.6312039	0.035810637	0.98968385	50.81606125
0.391311124	132.6231886	0.042313914	0.992297227	49.88204296
0.332084066	15.21973548	0.025463323	0.985287803	47.27132092
0.371167734	-18.32149183	0.0366566	0.987783846	52.66205591
0.317422442	-157.9710865	0.021949995	0.988937072	55.43785071
0.338359799	36.32266298	0.024906224	0.993113139	55.9792958
0.32186769	31.58995202	0.024774474	0.986884215	59.7301675
0.370459145	58.42715243	0.044267115	0.986860129	65.4332869
0.302471953	-169.2929122	0.016935142	0.980958254	69.10459114
0.293380097	77.80942964	0.023015828	0.984917675	72.83231659
0.364363577	150.4593981	0.032863394	0.988627286	54.08243075
0.27407953	-240.7412247	0.01699049	0.983063801	61.4689591
0.363591282	-121.7281723	0.042037783	0.988779002	48.31937147
0.344247518	-4.735432497	0.031954948	0.989767289	48.44656349
0.339266275	-238.0073863	0.021233742	0.989940844	58.9293395
0.342734876	26.35990583	0.02165292	0.990016668	54.25819632
0.360466806	-181.5478419	0.038486459	0.991121249	60.22580824
0.386914741	239.0867435	0.071293693	0.979467869	56.51015032
0.317890008	-164.9820871	0.028326438	0.980961361	60.56384631
0.375672515	8.85540969	0.042020495	0.989245827	42.4763836
0.299034655	-89.70683033	0.021035471	0.986565969	76.37819538
0.354192096	-81.13034157	0.032010804	0.990640463	42.42737579
0.448006148	65.73662729	0.053967519	0.990952447	31.05534929
0.399633825	259.393653	0.04966646	0.988645542	49.51635154
0.331873158	23.85547941	0.029510971	0.982015747	37.95562329
0.385423803	116.1113055	0.037057212	0.987342762	41.64316493
0.405156667	94.66146304	0.044875368	0.990643772	41.74378972
0.400791221	286.8327766	0.048487481	0.987499048	47.4270163
0.332140382	-268.4408346	0.02800351	0.98730231	76.99676171
0.335279761	-106.6394323	0.018344973	0.987962519	60.01906467
0.333393345	-82.54569737	0.024584587	0.99031042	63.26428605
0.351323107	-393.3566997	0.040660488	0.986600849	51.1617289
0.43089476	137.1894457	0.071798037	0.980275767	42.33325484
0.406059361	346.0799219	0.055507339	0.990193055	52.10929661
0.42453176	92.599512	0.051423377	0.992020449	43.3845991
0.442372426	-51.89700909	0.07739288	0.990960134	29.47026251
0.400201973	231.2525494	0.039364304	0.991124882	50.18020906
0.317299733	64.48699822	0.023179877	0.987018157	47.33804329
0.330712196	3.06151096	0.025539549	0.988039893	55.63275752
0.298522736	-3.802481236	0.02195266	0.98489094	68.9942269
0.322442998	-208.616438	0.032385587	0.991870451	72.92312495
0.450191053	350.4036867	0.078609566	0.990786634	50.22387827
0.318996922	-170.0951192	0.029159807	0.984365065	53.23781871
0.421102535	-20.99734428	0.042734849	0.982785404	19.68626526
0.379902109	67.74874547	0.038034269	0.989310516	59.35193606
0.368644336	-186.3339845	0.040722586	0.993266049	57.98399952
0.341032858	-85.92301485	0.026931133	0.987313793	54.56295292
0.295818806	-130.0808859	0.016053648	0.981562166	51.67305558
0.325498592	21.64041469	0.026146938	0.97362667	42.81239118
0.325580825	436.5442412	0.030281158	0.986540103	74.2129608
0.365405684	553.2031799	0.042150645	0.987203369	72.94395257
0.349351203	159.8938475	0.025193263	0.988432884	64.75200126
0.379261881	-26.05884738	0.032415127	0.986952418	42.68222282
0.336744879	-27.16050863	0.028829749	0.991809414	66.13135317
0.288239589	-311.8872423	0.013980227	0.986848489	92.7812026
0.39413079	211.1177206	0.04036722	0.990515134	43.69882187
0.354802183	-36.98623223	0.028636871	0.989997762	61.22840937
0.336885002	-135.8595647	0.023525067	0.990190964	45.71120907
0.322771484	94.47121037	0.022115056	0.991042381	81.93466625
0.330660403	-20.75185107	0.0342681	0.99027824	65.88227039
0.400684838	-37.87605497	0.038675247	0.989680825	36.97153008
0.332117191	-39.01407134	0.027060896	0.985389568	50.98799993
0.327572838	-146.3678734	0.029237044	0.987431371	52.22672371
0.360786841	-231.4754874	0.033890989	0.988369419	54.84044362
0.339335682	-348.5507628	0.032230156	0.989038627	73.53574454
0.292684438	-270.9380822	0.019592671	0.985759545	81.42698599
0.319029917	69.61867008	0.024883341	0.987577269	59.52836808
0.468486676	112.9013665	0.079371831	0.981096851	28.35831458
0.318753752	-132.0500838	0.022042326	0.98874667	62.59551233
0.384506244	163.9788313	0.028570814	0.987480655	47.13688124
0.326436561	-14.12906532	0.030400819	0.985893634	75.22995285
0.370196632	74.36129666	0.035885054	0.990352885	45.30932989
0.387871515	8.924589373	0.048158442	0.961425739	24.72456781
0.396853683	-21.63292918	0.06817094	0.985226776	47.04073915
0.352246655	165.6878678	0.031577452	0.989034176	50.75441393
0.303767037	-56.55764343	0.02380048	0.983119309	58.92039264
0.342114193	-207.9630771	0.030127056	0.983654886	46.11259949
0.355636586	-87.83639323	0.034474767	0.989480467	47.64252953
0.385432865	46.86705902	0.027770494	0.989932853	43.19157603
0.197436188	-1303.048881	0.014250068	0.974972206	169.5037172
0.43802884	36.14618032	0.058882641	0.991524411	31.96779198
0.410949327	143.982156	0.059428917	0.98325505	45.13846022
0.465852978	95.939284	0.10408641	0.985689942	28.1637987
0.221409644	-491.6875756	0.011823524	0.97681177	116.3878882

log.sigma.3.5.mm.3D_glcm_Contrast	log.sigma.3.5.mm.3D_glcm_DifferenceEntropy	log.sigma.3.5.mm.3D_glcm_InverseVariance	log.sigma.3.5.mm.3D_glcm_Entropy	log.sigma.3.5.mm.3D_glcm_Dissimilarity
15.05046971	3.020489801	0.328986286	7.601931421	2.77068594
11.13580416	2.874054406	0.341668752	7.328058669	2.449822474
16.26276382	3.110033192	0.302008026	7.654080414	3.032075721
10.00235585	2.792928612	0.349176575	7.271615722	2.337074035
7.291978355	2.546501499	0.395272988	6.465620867	1.894572001
8.90558463	2.723967578	0.363627499	6.970920366	2.190200138
4.350763732	2.23793894	0.42831394	5.879508482	1.458194517
10.23080215	2.781721538	0.365579311	7.248722473	2.301859627
12.15362063	2.901219947	0.347819425	7.457891846	2.512653063
13.14082236	2.97745995	0.316128131	7.683099103	2.759124293
6.580084498	2.515461235	0.400980699	6.619608847	1.854200426
13.67441711	2.991649678	0.314415651	7.586171981	2.797441747
6.928789331	2.555544837	0.39785407	6.696865133	1.879098779
10.00367175	2.804214331	0.338406674	7.260637054	2.413383028
16.87454724	3.06810171	0.318436123	7.704904533	2.929702508
13.98976969	2.990388556	0.323134876	7.548293185	2.587177205
11.12279428	2.828857323	0.357943898	7.170206681	2.400023275
8.678833369	2.697878131	0.373682666	7.073921689	2.143327676
12.29186296	2.892037887	0.330918442	7.23976087	2.599558632
9.40185837	2.753495984	0.360493142	7.277460336	2.252055457
14.19498385	3.025224751	0.32002307	7.700425292	2.795484247
11.35129561	2.860309613	0.338891951	7.413416821	2.507843114
12.41703334	2.942727737	0.324135976	7.667843306	2.659438487
10.08705984	2.775223706	0.344239026	7.1472155	2.342800583
14.83312038	3.064996898	0.301895896	7.854440301	2.930300647
18.60186687	3.184385954	0.295318757	7.794334697	3.174144574
9.354678025	2.757170974	0.35073672	7.28631404	2.28139743
17.26749465	3.159628366	0.281535928	7.870383766	3.196120127
11.39988201	2.851338701	0.342889825	7.218027637	2.430466341
14.00329821	2.981811737	0.333180428	7.416996468	2.662493183
12.98915866	2.9362635	0.341087243	7.567721433	2.597717955
11.36620742	2.873299168	0.340287114	7.524234341	2.493633806
10.74717088	2.812279726	0.353528089	7.287802593	2.374098396
13.51440707	2.834194906	0.3457398	6.671063802	2.476395098
16.38267988	3.062893703	0.319161356	7.467272195	2.920177058
10.19203914	2.797076821	0.362029296	7.160231384	2.300803725
17.44392285	3.162032966	0.293962926	7.963346616	3.089538698
10.64280925	2.833740152	0.349033851	7.193424658	2.400198236
5.421712747	2.392638594	0.410035674	6.426772978	1.696476947
8.078480305	2.657919368	0.375341811	6.930845242	2.067274994
11.07694388	2.861955967	0.334751056	7.175720283	2.524898793
9.098520525	2.724478762	0.369311479	7.045007384	2.185208906
8.27482358	2.659575117	0.380338288	6.954517149	2.052554109
9.919583271	2.711673919	0.379837621	6.888177525	2.163918549
19.73915062	3.169202069	0.311219125	7.909996081	3.066001759
10.59269309	2.845224743	0.336683103	7.577444244	2.473548092
13.89164912	2.992217919	0.331629513	7.64925789	2.702554511
13.81873702	2.94924973	0.348300924	7.166893918	2.617650047
6.936780597	2.486090153	0.400563565	6.184672294	1.898600083
8.691498758	2.67305035	0.385955926	6.74335972	2.088386154
7.551833589	2.583389721	0.394396567	6.843030899	1.924299851
8.13429586	2.580694644	0.397439582	6.405526504	1.909181122
7.796124143	2.630093478	0.384838502	7.050324927	2.02829531
14.36064702	2.951201723	0.331625947	7.362143321	2.745727139
12.07754241	2.921333308	0.326139126	7.560455214	2.598575556
17.48489674	3.149616751	0.302046919	7.863132819	3.104965253
16.52538281	3.073541696	0.317060712	7.73869128	2.884340213
8.15936094	2.617561997	0.391363798	6.631085889	1.935343368
14.06863011	3.003642906	0.323949924	7.435688757	2.773994045
5.312663847	2.385591966	0.417924277	6.273343205	1.746546428
10.18013937	2.795553451	0.357739996	7.348204314	2.280687648
12.96329375	2.915397537	0.352532396	7.417216734	2.499286915
12.97884922	2.931415984	0.338056704	7.47431057	2.605271525
13.2579282	2.968173288	0.303202812	7.552448842	2.824940292
12.9248496	2.908260088	0.325167095	7.117615952	2.682513162
13.74548309	2.987649903	0.325382987	7.495765215	2.726109799
10.67364568	2.822990007	0.352842617	7.20714208	2.374592362
12.57528198	2.920740782	0.344637034	7.597669675	2.543242855
9.406498482	2.726720028	0.369467137	7.023746408	2.218360834
14.37410451	3.008520161	0.331765871	7.639137826	2.724802958
18.94934992	3.217633046	0.286650271	8.225715064	3.245811229
9.080658452	2.655756801	0.390629643	6.817883386	2.118370462
11.40947401	2.878259052	0.342902625	7.535739848	2.462702373
11.837409	2.901625021	0.334512869	7.423732602	2.549832007
15.02663883	3.038046274	0.320051593	7.886471758	2.800763601
14.7163961	3.021690064	0.330483855	7.574468116	2.767115748
9.202991269	2.70126121	0.379552303	6.881739703	2.130401283
11.24713767	2.85364289	0.338093675	7.321301856	2.537929113
13.59344084	2.975823847	0.323370345	7.496048062	2.715826891
12.61291011	2.925791296	0.348353116	7.401296521	2.524857694
19.78609027	3.131903994	0.328310484	7.474817359	2.994340238
20.78873057	3.25761879	0.291681476	7.990845308	3.31088234
13.41454313	2.99081213	0.317579337	7.619570274	2.734541309
6.106658738	2.355520016	0.422549597	5.826612016	1.695302714
12.85378171	2.958276135	0.32441772	7.698046171	2.686856897
8.296902931	2.681502528	0.371896196	7.170100666	2.117014626
14.53854728	3.012356852	0.325448157	7.6316683	2.76452146
9.088167585	2.72613152	0.360962889	7.111299321	2.224826251
7.450119549	2.381609655	0.371209257	6.00448051	2.062291092
9.097576352	2.690778063	0.376205983	6.667969542	2.162615709
11.03843695	2.872820711	0.340804661	7.372826514	2.453274688
15.19191262	3.055160911	0.30746383	7.5585876	2.918208149
11.82315696	2.906702336	0.328472022	7.245986112	2.546009788
11.32599384	2.860382408	0.349677567	7.292275925	2.445308043
8.272148161	2.677256867	0.372261033	7.159217965	2.114827917
48.06743534	3.807209185	0.203937277	8.54639748	5.259636912
5.977938223	2.45915312	0.408313636	6.388872221	1.768598526
6.493569501	2.414795072	0.402145482	6.415436424	1.891547259
5.543502752	2.397447402	0.406611524	6.164003137	1.666155379
29.02681216	3.489750649	0.228764901	8.381778804	4.155609673

log.sigma.3.5.mm.3D_glcm_DifferenceVariance	log.sigma.3.5.mm.3D_glcm_Idn	log.sigma.3.5.mm.3D_glcm_Idm	log.sigma.3.5.mm.3D_glcm_Correlation	log.sigma.3.5.mm.3D_glcm_Autocorrelation
7.146957647	0.921051928	0.336745787	0.6861416	216.9927627
5.003788229	0.924208136	0.35663353	0.656232936	204.5096168
6.830537857	0.905496258	0.298256028	0.586861403	195.3512173
4.38850538	0.92934268	0.359697954	0.667401556	198.0504896
3.54506498	0.923004792	0.434801642	0.724521504	110.0141167
3.986057558	0.921372441	0.381096111	0.638966341	142.8436846
2.095985083	0.93323788	0.506287644	0.768687653	85.66512783
4.82190234	0.939770548	0.369530217	0.709452059	319.1188162
5.717505461	0.925132533	0.352832077	0.661475681	263.0366529
5.328549547	0.930290899	0.308994798	0.647807345	444.1543045
3.04206741	0.929686054	0.426617793	0.656120361	213.9812223
5.624720253	0.911587589	0.307588928	0.598561377	223.9097009
3.307364096	0.942223433	0.429414004	0.665974144	229.4737789
4.030755331	0.913694264	0.341765002	0.628808653	148.0988306
7.819861236	0.91721726	0.324286305	0.606976523	215.9771998
7.047772113	0.938593763	0.378824759	0.649687884	386.0550523
5.160342391	0.934062902	0.365273644	0.638183721	349.0642342
3.931646714	0.941898779	0.391311124	0.702762968	301.006975
5.18499393	0.919967364	0.332084066	0.587648645	271.0685228
4.204123	0.927281231	0.371167734	0.69577696	245.4469691
6.172812512	0.929849799	0.317422442	0.592922717	303.9573061
4.858913092	0.943464189	0.338359799	0.663637547	375.3302211
5.158691784	0.922832181	0.32186769	0.656044684	268.8334381
4.287947228	0.924709692	0.370459145	0.730752428	237.8751216
5.999479388	0.907931257	0.302471953	0.645775132	217.9221127
8.25842477	0.919739368	0.293380097	0.594316451	436.2797245
4.01788621	0.928527455	0.364363577	0.701522831	197.84725
6.76573688	0.911615783	0.27407953	0.563228915	301.9518555
5.236053415	0.931429324	0.363591282	0.61952207	305.2045629
6.67947717	0.935018911	0.344247518	0.553312401	344.9077595
6.069504717	0.934618707	0.339266275	0.638592539	420.6426913
4.967045148	0.933240197	0.342734876	0.651987445	288.136806
4.903033066	0.938017702	0.360466806	0.696703132	381.0122548
6.845376509	0.914538073	0.386914741	0.609750789	166.6408882
7.490907123	0.912137788	0.317890008	0.579128937	283.0527503
4.778847491	0.932741667	0.375672515	0.6100981	299.6269666
7.629059856	0.923455205	0.299034655	0.627717709	406.2924739
4.751664785	0.935537498	0.354192096	0.598978199	449.4751405
2.412008037	0.937318404	0.448006148	0.697360153	182.4828857
3.680639994	0.930446649	0.399633825	0.714047034	157.3073615
4.546969	0.910379352	0.331873158	0.54579199	194.1951513
4.188930259	0.927014457	0.385423803	0.635004373	174.3464789
3.956151818	0.937468051	0.405156667	0.66926512	292.5200946
5.058576298	0.930750857	0.400791221	0.647415126	150.5132894
9.879170301	0.930356428	0.332140382	0.58993948	515.9913397
4.34472869	0.92528529	0.335279761	0.699046212	243.6226644
6.363819144	0.935489499	0.333393345	0.641637734	342.1170324
6.779206257	0.927194363	0.351323107	0.574566234	389.8486019
3.036347128	0.911495534	0.43089476	0.715943288	96.280267
4.224351045	0.936607991	0.406059361	0.712418671	152.3626763
3.75462794	0.942913543	0.42453176	0.702128842	290.5434899
4.362330722	0.942093093	0.442372426	0.569309186	317.2780482
3.58079423	0.937908246	0.400201973	0.729899059	220.2604149
6.442221889	0.925554033	0.317299733	0.538460947	394.8424039
5.159419017	0.926670121	0.330712196	0.643160459	275.0179924
7.513916401	0.919044747	0.298522736	0.597902894	319.3400019
7.897694699	0.94163356	0.322442998	0.631275661	677.8871257
4.283968251	0.94134213	0.450191053	0.720449228	209.9719387
6.175056676	0.917922993	0.318996922	0.581893395	368.7746079
2.179104497	0.912220249	0.421102535	0.575978867	114.3356691
4.857432576	0.933356214	0.379902109	0.707040281	312.6439727
6.536990211	0.947852658	0.368644336	0.633682505	461.1757439
5.947562591	0.927029327	0.341032858	0.617492372	325.4399583
5.015833579	0.907338107	0.295818806	0.592635722	268.3372585
5.250963535	0.894868746	0.325498592	0.539430231	127.2082456
6.110843551	0.923804878	0.325580825	0.687148376	227.3094702
4.917515809	0.926381005	0.365405684	0.741440346	181.0221236
5.965921712	0.930639737	0.349351203	0.674508494	327.9316485
4.350277086	0.926061688	0.379261881	0.635011709	277.4967681
6.735815547	0.940794411	0.336744879	0.641273806	502.8324566
8.089819733	0.923765185	0.288239589	0.661574902	483.4931894
4.438253708	0.937732002	0.39413079	0.651545577	272.9713715
5.186418781	0.934180794	0.354802183	0.685410882	282.9532306
5.145779482	0.933662518	0.336885002	0.588566743	387.1537606
6.988280988	0.93788811	0.322771484	0.68931984	404.8635733
6.863045007	0.935734496	0.330660403	0.632039563	382.8261444
4.525496357	0.935581432	0.400684838	0.601112865	254.2994569
4.534833557	0.918744653	0.332117191	0.63713242	244.7100062
5.878859282	0.926118577	0.327572838	0.59030834	371.6352663
6.072011389	0.931253989	0.360786841	0.626199189	343.3666202
10.5552342	0.936506802	0.339335682	0.57761632	690.2868195
9.482859075	0.922893601	0.292684438	0.59682733	465.0592553
5.766421605	0.925433096	0.319029917	0.63066891	328.303926
2.925187972	0.916595597	0.468486676	0.641413218	73.31888703
5.452446034	0.92847699	0.318753752	0.658032789	439.998862
3.737174501	0.926280348	0.384506244	0.70037527	146.1870962
6.700169863	0.923099903	0.326436561	0.674119269	301.7541829
3.998808921	0.934363415	0.370196632	0.663944427	294.1645216
2.353730163	0.874185864	0.387871515	0.537066309	56.76752509
4.20917718	0.922705105	0.396853683	0.66869635	214.6659617
4.90524997	0.930501231	0.352246655	0.643376324	315.4872577
6.431650373	0.914008148	0.303767037	0.589743971	307.5628064
5.165248561	0.916253769	0.342114193	0.592303345	261.3221601
5.154149581	0.932826108	0.355636586	0.615410207	363.0015341
3.694054212	0.933349185	0.385432865	0.67731667	216.7535049
19.4571767	0.896196123	0.197436188	0.562801755	612.9952444
2.759833701	0.939489958	0.43802884	0.682139501	294.630194
2.568561396	0.914993625	0.410949327	0.741928748	102.443332
2.641229104	0.92474977	0.465852978	0.668707133	84.51930177
11.19286921	0.897720032	0.221409644	0.602141992	403.3191731

log.sigma.3.5.mm.3D_glcm_SumEntropy	log.sigma.3.5.mm.3D_glcm_AverageIntensity	log.sigma.3.5.mm.3D_glcm_Energy	log.sigma.3.5.mm.3D_glcm_SumSquares	log.sigma.3.5.mm.3D_glcm_ClusterProminence
5.079625792	14.22302639	0.008617034	24.77519677	18113.2633
4.812263192	13.93443955	0.010449981	16.60648971	9613.246428
4.929051758	13.5694467	0.007277374	19.81654201	12082.64205
4.791991985	13.72258569	0.010989589	15.22982855	8566.999891
4.490598251	10.00849677	0.021922427	13.47920439	6443.789795
4.592780893	11.6117925	0.013562463	12.45353847	6299.721475
4.200907742	8.828776263	0.035155491	9.866861459	3572.852478
4.888153717	17.56350818	0.011446103	17.95376232	13334.17846
4.901901645	15.90007856	0.008970301	18.42718516	10033.51188
4.969380397	20.90674436	0.007312406	18.20392085	13914.62996
4.436451423	14.41077765	0.016356112	9.586242774	3484.800062
4.881564314	14.63363361	0.007727572	17.26886965	10029.65224
4.498173266	14.93512908	0.017723572	10.73053902	5222.602773
4.733836438	11.81407976	0.009256599	13.513645	5821.521157
4.996106578	14.30216241	0.007179743	22.10581569	16100.55172
4.946075704	19.43068455	0.012663394	19.42957018	16498.32531
4.746743956	18.48540682	0.012140332	15.6520945	12565.42988
4.756901268	17.13017887	0.012133874	15.01241878	8181.913189
4.741961712	16.2413934	0.009395569	15.33378656	6856.746274
4.82627918	15.33008142	0.010834866	15.67367881	9470.356981
4.891957662	17.26874572	0.007681221	17.58821827	12085.74694
4.892504423	19.19377333	0.009019111	16.49777624	12509.55172
4.962825692	16.08723192	0.007591527	18.51345311	11436.09976
4.89496008	14.98323443	0.012006312	19.08488575	12395.99254
5.032289372	14.30594059	0.005914221	21.13762125	12141.37928
5.027262188	20.69396105	0.006735461	22.47398744	19747.00769
4.849884181	13.6777729	0.00985536	16.08739921	8609.081189
4.949591924	17.16930897	0.006282273	20.2263311	12115.17311
4.73450542	17.28430176	0.01241801	15.32798619	10932.57751
4.75578267	18.43718158	0.010325222	15.50686777	10998.89217
4.923029196	20.34078013	0.008353292	17.57360274	14306.63778
4.891005493	16.73469921	0.008278168	16.79279244	10669.87078
4.841731534	19.26820334	0.011435578	17.66758709	19898.14905
4.61075899	12.48493896	0.0189558	17.50613935	7543.456525
4.885762354	16.55366603	0.008790113	19.76083216	12283.08407
4.68552266	17.12098939	0.012395699	13.51453027	6625.258734
5.095867904	19.92008158	0.006649796	23.12881842	22634.55724
4.672602428	21.10759688	0.011574255	12.91029275	8103.445175
4.40041685	13.26920423	0.019701879	9.11926551	3488.407879
4.682294374	12.12496292	0.014516158	14.45488853	8057.133643
4.612774006	13.69179053	0.010093414	12.25814179	4376.227493
4.637367093	12.89193593	0.012240761	12.6948015	4940.735554
4.631009006	16.88754431	0.01378539	12.76305573	5351.614919
4.61973412	11.88850269	0.015872343	14.48191661	8450.057207
5.093832289	22.50452755	0.007835363	23.5124061	23602.28322
4.97060329	15.21947772	0.007431733	17.81293413	10855.78571
4.957915358	18.28475074	0.008454133	19.03845089	17335.59751
4.645519513	19.63853784	0.013051917	16.37452114	13298.65788
4.41988852	9.349782109	0.024094425	12.31750886	4310.573213
4.640283363	11.91113478	0.017925175	15.47135422	10151.53357
4.626587337	16.82080113	0.016038461	13.04872487	7364.413858
4.271150754	17.68228768	0.024343149	9.52075785	6056.649476
4.736289779	14.50026418	0.012990712	14.75490345	7920.861133
4.76562209	19.78072638	0.008492223	15.44002329	7264.368832
4.902226557	16.32094931	0.008181615	17.39412402	9948.867476
5.043472353	17.64015999	0.006744657	22.00040775	16019.33291
5.052602312	25.86922748	0.008145582	22.33882635	23990.95278
4.535781925	14.15315543	0.023527167	15.04307004	10386.59012
4.81903405	19.00852193	0.008721568	17.10465492	11703.38976
4.158245029	10.52325742	0.018248626	6.249732275	1240.420259
4.909108755	17.39429041	0.010159149	17.82971632	9393.81502
4.865594452	21.28722472	0.011523687	17.32717721	15638.36504
4.865136531	17.85016705	0.009086778	17.29253944	11524.90984
4.842962182	16.09173148	0.007113012	16.31039882	8038.138789
4.638388499	10.94109088	0.009357554	13.9343102	4288.328095
4.947072964	14.6313507	0.008840131	22.64614999	15789.37525
4.889613191	12.89696149	0.01160324	21.46917752	17659.16276
4.98161416	17.817379	0.008523897	19.6952058	12850.06177
4.675153304	16.41140658	0.011812125	13.08445315	5930.217341
4.992514411	22.1921577	0.008646035	19.91513424	16873.40831
5.269460265	21.66871782	0.004745708	27.28131546	25289.71146
4.566560794	16.31278425	0.015698442	13.65967633	6890.770226
4.952105458	16.53468866	0.009150921	18.62776351	13834.52526
4.751173014	19.56011881	0.009252158	14.2231471	8330.561927
5.157075597	19.80166315	0.007113047	23.85214749	24527.82671
4.931863619	19.3527291	0.009252585	19.77435945	19296.51107
4.546221644	15.74544188	0.014870304	11.72552039	6393.707209
4.800061843	15.34190859	0.009106022	15.7910515	8412.087905
4.804077485	19.13574007	0.009576036	16.55524664	11613.04742
4.808677874	18.33016894	0.011527305	17.05735496	13583.07228
4.937255487	26.13866284	0.010533583	23.0261737	32577.35353
5.13227033	21.3558729	0.006707029	24.63946063	27323.2868
4.933824948	17.86959337	0.007861698	18.53279905	12788.40932
4.113838427	8.230891784	0.029943766	8.616243329	2055.466424
4.989848981	20.78232934	0.007231552	18.58589384	13730.31134
4.75060592	11.68189051	0.010452559	13.85844604	6274.767032
5.07801119	17.01221869	0.00853023	22.96814443	19105.79535
4.707696734	16.9475571	0.011715086	14.08664629	6164.937334
4.126524756	7.241425683	0.020179822	8.04367184	1169.188719
4.604406419	14.32257696	0.018005244	14.03457888	8589.462721
4.797647126	17.56823186	0.009695078	15.91418635	7365.526778
4.915266938	17.28405503	0.007811782	19.00707903	10907.09308
4.719404596	15.89956253	0.010326136	14.51238274	8463.076085
4.734651879	18.89071799	0.01174779	14.82930367	10604.64538
4.729504068	14.43575871	0.011016746	13.03485386	6176.630299
5.569478285	24.4311414	0.003823455	53.1834269	85323.4141
4.376742728	16.98213117	0.021169609	9.573618544	4173.615685
4.540723526	9.630561937	0.017875277	12.90800743	4724.551791
4.249719919	8.880217922	0.028261766	8.426825363	2703.597863
5.391352861	19.6973113	0.003977446	35.95518904	42385.68374

log.sigma.3.5.mm.3D_glcm_SumAverage	log.sigma.3.5.mm.3D_glcm_Imc2	log.sigma.3.5.mm.3D_glcm_Imc1	log.sigma.3.5.mm.3D_glcm_DifferenceAverage	log.sigma.3.5.mm.3D_glcm_Id
28.31751394	0.90251653	-0.20482588	2.77068594	0.413377884
27.83503259	0.831137519	-0.154818974	2.449822474	0.431903333
27.11606729	0.832488105	-0.147703862	3.032075721	0.3816147
27.42131652	0.82529551	-0.153915289	2.337074035	0.435478785
20.01699355	0.891640418	-0.228064504	1.894572001	0.495963954
23.223585	0.81746793	-0.154088186	2.190200138	0.452824762
17.65755253	0.887451603	-0.247730615	1.458194517	0.554617779
35.01273601	0.881658186	-0.193452056	2.301859627	0.442284789
31.69466011	0.85390455	-0.164966531	2.512653063	0.427771786
41.57180551	0.809405921	-0.136344603	2.759124293	0.392635784
28.82155529	0.813407368	-0.161262034	1.854200426	0.489785038
29.23517741	0.801973958	-0.13407151	2.797441747	0.391234957
29.82889521	0.815091525	-0.160478213	1.879098779	0.491802974
23.62815952	0.795868851	-0.136978795	2.413383028	0.421134292
28.48985501	0.845037549	-0.162201536	2.929702508	0.40268741
38.63810969	0.845731286	-0.161962992	2.587177205	0.449198341
36.8304349	0.839482023	-0.166990092	2.400023275	0.438017958
34.09899054	0.853272397	-0.17791784	2.143327676	0.460422797
32.39112167	0.83052244	-0.160007127	2.599558632	0.411869183
30.63520152	0.839269103	-0.162670672	2.252055457	0.44463128
34.27196067	0.737015477	-0.105650356	2.795484247	0.398535908
38.16603203	0.844405488	-0.163635055	2.507843114	0.417348155
32.06236084	0.79820742	-0.131880926	2.659438487	0.403407705
29.93504043	0.907238139	-0.22663569	2.342800583	0.443758917
28.5899742	0.794821847	-0.127608089	2.930300647	0.386114209
41.11845763	0.852440947	-0.157980838	3.174144574	0.377052836
27.32335305	0.856194209	-0.173838551	2.28139743	0.439634566
34.11093599	0.734274322	-0.100648246	3.196120127	0.362176264
34.40744805	0.797353568	-0.143580011	2.430466341	0.437758202
36.6765833	0.778379505	-0.125524573	2.662493183	0.420546822
40.45448235	0.811653405	-0.140096669	2.597717955	0.416899006
33.31042479	0.797242692	-0.135449274	2.493633806	0.420827078
38.3996409	0.852589641	-0.172890062	2.374098396	0.435332786
24.96987791	0.91960119	-0.256324375	2.476395098	0.455391644
32.98423798	0.876393906	-0.183058684	2.920177058	0.397579775
34.14943973	0.795148097	-0.137978854	2.300803725	0.4471749
39.57531206	0.805221109	-0.130583988	3.089538698	0.382501071
42.02489695	0.771941402	-0.125211885	2.400198236	0.430289101
26.53840846	0.843896671	-0.193914357	1.696476947	0.50794294
24.24365354	0.865751825	-0.189592696	2.067274994	0.467967226
27.38358107	0.76499006	-0.122724652	2.524898793	0.412149758
25.78280761	0.811630179	-0.151430554	2.185208906	0.455759343
33.71301503	0.831070931	-0.162141159	2.052554109	0.472158469
23.75921268	0.851157591	-0.180855657	2.163918549	0.467487445
44.79604807	0.832777407	-0.149564613	3.066001759	0.408017903
30.41152382	0.830131005	-0.149911132	2.473548092	0.415436488
36.31913866	0.813639614	-0.140130704	2.702554511	0.41146379
39.01303007	0.807654976	-0.14336807	2.617650047	0.42538154
18.69956422	0.921651986	-0.279126109	1.898600083	0.491915046
23.79077838	0.889234327	-0.213767164	2.088386154	0.471583757
33.56040431	0.846393885	-0.176449965	1.924299851	0.487942178
35.3237826	0.799437614	-0.15489042	1.909181122	0.501804564
28.95798641	0.851370897	-0.17623452	2.02829531	0.468252484
39.32370344	0.824158849	-0.151641616	2.745727139	0.398649585
32.50366456	0.800766876	-0.133679222	2.598575556	0.411113749
35.0118598	0.820284704	-0.139231502	3.104965253	0.380965755
51.52796403	0.840823021	-0.154893289	2.884340213	0.402184365
28.24525405	0.87156988	-0.202463532	1.935343368	0.507376303
37.89316738	0.819962857	-0.144419976	2.773994045	0.399748751
21.04651484	0.732400764	-0.12428423	1.746546428	0.485813521
34.66083405	0.867426095	-0.179317495	2.280687648	0.451279084
42.42242018	0.813509954	-0.145098562	2.499286915	0.440126677
35.49775609	0.824032895	-0.149474195	2.605271525	0.418277235
32.16956007	0.782488371	-0.124349915	2.824940292	0.382440055
21.88218177	0.847758821	-0.171134854	2.682513162	0.405859172
29.13176869	0.861279608	-0.171172013	2.726109799	0.405503534
25.72457987	0.904741242	-0.216791828	2.374592362	0.438863801
35.48861832	0.840332683	-0.155353679	2.543242855	0.425105852
32.81115819	0.845717412	-0.173542655	2.218360834	0.450639124
44.26698857	0.838758732	-0.155954075	2.724802958	0.413647495
43.12878549	0.824186584	-0.136964556	3.245811229	0.372485084
32.515186	0.847481303	-0.178739428	2.118370462	0.461912986
32.89211198	0.832245355	-0.152913483	2.462702373	0.430372169
38.9195729	0.737675089	-0.109528674	2.549832007	0.41599728
39.39952723	0.853943665	-0.160300963	2.800763601	0.403104787
38.47041906	0.841949935	-0.156855698	2.767115748	0.408552944
31.45473119	0.805539064	-0.149311129	2.130401283	0.467693069
30.64420867	0.839360354	-0.163116998	2.537929113	0.411782879
38.05105929	0.771091485	-0.124101104	2.715826891	0.407327799
36.48588064	0.793746861	-0.133431277	2.524857694	0.433728227
52.03201886	0.887098165	-0.191540421	2.994340238	0.413602523
42.42148196	0.825683101	-0.138190129	3.31088234	0.375204821
35.59575443	0.808282016	-0.136603475	2.734541309	0.400839908
16.46178357	0.880674263	-0.246160412	1.695302714	0.522422146
41.3541947	0.806921182	-0.136400366	2.686856897	0.400751395
23.36378102	0.826836922	-0.154415613	2.117014626	0.455992507
33.85540063	0.883838475	-0.186473534	2.76452146	0.405809668
33.76883942	0.821400776	-0.154907524	2.224826251	0.444279773
14.48285137	0.859091097	-0.225133978	2.062291092	0.459141728
28.64515393	0.902943964	-0.236030364	2.162615709	0.464120868
34.96001399	0.799861751	-0.134998678	2.453274688	0.428578889
34.44482657	0.847136789	-0.159402268	2.918208149	0.387261938
31.79525783	0.791261391	-0.13365952	2.546009788	0.42055531
37.62508034	0.773056415	-0.127370458	2.445308043	0.430709935
28.84513164	0.809225173	-0.146776394	2.114827917	0.45666559
48.27416402	0.929777174	-0.210509595	5.259636912	0.285576752
33.94871756	0.835409897	-0.181089984	1.768598526	0.499257695
19.26112387	0.924642091	-0.276815733	1.891547259	0.476909427
17.76043584	0.857551642	-0.206374782	1.666155379	0.522395701
39.06072771	0.88289732	-0.168035478	4.155609673	0.313419919

log.sigma.3.5.mm.3D_glcm_ClusterTendency	log.sigma.3.5.mm.3D_firstorder_InterquartileRange	log.sigma.3.5.mm.3D_firstorder_Skewness	log.sigma.3.5.mm.3D_firstorder_Uniformity
81.08740024	159.4445572	0.371767347	0.064328649
54.3157391	131.9176903	0.226474982	0.077179
62.33337737	143.0646076	-0.31110029	0.068844186
50.25593974	116.6431217	0.271704282	0.079770372
46.62483921	107.0591221	0.799255771	0.103025646
40.90856924	95.45339084	0.437639985	0.092143103
35.11668211	109.3116407	0.816887913	0.126993177
60.75446884	125.0796852	0.136151755	0.075702611
59.65912271	148.7620602	0.283772012	0.071283115
61.51857473	139.8857803	-0.239472423	0.066588201
31.7648866	95.8433075	0.203784936	0.100776694
54.59128361	123.4206924	-0.158392106	0.071390256
35.00184837	86.74845505	0.457695539	0.10209288
44.05090827	115.5589104	-0.183315714	0.078321308
69.0963886	151.8266538	0.547509569	0.064800626
65.2741441	126.0441561	-0.07660995	0.075227076
50.81606125	102.5000233	-0.450321585	0.082740268
49.88204296	139.4855871	0.427710527	0.08020859
47.27132092	131.6565938	0.062091917	0.076899115
52.66205591	115.3335562	-0.068549809	0.078964201
55.43785071	128.9486175	-0.344716571	0.071134952
55.9792958	120.4713869	-0.012709642	0.07502199
59.7301675	127.2746699	0.051666724	0.070567764
65.4332869	134.1637036	0.062259732	0.076474691
69.10459114	156.4925337	-0.270751979	0.062115533
72.83231659	149.0319104	0.018385251	0.063738577
54.08243075	130.1686726	0.38446395	0.073880628
61.4689591	144.0723653	-0.419327266	0.066636401
48.31937147	105.4168692	-0.325453898	0.086057448
48.44656349	113.8950577	-0.081635423	0.077713114
58.9293395	127.4941587	-0.601640473	0.071534056
54.25819632	131.198966	0.095664897	0.07179288
60.22580824	113.4130735	-0.397712484	0.079604762
56.51015032	162.284914	0.459990041	0.088290593
60.56384631	124.4683315	-0.363771514	0.072290196
42.4763836	102.4435539	-0.045256956	0.087651097
76.37819538	143.0253668	-0.202523479	0.06189463
42.42737579	98.86332226	-0.461027282	0.087165612
31.05534929	91.79587159	0.180275482	0.102808211
49.51635154	114.8478212	0.701254244	0.088340128
37.95562329	114.4740868	-0.021556768	0.082040308
41.64316493	122.6842084	0.384329352	0.083414777
41.74378972	116.3553381	0.213678	0.087339154
47.4270163	107.2660961	0.846053785	0.092372465
76.99676171	126.2014425	-0.507913722	0.067442462
60.01906467	135.7866909	-0.248703528	0.069178855
63.26428605	125.4617026	-0.207495128	0.070267343
51.1617289	98.88477755	-1.016937958	0.089060575
42.33325484	119.105653	0.551273918	0.103258136
52.10929661	114.9664526	0.85668155	0.0936773
43.3845991	105.7560759	0.233384386	0.09161067
29.47026251	70.3176384	-0.335009878	0.123079424
50.18020906	125.0458641	0.569649592	0.081263509
47.33804329	127.5783424	0.042912793	0.073707685
55.63275752	141.1874561	0.03493686	0.070044721
68.9942269	141.9848394	0.018691038	0.063773094
72.92312495	133.5269489	-0.393397775	0.068861416
50.22387827	92.43755364	0.973089624	0.108249654
53.23781871	119.0631796	-0.530582973	0.075038887
19.68626526	79.35065079	-0.322619125	0.115639366
59.35193606	144.8379135	0.129687213	0.072191037
57.98399952	112.1845484	-0.4213772	0.081509457
54.56295292	123.7417917	-0.166887768	0.07386512
51.67305558	143.8461852	-0.29560121	0.069837887
42.81239118	145.8400393	0.068090379	0.073720195
74.2129608	163.8650465	0.675305462	0.07000486
72.94395257	138.5872898	0.873869368	0.076670324
64.75200126	140.0265608	0.281195179	0.069121846
42.68222282	109.314045	-0.204382046	0.08442449
66.13135317	128.6256888	-0.09660139	0.070219818
92.7812026	177.0322953	-0.343302846	0.053953061
43.69882187	98.85364532	0.677800652	0.09513784
61.22840937	128.994381	0.00167852	0.072938748
45.71120907	116.5272408	-0.420633598	0.077190519
81.93466625	145.7287426	-0.033272267	0.062908362
65.88227039	136.97859	-0.119650672	0.07051637
36.97153008	91.20271635	-0.246513623	0.09540076
50.98799993	121.1414223	-0.082448053	0.07664327
52.22672371	113.9488064	-0.357778331	0.077287588
54.84044362	101.3019167	-0.528953017	0.082752217
73.53574454	106.4287665	-0.656699831	0.07851458
81.42698599	129.2909336	-0.523688345	0.066317036
59.52836808	127.7566819	0.030268906	0.069864832
28.35831458	102.5322914	0.77518676	0.121482548
62.59551233	138.0949917	-0.384578103	0.066100777
47.13688124	125.7263424	0.46344745	0.078164829
75.22995285	135.6277986	-0.037604728	0.066622728
45.30932989	115.1635847	0.200302112	0.084173187
24.72456781	123.2414646	0.130015161	0.103853281
47.04073915	95.11804342	-0.121051919	0.09608362
50.75441393	133.2718859	0.432531776	0.075609273
58.92039264	131.1830215	-0.230644362	0.069172212
46.11259949	106.2478476	-0.735523405	0.084475073
47.64252953	99.91771889	-0.276058494	0.084850925
43.19157603	111.0999479	0.127981482	0.082154077
169.5037172	229.7536144	-0.642763848	0.045149055
31.96779198	78.21284199	0.089201503	0.113553993
45.13846022	140.0921664	0.488675013	0.085717093
28.1637987	80.90534043	0.634865039	0.119001603
116.3878882	187.7868862	-0.490940987	0.049022464

log.sigma.3.5.mm.3D_firstorder_MeanAbsoluteDeviation	log.sigma.3.5.mm.3D_firstorder_Energy	log.sigma.3.5.mm.3D_firstorder_RobustMeanAbsoluteDeviation	log.sigma.3.5.mm.3D_firstorder_Median
97.55857007	33542088.59	67.7506063	90.95928192
80.78671433	35790645.29	55.57123089	70.75906372
86.35290437	14235124.2	58.96068474	39.63533783
75.79255895	40569007.1	50.1431866	72.49908447
73.07364111	12327852.79	48.32065278	39.75455093
65.79636366	17176580.64	40.90248128	44.40237427
65.90708496	10879315.29	46.62289234	32.06729889
82.36419583	38838894.25	54.2812616	85.37471008
84.32666362	45455014.09	59.42727416	93.06378937
86.63302641	59620444.81	58.51257369	84.24765778
60.19134113	20357686.31	40.65739901	62.41169357
80.52727021	28199259	52.96909833	79.85158539
61.09711979	31655468.02	37.97272724	52.78038788
72.30323591	22488197.76	49.07569122	60.43235779
91.7281727	32307971.41	62.22214613	69.90768814
85.04259845	60063482.41	55.28999006	39.0812912
74.26478889	20163668.24	45.03704598	51.31418228
78.58358286	57598142.85	56.57949374	99.03631973
77.00534394	19888699.86	54.34748054	79.72202682
75.24757122	42291394.38	49.92034417	61.91091156
81.44712913	89194820.24	53.72268701	46.72055244
77.94352254	32777752.61	51.14452323	68.06335449
81.59371507	62074354.98	53.98261018	57.73151398
85.53055661	16735922.81	58.65443523	47.91526604
92.11529341	34627771.73	65.67903502	29.57473469
93.08979163	33978069.38	62.3806377	92.14130402
79.31613006	32521992.45	54.21555786	58.45519638
87.50971021	52499812.7	60.02100111	70.62361145
72.79831145	48159979.02	45.44572324	60.95070267
77.02212043	47241305.2	48.76981383	62.02251434
82.01965578	70033245.88	53.39042667	78.33899689
80.86530771	105442804.4	54.69781443	96.65000153
79.08558047	42808402.2	49.53583188	64.88784027
90.13055991	10184028.31	67.56597182	62.78485489
81.48362853	12374489.68	53.11095012	50.3175602
68.76419861	39848666.59	44.07514369	57.50198364
95.0332415	81553484.75	61.25945774	82.31538391
68.46862938	44884650.33	42.7638724	69.50869751
60.27236978	12588056.68	38.94361518	36.230299
74.87529531	28809372.65	49.93428062	70.6103363
70.20910827	18779306.77	48.07650239	73.60151672
72.95308161	30242257.14	51.07654029	70.86377335
70.50433377	44741263.27	48.71386732	68.349617
73.78475573	30571930.53	47.97724809	62.31748962
90.51366077	59923707.24	56.4198541	53.83592415
82.36467931	55086855.56	57.11426898	76.29389954
84.33154429	66226837.19	53.77822529	72.77885437
74.61782944	16931608.93	43.53633033	37.61011124
71.49638897	7245650.555	51.09353627	45.19065094
76.87522831	18832108.23	50.56621349	59.94028473
69.81574357	58912153.35	45.86747386	65.13095093
53.16432054	17353743.86	30.94741282	47.7912674
77.15187187	75972739.66	52.9355366	83.55934143
77.9667957	18243100.52	52.79392068	71.49394989
83.2075414	76976932.04	57.90996167	86.82301331
91.19490567	30842527.04	60.26769973	48.93991852
88.61383704	68607776.13	57.23205153	77.39227295
70.87382199	32473302.41	43.45435017	40.04969978
77.53613866	19677244.64	50.49751269	65.29502106
49.1606583	13456091.36	33.61247088	76.06764984
84.78849917	74276349.66	60.58479354	103.9509354
76.7976159	88671451.22	48.20921159	65.43890381
79.45604311	39927778.99	52.18942486	79.45638275
81.76696733	33312826.73	58.77253816	89.49505615
79.81478816	11664550.34	59.48637177	96.80197906
95.85340343	38355508.15	68.0909937	74.61985779
88.38328301	21283042.75	57.62047202	59.38552475
87.56790555	85395203.76	59.67796506	93.52014923
69.2041437	16919608.2	46.61791937	67.67376709
85.53047486	43867335.58	54.8542511	61.0961647
105.7869717	89650672.33	74.32907706	86.51261902
68.65617596	34486618.06	43.56235232	75.26584625
82.20204734	98135856.28	54.2352206	88.63629913
75.03480565	97105241.9	50.03705871	79.07852936
94.61610738	97746748.49	62.30013625	78.4240799
88.07594972	43487287.16	57.81778185	61.7232399
63.20548364	24079891.24	39.5090011	51.96622086
75.36211804	24888120.83	51.15679193	88.07365417
76.78696159	46884386.17	48.55383393	65.67141342
75.47123165	56508146.96	45.31121232	48.44072723
82.59455551	24300535.27	47.90073708	59.08687973
92.23021957	48021545.32	56.57056287	59.09270859
84.12608719	48075614.14	55.14153893	78.62028503
60.74785023	5320934.288	43.35407779	45.59421539
86.9348624	77110388.88	58.12574923	88.81528854
75.09928521	52470124.79	52.83762668	66.35375595
90.93857482	30795961.01	58.41467194	76.28829956
72.02036999	46665993.14	49.12729882	70.71224976
62.16801435	5521858.634	50.10690782	88.70490265
69.17468844	8240458.256	42.46795869	62.47488594
80.39354931	63902862.55	56.57218123	83.48623657
84.26734947	22107361.71	56.09485173	86.92156982
71.14566675	15708203.3	45.66024334	46.22352409
72.41035776	59221492.58	43.82372358	60.34932327
70.06284692	84725810.4	46.80905294	94.39949799
144.9151256	29514483.27	99.23954908	59.39618301
57.38873569	17945964.54	36.09297569	54.71253967
77.47449955	8307376.978	58.6875094	84.02301788
55.66269206	7770392.831	35.13996799	44.72021866
119.9205658	29762936.76	81.84817707	70.33759689

log.sigma.3.5.mm.3D_firstorder_TotalEnergy	log.sigma.3.5.mm.3D_firstorder_Maximum	log.sigma.3.5.mm.3D_firstorder_RootMeanSquared	log.sigma.3.5.mm.3D_firstorder_90Percentile	log.sigma.3.5.mm.3D_firstorder_Minimum
905636392.1	516.3563843	166.9791466	285.6572998	-222.6165924
966347422.9	435.7878723	133.7734004	233.9898178	-238.5141754
384348353.4	353.6335754	112.8391299	154.613829	-293.8450623
1095363192	452.1275635	128.6022051	218.7006592	-246.2638245
332852025.4	348.1614075	113.9752658	217.6989594	-150.5487518
463767677.3	355.9434814	102.4027	169.7620773	-221.3375397
293741512.9	302.5465088	101.4526249	187.1962219	-147.3024139
1048650145	500.2983704	146.6880804	250.719104	-318.217804
1227285380	441.8342285	149.7490321	256.7312378	-253.7595978
1609752010	446.9179077	140.9029026	227.8421021	-412.6029968
549657530.4	297.7153015	106.1414785	181.8921631	-258.0678711
761379993	392.2278442	129.3656264	210.0915833	-262.0558472
854697636.4	406.828064	103.95962	172.841983	-288.7877808
607181339.4	368.5623474	110.0156505	179.9264587	-212.8370819
872315228	489.2922058	144.467237	236.7776993	-230.6377869
1621714025	481.6451721	120.9030296	197.284726	-406.1455078
544419042.6	384.0179443	112.0849373	161.777005	-379.8379517
1555149857	507.6604309	150.056209	249.0912888	-297.5462341
536994896.3	396.9732056	132.3165082	222.3014908	-275.1664429
1141867648	356.5267944	120.7402686	205.7085419	-293.9191284
2408260146	484.1909485	113.2863058	167.2029526	-369.7488403
884999320.5	593.5861206	124.3137743	196.5326233	-377.5817871
1676007584	421.6460571	122.7311491	206.0413361	-323.9718018
451869915.9	360.8974609	127.4694816	224.3560486	-286.626709
934949836.8	338.7285767	115.3165878	158.8609787	-312.4771729
917407873.4	440.9083252	158.5298335	268.8059875	-385.5393982
878093796.2	431.8877869	121.2813999	213.1313019	-238.9877167
1417494943	415.9378052	125.0928867	186.6974487	-349.0810547
1300319434	419.8910217	119.0330501	190.1446472	-338.2400513
1275515240	502.8738403	122.2886445	190.3850891	-364.0466309
1890897639	435.1410522	130.0310533	202.2877075	-410.5212097
2846955720	523.4399414	145.2190101	234.9414413	-284.9714966
1155826859	433.4099426	130.0524196	208.8797455	-390.7802429
274968764.4	394.8201294	145.5081305	253.4772339	-180.0368805
334111221.4	331.6452637	118.0476011	188.7679779	-337.543335
1075913998	377.191803	111.2964639	184.9151611	-343.1784363
2201944088	471.5039368	151.163865	251.0676819	-376.9982605
1211885559	367.1322021	115.0495775	183.0355225	-425.5943909
339877530.3	312.491333	92.1005862	157.9761993	-264.5473938
777853061.6	444.5879211	132.7013225	234.8209	-194.8695526
507041282.7	344.6450806	119.4570632	199.2341766	-242.4570923
816540942.9	400.9747314	125.1133175	216.9019897	-211.0832672
1208014108	376.3211975	121.5760168	209.1624329	-317.4849854
825442124.4	451.5999451	127.3857894	225.5210022	-190.0681305
1617940095	433.0680847	130.9597648	198.1258041	-477.6160278
1487345100	416.3544312	124.1326333	195.355191	-276.5731812
1788124604	526.2504272	134.0415194	210.3477249	-356.056366
457153441	319.9180603	105.6816326	128.2686806	-440.2052612
195632565	287.7109985	113.344158	211.5292297	-144.5570679
508466922.1	524.6921387	131.4426244	230.804039	-193.7861633
1590628141	424.5657043	121.6025785	211.3797348	-313.1513367
468551084.1	328.0065308	94.19159828	145.7895813	-361.6616516
2051263971	453.699707	141.4518381	245.5278656	-233.184021
492563714	382.1001587	124.8161775	205.39711	-378.431488
2078377165	452.6970825	139.8291094	231.9400208	-286.1294556
832748230.1	426.0423279	127.812692	202.0453598	-371.1485291
1852409955	532.6290283	144.6491756	229.4194397	-544.1806641
876779165	446.280426	115.553058	205.7361588	-256.8420715
531285605.3	305.9004517	119.7581523	195.5823425	-399.7174988
363314466.8	225.4872131	97.86362566	155.2878326	-173.1645508
2005461441	427.2054138	158.91971	266.6705322	-290.7275696
2394129183	605.0255737	127.1230863	205.0248474	-435.4558105
1078050033	419.0455322	132.5955684	208.5432434	-329.7399902
899446321.7	333.645813	133.541825	215.1177979	-275.9642029
314942859.3	351.3881531	140.6073409	225.8760437	-143.384079
1035598720	508.2441101	157.6634071	274.2720459	-247.0268097
574642154.3	456.1304321	141.831934	252.2298553	-208.7562866
2305670502	466.4324951	154.7483654	267.2661133	-302.5081482
456829421.3	310.5964661	114.392062	192.8531097	-321.1216736
1184418061	527.5308838	132.2797793	218.8551453	-463.7661743
2420568153	451.3531189	153.4363665	241.8611847	-433.636261
931138687.5	429.2869873	129.3559196	226.108963	-276.1497192
2649668120	503.7487183	144.8229319	239.6940125	-285.7197876
2621841531	440.2043762	122.1230071	191.0616455	-379.6980286
2639162209	584.022522	151.4413377	248.9746841	-377.5349426
1174156753	538.5794067	136.587206	215.9019318	-383.3409119
650157063.5	396.9096985	101.1187762	157.7235779	-321.5861816
671979262.5	396.595459	135.3275829	225.4325165	-266.7753296
1265878427	380.9746399	119.6305696	179.3239975	-391.7632141
1525719968	381.6557922	112.6494785	163.6828033	-396.6478577
656114452.2	428.6453247	133.573211	201.6476974	-555.4124756
1296581724	432.1291809	134.438076	189.6739685	-454.258606
1298041582	429.3770447	139.3999355	230.1111893	-346.1313171
143665225.8	280.5978699	100.8656063	189.8557465	-102.8757401
2081980500	396.3397827	141.3026517	223.572345	-403.0774536
1416693369	421.9023438	122.8969604	214.3111725	-185.7117767
831490947.2	439.4253845	146.8529844	243.3133972	-316.5584106
1259981815	401.2317505	123.8366743	214.706134	-317.6810608
149090183.1	231.1008453	115.9102203	186.0976868	-60.33615494
222492372.9	313.5944214	119.4020443	205.3202621	-264.0323181
1725377289	442.8577881	141.0717337	241.7955322	-302.4863281
596898766.1	390.4748535	141.4449086	228.3793793	-316.35495
424121489.2	275.1652527	100.2819435	145.2939758	-330.0058289
1598980300	378.9699097	116.6394172	180.9963379	-388.8487244
2287596881	427.8490601	134.1781823	217.5462265	-227.104538
796891048.2	460.848938	182.7222606	222.5049149	-566.6408691
484541042.6	299.6064453	100.8921197	182.291452	-342.3648376
224299178.4	348.1047974	139.3189122	243.2967743	-106.5213852
209800606.4	313.9613953	93.54379924	165.5329086	-128.1853638
803599292.4	413.9619446	160.596097	234.5912796	-406.3211975

log.sigma.3.5.mm.3D_firstorder_Entropy	log.sigma.3.5.mm.3D_firstorder_StandardDeviation	log.sigma.3.5.mm.3D_firstorder_Range	log.sigma.3.5.mm.3D_firstorder_Variance	log.sigma.3.5.mm.3D_firstorder_10Percentile
4.252555501	121.9740487	738.9729767	14877.66855	-20.38945503
4.001390879	102.3036495	674.3020477	10466.0367	-20.53701916
4.12605145	109.8846241	647.4786377	12074.63061	-136.0508972
3.967392662	98.01051816	698.3913879	9606.061671	-25.29446182
3.690247134	92.77425462	498.7101593	8607.062321	-27.6224987
3.795029306	88.41898775	577.2810211	7817.917395	-49.33213768
3.425570657	80.21514674	449.8489227	6434.469767	-12.50668488
4.074580486	107.4121693	818.5161743	11537.37412	-12.70639496
4.065419752	104.7999786	695.5938263	10983.03552	-5.997833633
4.177467465	111.6825143	859.5209045	12472.984	-56.83436737
3.604950523	77.00771583	555.7831726	5930.188298	-10.14138241
4.091036164	104.5102836	654.2836914	10922.39938	-55.91650696
3.681520789	82.49295762	695.6158447	6805.088057	-23.49571228
3.91629648	92.41066574	581.3994293	8539.731143	-61.08339844
4.212618466	116.2985077	719.9299927	13525.3429	-48.85706444
4.118910239	111.5962981	887.7906799	12453.73375	-76.89470215
3.990406913	102.2450483	763.855896	10454.0499	-85.82291412
3.915895879	97.38520072	805.206665	9483.87732	11.45096941
3.947543303	96.22383931	672.1396484	9259.027252	-19.53972626
3.969448731	97.90729096	650.4459229	9585.837623	-39.90016174
4.105187429	106.2751958	853.9397888	11294.41723	-94.11574783
4.04416415	102.9933354	971.1679077	10607.62714	-54.23862457
4.112970069	106.1667381	745.6178589	11271.37629	-70.99049377
4.045651527	109.1864095	647.5241699	11921.67202	-52.8880928
4.195757708	113.8213758	651.2057495	12955.30558	-144.8555527
4.258320198	120.4782861	826.4477234	14515.01743	-27.88152733
4.020793658	101.0619581	670.8755035	10213.51938	-48.82852173
4.165390652	111.1601513	765.0188599	12356.57924	-98.37020874
3.933282906	99.23709536	758.131073	9848.001095	-40.88516769
4.044155809	103.397279	866.9204712	10690.99731	-57.91385269
4.108078196	108.3221736	845.662262	11733.6933	-60.53395042
4.080896584	103.7640616	808.411438	10766.98049	-17.00423908
4.042797198	108.2905414	824.1901855	11726.84136	-37.71080017
3.886008665	106.7603351	574.8570099	11397.76916	-14.14650536
4.098425595	107.2826291	669.1885986	11509.56252	-84.73291855
3.862908933	91.29707671	720.3702393	8335.156217	-40.47705765
4.326528008	125.3357627	848.5021973	15709.05341	-68.10826111
3.881255526	92.92648653	792.726593	8635.3319	-41.28416824
3.641363657	79.87428569	577.0387268	6379.901515	-40.33060875
3.853473327	95.45079636	639.4574738	9110.854526	-8.640185833
3.864108718	89.72280732	587.1021729	8050.182153	-28.24098778
3.854223941	90.8197741	612.0579987	8248.231367	-13.83069429
3.810378197	89.04015632	693.8061829	7928.149437	-11.9176897
3.827873278	96.04675101	641.6680756	9224.978379	-10.24266682
4.260124657	122.1585885	910.6841125	14922.72075	-107.7180794
4.089447204	103.7797296	692.9276123	10770.23227	-71.12582245
4.169668111	112.7690246	882.3067932	12716.85291	-59.85639572
3.909019225	103.534896	760.1233215	10719.4747	-123.5380325
3.62556134	86.83386381	432.2680664	7540.119905	-19.63499126
3.837891376	98.96247965	718.478302	9793.572378	-10.01662703
3.812236358	91.66812383	737.717041	8403.044926	-11.15575228
3.496184641	76.95957171	689.6681824	5922.775678	-16.21015358
3.923636248	97.32219041	686.883728	9471.608747	-0.897680211
4.016056108	99.97899297	760.5316467	9995.799036	-42.67922211
4.091350425	104.5487724	738.8265381	10930.44582	-28.21386795
4.263094742	118.5907252	797.1908569	14063.7601	-108.4421219
4.208970642	118.5081678	1076.809692	14044.18582	-42.44774017
3.710070086	94.81365604	703.1224976	8989.629372	-18.00523815
4.022736217	102.8756136	705.6179504	10583.39187	-59.21184425
3.353747399	62.77262515	398.6517639	3940.402468	-1.831173134
4.053100992	104.760091	717.9329834	10974.67666	1.844029665
4.010038524	105.0803444	1040.481384	11041.87878	-40.40106201
4.069527322	104.1397598	748.7855225	10845.08957	-41.69989014
4.033816644	101.0297125	609.6100159	10207.00281	-43.05936508
3.927186367	95.37688154	494.7722321	9096.749531	-11.7558754
4.129624718	117.8682593	755.2709198	13892.92655	-26.11090126
4.058909333	113.3289096	664.8867188	12843.44175	-33.313731
4.144934359	111.4065235	768.9406433	12411.41347	-12.69284153
3.839416126	89.34087986	631.7181396	7981.792814	-36.07846603
4.176752877	113.7602797	991.2970581	12941.40124	-64.85673981
4.425631733	132.5166108	884.9893799	17560.65215	-107.5107338
3.766041256	90.98825213	705.4367065	8278.862026	0.055689916
4.09610173	106.9297815	789.4685059	11433.97818	-17.62430458
3.981674097	97.41738547	819.9024048	9490.146991	-55.06221771
4.311737518	123.9325536	961.5574646	15359.27783	-53.9447525
4.174747949	116.585449	921.9203186	13592.16692	-56.95890808
3.751013299	85.77687244	718.4958801	7357.671845	-40.28392029
3.971358682	97.052949	663.3707886	9419.27491	-17.63873978
4.034170308	102.785652	772.737854	10564.89026	-67.81421661
4.004660072	104.1550183	778.3036499	10848.26784	-91.76119843
4.126145181	119.4852091	984.0578003	14276.71519	-57.62826958
4.290862784	125.1848511	886.3877869	15671.24694	-112.8674408
4.141757191	110.4105669	775.5083618	12190.49328	-40.66115189
3.372979236	73.6951595	383.4736099	5430.976534	-5.929821491
4.186629444	112.387913	799.4172363	12631.04298	-55.24537659
3.904897768	93.52509669	607.6141205	8746.943711	-30.83763771
4.248764277	120.4853957	755.9837952	14516.73058	-61.43719978
3.864553222	91.74550724	718.9128113	8417.2381	-14.5539835
3.396163763	70.88176316	291.4370003	5024.224349	4.791620255
3.820974868	94.40635629	577.6267395	8912.560108	-30.06598587
3.987984882	99.94019466	745.3441162	9988.042509	-10.70388412
4.135224615	109.5124019	706.8298035	11992.96617	-44.01959991
3.876900469	93.87905671	605.1710815	8813.277288	-82.59096985
3.96321301	99.84569002	767.818634	9969.161815	-56.9710434
3.885062977	90.29921121	654.953598	8153.947545	-6.586900473
4.77306117	181.6484171	1027.489807	32996.14743	-253.3205322
3.533236883	77.48601375	641.971283	6004.082327	-19.53773918
3.778634985	91.58409685	454.6261826	8387.646796	5.985861969
3.481478631	73.36740955	442.146759	5382.776783	-26.33435612
4.590910967	151.8833523	820.2831421	23068.55271	-163.856926

log.sigma.3.5.mm.3D_firstorder_Kurtosis	log.sigma.3.5.mm.3D_firstorder_Mean	log.sigma.3.5.mm.3D_glrlm_ShortRunLowGrayLevelEmphasis	log.sigma.3.5.mm.3D_glrlm_GrayLevelVariance
3.045110062	114.0366908	0.012409692	24.84554912
3.294190154	86.19330572	0.009534418	17.38982432
3.014692853	25.65226344	0.012293268	19.95990904
3.456787275	83.26142853	0.00833853	16.12145601
3.081141053	66.20648678	0.014585502	14.71980463
3.789692259	51.6565153	0.012820693	13.33438389
2.853807506	62.11413144	0.018206353	11.2964781
3.74875879	99.90004411	0.005007158	19.46275255
3.058124713	106.9660558	0.005668544	18.22949725
3.474661635	85.91067442	0.003119964	20.57965223
3.491882116	73.04673268	0.005908606	9.982372601
3.37675511	76.24346466	0.009620795	18.07497462
4.471365162	63.26542929	0.00524149	11.85872318
3.049255767	59.69683587	0.013662628	14.1996754
3.374821395	85.70554047	0.008282426	22.42820626
3.789383297	46.51675846	0.003484804	21.36878929
4.475882366	45.92366794	0.004797664	17.83782529
3.413067656	114.1621151	0.00423399	15.79291102
3.260894282	90.82197464	0.005593805	15.34275373
3.447247771	70.65673959	0.00668286	16.12525159
3.704996883	39.23480408	0.005069969	18.80553968
4.279623676	69.61528097	0.003614191	17.66694331
3.320314948	61.57563379	0.005925745	18.63381643
3.068560262	65.77839103	0.008593144	19.94654987
2.568243108	18.50972256	0.008507415	21.09438018
3.747214906	103.0373267	0.003971578	24.14411406
3.093574331	67.04967239	0.008568333	16.92407746
3.142495075	57.37291234	0.005473508	20.21774828
4.542076885	65.73329393	0.005371605	16.93020906
4.333751967	65.29559905	0.00429387	18.21917597
4.097720683	71.93317396	0.003291802	19.54910809
3.582694928	101.595179	0.004864713	17.8782661
4.949839833	72.01937577	0.004521897	19.80412495
2.539195736	98.86782529	0.013291532	18.94338008
3.588017176	49.25112802	0.007767246	19.30254812
3.942276738	63.65333196	0.004592737	14.21152082
3.625867778	84.50716339	0.003926707	26.14039927
4.610268224	67.8312125	0.002740287	14.75546608
3.821574691	45.85429602	0.008013709	11.04138238
3.409346184	92.18886299	0.009327381	15.45931363
3.24693223	78.86575804	0.009290629	13.47963627
2.956387797	86.05295369	0.007919466	13.8902203
3.420238152	82.78030222	0.004240466	13.45323376
3.870980209	83.67891592	0.011545864	15.70201781
4.143527062	47.19893276	0.003008784	25.41200879
3.090239268	68.10784368	0.006572714	17.64442656
4.175234157	72.45878837	0.004713349	21.29244898
4.76845133	21.19275289	0.004155059	18.2933917
2.621466109	72.84763718	0.020586156	12.76718696
3.940538941	86.50775186	0.011451445	16.72376997
4.14269939	79.90082703	0.004677311	14.36685213
6.912914026	54.3072878	0.004797238	10.65700459
3.225022213	102.6499574	0.005766599	15.96454926
3.539967941	74.72134313	0.003813333	16.48571272
3.155851249	92.85329302	0.0055944	18.21835192
3.282017357	47.66890111	0.005239794	23.29492
4.641120388	82.94093183	0.001985788	23.63551203
4.486725871	66.05209945	0.006476431	15.86278001
4.374549167	61.30761104	0.00531843	17.71490535
3.344749692	75.07920324	0.01303758	6.575079267
3.019190561	119.502291	0.004367833	18.20284203
5.104758841	71.54299602	0.002834999	19.00299024
3.891916814	82.07615479	0.004660665	18.21365222
2.868732081	87.32935478	0.005785584	16.60830773
2.406428904	103.3134782	0.018632732	14.77497922
3.031485511	104.7130526	0.007057171	22.65986174
3.493234664	85.2810398	0.009220701	21.5953215
3.203260644	107.4041112	0.004133274	20.66577126
3.468104436	71.44054196	0.005185619	13.24717038
3.978014981	67.50213897	0.002760696	21.81516246
2.89318335	77.34381957	0.002999355	28.55121499
3.952132816	91.9461359	0.004757728	14.06197759
3.665880901	97.67140543	0.005464792	19.17906728
3.68594805	73.64700855	0.003191148	15.81379793
3.763625211	87.03563025	0.003744286	25.62015934
4.450072323	71.1610703	0.0045246	22.75101506
4.707483216	53.54750275	0.006814445	12.67501655
3.459182192	94.30948938	0.007020973	15.67782957
4.0301116	61.20933692	0.003742229	17.83123598
4.28370103	42.91430012	0.004193356	18.57665919
6.410655061	59.70835374	0.002817978	24.65508028
4.216960852	49.01376685	0.003488406	26.38219006
3.722438871	85.09905251	0.005419351	20.34537975
2.729880621	68.86867211	0.019798601	9.315763451
3.515673061	85.64692871	0.002952498	20.77466758
2.876480697	79.72903583	0.010002709	14.54981632
3.503088698	83.95873063	0.007481425	24.39392905
3.313421538	83.17622135	0.004281116	14.10298346
1.846744409	91.71125782	0.035314241	8.0949098
4.008775979	73.10463792	0.01098195	15.40501357
3.043286067	99.56501166	0.003891891	16.58934592
3.425435959	89.5192494	0.005831322	19.79288996
4.020772204	35.25891228	0.007385509	14.94891038
4.420141114	60.29586902	0.003682435	16.95276341
3.332554518	99.24634537	0.005978945	13.70876988
3.038815435	19.78072485	0.004782431	53.50937989
4.425875182	64.61530385	0.004690666	10.35688537
2.371969041	104.9862491	0.017800223	13.76837009
3.503677008	58.03159134	0.019353757	9.636985996
3.006241564	52.17809566	0.006506082	37.33461519

log.sigma.3.5.mm.3D_glrlm_LowGrayLevelRunEmphasis	log.sigma.3.5.mm.3D_glrlm_GrayLevelNonUniformityNormalized	log.sigma.3.5.mm.3D_glrlm_RunVariance	log.sigma.3.5.mm.3D_glrlm_GrayLevelNonUniformity
0.013232438	0.06135576	0.142867614	66.03603738
0.010278572	0.073774967	0.162434563	130.6500908
0.012815757	0.066869163	0.113197984	68.45253491
0.008934107	0.076198833	0.160371713	166.0251048
0.016164912	0.092532069	0.23673304	74.84865708
0.013784702	0.087511342	0.180897071	125.2166476
0.020962644	0.109124228	0.347111672	93.61581803
0.00539161	0.07173042	0.170829507	114.5090888
0.006058723	0.069207482	0.159122484	125.3918115
0.003296496	0.064855679	0.119939888	176.9593738
0.006493483	0.096727703	0.216553568	149.7755205
0.01011771	0.069803271	0.114084263	107.2845674
0.00580634	0.095539085	0.232885723	238.4582097
0.014519924	0.076801318	0.13995866	127.827831
0.00878516	0.06343306	0.133707039	88.51884867
0.003796637	0.068141225	0.293241526	240.682097
0.005100869	0.078514422	0.182187152	110.7685124
0.004638456	0.076654938	0.212113869	170.1354501
0.005939297	0.075032341	0.130921628	76.96868555
0.00724351	0.07554366	0.173951959	193.0960331
0.00540025	0.069615008	0.136812691	434.1531325
0.003848759	0.07307188	0.144812524	138.766937
0.006322771	0.06886307	0.128305636	256.537347
0.009187708	0.071770021	0.188462418	64.93841653
0.009042019	0.061529549	0.117699717	145.7193619
0.004152135	0.062140209	0.116714892	76.69818287
0.00905408	0.071925535	0.129631566	144.2071735
0.00573591	0.065612954	0.100458274	203.0118812
0.005733203	0.080982214	0.221987668	240.9138765
0.004563813	0.074259889	0.163720486	209.2422803
0.003507042	0.069878872	0.142917306	258.3461116
0.005216195	0.070220153	0.151967571	314.5431081
0.004907186	0.075352836	0.174723291	168.3937251
0.014192835	0.078992372	0.202202303	33.17193918
0.008167127	0.069651938	0.125481381	56.04922503
0.004970803	0.082971142	0.192246646	233.4964513
0.004164815	0.059956541	0.131590099	193.3480351
0.002945151	0.083431447	0.168613628	249.8481798
0.00883393	0.096278434	0.268256749	120.834714
0.010257659	0.082573023	0.207702324	116.9728002
0.009888382	0.079554635	0.132941402	94.57576523
0.008652466	0.079778591	0.186310074	134.8167682
0.004663198	0.082614213	0.225518875	215.0714328
0.012570209	0.085999088	0.207939157	140.6675524
0.003203711	0.063955976	0.184767965	197.4843066
0.007023282	0.068466592	0.136999633	219.942416
0.005018958	0.068066649	0.153331572	224.3581736
0.00442244	0.084713652	0.165404435	113.2541779
0.022909671	0.092503552	0.232398442	44.7215232
0.012417474	0.08527342	0.208343712	80.58444203
0.005170393	0.086055097	0.240297112	292.3489866
0.005256974	0.112081813	0.304510232	183.6272731
0.006337461	0.077091365	0.214006406	254.2966389
0.004014228	0.072516922	0.117118252	77.15391556
0.005963373	0.068085616	0.159358788	240.1208748
0.005548999	0.06195123	0.127455665	106.0775372
0.002109336	0.065945195	0.147143321	194.2551857
0.007315577	0.095831555	0.306221358	193.5066667
0.00561358	0.072616963	0.129722408	90.2189329
0.014218842	0.112880394	0.184924284	137.5472076
0.004756174	0.069253034	0.203982699	177.6314282
0.003057079	0.076673989	0.200808285	368.589684
0.00496739	0.071469431	0.153815874	144.6679768
0.006067528	0.069252049	0.120697153	118.5516699
0.019564409	0.072684597	0.12761277	38.88464712
0.007610585	0.067185083	0.129318185	93.468087
0.009958404	0.072478054	0.157088209	67.87176803
0.004448592	0.0667775	0.1609754	211.5426102
0.005569457	0.081929075	0.167748136	92.89384025
0.002936461	0.067205215	0.15367611	150.6040863
0.003179973	0.053467255	0.118166086	185.711092
0.005176054	0.0900687	0.211123086	161.9503606
0.005915332	0.070218239	0.173267997	290.686306
0.003420671	0.075233305	0.16377338	437.0602465
0.003978481	0.060836513	0.137470287	233.2822366
0.004803208	0.067429197	0.154058677	140.3678043
0.007308987	0.090756723	0.224275199	184.7103337
0.007417792	0.074760369	0.135492232	91.65839465
0.003990324	0.07444497	0.166187886	217.4006191
0.004513251	0.077858315	0.185226147	304.3731206
0.002944844	0.074109287	0.163851629	89.83014484
0.003657719	0.064055195	0.11963436	154.7588181
0.005736111	0.067481358	0.135271934	150.4715064
0.02257959	0.109876011	0.268516137	48.31231459
0.003140901	0.064988267	0.123188736	227.1184236
0.010961295	0.075928274	0.176207871	231.4179674
0.007872093	0.063521804	0.139762293	81.80524784
0.004653445	0.080814816	0.183248587	215.968701
0.038836715	0.101134786	0.179239353	36.6097962
0.011642546	0.087651531	0.196829768	44.27187258
0.004227669	0.072734536	0.169521813	206.7639597
0.006098372	0.067521349	0.112004452	68.1047885
0.007775099	0.081456495	0.145623866	113.737234
0.003959528	0.080305626	0.174370644	308.5235937
0.006526305	0.079802514	0.186676602	328.710214
0.004897181	0.043921946	0.07112202	36.64109931
0.005160046	0.105696258	0.242981182	158.266957
0.019491927	0.080597298	0.191008909	30.26157562
0.021463764	0.105849409	0.289731726	78.73558228
0.006716037	0.048538629	0.069630879	52.68282048

log.sigma.3.5.mm.3D_glrlm_LongRunEmphasis	log.sigma.3.5.mm.3D_glrlm_ShortRunHighGrayLevelEmphasis	log.sigma.3.5.mm.3D_glrlm_RunLengthNonUniformity	log.sigma.3.5.mm.3D_glrlm_ShortRunEmphasis
1.398434166	209.2202569	874.8380399	0.919934587
1.44221413	198.1773874	1420.004158	0.914586231
1.310309802	188.0411284	870.9972144	0.937269392
1.435841721	192.4407213	1756.694035	0.916228301
1.626603708	109.7669867	608.7674328	0.889234876
1.501609851	133.1337237	1116.887071	0.903411245
1.888231169	85.83915211	597.6154631	0.858065724
1.454819057	301.4335843	1281.391143	0.91457634
1.415828151	249.7585702	1485.412602	0.92285766
1.335195514	426.6542708	2285.444133	0.93128367
1.587063455	195.4608747	1172.673394	0.891700539
1.320399592	214.2491277	1296.554718	0.933920065
1.622294845	215.0721646	1875.819195	0.888300096
1.39129036	142.8722338	1358.687233	0.92124362
1.37040602	200.6535822	1153.90036	0.925712064
1.65639904	364.4337249	2759.662885	0.903682191
1.488895143	317.1966886	1117.99403	0.908740141
1.549207271	284.1691416	1733.193021	0.903269898
1.361860155	259.4545643	852.2310731	0.927841049
1.469094366	231.3189757	2033.013149	0.911026853
1.382904269	284.9521925	5103.67604	0.922327931
1.400167376	357.5897278	1549.099033	0.920629046
1.355976254	253.2368468	3093.267052	0.928053833
1.500232457	230.8204284	719.1904237	0.909014962
1.330563655	208.7578178	1987.845215	0.931997579
1.319714301	422.8015385	1044.564228	0.935505861
1.350095471	177.8465457	1681.076599	0.931522943
1.279135907	282.2556602	2662.435968	0.941965961
1.544537899	284.8684045	2373.081082	0.910740576
1.426883212	319.8392902	2301.753368	0.921329397
1.403341998	395.2680765	2995.724913	0.918270702
1.403346676	272.2883025	3681.288672	0.923706724
1.464948968	364.331111	1787.448785	0.913144051
1.527789544	164.4433513	330.6587305	0.905997081
1.34918526	270.065621	671.8015385	0.92931559
1.505467859	278.7548891	2218.372736	0.907758659
1.360738277	392.2803893	2679.702854	0.928198406
1.456909207	421.8510587	2385.721602	0.912012401
1.690860154	170.3629529	938.9526679	0.884846707
1.549607833	153.0497697	1096.873446	0.900615938
1.364554934	185.835194	986.7289715	0.92748892
1.500379031	168.1435254	1326.406184	0.905870359
1.587810987	269.7147927	1996.271083	0.896496483
1.544515166	145.913224	1273.07159	0.902443026
1.473308285	478.9773176	2481.561341	0.914913224
1.380101157	228.3986073	2640.072313	0.923812295
1.408370664	332.1848451	2697.678858	0.922334122
1.460042062	352.4457598	1060.512318	0.909798716
1.616426648	96.84816553	369.3208275	0.891995652
1.550819651	150.1853861	734.2820722	0.901156222
1.626971719	267.0240745	2567.727253	0.89063151
1.751518221	287.7808751	1210.94765	0.88113238
1.548302762	213.1467604	2582.455838	0.904436843
1.332497914	369.1125214	889.8017086	0.930552588
1.409977653	262.5919556	2908.033532	0.924994262
1.349092487	304.5796693	1433.327536	0.93058792
1.393809169	640.6689417	2429.71302	0.924816676
1.779524932	198.3666575	1460.655416	0.872845208
1.354473025	348.6709939	1036.363253	0.929559818
1.523413235	104.0265325	936.9970846	0.897365613
1.525920635	293.015368	2015.681555	0.906197347
1.5133591	434.4089414	3812.710139	0.909456216
1.419660567	308.003034	1642.727335	0.918612294
1.316241106	253.6180145	1467.305452	0.939625019
1.348411371	119.1748401	447.2933397	0.930411586
1.363259436	227.5840465	1147.116127	0.925639187
1.43894374	176.5706131	747.1216885	0.912541747
1.432017065	313.1137803	2561.81898	0.917784529
1.474685233	252.5874707	890.357297	0.905831063
1.41090787	476.3186077	1830.158815	0.921541771
1.32395513	464.1423022	2935.989328	0.93481674
1.539451073	254.8248946	1417.399298	0.906597071
1.45834772	267.3736321	3325.931328	0.914733812
1.425696353	361.6358097	4748.848193	0.921524843
1.37615075	394.8594241	3159.421437	0.925231202
1.413350202	365.747754	1698.42484	0.920956683
1.575190131	231.7983798	1575.998245	0.899931911
1.37115988	231.5410549	1017.652099	0.926883127
1.437089244	344.6859638	2382.733903	0.91972697
1.491118036	316.9505035	3095.694451	0.90915299
1.434819975	636.5350577	983.2708088	0.918469744
1.332806149	450.8519544	2026.395842	0.93196843
1.371399143	317.0491306	1843.874475	0.926405071
1.710093318	73.7854919	325.581401	0.879228321
1.347037494	424.6823966	2901.625484	0.928262086
1.480761976	139.9743657	2401.304209	0.907736975
1.3746224	288.5916362	1068.162922	0.927491223
1.489461806	273.6543259	2118.48004	0.909270386
1.503607207	55.99210705	290.2634883	0.907153255
1.518121025	202.4067559	397.4638374	0.905893455
1.451468573	300.0907279	2286.071823	0.915296499
1.316997017	290.1431628	850.5868763	0.933589617
1.401456384	243.2318103	1136.745956	0.920294074
1.465558798	341.3517901	3067.480369	0.912409674
1.499826051	204.1291336	3241.03888	0.906700189
1.19722229	609.9413267	748.7313765	0.958223478
1.646790064	269.9423931	1118.76314	0.885584618
1.507324595	102.4687381	297.4882133	0.907240018
1.733404287	79.77677287	545.7276117	0.879475606
1.201403421	397.7980622	965.295694	0.955018463

log.sigma.3.5.mm.3D_glrlm_LongRunHighGrayLevelEmphasis	log.sigma.3.5.mm.3D_glrlm_RunPercentage	log.sigma.3.5.mm.3D_glrlm_LongRunLowGrayLevelEmphasis	log.sigma.3.5.mm.3D_glrlm_RunEntropy
300.5830887	0.894046934	0.017065951	4.786449777
295.5812944	0.885269231	0.014175974	4.582394768
266.678778	0.915439659	0.015228199	4.565219936
291.2609176	0.887704224	0.011938625	4.54243933
178.2562224	0.851341493	0.0250975	4.410273469
218.0969088	0.87292195	0.018557021	4.43279813
157.0093214	0.810130267	0.038025397	4.321670157
464.2429186	0.884125293	0.007361099	4.65929552
370.704865	0.893628325	0.008155926	4.603325165
611.6782069	0.908476139	0.004148159	4.660847568
342.4750311	0.856711081	0.009657866	4.297345516
302.7300687	0.911983565	0.012377361	4.555097628
376.5213536	0.851537674	0.008939019	4.424063497
215.9809529	0.895710855	0.018540464	4.445905863
293.0448439	0.901361558	0.011256064	4.710416118
629.4858293	0.858902597	0.005937435	4.805957644
529.9095075	0.878360891	0.006699572	4.599169356
461.166454	0.867444518	0.006962375	4.568278617
368.4982921	0.902965872	0.007655008	4.439868159
362.1438983	0.88067775	0.010060377	4.575577573
428.4561543	0.897255119	0.006983028	4.646730671
545.4590028	0.895042251	0.005017286	4.584634702
369.4215527	0.903832154	0.008199339	4.621044077
363.4633429	0.877669903	0.012332159	4.639048373
297.3032417	0.909517901	0.011633204	4.66172828
584.9327424	0.912778789	0.005045626	4.712739089
255.4020155	0.906620742	0.011503512	4.506482054
389.816933	0.922159807	0.006948715	4.586402618
471.2479732	0.874306923	0.00788316	4.572089915
489.6193813	0.891518738	0.006008379	4.610856839
613.9320575	0.89245255	0.004552294	4.665782126
404.4075078	0.895661538	0.007064979	4.621690776
571.1021566	0.88244233	0.006876303	4.644604668
248.9896983	0.871901487	0.019337004	4.48381872
391.3213644	0.906011781	0.010009727	4.575573403
453.0371232	0.874345425	0.00698579	4.503235016
568.273062	0.90335582	0.005323013	4.835784083
677.2343201	0.882766599	0.003998518	4.487420187
309.605985	0.844546962	0.013606271	4.391142946
242.758688	0.865337596	0.015371779	4.514940107
270.5332673	0.903086275	0.012718166	4.361831295
263.0710358	0.874343048	0.012550162	4.478478282
459.7596901	0.859342838	0.007066971	4.501049742
228.0490305	0.867426098	0.018200318	4.494930634
773.6477561	0.88318436	0.004261043	4.856304079
346.0811106	0.898396988	0.009194051	4.607989336
500.5763462	0.894027297	0.006566561	4.720858328
578.0998298	0.881367972	0.005747441	4.516901904
152.0532574	0.856110202	0.035383955	4.287492894
233.7097098	0.866266761	0.017743519	4.501587982
463.233912	0.852023479	0.007951224	4.545218497
556.7981951	0.836086204	0.00802431	4.304254797
341.9602846	0.868236057	0.009622239	4.582518156
526.249385	0.908559417	0.004977507	4.483947212
389.8748012	0.89562533	0.0079401	4.628265293
436.2400886	0.906779661	0.007054511	4.744840839
953.2386118	0.897881624	0.002754092	4.744131083
360.5499315	0.82882085	0.012393626	4.554297052
510.7293692	0.90530388	0.007001291	4.511435526
178.3787867	0.867068163	0.020103426	4.000938805
467.3911004	0.87186462	0.006996443	4.684184048
711.2424355	0.875509947	0.004304147	4.652551586
470.7402381	0.891000237	0.006516861	4.628401415
361.3275195	0.916323505	0.007475694	4.466132696
171.026431	0.906649283	0.02415572	4.377092267
312.8527672	0.901341044	0.010274863	4.637043339
259.3772484	0.884760797	0.013634494	4.63830704
474.7886895	0.888239355	0.006101624	4.719420504
415.3018837	0.876732703	0.007480722	4.441589174
719.8407197	0.893498205	0.003849071	4.730525604
652.3069275	0.912047511	0.004068914	4.882849487
412.3208314	0.87153361	0.007608055	4.411206759
412.2049795	0.884377004	0.008231196	4.687522906
561.968692	0.892064317	0.004644028	4.539695365
577.4520935	0.899559614	0.005161574	4.841497283
556.2016662	0.892815893	0.006206644	4.725270535
401.2525382	0.863563613	0.010045291	4.42995367
339.5884202	0.902190525	0.009380177	4.466797564
543.4730542	0.890673429	0.005302041	4.598380074
521.7227526	0.877420581	0.006174132	4.641541409
991.6305898	0.889359539	0.003618816	4.699890637
652.0679945	0.909151443	0.00446048	4.780351585
462.339857	0.901001182	0.007299804	4.660690038
121.3629105	0.839682306	0.039044511	4.113000115
618.9272127	0.904931681	0.004034309	4.68562856
217.9370474	0.877109074	0.015884721	4.520602357
418.0028701	0.901475975	0.009767343	4.754387738
435.3314066	0.877651103	0.006660613	4.480015165
86.57802599	0.880029946	0.058264511	3.929665393
333.2923498	0.873036998	0.015033691	4.422621102
455.7828803	0.884986704	0.006029139	4.569417635
409.9835598	0.912634876	0.007356406	4.583774185
379.0852143	0.893775239	0.009640305	4.425250408
546.8953085	0.882185584	0.005392253	4.577361835
329.360607	0.874987741	0.009418174	4.514754607
776.5999718	0.943351897	0.005407425	5.070892046
491.5850857	0.84829181	0.007729042	4.275982053
152.4856944	0.876527678	0.028837813	4.334888473
143.937204	0.836105336	0.033873533	4.262043692
511.7721825	0.940541261	0.007620015	4.908676737

log.sigma.3.5.mm.3D_glrlm_HighGrayLevelRunEmphasis	log.sigma.3.5.mm.3D_glrlm_RunLengthNonUniformityNormalized	log.sigma.3.5.mm.3D_glszm_GrayLevelVariance
224.9094562	0.811949353	34.12809206
214.3255662	0.800822015	23.811116
201.2394372	0.849583208	24.27040763
208.569253	0.804764997	25.26496645
120.451296	0.751003141	19.32639053
146.8484804	0.77874853	22.57952696
96.15567165	0.694189745	16.0576
327.6173531	0.801431422	29.53532079
269.0465587	0.818668976	25.30880911
458.2828907	0.836334478	27.81677178
218.5302109	0.75541339	16.48454964
229.2910268	0.842215479	24.77466439
239.8875093	0.74937627	23.73065173
155.0521389	0.814829927	19.48498172
216.3312105	0.824909638	28.92565911
400.1899049	0.780116652	30.78052577
350.8484586	0.790120976	28.90831025
311.2506257	0.778872726	25.41197357
277.7459746	0.829221628	21.65643599
252.5587862	0.793979235	24.25538462
309.1714074	0.817035835	26.774872
388.7453864	0.813888359	27.52279008
272.8129047	0.829233173	25.06338188
251.5997427	0.791765312	28.03582524
223.9684845	0.83787105	24.30875361
450.3853753	0.845400855	33.96610013
190.6411128	0.837233072	22.71678467
300.6722388	0.859516641	25.12692235
311.3844786	0.795174613	32.04644267
346.5529781	0.815705316	30.60989249
432.0602445	0.808731127	30.15789222
293.5922426	0.820493984	25.46301561
397.0327388	0.798478147	32.2674999
177.9035233	0.785287022	23.13923908
290.4216432	0.832908614	27.76203636
305.8345394	0.787211822	24.90495207
421.6710324	0.829801859	37.10072962
463.1471386	0.795502019	25.75745087
190.6049766	0.744422732	22.08599333
167.1139175	0.773033184	23.18786596
199.9932301	0.828471867	20.86368815
183.5076852	0.783430824	21.44835046
298.5542332	0.765243587	23.18843163
158.9601875	0.776961234	27.76851214
524.4139606	0.802289334	40.12518284
248.02661	0.820489196	21.62979315
359.4377909	0.817351868	31.36548209
389.3045662	0.791518887	30.3364112
105.4418549	0.759600036	13.92424684
163.1936928	0.774967135	25.54785474
296.7927081	0.753982523	26.41934256
324.734087	0.736759673	25.99145073
232.7176793	0.781258663	24.32408142
396.5225093	0.834759592	24.01689242
282.5652847	0.823387346	26.50494571
326.5516187	0.835332077	31.90273568
691.2004808	0.823199447	38.08349479
221.7429758	0.720918595	26.38720879
375.6962334	0.832800595	25.61262598
116.3628482	0.766687344	11.24979612
320.4918389	0.784303159	26.54428848
476.2591739	0.791428315	34.40058023
334.5878286	0.809837926	28.17317632
270.6813912	0.855469901	21.35337016
127.9030296	0.834620664	18.13705104
242.332464	0.823726167	26.63506419
190.650915	0.79669777	30.81141582
339.3223277	0.807668221	32.24582702
279.5464716	0.783423704	18.31538242
515.5093404	0.815643066	35.70190471
495.8713025	0.844052492	33.84134346
278.6075195	0.786125793	25.06258489
290.3510903	0.801821443	27.49927272
392.8452913	0.815970736	24.23009814
425.512253	0.823051751	37.94492858
396.6229527	0.814572847	35.26926336
257.01298	0.772273562	25.76719413
249.2600677	0.827637148	23.48725286
375.5405414	0.813436683	29.14528606
348.7925819	0.790332777	30.30642514
693.1502843	0.809748202	46.93309074
484.9822436	0.83763688	39.96933154
341.264702	0.825821701	30.23024442
81.07334473	0.736047976	11.42530612
457.8050874	0.829284959	28.31765385
152.7469681	0.786718433	19.98922176
309.7169138	0.828321882	34.67018769
299.3309049	0.790925186	22.18576003
61.05621661	0.793072706	7.411337868
222.9159218	0.784867241	24.29245542
325.1568252	0.802849265	22.38613566
311.0513364	0.841669155	29.29108247
265.6819184	0.812867234	23.69549464
373.8682136	0.796890025	29.28278199
224.0224936	0.785147749	21.92349062
639.0345782	0.896826687	62.42862229
303.4700034	0.744638299	19.00350728
110.668796	0.788504834	17.2915879
89.14093812	0.732521309	16.43339086
418.4878428	0.888719902	41.9694814

log.sigma.3.5.mm.3D_glszm_SmallAreaHighGrayLevelEmphasis	log.sigma.3.5.mm.3D_glszm_GrayLevelNonUniformityNormalized	log.sigma.3.5.mm.3D_glszm_SizeZoneNonUniformityNormalized
142.7648242	0.04881458	0.312591871
121.3258316	0.058768	0.272616
104.3665318	0.056725838	0.315410914
127.4214883	0.056390071	0.305902153
79.74377034	0.066719264	0.271900066
76.50966583	0.059832743	0.252960516
50.16419099	0.072711111	0.1976
191.1411539	0.055616427	0.321950329
157.5429984	0.056637301	0.3427309
239.6825093	0.05395138	0.287656378
106.7626585	0.074375411	0.272990631
130.3626692	0.056479182	0.316490545
147.1231714	0.057492087	0.28355166
81.41761104	0.063466046	0.275926503
119.9678449	0.054691066	0.328640464
235.4636309	0.05160612	0.329054563
166.1713557	0.053933518	0.284709141
187.0913255	0.057536679	0.269921728
173.736666	0.062785467	0.329913495
135.3741273	0.057448521	0.274840237
156.1251342	0.05464262	0.287792187
209.5056085	0.055960331	0.280429752
151.2613799	0.05601358	0.290285311
136.1154699	0.054068346	0.255535726
120.5175786	0.056144931	0.301689015
260.3123889	0.050378078	0.331612903
106.3412772	0.059741264	0.338842659
154.0765709	0.055783476	0.316199869
187.0770449	0.053066667	0.340999111
199.716051	0.051636331	0.334358736
207.2136013	0.052565952	0.284731612
163.8338522	0.056008619	0.308554398
222.571411	0.052620261	0.283087229
101.0543922	0.064277234	0.310079711
152.3609466	0.053963636	0.293804959
165.6013093	0.056927298	0.292992752
245.5564985	0.046203231	0.327106878
238.2866694	0.057044994	0.303262996
98.71984573	0.06189472	0.235273643
105.5971481	0.058886369	0.246016629
114.3016099	0.060858832	0.327189128
109.7800079	0.061103299	0.300962683
168.7456053	0.059714818	0.302197091
107.0222567	0.054280175	0.324903071
273.3095485	0.044997684	0.308484151
128.1197757	0.061226943	0.287373576
211.6151698	0.051735147	0.328874376
193.9956723	0.052939366	0.305957389
74.83689263	0.084901812	0.311695123
108.4563634	0.059571629	0.301888591
171.2170092	0.056012111	0.288533737
166.9058633	0.061465096	0.262418845
155.6834308	0.058393835	0.30491746
206.720597	0.058691626	0.295484557
156.8636882	0.055634221	0.285464682
184.1645584	0.049705529	0.305664854
387.1891615	0.047716161	0.323800362
155.5143703	0.056225032	0.313065532
197.8493659	0.057155755	0.317883085
49.09180658	0.089642708	0.242213846
187.4821274	0.055839887	0.328076149
264.1895084	0.051022641	0.318604671
199.3379671	0.054141705	0.323487681
126.700487	0.060862992	0.263887588
65.50694293	0.066398866	0.33217155
184.8833281	0.056512369	0.363176702
140.0422121	0.050765306	0.305127551
204.4831133	0.049028367	0.309606457
125.3141172	0.068749064	0.285494479
284.1044174	0.048557279	0.297395398
296.2592079	0.048443143	0.356503292
174.4851762	0.059697778	0.279488
169.2077412	0.0539147	0.298242392
193.3914037	0.057006018	0.280226898
249.8010618	0.047251531	0.306182717
205.8995187	0.0507264	0.29877504
133.0023711	0.056770962	0.289357831
136.7700392	0.059197595	0.293820095
193.9540168	0.05231434	0.300724509
174.6685284	0.051029503	0.293866407
371.6085605	0.045397352	0.318138923
269.8805985	0.045354826	0.34586928
211.637331	0.051957701	0.326971864
56.09937839	0.087709751	0.307755102
247.8632398	0.05316227	0.298549979
87.23653065	0.061747212	0.282411406
187.5043421	0.048301911	0.326435064
167.5924349	0.060885633	0.282995921
31.37181764	0.104761905	0.325714286
97.27547831	0.061947874	0.256735254
188.9517037	0.059903234	0.292510619
175.3323066	0.051310899	0.343301185
131.4482993	0.057585941	0.307346729
204.2131092	0.052313569	0.2900224
130.8558665	0.059006316	0.301624227
387.8917325	0.035599858	0.418009078
155.5285029	0.070629775	0.250339509
60.89500596	0.073251418	0.259451796
48.20838146	0.071581197	0.235453649
234.2068681	0.043571163	0.396016077

log.sigma.3.5.mm.3D_glszm_SizeZoneNonUniformity	log.sigma.3.5.mm.3D_glszm_GrayLevelNonUniformity	log.sigma.3.5.mm.3D_glszm_LargeAreaEmphasis	log.sigma.3.5.mm.3D_glszm_ZoneVariance
104.0930931	16.25525526	76.74174174	63.69077185
136.308	29.384	120.216	104.216
123.0102564	22.12307692	29.51794872	21.30017094
184.7649007	34.05960265	164.4751656	147.9813468
53.02051282	13.01025641	265.4307692	241.7463248
89.5480226	21.18079096	155.6440678	134.2338409
29.64	10.90666667	641.6333333	591.9778222
142.9459459	24.69369369	238.2094595	221.6826708
185.7601476	30.69741697	153.2158672	139.2293712
261.479648	49.04180418	61.58305831	50.66908473
85.17307692	23.20512821	450.2019231	416.6585203
175.6522523	31.34594595	34.1027027	24.88518789
157.3711712	31.90810811	499.2342342	471.3824657
136.3076923	31.35222672	63.17004049	49.0239145
151.5032538	25.21258134	56.23427332	44.95866291
351.1012184	55.06373008	211.940956	197.1108777
108.1894737	20.49473684	142.1552632	124.3157548
132.5315682	28.25050916	236.0570265	208.9152608
112.1705882	21.34705882	58.7	47.53653979
178.6461538	37.34153846	249.4661538	229.5470982
540.185935	102.5641982	94.62440064	80.9142867
154.2363636	30.77818182	79.85272727	64.98118678
337.3115318	65.08777969	81.10068847	68.52322765
62.86178862	13.30081301	86.82926829	69.29836737
239.8427673	44.63522013	45.05408805	34.32537321
154.2	23.42580645	38.83870968	30.38499711
232.4460641	40.98250729	54.83819242	44.4502567
369.9538462	65.26666667	35.57179487	27.34911608
255.7493333	39.8	428.044	407.504976
293.2326112	45.28506271	162.945268	149.9704861
293.8430233	54.24806202	102.2732558	86.16454465
400.8121632	72.7551963	181.4072363	166.5915523
156.8303249	29.15162455	373.3592058	352.4871724
41.2406015	8.54887218	81.33082707	68.25145571
80.79636364	14.84	44.18909091	33.76206281
205.6809117	39.96296296	427.6623932	406.6619772
350.3314659	49.48366013	64.24556489	53.14067762
229.8733509	43.24010554	195.3997361	175.386507
51.99547511	13.67873303	444.3529412	399.2626277
77.4952381	18.54920635	273.8222222	246.8482137
125.640625	23.36979167	79.984375	68.23947483
129.4139535	26.2744186	171.2837209	151.096463
168.0215827	33.20143885	562.2931655	532.6533662
128.6616162	21.49494949	388.7575758	366.1230487
288.124197	42.02783726	121.117773	107.123445
259.4983389	55.28792913	88.19601329	72.52213061
345.3180952	54.32190476	126.7428571	114.4194141
104.6374269	18.10526316	187.8362573	168.1869977
40.83206107	11.1221374	179.8778626	161.3418798
81.81180812	16.14391144	227.2250923	211.0474803
196.2029412	38.08823529	1043.744118	1009.418304
77.41355932	18.13220339	1094.705085	1050.741442
228.3831776	43.73698264	570.4365821	544.7375031
102.5331412	20.36599424	42.71181556	31.32362199
282.3245703	55.02224469	175.7300303	159.8833519
181.8705882	29.57478992	63.66722689	53.59860462
304.3723404	44.85319149	89.98404255	77.81582051
144.3232104	25.9197397	805.1279826	777.2972083
131.2857143	23.60532688	50.76513317	39.72922395
51.5915493	19.09389671	453.056338	409.5459014
213.577573	36.35176651	282.3671275	261.9578293
395.3883965	63.3190975	575.9911362	556.4420547
192.7986577	32.26845638	85.29362416	70.77445554
141.707635	32.68342644	40.58472998	28.48417826
61.11956522	12.2173913	38.59782609	28.31604442
169.2403433	26.33476395	125.2081545	114.2443819
85.43571429	14.21428571	82.15714286	67.87954082
252.019656	39.90909091	206.2014742	187.0097556
77.36900369	18.63099631	136.6678967	113.9033782
200.1471025	32.67904903	146.0698366	132.1933813
450.263658	61.18368963	35.71496437	26.62447678
104.808	22.38666667	396.0133333	365.8073173
346.5576592	62.64888124	164.7581756	148.5440387
419.499666	85.33800935	223.2004008	204.2834214
364.9697987	56.3238255	100.9295302	88.14530595
186.7344	31.704	139.3904	125.4804838
122.1090047	23.95734597	556.7037915	525.5610442
109.3010753	22.02150538	58.96505376	45.61899497
242.0832298	42.11304348	150.8149068	134.2535458
307.678128	53.42788921	395.6303725	377.5414779
118.9839572	16.97860963	98.81818182	85.55612114
295.3723653	38.73302108	65.46955504	55.78973312
228.226361	36.26647564	70.92836676	58.36550603
32.31428571	9.20952381	222.8285714	198.0186848
321.239777	57.20260223	48.6802974	35.79780199
205.877915	45.01371742	179.5555556	156.8462125
142.3256881	21.05963303	68.1146789	57.38755155
175.174475	37.68820679	329.8303716	305.6633791
34.2	11	73.66666667	58.34503401
34.65925926	8.362962963	135.1555556	116.8244719
234.5935162	48.04239401	176.69202	160.6620808
123.2451253	18.42061281	44.11420613	34.64015642
127.2415459	23.84057971	68.7826087	54.54748069
286.8321537	51.73811931	401.0485339	381.6760748
294.3852459	57.59016393	246.7233607	223.4743643
177.2358491	15.09433962	15.83962264	11.49279103
72.84879725	20.5532646	505.185567	468.4811233
23.86956522	6.739130435	106.2608696	84.61814745
36.73076923	11.16666667	270.0769231	237.6745562
206.3243762	22.70057582	12.01919386	7.1130964

log.sigma.3.5.mm.3D_glszm_ZonePercentage	log.sigma.3.5.mm.3D_glszm_LargeAreaLowGrayLevelEmphasis	log.sigma.3.5.mm.3D_glszm_LargeAreaHighGrayLevelEmphasis	log.sigma.3.5.mm.3D_glszm_HighGrayLevelZoneEmphasis
0.27680798	0.590839758	12528.003	252.3333333
0.25	0.910455148	18383.344	237.614
0.348837209	0.201774469	6288.520513	189.2282051
0.246229107	1.096606381	26654.84603	224.0562914
0.205479452	3.888807232	19671.27692	151.0461538
0.216117216	1.322787812	20295.78249	151.5367232
0.141911069	12.79003693	34927.42667	122.56
0.24598338	0.947087628	62708.81306	340.295045
0.267390232	0.84351218	30175.65129	273.400369
0.302697303	0.151968404	26741.67547	449.8954895
0.172661871	2.533680204	83192.58333	211.0512821
0.329376855	0.207353643	7344.169369	227.2342342
0.189484466	2.53952294	100844.8432	266.6972973
0.265877287	0.553937231	9421.477733	153.791498
0.297803618	0.374780112	10314.57267	212.0845987
0.259673887	0.671621659	70438.84536	403.158388
0.236760125	0.430306588	49792.86053	325.1105263
0.191946833	1.078992858	55907.12831	345.5376782
0.299295775	0.296190682	12918.41765	297.4117647
0.224060669	1.247070463	52915.95077	261.4153846
0.270071942	0.352703399	28141.64145	294.8129995
0.259311645	0.232554417	29549.25818	387.4181818
0.281970396	0.347566491	21315.57229	273.7633391
0.238834951	0.496593594	17931.38211	269.9268293
0.305299539	0.302255631	10238.73208	219.0779874
0.343934911	0.110761843	15208.25806	454.4365591
0.310266848	0.358537409	9660.476676	188.548105
0.348733234	0.130741183	11364.61709	279.3794872
0.220653133	1.589666116	118459.8573	315.3626667
0.2776195	0.532652618	51725.75143	343.6214367
0.249154998	0.247188509	45623.27422	401.9525194
0.2598	0.824013906	42587.04465	298.208622
0.218885816	1.131401239	130128.4116	413.433213
0.276507277	0.846880457	9238.503759	186.9022556
0.309684685	0.191588264	12596.02182	282.4836364
0.218215729	1.624967027	115321.6823	304.4900285
0.300084057	0.182175719	25415.92904	423.0709617
0.223532881	0.441099602	89017.9934	437.4511873
0.148921833	2.957141538	70145.45249	212.1764706
0.192542787	2.558964044	31627.85079	201.5587302
0.291793313	0.508965671	14159.96094	201.0104167
0.222567288	1.414705065	22950.77907	200.8837209
0.183680211	2.490198193	132059.5072	306.4082734
0.210191083	3.685212741	43463.41162	188.1489899
0.267315398	0.250908812	61897.89507	494.627409
0.252587413	0.386959941	24465.76301	232.0885936
0.284861639	0.420445034	41285.08095	361.7047619
0.225593668	0.485044343	76323.53216	352.4444444
0.232269504	3.332507359	10949.9313	136.778626
0.248623853	2.309554392	23737.15129	195.701107
0.170682731	4.351806665	256360.5279	316.6205882
0.150817996	3.715599087	327529.461	328.0440678
0.197260996	3.533314377	95984.31909	270.2149533
0.296327925	0.127426429	15855.48127	388.6397695
0.251206502	0.851310822	38840.34277	292.3367037
0.315148305	0.231923468	19691.25042	333.4941176
0.286672766	0.150745928	55946.14149	687.1404255
0.189555921	5.176343974	128164.8937	270.9262473
0.301020408	0.165578418	18281.51574	356.2711864
0.151601423	4.281207604	50916.84038	105.8779343
0.221353281	1.275340486	66696.78034	333.6820276
0.22617095	1.408924712	238960.6938	467.4496374
0.262439454	0.297792474	26888.34564	340.1459732
0.287473233	0.167718752	11859.03166	254.6405959
0.311864407	0.501401925	4394.570652	119.4456522
0.302009073	0.885873305	19213.80687	292.3390558
0.264650284	0.710339292	11127.54643	239.5107143
0.228266966	0.816659873	55344.957	363.1818182
0.209590101	0.52981642	38837.43911	244.6457565
0.268448345	0.318719073	69217.526	517.5468053
0.331670168	0.09627271	16457.07759	489.695962
0.181950509	1.831826653	88550.72267	323.8453333
0.248343663	0.772300127	38153.8537	306.2340792
0.229918599	0.596109287	86715.51169	371.3540414
0.279680901	0.301585518	36644.40772	440.9370805
0.268125268	0.429942522	48927.1488	378.9712
0.179193206	2.393175297	134646.8578	253.4052133
0.273730684	0.308347998	13025.37903	251.8870968
0.245726496	0.421102747	56523.28323	353.4136646
0.235122389	1.155948632	139053.4499	327.5148042
0.274596182	0.152344738	67270.85027	672.3342246
0.32141513	0.13984723	33106.1007	458.3969555
0.282134196	0.272533755	21061.37966	355.7793696
0.200764818	5.291183395	10522.0381	104.6857143
0.278612118	0.120468595	22027.52138	447.197026
0.20984456	1.834195344	20635.77092	164.8134431
0.305322129	0.293278336	18167.28211	321.4908257
0.20341768	1.388689505	81450.00162	311.0210016
0.255474453	3.142599924	2911.447619	59.11428571
0.233564014	0.756038127	26872.11852	210.0592593
0.249766428	0.73825576	44143.08728	347.1047382
0.324886878	0.165752617	13244.94986	300.6685237
0.265044814	0.279060011	19441.67391	240.2971014
0.227199632	1.134073049	144375.5177	373.4246714
0.207394815	1.350442038	48674.17725	236.2038934
0.479638009	0.034283466	11150.93632	601.0188679
0.165059558	1.919986828	136262.8213	310.814433
0.214953271	2.014825241	7117.858696	128.4565217
0.175675676	4.358640681	18417.97436	100.1089744
0.451473137	0.04671603	5578.046065	380.925144

log.sigma.3.5.mm.3D_glszm_SmallAreaEmphasis	log.sigma.3.5.mm.3D_glszm_LowGrayLevelZoneEmphasis	log.sigma.3.5.mm.3D_glszm_ZoneEntropy	log.sigma.3.5.mm.3D_glszm_SmallAreaLowGrayLevelEmphasis
0.574646898	0.017162767	6.382293606	0.007546423
0.529610689	0.011378498	6.5163172	0.005197822
0.578386462	0.018476216	6.1950564	0.012823494
0.570528845	0.012828384	6.496050982	0.00804662
0.533533094	0.021300731	5.988024578	0.014400138
0.5122124	0.022136449	6.413310248	0.013202674
0.43990377	0.031095491	6.027976008	0.016890217
0.584372018	0.007983796	6.331226908	0.005590517
0.607316638	0.00871568	6.342805304	0.006450221
0.548726012	0.004583679	6.616362435	0.003188403
0.538384747	0.011014264	6.158400513	0.008055964
0.579241511	0.012965982	6.386981915	0.00656078
0.545872694	0.008424904	6.549701796	0.00593794
0.538093418	0.020634767	6.433409195	0.012458133
0.591674671	0.012428868	6.319965984	0.008756984
0.593678225	0.005041127	6.636304288	0.003448163
0.545863933	0.0087027	6.4171047	0.006011235
0.533842466	0.00696829	6.568764373	0.005049264
0.593653515	0.008469189	6.1547515	0.006423166
0.538859895	0.009208422	6.62933861	0.004424975
0.551319447	0.007533354	6.819870187	0.004858099
0.544440276	0.005765417	6.602971083	0.004142488
0.554248396	0.007570373	6.701329688	0.003999774
0.517414775	0.01587976	6.36798469	0.011669498
0.566538517	0.0113765	6.545044195	0.007587056
0.594098418	0.006399967	6.407327778	0.005107798
0.603145789	0.012439394	6.29905107	0.009085188
0.579381868	0.007691433	6.517709137	0.00525486
0.606006177	0.010377822	6.554595964	0.008108646
0.598136094	0.006875801	6.532825194	0.005171046
0.548198376	0.005437022	6.809888071	0.003882895
0.573924786	0.006961122	6.669258573	0.004862411
0.547959414	0.006518837	6.676906661	0.00405275
0.573489615	0.025992354	5.741115756	0.022149143
0.553464656	0.010933745	6.217375945	0.004972202
0.554854874	0.007006395	6.50008737	0.003852436
0.592134723	0.005670791	6.770806662	0.003852302
0.570078492	0.004582724	6.603289777	0.003321415
0.496455735	0.014825335	6.355877687	0.007500083
0.506093372	0.013438663	6.45812532	0.007733766
0.592569579	0.012871663	6.163142669	0.007665465
0.566824532	0.012057571	6.374784261	0.008600759
0.56666888	0.007146389	6.423487529	0.005093228
0.589248207	0.017977913	6.30159764	0.011271288
0.57200528	0.005110041	6.856657917	0.003803215
0.551501923	0.009246142	6.587772985	0.005370757
0.592454667	0.006484232	6.641515039	0.003672264
0.571047937	0.007930419	6.437243545	0.0059867
0.573197821	0.022730792	5.250250882	0.008806869
0.562692694	0.019553146	6.138095087	0.015136998
0.553862474	0.007232498	6.625303966	0.003756631
0.521627608	0.009248986	6.388935626	0.00390317
0.570821958	0.008101339	6.506936012	0.005321059
0.558570473	0.006363061	6.306000667	0.005009323
0.550148634	0.008608652	6.726997676	0.005797101
0.566240875	0.007447235	6.557127598	0.005044386
0.588039732	0.003097642	6.734066261	0.002353188
0.578562746	0.009071528	6.398963715	0.004737018
0.581985117	0.007413728	6.274296499	0.003472369
0.506228326	0.023089813	5.937443092	0.012211995
0.594448919	0.006977901	6.461234662	0.005267358
0.582837738	0.004652109	6.727489551	0.00334255
0.588858005	0.007413217	6.492962684	0.00521577
0.523262417	0.0088106	6.488014117	0.006015074
0.594988404	0.030661179	5.663746838	0.023174546
0.624611631	0.007830616	6.122468291	0.004033196
0.560597853	0.012822062	6.289700881	0.008803507
0.576170377	0.006223686	6.745941288	0.004369221
0.550374597	0.01011956	6.095982584	0.007797654
0.55862359	0.004549685	6.666739818	0.003445826
0.62020415	0.003957806	6.636545905	0.002726859
0.544205585	0.00805627	6.371318414	0.006066404
0.564801227	0.007524013	6.778923807	0.004651217
0.545141632	0.00472234	6.803889244	0.003122693
0.571410516	0.005372841	6.863847739	0.00366536
0.560486629	0.007048981	6.661142323	0.003943437
0.550630633	0.014866966	6.4092071	0.010278948
0.558856562	0.011530078	6.338265197	0.008068763
0.56491423	0.005992124	6.667201745	0.004138059
0.556908708	0.006674533	6.750440557	0.004495881
0.580128108	0.005972276	6.598057138	0.005174126
0.607675298	0.005507253	6.631714482	0.004004426
0.592953124	0.008113837	6.591028909	0.005021474
0.57222774	0.028866266	5.369480189	0.022174642
0.565044002	0.004181617	6.76691058	0.002842206
0.549146297	0.013688419	6.525530652	0.008444336
0.590883928	0.011480169	6.462480054	0.007134119
0.547746603	0.006711347	6.520616008	0.004676292
0.59002258	0.052469104	5.083859419	0.039696061
0.509389713	0.018587889	5.890673772	0.008281937
0.554651769	0.00530483	6.522318838	0.003615244
0.607429285	0.009696086	6.236276597	0.007632869
0.570869157	0.01278886	6.332589413	0.008069594
0.551629948	0.005669834	6.721833572	0.003520012
0.568734523	0.008671902	6.633548989	0.005896729
0.668560252	0.00670869	6.406111326	0.005309837
0.512477156	0.007819551	6.239472163	0.003236069
0.522449889	0.029559618	5.550910397	0.021745041
0.493152579	0.033308354	6.036266883	0.021645974
0.652781689	0.008868162	6.321208296	0.006632117

log.sigma.3.5.mm.3D_ngtdm_Coarseness	log.sigma.3.5.mm.3D_ngtdm_Complexity	log.sigma.3.5.mm.3D_ngtdm_Strength	log.sigma.3.5.mm.3D_ngtdm_Busyness	log.sigma.3.5.mm.3D_ngtdm_Contrast
0.009680242	0.452029393	2.742904757	0.261446212	8.25E-05
0.005450389	0.239445975	1.535792487	0.42297334	3.33E-05
0.009469643	0.453254353	2.21767554	0.258482733	8.65E-05
0.004557358	0.189461284	1.305657607	0.492785127	2.39E-05
0.011579629	0.220314151	1.884694039	0.422775926	8.33E-05
0.006689253	0.186169787	1.351935517	0.479237783	3.71E-05
0.011576846	0.12502571	1.637661701	0.501311886	5.29E-05
0.006462559	0.357830729	2.870449861	0.217934321	2.75E-05
0.005265854	0.269707863	1.599702538	0.382305613	3.40E-05
0.003825945	0.282964782	1.940942864	0.286953592	1.75E-05
0.006261524	0.129426483	1.228333328	0.440237289	2.54E-05
0.006075238	0.290569681	1.462411692	0.38065429	5.10E-05
0.003796	0.142700025	1.387122559	0.455314649	1.07E-05
0.005705913	0.163136654	1.009385671	0.6041857	4.23E-05
0.006546649	0.441384854	2.055429981	0.360331693	6.04E-05
0.003127908	0.167507821	1.404148984	0.388776654	1.23E-05
0.007532861	0.362788227	3.107593631	0.173420748	2.87E-05
0.004184029	0.240037311	1.879776199	0.353993624	1.63E-05
0.00855931	0.434757249	2.292792579	0.241679969	6.02E-05
0.004002748	0.137590187	1.091513407	0.528000218	2.01E-05
0.001615525	0.112774999	0.717247876	0.827920611	7.35E-06
0.005208185	0.359196911	2.84070199	0.226533903	2.07E-05
0.002665485	0.139140802	0.873911354	0.681978415	1.57E-05
0.011702816	0.374797727	3.226806231	0.193029607	6.96E-05
0.004487361	0.166404103	0.959498829	0.595058758	4.04E-05
0.00737268	0.647445599	3.164530315	0.177829993	6.27E-05
0.005240333	0.186499546	1.394421291	0.506085921	2.91E-05
0.002873861	0.225092442	0.979353833	0.556798414	2.42E-05
0.003137766	0.172000203	1.215718209	0.463594223	1.46E-05
0.00365654	0.253063419	1.743905033	0.327769758	1.41E-05
0.002943522	0.183162984	1.490716534	0.364256157	1.10E-05
0.002131515	0.143540112	0.925491945	0.699679145	1.01E-05
0.004602352	0.27437944	2.270296404	0.253286304	1.87E-05
0.020942418	0.662981311	3.830033299	0.178226867	0.000237516
0.011486043	0.61334586	3.157083368	0.165713293	9.66E-05
0.003477715	0.15206837	1.138375318	0.460336189	1.40E-05
0.003092164	0.259458811	1.394962931	0.395679361	2.18E-05
0.003077866	0.177343883	1.384184707	0.374750296	1.25E-05
0.008284423	0.16638539	1.869380544	0.310324982	2.55E-05
0.006667147	0.220052908	1.789401815	0.433080135	3.56E-05
0.007221561	0.282896688	1.408440432	0.387907718	5.75E-05
0.00607169	0.175224204	1.298826575	0.470807456	3.04E-05
0.003908274	0.132337176	1.115562794	0.472584261	1.51E-05
0.005539473	0.223749648	1.646581656	0.498789419	3.12E-05
0.003471321	0.303941044	1.905410998	0.261685471	1.70E-05
0.003039013	0.146570381	0.948583463	0.63902666	1.79E-05
0.00302075	0.235273787	1.59136243	0.389821602	1.39E-05
0.007516238	0.330651734	2.534696635	0.181318602	4.04E-05
0.018732476	0.263497806	2.087759964	0.365718474	0.000168009
0.009760648	0.399321963	3.479882528	0.263847458	4.99E-05
0.003082547	0.112742608	1.170479921	0.504701215	9.02E-06
0.005604748	0.2155466	2.271792925	0.243233039	1.37E-05
0.003170822	0.120416971	1.014013043	0.643056153	1.27E-05
0.008932998	0.459913515	2.55321154	0.183069126	5.98E-05
0.002842592	0.158864384	0.992133631	0.601994297	1.49E-05
0.006350758	0.400238103	2.314792719	0.238365357	3.88E-05
0.003496028	0.330818688	2.309160742	0.216638864	1.52E-05
0.004623748	0.167225354	1.566278449	0.444468293	1.95E-05
0.007949547	0.391256139	2.510703384	0.203331339	5.22E-05
0.006935492	0.092785673	0.728923743	0.71705068	3.59E-05
0.00383932	0.189094091	1.34289632	0.441339734	2.00E-05
0.00208643	0.186738512	1.522129827	0.408311056	6.46E-06
0.004676032	0.277882271	1.740786669	0.3204419	2.60E-05
0.005227859	0.258769926	1.281971526	0.428040794	4.61E-05
0.016443333	0.482412205	2.150401697	0.303734827	0.000199054
0.007065969	0.373261317	2.286593647	0.337533425	5.82E-05
0.011245164	0.413414883	3.379107213	0.248120709	7.11E-05
0.003229463	0.185096495	1.241110828	0.476660737	1.74E-05
0.008517381	0.276109507	2.054282534	0.249120784	4.43E-05
0.004578039	0.405603103	2.7748283	0.198753637	1.90E-05
0.00298608	0.25662302	1.360153108	0.396092237	2.34E-05
0.005255875	0.2119802	1.753498012	0.342391942	2.19E-05
0.002429531	0.125968044	0.906694461	0.685556953	1.24E-05
0.001704149	0.109087239	0.778171218	0.695987901	6.51E-06
0.002788192	0.248892013	1.694564188	0.36805198	1.28E-05
0.004865261	0.408681612	2.770471023	0.222894968	2.26E-05
0.004692266	0.196982239	1.547366983	0.353994903	1.62E-05
0.007150847	0.355173529	1.934300267	0.299162901	5.05E-05
0.003212122	0.194639209	1.226493385	0.423981767	1.76E-05
0.002716603	0.134602326	1.074633493	0.484328664	1.12E-05
0.008922542	0.824636418	6.480164964	0.078505204	3.51E-05
0.004092618	0.388910446	2.206710936	0.242239251	2.63E-05
0.004470523	0.259546213	1.577095019	0.350408432	2.77E-05
0.018190175	0.250623324	1.904331947	0.429207983	0.000142065
0.002920113	0.166994639	1.137013715	0.457867871	1.77E-05
0.003207283	0.101193639	0.72466469	1.038010546	1.82E-05
0.008429998	0.397204027	2.621048234	0.21728625	6.10E-05
0.003600236	0.149944829	1.156025464	0.492187219	1.57E-05
0.018884329	0.205043741	1.064469574	0.80298395	0.000336524
0.017936324	0.523850652	4.140759344	0.141623929	0.0001191
0.003284912	0.158790157	0.992587832	0.568459028	2.01E-05
0.008831558	0.561530827	2.694074216	0.202885505	7.79E-05
0.006713763	0.237502301	1.561622162	0.312673247	4.43E-05
0.002450051	0.147317163	1.072051717	0.516845229	1.09E-05
0.002452137	0.0909507	0.722365731	0.832979039	9.54E-06
0.011017785	2.138470398	6.48242306	0.080445648	0.000207871
0.005843558	0.145598149	1.491498159	0.352718327	2.35E-05
0.029788166	0.355069363	3.474672782	0.250158822	0.000205323
0.012468045	0.164620424	1.690673232	0.446847152	5.90E-05
0.009085102	0.882174729	3.523984851	0.157958207	0.000131238

log.sigma.3.0.mm.3D_gldm_GrayLevelVariance	log.sigma.3.0.mm.3D_gldm_HighGrayLevelEmphasis	log.sigma.3.0.mm.3D_gldm_GrayLevelNonUniformityNormalized	log.sigma.3.0.mm.3D_gldm_DependenceEntropy
21.90286268	254.6492103	0.0721354	6.679728248
16.48519975	250.5905	0.0808705	6.72014166
19.57443493	226.234347	0.06808734	6.541847014
15.24496889	274.1492051	0.085769831	6.684820729
12.82265731	108.0958904	0.111322328	6.437396401
12.36988622	211.4072039	0.094306318	6.544964171
8.788296969	100.0567644	0.141158327	6.44473518
16.72214117	365.7390582	0.085546612	6.701437636
15.82169961	340.3369512	0.076944233	6.798235901
18.30049027	317.2460872	0.070485292	6.699984956
9.50381977	292.160487	0.102888631	6.483092786
17.98015867	273.4676558	0.072829381	6.627867796
10.56234957	253.045408	0.106383802	6.60639699
13.54319899	168.0355221	0.079806753	6.560940896
19.18443236	146.6059432	0.067326182	6.788569811
18.16358052	376.8829399	0.081767752	7.111017523
15.03344649	270.117134	0.092145457	6.681759132
13.72774921	310.5152463	0.086773103	6.72646893
14.917424	258.8380282	0.078092454	6.421990254
14.06331019	293.9593244	0.083658228	6.725958603
18.13697016	299.0876259	0.071774504	6.84934573
16.07578289	332.1810467	0.077602769	6.679135099
17.22352289	289.6457171	0.072191775	6.777909428
17.83903195	297.0533981	0.084550853	6.710421739
19.9073756	248.4581413	0.063472302	6.789941285
22.91098821	509.681213	0.065237562	6.708902031
15.13258345	203.3595658	0.0789521	6.513004359
20.4526415	361.1219076	0.067047914	6.575188927
14.50022518	288.3009709	0.092973627	6.790584682
16.3877598	363.9582146	0.082618878	6.736777774
17.87834559	451.2759536	0.075408932	6.790208673
15.86733244	299.0018	0.07559168	6.826101786
17.05594742	330.7838799	0.087414987	6.730501212
17.36119744	159.7193347	0.090866654	6.451289502
19.75068861	349.4921171	0.073763798	6.527283845
13.29621515	318.605844	0.095106825	6.704249084
24.23270259	392.1005884	0.064798594	6.891036275
14.24863376	484.2981421	0.088762115	6.628854706
9.373164155	176.3295148	0.107259102	6.619105361
13.90629838	224.5464548	0.095456896	6.645002857
13.29409085	272.8320669	0.085005913	6.404088123
13.25074344	166.3721532	0.086763092	6.70995721
12.44996573	340.9491245	0.0959737	6.725869873
13.71807145	165.6066879	0.102137905	6.582641571
22.06124228	507.1196337	0.071518465	6.994504165
17.0208471	266.5194406	0.071638515	6.725246921
19.74649692	376.0607705	0.072941596	6.829209679
17.32003571	383.1992084	0.089596633	6.621532935
11.66540918	133.4468085	0.11611589	6.138868604
14.10690683	190.2899083	0.103481188	6.476046098
12.01032538	269.4339859	0.099339124	6.768638178
9.618693047	336.8660532	0.130070655	6.368665002
13.62074407	293.4448249	0.091524441	6.720506254
15.79242161	337.9504697	0.07681436	6.493257871
16.44555263	320.9245618	0.07572241	6.750763875
21.83455612	311.3029661	0.065462511	6.82212401
21.71424094	709.6764257	0.073033911	6.878502389
13.00557516	227.7750822	0.116236637	6.691346782
18.1633434	440.298105	0.076798358	6.60423915
6.342638011	104.6135231	0.116114538	6.195860998
16.8011979	355.0309419	0.076895365	6.846488646
17.29442778	448.3921997	0.08828957	6.833259367
17.4717291	311.9167768	0.075887053	6.714436399
15.25493491	281.9860814	0.074000179	6.526180419
13.76066935	135.1711864	0.07638035	6.337345549
21.54481028	275.8470512	0.072271278	6.598423567
18.86688602	223.5623819	0.080454615	6.648917755
18.24529197	377.3084689	0.076131716	6.871600858
12.42175699	295.0672854	0.089398624	6.420124971
20.46840303	532.4515357	0.075251124	6.836429853
25.36071975	508.9217437	0.056546148	6.97997796
12.60354811	253.6438622	0.101693826	6.5789046
17.00646365	296.6943791	0.077910191	6.862264585
14.64807864	408.5526033	0.080595128	6.804013589
23.75946944	478.984045	0.066849425	6.968423837
20.38896922	410.4641785	0.075180347	6.793715003
11.50724961	274.0573248	0.102119807	6.555734026
15.16192543	263.7520235	0.076442933	6.551606927
16.38832535	357.2167277	0.081207017	6.726785092
16.98180175	301.9189311	0.086383224	6.827529887
21.9192666	575.0323054	0.08324223	6.700223081
25.30393792	470.5822356	0.069398493	6.803043785
19.23278295	385.0254648	0.073997382	6.802485146
8.402772649	55.75717017	0.131704499	6.151801439
19.55887197	471.8094252	0.068472477	6.788395435
12.62192216	183.4153713	0.084173442	6.725762784
22.04107525	322.5826331	0.071843443	6.731919785
13.03292462	380.4423267	0.089729217	6.60483545
8.157931814	65.41605839	0.108198507	5.996022682
13.62590247	299.1072664	0.110547048	6.425110166
14.94968625	260.8464653	0.080658873	6.762287818
20.66066297	402.6597285	0.070168096	6.612161038
14.96305993	294.5505762	0.084999697	6.486261084
16.44483666	319.7070986	0.086271399	6.733657705
12.51912172	259.5418615	0.086236359	6.81173015
52.25736958	587.3631222	0.045942446	6.80147295
8.828994176	352.6653432	0.123011007	6.462336237
12.0024893	131.9766355	0.097464844	6.192259324
7.904217139	114.5191441	0.125065944	6.488916291
36.33562111	413.1126516	0.050953807	6.690821481

log.sigma.3.0.mm.3D_gldm_DependenceNonUniformity	log.sigma.3.0.mm.3D_gldm_GrayLevelNonUniformity	log.sigma.3.0.mm.3D_gldm_SmallDependenceEmphasis
184.7672485	86.77888612	0.253709658
280.072	161.741	0.240939958
204.7406082	76.1216458	0.313022641
350.0917244	210.3933958	0.235727064
112.5869336	105.6448894	0.178045149
228.7155067	154.4737485	0.215150309
96.85430464	149.2043519	0.124365579
237.998338	154.4116343	0.21642557
281.2476566	155.9659595	0.243229716
516.7435897	211.6673327	0.258216128
233.6939679	185.9197565	0.162076859
289.5649852	122.7175074	0.296068587
340.6155685	311.5981564	0.181314096
316.9160388	148.2809473	0.231629116
242.879845	104.2209302	0.278181458
510.3550742	335.9836943	0.226917765
208.5588785	147.8934579	0.215168419
323.9460516	221.9655981	0.190910862
185.709507	88.71302817	0.252647887
408.202344	242.6925198	0.206539009
1110.774676	498.8328058	0.256148246
333.0358322	164.5954738	0.238766517
668.271536	297.5023053	0.265211398
131.7300971	87.08737864	0.211863412
463.2304147	165.281874	0.277440891
237.2529586	88.20118343	0.297712767
376.0194482	174.5630936	0.298565913
660.3758569	224.9457526	0.304020159
414.4663136	316.017358	0.209299899
444.4080405	260.9930358	0.252336128
632.064703	312.3437953	0.248797342
764.8152	377.9584	0.24895475
336.9107072	221.2473331	0.220095029
64.15176715	43.70686071	0.24366708
147.8783784	65.50225225	0.28657663
394.8035437	305.9586571	0.204133341
587.8517792	231.266181	0.280637224
493.3692126	300.9923326	0.22855263
171.4568733	159.1725067	0.151836345
212.0232274	156.1674817	0.191702112
212.0987842	111.8677812	0.272827736
251.0559006	167.626294	0.215637561
352.563264	290.5123885	0.195626739
224.6135881	192.4278132	0.211422124
507.30166	249.885518	0.242277125
596.313007	256.1076923	0.247392817
591.0911557	268.8627238	0.265292037
216.2823219	135.828496	0.243150384
62.96453901	65.4893617	0.182321388
131.6752294	112.7944954	0.227312121
443.9578313	395.7670683	0.18089003
212.2699387	254.4182004	0.160513504
467.7116144	347.5183039	0.197912868
195.6592656	89.94961571	0.265848986
584.8806198	298.1191262	0.245613196
314.3951271	123.5932203	0.280915119
488.3821287	239.4781946	0.267018776
239.984375	282.6875	0.160986746
221.0379009	105.3673469	0.258178779
196.2498221	163.1409253	0.167440796
395.8894934	226.149269	0.231024301
690.3289594	484.4448697	0.22190672
356.3104359	172.339498	0.248531838
323.1006424	138.232334	0.261562004
100.2271186	45.06440678	0.271202322
257.5029164	111.514582	0.262746087
148.6124764	85.12098299	0.244841298
494.8547392	271.4856983	0.231095673
194.9427688	115.5924207	0.194352393
369.4156362	188.6545672	0.274189618
676.397584	215.3277311	0.29546272
247.3474042	209.5909753	0.178574103
655.7869203	364.5417824	0.235539764
944.2293043	524.7548764	0.222736283
665.4720788	284.9122478	0.258563244
348.8249678	175.2453882	0.251151578
289.8203822	240.4921444	0.202849287
228.9190581	103.8859455	0.239815214
467.9871795	266.034188	0.227540722
572.9447563	384.6644958	0.22232355
195.9133627	113.3759178	0.253872718
444.1554385	184.3917953	0.305376682
379.5424414	183.069523	0.272621796
55.27533461	68.88145315	0.15341181
655.6271362	264.4407043	0.263740273
485.2907311	292.4185377	0.20383571
235.1680672	102.592437	0.271961894
416.4748603	273.0460072	0.189781534
53.99756691	44.46958637	0.21796435
68.2283737	63.89619377	0.199316897
443.7474307	258.99564	0.238006392
195.2497738	77.53574661	0.291152802
240.7362356	132.7695262	0.267138834
611.7769354	375.5393981	0.234887296
621.7161071	405.8283043	0.204944974
201.6923077	40.61312217	0.404037909
201.4117981	216.8684061	0.140396467
52.90186916	41.71495327	0.183381809
92.61936937	111.0585586	0.168333547
256.0745234	58.80069324	0.381044888

log.sigma.3.0.mm.3D_gldm_DependenceNonUniformityNormalized	log.sigma.3.0.mm.3D_gldm_DependenceVariance	log.sigma.3.0.mm.3D_gldm_LargeDependenceEmphasis
0.153588735	4.539177133	17.08478803
0.140036	6.621116	21.66
0.183131134	3.839244626	13.27370304
0.142719822	5.938793218	20.48308194
0.118637443	8.093641912	28.95995785
0.139630956	5.855237559	21.33211233
0.091631319	14.50403446	49.03216651
0.131855035	7.33515059	24.36620499
0.138750694	6.838809763	22.03996053
0.172075787	3.922630372	14.93406593
0.129327044	5.524950165	24.39236303
0.171848656	3.760788243	13.95905045
0.116290737	7.112136193	27.51143735
0.170568374	4.245621008	16.25941873
0.156899125	4.386573657	16.06459948
0.124204204	15.28775736	36.47821854
0.129943226	5.849930028	22.33707165
0.126640364	6.425705799	24.64972635
0.163476679	4.293291882	16.0915493
0.140710908	5.955194283	21.6997587
0.159823694	4.409719994	16.42417266
0.157018308	4.487487221	17.12164074
0.162162469	4.056426206	15.52026207
0.127893298	8.433269865	26.58252427
0.177891864	3.801449855	14.15284178
0.175482958	3.68204676	13.65680473
0.170067593	3.834815479	14.10900045
0.19683334	2.59409072	11.06557377
0.121937721	8.494377446	27.46131215
0.140679975	5.942807904	20.25989237
0.152598914	4.427584162	17.12264606
0.15296304	5.2631	18.2952
0.133113673	6.347708954	22.50296326
0.133371657	9.282221291	26.34719335
0.166529705	4.203488962	15.06081081
0.122724135	7.42622969	25.97482126
0.164710501	4.120361812	15.1686747
0.145493722	5.029883169	19.13388381
0.115536977	7.870523681	30.77358491
0.12959855	9.47740628	28.65281174
0.161169289	4.279060153	15.77507599
0.129946118	6.030386131	22.59006211
0.116472832	8.478772645	28.92996366
0.11922165	8.27473055	27.53397028
0.145192232	8.159506083	23.10589582
0.166800841	3.548034153	14.92615385
0.160361138	5.039551334	17.06836679
0.142666439	6.060579848	20.50659631
0.111639254	8.196946834	30.17375887
0.120802963	7.354613248	25.61651376
0.111435199	8.376105829	30.40763052
0.108522464	8.64772751	32.48568507
0.123179251	7.309925382	25.99499605
0.167087332	3.570969654	14.5439795
0.148559975	5.3725341	18.92024384
0.166522843	4.499837286	15.65572034
0.1489424	5.348349924	18.305276
0.098677786	11.65008156	39.22121711
0.161106342	4.857393603	16.9548105
0.139679589	4.455994225	21.43274021
0.134610504	6.65154152	22.51853111
0.125811729	7.989160788	25.75123018
0.156895833	4.560341844	16.97269925
0.172966083	3.126636373	13.62312634
0.169876472	3.904843436	14.73898305
0.166884586	4.137641656	15.47310434
0.140465479	5.848478243	20.40075614
0.138770258	6.012190285	21.09254066
0.150767803	4.405321294	18.94895592
0.147353664	5.704119729	18.88272836
0.177625416	3.758632865	13.68907563
0.120013297	6.871447065	26.67200388
0.140155358	6.225043839	21.1709767
0.145020627	5.419442036	19.93564737
0.156140797	4.72951961	17.0370718
0.149646061	5.211401142	18.43028743
0.12306598	7.180922553	25.62165605
0.168446695	3.74951391	15.53274467
0.142853229	4.783412795	18.995116
0.12866489	7.246196961	24.24769818
0.14384241	6.859675566	21.2907489
0.16716426	4.37708532	14.98795634
0.153412466	5.809585085	18.45028294
0.105688976	10.85019139	37.04971319
0.169763629	4.074161364	15.16571724
0.139692208	5.205953992	20.81232009
0.16468352	5.210652889	17.00420168
0.136863247	5.741511552	22.48340453
0.131380941	6.690192457	23.41849148
0.118042169	7.218591731	26.71280277
0.138196023	6.687909757	21.92245406
0.176696628	3.405635429	13.3638009
0.154120509	4.106542877	16.10243278
0.140541451	5.674698021	20.30668504
0.13211137	6.069835164	22.86485338
0.228158719	2.522097213	8.977375566
0.114243788	8.584923149	32.61883154
0.123602498	6.591732902	26.00934579
0.104301092	11.64241945	37.27702703
0.221901667	2.057919256	8.592720971

log.sigma.3.0.mm.3D_gldm_LargeDependenceLowGrayLevelEmphasis	log.sigma.3.0.mm.3D_gldm_SmallDependenceHighGrayLevelEmphasis	log.sigma.3.0.mm.3D_gldm_LargeDependenceHighGrayLevelEmphasis
0.09945739	70.73366763	3704.990025
0.116735941	64.16846666	4611.8385
0.083155994	67.32616903	3120.443649
0.09542538	67.9049788	5073.248267
0.432896944	24.34127214	2425.185458
0.117047655	44.69097623	4443.473138
0.720129191	15.09555635	3755.680227
0.081620641	80.16071737	8005.712465
0.082928764	81.70821604	6668.959546
0.060899491	79.98407884	4648.331668
0.09363118	45.85296113	6891.643608
0.063851055	76.94454	3876.443323
0.12631436	48.50229935	6433.105497
0.15095211	37.73866258	2738.438105
0.182436004	40.30703802	2311.360465
0.119904168	85.05274025	12060.86055
0.100116278	54.09978736	6117.825545
0.102282478	65.72544547	6636.929633
0.080759391	68.58186833	3777.915493
0.094659036	61.30173063	5896.612203
0.068072048	73.47503184	4919.95554
0.058955327	79.23425888	5660.859972
0.065790294	75.90950431	4470.859015
0.120747111	64.69048666	6885.160194
0.083685149	68.66774794	3442.528034
0.033133361	148.768947	6532.372041
0.085852748	58.71994154	2793.954772
0.036376021	103.9317384	4194.511475
0.119628452	60.08558756	7261.637246
0.064418041	91.13620916	6967.668883
0.041466792	105.0225051	7964.691212
0.077560022	76.28226895	5020.3254
0.101038812	74.39214122	6875.000395
0.27730343	44.08428107	3089.120582
0.058711053	96.41058222	5356.908784
0.095057054	65.19732615	7835.479329
0.049319247	108.1984886	5757.355282
0.041600851	103.2419849	9401.837511
0.214455288	29.91329814	4926.419811
0.167804401	48.49046052	5420.165037
0.066285847	70.1030634	4236.988602
0.191754615	37.94889248	3267.179607
0.099863379	68.03168327	8945.091179
0.246556479	40.99151462	3555.351911
0.052448016	114.602839	11597.06411
0.070619528	63.40643709	4132.038322
0.05622053	101.6669773	6159.380358
0.059010308	87.2497374	8203.961741
0.349676896	26.85075882	3143.315603
0.186471811	50.62481213	3993.897248
0.142731681	53.50773053	7245.634287
0.110794414	53.87482748	10449.85072
0.111632798	64.75629331	6570.816961
0.049917557	83.84048712	4831.47737
0.07423458	81.56782835	5456.495809
0.072202482	88.36413696	4627.458686
0.028865712	189.9514158	12456.7237
0.223863896	43.32476194	7355.418997
0.044534327	105.2488901	7619.478863
0.259761641	16.11580019	2314.700356
0.081049697	83.33833557	7015.122747
0.068846635	98.44607908	10875.10151
0.069999381	78.34170797	5119.011449
0.058633916	69.98005859	3869.110278
0.172526166	35.44334261	1759.147458
0.084316613	83.67012506	3416.723266
0.126462662	65.23449329	3820.681474
0.068938815	87.82737104	7206.162086
0.070591278	51.3034006	5643.183295
0.03955097	144.1872596	9783.330674
0.033730714	147.9216827	6799.380777
0.12975498	51.47488868	5972.202814
0.095526573	72.87959721	5617.242359
0.054405893	88.74084926	8059.604669
0.046541306	125.3412884	7731.24824
0.055617296	98.06428112	7374.995281
0.104221113	52.91862972	6826.723142
0.069321848	62.10148597	4341.204562
0.059770582	77.53016257	6913.521978
0.095100932	61.83566853	7345.619807
0.046796246	140.0783201	11836.68576
0.035758462	131.9558985	7298.182537
0.056294081	107.0516182	6857.166128
1.346453439	10.63252149	1317.787763
0.036175474	118.5031465	7201.015536
0.150367408	38.44357046	3460.647381
0.07032424	91.15634032	5063.928571
0.069186794	73.17196561	7890.618469
0.717527259	14.73806274	1168.199513
0.101498287	56.01463653	7697.750865
0.108809768	63.84438871	5003.043289
0.039955911	113.101766	5274.523982
0.060299865	69.37672817	5038.941101
0.080914742	72.76444479	6348.802435
0.107095577	54.92212535	5562.152996
0.032681826	223.0734491	5641.012443
0.103439743	50.75978328	11198.46228
0.303053772	27.7210995	2749.107477
0.396234877	20.54707497	3878.978604
0.038225835	141.9344199	3948.94974

log.sigma.3.0.mm.3D_gldm_SmallDependenceLowGrayLevelEmphasis	log.sigma.3.0.mm.3D_gldm_LowGrayLevelEmphasis	log.sigma.3.0.mm.3D_gldzm_DistanceZoneVariabilityNormalized
0.002961706	0.007487533	1
0.002698828	0.006766535	1
0.004808083	0.010264671	1
0.001913271	0.006079965	1
0.003688331	0.018052053	1
0.002525526	0.007445822	1
0.00333128	0.017082256	1
0.001502721	0.004217996	1
0.001508345	0.004294964	1
0.001608051	0.005264361	0.9978836
0.001411292	0.004639433	1
0.003109601	0.006701342	0.996575353
0.001579203	0.00535388	1
0.00399929	0.013109886	1
0.010281718	0.020583794	1
0.001283116	0.003894305	0.994555394
0.002543517	0.007738408	1
0.001285813	0.004437939	1
0.002482091	0.00648237	1
0.001292383	0.005190059	0.99705883
0.001885246	0.00542976	0.997177804
0.001696789	0.004540123	0.996715937
0.002128036	0.005540643	0.998474448
0.002400876	0.006166901	1
0.002618906	0.007105003	1
0.001843033	0.003494739	1
0.004001968	0.008548105	1
0.001884683	0.004414691	1
0.001887501	0.006089298	1
0.001742897	0.004156171	1
0.001170119	0.00313405	1
0.001769305	0.004912005	1
0.001601614	0.005017431	1
0.005777035	0.01289698	1
0.002542315	0.006843676	1
0.001320318	0.004773285	1
0.001931773	0.004458495	1
0.00103077	0.002791387	1
0.002250805	0.008425059	1
0.001938553	0.006818219	1
0.002707889	0.0058945	1
0.003844185	0.01088673	1
0.001155908	0.0038588	1
0.003131731	0.010619449	1
0.001126922	0.003146696	1
0.002684224	0.006734722	1
0.002377228	0.005034829	0.991274164
0.001957679	0.004463152	1
0.004049311	0.01380404	1
0.003188212	0.008619617	1
0.001220698	0.005438892	1
0.0010644	0.004867356	1
0.001179975	0.004471511	1
0.002104283	0.004617478	1
0.001529442	0.004530692	1
0.002478105	0.006084806	1
0.000823442	0.001993372	0.994082892
0.001539258	0.006326158	1
0.0019428	0.00419243	1
0.004430572	0.016839306	1
0.001402265	0.004052467	1
0.001110242	0.003333957	0.998546512
0.002237521	0.006228193	1
0.002181723	0.005614281	1
0.006402926	0.014909536	1
0.002074755	0.005846366	1
0.002716639	0.007466382	1
0.00158567	0.004075564	1
0.002031588	0.005169965	1
0.001140318	0.002745831	1
0.001116072	0.002894723	1
0.001985533	0.00593083	1
0.001516654	0.00523313	1
0.000978597	0.003206988	0.997571345
0.001185996	0.003677851	1
0.001629552	0.004542619	1
0.002317082	0.005805305	1
0.003167641	0.006985494	1
0.001541593	0.004346035	1
0.001999454	0.005606737	0.994709032
0.001426569	0.00405565	1
0.00196219	0.003831756	1
0.002108605	0.004544011	0.994858646
0.010328254	0.037893664	1
0.00113175	0.002975882	1
0.002077068	0.007866638	0.997506238
0.003048226	0.006273945	1
0.001021454	0.003456874	1
0.010980279	0.032938148	1
0.003264692	0.006449179	1
0.001773183	0.005405232	1
0.002298703	0.004484338	1
0.003005543	0.006053157	1
0.001851305	0.005678537	1
0.001403323	0.005163473	1
0.002673468	0.00652964	1
0.000776565	0.004320821	1
0.004530453	0.013388659	1
0.003752257	0.013479205	1
0.003570066	0.00681935	1

log.sigma.3.0.mm.3D_gldzm_LowIntensityEmphasis	log.sigma.3.0.mm.3D_gldzm_LargeDistanceEmphasis	log.sigma.3.0.mm.3D_gldzm_HighIntensitySmallDistanceEmphasis
0.011003964	1	283.5179063
0.010587876	1	270.7692308
0.015014834	1	214.1745283
0.00870138	1	291.9379728
0.023789025	1	140.8643216
0.011689164	1	207.559322
0.029009138	1	126.8543046
0.006822852	1	377.529148
0.006060544	1	341.4366197
0.00664502	1.003177966	311.3061441
0.00833452	1	281.5267857
0.010123558	1.005145798	262.130789
0.008349315	1	271.817734
0.017898547	1	163.2109228
0.035641232	1	147.1077844
0.005501449	1.008189263	379.5507279
0.013477404	1	251.5898734
0.006565053	1	347.6085343
0.009409981	1	276.9769452
0.006974586	1.004418262	300.3210604
0.007344031	1.004239284	287.5715968
0.006770957	1.004934211	332.7927632
0.007742103	1.002290076	286.9755725
0.010632354	1	314.8659004
0.009208739	1	246.3447099
0.00570748	1	506.4714588
0.012773401	1	200.5436767
0.006011274	1	342.0595432
0.009810955	1	291.5657568
0.006555889	1	364.2322792
0.004576337	1	424.507113
0.006786591	1	309.8079835
0.007268424	1	344.2727273
0.021125071	1	188.0141844
0.009822541	1	338.267101
0.007214222	1	317.1411126
0.006580699	1	389.7972973
0.004362665	1	453.5101351
0.014744609	1	198.1936759
0.010040898	1	259.5718232
0.009478428	1	260.9393204
0.017601021	1	183.7637131
0.00584959	1	349.7390625
0.015906014	1	201.8127854
0.004699811	1	474.7833501
0.010349837	1	255.5226179
0.00849013	1.013146363	382.4456617
0.007720577	1	352.9907407
0.022298149	1	155.4615385
0.013178228	1	231.4587814
0.007716978	1	299.5664516
0.008624807	1	337.1038576
0.005853191	1	335.0072993
0.00737144	1	320.3497268
0.006155679	1	336.6444043
0.008581765	1	317.6872038
0.002925022	1.008902077	717.5717112
0.009339253	1	277.220524
0.007162472	1	410.1882353
0.028692442	1	98.14552239
0.005817531	1	370.0205656
0.005066137	1.002181818	444.9934545
0.009517485	1	317.1437216
0.00805247	1	269.8353345
0.022566303	1	132.8465608
0.007393171	1	323.4569672
0.010489283	1	271.2565789
0.006627714	1	390.5703381
0.0100927	1	260.0638298
0.004008059	1	527.0894207
0.003655754	1	507.0447094
0.010540957	1	295.0479616
0.006532167	1	315.1207165
0.004414233	1.003647416	392.6244681
0.004704767	1	490.4037412
0.007035128	1	390.9925595
0.011239132	1	262.3598485
0.012217402	1	261.997416
0.006863325	1	338.8300117
0.009087206	1.00795756	277.617374
0.006534051	1	556.340796
0.006153967	1	437.5245203
0.00735531	1.007731959	395.8340851
0.059538886	1	76.83505155
0.004090192	1	454.9300412
0.010134952	1.003745318	193.4110487
0.010265974	1	340.2887931
0.005193253	1	392.1610429
0.048413272	1	70.03846154
0.015429491	1	282.0461538
0.007148412	1	277.8226545
0.007244658	1	394.6718346
0.010519526	1	263.5541922
0.008537026	1	312.2825342
0.006757211	1	274.1808015
0.007692425	1	554.2075472
0.00714664	1	364.1267606
0.022876815	1	158.6741573
0.022202044	1	124.2831325
0.009150122	1	377.3137996

log.sigma.3.0.mm.3D_gldzm_LowIntensityLargeDistanceEmphasis	log.sigma.3.0.mm.3D_gldzm_HighIntensityEmphasis	log.sigma.3.0.mm.3D_gldzm_DistanceZoneVariability	log.sigma.3.0.mm.3D_gldzm_ZonePercentage
0.011003964	283.5179063	363	0.301745636
0.010587876	270.7692308	559	0.2795
0.015014834	214.1745283	424	0.379248658
0.00870138	291.9379728	661	0.26946596
0.023789025	140.8643216	199	0.209694415
0.011689164	207.559322	413	0.252136752
0.029009138	126.8543046	151	0.142857143
0.006822852	377.529148	446	0.247091413
0.006060544	341.4366197	568	0.28021707
0.006661234	311.4618644	942.0021186	0.314352314
0.00833452	281.5267857	336	0.185943553
0.010146428	262.4202401	581.0034305	0.345994065
0.008349315	271.817734	609	0.207920792
0.017898547	163.2109228	531	0.285791173
0.035641232	147.1077844	501	0.323643411
0.005606636	379.9008189	1093.016379	0.26746167
0.013477404	251.5898734	395	0.246105919
0.006565053	347.6085343	539	0.210711493
0.009409981	276.9769452	347	0.305457746
0.006997128	300.5375552	677.0029455	0.234057222
0.007356436	287.9778615	2117.008479	0.305467626
0.006779523	333.5032895	606.0032895	0.286657237
0.007765004	287.0328244	1308.001527	0.317884009
0.010632354	314.8659004	261	0.253398058
0.009208739	246.3447099	879	0.337557604
0.00570748	506.4714588	473	0.349852071
0.012773401	200.5436767	767	0.346901854
0.006011274	342.0595432	1226	0.365424739
0.009810955	291.5657568	806	0.237128567
0.006555889	364.2322792	917	0.290281735
0.004576337	424.507113	1195	0.288507967
0.006786591	309.8079835	1453	0.2906
0.007268424	344.2727273	638	0.252074279
0.021125071	188.0141844	141	0.293139293
0.009822541	338.267101	307	0.345720721
0.007214222	317.1411126	737	0.229095431
0.006580699	389.7972973	1184	0.331745587
0.004362665	453.5101351	888	0.261869655
0.014744609	198.1936759	253	0.170485175
0.010040898	259.5718232	362	0.221271394
0.009478428	260.9393204	412	0.313069909
0.017601021	183.7637131	474	0.245341615
0.00584959	349.7390625	640	0.211430459
0.015906014	201.8127854	438	0.232484076
0.004699811	474.7833501	997	0.285346308
0.010349837	255.5226179	1039	0.290629371
0.008691503	383.283085	1131.043821	0.309549647
0.007720577	352.9907407	432	0.284960422
0.022298149	155.4615385	117	0.207446809
0.013178228	231.4587814	279	0.255963303
0.007716978	299.5664516	775	0.194528112
0.008624807	337.1038576	337	0.172290389
0.005853191	335.0072993	822	0.2164867
0.00737144	320.3497268	366	0.312553373
0.006155679	336.6444043	1108	0.281432563
0.008581765	317.6872038	633	0.335275424
0.002943723	718.6547972	1005.017804	0.308325709
0.009339253	277.220524	458	0.188322368
0.007162472	410.1882353	425	0.309766764
0.028692442	98.14552239	268	0.190747331
0.005817531	370.0205656	778	0.264535872
0.005077269	445.1003636	1373.001455	0.250592309
0.009517485	317.1437216	661	0.291061207
0.00805247	269.8353345	583	0.312098501
0.022566303	132.8465608	189	0.320338983
0.007393171	323.4569672	488	0.316267012
0.010489283	271.2565789	304	0.287334594
0.006627714	390.5703381	917	0.257150869
0.0100927	260.0638298	282	0.218097448
0.004008059	527.0894207	794	0.316713203
0.003655754	507.0447094	1342	0.352415966
0.010540957	295.0479616	417	0.202328967
0.006532167	315.1207165	1284	0.274417611
0.004426172	392.9039514	1641.004863	0.252649363
0.004704767	490.4037412	1283	0.301032379
0.007035128	390.9925595	672	0.288288288
0.011239132	262.3598485	528	0.224203822
0.012217402	261.997416	387	0.284768212
0.006863325	338.8300117	853	0.26037851
0.00912289	278.066313	1125.015915	0.253986077
0.006534051	556.340796	402	0.295154185
0.006153967	437.5245203	938	0.353029733
0.007402736	396.3569588	772.0103093	0.313662086
0.059538886	76.83505155	97	0.185468451
0.004090192	454.9300412	1215	0.314603832
0.010165905	193.5243446	799.0024969	0.230569948
0.010265974	340.2887931	464	0.324929972
0.005193253	392.1610429	652	0.214262241
0.048413272	70.03846154	104	0.253041363
0.015429491	282.0461538	130	0.224913495
0.007148412	277.8226545	874	0.272189349
0.007244658	394.6718346	387	0.350226244
0.010519526	263.5541922	489	0.313060179
0.008537026	312.2825342	1168	0.268320698
0.006757211	274.1808015	1073	0.2280068
0.007692425	554.2075472	424	0.479638009
0.00714664	364.1267606	284	0.161089053
0.022876815	158.6741573	89	0.207943925
0.022202044	124.2831325	166	0.186936937
0.009150122	377.3137996	529	0.458405546

log.sigma.3.0.mm.3D_gldzm_IntensityVariabilityNormalized	log.sigma.3.0.mm.3D_gldzm_LowIntensitySmallDistanceEmphasis	log.sigma.3.0.mm.3D_gldzm_IntensityVariability
0.048046202	0.011003964	17.44077135
0.055385767	0.010587876	30.96064401
0.056003026	0.015014834	23.74528302
0.053471909	0.00870138	35.34493192
0.061387339	0.023789025	12.2160804
0.062115625	0.011689164	25.65375303
0.072321389	0.029009138	10.9205298
0.054063022	0.006822852	24.11210762
0.060200109	0.006060544	34.19366197
0.054229657	0.006640967	51.19279661
0.071446287	0.00833452	24.00595238
0.054273601	0.01011784	31.64150943
0.058991968	0.008349315	35.92610837
0.064069144	0.017898547	34.02071563
0.055812527	0.035641232	27.96207585
0.053175151	0.005475153	58.43949045
0.055189873	0.013477404	21.8
0.059558517	0.006565053	32.10204082
0.061083474	0.009409981	21.19596542
0.058534924	0.006968951	39.74521355
0.054226759	0.00734093	115.1234103
0.055342149	0.006768815	33.64802632
0.05580444	0.007736378	73.10381679
0.0528031	0.010632354	13.7816092
0.055400891	0.009208739	48.69738339
0.049483974	0.00570748	23.40591966
0.06089354	0.012773401	46.7053455
0.052729203	0.006011274	64.64600326
0.054802997	0.009810955	44.17121588
0.052369576	0.006555889	48.02290076
0.05301938	0.004576337	63.358159
0.056717739	0.006786591	82.41087405
0.052819843	0.007268424	33.69905956
0.055781902	0.021125071	7.865248227
0.052764486	0.009822541	16.19869707
0.054839286	0.007214222	40.4165536
0.046160233	0.006580699	54.65371622
0.057069739	0.004362665	50.67792793
0.065443922	0.014744609	16.55731225
0.057858429	0.010040898	20.94475138
0.059206806	0.009478428	24.39320388
0.05876017	0.017601021	27.85232068
0.05809082	0.00584959	37.178125
0.054544317	0.015906014	23.89041096
0.048141415	0.004699811	47.99699097
0.057237423	0.010349837	59.46968239
0.050430877	0.008439787	57.54163015
0.054526749	0.007720577	23.55555556
0.080429542	0.022298149	9.41025641
0.058696574	0.013178228	16.37634409
0.058783767	0.007716978	45.55741935
0.065220263	0.008624807	21.97922849
0.056538263	0.005853191	46.47445255
0.059108961	0.00737144	21.63387978
0.054037587	0.006155679	59.87364621
0.049182783	0.008581765	31.13270142
0.04829076	0.002920347	48.82195846
0.056492058	0.009339253	25.87336245
0.054948097	0.007162472	23.35294118
0.084567832	0.028692442	22.6641791
0.055854772	0.005817531	43.45501285
0.050994248	0.005063354	70.11709091
0.051915564	0.009517485	34.31618759
0.061322961	0.00805247	35.75128645
0.063744016	0.022566303	12.04761905
0.054084923	0.007393171	26.39344262
0.049190616	0.010489283	14.95394737
0.051651288	0.006627714	47.36423119
0.063150747	0.0100927	17.80851064
0.04863301	0.004008059	38.61460957
0.049306927	0.003655754	66.16989568
0.058548844	0.010540957	24.41486811
0.055055755	0.006532167	70.69158879
0.056911891	0.004411249	93.62006079
0.047829127	0.004704767	61.36477007
0.052047902	0.007035128	34.97619048
0.06024736	0.011239132	31.81060606
0.059671895	0.012217402	23.09302326
0.053739027	0.006863325	45.83939039
0.051389153	0.009078285	58.12113174
0.046644885	0.006534051	18.75124378
0.045780843	0.006153967	42.9424307
0.049238097	0.007343453	38.20876289
0.085768945	0.059538886	8.319587629
0.053115887	0.004090192	64.53580247
0.062884877	0.010127214	50.37078652
0.048314878	0.010265974	22.41810345
0.061180323	0.005193253	39.88957055
0.093195266	0.048413272	9.692307692
0.058224852	0.015429491	7.569230769
0.057789484	0.007148412	50.50800915
0.050524474	0.007244658	19.55297158
0.057640274	0.010519526	28.18609407
0.051091316	0.008537026	59.67465753
0.059705783	0.006757211	64.06430568
0.035955856	0.007692425	15.24528302
0.072009522	0.00714664	20.45070423
0.076126752	0.022876815	6.775280899
0.07323269	0.022202044	12.15662651
0.043807019	0.009150122	23.17391304

log.sigma.3.0.mm.3D_gldzm_HighIntensityLargeDistanceEmphasis	log.sigma.3.0.mm.3D_gldzm_SmallDistanceEmphasis	log.sigma.3.0.mm.3D_glcm_SumVariance	log.sigma.3.0.mm.3D_glcm_Homogeneity1
283.5179063	1	723.4989559	0.398214913
270.7692308	1	711.288865	0.42017681
214.1745283	1	632.0037616	0.365987533
291.9379728	1	787.8236391	0.42056341
140.8643216	1	258.9644615	0.48032096
207.559322	1	602.9812833	0.427904369
126.8543046	1	244.8638112	0.55088443
377.529148	1	1105.275285	0.439020644
341.4366197	1	1030.176352	0.426732083
312.0847458	0.999205508	937.5144371	0.386916218
281.5267857	1	879.9296912	0.465337533
263.5780446	0.998713551	794.1488263	0.374539065
271.817734	1	731.8095195	0.472727654
163.2109228	1	438.1461534	0.403919565
147.1077844	1	385.1066195	0.394172962
381.3011829	0.997952684	1145.39824	0.443122055
251.5898734	1	793.6380222	0.437645409
347.6085343	1	922.0058336	0.449291586
276.9769452	1	739.4269829	0.409001641
301.4035346	0.998895434	871.1762386	0.431230524
289.6029204	0.998940179	879.9995961	0.379646055
336.3453947	0.998766447	975.5604146	0.401078534
287.2618321	0.999427481	847.3638335	0.385402302
314.8659004	1	857.5980757	0.441121111
246.3447099	1	711.4750042	0.370266597
506.4714588	1	1576.324815	0.367811506
200.5436767	1	592.0232577	0.420008704
342.0595432	1	1100.964084	0.346120541
291.5657568	1	855.8040828	0.440537377
364.2322792	1	1124.474744	0.412512049
424.507113	1	1374.614675	0.398531325
309.8079835	1	877.8234738	0.410465536
344.2727273	1	971.7077052	0.426829872
188.0141844	1	424.0259762	0.445589663
338.267101	1	1043.465331	0.374343645
317.1411126	1	958.240678	0.442465783
389.7972973	1	1163.542638	0.367138302
453.5101351	1	1504.4392	0.410091009
198.1936759	1	481.9302895	0.492626736
259.5718232	1	633.0100345	0.453912098
260.9393204	1	799.7714882	0.399469851
183.7637131	1	437.8770153	0.444314053
349.7390625	1	1043.20366	0.458287474
201.8127854	1	446.0854619	0.461617067
474.7833501	1	1586.281202	0.394381534
255.5226179	1	765.8114128	0.392287963
386.6327783	0.996713409	1104.910351	0.391763307
352.9907407	1	1200.047852	0.404478518
155.4615385	1	343.0677109	0.490282381
231.4587814	1	525.2330094	0.456750332
299.5664516	1	799.9553311	0.479106276
337.1038576	1	1048.40158	0.480889902
335.0072993	1	857.362927	0.455946322
320.3497268	1	1038.720626	0.386847514
336.6444043	1	946.3177288	0.405056732
317.6872038	1	918.9475694	0.369527459
722.9871414	0.997774481	2302.893109	0.394551201
277.220524	1	656.4268267	0.499046007
410.1882353	1	1353.134372	0.384409765
98.14552239	1	265.5377024	0.460619561
370.0205656	1	1062.908415	0.429063822
445.528	0.999454545	1369.987042	0.433702686
317.1437216	1	912.8325363	0.398304123
269.8353345	1	831.2482439	0.382152061
132.8465608	1	360.2377061	0.396187948
323.4569672	1	773.8946115	0.392296003
271.2565789	1	623.3809784	0.422507412
390.5703381	1	1126.917547	0.417649961
260.0638298	1	890.2874107	0.423816634
527.0894207	1	1672.443494	0.398053683
507.0447094	1	1572.813277	0.363089779
295.0479616	1	747.5752319	0.461919149
315.1207165	1	869.640199	0.420246547
394.0218845	0.999088146	1259.502716	0.409738813
490.4037412	1	1439.698676	0.391804325
390.9925595	1	1238.912185	0.395705855
262.3598485	1	824.037916	0.44927963
261.997416	1	761.5422574	0.398223766
338.8300117	1	1095.329971	0.402296658
279.862069	0.99801061	897.1658997	0.425238133
556.340796	1	1847.259141	0.406516453
437.5245203	1	1415.277274	0.361588528
398.4484536	0.99806701	1146.143409	0.38644612
76.83505155	1	116.5453176	0.515727429
454.9300412	1	1419.323773	0.383923652
193.9775281	0.99906367	498.9535555	0.438795305
340.2887931	1	946.2410102	0.39415397
392.1610429	1	1169.177271	0.433064583
70.03846154	1	144.0772666	0.462250815
282.0461538	1	897.9783058	0.467675965
277.8226545	1	746.8349737	0.424460023
394.6718346	1	1228.477338	0.364683677
263.5541922	1	872.0867907	0.397852795
312.2825342	1	940.6251471	0.411421276
274.1808015	1	746.5886636	0.446036568
554.2075472	1	1783.22426	0.285018371
364.1267606	1	1092.339713	0.48860173
158.6741573	1	335.9089499	0.48297048
124.2831325	1	296.4995713	0.509240728
377.3137996	1	1218.024063	0.305209993

log.sigma.3.0.mm.3D_glcm_Homogeneity2	log.sigma.3.0.mm.3D_glcm_ClusterShade	log.sigma.3.0.mm.3D_glcm_MaximumProbability	log.sigma.3.0.mm.3D_glcm_Idmn	log.sigma.3.0.mm.3D_glcm_SumVariance2
0.318906115	265.6799485	0.030755652	0.985018347	69.08645391
0.343516682	109.8963302	0.037212295	0.985015315	50.17482125
0.281105828	-153.8781564	0.025401022	0.976400157	59.22460484
0.343982377	55.11703589	0.034795811	0.988765214	45.08702248
0.415639475	246.6630747	0.066266509	0.983379015	40.34561154
0.352200239	75.03827595	0.036162385	0.985208905	37.54071508
0.50137734	144.1399497	0.12050606	0.988130749	27.80568756
0.366308772	57.37595859	0.054108731	0.990852026	49.77365234
0.351573954	76.63908742	0.034345697	0.986222676	50.3231126
0.302888594	-97.3845778	0.023948083	0.98552873	53.80159923
0.397340567	17.65800794	0.046189912	0.988409219	29.95371296
0.288827159	-106.4441865	0.021965415	0.979957958	52.52614795
0.407127538	77.59721629	0.046121201	0.990558705	31.79239621
0.322447569	-48.70327518	0.022849405	0.981885429	40.75481829
0.313464128	245.8646122	0.022561165	0.977479745	57.01784386
0.372349448	-57.53742996	0.07473869	0.988224015	55.88057852
0.36620104	-126.4937374	0.041808997	0.986556336	44.82217266
0.377824419	96.41940592	0.039077973	0.991280878	42.94323361
0.32984545	35.34053751	0.031639181	0.983399662	45.39436854
0.355303271	-28.63272107	0.037369837	0.986946692	45.65147216
0.296133974	-151.4346083	0.019314378	0.985366053	52.82756439
0.319293714	-13.34436188	0.030716755	0.990583319	47.20088565
0.301259102	-53.81773477	0.021690746	0.985614811	52.29314454
0.368109863	41.19959364	0.058350389	0.986679748	57.3129945
0.285274595	-128.540827	0.016408416	0.981415161	62.08324123
0.283616537	0.870762938	0.019090195	0.984258572	69.31654526
0.341769711	111.3602894	0.03249417	0.987136645	46.81235313
0.257768279	-233.6330836	0.018025848	0.97995427	57.24509825
0.36802388	-80.4376642	0.045859739	0.988601367	42.40334893
0.335410751	-3.650085101	0.036622364	0.988251281	44.2479597
0.318336527	-211.8963186	0.025147152	0.988834344	53.61616168
0.330180595	47.89061426	0.024873608	0.989668776	47.14082989
0.351081409	-110.1647959	0.050182672	0.989665207	51.14673217
0.374806607	268.2697579	0.063299116	0.980255933	51.95746492
0.291153276	-239.9669869	0.027098851	0.979616889	57.11894127
0.371273006	-34.74946894	0.04654001	0.987157245	39.80067492
0.282998188	-98.04618733	0.021997153	0.983904403	68.22705999
0.331541242	-107.1259105	0.031033041	0.988614984	41.46072351
0.428662543	50.06965772	0.05941743	0.990073149	26.86471626
0.382761071	208.5620626	0.066214498	0.988962225	44.82418347
0.317887571	-8.226335043	0.029027443	0.981443772	37.31889773
0.37237845	98.17323049	0.036219106	0.984719748	39.27809048
0.389485162	59.82287778	0.054728182	0.989420991	37.59106462
0.394104499	265.7879049	0.054038324	0.986874076	41.76456225
0.316744741	-212.7501447	0.033423211	0.985116306	66.98143835
0.308917412	-118.2480719	0.018221476	0.985595212	55.257314
0.311071423	-96.33305486	0.023170849	0.987542002	57.95743333
0.326769304	-367.4130695	0.041964755	0.983929491	48.54628346
0.429216476	129.4068518	0.081109281	0.981416946	38.53205519
0.388094533	268.0947435	0.056750662	0.990373372	44.35143678
0.414285428	71.93334977	0.049935143	0.99121184	36.8651935
0.41865832	-88.03129815	0.066050339	0.989459851	27.52835992
0.38568714	162.759411	0.050243767	0.991060337	41.51555199
0.30396167	32.50360068	0.022833308	0.982958142	45.51407395
0.325578401	16.38900533	0.029931526	0.987956608	47.37449019
0.286199865	-31.44244502	0.023427221	0.980495034	63.63853874
0.314560167	-184.1541528	0.032173041	0.991264223	65.89790701
0.440705701	272.6599207	0.082344757	0.990515364	42.80095498
0.302303913	-237.6448129	0.033875674	0.982118972	50.40742209
0.389284632	-21.99656862	0.037523583	0.978687186	18.21854224
0.35440988	46.11079019	0.037142391	0.988582008	51.68932046
0.362171536	-193.5097805	0.050719088	0.991481497	52.22141707
0.318556382	-102.688699	0.02571424	0.984736521	52.27844136
0.296852183	-118.9037744	0.018628466	0.981103173	46.09620811
0.313497664	44.10213337	0.023629489	0.97243938	38.92544618
0.310745303	413.4857712	0.029187955	0.985947785	67.28357387
0.345740941	440.5131917	0.039314569	0.985344526	62.82046767
0.340754263	107.9413819	0.030681972	0.988428652	55.23541355
0.34749173	-57.27869155	0.033321957	0.986076455	39.63109226
0.319988223	-85.65276128	0.034111287	0.990499312	61.69623357
0.278247182	-222.884097	0.013661879	0.984467468	79.03344684
0.393854947	190.4293686	0.050113752	0.990090491	40.0149131
0.343294029	-24.26594936	0.035769472	0.987424085	53.7658055
0.330800322	-126.6739896	0.026973895	0.988421443	41.60931345
0.310918676	2.344727666	0.026567635	0.988534769	75.81770137
0.316322745	-66.62041173	0.033062208	0.988060939	58.51760315
0.379779359	-35.90259461	0.045156055	0.988200839	33.63796128
0.31583123	-39.09980299	0.023537118	0.983315819	48.57919498
0.322971259	-162.9763351	0.030122185	0.983905514	49.06424121
0.351689949	-218.5684352	0.036598755	0.985076717	50.88681258
0.331833125	-283.1403261	0.03662985	0.985921993	66.96594086
0.27888676	-305.5230662	0.023704496	0.981477447	74.90028457
0.303137308	13.18975067	0.031874366	0.985584384	52.64840235
0.458173359	116.842362	0.097867364	0.978939559	26.07124304
0.299924883	-104.3305171	0.020723148	0.98752296	57.6413735
0.363913199	122.631374	0.028463196	0.987717409	39.79365994
0.313280974	7.54686591	0.033020395	0.984899982	66.85505635
0.357219257	46.07029324	0.036967795	0.989470802	39.2410307
0.393221476	26.46923394	0.051961157	0.966270633	24.27940851
0.402920058	-34.36336932	0.064420883	0.987560574	42.89591955
0.347742339	149.3962533	0.038469254	0.986574707	46.10575578
0.279411622	-118.9565671	0.02541188	0.980070033	54.67345213
0.316859043	-229.682593	0.029425059	0.980815639	45.42472175
0.334137879	-129.0393157	0.036310295	0.985536876	46.88712965
0.373198851	44.78385429	0.030225909	0.989351691	38.53129847
0.199556424	-1209.622773	0.015619235	0.96657406	162.1823885
0.424356231	1.393949766	0.064784684	0.99168322	27.63699632
0.41731151	154.3021081	0.072785121	0.985863201	37.54804441
0.449963885	62.59055935	0.089764014	0.985426043	23.62858257
0.214724033	-562.777568	0.013243762	0.971015118	105.892637

log.sigma.3.0.mm.3D_glcm_Contrast	log.sigma.3.0.mm.3D_glcm_DifferenceEntropy	log.sigma.3.0.mm.3D_glcm_InverseVariance	log.sigma.3.0.mm.3D_glcm_Entropy	log.sigma.3.0.mm.3D_glcm_Dissimilarity
16.46246637	3.094939076	0.316972385	7.522457565	2.935411186
13.50762473	2.992832604	0.328213639	7.349878879	2.65666773
20.25648987	3.252642372	0.282263699	7.759288945	3.353569665
12.94785144	2.946281922	0.337901543	7.294840945	2.599152474
7.993809001	2.617002637	0.387787803	6.434483052	2.000848299
11.52591294	2.88404888	0.344580068	7.069684895	2.480016121
4.537629455	2.27585127	0.423462294	5.782622575	1.487229049
11.85673224	2.865382513	0.356307413	7.116184759	2.423678412
13.29301735	2.964483595	0.335530011	7.381371864	2.601271224
14.7146021	3.056567752	0.307252233	7.665451961	2.890991623
8.313624116	2.654131441	0.385138987	6.714787787	2.074451425
17.00674479	3.145900245	0.289824267	7.660776509	3.105245672
8.341529308	2.662062722	0.38937756	6.719817175	2.053114033
12.13096227	2.923915208	0.326607394	7.334042344	2.636300205
16.81964642	3.099038876	0.309084563	7.647140673	2.981895133
16.29989417	3.073687555	0.31441128	7.505918473	2.746168574
12.11583826	2.876550191	0.36007243	7.089115791	2.465613193
9.258531229	2.750170929	0.364562308	7.013365996	2.237586019
13.01091082	2.927861329	0.334165315	7.257365649	2.656831746
10.85751361	2.848214088	0.345999704	7.263789509	2.415435609
17.9761592	3.188451128	0.293975236	7.829375565	3.136947698
13.4468543	2.969513299	0.323355615	7.3994355	2.716948401
15.58395153	3.094894755	0.298139768	7.731789977	2.965640057
11.88104682	2.882433799	0.33199682	7.101016954	2.484198643
17.89071176	3.189597391	0.287600135	7.91369137	3.203161174
21.95238907	3.287261936	0.285090812	7.836708448	3.397949328
11.40917591	2.891645954	0.334760536	7.297524893	2.517044206
22.08532528	3.325992242	0.265389256	7.947418244	3.570248665
12.40971955	2.902277357	0.341541571	7.144660178	2.487421395
16.24871047	3.075091796	0.321310214	7.404017239	2.837031167
16.2038079	3.082049248	0.320202914	7.633574387	2.889195012
12.49105814	2.946531546	0.325134851	7.495111888	2.626339867
12.59932981	2.925516295	0.339097488	7.278711194	2.547359297
13.99077517	2.877168745	0.339574539	6.691603696	2.546376843
21.69089481	3.245615089	0.288041849	7.528231979	3.340431535
12.38537804	2.910340049	0.351070063	7.129488944	2.469870086
21.20257578	3.285741909	0.284894708	7.979738617	3.378048752
13.89069578	3.003301544	0.328897357	7.307933321	2.711702854
5.951836816	2.466494244	0.396151056	6.429425344	1.80209537
9.78038254	2.785191003	0.356009191	6.937831862	2.254769104
13.50513427	2.990346731	0.321377917	7.233843383	2.746056428
10.31213561	2.800372343	0.36400316	7.067519257	2.312396521
10.12489512	2.77769057	0.368970482	6.955085616	2.239200214
10.35885456	2.769320714	0.372890513	6.796800494	2.236245051
22.14168924	3.259016691	0.299925936	7.90039345	3.270247082
13.7233361	3.01025871	0.315526793	7.671877965	2.793280802
17.15985779	3.126328709	0.31435083	7.70551642	2.993248462
16.81450532	3.079882379	0.328244321	7.272896864	2.893130843
7.254105051	2.536348502	0.399665092	6.129611675	1.928053194
9.703471452	2.75524519	0.372921395	6.704576896	2.227828262
8.376255832	2.646755871	0.389058134	6.784393438	2.016900275
10.30259474	2.70316284	0.393002859	6.419075157	2.121591976
8.967801463	2.725545504	0.371478155	6.986339177	2.173615616
16.72664159	3.088143649	0.309742281	7.454907336	2.980646808
13.81653502	3.011118836	0.325390743	7.500534903	2.742753357
21.67763822	3.291307136	0.282360032	7.945766114	3.40927715
18.67354499	3.150267222	0.31216461	7.712350344	3.038658597
8.398408838	2.640939602	0.393579738	6.554018059	1.981818133
18.74400197	3.181390443	0.301088496	7.477120558	3.127477314
6.685310626	2.531766218	0.388653159	6.378765226	1.961870201
12.39315721	2.923971289	0.344203882	7.382342238	2.527578678
15.88436278	3.024145958	0.341752232	7.380770683	2.696823156
15.83752084	3.058317193	0.325106642	7.551379546	2.871523724
14.74679552	3.050160746	0.300114483	7.518134126	2.927976488
13.53070649	2.951117618	0.315945699	7.121060517	2.766765336
15.30491238	3.066240175	0.315494323	7.535588224	2.894758856
12.25922256	2.928537199	0.333204615	7.237398405	2.567677932
14.21434319	3.003317171	0.33185653	7.521264166	2.686608337
11.66838903	2.867791008	0.350153112	7.084770223	2.488210835
18.53687517	3.158009609	0.317348188	7.676751003	3.036136184
21.42845417	3.296064234	0.280337029	8.202675232	3.428900038
9.458761368	2.718016842	0.378101982	6.784394323	2.168997119
12.82470794	2.954910299	0.334095552	7.507369105	2.60219995
14.1304048	3.015257843	0.323606956	7.443052066	2.733441317
18.60784766	3.169150056	0.305936528	7.928895186	3.063667245
17.33868995	3.128710198	0.317286559	7.589401035	2.986936855
11.37097781	2.835472651	0.367287344	6.910786799	2.355564679
12.93246567	2.962068666	0.316843197	7.414117597	2.728900524
15.60936494	3.062871535	0.318497821	7.509010627	2.863018407
15.53358899	3.044785011	0.334308957	7.437995671	2.742125755
22.30633761	3.21261749	0.319808429	7.47071619	3.16065208
26.14780187	3.40587104	0.277074194	8.02196914	3.670175504
16.78714929	3.135062618	0.297481304	7.611868853	3.019135551
5.984000316	2.388035959	0.405967178	5.796806591	1.720540955
16.15814544	3.10932976	0.303325147	7.771329343	2.983354176
9.439260229	2.772413884	0.356831678	7.165651713	2.281628686
16.68154861	3.102881029	0.313154922	7.61420713	2.952356947
10.70817731	2.823102773	0.349392016	7.083111256	2.381156655
7.451144229	2.439758559	0.383334188	6.034874021	2.046866172
10.43160179	2.746746107	0.379678649	6.519594853	2.231335198
11.94487706	2.928173649	0.333455111	7.337757737	2.5406301
20.90741872	3.255239858	0.284137193	7.632255277	3.36652037
15.21857528	3.058359316	0.314473626	7.358637423	2.86172971
14.93234668	3.036796407	0.328458348	7.431049737	2.770561645
9.448782677	2.756712828	0.364171192	7.143777308	2.24148253
57.2903458	3.923067356	0.200454238	8.584847398	5.635616275
6.81554563	2.547976591	0.389805389	6.348135602	1.883906008
6.638291292	2.460000678	0.391121721	6.248587165	1.889932655
6.249654158	2.484773458	0.397002747	6.17359765	1.775924177
34.96636845	3.625101779	0.219262075	8.377533192	4.511348632

log.sigma.3.0.mm.3D_glcm_DifferenceVariance	log.sigma.3.0.mm.3D_glcm_Idn	log.sigma.3.0.mm.3D_glcm_Idm	log.sigma.3.0.mm.3D_glcm_Correlation	log.sigma.3.0.mm.3D_glcm_Autocorrelation
7.619426914	0.92127378	0.318906115	0.613792799	246.1157489
6.322531006	0.921397549	0.343516682	0.571063409	241.2093488
8.761960982	0.900465915	0.281105828	0.491034057	217.5602393
6.027945158	0.931085781	0.343982377	0.553433631	263.4257733
3.859817286	0.919105584	0.415639475	0.659860412	99.92215738
5.236346909	0.921005668	0.352200239	0.5264051	206.9713413
2.223204046	0.932086429	0.50137734	0.7049473	94.15460319
5.879273189	0.938809312	0.366308772	0.613368202	355.354995
6.414093978	0.925271118	0.351573954	0.582783374	334.9801216
6.173429468	0.919360913	0.302888594	0.569247605	309.0605043
3.894720433	0.930359766	0.397340567	0.564972533	286.9495678
7.14123615	0.906402722	0.288827159	0.509166442	266.1234561
4.028943063	0.93751202	0.407127538	0.579924521	244.2096569
5.00368847	0.910239794	0.322447569	0.540294037	158.5623405
7.557397062	0.904647895	0.313464128	0.539417265	142.1322711
8.509040044	0.933960063	0.372349448	0.55156539	367.7128794
5.846771284	0.926667665	0.36620104	0.571290304	264.2691693
4.106856527	0.937803091	0.377824419	0.643094718	302.3613895
5.61774809	0.915981864	0.32984545	0.5520884	249.1355121
4.895270023	0.925131807	0.355303271	0.614231717	288.5680318
7.90621687	0.920466777	0.296133974	0.492771092	291.2449734
5.860630838	0.934888512	0.319293714	0.556581613	318.5684556
6.557190175	0.919867024	0.301259102	0.541770711	282.5490581
5.431342405	0.925944961	0.368109863	0.654648659	284.7612711
7.365244603	0.909302227	0.285274595	0.552138391	242.8900568
10.13684137	0.919247268	0.283616537	0.520032735	491.8332226
4.943972404	0.924464073	0.341769711	0.604626782	206.1735728
8.998734309	0.905728069	0.257768279	0.446184364	354.7233409
5.974818196	0.932234256	0.36802388	0.548296496	282.132173
7.9731541	0.93144617	0.335410751	0.465030564	359.2514075
7.665004531	0.931686435	0.318336527	0.536324851	433.7432983
5.42480528	0.931861566	0.330180595	0.578205987	291.0493748
5.918855268	0.934151654	0.351081409	0.603528149	317.2551672
7.067999556	0.915196667	0.374806607	0.567773626	151.5458752
10.13848466	0.909800434	0.291153276	0.45676221	337.1582221
6.165602339	0.928847479	0.371273006	0.522413216	311.6204726
9.513479991	0.917445376	0.282998188	0.525262521	374.5725788
6.381685322	0.930220024	0.331541242	0.498851809	466.9113194
2.58489998	0.933680203	0.428662543	0.630890391	169.5225879
4.604256629	0.93192792	0.382761071	0.634957737	216.9928622
5.811640237	0.910416858	0.317887571	0.46595938	265.2790937
4.847612437	0.920774346	0.37237845	0.575789933	158.046238
4.99920157	0.934635188	0.389485162	0.57637928	335.66951
5.220055113	0.928601603	0.394104499	0.590909005	159.1586699
11.02510131	0.924510028	0.316744741	0.502398694	494.5523471
5.775786289	0.919454907	0.308917412	0.600856142	259.4217912
7.954984947	0.927725688	0.311071423	0.545014773	357.0352015
8.253277934	0.920562729	0.326769304	0.485714284	379.3546195
3.344621514	0.914518081	0.429216476	0.675697537	125.9885467
4.651581309	0.936607681	0.388094533	0.638441179	183.2396106
4.221531196	0.940515992	0.414285428	0.627599377	265.0444804
5.668217205	0.938470346	0.41865832	0.458314083	330.938546
4.139810433	0.937786337	0.38568714	0.643574991	283.0828338
7.532912021	0.915426832	0.30396167	0.463099252	335.8903245
6.148407276	0.927433925	0.325578401	0.548453157	310.4031466
9.708848088	0.910260066	0.286199865	0.494495479	303.5438071
9.148485292	0.94017114	0.314560167	0.559845572	694.3340475
4.354839231	0.93997759	0.440705701	0.67083476	221.5961731
8.748898097	0.914563561	0.302303913	0.460133347	424.8308397
2.738884536	0.902996204	0.389284632	0.464390919	101.1561119
5.863108256	0.931009964	0.35440988	0.612705514	344.9562799
8.42254896	0.943267392	0.362171536	0.532524021	430.5499262
7.327221727	0.920615039	0.318556382	0.536687892	300.9424252
5.940360865	0.907903081	0.296852183	0.516341615	276.4342152
5.466560355	0.891861343	0.313497664	0.486207987	132.970834
6.752053377	0.921790708	0.310745303	0.627849439	260.54196
5.567022175	0.921053566	0.345740941	0.669202702	215.3188749
6.858171407	0.931093995	0.340754263	0.590014816	363.4365974
5.344931479	0.923259789	0.34749173	0.543216876	292.2765425
9.084805797	0.937743687	0.319988223	0.53673493	518.3825378
9.35738774	0.918233421	0.278247182	0.574286958	494.2011688
4.62819271	0.936409657	0.393854947	0.611201552	249.2706233
5.895291309	0.9270433	0.343294029	0.614079937	289.3793848
6.463787514	0.929742096	0.330800322	0.49396762	398.4217199
9.003382318	0.931469444	0.310918676	0.605247657	455.7836174
8.226598355	0.929696303	0.316322745	0.541107186	394.6603815
5.682975166	0.931775802	0.379779359	0.494997933	270.9051081
5.252451842	0.913420297	0.31583123	0.576288978	256.6435283
7.090351891	0.91850571	0.322971259	0.521079446	352.600776
7.843850427	0.924453534	0.351689949	0.532902206	295.1395359
12.08061196	0.929000998	0.331833125	0.501993649	564.8198625
12.31905346	0.914008374	0.27888676	0.486379102	446.1444165
7.483607555	0.921074234	0.303137308	0.515542346	367.8413114
2.79866196	0.910579096	0.458173359	0.617567309	50.84455939
7.062478297	0.925682804	0.299924883	0.561431522	447.8789973
4.152112828	0.926260664	0.363913199	0.615884826	177.4679945
7.762542982	0.920854649	0.313280974	0.598104824	312.3970184
4.89843563	0.932455732	0.357219257	0.569330237	372.1929427
2.60693922	0.882521074	0.393221476	0.523806313	60.85330065
5.252923848	0.931121163	0.402920058	0.599903032	292.3502671
5.385119993	0.924102317	0.347742339	0.587431409	251.6469123
9.276021945	0.908585836	0.279411622	0.447099879	390.5241371
6.824560414	0.910561939	0.316859043	0.499040862	287.1469163
7.03934847	0.923187593	0.334137879	0.516251825	307.5177517
4.322681477	0.932074178	0.373198851	0.604678415	251.43466
24.49291777	0.883904981	0.199556424	0.483031906	550.6074066
3.178676584	0.940098952	0.424356231	0.602699697	345.5595886
2.759720917	0.922334073	0.41731151	0.689441253	125.0158524
2.987161358	0.923865633	0.449963885	0.578579247	111.1414841
14.06109937	0.888063127	0.214724033	0.50611274	391.2401974

log.sigma.3.0.mm.3D_glcm_SumEntropy	log.sigma.3.0.mm.3D_glcm_AverageIntensity	log.sigma.3.0.mm.3D_glcm_Energy	log.sigma.3.0.mm.3D_glcm_SumSquares	log.sigma.3.0.mm.3D_glcm_ClusterProminence
4.944731581	15.34416208	0.009382243	22.07259427	15213.0284
4.753598297	15.26911307	0.01087755	16.31355643	8595.127824
4.899208247	14.43129013	0.006870667	20.0696078	10792.34511
4.708689917	16.04776007	0.011348723	14.87180962	8061.427306
4.380113832	9.582206512	0.022227117	12.08485513	5703.194403
4.53862848	14.1638046	0.01289191	12.35093522	5794.555575
4.064228508	9.397497567	0.038537132	8.085829254	2706.936865
4.707088645	18.68844174	0.014066018	15.347243	10296.88082
4.792991686	18.14629691	0.009851729	16.39993897	8060.776381
4.87274308	17.39697032	0.007621809	17.51415433	10607.29208
4.403816905	16.78409109	0.015398612	9.630872684	3705.404052
4.844962562	16.07533168	0.00755158	17.74763014	9649.8339
4.418833125	15.4608553	0.017426012	10.27736287	4688.809643
4.675133197	12.30405318	0.008978107	13.22144514	5319.66044
4.87239741	11.49961131	0.0072588	18.58444076	11296.66272
4.824318226	19.03748977	0.013240657	17.76282045	13236.43021
4.641739135	16.02494327	0.013817325	14.45369166	9619.944772
4.640050662	17.22068881	0.012863903	13.45197308	6219.044388
4.705807222	15.54199187	0.010020616	14.8111142	6637.336844
4.726644183	16.766891	0.010987394	14.46847939	7265.808526
4.857055932	16.93260398	0.006996531	17.96584309	10793.33138
4.752088919	17.74953455	0.009610952	15.05846421	9584.166933
4.87093933	16.61506092	0.007247362	17.39454532	9143.449002
4.780346083	16.57915728	0.013781014	17.71361966	11138.92162
4.969843046	15.28486185	0.005679986	20.50762695	9958.638179
4.997226908	22.03061371	0.006485814	22.04174598	18463.04378
4.74491669	14.06153579	0.010249568	14.74727232	7395.505844
4.892195359	18.76228941	0.006177284	20.01546944	11397.83713
4.623987012	16.64176986	0.014033858	14.04759863	8850.59995
4.669968866	18.8849083	0.011012701	14.88291625	9802.72717
4.855923818	20.72162584	0.008192501	16.75151049	11986.76206
4.787007162	16.88603033	0.008600853	15.18414698	8262.072684
4.7373086	17.61050394	0.012576816	16.04416166	13385.78683
4.548175693	11.91769676	0.017654173	16.48706002	7250.738147
4.831870696	18.26873423	0.008464571	20.08856877	13261.98656
4.609150135	17.51435279	0.01396115	13.39189709	6690.495659
4.999921089	19.21448738	0.006842105	22.02922199	18904.86879
4.647099971	21.54514326	0.010799325	13.29833375	8340.702301
4.303276222	12.81760624	0.01961888	8.204138268	2680.131634
4.607712133	14.45549002	0.015710325	13.9622731	7502.807923
4.594614387	16.11181068	0.010239089	12.8024456	4819.678262
4.594958164	12.2791967	0.012565781	12.39755652	4888.181553
4.554513561	18.19995457	0.015552314	12.21842412	5055.87237
4.493288924	12.30989359	0.017994742	13.18413578	7480.626337
4.992241229	22.12419494	0.007979295	21.42078217	18710.53722
4.905646873	15.81701932	0.007217843	17.57344258	9833.019689
4.890277549	18.75293502	0.008187357	18.44848092	15140.36698
4.611320774	19.42577703	0.012292665	16.50032455	12059.42886
4.290060358	10.87008189	0.026382154	11.44654006	4074.99346
4.496387611	13.24429367	0.019011812	13.87460402	7782.472385
4.495826526	16.09540657	0.017179804	11.61528157	5843.660551
4.194928687	18.12057184	0.024395332	9.64269773	5901.950433
4.599863137	16.62978174	0.014852376	12.96968835	6054.907648
4.745112991	18.23623034	0.008293967	15.95189919	7101.236447
4.774620709	17.48475447	0.009343776	15.6165899	8154.668094
4.992132981	17.24596822	0.006479921	21.84626642	13763.59179
4.953510904	26.27610956	0.008726682	21.0788212	21799.18222
4.417164076	14.62568356	0.025171317	13.22025638	8053.661933
4.743631046	20.57717898	0.009250773	17.06211073	12424.67829
4.100273442	9.913143263	0.017021018	6.225963216	1082.093569
4.813506806	18.37976749	0.010259079	16.2512737	8623.722183
4.758059357	20.61578437	0.013106566	16.35560234	14060.85108
4.831063558	17.17926558	0.008765685	17.47078869	11056.46084
4.7573682	16.40601512	0.00759499	15.37447645	7115.282662
4.581047882	11.25150027	0.009283193	13.11403817	3690.257309
4.884665202	15.79422007	0.008897938	21.14125053	14394.4036
4.796600353	14.27578901	0.011523267	19.3437566	14065.48911
4.849608151	18.87759295	0.009720275	17.40390845	11099.60604
4.614589851	16.92314371	0.011545177	13.11145162	5578.668998
4.92552506	22.63257409	0.009172314	19.82307025	15907.7845
5.159314554	22.01681636	0.004807771	24.44702742	19096.88128
4.488882097	15.58944356	0.01707673	12.80773873	6451.251391
4.851601723	16.79034999	0.00985823	17.16996949	10536.8566
4.677046781	19.90516685	0.009514343	13.64186255	7253.020319
5.092049776	21.1231123	0.007326424	22.94597499	21981.63206
4.853461493	19.74942881	0.009087813	18.3950807	16342.69557
4.464431001	16.32903278	0.015701389	11.54601121	5701.533761
4.777309038	15.76215846	0.008519324	15.63658943	7683.611541
4.761227572	18.6515046	0.009739544	16.50836853	10452.04973
4.75025735	17.00203767	0.011803889	17.00481881	11642.78777
4.866419282	23.64184762	0.011141543	21.55585709	26978.8015
5.051949041	21.00369676	0.00699528	24.23540083	24227.4813
4.819961329	19.07647743	0.008766421	17.33625345	11507.46244
4.026788142	6.768596557	0.032133278	8.01381084	1961.739455
4.932232347	21.04076448	0.007005355	17.84107529	11703.94584
4.639621229	13.03642794	0.01079674	12.3532589	4886.652587
4.97819625	17.40234421	0.009040182	21.29717825	16099.19352
4.598244337	19.20176222	0.012458385	12.67726378	5682.671933
4.107620055	7.525518358	0.02061326	7.932638185	1205.861192
4.476287733	16.88417714	0.019915325	13.57851137	8727.61738
4.712366853	15.62975102	0.010675283	14.89004822	6581.751719
4.833303117	19.72345747	0.007990097	18.9427337	11718.83129
4.690688143	16.73896046	0.009797373	15.33734829	8561.792679
4.718389422	17.37672006	0.011046606	15.73351522	10331.00608
4.642076223	15.64687344	0.011559405	12.21311556	5117.680641
5.536847769	23.20105508	0.003841636	53.18365495	77407.50938
4.267547429	18.48570673	0.022357176	8.870323815	3632.1983
4.386710273	10.82870012	0.021557077	11.04658393	3921.404124
4.150142115	10.33403452	0.027731217	7.469559183	2166.139142
5.30411012	19.54637181	0.004050524	34.7406247	35823.51375

log.sigma.3.0.mm.3D_glcm_SumAverage	log.sigma.3.0.mm.3D_glcm_Imc2	log.sigma.3.0.mm.3D_glcm_Imc1	log.sigma.3.0.mm.3D_glcm_DifferenceAverage	log.sigma.3.0.mm.3D_glcm_Id
30.52556449	0.867235581	-0.174578044	2.935411186	0.398214913
30.46474692	0.794813137	-0.133038052	2.65666773	0.42017681
28.83095381	0.804141804	-0.128321051	3.353569665	0.365987533
31.96152645	0.772842793	-0.123734654	2.599152474	0.42056341
19.16441302	0.853715327	-0.193226532	2.000848299	0.48032096
28.31676104	0.770979765	-0.125836182	2.480016121	0.427904369
18.79499513	0.845432497	-0.211757204	1.487229049	0.55088443
37.19492782	0.834589924	-0.159411829	2.423678412	0.439020644
36.09452529	0.816414409	-0.141548075	2.601271224	0.426732083
34.59956751	0.756325036	-0.110735671	2.890991623	0.386916218
33.55752545	0.77008401	-0.133504792	2.074451425	0.465337533
32.07743467	0.757999807	-0.111008284	3.105245672	0.374539065
30.87598738	0.762527575	-0.130306392	2.053114033	0.472727654
24.60810636	0.744453661	-0.111664622	2.636300205	0.403919565
22.98515256	0.802436399	-0.136034095	2.981895133	0.394172962
37.83177321	0.801043681	-0.134596929	2.746168574	0.443122055
32.00465456	0.807379585	-0.147041731	2.465613193	0.437645409
34.28828766	0.809778388	-0.149699108	2.237586019	0.449291586
31.0484142	0.811135866	-0.147677079	2.656831746	0.409001641
33.45842105	0.792141909	-0.134361413	2.415435609	0.431230524
33.61715951	0.664860143	-0.078808225	3.136947698	0.379646055
35.22067913	0.7836191	-0.128552847	2.716948401	0.401078534
33.06763129	0.716567132	-0.096589691	2.965640057	0.385402302
33.06936343	0.882165607	-0.199657966	2.484198643	0.441121111
30.45229619	0.74338625	-0.103023482	3.203161174	0.370266597
43.81536739	0.836189908	-0.145227926	3.397949328	0.367811506
28.09287551	0.807341163	-0.140596006	2.517044206	0.420008704
37.19818168	0.669164277	-0.077515866	3.570248665	0.346120541
33.14380674	0.758335523	-0.125492022	2.487421395	0.440537377
37.53657714	0.742521347	-0.108212905	2.837031167	0.412512049
41.2009103	0.747519855	-0.108063658	2.889195012	0.398531325
33.60778163	0.743076626	-0.109806274	2.626339867	0.410465536
35.07756744	0.795058129	-0.1356529	2.547359297	0.426829872
23.83539351	0.904165109	-0.236416769	2.546376843	0.445589663
36.2374073	0.844724173	-0.155678516	3.340431535	0.374343645
34.91456271	0.751442586	-0.11621925	2.469870086	0.442465783
38.09524515	0.744244854	-0.102701048	3.378048752	0.367138302
42.89581764	0.72224478	-0.101234822	2.711702854	0.410091009
25.63521249	0.796626208	-0.159832238	1.80209537	0.492626736
28.85958668	0.830827373	-0.16192694	2.254769104	0.453912098
32.20639066	0.741228413	-0.109362916	2.746056428	0.399469851
24.55839339	0.785252932	-0.134965635	2.312396521	0.444314053
36.26529291	0.786737967	-0.135324019	2.239200214	0.458287474
24.59999561	0.807244299	-0.152920888	2.236245051	0.461617067
43.96992118	0.788135529	-0.123090988	3.270247082	0.394381534
31.55911127	0.766771999	-0.114771917	2.793280802	0.392287963
37.24678325	0.763282085	-0.113310312	2.993248462	0.391763307
38.54426592	0.774726169	-0.122172506	2.893130843	0.404478518
21.74016378	0.887446566	-0.237012629	1.928053194	0.490282381
26.42519693	0.846044956	-0.176527311	2.227828262	0.456750332
32.11933224	0.797710143	-0.145360087	2.016900275	0.479106276
36.14588758	0.746205439	-0.124766146	2.121591976	0.480889902
33.1618999	0.791863213	-0.139078705	2.173615616	0.455946322
36.2595194	0.793012756	-0.129829936	2.980646808	0.386847514
34.75672289	0.739918775	-0.106213778	2.742753357	0.405056732
34.23732864	0.780153561	-0.116233167	3.40927715	0.369527459
52.25027884	0.806879144	-0.133666636	3.038658597	0.394551201
29.18828316	0.840925059	-0.178214455	1.981818133	0.499046007
40.83663344	0.787760983	-0.12455365	3.127477314	0.384409765
19.82628653	0.659020764	-0.092337097	1.961870201	0.460619561
36.61282598	0.80779463	-0.138960128	2.527578678	0.429063822
41.05897186	0.764811151	-0.119783718	2.696823156	0.433702686
34.16494694	0.789026642	-0.12747586	2.871523724	0.398304123
32.77705256	0.746742181	-0.107425043	2.927976488	0.382152061
22.50300054	0.81758989	-0.149233708	2.766765336	0.396187948
31.46589901	0.834880532	-0.151403042	2.894758856	0.392296003
28.47153511	0.873235983	-0.184521279	2.567677932	0.422507412
37.58513096	0.791525432	-0.128286133	2.686608337	0.417649961
33.7789104	0.798826378	-0.139633386	2.488210835	0.423816634
45.05958584	0.800202872	-0.131278482	3.036136184	0.398053683
43.8081962	0.773551022	-0.111522432	3.428900038	0.363089779
31.08807569	0.822330407	-0.160989368	2.168997119	0.461919149
33.41366209	0.782445297	-0.125790722	2.60219995	0.420246547
39.57527273	0.679365657	-0.087520117	2.733441317	0.409738813
42.02053576	0.811959869	-0.133492206	3.063667245	0.391804325
39.20876682	0.804023083	-0.131783031	2.986936855	0.395705855
32.5780595	0.753088724	-0.11966664	2.355564679	0.44927963
31.47816399	0.806720042	-0.139684739	2.728900524	0.398223766
37.10698552	0.737413702	-0.107612348	2.863018407	0.402296658
33.84066264	0.751192938	-0.111376493	2.742125755	0.425238133
47.05931583	0.862481181	-0.168695317	3.16065208	0.406516453
41.66304504	0.776689838	-0.112386229	3.670175504	0.361588528
37.88754098	0.756585506	-0.111198031	3.019135551	0.38644612
13.53719311	0.846885672	-0.217613391	1.720540955	0.515727429
41.83244284	0.752733762	-0.108396487	2.983354176	0.383923652
26.06710361	0.761848604	-0.11946152	2.281628686	0.438795305
34.6306994	0.850662314	-0.159937999	2.952356947	0.39415397
38.21180414	0.76520714	-0.124655675	2.381156655	0.433064583
15.05103672	0.844921842	-0.210116871	2.046866172	0.462250815
33.71600001	0.882156171	-0.218066019	2.231335198	0.467675965
31.18299294	0.766306827	-0.118581883	2.5406301	0.424460023
39.09329353	0.806225651	-0.131919645	3.36652037	0.364683677
33.44145792	0.748649351	-0.110625178	2.86172971	0.397852795
34.61322	0.713491654	-0.0998455	2.770561645	0.411421276
31.25076103	0.764085283	-0.122707826	2.24148253	0.446036568
45.79770282	0.923928871	-0.202025712	5.635616275	0.285018371
36.89710212	0.785864565	-0.148782853	1.883906008	0.48860173
21.65740023	0.906637296	-0.257862539	1.889932655	0.48297048
20.66806903	0.807016823	-0.166510954	1.775924177	0.509240728
38.65115188	0.851343858	-0.144273838	4.511348632	0.305209993

log.sigma.3.0.mm.3D_glcm_ClusterTendency	log.sigma.3.0.mm.3D_firstorder_InterquartileRange	log.sigma.3.0.mm.3D_firstorder_Skewness	log.sigma.3.0.mm.3D_firstorder_Uniformity
69.08645391	136.7718143	0.424220225	0.0721354
50.17482125	119.7251155	0.181397021	0.0808705
59.22460484	140.6420002	-0.380595514	0.06808734
45.08702248	104.8225183	0.084295868	0.085769831
40.34561154	86.2695303	0.900762529	0.111322328
37.54071508	92.96542215	0.130209467	0.094306318
27.80568756	80.77974224	0.862627709	0.141158327
49.77365234	109.172226	-0.001357825	0.085546612
50.3231126	132.2363176	0.132822316	0.076944233
53.80159923	128.9404421	-0.240925765	0.070485292
29.95371296	89.61224413	-0.069099828	0.102888631
52.52614795	119.7977619	-0.308775356	0.072829381
31.79239621	81.5558567	0.32286578	0.106383802
40.75481829	114.2655121	-0.273578835	0.079806753
57.01784386	142.7046816	0.412063872	0.067326182
55.88057852	113.0028839	-0.142320214	0.081767752
44.82217266	88.02883959	-0.419767422	0.092145457
42.94323361	125.3403077	0.36100411	0.086773103
45.39436854	126.9256086	0.041891999	0.078092454
45.65147216	108.1692076	-0.122532874	0.083658228
52.82756439	126.4303226	-0.385339894	0.071774504
47.20088565	112.1813424	-0.137230443	0.077602769
52.29314454	122.4320564	-0.129581953	0.072191775
57.3129945	109.8786478	0.028610119	0.084550853
62.08324123	152.9397984	-0.21876776	0.063472302
69.31654526	146.9302685	-0.135158775	0.065237562
46.81235313	113.5699656	0.288501344	0.0789521
57.24509825	142.2311015	-0.502482083	0.067047914
42.40334893	93.42218256	-0.280471181	0.092973627
44.2479597	106.9020181	-0.079733608	0.082618878
53.61616168	118.4680703	-0.640364468	0.075408932
47.14082989	122.9634013	0.104494645	0.07559168
51.14673217	100.7634625	-0.304697771	0.087414987
51.95746492	150.2681704	0.598483518	0.090866654
57.11894127	118.0170848	-0.54732973	0.073763798
39.80067492	91.77180004	-0.278591407	0.095106825
68.22705999	133.4228914	-0.229689888	0.064798594
41.46072351	94.38733482	-0.587067955	0.088762115
26.86471626	82.05610538	0.177141359	0.107259102
44.82418347	98.12833405	0.627633173	0.095456896
37.31889773	107.4275625	-0.260393435	0.085005913
39.27809048	115.6361065	0.321120236	0.086763092
37.59106462	100.4054184	0.108966814	0.0959737
41.76456225	95.30114555	0.897746733	0.102137905
66.98143835	119.3273444	-0.53180516	0.071518465
55.257314	130.3868841	-0.356504092	0.071638515
57.95743333	118.9548671	-0.309772866	0.072941596
48.54628346	98.5377903	-1.017513746	0.089596633
38.53205519	108.7522051	0.530046701	0.11611589
44.35143678	97.94870329	0.826880046	0.103481188
36.8651935	97.3017199	0.238457732	0.099339124
27.52835992	66.01639831	-0.577516624	0.130070655
41.51555199	109.6415997	0.541295871	0.091524441
45.51407395	116.7839826	-0.013644512	0.07681436
47.37449019	124.9048462	0.027838752	0.07572241
63.63853874	132.5634313	-0.060486202	0.065462511
65.89790701	124.6549277	-0.39110303	0.073033911
42.80095498	82.81015223	0.874692633	0.116236637
50.40742209	115.0539767	-0.814655287	0.076798358
18.21854224	78.95866203	-0.325276452	0.116114538
51.68932046	132.0547485	0.022239183	0.076895365
52.22141707	101.2146378	-0.480152051	0.08828957
52.27844136	119.0340109	-0.278706358	0.075887053
46.09620811	129.277683	-0.365152191	0.074000179
38.92544618	135.1504955	0.144967411	0.07638035
67.28357387	142.2918811	0.685762073	0.072271278
62.82046767	114.3793366	0.806751625	0.080454615
55.23541355	123.9167604	0.13172431	0.076131716
39.63109226	102.2346392	-0.360703238	0.089398624
61.69623357	115.1716636	-0.211112255	0.075251124
79.03344684	171.1832509	-0.329184405	0.056546148
40.0149131	89.52202225	0.635001883	0.101693826
53.7658055	118.4696932	-0.022713897	0.077910191
41.60931345	110.6211414	-0.48838087	0.080595128
75.81770137	135.8238821	-0.17887993	0.066849425
58.51760315	121.9768915	-0.277106048	0.075180347
33.63796128	84.69288659	-0.288655536	0.102119807
48.57919498	119.0888348	-0.186989049	0.076442933
49.06424121	109.163251	-0.480813674	0.081207017
50.88681258	96.0232892	-0.573787792	0.086383224
66.96594086	99.58060718	-0.728387613	0.08324223
74.90028457	123.0738916	-0.628871397	0.069398493
52.64840235	118.1469874	-0.140916929	0.073997382
26.07124304	89.31534004	0.897992245	0.131704499
57.6413735	131.103688	-0.437884974	0.068472477
39.79365994	111.1821803	0.398139238	0.084173442
66.85505635	125.253396	-0.075285621	0.071843443
39.2410307	104.0863442	0.057977054	0.089729217
24.27940851	122.3169394	0.228975303	0.108198507
42.89591955	72.7413187	-0.259942692	0.110547048
46.10575578	119.5209455	0.366682083	0.080658873
54.67345213	120.4333458	-0.447797304	0.070168096
45.42472175	102.6110902	-0.851411202	0.084999697
46.88712965	96.5685029	-0.440849827	0.086271399
38.53129847	103.8403625	0.105216709	0.086236359
162.1823885	226.593504	-0.683401159	0.045942446
27.63699632	67.34711981	-0.15292558	0.123011007
37.54804441	120.0514817	0.616764556	0.097464844
23.62858257	67.06279922	0.492332555	0.125065944
105.892637	196.0799036	-0.578774875	0.050953807

log.sigma.3.0.mm.3D_firstorder_MeanAbsoluteDeviation	log.sigma.3.0.mm.3D_firstorder_Energy	log.sigma.3.0.mm.3D_firstorder_RobustMeanAbsoluteDeviation	log.sigma.3.0.mm.3D_firstorder_Median
90.17894188	26974177.95	59.66005278	73.68908691
78.00716821	30296685.86	51.84273327	54.85644722
86.66791025	14213591.8	58.82070492	35.7374382
72.37901931	33582708.86	45.25537137	56.79737091
67.90739492	10587142.52	42.27217433	32.5684967
64.51862031	15237456.6	39.4390144	37.67439461
57.63119306	8533526.436	37.1644531	28.29708672
76.03828593	30138098.77	48.33520184	66.80271149
78.27226045	35706404.57	53.79550528	74.97232056
82.02370884	48836043.86	54.4084866	68.28872681
58.34978501	16709815.24	38.29767623	50.0015831
80.53878252	25422506.4	52.01194009	66.42816925
58.82594164	27151714.91	35.66624451	44.9012146
71.61784802	19949520.32	48.36429296	51.28198624
86.53336628	25970435.89	59.42032161	58.12778854
79.47039119	51672732.09	50.25386843	28.26630402
69.40487318	17248355.59	40.84782794	41.68304062
73.77864229	44002843.39	52.08042614	75.86946106
76.31694489	17396330.57	52.82998955	65.73889923
71.18647586	34550069.48	46.61434353	49.94517517
81.5479472	85537816.14	53.55592617	39.88487625
75.06561357	28027351.92	48.53232095	59.13462448
79.59196276	54612023.16	52.50353334	52.03152847
79.00461859	14521629.41	51.11738503	39.93913269
89.99883444	32878219.44	64.14367008	26.12232399
92.11796395	29986987.01	61.74625764	79.37692642
74.47273404	27522859.36	49.13849021	48.6937561
88.06410155	50634965.22	59.55903857	62.17973709
68.45731328	39502017.08	41.60886327	45.75611115
74.11765486	41226490.21	46.1253443	47.96235275
78.87763034	59976237.46	50.16951584	65.93499756
77.06084608	83775562.43	51.83751229	76.93600845
73.99322948	34763943.83	44.55577088	49.12811279
85.63497598	8725086.425	61.57788614	50.21499252
81.90614062	12432622.13	51.19117299	46.8390007
65.6957662	34294400.72	39.77114302	44.75906372
92.11809809	69915339.64	58.07002244	67.42542267
68.40943945	40101517.64	41.97025353	58.35152054
57.17513147	10581253.39	36.49879215	27.5019474
70.24496699	23342106.93	44.25202901	55.60609627
69.08010831	16525305.66	45.15207456	63.77729416
70.86608554	25781693.28	47.75625609	56.99333191
66.91382529	36443307.17	43.54627078	51.38957977
69.07232854	25574967.38	42.61573754	49.45948792
86.07580069	53123807.48	53.1474887	44.25444794
80.57524019	49771414.38	54.65659189	65.09072876
82.06042947	58366253.33	51.39576187	61.70377541
74.55543126	16568368.72	43.30543509	32.17973709
68.2827751	6340658.59	47.32896556	39.06951332
70.45045502	14782546.87	44.0932232	47.08527946
64.93026111	45994114.43	41.61672388	50.36803055
51.62215082	14678554.66	29.04581123	35.41944122
71.14880042	56742292.8	46.95824723	61.54330444
75.74832883	15924470.89	49.70396807	61.13622284
78.8240059	61714358.63	53.15502428	64.17206573
88.67837445	28233241.7	57.66898202	39.57718849
84.80547548	59502756.9	52.82202578	62.01583099
65.81038864	26715475.54	38.88231895	31.64491558
78.18241687	19050476.66	49.45853798	55.57816124
49.16056975	10667119.67	33.632022	61.90700912
80.53647452	58570829.87	55.71371762	81.86463928
73.59204588	76478289.65	44.3158956	50.98250198
78.39390436	34832489.06	50.18868972	68.39559937
76.80410007	27050025.16	53.21472841	73.17977142
76.1407481	9574279.202	55.72899616	80.3178978
92.11726063	32119604.61	63.38364757	58.12833023
81.64658432	17604308.15	50.93256208	49.38039589
81.7086104	66857819.66	53.85454145	71.70524216
66.4027475	14313215.25	43.13080354	54.93897247
82.28577981	38946846.61	50.44079281	51.55224228
100.2523821	74787428.69	70.69378216	67.71199417
65.09448576	27853935.87	39.77734891	60.65770721
78.42240554	79861764.49	51.06339207	70.82176971
72.48860762	81725611.41	47.36181352	63.92129517
91.17179914	84381730.11	58.49953458	62.18961334
82.93108164	36949749.02	52.63883569	48.98427582
60.76163897	21150012	36.26459627	42.15483856
75.0240647	21828096.88	50.44291266	80.16294098
74.28445358	41130657.45	46.29863948	53.88574982
73.71677935	52261650.4	43.40181769	41.60710144
80.03715716	22112463.85	45.37456435	49.46717834
91.46194807	46330486.96	54.83516111	53.20978546
81.03809101	40267377.75	50.82258286	62.01196861
58.01134359	4593631.142	39.66834022	36.30189133
84.66734032	65735549.03	55.85404929	72.92159653
69.267072	41440303.74	47.00919641	55.40379333
86.80736453	26720203.94	54.34634517	62.34774971
68.89742154	38259687.2	45.41634983	55.4962883
61.9032895	4466503.346	49.18874103	61.1231308
63.70633701	6977440.284	36.36845405	52.51483727
75.93802261	50179356.69	51.81389595	63.92102051
84.60076978	20606405.91	53.97467986	77.7966156
71.91236313	15927044.7	45.19344066	41.61627388
72.34013089	54358667.23	42.78481069	49.82942581
67.23080034	66921919.54	43.90586527	76.76749802
142.9452268	29002131.38	96.98970076	53.81008148
52.94778244	14252603.25	31.69043087	45.05644226
70.53534766	6413880.941	50.35338331	64.59293365
51.27153988	6200017.657	30.97100456	36.81630707
119.9646532	28834142.18	82.63240354	74.72933197

log.sigma.3.0.mm.3D_firstorder_TotalEnergy	log.sigma.3.0.mm.3D_firstorder_Maximum	log.sigma.3.0.mm.3D_firstorder_RootMeanSquared	log.sigma.3.0.mm.3D_firstorder_90Percentile	log.sigma.3.0.mm.3D_firstorder_Minimum
728302804.5	501.0353394	149.7411953	264.3860535	-268.5430603
818010518.3	413.4581299	123.0786047	213.7551773	-299.3637085
383766978.5	355.8731689	112.7537559	151.5675293	-311.7002869
906733139.2	477.2168579	117.0062574	197.2337585	-307.6593323
285852848	340.4241333	105.6224589	197.1098816	-165.3983765
411411328.3	370.9804382	96.44934711	145.6281677	-277.0391846
230405213.8	282.9786072	89.8517987	172.9321564	-174.9206696
813728666.8	477.8643494	129.2169015	221.5743225	-365.9974976
964072923.3	399.2312317	132.722999	224.4183411	-328.0645142
1318573184	414.1118164	127.5241893	205.0418121	-328.9738159
451165011.5	287.7497864	96.16272192	156.2917725	-340.8470459
686407672.7	374.2878723	122.8313514	191.509201	-319.824585
733096302.5	397.3670654	96.28063515	151.2607513	-308.5290527
538637048.7	355.6717224	103.619949	162.6995178	-240.328949
701201769	448.3185425	129.5251611	210.6178406	-195.9341125
1395163766	465.0244751	112.1405396	177.553717	-415.6235962
465705601	352.2263184	103.6659973	145.9771881	-349.072998
1188076772	463.0874023	131.1565851	223.7099884	-323.6015015
469700925.4	367.7661743	123.7484193	205.1114273	-291.9405518
932851876	346.6151123	109.131619	183.1950989	-341.9488831
2309521036	471.8776245	110.9396207	156.2983871	-360.7367249
756738501.9	539.4158936	114.953103	175.931427	-370.7989807
1474524625	433.7471008	115.1178913	181.4919586	-329.0127258
392083994	353.4741211	118.7378176	209.8864517	-326.3739319
887711925	379.9902954	112.3656724	154.8299164	-343.334198
809648649.4	434.5542297	148.9285873	250.9643829	-444.9841614
743117202.6	428.3608704	111.5712771	189.0735016	-254.0146332
1367144061	394.6083679	122.8510848	179.0920898	-378.4510498
1066554461	430.8102417	107.8037961	168.4084351	-348.6997986
1113115236	488.2976379	114.2387306	175.2047333	-383.6593018
1619358411	454.9340515	120.3329494	181.2140961	-435.0180054
2261940186	510.2778625	129.4415408	210.4373962	-309.9703674
938626483.5	462.5370483	117.1975269	186.6869202	-351.5621948
235577333.5	417.0652771	134.6828601	246.6535797	-189.3396454
335680797.4	374.0098877	118.3245562	178.236911	-380.1202087
925948819.4	365.0462036	103.2490534	163.2696228	-367.5168457
1887714170	470.4830017	139.9629095	227.5426016	-377.6416016
1082740976	367.5274963	108.7468058	165.8649292	-473.795929
285693841.5	316.3741455	84.4406576	142.3804077	-255.7303619
630236887.1	437.1991577	119.4478611	211.0949326	-252.0856018
446183252.7	321.7355957	112.0590163	185.0523224	-304.3544312
696105718.5	379.211853	115.518664	201.9064041	-224.5696564
983969293.5	356.9725952	109.724265	187.4246552	-370.1124573
690524119.3	433.1776428	116.5110443	209.7867447	-217.6557312
1434342802	421.8758545	123.3056982	178.5211838	-491.6614075
1343828188	402.0379944	117.9918385	180.4438629	-312.9962769
1575888840	477.5485535	125.8355184	191.051651	-398.5688477
447345955.5	305.6681824	104.5418725	122.1905403	-425.3721619
171197781.9	261.6470947	106.0297244	201.4757477	-181.4072266
399128765.5	516.8141479	116.4558698	207.6111572	-247.8688507
1241841090	413.1296387	107.4463003	184.9897217	-323.7263794
396320975.8	332.0806885	86.62778739	127.2524071	-388.2695007
1532041906	443.2108154	122.2455733	211.4008667	-305.0542603
429960714	370.9147339	116.6149037	185.2563934	-361.0478821
1666287683	465.4546204	125.201751	212.1787323	-336.6098633
762297526	417.1604309	122.286743	186.001976	-360.0920715
1606574436	537.067627	134.7093795	209.4140167	-567.1032104
721317839.6	420.8436584	104.8092635	189.9903992	-299.9789124
514362869.9	301.6211548	117.8354237	181.7671097	-435.9754333
288012231.1	228.3582306	87.13355263	138.8316711	-168.1200409
1581412407	437.3077698	141.1214976	238.7821198	-338.6295776
2064913821	598.9344482	118.0596804	185.9329102	-436.2715149
940477204.6	402.1911011	123.8464857	190.5857391	-338.0391846
730350679.4	341.4171753	120.3359527	194.0633652	-304.5856018
258505538.4	333.8693237	127.3875656	215.4041061	-163.3920288
867229324.5	494.4362183	144.2786707	258.330127	-282.2744141
475316319.9	424.069519	128.9931496	228.811705	-252.5718994
1805161131	454.3796997	136.9258468	235.3085175	-370.7043457
386456811.8	335.4218445	105.2129842	174.4630402	-332.2367859
1051564859	559.0706177	124.6404428	195.078064	-475.7581177
2019260575	402.0011292	140.1411997	218.386438	-452.1291809
752056268.4	437.8034973	116.2530323	199.7350616	-284.9918518
2156267641	436.7554321	130.6450406	217.2450745	-322.5096436
2206591508	405.6341248	112.0353929	171.1072083	-400.4417725
2278306713	509.2733459	140.7075788	231.0483612	-442.8220215
997643223.7	492.892334	125.9025742	194.2787018	-423.3386536
571050324	386.9375	94.76759369	138.371814	-343.3462524
589358615.7	374.5993347	126.7354782	210.2766144	-284.4051819
1110527751	347.9500122	112.0497467	161.133316	-387.1145325
1411064561	378.3794556	108.3341082	151.4147491	-362.6096191
597036523.9	412.4586487	127.4177742	187.7674774	-521.5371094
1250923148	409.8213806	132.0497719	179.4317139	-458.3456726
1087219199	417.4826965	127.5783047	205.2906113	-380.7720947
124028040.8	279.6341858	93.71890706	176.790036	-94.99166107
1774859824	414.3938293	130.4649982	207.5144867	-442.5511475
1118888201	402.2052002	109.2185881	191.6998093	-226.2785797
721445506.4	436.8870239	136.7904507	222.0075424	-336.1712036
1033011554	362.7395325	112.1294602	193.7152374	-377.7819214
120595590.3	228.5301666	104.2468452	178.5877991	-93.06378937
188390887.7	330.8799438	109.8712701	187.1047195	-334.7314453
1354842631	400.0856628	125.0093197	217.4476166	-279.723999
556372959.7	369.5473633	136.5588926	217.3916473	-394.6167297
430030206.9	280.3934021	100.9780744	140.4719009	-352.3638611
1467684015	382.219696	111.7480791	166.1684418	-374.7080383
1806891828	413.6063538	119.2499576	194.494278	-275.9122314
783057547.2	422.5521851	181.1293486	214.6825607	-548.2548218
384820287.8	285.6141052	89.9126798	155.2982788	-385.6330872
173174785.4	329.6795044	122.4161065	219.5101303	-156.6203613
167400476.7	298.7278137	83.558374	144.5425369	-179.3365479
778521839	392.3999634	158.0704223	223.8934631	-417.2298889

log.sigma.3.0.mm.3D_firstorder_Entropy	log.sigma.3.0.mm.3D_firstorder_StandardDeviation	log.sigma.3.0.mm.3D_firstorder_Range	log.sigma.3.0.mm.3D_firstorder_Variance	log.sigma.3.0.mm.3D_firstorder_10Percentile
4.15814205	116.521768	769.5783997	13577.32242	-25.9254879
3.972532894	101.1742205	712.8218384	10236.22288	-33.78089333
4.138713099	110.5500104	667.5734558	12221.30481	-141.1360321
3.927221519	97.44743065	784.8761902	9496.001739	-39.75670929
3.617973746	89.30281324	505.8225098	7974.992453	-34.9117485
3.785952821	87.74885498	648.0196228	7699.861551	-61.62312279
3.316897805	74.04255938	457.8992767	5482.3006	-16.7694828
3.958528739	101.9747119	843.8618469	10398.84187	-26.71433029
3.982367587	99.34698195	727.2957458	9869.822823	-20.12847633
4.112736127	106.6617277	743.0856323	11376.72416	-67.10753479
3.598516263	76.73985816	628.5968323	5889.00583	-25.60373459
4.086279649	105.9742972	694.1124573	11230.55166	-77.00816498
3.638815128	81.20444444	705.8961182	6594.161798	-31.15343857
3.905304044	91.76618615	596.0006714	8421.032921	-72.29800568
4.135916921	109.1952633	644.252655	11923.60552	-58.65760498
4.033497995	106.1817746	880.6480713	11274.56927	-78.8000351
3.87900342	96.98421967	701.2993164	9405.938865	-87.83596802
3.832495089	92.46689664	786.6889038	8550.126975	-1.328463709
3.948712049	96.22004762	659.7067261	9258.297565	-35.65764809
3.901512835	93.5895804	688.5639954	8759.00956	-54.3603096
4.097799645	106.357614	832.6143494	11311.94205	-104.339888
4.005512903	99.88178706	910.2148743	9976.371387	-68.00793457
4.079112977	103.6153544	762.7598267	10736.14166	-82.32216644
3.974816249	105.079892	679.848053	11041.78371	-56.90009689
4.174305986	111.3078535	723.3244934	12389.43826	-137.3584732
4.234107967	119.4739901	879.5383911	14274.03431	-38.96300316
3.965768704	96.96906031	682.3755035	9402.998658	-55.97247696
4.170012285	112.7685454	773.0594177	12716.74484	-111.1619049
3.856562467	94.90684847	779.5100403	9007.309887	-49.64270096
3.984645924	101.0061064	871.9569397	10202.23354	-62.08995514
4.057316281	105.4436242	889.9520569	11118.35789	-75.33273697
4.016434239	99.1846256	820.24823	9837.589956	-30.5025898
3.959840876	103.138999	814.0992432	10637.65311	-52.72066879
3.861426434	104.0284619	606.4049225	10821.92088	-20.69034958
4.104800218	110.8542961	754.1300964	12288.67497	-97.19583893
3.809098746	90.73201688	732.5630493	8232.298888	-51.92127228
4.283291174	122.8396012	848.1246033	15089.56762	-81.58929138
3.876513066	94.07279093	841.3234253	8849.689994	-56.02074814
3.585349589	76.35391144	572.1045074	5829.919792	-47.08653793
3.801365742	92.90728164	689.2847595	8631.762983	-22.38004208
3.862128935	90.80567182	626.0900269	8245.670035	-40.5523243
3.84221203	90.53766874	603.7815094	8197.069461	-27.16468315
3.766914259	87.84335239	727.0850525	7716.454559	-26.27427711
3.740325648	92.523823	650.833374	8560.657822	-15.63013372
4.192523608	117.2618858	913.537262	13750.34987	-111.4830833
4.066981177	103.023883	715.0342712	10613.92046	-79.17530518
4.135953726	111.0080895	876.1174011	12322.79593	-71.53858948
3.91658067	103.3214833	731.0403442	10675.32892	-122.1142921
3.519439585	84.78131418	443.0543213	7187.871234	-22.33445721
3.738750426	93.58465951	764.6829987	8758.088495	-18.64304962
3.718896318	86.62518941	736.8560181	7503.923441	-20.99023647
3.444353949	77.00250421	720.3501892	5929.385654	-29.19645119
3.823194895	91.86390548	748.2650757	8438.977129	-11.83618164
4.000597901	99.09305414	731.962616	9819.433378	-57.178936
4.026497068	101.1522477	802.0644836	10231.77721	-40.85793076
4.238417783	116.3255324	777.2525024	13531.62948	-110.2731552
4.168524962	116.0443912	1104.170837	13466.30073	-53.82342758
3.63217075	89.8553888	720.8225708	8073.990896	-26.15405598
4.03404328	106.2886686	737.5965881	11297.28106	-80.42756958
3.355743389	62.66958944	396.4782715	3927.47744	-20.36528778
4.007016858	102.3432814	775.9373474	10474.14724	-12.71632862
3.948121159	103.7236881	1035.205963	10758.60348	-50.24209747
4.060111102	104.5362423	740.2302856	10927.82595	-60.48406219
3.981719518	97.27039608	646.0027771	9461.529954	-52.77290802
3.884646803	92.54104088	497.2613525	8563.844247	-22.44675083
4.11938873	115.5658623	776.7106323	13355.46853	-41.53507996
4.010289989	108.4447887	676.6414185	11760.27219	-42.69986534
4.062711744	106.4259332	825.0840454	11326.47925	-24.00619316
3.795362435	87.97744464	667.6586304	7740.030766	-45.792836
4.137939707	112.7623833	1034.828735	12715.35508	-82.15071259
4.356373327	125.5944178	854.1303101	15773.95777	-104.4177818
3.718289728	88.35836585	722.7953491	7807.200815	-13.159729
4.028313646	102.8911386	759.2650757	10586.5864	-32.34909744
3.94186167	95.45446915	806.0758972	9111.55568	-63.49970245
4.262125456	121.6219205	952.0953674	14791.89155	-63.25186234
4.114913175	112.6403492	916.2309875	12687.84826	-69.73994446
3.703513568	84.72566508	730.2837524	7178.438323	-51.52155151
3.981839355	97.16603953	659.0045166	9441.239237	-37.91695862
3.988762587	100.779096	735.0645447	10156.4262	-80.1824646
3.963703131	102.7529256	740.9890747	10558.16372	-101.3331284
4.074103131	117.0279971	933.9957581	13695.55211	-67.48369293
4.260059003	125.7692153	868.1670532	15817.89551	-126.435173
4.114672483	109.3160613	798.2547913	11950.00126	-63.20900002
3.310463265	72.12646457	374.6258469	5202.226891	-12.00206337
4.154662195	110.5649269	856.9449768	12224.60306	-70.95828094
3.833232144	88.38343865	628.4837799	7811.632228	-41.3336544
4.188136673	117.10556	773.0582275	13713.71219	-63.41204948
3.817020253	90.03335633	740.5214539	8106.005253	-25.74472389
3.386990767	70.97982876	321.593956	5038.136091	-8.040036201
3.694209776	92.02806896	665.6113892	8469.165476	-26.39436798
3.935949504	96.36949567	679.8096619	9287.079696	-27.03912735
4.161937144	113.3287305	764.164093	12843.40115	-66.91323547
3.892461016	96.26472576	632.7572632	9266.897425	-93.07201004
3.962460774	101.0268134	756.9277344	10206.41702	-75.06413879
3.842371362	88.00383811	689.5185852	7744.675521	-22.20408535
4.750133178	180.438675	970.8070068	32558.11544	-261.9231384
3.450290532	74.05889965	671.2471924	5484.720617	-28.44204903
3.671419711	86.31325844	486.2998657	7449.978583	-3.509399319
3.42327015	69.97787683	478.0643616	4896.903246	-31.99754696
4.544274282	150.7631604	809.6298523	22729.53054	-171.4244354

log.sigma.3.0.mm.3D_firstorder_Kurtosis	log.sigma.3.0.mm.3D_firstorder_Mean	log.sigma.3.0.mm.3D_glrlm_ShortRunLowGrayLevelEmphasis	log.sigma.3.0.mm.3D_glrlm_GrayLevelVariance
3.437695406	94.04840846	0.007242333	22.83158823
3.622261598	70.08651829	0.006513981	17.38502217
3.018144554	22.18343239	0.010157295	20.11164019
4.123927569	64.76467041	0.005852093	16.16735727
3.495692847	56.40134184	0.016852253	13.6817402
4.074826049	40.03267425	0.007192803	13.09954075
3.47052207	50.90230967	0.015510852	9.716748688
4.279225604	79.36098388	0.00404633	17.77967633
3.502365199	88.00892929	0.004071213	16.42478621
3.424883631	69.89774469	0.005068289	18.8704521
4.393029189	57.95052422	0.004390364	10.01753292
3.534280882	62.10466348	0.0065902	18.62828648
4.80221015	51.72812492	0.005012657	11.41822156
3.183502062	48.12547041	0.012685753	13.92632412
3.172389273	69.66463845	0.020646576	19.75133527
4.087438983	36.06842612	0.00368424	19.56065297
4.500138577	36.61557228	0.007662673	16.00132894
3.571907779	93.0157128	0.0041354	14.31629834
3.287684784	77.81628178	0.006277952	15.48225988
3.651161966	56.13110284	0.004906058	14.68769162
3.676791252	31.5540392	0.00523872	18.79332402
4.294818957	56.9020607	0.004395441	16.81471956
3.361557027	50.15961755	0.005366333	17.8112266
3.681417606	55.28910941	0.005962677	18.98731363
2.655480782	15.38200481	0.006830321	20.24689688
3.962919652	88.91394609	0.003458747	23.70365335
3.470500701	55.18288873	0.008394251	15.69963387
3.267505826	48.7405806	0.004314475	20.93017784
4.764459361	51.13070082	0.005883202	15.61297781
4.716279924	53.36903621	0.004037628	17.43542044
4.266554172	57.97983107	0.003025228	18.62814989
3.679580195	83.17164499	0.004684416	16.48421138
4.649161175	55.65615145	0.004727788	17.98793767
2.831788689	85.54269073	0.012396291	18.14921932
4.119388118	41.37663136	0.006794762	20.58298274
4.607700004	49.27543138	0.004560687	14.34277247
3.681135107	67.08240017	0.00435486	25.1854317
5.08532134	54.55435623	0.002690704	15.05722992
3.87646184	36.05974022	0.00788864	10.06600939
3.942006693	75.07348759	0.006409812	14.83570597
3.781503545	65.66241777	0.005781627	13.92833062
3.306344644	71.74602621	0.010449289	13.94465198
3.933649391	65.74921876	0.003600156	13.32546952
4.355044964	70.8107734	0.0100317	14.71890249
4.340530861	38.13063524	0.003052099	23.34452052
3.374790103	57.51654974	0.006581995	17.4756116
4.392204942	59.26028817	0.004980016	20.68743746
4.75276471	15.92715276	0.004398336	18.29331469
2.809443828	63.67441583	0.012912457	12.29074102
4.358855775	69.31003615	0.008174353	15.02558034
4.515896105	63.56716131	0.005053557	12.84571371
7.535929972	39.68611714	0.004679559	10.66122247
3.699437719	80.65359921	0.004118551	14.45831599
3.71250399	61.47847098	0.004513166	16.3451467
3.485148605	73.78144232	0.004310334	17.21257941
3.457599123	37.71495769	0.005892071	22.70975074
4.945816723	68.41283651	0.001924874	22.92095127
4.669218196	53.95359879	0.005771683	14.36073903
4.991811794	50.87146557	0.004154864	19.00956461
3.31661059	60.5374145	0.016173375	6.596573762
3.559141222	97.16547655	0.003827875	17.54353613
5.582121169	56.38691924	0.003176724	18.58987699
4.083738238	66.40878015	0.006112393	18.32244844
3.231449638	70.84639418	0.005457846	15.6505481
2.438750852	87.54283306	0.01451155	14.11255075
3.214243861	86.37630626	0.005533299	22.222863
3.759249204	69.84955594	0.007116051	19.97837832
3.770806501	86.15223888	0.0039252	19.19178965
3.931157579	57.70391041	0.005045561	13.03750553
4.36757111	53.10258842	0.002661399	21.69167549
3.016540218	62.17393411	0.00278443	25.83243494
4.38835462	75.54843949	0.005636007	13.53646866
3.703567885	80.50801344	0.004932575	17.82104252
3.93915624	58.65469785	0.00304302	15.3178967
4.04333631	70.75825878	0.003550211	24.79790425
4.769935845	56.24597695	0.004451995	21.41606517
5.031099631	42.45537059	0.005672943	12.44761407
3.481317387	81.36732884	0.006911115	15.57638478
4.317398723	48.9767245	0.004220123	17.23100541
4.360763289	34.32368397	0.005447402	18.14560653
6.332956088	50.39580395	0.00405303	23.57334586
4.355258073	40.23986509	0.00381076	26.53137365
4.100895958	65.77402651	0.004476016	20.29616025
3.021030428	59.84151277	0.034386182	8.959283556
3.678688398	69.25686025	0.002870923	20.23258051
3.083266825	64.16438073	0.007335574	13.10069028
3.717611946	70.69593493	0.006199323	23.04503449
3.84300788	66.83569845	0.003248993	13.67249434
1.961684231	76.34964731	0.030714867	8.249932111
5.252935598	60.02108394	0.006492983	14.78265269
3.239891462	79.62568883	0.005081048	15.62975038
3.96855667	76.19009118	0.004440584	21.46413423
4.243592113	30.49055731	0.006018311	15.69136169
4.54844289	47.76207869	0.005495663	17.46346074
3.577111813	80.4728331	0.004811767	13.12137971
3.012955084	15.80270495	0.006466466	53.3426244
5.411124871	50.98597231	0.004106002	9.549153789
2.829191145	86.80855114	0.012583256	12.54750865
4.043696549	45.66288012	0.01270222	8.706715695
2.995534494	47.50502972	0.006743697	36.77900587

log.sigma.3.0.mm.3D_glrlm_LowGrayLevelRunEmphasis	log.sigma.3.0.mm.3D_glrlm_GrayLevelNonUniformityNormalized	log.sigma.3.0.mm.3D_glrlm_RunVariance	log.sigma.3.0.mm.3D_glrlm_GrayLevelNonUniformity
0.00766886	0.068514304	0.127821884	74.3994694
0.006948859	0.076136596	0.157338783	135.4491665
0.010575289	0.0661652	0.097808411	68.08677671
0.006240023	0.081297079	0.154223328	177.9479534
0.018469333	0.101111681	0.208919606	82.86522935
0.007677107	0.089622322	0.156063134	130.2866711
0.017620606	0.120906271	0.328284148	103.9715132
0.00434096	0.079125875	0.182528375	125.7103928
0.004372178	0.074022312	0.162588736	133.3442778
0.005361248	0.0685448	0.112037407	187.5000029
0.004765659	0.09873432	0.181089925	155.5123028
0.006889372	0.070589015	0.109854947	108.9295344
0.005481124	0.100009503	0.201267193	253.4712339
0.013429661	0.078352163	0.123550256	131.7847122
0.021489508	0.065926906	0.1220736	92.57397301
0.003998315	0.073696113	0.269915477	261.0629776
0.008117777	0.086979072	0.167116463	123.239635
0.004499287	0.082847741	0.200332285	185.3323713
0.006633396	0.07527615	0.123234153	77.52096685
0.005287369	0.080082599	0.163502465	205.8642109
0.005550027	0.070007497	0.123689621	440.4230151
0.004656259	0.075210811	0.132246539	143.8937422
0.005665895	0.070339021	0.120341178	263.3026875
0.006395296	0.077546661	0.193218576	69.93277075
0.007213882	0.062655585	0.109730385	149.233804
0.003601838	0.063695056	0.109638213	78.98294042
0.008777826	0.076698912	0.113424599	155.2319267
0.004500547	0.065735211	0.091937593	204.3547041
0.006319551	0.086232715	0.219497952	255.5812855
0.004280203	0.078314723	0.16326511	221.038712
0.003208889	0.073152496	0.129206124	273.1482856
0.004996282	0.073547861	0.137916885	330.8995181
0.00510446	0.081432842	0.170743375	182.2534517
0.013292043	0.082276286	0.172346442	34.83337604
0.007090215	0.070946042	0.110761773	57.44768916
0.004920172	0.088604962	0.191091732	248.8969415
0.004576395	0.062553057	0.11584949	203.339673
0.002864683	0.085066514	0.143272305	257.956194
0.008670865	0.100670649	0.227969793	127.7688545
0.006965415	0.087349619	0.203707007	124.3856996
0.006071964	0.081935844	0.119275428	97.94051891
0.011208471	0.082578005	0.166491668	140.7577036
0.003934328	0.088502859	0.215432644	231.7895334
0.010885807	0.093895411	0.201828401	153.9732023
0.003245525	0.067696865	0.167367789	210.5401439
0.006922562	0.070415319	0.115816319	228.8012948
0.005217036	0.070419767	0.126144297	234.9805225
0.004645574	0.0843952	0.147416611	114.2406865
0.01418664	0.103825448	0.200568083	50.30753031
0.008835544	0.094961806	0.191997942	90.54850841
0.005557232	0.092767379	0.222438752	317.2756464
0.005089799	0.118830945	0.243146588	197.9510799
0.004511624	0.085165127	0.198407014	282.2516081
0.004741696	0.074813958	0.109029144	79.82123358
0.004605748	0.07247473	0.147674929	255.9867146
0.006217232	0.06328472	0.123298347	108.7554853
0.002041881	0.069418699	0.135205469	204.9301489
0.006460569	0.102381379	0.284166923	208.6653384
0.004351383	0.073808728	0.127189968	91.63735526
0.017385194	0.113473009	0.161291142	140.3235431
0.004125045	0.073376184	0.166019979	191.0791548
0.003423178	0.081671964	0.198470303	392.9487999
0.006433891	0.073252592	0.132182104	150.2739374
0.005737194	0.072900423	0.117920279	124.4671189
0.015262823	0.075019636	0.115100714	40.36944151
0.005899806	0.069143609	0.116238263	96.99378612
0.007623035	0.075744874	0.14611944	71.49002497
0.004184962	0.072494099	0.162565765	229.915543
0.005350308	0.086133299	0.143926132	99.33349872
0.002819633	0.070991964	0.140378388	160.0484931
0.002935288	0.055933622	0.108274	195.3665104
0.00610747	0.094577379	0.212065858	169.2638481
0.005312371	0.074078264	0.162707392	308.534148
0.003263051	0.077988246	0.157267206	452.9731654
0.003762755	0.064197437	0.128096324	247.2457319
0.004696615	0.071638165	0.137291277	150.0878503
0.006059329	0.095568715	0.190828711	196.6833866
0.007237285	0.075153656	0.126180443	92.57793765
0.00447956	0.077717779	0.155787371	227.6430922
0.005815918	0.080522596	0.184034715	315.679224
0.004252434	0.077613491	0.161591542	94.37922878
0.003968544	0.066349882	0.114300823	161.0351621
0.004703973	0.070478676	0.136117848	157.2964523
0.038468082	0.117685928	0.265620927	51.86254003
0.003032972	0.066758222	0.116866866	234.7354049
0.007953378	0.081560312	0.155258196	251.2105641
0.006492405	0.06831539	0.128441602	88.45637172
0.003516983	0.085327382	0.169440397	228.8624708
0.033401238	0.103839461	0.173217352	37.62083014
0.006867075	0.102026773	0.188185463	51.25657082
0.005477683	0.076759009	0.159800894	219.0292099
0.004625959	0.068158904	0.102289841	69.08504344
0.00629368	0.081890097	0.123142085	115.8256115
0.0058518	0.081335203	0.154060969	315.7023932
0.005229837	0.083246121	0.172527007	345.1646743
0.006666196	0.044608178	0.079408206	37.11041588
0.00449338	0.113378842	0.234796209	170.047969
0.013707504	0.090427472	0.199884799	33.67169511
0.013972593	0.112809312	0.263501683	84.67422265
0.006959476	0.050326451	0.069332675	54.60180897

log.sigma.3.0.mm.3D_glrlm_LongRunEmphasis	log.sigma.3.0.mm.3D_glrlm_ShortRunHighGrayLevelEmphasis	log.sigma.3.0.mm.3D_glrlm_RunLengthNonUniformity	log.sigma.3.0.mm.3D_glrlm_ShortRunEmphasis
1.360491022	240.8320099	896.7815777	0.926100446
1.424377708	235.3835823	1439.662205	0.918388978
1.281136153	211.2658255	879.326149	0.939295388
1.417067878	255.6139201	1780.747223	0.919794392
1.560848714	101.6115318	630.0866019	0.897391869
1.43195029	193.663619	1168.281279	0.915179994
1.858358462	92.07916257	599.0437974	0.858193027
1.478622666	338.391248	1265.245326	0.911825084
1.43272004	316.8014971	1459.432995	0.918445133
1.319989277	295.7805494	2295.499577	0.932360252
1.506477235	264.3746818	1223.891908	0.901362601
1.306039889	255.4512593	1310.947875	0.936975124
1.546507159	230.3702476	1945.55745	0.897200684
1.347671867	155.8837664	1399.149909	0.928742619
1.342989162	136.5102046	1173.788119	0.930043263
1.624533758	346.145782	2765.878928	0.903593251
1.461195283	244.7949762	1128.118642	0.91080044
1.51644393	288.7155819	1773.21184	0.908989467
1.344930153	243.3783028	859.9735714	0.929836056
1.442109808	271.6339882	2064.666621	0.915111842
1.347185734	277.0527161	5227.949958	0.928497689
1.367810878	307.4883502	1580.93842	0.925721237
1.335893704	269.5655648	3132.565432	0.931154962
1.509286016	275.0399622	711.4090966	0.907481892
1.30871021	232.8036696	2018.795901	0.935815177
1.301334623	480.5974476	1056.438363	0.938333991
1.310326419	190.9018285	1721.585188	0.93740471
1.259205622	339.0896832	2695.628008	0.944987774
1.551263093	264.8281819	2343.406681	0.907306267
1.422963031	337.7086848	2314.209135	0.922650488
1.363001289	415.2851718	3081.898874	0.925817731
1.377841969	279.3910372	3712.435177	0.925413396
1.455294927	305.8606708	1792.917825	0.914096112
1.469590826	151.6509544	335.5689874	0.909932319
1.316727275	324.661636	681.3111779	0.933222823
1.508084394	290.9423022	2204.913023	0.906242015
1.324837971	366.5532547	2731.764249	0.932863783
1.397303626	443.8468371	2466.752475	0.920463551
1.611549638	160.0971589	958.2225666	0.889386211
1.528956548	209.0035397	1114.390068	0.905133008
1.336078016	253.7976801	999.2402989	0.930589183
1.456748781	154.790854	1355.231858	0.911310569
1.559529386	312.0128322	2030.009129	0.901094103
1.529871855	156.1145625	1281.479922	0.904322464
1.435721086	464.5578124	2529.305826	0.919535735
1.329564037	246.6804791	2716.177379	0.930816032
1.349859314	350.7368947	2777.489193	0.929186818
1.409356746	349.0876016	1097.632086	0.918593207
1.571210251	124.0762992	366.9996461	0.890101901
1.508661254	178.5715508	751.9051917	0.907189245
1.588452323	246.7017009	2609.917668	0.894609694
1.635929592	302.6084947	1256.896475	0.889849493
1.519269673	272.7346375	2602.217477	0.90614127
1.316667297	314.7527574	894.7736315	0.93205767
1.394171027	300.6644921	2904.2425	0.924295107
1.334440289	292.088012	1446.771008	0.933383328
1.373973328	659.8963245	2435.204367	0.925349365
1.730063618	209.7713084	1496.562822	0.878759031
1.352429066	406.5663011	1031.469616	0.928414666
1.460362979	94.16513859	975.6213061	0.907455844
1.446817423	330.235761	2090.357407	0.914682343
1.506560312	411.2442073	3820.323844	0.91027904
1.364079933	290.3083202	1702.23408	0.927398726
1.319625952	263.3872322	1450.085785	0.936415156
1.321510529	127.2283262	452.9289679	0.933291082
1.328612726	264.0338866	1172.432282	0.931115825
1.406900574	211.246678	763.8017383	0.918421127
1.431227906	351.120787	2571.636787	0.918841595
1.40604251	269.5438711	933.8772675	0.917979849
1.382008967	493.8249605	1856.455214	0.925047495
1.299921228	478.1829698	2980.530342	0.938603824
1.55299144	234.2222243	1392.693133	0.901637582
1.431728546	276.6443009	3379.708409	0.918676044
1.418406728	376.0347257	4733.747457	0.920654689
1.355228178	447.341626	3194.340169	0.927968024
1.378262783	379.7063106	1723.113495	0.924608335
1.509039129	248.9032097	1616.991513	0.906005559
1.348173274	243.8849248	1028.733406	0.929817208
1.415784433	327.5975208	2394.919271	0.921331523
1.481307615	274.0956326	3124.329535	0.911900697
1.422353249	531.1247296	994.0050084	0.921732718
1.316980965	437.0382393	2053.494169	0.935297002
1.370140292	358.7565652	1848.71921	0.92711173
1.700660917	53.39831782	327.6632408	0.881285338
1.326006013	439.0762094	2954.96403	0.932982987
1.431230417	170.0360394	2468.854186	0.914521397
1.349520419	303.2079675	1082.078838	0.93052213
1.463291687	350.4354586	2135.124824	0.911239659
1.479541031	61.62583849	290.0030822	0.910036428
1.522372484	269.8069256	388.9997326	0.899821715
1.430847118	243.4339702	2306.972591	0.91770287
1.294178506	377.4862786	861.3198957	0.937075253
1.347686425	270.4300908	1175.346655	0.928294507
1.416951343	294.3086914	3149.804523	0.9191957
1.466298345	238.82611	3295.747031	0.911022477
1.211385279	558.0987399	744.427015	0.957152819
1.629073372	314.4709781	1122.326601	0.886978239
1.539156012	123.4339538	290.333548	0.900329978
1.679392749	103.3572255	557.2060486	0.884155171
1.202122841	389.978069	964.1792282	0.954727365

log.sigma.3.0.mm.3D_glrlm_LongRunHighGrayLevelEmphasis	log.sigma.3.0.mm.3D_glrlm_RunPercentage	log.sigma.3.0.mm.3D_glrlm_LongRunLowGrayLevelEmphasis	log.sigma.3.0.mm.3D_glrlm_RunEntropy
338.9644456	0.9022316	0.009785412	4.666655843
348.8229953	0.889307692	0.009193412	4.546159052
291.5696129	0.920324756	0.012419698	4.561720953
382.3283201	0.891780865	0.00821511	4.498025794
160.9113493	0.862770528	0.027025665	4.301662168
302.2203136	0.88715131	0.010097084	4.368655302
173.6489933	0.812459064	0.030283064	4.216315441
531.296718	0.879735777	0.005928638	4.576573479
479.83667	0.88850518	0.005966112	4.545092682
418.4171633	0.910832757	0.006718503	4.588861086
438.2907174	0.871397557	0.006686546	4.241278239
358.2142186	0.9156357	0.008306095	4.538149558
385.4323004	0.864747748	0.007967254	4.331851374
227.0599045	0.905150286	0.016841841	4.397500515
196.5444527	0.907026436	0.02553242	4.612213274
595.5142319	0.861411161	0.006038552	4.725738269
396.1947171	0.882290918	0.010344939	4.485588698
459.0970171	0.874270764	0.006582233	4.461359863
343.5903611	0.906351571	0.008364364	4.433259452
419.250867	0.885848381	0.007233935	4.48591211
403.6602191	0.905146652	0.00702324	4.608403808
454.593486	0.901751714	0.005930632	4.521391969
386.8881044	0.908237358	0.007102988	4.571376788
437.9795745	0.874607916	0.008711016	4.595532264
324.493998	0.914717003	0.009044461	4.624935408
658.6406242	0.916989076	0.004301806	4.673725702
265.7164125	0.915179348	0.010661148	4.420936669
457.8134007	0.926516107	0.005358051	4.577308055
440.8289231	0.870957521	0.008784275	4.516543125
513.1709303	0.892931064	0.005607402	4.55077733
618.7829904	0.901422575	0.004094811	4.583939838
407.522478	0.899615385	0.006566022	4.54706752
475.4636501	0.883870772	0.007079355	4.563597757
223.3243263	0.879577803	0.018050759	4.452162497
461.547775	0.911729037	0.00843349	4.564334432
476.5903933	0.872815093	0.006814584	4.467520144
518.0789565	0.910619221	0.0056293	4.767051584
679.3396026	0.894018102	0.003726716	4.439463373
278.3631751	0.854395604	0.012938548	4.301108138
332.6403941	0.870039496	0.010047907	4.45888497
364.4341333	0.908054711	0.007465353	4.350511971
236.7785658	0.881947762	0.015077184	4.445263464
522.6209243	0.86452695	0.005828492	4.45230409
242.1781079	0.86967173	0.015559949	4.409367381
729.2124153	0.889767073	0.004258203	4.767730682
356.2622604	0.908725121	0.008518491	4.55079075
505.0527365	0.905066989	0.006366505	4.648621158
544.8661655	0.892277248	0.005842088	4.481726811
200.5187347	0.858156028	0.020758321	4.199166336
276.4669489	0.874100212	0.012474323	4.380139712
417.7330073	0.857931727	0.008325396	4.43613758
546.18535	0.85067642	0.007345659	4.203112048
433.610101	0.872206803	0.006699358	4.480099418
444.8448251	0.911055639	0.005802864	4.466251576
441.0083071	0.896895332	0.00613025	4.568208118
412.8863047	0.90999837	0.007773108	4.712529563
969.9400666	0.900016422	0.002624235	4.70529112
376.7345609	0.836506832	0.01057571	4.452887015
598.705447	0.90468715	0.005288661	4.529313231
153.5347175	0.87998905	0.023259092	3.953862828
503.3246776	0.885256192	0.005695312	4.5938687
669.2098174	0.876365115	0.004779315	4.59521846
423.7404365	0.902957017	0.00798847	4.573366365
373.4311538	0.913852743	0.007102923	4.430394851
176.3861213	0.911864407	0.018902376	4.320348972
356.2377055	0.908968543	0.007643987	4.600814903
305.4416741	0.891740585	0.010133579	4.567702061
531.7304575	0.889102205	0.005569657	4.642897503
416.8205193	0.891784163	0.006847451	4.346611666
733.9100231	0.898837102	0.003616499	4.677422434
660.3132177	0.917259211	0.003668898	4.797191783
383.5655067	0.86731609	0.008769506	4.389178135
417.7489986	0.889769346	0.007273249	4.603499525
579.7788602	0.891922545	0.004405255	4.508429702
644.8076108	0.903530304	0.004770905	4.782205725
564.738887	0.898623899	0.005886148	4.649354789
412.2617192	0.873297403	0.00812276	4.355632095
358.024752	0.906435728	0.008811431	4.459875776
507.8238739	0.893467643	0.005811893	4.547005837
448.9555839	0.879873551	0.00774829	4.594054981
815.4107416	0.892352875	0.005250677	4.640596828
623.8208637	0.913175646	0.004724521	4.737162096
524.716201	0.901716311	0.005838241	4.639442651
86.43188814	0.841594352	0.063356825	4.047856256
627.2637259	0.910369279	0.003810504	4.635666132
258.8708218	0.88651964	0.011043577	4.417977649
430.0467147	0.906377936	0.007942256	4.682877433
550.3991221	0.881089006	0.004913717	4.426180506
93.53044659	0.88115291	0.047515735	3.929753845
452.8243625	0.868645196	0.008815362	4.342894577
366.2376203	0.88834056	0.007566496	4.511535839
520.3202458	0.91709015	0.005485564	4.601407023
401.5437619	0.90524968	0.007592238	4.396454607
452.2950325	0.891339306	0.007587001	4.542935338
376.494899	0.880839517	0.007402619	4.454024551
717.7752808	0.940741385	0.007540797	5.06001384
571.3635505	0.849906191	0.006573068	4.194738229
194.5311831	0.868979152	0.019847257	4.274862453
187.9422817	0.843728344	0.021296352	4.184043227
502.8088705	0.940141314	0.007874732	4.868510945

log.sigma.3.0.mm.3D_glrlm_HighGrayLevelRunEmphasis	log.sigma.3.0.mm.3D_glrlm_RunLengthNonUniformityNormalized	log.sigma.3.0.mm.3D_glszm_GrayLevelVariance
257.7022149	0.824880628	34.43473048
253.8537542	0.808620371	27.09657227
225.4888448	0.853477911	25.24172303
276.3540143	0.812293182	28.67384722
110.9899653	0.767129084	21.95570819
211.5454775	0.802322196	22.12482925
104.1153893	0.694261462	16.95171264
368.9243443	0.795518299	30.5341149
343.0606563	0.809226568	22.61296122
317.2788374	0.838194117	26.1954898
292.8194563	0.774757286	18.52720203
273.0039032	0.848508099	27.22145171
255.1616539	0.766015071	23.27655501
167.8859481	0.830687806	18.89436482
146.7105653	0.834072757	26.63415684
380.8919077	0.779670436	29.54008814
269.4061975	0.794007792	26.81766384
314.6672126	0.790649841	23.67532123
260.5509186	0.833556289	22.35836192
295.6317851	0.802071744	23.33221991
298.7068226	0.82995616	26.78171625
332.1892428	0.824686238	27.25328947
289.5929814	0.835779578	25.16137113
300.5946082	0.787366946	30.66572716
248.6610159	0.846129372	25.08331554
511.2218006	0.85125972	35.31533239
203.561005	0.849708981	22.68476548
359.8130395	0.866232012	27.82174754
290.421014	0.788234573	28.77189072
365.2976145	0.818671335	30.78157997
449.7367637	0.824309236	29.17958019
300.6880819	0.823866645	25.05106884
332.9775283	0.800062806	30.90504221
163.7762779	0.79165107	26.85025904
348.6709217	0.840341046	30.47607932
320.0106695	0.784012801	27.6795988
392.5991931	0.839432481	37.34741371
483.1460274	0.812736167	26.35247621
178.3870528	0.752233087	19.39555375
228.2675511	0.781810164	25.10974177
272.7983665	0.83474629	22.71657908
168.3935981	0.794004573	23.10541402
344.0630463	0.774062327	24.78210693
169.6433422	0.78048278	28.15093513
506.1704484	0.811890914	36.05524095
265.6807393	0.834951625	24.15480199
376.9220691	0.831519174	33.28818072
381.3653317	0.809547817	29.32398834
136.8159594	0.754605603	16.54408649
194.1001065	0.786901055	25.60313973
273.0463645	0.761475017	24.90533028
338.658388	0.752670885	26.84228971
297.8020966	0.784066978	25.55554372
337.8583379	0.83757363	23.8702559
323.422642	0.821342992	27.60831302
312.2703784	0.84084213	33.0215404
711.6718465	0.823685377	38.12100916
233.8866765	0.731930082	26.15719094
438.9628468	0.829804463	29.70878893
104.1845608	0.786712603	11.1968562
358.5812948	0.801364739	26.9616048
450.4201665	0.792747719	35.00104463
312.5791612	0.828259905	30.04376535
281.5445309	0.847846366	21.0293537
135.8897864	0.840458806	18.61683603
280.1905223	0.835166975	29.06731641
227.1979128	0.808513516	32.83859938
380.1807839	0.809958643	31.3913727
294.2650696	0.808207166	21.89794276
533.2303369	0.822713606	35.02080624
509.2823232	0.852193241	32.441277
257.1847933	0.77608533	26.85824865
299.2882277	0.809965497	26.67652379
408.4766802	0.813812356	24.52068477
480.7338268	0.828711516	37.77231243
410.6551184	0.821694458	33.77130944
274.4644638	0.784128751	24.33572945
262.8864078	0.833348043	22.98741395
356.3002835	0.815938872	28.60765878
301.1869201	0.795815087	30.45619277
575.7736462	0.81636573	42.90127101
468.7500945	0.845055527	40.84383141
386.132756	0.827349234	33.54282967
58.54049297	0.739302696	12.99989372
471.1472718	0.839555681	28.97479991
184.837673	0.800489263	19.48862923
324.509987	0.834693333	34.94329667
382.822399	0.794510987	23.25007292
66.71558049	0.795269644	9.078402367
299.6756746	0.772096722	29.48071006
263.423033	0.807536959	24.05119286
402.8274779	0.848741438	32.59092336
292.7072039	0.829741561	24.66524479
319.9773605	0.810477754	30.57160114
261.0481522	0.793606305	22.04310323
584.8643468	0.894341089	62.28862585
353.7463286	0.746581789	19.52767308
134.9274017	0.775598792	18.42469385
115.9081333	0.741283862	16.79870083
410.4409468	0.888061603	42.67974314

log.sigma.3.0.mm.3D_glszm_SmallAreaHighGrayLevelEmphasis	log.sigma.3.0.mm.3D_glszm_GrayLevelNonUniformityNormalized	log.sigma.3.0.mm.3D_glszm_SizeZoneNonUniformityNormalized
166.5345659	0.048046202	0.325000569
159.7299999	0.055385767	0.339460639
129.6836287	0.056003026	0.356221075
174.6683748	0.053471909	0.336845791
80.90261399	0.061387339	0.291028004
116.1534986	0.062115625	0.305231314
55.85972236	0.072321389	0.21538529
215.5927393	0.054063022	0.332381508
197.3848492	0.060200109	0.344853452
172.9018229	0.054229657	0.305760288
134.4824846	0.071446287	0.24346301
158.2215291	0.054273601	0.366451989
151.7221967	0.058991968	0.296922733
82.20855548	0.064069144	0.258535755
90.17499332	0.055812527	0.368655902
218.3041265	0.053175151	0.330962634
142.4069389	0.055189873	0.3284025
204.4679729	0.059558517	0.313729472
156.5288317	0.061083474	0.310583096
164.8685128	0.058534924	0.298348737
164.059733	0.054226759	0.320144154
186.7329265	0.055342149	0.298541378
168.7247992	0.05580444	0.327434299
167.0950488	0.0528031	0.295606348
143.5039407	0.055400891	0.31828883
303.8651566	0.049483974	0.360950078
123.424577	0.06089354	0.381977225
199.9540491	0.052729203	0.335957729
167.6883526	0.054802997	0.339528598
222.9785254	0.052369576	0.364731849
246.2344823	0.05301938	0.337377147
181.630084	0.056717739	0.328520293
199.3597081	0.052819843	0.330293531
108.9382603	0.055781902	0.323977667
201.6112523	0.052764486	0.343356428
186.6867715	0.054839286	0.328260633
234.5443375	0.046160233	0.353624053
254.8716089	0.057069739	0.326419325
106.2195962	0.065443922	0.252511366
141.0987441	0.057858429	0.277113031
154.4978242	0.059206806	0.366469507
104.9703612	0.05876017	0.334018765
210.0882383	0.05809082	0.350063477
125.8774224	0.054544317	0.373490961
269.0179643	0.048141415	0.327442709
144.2047955	0.057237423	0.306635072
234.629614	0.050430877	0.344980071
207.6093643	0.054526749	0.334630058
82.57168344	0.080429542	0.314047776
149.5198436	0.058696574	0.388342904
184.8339798	0.058783767	0.346787097
184.716517	0.065220263	0.310639347
199.2423833	0.056538263	0.327484445
180.3141641	0.059108961	0.337857207
197.578335	0.054037587	0.334006699
190.4757615	0.049182783	0.345687054
445.4061223	0.04829076	0.37031917
148.3018179	0.056492058	0.279361568
229.4149203	0.054948097	0.323216609
45.40513395	0.084567832	0.240421029
215.1111624	0.055854772	0.344535788
267.4765254	0.050994248	0.353719537
185.9847392	0.051915564	0.323914392
149.4830417	0.061322961	0.311322226
75.54924857	0.063744016	0.332381512
189.766439	0.054084923	0.316002083
173.4993149	0.049190616	0.35359678
227.4625711	0.051651288	0.352806375
123.571561	0.063150747	0.264599366
332.7284726	0.04863301	0.38121554
301.7660471	0.049306927	0.346030237
165.9044871	0.058548844	0.290121859
184.4434548	0.055055755	0.333270252
226.9323353	0.056911891	0.315458652
291.1412423	0.047829127	0.340123165
229.0835762	0.052047902	0.346677473
151.4355323	0.06024736	0.346045684
144.7243091	0.059671895	0.295795525
197.4131816	0.053739027	0.327875274
160.703923	0.051389153	0.343228882
329.5159648	0.046644885	0.354199153
272.7700229	0.045780843	0.398491096
248.3482303	0.049238097	0.368148183
34.15469453	0.085768945	0.249229461
253.13259	0.053115887	0.31598232
105.1679122	0.062884877	0.300253584
200.4215934	0.048314878	0.3327419
207.6111614	0.061180323	0.279503369
37.62662489	0.093195266	0.319526627
161.409661	0.058224852	0.343786982
162.3615969	0.057789484	0.347993653
230.7633946	0.050524474	0.349972291
150.8560034	0.057640274	0.359244901
182.7406383	0.051091316	0.341930651
158.3666054	0.059705783	0.32068592
369.4163822	0.035955856	0.4313479
173.379894	0.072009522	0.226294386
84.40890598	0.076126752	0.295038505
68.43045461	0.07323269	0.292204964
235.7377279	0.043807019	0.399301746

log.sigma.3.0.mm.3D_glszm_SizeZoneNonUniformity	log.sigma.3.0.mm.3D_glszm_GrayLevelNonUniformity	log.sigma.3.0.mm.3D_glszm_LargeAreaEmphasis	log.sigma.3.0.mm.3D_glszm_ZoneVariance
117.9752066	17.44077135	60.73002755	49.74710288
189.7584973	30.96064401	163.4991055	150.6983273
151.0377358	23.74528302	27.39150943	20.43883499
222.6550681	35.34493192	228.785174	215.0133274
57.91457286	12.2160804	268.5477387	245.8058635
126.0605327	25.65375303	108.1840194	92.45405672
32.52317881	10.9205298	804.6291391	755.6291391
148.2421525	24.11210762	279.7825112	263.4036126
195.8767606	34.19366197	128.7130282	115.9776799
288.6377119	51.19279661	65.36122881	55.24155123
81.80357143	24.00595238	339.5744048	310.6517769
213.6415094	31.64150943	40.74614065	32.39275175
180.8259442	35.92610837	451.8111658	428.6796466
137.2824859	34.02071563	49.5480226	37.30461305
184.6966068	27.96207585	48.21956088	38.67257103
363.7279345	58.43949045	248.0828025	234.103777
129.7189873	21.8	242.0278481	225.5175132
169.1001855	32.10204082	279.2356215	256.7127609
107.7723343	21.19596542	59.95389049	49.23628632
202.5787923	39.74521355	261.6303387	243.3764286
679.6660386	115.1234103	68.93829487	58.22138395
181.5131579	33.64802632	63.74506579	51.57552534
428.9389313	73.10381679	52.02671756	42.13065031
77.1532567	13.7816092	106.9425287	91.36877028
279.7758817	48.69738339	33.74288965	24.96673617
170.7293869	23.40591966	34.61733615	26.44716599
292.9765319	46.7053455	42.42112125	34.11139423
411.8841762	64.64600326	44.50815661	37.01949669
273.6600496	44.17121588	379.4156328	361.6315183
334.4591058	48.02290076	195.0632497	183.1957131
403.1656904	63.358159	83.80920502	71.7952949
477.3399862	82.41087405	113.7123193	101.8707632
210.7272727	33.69905956	312.1175549	296.3797943
45.68085106	7.865248227	75.56737589	63.930084
105.4104235	16.19869707	36.93811075	28.57150739
241.9280868	40.4165536	398.9213026	379.8681368
418.6908784	54.65371622	63.16807432	54.08171952
289.8603604	50.67792793	141.6317568	127.0493352
63.88537549	16.55731225	451.5098814	417.1044697
100.3149171	20.94475138	250.2596685	229.8352614
150.9854369	24.39320388	76.16990291	65.96715053
158.3248945	27.85232068	131.8227848	115.2094216
224.040625	37.178125	586.5015625	564.1316187
163.5890411	23.89041096	287.0821918	268.5804091
326.4603811	47.99699097	152.8144433	140.5328282
318.5938402	59.46968239	61.96053898	50.12137605
393.6222612	57.54163015	76.86064855	66.42452114
144.5601852	23.55555556	134.3796296	122.0647291
36.74358974	9.41025641	240.2905983	217.0532544
108.3476703	16.37634409	274.5304659	259.2673013
268.76	45.55741935	1048.247742	1021.821509
104.6854599	21.97922849	1111.703264	1078.015057
269.1922141	46.47445255	582.9014599	561.5642534
123.6557377	21.63387978	50.58196721	40.34546418
370.0794224	59.87364621	270.8998195	258.2742403
218.8199052	31.13270142	51.1943128	42.29827622
374.3926805	48.82195846	97.34619189	86.82704494
127.9475983	25.87336245	813.0786026	784.8820389
137.3670588	23.35294118	57.84	47.41849689
64.43283582	22.6641791	197.0261194	169.5419219
268.0488432	43.45501285	188.9395887	174.6496339
486.3643636	70.11709091	660.9563636	645.0319106
214.107413	34.31618759	77.84720121	66.04314281
181.5008576	35.75128645	38.36363636	28.09727293
62.82010582	12.04761905	38.88888889	29.14392094
154.2090164	26.39344262	91.02254098	81.02502268
107.4934211	14.95394737	88.50657895	76.3943473
323.523446	47.36423119	207.2606325	192.1381181
74.61702128	17.80851064	154.0744681	133.0512675
302.6851385	38.61460957	130.9508816	120.9815128
464.3725782	66.16989568	30.0923994	22.04067599
120.9808153	24.41486811	295.4748201	271.0470473
427.9190031	70.69158879	204.3652648	191.0859385
518.9294833	93.62006079	155.8522796	140.1860825
436.3780203	61.36477007	106.9384256	95.90339404
232.9672619	34.97619048	124.3169643	112.2847377
182.7121212	31.81060606	487.4943182	467.6006909
114.4728682	23.09302326	47.70801034	35.37647978
279.6776084	45.83939039	157.080891	142.330969
388.1918656	58.12113174	452.4465075	436.9447771
142.3880597	18.75124378	81.60696517	70.12801663
373.7846482	42.9424307	73.05863539	65.03488459
285.6829897	38.20876289	70.82216495	60.65790068
24.17525773	8.319587629	264.628866	235.5578701
383.9185185	64.53580247	48.65349794	38.54999475
240.5031211	50.37078652	222.7265918	203.9163405
154.3922414	22.41810345	74.61637931	65.14484245
182.2361963	39.88957055	342.3634969	320.5809473
33.23076923	9.692307692	79.16346154	63.54576553
44.69230769	7.569230769	170.7692308	151.0009467
304.1464531	50.50800915	183.1521739	169.6545369
135.4392765	19.55297158	38.96382429	30.81110243
175.6707566	28.18609407	57.55828221	47.35489564
399.375	59.67465753	269.4340753	255.5444143
344.0959925	64.06430568	208.4641193	189.2285785
182.8915094	15.24528302	15.14622642	10.7993948
64.26760563	20.45070423	734.0316901	695.4955738
26.25842697	6.775280899	130.5617978	107.4354248
48.5060241	12.15662651	360.6987952	332.0827406
211.2306238	23.17391304	10.96030246	6.20147155

log.sigma.3.0.mm.3D_glszm_ZonePercentage	log.sigma.3.0.mm.3D_glszm_LargeAreaLowGrayLevelEmphasis	log.sigma.3.0.mm.3D_glszm_LargeAreaHighGrayLevelEmphasis	log.sigma.3.0.mm.3D_glszm_HighGrayLevelZoneEmphasis
0.301745636	0.360188548	11657.27548	283.5179063
0.2795	0.890997397	31568.15206	270.7692308
0.379248658	0.152202095	6451.391509	214.1745283
0.26946596	1.022360218	52963.2587	291.9379728
0.209694415	3.781673457	20629.71357	140.8643216
0.252136752	0.574896841	21810.97094	207.559322
0.142857143	11.78310097	57660	126.8543046
0.247091413	0.931102265	86152.8991	377.529148
0.28021707	0.506063339	34689.63556	341.4366197
0.314352314	0.243532116	19303.0572	311.4618644
0.185943553	1.32630529	89377.64286	281.5267857
0.345994065	0.172573723	10964.85935	262.4202401
0.207920792	2.147804388	97283.97373	271.817734
0.285791173	0.393770794	8090.561205	163.2109228
0.323643411	0.548363139	5752.251497	147.1077844
0.26746167	0.787088827	80845.30664	379.9008189
0.246105919	0.969757028	63471.66582	251.5898734
0.210711493	1.205644806	67574.82746	347.6085343
0.305457746	0.306561357	12870.3804	276.9769452
0.234057222	1.049840886	67717.8704	300.5375552
0.305467626	0.258928803	20497.8601	287.9778615
0.286657237	0.218361726	20063.88158	333.5032895
0.317884009	0.201651798	14916.52061	287.0328244
0.253398058	0.464525671	26508.75479	314.8659004
0.337557604	0.17739905	8408.05347	246.3447099
0.349852071	0.082964485	16159.74841	506.4714588
0.346901854	0.248091529	8164.573664	200.5436767
0.365424739	0.12782331	16960.31811	342.0595432
0.237128567	1.527377589	96863.1402	291.5657568
0.290281735	0.603537917	64666.7241	364.2322792
0.288507967	0.193608644	38277.49874	424.507113
0.2906	0.47529851	28834.81693	309.8079835
0.252074279	1.108664649	91305.04075	344.2727273
0.293139293	0.832278825	7797.340426	188.0141844
0.345720721	0.133195131	12557.83388	338.267101
0.229095431	1.41470598	114960.6201	317.1411126
0.331745587	0.19149896	22926.61571	389.7972973
0.261869655	0.29723259	69279.69932	453.5101351
0.170485175	3.095493702	68232.85771	198.1936759
0.221271394	1.453897871	44561.74586	259.5718232
0.313069909	0.318695636	19280.55583	260.9393204
0.245341615	1.09518013	17425.84599	183.7637131
0.211430459	2.085664034	167683.4797	349.7390625
0.232484076	2.3816192	36342.68265	201.8127854
0.285346308	0.326081449	74644.75627	474.7833501
0.290629371	0.264759268	17323.31376	255.5226179
0.309549647	0.243766573	26379.34093	383.283085
0.284960422	0.346464467	54346.39815	352.9907407
0.207446809	2.726028174	22622.94017	155.4615385
0.255963303	2.027486579	38481.13262	231.4587814
0.194528112	4.767190698	234964.6516	299.5664516
0.172290389	3.558576429	352227.3887	337.1038576
0.2164867	2.50737707	138002.7275	335.0072993
0.312553373	0.16717484	16647.90164	320.3497268
0.281432563	1.104239362	68443.79242	336.6444043
0.335275424	0.199884409	15073.1564	317.6872038
0.308325709	0.153788464	63560.83581	718.6547972
0.188322368	4.585391613	146553.19	277.220524
0.309766764	0.147769887	24704.83059	410.1882353
0.190747331	2.265418432	18730.00746	98.14552239
0.264535872	0.716940926	52087.96915	370.0205656
0.250592309	1.639627953	269592.9396	445.1003636
0.291061207	0.299432904	22356.77307	317.1437216
0.312098501	0.15603115	10825.97942	269.8353345
0.320338983	0.445002052	4494.185185	132.8465608
0.316267012	0.477911442	18389.57377	323.4569672
0.287334594	0.540173707	15640.52961	271.2565789
0.257150869	0.705255939	63342.43621	390.5703381
0.218097448	0.546820261	46086.04965	260.0638298
0.316713203	0.265910944	66253.5466	527.0894207
0.352415966	0.074427257	14267.12519	507.0447094
0.202328967	1.43365752	62870.80096	295.0479616
0.274417611	0.865120323	50316.55374	315.1207165
0.252649363	0.399809568	63029.80182	392.9039514
0.301032379	0.269667362	45465.66329	490.4037412
0.288288288	0.351970312	46810.72321	390.9925595
0.224203822	1.87769678	129200.5322	262.3598485
0.284768212	0.220776552	12042.37468	261.997416
0.26037851	0.467122479	55142.27902	338.8300117
0.253986077	1.567627739	133855.2706	278.066313
0.295154185	0.163229223	45175.30846	556.340796
0.353029733	0.159875423	35342.02026	437.5245203
0.313662086	0.210086594	25592.50387	396.3569588
0.185468451	9.638070845	8273.123711	76.83505155
0.314603832	0.114405538	22353.37613	454.9300412
0.230569948	1.627845277	32891.29713	193.5243446
0.324929972	0.28068319	21708.95259	340.2887931
0.214262241	1.054984782	113208.5368	392.1610429
0.253041363	2.513407079	3529.596154	70.03846154
0.224913495	0.651076749	47159.01538	282.0461538
0.272189349	0.920205814	37923.8833	277.8226545
0.350226244	0.114963395	14574.61757	394.6718346
0.313060179	0.204547428	17707.93047	263.5541922
0.268320698	0.933401497	80339.36729	312.2825342
0.2280068	0.937025117	48524.23113	274.1808015
0.479638009	0.043457402	9709.195755	554.2075472
0.161089053	2.280784726	240239.5246	364.1267606
0.207943925	1.586258417	12191.91011	158.6741573
0.186936937	3.863144155	35115.5241	124.2831325
0.458405546	0.043451923	5114.907372	377.3137996

log.sigma.3.0.mm.3D_glszm_SmallAreaEmphasis	log.sigma.3.0.mm.3D_glszm_LowGrayLevelZoneEmphasis	log.sigma.3.0.mm.3D_glszm_ZoneEntropy	log.sigma.3.0.mm.3D_glszm_SmallAreaLowGrayLevelEmphasis
0.587799495	0.011003964	6.371187772	0.008190166
0.603293427	0.010587876	6.33310697	0.008270417
0.61809421	0.015014834	6.176870582	0.010911447
0.600370995	0.00870138	6.456115653	0.005363986
0.550672678	0.023789025	5.932314615	0.011492329
0.56731672	0.011689164	6.281954634	0.008011805
0.461099732	0.029009138	6.013068691	0.01621208
0.596088228	0.006822852	6.336323662	0.005056687
0.609297968	0.006060544	6.222916998	0.004455012
0.565462086	0.00664502	6.545286392	0.003689641
0.498167647	0.00833452	6.360897907	0.006115095
0.628268211	0.010123558	6.266196552	0.007778282
0.560485388	0.008349315	6.500048788	0.006121213
0.516788742	0.017898547	6.447817155	0.009891846
0.630434126	0.035641232	6.190777513	0.027856167
0.59547063	0.005501449	6.59709743	0.003899496
0.59001765	0.013477404	6.233788506	0.007755159
0.580350609	0.006565053	6.44927191	0.004884868
0.571983853	0.009409981	6.147027516	0.006694117
0.563524175	0.006974586	6.510219384	0.00374675
0.585087708	0.007344031	6.686296213	0.004838455
0.560428804	0.006770957	6.537431666	0.004828859
0.591978657	0.007742103	6.582038855	0.005474524
0.561618153	0.010632354	6.267900132	0.008192051
0.581110189	0.009208739	6.49640603	0.006218499
0.622995852	0.00570748	6.342361009	0.004729235
0.642085402	0.012773401	6.16902818	0.010038171
0.59778313	0.006011274	6.460645044	0.004259378
0.602772743	0.009810955	6.43669544	0.006234257
0.627288948	0.006555889	6.476689562	0.005182662
0.60243649	0.004576337	6.628893981	0.0033337
0.593282003	0.006786591	6.597201256	0.004976121
0.594848527	0.007268424	6.498372652	0.005090527
0.585532584	0.021125071	5.946551357	0.017520612
0.606809823	0.009822541	6.215084798	0.005647676
0.592372363	0.007214222	6.471007907	0.00422652
0.615931926	0.006580699	6.639607931	0.004958088
0.590243083	0.004362665	6.50767003	0.003257452
0.51303434	0.014744609	6.304986139	0.010307276
0.540446081	0.010040898	6.432365062	0.006736437
0.627516952	0.009478428	6.038486207	0.007525751
0.599445412	0.017601021	6.290425114	0.01301499
0.614631342	0.00584959	6.36588643	0.004415712
0.634463027	0.015906014	6.147942207	0.01059689
0.590636385	0.004699811	6.664727228	0.003202689
0.570217008	0.010349837	6.563057086	0.007566369
0.607280447	0.00849013	6.588817605	0.006706088
0.597697334	0.007720577	6.300397896	0.005950106
0.577489618	0.022298149	5.438084452	0.016004577
0.647180911	0.013178228	5.92718835	0.010793356
0.611353519	0.007716978	6.420095339	0.004411036
0.572078485	0.008624807	6.184036551	0.004013925
0.592611157	0.005853191	6.475530906	0.004159909
0.602275062	0.00737144	6.133440583	0.005870537
0.598455611	0.006155679	6.547106702	0.004321095
0.606994261	0.008581765	6.447593916	0.006250566
0.631540618	0.002925022	6.5857122	0.002287337
0.539534016	0.009339253	6.506973417	0.006323687
0.58674489	0.007162472	6.39005505	0.005476583
0.499067645	0.028692442	6.073141117	0.017021172
0.609290479	0.005817531	6.45090112	0.004389496
0.616565768	0.005066137	6.613204803	0.003625833
0.587373573	0.009517485	6.548295923	0.005970246
0.575183837	0.00805247	6.318823527	0.005658778
0.596105178	0.022566303	5.837578719	0.016875261
0.576950244	0.007393171	6.363013349	0.005342521
0.616136277	0.010489283	6.204338083	0.007934847
0.616162454	0.006627714	6.490570701	0.005240657
0.524467507	0.0100927	6.317856381	0.007795323
0.640399154	0.004008059	6.388765218	0.003066987
0.609184743	0.003655754	6.608408761	0.002577498
0.551279689	0.010540957	6.366200209	0.008212009
0.597740524	0.006532167	6.565323073	0.004193987
0.580127987	0.004414233	6.651957414	0.002977272
0.604344889	0.004704767	6.71851111	0.002858079
0.609530535	0.007035128	6.479815299	0.004444211
0.607240844	0.011239132	6.178793623	0.008891805
0.562169699	0.012217402	6.353631167	0.009585819
0.592265267	0.006863325	6.541433806	0.004775103
0.605956346	0.009087206	6.516223978	0.006443144
0.616827358	0.006534051	6.441069086	0.003673
0.656136761	0.006153967	6.487325656	0.004942249
0.630086616	0.00735531	6.499641635	0.00584378
0.503252983	0.059538886	5.377197802	0.04628274
0.580535776	0.004090192	6.663639156	0.002930113
0.565890231	0.010134952	6.466905157	0.006674106
0.595738753	0.010265974	6.447127155	0.008263173
0.544020421	0.005193253	6.535078486	0.003725151
0.583247842	0.048413272	5.235672532	0.036737241
0.605330769	0.015429491	5.814600652	0.01328371
0.611555756	0.007148412	6.356495015	0.005262763
0.610917628	0.007244658	6.284856935	0.005862931
0.619770828	0.010519526	6.178031402	0.008541213
0.605005712	0.008537026	6.587636287	0.005445542
0.587628554	0.006757211	6.544963555	0.004654785
0.682321119	0.007692425	6.37438945	0.004315308
0.473911711	0.00714664	6.323035339	0.002640567
0.558346677	0.022876815	5.421256224	0.018576014
0.557507168	0.022202044	5.861047891	0.015517041
0.656196002	0.009150122	6.356634301	0.006889867

log.sigma.3.0.mm.3D_ngtdm_Coarseness	log.sigma.3.0.mm.3D_ngtdm_Complexity	log.sigma.3.0.mm.3D_ngtdm_Strength	log.sigma.3.0.mm.3D_ngtdm_Busyness	log.sigma.3.0.mm.3D_ngtdm_Contrast
0.008089355	0.634705098	3.146329993	0.224226831	6.75E-05
0.004954656	0.288079084	1.495147796	0.386298866	3.41E-05
0.008384153	0.575052148	2.072143443	0.25897009	9.50E-05
0.003922912	0.269573994	1.498903883	0.400749571	2.19E-05
0.009918739	0.242880271	1.716296617	0.479355715	8.47E-05
0.005893663	0.283546797	1.54467098	0.356192528	3.35E-05
0.009675283	0.121236502	1.300227463	0.570006537	5.34E-05
0.00551016	0.400236934	2.581108287	0.226370188	2.73E-05
0.004807272	0.273413689	1.420781728	0.365250234	3.48E-05
0.003389291	0.218401199	1.112303381	0.485427704	2.45E-05
0.005694287	0.178605778	1.427696914	0.351092463	2.39E-05
0.005255778	0.37261873	1.409722954	0.371452075	5.69E-05
0.003332338	0.150090363	1.135970556	0.51770553	1.25E-05
0.005078767	0.205908027	0.976657447	0.589526864	4.36E-05
0.005901592	0.356550482	1.330687757	0.610101372	7.73E-05
0.002743373	0.201253762	1.320251893	0.416337603	1.18E-05
0.006466413	0.293654814	1.956073031	0.273272335	3.68E-05
0.003745018	0.226327437	1.510435521	0.414009297	1.78E-05
0.00835298	0.421235326	2.028763475	0.269237449	6.88E-05
0.003553526	0.1553876	0.994170512	0.52483897	2.04E-05
0.001390479	0.11763029	0.526422883	1.070453499	9.88E-06
0.0046273	0.37600345	2.216086531	0.283047929	2.35E-05
0.002299905	0.166218225	0.759368457	0.739901411	1.75E-05
0.010107887	0.495213339	3.472909251	0.170457117	6.28E-05
0.003949231	0.230598706	1.000680525	0.554550999	3.80E-05
0.006768655	0.778335232	3.118795415	0.168542078	6.45E-05
0.004516823	0.232568946	1.307805061	0.49803569	2.88E-05
0.002573981	0.2625903	0.905895819	0.566970226	2.87E-05
0.002889403	0.185413943	1.117578558	0.506174394	1.43E-05
0.003281034	0.298247758	1.652309424	0.333345669	1.41E-05
0.002462261	0.205666315	1.183903631	0.435670801	1.31E-05
0.001936315	0.167280346	0.919668436	0.703771738	9.42E-06
0.003968036	0.286800985	1.805296804	0.325208499	2.01E-05
0.019446207	0.86372583	4.31100779	0.167639689	0.000204535
0.010001743	0.845863082	3.267319883	0.152522952	0.000105286
0.003041775	0.18113725	1.119954622	0.4719572	1.46E-05
0.002711498	0.276896929	1.125666402	0.477979201	2.54E-05
0.002683335	0.237022289	1.409799462	0.380674675	1.31E-05
0.007248082	0.185109629	1.650166911	0.357425112	2.58E-05
0.005953227	0.296185705	1.974937365	0.340835603	3.25E-05
0.006572703	0.386982258	1.665658381	0.30580668	5.63E-05
0.005588381	0.188668776	1.163386048	0.519266544	3.34E-05
0.003463309	0.156519133	1.085872837	0.448672141	1.53E-05
0.004855415	0.240106289	1.466842118	0.517777524	3.13E-05
0.003008132	0.321253475	1.589026935	0.311819691	1.83E-05
0.002627664	0.184428351	0.882248949	0.661564142	1.92E-05
0.00264297	0.269554666	1.36457394	0.425857267	1.56E-05
0.006649637	0.417836667	2.387733054	0.196794383	4.42E-05
0.015687831	0.304654646	2.105553257	0.33393492	0.00017095
0.00829602	0.443041412	2.996594622	0.264724992	4.95E-05
0.002704811	0.123815974	1.029594558	0.576666181	8.84E-06
0.004969264	0.259738304	2.167577179	0.251796018	1.46E-05
0.002725399	0.14399892	1.001709453	0.57856372	1.17E-05
0.007829985	0.465789627	1.988722469	0.234600762	7.20E-05
0.002525565	0.191259577	0.977248097	0.573121796	1.45E-05
0.005608052	0.429080799	1.820978905	0.285119474	4.66E-05
0.003112382	0.405710353	2.365334771	0.218490648	1.46E-05
0.004354876	0.185146454	1.553439815	0.413846573	1.68E-05
0.006991795	0.573523916	2.868426549	0.177626806	5.33E-05
0.006110705	0.105481248	0.594305402	0.856010316	4.21E-05
0.003291323	0.229898695	1.270583766	0.441113459	2.03E-05
0.00185355	0.206280317	1.35074875	0.462956813	6.99E-06
0.004167396	0.310444045	1.500097894	0.365273736	2.98E-05
0.004706689	0.308255614	1.259260935	0.419920518	4.35E-05
0.014706818	0.473196667	1.774821792	0.367301907	0.000226144
0.006492523	0.472806294	2.306460504	0.289733522	5.46E-05
0.00988417	0.39179217	2.452040413	0.274876978	8.63E-05
0.00284642	0.232776148	1.31070842	0.433762516	1.55E-05
0.007302289	0.321064232	1.810878545	0.272656935	4.99E-05
0.004031685	0.440449159	2.270259661	0.225114694	2.30E-05
0.002603288	0.270137908	1.107746073	0.457743099	2.49E-05
0.004806836	0.220221563	1.577865128	0.378660074	2.22E-05
0.00215182	0.139336088	0.793306364	0.732247513	1.27E-05
0.001532644	0.129159163	0.740965965	0.712959601	6.58E-06
0.002431165	0.265696818	1.435297263	0.400268174	1.47E-05
0.004290306	0.409765909	2.183372883	0.261036854	2.67E-05
0.004140175	0.224344862	1.384525522	0.379019956	1.80E-05
0.006676345	0.384556556	1.803163506	0.306901843	5.53E-05
0.002971781	0.198131141	1.06529571	0.482911587	1.98E-05
0.002317341	0.146073964	0.820018112	0.628731332	1.36E-05
0.007957737	0.821201766	4.799551694	0.102168677	4.21E-05
0.003503792	0.427279352	1.751818458	0.294652782	3.27E-05
0.003882839	0.340867484	1.632867796	0.329411024	2.75E-05
0.016544567	0.231544996	1.666255246	0.618549386	0.000158664
0.002561662	0.21889137	1.124300396	0.453086679	1.73E-05
0.002752935	0.114906412	0.644931911	1.005993212	1.82E-05
0.007080891	0.483222822	2.44714732	0.228612931	6.08E-05
0.003277056	0.17932353	1.18660678	0.433118834	1.55E-05
0.018915062	0.242871921	1.263992416	0.615404872	0.000286998
0.015214224	0.725105639	5.279927328	0.110496089	9.39E-05
0.002949185	0.169496488	0.848852667	0.672550194	2.06E-05
0.007874749	0.708311678	2.676984855	0.187402256	9.25E-05
0.00581296	0.337664522	1.649927343	0.302653066	4.75E-05
0.002151694	0.157120427	0.808297328	0.662790759	1.41E-05
0.002242023	0.09976193	0.662716225	0.819685099	9.96E-06
0.009876009	2.138560459	5.146273147	0.100688706	0.000258155
0.00518296	0.162026477	1.466419571	0.352224573	2.32E-05
0.026146642	0.38915186	3.391634119	0.213140821	0.000193113
0.010752133	0.186239711	1.464253809	0.418261617	5.98E-05
0.007719903	0.960150372	2.872740886	0.188786925	0.000157681

log.sigma.2.5.mm.3D_gldm_GrayLevelVariance	log.sigma.2.5.mm.3D_gldm_HighGrayLevelEmphasis	log.sigma.2.5.mm.3D_gldm_GrayLevelNonUniformityNormalized	log.sigma.2.5.mm.3D_gldm_DependenceEntropy
19.61543081	323.527847	0.079628443	6.585715627
15.983664	364.03	0.0890675	6.610159537
20.13372333	316.1091234	0.068064938	6.444870921
15.67350976	393.1858948	0.091750345	6.51140093
12.28371721	99.66385669	0.121137996	6.276353822
12.7628112	257.3663004	0.098015538	6.409988891
7.528175008	153.7161779	0.154598483	6.319633339
15.23147797	409.666482	0.097692621	6.551862074
14.19431021	383.6038481	0.085992773	6.625151435
16.67828963	294.2673993	0.076456677	6.598264957
9.856165946	379.189264	0.109326731	6.458610552
19.48540042	322.5139466	0.072629327	6.554189535
10.376298	238.9754182	0.110173969	6.508375381
13.46881203	181.4348762	0.081058721	6.491265433
16.73256941	231.1052972	0.070778165	6.538917321
16.40771218	360.0683865	0.087189965	6.985046527
13.46376918	198.576324	0.099563087	6.485286177
12.30337851	313.9053948	0.092840319	6.612791169
14.7261952	242.693662	0.083806536	6.534708721
12.88236545	343.1920028	0.091030551	6.603651641
18.47169815	323.0871942	0.071514932	6.757405043
15.13486139	279.3008015	0.082377989	6.553407372
16.83660345	273.8781849	0.07458516	6.600391266
16.63267132	315.3854369	0.095797907	6.5657759
18.72847988	274.0898618	0.065870833	6.692188206
22.70763235	524.5073964	0.066871127	6.567240091
14.49019161	278.1302578	0.083430755	6.38861031
21.16012891	384.7698957	0.067165007	6.519196818
13.23347962	373.819947	0.105883988	6.700240229
15.73587416	381.8300095	0.089884532	6.571355142
17.02705296	426.7327378	0.079535262	6.593798132
14.120716	378.0908	0.08189288	6.676046172
15.95637875	276.2603714	0.09557676	6.552355034
16.16209301	193.4781705	0.096476934	6.272053643
20.94490986	413.6362613	0.076776946	6.405260126
13.02280462	334.5990053	0.108993652	6.585293695
23.57305526	365.9008126	0.068137328	6.755208926
15.00133117	463.1144205	0.089337997	6.512294746
8.263567451	142.3955526	0.113355577	6.439382837
13.30381364	330.1699267	0.108283368	6.447779364
14.15712392	323.5	0.089501668	6.337961045
12.89713269	231.4073499	0.094805156	6.578904791
12.25750363	317.0465808	0.107631798	6.603697349
12.79188022	202.7165605	0.112418917	6.471141515
20.21611699	579.7945049	0.076221269	6.911783768
17.42582511	354.2176224	0.074325395	6.620134927
19.55298767	394.4785676	0.076208345	6.726252468
16.85733878	375.055409	0.091151725	6.498543374
11.94229101	172.8812057	0.129954479	6.172398916
12.67265718	288.293578	0.112675701	6.411820058
10.95007717	280.2098394	0.109083044	6.624502448
9.734152113	354.2009202	0.137540199	6.373410982
12.13186311	368.1651304	0.103491529	6.566066443
15.80914661	284.9820666	0.080755316	6.343902617
15.77583942	365.8658877	0.083659587	6.643725026
21.30222295	367.8575212	0.069313775	6.840007989
20.42584223	626.9460201	0.079095385	6.684504167
11.59421241	307.0185033	0.127201323	6.563205782
19.77715918	554.0204082	0.080263113	6.486074708
6.930801788	113.688968	0.113598865	6.078149395
16.48507535	358.1880313	0.082326905	6.713850024
17.08390018	466.1113541	0.095465668	6.711209068
18.02652711	291.1272567	0.077480867	6.612994296
14.1029018	258.9550321	0.081266134	6.519926381
12.94264866	140.2881356	0.080528584	6.273986751
20.11905333	252.014906	0.073432208	6.549638223
17.52444424	295.1342155	0.089102383	6.476358699
16.48739183	504.790802	0.087724659	6.667222823
12.33431249	310.103635	0.098732003	6.389122806
20.59669861	423.3777423	0.08081003	6.732567007
22.87749364	398.227416	0.0601349	6.804710791
11.72662235	231.8335759	0.111385611	6.559500634
15.7000995	340.2851037	0.083879942	6.694822531
14.11233459	384.7531869	0.086252413	6.706562169
23.0337771	539.4066166	0.07044399	6.796528496
18.93861135	425.2784213	0.082681873	6.665346048
11.676295	292.7333333	0.111383739	6.442676756
16.2291225	278.1258278	0.076977889	6.38921034
15.41503857	374.6193529	0.09020836	6.581612376
16.84585815	289.9005165	0.091900237	6.722627093
21.13180065	508.1732746	0.091064147	6.637791451
25.90647212	456.6759503	0.071490665	6.623901958
19.88595116	396.9426031	0.078321741	6.64295853
7.806397128	80.10516252	0.149530763	6.023937436
19.08648181	444.2661833	0.07207488	6.631696898
11.44818843	191.8995394	0.091090688	6.555333398
20.85957275	300.9530812	0.076785616	6.590344643
12.41965911	393.5994085	0.098984875	6.46528197
7.8230889	86.90754258	0.119055653	5.91271698
13.29619198	386.8287197	0.116174375	6.166317189
13.75274053	266.4251012	0.088896478	6.603834516
23.7051379	549.6705882	0.072182797	6.547646944
16.33959548	322.7912932	0.084493927	6.378176346
17.17106749	375.1656329	0.088407281	6.666825401
11.77602283	266.160221	0.093468493	6.67255014
49.64521099	576.6696833	0.049581806	6.724193857
8.16272796	370.4787294	0.133958289	6.195163334
10.44968993	185.3317757	0.117259149	6.119618353
7.508075643	147.8558559	0.137869288	6.235888776
35.56192537	402.347487	0.053259103	6.55406045

log.sigma.2.5.mm.3D_gldm_DependenceNonUniformity	log.sigma.2.5.mm.3D_gldm_GrayLevelNonUniformity	log.sigma.2.5.mm.3D_gldm_SmallDependenceEmphasis
191.5552785	95.79301746	0.288939452
280.676	178.135	0.257925857
216.2719141	76.09660107	0.338447267
365.2812882	225.0635956	0.260619467
114.6733404	114.9599579	0.201201488
234.7264957	160.5494505	0.246969101
92.01229896	163.410596	0.145358512
231.4742382	176.3351801	0.232653052
284.4721263	174.3073508	0.258793674
510.1202131	229.5994006	0.28801606
226.4775872	197.5534034	0.198861248
305.8534125	122.3804154	0.323561979
359.8767497	322.6995562	0.20447107
318.6501615	150.6071044	0.248030275
266.502584	109.5645995	0.289298001
520.4339255	358.2635678	0.238442471
215.7314642	159.7987539	0.223427998
326.1415168	237.4855356	0.207137971
176.2411972	95.20422535	0.251265784
395.6046191	264.0796277	0.22018396
1188.094676	497.028777	0.284009508
340.2390382	174.7237152	0.245784039
715.2819704	307.3654453	0.29462121
132.7378641	98.67184466	0.233787664
474.5675883	171.5276498	0.305453206
247.3002959	90.40976331	0.310441886
397.9443691	184.4654003	0.336016016
668.8801788	225.3385991	0.337024876
391.9455722	359.8996764	0.217336596
445.912314	283.9452358	0.261501337
683.9406084	329.4350555	0.260451242
759.7708	409.4644	0.26001415
342.600158	241.9047807	0.253896079
69.06652807	46.40540541	0.29453812
161.2274775	68.17792793	0.306701703
384.8607398	350.6325769	0.214940219
623.129448	243.1821238	0.310642278
529.0094367	302.9451489	0.25053839
181.3557951	168.2196765	0.163983682
199.8679707	177.1515892	0.230699095
219.7066869	117.7841945	0.301328082
245.2515528	183.1635611	0.220714404
341.3316815	325.8014536	0.217704246
220.9490446	211.7972399	0.218383719
504.2329708	266.3171151	0.260773246
625.0760839	265.7132867	0.284937219
608.1578947	280.9039609	0.290035389
218.9551451	138.1860158	0.267155047
61.75177305	73.29432624	0.197577098
133.9706422	122.8165138	0.241832591
451.125	434.5868474	0.203531296
198.5429448	269.0286299	0.179035182
458.7113511	392.9573347	0.214836477
206.3970965	94.56447481	0.289600536
600.7556515	329.3677927	0.262234404
301.6769068	130.8644068	0.302958099
508.6389143	259.3537664	0.286153503
244.7680921	309.3536184	0.1808468
232.4212828	110.1209913	0.28992023
207.3601423	159.6064057	0.208201173
418.0452227	242.1234274	0.261119685
699.8285037	523.8201203	0.238394304
358.6089828	175.9590489	0.284031652
302.5556745	151.8051392	0.27292074
98.86779661	47.51186441	0.303119872
262.4581983	113.3058976	0.300981816
160.2438563	94.27032136	0.277000053
493.5300056	312.8261357	0.24653999
180.1399845	127.6604795	0.23867663
375.15716	202.5907459	0.289701894
719.5672269	228.9936975	0.320610053
244.5769044	229.5657448	0.201774424
677.1812353	392.4742466	0.247987933
922.8596222	561.589464	0.238599054
699.1769122	300.2322853	0.294573141
354.3204633	192.7314457	0.269089381
297.3524416	262.3087049	0.222136367
245.5710081	104.6129507	0.282696526
465.041514	295.5225885	0.246094059
591.2030092	409.2317539	0.253441848
191.3069016	124.0293686	0.285187316
483.5796011	189.9506963	0.341292763
403.6046888	193.7679871	0.307758864
50.23135755	78.20458891	0.160367122
685.3557742	278.3531849	0.30373774
495.7938975	316.4490501	0.226314533
237.0154062	109.6498599	0.301912556
406.5018074	301.210976	0.217674236
54.37226277	48.93187348	0.205083535
73.75778547	67.14878893	0.211759604
452.2930551	285.4465898	0.262735258
192.7882353	79.76199095	0.326541365
263.0524968	131.9795134	0.302663818
634.4339536	384.8368941	0.266136146
618.4479388	439.8627284	0.227280092
188.7873303	43.83031674	0.391856852
203.8247306	236.1684628	0.16694062
51.96728972	50.18691589	0.219249468
91.93468468	122.4279279	0.193051621
269.6516464	61.4610052	0.41165829

log.sigma.2.5.mm.3D_gldm_DependenceNonUniformityNormalized	log.sigma.2.5.mm.3D_gldm_DependenceVariance	log.sigma.2.5.mm.3D_gldm_LargeDependenceEmphasis
0.15923132	4.852156116	16.48462178
0.140338	6.879036	21.64
0.193445361	3.871144166	12.61538462
0.148912062	5.17438068	18.21728496
0.120835975	8.154296964	27.54583772
0.143300669	5.343998831	19.23076923
0.087050425	13.95649314	47.68874172
0.128240575	7.028082964	23.72243767
0.140341454	7.107205172	21.7972373
0.169870201	3.999224884	14.54711955
0.125333474	6.389065581	24.32595462
0.181515378	3.481388055	12.68189911
0.122866763	6.61926129	24.81768522
0.171501702	4.0372659	15.39612487
0.172159292	3.336428099	13.4379845
0.126657076	14.4499899	34.63105378
0.134412127	5.911139838	21.65794393
0.127498638	6.306518788	23.57544957
0.155141899	5.726009224	18.85387324
0.136368362	5.854447604	21.45708376
0.170948874	4.65638085	15.47338129
0.160414445	4.359357859	16.5747289
0.17357	3.685222929	13.77020141
0.128871713	8.875336035	26.49902913
0.182245618	3.478324733	12.89784946
0.18291442	3.185515213	12.36390533
0.179983885	3.54983317	12.6969697
0.199368161	2.51616481	10.38122206
0.115312025	9.449741072	29.65607532
0.141156161	6.899989087	21.48179804
0.165123276	3.675026189	14.91743119
0.15195416	5.31738544	18.194
0.13536158	5.682378335	20.44606875
0.143589455	7.088290593	21.04573805
0.181562475	3.711341409	13.3018018
0.119633429	9.280918929	28.87193037
0.17459497	3.976937548	13.9111796
0.156003962	4.358907793	16.70923032
0.122207409	6.798108848	27.23045822
0.122168686	7.702391485	25.68704156
0.16695037	4.643704788	15.46960486
0.126941798	6.826513466	23.95548654
0.112762366	8.673351356	29.04096465
0.117276563	9.623913073	29.61677282
0.144313958	7.45159336	21.7847739
0.174846457	3.290620451	13.31160839
0.16499129	4.622951326	15.75094954
0.144429515	4.973047389	18.15435356
0.109488959	9.828429153	32.34042553
0.122908846	8.290157394	26.11559633
0.113234187	8.809754077	29.63303213
0.101504573	10.02044885	35.01022495
0.120808889	7.490259037	25.99815644
0.176257128	2.91850375	12.5969257
0.152592241	6.090684052	19.36677673
0.159786497	6.738060184	18.86970339
0.155120132	4.96614338	16.92436719
0.100644775	11.06430705	36.75986842
0.169403267	4.893388384	15.85276968
0.14758729	4.040658046	18.19003559
0.142143904	5.981316104	19.97313839
0.127543011	7.718026361	24.6872608
0.157907962	4.393875955	15.91677675
0.161967706	4.562567346	16.13276231
0.167572537	4.325699512	15.11525424
0.170096046	4.292467099	14.78872327
0.151459221	5.630872531	18.36862004
0.138398768	6.573086189	21.54907459
0.1393194	4.98016986	19.33565352
0.149643861	5.915666847	18.69764659
0.188961982	3.417784761	12.31722689
0.118669046	8.450093591	28.23338185
0.14472777	5.167225056	18.82987818
0.141738538	5.563083596	19.90892336
0.164049017	4.040000123	14.84561239
0.152003631	5.785294763	18.77434577
0.126264306	7.478607471	24.97622081
0.180699785	2.887330586	12.55261221
0.141954064	5.407126756	19.46275946
0.132765104	7.387616823	23.22097462
0.14046028	7.328964704	21.80469897
0.182002108	3.933606048	13.11516748
0.163138516	5.241393598	16.43168957
0.096044661	10.22230915	38.33460803
0.17746136	3.661628621	13.39047126
0.142715572	5.344086791	19.89982729
0.165977175	4.11146027	14.72408964
0.133585872	5.339016003	21.11633257
0.13229261	5.527802937	21.93917275
0.127608625	5.249146921	22.3183391
0.140857382	5.798449758	19.78106509
0.174468991	4.355712618	14.27963801
0.168407488	3.673800456	13.90396927
0.145746371	5.927723138	19.4599127
0.131416902	5.977041724	21.95325117
0.213560328	2.874306423	9.977375566
0.115612439	7.326380258	28.69143505
0.121418901	7.022512883	25.22429907
0.10353005	9.845928699	33.7545045
0.233666938	2.06260494	8.093587522

log.sigma.2.5.mm.3D_gldm_LargeDependenceLowGrayLevelEmphasis	log.sigma.2.5.mm.3D_gldm_SmallDependenceHighGrayLevelEmphasis	log.sigma.2.5.mm.3D_gldm_LargeDependenceHighGrayLevelEmphasis
0.065384311	99.8094354	4773.359933
0.070718276	98.35218524	7124.569
0.045220354	99.75661797	4211.22898
0.052048811	101.1049243	6893.144313
0.427795719	25.95273313	2167.86196
0.083497387	60.79917347	5030.647131
0.376503686	23.94749905	6344.077578
0.067096835	93.87506907	8920.139612
0.071320344	99.7106	7509.814998
0.059927278	85.44222465	4195.145854
0.06823176	71.14423018	9150.814056
0.050849419	98.55933898	4158.8273
0.118350269	50.39309212	5608.789689
0.117101266	41.8474664	2854.664155
0.071384197	62.50346238	3104.995478
0.115293576	85.48413694	11261.15235
0.147988956	43.72033705	4316.750779
0.091133908	67.01303511	6656.534011
0.097805987	62.02906727	4182.440141
0.071908769	73.23689286	7154.837642
0.057321903	88.107829	5008.490072
0.067948424	65.98724897	4725.695898
0.062208791	76.45581045	3838.017957
0.109875335	75.30667542	7522.874757
0.060237571	82.0511529	3564.605223
0.028136191	154.0015992	6256.300296
0.051737425	88.22674819	3532.06332
0.031443975	119.8567343	4202.423547
0.089298764	78.13507903	10582.46484
0.063688653	95.96066435	7734.716366
0.038631357	104.7751138	6509.967407
0.055768984	98.92782028	6454.6872
0.093146419	69.69522989	5360.031213
0.153819009	63.02002268	3249.008316
0.037046088	118.349013	5667.865991
0.099566887	69.32859411	9308.533105
0.055494766	112.1032671	4891.254693
0.038273462	108.6504032	7855.69596
0.233597104	24.48927351	3594.078841
0.091337647	79.20112709	7693.233496
0.052190588	87.49008753	5023.668693
0.126331935	54.35203418	5017.024327
0.103891235	68.30282833	8574.524612
0.18796221	46.39049791	5078.809979
0.040416733	142.9514491	12591.74585
0.044390661	94.28033937	4864.446993
0.046755624	110.9788669	6130.615844
0.052213443	91.18809862	7210.257256
0.247742617	39.66957619	4744.235816
0.106965671	73.71896541	6772.409174
0.124604855	60.58168043	7613.306225
0.105005535	58.25745272	12138.95961
0.081798145	82.76525123	8682.026073
0.052928904	79.23292226	3572.006832
0.061872516	96.67428968	6577.798832
0.06260526	110.5626229	6527.072564
0.030196076	180.5666719	10193.33455
0.140169964	59.70023819	10082.84087
0.031561373	150.5577619	8844.491254
0.187887653	21.86290631	2106.884698
0.069054322	93.96405694	6476.99864
0.060396774	108.9178931	11007.09185
0.070655155	78.30758524	4568.535447
0.073374342	64.57443876	4154.626338
0.157991285	40.53973142	1877.410169
0.081503152	85.51464448	3207.277382
0.075323498	84.98878912	4936.344045
0.04843042	121.5612506	10166.4341
0.067181735	67.24763947	6122.399072
0.049255759	118.4876258	7778.534503
0.040244005	123.9089505	4680.262605
0.14710643	50.33330159	5854.358564
0.069153445	85.17239747	6010.023082
0.05717029	88.09720128	7617.235141
0.037509047	158.8847483	7730.711168
0.051846461	104.834025	7908.357786
0.092114413	61.66601881	7215.51465
0.052354214	72.46176592	3633.467991
0.057266349	86.00673393	7323.828755
0.094388307	67.37175765	6713.181451
0.050886894	139.5013986	10765.17768
0.032797336	142.7143153	6289.363944
0.052225958	119.1252831	6409.052546
0.714107068	16.32560239	2444.460803
0.033159036	126.6036443	6093.799068
0.125310659	43.37260285	3591.83909
0.062551861	88.90578829	4260.77521
0.059992343	83.49600887	7912.025304
0.383267904	16.45822421	1595.306569
0.063099163	78.7124402	8689.015571
0.088795485	71.03560739	4871.322641
0.028891736	159.7098175	7933.875113
0.047552957	89.66649624	4747.213828
0.05846295	94.75666775	7281.460142
0.095637143	61.06704204	5530.940076
0.02558649	208.4021912	6233.753394
0.085345688	58.98424531	10478.69541
0.175229782	43.6316988	3980.883178
0.267626024	28.19186665	4729.094595
0.041221282	156.5930592	3457.832756

log.sigma.2.5.mm.3D_gldm_SmallDependenceLowGrayLevelEmphasis	log.sigma.2.5.mm.3D_gldm_LowGrayLevelEmphasis	log.sigma.2.5.mm.3D_gldzm_DistanceZoneVariabilityNormalized
0.002532723	0.005321097	1
0.001719582	0.004134583	1
0.002891985	0.005655769	1
0.00161201	0.003785897	1
0.008255845	0.023358681	1
0.003131604	0.006905616	1
0.00265391	0.009104937	1
0.001645058	0.003736553	1
0.001477898	0.00398779	1
0.002447716	0.005613231	1
0.001362399	0.00364596	1
0.002908656	0.006132243	1
0.002008887	0.005939581	1
0.005654973	0.012786842	1
0.003031526	0.006976141	1
0.001363878	0.004155101	0.99480523
0.004500765	0.012248613	1
0.001420109	0.004335766	1
0.002857588	0.007044672	1
0.001191548	0.004318405	0.997260279
0.001937415	0.004890737	0.998276606
0.00271001	0.006290901	0.996835451
0.002703623	0.006728666	0.99721449
0.003280233	0.006678555	1
0.002342704	0.005883228	1
0.001707786	0.003248202	1
0.002755016	0.005516825	1
0.00204405	0.004203075	1
0.001117016	0.003897652	0.997599043
0.001991341	0.004157241	1
0.001301283	0.003346532	1
0.001219129	0.003459949	1
0.003937895	0.007539474	1
0.005036428	0.009789032	1
0.002551127	0.004799155	1
0.001673777	0.005164543	1
0.002125762	0.005528778	0.998466259
0.001254515	0.003061127	1
0.00304506	0.010588004	1
0.001660365	0.004400779	1
0.002799335	0.005157473	1
0.002495997	0.006782367	1
0.001775836	0.004536627	1
0.00350435	0.008206807	1
0.00103474	0.002473889	1
0.001665796	0.004549826	1
0.002042341	0.004405137	0.990506545
0.002177501	0.004481009	1
0.00396129	0.010117133	1
0.002101461	0.005170268	1
0.001570343	0.005289847	1
0.001582956	0.004198146	1
0.001047061	0.003448519	1
0.003786883	0.006831601	1
0.001554856	0.003916409	0.998326361
0.002235925	0.004824702	1
0.000995869	0.002301803	0.996265186
0.001277823	0.004332562	1
0.00188454	0.003386267	1
0.00562428	0.014924381	1
0.002190573	0.004684229	1
0.001108941	0.003177816	0.998639456
0.00429241	0.008350462	1
0.003411706	0.006859288	1
0.007904392	0.014836416	1
0.002455257	0.006460974	1
0.002715707	0.00561358	1
0.001173434	0.002815698	1
0.002147538	0.004985227	1
0.001760818	0.003764513	1
0.002015412	0.004268657	1
0.002032143	0.006266428	1
0.00144082	0.004604561	1
0.001160673	0.003531296	0.994410329
0.00111513	0.003425894	1
0.001700798	0.004326814	1
0.00231143	0.005366137	1
0.002893529	0.006153503	1
0.001531914	0.004011854	1
0.003129224	0.006836538	0.998422714
0.002127064	0.004186046	1
0.002691972	0.004570609	0.996157555
0.002241448	0.005164959	1
0.00530846	0.020694038	1
0.001441127	0.003267794	0.998527247
0.002449416	0.0075165	0.993243321
0.004354492	0.007646727	1
0.001205902	0.003404022	1
0.007400423	0.020114521	1
0.002916596	0.005182449	1
0.002021284	0.005386846	1
0.0023887	0.003698005	1
0.003039587	0.005704469	1
0.001687725	0.004110118	1
0.001694474	0.0050838	0.998330552
0.004209048	0.006077052	1
0.000882817	0.004145758	1
0.004040277	0.009101804	1
0.003954967	0.012181535	1
0.005869803	0.009776132	1

log.sigma.2.5.mm.3D_gldzm_LowIntensityEmphasis	log.sigma.2.5.mm.3D_gldzm_LargeDistanceEmphasis	log.sigma.2.5.mm.3D_gldzm_HighIntensitySmallDistanceEmphasis
0.008173201	1	350.1141439
0.006340866	1	387.6575809
0.008056184	1	294.8471616
0.005891525	1	397.0611413
0.041910368	1	130.9409091
0.012552073	1	245.2378855
0.017418606	1	170.5688623
0.006722875	1	414.3866091
0.00568546	1	393.9461279
0.008221452	1	294.1908549
0.006521482	1	360.7444717
0.008645719	1	309.6135647
0.009717266	1	250.5631659
0.021606804	1	171.815331
0.009872766	1	223.2576336
0.005771173	1.0078125	361.0340712
0.021422152	1	198.6545012
0.006531883	1	331.312709
0.010893476	1	249.9650146
0.005994519	1.004115226	334.8353909
0.006770621	1.002587322	308.0018327
0.010447051	1.004754358	270.6739303
0.009505092	1.0041841	261.4306137
0.012883965	1	327.738676
0.007475871	1	269.4263323
0.00509045	1	509.3271222
0.007770611	1	267.0893271
0.005805164	1	358.9693572
0.005653171	1.003605769	361.9206731
0.007013614	1	373.5936214
0.0048029	1	405.6759333
0.004511027	1	385.0301609
0.014995038	1	280.2957143
0.015707937	1	223.8944099
0.007660143	1	388.7032641
0.008215655	1	323.7357513
0.007205179	1.002302379	363.7620875
0.004812104	1	436.0695297
0.018608184	1	152.738806
0.0070428	1	354.6323529
0.00862487	1	300.0285714
0.011060523	1	253.3235294
0.007985119	1	316.7892504
0.015369413	1	222.578125
0.00380442	1	551.146833
0.006144528	1	332.1748727
0.006739801	1.014308426	384.8624801
0.007699976	1	341.1571125
0.018842368	1	202.515625
0.008229039	1	313.4217687
0.008133495	1	301.0402235
0.008907354	1	327.2406417
0.004754507	1	396.011236
0.012087297	1	276.1725888
0.005632215	1.002512563	375.5175879
0.007191291	1	370.7401216
0.003346729	1.005612722	635.6587933
0.006651732	1	341.1905697
0.005931327	1	527.3873684
0.027011411	1	106.6291793
0.00797819	1	368.0197674
0.004713897	1.002042206	461.3548332
0.014797254	1	280.6276882
0.011546084	1	244.4323873
0.024906953	1	137.3366834
0.007935811	1	286.0455373
0.009067057	1	314.8189911
0.00452326	1	505.5622449
0.008741357	1	279.982808
0.005901636	1	414.6494465
0.005944397	1	392.7603591
0.00985298	1	255.77897
0.005938929	1	348.2228057
0.004915471	1.008408072	366.9764574
0.00382657	1	545.556172
0.006721981	1	395.3310153
0.009968668	1	278.1382799
0.009720175	1	262.1888889
0.006288016	1	349.3289037
0.012262373	1.002367798	269.390884
0.007070647	1	492.6741071
0.007528397	1.005774783	420.4381617
0.007706265	1	390.1651163
0.030211869	1	106.75
0.004570101	1.002210759	422.1519897
0.010753219	1.010169492	193.3983051
0.013419672	1	300.4851485
0.005364274	1	392.1856946
0.033179106	1	85.48979592
0.012788146	1	383.6102941
0.007598609	1	274.4967949
0.006611864	1	505.8305489
0.009564475	1	297.8379374
0.006297389	1	356.4529457
0.007229197	1.002506266	274.7719298
0.009856079	1	531.4596577
0.006622482	1	361.0449102
0.017265689	1	212.0490196
0.022728629	1	147.2222222
0.013913958	1	380.8188277

log.sigma.2.5.mm.3D_gldzm_LowIntensityLargeDistanceEmphasis	log.sigma.2.5.mm.3D_gldzm_HighIntensityEmphasis	log.sigma.2.5.mm.3D_gldzm_DistanceZoneVariability	log.sigma.2.5.mm.3D_gldzm_ZonePercentage
0.008173201	350.1141439	403	0.334995844
0.006340866	387.6575809	587	0.2935
0.008056184	294.8471616	458	0.409660107
0.005891525	397.0611413	736	0.300040766
0.041910368	130.9409091	220	0.231822972
0.012552073	245.2378855	454	0.277167277
0.017418606	170.5688623	167	0.157994324
0.006722875	414.3866091	463	0.256509695
0.00568546	393.9461279	594	0.293043907
0.008221452	294.1908549	1006	0.334998335
0.006521482	360.7444717	407	0.225235196
0.008645719	309.6135647	634	0.376261128
0.009717266	250.5631659	657	0.224308638
0.021606804	171.815331	574	0.308934338
0.009872766	223.2576336	524	0.338501292
0.005960964	361.2013889	1146.015625	0.280360185
0.021422152	198.6545012	411	0.256074766
0.006531883	331.312709	598	0.233776388
0.010893476	249.9650146	343	0.30193662
0.006015516	335.037037	727.0027435	0.251292658
0.006777744	308.2867615	2315.00345	0.333669065
0.010460221	271.1030111	629.0031696	0.297501179
0.009534761	261.5822873	1430.005579	0.347973793
0.012883965	327.738676	287	0.278640777
0.007475871	269.4263323	957	0.367511521
0.00509045	509.3271222	483	0.357248521
0.007770611	267.0893271	862	0.389868838
0.005805164	358.9693572	1338	0.39880775
0.005667257	362.1514423	830.0024038	0.244777876
0.007013614	373.5936214	972	0.307692308
0.0048029	405.6759333	1259	0.30395944
0.004511027	385.0301609	1492	0.2984
0.014995038	280.2957143	700	0.276570525
0.015707937	223.8944099	161	0.334719335
0.007660143	388.7032641	337	0.379504505
0.008215655	323.7357513	772	0.239975132
0.007210935	363.9923254	1301.001535	0.36508826
0.004812104	436.0695297	978	0.288410498
0.018608184	152.738806	268	0.180592992
0.0070428	354.6323529	408	0.249388753
0.00862487	300.0285714	455	0.345744681
0.011060523	253.3235294	476	0.246376812
0.007985119	316.7892504	707	0.233564585
0.015369413	222.578125	448	0.237791932
0.00380442	551.146833	1042	0.298225529
0.006144528	332.1748727	1178	0.32951049
0.006796105	385.9523052	1246.057234	0.341291373
0.007699976	341.1571125	471	0.310686016
0.018842368	202.515625	128	0.226950355
0.008229039	313.4217687	294	0.269724771
0.008133495	301.0402235	895	0.224648594
0.008907354	327.2406417	374	0.191206544
0.004754507	396.011236	890	0.234395575
0.012087297	276.1725888	394	0.33646456
0.005638496	375.7688442	1192.001675	0.303276607
0.007191291	370.7401216	658	0.348516949
0.003361519	636.1927035	1065.007484	0.326014029
0.006651732	341.1905697	509	0.209292763
0.005931327	527.3873684	475	0.346209913
0.027011411	106.6291793	329	0.234163701
0.00797819	368.0197674	860	0.292417545
0.004725981	461.4411164	1467.001361	0.267723711
0.014797254	280.6276882	744	0.327608983
0.011546084	244.4323873	599	0.320663812
0.024906953	137.3366834	199	0.337288136
0.007935811	286.0455373	549	0.355800389
0.009067057	314.8189911	337	0.31852552
0.00452326	505.5622449	980	0.274817723
0.008741357	279.982808	349	0.269914927
0.005901636	414.6494465	813	0.324291982
0.005944397	392.7603591	1448	0.380252101
0.00985298	255.77897	466	0.226103833
0.005938929	348.2228057	1333	0.284889934
0.005019163	367.4304933	1774.028027	0.27399785
0.00382657	545.556172	1442	0.338338808
0.006721981	395.3310153	719	0.308451308
0.009968668	278.1382799	593	0.251804671
0.009720175	262.1888889	450	0.331125828
0.006288016	349.3289037	903	0.275641026
0.012276383	269.4909234	1265.001579	0.284527285
0.007070647	492.6741071	448	0.328928047
0.007545134	421.0048123	1035.0077	0.391042529
0.007706265	390.1651163	860	0.347615198
0.030211869	106.75	100	0.191204589
0.004579927	422.2763449	1355.001474	0.351372346
0.010868093	193.6644068	879.020339	0.254749568
0.013419672	300.4851485	505	0.353641457
0.005364274	392.1856946	727	0.238908971
0.033179106	85.48979592	98	0.238442822
0.012788146	383.6102941	136	0.235294118
0.007598609	274.4967949	936	0.291497976
0.006611864	505.8305489	419	0.37918552
0.009564475	297.8379374	543	0.347631242
0.006297389	356.4529457	1307	0.300252699
0.00725426	274.8345865	1195.001671	0.254356141
0.009856079	531.4596577	409	0.462669683
0.006622482	361.0449102	334	0.189449801
0.017265689	212.0490196	102	0.238317757
0.022728629	147.2222222	189	0.212837838
0.013913958	380.8188277	563	0.487868284

log.sigma.2.5.mm.3D_gldzm_IntensityVariabilityNormalized	log.sigma.2.5.mm.3D_gldzm_LowIntensitySmallDistanceEmphasis	log.sigma.2.5.mm.3D_gldzm_IntensityVariability
0.050902351	0.008173201	20.51364764
0.053211403	0.006340866	31.2350937
0.052868938	0.008056184	24.2139738
0.052763912	0.005891525	38.83423913
0.060785124	0.041910368	13.37272727
0.061033593	0.012552073	27.7092511
0.070242748	0.017418606	11.73053892
0.052792148	0.006722875	24.44276458
0.058616468	0.00568546	34.81818182
0.053873578	0.008221452	54.19681909
0.065155842	0.006521482	26.51842752
0.051791738	0.008645719	32.83596215
0.062567039	0.009717266	41.1065449
0.063616166	0.021606804	36.51567944
0.058176971	0.009872766	30.48473282
0.054307726	0.005723725	62.5625
0.054623167	0.021422152	22.45012165
0.060888581	0.006531883	36.41137124
0.05819854	0.010893476	19.96209913
0.059120392	0.00598927	43.09876543
0.052763037	0.00676884	122.3574817
0.056899094	0.010443758	35.90332805
0.054617975	0.009497675	78.32217573
0.054717187	0.012883965	15.70383275
0.056440527	0.007475871	54.01358412
0.048982164	0.00509045	23.65838509
0.060868535	0.007770611	52.46867749
0.051186319	0.005805164	68.48729447
0.054077871	0.00564965	44.99278846
0.053946722	0.007013614	52.43621399
0.053878004	0.0048029	67.83240667
0.05912768	0.004511027	88.21849866
0.052338776	0.014995038	36.63714286
0.056903669	0.015707937	9.161490683
0.050744481	0.007660143	17.10089021
0.0550149	0.008215655	42.47150259
0.046439264	0.00720374	60.51036071
0.055013989	0.004812104	53.80368098
0.068305859	0.018608184	18.30597015
0.056456651	0.0070428	23.03431373
0.058422896	0.00862487	26.58241758
0.057058117	0.011060523	27.15966387
0.056335013	0.007985119	39.82885431
0.056491151	0.015369413	25.30803571
0.049312742	0.00380442	51.38387716
0.055002436	0.006144528	64.79286927
0.051144851	0.006725725	64.34022258
0.054854603	0.007699976	25.83651805
0.064697266	0.018842368	8.28125
0.057522329	0.008229039	16.91156463
0.060336444	0.008133495	54.00111732
0.063356115	0.008907354	23.69518717
0.056800909	0.004754507	50.55280899
0.057847407	0.012087297	22.79187817
0.053950658	0.005630645	64.41708543
0.046290223	0.007191291	30.45896657
0.047835899	0.003343031	51.13657624
0.05810152	0.006651732	29.57367387
0.05074349	0.005931327	24.10315789
0.07742907	0.027011411	25.47416413
0.054721471	0.00797819	47.06046512
0.051354496	0.004710876	75.43975494
0.050706729	0.014797254	37.72580645
0.060075083	0.011546084	35.98497496
0.064518573	0.024906953	12.83919598
0.054137179	0.007935811	29.72131148
0.05188916	0.009067057	17.48664688
0.052313619	0.00452326	51.26734694
0.060795888	0.008741357	21.21776504
0.047201911	0.005901636	38.37515375
0.050326608	0.005944397	72.87292818
0.057829395	0.00985298	26.94849785
0.054557713	0.005938929	72.72543136
0.057509828	0.004889548	102.5975336
0.047740559	0.00382657	68.84188627
0.049738762	0.006721981	35.76216968
0.058527111	0.009968668	34.70657673
0.057501235	0.009720175	25.87555556
0.05360868	0.006288016	48.40863787
0.051469237	0.01225887	65.21152328
0.049645249	0.007070647	22.24107143
0.045165402	0.007524212	46.92685274
0.047909681	0.007706265	41.20232558
0.0858	0.030211869	8.58
0.053252086	0.004567645	72.26308032
0.061638737	0.010724501	54.55028249
0.048712871	0.013419672	24.6
0.060577565	0.005364274	44.03988996
0.091420242	0.033179106	8.959183673
0.059364187	0.012788146	8.073529412
0.056795146	0.007598609	53.16025641
0.046656148	0.006611864	19.54892601
0.054967119	0.009564475	29.84714549
0.051384195	0.006297389	67.15914308
0.059220036	0.007222932	70.88638262
0.035503135	0.009856079	14.5207824
0.071587364	0.006622482	23.91017964
0.073817762	0.017265689	7.529411765
0.076677585	0.022728629	14.49206349
0.043357552	0.013913958	24.41030195

log.sigma.2.5.mm.3D_gldzm_HighIntensityLargeDistanceEmphasis	log.sigma.2.5.mm.3D_gldzm_SmallDistanceEmphasis	log.sigma.2.5.mm.3D_glcm_SumVariance	log.sigma.2.5.mm.3D_glcm_Homogeneity1
350.1141439	1	947.6442344	0.384107341
387.6575809	1	1091.303234	0.407955178
294.8471616	1	938.8351097	0.34586761
397.0611413	1	1168.330458	0.397147416
130.9409091	1	230.7315742	0.466610613
245.2378855	1	757.2488075	0.408042675
170.5688623	1	421.1390834	0.543671205
414.3866091	1	1253.019433	0.433963213
393.9461279	1	1175.965358	0.420292081
294.1908549	1	858.9478976	0.378855423
360.7444717	1	1187.545883	0.444546262
309.6135647	1	957.564683	0.351996951
250.5631659	1	685.4606298	0.453667897
171.815331	1	478.4372421	0.387814815
223.2576336	1	668.2367116	0.376047797
361.8706597	0.998046875	1092.5344	0.432676624
198.6545012	1	551.6242602	0.431378783
331.312709	1	928.3596279	0.442217564
249.9650146	1	672.268851	0.413855964
335.8436214	0.998971193	1039.645244	0.423481856
309.4264769	0.999353169	955.5335525	0.355580621
272.8193344	0.99881141	806.5820219	0.394305664
262.1889819	0.998953975	793.7156558	0.36747151
327.738676	1	920.0038215	0.433213877
269.4263323	1	801.8674098	0.350834151
509.3271222	1	1611.342495	0.355403417
267.0893271	1	843.9387226	0.395577945
358.9693572	1	1168.77825	0.332373637
363.0745192	0.999098558	1142.489991	0.440502118
373.5936214	1	1173.320005	0.407917044
405.6759333	1	1293.349875	0.378154199
385.0301609	1	1138.430049	0.403073059
280.2957143	1	791.6871689	0.41364699
223.8944099	1	533.5819757	0.424905732
388.7032641	1	1245.947913	0.355562344
323.7357513	1	1016.154763	0.444404873
364.9132771	0.999424405	1072.579211	0.349463986
436.0695297	1	1424.351892	0.384840712
152.738806	1	372.475871	0.474591067
354.6323529	1	992.6662503	0.442221487
300.0285714	1	972.8389067	0.383462542
253.3235294	1	653.7189305	0.440504518
316.7892504	1	955.3407916	0.454701908
222.578125	1	568.4616515	0.460181462
551.146833	1	1855.13174	0.388335088
332.1748727	1	1058.336658	0.363579211
390.3116057	0.996422893	1161.179512	0.371592132
341.1571125	1	1170.433912	0.38866801
202.515625	1	462.0556155	0.485566895
313.4217687	1	857.4651798	0.441698232
301.0402235	1	834.4098594	0.466392288
327.2406417	1	1111.85688	0.477425906
396.011236	1	1111.878145	0.446566846
276.1725888	1	847.7493588	0.368868187
376.7738693	0.999371859	1091.561215	0.397267384
370.7401216	1	1107.675557	0.364148383
638.3283442	0.998596819	1992.090751	0.388510977
341.1905697	1	928.0189101	0.486086192
527.3873684	1	1722.823157	0.371537011
106.6291793	1	291.1803458	0.429517239
368.0197674	1	1064.86098	0.407441557
461.7862491	0.999489449	1432.771008	0.424613033
280.6276882	1	830.270048	0.382880186
244.4323873	1	747.4385262	0.388601551
137.3366834	1	372.1565916	0.389499845
286.0455373	1	693.5749629	0.375917598
314.8189911	1	856.9479695	0.403652761
505.5622449	1	1561.739041	0.412683342
279.982808	1	942.2063074	0.407514469
414.6494465	1	1272.074573	0.385857121
392.7603591	1	1200.787428	0.346087891
255.77897	1	667.0233869	0.459662275
348.2228057	1	1010.711196	0.404555828
369.2466368	0.997897982	1181.570085	0.401622266
545.556172	1	1653.30733	0.373020394
395.3310153	1	1293.928119	0.386040026
278.1382799	1	888.4078783	0.433353378
262.1888889	1	801.1262097	0.372805581
349.3289037	1	1153.196889	0.398456729
269.8910813	0.999408051	850.2769271	0.411911788
492.6741071	1	1595.091224	0.396795199
423.2714148	0.998556304	1359.057223	0.341264887
390.1651163	1	1172.24861	0.366160375
106.75	1	184.3438991	0.522087175
422.7737657	0.99944731	1328.789322	0.3620697
194.7288136	0.997457627	527.1554402	0.423924577
300.4851485	1	869.3349758	0.377973953
392.1856946	1	1214.92555	0.423601688
85.48979592	1	204.6939371	0.461350208
383.6102941	1	1207.576569	0.443910868
274.4967949	1	766.493111	0.41538159
505.8305489	1	1695.772633	0.353351163
297.8379374	1	968.3704223	0.37131107
356.4529457	1	1120.630608	0.390025037
275.085213	0.999373434	772.0967554	0.434630074
531.4596577	1	1746.024304	0.291721207
361.0449102	1	1157.770898	0.475169033
212.0490196	1	507.6364952	0.477294108
147.2222222	1	404.7200358	0.496658814
380.8188277	1	1167.351198	0.293083636

log.sigma.2.5.mm.3D_glcm_Homogeneity2	log.sigma.2.5.mm.3D_glcm_ClusterShade	log.sigma.2.5.mm.3D_glcm_MaximumProbability	log.sigma.2.5.mm.3D_glcm_Idmn	log.sigma.2.5.mm.3D_glcm_SumVariance2
0.301909997	194.8971761	0.035204077	0.984093195	55.87493042
0.331210254	39.53424561	0.042964409	0.985480189	41.68401952
0.260634292	-194.836716	0.027289765	0.971248042	55.09228513
0.31812977	7.042296516	0.034077662	0.987647468	39.88015336
0.399436507	223.6824945	0.073165623	0.982566066	34.71956267
0.330608152	-19.96590063	0.036542194	0.982791286	34.63658039
0.492547072	98.89231098	0.124980626	0.989627601	21.32588657
0.363595267	15.2610797	0.065508104	0.988740681	40.78478473
0.345277672	15.75509887	0.044308959	0.985476822	40.11632396
0.294936994	-82.9108142	0.026142173	0.98317765	45.33012577
0.373704221	-36.89395931	0.047104375	0.98786833	27.14700259
0.264945034	-179.5333163	0.023389103	0.975896351	50.0412753
0.383646024	49.83897016	0.047400141	0.987672089	29.25395537
0.304868069	-59.18362595	0.026388514	0.977128134	36.9273558
0.29234491	118.7169578	0.020127836	0.976670455	45.97826095
0.361929095	-83.47013192	0.067801478	0.985520491	44.79445713
0.358735537	-78.13905957	0.04970691	0.981854797	36.83997887
0.368278397	64.92285806	0.041162882	0.988679503	36.21694229
0.335791507	43.3732644	0.041581283	0.981453597	41.37413854
0.348148503	-49.11804761	0.038609196	0.985029336	37.38306133
0.270179768	-145.5659164	0.023750548	0.981877814	46.90899877
0.313130132	-70.90510612	0.029490554	0.985935157	42.00913612
0.282946801	-103.9551278	0.023653502	0.980653193	47.0178543
0.360276306	30.51599819	0.068931991	0.984103564	48.34209634
0.263525971	-111.9919666	0.016771789	0.981126842	51.18188902
0.271477845	-63.72130525	0.022948751	0.981652894	63.3127776
0.31459778	35.10262338	0.030287578	0.984855723	40.31990678
0.244761054	-237.2676328	0.020526319	0.974643767	53.11744093
0.369867382	-44.8909291	0.060736448	0.989318391	34.38241886
0.332607708	-14.56198047	0.047136991	0.98771153	40.68008835
0.29557508	-175.1514034	0.02440631	0.985348776	45.61147906
0.32224343	46.87375195	0.032667315	0.989639856	38.64317291
0.337976251	-81.44425018	0.049515337	0.986174416	43.84860574
0.351199964	259.3010533	0.054417945	0.981971674	46.98060452
0.272856232	-268.4231144	0.034783217	0.975106722	53.73446262
0.37566	-98.28542501	0.0562926	0.98457702	35.92085817
0.263816473	-120.0263901	0.024198258	0.979904211	60.05800791
0.303179395	-122.2820893	0.029984844	0.984618133	39.10421426
0.405963998	33.27515767	0.061940752	0.987711506	21.84610587
0.371477031	111.5826688	0.06806682	0.986977205	38.23581064
0.30144505	-70.56792828	0.032991054	0.980027302	36.27494669
0.368137359	78.24714077	0.047825937	0.984727371	36.1907997
0.385944261	23.82117699	0.058118468	0.98647402	33.87246797
0.393285136	209.8398472	0.06570926	0.985427273	36.27038053
0.310842913	-188.819538	0.034111541	0.983985233	58.93380629
0.277411725	-151.3251966	0.021845008	0.981903158	47.70656471
0.288398302	-125.790455	0.026793663	0.983251709	51.09755964
0.309885579	-316.6789678	0.044393364	0.980571888	43.7965271
0.422765735	131.478674	0.098253598	0.984081465	35.34845117
0.370261775	151.3116603	0.067222167	0.988651701	35.2104245
0.399320495	37.50330337	0.054691123	0.990587197	30.17642345
0.415331096	-121.6038742	0.074668689	0.986738875	25.31707127
0.375351268	96.96227815	0.051980404	0.990896994	31.97980007
0.283621823	-10.04585672	0.024400309	0.979504587	43.12877775
0.317801026	18.28400117	0.03612825	0.98606518	40.79663717
0.28312467	-56.08405031	0.029893368	0.976823	54.04879821
0.308956679	-132.5901346	0.037670923	0.987939798	59.34360631
0.424434775	159.1875183	0.09008193	0.991320393	33.84432371
0.290345625	-344.1798745	0.03496494	0.98137279	54.68999186
0.351631595	-22.99387853	0.035775533	0.976364002	17.78426367
0.329527134	2.031248187	0.032234612	0.986153043	45.0442112
0.353291376	-179.5595823	0.048164551	0.988804959	46.81375124
0.302079487	-124.8142879	0.027113992	0.977583363	49.90028767
0.306819271	-116.4218195	0.027047642	0.978916186	39.1422636
0.304236871	54.22259215	0.029862998	0.97086272	34.33504787
0.291214784	325.0980515	0.02705435	0.983073578	58.56524359
0.325471766	258.7871284	0.047374997	0.98536108	50.1956754
0.336658577	27.30041563	0.042550069	0.987701794	47.59650697
0.328958073	-88.77519662	0.043514782	0.98394536	34.88259978
0.307712121	-97.22137473	0.037950388	0.985812495	53.74914829
0.259610796	-184.2987419	0.014747345	0.977350909	62.24046564
0.391813661	143.4817386	0.05982315	0.988331717	33.8700995
0.32533678	-30.08987211	0.03484609	0.986662145	43.27622474
0.323156228	-134.6366408	0.033915402	0.985491393	35.67312722
0.290927645	-83.05815691	0.026653575	0.986146081	68.85386488
0.306786908	-128.7801425	0.0373234	0.984494818	50.13218159
0.36198766	-46.53440178	0.052735197	0.985749276	30.46765678
0.2866067	-24.00135241	0.023969147	0.980667218	47.64114527
0.320339717	-158.5440278	0.038735883	0.983350078	40.92367128
0.33851571	-183.7950076	0.044416011	0.983721948	44.783252
0.322202539	-267.3462824	0.044196044	0.98192893	57.44885321
0.258001533	-321.3709308	0.024417138	0.978394728	69.36642354
0.282093402	-59.23940515	0.028719355	0.982757723	48.75121781
0.465461457	105.4931915	0.114207092	0.982132438	23.149893
0.27596436	-127.5332339	0.022257792	0.9828038	50.88328803
0.346773863	64.29554306	0.036651349	0.982949565	32.33445705
0.295884958	-31.72388677	0.030283227	0.981802563	58.57789494
0.347390697	3.593662113	0.040452477	0.987591078	34.23159824
0.391591664	35.92884343	0.061897433	0.970848007	21.84736445
0.373733518	-77.42959029	0.048581309	0.98751353	35.50318315
0.339233299	88.94101804	0.039973424	0.983512099	39.69848827
0.270836788	-226.2211235	0.026233358	0.974982476	59.8505328
0.288600173	-247.3150981	0.027743332	0.976881444	43.85591857
0.311547743	-161.8162384	0.040945795	0.982362118	42.47347994
0.360622102	28.04725177	0.035996295	0.988025701	32.9475919
0.209853316	-1098.448346	0.016967988	0.965138299	143.918213
0.40938737	-23.87460154	0.081106026	0.990226018	23.15940185
0.411781747	121.4642553	0.079161821	0.987083828	29.67233265
0.435553465	26.50358518	0.08362919	0.984922475	20.24921081
0.203688209	-537.6986872	0.014123532	0.968211941	94.80739438

log.sigma.2.5.mm.3D_glcm_Contrast	log.sigma.2.5.mm.3D_glcm_DifferenceEntropy	log.sigma.2.5.mm.3D_glcm_InverseVariance	log.sigma.2.5.mm.3D_glcm_Entropy	log.sigma.2.5.mm.3D_glcm_Dissimilarity
18.61716052	3.189442071	0.296295223	7.464956168	3.142559997
17.08263294	3.11947902	0.322348015	7.299572909	2.913201463
27.02307218	3.446367835	0.261866949	7.849706363	3.844283418
18.13031233	3.138382637	0.319677401	7.270515944	3.000146218
9.198972153	2.723830621	0.372964484	6.405302858	2.157167833
15.72137087	3.074527425	0.325002075	7.133364679	2.839733227
5.288303294	2.37442472	0.410391099	5.703159184	1.584809535
14.94999329	2.960272881	0.364219833	6.927392178	2.60773776
15.12583571	3.030697431	0.33501019	7.234822052	2.729607555
17.37559067	3.163387358	0.299123355	7.6250384	3.091606383
11.70485085	2.852944685	0.362393699	6.774495511	2.395330792
24.14372798	3.367705203	0.265068407	7.763404513	3.61690695
10.37807929	2.787507732	0.367381812	6.769058507	2.267285738
15.63419745	3.095022054	0.303127813	7.407324937	2.95767806
18.71335693	3.188460762	0.293959444	7.62146827	3.193551113
19.33702435	3.15824616	0.316255217	7.427353743	2.940801305
13.52492338	2.942060419	0.351335744	7.002732535	2.575804863
10.00792814	2.803254617	0.349452515	6.964819793	2.321041311
13.57199234	2.971702086	0.323866901	7.160143773	2.696845333
13.57358174	2.97661257	0.336917775	7.21470909	2.631368983
23.94236762	3.378929247	0.271000367	7.903983661	3.604346952
16.37479404	3.100910786	0.311352327	7.431976989	2.94146153
20.12447818	3.254342333	0.28598342	7.783517834	3.32728167
14.48364986	2.999150333	0.32424946	6.974461462	2.679247169
22.10097092	3.327598195	0.267766203	7.904940385	3.543797203
27.4852396	3.412484322	0.277582964	7.85676187	3.734490479
15.54046572	3.088229976	0.312237566	7.364056714	2.901189002
28.68629897	3.492879165	0.254583885	8.023911136	3.987713186
14.76644541	2.989442591	0.338974898	6.984908159	2.628409561
20.06741167	3.19637382	0.316090805	7.392110977	3.076659155
20.43835363	3.23894852	0.298583126	7.641355873	3.242117904
14.07366832	3.02581501	0.316683267	7.404066085	2.766539178
16.21824676	3.070845352	0.331937502	7.259160502	2.825533995
14.65010425	2.942774194	0.342852879	6.70632697	2.670096884
28.8770238	3.423557395	0.280940272	7.548146805	3.797568247
15.35307334	2.997058928	0.350571139	6.981414299	2.619931583
27.06482552	3.438756263	0.266947342	8.001454031	3.768577163
19.17437129	3.206692319	0.301663295	7.434659098	3.148788575
6.816354912	2.564228872	0.377469035	6.420157038	1.945941552
12.59453376	2.919168006	0.352638579	6.873921039	2.483777973
18.36451982	3.178783189	0.30368869	7.280124673	3.119736313
12.07868266	2.899589733	0.348989408	6.995393813	2.454357011
12.4514173	2.882732539	0.35719397	6.876027228	2.398154587
11.52909014	2.84866655	0.363972721	6.701229118	2.340248742
25.34996893	3.345159462	0.29329932	7.868972307	3.461842064
20.02129919	3.248309511	0.286661883	7.738079527	3.31525253
22.31097482	3.294053389	0.290570244	7.725504785	3.380037063
20.68900049	3.216016423	0.315038755	7.309503018	3.192086741
8.256915564	2.639931739	0.378210351	6.078621793	2.039702621
12.26075362	2.911790283	0.34594439	6.692338709	2.485621719
10.28690529	2.769636788	0.37280637	6.748640718	2.201614826
13.36228339	2.823528404	0.377603401	6.389119091	2.317657726
11.04350438	2.8485404	0.357455497	6.864889724	2.360245096
20.31852138	3.225395322	0.28865212	7.486692625	3.29096378
17.2564065	3.143715762	0.313306179	7.447232573	2.981093256
28.09577452	3.450959194	0.273208159	7.895214085	3.770154357
21.86366194	3.242514488	0.307508953	7.681768314	3.230810223
9.343991466	2.711716796	0.382121651	6.476110537	2.092149253
26.90944681	3.372849923	0.287145373	7.586533782	3.589758964
9.37180476	2.755451504	0.35119067	6.57163801	2.30916338
16.30675401	3.084345526	0.323204016	7.401347128	2.854688368
20.35012212	3.147414019	0.334001174	7.34870213	2.963647781
21.12616305	3.250480403	0.298601669	7.641395386	3.266135503
16.7466758	3.137617665	0.302081576	7.422277108	3.025391795
14.31423489	3.010038644	0.294664242	7.076094866	2.867067196
17.42500888	3.169291169	0.291087607	7.600630416	3.127707485
16.07335953	3.090056747	0.321166902	7.206902823	2.886150965
17.14520777	3.104173064	0.325932316	7.396198976	2.875558026
15.66316747	3.060334221	0.320264631	7.088778096	2.830351407
24.67847936	3.322807422	0.301749249	7.651268156	3.412363582
25.4505121	3.408435658	0.268053796	8.146995123	3.728366698
10.49765166	2.787402723	0.370896247	6.709052487	2.259140583
15.55783968	3.068463657	0.320353746	7.443302932	2.834010123
17.0426918	3.114626686	0.322373071	7.365902918	2.928156186
24.0336485	3.326549502	0.29081411	7.961994427	3.442077491
21.69919721	3.275447194	0.300104317	7.572619502	3.294863318
14.89850797	3.005451363	0.346272358	6.931149346	2.654469739
16.31048152	3.120636503	0.294246837	7.522311637	3.067324221
18.59949485	3.149484501	0.320374192	7.36604781	3.030005657
20.66912694	3.198348352	0.324898992	7.441513245	3.080780714
27.79553569	3.351367218	0.304562153	7.416096246	3.488584911
34.53673412	3.583491446	0.261834304	8.089329458	4.17791819
23.04825434	3.326120789	0.281889402	7.65171143	3.459704058
5.560875366	2.397332459	0.401639711	5.716034215	1.683481077
21.49501844	3.295678558	0.279032325	7.8024155	3.406414891
11.43223742	2.903769733	0.337359718	7.147388319	2.501779621
20.4124186	3.234067242	0.298793029	7.603733957	3.251587118
12.82752115	2.924113471	0.344230193	7.025996315	2.552133705
7.240600181	2.48903224	0.381956176	5.991387972	2.033571495
13.00958328	2.856808195	0.362284667	6.407480552	2.464407253
13.90468203	3.012617415	0.335834327	7.270616487	2.701607513
33.04256616	3.517931794	0.262871848	7.750991791	4.016241771
21.60809217	3.280910712	0.292664157	7.470866522	3.365731544
21.10716832	3.236538358	0.312500316	7.457154814	3.211177687
11.50346125	2.873880407	0.352648307	7.1145361	2.430509076
67.03782413	4.000645009	0.208903376	8.486235925	5.916622462
8.122464208	2.63998808	0.386890603	6.289583936	2.024414241
7.261343159	2.542029561	0.395003212	6.067603448	1.953924252
7.926114858	2.61639591	0.386090604	6.103818775	1.946017787
43.74807681	3.769333943	0.210055683	8.370059449	4.979246539

log.sigma.2.5.mm.3D_glcm_DifferenceVariance	log.sigma.2.5.mm.3D_glcm_Idn	log.sigma.2.5.mm.3D_glcm_Idm	log.sigma.2.5.mm.3D_glcm_Correlation	log.sigma.2.5.mm.3D_glcm_Autocorrelation
8.531866138	0.918557195	0.301909997	0.498050774	309.5959265
8.480480886	0.924313088	0.331210254	0.415394051	348.7617373
11.95638533	0.891970582	0.260634292	0.343808091	305.6836194
8.9577814	0.929678166	0.31812977	0.377345885	369.3139459
4.441967222	0.917016709	0.399436507	0.566098475	89.71930675
7.52195794	0.91694214	0.330608152	0.372047306	250.6686109
2.7001174	0.937045274	0.492547072	0.584108415	146.6572939
8.055513014	0.935307493	0.363595267	0.462611035	392.7625308
7.574521859	0.924545576	0.345277672	0.452933501	373.3110177
7.656852471	0.914853288	0.294936994	0.444338678	283.6213491
5.83557771	0.930729392	0.373704221	0.399686835	372.3076703
10.82086754	0.900145772	0.264945034	0.347777963	311.1899025
5.127624985	0.929779618	0.383646024	0.471245265	229.1657587
6.664141104	0.90135247	0.304868069	0.404428786	169.3565058
8.189595782	0.901733045	0.29234491	0.415337386	227.0202092
10.46278953	0.928478569	0.361929095	0.401265281	349.0728999
6.722843942	0.916575383	0.358735537	0.45894294	190.324094
4.497404809	0.929901368	0.368278397	0.562323603	302.214011
5.968875195	0.912283934	0.335791507	0.49985334	227.9282697
6.496133995	0.922250592	0.348148503	0.46621595	334.2351667
10.70952109	0.91266659	0.270179768	0.327811734	310.2575322
7.517047227	0.923102167	0.313130132	0.437989862	267.5881393
8.770795541	0.909358192	0.282946801	0.401927957	264.4660537
7.061989181	0.921368855	0.360276306	0.535253597	299.984546
9.285718119	0.90892837	0.263525971	0.398533018	266.9971484
13.28269263	0.914867918	0.271477845	0.396400437	499.1347545
6.98170733	0.919571955	0.31459778	0.441494678	278.2767721
12.42373607	0.897096756	0.244761054	0.303003023	371.5444616
7.606217372	0.936316791	0.369867382	0.402523163	360.493112
10.40159953	0.931793199	0.332607708	0.34195292	371.0107119
9.719967822	0.922652679	0.29557508	0.38269018	407.2745976
6.271045872	0.932275872	0.32224343	0.463179075	363.1434338
8.051548743	0.926373103	0.337976251	0.458237298	262.4194152
7.215462016	0.916712935	0.351199964	0.510981136	184.4763318
14.08774483	0.902657759	0.272856232	0.309469997	391.9430155
8.380253562	0.92599016	0.37566	0.398812238	325.142737
12.58503893	0.909635746	0.263816473	0.379040399	345.1336718
9.080777755	0.920719766	0.303179395	0.344639961	442.0290615
2.926148806	0.926171325	0.405963998	0.518163061	134.8226339
6.351595474	0.92853879	0.371477031	0.498260834	319.3879359
8.476242881	0.909617636	0.30144505	0.326780215	313.4088599
5.964749208	0.922336814	0.368137359	0.489258736	221.54839
6.596433208	0.929057037	0.385944261	0.46203068	307.876196
5.950332979	0.925944289	0.393285136	0.50262192	194.2539425
13.02350725	0.922740741	0.310842913	0.396992043	566.9518043
8.846150866	0.912038186	0.277411725	0.410208466	341.1125213
10.64002966	0.917901635	0.288398302	0.396000589	370.1114609
10.31499592	0.913743843	0.309885579	0.360072153	368.7967053
3.941463514	0.921051135	0.422765735	0.605273463	161.2487913
6.001588898	0.932242597	0.370261775	0.481611616	279.0435872
5.361197194	0.939466401	0.399320495	0.489264408	272.4679485
7.859789548	0.934217633	0.415331096	0.312901491	346.5275736
5.369708683	0.938601692	0.375351268	0.484860578	352.1225839
9.228302021	0.907968023	0.283621823	0.357018391	278.733688
8.235639796	0.924433805	0.317801026	0.404802368	348.9431897
13.55392245	0.905608774	0.28312467	0.321834898	354.3691378
11.18419594	0.931700472	0.308956679	0.462263979	605.2346482
4.860590286	0.942254103	0.424434775	0.564328226	298.1952735
13.77979464	0.916340187	0.290345625	0.344800108	527.4520608
3.928945711	0.898806327	0.351631595	0.311103638	108.3549354
8.006925776	0.925534701	0.329527134	0.46839541	342.720926
11.37632252	0.937432406	0.353291376	0.393106294	444.8845977
10.16480552	0.906642221	0.302079487	0.405419226	274.7285768
7.404976001	0.906089131	0.306819271	0.401518079	249.5162881
5.785768028	0.888402938	0.304236871	0.411355076	135.7301686
7.495409787	0.913793204	0.291214784	0.538291069	235.4384742
7.643467805	0.922516619	0.325471766	0.509790267	281.93861
8.740491199	0.930831321	0.336658577	0.471139942	483.4531501
7.519305757	0.919552351	0.328958073	0.380863602	304.6012351
12.77628404	0.926979266	0.307712121	0.371899706	401.3481961
11.24423016	0.902930162	0.259610796	0.421304666	384.0694399
5.293853968	0.932284336	0.391813661	0.518053272	223.7075675
7.368109419	0.925738909	0.32533678	0.471058778	327.1827552
8.295776017	0.923849897	0.323156228	0.356369506	372.9633331
11.94058875	0.925984647	0.290927645	0.482133601	513.0463927
10.65592829	0.921885892	0.306786908	0.39585097	407.4978171
7.723275786	0.926722963	0.36198766	0.344192008	287.0809029
6.706675073	0.907169018	0.2866067	0.481428584	267.1937752
9.150507118	0.919679017	0.320339717	0.380339769	365.3728679
10.99247788	0.923409734	0.33851571	0.370266404	278.5699164
15.41773821	0.921255106	0.322202539	0.349617682	490.4537468
16.73279866	0.908583933	0.258001533	0.340056262	426.8357658
10.8677263	0.916072774	0.282093402	0.360197122	372.2718168
2.572942979	0.91637792	0.465461457	0.599445741	73.65180627
9.677647641	0.914695133	0.27596436	0.406308911	418.8880527
5.095869478	0.91469747	0.346773863	0.476596475	184.0323433
9.646484955	0.914110535	0.295884958	0.48076659	287.4831784
6.18822807	0.928518235	0.347390697	0.453417877	382.4136627
2.676988563	0.889309618	0.391591664	0.491296507	80.83098962
6.752634623	0.931356868	0.373733518	0.45617381	376.2791378
6.510748573	0.917776226	0.339233299	0.478905603	255.3684102
16.553903	0.903345652	0.270836788	0.289365884	520.1721855
10.06124544	0.903782237	0.288600173	0.342782623	312.6740766
10.55152581	0.917891602	0.311547743	0.338369742	355.7423602
5.49790526	0.929457497	0.360622102	0.480847075	256.6052392
31.00753634	0.885278205	0.209853316	0.370526509	535.8413969
3.943129425	0.936312495	0.40938737	0.479557222	361.2414803
3.264613897	0.926323495	0.411781747	0.594388533	175.7193493
4.045771295	0.924596556	0.435553465	0.434091984	142.6754597
18.42624946	0.88482839	0.203688209	0.37290131	373.0694078

log.sigma.2.5.mm.3D_glcm_SumEntropy	log.sigma.2.5.mm.3D_glcm_AverageIntensity	log.sigma.2.5.mm.3D_glcm_Energy	log.sigma.2.5.mm.3D_glcm_SumSquares	log.sigma.2.5.mm.3D_glcm_ClusterProminence
4.794779068	17.41666698	0.00974616	18.97266343	11922.35832
4.621535652	18.64235078	0.012222789	14.82632929	7254.139813
4.836167649	17.38092091	0.006708543	21.00484774	9680.826239
4.558540443	19.24068775	0.012361946	13.80917627	8152.349002
4.257390332	9.127816984	0.024069177	10.97963371	5238.974668
4.48374081	15.70297629	0.012864189	12.77182958	5453.325935
3.89116582	11.94261299	0.041155188	6.653547467	1958.284215
4.492087143	19.78500209	0.018257053	13.47917513	8192.665154
4.615364174	19.28197143	0.011889171	13.97877213	6584.854938
4.740606917	16.7201395	0.008300877	16.07915001	7778.377385
4.325286041	19.27652819	0.015709948	9.869899499	3998.7785
4.787103826	17.55742682	0.007115871	18.98907537	9912.387847
4.346175246	14.99984839	0.017160369	10.11822651	4313.079352
4.603321465	12.80692738	0.008705723	13.14038831	4609.426996
4.733578074	14.86431365	0.007352727	16.45580878	7001.656872
4.655992459	18.68686486	0.013663492	15.79673773	9549.931062
4.477418792	13.58587684	0.015262383	12.6471344	6791.806031
4.520439404	17.23633669	0.013701703	11.88111575	4812.325972
4.611967944	14.86493035	0.011839587	13.7582133	6000.304632
4.57860548	18.1731609	0.01216128	13.01967192	5504.740346
4.757585862	17.67749884	0.006848154	17.64893895	8844.790783
4.671342894	16.25537689	0.009524774	14.8890694	7098.697636
4.782972343	16.13874218	0.007240569	17.26281438	7765.404197
4.623765266	17.11654478	0.016204145	16.06901548	9899.609203
4.833781974	16.28245485	0.005804081	18.94598554	7434.838291
4.934787334	22.31222489	0.006641454	21.51857918	16329.43614
4.64171065	16.55334812	0.009934923	14.31875218	6124.904414
4.831461243	19.33740842	0.006055256	20.3184126	10526.54132
4.42583115	18.98024636	0.017675164	11.96611171	6687.277321
4.600049281	19.27495752	0.012341581	14.5665457	8801.031105
4.725695892	20.17921882	0.008436734	15.81334265	9226.414888
4.625606559	19.01632281	0.009621981	12.92313484	6089.810732
4.622732998	16.05383445	0.013570764	15.26937401	9490.04462
4.50294343	13.29993929	0.017047467	15.66817647	7098.970281
4.751370862	19.86637002	0.008920792	20.21733284	13623.59662
4.468215356	17.95428242	0.017647802	13.11439497	6766.025467
4.888693492	18.56813992	0.007198813	21.46817677	15716.38608
4.592254737	21.05522058	0.010178323	13.87355071	8061.210395
4.171681137	11.44823034	0.019409743	7.165615195	1884.368533
4.488206108	17.74117338	0.018394802	13.02624265	6554.933234
4.549498493	17.63135681	0.010616036	13.98652556	5392.475299
4.510378927	14.69164279	0.014524583	12.22779708	4971.336816
4.43056253	17.43664065	0.018343807	11.81484736	5155.334566
4.357745397	13.72696451	0.020578958	12.13394974	6308.105081
4.886273199	23.75141125	0.008525263	20.30178788	14965.91671
4.771249465	18.43844816	0.007452488	17.09697864	8691.97394
4.782602426	19.2527873	0.008464841	17.70691902	12504.83423
4.541181695	19.23562617	0.012269311	16.23451445	9964.216242
4.200328821	12.42785082	0.029137109	10.90134168	4204.828883
4.388838048	16.60395728	0.019589737	12.26474158	5454.673119
4.351496448	16.39896245	0.018689236	10.36125256	4593.536515
4.106977896	18.59985942	0.026330527	9.787068104	5309.020327
4.387941164	18.69946204	0.01733124	10.74682802	4740.739539
4.68174426	16.60131299	0.008513095	16.26809877	7182.988618
4.628015054	18.64517433	0.010860452	14.47479611	7079.819805
4.843658499	18.87963504	0.0076055	20.53008425	11447.44319
4.85751007	24.51940838	0.009698604	19.9613767	18173.24332
4.277230496	17.14789971	0.026279771	11.11919117	5599.170471
4.786387316	22.9676794	0.008842322	18.94767527	15252.53601
4.081156427	10.30777975	0.015315731	6.789017108	1100.878113
4.700635425	18.42561966	0.010328782	15.47690251	8163.286598
4.641104371	21.03782641	0.014380915	15.98783457	12461.75757
4.777901912	16.40586713	0.008702164	18.11192534	10594.47109
4.621882662	15.62258475	0.009082536	14.02845412	5974.533596
4.487651868	11.43257394	0.009955568	12.16232069	3093.187938
4.810306318	15.06662189	0.008465628	19.57750395	11422.19606
4.666350207	16.58231033	0.01262564	16.87726597	10378.64391
4.715441204	21.90812711	0.011772718	15.42860757	9721.029658
4.507271514	17.38805404	0.012520402	12.91917455	5073.558858
4.79835048	20.00851312	0.009979083	18.75909353	13712.10861
4.980903477	19.5532206	0.005182564	21.89059933	13439.64274
4.357650886	14.78227739	0.019471598	11.33770296	5379.909872
4.687401969	18.02684445	0.010502666	14.92104915	7694.657133
4.531524057	19.36682943	0.011074471	12.94974334	6124.273757
4.998209265	22.53780955	0.007501305	22.45500176	19565.4844
4.751892863	20.16749962	0.009861395	17.17543597	12679.19548
4.365884907	16.89691266	0.017077863	11.63884212	5072.645374
4.758978909	16.1414179	0.008053731	16.35105975	7826.263987
4.585559425	19.11449976	0.011867834	14.83598761	8365.825595
4.636635257	16.62528332	0.012820066	16.5643379	9506.45997
4.743697778	22.14282301	0.012538637	20.11843628	20527.96588
4.98398379	20.67779416	0.006836979	24.55580445	21289.78578
4.735460639	19.3230224	0.009073665	17.30782137	11345.23788
3.947480479	8.321309228	0.036553415	7.177692093	1723.515636
4.824953627	20.44731258	0.007198902	17.46225574	9990.126312
4.499150536	13.37173837	0.011695324	10.94167362	3554.596653
4.869413188	16.76676077	0.009297771	20.22063512	13281.53037
4.474652816	19.52847106	0.014037261	11.86691128	5196.739848
4.047515672	8.784324205	0.023130134	7.271991158	1110.334432
4.268855672	19.33168047	0.019760902	12.2259949	8618.87974
4.595080358	15.80787638	0.012103244	13.73368713	5534.641615
4.873234071	22.84685839	0.00763505	21.73110655	15856.5436
4.643542247	17.60117415	0.00928733	16.7465825	8456.063491
4.602594771	18.90177604	0.011762498	15.58870677	9338.418695
4.513943494	15.88410998	0.012666912	11.3846054	4134.613426
5.432357441	23.06746284	0.004279492	50.9179632	62281.9376
4.13031462	18.93827455	0.024707492	8.012067759	3082.204014
4.223062533	13.04193321	0.02560104	9.233418953	3111.160563
4.022449815	11.81487152	0.029702337	7.043831416	1977.953274
5.213682214	19.25777354	0.004181633	33.66118521	28516.87718

log.sigma.2.5.mm.3D_glcm_SumAverage	log.sigma.2.5.mm.3D_glcm_Imc2	log.sigma.2.5.mm.3D_glcm_Imc1	log.sigma.2.5.mm.3D_glcm_DifferenceAverage	log.sigma.2.5.mm.3D_glcm_Id
34.656886	0.812316999	-0.13823841	3.142559997	0.384107341
37.01884988	0.737571627	-0.10565774	2.913201463	0.407955178
34.56348652	0.771116972	-0.110050724	3.844283418	0.34586761
38.15072509	0.715024113	-0.096905952	3.000146218	0.397147416
18.25563397	0.811361442	-0.161420059	2.157167833	0.466610613
31.36460205	0.716204155	-0.099402706	2.839733227	0.408042675
23.88522599	0.787948652	-0.170434191	1.584809535	0.543671205
39.30881765	0.795937978	-0.136979655	2.60773776	0.433963213
38.3170095	0.761302555	-0.115616917	2.729607555	0.420292081
33.26424403	0.686028504	-0.083606659	3.091606383	0.378855423
38.38956949	0.711266661	-0.103591562	2.395330792	0.444546262
34.91209863	0.71315119	-0.090815008	3.61690695	0.351996951
29.96205534	0.706020147	-0.104296441	2.267285738	0.453667897
25.61385476	0.673850647	-0.084668635	2.95767806	0.387814815
29.67824764	0.736269804	-0.104978979	3.193551113	0.376047797
37.02448141	0.747774648	-0.109012956	2.940801305	0.432676624
27.16494459	0.749169868	-0.117479503	2.575804863	0.431378783
34.38849814	0.763750484	-0.124892913	2.321041311	0.442217564
29.72719439	0.788489733	-0.136210282	2.696845333	0.413855964
36.23700745	0.712291823	-0.100343486	2.631368983	0.423481856
34.90061559	0.573695575	-0.053530073	3.604346952	0.355580621
32.32183182	0.723550861	-0.101249034	2.94146153	0.394305664
32.10853065	0.635546339	-0.071001227	3.32728167	0.36747151
34.14741046	0.842962947	-0.170380337	2.679247169	0.433213877
32.23176743	0.665269927	-0.075430664	3.543797203	0.350834151
44.27778143	0.819376176	-0.133359262	3.734490479	0.355403417
32.98892506	0.733128792	-0.103288836	2.901189002	0.395577945
38.23250764	0.617680708	-0.061611634	3.987713186	0.332373637
37.71395465	0.705707944	-0.103606288	2.628409561	0.440502118
38.25468116	0.713330243	-0.095081271	3.076659155	0.407917044
40.04867803	0.659309337	-0.076132783	3.242117904	0.378154199
37.7882323	0.671517976	-0.084098433	2.766539178	0.403073059
31.9688323	0.730929754	-0.105436873	2.825533995	0.41364699
26.55987973	0.892508829	-0.219626388	2.670096884	0.424905732
39.27895594	0.834529687	-0.146895613	3.797568247	0.355562344
35.77662336	0.708873562	-0.099098496	2.619931583	0.444404873
36.70871146	0.67929977	-0.078554172	3.768577163	0.349463986
41.81103224	0.653140693	-0.074862256	3.148788575	0.384840712
22.89646068	0.722717842	-0.118827258	1.945941552	0.474591067
35.38147047	0.779598198	-0.131411782	2.483777973	0.442221487
35.1519165	0.713035934	-0.09589327	3.119736313	0.383462542
29.35907121	0.763014648	-0.123187317	2.454357011	0.440504518
34.78571197	0.741825557	-0.113923453	2.398154587	0.454701908
27.4258902	0.760765	-0.129199174	2.340248742	0.460181462
47.26745979	0.750140392	-0.104872599	3.461842064	0.388335088
36.56030729	0.655766713	-0.073968947	3.31525253	0.363579211
38.10035508	0.694894954	-0.085119232	3.380037063	0.371592132
38.10594193	0.744938421	-0.10706176	3.192086741	0.38866801
24.85570165	0.854485793	-0.207570351	2.039702621	0.485566895
33.06373328	0.789130692	-0.138832309	2.485621719	0.441698232
32.71013735	0.724727486	-0.109201286	2.201614826	0.466392288
37.06957398	0.714083103	-0.108760516	2.317657726	0.477425906
37.24929754	0.701333383	-0.099754602	2.360245096	0.446566846
33.04709246	0.764664751	-0.114614756	3.29096378	0.368868187
37.0433369	0.665839476	-0.079372267	2.981093256	0.397267384
37.30296947	0.747337167	-0.101615962	3.770154357	0.364148383
48.82032885	0.774228841	-0.116248008	3.230810223	0.388510977
34.17975864	0.776820334	-0.139398128	2.092149253	0.486086192
45.62885086	0.790498961	-0.122428939	3.589758964	0.371537011
20.6155595	0.582648011	-0.065337299	2.30916338	0.429517239
36.63493211	0.737636314	-0.104095662	2.854688368	0.407441557
41.86944351	0.712503466	-0.097248689	2.963647781	0.424613033
32.71179581	0.735208284	-0.101705237	3.266135503	0.382880186
31.23500384	0.694776542	-0.088496471	3.025391795	0.388601551
22.86514787	0.787404715	-0.133470308	2.867067196	0.389499845
30.00902698	0.791251846	-0.124883004	3.127707485	0.375917598
33.06866698	0.820730585	-0.146867362	2.886150965	0.403652761
43.62669193	0.745506551	-0.106870627	2.875558026	0.412683342
34.62797813	0.735547179	-0.108270391	2.830351407	0.407514469
39.70283622	0.754447433	-0.107475344	3.412363582	0.385857121
38.72275013	0.684386325	-0.079625627	3.728366698	0.346087891
29.51915572	0.767597108	-0.131035552	2.259140583	0.459662275
35.79015088	0.700001347	-0.091678462	2.834010123	0.404555828
38.38252728	0.599221162	-0.0638387	2.928156186	0.401622266
44.80168407	0.761671467	-0.108170081	3.442077491	0.373020394
40.01829617	0.749071338	-0.104829339	3.294863318	0.386040026
33.65616512	0.693278368	-0.092785033	2.654469739	0.433353378
32.20737481	0.767841193	-0.117897194	3.067324221	0.372805581
37.936178	0.682508507	-0.086872945	3.030005657	0.398456729
33.01752889	0.688524745	-0.086665579	3.080780714	0.411911788
43.95614789	0.829877092	-0.14589008	3.488584911	0.396795199
40.89621766	0.730975459	-0.091393795	4.17791819	0.341264887
38.25373765	0.708290867	-0.089323505	3.459704058	0.366160375
16.64261846	0.810615129	-0.188846764	1.683481077	0.522087175
40.57218585	0.676605333	-0.080046565	3.406414891	0.3620697
26.74347675	0.666894298	-0.083100863	2.501779621	0.423924577
33.34134598	0.806900637	-0.131774735	3.251587118	0.377973953
38.83502196	0.700327192	-0.097214172	2.552133705	0.423601688
17.56864841	0.809711149	-0.177619728	2.033571495	0.461350208
38.5033041	0.846430445	-0.183946042	2.464407253	0.443910868
31.55344928	0.712619088	-0.096898656	2.701607513	0.41538159
45.31922517	0.815213757	-0.132701108	4.016241771	0.353351163
35.04862068	0.701788335	-0.089110484	3.365731544	0.37131107
37.43758551	0.643555209	-0.07438948	3.211177687	0.390025037
31.7007624	0.688877769	-0.091961962	2.430509076	0.434630074
45.45789991	0.918545088	-0.198107275	5.916622462	0.291721207
37.81413431	0.724241303	-0.117742647	2.024414241	0.475169033
26.08386642	0.861325703	-0.213846968	1.953924252	0.477294108
23.62974303	0.753253047	-0.13470871	1.946017787	0.496658814
37.96226477	0.820864134	-0.125672593	4.979246539	0.293083636

log.sigma.2.5.mm.3D_glcm_ClusterTendency	log.sigma.2.5.mm.3D_firstorder_InterquartileRange	log.sigma.2.5.mm.3D_firstorder_Skewness	log.sigma.2.5.mm.3D_firstorder_Uniformity
55.87493042	116.5642338	0.294560423	0.079628443
41.68401952	104.7006336	-0.059633851	0.0890675
55.09228513	144.2699013	-0.471547567	0.068064938
39.88015336	93.93331718	-0.258227973	0.091750345
34.71956267	71.00373411	0.967098777	0.121137996
34.63658039	85.71079636	-0.342824102	0.098015538
21.32588657	56.53668785	0.754003898	0.154598483
40.78478473	90.75364113	-0.297695725	0.097692621
40.11632396	111.2465324	-0.102765968	0.085992773
45.33012577	113.8042393	-0.306829051	0.076456677
27.14700259	82.83244705	-0.471119974	0.109326731
50.0412753	121.3293591	-0.560280541	0.072629327
29.25395537	77.54334322	0.110912813	0.110173969
36.9273558	116.5556331	-0.366075341	0.081058721
45.97826095	134.9262104	0.211214591	0.070778165
44.79445713	99.91699982	-0.268149225	0.087189965
36.83997887	82.4424715	-0.332976072	0.099563087
36.21694229	110.2601156	0.212082658	0.092840319
41.37413854	120.9118078	0.045788443	0.083806536
37.38306133	95.22075701	-0.35023663	0.091030551
46.90899877	128.1433792	-0.46685377	0.071514932
42.00913612	108.0517006	-0.302401334	0.082377989
47.0178543	116.4629984	-0.387588807	0.07458516
48.34209634	85.43304534	-0.06793954	0.095797907
51.18188902	149.5490398	-0.19707023	0.065870833
63.3127776	140.1884418	-0.321824712	0.066871127
40.31990678	103.7031326	-0.009371104	0.083430755
53.11744093	136.8678074	-0.628474358	0.067165007
34.38241886	81.51426327	-0.359605317	0.105883988
40.68008835	98.11083734	-0.176528458	0.089884532
45.61147906	110.4249493	-0.669856294	0.079535262
38.64317291	112.5901437	-0.000438178	0.08189288
43.84860574	88.14529689	-0.370748876	0.09557676
46.98060452	115.9244947	0.642291746	0.096476934
53.73446262	111.4045844	-0.802892537	0.076776946
35.92085817	76.91664147	-0.687176599	0.108993652
60.05800791	125.2861233	-0.320122274	0.068137328
39.10421426	95.68353552	-0.725620059	0.089337997
21.84610587	77.78974056	0.193865557	0.113355577
38.23581064	82.61163116	0.317732237	0.108283368
36.27494669	94.02823281	-0.677854502	0.089501668
36.1907997	96.90243411	0.130692482	0.094805156
33.87246797	82.65222311	-0.148274999	0.107631798
36.27038053	77.50979376	0.75627966	0.112418917
58.93380629	110.764122	-0.595102681	0.076221269
47.70656471	121.2164502	-0.524628826	0.074325395
51.09755964	111.8959219	-0.516202533	0.076208345
43.7965271	96.24670458	-1.016842559	0.091151725
35.34845117	83.18480825	0.528557138	0.129954479
35.2104245	82.36848915	0.535238352	0.112675701
30.17642345	83.02666265	0.171602905	0.109083044
25.31707127	60.43767138	-1.052217853	0.137540199
31.97980007	91.44367886	0.300885972	0.103491529
43.12877775	107.1345558	-0.23219118	0.080755316
40.79663717	108.0401125	-0.102119599	0.083659587
54.04879821	126.9048128	-0.208352422	0.069313775
59.34360631	110.7049119	-0.422682165	0.079095385
33.84432371	69.98387501	0.626730925	0.127201323
54.68999186	107.2019355	-1.133767898	0.080263113
17.78426367	78.62155056	-0.399444648	0.113598865
45.0442112	118.8790169	-0.213603969	0.082326905
46.81375124	88.77050805	-0.590984865	0.095465668
49.90028767	111.2631412	-0.495290677	0.077480867
39.1422636	109.51999	-0.513231893	0.081266134
34.33504787	123.679395	0.148024581	0.080528584
58.56524359	129.6937751	0.583057726	0.073432208
50.1956754	95.75273534	0.510953173	0.089102383
47.59650697	104.7907724	-0.215352384	0.087724659
34.88259978	89.37814474	-0.650980712	0.098732003
53.74914829	103.5390851	-0.406815834	0.08081003
62.24046564	159.0713305	-0.34572973	0.0601349
33.8700995	78.03313255	0.474846861	0.111385611
43.27622474	109.2979417	-0.153107001	0.083879942
35.67312722	100.3697878	-0.637820741	0.086252413
68.85386488	124.0397534	-0.35000975	0.07044399
50.13218159	105.6036401	-0.566308195	0.082681873
30.46765678	74.53847408	-0.426945887	0.111383739
47.64114527	114.9830213	-0.239910312	0.076977889
40.92367128	94.30166101	-0.697159153	0.09020836
44.783252	89.37327003	-0.618656862	0.091900237
57.44885321	91.92285067	-0.827585833	0.091064147
69.36642354	120.2967968	-0.765766049	0.071490665
48.75121781	109.5168782	-0.448905602	0.078321741
23.149893	73.58644295	0.852854901	0.149530763
50.88328803	124.3378269	-0.576962675	0.07207488
32.33445705	98.03409404	0.204243037	0.091090688
58.57789494	110.2901295	-0.22797372	0.076785616
34.23159824	91.42190576	-0.246866955	0.098984875
21.84736445	113.5503182	0.267798814	0.119055653
35.50318315	69.34263158	-0.57835309	0.116174375
39.69848827	103.9705153	0.111258403	0.088896478
59.8505328	120.0562782	-0.856454536	0.072182797
43.85591857	102.6989641	-0.943186082	0.084493927
42.47347994	95.46411467	-0.671874734	0.088407281
32.9475919	93.83029866	-0.027647676	0.093468493
143.918213	204.5580254	-0.684406583	0.049581806
23.15940185	59.24034691	-0.474463702	0.133958289
29.67233265	91.22656107	0.617307056	0.117259149
20.24921081	57.65813881	0.06953052	0.137869288
94.80739438	194.0737734	-0.639834825	0.053259103

log.sigma.2.5.mm.3D_firstorder_MeanAbsoluteDeviation	log.sigma.2.5.mm.3D_firstorder_Energy	log.sigma.2.5.mm.3D_firstorder_RobustMeanAbsoluteDeviation	log.sigma.2.5.mm.3D_firstorder_Median
82.01873115	21070663.67	50.84820401	56.91096497
73.46899908	25677356.51	45.74901897	42.29846001
87.3824632	14348087.18	58.93277897	31.70744038
69.95260573	29603528.26	41.13942553	44.08588409
62.90433431	9231735.33	36.02856993	27.32805824
63.88936759	14296893.97	37.99324665	31.23241043
49.89395815	6626026.284	29.28117582	24.62643623
69.37932545	23488153.69	41.64055762	48.4382782
71.47774042	27250504.96	47.01022603	58.74886703
77.10734212	39838253.18	49.69045817	54.84773254
56.03002733	14403815.84	34.37683931	40.89497375
82.41088227	24305130.25	52.26637522	55.73562241
57.11367952	23719785.02	33.82327648	35.53657532
71.07228769	18012995.02	47.94256352	41.42301559
80.94525158	20522732.2	55.82772393	47.11515808
73.81240304	44728814.39	45.30202988	20.49387741
64.76740745	14632504.4	37.27173432	33.41797256
68.07186653	32777819.92	46.11720673	56.97930527
74.12120399	15173560.3	49.58442809	52.48661995
66.36205596	28251716.71	42.10556517	39.11914063
82.51623033	84024456.73	54.28223433	34.39284897
72.59885182	24218396.38	46.13579699	48.71294403
77.9007934	48912534.73	50.82421211	44.64292526
72.81507157	12664219.59	43.56045557	31.0857811
86.95356573	30812475.86	61.6509805	20.85311222
90.11748402	26284341.09	58.6408906	65.30604935
70.97726828	23978091.48	45.14727385	40.15211105
88.82930065	49450802.46	59.21473971	53.32845688
63.17630077	32454012.65	36.34650803	34.08582687
70.66919979	36239091.3	42.52358236	36.57650375
76.2271338	51702004.93	47.6229733	53.70807648
71.55023441	64623178.96	47.14772487	59.84025764
69.56921601	29400357.74	39.67533958	38.19972229
78.60994741	7186538.598	52.28084788	42.37229919
82.37636473	12547372.47	49.10117591	44.90900803
61.54999783	30297376.97	34.28563328	35.56521988
89.31502633	61285169.65	54.97426626	50.31303406
69.15673733	37558091	41.50676269	48.08010864
53.60045068	8752217.264	33.73079649	21.76873302
65.15439617	18896521.6	37.95023991	42.37727356
68.02102475	15165446.4	41.58446282	55.68171692
66.90781442	21668378.56	42.28850532	44.53098869
62.27454068	30276540.67	37.01365823	39.21526718
63.55197642	21066793.6	36.4660329	39.59796333
81.60579264	46896747.91	49.93182372	35.28829193
79.52047538	46480319.26	51.89481043	54.8422966
80.36555377	52830686.12	49.19667312	51.67080498
74.22429143	16066005.15	43.43429967	28.32063675
66.25263964	5832501.844	43.83103489	31.63929844
63.78815265	11565678.6	37.58267924	36.63831711
59.51839476	36011978	36.24494255	38.16235352
49.94530644	13145621.91	26.4773492	26.93957615
64.27013892	42058409.34	39.86958575	44.93655777
73.55861849	13994552.91	46.76288786	48.972229
73.69815768	50360352.9	46.8415043	46.44612885
85.98826035	26561498.82	54.28003465	32.18721199
80.3302463	50871524.15	48.28583759	48.67896271
59.85740709	21724014.17	33.62634942	24.88337326
78.28295003	19021931.68	46.69143618	47.32681847
50.27153066	8909064.247	33.50047007	48.93054581
75.94265387	46408291.9	49.62933382	62.52587128
70.50015922	67961474.61	40.15351654	38.82564545
78.20261519	31449460.44	48.65125838	54.04188538
71.28980436	21681685.09	47.03366037	57.00971603
72.09686663	7606355.902	51.22994793	64.63955688
86.84730349	26494378.19	57.36375094	47.69981766
75.38682323	14667018.97	44.41042828	39.67847061
74.25901647	51630344.86	46.09277603	53.1873703
63.43461312	12405949.69	38.99232449	45.48058701
79.76710944	35939433.18	46.0765042	42.29793549
94.04250681	62514420.4	65.13549451	52.46802711
60.53326696	21922899.91	34.90005988	46.5365715
73.74683533	64228518.27	46.88168987	55.73242569
69.34107452	69446036.99	43.65125734	49.71149445
87.76727144	74140007.28	54.6408892	50.32950783
77.18786183	31512101.91	45.90292679	42.11231232
58.646916	19266102.7	32.80901903	32.98774719
76.10580243	19823689.25	49.31562437	66.9148407
69.9454516	35637222.29	41.51910046	43.66571426
72.28960385	49567269.31	41.52478658	34.31846619
77.04515142	20025573.99	42.00513737	41.06142998
91.52323673	45606182.11	53.80849533	47.39632416
79.46294948	36244838.6	47.16622418	50.39291382
54.46479628	3846530.673	35.61957672	27.99038315
82.07850041	56761232.88	52.78072284	58.30492783
64.65207976	33007424.77	42.12565884	42.38806725
82.44883487	22974303.59	49.69767828	53.81100845
64.39444291	31230673.13	40.19585212	42.69667053
58.92530709	3483692.953	44.94202065	41.76727295
59.65408889	5971175.225	32.21441438	42.10612679
70.06825668	39059397.1	45.24052878	48.48496246
87.1081037	20428726.36	52.47184085	68.28106689
73.78928691	16789764.82	45.00609138	37.5825901
73.05916294	51972012.81	42.05023442	42.77817535
63.66342414	52091777.54	40.15712741	59.41485405
138.3952462	27575304.89	92.77945181	48.32513428
48.86611295	11526224.78	27.62929616	36.1936264
61.91263605	4721512.943	39.65494855	47.74157715
47.40781866	5129552.537	26.95261467	30.33120155
118.8987676	27537179.07	82.3445391	71.34189987

log.sigma.2.5.mm.3D_firstorder_TotalEnergy	log.sigma.2.5.mm.3D_firstorder_Maximum	log.sigma.2.5.mm.3D_firstorder_RootMeanSquared	log.sigma.2.5.mm.3D_firstorder_90Percentile	log.sigma.2.5.mm.3D_firstorder_Minimum
568907919.2	459.046814	132.3446208	229.5743561	-334.0886536
693288625.7	407.401123	113.3078914	192.4687408	-384.4693298
387398353.9	319.6045837	113.2859632	149.4284744	-379.5233765
799295263	475.3623047	109.855793	172.8478638	-414.1976318
249256853.9	357.6290894	98.62989398	182.3479553	-173.6331482
386016137.2	353.7434387	93.42517057	132.9239594	-334.9137573
178902709.7	281.7668762	79.1751844	147.5595001	-230.6373596
634180149.7	431.2877197	114.0737811	187.6347198	-423.4701843
735763633.9	356.8924866	115.9472367	195.8138519	-396.1617126
1075632836	407.706604	115.1787809	178.351059	-346.2290649
388903027.6	307.9756165	89.28113521	131.866925	-417.1820679
656238516.8	357.5263367	120.1016638	177.2810059	-365.1472473
640434195.5	358.9458008	89.99029768	136.9819031	-319.5956726
486350865.5	326.2848206	98.46233022	149.5673676	-265.5514832
554113769.5	364.760498	115.1415604	188.6909241	-278.3435364
1207677988	431.6256409	104.3339435	154.5770233	-401.5324097
395077618.7	329.0154419	95.48206727	125.7540665	-294.17099
885001137.8	369.272522	113.1982629	192.0922562	-328.9292297
409686128.1	340.9716797	115.5725197	200.3549652	-284.213562
762796351.1	318.9046631	98.68441182	158.3900909	-390.3569336
2268660332	453.708374	109.9538535	147.53582	-383.1461182
653896702.4	471.1507263	106.8568475	157.8078156	-328.323761
1320638438	403.0635071	108.9453698	163.4706726	-346.6038818
341933929	336.2264404	110.8844391	192.813205	-363.2559509
831936848.2	437.7958984	108.7784355	144.6526733	-351.8852844
709677209.4	433.8322144	139.4312759	224.7949997	-452.6926575
647408469.9	416.6443787	104.1388878	160.3816376	-331.100647
1335171667	355.9454346	121.4060724	171.0205872	-415.669281
876258341.6	452.8272095	97.71441094	144.9159637	-408.9797058
978455465	541.0551758	107.1060089	159.3983002	-413.0997314
1395954133	438.3130493	111.724558	163.0479813	-440.8524475
1744825832	476.5383301	113.6865682	181.8929108	-377.1451416
793809658.9	466.5293579	107.7780284	163.9349976	-343.4397888
194036542.1	408.0471802	122.2326826	221.4420776	-230.193512
338779056.6	346.8690186	118.8693566	159.7242844	-427.7980957
818029178.1	346.639679	97.0458537	143.3533325	-386.031311
1654699580	456.2322083	131.0401623	203.9847046	-393.3217773
1014068457	372.6642151	105.2417013	150.1616516	-467.4678345
236309866.1	303.5973511	76.79661795	120.0144241	-232.9432678
510206083.2	373.3424988	107.4729783	180.3872147	-354.0160522
409467052.7	345.334259	107.3494082	167.6450806	-368.8198547
585046221.1	366.5548706	105.9033373	185.6199493	-289.4545898
817466598.1	348.6671448	100.0108033	163.8154205	-359.0899048
568803427.1	399.6521301	105.7447393	187.5848465	-264.2302856
1266212194	382.5618896	115.8537013	161.1444992	-538.8610229
1254968620	392.4825134	114.0240672	167.1687866	-392.5503235
1426428525	439.8609924	119.7196559	174.0645828	-422.8883667
433782138.9	322.3044739	102.9447848	116.4560394	-439.8808594
157477549.8	297.4915466	101.6922579	190.3843018	-225.4990692
312273322.3	446.9953308	103.0083216	176.3367569	-329.9104614
972323406	435.3272095	95.07445033	156.0906036	-329.3478088
354931791.5	302.1018677	81.97966575	108.4621201	-406.6520386
1135577052	442.9900513	105.246127	176.7602844	-383.5038452
377852928.5	375.4718018	109.3203634	173.366745	-330.3662415
1359729528	427.8041992	113.0997578	185.8259369	-387.4370728
717160468.2	396.0154419	118.6110892	168.0221573	-422.7272644
1373531152	459.9878235	124.5565775	188.1845215	-533.2773438
586548382.6	400.7520752	94.51228288	167.2121384	-350.1594543
513592155.4	353.3271179	117.7471091	167.8707733	-500.0527649
240544734.7	267.7799377	79.63021408	125.8924576	-198.5992584
1253023881	425.0584412	125.6175387	212.223114	-354.5346375
1834959814	533.9772339	111.2919922	165.1978821	-454.6019897
849135432	371.1893921	117.6787461	174.8422852	-348.2868347
585405497.4	341.6872864	107.7353136	168.4610565	-313.1228333
205371609.4	313.0840149	113.5435099	196.8859787	-184.3462067
715348211.2	455.0856323	131.0369882	236.6962433	-299.1513062
396009512.3	430.3987122	117.7411011	203.5271164	-327.0010986
1394019311	406.4851379	120.3266549	205.2361145	-457.2219543
334960641.7	370.1676331	97.95254976	152.5393585	-369.2591858
970364695.8	525.8381348	119.7315059	173.2298004	-426.2108765
1687889351	369.1616821	128.1272883	199.2363022	-411.7367859
591918297.5	413.1837769	103.1359337	172.4850769	-297.8206482
1734169993	444.1269226	117.162176	193.2772461	-357.6528015
1875042999	376.038269	103.2761135	152.4175415	-401.6908875
2001780197	476.9885559	131.8923449	211.8653091	-496.9798584
850826751.7	430.2982483	116.2699671	171.2505951	-440.6558838
520184772.9	384.9625549	90.44852309	127.3087921	-372.0683899
535239609.8	372.0377808	120.7765201	200.9173859	-311.9629822
962205001.8	359.7064209	104.2989596	143.9737854	-413.9267578
1338316271	470.4471436	105.5045403	137.5781891	-366.0831909
540690497.7	424.178772	121.2561941	169.0062347	-492.8843689
1231366917	450.0881958	131.0135098	166.0606018	-474.1296082
978610642.2	443.7271118	121.0384176	180.7071411	-414.7565002
103856328.2	259.2991943	85.75979921	171.0428406	-141.2507172
1532553288	390.726593	121.2326994	185.6335068	-437.624176
891200468.7	342.5426025	97.47447707	166.2674423	-259.6386719
620306196.9	435.32547	126.8402455	201.2993622	-333.8218384
843228174.4	328.4163208	101.3070563	171.3498138	-416.775177
94059709.74	213.2324524	92.06594687	162.7468567	-137.5718994
161221731.1	335.7442322	101.6403118	152.9402618	-408.0323181
1054603722	372.6315613	110.2916452	189.5050812	-319.6971436
551575611.8	347.9463806	135.9688757	206.894519	-497.0543213
453323650.1	300.8061829	103.6768495	138.5885132	-380.7801819
1403244346	379.5932312	109.2673561	152.0877838	-400.3013
1406477994	420.6109009	105.2103892	168.8537521	-311.6246338
744533232.1	469.6720581	176.6176203	209.1449768	-547.3929443
311208069.2	265.5622559	80.85695577	133.100946	-422.2164612
127480849.5	317.8782959	105.031292	190.1505249	-231.4586792
138497918.5	283.2507629	76.00344155	121.7454414	-233.4869537
743503834.9	432.5391541	154.4745078	205.083812	-416.5273438

log.sigma.2.5.mm.3D_firstorder_Entropy	log.sigma.2.5.mm.3D_firstorder_StandardDeviation	log.sigma.2.5.mm.3D_firstorder_Range	log.sigma.2.5.mm.3D_firstorder_Variance	log.sigma.2.5.mm.3D_firstorder_10Percentile
4.055867826	110.4162176	793.1354675	12191.74112	-39.86465225
3.919715921	99.73683315	791.8704529	9947.435888	-51.62320404
4.148464206	111.8884219	699.1279602	12519.01894	-141.5204514
3.896954401	98.78853691	889.5599365	9759.175025	-56.1326561
3.571048507	87.26091698	531.2622375	7614.467633	-38.94376831
3.771383694	88.94385273	688.657196	7911.008939	-75.59813004
3.219478492	68.49690204	512.4042358	4691.825589	-23.13508034
3.835244428	97.55502779	854.7579041	9516.983448	-37.74362488
3.879087513	93.87469164	753.0541992	8812.457731	-32.1536911
4.03190198	101.8985982	753.9356689	10383.32431	-79.55122833
3.587931483	78.10512411	725.1576843	6100.410413	-40.44133759
4.118171265	110.1526626	722.673584	12133.60909	-95.95290222
3.613739148	80.33698483	678.5414734	6454.031131	-42.85690155
3.888869296	91.41030739	591.8363037	8355.844296	-84.74844818
4.055331764	102.0753893	643.1040344	10419.3851	-73.81481171
3.954456849	101.0164507	833.1580505	10204.32331	-89.31155243
3.781894693	91.43969023	623.1864319	8361.21695	-84.3160553
3.76580875	87.49083252	698.2017517	7654.645775	-17.64641953
3.90705488	95.78257744	625.1852417	9174.30214	-45.68136597
3.818419462	89.35308058	709.2615967	7983.973009	-65.55685425
4.104290089	107.3266694	836.8544922	11519.01395	-117.2903152
3.944292373	97.40910869	799.4744873	9488.534456	-82.78082275
4.047562539	102.0145207	749.6673889	10406.96244	-95.26411438
3.871369727	101.4256722	699.4823914	10287.16699	-61.04708405
4.135134794	108.1375831	789.6811829	11693.73688	-137.2539841
4.22067107	119.0091993	886.5248718	14163.18951	-58.85548553
3.927758429	94.68484412	747.7450256	8965.219706	-68.06993103
4.180805389	114.8115686	771.6147156	13181.69628	-125.1694611
3.744416257	90.37841958	861.8069153	8168.258726	-62.90043564
3.912534455	98.96190306	954.1549072	9793.458258	-67.79279633
4.006190929	102.8154488	879.1654968	10571.01651	-90.33600082
3.923187908	93.59841061	853.6834717	8760.662468	-43.86346016
3.877250743	99.78366516	809.9691467	9956.779833	-71.66571808
3.822492548	100.006341	638.2406921	10001.26823	-25.16790962
4.105091316	114.2243968	774.6671143	13047.21283	-116.5055107
3.702110668	90.19354453	732.67099	8134.875476	-55.9240921
4.247040339	121.1616048	849.5539856	14680.13447	-98.60441589
3.893238669	96.61260067	840.1320496	9333.994609	-73.77113342
3.508166324	71.90435382	536.5406189	5170.236098	-51.96987038
3.722375182	90.87564824	727.358551	8258.383443	-34.26169395
3.854836542	93.88659667	714.1541138	8814.693035	-53.88247299
3.789514118	89.2978146	656.0094604	7974.099692	-38.36768608
3.694445268	87.21026971	707.7570496	7605.631144	-35.29164963
3.654160799	88.98691238	663.8824158	7918.670574	-25.54428463
4.119187121	112.1787895	921.4229126	12584.08081	-111.9545197
4.057394378	104.2622827	785.0328369	10870.62359	-93.04954681
4.103265325	110.3468665	862.7493591	12176.43094	-90.01961136
3.897755054	102.4120561	762.1853333	10488.22924	-131.166275
3.463171592	85.81850957	522.9906158	7364.816584	-30.12800617
3.659056397	88.68291558	776.9057922	7864.659516	-32.4860981
3.637915128	82.26018932	764.6750183	6766.738746	-32.33154259
3.399572148	77.63216464	708.7539063	6026.752987	-39.69818115
3.7136214	86.98000857	826.4938965	7565.521892	-25.20418701
3.976814876	98.61537432	705.8380432	9724.992053	-70.73446655
3.958811007	98.77454424	815.241272	9756.410591	-52.44814301
4.19704036	115.3048372	818.7427063	13295.20548	-116.5875221
4.103847077	112.5652498	993.2651672	12670.93546	-68.62847595
3.529806738	84.58979237	750.9115295	7155.432974	-31.01432705
4.033852669	110.6441911	853.3798828	12242.13702	-87.78595123
3.413538902	65.2249393	466.3791962	4254.292707	-35.4532486
3.965413353	101.1498025	779.5930786	10231.28255	-26.93052292
3.89420395	103.0011454	988.5792236	10609.23595	-58.2880806
4.05230233	106.1309004	719.4762268	11263.76802	-81.25757599
3.90585114	93.39124989	654.8101196	8721.925556	-64.38508682
3.840500859	89.62568456	497.4302216	8032.763332	-43.80362968
4.107158991	111.981449	754.2369385	12539.84491	-58.28235855
3.948961485	104.6174695	757.3998108	10944.81492	-56.88687057
3.93320996	101.3180045	863.7070923	10265.33804	-35.22619057
3.74049026	87.66613273	739.4268188	7685.350828	-60.09094086
4.094042845	113.195649	952.0490112	12813.25496	-99.19763641
4.283280833	119.1927063	780.898468	14206.90124	-107.3280075
3.646348243	85.15133388	711.004425	7250.749661	-24.42478561
3.956132141	98.83143343	801.7797241	9767.652234	-48.79223633
3.885058282	93.538983	777.7291565	8749.541341	-76.8312912
4.215922694	119.6642488	973.9684143	14319.53245	-75.44026718
4.03842429	108.6402509	870.9541321	11802.70412	-83.09819031
3.660855161	84.87259046	757.0309448	7203.356612	-66.92667389
4.014877258	100.3390019	684.0007629	10067.91531	-56.331604
3.8962388	97.8030752	773.6331787	9565.441518	-86.42910767
3.921987566	102.3503056	836.5303345	10475.58506	-114.3141388
4.001763734	114.7073653	917.0631409	13157.77966	-79.87821732
4.245095088	127.1954937	924.217804	16178.69361	-137.7606506
4.094134487	111.1830267	858.4836121	12361.66543	-80.05802917
3.215251802	69.52688706	400.5499115	4833.988025	-14.76382351
4.113880078	109.1484409	828.350769	11913.38215	-86.91160965
3.766596553	84.48366983	602.1812744	7137.490468	-50.3567627
4.12093097	113.6383417	769.1473083	12913.67271	-82.74978027
3.742364087	87.78184368	745.1914978	7705.65208	-38.49429855
3.335833354	69.47520055	350.8043518	4826.803491	-15.87420177
3.601471082	90.50798568	743.7765503	8191.695471	-30.81772251
3.860211243	92.54243509	692.3287048	8564.102292	-41.21069717
4.180856992	121.4033683	845.0007019	14738.77784	-84.44365082
3.925442832	100.5574761	681.5863647	10111.80599	-107.1655853
3.953615311	103.3119361	779.8945313	10673.35614	-87.53730774
3.776737644	85.58283841	732.2355347	7324.422231	-37.07747269
4.680627239	176.2508857	1017.065002	31064.3747	-250.3771927
3.378632365	71.21274016	687.778717	5071.254361	-37.05777817
3.516681832	80.42083381	549.3369751	6467.510511	-12.01035471
3.345152084	67.93200293	516.7377167	4614.757022	-40.02397881
4.50169265	148.9027709	849.0664978	22172.03518	-180.5353531

log.sigma.2.5.mm.3D_firstorder_Kurtosis	log.sigma.2.5.mm.3D_firstorder_Mean	log.sigma.2.5.mm.3D_glrlm_ShortRunLowGrayLevelEmphasis	log.sigma.2.5.mm.3D_glrlm_GrayLevelVariance
3.974177892	72.96134271	0.005195942	20.58799172
4.418390507	53.77027399	0.003984463	17.09203791
3.136581823	17.73951863	0.005612257	20.85478868
5.163646139	48.05330613	0.003679528	16.73002609
4.179035318	45.97160377	0.022814607	13.30279019
4.654182655	28.58764694	0.006843589	13.55544513
4.245795745	39.7100017	0.008318592	8.509375891
5.340496171	59.12566349	0.003627354	16.42213289
4.388452126	68.05368443	0.003811451	14.94166355
3.635104776	53.69196646	0.005482005	17.37665173
6.030210537	43.25171317	0.003506354	10.58316519
3.962911118	47.86230835	0.006053205	20.21468324
5.108046262	40.54901411	0.00566089	11.19446112
3.282558355	36.59216003	0.012701989	13.91134486
2.972643662	53.27470168	0.006792972	17.20316891
4.467685381	26.10073661	0.003961812	17.77332299
4.56379928	27.48832878	0.012134531	14.44827804
3.832692072	71.82757791	0.004080422	12.98169829
3.38305245	64.6738368	0.006862127	15.43609108
4.220692832	41.88842474	0.004128829	13.66909924
3.615801137	23.89217307	0.004748745	19.18211938
4.230722566	43.93007389	0.006205092	15.85659173
3.567178028	38.23782375	0.006603402	17.49575129
4.501133685	44.81285349	0.006634036	17.78271736
2.849786042	11.7902986	0.005688772	19.15686817
4.108471827	72.64909621	0.003210456	23.57096192
3.987638341	43.35537157	0.005414153	15.0588847
3.451198686	39.46819147	0.004133102	21.74507775
5.185996095	37.14629699	0.003715011	14.51484735
5.308200491	40.96631411	0.004086159	16.94000869
4.30435728	43.71910735	0.003238231	17.74909471
4.031604272	64.52885652	0.003285894	14.78757818
4.81532863	40.73479569	0.007537535	17.03393401
3.340287942	70.2820066	0.009568097	17.18075246
4.547617491	32.90457601	0.004802102	21.94502912
5.8079021	35.81929989	0.005050395	14.33747295
3.862819525	49.91382244	0.00536567	24.58953964
5.343549526	41.73513004	0.00297676	15.86009718
3.975731878	26.97191929	0.010004254	8.849504484
4.780349666	57.37645538	0.00420002	14.40489521
4.842560941	52.04999908	0.005124275	14.97904088
3.959496329	56.93344501	0.006506622	13.88837315
4.943137664	48.95436276	0.004346601	13.4051388
4.87657749	57.12511985	0.007936341	13.85974234
4.585970136	28.94821761	0.002402682	21.47143874
3.80915144	46.16128587	0.00442672	18.03327683
4.541833127	46.43667817	0.004339772	20.53110115
4.736877917	10.45942075	0.004437367	17.79062274
3.30846025	54.55729779	0.009748726	12.85175369
4.744572758	52.4028129	0.004957513	13.73184144
5.139448997	47.6698265	0.005022662	11.92403667
8.349611854	26.34222104	0.004072968	11.00613999
4.601103746	59.25559354	0.003209542	13.0835438
4.051370164	47.17997241	0.006826282	16.46065365
4.107847591	55.09214671	0.003766856	16.68469815
3.875430419	27.80980045	0.004748529	22.54098202
5.222969068	53.32359276	0.002231903	21.66141324
4.972977272	42.15612224	0.004003923	12.79754722
6.009127247	40.27709864	0.00339576	20.94880575
3.669868236	45.68017389	0.01458311	7.274679999
4.372010733	74.4881432	0.004577408	17.39481943
6.066251681	42.15058221	0.003049857	18.49869191
4.260795786	50.83816746	0.008409058	19.00809544
3.747191937	53.71193763	0.006792576	14.70978051
2.603424996	69.709148	0.014645182	13.39198689
3.314796038	68.05032969	0.006179627	20.87629808
4.360090664	54.01992202	0.005492431	18.65544028
4.862806567	64.90890425	0.002716646	17.60736697
4.75213226	43.69612312	0.004883503	13.07718161
4.69476336	39.01766953	0.003693405	21.98795756
3.216916242	47.00745428	0.004174331	23.45209137
4.812886221	58.19167602	0.005922727	12.74085158
4.084737117	62.92315349	0.004403729	16.52250185
4.416571786	43.77686914	0.003373806	14.91204339
4.452992804	55.46222304	0.003318892	24.08401871
5.016323671	41.4246439	0.004266754	20.10926585
5.290083921	31.26625526	0.005266422	12.70943817
3.664356093	67.22389828	0.006071533	16.75291315
4.87765548	36.2330162	0.003894024	16.43011008
4.577301398	25.60513548	0.006813337	18.08526661
6.185062465	39.31011251	0.004204297	22.83659815
4.511611507	31.3981868	0.004601128	27.11700143
4.858606339	47.83966046	0.005109402	21.07494568
3.199540739	50.20712236	0.018802895	8.355270091
3.960422166	52.76348404	0.003186822	19.79450396
3.385002138	48.61875371	0.00714791	12.04188541
3.978932133	56.34514339	0.007698565	21.88345701
4.718999978	50.57141069	0.003239733	13.21241575
2.258971787	60.40972671	0.019039199	8.075005084
7.233078322	46.24994609	0.005256383	14.35580536
3.734601935	60.00120583	0.005148202	14.54916033
5.10683147	61.22709639	0.003743042	24.97411641
4.553041489	25.24050584	0.005700765	17.12092366
4.749162963	35.58087927	0.00399668	18.26336041
4.074361757	61.19480179	0.00480497	12.5249911
3.087631676	11.37581268	0.006206221	50.93909157
6.563630532	38.29612169	0.003970785	8.90966677
3.7800623	67.55784031	0.008814748	11.17199603
5.004872552	34.08469019	0.011912002	8.291412699
3.045256061	41.1137248	0.009829623	36.12970343

log.sigma.2.5.mm.3D_glrlm_LowGrayLevelRunEmphasis	log.sigma.2.5.mm.3D_glrlm_GrayLevelNonUniformityNormalized	log.sigma.2.5.mm.3D_glrlm_RunVariance	log.sigma.2.5.mm.3D_glrlm_GrayLevelNonUniformity
0.005467645	0.075351813	0.118289222	82.26083971
0.004247439	0.082448698	0.150105844	146.9212005
0.005820119	0.065581988	0.097229997	67.82478863
0.003893488	0.086688136	0.13445954	191.3464977
0.024445008	0.109069365	0.188563579	89.99349476
0.007199271	0.092666451	0.137269945	135.9147449
0.009410599	0.133439486	0.306966827	115.0729541
0.00386971	0.089798822	0.169302681	142.9054811
0.004074397	0.081299876	0.158794751	146.87406
0.005753048	0.073804895	0.111864316	202.521787
0.003770275	0.103651135	0.171032737	164.0527132
0.006301147	0.070387498	0.095903409	109.3337307
0.006111187	0.103378768	0.184128621	264.9000922
0.013266853	0.07895385	0.116211823	133.3419685
0.007131742	0.069303845	0.103170711	98.2991068
0.004280375	0.079184527	0.24653429	281.8374937
0.012835625	0.092816542	0.154197155	132.0366817
0.004415332	0.088107792	0.189310697	198.1047859
0.007255296	0.079359242	0.137720596	81.07952934
0.004426271	0.086207899	0.159100812	221.7830824
0.004999117	0.069625573	0.115349623	441.3051737
0.006514435	0.079120465	0.126365312	151.7604856
0.006908488	0.072304938	0.107615443	273.0819373
0.007028746	0.086447536	0.181048589	78.14974421
0.005980514	0.064826498	0.097138975	155.3785011
0.003337835	0.064787504	0.101095626	80.77305303
0.005644765	0.080794714	0.103095732	164.7659349
0.004291947	0.065720709	0.083873634	205.2065026
0.004022985	0.095810052	0.229015969	282.352511
0.004313245	0.083375653	0.162565086	234.9897019
0.003419276	0.076811849	0.113231717	289.3903527
0.003508691	0.078908484	0.138768928	355.3566669
0.007911398	0.088603619	0.149537125	199.8182857
0.010119304	0.088262724	0.143961417	38.01463826
0.004976137	0.073460467	0.096775344	59.98449497
0.005396372	0.099018998	0.199990344	276.7594023
0.00564003	0.065356178	0.105837984	213.9989986
0.003144606	0.08526242	0.124735696	261.2182875
0.010898096	0.10664848	0.201869667	136.9200287
0.004525922	0.097559031	0.181812124	139.7741689
0.005341108	0.085372509	0.112334677	102.4767661
0.006989209	0.088105693	0.167333925	149.7301966
0.004698817	0.097773565	0.204879458	256.1167885
0.0085324	0.102140605	0.212690338	166.8923175
0.002548723	0.071838665	0.161974472	224.1889764
0.004648044	0.072527763	0.104649947	237.7261465
0.004544646	0.073074591	0.11555541	245.2006783
0.004661201	0.085945847	0.133693613	117.1551961
0.010566641	0.114903232	0.212881741	55.51103655
0.005330175	0.101775134	0.181571953	97.26790642
0.005457116	0.100501839	0.206694522	345.6994215
0.004418271	0.123523468	0.252972625	204.6539652
0.003501014	0.095323925	0.18818298	316.1572062
0.007077179	0.078140109	0.100039727	84.08028703
0.004003475	0.078767505	0.144032395	278.6471475
0.004980885	0.066112104	0.13820219	112.9268098
0.002357757	0.074502745	0.125541793	221.2791999
0.004441955	0.112540147	0.252923009	231.1412871
0.003526833	0.076430354	0.116284178	95.57052073
0.015454837	0.110230274	0.135263415	138.439237
0.00484071	0.07833239	0.145664023	206.1210097
0.003271813	0.087512989	0.17656174	422.7334718
0.008734206	0.074053657	0.119735808	152.7219911
0.00710331	0.078797409	0.121877842	133.623238
0.015306448	0.078091948	0.113371837	42.03592505
0.006547527	0.070151268	0.104797224	98.93892307
0.005790203	0.083201782	0.131718597	79.35019798
0.002893418	0.081745952	0.161613837	259.4404789
0.005169023	0.093576357	0.140471918	108.0302504
0.003889314	0.075510324	0.132502062	170.6059105
0.004356991	0.059265761	0.096197477	208.470594
0.006434932	0.102222812	0.203233039	182.9407787
0.004700422	0.079749899	0.144871752	334.5056353
0.003606866	0.082545866	0.152390911	479.9352058
0.003492346	0.067507507	0.111991051	262.4456107
0.004486275	0.077833335	0.134794764	163.2928662
0.005603387	0.102423597	0.178061107	211.7976105
0.006329269	0.075051705	0.100550109	93.72357634
0.004132943	0.084985424	0.147952726	249.124084
0.0071773	0.084680664	0.164377031	334.044013
0.004401789	0.083529805	0.154994344	101.5568823
0.004748088	0.068139683	0.097375118	166.9311489
0.005342048	0.074259872	0.12083587	167.2173452
0.021160743	0.131262494	0.262007712	57.38359372
0.003338199	0.069938038	0.103823876	248.0981597
0.007664835	0.087225114	0.144233441	270.265077
0.007976603	0.072834796	0.111505467	95.00434815
0.003483152	0.093089693	0.15302037	250.9760088
0.020561914	0.112388271	0.158654331	40.79451408
0.005516731	0.110142944	0.160812418	56.0117951
0.005497895	0.083854071	0.145030856	240.997238
0.003861371	0.068857601	0.104132893	69.81313391
0.005918674	0.081268202	0.107784327	116.225779
0.00423204	0.082926628	0.145385254	323.8867474
0.005182285	0.089068365	0.159742983	370.9397251
0.006326955	0.047789737	0.078414844	39.55407983
0.004308912	0.122012541	0.205604691	185.2550837
0.009447355	0.107214547	0.182307427	40.15625986
0.012871046	0.124324592	0.224198023	93.94300419
0.010051136	0.052230096	0.064450073	56.90229452

log.sigma.2.5.mm.3D_glrlm_LongRunEmphasis	log.sigma.2.5.mm.3D_glrlm_ShortRunHighGrayLevelEmphasis	log.sigma.2.5.mm.3D_glrlm_RunLengthNonUniformity	log.sigma.2.5.mm.3D_glrlm_ShortRunEmphasis
1.335629139	305.2369552	909.9605685	0.929972511
1.412630387	339.3246634	1440.909285	0.91839884
1.269258463	295.8498088	894.3211491	0.944040519
1.3740743	365.0662665	1817.026093	0.924636488
1.519006683	93.88149764	640.2401533	0.901241704
1.388803716	235.8205055	1193.425003	0.920365468
1.821810349	137.4538294	600.3201243	0.859045041
1.459840844	377.9972689	1266.267291	0.911780645
1.421498599	357.6342341	1471.981741	0.920627228
1.31266615	275.6848542	2320.920415	0.935373079
1.481125762	342.9694298	1240.214037	0.90535119
1.274261049	302.3730876	1332.460347	0.941173602
1.498900691	218.4910062	2010.758437	0.905757539
1.330076898	168.046308	1412.463943	0.931000178
1.297848216	215.8936927	1202.965035	0.936188716
1.586957629	330.2325769	2793.159666	0.9057003
1.434795375	181.1779055	1137.941955	0.913199186
1.491649948	290.8152858	1791.707485	0.911340779
1.379256602	226.7631911	842.3014801	0.924942413
1.435785931	314.7392563	2068.15293	0.915373822
1.319790594	300.8347963	5340.649344	0.934082815
1.354445964	257.871412	1590.583455	0.927407726
1.301713992	255.7177711	3211.207228	0.93729161
1.488550565	291.0142936	714.4066225	0.908238317
1.280037221	257.1358893	2048.97245	0.939520263
1.279435396	494.832106	1072.589303	0.942177011
1.281836122	261.5481813	1758.150077	0.942601138
1.240574889	362.0281253	2726.218225	0.947793301
1.579529936	339.3643295	2303.115943	0.902887949
1.425170997	353.6917717	2301.569318	0.921303902
1.324696751	395.8518038	3153.671507	0.931438955
1.376034506	352.6257951	3723.020506	0.926245783
1.413162568	255.4860119	1824.754097	0.918257792
1.397458219	183.8675706	351.1879854	0.920976948
1.28275372	385.9010326	696.458861	0.938406295
1.531326738	303.0493305	2172.32733	0.902484628
1.296899359	344.2634903	2788.594333	0.938041639
1.352811576	427.5391613	2535.305402	0.927006943
1.550534153	129.5527716	988.9791594	0.89746428
1.489203446	304.7961943	1127.650791	0.907826867
1.317033481	300.8249527	1010.83249	0.93388833
1.4642421	214.0514859	1342.799553	0.909100772
1.546683099	288.3063059	2020.585203	0.899487347
1.550464539	187.8984534	1269.494814	0.902197561
1.420478004	533.0899932	2550.761572	0.921853764
1.296888684	330.1889765	2785.173249	0.937260009
1.325145894	367.8022536	2815.635898	0.932434851
1.376035816	342.5859311	1119.036341	0.923475327
1.586713293	158.8558801	365.5148529	0.890592862
1.490217813	266.8309056	754.0219217	0.908136675
1.554740299	255.6970878	2646.242849	0.898188411
1.65850269	314.2948326	1234.796313	0.885565843
1.505938555	338.8824487	2600.029953	0.905555366
1.286627727	267.2798931	917.0609971	0.938526988
1.385597343	341.3139465	2911.929927	0.925042968
1.363008644	344.3785101	1428.959432	0.931085784
1.348352846	585.3770412	2472.406228	0.929207379
1.671214947	277.8700133	1523.737316	0.882893637
1.323345095	515.6225007	1052.371048	0.933573776
1.392002152	103.855674	1016.466225	0.917986257
1.397963318	334.1939196	2149.995879	0.92191765
1.47215024	426.8075737	3839.378407	0.911053981
1.335998571	270.7903501	1723.547799	0.93073528
1.338963488	240.3498725	1419.712406	0.931245802
1.317629733	132.3490872	453.3419122	0.933897812
1.304213308	240.0557693	1188.351938	0.934249457
1.36533346	276.7902771	786.4772592	0.925872356
1.428548205	467.6687014	2575.798899	0.919248337
1.398233482	282.7092554	933.4035039	0.918022638
1.366950866	392.2854983	1865.483783	0.926185038
1.270502145	376.4999521	3035.126813	0.943048659
1.54112449	213.2503026	1389.519948	0.901327679
1.393131037	316.4580567	3435.157745	0.922738728
1.411016059	353.5467106	4733.799558	0.92039885
1.316053045	505.5667903	3274.411462	0.933973409
1.371851756	392.5132839	1729.354194	0.925590905
1.481052278	265.9473644	1634.682346	0.908983404
1.287388283	259.4905349	1065.205948	0.938687462
1.40384031	343.9150263	2394.020196	0.921282237
1.444232379	264.3491285	3168.121859	0.915127961
1.415919724	468.4013192	989.1224883	0.920060112
1.275482272	426.342967	2104.293814	0.941621895
1.331774867	370.5957283	1893.301984	0.933136282
1.716630867	73.69313711	315.7181565	0.871721849
1.290777388	415.0935035	3028.10193	0.939175437
1.404788457	177.5163589	2507.778423	0.918370773
1.313458024	282.1045731	1100.910361	0.934596684
1.432417608	361.5533148	2154.395271	0.913033418
1.454117435	80.83806022	289.4267973	0.9095747
1.460553591	350.3470336	400.8121776	0.906755314
1.397885062	248.0635929	2346.74657	0.921728456
1.295062221	512.5451935	862.5189486	0.937710777
1.303577271	299.4538651	1213.413833	0.936625312
1.391751473	346.0938904	3211.170858	0.924219049
1.44179603	245.0953139	3329.863417	0.913304124
1.222298215	543.1771989	730.9743283	0.952067072
1.562934907	331.8597744	1158.041755	0.894676745
1.497792639	172.5490771	294.1448615	0.905618661
1.61341869	132.0339771	563.3941433	0.886125092
1.187541259	382.8970593	975.8163746	0.957881026

log.sigma.2.5.mm.3D_glrlm_LongRunHighGrayLevelEmphasis	log.sigma.2.5.mm.3D_glrlm_RunPercentage	log.sigma.2.5.mm.3D_glrlm_LongRunLowGrayLevelEmphasis	log.sigma.2.5.mm.3D_glrlm_RunEntropy
425.6429883	0.907283074	0.006778626	4.55126094
506.2562293	0.890692308	0.005575371	4.500708519
404.8528612	0.924728223	0.006791285	4.551288385
537.5184581	0.899557841	0.004951017	4.443397407
144.6949766	0.869092972	0.032765284	4.2417379
358.6986323	0.89513478	0.008904	4.32676071
269.7940634	0.815078961	0.015817806	4.124196283
589.8292215	0.881312593	0.005138848	4.461405055
536.8647938	0.891047778	0.005451404	4.440452383
385.6740772	0.91354799	0.007038786	4.503983627
561.8136232	0.875569367	0.005106013	4.227769632
412.2492218	0.921798676	0.007427367	4.546387833
354.5969987	0.874386112	0.008481253	4.26774942
242.5100467	0.908834976	0.015881828	4.368236947
300.5111148	0.916219439	0.008705015	4.496056014
559.9210921	0.865679465	0.006244624	4.635997847
285.094873	0.885837527	0.016102097	4.381014786
460.405866	0.878631142	0.006265992	4.38369012
331.2727887	0.899106176	0.009208518	4.427187339
490.8126907	0.886537799	0.005933938	4.412176597
427.2682503	0.911964582	0.006202127	4.5864117
379.6388084	0.904036558	0.007991671	4.456830215
357.6856337	0.916319788	0.008329589	4.508309835
461.8093222	0.87699776	0.00905837	4.492971007
351.6647916	0.920418291	0.007301594	4.566752003
669.4266037	0.921939008	0.00395019	4.641878875
356.8692196	0.922137564	0.006776506	4.356916287
480.6259023	0.930597272	0.005015003	4.573465111
586.3800515	0.865571322	0.005802234	4.444861545
539.6485962	0.891591789	0.005543422	4.492294256
567.663081	0.90950117	0.00426554	4.50220566
515.7922446	0.900446154	0.004647014	4.454521405
387.4552897	0.890678662	0.009770159	4.464661272
261.2532502	0.89477051	0.012917362	4.356645312
533.5247972	0.919352044	0.005762842	4.538822635
509.3886464	0.868224098	0.007363361	4.394210846
472.8335543	0.917236028	0.006889339	4.707530471
628.888802	0.903296055	0.003947911	4.422376501
217.6810829	0.864607091	0.015623572	4.185580877
483.2496224	0.875352642	0.006247295	4.375911264
427.6342408	0.911912556	0.006374512	4.333255865
332.8927775	0.87928014	0.009435739	4.415412981
484.488003	0.864882722	0.006618886	4.396851206
304.7825426	0.866487016	0.011914346	4.341207213
825.0732082	0.89284928	0.003325405	4.684028919
461.9492444	0.916707907	0.005667708	4.51374372
522.4155377	0.910159022	0.0055132	4.597688766
521.31933	0.898822813	0.005743813	4.43721152
265.9356583	0.855973813	0.014964881	4.170422135
421.1751361	0.876076217	0.007315274	4.304158596
428.1598021	0.862951807	0.007805424	4.348222464
585.4966287	0.846193173	0.00638883	4.187694545
544.8211399	0.8729969	0.005067762	4.379080219
366.9615414	0.918807068	0.008241158	4.412848777
501.7805296	0.898321643	0.005218239	4.505645383
497.7934349	0.904498044	0.006192414	4.702688345
841.1840755	0.905458981	0.002972352	4.623467826
500.3011482	0.843496964	0.006968152	4.328230459
735.5510837	0.911134784	0.004154828	4.513345743
159.0474815	0.893785929	0.019536961	3.965292821
494.0446697	0.894593676	0.006178593	4.524396262
681.7844354	0.880024113	0.004450507	4.537180574
388.8394118	0.907902313	0.010255542	4.551284729
347.5066279	0.907634657	0.008613711	4.394577895
182.4191678	0.912125163	0.018475827	4.287461363
322.6380375	0.91385413	0.008224409	4.571228275
397.290233	0.90119238	0.007257593	4.475594681
713.725545	0.889619915	0.003836362	4.523379712
435.9380707	0.892736034	0.006544974	4.301216336
577.9512553	0.900954251	0.00485233	4.635295983
503.984675	0.923723335	0.00522453	4.701166463
349.6456791	0.867390736	0.009236509	4.327598185
470.2694864	0.896296053	0.006168565	4.510228142
543.0576614	0.892784991	0.004796961	4.455608353
707.3256943	0.912031188	0.004380946	4.703209137
584.3836346	0.8998449	0.005551531	4.578049146
433.459437	0.877576351	0.007366614	4.308959373
360.1146184	0.918888323	0.007524291	4.447618807
527.3128687	0.89426599	0.005334553	4.463173422
419.5943486	0.885418646	0.009033576	4.537415568
717.0526928	0.892126963	0.005392782	4.581481932
587.2527538	0.921918879	0.005426529	4.68720958
527.8290717	0.909800386	0.006445579	4.590887515
130.7211654	0.83453449	0.034370267	4.003285161
576.0277551	0.918495797	0.004056167	4.564113132
267.5079038	0.89172313	0.010206919	4.337319305
393.6680063	0.913165266	0.009284124	4.594097418
560.2590118	0.885689729	0.004691938	4.345142251
123.3419177	0.882650197	0.028211447	3.89599221
565.5681914	0.879558158	0.006790909	4.201250035
368.4015267	0.894641018	0.007264546	4.421453638
714.8751043	0.917298991	0.00442269	4.629927564
424.9539117	0.915443711	0.006943962	4.387777337
522.9977418	0.896976444	0.00541553	4.514878047
380.5537898	0.884729805	0.00709125	4.384420875
712.1798307	0.935955447	0.006864383	5.023451033
578.1728751	0.860683276	0.006056429	4.089000745
269.5605785	0.874370956	0.01280915	4.1308746
236.0397745	0.850398475	0.017909539	4.09210091
480.897702	0.944007466	0.011005454	4.813924992

log.sigma.2.5.mm.3D_glrlm_HighGrayLevelRunEmphasis	log.sigma.2.5.mm.3D_glrlm_RunLengthNonUniformityNormalized	log.sigma.2.5.mm.3D_glszm_GrayLevelVariance
326.1996781	0.832846294	33.04442488
367.1342657	0.808234515	30.78879412
314.5628453	0.864081167	27.54608036
394.1623266	0.822056465	32.59361524
102.2117197	0.774323416	25.13163223
256.7591752	0.812732169	24.70884356
157.2244893	0.694768004	19.14625838
412.5662175	0.795018768	32.74549025
386.5092267	0.813915632	24.19337029
294.5209997	0.844983737	26.93661095
379.0172335	0.782155145	21.98552361
321.8491284	0.857273185	30.23868533
240.2628216	0.783180288	23.87049663
180.9180307	0.835312811	19.58960288
230.8304162	0.846804802	22.90549429
362.9893283	0.783687403	28.07551782
198.4820025	0.798402257	27.36945673
316.8515269	0.795123302	22.9520615
244.103286	0.823071363	23.47567765
343.6652706	0.802741937	23.84614285
322.5405499	0.841835004	28.27654446
278.6402377	0.827982411	25.74132072
273.3085996	0.849234949	26.10748382
318.2670864	0.788699258	29.4243951
273.8374823	0.853865718	24.30919071
524.9726762	0.859805406	35.92682895
277.8187144	0.861235559	22.21402905
383.219889	0.872371032	30.13531619
375.057425	0.779264864	28.82566915
383.1542696	0.815647059	30.84858656
425.7414635	0.836175642	28.03898223
379.6469421	0.825567269	23.54608448
277.3914967	0.808395435	32.23321429
197.0136681	0.814412763	28.41580186
412.3020998	0.85183768	33.09543978
335.3909081	0.776410174	30.31482724
366.5494532	0.850845522	36.94605813
462.059436	0.826719104	28.59113587
143.5165937	0.767668355	18.42108487
333.3530048	0.786739225	28.33417436
322.7563873	0.841484107	25.78041299
233.7053684	0.789372015	27.44031142
319.0692843	0.770419608	28.31800404
205.947055	0.776106666	27.51450395
578.988864	0.816354212	34.82806669
353.1321364	0.848922551	26.37038115
394.5001208	0.838337329	31.98606818
372.7955017	0.819640588	29.79572757
175.9729489	0.754507991	22.78808594
291.4671868	0.788055611	25.34619834
282.8106007	0.768142766	24.81210948
354.6793454	0.743858132	28.52394978
371.6560385	0.782760361	26.42141144
284.8688647	0.851527951	26.62882321
367.6910954	0.822549771	28.32423985
369.089241	0.835967951	36.31719265
628.66317	0.831595756	38.25525197
311.4464036	0.739592869	25.62221853
553.0422382	0.840923069	36.22845873
113.3789429	0.80776537	13.38051201
360.6920935	0.816197501	30.35777582
467.536708	0.793775834	35.35170654
291.0613926	0.834807944	31.67527857
258.5142992	0.836179127	22.32785304
141.1484358	0.841499038	19.08472008
254.7409617	0.841957946	29.17047389
297.315412	0.824071961	33.93332688
507.1986961	0.810824489	32.76603394
308.9571114	0.807549087	23.06508157
423.5305515	0.825034011	39.53878926
398.8873825	0.861904869	31.10398368
234.5653822	0.774887163	25.92474074
341.8022173	0.817904147	27.85846049
384.4556249	0.813067052	25.22197184
540.4875459	0.841631237	37.9633965
424.6714501	0.82371249	34.11957962
292.6759789	0.789563497	25.2585618
277.1900645	0.852019989	25.0973284
373.9157137	0.815389514	29.54103769
289.3726361	0.802187574	31.47446098
508.8009766	0.812554672	39.59247449
454.5087471	0.858429964	41.67389055
397.1173546	0.840141186	36.3752623
82.54548065	0.719271873	12.4659
443.0328062	0.853004613	29.64080569
192.7061974	0.808484386	20.22889974
301.4985254	0.843191921	34.31076953
394.9723372	0.797779414	25.72919178
88.04049633	0.793509138	10.2607247
386.568489	0.785936605	33.42511894
267.9812934	0.81577981	25.05256844
548.0474281	0.849945275	40.65110133
321.0505381	0.847617409	28.2251661
374.7817756	0.821254884	32.01209191
267.4090631	0.7983113	23.70344407
573.2456851	0.88245443	63.35628075
370.7583996	0.76120945	19.01643121
188.3139011	0.783613764	19.60140331
148.5734777	0.744398145	17.45516643
400.8866245	0.895305334	43.18636207

log.sigma.2.5.mm.3D_glszm_SmallAreaHighGrayLevelEmphasis	log.sigma.2.5.mm.3D_glszm_GrayLevelNonUniformityNormalized	log.sigma.2.5.mm.3D_glszm_SizeZoneNonUniformityNormalized
224.0952618	0.050902351	0.381635254
242.7911296	0.053211403	0.375765086
183.0096177	0.052868938	0.377967621
240.1434202	0.052763912	0.370484552
81.76385985	0.060785124	0.327933884
152.1572527	0.061033593	0.373022958
90.54715577	0.070242748	0.311556528
246.3941768	0.052792148	0.375348115
242.0473645	0.058616468	0.380630094
185.4489555	0.053873578	0.361953527
200.8027232	0.065155842	0.324777089
194.4894746	0.051791738	0.398197813
152.4340558	0.062567039	0.354563546
86.92589378	0.063616166	0.278108269
129.2889258	0.058176971	0.36011159
214.8356791	0.054307726	0.345243478
116.3249541	0.054623167	0.333925326
187.1107501	0.060888581	0.322927037
140.7836615	0.05819854	0.312633342
191.5725439	0.059120392	0.326645855
187.0561924	0.052763037	0.353113684
150.9612567	0.056899094	0.306933627
156.3266046	0.054617975	0.357562601
188.732654	0.054717187	0.328509512
163.7043538	0.056440527	0.350703009
306.9763945	0.048982164	0.38275701
172.0376528	0.060868535	0.42045424
221.3475691	0.051186319	0.384327499
219.1445776	0.054077871	0.369779147
221.8622723	0.053946722	0.365226337
232.8968221	0.053878004	0.33403151
235.5453725	0.05912768	0.360747939
181.1539107	0.052338776	0.419959184
148.1560067	0.056903669	0.437907488
226.6085955	0.050744481	0.350157173
192.5378399	0.0550149	0.35325445
228.380452	0.046439264	0.382649049
250.2958457	0.055013989	0.336701921
80.14225873	0.068305859	0.270605926
225.5157788	0.056456651	0.404880334
176.7087268	0.058422896	0.389919092
152.7297492	0.057058117	0.354291717
203.5380339	0.056335013	0.410489968
136.9625254	0.056491151	0.397022481
337.29194	0.049312742	0.373364746
199.1614962	0.055002436	0.360484087
235.0866274	0.051144851	0.366904593
209.679556	0.054854603	0.378203308
128.8248895	0.064697266	0.352905273
201.2178764	0.057522329	0.405085844
189.3923497	0.060336444	0.367762554
188.913173	0.063356115	0.36357917
246.2153466	0.056800909	0.378661785
166.5295784	0.057847407	0.365044191
225.0134024	0.053950658	0.352813762
238.5408113	0.046290223	0.404781922
412.6521094	0.047835899	0.401785675
197.8850242	0.05810152	0.333806802
308.122345	0.05074349	0.35632133
56.49659258	0.07742907	0.303018265
229.8785648	0.054721471	0.392322877
286.3746189	0.051354496	0.379968405
172.0042818	0.050706729	0.384090646
137.7610416	0.060075083	0.34753805
85.82042889	0.064518573	0.428953814
187.2981877	0.054137179	0.374693515
197.6368692	0.05188916	0.387456084
302.2249054	0.052313619	0.375237401
164.6424977	0.060795888	0.354668681
271.6776228	0.047201911	0.423894313
238.6099473	0.050326608	0.372538041
151.5185414	0.057829395	0.338595296
209.0821661	0.054557713	0.351288685
218.892836	0.057509828	0.345663496
349.8733613	0.047740559	0.394222464
233.2041382	0.049738762	0.370157517
164.6600035	0.058527111	0.354885127
150.1760948	0.057501235	0.348098765
213.0835814	0.05360868	0.374627947
165.2908763	0.051469237	0.393088721
317.3532703	0.049645249	0.411013233
276.6800354	0.045165402	0.435584857
255.9429209	0.047909681	0.418729043
62.42375015	0.0858	0.3098
259.4037819	0.053252086	0.380736583
112.6390552	0.061638737	0.333394618
187.2928593	0.048712871	0.388601118
230.0113293	0.060577565	0.359554537
37.60972878	0.091420242	0.292794669
228.2261705	0.059364187	0.375108131
177.2897095	0.056795146	0.406708854
305.879727	0.046656148	0.408741121
187.5634043	0.054967119	0.393499724
222.5049497	0.051384195	0.386093011
165.8196808	0.059220036	0.36209083
359.7229094	0.035503135	0.443009069
181.2693268	0.071587364	0.276255871
126.3182631	0.073817762	0.387543253
91.50629601	0.076677585	0.377509028
255.6764279	0.043357552	0.43432323

log.sigma.2.5.mm.3D_glszm_SizeZoneNonUniformity	log.sigma.2.5.mm.3D_glszm_GrayLevelNonUniformity	log.sigma.2.5.mm.3D_glszm_LargeAreaEmphasis	log.sigma.2.5.mm.3D_glszm_ZoneVariance
153.7990074	20.51364764	78.97022333	70.05933169
220.5741056	31.2350937	207.407155	195.7984497
173.1091703	24.2139738	24.48908297	18.53036746
272.6766304	38.83423913	132.6345109	121.5264189
72.14545455	13.37272727	346.2045455	327.5970868
169.3524229	27.7092511	176.6740088	163.6568534
52.02994012	11.73053892	781.6826347	741.6220732
173.7861771	24.44276458	344.0410367	328.8428271
226.0942761	34.81818182	151.9208754	140.2760064
364.1252485	54.19681909	57.64910537	48.73834626
132.1842752	26.51842752	287.9336609	268.2218063
252.4574132	32.83596215	42.39274448	35.3292226
232.9482496	41.1065449	497.5022831	477.6272434
159.6341463	36.51567944	61.6445993	51.16685889
188.6984733	30.48473282	35.8740458	27.14675718
397.7204861	62.5625	241.8255208	229.1031713
137.243309	22.45012165	176.9367397	161.6868595
193.1103679	36.41137124	273.8729097	255.5751166
107.2332362	19.96209913	71.72594752	60.75691251
238.1248285	43.09876543	208.7750343	192.93922
818.8706339	122.3574817	63.03837861	54.05648075
193.6751189	35.90332805	69.47860539	58.18005782
512.7447699	78.32217573	66.00488145	57.74627205
94.28222997	15.70383275	119.261324	106.3814785
335.6227795	54.01358412	25.23092999	17.82707193
184.8716356	23.65838509	50.16149068	42.32612768
362.4315545	52.46867749	58.94083527	52.3617888
514.2301943	68.48729447	26.89013453	20.60270946
307.65625	44.99278846	427.3353365	410.6453619
355	52.43621399	271.3364198	260.7739198
420.5456712	67.83240667	95.12946783	84.30594272
538.2359249	88.21849866	157.844504	146.6139195
293.9714286	36.63714286	405.8814286	392.8080388
70.50310559	9.161490683	71.59627329	62.67065314
118.0029674	17.10089021	31.82789318	24.88458998
272.7124352	42.47150259	405.7603627	388.3956532
498.5917114	60.51036071	51.3791251	43.87665515
329.2944785	53.80368098	101.0030675	88.98103575
72.52238806	18.30597015	418.7462687	388.0844286
165.1911765	23.03431373	369.7647059	353.6861784
177.4131868	26.58241758	80.0967033	71.73125951
168.6428571	27.15966387	369.8907563	353.4167079
290.2164074	39.82885431	754.1287129	735.7977189
177.8660714	25.30803571	407.7857143	390.1006856
389.0460653	51.38387716	148.646833	137.4031042
424.6502547	64.79286927	53.4762309	44.26619101
461.5659777	64.34022258	75.49284579	66.90766629
178.133758	25.83651805	119.5159236	109.1559991
45.171875	8.28125	250.90625	231.4912109
119.0952381	16.91156463	245.0340136	231.2885835
329.147486	54.00111732	909.5039106	889.6889785
135.9786096	23.69518717	979.973262	952.6209214
337.0089888	50.55280899	867.0438202	848.8425716
143.8274112	22.79187817	49.10913706	40.27587029
421.2596315	64.41708543	311.7077052	300.8353865
266.3465046	30.45896657	55.36474164	47.13185392
429.5088868	51.13657624	215.5369504	206.128298
169.9076621	29.57367387	690.1807466	667.3515001
169.2526316	24.10315789	35.33894737	26.99597119
99.69300912	25.47416413	181.0547112	162.8173982
337.3976744	47.06046512	166.0290698	154.3342604
558.1735875	75.43975494	798.0428863	784.091212
285.7634409	37.72580645	74.52016129	65.20289737
208.1752922	35.98497496	46.50083472	36.77559985
85.36180905	12.83919598	53.75879397	44.9686119
205.7067395	29.72131148	32.71948998	24.82021626
130.5727003	17.48664688	127.7804154	117.9241694
367.7326531	51.26734694	254.2326531	240.9919658
123.7793696	21.21776504	107.6762178	93.95014819
344.6260763	38.37515375	137.5879459	128.0791051
539.4350829	72.87292818	28.5980663	21.68203809
157.7854077	26.94849785	281.1866953	261.6260062
468.267817	72.72543136	159.9182296	147.5972343
616.6636771	102.5975336	187.1070628	173.7870178
568.4687933	68.84188627	74.28710125	65.55142823
266.1432545	35.76216968	152.3184979	141.8079159
210.4468803	34.70657673	383.6812816	367.9098021
156.6444444	25.87555556	38.98888889	29.86848889
338.2890365	48.40863787	208.2768549	195.1151459
498.0434096	65.21152328	417.6961326	405.3437095
184.1339286	22.24107143	103.1830357	93.94034997
452.572666	46.92685274	85.73532243	79.19570995
360.1069767	41.20232558	87.44418605	79.16852893
30.98	8.58	331.53	304.1771
516.6595431	72.26308032	46.91230656	38.81268284
295.0542373	54.55028249	168.5175141	153.1085627
196.2435644	24.6	63.17227723	55.17626115
261.3961486	44.03988996	299.2145805	281.6945409
28.69387755	8.959183673	123.3367347	105.7481258
51.01470588	8.073529412	186.9264706	168.8639706
380.6794872	53.16025641	173.7190171	161.9503057
171.2625298	19.54892601	34.63245823	27.67746823
213.6703499	29.84714549	49.23756906	40.96267581
504.6235654	67.15914308	208.3022188	197.2098026
433.4227235	70.88638262	230.1637427	214.7070866
181.190709	14.5207824	20.78728606	16.11576927
92.26946108	23.91017964	630.8113772	602.9494155
39.52941176	7.529411765	148.6078431	131.0007689
71.34920635	14.49206349	388.2751323	366.2000504
244.5239787	24.41030195	9.658969805	5.457562096

log.sigma.2.5.mm.3D_glszm_ZonePercentage	log.sigma.2.5.mm.3D_glszm_LargeAreaLowGrayLevelEmphasis	log.sigma.2.5.mm.3D_glszm_LargeAreaHighGrayLevelEmphasis	log.sigma.2.5.mm.3D_glszm_HighGrayLevelZoneEmphasis
0.334995844	0.309348245	21321.20596	350.1141439
0.2935	0.6827684	64373.48211	387.6575809
0.409660107	0.085627024	8071.270742	294.8471616
0.300040766	0.374627409	48192.74864	397.0611413
0.231822972	5.003104509	25241.23182	130.9409091
0.277167277	0.723778303	44847.93392	245.2378855
0.157994324	6.139899788	101433.2096	170.5688623
0.256509695	0.980301617	122433.5724	414.3866091
0.293043907	0.496578293	48259.20707	393.9461279
0.334998335	0.226665618	15893.90755	294.1908549
0.225235196	0.823333535	102507.4324	360.7444717
0.376261128	0.155552094	13254.38486	309.6135647
0.224308638	2.389957731	105394.175	250.5631659
0.308934338	0.40906739	10832.83275	171.815331
0.338501292	0.191864064	7818.505725	223.2576336
0.280360185	0.766519229	78801.88976	361.2013889
0.256074766	0.992078396	34248.39659	198.6545012
0.233776388	1.1116156	69354.23411	331.312709
0.30193662	0.370193595	14931.34111	249.9650146
0.251292658	0.6795216	66089.49246	335.037037
0.333669065	0.210030409	20688.96248	308.2867615
0.297501179	0.275017064	18932.87956	271.1030111
0.347973793	0.260693682	18154.73501	261.5822873
0.278640777	0.45909214	33002.70732	327.738676
0.367511521	0.111682253	6975.882968	269.4263323
0.357248521	0.113412757	23838.04555	509.3271222
0.389868838	0.235285669	15420.4594	267.0893271
0.39880775	0.077403905	10474.89163	358.9693572
0.244777876	1.240195465	149427.9399	362.1514423
0.307692308	0.781155086	95611.68004	373.5936214
0.30395944	0.232539257	40540.78872	405.6759333
0.2984	0.480226346	53225.13941	385.0301609
0.276570525	1.641755079	103020.4357	280.2957143
0.334719335	0.528401223	10374.24224	223.8944099
0.379504505	0.085053944	13466.77448	388.7032641
0.239975132	1.309018976	128601.1386	323.7357513
0.36508826	0.169735623	17661.92786	363.9923254
0.288410498	0.225533878	46656.95808	436.0695297
0.180592992	3.492597783	51972.60821	152.738806
0.249388753	1.298296783	106661.6275	354.6323529
0.345744681	0.27472317	24286.02198	300.0285714
0.246376812	1.972284048	70953.84664	253.3235294
0.233564585	2.721170365	211172.0566	316.7892504
0.237791932	2.487412714	68498.02232	222.578125
0.298225529	0.274110402	82731.83685	551.146833
0.32951049	0.165877379	19190.85059	332.1748727
0.341291373	0.218520479	27696.75676	385.9523052
0.310686016	0.314050096	47174.82378	341.1571125
0.226950355	1.947089573	33813.97656	202.515625
0.269724771	0.988917847	61870.28571	313.4217687
0.224648594	3.764761551	222283.9598	301.0402235
0.191206544	2.887477386	335205.2433	327.2406417
0.234395575	2.79396931	271346.4236	396.011236
0.33646456	0.194974671	13583.21827	276.1725888
0.303276607	1.026564138	95980.98157	375.7688442
0.348516949	0.171025682	19501.5152	370.7401216
0.326014029	0.376510574	124962.4247	636.1927035
0.209292763	2.678435232	179640.8664	341.1905697
0.346209913	0.070202714	19593.86105	527.3873684
0.234163701	1.713342724	20339.50456	106.6291793
0.292417545	0.564462186	50696.21744	368.0197674
0.267723711	1.87790968	341966.5221	461.4411164
0.327608983	0.303577779	20314.36828	280.6276882
0.320663812	0.20909768	11488.38063	244.4323873
0.337288136	0.535606639	6328.512563	137.3366834
0.355800389	0.180306004	6726.001821	286.0455373
0.31852552	0.513178243	32849.67359	314.8189911
0.274817723	0.581110408	113028.9367	505.5622449
0.269914927	0.372762906	32525.54155	279.982808
0.324291982	0.352252587	55179.89176	414.6494465
0.380252101	0.08986015	10674.9047	392.7603591
0.226103833	1.442970408	56233.8176	255.77897
0.284889934	0.568445639	47024.21305	348.2228057
0.27399785	0.510461131	70397.44731	367.4304933
0.338338808	0.168617997	36480.17268	545.556172
0.308451308	0.407699946	59139.61892	395.3310153
0.251804671	1.364602182	109569.4081	278.1382799
0.331125828	0.163456458	10424.44222	262.1888889
0.275641026	0.577295388	77021.25249	349.3289037
0.284527285	1.553948299	114936.7806	269.4909234
0.328928047	0.225379565	50211.63839	492.6741071
0.391042529	0.192828633	40063.17421	421.0048123
0.347615198	0.244992039	33387.59767	390.1651163
0.191204589	6.383650774	18571.89	106.75
0.351372346	0.116022141	20159.1776	422.2763449
0.254749568	1.079534512	27613.48814	193.6644068
0.353641457	0.251099367	17382.04554	300.4851485
0.238908971	0.8612716	105635.5585	392.1856946
0.238442822	2.193092288	7805.234694	85.48979592
0.235294118	0.519674185	69647.28676	383.6102941
0.291497976	0.785568364	39545.36859	274.4967949
0.37918552	0.071467357	18477.10263	505.8305489
0.347631242	0.158248912	16734.07366	297.8379374
0.300252699	0.584710391	75771.78577	356.4529457
0.254356141	0.965782904	56513.8914	274.8345865
0.462669683	0.047141012	12883.89731	531.4596577
0.189449801	1.792535372	224710.7844	361.0449102
0.238317757	1.068408436	21622.80392	212.0490196
0.212837838	2.98464593	52027.60847	147.2222222
0.487868284	0.047313057	4200.119005	380.8188277

log.sigma.2.5.mm.3D_glszm_SmallAreaEmphasis	log.sigma.2.5.mm.3D_glszm_LowGrayLevelZoneEmphasis	log.sigma.2.5.mm.3D_glszm_ZoneEntropy	log.sigma.2.5.mm.3D_glszm_SmallAreaLowGrayLevelEmphasis
0.640078324	0.008173201	6.218259919	0.006690919
0.634865519	0.006340866	6.245627648	0.005054351
0.636109916	0.008056184	6.123523005	0.006178321
0.629509578	0.005891525	6.314627627	0.004641347
0.588342801	0.041910368	5.966324075	0.029199505
0.632119199	0.012552073	6.043066023	0.009844409
0.576556868	0.017418606	5.779588087	0.014382014
0.636606151	0.006722875	6.273687992	0.005687605
0.640626552	0.00568546	6.134774747	0.004253835
0.624284871	0.008221452	6.383389276	0.006201091
0.586888162	0.006521482	6.147688795	0.005174595
0.654888659	0.008645719	6.199603024	0.00656247
0.618226552	0.009717266	6.25630871	0.007431292
0.537771263	0.021606804	6.374048716	0.015479986
0.622304483	0.009872766	6.172049704	0.007579631
0.60815391	0.005771173	6.513967507	0.003857717
0.597140431	0.021422152	6.282452058	0.014278784
0.589049824	0.006531883	6.379848196	0.005018039
0.575402228	0.010893476	6.233628738	0.00793214
0.591324682	0.005994519	6.374858601	0.003386487
0.614896601	0.006770621	6.560619732	0.004760694
0.569162388	0.010447051	6.461857551	0.007721967
0.619907615	0.009505092	6.412515333	0.006289603
0.59307711	0.012883965	6.207157157	0.010722943
0.613630001	0.007475871	6.375977523	0.005208397
0.642670599	0.00509045	6.299485684	0.004254972
0.674205116	0.007770611	6.010066376	0.006265222
0.64376342	0.005805164	6.388486443	0.004431946
0.631098948	0.005653171	6.344348725	0.003454464
0.626134408	0.007013614	6.363050624	0.005797586
0.598671516	0.0048029	6.565053396	0.003527286
0.623969535	0.004511027	6.415714734	0.003328979
0.673866589	0.014995038	6.200832396	0.012987582
0.687609303	0.015707937	5.540861558	0.013815951
0.606583694	0.007660143	6.199286814	0.005979378
0.615332891	0.008215655	6.406982053	0.005239553
0.641753599	0.007205179	6.515289405	0.00479074
0.600496561	0.004812104	6.486629219	0.003668605
0.534670922	0.018608184	6.21796419	0.013319302
0.661407642	0.0070428	6.037438672	0.005721105
0.648340187	0.00862487	6.011853393	0.007314644
0.616938601	0.011060523	6.197762726	0.008539604
0.665804844	0.007985119	6.191341632	0.006639853
0.654966084	0.015369413	6.101613383	0.013157545
0.634787031	0.00380442	6.526931108	0.002978368
0.62275897	0.006144528	6.433110846	0.003947317
0.626587882	0.006739801	6.471337508	0.005159792
0.63838166	0.007699976	6.188976541	0.00625551
0.611901859	0.018842368	5.477943215	0.015302402
0.661460861	0.008229039	5.903844782	0.006840898
0.62950653	0.008133495	6.260013476	0.005481737
0.624859143	0.008907354	6.084709429	0.007167084
0.639126337	0.004754507	6.246550668	0.003615681
0.626033197	0.012087297	6.103156411	0.010204751
0.61458196	0.005632215	6.435787625	0.00430607
0.660810075	0.007191291	6.3332824	0.00554884
0.657762076	0.003346729	6.439509789	0.002638453
0.596700238	0.006651732	6.282989818	0.005096437
0.614925557	0.005931327	6.367540548	0.004937044
0.566370114	0.027011411	5.946972521	0.01963911
0.650628064	0.00797819	6.282554182	0.006679909
0.639771895	0.004713897	6.497612307	0.003388712
0.643794778	0.014797254	6.31761986	0.011520547
0.610885634	0.011546084	6.214000517	0.00941198
0.678977686	0.024906953	5.536561411	0.021325966
0.635932935	0.007935811	6.208521038	0.005673485
0.645735014	0.009067057	6.09736515	0.007636669
0.635535331	0.00452326	6.411161908	0.003705299
0.618562597	0.008741357	6.122682891	0.006881495
0.677043102	0.005901636	6.362712099	0.004773133
0.632780754	0.005944397	6.457777163	0.00458475
0.598813167	0.00985298	6.280936197	0.007385252
0.614444714	0.005938929	6.500395734	0.003760771
0.607738788	0.004915471	6.464098643	0.003356963
0.652808461	0.00382657	6.550090979	0.002344338
0.630546464	0.006721981	6.399394398	0.004460441
0.615127151	0.009968668	6.208157488	0.008028961
0.611859245	0.009720175	6.218782526	0.007545902
0.634355721	0.006288016	6.325381688	0.004624788
0.650934242	0.012262373	6.360397118	0.009568586
0.66657445	0.007070647	6.202635206	0.005699836
0.685508191	0.007528397	6.314094705	0.006297547
0.672990579	0.007706265	6.357966382	0.00543112
0.571434119	0.030211869	5.270273725	0.022714478
0.641354484	0.004570101	6.440539718	0.003488293
0.598381816	0.010753219	6.363079268	0.007633795
0.647578098	0.013419672	6.292216656	0.011232889
0.620933899	0.005364274	6.250014361	0.004220857
0.556671126	0.033179106	5.196243202	0.026685838
0.634386015	0.012788146	5.729233159	0.011620159
0.662885803	0.007598609	6.172171001	0.005818675
0.664690862	0.006611864	6.18404198	0.005908366
0.651813946	0.009564475	6.163808462	0.007805825
0.645765222	0.006297389	6.415837559	0.004780445
0.625076854	0.007229197	6.375350179	0.005474169
0.690446541	0.009856079	6.337283834	0.008514892
0.537117157	0.006622482	6.139116513	0.002868958
0.645771263	0.017265689	5.260441314	0.015286115
0.636909374	0.022728629	5.508623621	0.014846104
0.685165403	0.013913958	6.208701366	0.010862528

log.sigma.2.5.mm.3D_ngtdm_Coarseness	log.sigma.2.5.mm.3D_ngtdm_Complexity	log.sigma.2.5.mm.3D_ngtdm_Strength	log.sigma.2.5.mm.3D_ngtdm_Busyness	log.sigma.2.5.mm.3D_ngtdm_Contrast
0.006647526	0.781692246	2.911312818	0.212417566	6.43E-05
0.004442525	0.376173036	1.65609486	0.303746254	3.24E-05
0.007172413	0.634250527	1.642184183	0.273932087	0.000127519
0.003299932	0.392874127	1.628651806	0.335003222	2.24E-05
0.008467053	0.304480129	1.789294983	0.524898121	8.30E-05
0.004980349	0.368981738	1.480997997	0.34925558	3.86E-05
0.007891956	0.202212129	1.614138084	0.389682422	3.85E-05
0.004629287	0.485468489	2.428307642	0.230770999	2.67E-05
0.004195142	0.361426721	1.566070523	0.326271855	3.01E-05
0.002910528	0.266935401	1.001171358	0.531002261	2.39E-05
0.004820308	0.283994789	1.75300656	0.279932184	2.17E-05
0.004549677	0.523843447	1.415049131	0.349130248	6.56E-05
0.002855813	0.171283567	0.953222868	0.610555765	1.43E-05
0.004266962	0.248673495	0.811379035	0.660892972	5.23E-05
0.005156568	0.396716522	1.089348798	0.500487898	7.59E-05
0.002415506	0.231061434	1.103624112	0.467080076	1.23E-05
0.005425597	0.282478173	1.323437334	0.410848514	4.41E-05
0.003461413	0.208741225	1.170699892	0.47357516	1.98E-05
0.007845352	0.433512514	1.889535323	0.285638145	7.11E-05
0.003056095	0.19727715	0.981252578	0.504476198	2.00E-05
0.001171363	0.16380804	0.491483674	1.106651647	1.09E-05
0.003978091	0.39906778	1.636845755	0.364660173	2.74E-05
0.001957401	0.197719115	0.619839921	0.863821004	2.05E-05
0.008431093	0.541334867	2.811059357	0.201790583	7.37E-05
0.003363677	0.323890755	1.035880615	0.53495507	3.64E-05
0.006204841	0.880335558	2.695350488	0.176277966	7.34E-05
0.003769234	0.326752052	1.279689385	0.432523133	2.90E-05
0.002293191	0.326938796	0.838029397	0.583142575	3.27E-05
0.002528842	0.222988261	1.125083564	0.465311029	1.35E-05
0.002826007	0.376165173	1.607050447	0.353090329	1.48E-05
0.002033729	0.250166355	0.979085439	0.530169855	1.50E-05
0.001743796	0.195624728	0.927723329	0.633226469	8.53E-06
0.003302259	0.295028367	1.303315741	0.452859699	2.52E-05
0.01785079	0.911659045	4.094134081	0.15412626	0.000200855
0.008467903	1.067076373	3.125924769	0.153871597	0.000124692
0.002607615	0.199211604	1.022044535	0.512242701	1.59E-05
0.00233476	0.322001846	0.936061088	0.559758109	2.92E-05
0.002349459	0.300504675	1.246014152	0.425245499	1.58E-05
0.006118715	0.193748551	1.262940933	0.484576855	2.83E-05
0.005054913	0.335911977	1.728766176	0.309604348	3.60E-05
0.005608843	0.525873742	1.681687062	0.283385399	6.12E-05
0.004898466	0.248632775	1.273850685	0.41443294	3.10E-05
0.002962529	0.187567172	1.056836186	0.494673567	1.60E-05
0.004279846	0.256765716	1.276655481	0.498039029	3.19E-05
0.00258772	0.371534953	1.432226207	0.32930254	1.91E-05
0.002212462	0.243125428	0.81682965	0.634315242	2.26E-05
0.002242032	0.299549493	1.078782761	0.499418057	1.94E-05
0.005619976	0.461284305	1.854039602	0.245732188	5.51E-05
0.014095669	0.494849997	3.046982043	0.208573327	0.000125794
0.006855957	0.441013321	2.125542796	0.270415337	6.05E-05
0.002302811	0.153303996	0.970586943	0.609460833	8.73E-06
0.00438128	0.274929693	1.722247378	0.286169425	1.85E-05
0.002311121	0.187379719	1.050854531	0.518885049	1.03E-05
0.006678947	0.682284732	2.152920606	0.242926762	6.76E-05
0.002151447	0.23645108	0.925225384	0.574344591	1.52E-05
0.004939268	0.53526952	1.709622537	0.27244679	5.01E-05
0.002725689	0.432275943	1.963779103	0.268643017	1.63E-05
0.00376893	0.219237731	1.605560658	0.368667162	1.50E-05
0.005909515	0.88145421	3.377919303	0.153462541	5.44E-05
0.005325884	0.163781922	0.637454531	0.794921931	4.53E-05
0.002808363	0.285910349	1.195525816	0.470485137	2.19E-05
0.001628914	0.242251224	1.183935351	0.490879747	7.59E-06
0.003607175	0.314558034	1.065493784	0.478360967	4.23E-05
0.004205919	0.334121408	1.087974461	0.46142083	4.43E-05
0.013651666	0.514092141	1.61453927	0.362665881	0.000228918
0.005629347	0.49671618	1.818426491	0.35801048	6.25E-05
0.007830324	0.703358552	3.020557464	0.20017027	6.82E-05
0.00238947	0.30577138	1.464990907	0.381688612	1.39E-05
0.005922427	0.412685069	1.623832735	0.293509125	5.51E-05
0.003395311	0.515062174	1.838023572	0.292412195	2.77E-05
0.002247164	0.270597374	0.810118796	0.6400905	2.92E-05
0.004165915	0.263628931	1.491295385	0.416670294	2.16E-05
0.001859094	0.183062018	0.818108882	0.687296789	1.18E-05
0.001334081	0.130180493	0.566314347	0.898432125	8.23E-06
0.002085636	0.331801007	1.327524488	0.410598562	1.59E-05
0.003594091	0.495175894	1.882908668	0.281197695	2.74E-05
0.003560003	0.281399293	1.288841223	0.398132098	2.01E-05
0.005984916	0.468284898	1.732381706	0.314466855	6.34E-05
0.002593253	0.254998071	1.074242509	0.468314749	1.84E-05
0.001992437	0.21994205	0.876017364	0.640369262	1.34E-05
0.006680184	0.851630612	3.512942846	0.136769815	5.31E-05
0.002915026	0.552858745	1.514201724	0.339634092	3.87E-05
0.003322843	0.425636264	1.477198098	0.351765118	3.22E-05
0.015281785	0.255382445	1.829432326	0.417790784	0.000125011
0.002152493	0.258911032	0.930840444	0.538973822	2.02E-05
0.002353288	0.129147396	0.526421386	1.071995541	1.91E-05
0.005816985	0.529641755	1.945096914	0.28971808	7.16E-05
0.00283397	0.199520742	1.069042042	0.470430119	1.65E-05
0.017173117	0.309065138	1.430252131	0.459361887	0.000252448
0.013325022	0.94537457	5.787207974	0.101255012	9.67E-05
0.002556843	0.173334023	0.683654598	0.761570915	2.29E-05
0.006949876	1.109797618	3.201750726	0.152856324	0.000107955
0.004908929	0.486857473	1.579705494	0.308357835	5.61E-05
0.001899268	0.2161928	0.816187728	0.622327488	1.55E-05
0.00191674	0.12409885	0.630571064	0.861397708	1.01E-05
0.008669931	2.72355238	4.929474698	0.102046084	0.000249343
0.004556079	0.194748554	1.46153816	0.352532801	2.19E-05
0.021619032	0.477702582	3.46794078	0.161616471	0.000162736
0.009118611	0.265897067	1.602079254	0.343271051	5.37E-05
0.006495409	1.219965903	2.574359305	0.20775668	0.000170297

log.sigma.2.0.mm.3D_gldm_GrayLevelVariance	log.sigma.2.0.mm.3D_gldm_HighGrayLevelEmphasis	log.sigma.2.0.mm.3D_gldm_GrayLevelNonUniformityNormalized	log.sigma.2.0.mm.3D_gldm_DependenceEntropy
19.84956285	404.276808	0.079711362	6.360710515
17.026375	502.127	0.0941865	6.47928713
22.87796698	384.6261181	0.06677686	6.426872448
19.23117046	496.9735018	0.086799877	6.341638822
13.31654084	156.3319283	0.118402045	6.028978465
14.83129796	384.3485958	0.096345047	6.227162574
7.5497548	194.8278146	0.147078225	6.085384509
15.86305937	598.2897507	0.104155432	6.420280733
14.34240759	474.5263937	0.094556492	6.453241603
17.21554591	459.8814519	0.078131326	6.429830859
11.88386001	440.6740454	0.115151096	6.322314897
25.83289313	505.6118694	0.066915091	6.427437322
11.49544151	325.9310345	0.106876398	6.374848955
14.47032441	197.7389666	0.079202494	6.360125507
16.61427056	340.9166667	0.07263853	6.322396393
16.41441396	423.9944025	0.090112161	6.820206689
13.29212876	246.3632399	0.100950495	6.330086563
12.39842763	321.7759969	0.099577221	6.442140461
16.32689369	229.8829225	0.090634919	6.466704351
13.12580395	399.1582213	0.096560149	6.519640242
21.46748686	469.4201439	0.066793023	6.624381879
16.3821892	368.9222065	0.08290437	6.381697385
18.13125603	362.4899296	0.075267151	6.467731086
17.44285041	375.1300971	0.096303139	6.372124983
18.95733065	269.4450845	0.066909646	6.588084101
25.06762718	414.0207101	0.069855966	6.493605107
16.4913122	407.7114428	0.081341371	6.221619121
24.1632969	412.7290611	0.064944507	6.538691821
13.44504956	475.8152398	0.115789468	6.456977078
16.98539424	403.0164609	0.093738517	6.540186029
18.08630554	331.5523901	0.079032236	6.475399363
13.26300736	470.3332	0.08913424	6.49814877
17.81209096	443.6286053	0.099524021	6.412880238
15.85261993	327.9147609	0.114690894	6.132196701
24.87372043	404.6407658	0.075752273	6.230272523
14.36538926	519.9092322	0.116929616	6.393741438
25.97796834	548.9504063	0.069216325	6.586172522
18.29574826	447.1801828	0.086038024	6.429812032
8.184519874	135.3086253	0.113619852	6.206779793
14.93149999	460.8227384	0.116366324	6.33163696
18.36256594	461.9194529	0.083547362	6.208868411
13.62602475	382.2779503	0.102954919	6.4778627
13.67575752	535.436736	0.118015823	6.367946888
13.23818749	383.8619958	0.121225337	6.346171918
20.85849558	822.4095592	0.0789498	6.673492103
21.16856179	458.2416783	0.071099888	6.489352751
22.45577744	501.8827998	0.07345313	6.535560942
18.32869228	368.951847	0.085314256	6.303566671
15.17107728	193.7535461	0.124779941	6.013355413
12.9657512	412.2688073	0.11767528	6.269846656
11.46435718	366.1777108	0.109651205	6.44363191
11.44025514	592.4079755	0.141620769	6.209784218
13.04270404	458.3560706	0.109901923	6.385681303
17.1562023	370.9880444	0.07955786	6.241690215
17.60955122	459.6022352	0.087199207	6.530121997
23.68899809	518.3273305	0.073426503	6.805394125
21.22047341	508.0021348	0.079923336	6.511700475
11.77904191	363.8042763	0.127785976	6.336363919
23.84994507	737.308309	0.081300096	6.392160254
9.133631033	171.8640569	0.105721559	6.070156589
18.24323032	486.4410065	0.085012153	6.500155805
19.15506284	534.4541644	0.098485082	6.587517141
21.6191336	380.8476442	0.075172746	6.482122677
14.49007057	269.9421842	0.090097965	6.372913743
13.31860098	196.9440678	0.087291008	6.132515716
20.93025135	294.4756967	0.071715174	6.407009695
19.7404955	462.8071834	0.086207882	6.394934257
16.79521335	762.6702187	0.098436061	6.410860003
14.53583308	486.1276102	0.096164785	6.219491042
23.51942905	488.1308337	0.082162446	6.575671396
23.20975571	456.3589811	0.062918872	6.645953955
12.13807263	306.2081514	0.118462818	6.334943519
16.38254759	391.0147467	0.086766064	6.532534664
15.27123309	486.3965597	0.088896946	6.537600388
24.86172638	610.0316753	0.069717744	6.612510762
20.32370079	445.6988417	0.086055351	6.464400404
13.32212963	313.6046709	0.110939096	6.301647653
20.45221972	331.2906549	0.079579031	6.329969726
16.30342132	395.3757631	0.097456098	6.451212
18.85045258	349.9629463	0.093093829	6.619944352
23.03709669	535.0190896	0.086386091	6.420251971
29.87565586	675.8618743	0.067025854	6.499384939
24.78580993	591.4721099	0.075679549	6.52760021
8.561688157	132.9502868	0.150788399	5.955005702
21.12992419	381.4513206	0.072371359	6.480300842
12.02530162	263.4677605	0.092644794	6.367391579
22.410448	430.4334734	0.077272085	6.399391532
13.36575575	410.9638515	0.104398355	6.313292548
8.136158323	114.7128954	0.118759657	5.787408694
14.56768058	489.8529412	0.12094563	5.960479152
14.69192197	422.2388664	0.095380728	6.382214324
32.7874073	836.9846154	0.070498966	6.378282142
21.05901402	393.5588988	0.077180344	6.306353148
20.67855081	482.4594533	0.086991663	6.565521023
12.61232101	308.7067573	0.094937714	6.436941059
50.27115538	616.3235294	0.048540161	6.752570059
8.666063525	392.9194555	0.139424529	6.015102352
9.983726745	280.0023364	0.139542755	5.792246305
8.711608991	314.606982	0.145252516	6.081342619
38.25710362	559.8292894	0.051859405	6.488070703

log.sigma.2.0.mm.3D_gldm_DependenceNonUniformity	log.sigma.2.0.mm.3D_gldm_GrayLevelNonUniformity	log.sigma.2.0.mm.3D_gldm_SmallDependenceEmphasis
229.3275145	95.89276808	0.318628288
293.413	188.373	0.285681025
237.0948122	74.65652952	0.393684784
423.847126	212.9200978	0.322911327
135.7544784	112.3635406	0.264222113
260.3516484	157.8131868	0.28768273
113.4635762	155.461684	0.200884399
241.5573407	188.000554	0.273842192
288.7740503	191.6660089	0.281309113
548.2434232	234.6283716	0.323359258
236.9524073	208.0780299	0.245188429
358.7827893	112.7519288	0.389545213
415.2219187	313.0409696	0.248254001
345.3110872	147.1582347	0.306078683
314.7661499	112.4444444	0.34637397
564.5366269	370.2708688	0.279368912
239.4735202	162.0255452	0.256643823
342.9311962	254.7185301	0.248745764
177.3362676	102.9612676	0.307430971
399.4026198	280.1209928	0.247436384
1352.22705	464.2115108	0.346515866
369.7421028	175.8401697	0.308309793
781.2237321	310.1759282	0.331302595
161.2019417	99.19223301	0.287360553
496.0660522	174.2327189	0.340224636
264.1079882	94.44526627	0.364047716
460.7928539	179.8457711	0.395664273
706.1469449	217.8888227	0.384897733
414.3403942	393.5684025	0.248205791
466.2079772	296.1199747	0.30007067
738.3978754	327.351521	0.32100057
791.142	445.6712	0.286455009
371.5681549	251.8952983	0.285595525
66.63825364	55.16632017	0.301644881
174.7612613	67.26801802	0.355745083
399.9039478	376.1625738	0.249225254
692.5186327	247.0330625	0.364674026
565.4565025	291.7549395	0.306649989
210.1752022	168.6118598	0.227279064
205.0831296	190.3753056	0.255878751
248.4072948	109.9483283	0.353684169
251.5496894	198.9089027	0.253838384
377.6970598	357.2338949	0.235841175
231.6316348	228.388535	0.259718573
566.9793932	275.850601	0.301699836
710.8623776	254.1820979	0.356259148
698.6679327	270.7482366	0.348696345
260.8021108	129.3364116	0.307497057
75.15248227	70.37588652	0.271080274
143.6165138	128.266055	0.270051869
514.8629518	436.8504016	0.245233869
208.9591002	277.0102249	0.205545413
500.0982354	417.2976034	0.256193109
234.0913749	93.16225448	0.351773413
632.6530353	343.3032766	0.316939854
301.7309322	138.6292373	0.319116317
570.8450747	262.0686185	0.32219435
285.1800987	310.7754934	0.215772185
245.7827988	111.5437318	0.343395615
235.4113879	148.53879	0.281749603
467.5426726	250.0207412	0.299339299
756.7590669	540.3876435	0.282768314
428.2073976	170.7173052	0.346429619
306.6713062	168.3029979	0.314451095
101.1525424	51.50169492	0.338194777
310.0680493	110.6565133	0.343281063
170.9584121	91.20793951	0.31777695
508.4324173	351.022995	0.282584955
209.4098995	124.3410673	0.294457635
409.8671719	205.9812525	0.322202845
749.6018908	239.595063	0.356949248
259.7918486	244.151868	0.238779756
740.0844197	405.9784142	0.292216056
990.5896176	578.8080172	0.280080123
815.2510558	297.1370249	0.351009971
377.8803089	200.5950236	0.317805857
320.4369427	261.2615711	0.266720007
261.1883738	108.1479029	0.350021828
486.3137973	319.2661783	0.281770826
632.6568605	414.5468224	0.27681209
225.2276065	117.6578561	0.331008406
572.5287919	178.0876929	0.408784523
458.8997575	187.2312045	0.358639432
58.53728489	78.8623327	0.211399784
766.8011393	279.4981875	0.360222379
545.566494	321.8480138	0.277681091
269.9915966	110.3445378	0.348759041
434.2757148	317.6841932	0.262594367
56.88807786	48.81021898	0.264143479
83.35640138	69.90657439	0.256796733
486.0769231	306.2675179	0.293271627
232.0950226	77.90135747	0.39797298
307.8617157	120.5556978	0.375620549
681.4516425	378.6747071	0.311840719
719.5852104	446.7768806	0.266600706
194.8868778	42.90950226	0.429192664
215.9279637	245.8054453	0.218306212
54.71028037	59.72429907	0.241312476
101.9481982	128.9842342	0.24202646
293.9896014	59.8457539	0.434354051

log.sigma.2.0.mm.3D_gldm_DependenceNonUniformityNormalized	log.sigma.2.0.mm.3D_gldm_DependenceVariance	log.sigma.2.0.mm.3D_gldm_LargeDependenceEmphasis
0.190629688	3.358135556	12.28844555
0.1467065	5.9861	19.163
0.212070494	3.652714885	11.15384615
0.172787251	3.537187091	13.20464737
0.14305003	4.775895208	18.13804004
0.15894484	4.413208281	15.81929182
0.107344916	10.00915284	32.75402081
0.133826782	7.602963145	23.15844875
0.142463764	7.133856723	21.35125802
0.182565243	3.826275844	13.13253413
0.131130275	6.879333245	22.839513
0.212927471	2.634035696	9.624332344
0.141762348	5.689949961	19.86855582
0.185850962	3.463068383	12.72981701
0.203337306	2.773300883	10.51808786
0.137390272	11.58441782	28.46653687
0.149204686	5.553081201	18.8517134
0.134062235	6.207966422	21.4112588
0.156105869	5.775512671	17.81690141
0.137677566	6.26685612	21.11099621
0.194565043	3.894129703	12.38359712
0.174324424	3.609658117	13.40735502
0.189571398	3.28529797	11.9478282
0.15650674	6.640433594	19.3223301
0.190501556	3.36211394	11.91167435
0.195346145	3.403108697	11.54585799
0.208409251	2.796852463	9.987788331
0.21047599	2.897563216	10.14336811
0.121900675	8.294789643	26.21565166
0.147580873	7.288553955	20.8493194
0.178270854	3.498715447	12.87928537
0.1582284	5.00618736	16.7656
0.146806857	5.701345356	18.73370209
0.138541068	9.032913931	24.88357588
0.196803222	2.861450978	10.77477477
0.124309589	7.800932043	25.06589991
0.194037162	3.526271061	11.78565424
0.166752139	4.055111971	14.50339133
0.141627495	6.020646101	20.99191375
0.125356436	8.504718109	25.99511002
0.188759343	3.231358727	11.72796353
0.130201703	7.005137405	22.95548654
0.124776036	7.441183516	24.72117608
0.122946728	9.20141678	27.16029724
0.162272293	5.277136142	16.64796795
0.198842623	2.644874566	10.36783217
0.189546373	3.342448101	11.84807379
0.172033055	3.754690513	13.77968338
0.133249082	6.877948795	22.31560284
0.13175827	7.750458716	23.75045872
0.129232669	7.005678336	23.23945783
0.106829806	8.965140034	31.27096115
0.131708779	6.581696638	22.09454833
0.199907237	2.59895817	10.30657558
0.160694192	5.663350294	17.18745237
0.159815112	10.18873438	23.57627119
0.174091209	3.891044334	13.65690759
0.117261554	8.087125065	27.41529605
0.179141982	3.763978019	12.98396501
0.167552589	3.927282583	14.73523132
0.158974047	4.7645594	16.16695002
0.137918547	7.415131592	22.16201932
0.188554556	3.859228249	12.69616909
0.164170935	5.244928676	16.38222698
0.171444987	4.137075553	14.05423729
0.200951425	2.920584212	10.85742061
0.161586401	5.093353011	16.27599244
0.142577795	6.328429937	20.06786315
0.161956612	3.943982979	14.83449343
0.163489099	4.912487078	15.82329477
0.196849236	3.086410024	11.09138655
0.126051358	7.682495154	24.86414362
0.158171494	5.011111887	16.68241077
0.152140933	5.693835847	18.30609737
0.191283683	3.257973897	11.61661192
0.162110815	5.300430418	16.55212355
0.136066642	6.176038875	20.80382166
0.192191592	2.9868194	11.19278882
0.148447435	5.427937074	18.21794872
0.142074301	6.533946463	20.55311026
0.16536535	4.919042352	15.69016153
0.21547941	2.999317105	9.989838163
0.185488988	4.130147716	13.11560226
0.111925975	8.88419875	29.95984704
0.198550269	3.267722174	11.29207664
0.157042744	4.619226832	16.4398388
0.189069746	3.121483888	11.52661064
0.142713018	5.140283605	18.68452185
0.138413815	5.190236856	19.24817518
0.144215227	4.527424241	18.03114187
0.151378674	5.661950545	18.16723762
0.210040744	3.131799922	10.43891403
0.197094568	3.216013811	11.12932138
0.156547586	5.746486088	17.66666667
0.152908035	4.59290461	16.89800255
0.220460269	2.724509121	9.411764706
0.122477574	6.482016904	24.26715825
0.127827758	6.185147174	22.52336449
0.11480653	8.62636454	28.29279279
0.25475702	1.494066302	6.688041594

log.sigma.2.0.mm.3D_gldm_LargeDependenceLowGrayLevelEmphasis	log.sigma.2.0.mm.3D_gldm_SmallDependenceHighGrayLevelEmphasis	log.sigma.2.0.mm.3D_gldm_LargeDependenceHighGrayLevelEmphasis
0.043438674	134.6393786	4666.99335
0.042499426	142.3263554	9228.701
0.033197412	144.5570595	4399.529517
0.030061699	155.344065	6484.379943
0.149330399	49.53121664	2493.429926
0.044252345	101.923801	6238.197192
0.189473077	37.63788188	5912.458846
0.042001833	158.1438782	13331.48421
0.053889938	130.0058509	9525.939319
0.030901363	145.1917675	6063.270729
0.05529271	102.6274118	10064.73381
0.022831409	188.7989423	4997.697329
0.06635597	81.41905251	6322.607033
0.092035318	58.28387798	2546.563509
0.035595899	115.3391449	3513.531654
0.075784838	118.6303846	11280.43101
0.087724788	62.98183563	4611.46729
0.077044709	78.99003304	6387.281079
0.107916087	76.62196211	3658.275528
0.058112758	92.44166773	8239.717339
0.029785741	157.0786885	5766.846331
0.039707582	109.1576126	5033.327676
0.037128068	110.5957024	4457.914341
0.061070668	109.3830907	6694.485437
0.054605925	89.71521899	3232.421275
0.035570063	153.6145338	4564.08432
0.026734786	153.4915814	4121.238354
0.029737679	152.318373	4285.73532
0.057984418	113.450902	12267.01147
0.058718126	118.8613199	8065.395062
0.045346822	102.0840309	4371.556253
0.039052956	133.6651982	7595.8592
0.045716233	120.4030077	8249.123667
0.091582372	102.5861846	7142.143451
0.041295139	128.8314029	4530.474099
0.050657591	121.5932443	12955.05875
0.024180123	195.5469087	6435.732138
0.035354279	130.4963787	6592.933648
0.183231558	31.47377692	2713.238544
0.061939698	114.1489481	11385.00856
0.028468811	148.8714319	5562.340426
0.066230818	94.98978654	8365.557453
0.049458843	120.9535535	12817.44334
0.080566894	102.2535553	9556.299363
0.021164312	236.77303	13784.45879
0.027374213	153.9970655	4860.613706
0.026745779	170.6256336	5971.886598
0.041534821	101.7865742	5388.625989
0.164930912	63.71730975	3597.398936
0.062536054	110.5891537	9432.454128
0.069911912	91.35468853	8115.545181
0.054295984	111.1486795	18616.02352
0.05251053	117.6496077	9658.822755
0.032131657	130.7066726	3849.076857
0.043800981	144.6362683	7543.634747
0.050594631	156.8390429	11822.77966
0.032179368	162.4798638	6792.200976
0.084608893	81.59814228	9300.42023
0.024211809	239.3171327	9761.738338
0.097357579	46.26395073	2564.14306
0.038001966	143.8035746	7465.520911
0.046212324	148.5864128	11489.5965
0.042420955	127.4784871	4816.177455
0.069565735	78.02953263	4365.889722
0.088583863	64.30616915	2564.469492
0.046455791	107.1526969	2970.995463
0.039252131	144.9757924	7446.408318
0.028378222	209.2192305	14751.92092
0.032153354	130.5580622	7427.49652
0.035680032	148.2959139	7733.532509
0.029089757	159.010571	4924.015494
0.091661552	74.06911501	7071.36196
0.050132369	114.1006318	6219.00919
0.039975074	131.3744867	8861.984334
0.022870694	212.220016	6885.183717
0.041865657	129.7033569	7379.993136
0.071008807	79.10119213	6522.940552
0.040092704	113.7957768	3687.467255
0.04960567	105.6987781	7279.867827
0.067154172	89.6662311	7241.860993
0.037980067	173.0910969	8210.725404
0.016061348	258.0261932	6997.435077
0.025088261	203.719872	7839.029103
0.284391749	32.13547516	3390.797323
0.035122895	128.4779753	4447.112636
0.068385113	69.75504441	4302.875936
0.031328552	146.0278199	4903.404062
0.050468872	102.8421162	7534.236937
0.220997867	30.53681085	1901.180049
0.042853786	123.9318755	8721.586505
0.046569119	117.1308234	7448.475553
0.021585122	316.9612013	8740.655204
0.031973335	135.2254744	4533.050576
0.039296563	139.6077918	8663.312198
0.061346058	82.30891708	5042.459839
0.02496685	265.8470298	5683.696833
0.06438546	80.46502625	9455.452638
0.093111614	71.8811768	5762.343458
0.094510502	71.8014047	8841.492117
0.022873805	231.0355037	3992.194974

log.sigma.2.0.mm.3D_gldm_SmallDependenceLowGrayLevelEmphasis	log.sigma.2.0.mm.3D_gldm_LowGrayLevelEmphasis	log.sigma.2.0.mm.3D_gldzm_DistanceZoneVariabilityNormalized
0.002590055	0.005934027	1
0.001676339	0.003239281	1
0.002839465	0.004769109	1
0.002368354	0.003920645	1
0.004526566	0.011180353	1
0.002639317	0.004745124	1
0.002729894	0.007212118	1
0.001337022	0.002620618	1
0.001270523	0.003663017	1
0.001413797	0.003077205	0.996466442
0.001615838	0.003508199	1
0.002499381	0.004070979	1
0.001603667	0.004189223	1
0.005080245	0.011947624	1
0.002187953	0.004400824	1
0.001500329	0.003473662	0.993902496
0.003443733	0.007217078	1
0.001751212	0.004358361	1
0.004510749	0.010015351	1
0.001525216	0.003730294	1
0.001375755	0.0030137	0.995049535
0.002180315	0.004330749	1
0.002438293	0.00462724	0.992528125
0.003514711	0.005729989	1
0.003027167	0.006344482	1
0.002888783	0.005023867	1
0.002048344	0.003673253	1
0.002742853	0.00464069	1
0.001186154	0.002878129	0.993464123
0.002436844	0.004581336	1
0.003060736	0.005707193	1
0.001060237	0.002709601	0.998766955
0.00208113	0.003864123	1
0.00346215	0.005853442	1
0.004123068	0.007864523	1
0.00129242	0.002876613	1
0.001481136	0.002863986	1
0.001562857	0.003440828	1
0.003863595	0.011553841	1
0.001761682	0.003491872	1
0.003414568	0.004892261	1
0.00177246	0.003899605	1
0.001102226	0.002644682	1
0.002072611	0.004203329	1
0.000826951	0.001724952	1
0.00154698	0.003593466	1
0.001647146	0.003205208	0.986468828
0.002638903	0.005042023	1
0.003507653	0.011564094	1
0.001945808	0.003850511	1
0.001634621	0.00391101	1
0.001337456	0.002715625	1
0.001125981	0.002896581	0.996219295
0.002847795	0.004929041	1
0.001440991	0.003538967	0.998559079
0.00221837	0.003730309	1
0.001926684	0.00374361	0.998326361
0.002121297	0.004484549	1
0.001349828	0.003390977	1
0.005020372	0.010230712	1
0.00174893	0.003393184	1
0.001741881	0.00332989	0.997678471
0.003509328	0.006241592	1
0.004831701	0.007773534	0.997023816
0.005041146	0.009104583	1
0.003052714	0.005880592	1
0.0028219	0.004418408	1
0.000878983	0.001867671	1
0.001764027	0.003306203	1
0.00194304	0.003539741	1
0.001843107	0.003537192	1
0.001874546	0.004674292	1
0.001619575	0.003910582	1
0.001141728	0.002754226	0.996049398
0.001315943	0.002677643	1
0.002120314	0.004078232	1
0.00218259	0.004863714	1
0.003584418	0.006006128	1
0.001997413	0.004138044	1
0.002645373	0.005610208	0.998542275
0.00210144	0.004078431	1
0.001542682	0.002496832	1
0.002627376	0.003846797	0.99598798
0.00446689	0.011869542	1
0.002779031	0.005076811	0.996266348
0.002173246	0.005332535	0.996343705
0.002329183	0.004131746	1
0.001571969	0.00370477	0.99772469
0.00927816	0.017112364	1
0.004649452	0.006293776	1
0.001528946	0.003306553	1
0.002338093	0.004463447	1
0.003556774	0.005333929	1
0.00211915	0.003727522	1
0.001900043	0.004606778	0.998577526
0.003048128	0.004848304	1
0.001584515	0.003631857	1
0.003554353	0.006548321	1
0.002604537	0.00515109	1
0.003238339	0.005843739	0.996661111

log.sigma.2.0.mm.3D_gldzm_LowIntensityEmphasis	log.sigma.2.0.mm.3D_gldzm_LargeDistanceEmphasis	log.sigma.2.0.mm.3D_gldzm_HighIntensitySmallDistanceEmphasis
0.008486168	1	425.2056893
0.005484631	1	502.9814815
0.006728537	1	370.6015474
0.006898878	1	488.4459459
0.017303935	1	191.2380952
0.008682852	1	356.5357143
0.013041317	1	194.2576419
0.004602004	1	590.0437956
0.005115227	1	470.3510972
0.004184024	1.005309735	451.9285398
0.00628593	1	419.2271805
0.0060945	1	489.5033201
0.006291199	1	330.1204969
0.017183496	1	192.2163743
0.005925828	1	337.7902208
0.005185755	1.009174312	423.2155963
0.012732064	1	246.4810127
0.006727701	1	325.415493
0.015389917	1	251.2179487
0.006014249	1	378.6237745
0.003842076	1.007444169	454.8204537
0.006610751	1	357.2075718
0.006903693	1.01125	338.8889063
0.011048134	1	391.819242
0.008567066	1	264.9808795
0.007599786	1	426.2756757
0.004945016	1	392.9646465
0.006724675	1	397.4175017
0.004658379	1.009836066	461.8243169
0.00780276	1	400.1019645
0.009079296	1	319.0241672
0.003576026	1.001850709	470.2720543
0.006875414	1	428.3300248
0.010457545	1	351.7030303
0.011983916	1	372
0.005126266	1	488.6102088
0.003938496	1	538.6856369
0.00507024	1	427.7950043
0.017249762	1	140.4134367
0.006473793	1	454.3819742
0.008873108	1	428.1140684
0.00657158	1	381.7142857
0.004543745	1	520.8754814
0.007714386	1	398.8549618
0.002605032	1	788.5798319
0.004549163	1	435.9354396
0.004539884	1.020435967	488.7648161
0.008270908	1	332.5505618
0.015893391	1	235.9651163
0.006801866	1	416.1353846
0.006478731	1	375.8265403
0.006301849	1	539.1830986
0.004311676	1.005681818	464.8269413
0.007545266	1	370.9037657
0.004795782	1.002162942	461.5243331
0.006394263	1	498.831669
0.005796685	1.002512563	509.4974874
0.0092918	1	387.5951134
0.004595846	1	702.7063197
0.01688656	1	165.3534483
0.005582449	1	489.5404299
0.005904244	1.003486345	532.2424463
0.009922382	1	372.210643
0.01415061	1.004470939	253.6125186
0.014303314	1	193.8090909
0.008325329	1	313.97411
0.008226586	1	460.8803191
0.002963455	1	749.456912
0.005734577	1	443.9642005
0.005766575	1	467.5395764
0.004905408	1	448.0196825
0.007604089	1	318.7546468
0.005498834	1	394.9677211
0.003993128	1.005937655	467.2742454
0.003624997	1	611.4722222
0.006549557	1	414.1368039
0.008141121	1	296.4460857
0.009542693	1	329.4981752
0.006899073	1	375.986421
0.009719943	1.002188184	327.3014223
0.006349044	1	523.3111546
0.003571789	1	638.5996732
0.006819371	1.006030151	570.2371859
0.019659038	1	161.0564516
0.007451419	1.005610973	359.8095387
0.007627542	1.005494505	254.2042125
0.006378446	1	422.1592129
0.00593279	1.003416856	399.1560364
0.033285689	1	123.887931
0.017216169	1	487.9746835
0.004961596	1	410.3572111
0.006298764	1	811.4503968
0.00882049	1	366.5619335
0.006502384	1	450.1211718
0.006888881	1.002135231	312.3281139
0.006819386	1	617.7283105
0.006842943	1	377.9198113
0.013751587	1	308.7433628
0.010319779	1	301.2121212
0.007510969	1.005016722	536.7127926

log.sigma.2.0.mm.3D_gldzm_LowIntensityLargeDistanceEmphasis	log.sigma.2.0.mm.3D_gldzm_HighIntensityEmphasis	log.sigma.2.0.mm.3D_gldzm_DistanceZoneVariability	log.sigma.2.0.mm.3D_gldzm_ZonePercentage
0.008486168	425.2056893	457	0.379883624
0.005484631	502.9814815	648	0.324
0.006728537	370.6015474	517	0.462432916
0.006898878	488.4459459	888	0.362005707
0.017303935	191.2380952	273	0.287671233
0.008682852	356.5357143	532	0.324786325
0.013041317	194.2576419	229	0.216650899
0.004602004	590.0437956	548	0.303601108
0.005115227	470.3510972	638	0.314750863
0.004204018	452.2929204	1126.00708	0.376290376
0.00628593	419.2271805	493	0.272827892
0.0060945	489.5033201	753	0.446884273
0.006291199	330.1204969	805	0.274837829
0.017183496	192.2163743	684	0.368137783
0.005925828	337.7902208	634	0.409560724
0.005354102	423.7041284	1300.024465	0.318325627
0.012732064	246.4810127	474	0.295327103
0.006727701	325.415493	710	0.277560594
0.015389917	251.2179487	390	0.343309859
0.006014249	378.6237745	816	0.281282316
0.003861095	455.7398086	2807.034739	0.405899281
0.006610751	357.2075718	766	0.361150401
0.006973883	339.519375	1588.045	0.388255278
0.011048134	391.819242	343	0.333009709
0.008567066	264.9808795	1046	0.401689708
0.007599786	426.2756757	555	0.410502959
0.004945016	392.9646465	990	0.447761194
0.006724675	397.4175017	1497	0.446199702
0.004690472	462.7628415	909.0196721	0.269196823
0.00780276	400.1019645	1069	0.338398227
0.009079296	319.0241672	1531	0.369628199
0.003578763	470.5848242	1619.001234	0.3242
0.006875414	428.3300248	806	0.318451205
0.010457545	351.7030303	165	0.343035343
0.011983916	372	365	0.411036036
0.005126266	488.6102088	862	0.267951508
0.003938496	538.6856369	1476	0.413561222
0.00507024	427.7950043	1161	0.34237688
0.017249762	140.4134367	387	0.260781671
0.006473793	454.3819742	466	0.284841076
0.008873108	428.1140684	526	0.399696049
0.00657158	381.7142857	553	0.286231884
0.004543745	520.8754814	779	0.257350512
0.007714386	398.8549618	524	0.278131635
0.002605032	788.5798319	1190	0.340583858
0.004549163	435.9354396	1456	0.407272727
0.004598669	490.979564	1448.13624	0.3982637
0.008270908	332.5505618	534	0.352242744
0.015893391	235.9651163	172	0.304964539
0.006801866	416.1353846	325	0.298165138
0.006478731	375.8265403	1055	0.264809237
0.006301849	539.1830986	426	0.217791411
0.004325814	465.4327652	1052.007576	0.278114301
0.007545266	370.9037657	478	0.408198121
0.004800687	461.7627974	1385.001442	0.352298705
0.006394263	498.831669	701	0.371292373
0.005806499	509.6582915	1192.001675	0.364135407
0.0092918	387.5951134	573	0.235608553
0.004595846	702.7063197	538	0.39212828
0.01688656	165.3534483	464	0.33024911
0.005582449	489.5404299	977	0.332199932
0.005917585	532.5671121	1717.004648	0.313650447
0.009922382	372.210643	902	0.397181858
0.014173421	253.8315946	669.0029806	0.359207709
0.014303314	193.8090909	220	0.372881356
0.008325329	313.97411	618	0.400518471
0.008226586	460.8803191	376	0.355387524
0.002963455	749.456912	1114	0.31239484
0.005734577	443.9642005	419	0.324052591
0.005766575	467.5395764	897	0.357798165
0.004905408	448.0196825	1575	0.413602941
0.007604089	318.7546468	538	0.261038331
0.005498834	394.9677211	1549	0.331053644
0.004052617	467.6249381	2013.015834	0.310397788
0.003624997	611.4722222	1692	0.396996715
0.006549557	414.1368039	826	0.354354354
0.008141121	296.4460857	677	0.287473461
0.009542693	329.4981752	548	0.403237675
0.006899073	375.986421	1031	0.314713065
0.009726005	327.4989059	1369.001459	0.307882327
0.006349044	523.3111546	511	0.375183554
0.003571789	638.5996732	1224	0.460669928
0.006832522	571.0723618	991.0080402	0.4021827
0.019659038	161.0564516	124	0.23709369
0.0074779	360.1508728	1598.011222	0.415328845
0.007689546	254.3470696	1088.007326	0.31433506
0.006378446	422.1592129	559	0.391456583
0.005946137	399.3747153	876.0022779	0.288531055
0.033285689	123.887931	116	0.282238443
0.017216169	487.9746835	158	0.273356401
0.004961596	410.3572111	1047	0.326066646
0.006298764	811.4503968	504	0.456108597
0.00882049	366.5619335	662	0.423815621
0.006502384	450.1211718	1502	0.345049391
0.006922244	312.3622776	1403.001423	0.298555036
0.006819386	617.7283105	438	0.495475113
0.006842943	377.9198113	424	0.240499149
0.013751587	308.7433628	113	0.264018692
0.010319779	301.2121212	231	0.260135135
0.007524866	537.1655518	596.0033445	0.518197574

log.sigma.2.0.mm.3D_gldzm_IntensityVariabilityNormalized	log.sigma.2.0.mm.3D_gldzm_LowIntensitySmallDistanceEmphasis	log.sigma.2.0.mm.3D_gldzm_IntensityVariability
0.053909763	0.008486168	24.63676149
0.051183128	0.005484631	33.16666667
0.048460655	0.006728537	25.05415861
0.049630712	0.006898878	44.07207207
0.057172376	0.017303935	15.60805861
0.055846854	0.008682852	29.71052632
0.077973341	0.013041317	17.8558952
0.055916671	0.004602004	30.64233577
0.057630133	0.005115227	36.76802508
0.052511551	0.004179026	59.3380531
0.063612687	0.00628593	31.36105477
0.045905797	0.0060945	34.56706507
0.060011574	0.006291199	48.30931677
0.059377244	0.017183496	40.61403509
0.055647882	0.005925828	35.2807571
0.052040373	0.005143669	68.06880734
0.05761185	0.012732064	27.30801688
0.0581829	0.006727701	41.30985915
0.058737673	0.015389917	22.90769231
0.058808511	0.006014249	47.9877451
0.048851596	0.003837321	137.8103509
0.054499656	0.006610751	41.74673629
0.053759375	0.006886146	86.015
0.052588632	0.011048134	18.03790087
0.052392982	0.008567066	54.80305927
0.04531775	0.007599786	25.15135135
0.057206408	0.004945016	56.63434343
0.047338052	0.006724675	70.86506346
0.052166383	0.004650356	47.73224044
0.051810484	0.00780276	55.38540692
0.052651473	0.009079296	80.60940562
0.059262662	0.003575341	96.06477483
0.050763812	0.006875414	40.91563275
0.052708907	0.010457545	8.696969697
0.04792644	0.011983916	17.49315068
0.053644199	0.005126266	46.2412993
0.043627581	0.003938496	64.39430894
0.050468091	0.00507024	58.59345392
0.073453118	0.017249762	28.42635659
0.054053307	0.006473793	25.1888412
0.05284882	0.008873108	27.79847909
0.054409779	0.00657158	30.08860759
0.055370352	0.004543745	43.13350449
0.056530797	0.007714386	29.6221374
0.047581385	0.002605032	56.62184874
0.050640208	0.004549163	73.73214286
0.048175798	0.004525188	70.72207084
0.055373199	0.008270908	29.56928839
0.055029746	0.015893391	9.465116279
0.058215385	0.006801866	18.92
0.060087599	0.006478731	63.39241706
0.061804316	0.006301849	26.3286385
0.054784349	0.004308141	57.85227273
0.056844243	0.007545266	27.17154812
0.050974415	0.004794556	70.70151406
0.04313992	0.006394263	30.24108417
0.047787794	0.005794231	57.05862647
0.061094207	0.0092918	35.0069808
0.0489352	0.004595846	26.32713755
0.070767687	0.01688656	32.8362069
0.051377171	0.005582449	50.19549642
0.048738943	0.005900909	83.87972109
0.047288361	0.009922382	42.654102
0.057349286	0.014144907	38.48137109
0.060578512	0.014303314	13.32727273
0.048171888	0.008325329	29.77022654
0.049527501	0.008226586	18.62234043
0.053329745	0.002963455	59.40933573
0.058412745	0.005734577	24.47494033
0.044365648	0.005766575	39.79598662
0.046764424	0.004905408	73.65396825
0.059106425	0.007604089	31.79925651
0.052962802	0.005498834	82.03938025
0.054184404	0.003978255	109.5066799
0.045981926	0.003624997	77.80141844
0.047957132	0.006549557	39.6125908
0.05726236	0.008141121	38.76661743
0.048917097	0.009542693	26.80656934
0.053062154	0.006899073	54.7070805
0.049362086	0.009718428	67.6754194
0.046549301	0.006349044	23.78669276
0.043965462	0.003571789	53.81372549
0.045502891	0.006816083	45.27537688
0.071540062	0.019659038	8.870967742
0.050730406	0.007444798	81.37157107
0.060860538	0.007612041	66.45970696
0.049049382	0.006378446	27.41860465
0.059907846	0.005929453	52.59908884
0.075505351	0.033285689	8.75862069
0.055439833	0.017216169	8.759493671
0.055121788	0.004961596	57.71251194
0.043390967	0.006298764	21.86904762
0.051190661	0.00882049	33.88821752
0.045996372	0.006502384	69.08655126
0.057856917	0.006880541	81.28896797
0.035653969	0.006819386	15.61643836
0.073535956	0.006842943	31.17924528
0.068525335	0.013751587	7.743362832
0.073068346	0.010319779	16.87878788
0.042225478	0.007507495	25.25083612

log.sigma.2.0.mm.3D_gldzm_HighIntensityLargeDistanceEmphasis	log.sigma.2.0.mm.3D_gldzm_SmallDistanceEmphasis	log.sigma.2.0.mm.3D_glcm_SumVariance	log.sigma.2.0.mm.3D_glcm_Homogeneity1
425.2056893	1	1207.780269	0.345877895
502.9814815	1	1547.503953	0.390770185
370.6015474	1	1162.529521	0.313967255
488.4459459	1	1512.823528	0.357644862
191.2380952	1	403.2968693	0.416470662
356.5357143	1	1194.92619	0.369277784
194.2576419	1	557.2856322	0.485998547
590.0437956	1	1927.851807	0.417415425
470.3510972	1	1475.82268	0.405574115
453.7504425	0.998672566	1403.478414	0.347868459
419.2271805	1	1367.131729	0.409981953
489.5033201	1	1534.844828	0.308857005
330.1204969	1	976.6931481	0.406713656
192.2163743	1	527.8935144	0.358032734
337.7902208	1	1053.602146	0.330237665
425.6582569	0.997706422	1303.648786	0.409698227
246.4810127	1	714.0639024	0.400491052
325.415493	1	956.4570707	0.418280431
251.2179487	1	622.0138818	0.395473892
378.6237745	1	1234.91809	0.402563372
459.4172279	0.998138958	1430.348436	0.322355012
357.2075718	1	1090.338893	0.355574514
342.04125	0.9971875	1089.68858	0.33799813
391.819242	1	1118.848375	0.391204046
264.9808795	1	768.6957484	0.328876173
426.2756757	1	1216.679686	0.328604542
392.9646465	1	1254.060739	0.347570782
397.4175017	1	1236.812198	0.311016366
466.5169399	0.997540984	1497.019639	0.425066273
400.1019645	1	1237.207404	0.388638518
319.0241672	1	977.8729337	0.345838589
471.8359038	0.999537323	1450.323278	0.391040014
428.3300248	1	1337.48348	0.382758023
351.7030303	1	988.6294699	0.418149891
372	1	1203.046959	0.326116146
488.6102088	1	1643.686675	0.424248674
538.6856369	1	1685.458228	0.324280906
427.7950043	1	1350.227441	0.351631021
140.4134367	1	348.4928795	0.432271963
454.3819742	1	1421.493667	0.417721665
428.1140684	1	1408.449691	0.333715611
381.7142857	1	1170.050437	0.41746148
520.8754814	1	1710.304332	0.42829275
398.8549618	1	1182.465161	0.432828611
788.5798319	1	2732.76591	0.352149035
435.9354396	1	1367.957753	0.321143569
499.8385559	0.994891008	1510.619738	0.336133857
332.5505618	1	1139.13917	0.35215209
235.9651163	1	516.9750671	0.429544694
416.1353846	1	1285.443552	0.417260546
375.8265403	1	1111.789232	0.420491607
539.1830986	1	1943.618785	0.454729555
467.8560606	0.998579545	1410.523289	0.415480108
370.9037657	1	1136.70533	0.333554689
462.7166547	0.999459265	1395.083313	0.372197957
498.831669	1	1607.662528	0.357300661
510.3015075	0.999371859	1549.336727	0.355629824
387.5951134	1	1122.453549	0.442929714
702.7063197	1	2390.991727	0.339378904
165.3534483	1	473.8570165	0.385804849
489.5404299	1	1483.147942	0.37940619
533.8657757	0.999128414	1669.446542	0.39820435
372.210643	1	1099.412092	0.332274551
254.7078987	0.998882265	784.1277837	0.381561375
193.8090909	1	552.5761459	0.376536011
313.97411	1	820.8660836	0.338799261
460.8803191	1	1406.559656	0.369134059
749.456912	1	2515.911227	0.395050595
443.9642005	1	1520.440914	0.364191026
467.5395764	1	1492.30618	0.36014187
448.0196825	1	1374.385947	0.322690573
318.7546468	1	922.3801512	0.431933628
394.9677211	1	1155.052163	0.377829457
469.0277091	0.998515586	1519.138925	0.380069598
611.4722222	1	1896.805515	0.335802679
414.1368039	1	1361.158098	0.358673633
296.4460857	1	953.9316877	0.39827521
329.4981752	1	964.1761211	0.331736187
375.986421	1	1211.869351	0.37780687
328.2888403	0.999452954	1030.800303	0.38263554
523.3111546	1	1676.587167	0.360726857
638.5996732	1	2136.243262	0.303173387
574.4130653	0.998492462	1843.016239	0.334877867
161.0564516	1	342.5834907	0.487381119
361.5162095	0.998597257	1118.415091	0.328075856
254.9184982	0.998626374	772.1921017	0.389365292
422.1592129	1	1271.199866	0.337349186
400.2494305	0.999145786	1269.311926	0.396834491
123.887931	1	286.636846	0.441100548
487.9746835	1	1547.415243	0.409768463
410.3572111	1	1279.806011	0.384712167
811.4503968	1	2709.43909	0.31252067
366.5619335	1	1189.981762	0.32153201
450.1211718	1	1456.963709	0.362644562
312.4989324	0.999466192	918.7604501	0.392825968
617.7283105	1	1882.059753	0.279764919
377.9198113	1	1240.833646	0.441233539
308.7433628	1	832.5060054	0.457485849
301.2121212	1	975.4046645	0.466371232
538.9765886	0.998745819	1701.759774	0.270320816

log.sigma.2.0.mm.3D_glcm_Homogeneity2	log.sigma.2.0.mm.3D_glcm_ClusterShade	log.sigma.2.0.mm.3D_glcm_MaximumProbability	log.sigma.2.0.mm.3D_glcm_Idmn	log.sigma.2.0.mm.3D_glcm_SumVariance2
0.259009385	71.99208656	0.030846052	0.977828658	46.3368613
0.314494738	-42.67280909	0.043411209	0.984519668	42.16812051
0.228466738	-209.8930917	0.026423216	0.963466889	51.05973345
0.275271228	-116.7973812	0.027596792	0.980539169	44.68199109
0.339736895	231.0890157	0.053708129	0.982548103	32.88181468
0.28736027	-117.0024871	0.039010094	0.978393265	32.34184819
0.422849109	58.8483223	0.101428843	0.985689853	17.9380797
0.34543447	-87.61931816	0.056422024	0.986359114	39.77982637
0.329972063	-8.622499439	0.047215887	0.983030836	34.82800814
0.261364064	-69.24359136	0.022375526	0.979212114	39.15928218
0.334689583	-77.71718488	0.045857836	0.984818062	28.00006568
0.221828383	-267.2601103	0.017939036	0.974548434	58.77820418
0.327796589	19.818662	0.041926861	0.98821682	26.58207416
0.27265459	-56.45792464	0.024199335	0.97095445	34.52229892
0.240773623	11.24404348	0.018769916	0.974602353	36.39673307
0.337479667	-89.35542919	0.057251308	0.983237605	40.39203275
0.321876937	-25.12118472	0.045185926	0.980260924	31.5160004
0.342620129	20.94313773	0.048358839	0.986049469	31.03910041
0.317139302	113.0784651	0.04986353	0.980039402	41.00621574
0.325295169	-94.63948673	0.037479543	0.981315071	30.97181576
0.236025564	-138.0018096	0.015412478	0.98107958	51.80872285
0.270402758	-71.18194057	0.030505123	0.98230274	37.40843513
0.25209855	-162.2081091	0.020667051	0.97338615	41.6525023
0.313549158	11.85617701	0.047695832	0.980693965	41.04598335
0.24080654	-71.37081129	0.019578324	0.982453348	42.73630636
0.242573084	-108.8307406	0.027823312	0.972561075	55.91988106
0.262001261	-30.88034308	0.023404725	0.980161146	38.13632938
0.223951501	-228.6122136	0.018984588	0.966687346	51.91582225
0.355145054	-60.71331526	0.059998163	0.986643184	31.57457573
0.312304339	-56.86129812	0.049421068	0.98286844	38.83041953
0.259769144	-167.5154116	0.023355254	0.977354489	40.15533819
0.310029036	12.45356509	0.031569875	0.988407272	34.25054699
0.305647597	-107.6872143	0.044491184	0.984047365	42.50421288
0.344030212	179.8220374	0.070925994	0.98292599	40.33375533
0.242322178	-350.9647422	0.034248008	0.963879892	54.64039484
0.354733788	-176.1917016	0.058265629	0.98358206	35.97883504
0.239633567	-163.9730151	0.021890413	0.975451012	64.11053552
0.268422185	-143.6027588	0.029938216	0.9783374	41.52919524
0.356170361	18.0634723	0.051304458	0.982367535	19.06239566
0.345760744	17.67106766	0.060663459	0.983397858	35.76219306
0.247537885	-158.5675501	0.02317596	0.975446374	40.01540833
0.343108734	10.61758849	0.048023847	0.984324382	31.73730761
0.35750504	-39.77000091	0.059069813	0.988597653	33.82783078
0.36173361	122.5611373	0.07120546	0.986957712	31.40514495
0.271699277	-184.5457364	0.027871834	0.98458258	49.32298261
0.233303723	-147.9523393	0.018539811	0.975073932	50.54785316
0.251867517	-178.4344811	0.022716511	0.9784583	55.01242046
0.269067839	-282.8488185	0.031397816	0.970868201	43.11748292
0.357465325	212.6991217	0.070578193	0.981851475	38.26241586
0.343827584	48.03757974	0.059715864	0.983796683	29.07399408
0.345865488	11.10905485	0.044484857	0.989613973	25.74849714
0.390698988	-196.5175975	0.083371858	0.983273985	27.05333116
0.34164857	19.85899067	0.050045106	0.987450447	30.02084983
0.244829023	-87.65556384	0.023027339	0.974605847	38.94652819
0.291832134	-13.6354092	0.035642999	0.981116905	39.93478105
0.280282055	-92.9714368	0.040776422	0.97583551	57.95606003
0.272033416	-98.55717458	0.03286436	0.98104758	52.24562214
0.373016283	100.2095025	0.073648907	0.988277578	27.88466562
0.254058225	-500.4681021	0.025667935	0.982114806	55.64628548
0.301504493	-38.73330547	0.036201621	0.977056418	19.98274433
0.298783995	-56.99743905	0.036771761	0.985753144	45.44672551
0.325086323	-180.2503614	0.050389478	0.98361079	46.06192882
0.247595235	-171.095596	0.024141199	0.975875246	48.22045997
0.30207355	-130.1402598	0.03570474	0.972957403	35.36197641
0.293533856	42.90588358	0.03662279	0.972070241	31.27788099
0.250671221	197.3174954	0.025440774	0.97862251	50.77940879
0.290061462	109.6524397	0.035446288	0.982047518	48.28064228
0.319093647	-144.9502664	0.044022189	0.986883699	42.96595066
0.282084648	-139.7572163	0.031633464	0.980309756	34.85159621
0.281341449	-173.1131541	0.034772465	0.979851004	54.14209457
0.236205456	-111.1465192	0.015185067	0.973452633	55.16054029
0.360392143	94.52200072	0.057683669	0.985391418	29.48779887
0.296796786	-20.25912885	0.037748245	0.986511848	39.37439237
0.300816379	-148.9596486	0.033538049	0.980008211	35.68050751
0.250536503	-169.3201813	0.021558258	0.981510914	64.92137897
0.278547575	-201.2274607	0.035616995	0.974680615	46.0722426
0.322040282	-72.0742273	0.045697331	0.983495651	28.48610665
0.243821815	1.461774459	0.026514184	0.976456307	47.67273352
0.298761876	-163.3782543	0.03823816	0.979128547	37.171642
0.307474465	-137.8164183	0.040484616	0.981004735	42.59997447
0.282864065	-282.9823143	0.036885978	0.976032573	54.61173103
0.217435283	-451.296333	0.021462174	0.97593035	70.84094065
0.251193399	-259.114362	0.029100388	0.980304153	58.853515
0.425551315	113.3828604	0.090004712	0.9842257	21.94758809
0.240796853	-180.1727549	0.028624897	0.973180063	46.82438075
0.30798028	11.08754793	0.035527291	0.979908307	29.73051802
0.252013353	-41.69742183	0.028597559	0.977350721	53.13124889
0.317541529	-49.17486228	0.042449116	0.98340059	30.93743505
0.366437527	37.93615479	0.063741779	0.975083666	19.19924431
0.333180687	-140.2496925	0.051579109	0.984804502	35.14996592
0.305993443	-5.353646908	0.046567679	0.98203931	34.36786535
0.227509649	-617.159545	0.026429527	0.973362507	69.21034159
0.234391072	-265.6654701	0.025890079	0.968133638	44.58330434
0.284366079	-215.1147174	0.040743796	0.977734426	47.66234531
0.31163205	-16.51162137	0.031897557	0.985117148	28.68423634
0.198475604	-874.8554655	0.01414689	0.963467584	126.9655156
0.370191697	-45.42851098	0.063055572	0.988489129	19.68169355
0.39018447	70.56786311	0.088164955	0.986712842	24.12651511
0.402227166	-30.01948729	0.0762806	0.98523198	19.99660713
0.180971207	-523.9873486	0.011905583	0.970046723	95.97876936

log.sigma.2.0.mm.3D_glcm_Contrast	log.sigma.2.0.mm.3D_glcm_DifferenceEntropy	log.sigma.2.0.mm.3D_glcm_InverseVariance	log.sigma.2.0.mm.3D_glcm_Entropy	log.sigma.2.0.mm.3D_glcm_Dissimilarity
26.90234534	3.414811621	0.267812289	7.526935414	3.757742163
25.96253355	3.340095609	0.305924781	7.371082968	3.434512037
40.37714795	3.709210804	0.234257329	7.972271208	4.686559265
31.46679998	3.479402806	0.279173185	7.583577202	3.863985652
13.80282728	2.989425229	0.333914485	6.589013162	2.68013973
24.75422517	3.347707967	0.293016685	7.246132302	3.507815649
8.127417903	2.653513665	0.382701026	5.988912305	2.008781225
23.09698381	3.205266481	0.328540463	7.030249225	3.11191702
20.56251676	3.19666858	0.320340543	7.178142313	3.086257718
26.39986687	3.442532591	0.259317021	7.730244574	3.766363035
20.43025675	3.190847969	0.316507853	6.925121301	3.065725916
46.03709895	3.772700882	0.226812424	8.100318985	4.859296014
16.43581173	3.075109412	0.320942241	6.968063546	2.847075225
21.97685557	3.315140987	0.275820375	7.558057888	3.495660186
26.8015208	3.448353565	0.249025491	7.714692858	3.91550063
26.87492889	3.351798775	0.30089403	7.549874281	3.417577762
18.46543018	3.158870441	0.311563121	7.087613144	3.021864725
14.39362136	3.014836252	0.333540558	7.027529802	2.709828829
18.30584765	3.162303322	0.309757178	7.170680494	3.082000922
20.11426046	3.182010863	0.315744195	7.20668657	3.075431814
37.9149544	3.687164263	0.234809049	8.28658578	4.490916364
24.85374391	3.380713661	0.273502421	7.552055295	3.629107018
30.62687121	3.5144165	0.259264819	7.862261973	4.023635389
21.90527452	3.246425604	0.307555369	7.099585431	3.265246838
30.23725775	3.533113975	0.245007901	7.966282612	4.088594671
38.5134179	3.629853529	0.249398174	7.856352526	4.386905586
28.11913447	3.465750884	0.261854751	7.608257638	3.846786495
41.73615592	3.739100183	0.230117616	8.216052239	4.717349336
21.03547535	3.167681771	0.33295593	6.989799044	3.026342187
28.80161635	3.414415966	0.290931686	7.489678955	3.609824489
29.113621	3.485393424	0.261665752	7.773154869	3.882814582
17.75660053	3.165449039	0.30697844	7.399898224	3.03725844
27.90175073	3.395430556	0.298281979	7.405199375	3.593713472
17.93208146	3.118208608	0.310473264	6.608585605	2.956905131
44.01460119	3.690539899	0.252030547	7.647855461	4.640188845
24.04835653	3.20845522	0.336007608	6.997344786	3.123825669
41.96587197	3.720445699	0.242274615	8.238720158	4.603383069
31.29707316	3.524336113	0.265217519	7.709325458	3.955705144
9.960619407	2.818349091	0.345886495	6.597815276	2.367002442
21.24854347	3.21144867	0.320620064	6.915192748	3.093296123
33.79071159	3.572560059	0.250148745	7.586180889	4.183726589
17.57145183	3.113949296	0.328365544	7.022140391	2.883180989
19.94263789	3.120781619	0.339755221	6.92015288	2.903920369
16.31704255	3.053899458	0.339387065	6.715490276	2.741469879
35.5333098	3.575132295	0.271897211	7.91865633	4.131974514
35.9495964	3.642557421	0.238792124	8.116974748	4.381363042
36.34137175	3.617644482	0.256322986	8.051734544	4.274333231
30.23878252	3.479544538	0.275528985	7.574804469	3.886988217
14.38582207	2.983754044	0.337956863	6.394343251	2.685481603
18.19267025	3.121258744	0.329571144	6.724092334	2.926515934
16.084388	3.049434685	0.338782571	6.887738812	2.753057733
22.65232833	3.072234747	0.353988883	6.501312559	2.858427496
18.52891259	3.141540208	0.330236627	7.008188902	2.939477815
29.36476619	3.465615656	0.257288164	7.587762966	3.943971833
27.30641964	3.406900597	0.288871079	7.589386089	3.611813852
43.51862401	3.715748355	0.247427914	8.042216097	4.529261563
30.58303144	3.480014618	0.271909185	7.807329062	3.84427799
13.80497138	2.931096974	0.357827604	6.586758919	2.52239374
40.69277367	3.602527139	0.254568248	7.625055545	4.313622109
15.8360443	3.092714013	0.303079106	6.913434819	2.950589994
26.43642048	3.343376444	0.294956702	7.543792281	3.474223228
30.91067681	3.394428117	0.309599217	7.479815032	3.584343686
36.94271733	3.627512821	0.251269755	7.887495124	4.340286552
22.46304106	3.295814608	0.300916908	7.370206915	3.354952303
17.80151214	3.171702127	0.293893333	7.086684142	3.171305485
25.42613063	3.418778471	0.257893497	7.794584266	3.780781095
30.27566509	3.451431827	0.285987987	7.420965463	3.779480184
25.42787466	3.305737905	0.313273625	7.35866747	3.351181184
26.72302521	3.394260949	0.284900978	7.311953591	3.643405208
38.02732773	3.580592284	0.276836813	7.797862825	4.153230078
36.38069958	3.652188016	0.240844856	8.27250716	4.410943173
15.30377432	3.022261456	0.343970974	6.786038529	2.685873749
23.27127005	3.31688793	0.294405612	7.567390064	3.389998806
25.80575294	3.373066145	0.293553434	7.557601861	3.508043893
36.09508034	3.601440624	0.254880788	8.169815887	4.228328146
33.25368293	3.541830504	0.27356574	7.661638607	4.006971779
22.28061139	3.241428093	0.318179132	7.035130737	3.21163304
28.07889084	3.487884076	0.250210754	7.686917766	3.977537312
27.08884788	3.381441816	0.2955672	7.406603161	3.571143422
32.16929805	3.471781189	0.295711703	7.61129168	3.793611499
40.05390362	3.586660638	0.281137149	7.597461133	4.206551352
52.05618721	3.861179025	0.225180155	8.319727152	5.148561799
40.47618434	3.676315193	0.25017143	7.969322993	4.43790371
7.45161448	2.565780157	0.398597309	5.773322085	1.947962956
32.89918427	3.5787217	0.249554351	7.943177699	4.185780144
17.15413585	3.167482607	0.303281352	7.30923609	3.039116553
32.59413834	3.552336754	0.256867608	7.751306543	4.112541629
18.8438006	3.149854541	0.319728601	7.050877554	3.016032315
8.871692065	2.675631037	0.359527399	6.111230077	2.236085802
20.87522759	3.112468328	0.32196134	6.554222114	3.02021334
22.72513864	3.300991685	0.304007032	7.307644181	3.344023145
58.24436627	3.816215653	0.237795471	7.856416728	5.129491862
37.61101367	3.651314372	0.239895546	7.776083266	4.441056372
36.19845851	3.574825809	0.276000728	7.782582954	4.100032873
17.62315994	3.137277802	0.313314884	7.257478294	2.987253298
85.83365706	4.140694329	0.200228326	8.520598289	6.58312234
11.88009267	2.859047321	0.366934411	6.309713435	2.408407258
10.46880135	2.755929349	0.380212916	6.0135736	2.257421613
12.86961147	2.847366618	0.374347223	6.152802853	2.362328042
62.17030307	3.994640201	0.185256238	8.532444154	5.849489815

log.sigma.2.0.mm.3D_glcm_DifferenceVariance	log.sigma.2.0.mm.3D_glcm_Idn	log.sigma.2.0.mm.3D_glcm_Idm	log.sigma.2.0.mm.3D_glcm_Correlation	log.sigma.2.0.mm.3D_glcm_Autocorrelation
12.58812436	0.905206178	0.259009385	0.267126207	379.6789193
14.06092685	0.925301151	0.314494738	0.234774625	475.0848942
18.09856119	0.879889507	0.228466738	0.123029427	365.7039242
16.35963389	0.915057882	0.275271228	0.176970928	464.392938
6.519851179	0.915812338	0.339736895	0.384098924	142.3084342
12.31580924	0.908941604	0.28736027	0.135612273	372.1855672
4.013546504	0.925082623	0.422849109	0.360233837	185.8585721
13.32484051	0.932016318	0.34543447	0.263885179	578.0626111
10.93976803	0.92113077	0.329972063	0.25963108	453.1754792
12.05784125	0.907138217	0.261364064	0.197925691	435.0365442
10.87837863	0.925526766	0.334689583	0.163906086	419.9568026
22.06621999	0.900328567	0.221828383	0.123916013	471.7916621
8.199393403	0.931208728	0.327796589	0.236883099	310.2559761
9.471509454	0.890371316	0.27265459	0.221713344	182.0774624
11.16764326	0.895450802	0.240773623	0.150703597	334.9551762
14.97690293	0.924480681	0.337479667	0.204071991	405.9900735
9.187040477	0.912805445	0.321876937	0.257187953	235.6205484
6.921882022	0.924719634	0.342620129	0.361676445	307.56035
8.484767651	0.910879374	0.317139302	0.369072409	211.0862477
10.43569036	0.916899941	0.325295169	0.215489993	385.1229337
17.50406833	0.911859902	0.236025564	0.157436568	443.9856395
11.43976605	0.914477622	0.270402758	0.203574184	345.6249734
14.0267926	0.897033212	0.25209855	0.159141613	345.6225913
11.00574865	0.914574876	0.313549158	0.300901279	352.6135478
13.29429085	0.912981772	0.24080654	0.176456615	253.6169476
19.03403325	0.898136307	0.242573084	0.187843792	382.2189869
13.13664969	0.910219811	0.262001261	0.15645598	391.6738139
19.1385707	0.885470799	0.223951501	0.114867111	387.3575554
11.59098521	0.931908539	0.355145054	0.205423209	456.8709906
15.59992741	0.922172216	0.312304339	0.151299152	385.7810738
13.84445205	0.905229489	0.259769144	0.16266409	312.9663484
8.406952697	0.930217016	0.310029036	0.315107234	448.651431
14.78328564	0.923898249	0.305647597	0.210181392	415.0333414
8.987410736	0.919359374	0.344030212	0.373350995	315.4050087
22.1158398	0.885846213	0.242322178	0.117015488	375.2963679
14.17967365	0.927348656	0.354733788	0.20025621	497.9753878
20.51985843	0.902981117	0.239633567	0.209506047	516.5414946
15.46932196	0.908718792	0.268422185	0.145106037	418.718844
4.285593853	0.912355025	0.356170361	0.309121866	125.9221619
11.60372203	0.923332483	0.345760744	0.254865838	436.692337
16.0751639	0.901523084	0.247537885	0.090602551	433.7952486
9.189493275	0.92344328	0.343108734	0.282547749	366.9799666
11.4077049	0.937306855	0.35750504	0.258501413	516.7197493
8.714251506	0.930678168	0.36173361	0.306955121	368.0391394
18.17992546	0.923514698	0.271699277	0.165018658	802.2780067
16.55952991	0.8996358	0.233303723	0.172571685	426.2177629
17.83244185	0.908875728	0.251867517	0.20792354	466.9381125
14.96938128	0.895351908	0.269067839	0.178902722	358.2094755
7.023937311	0.916167471	0.357465325	0.415192482	176.4345363
9.550720703	0.922529155	0.343827584	0.232912048	396.6180592
8.424656598	0.936636371	0.345865488	0.233690293	348.0535922
14.34537658	0.930356809	0.390698988	0.094501799	574.3682319
9.7840428	0.931452187	0.34164857	0.238166366	433.404191
13.6062508	0.898556545	0.244829023	0.141816773	357.9958584
14.12168382	0.915945341	0.291832134	0.188383509	431.5643687
22.69777275	0.907412341	0.280282055	0.148323234	493.147362
15.60335432	0.915175475	0.272033416	0.262034623	477.2098328
7.334842901	0.933772106	0.373016283	0.334138806	349.9530734
21.87048563	0.919556084	0.254058225	0.160521545	706.899471
7.01843832	0.901711866	0.301504493	0.117182058	162.8727984
14.21538941	0.927606413	0.298783995	0.265637045	458.303796
17.86439321	0.926668795	0.325086323	0.196732066	507.3014615
17.74586134	0.903348454	0.247595235	0.138548235	347.7922391
11.05878411	0.898880832	0.30207355	0.223467262	257.1245309
7.536753264	0.89173088	0.293533856	0.27132531	189.0484519
10.98800407	0.90386365	0.250671221	0.329219248	270.8184575
15.82518019	0.918554115	0.290061462	0.230697472	435.3153462
14.08121158	0.931585846	0.319093647	0.258314396	741.7296908
13.31268181	0.912842699	0.282084648	0.137858408	465.0736633
20.49853027	0.915974682	0.281341449	0.177535786	459.6409642
16.64738047	0.896681664	0.236205456	0.208167317	429.408965
8.006718196	0.926008061	0.360392143	0.309189737	294.9110756
11.62170048	0.92696886	0.296796786	0.258952833	365.0225316
13.3488744	0.914001671	0.300816379	0.16373897	465.5708044
17.93508673	0.915269644	0.250536503	0.286008736	577.2539278
17.0312958	0.903798716	0.278547575	0.164515044	422.0625349
11.84618206	0.922460591	0.322040282	0.127094825	302.4372358
12.05975508	0.899686526	0.243821815	0.246899069	310.8406039
14.11744789	0.912872946	0.298761876	0.163066123	377.8631635
17.58808328	0.919007723	0.307474465	0.145384458	327.2688493
22.17957835	0.909843997	0.282864065	0.154888838	509.4666188
25.21728919	0.903580648	0.217435283	0.157967783	640.1907497
20.56284102	0.913732193	0.251193399	0.18833226	558.7000633
3.520646201	0.920879153	0.425551315	0.475207923	122.4203474
15.16805479	0.896124481	0.240796853	0.178147883	354.4777559
7.848211824	0.908764653	0.30798028	0.267161443	254.0015703
15.47447514	0.905195372	0.252013353	0.241435604	398.9800327
9.642298368	0.920116233	0.317541529	0.243942901	393.8451191
3.666589606	0.897150997	0.366437527	0.355152348	105.9608616
11.61399304	0.926849232	0.333180687	0.254049182	468.7899949
11.4377361	0.917106043	0.305993443	0.205870984	398.0993917
31.51785856	0.903619711	0.227509649	0.084816371	793.4713287
17.6427777	0.888445152	0.234391072	0.093523232	371.7621662
19.11987915	0.911155491	0.284366079	0.140630065	448.8622426
8.602122202	0.922399664	0.31163205	0.239673935	295.7545904
41.54009278	0.884706172	0.198475604	0.201742719	567.7956665
6.000664642	0.932369838	0.370191697	0.249377306	380.6226228
5.289514355	0.927950274	0.39018447	0.381911118	267.141033
7.186338049	0.928401394	0.402227166	0.214985745	305.9219905
27.45022384	0.889496737	0.180971207	0.219458205	520.8417786

log.sigma.2.0.mm.3D_glcm_SumEntropy	log.sigma.2.0.mm.3D_glcm_AverageIntensity	log.sigma.2.0.mm.3D_glcm_Energy	log.sigma.2.0.mm.3D_glcm_SumSquares	log.sigma.2.0.mm.3D_glcm_ClusterProminence
4.640521026	19.56589243	0.009390574	17.78940102	10168.86
4.607745386	21.84658223	0.01258293	16.01059374	8682.545783
4.768140619	19.34637418	0.006479002	22.86431391	9401.109084
4.629537309	21.64679597	0.009726232	17.7143449	11552.21565
4.208531618	11.73769001	0.01974303	11.88049775	6245.935328
4.389216075	19.43788603	0.012137913	13.98968967	5991.929059
3.861058169	13.54209653	0.03211356	6.5163744	1628.259963
4.460059757	24.05240142	0.017500688	14.95126491	9276.481658
4.447366812	21.38681331	0.014028705	13.16049615	6709.769287
4.625068547	20.99392933	0.007849249	15.49946213	6411.318611
4.29901895	20.6333133	0.015383614	11.10363964	5235.562182
4.87492971	21.88875901	0.005642012	24.47540625	15126.26467
4.259716684	17.68313035	0.014735753	10.62578074	4371.169572
4.54213523	13.37807679	0.007997794	14.15118318	4387.849107
4.578910757	18.46650435	0.006824585	16.24295814	4879.279955
4.587982482	20.24515611	0.012475321	15.99281041	8280.922391
4.36117507	15.26973355	0.013959927	12.7455576	5260.891156
4.415580965	17.51880736	0.014161234	11.78897183	4142.4284
4.558609318	14.36385564	0.013035231	15.24120854	7392.823706
4.412579174	19.71697417	0.012856318	12.67039964	4981.64268
4.848223654	21.20470882	0.005154028	21.37526602	9861.224777
4.563883322	18.72413792	0.009238241	15.00197314	6150.419454
4.65989176	18.74297838	0.007148908	17.9263935	7297.485872
4.470170574	18.77417358	0.014066394	15.6524716	9306.183899
4.710449805	16.10739008	0.005821028	18.19812833	6302.235122
4.807522217	19.74340676	0.007541696	22.43048968	14644.16234
4.584570171	19.94407889	0.008292378	15.52150929	6526.135445
4.810974959	19.94111208	0.0055767	22.68081981	10923.55433
4.344481489	21.4482665	0.019049741	12.30898712	6203.296332
4.53714344	19.76419128	0.012436845	15.94998431	8785.051676
4.603218258	17.82492737	0.008104688	17.19305745	7616.89698
4.536637638	21.20782753	0.010216239	12.21769545	5268.362193
4.579247702	20.46747924	0.013157951	16.38490784	9713.403538
4.407611848	17.69078789	0.020251751	15.00787351	6611.143922
4.719440528	19.60932798	0.00862413	23.76481403	15063.59661
4.40042068	22.37783825	0.018551385	13.88573074	8362.675769
4.944843048	22.82940055	0.006348383	25.17462465	17729.14537
4.623949007	20.61756641	0.008884382	17.00315024	9216.850316
4.089072355	11.11951893	0.017165013	7.255753766	1530.714041
4.394732639	20.96952956	0.019144824	13.27968499	7471.904477
4.58826768	21.04272764	0.008243362	16.93960247	8097.026636
4.388746942	19.17601952	0.01545019	12.29966125	5056.78722
4.365116979	22.75740619	0.019339038	12.78143169	6851.93251
4.243749257	19.20095345	0.021590075	11.70642873	5795.235975
4.725452413	28.55844274	0.008326259	20.32434603	13074.4458
4.816598798	20.80115422	0.005873241	20.29410734	10206.24395
4.848387211	21.71609	0.006831514	21.3074661	13703.56546
4.576418123	19.04923195	0.009581925	18.39402505	9180.363168
4.230935963	13.07180392	0.024041543	13.44348975	7102.337291
4.24832349	19.99347833	0.019581433	11.62077041	4665.303839
4.227392494	18.75611679	0.016902148	9.887685956	4084.342477
4.107468181	24.06130413	0.025749792	11.35409659	6404.485025
4.343229267	20.88157644	0.01667051	11.35565133	5180.008156
4.5823861	19.07045286	0.008001007	17.02403979	7157.334296
4.583466852	20.87437125	0.010785628	15.76836977	7939.273114
4.884729748	22.38473224	0.007904757	24.26160323	13971.99129
4.749140675	21.88707546	0.008754725	19.73267859	15050.94543
4.141023171	18.7448744	0.022487429	10.43146627	5294.080359
4.708933867	26.81961431	0.008642676	22.57242395	20324.00101
4.138766213	12.72144092	0.012899293	8.954697157	1675.462442
4.67631943	21.4243863	0.010193787	16.99682177	10425.14373
4.5868089	22.5874603	0.014374811	18.26133211	13311.89497
4.724670313	18.87446596	0.007417108	20.11240556	11043.55117
4.504856167	15.9490811	0.011022799	14.57679534	5862.149543
4.420756096	13.62586807	0.011362327	12.26984828	3119.415899
4.774817696	16.35441244	0.007216333	19.41718193	9789.206329
4.656875838	20.93089061	0.010542981	18.42786943	11478.95039
4.579402643	27.30274924	0.013433502	16.61779611	10971.44847
4.493289772	21.71944882	0.010543061	14.16303255	6154.071814
4.766930436	21.53394526	0.009718204	21.82138687	15054.0676
4.895958531	20.86783908	0.005015198	21.8827671	11518.71255
4.257169587	17.13241655	0.019422045	11.52192857	5074.705653
4.597755105	19.18285356	0.010152762	14.7968452	7492.473662
4.523931504	21.70246006	0.010218011	14.39538429	6497.496102
4.95050331	24.06587238	0.00651333	24.45301379	18861.44916
4.66790427	20.69721403	0.009868795	18.58099173	11383.65207
4.271069476	17.54051159	0.016201454	12.54992238	4867.688698
4.707825429	17.69175668	0.008002777	19.25973452	10431.10807
4.473619943	19.57972936	0.012368246	15.42307824	7995.363024
4.601106313	18.26360716	0.011897742	17.78459601	9372.629113
4.707530916	22.68716061	0.010546098	22.2111969	18295.18986
4.971338039	25.52148849	0.005686595	29.31046163	22471.75433
4.839484661	23.75367211	0.007577657	23.44328224	17500.68768
3.891900981	10.89883284	0.034883265	7.349800643	2246.675847
4.734532367	18.99408445	0.00721203	19.41620284	9587.222076
4.428730697	15.86317268	0.011014332	11.93512126	3491.970994
4.790092329	20.08229347	0.008159063	20.25338489	12354.45247
4.347578687	19.92729847	0.014942057	12.17100415	5511.255956
3.980823485	10.16669879	0.022312155	7.017734093	1156.974316
4.249975038	21.68757026	0.017687648	13.02355034	10223.96412
4.467683699	20.06648513	0.01295968	13.51818488	5541.546207
4.85682768	28.51448132	0.007458861	29.70482693	30024.44585
4.627995503	19.52206964	0.007911387	20.09083245	9643.174433
4.69646619	21.34837378	0.010071279	19.38729532	11328.55564
4.39961156	17.23208524	0.011570562	11.77059729	3817.747212
5.329744481	24.08817068	0.004042392	51.46431371	52729.16815
3.973925846	19.57132036	0.024946066	7.914630954	2870.609019
4.046841388	16.24399733	0.029821985	8.712069896	2903.986681
3.969682031	17.4531911	0.028919161	8.318314639	2461.490244
5.19997415	22.99163138	0.003733049	37.93389046	27949.93294

log.sigma.2.0.mm.3D_glcm_SumAverage	log.sigma.2.0.mm.3D_glcm_Imc2	log.sigma.2.0.mm.3D_glcm_Imc1	log.sigma.2.0.mm.3D_glcm_DifferenceAverage	log.sigma.2.0.mm.3D_glcm_Id
38.720168	0.772058103	-0.114668669	3.757742163	0.345877895
43.40606555	0.736734776	-0.102856775	3.434512037	0.390770185
38.1066177	0.759202116	-0.102650441	4.686559265	0.313967255
42.94558446	0.699501285	-0.085297864	3.863985652	0.357644862
23.45242748	0.758910376	-0.129195738	2.68013973	0.416470662
38.48569073	0.668627258	-0.079091094	3.507815649	0.369277784
27.08419307	0.700857513	-0.114431341	2.008781225	0.485998547
47.91177771	0.766471689	-0.119255502	3.11191702	0.417415425
42.40743637	0.721217656	-0.09801424	3.086257718	0.405574115
41.56168293	0.601478031	-0.057260849	3.766363035	0.347868459
40.89297746	0.667644915	-0.082001417	3.065725916	0.409981953
43.29448342	0.742652784	-0.095345815	4.859296014	0.308857005
35.08321222	0.630077337	-0.072624239	2.847075225	0.406713656
26.7528374	0.626486138	-0.068634482	3.495660186	0.358032734
36.47204027	0.661496128	-0.074041931	3.91550063	0.330237665
40.13014328	0.711708098	-0.09077561	3.417577762	0.409698227
30.48595599	0.695973868	-0.091413689	3.021864725	0.400491052
34.8357305	0.683606126	-0.090007841	2.709828829	0.418280431
28.65948631	0.770201031	-0.124924351	3.082000922	0.395473892
39.11041668	0.630121153	-0.073116614	3.075431814	0.402563372
41.97663815	0.539997589	-0.042067522	4.490916364	0.322355012
37.01258209	0.66414389	-0.076495956	3.629107018	0.355574514
37.03318869	0.552140774	-0.050510933	4.023635389	0.33799813
37.29988799	0.799879171	-0.137462209	3.265246838	0.391204046
31.65372756	0.601988611	-0.056377808	4.088594671	0.328876173
38.87580894	0.789663452	-0.116854214	4.386905586	0.328604542
39.45431946	0.683178542	-0.079697805	3.846786495	0.347570782
39.23307168	0.609303081	-0.055803596	4.717349336	0.311016366
42.62551061	0.660930013	-0.085576351	3.026342187	0.425066273
39.15467375	0.684197069	-0.081508264	3.609824489	0.388638518
35.22517031	0.576479078	-0.05202754	3.882814582	0.345838589
42.16710515	0.610743071	-0.064560449	3.03725844	0.391040014
40.56482111	0.692525283	-0.0856795	3.593713472	0.382758023
35.20040949	0.866813167	-0.195694603	2.956905131	0.418149891
38.60681006	0.827649248	-0.1397984	4.640188845	0.326116146
44.49647691	0.67637589	-0.084542546	3.123825669	0.424248674
45.21069632	0.671495313	-0.071100264	4.603383069	0.324280906
40.79978615	0.613941848	-0.059842806	3.955705144	0.351631021
22.23903785	0.610295311	-0.071881534	2.367002442	0.432271963
41.61982705	0.736964364	-0.107703643	3.093296123	0.417721665
41.58009567	0.708499578	-0.087339092	4.183726589	0.333715611
38.12695569	0.699547739	-0.093415646	2.883180989	0.41746148
45.30967417	0.697806852	-0.093329764	2.903920369	0.42829275
38.17041303	0.704489434	-0.100698681	2.741469879	0.432828611
56.52712005	0.679860979	-0.076524436	4.131974514	0.352149035
41.11206473	0.595656979	-0.052995164	4.381363042	0.321143569
43.00067896	0.653465976	-0.067201279	4.274333231	0.336133857
37.6821916	0.721452787	-0.093035145	3.886988217	0.35215209
26.10651718	0.84053356	-0.184786256	2.685481603	0.429544694
39.69353685	0.735423247	-0.110491734	2.926515934	0.417260546
37.18237749	0.623669703	-0.069788855	2.753057733	0.420491607
47.88600358	0.702833675	-0.099858565	2.858427496	0.454729555
41.49817378	0.640775787	-0.074472483	2.939477815	0.415480108
37.71462635	0.732890724	-0.097733052	3.943971833	0.333554689
41.39565897	0.631608564	-0.065703467	3.611813852	0.372197957
44.25089439	0.775564558	-0.108524203	4.529261563	0.357300661
43.4412187	0.697890768	-0.08292016	3.84427799	0.355629824
37.22509676	0.689167206	-0.097074816	2.52239374	0.442929714
53.0341888	0.775122244	-0.113610292	4.313622109	0.339378904
25.44288184	0.567615389	-0.0559664	2.950589994	0.385804849
42.59314019	0.710000206	-0.089900592	3.474223228	0.37940619
44.87797294	0.667550414	-0.078335444	3.584343686	0.39820435
37.14605204	0.686897585	-0.078547677	4.340286552	0.332274551
31.86789099	0.66868885	-0.078066444	3.354952303	0.381561375
27.25173615	0.776520268	-0.124088425	3.171305485	0.376536011
32.52487017	0.741927206	-0.098796363	3.780781095	0.338799261
41.51102944	0.808161335	-0.132877571	3.779480184	0.369134059
54.30789705	0.671692783	-0.079928457	3.351181184	0.395050595
43.03620094	0.698717002	-0.087151966	3.643405208	0.364191026
42.69002264	0.730744083	-0.09435805	4.153230078	0.36014187
41.21680553	0.618800187	-0.058590587	4.410943173	0.322690573
34.13773916	0.693989206	-0.095321692	2.685873749	0.431933628
37.99896217	0.631323445	-0.066949194	3.389998806	0.377829457
43.03955042	0.55078652	-0.048283022	3.508043893	0.380069598
47.7499927	0.681082955	-0.076637519	4.228328146	0.335802679
40.93137178	0.694414847	-0.082072651	4.006971779	0.358673633
34.69200474	0.642112679	-0.073407071	3.21163304	0.39827521
34.98042122	0.726918821	-0.095811611	3.977537312	0.331736187
38.74740929	0.638197989	-0.069059087	3.571143422	0.37780687
36.03662313	0.645636388	-0.068883835	3.793611499	0.38263554
44.9811035	0.807278554	-0.129104406	4.206551352	0.360726857
50.41796892	0.699200474	-0.078199908	5.148561799	0.303173387
47.07860327	0.716410602	-0.087094791	4.43790371	0.334877867
21.79766568	0.80043731	-0.175297296	1.947962956	0.487381119
37.46937236	0.579775157	-0.051738907	4.185780144	0.328075856
31.67678723	0.568203747	-0.053345124	3.039116553	0.389365292
39.69014515	0.767033612	-0.107615429	4.112541629	0.337349186
39.53757734	0.634489076	-0.073450132	3.016032315	0.396834491
20.33339759	0.784770913	-0.152806194	2.236085802	0.441100548
43.13662895	0.818487093	-0.157486029	3.02021334	0.409768463
39.75815006	0.651713091	-0.073969681	3.344023145	0.384712167
56.23941736	0.827753157	-0.13798885	5.129491862	0.31252067
38.47138286	0.691003608	-0.080165756	4.441056372	0.32153201
42.23696334	0.636347061	-0.065889278	4.100032873	0.362644562
34.23349261	0.561255061	-0.053120458	2.987253298	0.392825968
47.2230766	0.920033148	-0.198343271	6.58312234	0.279764919
38.91880437	0.626066901	-0.078930006	2.408407258	0.441233539
32.47811063	0.811946775	-0.169322315	2.257421613	0.457485849
34.87907969	0.715158818	-0.114007404	2.362328042	0.466371232
45.27150256	0.8314916	-0.129219361	5.849489815	0.270320816

log.sigma.2.0.mm.3D_glcm_ClusterTendency	log.sigma.2.0.mm.3D_firstorder_InterquartileRange	log.sigma.2.0.mm.3D_firstorder_Skewness	log.sigma.2.0.mm.3D_firstorder_Uniformity
46.3368613	106.3442962	-0.200051687	0.079711362
42.16812051	90.26536584	-0.570637096	0.0941865
51.05973345	145.3834057	-0.514450219	0.06677686
44.68199109	94.99416542	-0.736108856	0.086799877
32.88181468	68.25151157	1.00440907	0.118402045
32.34184819	90.50059795	-0.914415354	0.096345047
17.9380797	54.98707938	0.302350483	0.147078225
39.77982637	80.24375582	-0.830049413	0.104155432
34.82800814	95.00056529	-0.345700875	0.094556492
39.15928218	110.4363751	-0.46569407	0.078131326
28.00006568	71.17170469	-0.959622379	0.115151096
58.77820418	134.0330601	-0.799612795	0.066915091
26.58207416	78.73082638	-0.080691649	0.106876398
34.52229892	117.8424234	-0.406508664	0.079202494
36.39673307	127.0939164	-0.009606727	0.07263853
40.39203275	93.31952286	-0.404685344	0.090112161
31.5160004	84.7043438	-0.178607146	0.100950495
31.03910041	91.84847164	-0.007087061	0.099577221
41.00621574	101.7046144	0.35529205	0.090634919
30.97181576	85.98549747	-0.813270744	0.096560149
51.80872285	136.2168064	-0.533722472	0.066793023
37.40843513	105.7224522	-0.466295037	0.08290437
41.6525023	118.6572552	-0.724343194	0.075267151
41.04598335	85.91764891	-0.164501473	0.096303139
42.73630636	143.0360832	-0.210027561	0.066909646
55.91988106	125.7580265	-0.51568485	0.069855966
38.13632938	105.458787	-0.485037713	0.081341371
51.91582225	140.0001421	-0.674932712	0.064944507
31.57457573	70.33183074	-0.563193639	0.115789468
38.83041953	91.10175514	-0.436428014	0.093738517
40.15533819	113.8375993	-0.693325734	0.079032236
34.25054699	98.23483528	-0.247641176	0.08913424
42.50421288	79.06564021	-0.717691729	0.099524021
40.33375533	79.98017693	0.445582775	0.114690894
54.64039484	117.8246212	-1.069376362	0.075752273
35.97883504	68.18409204	-1.206155184	0.116929616
64.11053552	122.4248142	-0.509576575	0.069216325
41.52919524	97.97615004	-0.778522678	0.086038024
19.06239566	74.93492031	0.067484384	0.113619852
35.76219306	69.22714996	-0.386058781	0.116366324
40.01540833	103.2668192	-1.089550309	0.083547362
31.73730761	82.38390732	-0.285335667	0.102954919
33.82783078	70.24966806	-0.624363561	0.118015823
31.40514495	69.07880676	0.244193691	0.121225337
49.32298261	108.1053519	-0.744582946	0.0789498
50.54785316	123.7454042	-0.679864889	0.071099888
55.01242046	119.0604634	-0.720585104	0.07345313
43.11748292	105.441165	-1.003125346	0.085314256
38.26241586	69.14153829	0.817106623	0.124779941
29.07399408	68.65343386	-0.207189779	0.11767528
25.74849714	76.59127057	-0.139391277	0.109651205
27.05333116	57.97027636	-1.786305628	0.141620769
30.02084983	75.42519855	-0.247053556	0.109901923
38.94652819	110.1769371	-0.565377062	0.07955786
39.93478105	93.01091337	-0.40230673	0.087199207
57.95606003	115.8207655	-0.451137586	0.073426503
52.24562214	105.151751	-0.489150618	0.079923336
27.88466562	65.5113709	0.338466761	0.127785976
55.64628548	102.8339837	-1.417620948	0.081300096
19.98274433	84.99172354	-0.518750189	0.105721559
45.44672551	103.0245972	-0.575878569	0.085012153
46.06192882	82.39048004	-0.692324578	0.098485082
48.22045997	114.3067322	-0.696890375	0.075172746
35.36197641	93.68446281	-0.751026209	0.090097965
31.27788099	103.4588269	-0.019526082	0.087291008
50.77940879	122.4628115	0.261853558	0.071715174
48.28064228	94.74905252	-0.12716492	0.086207882
42.96595066	85.48606837	-0.845341565	0.098436061
34.85159621	88.39842176	-1.095254048	0.096164785
54.14209457	98.94448328	-0.680504288	0.082162446
55.16054029	142.95856	-0.375009697	0.062918872
29.48779887	69.85705256	0.240443802	0.118462818
39.37439237	102.2823401	-0.316343374	0.086766064
35.68050751	96.37948036	-0.841721407	0.088896946
64.92137897	120.9364519	-0.494527109	0.069717744
46.0722426	97.85005379	-0.887037555	0.086055351
28.48610665	73.1777072	-0.608848778	0.110939096
47.67273352	102.9567007	-0.089111868	0.079579031
37.171642	85.74577951	-0.929709437	0.097456098
42.59997447	86.59025764	-0.632761477	0.093093829
54.61173103	91.1530664	-0.894308347	0.086386091
70.84094065	129.3001213	-0.875487342	0.067025854
58.853515	110.2759681	-0.84152226	0.075679549
21.94758809	61.15396351	0.857779401	0.150788399
46.82438075	123.5347781	-0.762570674	0.072371359
29.73051802	95.55238461	-0.171053293	0.092644794
53.13124889	108.0779892	-0.487582307	0.077272085
30.93743505	81.09810632	-0.721634403	0.104398355
19.19924431	84.78182209	0.2386829	0.118759657
35.14996592	70.7999377	-1.083519228	0.12094563
34.36786535	89.11411483	-0.461085992	0.095380728
69.21034159	122.7948483	-1.320235575	0.070498966
44.58330434	110.3166327	-0.9965859	0.077180344
47.66234531	94.95901775	-0.901826592	0.086991663
28.68423634	90.28054237	-0.312704354	0.094937714
126.9655156	211.8375702	-0.550513325	0.048540161
19.68169355	57.49474852	-0.76792587	0.139424529
24.12651511	65.8137877	0.114150178	0.139542755
19.99660713	55.81184095	-0.753014949	0.145252516
95.97876936	200.4753904	-0.642635566	0.051859405

log.sigma.2.0.mm.3D_firstorder_MeanAbsoluteDeviation	log.sigma.2.0.mm.3D_firstorder_Energy	log.sigma.2.0.mm.3D_firstorder_RobustMeanAbsoluteDeviation	log.sigma.2.0.mm.3D_firstorder_Median
79.89328622	18247391.78	47.51426079	46.92956924
71.18223929	24200805.9	40.92636705	35.37108994
91.84359135	16180600.19	60.56999255	26.43486786
74.76246113	32156507.03	42.21976663	38.34294128
61.21051567	9004044.2	32.01985108	25.92497253
67.5767853	15734338.88	39.08840077	29.20950031
47.29626724	5859591.864	26.14471908	22.38938141
66.73037637	20822096.65	37.33547415	36.80317307
67.82106827	22809615.88	41.39376842	39.90931702
77.10015869	36633590.16	48.3378046	45.9368515
57.53860747	14983501.72	32.2656857	33.85533142
92.63593371	29116988.97	56.84921034	48.53727341
59.10553818	23715462.73	34.38307514	28.62988281
73.80740229	18098637.89	49.58379954	35.07472992
79.18749859	18211236.62	53.3745644	33.72694397
71.58946637	43045630.09	42.39743137	14.63436127
64.02256614	13811443.28	36.89713138	24.99701309
64.42473993	26675036.15	39.71699869	42.5052681
73.17859798	14636980.73	43.91247688	40.59467316
64.29875988	26073826.22	38.47010278	32.24647522
88.72360081	94659503.78	58.51131747	29.11057377
74.01312059	23689900.46	45.79052105	40.06553268
79.78993054	49336067.58	51.00079738	38.23395157
71.32603831	12399008.13	39.28089647	30.29096699
85.97221437	30872612.68	59.50157726	18.53811073
90.8006201	25285146.87	55.29215008	57.10404205
74.46661948	24903388.66	46.42826311	35.72042084
93.3813216	53690319.77	60.30406229	46.21909714
60.77838096	30580153.87	32.04910912	26.63341141
70.72340784	35961547.53	40.52029116	26.70719719
78.76756002	50455150.53	48.86512303	43.42965508
66.97942666	52184463.69	42.17902868	45.18401337
70.73740581	30136822.31	37.62865883	34.16951752
70.40928625	6152743.804	40.1671612	37.60403442
89.17197105	14335479.89	51.54112065	43.30911064
60.99700606	30762904.64	31.07391861	29.32106781
91.55934033	61937524.2	54.25231883	41.30029678
75.19714489	41702685.71	43.88929154	39.46047211
52.42810433	8045508.855	32.70424509	19.48151207
63.52379191	17917941.53	33.33178788	36.0751915
75.69945628	17036731.93	44.85472549	49.75575066
64.10675562	19899137.26	36.65455826	37.10127068
60.44524639	29173984.31	31.86170041	31.22804451
61.07204927	19081073.35	32.60605628	32.07767105
80.8473702	46762681.38	47.78024318	30.26018429
85.32265307	51410734.93	53.46203947	45.81694412
85.28808678	56084443.72	51.12304343	45.74675941
78.71060764	17300712.64	47.56939586	26.28352451
67.45340152	6557006.571	35.44628939	26.22973537
60.37464709	10308528.33	32.51170306	31.97469234
58.38119549	32658385.32	33.64490479	29.65607357
52.21131007	14339549.39	25.39057954	22.60255241
60.96572879	36806612.41	33.81037921	35.3332901
76.19888717	13823812.5	47.80284084	37.41058731
73.59873676	48697741.51	43.07885135	35.47810745
87.06227459	28549966.65	51.68808829	25.37719917
80.0048902	48219001.6	46.16398773	38.95044327
56.93742028	20231536.31	29.99423535	20.82862949
82.27347244	21571891.76	45.78404742	42.00038528
56.2400148	9367010.313	35.96740855	37.17414856
75.15444166	41790862.78	45.45049235	48.92417908
71.86284805	70387922.17	38.02183662	31.24565125
84.19722028	33731249.12	50.7442285	46.59053421
68.78095991	19305027.07	42.17578779	43.60896683
69.32987606	6413197.826	45.67267147	47.54693604
85.37174679	24185269.26	54.19034783	42.9331131
75.78518418	14521732.08	42.39549915	36.92107201
69.23630095	44592136.31	39.20143431	40.15073013
66.88562845	12903198.71	38.9079842	40.4149704
83.54056395	38601367.22	45.98319527	36.47256088
92.25471716	59142170.57	61.07909942	38.68026352
58.25415283	19033960.19	31.0226827	34.91817856
72.30300313	57537530.43	43.73863541	42.87083435
69.98231551	67648911.82	42.03563159	38.86584091
89.49335857	73574128.97	54.09300405	42.91080284
77.70702176	31302575.32	43.52888526	35.47587585
61.428378	20429232.57	32.84259576	27.15296936
80.39961819	21143201.82	47.21717622	56.63111496
68.88317072	35080344.01	38.13712589	32.6849308
75.10491633	53724474.37	41.66853648	28.8664856
80.42513103	20606509.37	42.8909997	33.25116348
98.20540805	50947641.33	57.43780933	41.31230545
85.76101017	40697965.75	48.63833108	40.31342888
52.0811072	3634536.766	29.12912503	25.48588181
85.03888292	56244633.9	53.06805958	49.95602989
64.10141794	29894513.22	40.17835573	34.41682816
83.78634025	22346141.64	49.05465381	48.32502747
62.71178727	29257759.55	35.73531418	35.16805649
55.52021254	2909323.651	37.86222794	30.67595291
59.50899266	5873573.234	30.0405161	36.08707809
67.50859988	35039473.23	39.89645721	40.82495117
96.71732568	24988382.44	53.54722958	56.58038712
82.6700974	21115306.52	49.2701077	33.28293037
78.87783038	58715221.92	43.80044007	37.58942413
63.98934985	45421487.56	38.95895537	43.92514038
136.989992	27856360.53	90.75635285	39.60772896
48.6834681	10842452.26	26.16700432	28.21368408
54.28611972	3668902.219	30.16738071	38.36596489
47.82895975	5282076.223	24.91758118	27.00154495
121.1809644	28838708.04	82.07859452	62.94493675

log.sigma.2.0.mm.3D_firstorder_TotalEnergy	log.sigma.2.0.mm.3D_firstorder_Maximum	log.sigma.2.0.mm.3D_firstorder_RootMeanSquared	log.sigma.2.0.mm.3D_firstorder_90Percentile	log.sigma.2.0.mm.3D_firstorder_Minimum
492679578.1	387.3197632	123.1594057	193.6515411	-423.8396606
653421759.2	451.1410522	110.0018316	167.2265747	-484.3484192
436876205.1	319.930481	120.3029873	152.7493027	-425.6919861
868225689.9	433.4772034	114.4947729	156.852774	-478.5336914
243109193.4	404.7761536	97.40599856	164.8902527	-243.4085083
424827149.7	332.3538208	98.00930308	115.857225	-448.012146
158208980.3	265.6838379	74.45539809	126.0896469	-296.2915039
562196609.6	408.9588013	107.404784	163.7118195	-539.8084717
615859628.7	345.7139282	106.0796581	168.1868225	-451.2470093
989106934.3	371.212616	110.449073	162.7342773	-452.2300415
404554546.6	388.462738	91.05998717	117.5656845	-456.972168
786158702.2	494.561615	131.4538404	171.7602539	-494.5368347
640317493.6	481.5785828	89.98209815	125.769693	-399.2616577
488663223.1	347.5612183	98.69612248	137.3295456	-299.2077332
491703388.8	371.862915	108.4636563	169.9356506	-383.4338074
1162232012	460.3030701	102.3520308	135.664624	-469.3490906
372908968.5	370.6731567	92.76454392	115.2914536	-343.1166687
720225976	378.4325562	102.1179854	170.1754898	-361.861969
395198479.7	405.7362366	113.5106433	181.7157059	-293.7168884
703993308.1	316.9549255	94.80440545	135.1832581	-426.2193604
2555806602	565.3598633	116.7050662	148.2736084	-477.9921875
639627312.4	466.11203	105.6844967	144.8092499	-403.6888123
1332073825	369.7541809	109.4160312	147.2738647	-403.467804
334773219.5	370.5891113	109.7172364	175.3327759	-424.5262146
833560542.3	606.0415039	108.8845354	138.4906235	-350.4043884
682698965.5	447.8752136	136.7553718	208.9387207	-407.1513977
672391493.9	434.0151062	106.1291906	149.1325531	-445.5655823
1449638634	367.5916748	126.5032645	170.5873138	-433.1013489
825664154.6	425.9787903	94.85151039	125.7457367	-482.5794983
970961783.4	507.4454651	106.6950751	147.9896454	-448.9926147
1362289064	439.8995972	110.369152	145.3040237	-397.1023254
1408980520	466.023407	102.1611117	158.71241	-451.3510742
813694202.4	519.7908936	109.1195726	143.2079315	-463.5065002
166124082.7	395.2803345	113.0998106	198.0075226	-372.009552
387057956.9	345.2225952	127.0573037	149.7485535	-435.8050232
830598425.4	363.2124329	97.78857988	120.437027	-503.4490662
1672313153	436.8952026	131.7357495	184.4812317	-517.1470947
1125972514	414.1842651	110.896571	142.9243164	-462.462677
217228739.1	324.2862549	73.630849	107.0180649	-226.524704
483784421.2	367.6111755	104.6531748	160.8366928	-473.1305237
459991762.1	376.8148193	113.779809	154.4785614	-463.9205627
537276706	364.6217957	101.4877356	165.2005524	-415.2120972
787697576.3	452.0031128	98.17290947	139.7175812	-504.3693542
515188980.5	406.2378845	100.6377576	161.528035	-424.9401245
1262592397	470.837738	115.6879839	145.6870422	-662.4993286
1388089843	405.8132324	119.9192443	163.8709137	-471.0488281
1514279980	454.0639343	123.3512466	165.9854813	-491.4386597
467119241.2	290.9673767	106.8273353	114.9456253	-434.2645264
177039177.4	385.3766479	107.8234584	200.7703339	-267.8404846
278330264.9	328.9763184	97.24898683	147.3017578	-441.7932739
881776403.6	513.6334839	90.53941613	133.8805664	-418.7561035
387167833.5	263.1122742	85.62159756	93.92907333	-551.1862183
993778535.1	445.6566467	98.45609925	144.1172607	-467.3673706
373242937.6	364.5845947	108.6514358	158.6134338	-403.0717468
1314839021	424.9805603	111.2171348	166.5648254	-460.9550476
770849099.5	474.1569824	122.970746	157.8436813	-503.6309204
1301913043	446.4628906	121.2658174	173.0169403	-489.9346619
546251480.4	388.1015015	91.20793982	135.7955765	-420.9075317
582441077.7	475.1938782	125.3912006	153.6994904	-616.2006836
252909278.4	339.1946716	81.65115587	120.3755463	-269.1517639
1128353295	543.5023804	119.2046389	184.6965485	-469.6960144
1900473899	488.010376	113.2613131	150.2024292	-524.7883911
910743726.1	491.4926453	121.8730402	167.7038269	-421.0623779
521235731	311.8879395	101.6592196	146.9289307	-341.7573547
173156341.3	313.2737427	104.2584616	173.0134827	-255.9905853
653002270.1	455.5568848	125.1965901	210.5058838	-348.1002808
392086766.2	485.8113403	117.1564966	163.7916763	-458.604126
1203987680	410.9232788	111.8248875	173.8671341	-621.579895
348386365.1	361.7166138	99.89630628	131.1444427	-487.8927917
1042236915	519.0871582	124.0863976	157.6808899	-492.5568542
1596838605	389.6427307	124.6235619	180.8054886	-460.5946045
513916925.1	382.6321106	96.10048566	142.2383423	-355.8691101
1553513322	554.6135254	110.8917114	170.3857819	-400.1611938
1826520619	344.8093872	101.9310645	136.2658691	-484.6620483
1986501482	485.5097656	131.3880424	197.9499359	-533.3604126
845169533.7	358.0968018	115.882778	148.4470825	-452.3608398
551589279.3	467.7941284	93.13878548	114.2736053	-393.2970581
570866449.2	434.9396973	124.7313576	178.983728	-350.5149536
947169288.2	375.4199524	103.4808471	128.3827438	-443.7070923
1450560808	538.6044312	109.8398017	129.959256	-417.98526
556375753	424.1033325	123.0024216	155.3573059	-517.7671509
1375586316	476.0331421	138.4733708	168.4109436	-592.1066284
1098845075	501.8556519	128.2586024	169.6203064	-549.1275024
98132492.69	280.4049988	83.36306786	152.3005951	-204.9251404
1518605115	394.0406494	120.6797529	169.7226273	-419.0942383
807151857	335.2323608	92.76429237	142.188739	-327.0715942
603345824.3	434.3475647	125.0941988	184.2049301	-433.6569214
789959507.8	345.6127014	98.05495778	148.3409576	-438.5297852
78551738.57	236.0751495	84.13469173	154.4574127	-190.9763031
158586477.3	351.0432434	100.8062072	119.5687416	-483.8813782
946065777.2	382.4125366	104.462071	164.6441345	-440.7095642
674686325.8	411.9451294	150.3792564	199.0399933	-634.4525757
570113276.1	342.8233032	116.2674588	144.5088394	-429.6881104
1585310992	434.4559326	116.1397855	145.9702759	-489.5237427
1226380164	440.4944458	98.24370113	145.9916916	-360.4081421
752121734.4	540.2027588	177.5154079	206.1020401	-559.9356689
292746210.9	303.9988403	78.42194386	109.3929642	-435.9169617
99060359.91	304.1129456	92.58618389	158.5083755	-334.6357727
142616058	275.8529358	77.12511935	101.9222252	-378.4915771
778645117.2	535.3544922	158.0829369	202.0404846	-524.1537476

log.sigma.2.0.mm.3D_firstorder_Entropy	log.sigma.2.0.mm.3D_firstorder_StandardDeviation	log.sigma.2.0.mm.3D_firstorder_Range	log.sigma.2.0.mm.3D_firstorder_Variance	log.sigma.2.0.mm.3D_firstorder_10Percentile
4.070691028	111.2726663	811.1594238	12381.60627	-67.32965088
3.908921966	103.0663603	935.4894714	10622.67462	-71.31615524
4.218593726	119.5984371	745.622467	14303.78616	-158.726445
3.996776162	109.2542781	912.0108948	11936.49729	-77.98970032
3.618654402	90.49011351	648.1846619	8188.460643	-57.80958023
3.820240397	96.33045057	780.3659668	9279.555708	-91.36494598
3.288444228	68.36010217	561.9753418	4673.103569	-36.69380722
3.799796945	99.4131371	948.7672729	9882.971828	-51.68312759
3.837001563	94.18078007	796.9609375	8870.019335	-54.33162537
4.0328624	103.5469155	823.4426575	10721.9637	-98.55152893
3.607402408	85.94274929	845.434906	7386.156155	-63.34638596
4.270237681	126.7376697	989.0984497	16062.43692	-127.8943237
3.673076324	84.62487951	880.8402405	7161.370233	-59.81686935
3.93699744	95.20305278	646.7689514	9063.621259	-101.6123642
4.049504768	101.7479516	755.2967224	10352.64565	-92.70465698
3.935087095	100.9035176	929.6521606	10181.51987	-100.1028214
3.765037867	90.74433446	713.7898254	8234.534236	-94.75673523
3.744471522	87.69044667	740.2945251	7689.614437	-35.22115135
3.918595611	100.6392415	699.453125	10128.25693	-57.69007683
3.781914802	90.31390569	743.1742859	8156.601561	-75.72386169
4.207781024	115.48903	1043.352051	13337.71606	-137.5982315
3.976149149	100.7704914	869.8008423	10154.69193	-98.43679047
4.061145375	106.1121296	773.2219849	11259.78406	-115.6879272
3.88943827	103.9435278	795.1153259	10804.25698	-70.91299667
4.140404319	108.5843478	956.4458923	11790.56059	-138.8388504
4.246734356	125.0377866	855.0266113	15634.44808	-87.83135147
3.98944577	101.1487876	879.5806885	10231.07724	-87.24651337
4.265013219	122.8100047	800.6930237	15082.29726	-139.5545959
3.6832247	91.49531888	908.5582886	8371.393376	-77.46309204
3.915296154	102.7249973	956.4380798	10552.42506	-84.00775146
4.03743458	106.1822183	837.0019226	11274.66348	-113.3981873
3.850421725	90.71274291	917.3744812	8228.801726	-59.79064407
3.888266836	105.3512281	983.2973938	11098.88126	-92.46858215
3.727494404	99.19936398	767.2898865	9840.513813	-37.73431396
4.154192915	124.6949544	781.0276184	15548.83167	-137.6866455
3.667657959	94.68503483	866.661499	8965.255821	-68.62389069
4.28226559	127.2073409	954.0422974	16181.70759	-128.3776642
3.9903634	106.7158455	876.6469421	11388.27169	-100.5370102
3.506107284	71.07670324	550.8109589	5051.897743	-69.17825241
3.713824259	96.45764852	840.7416992	9304.077959	-52.80405807
3.984316598	106.947942	840.7353821	11437.86229	-84.10374451
3.766846763	92.04445087	779.8338928	8472.180937	-52.09969978
3.664028752	92.24066519	956.372467	8508.340314	-49.63268661
3.629292305	90.62824475	831.178009	8213.478746	-38.92647247
4.105216958	113.869431	1133.337067	12966.24731	-120.165062
4.165642225	114.6678435	876.8620605	13148.71432	-118.4200684
4.173611138	118.2615967	945.502594	13985.80526	-110.875351
3.975655713	106.6869318	725.2319031	11382.10142	-139.06987
3.626049924	97.2029942	653.2171326	9448.422081	-43.95885468
3.649274168	89.99420898	770.7695923	8098.95765	-50.33706207
3.655430088	84.15958852	932.3895874	7082.83634	-52.45534134
3.408082754	84.27717561	814.2984924	7102.642328	-61.41657448
3.708008009	89.91651326	913.0240173	8084.979357	-50.39782867
4.010057579	103.527449	767.6563416	10717.93269	-90.86621094
3.982468499	104.4858971	885.9356079	10917.30269	-77.41867218
4.210397519	121.5337157	977.7879028	14770.44405	-137.414444
4.10846634	114.8085116	936.3975525	13180.99433	-85.65335236
3.535483844	85.54769004	809.0090332	7318.407271	-42.66560249
4.075204877	121.6803987	1091.394562	14806.11942	-105.745118
3.574866326	75.28603055	608.3464355	5667.986395	-66.0483429
3.987776358	106.6006011	1013.198395	11363.68817	-49.70383835
3.923801427	109.2192182	1012.798767	11928.83762	-79.22779846
4.141740803	116.2868869	912.5550232	13522.64006	-113.1206894
3.859421232	94.63727437	653.6452942	8956.213699	-83.20385437
3.846024042	90.96135505	569.264328	8273.968112	-69.24705429
4.170653461	114.2521325	803.6571655	13053.54979	-81.19399261
4.006342291	110.6107239	944.4154663	12234.73225	-78.22269287
3.850676698	102.2464467	1032.503174	10454.33586	-51.01469994
3.803414648	95.17012727	849.6094055	9057.353124	-83.06627655
4.1262957	121.2630548	1011.644012	14704.72847	-127.1738724
4.281350814	120.1599234	850.2373352	14438.4072	-115.4975578
3.620399314	86.78605025	738.5012207	7531.818518	-39.03738785
3.957353288	100.7870751	954.7747192	10158.0345	-67.04750671
3.895614406	97.40517078	829.4714355	9487.767295	-89.74649048
4.256430043	124.3961926	1018.870178	15474.41274	-103.0650719
4.024741248	112.4983269	810.4576416	12655.87355	-113.378212
3.697291522	90.78468014	861.0911865	8241.858148	-86.92370758
4.099763841	112.8159355	785.4546509	12727.4353	-87.23499298
3.856707889	100.6642837	819.1270447	10133.29802	-94.63752747
3.955385629	108.4474558	956.5896912	11760.85068	-130.3636688
4.06079308	119.802495	941.8704834	14352.63781	-101.7590134
4.331924594	136.5066337	1068.139771	18634.06104	-156.5825745
4.188085075	124.0601257	1050.983154	15390.91479	-105.5405396
3.270702548	72.5087955	485.3301392	5257.525425	-23.22230415
4.148807774	114.7681122	813.1348877	13171.71958	-113.7133789
3.787482176	86.32203472	662.3039551	7451.493679	-69.0167244
4.149016686	117.7400303	868.0044861	13862.71474	-112.7247261
3.732232348	91.2548609	784.1424866	8327.449639	-54.81543884
3.414192006	71.13163702	427.0514526	5059.709785	-30.17441177
3.592432676	95.28343859	834.9246216	9078.93367	-44.19892578
3.851204263	95.52385043	823.1221008	9124.806001	-62.590168
4.295961396	143.1344888	1046.397705	20487.48188	-108.8990921
4.090224659	114.5331541	772.5114136	13117.84339	-123.1470764
4.033617771	113.4850111	923.9796753	12878.84774	-121.4592819
3.792426106	88.46575517	800.9025879	7826.189838	-57.12288284
4.716622645	177.3676327	1100.138428	31459.27714	-256.2161377
3.369061623	73.47547882	739.915802	5398.645988	-43.72452927
3.42154791	78.69674124	638.7487183	6193.177082	-27.17819653
3.336638815	73.20029427	654.3445129	5358.283081	-48.51959305
4.558136929	154.4198343	1059.50824	23845.48524	-191.7672531

log.sigma.2.0.mm.3D_firstorder_Kurtosis	log.sigma.2.0.mm.3D_firstorder_Mean	log.sigma.2.0.mm.3D_glrlm_ShortRunLowGrayLevelEmphasis	log.sigma.2.0.mm.3D_glrlm_GrayLevelVariance
4.703094543	52.78856834	0.005885788	20.71578526
5.931752717	38.44123218	0.003197132	18.2770704
3.342099177	13.00086913	0.004755558	23.76727048
6.353804681	34.24260092	0.003941497	20.40776305
5.320291136	36.04813328	0.010851245	14.33916082
5.879008665	18.0628841	0.004756242	15.81855032
5.060336024	29.50428334	0.006866386	8.342911285
7.16965374	40.65483725	0.002582903	17.27712217
5.64626541	48.81469591	0.003573049	15.37595543
4.119337107	38.43220032	0.003001156	18.03996385
7.874283301	30.09593177	0.003444094	12.91216985
4.843470548	34.89520353	0.004080701	26.87879885
5.753277706	30.58443648	0.004023431	12.33066979
3.456979894	26.02505203	0.011825553	15.02213319
3.281200819	37.57285052	0.004312705	17.17801164
5.124072561	17.1586229	0.003361879	17.8728699
4.998088727	19.25425596	0.007138567	14.16401206
4.555518934	52.33037835	0.004178451	13.23224403
4.297585912	52.50151619	0.009933343	17.21630994
5.505607003	28.83181804	0.003624573	14.08409909
3.736470298	16.80346472	0.002931639	22.31441399
4.501510474	31.85154511	0.00427426	17.21458927
4.14407502	26.68489895	0.00458049	18.86387666
5.352684987	35.12285569	0.005807312	18.74354663
3.353461557	8.079693801	0.006220035	19.59797221
4.25275551	55.38577099	0.005012561	26.21053757
4.680789686	32.12985928	0.003623955	17.17258925
3.743633814	30.34433524	0.004624267	24.99567512
6.15498822	25.00831158	0.002776398	14.84203345
5.899745582	28.8342501	0.0045756	18.31604646
4.259502153	30.1112311	0.005685839	18.909912
4.803158139	46.99032893	0.002594118	14.00012241
5.833860008	28.42885612	0.003848316	19.1991483
4.564533735	54.32359844	0.005848703	17.35339992
4.856826882	24.38702043	0.007922178	25.99585111
7.780988842	24.44075559	0.002816496	15.80208096
4.337081818	34.24324924	0.002818736	27.20071517
5.316037039	30.16252264	0.003373685	19.36328624
4.164029252	19.22509248	0.011125333	8.742843975
6.4480708	40.59814083	0.003444511	16.44541619
6.066905381	38.83275214	0.00498287	19.34515747
5.370287375	42.75019941	0.003795603	14.80007584
7.089753446	33.60922254	0.002558679	15.02306847
6.034664613	43.75476555	0.00413071	14.57437276
5.555124291	20.43189423	0.001687405	22.17135993
4.248495036	35.09858732	0.003516436	21.9169437
4.744889015	35.06743185	0.003157926	23.45175137
4.564843602	5.47523115	0.005031035	19.21432479
4.787104573	46.66343424	0.011317409	16.46465067
6.04293308	36.85658407	0.003794373	14.19114772
6.23032686	33.38486981	0.003775686	12.44669406
10.56301276	15.11342585	0.00270261	12.88423441
6.337567764	40.10765666	0.00276789	14.2013258
4.637054524	32.97274344	0.004912326	17.82430698
5.240576918	38.10444057	0.003450386	18.77989875
4.63091106	18.74460772	0.003765873	25.56771685
5.511571738	39.04361844	0.003722047	22.37038936
6.715603041	31.6303812	0.004413335	12.95746474
7.303163429	30.27926291	0.00336856	25.2469432
4.238853426	31.60577256	0.01014292	9.61551201
5.990886575	53.34845607	0.003348722	19.41286825
6.599105551	29.98812158	0.003317472	20.79779699
4.558408471	36.47461939	0.006278915	22.73659863
4.604314764	37.12658394	0.007868059	15.3846642
3.219364875	50.94957029	0.009004526	13.98694779
3.668995889	51.19215161	0.005786391	21.79419012
5.950592877	38.6123353	0.004475619	21.06689274
7.27973943	45.2821114	0.001819482	18.14528548
6.014627928	30.36311717	0.003281156	15.35758459
5.113768086	26.31930102	0.003521707	25.04312427
3.690803021	33.05487843	0.003476924	24.06138326
6.019614404	41.27329434	0.004487523	13.28801232
4.986523465	46.24864499	0.003787309	17.36569346
5.323690354	30.03622183	0.002661309	16.31314194
4.804800975	42.28953709	0.002625777	25.92530393
5.32620893	27.80188283	0.004059534	21.69013364
5.63244662	20.80805644	0.004762526	14.46448147
4.474446865	53.20222058	0.006018802	21.43700648
5.797178743	23.97890102	0.004091695	17.48540446
5.098475276	17.43362695	0.005599406	20.2726462
5.951862663	27.87396468	0.004054721	24.55421078
4.661097617	23.25539464	0.002488501	31.15784117
5.925756756	32.54772358	0.003915377	26.20072679
4.376203508	41.13241615	0.011315379	9.381375489
4.280024186	37.30795077	0.005058594	21.9880165
3.999233805	33.96645785	0.005159207	12.70605943
4.443218924	42.25924548	0.004102346	23.44700963
6.259405212	35.87931306	0.003608163	14.29040105
3.172475857	44.93257802	0.01699051	8.649422774
9.616165321	32.90832325	0.006598639	15.78129124
5.088524717	42.27905237	0.003210962	15.75746831
6.814451436	46.11332643	0.004492423	34.42254184
4.76916323	20.00696343	0.005393465	22.04978187
5.216927752	24.69012041	0.003734903	22.14538537
4.886680137	42.72744988	0.004451326	13.41041179
3.331376589	7.241748222	0.004871685	51.67216951
7.703907662	27.41086082	0.00353119	9.526630984
5.768239545	48.77524336	0.006547267	10.89930681
7.643669445	24.28993525	0.005112039	9.641046718
3.452104691	33.83385446	0.005829271	38.93692008

log.sigma.2.0.mm.3D_glrlm_LowGrayLevelRunEmphasis	log.sigma.2.0.mm.3D_glrlm_GrayLevelNonUniformityNormalized	log.sigma.2.0.mm.3D_glrlm_RunVariance	log.sigma.2.0.mm.3D_glrlm_GrayLevelNonUniformity
0.006110187	0.076138072	0.094718353	84.60127551
0.003360749	0.087112761	0.134978694	156.6396691
0.004901616	0.064003475	0.0848604	66.78658774
0.004075328	0.082596185	0.100825719	186.208058
0.01150405	0.109939709	0.130404281	93.71884245
0.004947311	0.09094639	0.115684372	135.3753721
0.007475788	0.130820762	0.223679688	118.3060742
0.002730341	0.094601946	0.160603117	151.4795457
0.003784641	0.087421203	0.152727813	158.3749432
0.003140422	0.074958593	0.097800768	207.3629707
0.003652907	0.107130106	0.159898148	171.3505495
0.004190625	0.064879039	0.076441456	102.4191335
0.00429143	0.100721152	0.143453179	263.6977221
0.012299636	0.076827075	0.098551262	131.5295075
0.004481945	0.070843307	0.08544943	102.1438155
0.003587873	0.082436614	0.198812789	298.3895746
0.007504638	0.094630792	0.134119901	136.4469931
0.004466188	0.093114007	0.163117167	211.6947482
0.010398783	0.084315997	0.125107024	86.7078721
0.003852619	0.090798121	0.150755717	234.5745336
0.003063434	0.064937344	0.092532009	418.1106971
0.004456705	0.079227737	0.101719745	154.3060876
0.004753751	0.072856401	0.094484515	277.8307758
0.006027483	0.089081604	0.137016268	82.7585229
0.006482358	0.065427381	0.090013047	157.775801
0.005176981	0.06671444	0.08549591	83.78103505
0.003747816	0.078833014	0.081413886	163.0531937
0.0047651	0.063054253	0.078406443	197.7967914
0.002977138	0.104208925	0.200091873	310.6385259
0.004787204	0.086133404	0.150855055	244.1346783
0.005896711	0.075867965	0.097590939	289.3477443
0.002752634	0.085344659	0.125913624	386.9079944
0.004028897	0.091518464	0.134779183	208.4469182
0.00615558	0.101015042	0.152126379	43.0589432
0.008145679	0.072538266	0.08475181	59.93427417
0.002997482	0.106346825	0.174464046	300.7392859
0.002928438	0.065929651	0.089799753	218.3658753
0.003533504	0.081425251	0.108350113	252.4439995
0.011875089	0.106918475	0.152298559	141.1963423
0.003655863	0.103576278	0.175213505	148.7733382
0.005106367	0.080267283	0.088545026	97.86803416
0.004039093	0.094711938	0.161939594	161.9577372
0.00273786	0.107070081	0.172615378	284.8524086
0.004392551	0.108269305	0.190742928	178.7092127
0.001772626	0.074559448	0.121914979	236.8007432
0.003657252	0.069135793	0.083737456	230.2718131
0.003282187	0.070513701	0.091809841	240.7803752
0.005224619	0.080756622	0.104334216	112.2545909
0.012023168	0.111911196	0.145921098	56.03820289
0.004017399	0.106834498	0.159732376	103.0693888
0.004035254	0.101839682	0.159240125	358.5348985
0.002886154	0.126875977	0.217844286	212.7976708
0.002966818	0.101004985	0.155177143	340.2561792
0.005069853	0.077108004	0.079841477	84.13102035
0.003631007	0.081507766	0.124887406	291.395691
0.003917614	0.067892434	0.160862482	115.1146747
0.003865617	0.075734607	0.102394294	228.0663165
0.004708085	0.115526057	0.19160541	244.401166
0.003498498	0.077675191	0.09601888	98.23661442
0.010585048	0.101582438	0.110839097	130.1841486
0.003504941	0.080351816	0.119137939	214.7455822
0.003484856	0.089534774	0.157610221	437.7681388
0.006475794	0.071675929	0.094640139	150.444231
0.008141606	0.085367993	0.119490052	145.1789916
0.009382766	0.082961202	0.101473281	44.90784532
0.006008745	0.068945991	0.083598065	98.958883
0.004630355	0.081206485	0.117064365	78.18134724
0.0019249	0.090759951	0.145120982	290.0782662
0.003422623	0.091586784	0.110909989	107.9555164
0.003668342	0.07660303	0.115550735	175.0599184
0.003611695	0.061327154	0.087303836	217.1117937
0.004817346	0.108077651	0.172602653	195.9008861
0.00400048	0.081746262	0.121376478	347.0021222
0.002818569	0.084243222	0.135974058	494.7438352
0.002733642	0.066904509	0.088496534	264.4145353
0.004231881	0.080312072	0.118898592	170.2868039
0.005041072	0.102338509	0.147792548	214.9315446
0.006203983	0.076063802	0.089572288	95.97493176
0.004294511	0.091022536	0.134288238	268.7587783
0.005871976	0.085703379	0.145625959	341.4545872
0.004226348	0.080618455	0.116376271	100.2152118
0.002561628	0.064236596	0.077016427	159.9105448
0.004018445	0.071766613	0.096956447	163.9341043
0.01227947	0.134563629	0.209551736	60.70052363
0.005224594	0.069630523	0.086419201	249.9768487
0.005454047	0.088209816	0.119181417	277.7352972
0.004249182	0.073642973	0.087996998	97.49063747
0.003820035	0.097505446	0.134361735	266.1830141
0.017859039	0.110683155	0.143279616	40.69817906
0.006769877	0.113635311	0.130776957	58.9491366
0.003393326	0.088405465	0.128310856	256.245097
0.004612098	0.067110702	0.080355699	69.3153685
0.00553284	0.073968326	0.087936161	107.4987018
0.003888877	0.080514657	0.126824174	317.5111194
0.00471493	0.090545859	0.12487913	385.1042396
0.004993742	0.04717543	0.073447671	39.1678444
0.003777157	0.12776579	0.167382512	197.4519596
0.006890191	0.124790163	0.176186811	47.21743276
0.005438243	0.131689546	0.190315074	101.5555996
0.005964147	0.051004481	0.053995139	55.96675563

log.sigma.2.0.mm.3D_glrlm_LongRunEmphasis	log.sigma.2.0.mm.3D_glrlm_ShortRunHighGrayLevelEmphasis	log.sigma.2.0.mm.3D_glrlm_RunLengthNonUniformity	log.sigma.2.0.mm.3D_glrlm_ShortRunEmphasis
1.26908804	383.8185681	957.2839232	0.942722423
1.37441658	466.4635474	1477.578198	0.924566678
1.235146836	364.2067727	917.9679948	0.950667068
1.287126741	466.7567813	1922.69217	0.938879063
1.373802556	147.5463062	697.4796612	0.92259164
1.328861856	355.7993432	1243.048504	0.930901474
1.598359007	176.0721608	685.8470841	0.892263001
1.435053994	550.7104442	1289.524266	0.916486279
1.408437633	440.1241856	1479.178278	0.921677322
1.277633442	432.0452849	2370.646915	0.940849475
1.442015682	401.4769038	1279.209906	0.913433845
1.217103514	480.2014673	1396.252234	0.952934245
1.399141175	300.8293579	2128.478007	0.920083903
1.279156887	185.590078	1468.563624	0.940871128
1.240135988	323.7714318	1262.86077	0.948871319
1.493199976	390.937888	2903.226747	0.914545773
1.377581066	227.6552508	1181.946812	0.923070311
1.434840609	298.1953752	1841.133786	0.917932642
1.349802992	216.727006	855.8943932	0.928734357
1.416536338	366.203625	2085.164751	0.917258057
1.258983107	442.9481265	5577.576159	0.944973698
1.291124209	344.9143126	1658.648479	0.938038003
1.264993425	340.3682687	3299.049468	0.944155674
1.371065134	351.5971688	770.6727733	0.927660707
1.258055975	253.9907085	2084.235777	0.943994329
1.246185323	393.4610667	1091.737353	0.946341372
1.225952891	387.1952739	1827.517783	0.95241302
1.223528619	391.3506144	2762.939382	0.951358234
1.51657089	432.9339141	2361.436529	0.908854847
1.397559381	374.3341378	2335.517004	0.925041795
1.278910726	310.5036874	3263.649733	0.940254667
1.345107973	439.2034002	3788.974545	0.930806164
1.372587983	410.0460017	1873.400791	0.924943337
1.431982849	306.3266597	340.4460663	0.913173501
1.241710329	380.8273723	720.8020984	0.947697472
1.473064931	472.3178584	2241.460459	0.910204928
1.252187633	519.389857	2880.567224	0.946527003
1.306276653	416.827524	2620.757074	0.935628917
1.41917696	124.9885833	1065.924918	0.917286429
1.476096122	421.7726226	1134.075339	0.908942522
1.255248164	433.6526134	1054.23079	0.944309784
1.441587033	351.658647	1368.04884	0.914019714
1.470646682	488.5777168	2102.449662	0.909309419
1.49968444	354.0470313	1304.966225	0.90917048
1.334760845	764.4115071	2661.079375	0.932065479
1.236997264	433.1694003	2915.464229	0.949035992
1.258586421	473.5530426	2958.187695	0.945009458
1.29620986	342.3626396	1180.829681	0.937320556
1.420384014	182.9206576	400.5145124	0.913593447
1.440829379	378.6622134	771.0746249	0.913572881
1.442458167	336.6923221	2806.78755	0.912757552
1.586271335	526.1441365	1270.492255	0.892343751
1.428708969	422.3859345	2703.991891	0.915349261
1.233483621	351.4911372	953.0398105	0.948076622
1.339550999	430.4709264	2998.889964	0.932411103
1.40448553	481.0365719	1407.328959	0.92784671
1.289923543	477.7300867	2567.336604	0.938734788
1.521876968	332.4853068	1645.727522	0.902204363
1.274594079	690.8090687	1083.66118	0.940837162
1.316065624	159.8678664	1078.340598	0.933343796
1.332142894	455.6474059	2237.925086	0.931786528
1.420956742	493.0516598	3970.639407	0.919662658
1.267109276	358.8249822	1810.281542	0.943221607
1.3292532	251.4040355	1430.115822	0.933245953
1.291963807	185.6595939	460.0581869	0.937364249
1.240474624	281.2039315	1249.628392	0.947113623
1.326855059	431.6961511	807.6305439	0.932438143
1.393515323	706.4652825	2619.721716	0.9232722
1.315759905	450.6322344	989.9711034	0.93317263
1.320758857	455.0026986	1925.462057	0.934271724
1.245895566	432.9172365	3087.733301	0.94751787
1.470780365	282.0500054	1435.15087	0.9097052
1.338882724	365.6942031	3542.232713	0.93038343
1.367245435	450.3904573	4863.373005	0.927494278
1.252470318	578.0207493	3427.001379	0.945452299
1.329234396	414.2263626	1779.667785	0.932812426
1.409464644	287.3788615	1700.242489	0.918628824
1.251732371	313.5708658	1098.63043	0.946741549
1.369661954	364.8480043	2438.613283	0.926216989
1.398098505	321.2911171	3250.525042	0.921636359
1.320993754	500.6865234	1048.906802	0.934563485
1.217828361	640.4720326	2200.852059	0.952804436
1.271533448	556.1230777	1967.653818	0.942832448
1.567314348	123.0945964	347.4429324	0.896702119
1.244724595	359.4870892	3128.323587	0.947340215
1.338101373	244.644915	2619.380856	0.929428248
1.25286237	406.7523112	1145.855096	0.944796733
1.380298058	379.2892215	2231.065869	0.922159675
1.401448922	107.5839601	300.0578902	0.920096648
1.376014017	452.1004729	423.0033021	0.92145707
1.358300346	392.09562	2398.763709	0.92717505
1.227218578	794.7827868	910.2416236	0.951382112
1.244656492	370.9733506	1270.731	0.948561967
1.348080595	446.5609986	3288.868795	0.930166
1.352242182	287.3785931	3517.418654	0.926966886
1.20945443	589.323627	737.4005975	0.954318986
1.47376658	355.8326047	1211.169325	0.905820457
1.464197128	260.0748622	303.0965331	0.91403213
1.523304425	282.7917284	596.6785982	0.900635457
1.160903302	535.7155217	994.2826893	0.962462376

log.sigma.2.0.mm.3D_glrlm_LongRunHighGrayLevelEmphasis	log.sigma.2.0.mm.3D_glrlm_RunPercentage	log.sigma.2.0.mm.3D_glrlm_LongRunLowGrayLevelEmphasis	log.sigma.2.0.mm.3D_glrlm_RunEntropy
509.236659	0.923524522	0.007118645	4.493900888
686.4939536	0.898846154	0.004170177	4.462151051
476.9423004	0.933122334	0.005586259	4.59136059
639.3856442	0.918874847	0.004699114	4.458954716
210.1581298	0.897868201	0.014606896	4.172624175
513.5223055	0.908565793	0.005845713	4.322870357
306.8213497	0.855032385	0.010879848	4.023628546
853.9604438	0.886767526	0.00349969	4.405486006
662.9315449	0.893438579	0.004851962	4.405114714
588.3508248	0.921130152	0.003788049	4.47675049
636.8758748	0.884806947	0.004715725	4.219808291
617.595746	0.936772426	0.004686879	4.634777283
454.5832611	0.893636579	0.005619021	4.24910765
253.6372931	0.921379482	0.014455341	4.366312485
422.4275515	0.931425164	0.005275853	4.4274808
625.6330028	0.880431323	0.00489046	4.566091673
339.1006331	0.898202732	0.009264503	4.312342534
456.4803912	0.888494617	0.005995196	4.332721107
305.3459539	0.904997291	0.012604034	4.422737556
564.7200949	0.890276562	0.004993529	4.366158995
591.4824784	0.926397344	0.003682005	4.627214304
477.8008862	0.918072027	0.00530781	4.432008534
461.0949255	0.925260859	0.00556283	4.484673779
508.6211185	0.901493652	0.00715048	4.420209519
339.687631	0.926001418	0.007665951	4.552475923
513.3521805	0.928709604	0.005920473	4.648548085
500.840555	0.935323383	0.004335641	4.363407834
506.98089	0.934930643	0.005398757	4.641998161
720.0730383	0.875642157	0.004109903	4.352372516
560.0222324	0.896827136	0.005898393	4.479263816
425.6919236	0.92066263	0.006875925	4.487519635
629.832837	0.906569231	0.003546059	4.361728544
609.1107401	0.899614017	0.004918523	4.445954531
459.2265238	0.885335039	0.007687925	4.32667908
505.8338377	0.930266805	0.009119104	4.539049953
766.5730002	0.878649482	0.00394466	4.32339948
687.7736122	0.927926375	0.003439493	4.699127484
586.3849175	0.914139237	0.004280503	4.476328108
190.6103762	0.889643375	0.015612977	4.084246829
674.9754512	0.877609554	0.004762038	4.369949784
582.9844378	0.926350245	0.005670445	4.400139584
546.905628	0.884854276	0.005291747	4.382103176
784.0242657	0.87857996	0.003666471	4.322627959
566.9682396	0.875469541	0.005835384	4.292919846
1100.187986	0.908766677	0.002191728	4.611413093
569.1011067	0.931576116	0.004297379	4.559006041
632.4659162	0.926290747	0.003858663	4.597125848
482.8760133	0.91668358	0.006116777	4.436483884
267.2375411	0.887343153	0.015322092	4.218544771
590.494031	0.884615385	0.005154889	4.26543114
524.120498	0.883495521	0.00535029	4.283359121
942.3941113	0.856811389	0.003886595	4.160585939
650.207066	0.886975547	0.003973365	4.324351303
457.8318983	0.931682323	0.005772867	4.394492189
612.2909682	0.907895508	0.004516697	4.497526473
725.8182759	0.897734681	0.004764695	4.750067194
653.9546904	0.918267765	0.004562352	4.57174264
546.3300689	0.869369939	0.006283862	4.228884488
943.3699168	0.921675264	0.004069653	4.518251438
226.7940294	0.91201752	0.01265793	4.052371784
644.1392701	0.908586823	0.004264418	4.494426254
756.1212352	0.890762782	0.004356067	4.529045799
482.9376882	0.924126952	0.007372015	4.572108013
359.2334255	0.910105419	0.009477639	4.349169874
252.6957739	0.917340287	0.011148059	4.280201447
362.3091562	0.930106187	0.007010235	4.565752181
613.2919294	0.909844409	0.005379396	4.496407659
1057.994181	0.895897148	0.002468628	4.424011635
643.3160888	0.911535487	0.004088402	4.286603471
645.7691769	0.911417262	0.004378945	4.626101312
567.5573688	0.929642049	0.00423928	4.680180913
444.8464472	0.879035569	0.006529674	4.266265926
520.8498973	0.907064297	0.005023327	4.472753214
665.4266213	0.901870208	0.00361344	4.435303979
762.1936798	0.927264195	0.003228101	4.676522988
593.8905513	0.909447909	0.0050625	4.5284331
442.6111375	0.891360444	0.006439258	4.289512305
414.625956	0.928284372	0.007072317	4.505883495
543.4872975	0.900911055	0.005296508	4.403198467
491.026621	0.894453178	0.007205643	4.539108135
705.5035213	0.912233141	0.005028515	4.548684565
827.4570867	0.936770794	0.002896441	4.704819013
754.0777007	0.923170201	0.004503372	4.628203609
201.4440384	0.861891455	0.017606599	3.965505344
477.5290364	0.929510417	0.005987657	4.555121696
352.7382764	0.906226474	0.0068575	4.301078838
538.9676726	0.926955398	0.004916108	4.564428308
566.6929564	0.896913471	0.004846313	4.29332273
157.4202023	0.894254164	0.02226127	3.940281661
673.0587295	0.897125366	0.007583876	4.139556229
571.9939754	0.90245071	0.00429334	4.390443692
1028.781925	0.934493561	0.005130701	4.672755387
492.9168254	0.930266916	0.006188471	4.487314979
653.5387238	0.905670713	0.004645769	4.564358375
415.6341273	0.903543758	0.005980243	4.323695992
744.1629818	0.939001044	0.005537425	5.045028275
578.861783	0.876259872	0.005014193	4.024705554
402.8161009	0.882997843	0.008748806	4.025936514
479.2260941	0.867896743	0.007157233	4.021913769
654.0549325	0.950806559	0.006523038	4.839498625

log.sigma.2.0.mm.3D_glrlm_HighGrayLevelRunEmphasis	log.sigma.2.0.mm.3D_glrlm_RunLengthNonUniformityNormalized	log.sigma.2.0.mm.3D_glszm_GrayLevelVariance
405.905675	0.860913296	32.09046728
503.5155809	0.821325237	35.14060357
383.7819062	0.879160188	33.30736394
497.075923	0.852264588	38.50923221
158.327814	0.817483546	30.60800494
383.1511008	0.834624087	29.75533877
196.3236061	0.756924329	17.91315955
599.8554271	0.804773623	34.87388779
476.3270007	0.815777724	27.24199841
459.5102159	0.856512673	29.13525335
440.0436287	0.798593403	26.89795885
504.6426788	0.884055847	42.06565328
326.5092607	0.812065264	25.28191042
197.4626015	0.856879291	22.07409972
341.0826844	0.875009988	24.99138463
426.1429642	0.801288471	31.34314533
246.509647	0.818546198	26.39064253
323.5819194	0.808459266	25.24766713
231.86358	0.831067739	26.90972387
399.2656869	0.806137802	26.18757059
469.0705869	0.865767882	33.05186264
368.2470095	0.8507092	28.72743866
361.3643117	0.864299872	28.37182461
377.3675336	0.82874017	34.90673104
269.1992393	0.863594035	28.12701395
415.0546854	0.868913346	42.0450418
407.10507	0.883099849	26.22280278
411.9264201	0.880351924	36.13822256
476.1950525	0.790519981	32.12096629
404.040195	0.823235353	33.24868105
330.8144082	0.855222445	30.49034092
471.2689281	0.835008174	24.25729466
443.4827312	0.822057981	36.91865599
332.1483137	0.798086155	31.83522498
403.0817665	0.871975839	41.05911053
519.4165051	0.791974575	34.65330182
548.8128068	0.869375532	43.05013917
446.2302038	0.844961903	34.58762494
135.8522774	0.806285777	16.26662393
462.6934452	0.789048607	34.74762843
460.4380366	0.864196905	33.26845841
383.7274447	0.799409237	29.79568293
536.5675536	0.789727078	33.48058882
387.0487791	0.790021709	30.85956529
821.1735352	0.837381265	39.01666549
457.227141	0.874990833	32.6871132
501.4655987	0.865886372	37.12704267
366.8886296	0.848952423	30.02512309
197.2791589	0.798813201	31.07585181
413.557653	0.798641675	29.31726391
367.727392	0.796711394	26.47323645
590.9972491	0.756440857	32.82745928
460.1275267	0.8020797	31.12874394
370.8633562	0.872830309	27.95675846
460.8337182	0.838435586	33.38919902
518.5969194	0.829434849	43.33499525
508.5310361	0.852001357	38.76102175
366.5087029	0.776317253	29.83736435
735.6280525	0.856402204	44.37373723
171.5534925	0.840408395	16.76575505
487.9327098	0.836853085	36.76998394
535.4865297	0.811422397	40.50593263
380.6289544	0.86195419	37.50726889
269.6635904	0.840291052	25.69105435
197.6028792	0.849109774	22.67512397
295.8253733	0.870310225	35.00844147
462.9629123	0.838273116	40.52126952
764.2243064	0.819101029	36.02993483
484.3682009	0.839448342	27.47306065
487.5433568	0.842193297	44.79480344
456.6956856	0.871617787	35.81367438
308.3695882	0.790895796	28.61598098
392.1644307	0.833826324	30.77152339
486.0061602	0.827602922	31.24710236
610.7989874	0.866748878	40.93438843
444.8543235	0.839011546	38.03041731
313.2217384	0.808983845	30.230952
331.263887	0.870154782	35.57593305
394.5597804	0.825165746	33.21585458
349.2105997	0.815287709	36.85982696
535.4584814	0.843159835	42.57764025
673.8149429	0.883676016	47.68077182
590.5637215	0.861046892	45.39576879
135.4383636	0.767438229	18.40810354
380.3113851	0.871063701	32.5047621
263.3442477	0.831515514	21.7845696
430.5031631	0.865185432	39.07838237
411.1712632	0.816419104	27.78103191
115.9849269	0.813737983	15.62031807
490.2796665	0.81474575	38.42733536
422.6832912	0.827033976	29.97682741
836.1307851	0.88075325	55.74593333
392.1748832	0.874056744	35.34392713
481.1242761	0.833490214	39.82944534
309.4503638	0.826344307	26.35520016
616.9709993	0.88749096	68.47879006
392.9079175	0.782753647	23.16614009
282.6348054	0.80039939	23.64163208
314.3560219	0.772745239	21.12074361
557.9880057	0.905796815	48.3711284

log.sigma.2.0.mm.3D_glszm_SmallAreaHighGrayLevelEmphasis	log.sigma.2.0.mm.3D_glszm_GrayLevelNonUniformityNormalized	log.sigma.2.0.mm.3D_glszm_SizeZoneNonUniformityNormalized
267.7273326	0.053909763	0.366642886
329.4352215	0.051183128	0.417519242
251.9301983	0.048460655	0.44922537
323.943177	0.049630712	0.438197488
136.6586625	0.057172376	0.437427042
221.486031	0.055846854	0.396348013
112.4573938	0.077973341	0.379035488
385.1964911	0.055916671	0.436997176
304.8618862	0.057630133	0.423934513
289.9359077	0.052511551	0.396328608
267.0852059	0.063612687	0.3983024
333.2120223	0.045905797	0.4527459
209.8955888	0.060011574	0.381823232
113.5971813	0.059377244	0.346486953
214.0805827	0.055647882	0.396232423
278.9675316	0.052040373	0.408189079
151.3728038	0.05761185	0.360973135
199.2524638	0.0581829	0.38930371
176.2737594	0.058737673	0.444549638
223.4266461	0.058808511	0.363132569
295.0392628	0.048851596	0.405531839
223.3310534	0.054499656	0.382625827
208.9099568	0.053759375	0.392569531
242.9296641	0.052588632	0.386905116
171.2222342	0.052392982	0.396914038
297.7351871	0.04531775	0.456122068
273.4396574	0.057206408	0.48040404
269.2304365	0.047338052	0.443805
305.6858447	0.052166383	0.436519454
267.670868	0.051810484	0.437066893
207.0255991	0.052651473	0.405776803
301.8685768	0.059262662	0.39523017
276.8151635	0.050763812	0.424865617
232.5354013	0.052708907	0.450137741
234.132437	0.04792644	0.430617377
329.3892898	0.053644199	0.452508869
371.7197717	0.043627581	0.456154846
281.6997236	0.050468091	0.422912767
80.81566552	0.073453118	0.324720069
289.8654349	0.054053307	0.418399676
281.7303155	0.05284882	0.452204022
239.7093646	0.054409779	0.401835786
326.6206794	0.055370352	0.405406029
275.7397354	0.056530797	0.463114038
515.4697017	0.047581385	0.417991667
288.295093	0.050640208	0.432930051
331.2403512	0.048175798	0.433838324
207.8581271	0.055373199	0.403519477
169.7655451	0.055029746	0.440305571
279.0873579	0.058215385	0.443370414
249.5515515	0.060087599	0.418907931
349.8478105	0.061804316	0.427902312
309.7364195	0.054784349	0.430939365
249.5597973	0.056844243	0.41529385
314.5410644	0.050974415	0.449643902
322.9180122	0.04313992	0.422321078
340.2578608	0.047787794	0.426940678
245.3186784	0.061094207	0.390355406
470.9425853	0.0489352	0.443104711
99.74960932	0.070767687	0.364447087
320.6401629	0.051377171	0.427455845
358.8553082	0.048738943	0.443125407
246.7441608	0.047288361	0.429929056
159.3390211	0.057349286	0.418218243
131.8868695	0.060578512	0.47214876
208.1520051	0.048171888	0.395984541
314.6853196	0.049527501	0.447020711
498.2490259	0.053329745	0.439026395
287.7466629	0.058412745	0.429189854
313.9860485	0.044365648	0.456404291
296.2737904	0.046764424	0.424357974
198.9160647	0.059106425	0.396491204
255.3830117	0.052962802	0.404429689
306.8651957	0.054184404	0.411121865
413.9384638	0.045981926	0.44077609
269.4864003	0.047957132	0.443116862
200.7389801	0.05726236	0.449234065
219.0443944	0.048917097	0.428718898
245.5730287	0.053062154	0.414936202
210.0404239	0.049362086	0.423129204
359.1631346	0.046549301	0.453724519
447.6464173	0.043965462	0.491234675
396.9625734	0.045502891	0.467401328
99.19973504	0.071540062	0.380593132
235.5523629	0.050730406	0.427864255
157.2783817	0.060860538	0.385678729
286.2785117	0.049049382	0.448996899
253.881875	0.059907846	0.414389195
77.70499865	0.075505351	0.451545779
322.6793717	0.055439833	0.42789617
262.7137329	0.055121788	0.429803988
556.8975523	0.043390967	0.471694696
247.7629788	0.051190661	0.471198693
302.4351993	0.045996372	0.45267207
196.4923636	0.057856917	0.383822393
454.9905623	0.035653969	0.500156377
227.3285218	0.073535956	0.384567462
198.3911316	0.068525335	0.40966403
204.0757539	0.073068346	0.465152452
361.554084	0.042225478	0.438966007

log.sigma.2.0.mm.3D_glszm_SizeZoneNonUniformity	log.sigma.2.0.mm.3D_glszm_GrayLevelNonUniformity	log.sigma.2.0.mm.3D_glszm_LargeAreaEmphasis	log.sigma.2.0.mm.3D_glszm_ZoneVariance
167.5557987	24.63676149	50.6761488	43.74669737
270.5524691	33.16666667	222.4506173	212.9246304
232.2495164	25.05415861	18.90522244	14.22892076
389.1193694	44.07207207	55.95157658	48.3207925
119.4175824	15.60805861	163.0586081	150.9747078
210.8571429	29.71052632	77.7593985	68.2794816
86.79912664	17.8558952	523.1746725	501.8697965
239.4744526	30.64233577	284.870438	274.0213484
270.4702194	36.76802508	238.7037618	228.6096958
447.8513274	59.3380531	47.67699115	40.61456731
196.3630832	31.36105477	243.3732252	229.9386955
340.9176627	34.56706507	16.30942895	11.30205693
307.3677019	48.30931677	237.1540373	223.9152872
236.997076	40.61403509	39.99707602	32.61838685
251.2113565	35.2807571	23.70031546	17.73870772
533.911315	68.06880734	284.5925076	274.7238793
171.1012658	27.30801688	181.5084388	170.0429285
276.4056338	41.30985915	295.1211268	282.1408371
173.374359	22.90769231	99.21538462	90.73086128
296.3161765	47.9877451	306.4767157	293.8376451
1144.005317	137.8103509	31.9213045	25.85165746
293.0913838	41.74673629	44.87075718	37.20378658
628.11125	86.015	35.030625	28.39678086
132.7084548	18.03790087	69.46938776	60.45188654
415.1720841	54.80305927	20.61759082	14.42006149
253.1477477	25.15135135	33.48828829	27.55401672
475.6	56.63434343	26.31616162	21.32838384
664.3760855	70.86506346	22.61389446	17.59114577
399.4153005	47.73224044	842.9606557	829.1612577
467.2245089	55.38540692	288.1992516	279.4666461
621.2442848	80.60940562	53.75571522	46.43641084
640.6681061	96.06477483	199.4682295	189.9539922
342.4416873	40.91563275	258.9416873	249.0808407
74.27272727	8.696969697	83.99393939	75.49583104
157.1753425	17.49315068	38.59178082	32.67289172
390.062645	46.2412993	607.9733179	594.0453553
673.2845528	64.39430894	55.1700542	49.32322535
491.0017227	58.59345392	112.6175711	104.086744
125.6666667	28.42635659	214.6459948	199.9416435
194.9742489	25.1888412	375.0042918	362.6790694
237.8593156	27.79847909	31.47908745	25.21957813
222.2151899	30.08860759	242.9981917	230.7924554
315.8112965	43.13350449	842.9922978	827.8932373
242.6717557	29.6221374	517.8244275	504.8973836
497.410084	56.62184874	169.2487395	160.627854
630.3461538	73.73214286	30.40041209	24.37163339
636.8746594	70.72207084	61.01634877	54.71173407
215.4794007	29.56928839	70.44569288	62.38604834
75.73255814	9.465116279	94.20930233	83.45700379
144.0953846	18.92	207.9261538	196.6778698
441.9478673	63.39241706	790.9402844	776.6798176
182.286385	26.3286385	911.5352113	890.4528863
455.0719697	57.85227273	692.1922348	679.2635795
198.5104603	27.17154812	24.01464435	18.01316941
623.6560923	70.70151406	285.1586157	277.1015314
296.0470756	30.24108417	56.90727532	49.65343579
509.7671692	57.05862647	75.32579564	67.78401008
223.6736475	35.0069808	690.9109948	672.8966738
238.3903346	26.32713755	32.89591078	26.39246279
169.1034483	32.8362069	80.16163793	70.99274952
417.6243603	50.19549642	135.7205732	126.6590559
762.6188263	83.87972109	665.6077862	655.4427675
387.7960089	42.654102	53.50886918	47.16986273
280.6244411	38.48137109	61.13263785	53.38251292
103.8727273	13.32727273	57.57272727	50.38057851
244.7184466	29.77022654	31.82686084	25.5930316
168.0797872	18.62234043	70.97340426	63.05576618
489.0754039	59.40933573	380.02693	369.7800347
179.8305489	24.47494033	93.2052506	83.68235542
409.3946488	39.79598662	120.1884058	112.3770974
668.3638095	73.65396825	25.03619048	19.19054109
213.3122677	31.79925651	438.1988848	423.5234346
626.4615881	82.03938025	202.0858618	192.9614841
830.8772885	109.5066799	218.8307768	208.4516035
745.7931442	77.80141844	52.33806147	45.99314113
366.0145278	39.6125908	116.3038741	108.3399988
304.1314623	38.76661743	478.9881832	466.8876506
234.9379562	26.80656934	23.03467153	16.88463357
427.7992241	54.7070805	179.0242483	168.9277575
580.1101386	67.6754194	302.5609044	292.0114384
231.853229	23.78669276	88.43444227	81.33028749
601.2712418	53.81372549	33.88643791	29.17427525
465.0643216	45.27537688	49.05929648	42.87695159
47.19354839	8.870967742	257.9112903	240.1219433
686.2942643	81.37157107	40.90773067	35.11056368
421.1611722	66.45970696	122.6501832	112.5293946
250.9892665	27.41860465	85.31305903	78.78727347
363.833713	52.59908884	391.1514806	379.1394931
52.37931034	8.75862069	118.7672414	106.2136593
67.60759494	8.759493671	127.6708861	114.2882551
450.0047755	57.71251194	299.974212	290.568596
237.734127	21.86904762	22.83928571	18.03240347
311.9335347	33.88821752	37.56495468	31.99763602
679.9134487	69.08655126	167.786285	159.3870937
539.2704626	81.28896797	136.4241993	125.2052755
219.0684932	15.61643836	14.28767123	10.21427827
163.0566038	31.17924528	709.7617925	692.4726715
46.2920354	7.743362832	178.0353982	163.689404
107.4502165	16.87878788	314.978355	300.2008208
262.5016722	25.25083612	7.511705686	3.78770931

log.sigma.2.0.mm.3D_glszm_ZonePercentage	log.sigma.2.0.mm.3D_glszm_LargeAreaLowGrayLevelEmphasis	log.sigma.2.0.mm.3D_glszm_LargeAreaHighGrayLevelEmphasis	log.sigma.2.0.mm.3D_glszm_HighGrayLevelZoneEmphasis
0.379883624	0.155362616	18511.40263	425.2056893
0.324	0.477138094	105163.9599	502.9814815
0.462432916	0.055542339	7365.040619	370.6015474
0.362005707	0.123943882	26821.30405	488.4459459
0.287671233	1.280804388	21549.38095	191.2380952
0.324786325	0.20866106	30386.90789	356.5357143
0.216650899	2.962856034	93591.27074	194.2576419
0.303601108	0.514780519	159270.9617	590.0437956
0.314750863	0.570294997	102046.2085	470.3510972
0.376290376	0.110416177	21488.44956	452.2929204
0.272827892	0.574713447	104774.3976	419.2271805
0.446884273	0.038549314	8358.682603	489.5033201
0.274837829	0.790009721	72482.07702	330.1204969
0.368137783	0.246767272	7766.796784	192.2163743
0.409560724	0.082312297	7499.35489	337.7902208
0.318325627	0.728227513	113259.1491	423.7041284
0.295327103	0.773699283	43914.44093	246.4810127
0.277560594	1.086756424	81524.10282	325.415493
0.343309859	0.559658026	18874.97692	251.2179487
0.281282316	0.802837823	118633.2279	378.6237745
0.405899281	0.073533872	14895.1301	455.7398086
0.361150401	0.129369929	16524.01567	357.2075718
0.388255278	0.104066892	12823.26563	339.519375
0.333009709	0.216300208	23691.00875	391.819242
0.401689708	0.092070243	5562.159656	264.9808795
0.410502959	0.100164679	12525.91351	426.2756757
0.447761194	0.069162774	10663.68081	392.9646465
0.446199702	0.061987986	9452.070808	397.4175017
0.269196823	1.836698443	388753.224	462.7628415
0.338398227	0.770098463	109495.3873	400.1019645
0.369628199	0.174450142	17902.4128	319.0241672
0.3242	0.459384828	87578.71561	470.5848242
0.318451205	0.603315446	112661.9504	428.3300248
0.343035343	0.301189324	24310.04242	351.7030303
0.411036036	0.118525156	15494.39178	372
0.267951508	1.17634542	316397.3573	488.6102088
0.413561222	0.106960195	29740.5122	538.6856369
0.34237688	0.263950838	49392.79587	427.7950043
0.260781671	1.775315364	26831.7261	140.4134367
0.284841076	0.88213079	160966.382	454.3819742
0.399696049	0.077167356	14374.36502	428.1140684
0.286231884	0.705604421	84954.8264	381.7142857
0.257350512	1.666094039	428717.4467	520.8754814
0.278131635	1.521905086	177796.6031	398.8549618
0.340583858	0.208972219	138863.7655	788.5798319
0.407272727	0.072900482	14120.54533	435.9354396
0.3982637	0.129955804	29921.69619	490.979564
0.352242744	0.188708482	28015.23034	332.5505618
0.304964539	0.639791698	15038.49419	235.9651163
0.298165138	0.545417626	80615.47385	416.1353846
0.264809237	2.317779538	271892.963	375.8265403
0.217791411	1.541927843	541981.3803	539.1830986
0.278114301	1.646926609	292565.9432	465.4327652
0.408198121	0.074258689	8706.780335	370.9037657
0.352298705	0.68808193	119803.2675	461.7627974
0.371292373	0.117793499	29268.8331	498.831669
0.364135407	0.166008512	36075.97236	509.6582915
0.235608553	2.125513841	226288.2199	387.5951134
0.39212828	0.053875175	24423.08736	702.7063197
0.33024911	0.50305591	13484.87716	165.3534483
0.332199932	0.320198427	58988.24053	489.5404299
0.313650447	1.342078846	332539.6932	532.5671121
0.397181858	0.160338287	19627.06984	372.210643
0.359207709	0.254907346	15768.76304	253.8315946
0.372881356	0.35184025	10028.27727	193.8090909
0.400518471	0.130546848	8478.592233	313.97411
0.355387524	0.169156089	31357.34574	460.8803191
0.31239484	0.525333678	276599.2406	749.456912
0.324052591	0.193361831	46447.71599	443.9642005
0.357798165	0.259329608	57225.11706	467.5395764
0.413602941	0.063155323	11055.50921	448.0196825
0.261038331	1.574408264	123143.9461	318.7546468
0.331053644	0.570514253	72859.67721	394.9677211
0.310397788	0.453775338	106885.8318	467.6249381
0.396996715	0.09603555	30204.70626	611.4722222
0.354354354	0.278769295	50190.67797	414.1368039
0.287473461	1.556629476	148950.4535	296.4460857
0.403237675	0.081975823	7446.90146	329.4981752
0.314713065	0.458842802	71295.55868	375.986421
0.307882327	0.887891899	105454.8512	327.4989059
0.375183554	0.181925552	45669.87476	523.3111546
0.460669928	0.052424035	23205.75245	638.5996732
0.4021827	0.090075254	28800.1407	571.0723618
0.23709369	2.449666423	27915.91129	161.0564516
0.415328845	0.117406593	15624.23504	360.1508728
0.31433506	0.506186182	30611.46795	254.3470696
0.391456583	0.214361227	35175.02504	422.1592129
0.288531055	0.998590844	154746.7483	399.3747153
0.282238443	1.394471045	10767.73276	123.887931
0.273356401	0.28388696	61268.38608	487.9746835
0.326066646	0.758121948	119609.4222	410.3572111
0.456108597	0.039493984	18654.58333	811.4503968
0.423815621	0.104766473	14717.11631	366.5619335
0.345049391	0.353892487	81286.82024	450.1211718
0.298555036	0.483484091	39641.87758	312.3622776
0.495475113	0.034117308	8882.502283	617.7283105
0.240499149	1.865301245	271672.5637	377.9198113
0.264018692	0.735170175	44165.79646	308.7433628
0.260135135	1.024343668	98124.87013	301.2121212
0.518197574	0.025573573	4471.72408	537.1655518

log.sigma.2.0.mm.3D_glszm_SmallAreaEmphasis	log.sigma.2.0.mm.3D_glszm_LowGrayLevelZoneEmphasis	log.sigma.2.0.mm.3D_glszm_ZoneEntropy	log.sigma.2.0.mm.3D_glszm_SmallAreaLowGrayLevelEmphasis
0.62402308	0.008486168	6.203617785	0.005580085
0.670940803	0.005484631	6.158386498	0.004713338
0.696971906	0.006728537	6.031181233	0.005613766
0.688522264	0.006898878	6.278144546	0.006057314
0.686712789	0.017303935	5.774181991	0.013466517
0.654074051	0.008682852	6.106448356	0.007398929
0.639009921	0.013041317	5.534256178	0.011000291
0.686540712	0.004602004	5.974185546	0.004022142
0.676541166	0.005115227	6.028648619	0.003139134
0.653005688	0.004184024	6.253863978	0.003228527
0.655324646	0.00628593	6.035895872	0.005282835
0.699693478	0.0060945	6.254465069	0.005122287
0.641782133	0.006291199	6.211263486	0.004914079
0.608694387	0.017183496	6.204263943	0.011247075
0.652438672	0.005925828	6.078248885	0.004673099
0.664206658	0.005185755	6.329932301	0.0041096
0.621214835	0.012732064	6.17098335	0.010315358
0.648544506	0.006727701	6.130903938	0.005468797
0.693170622	0.015389917	5.867909086	0.011219637
0.62556679	0.006014249	6.272269029	0.004609322
0.661539814	0.003842076	6.416896656	0.00288709
0.641678892	0.006610751	6.254332712	0.005336096
0.650870777	0.006903693	6.310083845	0.005584109
0.64355346	0.011048134	6.137593321	0.009851401
0.654961339	0.008567066	6.278332706	0.006511599
0.702275615	0.007599786	6.186071043	0.006351528
0.720638144	0.004945016	5.918544931	0.004125218
0.692415984	0.006724675	6.25945704	0.005599042
0.687209575	0.004658379	6.171833972	0.003859911
0.687254807	0.00780276	6.15920623	0.006531498
0.662241224	0.009079296	6.332007505	0.007406724
0.653583195	0.003576026	6.247607055	0.002731923
0.677575772	0.006875414	6.22771937	0.005959841
0.697278059	0.010457545	5.656728101	0.009505345
0.681828584	0.011983916	5.995115403	0.008683222
0.699550091	0.005126266	6.133081266	0.004298935
0.702268601	0.003938496	6.341372296	0.003170585
0.676246169	0.00507024	6.290521661	0.003914845
0.583957636	0.017249762	5.901908432	0.011629889
0.672091683	0.006473793	6.068689949	0.005658063
0.698882516	0.008873108	6.014615686	0.008106713
0.658809639	0.00657158	6.144478021	0.005537952
0.660606923	0.004543745	6.194927354	0.003746002
0.707276411	0.007714386	5.937993857	0.006798264
0.672468235	0.002605032	6.424025264	0.002145983
0.684213159	0.004549163	6.294524078	0.003123799
0.685027541	0.004539884	6.338934757	0.003656671
0.658137109	0.008270908	6.087838529	0.006642502
0.689252652	0.015893391	5.657767575	0.008388683
0.690765463	0.006801866	5.743811814	0.005950299
0.672801404	0.006478731	6.121205546	0.005395369
0.680273298	0.006301849	5.930518682	0.005665872
0.682357797	0.004311676	6.193323012	0.003467977
0.669589494	0.007545266	6.065411632	0.00632444
0.697398276	0.004795782	6.196494361	0.003376378
0.674314031	0.006394263	6.358265572	0.005537992
0.67939488	0.005796685	6.392319073	0.004653154
0.64931627	0.0092918	6.124074672	0.008200091
0.692228796	0.004595846	6.197535329	0.002703118
0.620515	0.01688656	5.821052128	0.01335216
0.679851809	0.005582449	6.239956701	0.004731593
0.692333673	0.005904244	6.318997524	0.005054668
0.681256118	0.009922382	6.281405488	0.007910441
0.672240156	0.01415061	6.032858174	0.012515967
0.713449989	0.014303314	5.582912517	0.012301778
0.653064344	0.008325329	6.278107805	0.006719624
0.695152524	0.008226586	6.064555578	0.00746187
0.688958452	0.002963455	6.170470669	0.002513358
0.681214165	0.005734577	5.914382484	0.004933276
0.702628881	0.005766575	6.300425347	0.004934945
0.677247968	0.004905408	6.375072043	0.003963474
0.654120856	0.007604089	6.127619813	0.006221377
0.66103523	0.005498834	6.329733886	0.004149355
0.666477999	0.003993128	6.321501187	0.003128188
0.690622094	0.003624997	6.421126402	0.002869492
0.692337757	0.006549557	6.217447376	0.005320904
0.696863484	0.008141121	5.989269303	0.006671927
0.679997449	0.009542693	6.169401935	0.008140812
0.669963815	0.006899073	6.289519654	0.005600585
0.675430789	0.009719943	6.308277717	0.007499455
0.699859724	0.006349044	6.132416178	0.005058728
0.728631876	0.003571789	6.23911171	0.003084713
0.710695078	0.006819371	6.293605416	0.006171979
0.637168221	0.019659038	5.391111836	0.016363388
0.680140881	0.007451419	6.250988505	0.005946224
0.645047154	0.007627542	6.146556201	0.005812763
0.696948302	0.006378446	6.156571354	0.00540553
0.669559135	0.00593279	6.114759335	0.004702948
0.695837699	0.033285689	5.082788407	0.03034835
0.679024932	0.017216169	5.774010572	0.016436524
0.681551516	0.004961596	6.107538958	0.004134509
0.713896159	0.006298764	6.181809344	0.004422805
0.713726252	0.00882049	5.997644107	0.00787866
0.699777638	0.006502384	6.316425152	0.00563435
0.642974572	0.006888881	6.295037754	0.005419027
0.734905596	0.006819386	6.28395778	0.005667498
0.644303933	0.006842943	5.843144243	0.005890743
0.664472442	0.013751587	5.398193981	0.012502728
0.70560329	0.010319779	5.510593557	0.009209638
0.688742322	0.007510969	6.30076215	0.005471158

log.sigma.2.0.mm.3D_ngtdm_Coarseness	log.sigma.2.0.mm.3D_ngtdm_Complexity	log.sigma.2.0.mm.3D_ngtdm_Strength	log.sigma.2.0.mm.3D_ngtdm_Busyness	log.sigma.2.0.mm.3D_ngtdm_Contrast
0.005927227	0.830383278	2.118536985	0.228931688	9.22E-05
0.003756166	0.642256092	2.09297549	0.238906189	3.15E-05
0.006217594	0.935611837	1.615634824	0.260268645	0.000160717
0.002723815	0.65710351	1.680662179	0.312855195	2.98E-05
0.006598976	0.62304975	2.274517217	0.371717832	7.82E-05
0.004174054	0.581964332	1.588691005	0.291916322	4.68E-05
0.006771122	0.284632662	1.399413524	0.371710114	4.63E-05
0.003951589	0.743914015	2.751852699	0.194002245	2.93E-05
0.003700982	0.495853932	1.699594275	0.291702177	3.15E-05
0.002411195	0.373921003	0.953339501	0.482059895	2.96E-05
0.00372808	0.535243166	2.025173556	0.265433095	2.53E-05
0.003816297	1.260092332	2.147514337	0.230546755	7.00E-05
0.00243643	0.320343892	1.255035096	0.487258074	1.44E-05
0.003702791	0.364543571	0.793818538	0.644477349	5.98E-05
0.004343309	0.584456246	1.09504162	0.408825869	8.01E-05
0.002099953	0.299663199	1.00082046	0.468969487	1.43E-05
0.004460838	0.460107686	1.391942095	0.37992954	4.41E-05
0.003047541	0.302151798	1.22937212	0.44190887	1.95E-05
0.006823247	0.630453388	2.070303178	0.285621026	7.55E-05
0.002566399	0.284242708	0.993676518	0.485917307	2.19E-05
0.001000225	0.245234572	0.47359419	1.019715968	1.50E-05
0.003339559	0.530389603	1.450125784	0.361858544	3.55E-05
0.001669484	0.265184281	0.577811002	0.822879518	2.60E-05
0.007249286	0.907137715	3.011082398	0.171935832	7.65E-05
0.002878466	0.43585143	0.995190274	0.591683395	4.25E-05
0.005320604	1.105071188	2.184790771	0.217876262	9.60E-05
0.003134313	0.576070677	1.369462974	0.354457014	3.47E-05
0.002095025	0.418327351	0.746374028	0.604172769	4.39E-05
0.002169749	0.346382334	1.290482978	0.400419645	1.31E-05
0.002424284	0.452672238	1.266263378	0.398451858	2.01E-05
0.001696958	0.295282971	0.666671381	0.758517888	2.21E-05
0.001507248	0.239076859	0.883153632	0.607804861	8.70E-06
0.002714684	0.523616444	1.576056476	0.347858356	2.70E-05
0.01334128	1.279153052	4.455431235	0.128189356	0.000195676
0.007145309	1.441839132	2.691562639	0.175252256	0.000180026
0.002148372	0.354075821	1.410337081	0.396672012	1.59E-05
0.00202131	0.515284526	1.038265592	0.442232635	3.25E-05
0.002049603	0.397408279	0.967270107	0.488495215	2.41E-05
0.004970204	0.250622231	0.97909806	0.605161201	3.64E-05
0.004123221	0.614258551	2.254081785	0.246055409	3.73E-05
0.004648141	1.087688643	2.276847435	0.219688453	7.27E-05
0.004002035	0.419679702	1.608867162	0.308272258	3.05E-05
0.002382657	0.401751648	1.762507507	0.330157578	1.36E-05
0.003701724	0.41574303	1.64716349	0.325654239	2.96E-05
0.002137612	0.56008501	1.489600428	0.295984838	2.11E-05
0.001882216	0.42068463	0.849561712	0.559814554	2.93E-05
0.001880777	0.475824278	1.064163646	0.473091244	2.51E-05
0.004598398	0.643760187	1.546505692	0.29507113	7.37E-05
0.011749234	0.979878425	3.832137474	0.187973352	0.000145983
0.005730567	0.690075678	2.306865366	0.221650508	6.26E-05
0.001873029	0.249970949	1.037168049	0.571024868	9.90E-06
0.003641695	0.476666908	2.305872826	0.232115126	2.30E-05
0.001875988	0.303458058	1.142248118	0.476999981	1.13E-05
0.005869383	0.970007941	2.195945901	0.218711439	7.92E-05
0.001826541	0.35567585	0.940199599	0.526532375	1.82E-05
0.004179368	0.814909483	1.771927454	0.233728964	5.95E-05
0.002280816	0.476821343	1.318278425	0.384771383	2.40E-05
0.003046407	0.290615105	1.383027326	0.395372634	1.81E-05
0.00481583	1.495983236	3.831635928	0.135689433	6.49E-05
0.004751748	0.330869213	0.859402257	0.558887724	5.45E-05
0.002502012	0.505018615	1.564833825	0.345871702	2.13E-05
0.00136554	0.300669439	0.931366651	0.551342609	1.11E-05
0.003024282	0.602097732	1.265569085	0.395292424	4.93E-05
0.003713785	0.376683505	0.945680056	0.492371365	5.34E-05
0.012181218	0.806454752	1.967960747	0.242666298	0.000207392
0.004663183	0.756342531	1.802660647	0.332549935	6.82E-05
0.006377807	1.358841768	3.533076943	0.152788276	7.86E-05
0.0019697	0.460113434	1.693824016	0.324664807	1.47E-05
0.004919433	0.765453668	2.099565959	0.229112959	6.41E-05
0.002788788	0.729088468	1.640440244	0.307736685	3.71E-05
0.001900433	0.406382607	0.805154942	0.596994265	3.11E-05
0.003463375	0.341779367	1.34052259	0.405567969	2.60E-05
0.001590883	0.313558743	0.97658404	0.60304921	1.16E-05
0.001127532	0.171703216	0.516346989	0.899686316	1.05E-05
0.001720117	0.478429552	1.185451831	0.436420392	2.03E-05
0.002957861	0.553223928	1.338387992	0.356963379	4.28E-05
0.00304264	0.406261413	1.250061901	0.413530277	2.54E-05
0.004840041	0.867711472	1.98587229	0.282109776	7.77E-05
0.00212565	0.335175571	0.940534679	0.522326212	2.30E-05
0.001697518	0.336037158	0.899307545	0.612685476	1.63E-05
0.005586141	1.159540834	2.902645588	0.155043508	7.18E-05
0.002508744	0.966129903	1.680114114	0.275849374	4.54E-05
0.002780862	0.858618688	1.909987427	0.265006442	3.72E-05
0.013804819	0.416029198	2.442907342	0.268587357	0.000115714
0.001732253	0.332846894	0.684450844	0.721269191	2.99E-05
0.00196605	0.193291257	0.521018804	0.940032966	2.19E-05
0.004638729	0.981814431	2.202876992	0.234861956	7.45E-05
0.002314801	0.287576923	1.074048785	0.501760979	1.96E-05
0.016568073	0.521094182	1.898779218	0.298107856	0.000213265
0.010636054	1.558749125	6.088857014	0.092585557	0.000104586
0.002125206	0.338529064	0.972885286	0.513612493	2.04E-05
0.005929109	2.302867965	4.214912441	0.107137973	0.000130782
0.004308797	0.799904123	1.552915178	0.289716033	8.19E-05
0.001597249	0.344865122	0.841949678	0.584918684	2.09E-05
0.001583109	0.195039675	0.641467637	0.830397091	1.14E-05
0.007787855	3.633468212	4.699850172	0.104844467	0.00027829
0.003726453	0.361780908	1.908257055	0.317445341	2.00E-05
0.01606309	0.807835375	4.040536404	0.128307919	0.000146641
0.007413475	0.60342635	2.753305742	0.195548208	4.81E-05
0.005668803	2.023600392	3.005570281	0.172640406	0.000182994

log.sigma.1.5.mm.3D_gldm_GrayLevelVariance	log.sigma.1.5.mm.3D_gldm_HighGrayLevelEmphasis	log.sigma.1.5.mm.3D_gldm_GrayLevelNonUniformityNormalized	log.sigma.1.5.mm.3D_gldm_DependenceEntropy
21.16875448	500.6076475	0.078659682	6.187691828
17.5049	523.305	0.0932285	6.281654339
24.00628518	341.8533095	0.068162544	6.285790534
22.9996063	774.6490012	0.078144037	6.28357526
12.66456067	195.5837724	0.114953237	5.978837229
16.38139703	495.7454212	0.096403935	6.22818069
7.941549288	212.7265847	0.138397976	5.857393926
16.04722616	616.2022161	0.104181828	6.234462226
14.23415416	404.5061667	0.100046754	6.241936126
17.56622199	522.8501499	0.07860948	6.270132901
13.17005104	464.3386829	0.110132795	6.12652064
30.74032667	627.2593472	0.063222534	6.324724242
12.33902297	349.4356436	0.105247545	6.196938739
13.84668892	214.9886975	0.084329713	6.174297712
15.79957468	393.3837209	0.075684888	6.043512553
15.10062171	451.5943052	0.100162101	6.588434176
11.73710038	266.6218069	0.099130637	6.131976891
11.93986289	296.5570758	0.111479355	6.287812184
15.46285149	212.6971831	0.09397162	6.297288225
12.26969126	420.5018959	0.106457246	6.318683714
22.96048809	503.6866187	0.064485399	6.497193807
15.7504313	389.4917492	0.085272641	6.279866261
17.38939332	423.8701771	0.079988678	6.396621489
17.59747856	438.2456311	0.100684325	6.229404742
17.28272821	330.3172043	0.073321559	6.449629626
25.63901824	386.8639053	0.070498232	6.40098797
18.31743139	474.0655812	0.079291262	6.058947472
24.13523993	398.0590164	0.065802715	6.399708084
12.40559512	501.3892321	0.121382031	6.247981591
17.54168021	603.7211143	0.098641876	6.321355315
18.31879341	427.0574602	0.078292677	6.27124504
11.64519836	441.6186	0.10071896	6.254301965
20.15827321	607.9190043	0.094191176	6.259110373
16.68573355	461.0977131	0.116670485	5.980826638
27.14809954	561.393018	0.074656582	6.173152817
15.22209018	597.4308362	0.117195919	6.18161395
27.20238196	775.1919305	0.070459557	6.498038665
20.63754673	432.5443822	0.08103232	6.30282733
7.943279982	152.2021563	0.114636082	6.036509916
18.1027956	670.4743276	0.113757689	6.221935245
22.41772237	624.1952888	0.073181604	6.203400741
14.50695355	485.1858178	0.111994136	6.184883651
14.29820504	656.0759828	0.115435587	6.236383469
14.13698409	626.2266454	0.115920299	6.139396086
21.05550279	982.339439	0.082905227	6.492951093
24.60270135	486.1012587	0.065841459	6.345829417
25.13019862	623.8524145	0.070675245	6.397098646
19.45802165	443.1497361	0.080939634	6.205507238
15.67360797	209.2978723	0.129017655	5.881242828
13.49401902	472.5541284	0.109157478	6.111336414
12.08926671	511.5848394	0.109649189	6.211573246
12.63278528	730.4672802	0.138226045	6.066894287
14.17055187	475.5206742	0.108369172	6.174951756
18.04730022	430.0922289	0.078711911	6.156633535
18.86301256	666.014224	0.087936111	6.360297857
24.79761563	647.4692797	0.079551269	6.64393268
21.1535351	724.462641	0.08028346	6.341804421
11.50395968	604.7228618	0.127749118	6.124341702
26.15354731	772.7733236	0.078857449	6.223836001
11.22333101	214.4227758	0.097341726	5.989150812
19.59639336	639.7266236	0.086354199	6.312214915
19.8675097	764.688901	0.09633762	6.43662798
23.67533356	483.2122413	0.072465201	6.348394955
14.70350035	318.0562099	0.094735406	6.285655189
13.28464234	203.4440678	0.096202241	5.956601468
22.16061077	502.7731692	0.069820052	6.224834299
21.61650098	723.0652174	0.083429519	6.236414135
17.22215578	727.296691	0.10551246	6.270866934
16.93611325	604.1268368	0.091658397	6.181073813
24.76595425	560.1336258	0.080438673	6.436966317
22.7318217	664.5317752	0.066013446	6.474966843
12.25897887	355.506065	0.119407325	6.155240039
16.27630077	366.5396452	0.089643234	6.337720069
15.88191589	555.6751651	0.091032057	6.350088064
25.13748643	635.1417175	0.068153276	6.466515759
21.416435	604.8326898	0.085272071	6.276436069
14.54347826	303.3125265	0.106880333	6.229741138
21.3109085	460.5202355	0.078456056	6.180743166
16.77331643	503.0323565	0.097831232	6.24957737
19.40955607	416.1196946	0.091349332	6.421763613
24.31855471	819.417768	0.08091021	6.277712594
31.56702437	663.4734663	0.063763368	6.401958808
28.69689165	831.0929669	0.072623024	6.417213707
9.325512103	146.7036329	0.156308837	5.684369224
22.2465693	526.2967374	0.071849201	6.308576383
12.51255332	279.4219919	0.092515699	6.184721
23.6001278	537.2191877	0.073898187	6.270882111
14.55518961	474.1876438	0.105122341	6.217549005
9.026243037	145.9902676	0.127308032	5.620399565
16.31105051	560.1366782	0.112019732	5.862001157
15.54851593	438.9134226	0.095750835	6.25206514
38.7067947	923.3520362	0.065696444	6.277184972
25.67222372	470.2016645	0.070680748	6.238731878
23.68152206	706.4477372	0.08206043	6.446661403
13.11547614	395.4235019	0.09585723	6.153936385
46.72936032	654.0395928	0.05138869	6.549344249
9.068603412	378.6108905	0.13490354	5.854045534
9.750562276	290.1565421	0.129607389	5.598807476
10.42491351	458.3096847	0.135543483	5.930224984
39.48680349	691.0190641	0.050461209	6.381614756

log.sigma.1.5.mm.3D_gldm_DependenceNonUniformity	log.sigma.1.5.mm.3D_gldm_GrayLevelNonUniformity	log.sigma.1.5.mm.3D_gldm_SmallDependenceEmphasis
249.0282627	94.62759767	0.370126642
340.775	186.457	0.329599122
249.9552773	76.20572451	0.398734454
503.3253159	191.6873216	0.385550278
149.2781876	109.0906217	0.294220981
279.037851	157.9096459	0.32994896
138.1296121	146.2866604	0.248693337
267.3811634	188.0481994	0.306464411
318.146522	202.7947706	0.325371492
613.3056943	236.0642691	0.37855978
279.4571112	199.0099613	0.287553461
418.8908012	106.5299703	0.438197104
462.1928986	308.270058	0.290495619
362.6469322	156.6846071	0.357525118
356.5245478	117.1602067	0.409539412
589.1445607	411.5660745	0.305855151
272.6037383	159.1046729	0.294424907
353.1501173	285.1641908	0.285873059
190.5809859	106.7517606	0.324518575
414.3560841	308.8324716	0.273714999
1549.235108	448.1735252	0.402162798
389.1037247	180.863272	0.345852611
770.8767289	329.6333414	0.341492318
165.1572816	103.7048544	0.329796793
500.6482335	190.9293395	0.363401139
286.2381657	95.31360947	0.398980379
542.4545455	175.3129806	0.450831836
771.7910581	220.7681073	0.41837188
441.4030597	412.5775228	0.271722957
524.354226	311.6096866	0.339714953
837.1931434	324.2882665	0.377785675
808.8312	503.5948	0.305764081
431.9118925	238.3978665	0.321162732
72.72557173	56.11850312	0.324298036
192.259009	66.29504505	0.413539443
456.131489	377.0192726	0.286990007
737.7027178	251.4701597	0.395977501
647.1598349	274.7805957	0.356398542
223.0727763	170.1199461	0.27297489
237.3471883	186.1075795	0.320004965
296.9072948	96.30699088	0.416340006
273.9120083	216.3726708	0.290487272
429.8794186	349.4235216	0.281962488
264.6719745	218.3938429	0.291780285
613.4018317	289.6708643	0.347075476
833.5057343	235.3832168	0.415859623
788.0819316	260.5089528	0.398111806
297.2915567	122.7044855	0.360876232
84.39361702	72.76595745	0.323080503
163.4972477	118.9816514	0.294426784
586	436.8423695	0.286775844
241.4488753	270.3701431	0.253940935
567.4129576	411.4777456	0.299286188
249.1417592	92.17164816	0.378887999
689.487173	346.2044704	0.347334612
329.8400424	150.1927966	0.356807458
641.0042696	263.2494663	0.361107647
328.6324013	310.6858553	0.252519871
291.9198251	108.1924198	0.386017568
258.1074733	136.7651246	0.32479186
519.2114927	253.9676981	0.330967155
854.2323674	528.6045198	0.327082796
486.2373404	164.568472	0.390400474
303.9464668	176.9657388	0.314763036
108.5728814	56.75932203	0.353096222
348.5683733	107.7323396	0.406855914
196.2570888	88.268431	0.359979242
535.5053281	376.2574313	0.309791713
230.136891	118.5143078	0.329512333
466.1958516	201.6597527	0.370418308
833.4585084	251.3792017	0.403166546
288.1062591	246.0984959	0.283829745
807.3556315	419.4406925	0.328896293
1098.115343	592.709722	0.321704159
917.3688409	290.4692633	0.395026162
431.3337623	198.7691978	0.365843611
359.1944798	251.7031847	0.312979173
304.0831494	106.6217807	0.391378328
538.6141636	320.495116	0.313133671
713.1446216	406.7785762	0.321064585
264.5550661	110.1997063	0.365255416
639.7376741	169.4192699	0.448085836
515.9725141	179.6693614	0.40642219
65.24091778	81.74952199	0.236914728
847.4583118	277.4816157	0.408053092
617.3235463	321.3995394	0.329607217
306.7787115	105.5266106	0.40273911
481.5238252	319.8872823	0.313560486
61.06326034	52.32360097	0.295303887
98.48788927	64.74740484	0.31611792
529.7985051	307.4559327	0.318567278
253.7945701	72.59457014	0.436506553
350.6504481	110.4033291	0.432961338
795.5274523	357.2090512	0.363945756
808.0875478	451.1041224	0.318574258
229.1085973	45.42760181	0.481303539
242.3647192	237.8349404	0.257681386
60.79439252	55.47196262	0.277659142
119.6148649	120.3626126	0.267048758
331.8353553	58.2322357	0.508546257

log.sigma.1.5.mm.3D_gldm_DependenceNonUniformityNormalized	log.sigma.1.5.mm.3D_gldm_DependenceVariance	log.sigma.1.5.mm.3D_gldm_LargeDependenceEmphasis
0.207006037	2.494952699	9.753117207
0.1703875	5.425039	16.19
0.223573593	2.934770434	9.765652952
0.205187654	2.906890886	10.32490828
0.157300514	4.453999052	15.91675448
0.170352778	3.856431725	13.75702076
0.130680806	7.136162127	23.20435194
0.148133609	5.662791415	18.38060942
0.156954377	5.854054624	17.70843611
0.204231	3.298000701	10.95004995
0.154652524	4.594972083	16.42446043
0.248599882	1.877450361	7.347774481
0.157798873	4.439912806	15.90815978
0.195181341	3.231528977	11.37459634
0.230313015	2.133293605	8.280361757
0.143379061	9.400957991	24.30104648
0.169846566	4.004847003	14.44922118
0.138057122	6.598654148	20.9781079
0.167764952	5.440683272	16.36091549
0.142832156	5.893490352	19.54601861
0.222911526	3.085400052	9.861870504
0.183452958	3.75011698	12.75577558
0.187060599	3.438603925	12.15263286
0.160346875	5.415345461	16.76504854
0.192261226	3.548443727	11.89708141
0.21171462	3.021742936	10.1908284
0.24534353	2.156112247	7.787878788
0.230042044	2.546758381	8.849776453
0.129862624	7.53544944	23.54721977
0.165987409	5.516204825	16.46122191
0.202122922	2.882872418	10.43408981
0.16176624	4.78724096	15.9108
0.170648713	3.946635329	13.9498222
0.151196615	5.459848462	17.79002079
0.216507893	2.830345548	9.691441441
0.141787842	6.17106298	19.87970159
0.206697315	3.932377912	11.6657327
0.190846309	3.406627828	11.79799469
0.150318582	4.778603396	17.25606469
0.145077743	7.211205995	21.03178484
0.225613446	2.653964302	9.118541033
0.141776402	6.107996948	19.82712215
0.142015004	6.229013212	20.0049554
0.140484063	6.031127023	19.84394904
0.175558624	4.884205781	14.80652547
0.233148457	2.086255563	8.111048951
0.213804105	2.712754247	9.672273467
0.19610261	2.863143183	10.75197889
0.149634073	6.744479654	20.08510638
0.149997475	4.859166737	17.08990826
0.147088353	5.294755075	18.06676707
0.12344012	7.106783804	24.3997955
0.149437176	5.162509887	17.53726626
0.212759829	2.334842672	9.266438941
0.175130092	4.587509949	14.42291085
0.174703412	11.37810166	24.09216102
0.195487731	3.425675845	11.62458066
0.135128454	7.383531396	22.89391447
0.212769552	2.761719182	9.846938776
0.183706387	3.053038335	11.94234875
0.1765425	3.750429419	13.22577355
0.155682954	6.05579309	18.08292327
0.214107151	2.641885772	9.57331572
0.162712241	5.529238063	16.9614561
0.184021833	3.874392416	12.9559322
0.225903029	2.306502428	8.672067401
0.185498194	3.603339039	12.36672968
0.15016975	6.218587149	18.94223219
0.177986768	3.838029631	13.29853055
0.185957659	4.508033907	13.57997607
0.218870407	3.029778088	9.948004202
0.139789548	6.401403953	20.48665696
0.172548756	4.323099381	14.31053644
0.168655405	4.565874213	14.9576102
0.215243745	2.65927722	9.555138433
0.185042369	3.741421878	12.53667954
0.152524195	5.361235479	17.46454352
0.223755077	2.493351764	9.114790287
0.164412138	4.411923174	15.1037851
0.160149253	5.134697405	16.38648102
0.194240137	3.531400872	11.81497797
0.240774435	2.395081399	8.257056831
0.208558009	3.447118586	10.93532741
0.124743629	7.225076683	24.83938815
0.219435088	2.662952386	9.370274469
0.1776982	3.625521805	12.99539436
0.21483103	2.866846739	9.851540616
0.158239837	4.880151717	16.22839303
0.14857241	5.139550441	18.12408759
0.170394272	3.664048563	13.8200692
0.164994863	4.153968553	14.6543133
0.229678344	2.300427919	8.471493213
0.224488123	2.98574663	9.615877081
0.182753837	4.447060045	13.70526074
0.171714311	3.427079644	13.15257119
0.259172621	2.460924224	7.954751131
0.137472898	5.175932197	19.45944413
0.142042973	5.117302821	18.60747664
0.134701424	5.921166099	20.63288288
0.287552301	1.678024444	6.155979203

log.sigma.1.5.mm.3D_gldm_LargeDependenceLowGrayLevelEmphasis	log.sigma.1.5.mm.3D_gldm_SmallDependenceHighGrayLevelEmphasis	log.sigma.1.5.mm.3D_gldm_LargeDependenceHighGrayLevelEmphasis
0.022916163	186.1991331	4754.143807
0.034872895	171.538748	8272.832
0.035895478	134.9361065	3364.457961
0.014275822	289.7812719	8077.576845
0.096897673	63.77918443	2840.940991
0.029330306	154.2001366	7006.868742
0.120517096	53.02989839	4653.869442
0.031805288	182.1900306	11138.73518
0.04969566	131.4725714	6821.965466
0.022654304	194.5832965	5749.871795
0.037275061	127.7490755	7706.489209
0.014987339	267.4988589	4692.651632
0.049595469	103.2738884	5425.884944
0.060967341	76.53861936	2478.812164
0.023964753	163.2009771	3186.124031
0.058103743	136.9801802	10567.69214
0.058831337	78.32775775	3900.775078
0.080840865	86.8647699	5813.59226
0.099565354	73.98904726	3090.661972
0.049983217	111.5173438	8116.477077
0.022320568	200.0320304	4953.712518
0.035359931	131.1837694	4986.779821
0.030864135	135.0992672	5322.966028
0.043616328	154.6793979	6879.826214
0.041642163	118.6827132	3954.520737
0.037170083	156.8174815	3776.27071
0.018289215	208.1103989	3724.207598
0.026158558	161.789982	3577.549329
0.048705555	130.890688	11721.11268
0.029048809	204.6517277	9763.779994
0.026548323	157.540852	4549.727185
0.038598857	136.1800529	6835.1562
0.024525136	188.0996089	8537.022916
0.044681432	162.8988217	7620.698545
0.019637755	216.5714931	5608.582207
0.035247969	166.3090183	11835.10445
0.016173965	301.9841263	9002.605212
0.030756615	148.27479	5145.091713
0.126416095	41.40229319	2579.506739
0.033577289	215.3233526	13735.72616
0.016343749	249.4639266	5766.028116
0.043775584	137.1283815	9357.970497
0.03214254	183.1301211	12905.59234
0.034068463	183.3426721	11989.1311
0.015714386	334.1875291	14595.95879
0.019490759	198.2296236	3982.21035
0.017326758	244.0348638	6066.641888
0.026946129	148.3944672	4982.098285
0.13605583	82.02787173	3530.638298
0.039197046	131.5962491	8100.66422
0.037398109	146.0189605	9095.932731
0.034236329	171.2366263	18068.72699
0.040118439	140.4324436	8174.679484
0.025069547	161.2145164	3965.243382
0.023191294	229.5345681	9433.085598
0.040325701	225.7376222	15221.47034
0.017328605	259.6292861	8355.089052
0.04018302	155.8254786	13390.24836
0.014418259	285.2383247	7712.643586
0.066303017	71.02828614	2515.547331
0.023270695	211.9344104	8165.693302
0.024913482	249.0300392	13603.49371
0.022811362	184.0908356	4637.567151
0.05978947	94.36096963	5358.389186
0.072313312	68.3369017	2606.518644
0.020199896	210.354631	4156.38302
0.018874978	257.0244062	8861.216446
0.027757486	219.7461842	13494.36736
0.023147671	185.0517894	8230.966744
0.027847496	201.1011851	7671.766653
0.016674397	265.2443085	6470.35688
0.063112237	104.903515	6943.410966
0.044479103	120.8388973	5039.321009
0.028250511	172.4198173	8310.905084
0.0171478	250.2529066	5980.10183
0.021912526	208.103808	7703.585157
0.063744053	91.93206247	5333.265817
0.022626749	177.5580673	4148.169978
0.032175753	150.5528853	7700.413919
0.042789225	130.067809	6838.112508
0.016019811	289.1778219	9552.98605
0.014123026	288.7082778	5599.188182
0.014533259	327.260356	9117.381568
0.206609915	39.23064	3160.107075
0.019405081	206.7159787	5041.457794
0.051380082	88.22267189	3656.286126
0.021341391	217.8451538	5239.787815
0.037019862	145.1156052	7577.696681
0.155521584	47.89657729	2329.474453
0.027897969	177.0230187	7721.375433
0.035801354	134.0817112	6440.380878
0.013734424	392.4726637	7832.899548
0.024343996	193.1624094	4599.264405
0.020246865	243.1655676	9856.662302
0.035854163	126.98004	5112.528474
0.024523118	323.6829858	5035.007919
0.053780466	94.95616654	7320.360182
0.071675702	78.20219942	5192.941589
0.046949551	114.3771968	9538.593468
0.012502175	349.3609402	4138.171577

log.sigma.1.5.mm.3D_gldm_SmallDependenceLowGrayLevelEmphasis	log.sigma.1.5.mm.3D_gldm_LowGrayLevelEmphasis	log.sigma.1.5.mm.3D_gldzm_DistanceZoneVariabilityNormalized
0.002169866	0.00365641	1
0.002106655	0.0036696	1
0.003870014	0.006349255	1
0.001235545	0.002060755	1
0.003801173	0.008109498	1
0.001842052	0.00331903	1
0.002966519	0.006796163	1
0.001448924	0.002614036	1
0.002794341	0.004690956	1
0.001439839	0.002765458	0.996884743
0.001985703	0.003610629	1
0.002858412	0.003955263	1
0.00160186	0.003914644	1
0.00442495	0.008110093	1
0.002119439	0.00380731	1
0.00137743	0.00306432	0.994285761
0.003031885	0.006068966	1
0.002900101	0.005605976	1
0.006584732	0.01069561	1
0.001812625	0.00370374	0.995370395
0.00175981	0.003155221	0.995027999
0.002135444	0.003957743	0.997599043
0.001875614	0.003626111	0.995021811
0.002302819	0.004021766	1
0.002595519	0.004948787	1
0.005147329	0.007279114	1
0.001896372	0.003196606	1
0.002852395	0.004593684	1
0.001192468	0.002706427	0.993865089
0.001148575	0.002332931	1
0.001761022	0.003411138	1
0.001150941	0.002844546	0.998824912
0.001640537	0.002822294	1
0.003016066	0.004629725	1
0.002825154	0.003923991	1
0.001781837	0.003037147	1
0.001065004	0.00190569	1
0.002439092	0.004174527	1
0.004732577	0.010186153	1
0.001470667	0.002527691	1
0.001994202	0.002980928	1
0.001623179	0.003148091	1
0.001102039	0.002241524	1
0.001267704	0.002442195	1
0.000782791	0.001470441	1
0.002205798	0.003616599	1
0.001522592	0.00261203	0.983204564
0.002819169	0.004404047	1
0.004403238	0.011304965	1
0.00307749	0.004611177	1
0.001366677	0.002827883	1
0.001294651	0.002325456	1
0.002130796	0.003725274	0.996732035
0.002321791	0.004035052	1
0.001089881	0.002131317	0.997350998
0.001975447	0.003044796	1
0.001187137	0.002123018	0.996992488
0.000966908	0.00226232	0.996946572
0.001953012	0.002780852	1
0.003827017	0.007755639	1
0.001660843	0.002797332	1
0.000845268	0.001777254	0.998960499
0.002240085	0.003712861	1
0.002773707	0.005471565	1
0.005966074	0.009530417	1
0.001931755	0.003246599	1
0.001764567	0.002685303	1
0.001107992	0.002114799	1
0.001709123	0.002841306	1
0.002492762	0.00386531	1
0.001181158	0.002169235	1
0.002000652	0.004195501	1
0.002472379	0.004601839	1
0.001174263	0.002452951	0.999125874
0.001617463	0.00267576	1
0.001469501	0.002574575	1
0.004090681	0.006838272	1
0.002211723	0.003701584	1
0.002177962	0.003650113	1
0.002111726	0.003995763	0.997448984
0.001379198	0.002205898	1
0.00169947	0.002655062	0.998523986
0.001358994	0.002144638	1
0.004733312	0.010955626	1
0.001668174	0.002920268	0.996621631
0.002864245	0.005667947	0.993675953
0.001939762	0.003247883	1
0.001959979	0.003492279	0.994269388
0.008438608	0.014284682	1
0.003148093	0.004448403	1
0.002036659	0.003699195	1
0.004748282	0.005392065	1
0.003816744	0.005160936	1
0.001082357	0.002035156	1
0.001578589	0.003435411	0.998772253
0.002189561	0.004982464	1
0.001619328	0.003707858	1
0.004529442	0.007123717	1
0.002193898	0.003835008	1
0.002764127	0.003646358	1

log.sigma.1.5.mm.3D_gldzm_LowIntensityEmphasis	log.sigma.1.5.mm.3D_gldzm_LargeDistanceEmphasis	log.sigma.1.5.mm.3D_gldzm_HighIntensitySmallDistanceEmphasis
0.005569661	1	506.794
0.006151252	1	521.5820896
0.009103188	1	339.3934741
0.003060884	1	756.8183575
0.012409724	1	217.5798046
0.005372949	1	468.9681208
0.011893966	1	215.8777778
0.004484237	1	603.3692053
0.008262282	1	408.7563025
0.003671471	1.004680187	516.2817863
0.006605894	1	444.9417989
0.006036101	1	614.8669834
0.005481582	1	357.2796976
0.011881013	1	213.0464807
0.004958272	1	398.3132867
0.004364802	1.008595989	449.0313395
0.009861574	1	264.1635338
0.00972799	1	309.9772152
0.018930041	1	231.678744
0.006412608	1.006960557	409.7810325
0.00418097	1.007476636	495.989486
0.005816714	1.003605769	382.0724159
0.00526779	1.007485964	397.4602308
0.006600559	1	470.5146667
0.006847236	1	326.8897127
0.01194317	1	394.3513072
0.004046058	1	464.8251996
0.006421021	1	388.7559153
0.004305573	1.009230769	485.9971795
0.00327708	1	604.1261643
0.004522754	1	418.2709641
0.003666604	1.001763668	447.8018812
0.004838185	1	588.2516704
0.009050649	1	506.3641975
0.006311838	1	530.4866828
0.006035242	1	579.5364103
0.002573546	1	764.3259773
0.006551397	1	419.1349739
0.016513655	1	152.8691796
0.00450559	1	675.2891344
0.004484187	1	602.7996743
0.005317275	1	482.2836066
0.003815002	1	654.623608
0.004171749	1	635.8333333
0.002152683	1	963.9813711
0.005026415	1	478.9638626
0.003649966	1.025408348	610.522686
0.00732656	1	413.1235772
0.015713206	1	254.9447236
0.009867863	1	454.5751445
0.004629613	1	510.4725185
0.005044383	1	677.2623274
0.006856217	1.004909984	473.1440262
0.005727159	1	426.9230769
0.003012424	1.00397878	663.5008289
0.00507993	1	635.7522698
0.00310615	1.004518072	722.3503389
0.003708908	1.004587156	624.7725535
0.004625204	1	747.7043189
0.01134594	1	217.377907
0.004763769	1	645.8200562
0.002529533	1.001560062	763.8866355
0.005490672	1	472.8097166
0.00847554	1	303.1488095
0.016050506	1	197.9254386
0.004417827	1	517.7103448
0.004582533	1	718.2232779
0.003447287	1	716.8306922
0.004995965	1	563.5912088
0.00641705	1	545.2944606
0.002804215	1	660.6513234
0.006834155	1	373.0318979
0.007214396	1	371.3315789
0.003536722	1.001311762	538.4499344
0.003836508	1	637.3896444
0.003899143	1	573.4152174
0.013028369	1	291.7747748
0.005291206	1	457.9967267
0.006655245	1	482.2923767
0.006413538	1.003831418	404.526341
0.003520953	1	801.1639929
0.003595637	1.002215657	646.6410635
0.003162575	1	811.3756661
0.019052925	1	174.3432836
0.003931451	1.005076142	508.427101
0.008488606	1.009516257	269.9781919
0.00458415	1	538.3225806
0.005935596	1.00862069	467.9748563
0.027628513	1	168.1875
0.009277879	1	559.0945274
0.006141983	1	423.6202873
0.009786634	1	907.9411765
0.008163086	1	451.823219
0.002876098	1	673.8784562
0.004813907	1.001842752	401.3330774
0.00536447	1	670.7738589
0.006183894	1	372.1656315
0.014678137	1	290.4632353
0.00790458	1	432.3015873
0.004997262	1	688.805638

log.sigma.1.5.mm.3D_gldzm_LowIntensityLargeDistanceEmphasis	log.sigma.1.5.mm.3D_gldzm_HighIntensityEmphasis	log.sigma.1.5.mm.3D_gldzm_DistanceZoneVariability	log.sigma.1.5.mm.3D_gldzm_ZonePercentage
0.005569661	506.794	500	0.415627598
0.006151252	521.5820896	737	0.3685
0.009103188	339.3934741	521	0.466010733
0.003060884	756.8183575	1035	0.421932328
0.012409724	217.5798046	307	0.323498419
0.005372949	468.9681208	596	0.363858364
0.011893966	215.8777778	270	0.255439924
0.004484237	603.3692053	604	0.334626039
0.008262282	408.7563025	714	0.352244697
0.00368871	516.600624	1278.00624	0.426906427
0.006605894	444.9417989	567	0.313779745
0.006036101	614.8669834	842	0.499703264
0.005481582	357.2796976	926	0.316148856
0.011881013	213.0464807	753	0.405274489
0.004958272	398.3132867	715	0.461886305
0.004495239	449.3467049	1388.022923	0.33974203
0.009861574	264.1635338	532	0.331464174
0.00972799	309.9772152	790	0.308835027
0.018930041	231.678744	414	0.36443662
0.006431772	410.5475638	858.0092807	0.297138918
0.004208298	496.7523364	3194.039875	0.461870504
0.005829191	382.3329327	830.0024038	0.392267798
0.005304231	398.1522146	1595.019963	0.388983256
0.006600559	470.5146667	375	0.36407767
0.006847236	326.8897127	1079	0.414362519
0.01194317	394.3513072	612	0.452662722
0.004046058	464.8251996	1127	0.509724107
0.006421021	388.7559153	1606	0.478688525
0.004330041	487.0748718	969.0184615	0.286849073
0.00327708	604.1261643	1181	0.373852485
0.004522754	418.2709641	1753	0.423225495
0.003669666	448.0558495	1699.001176	0.3402
0.004838185	588.2516704	898	0.354800474
0.009050649	506.3641975	162	0.336798337
0.006311838	530.4866828	413	0.46509009
0.006035242	579.5364103	975	0.303077401
0.002573546	764.3259773	1586	0.44438218
0.006551397	419.1349739	1341	0.395458567
0.016513655	152.8691796	451	0.303908356
0.00450559	675.2891344	543	0.33190709
0.004484187	602.7996743	614	0.46656535
0.005317275	482.2836066	610	0.31573499
0.003815002	654.623608	898	0.296663363
0.004171749	635.8333333	588	0.312101911
0.002152683	963.9813711	1342	0.384087006
0.005026415	478.9638626	1688	0.472167832
0.003707205	614.4047187	1625.237145	0.448453608
0.00732656	413.1235772	615	0.405672823
0.015713206	254.9447236	199	0.352836879
0.009867863	454.5751445	346	0.317431193
0.004629613	510.4725185	1219	0.305973896
0.005044383	677.2623274	507	0.259202454
0.006868056	473.6963993	1218.006547	0.321833026
0.005727159	426.9230769	520	0.444064902
0.003016788	664.4323607	1504.005305	0.383032766
0.00507993	635.7522698	771	0.408368644
0.00311594	722.872741	1324.006024	0.405001525
0.00371931	625.2782875	652.0030581	0.268914474
0.004625204	747.7043189	602	0.43877551
0.01134594	217.377907	516	0.367259786
0.004763769	645.8200562	1067	0.362801768
0.002534348	764.0130005	1921.00104	0.350464735
0.005490672	472.8097166	988	0.435050638
0.00847554	303.1488095	672	0.359743041
0.016050506	197.9254386	228	0.386440678
0.004417827	517.7103448	725	0.469863901
0.004582533	718.2232779	421	0.397920605
0.003447287	716.8306922	1199	0.336231071
0.004995965	563.5912088	455	0.351894818
0.00641705	545.2944606	1029	0.410450738
0.002804215	660.6513234	1738	0.456407563
0.006834155	373.0318979	627	0.304221252
0.007214396	371.3315789	1710	0.365462706
0.003557218	538.4709226	2285.000875	0.351251728
0.003836508	637.3896444	1912	0.448615673
0.003899143	573.4152174	920	0.394680395
0.013028369	291.7747748	777	0.329936306
0.005291206	457.9967267	611	0.449595291
0.006655245	482.2923767	1115	0.34035409
0.006426079	404.8199234	1562.005109	0.351673029
0.003520953	801.1639929	561	0.411894273
0.003602475	646.8205318	1352.001477	0.50959729
0.003162575	811.3756661	1126	0.455133387
0.019052925	174.3432836	134	0.256214149
0.003946074	508.9892837	1767.010152	0.459088555
0.008569042	270.4385408	1253.025377	0.362982153
0.00458415	538.3225806	651	0.455882353
0.00596008	468.8390805	1038.017241	0.343082484
0.027628513	168.1875	128	0.311435523
0.009277879	559.0945274	201	0.347750865
0.006141983	423.6202873	1114	0.34693242
0.009786634	907.9411765	544	0.492307692
0.008163086	451.823219	758	0.485275288
0.002876098	673.8784562	1736	0.398805422
0.004851514	401.3556511	1626.001229	0.345941351
0.00536447	670.7738589	482	0.545248869
0.006183894	372.1656315	483	0.273964833
0.014678137	290.4632353	136	0.317757009
0.00790458	432.3015873	252	0.283783784
0.004997262	688.805638	674	0.584055459

log.sigma.1.5.mm.3D_gldzm_IntensityVariabilityNormalized	log.sigma.1.5.mm.3D_gldzm_LowIntensitySmallDistanceEmphasis	log.sigma.1.5.mm.3D_gldzm_IntensityVariability
0.049856	0.005569661	24.928
0.05270367	0.006151252	38.84260516
0.047689922	0.009103188	24.84644914
0.047214171	0.003060884	48.86666667
0.065539157	0.012409724	20.12052117
0.057914058	0.005372949	34.51677852
0.074211248	0.011893966	20.03703704
0.055858296	0.004484237	33.7384106
0.057171888	0.008262282	40.82072829
0.052127015	0.003667162	66.82683307
0.058916479	0.006605894	33.40564374
0.044930349	0.006036101	37.83135392
0.062292122	0.005481582	57.6825054
0.05733066	0.011881013	43.16998672
0.056489804	0.004958272	40.39020979
0.053411918	0.004332193	74.56303725
0.063634179	0.009861574	33.85338346
0.060483897	0.00972799	47.78227848
0.057632617	0.018930041	23.85990338
0.059501187	0.006407818	51.2900232
0.048655778	0.004174139	156.1850467
0.054187662	0.005813595	45.08413462
0.053589865	0.005258679	85.90455396
0.051377778	0.006600559	19.26666667
0.054247359	0.006847236	58.53290083
0.044833184	0.01194317	27.4379085
0.056169885	0.004046058	63.30346051
0.047224062	0.006421021	75.84184309
0.057325444	0.004299457	55.89230769
0.051166472	0.00327708	60.42760373
0.052168087	0.004522754	91.45065602
0.063008549	0.003665839	107.1775426
0.049679813	0.004838185	44.61247216
0.054945892	0.009050649	8.901234568
0.046661468	0.006311838	19.27118644
0.052802104	0.006035242	51.48205128
0.043030998	0.002573546	68.24716267
0.049050732	0.006551397	65.77703207
0.071479491	0.016513655	32.23725055
0.047851612	0.00450559	25.98342541
0.050568176	0.004484187	31.04885993
0.055694706	0.005317275	33.97377049
0.055810735	0.003815002	50.11804009
0.057395067	0.004171749	33.74829932
0.048116453	0.002152683	64.57228018
0.046673901	0.005026415	78.78554502
0.045655317	0.003635657	75.46823956
0.051776059	0.00732656	31.84227642
0.054013788	0.015713206	10.74874372
0.060509873	0.009867863	20.93641618
0.0592243	0.004629613	72.19442166
0.061781995	0.005044383	31.3234714
0.056044262	0.006853257	68.48608838
0.055428994	0.005727159	28.82307692
0.050102372	0.003011333	75.55437666
0.042717444	0.00507993	32.93514916
0.049324557	0.003103702	65.50301205
0.063649711	0.003706308	41.62691131
0.048950895	0.004625204	29.46843854
0.064314044	0.01134594	33.18604651
0.050288584	0.004763769	53.6579194
0.046971967	0.002528329	90.32709308
0.047173368	0.005490672	46.60728745
0.058646896	0.00847554	39.41071429
0.058864266	0.016050506	13.42105263
0.048055172	0.004417827	34.84
0.048357886	0.004582533	20.35866983
0.054331487	0.003447287	65.14345288
0.052983939	0.004995965	24.10769231
0.044719651	0.00641705	46.01652089
0.048543291	0.002804215	84.36823936
0.059850583	0.006834155	37.52631579
0.052438015	0.007214396	89.66900585
0.053447281	0.003531598	122.2339309
0.045149223	0.003836508	86.32531381
0.047403119	0.003899143	43.61086957
0.054539371	0.013028369	42.37709138
0.050581135	0.005291206	30.90507365
0.053444067	0.006655245	59.59013453
0.04772635	0.006410402	74.7394636
0.045630892	0.003520953	25.59893048
0.043238591	0.003593927	58.5450517
0.044048472	0.003162575	49.59857904
0.071842281	0.019052925	9.626865672
0.048205377	0.003927796	85.46813311
0.060616142	0.008468497	76.4369548
0.04789748	0.00458415	31.1812596
0.056836365	0.005929475	59.33716475
0.075439453	0.027628513	9.65625
0.059726244	0.009277879	12.00497512
0.054970363	0.006141983	61.23698384
0.040576341	0.009786634	22.07352941
0.0467833	0.008163086	35.46174142
0.045021794	0.002876098	78.1578341
0.057015134	0.004804505	92.82063882
0.03656101	0.00536447	17.62240664
0.073681142	0.006183894	35.58799172
0.077962803	0.014678137	10.60294118
0.069601915	0.00790458	17.53968254
0.040455582	0.004997262	27.26706231

log.sigma.1.5.mm.3D_gldzm_HighIntensityLargeDistanceEmphasis	log.sigma.1.5.mm.3D_gldzm_SmallDistanceEmphasis	log.sigma.1.5.mm.3D_glcm_SumVariance	log.sigma.1.5.mm.3D_glcm_Homogeneity1
506.794	1	1528.368072	0.318160241
521.5820896	1	1633.251353	0.364825186
339.3934741	1	1002.458519	0.299010308
756.8183575	1	2518.828837	0.315038988
217.5798046	1	532.1439594	0.39590695
468.9681208	1	1564.232715	0.351653211
215.8777778	1	617.8361858	0.444272035
603.3692053	1	2000.022955	0.390062381
408.7563025	1	1236.979586	0.383794683
517.875975	0.998829953	1622.626026	0.327086684
444.9417989	1	1447.537197	0.378596845
614.8669834	1	1968.512255	0.279242091
357.2796976	1	1043.484157	0.376332941
213.0464807	1	590.0693251	0.343931554
398.3132867	1	1223.586662	0.305291899
450.6081662	0.997851003	1411.227273	0.395697824
264.1635338	1	792.9554598	0.372476128
309.9772152	1	875.7483316	0.410719578
231.678744	1	566.4409396	0.384392129
413.6136891	0.998259861	1297.711658	0.392487401
499.8037383	0.998130841	1568.729977	0.29256578
383.375	0.999098558	1157.973616	0.339639018
400.9201497	0.998128509	1291.873858	0.327568852
470.5146667	1	1323.647904	0.369719718
326.8897127	1	966.9304602	0.326532199
394.3513072	1	1129.498377	0.307214261
464.8251996	1	1466.760707	0.316919193
388.7559153	1	1168.678196	0.2957357
491.385641	0.997692308	1600.500491	0.413061507
604.1261643	1	1974.691939	0.367017066
418.2709641	1	1290.183154	0.318106369
449.0717225	0.999559083	1364.778693	0.380091714
588.2516704	1	1938.346882	0.350644951
506.3641975	1	1426.914587	0.389562226
530.4866828	1	1725.732582	0.303721562
579.5364103	1	1927.218633	0.398042597
764.3259773	1	2512.711796	0.305810543
419.1349739	1	1288.536788	0.322277821
152.8691796	1	405.6778009	0.405726435
675.2891344	1	2176.8811	0.390695722
602.7996743	1	1975.0352	0.299849723
482.2836066	1	1509.616301	0.393737895
654.623608	1	2158.752685	0.398488945
635.8333333	1	2045.273702	0.406977541
963.9813711	1	3316.51928	0.335246706
478.9638626	1	1449.887031	0.28872338
629.9328494	0.993647913	1954.29989	0.304083948
413.1235772	1	1359.262133	0.316871512
254.9447236	1	566.9001902	0.407728261
454.5751445	1	1481.646857	0.386744958
510.4725185	1	1630.693692	0.396154562
677.2623274	1	2455.348626	0.427635137
475.905892	0.998772504	1476.00327	0.385762577
426.9230769	1	1321.243555	0.310120263
668.1584881	0.999005305	2158.86987	0.349949553
635.7522698	1	2088.443383	0.344679893
724.9623494	0.998870482	2353.083097	0.328449484
627.3012232	0.998853211	1988.133482	0.422462165
747.7043189	1	2500.9171	0.310219713
217.377907	1	611.0129835	0.357557917
645.8200562	1	2039.919897	0.347899233
764.5184607	0.999609984	2489.17304	0.364525056
472.8097166	1	1448.374249	0.308257521
303.1488095	1	961.0719251	0.367258491
197.9254386	1	572.1635235	0.366589764
517.7103448	1	1511.841883	0.306919261
718.2232779	1	2359.056973	0.340086178
716.8306922	1	2393.784532	0.377023293
563.5912088	1	1942.284558	0.34208558
545.2944606	1	1749.526458	0.334038894
660.6513234	1	2141.452903	0.307361369
373.0318979	1	1090.457869	0.398036796
371.3315789	1	1075.558077	0.356680318
538.5548754	0.999672059	1764.866063	0.359038131
637.3896444	1	1988.587557	0.307466795
573.4152174	1	1931.72529	0.335671811
291.7747748	1	897.1038767	0.371943783
457.9967267	1	1372.697333	0.30544777
482.2923767	1	1585.799862	0.360263842
405.9942529	0.999042146	1256.233496	0.357585976
801.1639929	1	2711.341461	0.32393306
647.5384047	0.999446086	2094.566282	0.276663716
811.3756661	1	2670.984911	0.303755447
174.3432836	1	384.0649881	0.460329132
511.2380147	0.998730964	1598.242469	0.305185831
272.2799366	0.997620936	829.864092	0.358794156
538.3225806	1	1646.393173	0.31209198
472.295977	0.997844828	1466.214002	0.368061651
168.1875	1	387.633527	0.425346308
559.0945274	1	1799.822513	0.379116852
423.6202873	1	1330.971872	0.357633281
907.9411765	1	2959.802771	0.284799588
451.823219	1	1409.841995	0.294682102
673.8784562	1	2264.465904	0.327816976
401.4459459	0.999539312	1200.061309	0.360164279
670.7738589	1	2025.910973	0.261565625
372.1656315	1	1190.265803	0.409159755
290.4632353	1	872.1453243	0.424871567
432.3015873	1	1456.243285	0.423236533
688.805638	1	2154.470462	0.253132619

log.sigma.1.5.mm.3D_glcm_Homogeneity2	log.sigma.1.5.mm.3D_glcm_ClusterShade	log.sigma.1.5.mm.3D_glcm_MaximumProbability	log.sigma.1.5.mm.3D_glcm_Idmn	log.sigma.1.5.mm.3D_glcm_SumVariance2
0.229668609	-4.991706868	0.02497709	0.979548633	47.26616654
0.284755865	-112.9137091	0.043943938	0.984549867	39.31715426
0.21261813	-169.5395216	0.024288183	0.962965923	47.82978192
0.226482486	-267.6962585	0.022191398	0.978589013	47.18331531
0.317006244	150.0007427	0.046215381	0.981319252	27.00862931
0.268919886	-177.1116687	0.033088335	0.980086163	35.83229471
0.37308294	24.38958756	0.075002288	0.981910991	16.05861924
0.313693594	-133.3289081	0.047306671	0.984462209	34.955077
0.304891044	15.87340094	0.047962222	0.98359925	30.14413072
0.239668193	-69.31852699	0.022639879	0.978082399	37.19260913
0.299055831	-120.1380549	0.040251715	0.982250505	28.37744981
0.191162587	-330.2014922	0.016281779	0.970713356	64.34858852
0.292938538	31.66584213	0.037981241	0.986866602	25.64517528
0.257329736	-30.57110296	0.025717965	0.969829336	29.607849
0.212189746	-15.76848751	0.023280779	0.974440113	29.31207374
0.322272796	-72.80135879	0.051679777	0.982294386	33.55981346
0.288952592	-18.82108742	0.035162281	0.980403964	24.59244964
0.336054651	12.66347563	0.053922688	0.981835396	26.82577205
0.304824693	110.5464901	0.046772404	0.97717024	34.27710167
0.314772116	-87.49075356	0.041044557	0.983959488	27.11436458
0.203840071	-149.190652	0.013004757	0.981705322	49.38911115
0.253186397	-73.84191065	0.03024644	0.982021824	33.06203828
0.241189947	-138.283673	0.021767054	0.971224662	35.53318331
0.290223307	18.36543123	0.03900511	0.979788728	36.08989019
0.239154468	-35.26269669	0.019274086	0.983674296	36.48858249
0.219886564	-101.7987129	0.026768454	0.965243593	50.81335683
0.229735522	-87.67183433	0.020615691	0.974853477	38.79832287
0.207919073	-143.3212111	0.018973524	0.970267424	47.7342497
0.341716505	-58.76392452	0.06816084	0.984424482	25.97581035
0.289421758	-99.58687858	0.04795284	0.983445195	37.69700875
0.229804783	-106.3813349	0.02106982	0.973719612	35.97140215
0.297374694	7.764764578	0.035133779	0.985384799	25.43400146
0.269004751	-205.2854811	0.033188965	0.984261602	43.03557261
0.313921918	183.6468448	0.060632475	0.984788196	39.01216734
0.220213733	-325.78292	0.024476294	0.966917223	59.22837099
0.324610157	-186.1087581	0.05637507	0.98187692	35.51606693
0.220007888	-236.8187534	0.022584632	0.975815813	57.09751658
0.237002941	-120.575009	0.024666676	0.975625355	43.21347689
0.32460089	-4.549395891	0.045667798	0.9777663	16.60480816
0.317392886	-89.42249515	0.058956144	0.983416973	39.60236115
0.21145111	-227.3496044	0.01777637	0.971194141	47.73901867
0.317458821	-16.1255828	0.050263308	0.982126304	31.21620472
0.322345931	-88.27871027	0.050249472	0.986980461	31.48257778
0.332600495	66.24724275	0.052448177	0.988008795	32.04975024
0.25335465	-172.8366327	0.030818112	0.983339125	47.89994235
0.199874168	-132.21393	0.016025193	0.970869989	53.51082862
0.217245326	-219.7965178	0.019940326	0.976145688	55.40064146
0.229384715	-187.9163835	0.02334864	0.971321558	41.00017685
0.334292861	171.2012574	0.074591871	0.978498459	34.38385674
0.308496511	-49.05693685	0.046003358	0.980706744	28.06861062
0.318431567	-28.56388759	0.043726777	0.988068955	26.1262285
0.359267518	-217.7292177	0.072223896	0.983389194	27.9203282
0.307751961	-48.70832903	0.045819891	0.985245866	29.4463827
0.218862624	-78.33995515	0.020352974	0.971789413	37.04525265
0.267719253	-84.14490074	0.034457583	0.982748606	39.19956818
0.26828959	-165.6040986	0.061729312	0.973317277	55.00194419
0.241491353	-163.1433307	0.026521391	0.982309604	45.17272532
0.349236914	29.5321439	0.066919232	0.988737753	26.41818085
0.222295319	-478.6789745	0.026757714	0.978772608	53.60508139
0.271165071	-38.34557357	0.029568289	0.974763984	22.59217334
0.262675188	-113.670829	0.030896928	0.987596792	39.95868718
0.286188382	-120.2088777	0.044511048	0.98615173	40.67911272
0.223120125	-211.7291332	0.020888268	0.973731722	51.93961683
0.286266094	-123.0316308	0.039867256	0.974115055	31.22676481
0.283675928	14.70732591	0.044494619	0.970204373	27.29952598
0.216134472	105.1817151	0.01944135	0.980422492	48.75705597
0.258044495	-85.424875	0.029035867	0.980615591	45.19536671
0.298663128	-206.3839124	0.04768812	0.98632928	37.03999387
0.257745016	-208.3331125	0.032181906	0.975304008	37.39654883
0.252256085	-190.3057498	0.034053194	0.976363055	52.69559936
0.220547101	-144.5624938	0.016667379	0.976422818	51.27492917
0.320757336	45.93038996	0.061384382	0.98523796	23.41106732
0.273355472	-20.81502042	0.031857784	0.986712996	35.32527164
0.277601882	-144.0947254	0.032383903	0.979219825	34.54645036
0.219344526	-168.9360313	0.018144612	0.979072187	56.52218656
0.253646404	-223.4377232	0.029984721	0.97625217	46.65568391
0.292062399	-59.71846905	0.041102241	0.984294802	28.70137985
0.216234214	8.758295759	0.019855778	0.974793319	46.13450623
0.279867728	-157.2174323	0.03538425	0.980012998	36.41903934
0.279618206	-123.6179378	0.039235014	0.977959445	40.52764929
0.239728192	-247.9806074	0.03325087	0.979047595	48.11941857
0.188337207	-392.7716539	0.016980152	0.97363628	64.84220117
0.218318865	-319.2919214	0.02297172	0.979438352	57.98621371
0.394122685	121.3174971	0.101053071	0.981401401	20.18996557
0.218446451	-172.1228559	0.020035699	0.972605758	48.33030675
0.273337412	-31.10118433	0.031137602	0.977257455	26.861877
0.225765434	-91.73853929	0.022507121	0.977264493	53.09868678
0.28587551	-75.12093448	0.039043983	0.980555626	31.56743772
0.350174484	37.85738426	0.061559539	0.972747252	17.45670283
0.299523002	-165.0125641	0.044573047	0.984469712	35.78683739
0.275152084	-80.71886697	0.035511437	0.978016693	32.60939136
0.199133479	-722.5698569	0.020192178	0.970292801	79.01875888
0.209196207	-242.2593316	0.02223751	0.96965847	53.43014747
0.245634785	-238.2819397	0.038479032	0.981538083	46.65939881
0.274262161	-22.3182758	0.030936528	0.98506668	25.85476553
0.178543104	-471.2740965	0.019051278	0.966412927	101.6231503
0.332288511	-43.06956179	0.069472096	0.986754527	17.51632789
0.350378062	-6.669694836	0.065414773	0.983242661	20.5659194
0.353024363	-85.15506696	0.062342864	0.987143182	21.44894655
0.165099439	-450.628901	0.014582342	0.970735952	81.93784268

log.sigma.1.5.mm.3D_glcm_Contrast	log.sigma.1.5.mm.3D_glcm_DifferenceEntropy	log.sigma.1.5.mm.3D_glcm_InverseVariance	log.sigma.1.5.mm.3D_glcm_Entropy	log.sigma.1.5.mm.3D_glcm_Dissimilarity
37.90987321	3.658743415	0.232340889	7.767639135	4.4619484
32.92869165	3.498146924	0.281975872	7.466976332	3.886187812
49.62273319	3.852964566	0.214969992	8.006434683	5.162308243
44.63806598	3.733095128	0.229892731	7.8724531	4.680283292
17.11104854	3.128008897	0.3193513	6.716651385	2.984643234
33.66818883	3.538045131	0.269158027	7.413113496	4.028114787
11.41221262	2.861093887	0.358470266	6.210616668	2.393342564
29.20565487	3.372628165	0.303808978	7.137837957	3.540465172
24.74250953	3.329106297	0.301188946	7.16147024	3.414600086
33.18858379	3.594069278	0.244915785	7.831603461	4.212662291
27.11321582	3.372138482	0.295061922	7.107505304	3.541180153
62.21863721	3.979440873	0.20098212	8.318350891	5.680165037
21.63483796	3.263146025	0.294063579	7.117080343	3.284651736
24.63057996	3.396845653	0.266237687	7.518215868	3.723580742
30.46746258	3.543437233	0.229027403	7.648318532	4.250810659
29.99015111	3.43777314	0.294412545	7.456825577	3.639649315
20.7020639	3.265023295	0.292527766	7.136305178	3.299157917
18.04995868	3.145868192	0.326832718	6.947652881	2.958263664
21.31462571	3.257528936	0.300119167	7.150339922	3.292979203
24.31170835	3.292004261	0.305282108	7.159865198	3.333300512
47.34528982	3.859072553	0.206799279	8.442342354	5.110642422
29.51648042	3.515945675	0.251287851	7.622111798	3.986228642
35.55867161	3.626090711	0.241860226	7.819718137	4.332321262
29.05395791	3.437370097	0.2825107	7.163643767	3.752405797
34.0752621	3.612464232	0.23859859	7.921846576	4.274120176
46.94789773	3.799760165	0.221121024	7.953443593	4.931876852
38.29871665	3.682887829	0.236204719	7.815896303	4.524158743
48.53244767	3.852226216	0.21459758	8.286246129	5.11427242
23.55792718	3.239625082	0.327583895	6.920540186	3.204714932
35.27307478	3.553041493	0.283366955	7.554220867	4.009673438
36.04777389	3.650555265	0.238493181	7.907804283	4.400203512
19.22677762	3.223542591	0.298451747	7.213799001	3.177230289
38.09569387	3.589652564	0.271764272	7.593747137	4.186542752
23.67826769	3.291670415	0.30364458	6.673490253	3.411008511
58.25713061	3.906703345	0.223587083	7.859970967	5.405002467
29.87065236	3.353923343	0.319859528	7.103791505	3.505275572
52.53546358	3.881663217	0.218682942	8.278636982	5.151362648
41.55309349	3.729968589	0.238089167	7.951134152	4.60927838
12.79115582	2.987843063	0.318080928	6.68445294	2.685521866
32.67454394	3.442225113	0.305688193	7.079319866	3.725967846
47.296111	3.814629208	0.212735849	7.912775347	5.010218597
24.29849433	3.315703916	0.307621669	7.069375027	3.355023892
25.32759204	3.286999676	0.314067579	7.033137089	3.308478627
21.85180353	3.217076605	0.32511258	6.874869596	3.130200048
43.94519663	3.72857033	0.249680953	7.99537798	4.586440973
49.80308532	3.878825719	0.207329078	8.398395081	5.221241069
49.15161932	3.847769501	0.220083641	8.280949928	5.043709316
40.05872842	3.69672515	0.233662288	7.785934561	4.571128813
20.05026688	3.174651209	0.32418265	6.458247291	3.107429892
25.13864355	3.317171541	0.302576207	6.9392327	3.408597153
21.72649772	3.241034578	0.313990333	7.074337891	3.177817205
28.12333559	3.198833265	0.343885089	6.621504613	3.19629932
25.87951041	3.348781029	0.302765224	7.162549847	3.448405475
37.14348119	3.646499421	0.221278262	7.705938067	4.480153283
35.29711492	3.576058816	0.267889297	7.712039763	4.099202337
53.4285235	3.854770798	0.234865552	8.032557934	5.013405614
37.89792425	3.644837584	0.242634611	7.862972888	4.345323664
18.71888903	3.085630924	0.339498968	6.658197662	2.854107846
51.26302783	3.766519329	0.234337652	7.73016695	4.896387739
22.04067305	3.318390206	0.277711153	7.192798982	3.467499927
33.78900419	3.512663752	0.268677233	7.565956153	3.967567901
37.85952626	3.574191513	0.281528452	7.611420949	4.08404938
49.7043958	3.831467758	0.230441168	8.1181396	5.033573686
28.35042201	3.447506244	0.276823126	7.358601981	3.730498465
20.84975284	3.27381627	0.288568638	7.005982129	3.395540998
35.69536011	3.650769031	0.22322002	7.946667745	4.482198725
42.19436032	3.650424964	0.261673937	7.572142945	4.42106327
32.05178264	3.439131068	0.296638815	7.290450932	3.716058088
36.46769657	3.599248475	0.25209696	7.480111176	4.222738254
47.41191934	3.751027764	0.250095366	7.925418005	4.704740581
43.91450677	3.786490811	0.225414897	8.304965913	4.837453331
19.69999175	3.182282413	0.32540112	6.754655139	3.07066141
28.83937385	3.475998196	0.271468112	7.630338911	3.799735332
31.93310813	3.516649841	0.276792329	7.646441408	3.904275892
44.87112311	3.781385327	0.222268325	8.288754751	4.822621136
42.95975963	3.698784991	0.258111329	7.804683821	4.529266731
28.90654159	3.43169979	0.287971907	7.219922826	3.687814833
38.07926333	3.696141831	0.223700503	7.833173964	4.622508378
34.0505016	3.530615814	0.279601387	7.506872179	3.979215536
39.66537899	3.628245057	0.27555955	7.75573136	4.266688143
47.95048992	3.74184952	0.249707478	7.684558174	4.736067718
62.57169304	4.011463842	0.196398757	8.422070771	5.752904789
54.97677667	3.89148579	0.222485224	8.143308323	5.215751799
10.76590419	2.788320023	0.374654686	5.875025006	2.293300796
44.34703504	3.796955446	0.224765068	8.182515766	4.882656877
22.51414721	3.350224046	0.276431726	7.418937324	3.502312537
44.31632465	3.766963151	0.228921992	7.937156594	4.808761949
26.63761399	3.398956356	0.284195097	7.279451835	3.596856253
12.2510111	2.891648556	0.344899403	6.084417881	2.544446871
29.30969188	3.316832001	0.302875051	6.736014085	3.558217606
30.16839918	3.488586464	0.275058269	7.435981683	3.846744888
81.35435198	4.071490579	0.201075879	8.116040301	6.129445241
55.15872924	3.924060575	0.212197869	8.134926883	5.394848945
46.79640973	3.762269442	0.253212726	7.924453447	4.731984471
23.3619525	3.332261272	0.28357807	7.343273591	3.470321566
92.54586394	4.228935871	0.186786099	8.516480676	6.928902507
15.59183964	3.032707626	0.343105321	6.418234789	2.772242739
14.50481003	2.97887313	0.340550368	6.228974961	2.657681937
20.45415735	3.110407303	0.348565661	6.420257504	2.949167737
75.57327919	4.140203028	0.177646088	8.572370502	6.457783503

log.sigma.1.5.mm.3D_glcm_DifferenceVariance	log.sigma.1.5.mm.3D_glcm_Idn	log.sigma.1.5.mm.3D_glcm_Idm	log.sigma.1.5.mm.3D_glcm_Correlation	log.sigma.1.5.mm.3D_glcm_Autocorrelation
17.89636904	0.908780801	0.229668609	0.111962882	468.5435488
17.78313165	0.925015933	0.284755865	0.087873298	496.0649715
22.83814222	0.879693142	0.21261813	-0.012913387	316.6989362
22.6619178	0.909565504	0.226482486	0.032862886	739.809425
8.153581348	0.913182235	0.317006244	0.203667911	179.6968405
17.38746103	0.913809206	0.268919886	0.032402475	474.4831136
5.649229711	0.915878659	0.37308294	0.160532883	202.4950215
16.62621482	0.927053995	0.313693594	0.090149118	594.9150738
13.04536198	0.921869289	0.304891044	0.100510471	382.7451063
15.36426262	0.90501014	0.239668193	0.060165254	493.9028144
14.48545732	0.919583921	0.299055831	0.027774794	440.5719245
29.71002382	0.892977531	0.191162587	0.020242685	590.037706
10.78986441	0.927220895	0.292938538	0.087991772	327.5941376
10.52788313	0.887844851	0.257329736	0.091443319	198.6182773
12.25228147	0.89311327	0.212189746	-0.015411656	379.3118314
16.60732075	0.921897667	0.322272796	0.057657934	432.5768222
9.741908176	0.910482122	0.288952592	0.084402474	256.2206895
9.218975215	0.917056177	0.336054651	0.189712765	281.258201
10.26773037	0.906005469	0.304824693	0.217427865	192.1398024
12.99511456	0.923755535	0.314772116	0.059464729	399.7864157
21.14136427	0.911506837	0.203840071	0.025107422	479.9321033
13.44059532	0.913312558	0.253186397	0.059109591	362.3994576
16.41920409	0.893321179	0.241189947	0.008661331	399.6762933
14.817491	0.913123634	0.290223307	0.108698688	407.0742248
15.68227287	0.917019267	0.239154468	0.038696467	308.6426535
22.51104974	0.884624712	0.219886564	0.042835083	354.0839457
17.72944962	0.899247104	0.229735522	0.011643774	449.4252469
22.20391924	0.89079461	0.207919073	-0.002917651	365.6695783
13.05760037	0.926805798	0.341716505	0.052800497	481.795744
19.11759339	0.923120135	0.289421758	0.035822703	588.8439015
16.60131261	0.896774815	0.229804783	0.004626212	399.5083317
9.044908375	0.921921846	0.297374694	0.139390032	419.5855952
20.46297476	0.924481579	0.269004751	0.063741995	579.6867733
11.94518951	0.923401969	0.313921918	0.233070667	436.4657788
28.83003683	0.88957461	0.220213733	0.013746398	520.8567848
17.53931775	0.923331036	0.324610157	0.08701416	573.7847913
25.90049391	0.903629522	0.220007888	0.043903565	740.7210303
20.2339942	0.90269015	0.237002941	0.023470219	399.405688
5.549671032	0.902356537	0.32460089	0.12719633	141.6770328
18.75878051	0.925553522	0.317392886	0.096753341	643.4575638
22.06606108	0.892799831	0.21145111	0.007685883	590.9153958
13.01062289	0.919525837	0.317458821	0.123923797	459.4171417
14.33617368	0.932511495	0.322345931	0.108623671	637.23813
12.00807597	0.934069048	0.332600495	0.182366382	606.2635978
22.76863129	0.920791007	0.25335465	0.045014991	958.1985589
22.45348025	0.89099255	0.199874168	0.03913637	446.7777886
23.61174427	0.903133821	0.217245326	0.063733213	587.9392515
19.09054526	0.893927061	0.229384715	0.016733928	418.4837476
10.28673249	0.911244918	0.334292861	0.221017969	189.1232402
13.46724197	0.916366144	0.308496511	0.060749027	450.150858
11.58805051	0.932882144	0.318431567	0.093017819	491.9185517
17.83952255	0.930224832	0.359267518	-0.000462154	712.2705605
13.92030098	0.926542485	0.307751961	0.066952284	449.4184972
16.98317865	0.892440606	0.218862624	0.001966114	407.638551
18.41750764	0.919558066	0.267719253	0.053549486	641.1178851
28.14071969	0.903374853	0.26828959	0.019531052	623.0148566
18.93188169	0.916535888	0.241491353	0.089830792	695.7819937
10.52884692	0.936617731	0.349236914	0.166620653	587.2135231
27.19868117	0.911817792	0.222295319	0.027445241	732.9247854
9.958270843	0.897805525	0.271165071	0.013771467	202.8314956
17.98343671	0.931430895	0.262675188	0.088113886	607.8981013
21.07839822	0.930279665	0.286188382	0.038001771	729.1895442
24.1575265	0.899666954	0.223120125	0.025251127	444.8391869
14.35222091	0.9018246	0.286266094	0.051212859	304.9820433
9.233222418	0.889603155	0.283675928	0.13018451	192.842117
15.54829816	0.90759043	0.216134472	0.154809128	465.7039442
22.53238883	0.916198482	0.258044495	0.037755673	695.2683765
18.19105322	0.931047142	0.298663128	0.07667685	702.3472615
18.5780886	0.903880539	0.257745016	0.014841372	579.7341239
25.1098068	0.908214225	0.252256085	0.055981854	528.8649297
20.40208414	0.902235612	0.220547101	0.078613551	640.6632505
10.24027046	0.92503831	0.320757336	0.084470296	338.3831761
14.32743712	0.927108234	0.273355472	0.102613174	339.7195866
16.63738514	0.912331927	0.277601882	0.040892775	531.6055631
21.4477589	0.908398972	0.219344526	0.11700468	598.8630007
22.37861426	0.907082073	0.253646404	0.043222184	578.8906369
15.25121102	0.923612993	0.292062399	0.001188908	285.0008239
16.60375106	0.896674608	0.216234214	0.08740514	424.429299
18.11784731	0.915126691	0.279867728	0.036593643	480.6909209
21.36080673	0.912178305	0.279618206	0.015950286	389.256532
25.47175487	0.912750228	0.239728192	0.006304548	789.5572407
29.34006908	0.897478347	0.188337207	0.024664846	624.7869998
27.64880764	0.911231267	0.218318865	0.033501459	782.6858548
5.408965054	0.916272944	0.394122685	0.28368833	133.452066
20.39803323	0.894930182	0.218446451	0.046035487	488.2480387
10.21880818	0.902790371	0.273337412	0.08836391	268.4062699
21.06847997	0.904988428	0.225765434	0.092553409	501.8917361
13.65159474	0.913992571	0.28587551	0.08713163	447.7299914
5.692930936	0.896083526	0.350174484	0.161665737	134.7117217
16.57157724	0.92661647	0.299523002	0.098375167	537.0392971
15.32628711	0.908949366	0.275152084	0.043086901	410.1439651
43.48194147	0.897804409	0.199133479	-0.015135539	859.0261566
25.90113934	0.891013888	0.209196207	-0.009842042	432.9029798
24.27952706	0.917272967	0.245634785	0.004368073	669.0890138
11.27839508	0.921636865	0.274262161	0.054167024	372.6386189
44.03535861	0.887159964	0.178543104	0.054617578	603.9374152
7.8715332	0.927509647	0.332288511	0.062573992	364.0096052
7.412003533	0.919584831	0.350378062	0.168675888	276.3958591
11.66388614	0.933522987	0.353024363	0.025928776	439.1635401
33.665416	0.890430381	0.165099439	0.047428247	642.0987109

log.sigma.1.5.mm.3D_glcm_SumEntropy	log.sigma.1.5.mm.3D_glcm_AverageIntensity	log.sigma.1.5.mm.3D_glcm_Energy	log.sigma.1.5.mm.3D_glcm_SumSquares	log.sigma.1.5.mm.3D_glcm_ClusterProminence
4.698386344	21.77060339	0.007507977	20.1261378	11145.63293
4.54901868	22.40132332	0.011437742	17.14585529	8342.220014
4.720165624	18.1404913	0.006262185	24.04305251	8964.67217
4.659846159	27.49040683	0.007288083	21.83246656	13791.728
4.149008178	13.34087943	0.016892562	11.40962589	4580.925592
4.445742454	21.94161321	0.010580552	16.00154759	7384.686671
3.846872958	14.18882935	0.025456373	6.867707965	1420.007575
4.393649344	24.47712443	0.015124525	15.37043051	8103.427564
4.319015263	19.73427226	0.014080334	12.96469069	5973.690619
4.585246793	22.39960233	0.007359121	16.36529534	6140.175901
4.292783013	21.16020072	0.013353552	12.58880349	5255.657675
4.92270828	24.59076177	0.004814163	30.25112002	19390.39362
4.239923157	18.27871406	0.013113566	11.25527621	4455.470482
4.424031387	14.05730998	0.008552214	13.6637208	3466.35541
4.408585767	19.80349552	0.007349991	14.71086052	3662.175545
4.43710433	20.95375587	0.013750062	15.04296304	6160.193235
4.233625451	16.04885025	0.012140626	11.69865657	3142.745713
4.273811739	16.78585031	0.016355839	11.71665373	3681.533522
4.417875072	13.7790886	0.013113374	14.33277003	5988.078392
4.308786698	20.15646397	0.013524028	11.94246832	4185.553187
4.812730345	22.20125399	0.004492776	23.77636209	9516.258267
4.487254561	19.24591467	0.00863508	14.64237191	4858.359323
4.539221327	20.30559674	0.007417556	16.79388248	5795.930689
4.382170305	20.32461781	0.013873052	15.33392897	7995.240193
4.598974291	17.79202344	0.006195711	16.91085818	5270.489041
4.739342144	19.11792067	0.007051434	23.55949211	12281.86721
4.604958	21.44246765	0.006989781	17.79898944	6553.233555
4.774866781	19.46276981	0.005257028	22.85150412	9098.116249
4.191868872	22.07823241	0.01979594	11.52443845	4515.099271
4.495912829	24.44121511	0.012588211	17.9642054	8282.434796
4.561499069	20.28529683	0.00713025	17.00910119	5798.170139
4.294501953	20.63392406	0.011989233	10.44111649	3506.606186
4.566841071	24.27156955	0.010542536	19.48160727	11303.23641
4.344703759	20.95233759	0.018668457	15.02100813	8616.400549
4.811086055	23.12161189	0.007072839	27.19961854	15143.05826
4.354805053	24.05619864	0.01737268	15.22304139	8780.972951
4.836314707	27.51741433	0.006255701	26.44791692	16215.48342
4.6602841	20.24295454	0.007417216	19.66753803	9415.773105
4.000403957	11.86260905	0.015774406	7.348990996	1181.770396
4.433720071	25.48296977	0.01743379	17.08925122	10578.52067
4.712010229	24.505432	0.006154066	22.03598281	10922.69675
4.337050492	21.56550297	0.016224032	12.83066054	5783.127803
4.303764238	25.33176436	0.016525796	13.75886757	6881.089061
4.271816113	24.65885	0.018229113	13.19505401	6832.72864
4.705852075	31.21489754	0.00814574	21.86444014	12926.85803
4.86211259	21.40081865	0.004824421	24.24471726	11202.6332
4.854043231	24.50413005	0.005754908	25.03525564	13705.81557
4.594450857	20.7559176	0.00761092	18.96055253	7921.824243
4.164432754	13.66330129	0.023034795	14.1231258	6690.083029
4.273185526	21.37801212	0.01509252	12.22418961	4613.126233
4.251726486	22.24686869	0.014946203	11.35902074	4342.600442
4.10976994	26.79098862	0.022895798	13.07216151	6996.488423
4.323224655	21.32028682	0.014665149	12.87399465	5681.773289
4.545628383	20.46197283	0.007260348	17.68415865	6661.127741
4.562093912	25.49722793	0.010068099	18.23121977	8222.182468
4.811071313	25.24771603	0.009662256	26.39692772	13807.01205
4.645573465	26.58359237	0.007969115	20.46787521	12204.89987
4.094123514	24.27888477	0.020723793	11.0905874	5390.088542
4.653221323	27.45055507	0.008188119	24.24624791	20623.14789
4.216729703	14.24672663	0.010948442	11.25316098	2303.636614
4.527606542	24.89959989	0.010151576	17.59098046	10822.56859
4.498562169	27.2735531	0.012135374	18.43811935	11710.19951
4.787000936	21.34263709	0.006356236	23.7435436	12086.10081
4.392924898	17.61055918	0.011526823	15.09995836	5102.899392
4.314604893	13.8282224	0.012856268	12.03731971	2793.920472
4.758305763	21.67527213	0.006112127	20.21412212	10020.84649
4.604546423	26.55991771	0.009255708	20.98488666	11536.37603
4.410238971	26.71690622	0.014457236	16.26149273	10665.57739
4.500587677	24.21983444	0.009679596	17.05126399	7831.810215
4.743964934	23.18966753	0.008629633	23.74970637	14632.99248
4.83140179	25.48026828	0.005078077	23.44331809	10666.77755
4.07397966	18.5773688	0.019306142	10.44779965	4147.131449
4.52201571	18.57931825	0.009833323	15.11549874	6540.428439
4.487710818	23.2043477	0.010053958	15.46398786	6398.352741
4.868703588	24.63605435	0.005672528	24.64698892	14737.0145
4.664498182	24.25449624	0.008934029	21.02443838	11009.32663
4.298323893	17.11653158	0.013632115	13.65799559	4964.620223
4.682755355	20.82619801	0.006897985	19.71436131	10121.49774
4.460138124	22.08445687	0.011497618	16.44064516	7871.822547
4.581266313	19.98480016	0.01057352	18.80473039	8576.430874
4.590233572	28.46808052	0.009340924	22.85612874	15527.90648
4.91632684	25.42016307	0.005031276	30.37312524	18935.73635
4.808651443	28.34452973	0.006556645	25.92727659	19500.80214
3.82352305	11.44916697	0.033176512	7.738967439	2734.628033
4.778606912	22.31124757	0.005913597	21.45102826	9356.384097
4.362465465	16.41171122	0.01005074	12.66832645	3140.849253
4.791554349	22.58944203	0.006869446	23.18136981	12324.11582
4.384062097	21.32525854	0.01283353	13.32359542	6033.699326
3.860335247	11.54956965	0.024504771	7.426928484	1320.285434
4.27788894	23.25117446	0.015468701	15.28802984	11489.3546
4.44096564	20.48806742	0.010997195	14.3820939	5563.335341
4.965224236	29.76120466	0.005718463	36.66834196	38166.96141
4.803903362	21.13761456	0.005962188	25.19043406	11985.27283
4.641218873	26.24019276	0.009343395	21.97254251	12303.17864
4.30866221	19.48999344	0.010873649	11.51522473	3531.410824
5.191214881	25.07180122	0.004385819	47.28712689	38919.08619
3.887363121	19.25249575	0.023012526	8.032551869	2403.96288
3.976847763	16.59059244	0.023518047	8.881290679	2320.659105
4.021898922	21.0868159	0.022319051	9.47162781	3344.184621
5.091540256	25.79894781	0.00372152	37.69170149	25510.28686

log.sigma.1.5.mm.3D_glcm_SumAverage	log.sigma.1.5.mm.3D_glcm_Imc2	log.sigma.1.5.mm.3D_glcm_Imc1	log.sigma.1.5.mm.3D_glcm_DifferenceAverage	log.sigma.1.5.mm.3D_glcm_Id
43.18321999	0.761550137	-0.105575744	4.4619484	0.318160241
44.47286649	0.702608851	-0.087863195	3.886187812	0.364825186
35.61708805	0.778710127	-0.110072916	5.162308243	0.299010308
54.3752767	0.666597839	-0.072166395	4.680283292	0.315038988
26.62283229	0.715317325	-0.10468166	2.984643234	0.39590695
43.54036914	0.67976898	-0.079715766	4.028114787	0.351653211
28.3776587	0.648080227	-0.087022796	2.393342564	0.444272035
48.72258975	0.704775319	-0.092028606	3.540465172	0.390062381
39.05829031	0.68204515	-0.083044887	3.414600086	0.383794683
44.40267506	0.578012599	-0.050260411	4.212662291	0.327086684
41.96442865	0.637678847	-0.070361467	3.541180153	0.378596845
48.55929498	0.755664614	-0.097047598	5.680165037	0.279242091
36.14327709	0.597491857	-0.060014864	3.284651736	0.376332941
28.09739349	0.586152502	-0.058522635	3.723580742	0.343931554
38.96657414	0.62838053	-0.06389467	4.250810659	0.305291899
41.55391225	0.65343075	-0.072520591	3.639649315	0.395697824
31.95263618	0.636624591	-0.070319022	3.299157917	0.372476128
33.40962781	0.614813777	-0.06822654	2.958263664	0.410719578
27.48403107	0.733672378	-0.106116279	3.292979203	0.384392129
39.95416134	0.59634767	-0.062733746	3.333300512	0.392487401
43.79130734	0.484600754	-0.03130867	5.110642422	0.29256578
38.02683019	0.609707724	-0.060181673	3.986228642	0.339639018
39.98405244	0.533948632	-0.045390321	4.332321262	0.327568852
40.2645317	0.77818848	-0.121931468	3.752405797	0.369719718
35.10200347	0.591714097	-0.053161431	4.274120176	0.326532199
37.58175532	0.760642867	-0.102970601	4.931876852	0.307214261
42.39314059	0.657199555	-0.069263044	4.524158743	0.316919193
38.2552771	0.608783937	-0.054031294	5.11427242	0.2957357
43.87208191	0.6315944	-0.075168112	3.204714932	0.413061507
48.50716695	0.659952421	-0.073111804	4.009673438	0.367017066
39.97630901	0.525708269	-0.039792587	4.400203512	0.318106369
40.89141319	0.514402574	-0.04341442	3.177230289	0.380091714
48.10186995	0.669987348	-0.076063095	4.186542752	0.350644951
41.59777852	0.84358288	-0.172389044	3.411008511	0.389562226
45.63334628	0.838185503	-0.142777491	5.405002467	0.303721562
47.84838217	0.6408881	-0.071897464	3.505275572	0.398042597
54.39039129	0.631375005	-0.059709922	5.151362648	0.305810543
39.94929339	0.601793772	-0.054407844	4.60927838	0.322277821
23.7252181	0.525294243	-0.048280597	2.685521866	0.405726435
50.66406352	0.740315023	-0.10664939	3.725967846	0.390695722
48.61246666	0.731422324	-0.09189387	5.010218597	0.299849723
42.78652628	0.671287871	-0.081310222	3.355023892	0.393737895
50.42593942	0.637673024	-0.072033703	3.308478627	0.398488945
49.14028823	0.692004117	-0.092476065	3.130200048	0.406977541
61.87757193	0.660743686	-0.069275456	4.586440973	0.335246706
42.22985017	0.5867869	-0.04876524	5.221241069	0.28872338
48.43032033	0.612878656	-0.055349786	5.043709316	0.304083948
40.90219594	0.70669396	-0.084262615	4.571128813	0.316871512
27.23689124	0.799191373	-0.152524311	3.107429892	0.407728261
42.39884499	0.726852166	-0.101462248	3.408597153	0.386744958
44.30869977	0.571936971	-0.054622123	3.177817205	0.396154562
53.37868559	0.689455711	-0.092989609	3.19629932	0.427635137
42.35676494	0.586443444	-0.057457013	3.448405475	0.385762577
40.38127583	0.725752419	-0.092082684	4.480153283	0.310120263
50.60186378	0.602482333	-0.05732234	4.099202337	0.349949553
49.90466217	0.770369335	-0.106295466	5.013405614	0.344679893
52.68619745	0.64523255	-0.066298274	4.345323664	0.328449484
48.38514513	0.648850146	-0.080805287	2.854107846	0.422462165
54.12343054	0.778321662	-0.112697436	4.896387739	0.310219713
28.47403999	0.591322199	-0.058138962	3.467499927	0.357557917
49.24846744	0.649156088	-0.06990366	3.967567901	0.347899233
53.98078637	0.625100427	-0.062996884	4.08404938	0.364525056
42.15540592	0.687259236	-0.07552845	5.033573686	0.308257521
34.88593165	0.651037755	-0.072066048	3.730498465	0.367258491
27.6564448	0.761756684	-0.117942861	3.395540998	0.366589764
43.0082553	0.721598294	-0.088209025	4.482198725	0.306919261
52.70675717	0.79817754	-0.1256874	4.42106327	0.340086178
52.95642967	0.604040416	-0.060748967	3.716058088	0.377023293
48.14543269	0.707534284	-0.088067082	4.222738254	0.34208558
45.93647232	0.722995389	-0.089363863	4.704740581	0.334038894
50.5496843	0.597947305	-0.052352419	4.837453331	0.307361369
36.73937186	0.62000856	-0.07023534	3.07066141	0.398036796
36.77425871	0.56188274	-0.048849513	3.799735332	0.356680318
46.08476011	0.5149237	-0.039530474	3.904275892	0.359038131
48.82352455	0.614961103	-0.056624227	4.822621136	0.307466795
48.08157919	0.678666351	-0.075857586	4.529266731	0.335671811
33.76690273	0.636969318	-0.06870766	3.687814833	0.371943783
41.10371747	0.721981273	-0.090484265	4.622508378	0.30544777
43.82224555	0.618343863	-0.061894241	3.979215536	0.360263842
39.448193	0.619580942	-0.060412061	4.266688143	0.357585976
56.19660041	0.795073352	-0.121601	4.736067718	0.32393306
49.96873153	0.676972089	-0.070125693	5.752904789	0.276663716
55.92604328	0.70369205	-0.079402194	5.215751799	0.303755447
22.89833395	0.744457272	-0.136975158	2.293300796	0.460329132
44.14738868	0.562063007	-0.045225496	4.882656877	0.305185831
32.69969097	0.495491083	-0.037384383	3.502312537	0.358794156
44.70713586	0.761243201	-0.103303387	4.808761949	0.31209198
42.26053829	0.585527356	-0.055972681	3.596856253	0.368061651
23.0991393	0.765055589	-0.138589287	2.544446871	0.425346308
46.27756802	0.817398491	-0.152468322	3.558217606	0.379116852
40.47352149	0.591003773	-0.055665519	3.846744888	0.357633281
58.63777378	0.854188289	-0.147850113	6.129445241	0.284799588
41.63313625	0.736764279	-0.090863507	5.394848945	0.294682102
51.73472929	0.628888431	-0.061216682	4.731984471	0.327816976
38.57521273	0.503647893	-0.038844561	3.470321566	0.360164279
49.05764041	0.90732925	-0.18492029	6.928902507	0.261565625
38.13272084	0.574091303	-0.061053001	2.772242739	0.409159755
33.15818623	0.77386308	-0.138488788	2.657681937	0.424871567
41.90039666	0.704824293	-0.101672784	2.949167737	0.423236533
50.61632329	0.831337434	-0.127734304	6.457783503	0.253132619

log.sigma.1.5.mm.3D_glcm_ClusterTendency	log.sigma.1.5.mm.3D_firstorder_InterquartileRange	log.sigma.1.5.mm.3D_firstorder_Skewness	log.sigma.1.5.mm.3D_firstorder_Uniformity
47.26616654	105.2025652	-0.386277846	0.078659682
39.31715426	89.96993446	-0.777739173	0.0932285
47.82978192	133.3871107	-0.45698039	0.068162544
47.18331531	108.770586	-1.003616002	0.078144037
27.00862931	69.4198513	0.863917685	0.114953237
35.83229471	89.6958046	-1.157846179	0.096403935
16.05861924	59.00881004	-0.089043567	0.138397976
34.955077	79.88395786	-1.097771195	0.104181828
30.14413072	80.98667622	-0.219299198	0.100046754
37.19260913	112.1323051	-0.532396366	0.07860948
28.37744981	76.11240721	-1.24588093	0.110132795
64.34858852	141.3228607	-0.790676308	0.063222534
25.64517528	80.54511452	-0.008120489	0.105247545
29.607849	109.5160208	-0.315638849	0.084329713
29.31207374	119.798007	-0.179505958	0.075684888
33.55981346	84.2405262	-0.428568878	0.100162101
24.59244964	87.44741631	-0.113449607	0.099130637
26.82577205	73.55230379	-0.108984406	0.111479355
34.27710167	87.56133604	0.44677799	0.09397162
27.11436458	78.0295496	-0.973188754	0.106457246
49.38911115	142.4409924	-0.475516735	0.064485399
33.06203828	102.9673843	-0.52051021	0.085272641
35.53318331	111.9608479	-0.872342522	0.079988678
36.08989019	80.15215397	-0.101392152	0.100684325
36.48858249	123.3434916	-0.223854937	0.073321559
50.81335683	117.5230501	-0.512648427	0.070498232
38.79832287	109.1424503	-0.651636726	0.079291262
47.7342497	135.3814659	-0.520704114	0.065802715
25.97581035	67.200809	-0.686872922	0.121382031
37.69700875	82.87943649	-0.532529716	0.098641876
35.97140215	111.2529573	-0.643210795	0.078292677
25.43400146	85.79003072	-0.244513282	0.10071896
43.03557261	87.30900764	-0.91694082	0.094191176
39.01216734	68.43975925	0.386393302	0.116670485
59.22837099	118.5667329	-1.066844297	0.074656582
35.51606693	68.55123043	-1.24313062	0.117195919
57.09751658	119.8369637	-0.608002305	0.070459557
43.21347689	104.1696854	-0.672978951	0.08103232
16.60480816	76.80818558	-0.214559875	0.114636082
39.60236115	70.16378474	-0.733075689	0.113757689
47.73901867	124.9930258	-1.046377928	0.073181604
31.21620472	72.96442747	-0.484625416	0.111994136
31.48257778	70.13694954	-0.883026049	0.115435587
32.04975024	71.39839959	-0.060206505	0.115920299
47.89994235	99.63108587	-0.862978148	0.082905227
53.51082862	134.2323799	-0.580491059	0.065841459
55.40064146	120.345757	-0.748647613	0.070675245
41.00017685	108.9295845	-0.87787364	0.080939634
34.38385674	62.05906713	0.736400932	0.129017655
28.06861062	72.94749665	-0.924683653	0.109157478
26.1262285	75.4737668	-0.432745155	0.109649189
27.9203282	58.69244003	-1.956731775	0.138226045
29.4463827	76.21212959	-0.624762808	0.108369172
37.04525265	112.0259409	-0.582741275	0.078711911
39.19956818	93.06258583	-0.560709923	0.087936111
55.00194419	108.6341791	-0.61717904	0.079551269
45.17272532	105.812149	-0.591277199	0.08028346
26.41818085	65.97233582	-0.056848432	0.127749118
53.60508139	107.0472426	-1.403455654	0.078857449
22.59217334	91.621521	-0.448757637	0.097341726
39.95868718	97.00253963	-0.634775677	0.086354199
40.67911272	82.53964996	-0.588347505	0.09633762
51.93961683	119.5623608	-0.755235729	0.072465201
31.22676481	90.31500649	-0.819060822	0.094735406
27.29952598	86.64395738	-0.283035582	0.096202241
48.75705597	125.9648314	-0.006736657	0.069820052
45.19536671	99.05570412	-0.544620031	0.083429519
37.03999387	79.48762894	-1.216286665	0.10551246
37.39654883	91.61945534	-1.22671961	0.091658397
52.69559936	103.3558865	-0.772189315	0.080438673
51.27492917	132.790164	-0.480929333	0.066013446
23.41106732	69.94375801	0.0674967	0.119407325
35.32527164	93.99161625	-0.317579773	0.089643234
34.54645036	94.75384521	-0.9084446	0.091032057
56.52218656	127.3016863	-0.522616165	0.068153276
46.65568391	98.03857613	-0.939600731	0.085272071
28.70137985	78.14977264	-0.588027633	0.106880333
46.13450623	106.1621904	-0.236697012	0.078456056
36.41903934	83.6724577	-0.980700857	0.097831232
40.52764929	89.17201805	-0.631599852	0.091349332
48.11941857	100.1720371	-0.861067115	0.08091021
64.84220117	133.7350044	-0.817642754	0.063763368
57.98621371	116.5781465	-0.983153682	0.072623024
20.18996557	51.08955336	1.082704481	0.156308837
48.33030675	122.8601317	-0.780878396	0.071849201
26.861877	93.01805639	-0.413811908	0.092515699
53.09868678	116.5594482	-0.504688823	0.073898187
31.56743772	77.95716953	-0.862509148	0.105122341
17.45670283	69.31156063	0.229039896	0.127308032
35.78683739	73.89347482	-1.082774665	0.112019732
32.60939136	86.53939915	-0.847459108	0.095750835
79.01875888	130.8794975	-1.378813676	0.065696444
53.43014747	122.4801407	-0.921524101	0.070680748
46.65939881	102.5157375	-0.838341102	0.08206043
25.85476553	89.54659224	-0.46542959	0.09585723
101.6231503	190.0169239	-0.352344963	0.05138869
17.51632789	61.82080603	-0.801049927	0.13490354
20.5659194	60.99395639	-0.628842505	0.129607389
21.44894655	60.30952859	-1.121855796	0.135543483
81.93784268	185.3491011	-0.613948762	0.050461209

log.sigma.1.5.mm.3D_firstorder_MeanAbsoluteDeviation	log.sigma.1.5.mm.3D_firstorder_Energy	log.sigma.1.5.mm.3D_firstorder_RobustMeanAbsoluteDeviation	log.sigma.1.5.mm.3D_firstorder_Median
80.8865527	17259624.26	47.39844868	38.46511459
70.99506236	23036040.49	40.02108337	26.66234207
91.25383377	16757698.82	57.77359183	21.91469002
82.31990535	36316225.21	47.43390753	31.37704659
59.56068115	8100777.984	31.39112794	20.147686
68.90618413	16797943.27	38.8920063	21.68232632
47.77998669	5643660.496	26.00813979	18.06536293
65.88045312	19187091.41	35.6630121	27.41666794
65.00832552	19941283.71	37.28985113	27.85491562
77.11008979	34659242.06	48.08733424	31.44034195
60.34156674	15450786.05	33.55009031	26.44670296
100.6419592	33462237.4	61.00205915	38.36181259
60.25533104	23617410.36	34.95948492	22.1072197
70.73322094	16535865.71	46.15430812	26.16593456
76.31895247	15969526.95	50.9597792	24.43003368
66.5797584	39124757.63	37.57735669	7.693273544
61.79836885	11964001.87	37.74210455	16.5407238
59.52427216	21926570.73	33.46376821	29.00447273
69.00944509	12531702.36	39.89629311	30.8308115
60.16181683	23026249.73	34.43312093	22.63739014
91.29887739	100276735.4	60.11473408	21.27825928
71.96936164	21621177.43	44.41867101	27.29194069
77.08464339	45686768.99	48.35084938	28.85230064
69.91108669	11906539.09	37.16454689	23.08880329
79.49744499	28019809.71	52.85656173	12.69759941
90.45459343	23397968.74	53.63636032	41.83751488
77.39377381	26165346.19	47.33259323	26.7680397
92.20774952	52063459.85	58.53543621	33.37549591
57.60503661	26986976.17	30.00008229	18.22816277
69.93424659	35314692.56	38.25419027	18.35046005
79.09358155	48508740.07	49.20136474	31.01498699
60.70159061	40947756.53	36.61111928	30.63302898
74.25297318	32737201.26	39.35072385	27.59041977
65.79636177	5652323.509	32.81321977	29.37537193
92.80959372	15296663.38	53.31042881	37.04669571
61.92309621	31295480.97	31.42752012	21.69421387
91.95854187	62146073.44	53.03017674	28.72706223
79.87853018	44937774.83	46.29363199	29.68431091
51.84516333	7496928.429	32.48633697	16.31516552
66.58117355	19395304.49	33.05607857	27.2005024
85.34407045	19224515.12	52.33645132	40.15325928
63.11357792	19253938.69	33.46863632	28.41354752
60.58588149	28383661.26	31.19799459	23.71637154
60.6242506	18290635.74	31.51399023	27.22739887
78.88959274	46301531.1	45.10407428	21.61164665
92.10545403	56921182.12	57.65387838	36.87055206
89.77681115	59964411.39	53.27135356	36.10116768
80.27598152	18314649.27	48.42393472	19.10640812
65.54617574	6221963.726	31.82798738	19.73734188
60.44316295	9695270.896	32.88014182	26.08887291
59.10367547	31818336.63	33.47142097	22.21613693
54.95696363	15550335.6	26.45032547	17.99483013
62.12772315	35809118.81	33.66080741	26.35317039
77.64035666	13677194.34	48.25400915	27.41375923
74.61387456	48363415.69	42.19801819	25.14894104
86.7012528	29496004.49	49.83026334	17.03159142
79.6972223	45274727.71	46.11618147	27.69066238
54.37228832	18503112.06	28.45870531	15.79221678
85.87341119	23042185.37	47.1765793	33.70910835
61.88155503	10300616.31	39.06670498	24.04094887
74.91433666	39346540.44	42.92630044	33.72723389
72.91574328	69987056.75	38.27818641	23.13215637
87.31621164	34675221.41	52.10333413	35.4705162
67.33891107	17966186.29	39.91072006	28.29554462
66.14999544	5489938.075	40.12128227	37.49323654
86.01939544	23190435.7	53.60468143	35.59176254
78.66700884	14864322.81	44.06577047	28.65877724
66.67600377	41252421.96	35.4753065	29.49487209
71.36431314	14068840.64	40.92274081	29.58734322
85.45526935	39366848.92	47.14937354	28.50662613
89.15499238	55519789.05	57.48729885	22.55579662
57.28704414	17022670.95	30.70033594	23.21177101
70.81562146	51823962.74	41.46540533	30.56067848
69.84606884	66398781.76	41.13391473	26.29866982
90.35238244	70383313.33	54.80394315	36.63399315
79.6096162	31802886.12	44.16204817	27.96549606
64.15721485	21703213.16	34.67170913	20.67686844
81.42600782	19864795.46	47.2295845	44.27522659
69.07068913	34799465.35	37.86270904	22.27654839
75.686211	54412735.7	41.71755751	21.86319542
83.30983186	21036609.45	45.52873403	25.19069767
101.1580404	52988512.93	59.82400091	33.56036377
91.69118863	45223020.57	51.53828182	32.03273773
50.32687807	3486139.195	25.11068704	17.2684803
86.75871951	55447889.85	53.70979074	38.68398285
65.07639053	28487338.84	40.59220606	26.32829094
86.8424766	22211337.49	51.532804	38.78338242
63.46326905	29092965.55	34.8021338	26.84366608
53.64177872	2664986.492	31.585935	23.86574173
63.25839082	6114922.259	32.92225339	28.33395386
67.74981222	33368916.35	38.5432691	33.03665924
104.4751202	27828987.13	57.57385915	42.24358368
91.37587416	25202667.79	54.75571261	30.11836052
84.3892915	65302300.03	47.10825899	30.33463478
64.36515379	41668330.99	38.49391023	29.78422832
127.127012	25772157.38	79.72297356	21.18030739
49.37441144	10489534.93	26.95825111	20.79009247
51.93897077	3006560.458	28.69114258	29.07574654
51.41491524	5971507.276	27.24380027	21.84807777
119.5612116	29063131.8	78.46894141	45.13996124

log.sigma.1.5.mm.3D_firstorder_TotalEnergy	log.sigma.1.5.mm.3D_firstorder_Maximum	log.sigma.1.5.mm.3D_firstorder_RootMeanSquared	log.sigma.1.5.mm.3D_firstorder_90Percentile	log.sigma.1.5.mm.3D_firstorder_Minimum
466009855	509.9192505	119.779599	167.9442688	-484.0392761
621973093.3	554.3299561	107.3220399	143.8846954	-521.6502075
452457868.1	409.1960754	122.42956	151.1549942	-405.657074
980538080.6	421.3631287	121.675062	151.1275909	-631.472168
218721005.6	417.9642944	92.39112571	118.7627487	-297.283844
453544468.2	435.0768738	101.2677373	109.3821259	-523.4650879
152378833.4	260.7318726	73.07064593	100.7856766	-304.1868286
518051468	428.2763062	103.1017339	137.1999634	-571.0515747
538414660.3	473.1305542	99.18584141	150.5131439	-448.2110901
935799535.6	389.391449	107.4315556	140.8884094	-502.2574463
417171223.3	405.6163635	92.46901109	106.5647675	-477.0150146
903480409.7	479.3664856	140.9215902	167.9591858	-562.4645386
637670079.8	541.4365234	89.7958885	113.5210205	-406.5259705
446468374.2	348.7356873	94.33885775	122.7525513	-303.579834
431177227.6	353.8087158	101.5688529	151.2361404	-434.4176636
1056368456	458.4427185	97.57931375	118.4365326	-498.3155518
323028050.4	381.0627747	86.33774824	105.2337677	-374.1992188
592017409.7	360.9131775	92.58381814	138.008197	-372.510498
338355963.6	413.2810669	105.0306057	157.9863663	-293.1833801
621708742.8	431.5669861	89.09180152	114.4039078	-464.2019958
2707471855	693.6217041	120.1178889	149.2909836	-524.0493774
583771790.7	521.1447144	100.9646481	132.6760559	-425.7703247
1233542763	348.3984985	105.2916461	131.6888275	-454.9121399
321476555.4	414.4177246	107.5162624	144.005307	-466.5099182
756534862.1	665.253418	103.7318446	126.3601593	-404.7236633
631745156	409.0149536	131.5529927	192.8371246	-413.8868713
706464347	418.965271	108.7849637	139.5351715	-491.9118347
1405713416	489.617218	124.5719465	162.3882507	-442.1903992
728648356.6	376.133606	89.10488838	107.8448349	-512.2352905
953496699.2	524.8863525	105.7311369	133.9197784	-551.2346191
1309735982	397.7638855	108.2193572	137.0746567	-470.4050903
1105589426	395.8622742	90.49613973	127.4756355	-459.4873352
883904434.1	578.4034424	113.7299116	127.3196487	-561.9580688
152612734.7	467.1108704	108.4029166	168.0669403	-458.4941406
413009911.1	424.2615662	131.2477515	149.6422363	-530.6646729
844977986.1	374.4470825	98.63141978	112.9832428	-564.3320923
1677943983	446.7570801	131.9573462	167.9176147	-638.1190796
1213319921	490.9694519	115.1176425	141.3600769	-453.4998474
202417067.6	279.9291382	71.07628693	94.03907928	-250.3119507
523673221.3	448.8715515	108.8821408	135.9349213	-576.4365845
519061908.3	360.3200378	120.8647764	152.001709	-553.0036621
519856344.6	365.5545959	99.82888747	136.1104996	-487.8092957
766358854	448.6876526	96.8340279	114.4963791	-588.5229492
493847165	442.3670044	98.53124064	132.8479065	-555.0946655
1250141340	469.0193481	115.1161419	134.6368103	-737.1751709
1536871917	471.4505005	126.1824433	167.3828766	-491.7447205
1619039108	477.1841125	127.5466693	163.2886887	-571.9152222
494495530.2	369.4463501	109.9131621	113.8798332	-495.9271545
167993020.6	402.9159851	105.0326167	186.6181427	-282.8691101
261772314.2	345.0729675	94.31195015	123.6112312	-489.6835327
859095088.9	492.7337036	89.36738934	113.9515221	-520.6322632
419859061.1	281.0072937	89.16316124	85.23464966	-645.5473022
966846207.8	498.2166443	97.11280947	121.2644562	-490.4131165
369284247.1	368.4798889	108.0737102	143.3610535	-451.9549866
1305812224	459.974823	110.8347065	149.574292	-593.0529785
796392121.4	444.4587097	124.9915347	148.8422852	-591.588501
1222417648	467.7594299	117.505235	155.0171783	-612.0195313
499584025.6	388.234375	87.2249249	105.2359871	-554.6463623
622139005	489.3745728	129.593965	148.8141525	-627.440918
278116640.4	363.6121826	85.62359202	112.0153351	-324.9390564
1062356592	666.2866821	115.6660126	159.3907318	-574.0888062
1889650532	594.631897	112.9383361	135.3461334	-634.9919434
936230977.9	518.4398804	123.5665878	153.3415833	-479.6368713
485087029.7	361.3642273	98.07075376	129.4334229	-388.0359192
148228328	309.5166931	96.46232315	146.7494278	-294.2055054
626141764	516.4693604	122.5946424	176.1550781	-491.6906433
401336715.8	473.6087341	118.5303946	148.7303986	-602.5939941
1113815393	512.0269775	107.5558548	139.0633163	-610.8825684
379858697.3	319.8899536	104.3109481	124.3941299	-558.8448486
1062904921	477.8344421	125.3107011	144.1723633	-536.9112549
1499034304	403.0591736	120.7467454	164.9319641	-597.0228271
459612115.6	449.0359497	90.88136812	112.7899399	-420.414032
1399246994	690.8846436	105.2419258	148.3405518	-423.8624268
1792767108	364.4858704	100.9848461	124.0970535	-546.8762817
1900349460	512.1628418	128.5073983	174.9566711	-567.1097412
858677925.2	400.9706421	116.8051873	139.1820831	-565.4677124
585986755.4	614.8638916	95.99896753	105.5023499	-398.9648743
536349477.5	440.7368774	120.9016758	159.5165192	-457.822113
939585564.3	433.8003235	103.0657432	115.1081886	-518.9121094
1469143864	521.458252	110.5411385	121.1468201	-462.3236694
567988455.2	439.3441162	124.2794488	145.9151993	-666.3197021
1430689849	535.0513916	141.2196363	166.0227875	-586.9105835
1221021555	526.7636719	135.2010099	162.605275	-672.6675415
94125758.27	316.1481323	81.64348178	125.0695267	-227.6200562
1497093026	414.96521	119.8219483	155.058197	-504.5141602
769158148.6	361.016449	90.55470781	123.1361557	-362.1162415
599706112.2	508.791687	124.7163097	164.2535812	-512.4695435
785510069.9	369.4164124	97.77842111	126.997583	-495.9987488
71954635.29	233.3659515	80.52423268	141.1967773	-241.0857391
165102901	462.4831848	102.8564547	113.7509521	-544.5003662
900960741.4	394.2626953	101.9414716	137.9369659	-463.5218811
751382652.5	487.0530396	158.6965776	191.6418701	-695.0651855
680472030.2	479.5874023	127.0231103	153.6519226	-485.6977539
1763162101	553.0141602	122.4813333	140.2491302	-605.5523682
1125044937	489.0289001	94.09728464	128.0102425	-444.4988403
695848249.2	614.0931396	170.7454954	198.6074356	-599.0723877
283217443	359.7424622	77.13508466	89.73101044	-444.4322205
81177132.36	313.6013794	83.8133281	123.3069077	-360.1373291
161230696.5	427.9577026	82.00408743	94.05677795	-483.5751343
784704558.5	593.21875	158.6968481	207.5554932	-592.2634277

log.sigma.1.5.mm.3D_firstorder_Entropy	log.sigma.1.5.mm.3D_firstorder_StandardDeviation	log.sigma.1.5.mm.3D_firstorder_Range	log.sigma.1.5.mm.3D_firstorder_Variance	log.sigma.1.5.mm.3D_firstorder_10Percentile
4.116276021	114.6207908	993.9585266	13137.92568	-97.8016449
3.917540776	104.3798408	1075.980164	10895.15117	-87.67025986
4.239295553	122.1379077	814.8531494	14917.6685	-162.5916473
4.120881917	119.5183945	1052.835297	14284.64662	-104.5618851
3.634072017	88.75133474	715.2481384	7876.799418	-70.7539856
3.836165222	100.8074452	958.5419617	10162.141	-103.1077911
3.348830085	70.20343552	564.9187012	4928.522358	-52.14277954
3.789089359	100.0258256	999.3278809	10005.16579	-73.28527222
3.799815794	94.10464041	921.3416443	8855.683348	-68.93021698
4.037469223	104.6046266	891.6488953	10942.12791	-108.4641342
3.664151895	90.56665419	882.6313782	8202.318851	-72.97605438
4.379902853	138.9650849	1041.831024	19311.29481	-140.4329712
3.710116192	87.18534598	947.9624939	7601.284554	-71.71808014
3.89329626	92.82359717	652.3155212	8616.220191	-108.6324181
4.007071269	98.8445206	788.2263794	9770.239252	-99.83181915
3.831876226	97.08641588	956.7582703	9425.772149	-97.84378815
3.728896203	85.52810244	755.2619934	7315.056307	-94.05143738
3.654773268	86.03279611	733.4236755	7401.642006	-49.43124809
3.874512357	97.7678373	706.464447	9558.550009	-66.70610428
3.681128576	87.29543436	895.7689819	7620.49286	-77.23822021
4.26108198	119.6478109	1217.671082	14315.59866	-144.6819214
3.94217253	98.80007435	946.9150391	9761.454692	-105.4277344
4.003651351	104.0063237	803.3106384	10817.31538	-120.9824524
3.866647368	104.5115209	880.9276428	10922.658	-81.08106918
4.060988249	103.6221361	1069.977081	10737.5471	-129.9806427
4.250845769	126.0624071	822.901825	15891.73049	-114.6500153
4.036857335	106.6802362	910.8771057	11380.6728	-103.172348
4.271375853	122.787621	931.8076172	15076.79987	-146.1395691
3.614268158	87.78791283	888.3688965	7706.71764	-75.50665283
3.900308642	104.23062	1076.120972	10864.02214	-99.07753143
4.055145024	106.702181	868.1689758	11385.35544	-123.1000374
3.724085204	85.06255859	855.3496094	7235.638874	-62.60617905
3.950560824	112.1902801	1140.361511	12586.65895	-99.03891754
3.715699475	101.056071	925.605011	10212.32949	-45.90304947
4.201075965	130.3098224	954.926239	16980.64981	-160.7596695
3.683543977	97.38114134	938.7791748	9483.086688	-81.14966431
4.289240082	130.2459985	1084.87616	16964.02013	-138.1280304
4.077441521	113.3847108	944.4692993	12856.09265	-119.8067245
3.484419045	69.92424331	530.2410889	4889.399802	-70.96643753
3.784300745	105.6928119	1025.308136	11170.9705	-75.43876266
4.145981216	118.0479978	913.3237	13935.32978	-106.0616646
3.730817763	95.33217184	853.3638916	9088.222987	-66.73143234
3.674795004	94.57964732	1037.210602	8945.309688	-67.02754059
3.678585384	93.52989481	997.4616699	8747.841224	-55.36412582
4.073801134	114.4002368	1206.194519	13087.41419	-128.4375969
4.281866316	123.7886444	963.1952209	15323.62847	-134.5881104
4.246378967	125.1976501	1049.099335	15674.4516	-129.0042267
4.035713756	109.8921294	865.3735046	12076.28009	-141.5334167
3.602897568	98.9266932	685.7850952	9786.490627	-52.09288139
3.700150552	91.39192557	834.7565002	8352.484059	-69.0007225
3.673195818	86.80224176	1013.365967	7534.629175	-69.23502579
3.442573446	88.86388693	926.5545959	7896.7904	-74.51470184
3.735528917	93.94178762	988.6297607	8825.059461	-72.19088593
4.036510275	106.1917245	820.4348755	11276.68235	-106.232399
4.002202198	108.3024601	1053.027802	11729.42286	-97.72134705
4.183674723	124.4949889	1036.047211	15499.00226	-142.7948593
4.103982257	114.687615	1079.778961	13153.24904	-101.6628952
3.507963048	84.59756354	942.8807373	7156.747757	-53.33063889
4.133122662	127.9438177	1116.815491	16369.62049	-112.8177704
3.714911911	83.47335731	688.551239	6967.80138	-84.33850098
4.006007482	110.2773478	1240.375488	12161.09343	-77.75747681
3.954156083	111.2110633	1229.62384	12367.9006	-97.3314621
4.199592818	121.3430719	998.0767517	14724.14109	-132.7012939
3.830659662	95.40668095	749.4001465	9102.434769	-93.32359085
3.806480262	90.87887487	603.7221985	8258.969898	-81.94290924
4.204146678	117.2647642	1008.160004	13751.02491	-102.8993088
4.069340147	115.9798361	1076.202728	13451.32239	-94.65927811
3.806003211	103.7051549	1122.909546	10754.75916	-64.05866432
3.887627283	102.6352146	878.7348022	10533.98728	-107.8777603
4.159718877	124.2720552	1014.745697	15443.54371	-135.4227478
4.246680521	118.9517497	1000.082001	14149.51876	-123.42379
3.602766851	87.10675465	869.4499817	7587.586706	-54.52438736
3.931658677	100.6805298	1114.74707	10136.56908	-81.41912384
3.897722025	99.31084812	911.3621521	9862.644554	-99.86016083
4.280586685	124.9610768	1079.272583	15615.27072	-120.7294609
4.048494419	115.6255246	966.4383545	13369.26194	-122.2679901
3.745403589	95.20086667	1013.828766	9063.205014	-97.1913208
4.111203649	115.1701918	898.5589905	13264.17307	-107.3581528
3.855325653	102.1403239	952.7124329	10432.64576	-98.8053894
3.982052951	110.0446513	983.7819214	12109.82528	-130.8286285
4.125428759	123.0680369	1105.663818	15145.74171	-127.1037971
4.386732305	140.3181234	1121.961975	19689.17576	-167.7637939
4.268858258	133.596399	1199.431213	17847.99782	-129.0871231
3.276168845	75.6362387	543.7681885	5720.840605	-32.0294529
4.181203862	117.4381999	919.4793701	13791.7308	-133.1414902
3.803721804	88.1220612	723.1326904	7765.49767	-88.96330872
4.190345261	121.351052	1021.26123	14726.07783	-125.5472626
3.761116196	94.93387897	865.4151611	9012.441377	-74.61401062
3.42215805	74.62836361	474.4516907	5569.392655	-43.82201004
3.666419933	100.7151409	1006.983551	10143.53961	-68.60932083
3.85676152	98.29286876	857.7845764	9661.48805	-82.79006958
4.397126593	155.630982	1182.118225	24221.00256	-120.0465118
4.231768542	126.1943171	965.2851563	15925.00567	-154.5758606
4.131013464	121.4697487	1158.566528	14754.89985	-135.1958282
3.795804219	90.30778451	933.5277405	8155.495943	-73.28653717
4.684063637	170.7007434	1213.165527	29138.74378	-242.7322906
3.398895073	75.02620194	804.1746826	5628.930978	-58.08793411
3.448121478	77.70378275	673.7387085	6037.877854	-38.66202431
3.418817459	80.36426109	911.5328369	6458.414461	-65.38835754
4.609567706	156.6416257	1185.482178	24536.5989	-183.4790009

log.sigma.1.5.mm.3D_firstorder_Kurtosis	log.sigma.1.5.mm.3D_firstorder_Mean	log.sigma.1.5.mm.3D_glrlm_ShortRunLowGrayLevelEmphasis	log.sigma.1.5.mm.3D_glrlm_GrayLevelVariance
5.378538125	34.7739364	0.003644896	22.13195385
6.667598056	24.95734507	0.003680286	18.76528456
3.7645392	8.445629278	0.00635895	24.95060526
6.809642094	22.80732532	0.002047819	24.20790542
5.879110076	25.67724073	0.007910046	13.58542842
6.951744653	9.644356765	0.003300453	17.4091502
5.516572597	20.26812616	0.006580424	8.716782934
7.942489025	24.99603471	0.002593117	17.40705966
6.235908393	31.33923721	0.004708466	15.28270358
4.702566923	24.4828764	0.002714644	18.39352151
8.559664548	18.66009543	0.003598868	14.18009171
5.23874699	23.40085017	0.004001396	31.7763561
6.459891161	21.4945816	0.003780413	13.18075712
3.678409438	16.84042429	0.008040646	14.47381095
3.710925244	23.3664851	0.003749817	16.36004936
5.846228043	9.795423537	0.002964104	16.46801491
5.09685286	11.79620547	0.00599372	12.40803415
5.455245844	34.20703693	0.005529129	12.9124614
4.871650857	38.3780943	0.010776872	16.41256796
6.585863868	17.80045613	0.003645311	13.22144594
4.018979467	10.61643006	0.003114986	23.78207344
4.888775257	20.79436184	0.003905313	16.61599435
4.643378146	16.40168784	0.003574274	18.18085904
5.906791772	25.24061595	0.004002876	18.9655639
4.223871662	4.769537733	0.004874873	17.994445
4.307347047	37.60929945	0.007385202	26.71244553
4.854494459	21.29543456	0.003158422	18.95590543
3.860036986	21.00880722	0.004578879	24.99105472
6.832860704	15.26314166	0.002620372	13.65232646
6.353534069	17.7496806	0.002276781	18.8018071
4.340245045	18.05751475	0.003347539	19.12677726
5.584210134	30.88547283	0.002728553	12.39538425
7.176375038	18.65030406	0.002818703	21.52002037
6.56751116	39.2283423	0.004695145	18.12710624
4.986685318	15.66277289	0.003979007	28.42540263
8.701494516	15.65472069	0.003057776	16.62902503
4.92842612	21.18303803	0.00187802	28.61523174
5.151283864	19.89922022	0.004164926	21.70356918
4.18116115	12.74514658	0.009961628	8.48015128
7.999476276	26.16008599	0.002524205	19.8804835
5.828941694	25.94155711	0.002991102	23.3801061
6.565987573	29.62404069	0.003096354	15.80350876
8.532305606	20.77304193	0.002196822	15.58608161
7.687048338	30.9929695	0.002403732	15.42442175
6.872753534	12.81842186	0.001445842	22.4186386
4.165011297	24.46181745	0.003597869	25.46414178
5.003087087	24.36598539	0.002588449	26.232666
4.774980052	2.150143544	0.0044322	20.32219079
5.242822976	35.28965785	0.011182376	17.02852716
7.844973425	23.28647424	0.004716878	14.56474976
7.260350672	21.25796563	0.002766089	13.05873207
11.56558244	7.299241261	0.002335079	14.00142924
7.453884222	24.61378277	0.003727792	15.34426963
4.867496477	20.08094857	0.00400635	18.73323175
5.971172988	23.55651286	0.002086068	20.06283236
5.169535022	11.13020575	0.00308896	26.90785121
6.261696882	25.57794361	0.002099026	22.28868609
8.800141589	21.24711196	0.002184747	12.5983002
7.418770159	20.61492811	0.002816363	27.45399463
4.469646127	19.06824926	0.007617869	11.78334863
7.282899905	34.89316607	0.002794692	20.82612204
6.902082597	19.67656339	0.001726335	21.49023727
4.852185446	23.33582094	0.003700848	24.68440503
5.275110776	22.70325912	0.005424002	15.67321366
3.860681211	32.34207615	0.0095733	14.06620876
4.537733586	35.75501964	0.003212754	23.07056696
6.853405502	24.45673859	0.002708488	22.97300237
9.16591519	28.52196956	0.002085589	18.70079001
6.586526278	18.62220743	0.002846152	17.95966239
5.527246448	16.10056208	0.003906637	26.24427588
4.399314344	20.74265589	0.002129699	23.62028426
7.413295518	25.91980642	0.004097269	13.39502699
5.551080684	30.64790153	0.004555458	17.29199825
6.071028227	18.31105101	0.002391609	16.91964382
4.90364971	29.98133915	0.002659414	26.20881915
5.629720212	16.55867907	0.002551381	22.68787137
6.371492131	12.35300585	0.00692613	15.67989431
4.840764623	36.78372101	0.003678592	22.19123039
6.596907103	13.78048103	0.003668411	17.92578799
5.529224443	10.46508568	0.003960718	20.78507463
6.236350995	17.3101033	0.002208195	25.66180165
4.671980692	15.93141284	0.002647176	32.74798412
6.355414839	20.76813106	0.002143497	30.21214425
6.022834856	30.73788399	0.010595041	10.24226375
4.451596765	23.78168389	0.002887348	23.17630441
4.455091707	20.84843965	0.005583977	13.17150925
4.955095359	28.77638057	0.003225244	24.61188942
7.1062816	23.41320692	0.003470154	15.62703796
4.201240076	30.24498956	0.014326774	9.748185342
10.10338763	20.87847362	0.004569544	17.52060365
6.191862296	27.02916185	0.00367167	16.58551993
7.157998824	31.04192608	0.005603004	40.56666585
4.692547837	14.48671351	0.005245026	26.81643478
5.4394694	15.70914243	0.002000979	25.11507059
5.498393167	26.43488289	0.003336668	13.91710234
3.7673325	3.909015639	0.004910788	48.25160891
8.300832814	17.91341137	0.003604912	9.887978907
7.139407566	31.41331107	0.007245303	10.60781097
10.25443188	16.3173495	0.003856244	11.38625055
4.11892429	25.45762545	0.003676304	40.52522062

log.sigma.1.5.mm.3D_glrlm_LowGrayLevelRunEmphasis	log.sigma.1.5.mm.3D_glrlm_GrayLevelNonUniformityNormalized	log.sigma.1.5.mm.3D_glrlm_RunVariance	log.sigma.1.5.mm.3D_glrlm_GrayLevelNonUniformity
0.003754824	0.075365236	0.079393399	84.76896365
0.003818788	0.086601439	0.115554371	158.0370702
0.006530134	0.065236359	0.073737379	68.41722823
0.002113594	0.07497699	0.079193029	171.7456315
0.008324839	0.10773224	0.115212465	92.88501854
0.003430316	0.091654931	0.103918093	137.751056
0.007019594	0.126477455	0.160382044	118.2367116
0.002716854	0.095917417	0.131244545	156.0613324
0.004896836	0.092693312	0.125916468	170.262091
0.002818481	0.075576076	0.082515826	211.5575111
0.003756668	0.103725986	0.117041801	169.8834254
0.004071988	0.06150899	0.058663318	98.30948898
0.003995324	0.099194611	0.118813338	263.9047302
0.008326037	0.081173288	0.087775121	140.0771208
0.003868757	0.073694135	0.066649545	107.5912443
0.003151408	0.091667467	0.16910813	335.3753496
0.006260234	0.094430905	0.105153705	138.5726366
0.005829262	0.103113555	0.150974266	235.5002926
0.011165315	0.087937403	0.118023718	91.06421317
0.003841159	0.099741979	0.141812829	259.4259761
0.003217336	0.062745993	0.074155836	409.2079629
0.004062316	0.081152196	0.094935135	158.8868919
0.003717021	0.076956452	0.092422296	293.3419503
0.004173271	0.093053362	0.119947478	87.13486325
0.005063567	0.071097419	0.090325204	171.6966109
0.007550088	0.067390803	0.074338157	85.23943363
0.003248499	0.077040715	0.060529971	161.351025
0.004702413	0.063730868	0.069415582	201.3987296
0.002794121	0.109715228	0.175076378	330.190604
0.002390657	0.090978248	0.119420837	261.9675131
0.003475479	0.075161823	0.079955497	290.4081133
0.002888122	0.095511839	0.120087376	434.7250128
0.002925282	0.088551257	0.102875034	205.5187198
0.004870688	0.106181914	0.120743343	46.15646989
0.004063841	0.071244845	0.075012011	59.33238837
0.003190901	0.107276092	0.140043011	309.3037347
0.001946482	0.066776152	0.086259173	222.0213008
0.004303763	0.077020067	0.088932317	242.1384136
0.010502019	0.108726066	0.12255049	145.6585433
0.002642575	0.102148552	0.147026213	149.6981089
0.00306467	0.070944874	0.069713023	87.83085808
0.00326398	0.101939505	0.137105236	176.492429
0.002319273	0.106596122	0.139343648	289.0783783
0.00253275	0.106420783	0.142129962	179.5975822
0.001509662	0.07797473	0.10645	249.954322
0.003695095	0.063903084	0.063966239	215.6832553
0.002671096	0.067871457	0.074187831	234.4241707
0.004557254	0.07773717	0.081774938	109.6609535
0.011755786	0.115884367	0.137503486	58.71796089
0.004872813	0.101930253	0.123000677	100.4341094
0.002916218	0.102596864	0.131163501	368.3515969
0.00245869	0.125607593	0.171883509	215.903326
0.003887998	0.100549163	0.126576766	344.8642876
0.004130963	0.076341556	0.072353619	83.78726507
0.002181332	0.0822619	0.106980121	297.2823882
0.00320132	0.070902779	0.155820939	120.7158708
0.002175864	0.076364599	0.089117793	232.47075
0.002330432	0.117414886	0.158192933	253.3260174
0.002880559	0.075729288	0.073346835	97.27443573
0.007940836	0.093739615	0.090203292	121.6799861
0.002895018	0.081963599	0.099446938	221.8044904
0.001821156	0.088511051	0.12937879	439.6387236
0.00380897	0.069898351	0.074432015	148.7791835
0.005667246	0.088483355	0.120325543	150.1767654
0.009884445	0.090151779	0.09477775	49.07576715
0.003311255	0.06760297	0.065894388	98.20887342
0.002788262	0.079029349	0.095259182	77.31472187
0.00218991	0.096590082	0.134195789	310.4875345
0.002944292	0.087145732	0.10183306	103.6886721
0.004022759	0.075265978	0.096612582	174.1043808
0.002205582	0.064003617	0.077002307	228.4587165
0.004334577	0.1103404	0.146595255	203.3833758
0.004744388	0.084529428	0.105267768	362.6945258
0.002509056	0.086084746	0.111366291	512.6066402
0.002740162	0.065710685	0.073337557	262.5516412
0.002646988	0.080381158	0.093568602	173.2205258
0.007182141	0.099586788	0.127236357	212.2168083
0.003787731	0.075640572	0.068465016	96.57686969
0.003803291	0.091955791	0.111038678	274.9927148
0.004136285	0.084772472	0.117943662	343.3518811
0.002278085	0.07680638	0.086131949	97.06559161
0.002714557	0.061467804	0.062457004	154.4055796
0.002207419	0.068974764	0.079972563	159.2638683
0.01134663	0.139598165	0.1765352	64.0260027
0.002979322	0.069109736	0.073554134	250.5947765
0.005818463	0.088431234	0.098306688	282.8737161
0.003325546	0.070794802	0.07525993	94.71359084
0.003621454	0.098260632	0.115982455	271.8369292
0.014962815	0.117620553	0.126143404	43.53739317
0.004687488	0.106083073	0.102076125	56.15997226
0.003824547	0.090100018	0.109708791	264.4022931
0.005651562	0.062930187	0.065673526	65.57366002
0.005348074	0.067592626	0.07117361	99.19542625
0.002085055	0.076695112	0.10254624	307.6689055
0.003502151	0.091380677	0.099426143	395.022411
0.005055853	0.049227799	0.061521205	41.27762563
0.003824924	0.125009267	0.142616964	196.873912
0.007526463	0.118810645	0.134139009	45.63985895
0.004035966	0.125260356	0.147680768	99.11277276
0.003732488	0.049513861	0.047471252	54.68934932

log.sigma.1.5.mm.3D_glrlm_LongRunEmphasis	log.sigma.1.5.mm.3D_glrlm_ShortRunHighGrayLevelEmphasis	log.sigma.1.5.mm.3D_glrlm_RunLengthNonUniformity	log.sigma.1.5.mm.3D_glrlm_ShortRunEmphasis
1.224365867	477.3633027	990.0651882	0.951325153
1.31820667	490.4489462	1541.416221	0.935285085
1.211388895	325.5115689	928.1592065	0.953350046
1.226892735	734.7154665	2010.163671	0.950227043
1.329401574	184.574633	720.2507893	0.930868045
1.293004686	462.8164197	1278.301715	0.938070326
1.440341967	196.1257475	746.5771479	0.913738165
1.363305222	571.3750927	1342.280865	0.926242433
1.345252396	378.6200189	1533.594661	0.930754262
1.234394737	495.8017694	2451.724912	0.949321324
1.336403041	429.9894148	1359.30141	0.928641919
1.170624519	601.8485537	1443.867217	0.961323414
1.332192968	326.5635977	2223.982896	0.931368048
1.248979776	203.2066206	1502.154422	0.946647136
1.192063868	377.2458644	1306.282071	0.957354556
1.43466202	417.3618207	2980.065096	0.920735948
1.303729357	248.7759765	1237.150895	0.934514454
1.408492192	275.8006132	1857.613301	0.920453693
1.323865189	201.8969145	873.1233818	0.934386531
1.389528965	388.2612537	2129.372029	0.922803288
1.210379045	480.0593189	5779.40767	0.953960677
1.27036659	366.4819334	1684.210905	0.942144101
1.263188132	397.7660601	3291.52281	0.943600849
1.332613654	412.6239055	785.8070581	0.932426566
1.254535424	312.1305597	2097.563534	0.945937584
1.217532572	369.2836721	1112.415247	0.951133353
1.175554188	454.5468548	1887.1067	0.960366516
1.197029194	379.9879431	2820.668019	0.956703909
1.461322605	458.6300631	2421.371589	0.915203496
1.325344989	565.0871562	2428.053653	0.934571099
1.229864548	404.4095209	3383.688341	0.949419017
1.328841316	413.6166787	3830.274236	0.933668643
1.29364981	568.4692039	1968.874483	0.937113773
1.347458794	432.6381249	358.7957216	0.926478541
1.213104271	532.5790565	737.4303701	0.953533991
1.386723938	550.6674104	2355.56013	0.922516648
1.239320132	735.8126601	2913.656521	0.949559927
1.252910908	408.0166803	2724.806276	0.94547522
1.351973237	141.2296526	1104.579203	0.925833403
1.395281637	621.310084	1200.717012	0.923513602
1.200311708	594.947874	1101.870388	0.955643794
1.383747336	448.7202572	1412.705378	0.922069255
1.386849639	606.2795509	2214.293246	0.922314819
1.390316065	580.5750841	1379.105349	0.922562059
1.29594191	920.7200325	2730.221498	0.938535368
1.186361202	464.9925462	3020.764832	0.957828615
1.213647855	593.6418123	3049.096138	0.95255099
1.237748343	417.0138547	1228.605275	0.947272664
1.380339047	199.354239	416.2523043	0.924203851
1.347435048	437.3175453	815.1416143	0.9275334
1.364306154	474.4395404	2960.460576	0.925983138
1.469636204	660.9818166	1358.792795	0.90955179
1.353622368	441.9011588	2840.322856	0.927703777
1.211601177	409.5718298	968.752759	0.952403991
1.294743658	626.5585905	3085.968628	0.939515296
1.388760985	603.4055262	1423.195187	0.930861078
1.250236493	686.136864	2648.481967	0.946763575
1.431811997	556.961288	1734.273854	0.915819013
1.215206409	733.3731385	1130.872726	0.95150044
1.263331382	202.4260486	1115.770109	0.942039107
1.281350611	603.5044632	2313.6975	0.940169756
1.351134921	712.4728806	4141.345748	0.930286126
1.213555086	459.820485	1880.887874	0.952865585
1.333512844	295.8360165	1421.317108	0.931964016
1.270423059	191.5129164	467.3488252	0.941750828
1.194716236	482.5117582	1292.059502	0.955444785
1.265743816	682.7522554	845.0797931	0.944012396
1.366270241	675.3534527	2658.474398	0.927145997
1.283521231	565.1658731	1018.59543	0.940634234
1.272085028	526.8475389	1989.44103	0.942343915
1.215877022	634.2260918	3159.265592	0.953436376
1.399240043	330.1686263	1503.620057	0.921680264
1.295761031	345.1516931	3648.707415	0.93793838
1.307861238	519.6854725	5046.824344	0.936714235
1.211657655	605.9827448	3531.450655	0.952981944
1.264494278	568.5503998	1859.189246	0.943604453
1.350529933	281.184313	1772.997231	0.929388643
1.201735067	439.496949	1131.178939	0.954010228
1.312610662	468.6588177	2521.087012	0.934613916
1.327995959	387.3142762	3395.352639	0.932458771
1.248690567	774.8409104	1095.714486	0.945563088
1.182061737	634.4508519	2254.824988	0.958841579
1.228844036	788.1718754	2023.554311	0.949739968
1.478975534	136.8479691	361.3874117	0.908059305
1.2083839	500.722101	3217.129993	0.954443507
1.278455338	262.2978388	2737.433059	0.940572281
1.215185733	512.2951572	1180.961707	0.952516477
1.32808253	442.2335108	2314.881745	0.931633975
1.366084833	137.63396	303.9227924	0.923316423
1.294708032	524.6169202	448.012779	0.936341739
1.307895575	410.0637455	2480.578244	0.93573972
1.191209523	882.5136854	930.2811619	0.956777558
1.204848206	447.255365	1302.947021	0.954669818
1.281124105	663.1572992	3450.463653	0.942333517
1.285544868	371.8964622	3679.922116	0.938498123
1.174132362	631.1337427	758.278927	0.961694784
1.398328178	348.159314	1277.776286	0.919626163
1.379498831	268.83995	314.287578	0.922487402
1.40924815	419.1487471	639.7578432	0.918282492
1.13986003	669.2694197	1014.312889	0.967552039

log.sigma.1.5.mm.3D_glrlm_LongRunHighGrayLevelEmphasis	log.sigma.1.5.mm.3D_glrlm_RunPercentage	log.sigma.1.5.mm.3D_glrlm_LongRunLowGrayLevelEmphasis	log.sigma.1.5.mm.3D_glrlm_RunEntropy
611.2698617	0.934842381	0.004257664	4.489033373
688.0860726	0.912269231	0.00449055	4.410758597
414.6343586	0.937938627	0.007289545	4.593741089
952.2107326	0.933707548	0.00241231	4.512120219
256.4663881	0.908243495	0.010295154	4.140041515
644.0264764	0.917441533	0.004039392	4.302311131
303.5383132	0.884287898	0.00926377	3.964634007
838.5965176	0.901299808	0.003326595	4.337826306
540.8330463	0.906037722	0.005831389	4.320517147
646.101936	0.932067932	0.003295201	4.434105864
622.0636841	0.906176834	0.004497768	4.188989341
736.1309217	0.948504908	0.004378954	4.689427155
463.8891089	0.908212307	0.005028124	4.224603135
268.9868485	0.92870746	0.009642862	4.298315836
467.864692	0.943102763	0.00440622	4.338610711
644.2610895	0.889997566	0.004180569	4.432025449
348.3354273	0.914162473	0.007482392	4.202217189
413.4653388	0.892614422	0.007362128	4.239981375
276.9425147	0.911362405	0.013066194	4.35656015
584.027615	0.896348739	0.004820622	4.246531422
610.220419	0.93833979	0.003680689	4.626928824
495.2315958	0.923040656	0.004794593	4.379419823
538.5404888	0.924924869	0.004374964	4.432953692
578.0481447	0.908887229	0.005011296	4.375222244
414.7408841	0.927330734	0.005954186	4.470802046
469.0082983	0.9354802	0.008278213	4.624362587
558.0699306	0.947187141	0.003646093	4.357507362
477.7474546	0.941900722	0.005257872	4.618082786
732.4764949	0.884558807	0.003737404	4.246887571
798.236062	0.911218253	0.002958437	4.408875775
526.678002	0.932771236	0.00405304	4.450960942
584.6323721	0.910184615	0.003679004	4.226769861
787.938791	0.916816096	0.003421093	4.430006899
613.938581	0.903406365	0.005698661	4.244676232
684.4239659	0.937716563	0.004453284	4.559183562
828.7174975	0.896057005	0.003853911	4.268271147
961.2296385	0.931504192	0.002269201	4.692700119
542.8978967	0.927046707	0.004942674	4.504044727
205.2547044	0.902602115	0.013059489	4.010049395
932.0821687	0.895476773	0.003253533	4.371603092
751.1283948	0.940671031	0.00339613	4.492869699
668.9262711	0.896002548	0.004090243	4.307874805
907.8125692	0.89570786	0.00292852	4.258110588
865.9245989	0.895516903	0.003184638	4.257368468
1274.515448	0.917308793	0.00181624	4.547973743
577.5401605	0.944055944	0.004121015	4.619086933
757.7630623	0.936996536	0.003040744	4.62287152
552.0366362	0.930434341	0.005117118	4.434915697
280.424156	0.897981451	0.01450144	4.161675681
637.5936225	0.903952011	0.005621381	4.229444966
696.6752625	0.901007878	0.003654234	4.226806329
1077.886584	0.878519742	0.003100082	4.102567266
642.2795579	0.903162416	0.00466941	4.283695789
521.0308392	0.937200289	0.004681503	4.397314176
860.7280697	0.917840605	0.002645071	4.475320138
896.7113525	0.901320078	0.003831537	4.717855108
905.4244945	0.928331808	0.002535789	4.522489223
860.81547	0.886987601	0.003078032	4.122674408
941.3727143	0.936084324	0.003165817	4.507876575
270.3871599	0.923788667	0.009384748	4.14360237
816.6483628	0.92006905	0.003366003	4.466584132
1031.146763	0.905076334	0.002298606	4.498609637
587.1476627	0.937201504	0.004294469	4.569785848
424.6750305	0.908417065	0.006838238	4.334252657
258.514185	0.922555411	0.011320645	4.230494257
597.9660139	0.941422803	0.003741493	4.547308009
914.5265186	0.924603752	0.003167537	4.497473807
991.5143082	0.901268389	0.002713865	4.36327967
778.7786541	0.920161818	0.003415041	4.334884264
713.9761238	0.922616673	0.0045605	4.608109866
806.7891181	0.937297996	0.002556731	4.61473238
493.5057382	0.894114134	0.005541754	4.188389604
472.836062	0.916911898	0.005639775	4.408118378
727.5314647	0.914476094	0.003079766	4.387359782
768.6693011	0.937461647	0.003101756	4.654510395
767.5040395	0.924396924	0.003093606	4.487333882
410.6511545	0.904654581	0.008430342	4.2802864
553.237784	0.939491708	0.004263936	4.468584854
662.3346168	0.912698413	0.0044434	4.353605579
553.3437278	0.909447391	0.004982435	4.499430533
1023.016476	0.9277646	0.00259636	4.541676629
786.4322844	0.94534032	0.003010161	4.718265013
1022.375366	0.933213109	0.002497518	4.664350552
211.1904084	0.877040741	0.015268047	3.915428744
637.8658999	0.93885193	0.003398708	4.547381549
357.872061	0.920729817	0.006905599	4.255193413
652.0172041	0.936813187	0.003774979	4.55775742
628.9376747	0.908895574	0.004345468	4.277610529
195.4974906	0.899868987	0.0179842	3.945069488
725.0059102	0.915890338	0.005231818	4.133563812
574.8719756	0.913829864	0.004553614	4.34742401
1100.695522	0.942916812	0.005866318	4.73674937
568.3414423	0.93942677	0.00581132	4.586706808
908.5454407	0.921433494	0.002491702	4.589112618
507.2728639	0.91851646	0.004267782	4.264566471
765.4195379	0.948311869	0.005669224	4.976971576
529.0984442	0.893101793	0.004913365	3.983152606
398.1315387	0.897196262	0.008909435	3.988326551
647.6815829	0.890939016	0.004936896	4.006680547
786.3387376	0.95707239	0.003975626	4.863631841

log.sigma.1.5.mm.3D_glrlm_HighGrayLevelRunEmphasis	log.sigma.1.5.mm.3D_glrlm_RunLengthNonUniformityNormalized	log.sigma.1.5.mm.3D_glszm_GrayLevelVariance
501.2888245	0.880036807	37.725036
523.9455995	0.844432761	35.66975656
341.6188902	0.88469884	36.45978316
773.8318666	0.877381181	43.69616467
197.1034441	0.834888572	28.1812433
494.4127207	0.850231065	32.53676355
213.8189538	0.798417853	21.22360768
616.6381583	0.824668847	36.15662416
405.8208878	0.834625731	28.67015041
522.5259177	0.875574255	30.40156396
463.6255868	0.829573075	30.56443611
626.5401859	0.903195726	47.49947811
350.1107815	0.835640433	26.75473249
214.7978459	0.869818218	23.7556582
393.7919107	0.894232983	25.1481559
452.6557748	0.813958649	30.97425924
266.367496	0.842444533	23.0386787
298.2826433	0.81277723	25.69200449
214.6194497	0.84265704	28.09961143
420.6332428	0.817912069	26.80582577
503.4310046	0.885998333	34.52912734
389.2358194	0.859583203	28.82966063
422.5438006	0.862846196	29.21511172
440.6870868	0.83857543	37.54180267
330.1307082	0.868094634	27.5580245
387.6822751	0.879320707	41.43947574
473.6526686	0.90090352	27.65535627
397.5437534	0.892393578	36.92483976
501.2925595	0.80319226	30.47468139
604.3154899	0.842948503	34.73200928
426.430094	0.875539707	31.05926862
442.4354092	0.841036175	23.20159632
607.2083645	0.848044508	43.0447716
464.3480749	0.825127463	39.32407407
559.7959702	0.885226244	45.95384859
597.1879132	0.816757687	37.86449546
775.004787	0.87617431	46.8225349
432.0171649	0.866611548	37.18172855
152.4013194	0.823866696	16.74082232
671.7946114	0.819227025	44.84454755
623.2874826	0.889901525	37.6148739
486.0628015	0.815729554	34.16329213
656.8352925	0.816334376	36.06571892
628.0227368	0.8170173	34.24925957
981.6196442	0.851430063	40.8993278
485.7358306	0.89486237	38.53595178
623.5116874	0.882618353	42.26031388
441.5200552	0.870648126	33.72919294
212.8781282	0.820965718	32.50023989
472.0157333	0.827073106	31.23857296
512.0450019	0.824212619	28.7078315
728.4612106	0.790117002	34.28477839
476.013852	0.82783606	33.898525
430.0759283	0.882378094	28.46005917
666.5572776	0.853703786	36.57764953
648.0376224	0.835758922	46.28929364
724.6063316	0.869748837	39.60935856
606.6746181	0.803126884	29.88843999
771.6650798	0.880237037	47.12608029
214.6703474	0.85908786	21.10649525
640.9861958	0.854796194	41.13029287
765.3979146	0.833568491	43.69216121
482.8558964	0.883448548	40.18655854
317.7356015	0.837097315	26.56023243
203.4026003	0.858314259	25.14133195
504.0283838	0.889231558	36.86106254
723.2194554	0.863568074	43.70465073
727.9971401	0.826807831	38.92108867
602.3739762	0.855888362	34.49010506
559.5294727	0.859808533	45.34398271
664.9366858	0.884887111	35.53624326
357.0574657	0.815351629	30.81140287
367.3770329	0.850046399	32.06103109
555.2182289	0.847355009	32.70288043
635.5562052	0.883659936	42.22870721
603.5624546	0.862552495	41.00975425
302.8794099	0.831698965	32.17922611
460.6093121	0.885822967	35.42848112
502.1437352	0.842735247	35.66196304
415.7240818	0.838026974	39.87818734
819.4534055	0.866814069	44.57081669
662.4684226	0.897400905	48.37862158
830.4649759	0.876203068	50.13522221
148.9555262	0.787133044	23.08999777
525.3703108	0.887124085	36.38669724
279.1203286	0.855598286	23.0687564
537.5615811	0.882573131	39.33894446
474.4904209	0.836374564	31.61258276
147.6984697	0.819759372	20.41308594
560.221342	0.846102615	38.52830375
438.5349616	0.845108932	32.36904631
922.8457089	0.892490729	67.58076071
469.2739793	0.887694597	41.30643758
704.9216169	0.859954556	43.65445188
395.8935159	0.851010895	27.94988009
655.271788	0.904258276	65.02168093
378.63762	0.810841028	23.72118703
290.9194317	0.817339529	23.28498054
457.3447638	0.808033541	25.92718569
691.4699591	0.918047285	54.64770536

log.sigma.1.5.mm.3D_glszm_SmallAreaHighGrayLevelEmphasis	log.sigma.1.5.mm.3D_glszm_GrayLevelNonUniformityNormalized	log.sigma.1.5.mm.3D_glszm_SizeZoneNonUniformityNormalized
351.2579033	0.049856	0.453144
358.7820281	0.05270367	0.446820419
233.234408	0.047689922	0.44156557
544.244861	0.047214171	0.499018413
153.1528026	0.065539157	0.433967469
323.8904546	0.057914058	0.468498041
145.8550426	0.074211248	0.455884774
418.3803238	0.055858296	0.48008311
292.6632484	0.057171888	0.496069016
362.3517284	0.052127015	0.465030021
298.0602686	0.058916479	0.436297976
434.011959	0.044930349	0.477454426
244.34135	0.062292122	0.434762022
147.9594372	0.05733066	0.44034398
286.2978156	0.056489804	0.474638369
314.2136346	0.053411918	0.463053464
174.1932235	0.063634179	0.394758042
216.7411899	0.060483897	0.476811408
158.9211715	0.057632617	0.439088427
272.3629638	0.059501187	0.431923278
348.2261257	0.048655778	0.457098048
259.598172	0.054187662	0.444471732
261.0187858	0.053589865	0.42835661
347.1801674	0.051377778	0.487402667
227.1999658	0.054247359	0.45048319
282.9702502	0.044833184	0.476088257
338.7746004	0.056169885	0.509238825
273.9372321	0.047224062	0.47348207
342.2905414	0.057325444	0.487204734
435.3361674	0.051166472	0.486465423
295.6524479	0.052168087	0.47215384
304.1352885	0.063008549	0.431704765
400.4780597	0.049679813	0.448529521
396.8853134	0.054945892	0.557765585
375.1373499	0.046661468	0.500894066
412.249581	0.052802104	0.484863116
549.0320896	0.043030998	0.487462014
290.5178533	0.049050732	0.46720785
99.76419038	0.071479491	0.407121892
520.0017937	0.047851612	0.561524713
429.7710261	0.050568176	0.492811595
328.2480581	0.055694706	0.46826122
463.1891252	0.055810735	0.480419244
455.8982745	0.057395067	0.49542436
683.9695066	0.048116453	0.474656906
337.5834523	0.046673901	0.471361829
437.7334291	0.045655317	0.480329629
280.5933084	0.051776059	0.457323022
198.908119	0.054013788	0.526072574
307.2837249	0.060509873	0.46421531
360.7270485	0.0592243	0.468531139
489.329208	0.061781995	0.525958086
335.7790886	0.056044262	0.483342753
287.5013569	0.055428994	0.427463018
475.0348006	0.050102372	0.483713739
440.5341357	0.042717444	0.458321347
501.7020198	0.049324557	0.454270395
424.0549278	0.063649711	0.436710341
507.9723882	0.048950895	0.454663856
147.9881068	0.064314044	0.418138934
446.8130535	0.050288584	0.456635945
564.4980775	0.046971967	0.513387088
334.78935	0.047173368	0.476331361
196.6933832	0.058646896	0.422836062
136.8312184	0.058864266	0.490958757
365.4227428	0.048055172	0.455398811
511.0373438	0.048357886	0.480865037
503.3466156	0.054331487	0.481800583
390.5840626	0.052983939	0.479917884
390.7812719	0.044719651	0.493198696
467.0286359	0.048543291	0.472850823
267.3229739	0.059850583	0.475714079
254.7187998	0.052438015	0.449667248
370.7747239	0.053447281	0.458685611
448.4673535	0.045149223	0.466886084
411.93846	0.047403119	0.514409263
216.7927322	0.054539371	0.518244113
311.4990007	0.050581135	0.445742404
333.6437865	0.053444067	0.463931308
287.4214812	0.04772635	0.476274244
545.3590516	0.045630892	0.457195421
471.3312766	0.043238591	0.505884419
585.6187161	0.044048472	0.503938871
114.869527	0.071842281	0.43049677
362.7953938	0.048205377	0.488305659
181.8524041	0.060616142	0.446635822
390.025222	0.04789748	0.482540154
324.4674758	0.056836365	0.469807402
121.6127019	0.075439453	0.502685547
395.4716547	0.059726244	0.464369694
292.9836704	0.054970363	0.468807313
662.7803893	0.040576341	0.51555066
328.2697348	0.0467833	0.522013213
479.0914023	0.045021794	0.498199553
280.971808	0.057015134	0.458966701
514.9085207	0.03656101	0.535476318
251.2631832	0.073681142	0.444457304
184.4315207	0.077962803	0.419442042
296.7734284	0.069601915	0.472190728
514.3292732	0.040455582	0.524249575

log.sigma.1.5.mm.3D_glszm_SizeZoneNonUniformity	log.sigma.1.5.mm.3D_glszm_GrayLevelNonUniformity	log.sigma.1.5.mm.3D_glszm_LargeAreaEmphasis	log.sigma.1.5.mm.3D_glszm_ZoneVariance
226.572	24.928	37.626	31.837164
329.3066486	38.84260516	133.3731343	126.0089438
230.0556622	24.84644914	28.87907869	24.27430639
516.484058	48.86666667	35.19710145	29.57997246
133.228013	20.12052117	161.1954397	151.6398901
279.2248322	34.51677852	117.238255	109.6849804
123.0888889	20.03703704	384.4481481	369.1223731
289.9701987	33.7384106	188.3725166	179.4419187
354.1932773	40.82072829	208.452381	200.3928258
596.1684867	66.82683307	36.91809672	31.4311011
247.3809524	33.40564374	140.664903	130.50826
402.0166271	37.83135392	11.20783848	7.203086475
402.5896328	57.6825054	258.2948164	248.2898239
331.5790173	43.16998672	45.97078353	39.88240751
339.3664336	40.39020979	17.35664336	12.66926696
646.4226361	74.56303725	226.4491404	217.7854795
210.0112782	33.85338346	74.10338346	65.0015935
376.6810127	47.78227848	333.0683544	322.5838744
181.7826087	23.85990338	88.70531401	81.1759901
372.3178654	51.2900232	312.0104408	300.6843269
1467.284735	156.1850467	20.94890966	16.26121253
369.8004808	45.08413462	50.04447115	43.54564852
686.6556457	85.90455396	49.51403618	42.90499917
182.776	19.26666667	114.3733333	106.8291556
486.0713624	58.53290083	29.11955514	23.29531772
291.3660131	27.4379085	20.23856209	15.35821693
573.9121562	63.30346051	14.00621118	10.15737299
760.4122042	75.84184309	20.32814446	15.96405176
475.0246154	55.89230769	701.2420513	689.0887826
574.5156647	60.42760373	241.3878069	234.2329747
827.6856817	91.45065602	37.20821449	31.62535938
734.329806	107.1775426	203.0099941	194.3696432
402.77951	44.61247216	188.5356347	180.5917741
90.35802469	8.901234568	119.3888889	110.5731215
206.8692494	19.27118644	23.89830508	19.27528449
472.7415385	51.48205128	428.7702564	417.8836408
773.1147541	68.24716267	33.34867591	28.28475715
626.5257271	65.77703207	63.70246085	57.308087
183.6119734	32.23725055	198.1685144	187.3413503
304.907919	25.98342541	260.1031308	251.0256165
302.5863192	31.04885993	16.99674267	12.40291144
285.6393443	33.97377049	259.7639344	249.7326955
431.4164811	50.11804009	585.2126949	573.8502401
291.3095238	33.74829932	390.9319728	380.6658337
636.9895678	64.57228018	136.6467958	129.8681839
795.6587678	78.78554502	19.41291469	14.92745242
793.984876	75.46823956	54.03024803	49.05786066
281.2536585	31.84227642	47.68455285	41.60812744
104.6884422	10.74874372	116.4824121	108.4498876
160.6184971	20.93641618	166.4277457	156.5034248
571.1394586	72.19442166	582.5939295	571.912454
266.6607495	31.3234714	579.7988166	564.9147439
590.6448445	68.48608838	504.9664484	495.3117485
222.2807692	28.82307692	16.07115385	10.9999963
729.4403183	75.55437666	216.8799735	210.063996
353.3657588	32.93514916	53.00389105	47.00742715
603.2710843	65.50301205	86.95406627	80.85748056
285.6085627	41.62691131	595.3883792	581.5599884
273.7076412	29.46843854	21.91362126	16.71946226
215.7596899	33.18604651	48.79651163	41.3825003
487.230553	53.6579194	80.81818182	73.22084798
987.2433697	90.32709308	492.3718149	484.2301851
470.6153846	46.60728745	25.38562753	20.10214374
284.1458333	39.41071429	82.45535714	74.7282809
111.9385965	13.42105263	63.42982456	56.73353339
330.1641379	34.84	13.24827586	8.718717717
202.4441805	20.35866983	42.29928741	35.98379607
577.6788991	65.14345288	567.1259383	558.2803991
218.3626374	24.10769231	64.41978022	56.34419032
507.5014577	46.01652089	70.89504373	64.95926206
821.8147296	84.36823936	22.56156502	17.76097812
298.2727273	37.52631579	326.9106858	316.1057821
768.9309942	89.66900585	107.8964912	100.4093872
1049.013992	122.2339309	196.6055969	188.5004094
892.6861925	86.32531381	26.96338912	21.99459371
473.2565217	43.61086957	128.0684783	121.6488646
402.6756757	42.37709138	236.2870013	227.100719
272.3486088	30.90507365	19.49754501	14.5503789
517.2834081	59.59013453	130.3246637	121.6921346
745.8454662	74.7394636	318.3467433	310.2609642
256.486631	25.59893048	56.22103387	50.32678468
684.9675037	58.5450517	12.37592319	8.525169147
567.4351687	49.59857904	36.38365897	31.55615533
57.68656716	9.626865672	247.9477612	232.7144687
865.7659334	85.46813311	30.9216018	26.17692027
563.2077716	76.4369548	85.71134021	78.12155553
314.1336406	31.1812596	23.40399386	18.59233933
490.4789272	59.33716475	142.4741379	133.9783648
64.34375	9.65625	127.2265625	116.9164429
93.33830846	12.00497512	74.34825871	66.07905745
522.2513465	61.23698384	249.6651706	241.3569077
280.4595588	22.07352941	20.81066176	16.6846852
395.6860158	35.46174142	22.4591029	18.21267605
864.874424	78.1578341	116.7528802	110.4653817
747.1977887	92.82063882	132.1732187	123.8172838
258.0995851	17.62240664	19.36099585	15.99734853
214.6728778	35.58799172	539.6749482	526.3516925
57.04411765	10.60294118	113.6029412	103.6989619
118.9920635	17.53968254	216.9920635	204.5748299
353.3442136	27.26706231	6.362017804	3.430504803

log.sigma.1.5.mm.3D_glszm_ZonePercentage	log.sigma.1.5.mm.3D_glszm_LargeAreaLowGrayLevelEmphasis	log.sigma.1.5.mm.3D_glszm_LargeAreaHighGrayLevelEmphasis	log.sigma.1.5.mm.3D_glszm_HighGrayLevelZoneEmphasis
0.415627598	0.084656712	17883.778	506.794
0.3685	0.275515261	66178.93351	521.5820896
0.466010733	0.097983655	9602.238004	339.3934741
0.421932328	0.047644506	27300.23961	756.8183575
0.323498419	0.913853317	29081.35179	217.5798046
0.363858364	0.238369256	58932.27685	468.9681208
0.255439924	1.951969309	76726.46667	215.8777778
0.334626039	0.316640802	113543.4222	603.3692053
0.352244697	0.56851341	77747.65546	408.7563025
0.426906427	0.074329438	19162.83853	516.600624
0.313779745	0.30923408	65386.30335	444.9417989
0.499703264	0.023359955	7086.470309	614.8669834
0.316148856	0.789514492	85464.83477	357.2796976
0.405274489	0.222696036	10156.29349	213.0464807
0.461886305	0.049680383	6612.12028	398.3132867
0.33974203	0.520245376	100032.8288	449.3467049
0.331464174	0.287644505	19945.87218	264.1635338
0.308835027	1.277010945	88009.70759	309.9772152
0.36443662	0.523034094	15871.99517	231.678744
0.297138918	0.747305564	131796.3643	410.5475638
0.461870504	0.046371671	10399.18879	496.7523364
0.392267798	0.133090004	19641.25	382.3329327
0.388983256	0.118476196	21706.6257	398.1522146
0.36407767	0.283096975	47441.912	470.5146667
0.414362519	0.095823923	9787.180723	326.8897127
0.452662722	0.068484286	7530.444444	394.3513072
0.509724107	0.032440034	6641.212955	464.8251996
0.478688525	0.056782171	8186.034869	388.7559153
0.286849073	1.423234877	347061.8626	487.0748718
0.373852485	0.419427938	139994.5478	604.1261643
0.423225495	0.089082688	16350.92071	418.2709641
0.3402	0.480847157	86492.65785	448.0558495
0.354800474	0.311481715	115762.8719	588.2516704
0.336798337	0.287398451	51176.2716	506.3641975
0.46509009	0.046299632	13931.19855	530.4866828
0.303077401	0.728721843	254550.5569	579.5364103
0.44438218	0.04539083	25580.57125	764.3259773
0.395458567	0.157656017	26954.6085	419.1349739
0.303908356	1.376878385	29250.27494	152.8691796
0.33190709	0.405914805	168393.2744	675.2891344
0.46656535	0.029844954	10966.53257	602.7996743
0.31573499	0.55707921	122317.218	482.2836066
0.296663363	0.911783931	377543.9465	654.623608
0.312101911	0.648565239	237240.2228	635.8333333
0.384087006	0.139364239	135591.4858	963.9813711
0.472167832	0.044258859	9547.277251	478.9638626
0.448453608	0.090990376	33261.9637	614.4047187
0.405672823	0.110863832	21880.86341	413.1235772
0.352836879	0.711699108	20072.72362	254.9447236
0.317431193	0.362144551	78533.54335	454.5751445
0.305973896	1.165601205	292918.7137	510.4725185
0.259202454	0.792007391	427260.7416	677.2623274
0.321833026	1.094835799	234781.1342	473.6963993
0.444064902	0.043028803	6870.638462	426.9230769
0.383032766	0.339706307	139641.0862	664.4323607
0.408368644	0.088333193	33492.76524	635.7522698
0.405001525	0.127590607	60415.72214	722.872741
0.268914474	1.04768484	340068.682	625.2782875
0.43877551	0.031835448	17134.94186	747.7043189
0.367259786	0.252065456	10112.86047	217.377907
0.362801768	0.139141614	48510.89784	645.8200562
0.350464735	0.656239491	371006.7374	764.0130005
0.435050638	0.058325653	12157.08603	472.8097166
0.359743041	0.274356681	25870.18006	303.1488095
0.386440678	0.34009497	12476.59649	197.9254386
0.469863901	0.030990006	6392.128276	517.7103448
0.397920605	0.063224564	30075.84561	718.2232779
0.336231071	0.812736516	397640.362	716.8306922
0.351894818	0.110077627	39240.10549	563.5912088
0.410450738	0.131407378	40332.73372	545.2944606
0.456407563	0.037071221	14618.94419	660.6513234
0.304221252	0.98610186	109397.1834	373.0318979
0.365462706	0.318632277	37528.75497	371.3315789
0.351251728	0.355139322	109924.6826	538.4709226
0.448615673	0.046773793	16715.36506	637.3896444
0.394680395	0.214792331	77597.65326	573.4152174
0.329936306	0.778821145	73344.95367	291.7747748
0.449595291	0.048502028	8634.8527	457.9967267
0.34035409	0.265158537	65751.18386	482.2923767
0.351673029	0.776072487	132053.8755	404.8199234
0.411894273	0.073260151	44910.36898	801.1639929
0.50959729	0.021119175	8380.003693	646.8205318
0.455133387	0.046606047	30046.39254	811.3756661
0.256214149	2.006319456	31284.47761	174.3432836
0.459088555	0.060579721	16671.20474	508.9892837
0.362982153	0.317969954	23915.53529	270.4385408
0.455882353	0.048304432	12434.84332	538.3225806
0.343082484	0.305641458	67630.95498	468.8390805
0.311435523	1.040735431	16267.90625	168.1875
0.347750865	0.141003687	41723.44776	559.0945274
0.34693242	0.580439078	108599.947	423.6202873
0.492307692	0.032538992	18411.68382	907.9411765
0.485275288	0.055710088	10441.19393	451.823219
0.398805422	0.165674156	83503.87039	673.8784562
0.345941351	0.344286653	51539.89742	401.3556511
0.545248869	0.042126095	12320.32988	670.7738589
0.273964833	1.441592951	203325.4679	372.1656315
0.317757009	0.429165191	31120.16912	290.4632353
0.283783784	0.484843414	98743.36111	432.3015873
0.584055459	0.013616258	4408.694362	688.805638

log.sigma.1.5.mm.3D_glszm_SmallAreaEmphasis	log.sigma.1.5.mm.3D_glszm_LowGrayLevelZoneEmphasis	log.sigma.1.5.mm.3D_glszm_ZoneEntropy	log.sigma.1.5.mm.3D_glszm_SmallAreaLowGrayLevelEmphasis
0.699925403	0.005569661	6.056515507	0.004757285
0.694495413	0.006151252	6.06496423	0.00521557
0.690029514	0.009103188	6.065345539	0.007545289
0.733308926	0.003060884	6.172303499	0.002682233
0.684281661	0.012409724	5.767002446	0.010344976
0.711282369	0.005372949	5.979215736	0.004609765
0.701267616	0.011893966	5.403117276	0.010237383
0.720291542	0.004484237	5.881187637	0.004002393
0.731631165	0.008262282	5.818398678	0.007373314
0.709137064	0.003671471	6.043963285	0.002979004
0.686733074	0.006605894	6.029686181	0.0057864
0.718487262	0.006036101	6.219331138	0.005399592
0.685569149	0.005481582	5.991696756	0.004273337
0.689983054	0.011881013	5.929231107	0.009749192
0.7160518	0.004958272	5.82461093	0.004094773
0.707692428	0.004364802	6.162568534	0.003565156
0.652816059	0.009861574	5.998354421	0.008073349
0.717440696	0.00972799	5.845720133	0.00859445
0.688959751	0.018930041	5.95979289	0.016819822
0.683576166	0.006412608	6.080138307	0.005466064
0.703136309	0.00418097	6.27252763	0.003402771
0.69297553	0.005816714	6.036756792	0.004902653
0.680721876	0.00526779	6.200159514	0.004278048
0.725437036	0.006600559	5.863001513	0.005847445
0.698043639	0.006847236	6.065714269	0.005573576
0.717377642	0.01194317	6.187062197	0.010774546
0.741552061	0.004046058	5.830600142	0.003372865
0.715598037	0.006421021	6.177886415	0.005467988
0.725651127	0.004305573	5.915203911	0.003681721
0.725098661	0.00327708	6.060916041	0.00273128
0.714474775	0.004522754	6.114216329	0.003668381
0.683421008	0.003666604	6.043941548	0.002849253
0.696267986	0.004838185	6.232258585	0.004250274
0.772867366	0.009050649	5.509211518	0.008498712
0.735160513	0.006311838	5.953648119	0.00577942
0.723755262	0.006035242	6.086137679	0.005474431
0.725797588	0.002573546	6.301448614	0.002161209
0.710709707	0.006551397	6.180852085	0.00562563
0.663271928	0.016513655	5.690219349	0.01374497
0.774790473	0.00450559	5.932269226	0.004133929
0.729512625	0.004484187	6.000318327	0.003995614
0.711216129	0.005317275	5.938572723	0.004693449
0.720476827	0.003815002	6.058119436	0.003347702
0.731221474	0.004171749	5.900053015	0.003726444
0.716375433	0.002152683	6.230778907	0.001837664
0.713978681	0.005026415	6.207568851	0.004255726
0.720495217	0.003649966	6.235707531	0.003085958
0.702903657	0.00732656	6.033590108	0.006469292
0.751515263	0.015713206	5.527593763	0.010149967
0.707961898	0.009867863	5.805984174	0.009210527
0.711803995	0.004629613	5.986232641	0.003997205
0.752146961	0.005044383	5.701474462	0.004672316
0.722556024	0.006856217	6.003473491	0.006131256
0.679656606	0.005727159	6.016638721	0.004728085
0.723136074	0.003012424	6.109740942	0.002546366
0.704099358	0.00507993	6.305703993	0.004548693
0.701017761	0.00310615	6.287416396	0.002648183
0.687170641	0.003708908	5.936994584	0.003159368
0.701212948	0.004625204	6.18525408	0.004205462
0.67221235	0.01134594	5.864034446	0.009136091
0.702498022	0.004763769	6.251433128	0.004219004
0.744153975	0.002529533	6.198556348	0.002142708
0.717501971	0.005490672	6.192056611	0.00469358
0.675370064	0.00847554	5.978378851	0.006813238
0.726983265	0.016050506	5.485389985	0.014378655
0.701877024	0.004417827	6.140509078	0.003747783
0.720387362	0.004582533	6.038770068	0.004166394
0.721622887	0.003447287	6.059768151	0.002997384
0.719300978	0.004995965	5.972970965	0.004481833
0.729800726	0.00641705	6.225572477	0.005671591
0.715056176	0.002804215	6.210382021	0.002306169
0.717062449	0.006834155	5.936732862	0.005903541
0.697275535	0.007214396	6.181307763	0.006096215
0.704372546	0.003536722	6.172933628	0.002937096
0.710608593	0.003836508	6.345745532	0.003303869
0.744324602	0.003899143	6.059834251	0.00339829
0.74727522	0.013028369	5.896166115	0.011493095
0.693072343	0.005291206	6.099436734	0.004477259
0.708202772	0.006655245	6.115119615	0.005923365
0.717544805	0.006413538	6.209532366	0.00542136
0.702821388	0.003520953	6.160154018	0.003107941
0.739026811	0.003595637	6.217639995	0.003083708
0.737449116	0.003162575	6.216132916	0.002773997
0.678032042	0.019052925	5.309445375	0.016437582
0.726297229	0.003931451	6.125341608	0.003278142
0.694957957	0.008488606	5.97325144	0.006945999
0.721828123	0.00458415	6.037510361	0.003897483
0.712566126	0.005935596	6.019494058	0.005262087
0.735489878	0.027628513	4.985308173	0.025365421
0.707925471	0.009277879	5.70751212	0.00866049
0.711893883	0.006141983	6.059444782	0.005353555
0.745763713	0.009786634	6.149902927	0.009482573
0.749757438	0.008163086	5.960827628	0.007509451
0.733600208	0.002876098	6.221457987	0.002436381
0.704458195	0.004813907	6.065102878	0.003964165
0.760046646	0.00536447	6.1442933	0.003225358
0.69322304	0.006183894	5.701579289	0.00518394
0.671433696	0.014678137	5.314002986	0.013535421
0.713965856	0.00790458	5.51816075	0.007201491
0.752299829	0.004997262	6.12358122	0.004518217

log.sigma.1.5.mm.3D_ngtdm_Coarseness	log.sigma.1.5.mm.3D_ngtdm_Complexity	log.sigma.1.5.mm.3D_ngtdm_Strength	log.sigma.1.5.mm.3D_ngtdm_Busyness	log.sigma.1.5.mm.3D_ngtdm_Contrast
0.005483371	1.466463334	2.895326493	0.168361549	8.16E-05
0.00345436	0.891328535	2.231212141	0.226627335	3.21E-05
0.005994105	1.35902088	1.915999441	0.232201827	0.000145737
0.002570793	0.968960701	1.873113993	0.245328699	3.77E-05
0.006467387	0.769135724	2.174026559	0.312549223	7.92E-05
0.003906261	0.994062929	2.204316181	0.214424949	4.18E-05
0.006336435	0.370978585	1.318505905	0.355650828	5.37E-05
0.003779236	0.87391857	2.477431261	0.197968042	3.43E-05
0.003457013	0.616948707	1.620788865	0.306368088	3.12E-05
0.002227237	0.516383771	1.016917377	0.426847561	2.91E-05
0.00347743	0.762532705	2.137713433	0.248692822	2.82E-05
0.003647342	1.791713099	2.418315089	0.194495723	8.13E-05
0.002313561	0.389081232	1.162239763	0.479871732	1.71E-05
0.00345322	0.430298539	0.795385832	0.611290924	5.78E-05
0.004013143	0.760201177	1.218518328	0.35207687	7.23E-05
0.001879394	0.348336373	0.962531323	0.474816626	1.37E-05
0.004039599	0.574469862	1.357047007	0.376973703	4.07E-05
0.002752061	0.309644127	0.985881409	0.518067938	2.27E-05
0.00632471	0.695867439	1.845942821	0.313078227	8.01E-05
0.002353207	0.385878747	1.170574636	0.43672447	1.84E-05
0.000943426	0.319493794	0.48998814	0.983377909	1.67E-05
0.003192768	0.603235892	1.317964499	0.363484966	3.83E-05
0.001563459	0.336861663	0.630492019	0.717415514	2.40E-05
0.006597474	1.152727855	2.845831172	0.166154771	8.56E-05
0.002734646	0.501150253	1.018178774	0.511861275	3.89E-05
0.004915204	1.163727095	1.764374965	0.255953343	0.000119639
0.002916575	0.727242356	1.253552056	0.341409033	4.37E-05
0.002014862	0.596130513	0.858896674	0.538023286	3.86E-05
0.002054526	0.385948882	1.241864455	0.404799029	1.33E-05
0.00229931	0.672484116	1.578549589	0.286956366	1.82E-05
0.001581294	0.357091973	0.630563284	0.692074389	2.43E-05
0.001363331	0.224686714	0.702078444	0.723483011	9.17E-06
0.002586668	0.908409143	2.138259244	0.247173026	2.57E-05
0.011804434	2.067376068	5.590804916	0.09642803	0.000184068
0.006830643	2.061753961	3.052564592	0.138668628	0.000191174
0.002086426	0.445330832	1.395899256	0.355552296	1.73E-05
0.001936678	0.695143403	1.168343395	0.345756075	3.33E-05
0.001972985	0.521766175	0.954793884	0.490150687	2.89E-05
0.00437903	0.264162809	0.747612892	0.677017438	4.54E-05
0.003996083	1.108085331	3.013290145	0.163384167	3.80E-05
0.004437206	1.47520987	2.311515635	0.192664702	9.62E-05
0.003542288	0.596954933	1.846311662	0.266766593	3.15E-05
0.002170728	0.548188122	1.801423338	0.295320462	1.43E-05
0.003467569	0.720358014	2.412299063	0.21229946	2.57E-05
0.001991506	0.879929853	2.012496079	0.230580775	1.73E-05
0.001797679	0.634925816	0.930332183	0.504387672	3.34E-05
0.001795676	0.702421857	1.190279245	0.392665386	2.75E-05
0.004308227	0.911645543	1.605354013	0.257052151	7.57E-05
0.00990412	1.200708244	3.403312348	0.212291789	0.00018296
0.005711338	0.999591162	2.741663752	0.179201781	6.16E-05
0.001698593	0.389042181	1.268664923	0.442143216	9.34E-06
0.00336213	0.594500427	2.22836034	0.219063538	2.77E-05
0.001766111	0.401252627	1.113612732	0.462454173	1.31E-05
0.005632548	1.161929292	2.128558663	0.203188082	8.90E-05
0.001742817	0.554771775	1.215013972	0.381286962	1.74E-05
0.00397442	1.162639707	2.07103117	0.184839636	5.73E-05
0.002079772	0.779027312	1.759891046	0.284950801	2.06E-05
0.002704885	0.466098707	1.862856774	0.286823941	1.62E-05
0.004584627	2.004631415	3.873859094	0.127989299	7.23E-05
0.004618723	0.541341144	1.106436699	0.425897762	5.77E-05
0.002376803	0.798857014	2.040922316	0.259253466	1.97E-05
0.001286277	0.483348765	1.204754999	0.408456532	1.01E-05
0.00287289	0.844920038	1.409108314	0.330590352	5.34E-05
0.003642942	0.528912656	1.136635319	0.388191624	4.82E-05
0.011091459	1.050924886	2.174338708	0.215301399	0.000192801
0.004257336	1.174336539	2.205270395	0.227428777	6.79E-05
0.006189369	2.110046435	4.296822435	0.105141016	7.88E-05
0.001869073	0.554500438	1.63220518	0.322847157	1.54E-05
0.004849365	1.082098752	2.286456025	0.186304877	7.31E-05
0.002664948	0.919102877	1.647049501	0.282029308	4.07E-05
0.001767282	0.590553881	0.991374434	0.439469348	2.75E-05
0.003206681	0.559152779	1.736640021	0.323802467	2.24E-05
0.001481153	0.347594025	0.869426105	0.687986241	1.39E-05
0.001063762	0.251235307	0.616137068	0.758633361	9.99E-06
0.001594947	0.642847828	1.182186087	0.417528162	2.08E-05
0.002878956	0.722175719	1.364945664	0.291610022	4.65E-05
0.002923002	0.502610846	1.280666375	0.424246807	2.99E-05
0.004595083	1.223569866	2.229506414	0.21890901	7.83E-05
0.002023258	0.492397556	1.127164836	0.414980417	2.14E-05
0.001610546	0.444276843	0.934981009	0.534754971	1.64E-05
0.005272362	1.404428525	2.724673745	0.130536194	8.34E-05
0.002376368	1.111744384	1.469716041	0.300021153	5.58E-05
0.002679818	1.441288309	2.541841461	0.189359617	3.73E-05
0.012351331	0.614518414	2.573539909	0.245658214	0.000128973
0.001628133	0.534689172	0.879050926	0.532399469	2.77E-05
0.001834863	0.281020687	0.589466549	0.826589342	2.14E-05
0.004366915	1.506144121	2.546121127	0.191738749	7.52E-05
0.002126063	0.429451425	1.188214126	0.430910769	2.00E-05
0.015692098	0.677925126	1.935081707	0.251744773	0.000255049
0.010450244	2.272955773	6.658936926	0.082041685	0.00012648
0.001940959	0.414984159	0.947956463	0.522601281	2.35E-05
0.005725618	3.289402489	4.560964391	0.094954273	0.000152031
0.004169387	1.176938679	1.720239833	0.251606066	0.000102092
0.00152034	0.548634329	1.028712586	0.435458948	2.22E-05
0.001445564	0.282259301	0.722985111	0.703705011	1.16E-05
0.00802268	4.136889368	4.590156616	0.096072792	0.000266355
0.003631378	0.432229487	1.587797315	0.319233282	2.31E-05
0.015184036	1.149261005	4.140199554	0.121847629	0.000161494
0.007103201	0.961828672	3.436996503	0.148547913	5.63E-05
0.005489545	3.076430813	3.65644267	0.130285217	0.000161137

log.sigma.1.0.mm.3D_gldm_GrayLevelVariance	log.sigma.1.0.mm.3D_gldm_HighGrayLevelEmphasis	log.sigma.1.0.mm.3D_gldm_GrayLevelNonUniformityNormalized	log.sigma.1.0.mm.3D_gldm_DependenceEntropy
8.911533856	209.9476309	0.117763917	5.858747796
7.10549775	203.0635	0.1446385	5.931537198
9.49423565	145.59839	0.108987426	6.084857723
10.07905924	369.1916021	0.114673597	5.956223754
4.719384056	86.04214963	0.174973157	5.667177362
6.790181972	221.9908425	0.148026644	5.919494199
3.484734379	83.29706717	0.206250352	5.615440985
6.461087315	261.4692521	0.157054964	5.872006635
5.716954318	204.4893932	0.157191433	5.875280731
7.120145478	261.7898768	0.12450165	6.004135476
5.413399101	226.0846707	0.16901345	5.726379615
12.81315993	267.504451	0.09561729	6.043700995
4.970029168	124.8385114	0.163703262	5.848634678
4.967854656	101.6905274	0.139577957	5.92404223
5.8410064	173.0820413	0.125322997	5.714525122
5.566011393	166.16257	0.163069083	6.116214809
4.227232267	119.1109034	0.157125416	5.724828348
4.462833491	128.8577013	0.186035195	5.964408252
5.326569009	91.44366197	0.152298031	5.920395019
4.365340863	144.5032747	0.177523328	5.963564309
9.332446478	225.5789928	0.100855277	6.145486771
5.721006855	172.3512494	0.140020731	5.96898523
6.236684977	170.4011162	0.133093846	6.049526016
6.924555566	203.6592233	0.155664059	5.914352285
6.31482037	164.8291091	0.124941895	6.04724174
10.02661792	159.4193787	0.110668832	6.067329373
7.639892311	201.1781999	0.120738563	5.777636218
9.079193943	176.0625931	0.106554756	6.053818545
4.320008455	194.6528391	0.197172628	5.898494397
6.990042469	257.4289332	0.154745918	6.016471853
7.269039454	198.5589087	0.121936232	5.93133284
4.22311744	175.7912	0.17211536	5.90812559
8.748332998	293.5989727	0.13690764	5.901690884
7.356339227	185.3929314	0.173222799	5.722031091
10.92488308	230.1182432	0.11379961	5.858989841
6.296755782	256.1961455	0.175410512	5.900396979
10.99568912	368.7979826	0.110351183	6.208277012
8.586686305	201.3550575	0.122145364	5.966959992
3.169642403	79.74393531	0.177101118	5.690021267
8.111400503	297.8392421	0.158558802	5.911218563
9.524521092	266.2788754	0.108033231	5.921344297
6.296956511	205.51294	0.171109118	5.852732235
5.700887148	290.8833829	0.173278834	5.838372897
6.130461682	266.9426752	0.173962433	5.883335616
8.295022393	397.8546079	0.132853966	6.081094522
10.66712903	235.6083916	0.098245195	6.044279704
10.6988108	334.4449267	0.106455804	6.066919664
7.958247906	191.2605541	0.125198411	5.925369661
5.641906846	90.84397163	0.189735174	5.618730981
5.527168589	200.2192661	0.165115731	5.766204531
5.078970248	291.3714859	0.165138213	5.875592494
5.320017899	281.4642127	0.199780655	5.771228419
5.869059539	230.1506452	0.160773351	5.813085911
7.40652883	173.2023911	0.121674454	5.839261645
7.611727094	261.6647193	0.13437143	6.063375757
10.11953254	289.3882415	0.120890638	6.369300816
8.277522147	369.231168	0.124794721	5.965691074
4.383146418	257.4050164	0.199860549	5.787221824
10.96742216	368.0451895	0.119537778	5.933286981
4.934496777	101.7572954	0.147015869	5.796013275
8.027245797	303.5141108	0.133725827	5.961662596
7.681230607	327.8022599	0.14964964	6.082878103
9.386714846	203.8608542	0.112321013	6.060476187
5.902469577	148.2243041	0.149334675	5.946099715
5.420166619	88.26101695	0.144900891	5.668321245
9.33050185	241.1276734	0.108416367	5.914837542
8.88264854	331.6928166	0.126164501	6.008071114
7.11039326	297.9439148	0.159792161	5.921208918
7.291603488	258.9543697	0.135677923	5.77793304
9.815565479	292.3960909	0.121787276	6.113049518
8.888298449	329.0144433	0.108128454	6.148122062
4.829190053	148.9340126	0.179104277	5.881918839
6.385384653	177.7608463	0.143065598	5.965951553
6.343767094	257.3406543	0.142403269	6.037735955
10.01471193	302.7200845	0.106233956	6.082735005
8.789821629	258.8258258	0.128398117	6.001583824
6.141854031	168.2904459	0.159401914	5.934351987
7.942060154	210.004415	0.12391107	5.80440124
6.755722605	254.7893773	0.152208462	5.958690684
7.582913368	223.3752526	0.138010296	6.075113666
9.915682863	444.0352423	0.123019184	6.025089912
12.44744491	295.4644336	0.097903593	6.06236356
11.91998743	370.1758286	0.109729056	6.154101274
3.676158652	54.56787763	0.220696891	5.457986787
8.8219784	263.6136717	0.112150926	5.99835459
5.182436665	123.6312608	0.140908414	5.855752563
9.701772866	207.7857143	0.10994692	5.967500118
6.098899453	230.0059152	0.157055369	5.876521954
3.845892459	86.20924574	0.188685835	5.361178668
6.897103722	228.0726644	0.16107925	5.604195629
6.378013875	203.1111803	0.144865133	5.912444388
15.68793923	461.5665158	0.099658074	5.987179746
11.0262693	260.4923175	0.10457226	6.033312078
9.979186652	327.2085918	0.121964573	6.133616689
5.306574521	201.098385	0.148792067	5.859271749
17.49702095	259.081448	0.084447493	6.294615366
3.720115605	168.8054453	0.197429097	5.516956531
3.953642239	127.5373832	0.188324308	5.394696831
4.563061795	195.893018	0.200927279	5.720462495
16.08291332	306.2331023	0.079787282	6.034524329

log.sigma.1.0.mm.3D_gldm_DependenceNonUniformity	log.sigma.1.0.mm.3D_gldm_GrayLevelNonUniformity	log.sigma.1.0.mm.3D_gldm_SmallDependenceEmphasis
187.2011638	141.6699917	0.287017674
249.408	289.277	0.242552786
176.5277281	121.8479428	0.29979419
394.2221769	281.2943335	0.289606111
107.345627	166.0495258	0.211152449
204.002442	242.4676435	0.245439497
100.6423841	218.0066225	0.176713861
205.0554017	283.4842105	0.234620633
230.3369512	318.627035	0.22488859
430.8235098	373.8784549	0.265396228
213.71057	305.4073049	0.207638477
308.3091988	161.1151335	0.34383822
334.080915	479.4868556	0.205632054
250.3261572	259.335845	0.219910231
251.7067183	194	0.274605603
431.3339012	670.050864	0.211749428
198.8903427	252.1862928	0.192979405
238.0625489	475.8780297	0.18503593
131.096831	173.0105634	0.213354215
284.1003102	514.9951741	0.178979835
1161.333525	700.9441727	0.296088472
273.038661	296.9839698	0.241855808
545.9895656	548.4797379	0.229574616
117.7902913	160.3339806	0.23707377
363.922427	325.3486943	0.237472568
208.7633136	149.6242604	0.297037852
399.3202171	266.9529625	0.332316521
547.466766	357.4912072	0.307092515
296.4939688	670.1897617	0.179305146
375.5805635	488.8423552	0.241393691
604.6310961	505.0598745	0.259838045
544.7364	860.5768	0.200442524
332.7992888	346.5132359	0.254844973
53.52806653	83.32016632	0.233743806
146.8220721	101.0540541	0.326479484
331.548337	564.295617	0.210543145
527.430933	393.8433735	0.290274226
502.6189914	414.1949277	0.272806855
165.245283	262.8180593	0.185815743
180.5195599	259.4022005	0.235615539
231.6641337	142.1717325	0.312084397
205	330.5828157	0.218689231
323.7472745	524.5150314	0.205456447
197.0902335	327.7452229	0.214707077
445.0246136	464.1917573	0.24923647
631.0716084	351.2265734	0.315422372
601.6093326	392.3960933	0.311805852
222.1451187	189.8007916	0.261417392
59.63475177	107.0106383	0.230596411
122.9357798	179.9761468	0.216311136
441.6817269	657.9106426	0.209754504
184.0961145	390.7709611	0.189884304
436.3436924	610.456413	0.22732174
185.6592656	142.4807857	0.275721193
504.2943866	529.02032	0.251735907
241.4385593	228.2415254	0.282392975
476.4016468	409.2018908	0.271740974
230.7697368	486.0608553	0.175142928
214.5218659	164.0058309	0.299946032
188.3537367	206.5572954	0.232613528
387.4848691	393.2876573	0.25274031
623.7060324	821.1275743	0.239012436
345.6930867	255.0810216	0.293012251
224.2997859	278.9571734	0.231547301
77.90169492	85.49152542	0.242900426
257.4147764	167.286455	0.287864631
146.5274102	133.4820416	0.29463716
397.4116657	569.8188446	0.230865748
182.232792	175.4315545	0.270560141
344.1719186	305.320702	0.270991276
583.5120798	411.7531513	0.288679678
209.0329937	369.1339156	0.187924634
586.5516136	669.4039325	0.238171049
796.7920442	927.1876824	0.229226951
693.8282496	452.7691225	0.295307087
323.00858	299.2960103	0.261683619
264.9575372	375.3915074	0.239726599
216.32156	168.3951435	0.26707741
387.1794872	498.6349206	0.233946822
541.0076353	614.5598473	0.242268638
194.9060206	167.5521292	0.278056987
483.1761385	260.1298457	0.334596295
380.279709	271.4696847	0.300004838
50.96175908	115.4244742	0.17376723
612.0823408	433.1268773	0.291019164
458.388601	489.5158319	0.240305529
234.9131653	157.0042017	0.295573506
360.2014459	477.9194873	0.22246019
45.76885645	77.54987835	0.21953858
71.83391003	93.10380623	0.217418734
405.0267829	465.1619433	0.237107481
191.7493213	110.1221719	0.33129911
265.1370038	163.3418694	0.318149089
605.2880772	530.911785	0.281810555
581.1283468	700.2154696	0.225637024
154.9932127	74.65158371	0.342440206
180.8502552	348.0674986	0.179759649
47.92990654	80.60280374	0.181726588
84.08333333	178.4234234	0.197776233
246.2183709	92.0745234	0.372751794

log.sigma.1.0.mm.3D_gldm_DependenceNonUniformityNormalized	log.sigma.1.0.mm.3D_gldm_DependenceVariance	log.sigma.1.0.mm.3D_gldm_LargeDependenceEmphasis
0.15561194	4.153682018	15.71820449
0.124704	8.718476	26.51
0.157896	5.399582695	17.19677996
0.160710223	4.138445249	15.3212393
0.113114465	7.489827349	28.08746048
0.124543615	7.539212924	24.79365079
0.095215122	10.08119945	37.80037843
0.113604101	9.383424007	29.54626039
0.113634411	9.536034136	30.00641342
0.143464372	6.083284237	19.90642691
0.118268163	7.349013199	26.6613171
0.182972818	3.400084882	12.2652819
0.114059718	7.828641919	28.0126323
0.134728825	6.96213969	23.24757804
0.162601239	3.614018922	14.65633075
0.104972962	11.36589176	34.8797761
0.123919217	7.441656816	26.41433022
0.093065891	11.57009391	39.77873339
0.11540214	8.96830676	29.20246479
0.097931855	10.97671273	37.46673561
0.167098349	4.524591895	15.31510791
0.128731099	6.848073896	23.28477133
0.132489582	6.187812367	22.06187818
0.114359506	8.618833066	28.30485437
0.139755156	5.825681157	20.59677419
0.154410735	5.38701376	17.47189349
0.180606159	3.231096685	12.25825418
0.163179364	4.901511502	15.96035768
0.087229764	14.34474878	46.36922624
0.118892233	9.846285719	29.18233618
0.145975639	4.797050669	17.90487687
0.10894728	9.05660016	30.9328
0.131489249	5.964686641	21.41011458
0.111284962	10.60626467	31.92723493
0.165340171	4.082359386	14.51351351
0.103061342	10.77145341	34.53745726
0.147781152	6.131866503	19.18604651
0.148221466	5.439325792	18.3895606
0.111351269	7.955650932	29.68733154
0.110342029	9.901314256	31.26650367
0.176036576	4.52165538	14.59118541
0.10610766	10.01952621	32.54761905
0.10695318	9.068974756	31.36471754
0.104612651	10.90664485	34.54564756
0.127368235	7.468678172	24.1161992
0.176523527	3.368498489	12.84111888
0.163214686	4.42245519	15.20564297
0.146533719	5.251919717	18.49076517
0.105735375	12.81604296	37.0070922
0.112785119	8.029795472	28.23853211
0.110863887	8.160291517	29.04317269
0.094118668	12.19612142	40.32515337
0.114918012	8.375886345	28.010798
0.158547622	4.306261263	15.98035867
0.12809103	7.681710847	24.28676657
0.127880593	12.65593131	31.39088983
0.1452887	5.43183107	18.74687405
0.094888872	11.26901118	39.18667763
0.156357045	4.755038292	16.33090379
0.134059599	5.529481136	20.85907473
0.131752761	6.322272747	21.79088745
0.113669771	10.29358111	30.74995444
0.152220646	4.682685464	16.66446499
0.120074832	9.883318278	29.28479657
0.132036771	5.940982476	21.6440678
0.166827464	3.595203224	14.12508101
0.138494717	7.532441637	22.28733459
0.111444662	10.29715431	31.40661806
0.140937967	5.820906075	19.68832173
0.137284371	6.57034973	21.16354208
0.153233214	5.416469253	17.69957983
0.101423093	11.05053086	36.25473071
0.125358327	7.169128309	24.19555461
0.122376293	7.961197667	25.99401014
0.162794052	4.480190859	15.53402159
0.138570819	6.078973991	20.45774346
0.112508508	9.675263995	30.15329087
0.159177012	3.487346505	14.97939662
0.118186657	8.038377678	26.73687424
0.121492844	8.100156592	26.04783292
0.143102805	6.259689797	20.00734214
0.181850259	3.491797113	12.55513737
0.153710473	5.586160091	17.75424414
0.097441222	10.25259479	37.24091778
0.158488436	4.497543286	15.91921284
0.13194836	5.94746234	21.55382844
0.164505018	4.319131574	15.20728291
0.118370505	7.680724383	26.46040092
0.111359748	8.396481195	29.52068127
0.124280121	5.681062248	23.5017301
0.126137273	6.614334426	23.34755528
0.173528798	3.571990745	13.17918552
0.169742	4.779321957	15.24839949
0.139050787	7.309570006	21.93452791
0.123486687	6.513389694	23.84912877
0.175331689	5.351277615	15.62895928
0.102580973	8.62857972	33.16562677
0.111985763	8.339876845	30.6588785
0.094688438	13.3368436	41.44594595
0.213360807	2.337987979	9.305025997

log.sigma.1.0.mm.3D_gldm_LargeDependenceLowGrayLevelEmphasis	log.sigma.1.0.mm.3D_gldm_SmallDependenceHighGrayLevelEmphasis	log.sigma.1.0.mm.3D_gldm_LargeDependenceHighGrayLevelEmphasis
0.083074249	62.04774494	3206.64921
0.140212787	48.09136508	5244.1545
0.140661256	44.87019093	2504.829159
0.043336365	102.2278097	5678.959234
0.374385443	20.43216527	2223.43098
0.114374284	51.48542607	5588.923687
0.493971441	14.50382143	3008.56859
0.116713678	56.6006173	7669.166759
0.156370873	45.41073058	5916.013814
0.078731157	68.65157043	5220.539794
0.120210844	44.41042094	6095.57554
0.052489807	90.88372353	3300.681899
0.243423684	26.72461886	3398.288836
0.246816171	22.18730776	2366.306243
0.091145093	47.95347743	2512.48385
0.225094211	35.36778753	5597.095644
0.229833942	22.834901	3180.253583
0.334564772	24.13406077	4901.856919
0.374041069	21.18533465	2443.183099
0.271070381	24.12584223	5364.217166
0.074209237	66.53234603	3423.515971
0.141487198	40.52275823	4007.88166
0.134846938	36.06624908	3867.909003
0.151702607	51.10616808	5461.486408
0.135552868	39.40956662	3335.003456
0.130477983	47.70784134	2673.823225
0.065299104	65.78116452	2491.823157
0.099941591	53.47754973	2789.396721
0.241592512	33.0385663	9048.46749
0.118126906	62.1636646	7393.938588
0.094618618	50.64712039	3604.047562
0.186123244	36.16449393	5270.4656
0.076657894	73.49279756	6281.140656
0.193720239	50.13674434	5431.983368
0.066955095	68.97769842	3465.503378
0.139475108	52.13906284	8780.988809
0.054122875	105.717475	7080.185486
0.097595381	53.6022454	3707.89826
0.391609804	14.59726268	2374.478437
0.110195125	68.65447188	9121.830685
0.058437534	79.90170907	3893.739362
0.168069414	44.07247205	6460.557453
0.111042281	57.72345219	9026.144037
0.136580174	57.32806153	8906.901274
0.06253206	95.97649263	9575.080137
0.059895247	74.31190448	3015.561958
0.04774824	101.9061815	5086.094411
0.101463596	47.78347838	3633.478232
0.485123986	23.45782137	3027.946809
0.147014111	41.161766	5633.250459
0.102411633	60.2982732	8393.115964
0.146927906	48.87900526	11517.47955
0.126270966	50.59642232	6359.738214
0.101021558	47.81332181	2753.22801
0.09774691	64.78602399	6252.747269
0.113526346	77.62401874	8987.757945
0.052962378	99.63289285	6853.361086
0.15741878	45.23784591	9905.682155
0.046917733	108.011741	6015.285714
0.227132413	23.97149178	2095.589324
0.076767874	77.86253183	6412.466848
0.096910563	77.15300553	9947.272644
0.090372138	60.48229741	3377.437252
0.210369328	32.5021243	4277.89561
0.266882769	19.19869402	1906.79322
0.065565213	71.82140934	3254.530136
0.071046967	97.72176064	7253.488658
0.11025571	67.66430598	9177.450365
0.077668195	63.83219622	5201.353442
0.076142148	76.41027853	6183.685281
0.056750407	93.42131288	5763.324842
0.259267674	28.66451414	5223.422125
0.145869188	41.95343368	4181.389827
0.103941252	56.93144846	6697.09338
0.05545854	89.4594782	4622.254106
0.082632524	63.96507564	5310.323895
0.186450154	39.37148845	5077.130361
0.076758353	54.10325906	3153.649742
0.107433618	57.15118326	6876.266178
0.120130406	52.44634136	5861.358635
0.04711483	121.5308855	8835.011747
0.045719305	95.00449688	3750.652992
0.051015188	108.3020791	6541.910671
0.820906809	12.01918156	1781.565966
0.062757664	73.72792214	4270.120663
0.189054035	27.8704758	2651.989925
0.079511915	59.39722026	3184.044818
0.120608151	49.15680836	5985.82583
0.387069267	20.69651885	2349.639903
0.110719104	45.68193243	5311.619377
0.121233436	45.74256085	4723.910308
0.030867055	147.0637818	6111.470588
0.06225141	78.46812435	4049.229834
0.068761014	88.21347722	7231.254997
0.124258251	45.10348433	4722.616447
0.080321956	95.88179015	3913.669683
0.202244138	30.26131681	5550.898469
0.25602099	22.34887074	3803.219626
0.215556721	37.24416382	8169.974099
0.036955611	115.0480087	2794.588388

log.sigma.1.0.mm.3D_gldm_SmallDependenceLowGrayLevelEmphasis	log.sigma.1.0.mm.3D_gldm_LowGrayLevelEmphasis	log.sigma.1.0.mm.3D_gldzm_DistanceZoneVariabilityNormalized
0.003459761	0.007342493	1
0.004234007	0.008282315	1
0.007082531	0.014496723	1
0.001727165	0.003734217	1
0.005335602	0.016065137	1
0.002888419	0.00648617	1
0.005544623	0.016556625	1
0.002215578	0.005226414	1
0.002121903	0.006186198	1
0.001835174	0.004814026	0.995327128
0.002422482	0.006100685	1
0.003742965	0.006858945	1
0.003387984	0.011025559	1
0.004475559	0.013582099	1
0.003033959	0.007636356	1
0.002528861	0.007873091	0.997804613
0.003201846	0.010779834	1
0.004037624	0.011008045	1
0.005905388	0.016584271	1
0.003213375	0.009437703	0.996212135
0.002794422	0.006328646	0.99382722
0.002901578	0.007631842	0.996289438
0.002998765	0.008161781	0.998052582
0.003015009	0.007094499	1
0.002793537	0.008019459	1
0.006660289	0.012226604	1
0.003197529	0.006908579	1
0.003890975	0.008486538	1
0.001794465	0.006202744	1
0.001722027	0.00490898	1
0.002389276	0.00658627	1
0.001782488	0.006649099	0.998082456
0.002381632	0.005199911	1
0.003917772	0.008620396	1
0.004493796	0.007710635	1
0.002348692	0.005621817	1
0.001442219	0.003524275	0.998207887
0.003224681	0.007349133	1
0.005071983	0.016523399	1
0.001994023	0.004768269	1
0.002978388	0.005740061	1
0.002626962	0.006637955	1
0.001482066	0.004301076	1
0.001775069	0.004845207	1
0.001287081	0.003280822	1
0.002675092	0.006018229	1
0.00173655	0.003945314	0.985623757
0.003971278	0.008285499	1
0.005668714	0.019063928	1
0.003996477	0.00809252	1
0.001256377	0.004072122	1
0.001444802	0.0051408	1
0.00200756	0.005507026	0.995444215
0.003869859	0.008814356	1
0.001899681	0.004979235	1
0.002744439	0.005355056	1
0.001434661	0.003497039	1
0.001463185	0.004780108	1
0.002201946	0.004235951	1
0.00575951	0.014806718	1
0.002076499	0.004685674	1
0.001278756	0.003720285	0.99853908
0.003948727	0.008027305	1
0.003766594	0.009708988	1
0.009767552	0.019173339	1
0.002603932	0.005884887	1
0.002260655	0.004488179	1
0.002017495	0.004770086	1
0.002736662	0.005614493	1
0.002448521	0.005142604	1
0.001537663	0.003851708	1
0.003197049	0.009149381	1
0.002636732	0.007343725	1
0.001863486	0.005017677	0.997392443
0.001813468	0.004432169	1
0.002420664	0.005495531	1
0.003135033	0.008282805	1
0.002793543	0.006736463	1
0.002317569	0.005496146	1
0.002259716	0.005950976	0.996469561
0.001565872	0.003277428	1
0.002420359	0.004925332	1
0.00224693	0.004280965	1
0.010308931	0.028754272	1
0.001972764	0.004929785	0.996737366
0.004460706	0.011760966	0.995321663
0.003409057	0.007415173	1
0.002049115	0.005647155	0.994143535
0.006532928	0.016876579	1
0.003866835	0.007552502	1
0.002595647	0.00679855	1
0.002501611	0.003997433	1
0.00289813	0.005898002	1
0.001599327	0.003927701	1
0.001939777	0.006035563	0.998154983
0.005499249	0.010470302	1
0.002305873	0.007380591	1
0.005400822	0.012121855	1
0.003979251	0.008225183	1
0.004741749	0.00710355	1

log.sigma.1.0.mm.3D_gldzm_LowIntensityEmphasis	log.sigma.1.0.mm.3D_gldzm_LargeDistanceEmphasis	log.sigma.1.0.mm.3D_gldzm_HighIntensitySmallDistanceEmphasis
0.011696375	1	217.2553191
0.017179977	1	198.5570866
0.024094175	1	147.5268817
0.005844458	1	356.8173333
0.025659524	1	99.86
0.011741923	1	207.7708831
0.032415402	1	83.98378378
0.009373099	1	243.8738532
0.009421308	1	204.6807611
0.006821127	1.007025761	258.4077869
0.011842582	1	212.0947368
0.010789134	1	264.2389241
0.017716933	1	130.9356436
0.020546324	1	100.5575621
0.010906711	1	175.5484581
0.01224075	1.003296703	166.7662088
0.016960304	1	118.415625
0.021908683	1	132.088843
0.027931557	1	102.9490196
0.018239442	1.0056926	135.2035104
0.009229917	1.009287926	223.7344096
0.011849203	1.005576208	169.1510223
0.013203011	1.002923977	156.0031676
0.012397307	1	218.6085271
0.011798513	1	165.7322239
0.021347468	1	163.3363229
0.009497821	1	197.1324257
0.012405887	1	173.3412073
0.009963238	1	186.7817896
0.007027215	1	257.8028674
0.009307706	1	193.5553633
0.008897941	1.002879079	182.5203935
0.009501684	1	288.7969697
0.01622069	1	217.375
0.013474816	1	210.6634304
0.011218028	1	248.2471098
0.004931247	1.002690583	361.9560538
0.011872915	1	196.2024415
0.028555649	1	77.65185185
0.008536534	1	293.7525253
0.009141418	1	256.4251101
0.012022267	1	205.2295082
0.007344476	1	281.3631922
0.008352926	1	270.92
0.005157696	1	385.2736264
0.008381829	1	235.1159184
0.005490645	1.021721641	324.6202735
0.014865366	1	180.75
0.028312558	1	104.7676056
0.018305601	1	191.607438
0.006050131	1	287.3890866
0.009110924	1	254.4432432
0.008882123	1.006849315	223.4583333
0.014127007	1	173.2412791
0.007465122	1	259.3051331
0.009320081	1	273.9604811
0.005212249	1	369.5635776
0.008495339	1	259.3914081
0.007080347	1	359.5369128
0.02494682	1	104.5747801
0.008116958	1	310.3810143
0.005347419	1.002192982	323.349598
0.013309349	1	204.743338
0.016395727	1	139.5606061
0.040627343	1	79.375
0.008648975	1	252.885947
0.007492677	1	335.2638037
0.008700285	1	294.6651109
0.010034977	1	236.6211699
0.008849664	1	282.5523416
0.005243822	1	323.2883333
0.017187246	1	153.6954315
0.011135626	1	177.9015544
0.008184346	1.003916449	246.4616514
0.006128601	1	302.9948643
0.009155278	1	245.2569659
0.013514999	1	163.1993127
0.0103083	1	205.2562814
0.01011934	1	242.3359477
0.009385304	1.00530504	215.2318744
0.00550818	1	434.5771144
0.007096974	1	284.201232
0.007302589	1	362.1504425
0.058504656	1	72.94623656
0.006783219	1.004901961	251.6764706
0.019633302	1.007033998	115.0026377
0.011649811	1	199.5809313
0.009390255	1.008810573	221.8061674
0.028776253	1	97.20618557
0.017061813	1	215.1492537
0.011278302	1	193.6740548
0.007118497	1	446.6683292
0.008926715	1	246.5154265
0.005639856	1	313.08659
0.008665942	1.002770083	201.5099261
0.016161	1	275.8803419
0.013116914	1	167.7699681
0.030325859	1	125.0263158
0.020128041	1	186.1741573
0.011734777	1	307.277551

log.sigma.1.0.mm.3D_gldzm_LowIntensityLargeDistanceEmphasis	log.sigma.1.0.mm.3D_gldzm_HighIntensityEmphasis	log.sigma.1.0.mm.3D_gldzm_DistanceZoneVariability	log.sigma.1.0.mm.3D_gldzm_ZonePercentage
0.011696375	217.2553191	376	0.312551953
0.017179977	198.5570866	508	0.254
0.024094175	147.5268817	372	0.33273703
0.005844458	356.8173333	750	0.305748064
0.025659524	99.86	200	0.210748156
0.011741923	207.7708831	419	0.255799756
0.032415402	83.98378378	185	0.175023652
0.009373099	243.8738532	436	0.241551247
0.009421308	204.6807611	473	0.233349778
0.006874554	258.6405152	850.0093677	0.284382284
0.011842582	212.0947368	380	0.210293304
0.010789134	264.2389241	632	0.375074184
0.017716933	130.9356436	606	0.206896552
0.020546324	100.5575621	443	0.238428418
0.010906711	175.5484581	454	0.293281654
0.012255402	166.9516484	908.0021978	0.221465077
0.016960304	118.415625	320	0.199376947
0.021908683	132.088843	484	0.189210321
0.027931557	102.9490196	255	0.224471831
0.018264742	135.5237192	525.0037951	0.181661496
0.009304563	224.1676249	2247.043344	0.325323741
0.011882198	169.3866171	536.0037175	0.253653937
0.013216006	156.1676413	1024.001949	0.248968697
0.012397307	218.6085271	258	0.250485437
0.011798513	165.7322239	661	0.253840246
0.021347468	163.3363229	446	0.329881657
0.009497821	197.1324257	808	0.3654455
0.012405887	173.3412073	1143	0.340685544
0.009963238	186.7817896	637	0.187408061
0.007027215	257.8028674	837	0.264957265
0.009307706	193.5553633	1156	0.279092226
0.008910737	182.6823417	1040.001919	0.2084
0.009501684	288.7969697	660	0.260766495
0.01622069	217.375	120	0.249480249
0.013474816	210.6634304	309	0.347972973
0.011218028	248.2471098	692	0.215107243
0.004936806	362.2816143	1113.001794	0.31241244
0.011872915	196.2024415	983	0.28988499
0.028555649	77.65185185	270	0.181940701
0.008536534	293.7525253	396	0.24205379
0.009141418	256.4251101	454	0.344984802
0.012022267	205.2295082	427	0.221014493
0.007344476	281.3631922	614	0.202841097
0.008352926	270.92	400	0.212314225
0.005157696	385.2736264	910	0.26044648
0.008381829	235.1159184	1225	0.342657343
0.005574964	326.4352373	1225.13033	0.337221921
0.014865366	180.75	428	0.2823219
0.028312558	104.7676056	142	0.25177305
0.018305601	191.607438	242	0.222018349
0.006050131	287.3890866	843	0.211596386
0.009110924	254.4432432	370	0.189161554
0.008914238	223.8504566	872.0091324	0.230708454
0.014127007	173.2412791	344	0.293766012
0.007465122	259.3051331	1052	0.267208534
0.009320081	273.9604811	582	0.308262712
0.005212249	369.5635776	928	0.283013114
0.008495339	259.3914081	419	0.172286184
0.007080347	359.5369128	447	0.325801749
0.02494682	104.5747801	341	0.242704626
0.008116958	310.3810143	769	0.261475689
0.005360395	323.4422515	1366.001462	0.249316566
0.013309349	204.743338	713	0.313958609
0.016395727	139.5606061	462	0.24732334
0.040627343	79.375	144	0.244067797
0.008648975	252.885947	491	0.318211277
0.007492677	335.2638037	326	0.308128544
0.008700285	294.6651109	857	0.240325294
0.010034977	236.6211699	359	0.277648879
0.008849664	282.5523416	726	0.28958915
0.005243822	323.2883333	1200	0.31512605
0.017187246	153.6954315	394	0.191169335
0.011135626	177.9015544	1158	0.24748878
0.008215515	246.593342	1528.005222	0.235294118
0.006128601	302.9948643	1363	0.319802909
0.009155278	245.2569659	646	0.277134277
0.013514999	163.1993127	582	0.247133758
0.0103083	205.2562814	398	0.292862399
0.01011934	242.3359477	765	0.233516484
0.009466332	215.495137	1127.007073	0.253986077
0.00550818	434.5771144	402	0.295154185
0.007096974	284.201232	974	0.366578848
0.007302589	362.1504425	791	0.319725141
0.058504656	72.94623656	93	0.177820268
0.006812745	251.8848039	1220.006536	0.316934231
0.019709066	115.2075029	849.0093787	0.245538284
0.011649811	199.5809313	451	0.315826331
0.009434444	222.3215859	677.0117474	0.22379231
0.028776253	97.20618557	97	0.236009732
0.017061813	215.1492537	134	0.23183391
0.011278302	193.6740548	767	0.238866397
0.007118497	446.6683292	401	0.362895928
0.008926715	246.5154265	551	0.352752881
0.005639856	313.08659	1305	0.299793246
0.008776745	201.5272392	1081.001847	0.230131747
0.016161	275.8803419	351	0.397058824
0.013116914	167.7699681	313	0.177538287
0.030325859	125.0263158	76	0.177570093
0.020128041	186.1741573	178	0.20045045
0.011734777	307.277551	490	0.424610052

log.sigma.1.0.mm.3D_gldzm_IntensityVariabilityNormalized	log.sigma.1.0.mm.3D_gldzm_LowIntensitySmallDistanceEmphasis	log.sigma.1.0.mm.3D_gldzm_IntensityVariability
0.070690923	0.011696375	26.57978723
0.0751519	0.017179977	38.17716535
0.065556712	0.024094175	24.38709677
0.065297778	0.005844458	48.97333333
0.08505	0.025659524	17.01
0.073666703	0.011741923	30.86634845
0.109306063	0.032415402	20.22162162
0.07986491	0.009373099	34.82110092
0.083435764	0.009421308	39.46511628
0.074499942	0.00680777	63.62295082
0.087132964	0.011842582	33.11052632
0.060006409	0.010789134	37.92405063
0.088994543	0.017716933	53.93069307
0.088902364	0.020546324	39.38374718
0.086689049	0.010906711	39.35682819
0.079067745	0.012237087	71.95164835
0.10921875	0.016960304	34.95
0.090456594	0.021908683	43.78099174
0.086720492	0.027931557	22.11372549
0.089594533	0.018233117	47.21631879
0.067561194	0.009211256	152.7558602
0.082399359	0.011840954	44.33085502
0.080769392	0.013199762	82.86939571
0.076197344	0.012397307	19.65891473
0.077920265	0.011798513	51.50529501
0.063795773	0.021347468	28.4529148
0.075544064	0.009497821	61.03960396
0.067831963	0.012405887	77.53193351
0.089546022	0.009963238	57.04081633
0.072491075	0.007027215	60.67502987
0.0758881	0.009307706	87.7266436
0.090524276	0.008894742	94.32629559
0.069292929	0.009501684	45.73333333
0.07125	0.01622069	8.55
0.064358354	0.013474816	19.88673139
0.076029938	0.011218028	52.61271676
0.061606708	0.004929857	68.69147982
0.071019126	0.011872915	69.81180061
0.112455418	0.028555649	30.36296296
0.070260688	0.008536534	27.82323232
0.068456597	0.009141418	31.07929515
0.078127999	0.012022267	33.36065574
0.082886821	0.007344476	50.89250814
0.0815625	0.008352926	32.625
0.071121845	0.005157696	64.72087912
0.064571095	0.008381829	79.09959184
0.062423263	0.005469565	77.59211585
0.072298891	0.014865366	30.94392523
0.085002976	0.028312558	12.07042254
0.086571955	0.018305601	20.95041322
0.084735221	0.006050131	71.43179122
0.086822498	0.009110924	32.12432432
0.082186568	0.008874095	71.99543379
0.073874392	0.014127007	25.4127907
0.074617965	0.007465122	78.49809886
0.061843861	0.009320081	35.99312715
0.072943761	0.005212249	67.69181034
0.098410239	0.008495339	41.23389021
0.068740647	0.007080347	30.72706935
0.092560263	0.02494682	31.56304985
0.072742369	0.008116958	55.93888166
0.071037883	0.005344175	97.17982456
0.068279144	0.013309349	48.68302945
0.0854463	0.016395727	39.47619048
0.093074846	0.040627343	13.40277778
0.068288252	0.008648975	33.52953157
0.065847416	0.007492677	21.46625767
0.076766392	0.008700285	65.78879813
0.077971152	0.010034977	27.99164345
0.064146347	0.008849664	46.57024793
0.067326389	0.005243822	80.79166667
0.088278492	0.017187246	34.78172589
0.077597907	0.011135626	89.85837651
0.07690079	0.008176554	117.8120104
0.064976324	0.006128601	88.56272927
0.068394214	0.009155278	44.18266254
0.081163425	0.013514999	47.2371134
0.077359158	0.0103083	30.78894472
0.078528771	0.01011934	60.0745098
0.070617381	0.009365046	79.86825818
0.066037969	0.00550818	26.54726368
0.062035932	0.007096974	60.42299795
0.060764191	0.007302589	48.06447535
0.09931784	0.058504656	9.23655914
0.070005126	0.006775838	85.68627451
0.094510926	0.019614361	80.61781946
0.068785306	0.011649811	31.02217295
0.08428695	0.009379208	57.39941263
0.110001063	0.028776253	10.67010309
0.083092003	0.017061813	11.13432836
0.079772017	0.011278302	61.1851369
0.055665077	0.007118497	22.32169576
0.066024157	0.008926715	36.37931034
0.064584489	0.005639856	84.28275862
0.087734645	0.008638241	95.0166205
0.052799896	0.016161	18.53276353
0.100409313	0.013116914	31.42811502
0.08933518	0.030325859	6.789473684
0.103459159	0.020128041	18.41573034
0.056251562	0.011734777	27.56326531

log.sigma.1.0.mm.3D_gldzm_HighIntensityLargeDistanceEmphasis	log.sigma.1.0.mm.3D_gldzm_SmallDistanceEmphasis	log.sigma.1.0.mm.3D_glcm_SumVariance	log.sigma.1.0.mm.3D_glcm_Homogeneity1
217.2553191	1	598.6365758	0.396411506
198.5570866	1	587.4532857	0.44720755
147.5268817	1	387.9446315	0.394059453
356.8173333	1	1154.507728	0.38420949
99.86	1	213.4250689	0.485256893
207.7708831	1	667.7432162	0.440725219
83.98378378	1	215.8941816	0.527036949
243.8738532	1	802.735418	0.469032553
204.6807611	1	610.0347319	0.480015466
259.5714286	0.99824356	792.7519571	0.41961127
212.0947368	1	685.3718898	0.467677882
264.2389241	1	785.7289066	0.3578085
130.9356436	1	337.5070176	0.475528497
100.5575621	1	261.1148041	0.441930136
175.5484581	1	503.0516141	0.406959823
167.6934066	0.999175824	481.3276285	0.487359642
118.415625	1	328.0546652	0.466995546
132.088843	1	353.0167663	0.515319215
102.9490196	1	221.7198413	0.479028783
136.8045541	0.99857685	410.7780193	0.503924154
225.9004865	0.997678019	657.0428197	0.373722627
170.3289963	0.998605948	490.426139	0.444238852
156.8255361	0.999269006	484.7122816	0.434559754
218.6085271	1	592.446345	0.46579718
165.7322239	1	466.63159	0.429738127
163.3363229	1	425.8984427	0.397806399
197.1324257	1	585.7579437	0.402131483
173.3412073	1	487.6947283	0.390767321
186.7817896	1	589.756446	0.523926027
257.8028674	1	794.8610072	0.456069287
193.5553633	1	573.2688021	0.411630492
183.3301344	0.99928023	514.3900261	0.495231354
288.7969697	1	898.1073995	0.419729728
217.375	1	533.9788483	0.47872777
210.6634304	1	665.6219778	0.383419432
248.2471098	1	786.5012841	0.482111184
363.5838565	0.999327354	1148.395897	0.380134598
196.2024415	1	573.3319599	0.402709145
77.65185185	1	202.3823732	0.492550455
293.7525253	1	922.5537051	0.459676524
256.4251101	1	795.1432135	0.374339991
205.2295082	1	604.8102324	0.48081283
281.3631922	1	915.9699374	0.480499756
270.92	1	825.4066709	0.490202781
385.2736264	1	1273.673559	0.413695602
235.1159184	1	674.4375978	0.364191957
333.6950925	0.99456959	1028.664736	0.37153637
180.75	1	551.9522345	0.408330531
104.7676056	1	222.9655043	0.496319033
191.607438	1	595.3113587	0.476601078
287.3890866	1	916.7510698	0.476230256
254.4432432	1	893.2668478	0.505361121
225.4189498	0.998287671	691.6886707	0.466569952
173.2412791	1	490.3972311	0.402289348
259.3051331	1	789.9514834	0.436718775
273.9604811	1	883.3801654	0.423169417
369.5635776	1	1169.68855	0.407153548
259.3914081	1	808.8878611	0.517007852
359.5369128	1	1156.10074	0.384465441
104.5747801	1	266.6000345	0.437641084
310.3810143	1	933.2214085	0.429412212
323.8128655	0.999451754	1030.743174	0.446177296
204.743338	1	575.3765223	0.394774501
139.5606061	1	414.5986121	0.459011299
79.375	1	222.7723746	0.449715081
252.885947	1	695.6606855	0.389116363
335.2638037	1	1036.33174	0.41975611
294.6651109	1	927.682044	0.464262505
236.6211699	1	787.9890651	0.415675103
282.5523416	1	890.1004648	0.412551135
323.2883333	1	1023.559283	0.393202635
153.6954315	1	424.831282	0.497248677
177.9015544	1	503.7343387	0.448952142
247.1201044	0.999020888	786.6632034	0.448181482
302.9948643	1	911.9266914	0.382861863
245.2569659	1	781.6315961	0.416937886
163.1993127	1	485.5907422	0.463901394
205.2562814	1	601.9894211	0.399847465
242.3359477	1	781.4617728	0.449323701
216.5481874	0.99867374	662.8474273	0.44324653
434.5771144	1	1421.107141	0.400511686
284.201232	1	884.8019314	0.355195272
362.1504425	1	1147.057687	0.376757215
72.94623656	1	123.2543808	0.53328717
252.7181373	0.99877451	781.4078639	0.390462788
116.0269637	0.998241501	335.6391155	0.440189217
199.5809313	1	586.1049658	0.388350757
224.3832599	0.997797357	689.5689722	0.45665471
97.20618557	1	223.7469216	0.505670813
215.1492537	1	687.6679508	0.461822581
193.6740548	1	591.9611662	0.445303708
446.6683292	1	1429.125038	0.354990974
246.5154265	1	769.3231692	0.374648764
313.08659	1	1008.271768	0.400610277
201.5964912	0.999307479	595.0765272	0.455851534
275.8803419	1	748.4127139	0.346259141
167.7699681	1	499.6758329	0.50501345
125.0263158	1	354.4912538	0.503764545
186.1741573	1	591.7759038	0.518401517
307.277551	1	910.855627	0.322547913

log.sigma.1.0.mm.3D_glcm_Homogeneity2	log.sigma.1.0.mm.3D_glcm_ClusterShade	log.sigma.1.0.mm.3D_glcm_MaximumProbability	log.sigma.1.0.mm.3D_glcm_Idmn	log.sigma.1.0.mm.3D_glcm_SumVariance2
0.31454247	1.137969314	0.049574862	0.983057754	18.28192568
0.377121163	-23.15957895	0.093596156	0.985179759	14.24413604
0.31541421	-37.2119034	0.052849411	0.969155339	19.11005054
0.302342514	-63.75496873	0.041066139	0.979996487	19.93233582
0.42145204	24.08590527	0.095854991	0.982318663	9.050114216
0.369736969	-41.31635166	0.078447346	0.983556168	13.50714379
0.473852641	6.138959132	0.132420948	0.980735898	6.648529596
0.404317667	-29.14710987	0.101335024	0.985767108	12.74699404
0.416914991	10.27453433	0.11034086	0.987831352	11.47733228
0.342204912	-14.46241226	0.053903233	0.983457555	14.20209643
0.402013057	-32.90486861	0.090901337	0.984885646	10.77316211
0.272564396	-60.40860725	0.031404702	0.972408085	25.23267907
0.409888307	5.186337867	0.094152583	0.986419695	10.00401247
0.367510105	-4.830118433	0.067819134	0.974446533	10.10477876
0.324877751	-8.330516229	0.041588592	0.9769613	11.29178447
0.426533166	-15.65325555	0.085689305	0.983711371	11.38140247
0.398402827	-3.642310686	0.083441488	0.983818766	8.251376192
0.459392211	2.60293894	0.12763382	0.982246503	9.187899393
0.413746587	12.20840459	0.099170657	0.981707047	10.94730611
0.445623925	-16.61195951	0.11334645	0.986072178	9.099424242
0.289035567	-23.51702841	0.032017846	0.983042848	18.92529549
0.372075155	-19.2126289	0.073215244	0.985604091	11.63055864
0.361257797	-31.401591	0.056853325	0.975306204	13.02236652
0.400962228	2.360333448	0.089692444	0.985833165	13.48631397
0.355320004	-10.90564925	0.045935021	0.985792753	13.01093064
0.319069173	-10.34146696	0.053823688	0.974873659	20.21416432
0.322370637	-27.09161486	0.04147542	0.976536473	15.29733936
0.309871305	-30.7336564	0.046123593	0.976881887	17.92067808
0.470443589	-13.90660708	0.151901363	0.985481951	8.419218171
0.390100589	-14.11308565	0.101456714	0.986717188	14.26389166
0.333429444	-26.80388932	0.047657535	0.978385426	14.25053743
0.433799348	4.630353456	0.103065922	0.98894326	8.800342918
0.345876426	-49.68388699	0.063582961	0.986488524	17.06107008
0.415931173	58.97741793	0.128874504	0.985726497	15.84211458
0.304530777	-67.78939853	0.047859104	0.976936942	22.0470119
0.420734191	-33.58206348	0.11598914	0.985731406	13.0315369
0.299356189	-45.92773957	0.051871778	0.981673947	20.95619041
0.324254746	-29.68850499	0.05564149	0.979628608	17.20778253
0.428644934	-4.058515449	0.090492429	0.973778619	6.441156579
0.394587565	-14.05548799	0.104173383	0.986312716	16.12241049
0.288582095	-54.64525719	0.035409103	0.973149501	18.63536525
0.418562017	-1.629630285	0.118969745	0.98518904	12.6870295
0.418325759	-15.40527168	0.09890433	0.9883849	11.37380098
0.429758359	26.75743564	0.119304763	0.989603326	12.5465491
0.339223937	-36.38920649	0.064964885	0.985749002	16.66491938
0.278461032	-29.30175312	0.029994824	0.976298765	21.94705777
0.287770467	-51.93566339	0.041499625	0.979934945	21.24532617
0.330606333	-42.57179612	0.055915161	0.977816099	16.42268264
0.435732268	21.79260465	0.155401135	0.977579594	11.31989086
0.413010868	-19.6977039	0.098750746	0.981815392	10.79202437
0.411529922	-4.967956193	0.091709574	0.989272994	10.14743839
0.450091742	-42.58321061	0.136773326	0.984797014	10.53610623
0.400490957	-13.23303585	0.094203031	0.986919819	11.22389283
0.32171611	-16.83445944	0.048878729	0.973689835	15.10430401
0.364842633	-16.33140834	0.077349256	0.983040521	14.50502126
0.351921319	-35.32373197	0.086121961	0.975035974	20.81019849
0.328516936	-36.21981602	0.054365235	0.984276907	16.57609069
0.462209427	-1.961216165	0.126399793	0.991404169	8.863105762
0.304248802	-109.7210491	0.046783233	0.979682746	21.42893867
0.363029168	-9.763670614	0.057113058	0.975391254	9.539268026
0.356114987	-20.93301952	0.06381156	0.987900922	15.87870511
0.377885365	-23.44118812	0.093460778	0.987283192	14.78070669
0.315594928	-52.24870548	0.041587316	0.973786574	19.43932523
0.391081313	-24.3290297	0.103430176	0.977298192	12.11959562
0.378489332	0.371258543	0.073213246	0.971912308	10.51257683
0.306167956	19.56196761	0.037980737	0.982170945	19.0135869
0.345785957	-14.66019547	0.072151403	0.980503555	17.88655366
0.399228109	-40.98763133	0.106000793	0.98669659	14.84965928
0.339707618	-48.4507628	0.065042211	0.978912304	14.77174072
0.337727085	-41.45636052	0.06469816	0.980506151	19.35932978
0.312045064	-31.59480445	0.039852294	0.980018905	18.68093321
0.437006154	7.125678116	0.113679656	0.987099778	9.333020838
0.378309311	-5.024560634	0.077736285	0.989313773	12.87082196
0.378170083	-31.04565587	0.078160109	0.982631944	12.62892055
0.299726369	-31.18475945	0.042990151	0.983571016	20.61519336
0.342693845	-47.39633533	0.060752112	0.979554425	17.26252481
0.397734017	-15.37993104	0.098176812	0.988242102	11.90301328
0.318512169	-18.90725155	0.045262875	0.97930963	16.39734646
0.380570081	-37.28946268	0.083261553	0.983953842	13.43857932
0.374773907	-30.85154086	0.082541125	0.981990293	15.19054197
0.324579069	-51.49479246	0.057585037	0.983360149	19.58585392
0.27007605	-70.56997471	0.032881963	0.975389605	25.00035393
0.296086727	-78.93934661	0.050809036	0.9808781	21.66843213
0.48289253	24.29540986	0.143616527	0.980047085	7.499980938
0.308839768	-41.42955165	0.044805811	0.978842628	17.95132222
0.366179859	-9.602573404	0.061710591	0.975948487	10.3838999
0.307024222	-19.10047021	0.04155971	0.977915293	19.81241409
0.387987986	-16.39802077	0.085363539	0.984278375	12.34821528
0.447333083	7.814595788	0.126066514	0.976843693	6.860302624
0.395435995	-38.13953772	0.082803538	0.985986134	13.69530123
0.374851755	-26.47758637	0.074853717	0.979784558	12.91571601
0.272391805	-158.7486558	0.033170018	0.973500354	29.64681233
0.293398165	-64.01931527	0.044782074	0.976174645	21.92717527
0.324476382	-48.07167861	0.065118401	0.981841238	19.53920744
0.385914935	-8.358309405	0.07184501	0.987148711	10.20701951
0.265492517	-79.13618989	0.045262414	0.967819759	37.14203329
0.447107665	-11.32716896	0.118860466	0.98727308	7.340370685
0.443742222	-6.811876225	0.124371505	0.98156676	7.764865821
0.462205406	-22.33128079	0.15591443	0.988725622	8.513804675
0.233453535	-105.5238349	0.023852956	0.970930416	32.5268039

log.sigma.1.0.mm.3D_glcm_Contrast	log.sigma.1.0.mm.3D_glcm_DifferenceEntropy	log.sigma.1.0.mm.3D_glcm_InverseVariance	log.sigma.1.0.mm.3D_glcm_Entropy	log.sigma.1.0.mm.3D_glcm_Dissimilarity
15.4339502	3.074120871	0.312032862	6.695522714	2.868203168
12.67777831	2.917372569	0.35411397	6.284654809	2.454820289
18.67467694	3.205436348	0.306652396	6.894180949	3.110393951
20.09881797	3.218179908	0.30481047	6.901255618	3.167007701
6.910457824	2.572105992	0.396975042	5.576088724	1.904011867
13.14008347	2.931584139	0.354882002	6.246304308	2.507909975
5.43020184	2.402622831	0.427349714	5.173607669	1.630335618
11.42462176	2.815707927	0.376846076	6.031186964	2.265133525
9.548012622	2.731285095	0.385020709	5.940274311	2.110199127
13.08161044	2.983190727	0.330256027	6.567016739	2.618572858
10.41689603	2.760864768	0.383206503	5.884234077	2.196554745
24.68396898	3.371322099	0.277622148	7.239483534	3.582489964
8.48289984	2.67066492	0.387355319	5.889615475	2.040192879
9.087062389	2.758497661	0.353504116	6.152898425	2.262634487
11.08434393	2.880294106	0.332692721	6.384777321	2.535356521
10.37455145	2.773177594	0.378092448	6.046875172	2.154162649
7.671407976	2.646016356	0.382246993	5.840642778	2.030123368
7.099625171	2.556712208	0.402798613	5.568897206	1.81517929
7.928103509	2.655109531	0.379456745	5.894220493	2.023090232
8.063919579	2.604724192	0.399150424	5.694418665	1.910036203
18.65804255	3.236293332	0.287567364	7.174153177	3.205705262
10.37975697	2.834252054	0.356505188	6.246317415	2.336982417
12.22048122	2.922985755	0.345149648	6.422798166	2.504232887
11.17053907	2.82618403	0.372909628	6.002613021	2.29579353
11.96671466	2.911998582	0.349624535	6.510752816	2.492678999
17.54904751	3.166887238	0.310182774	6.852838821	3.020254735
14.93361915	3.071119083	0.319067357	6.686658291	2.829248184
17.26630918	3.170364527	0.305704787	6.976369971	3.034823574
7.723202206	2.568739298	0.401051926	5.485089363	1.829003665
13.78779336	2.960412867	0.356403683	6.333763526	2.509284755
13.70726922	3.01761659	0.329662059	6.654061588	2.698634577
6.73800357	2.54965019	0.403348923	5.731372738	1.846075819
16.90649876	3.083822607	0.338231255	6.559767078	2.818634592
10.39355323	2.774713138	0.36982576	5.715640511	2.212331197
21.61898801	3.265398189	0.30855616	6.846993163	3.299714482
11.47584994	2.8002086	0.38075124	5.947907116	2.220355552
21.80107355	3.300884204	0.29668501	7.056965697	3.327024273
16.34470721	3.11942142	0.320122189	6.780733438	2.903372208
5.677420334	2.470221164	0.401941513	5.506715685	1.774247422
14.39571374	2.953790492	0.359094108	6.15578677	2.509256151
18.8112061	3.224105914	0.281317472	6.875133546	3.203742454
10.14624042	2.757375217	0.383229682	5.90952424	2.15150521
9.912387995	2.722211219	0.394224611	5.854009105	2.107759062
9.372676626	2.699617053	0.391659461	5.773492186	2.053808753
17.92647836	3.143797677	0.327079399	6.708404656	2.927515838
20.46078151	3.291552513	0.280532206	7.25823074	3.359605898
21.12278146	3.295466222	0.286856494	7.158197974	3.341284334
15.48236976	3.066838258	0.328241866	6.642947724	2.809801272
8.057855281	2.654227644	0.380180789	5.494318685	1.984677295
9.909472094	2.723443338	0.388308626	5.813120187	2.130535438
9.012810622	2.701264151	0.387370092	5.914759671	2.075156658
10.82212428	2.694657901	0.401682942	5.607240012	2.061582648
10.34504683	2.778735309	0.379343102	5.993834366	2.206408246
14.37249609	3.035968466	0.320610373	6.615663629	2.777300697
13.6163107	2.974697221	0.346944566	6.472911058	2.563271538
20.52025295	3.244304853	0.302444788	6.895763325	3.113164056
15.49274496	3.059979547	0.327314264	6.674236426	2.795050658
7.236269439	2.511697087	0.419424821	5.412241142	1.776092125
22.09766522	3.236726087	0.312851463	6.774797045	3.242606175
9.856740954	2.801901814	0.356847125	6.105398028	2.315690314
14.26515616	2.974238061	0.350865807	6.480789273	2.600435319
14.99592057	3.000818046	0.351853605	6.398979628	2.60028715
18.54504987	3.190488641	0.310490761	6.918166474	3.071407637
11.24229456	2.861939207	0.35830301	6.171420749	2.336279464
9.055941462	2.747200509	0.366598917	5.98382258	2.224259908
15.07197699	3.080209178	0.311190586	6.832956199	2.895490659
17.08310853	3.103054254	0.328773445	6.558689627	2.851767598
13.22549084	2.885118819	0.370174328	6.164168117	2.385677613
14.70956183	3.026635337	0.338466957	6.44115668	2.721648983
18.27738789	3.154074734	0.324389152	6.781741379	2.95526744
17.15169647	3.167958391	0.301691984	7.010038748	3.013968234
7.997935435	2.635186374	0.39243216	5.66120861	1.942652919
10.86423491	2.847630519	0.361377088	6.294832856	2.333840045
11.97801209	2.896424197	0.35830825	6.340597593	2.407311776
18.19967989	3.194704117	0.298951072	7.076686422	3.104117588
16.64577985	3.092025188	0.336227165	6.643006338	2.833035171
11.400366	2.838639879	0.369169869	6.074310107	2.301052917
14.12025185	3.036790765	0.318417609	6.617378171	2.781029884
12.88136016	2.916625537	0.360701992	6.251869883	2.45342464
14.53871604	2.993258839	0.355368059	6.502677174	2.595672836
21.18660773	3.234165508	0.316449668	6.772024629	3.16399589
24.65642126	3.394397499	0.276379597	7.336967747	3.620587165
23.0434191	3.31525967	0.300656105	7.000735453	3.383041066
4.940438468	2.318290987	0.445248521	4.944575908	1.561487365
16.88179033	3.164324727	0.302102727	6.923165464	3.016942214
9.586149434	2.797141413	0.358640668	6.208568781	2.290456378
17.71298103	3.178314994	0.303686869	6.877880231	3.070244755
10.7659787	2.820135363	0.369199131	6.098503322	2.282414131
5.750995826	2.432919143	0.419305237	5.159271462	1.733841613
12.16939482	2.803738939	0.376533034	5.843784422	2.31688096
11.91415661	2.889102691	0.360668595	6.264237285	2.413796619
33.54883939	3.502694393	0.275421798	7.131730152	3.966484388
22.28649098	3.319559425	0.291237861	7.073918636	3.403833255
20.3616545	3.241600952	0.312319239	6.937471534	3.152412055
9.304987223	2.743713502	0.376570743	6.093904474	2.179238199
35.46134473	3.613361586	0.2538103	7.566977621	4.266376027
6.629780032	2.514594826	0.417352398	5.400322104	1.79219892
6.595745458	2.509725115	0.40170344	5.312226816	1.805158872
8.331896436	2.590652883	0.39011271	5.357961115	1.872797216
31.32106767	3.556169769	0.242769051	7.589040341	4.172955998

log.sigma.1.0.mm.3D_glcm_DifferenceVariance	log.sigma.1.0.mm.3D_glcm_Idn	log.sigma.1.0.mm.3D_glcm_Idm	log.sigma.1.0.mm.3D_glcm_Correlation	log.sigma.1.0.mm.3D_glcm_Autocorrelation
7.185407121	0.915764701	0.31454247	0.083330037	198.3882207
6.643006581	0.925161084	0.377121163	0.057065289	193.2625309
8.979648599	0.891102258	0.31541421	0.012317116	135.6525962
10.04638751	0.911669908	0.302342514	-0.001168559	356.6080242
3.275328443	0.915077906	0.42145204	0.121118106	79.25817789
6.841199079	0.9213021	0.369736969	0.013358483	215.2310114
2.760349523	0.914688649	0.473852641	0.094115452	78.79261362
6.286506183	0.928432225	0.404317667	0.054132618	253.3303402
5.089543079	0.932426627	0.416914991	0.090400487	197.8121012
6.213681992	0.917357242	0.342204912	0.041459192	252.8514273
5.577400327	0.925219259	0.402013057	0.017755621	218.7203265
11.79154585	0.895706596	0.272564396	0.010485392	253.6311637
4.312555153	0.926813717	0.409888307	0.080942617	117.7981568
3.893605721	0.896191485	0.367510105	0.052639912	95.35978571
4.632243983	0.899103265	0.324877751	0.007632856	167.9821103
5.707093254	0.924293311	0.426533166	0.044965533	160.6790306
3.542884178	0.917373738	0.398402827	0.035621422	114.3996903
3.784112593	0.91978185	0.459392211	0.121511256	121.8868327
3.778672028	0.914619438	0.413746587	0.15076775	83.2812226
4.364735592	0.929024375	0.445623925	0.059109755	138.9602092
8.375983923	0.914517953	0.289035567	0.007244991	216.0679143
4.878123947	0.922630781	0.372075155	0.054276596	164.3538084
5.84772993	0.901926772	0.361257797	0.031956732	163.2508529
5.868010849	0.927240716	0.400962228	0.091316797	193.2133578
5.732676457	0.923342557	0.355320004	0.042281035	158.3705434
8.410115015	0.900698954	0.319069173	0.068598401	147.6326247
6.912865775	0.902241174	0.322370637	0.012639071	193.6527675
8.033928362	0.90311827	0.309871305	0.018614564	165.8750235
4.323432776	0.929407723	0.470443589	0.042308801	189.2578137
7.478925968	0.930562193	0.390100589	0.017827673	252.0547267
6.413782813	0.906143924	0.333429444	0.01983844	189.791791
3.308717918	0.932763772	0.433799348	0.127762226	169.5121908
8.940866472	0.928802281	0.345876426	0.005943077	282.4708648
5.464244753	0.927263175	0.415931173	0.190975927	176.3406955
10.69881688	0.905970923	0.304530777	0.010699636	217.5664098
6.541251724	0.930086556	0.420734191	0.062221069	248.0599874
10.7179413	0.914968487	0.299356189	-0.015865197	355.1909087
7.908883664	0.909991242	0.324254746	0.025214536	190.4904359
2.522983068	0.895741243	0.428644934	0.06221798	75.31706222
8.087188893	0.930896384	0.394587565	0.055413888	288.2291725
8.523170742	0.894860066	0.288582095	-0.00446933	254.758841
5.50633479	0.926751787	0.418562017	0.107307166	196.7076959
5.46446252	0.934841409	0.418325759	0.067798166	283.8673828
5.142768413	0.93825264	0.429758359	0.136175978	258.702147
9.325144436	0.926263638	0.339223937	-0.033604507	387.589748
9.1576268	0.900355329	0.278461032	0.034466332	221.9350562
9.939005874	0.909493217	0.287770467	0.005908321	322.4719805
7.57323341	0.906677006	0.330606333	0.029626031	183.6273134
4.100230564	0.908947434	0.435732268	0.138441974	82.43578041
5.354593891	0.918860304	0.413010868	0.04388941	193.171759
4.698536786	0.935276825	0.411529922	0.05970537	284.17519
6.559021087	0.93067446	0.450091742	-0.012710108	275.1909937
5.46307575	0.929644055	0.400490957	0.040721523	221.2032777
6.642076896	0.896366058	0.32171611	0.023619506	165.6449228
7.030141227	0.91971718	0.364842633	0.031183484	251.5203921
10.80556191	0.906012357	0.351921319	0.007860579	280.3512285
7.667400296	0.920479518	0.328516936	0.035879767	359.7055586
4.076580633	0.944111936	0.462209427	0.098971432	250.8448896
11.56421926	0.912903441	0.304248802	-0.012309276	356.0484123
4.479304212	0.899245029	0.363029168	-0.014952951	96.37083246
7.487753438	0.931738585	0.356114987	0.055071087	292.636338
8.216005479	0.932227292	0.377885365	-0.005597699	318.3952752
9.081066088	0.899583769	0.315594928	0.022546578	191.4732033
5.772094055	0.908222958	0.391081313	0.037266176	141.6288939
4.079886813	0.893173229	0.378489332	0.072441478	83.52005662
6.675724815	0.912031628	0.306167956	0.114600997	227.4538531
8.917606343	0.914940639	0.345785957	0.022263989	322.1884554
7.523657405	0.932004479	0.399228109	0.058877334	289.4281081
7.295974666	0.90928856	0.339707618	0.001598542	250.7042079
9.51361562	0.914871864	0.337727085	0.029358112	281.7486002
8.052973776	0.909578945	0.312045064	0.04279453	320.9028075
4.218599172	0.930262575	0.437006154	0.072390308	142.6934068
5.410018628	0.934073247	0.378309311	0.083379595	168.7616002
6.178313788	0.918709283	0.378170083	0.026288964	249.6720604
8.53003047	0.917230868	0.299726369	0.063595322	289.9222799
8.611785782	0.912642187	0.342693845	0.018650619	249.9451098
6.094852546	0.9335441	0.397734017	0.02258006	161.9791751
6.373190165	0.90658809	0.318512169	0.068042538	198.8829442
6.847697437	0.922996014	0.380570081	0.021608262	247.7270184
7.787786808	0.919355265	0.374773907	0.02274386	215.1844231
11.17234922	0.921304762	0.324579069	-0.036552521	429.424676
11.52815066	0.900397411	0.27007605	0.008186303	282.4232675
11.57048651	0.913954111	0.296086727	-0.02411351	354.160686
2.481259185	0.913049907	0.48289253	0.187862696	49.34398501
7.76853038	0.90646701	0.308839768	0.030719563	251.4743788
4.333532771	0.900101638	0.366179859	0.040308715	118.1261883
8.266078522	0.905252646	0.307024222	0.055416696	195.1525868
5.547252431	0.922315374	0.387987986	0.067488988	221.4171278
2.71880889	0.904280083	0.447333083	0.075504266	81.1182982
6.779249495	0.929386971	0.395435995	0.055026502	219.6489789
6.082243717	0.912443262	0.374851755	0.040373613	194.099576
17.74645818	0.90252226	0.272391805	-0.059649572	433.7772236
10.66567022	0.903043784	0.293398165	-0.006855929	248.1371598
10.39577251	0.917108717	0.324476382	-0.01856985	315.1388039
4.550376975	0.927387427	0.385914935	0.045955308	193.7903249
17.16175868	0.889796741	0.265492517	0.025301696	244.0037304
3.411822024	0.9296196	0.447107665	0.049779551	162.9724584
3.328406968	0.915642259	0.443742222	0.081740291	121.3075973
4.799675803	0.937778063	0.462205406	0.009701856	189.3944981
13.87169197	0.890222684	0.233453535	0.021606733	291.1286341

log.sigma.1.0.mm.3D_glcm_SumEntropy	log.sigma.1.0.mm.3D_glcm_AverageIntensity	log.sigma.1.0.mm.3D_glcm_Energy	log.sigma.1.0.mm.3D_glcm_SumSquares	log.sigma.1.0.mm.3D_glcm_ClusterProminence
4.028954922	14.06495814	0.016235519	8.496259062	1899.187092
3.833766485	13.89092648	0.02679251	6.765319451	1171.290806
4.079607759	11.6422862	0.014820645	9.44618187	1491.426307
4.086956247	19.01959312	0.014617632	10.13180342	2319.298545
3.448865396	8.872275586	0.037217781	3.99014301	526.696523
3.757289337	14.67013751	0.025283286	6.690054369	1154.633595
3.253169299	8.859175801	0.052604123	3.019682859	254.1622095
3.705209306	15.90673554	0.032380389	6.053476694	1136.875618
3.629384503	14.0507255	0.034598016	5.299914093	962.246395
3.882492146	15.8984389	0.018527661	6.869200149	1036.875762
3.5993633	14.78616763	0.031960585	5.297514534	779.9504259
4.265926212	15.95106726	0.010959878	12.69783656	3190.11369
3.574769753	10.83585542	0.032246627	4.621728077	677.3188541
3.661244813	9.752103283	0.022337092	4.797960288	416.3788295
3.742041568	12.95876698	0.017995728	5.594032099	543.9136859
3.653831196	12.66596183	0.034121265	5.438988481	773.0865938
3.495009588	10.68895846	0.029355853	3.980696042	325.3922963
3.490408691	11.01643294	0.044359419	4.071881141	511.1741475
3.649855573	9.083657904	0.031335143	4.718852405	591.7600246
3.512606628	11.77713301	0.038921174	4.290835955	500.6996281
4.13299374	14.73208251	0.011195449	9.571840875	1482.309929
3.734365222	12.80788605	0.023371459	5.503747224	642.0175125
3.820058253	12.76910575	0.020273048	6.310711935	785.77272
3.696904104	13.88132651	0.032551003	6.192075777	1218.233604
3.849992123	12.57577906	0.018071808	6.262454109	750.1048463
4.104298107	12.12266501	0.016294146	9.440802958	1963.919476
3.940997658	13.91259319	0.0161005	7.557739627	1043.933243
4.071543839	12.87317955	0.013790115	8.799453237	1311.528853
3.390748111	13.75076385	0.052300767	4.035605094	502.0755053
3.805835937	15.92095724	0.030038551	7.242840042	1203.110843
3.899572478	13.7715588	0.017612471	6.989451664	907.3431
3.514358917	12.99978376	0.035622985	3.884586622	467.8945598
3.929028401	16.90407123	0.021369301	8.582768185	1885.76272
3.693018622	13.23539646	0.039940052	6.675603581	1744.372742
4.124328985	14.75712071	0.01571495	11.00825262	2272.99622
3.663747673	15.74238778	0.038128796	6.170768665	1248.241729
4.126880906	19.08525671	0.015439325	10.45165493	2321.255188
4.005741731	13.79718037	0.01732067	8.41988774	1510.380241
3.337187529	8.667499043	0.035524455	3.029644228	170.0016189
3.82209968	17.01002752	0.032047695	7.792431786	1891.16911
4.059352798	15.96247464	0.013232783	9.361642838	1684.943878
3.671415601	14.00235293	0.037729003	5.708317478	1049.99089
3.598514131	16.85139705	0.036378258	5.408838557	939.2632174
3.608100307	16.07514037	0.039747562	5.625348691	1220.276027
3.936385591	19.87291587	0.020131837	8.279042798	1820.262542
4.226067753	14.89759496	0.010955887	10.71542431	1941.878475
4.173445154	18.10928309	0.012642804	10.8390839	2138.185754
3.943034023	13.54222856	0.018298037	7.976263099	1347.848754
3.519586807	9.033307792	0.04893731	4.844436535	729.654428
3.60468647	13.89065643	0.034149856	5.175374116	715.7089935
3.588297088	16.8581532	0.032459347	4.855288513	679.418954
3.471343309	16.59103398	0.047755486	5.339557629	959.4013608
3.645356889	14.8674471	0.031751048	5.410814091	866.0146021
3.925226911	12.86318046	0.016726952	7.369200027	1068.843449
3.857924025	15.85748167	0.023518614	7.068760733	1172.41566
4.113534949	16.77280997	0.020036138	10.53682282	2086.732814
3.96000122	19.0786677	0.018039084	8.248984865	1663.589629
3.365775125	15.83043573	0.048763408	4.070703782	675.3810183
4.062491503	19.06111807	0.0165063	10.87295631	3174.356596
3.608792148	9.820891962	0.024082309	4.849002245	407.4473988
3.901950417	17.18991583	0.021921446	7.718600925	1648.417185
3.816084688	17.98247297	0.027466991	7.512811516	1537.519753
4.08043661	13.82921198	0.015032635	9.496093775	1790.92107
3.721377473	11.89151621	0.028798357	5.840472545	773.5474206
3.668709697	9.118817502	0.026151604	4.892129574	429.7898601
4.08531907	15.05900452	0.014168898	8.640301096	1690.252308
3.974776603	17.98533466	0.020359163	8.982245719	1736.680231
3.788218365	17.03532168	0.031860592	7.159803691	1652.603854
3.859527785	15.83310348	0.02088629	7.370325638	1268.707615
4.0462819	16.81128671	0.018898344	9.539069016	2118.651698
4.10634558	17.95405697	0.013260592	9.212794332	1540.294396
3.478955583	11.93128835	0.040193691	4.332739068	614.8629365
3.787482374	12.97580204	0.025292967	5.977955808	938.2028481
3.770289758	15.79585804	0.024628285	6.151733162	923.3409226
4.163785015	17.09880696	0.013168219	9.944082695	2000.878879
3.962341506	15.81240439	0.02031106	8.535627221	1642.328315
3.680000632	12.72647255	0.031306933	5.871256836	874.8779163
3.965356255	14.08204219	0.017125429	7.629399577	1252.456784
3.756624179	15.73948993	0.027764391	6.621277472	1179.616478
3.87814651	14.6701819	0.024571884	7.49324605	1292.265203
4.027562173	20.90683203	0.017935416	9.599497606	2498.085883
4.283417774	16.88867467	0.010903205	12.71601857	2709.466385
4.109785218	19.06492565	0.015582349	10.84263585	2866.489174
3.198528897	6.978542797	0.060364054	3.110104851	397.5703285
4.068319835	15.85389525	0.014552312	8.741007316	1367.745091
3.684025371	10.85939055	0.022831395	4.992512334	481.945377
4.104842944	13.95654427	0.014559206	9.455838435	1839.857121
3.710049941	14.86670842	0.029353745	5.778548495	1007.001831
3.255188612	8.990828462	0.049054058	3.152824612	218.3333584
3.654499494	14.80756922	0.0307886	6.466174013	1758.566803
3.782590869	13.92288581	0.024964379	6.207468154	907.6531463
4.291859404	21.11085351	0.012241737	14.56466248	5367.51207
4.17194667	15.77608334	0.013209522	11.19623851	2119.832037
4.071775547	17.88765878	0.017809564	10.09771861	2018.782646
3.641459257	13.91284838	0.026670842	4.879714642	561.9628378
4.544789865	15.75920122	0.009742947	18.79550877	5355.141376
3.329580005	12.75907492	0.04488527	3.492537679	372.0706916
3.380821424	11.00055377	0.043882425	3.59015282	317.6037624
3.370084421	13.76054993	0.050481786	4.214518477	578.2110395
4.470774608	17.24123974	0.007616112	16.47694561	4140.785644

log.sigma.1.0.mm.3D_glcm_SumAverage	log.sigma.1.0.mm.3D_glcm_Imc2	log.sigma.1.0.mm.3D_glcm_Imc1	log.sigma.1.0.mm.3D_glcm_DifferenceAverage	log.sigma.1.0.mm.3D_glcm_Id
28.1193402	0.59882999	-0.064570332	2.868203168	0.396411506
27.7754452	0.525416647	-0.05101325	2.454820289	0.44720755
23.28457241	0.618677441	-0.067813894	3.110393951	0.394059453
37.77024042	0.530178951	-0.046537684	3.167007701	0.38420949
17.74455117	0.537485359	-0.062593523	1.904011867	0.485256893
29.33522391	0.529637541	-0.051526446	2.507909975	0.440725219
17.7183516	0.508614587	-0.0593089	1.630335618	0.527036949
31.81185758	0.543964712	-0.057043984	2.265133525	0.469032553
28.09468233	0.529936548	-0.05435271	2.110199127	0.480015466
31.78493564	0.451065482	-0.034141142	2.618572858	0.41961127
29.57233526	0.468885099	-0.041936653	2.196554745	0.467677882
31.84291636	0.605296174	-0.061286287	3.582489964	0.3578085
21.67171084	0.46099176	-0.040478062	2.040192879	0.475528497
19.50420657	0.441016378	-0.038571713	2.262634487	0.441930136
25.91753396	0.458490614	-0.036664405	2.535356521	0.406959823
25.33192367	0.503648497	-0.048259226	2.154162649	0.487359642
21.37791691	0.45383097	-0.039134212	2.030123368	0.466995546
22.03286589	0.475779153	-0.047732459	1.81517929	0.515319215
18.16731581	0.549981003	-0.063125676	2.023090232	0.479028783
23.55426602	0.468094682	-0.047315965	1.910036203	0.503924154
29.39392539	0.37184788	-0.020552327	3.205705262	0.373722627
25.61566217	0.448942456	-0.037750125	2.336982417	0.444238852
25.5382115	0.415463481	-0.034040523	2.504232887	0.434559754
27.75842062	0.638232818	-0.084787414	2.29579353	0.46579718
25.14827784	0.460332291	-0.036539107	2.492678999	0.429738127
24.24533002	0.600642647	-0.063934121	3.020254735	0.397806399
27.82518637	0.484851411	-0.039728554	2.829248184	0.402131483
25.74575496	0.453769744	-0.032823302	3.034823574	0.390767321
27.5015277	0.489484249	-0.053786813	1.829003665	0.523926027
31.74492952	0.542506564	-0.053689796	2.509284755	0.456069287
27.54311759	0.389654366	-0.024662238	2.698634577	0.411630492
25.99956753	0.392121689	-0.03176604	1.846075819	0.495231354
33.6113854	0.534329473	-0.050044276	2.818634592	0.419729728
26.4548869	0.718538805	-0.124077653	2.212331197	0.47872777
29.49280902	0.684240524	-0.088320689	3.299714482	0.383419432
31.47500264	0.488994226	-0.045473357	2.220355552	0.482111184
37.70419255	0.492189248	-0.038626791	3.327024273	0.380134598
27.58795863	0.446670827	-0.032390324	2.903372208	0.402709145
17.33499809	0.38236608	-0.028956937	1.774247422	0.492550455
33.92892215	0.599822811	-0.070294607	2.509256151	0.459676524
31.92494929	0.557545997	-0.052871385	3.203742454	0.374339991
28.00470587	0.541315061	-0.058603325	2.15150521	0.48081283
33.67494019	0.487489719	-0.045729615	2.107759062	0.480499756
32.11859907	0.553977733	-0.063912937	2.053808753	0.490202781
39.39060577	0.526376627	-0.04690071	2.927515838	0.413695602
29.76981249	0.448671334	-0.030597298	3.359605898	0.364191957
35.91326453	0.460485848	-0.032646401	3.341284334	0.37153637
27.08445712	0.55735465	-0.054777605	2.809801272	0.408330531
18.06661558	0.65553647	-0.101935926	1.984677295	0.496319033
27.78131286	0.555654699	-0.061835146	2.130535438	0.476601078
33.69810754	0.436308615	-0.035486972	2.075156658	0.476230256
33.18206795	0.547593891	-0.062173657	2.061582648	0.505361121
29.73098578	0.447109565	-0.037315744	2.206408246	0.466569952
25.72636093	0.566560066	-0.057325585	2.777300697	0.402289348
31.70467039	0.463410529	-0.037098492	2.563271538	0.436718775
33.48302095	0.611749314	-0.065733904	3.113164056	0.423169417
37.9174542	0.480183115	-0.038323017	2.795050658	0.407153548
31.65036533	0.49691522	-0.052880783	1.776092125	0.517007852
37.74730762	0.632451127	-0.072466025	3.242606175	0.384465441
19.64178392	0.458553548	-0.038177426	2.315690314	0.437641084
34.18958403	0.51338758	-0.045941845	2.600435319	0.429412212
35.69021878	0.484995882	-0.041305185	2.60028715	0.446177296
27.65842396	0.519004892	-0.044744264	3.071407637	0.394774501
23.78303242	0.514193572	-0.04872206	2.336279464	0.459011299
18.237635	0.575025161	-0.065992762	2.224259908	0.449715081
30.09751547	0.560774188	-0.053857282	2.895490659	0.389116363
35.8876498	0.656921797	-0.082383696	2.851767598	0.41975611
34.00121307	0.484731852	-0.042723831	2.385677613	0.464262505
31.66620697	0.547453516	-0.053852071	2.721648983	0.415675103
33.55452575	0.562830091	-0.054876615	2.95526744	0.412551135
35.80605319	0.438695226	-0.030148985	3.013968234	0.393202635
23.86257669	0.478169615	-0.046032376	1.942652919	0.497248677
25.94278297	0.425825376	-0.031853122	2.333840045	0.448952142
31.59171608	0.380352842	-0.024596225	2.407311776	0.448181482
34.0184685	0.460364307	-0.033281767	3.104117588	0.382861863
31.60947304	0.527641311	-0.048055864	2.833035171	0.416937886
25.44428996	0.507241836	-0.048074373	2.301052917	0.463901394
28.16408439	0.538672279	-0.051440528	2.781029884	0.399847465
31.46982407	0.470665933	-0.039399176	2.45342464	0.449323701
29.32720697	0.491629437	-0.042219961	2.595672836	0.44324653
41.46442703	0.664151681	-0.081563184	3.16399589	0.400511686
33.60576976	0.515233633	-0.04096982	3.620587165	0.355195272
37.65652979	0.55418733	-0.050973304	3.383041066	0.376757215
13.95708559	0.602273561	-0.094590912	1.561487365	0.53328717
31.69896394	0.404387579	-0.025536986	3.016942214	0.390462788
21.71878109	0.355023764	-0.021646781	2.290456378	0.440189217
27.90158883	0.578965581	-0.057817223	3.070244755	0.388350757
29.73341685	0.454623261	-0.038044577	2.282414131	0.45665471
17.98165692	0.616729655	-0.09310272	1.733841613	0.505670813
29.61513844	0.67874651	-0.102166212	2.31688096	0.461822581
27.84577162	0.435720627	-0.033384488	2.413796619	0.445303708
41.70127347	0.735735725	-0.102140986	3.966484388	0.354990974
31.51036547	0.591410208	-0.059009215	3.403833255	0.374648764
35.51582796	0.487723409	-0.03846193	3.152412055	0.400610277
27.82546246	0.379220995	-0.025447139	2.179238199	0.455851534
31.21423197	0.76779442	-0.110851706	4.266376027	0.346259141
25.51814985	0.426302314	-0.037352856	1.79219892	0.50501345
22.00110755	0.608548029	-0.08452678	1.805158872	0.503764545
27.52076273	0.566273181	-0.071335884	1.872797216	0.518401517
34.1072302	0.681407572	-0.078739347	4.172955998	0.322547913

log.sigma.1.0.mm.3D_glcm_ClusterTendency	log.sigma.1.0.mm.3D_firstorder_InterquartileRange	log.sigma.1.0.mm.3D_firstorder_Skewness	log.sigma.1.0.mm.3D_firstorder_Uniformity
18.28192568	72.60802078	-0.252469491	0.117763917
14.24413604	58.49832726	-0.649229971	0.1446385
19.11005054	80.9298296	-0.41610438	0.108987426
19.93233582	74.20653915	-0.984436655	0.114673597
9.050114216	44.80740261	0.719576604	0.174973157
13.50714379	56.328897	-1.097684285	0.148026644
6.648529596	38.61639118	-0.027712337	0.206250352
12.74699404	52.51021194	-1.009017187	0.157054964
11.47733228	52.61756563	-0.009503762	0.157191433
14.20209643	68.85862446	-0.469118355	0.12450165
10.77316211	49.94780588	-1.265136012	0.16901345
25.23267907	93.50363541	-0.669825231	0.09561729
10.00401247	51.5570116	0.110216996	0.163703262
10.10477876	63.5810833	-0.246997359	0.139577957
11.29178447	73.01061916	-0.274215594	0.125322997
11.38140247	48.90610313	-0.417794537	0.163069083
8.251376192	56.75199509	-0.14957036	0.157125416
9.187899393	42.93215537	-0.110593706	0.186035195
10.94730611	55.57455182	0.286529903	0.152298031
9.099424242	45.49724197	-0.886127568	0.177523328
18.92529549	90.33400631	-0.3808245	0.100855277
11.63055864	61.49474716	-0.507634204	0.140020731
13.02236652	64.75500298	-0.840022017	0.133093846
13.48631397	50.01781058	-0.011213832	0.155664059
13.01093064	68.85142374	-0.233844883	0.124941895
20.21416432	75.84664726	-0.296894677	0.110668832
15.29733936	71.78352642	-0.620887103	0.120738563
17.92067808	83.16019344	-0.400355054	0.106554756
8.419218171	39.46286345	-0.711461191	0.197172628
14.26389166	50.73080873	-0.417388651	0.154745918
14.25053743	71.13949394	-0.56509075	0.121936232
8.800342918	49.67664647	-0.051644629	0.17211536
17.06107008	59.35141945	-0.848212414	0.13690764
15.84211458	47.69301128	0.649394156	0.173222799
22.0470119	77.18379211	-0.9278055	0.11379961
13.0315369	44.7225523	-0.981364877	0.175410512
20.95619041	75.49674988	-0.549115762	0.110351183
17.20778253	68.22703838	-0.562567247	0.122145364
6.441156579	51.07601595	-0.340156303	0.177101118
16.12241049	48.66541195	-0.576606953	0.158558802
18.63536525	82.06168842	-0.869149868	0.108033231
12.6870295	46.68915868	-0.352388905	0.171109118
11.37380098	46.67434216	-0.845827808	0.173278834
12.5465491	47.24007249	0.12424053	0.173962433
16.66491938	61.34524775	-0.862345891	0.132853966
21.94705777	88.27598953	-0.433971339	0.098245195
21.24532617	80.41052294	-0.687527769	0.106455804
16.42268264	69.66470814	-0.734011332	0.125198411
11.31989086	42.26740229	0.444746324	0.189735174
10.79202437	48.84807277	-1.056439031	0.165115731
10.14743839	48.82770371	-0.446071743	0.165138213
10.53610623	40.08607626	-1.637307384	0.199780655
11.22389283	50.5107851	-0.655888487	0.160773351
15.10430401	72.64860439	-0.453028334	0.121674454
14.50502126	59.38912582	-0.511519578	0.13437143
20.81019849	67.89757156	-0.615347461	0.120890638
16.57609069	66.44168091	-0.64037083	0.124794721
8.863105762	41.0337534	-0.322034703	0.199860549
21.42893867	72.23744059	-1.260037622	0.119537778
9.539268026	57.5986042	-0.326669089	0.147015869
15.87870511	62.53104687	-0.50214914	0.133725827
14.78070669	52.97128487	-0.482228209	0.14964964
19.43932523	77.08412266	-0.71146282	0.112321013
12.11959562	56.54888916	-0.71812898	0.149334675
10.51257683	57.55521393	-0.358375127	0.144900891
19.0135869	80.4656086	-0.011293531	0.108416367
17.88655366	69.3050127	-0.556411872	0.126164501
14.84965928	49.96817231	-1.159710799	0.159792161
14.77174072	61.92223454	-1.130073751	0.135677923
19.35932978	67.24925518	-0.72205337	0.121787276
18.68093321	82.07046986	-0.5352197	0.108128454
9.333020838	45.9222374	-0.033345767	0.179104277
12.87082196	58.19063854	-0.265738565	0.143065598
12.62892055	59.21998215	-0.842952666	0.142403269
20.61519336	82.41932583	-0.478044136	0.106233956
17.26252481	64.99386215	-0.858413654	0.128398117
11.90301328	49.66323662	-0.486424652	0.159401914
16.39734646	68.62045479	-0.432638905	0.12391107
13.43857932	54.69571877	-0.964110123	0.152208462
15.19054197	58.0470047	-0.626973856	0.138010296
19.58585392	66.02676201	-0.801729715	0.123019184
25.00035393	87.8295536	-0.712799476	0.097903593
21.66843213	75.42008781	-0.92010744	0.109729056
7.499980938	34.38419557	1.120926206	0.220696891
17.95132222	77.79533625	-0.706808234	0.112150926
10.3838999	60.24989319	-0.427011154	0.140908414
19.81241409	78.95001316	-0.402399094	0.10994692
12.34821528	52.04273081	-0.660154784	0.157055369
6.860302624	43.29683161	0.197798593	0.188685835
13.69530123	51.58059931	-0.843373248	0.16107925
12.91571601	57.19524336	-0.866790174	0.144865133
29.64681233	85.03366661	-1.272557288	0.099658074
21.92717527	82.47158861	-0.810313396	0.10457226
19.53920744	67.92900085	-0.662757044	0.121964573
10.20701951	56.86565208	-0.429963819	0.148792067
37.14203329	104.4580564	-0.264215871	0.084447493
7.340370685	40.52089405	-0.761632088	0.197429097
7.764865821	44.5160321	-0.783958201	0.188324308
8.513804675	38.23105693	-0.967005262	0.200927279
32.5268039	116.0933132	-0.58706988	0.079787282

log.sigma.1.0.mm.3D_firstorder_MeanAbsoluteDeviation	log.sigma.1.0.mm.3D_firstorder_Energy	log.sigma.1.0.mm.3D_firstorder_RobustMeanAbsoluteDeviation	log.sigma.1.0.mm.3D_firstorder_Median
52.64211069	7007873.809	31.44020892	19.49815178
45.3491808	9067245.907	25.89704768	12.99996138
56.7750216	6671214.56	35.25699593	11.00607252
54.9675943	15677407.49	32.25055339	16.35048866
36.72504005	2912770.939	20.15939286	10.00345421
44.13455342	6882330.56	24.92595979	11.53824663
31.32899871	2374554.237	17.11399914	9.766117096
42.00129799	7471735.091	23.021866	13.44754124
40.92303723	7641927.183	23.37748601	13.05174255
48.26626127	13590827.49	29.87554513	16.18202209
38.61512222	6154248.154	21.70863219	13.49452686
64.47407568	13628402.81	39.41092093	19.39138794
38.12985475	9325942.122	22.19759988	11.67243099
41.85594843	5802278.278	27.19668007	12.42115545
46.0288315	5779108.886	30.41392979	13.53524303
39.64709257	14197884.12	21.98184511	3.21696806
37.48665145	4181304.566	23.63083193	7.625928879
35.57938985	7738674.294	19.49868202	13.85579252
40.99794835	4147760.825	24.36052579	14.87404919
35.5015284	8030645.892	20.16233685	10.64804173
57.62215523	40319743.16	37.73028234	9.453203678
43.18286035	7722589.803	26.64658017	13.44488621
45.72405759	16090120.47	28.5306374	14.26492119
43.73000451	4594588.325	23.46995699	10.20904446
46.60737168	10085065.26	30.08690142	5.580407858
56.86938686	8938865.376	33.88679955	20.81416512
50.03974293	10699965.44	30.71057688	13.4585619
56.14859915	19246705.45	35.49056642	16.77742195
33.80251788	9135480.693	17.79746189	8.99938488
43.92561442	13911903.21	23.81588298	8.339655876
49.49587522	18770870.87	30.976295	14.89685774
35.84044468	14131481.01	21.22808894	14.32590866
48.65802899	13881830.74	26.21238272	14.14214134
43.39986184	2411538.734	21.58699	14.22546005
59.05433094	6124038.782	34.34435099	19.38891602
39.77550943	12698605.65	20.37760898	10.76991367
57.9985931	24666179.45	33.60037599	14.03911018
51.77757613	18333573.32	30.49504542	15.58749104
32.77824588	2907465.434	20.84442648	8.505861759
44.75320019	8432504.157	22.53703697	13.43653727
56.41258425	8070610.275	35.08292207	18.47725105
40.78051604	7958743	21.29076389	14.32135105
38.66858042	11038973.41	20.45289259	11.63044167
39.43261307	7555243.485	20.69261099	13.88836145
48.71882318	18041877.66	27.54547122	10.31275082
60.71036044	24144371.76	38.3687335	18.61291695
58.4113983	25099628.86	34.91035906	18.10816002
50.73521501	7492703.998	30.41311016	9.292110443
39.55687115	2125363.883	20.45982899	10.82364082
39.00772394	3854480.44	21.4880049	14.42123032
38.04617348	12911730.63	21.74854361	11.2525115
35.76348449	6379151.296	17.72611942	8.724117279
40.34328343	14286365.4	22.43154065	13.75146866
49.62549767	5483562.299	30.99804421	12.01204586
47.37138893	18960015.54	27.09131659	11.73114491
54.30272916	11809390.91	30.74521849	6.116834641
50.0663764	17331064.66	29.53352838	14.67692471
33.40439768	6811050.243	17.97251173	8.418585777
55.24698949	9332775.98	30.64337646	16.98954201
40.45177644	4330347.411	25.29678969	11.9736948
47.91076935	15498055.07	27.3912879	17.17688179
45.56406913	26624277.72	24.21411837	11.81468296
55.10169263	13526147.92	33.22650753	17.58074951
42.39228707	7006306.925	24.87315433	13.85971975
42.0272977	2096280.785	25.40482859	19.06975365
55.48793405	9458904.519	34.34317027	18.1622448
51.16636332	6007115.853	29.25385101	13.45046139
42.53724291	16313383.08	22.65579195	14.70699024
46.9365625	5931663.067	27.34687602	14.58262157
53.59153762	15300601.77	30.07654908	13.93048668
54.8418523	21317481.14	34.80240306	10.93879032
36.06257558	6377055.078	19.91361725	11.07335567
43.95471243	19516262.78	25.48107924	15.87811184
43.86864853	25996411.93	25.80676889	12.67949963
57.01684465	27372203.75	34.90214227	19.107337
50.86152012	12791763.09	28.65785023	12.89737701
41.35287419	8900487.431	22.75335063	10.29831123
50.31137255	7117673.038	29.98648443	21.16438103
43.55362331	13710548.47	24.01197022	11.25704765
47.32055033	21009275.2	26.66949306	10.98430443
53.08081889	8447560.544	29.46678556	11.94073582
63.74460513	20729248.16	38.4178304	16.67180634
59.28934343	18561950.84	33.8318659	16.84456539
31.02496701	1284451.862	15.51949225	8.489639282
54.55986658	21648318.71	33.85383273	19.24924088
41.78742935	11370507.79	26.32539459	12.60774279
56.07610118	8859382.68	34.14543053	20.91652107
41.21806822	11870187.38	22.97679834	13.75051403
34.4187832	1067719.996	19.80182514	10.64128304
41.64403916	2522598.929	22.13881692	13.52254629
43.3450068	13108254.88	25.17271618	16.53630066
66.65025421	10999567.67	37.02674015	22.11985207
60.08878822	10766959.09	36.50494823	16.33208847
54.74319178	27201128.77	30.83509781	15.32168007
40.78964654	16111717.51	24.51420484	14.64800787
75.98677904	9563856.365	46.35995501	9.048953533
31.60991125	4094013.297	17.72371882	10.79489613
33.65814852	1132797.133	19.15407177	15.9163146
33.95164378	2566397.522	18.04849802	12.40427208
75.00255221	11749450.19	48.49377913	20.38480091

log.sigma.1.0.mm.3D_firstorder_TotalEnergy	log.sigma.1.0.mm.3D_firstorder_Maximum	log.sigma.1.0.mm.3D_firstorder_RootMeanSquared	log.sigma.1.0.mm.3D_firstorder_90Percentile	log.sigma.1.0.mm.3D_firstorder_Minimum
189212592.9	387.4711609	76.32385939	104.5725021	-315.6851196
244815639.5	357.3099365	67.33218364	86.67888031	-317.066803
180122793.1	281.7272949	77.24698766	89.75660172	-272.1857605
423290002.2	292.8328552	79.94445556	97.41013947	-428.5405273
78644815.35	251.4931183	55.40131335	70.89849548	-196.4250031
185822925.1	304.9252625	64.82026696	70.70976791	-332.7148438
64112964.4	196.492691	47.3972946	57.47470398	-178.9125366
201736847.5	299.1663208	64.33867752	82.1675354	-366.673645
206332033.9	349.1261292	61.40087685	87.04933472	-306.8392944
366952342.3	298.5808716	67.27369532	85.19170837	-353.2461548
166164700.2	263.2749939	58.35907851	64.63918152	-336.2301331
367966875.8	306.5415955	89.93371382	107.0936111	-374.6552734
251800437.3	342.8436584	56.42696244	68.58470306	-235.4813385
156661513.5	216.8737488	55.8825766	71.22757721	-201.6632385
156035939.9	202.2140656	61.10052761	83.63604889	-276.5809021
383342871.2	288.9174194	58.78191669	68.70351868	-299.5776978
112895223.3	252.4642487	51.04090697	60.47011414	-228.1849518
208944205.9	220.0454559	55.00257402	79.37875824	-238.6519775
111989542.3	286.5133362	60.42514303	90.76281738	-178.7657471
216827439.1	279.2937622	52.61400645	64.4407959	-271.657135
1088633065	447.4273376	76.16693438	94.05575867	-345.0090027
208509924.7	331.4450684	60.34081189	78.77934265	-275.6626282
434433252.6	201.3442383	62.48536925	77.00469971	-284.9559326
124053884.8	340.6137085	66.78896139	87.96917725	-304.2019043
272296762	397.6482544	62.23273196	74.90342865	-291.5201111
241349365.2	330.7423401	81.3116647	115.0615143	-273.0599976
288899067	261.4934692	69.56596537	89.32962036	-301.2463989
519661047.2	325.2924805	75.74115748	95.86138763	-293.2507935
246657978.7	210.548111	51.84299938	61.59358444	-306.1022034
375621386.6	376.5343018	66.36184636	79.90628662	-359.0536804
506813513.6	265.7746582	67.31892369	83.20177155	-322.524292
381549987.2	277.3856201	53.1629213	71.4145134	-287.5387268
374809430.1	438.5128784	74.05890728	83.63726044	-387.9630127
65111545.82	337.7799072	70.80673726	100.5239487	-297.1434326
165349047.1	350.3753967	83.0448076	90.75596161	-343.4512939
342862352.6	291.9115601	62.82789191	69.3991333	-370.224884
665986845.2	353.8683777	83.1338071	101.7909485	-430.9157715
495006479.6	332.8068237	73.52917022	88.46666718	-311.7457581
78501566.72	148.7810974	44.26294741	56.27418556	-187.60672
227677612.2	357.125061	71.79374899	85.88311768	-380.5112
217906477.4	243.8418884	78.31144603	97.90583038	-355.8488464
214886061	280.0335388	64.18280295	86.85516663	-305.6187439
298052282.2	276.5837708	60.38904104	68.58416443	-386.2288818
203991574.1	334.2724609	63.32625178	77.00811005	-367.423584
487130696.8	361.7710571	71.85870999	80.67640686	-463.0154419
651898037.6	326.3875732	82.18071807	106.9879532	-335.0855103
677689979.3	345.2111206	82.51938535	101.1100922	-402.9944153
202303008	277.6985474	70.30232489	73.57274628	-313.4804077
57384824.84	239.3348083	61.38709701	104.5628639	-195.4401855
104070971.9	208.3543549	59.46612975	71.11531143	-319.6821899
348616727	292.46875	56.92887002	69.51438446	-382.2910767
172237085	210.1162872	57.10800989	54.99172211	-386.9950256
385731865.7	309.8413391	61.33954897	74.09333191	-346.7888794
148056182.1	248.5604553	68.43100896	89.509552	-293.8186035
511920419.6	295.2165527	69.39635133	91.13517914	-368.2958679
318853554.6	274.2611084	79.08839358	94.0469162	-390.8244934
467938745.8	297.4765015	72.70125584	92.91249084	-426.0569153
183898356.6	320.7892151	52.92066059	61.22141228	-364.8858643
251984951.4	321.8248596	82.47614718	93.46879196	-428.7754211
116919380.1	242.2658386	55.51664437	69.62420654	-203.225708
418447487	423.8612671	72.59238875	96.11777496	-390.1729431
718855498.4	395.440094	69.65806998	82.65044556	-415.5994263
365205993.9	298.2905884	77.1753307	93.73206329	-308.0210266
189170287	227.1293945	61.24295583	79.34949493	-265.7380676
56599581.19	211.3493347	59.6071999	83.30748672	-188.4399414
255390422	345.0290833	78.29561824	106.4919022	-332.6635742
162192128	272.5860901	75.35119949	87.5645462	-419.578125
440461343.3	333.2165222	67.63653457	83.46941757	-379.7304382
160154902.8	235.809433	67.73123189	80.08257294	-360.647583
413116247.7	313.5614319	78.12267201	89.75895233	-379.6959839
575571990.8	259.3541565	74.82030494	96.84822464	-422.5763855
172180487.1	317.8051453	55.62513629	67.29118347	-270.4576111
526939094.9	461.3166199	64.58353401	87.42329102	-293.414032
701903122.2	248.7003784	63.18774508	77.31378937	-369.6643372
739049501.3	376.8198853	80.13978249	106.1934776	-385.0321045
345377603.3	295.5075684	74.07882188	87.85605621	-372.7382202
240313160.6	426.0732727	61.47682639	65.78447113	-284.666748
192177172	287.6713257	72.37011932	90.98389282	-308.7610779
370184808.7	276.1455688	64.69272839	72.64882278	-358.8304138
567250430.4	307.1285706	68.6877337	75.79524536	-328.6771545
228084134.7	342.8317261	78.75481266	87.87976761	-478.9613647
559689700.4	340.1858215	88.32751215	102.2972549	-389.276062
501172672.8	371.0576172	86.61875985	105.7240417	-434.025116
34680200.27	224.4296722	49.55734963	70.37180786	-135.0307007
584504605.1	283.0422974	74.86967567	96.31684723	-350.9107666
307003710.3	215.3105316	57.2104051	74.48565598	-235.0428009
239203332.4	346.6518555	78.76578887	100.6239548	-318.178894
320495059.2	265.9486084	62.45652416	77.50653839	-334.1618958
28828439.88	160.2180328	50.96919531	82.65687561	-180.103363
68110171.08	348.5171509	66.0632879	70.48061218	-335.7558594
353922881.9	234.8352356	63.89285372	82.67012787	-303.4981995
296988327.1	301.5710754	99.77153844	114.3310211	-478.1740112
290707895.3	340.6220703	83.02445178	99.03467102	-351.8858948
734430476.7	374.7527771	79.04950042	89.84260864	-401.6929626
435016372.8	312.6048279	58.51200201	78.14875412	-315.1452637
258224121.8	380.0305176	104.0136628	125.4745377	-374.4556274
110538359	248.6178894	48.1890615	57.48488617	-294.8751831
30585522.6	187.574585	51.44630477	68.93471375	-238.0727692
69292733.1	319.2892456	53.75953218	59.87220421	-314.9342041
317235155.1	371.5048218	100.9034157	130.1429428	-389.59198

log.sigma.1.0.mm.3D_firstorder_Entropy	log.sigma.1.0.mm.3D_firstorder_StandardDeviation	log.sigma.1.0.mm.3D_firstorder_Range	log.sigma.1.0.mm.3D_firstorder_Variance	log.sigma.1.0.mm.3D_firstorder_10Percentile
3.508098411	74.35663714	703.1562805	5528.909486	-66.2370575
3.281224649	66.1951953	674.3767395	4381.80388	-58.62455673
3.579876387	77.1389386	553.9130554	5950.415848	-99.21337204
3.550263695	79.1173549	721.3733826	6259.555846	-71.79260864
2.978493318	53.87694733	447.9181213	2902.725453	-45.80726089
3.221397491	64.68748257	637.6401062	4184.470402	-68.29458542
2.766859206	46.25479551	375.4052277	2139.506107	-34.65855637
3.174493575	63.23914917	665.8399658	3999.189988	-53.03120117
3.151995502	59.52045409	655.9654236	3542.684456	-49.47056885
3.384472441	66.25782994	651.8270264	4390.100028	-70.49717102
3.0328821	57.70550731	599.505127	3329.925574	-50.8766449
3.771545655	89.17062194	681.1968689	7951.399817	-90.30279694
3.061497738	55.33470194	578.3249969	3061.929239	-48.19033813
3.163350149	55.27546873	418.5369873	3055.377443	-62.62110596
3.290844569	60.11605087	478.7949677	3613.939573	-63.1291626
3.113557871	58.62125234	588.4951172	3436.451226	-58.69541321
3.024141798	50.76443426	480.6492004	2577.027786	-54.65931168
2.933339617	52.50139166	458.6974335	2756.396127	-34.19351387
3.142524513	57.24839595	465.2790833	3277.378839	-41.90359306
2.946908935	51.92346964	550.9508972	2696.046699	-46.92086411
3.621550034	75.99463354	792.4363403	5775.184328	-91.48638077
3.218865952	59.50011653	607.1076965	3540.263867	-62.57843018
3.270106814	62.01047728	486.3001709	3845.299292	-73.37667084
3.217879502	65.53928691	644.8156128	4295.398128	-51.35797768
3.329482784	62.19427175	689.1683655	3868.127439	-76.34468002
3.603414751	79.14991371	603.8023376	6264.70884	-77.76124191
3.426576889	68.79695475	562.7398682	4733.020982	-69.92088318
3.578947298	75.01806028	618.5432739	5627.709369	-89.22626801
2.882124162	51.33836336	516.6503143	2635.627552	-46.2531868
3.244836316	65.8511978	735.5879822	4336.380251	-64.55532532
3.40503592	66.81528773	588.2989502	4464.282675	-77.4712265
2.973662181	50.9792902	564.9243469	2598.88803	-40.16707802
3.378304116	73.46055644	826.4758911	5396.453352	-65.4931488
3.138913019	67.57111428	634.9233398	4565.855485	-39.77873611
3.585857425	82.72764136	693.8266907	6843.862644	-100.719973
3.083996831	62.32840554	662.1364441	3884.830137	-53.72125931
3.644128497	82.50998881	784.7841492	6807.898254	-88.78395538
3.471029483	72.86893403	644.5525818	5309.881547	-81.33841705
2.832714483	43.79966713	336.3878174	1918.410841	-48.82489815
3.250972352	70.61709543	737.636261	4986.774167	-54.47667694
3.565458937	77.27022448	599.6907349	5970.687592	-78.67006683
3.126586556	62.32946365	585.6522827	3884.962038	-44.53433189
3.054435071	59.56460399	662.8126526	3547.942049	-45.77383423
3.079814868	61.21262134	701.6960449	3746.985012	-40.95240402
3.394692871	71.60745294	824.786499	5127.627317	-76.04525833
3.697226232	81.22357913	661.4730835	6597.269807	-90.92375336
3.646040398	81.60763646	748.2055359	6659.806328	-86.47671509
3.414894243	70.29745354	591.1789551	4941.731974	-88.28335571
3.010792468	58.78102496	434.7749939	3455.208896	-36.73392982
3.075860618	58.40294568	528.0365448	3410.904064	-46.79293213
3.060991397	55.98041664	674.7598267	3133.807047	-47.38549805
2.897430707	57.02562887	597.1113129	3251.922348	-49.39606857
3.130888635	60.18593824	656.6302185	3622.347162	-51.44541473
3.413201902	67.78950558	542.3790588	4595.417067	-71.06228638
3.369145564	68.50091402	663.5124207	4692.375222	-65.24367065
3.551466555	78.92444357	665.0856018	6229.067794	-93.95272446
3.438909638	71.6505349	723.5334167	5133.799151	-67.5109024
2.843695829	51.92877007	685.6750793	2696.597161	-36.24674149
3.535395715	81.84248832	750.6002808	6698.192894	-76.25287247
3.128078403	54.83517945	445.4915466	3006.896905	-59.37284317
3.374495835	70.4864725	814.0342102	4968.342805	-53.7743988
3.294192382	68.98206604	811.0395203	4758.525436	-63.87293396
3.549083388	76.37330541	606.311615	5832.881779	-78.95638275
3.192076908	60.29624481	492.8674622	3635.637138	-59.73107948
3.174212138	57.6398289	399.7892761	3322.349875	-58.45367966
3.580349635	76.0926659	677.6926575	5790.093804	-70.84978333
3.449312702	74.44382277	692.1642151	5541.882748	-65.23275375
3.183292459	66.2147698	712.9469604	4384.39574	-45.57172203
3.315955974	67.16056535	596.457016	4510.541538	-75.11971893
3.527492267	77.73291504	693.2574158	6042.406081	-83.88631439
3.559075507	74.17823486	681.930542	5502.410527	-78.25128021
2.976997445	54.26511457	588.2627563	2944.702659	-40.05021286
3.259501995	62.81669612	754.7306519	3945.937312	-53.96414261
3.246650916	62.60156687	618.3647156	3918.956175	-64.46700287
3.629287482	78.64659163	761.8519897	6185.286375	-78.15788879
3.437956051	73.67307018	668.2457886	5427.721269	-78.90962219
3.152311947	61.19938782	710.7400208	3745.36507	-62.85838318
3.426591563	70.2180095	596.4324036	4930.568858	-70.75173187
3.216097939	64.39157382	634.9759827	4146.274779	-66.58179855
3.346735527	68.51664414	635.8057251	4694.530525	-77.78372345
3.511488444	78.33888313	821.7930908	6136.98061	-84.09751129
3.752205181	87.95503313	729.4618835	7736.087853	-105.0750381
3.655865666	85.98442786	805.0827332	7393.321834	-86.61840897
2.698932623	46.99117242	359.4603729	2208.170286	-26.20723228
3.531470414	73.97247759	633.953064	5471.927441	-86.64614258
3.177184284	56.38513149	450.3533325	3179.283053	-60.44796715
3.583093446	77.44395801	664.8307495	5997.566631	-81.41435318
3.161705178	61.36175742	600.1105042	3765.265274	-52.91500092
2.85923916	48.70354151	340.3213959	2372.034956	-33.6758728
3.123056305	65.31418958	684.2730103	4265.943361	-47.03272095
3.235691996	62.53182739	538.3334351	3910.229436	-54.20528412
3.785490301	98.60739316	779.7450867	9723.417985	-81.70662842
3.648784533	82.68915513	692.5079651	6837.496377	-100.1566986
3.539182701	78.66895399	776.4457397	6188.804322	-88.14658203
3.15240982	57.16480242	627.7500916	3267.814636	-49.94631195
3.986108042	104.0013195	754.486145	10816.27446	-136.1801071
2.802926573	47.40303194	543.4930725	2247.047437	-38.42815094
2.853406772	49.17470331	425.6473541	2418.151445	-31.70563049
2.844186799	53.11670504	634.2234497	2821.384354	-47.19903641
3.967162587	99.99909742	761.0968018	9999.819485	-116.5718628

log.sigma.1.0.mm.3D_firstorder_Kurtosis	log.sigma.1.0.mm.3D_firstorder_Mean	log.sigma.1.0.mm.3D_glrlm_ShortRunLowGrayLevelEmphasis	log.sigma.1.0.mm.3D_glrlm_GrayLevelVariance
5.831645379	17.21691105	0.007195686	9.555344485
6.357550241	12.32148827	0.008234779	7.859910604
4.163221594	4.084269097	0.014501424	10.13647089
6.684798935	11.46996642	0.003638687	10.79179877
5.86132938	12.90659007	0.015110702	5.25811848
7.061796005	4.146879198	0.006327766	7.484080397
5.792011157	10.34395612	0.015618341	3.982775948
7.732967708	11.84379317	0.005045906	7.238529909
6.589423444	15.07923149	0.005828168	6.367178166
5.244312844	11.64689028	0.004607166	7.682206776
8.612410513	8.709562032	0.005898442	6.013422966
5.251060741	11.69072559	0.006827176	13.52127695
6.836689444	11.04865835	0.010469073	5.519309417
3.830732145	8.21492082	0.012988332	5.352492928
4.032557388	10.92405153	0.007363471	6.179523704
6.11487067	4.343098406	0.007415543	6.272076536
4.892943886	5.305317938	0.010193301	4.621287586
6.009989167	16.39777491	0.010487568	5.088243741
4.788700189	19.33440123	0.015855	5.867866844
6.815726853	8.496291886	0.008988526	4.946906291
4.179443144	5.120309164	0.00615623	9.87065823
5.201396383	10.03741563	0.007302251	6.224392235
4.751343341	7.68908821	0.007881466	6.707496343
6.537258284	12.85951926	0.006850001	7.747130915
4.789306817	2.187575768	0.007655285	6.781218736
4.364574307	18.62466043	0.012204627	10.73143875
4.747236827	10.31516146	0.006737338	8.040118458
3.965726986	10.44095628	0.008285165	9.64846036
6.962369762	7.215887537	0.005714173	5.048430533
6.472844238	8.216714707	0.004645156	7.788234975
4.456089884	8.219173455	0.006317873	7.783162743
6.060265017	15.08005874	0.006123425	4.702696898
7.684925767	9.395126173	0.005104875	9.602467391
7.42581237	21.15983358	0.008408778	8.332046661
5.076181988	7.251029236	0.007723618	11.63347751
8.455457276	7.906570982	0.005453785	7.168001939
5.278346147	10.16521664	0.003392232	11.84766047
5.036083068	9.83144576	0.007175395	9.245150233
4.176064576	6.387305603	0.015627115	3.492756263
8.267621693	12.94481469	0.004611325	9.176304156
5.304128634	12.72772517	0.005662004	10.12115319
6.840653084	15.31241837	0.006349018	7.128975847
8.476291283	9.944557783	0.004058015	6.435047247
8.35513269	16.22433826	0.004580102	6.955586368
7.595920447	6.003905804	0.003146358	9.112587448
4.089834878	12.50602321	0.005853889	11.22845357
5.169900673	12.23285047	0.003820108	11.41331787
5.073617531	0.827593806	0.008178293	8.546535507
5.227067662	17.69651896	0.018422619	6.397105157
7.989922241	11.19448628	0.008035657	6.183227159
7.748536087	10.34839096	0.003798971	5.664977787
10.11510822	3.06634071	0.004932997	6.154069127
7.514184157	11.84031695	0.0052226	6.565465029
4.808404079	9.348043703	0.008622787	7.896612848
5.947961709	11.11208156	0.00475612	8.328538672
5.282898427	5.089813958	0.005286119	11.26029738
6.700624455	12.31557756	0.00336267	8.947809662
10.00556266	10.19799767	0.004438667	5.03619317
7.239519168	10.20401684	0.004186084	11.79238288
4.560558073	8.671845031	0.014347926	5.314480746
7.418286375	17.35834379	0.004548588	8.793411844
7.021750494	9.680975043	0.00349862	8.615828035
4.960348632	11.09729199	0.00792012	10.0357792
5.320510157	10.72671899	0.009378705	6.529404569
4.049593562	15.18777154	0.019049387	5.885490571
4.982392063	18.44207234	0.005711535	9.903152457
6.736991515	11.65849542	0.004398616	9.718666237
9.185674942	13.79511033	0.00461813	8.028174403
6.457045678	8.77372412	0.005513485	7.914271762
5.762872826	7.793959251	0.00504434	10.67816036
4.836569569	9.780976663	0.003695998	9.498583068
7.612371508	12.22510239	0.008700376	5.492623984
5.756161906	15.00318477	0.006983979	7.003431338
6.253472415	8.586905907	0.004797731	6.983581605
5.06669834	15.39799865	0.004277176	10.6594628
5.832312201	7.742776131	0.005349927	9.551043712
6.829241708	5.833962081	0.007970156	6.867607023
4.946185499	17.51757155	0.006539722	8.439216089
6.964607159	6.234927959	0.00533255	7.49222711
5.787978689	4.845021784	0.0057076	8.366065277
6.766375058	8.083310418	0.003208206	10.74984461
4.620758905	8.103181495	0.004827539	13.11976907
6.328883561	10.46363815	0.004232998	12.8375518
6.700731393	15.74041349	0.027340177	4.193470051
4.518165679	11.55598954	0.00475409	9.392094091
4.478720606	9.682324034	0.011374034	5.601421808
4.966780604	14.36951163	0.007277789	10.32639711
7.202588527	11.64268594	0.005373405	6.750200782
4.577876887	15.02743872	0.016130457	4.293173856
9.630080533	9.920415683	0.007530709	7.602705301
6.386484992	13.11744335	0.006548257	6.98364742
6.95969864	15.19677259	0.004019595	16.75438913
4.731299928	7.45407387	0.005804231	11.71681273
5.403871091	7.747205579	0.003775092	10.87210348
5.709062323	12.48357898	0.005673786	5.815675699
4.064307137	1.602366858	0.010563187	18.59486135
8.324681102	8.668229976	0.006905379	4.231162999
6.939050064	15.11855908	0.01189804	4.455513257
10.68060698	8.288724	0.008189219	5.286798465
4.37483346	13.4788654	0.00716249	16.76246728

log.sigma.1.0.mm.3D_glrlm_LowGrayLevelRunEmphasis	log.sigma.1.0.mm.3D_glrlm_GrayLevelNonUniformityNormalized	log.sigma.1.0.mm.3D_glrlm_RunVariance	log.sigma.1.0.mm.3D_glrlm_GrayLevelNonUniformity
0.007557917	0.110764531	0.119221574	120.9705818
0.008718123	0.129622912	0.171858873	227.2160911
0.015111092	0.102417164	0.122258884	103.8082729
0.003828823	0.108593675	0.11236963	242.387194
0.016499189	0.15941043	0.186702935	130.7462671
0.006748389	0.136210896	0.169334627	196.0943139
0.017248993	0.183330678	0.252681347	162.0810806
0.005431728	0.140363997	0.203836407	219.39883
0.006342213	0.140016637	0.21203326	245.4201091
0.00491491	0.11698957	0.142207656	314.6423384
0.00634315	0.154753088	0.181099419	243.1870523
0.00707422	0.091594916	0.090609844	142.6178411
0.011363657	0.14948877	0.197287619	379.0963254
0.013946545	0.130994921	0.163665311	215.0060717
0.007779227	0.120312714	0.113089047	169.6066204
0.008119838	0.146381036	0.243491637	512.7332519
0.011064236	0.145845113	0.181283315	203.9129496
0.011531783	0.164173223	0.269345833	350.4230513
0.017158602	0.138128992	0.198128571	135.8071241
0.009854071	0.159233179	0.261165548	388.4320456
0.006474268	0.096741097	0.111877275	613.3024064
0.007839738	0.130293982	0.164229083	243.9383948
0.008417498	0.125252791	0.161710992	456.9805047
0.00736974	0.139883667	0.196853524	125.1248803
0.008194313	0.118615301	0.152030555	275.1418008
0.012732283	0.103037382	0.126107468	126.054784
0.007043255	0.116178101	0.095735045	237.1014845
0.00870484	0.100918355	0.117047928	308.3194685
0.006417426	0.168402273	0.316766983	470.5683441
0.005036524	0.137130449	0.198290188	376.7664571
0.006725543	0.114808748	0.130665382	427.6540793
0.006753922	0.157091183	0.21934631	674.6358018
0.005407763	0.126269362	0.151225683	283.598886
0.009042768	0.150592934	0.199484247	62.39398389
0.008014328	0.107110324	0.107460321	86.97844414
0.005901464	0.154094391	0.230123806	421.9820582
0.00360289	0.102119991	0.135750289	327.8883512
0.007574444	0.113666009	0.132217935	346.9603346
0.017043926	0.164429563	0.208060507	209.7185107
0.004972681	0.139273069	0.2119904	196.1711833
0.00590728	0.103363079	0.104131083	124.6665383
0.006894833	0.149464717	0.222429916	247.2210014
0.004431843	0.15607159	0.2189682	404.8908097
0.005007628	0.153408871	0.232156926	246.1713136
0.00337479	0.121712945	0.167842678	374.9626117
0.006136884	0.094107586	0.096053216	309.5637668
0.00402525	0.100444954	0.111529786	337.7540189
0.008602964	0.117238935	0.129507615	159.7370814
0.019992655	0.159443645	0.240747083	76.40817034
0.008555103	0.149549559	0.192618137	141.1192527
0.004160439	0.150682404	0.202202495	518.0136992
0.005407842	0.173193692	0.270942465	282.8182651
0.005663947	0.145142491	0.191873864	478.4722269
0.009067519	0.115228456	0.119328011	122.3988258
0.005108345	0.12239284	0.167849269	424.9204315
0.005617415	0.106918648	0.200829242	176.0830392
0.003576451	0.116626537	0.134542095	343.5223055
0.004933556	0.177361035	0.26277238	360.4192621
0.004381292	0.111993896	0.117861847	139.483914
0.01528677	0.138901074	0.150094277	173.3099237
0.004841118	0.123607663	0.153476232	322.5482751
0.003809972	0.132393397	0.214680662	628.1723388
0.008302883	0.106330578	0.124639764	218.6585892
0.010082426	0.133519666	0.198194585	216.8376891
0.020089741	0.134246347	0.151296834	70.18167378
0.006010277	0.103663006	0.106805872	146.1545983
0.00465408	0.115978511	0.149503805	109.3086788
0.004976423	0.141407839	0.208148797	434.6662847
0.005818133	0.125657449	0.1437757	145.479523
0.00533452	0.111891859	0.146412506	250.1147539
0.003923485	0.102597398	0.128525737	353.1014764
0.009517652	0.160169696	0.247257711	279.148044
0.007525676	0.13117939	0.169098707	540.1036656
0.005164919	0.130759177	0.183589597	745.1746754
0.004517342	0.100556908	0.11367118	390.2726208
0.005674445	0.118768157	0.143166887	247.1676051
0.008601932	0.14272171	0.203098309	290.6342066
0.00690154	0.11779001	0.110479296	145.3705125
0.005715179	0.138383351	0.183653395	395.4967449
0.006138148	0.124863458	0.179741956	486.8903776
0.003389188	0.113741701	0.139286598	138.8057702
0.005036622	0.093282952	0.095631371	228.7062336
0.004435838	0.10220374	0.123364114	228.6789372
0.030043019	0.195070357	0.251326105	85.6114014
0.005027913	0.105910002	0.115483704	371.6149536
0.012134974	0.132345869	0.151169783	407.6455286
0.007627093	0.104284992	0.111567841	135.7645594
0.005811401	0.143325308	0.179549066	380.3040647
0.017488169	0.16739421	0.203932663	59.30797218
0.007968517	0.149125466	0.168540798	75.55008585
0.007012909	0.133556738	0.166917707	377.9446628
0.004155017	0.094608943	0.100619783	96.10962488
0.006069818	0.098607214	0.106961158	140.7972914
0.004024767	0.110976312	0.155382296	430.6839918
0.0061513	0.138159944	0.168076834	571.1608492
0.010925251	0.078337741	0.111223434	63.3991883
0.007612534	0.177687216	0.222954778	265.70095
0.012782829	0.170198223	0.213458717	62.4410221
0.008834686	0.173141964	0.262562553	128.3429712
0.007336629	0.077348157	0.073199342	83.63864358

log.sigma.1.0.mm.3D_glrlm_LongRunEmphasis	log.sigma.1.0.mm.3D_glrlm_ShortRunHighGrayLevelEmphasis	log.sigma.1.0.mm.3D_glrlm_RunLengthNonUniformity	log.sigma.1.0.mm.3D_glrlm_ShortRunEmphasis
1.334126806	196.2586149	911.9833659	0.930916421
1.475406084	184.8935167	1376.719651	0.907320364
1.340868793	135.5105302	844.9436587	0.930249022
1.321159251	343.4063448	1867.096584	0.93178481
1.529501786	78.58016464	625.7535245	0.89575177
1.466784934	200.8916828	1136.204723	0.908894069
1.687283455	73.73030061	639.9752897	0.875297042
1.539370109	235.3713041	1208.983916	0.901206916
1.552066938	185.5451881	1356.459199	0.901275795
1.390372577	241.2340228	2196.010903	0.922283758
1.505600724	203.0072927	1215.530578	0.90132934
1.262838055	251.8954733	1339.298517	0.942490469
1.533080342	113.070225	1953.66666	0.899777286
1.449259151	92.71684334	1308.632946	0.912515622
1.320599332	161.6464768	1183.38829	0.932785394
1.625526695	148.9813014	2643.551628	0.891200207
1.501965105	107.352767	1085.412268	0.902554498
1.710809824	114.1990848	1549.668143	0.875999666
1.537280488	83.77362456	759.1150799	0.899511161
1.683323454	127.3508037	1796.75779	0.881483491
1.314428511	210.7717903	5335.13245	0.934124463
1.451198532	156.9987801	1489.355883	0.911764074
1.442050116	154.6481575	2922.672804	0.914154407
1.52854062	185.8464528	694.1854466	0.902000977
1.414495486	151.7280408	1876.194058	0.918531959
1.34839507	148.8810933	1017.356297	0.929405316
1.270611943	189.2731073	1755.395846	0.94243466
1.323785076	164.3116415	2565.534487	0.933265804
1.818402092	168.2525076	1985.005928	0.866199832
1.523758598	233.3893628	2140.769057	0.903932844
1.36807136	183.0177532	3054.117851	0.924060341
1.580481548	158.4649108	3277.337813	0.89534081
1.421830103	268.5385242	1800.028844	0.915318794
1.551883656	169.6464514	315.5412874	0.895003786
1.305316753	213.6850475	686.3570051	0.93550944
1.61261787	228.0205978	2053.056303	0.889232534
1.372630279	341.0780333	2644.401301	0.925635546
1.367309794	186.1693407	2513.131768	0.925584265
1.565811153	71.21777834	969.5535145	0.894298425
1.563112156	268.0969492	1077.786652	0.897197003
1.295359582	249.1532389	1023.706829	0.937413063
1.588738263	184.8986658	1253.713403	0.893457033
1.582255711	260.3209088	1968.256789	0.893864461
1.613328693	239.550558	1208.775208	0.890830943
1.456357398	362.4283876	2448.937666	0.911773906
1.277781672	221.385616	2805.972614	0.939431262
1.314047983	312.1529548	2831.374979	0.934296552
1.36975925	175.5666236	1114.091779	0.922886584
1.630177487	82.99521701	359.5666459	0.888976962
1.528771184	179.7213943	724.7070711	0.898644725
1.547275307	261.730963	2632.168155	0.897358791
1.709303478	245.3274021	1186.130903	0.876998652
1.520225286	207.7433054	2550.559136	0.901483208
1.335283264	161.2442435	885.1314516	0.930353369
1.45468928	239.2681097	2764.088847	0.91259515
1.517735371	262.3979715	1294.442659	0.907362963
1.37575279	341.4323882	2414.348656	0.923732609
1.699911012	226.8057961	1476.271155	0.87665628
1.332657096	342.1329251	1039.102659	0.930734527
1.421238494	93.29511008	1000.807183	0.915165262
1.424544762	279.2171929	2093.226338	0.915603044
1.554153073	296.0188145	3670.384085	0.901258583
1.346036902	189.5855064	1710.233969	0.929368738
1.524371436	133.9552994	1263.443808	0.903463904
1.426116354	80.34407241	417.7953537	0.913919229
1.305330048	226.7331509	1187.757299	0.934619192
1.410939691	305.6252134	762.5125299	0.91868169
1.555975938	268.1920035	2353.401431	0.897399451
1.392363122	237.303539	943.1862859	0.921595952
1.405438429	268.4271551	1809.698356	0.919022019
1.353987758	305.5191335	2855.779834	0.928570352
1.648692561	132.9612838	1296.18937	0.885842329
1.462673622	162.4938778	3259.554868	0.910264605
1.490144723	233.2763584	4485.441801	0.908109384
1.320285197	282.9188343	3255.977341	0.932914422
1.399213196	237.314083	1685.055679	0.919198209
1.54314546	151.1427382	1568.889302	0.89968257
1.32389205	194.966547	1026.055125	0.929537317
1.50013778	229.8146434	2231.009729	0.904822223
1.485485157	202.1747565	3065.236765	0.907561513
1.386296048	409.2810792	995.4050548	0.922021603
1.2709663	277.7274277	2107.445148	0.942204595
1.346769325	343.4380427	1853.94828	0.92818577
1.678347242	49.84982106	320.5432775	0.878326485
1.32759758	244.5349529	2929.219458	0.931063829
1.424667574	112.8742593	2463.775548	0.914407726
1.316029982	193.5203602	1094.960187	0.933714588
1.497254463	208.1886739	2065.365563	0.903574634
1.554143714	78.8345872	271.111858	0.896619969
1.475043345	206.6505336	397.249786	0.906035547
1.455856689	184.9054112	2245.708153	0.911382577
1.284892415	432.4903068	865.2433593	0.938790296
1.304731391	242.5087038	1204.925395	0.935148454
1.414670601	300.321763	3154.39329	0.920541029
1.465694125	183.0930414	3255.224576	0.908326309
1.306745321	244.3874199	686.3918261	0.936930296
1.615294371	149.5126786	1105.923828	0.884084985
1.579004517	114.1765654	277.0786725	0.891921702
1.700948115	170.8132355	536.4306019	0.87520406
1.212896229	292.2057012	954.4905351	0.952477119

log.sigma.1.0.mm.3D_glrlm_LongRunHighGrayLevelEmphasis	log.sigma.1.0.mm.3D_glrlm_RunPercentage	log.sigma.1.0.mm.3D_glrlm_LongRunLowGrayLevelEmphasis	log.sigma.1.0.mm.3D_glrlm_RunEntropy
278.8570317	0.907666731	0.0093018	4.017423713
298.4790258	0.876230769	0.01118801	3.938759364
195.181339	0.906357507	0.01798925	4.088061528
488.5227767	0.90984352	0.004720848	4.057698742
129.2742963	0.863905325	0.023556658	3.678636813
326.9869572	0.878698225	0.008886233	3.865461387
139.1004807	0.835965359	0.026201339	3.590332318
402.4175774	0.865757511	0.007538865	3.881062088
315.2142876	0.864445372	0.009204897	3.856601829
364.2004085	0.895463511	0.006455252	3.952178524
341.5555048	0.86943936	0.008608627	3.719813192
338.275002	0.923944305	0.008183205	4.204726813
190.2515434	0.865666938	0.016052703	3.765292408
147.5338233	0.883249151	0.018782827	3.770442191
228.2174291	0.910653946	0.009762921	3.779156636
268.1545967	0.851957242	0.012157931	3.882690001
179.2705999	0.870932183	0.015490011	3.684619956
218.3114636	0.834185361	0.017476236	3.774458682
138.0441162	0.865452329	0.024074762	3.825369553
243.1207691	0.840505926	0.014796461	3.75459197
296.2870696	0.912119535	0.007987812	4.112817842
250.1407979	0.882530011	0.010578854	3.838189872
247.346129	0.885222033	0.011148064	3.879399869
307.7755263	0.8678118	0.010169242	3.910147062
232.4958456	0.890641616	0.010925436	3.910591635
213.7979203	0.904756486	0.015308339	4.123291112
255.9472932	0.922902968	0.008474793	3.867263376
233.0171143	0.910558294	0.010727336	4.074492114
354.4159945	0.820807025	0.010694722	3.78365259
391.0319455	0.869335476	0.007160813	3.945908379
272.2155858	0.899212569	0.008686497	3.957178631
275.9557647	0.858569231	0.010243656	3.708270684
417.60067	0.887305109	0.006907727	3.994456216
282.1938383	0.860866784	0.012359062	3.85717362
302.4069119	0.914241164	0.009380557	4.061429439
412.6999461	0.850960044	0.008363748	3.866950717
506.6798652	0.899497812	0.004651393	4.198882916
275.6514117	0.900052174	0.009519072	4.023661049
124.9578785	0.85916442	0.024585927	3.541800923
463.7544146	0.860682716	0.00694522	3.988741314
345.4561658	0.916413374	0.007059968	4.031568294
324.3327085	0.855908584	0.009903189	3.883132912
459.4719788	0.856852431	0.006480762	3.806390005
427.5592206	0.851461702	0.00741061	3.852288737
579.6996775	0.881533178	0.004552372	4.033064779
301.06861	0.920086068	0.007425468	4.151195036
439.7391984	0.912162444	0.005003046	4.143252604
263.1486653	0.898518368	0.010625709	3.966285
144.3678511	0.84929078	0.028588159	3.774017721
306.1551388	0.865561044	0.01126231	3.772765445
450.2173447	0.862700803	0.006084883	3.782592869
483.5036419	0.834473808	0.008069262	3.749096369
349.2414659	0.868033468	0.007991929	3.831678177
231.153861	0.907048545	0.011185896	3.920240515
379.8126684	0.881733456	0.006931419	4.005242738
438.9459143	0.871985007	0.007466038	4.223893251
507.3528558	0.898116217	0.004627571	3.999091384
435.7563282	0.83524165	0.007733898	3.682387096
490.9678012	0.907602601	0.005314987	4.049807495
144.2094758	0.887872981	0.019918111	3.719069762
430.2796706	0.887191693	0.006311696	3.988388813
508.2944991	0.864504914	0.005553233	4.017297544
274.1982311	0.905328049	0.010166759	4.068494649
225.7319255	0.86904958	0.013858715	3.871120699
126.2255668	0.886049544	0.025223562	3.762528432
312.9126828	0.913654719	0.007400071	4.05734382
466.4396193	0.890722699	0.005943517	4.034944067
462.0155138	0.86174986	0.006912741	3.914632305
362.4073763	0.895234696	0.007337337	3.878725348
411.3015195	0.891534473	0.006776981	4.120471874
445.2530764	0.90366435	0.005048254	4.088286754
243.6823466	0.845370059	0.014142199	3.771015698
258.979881	0.879757345	0.010302198	3.905495034
383.9241868	0.875134388	0.007116828	3.912543722
398.9312095	0.910587301	0.005656086	4.130720382
362.6891554	0.892617893	0.007275246	4.02440495
260.0187386	0.86441287	0.011980938	3.865170578
278.3978409	0.908077206	0.008550388	3.931919512
383.2613748	0.872147084	0.00771457	3.900160477
332.5386483	0.875520392	0.008381963	4.015787442
615.4615647	0.895854513	0.004283986	4.078299026
376.3928151	0.922671608	0.006008867	4.195956582
498.5350054	0.904296996	0.005412362	4.189808858
88.60130605	0.838652743	0.044993037	3.502971615
351.1743546	0.908477074	0.006320308	4.041685486
176.2648905	0.88651964	0.015877765	3.781145444
273.988972	0.911549235	0.009275	4.072064351
343.6983754	0.871786446	0.008062768	3.842747322
131.6876891	0.86168819	0.024720786	3.552199769
336.3811551	0.87609795	0.010160018	3.762066505
295.8721203	0.88112977	0.009379202	3.873882767
594.2056946	0.919248173	0.004783508	4.239273496
341.1669074	0.914015562	0.007307425	4.12560845
464.0010361	0.891374649	0.005320554	4.136680141
293.9973328	0.878322273	0.00857161	3.800420762
336.2327593	0.915158371	0.012599297	4.448789263
272.1928185	0.847942755	0.011354721	3.580786979
200.4577043	0.856757728	0.017526727	3.566846093
334.0399148	0.834546085	0.012447302	3.670892105
370.7729971	0.936941741	0.008111249	4.327459836

log.sigma.1.0.mm.3D_glrlm_HighGrayLevelRunEmphasis	log.sigma.1.0.mm.3D_glrlm_RunLengthNonUniformityNormalized	log.sigma.1.0.mm.3D_glszm_GrayLevelVariance
210.4532426	0.834772362	20.50993096
203.4787118	0.785259311	18.5853548
145.6341464	0.833378557	18.83859406
368.8379336	0.836266321	23.77916622
86.97375652	0.762383628	14.4824
221.4476121	0.788687372	18.00217588
83.81876772	0.723333349	11.68783053
261.3535741	0.773271194	17.97846877
205.2783119	0.773641213	15.25471441
261.6717217	0.816279485	16.57209906
225.6074828	0.773220357	16.5365928
267.3494798	0.859921285	24.72412023
125.2933265	0.770150477	15.07137917
101.6378214	0.796386526	11.05656589
173.21149	0.838844755	12.62066506
166.762655	0.754071432	15.58053858
119.000788	0.775794986	11.22842773
129.6814823	0.725123017	13.39592156
92.39882428	0.770825237	13.77617839
144.505038	0.735498958	13.01620645
225.6252683	0.841390596	18.42723637
172.2830035	0.794734838	14.20356269
169.7203013	0.799951309	13.47460377
205.0070228	0.775471577	19.66734271
165.0443712	0.808429265	14.71378121
159.8706796	0.831466486	20.624907
200.9990539	0.859939029	14.5815591
176.0689927	0.839577483	17.71394521
194.4423387	0.708443594	14.04709576
257.8699861	0.778895817	17.57138555
198.2890939	0.819744347	16.17720693
176.4742946	0.762227188	12.20696671
293.4940874	0.801374277	24.29110193
187.6624522	0.761195927	21.60826389
229.179777	0.844829334	21.75664268
256.3372926	0.749593272	19.72184796
368.6193317	0.823428945	23.65560216
201.2277246	0.82318353	19.76946028
79.69069106	0.759613282	9.068916324
298.4260444	0.765171622	24.16266197
266.0149693	0.848660903	19.7126521
206.3188508	0.757847349	19.26167532
291.1022648	0.758553696	18.59592409
268.0599408	0.753079884	19.538975
397.6769808	0.794555085	21.47040333
235.6288253	0.852868897	20.52331529
334.2720578	0.841865218	22.36357035
190.7101311	0.817330306	17.82982684
92.24742918	0.74986404	13.71300337
200.1182611	0.767761602	16.86525852
291.5555454	0.76541037	15.23694257
280.5318394	0.726298725	17.28324324
230.3391207	0.773525693	17.84931898
173.2608802	0.833186279	16.33652481
261.9635727	0.796081564	18.43591331
289.3383574	0.785873461	22.72164063
369.4595766	0.819343617	20.46173524
258.1174817	0.725932612	16.13564516
367.8035193	0.83417468	24.57321742
101.8805276	0.801451258	11.82827805
304.3334715	0.802053229	22.08799363
328.1879117	0.773412638	20.89081202
203.9369865	0.831373128	20.27131867
148.2641424	0.777741937	14.09151346
88.07866785	0.798804856	13.58487654
241.9446549	0.842292917	19.8728726
332.2679519	0.808775724	21.77831307
298.4788496	0.765495218	21.20080768
258.1490425	0.814551173	17.59765986
292.2234911	0.809368349	23.23588439
329.038118	0.829613476	18.55312222
149.5966337	0.743400296	15.96078229
178.1806047	0.791364275	16.62471476
257.1000175	0.786982604	16.96798456
303.0299318	0.838752317	21.15900416
258.4948288	0.809531588	21.18480001
168.137582	0.770221645	16.38153777
209.8524966	0.831165217	17.19433853
254.3503311	0.78036165	19.13625358
223.0493397	0.785858889	19.47037941
443.9909746	0.815516109	22.9515606
295.0717794	0.859368354	23.64727684
370.1077822	0.828511005	26.81146782
55.74738306	0.729530676	12.34084865
263.0762297	0.834692703	17.99975904
123.5380161	0.799623986	12.18381301
207.506261	0.840800145	20.51974179
230.2455547	0.777916759	17.29787541
87.15058209	0.764527118	10.15920927
228.0798925	0.783308442	19.14925373
202.995479	0.793460154	16.82220133
461.0714702	0.8514854	34.4061915
259.8619804	0.843658011	21.02514155
326.6905155	0.812756357	22.6518636
201.373014	0.787148481	14.72422199
260.0750398	0.847919466	30.70363065
168.9800411	0.739395048	13.94657494
127.8240366	0.75490196	14.77631579
195.5178864	0.723294919	15.56359677
306.539333	0.882535772	27.77374427

log.sigma.1.0.mm.3D_glszm_SmallAreaHighGrayLevelEmphasis	log.sigma.1.0.mm.3D_glszm_GrayLevelNonUniformityNormalized	log.sigma.1.0.mm.3D_glszm_SizeZoneNonUniformityNormalized
150.8925399	0.070690923	0.441574242
133.5277529	0.0751519	0.442394135
103.4472851	0.065556712	0.427881836
242.7794168	0.065297778	0.460295111
72.62798237	0.08505	0.48875
145.5140084	0.073666703	0.471021468
56.04286171	0.109306063	0.469685902
164.6098888	0.07986491	0.478863311
135.9360352	0.083435764	0.442066071
174.1823547	0.074499942	0.423876619
142.0434341	0.087132964	0.434833795
188.3768323	0.060006409	0.476155664
90.87162123	0.088994543	0.442146195
62.10034137	0.088902364	0.353341928
118.6143899	0.086689049	0.424964583
114.4755801	0.079067745	0.438014733
72.07305297	0.10921875	0.353222656
88.65362964	0.090456594	0.443719691
67.14427749	0.086720492	0.402968089
85.64476992	0.089594533	0.414083513
150.1527752	0.067561194	0.414496644
114.596662	0.082399359	0.446829093
96.57279593	0.080769392	0.381027021
155.1494489	0.076197344	0.469563127
108.8035072	0.077920265	0.396348539
109.6334862	0.063795773	0.432303083
138.3061315	0.075544064	0.458205691
118.6464268	0.067831963	0.433983263
121.5401846	0.089546022	0.428467921
171.5764439	0.072491075	0.416465759
129.5137399	0.0758881	0.415199471
121.2133463	0.090524276	0.414487863
207.8403535	0.069292929	0.489320478
157.3471167	0.07125	0.454444444
150.8034677	0.064358354	0.505220934
171.1586522	0.076029938	0.465973638
254.7282016	0.061606708	0.456984858
133.352307	0.071019126	0.439627275
53.16561766	0.112455418	0.428230453
209.7775462	0.070260688	0.498813897
169.6244961	0.068456597	0.424828737
140.4970636	0.078127999	0.479227111
196.8568476	0.082886821	0.481087332
195.3422012	0.0815625	0.5136625
266.6026046	0.071121845	0.460548243
162.253221	0.064571095	0.438264723
227.8140859	0.062423263	0.461079875
119.3447976	0.072298891	0.409271552
72.00449657	0.085002976	0.455762745
129.1607075	0.086571955	0.455091865
201.3115394	0.084735221	0.46363676
175.9721462	0.086822498	0.478276114
156.4741759	0.082186568	0.480502387
118.0298917	0.073874392	0.428846674
175.4841814	0.074617965	0.442891686
190.0448035	0.061843861	0.468741512
259.2795575	0.072943761	0.46875929
178.1860438	0.098410239	0.450527167
250.0455046	0.068740647	0.454058626
69.59878442	0.092560263	0.417256474
219.1803523	0.072742369	0.467414997
226.8348148	0.071037883	0.469586796
146.5038242	0.068279144	0.459164505
89.86539875	0.0854463	0.409690598
53.73711568	0.093074846	0.490354938
166.1885919	0.068288252	0.402167736
246.9349448	0.065847416	0.521528849
204.7687424	0.076766392	0.467064425
162.2166394	0.077971152	0.484004624
194.6533042	0.064146347	0.459262042
218.6038748	0.067326389	0.429220833
103.8147434	0.088278492	0.429281868
120.5744227	0.077597907	0.447446762
167.6591963	0.07690079	0.440008283
206.8402394	0.064976324	0.434457136
164.6295486	0.068394214	0.44802979
119.5651055	0.081163425	0.497526009
132.0328157	0.077359158	0.409547739
175.1030398	0.078528771	0.497311291
149.9086332	0.070617381	0.454984947
304.393531	0.066037969	0.457265909
195.6160141	0.062035932	0.457791701
252.8243368	0.060764191	0.468323315
49.21226893	0.09931784	0.411723899
170.5712174	0.070005126	0.434511032
78.25969831	0.094510926	0.458489381
136.8051999	0.068785306	0.44266744
151.418336	0.08428695	0.464599222
65.34527035	0.110001063	0.443086407
134.2045344	0.083092003	0.432724438
133.0505478	0.079772017	0.470311361
310.9492718	0.055665077	0.471558013
166.0004984	0.066024157	0.440047958
218.7613369	0.064584489	0.471283158
136.9463796	0.087734645	0.443899636
196.0525936	0.052799896	0.429566318
115.9001337	0.100409313	0.446273821
81.04631474	0.08933518	0.431094183
133.7820525	0.103459159	0.481062997
210.7645272	0.056251562	0.432153269

log.sigma.1.0.mm.3D_glszm_SizeZoneNonUniformity	log.sigma.1.0.mm.3D_glszm_GrayLevelNonUniformity	log.sigma.1.0.mm.3D_glszm_LargeAreaEmphasis	log.sigma.1.0.mm.3D_glszm_ZoneVariance
166.0319149	26.57978723	207.9441489	197.7075529
224.7362205	38.17716535	644.1968504	628.6968194
159.172043	24.38709677	108.0860215	99.05373454
345.2213333	48.97333333	264.4813333	253.7840729
97.75	17.01	572.725	550.209975
197.3579952	30.86634845	495.8902148	480.6075267
86.89189189	20.22162162	1083.12973	1050.485493
208.7844037	34.82110092	770.1123853	752.9735449
209.0972516	39.46511628	844.6575053	826.292747
361.9906323	63.62295082	330.3126464	317.9476235
165.2368421	33.11052632	931.5078947	908.895367
300.9303797	37.92405063	51.3085443	44.20024585
267.9405941	53.93069307	1312.5033	1289.142189
156.530474	39.38374718	387.255079	369.6643448
192.9339207	39.35682819	242.2643172	230.6383202
398.5934066	71.95164835	1049.012088	1028.62339
113.03125	34.95	708.471875	683.3153809
214.7603306	43.78099174	1704.673554	1676.741018
102.7568627	22.11372549	471.5529412	451.7067897
218.2220114	47.21631879	1760.897533	1730.595329
937.1769129	152.7558602	104.9429456	95.49432339
240.394052	44.33085502	577.5260223	561.9836687
390.9337232	82.86939571	683.67154	667.5387118
121.1472868	19.65891473	416.3488372	400.4107926
261.9863843	51.50529501	414.2178517	398.6983047
192.8071749	28.4529148	100.3408072	91.15148103
370.230198	61.03960396	73.06064356	65.57283446
496.0428696	77.53193351	133.6491689	125.0334288
272.9340659	57.04081633	2697.546311	2669.073951
348.5818399	60.67502987	887.6642772	873.4197402
479.9705882	87.7266436	433.6193772	420.7811658
431.8963532	94.32629559	2203.21881	2180.193549
322.9515152	45.73333333	766.519697	751.8136341
54.53333333	8.55	261.575	245.5082639
156.1132686	19.88673139	132.2459547	123.9873064
322.4537572	52.61271676	1864.359827	1842.748077
509.5381166	68.69147982	428.935426	418.6896853
432.1536114	69.81180061	346.2024415	334.3023981
115.6222222	30.36296296	884.3333333	854.1240604
197.530303	27.82323232	786.5909091	769.5231609
192.8722467	31.07929515	61.30396476	52.90162821
204.6299766	33.36065574	1030.018735	1009.546819
295.3876221	50.89250814	1883.737785	1859.433206
205.465	32.625	1137.085	1114.9009
419.0989011	64.72087912	722.5494505	707.8072262
536.8742857	79.09959184	102.6522449	94.13537693
573.1222848	77.59211585	178.616251	169.8226166
175.1682243	30.94392523	245.7990654	233.2529042
64.71830986	12.07042254	332.3239437	316.5485023
110.1322314	20.95041322	509.4132231	489.126016
390.8457888	71.43179122	2014.785291	1992.450416
176.9621622	32.12432432	1652.918919	1624.97198
420.9200913	71.99543379	1631.697489	1612.909816
147.5232558	25.4127907	181.9040698	170.3163788
465.9220532	78.49809886	973.4096958	959.4041722
272.8075601	35.99312715	155.4020619	144.878615
435.0086207	67.69181034	400.4170259	387.9320737
188.7708831	41.23389021	2222.171838	2188.481986
202.9642058	30.72706935	181.5346756	172.1137586
142.2844575	31.56304985	422.9648094	405.9884762
359.4421326	55.93888166	579.3927178	564.7663204
642.3947368	97.17982456	1858.679094	1842.591254
327.3842917	48.68302945	188.2622721	178.1171983
189.2770563	39.47619048	281.004329	264.6561346
70.61111111	13.40277778	252.4166667	235.6294367
197.4643585	33.52953157	81.13441955	71.25869728
170.0184049	21.46625767	188.9815951	178.4489819
400.2742124	65.78879813	1388.898483	1371.584339
173.7576602	27.99164345	388.7827298	375.8106936
333.4242424	46.57024793	525.3953168	513.4709473
515.065	80.79166667	120.1866667	110.1166222
169.1370558	34.78172589	1433.423858	1406.060869
518.1433506	89.85837651	1234.287565	1217.96122
674.0926893	117.8120104	1914.542428	1896.479928
592.165077	88.56272927	133.3206163	123.5429507
289.4272446	44.18266254	565.0789474	552.0586917
289.5601375	47.2371134	1033.06701	1016.693724
163	30.78894472	215.8115578	204.1522499
380.4431373	60.0745098	1366.10719	1347.768643
514.5879752	79.86825818	1216.158267	1200.656537
183.8208955	26.54726368	237.3432836	225.864335
445.889117	60.42299795	94.37063655	86.92905586
370.4437421	48.06447535	304.1719343	294.3895116
38.29032258	9.23655914	566.311828	534.6863221
531.8415033	85.68627451	382.5343137	372.5788452
391.091442	80.61781946	994.1148886	977.5281284
199.6430155	31.02217295	147.2062084	137.180771
316.3920705	57.39941263	1290.259912	1270.293056
42.97938144	10.67010309	265.3505155	247.3973855
57.98507463	11.13432836	242.2537313	223.6480285
360.7288136	61.1851369	1124.473272	1106.946987
189.0947631	22.32169576	66.70074813	59.1073563
242.4664247	36.37931034	94.99818512	86.96183478
615.0245211	84.28275862	814.932567	803.806125
480.7433056	95.0166205	1576.581717	1557.699764
150.7777778	18.53276353	62.37606838	56.03313285
139.6837061	31.42811502	1333.747604	1302.021558
32.76315789	6.789473684	348.8421053	317.1274238
85.62921348	18.41573034	715.3370787	690.449312
211.755102	27.56326531	21.43673469	15.89022907

log.sigma.1.0.mm.3D_glszm_ZonePercentage	log.sigma.1.0.mm.3D_glszm_LargeAreaLowGrayLevelEmphasis	log.sigma.1.0.mm.3D_glszm_LargeAreaHighGrayLevelEmphasis	log.sigma.1.0.mm.3D_glszm_HighGrayLevelZoneEmphasis
0.312551953	1.030210578	42732.89096	217.2553191
0.254	3.33672527	125435.6083	198.5570866
0.33273703	0.78707642	15737.77688	147.5268817
0.305748064	0.718110118	98441.85733	356.8173333
0.210748156	7.201839942	46603.42	99.86
0.255799756	2.222024752	112071.6778	207.7708831
0.175023652	14.03410959	85258.49189	83.98378378
0.241551247	3.060640528	195117.3945	243.8738532
0.233349778	4.340137737	165515.5899	204.6807611
0.284382284	1.263602533	87189.76698	258.6405152
0.210293304	4.122159496	212123.6658	212.0947368
0.375074184	0.202252125	13967.19146	264.2389241
0.206896552	11.10253723	156658.7756	130.9356436
0.238428418	3.816593035	40145.65914	100.5575621
0.293281654	1.421609603	42322.1652	175.5484581
0.221465077	6.509206795	171300.0835	166.9516484
0.199376947	5.821872214	87524.19063	118.415625
0.189210321	14.20347019	206395.8058	132.088843
0.224471831	5.810399484	39163.19216	102.9490196
0.181661496	12.64206933	247623.2334	135.5237192
0.325323741	0.484119835	23468.7395	224.1676249
0.253653937	3.370018366	100047.697	169.3866171
0.248968697	3.902952116	121099.96	156.1676413
0.250485437	2.175195914	80601.18217	218.6085271
0.253840246	2.555489044	68621.26324	165.7322239
0.329881657	0.699519425	15177.48206	163.3363229
0.3654455	0.364663069	15112.81312	197.1324257
0.340685544	0.785184895	23360.54593	173.3412073
0.187408061	14.1369433	517173.4364	186.7817896
0.264957265	3.576311534	221435.2115	257.8028674
0.279092226	2.128354328	89298.01125	193.5553633
0.2084	13.02297934	374814.2284	182.6823417
0.260766495	2.613281827	227033.5742	288.7969697
0.249480249	1.598468882	43470.21667	217.375
0.347972973	0.569365979	31626.59223	210.6634304
0.215107243	7.501180532	465613.9408	248.2471098
0.31241244	1.177112173	157256.8825	362.2816143
0.28988499	1.721035394	70686.22482	196.2024415
0.181940701	11.20665967	71302.02963	77.65185185
0.24205379	2.671368818	233063.154	293.7525253
0.344984802	0.237050335	16534.29736	256.4251101
0.221014493	5.291136823	201658.2927	205.2295082
0.202841097	6.568586104	543415.8469	281.3631922
0.212314225	4.567845249	284629.55	270.92
0.26044648	1.824256131	287639.1187	385.2736264
0.342657343	0.445691357	24440.62857	235.1159184
0.337221921	0.552701632	58421.15366	326.4352373
0.2823219	1.218172557	50625.17991	180.75
0.25177305	4.211882321	26806.67606	104.7676056
0.222018349	2.603476418	100753.1446	191.607438
0.211596386	7.047631069	579291.8814	287.3890866
0.189161554	5.841208936	470672.8432	254.4432432
0.230708454	7.178840953	372936.863	223.8504566
0.293766012	1.089554184	30988.02326	173.2412791
0.267208534	3.881001751	245650.0798	259.3051331
0.308262712	0.544741015	45211.03952	273.9604811
0.283013114	1.120323397	143976.4881	369.5635776
0.172286184	9.057699195	547961.9403	259.3914081
0.325801749	0.503632224	66360.96197	359.5369128
0.242704626	4.272437234	43132.50147	104.5747801
0.261475689	1.992583689	169993.6268	310.3810143
0.249316566	5.84063966	593495.489	323.4422515
0.313958609	0.903381821	40264.00842	204.743338
0.24732334	1.92436734	41874.32684	139.5606061
0.244067797	2.983712552	22060.81944	79.375
0.318211277	0.367808855	18491.72301	252.885947
0.308128544	0.591117465	61201.7454	335.2638037
0.240325294	4.839016483	400196.8518	294.6651109
0.277648879	1.478618132	103234.117	236.6211699
0.28958915	1.793442344	155206.332	282.5523416
0.31512605	0.371875832	39594.91083	323.2883333
0.191169335	9.929581736	209232.6827	153.6954315
0.24748878	7.316124853	209365.2237	177.9015544
0.235294118	7.454182501	493983.4589	246.593342
0.319802909	0.457246451	39562.48789	302.9948643
0.277134277	2.208914825	146083.0387	245.2569659
0.247133758	6.109148488	176402.3024	163.1993127
0.292862399	1.085277093	43554.97487	205.2562814
0.233516484	5.440100417	345363.9817	242.3359477
0.253986077	5.393324362	275840.122	215.495137
0.295154185	0.54236159	104938.5622	434.5771144
0.366578848	0.322396622	28326.68686	284.201232
0.319725141	0.82661279	113224.7295	362.1504425
0.177820268	12.70049903	26063.58065	72.94623656
0.316934231	1.438003325	102656.2778	251.8848039
0.245538284	8.353746102	120351.0633	115.2075029
0.315826331	0.69906601	31867.1796	199.5809313
0.22379231	5.805742049	288524.0206	222.3215859
0.236009732	3.457713632	20839.46392	97.20618557
0.23183391	1.12124131	53516.31343	215.1492537
0.238866397	5.599830949	227570.5711	193.6740548
0.362895928	0.15254465	30500.87531	446.6683292
0.352752881	0.371061339	25025.90381	246.5154265
0.299793246	2.480289094	269002.1785	313.08659
0.230131747	8.23853365	303862.699	201.5272392
0.397058824	0.276767169	15600.39316	275.8803419
0.177538287	8.092145362	221219.0479	167.7699681
0.177570093	2.894221336	42949.21053	125.0263158
0.20045045	3.622624481	142542.6124	186.1741573
0.424610052	0.0831364	6406.985714	307.277551

log.sigma.1.0.mm.3D_glszm_SmallAreaEmphasis	log.sigma.1.0.mm.3D_glszm_LowGrayLevelZoneEmphasis	log.sigma.1.0.mm.3D_glszm_ZoneEntropy	log.sigma.1.0.mm.3D_glszm_SmallAreaLowGrayLevelEmphasis
0.690678647	0.011696375	5.635402557	0.009747506
0.691498236	0.017179977	5.617162607	0.015220174
0.68000167	0.024094175	5.722951365	0.018507391
0.705495866	0.005844458	5.809653668	0.004954656
0.725612052	0.025659524	5.10427605	0.021370003
0.713425297	0.011741923	5.542237144	0.010063568
0.711282545	0.032415402	4.844578901	0.027484569
0.719172584	0.009373099	5.386061237	0.008170364
0.691137126	0.009421308	5.458799786	0.007665606
0.676967583	0.006821127	5.734102112	0.005380985
0.685382091	0.011842582	5.432019319	0.010042679
0.716839162	0.010789134	5.8373144	0.008942066
0.691312166	0.017716933	5.481798037	0.013092431
0.615748299	0.020546324	5.682197917	0.014828229
0.676057895	0.010906711	5.362943833	0.008639463
0.688297461	0.01224075	5.674757381	0.009428888
0.614198875	0.016960304	5.341683593	0.01249279
0.692387114	0.021908683	5.379011303	0.018823516
0.659810921	0.027931557	5.418999989	0.022065032
0.668870981	0.018239442	5.516225259	0.01502459
0.669694552	0.009229917	6.028136063	0.007330936
0.694812494	0.011849203	5.530017302	0.009760892
0.641359286	0.013203011	5.800065423	0.009976575
0.711903979	0.012397307	5.34294542	0.010759523
0.654464416	0.011798513	5.762538119	0.008981462
0.683672686	0.021347468	5.736820669	0.0183774
0.703888553	0.009497821	5.583589966	0.007563073
0.684921779	0.012405887	5.77905473	0.009899085
0.680580223	0.009963238	5.455671913	0.008009536
0.670843381	0.007027215	5.801941997	0.005441052
0.670015777	0.009307706	5.762090405	0.006985145
0.669138484	0.008897941	5.581518967	0.006733429
0.726625836	0.009501684	5.675679354	0.008102307
0.70079618	0.01622069	5.294571509	0.014205225
0.738393978	0.013474816	5.467584396	0.011884779
0.709660646	0.011218028	5.620359703	0.009758554
0.702892085	0.004931247	5.904232139	0.003920949
0.68905157	0.011872915	5.736330646	0.009605778
0.680296954	0.028555649	5.0094222	0.022636959
0.733765042	0.008536534	5.486216601	0.007316134
0.67684029	0.009141418	5.725464394	0.007699457
0.719539009	0.012022267	5.459063935	0.010500313
0.720990917	0.007344476	5.455499221	0.006255194
0.742901729	0.008352926	5.412242186	0.007315589
0.705681983	0.005157696	5.717958257	0.004247218
0.688606454	0.008381829	5.907187341	0.006616626
0.706075579	0.005490645	5.913878055	0.004429491
0.665251897	0.014865366	5.702734588	0.012420897
0.69690948	0.028312558	5.01007811	0.017321321
0.70124338	0.018305601	5.267321652	0.016521713
0.708062097	0.006050131	5.516191397	0.00490233
0.718365345	0.009110924	5.304808739	0.005885919
0.720371516	0.008882123	5.480454412	0.007474036
0.680991417	0.014127007	5.623217055	0.01128221
0.692008036	0.007465122	5.729009195	0.00604833
0.711558567	0.009320081	5.783995861	0.008145642
0.711341083	0.005212249	5.753018101	0.004353038
0.697771418	0.008495339	5.418380275	0.007207055
0.700737884	0.007080347	5.720829131	0.006088143
0.671548936	0.02494682	5.360020061	0.020002104
0.710866174	0.008116958	5.708759487	0.007036767
0.712606468	0.005347419	5.738907828	0.004315137
0.704629252	0.013309349	5.735562237	0.011148401
0.665281471	0.016395727	5.571314686	0.01293321
0.725749802	0.040627343	4.807418117	0.036498721
0.659142976	0.008648975	5.84471794	0.007029292
0.748131885	0.007492677	5.482747155	0.006675312
0.710208698	0.008700285	5.601578729	0.007461543
0.722945343	0.010034977	5.360378511	0.008838985
0.704738231	0.008849664	5.800161874	0.007565337
0.681459091	0.005243822	5.894376643	0.004095198
0.679886589	0.017187246	5.398851256	0.014370249
0.695594637	0.011135626	5.655989552	0.008947411
0.690000948	0.008184346	5.73451273	0.006713697
0.685538257	0.006128601	5.96714843	0.004753752
0.695645757	0.009155278	5.714299662	0.007642306
0.733149084	0.013514999	5.393822028	0.010845279
0.664799876	0.0103083	5.635757205	0.008118694
0.7328181	0.01011934	5.488710578	0.008749161
0.701461109	0.009385304	5.747129798	0.007574399
0.702470792	0.00550818	5.685125514	0.004716686
0.703407617	0.007096974	5.838167712	0.00578911
0.711205045	0.007302589	5.898510072	0.006348217
0.666578162	0.058504656	4.935859323	0.051088752
0.685152702	0.006783219	5.782487451	0.005197697
0.704024219	0.019633302	5.355509151	0.015247561
0.691512023	0.011649811	5.666695263	0.009281754
0.708433313	0.009390255	5.464009707	0.007792904
0.684599454	0.028776253	4.560040772	0.024401356
0.681365841	0.017061813	5.268760647	0.015447316
0.712769866	0.011278302	5.559152015	0.009176755
0.71342179	0.007118497	5.835161887	0.006439522
0.689873691	0.008926715	5.795022951	0.007213134
0.713795396	0.005639856	5.771934754	0.004568772
0.692680026	0.008665942	5.474071651	0.006872136
0.681161836	0.016161	5.948868243	0.012161867
0.694295648	0.013116914	5.190494645	0.010966213
0.677164423	0.030325859	4.765753791	0.027484003
0.720168915	0.020128041	5.030583816	0.018366146
0.68373372	0.011734777	5.949655937	0.010428003

log.sigma.1.0.mm.3D_ngtdm_Coarseness	log.sigma.1.0.mm.3D_ngtdm_Complexity	log.sigma.1.0.mm.3D_ngtdm_Strength	log.sigma.1.0.mm.3D_ngtdm_Busyness	log.sigma.1.0.mm.3D_ngtdm_Contrast
0.005552856	0.547589341	1.577102023	0.336432857	4.42E-05
0.003464012	0.231526209	0.825465444	0.601395771	2.35E-05
0.006112821	0.433523225	0.95765273	0.47394213	7.73E-05
0.002651349	0.339078764	0.939095844	0.478541706	2.29E-05
0.00653116	0.216888003	0.888503862	0.712299708	4.81E-05
0.003979845	0.331590654	1.116439679	0.437203592	2.37E-05
0.006186666	0.123939129	0.59502427	0.825296907	3.48E-05
0.003758722	0.273082274	1.10999677	0.447093947	2.07E-05
0.003399532	0.213146991	0.860966321	0.596513876	1.70E-05
0.002210632	0.192332973	0.590730783	0.764159217	1.42E-05
0.003583104	0.213002006	0.911313364	0.543305458	1.91E-05
0.003793503	0.494590861	1.006570073	0.443899058	5.33E-05
0.00227955	0.108978638	0.471247857	1.257100443	1.12E-05
0.003452532	0.122583039	0.368224137	1.266385903	2.94E-05
0.003989807	0.241372737	0.654735127	0.700234461	3.44E-05
0.001772047	0.09505065	0.391758102	1.252304575	7.83E-06
0.004034813	0.167447134	0.626552811	0.817823889	2.12E-05
0.002653633	0.080574815	0.388596459	1.263688123	1.40E-05
0.006155349	0.177246213	0.723013303	0.765127752	4.63E-05
0.002370356	0.096212911	0.453551339	1.143146982	1.02E-05
0.000946549	0.116272515	0.273193521	1.8653763	8.28E-06
0.003237093	0.188896232	0.684593399	0.728936266	1.81E-05
0.001577006	0.084634957	0.255461859	1.724115851	1.33E-05
0.006623529	0.413312157	1.586658366	0.314378839	4.23E-05
0.002739478	0.165014042	0.558223626	0.918739139	1.73E-05
0.004983361	0.393600535	0.914914526	0.530366892	6.16E-05
0.002939193	0.230973255	0.596377694	0.743211332	2.62E-05
0.002002218	0.178222724	0.408023573	1.159023108	2.05E-05
0.002026834	0.089379987	0.457520313	1.072512568	7.76E-06
0.002295791	0.198806187	0.72897835	0.650094942	1.07E-05
0.001602811	0.108608677	0.292578876	1.447046106	1.40E-05
0.00134947	0.065155178	0.327540419	1.640344432	4.62E-06
0.002631712	0.331331034	1.132391481	0.474976925	1.49E-05
0.012683162	0.783717011	3.215253458	0.183563883	0.000101847
0.007232569	0.711872568	1.631990143	0.27495646	9.92E-05
0.002125382	0.160430174	0.721533839	0.710987527	9.11E-06
0.001961807	0.253302514	0.659484528	0.636237173	1.66E-05
0.00200583	0.178113748	0.477320612	0.980352224	1.65E-05
0.004278468	0.072943664	0.301309708	1.500203685	3.08E-05
0.004186771	0.439897788	1.655445654	0.304035712	2.13E-05
0.00461549	0.487342192	1.126232856	0.402853528	5.75E-05
0.003617075	0.202692765	0.890808529	0.556121121	1.90E-05
0.00217831	0.17023256	0.825563353	0.648155507	8.48E-06
0.00355819	0.267144772	1.318245227	0.421632513	1.45E-05
0.002005802	0.267979883	0.96517841	0.515060985	9.60E-06
0.001812644	0.227801341	0.486471516	0.972126211	1.94E-05
0.001833693	0.24719117	0.613756283	0.712420363	1.57E-05
0.004459229	0.326867201	0.908840331	0.495886787	3.89E-05
0.010724703	0.347072144	1.358465627	0.432133105	0.000100245
0.006081459	0.311285789	1.289135532	0.366789794	3.56E-05
0.001697713	0.129914016	0.635370282	0.832207663	5.63E-06
0.003473795	0.205963069	1.060125638	0.462581649	1.52E-05
0.001770572	0.130851275	0.54168028	0.954856628	7.94E-06
0.005813348	0.340204598	0.925933818	0.46145612	5.51E-05
0.001750533	0.157475165	0.501191854	0.9125731	1.09E-05
0.003987296	0.330706273	0.886091725	0.422539667	3.71E-05
0.002067996	0.248365912	0.877318792	0.571907103	1.16E-05
0.002628079	0.175622814	1.09080908	0.579140109	7.49E-06
0.004869565	0.604783452	1.710448378	0.258192549	4.48E-05
0.004632768	0.182460368	0.552350124	0.855527947	3.52E-05
0.002328714	0.272266578	1.0296431	0.530617277	1.11E-05
0.001272777	0.165063081	0.633802206	0.847668317	4.99E-06
0.002932052	0.249051027	0.621839732	0.721103466	3.04E-05
0.003776071	0.171677225	0.560874245	0.758551718	2.62E-05
0.011112722	0.293430395	0.878880802	0.510620729	0.00013208
0.004326909	0.457460457	1.28880822	0.394199671	3.38E-05
0.006426397	0.572180264	1.716445511	0.239104467	5.45E-05
0.001933486	0.172403447	0.738355822	0.703380768	9.20E-06
0.005010381	0.398142443	1.251482699	0.367165098	4.08E-05
0.00275821	0.311609465	0.872382889	0.514354999	2.13E-05
0.001767972	0.18883228	0.517809355	0.851648521	1.46E-05
0.00317689	0.16578227	0.740125158	0.761862893	1.39E-05
0.001465839	0.114712311	0.444190132	1.360232444	7.32E-06
0.001065403	0.07655979	0.285281626	1.615405548	5.81E-06
0.001591958	0.203762663	0.568257734	0.86412973	1.20E-05
0.002931491	0.259794126	0.750724426	0.583293519	2.46E-05
0.002978734	0.187923849	0.746703636	0.742415657	1.62E-05
0.004694805	0.327170263	0.933001275	0.486793604	4.64E-05
0.002093257	0.175190883	0.639855506	0.740388907	1.07E-05
0.001615115	0.133359	0.433413251	1.058893374	9.33E-06
0.005373484	0.538180845	1.65758728	0.224218035	4.18E-05
0.002436946	0.320700677	0.655841042	0.650394324	3.24E-05
0.002761555	0.37757815	0.977460966	0.449235704	2.67E-05
0.011765963	0.205967714	1.11404246	0.609274776	7.96E-05
0.001687779	0.16568032	0.418358796	1.033154757	1.55E-05
0.001854824	0.074438715	0.22851848	1.967198313	1.50E-05
0.00437733	0.486931326	1.168284889	0.435432781	4.41E-05
0.002147058	0.150161051	0.600626029	0.836509645	1.13E-05
0.015932358	0.278311639	1.080871303	0.407683137	0.000145494
0.011401077	0.732713521	3.070008941	0.175087598	8.16E-05
0.002002057	0.127061147	0.430676152	1.084113568	1.42E-05
0.00594569	1.09329192	2.412958698	0.176708476	8.00E-05
0.004218565	0.451820531	0.961575066	0.429598138	5.41E-05
0.001590392	0.186037859	0.512193249	0.846815984	1.27E-05
0.001452964	0.097266188	0.38310834	1.321067262	6.17E-06
0.008302874	1.224597077	2.162515889	0.204573696	0.000135966
0.003704327	0.132907152	0.697521015	0.702355103	1.47E-05
0.015726059	0.356454709	1.771460265	0.263159667	0.00010347
0.007602791	0.348523518	1.794898444	0.281428966	3.16E-05
0.005633305	0.952199326	1.68258519	0.271863711	9.41E-05

log.sigma.0.5.mm.3D_gldm_GrayLevelVariance	log.sigma.0.5.mm.3D_gldm_HighGrayLevelEmphasis	log.sigma.0.5.mm.3D_gldm_GrayLevelNonUniformityNormalized	log.sigma.0.5.mm.3D_gldm_DependenceEntropy
0.645593	22.6957606	0.392216328	5.050565348
0.55719375	22.2495	0.423809	5.002898243
0.716673974	13.40250447	0.366156662	5.17871757
0.712559594	32.47655932	0.386513581	5.026583649
0.409260039	7.561643836	0.44431996	4.79180998
0.530785183	21.41758242	0.439763194	4.933755083
0.361183586	7.45884579	0.473046742	4.671654607
0.526435494	22.28698061	0.439726828	4.931986053
0.466652826	22.48297977	0.444170204	4.996471674
0.532144512	21.94472194	0.418316615	5.107317107
0.45690298	21.9994466	0.472821828	4.800226018
0.883052241	22.25103858	0.348599706	5.208626288
0.425306978	13.78559235	0.455923803	4.940917012
0.408619347	13.4547901	0.450110713	5.00112994
0.456190867	13.60852713	0.420018495	4.930910556
0.474160414	13.05256753	0.419472099	5.230657627
0.375271979	13.20996885	0.457419086	4.883241018
0.406439868	14.20484754	0.472650765	4.974182906
0.446401229	7.907570423	0.438932008	5.038504796
0.394146677	13.55946225	0.47589564	5.034609281
0.672858009	21.44546763	0.36175759	5.235979448
0.440961615	13.6581801	0.447084575	4.949666336
0.482154807	13.44285368	0.442380369	5.053521407
0.566866811	22.20291262	0.407478556	4.958211454
0.488663754	12.95737327	0.409356042	5.220506665
0.717669528	14.60872781	0.362921685	5.243068201
0.580502365	21.84215287	0.397330604	5.084311362
0.650724034	13.74843517	0.375983795	5.188663327
0.396319427	21.88673139	0.46975665	4.908024596
0.562957191	21.85596708	0.40665104	5.111637421
0.546500546	21.7549493	0.411476015	5.100035268
0.373119	13.9966	0.4818608	5.059595298
0.665540424	21.97629395	0.409518104	5.00631611
0.563085395	14.78586279	0.425080286	4.826093809
0.77635617	22.0213964	0.391757467	5.053724625
0.52024869	21.91482748	0.442504166	4.899121127
0.782048902	32.43961894	0.365282172	5.27048054
0.635582367	21.97611324	0.399699849	5.053788125
0.315792533	7.067385445	0.484673898	4.788345729
0.62668205	22.57212714	0.430600307	4.9938012
0.695318779	22.12462006	0.380909267	5.034968197
0.524024654	22.72412008	0.436660108	4.971390308
0.481959032	21.99537496	0.446640188	4.877023124
0.493058091	22.68895966	0.454860689	4.894342309
0.617633909	31.73640527	0.403165751	5.097163089
0.7623599	22.23748252	0.349663103	5.266730686
0.755818455	32.51329354	0.373902043	5.217525525
0.600829063	21.19063325	0.400843074	5.007952494
0.468374327	8.09929078	0.425915447	4.834004761
0.455602222	22.11651376	0.463200067	4.857061003
0.434635883	21.98343373	0.453488401	4.933207733
0.478731218	21.51380368	0.459560223	4.847246136
0.474380166	22.25625494	0.452037076	4.859205359
0.564927682	13.62596072	0.400421954	4.986558473
0.577046832	22.1003302	0.402154288	5.1160413
0.750699108	21.68326271	0.365945265	5.429925874
0.615502406	32.44037816	0.400298795	5.075787551
0.406568533	21.95723684	0.45914026	4.820360589
0.745392545	32.41472303	0.402352549	5.061964013
0.417935943	13.37793594	0.447382885	4.872329554
0.609550561	22.70690241	0.412095593	5.077624107
0.595135996	32.39511573	0.417080231	5.135515863
0.669794962	22.04667547	0.390782754	5.077297696
0.480047996	13.80567452	0.445519375	4.954918913
0.441183568	7.722033898	0.452559609	4.797808043
0.676856869	22.6992871	0.372307106	5.04430618
0.66825894	32.34877127	0.398333339	5.09400357
0.559709401	22.49130679	0.445316253	4.933484907
0.554545297	21.8870843	0.432699963	4.855307037
0.714966264	21.93298763	0.386816555	5.238647965
0.645072735	31.96743697	0.37279489	5.283300242
0.426815226	13.87481805	0.462692112	4.900546435
0.507192765	14.06262022	0.427884596	5.027129228
0.497029978	21.79204423	0.43948142	5.036272166
0.702418886	22.5408259	0.37446272	5.192413558
0.656844379	21.86186186	0.394068089	5.025245533
0.49477707	13.45477707	0.441637569	4.912750876
0.560041497	22.62030905	0.4219411	4.945886402
0.534710109	21.61446886	0.434603756	4.909491893
0.584824236	21.61733663	0.409077286	5.152136483
0.722773692	32.17988253	0.387863576	5.067548163
0.862739776	21.93902898	0.349819517	5.208535391
0.860509525	32.51818917	0.358533927	5.262060218
0.378248741	7.718929254	0.466308874	4.684802598
0.644941912	22.14888659	0.392902227	5.134082877
0.429553084	13.48215314	0.443966679	4.896781785
0.709838347	22.52030812	0.381788009	5.086568793
0.496323212	22.15872494	0.447455785	4.848561209
0.372221334	7.639902676	0.452773782	4.761144565
0.550592067	22.01730104	0.444768382	4.734791601
0.493312996	22.31859234	0.447928771	4.923334929
1.076975492	33.10950226	0.345605536	5.144916643
0.789634091	21.87195903	0.361898548	5.134337063
0.723800465	32.25178038	0.385669629	5.167974387
0.43547201	22.02656184	0.449394024	4.961529776
1.202999273	21.83144796	0.280514834	5.538710631
0.352021399	13.53431651	0.491806269	4.605030603
0.340919513	14.19392523	0.515973011	4.530358696
0.402563459	22.04391892	0.50592738	4.674626223
1.094849803	22.69237435	0.292165897	5.319326252

log.sigma.0.5.mm.3D_gldm_DependenceNonUniformity	log.sigma.0.5.mm.3D_gldm_GrayLevelNonUniformity	log.sigma.0.5.mm.3D_gldm_SmallDependenceEmphasis
81.16791355	471.8362427	0.06436231
125.435	847.618	0.058173998
72.37209302	409.3631485	0.073145513
169.8487566	948.1178149	0.074031851
74.55742887	421.6596417	0.041731746
108.3736264	720.3321123	0.057847526
78.01608325	500.0104068	0.036402168
112.3207756	793.7069252	0.055987261
125.9412926	900.3330044	0.055278288
189.5154845	1256.204795	0.063081317
115.9883785	854.3890426	0.049556022
111.7454006	587.3905045	0.094861608
200.3861386	1335.400819	0.044145565
124.3616792	836.3057051	0.042723967
108.7390181	650.1886305	0.055106018
267.6439523	1723.610854	0.0497017
114.6897196	734.1576324	0.042848444
152.4949179	1209.040657	0.043908577
74.33450704	498.6267606	0.049180659
178.2492244	1380.573251	0.037222726
467.8661871	2514.215252	0.070179104
143.484677	948.2663838	0.059626065
251.6789614	1823.049503	0.050354285
74.40582524	419.7029126	0.064517196
168.8970814	1065.963134	0.054276947
89.4112426	490.6701183	0.068707651
156.070104	878.4979647	0.11266391
213.9651267	1261.425633	0.069163704
207.5742866	1596.702854	0.03831596
204.4542577	1284.610636	0.06005534
256.8140995	1704.333655	0.062891491
306.8572	2409.304	0.042322291
164.2722244	1036.49032	0.060351701
31.2993763	204.4636175	0.055104089
56.73873874	347.8806306	0.092199364
211.4824371	1423.535903	0.047518704
221.5968058	1303.692071	0.070733257
211.6759068	1355.382188	0.067413849
113.5754717	719.2560647	0.039584427
95.33618582	704.4621027	0.065581867
89	501.2765957	0.087271031
118.7142857	843.6273292	0.050809105
200.5011563	1351.979848	0.044011494
118.2123142	856.9575372	0.054760116
229.583858	1408.661133	0.054746165
243.3048951	1250.045594	0.079066203
237.611503	1378.20293	0.078612748
104.1754617	607.6781003	0.05666609
39.92553191	240.2163121	0.047127323
68.46055046	504.8880734	0.051434235
256.8323293	1806.697791	0.050159198
132.8619632	898.8997955	0.043684742
244.2241243	1716.384777	0.052946223
85.9735269	468.8941076	0.063727437
252.4432309	1583.281433	0.062650117
108.782839	690.904661	0.07553698
215.4989326	1312.57975	0.065896223
182.0041118	1116.629112	0.037603275
88.24781341	552.0276968	0.080804693
99.9366548	628.5729537	0.048815736
189.9187351	1211.973138	0.062262714
338.0533989	2288.519227	0.05806528
145.660502	887.4676354	0.068967744
113.5556745	832.2301927	0.053504664
36.93898305	267.0101695	0.061245293
115.022035	574.4698639	0.070314704
69.4442344	421.436673	0.069104633
213.996074	1587.997757	0.057922214
79.77184841	559.4810518	0.066237563
150.474272	969.7491025	0.069981565
242.3151261	1419.602941	0.071929561
131.927705	953.6084425	0.045579832
296.3990169	2002.072024	0.053345178
392.6940562	2861.463523	0.052030893
279.7602065	1595.960113	0.071691979
150.9073359	918.5727156	0.069673248
150.9524416	1040.056476	0.051443236
88.01398087	573.4179544	0.061947601
212.2405372	1423.761905	0.051099922
269.6934651	1821.621154	0.05796812
93.19676946	528.2701909	0.069502388
179.6443357	929.4704554	0.088084456
158.5828618	887.0129345	0.082845905
39.21414914	243.8795411	0.039005222
237.9989643	1517.3884	0.072512782
233.8324698	1542.340242	0.052608903
93.43557423	545.1932773	0.078035425
204.7778508	1361.607953	0.05402735
29.28223844	186.0900243	0.053756284
42.01384083	257.0761246	0.054116048
195.0504516	1438.299284	0.059075809
74.24886878	381.8941176	0.11293269
105.815621	565.2855314	0.079721651
266.6453021	1678.819894	0.067026289
309.6719082	2114.848279	0.051770751
64.24434389	247.9751131	0.083831298
128.2694271	867.0544526	0.040225475
27.59813084	220.8364486	0.055613202
54.67792793	449.2635135	0.054791476
87.11265165	337.1594454	0.095929835

log.sigma.0.5.mm.3D_gldm_DependenceNonUniformityNormalized	log.sigma.0.5.mm.3D_gldm_DependenceVariance	log.sigma.0.5.mm.3D_gldm_LargeDependenceEmphasis
0.06747125	16.71584685	91.15128845
0.0627175	18.990399	103.612
0.064733536	18.26861793	85.54382826
0.069241238	15.73810948	83.96045658
0.078564203	13.44506391	101.971549
0.066162165	17.52245733	105.0671551
0.073808972	13.87973496	106.5818354
0.062227577	19.14972905	105.9130194
0.062131866	19.15691982	104.0567341
0.063108719	18.80020945	99.47685648
0.064188367	18.41786645	113.2080797
0.066317745	18.07723375	79.43679525
0.068414523	16.74621161	110.6223967
0.066933089	17.309443	111.1636168
0.070244844	15.33998691	90.00387597
0.065136031	17.62692191	102.1603797
0.071457769	15.48508419	111.4274143
0.059614901	20.80900222	123.4057858
0.065435305	18.01625422	106.2042254
0.061444062	19.7326788	127.6963116
0.067318876	16.3343157	85.72920863
0.067649541	17.53839837	106.534182
0.061072303	19.84168434	109.8497937
0.072238665	14.73091149	89.1592233
0.06486063	18.48700687	106.4316436
0.066132576	18.43423506	84.12573964
0.070588016	16.51991103	65.98959747
0.063775	18.6793647	87.91862891
0.061069222	19.78859539	121.6625478
0.064721196	17.68087821	91.21462488
0.062002438	19.32737202	101.583776
0.06137144	19.71399216	119.658
0.064904079	17.47099553	98.12998815
0.065071468	18.5649699	95.57380457
0.063894976	19.79246814	86.78153153
0.065739023	17.11718297	107.8728629
0.062089326	19.72548237	91.95881199
0.062422857	19.05672126	96.33176054
0.076533337	13.82320493	111.8652291
0.058273952	21.89303627	109.6748166
0.067629179	17.01554864	78.89361702
0.061446318	19.73157436	111.0072464
0.066237581	16.94492503	109.7135778
0.06274539	18.88438566	110.9384289
0.06570803	17.17999423	98.4997138
0.068057313	16.40818301	80.0551049
0.06446324	18.02467255	85.45523603
0.068717323	15.63445848	93.0883905
0.07078995	15.78009154	96.65248227
0.062807844	19.2423668	110.9449541
0.064465946	17.89660424	111.8950803
0.067925339	16.55683211	111.4897751
0.064320286	18.05943328	108.697656
0.073418896	14.96942551	86.28608027
0.064120709	17.92008371	97.82651765
0.057618029	24.05578498	101.809322
0.065720931	17.28075741	93.13906679
0.074837217	14.31635215	108.7384868
0.064320564	18.51228655	93.03498542
0.071129292	15.59247527	100.8491103
0.064576245	17.98371255	100.6817409
0.061609878	19.44469957	106.2057591
0.064139367	18.14246872	90.55350066
0.060789976	20.4375129	109.1884368
0.062608446	19.55908072	106.2067797
0.074544417	13.96431441	82.01879456
0.065637273	17.84139922	92.08128544
0.060010116	20.67537776	112.3213685
0.061695165	19.73888072	104.0007734
0.060021648	20.81007897	98.72397288
0.063633174	18.53080396	89.97058824
0.064011502	18.19397366	113.9412906
0.063346659	18.34137414	104.3007053
0.060312403	20.16031672	113.2171709
0.065640593	17.3580003	88.10417644
0.064739312	17.58754599	93.51136851
0.064098701	17.94312611	107.1834395
0.064763783	17.91885454	96.73509934
0.064786489	17.63280699	106.3351648
0.060564443	19.97750914	105.8758141
0.068426409	16.59229862	87.56240822
0.067611718	16.96566671	77.9725254
0.064099782	18.58120247	86.36054972
0.074979253	14.13897612	99.7456979
0.061625832	20.07337209	92.42465044
0.067309289	16.61074869	103.5607369
0.065431074	17.78761897	85.03221289
0.067294726	16.97883605	106.2481104
0.071246322	15.86095275	94.85888078
0.072688306	14.7127908	95.65051903
0.060744457	19.94389071	108.1142946
0.067193546	18.05632481	75.41809955
0.067743675	17.02381464	79.37516005
0.061255525	19.87917023	96.4909258
0.065803635	17.46934486	109.761581
0.072674597	16.34077926	70.60633484
0.07275634	14.86758796	113.0045377
0.064481614	18.51235916	112.4392523
0.061574243	20.28061338	119.1576577
0.075487566	14.29462438	63.27036395

log.sigma.0.5.mm.3D_gldm_LargeDependenceLowGrayLevelEmphasis	log.sigma.0.5.mm.3D_gldm_SmallDependenceHighGrayLevelEmphasis	log.sigma.0.5.mm.3D_gldm_LargeDependenceHighGrayLevelEmphasis
4.0356514	1.379256791	2141.713217
4.533422353	1.113634847	2448.4795
6.389284436	0.916032819	1249.951699
2.550089747	2.109047461	2842.818997
14.8223437	0.322647548	799.1443625
4.690809873	1.08134225	2444.783883
14.74520892	0.218271603	860.6263009
4.581177396	1.07326677	2522.595014
4.533749497	1.103509001	2463.813024
4.431092328	1.366111669	2318.775891
4.905711647	0.914101021	2686.337023
3.57501415	2.046963455	1855.254006
8.03103234	0.666224052	1617.754182
8.267953146	0.581934052	1595.853068
6.754631065	0.761583624	1284.374677
8.170844555	0.695725658	1392.381358
8.40075251	0.558442695	1580.82866
8.499972852	0.576755087	1871.574668
14.70895246	0.444516065	864.2086268
9.237579187	0.450015515	1872.743192
4.074469512	1.558707173	1906.775252
7.739719533	0.752875243	1555.243281
7.888541886	0.634707697	1628.123271
4.069040581	1.512845224	2039.191262
8.705223531	0.742471116	1417.121736
5.954901066	0.9530295	1275.713757
2.933752704	2.434084598	1542.35052
6.465638804	1.027059754	1294.115052
5.302303494	0.753308597	2875.694322
4.093687134	1.252451463	2117.591959
4.550522893	1.322062399	2358.342588
8.507762722	0.57198886	1772.9806
4.358112576	1.236290666	2300.674042
6.569385504	0.914530713	1453.455301
3.760743615	1.653599104	2082.390766
4.751620106	0.922911981	2535.435499
2.773064908	2.188952163	3141.922387
4.237244823	1.414190699	2272.630198
16.77435235	0.263419066	852.0673854
4.68965708	1.2818805	2636.678484
3.511380717	1.740771807	1845.788754
4.759654838	0.971191914	2662.735507
4.828639971	0.770201946	2578.346217
4.788340611	1.187872082	2642.043524
3.021660752	1.525991115	3306.821694
3.658394909	1.785535572	1850.351049
2.581176848	2.487620614	2914.56484
4.263443257	1.051529142	2134.470317
12.80632634	0.403290143	819.1914894
4.794939977	0.989417003	2638.709174
4.958093299	1.069426581	2611.980422
4.953135642	0.767023106	2607.064928
4.730927239	1.074061245	2572.657624
6.537893538	0.871646023	1231.453459
4.377417317	1.211181483	2277.13462
4.652155159	1.575625227	2336.938559
2.82572169	2.046031996	3154.186032
4.898982595	0.7740019	2506.250822
2.78076594	2.421216386	3191.754373
7.459668446	0.675016459	1455.091815
4.440842026	1.377217234	2369.212853
3.195922998	1.756169323	3619.915436
4.020407524	1.494146826	2125.079701
7.637540893	0.680964654	1645.044433
13.51352119	0.326197956	904.1559322
3.789196627	1.70783394	1863.248866
2.78222627	1.923029654	3127.97448
4.81998313	1.120431507	2694.461582
4.48597243	1.088986861	2482.273782
4.354566457	1.360210908	2333.343438
2.813330206	2.138496751	2972.695903
8.052084273	0.607963439	1697.702086
7.414071544	0.697881033	1555.261594
4.993109268	1.055608824	2655.385041
3.926472503	1.581365833	2066.376115
4.149303494	1.364954823	2194.290004
7.688734165	0.64425305	1586.147771
4.177412217	1.361204489	2310.617366
4.691166354	0.935065842	2499.06746
4.750362866	1.150440038	2457.496968
2.661440423	2.02785325	2963.27533
3.53477549	1.779097107	1818.172375
2.600526853	2.408829015	2957.163298
13.55198162	0.384101762	815.3824092
4.048175039	1.488214924	2189.685914
7.568781424	0.693656559	1505.831031
3.754048408	1.855050628	2002.647759
4.668178707	1.047906596	2499.301676
13.1588943	0.388325814	762.9051095
4.239379085	1.012046385	2246.788927
4.651831382	1.153167875	2581.187481
2.248644169	3.311899041	2604.182805
3.548263006	1.635348712	1864.209987
2.882500684	1.921554317	3315.935677
4.901658677	1.069189913	2546.615597
3.540664519	1.977531041	1565.197964
8.041358795	0.51456941	1670.740216
7.555420561	0.697098616	1725.39486
5.054953616	1.018636995	2867.197072
2.97687761	2.061693497	1450.870884

log.sigma.0.5.mm.3D_gldm_SmallDependenceLowGrayLevelEmphasis	log.sigma.0.5.mm.3D_gldm_LowGrayLevelEmphasis	log.sigma.0.5.mm.3D_gldzm_DistanceZoneVariabilityNormalized
0.005650281	0.05174799	1
0.006526837	0.052766407	1
0.019444451	0.107918903	1
0.004791605	0.035282648	1
0.016699208	0.173820103	1
0.006844529	0.054969078	1
0.016419	0.176250657	1
0.006588327	0.052670163	1
0.004386982	0.049478645	1
0.005498386	0.052080822	1
0.005730303	0.052161872	1
0.01295115	0.061412698	1
0.006232163	0.085053622	1
0.005641711	0.086546615	1
0.008273809	0.08787683	1
0.008479666	0.095340513	1
0.006513061	0.087345102	1
0.007543395	0.082637043	1
0.014203614	0.173503032	1
0.007080221	0.088395381	1
0.006201066	0.055415534	0.990588446
0.011354687	0.088326323	1
0.009022265	0.094488123	1
0.00557059	0.051655721	1
0.009089788	0.094960474	1
0.014436691	0.093515465	1
0.007966317	0.052844208	1
0.01187143	0.097302158	1
0.003009428	0.050146814	1
0.005399	0.052783711	1
0.005139248	0.052460207	1
0.006011571	0.0821565	1
0.007933922	0.056708984	1
0.009694976	0.082748867	1
0.012235963	0.058307429	1
0.005715339	0.053273158	1
0.004002748	0.035288354	1
0.006206495	0.053576874	1
0.017612323	0.180826969	1
0.007641685	0.054330861	1
0.009802605	0.056420822	1
0.004854482	0.049973043	1
0.004777386	0.05196745	1
0.005174175	0.049649172	1
0.00407925	0.036176847	1
0.006969153	0.054504469	1
0.004285782	0.035067345	1
0.006397236	0.055969904	1
0.014754538	0.169548365	1
0.005859335	0.051796756	1
0.004236881	0.050538049	1
0.004435787	0.054087415	1
0.004653895	0.050526798	1
0.012411434	0.095719233	1
0.006446567	0.053138316	1
0.009166647	0.057825582	1
0.003863441	0.034516661	1
0.003877182	0.050635919	1
0.005562972	0.036141958	1
0.007081804	0.087943654	1
0.006022746	0.052109242	1
0.003190485	0.034125043	0.99248131
0.006513702	0.05427895	1
0.010991711	0.091697151	1
0.033811778	0.186261299	1
0.006044668	0.051548011	1
0.004641004	0.035293485	1
0.007747604	0.054733389	1
0.007492063	0.054240885	1
0.007686897	0.056359369	1
0.003797337	0.034767063	1
0.007839823	0.086109287	1
0.009852019	0.088026093	1
0.00518793	0.052539636	1
0.006928014	0.053823754	1
0.007683216	0.055190616	1
0.011149471	0.094227442	1
0.005095704	0.050676906	1
0.006143475	0.054354624	1
0.005612163	0.053858364	1
0.004550119	0.035608245	1
0.011101193	0.059339074	1
0.005926121	0.036682431	1
0.010322262	0.165847143	1
0.006222171	0.053383651	0.992094986
0.008699372	0.088590162	0.986842682
0.00678964	0.053002982	1
0.005361633	0.051528461	1
0.018875555	0.16693701	1
0.007232654	0.054466792	1
0.005347492	0.050694075	1
0.011834215	0.040867749	1
0.008769813	0.057963532	1
0.003899903	0.035098593	1
0.004121284	0.050169465	0.989690998
0.010596202	0.066431893	1
0.00763391	0.086275288	1
0.009240055	0.08086189	1
0.006665385	0.051564602	1
0.014924549	0.063858141	1

log.sigma.0.5.mm.3D_gldzm_LowIntensityEmphasis	log.sigma.0.5.mm.3D_gldzm_LargeDistanceEmphasis	log.sigma.0.5.mm.3D_gldzm_HighIntensitySmallDistanceEmphasis
0.097368651	1	21.32
0.128545464	1	18.27184466
0.312496246	1	12.44594595
0.072483849	1	27.59756098
0.529771825	1	7.928571429
0.13835298	1	17.7375
0.653744856	1	4.962962963
0.137649168	1	18.40229885
0.088427364	1	20.87640449
0.096021297	1	22.21118012
0.135939971	1	18.35135135
0.14787534	1	22.04487179
0.166732188	1	16.47368421
0.149922386	1	14.47058824
0.176420635	1	14.65714286
0.217432292	1	14.74375
0.176792929	1	13.36363636
0.207895913	1	13.05747126
0.335653595	1	10.35294118
0.240664112	1	12.09195402
0.099937857	1.014184397	22.28900709
0.206349304	1	13.12068966
0.231096825	1	12.68571429
0.092714328	1	24.26229508
0.195092164	1	14.1328125
0.264697708	1	14.18918919
0.074999792	1	21.76734694
0.19634964	1	15.55502392
0.089968599	1	20.3627451
0.100972453	1	21.46875
0.090613048	1	21.22746781
0.171830484	1	14.02564103
0.155934284	1	20.3381295
0.197574985	1	18.45454545
0.143241413	1	17.78205128
0.144234554	1	18.7295082
0.061541079	1	30.99145299
0.102507324	1	21.2955665
0.564393939	1	7.136363636
0.140961362	1	19.26315789
0.131192788	1	19.02912621
0.117410204	1	17.52
0.138023028	1	15.86407767
0.108585408	1	22.09876543
0.091645584	1	26.16883117
0.102478838	1	22.43162393
0.060730505	1	31.16862745
0.132966037	1	17.70149254
0.392111111	1	9.15
0.12682943	1	18.61538462
0.094524609	1	21.85889571
0.129982644	1	15.96923077
0.097893975	1	20.59770115
0.219095118	1	14.53030303
0.118348089	1	18.88038278
0.13923403	1	20.3125
0.064099206	1	31.65789474
0.130551884	1	19.90909091
0.073726208	1	30.20192308
0.16581384	1	14.70175439
0.111873386	1	22.23417722
0.060719486	1.011320755	30.69811321
0.103103721	1	22.4676259
0.244000639	1	13.66666667
0.656084229	1	5.709677419
0.092411539	1	25.06122449
0.07171192	1	28.15277778
0.160320303	1	18.82954545
0.13383578	1	15.43421053
0.129028405	1	18.59615385
0.05821485	1	29.10788382
0.218157675	1	13.23611111
0.221809834	1	13.26315789
0.114678207	1	20.425
0.112124365	1	22.04460967
0.126527049	1	18.92142857
0.273793226	1	12.37755102
0.089689517	1	22.88461538
0.144779449	1	17.45985401
0.111608535	1	19.49777778
0.06934903	1	29.95505618
0.139936633	1	19.87387387
0.080923469	1	27.8423913
0.305359477	1	11.70588235
0.095962999	1.011904762	20.56448413
0.195263061	1.01986755	13.45860927
0.096041867	1	24.43434343
0.115137356	1	18.94814815
0.328914141	1	9
0.152532242	1	18.10714286
0.099960387	1	19.78527607
0.112189975	1	28.80508475
0.128334699	1	20.43119266
0.065120516	1	28.23015873
0.089843216	1.015544041	20.9119171
0.153216634	1	23.92537313
0.230745614	1	13.10526316
0.176944444	1	13.14285714
0.137491107	1	19.05
0.189894304	1	20.31578947

log.sigma.0.5.mm.3D_gldzm_LowIntensityLargeDistanceEmphasis	log.sigma.0.5.mm.3D_gldzm_HighIntensityEmphasis	log.sigma.0.5.mm.3D_gldzm_DistanceZoneVariability	log.sigma.0.5.mm.3D_gldzm_ZonePercentage
0.097368651	21.32	75	0.06234414
0.128545464	18.27184466	103	0.0515
0.312496246	12.44594595	74	0.066189624
0.072483849	27.59756098	164	0.06685691
0.529771825	7.928571429	28	0.029504742
0.13835298	17.7375	80	0.048840049
0.653744856	4.962962963	27	0.025543992
0.137649168	18.40229885	87	0.048199446
0.088427364	20.87640449	89	0.043907252
0.096021297	22.21118012	161	0.053613054
0.135939971	18.35135135	74	0.040951854
0.14787534	22.04487179	156	0.092581602
0.166732188	16.47368421	95	0.032434278
0.149922386	14.47058824	68	0.036598493
0.176420635	14.65714286	70	0.045219638
0.217432292	14.74375	160	0.038938915
0.176792929	13.36363636	55	0.034267913
0.207895913	13.05747126	87	0.034010946
0.335653595	10.35294118	51	0.044894366
0.240664112	12.09195402	87	0.029989659
0.100922884	22.36879433	419.0189125	0.060863309
0.206349304	13.12068966	116	0.054691183
0.231096825	12.68571429	175	0.042465421
0.092714328	24.26229508	61	0.059223301
0.195092164	14.1328125	128	0.049155146
0.264697708	14.18918919	74	0.054733728
0.074999792	21.76734694	245	0.110809588
0.19634964	15.55502392	209	0.062295082
0.089968599	20.3627451	102	0.030008826
0.100972453	21.46875	160	0.05064894
0.090613048	21.22746781	233	0.056253018
0.171830484	14.02564103	156	0.0312
0.155934284	20.3381295	139	0.054919004
0.197574985	18.45454545	22	0.045738046
0.143241413	17.78205128	78	0.087837838
0.144234554	18.7295082	122	0.037923531
0.061541079	30.99145299	234	0.065564584
0.102507324	21.2955665	203	0.059864347
0.564393939	7.136363636	44	0.029649596
0.140961362	19.26315789	95	0.05806846
0.131192788	19.02912621	103	0.078267477
0.117410204	17.52	75	0.038819876
0.138023028	15.86407767	103	0.03402709
0.108585408	22.09876543	81	0.042993631
0.091645584	26.16883117	154	0.044075558
0.102478838	22.43162393	234	0.065454545
0.060730505	31.16862745	255	0.069180684
0.132966037	17.70149254	67	0.044195251
0.392111111	9.15	20	0.035460993
0.12682943	18.61538462	52	0.047706422
0.094524609	21.85889571	163	0.040913655
0.129982644	15.96923077	65	0.033231084
0.097893975	20.59770115	174	0.045825652
0.219095118	14.53030303	66	0.056362084
0.118348089	18.88038278	209	0.053086106
0.13923403	20.3125	128	0.06779661
0.064099206	31.65789474	190	0.057944495
0.130551884	19.90909091	66	0.027138158
0.073726208	30.20192308	104	0.075801749
0.16581384	14.70175439	57	0.040569395
0.111873386	22.23417722	158	0.053723223
0.061427034	30.74339623	263.0075472	0.048295972
0.103103721	22.4676259	139	0.061206517
0.244000639	13.66666667	87	0.046573876
0.656084229	5.709677419	31	0.052542373
0.092411539	25.06122449	98	0.063512638
0.07171192	28.15277778	72	0.06805293
0.160320303	18.82954545	176	0.04935502
0.13383578	15.43421053	76	0.058778036
0.129028405	18.59615385	156	0.062225768
0.05821485	29.10788382	241	0.063287815
0.218157675	13.23611111	72	0.034934498
0.221809834	13.26315789	209	0.044667664
0.114678207	20.425	280	0.043004147
0.112124365	22.04460967	269	0.063115908
0.126527049	18.92142857	140	0.06006006
0.273793226	12.37755102	98	0.041613588
0.089689517	22.88461538	78	0.057395143
0.144779449	17.45985401	137	0.041819292
0.111608535	19.49777778	225	0.050527734
0.06934903	29.95505618	89	0.065345081
0.139936633	19.87387387	222	0.083552879
0.080923469	27.8423913	184	0.074373484
0.305359477	11.70588235	17	0.03250478
0.09728575	20.59126984	250.0079365	0.065251165
0.196057763	13.58278146	149.013245	0.043465746
0.096041867	24.43434343	99	0.069327731
0.115137356	18.94814815	135	0.044364114
0.328914141	9	22	0.053527981
0.152532242	18.10714286	28	0.048442907
0.099960387	19.78527607	163	0.050763002
0.112189975	28.80508475	118	0.10678733
0.128334699	20.43119266	109	0.06978233
0.065120516	28.23015873	252	0.05789111
0.093729226	20.92746114	191.0103627	0.041011475
0.153216634	23.92537313	67	0.075791855
0.230745614	13.10526316	57	0.032331254
0.176944444	13.14285714	21	0.049065421
0.137491107	19.05	40	0.045045045
0.189894304	20.31578947	95	0.082322357

log.sigma.0.5.mm.3D_gldzm_IntensityVariabilityNormalized	log.sigma.0.5.mm.3D_gldzm_LowIntensitySmallDistanceEmphasis	log.sigma.0.5.mm.3D_gldzm_IntensityVariability
0.265066667	0.097368651	19.88
0.262324442	0.128545464	27.01941748
0.256391527	0.312496246	18.97297297
0.227245687	0.072483849	37.26829268
0.301020408	0.529771825	8.428571429
0.2665625	0.13835298	21.325
0.410150892	0.653744856	11.07407407
0.23952966	0.137649168	20.83908046
0.269284181	0.088427364	23.96629213
0.301724471	0.096021297	48.57763975
0.298392988	0.135939971	22.08108108
0.227071006	0.14787534	35.42307692
0.256288089	0.166732188	24.34736842
0.37283737	0.149922386	25.35294118
0.348163265	0.176420635	24.37142857
0.274921875	0.217432292	43.9875
0.350082645	0.176792929	19.25454545
0.275994187	0.207895913	24.01149425
0.291810842	0.335653595	14.88235294
0.289998679	0.240664112	25.22988506
0.3154715	0.0996916	133.4444444
0.309601665	0.206349304	35.9137931
0.280914286	0.231096825	49.16
0.262563827	0.092714328	16.01639344
0.323364258	0.195092164	41.390625
0.246530314	0.264697708	18.24324324
0.226222407	0.074999792	55.4244898
0.295483162	0.19634964	61.75598086
0.324682814	0.089968599	33.11764706
0.238203125	0.100972453	38.1125
0.324356684	0.090613048	75.5751073
0.26035503	0.171830484	40.61538462
0.236996015	0.155934284	32.94244604
0.194214876	0.197574985	4.272727273
0.240302433	0.143241413	18.74358974
0.272104273	0.144234554	33.19672131
0.213565637	0.061541079	49.97435897
0.288747604	0.102507324	58.61576355
0.412190083	0.564393939	18.13636364
0.221274238	0.140961362	21.02105263
0.2442266	0.131192788	25.15533981
0.249066667	0.117410204	18.68
0.29965124	0.138023028	30.86407767
0.222069806	0.108585408	17.98765432
0.257969303	0.091645584	39.72727273
0.248228505	0.102478838	58.08547009
0.229342561	0.060730505	58.48235294
0.249721542	0.132966037	16.73134328
0.225	0.392111111	4.5
0.23964497	0.12682943	12.46153846
0.245737514	0.094524609	40.05521472
0.273846154	0.129982644	17.8
0.299841459	0.097893975	52.17241379
0.318640955	0.219095118	21.03030303
0.244477004	0.118348089	51.09569378
0.233642578	0.13923403	29.90625
0.273185596	0.064099206	51.90526316
0.261248852	0.130551884	17.24242424
0.217455621	0.073726208	22.61538462
0.375192367	0.16581384	21.38596491
0.198686108	0.111873386	31.39240506
0.227141331	0.0605426	60.19245283
0.267325708	0.103103721	37.15827338
0.278372308	0.244000639	24.2183908
0.438085328	0.656084229	13.58064516
0.225739275	0.092411539	22.12244898
0.247299383	0.07171192	17.80555556
0.235020661	0.160320303	41.36363636
0.326177285	0.13383578	24.78947368
0.234138725	0.129028405	36.52564103
0.273703965	0.05821485	65.9626556
0.273533951	0.218157675	19.69444444
0.274696092	0.221809834	57.41148325
0.283647959	0.114678207	79.42142857
0.252387336	0.112124365	67.89219331
0.240306122	0.126527049	33.64285714
0.303831737	0.273793226	29.7755102
0.310979619	0.089689517	24.25641026
0.288720763	0.144779449	39.55474453
0.266844444	0.111608535	60.04
0.240499937	0.06934903	21.40449438
0.221897573	0.139936633	49.26126126
0.204985822	0.080923469	37.7173913
0.307958478	0.305359477	5.235294118
0.289902998	0.095632311	73.05555556
0.344414719	0.195064386	52.00662252
0.2241608	0.096041867	22.19191919
0.292400549	0.115137356	39.47407407
0.334710744	0.328914141	7.363636364
0.270408163	0.152532242	7.571428571
0.271782905	0.099960387	44.3006135
0.163602413	0.112189975	19.30508475
0.233734534	0.128334699	25.47706422
0.258471907	0.065120516	65.13492063
0.327713496	0.088871713	63.24870466
0.170416574	0.153216634	11.41791045
0.316097261	0.230745614	18.01754386
0.315192744	0.176944444	6.619047619
0.23375	0.137491107	9.35
0.172742382	0.189894304	16.41052632

log.sigma.0.5.mm.3D_gldzm_HighIntensityLargeDistanceEmphasis	log.sigma.0.5.mm.3D_gldzm_SmallDistanceEmphasis	log.sigma.0.5.mm.3D_glcm_SumVariance	log.sigma.0.5.mm.3D_glcm_Homogeneity1
21.32	1	52.37977222	0.688973577
18.27184466	1	52.8393707	0.706504418
12.44594595	1	25.06403455	0.672830715
27.59756098	1	82.16998189	0.676326499
7.928571429	1	12.64757371	0.730900192
17.7375	1	52.05912206	0.705300282
4.962962963	1	13.08045037	0.736037412
18.40229885	1	54.40240591	0.713800475
20.87640449	1	56.09014508	0.722993672
22.21118012	1	52.48758488	0.705403117
18.35135135	1	54.87431678	0.725439187
22.04487179	1	48.6315101	0.653275792
16.47368421	1	29.71008884	0.728392011
14.47058824	1	28.89068011	0.726177824
14.65714286	1	29.21042816	0.701539845
14.74375	1	27.0614618	0.716882013
13.36363636	1	29.42201903	0.731370337
13.05747126	1	31.14825728	0.742271091
10.35294118	1	12.65542871	0.730390275
12.09195402	1	29.85526575	0.743665108
22.68794326	0.996453901	48.37920743	0.66616306
13.12068966	1	28.83866042	0.721699124
12.68571429	1	28.08592062	0.717342102
24.26229508	1	52.49045616	0.69778631
14.1328125	1	26.27451708	0.713021569
14.18918919	1	27.82857644	0.67261726
21.76734694	1	51.68234969	0.684655828
15.55502392	1	26.51254279	0.679898115
20.3627451	1	56.15330082	0.734998672
21.46875	1	52.33460943	0.696080359
21.22746781	1	51.86686969	0.699849837
14.02564103	1	31.16430625	0.74900848
20.3381295	1	50.79913417	0.690932217
18.45454545	1	30.83977701	0.711506475
17.78205128	1	49.2878108	0.670734248
18.7295082	1	52.91890876	0.713537781
30.99145299	1	80.77971454	0.670842262
21.2955665	1	50.71836497	0.683358193
7.136363636	1	12.60039552	0.743136696
19.26315789	1	53.57177022	0.706199074
19.02912621	1	50.35265037	0.661539157
17.52	1	55.13367696	0.718802097
15.86407767	1	54.28507259	0.718279097
22.09876543	1	56.20956328	0.725370676
26.16883117	1	82.66160963	0.688245276
22.43162393	1	48.79353566	0.656300813
31.16862745	1	81.08619402	0.668609701
17.70149254	1	49.42982039	0.687383785
9.15	1	13.29493088	0.71828262
18.61538462	1	55.63983512	0.726800761
21.85889571	1	55.02034473	0.725939
15.96923077	1	53.37630417	0.717421306
20.59770115	1	54.99483756	0.718996558
14.53030303	1	27.2703873	0.68733093
18.88038278	1	52.45112625	0.696650729
20.3125	1	48.48020732	0.679378546
31.65789474	1	84.04910784	0.693288091
19.90909091	1	55.74231775	0.728184884
30.20192308	1	82.37586471	0.684669691
14.70175439	1	29.10602046	0.711482257
22.23417722	1	53.27878142	0.704509384
30.9245283	0.997169811	84.89363047	0.704783159
22.4676259	1	49.94983174	0.678726409
13.66666667	1	29.22573947	0.717443883
5.709677419	1	13.21061715	0.726901831
25.06122449	1	51.15321822	0.679791872
28.15277778	1	83.13809996	0.68893319
18.82954545	1	54.24072503	0.713710553
15.43421053	1	52.74413215	0.696681667
18.59615385	1	50.04669488	0.68599893
29.10788382	1	81.62407863	0.680704054
13.23611111	1	30.35659693	0.728417738
13.26315789	1	29.00514698	0.714703896
20.425	1	53.33329152	0.716924801
22.04460967	1	50.45464138	0.679329691
18.92142857	1	50.7848137	0.683389916
12.37755102	1	28.40499915	0.712236036
22.88461538	1	53.58344414	0.698679183
17.45985401	1	52.33234328	0.705669419
19.49777778	1	51.05178953	0.701023925
29.95505618	1	82.08313335	0.677950188
19.87387387	1	47.49511832	0.648898965
27.8423913	1	80.20582567	0.663295749
11.70588235	1	13.34916661	0.731393976
20.6984127	0.99702381	50.71526018	0.681989793
14.0794702	0.995033113	28.93604086	0.71513729
24.43434343	1	50.66908486	0.676074603
18.94814815	1	53.91777906	0.712900972
9	1	13.65945072	0.73094111
18.10714286	1	53.54811249	0.712062999
19.78527607	1	54.45593957	0.714422302
28.80508475	1	79.66488131	0.646516642
20.43119266	1	48.42430489	0.657144827
28.23015873	1	81.46923598	0.678527789
20.98963731	0.99611399	55.00475389	0.723509846
23.92537313	1	43.39625798	0.624342384
13.10526316	1	30.82691897	0.736081732
13.14285714	1	33.06793192	0.750244836
19.05	1	57.63782039	0.74481673
20.31578947	1	46.57523866	0.623118197

log.sigma.0.5.mm.3D_glcm_Homogeneity2	log.sigma.0.5.mm.3D_glcm_ClusterShade	log.sigma.0.5.mm.3D_glcm_MaximumProbability	log.sigma.0.5.mm.3D_glcm_Idmn	log.sigma.0.5.mm.3D_glcm_SumVariance2
0.67207318	-0.242116558	0.305188053	0.98336407	1.303024468
0.692628119	-0.686299723	0.340908831	0.98511294	1.116497624
0.649551372	-0.827551347	0.288293808	0.966022251	1.435233464
0.657389807	-1.023727685	0.286147468	0.980866349	1.401960996
0.72494485	0.218058367	0.324388104	0.975063117	0.802089716
0.691581351	-0.822298042	0.329514847	0.98492543	1.058328935
0.730403297	-0.043437531	0.357092975	0.975872829	0.709068774
0.701458396	-0.723357753	0.356480074	0.985738332	1.041321428
0.713045628	-0.122502865	0.358678974	0.987548506	0.937223682
0.691497999	-0.380996682	0.32506963	0.985549357	1.069672565
0.713991715	-0.696453065	0.377978788	0.982939436	0.918273761
0.627840766	-1.051682091	0.268066855	0.976523038	1.720645641
0.720056214	-0.054127206	0.347132765	0.985302729	0.853810576
0.71734007	-0.215186042	0.33254661	0.98061895	0.85002611
0.690059422	-0.149489667	0.292231801	0.97742308	0.894827178
0.705927384	-0.343839175	0.281127973	0.977568578	0.969888997
0.724603839	-0.211499656	0.325403321	0.981750692	0.746767948
0.734347343	-0.259318674	0.398711156	0.981518508	0.83549
0.721602335	0.008105944	0.354946602	0.97310688	0.919459825
0.735093517	-0.441982862	0.380746449	0.980948443	0.829322537
0.644892382	-0.443723247	0.241795569	0.984783432	1.342495612
0.711316086	-0.384268372	0.342795238	0.984330758	0.906974185
0.70383473	-0.668202645	0.355607201	0.97631634	1.005986927
0.684673028	-0.144175884	0.292627859	0.985711819	1.106272989
0.700143489	-0.214757501	0.267893683	0.982344103	1.0105329
0.651906284	-0.185003907	0.289532006	0.976132718	1.4517641
0.667092099	-0.563434266	0.303240144	0.978751299	1.152607685
0.659129264	-0.617754206	0.29990448	0.97664422	1.291436018
0.726586416	-0.415462689	0.37867497	0.98560814	0.794228901
0.681005543	-0.400036803	0.306156801	0.984326368	1.112405094
0.684191763	-0.573649801	0.319697206	0.980346654	1.0840767
0.74293145	-0.111277564	0.3889096	0.983135201	0.781092708
0.67341846	-0.872299361	0.318108528	0.985841102	1.293576689
0.699245813	0.59811373	0.352879003	0.98298815	1.133557566
0.646737221	-1.373902249	0.32544508	0.978442311	1.558854556
0.701145192	-0.727977477	0.344397661	0.981752156	1.067335524
0.648140742	-0.9928946	0.291701443	0.983280523	1.596585886
0.663966884	-0.687819151	0.314674448	0.98229665	1.269637584
0.739143811	-0.154701765	0.335229169	0.96610198	0.641632676
0.690530071	-0.551053017	0.363346106	0.98374312	1.223354147
0.639435226	-0.946491141	0.277685091	0.974437082	1.384736353
0.707033035	-0.281965021	0.364369486	0.982834093	1.053111329
0.708075109	-0.445515754	0.34451364	0.983488191	0.941150202
0.71602387	0.119318853	0.369028537	0.987857321	0.977786873
0.671190035	-0.826533489	0.294211594	0.986072526	1.253893344
0.631986242	-0.615796096	0.255419605	0.978985003	1.546982849
0.646032247	-0.958454472	0.290482076	0.983478547	1.525083374
0.670726245	-0.892828601	0.290549652	0.978242924	1.244333121
0.709270347	0.152115708	0.344644094	0.971986537	0.922534015
0.716643888	-0.553887544	0.376618495	0.983708278	0.883093119
0.716739915	-0.227216941	0.349817034	0.984738984	0.857019221
0.70606972	-0.842965987	0.348326697	0.98198646	0.957217928
0.708300433	-0.337949475	0.358891624	0.98710202	0.912228051
0.671484255	-0.315400533	0.281943268	0.972555295	1.140636651
0.681833	-0.44549823	0.306188848	0.980050567	1.10326446
0.65535967	-0.829714783	0.293443376	0.973490902	1.530830447
0.67792993	-0.698429012	0.305034049	0.983931738	1.22172413
0.721804737	-0.225044787	0.330660233	0.988995825	0.804217256
0.665722536	-1.741864029	0.32170716	0.98394708	1.497209442
0.700537867	-0.256629542	0.319571793	0.978254802	0.817770868
0.690734589	-0.391846122	0.328334299	0.987659652	1.203187515
0.689933649	-0.614283729	0.333039409	0.987306605	1.175908374
0.657549656	-0.925017164	0.306888854	0.975598809	1.391276316
0.704759821	-0.627626487	0.366187144	0.976595816	0.991104911
0.716857308	-0.091177055	0.3830164	0.971850155	0.848092026
0.662671736	0.258888149	0.262882241	0.983581546	1.349626605
0.671075563	-0.624384583	0.306878821	0.981689123	1.329814883
0.700690882	-1.025148273	0.368487786	0.98494371	1.134130172
0.679902624	-0.965022492	0.344173427	0.97909089	1.112611006
0.666407026	-0.903788031	0.316924477	0.981392588	1.403719281
0.661670183	-0.615307286	0.267093194	0.982341654	1.318975802
0.719792947	-0.12670849	0.363300825	0.98525713	0.838723865
0.70274783	-0.295639707	0.34491713	0.986714792	1.014401968
0.704367968	-0.664183405	0.349642327	0.982164779	0.994963302
0.660292041	-0.627958367	0.292079695	0.981976743	1.436065985
0.664806469	-0.84943637	0.306990476	0.977013171	1.285330663
0.699696672	-0.454908857	0.343247472	0.986235502	0.961641543
0.682658664	-0.436834151	0.339542533	0.980370314	1.147075637
0.69142709	-0.795581951	0.337050206	0.980779977	1.065159382
0.684423716	-0.673443658	0.323392664	0.983704595	1.168562883
0.658046121	-0.885554086	0.294253198	0.984164286	1.426198153
0.622502134	-1.199844857	0.265212242	0.976549667	1.692424619
0.639746365	-1.479268681	0.28023466	0.981977531	1.667353963
0.726128871	0.133186374	0.351653868	0.975794897	0.749105046
0.661355878	-0.826457313	0.318489024	0.97679071	1.309478914
0.704934093	-0.255849758	0.32617009	0.978902917	0.867367942
0.655283156	-0.42422324	0.301672583	0.981106083	1.430698755
0.70122114	-0.43482455	0.34510207	0.982616617	1.005833623
0.725472977	0.008495361	0.345429341	0.963254474	0.693527288
0.700017456	-0.785436258	0.341399066	0.985354655	1.101275387
0.701349241	-0.527979042	0.364633352	0.982125239	0.991036528
0.619768455	-2.749910726	0.277909243	0.977450055	2.05530463
0.631849752	-1.198713156	0.278655577	0.977683117	1.567064501
0.657404721	-1.000548881	0.311317945	0.984131257	1.435270193
0.714413122	-0.275312947	0.342619081	0.988090405	0.851443046
0.589675923	-1.45187694	0.202343922	0.968728466	2.545702538
0.729670518	-0.334275687	0.369361994	0.982110449	0.69979873
0.743986155	-0.256306614	0.432731224	0.976431818	0.664340843
0.73650467	-0.656123892	0.42306145	0.988538945	0.769881677
0.589578012	-1.620418958	0.209425591	0.971148706	2.224575435

log.sigma.0.5.mm.3D_glcm_Contrast	log.sigma.0.5.mm.3D_glcm_DifferenceEntropy	log.sigma.0.5.mm.3D_glcm_InverseVariance	log.sigma.0.5.mm.3D_glcm_Entropy	log.sigma.0.5.mm.3D_glcm_Dissimilarity
1.140762367	1.531573545	0.478230925	3.218856227	0.7350489
1.018680049	1.471672565	0.468771029	3.003795246	0.680430533
1.400391437	1.662120832	0.4551697	3.446287439	0.814821742
1.342827421	1.608012833	0.482714722	3.311767916	0.789697799
0.67260193	1.228854795	0.489240055	2.574164977	0.570496237
1.031624439	1.472457031	0.471845858	2.917338806	0.684720647
0.647218965	1.214153574	0.481898022	2.477292938	0.557197666
0.97754343	1.441573984	0.466545248	2.888104131	0.658648957
0.839931406	1.365255928	0.47003244	2.792235932	0.617605491
0.981621035	1.456786035	0.472139078	3.016800039	0.676665772
0.904294369	1.409884096	0.451561743	2.727088388	0.626355536
1.669523889	1.740426478	0.470924439	3.624297967	0.890796084
0.763113935	1.311446371	0.473615359	2.678438848	0.593180866
0.74189121	1.293306713	0.475176264	2.683155703	0.594748419
0.875379888	1.374560351	0.501817267	2.877238406	0.66235279
0.885812	1.390945585	0.473572914	2.897917133	0.637095443
0.697793405	1.259275167	0.481340894	2.567351157	0.575231264
0.714234347	1.291421428	0.449613126	2.616707876	0.561509161
0.733174668	1.291735249	0.467129455	2.734593352	0.586191886
0.741325612	1.307283187	0.440912916	2.59421655	0.564828305
1.307495469	1.611970024	0.487230144	3.434020499	0.807593308
0.811968645	1.352031263	0.470457306	2.778235563	0.616361173
0.937726182	1.432814536	0.451807678	2.868409178	0.649370098
0.966130669	1.431094187	0.494285117	3.00358703	0.685798611
0.930006463	1.428524693	0.46581007	2.988702654	0.653862834
1.282264964	1.603704029	0.478797974	3.434631068	0.792115429
1.127325158	1.530556399	0.482653757	3.16450457	0.741917648
1.251544788	1.598916696	0.464345449	3.337323661	0.775327758
0.745146647	1.300972579	0.459933347	2.586498821	0.579634853
1.072889397	1.496821801	0.479519103	3.103943985	0.708889162
1.040248668	1.494495071	0.468454698	3.070675546	0.698915716
0.643315532	1.231945698	0.451863008	2.521861375	0.53564624
1.225680333	1.569554742	0.466796467	3.171392125	0.745445839
0.886549471	1.395369575	0.474989333	2.903303185	0.648750713
1.513398524	1.699242443	0.450311915	3.42021122	0.836876828
0.971534177	1.441299929	0.466801807	2.897769156	0.658736808
1.457964004	1.677455041	0.461799116	3.512192074	0.82499331
1.216076371	1.581050201	0.468702034	3.246757803	0.761116507
0.594858841	1.163611237	0.48123623	2.352670767	0.533903455
1.127696879	1.52601278	0.4523848	3.02635524	0.700719411
1.385581329	1.637353793	0.487870223	3.37215295	0.829022336
0.90492858	1.411341991	0.462890345	2.88594737	0.638308656
0.870404803	1.377672892	0.476921587	2.783773249	0.630412373
0.818391372	1.34812835	0.470353724	2.72950598	0.609005364
1.210880411	1.555846549	0.476792388	3.217955487	0.745241891
1.456326353	1.675335885	0.479699758	3.583351046	0.853591639
1.437496484	1.668153509	0.468063113	3.484142518	0.825434568
1.171195154	1.539072648	0.482299186	3.194515908	0.741898069
0.763651385	1.306839077	0.489454956	2.742196502	0.611824653
0.860675271	1.375029827	0.459910799	2.687426125	0.614579844
0.795426593	1.337605492	0.471143687	2.705647452	0.603968169
0.967254585	1.422620678	0.466798028	2.775814676	0.648896162
0.871605666	1.384920139	0.471626659	2.764078824	0.630750558
1.101949044	1.505815719	0.49083349	3.163137399	0.729859728
1.06601477	1.491977881	0.480570311	3.083176534	0.706084262
1.451419985	1.689007483	0.434313313	3.504447579	0.813083044
1.110063737	1.507354974	0.482262668	3.149223664	0.718498131
0.738068832	1.268244608	0.489430188	2.577115765	0.585905257
1.420550915	1.631305522	0.463086473	3.28426551	0.786093837
0.842821483	1.365435046	0.486360162	2.760776585	0.639469201
1.062562913	1.483108433	0.472242417	3.074764847	0.689929739
1.094811576	1.507790895	0.462583617	3.069739443	0.696489095
1.321471571	1.626188856	0.461344254	3.350473066	0.788649957
0.927496752	1.430036284	0.458061051	2.875589177	0.646096086
0.772646729	1.329968263	0.463626073	2.702633493	0.600639765
1.118480113	1.515634691	0.496494765	3.295104396	0.747260633
1.28548214	1.585961064	0.466637155	3.231928885	0.757541828
1.044156838	1.470780831	0.459524219	2.929637364	0.669671969
1.120101263	1.531265111	0.463971052	3.023616038	0.718403752
1.296914803	1.608787817	0.459175466	3.300227301	0.768540281
1.214143992	1.573836008	0.477020958	3.338943963	0.764262598
0.762237392	1.316001488	0.471339263	2.654935932	0.593990685
0.896114147	1.408263053	0.470282494	2.920640139	0.644296873
0.945879411	1.437467409	0.459513737	2.887458704	0.649222201
1.245218682	1.58168776	0.478824334	3.37887078	0.771050346
1.242723427	1.581636296	0.473764533	3.247703532	0.763381823
0.934381832	1.426672731	0.469826504	2.857303003	0.655141088
1.035995417	1.493505365	0.468336085	3.082596607	0.700913795
1.024899384	1.47702712	0.467574983	2.95888694	0.683493715
1.120462853	1.533077251	0.456247997	3.125117161	0.711019658
1.388726014	1.631668251	0.470734767	3.357290502	0.795376116
1.65137966	1.744023942	0.472973863	3.64586566	0.899234121
1.590813117	1.713121907	0.467011645	3.547925286	0.857909116
0.647504985	1.198083153	0.494244375	2.491243767	0.564369379
1.244300731	1.598991486	0.461293823	3.29149657	0.770314398
0.816075471	1.35001196	0.485256411	2.773587176	0.627642426
1.30469489	1.614164941	0.470238264	3.378165773	0.789997405
0.916328428	1.411113892	0.475389034	2.853919309	0.649812735
0.651010044	1.201423103	0.493555752	2.484707293	0.566046713
1.011257946	1.437194357	0.471230651	2.850658979	0.665813402
0.941639858	1.439019547	0.460910265	2.860534345	0.654236866
2.065303017	1.829863772	0.463817588	3.711882363	0.957111244
1.565244748	1.716756469	0.46706593	3.548466234	0.870052087
1.379847332	1.64502724	0.460266682	3.372764218	0.7972223
0.798678521	1.337309251	0.476741522	2.733914585	0.608694249
2.281283941	1.931509483	0.441670999	4.211886911	1.048896225
0.684911417	1.251774546	0.474676155	2.44421482	0.564593775
0.636554316	1.228717328	0.448209747	2.380637163	0.532782127
0.777567281	1.321359569	0.439255313	2.426467184	0.567605881
2.062834158	1.874318097	0.459115599	4.042117891	1.01897494

log.sigma.0.5.mm.3D_glcm_DifferenceVariance	log.sigma.0.5.mm.3D_glcm_Idn	log.sigma.0.5.mm.3D_glcm_Idm	log.sigma.0.5.mm.3D_glcm_Correlation	log.sigma.0.5.mm.3D_glcm_Autocorrelation
0.598901066	0.922546723	0.67207318	0.065836703	21.81258678
0.555076563	0.927900315	0.692628119	0.045420107	21.46231293
0.735336703	0.893170868	0.649551372	0.012763909	12.65585387
0.71796017	0.917823959	0.657389807	0.021548956	31.3952728
0.34572088	0.907285497	0.72494485	0.083276233	7.009614253
0.561989358	0.9275129	0.691581351	0.012640117	20.88829579
0.335541826	0.90925085	0.730403297	0.04374741	7.005197124
0.542942562	0.930093435	0.701458396	0.031531194	21.65804877
0.457980176	0.933712051	0.713045628	0.05393605	21.96881927
0.523050075	0.928017178	0.691497999	0.043340183	21.28853775
0.510923062	0.925292244	0.713991715	0.008202056	21.37998204
0.872489343	0.90881807	0.627840766	0.014190429	21.21706486
0.41056384	0.928091205	0.720056214	0.055441812	13.18067963
0.383047179	0.917617215	0.71734007	0.067646095	12.91640896
0.435232785	0.909047197	0.690059422	0.010214192	13.21812999
0.478054629	0.913127022	0.705927384	0.044575084	12.62026507
0.366431197	0.919942854	0.724603839	0.034020118	12.82620269
0.397352154	0.922390113	0.734347343	0.076090908	13.6139526
0.385070225	0.905728734	0.721602335	0.109091901	7.26333545
0.417568385	0.922298106	0.735093517	0.05532235	13.10026571
0.65501821	0.923520097	0.644892382	0.013214312	20.69639748
0.429314584	0.925579256	0.711316086	0.054068424	13.05787419
0.509206089	0.912084159	0.70383473	0.034960447	12.91629312
0.494089671	0.926759082	0.684673028	0.066525358	21.33219363
0.500901924	0.921884719	0.700143489	0.041666575	12.46738469
0.653677142	0.907331318	0.651906284	0.06040075	13.76885381
0.576036553	0.912313109	0.667092099	0.011446808	21.25404368
0.64904119	0.909282338	0.659129264	0.015766877	13.02740316
0.404024851	0.929748135	0.726586416	0.031254812	21.43412557
0.569481215	0.925013077	0.681005543	0.018283756	21.34336363
0.551232314	0.91711076	0.684191763	0.020842725	21.1104801
0.354314514	0.925348344	0.74293145	0.094674016	13.48433326
0.668600703	0.929491782	0.67341846	0.026773857	21.06274276
0.462781205	0.92207979	0.699245813	0.114635702	14.0997029
0.811011381	0.913904984	0.646737221	0.015364059	21.10432988
0.537296939	0.921674549	0.701145192	0.046451961	21.24759442
0.776671709	0.922806346	0.648140742	0.044234068	31.49848564
0.636334744	0.920177739	0.663966884	0.021249612	21.13944838
0.309192526	0.895243063	0.739143811	0.037529965	6.658960906
0.63539102	0.926493982	0.690530071	0.040088575	21.76204757
0.696280172	0.903498997	0.639435226	-0.000115136	21.27219394
0.496696348	0.923685778	0.707033035	0.074230649	21.97456329
0.472498131	0.924294467	0.708075109	0.03867028	21.38479636
0.446322762	0.934528918	0.71602387	0.085195932	22.0078341
0.654190672	0.929351489	0.671190035	0.016301398	31.16594317
0.726406152	0.91140111	0.631986242	0.029809901	21.21118683
0.755102929	0.922601865	0.646032247	0.029974828	31.44261986
0.619724564	0.912750779	0.670726245	0.030180627	20.60997266
0.388141283	0.901564896	0.709270347	0.081026033	7.442574237
0.48113009	0.926346707	0.716643888	0.013655144	21.56422158
0.430063772	0.927018562	0.716739915	0.037437786	21.40570579
0.544925793	0.922900473	0.70606972	-0.004782494	20.96533697
0.472723769	0.932444453	0.708300433	0.02269609	21.54091663
0.567684688	0.901886367	0.671484255	0.016580138	13.05064535
0.566474032	0.916364152	0.681833	0.01702196	21.36269997
0.789111378	0.906496298	0.65535967	0.027033393	20.95065557
0.593043372	0.924164526	0.67792993	0.047776408	31.5535548
0.394447831	0.936478121	0.721804737	0.042439437	21.38360376
0.801497503	0.92656264	0.665722536	0.025591637	31.43321937
0.432892598	0.912149041	0.700537867	-0.01453484	12.92221958
0.585662599	0.934137719	0.690734589	0.061914909	21.78621648
0.608814168	0.933700209	0.689933649	0.03445798	31.55486242
0.697693036	0.908255757	0.657549656	0.02487973	21.12760171
0.508972399	0.912408914	0.704759821	0.033013563	13.2965513
0.409995332	0.903884425	0.716857308	0.045629918	7.336897616
0.558943893	0.920873111	0.662671736	0.093051611	21.60260332
0.709585242	0.921136598	0.671075563	0.016438878	31.52200004
0.594941996	0.929375621	0.700690882	0.041746135	21.76405078
0.60345783	0.915380909	0.679902624	-0.003818311	21.27296953
0.704298662	0.919998505	0.666407026	0.039755068	21.10722959
0.629302705	0.919760473	0.661670183	0.041293026	31.25870695
0.408740873	0.927999386	0.719792947	0.046039717	13.35721743
0.480404193	0.931079496	0.70274783	0.061398748	13.37299577
0.524091391	0.922685324	0.704367968	0.025205248	21.23505388
0.64847862	0.919218228	0.660292041	0.070993881	21.5138446
0.659467027	0.910701965	0.664806469	0.017138917	21.18264429
0.504357991	0.930130459	0.699696672	0.014805938	12.95323422
0.54426094	0.916805879	0.682658664	0.046984139	21.78899917
0.556875565	0.918962477	0.69142709	0.019548686	21.05546486
0.613906395	0.925241748	0.684423716	0.021581062	20.98895697
0.755730043	0.92536715	0.658046121	0.012968533	31.38518576
0.841670821	0.907724227	0.622502134	0.012610227	20.880765
0.853141835	0.920246192	0.639746365	0.024521889	31.36398901
0.328175745	0.907898051	0.726128871	0.068552953	7.164135346
0.650304596	0.909875003	0.661355878	0.025437276	21.27973669
0.421702106	0.91358211	0.704934093	0.030587127	13.02796064
0.67930614	0.917559806	0.655283156	0.045535428	21.52010046
0.493391346	0.922240343	0.70122114	0.0459584	21.46528515
0.328779937	0.889600358	0.725472977	0.028983785	7.190784077
0.566308243	0.92951966	0.700017456	0.040251755	21.34902311
0.513044655	0.921992899	0.701349241	0.025575455	21.61654406
1.145321012	0.913040788	0.619768455	-0.005335836	31.62372097
0.805655691	0.910419897	0.631849752	0.00164368	20.95623018
0.742825416	0.925173139	0.657404721	0.019381791	31.23688723
0.427755496	0.934381063	0.714413122	0.031925929	21.43960788
1.176667719	0.895569378	0.589675923	0.05485559	20.61724705
0.36559817	0.921410375	0.729670518	0.010468591	13.047675
0.351571749	0.913626641	0.743986155	0.022552066	13.69716063
0.453515772	0.939096881	0.73650467	-0.005351961	21.50620851
1.022500816	0.897261735	0.589578012	0.037793173	21.38355249

log.sigma.0.5.mm.3D_glcm_SumEntropy	log.sigma.0.5.mm.3D_glcm_AverageIntensity	log.sigma.0.5.mm.3D_glcm_Energy	log.sigma.0.5.mm.3D_glcm_SumSquares	log.sigma.0.5.mm.3D_glcm_ClusterProminence
2.185480274	4.666039339	0.167698548	0.610946709	8.009598871
2.068433929	4.630093801	0.193362446	0.533794418	5.790651199
2.251689693	3.556267244	0.147176099	0.708906225	7.755912564
2.216751517	5.601822915	0.161754188	0.686197104	10.15692764
1.841418464	2.641393246	0.218489445	0.368672911	2.514727275
1.997944156	4.569637093	0.199862187	0.522488343	5.707913086
1.77054268	2.643772689	0.23381705	0.339071935	1.732972877
1.999441343	4.652102445	0.207698703	0.504716214	5.896705261
1.942636143	4.684487349	0.214197933	0.444288772	4.782593262
2.052577849	4.611559596	0.187057152	0.5128234	4.826821194
1.901538601	4.623466136	0.226644858	0.455642033	4.0901913
2.360806641	4.604804805	0.13702417	0.847542383	14.25734126
1.883048397	3.627378928	0.219518548	0.404231128	3.375256366
1.885308389	3.590162829	0.210682919	0.39797933	2.527812374
1.949174542	3.634998662	0.186022689	0.442551767	2.852625916
1.991350117	3.54953463	0.187432953	0.463925249	3.790155986
1.80452848	3.579653777	0.219580422	0.361140338	1.914379284
1.865790963	3.685575934	0.239109408	0.387431087	2.900861684
1.947163973	2.68625454	0.211740973	0.413158623	3.167895651
1.845494888	3.616385695	0.234854828	0.392662037	2.769821447
2.238456402	4.548363896	0.137941005	0.66249777	6.822925876
1.935815296	3.610275074	0.207220282	0.429735708	3.068463975
1.979769945	3.591546916	0.205912385	0.485928277	4.157591465
2.061623214	4.614871258	0.182226721	0.518100914	5.996503105
2.029985362	3.528061501	0.175537195	0.485134841	3.621819749
2.274186455	3.704868646	0.148208256	0.683507266	8.795774116
2.110683589	4.609513764	0.169316799	0.569983211	5.297608511
2.194027341	3.607962144	0.154751103	0.635745202	6.096084507
1.816625643	4.628373122	0.236301677	0.384843887	2.673199233
2.080759608	4.618815788	0.178110566	0.546323623	5.544249219
2.060707982	4.593420729	0.181228517	0.531081342	4.683649091
1.822921301	3.667392647	0.242460548	0.35610206	2.496447055
2.13925623	4.587561407	0.180577358	0.629814256	9.070155826
2.043133669	3.746635309	0.203170455	0.505026759	6.693307313
2.276870263	4.59268464	0.161549219	0.76806327	10.41884785
2.013084582	4.606901229	0.20539144	0.509717425	5.857138884
2.320181709	5.609260148	0.148896145	0.763637473	11.81340577
2.16071547	4.596305142	0.169826404	0.621428489	7.055908102
1.698519687	2.578218522	0.236464568	0.309122879	1.157785291
2.089683188	4.662400027	0.203825969	0.587762756	8.793142308
2.226947847	4.612184242	0.151083754	0.69257942	8.469242764
2.013564521	4.683710867	0.210822855	0.489509977	5.451014498
1.941275901	4.62244829	0.212661249	0.452888751	4.450120711
1.942212207	4.686968998	0.225079225	0.449044561	5.441259734
2.140990033	5.581682684	0.16901417	0.616193439	8.56923709
2.332673881	4.603082135	0.130689917	0.7508273	9.002654746
2.291175045	5.605415315	0.149593955	0.740644964	10.39976473
2.134233168	4.537803877	0.169023999	0.603882069	6.867893222
1.924299271	2.720613287	0.20808508	0.42154635	3.362840295
1.886753934	4.643121864	0.228704805	0.435942097	3.73619708
1.890409922	4.624959684	0.218429261	0.413111453	3.253728454
1.918118793	4.579065207	0.216275295	0.481118128	4.938392808
1.926273714	4.640119748	0.217582091	0.445958429	4.069763036
2.111078697	3.611225726	0.16590575	0.560646424	5.290766375
2.076305983	4.620963299	0.178792287	0.542319808	5.348673417
2.298149598	4.575015057	0.146781975	0.745562608	9.570931419
2.128618464	5.614762503	0.175553372	0.582946967	7.693291602
1.832925687	4.622443399	0.223881759	0.385571522	3.256881669
2.216518615	5.604815745	0.174163735	0.729440089	13.49613952
1.872695708	3.595615652	0.203533245	0.415148088	2.42034112
2.111304728	4.663795209	0.188763001	0.566437607	7.711918673
2.081484991	5.615561887	0.190925589	0.567679987	7.575603492
2.221047743	4.594560911	0.161160839	0.678186972	8.204839673
1.975100445	3.644253841	0.210654281	0.479650416	4.311027926
1.894749349	2.705154794	0.223644585	0.405184689	2.526759928
2.226283093	4.641617427	0.15326474	0.617026679	7.546545648
2.182297475	5.613438175	0.171768349	0.653824256	8.642587547
2.038187229	4.662773037	0.210976436	0.544571752	7.86963614
2.039511084	4.612456883	0.193796032	0.558178067	6.135034771
2.208422958	4.591349566	0.169939195	0.675158521	9.503488752
2.215984129	5.58859505	0.149142361	0.633279949	6.971735616
1.871346549	3.652101805	0.226247258	0.400240314	3.106302303
2.015146735	3.652843693	0.199216548	0.477629029	4.506118395
1.97918408	4.60681863	0.205490619	0.485210678	4.560049833
2.265063738	4.633123299	0.154690985	0.670321167	8.829020288
2.167047507	4.601295718	0.169507367	0.632013523	7.43360899
1.957710505	3.598107901	0.205672829	0.474005844	4.111080189
2.088577458	4.66483729	0.185385046	0.545767764	5.467700958
2.014979197	4.587524642	0.197874061	0.522514691	5.593073454
2.097369481	4.580056317	0.181038143	0.572256434	6.0131712
2.221950845	5.601411141	0.162435655	0.703731042	11.02675905
2.369144799	4.568420946	0.133264339	0.83595107	11.84256797
2.335200587	5.598641379	0.14420387	0.81454177	14.08677459
1.794583178	2.6718086	0.231417472	0.349152508	2.225710794
2.193587304	4.611224058	0.164161874	0.638444911	6.660747306
1.917409902	3.607646149	0.20434823	0.420860853	2.840356757
2.254284767	4.635561553	0.157370391	0.683848411	8.654754929
1.987291847	4.630635727	0.207826709	0.480540513	5.021670398
1.759101009	2.679504686	0.2263205	0.336134333	1.467940398
1.994463068	4.618048337	0.205967111	0.528133333	8.393936563
1.984132207	4.648016108	0.211333851	0.483169097	4.299305646
2.438127955	5.623702655	0.137337942	1.030151912	24.23029535
2.310494084	4.577740569	0.140313007	0.783077312	10.00745293
2.229474	5.587751688	0.163830275	0.703779381	9.677211216
1.898889361	4.628863222	0.213003873	0.412530392	2.942317256
2.675636215	4.533322966	0.088164526	1.20674662	23.30149887
1.734594458	3.611637515	0.242969858	0.346177537	1.987751302
1.707740859	3.699998286	0.269077212	0.32522379	1.616443871
1.734387695	4.637679137	0.26630588	0.386862239	3.200777642
2.580248446	4.619846528	0.093161753	1.071852398	17.9231078

log.sigma.0.5.mm.3D_glcm_SumAverage	log.sigma.0.5.mm.3D_glcm_Imc2	log.sigma.0.5.mm.3D_glcm_Imc1	log.sigma.0.5.mm.3D_glcm_DifferenceAverage	log.sigma.0.5.mm.3D_glcm_Id
9.332078679	0.289298464	-0.02888614	0.7350489	0.688973577
9.260187602	0.223560229	-0.018364999	0.680430533	0.706504418
7.112534488	0.262090961	-0.020805134	0.814821742	0.672830715
11.20364583	0.244988613	-0.019047858	0.789697799	0.676326499
5.282786493	0.25822465	-0.034453555	0.570496237	0.730900192
9.139274187	0.226733121	-0.018185038	0.684720647	0.705300282
5.287545377	0.215972475	-0.023845956	0.557197666	0.736037412
9.304204889	0.234551467	-0.020307019	0.658648957	0.713800475
9.368974698	0.249840791	-0.022998465	0.617605491	0.722993672
9.223119191	0.205330289	-0.014720839	0.676665772	0.705403117
9.246932272	0.191470018	-0.014836576	0.626355536	0.725439187
9.209609609	0.274978285	-0.023476148	0.890796084	0.653275792
7.254757855	0.22793854	-0.021277398	0.593180866	0.728392011
7.180325657	0.216326115	-0.025670246	0.594748419	0.726177824
7.269997325	0.190025532	-0.013189989	0.66235279	0.701539845
7.09906926	0.22577193	-0.019985959	0.637095443	0.716882013
7.159307554	0.17363153	-0.01300349	0.575231264	0.731370337
7.371151868	0.228140338	-0.025081285	0.561509161	0.742271091
5.372509081	0.266362919	-0.034878804	0.586191886	0.730390275
7.23277139	0.213876867	-0.026181476	0.564828305	0.743665108
9.096727792	0.1752497	-0.009263383	0.807593308	0.66616306
7.220550149	0.17116669	-0.015188798	0.616361173	0.721699124
7.183093833	0.207964205	-0.024482896	0.649370098	0.717342102
9.229742515	0.284973676	-0.030085989	0.685798611	0.69778631
7.056123002	0.221816894	-0.018603478	0.653862834	0.713021569
7.409737292	0.269201629	-0.024151515	0.792115429	0.67261726
9.219027528	0.187377489	-0.012067568	0.741917648	0.684655828
7.215924287	0.213903058	-0.014915746	0.775327758	0.679898115
9.256746243	0.214477617	-0.025124019	0.579634853	0.734998672
9.237631576	0.237743237	-0.018942072	0.708889162	0.696080359
9.186841458	0.17794211	-0.010944736	0.698915716	0.699849837
7.334785294	0.204474929	-0.022710383	0.53564624	0.74900848
9.175122815	0.25079568	-0.022145952	0.745445839	0.690932217
7.493270618	0.352745425	-0.051217743	0.648750713	0.711506475
9.185369279	0.286834322	-0.025179712	0.836876828	0.670734248
9.213802458	0.223942559	-0.018559091	0.658736808	0.713537781
11.2185203	0.236089758	-0.017163134	0.82499331	0.670842262
9.192610285	0.201497595	-0.013171419	0.761116507	0.683358193
5.156437043	0.125520006	-0.007533498	0.533903455	0.743136696
9.324800054	0.274512926	-0.027771552	0.700719411	0.706199074
9.224368483	0.210393861	-0.01384664	0.829022336	0.661539157
9.367421734	0.277018648	-0.031247907	0.638308656	0.718802097
9.244896579	0.233475261	-0.021080184	0.630412373	0.718279097
9.373937996	0.278691216	-0.034131485	0.609005364	0.725370676
11.16336537	0.245295554	-0.019546742	0.745241891	0.688245276
9.20616427	0.209259687	-0.013263018	0.853591639	0.656300813
11.21083063	0.221801754	-0.01488095	0.825434568	0.668609701
9.075607754	0.267390116	-0.023763079	0.741898069	0.687383785
5.441226573	0.318597175	-0.050943095	0.611824653	0.71828262
9.286243728	0.229616527	-0.021097284	0.614579844	0.726800761
9.249919368	0.194358825	-0.014922076	0.603968169	0.725939
9.158130413	0.261765233	-0.025975891	0.648896162	0.717421306
9.280239496	0.216222113	-0.018709402	0.630750558	0.718996558
7.222451452	0.231747012	-0.018445897	0.729859728	0.68733093
9.241926599	0.221102418	-0.016949663	0.706084262	0.696650729
9.150030113	0.266624785	-0.021443651	0.813083044	0.679378546
11.22952501	0.223171726	-0.016415069	0.718498131	0.693288091
9.244886797	0.224583513	-0.023723739	0.585905257	0.728184884
11.20963149	0.308427945	-0.03070352	0.786093837	0.684669691
7.191231303	0.182048779	-0.012956198	0.639469201	0.711482257
9.327590417	0.264250974	-0.023986087	0.689929739	0.704509384
11.23112377	0.252856619	-0.022944031	0.696489095	0.704783159
9.189121822	0.218306063	-0.015623684	0.788649957	0.678726409
7.288507683	0.213152008	-0.016465196	0.646096086	0.717443883
5.410309588	0.244998106	-0.025214776	0.600639765	0.726901831
9.283234854	0.268381972	-0.023087877	0.747260633	0.679791872
11.22687635	0.294557913	-0.028831599	0.757541828	0.68893319
9.325546074	0.208373631	-0.015998612	0.669671969	0.713710553
9.224913765	0.20750437	-0.014982593	0.718403752	0.696681667
9.182699132	0.283563761	-0.026219929	0.768540281	0.68599893
11.1771901	0.196249202	-0.012368011	0.764262598	0.680704054
7.30420361	0.21137886	-0.019317306	0.593990685	0.728417738
7.305687385	0.227138479	-0.01922814	0.644296873	0.714703896
9.21363726	0.18246336	-0.012063636	0.649222201	0.716924801
9.266246599	0.227791616	-0.017452724	0.771050346	0.679329691
9.202591437	0.226162951	-0.016559572	0.763381823	0.683389916
7.196215802	0.2162673	-0.017011715	0.655141088	0.712236036
9.32967458	0.236412721	-0.020512097	0.700913795	0.698679183
9.175049283	0.217374313	-0.016832956	0.683493715	0.705669419
9.160112633	0.233251676	-0.018381967	0.711019658	0.701023925
11.20282228	0.29424728	-0.027107124	0.795376116	0.677950188
9.136841892	0.225704826	-0.014744271	0.899234121	0.648898965
11.19728276	0.268895794	-0.021301848	0.857909116	0.663295749
5.3436172	0.293546723	-0.04237656	0.564369379	0.731393976
9.222448115	0.166154189	-0.008987035	0.770314398	0.681989793
7.215292297	0.14454328	-0.007942142	0.627642426	0.71513729
9.271123106	0.246559336	-0.019030891	0.789997405	0.676074603
9.261271454	0.214454238	-0.018190532	0.649812735	0.712900972
5.359009372	0.225669573	-0.02381467	0.566046713	0.73094111
9.236096674	0.343908833	-0.046853843	0.665813402	0.712062999
9.296032216	0.184137188	-0.012641117	0.654236866	0.714422302
11.24740531	0.332869276	-0.032592113	0.957111244	0.646516642
9.155481139	0.249379073	-0.018299752	0.870052087	0.657144827
11.17550338	0.254818482	-0.020396959	0.7972223	0.678527789
9.257726443	0.178195449	-0.012400636	0.608694249	0.723509846
9.066645933	0.385397494	-0.038587625	1.048896225	0.624342384
7.22327503	0.17101037	-0.01321589	0.564593775	0.736081732
7.399996572	0.196594668	-0.017618743	0.532782127	0.750244836
9.275358274	0.218057873	-0.02209051	0.567605881	0.74481673
9.239693055	0.293349089	-0.022251618	1.01897494	0.623118197

log.sigma.0.5.mm.3D_glcm_ClusterTendency	log.sigma.0.5.mm.3D_firstorder_InterquartileRange	log.sigma.0.5.mm.3D_firstorder_Skewness	log.sigma.0.5.mm.3D_firstorder_Uniformity
1.303024468	18.47120714	-0.245591537	0.392216328
1.116497624	14.89733231	-0.631572571	0.423809
1.435233464	20.14582026	-0.415927234	0.366156662
1.401960996	18.66713095	-0.98026913	0.386513581
0.802089716	11.13166857	0.708847155	0.44431996
1.058328935	14.14715886	-1.08930026	0.439763194
0.709068774	9.86817503	-0.007176752	0.473046742
1.041321428	13.13134742	-0.996630436	0.439726828
0.937223682	13.23549068	0.003731045	0.444170204
1.069672565	17.18609929	-0.466627166	0.418316615
0.918273761	12.66607225	-1.264319403	0.472821828
1.720645641	23.40407276	-0.664054891	0.348599706
0.853810576	12.86226225	0.11486324	0.455923803
0.85002611	15.88649833	-0.248395309	0.450110713
0.894827178	18.21206331	-0.283351912	0.420018495
0.969888997	12.21713257	-0.420485486	0.419472099
0.746767948	14.06684923	-0.16188628	0.457419086
0.83549	10.68924063	-0.11379998	0.472650765
0.919459825	13.91392034	0.257704683	0.438932008
0.829322537	11.35751414	-0.875369645	0.47589564
1.342495612	22.72031474	-0.37476522	0.36175759
0.906974185	15.34833336	-0.508094238	0.447084575
1.005986927	16.16918659	-0.836100847	0.442380369
1.106272989	12.48086709	-0.012608748	0.407478556
1.0105329	17.26301956	-0.234388234	0.409356042
1.4517641	18.90465987	-0.273599811	0.362921685
1.152607685	17.95208526	-0.615933519	0.397330604
1.291436018	20.96063995	-0.396923106	0.375983795
0.794228901	9.856416941	-0.7157632	0.46975665
1.112405094	12.69214511	-0.406488771	0.40665104
1.0840767	17.88296187	-0.561475823	0.411476015
0.781092708	12.39657766	-0.033437393	0.4818608
1.293576689	14.76517951	-0.841347788	0.409518104
1.133557566	12.32097435	0.681730989	0.425080286
1.558854556	19.45486927	-0.916442135	0.391757467
1.067335524	11.25335789	-0.95744068	0.442504166
1.596585886	18.85717249	-0.54191433	0.365282172
1.269637584	17.17609882	-0.555665974	0.399699849
0.641632676	12.78226268	-0.344083228	0.484673898
1.223354147	12.17908829	-0.558332037	0.430600307
1.384736353	20.69524992	-0.855592765	0.380909267
1.053111329	11.80744272	-0.335167209	0.436660108
0.941150202	11.69844067	-0.83623176	0.446640188
0.977786873	11.94653291	0.154373258	0.454860689
1.253893344	15.36139739	-0.865137642	0.403165751
1.546982849	22.16788626	-0.424113639	0.349663103
1.525083374	20.15147734	-0.68497157	0.373902043
1.244333121	17.33910322	-0.726169177	0.400843074
0.922534015	10.62923086	0.410265853	0.425915447
0.883093119	12.32125419	-1.050587596	0.463200067
0.857019221	12.33912367	-0.439000231	0.453488401
0.957217928	10.12893653	-1.606577859	0.459560223
0.912228051	12.72688699	-0.651876491	0.452037076
1.140636651	18.22521567	-0.442777688	0.400421954
1.10326446	14.9160409	-0.503795386	0.402154288
1.530830447	16.92788899	-0.61219	0.365945265
1.22172413	16.61977553	-0.64491689	0.400298795
0.804217256	10.24309963	-0.342949886	0.45914026
1.497209442	18.07847536	-1.252172091	0.402352549
0.817770868	14.53892946	-0.319920469	0.447382885
1.203187515	15.68532991	-0.489606533	0.412095593
1.175908374	13.27368975	-0.479990646	0.417080231
1.391276316	19.29646587	-0.707479231	0.390782754
0.991104911	14.19841582	-0.70964965	0.445519375
0.848092026	14.57479417	-0.355191103	0.452559609
1.349626605	20.05685925	-0.006266049	0.372307106
1.329814883	17.54569018	-0.546190666	0.398333339
1.134130172	12.47206521	-1.147589007	0.445316253
1.112611006	15.55811882	-1.121760667	0.432699963
1.403719281	16.91382146	-0.715913697	0.386816555
1.318975802	20.5724268	-0.539215111	0.37279489
0.838723865	11.62996721	-0.04212237	0.462692112
1.014401968	14.51928031	-0.262909896	0.427884596
0.994963302	14.86757231	-0.837844852	0.43948142
1.436065985	20.76000273	-0.475641142	0.37446272
1.285330663	16.3556397	-0.854994367	0.394068089
0.961641543	12.56655097	-0.479119541	0.441637569
1.147075637	17.15081453	-0.453027635	0.4219411
1.065159382	13.66237688	-0.967419317	0.434603756
1.168562883	14.54655457	-0.629323918	0.409077286
1.426198153	16.66042352	-0.801568535	0.387863576
1.692424619	22.15061474	-0.706505425	0.349819517
1.667353963	18.92276907	-0.914856173	0.358533927
0.749105046	8.482336521	1.106826917	0.466308874
1.309478914	19.6725322	-0.700984937	0.392902227
0.867367942	15.24938095	-0.42331992	0.443966679
1.430698755	19.5202049	-0.39787759	0.381788009
1.005833623	13.20324862	-0.642145378	0.447455785
0.693527288	10.86404824	0.193138903	0.452773782
1.101275387	13.03900605	-0.837374476	0.444768382
0.991036528	14.38405883	-0.861756981	0.447928771
2.05530463	21.2673192	-1.26592833	0.345605536
1.567064501	20.56441867	-0.805567986	0.361898548
1.435270193	17.0125103	-0.650551571	0.385669629
0.851443046	14.29035151	-0.423575397	0.449394024
2.545702538	25.81498027	-0.264260878	0.280514834
0.69979873	10.16903198	-0.767628017	0.491806269
0.664340843	11.24395341	-0.774095093	0.515973011
0.769881677	9.643591911	-0.949675457	0.50592738
2.224575435	29.14483571	-0.586708487	0.292165897

log.sigma.0.5.mm.3D_firstorder_MeanAbsoluteDeviation	log.sigma.0.5.mm.3D_firstorder_Energy	log.sigma.0.5.mm.3D_firstorder_RobustMeanAbsoluteDeviation	log.sigma.0.5.mm.3D_firstorder_Median
13.2271711	441528.9076	7.910097261	4.953342438
11.39408414	571033.3904	6.513882601	3.266896009
14.23546346	419606.0639	8.838103776	2.788058996
13.8231875	990691.6103	8.120076733	4.141353607
9.188714058	181811.8785	5.052120917	2.438078403
11.07611244	433699.1529	6.252203293	2.899500728
7.888556562	150619.8253	4.310544139	2.498163939
10.54612221	470314.7345	5.786147343	3.370844841
10.27446394	481233.6165	5.870592309	3.183873653
12.0956809	854257.3712	7.479140317	4.008183479
9.674731378	386230.6992	5.446502152	3.424237967
16.14546407	854460.6889	9.871764504	4.758594036
9.554490034	585506.5581	5.564757167	2.947534084
10.43478298	360783.242	6.784337031	3.117317438
11.49038177	360544.7146	7.590489285	3.381053448
9.90932503	886963.5383	5.496043418	0.829544127
9.364183104	260187.5732	5.914274726	1.931486845
8.884239263	482914.2427	4.866622584	3.477119923
10.22070452	256778.4315	6.09448057	3.728919625
8.851585854	499265.2646	5.027455594	2.669549704
14.44304235	2535406.786	9.453475357	2.298009157
10.77476022	481191.5754	6.644535937	3.378742695
11.3994206	999812.4396	7.114552653	3.562192202
10.9510895	288106.482	5.886355592	2.517282963
11.63728251	629765.9017	7.50766929	1.377934039
14.25257479	561542.8781	8.490414381	5.155616999
12.56184792	674005.6714	7.711612371	3.377356052
14.03286198	1201939.6	8.870226828	4.266103745
8.412173854	565701.3104	4.43271003	2.252379656
11.01649457	874757.749	5.969016857	2.104815245
12.39986918	1177948.612	7.7643877	3.668031573
8.956324463	883491.9361	5.300058643	3.577769041
12.2326788	876587.4356	6.602375924	3.478317976
10.95971279	153690.7004	5.437688408	3.51095891
14.79741657	384747.7753	8.612139523	4.780709028
9.995531088	800836.2785	5.125686183	2.687288046
14.54337764	1551132.658	8.431898106	3.483972311
12.99997443	1154275.566	7.667835623	3.834733486
8.223274542	182656.1566	5.234504236	2.137717605
11.26852926	533763.3745	5.683467664	3.379569054
14.16636553	508586.0585	8.812742359	4.639505625
10.26511075	503973.2554	5.362056514	3.590352654
9.695666963	693053.83	5.134811097	2.883692741
9.925752659	478705.3609	5.211215332	3.44592905
12.1873983	1130946.714	6.88354457	2.573317885
15.26356945	1525100.421	9.653332937	4.674637794
14.67476083	1584067.745	8.778547385	4.526601315
12.72333912	471577.6343	7.623227686	2.36696887
9.854450876	131512.2959	5.131069506	2.72999382
9.796413632	242758.1248	5.406124503	3.560360909
9.55761999	814437.047	5.467738393	2.821696162
8.985096234	401339.4777	4.462095404	2.115698338
10.13140602	898460.3429	5.644909742	3.501148939
12.45716814	345309.1749	7.788027648	3.038534164
11.87303466	1189990.372	6.7948933	2.909879923
13.62061296	742990.0214	7.712504386	1.476193428
12.5451027	1087045.606	7.406233813	3.71475935
8.354047909	425220.6548	4.503667224	2.109630704
13.86297718	587294.5421	7.697645317	4.390445471
10.15283524	272960.7551	6.34180784	3.087536335
12.03329044	975628.384	6.888371807	4.196013927
11.40340551	1665173.662	6.067166307	2.935845852
13.79958107	847535.9899	8.327383421	4.35042429
10.63635781	441136.7242	6.229732318	3.512930512
10.5712543	132235.3038	6.395287678	4.763033628
13.94538551	597584.13	8.625734454	4.539243221
12.84992372	377802.2383	7.359848445	3.369620919
10.68468873	1027882.19	5.696414148	3.698389053
11.80994635	374449.313	6.897708605	3.700404644
13.42424746	959481.8978	7.543751952	3.434366941
13.73095015	1337769.754	8.704331858	2.767515779
9.039086291	399406.0744	5.003904048	2.766714573
11.00990324	1224577.223	6.380033834	4.009845734
10.9976819	1632569.501	6.474869543	3.168661356
14.29760556	1720761.693	8.758057741	4.703510523
12.76460962	804848.0151	7.202975279	3.257995129
10.38319907	560718.3813	5.720577293	2.56237793
12.57663616	442978.1678	7.515409032	5.410303116
10.91492808	861114.7118	6.017906538	2.869104505
11.85451559	1316672.353	6.689435475	2.717813969
13.30901812	531458.0465	7.398044861	2.934917212
15.97377068	1301037.562	9.637466755	4.085735321
14.87272138	1168238.561	8.493252215	4.159601212
7.741962827	79864.2298	3.886801993	2.136856556
13.67288321	1359475.623	8.488698803	4.763581991
10.49489316	716919.9492	6.612481044	3.147698045
14.07573508	557048.0635	8.582682244	5.199855328
10.35688225	748612.6669	5.78288626	3.407601118
8.641496385	67339.42778	4.974138217	2.654719114
10.4615487	158850.8307	5.560847294	3.322865486
10.87470795	823950.1738	6.329298063	4.085979462
16.68318609	688574.7377	9.270098619	5.571342468
15.08712409	678963.8917	9.165068849	3.891278744
13.74228528	1713932.752	7.744351167	3.829221487
10.22799343	1012266.989	6.151943432	3.674384475
19.01319146	598790.1392	11.59667053	2.274914026
7.924166869	256995.3029	4.445490784	2.673403502
8.467906672	71327.28439	4.832997107	3.892305255
8.529235002	161914.9694	4.532194982	3.058830857
18.81931459	741128.1802	12.15389709	5.111952782

log.sigma.0.5.mm.3D_firstorder_TotalEnergy	log.sigma.0.5.mm.3D_firstorder_Maximum	log.sigma.0.5.mm.3D_firstorder_RootMeanSquared	log.sigma.0.5.mm.3D_firstorder_90Percentile	log.sigma.0.5.mm.3D_firstorder_Minimum
11921280.5	97.66710663	19.15784952	26.07017136	-79.00727844
15417901.54	89.36591339	16.89723928	21.60817871	-78.89109802
11329363.73	70.81447601	19.37313818	22.43606586	-69.01734924
26748673.48	74.3975296	20.0965019	24.41271935	-107.31707
4908920.721	63.34180832	13.8413363	17.69512558	-49.31306839
11709877.13	76.91685486	16.27186522	17.55900631	-83.28283691
4066735.284	50.11273193	11.9372304	14.51773643	-44.82675552
12698497.83	75.67657471	16.14193854	20.44106522	-92.30934143
12993307.65	87.87315369	15.40817152	21.7013443	-77.24668884
23064949.02	75.31994629	16.86617885	21.0756897	-89.23868561
10428228.88	65.90568542	14.6198972	16.14525261	-84.70534515
23070438.6	76.11013794	22.51884392	26.81935349	-94.18399048
15808677.07	85.90402985	14.13859311	17.20911102	-58.54337692
9741147.534	54.16765213	13.93478664	17.7304966	-50.62160873
9734707.293	50.41027451	15.26138992	20.83163834	-69.41207123
23948015.53	72.11056519	14.69213171	17.07142487	-74.76027679
7025064.475	63.30325699	12.73226757	15.02519875	-56.97441864
13038684.55	55.70259476	13.73993686	19.67677574	-59.71034622
6933017.651	72.15096283	15.03453863	22.57473087	-44.69906235
13480162.14	69.48221588	13.11873054	16.07435608	-67.29396057
68455983.22	112.0979996	19.09991444	23.610676	-86.36553192
12992172.54	83.14252472	15.06220913	19.56790352	-69.52141571
26994935.87	50.18776321	15.57607253	19.21860886	-70.99777985
7778875.015	86.43849182	16.72468329	22.01615639	-76.93400574
17003679.34	99.00697327	15.55138529	18.59940643	-73.41369629
15161657.71	84.51838684	20.37995009	28.77735062	-68.61215973
18198153.13	65.75054169	17.45972521	22.35484695	-75.21931458
32452369.2	81.41798401	18.92757866	24.00075455	-74.97588348
15273935.38	52.24079895	12.90084063	15.26660595	-75.92798615
23618459.22	94.60811615	16.64060402	19.99642754	-89.81391907
31804612.51	66.78856659	16.86390415	20.95980453	-81.14704895
23854282.27	69.53042603	13.29279456	17.7716074	-71.82748413
23667860.76	110.514534	18.61022176	21.08265877	-97.44425201
4149648.912	85.4388504	17.87521429	25.18381119	-74.3822937
10388189.93	89.13349915	20.81524734	23.00030041	-86.02394104
21622579.52	74.15648651	15.77779609	17.39583817	-92.97033691
41880581.76	90.52780151	20.84736628	25.5330719	-108.4986801
31165440.28	83.87335968	18.44976575	22.27981567	-78.53922272
4931716.229	37.47291565	11.09430771	14.24576349	-47.42616653
14411611.11	90.93075562	18.06270277	21.44716072	-95.0900116
13731823.58	62.20855331	19.65867672	24.49331093	-89.06163025
13607277.9	71.4517746	16.15102852	21.82773361	-76.31009674
18712453.41	69.53587341	15.13133594	17.35425415	-97.19094086
12925044.74	86.36394501	15.94019744	19.39894619	-92.07681274
30535561.27	91.64272308	17.99117886	20.10302143	-115.884491
41177711.36	81.90566254	20.65433408	26.81098366	-84.25039673
42769829.11	87.46957397	20.73047293	25.33083153	-101.818985
12732596.13	69.92598724	17.63709276	18.43015575	-78.94913483
3550831.988	59.53362274	15.27016141	25.76708374	-49.46231842
6554469.368	51.96307755	14.92360131	17.91255169	-80.34246063
21989800.27	73.21826172	14.29779597	17.51199398	-97.10061646
10836165.9	53.72447586	14.32423757	13.82716703	-95.76268768
24258429.26	77.88840485	15.38257913	18.52527237	-87.55671692
9323347.721	62.98166656	17.17218708	22.29946136	-73.79813385
32129740.04	74.07030487	17.3855733	22.72600861	-91.46013641
20060730.58	68.65274811	19.83766242	23.7488884	-97.87358093
29350231.35	74.49880219	18.20761971	23.06809998	-107.5173035
11480957.68	81.83666992	13.22285972	15.35359402	-91.26811218
15856952.64	81.72773743	20.68954437	23.39470348	-108.1352921
7369940.388	61.38233566	13.93836854	17.44863625	-50.69207001
26341966.37	105.7374878	18.21355414	24.04880333	-98.3923645
44959688.88	98.93731689	17.42056701	20.72287445	-104.1398315
22883471.73	74.2333374	19.31837123	23.54002571	-77.56019592
11910691.55	56.71396637	15.36732128	20.06022186	-66.73426056
3570353.202	53.28085327	14.97089288	21.15950832	-47.244133
16134771.51	87.76860046	19.67961349	26.63195686	-83.28842163
10200660.43	68.28821564	18.8968506	22.30333157	-105.7941208
27752819.13	83.40450287	16.97778389	20.81288338	-95.60677338
10110131.45	59.26209641	17.01755867	20.37273827	-90.56073761
25906011.24	79.42712402	19.56325995	22.45726967	-95.40904999
36119783.37	64.82632446	18.74313428	24.14154434	-106.4611206
10783964.01	80.07022095	13.9209328	16.90646935	-68.1472168
33063585.03	115.9324493	16.17769212	21.93888969	-74.47927856
44079376.52	62.39417267	15.83477846	19.31164169	-92.62423706
46460565.72	95.50557709	20.09340982	26.54534054	-96.79850006
21730896.41	74.48792267	18.58171531	21.82359886	-93.44269562
15139396.3	107.401535	15.43039135	16.50136604	-72.01354218
11960410.53	72.56975555	18.05433235	22.69591827	-77.5210495
23250097.22	68.88348389	16.21281989	18.07194805	-90.32733917
35550153.52	77.47042084	17.19540902	18.98608284	-82.75189209
14349367.26	86.64228821	19.75358564	21.96151848	-120.9342041
35128014.17	85.34309387	22.12835552	25.58452797	-98.17543793
31542441.15	93.67268372	21.73030996	26.54791546	-109.6750031
2156334.205	56.30635071	12.35734892	17.45199013	-33.54951096
36705841.81	70.92028046	18.76201926	24.12023335	-88.2835083
19356838.63	53.54275513	14.36548939	18.68823395	-59.30985641
15040297.71	87.09031677	19.7506881	25.3025362	-79.37723541
20212542.01	68.22652435	15.68475031	19.28571625	-84.12458801
1818164.55	40.48775482	12.80011289	20.53523254	-45.5288353
4288972.428	88.04217529	16.57795002	17.61881485	-83.63440704
22246654.69	58.55315399	16.01881265	20.65686035	-76.1800766
18591517.92	75.14113617	24.9628636	28.76412811	-119.9674759
18332025.08	85.70507813	20.84888455	24.96054516	-88.69911194
46276184.3	94.32474518	19.84278174	22.7056282	-100.8807983
27331208.71	78.52394867	14.66633416	19.54761887	-79.29531097
16167333.76	95.86799622	26.02622544	31.48339329	-93.63381195
6938873.178	62.22396851	12.07359036	14.42750015	-74.19218445
1925836.679	46.90821075	12.90939711	17.3510252	-60.00680542
4371704.174	80.56890869	13.50320989	14.85283823	-78.70104218
20010460.87	92.79821014	25.34216762	33.07651787	-98.43964386

log.sigma.0.5.mm.3D_firstorder_Entropy	log.sigma.0.5.mm.3D_firstorder_StandardDeviation	log.sigma.0.5.mm.3D_firstorder_Range	log.sigma.0.5.mm.3D_firstorder_Variance	log.sigma.0.5.mm.3D_firstorder_10Percentile
1.679979732	18.66760748	176.6743851	348.4795689	-16.8695282
1.557819288	16.61302975	168.2570114	275.9927574	-14.64449768
1.756578871	19.34632577	139.8318253	374.2803207	-25.30708447
1.709054283	19.89027754	181.7145996	395.6231408	-18.01531029
1.388888579	13.46329082	112.6548767	181.2601998	-11.38687763
1.482951964	16.2385955	160.1996918	263.6919837	-16.9914032
1.301732538	11.65284892	94.93948746	135.788888	-8.868139839
1.496813945	15.86754848	167.9859161	251.7790949	-13.43880634
1.457819148	14.94094657	165.1198425	223.2318844	-12.39113197
1.55110241	16.61418783	164.5586319	276.0312373	-17.56706848
1.383035444	14.45789439	150.6110306	209.0307101	-12.74096489
1.878105349	22.32850914	170.2941284	498.5623204	-22.67212296
1.393665599	13.86582985	144.4474068	192.2612373	-12.25770988
1.38098487	13.7825909	104.7892609	189.9598118	-15.52711782
1.472241639	15.01606281	119.8223457	225.4821424	-15.72869158
1.482960306	14.65210014	146.870842	214.6840386	-14.44941959
1.323566877	12.66418676	120.2776756	160.3816262	-13.79058342
1.369159062	13.11951242	115.412941	172.1216061	-8.610306644
1.454023622	14.2455373	116.8500252	202.9353329	-10.4832468
1.323549962	12.9451255	136.7761765	167.5762742	-11.77733135
1.738267239	19.0568971	198.4635315	363.165327	-22.97411022
1.422769874	14.85278614	152.6639404	220.605256	-15.62870502
1.456967872	15.45705437	121.1855431	238.9205299	-18.22722054
1.593366604	16.41432407	163.3724976	269.4300348	-12.71820259
1.515408739	15.54172571	172.4206696	241.545238	-19.11050892
1.779345053	19.84150223	153.1305466	393.6852109	-19.51978683
1.618663163	17.26908452	140.9698563	298.2212801	-17.64616585
1.703882084	18.74783087	156.3938675	351.4811624	-22.32323685
1.340457156	12.7741462	128.1687851	163.1788112	-11.46688271
1.590680326	16.51396667	184.4220352	272.711095	-16.23903885
1.569688792	16.73889011	147.9356155	280.1904422	-19.51069183
1.310437294	12.74802344	141.3579102	162.5121017	-10.03670263
1.645290629	18.46069692	207.958786	340.7973307	-16.29628372
1.568243277	17.06302072	159.8211441	291.1466762	-10.25346375
1.735656443	20.7372002	175.1574402	430.0314721	-25.49344463
1.487837909	15.65225202	167.1268234	244.9929934	-13.61111546
1.803147855	20.69150505	199.0264816	428.1383813	-22.2048336
1.656584959	18.28511245	162.4125824	334.3453371	-20.3464489
1.199974132	10.97827738	84.89908218	120.5225742	-12.28708229
1.595655429	17.76875754	186.0207672	315.7287447	-13.7772646
1.714137182	19.39987796	151.2701836	376.3552649	-19.93186951
1.523666012	15.68764877	147.7618713	246.1023238	-11.14907293
1.453593655	14.92506575	166.7268143	222.7575876	-11.39202099
1.458715072	15.41170609	178.4407578	237.5206845	-10.36051731
1.619652865	17.92889131	207.5272141	321.4451437	-18.99115658
1.818750154	20.41593139	166.1560593	416.8102545	-22.73460732
1.772431703	20.50492977	189.288559	420.4521447	-21.80484009
1.613279577	17.63579364	148.8751221	311.0212172	-22.15998745
1.493130053	14.6277017	108.9959412	213.9696571	-9.104619503
1.40100174	14.6581915	132.3055382	214.8625781	-11.76865025
1.405313729	14.06139006	170.3188782	197.7226906	-11.98775988
1.406793048	14.30333179	149.4871635	204.5853002	-12.28629351
1.449472036	15.09356475	165.4451218	227.8156968	-12.85936298
1.601119413	17.01239702	136.7798004	289.4216525	-17.85771561
1.607323639	17.16275689	165.5304413	294.5602242	-16.43554153
1.778795151	19.79698705	166.526329	391.9206963	-23.43342724
1.63554524	17.94715011	182.0161057	322.1001969	-16.99775963
1.351763301	12.9781168	173.1047821	168.4315156	-9.097297382
1.690789947	20.53234957	189.8630295	421.577379	-19.20252609
1.393233249	13.77000232	112.0744057	189.6129639	-14.99628029
1.617102169	17.68651927	204.1298523	312.812964	-13.67520809
1.596234847	17.25189123	203.0771484	297.6277511	-15.86673164
1.690754514	19.11915164	151.7935333	365.5419596	-19.68513298
1.461562515	15.13077963	123.4482269	228.9404923	-15.01566124
1.427109635	14.48355905	100.5249863	209.7734827	-14.45408602
1.732078958	19.12991252	171.0570221	365.9535532	-17.99018211
1.667796033	18.67076686	174.0823364	348.5975351	-16.37489185
1.510809686	16.62264108	179.0112762	276.3121965	-11.37761784
1.529490354	16.87531804	149.822834	284.776359	-18.83071823
1.723120355	19.4652211	174.836174	378.8948325	-20.94821472
1.699627974	18.5838205	171.2874451	345.3583843	-19.62391357
1.388801101	13.58260173	148.2174377	184.4870697	-9.94304657
1.522995197	15.7382715	190.4117279	247.6931898	-13.50379562
1.478296982	15.68874465	155.0184097	246.1367088	-16.06791687
1.746395063	19.72163323	192.3040771	388.9428171	-19.66012402
1.673364222	18.4802244	167.9306183	341.5186939	-19.75742531
1.469454791	15.36077149	179.4150772	235.9533008	-15.75456505
1.579111263	17.52649809	150.0908051	307.1781354	-17.63274574
1.510596891	16.13755806	159.2108231	260.4207801	-16.71901417
1.598378675	17.15273584	160.2223129	294.2163467	-19.39038773
1.723723894	19.65036055	207.5764923	386.1366699	-20.79160728
1.866039966	22.03519517	183.5185318	485.549826	-25.97159729
1.840378151	21.57067662	203.3476868	465.2940899	-21.91739235
1.331244165	11.72102225	89.85586166	137.3823625	-6.511954784
1.672058451	18.53771182	159.2037888	343.6467594	-21.61318398
1.413288414	14.16049874	112.8526115	200.5197246	-15.37206993
1.739501267	19.42152806	166.4675522	377.1957523	-20.2839695
1.468862746	15.41126745	152.3511124	237.5071645	-13.25314255
1.332486891	12.23649937	86.01659012	149.7319168	-8.804395676
1.48373631	16.39202589	171.6765823	268.6985128	-11.72101822
1.472913965	15.67943349	134.7332306	245.8446345	-13.65815926
1.939624805	24.67359636	195.1086121	608.7863575	-20.61619682
1.800634193	20.76559054	174.4041901	431.2097505	-24.76737461
1.732406613	19.74759493	195.2055435	389.9675057	-22.20127678
1.415616199	14.33036632	157.8192596	205.359399	-12.58754635
2.144834976	26.02320829	189.5018082	677.2073699	-33.92599754
1.246099342	11.878611	136.416153	141.1013994	-9.58827858
1.232957752	12.34430908	106.9150162	152.3819667	-8.123756027
1.267239665	13.34260149	159.2699509	178.0250146	-11.63329525
2.065503674	25.11704075	191.237854	630.8657358	-29.31989136

log.sigma.0.5.mm.3D_firstorder_Kurtosis	log.sigma.0.5.mm.3D_firstorder_Mean	log.sigma.0.5.mm.3D_glrlm_ShortRunLowGrayLevelEmphasis	log.sigma.0.5.mm.3D_glrlm_GrayLevelVariance
5.842319715	4.306231464	0.043477367	0.796522259
6.309742032	3.086087787	0.044607877	0.695927827
4.181509245	1.018902474	0.100225604	0.858228281
6.671045444	2.871628081	0.029944427	0.875728652
5.849191795	3.212847787	0.142451566	0.485335039
7.058186704	1.040006679	0.046612344	0.651165509
5.82716705	2.590092971	0.148895995	0.427122932
7.714404551	2.963626989	0.044913519	0.659586974
6.598813971	3.765616191	0.04016829	0.573335409
5.268691261	2.904608706	0.043188353	0.649290405
8.616840636	2.17041103	0.043389342	0.573713953
5.246320947	2.921645305	0.056689568	1.078106851
6.864664528	2.763797687	0.068724789	0.51846021
3.842199082	2.053890688	0.069654272	0.487536173
4.066710891	2.725413719	0.073675816	0.538433016
6.116603099	1.083833676	0.079575379	0.559306363
4.877578719	1.314918732	0.069302346	0.438998158
6.050340567	4.082188006	0.067554589	0.512136281
4.775776525	4.806455946	0.145719849	0.544305554
6.797207927	2.127161634	0.072442404	0.480822409
4.189726784	1.281173194	0.046842894	0.803698969
5.234878328	2.502975835	0.073962243	0.539289951
4.744039397	1.921849485	0.082419728	0.583713807
6.607347945	3.207022969	0.042375752	0.688425647
4.811776719	0.548038602	0.078103601	0.586011815
4.384419161	4.653724807	0.084493824	0.866053428
4.743778173	2.573076777	0.046651376	0.671681503
3.973521478	2.602320404	0.086718499	0.779895519
6.966841081	1.803573561	0.039518986	0.483376209
6.462307867	2.049050286	0.044451356	0.676195876
4.464998646	2.049590423	0.043418046	0.667603953
6.078205321	3.766468576	0.06554569	0.456509141
7.69823385	2.354362586	0.049798152	0.831944279
7.455156524	5.326970019	0.069992459	0.710903065
5.081540086	1.800846898	0.053352039	0.95193279
8.418874673	1.986417881	0.044635861	0.646180604
5.305247791	2.544464466	0.029881226	0.960943711
5.030071119	2.459373686	0.045798247	0.783841516
4.176214016	1.600340381	0.148174761	0.358734626
8.264748093	3.245379283	0.047544577	0.810016896
5.274798435	3.179356131	0.050550084	0.843435529
6.842391827	3.841015291	0.041055153	0.662904953
8.450206719	2.489927675	0.042763622	0.595512617
8.395493122	4.07052943	0.040253013	0.628308425
7.647862749	1.495785096	0.030385888	0.757938302
4.088227922	3.129099196	0.047046094	0.910693046
5.188590425	3.049649723	0.02978219	0.921801283
5.084076281	0.214065241	0.047557427	0.716983733
5.246763726	4.382712906	0.144381161	0.559892001
7.955281384	2.802016744	0.043146484	0.568410318
7.775419475	2.589262265	0.040436225	0.533200767
9.944852042	0.77361608	0.044927512	0.582612162
7.507419957	2.967834857	0.04115079	0.594260087
4.805076717	2.337168547	0.083741556	0.67731251
5.93089735	2.774515229	0.044872033	0.70542585
5.282034544	1.269706244	0.051101255	0.930005439
6.713756971	3.068748713	0.028475968	0.756863937
10.10832977	2.532292161	0.039913133	0.488053647
7.235673845	2.545558379	0.031090944	0.937085906
4.565647255	2.159896659	0.072553541	0.493972167
7.406786483	4.349780517	0.043769501	0.767626831
7.027268915	2.41834734	0.027657968	0.743403936
4.962291391	2.76722019	0.046790999	0.820329996
5.309722921	2.685902268	0.080059274	0.59345717
4.056807295	3.788687209	0.173604414	0.545900256
4.999947832	4.618834716	0.042576523	0.81961097
6.710015922	2.914348523	0.029809119	0.826044354
9.156152927	3.45441013	0.047870156	0.720767659
6.445328237	2.195664869	0.04710769	0.690624333
5.781682164	1.956094946	0.049579338	0.882290606
4.863180673	2.438585301	0.028701743	0.778287269
7.606028819	3.050459016	0.071556598	0.528337892
5.774694627	3.744934268	0.075048178	0.625099568
6.25781959	2.145576903	0.04357428	0.616743616
5.086174751	3.847375852	0.046399542	0.857803327
5.846783785	1.939445768	0.047861233	0.806144874
6.858648847	1.464129919	0.082575286	0.60509525
4.958552252	4.333679885	0.042314879	0.693978965
6.991356249	1.560368074	0.046048124	0.656833705
5.804412368	1.210679395	0.045210125	0.719525992
6.811137724	2.016798423	0.030068961	0.890033722
4.618474768	2.028371789	0.053780423	1.03850147
6.329559764	2.629121694	0.032046017	1.060678327
6.715550448	3.914295562	0.136705203	0.45053069
4.517790808	2.892508837	0.04597791	0.789209069
4.474791021	2.418173016	0.074196556	0.51600936
4.948811322	3.590811615	0.045501786	0.873133483
7.219370125	2.916200924	0.042304391	0.6188843
4.59860625	3.756457532	0.138911202	0.438354035
9.607044984	2.475866303	0.046418801	0.674831037
6.38224215	3.279896975	0.042026482	0.620096074
6.948869491	3.789221761	0.038316102	1.33541504
4.741965932	1.861783157	0.051924046	0.947195562
5.40325542	1.941257681	0.02956414	0.89459052
5.716290577	3.121211089	0.039868535	0.533044292
4.077393378	0.396283816	0.060163019	1.40928037
8.330688964	2.161061033	0.07062558	0.42077224
6.879332264	3.777640386	0.066726098	0.429698861
10.66435103	2.076454344	0.042864807	0.508320566
4.37879337	3.370418911	0.059654756	1.283608303

log.sigma.0.5.mm.3D_glrlm_LowGrayLevelRunEmphasis	log.sigma.0.5.mm.3D_glrlm_GrayLevelNonUniformityNormalized	log.sigma.0.5.mm.3D_glrlm_RunVariance	log.sigma.0.5.mm.3D_glrlm_GrayLevelNonUniformity
0.054291107	0.340412085	0.61088085	289.6218491
0.056200328	0.364272426	0.702836516	499.1599373
0.118492363	0.319305346	0.578190837	258.5306508
0.036903682	0.338967957	0.558653807	599.8018542
0.184225908	0.407890915	0.672795587	262.0680908
0.059004651	0.38879637	0.721281758	432.5553202
0.191058499	0.428874915	0.722409501	303.1317899
0.056366955	0.379650978	0.773174031	466.407437
0.051536687	0.390521932	0.755492184	542.0228485
0.054692608	0.367553632	0.690710678	765.6287769
0.055874011	0.410621919	0.82458845	493.2924298
0.066542858	0.300452068	0.533098308	373.5275412
0.089647667	0.408904289	0.798714435	797.8032148
0.09113912	0.406512549	0.799940551	503.3526432
0.092041795	0.384036524	0.661193424	420.0157192
0.101002559	0.388443139	0.739143488	1093.93576
0.091920201	0.422834849	0.753409304	449.3722477
0.088506185	0.406499069	0.96245956	675.4782838
0.186029386	0.387182097	0.729469609	298.0066358
0.095621301	0.422312935	0.978121279	783.412493
0.057938203	0.323803779	0.583828765	1616.870026
0.094520325	0.396844577	0.744416353	569.3844193
0.103242153	0.386399708	0.807460623	1073.807341
0.053684457	0.365242325	0.604157078	266.0560428
0.100308353	0.374843657	0.745704217	662.3575809
0.100641323	0.314649622	0.567602541	309.3292055
0.054822306	0.359710673	0.453841898	611.0414827
0.104589256	0.327104244	0.618062757	789.1553437
0.052806288	0.413250499	0.955321833	915.094362
0.055312936	0.363336955	0.644691139	813.9136816
0.055128549	0.359055037	0.707333822	1026.323315
0.086603891	0.428708455	0.903533334	1404.01778
0.061303199	0.353344764	0.692714392	620.086485
0.087552529	0.366825266	0.630604584	123.7799982
0.063330697	0.326722696	0.618644349	210.4273685
0.056943291	0.387027034	0.779014227	837.3098635
0.036937149	0.312836461	0.652726808	794.9922036
0.056845649	0.341431673	0.68111002	811.4347326
0.193578244	0.455279204	0.770112165	444.5767696
0.059013681	0.358812213	0.790191152	398.3346005
0.060110996	0.333406718	0.544395326	323.2305903
0.052783062	0.373015309	0.793516502	483.7352774
0.055135032	0.393836801	0.788624497	796.8889738
0.052211981	0.394144249	0.790510007	497.4544449
0.038223994	0.356433611	0.712808172	862.1009737
0.05709042	0.30762609	0.567975107	805.4550997
0.036560819	0.322162411	0.597464547	858.7438466
0.059457929	0.357233556	0.638289755	379.4438448
0.181575361	0.378587415	0.65913056	147.754258
0.05529227	0.403675831	0.776054573	295.09638
0.052903526	0.403940326	0.809772285	1071.727361
0.057947987	0.407418465	0.781447505	529.2137696
0.053215436	0.392069031	0.763998873	1001.593752
0.102215335	0.359277437	0.583510949	300.4082563
0.056152904	0.354396902	0.687684945	969.5606683
0.062400752	0.314388402	0.681041917	415.5142926
0.035931406	0.35152235	0.64383867	811.713541
0.052817116	0.421356098	0.777077165	681.5799402
0.038374553	0.342396021	0.640420737	332.2186725
0.092806549	0.405836267	0.718364903	389.9941495
0.055096574	0.356554696	0.723516204	722.7190723
0.035615497	0.360336593	0.763784896	1345.592807
0.057468171	0.336324349	0.644727356	543.782914
0.100137637	0.385234195	0.747338984	487.3046292
0.209801774	0.387844385	0.740330768	155.8142556
0.053285958	0.331588107	0.530024617	369.2957776
0.037086542	0.348581427	0.610583723	260.9133009
0.059727786	0.376256602	0.829774464	899.9943575
0.058518938	0.369100105	0.731074591	327.3316125
0.060709264	0.328984388	0.685061371	576.9269993
0.035926757	0.332803023	0.617977395	905.0406015
0.092249583	0.408230085	0.821539808	557.4730493
0.094404273	0.371822833	0.733757646	1187.293406
0.055867274	0.38301665	0.837419485	1665.46912
0.056852901	0.324515233	0.616559405	989.3097742
0.058954452	0.340010396	0.643685719	557.8504645
0.10299348	0.386937336	0.775685707	615.669048
0.0532746	0.361219196	0.692194526	342.4313845
0.0583263	0.378856848	0.750871305	840.0191796
0.057220364	0.355568927	0.743941685	1080.383574
0.037347067	0.336834724	0.590615804	328.1064486
0.063602242	0.301984623	0.53563176	592.6635563
0.038854657	0.307791576	0.600501341	550.1483028
0.175743528	0.42313705	0.679569878	151.1426663
0.056493266	0.3347171	0.646890376	920.3561876
0.094281865	0.399834449	0.729172582	945.0362497
0.055709597	0.328556814	0.57135051	339.5814926
0.054469295	0.390778967	0.726120436	803.2558544
0.176150198	0.413325427	0.622317546	118.3776427
0.058260393	0.394225089	0.673068572	158.0791263
0.053632725	0.38376822	0.770534842	835.4124846
0.044391644	0.29267	0.505685639	241.8851852
0.061912219	0.313283859	0.53472054	359.8085155
0.036869083	0.326913705	0.688958027	999.5355152
0.052264365	0.399606823	0.799621734	1258.870561
0.070724852	0.250302346	0.459150246	167.2413141
0.092370603	0.444761263	0.783739852	515.7472627
0.086436781	0.444430691	0.78899328	126.8794039
0.055596411	0.439068134	0.817938839	255.9913706
0.068463196	0.261649257	0.424584938	232.4938492

log.sigma.0.5.mm.3D_glrlm_LongRunEmphasis	log.sigma.0.5.mm.3D_glrlm_ShortRunHighGrayLevelEmphasis	log.sigma.0.5.mm.3D_glrlm_RunLengthNonUniformity	log.sigma.0.5.mm.3D_glrlm_ShortRunEmphasis
2.630628563	17.14896782	468.2574941	0.767329412
2.847770927	16.10245607	718.7200332	0.749528071
2.511554331	10.07300037	462.0412616	0.781229342
2.494389071	24.77943579	1000.786596	0.778725319
2.882081538	5.493808008	325.4409534	0.732253277
2.913487252	14.99736615	571.0837748	0.739165245
2.99309072	5.131587559	350.9747182	0.722449903
2.946726277	16.03930659	644.8591599	0.749917923
2.909680851	16.48241056	734.8279806	0.752858693
2.790322944	16.09889397	1114.291998	0.756251845
3.117308875	15.17299317	598.8064387	0.727910844
2.388300063	17.40157567	736.4208485	0.796296064
3.072381819	9.788860007	978.437394	0.731007955
3.12350352	9.437174636	625.5264278	0.728948966
2.692516601	10.27525001	605.3316007	0.768698964
2.906064378	9.59958276	1485.177996	0.748308553
3.071513428	9.072167519	519.7943767	0.71779871
3.377212066	9.750863186	809.6733329	0.717298132
2.961827964	5.837561381	398.1941582	0.738120995
3.517010464	8.974398166	879.6514081	0.703303969
2.532974629	16.42800109	2813.406279	0.777286985
2.986141683	9.63995577	731.7216474	0.73407651
3.084433819	9.305281157	1436.36588	0.738364417
2.631847345	16.74882219	397.2223147	0.761768548
2.962054732	9.444384593	909.4532646	0.73875946
2.470222672	11.36547095	571.0864704	0.789039537
2.157267875	17.63442587	1085.557832	0.82642824
2.574863457	10.44327867	1373.511313	0.780477802
3.450345704	14.76623192	1080.448977	0.713555489
2.649469084	16.40005288	1245.057237	0.771635407
2.823953951	15.83591927	1516.527575	0.753794049
3.299652557	9.751453207	1618.343553	0.721444614
2.783030361	16.07823745	939.4160963	0.757615358
2.684832465	11.18466351	182.8982928	0.760111232
2.552344249	16.47670431	371.2207922	0.784924861
3.005651275	15.44830829	1100.47143	0.736787412
2.638875233	24.6565327	1434.212858	0.77833371
2.72942089	16.21466006	1297.543131	0.76544388
3.109540068	4.843117891	474.5456964	0.717587746
2.97398988	16.25110745	579.7641141	0.748366641
2.4058184	17.19955296	574.4651349	0.796763338
3.02532154	16.25817661	664.2623014	0.740453276
3.043870165	15.46398489	1020.746018	0.734068329
3.039547898	16.25373939	640.5286016	0.735998089
2.823481688	23.24364531	1292.815401	0.755999237
2.45185712	17.40918006	1536.102955	0.792660082
2.522070305	25.05875877	1535.097402	0.786417887
2.698657815	15.36275595	571.0155346	0.757189913
2.771956551	5.980586167	207.4747956	0.750667298
3.024344907	15.45636179	370.2676924	0.734188431
3.084068376	15.58140766	1336.256598	0.733485243
3.066693766	14.69960239	642.4953013	0.725290577
2.994339496	15.80138837	1300.689467	0.737245587
2.558942103	10.3847132	466.9425785	0.773565698
2.768714727	16.36400672	1474.619273	0.760610741
2.742609481	15.97960429	720.2791149	0.764200736
2.683809618	24.32440615	1260.742688	0.764309785
3.063795824	15.51507702	802.6488079	0.725959091
2.656327023	24.10894321	533.3128427	0.767238749
2.890035455	9.564257249	501.0254706	0.74500804
2.84396236	16.88866142	1079.647069	0.755619723
2.94062336	23.55073923	1950.114882	0.747723019
2.639855696	16.61491899	906.0552034	0.775164712
2.963135831	9.649334852	647.5910946	0.738011014
2.917611354	5.366292647	208.4530813	0.743835023
2.464543298	17.71238022	624.3759234	0.774900124
2.62518239	24.19746004	413.0855108	0.768264346
3.066870849	15.9649813	1224.78001	0.740563408
2.871759117	15.52402086	467.8501828	0.751759731
2.745466627	16.1111435	959.9318967	0.766145421
2.601004073	24.40796567	1527.244862	0.775831566
3.118985394	9.693082889	680.133801	0.728891861
2.902965432	10.16776018	1664.797141	0.74658906
3.094738611	15.36015552	2212.582969	0.738175843
2.584320809	17.26768078	1723.085939	0.778251948
2.677168026	16.11088479	897.0541759	0.765673562
2.986582292	9.376335124	816.5713609	0.740337087
2.766997157	16.71322893	511.585183	0.760011617
2.956745327	15.14083287	1135.579337	0.739691691
2.906746805	15.49443612	1589.637044	0.748445823
2.563708052	24.19138989	540.9514858	0.770777299
2.384259511	17.05549906	1165.397326	0.797464795
2.531396028	24.86259258	1024.672202	0.784520908
2.852766019	5.599662564	184.4397618	0.739570668
2.635570795	16.59724952	1541.316018	0.775452686
2.910854906	9.62340232	1224.338851	0.744001117
2.499064582	17.34861063	590.5841322	0.782329598
2.936003433	15.76274511	1047.981779	0.737591095
2.710789649	5.654729881	153.0076511	0.752425059
2.793120531	15.89269	212.3580691	0.749196736
2.965170936	15.9337406	1128.380068	0.744938406
2.311324902	26.12503965	499.83792	0.804186046
2.397500473	16.81070853	674.0265411	0.793095783
2.7317975	23.96027162	1683.910128	0.768800613
3.050643868	15.67997313	1590.878576	0.734365994
2.230367751	17.6434396	407.4498754	0.806802368
3.112489116	9.127633553	562.1630478	0.716237125
3.077590892	9.730514487	141.6982458	0.724633596
3.166250416	14.71208663	282.9534457	0.715996955
2.124645287	18.57395618	560.576464	0.821551265

log.sigma.0.5.mm.3D_glrlm_LongRunHighGrayLevelEmphasis	log.sigma.0.5.mm.3D_glrlm_RunPercentage	log.sigma.0.5.mm.3D_glrlm_LongRunLowGrayLevelEmphasis	log.sigma.0.5.mm.3D_glrlm_RunEntropy
60.56876925	0.706630859	0.12775099	2.988623438
65.03171328	0.684653846	0.138702835	2.946576374
34.75384228	0.722994358	0.236933325	2.988167338
82.2821249	0.720781461	0.082998397	2.971387817
22.10570833	0.676582638	0.46526425	2.763614492
64.70274325	0.678594909	0.146479814	2.89215986
23.17767918	0.668146423	0.475093099	2.70920948
67.65819846	0.68020456	0.142256208	2.90267831
66.96920515	0.684072711	0.136120592	2.839139265
62.8403303	0.692999308	0.13600071	2.909602019
71.10503044	0.663998978	0.149215429	2.845980705
54.14280068	0.737183291	0.130727248	3.071457793
43.40747768	0.665808756	0.244235027	2.844985174
43.25320338	0.665852447	0.253046508	2.822010533
37.33185074	0.706122043	0.221986049	2.752944501
38.63574965	0.68483816	0.256819498	2.861075315
41.83997269	0.66173017	0.251845463	2.764309096
49.66031308	0.648884345	0.25520711	2.872255305
23.79576501	0.67727519	0.466728201	2.840606984
49.57959817	0.638824808	0.282481452	2.874727066
55.04451181	0.718063088	0.132219222	3.000868889
41.9907241	0.675624705	0.242142042	2.834131093
43.34799785	0.673566909	0.259294567	2.880593343
59.11649216	0.706646751	0.129207686	2.890264367
38.76886988	0.677773839	0.263528414	2.917238094
36.63615797	0.726729631	0.208252093	2.996848561
48.1414863	0.767943499	0.107764491	2.686828348
36.3327655	0.718422561	0.225576945	2.957555648
78.34679686	0.650259126	0.16206894	2.838023715
59.32007866	0.708646845	0.131032697	2.888109508
63.14618554	0.689633399	0.138618915	2.938588651
47.45947564	0.653953846	0.253322065	2.809221083
62.86151745	0.693037109	0.142431398	3.020171126
40.12193645	0.700943547	0.205983388	2.87646675
58.23133249	0.723665974	0.132371096	2.975939232
67.99851013	0.672054709	0.147221242	2.926527172
87.43881784	0.711576179	0.087345659	3.100628651
61.84293046	0.700360683	0.134731978	2.992599819
22.77072501	0.657630106	0.516053811	2.650415665
69.09826678	0.678107956	0.145234278	3.023017882
54.3238092	0.735913023	0.124216219	2.909743173
70.53622541	0.67100653	0.14105343	2.957481876
68.98575204	0.668013519	0.146896924	2.902454879
70.4942782	0.669443083	0.141646712	2.903472749
91.76447834	0.691625204	0.095165805	2.989558274
55.34578539	0.73161915	0.124918049	3.029455101
83.55995329	0.722630327	0.083374659	3.034663216
59.11363032	0.699969556	0.139239855	2.936170617
22.99201203	0.692580469	0.425911795	2.77471974
69.16496005	0.6701482	0.144397057	2.830027445
69.658834	0.665566111	0.14689707	2.862920771
68.51161005	0.663717162	0.152542845	2.868461972
68.4755817	0.67229189	0.141703588	2.887323509
35.45488959	0.713656966	0.223971152	2.876581765
62.57725646	0.694652312	0.13685885	2.958254434
60.88141183	0.699315515	0.14384914	3.111592938
88.55268123	0.703474324	0.087824755	2.957383362
68.74727643	0.664726721	0.147172795	2.792460506
88.18389091	0.706436421	0.088703087	3.008252028
39.93751159	0.683328771	0.236287227	2.765872226
65.56564211	0.688698245	0.137868318	3.004836598
97.51680627	0.680209166	0.0948347	3.017335348
59.63401037	0.711174339	0.132402326	2.983337867
42.50644651	0.676124197	0.242305877	2.86065629
23.58688087	0.680443286	0.463883181	2.784496495
55.96528716	0.721172541	0.121613881	2.982055924
86.5965986	0.707067035	0.086957133	2.976266868
71.15795904	0.670261875	0.150198509	2.964080153
65.32277228	0.685406627	0.141228775	2.90590712
62.2013663	0.698965972	0.139873452	3.067165363
84.19606681	0.713376697	0.086658791	2.972993008
44.73342291	0.662113239	0.245743215	2.849001274
41.92145976	0.681868249	0.233096488	2.920044668
69.75046553	0.667438536	0.150236184	2.934793517
59.16340248	0.714958669	0.129063892	3.031197364
60.24808004	0.703329703	0.135319153	2.993548039
41.96553643	0.675126572	0.250074465	2.889939151
64.14957463	0.697005717	0.130976279	2.906995729
66.28708254	0.676223349	0.146952972	2.933139396
64.84752816	0.681994852	0.144661777	3.002869774
84.18380071	0.71444708	0.085645581	3.010999214
53.51285441	0.738050433	0.128049709	3.062912218
84.04200478	0.721814564	0.085803359	3.1047955
22.54324705	0.682600382	0.436612926	2.683557476
60.02203708	0.711309405	0.129968187	2.960567404
40.5250897	0.679819317	0.237779618	2.807645495
57.19355458	0.72306615	0.123556287	2.990594778
66.81969175	0.675067621	0.141083498	2.89651079
21.17293939	0.69661239	0.419335704	2.606688568
63.30660963	0.692440777	0.139222125	2.824031596
68.25003963	0.677311166	0.139776803	2.886596816
77.81617478	0.747163244	0.083974385	3.104127078
53.84116591	0.7347582	0.126421506	3.000749508
90.55556921	0.70181484	0.089604259	3.069232445
68.76036305	0.668965968	0.145290438	2.859946152
48.91309416	0.755134006	0.134876907	3.244637529
43.8336366	0.657445787	0.246675316	2.708204856
45.31334896	0.665708124	0.228740876	2.65439267
72.69696946	0.656011781	0.149338611	2.73387752
48.40924707	0.769297427	0.12247217	3.11918662

log.sigma.0.5.mm.3D_glrlm_HighGrayLevelRunEmphasis	log.sigma.0.5.mm.3D_glrlm_RunLengthNonUniformityNormalized	log.sigma.0.5.mm.3D_glszm_GrayLevelVariance
22.49427486	0.54882732	3.0016
21.82981734	0.523806599	2.734470732
13.12017606	0.569223183	3.035975164
32.12305332	0.564883666	3.563652588
7.51380592	0.50441461	2.37244898
20.82389709	0.511816747	3.01109375
7.264611239	0.494020944	1.533607682
21.79725199	0.524037424	2.858766019
22.14276051	0.528226661	2.453983083
21.57401003	0.533578614	2.873037306
21.39039108	0.497370625	2.993425858
22.01304754	0.590998137	3.763765615
13.55845821	0.500339208	2.822603878
13.17787187	0.500534025	2.220588235
13.46413006	0.551073147	2.306938776
12.91418804	0.524402061	2.624648438
12.9061393	0.486477106	2.292892562
13.86498826	0.485050948	2.401374026
7.879228742	0.512856878	1.931564783
13.14163116	0.469721337	2.317347074
21.26481015	0.562609201	3.116822874
13.37440112	0.506647898	2.38941736
12.99036503	0.512416257	2.298906122
22.06795852	0.543051252	3.192690137
12.8614336	0.51178765	2.476989746
14.48413267	0.580153511	3.317019722
21.55272093	0.638112806	1.93759267
13.53362541	0.567682646	2.643208718
21.25838633	0.482375432	2.341887735
21.51671554	0.554768431	2.924960938
21.34198201	0.529615191	2.73377664
13.73131449	0.490254433	2.220249836
21.56031856	0.534763626	3.932715698
14.72440719	0.54016188	3.876033058
21.46499269	0.575119391	3.087606838
21.40440534	0.50801905	3.185232464
32.00411993	0.563542278	3.630506246
21.53223787	0.545423084	3.13873183
6.892912869	0.484128461	1.97107438
22.1016952	0.521855753	3.263157895
21.82491321	0.591400926	3.029126214
22.29958185	0.511389604	2.7744
21.49994472	0.503748005	2.538033745
22.34879242	0.50628923	3.427831123
31.19861496	0.533167813	3.451846854
22.06597382	0.585294298	3.393692015
32.13674165	0.575267811	3.637001153
20.70229853	0.536094303	2.873245712
8.005046228	0.528245976	2.1275
21.55105282	0.505006271	2.615384615
21.57119938	0.502492847	2.72498024
20.86787338	0.493721228	2.562366864
21.8110216	0.507988383	2.802880169
13.50073088	0.557306126	2.70087236
21.7705242	0.53837927	2.726906435
21.28202851	0.543942898	3.553710938
32.09178247	0.544604188	3.456204986
21.63351735	0.494656644	3.173553719
31.87668545	0.549095977	3.936298077
13.08311611	0.519140125	2.39027393
22.49637606	0.531891473	3.547708701
31.88758683	0.521161226	3.427981488
21.69934095	0.559247922	3.271880337
13.41336684	0.510192229	2.63205179
7.452616142	0.517782151	1.837669095
22.75767464	0.559797682	3.410245731
31.88860269	0.550491883	3.291473765
21.98114149	0.511517964	3.280862603
21.23304139	0.526862423	2.341239612
21.43243072	0.546324557	3.054363905
31.68510654	0.560377062	3.017406725
13.55344147	0.497233412	2.763695988
13.81393162	0.520148082	2.614958449
21.25544484	0.508182007	2.994375
22.33249635	0.564009291	3.256090988
21.41334205	0.546000099	3.092397959
13.02988387	0.512216422	2.943877551
22.26248994	0.538746854	2.864727153
21.00879695	0.511123963	3.053119506
21.12045503	0.522262206	3.032217284
31.74671768	0.554569714	3.703825275
21.62550595	0.592555994	3.58428699
32.0626806	0.572641564	4.076766304
7.622199276	0.513664778	1.98615917
21.73355938	0.559808247	2.7305839
13.17902633	0.516642896	2.486382176
22.31347128	0.570205571	3.681257015
21.73090896	0.508683617	2.829410151
7.557196747	0.53119034	1.561983471
21.57624105	0.527313356	2.952806122
21.81228462	0.517695777	2.738002936
32.75786568	0.603492652	4.894642344
21.49397034	0.586015484	3.462839828
31.64627624	0.550037737	3.467687075
21.66209214	0.504123932	2.655158528
21.83200768	0.607911861	4.80106928
13.11923127	0.483752602	2.571252693
13.79414231	0.494855167	2.031746032
21.28555166	0.483979863	3.05
22.64385745	0.630111988	4.483767313

log.sigma.0.5.mm.3D_glszm_SmallAreaHighGrayLevelEmphasis	log.sigma.0.5.mm.3D_glszm_GrayLevelNonUniformityNormalized	log.sigma.0.5.mm.3D_glszm_SizeZoneNonUniformityNormalized
11.63952568	0.265066667	0.318755556
9.86925046	0.262324442	0.329814309
6.990616298	0.256391527	0.324324324
16.50475496	0.227245687	0.387938727
4.937532363	0.301020408	0.482142857
13.00789937	0.2665625	0.464375
3.005865617	0.410150892	0.390946502
10.96137086	0.23952966	0.370854802
11.08984924	0.269284181	0.378866305
15.02076498	0.301724471	0.416843486
11.04587552	0.298392988	0.390796202
13.48838784	0.227071006	0.372123603
11.39338861	0.256288089	0.440443213
8.3791823	0.37283737	0.299307958
8.374877069	0.348163265	0.333061224
9.360439537	0.274921875	0.35078125
9.392238144	0.350082645	0.454545455
8.421782244	0.275994187	0.391729423
5.072749078	0.291810842	0.291810842
6.824894735	0.289998679	0.310873299
15.15205815	0.3154715	0.395771507
8.362997709	0.309601665	0.429250892
7.703571593	0.280914286	0.292538776
16.42486185	0.262563827	0.418435904
8.551937536	0.323364258	0.334960938
8.042901217	0.246530314	0.336742148
14.19722219	0.226222407	0.385189504
9.219561852	0.295483162	0.287378952
10.35978114	0.324682814	0.281045752
11.58979687	0.238203125	0.306171875
13.46520922	0.324356684	0.380279615
8.886629506	0.26035503	0.393902038
12.50152897	0.236996015	0.361834274
9.423746739	0.194214876	0.260330579
10.70196522	0.240302433	0.406969099
11.9914325	0.272104273	0.38081161
18.09343553	0.213565637	0.334721309
13.30910381	0.288747604	0.369749327
5.045455109	0.412190083	0.522727273
11.34227308	0.221274238	0.387922438
11.95882181	0.2442266	0.391460081
11.02663302	0.249066667	0.425066667
8.68584244	0.29965124	0.373927797
15.68424063	0.222069806	0.515622619
15.85897402	0.257969303	0.37620172
15.95374508	0.248228505	0.444809701
20.59491237	0.229342561	0.39881584
12.43236436	0.249721542	0.468478503
5.965001852	0.225	0.445
10.08992935	0.23964497	0.317307692
14.54840292	0.245737514	0.430689902
11.40582793	0.273846154	0.460828402
11.75705027	0.299841459	0.357180605
7.975718764	0.318640955	0.348484848
11.8572619	0.244477004	0.424372153
13.61041207	0.233642578	0.362304688
19.76662256	0.273185596	0.405373961
14.20770615	0.261248852	0.484389348
19.97011479	0.217455621	0.426960059
9.698255783	0.375192367	0.385041551
14.7653913	0.198686108	0.429258132
18.93373271	0.227141331	0.390331079
13.45394942	0.267325708	0.358728844
7.318438019	0.278372308	0.323820848
2.787193397	0.438085328	0.411030177
15.90475366	0.225739275	0.394210746
15.33566479	0.247299383	0.355709877
11.74670614	0.235020661	0.402182335
9.832601313	0.326177285	0.459141274
11.85199112	0.234138725	0.370726496
18.37199037	0.273703965	0.393708786
9.038248176	0.273533951	0.426697531
7.591393134	0.274696092	0.347313477
12.60421577	0.283647959	0.364285714
14.22727293	0.252387336	0.391882368
13.1029299	0.240306122	0.448469388
8.458588279	0.303831737	0.387338609
13.43649188	0.310979619	0.343852728
11.20551994	0.288720763	0.412648516
11.90874554	0.266844444	0.349175309
16.15803411	0.240499937	0.334174978
11.93393812	0.221897573	0.35889944
18.50005813	0.204985822	0.422081758
6.341783666	0.307958478	0.280276817
11.74032214	0.289902998	0.327317964
9.059182709	0.344414719	0.419323714
16.30016915	0.2241608	0.397204367
12.64051013	0.292400549	0.465569273
3.032946826	0.334710744	0.26446281
10.80253875	0.270408163	0.395408163
11.91948096	0.271782905	0.384696451
19.78709042	0.163602413	0.480034473
13.0388062	0.233734534	0.380860197
16.93780366	0.258471907	0.374779541
14.13266886	0.327713496	0.465059465
13.9720104	0.170416574	0.31521497
9.426597843	0.316097261	0.45829486
7.791710278	0.315192744	0.419501134
14.08288282	0.23375	0.54625
13.50796075	0.172742382	0.421163435

log.sigma.0.5.mm.3D_glszm_SizeZoneNonUniformity	log.sigma.0.5.mm.3D_glszm_GrayLevelNonUniformity	log.sigma.0.5.mm.3D_glszm_LargeAreaEmphasis	log.sigma.0.5.mm.3D_glszm_ZoneVariance
23.90666667	19.88	7301.053333	7043.771733
33.97087379	27.01941748	15840.3301	15463.29173
24	18.97297297	5891.378378	5663.123448
63.62195122	37.26829268	13706.71341	13482.99208
13.5	8.428571429	14128.17857	12979.45281
37.15	21.325	14569.975	14150.74938
10.55555556	11.07407407	19384.18519	17851.60768
32.26436782	20.83908046	16007.96552	15577.52226
33.71910112	23.96629213	19928.52809	19409.81467
67.11180124	48.57763975	22851.93168	22504.02809
28.91891892	22.08108108	20746.82432	20150.54072
58.05128205	35.42307692	5964.916667	5848.248973
41.84210526	24.34736842	40607.71579	39657.12953
20.35294118	25.35294118	22533.29412	21786.71886
23.31428571	24.37142857	13948.71429	13459.67265
56.125	43.9875	43223.24375	42563.71715
25	19.25454545	21239.58182	20388.00331
34.08045977	24.01149425	34912.73563	34048.24045
14.88235294	14.88235294	10752.03922	10255.88543
27.04597701	25.22988506	45336.83908	44224.96155
167.4113475	133.4444444	39781.59338	39511.63994
49.79310345	35.9137931	16938.80172	16604.47941
51.19428571	49.16	41477.54857	40923.01336
25.52459016	16.01639344	6951.836066	6666.724536
42.875	41.390625	21001.625	20587.75684
24.91891892	18.24324324	8476.621622	8142.818846
94.37142857	55.4244898	5724.942857	5643.501441
60.06220096	61.75598086	18928.10048	18670.4135
28.66666667	33.11764706	52456.06863	51345.61101
48.9875	38.1125	23590.78125	23200.96559
88.60515021	75.5751073	29475.57082	29159.55534
61.44871795	40.61538462	74171.01282	73143.72814
50.29496403	32.94244604	18535.27338	18203.71906
5.727272727	4.272727273	4391.409091	3913.390496
31.74358974	18.74358974	3798.769231	3669.159763
46.45901639	33.19672131	37147.04098	36451.7246
78.32478632	49.97435897	18733.33761	18500.70993
75.0591133	58.61576355	22042.29064	21763.25254
23	18.13636364	24081.90909	22944.38017
36.85263158	21.02105263	11841.55789	11544.99324
40.32038835	25.15533981	6111.203883	5947.959845
31.88	18.68	21342.50667	20678.92907
38.51456311	30.86407767	39333.64078	38469.96569
41.7654321	17.98765432	19663.11111	19122.11797
57.93506494	39.72727273	31313.5974	30798.83792
104.0854701	58.08547009	18075.41453	17842.00404
101.6980392	58.48235294	19008.72157	18799.77738
31.3880597	16.73134328	13471.49254	12959.51749
8.9	4.5	6643	5847.76
16.5	12.46153846	10298.30769	9858.921598
70.20245399	40.05521472	42912.78528	42315.38771
29.95384615	17.8	26829.53846	25923.99148
62.14942529	52.17241379	36798.39655	36322.20389
23	21.03030303	8136.015152	7821.221534
88.6937799	51.09569378	28606.25359	28251.40894
46.375	29.90625	9831.890625	9614.328125
77.02105263	51.90526316	22015.86842	21718.03349
31.96969697	17.24242424	40825.15152	39467.34068
44.40384615	22.61538462	6925.634615	6751.597633
21.94736842	21.38596491	15360.36842	14752.78917
67.82278481	31.39240506	21772.08228	21425.60411
103.4377358	60.19245283	46111.1434	45682.41902
49.86330935	37.15827338	13958.98561	13692.05114
28.17241379	24.2183908	17135.58621	16674.57101
12.74193548	13.58064516	5003.935484	4641.708637
38.63265306	22.12244898	8684.316327	8436.414515
25.61111111	17.80555556	6032.666667	5816.739969
70.78409091	41.36363636	31427.46591	31016.94305
34.89473684	24.78947368	9375.644737	9086.197195
57.83333333	36.52564103	14924.39103	14666.12964
94.88381743	65.9626556	21368.75519	21119.08896
30.72222222	19.69444444	26929.40278	26110.01215
72.58851675	57.41148325	43104.83732	42603.63449
102	79.42142857	65219.21786	64678.48927
105.4163569	67.89219331	24118.95911	23867.93101
62.78571429	33.64285714	14822.57857	14545.35607
37.95918367	29.7755102	24670.31633	24092.846
26.82051282	24.25641026	9768.628205	9465.064596
56.53284672	39.55474453	33663.9708	33092.16751
78.56444444	60.04	35164.15556	34772.46748
29.74157303	21.40449438	7854.337079	7620.143921
79.67567568	49.26126126	10525.81532	10382.57108
77.66304348	37.7173913	11351.91304	11171.12748
4.764705882	5.235294118	7444.764706	6498.297578
82.48412698	73.05555556	22202.92857	21968.06078
63.31788079	52.00662252	34955.23179	34425.92711
39.32323232	22.19191919	7535.69697	7327.6382
62.85185185	39.47407407	30337.8	29829.71501
5.818181818	7.363636364	3332.045455	2983.035124
11.07142857	7.571428571	5252.214286	4826.086735
62.70552147	44.3006135	27599.45399	27211.38816
56.6440678	19.30508475	3322.822034	3235.12992
41.51376147	25.47706422	7729.321101	7523.964313
94.44444444	65.13492063	28180.26587	27881.88138
89.75647668	63.24870466	50878.42487	50283.87371
21.11940299	11.41791045	2975.074627	2800.992203
26.12280702	18.01754386	26684.82456	25728.17051
8.80952381	6.619047619	4442	4026.61678
21.85	9.35	9901.1	9408.26
40.01052632	16.41052632	3521.621053	3374.062493

log.sigma.0.5.mm.3D_glszm_ZonePercentage	log.sigma.0.5.mm.3D_glszm_LargeAreaLowGrayLevelEmphasis	log.sigma.0.5.mm.3D_glszm_LargeAreaHighGrayLevelEmphasis	log.sigma.0.5.mm.3D_glszm_HighGrayLevelZoneEmphasis
0.06234414	327.7751794	168505.96	21.32
0.0515	702.7800568	368526.5534	18.27184466
0.066189624	444.4226727	83608.27027	12.44594595
0.06685691	423.2185007	455399.6524	27.59756098
0.029504742	2134.794177	107116.5714	7.928571429
0.048840049	664.9831655	331532.4	17.7375
0.025543992	2773.120566	152391.037	4.962962963
0.048199446	712.2533739	371618.8276	18.40229885
0.043907252	893.6937886	459736.9551	20.87640449
0.053613054	1038.49877	521698.2236	22.21118012
0.040951854	920.744675	482429.0405	18.35135135
0.092581602	274.0166058	135135.0705	22.04487179
0.032434278	3023.339466	580163.4105	16.47368421
0.036598493	1699.298697	318748.3676	14.47058824
0.045219638	1087.257183	192331.4	14.65714286
0.038938915	3517.724057	574360.4563	14.74375
0.034267913	1657.015051	292461.1636	13.36363636
0.034010946	2466.123193	518118.6897	13.05747126
0.044894366	1507.697092	85677.98039	10.35294118
0.029989659	3312.928295	656564.5287	12.09195402
0.060863309	1901.950305	870638.7991	22.36879433
0.054691183	1252.192573	243216.681	13.12068966
0.042465421	3023.375537	601850.7771	12.68571429
0.059223301	323.0543329	155924.541	24.26229508
0.049155146	1729.058731	276179.4297	14.1328125
0.054733728	619.3473967	123332.7432	14.18918919
0.110809588	259.6358385	130922.2082	21.76734694
0.062295082	1391.448359	273167.9809	15.55502392
0.030008826	2357.08804	1208042.461	20.3627451
0.05064894	1095.703358	529192.4813	21.46875
0.056253018	1345.106169	670593.1974	21.22746781
0.0312	5276.570091	1094617.571	14.02564103
0.054919004	837.5994436	425128.8921	20.3381295
0.045738046	316.9343932	64325.81818	18.45454545
0.087837838	165.1702179	89858.70513	17.78205128
0.037923531	1675.391838	853246.8525	18.7295082
0.065564584	576.3444707	624407.8077	30.99145299
0.059864347	990.905721	507614.399	21.2955665
0.029649596	3670.111742	180974.1364	7.136363636
0.05806846	520.0309547	277712.3789	19.26315789
0.078267477	278.2854997	139400.0583	19.02912621
0.038819876	942.8581856	498568.8933	17.52
0.03402709	1782.20523	900124.835	15.86407767
0.042993631	875.9427848	456016.9136	22.09876543
0.044075558	978.9579745	1029460.188	26.16883117
0.065454545	830.6654393	409497.9103	22.43162393
0.069180684	578.2563523	639489.2941	31.16862745
0.044195251	628.4252328	301530.0448	17.70149254
0.035460993	924.2769306	53834.6	9.15
0.047706422	456.0142333	239948.5577	18.61538462
0.040913655	1953.628622	978100.2209	21.85889571
0.033231084	1211.598384	615703.3692	15.96923077
0.045825652	1638.606	853420.9023	20.59770115
0.056362084	626.8785859	113299.2424	14.53030303
0.053086106	1312.858568	648015.6411	18.88038278
0.06779661	453.5415509	222048.5156	20.3125
0.057944495	681.888657	729454.5579	31.65789474
0.027138158	1888.909236	918872.3636	19.90909091
0.075801749	208.2152634	235230.375	30.20192308
0.040569395	1168.121369	215894.1754	14.70175439
0.053723223	974.9838786	503028.8797	22.23417722
0.048295972	1407.430739	1546431.094	30.74339623
0.061206517	628.1445275	321377.7266	22.4676259
0.046573876	1227.184371	251851.7816	13.66666667
0.052542373	678.668629	40722.70968	5.709677419
0.063512638	404.3064959	194529.1531	25.06122449
0.06805293	186.4370395	200321.3194	28.15277778
0.04935502	1381.048285	736391.625	18.82954545
0.058778036	417.9994315	217354.0526	15.43421053
0.062225768	669.9410921	344272.5897	18.59615385
0.063287815	677.8115496	693644.249	29.10788382
0.034934498	1966.226368	390440.75	13.23611111
0.044667664	3120.979209	628502.0239	13.26315789
0.043004147	2955.911722	1491802.721	20.425
0.063115908	1087.594184	554220.5316	22.04460967
0.06006006	670.9761735	339632.8714	18.92142857
0.041613588	1820.605517	354830.1429	12.37755102
0.057395143	431.1012369	228273.4744	22.88461538
0.041819292	1522.169703	771644.4745	17.45985401
0.050527734	1606.084224	799507.8044	19.49777778
0.065345081	241.7854008	261681.8315	29.95505618
0.083552879	477.0586831	240950.4414	19.87387387
0.074373484	347.7135219	379959.413	27.8423913
0.03250478	1068.995016	58395.82353	11.70588235
0.065251165	985.5803301	516332.9167	20.59126984
0.043465746	2659.608436	491012.7351	13.58278146
0.069327731	338.9256604	173599.1818	24.43434343
0.044364114	1361.820044	699283.6815	18.94814815
0.053527981	483.6868687	25933.18182	9
0.048442907	236.3949504	120984.1429	18.10714286
0.050763002	1216.876558	644963.2822	19.78527607
0.10678733	100.3408689	112600.7119	28.80508475
0.06978233	349.7367955	177336.5872	20.43119266
0.05789111	857.9772364	947375.3571	28.23015873
0.041011475	2313.799431	1160642.658	20.92746114
0.075791855	144.4929882	64872.43284	23.92537313
0.032331254	1963.088109	384493.3158	13.10526316
0.049065421	306.7569841	66907.57143	13.14285714
0.045045045	434.2236508	232306.625	19.05
0.082322357	163.869924	79410.93684	20.31578947

log.sigma.0.5.mm.3D_glszm_SmallAreaEmphasis	log.sigma.0.5.mm.3D_glszm_LowGrayLevelZoneEmphasis	log.sigma.0.5.mm.3D_glszm_ZoneEntropy	log.sigma.0.5.mm.3D_glszm_SmallAreaLowGrayLevelEmphasis
0.563243069	0.097368651	3.997030984	0.060883386
0.595285409	0.128545464	4.233702612	0.094808765
0.588441494	0.312496246	4.018009018	0.225111643
0.646739222	0.072483849	4.162966944	0.053926223
0.708521658	0.529771825	3.022563192	0.423851121
0.705886329	0.13835298	3.630107053	0.106781753
0.642149279	0.653744856	2.922148856	0.446708466
0.622825018	0.137649168	3.973097332	0.103141138
0.636946663	0.088427364	3.940683451	0.067298577
0.67110375	0.096021297	3.85763605	0.072190581
0.64389353	0.135939971	3.605488429	0.103118008
0.632370419	0.14787534	4.30408089	0.110602434
0.685225718	0.166732188	3.818208779	0.122199025
0.548812472	0.149922386	3.620571577	0.083419345
0.594197347	0.176420635	3.691779842	0.117339706
0.610291351	0.217432292	4.145102644	0.13540862
0.699260029	0.176792929	3.336446181	0.133347869
0.647829517	0.207895913	3.716765878	0.157407808
0.533523437	0.335653595	3.825742803	0.192753365
0.561126624	0.240664112	3.999974964	0.151406075
0.653589437	0.099937857	4.042063291	0.069159855
0.680726723	0.206349304	3.621677806	0.165771571
0.551115502	0.231096825	4.241923047	0.123171289
0.671041295	0.092714328	3.817478219	0.069974205
0.596318788	0.195092164	3.950719396	0.123085749
0.600752196	0.264697708	4.142754758	0.18794099
0.642311298	0.074999792	4.188089965	0.049670972
0.553129367	0.19634964	4.437687486	0.118619994
0.535106043	0.089968599	4.055273835	0.055097887
0.568883409	0.100972453	4.479580373	0.069478845
0.637129995	0.090613048	3.88916345	0.06176886
0.651635745	0.171830484	4.017574238	0.123728154
0.621938165	0.155934284	4.202663442	0.109782443
0.512178861	0.197574985	3.732158891	0.149053481
0.661118683	0.143241413	3.87415894	0.113914128
0.639323938	0.144234554	3.898136619	0.109482404
0.59552494	0.061541079	4.54353381	0.04184546
0.628411488	0.102507324	4.040808188	0.07080042
0.749053133	0.564393939	2.644266043	0.456695886
0.645760949	0.140961362	4.155148436	0.097783316
0.64655682	0.131192788	3.961038456	0.094833587
0.676721176	0.117410204	3.846705739	0.088456269
0.63365357	0.138023028	3.798315264	0.098042055
0.743055062	0.108585408	3.667234842	0.092139672
0.632982287	0.091645584	4.21732384	0.067010319
0.692045792	0.102478838	4.065706372	0.073713137
0.653729768	0.060730505	4.276556257	0.043185945
0.701285868	0.132966037	3.757301293	0.104575748
0.681359127	0.392111111	3.321928095	0.279935791
0.560899005	0.12682943	3.885429326	0.092157858
0.682142672	0.094524609	4.032506545	0.07277511
0.701512063	0.129982644	3.556950899	0.083686179
0.615025913	0.097893975	3.939441718	0.069514306
0.604178465	0.219095118	3.732524752	0.153155566
0.676781604	0.118348089	4.142828431	0.089823604
0.623235073	0.13923403	4.165328656	0.100403153
0.659737528	0.064099206	4.060854598	0.049178058
0.721384107	0.130551884	3.495649187	0.10600236
0.676203745	0.073726208	4.12286072	0.056905336
0.642798291	0.16581384	3.445070429	0.113717437
0.680545675	0.111873386	4.298027558	0.081203485
0.649443418	0.060719486	4.364509072	0.045028908
0.620720287	0.103103721	4.128255448	0.073663616
0.582656768	0.244000639	4.006688464	0.166808153
0.659961675	0.656084229	2.783132797	0.50813796
0.648554055	0.092411539	4.294842955	0.070216772
0.604273372	0.07171192	4.075341669	0.0519504
0.657460517	0.160320303	4.110316154	0.119524215
0.701822961	0.13383578	3.315499083	0.098169225
0.632005037	0.129028405	4.323999749	0.087896178
0.65204733	0.05821485	4.131166955	0.041985696
0.678151286	0.218157675	3.734754865	0.154292887
0.610888327	0.221809834	4.24836393	0.157577013
0.626604143	0.114678207	4.134238932	0.083984069
0.650601214	0.112124365	4.265707479	0.078513064
0.695352618	0.126527049	3.996770904	0.09682965
0.644891894	0.273793226	3.845349166	0.189148326
0.598688024	0.089689517	3.751775181	0.059689312
0.667039083	0.144779449	3.909888791	0.110096989
0.612516383	0.111608535	4.260871207	0.076105874
0.592590936	0.06934903	4.254156424	0.050947522
0.620942912	0.139936633	4.394490857	0.10303828
0.674677479	0.080923469	4.224778797	0.061923715
0.529328923	0.305359477	3.499227547	0.172666793
0.587825963	0.095962999	4.220645077	0.060959512
0.668748517	0.195263061	3.539466343	0.139210769
0.654548094	0.096041867	4.136128261	0.069070875
0.70679652	0.115137356	3.682360407	0.089793628
0.491644769	0.328914141	3.11353887	0.266050282
0.635774492	0.152532242	3.142026691	0.119954936
0.643690209	0.099960387	3.92778571	0.073681218
0.717617235	0.112189975	4.125632455	0.097640976
0.63898127	0.128334699	4.192566525	0.088989031
0.635241375	0.065120516	4.230009733	0.046587879
0.707125315	0.089843216	3.632102846	0.069323481
0.578962244	0.153216634	4.531611579	0.100495786
0.702785004	0.230745614	3.324636228	0.175779484
0.662623434	0.176944444	2.713216236	0.146412628
0.763153224	0.137491107	3.358294659	0.121144682
0.66785755	0.189894304	4.260541778	0.147497559

log.sigma.0.5.mm.3D_ngtdm_Coarseness	log.sigma.0.5.mm.3D_ngtdm_Complexity	log.sigma.0.5.mm.3D_ngtdm_Strength	log.sigma.0.5.mm.3D_ngtdm_Busyness	log.sigma.0.5.mm.3D_ngtdm_Contrast
0.004675127	0.014392016	0.127198276	4.031068408	1.19E-05
0.002687974	0.008368889	0.079583979	6.80508945	5.83E-06
0.005046463	0.008404131	0.057471845	7.675350679	2.90E-05
0.002136811	0.007986969	0.070612651	7.377998288	6.95E-06
0.005140018	0.005967028	0.04974122	12.16590006	2.30E-05
0.002884346	0.010649214	0.09323477	6.354546074	7.02E-06
0.004538151	0.005403695	0.044968496	13.01778556	1.78E-05
0.002889022	0.009321315	0.088329886	6.232829251	6.07E-06
0.002649775	0.00788158	0.082278392	6.771209598	4.54E-06
0.00178989	0.005568411	0.052929163	10.32999674	3.69E-06
0.002698938	0.006762161	0.067675884	7.827744663	6.71E-06
0.003378536	0.011455902	0.079621778	5.940594896	1.37E-05
0.001678557	0.004095581	0.040320918	16.22546625	3.72E-06
0.002687032	0.004502035	0.040627494	12.71784698	7.78E-06
0.00325401	0.005837555	0.045115069	10.88506246	1.14E-05
0.001244683	0.002080933	0.017609201	29.17065415	4.33E-06
0.002950234	0.005266717	0.046457604	11.59670595	8.28E-06
0.002129148	0.003034251	0.031530294	15.45273837	5.31E-06
0.004995119	0.004597649	0.043437083	12.46011401	2.01E-05
0.001744309	0.002745461	0.027115547	18.96204068	4.60E-06
0.00078578	0.003588962	0.029116663	21.34342315	1.80E-06
0.002454923	0.0056531	0.056536433	11.14120619	5.29E-06
0.001242778	0.002098264	0.017582946	27.64772176	4.40E-06
0.00502646	0.016487329	0.15138542	3.72831404	1.18E-05
0.00205819	0.004796138	0.044903342	14.25019665	5.04E-06
0.004411909	0.010066594	0.07525302	6.779719566	1.67E-05
0.002365549	0.00617078	0.049508151	9.650465654	7.92E-06
0.001715972	0.004120716	0.030865984	17.25101127	5.99E-06
0.001461869	0.003513324	0.037507392	14.45601413	2.93E-06
0.001668552	0.00549837	0.048192684	11.24302652	3.90E-06
0.001287616	0.003153385	0.027544997	17.46817081	3.79E-06
0.001062299	0.001514913	0.016562155	30.80792805	2.40E-06
0.002005819	0.009336181	0.082580372	7.992530396	4.70E-06
0.011152705	0.025206092	0.233019169	2.503398601	3.21E-05
0.006187809	0.021482102	0.157519976	3.080760561	2.16E-05
0.001535797	0.00388776	0.036832737	14.19466272	4.47E-06
0.001611562	0.006821838	0.058285528	8.509919064	4.13E-06
0.001567529	0.005332177	0.043306598	12.04591672	4.30E-06
0.003085567	0.002153729	0.017051345	27.56470165	1.77E-05
0.003208178	0.010348861	0.093575373	5.722538143	8.45E-06
0.003999118	0.010788989	0.0790711	5.80825551	1.72E-05
0.002795397	0.006286241	0.064964213	7.782715815	7.25E-06
0.001623635	0.004114738	0.039712072	13.33039345	4.32E-06
0.002676469	0.008395881	0.088711006	6.689911184	5.18E-06
0.001499185	0.006600988	0.062437526	8.872119634	3.09E-06
0.00158718	0.005382876	0.038765747	12.48834366	5.30E-06
0.001525045	0.00664124	0.056360224	8.896806234	3.86E-06
0.003374732	0.008815616	0.071566299	6.827479698	1.19E-05
0.009373625	0.010013525	0.081050734	6.769669795	4.59E-05
0.004560814	0.011309213	0.112323278	4.651105821	1.11E-05
0.001238143	0.003054959	0.031163451	17.38331865	2.84E-06
0.002377327	0.006544175	0.0590416	9.047027171	6.81E-06
0.001318959	0.004322843	0.042712006	13.5718762	2.53E-06
0.004613397	0.007576549	0.058602888	7.92314744	1.97E-05
0.001333564	0.003394367	0.028427211	16.95714056	4.35E-06
0.003073096	0.007151784	0.056108945	7.811567628	1.27E-05
0.001632717	0.005650477	0.056674863	9.505978353	4.14E-06
0.001935923	0.006543901	0.069266856	9.220577239	3.22E-06
0.003954351	0.017238354	0.155515082	3.349289283	9.94E-06
0.00337682	0.006375196	0.049778567	10.18386479	1.14E-05
0.001869874	0.007446817	0.075412173	8.439212387	3.44E-06
0.000955025	0.00410567	0.041202401	13.70445203	1.84E-06
0.002456501	0.005857832	0.048740802	9.394417747	8.99E-06
0.00288833	0.004485217	0.039527504	11.80054231	9.52E-06
0.009432572	0.009579223	0.079468913	6.408731657	4.04E-05
0.003580263	0.011354672	0.098153312	5.416558805	9.87E-06
0.004897949	0.018119526	0.166063378	3.197193225	1.48E-05
0.001483368	0.004627457	0.045062418	12.10564981	3.26E-06
0.003884532	0.010303524	0.086424243	5.642436447	1.26E-05
0.00214475	0.00731296	0.057340908	8.949412243	6.77E-06
0.001473587	0.005034866	0.046769827	10.86132405	3.86E-06
0.002372645	0.00589257	0.056455804	11.36679709	5.38E-06
0.001160021	0.003717946	0.038940881	19.83587856	2.18E-06
0.000794177	0.001904861	0.018378839	27.50042661	2.07E-06
0.001355978	0.004128005	0.035283086	14.17057147	3.75E-06
0.002283041	0.00582899	0.046141837	10.07983468	8.77E-06
0.002092944	0.006018606	0.058405814	13.24960542	5.87E-06
0.004106214	0.00926275	0.088874929	5.381493069	1.15E-05
0.00150423	0.003945385	0.034659444	14.66215468	4.68E-06
0.001186806	0.003916209	0.034081241	15.83987809	2.89E-06
0.003996047	0.017609525	0.153351187	3.377564921	9.86E-06
0.002079376	0.007496997	0.04984554	9.636401915	8.58E-06
0.002257133	0.010237938	0.080516863	6.103850143	6.91E-06
0.009512117	0.010485692	0.092030129	6.296667837	3.70E-05
0.001454325	0.003482079	0.028788207	15.73552181	5.06E-06
0.001409889	0.002489249	0.020783	24.42771352	4.60E-06
0.003922683	0.012577681	0.10241871	4.895464451	1.16E-05
0.001643845	0.004069528	0.03973406	13.16530786	4.43E-06
0.012483191	0.007866882	0.06135337	7.239286164	7.95E-05
0.008612804	0.023663547	0.238215553	2.522909277	2.66E-05
0.001596549	0.003913174	0.037496784	13.48395768	4.21E-06
0.005245706	0.024146975	0.169877374	2.668463066	2.12E-05
0.003553061	0.012593695	0.086608849	5.522598085	1.29E-05
0.001248372	0.005588962	0.047907226	10.79882526	3.10E-06
0.001087541	0.00340523	0.03550926	16.49145023	1.81E-06
0.007813729	0.022589558	0.145624723	2.906391637	3.90E-05
0.002601485	0.004718278	0.042931462	12.52800311	6.93E-06
0.012423375	0.012290897	0.13892513	3.312613885	3.83E-05
0.005285129	0.017410387	0.193820732	3.273557256	8.54E-06
0.005515818	0.017728813	0.112517334	3.904020711	2.66E-05

original_gldm_GrayLevelVariance	original_gldm_HighGrayLevelEmphasis	original_gldm_GrayLevelNonUniformityNormalized	original_gldm_DependenceEntropy	original_gldm_DependenceNonUniformity
50.72935008	190.1970075	0.070055534	6.362711131	250.527847
56.50289975	164.6525	0.072788	6.697563738	328.036
101.8674031	324.2987478	0.046903652	6.839754907	260.1449016
42.41461847	139.3644517	0.065021674	6.509919049	515.1842642
23.85506345	135.2476291	0.073098964	6.490922914	155.8956797
41.02323667	154.0079365	0.070777818	6.48880025	305.5103785
11.75518439	53.31315043	0.135558859	6.021327353	130.4796594
53.96271054	120.0177285	0.093231022	6.477607344	271.9418283
60.70153763	149.5742477	0.0864406	6.538686283	317.0167736
61.15556993	250.2707293	0.046027344	6.867805923	710.3972694
25.73890628	91.03818484	0.079212642	6.464936749	285.8577753
83.54987717	332.4777448	0.03562134	6.987594995	446.2724036
36.33548925	217.954592	0.059255691	6.785157216	533.9675657
47.89103907	230.7518837	0.050323218	6.692321339	430.0376749
80.63698095	356.3514212	0.044018622	6.819288111	375.5671835
193.6675308	718.1321489	0.039146864	7.517071969	682.4981747
57.97578673	159.188162	0.058945468	6.712440425	303.553271
24.56009172	112.426896	0.100726171	6.34711681	375.4480063
29.79175681	133.3617958	0.071330713	6.547714095	195.709507
79.82362748	288.1437435	0.054765672	7.046842048	474.9027921
100.1748726	433.2820144	0.033948636	7.178047035	1654.245468
58.77166415	363.233852	0.047636386	6.826132151	483.9156058
79.40325901	284.467605	0.044347343	6.95438938	938.3584082
34.09039118	107.8834951	0.089669149	6.368223451	169.7320388
105.2176498	671.6539939	0.03395491	7.110428985	645.6144393
63.255311	328.6656805	0.062329313	6.606534234	271.5473373
115.9823509	358.6852103	0.03764431	6.990055232	553.9823609
91.65019054	367.1508197	0.039979744	6.984586131	829.6670641
58.02136984	144.0102971	0.092664102	6.716512094	453.8114151
123.0822064	322.804685	0.066236936	6.96915419	508.0788224
74.68857374	356.8831482	0.040037272	7.118882678	886.6508933
72.80347824	318.4276	0.04670592	7.180988314	939.598
56.84684843	158.1469775	0.08213834	6.550615066	415.3753457
30.51552336	75.19126819	0.130786952	5.859996083	66.04781705
81.93263407	278.7849099	0.047304906	6.594630064	220.8963964
40.97689449	144.0400995	0.089590011	6.628443041	457.0360584
100.1162773	241.0728495	0.048796252	7.011534935	736.9097786
48.02184891	221.9256856	0.061394235	6.633915502	695.6240047
36.4507051	274.4083558	0.056518407	6.839243395	271.4110512
32.22663176	83.55562347	0.108778791	6.319058934	217.5061125
51.68586476	184.3381459	0.057292292	6.405037781	312.2993921
27.97339566	91.54347826	0.093417392	6.436293063	283.5403727
31.85885559	185.8909812	0.099988224	6.436366699	429.0488933
30.58384609	79.52441614	0.116570539	6.229825964	248.2653928
151.2768653	384.9361763	0.049746741	7.1718355	674.2215226
67.85372969	398.1678322	0.043611404	6.876590222	895.9577622
65.55468323	441.0583288	0.046701764	6.856067079	834.8030385
79.83383009	217.2460422	0.059355442	6.632793197	319.6187335
16.66788328	76.56560284	0.124729641	5.937001183	76.40780142
37.01791853	166.4137615	0.099385574	6.288872797	170.346789
66.17836985	309.5978916	0.077397063	6.712308455	620.9628514
34.79027851	111.9084867	0.105429364	6.486908492	244.4611452
24.34978212	172.3273637	0.093674511	6.408886522	579.0089544
86.18287522	422.8787361	0.035796042	6.944433919	295.823228
66.7653845	231.1155702	0.069283816	6.792227702	673.1051562
183.5945209	378.7944915	0.077209315	7.066521009	327.1588983
69.47284619	266.0795974	0.063081383	6.779900947	635.5965233
23.00477663	99.15049342	0.105895489	6.408646297	342.8708882
56.58884213	280.7368805	0.063533264	6.509114533	301.2842566
49.47205127	277.1060498	0.042205646	6.778795116	319.7758007
39.57080824	186.2845971	0.067039282	6.629326433	535.8371302
59.10742056	183.8039001	0.079389497	6.803123532	838.5556771
62.08538188	332.3989432	0.04998506	6.789763118	528.1994716
58.45029867	154.3078158	0.073620747	6.630126528	305.2987152
60.06355645	192.3525424	0.075380638	6.328632466	109.5152542
39.65654857	190.8684381	0.052815193	6.624022959	352.889825
47.44880932	243.1049149	0.062480123	6.649727195	199.6786389
47.49514035	148.625631	0.092878652	6.498202646	530.9310151
39.34102183	138.7695282	0.077504009	6.407856666	237.8553751
79.92588729	301.7778221	0.066204257	6.691814076	475.750698
141.3712931	483.8253676	0.035995097	7.235859115	881.4138655
30.73504027	107.8951965	0.0875832	6.56559393	299.3202329
60.66608928	215.9425091	0.068785465	6.753031271	799.5458431
94.22672301	325.9666718	0.052568741	7.121450544	1160.197358
67.9496926	299.1295167	0.052905854	6.82524716	961.8831534
63.19305001	232.1818962	0.063223547	6.770648256	437.2539683
54.19754509	242.3707006	0.077595936	6.621108838	390.4394904
36.5443426	157.2531273	0.065333392	6.323813289	304.8999264
61.10572646	172.7640415	0.068743002	6.837446849	537.4346764
79.90531312	267.6377723	0.070371289	6.861409713	702.8306759
98.84002374	249.9566814	0.069216687	6.652721339	265.8208517
84.326627	310.6496048	0.046395933	6.79160137	681.8039142
67.56927307	172.0153597	0.055655944	6.644235919	555.672595
12.93519152	59.45124283	0.097521652	6.317777134	68.37284895
63.60902435	300.8982393	0.048257719	6.814327562	877.736406
47.8859686	217.7852619	0.053664876	6.77760081	689.3920553
65.90346089	218.6197479	0.053045728	6.680174093	318.1428571
27.7116994	116.9871837	0.084337984	6.530510474	470.5241538
25.61583225	77.9756691	0.115373459	5.845726539	61.96350365
25.94194574	86.96020761	0.095490954	6.068140878	97.57093426
40.20521194	137.4219869	0.083088818	6.450492112	544.0563687
74.20660019	205.4904977	0.061429537	6.379805089	268.3846154
57.37190943	201.5409731	0.058103715	6.546669954	376.9705506
55.884317	267.9937974	0.060653265	6.851069382	783.6028027
38.76170486	201.5979601	0.06152105	6.721271142	900.7492563
192.3882036	713.4049774	0.038351487	6.825744099	235.0633484
19.82941275	85.92456041	0.086126913	6.432528113	265.2166761
19.90725718	70.9228972	0.10696349	5.988550455	63.24299065
46.93876182	108.9898649	0.096032688	6.42630821	121.3423423
143.6391603	581.5147314	0.03296799	6.875487539	351.1507799

original_gldm_GrayLevelNonUniformity	original_gldm_SmallDependenceEmphasis	original_gldm_DependenceNonUniformityNormalized	original_gldm_DependenceVariance	original_gldm_LargeDependenceEmphasis
84.27680798	0.390514878	0.208252574	2.651925188	9.802992519
145.576	0.343799394	0.164018	4.983975	15.841
52.43828265	0.452096978	0.232687747	3.706295743	10.43470483
159.4981655	0.376998195	0.210022122	2.46086915	9.549531186
69.37091675	0.290277601	0.164273635	4.080246413	15.02107482
115.9340659	0.343840024	0.186514273	3.382109276	12.08424908
143.2857143	0.234775454	0.123443386	6.620629779	24.01986755
168.2819945	0.323139412	0.150660293	5.620108501	18.03157895
175.2150962	0.340087732	0.156397027	5.706093052	17.62851505
138.2201132	0.42405436	0.236562527	2.720184245	8.952713953
143.1372441	0.289680808	0.158194674	4.271753241	15.62645268
60.02195846	0.488726543	0.264850091	2.307564916	7.547181009
173.5599181	0.328713351	0.18230371	3.506024741	12.60874018
93.50053821	0.410691429	0.231451924	2.770523069	9.218514532
68.14082687	0.433889159	0.24261446	1.91258872	7.551679587
160.8544658	0.351334646	0.166098363	10.523679	23.39376977
94.60747664	0.358676401	0.189129764	3.585499364	12.13520249
257.657545	0.295339574	0.146774045	5.17057648	17.88741204
81.03169014	0.326565194	0.172279496	3.635733857	13.35739437
158.8752154	0.323966748	0.163703134	8.032932338	20.11340917
235.9430216	0.43165856	0.238020931	4.142719321	10.68748201
101.0367751	0.406747098	0.228154458	2.596436124	9.042432815
182.7553992	0.425831709	0.227701628	3.603727241	10.36423198
92.3592233	0.312647082	0.164788387	4.851544915	15.7223301
88.41858679	0.454257043	0.247931812	2.73338725	8.582949309
84.26923077	0.405077241	0.200848622	3.386442614	11.21153846
83.23156943	0.464672742	0.250557377	2.096479896	7.553595658
134.1320417	0.458114038	0.247292717	2.58491288	8.383606557
314.9652839	0.289256761	0.133513214	7.550058897	22.93704031
209.2424818	0.34315979	0.160835335	5.536636357	16.92592593
165.8343795	0.399553977	0.21406347	2.576150706	9.448092709
233.5296	0.364476187	0.1879196	4.46994816	13.4268
207.8921375	0.329548506	0.164115111	4.265975706	14.82141446
62.90852391	0.277935247	0.137313549	5.372634109	19.83160083
42.00675676	0.462988577	0.248757203	2.140065741	7.668918919
288.2110662	0.303895631	0.142069027	6.27402238	19.96425241
174.1538246	0.41215946	0.206475141	5.832758049	14.20201737
208.1878502	0.39921189	0.205138309	3.283664558	10.82954881
83.87331536	0.322135842	0.182891544	3.55699065	12.74393531
177.9621027	0.295559748	0.132949946	7.622058094	23.21393643
75.39665653	0.44210981	0.237309568	2.180162785	8.048632219
180.4824017	0.300422125	0.146760027	5.323112106	18.04865424
302.6643541	0.282773904	0.141740632	5.687720547	19.28278824
219.618896	0.287027799	0.131775686	6.763816202	22.21656051
173.8151116	0.379455655	0.192965519	4.123568771	12.67258157
155.9107692	0.450148052	0.250617556	1.949105462	7.361398601
172.1427021	0.41722552	0.226479392	2.354513816	8.676614216
89.9828496	0.394824707	0.210830299	2.734222819	9.821899736
70.34751773	0.254213191	0.135474825	5.408279262	20.18439716
108.3302752	0.326740495	0.156281458	5.550344247	17.34862385
308.3498996	0.30505224	0.15586417	4.705782971	16.26305221
206.2198364	0.249682904	0.124980136	6.61180532	23.37321063
355.6821175	0.309809101	0.152491165	4.897574281	16.80616276
41.91716482	0.448638456	0.252624447	2.174472613	7.669513237
272.7703835	0.348275081	0.170969052	4.646074066	14.78003556
145.7711864	0.401431729	0.173283315	22.52759511	42.01271186
206.8438548	0.382971611	0.193838525	3.491316882	11.67276609
257.5378289	0.275416614	0.140983095	6.015917157	19.90625
87.16763848	0.411469459	0.219594939	2.751226105	9.483965015
59.29893238	0.382682931	0.227598435	2.112053292	8.543060498
197.1625298	0.351561919	0.182195556	3.510081588	12.4083645
435.6101695	0.323328317	0.152825893	5.980251614	18.26152725
113.5160722	0.430414363	0.232584532	2.449492684	8.597534126
137.5235546	0.357000223	0.163436143	6.423655022	18.17665953
44.47457627	0.364904509	0.185619075	3.887721919	12.76610169
81.49384316	0.400626645	0.22870371	2.174766228	8.462086844
66.10396975	0.380504216	0.188732173	4.115472715	12.86767486
331.205272	0.318120304	0.148886992	5.845499135	18.54514863
100.2126837	0.354256297	0.183956207	3.761504777	12.65970611
165.9740726	0.388089397	0.189768926	4.001395057	12.65536498
137.0693277	0.437077665	0.231463725	3.100080933	9.56460084
180.5089762	0.290723352	0.145230584	6.290074136	19.67054828
321.8471896	0.343179524	0.170879642	4.439969395	14.48151314
342.275073	0.353666661	0.178190348	4.334115056	13.81400706
225.4847489	0.424091135	0.225688211	2.626937473	9.057719381
147.3740884	0.37021096	0.18758214	3.649973562	12.26297726
182.7384289	0.342814253	0.165791716	5.103055973	15.82972399
88.78807947	0.415173341	0.22435609	2.342107586	8.715967623
225.2020757	0.331086504	0.164052099	4.846328275	15.64224664
313.3633506	0.340352235	0.157833073	5.965712702	17.8560521
94.27312775	0.392701042	0.195169495	3.973999539	12.30837004
123.2739932	0.475917036	0.256606667	2.39752033	7.851336093
137.6928052	0.437363031	0.224604929	3.706370342	10.69442199
51.00382409	0.226888436	0.130732025	5.436257216	21.91395793
186.3713102	0.421959593	0.227275092	2.668873924	9.087001554
186.4317789	0.371977796	0.198443309	3.136248251	10.99366724
75.74929972	0.417774708	0.222789116	2.562142504	9.088235294
256.6404864	0.305559501	0.154625092	4.430551513	16.05948078
47.41849148	0.277630042	0.150762783	3.928404402	16.32360097
55.19377163	0.296044045	0.168807845	2.991463225	13.16955017
266.7981937	0.355482867	0.169435182	4.60776017	14.88726254
67.87963801	0.447291896	0.242882005	2.115419422	7.801809955
90.75800256	0.463821267	0.241338381	2.969334105	9.139564661
264.0236618	0.362632262	0.180014427	4.554924322	14.00987824
289.518062	0.352479337	0.191404432	3.000692662	11.22269443
33.90271493	0.49581249	0.265908765	2.496324809	7.800904977
151.841747	0.273297243	0.15043487	4.454087278	16.77765173
45.78037383	0.301316661	0.147763997	5.00991353	17.55607477
85.27702703	0.276711292	0.136646782	6.963715405	21.77927928
38.04506066	0.535663531	0.304290104	1.549672753	5.703639515

original_gldm_LargeDependenceLowGrayLevelEmphasis	original_gldm_SmallDependenceHighGrayLevelEmphasis	original_gldm_LargeDependenceHighGrayLevelEmphasis	original_gldm_SmallDependenceLowGrayLevelEmphasis
0.1594559	111.5835625	1112.163757	0.006036588
0.509451345	93.92750261	1232.0235	0.005983869
0.247328454	193.6287607	1413.561717	0.005552556
0.465703143	88.74233866	672.3599674	0.011985182
0.310609435	61.20240232	1302.613277	0.005015849
0.2991121	91.44087267	850.2936508	0.005179947
1.06837315	26.92264895	710.2005676	0.008932142
1.302068276	77.86851186	670.0132964	0.013952572
0.904664904	98.1239749	751.046374	0.008818438
0.174785273	142.252818	1379.25974	0.005420395
0.962319341	46.62158093	673.3458771	0.011294301
0.247056361	220.1062737	1479.12819	0.007417195
0.146308603	101.6656159	1791.506658	0.002686425
0.15375197	126.5602001	1239.344995	0.004346102
0.077621151	211.129275	1647.970284	0.003976937
0.115113804	230.6488977	29198.6622	0.002563303
0.815522936	87.095707	936.9040498	0.011736375
0.414986978	59.8941378	1032.499609	0.00450237
0.32706818	73.27844228	964.1223592	0.006586806
0.224496684	124.8367508	11717.99	0.002613646
0.085257534	202.6210247	7929.249496	0.002898768
0.061820139	191.0337728	2151.648751	0.002900541
0.199741141	152.7731531	3448.633584	0.004339943
0.696471032	66.2198528	695.8466019	0.009598141
0.028558745	322.3413006	6698.63172	0.001555217
0.098147347	198.6033642	2012.449704	0.003632398
0.1857907	208.869462	2000.341474	0.007262333
0.124521522	219.0862672	1640.95231	0.003253253
0.954764217	84.03058716	1109.235363	0.005753402
0.366423476	161.2934169	2905.209877	0.004108263
0.097059937	179.8327966	2551.428296	0.002673279
0.157460436	153.4232116	3558.1204	0.003015496
0.426488777	87.66133271	1339.792967	0.006532507
1.358189131	53.8804516	451.4033264	0.011686714
0.156806309	187.510794	992.4222973	0.005408502
0.496683745	84.33103335	1177.778365	0.003923296
1.803352983	149.8807903	1286.92659	0.013743316
0.150393602	129.585811	1257.56591	0.003752804
0.101175226	115.1622702	2406.703504	0.002462416
1.774191692	56.17857535	531.7793399	0.010665713
0.210385222	128.8911497	672.8639818	0.008047481
0.845649556	54.19067542	672.2846791	0.00827377
0.212158515	88.73329581	2072.096465	0.002360618
1.376966518	54.99150619	557.5286624	0.014086227
0.282051729	222.5430732	2299.076989	0.004718052
0.04533998	214.6406974	2320.426294	0.002532045
0.046533762	233.7847794	2794.221649	0.001461008
0.318958515	127.5307645	1109.871372	0.008071968
0.664158477	38.72808103	850.2996454	0.00671248
0.241583189	101.4759345	1500.518349	0.005804588
0.125847799	135.2182752	3771.11747	0.001736637
0.704134227	59.38122466	1180.787832	0.005139747
0.182921402	86.16392268	1817.073742	0.002454604
0.065915463	235.4533759	2133.92912	0.00346531
0.243933327	127.0104484	1673.308611	0.003383388
3.843017665	167.2532881	11752.76907	0.011337066
0.152333687	149.4690656	1871.698384	0.003515355
0.495392639	50.80304449	1088.820724	0.005034376
0.081767361	169.7905635	1523.658163	0.003373358
0.114476495	139.254241	1648.402847	0.003951667
0.183635035	106.0999111	1275.323019	0.0040635
0.379218727	101.0751798	1517.002369	0.005292772
0.063975642	184.4716049	1867.787759	0.003159598
1.245307117	101.3504307	721.874197	0.008330919
0.329314736	121.7698084	928.0779661	0.007485666
0.146931701	103.9207402	1158.404407	0.005785834
0.209236814	136.8139871	1845.023629	0.004660699
0.496075165	93.78539989	1012.940269	0.004753983
0.34698857	84.11296521	786.6798144	0.007111457
0.145866913	174.8933626	2487.571201	0.003145247
0.09750841	236.2628311	5793.678834	0.004098353
0.741277235	61.46511845	848.0164968	0.006826803
0.232268391	110.054686	3024.59393	0.003459402
0.177315021	154.145816	4186.041315	0.002761992
0.091124624	172.6840787	1601.970436	0.00330626
0.206060942	143.8324992	1294.972973	0.003821419
0.178617195	136.9983714	1890.682378	0.002486097
0.1757346	96.91065415	781.9816041	0.00772131
0.55401338	104.4476593	970.4343712	0.00680963
0.245836983	128.6187227	4278.503256	0.002800045
0.274615647	178.6278938	1071.159325	0.005917082
0.10830038	210.2283682	1184.024087	0.004695237
0.981428674	126.1881223	549.3791431	0.016857508
1.203200327	24.06557888	799.8795411	0.015353657
0.108167897	167.3591907	2015.26696	0.00277037
0.187657006	123.6363914	1214.761082	0.003525691
0.274270698	129.8988961	1003.235994	0.00673022
0.469166736	64.35354688	989.0647387	0.006423295
0.699729946	48.80325692	580.4476886	0.012420222
0.560141613	52.09817217	570.399654	0.010362125
0.445674637	92.25867849	776.6689505	0.005850987
0.203708006	153.2527728	657.6968326	0.011401752
0.207187279	133.7357159	873.4513444	0.007789274
0.160742851	146.4500739	1939.164484	0.002367446
0.147699066	104.5505575	1386.675733	0.003349628
0.044940201	413.3523133	5352.579186	0.003797563
0.658196805	42.37105542	788.1389677	0.008720193
0.85673016	45.85202418	589.1565421	0.009109389
1.784891681	57.3412997	1577.89527	0.010428953
0.043656271	364.817591	2421.908146	0.003307801

original_gldm_LowGrayLevelEmphasis	original_gldzm_DistanceZoneVariabilityNormalized	original_gldzm_LowIntensityEmphasis	original_gldzm_LargeDistanceEmphasis	original_gldzm_HighIntensitySmallDistanceEmphasis
0.015631513	1	0.015354596	1	283.0764818
0.024161423	1	0.017568642	1	275.1686587
0.015915476	1	0.012275649	1	423.9571184
0.039754238	1	0.032564141	1	225.2509542
0.017422068	1	0.01702152	1	203.5093168
0.019808149	1	0.01512789	1	259.7021944
0.042827112	1	0.039861398	1	115.7722008
0.056066081	1	0.041276321	1	244.3634895
0.039666252	1	0.026018343	1	292.8355615
0.015485561	0.997289978	0.013199383	1.004070556	326.9721845
0.042284455	1	0.036164635	1	162.3086207
0.020354191	0.997872343	0.014872761	1.003194888	432.1546858
0.009504382	1	0.008118	1	301.4536542
0.012136266	1	0.010461857	1	301.570634
0.010136897	1	0.009885574	1	469.5949045
0.007386402	0.995180751	0.007039955	1.007246377	656.2080314
0.044616224	1	0.032506332	1	244.5843558
0.019641955	1	0.01482864	1	204.9095355
0.02271488	1	0.020926407	1	218.3182898
0.010560705	0.996292877	0.007933776	1.005571031	377.3737233
0.008113594	0.995407602	0.006705677	1.006904488	463.7275604
0.007164617	0.998025668	0.007390791	1.002964427	461.4804842
0.014110909	0.996061069	0.010371245	1.005920079	350.7894672
0.034681972	1	0.030914587	1	201.3016304
0.003384125	0.998549674	0.003338255	1.002177068	709.1634615
0.008682649	1	0.009077524	1	478.1940789
0.019801409	1	0.01612417	1	447.5332757
0.010091612	1	0.007523964	1	472.3178995
0.031169202	0.992613213	0.019450658	1.011121409	290.2069045
0.016151657	1	0.011514796	1	475.5083752
0.008209131	1	0.006816562	1	446.0792079
0.009814463	0.999029126	0.008084906	1.001457018	422.1079407
0.02477368	1	0.019449168	1	272.0113122
0.055941603	1	0.042066228	1	192.7857143
0.015060779	1	0.011811483	1	394.2291221
0.020299209	1	0.012778391	1	282.1104762
0.049229033	1	0.033690486	1	357.6559271
0.011219042	1	0.00934422	1	319.8034076
0.007304694	1	0.007377836	1	353.6352313
0.054518367	1	0.03405572	1	194.2732673
0.020769279	1	0.017782332	1	281.6594427
0.037779682	1	0.027192734	1	179.4414557
0.009485468	1	0.008088134	1	324.0941307
0.056045898	1	0.048638804	1	192.4698582
0.016029507	1	0.012062467	1	578.2338009
0.005691817	1	0.005555386	1	469.2826205
0.004524983	0.984154944	0.003747626	1.023958928	553.8800627
0.025665499	1	0.020520249	1	321.7339181
0.028623549	1	0.026106117	1	148.164557
0.015539777	1	0.017931011	1	305.5526316
0.006644558	1	0.005388703	1	461.7905199
0.026993588	1	0.021579388	1	250.5648703
0.009483708	1	0.007783121	1	277.6225218
0.007579556	1	0.007526179	1	509.070626
0.012620768	0.99734043	0.009436729	1.003994674	369.9182756
0.052327823	1	0.02939263	1	408.2970414
0.010765204	0.991537529	0.009214976	1.012747875	383.5385977
0.023208865	0.997260279	0.019271044	1.004115226	183.7918381
0.008113797	1	0.008083343	1	397.2593168
0.010784913	1	0.009875961	1	356.0919003
0.012874026	1	0.011298358	1	298.9196113
0.018667774	0.998963194	0.016266902	1.001556017	319.6790716
0.007106669	1	0.007153916	1	426.4533214
0.040886165	1	0.021994557	1	282.7853261
0.021995182	1	0.020805864	1	329.3277311
0.016788513	1	0.015365042	1	248.8260274
0.013896547	1	0.012575828	1	347.7657267
0.020633336	1	0.014329462	1	295.6342669
0.022874775	1	0.019399041	1	235.2259332
0.009599133	1	0.008065085	1	449.9538606
0.010236067	1	0.009416081	1	531.3731579
0.028933366	1	0.022363253	1	208.9772727
0.013173403	1	0.0099006	1	316.3512304
0.010202502	0.994546204	0.007520357	1.008203125	437.540332
0.008690652	0.998054477	0.007861045	1.00292113	401.1937683
0.0132351	1	0.010178076	1	387.0010571
0.009161385	1	0.007056315	1	400.8463277
0.019001218	1	0.018204772	1	227.5211931
0.026870048	1	0.019842833	1	316.9591667
0.011296892	0.992753719	0.007926281	1.010909091	386.0269697
0.018357504	1	0.014904174	1	440.4940978
0.011150455	0.997191017	0.009796122	1.004219409	429.2071027
0.05025836	0.998353911	0.03748837	1.00247117	276.9623147
0.055525814	1	0.066508759	1	110.6774194
0.00846917	0.995689675	0.006515489	1.006479482	389.2583693
0.012405607	0.994554159	0.009307069	1.008191126	328.502901
0.021642708	1	0.01653876	1	304.8981077
0.024689117	0.997993984	0.021165706	1.003012048	211.2791165
0.045007044	1	0.045196852	1	173.7459016
0.041646775	1	0.036360628	1	172.4918919
0.022547111	1	0.015288017	1	259.4024691
0.025671441	1	0.025415593	1	319.5061947
0.018297253	1	0.016407795	1	282.9556104
0.008316802	1	0.00637312	1	403.5814878
0.011108224	0.998943476	0.009462391	1.001585624	290.4339323
0.006532079	1	0.007246016	1	839.7600806
0.034045702	1	0.031813716	1	156.3678161
0.04526999	1	0.035566749	1	150.1865672
0.057910828	1	0.036472578	1	205.7563636
0.006503471	1	0.006164884	1	660.002809

original_gldzm_LowIntensityLargeDistanceEmphasis	original_gldzm_HighIntensityEmphasis	original_gldzm_DistanceZoneVariability	original_gldzm_ZonePercentage	original_gldzm_IntensityVariabilityNormalized
0.015354596	283.0764818	523	0.434746467	0.0437577
0.017568642	275.1686587	753	0.3765	0.041038149
0.012275649	423.9571184	583	0.521466905	0.032184037
0.032564141	225.2509542	1048	0.427231961	0.044448532
0.01702152	203.5093168	322	0.339304531	0.053836658
0.01512789	259.7021944	638	0.389499389	0.042521202
0.039861398	115.7722008	259	0.245033113	0.073329259
0.041276321	244.3634895	619	0.342936288	0.04342561
0.026018343	292.8355615	748	0.369018254	0.0383554
0.013220151	327.1716418	1470.005427	0.490842491	0.037599716
0.036164635	162.3086207	580	0.32097399	0.054476813
0.014877873	432.6538871	937.0021299	0.55727003	0.030121773
0.008118	301.4536542	1122	0.383065893	0.044819062
0.010461857	301.570634	899	0.483853606	0.04059015
0.009885574	469.5949045	785	0.507105943	0.033041503
0.007055222	657.6727053	1648.019324	0.403017766	0.026796862
0.032506332	244.5843558	652	0.40623053	0.040879408
0.01482864	204.9095355	818	0.319781079	0.052229482
0.020926407	218.3182898	421	0.370598592	0.047872671
0.007975882	377.6815227	1073.007428	0.371251293	0.038767028
0.006759312	464.0572497	3460.036824	0.500143885	0.031551384
0.007403966	461.6472332	1010.001976	0.477133428	0.035909013
0.010401502	351.5564874	2019.015787	0.491870905	0.035656039
0.030914587	201.3016304	368	0.357281553	0.051098771
0.003341738	709.5036284	1376.001451	0.529185868	0.030777025
0.009077524	478.1940789	608	0.449704142	0.037385301
0.01612417	447.5332757	1157	0.523292628	0.029603727
0.007523964	472.3178995	1771	0.527868852	0.031345401
0.019497296	291.1112141	1071.029657	0.317446308	0.042693051
0.011514796	475.5083752	1194	0.377967711	0.031086476
0.006816562	446.0792079	1919	0.463302752	0.033856058
0.008096948	422.1520155	2057.000971	0.4118	0.035916425
0.019449168	272.0113122	884	0.349269064	0.042553899
0.042066228	192.7857143	140	0.291060291	0.055816327
0.011811483	394.2291221	467	0.525900901	0.034265827
0.012778391	282.1104762	1050	0.326391048	0.044166893
0.033690486	357.6559271	1645	0.460913421	0.033275007
0.00934422	319.8034076	1526	0.450014745	0.040448736
0.007377836	353.6352313	562	0.378706199	0.043768443
0.03405572	194.2732673	505	0.308679707	0.050649936
0.017782332	281.6594427	646	0.490881459	0.039782802
0.027192734	179.4414557	632	0.327122153	0.051749519
0.008088134	324.0941307	903	0.298315164	0.046912654
0.048638804	192.4698582	564	0.299363057	0.050777124
0.012062467	578.2338009	1497	0.428448769	0.029542496
0.005555386	469.2826205	1847	0.516643357	0.035983665
0.003837265	556.2515687	1725.223617	0.475583288	0.037119644
0.020520249	321.7339181	684	0.451187335	0.037866352
0.026106117	148.164557	158	0.280141844	0.065614485
0.017931011	305.5526316	380	0.348623853	0.041454294
0.005388703	461.7905199	1308	0.328313253	0.040962929
0.021579388	250.5648703	501	0.256134969	0.047191047
0.007783121	277.6225218	1261	0.332104293	0.052297278
0.007526179	509.070626	623	0.532023911	0.031394201
0.009532973	369.960719	1498.005326	0.381508763	0.037851883
0.02939263	408.2970414	845	0.447563559	0.031235601
0.009268911	384.5637394	1400.050992	0.430619091	0.038555602
0.019321849	183.8751715	727.0027435	0.299753289	0.058653736
0.008083343	397.2593168	644	0.469387755	0.040179777
0.009875961	356.0919003	642	0.456939502	0.037684029
0.011298358	298.9196113	1132	0.384903094	0.044250771
0.016270431	319.8506224	1926.001037	0.35137598	0.040600089
0.007153916	426.4533214	1114	0.490532805	0.036507773
0.021994557	282.7853261	736	0.394004283	0.037559812
0.020805864	329.3277311	238	0.403389831	0.039933621
0.015365042	248.8260274	730	0.473104342	0.043824357
0.012575828	347.7657267	461	0.435727788	0.039276119
0.014329462	295.6342669	1214	0.340437465	0.043437491
0.019399041	235.2259332	509	0.393658159	0.045649816
0.008065085	449.9538606	1062	0.423613881	0.035847156
0.009416081	531.3731579	1900	0.49894958	0.028527978
0.022363253	208.9772727	660	0.320232897	0.049343434
0.0099006	316.3512304	1788	0.382132934	0.042545631
0.007549641	438.7605469	2546.038281	0.393180771	0.034096069
0.007924438	401.2302824	2050.003895	0.481933365	0.037813979
0.010178076	387.0010571	946	0.405834406	0.03707834
0.007056315	400.8463277	885	0.375796178	0.040666475
0.018204772	227.5211931	637	0.468727005	0.043490262
0.019842833	316.9591667	1200	0.366300366	0.037102778
0.008017132	386.7078788	1638.043636	0.370536717	0.039916253
0.014904174	440.4940978	593	0.435389134	0.036806588
0.010037833	429.3642757	1418.005626	0.535190064	0.033894734
0.03995954	276.9629325	1212.001647	0.490703314	0.038170819
0.066508759	110.6774194	124	0.23709369	0.069719043
0.006535103	390.312095	1844.017279	0.479544278	0.039016835
0.009573567	328.8361775	1457.021843	0.421704088	0.039874198
0.01653876	304.8981077	687	0.481092437	0.040013221
0.021186623	211.3875502	994.002008	0.327308577	0.048158497
0.045196852	173.7459016	122	0.296836983	0.058586402
0.036360628	172.4918919	185	0.320069204	0.058758218
0.015288017	259.4024691	1215	0.378386795	0.044900337
0.025415593	319.5061947	565	0.511312217	0.037894902
0.016407795	282.9556104	811	0.519206146	0.039592776
0.00637312	403.5814878	1761	0.404548587	0.040027784
0.009466355	290.5924947	1890.001057	0.402039949	0.042695069
0.007246016	839.7600806	496	0.561085973	0.02471384
0.031813716	156.3678161	522	0.296086217	0.052494825
0.035566749	150.1865672	134	0.313084112	0.059144576
0.036472578	205.7563636	275	0.309684685	0.048780165
0.006164884	660.002809	712	0.616984402	0.027655915

original_gldzm_LowIntensitySmallDistanceEmphasis	original_gldzm_IntensityVariability	original_gldzm_HighIntensityLargeDistanceEmphasis	original_gldzm_SmallDistanceEmphasis	original_glcm_SumVariance
0.015354596	22.88527725	283.0764818	1	460.8045581
0.017568642	30.90172643	275.1686587	1	399.3637793
0.012275649	18.76329331	423.9571184	1	914.6097282
0.032564141	46.58206107	225.2509542	1	324.174179
0.01702152	17.33540373	203.5093168	1	320.3818583
0.01512789	27.12852665	259.7021944	1	386.9002027
0.039861398	18.99227799	115.7722008	1	95.47818191
0.041276321	26.88045234	244.3634895	1	238.8842643
0.026018343	28.68983957	292.8355615	1	307.034063
0.013194191	55.421981	327.9694708	0.998982361	647.4656357
0.036164635	31.59655172	162.3086207	1	207.8491415
0.014871483	28.28434505	434.6506922	0.999201278	841.5570714
0.008118	50.28698752	301.4536542	1	574.526364
0.010461857	36.49054505	301.570634	1	610.3722717
0.009885574	25.93757962	469.5949045	1	859.3565079
0.007036138	44.37560386	663.531401	0.998188406	2067.850509
0.032506332	26.65337423	244.5843558	1	396.4697935
0.01482864	42.72371638	204.9095355	1	253.9150081
0.020926407	20.1543943	218.3182898	1	303.1124068
0.00792325	41.75208914	378.9127205	0.998607242	767.8361605
0.006692268	109.6726122	465.3760069	0.998273878	1236.661832
0.007387497	36.33992095	462.3142292	0.999258893	1002.986446
0.010363681	72.27479033	354.6245683	0.99851998	706.2222125
0.030914587	18.80434783	201.3016304	1	237.3217314
0.003337384	42.4107402	710.8642961	0.999455733	2055.151178
0.009077524	22.73026316	478.1940789	1	889.4391512
0.01612417	34.25151253	447.5332757	1	697.9030868
0.007523964	55.51270469	472.3178995	1	1008.047545
0.019438998	46.06580167	294.7284523	0.997219648	334.4150618
0.011514796	37.11725293	475.5083752	1	702.2775689
0.006816562	64.96977592	446.0792079	1	993.2419919
0.008081896	73.95191841	422.3283147	0.999635746	807.3666107
0.019449168	37.61764706	272.0113122	1	406.709244
0.042066228	7.814285714	192.7857143	1	132.3998978
0.011811483	16.00214133	394.2291221	1	767.6789642
0.012778391	46.3752381	282.1104762	1	366.8592069
0.033690486	54.73738602	357.6559271	1	594.5043213
0.00934422	61.72477064	319.8034076	1	581.2866208
0.007377836	24.59786477	353.6352313	1	753.8255829
0.03405572	25.57821782	194.2732673	1	162.5259899
0.017782332	25.6996904	281.6594427	1	412.8409126
0.027192734	32.7056962	179.4414557	1	193.4560751
0.008088134	42.36212625	324.0941307	1	487.5300995
0.048638804	28.63829787	192.4698582	1	145.8690534
0.012062467	44.2251169	578.2338009	1	1119.91082
0.005555386	66.46182999	469.2826205	1	1140.931046
0.003725216	65.07073588	565.7375927	0.994010268	1274.960513
0.020520249	25.9005848	321.7339181	1	597.6237587
0.026106117	10.36708861	148.164557	1	158.8882889
0.017931011	15.75263158	305.5526316	1	372.3127144
0.005388703	53.5795107	461.7905199	1	787.2751675
0.021579388	23.64271457	250.5648703	1	290.3837745
0.007783121	65.94686757	277.6225218	1	441.4358612
0.007526179	19.55858748	509.070626	1	1114.259553
0.009412668	56.85352863	370.1304927	0.999001332	567.7048134
0.02939263	26.39408284	408.2970414	1	1138.524832
0.009201492	54.44050992	388.6643059	0.996813031	709.5652077
0.019258343	42.75857339	184.2085048	0.998971193	227.4038395
0.008083343	25.8757764	397.2593168	1	761.4934127
0.009875961	24.19314642	356.0919003	1	709.6688864
0.011298358	50.09187279	298.9196113	1	462.8652125
0.01626602	78.27697095	320.5368257	0.999610996	470.3563963
0.007153916	40.66965889	426.4533214	1	898.3632514
0.021994557	27.64402174	282.7853261	1	339.7633569
0.020805864	9.504201681	329.3277311	1	408.6141252
0.015365042	31.99178082	248.8260274	1	467.3334939
0.012575828	18.10629067	347.7657267	1	626.4100909
0.014329462	52.73311367	295.6342669	1	343.4927705
0.019399041	23.23575639	235.2259332	1	343.9623562
0.008065085	38.06967985	449.9538606	1	785.467838
0.009416081	54.20315789	531.3731579	1	1354.562076
0.022363253	32.56666667	208.9772727	1	233.4606366
0.0099006	76.07158837	316.3512304	1	562.132606
0.007513035	87.2859375	443.6414063	0.997949219	857.0468695
0.007845197	77.66991237	401.3763389	0.999269718	811.9083606
0.010178076	35.07610994	387.0010571	1	603.8083311
0.007056315	35.98983051	400.8463277	1	656.1663402
0.018204772	27.7032967	227.5211931	1	388.7954464
0.019842833	44.52333333	316.9591667	1	446.9885497
0.007903568	65.86181818	389.4315152	0.997272727	752.8474969
0.014904174	21.82630691	440.4940978	1	688.7843525
0.009735694	48.19831224	429.9929677	0.998945148	851.4617767
0.036870578	46.33937397	276.9654036	0.999382208	394.1496353
0.066508759	8.64516129	110.6774194	1	106.6126262
0.006510586	72.25917927	394.5269978	0.99838013	828.4236505
0.009240444	58.41569966	330.1692833	0.997952218	520.5936404
0.01653876	27.48908297	304.8981077	1	558.2712242
0.021160477	47.96586345	211.8212851	0.999246988	277.0490464
0.045196852	7.147540984	173.7459016	1	126.6831834
0.036360628	10.87027027	172.4918919	1	198.8049954
0.015288017	54.55390947	259.4024691	1	292.837197
0.025415593	21.41061947	319.5061947	1	493.5022929
0.016407795	32.10974106	282.9556104	1	524.7185199
0.00637312	70.48892675	403.5814878	1	737.8299588
0.0094614	80.77906977	291.2267442	0.999603594	509.5959263
0.007246016	12.25806452	839.7600806	1	2223.760292
0.031813716	27.40229885	156.3678161	1	185.2313829
0.035566749	7.925373134	150.1865672	1	126.0256369
0.036472578	13.41454545	205.7563636	1	226.0525849
0.006164884	19.69101124	660.002809	1	1713.321575

original_glcm_Homogeneity1	original_glcm_Homogeneity2	original_glcm_ClusterShade	original_glcm_MaximumProbability	original_glcm_Idmn	original_glcm_SumVariance2	original_glcm_Contrast	original_glcm_DifferenceEntropy
0.305786459	0.221857163	2867.717464	0.020929603	0.971283366	124.3856766	62.03997131	3.918231025
0.34935361	0.273609344	4308.281508	0.026851944	0.974110658	160.8099415	56.09958429	3.784221659
0.272351931	0.194308855	3982.034106	0.019121878	0.94816332	306.5068132	100.9569073	4.283759405
0.307842237	0.223259202	1685.796876	0.015868206	0.97054351	110.6724724	64.9417418	3.925892323
0.373376518	0.291947482	357.3118626	0.023475479	0.979715609	57.70979676	28.47471454	3.373389142
0.325650433	0.244335004	1676.484922	0.021985716	0.968365481	116.2959882	54.0582369	3.821834112
0.45065798	0.383855971	212.6135175	0.0533321	0.987542742	22.17157299	14.97101642	2.92542029
0.375228908	0.30244966	3285.22888	0.03496848	0.988137265	126.636993	51.7934295	3.624801384
0.37383545	0.302969946	3078.120354	0.04101838	0.977817178	148.8508819	54.24437755	3.674396569
0.286506929	0.200143768	2682.784424	0.011315381	0.971561605	178.667698	59.78086072	3.98276189
0.367934201	0.289544661	961.8645049	0.02469869	0.976124908	74.50692217	38.3835309	3.544071567
0.256097726	0.171506178	2128.760052	0.008388283	0.960103527	216.3782581	96.80906799	4.274764969
0.341827379	0.258125588	1098.038244	0.020185588	0.978556222	106.7354752	36.7072281	3.600741859
0.28955654	0.20262767	1580.91599	0.012765043	0.970454169	141.5224253	50.18950356	3.874243267
0.277138768	0.192547682	2791.488124	0.01066187	0.963563536	179.8843241	83.53795786	4.099418916
0.348590415	0.275471918	9015.062817	0.038398907	0.974449824	667.028282	68.7356408	3.926169315
0.32545522	0.242127365	3501.710186	0.016326967	0.974042975	175.3334571	50.14370642	3.771468557
0.387147589	0.312905498	908.0267791	0.039113943	0.979996008	61.62810129	32.7408305	3.430902983
0.359561428	0.27985074	821.9300412	0.023812837	0.975976318	75.09256438	35.64647218	3.526013731
0.352130789	0.273745977	6625.696237	0.020160352	0.97688121	240.261702	49.0159541	3.735567823
0.266980937	0.183528349	4199.437594	0.013040215	0.960974545	301.8489192	84.93156512	4.235083705
0.292589409	0.204982958	2494.231418	0.012040881	0.974748589	167.7718976	54.95087397	3.905893876
0.282952196	0.199927979	3479.50868	0.012533473	0.971823877	209.0318544	69.85735804	4.083538141
0.366677845	0.291296789	1351.021255	0.034619745	0.983186143	82.86982987	43.75622799	3.572301568
0.265024732	0.180975312	4070.242249	0.009341088	0.970414314	337.9441533	76.35152058	4.154776054
0.303817473	0.222529753	3334.811716	0.021795004	0.970307229	164.0051286	76.61416956	4.036600145
0.295631056	0.210276656	5276.289326	0.012899806	0.983410932	254.452679	58.07933393	3.944129209
0.26271665	0.18187303	3226.752948	0.010610538	0.955852804	260.2453039	97.04949529	4.301808067
0.390843722	0.32213148	4905.181592	0.037991291	0.979425098	157.5826766	50.72252449	3.599377871
0.347436189	0.275846938	7955.916067	0.031972355	0.969113072	284.7750435	75.13230036	3.935415014
0.290343523	0.205580019	3112.161165	0.0090538	0.969019487	222.2618735	66.53385144	4.026835478
0.329130323	0.247014014	4266.765045	0.017477187	0.986512945	209.7048853	45.2854794	3.757826852
0.349072631	0.273161788	5219.315134	0.029147514	0.972088064	171.4925613	62.28660179	3.794554324
0.411127071	0.343262779	1094.129769	0.058986789	0.979130513	58.61878478	40.29991688	3.348632716
0.262817612	0.180419878	4311.081995	0.013938954	0.962663967	240.4984183	100.7178134	4.262151942
0.377372594	0.305916558	2418.982296	0.03574561	0.975000119	121.8149795	52.52309965	3.658363741
0.295140537	0.21598394	6752.75215	0.022240804	0.971115027	274.4574644	94.27397859	4.207457961
0.299220218	0.216408908	1928.187026	0.019656495	0.967901271	130.1199452	66.67855691	4.010055535
0.354737023	0.268766009	957.713992	0.022199914	0.982643906	113.3375764	22.81038674	3.345214759
0.398244404	0.32922526	1272.604117	0.050592602	0.975747857	74.50393775	44.2209998	3.536428901
0.281517585	0.196234318	1401.593328	0.015744548	0.961215074	115.8030279	74.50253191	4.073365681
0.380751916	0.30566404	1027.135314	0.038304206	0.982850323	69.94794746	37.33566212	3.509221858
0.381087674	0.306102935	1709.386897	0.037426553	0.983510482	81.56456611	39.49206846	3.521552883
0.412242526	0.343461784	1234.232915	0.053047059	0.980879739	66.13569638	35.86534079	3.369506898
0.299656224	0.221304476	16615.73627	0.01884725	0.985888752	491.1843816	98.7720861	4.158871409
0.265558244	0.178569412	2776.55356	0.008964954	0.967379786	196.3334354	76.13482591	4.135126594
0.283487673	0.198592722	3424.731453	0.013916882	0.972605848	191.7872836	75.53862941	4.091685794
0.297854037	0.215757438	6073.874473	0.017765986	0.964524702	252.3628065	75.35506135	4.035449861
0.424403726	0.354424644	447.3561095	0.049714005	0.983773585	36.29766149	23.97956547	3.131520153
0.371464172	0.296263154	1189.08211	0.047854142	0.978019199	71.54008609	44.77077553	3.590569391
0.366866508	0.287950734	5354.918795	0.027526334	0.983636163	167.8760296	37.38096128	3.534527611
0.410813518	0.343308814	2817.744516	0.04226194	0.976669883	110.896937	46.32628717	3.432453353
0.373433471	0.296533379	731.9323132	0.031098585	0.979612272	57.59312397	37.35932793	3.522374769
0.271032826	0.182569653	2838.771552	0.010787154	0.983087089	239.8168903	71.20533817	4.041027077
0.338988137	0.260890321	4504.874888	0.023646848	0.979476061	177.1042743	58.26659666	3.829366858
0.349131466	0.283425711	18032.78024	0.113106474	0.960237471	619.2946535	101.8545783	4.20117204
0.311926136	0.228379526	6752.960982	0.018221418	0.978609858	199.7854632	66.7536414	3.932633734
0.398594781	0.323754796	1084.611134	0.040540257	0.984174512	59.95479373	32.06639092	3.33548462
0.291776772	0.207666104	3249.574648	0.019628878	0.975029998	148.0228434	81.53548333	4.054070152
0.300308059	0.210806506	631.2215367	0.010322149	0.971687493	139.9058941	41.15321491	3.732340001
0.336652975	0.255435004	1714.243537	0.018947267	0.975905956	101.6255754	52.64530407	3.748343945
0.362355583	0.289562517	4754.8592	0.033841516	0.971554857	172.1559501	64.52833195	3.785834912
0.27717184	0.193298208	2510.323865	0.01272307	0.964573618	159.3556779	80.58067967	4.143706102
0.35056846	0.278754924	2156.464648	0.034717657	0.956381433	153.7877425	62.29115569	3.862707233
0.334085539	0.255156169	2516.908951	0.033918981	0.970236771	131.8373157	56.50149212	3.771469757
0.292324723	0.203985597	810.0263171	0.011430174	0.976175329	95.81754446	51.41244987	3.875402027
0.317615007	0.237703724	1200.267349	0.028516564	0.972166269	116.5320164	62.92362255	3.924768466
0.370525675	0.298023265	2696.898134	0.034945587	0.978782897	120.0117017	57.43667856	3.704287909
0.320635121	0.239383486	1809.762853	0.023384311	0.972507187	108.4809172	58.86189223	3.886744673
0.315120294	0.237479039	5698.96646	0.022535461	0.973818811	212.8129217	78.99354018	4.027119656
0.276461633	0.194960636	9076.901397	0.008934067	0.96927906	440.6640157	91.61945054	4.225070863
0.387119867	0.31183712	990.3249593	0.041840959	0.986341249	76.75373149	34.9931346	3.441847799
0.342515137	0.263873257	5329.066465	0.023338649	0.978638832	182.4674211	51.39075251	3.775250716
0.327843473	0.248483473	6912.15327	0.018298711	0.974580331	276.7863991	62.28718645	3.896985701
0.287110789	0.200960898	5236.791323	0.012867051	0.976876662	196.9919745	71.75439894	4.037588693
0.314905653	0.235868946	3781.906284	0.019114851	0.976213549	173.0290989	73.17897806	3.991914265
0.345195452	0.266938778	3550.963728	0.032499024	0.980168346	157.3057699	51.52724887	3.747910926
0.288889776	0.202895562	1015.20886	0.018158544	0.964701565	93.08488714	55.64327675	3.933331734
0.341744048	0.26595808	3754.949871	0.028203908	0.974978181	183.2341712	64.02738773	3.861818056
0.349032289	0.277150416	6505.393016	0.025500231	0.964949694	252.5538645	71.94274327	3.909219515
0.31263042	0.234506094	11942.3482	0.021693373	0.978581473	302.321162	95.8578539	4.063483803
0.257063862	0.1738834	4913.882766	0.011568204	0.966675705	237.1825301	109.0266448	4.344398462
0.286910241	0.205061415	3596.321673	0.017284246	0.9749624	174.0690138	83.69862351	4.148923003
0.455308857	0.388242114	157.4613384	0.042457157	0.984954958	30.63145132	12.07019173	2.799043085
0.282875594	0.198784153	3173.577774	0.009918114	0.965562377	182.1941284	70.80810668	4.085301629
0.319774614	0.234906729	1315.250651	0.014111499	0.974270596	118.2924615	46.90914039	3.77934392
0.289732296	0.206066387	4080.475034	0.015520854	0.968222543	189.4750372	71.74526132	4.051377907
0.367603821	0.289769543	1141.214151	0.026654182	0.978148958	75.10133102	40.78404856	3.570081743
0.419139256	0.347429886	648.1658816	0.046505175	0.97888908	43.19174142	26.15571824	3.055478257
0.376415445	0.298365878	1326.663575	0.03149427	0.977444298	72.59227113	42.40612053	3.393777502
0.350099747	0.273747283	1399.039731	0.029661611	0.976791377	90.94811767	50.95845579	3.742963274
0.26823536	0.187281196	3927.425288	0.018327314	0.964908963	177.8027877	123.9157769	4.322361283
0.274493137	0.192292618	2381.693213	0.016575544	0.959050876	156.36788	86.08956219	4.198253789
0.317325094	0.239303931	2851.784245	0.017732789	0.967915592	155.7023438	73.78129342	4.020575303
0.328403985	0.243096715	1369.568641	0.014845583	0.978961869	103.6985207	41.97657875	3.688685212
0.233889623	0.160027396	10930.76186	0.014512547	0.946926922	577.9387087	211.7715358	4.697365936
0.39034234	0.312685122	583.708234	0.027149131	0.984865584	52.48598077	25.72395109	3.293911104
0.412026594	0.337582896	495.7842226	0.040920394	0.98151169	38.32815378	21.88506247	3.110709983
0.410983631	0.341574455	3131.802259	0.054132492	0.975291914	123.8728254	34.58107816	3.345759095
0.222870858	0.142615443	7422.996368	0.010820726	0.952687654	422.1203028	152.9572686	4.581850464

original_glcm_InverseVariance	original_glcm_Entropy	original_glcm_Dissimilarity	original_glcm_DifferenceVariance	original_glcm_Idn	original_glcm_Idm	original_glcm_Correlation	original_glcm_Autocorrelation
0.224686526	7.969719996	5.413748355	32.42609393	0.898686111	0.221857163	0.332747063	151.7884782
0.267353071	7.941858211	4.875667223	32.17419262	0.90875954	0.273609344	0.484950442	131.8832173
0.18871997	8.713123787	7.215757348	48.14600555	0.863652886	0.194308855	0.509731628	282.5308746
0.22694611	8.300134042	5.414990159	35.42528418	0.899350175	0.223259202	0.266717406	109.0756549
0.283699483	7.473672303	3.613871182	15.26527482	0.914258187	0.291947482	0.340843136	117.6657611
0.245921067	8.054720664	5.005414354	28.77852708	0.895327089	0.244335004	0.367366609	131.6835121
0.369022992	6.209227023	2.528548964	8.547561565	0.934194572	0.383855971	0.189992869	40.92366516
0.296687083	7.356233742	4.430819477	32.01999794	0.939906346	0.30244966	0.417463825	77.95743207
0.296160971	7.523371635	4.607151947	32.84012438	0.918763634	0.302969946	0.467129686	100.1275581
0.204108028	8.935305458	5.596465576	28.15407224	0.894185304	0.200143768	0.501398973	214.8150208
0.280411539	7.626688736	4.059825345	21.66115163	0.910799822	0.289544661	0.325757835	76.34386333
0.176527113	9.221092921	7.025820117	46.82152988	0.87797895	0.171506178	0.385763633	266.844058
0.260231347	8.242487133	4.214735774	18.71696746	0.910356418	0.258125588	0.492228101	197.1616152
0.207175623	8.649878407	5.291229491	21.63327033	0.889536487	0.20262767	0.477032971	205.8578129
0.203533742	8.758462278	6.3707644	41.76605521	0.886226268	0.192547682	0.365217618	273.5606363
0.247774748	8.961961204	5.360226007	39.35457603	0.910169388	0.275471918	0.814714124	620.2615589
0.240886559	8.265903551	4.860434578	26.00329097	0.903658243	0.242127365	0.555064382	133.4477525
0.306743823	7.242293456	3.744586377	18.4975497	0.918597174	0.312905498	0.311122344	92.27581067
0.279064099	7.687351101	4.054376832	18.84701472	0.907950066	0.27985074	0.363590352	109.8660931
0.253456724	8.341213154	4.643010101	27.00205467	0.91181152	0.273745977	0.66200489	249.2612303
0.180588323	9.607823429	6.670706599	40.00857751	0.878126545	0.183528349	0.564251668	387.3914441
0.208686745	8.726279776	5.342743566	25.93316495	0.900207725	0.204982958	0.509075539	323.7215827
0.199506701	9.030519809	6.058498108	32.43506066	0.895130772	0.199927979	0.501097893	229.7229903
0.292662106	7.425858697	4.253032123	25.40302583	0.925867165	0.291296789	0.313369128	83.92650099
0.18623452	9.381459051	6.458814279	33.93382254	0.890676737	0.180975312	0.635287762	626.9289896
0.221090692	8.190976996	5.917056922	41.32923756	0.899420827	0.222529753	0.369505277	281.0482987
0.212627241	8.777295636	5.459121364	28.05522914	0.918543676	0.210276656	0.616282839	227.1428006
0.183270822	9.318314206	7.128619094	45.49829056	0.871745497	0.18187303	0.462857278	315.6217101
0.303331356	7.541924606	4.353090536	31.24129814	0.922983045	0.32213148	0.517676644	109.8435795
0.263553737	8.278850321	5.531070918	44.14417225	0.903470663	0.275846938	0.579060526	219.0380315
0.209083975	9.214047505	5.754481591	33.05944247	0.892653222	0.205580019	0.543065664	319.7386307
0.245848538	8.719957803	4.710047633	22.77177359	0.927595031	0.247014014	0.645534493	266.2787968
0.272906612	7.891799544	4.98987038	37.07914251	0.907895904	0.273161788	0.472143252	131.2099753
0.325851238	6.353124436	3.780064078	25.82047124	0.923509752	0.343262779	0.18800679	45.68469659
0.185352566	8.543417427	7.129553724	49.05627914	0.883199648	0.180419878	0.421789696	239.1154014
0.294323234	7.711305115	4.499332852	32.06965424	0.914276744	0.305916558	0.403530801	123.7341113
0.206092718	8.945200531	6.564798283	50.74366575	0.900773594	0.21598394	0.492907035	183.8154286
0.217682942	8.559695498	5.679684068	34.09192802	0.892245509	0.216408908	0.32934448	191.6614883
0.273071057	8.125536264	3.549458037	10.03860848	0.913606327	0.268766009	0.665297076	256.35174
0.304310389	7.041376947	4.089540442	27.36395922	0.915967952	0.32922526	0.257897604	56.30725733
0.201808955	8.324153803	6.076269742	37.28812977	0.881245657	0.196234318	0.223183905	136.0407159
0.295422033	7.365673675	3.946440515	21.65510225	0.92504101	0.30566404	0.308594498	70.74029123
0.29694344	7.351490229	3.988896499	23.45255721	0.927469171	0.306102935	0.351419041	164.3543721
0.32624386	6.844861133	3.633598061	22.57874966	0.925580906	0.343461784	0.294719265	52.1681875
0.224406099	8.971794554	6.517196996	55.18485691	0.93072119	0.221304476	0.663821552	337.2527214
0.18824713	9.164470108	6.303213838	36.14294261	0.887743489	0.178569412	0.447915896	360.6208873
0.204625659	8.969441224	6.062167252	38.41096635	0.899701496	0.198592722	0.440626205	399.0260525
0.221788239	8.457448032	5.920322553	39.86427693	0.889407653	0.215757438	0.543873231	189.0371275
0.341776031	6.353228223	3.081253185	14.39924174	0.928377462	0.354424644	0.205473675	61.18705412
0.286632178	7.177442978	4.285837196	26.29711899	0.916754779	0.296263154	0.223693055	127.0547821
0.285950948	7.756930592	3.995668829	21.28746803	0.925326494	0.287950734	0.630250587	255.4706559
0.326510869	7.172579643	3.938082832	30.58358408	0.921691568	0.343308814	0.418519623	98.06731207
0.293051276	7.458761961	3.979227775	21.41754998	0.918005428	0.296533379	0.217691739	151.8924441
0.187426773	8.921050164	6.02262229	34.48835132	0.917494088	0.182569653	0.544727765	354.1065993
0.258914306	8.203444882	4.998161476	33.11025801	0.917609658	0.260890321	0.507763945	185.6706343
0.195359498	8.330733895	6.690356586	56.37483873	0.888451418	0.283425711	0.72028952	332.6168263
0.228682654	8.404925918	5.41391602	37.09541682	0.914283975	0.228379526	0.505175307	227.26518
0.314711461	7.087482979	3.525242916	19.44360885	0.929710674	0.323754796	0.306190313	82.67744836
0.215530591	8.240483486	6.044017102	44.6764573	0.907358127	0.207666104	0.295609114	241.1180434
0.217671564	8.641887063	4.779521121	17.98805075	0.891462458	0.210806506	0.547495317	240.1939396
0.260411757	8.121997744	4.74402775	29.90704426	0.910411797	0.255435004	0.323533317	157.1525175
0.280315202	8.048110493	4.952116432	39.7211354	0.909283937	0.289562517	0.458881797	152.1992852
0.198264933	8.823665404	6.347461454	39.65990758	0.885945632	0.193298208	0.335643132	285.9507058
0.26058198	7.915138921	5.24087329	34.43972742	0.883065443	0.278754924	0.427110967	111.1877052
0.249823115	7.385718201	5.023308974	30.78008209	0.899119811	0.255156169	0.397736079	135.0985207
0.207013305	8.472775709	5.22820795	23.89467732	0.901634655	0.203985597	0.304027511	160.844575
0.231238319	8.127202424	5.405938787	33.49206993	0.901003024	0.237703724	0.304104461	206.4519279
0.2897371	7.619045593	4.660271027	35.54589612	0.921108721	0.298023265	0.357657486	113.8487299
0.235473685	7.894354571	5.229734723	31.33807898	0.90187871	0.239383486	0.302539408	115.8896881
0.241914825	8.312289863	5.866693357	44.14701023	0.907314551	0.237479039	0.462242107	247.290441
0.198375038	9.429212409	6.71579894	45.88589161	0.89445065	0.194960636	0.659300608	415.4635727
0.302438625	7.443276273	3.767948143	20.63436605	0.933286395	0.31183712	0.377539722	85.29222565
0.260111787	8.209506696	4.764015909	28.44764355	0.914737875	0.263873257	0.563626956	185.0424753
0.246804294	8.798139714	5.241241151	34.49132484	0.907786285	0.248483473	0.633641378	273.8511646
0.206671284	8.819267452	5.827847554	37.43365557	0.907810539	0.200960898	0.47109617	259.7327524
0.240340307	8.398435599	5.707080047	40.31422111	0.910541324	0.235868946	0.410895003	195.4438017
0.265913637	7.923512143	4.731829696	28.90702528	0.918405595	0.266938778	0.511367399	214.7130523
0.205249589	8.193584951	5.488331731	25.15315012	0.881752589	0.202895562	0.257070996	133.329452
0.261397573	8.307155266	5.196190431	36.55121834	0.910787939	0.26595808	0.488982862	145.9222908
0.266097411	8.301367325	5.430904398	42.11978135	0.897474193	0.277150416	0.560517947	237.3887131
0.238060998	8.123331808	6.185640212	57.24990099	0.918596555	0.234506094	0.520013911	207.615932
0.179242094	9.099480869	7.346159608	54.5096052	0.889784588	0.1738834	0.378561264	263.6601862
0.205763498	8.594101896	6.296911241	43.72587278	0.904858495	0.205061415	0.354604433	124.8451563
0.380758772	6.53791895	2.344827047	6.508430761	0.926143295	0.388242114	0.433362424	47.23025655
0.202922571	8.954053468	6.012778275	34.33198436	0.886212766	0.198784153	0.445826352	267.6117529
0.238198528	8.493194893	4.796017676	23.75541141	0.902121812	0.234906729	0.434068222	178.6272961
0.207272137	8.51533365	5.942089198	36.07988276	0.892100511	0.206066387	0.454565734	181.5311074
0.28412841	7.689127967	4.130710506	23.579065	0.915518115	0.289769543	0.301278362	99.28017309
0.351126373	6.264725909	3.11077046	16.29531137	0.920986554	0.347429886	0.246468987	48.11791466
0.304108487	7.014108178	3.950126932	26.65837996	0.918275705	0.298365878	0.269419791	70.31590774
0.272539605	7.764749341	4.726488076	28.49594854	0.912360573	0.273747283	0.284167451	101.1663554
0.194017288	8.304922627	7.476512221	67.24790068	0.891608726	0.187281196	0.181371555	146.3416136
0.193256627	8.551469263	6.611302508	42.03188555	0.877482716	0.192292618	0.298312333	168.1689086
0.233935056	8.549824622	5.749859821	40.26412641	0.896720241	0.239303931	0.363566012	237.7222148
0.24830813	8.341432455	4.4912696	21.6099311	0.911178167	0.243096715	0.426762392	175.4352682
0.16303399	8.917601428	10.24961513	104.3933807	0.865257961	0.160027396	0.473776092	635.330482
0.31018573	7.316651592	3.371351892	14.22309551	0.926658459	0.312685122	0.346964948	72.13757596
0.332552559	6.477174622	3.047272762	12.54525671	0.920752919	0.337582896	0.265875444	50.27922841
0.321941111	6.962334848	3.61610554	21.13077196	0.914893757	0.341574455	0.557469629	76.9468991
0.150635857	9.216671558	9.020401274	70.03540125	0.866708865	0.142615443	0.477831294	507.7024259

original_glcm_SumEntropy	original_glcm_AverageIntensity	original_glcm_Energy	original_glcm_SumSquares	original_glcm_ClusterProminence	original_glcm_SumAverage	original_glcm_Imc2	original_glcm_Imc1
4.999697537	11.95133017	0.006209349	50.6649559	134533.0264	23.33837972	0.811918666	-0.125700951
5.117696271	10.56577222	0.007825765	58.56758944	221035.1543	20.56048397	0.822379584	-0.13187199
5.747696198	15.72497528	0.004065629	107.3570289	230736.8687	30.40248088	0.925124811	-0.198691268
5.15174728	10.20429974	0.005229081	48.59658116	70925.41851	19.75982505	0.744115038	-0.091922735
4.804191058	10.60841316	0.008080292	22.83018528	13489.4366	21.00622179	0.82048544	-0.140415416
5.102018585	11.03917762	0.006673303	46.25204798	63356.51694	21.55112092	0.801525187	-0.119580335
3.947786216	6.269381381	0.022477597	9.54509739	5408.26217	12.50947125	0.623962569	-0.076989795
4.800434341	7.946723363	0.012197884	48.28070188	148132.6042	15.38354505	0.780162866	-0.118835415
4.913405348	8.987290923	0.013059483	54.81215388	121051.1083	17.48551919	0.811246352	-0.133068219
5.558202704	13.93301725	0.003170887	62.900973	136930.8684	27.20709228	0.804878593	-0.110430689
4.862091009	8.35585344	0.008282137	30.61782689	35615.86621	16.40761154	0.768922398	-0.111251295
5.783252529	15.89329023	0.002290315	82.65139879	154805.6258	30.78422111	0.882064178	-0.150733591
5.178850271	13.60685223	0.005500169	37.67586198	44806.69258	26.80472991	0.776667494	-0.107233317
5.404355469	13.82160858	0.003693318	50.11296363	72779.73621	27.05625196	0.807120884	-0.117474737
5.522284539	16.29043907	0.003345919	70.02100285	142356.8559	31.58591593	0.87577078	-0.154169433
5.966108675	22.11524497	0.00506505	184.6249982	769075.7682	43.36772788	0.93563249	-0.210077366
5.343514712	10.38361387	0.005324434	60.1736305	172601.2803	20.20843727	0.866511637	-0.15591438
4.578366432	9.376308951	0.013193923	25.83238471	28803.71297	18.44357927	0.666712648	-0.078781204
4.9006545	10.15795324	0.007691102	29.79427768	28606.02391	19.99729612	0.825358122	-0.138709693
5.418167186	14.48318906	0.005438576	75.50550026	360976.8707	28.38077862	0.860918701	-0.152221927
5.930870502	18.75010975	0.001935332	98.94718597	273447.4779	36.50381783	0.772260037	-0.091467974
5.480952347	17.52046493	0.003722111	57.79660952	119428.4444	34.37927043	0.844310465	-0.134388531
5.626054037	14.39337916	0.003195823	72.83029518	189352.2339	27.91951315	0.783475177	-0.103288516
4.794356987	8.821663595	0.010547723	34.50971431	50958.55886	17.21967562	0.826064221	-0.14285645
5.954212491	24.21438808	0.002212388	105.3733042	272793.6784	47.39177311	0.878339259	-0.147483511
5.265375587	16.55211383	0.005926871	64.29248944	161776.452	32.19798297	0.868587556	-0.155694829
5.631842181	13.73325059	0.00349487	81.08511439	310848.3238	26.65484203	0.893082071	-0.168591097
5.809858773	17.14104503	0.002615484	94.33082743	190402.5269	33.15222893	0.80319328	-0.105554186
4.937605084	9.36620109	0.012866136	56.05645159	268300.408	18.22929134	0.789228654	-0.125316975
5.385166824	13.34545244	0.008409167	95.6585713	440605.698	25.80815319	0.853304521	-0.146147537
5.748321691	17.18063683	0.002638747	75.22576311	176436.9162	33.51168475	0.812232324	-0.111093691
5.564267315	15.37459989	0.004418544	66.42499341	225232.5378	30.00720534	0.848312088	-0.137512251
5.050509613	10.48758496	0.008906156	62.90402201	278893.7449	20.38189345	0.785440361	-0.113863331
4.233050819	6.519585803	0.021676698	26.81456835	39328.36466	12.81910773	0.79275135	-0.144732157
5.618650691	14.91464713	0.003820204	92.12314036	231930.5291	28.56971248	0.936431127	-0.21527018
4.977292868	10.61356933	0.011212722	48.16759345	99026.85905	20.62845784	0.762613689	-0.106550259
5.671965989	12.27050936	0.003838947	98.79825082	387989.5902	23.5496915	0.823980504	-0.119123438
5.277460534	13.61648469	0.004912033	52.92315447	86154.54072	26.51685596	0.70980566	-0.0782957
5.267992343	15.40753437	0.005784304	34.7431918	40676.73401	30.57513926	0.86841863	-0.161244639
4.581358009	7.106231122	0.016293318	31.96915084	44287.8785	13.95797439	0.721620907	-0.099337215
5.19027009	11.56828092	0.004722326	51.78093756	58234.20757	22.42162213	0.803986881	-0.116454137
4.709148969	8.084791475	0.01134479	29.25608055	35708.05611	15.82136091	0.717068398	-0.093061739
4.65756859	12.66435108	0.012406593	33.13661861	69234.91141	24.80489181	0.699831369	-0.086663107
4.427954879	6.799437287	0.018235206	27.70635584	42644.89758	13.3508533	0.691502112	-0.091047784
5.857892479	15.99419533	0.004323828	154.0198416	1186694.058	30.90414194	0.896869449	-0.167300193
5.629119075	18.71183238	0.002719766	71.19139905	139400.7209	36.36116949	0.766007339	-0.091590622
5.557391013	19.67294748	0.00332369	70.09451978	180254.3228	38.4673808	0.775892277	-0.097816632
5.484707371	12.41116752	0.004912393	87.19414178	308634.8353	24.05948106	0.879141658	-0.159911372
4.173474051	7.691823667	0.019162781	16.29696469	12076.14403	15.243845	0.751881648	-0.123110469
4.598614246	11.15569914	0.013772043	31.14838484	41940.22196	21.93710993	0.789783917	-0.126804781
4.96824663	15.16662978	0.00906117	53.49478966	272254.7185	29.85264485	0.772158848	-0.110569346
4.705024845	9.259179423	0.014360322	43.31053351	121757.5689	18.09847672	0.797896744	-0.131685107
4.643061878	12.35701058	0.010606969	26.41471926	23174.62248	24.23451049	0.602962536	-0.05888013
5.753749437	18.07366747	0.002819348	79.93338737	188385.006	35.31690598	0.928097555	-0.199152886
5.213407786	12.83785249	0.007073178	62.92803919	220047.8422	24.97091551	0.763972353	-0.100645247
5.728660311	14.77589645	0.016671429	186.3092076	1243163.612	28.50035295	0.939823251	-0.227415969
5.279036953	14.3773436	0.005472126	71.02673563	404875.4076	27.85667411	0.802467988	-0.114194292
4.461540464	8.87099418	0.013970128	25.67381904	37670.0757	17.40127114	0.693817117	-0.090012561
5.198130637	15.47651467	0.005460549	62.12485072	157218.4038	29.96494872	0.848110211	-0.141250652
5.491339993	14.90108314	0.003283845	46.66782928	49746.56326	29.35499634	0.871649911	-0.152885851
5.069590266	12.32201764	0.005961594	42.101763	73640.96217	24.07295716	0.74675833	-0.095056027
5.118405737	11.49718974	0.009095784	63.81754366	237370.0409	22.38479282	0.728696584	-0.090202358
5.450187634	16.80489793	0.003515647	63.81473918	120177.1875	32.63339125	0.796475264	-0.107368931
5.158040949	9.669266954	0.009560459	58.20695148	81581.79702	18.79376899	0.814791252	-0.129125271
4.928781098	10.95450854	0.010013296	49.54192322	97005.30365	21.55994431	0.909435728	-0.211748456
5.199793619	12.49863603	0.003739579	38.91715545	41850.92051	24.47228981	0.775323509	-0.103252756
5.208160955	14.22989359	0.006402973	47.8232295	66911.11405	27.78619889	0.881229451	-0.167065782
4.870469016	10.16562002	0.01158732	48.19422958	113961.9992	19.81631822	0.715821897	-0.089517729
5.000399121	10.48084412	0.007240647	46.053253	70694.96367	20.34392784	0.798258071	-0.119154497
5.316086653	15.08238644	0.006304411	77.74748784	292201.5568	29.24150271	0.81997298	-0.124751422
6.003145201	18.66311766	0.002376411	136.6382507	559230.7235	36.22473418	0.877621019	-0.144781609
4.785723561	8.846801109	0.011349521	30.48949166	32753.68924	17.29726858	0.763090214	-0.110575929
5.194913619	12.65847846	0.006695788	61.82409444	277585.0828	24.67848228	0.759673009	-0.099628005
5.592043295	15.19002138	0.004804001	88.19555405	370145.5092	29.67381558	0.807298262	-0.114011986
5.4300154	15.52112026	0.003930185	70.38815793	288601.1802	30.22168773	0.765087595	-0.094823155
5.358565325	13.50412848	0.005558731	66.93554836	189188.7421	26.1120305	0.829107147	-0.127915316
5.106988514	14.06693683	0.008511264	56.138532	155477.9106	27.44052111	0.815894181	-0.127669157
5.073256811	11.42616469	0.005279741	40.2689313	38625.33735	22.26492844	0.767026927	-0.102036131
5.312226349	11.12311594	0.007095599	67.02534543	176152.8389	21.54492203	0.809098699	-0.120084403
5.36296639	14.22652547	0.007296363	86.12110361	319074.4952	27.72499589	0.786349081	-0.109754964
5.321495526	12.98012254	0.006545387	107.331079	790166.2451	24.97615399	0.904996216	-0.18724531
5.653701953	15.83680069	0.003030102	92.79660848	271682.0585	30.43558186	0.826379197	-0.117377338
5.389552492	10.52416842	0.004516608	69.7341607	200998.3665	20.21925222	0.79306723	-0.108322743
4.335880607	6.529403304	0.015734089	10.77603511	4314.190336	13.05146689	0.82457254	-0.163097343
5.548093748	15.94540691	0.003174585	67.2061354	162527.9176	30.96610019	0.765289439	-0.093625103
5.302542071	12.91599059	0.004469484	43.63649978	58583.8313	25.35790072	0.75983413	-0.096680352
5.461920638	12.71793789	0.004133364	70.40391746	216347.8185	24.6636624	0.87624826	-0.156279276
4.836642586	9.729945782	0.008978614	31.86109141	43292.73928	19.04712149	0.709335782	-0.086864699
4.108086232	6.680471749	0.01923202	18.57168214	17869.84562	13.23809362	0.800475634	-0.152912273
4.611305051	8.111318134	0.01130198	32.30248577	52124.93026	15.84417887	0.848641077	-0.165204674
4.887912764	9.798233363	0.009368434	38.8147795	50371.19073	19.09385138	0.669919807	-0.07371736
5.28595665	12.16920039	0.005036296	83.71852355	204296.2258	23.05059579	0.875690758	-0.158251931
5.351694827	12.75506114	0.004354382	66.06288108	106021.7344	24.53928424	0.820848386	-0.12129413
5.351254319	15.14597944	0.005037666	61.45663675	131939.0255	29.47473558	0.733705313	-0.086788474
5.151854426	12.88840848	0.005223687	38.57361404	53542.96898	25.2978466	0.699037055	-0.078248
6.070338896	24.29407436	0.003225427	206.7464586	835239.6809	46.6316474	0.971151111	-0.275787199
4.658867784	8.195419827	0.00982773	21.10937357	21196.48496	16.17749431	0.721204095	-0.096441222
4.226672623	6.826286497	0.016486303	15.748129	14863.78838	13.5848832	0.80153923	-0.148077273
4.676015627	7.485195667	0.015721699	41.46670938	135457.8286	14.76536474	0.853159599	-0.173195558
6.039474318	21.86422503	0.002333119	150.146506	518690.9692	41.96534272	0.948809269	-0.220189564

original_glcm_DifferenceAverage	original_glcm_Id	original_glcm_ClusterTendency	original_firstorder_InterquartileRange	original_firstorder_Skewness	original_firstorder_Uniformity	original_firstorder_MeanAbsoluteDeviation
5.413748355	0.305786459	124.3856766	156.5	2.079646873	0.070055534	124.3489821
4.875667223	0.34935361	160.8099415	161.25	2.055876685	0.072788	132.829857
7.215757348	0.272351931	306.5068132	374.25	0.888146631	0.046903652	213.1109187
5.414990159	0.307842237	110.6724724	153	1.858610727	0.065021674	115.9755425
3.613871182	0.373376518	57.70979676	135	1.456359978	0.073098964	89.06023644
5.005414354	0.325650433	116.2959882	164	1.647734319	0.070777818	119.1494631
2.528548964	0.45065798	22.17157299	71	2.840393001	0.135558859	55.61309968
4.430819477	0.375228908	126.636993	117	2.414302124	0.093231022	123.0633276
4.607151947	0.37383545	148.8508819	169	1.688824857	0.0864406	146.815146
5.596465576	0.286506929	178.667698	234.5	1.189101297	0.046027344	151.445419
4.059825345	0.367934201	74.50692217	136	1.882511813	0.079212642	92.17059342
7.025820117	0.256097726	216.3782581	277	0.85294974	0.03562134	178.5243065
4.214735774	0.341827379	106.7354752	199	1.109376599	0.059255691	120.2378112
5.291229491	0.28955654	141.5224253	227	1.000723763	0.050323218	138.50234
6.3707644	0.277138768	179.8843241	238	1.193826171	0.044018622	172.4578401
5.360226007	0.348590415	667.028282	725	0.312350565	0.039146864	319.7133344
4.860434578	0.32545522	175.3334571	193	1.61735009	0.058945468	142.0002609
3.744586377	0.387147589	61.62810129	103	2.06284694	0.100726171	86.80361906
4.054376832	0.359561428	75.09256438	145.25	1.582006553	0.071330713	99.69978365
4.643010101	0.352130789	240.261702	211	1.646739646	0.054765672	163.9676492
6.670706599	0.266980937	301.8489192	337	0.755905224	0.033948636	203.9976041
5.342743566	0.292589409	167.7718976	225	1.159780173	0.047636386	148.2340373
6.058498108	0.282952196	209.0318544	288	1.14587766	0.044347343	177.7300372
4.253032123	0.366677845	82.86982987	116.75	2.581121261	0.089669149	97.78386276
6.458814279	0.265024732	337.9441533	397.25	0.622474711	0.03395491	217.4928955
5.917056922	0.303817473	164.0051286	187.5	1.582248841	0.062329313	147.1093055
5.459121364	0.295631056	254.452679	367	0.956104148	0.03764431	221.0989962
7.128619094	0.26271665	260.2453039	337	0.898024118	0.039979744	198.3778352
4.353090536	0.390843722	157.5826766	124	2.505352453	0.092664102	127.0865134
5.531070918	0.347436189	284.7750435	279.5	1.45471192	0.066236936	217.3762929
5.754481591	0.290343523	222.2618735	280	1.001488125	0.040037272	171.7210789
4.710047633	0.329130323	209.7048853	258	1.233435946	0.04670592	167.032224
4.98987038	0.349072631	171.4925613	144	2.299181944	0.08213834	128.7501738
3.780064078	0.411127071	58.61878478	86	2.987061469	0.130786952	86.65913443
7.129553724	0.262817612	240.4984183	249.25	1.370407355	0.047304906	174.1371743
4.499332852	0.377372594	121.8149795	127	2.137532915	0.089590011	112.450968
6.564798283	0.295140537	274.4574644	235	1.600745146	0.048796252	186.1108592
5.679684068	0.299220218	130.1199452	194	1.496305893	0.061394235	132.2054329
3.549458037	0.354737023	113.3375764	205.5	0.832628654	0.056518407	121.1774217
4.089540442	0.398244404	74.50393775	111	2.377369471	0.108778791	97.32477388
6.076269742	0.281517585	115.8030279	204.25	1.415882661	0.057292292	137.4041826
3.946440515	0.380751916	69.94794746	116	2.233372216	0.093417392	91.98572854
3.988896499	0.381087674	81.56456611	105	2.452569308	0.099988224	93.91690772
3.633598061	0.412242526	66.13569638	95	2.612371669	0.116570539	90.89063676
6.517196996	0.299656224	491.1843816	300.75	1.612490958	0.049746741	236.1601829
6.303213838	0.265558244	196.3334354	238	1.054841709	0.043611404	160.8255966
6.062167252	0.283487673	191.7872836	240.75	1.33634613	0.046701764	154.7198215
5.920322553	0.297854037	252.3628065	211.5	1.641663515	0.059355442	166.2962533
3.081253185	0.424403726	36.29766149	75.75	2.615222231	0.124729641	65.78168855
4.285837196	0.371464172	71.54008609	95.75	2.158634074	0.099385574	101.9825604
3.995668829	0.366866508	167.8760296	147	2.07297138	0.077397063	140.7406483
3.938082832	0.410813518	110.896937	97	2.809140471	0.105429364	92.16515488
3.979227775	0.373433471	57.59312397	111	2.138545516	0.093674511	85.47471428
6.02262229	0.271032826	239.8168903	315	0.909394543	0.035796042	187.6285598
4.998161476	0.338988137	177.1042743	172	1.858624981	0.069283816	147.3326512
6.690356586	0.349131466	619.2946535	422	1.150007296	0.077209315	278.7222843
5.41391602	0.311926136	199.7854632	164	2.195307954	0.063081383	142.0791031
3.525242916	0.398594781	59.95479373	90	2.696981497	0.105895489	77.28648448
6.044017102	0.291776772	148.0228434	165	1.977574405	0.063533264	133.2240191
4.779521121	0.300308059	139.9058941	262	0.527425249	0.042205646	146.5806775
4.74402775	0.336652975	101.6255754	150	1.944429531	0.067039282	110.8364752
4.952116432	0.362355583	172.1559501	141	2.198239012	0.079389497	131.9057386
6.347461454	0.27717184	159.3556779	208	1.362181136	0.04998506	148.8406576
5.24087329	0.35056846	153.7877425	215.25	1.300800421	0.073620747	152.9207697
5.023308974	0.334085539	131.8373157	169	1.621082804	0.075380638	144.9711117
5.22820795	0.292324723	95.81754446	191	1.266793657	0.052815193	118.5920249
5.405938787	0.317615007	116.5320164	148.75	1.374618496	0.062480123	122.8124792
4.660271027	0.370525675	120.0117017	121	2.312378803	0.092878652	117.4459133
5.229734723	0.320635121	108.4809172	145	1.954481523	0.077504009	112.2372834
5.866693357	0.315120294	212.8129217	198	1.711031994	0.066204257	165.6939766
6.71579894	0.276461633	440.6640157	416.5	0.872335443	0.035995097	248.8828434
3.767948143	0.387119867	76.75373149	134	2.050178466	0.0875832	99.35240756
4.764015909	0.342515137	182.4674211	166	1.977913405	0.068785465	137.9190995
5.241241151	0.327843473	276.7863991	261.5	1.394046879	0.052568741	188.5856105
5.827847554	0.287110789	196.9919745	200	1.782090104	0.052905854	149.0642709
5.707080047	0.314905653	173.0290989	170	1.857842341	0.063223547	143.1876948
4.731829696	0.345195452	157.3057699	155	1.910272104	0.077595936	132.4897399
5.488331731	0.288889776	93.08488714	173.5	1.332240549	0.065333392	116.27363
5.196190431	0.341744048	183.2341712	188	1.784456302	0.068743002	144.1221829
5.430904398	0.349032289	252.5538645	178	1.686637104	0.070371289	165.5207872
6.185640212	0.31263042	302.321162	155.75	2.393211126	0.069216687	166.9328749
7.346159608	0.257063862	237.1825301	247	1.487118152	0.046395933	173.2100162
6.296911241	0.286910241	174.0690138	224	1.804706388	0.055655944	151.7457768
2.344827047	0.455308857	30.63145132	98.5	1.464120577	0.097521652	66.98967203
6.012778275	0.282875594	182.1941284	216	1.3497968	0.048257719	150.4053336
4.796017676	0.319774614	118.2924615	200.75	1.267765973	0.053664876	133.1043074
5.942089198	0.289732296	189.4750372	199.25	1.652153254	0.053045728	149.0360762
4.130710506	0.367603821	75.10133102	112.5	2.092635977	0.084337984	90.72014241
3.11077046	0.419139256	43.19174142	77.5	2.596725206	0.115373459	78.59465667
3.950126932	0.376415445	72.59227113	99	2.75471241	0.095490954	81.26554998
4.726488076	0.350099747	90.94811767	141	1.912410077	0.083088818	114.0853497
7.476512221	0.26823536	177.8027877	192	1.992389662	0.061429537	154.0461989
6.611302508	0.274493137	156.36788	210	1.471596392	0.058103715	144.708019
5.749859821	0.317325094	155.7023438	181	1.670024777	0.060653265	137.1006659
4.4912696	0.328403985	103.6985207	168	1.466825469	0.06152105	116.437057
10.24961513	0.233889623	577.9387087	512.25	0.908126412	0.038351487	294.1914682
3.371351892	0.39034234	52.48598077	110	1.924884867	0.086126913	79.0954829
3.047272762	0.412026594	38.32815378	89	2.506288494	0.10696349	73.50545899
3.61610554	0.410983631	123.8728254	128.25	2.087998904	0.096032688	119.8764
9.020401274	0.222870858	422.1203028	400.5	0.909922442	0.03296799	247.3232581

original_firstorder_Energy	original_firstorder_RobustMeanAbsoluteDeviation	original_firstorder_Median	original_firstorder_TotalEnergy	original_firstorder_Maximum	original_firstorder_RootMeanSquared
658321686	69.52460347	-771	17774685522	64	739.7522029
1059105846	74.24039941	-767	28595857842	124	727.7038704
397484608	160.9440337	-644.5	10732084416	94	596.2649157
1508737438	68.21275271	-811	40735910826	61	784.2563783
478336480	57.00851454	-716	12915084960	-92	709.9596153
781674890	72.71952719	-726	21105222030	18	690.8060492
605360819	30.96208801	-772	16344742113	-91	756.7800253
1035449741	59.35163558	-800	27957143007	642	757.4010603
1007164639	87.03153552	-761	27193445253	237	704.893264
1360256846	100.8237245	-694	36726934842	85	673.0274754
879025312	58.16430422	-720	23733683424	27	697.4637078
785317455	119.9184989	-688	21203571285	80	682.6886344
1213458550	84.30624702	-667	32763380850	22	643.6544663
669878090	95.68937284	-620	18086708430	59	600.4474874
678427908	109.9440263	-692	18317553516	62	662.0127812
1294197488	283.4762156	-567	34943332176	190	561.2187933
760235503	87.91030026	-724	20526358581	109	688.2346839
1397677656	48.13197513	-767	37737296712	-9	739.185163
551475345	60.89683964	-716.5	14889834315	-32	696.7450386
1285366001	97.22162942	-698	34704882027	83	665.6401959
2218697657	147.080895	-566	59904836739	103	565.0106839
846287686	97.31615502	-653	22849767522	74	631.6677095
1591773125	122.2412463	-652	42977874375	231	621.4973436
719828930	53.06227952	-865	19435381110	170	835.9802862
681956123	167.8353246	-519	18412815321	159	511.749853
634617368	88.50234933	-720.5	17134668936	120	685.1215545
656074015	162.8344141	-565	17713998405	573	544.7309449
1293877200	144.7349746	-656	34934684400	99	621.012394
2333068683	62.10049397	-874	62992854441	105	828.4917341
1723920911	137.6409617	-814	46545864597	87	738.7268016
1529598090	117.4884216	-623	41299148430	90	607.6921399
2004484850	111.3236025	-660	54121090950	401	633.164252
1461999106	66.88638488	-802	39473975862	95	760.0243051
276558112	40.26749874	-791	7467069024	76	758.2643931
464955873	111.8625471	-762.5	12553808571	158	723.6013793
1817078950	60.73367466	-790	49061131650	56	751.5560379
2183638579	110.6183079	-826	58958241633	293	782.1987278
1571782766	85.80444372	-713	42438134682	65	680.8200959
665020208	84.11467401	-689	17955545616	-160	669.4227549
975426886	52.2148393	-808	26336525922	34	772.157175
642358855	88.28157484	-733	17343689085	27	698.6520103
1017365683	52.12113978	-756	27468873441	177	725.663002
1662862628	48.10558605	-771	44897290956	104	741.1770717
1061723597	44.40063252	-785	28666537119	96	750.6980464
1510143523	151.6382287	-708	40773875121	1025	657.4271901
987487025	106.2983051	-538	26662149675	197	525.5665009
1384550181	101.3025048	-630	37382854887	174	612.8817437
706136967	97.51187929	-727	19065698109	102	682.4877699
361630176	35.05131569	-818	9764014752	-100	800.7423152
604063586	48.85936985	-777.5	16309716822	29	744.4372211
1837552378	70.61673541	-720	49613914206	92	679.1413893
1197562098	44.03972059	-810.5	32334176646	22	782.4644154
1966432693	48.41676367	-744	53093682711	-14	719.6465445
360861829	135.605885	-549	9743269383	591	555.1265841
1918140000	81.84510056	-742	51789780000	260	698.0032481
1181174457	200.1793629	-871.5	31891710339	97	790.9627451
1692256630	76.06363602	-753	45690929010	256	718.3935159
1875767272	40.45360907	-901	50645716344	-37	878.228834
699351399	75.33313758	-747	18882487773	256	713.9547187
485648968	108.6318037	-590	13112522136	-34	587.9265502
1355119657	65.38350315	-700	36588230739	121	678.799182
3157347502	67.72191411	-800	85248382554	73	758.5665914
718595457	93.85669297	-583	19402077339	170	562.5144284
725701451	102.4226132	-677	19593939177	21	623.2905749
271856177	86.73192218	-729	7340116779	63	678.8027559
919376940	78.25322054	-783	24823177380	29	771.9049754
569733148	70.08258054	-751	15382794996	35	733.8256877
2040205976	59.21315731	-797	55085561352	203	756.3909748
601456631	64.63998144	-715	16239329037	152	682.0290862
1235384695	94.56803958	-752	33355386765	241	701.9787109
1289962371	186.7872554	-617	34828984017	308	582.0228712
1030728509	59.5561034	-739	27829669743	282	707.1851758
2318438485	77.2372538	-743	62597839095	173	703.9166957
2986089834	119.3384943	-727	80624425518	133	677.2165872
2026445419	88.3586946	-719	54714026313	250	689.5420135
1194910210	81.15427438	-755	32262575670	287	715.9727598
1137751325	73.44526077	-737	30719285775	186	695.0694798
585635819	75.83790253	-682	15812167113	12	656.4536801
1841661519	85.43325743	-795	49724861013	191	749.7784612
1937068677	92.17882596	-709	52300854279	58	659.5476263
762257285	80.47656478	-793	20580946695	528	748.1044499
1394383184	109.4638604	-756	37648345968	261	724.4281053
1618597189	95.88043156	-843	43702124103	307	808.8528932
255148831	43.64874887	-711	6889018437	-182	698.4670883
1129867834	94.21285798	-553	30506431518	166	540.8884251
1300787820	87.32400674	-633	35121271140	81	611.9110894
624495403	89.64562384	-692.5	16861375881	167	661.3030401
1772239814	50.77659815	-785	47850474978	17	763.1506321
227106326	36.39818914	-765	6131870802	-116	743.3506146
267185532	43.15170742	-698	7214009364	121	679.8960993
1728279161	64.78090708	-771	46663537347	149	733.6464053
635207051	88.20197938	-800	17150590377	326	758.1873185
742340692	92.35583689	-726.5	20043198684	85	689.383872
1983938987	82.28982356	-708	53566352649	86	675.102665
1917677843	73.14677016	-660	51777301761	114	638.3544111
342793312	220.7710438	-674.5	9255419424	338	622.7160186
1039755099	48.13786027	-784	28073387673	11	767.9613675
205856666	39.70719196	-712	5558129982	-52	693.5225438
450193586	63.62595869	-755.5	12155226822	-31	712.0216001
380915700	179.5031231	-592.5	10284723900	297	574.5284405

original_firstorder_90Percentile	original_firstorder_Minimum	original_firstorder_Entropy	original_firstorder_StandardDeviation	original_firstorder_Range	original_firstorder_Variance	original_firstorder_10Percentile
-496.6	-997	4.294241271	177.8229046	1061	31620.98541	-863
-462.5	-927	4.28537633	187.7715454	1051	35258.15328	-854
-114.7	-879	4.822596991	252.0666265	973	63537.58417	-780
-561	-1000	4.347174773	162.7405762	1061	26484.49515	-915
-543	-940	4.076319584	121.7668563	848	14827.1673	-829.2
-442.4	-905	4.260324576	160.0290756	923	25609.30504	-813
-664.6	-890	3.305631954	85.1494224	799	7250.424135	-824
-501.4	-920	4.003656913	183.4116507	1562	33639.83362	-865
-368	-897	4.174207865	194.5968379	1134	37867.92933	-827
-361	-970	4.751540979	195.3493157	1055	38161.35514	-839
-537	-864	4.010097743	126.5064182	891	16003.87384	-806
-283	-1019	5.044313512	228.2413242	1099	52094.10205	-904
-411.8	-931	4.377942495	150.4314541	953	22629.62238	-781
-321.1	-890	4.594749001	173.062297	949	29950.55863	-760
-235.7	-1010	4.841954922	224.5010372	1072	50400.71572	-839
50	-994	5.015808718	347.7662999	1184	120941.3994	-825
-375	-896	4.485642918	189.8871128	1005	36057.11561	-835
-565	-929	3.818386454	123.4772562	920	15246.63281	-831
-504.5	-913	4.183014707	136.2149704	881	18554.51817	-814.5
-322	-961	4.548393921	223.2908839	1044	49858.81881	-821
-72	-935	5.041235494	250.1487855	1038	62574.41487	-787
-328	-1024	4.731532831	191.7116229	1098	36753.34636	-793
-252	-903	4.815727508	222.932196	1134	49698.764	-798
-664.6	-1010	3.979580276	145.6516031	1180	21214.38948	-936
-7	-1013	5.072697408	256.1803355	1172	65628.3643	-722
-363	-1050	4.51398091	198.921367	1170	39569.71023	-819
-4	-850	5.032944813	269.0692387	1423	72398.25521	-744
-182.4	-975	4.937727178	239.3322213	1074	57279.91214	-816
-573.6	-1007	4.059583535	190.4610197	1112	36275.40003	-939.2
-175.4	-1021	4.553696266	277.2488295	1108	76866.91348	-900
-240.1	-966	4.901768442	215.8726647	1056	46601.00738	-792.9
-284	-975	4.795761045	213.2217529	1376	45463.51593	-797
-519	-952	4.1676322	188.1641595	1047	35405.75092	-875
-600	-878	3.503101785	137.8437011	954	19000.88592	-836
-344.1	-1005	4.790283506	226.023668	1163	51086.69849	-897.3
-523.2	-962	4.058240589	160.0637483	1018	25620.40353	-859
-362.8	-1025	4.768279128	250.0774544	1318	62538.73321	-966
-408	-954	4.447964357	173.1422008	1019	29978.2217	-817
-435.3	-1008	4.462630771	150.5698166	848	22671.26967	-805.7
-586	-912	3.766263866	141.7851191	946	20103.01999	-864
-412	-936	4.483318449	179.4301229	963	32195.169	-844.5
-545.1	-883	3.888912413	131.9721324	1060	17416.64372	-822
-560	-1006	3.862622973	140.8898947	1110	19849.96244	-833
-581.3	-900	3.688214789	138.1523901	996	19086.0829	-836
-31	-937	4.886214244	307.4455874	1962	94522.7892	-830
-155.4	-901	4.8214494	205.8012703	1098	42354.16284	-694
-309	-1035	4.741233869	202.2163856	1209	40891.4666	-777
-302.5	-908	4.550808033	223.3941508	1010	49904.9466	-830
-694.3	-961	3.433811419	101.4694297	861	10296.04516	-875
-515.6	-994	3.96540205	151.498346	1023	22951.74885	-840
-388.3	-1009	4.226011976	203.1737664	1101	41279.57937	-799.7
-630.5	-972	3.787518335	147.1195673	994	21644.16709	-873
-563	-996	3.860781375	123.2173412	982	15182.51318	-814
-158	-929	5.032517619	232.1281255	1520	53883.46666	-756
-368	-954	4.379056285	204.1914815	1214	41694.16113	-829
-53.4	-1050	4.642453734	339.6614188	1147	115369.8795	-998
-443.8	-1006	4.503110778	208.1345144	1262	43319.97609	-852.2
-748	-1057	3.719578181	119.7262329	1020	14334.37083	-965
-451	-1031	4.458290311	188.183444	1287	35413.0086	-849
-315.6	-906	4.740564336	175.699582	872	30870.34313	-770.6
-473	-943	4.289992933	157.2368546	1064	24723.42845	-805
-487.6	-1000	4.228074597	192.1034988	1073	36903.75425	-877
-246	-911	4.693152784	196.6003826	1081	38651.71045	-716
-265.7	-812	4.301868888	190.9894669	833	36476.97645	-756
-314	-913	4.294927695	193.5907623	976	37477.38325	-807
-571	-1041	4.503238041	157.3088784	1070	24746.08323	-929
-489	-1030	4.489974399	172.3204876	1065	29694.35045	-907.6
-510	-966	4.024482871	172.1510571	1169	29635.98647	-862
-458.2	-876	4.161796599	156.638752	1028	24535.69862	-794
-320	-1016	4.476146836	223.2298456	1257	49831.56397	-842
11.6	-950	5.118646716	296.8682428	1258	88130.75358	-790
-512	-883	3.982381148	138.0479597	1165	19057.23918	-809
-430.8	-964	4.352106936	194.4126875	1137	37796.29308	-833
-227	-984	4.712007873	242.504416	1117	58808.39176	-843
-393.1	-1006	4.665983679	206.2051244	1256	42520.55334	-841
-409	-990	4.501288169	198.715473	1277	39487.83919	-853
-401	-983	4.242352335	183.9464179	1169	33836.28466	-815
-409.6	-883	4.312273282	150.892033	895	22768.40562	-783
-442.5	-965	4.387876336	195.2782989	1156	38133.61403	-884
-227	-950	4.364347175	223.4546018	1008	49931.95908	-794
-392.1	-995	4.464404104	248.2905405	1523	61648.1925	-884
-351.6	-1026	4.831511251	229.5566122	1287	52696.2382	-897
-509.6	-1023	4.574011869	205.3645037	1330	42174.57937	-967
-569	-840	3.653115838	89.82360141	658	8068.279371	-785
-217.1	-874	4.703319915	199.2649361	1040	39706.51477	-695
-336	-880	4.538274675	172.7498921	961	29842.52521	-759.7
-358	-905	4.635046985	202.9050885	1072	41170.47496	-818
-586	-974	4.017432966	131.5373529	991	17302.07521	-868
-630	-882	3.584843185	125.9459618	766	15862.38529	-827
-553.7	-838	3.803294089	127.2109446	959	16182.62442	-770
-500	-934	4.103035168	158.4627554	1083	25110.44484	-845
-418.4	-1000	4.528827646	215.3102049	1326	46358.48433	-901
-386.1	-935	4.522656318	188.9708587	1020	35709.98544	-836
-390.2	-982	4.452393425	186.7103123	1068	34860.7407	-816
-400	-903	4.380170665	155.5985629	1017	24210.91276	-770
100.4	-1057	5.15700575	347.0371324	1395	120434.7713	-833.7
-629	-941	3.873721652	111.2469033	952	12375.87348	-868.8
-567.3	-834	3.652214287	111.4758784	782	12426.87147	-781.3
-467.7	-866	3.919299839	170.9198139	835	29213.58279	-817
11	-980	5.213810042	299.8356165	1277	89901.39689	-795

original_firstorder_Kurtosis	original_firstorder_Mean	original_glrlm_ShortRunLowGrayLevelEmphasis	original_glrlm_GrayLevelVariance	original_glrlm_LowGrayLevelRunEmphasis	original_glrlm_GrayLevelNonUniformityNormalized
7.853888583	-718.0615129	0.0148317	52.07493048	0.015604462	0.067338959
7.378565434	-703.061	0.021524055	58.24581471	0.02353217	0.068695692
2.492331139	-540.3649374	0.014489245	102.7325361	0.015487649	0.044298094
7.264145411	-767.1854872	0.036815333	43.92952905	0.039188811	0.063175878
6.207833573	-699.4394099	0.01598349	24.72308525	0.017249602	0.071288469
5.636787138	-672.014652	0.018070524	42.59893386	0.019445608	0.067612424
15.63461089	-751.974456	0.038591461	12.89516402	0.042631273	0.12741365
9.36300464	-734.8581717	0.04988117	57.05472392	0.054725811	0.087202073
5.070012385	-677.5002467	0.035006997	63.78635219	0.038525123	0.078663026
4.171166229	-644.0532801	0.014457208	61.70835767	0.015265259	0.045041149
7.972584892	-685.8948533	0.037467739	26.81044842	0.041083056	0.076779346
3.279079768	-643.4047478	0.018713906	84.91638686	0.019823888	0.035055101
4.038680954	-625.8286104	0.008726777	37.19579432	0.009370486	0.057313893
3.501601313	-574.9666308	0.011273902	48.39624522	0.011932447	0.049269615
3.717885783	-622.7842377	0.009700405	82.35840541	0.010120108	0.043113692
1.448269928	-440.4828425	0.007008059	183.739744	0.007490651	0.035792176
5.487173545	-661.5208723	0.040005611	58.85886218	0.04330332	0.056918421
7.921967962	-728.7990618	0.017605729	26.08751126	0.019284525	0.094462662
6.054114761	-683.3001761	0.021081455	31.13522821	0.022575967	0.068915358
5.160563285	-627.07101	0.009511243	72.91839572	0.01038007	0.053524365
2.591211106	-506.6188489	0.007638082	96.04940049	0.008044531	0.033695957
4.133914486	-601.8727016	0.006871539	60.08627151	0.007181544	0.046384301
3.646580494	-580.1380733	0.012921074	77.53599102	0.013777852	0.043318631
12.13911649	-823.1941748	0.031205061	36.16194956	0.033996596	0.085340445
2.187706371	-443.0119048	0.003240168	102.8869503	0.003381264	0.033575117
5.338622588	-655.6079882	0.008240422	65.2796116	0.008684582	0.059095609
2.995257742	-473.6386251	0.018599293	116.0470715	0.019545431	0.036787905
2.755170106	-573.0414307	0.009263076	92.2256997	0.00984353	0.038861193
9.711069853	-806.3021477	0.026781706	61.74743061	0.030052541	0.085136378
3.929006936	-684.7264957	0.014293017	124.7655453	0.015680771	0.060659838
3.38963149	-568.0569773	0.007620868	74.65871642	0.008090026	0.039360842
4.108138209	-596.1824	0.009031951	72.29081896	0.009674952	0.045187751
8.491680139	-736.3634927	0.022609568	58.45639369	0.024432785	0.077367076
13.52572369	-745.6299376	0.049053625	33.21678314	0.054617776	0.121328497
4.365943847	-687.3952703	0.014002778	83.3307416	0.014776478	0.045917269
7.955659908	-734.3133354	0.017889837	43.68750053	0.019787211	0.082831327
5.013004912	-741.1451387	0.041151327	101.5134554	0.045751941	0.046949607
5.295888263	-658.4358596	0.010378905	49.28391131	0.011052813	0.058564418
3.205064983	-652.2695418	0.006819341	37.16864204	0.007272386	0.054544172
9.419602543	-759.0281174	0.046335899	34.68196067	0.052271158	0.099392262
4.677188875	-675.2180851	0.019466872	53.05709856	0.020503907	0.055465247
9.330592216	-713.5615942	0.033540854	29.68596742	0.036910285	0.087979614
10.08794596	-727.6630327	0.008532295	34.07575694	0.009350534	0.093564357
10.87267949	-737.876327	0.050261731	33.28076217	0.055396377	0.106808349
5.215091961	-581.1090441	0.014496257	154.6297916	0.015629836	0.047015667
3.48936724	-483.5969231	0.005442621	68.28769845	0.005672922	0.042970191
4.664664095	-578.5607705	0.004239853	66.01305824	0.004473579	0.045729523
5.000461051	-644.8911609	0.023733338	81.21027464	0.025242509	0.056923929
12.0553481	-794.287234	0.025619542	17.88841752	0.028241235	0.118813924
8.025499728	-728.8587156	0.014708096	39.78527976	0.015691584	0.091537072
6.786913412	-648.0381526	0.006025665	67.13619672	0.006549263	0.07332113
12.1245211	-768.5092025	0.02389188	37.76259886	0.026579886	0.098540712
8.664633947	-709.0194891	0.008597174	25.87756328	0.009354896	0.088825234
3.700942793	-504.263877	0.007222226	86.94552959	0.007545463	0.035181249
5.987432824	-667.4686309	0.011356673	68.92949709	0.012331838	0.065431649
2.970599202	-714.3193856	0.040436907	175.7873387	0.047420707	0.054832622
8.406370224	-687.5821897	0.009930407	71.27693629	0.010616639	0.060453278
12.65812599	-870.0296053	0.021023846	24.73371387	0.022991826	0.099973695
7.56955678	-688.7077259	0.007704093	58.66783373	0.008092782	0.061078554
2.61942397	-561.0590747	0.010104159	49.88690686	0.010664415	0.041745214
7.794753825	-660.3369602	0.011922228	41.29697849	0.012741039	0.064794571
7.817785096	-733.8388919	0.016998331	62.01053156	0.018466805	0.073759459
4.625494164	-527.0396301	0.006788616	63.3329506	0.007096697	0.048582841
3.606716066	-593.3078158	0.034257191	60.59078494	0.038573653	0.067344366
4.879843036	-650.6118644	0.020312664	62.30344317	0.02174601	0.0707184
5.587333018	-755.705768	0.015930799	40.5838579	0.016717808	0.052114818
5.933641056	-713.3062382	0.012785045	49.32143686	0.013709009	0.058863288
8.677578679	-736.540101	0.018219593	50.60562066	0.020083422	0.086125861
7.252893099	-663.7981439	0.021068138	41.04702709	0.022573426	0.073806583
5.217118055	-665.53929	0.008819907	81.21662959	0.009454584	0.062675075
2.453553624	-500.6194853	0.009711412	138.7363143	0.010196723	0.035346952
8.347194102	-693.5803008	0.025427437	32.6042412	0.02815289	0.081720514
6.805850123	-676.5370806	0.011936253	60.12486519	0.012925635	0.065831687
4.020426461	-632.3084012	0.009232291	93.2974848	0.009985812	0.050267228
6.515039877	-657.9875645	0.008183802	69.53224279	0.008617074	0.051361299
6.57769679	-687.8438438	0.012089119	65.81052405	0.012986612	0.060505888
6.407475988	-670.2874735	0.008276392	56.74906083	0.008984663	0.072430527
4.628808017	-638.8763797	0.018064667	37.52587113	0.018934522	0.063021673
5.963609421	-723.9020147	0.023989879	63.9449275	0.026195331	0.064465606
4.840503132	-620.5409836	0.010076504	79.47956543	0.011040256	0.06632076
8.924508979	-705.6997063	0.016888506	103.604777	0.018071376	0.06567068
4.949625843	-687.0952202	0.010519076	86.19060346	0.011028223	0.044956305
6.92969549	-782.3480194	0.044730652	69.39311109	0.048295055	0.053386211
6.567033266	-692.667304	0.050914533	13.72482346	0.055750639	0.093708485
4.519289715	-502.8456758	0.007808735	63.75427715	0.00829661	0.047424568
4.323891562	-587.0201497	0.011287466	49.13795233	0.012125654	0.052092704
5.771440512	-629.4054622	0.019847306	67.19632054	0.021169664	0.051554366
8.910611556	-751.7292146	0.022371775	29.33065142	0.024339262	0.080586542
10.15974472	-732.6034063	0.042030516	27.61911771	0.045166789	0.109825695
13.10021574	-667.8892734	0.038689264	27.52606742	0.041506512	0.09239952
6.668761042	-716.3285581	0.020149325	42.31999463	0.021962458	0.078092955
7.369037532	-726.9728507	0.024603973	76.69672349	0.025648828	0.058968836
4.855189274	-662.978233	0.017172175	58.64915321	0.018084	0.055884249
5.594192862	-648.7702734	0.00747909	57.50936469	0.00810539	0.058249203
5.365184131	-619.10051	0.01027342	39.9553442	0.010972683	0.059698504
2.448085612	-517.0497738	0.006362537	190.724263	0.006576266	0.036747968
9.068860797	-759.8610323	0.030896755	20.96090297	0.033655421	0.08319447
11.43988292	-684.5046729	0.041171295	21.39737039	0.044810841	0.102034838
6.980304373	-691.2027027	0.049441352	47.78645762	0.055619409	0.089054379
2.722649415	-490.0831889	0.006240936	143.3845228	0.00646467	0.032387745

original_glrlm_RunVariance	original_glrlm_GrayLevelNonUniformity	original_glrlm_LongRunEmphasis	original_glrlm_ShortRunHighGrayLevelEmphasis	original_glrlm_RunLengthNonUniformity	original_glrlm_ShortRunEmphasis
0.076427007	75.80044996	1.2194754	190.0123356	991.8511168	0.95172626
0.111365456	125.2967202	1.315786632	166.5666162	1534.010989	0.933679091
0.075744672	46.5096995	1.212196377	327.4694132	932.9303205	0.954699749
0.074145701	145.0752597	1.21597193	140.8027373	2021.972681	0.95159244
0.112472375	61.65470343	1.319660409	133.2122193	727.537833	0.933181275
0.090091148	102.4547957	1.259772432	156.3139087	1306.24883	0.943268868
0.169176557	118.2718827	1.469277819	53.69059895	731.7719032	0.908339897
0.126584038	142.1537491	1.354364595	125.2352509	1349.881773	0.927597509
0.125603342	144.4565226	1.346393666	156.6917021	1530.992308	0.930143342
0.069518971	127.4821797	1.196292473	248.6527015	2530.742525	0.957197074
0.116617291	126.1091161	1.328853759	92.00371239	1373.461073	0.931411401
0.058263312	56.14409493	1.166287652	332.8353572	1454.285268	0.963073864
0.09468797	154.8606457	1.271135425	214.7439239	2317.754988	0.941328207
0.072613705	86.12983233	1.203426783	229.6950252	1560.14702	0.956385068
0.06202727	63.21707123	1.177912412	355.5951928	1322.67415	0.960506829
0.162664314	132.4890363	1.398618153	621.7976908	3092.715022	0.930433743
0.089142725	84.60641973	1.256785984	159.8821276	1284.522881	0.944077013
0.133734128	217.8481121	1.367349012	113.6299767	1905.342718	0.926433289
0.099487268	71.92142687	1.286563315	133.9183646	888.8166559	0.938306766
0.136497711	140.4983164	1.36057999	256.9182152	2192.512377	0.930522602
0.079544516	220.150809	1.211719794	404.4482662	5827.396613	0.956303581
0.06940812	92.56838131	1.200254885	359.4142058	1775.885238	0.955416362
0.078720914	167.5477063	1.215519341	275.2414679	3438.5413	0.954859007
0.109808682	80.15531584	1.314899237	110.6623244	788.7375897	0.932993166
0.066328887	82.66057407	1.186131084	642.5103989	2215.861262	0.959643686
0.082104436	74.37758184	1.236556369	329.8086538	1097.780745	0.947944461
0.059792408	77.15887926	1.171749205	354.4652882	1897.060099	0.961657741
0.062732678	123.324316	1.181183951	367.4022979	2853.193084	0.959409819
0.164620042	257.0737014	1.439599864	150.2965438	2435.840161	0.916823942
0.123956597	174.181272	1.337403632	323.2813563	2412.855634	0.932864829
0.071767524	152.8681212	1.210128052	349.7133571	3431.162791	0.952658168
0.100158824	208.6412946	1.274898191	309.9732904	3987.613865	0.943546386
0.109482958	178.9079071	1.308880249	158.3887899	1953.134045	0.935259065
0.138932785	52.10342314	1.399604401	79.71826692	346.3622987	0.91688632
0.060989415	38.65909607	1.1742119	281.9548249	760.7059847	0.961319522
0.138611861	238.8477253	1.385612858	148.386676	2355.165233	0.92221048
0.09611755	155.37552	1.260186819	245.0977622	2882.221225	0.947021063
0.081354063	185.2703138	1.231274589	222.8091803	2773.129484	0.949757491
0.093043376	74.63116872	1.271065434	268.2627151	1171.251431	0.940472323
0.159715471	144.2166486	1.433872449	88.76415271	1167.252073	0.916292909
0.063393892	69.00782539	1.183272098	187.0416502	1116.944919	0.958899183
0.12571123	153.215878	1.358257028	94.11332159	1432.997036	0.925234486
0.136499523	254.0149464	1.38183052	185.3536933	2217.318707	0.922359439
0.153680187	178.5826707	1.425491773	84.12516365	1342.063057	0.915591891
0.093775803	152.1413003	1.26126852	388.468937	2803.818339	0.945038317
0.05796303	145.7861783	1.168758663	390.5379523	3066.882203	0.961620313
0.067748717	158.7441195	1.195794229	434.174387	3094.59703	0.956226655
0.075952738	80.7862138	1.218060551	218.182169	1251.431709	0.95197987
0.146713117	59.7059145	1.410548248	76.86819228	405.2681684	0.917091665
0.124317388	90.45810168	1.343236562	168.7710113	824.4201206	0.930447444
0.116790689	265.185857	1.331603219	301.5529995	3015.200862	0.930373864
0.164132234	169.8413068	1.454925378	114.7393628	1367.19177	0.910691499
0.120855889	305.5464066	1.341330913	170.8766507	2859.026779	0.929092361
0.057254569	39.06954397	1.170280863	418.6696254	1001.45139	0.960525812
0.10670687	236.0000245	1.299273326	232.9631219	3062.676656	0.937414072
0.266887179	90.11196522	1.595511657	357.7910984	1315.091684	0.91399384
0.0851975	184.0642014	1.246200029	264.8068827	2642.881398	0.945952776
0.141544977	217.6703662	1.392305381	99.58481426	1776.312434	0.921459694
0.070581978	78.66540912	1.206145986	281.0231643	1140.250549	0.953638967
0.066901328	55.18958366	1.197385755	273.2837573	1175.143707	0.955009748
0.095618174	176.0436666	1.26849723	186.9785465	2342.489297	0.943260436
0.128372967	365.8005279	1.354273153	186.8326973	4116.222342	0.928512895
0.067082735	104.0583977	1.191997884	329.4045687	1916.39855	0.957562824
0.123953357	114.0875651	1.34208294	161.8352032	1413.992279	0.930719607
0.093804987	38.55745426	1.26672726	197.2879392	469.6283143	0.942879977
0.066387334	75.75244482	1.194034865	188.4748843	1295.627539	0.956138898
0.090876307	57.59532007	1.26139088	242.6581824	844.1006322	0.943448003
0.130160389	276.8936893	1.361977059	154.0519083	2653.126666	0.926382745
0.093180608	88.16154254	1.265730797	140.9962385	1027.951509	0.942692736
0.091948233	145.410467	1.26053295	300.2432413	2005.300507	0.94429691
0.071583442	126.6159004	1.202359976	459.1473981	3191.85868	0.955954426
0.134401335	151.2525832	1.377075639	110.9758768	1514.354612	0.923004209
0.104305847	282.3727929	1.295769846	208.835346	3641.329288	0.937331491
0.10115562	301.1559576	1.283629584	315.4273931	5129.189422	0.940494486
0.069760081	205.9824238	1.199735164	298.1464492	3572.331541	0.955904231
0.091807093	130.5584674	1.260055205	235.9024815	1866.611324	0.944543618
0.113784047	155.701112	1.316733281	244.9571055	1814.131967	0.934816614
0.068678863	80.63298803	1.198162623	157.5957583	1140.829292	0.956103491
0.11136274	192.6661328	1.314748873	178.036691	2514.746702	0.933912269
0.123967416	267.5593999	1.34408463	258.7591168	3361.219255	0.92995919
0.087681147	82.96229596	1.251428073	257.9462488	1095.774844	0.945539754
0.059288377	113.3207127	1.170913699	313.4800385	2279.183651	0.961660792
0.078388061	123.741957	1.218598751	177.348808	2050.367693	0.953198825
0.158779867	43.24912644	1.448508597	58.73553344	366.1281263	0.910039159
0.068831904	172.3551143	1.198769498	296.8847153	3236.302839	0.955753725
0.081893329	168.4292902	1.237184079	219.0374086	2817.711423	0.94753212
0.068043219	69.22041246	1.200072253	219.7001037	1192.286522	0.954669726
0.116013998	222.5230036	1.332304765	117.9689292	2299.714519	0.92984417
0.128494683	40.77909699	1.359126648	80.06448653	308.0901482	0.927396003
0.097532056	48.91498637	1.291453839	88.21622428	446.3641896	0.93475946
0.108032962	229.5323288	1.303299724	141.7896529	2491.614695	0.936712765
0.060892836	61.70010605	1.177386261	209.7003168	942.1454948	0.959938582
0.067656182	82.31693111	1.192793622	203.5262964	1317.691912	0.957561829
0.101940405	233.3625808	1.284216743	269.0546502	3431.740723	0.940658646
0.085250294	260.7846319	1.247168418	200.2926178	3788.576671	0.945523158
0.057170294	30.85942934	1.166188853	696.2801286	760.8484819	0.962370826
0.119949244	132.5198888	1.346922703	86.11633618	1314.27121	0.926222613
0.126316029	39.41188614	1.359075925	72.62923381	318.6085299	0.925524613
0.148398773	70.4522713	1.412374074	108.6573989	638.7938393	0.917823009
0.043768446	35.88437161	1.129137648	575.1814616	1023.499768	0.969861667

original_glrlm_LongRunHighGrayLevelEmphasis	original_glrlm_RunPercentage	original_glrlm_LongRunLowGrayLevelEmphasis	original_glrlm_RunEntropy	original_glrlm_HighGrayLevelRunEmphasis
220.4304466	0.935609694	0.019124495	4.662999639	195.482019
198.501881	0.911730769	0.033292086	4.77268276	172.3976873
367.7084694	0.938695473	0.020313652	5.161761369	335.0937689
158.9692938	0.936059456	0.049372653	4.720010929	144.1788171
168.4225641	0.911242604	0.023436414	4.537292266	139.4934058
179.4044169	0.925002348	0.025755853	4.685540803	160.5547963
70.61332398	0.878029256	0.063446889	3.943812579	56.51811181
142.7564316	0.902961858	0.079400012	4.544089991	128.4291853
176.1667044	0.905658229	0.056094614	4.710196597	160.208647
286.4297288	0.942442173	0.019042404	5.078999466	255.7581508
110.7549293	0.908858712	0.059313611	4.493815393	95.38623553
371.3904493	0.950422278	0.024914809	5.323977026	339.9318023
263.7685392	0.922420359	0.012408921	4.807737318	223.6893532
265.1140713	0.940796555	0.015097704	4.924231367	236.1719271
403.0259657	0.947127808	0.011949884	5.143673206	364.136685
1118.7012	0.90048112	0.009861181	5.523559112	686.1125417
186.5573805	0.926000479	0.059375564	4.890511459	164.9732964
140.0393844	0.901305106	0.027698878	4.362028654	118.1593712
159.5886805	0.918540087	0.029534665	4.624057612	138.617522
431.2819956	0.904780845	0.014701729	5.01799722	279.3169774
552.2295046	0.940066408	0.009942708	5.36334537	426.3255853
419.6624667	0.940811664	0.008553798	5.07123942	370.5592399
345.2760035	0.938457805	0.017936898	5.15316447	286.2364183
128.2629232	0.911650485	0.047107804	4.459536441	113.8589039
805.1705874	0.94543897	0.004008462	5.368969441	669.8982554
382.4091508	0.930871643	0.010764281	4.906685803	339.4709361
410.1063695	0.948613575	0.023739607	5.326021041	364.0453153
413.5900127	0.94584432	0.012470511	5.251404834	376.1459436
178.9597989	0.887591373	0.047797322	4.675254442	155.2309011
402.8283476	0.908661456	0.022736386	5.068388721	335.9201102
418.808289	0.937636965	0.010204942	5.250971147	362.6754104
392.9658548	0.923353846	0.012854156	5.206363195	324.1069978
192.1988413	0.913503328	0.033244245	4.657737817	164.4608971
90.52374762	0.892211738	0.081210166	4.079513339	81.61996369
308.9946887	0.948024948	0.018333116	5.078258404	287.0474545
177.4077768	0.896152651	0.029384264	4.632661398	153.4336478
280.1194582	0.927193569	0.071510251	5.167491653	251.2083647
256.977565	0.932808566	0.014198076	4.838906633	229.0974718
334.6361835	0.921884719	0.009370694	4.881701503	280.5279748
101.644617	0.886590182	0.083879615	4.380127572	91.01224476
204.8480637	0.945288754	0.025163032	4.803698607	190.3957771
111.2018399	0.901258162	0.05343287	4.427229408	97.20464358
236.8375965	0.896648116	0.013452047	4.437341919	194.3079665
97.12423487	0.887269312	0.081505638	4.309610549	86.36911655
450.7865587	0.92600502	0.021223761	5.310003228	399.761801
455.3491334	0.948983324	0.006698558	5.121866185	402.6899042
511.2759036	0.941754664	0.005524053	5.076441512	448.3923062
248.9598966	0.936066572	0.032264073	4.91696162	224.0569821
98.33004031	0.890616476	0.041304573	3.999898684	80.5933337
203.9605452	0.906351447	0.020507945	4.492751478	174.791165
393.3961121	0.907707754	0.009078993	4.739667463	317.7203526
143.792209	0.880997326	0.040246688	4.426883176	119.7379378
216.2862287	0.905735297	0.013019043	4.3877903	178.7816884
479.9076956	0.948301912	0.008942937	5.31398195	430.1129652
277.2598398	0.916023524	0.017098246	4.856430835	240.9447248
565.5707619	0.868684811	0.099667897	5.327160491	387.164619
313.7020676	0.928449105	0.01373718	4.915888453	273.920587
126.0913082	0.895116397	0.032813588	4.290253088	104.1379254
321.3641603	0.938663377	0.009839924	4.816769523	288.5928461
320.5996406	0.940925267	0.0132056	5.060600321	282.2123912
220.7846479	0.923730808	0.016639643	4.72299814	193.0304713
225.377669	0.903674419	0.025750784	4.773973281	193.6196727
381.5526198	0.943095214	0.008494216	5.024441596	338.9860462
180.8367679	0.906605172	0.060430537	4.811841926	165.3428408
221.9748276	0.923859192	0.028387147	4.709641293	201.8676669
220.280657	0.942021038	0.020191608	4.832414778	194.3838764
288.9923651	0.924676458	0.017909261	4.904630632	251.0155022
179.1174193	0.901397817	0.029463166	4.583471258	158.5098993
161.7934845	0.923731334	0.029679387	4.59520553	144.8475898
359.7698881	0.925316805	0.012418872	4.903125352	310.8779151
588.9715303	0.940671461	0.012366349	5.448331338	481.516356
132.3849385	0.897771806	0.041742394	4.537195596	114.7677194
273.0991947	0.916583096	0.017644769	4.812045134	219.5970197
408.8889664	0.920040641	0.013587231	5.15519102	330.994407
342.4511832	0.940926975	0.010590911	5.017113153	306.406544
269.969381	0.925584926	0.017258359	4.924092074	242.1065692
292.8205753	0.912493876	0.01250094	4.733636767	253.484325
178.8033083	0.941359597	0.022945278	4.652426726	161.5624894
203.7676572	0.912087912	0.036980091	4.885973323	182.7986644
348.2583708	0.905837033	0.015769912	4.878197973	273.3408899
284.1392486	0.927425731	0.023659413	4.886347369	262.7895647
344.6809215	0.948640746	0.013335761	5.140303329	319.349013
191.2937179	0.936788757	0.066320108	4.939871038	180.0017747
79.62105541	0.882335638	0.079846112	4.221713671	62.23425773
348.862919	0.941022985	0.010556546	5.037867105	306.3616141
252.6869235	0.930649661	0.015992638	4.930534274	225.2994391
248.14312	0.940206852	0.026964249	4.971751865	225.1099537
143.3629748	0.907303016	0.033674627	4.528140055	122.4812
94.98236286	0.903050721	0.060938389	4.094122634	82.65262856
103.320501	0.915757253	0.054086207	4.257560234	91.05141712
161.1757668	0.91514745	0.030748304	4.588065401	145.2418752
226.0877756	0.946745562	0.030273381	4.848513853	212.8061724
226.2124671	0.942923274	0.022342165	4.859243158	207.8227753
319.9287169	0.920196505	0.011208246	4.898565917	278.2368621
238.839401	0.928176796	0.014151626	4.791181255	207.3119798
818.5486447	0.949878176	0.007526594	5.431986374	719.2338414
106.8131275	0.903442559	0.046813447	4.389936132	89.82906893
86.67447744	0.902228613	0.0628749	4.15937159	75.10621486
141.1767074	0.890419265	0.087589823	4.489331516	114.2225185
643.7978653	0.96007199	0.00742467	5.436237443	587.9415211

original_glrlm_RunLengthNonUniformityNormalized	original_glszm_GrayLevelVariance	original_glszm_SmallAreaHighGrayLevelEmphasis	original_glszm_GrayLevelNonUniformityNormalized
0.880924792	69.62605793	232.8957171	0.0437577
0.840721636	72.34264359	227.1322651	0.041038149
0.888303245	101.622212	332.9502657	0.032184037
0.880355435	63.19902595	191.0375822	0.044448532
0.840203773	38.20319432	156.0569089	0.053836658
0.861707418	57.77720345	212.1671698	0.042521202
0.787627073	27.96299996	99.60270487	0.073329259
0.827737079	82.5930353	211.3021726	0.04342561
0.833390578	81.45972683	246.6071405	0.0383554
0.893788158	67.97037984	258.3066434	0.037599716
0.835731104	41.04295779	127.4983149	0.054476813
0.90764198	97.83014582	365.9571931	0.030121773
0.857383473	46.39396561	226.5247477	0.044819062
0.892102976	53.90216543	230.5220577	0.04059015
0.901617622	100.4655702	373.9999658	0.033041503
0.835000717	135.594359	474.8871705	0.026796862
0.863684385	65.91931151	191.255767	0.040879408
0.825496071	40.20463173	168.101931	0.052229482
0.8510283	45.90454804	177.2939138	0.047872671
0.834498023	75.33497481	296.1380834	0.038767028
0.891682413	84.33439715	350.9517478	0.031551384
0.889497925	74.62325903	348.6048591	0.035909013
0.88858114	72.46707339	276.7365609	0.035656039
0.839265828	62.40771946	169.8092611	0.051098771
0.899598561	90.72897765	527.6246214	0.030777025
0.872085516	81.345741	404.362326	0.037385301
0.904167401	114.6363774	360.2345965	0.029603727
0.898802801	91.92474655	372.7249967	0.031345401
0.805825638	87.16952074	241.7787484	0.042693051
0.839702514	122.3265525	375.7922533	0.031086476
0.883038821	74.64849544	337.4296836	0.033856058
0.862999059	73.22993734	324.3813047	0.035916425
0.844287542	75.18691775	227.9310686	0.042553899
0.805561419	64.12530612	176.6755493	0.055816327
0.903344999	92.71973369	330.3445187	0.034265827
0.816289762	64.70247256	235.9906841	0.044166893
0.870656819	114.5992975	297.7936849	0.033275007
0.876411207	59.33849793	259.6398182	0.040448736
0.855540042	43.88775788	249.0027003	0.043768443
0.804136505	54.79950593	170.945647	0.050649936
0.897534255	67.75749792	245.4274454	0.039782802
0.822433931	46.20703563	151.352946	0.051749519
0.816417401	59.19417372	260.5483904	0.046912654
0.802341481	60.42368027	174.1632492	0.050777124
0.866061481	183.1883031	467.0275466	0.029542496
0.90379968	74.48486945	365.3316878	0.035983665
0.891229961	72.07585399	432.0292593	0.037119644
0.881504082	93.65326938	255.3802288	0.037866352
0.80576281	34.02643807	124.3779384	0.065614485
0.833939849	69.68770083	270.3708688	0.041454294
0.833362389	78.61288799	352.9703902	0.040962929
0.792573907	71.24016239	209.2005312	0.047191047
0.830782085	42.83555654	230.5375189	0.052297278
0.901341341	94.10168269	391.2060749	0.031394201
0.84890098	85.80707348	298.4017038	0.037851883
0.801356764	131.6358251	336.4193826	0.031235601
0.867723463	91.45575962	313.7462271	0.038555602
0.81500923	45.96574973	152.0575901	0.058653736
0.885140375	82.85796024	328.375126	0.040179777
0.888439215	54.94507284	260.8088515	0.037684029
0.861888217	61.48200361	248.7504832	0.044250771
0.829672244	84.81373319	259.2302168	0.040600089
0.894497023	76.38720592	333.9010705	0.036507773
0.834445745	67.14248582	237.5159112	0.037559812
0.861052488	79.2095191	275.178856	0.039933621
0.891015043	52.63419966	194.1214969	0.043824357
0.862382033	68.92644021	283.8837751	0.039276119
0.824989798	77.45167988	254.7837979	0.043437491
0.860391102	58.94683902	194.914283	0.045649816
0.864160971	95.33034799	377.3456976	0.035847156
0.89078481	126.4952186	417.2647207	0.028527978
0.81778748	51.62685721	172.8988274	0.049343434
0.848605738	68.6937663	258.6125878	0.042545631
0.855794114	92.98942734	341.136574	0.034096069
0.890582393	85.67501439	321.3939838	0.037813979
0.864774527	91.53184992	325.219048	0.03707834
0.84348101	77.29363082	329.9607734	0.040666475
0.891283482	47.74235094	188.2161226	0.043490262
0.841133304	87.41141597	258.681994	0.037102778
0.832787434	81.79278457	309.4720013	0.039916253
0.867023275	152.2962386	387.944031	0.036806588
0.904045831	103.843508	364.0871306	0.033894734
0.8844067	86.2545678	242.95761	0.038170819
0.79171115	23.76690947	86.61626686	0.069719043
0.890211403	66.93382672	311.2803284	0.039016835
0.871207516	60.30140176	261.6240064	0.039874198
0.887657261	77.27039276	242.7116418	0.040013221
0.832365767	48.03040677	177.9182475	0.048158497
0.828478618	53.28628057	153.8815391	0.058586402
0.842690001	51.49189189	149.2139793	0.058758218
0.847435532	60.67369541	228.4972649	0.044900337
0.90016254	105.2076874	284.3515501	0.037894902
0.894466697	70.7881883	238.7992498	0.039592776
0.856212671	70.46198649	323.030817	0.040027784
0.866826686	53.20127559	228.0286819	0.042695069
0.905773796	180.9478042	667.7060032	0.02471384
0.82448025	36.79652383	125.4207592	0.052494825
0.823654263	38.99426376	137.7155668	0.059144576
0.807110673	61.14480661	166.5957135	0.048780165
0.923563874	137.9573286	540.4682109	0.027655915

original_glszm_SizeZoneNonUniformityNormalized	original_glszm_SizeZoneNonUniformity	original_glszm_GrayLevelNonUniformity	original_glszm_LargeAreaEmphasis	original_glszm_ZoneVariance	original_glszm_ZonePercentage
0.485517075	253.9254302	22.88527725	24.47227533	19.18139576	0.434746467
0.50843461	382.8512616	30.90172643	68.85524568	61.80068394	0.3765
0.511393426	298.1423671	18.76329331	12.89879931	9.221351677	0.521466905
0.45041992	472.0400763	46.58206107	21.36545802	15.88682095	0.427231961
0.384630223	123.8509317	17.33540373	46.32608696	37.64006983	0.339304531
0.439407042	280.3416928	27.12852665	29.34169279	22.75015969	0.389499389
0.462768891	119.8571429	18.99227799	258.2895753	241.6343525	0.245033113
0.533047466	329.9563813	26.88045234	120.7738288	112.2708104	0.342936288
0.529676285	396.197861	28.68983957	121.3729947	114.0294743	0.369018254
0.466284895	687.3039349	55.421981	18.84599729	14.69535126	0.490842491
0.417449465	242.1206897	31.59655172	74.99827586	65.29182818	0.32097399
0.529937475	497.6112886	28.28434505	8.706070288	5.485975722	0.55727003
0.404717194	454.0926916	50.28698752	52.59982175	45.78502308	0.383065893
0.439975947	395.538376	36.49054505	12.60066741	8.329249778	0.483853606
0.46357905	363.9095541	25.93757962	9.070063694	5.181380178	0.507105943
0.467431416	774.0664251	44.37560386	93.56099034	87.40423894	0.403017766
0.456227182	297.4601227	26.65337423	29.13343558	23.07368315	0.40623053
0.474874612	388.4474328	42.72371638	153.6552567	143.8762561	0.319781079
0.435446652	183.3230404	20.1543943	32.53206651	25.25104237	0.370598592
0.431372093	464.5877437	41.75208914	56.14206128	48.88661634	0.371251293
0.469985208	1633.668585	109.6726122	20.66340621	16.66570738	0.500143885
0.443646597	448.9703557	36.33992095	13.48517787	9.092591472	0.477133428
0.479575314	972.0991613	72.27479033	15.42920572	11.29589807	0.491870905
0.419187146	154.2608696	18.80434783	39.32065217	31.48673795	0.357281553
0.486201369	669.9854862	42.4107402	10.45428157	6.883333579	0.529185868
0.531650364	323.2434211	22.73026316	29.44736842	24.50259695	0.449704142
0.525067437	607.5030251	34.25151253	11.77787381	8.126042002	0.523292628
0.501951416	888.9559571	55.51270469	12.00169396	8.412904945	0.527868852
0.476977705	514.6589435	46.06580167	173.4022243	163.4788485	0.317446308
0.510574929	609.6264657	37.11725293	110.9840871	103.984207	0.377967711
0.458518678	879.8973424	64.96977592	15.20375195	10.54499301	0.463302752
0.464607578	956.6270034	73.95191841	49.3890238	43.49207556	0.4118
0.518928769	458.7330317	37.61764706	192.5531674	184.3556988	0.349269064
0.522959184	73.21428571	7.814285714	117.3642857	105.5601531	0.291060291
0.5180408	241.9250535	16.00214133	11.46466809	7.848969916	0.525900901
0.505736054	531.0228571	46.3752381	225.7895238	216.402595	0.326391048
0.516642307	849.8765957	54.73738602	30.66808511	25.96089984	0.460913421
0.493510475	753.0969856	61.72477064	34.89187418	29.95392618	0.450014745
0.383936374	215.772242	24.59786477	45.19928826	38.22668153	0.378706199
0.542672287	274.049505	25.57821782	242.1940594	231.6990256	0.308679707
0.524370022	338.7430341	25.6996904	20.58823529	16.43824823	0.490881459
0.475519748	300.528481	32.7056962	72.77531646	63.43029963	0.327122153
0.481742291	435.013289	42.36212625	215.6888151	204.451842	0.298315164
0.530349329	299.1170213	28.63829787	401.2765957	390.118153	0.299363057
0.481521047	720.8370073	44.2251169	66.80961924	61.362057	0.428448769
0.487346138	900.1283162	66.46182999	10.34704927	6.600613412	0.516643357
0.481309687	843.7358813	65.07073588	20.36508842	15.94382054	0.475583288
0.473051537	323.5672515	25.9005848	22.86842105	17.95610615	0.451187335
0.410350905	64.83544304	10.36708861	80.11392405	67.3717353	0.280141844
0.518504155	197.0315789	15.75263158	104.4526316	96.22479224	0.348623853
0.474646728	620.8379205	53.5795107	141.7553517	132.4780181	0.328313253
0.498456181	249.7265469	23.64271457	397.6167665	382.3740543	0.256134969
0.496388011	625.9452815	65.94686757	165.2775575	156.2108204	0.332104293
0.461112156	287.2728732	19.55858748	7.680577849	4.147626176	0.532023911
0.494811179	743.2063915	56.85352863	111.9207723	105.0502309	0.381508763
0.566113231	478.3656805	26.39408284	193.5976331	188.6054494	0.447563559
0.488362598	689.5679887	54.44050992	29.46529745	24.07250831	0.430619091
0.445274264	324.6049383	42.75857339	156.7846365	145.655228	0.299753289
0.479490567	308.7919255	25.8757764	17.41925466	12.8805023	0.469387755
0.401315981	257.6448598	24.19314642	11.19158879	6.402172436	0.456939502
0.482837843	546.5724382	50.09187279	41.8065371	35.05663933	0.384903094
0.504191883	972.0819502	78.27697095	235.8895228	227.7900666	0.35137598
0.490825111	546.7791741	40.66965889	15.09425494	10.93836644	0.490532805
0.519571863	382.4048913	27.64402174	89.66304348	83.22137878	0.394004283
0.494350681	117.6554622	9.504201681	43.87394958	37.72855024	0.403389831
0.428245449	312.6191781	31.99178082	13.42054795	8.952826046	0.473104342
0.480070205	221.3123644	18.10629067	32.87201735	27.60494257	0.435727788
0.524448431	636.6803954	52.73311367	269.5304778	260.9021764	0.340437465
0.471400836	239.9430255	23.23575639	60.96856582	54.51556849	0.393658159
0.534355815	567.4858757	38.06967985	56.30602637	50.73340373	0.423613881
0.496055402	942.5052632	54.20315789	14.16315789	10.14629806	0.49894958
0.444251607	293.2060606	32.56666667	91.56212121	81.81069559	0.320232897
0.476221667	851.48434	76.07158837	48.01733781	41.16922255	0.382132934
0.48040863	1229.846094	87.2859375	68.50273438	62.03405746	0.393180771
0.492699547	1012.004869	77.66991237	15.63680623	11.33128225	0.481933365
0.506568214	479.2135307	35.07610994	54.88900634	48.81741862	0.405834406
0.499180951	441.7751412	35.98983051	96.38305085	89.30203964	0.375796178
0.485421508	309.2135008	27.7032967	13.73155416	9.1799965	0.468727005
0.472888889	567.4666667	44.52333333	72.905	65.4521	0.366300366
0.520788246	859.3006061	65.86181818	152.7351515	145.4516955	0.370536717
0.516006017	305.9915683	21.82630691	35.06913997	29.79386832	0.435389134
0.53506976	760.8691983	48.19831224	14.15822785	10.66695399	0.535190064
0.522193031	633.9423394	46.33937397	19.68863262	15.5356318	0.490703314
0.445369407	55.22580645	8.64516129	122.7177419	104.9283949	0.23709369
0.486064916	900.1922246	72.25917927	14.43412527	10.08559423	0.479544278
0.460319398	674.3679181	58.41569966	30.86143345	25.23822246	0.421704088
0.47962684	329.503639	27.48908297	15.93886463	11.61827578	0.481092437
0.493320672	491.3473896	47.96586345	98.54718876	89.21281411	0.327308577
0.475947326	58.06557377	7.147540984	69.86065574	58.51148885	0.296836983
0.44531775	82.38378378	10.87027027	64.91891892	55.15751644	0.320069204
0.548307338	666.1934156	54.55390947	134.2592593	127.2748761	0.378386795
0.497363928	281.0106195	21.41061947	13.58053097	9.755564257	0.511312217
0.541127621	438.8545006	32.10974106	15.47472256	11.76518007	0.519206146
0.48839855	860.0698467	70.48892675	47.62123793	41.51099296	0.404548587
0.439777588	832.0591966	80.77906977	27.04862579	20.86188983	0.402039949
0.550240635	272.9193548	12.25806452	9.10483871	5.928394901	0.561085973
0.4250745	221.8888889	27.40229885	99.57662835	88.16983383	0.296086217
0.518712408	69.50746269	7.925373134	73.88059701	63.67877033	0.313084112
0.431828099	118.7527273	13.41454545	74.25454545	63.82751736	0.309684685
0.540970995	385.1713483	19.69101124	4.966292135	2.339343202	0.616984402

original_glszm_LargeAreaLowGrayLevelEmphasis	original_glszm_LargeAreaHighGrayLevelEmphasis	original_glszm_HighGrayLevelZoneEmphasis	original_glszm_SmallAreaEmphasis	original_glszm_LowGrayLevelZoneEmphasis
0.422948518	2060.162524	283.0764818	0.723953857	0.015354596
2.246322298	3370.450199	275.1686587	0.739472144	0.017568642
0.325925162	1574.696398	423.9571184	0.743009324	0.012275649
0.933954676	1233.621183	225.2509542	0.69787855	0.032564141
0.954585674	3742.726708	203.5093168	0.643943322	0.01702152
0.735141426	1779.299373	259.7021944	0.689255241	0.01512789
11.56769775	6595.528958	115.7722008	0.706855795	0.039861398
7.756488753	2875.844911	244.3634895	0.756851708	0.041276321
5.815985402	3367.934492	292.8355615	0.755494296	0.026018343
0.375034723	2036.475577	327.1716418	0.709368787	0.013199383
4.896440918	2365.337931	162.3086207	0.671779984	0.036164635
0.478676963	1574.220447	432.6538871	0.75594416	0.014872761
0.699261271	5272.988414	301.4536542	0.660753508	0.008118
0.222227431	1492.686318	301.570634	0.689282587	0.010461857
0.084138488	2006.028025	469.5949045	0.707770374	0.009885574
0.495588793	117597.3134	657.6727053	0.710955831	0.007039955
2.024204476	1387.756135	244.5843558	0.702136457	0.032506332
3.268981091	7961.288509	204.9095355	0.716081923	0.01482864
0.769473145	2134.743468	218.3182898	0.68595557	0.020926407
0.850143292	16085.65552	377.6815227	0.68310788	0.007933776
0.164055443	19023.63579	464.0572497	0.713006375	0.006705677
0.093601839	2950.576087	461.6472332	0.692532795	0.007390791
0.322042488	2990.940799	351.5564874	0.720099332	0.010371245
1.533649606	1552.869565	201.3016304	0.672544546	0.030914587
0.034879544	8063.305515	709.5036284	0.724864948	0.003338255
0.248457302	4461.217105	478.1940789	0.756126999	0.009077524
0.33385075	2115.828003	447.5332757	0.75229261	0.01612417
0.189353976	1902.457933	472.3178995	0.736276184	0.007523964
7.099762049	5722.97405	291.1112141	0.717971534	0.019450658
2.498129611	12121.93802	475.5083752	0.74226183	0.011514796
0.172844948	3095.271496	446.0792079	0.704286743	0.006816562
0.681070068	6741.835843	422.1520155	0.708658959	0.008084906
6.206558236	7499.391403	272.0113122	0.74732878	0.019449168
6.814734899	2487.621429	192.7857143	0.749007519	0.042066228
0.244002248	1267.265525	394.2291221	0.747635993	0.011811483
4.993939465	11451.57048	282.1104762	0.738775992	0.012778391
2.645111107	2255.81459	357.6559271	0.746169246	0.033690486
0.519334855	3031.899083	319.8034076	0.729906092	0.00934422
0.365783335	6806.72242	353.6352313	0.643549521	0.007377836
16.49828765	4254.045545	194.2732673	0.763825897	0.03405572
0.538838308	1301.825077	281.6594427	0.75147124	0.017782332
3.337394108	2259.591772	179.4414557	0.716080235	0.027192734
2.420187741	20417.03433	324.0941307	0.721364077	0.008088134
24.03131986	7665.184397	192.4698582	0.755775876	0.048638804
1.851644689	4795.419506	578.2338009	0.721370014	0.012062467
0.069090572	2853.84732	469.2826205	0.725833884	0.005555386
0.119917956	4781.649173	556.2515687	0.721360645	0.003747626
0.723860164	1646.568713	321.7339181	0.714514786	0.020520249
2.193326165	3592.512658	148.164557	0.66546624	0.026106117
1.475679624	8014.339474	305.5526316	0.746866541	0.017931011
1.238831794	18963.2156	461.7905199	0.716054946	0.005388703
10.9320146	16480.51297	250.5648703	0.733475454	0.021579388
1.827940638	15895.37431	277.6225218	0.731608577	0.007783121
0.066403046	2197.725522	509.070626	0.705786757	0.007526179
2.046388354	7503.090546	369.960719	0.730898434	0.009436729
20.36574844	17309.13846	408.2970414	0.780270853	0.02939263
0.356748701	3588.424221	384.5637394	0.725832617	0.009214976
3.484075204	7952.00823	183.8751715	0.69374802	0.019271044
0.149786028	2623.135093	397.2593168	0.719636602	0.008083343
0.162544631	1960.242991	356.0919003	0.657445112	0.009875961
0.698928884	3606.771201	298.9196113	0.722069231	0.011298358
4.969420445	12500.98963	319.8506224	0.737696878	0.016266902
0.114166	2863.240575	426.4533214	0.728070274	0.007153916
5.445335519	2385.512228	282.7853261	0.748505307	0.021994557
1.00055081	2641.05042	329.3277311	0.729303551	0.020805864
0.211543084	1757.460274	248.8260274	0.680090976	0.015365042
0.370525084	4517.559653	347.7657267	0.719818705	0.012575828
7.200045255	11064.65486	295.6342669	0.751693411	0.014329462
1.797386191	2745.660118	235.2259332	0.713701608	0.019399041
0.695217504	6300.5	449.9538606	0.758418438	0.008065085
0.141237092	7854.379474	531.3731579	0.732054991	0.009416081
3.596298556	3078.193939	208.9772727	0.693043601	0.022363253
0.854875819	5641.244407	316.3512304	0.717155349	0.0099006
1.03703018	11373.17852	438.7605469	0.720552143	0.007520357
0.156341386	2436.262902	401.2302824	0.72948331	0.007861045
0.898070652	4535.824524	387.0010571	0.739342233	0.010178076
1.191366547	8744.688136	400.8463277	0.733702327	0.007056315
0.280523009	1104.808477	227.5211931	0.723761904	0.018204772
2.851537376	2809.0675	316.9591667	0.714819685	0.019842833
2.475814546	13940.89636	386.7078788	0.749485493	0.007926281
0.824083352	2306.349073	440.4940978	0.745759636	0.014904174
0.222943172	1698.848101	429.3642757	0.759493548	0.009796122
1.683204199	807.1087315	276.9629325	0.750326855	0.03748837
6.384623466	3775.766129	110.6774194	0.691556601	0.066508759
0.190647009	2746.99406	390.312095	0.724704017	0.006515489
0.530218385	2782.565188	328.8361775	0.705272465	0.009307069
0.434561151	1539.369723	304.8981077	0.719855398	0.01653876
2.436995002	5213.62249	211.3875502	0.729489961	0.021165706
3.17178985	2122.393443	173.7459016	0.715618762	0.045196852
2.630700936	2299.627027	172.4918919	0.693177668	0.036360628
4.226799349	5001.516049	259.4024691	0.767175823	0.015288017
0.337375464	1038.953982	319.5061947	0.732940564	0.025415593
0.425167446	1127.071517	282.9556104	0.763243454	0.016407795
0.585625985	5130.819421	403.5814878	0.726362944	0.00637312
0.360578443	2958.682875	290.5924947	0.689715074	0.009462391
0.058688017	3822.91129	839.7600806	0.769031008	0.007246016
3.44239767	4262.718391	156.3678161	0.677869953	0.031813716
3.337592652	2237.828358	150.1865672	0.744472966	0.035566749
6.470932826	2458.752727	205.7563636	0.682472544	0.036472578
0.039021214	2175.477528	660.002809	0.763827849	0.006164884

original_glszm_ZoneEntropy	original_glszm_SmallAreaLowGrayLevelEmphasis	original_ngtdm_Coarseness	original_ngtdm_Complexity	original_ngtdm_Strength	original_ngtdm_Busyness	original_ngtdm_Contrast
6.173833641	0.01116672	0.00443511	2.567425977	5.452674268	0.480279691	0.000194997
6.270152958	0.011596574	0.002921334	1.517375542	4.440269686	0.847413708	0.00010455
6.361345777	0.008638864	0.00503211	2.712356594	4.150246315	0.658907923	0.000536384
6.383907646	0.020580382	0.002393293	1.39529028	3.302489532	0.969210244	7.00E-05
6.111953923	0.011330596	0.007380496	1.446104086	4.687624186	0.337893912	0.00011627
6.381891714	0.009458265	0.003603123	1.345783379	3.393221403	0.752815344	0.000116893
5.525997475	0.025374532	0.005519082	0.805021975	4.595207562	0.781461121	5.64E-05
6.09710997	0.029778329	0.00281782	1.609832603	5.839923054	1.189308242	0.000115361
6.234470201	0.017125592	0.002740198	1.195440125	3.581597768	1.381996594	0.000135695
6.468046894	0.008569954	0.002431212	0.987865994	2.205444158	0.879396688	8.29E-05
6.173586377	0.027126138	0.003395283	1.043764481	3.745117005	0.94216285	6.09E-05
6.481874826	0.010731463	0.004426651	2.270911811	3.397999069	0.452593155	0.000239634
6.457492705	0.005188784	0.002614802	0.624362692	1.925617062	0.779447621	4.91E-05
6.386496249	0.007083977	0.003746818	1.155848577	2.421031995	0.657255416	0.000127106
6.579009375	0.006247631	0.004488127	2.192255227	3.558865169	0.385322656	0.000243327
6.863424298	0.005176778	0.002401332	0.763116127	2.76638352	0.366473327	0.000136969
6.359094086	0.021191459	0.004384792	1.570318529	5.382411247	0.738074801	0.000132821
6.060799969	0.010028861	0.002189565	0.665630694	2.329782905	1.161471687	4.03E-05
6.199795019	0.013160197	0.006196874	1.368896099	4.252066248	0.452923877	0.000124195
6.552727619	0.005077756	0.002708557	0.792469859	2.775727454	0.702775737	0.000100608
6.723259942	0.004489421	0.001092533	0.46027833	0.76201423	1.772560818	6.95E-05
6.5863138	0.004918329	0.003814059	1.256179933	2.977898204	0.381902996	0.000100204
6.508080458	0.006648822	0.00166746	0.928727383	1.954774152	1.209176349	7.34E-05
6.163709427	0.020815916	0.005587989	2.596088006	10.16839039	0.453503811	0.000115595
6.593118766	0.002513838	0.003139313	1.173234211	2.362387568	0.471053458	0.000169579
6.259826951	0.006911599	0.004023931	2.79138343	4.708624145	0.33198348	0.000191268
6.589909852	0.010875343	0.00390815	2.349807637	6.75357227	0.364137554	0.000134011
6.559852819	0.004784981	0.001907677	0.990775357	1.463325377	1.558417216	0.000148806
6.416340431	0.01216859	0.001708175	0.928632875	4.108533806	1.428021943	4.74E-05
6.591984763	0.008390791	0.00200816	1.013520865	2.951286522	1.037349182	0.000140716
6.694673395	0.004454785	0.001970713	0.630365615	1.390577669	1.138305787	7.78E-05
6.655316027	0.00555672	0.001750167	0.79462699	2.913178198	0.736307638	3.38E-05
6.239129787	0.013592273	0.002099994	1.219886465	3.619066902	1.154959251	8.25E-05
5.508218978	0.029641473	0.008609655	3.441583309	12.9593939	0.553757721	0.000391782
6.300181012	0.008225978	0.006557757	5.006477338	8.169081604	0.332118822	0.000418087
6.227658867	0.007753706	0.001755097	0.788339772	2.833202895	1.256729484	4.32E-05
6.601927531	0.023487635	0.00178903	1.432082262	3.021394561	1.398361336	0.000104802
6.341461856	0.006548946	0.001709833	0.877772609	1.74815603	1.106653861	6.15E-05
6.426809661	0.00515743	0.006294023	0.71966042	2.848561607	0.349259108	0.000100694
5.819356771	0.024872391	0.002822734	1.324320847	4.429457821	1.302508688	8.80E-05
6.121208846	0.013352072	0.004129934	1.939413807	3.028751445	0.777671039	0.000250388
6.092439682	0.017741032	0.002886786	0.960415085	3.595038347	1.129134928	6.25E-05
6.217661507	0.005868469	0.001884136	0.817334306	3.166548772	0.781216516	3.15E-05
5.886224068	0.035869155	0.002637603	1.11806135	4.777694728	1.314570446	6.18E-05
6.885520555	0.008449614	0.001940034	3.387795647	10.88403775	0.457965775	6.98E-05
6.529391329	0.004107812	0.001919692	0.967123053	1.579034282	0.760657278	7.94E-05
6.508975405	0.002278172	0.001776976	0.978826835	1.765202644	0.741914754	7.34E-05
6.405995686	0.013748917	0.003844289	2.051669327	4.785968439	0.75441285	0.000228259
5.573943954	0.017448862	0.009343188	1.486731178	5.876806927	0.425392051	0.000190091
6.087245137	0.013866915	0.004973377	2.206050222	6.505621457	0.367020399	0.000129849
6.451880911	0.003921444	0.001670508	0.600412948	2.315307112	0.836553328	5.04E-05
6.090207971	0.01378801	0.002953773	1.082455186	4.824238449	0.846338403	5.90E-05
6.081341597	0.00548169	0.001492906	0.47175881	1.531577703	1.267340198	2.80E-05
6.564039654	0.005567244	0.007342272	3.39262375	7.8300325	0.205275847	0.000257894
6.475423545	0.006556863	0.001517885	0.77282059	2.080801463	1.393755576	6.59E-05
6.36261825	0.019088152	0.002435331	2.167425192	5.163528716	0.95657915	0.000399563
6.526624028	0.006383122	0.001940919	1.409626886	4.028000504	0.662768369	5.67E-05
6.082671761	0.01113624	0.002530194	0.858734415	4.212689479	0.87488546	2.88E-05
6.370846692	0.005974961	0.004167131	3.468673512	7.411796213	0.289487935	0.000135074
6.492605268	0.006925163	0.006378943	1.083755547	2.815290921	0.388806778	0.000171968
6.346532597	0.008124655	0.002198797	0.962768793	2.598959085	0.802852657	4.88E-05
6.438819526	0.011679313	0.001023967	0.533670827	1.545148216	2.197588364	4.00E-05
6.455695711	0.00544198	0.002900742	1.452380291	2.440384332	0.536935177	0.000119326
6.158650779	0.01542575	0.002820598	0.975338372	2.153410687	1.925217344	0.000201901
6.004717579	0.015144569	0.009283905	3.918205989	9.465375359	0.30694366	0.000519794
6.356927529	0.009220595	0.004402156	1.908892293	3.946784949	0.439934717	0.000115133
6.273441612	0.008733366	0.005963563	2.976601041	6.219015134	0.249468767	0.000178268
6.248895644	0.010226818	0.001457971	0.977809453	3.357318035	1.368970344	4.11E-05
6.1477061	0.013852263	0.004063912	2.011026711	4.793241635	0.641422969	0.000142588
6.372341415	0.00595084	0.002169707	1.646689309	3.427985055	0.701650429	0.000119859
6.787208552	0.006709201	0.001989037	1.197978882	2.415858198	0.764153771	0.000132121
6.23249328	0.015941843	0.003086194	0.874924437	3.732205799	0.953740499	5.80E-05
6.454972797	0.006647465	0.001331851	0.670856465	2.061589211	1.349710885	4.19E-05
6.688710183	0.005228149	0.00114436	0.469051244	1.378834971	1.761904535	4.99E-05
6.556609923	0.005522146	0.001528375	1.115330307	2.452861471	0.85135182	4.91E-05
6.430414888	0.007048469	0.002567263	1.948316801	4.898872754	0.599613475	8.46E-05
6.329485598	0.004958796	0.002578191	1.212389256	3.948307039	0.657116445	8.05E-05
6.125449057	0.013503129	0.004014765	1.4033221	2.527727528	0.782598597	0.000175497
6.562148816	0.01375102	0.00189025	1.026427069	3.167836429	1.310562911	6.33E-05
6.305491439	0.005599623	0.001284821	0.602958199	1.414114749	1.585970425	7.78E-05
6.396301435	0.010605616	0.004093941	4.996806797	14.57773952	0.373429375	0.000180561
6.468709011	0.007270133	0.002256289	2.122249236	3.376392872	0.702325323	0.00011998
6.340614814	0.027996457	0.002307091	2.384173154	5.377259333	0.948438286	9.02E-05
5.361139408	0.051748469	0.016171608	0.745145612	4.501055579	0.422278624	0.000181459
6.437373916	0.004393532	0.001691546	0.718349003	1.334097913	1.214636962	7.92E-05
6.463607295	0.006180719	0.002111387	0.592832638	1.480684271	1.20304013	6.54E-05
6.313545481	0.010641108	0.004389668	2.433805011	5.159655352	0.516424689	0.000192291
6.151966238	0.014577061	0.002020594	0.701905391	2.458280009	1.154350335	3.42E-05
5.50837773	0.031451339	0.014186419	2.636497818	10.97053053	0.29728861	0.000354799
5.752745749	0.021259912	0.009821416	2.700867806	10.96083836	0.362883443	0.00022133
6.105441438	0.011508089	0.001596176	0.887836529	2.36110399	1.516922908	5.15E-05
6.303855418	0.018640689	0.004146536	5.612688744	8.574231934	0.494513727	0.000290585
6.1924039	0.012682204	0.003484779	2.175481632	3.741297147	0.691031853	0.000180524
6.452574562	0.004494398	0.001288091	0.773695698	1.473672225	1.252686771	5.21E-05
6.527278302	0.00626971	0.001575984	0.522008836	1.564502096	1.13137726	2.86E-05
6.515555143	0.006179321	0.00573202	9.111964579	8.867383157	0.185285615	0.000987974
6.121197563	0.021770695	0.004105965	0.643431447	3.169027763	0.8780968	5.33E-05
5.315899961	0.017235583	0.013706546	2.242995684	9.949508609	0.346916511	0.000281904
6.000614216	0.022496338	0.006659626	1.559455652	6.84868147	0.677613648	0.000250347
6.495911631	0.004681447	0.005428314	5.208614342	6.14624663	0.293040328	0.000558713

wavelet.LHL_gldm_GrayLevelVariance	wavelet.LHL_gldm_HighGrayLevelEmphasis	wavelet.LHL_gldm_GrayLevelNonUniformityNormalized	wavelet.LHL_gldm_DependenceEntropy	wavelet.LHL_gldm_DependenceNonUniformity
73.21631499	1276.320033	0.038326876	6.733969583	371.394015
36.79639775	804.1695	0.059226	6.544737102	479.119
55.51762907	625.3944544	0.040804081	6.564583665	349.9051878
58.30890833	1596.336323	0.04119468	6.613747298	786.9657562
18.35029275	231.4056902	0.078899535	6.241931985	185.2444679
26.47532914	642.5634921	0.066757012	6.399592701	362.1465201
41.13173876	797.4597919	0.051421841	6.626803818	256.5061495
42.88265928	1075.65928	0.060808926	6.645191804	388.9900277
57.24232336	1566.796744	0.047104348	6.960266449	450.5934879
66.40640301	1470.571762	0.038845271	6.883757335	864.8694639
41.81534333	1385.664638	0.05140343	6.61609142	456.2197012
91.93873117	1993.31276	0.03190906	6.702241595	653.2587537
61.73338395	1461.320929	0.048658469	6.85039569	730.3820417
20.20836438	480.6318622	0.070272444	6.395706922	382.0764263
102.0229537	3891.586563	0.04126438	6.902865394	441.3901809
56.05963096	2219	0.056641657	6.956166395	902.9644682
27.00909735	976.8392523	0.073135548	6.472677766	311.1831776
39.1835643	837.8756841	0.059962123	6.786773177	511.6356529
17.34286274	387.8036972	0.073342343	6.202411768	245.0457746
32.13928728	982.0155119	0.055134265	6.602824029	703.0896243
46.19106464	1418.280576	0.049389949	6.736694808	1851.052086
62.24162097	1520.033946	0.039990077	6.833176764	599.8722301
46.29391989	903.2446008	0.047414475	6.675894124	1136.497209
169.6377123	3142.639806	0.031365821	7.132838755	309.223301
39.10897921	1040.039171	0.053608002	6.650608885	635.1666667
73.72597631	1323.338018	0.034331125	6.618457878	481.4245562
36.35763005	952.6861149	0.054719413	6.379303	687.6268657
52.55755953	1151.000894	0.041282869	6.652363559	1043.085842
48.50540435	1575.362754	0.043471329	6.929735617	835.7566931
23.73273085	756.2412156	0.08104622	6.515480067	582.2434315
29.47805185	637.6446161	0.05647393	6.58425974	977.2071463
18.37779484	523.5214	0.0922952	6.464775762	828.1248
31.29145276	1153.157645	0.061870655	6.559677177	578.576452
55.86114341	691.2993763	0.062573208	6.336882995	116.7671518
35.17484249	628.3277027	0.053744623	6.350164703	248.8963964
18.1855574	553.7979484	0.086734301	6.342825687	566.6916382
71.63735212	1991.717288	0.036610908	6.899278906	1096.745027
42.51076083	2094.592746	0.051095841	6.574088673	916.6478915
18.91622545	556.1623989	0.071098001	6.194429534	343.7075472
36.01649022	1028.035452	0.056677088	6.474059242	400.4755501
76.70816973	1233.121581	0.033933537	6.674211892	467.0531915
25.64881327	769.5942029	0.076702609	6.440943347	379.8312629
29.01330924	1676.402379	0.074288894	6.496656474	587.299967
35.33602755	546.8784501	0.066699799	6.460048712	407.5774947
75.56258419	2285.47739	0.040427142	6.976858938	966.1757298
41.74036982	1325.303776	0.052001917	6.625894847	928.2206993
90.59022032	3294.316061	0.033983494	6.881136518	1262.195876
54.48678781	949.8621372	0.043007393	6.559283199	465.5211082
15.11455661	458.2021277	0.10084377	5.927248353	99.46453901
27.93473277	536.7889908	0.058057403	6.381362433	276.053211
46.1874833	1172.561496	0.052078167	6.879564513	897.5818273
31.85457572	755.3435583	0.061989014	6.625533849	424.3568507
26.32963842	1545.009218	0.067143856	6.540390716	775.2133263
29.41284719	416.1682323	0.054565901	6.39913376	309.3091375
21.80014437	919.3025146	0.075593764	6.430485778	769.9550419
34.65015413	916.9729873	0.05712035	6.619333267	437.9798729
65.1987707	1448.226593	0.039808717	6.83977526	969.7929247
14.70470464	585.6435033	0.102431871	6.326120553	367.1743421
63.48809807	2583.791545	0.051769458	6.663161648	336.0758017
31.64685554	1819.883274	0.058584364	6.515369131	332.9871886
47.39224423	1278.230534	0.052382449	6.661339644	732.8395104
43.217647	1847.028613	0.062355547	6.808148239	1128.023875
47.28079565	2098.760458	0.048217129	6.54223294	665.3663584
41.48141441	893.2275161	0.049787587	6.588610765	481.5642398
20.10596093	282.9305085	0.071496696	6.048214166	136.2169492
33.09225827	871.2754375	0.053166328	6.434514067	411.3421905
48.6107754	971.7826087	0.044269782	6.447862665	323.9017013
70.46887961	2016.253505	0.04483407	6.923440584	929.7285474
18.83383248	415.5305491	0.078641672	6.236005194	262.9396752
42.15108235	1168.18588	0.051633169	6.683317528	620.0434783
30.78392619	1121.222952	0.057589672	6.587507611	909.595063
19.93623734	405.6841339	0.079995602	6.389673678	382.7962154
47.36332006	1540.238513	0.045869598	6.748752279	1243.553537
36.48331228	1678.444325	0.06121722	6.647077378	1480.292889
55.27221436	1785.086579	0.046135228	6.69616616	1217.645237
22.42835812	562.5770056	0.073757339	6.353334116	489.1578722
35.0371778	949.6437367	0.056701331	6.566691955	570.4717622
63.15863123	1172.700515	0.039719397	6.619674053	422.7218543
46.31164481	1084.327534	0.051185146	6.700856735	836.0750916
51.27913705	1513.306759	0.04298936	6.819614057	1235.523243
48.9061936	1258.689427	0.066933183	6.55243092	296.3773862
46.84535053	1069.665412	0.050218219	6.636570494	713.0436583
30.26268879	729.0464834	0.06190558	6.527426415	565.2748585
26.66874079	1208.950287	0.096512618	6.154952812	87.6959847
58.82640809	1887.950026	0.044613613	6.680952261	1152.430865
43.06947382	1084.367012	0.047479939	6.706778872	921.4484744
46.50912669	1111.196779	0.050802674	6.512534248	397.219888
31.58789587	992.791653	0.05926047	6.498229945	752.7817943
12.61467787	316.3576642	0.090841281	5.907128266	75.32603406
75.59986411	2300.403114	0.053429676	6.435284187	152.6920415
43.04582921	1358.039863	0.052660675	6.662390107	817.3980069
57.07968305	1087.460633	0.041836162	6.565851455	329.5502262
32.81165681	757.0486556	0.057363504	6.42315101	397.8911652
83.24620567	3411.685275	0.038240432	6.854270466	1374.245118
36.3751652	1328.980875	0.054003633	6.607723279	1164.262643
171.1175191	2381.180995	0.025137692	6.984641919	349.7737557
59.04760134	1601.714691	0.044950902	6.796236799	455.1565513
15.40979016	671.5116822	0.089647567	5.8176582	92.46261682
51.87918493	1577.626126	0.058107094	6.603809774	203.3513514
79.41436162	1443.161179	0.036202914	6.675126543	408.8734835

wavelet.LHL_gldm_GrayLevelNonUniformity	wavelet.LHL_gldm_SmallDependenceEmphasis	wavelet.LHL_gldm_DependenceNonUniformityNormalized	wavelet.LHL_gldm_DependenceVariance
46.10723192	0.524659845	0.308723204	1.427247896
118.452	0.422071385	0.2395595	2.087111
45.61896243	0.530584672	0.312974229	1.258671727
101.0505503	0.533296849	0.320817675	1.15102201
74.87565859	0.335763665	0.19519965	2.469646381
109.3479853	0.393963327	0.221090672	2.596356974
54.35288553	0.436960314	0.242673746	2.244067348
109.7601108	0.409150513	0.215506941	2.993851797
95.48051307	0.421798915	0.222295751	2.97257327
116.6523477	0.499263489	0.288001819	1.459317461
92.88599889	0.438876569	0.252473548	1.951177383
53.76676558	0.622406528	0.387690655	0.832549727
142.5206555	0.43874486	0.249362254	1.800039888
130.5662002	0.350124651	0.20563855	2.447573174
63.87726098	0.511419743	0.285135776	1.871710768
232.7405695	0.431402869	0.219752852	5.607472713
117.3825545	0.35239034	0.1938836	3.246465388
153.3831118	0.373150586	0.200013938	2.985181323
83.31690141	0.375128572	0.215709309	2.490726046
159.9445019	0.426884275	0.242361125	2.094436168
343.2601439	0.469612402	0.26633843	2.542887014
84.81895332	0.497001969	0.282825191	1.601765511
195.3950497	0.484735845	0.2757819	1.796225687
32.30679612	0.530245864	0.300216797	1.875860119
139.5952381	0.436043923	0.243919611	2.314184606
46.41568047	0.584122699	0.356083252	0.950052082
120.9846223	0.532632158	0.311002653	1.602098467
138.5040238	0.515771931	0.310904871	1.226943792
147.7590468	0.429753899	0.245883111	2.194704654
256.0250079	0.353141916	0.184312577	4.249900569
233.9150169	0.415339799	0.235926399	1.959489079
461.476	0.293311713	0.16562496	4.32189296
156.5946266	0.402652265	0.228595991	2.525320089
30.0977131	0.443023381	0.242759151	2.252721937
47.72522523	0.494181088	0.280288735	1.602122393
279.0242462	0.320074389	0.176155312	3.680746006
130.6643317	0.530172839	0.30729757	1.391649129
173.2659982	0.476108197	0.270317868	1.629250533
105.509434	0.401005187	0.231608859	2.074332866
92.72371638	0.432588181	0.244789456	2.174912273
44.65653495	0.576793089	0.354903641	0.991267172
148.189441	0.367732931	0.196600033	3.442995598
224.872481	0.361277361	0.194020471	3.169178091
125.6624204	0.394015622	0.21633625	2.453392294
141.2524327	0.495273593	0.27652425	2.234077619
185.9068531	0.463051112	0.259642154	1.926980566
125.2631579	0.56888562	0.342429701	1.210388827
65.19920844	0.523197903	0.307071971	1.282320159
56.87588652	0.305448499	0.176355566	3.114254816
63.28256881	0.441979704	0.253259827	2.211123643
207.4794177	0.41653642	0.225296643	2.659921816
121.2505112	0.409199718	0.216951355	3.418390689
254.9452199	0.369195643	0.20416469	2.863375984
63.89666951	0.463252808	0.264141023	1.64997546
297.6126492	0.355336349	0.195568972	3.095342191
107.8432203	0.423681465	0.231980865	4.028591595
130.5327844	0.500993669	0.295758745	1.438109436
249.1143092	0.271262097	0.150976292	5.153479917
71.02769679	0.449194849	0.244953208	2.119841648
82.31103203	0.423827635	0.237001558	2.269888173
154.0567834	0.444508998	0.249180384	2.146103101
342.1448879	0.397708942	0.205581169	4.124628257
109.5011008	0.515352426	0.292983865	1.700708937
93.00321199	0.454681618	0.257796702	1.713723526
42.18305085	0.401627208	0.230876185	1.766848607
82.03564485	0.447568146	0.266585995	1.440602911
46.83742911	0.52794321	0.306145275	1.430773189
159.878295	0.463258214	0.260720288	2.113106931
101.6836814	0.366003439	0.203356284	2.95411966
129.4443558	0.440654866	0.247324882	2.055638071
219.3014706	0.421984373	0.23886425	2.166566351
164.8709364	0.340261124	0.185733244	3.245488581
214.6238513	0.460191819	0.265773357	1.595497948
398.5853172	0.4161741	0.227352617	2.72506188
196.6283435	0.504195407	0.285698085	1.640902844
171.9283569	0.38175177	0.209848937	2.717517297
133.5316348	0.437502966	0.24223854	2.202885851
53.97866078	0.508486728	0.311053609	1.101089892
167.6825397	0.444425079	0.255212177	1.927707857
191.4316191	0.478203064	0.277458622	1.70941443
91.16299559	0.419857698	0.217604542	3.566315408
133.4298081	0.477790523	0.268364192	1.982985273
153.1544058	0.428680333	0.2284862	3.088933967
50.47609943	0.31077576	0.167678747	4.970302966
172.2977732	0.519990918	0.298402606	1.522389341
164.945308	0.451411122	0.265241357	1.684211922
72.54621849	0.489241343	0.278165188	1.673387002
180.3296089	0.4397092	0.247381464	2.165846333
37.33576642	0.301677401	0.183275022	2.72484771
30.88235294	0.454440274	0.264173082	1.804707798
169.0934288	0.45424163	0.254561821	1.944301166
46.22895928	0.508000621	0.298235499	1.284727176
89.60179257	0.459269782	0.25473186	1.906636654
166.4606019	0.54084031	0.315700693	1.303936558
254.1410965	0.444082364	0.247399627	1.968688596
22.22171946	0.633710231	0.395671669	1.041875678
79.24844016	0.451434646	0.258171612	1.683095096
38.36915888	0.365109077	0.216034151	2.067014586
51.5990991	0.432063044	0.22899927	3.23117036
41.77816291	0.59274098	0.354309778	1.311054309

wavelet.LHL_gldm_LargeDependenceEmphasis	wavelet.LHL_gldm_LargeDependenceLowGrayLevelEmphasis	wavelet.LHL_gldm_SmallDependenceHighGrayLevelEmphasis	wavelet.LHL_gldm_LargeDependenceHighGrayLevelEmphasis
5.443890274	0.006486388	644.1342262	6942.697423
7.929	0.012776165	337.2941275	6431.0635
5.152057245	0.014228879	335.291077	3156.315742
4.905421932	0.003820787	855.5117087	7835.337546
10.49841939	0.059942992	86.97831771	2287.285564
9.328449328	0.016514242	247.539022	6158.857753
8.059602649	0.013061271	347.8464247	6455.486282
10.00387812	0.011174238	449.4592907	10282.29972
9.693635915	0.007561954	678.7849192	14197.73014
5.845154845	0.004898691	722.9645999	8696.120213
7.397343664	0.006280757	606.0388912	10185.3171
3.727596439	0.002812426	1247.369768	7397.820772
7.213724821	0.006082875	625.6580008	10635.98942
9.917115178	0.023896358	167.9593342	4724.332616
6.510335917	0.002433227	1985.278455	25360.94444
13.54563154	0.006836543	962.1974854	28746.35799
11.50716511	0.013196444	338.6652502	11223.22305
10.69351056	0.015411151	325.1541416	8361.329163
9.464788732	0.028850965	144.0092641	3654.449824
7.852119959	0.009552538	420.8960973	7663.845915
7.796546763	0.006082143	665.400689	10941.49324
6.137199434	0.005030388	739.5117031	9543.477605
6.57534579	0.010759797	424.0466473	5997.415918
6.233009709	0.003461495	1646.910816	19141.49223
8.104454685	0.008844367	447.5542308	8377.205069
4.180473373	0.004612101	773.7222578	5557.123521
5.676616915	0.007223501	504.5318154	5333.936228
5.173472429	0.005563974	594.2487334	6005.7693
7.889085025	0.005695243	671.5690414	12400.56899
13.42165242	0.019216529	267.5966291	9995.799937
7.85610816	0.01429354	261.6253748	5045.328344
15.1776	0.032730006	164.0418564	7530.3054
8.953378111	0.008552377	457.2508776	10313.6855
8.018711019	0.019759046	322.1616416	5041.885655
6.18018018	0.012638269	313.0849512	3890.057432
13.27976376	0.026758712	178.9729596	7216.355611
5.421966938	0.003294362	1035.22897	11050.74447
6.431436155	0.003514051	981.8499741	13549.63963
8.216981132	0.0165118	218.180875	4602.980458
7.927872861	0.008832355	428.8215534	8227.112469
4.267477204	0.005223197	699.7934225	5499.907295
11.55900621	0.016945049	283.8718717	8622.305383
11.29930624	0.009698545	610.795931	18563.5038
9.239915074	0.021543044	235.7770143	4488.095011
7.136233543	0.003654596	1119.597062	16424.21694
7.12027972	0.006032933	608.2889489	9416.153007
4.706456864	0.001795587	1857.096659	15629.22491
5.277044855	0.007542563	487.3487269	5074.909631
12.71985816	0.031126252	135.1507394	5855.046099
7.735779817	0.017706247	235.5712941	4021.544954
9.148092369	0.009624081	500.2928781	10190.91591
10.59509202	0.016656438	315.4273611	7519.887526
10.40163287	0.007352884	573.7209806	15681.99631
6.614005124	0.02124147	192.9257292	2802.605465
11.17526035	0.012899961	318.2055793	10356.07366
10.90254237	0.013189299	385.436488	9930.716631
5.717291857	0.004962824	724.470457	8147.581885
17.77467105	0.032175573	160.0644541	10301.5111
7.744897959	0.004113799	1140.571895	20055.17055
8.254092527	0.005446276	766.1100136	15079.70819
7.693641618	0.007217375	571.9991214	9575.481129
12.01512666	0.007035825	724.4926951	21744.66557
6.110523998	0.003532402	1092.424386	12697.14091
6.85117773	0.009555325	404.4334545	6140.242505
7.806779661	0.037320653	114.9787415	2124.564407
6.338950097	0.008782089	387.9294789	5463.730395
5.491493384	0.007321188	503.2331764	5469.15879
7.354458777	0.004315791	939.2221825	14462.61526
10.57308585	0.030386895	146.1345815	4511.95437
7.611088951	0.007589603	510.7846999	8936.632629
8.06197479	0.007954045	470.5466301	9053.560137
11.95099466	0.034325534	140.8654136	4676.086851
6.517845694	0.00478	698.3409331	10136.29365
9.199354938	0.0061511	693.2819233	15364.99294
6.13984045	0.005641361	894.4823827	10922.75903
9.904332904	0.023216421	220.0774632	5437.591163
7.969002123	0.009623401	415.1062289	7459.366879
5.016188374	0.005892273	593.6482397	5761.933775
7.272893773	0.008141952	476.620615	7883.251221
6.44172468	0.004846356	726.190667	9806.547272
10.69309838	0.010207278	533.0271614	13286.49266
6.969138126	0.007925265	510.1007389	7448.281144
9.668552951	0.015189486	316.3623659	6883.654406
15.84894837	0.015979203	366.8546012	18870.38432
5.753495598	0.003609288	971.9034401	10921.94899
6.715025907	0.009494919	489.7543826	7199.316926
6.337535014	0.007118653	537.5520431	6918.47409
7.803154781	0.008717387	437.1498148	7749.899113
11.81265207	0.040807807	87.34255279	3910.065693
6.878892734	0.004931177	1058.029008	15616.25433
7.274057926	0.006200589	622.3303902	9748.976643
5.427149321	0.006908998	544.0878433	5908.101357
7.183098592	0.011186791	347.1449945	5456.943662
5.169997703	0.001831432	1819.735576	17813.27728
7.478113047	0.006318583	586.4737416	9802.80408
3.975113122	0.003373639	1533.146147	9258.4819
6.812819058	0.005518787	705.0895297	11042.16733
8.939252336	0.016245652	246.6688059	5954.077103
9.986486486	0.007850621	661.5385703	16050.66892
4.743500867	0.004845328	866.8713225	6758.564991

wavelet.LHL_gldm_SmallDependenceLowGrayLevelEmphasis	wavelet.LHL_gldm_LowGrayLevelEmphasis	wavelet.LHL_gldzm_DistanceZoneVariabilityNormalized	wavelet.LHL_gldzm_LowIntensityEmphasis
0.001910287	0.002385402	1	0.003348477
0.001973794	0.002932653	1	0.004303266
0.004001965	0.005146868	1	0.00695184
0.000869035	0.001195543	0.998680739	0.001508363
0.004079004	0.008020615	1	0.011513476
0.002260876	0.00328399	1	0.005214061
0.002214792	0.00313135	1	0.004689526
0.001362464	0.00198387	1	0.003075273
0.000830983	0.001250935	1	0.001823419
0.000928787	0.001318955	0.997690534	0.001726351
0.001003773	0.001437802	1	0.002053224
0.001056135	0.001276645	0.998302208	0.001579094
0.000984308	0.001442576	1	0.002067618
0.002227589	0.003755882	1	0.005822711
0.000848523	0.000980119	1	0.001488731
0.000504825	0.000783049	0.987373021	0.001084352
0.00126024	0.00198234	1	0.003276041
0.001058448	0.001958992	1	0.002675759
0.002656079	0.00462024	1	0.006632986
0.001406684	0.002053391	0.998632947	0.002975263
0.000615083	0.001022558	0.994724384	0.001243001
0.001083337	0.001453668	0.991830203	0.001994333
0.001187671	0.002289291	0.992288033	0.002568867
0.001425987	0.001601926	1	0.002392098
0.001024503	0.001614801	0.998501874	0.002165885
0.001429225	0.00181315	1	0.00225967
0.001338415	0.001881117	1	0.002335566
0.000959101	0.001468667	1	0.001756715
0.000639419	0.001037149	0.994252921	0.001370257
0.001032177	0.001969416	1	0.002774938
0.00129434	0.002387329	0.999005964	0.002987739
0.001011115	0.002597852	0.997603359	0.003375399
0.000861784	0.00140836	1	0.001974253
0.006475184	0.007480907	1	0.012917459
0.0024742	0.003449212	1	0.004620362
0.001809183	0.003160757	1	0.005273553
0.000665528	0.000924587	0.99816598	0.001166496
0.000607195	0.000864468	1	0.001168589
0.001751009	0.002904089	1	0.00404287
0.001292845	0.001878283	1	0.002731296
0.001678059	0.002128182	1	0.002688614
0.001326682	0.002229941	1	0.003380367
0.000439021	0.00133128	1	0.001557747
0.001639423	0.002995004	0.995283045	0.003929577
0.000620499	0.000862317	1	0.001160519
0.000763361	0.001198247	1	0.001541287
0.000486081	0.000625559	0.981024844	0.000788401
0.001695958	0.00234264	1	0.003014749
0.003292974	0.00487935	1	0.009740836
0.002398185	0.003596318	1	0.004991954
0.000798036	0.001456376	1	0.001866851
0.001431812	0.002327272	1	0.003266017
0.000540811	0.000967952	1	0.001365368
0.002878228	0.004699849	1	0.005948441
0.000884146	0.001614398	0.995043401	0.002321253
0.001187564	0.00188108	1	0.002597127
0.000849622	0.001256446	0.994794413	0.001575316
0.001061535	0.002406272	0.997297302	0.00374668
0.001233101	0.001453224	1	0.002443369
0.001052121	0.001380924	1	0.002204657
0.00102068	0.001509525	1	0.002113854
0.000479952	0.000819056	0.999195818	0.001129012
0.000746719	0.000993062	1	0.001341916
0.001603524	0.002327677	1	0.003246152
0.00546802	0.008206664	1	0.012751644
0.001369182	0.002070466	1	0.002796054
0.001922343	0.002462141	1	0.003338175
0.000594574	0.000891563	1	0.001190439
0.004117095	0.005934285	1	0.01032902
0.000991956	0.001538053	1	0.002099176
0.000764569	0.001326733	1	0.001705788
0.002383752	0.004296557	1	0.006635024
0.000630775	0.001009871	1	0.001278115
0.000632146	0.001020807	0.996788705	0.001425227
0.000568901	0.001217293	1	0.001265797
0.001750493	0.003615076	1	0.00481913
0.00115532	0.001796751	1	0.002451805
0.001390254	0.001890758	1	0.002490781
0.001373734	0.001947517	1	0.002818657
0.000629004	0.001013206	0.996814026	0.001234601
0.00161729	0.002123761	1	0.003521499
0.001288929	0.001858249	0.99726963	0.002504828
0.001299742	0.002220735	0.998352555	0.002893844
0.002774796	0.00335773	1	0.007874906
0.000698226	0.000973628	0.993926304	0.001250248
0.000985466	0.001985909	0.996749738	0.002291749
0.001440466	0.001942832	1	0.002692295
0.000940633	0.001555154	0.994861948	0.002007311
0.004125235	0.006453732	1	0.012627249
0.002034033	0.002282044	1	0.00396271
0.000799347	0.001244374	1	0.001639195
0.001893408	0.00240114	1	0.003354686
0.001635058	0.002439036	1	0.003331712
0.000428744	0.000569757	1	0.000735892
0.000730071	0.001171806	0.999168399	0.001533537
0.001662688	0.001866225	1	0.002414821
0.00127273	0.001650561	1	0.002524361
0.003016035	0.004003722	1	0.007379138
0.001736783	0.002116844	1	0.003546384
0.001548214	0.001892966	1	0.002413548

wavelet.LHL_gldzm_LargeDistanceEmphasis	wavelet.LHL_gldzm_HighIntensitySmallDistanceEmphasis	wavelet.LHL_gldzm_LowIntensityLargeDistanceEmphasis	wavelet.LHL_gldzm_HighIntensityEmphasis
1	1246.90795	0.003348477	1246.90795
1	799.4301619	0.004303266	799.4301619
1	629.8235294	0.00695184	629.8235294
1.001980198	1599.726073	0.001510563	1600.171617
1	253.93531	0.011513476	253.93531
1	629.6336898	0.005214061	629.6336898
1	794.9605263	0.004689526	794.9605263
1	1107.930622	0.003075273	1107.930622
1	1618.188465	0.001823419	1618.188465
1.003468208	1450.636416	0.001728187	1452.328902
1	1384.12845	0.002053224	1384.12845
1.002548853	1999.496177	0.001581926	2000.069669
1	1439.494307	0.002067618	1439.494307
1	482.8948752	0.005822711	482.8948752
1	3883.709856	0.001488731	3883.709856
1.019061584	2226.382209	0.001095424	2237.325024
1	963.5760369	0.003276041	963.5760369
1	879.452882	0.002675759	879.452882
1	384.158	0.006632986	384.158
1.002051984	982.252565	0.002976939	982.880985
1.007934409	1414.758992	0.00124978	1417.315261
1.012305168	1487.35685	0.002010508	1493.465135
1.011612903	881.5934409	0.00258242	884.7944086
1	3129.990446	0.002392098	3129.990446
1.002248876	1031.601949	0.00216729	1032.501499
1	1324.525967	0.00225967	1324.525967
1	949.3872153	0.002335566	949.3872153
1	1151.707547	0.001756715	1151.707547
1.008645533	1562.740922	0.001380032	1565.375216
1	760.5421592	0.002774938	760.5421592
1.001491795	630.5491049	0.002988957	631.0059672
1.00359928	559.8941212	0.003381158	560.4565087
1	1144.002532	0.001974253	1144.002532
1	732.5284553	0.012917459	732.5284553
1	631.183432	0.004620362	631.183432
1	561.979346	0.005273553	561.979346
1.002753557	1958.127581	0.001168109	1959.434144
1	2071.896423	0.001168589	2071.896423
1	547.1893064	0.00404287	547.1893064
1	1005.263736	0.002731296	1005.263736
1	1216.126437	0.002688614	1216.126437
1	780.7303371	0.003380367	780.7303371
1	1698.742067	0.001557747	1698.742067
1.007092199	599.3906619	0.00395167	599.9669031
1	2266.910146	0.001160519	2266.910146
1	1319.672594	0.001541287	1319.672594
1.028738026	3252.193565	0.000800609	3274.387755
1	936.9227421	0.003014749	936.9227421
1	448.5888325	0.009740836	448.5888325
1	536.7839286	0.004991954	536.7839286
1	1200.998429	0.001866851	1200.998429
1	774.6360656	0.003266017	774.6360656
1	1563.77335	0.001365368	1563.77335
1	413.9031746	0.005948441	413.9031746
1.007453416	901.0754658	0.002333308	902.563354
1	911.5629709	0.002597127	911.5629709
1.00782881	1447.47834	0.001580298	1450.712422
1.00405954	591.2033153	0.003757925	591.5696888
1	2555.45339	0.002443369	2555.45339
1	1810.001433	0.002204657	1810.001433
1	1288.705416	0.002113854	1288.705416
1.001206758	1838.780068	0.001129608	1839.39099
1	2113.529057	0.001341916	2113.529057
1	891.3961499	0.003246152	891.3961499
1	289.2359551	0.012751644	289.2359551
1	872.0899015	0.002796054	872.0899015
1	957.2018779	0.003338175	957.2018779
1	2034.558005	0.001190439	2034.558005
1	400.6025878	0.01032902	400.6025878
1	1159.887412	0.002099176	1159.887412
1	1115.197221	0.001705788	1115.197221
1	415.7193426	0.006635024	415.7193426
1	1523.972255	0.001278115	1523.972255
1.004824702	1670.732711	0.001430879	1672.071084
1	1779.884023	0.001265797	1779.884023
1	575.6450648	0.00481913	575.6450648
1	950.5888795	0.002451805	950.5888795
1	1177.397277	0.002490781	1177.397277
1	1078.741267	0.002818657	1078.741267
1.004786598	1511.76795	0.001237163	1514.077782
1	1276.606202	0.003521499	1276.606202
1.004101162	1067.889952	0.002512098	1068.484621
1.002473207	737.2359852	0.002898519	737.5630668
1	1193.818182	0.007874906	1193.818182
1.009138381	1871.822454	0.001269495	1875.285901
1.004883342	1086.380494	0.002297219	1087.585458
1	1109.266169	0.002692295	1109.266169
1.00772698	992.9294913	0.002068303	994.2247263
1	292.6546763	0.012627249	292.6546763
1	2335.299003	0.00396271	2335.299003
1	1369.08881	0.001639195	1369.08881
1	1077.51608	0.003354686	1077.51608
1	752.7013464	0.003331712	752.7013464
1	3377.201571	0.000735892	3377.201571
1.00124792	1329.756344	0.001534358	1330.230865
1	2409.7104	0.002414821	2409.7104
1	1576.298056	0.002524361	1576.298056
1	678.5611111	0.007379138	678.5611111
1	1544.901786	0.003546384	1544.901786
1	1456.887451	0.002413548	1456.887451

wavelet.LHL_gldzm_DistanceZoneVariability	wavelet.LHL_gldzm_ZonePercentage	wavelet.LHL_gldzm_IntensityVariabilityNormalized	wavelet.LHL_gldzm_LowIntensitySmallDistanceEmphasis	wavelet.LHL_gldzm_IntensityVariability
717	0.596009975	0.031506218	0.003348477	22.58995816
988	0.494	0.042837942	0.004303266	42.32388664
680	0.60822898	0.03407872	0.00695184	23.17352941
1513.00132	0.617611088	0.034556525	0.001507812	52.35313531
371	0.390937829	0.054990882	0.011513476	20.40161725
748	0.456654457	0.044961251	0.005214061	33.63101604
532	0.503311258	0.03880943	0.004689526	20.64661654
836	0.463157895	0.038125844	0.003075273	31.87320574
971	0.479033054	0.031904637	0.001823419	30.97940268
1726.004624	0.576090576	0.032075913	0.001725892	55.49132948
942	0.521306032	0.038450963	0.002053224	36.22080679
1175.001699	0.69851632	0.028126893	0.001578386	33.10535259
1493	0.509730283	0.035292629	0.002067618	52.69189551
761	0.409580194	0.05044369	0.005822711	38.38764783
903	0.583333333	0.030398242	0.001488731	27.4496124
2020.1652	0.49793137	0.03419781	0.001081585	69.96871945
651	0.405607477	0.047586013	0.003276041	30.97849462
1093	0.427286943	0.039188917	0.002675759	42.83348582
500	0.440140845	0.05596	0.006632986	27.98
1460.001368	0.50396415	0.043363943	0.002974845	63.39808482
3761.052896	0.544028777	0.036994715	0.001241307	139.8770167
1209.041017	0.574728901	0.032747158	0.00199029	39.91878589
2307.069677	0.564183451	0.037074899	0.002565479	86.19913978
628	0.609708738	0.022074932	0.002392098	13.86305732
1332.001499	0.512288786	0.03907042	0.002165534	52.11994003
905	0.669378698	0.030471597	0.00225967	27.57679558
1361	0.615558571	0.046330523	0.002335566	63.05584129
2014	0.600298063	0.035504201	0.001756715	71.50546177
1725.028818	0.510444248	0.035255504	0.001367813	61.16829971
1269	0.401709402	0.046003971	0.002774938	58.37903861
2009.000995	0.485514244	0.043997942	0.002987434	88.47986077
1663.004799	0.3334	0.053083444	0.003373959	88.49010198
1185	0.46819439	0.043083551	0.001974253	51.05400844
246	0.511434511	0.042501157	0.012917459	10.45528455
507	0.570945946	0.042867313	0.004620362	21.73372781
1162	0.361206093	0.050461991	0.005273553	58.63683305
2175.003671	0.610535164	0.031344506	0.001166093	68.29967875
1873	0.552344441	0.039686397	0.001168589	74.33262146
692	0.466307278	0.053948511	0.00404287	37.33236994
819	0.500611247	0.040370564	0.002731296	33.06349206
870	0.661094225	0.030384463	0.002688614	26.43448276
801	0.414596273	0.046765825	0.003380367	37.45942572
1229	0.406012554	0.0451345	0.001557747	55.47030106
842.0094563	0.449044586	0.041734431	0.003924053	35.30732861
1981	0.566971952	0.030160069	0.001160519	59.74709743
1912	0.534825175	0.038072141	0.001541287	72.79393305
2355.44065	0.651383614	0.028658231	0.000785349	68.80841316
919	0.606200528	0.035006352	0.003014749	32.17083787
197	0.34929078	0.059805715	0.009740836	11.78172589
560	0.513761468	0.04653699	0.004991954	26.06071429
1910	0.479417671	0.036893726	0.001866851	70.46701571
915	0.467791411	0.042357789	0.003266017	38.75737705
1606	0.422965499	0.045358393	0.001365368	72.84557908
630	0.538001708	0.046621315	0.005948441	29.37142857
1602.019876	0.408940818	0.050321361	0.002318239	81.0173913
929	0.492055085	0.039519559	0.002597127	36.71367061
1906.026096	0.584324489	0.032508357	0.001574071	62.28601253
737.0027064	0.303865132	0.054638075	0.003743868	40.37753721
708	0.516034985	0.035079319	0.002443369	24.83615819
698	0.496797153	0.044002102	0.002204657	30.71346705
1514	0.514790887	0.037097177	0.002113854	56.1651255
2484.000805	0.453070895	0.037079083	0.001128863	92.17860016
1325	0.583443417	0.037169099	0.001341916	49.2490566
987	0.528372591	0.040302042	0.003246152	39.7781155
267	0.452542373	0.051831278	0.012751644	13.83895131
812	0.52624757	0.043704045	0.002796054	35.48768473
639	0.603969754	0.036909686	0.003338175	23.58528951
1905	0.534212002	0.031431307	0.001190439	59.87664042
541	0.418406806	0.053549769	0.01032902	28.97042514
1279	0.51017152	0.038810007	0.002099176	49.63799844
1871	0.491334034	0.0442259	0.001705788	82.74665954
791	0.383794275	0.04761372	0.006635024	37.66245259
2523	0.539217782	0.036774477	0.001278115	92.78200555
3099.016082	0.477499616	0.040586368	0.001423814	126.183017
2466	0.578601595	0.035020973	0.001265797	86.36171938
1003	0.43028743	0.049046281	0.00481913	49.19341974
1187	0.50403397	0.040902958	0.002451805	48.55181129
808	0.59455482	0.033642535	0.002490781	27.18316832
1689	0.515567766	0.036596288	0.002818657	61.81113085
2499.012764	0.562991242	0.035537511	0.00123396	89.09254089
645	0.473568282	0.040399014	0.003521499	26.05736434
1459.005468	0.550621001	0.037294971	0.00250301	54.56254272
1211.001649	0.490299111	0.043015042	0.002892675	52.1772465
187	0.357552581	0.05053047	0.007874906	9.449197861
2284.042646	0.595028483	0.034082689	0.001245437	78.32201915
1837.009767	0.530512378	0.038956925	0.002290381	71.79761259
804	0.56302521	0.03783941	0.002692295	30.42288557
1545.020605	0.510351627	0.042662168	0.001992063	66.25434643
139	0.338199513	0.062160344	0.012627249	8.64028777
301	0.520761246	0.032571384	0.00396271	9.803986711
1689	0.52600436	0.038951233	0.001639195	65.78863233
653	0.590950226	0.034401244	0.003354686	22.46401225
817	0.523047375	0.042761753	0.003331712	34.93635251
2674	0.614288996	0.030233798	0.000735892	80.84517577
2402.000832	0.510837229	0.040832182	0.001533332	98.16056572
625	0.707013575	0.02108672	0.002414821	13.1792
926	0.525241066	0.034788146	0.002524361	32.21382289
180	0.420560748	0.062592593	0.007379138	11.26666667
448	0.504504505	0.036800462	0.003546384	16.48660714
773	0.669844021	0.030273008	0.002413548	23.40103493

wavelet.LHL_gldzm_HighIntensityLargeDistanceEmphasis	wavelet.LHL_gldzm_SmallDistanceEmphasis	wavelet.LHL_glcm_SumVariance	wavelet.LHL_glcm_Homogeneity1	wavelet.LHL_glcm_Homogeneity2
1246.90795	1	4298.656305	0.235192766	0.146795862
799.4301619	1	2545.362153	0.275546192	0.187213036
629.8235294	1	1977.782929	0.230934354	0.142874326
1601.953795	0.99950495	5440.649163	0.222569928	0.134116426
253.93531	1	635.8570482	0.344034576	0.254512843
629.6336898	1	2061.542797	0.293605438	0.20518502
794.9605263	1	2611.682224	0.283591315	0.195879854
1107.930622	1	3578.175984	0.300289433	0.213877797
1618.188465	1	5410.632393	0.294994382	0.20812386
1459.098844	0.999132948	5020.259497	0.236942813	0.148618953
1384.12845	1	4734.768942	0.263747637	0.176158957
2002.363636	0.999362787	6979.871838	0.184777349	0.101191447
1439.494307	1	5047.714861	0.267262707	0.178178561
482.8948752	1	1507.112573	0.323728313	0.234304572
3883.709856	1	14233.61554	0.243764957	0.158566161
2281.096285	0.995234604	7819.605548	0.294055362	0.214076098
963.5760369	1	3276.946238	0.324381482	0.238004015
879.452882	1	2760.66061	0.318119325	0.230646777
384.158	1	1204.494302	0.319588734	0.228408283
985.3946648	0.999487004	3259.759644	0.280261885	0.190642462
1427.540333	0.998016398	4836.198772	0.249765725	0.162417613
1517.898277	0.996923708	5272.822478	0.240147673	0.151322598
897.5982796	0.997096774	2904.385716	0.245394887	0.157706184
3129.990446	1	11374.65315	0.226549005	0.145347168
1036.0997	0.999437781	3460.215619	0.269246657	0.181634082
1324.525967	1	4395.284817	0.203744402	0.117444886
949.3872153	1	3166.774847	0.263580444	0.174812689
1151.707547	1	3872.131465	0.230651325	0.141245917
1575.912392	0.997838617	5465.262378	0.266297657	0.178064923
760.5421592	1	2509.359339	0.342455385	0.2599095
632.8334162	0.999627051	2044.964171	0.286400731	0.195715801
562.7060588	0.99910018	1656.163258	0.380305111	0.29980888
1144.002532	1	3892.577424	0.28709711	0.197495658
732.5284553	1	2086.979237	0.285567878	0.196885385
631.183432	1	1964.313632	0.258731752	0.169161823
561.979346	1	1767.040011	0.353757375	0.269916134
1964.660395	0.999311611	6944.835857	0.220223662	0.133258328
2071.896423	1	7373.18151	0.252366166	0.161708095
547.1893064	1	1751.821027	0.306377721	0.214307267
1005.263736	1	3464.06908	0.277286073	0.189892546
1216.126437	1	4137.548844	0.200680582	0.115275173
780.7303371	1	2466.616921	0.326541759	0.240475599
1698.742067	1	5888.243775	0.31826149	0.231140493
602.2718676	0.99822695	1645.450205	0.305770162	0.216764293
2266.910146	1	8048.311661	0.239509393	0.153503222
1319.672594	1	4458.361514	0.256764802	0.169618173
3363.164515	0.992815494	11852.33487	0.204368721	0.118724469
936.9227421	1	3107.519386	0.223454495	0.135669537
448.5888325	1	1439.034446	0.356542551	0.271879771
536.7839286	1	1687.183844	0.276889849	0.186140558
1200.998429	1	3956.734444	0.287529544	0.19889648
774.6360656	1	2458.856887	0.297392251	0.211110079
1563.77335	1	5403.043719	0.312541065	0.223311222
413.9031746	1	1246.017531	0.264533896	0.17329387
908.5149068	0.998136646	3051.675355	0.325486331	0.238023881
911.5629709	1	3015.870665	0.284955094	0.200167474
1463.648747	0.998042797	4909.350529	0.234530255	0.14557414
593.0351827	0.998985115	1902.815287	0.389880483	0.31178384
2555.45339	1	9196.049348	0.263741853	0.178339314
1810.001433	1	6463.082971	0.280797111	0.191510528
1288.705416	1	4325.183069	0.268069787	0.179706063
1841.834674	0.999698311	6479.294375	0.303287518	0.220490964
2113.529057	1	7387.477788	0.238133764	0.149441433
891.3961499	1	2860.485142	0.26254416	0.173435894
289.2359551	1	811.9616656	0.311357762	0.219234852
872.0899015	1	2870.353005	0.260554612	0.168637329
957.2018779	1	3133.016881	0.23064091	0.14118068
2034.558005	1	6964.585142	0.25649191	0.168790865
400.6025878	1	1267.932554	0.315364685	0.225095889
1159.887412	1	3952.62478	0.264795427	0.175523911
1115.197221	1	3794.700451	0.282534337	0.193138249
415.7193426	1	1231.169861	0.337089112	0.250546922
1523.972255	1	5300.741606	0.255264782	0.165877592
1677.424574	0.998793824	5822.166901	0.286320175	0.199371922
1779.884023	1	6143.959771	0.241769274	0.153785669
575.6450648	1	1801.834528	0.316597807	0.227730071
950.5888795	1	3155.180246	0.273319447	0.185132832
1177.397277	1	3861.981233	0.225137462	0.136707768
1078.741267	1	3590.953824	0.259380379	0.171221277
1523.317112	0.998803351	5186.28881	0.240972385	0.152729251
1276.606202	1	4244.27606	0.293167547	0.209279567
1070.863295	0.99897471	3535.69613	0.248905005	0.161196366
738.8713932	0.999381698	2345.114114	0.289852334	0.202084295
1193.818182	1	4271.002869	0.370197701	0.291165481
1889.139687	0.997715405	6512.348146	0.234591617	0.14791526
1092.405317	0.998779164	3617.33411	0.258638606	0.167936184
1109.266169	1	3656.038481	0.255650977	0.167720346
999.4056665	0.998068255	3319.917787	0.274541733	0.184572124
292.6546763	1	1001.321897	0.380844558	0.295777341
2335.299003	1	8136.915705	0.257811524	0.171479079
1369.08881	1	4561.527793	0.264155852	0.17588375
1077.51608	1	3650.398864	0.239483448	0.150209642
752.7013464	1	2446.38077	0.266576013	0.176630322
3377.201571	1	12303.45716	0.217111411	0.130843369
1332.128952	0.99968802	4541.063781	0.273542577	0.183854735
2409.7104	1	8074.126843	0.172070061	0.095288257
1576.298056	1	5601.64402	0.259639545	0.171120732
678.5611111	1	2224.495888	0.338797073	0.249763338
1544.901786	1	5534.608876	0.283300931	0.198909913
1456.887451	1	4812.707468	0.206298703	0.122410452

wavelet.LHL_glcm_ClusterShade	wavelet.LHL_glcm_MaximumProbability	wavelet.LHL_glcm_Idmn	wavelet.LHL_glcm_SumVariance2	wavelet.LHL_glcm_Contrast	wavelet.LHL_glcm_DifferenceEntropy
-1058.247013	0.010795599	0.974762078	192.9671311	95.63856244	4.254160207
-440.336386	0.012162495	0.97711044	83.89708038	69.59496946	4.035343975
-249.7432207	0.009009107	0.964663449	139.0873044	88.88442854	4.246213598
-588.4544471	0.007887172	0.978651841	127.0923667	99.4648361	4.320036027
200.9821033	0.021026534	0.979601909	43.82036517	24.29763601	3.358718005
-272.519045	0.018433334	0.980535742	58.15461291	51.66297526	3.830942249
-521.1262965	0.016733682	0.973971035	88.6672975	66.15976124	3.948199247
120.4845239	0.029054228	0.985662453	106.1331359	58.68387236	3.873418562
379.8396258	0.014885643	0.98635976	152.0514168	62.4622727	3.925946247
-606.2188183	0.007735125	0.98345916	166.1176361	96.08023813	4.273562737
-199.644269	0.010775337	0.97884963	95.77188995	76.53620022	4.113900659
-932.4007633	0.005657566	0.97616315	200.3350557	164.3264244	4.660030575
-976.6755286	0.012301426	0.984020069	157.6257371	80.36178489	4.07829776
64.24222359	0.017216025	0.982065387	48.22607812	32.92769411	3.548379628
-1710.097394	0.010471384	0.984468529	236.1821326	164.8484458	4.385521998
-596.4117469	0.028086109	0.987748096	131.8724705	100.5803088	4.155043465
-448.4628457	0.022218208	0.984290317	67.33795559	45.46980314	3.659213355
811.6131916	0.016602976	0.989315552	100.1605893	46.1295146	3.700071299
-114.0498029	0.020963856	0.971865549	35.96243724	30.73945806	3.487641689
-164.2081685	0.01092722	0.979616659	80.03021501	56.64803715	3.900006456
-466.4770865	0.00998573	0.980749186	102.4556712	90.0043306	4.223018704
-1360.279393	0.00852493	0.980934758	145.3486506	87.97953558	4.208459929
-363.2259745	0.009456129	0.982647684	101.7583817	89.47652516	4.215322415
-3669.985902	0.013347086	0.979744508	421.2423105	213.7923779	4.574722986
-151.0159951	0.013997253	0.984400277	88.82204769	71.43304186	4.051384456
-231.7831087	0.00674848	0.972727527	166.5802948	120.9187597	4.438295092
-283.3564563	0.012047845	0.97784133	101.149248	65.90110931	4.041025282
-291.2862822	0.007075272	0.978834309	124.3980144	86.3384563	4.23293618
-538.6922034	0.009437268	0.983092512	128.384297	68.03925847	4.041505282
-76.9240684	0.0366103	0.985003614	55.0412979	39.74916156	3.605777015
-105.7475233	0.010823883	0.978624119	72.89150529	47.56036578	3.822327408
201.4192336	0.03455457	0.989017098	44.67545435	25.25829433	3.299228735
-242.9534128	0.016082504	0.983505793	70.84817587	54.14176221	3.883242256
1819.311253	0.020116968	0.982566149	131.3985036	62.75860717	3.882722821
-224.5164928	0.014115262	0.967724362	83.30657251	64.44890452	4.026022851
-111.1729103	0.029144703	0.983758188	45.50594462	32.01805342	3.475232654
-1205.482536	0.006407702	0.979569542	173.0078555	109.4945919	4.396922566
-742.9615443	0.010469579	0.984879287	99.43079367	74.01557979	4.099387006
-96.03978979	0.01617654	0.978240071	46.04185124	34.48307675	3.601332485
-363.4813604	0.015803056	0.978158001	80.58317897	63.34234463	3.982821554
-962.1314365	0.007378601	0.965279774	184.066119	123.7670295	4.471542103
-159.261143	0.024904488	0.985950225	56.58311055	41.49253515	3.657064674
-204.3509095	0.025879648	0.989199105	65.8028666	50.37088155	3.713328814
957.9048147	0.02120299	0.984639262	80.81793858	41.16533574	3.717717496
-1757.175465	0.010228062	0.982231832	199.000464	113.3265012	4.354256317
-412.646692	0.01155215	0.978165733	99.11378914	81.51575487	4.182091402
-1515.234267	0.006189661	0.9848666	216.2559415	137.9198973	4.538917033
-674.26244	0.009210712	0.97696139	130.9387235	97.2628832	4.319896738
-79.74080682	0.035715394	0.978211916	33.26227531	30.60014083	3.388440923
126.599179	0.013574378	0.971081154	63.22225699	51.93950697	3.874239279
393.6551293	0.01400198	0.988196819	125.0680897	56.66346888	3.915629983
-6.742774959	0.02153876	0.977946171	73.58245156	57.56404068	3.888792179
21.61279463	0.018412997	0.990402862	60.10085661	40.55265738	3.67695577
3.864899138	0.011414076	0.972577873	65.90426675	53.64054963	3.915935327
-236.7138856	0.020531563	0.983792635	49.45192495	39.805815	3.628043957
-231.3264904	0.024872793	0.97805027	84.1501572	62.98118035	4.001377568
-491.6692425	0.008111466	0.981537756	171.0864865	87.25170884	4.242328089
-71.25701613	0.044278526	0.985464794	33.51813838	25.98515369	3.287489427
-2000.384612	0.014363065	0.984724429	146.1438567	128.8005765	4.242847409
-218.1778945	0.014901354	0.98617821	61.54965463	57.44145695	3.880288323
-334.8325036	0.013198463	0.98465354	103.0226776	83.66122474	4.095361567
-307.1100946	0.024391957	0.986848305	101.7201282	70.93600897	3.987791218
-377.0624985	0.011290058	0.981714118	94.13902676	96.3304298	4.253557531
-387.1852624	0.010243884	0.972806698	101.8568869	74.92522622	4.081723793
72.58226671	0.019651848	0.976516252	45.5576526	29.55955704	3.488994042
138.1073135	0.011536097	0.978328018	72.79595216	56.88482709	3.943598017
-318.244766	0.009201021	0.972928726	107.390947	88.39303023	4.220586673
-399.5186516	0.010449934	0.986432413	187.3475565	91.93153263	4.209317713
-154.3418227	0.019183603	0.977733007	39.93937804	35.55313611	3.605972713
-175.2170368	0.010142531	0.985568467	97.69272491	72.53737796	4.066953281
-246.7738722	0.014656603	0.984904172	72.70119318	52.81147221	3.881653923
68.8345287	0.026332175	0.978938192	44.77863139	32.22508741	3.515144615
-490.1464911	0.008316808	0.98220572	115.9898711	74.67440885	4.119837236
-427.578921	0.014557705	0.986206296	84.3677938	67.83306509	3.980104976
-690.1803949	0.008768878	0.982069329	121.9296785	96.89952264	4.259038694
57.48272724	0.018728887	0.984857481	50.74137218	38.00427693	3.634466796
-161.8512553	0.012135696	0.97866231	80.50946285	66.75820258	4.03149866
686.8327699	0.007922191	0.98009283	157.6578874	91.06992696	4.262965809
-525.1172015	0.010988997	0.982962616	115.4395631	78.7582774	4.136787403
-251.0946419	0.008780126	0.982966028	125.9667541	84.98147516	4.226350582
-853.9984695	0.0261755	0.980936107	117.4980044	87.01553464	4.059149091
-642.8378915	0.010977499	0.976877334	105.4503253	87.0264155	4.207776083
-113.1324235	0.017113363	0.983582234	69.52047357	53.67935252	3.890527625
-301.0304796	0.046397423	0.98648409	43.28786288	44.3601889	3.386709888
-528.9625006	0.009452607	0.980598393	132.217636	112.1710468	4.339129899
-284.4001591	0.009798279	0.982246299	105.3338289	70.95408089	4.053400191
-401.0549408	0.014589536	0.978869679	100.6512022	81.56821276	4.129316198
-206.1940101	0.013369616	0.980130677	77.17963294	56.4574756	3.948884984
-42.82574969	0.035758741	0.977216192	25.28599997	17.38486556	3.060173523
-60.91934144	0.017062243	0.984391233	156.0572067	126.5390218	4.194170262
-315.7226685	0.010750332	0.980070385	102.54655	77.13895651	4.117740315
-136.9604007	0.010061992	0.977784971	137.5507389	79.05719624	4.162140801
-387.8146837	0.01526061	0.974289273	71.07369883	60.50559045	3.978998214
-1085.343441	0.008600295	0.98958346	195.5497044	132.5431195	4.465816247
-153.1607302	0.011276373	0.984449927	83.56582419	61.07357404	3.971770301
-3200.888038	0.006728891	0.968398948	409.8969412	301.1362785	4.968343933
-1078.411168	0.01006956	0.978276293	142.8363088	85.47903943	4.131252971
-105.0201933	0.028831224	0.981914079	35.97433284	29.51909457	3.369353439
-1009.425145	0.022269444	0.979827592	103.1217353	89.93832233	4.069726188
-863.8864338	0.008321292	0.969420835	179.9595984	143.166122	4.523165987

wavelet.LHL_glcm_InverseVariance	wavelet.LHL_glcm_Entropy	wavelet.LHL_glcm_Dissimilarity	wavelet.LHL_glcm_DifferenceVariance	wavelet.LHL_glcm_Idn	wavelet.LHL_glcm_Idm	wavelet.LHL_glcm_Correlation
0.157813773	9.054463038	7.154095314	42.82820024	0.898327312	0.146795862	0.344437967
0.190530195	8.562168498	5.93474413	33.65179763	0.905529731	0.187213036	0.099262548
0.1499039	8.993572662	7.166677101	36.235016	0.87765067	0.142874326	0.229248632
0.142951514	9.2399207	7.455810899	42.72377817	0.903722831	0.134116426	0.127665973
0.258466172	7.485996158	3.656486296	10.68932277	0.906783967	0.254512843	0.28254394
0.214019691	8.163165661	5.171703524	23.96778214	0.911254905	0.20518502	0.067346899
0.208632915	8.360568136	5.694983478	32.63120397	0.901156098	0.195879854	0.135303476
0.222183802	8.3218814	5.273774148	30.01792925	0.925989737	0.213877797	0.291387465
0.208884535	8.764271024	5.491699082	31.00327971	0.927344855	0.20812386	0.409844033
0.154011817	9.4040785	7.144102962	43.41842026	0.9164853	0.148618953	0.267375563
0.18155211	8.766073476	6.298147479	35.96449651	0.907733572	0.176158957	0.109009129
0.107416133	9.597346043	9.698909575	67.29207399	0.897738421	0.101191447	0.099449273
0.1866246	8.971751083	6.181669283	40.67720389	0.921608797	0.178178561	0.322831412
0.238806498	7.951104144	4.198500225	14.78308312	0.913124907	0.234304572	0.189363393
0.167793389	9.052360936	8.183918296	92.58831972	0.928215186	0.158566161	0.175176825
0.21285829	8.881420317	6.47190385	56.6639881	0.935391503	0.214076098	0.126366245
0.23949634	7.955591539	4.607468493	22.83311159	0.923263179	0.238004015	0.217388166
0.236154265	8.349738563	4.651934839	23.6600038	0.935519038	0.230646777	0.363194287
0.238580704	7.622786998	4.120283426	13.05691691	0.891275575	0.228408283	0.091483205
0.198895099	8.658932175	5.463249394	25.40833821	0.908344632	0.190642462	0.177376233
0.166360119	9.179653006	6.836619787	41.30819012	0.911451756	0.162417613	0.075772099
0.154889298	9.153233088	6.926268337	37.62057557	0.909912036	0.151322598	0.252301996
0.161433093	9.112322422	6.920448663	39.09814162	0.91470419	0.157706184	0.069663101
0.152503238	9.17344779	9.528511903	112.7173985	0.917210962	0.145347168	0.337860816
0.186194307	8.766698424	6.089103051	32.61395484	0.919852405	0.181634082	0.12041972
0.124412895	9.318700132	8.375452627	48.50244222	0.890486003	0.117444886	0.165683132
0.182269179	8.761116363	6.018120766	28.85121042	0.903328967	0.174812689	0.216321636
0.147952854	9.312528824	7.01450434	35.90011511	0.903289333	0.141245917	0.188592053
0.184008418	9.149318817	6.046061395	29.8104726	0.915526841	0.178064923	0.317876001
0.266616068	7.866709197	4.284123532	20.78901045	0.925552286	0.2599095	0.168250509
0.203071801	8.637340941	5.120022467	20.55942482	0.904639574	0.195715801	0.216887458
0.300195163	7.501146008	3.405588818	13.17273606	0.935559237	0.29980888	0.276852454
0.203520988	8.428027085	5.319719087	25.04996207	0.917551237	0.197495658	0.138439355
0.201724425	7.706356084	5.574520812	30.5523372	0.917285396	0.196885385	0.339292064
0.17735629	8.40738063	6.043293899	27.47227247	0.883663024	0.169161823	0.128390378
0.273394942	7.697005249	3.879033416	16.55702274	0.921722094	0.269916134	0.177071098
0.138124093	9.62779375	7.815026826	46.62130449	0.905989027	0.133258328	0.233137219
0.17043258	8.924991228	6.268511414	34.06247661	0.919612822	0.161708095	0.152822488
0.223416917	7.943961105	4.408594724	14.73795917	0.902747447	0.214307267	0.14533295
0.200175104	8.532057637	5.747170328	29.55694716	0.906119959	0.189892546	0.125295805
0.121251185	9.346899142	8.558850843	48.0797898	0.876840665	0.115275173	0.200605701
0.247702583	7.881840539	4.466339205	21.09116858	0.9263015	0.240475599	0.156268741
0.24067514	8.021808688	4.72593788	27.23878246	0.936742866	0.231140493	0.143573555
0.222126503	8.122643423	4.717738149	18.44413796	0.918989496	0.216764293	0.312174006
0.158944923	9.518415588	7.531400737	53.98680643	0.91625022	0.153503222	0.272028052
0.17517941	9.019024895	6.552094771	37.78006777	0.905817477	0.169618173	0.10257979
0.123956677	9.815444218	8.696405169	59.81967269	0.918642183	0.118724469	0.232417188
0.141727092	9.068374993	7.46893081	40.39686063	0.899371789	0.135669537	0.156997127
0.269790657	6.968633095	3.811869167	15.43639953	0.909887395	0.271879771	0.05312986
0.188263564	8.283449489	5.415387066	21.83058904	0.889375152	0.186140558	0.101518332
0.203178739	8.876277991	5.389906442	26.94294802	0.929873792	0.19889648	0.37331313
0.219298508	8.352414582	5.317866511	28.4691476	0.908200806	0.211110079	0.124686337
0.227752631	8.206473927	4.561397965	19.21514929	0.936061189	0.223311222	0.195228102
0.179839234	8.419545674	5.611094075	21.75679191	0.889932537	0.17329387	0.107672418
0.244232522	7.983784838	4.393473048	19.77776485	0.920581014	0.238023881	0.125282482
0.199414539	8.620872107	5.768371048	28.66828461	0.905762233	0.200167474	0.153301005
0.151570526	9.405706551	6.994557746	37.04575055	0.91000434	0.14557414	0.332219238
0.307071649	7.20794504	3.378236386	14.1800872	0.928142367	0.31178384	0.129962532
0.183922658	8.648445957	7.179880765	75.43762907	0.928822307	0.178339314	0.057481035
0.197988583	8.272005192	5.487665118	25.84036043	0.923554434	0.191510528	0.036748382
0.186805489	8.785406526	6.275932312	42.69985877	0.923647747	0.179706063	0.109394256
0.222948228	8.704642096	5.638046989	38.10696447	0.930880674	0.220490964	0.175167081
0.153700714	8.937973082	7.111416677	43.97283455	0.91278908	0.149441433	-0.007330766
0.179449829	8.816958772	6.223130971	35.08303853	0.896353955	0.173435894	0.162926092
0.230847753	7.569543849	4.177398131	11.66998273	0.897337754	0.219234852	0.211311502
0.175052931	8.534325403	5.730736073	23.3418833	0.901751091	0.168637329	0.129864183
0.143716763	8.818139099	7.090853389	36.58813789	0.891931746	0.14118068	0.108164034
0.17532172	9.284038222	6.714535188	45.59052237	0.926793148	0.168790865	0.344127111
0.226553592	7.705560626	4.392441533	15.68500713	0.903570276	0.225095889	0.067474649
0.178581124	8.835446418	6.116488148	33.91872575	0.922616529	0.175523911	0.160299892
0.2038825	8.593142754	5.333060597	23.53531741	0.919567729	0.193138249	0.165812956
0.257791439	7.761462936	4.052527712	15.28520665	0.908619474	0.250546922	0.169889951
0.170970758	9.166342899	6.3283318	33.50046868	0.913331791	0.165877592	0.222606337
0.207504999	8.708109637	5.695218312	34.00859082	0.926986002	0.199371922	0.111477824
0.160198486	9.204898131	7.077052755	44.73992374	0.914414683	0.153785669	0.123394307
0.238105587	7.970587475	4.428212533	17.96830403	0.920909183	0.227730071	0.143049583
0.190577825	8.67828931	5.892307007	31.3529988	0.907208978	0.185132832	0.097495779
0.143164537	9.110383865	7.273606879	36.64447111	0.90531105	0.136707768	0.266737826
0.178558653	9.02503765	6.381617976	36.92357879	0.916566379	0.171221277	0.19588912
0.158907258	9.359901393	6.863100848	36.53359056	0.913843899	0.152729251	0.205886524
0.208445967	8.221948201	6.12233769	48.17471063	0.919264304	0.209279567	0.157116937
0.165016763	8.945047577	6.785810418	39.28275685	0.90289408	0.161196366	0.109921287
0.205905164	8.446856251	5.335771462	24.37595722	0.917214134	0.202084295	0.135949554
0.293049169	6.948029812	3.985287632	27.84296929	0.93526656	0.291165481	-0.005033496
0.157485558	9.298244973	7.544917196	52.43554462	0.911970993	0.14791526	0.088189184
0.174311454	8.988322118	6.098701815	32.88432613	0.913778629	0.167936184	0.198224067
0.180460634	8.634697929	6.513606868	37.55813634	0.907781523	0.167720346	0.112097725
0.191207621	8.662088391	5.564471319	24.95880679	0.908039342	0.184572124	0.161421535
0.303192181	6.760649853	3.026067178	7.846688176	0.903288765	0.295777341	0.18441496
0.179882309	8.139446582	7.277063569	71.17675048	0.926919047	0.171479079	0.096019281
0.181452392	8.924252427	6.272272168	36.91613864	0.910875031	0.17588375	0.149103634
0.158291749	8.845997442	6.71345338	33.11210596	0.901360511	0.150209642	0.272386675
0.184015441	8.446132	5.801363054	25.87822885	0.895928871	0.176630322	0.091350825
0.140028804	9.647302961	8.234265334	62.17556411	0.933612046	0.130843369	0.198104884
0.192371136	8.802384756	5.661265807	28.35445027	0.919170327	0.183854735	0.157848797
0.099088983	9.474971585	12.54025815	135.3792401	0.889165279	0.095288257	0.165228051
0.177600576	8.941720436	6.512156527	40.97718952	0.908332382	0.171120732	0.259177883
0.260602508	7.03412912	3.844778415	14.31610883	0.914557111	0.249763338	0.103957777
0.194996797	8.222504601	6.339158336	47.19349636	0.915228036	0.198909913	0.071663977
0.129034467	9.199578104	8.865646608	61.02761836	0.887675585	0.122410452	0.125234112

wavelet.LHL_glcm_Autocorrelation	wavelet.LHL_glcm_SumEntropy	wavelet.LHL_glcm_AverageIntensity	wavelet.LHL_glcm_Energy	wavelet.LHL_glcm_SumSquares	wavelet.LHL_glcm_ClusterProminence	wavelet.LHL_glcm_SumAverage
1240.436822	5.670076573	35.49216072	0.002585278	69.24773772	155941.9445	69.74456007
752.391756	5.116190261	27.77694674	0.00411111	36.33470244	34415.19743	54.72872098
597.7746058	5.502881725	24.72612977	0.002568354	56.96194977	66559.49633	48.38194758
1540.939201	5.439492419	39.82126856	0.00230103	54.99592534	74107.66632	78.333388
214.3676045	4.615103655	14.54942233	0.007978758	17.64539072	10375.05663	28.94549163
616.2995799	4.826302559	25.17749504	0.005647777	26.82693633	17890.38498	49.5852557
770.3794932	5.081746568	28.13661507	0.004448785	36.66934054	41020.52248	55.30539706
1037.604518	5.129861334	32.43485557	0.006120428	40.48929453	73250.96902	64.05349556
1543.216517	5.479424567	39.48416379	0.003919775	53.16212378	96911.83328	77.99433227
1435.13483	5.631060789	38.29395924	0.002152817	63.79239731	120100.2876	75.30231843
1348.554907	5.204361983	37.16374767	0.003362949	41.31338842	44987.42861	73.31405638
1947.654185	5.723831374	44.96762473	0.001653441	88.14935503	177907.833	88.05942537
1440.429122	5.46697146	38.21737314	0.003400087	58.25830716	155574.5726	75.39451041
465.115354	4.75988694	21.64634252	0.006121244	19.78579913	10114.92167	42.95508151
3859.777787	5.656793443	62.78509897	0.0029273	97.34246301	439234.113	123.9662851
2170.321303	5.325754306	47.08859151	0.005190741	56.50594568	125542.0409	93.00520796
951.4523762	4.860282502	31.02118877	0.006866619	27.49455191	31512.03571	61.51319538
817.4022389	5.125918239	28.67882841	0.005207088	37.37551164	67899.10758	56.70561643
375.916909	4.526273918	19.64484963	0.007115422	16.62796516	6163.246869	38.70965646
952.0077125	5.130309357	31.10422222	0.003611519	33.0624026	26847.97621	61.51908117
1375.193191	5.281163998	37.61644383	0.002920514	46.75547404	56214.21853	74.08317169
1500.286076	5.490271949	39.14648866	0.002457822	56.79733284	98556.44291	77.09536538
850.3786287	5.277295929	29.66764753	0.002798842	46.36074328	49208.21045	58.21680067
3115.300507	6.04049244	56.32103321	0.002548237	153.1203886	955251.6501	110.6926249
1003.921967	5.168954424	32.1062461	0.003748024	39.26959366	37477.85318	63.23198601
1259.703005	5.634112487	36.0535908	0.001957319	70.35362666	98847.86652	70.66168054
928.2547667	5.276378457	30.74471415	0.003343524	40.60249021	45359.64659	60.64417514
1121.262462	5.467245846	33.93813868	0.002164602	51.54213289	59441.61435	66.6855277
1557.299311	5.487822882	39.75795996	0.002597267	48.03811088	59850.21971	78.54060112
739.6200756	4.709965661	27.43749534	0.009183769	23.07512928	20495.62374	54.25084231
618.8764272	5.091186839	25.05774832	0.003788954	29.98412972	20667.80625	49.49872654
504.9713151	4.58286565	22.49318809	0.010952263	17.74990091	15637.35998	44.72607327
1120.12299	4.991606337	33.81307072	0.004912195	30.26242027	26197.31555	66.81095557
623.3862185	5.020308402	24.99177846	0.006650029	48.01240122	130006.7172	49.2385842
592.4890296	5.11798251	24.59856036	0.003933158	36.38872838	26203.67763	48.48740497
535.4172056	4.640781078	23.21273529	0.009238115	18.88367628	12621.32302	46.13195933
1950.463813	5.676935871	44.68479498	0.001804664	68.17419507	126746.4132	87.96757509
2055.348838	5.245418187	45.76926555	0.003141848	41.79205076	64231.03895	90.53159873
532.728746	4.735578919	23.17859072	0.005706374	18.91122708	8160.729861	46.03627317
1004.978582	5.099052788	32.05768508	0.004133411	34.4415983	31243.32233	63.26650837
1190.223557	5.685027134	35.03548581	0.00193725	73.93674722	117111.5177	68.56012584
728.2755883	4.740934932	27.20159204	0.007566974	23.32481455	22589.69004	53.83288702
1648.669737	4.807709194	40.94953154	0.007506092	28.00247877	39197.02432	81.11209547
504.2798596	4.912778146	22.47639322	0.006190734	30.74174551	41039.52755	44.46502812
2245.046804	5.714915552	47.81898001	0.002248224	75.49094721	204021.2926	94.31009176
1274.551266	5.254141982	36.15322417	0.003202034	43.66715399	48030.44726	71.27827358
3250.669227	5.815041409	57.67445085	0.001607771	85.9638478	228709.8187	113.6849002
907.6927926	5.417736546	30.60648672	0.002587188	55.58845613	76807.36815	59.97516053
437.0181648	4.284167766	21.08776161	0.012536068	14.95652193	8277.941979	41.77747403
514.0895126	4.92456403	22.89163271	0.004398263	28.33564327	15453.38789	45.22212602
1148.836275	5.381614674	34.04534844	0.003665934	44.72477556	81189.04994	67.2816328
727.8182144	4.968103141	27.30379832	0.005529069	32.32378017	28681.35527	53.80691439
1524.891389	4.878869744	39.32552785	0.005719416	24.86710862	21354.10014	77.97387616
390.3082532	5.004209387	20.04326638	0.003780329	28.63408468	14071.66485	39.35596451
887.7927194	4.718353354	30.04102102	0.006847886	21.04041011	16349.0332	59.51064775
884.1850603	5.147020313	30.05641029	0.004382877	35.66531134	29619.44054	59.29203865
1407.889265	5.648040685	37.84409741	0.002200129	63.80707643	132650.7802	74.48256347
568.4857342	4.371619243	23.91090175	0.013421455	14.47350464	7798.069859	47.60671889
2526.6124	5.313585427	50.84613086	0.003928884	65.13867815	207300.1405	100.4442097
1801.575507	4.856743047	42.86376239	0.004803482	28.39049859	23986.6437	84.8656814
1236.916926	5.223708796	35.62241079	0.003787351	45.2599841	66094.04379	70.20140914
1815.702665	5.18198683	42.96796944	0.00540783	41.92709213	66172.91474	85.04106996
2050.999749	5.18748775	45.896833	0.003053456	46.19726614	51581.68475	90.58784066
842.4945519	5.296336362	29.34960671	0.003166871	42.35628265	39582.7056	57.81880649
265.4147572	4.652934613	16.31044622	0.006936986	19.16758037	9271.522005	32.33612766
843.5237313	5.057642791	29.3876724	0.003690956	31.40653573	20331.17945	57.94942719
914.4669284	5.317255447	30.70987154	0.002833032	46.47811635	41308.64157	60.32263377
1958.284754	5.640233143	44.56093519	0.002804566	67.63097929	188122.2314	87.96399751
393.509846	4.576257067	20.05392031	0.007111556	17.87583235	8731.81708	39.61875148
1139.522176	5.238886434	34.15918647	0.003264912	41.64905292	52086.50336	67.32671577
1095.454357	5.036744129	33.448106	0.004157579	30.75907013	27499.68992	66.04480118
385.3904585	4.658225429	19.7867553	0.008210082	18.36238166	9863.954992	39.10181202
1508.281933	5.401641891	39.19819557	0.002660784	46.27836878	58275.25536	77.40630464
1638.140542	5.097479998	40.87215708	0.00449957	36.59882178	46415.67453	80.84550299
1727.833293	5.382032932	42.07878328	0.002706813	52.40400627	84654.18625	82.98353735
545.822751	4.743114395	23.5412918	0.006533409	22.3199491	16122.94948	46.58891164
920.4802303	5.1156542	30.68990375	0.003784151	35.92055961	29284.6222	60.56516311
1119.539284	5.55541893	33.83071579	0.002348693	61.28044742	105402.7344	66.41650079
1043.119717	5.356688784	32.68201119	0.003195319	47.33901537	62720.16686	64.30998312
1477.467393	5.472757443	38.90599168	0.002303534	51.7027273	62554.82523	76.60833264
1210.245955	5.117707071	35.15870336	0.006160121	49.96002916	99834.65249	69.35716718
1023.136776	5.26083977	32.46276115	0.003235784	46.51265141	59453.3358	63.82868948
698.2648918	4.996126002	26.73274148	0.004769573	29.48247385	22520.99794	52.69914052
1203.482713	4.369684092	34.96524981	0.013182358	20.89977541	21983.41388	69.3895266
1824.604223	5.437507224	43.31008478	0.002587236	58.87703953	92776.48491	85.31307521
1051.059666	5.312167778	32.76405665	0.002862734	42.84700285	54406.63711	64.57407335
1055.359145	5.197879189	32.94024884	0.003926162	43.94049093	48959.61803	64.82444673
967.7737806	5.106422292	31.40088087	0.003931311	32.6537247	26118.40599	62.05127009
316.1823809	4.211152066	17.74946907	0.012659902	10.79487025	2946.328466	35.45137497
2243.399459	5.237633836	47.95428193	0.005083537	67.67923056	177526.1096	94.57189576
1304.78184	5.292000311	36.51769543	0.003274961	43.21057476	50581.98728	72.06737354
1062.080294	5.459904972	32.90719781	0.00291234	53.62069895	72760.40834	64.72691714
724.3589996	4.992506691	27.31105224	0.004314887	31.91931591	23380.94279	53.72945212
3363.681153	5.686899556	58.65312166	0.001983768	79.43291622	254331.019	115.7223364
1298.683125	5.15375203	36.36911541	0.003468227	34.93846432	35038.00661	71.91806425
2222.004727	6.1518165	48.02850564	0.001714224	174.4882352	606088.7535	93.69660872
1588.238136	5.462293792	40.19301735	0.002984287	55.05527115	109024.6687	79.34340597
656.3360521	4.393121412	25.68680234	0.010487039	15.88967653	8482.531393	51.17410073
1555.285593	5.093041174	39.93235535	0.005450329	44.81682027	80276.91633	78.79006167
1367.458006	5.644966361	37.66607033	0.002241133	78.28242594	128474.6028	73.70825634

wavelet.LHL_glcm_Imc2	wavelet.LHL_glcm_Imc1	wavelet.LHL_glcm_DifferenceAverage	wavelet.LHL_glcm_Id	wavelet.LHL_glcm_ClusterTendency	wavelet.LHL_firstorder_InterquartileRange	wavelet.LHL_firstorder_Skewness
0.926547579	-0.19517105	7.154095314	0.235192766	192.9671311	236.2542993	-0.584649075
0.781707905	-0.104807709	5.93474413	0.275546192	83.89708038	147.6857559	-0.759629568
0.895626086	-0.16542205	7.166677101	0.230934354	139.0873044	225.8300199	-0.124186032
0.803396443	-0.106664294	7.455810899	0.222569928	127.0923667	223.9636726	-0.532379937
0.776893083	-0.11859235	3.656486296	0.344034576	43.82036517	110.2871789	0.67957075
0.76353316	-0.103314279	5.171703524	0.293605438	58.15461291	130.5819343	-0.578991124
0.885857915	-0.168572895	5.694983478	0.283591315	88.6672975	170.1092556	-0.664157387
0.857665051	-0.149019156	5.273774148	0.300289433	106.1331359	143.2109747	-0.133528378
0.888731424	-0.166721215	5.491699082	0.294994382	152.0514168	191.0130399	0.092177858
0.827312086	-0.117013588	7.144102962	0.236942813	166.1176361	229.2404479	-0.321696562
0.827313944	-0.123695857	6.298147479	0.263747637	95.77188995	171.7094393	-0.506957904
0.907463583	-0.16608864	9.698909575	0.184777349	200.3350557	291.2019596	-0.30700425
0.854484429	-0.137670957	6.181669283	0.267262707	157.6257371	173.3956555	-0.575741486
0.718125522	-0.090075078	4.198500225	0.323728313	48.22607812	126.7989238	-0.125557156
0.9470127	-0.227205519	8.183918296	0.243764957	236.1821326	203.3777721	-0.837983491
0.840113096	-0.133101227	6.47190385	0.294055362	131.8724705	155.2521767	-0.378596191
0.822608848	-0.135913718	4.607468493	0.324381482	67.33795559	116.1360231	-0.838887739
0.826717966	-0.131250453	4.651934839	0.318119325	100.1605893	142.4576044	0.621598794
0.738382385	-0.099540605	4.120283426	0.319588734	35.96243724	123.9390925	-0.597868624
0.738572171	-0.090024636	5.463249394	0.280261885	80.03021501	163.1847261	-0.420321102
0.671491001	-0.0666778	6.836619787	0.249765725	102.4556712	176.2310395	-0.498342353
0.846956504	-0.130850992	6.926268337	0.240147673	145.3486506	229.5794272	-0.677426872
0.73710741	-0.087134557	6.920448663	0.245394887	101.7583817	185.3339042	-0.40744607
0.984071478	-0.318324802	9.528511903	0.226549005	421.2423105	278.3051381	-0.492508455
0.780694082	-0.105026801	6.089103051	0.269246657	88.82204769	161.9103751	-0.256193647
0.900005413	-0.164194451	8.375452627	0.203744402	166.5802948	279.3460321	-0.17587678
0.811665099	-0.116053846	6.018120766	0.263580444	101.149248	158.1228708	-0.373077088
0.752358309	-0.086707221	7.01450434	0.230651325	124.3980144	222.3510461	-0.245125825
0.765255035	-0.096589167	6.046061395	0.266297657	128.384297	210.8451789	-0.354306619
0.743062072	-0.099256573	4.284123532	0.342455385	55.0412979	103.2581977	-0.310382462
0.646649187	-0.064489057	5.120022467	0.286400731	72.89150529	160.5198845	-0.18331551
0.67052788	-0.082064362	3.405588818	0.380305111	44.67545435	91.00293074	0.615392007
0.732339528	-0.088996246	5.319719087	0.28709711	70.84817587	139.0321712	-0.512001121
0.947369598	-0.258346194	5.574520812	0.285567878	131.3985036	134.0791731	0.885065864
0.872222474	-0.157104395	6.043293899	0.258731752	83.30657251	168.2296847	-0.438026556
0.685929896	-0.081805047	3.879033416	0.353757375	45.50594462	98.36390529	-0.631189093
0.783577228	-0.095074581	7.815026826	0.220223662	173.0078555	247.3690556	-0.555780871
0.723473925	-0.080314403	6.268511414	0.252366166	99.43079367	174.8239038	-0.862007637
0.706848394	-0.084117007	4.408594724	0.306377721	46.04185124	128.6335923	-0.543054836
0.80190047	-0.114927176	5.747170328	0.277286073	80.58317897	154.1086185	-0.68577721
0.904238587	-0.167317401	8.558850843	0.200680582	184.066119	282.0300332	-0.44224595
0.763586713	-0.105843094	4.466339205	0.326541759	56.58311055	112.4725811	-0.365823416
0.753513831	-0.101318167	4.72593788	0.31826149	65.8028666	113.512878	-0.657051501
0.799094227	-0.120453018	4.717738149	0.305770162	80.81793858	135.256591	1.056365539
0.839957626	-0.12350792	7.531400737	0.239509393	199.000464	213.0971167	-0.708385185
0.729452733	-0.081620422	6.552094771	0.256764802	99.11378914	166.855789	-0.468497767
0.823058641	-0.109709825	8.696405169	0.204368721	216.2559415	260.3791349	-0.579124027
0.865667105	-0.141806063	7.46893081	0.223454495	130.9387235	214.7297664	-0.413360151
0.830217613	-0.154308802	3.811869167	0.356542551	33.26227531	85.55091134	-0.677074349
0.816202263	-0.125318243	5.415387066	0.276889849	63.22225699	161.3589973	-0.018687098
0.794190136	-0.107640695	5.389906442	0.287529544	125.0680897	167.25094	0.161044281
0.79712562	-0.114807568	5.317866511	0.297392251	73.58245156	147.503609	-0.154702988
0.676245795	-0.074589936	4.561397965	0.312541065	60.10085661	130.255657	-0.278719076
0.783869641	-0.107074841	5.611094075	0.264533896	65.90426675	173.831063	-0.09270132
0.662671385	-0.071755692	4.393473048	0.325486331	49.45192495	115.8385773	-0.846371503
0.792109951	-0.109356777	5.768371048	0.284955094	84.1501572	158.4758895	-0.389706973
0.818637657	-0.112009705	6.994557746	0.234530255	171.0864865	221.4118755	-0.21776455
0.702292444	-0.094106921	3.378236386	0.389880483	33.51813838	82.78142631	-0.581282158
0.906141445	-0.181911741	7.179880765	0.263741853	146.1438567	163.3056286	-1.02366636
0.802108601	-0.119518863	5.487665118	0.280797111	61.54965463	151.4355408	-0.800919859
0.80587007	-0.114969806	6.275932312	0.268069787	103.0226776	163.4815632	-0.559688588
0.747211812	-0.092756462	5.638046989	0.303287518	101.7201282	135.9139381	-0.479779837
0.807573953	-0.112565617	7.111416677	0.238133764	94.13902676	183.4918249	-0.575810828
0.818559774	-0.118768524	6.223130971	0.26254416	101.8568869	183.9068648	-0.636641074
0.835255216	-0.147484457	4.177398131	0.311357762	45.5576526	127.5573348	0.097470179
0.778556803	-0.104766278	5.730736073	0.260554612	72.79595216	173.1726966	-0.143423453
0.87499507	-0.151909389	7.090853389	0.23064091	107.390947	206.5936721	-0.483554608
0.843435865	-0.126322885	6.714535188	0.25649191	187.3475565	186.2941705	-0.248963036
0.718438058	-0.09059221	4.392441533	0.315364685	39.93937804	111.9085123	-0.837864959
0.785837567	-0.104772568	6.116488148	0.264795427	97.69272491	169.1787641	-0.257753145
0.682658506	-0.07281395	5.333060597	0.282534337	72.70119318	154.9988853	-0.356874722
0.710171261	-0.088351557	4.052527712	0.337089112	44.77863139	107.0913575	-0.119399942
0.718136743	-0.077830189	6.3283318	0.255264782	115.9898711	196.7187792	-0.478649368
0.677037934	-0.073017277	5.695218312	0.286320175	84.3677938	137.9156349	-0.642206111
0.760413326	-0.092650799	7.077052755	0.241769274	121.9296785	188.6764997	-0.62268912
0.700044068	-0.08297626	4.428212533	0.316597807	50.74137218	117.0880554	0.075237721
0.759935562	-0.095049879	5.892307007	0.273319447	80.50946285	154.8076846	-0.369026092
0.892294011	-0.161219156	7.273606879	0.225137462	157.6578874	226.3689761	0.124257849
0.77386075	-0.097474368	6.381617976	0.259380379	115.4395631	169.7503347	-0.424572263
0.715305903	-0.075079832	6.863100848	0.240972385	125.9667541	206.9958483	-0.219132019
0.90866038	-0.192908544	6.12233769	0.293167547	117.4980044	121.9871913	-0.659005796
0.788498457	-0.104614561	6.785810418	0.248905005	105.4503253	172.0150647	-0.545652794
0.729635319	-0.087117781	5.335771462	0.289852334	69.52047357	139.1468844	-0.306170927
0.866646766	-0.185549805	3.985287632	0.370197701	43.28786288	88.74887122	-1.819467412
0.790610852	-0.103128608	7.544917196	0.234591617	132.217656	198.3149385	-0.584628977
0.756412546	-0.091349741	6.098701815	0.258638606	105.3338289	192.2137999	-0.358436261
0.864765328	-0.14838587	6.513606868	0.255650977	100.6512022	167.3402439	-0.440926908
0.686970941	-0.071594258	5.564471319	0.274541733	77.17963294	148.7552764	-0.419796768
0.823478838	-0.157848784	3.026067178	0.380844558	25.28599997	101.903939	-0.728992488
0.966850878	-0.288402939	7.277063569	0.257811524	156.0572067	159.2527365	-0.388718247
0.754513944	-0.090991478	6.272272168	0.264155852	102.54655	163.6183574	-0.530155397
0.903971388	-0.176589785	6.71345338	0.239483448	137.5507389	208.9928953	-0.254365128
0.782176645	-0.107220548	5.801363054	0.266576013	71.07369883	155.208939	-0.635769341
0.815638874	-0.109084962	8.234265334	0.217111411	195.5497044	230.7225202	-0.495289182
0.684542644	-0.070756744	5.661265807	0.273542577	83.56582419	163.1393664	-0.480867489
0.986118933	-0.319438305	12.54025815	0.172070061	409.8969412	384.5140639	-0.292080914
0.874432249	-0.151825679	6.512156527	0.259639545	142.8363088	196.0534487	-0.785291275
0.860838202	-0.176379494	3.844778415	0.338797073	35.97433284	101.959612	-0.823627597
0.91863875	-0.204156628	6.339158336	0.283300931	103.1217353	143.8381076	-1.184237318
0.931914732	-0.199903198	8.865646608	0.206298703	179.9595984	257.9895794	-0.356808471

wavelet.LHL_firstorder_Uniformity	wavelet.LHL_firstorder_MeanAbsoluteDeviation	wavelet.LHL_firstorder_Energy	wavelet.LHL_firstorder_RobustMeanAbsoluteDeviation	wavelet.LHL_firstorder_Median
0.038326876	159.3228885	58832111.89	101.0128638	58.72463057
0.059226	107.4066287	45892798.29	63.42071544	16.27694657
0.040804081	143.5449028	38658207.46	95.35120388	16.10719094
0.04119468	143.717859	89992048.6	93.35464175	31.39099374
0.078899535	77.07625586	11512762.72	47.5982939	25.59317774
0.066757012	93.50189018	27211225.2	57.15134617	20.95263842
0.051421841	118.2993486	27892567.94	73.96486571	33.52917278
0.060808926	111.8622167	51309494.98	62.80745559	33.50367606
0.047104348	138.0737119	79246186.78	83.36186203	43.0706464
0.038845271	152.3596775	131574150.6	97.73754836	54.40951434
0.05140343	117.9904945	48559377.79	73.40971779	34.17786935
0.03190906	183.29472	96687999.21	121.428136	9.629163002
0.048658469	135.2599352	114123636.7	76.68635723	33.54648494
0.070272444	83.85325093	24357899.88	54.00971663	24.5506287
0.04126438	167.4069782	99532152.85	90.75483139	34.05966274
0.056641657	125.6694933	146256362.7	70.39661934	17.80470089
0.073135548	89.15646004	27016817.77	50.56941826	17.02456866
0.059962123	108.7206185	67533336.1	62.77525914	31.14680976
0.073342343	79.44394021	12659398.49	52.92031451	24.63238952
0.055134265	106.4683908	61380515.12	69.31071706	35.65834684
0.049389949	123.5556165	201799416.5	76.03770572	22.61254905
0.039990077	149.4231117	82999249.38	97.63982896	31.50399644
0.047414475	126.4331699	120406017.5	80.36584778	29.03094938
0.031365821	228.1737435	111761402.6	131.7135578	50.97730244
0.053608002	113.9937278	63587687.24	70.52192121	8.464069616
0.034331125	168.6593318	62991142.94	115.2529239	27.02257486
0.054719413	110.4300093	50886532.19	68.72245493	21.29780995
0.041282869	140.0688932	110889074.4	94.26316161	24.5717999
0.043471329	133.1690507	108090775.7	88.00984305	42.3956497
0.08104622	82.45909375	47318598.11	45.9908396	13.62559424
0.05647393	103.2658733	79700436.77	67.36963175	31.43052017
0.0922952	72.02527749	60171305.76	40.1886295	16.67336081
0.061870655	100.2184819	50892156.19	60.57476356	27.94715882
0.062573208	121.1008052	17777695.63	61.93653181	28.09052598
0.053744623	112.0593944	19899202.5	72.05735495	36.33752673
0.086734301	73.40452222	37197491.69	42.56689991	18.49428958
0.036610908	160.7507166	163265677.7	105.2310495	48.07324935
0.051095841	118.2072963	91018455.24	74.34148099	31.26065182
0.071098001	82.18934923	17914587.86	53.80937615	22.83923049
0.056677088	108.6835168	37846398.04	66.55761461	31.06133561
0.033933537	171.6432277	63172365.4	117.5398213	25.41813754
0.076702609	85.45527528	31513510.76	48.24178592	18.33453219
0.074288894	89.19882691	56808665.55	49.9627935	23.54900647
0.066699799	105.4396668	46641975.64	60.31988033	25.30380882
0.040427142	155.4745339	165316901.8	92.77347614	25.75236074
0.052001917	117.7445431	93419526.73	72.40205917	16.77781078
0.033983494	176.6177572	211209403.3	111.8969102	43.3416212
0.043007393	139.5627332	51754237.93	90.36181149	27.23100768
0.10084377	65.45259869	5407588.41	36.98770536	14.74302749
0.058057403	101.0895256	19825483.77	66.50683085	27.38276565
0.052078167	122.0219292	125181907.4	73.61880348	38.55925419
0.061989014	102.2175212	41146617.15	62.86918365	25.50359893
0.067143856	91.82144968	67353693.04	56.34044356	31.26825449
0.054565901	105.671971	22461546.33	72.19927114	35.75220704
0.075593764	81.88497269	53931138.7	49.21764301	16.21278702
0.05712035	108.2352327	40954605.32	67.85242738	8.937247953
0.039808717	150.1533225	139404790.2	94.77405073	41.74129707
0.102431871	64.37944674	22590603.84	36.01814011	14.19282453
0.051769458	131.0708026	54918082.89	72.66597743	29.92101499
0.058584364	102.7037343	28250470.24	64.52435371	26.32548313
0.052382449	120.7613075	91640777.25	71.48573801	43.65903034
0.062355547	110.4312778	150961605	60.83522995	20.33569322
0.048217129	125.636727	67955647.97	78.22756721	27.15648499
0.049787587	120.2042186	48715762.61	77.68763204	23.98353285
0.071496696	83.50684692	8498663.133	53.34140557	40.28406004
0.053166328	110.2104309	33850832.68	73.19526506	32.80856234
0.044269782	133.3237966	34426982.64	88.67553763	58.95810028
0.04483407	146.5828987	162635418.5	83.68126631	39.10967369
0.078641672	78.94129724	15255632.65	49.02217363	20.94407114
0.051633169	117.8009813	67718057.71	72.9738186	30.33124943
0.057589672	102.6643129	73487544.07	65.70972757	15.66440225
0.079995602	79.70248039	27331554.79	47.27244663	23.72538781
0.045869598	129.0915968	141978508.6	83.24220916	38.2454149
0.06121722	104.9839615	152559344.5	60.73801456	29.87353561
0.046135228	133.800122	150027801.6	81.23277529	36.71357862
0.073757339	83.81804255	33280714.84	50.60707851	17.77816742
0.056701331	107.3394507	52183533.41	66.19844052	22.34476933
0.039719397	149.7580964	56390827.47	96.53056519	42.27902599
0.051185146	122.324821	95719899	73.58343518	28.99399237
0.04298936	135.8482482	143872445.2	88.78111182	25.57999229
0.066933183	111.4588144	41677215.49	55.50461699	15.34667661
0.050218219	123.5122836	78037977.58	75.33492069	22.1981082
0.06190558	99.4563719	48029865.11	60.74878539	27.33977489
0.096512618	77.43173788	8974053.082	39.6988676	21.89453624
0.044613613	138.4361164	145748154.9	84.3493727	45.39682382
0.047479939	123.2750842	100166646.2	80.69832224	46.78308648
0.050802674	122.5720186	42534946.68	73.42419029	33.4426585
0.05926047	102.4783119	60461158.79	63.30143257	16.34134096
0.090841281	66.28199254	3428495.523	43.32270488	25.53219905
0.053429676	139.4889096	28337582.01	71.83766722	34.61282545
0.052660675	117.7764341	87600979.69	71.11519546	26.58253346
0.041836162	142.4628851	41232892.84	92.04422199	43.26943908
0.057363504	105.7506478	32104831.23	66.27094044	21.33347303
0.038240432	163.1040835	230286252.7	99.19303435	41.89741324
0.054003633	110.3450923	112742765.3	69.99162278	37.59072391
0.025137692	248.7504654	94794304.84	162.682876	37.71271464
0.044950902	138.4198781	65652036.44	83.70728397	29.35134363
0.089647567	69.48175723	4460923.718	42.93397128	31.06372669
0.058107094	121.7622871	28958520.2	67.0863427	31.3292377
0.036202914	168.9446578	57359146.67	108.6500742	28.56796803

wavelet.LHL_firstorder_TotalEnergy	wavelet.LHL_firstorder_Maximum	wavelet.LHL_firstorder_RootMeanSquared	wavelet.LHL_firstorder_90Percentile	wavelet.LHL_firstorder_Minimum	wavelet.LHL_firstorder_Entropy
1588467021	644.0195434	221.1436155	321.530787	-793.1082098	5.042392858
1239105554	601.0690804	151.480689	163.0100308	-668.3339284	4.493711501
1043771601	584.3822534	185.9516149	240.3337106	-570.6442958	4.895686181
2429785312	665.2021451	191.5372709	236.6187948	-937.0790479	4.911491913
310844593.4	492.686825	110.1429415	141.2113805	-302.1725665	4.020236606
734703080.5	616.7948942	128.8893696	142.0378051	-590.2236926	4.296860727
753099334.4	515.6940494	162.4451525	212.7913619	-627.5955114	4.628353219
1385356365	756.0542802	168.6010469	226.9805841	-739.4657992	4.552243361
2139647043	700.8885354	197.7253316	322.370877	-888.6509281	4.835763285
3552502067	934.084861	209.3185037	291.4312338	-850.0893275	5.014685554
1311103200	543.366046	163.9296382	214.2624474	-853.9655841	4.647180441
2610575979	878.3174685	239.5445719	284.1893021	-1067.138313	5.256563808
3081338191	766.2079437	197.3913492	225.9454916	-893.5889131	4.839610138
657663296.7	505.9793354	114.4977799	158.6244958	-494.4605389	4.162535363
2687368127	875.3616401	253.5690367	297.7998652	-1486.895099	5.1078937
3948921794	1017.851802	188.6641198	231.3509706	-1107.290535	4.717108621
729454079.9	501.3563103	129.7416983	140.4259691	-729.3134857	4.241792581
1823400075	906.26467	162.4833378	225.487438	-632.0562024	4.52183817
341803759.2	314.8027465	105.5643723	145.6805729	-446.9459596	4.050687948
1657273908	517.9912824	145.4592688	202.847636	-705.8134441	4.497907996
5448584246	717.5295335	170.3991997	204.9930861	-898.0769509	4.726425369
2240979733	686.6551699	197.8184288	242.5100681	-919.1709649	4.950412078
3250962472	1009.38712	170.9317696	210.4998934	-685.1134562	4.745485149
3017557870	1059.927799	329.4028174	439.8990611	-1288.296255	5.508797075
1716867556	825.375213	156.2665503	181.3334163	-766.2266914	4.607303909
1700760860	719.3290541	215.8496747	282.8862189	-847.6367526	5.110243163
1373936369	561.0732019	151.7075017	199.5050022	-706.9463384	4.558685416
2994005008	715.9513958	181.8017741	238.3795512	-779.430473	4.87762573
2918450944	579.9809077	178.3276697	253.4880492	-912.379581	4.810073132
1277602149	570.5030904	122.3886436	144.0556695	-648.167441	4.146001724
2151911793	534.1903796	138.7155996	190.0298922	-568.5641732	4.457278314
1624625256	611.1983616	109.7007801	140.9326745	-509.2661195	3.935032258
1374088217	557.1624932	141.8010194	184.0984722	-779.4953691	4.421570128
479997782	837.6874793	192.2494894	227.0242486	-568.4431708	4.503850015
537278467.5	454.7977405	149.6963988	191.3063522	-561.6145925	4.54394625
1004332276	483.07566	107.5304089	131.9597911	-541.488438	3.984623479
4408173297	697.9973834	213.8819691	282.8491269	-1026.169528	5.074170398
2457498292	553.6632079	163.8327931	203.7120058	-1084.95383	4.646471524
483693872.3	375.3355025	109.8718558	145.855017	-529.66449	4.113526508
1021852747	502.5823521	152.0969925	199.676041	-745.6644538	4.527420279
1705653866	559.4632453	219.0965932	278.1028195	-802.3219592	5.122525099
850864790.5	627.7663745	127.7158603	158.4857281	-630.3969591	4.187526026
1533833970	639.5193995	136.9938539	175.7987477	-962.5997354	4.246854651
1259333342	738.6509793	157.3432149	259.6959546	-478.1614893	4.393457587
4463556348	728.4959181	217.5189902	256.3274631	-1141.943155	5.045976473
2522327222	562.5620702	161.6518981	195.7907954	-851.4211913	4.654718829
5702653888	902.611805	239.3750926	305.99724	-1353.059316	5.21841448
1397364424	809.3233732	184.7665537	221.5774397	-718.8926079	4.862483755
146004887.1	369.5903584	97.91793867	114.2260344	-482.7179739	3.738086549
535288061.7	467.6374678	134.8648109	196.0179809	-524.3651103	4.408283826
3379911501	893.7898051	177.2601521	257.623319	-759.0550929	4.69237765
1110958663	566.6125349	145.0382806	209.5582741	-604.5890591	4.430925221
1818549712	645.5311125	133.1865619	187.9733721	-920.4775035	4.29629964
606461750.9	589.1399114	138.4973227	195.3615763	-441.3975859	4.451240556
1456140745	440.4391853	117.0407477	137.8735123	-711.4390976	4.134060282
1105774344	531.4676217	147.2822389	178.012428	-707.8272623	4.52002438
3763929335	760.9152158	206.1902571	283.608485	-857.485184	4.999675921
609946303.8	423.3067667	96.37893757	109.6477551	-563.73856	3.776883182
1482788238	897.8252306	200.069381	211.7362233	-1222.065579	4.751302372
762762696.5	510.1291601	141.7994931	186.7405479	-1019.30081	4.437421306
2474300986	922.9750014	176.5211994	232.2011468	-818.7691876	4.684696372
4075963335	702.3577727	165.8692085	210.0741903	-1020.288129	4.552914212
1834802495	613.6388305	172.9833135	220.703883	-1093.28551	4.733166805
1315325590	524.5352729	161.4902537	202.6218583	-688.4377572	4.650296375
229463904.6	496.4596766	120.018806	167.9315775	-345.3875746	4.136860423
913972482.3	536.9313187	148.1159113	215.5374171	-669.9314567	4.523884493
929528531.3	636.1595947	180.3875859	255.7561761	-699.9901107	4.781180587
4391156300	914.5469185	213.558517	286.4755949	-1030.707311	4.96125789
411902081.4	463.7343164	108.6215132	129.0502074	-473.9023805	4.028634577
1828387558	897.4201056	164.3520326	209.7958762	-786.0234482	4.648506794
1984163690	601.2199295	138.91796	177.5739016	-783.9910988	4.459140087
737951979.4	458.0681405	115.1577506	163.4323327	-448.6262901	4.08999844
3833419731	616.3341691	174.1946379	229.320364	-924.9456484	4.774549923
4119102301	681.8355907	153.0719305	193.5134799	-968.1848723	4.491571385
4050750643	733.1231631	187.620008	236.139918	-994.308227	4.836875766
898579300.6	633.5056873	119.488246	144.3823476	-531.4170434	4.168895986
1408955402	588.3140305	148.8576935	194.0253782	-713.2961532	4.523802959
1522552342	829.0545454	203.7016305	284.311874	-761.7659719	4.970919528
2584437273	836.4899597	170.9342809	210.2504856	-760.1298164	4.708095065
3884556022	774.1279831	179.747337	237.9578231	-906.8461795	4.851499356
1125284818	722.7261815	174.9285893	172.4542424	-842.729867	4.50447309
2107025395	651.9022252	171.3788506	203.8379947	-750.054192	4.714494686
1296806358	727.8250364	139.3335928	183.4986316	-601.3825848	4.411317912
242299433.2	470.1593623	130.9916078	156.2944804	-818.8500301	3.910989419
3935200183	773.8934922	194.2653738	246.0320223	-1013.637637	4.884616725
2704499448	734.7719432	169.8035085	242.3908961	-737.8369214	4.709351066
1148443560	680.9737054	172.5873032	226.9038926	-752.0482106	4.697958717
1632451287	509.7700233	140.9571977	178.8228415	-744.0949707	4.453654318
92569379.12	241.3301321	91.33366441	125.1287017	-379.4740791	3.77594524
765114714.4	952.7608667	221.4203209	283.8846717	-1118.635745	4.779942498
2365226452	594.5875864	165.1712018	200.8542135	-869.9842268	4.652080302
1113288107	652.8966236	193.1704821	273.7645569	-735.9858771	4.89444461
866830443.3	486.8779373	143.365509	175.296667	-646.6871647	4.477632944
6217728823	1318.256268	230.0062688	279.5544991	-1376.491151	5.113256302
3044054663	647.5045956	154.7812608	212.8970237	-838.3072245	4.563822738
2559446231	1124.447114	327.4650768	405.6919416	-1140.311031	5.633543185
1772604984	514.7188226	192.9736204	239.6229393	-933.1474213	4.861528215
120444940.4	339.6130899	102.0917184	130.1020211	-586.0483628	3.843718623
781880045.4	579.5103773	180.5850109	202.8319517	-948.4556137	4.615463541
1548696960	680.2953809	222.9453591	276.3839176	-896.1422147	5.119755394

wavelet.LHL_firstorder_StandardDeviation	wavelet.LHL_firstorder_Range	wavelet.LHL_firstorder_Variance	wavelet.LHL_firstorder_10Percentile	wavelet.LHL_firstorder_Kurtosis	wavelet.LHL_firstorder_Mean
214.2471492	1437.127753	45901.84092	-209.6518948	4.155591525	54.79651213
151.3969925	1269.403009	22921.04934	-171.1239003	5.481230746	5.034858951
185.7218221	1155.026549	34492.59521	-234.6395564	3.186572595	9.241638975
190.7122827	1602.281193	36371.17478	-211.5463852	4.491681491	17.75813475
106.7011891	794.8593915	11385.14376	-95.72176444	5.384906483	27.31892757
128.6312693	1207.018587	16546.00345	-152.5080778	5.1871468	8.152677381
160.4602766	1143.289561	25747.50037	-173.6780075	4.484292136	25.31653995
163.5378227	1495.520079	26744.61946	-127.3587378	6.091028756	41.00845703
188.7919649	1589.539463	35642.40602	-161.8844327	4.042959789	58.76138812
203.4691327	1784.174188	41399.68795	-202.7299569	4.253843548	49.1380507
161.4228031	1397.33163	26057.32135	-162.815502	5.285658098	28.55879784
239.5321255	1945.455782	57375.63913	-306.8090192	3.988230241	2.441878625
196.0540965	1659.796857	38437.20874	-209.1886255	5.731780563	22.93765435
111.9909111	1000.439874	12541.96416	-111.4653315	4.507907321	23.82808075
252.1804783	2362.256739	63594.99362	-232.0003128	7.928947593	26.50024038
186.9922248	2125.142337	34966.09213	-174.5280699	7.150860217	25.0610846
129.5026552	1230.669796	16770.93769	-136.0514344	6.590710208	7.872139358
156.3561766	1538.320872	24447.25395	-112.1062212	6.2282634	44.19933388
103.9687005	761.7487061	10809.49068	-105.2882674	4.234138986	18.285131
141.7349299	1223.804727	20088.79036	-141.6720266	4.732044997	32.70486972
169.8581865	1615.606484	28851.80353	-194.3335942	5.002663138	13.5677461
197.1069283	1605.826135	38851.14119	-241.8420715	4.218988444	16.7627442
169.8415386	1694.500577	28846.14824	-189.9679756	4.491524835	19.27489594
325.484838	2348.224054	105940.3797	-365.8062908	4.699584844	50.65408538
156.2323356	1591.601904	24408.54269	-194.1704194	4.911577673	3.269868682
214.8140756	1566.965807	46145.08709	-255.8570719	3.439997393	21.11859293
150.4660597	1268.01954	22640.03511	-165.6945586	4.612999898	19.36829787
181.103981	1495.381869	32798.65194	-211.3981056	3.75657424	15.91329988
173.9063025	1492.360489	30243.40205	-184.9709222	3.698559088	39.46334668
121.5720369	1218.670531	14779.76016	-114.681007	6.212317335	14.1145291
135.6381433	1102.754553	18397.70592	-137.8152373	3.878603414	29.05704122
106.9456959	1120.464481	11437.38187	-80.95892608	7.334635507	24.43111306
139.6163267	1336.657862	19492.71868	-130.9953195	5.357384326	24.79537127
187.502881	1406.13065	35157.33038	-125.4040184	6.759939455	42.45628103
148.1817919	1016.412333	21957.84346	-169.3503009	3.877785298	21.24072406
106.3164952	1024.564098	11303.19714	-97.14659183	6.603144118	16.11184948
211.3328312	1724.166911	44661.56553	-238.0668414	4.084259927	32.92310982
162.6092474	1638.617038	26441.76735	-166.2312429	6.591680684	19.98541322
108.5598391	904.9999925	11785.23867	-116.6780422	4.557502091	16.92885222
150.0886302	1248.246806	22526.59691	-158.6173393	5.106734313	24.63530439
218.738711	1361.785205	47846.62369	-273.7730327	3.297324833	12.51772665
126.2363771	1258.163334	15935.62291	-109.3499433	7.096337592	19.38344824
134.2775193	1602.119135	18030.45218	-100.1003286	9.376732702	27.14523585
148.287495	1216.812469	21989.18117	-90.42889788	5.483421693	52.60899268
217.1454629	1870.439073	47152.15205	-254.5654602	5.112042742	12.74201898
161.4364833	1413.983261	26061.73813	-191.3934269	4.720834298	8.342542673
237.7854529	2255.671121	56541.92159	-261.9113162	4.693967843	27.54112132
184.4774535	1528.215981	34031.93084	-221.5086298	4.166557918	10.33191807
96.98489171	852.3083323	9406.069221	-89.72372475	7.401053993	13.48530652
132.2634066	992.0025781	17493.60871	-132.8220783	3.776619646	26.36111727
169.616913	1652.844898	28769.89717	-127.0224934	5.198034621	51.49042955
140.8313469	1171.201594	19833.46826	-113.483319	4.71756714	34.67902219
128.0246477	1566.008616	16390.31041	-98.07398961	6.379170721	36.71988363
135.3435778	1030.537497	18317.88404	-145.79764	3.513522295	29.38748635
116.481628	1151.878283	13567.96966	-112.7255106	6.941113328	11.42659017
147.2051509	1239.294884	21669.35646	-186.0308916	4.203206966	4.764602551
201.7971011	1618.4004	40722.07	-192.2593871	4.214207238	42.33617996
95.88870868	987.0453267	9194.644453	-86.7047089	7.236259993	9.708509342
199.2721839	2119.89081	39709.40328	-199.0043977	9.426791277	17.84247525
140.3810005	1529.42997	19706.8253	-151.7599303	7.158497277	20.00677262
172.0231641	1741.744189	29591.96899	-141.5122011	6.061463184	39.5950105
164.1144404	1722.645902	26933.54956	-146.3390535	6.619324622	24.06334882
171.8663111	1706.924341	29538.02888	-184.8779053	5.579200378	19.6264586
160.5840115	1212.97303	25787.22476	-174.7546175	4.574469652	17.08441636
112.246124	841.8472512	12599.19236	-85.46489384	4.494145642	42.48907419
143.6496177	1206.862775	20635.21266	-137.1009577	4.039505734	36.09862221
174.1892729	1336.149705	30341.90279	-173.7328535	3.940673298	46.88046871
209.7315272	1945.25423	43987.31351	-188.7509225	5.049403269	40.24831274
108.1292879	937.636697	11691.94291	-110.7398484	5.510216214	10.32909624
162.2098176	1683.443554	26312.02493	-164.6636883	5.469108969	26.44930424
138.2969896	1385.211028	19126.05732	-154.0621576	4.88187568	13.12030006
111.5597765	906.6944307	12445.58374	-95.70084866	4.812432616	28.56087842
171.8492769	1541.279818	29532.17396	-187.2119208	4.386159377	28.48855747
150.9661828	1650.020463	22790.78836	-147.2445822	6.617618012	25.30271793
185.6421455	1727.43139	34463.0062	-190.6308417	5.443108016	27.1709622
118.0620338	1164.922731	13938.64383	-114.363309	6.119896373	18.40644203
147.6927758	1301.610184	21813.15601	-150.6616671	5.059536268	18.58647092
198.7222766	1590.820517	39490.54323	-201.8660452	4.054964087	44.76394818
169.9914785	1596.619776	28897.10276	-189.5878814	4.972225906	17.92834698
178.8092135	1680.974163	31972.73482	-210.6901443	3.893075517	18.34040175
174.7864874	1565.456048	30550.31619	-162.8399106	7.242766148	7.049481072
170.9505161	1401.956417	29224.07894	-196.367866	5.077933171	12.10914835
137.386396	1329.207621	18875.02181	-140.780063	4.873309211	23.21267483
129.0765393	1289.009392	16660.75299	-72.79297796	13.7831038	22.31699611
191.6513711	1787.531129	36730.24803	-199.5241071	5.265007338	31.76141407
163.9164197	1472.608865	26868.59265	-149.2839475	4.751644722	44.32424668
170.2680688	1433.021916	28991.21525	-172.1070435	4.822516164	28.19861659
140.3921592	1253.864994	19709.95837	-148.5375415	4.814361461	12.6084578
88.27635349	620.8042112	7792.714586	-86.71391872	4.692330346	23.43338791
217.5261787	2071.396612	47317.63842	-147.9573814	7.7572748	41.34392439
164.0382624	1464.571813	26908.55152	-168.2455589	5.298156458	19.31254483
188.9546417	1388.882501	35703.85662	-196.2634879	3.966972968	40.13699694
143.0039732	1133.565102	20450.13636	-170.0205238	4.451604451	10.17510708
228.067781	2694.747419	52014.91274	-240.7706996	5.872243418	29.79884142
150.4794683	1485.81182	22644.07037	-139.1539428	5.596900085	36.23766444
327.2046417	2264.758145	107062.8776	-443.9374976	3.489498102	13.05752442
191.981899	1447.866244	36857.04954	-214.2661319	5.247069115	19.53890068
98.15440778	925.6614527	9634.287767	-79.75826459	8.279753845	28.07901708
180.049937	1527.965991	32417.9798	-203.540454	7.166625253	13.89123367
222.6899878	1576.437596	49590.83065	-276.5686482	3.721329778	10.66782599

wavelet.LHL_glrlm_ShortRunLowGrayLevelEmphasis	wavelet.LHL_glrlm_GrayLevelVariance	wavelet.LHL_glrlm_LowGrayLevelRunEmphasis	wavelet.LHL_glrlm_GrayLevelNonUniformityNormalized	wavelet.LHL_glrlm_RunVariance
0.002415728	74.56687585	0.002443309	0.037720225	0.042684093
0.002953731	38.06328846	0.003015255	0.057726772	0.062334166
0.005194273	56.4792793	0.005258352	0.04018077	0.044437845
0.001196255	59.48451688	0.001214774	0.040634985	0.039864114
0.007896012	19.10332089	0.008202491	0.077056349	0.081742218
0.003316982	27.60525549	0.003392941	0.064621302	0.070112338
0.00316605	42.43483224	0.003226719	0.050177166	0.064052111
0.001999761	44.8675474	0.002048707	0.05785917	0.075052398
0.00125254	59.30140689	0.001285339	0.045204506	0.075283466
0.001319928	67.78765028	0.001343916	0.038266966	0.046834781
0.001444523	43.19484598	0.001474395	0.050173307	0.056998873
0.00128457	93.21256542	0.001296256	0.031609406	0.028275764
0.001449402	63.52700817	0.00147894	0.047491075	0.058038318
0.003746215	20.95810989	0.003861028	0.068475509	0.081299819
0.001004882	105.2296203	0.001013703	0.040048891	0.05165272
0.000781538	59.25114039	0.000805868	0.052514437	0.096837299
0.001993553	28.29828795	0.002052878	0.070158486	0.085532965
0.001927963	40.92069828	0.002000251	0.057767967	0.081622349
0.004592596	17.97008647	0.004733341	0.071553261	0.076728938
0.002062847	33.0575006	0.002107757	0.054070491	0.06183408
0.001009081	47.70440663	0.001036815	0.048071883	0.058997217
0.001464072	63.6901182	0.001487531	0.039339463	0.047693977
0.002254274	47.52373151	0.002315217	0.046427788	0.05358301
0.001644941	174.5008302	0.001656381	0.03018509	0.046313915
0.001607081	40.45583509	0.001648724	0.051981141	0.064463123
0.001821547	74.79034897	0.00184217	0.034023859	0.033450611
0.001877481	37.12791955	0.001911153	0.053815779	0.04360888
0.001457904	53.44240913	0.001486777	0.040787997	0.042575533
0.001029972	49.75095861	0.001057311	0.042628469	0.063616957
0.001936288	25.14069047	0.002017361	0.076502862	0.101058344
0.002348334	30.26566343	0.002422832	0.055437359	0.061884876
0.002498073	19.51655516	0.002640022	0.087564578	0.118661356
0.001400141	32.49494244	0.001440144	0.060001366	0.069834445
0.007737077	57.8670699	0.007807894	0.060347475	0.063922419
0.003462368	36.06676843	0.003523618	0.052622865	0.047730488
0.003149476	19.28351814	0.00326821	0.082498274	0.099169236
0.000924224	72.82482835	0.000940075	0.03616599	0.042468591
0.000865648	43.72387459	0.000882532	0.050059236	0.051247332
0.002883606	19.53973064	0.002965193	0.069613712	0.064925806
0.001886586	37.29838114	0.001927682	0.054858704	0.061174216
0.00214151	77.63357816	0.002164767	0.033631283	0.033309164
0.002218896	27.02005539	0.002293645	0.073223213	0.087145615
0.001301538	30.52219073	0.001356818	0.071014387	0.087356164
0.002940246	36.72502638	0.003044822	0.063933922	0.071698394
0.000865792	77.83261278	0.000881939	0.039250889	0.054674738
0.001189547	43.05292172	0.001219085	0.050587095	0.055318021
0.000628191	92.23385176	0.000636202	0.033483982	0.036696464
0.002349112	55.72605164	0.002385799	0.042302646	0.043446423
0.004978027	16.01630547	0.005114627	0.096501266	0.105438682
0.003597486	28.7588184	0.003678261	0.05681652	0.062149606
0.001436666	47.85634423	0.001482984	0.050408699	0.072188372
0.002313151	33.22488715	0.002384996	0.059272427	0.079387967
0.000954735	27.4931034	0.000988956	0.064791242	0.079685813
0.004666167	29.97871091	0.004778495	0.053889253	0.053601515
0.001592884	22.88402997	0.001652545	0.072842492	0.08558947
0.001875935	36.16296955	0.00192905	0.054208125	0.079037331
0.001251439	66.54903609	0.001276189	0.03916721	0.045452373
0.002339453	15.80005608	0.002473107	0.096263092	0.127597997
0.001496622	66.10337127	0.001512426	0.049948593	0.060121859
0.001405795	32.72670653	0.001429508	0.056991544	0.064418835
0.001511953	49.08791572	0.001545456	0.05084738	0.061655571
0.00080939	45.47752332	0.00083832	0.05872837	0.089661366
0.000998859	48.59198532	0.001014856	0.047107801	0.0471983
0.002335277	42.58873183	0.002382181	0.048902213	0.053966116
0.008250195	20.79560155	0.008436381	0.069904776	0.06366917
0.002066149	33.82239816	0.002110447	0.052410786	0.053104824
0.002485083	49.70614234	0.00251754	0.043538213	0.043252924
0.000889645	72.68856408	0.000909786	0.043435708	0.057014369
0.006037838	19.64153389	0.006174183	0.076165209	0.080100257
0.001534152	43.44908747	0.001571341	0.050490031	0.058372201
0.001309629	31.73843812	0.001348762	0.056338394	0.064919078
0.004263115	20.97401463	0.004420142	0.07656336	0.094395756
0.001001236	48.50013267	0.001025613	0.04507959	0.052702613
0.001016763	37.99014578	0.001045176	0.058999509	0.070908909
0.001192595	56.81904978	0.001226272	0.045086813	0.048604438
0.003573626	23.43387144	0.003695237	0.071230654	0.075930218
0.001789251	36.29061313	0.001834963	0.055214019	0.062458187
0.001897018	64.16920529	0.001925471	0.03918802	0.041589285
0.001959721	47.7751886	0.001997857	0.049757902	0.057836213
0.001002716	52.39673012	0.00102726	0.042286571	0.050569404
0.002171028	51.35053662	0.002212016	0.063226305	0.077320584
0.001861624	48.27445767	0.001898973	0.048930033	0.053529709
0.002195885	31.54131419	0.002265027	0.059544646	0.07116439
0.003547875	28.73972755	0.003603619	0.089768584	0.116690311
0.000973998	60.34605272	0.000991172	0.043590446	0.045675819
0.001955429	44.1183017	0.002010561	0.046775686	0.053147291
0.001955742	47.89548618	0.001988324	0.049567102	0.050638893
0.001540293	32.6726979	0.001582423	0.057582335	0.062346775
0.006538282	13.21696035	0.006728602	0.087919612	0.093188443
0.002358781	78.65246555	0.002375296	0.051566116	0.053694411
0.001237964	44.39725832	0.001268071	0.051407039	0.057672448
0.002427801	58.24615847	0.002458492	0.041205376	0.043803615
0.002437214	33.84836257	0.002491737	0.055952496	0.056219312
0.000571553	85.04926078	0.000580048	0.037516174	0.041398458
0.001161512	37.56417488	0.001192509	0.052757999	0.059120225
0.001893312	173.9448571	0.001904145	0.024750594	0.030639568
0.001675988	60.57265762	0.001700763	0.04400458	0.055560425
0.004095639	16.09168438	0.004168383	0.087196917	0.072487328
0.002184516	54.38189349	0.00221427	0.055026752	0.075954114
0.001909774	80.94584857	0.001929751	0.035573824	0.036367

wavelet.LHL_glrlm_GrayLevelNonUniformity	wavelet.LHL_glrlm_LongRunEmphasis	wavelet.LHL_glrlm_ShortRunHighGrayLevelEmphasis	wavelet.LHL_glrlm_RunLengthNonUniformity	wavelet.LHL_glrlm_ShortRunEmphasis
43.62696749	1.124924006	1237.303974	1071.485034	0.970997685
109.1665444	1.181808006	770.5202177	1698.147363	0.958897757
43.24160094	1.124041347	608.963649	1000.82462	0.972396837
96.08363537	1.116524216	1553.488468	2202.591657	0.972983902
67.97392143	1.241205427	220.9350622	766.5851452	0.946046709
99.36563791	1.20620739	610.9004134	1361.466164	0.953434733
50.15848891	1.183174471	764.6631362	898.9083085	0.959339119
97.8249011	1.215838234	1028.94976	1493.013807	0.952457841
86.02207642	1.210879137	1504.943201	1691.223032	0.954816956
110.082463	1.137180303	1422.22945	2646.639658	0.968274934
86.01625123	1.16854021	1331.976989	1546.697728	0.960906448
51.82451799	1.084700406	1953.256377	1553.294549	0.979559406
132.0070767	1.169261648	1403.034724	2513.703164	0.961581428
118.7502194	1.230549802	456.9721395	1522.476582	0.950043968
59.250174	1.148506879	3762.078526	1358.144966	0.966883351
200.8076822	1.255563534	2114.874758	3357.003098	0.949640422
104.5036626	1.249293376	922.7233908	1291.750785	0.945135576
137.6976947	1.235249272	800.3045859	2084.056732	0.948479345
76.16342862	1.218166557	369.5286641	941.9391378	0.952787624
148.4195902	1.180061058	942.3730124	2468.978405	0.959390506
317.5039259	1.167110206	1366.262962	5997.958766	0.963130609
79.81341505	1.141110723	1467.022033	1860.77234	0.967104544
182.6097621	1.152997237	870.8797214	3600.456616	0.965960443
29.79536554	1.136261194	3044.975555	908.3886039	0.968367079
128.0594499	1.183758321	997.8082804	2217.498871	0.959565612
44.58923667	1.097936401	1292.671767	1233.497162	0.977022211
114.3301188	1.127216287	924.9882105	1966.83286	0.970666163
131.6524847	1.12339049	1117.871709	2995.010672	0.971559959
137.1737238	1.181767845	1512.042565	2903.038951	0.960103868
222.8517481	1.279003119	713.6755599	2508.06416	0.942442389
217.0095729	1.182261276	610.3794625	3508.941554	0.958298047
399.2494409	1.32486894	493.2487232	3851.147695	0.934600738
142.9005516	1.200122678	1101.85655	2122.063527	0.95585584
27.465044	1.181942427	668.8387882	409.761616	0.959847655
44.68235401	1.141873823	607.4073967	777.6455348	0.966663007
244.0170765	1.284087592	521.0741979	2523.390184	0.939035706
124.0750002	1.125180382	1929.899088	3177.419741	0.970814773
161.9726926	1.149951014	2020.61779	2954.67659	0.965492643
97.43507631	1.189932215	531.4368206	1250.485945	0.956993908
84.92345019	1.178662083	984.4130879	1391.654872	0.95941748
42.88027803	1.099021927	1201.082182	1198.244451	0.976433858
131.4180096	1.24723177	730.5311633	1561.496749	0.94668887
199.6956853	1.248669714	1589.986958	2446.568465	0.946480718
113.0285269	1.208787875	527.6277678	1565.294772	0.953414337
130.7433674	1.155670043	2204.019911	3041.125607	0.965270844
171.9554942	1.161791057	1275.817476	3083.030125	0.96292171
119.2927803	1.107369264	3209.422926	3334.845535	0.974919405
61.67058523	1.125629378	921.2825758	1352.031257	0.971244787
50.0509845	1.294483636	429.7748629	444.3627675	0.93948608
58.71607896	1.175387844	516.8440704	934.48815	0.961569328
188.8975095	1.203707598	1125.337517	3337.031319	0.955642104
108.4586973	1.223256995	722.8334721	1613.767734	0.951943523
229.5066299	1.229730133	1469.95315	3103.365778	0.949430186
60.12305757	1.155815483	401.0677815	1016.245517	0.964410082
266.4872878	1.245537001	869.0307465	3184.521569	0.946720938
95.97423065	1.21740975	875.1529775	1571.923961	0.954450723
123.1533599	1.133470582	1404.433466	2898.240525	0.969024135
211.1705226	1.360646901	543.0405682	1810.788319	0.925810687
64.91879748	1.175584364	2478.632173	1170.591786	0.960061713
75.62213525	1.187566796	1742.634395	1190.157472	0.958052188
141.760864	1.175812487	1230.772903	2519.250595	0.961140603
299.8678067	1.246445511	1754.361658	4467.788159	0.948686262
102.4572387	1.137847589	2033.657047	1999.722895	0.968115997
86.90093947	1.159567497	859.9200781	1612.834849	0.963088786
38.93347824	1.186924789	271.7315849	498.2576072	0.957487296
77.09762301	1.154740148	841.045606	1342.595307	0.964886156
44.26630726	1.126692799	941.3089978	941.20691	0.970633329
147.2152201	1.164576976	1944.458331	3074.284428	0.962863155
91.8196567	1.231705706	392.6371996	1054.538192	0.948865335
119.9717513	1.172205128	1120.965797	2141.571146	0.96037341
202.7625759	1.185683496	1074.627774	3232.212308	0.958832255
145.9848747	1.265424881	384.3731247	1648.153582	0.943908415
201.0408651	1.153924411	1484.285755	4065.009942	0.964733103
361.3555287	1.202483998	1603.77049	5454.59158	0.955555277
183.8814443	1.141461814	1727.260471	3747.126775	0.967658847
155.308271	1.219764132	536.6720761	1920.012082	0.951382781
123.0299834	1.180354776	912.0464146	2004.042928	0.959551443
51.25182106	1.121635767	1140.510438	1213.858536	0.971699883
154.7905093	1.167657586	1042.840999	2816.405108	0.962081094
179.7928618	1.148227137	1461.363373	3887.938502	0.965880226
80.59919767	1.221083728	1199.631542	1124.534223	0.951800085
123.8437952	1.156103606	1031.301377	2304.121063	0.964253622
138.4551991	1.204167834	697.5667496	2067.144549	0.955024688
42.81596126	1.323200545	1130.158541	402.1899605	0.934128694
161.5101905	1.133001292	1829.285541	3421.22873	0.969524048
154.7327726	1.157036549	1045.788055	3008.662392	0.963728534
67.62691666	1.146889746	1074.630336	1249.860439	0.96655455
165.9707503	1.177698437	953.6025509	2599.517147	0.960616092
33.33978251	1.271855666	295.4778334	325.8393725	0.940906784
28.37357444	1.158587405	2218.856506	500.0639019	0.963443812
156.7629495	1.166904981	1308.289447	2762.016215	0.962308767
43.71992776	1.128776783	1054.125016	980.3941114	0.969989245
83.04193572	1.164935401	728.3101739	1344.732244	0.962309003
157.2415581	1.120681414	3313.434177	3895.872182	0.972128788
235.4209389	1.172078033	1278.04715	4028.076413	0.960980056
21.28046292	1.088229848	2335.908909	814.9939446	0.979606395
73.80691748	1.161477306	1540.863237	1524.04787	0.963351332
34.99280766	1.211227342	640.1243065	354.857434	0.95281575
45.87566042	1.21386353	1501.055741	740.1946491	0.954074657
39.70781949	1.105801475	1409.397921	1046.7307	0.975528949

wavelet.LHL_glrlm_LongRunHighGrayLevelEmphasis	wavelet.LHL_glrlm_RunPercentage	wavelet.LHL_glrlm_LongRunLowGrayLevelEmphasis	wavelet.LHL_glrlm_RunEntropy	wavelet.LHL_glrlm_HighGrayLevelRunEmphasis
1437.725425	0.961378605	0.002561228	5.267261933	1275.220939
951.1929851	0.9455	0.003290248	4.818187025	803.8148744
701.9786698	0.962570524	0.005538066	5.113885984	625.8651434
1782.15731	0.963937408	0.001294214	5.138141249	1596.48286
284.8371788	0.929480425	0.009555773	4.410636319	232.568349
777.35989	0.938668169	0.003727301	4.654548525	641.5270549
944.0975014	0.945709919	0.003495808	4.935147879	797.2226515
1301.295677	0.936629022	0.002270911	4.923869084	1078.3684
1884.262964	0.938749953	0.001438413	5.187803071	1572.214499
1674.497304	0.957913881	0.001446893	5.271878062	1469.569243
1618.724367	0.948703759	0.001602762	4.95347985	1385.940526
2161.412154	0.973019858	0.00134458	5.427217142	1993.699315
1710.364443	0.94897182	0.001609238	5.14755001	1460.187625
590.9407897	0.933344374	0.004388103	4.54037968	480.8435166
4471.217056	0.955625124	0.001053079	5.372140828	3891.11994
2772.811517	0.93009716	0.000929225	5.130587834	2223.826949
1220.835925	0.927917565	0.002322933	4.651013413	976.5601001
1027.290282	0.931677392	0.002329055	4.91703342	841.2291601
472.3987597	0.936890574	0.005374607	4.405308109	387.8183019
1158.588279	0.946172407	0.002306286	4.816694341	982.1466244
1654.555144	0.950304372	0.001162216	5.03404718	1418.48419
1738.493845	0.956551699	0.001587562	5.213829472	1518.249995
1043.179305	0.954380005	0.002578476	5.02500736	902.3282346
3567.52225	0.958177745	0.001705518	5.75054634	3143.797543
1231.227786	0.945911615	0.001836962	4.929244076	1039.950949
1452.669034	0.969333182	0.001930158	5.297528648	1323.254239
1073.426179	0.960825244	0.002056937	4.80426104	952.8480414
1293.53928	0.962054339	0.001610994	5.114524022	1150.77193
1862.186352	0.946681151	0.001181359	5.122676886	1575.098392
965.3968429	0.921981153	0.002409021	4.59775413	756.8769284
754.75362	0.945065557	0.002745923	4.78570123	637.2469574
688.0235642	0.911738462	0.003342431	4.435080312	525.8990952
1384.613171	0.940947634	0.001620718	4.771591771	1152.90203
809.3237649	0.946105869	0.008130133	4.79892853	694.5235759
717.4246877	0.956167706	0.003782441	4.799410551	628.3437665
709.4803356	0.919298917	0.003819456	4.448063076	554.4455069
2245.649326	0.961247494	0.001007773	5.314934328	1989.558342
2410.54591	0.954177347	0.000955874	4.931636053	2093.629104
662.7660272	0.943137052	0.003324743	4.444645219	555.6955172
1213.769243	0.946210269	0.002108393	4.845452873	1026.962648
1358.391195	0.968844985	0.002264221	5.307641533	1231.4365
956.7163264	0.928889951	0.002639977	4.599758295	770.8226041
2088.670232	0.928794694	0.001599289	4.662414422	1678.388785
653.0790501	0.938265556	0.00351251	4.756429138	550.5429657
2643.916295	0.953304566	0.00095387	5.333432354	2284.243191
1539.886068	0.950812265	0.001347849	4.957507089	1325.136101
3650.899005	0.966547018	0.000670372	5.433196872	3292.985421
1070.685172	0.961589202	0.002544258	5.096060689	949.1362657
594.0719562	0.919258047	0.005776309	4.174058963	457.7885436
629.3642913	0.94805928	0.004048472	4.70473659	537.2676671
1404.268045	0.94049274	0.00169338	5.045894016	1175.473501
918.3269548	0.935425515	0.002718602	4.804752294	757.6670403
1895.222474	0.932861976	0.001144654	4.690248265	1546.910127
481.7103261	0.952768837	0.005259661	4.723422618	415.9590488
1146.773886	0.929133858	0.001926453	4.547475515	918.5317135
1115.927084	0.937622229	0.002181338	4.881432741	916.997331
1639.990765	0.958899289	0.001382404	5.254126709	1448.88752
795.4509711	0.901821862	0.003120455	4.318958474	586.1833771
3039.011721	0.947241534	0.001582014	5.064502347	2582.751219
2162.663615	0.944374487	0.00153517	4.759569227	1819.308192
1499.841313	0.947872257	0.001696504	5.000286186	1279.558107
2298.808852	0.930422958	0.000976997	4.966924575	1848.33277
2385.966137	0.957694001	0.001083821	4.995035727	2099.796255
1036.208011	0.951284797	0.00258391	4.939555451	893.0252973
334.7257547	0.943937419	0.009248057	4.45034016	283.419316
1005.610292	0.953337654	0.002305359	4.79811984	871.4958398
1097.242296	0.960956813	0.002657272	5.011079123	970.6700868
2343.415307	0.950407697	0.000999333	5.266196392	2018.149054
514.0459002	0.932298174	0.006783489	4.41304005	414.5485183
1370.389515	0.947807677	0.001732136	4.961717084	1167.65597
1329.596641	0.945075145	0.00152511	4.788138052	1121.003196
511.4695734	0.924980405	0.005156394	4.517470073	406.5405792
1779.041462	0.953129367	0.00113186	5.063241646	1539.321246
2018.187207	0.940597569	0.001173929	4.851999629	1678.421373
2037.763123	0.956881926	0.001367921	5.10836058	1785.028337
684.4279148	0.935352935	0.004228892	4.548740816	563.4165002
1119.778307	0.946104851	0.002038241	4.846467941	950.1384098
1313.503963	0.962359201	0.002047783	5.194365698	1173.349575
1266.356597	0.949539776	0.002166023	5.017410392	1084.135556
1738.17332	0.954792793	0.001133493	5.129862992	1513.098284
1534.679075	0.93578448	0.002399086	4.879279354	1259.661067
1236.527438	0.952578096	0.002061628	5.004460993	1069.618015
875.9025038	0.939804739	0.002576953	4.767058228	729.8553649
1597.706376	0.911604648	0.00387735	4.387333175	1209.538969
2140.502798	0.959347488	0.001065757	5.141887876	1887.286989
1253.647942	0.952194323	0.002243055	4.998986579	1084.811815
1273.439607	0.955397544	0.002131086	4.966308115	1111.577805
1169.055171	0.947142243	0.001772052	4.774032426	992.7669048
405.0228995	0.922515441	0.007605888	4.172387007	315.0839617
2661.293645	0.951823263	0.002447086	5.055810171	2301.978131
1582.821431	0.949668208	0.001401242	4.960082185	1358.894455
1228.243471	0.960180995	0.002589872	5.127840492	1087.075494
882.3530818	0.950113267	0.002728696	4.77539741	756.9040632
3827.356291	0.962837301	0.000616765	5.350502698	3409.827888
1556.215518	0.948183988	0.001329235	4.8812302	1329.55271
2587.313391	0.972589628	0.001950374	5.788405497	2383.081324
1863.022866	0.951350408	0.001809614	5.145354951	1600.470852
812.7037592	0.937634795	0.004494473	4.181317349	671.7199594
1919.717242	0.938496188	0.002352764	4.964445562	1575.278632
1594.054124	0.967204373	0.002015471	5.317339576	1444.049915

wavelet.LHL_glrlm_RunLengthNonUniformityNormalized	wavelet.LHL_glszm_GrayLevelVariance	wavelet.LHL_glszm_SmallAreaHighGrayLevelEmphasis	wavelet.LHL_glszm_GrayLevelNonUniformityNormalized
0.926304344	93.0882318	917.5482586	0.031506218
0.897606117	56.09519395	555.9853929	0.042837942
0.929845513	69.52678201	478.381531	0.03407872
0.93127683	76.13247677	1203.079007	0.034556525
0.868228044	31.91727756	177.9856765	0.054990882
0.884955257	44.48535131	431.6208204	0.044961251
0.898779317	59.55176593	570.8728339	0.03880943
0.882717459	71.49115748	801.2048368	0.038125844
0.888402209	83.98589158	1178.757632	0.031904637
0.919775054	86.10783688	1066.289111	0.032075913
0.901983246	62.08811378	958.7260655	0.038450963
0.947283642	110.88597	1614.58796	0.028126893
0.903965456	88.4763933	1003.128916	0.035292629
0.877341461	32.90869093	315.2808052	0.05044369
0.917229789	144.9900283	2954.122005	0.030398242
0.87734105	91.13599743	1636.059218	0.03419781
0.866377036	45.79320011	648.2785612	0.047586013
0.873785628	65.24607814	615.740799	0.039188917
0.884055762	27.023596	256.1202462	0.05596
0.898925807	45.73384285	686.6521203	0.043363943
0.90776889	67.039475	1029.817258	0.036994715
0.91689562	82.93583883	1093.421693	0.032747158
0.914747134	63.33717304	634.1286945	0.037074899
0.920050503	231.8483711	2361.3063	0.022074932
0.899545912	58.02278153	720.5073349	0.03907042
0.94109051	89.838381	1030.658037	0.030471597
0.925620839	46.12195186	713.7006919	0.046330523
0.927711102	65.68763073	849.8942115	0.035504201
0.901171528	65.47616009	1077.994077	0.035255504
0.860496404	44.04130999	526.70074	0.046003971
0.896046046	42.02061659	436.66292	0.043997942
0.843576551	36.85120276	373.9244392	0.053083444
0.890651259	49.87245349	779.1798996	0.043083551
0.900006026	83.72886509	533.9479164	0.042501157
0.915681247	47.77514793	471.5469359	0.042867313
0.852571146	36.9306318	379.0912406	0.050461991
0.925933115	88.82202239	1467.681944	0.031344506
0.912995972	61.14250132	1493.355511	0.039686397
0.893075762	29.49529512	369.9405806	0.053948511
0.898716483	54.98336964	694.0537005	0.040370564
0.939601962	89.94541419	934.0585559	0.030384463
0.869619026	47.27410961	545.9096514	0.046765825
0.869384996	53.380279	1193.769886	0.0451345
0.884973367	57.50646346	435.5912635	0.041734431
0.912719353	105.4816231	1692.191019	0.030160069
0.906835729	60.90751212	955.2703196	0.038072141
0.935904666	113.1945023	2518.573659	0.028658231
0.927112275	72.72908884	694.2953019	0.035006352
0.854978935	31.76582751	288.4011985	0.059805715
0.904005073	39.41428253	375.8865427	0.04653699
0.890160021	69.75036019	862.863993	0.036893726
0.881568835	50.05557646	554.4466406	0.042357789
0.875726553	45.00665158	1070.890162	0.045358393
0.91054422	37.97416226	300.6434335	0.046621315
0.869881837	39.33515721	604.9277053	0.050321361
0.887634945	52.92788408	643.4236798	0.039519559
0.92153349	84.29888158	1057.563796	0.032508357
0.824388237	33.10133469	386.7771035	0.054638075
0.90027884	102.8256946	1843.861646	0.035079319
0.896135398	47.32496654	1266.936463	0.044002102
0.903167478	72.84287033	923.1802278	0.037097177
0.874491277	73.43067048	1300.707005	0.037079083
0.919267585	65.84702656	1621.463586	0.037169099
0.907328904	58.54450306	637.3638473	0.040302042
0.894227706	32.75632987	202.078473	0.051831278
0.91207216	45.10247003	602.3058345	0.043704045
0.925491254	64.32388244	721.9086338	0.036909686
0.906859984	101.7440949	1479.006195	0.031431307
0.874359135	31.62180668	270.5911743	0.053549769
0.900933545	62.25269326	829.4987401	0.038810007
0.897602194	45.40731198	779.9328352	0.0442259
0.863690979	37.4635605	286.7222511	0.04761372
0.911266151	64.75042404	1076.48427	0.036774477
0.890077716	58.79179518	1184.02618	0.040586368
0.918457509	77.60216446	1327.260432	0.035020973
0.880231424	38.95831946	411.3714291	0.049046281
0.899111188	54.37595717	680.5277948	0.040902958
0.927977404	78.49118806	846.4156878	0.033642535
0.905061633	68.71340801	759.2457609	0.036596288
0.914085457	66.95363712	1092.478646	0.035537511
0.881538799	82.87691845	941.8441441	0.040399014
0.910096661	67.66836466	786.0375029	0.037294971
0.888693519	47.68880682	539.4988209	0.043015042
0.842424362	59.53341531	772.8902557	0.05053047
0.923012957	80.41045208	1411.364173	0.034082689
0.9090447	59.34450572	762.54521	0.038956925
0.915769402	67.03649662	810.8002908	0.03783941
0.901643937	47.86014067	709.2283174	0.042662168
0.857754516	23.83624036	176.7155276	0.062160344
0.908413503	128.1723601	1683.182528	0.032571384
0.905549326	63.73629598	991.0747164	0.038951233
0.923849622	75.35246676	784.6620381	0.034401244
0.905658235	48.73120006	553.7753377	0.042761753
0.929224147	109.8863777	2572.434534	0.030233798
0.902406404	55.14084454	950.5377283	0.040832182
0.947686038	208.5242778	1981.532729	0.02108672
0.908176593	81.9663011	1103.523585	0.034788146
0.883656517	28.59441358	453.9694442	0.062592593
0.887116076	83.20503827	1091.367886	0.036800462
0.937563076	98.49296352	1168.205181	0.030273008

wavelet.LHL_glszm_SizeZoneNonUniformityNormalized	wavelet.LHL_glszm_SizeZoneNonUniformity	wavelet.LHL_glszm_GrayLevelNonUniformity	wavelet.LHL_glszm_LargeAreaEmphasis	wavelet.LHL_glszm_ZoneVariance
0.533742212	382.693166	22.58995816	5.761506276	2.946412003
0.449781999	444.3846154	42.32388664	10.71052632	6.612770247
0.528767301	359.5617647	23.17352941	5.347058824	2.643935986
0.51903016	786.3306931	52.35313531	4.812541254	2.190920716
0.398347876	147.787062	20.40161725	19.54986523	13.00674944
0.443539278	331.7673797	33.63101604	18.30748663	13.51208928
0.476305896	253.3947368	20.64661654	10.98684211	7.039300554
0.488204254	408.138756	31.87320574	35.06578947	30.40411592
0.492758588	478.4685891	30.97940268	18.56333677	14.20551928
0.507504427	877.982659	55.49132948	7.147398844	4.13426476
0.449695953	423.6135881	36.22080679	9.196390658	5.516672977
0.602784609	709.4774851	33.10535259	3.057774002	1.0082789
0.466240536	696.0971199	52.69189551	11.08171467	7.232969755
0.403651396	307.1787122	38.38764783	22.67017083	16.70912987
0.537856462	485.6843854	27.4496124	7.291251384	4.352475874
0.50007501	1023.15347	69.96871945	43.78054741	39.74724279
0.42809479	278.6897081	30.97849462	28.38248848	22.30410499
0.453138118	495.2799634	42.83348582	28.99176578	23.51453858
0.41776	208.88	27.98	16.384	11.222016
0.4469151	653.3898769	63.39808482	9.997948016	6.060628021
0.488712371	1847.821476	139.8770167	9.697963502	6.319211979
0.506942645	617.9630845	39.91878589	6.233798195	3.206369481
0.497620719	1156.968172	86.19913978	8.36172043	5.220059475
0.540772445	339.6050955	13.86305732	5.942675159	3.252657309
0.461433077	615.5517241	52.11994003	11.94452774	8.134130086
0.561283233	507.961326	27.57679558	3.474033149	1.242228259
0.528412192	719.1689934	63.05584129	5.13519471	2.496061968
0.502261725	1011.555114	71.50546177	5.036246276	2.261226282
0.444081422	770.481268	61.16829971	8.824783862	4.986797997
0.448469008	569.107171	58.37903861	86.34121355	80.14429187
0.447097651	899.1133764	88.47986077	11.54748881	7.305240867
0.393852003	656.5512897	88.49010198	130.0923815	121.0959804
0.442210472	524.0194093	51.05400844	16.47848101	11.9165618
0.48476436	119.2520325	10.45528455	11.40243902	7.579301342
0.508708456	257.9151874	21.73372781	7.076923077	4.009243374
0.421769695	490.0963855	58.63683305	67.81841652	60.15380998
0.52980524	1154.445617	68.29967875	4.815511703	2.132771389
0.491196589	920.011212	74.33262146	7.543513081	4.265731391
0.434741388	300.8410405	37.33236994	12.08381503	7.484897591
0.467780625	383.1123321	33.06349206	13.17460317	9.184365221
0.552958119	481.0735632	26.43448276	3.659770115	1.371679218
0.463925399	371.6042447	37.45942572	54.96379526	49.14612353
0.460478099	565.9275834	55.47030106	42.13100081	36.06474003
0.464144996	392.6666667	35.30732861	21.04491726	16.08560938
0.522608649	1035.287733	59.74709743	8.882382635	5.77154854
0.49240647	941.4811715	72.79393305	11.79341004	8.297370658
0.554897038	1332.307788	68.80841316	4.192419825	1.835600223
0.52132528	479.0979325	32.17083787	5.251360174	2.53011683
0.406529413	80.08629442	11.78172589	33.66497462	25.46852534
0.46471301	260.2392857	26.06071429	10.38928571	6.600701531
0.472694827	902.8471204	70.46701571	26.30366492	21.95283682
0.475935382	435.4808743	38.75737705	21.74863388	17.17885156
0.439361113	705.6139477	72.84557908	32.59838107	27.00866032
0.483214916	304.4253968	29.37142857	7.915873016	4.460995213
0.433597469	698.0919255	81.0173913	58.18571429	52.20601867
0.466500433	433.378902	36.71367061	15.01614639	10.88593242
0.497744518	953.6784969	62.28601253	6.421189979	3.492375763
0.396976128	293.3653586	40.37753721	180.308525	169.4782805
0.494996648	350.4576271	24.83615819	11.9180791	8.16280443
0.456942061	318.9455587	30.71346705	13.12750716	9.075764977
0.475906074	720.5217966	56.1651255	9.884412153	6.110964769
0.479643688	1192.394208	92.17860016	77.89259855	73.02104286
0.539559986	714.9169811	49.2490566	6.76	3.822333357
0.475797321	469.6119554	39.7781155	8.411347518	4.829398185
0.462301337	123.4344569	13.83895131	12.68913858	7.80619731
[truncated: 1,148,199 more chars]
