# Supplementary material for: Peritumoral radiomics features predict distant metastasis in locally advanced NSCLC
Source: PLoS One. 2018 Nov 2;13(11):e0206108. doi: 10.1371/journal.pone.0206108 (PMC6214508; doi:10.1371/journal.pone.0206108)
Supplement: S10 File — (PDF) [file pone.0206108.s010.pdf]

X	general_info_BoundingBox
97	(6, 5, 6, 21, 20, 15)
98	(5, 6, 6, 13, 12, 10)
99	(6, 6, 6, 15, 18, 17)
100	(6, 6, 6, 12, 12, 11)
102	(6, 6, 5, 19, 20, 18)
103	(6, 5, 6, 11, 12, 11)
104	(5, 6, 6, 38, 39, 44)
105	(6, 6, 6, 26, 39, 35)
106	(6, 6, 6, 26, 35, 30)
109	(5, 6, 6, 21, 18, 17)
110	(7, 6, 6, 15, 38, 24)
111	(5, 6, 6, 17, 27, 29)
113	(6, 6, 6, 18, 18, 13)
115	(5, 5, 5, 17, 18, 14)
116	(6, 5, 6, 20, 18, 19)
118	(6, 5, 6, 31, 45, 30)
119	(6, 6, 5, 16, 15, 17)
120	(5, 6, 6, 13, 13, 14)
121	(6, 5, 6, 16, 21, 15)
122	(6, 6, 5, 35, 37, 42)
123	(6, 6, 6, 18, 21, 25)
124	(6, 6, 5, 14, 18, 12)
125	(6, 6, 5, 15, 14, 15)
127	(6, 6, 6, 28, 29, 22)
128	(6, 6, 6, 14, 24, 16)
129	(6, 6, 6, 21, 22, 27)
130	(5, 6, 6, 24, 28, 23)
131	(6, 6, 5, 20, 24, 26)
132	(5, 5, 6, 12, 10, 9)
133	(6, 6, 6, 21, 22, 16)
134	(6, 5, 6, 28, 29, 28)
137	(5, 6, 6, 14, 16, 18)
210	(5, 6, 5, 19, 30, 29)
310	(6, 5, 5, 23, 31, 23)
410	(6, 6, 6, 28, 29, 39)
510	(6, 6, 6, 20, 26, 23)
610	(6, 5, 6, 26, 28, 23)
710	(6, 5, 6, 27, 37, 40)
810	(5, 6, 6, 18, 13, 13)
910	(5, 6, 5, 34, 47, 43)
1010	(6, 6, 6, 19, 17, 19)
1110	(6, 5, 6, 36, 31, 28)
1210	(5, 5, 6, 31, 32, 16)
138	(5, 6, 6, 18, 23, 19)
142	(6, 6, 6, 15, 20, 18)
151	(6, 6, 5, 23, 32, 19)
161	(6, 5, 5, 33, 32, 32)
171	(6, 5, 6, 16, 10, 12)
181	(6, 6, 5, 16, 12, 10)
191	(6, 6, 6, 21, 12, 21)
201	(6, 6, 6, 20, 29, 29)
221	(6, 5, 5, 27, 27, 28)
231	(6, 7, 6, 32, 39, 31)
241	(6, 5, 6, 13, 22, 18)
251	(6, 6, 6, 13, 19, 14)
261	(6, 5, 6, 18, 19, 16)
271	(6, 6, 6, 31, 39, 27)
281	(6, 6, 5, 18, 21, 22)
291	(5, 5, 6, 18, 16, 17)
301	(6, 5, 5, 41, 44, 48)
311	(6, 6, 5, 17, 19, 19)
321	(7, 6, 5, 20, 27, 18)
331	(5, 6, 5, 29, 44, 44)
341	(6, 6, 5, 37, 43, 35)
351	(5, 6, 6, 11, 16, 16)
361	(6, 6, 6, 26, 26, 27)
371	(5, 6, 6, 13, 15, 12)
381	(6, 6, 6, 26, 37, 42)
391	(6, 6, 5, 34, 30, 36)
401	(6, 6, 6, 26, 23, 19)
411	(6, 5, 5, 17, 17, 11)
421	(6, 6, 5, 31, 30, 32)
431	(5, 6, 6, 18, 20, 18)
481	(6, 6, 6, 16, 19, 24)
491	(6, 6, 6, 19, 35, 35)
501	(6, 6, 6, 21, 10, 17)
541	(5, 6, 6, 22, 21, 23)
551	(5, 5, 6, 13, 13, 11)
561	(6, 6, 5, 25, 17, 17)
601	(5, 6, 5, 20, 23, 22)
611	(6, 5, 6, 20, 20, 15)
621	(6, 5, 6, 15, 16, 17)
641	(5, 6, 6, 17, 27, 31)
661	(5, 5, 5, 23, 23, 25)
681	(6, 6, 7, 17, 25, 26)
691	(5, 6, 5, 15, 14, 15)
701	(5, 6, 6, 24, 25, 14)
711	(6, 5, 5, 26, 32, 27)
721	(5, 6, 6, 22, 18, 16)
751	(5, 5, 6, 19, 18, 16)
771	(5, 6, 5, 16, 16, 17)
781	(6, 6, 6, 25, 28, 19)
791	(6, 6, 6, 32, 29, 33)
801	(5, 5, 6, 27, 32, 24)
811	(6, 5, 6, 31, 33, 27)
821	(6, 6, 5, 23, 23, 26)
841	(6, 6, 5, 23, 21, 23)
861	(5, 6, 5, 13, 12, 10)
871	(5, 5, 6, 12, 13, 10)
901	(5, 5, 6, 33, 44, 36)

general_info_GeneralSettings
{'distances': [1], 'verbose': True, 'additionalInfo': True, 'enableCExtensions': True, 'force2D': False, 'interpolator': 3, 'resampledPixelSpacing': [3, 3, 3], 'label': 1, 'normalizeScale': 1, 'normalize': False, 'force2Ddimension': 0, 'removeOutliers': None, 'minimumROISi
{'distances': [1], 'verbose': True, 'additionalInfo': True, 'enableCExtensions': True, 'force2D': False, 'interpolator': 3, 'resampledPixelSpacing': [3, 3, 3], 'label': 1, 'normalizeScale': 1, 'normalize': False, 'force2Ddimension': 0, 'removeOutliers': None, 'minimumROISi
{'distances': [1], 'verbose': True, 'additionalInfo': True, 'enableCExtensions': True, 'force2D': False, 'interpolator': 3, 'resampledPixelSpacing': [3, 3, 3], 'label': 1, 'normalizeScale': 1, 'normalize': False, 'force2Ddimension': 0, 'removeOutliers': None, 'minimumROISi
{'distances': [1], 'verbose': True, 'additionalInfo': True, 'enableCExtensions': True, 'force2D': False, 'interpolator': 3, 'resampledPixelSpacing': [3, 3, 3], 'label': 1, 'normalizeScale': 1, 'normalize': False, 'force2Ddimension': 0, 'removeOutliers': None, 'minimumROISi
{'distances': [1], 'verbose': True, 'additionalInfo': True, 'enableCExtensions': True, 'force2D': False, 'interpolator': 3, 'resampledPixelSpacing': [3, 3, 3], 'label': 1, 'normalizeScale': 1, 'normalize': False, 'force2Ddimension': 0, 'removeOutliers': None, 'minimumROISi
{'distances': [1], 'verbose': True, 'additionalInfo': True, 'enableCExtensions': True, 'force2D': False, 'interpolator': 3, 'resampledPixelSpacing': [3, 3, 3], 'label': 1, 'normalizeScale': 1, 'normalize': False, 'force2Ddimension': 0, 'removeOutliers': None, 'minimumROISi
{'distances': [1], 'verbose': True, 'additionalInfo': True, 'enableCExtensions': True, 'force2D': False, 'interpolator': 3, 'resampledPixelSpacing': [3, 3, 3], 'label': 1, 'normalizeScale': 1, 'normalize': False, 'force2Ddimension': 0, 'removeOutliers': None, 'minimumROISi
{'distances': [1], 'verbose': True, 'additionalInfo': True, 'enableCExtensions': True, 'force2D': False, 'interpolator': 3, 'resampledPixelSpacing': [3, 3, 3], 'label': 1, 'normalizeScale': 1, 'normalize': False, 'force2Ddimension': 0, 'removeOutliers': None, 'minimumROISi
{'distances': [1], 'verbose': True, 'additionalInfo': True, 'enableCExtensions': True, 'force2D': False, 'interpolator': 3, 'resampledPixelSpacing': [3, 3, 3], 'label': 1, 'normalizeScale': 1, 'normalize': False, 'force2Ddimension': 0, 'removeOutliers': None, 'minimumROISi
{'distances': [1], 'verbose': True, 'additionalInfo': True, 'enableCExtensions': True, 'force2D': False, 'interpolator': 3, 'resampledPixelSpacing': [3, 3, 3], 'label': 1, 'normalizeScale': 1, 'normalize': False, 'force2Ddimension': 0, 'removeOutliers': None, 'minimumROISi
{'distances': [1], 'verbose': True, 'additionalInfo': True, 'enableCExtensions': True, 'force2D': False, 'interpolator': 3, 'resampledPixelSpacing': [3, 3, 3], 'label': 1, 'normalizeScale': 1, 'normalize': False, 'force2Ddimension': 0, 'removeOutliers': None, 'minimumROISi
{'distances': [1], 'verbose': True, 'additionalInfo': True, 'enableCExtensions': True, 'force2D': False, 'interpolator': 3, 'resampledPixelSpacing': [3, 3, 3], 'label': 1, 'normalizeScale': 1, 'normalize': False, 'force2Ddimension': 0, 'removeOutliers': None, 'minimumROISi
{'distances': [1], 'verbose': True, 'additionalInfo': True, 'enableCExtensions': True, 'force2D': False, 'interpolator': 3, 'resampledPixelSpacing': [3, 3, 3], 'label': 1, 'normalizeScale': 1, 'normalize': False, 'force2Ddimension': 0, 'removeOutliers': None, 'minimumROISi
{'distances': [1], 'verbose': True, 'additionalInfo': True, 'enableCExtensions': True, 'force2D': False, 'interpolator': 3, 'resampledPixelSpacing': [3, 3, 3], 'label': 1, 'normalizeScale': 1, 'normalize': False, 'force2Ddimension': 0, 'removeOutliers': None, 'minimumROISi
{'distances': [1], 'verbose': True, 'additionalInfo': True, 'enableCExtensions': True, 'force2D': False, 'interpolator': 3, 'resampledPixelSpacing': [3, 3, 3], 'label': 1, 'normalizeScale': 1, 'normalize': False, 'force2Ddimension': 0, 'removeOutliers': None, 'minimumROISi
{'distances': [1], 'verbose': True, 'additionalInfo': True, 'enableCExtensions': True, 'force2D': False, 'interpolator': 3, 'resampledPixelSpacing': [3, 3, 3], 'label': 1, 'normalizeScale': 1, 'normalize': False, 'force2Ddimension': 0, 'removeOutliers': None, 'minimumROISi
{'distances': [1], 'verbose': True, 'additionalInfo': True, 'enableCExtensions': True, 'force2D': False, 'interpolator': 3, 'resampledPixelSpacing': [3, 3, 3], 'label': 1, 'normalizeScale': 1, 'normalize': False, 'force2Ddimension': 0, 'removeOutliers': None, 'minimumROISi
{'distances': [1], 'verbose': True, 'additionalInfo': True, 'enableCExtensions': True, 'force2D': False, 'interpolator': 3, 'resampledPixelSpacing': [3, 3, 3], 'label': 1, 'normalizeScale': 1, 'normalize': False, 'force2Ddimension': 0, 'removeOutliers': None, 'minimumROISi
{'distances': [1], 'verbose': True, 'additionalInfo': True, 'enableCExtensions': True, 'force2D': False, 'interpolator': 3, 'resampledPixelSpacing': [3, 3, 3], 'label': 1, 'normalizeScale': 1, 'normalize': False, 'force2Ddimension': 0, 'removeOutliers': None, 'minimumROISi
{'distances': [1], 'verbose': True, 'additionalInfo': True, 'enableCExtensions': True, 'force2D': False, 'interpolator': 3, 'resampledPixelSpacing': [3, 3, 3], 'label': 1, 'normalizeScale': 1, 'normalize': False, 'force2Ddimension': 0, 'removeOutliers': None, 'minimumROISi
{'distances': [1], 'verbose': True, 'additionalInfo': True, 'enableCExtensions': True, 'force2D': False, 'interpolator': 3, 'resampledPixelSpacing': [3, 3, 3], 'label': 1, 'normalizeScale': 1, 'normalize': False, 'force2Ddimension': 0, 'removeOutliers': None, 'minimumROISi
{'distances': [1], 'verbose': True, 'additionalInfo': True, 'enableCExtensions': True, 'force2D': False, 'interpolator': 3, 'resampledPixelSpacing': [3, 3, 3], 'label': 1, 'normalizeScale': 1, 'normalize': False, 'force2Ddimension': 0, 'removeOutliers': None, 'minimumROISi
{'distances': [1], 'verbose': True, 'additionalInfo': True, 'enableCExtensions': True, 'force2D': False, 'interpolator': 3, 'resampledPixelSpacing': [3, 3, 3], 'label': 1, 'normalizeScale': 1, 'normalize': False, 'force2Ddimension': 0, 'removeOutliers': None, 'minimumROISi
{'distances': [1], 'verbose': True, 'additionalInfo': True, 'enableCExtensions': True, 'force2D': False, 'interpolator': 3, 'resampledPixelSpacing': [3, 3, 3], 'label': 1, 'normalizeScale': 1, 'normalize': False, 'force2Ddimension': 0, 'removeOutliers': None, 'minimumROISi
{'distances': [1], 'verbose': True, 'additionalInfo': True, 'enableCExtensions': True, 'force2D': False, 'interpolator': 3, 'resampledPixelSpacing': [3, 3, 3], 'label': 1, 'normalizeScale': 1, 'normalize': False, 'force2Ddimension': 0, 'removeOutliers': None, 'minimumROISi
{'distances': [1], 'verbose': True, 'additionalInfo': True, 'enableCExtensions': True, 'force2D': False, 'interpolator': 3, 'resampledPixelSpacing': [3, 3, 3], 'label': 1, 'normalizeScale': 1, 'normalize': False, 'force2Ddimension': 0, 'removeOutliers': None, 'minimumROISi
{'distances': [1], 'verbose': True, 'additionalInfo': True, 'enableCExtensions': True, 'force2D': False, 'interpolator': 3, 'resampledPixelSpacing': [3, 3, 3], 'label': 1, 'normalizeScale': 1, 'normalize': False, 'force2Ddimension': 0, 'removeOutliers': None, 'minimumROISi
{'distances': [1], 'verbose': True, 'additionalInfo': True, 'enableCExtensions': True, 'force2D': False, 'interpolator': 3, 'resampledPixelSpacing': [3, 3, 3], 'label': 1, 'normalizeScale': 1, 'normalize': False, 'force2Ddimension': 0, 'removeOutliers': None, 'minimumROISi
{'distances': [1], 'verbose': True, 'additionalInfo': True, 'enableCExtensions': True, 'force2D': False, 'interpolator': 3, 'resampledPixelSpacing': [3, 3, 3], 'label': 1, 'normalizeScale': 1, 'normalize': False, 'force2Ddimension': 0, 'removeOutliers': None, 'minimumROISi
{'distances': [1], 'verbose': True, 'additionalInfo': True, 'enableCExtensions': True, 'force2D': False, 'interpolator': 3, 'resampledPixelSpacing': [3, 3, 3], 'label': 1, 'normalizeScale': 1, 'normalize': False, 'force2Ddimension': 0, 'removeOutliers': None, 'minimumROISi
{'distances': [1], 'verbose': True, 'additionalInfo': True, 'enableCExtensions': True, 'force2D': False, 'interpolator': 3, 'resampledPixelSpacing': [3, 3, 3], 'label': 1, 'normalizeScale': 1, 'normalize': False, 'force2Ddimension': 0, 'removeOutliers': None, 'minimumROISi
{'distances': [1], 'verbose': True, 'additionalInfo': True, 'enableCExtensions': True, 'force2D': False, 'interpolator': 3, 'resampledPixelSpacing': [3, 3, 3], 'label': 1, 'normalizeScale': 1, 'normalize': False, 'force2Ddimension': 0, 'removeOutliers': None, 'minimumROISi
{'distances': [1], 'verbose': True, 'additionalInfo': True, 'enableCExtensions': True, 'force2D': False, 'interpolator': 3, 'resampledPixelSpacing': [3, 3, 3], 'label': 1, 'normalizeScale': 1, 'normalize': False, 'force2Ddimension': 0, 'removeOutliers': None, 'minimumROISi
{'distances': [1], 'verbose': True, 'additionalInfo': True, 'enableCExtensions': True, 'force2D': False, 'interpolator': 3, 'resampledPixelSpacing': [3, 3, 3], 'label': 1, 'normalizeScale': 1, 'normalize': False, 'force2Ddimension': 0, 'removeOutliers': None, 'minimumROISi
{'distances': [1], 'verbose': True, 'additionalInfo': True, 'enableCExtensions': True, 'force2D': False, 'interpolator': 3, 'resampledPixelSpacing': [3, 3, 3], 'label': 1, 'normalizeScale': 1, 'normalize': False, 'force2Ddimension': 0, 'removeOutliers': None, 'minimumROISi
{'distances': [1], 'verbose': True, 'additionalInfo': True, 'enableCExtensions': True, 'force2D': False, 'interpolator': 3, 'resampledPixelSpacing': [3, 3, 3], 'label': 1, 'normalizeScale': 1, 'normalize': False, 'force2Ddimension': 0, 'removeOutliers': None, 'minimumROISi
{'distances': [1], 'verbose': True, 'additionalInfo': True, 'enableCExtensions': True, 'force2D': False, 'interpolator': 3, 'resampledPixelSpacing': [3, 3, 3], 'label': 1, 'normalizeScale': 1, 'normalize': False, 'force2Ddimension': 0, 'removeOutliers': None, 'minimumROISi
{'distances': [1], 'verbose': True, 'additionalInfo': True, 'enableCExtensions': True, 'force2D': False, 'interpolator': 3, 'resampledPixelSpacing': [3, 3, 3], 'label': 1, 'normalizeScale': 1, 'normalize': False, 'force2Ddimension': 0, 'removeOutliers': None, 'minimumROISi
{'distances': [1], 'verbose': True, 'additionalInfo': True, 'enableCExtensions': True, 'force2D': False, 'interpolator': 3, 'resampledPixelSpacing': [3, 3, 3], 'label': 1, 'normalizeScale': 1, 'normalize': False, 'force2Ddimension': 0, 'removeOutliers': None, 'minimumROISi
{'distances': [1], 'verbose': True, 'additionalInfo': True, 'enableCExtensions': True, 'force2D': False, 'interpolator': 3, 'resampledPixelSpacing': [3, 3, 3], 'label': 1, 'normalizeScale': 1, 'normalize': False, 'force2Ddimension': 0, 'removeOutliers': None, 'minimumROISi
{'distances': [1], 'verbose': True, 'additionalInfo': True, 'enableCExtensions': True, 'force2D': False, 'interpolator': 3, 'resampledPixelSpacing': [3, 3, 3], 'label': 1, 'normalizeScale': 1, 'normalize': False, 'force2Ddimension': 0, 'removeOutliers': None, 'minimumROISi
{'distances': [1], 'verbose': True, 'additionalInfo': True, 'enableCExtensions': True, 'force2D': False, 'interpolator': 3, 'resampledPixelSpacing': [3, 3, 3], 'label': 1, 'normalizeScale': 1, 'normalize': False, 'force2Ddimension': 0, 'removeOutliers': None, 'minimumROISi
{'distances': [1], 'verbose': True, 'additionalInfo': True, 'enableCExtensions': True, 'force2D': False, 'interpolator': 3, 'resampledPixelSpacing': [3, 3, 3], 'label': 1, 'normalizeScale': 1, 'normalize': False, 'force2Ddimension': 0, 'removeOutliers': None, 'minimumROISi
{'distances': [1], 'verbose': True, 'additionalInfo': True, 'enableCExtensions': True, 'force2D': False, 'interpolator': 3, 'resampledPixelSpacing': [3, 3, 3], 'label': 1, 'normalizeScale': 1, 'normalize': False, 'force2Ddimension': 0, 'removeOutliers': None, 'minimumROISi
{'distances': [1], 'verbose': True, 'additionalInfo': True, 'enableCExtensions': True, 'force2D': False, 'interpolator': 3, 'resampledPixelSpacing': [3, 3, 3], 'label': 1, 'normalizeScale': 1, 'normalize': False, 'force2Ddimension': 0, 'removeOutliers': None, 'minimumROISi
{'distances': [1], 'verbose': True, 'additionalInfo': True, 'enableCExtensions': True, 'force2D': False, 'interpolator': 3, 'resampledPixelSpacing': [3, 3, 3], 'label': 1, 'normalizeScale': 1, 'normalize': False, 'force2Ddimension': 0, 'removeOutliers': None, 'minimumROISi
{'distances': [1], 'verbose': True, 'additionalInfo': True, 'enableCExtensions': True, 'force2D': False, 'interpolator': 3, 'resampledPixelSpacing': [3, 3, 3], 'label': 1, 'normalizeScale': 1, 'normalize': False, 'force2Ddimension': 0, 'removeOutliers': None, 'minimumROISi
{'distances': [1], 'verbose': True, 'additionalInfo': True, 'enableCExtensions': True, 'force2D': False, 'interpolator': 3, 'resampledPixelSpacing': [3, 3, 3], 'label': 1, 'normalizeScale': 1, 'normalize': False, 'force2Ddimension': 0, 'removeOutliers': None, 'minimumROISi
{'distances': [1], 'verbose': True, 'additionalInfo': True, 'enableCExtensions': True, 'force2D': False, 'interpolator': 3, 'resampledPixelSpacing': [3, 3, 3], 'label': 1, 'normalizeScale': 1, 'normalize': False, 'force2Ddimension': 0, 'removeOutliers': None, 'minimumROISi
{'distances': [1], 'verbose': True, 'additionalInfo': True, 'enableCExtensions': True, 'force2D': False, 'interpolator': 3, 'resampledPixelSpacing': [3, 3, 3], 'label': 1, 'normalizeScale': 1, 'normalize': False, 'force2Ddimension': 0, 'removeOutliers': None, 'minimumROISi
{'distances': [1], 'verbose': True, 'additionalInfo': True, 'enableCExtensions': True, 'force2D': False, 'interpolator': 3, 'resampledPixelSpacing': [3, 3, 3], 'label': 1, 'normalizeScale': 1, 'normalize': False, 'force2Ddimension': 0, 'removeOutliers': None, 'minimumROISi
{'distances': [1], 'verbose': True, 'additionalInfo': True, 'enableCExtensions': True, 'force2D': False, 'interpolator': 3, 'resampledPixelSpacing': [3, 3, 3], 'label': 1, 'normalizeScale': 1, 'normalize': False, 'force2Ddimension': 0, 'removeOutliers': None, 'minimumROISi
{'distances': [1], 'verbose': True, 'additionalInfo': True, 'enableCExtensions': True, 'force2D': False, 'interpolator': 3, 'resampledPixelSpacing': [3, 3, 3], 'label': 1, 'normalizeScale': 1, 'normalize': False, 'force2Ddimension': 0, 'removeOutliers': None, 'minimumROISi
{'distances': [1], 'verbose': True, 'additionalInfo': True, 'enableCExtensions': True, 'force2D': False, 'interpolator': 3, 'resampledPixelSpacing': [3, 3, 3], 'label': 1, 'normalizeScale': 1, 'normalize': False, 'force2Ddimension': 0, 'removeOutliers': None, 'minimumROISi
{'distances': [1], 'verbose': True, 'additionalInfo': True, 'enableCExtensions': True, 'force2D': False, 'interpolator': 3, 'resampledPixelSpacing': [3, 3, 3], 'label': 1, 'normalizeScale': 1, 'normalize': False, 'force2Ddimension': 0, 'removeOutliers': None, 'minimumROISi
{'distances': [1], 'verbose': True, 'additionalInfo': True, 'enableCExtensions': True, 'force2D': False, 'interpolator': 3, 'resampledPixelSpacing': [3, 3, 3], 'label': 1, 'normalizeScale': 1, 'normalize': False, 'force2Ddimension': 0, 'removeOutliers': None, 'minimumROISi
{'distances': [1], 'verbose': True, 'additionalInfo': True, 'enableCExtensions': True, 'force2D': False, 'interpolator': 3, 'resampledPixelSpacing': [3, 3, 3], 'label': 1, 'normalizeScale': 1, 'normalize': False, 'force2Ddimension': 0, 'removeOutliers': None, 'minimumROISi
{'distances': [1], 'verbose': True, 'additionalInfo': True, 'enableCExtensions': True, 'force2D': False, 'interpolator': 3, 'resampledPixelSpacing': [3, 3, 3], 'label': 1, 'normalizeScale': 1, 'normalize': False, 'force2Ddimension': 0, 'removeOutliers': None, 'minimumROISi
{'distances': [1], 'verbose': True, 'additionalInfo': True, 'enableCExtensions': True, 'force2D': False, 'interpolator': 3, 'resampledPixelSpacing': [3, 3, 3], 'label': 1, 'normalizeScale': 1, 'normalize': False, 'force2Ddimension': 0, 'removeOutliers': None, 'minimumROISi
{'distances': [1], 'verbose': True, 'additionalInfo': True, 'enableCExtensions': True, 'force2D': False, 'interpolator': 3, 'resampledPixelSpacing': [3, 3, 3], 'label': 1, 'normalizeScale': 1, 'normalize': False, 'force2Ddimension': 0, 'removeOutliers': None, 'minimumROISi
{'distances': [1], 'verbose': True, 'additionalInfo': True, 'enableCExtensions': True, 'force2D': False, 'interpolator': 3, 'resampledPixelSpacing': [3, 3, 3], 'label': 1, 'normalizeScale': 1, 'normalize': False, 'force2Ddimension': 0, 'removeOutliers': None, 'minimumROISi
{'distances': [1], 'verbose': True, 'additionalInfo': True, 'enableCExtensions': True, 'force2D': False, 'interpolator': 3, 'resampledPixelSpacing': [3, 3, 3], 'label': 1, 'normalizeScale': 1, 'normalize': False, 'force2Ddimension': 0, 'removeOutliers': None, 'minimumROISi
{'distances': [1], 'verbose': True, 'additionalInfo': True, 'enableCExtensions': True, 'force2D': False, 'interpolator': 3, 'resampledPixelSpacing': [3, 3, 3], 'label': 1, 'normalizeScale': 1, 'normalize': False, 'force2Ddimension': 0, 'removeOutliers': None, 'minimumROISi
{'distances': [1], 'verbose': True, 'additionalInfo': True, 'enableCExtensions': True, 'force2D': False, 'interpolator': 3, 'resampledPixelSpacing': [3, 3, 3], 'label': 1, 'normalizeScale': 1, 'normalize': False, 'force2Ddimension': 0, 'removeOutliers': None, 'minimumROISi
{'distances': [1], 'verbose': True, 'additionalInfo': True, 'enableCExtensions': True, 'force2D': False, 'interpolator': 3, 'resampledPixelSpacing': [3, 3, 3], 'label': 1, 'normalizeScale': 1, 'normalize': False, 'force2Ddimension': 0, 'removeOutliers': None, 'minimumROISi
{'distances': [1], 'verbose': True, 'additionalInfo': True, 'enableCExtensions': True, 'force2D': False, 'interpolator': 3, 'resampledPixelSpacing': [3, 3, 3], 'label': 1, 'normalizeScale': 1, 'normalize': False, 'force2Ddimension': 0, 'removeOutliers': None, 'minimumROISi
{'distances': [1], 'verbose': True, 'additionalInfo': True, 'enableCExtensions': True, 'force2D': False, 'interpolator': 3, 'resampledPixelSpacing': [3, 3, 3], 'label': 1, 'normalizeScale': 1, 'normalize': False, 'force2Ddimension': 0, 'removeOutliers': None, 'minimumROISi
{'distances': [1], 'verbose': True, 'additionalInfo': True, 'enableCExtensions': True, 'force2D': False, 'interpolator': 3, 'resampledPixelSpacing': [3, 3, 3], 'label': 1, 'normalizeScale': 1, 'normalize': False, 'force2Ddimension': 0, 'removeOutliers': None, 'minimumROISi
{'distances': [1], 'verbose': True, 'additionalInfo': True, 'enableCExtensions': True, 'force2D': False, 'interpolator': 3, 'resampledPixelSpacing': [3, 3, 3], 'label': 1, 'normalizeScale': 1, 'normalize': False, 'force2Ddimension': 0, 'removeOutliers': None, 'minimumROISi
{'distances': [1], 'verbose': True, 'additionalInfo': True, 'enableCExtensions': True, 'force2D': False, 'interpolator': 3, 'resampledPixelSpacing': [3, 3, 3], 'label': 1, 'normalizeScale': 1, 'normalize': False, 'force2Ddimension': 0, 'removeOutliers': None, 'minimumROISi
{'distances': [1], 'verbose': True, 'additionalInfo': True, 'enableCExtensions': True, 'force2D': False, 'interpolator': 3, 'resampledPixelSpacing': [3, 3, 3], 'label': 1, 'normalizeScale': 1, 'normalize': False, 'force2Ddimension': 0, 'removeOutliers': None, 'minimumROISi
{'distances': [1], 'verbose': True, 'additionalInfo': True, 'enableCExtensions': True, 'force2D': False, 'interpolator': 3, 'resampledPixelSpacing': [3, 3, 3], 'label': 1, 'normalizeScale': 1, 'normalize': False, 'force2Ddimension': 0, 'removeOutliers': None, 'minimumROISi
{'distances': [1], 'verbose': True, 'additionalInfo': True, 'enableCExtensions': True, 'force2D': False, 'interpolator': 3, 'resampledPixelSpacing': [3, 3, 3], 'label': 1, 'normalizeScale': 1, 'normalize': False, 'force2Ddimension': 0, 'removeOutliers': None, 'minimumROISi
{'distances': [1], 'verbose': True, 'additionalInfo': True, 'enableCExtensions': True, 'force2D': False, 'interpolator': 3, 'resampledPixelSpacing': [3, 3, 3], 'label': 1, 'normalizeScale': 1, 'normalize': False, 'force2Ddimension': 0, 'removeOutliers': None, 'minimumROISi
{'distances': [1], 'verbose': True, 'additionalInfo': True, 'enableCExtensions': True, 'force2D': False, 'interpolator': 3, 'resampledPixelSpacing': [3, 3, 3], 'label': 1, 'normalizeScale': 1, 'normalize': False, 'force2Ddimension': 0, 'removeOutliers': None, 'minimumROISi
{'distances': [1], 'verbose': True, 'additionalInfo': True, 'enableCExtensions': True, 'force2D': False, 'interpolator': 3, 'resampledPixelSpacing': [3, 3, 3], 'label': 1, 'normalizeScale': 1, 'normalize': False, 'force2Ddimension': 0, 'removeOutliers': None, 'minimumROISi
{'distances': [1], 'verbose': True, 'additionalInfo': True, 'enableCExtensions': True, 'force2D': False, 'interpolator': 3, 'resampledPixelSpacing': [3, 3, 3], 'label': 1, 'normalizeScale': 1, 'normalize': False, 'force2Ddimension': 0, 'removeOutliers': None, 'minimumROISi
{'distances': [1], 'verbose': True, 'additionalInfo': True, 'enableCExtensions': True, 'force2D': False, 'interpolator': 3, 'resampledPixelSpacing': [3, 3, 3], 'label': 1, 'normalizeScale': 1, 'normalize': False, 'force2Ddimension': 0, 'removeOutliers': None, 'minimumROISi
{'distances': [1], 'verbose': True, 'additionalInfo': True, 'enableCExtensions': True, 'force2D': False, 'interpolator': 3, 'resampledPixelSpacing': [3, 3, 3], 'label': 1, 'normalizeScale': 1, 'normalize': False, 'force2Ddimension': 0, 'removeOutliers': None, 'minimumROISi
{'distances': [1], 'verbose': True, 'additionalInfo': True, 'enableCExtensions': True, 'force2D': False, 'interpolator': 3, 'resampledPixelSpacing': [3, 3, 3], 'label': 1, 'normalizeScale': 1, 'normalize': False, 'force2Ddimension': 0, 'removeOutliers': None, 'minimumROISi
{'distances': [1], 'verbose': True, 'additionalInfo': True, 'enableCExtensions': True, 'force2D': False, 'interpolator': 3, 'resampledPixelSpacing': [3, 3, 3], 'label': 1, 'normalizeScale': 1, 'normalize': False, 'force2Ddimension': 0, 'removeOutliers': None, 'minimumROISi
{'distances': [1], 'verbose': True, 'additionalInfo': True, 'enableCExtensions': True, 'force2D': False, 'interpolator': 3, 'resampledPixelSpacing': [3, 3, 3], 'label': 1, 'normalizeScale': 1, 'normalize': False, 'force2Ddimension': 0, 'removeOutliers': None, 'minimumROISi
{'distances': [1], 'verbose': True, 'additionalInfo': True, 'enableCExtensions': True, 'force2D': False, 'interpolator': 3, 'resampledPixelSpacing': [3, 3, 3], 'label': 1, 'normalizeScale': 1, 'normalize': False, 'force2Ddimension': 0, 'removeOutliers': None, 'minimumROISi
{'distances': [1], 'verbose': True, 'additionalInfo': True, 'enableCExtensions': True, 'force2D': False, 'interpolator': 3, 'resampledPixelSpacing': [3, 3, 3], 'label': 1, 'normalizeScale': 1, 'normalize': False, 'force2Ddimension': 0, 'removeOutliers': None, 'minimumROISi
{'distances': [1], 'verbose': True, 'additionalInfo': True, 'enableCExtensions': True, 'force2D': False, 'interpolator': 3, 'resampledPixelSpacing': [3, 3, 3], 'label': 1, 'normalizeScale': 1, 'normalize': False, 'force2Ddimension': 0, 'removeOutliers': None, 'minimumROISi
{'distances': [1], 'verbose': True, 'additionalInfo': True, 'enableCExtensions': True, 'force2D': False, 'interpolator': 3, 'resampledPixelSpacing': [3, 3, 3], 'label': 1, 'normalizeScale': 1, 'normalize': False, 'force2Ddimension': 0, 'removeOutliers': None, 'minimumROISi
{'distances': [1], 'verbose': True, 'additionalInfo': True, 'enableCExtensions': True, 'force2D': False, 'interpolator': 3, 'resampledPixelSpacing': [3, 3, 3], 'label': 1, 'normalizeScale': 1, 'normalize': False, 'force2Ddimension': 0, 'removeOutliers': None, 'minimumROISi
{'distances': [1], 'verbose': True, 'additionalInfo': True, 'enableCExtensions': True, 'force2D': False, 'interpolator': 3, 'resampledPixelSpacing': [3, 3, 3], 'label': 1, 'normalizeScale': 1, 'normalize': False, 'force2Ddimension': 0, 'removeOutliers': None, 'minimumROISi
{'distances': [1], 'verbose': True, 'additionalInfo': True, 'enableCExtensions': True, 'force2D': False, 'interpolator': 3, 'resampledPixelSpacing': [3, 3, 3], 'label': 1, 'normalizeScale': 1, 'normalize': False, 'force2Ddimension': 0, 'removeOutliers': None, 'minimumROISi
{'distances': [1], 'verbose': True, 'additionalInfo': True, 'enableCExtensions': True, 'force2D': False, 'interpolator': 3, 'resampledPixelSpacing': [3, 3, 3], 'label': 1, 'normalizeScale': 1, 'normalize': False, 'force2Ddimension': 0, 'removeOutliers': None, 'minimumROISi
{'distances': [1], 'verbose': True, 'additionalInfo': True, 'enableCExtensions': True, 'force2D': False, 'interpolator': 3, 'resampledPixelSpacing': [3, 3, 3], 'label': 1, 'normalizeScale': 1, 'normalize': False, 'force2Ddimension': 0, 'removeOutliers': None, 'minimumROISi
{'distances': [1], 'verbose': True, 'additionalInfo': True, 'enableCExtensions': True, 'force2D': False, 'interpolator': 3, 'resampledPixelSpacing': [3, 3, 3], 'label': 1, 'normalizeScale': 1, 'normalize': False, 'force2Ddimension': 0, 'removeOutliers': None, 'minimumROISi
{'distances': [1], 'verbose': True, 'additionalInfo': True, 'enableCExtensions': True, 'force2D': False, 'interpolator': 3, 'resampledPixelSpacing': [3, 3, 3], 'label': 1, 'normalizeScale': 1, 'normalize': False, 'force2Ddimension': 0, 'removeOutliers': None, 'minimumROISi
{'distances': [1], 'verbose': True, 'additionalInfo': True, 'enableCExtensions': True, 'force2D': False, 'interpolator': 3, 'resampledPixelSpacing': [3, 3, 3], 'label': 1, 'normalizeScale': 1, 'normalize': False, 'force2Ddimension': 0, 'removeOutliers': None, 'minimumROISi
{'distances': [1], 'verbose': True, 'additionalInfo': True, 'enableCExtensions': True, 'force2D': False, 'interpolator': 3, 'resampledPixelSpacing': [3, 3, 3], 'label': 1, 'normalizeScale': 1, 'normalize': False, 'force2Ddimension': 0, 'removeOutliers': None, 'minimumROISi
{'distances': [1], 'verbose': True, 'additionalInfo': True, 'enableCExtensions': True, 'force2D': False, 'interpolator': 3, 'resampledPixelSpacing': [3, 3, 3], 'label': 1, 'normalizeScale': 1, 'normalize': False, 'force2Ddimension': 0, 'removeOutliers': None, 'minimumROISi

general_info_ImageHash	general_info_ImageSpacing	general_info_InputImages	general_info_MaskHash	general_info_Version	general_info_VolumeNum
ed65c08c80974cb4fd975d41e064408072eb9fc5	(1.0, 1.0, 1.0)	{'LoG': {}, 'Original': {}, 'Wavelet': {}}	f321f0cb914f9763db9046e3649711c8adcf6b0c	1.1.1-beta1	1
65ad1ca0547df3f3064fed30066db1a82774efcf	(1.0, 1.0, 1.0)	{'LoG': {}, 'Original': {}, 'Wavelet': {}}	94dc12d25621abfff4548ea4a75ba50c99bb5d1c	1.1.1-beta1	1
7da59d8ecc6ae1e315f9ebbade22cbdcbf329bff	(1.0, 1.0, 1.0)	{'LoG': {}, 'Original': {}, 'Wavelet': {}}	febe19aaa07c67848a4df79325ecd8212d0e1ab9	1.1.1-beta1	1
1ad8fc837aa7f23c48e4dbce3b8ae95447fca758	(1.0, 1.0, 1.0)	{'LoG': {}, 'Original': {}, 'Wavelet': {}}	2b40a9941312c32b9a52840a173869f891bc7534	1.1.1-beta1	1
314fd58bf855390c8419fda7f6ac787be3a080af	(1.0, 1.0, 1.0)	{'LoG': {}, 'Original': {}, 'Wavelet': {}}	9248c02cb620be7004ab1036161d2fe2d7eadf13	1.1.1-beta1	2
f7303295530dfe4c40be14bae21df46c9dac8f12	(1.0, 1.0, 1.0)	{'LoG': {}, 'Original': {}, 'Wavelet': {}}	070bbab21d2b8a0fc08a371d2c7412b01e25444b	1.1.1-beta1	1
aa1d92a0b4bfd52bb01fba1ce0891d056f607af6	(1.0, 1.0, 1.0)	{'LoG': {}, 'Original': {}, 'Wavelet': {}}	e5dac524633f757a698f523476a48ddf4092b7c1	1.1.1-beta1	1
ed7f9b7a46fb4f2c715cd36f3f6ca2aba60dbe72	(1.0, 1.0, 1.0)	{'LoG': {}, 'Original': {}, 'Wavelet': {}}	fc74ead87d4be0ce1dedb2f0b92f687849d04c38	1.1.1-beta1	1
a2885c5a291b9e749465e2a0ec811843c1356957	(1.0, 1.0, 1.0)	{'LoG': {}, 'Original': {}, 'Wavelet': {}}	96e70ff4e3c57115c401e1430fb3d9d70638c238	1.1.1-beta1	1
45186d25cd3d063f3db17aa6ad67e7f04c255dfd	(1.0, 1.0, 1.0)	{'LoG': {}, 'Original': {}, 'Wavelet': {}}	d4927113984fb34f7944ad993b92e47ba3bdfe9b	1.1.1-beta1	1
73bb392ea6f27824996321b54d01efa59d755779	(1.0, 1.0, 1.0)	{'LoG': {}, 'Original': {}, 'Wavelet': {}}	52e6da1a69edec614dc392d31d24786646ba4e04	1.1.1-beta1	1
df2a327f0fd4e4a0a788f198a38a51886ce4e7fe	(1.0, 1.0, 1.0)	{'LoG': {}, 'Original': {}, 'Wavelet': {}}	aa2d1bfd83d067314de3f18e4360eb6d15524a0c	1.1.1-beta1	1
cb57eb7e4f3b9b6a9d2032f40065cf95023e077a	(1.0, 1.0, 1.0)	{'LoG': {}, 'Original': {}, 'Wavelet': {}}	3ddc3a49808dfcac0d60bd0bd2752bffa7e2781d	1.1.1-beta1	1
8ef38ffe29df9cac94a667a31964ea2ca652e5bb	(1.0, 1.0, 1.0)	{'LoG': {}, 'Original': {}, 'Wavelet': {}}	95ae5b238602a539c9fbe9292ef943968a4da745	1.1.1-beta1	1
89c8f005ffea423f2786070d8f173966baa33df8	(1.0, 1.0, 1.0)	{'LoG': {}, 'Original': {}, 'Wavelet': {}}	0ce521e522b1945f7ae2a528d9d7a1f482fdd136	1.1.1-beta1	1
019cb604b3fbc1d0b89d44e8dcdeebed40cab556	(1.0, 1.0, 1.0)	{'LoG': {}, 'Original': {}, 'Wavelet': {}}	c3b4c5b3cde4ba2668d3c817d2d5533306ed0e90	1.1.1-beta1	1
e48cf6ca33cd38279eacb0fa088efc1e13829fcb	(1.0, 1.0, 1.0)	{'LoG': {}, 'Original': {}, 'Wavelet': {}}	c352fab5a9416219c8deb5c6f26094949b1c298f	1.1.1-beta1	1
16dd0656efdc9e17c85f904f727385322aedba74	(1.0, 1.0, 1.0)	{'LoG': {}, 'Original': {}, 'Wavelet': {}}	7c890d3de8f65ed1da126fc535d413ac461b2825	1.1.1-beta1	1
16010807008a65eb9589d163a02dca4259c592eb	(1.0, 1.0, 1.0)	{'LoG': {}, 'Original': {}, 'Wavelet': {}}	437160933b3cbab3fb32a92a7e4b769e9feed753	1.1.1-beta1	1
7f89bd9f0d38399c5078bdaf8c23dbb400da36d5	(1.0, 1.0, 1.0)	{'LoG': {}, 'Original': {}, 'Wavelet': {}}	089cf4b238fae8a34bb96590061549a709ffbf8a	1.1.1-beta1	1
e4c2a9d419938e931e307ed2fef3aa78ae25b852	(1.0, 1.0, 1.0)	{'LoG': {}, 'Original': {}, 'Wavelet': {}}	69eddedaa73dbe3fcb1e33a4967a17d8cc1f58b7	1.1.1-beta1	2
b72f76ef35094b561f82869dbd9ce66688dfdc0d	(1.0, 1.0, 1.0)	{'LoG': {}, 'Original': {}, 'Wavelet': {}}	88d04b9a50464d32edb0636541ef8e821c330359	1.1.1-beta1	1
598b8f00d15ac8a578c0f506c2fa81a09ca45dae	(1.0, 1.0, 1.0)	{'LoG': {}, 'Original': {}, 'Wavelet': {}}	2425824029182dd10df8fc77ec20617b80145285	1.1.1-beta1	1
585288372551a5a7b62426ba406e35cc42ba2307	(1.0, 1.0, 1.0)	{'LoG': {}, 'Original': {}, 'Wavelet': {}}	1c0bbadf4abf7032f0c991b7246b1f4926d8fe9c	1.1.1-beta1	2
88dfd708f1296121cc8bc50d5a588774b97fcaee	(1.0, 1.0, 1.0)	{'LoG': {}, 'Original': {}, 'Wavelet': {}}	5fa7f7213e1e6ff3e0290ab213567c966fbf3b1a	1.1.1-beta1	1
0899aa8ed9ab2538553f22b4ca145c56d5b05c5d	(1.0, 1.0, 1.0)	{'LoG': {}, 'Original': {}, 'Wavelet': {}}	0307228a7088de3faa7f97dc3d330481cf233729	1.1.1-beta1	2
75992e6c089e7148cd27cfcb442e09ecc06d0933	(1.0, 1.0, 1.0)	{'LoG': {}, 'Original': {}, 'Wavelet': {}}	4762e75a5bf86a94b33d427ccd438624c3083d07	1.1.1-beta1	2
4618b426281c2e2b2187e4f17e263612b29add3d	(1.0, 1.0, 1.0)	{'LoG': {}, 'Original': {}, 'Wavelet': {}}	27420719f87a1afa48d64d8a333f6085e6c22ea1	1.1.1-beta1	1
8fe9f25f5324cac50cf02b6ee52d869af931a01a	(1.0, 1.0, 1.0)	{'LoG': {}, 'Original': {}, 'Wavelet': {}}	a8e2377739a1a8102f124b3ca289123f5745892e	1.1.1-beta1	1
c1d572ada6df0c9ad45d058195f67c5e18373f83	(1.0, 1.0, 1.0)	{'LoG': {}, 'Original': {}, 'Wavelet': {}}	398eaecad06a4cac00bf668816619079d1b36301	1.1.1-beta1	1
10d556d912f25c09216bafa5a66952f7d81fe841	(1.0, 1.0, 1.0)	{'LoG': {}, 'Original': {}, 'Wavelet': {}}	d1915955b0c8b814cd5b98bf53dfe3d0c5a17043	1.1.1-beta1	1
eb139341e5bdcb3228c5d66ca8cb59b543f7c121	(1.0, 1.0, 1.0)	{'LoG': {}, 'Original': {}, 'Wavelet': {}}	6e197f6d2a4de3a7c724906d41ccce1fa889dd3a	1.1.1-beta1	1
0f8bb6a679f462279fdc7030b67755129892e2a4	(1.0, 1.0, 1.0)	{'LoG': {}, 'Original': {}, 'Wavelet': {}}	18b5c2c935a7fbe6ed5ac24dce0714a3b6878bb1	1.1.1-beta1	2
488004ba1d23d972f102431a393957dd51c2b9bb	(1.0, 1.0, 1.0)	{'LoG': {}, 'Original': {}, 'Wavelet': {}}	2ea78f4a1a248feb88bcd521f6322246d099fb3d	1.1.1-beta1	1
e8f2c5199d3012336ad8ba7b56d5e4f689a00ae8	(1.0, 1.0, 1.0)	{'LoG': {}, 'Original': {}, 'Wavelet': {}}	7d484621c5ae3552e79fbbe1711dbb98095924db	1.1.1-beta1	1
a910d2fc98f00540b1f7953fcd1ae06a9ad06bc2	(1.0, 1.0, 1.0)	{'LoG': {}, 'Original': {}, 'Wavelet': {}}	db236094228a9defeb8f8987167a99a0f4726c28	1.1.1-beta1	1
41230a7cf76b04c1c8be28f95e81055d7454f454	(1.0, 1.0, 1.0)	{'LoG': {}, 'Original': {}, 'Wavelet': {}}	23b22786cb980cd4d112196b5358c629e751d558	1.1.1-beta1	1
c9833ed94573172487d3a2392d8edbd9ce169831	(1.0, 1.0, 1.0)	{'LoG': {}, 'Original': {}, 'Wavelet': {}}	cc24e57f3fb6b9ffcb0871d2a75d284db1ae46ad	1.1.1-beta1	1
b3585b13296efcf884530d0066f687afb24148d1	(1.0, 1.0, 1.0)	{'LoG': {}, 'Original': {}, 'Wavelet': {}}	c4dd5c9c08990a2ca49149df3687a4b4cb2f5b6d	1.1.1-beta1	1
6baca2b585341fcdca50a5175b832a8667da594c	(1.0, 1.0, 1.0)	{'LoG': {}, 'Original': {}, 'Wavelet': {}}	8b5a72d3d2ab2358539690421a2d065e23c3a263	1.1.1-beta1	1
0040252eb47789872b18d46df6e5ebd5f36ed66a	(1.0, 1.0, 1.0)	{'LoG': {}, 'Original': {}, 'Wavelet': {}}	ee5e84d1f5aec895082261ae1b181f49a6a1d0f6	1.1.1-beta1	1
4c13260d78c556a515754d7d4d7105016427b20b	(1.0, 1.0, 1.0)	{'LoG': {}, 'Original': {}, 'Wavelet': {}}	95dc61c6129aa89f1c5d1d44080c16b96bc28a90	1.1.1-beta1	1
0a3248d12a3ad904aff856fe2235bba407b54a68	(1.0, 1.0, 1.0)	{'LoG': {}, 'Original': {}, 'Wavelet': {}}	1886bc8d3f2038a2e4e694a61adab33e2128c78c	1.1.1-beta1	1
300ec0022da2f22a510eeaf86521db72d2680cfe	(1.0, 1.0, 1.0)	{'LoG': {}, 'Original': {}, 'Wavelet': {}}	231b2de446a28088ace02ebb19c02c0963b85f6b	1.1.1-beta1	1
fa63779c19a9655e95a0cbbf52490f5fd7bb8592	(1.0, 1.0, 1.0)	{'LoG': {}, 'Original': {}, 'Wavelet': {}}	eedb3b7ed6a952d2599c4493be325d4b4e978ac4	1.1.1-beta1	1
26420ffcbcae814458ecb352f9922a16cdcf7531	(1.0, 1.0, 1.0)	{'LoG': {}, 'Original': {}, 'Wavelet': {}}	7a7b7971a6d80299afd7bd439b1971c286375b0f	1.1.1-beta1	1
afd835f657fc4009b364a8267baa496797ba8a7a	(1.0, 1.0, 1.0)	{'LoG': {}, 'Original': {}, 'Wavelet': {}}	5fe9768ec217dc1b41c12f2d22d90e37c26786f8	1.1.1-beta1	2
3ad47ff43e11e3e7c5d94798ba0a1dcc8d191316	(1.0, 1.0, 1.0)	{'LoG': {}, 'Original': {}, 'Wavelet': {}}	f022a561ee29004faa41d133aee08aa3b6ba11e4	1.1.1-beta1	1
be465182b41363fb0a24c0f39e83be527145cef5	(1.0, 1.0, 1.0)	{'LoG': {}, 'Original': {}, 'Wavelet': {}}	885f6554ce52b44d6ffe76d5a6ba03f1de8d8a17	1.1.1-beta1	1
5ccee5534ebee2035cdc08fb25c930f46d416e76	(1.0, 1.0, 1.0)	{'LoG': {}, 'Original': {}, 'Wavelet': {}}	eb143341d37edd8a91dad7720d7b9ac8c95635ec	1.1.1-beta1	1
6b086e9648536757c76fe9546dc229c2078bc31e	(1.0, 1.0, 1.0)	{'LoG': {}, 'Original': {}, 'Wavelet': {}}	7367f40b8d146d1f780574de3a3ca9d4354401a5	1.1.1-beta1	1
deb3739483bf9882150472db24506ccdffc88835	(1.0, 1.0, 1.0)	{'LoG': {}, 'Original': {}, 'Wavelet': {}}	ce632d047a519dc7c6093b17062165c821bee9b9	1.1.1-beta1	2
ae2fb26d74deda60fcd9b5512d2d8c4cdc02f09b	(1.0, 1.0, 1.0)	{'LoG': {}, 'Original': {}, 'Wavelet': {}}	332399df6693ceeecfa744ca5f9aa348294439e1	1.1.1-beta1	1
e4c1f1b50a2622af66579a0cc705ed95224707ba	(1.0, 1.0, 1.0)	{'LoG': {}, 'Original': {}, 'Wavelet': {}}	9ecb37946e268e1d08bf6cbd73da9d5b413e8da2	1.1.1-beta1	1
81301f5e7f57e66819369a50fb8580038cd4307f	(1.0, 1.0, 1.0)	{'LoG': {}, 'Original': {}, 'Wavelet': {}}	07ee41daaffdb42fb60f39e8c010423705ec5b47	1.1.1-beta1	1
0988a7b4d40284e7aa00cdda8fdfd6e8170db508	(1.0, 1.0, 1.0)	{'LoG': {}, 'Original': {}, 'Wavelet': {}}	ba99f148301c16bbc474611d41ce4329d9c28ef6	1.1.1-beta1	1
bba9fd993814f7c2d8700f76c15be963fa6a13f9	(1.0, 1.0, 1.0)	{'LoG': {}, 'Original': {}, 'Wavelet': {}}	8608ee7fe6aa908127242d008313b2422dfed846	1.1.1-beta1	2
d31fc0b625e1403fbcef8e23f7659a794f8d2a92	(1.0, 1.0, 1.0)	{'LoG': {}, 'Original': {}, 'Wavelet': {}}	6ec78144391c1861e8891b697e51c1e1b9f6b8e3	1.1.1-beta1	1
ad39cbbd4fab07012ddc60e4825c63227300facc	(1.0, 1.0, 1.0)	{'LoG': {}, 'Original': {}, 'Wavelet': {}}	1fcb457baac71477b24b8a7ff8e846962da5a851	1.1.1-beta1	1
fbb72fe0721ecb66f8f14a631d06f617d8749d63	(1.0, 1.0, 1.0)	{'LoG': {}, 'Original': {}, 'Wavelet': {}}	99b64ec5056efb5b453d701f33da4424801699ec	1.1.1-beta1	2
1af4acb389de9db01f9f800373b5b5ee9f832914	(1.0, 1.0, 1.0)	{'LoG': {}, 'Original': {}, 'Wavelet': {}}	a9afbdedd44e6a2349deeb0f86db4fd6464bc4e4	1.1.1-beta1	1
934ce4d2a7a6080c14f4281770c6b6b8e7762379	(1.0, 1.0, 1.0)	{'LoG': {}, 'Original': {}, 'Wavelet': {}}	8487608900e0bdefb433d6346936aa2ad124dc08	1.1.1-beta1	1
69b1efb62fc712b7b2c2e9f857250ca3efbaa6a1	(1.0, 1.0, 1.0)	{'LoG': {}, 'Original': {}, 'Wavelet': {}}	10c530b2a5b5ddb004eede2ebad40a8b4b2c6b03	1.1.1-beta1	2
6f2d3c3db98af76d85136544b1253c8130449a21	(1.0, 1.0, 1.0)	{'LoG': {}, 'Original': {}, 'Wavelet': {}}	738212faed774b7a30e703369859df6213643056	1.1.1-beta1	1
cf2cdbe1128946803077cd3d274cbc70fc76011f	(1.0, 1.0, 1.0)	{'LoG': {}, 'Original': {}, 'Wavelet': {}}	bc8edb8674a827a14ad766015de1410336b432e7	1.1.1-beta1	1
ef087ab4b8bf4d7fd7bef824258b7d926e5e1fe4	(1.0, 1.0, 1.0)	{'LoG': {}, 'Original': {}, 'Wavelet': {}}	0af7e5a5203fd57d8fd50c6c945cf61657a4e93f	1.1.1-beta1	1
054fba93d28e56e01c378e31bf15e0717dfbf45e	(1.0, 1.0, 1.0)	{'LoG': {}, 'Original': {}, 'Wavelet': {}}	5229d15a6a293dece5f12cffad011158bec0c8b0	1.1.1-beta1	1
60e228850daf01015d767e6d5ac6f1d54dc80fbd	(1.0, 1.0, 1.0)	{'LoG': {}, 'Original': {}, 'Wavelet': {}}	28225b8cb3b9ff11ef2e5ea02c4b86a5e82b8fd8	1.1.1-beta1	2
050ceb7940625f4865e6256707da469e33ecd8d7	(1.0, 1.0, 1.0)	{'LoG': {}, 'Original': {}, 'Wavelet': {}}	b93697dee17dedf44d2a6ca27c320fb979dbd6c7	1.1.1-beta1	2
f218a4e109f512b25c86d77cc7e1ccf678b55635	(1.0, 1.0, 1.0)	{'LoG': {}, 'Original': {}, 'Wavelet': {}}	6ecd957434ea666a24e5ecb6e94605ec481b2a14	1.1.1-beta1	1
8bf3c8a5059fea0ae370db32a372863938187979	(1.0, 1.0, 1.0)	{'LoG': {}, 'Original': {}, 'Wavelet': {}}	bf16bbdfa93e576d49b18948b58d3cd9851ee7ab	1.1.1-beta1	1
a4207feed93d67307a4d9c541fc4036fe9faf03f	(1.0, 1.0, 1.0)	{'LoG': {}, 'Original': {}, 'Wavelet': {}}	be57e9c52517daafd9f65342a6fbe93b16fb1fb4	1.1.1-beta1	3
9a847ecddf2df6803b4253a70439f6719bd63f43	(1.0, 1.0, 1.0)	{'LoG': {}, 'Original': {}, 'Wavelet': {}}	55fcc85d7ed4ce202bf6ae29075a73769d16b3c8	1.1.1-beta1	1
2b60599e673e3162bc243e1fa284da7c7222285a	(1.0, 1.0, 1.0)	{'LoG': {}, 'Original': {}, 'Wavelet': {}}	33bac9f79e1840fa9dd25c1d0abf3232556fc221	1.1.1-beta1	1
6871c8e2428a7f0df42ad9c0b267ce065b97fddd	(1.0, 1.0, 1.0)	{'LoG': {}, 'Original': {}, 'Wavelet': {}}	376e6fb351efd2fe45d2430340545f1ca2043c4d	1.1.1-beta1	1
78520834edfc54938eb50245deb0b7bda563bc0d	(1.0, 1.0, 1.0)	{'LoG': {}, 'Original': {}, 'Wavelet': {}}	28da55365af2a45b57767ed0e94a93e5be95ec6b	1.1.1-beta1	1
19d2879a142774e357d38ac0e8fbb7492a584776	(1.0, 1.0, 1.0)	{'LoG': {}, 'Original': {}, 'Wavelet': {}}	d01884f8e08096d215f2deceb3b73a9b666a7b9a	1.1.1-beta1	1
c5454410a0192ace0171bad6421cfd2e11cd6772	(1.0, 1.0, 1.0)	{'LoG': {}, 'Original': {}, 'Wavelet': {}}	11bb95b43374a9403343907860b0cb98a4c02df5	1.1.1-beta1	1
e2e99cdda7f3e61d56e946a0b0bcc16d6fed45a6	(1.0, 1.0, 1.0)	{'LoG': {}, 'Original': {}, 'Wavelet': {}}	dceb3cb907d73234550207e1d76e4102dab4ed95	1.1.1-beta1	1
d6df8d2df93ef59b1bba950be2acb73a3f2f84c7	(1.0, 1.0, 1.0)	{'LoG': {}, 'Original': {}, 'Wavelet': {}}	296872bcfc4bb0557be793e2a5497840cb8934cb	1.1.1-beta1	2
ed65c08c80974cb4fd975d41e064408072eb9fc5	(1.0, 1.0, 1.0)	{'LoG': {}, 'Original': {}, 'Wavelet': {}}	12062dbc14a4235242d0cbbb0bbe0bb3c914c5a5	1.1.1-beta1	1
b54fcec088350813b092acb7a2f155e1fbdb8574	(1.0, 1.0, 1.0)	{'LoG': {}, 'Original': {}, 'Wavelet': {}}	8a17200ded8167649240df397a6cacd3e9fc7ac5	1.1.1-beta1	1
f7bab373d06471ab6d846c8ae3aa0b8840feb195	(1.0, 1.0, 1.0)	{'LoG': {}, 'Original': {}, 'Wavelet': {}}	787ddddd03f7ee5571616629f1e9acf5bfd4738e	1.1.1-beta1	1
8ea1a010ff3e496743303208f45e2b6a46e9638a	(1.0, 1.0, 1.0)	{'LoG': {}, 'Original': {}, 'Wavelet': {}}	c5573d613408c9fab44752d26d108ce6fca23e55	1.1.1-beta1	1
6dff4408189ff68146aa5b8501b7c858b599c1a0	(1.0, 1.0, 1.0)	{'LoG': {}, 'Original': {}, 'Wavelet': {}}	7f8d8527e887b1257774e81f9753ee506b3e5e4c	1.1.1-beta1	1
c88b281b945d81f98496f84645bb20b68ffee8f9	(1.0, 1.0, 1.0)	{'LoG': {}, 'Original': {}, 'Wavelet': {}}	153f497bad6f5583f03a3fc3bdd31a01c03caac0	1.1.1-beta1	1
9e3af65703fe371a72314f30f8610cee46c07806	(1.0, 1.0, 1.0)	{'LoG': {}, 'Original': {}, 'Wavelet': {}}	19192b6fffc00c404ecc3d7354fae72c3a049bf0	1.1.1-beta1	1
6dc8bb58620b346a123fda450f59eb23c3f875b2	(1.0, 1.0, 1.0)	{'LoG': {}, 'Original': {}, 'Wavelet': {}}	241c2e5ec730900266def224fa5698f867d48413	1.1.1-beta1	1
8a6ee45d54882e971c721576805518b0223edd45	(1.0, 1.0, 1.0)	{'LoG': {}, 'Original': {}, 'Wavelet': {}}	d31a5809bbb199861edd5fd56a38a3ad2ec85473	1.1.1-beta1	1
62ec0bfda20ae2f050345ff4ab504a7f5d0ee9d6	(1.0, 1.0, 1.0)	{'LoG': {}, 'Original': {}, 'Wavelet': {}}	059a9be7124bfde51a0b303d630a4e87512f380c	1.1.1-beta1	1
0f7b3f928363b4c91b7257db151003acc51dc16d	(1.0, 1.0, 1.0)	{'LoG': {}, 'Original': {}, 'Wavelet': {}}	e758386d49196b3f84ba9c4441dc12669b03988d	1.1.1-beta1	1
21632cf06e539ea4939f3b3dd4814f74dfb8d133	(1.0, 1.0, 1.0)	{'LoG': {}, 'Original': {}, 'Wavelet': {}}	92dfe5de3596ed4b4276572daaa310d8be07744c	1.1.1-beta1	1
285ebc6336a666cb2c34c76f74d2f91004ce6428	(1.0, 1.0, 1.0)	{'LoG': {}, 'Original': {}, 'Wavelet': {}}	4992df95b143dc06f55128a8fdc712f1c8af705b	1.1.1-beta1	1
8652292d9b0a653fb4d837fd11c908a3093e7e67	(1.0, 1.0, 1.0)	{'LoG': {}, 'Original': {}, 'Wavelet': {}}	804d7801063289c5eb9bfde5dd6f6b021a26b9e6	1.1.1-beta1	1
38de3f3af0ee7c556478b9c3d7d7f92423576c6b	(1.0, 1.0, 1.0)	{'LoG': {}, 'Original': {}, 'Wavelet': {}}	e2b693323a85bec6c8728cd6d0f6b2c7b7a23884	1.1.1-beta1	2
5a8ac6e079824e746b392f693cae4de3bf2660e8	(1.0, 1.0, 1.0)	{'LoG': {}, 'Original': {}, 'Wavelet': {}}	409a4e04e6ee1a5b14b58897f0f1b3a21cfaadd1	1.1.1-beta1	1
e7e5d7043754b07480d07b9ad918e2a543ea5b0d	(1.0, 1.0, 1.0)	{'LoG': {}, 'Original': {}, 'Wavelet': {}}	8076c2725819969590dce2e639d6bb8f3b40477e	1.1.1-beta1	1
53184def7583c0f3c45375d7b7c7a6817f6be21a	(1.0, 1.0, 1.0)	{'LoG': {}, 'Original': {}, 'Wavelet': {}}	59b57ca70ea867587979f16806d7ebd93bc88fb4	1.1.1-beta1	1
d76dda1554fb0894e0ccc0d972f7c221c9e3eb53	(1.0, 1.0, 1.0)	{'LoG': {}, 'Original': {}, 'Wavelet': {}}	a150e2f1613e6010ab6829404f1b3a9176a19ffc	1.1.1-beta1	1
014c1dd75c7dc2418eda6e673725cd7aa7f1ef8e	(1.0, 1.0, 1.0)	{'LoG': {}, 'Original': {}, 'Wavelet': {}}	abe9dd79495f44734ab5238b605f91f7302eb4a4	1.1.1-beta1	1

general_info_VoxelNum	original_shape_Maximum3DDiameter	original_shape_Compactness2	original_shape_Maximum2DDiameterSlice	original_shape_Sphericity	original_shape_Compactness1	original_shape_Elongation
2122	75.29940239	0.062623631	67.41661516	0.39711176	0.013276023	1.37134324
935	50.64582905	0.12526579	45	0.500354136	0.018776521	1.084464496
1534	72.31182476	0.082081686	56.36488268	0.434592362	0.015199245	1.380171384
977	47.52893855	0.148610242	42.63801121	0.529683259	0.02045141	1.069626577
2368	72.31182476	0.067607347	66.61080993	0.407378369	0.013794178	1.052796329
660	50.64582905	0.178251522	44.59820624	0.562787464	0.022398323	1.252392744
5651	159.4804063	0.026712429	110.6345335	0.298931118	0.008670728	1.59166319
5108	123.2923355	0.030995535	112.0892502	0.314122981	0.009340034	1.241208092
4546	132.7478813	0.032050915	102.396289	0.3176485	0.009497714	1.279671513
1072	71.18286311	0.098996848	56.92099788	0.462601592	0.016692046	1.233173163
1508	128.8953064	0.070497112	81.49846624	0.41310182	0.014085898	3.328643376
2305	92.36882591	0.055713602	80.04998438	0.381932907	0.012522167	1.072567913
1867	66.54321904	0.095325096	60.74537019	0.456810155	0.01637957	1.15252036
1717	65.8634952	0.096278065	55.07267925	0.458327346	0.016461239	1.155014047
1984	74.21590126	0.069458376	61.77378085	0.41106283	0.013981739	1.104529673
4376	157.1496102	0.036500973	121.4907404	0.331717318	0.010135638	1.804453217
1194	63	0.10162913	57.62811814	0.466665901	0.016912507	1.032722034
1233	54.74486277	0.138954403	43.26661531	0.517953498	0.019775844	1.153356446
1298	72.56031973	0.10626906	69.97142274	0.473662439	0.017294272	1.231602364
5199	155.3351216	0.028694781	104.1393297	0.30615003	0.008986702	1.239022226
3024	84	0.054923716	65.7951366	0.380119345	0.012433083	1.228603048
1079	59.09314681	0.114256089	56.36488268	0.485243565	0.017932406	1.118748893
1024	57.31491952	0.136920728	53.41348144	0.515414224	0.019630596	1.210314268
2647	107.5825265	0.042311806	97.9489663	0.348460739	0.010912636	1.340837538
1273	79.88116173	0.102859108	71.30918594	0.468540983	0.017014542	1.696193855
2369	90.74690077	0.044223122	61.18823416	0.353630568	0.011156387	1.732878315
3233	109.4486181	0.050478069	84.95881355	0.369573576	0.011919286	1.765050642
2762	85.74963557	0.058286907	75.17978452	0.387724879	0.01280809	1.023909924
447	41.89272013	0.229140601	36.61966685	0.611928503	0.025395098	1.185071157
2054	74.75961477	0.062023837	68.47627326	0.395839878	0.013212293	1.088643779
4758	108.2081328	0.034887359	83.57032966	0.326755342	0.009909071	1.217189755
2069	66.2721661	0.096326682	55.80322571	0.458404495	0.016465396	1.144473142
3229	97.34988444	0.044814449	90.24965374	0.35519978	0.011230728	1.076333095
2350	100.9801961	0.061202265	88.63972022	0.394084328	0.013124496	1.703164973
4404	136.7552558	0.027473077	84.90583019	0.301742002	0.008793313	1.620972656
2999	103.4891299	0.051482769	74.09453421	0.372009445	0.012037321	1.587316569
3721	109.5718942	0.051937716	75.95393341	0.373102032	0.01209039	1.523897976
5161	138.1955137	0.033368297	104.1393297	0.321942279	0.00969094	1.383380029
1230	59.62382074	0.118952019	55.15432893	0.491802357	0.018297206	1.302046367
7270	169.864652	0.020369271	138.7155363	0.273102175	0.007571582	1.931486384
2594	71.37226352	0.070721392	62.42595614	0.413539437	0.014108287	1.081675111
3867	140.0392802	0.031677115	107.2054103	0.316408792	0.009442167	1.348571065
3653	107.2473776	0.037487146	100.8017857	0.334678228	0.010271647	1.144801578
2488	86.37708029	0.058254595	72.49827584	0.38765322	0.01280454	1.497122385
1278	69.45502142	0.101168582	64.41273166	0.465959911	0.016874142	1.25165878
2745	101.5135459	0.042035716	95.85927185	0.347701167	0.010876974	1.207567386
4562	113.6045774	0.030615753	98.27003612	0.312834744	0.009282637	1.224953626
871	52.39274759	0.151392993	46.57252409	0.532968971	0.020642	1.162285029
851	54.16641026	0.122987182	46.57252409	0.497301707	0.018604963	1.215071511
1352	87.05170877	0.091782305	52.39274759	0.451079393	0.016072312	1.752314885
2569	114.9826074	0.05871586	88.23264702	0.388673688	0.012855133	1.454575513
3371	95.85927185	0.035813095	84.69356528	0.329620301	0.010039678	1.293397983
4512	134.7998516	0.03245298	106.0660172	0.318971242	0.009557101	1.414285437
1104	71.62401832	0.129824365	67.08203932	0.506351463	0.019115118	1.181422404
1330	65.8634952	0.091616369	60.37383539	0.450807389	0.016057777	1.247025457
1950	69.97142274	0.075651511	60.37383539	0.422933938	0.01459176	1.012331435
4045	130.5258595	0.032432773	108.7060256	0.318905023	0.009554125	1.561005115
3458	78.2879301	0.054345653	68.47627326	0.378781073	0.012367482	1.043988825
1648	69.13031173	0.084278638	56.60388679	0.438435626	0.015401309	1.194810307
9129	182.0384575	0.016046155	138.4232639	0.252226276	0.006720234	1.173588073
1766	73.05477397	0.069224126	70.29224708	0.410600204	0.013958142	1.366404256
2670	89.19641248	0.055130026	77.82673063	0.380594697	0.012456412	1.275404315
5789	165.2180378	0.020664851	112.4499889	0.274416841	0.00762632	1.646006303
7623	169.6260593	0.021297894	146.6321929	0.277190848	0.00774225	1.629546298
790	60.07495318	0.141667007	47.4341649	0.521302218	0.019967939	1.218521775
3862	87.51571287	0.044749845	80.49844719	0.355029065	0.011222632	1.012293283
1304	51.35172831	0.156263378	46.95742753	0.538624044	0.020971403	1.157707207
6250	129.034879	0.025421706	111.3238519	0.294036718	0.008458653	1.147599939
5857	125.463142	0.013489791	106.0235823	0.238050124	0.006161713	1.241800574
2898	87.82368701	0.049217334	80.77747211	0.366470788	0.011769497	1.156910825
1299	63	0.115787806	61.84658438	0.487402338	0.018052206	1.227731265
4290	115.0217371	0.036703628	88.84255737	0.33233009	0.010163736	1.262364395
2279	70.61161378	0.061091921	62.42595614	0.393847348	0.013112659	1.039582073
2259	83.73171442	0.067216806	59.16924877	0.406592432	0.013754278	1.35820013
3131	128.7206277	0.046249784	80.77747211	0.358952159	0.011409162	2.32670799
1342	68.60757976	0.093968261	62.64183905	0.454632415	0.016262581	1.122263764
2396	84.21401309	0.04679019	78.74642849	0.360344809	0.011475623	1.017439272
958	49.65883607	0.150349514	41.67733197	0.531741647	0.020570739	1.071856034
1707	76.72027112	0.071972244	75.89466384	0.415963299	0.014232507	1.260131545
2339	77.88452991	0.061746052	69.26037828	0.395248046	0.013182673	1.282881672
1989	75.29940239	0.051115738	67.88225099	0.371123292	0.011994336	1.304387857
1799	63	0.078645585	55.31726674	0.428441419	0.014877709	1.111755813
3269	105	0.041245833	84.69356528	0.345509527	0.010774296	1.127928057
3073	82.54089895	0.052071076	74.09453421	0.373421097	0.012105902	1.071246961
2439	90.64766958	0.061229139	75.89466384	0.394142002	0.013127377	1.132754011
1532	54.41507144	0.115967818	47.4341649	0.487654791	0.018066234	1.017688366
2385	88.02840451	0.063968145	84.95881355	0.399933623	0.013417783	1.609351469
2742	97.5807358	0.047203068	89.49860334	0.361401605	0.011526143	1.499304299
1464	80.77747211	0.073833493	75.71657678	0.419518521	0.014415363	1.669785871
2025	68.01470429	0.073239808	63.28506933	0.418391063	0.01435729	1.076690928
1304	67.08203932	0.115228949	47.4341649	0.486616914	0.018008589	1.18555901
2382	103.6194962	0.040903759	100.8017857	0.344551709	0.010729525	1.412129602
5183	112.4899996	0.029840236	97.34988444	0.310170686	0.009164315	1.182290357
3300	106.0660172	0.039430137	103.3150521	0.340363329	0.010534478	1.281418137
4352	117.9576195	0.03346594	93.96275858	0.322255997	0.009705108	1.20904944
3422	89.69949833	0.041936427	87.20665112	0.347427193	0.010864121	1.051788815
2817	82.43178998	0.04570773	69.97142274	0.357544321	0.011342106	1.068982668
1047	51.35172831	0.155332305	46.86149806	0.53755214	0.020908832	1.137292599
834	45.29900661	0.161587626	39.11521443	0.544673234	0.021325682	1.140484171
5774	135.1332676	0.025896488	130.2497601	0.295855937	0.008537276	1.168917102

original_shape_SurfaceVolumeRatio	original_shape_Volume	original_shape_SphericalDisproportion	original_shape_Flatness	original_shape_SurfaceArea	original_shape_Maximum2DDiameterColumn
0.315889358	57294	2.518182791	1.060870444	18098.56487	63.78087488
0.329469213	25245	1.998584459	1.177446459	8317.450286	42.63801121
0.321616998	41418	2.301006847	1.098803186	13320.73283	58.24946352
0.306700936	26379	1.88792072	1.008726864	8090.46399	42.63801121
0.296873208	63936	2.454720416	1.215195865	18980.88541	62.42595614
0.328980742	17820	1.776869712	1.387353145	5862.436824	42.42640687
0.302749412	152577	3.345252267	1.41362668	46192.59697	129.5221989
0.297974795	137916	3.183466541	1.333219739	41095.49185	106.3202709
0.306341793	122742	3.148133865	1.589884282	37601.0044	101.5135459
0.340479382	28944	2.161687331	2.03891662	9854.835246	58.94064811
0.340281742	40716	2.420710712	1.151838123	13854.91142	51.0881591
0.319510755	62235	2.618260912	2.11767253	19884.75186	88.23264702
0.28657987	50409	2.189093191	1.130376851	14446.20464	55.07267925
0.293717846	46359	2.181846686	1.21738601	13616.46564	51.26402247
0.31208587	53568	2.432718131	1.336407572	16717.81588	66.61080993
0.29709975	118152	3.014614991	1.746993665	35102.92967	91.83136719
0.325603221	32238	2.142860658	1.822738775	10496.79663	55.80322571
0.290235862	33291	1.93067525	1.155041687	9662.242081	49.2036584
0.311986421	35046	2.111208148	1.758471733	10933.87612	55.31726674
0.303940535	140373	3.266372372	2.108168158	42665.04477	152.4106296
0.293257393	81648	2.630752721	1.33215662	23943.87961	80.49844719
0.323888805	29133	2.060820735	1.56723125	9435.852552	47.4341649
0.310293838	27648	1.940187043	1.46677384	8579.004025	48.83646179
0.334419071	71469	2.869763758	1.421949849	23900.59658	98.4073168
0.317447919	34371	2.134285017	1.657795676	10911.00241	52.47856705
0.341946412	63963	2.82780984	1.082443886	21871.91834	87.46427842
0.294980556	87291	2.705821153	1.028080536	25749.14767	77.4209274
0.296322509	74574	2.579148396	1.554712328	22097.95479	79.71198153
0.344529446	12069	1.634177842	1.663756938	4158.125887	39.11521443
0.320363611	55458	2.526274022	1.299218261	17766.72511	70.80254233
0.293313483	128466	3.060393732	1.106382081	37680.80996	87.46427842
0.275969131	55863	2.181479482	1.255523351	15416.46354	59.16924877
0.307044154	87183	2.815317061	1.854019857	26769.03052	92.61209424
0.30766948	63450	2.537527955	1.241193815	19521.62852	78.23042886
0.325920241	118908	3.314089501	1.629960964	38754.52399	126.1427762
0.300480815	80973	2.688103796	1.311578534	24330.83304	73.79024326
0.278814506	100467	2.680231982	1.66734344	28011.65695	79.71198153
0.289738963	139347	3.106146858	1.462739692	40374.25533	125.0319959
0.305917199	33210	2.033337144	1.284422305	10159.51016	56.60388679
0.304690963	196290	3.661633227	1.082166628	59807.7891	119.7747887
0.283697786	70038	2.418149056	1.077763371	19869.62554	66.61080993
0.324580883	104409	3.160468439	1.32541121	33889.16537	121.3795699
0.312741502	98631	2.987944588	1.994485559	30846.00712	93.04837452
0.306880539	67176	2.579625161	1.067699126	20615.00711	64.20280368
0.318789517	34506	2.146107372	1.935540431	11000.15107	58.24946352
0.331112776	74115	2.876032912	1.646524206	24540.4234	74.27651042
0.310691565	123174	3.196575893	1.256197167	38269.12282	109.2016483
0.31670498	23517	1.876281839	1.677687061	7447.951023	51
0.342057985	22977	2.010851732	1.242279258	7859.466315	48.46648326
0.323184844	36504	2.216904644	1.530863859	11797.53954	69.97142274
0.302823583	69363	2.57285232	1.689564594	21004.75219	79.71198153
0.326158975	91017	3.033793718	1.227442384	29686.01141	94.15412896
0.305835792	121824	3.135078868	1.436126259	37258.13957	113.406349
0.308026284	29808	1.974912829	3.431217186	9181.647468	60.74537019
0.325153148	35910	2.218242257	1.399265805	11676.24956	45.79301257
0.30507887	52650	2.364435459	1.401147935	16062.40253	61.55485359
0.3172454	109215	3.135729846	1.104763509	34647.9564	99.36297097
0.28142733	93366	2.640047435	1.163962953	26275.74408	71.11961755
0.311270501	44496	2.280836551	1.216860815	13850.29221	63.78087488
0.30579577	246483	3.964693995	1.598483487	75373.4588	144.1561653
0.324798097	47682	2.435459092	1.256177836	15487.02285	64.89992296
0.305302047	72090	2.627466983	1.473325217	22009.22459	69.0651866
0.327153014	156303	3.644091214	1.794150241	51134.99754	120.9338662
0.295489471	205821	3.60762272	1.218869348	60817.93831	106.0660172
0.334501138	21330	1.918273057	1.740365755	7134.909275	48.46648326
0.289397564	104274	2.816670799	1.286712951	30176.64161	84.95881355
0.273937362	35208	1.856582548	1.064679345	9644.786652	42.63801121
0.29762401	168750	3.400935797	1.611525663	50224.05171	126.6056871
0.375666714	158139	4.200795964	1.233973444	59407.55854	119.4738465
0.308525251	78246	2.728730454	1.459312009	24140.86677	76.4852927
0.303113685	35073	2.051693073	1.596365791	10631.10629	53.66563146
0.298520464	115830	3.009056451	1.285385926	34577.62532	102.0441081
0.311018939	61533	2.539054798	1.096574605	19137.92837	61.55485359
0.302156194	60993	2.459465352	1.399350443	18429.41275	75.23961722
0.306971892	84537	2.785886572	1.362375943	25950.48282	81.88406438
0.321453605	36234	2.199579194	2.331781831	11647.54993	67.08203932
0.334309714	64692	2.775119763	2.066616256	21627.16399	75.17978452
0.30752023	25866	1.880612523	1.164591426	7954.318263	45
0.324262467	46089	2.404058248	1.576808206	14944.93283	73.54590403
0.307243755	63153	2.530056785	1.196759338	19403.36487	71.11961755
0.345382024	53703	2.694522334	1.069422656	18548.05082	63.78087488
0.309357773	48573	2.334041376	1.077864207	15026.43511	56.60388679
0.314362839	88263	2.894276198	2.027983133	27746.60729	92.4175308
0.296922539	82971	2.677941895	1.353344217	24635.96	78
0.303836225	65853	2.537156644	1.424935409	20008.5269	74.09453421
0.286746056	41364	2.050630937	1.025575023	11860.96386	48.83646179
0.301679285	64395	2.500414922	1.138926958	19426.63758	62.42595614
0.318676699	74034	2.767004869	1.212123551	23592.91072	91.58602513
0.338400793	39528	2.383684985	1.178872823	13376.30653	67.41661516
0.304536109	54675	2.390108411	1.2045207	16650.51175	61.55485359
0.303214388	35208	2.055004607	1.348123981	10675.57218	55.80322571
0.350316997	64314	2.902321987	1.621650897	22530.28734	79.88116173
0.300309025	139941	3.224031304	1.303256396	42025.54522	104.7854952
0.31811352	89100	2.938036844	1.773667001	28343.91464	86.53323061
0.306383636	117504	3.103123011	1.514004814	36001.30277	112.6099463
0.307897191	92394	2.878300894	1.146789227	28447.85309	79.71198153
0.319230249	76059	2.796856056	1.129231199	24280.33348	81.05553652
0.295320354	28269	1.86028466	1.130995974	8348.411094	42.63801121
0.314416131	22518	1.835963175	1.082908012	7080.022433	40.80441153
0.303708528	155898	3.380023428	1.978124981	47347.55213	127.2792206

original_shape_Maximum2DDiameterRow	log.sigma.5.0.mm.3D_gldm_GrayLevelVariance	log.sigma.5.0.mm.3D_gldm_HighGrayLevelEmphasis	log.sigma.5.0.mm.3D_gldm_GrayLevelNonUniformityNormalized
68.41052551	29.79583773	473.0749293	0.056881767
42.63801121	11.04742372	89.73475936	0.098442621
65.7951366	21.79449599	212.1949153	0.060534023
40.36087214	27.317917	254.9355169	0.059370642
64.41273166	13.69204754	159.7466216	0.079170043
38.41874542	16.24443526	96.1030303	0.079889807
154.2011673	20.11847991	239.5735268	0.063423862
121.823643	20.74107389	533.6642522	0.071568452
121.823643	21.23469746	304.6451826	0.061904617
68.01470429	18.70794929	437.6585821	0.066988402
127.6322843	13.33828599	143.6432361	0.076575681
91.38927727	18.63949144	248.6650759	0.066975593
55.15432893	17.22762989	236.7498661	0.089019703
61.84658438	20.82261899	361.5765871	0.072867882
66.61080993	18.88880373	222.1668347	0.073899665
150.359569	23.82243348	341.4373857	0.059629724
55.80322571	14.40773171	233.938861	0.089129343
49.2036584	14.3364505	98.22303325	0.091217525
64.41273166	10.18744317	61.66640986	0.094368247
120.9338662	23.75879385	297.4420081	0.058465791
78	11.60540675	181.6904762	0.087751909
53.16013544	13.2745179	106.6413346	0.075976538
49.92995093	16.73999691	122.8662109	0.072454453
88.58893836	14.07673812	202.420476	0.080689104
71.30918594	15.47949589	327.3778476	0.074968729
85.90692638	22.52481958	431.3360068	0.069461478
85.27602242	27.28671126	601.2100217	0.064813459
78.74642849	14.13586438	248.429761	0.08371029
34.20526275	11.89695159	74.57941834	0.087898943
69.7782201	10.95302988	187.4133398	0.089007698
104.4844486	18.45629166	334.3064313	0.068099019
62.42595614	9.370883822	267.9758337	0.125010249
92.61209424	29.31431233	338.5469186	0.055956294
98.08669635	24.50556161	418.4689362	0.063487913
116.498927	17.3629412	389.5006812	0.068693022
99.90495483	14.33167219	299.3547849	0.075403483
104.4844486	18.37302797	644.7847353	0.094384227
137.3790377	34.44972246	512.5934896	0.049846183
45	4.631016591	48.91300813	0.129372728
167.9196236	30.25077572	283.6341128	0.051294669
62.42595614	16.84464832	401.1195066	0.08960361
94.86832981	25.94560138	332.7887251	0.058427121
103.3150521	21.78207248	349.111689	0.064019845
80.72174428	14.94837665	207.9662379	0.078002063
64.20280368	15.88361118	129.4945227	0.078686866
97.71898485	22.19028285	367.08051	0.062361439
107.4150827	14.72780826	255.2021043	0.076258115
40.80441153	9.621280685	51.31343284	0.095762027
42.63801121	18.07785684	171.5640423	0.072481259
67.08203932	11.46990159	99.88831361	0.087351415
112.4499889	21.9093249	266.4682756	0.060192634
89.89994438	22.40328588	247.7772174	0.060185727
119.7747887	16.36400052	195.6283245	0.07322249
70.03570518	14.94378069	193.2291667	0.07274765
61.84658438	19.56097236	134.0992481	0.063947086
59.54829972	12.76839343	284.2235897	0.084172518
128.8254633	22.89474475	295.0998764	0.059256052
73.23933369	20.71552909	479.2301909	0.074213206
58.24946352	9.225194631	226.3404126	0.099912221
177.1016657	18.84905236	299.9154343	0.069456652
67.88225099	15.74367344	174.3952435	0.084055951
87.20665112	20.19845264	273.7951311	0.063993884
144.7791421	21.66190762	665.6939022	0.064268844
150.7481343	21.20392009	331.4406402	0.068993084
53.41348144	10.83832879	177.1177215	0.087309726
80.77747211	19.21978111	402.0390989	0.075322607
46.95742753	6.433954397	136.1388037	0.129505956
128.1600562	23.149056	443.8912	0.065938074
115.4512884	20.07015504	343.1181492	0.067528996
76.83749085	11.64988919	183.2436163	0.083832357
51.26402247	17.78842611	316.7136259	0.079586891
102.396289	22.23720633	447.2216783	0.063826104
63.07138812	17.8398145	388.3896446	0.074735249
81.05553652	18.26712137	251.1571492	0.070589317
128.4056074	26.97179083	243.3557969	0.057322633
53.66563146	12.51291197	264.8539493	0.087237724
79.88116173	11.11226331	209.6798831	0.0883627
41.67733197	16.75575965	112.1951983	0.089506671
56.60388679	13.32538371	164.6139426	0.079007869
72.56031973	35.48248348	576.8255665	0.059562001
68.41052551	32.57671997	559.8205128	0.056269007
58.24946352	13.6486931	352.032796	0.094783372
103.4456379	21.01016876	319.933007	0.068161931
80.49844719	16.30519894	342.1588025	0.077926862
82.37718131	28.56190062	383.8511685	0.055907648
48.83646179	13.82106702	138.5835509	0.085769894
74.21590126	23.12077775	313.5052411	0.066502819
96.84007435	17.42013874	354.3340627	0.069621805
55.80322571	13.73179066	137.6038251	0.078810393
65.7951366	17.14204932	211.4587654	0.075050267
60	16.60145988	121.4125767	0.095139072
84.69356528	24.74589314	238.6150294	0.058626446
107.3312629	16.66468053	433.2010419	0.077400854
94.15412896	14.75095418	225.8542424	0.077342516
104.4844486	14.15972013	211.1001838	0.075937977
84.53401682	26.80623102	643.8103448	0.066386373
71.56116265	25.93641854	256.055733	0.057626064
40.80441153	20.41231189	421.1002865	0.104055887
40.80441153	7.673091742	101.9772182	0.102369903
131.041978	16.41613934	240.8294077	0.068879054

log.sigma.5.0.mm.3D_gldm_DependenceEntropy	log.sigma.5.0.mm.3D_gldm_DependenceNonUniformity	log.sigma.5.0.mm.3D_gldm_GrayLevelNonUniformity	log.sigma.5.0.mm.3D_gldm_SmallDependenceEmphasis
7.114493207	338.1225259	120.7031103	0.241218763
6.517927354	110.2898396	92.04385027	0.147026362
7.075326457	205.4146023	92.85919166	0.184039333
7.090119461	133.6509724	58.00511771	0.23050635
7.002184295	282.5869932	187.4746622	0.143252056
6.778930508	75.80606061	52.72727273	0.210791297
7.157187645	780.3218899	358.4082463	0.191015496
7.233213546	592.6002349	365.5716523	0.165050068
7.334505428	577.133304	281.4183898	0.176341243
6.80789156	155.1399254	71.81156716	0.2209211
6.85684351	191.4416446	115.4761273	0.159749976
7.068272894	299.0633406	154.3787419	0.171153774
7.13958805	182.3411891	166.1997858	0.122036129
7.094172292	210.0879441	125.1141526	0.172385109
6.909352487	280.3014113	146.6169355	0.197630141
7.046107753	685.5	260.9396709	0.184313044
6.55686931	167.9564489	106.4204355	0.166503881
6.930070715	119.8434712	112.4712084	0.147031051
6.768294452	136.3281972	122.4899846	0.121527384
7.193104105	770.6268513	303.9636469	0.187661366
7.039828074	301.8756614	265.3617725	0.12873655
6.677319899	144.6589435	81.97868397	0.154244464
6.874496713	122.5097656	74.19335938	0.145491439
7.058500332	313.3460521	213.5840574	0.172494526
6.710397382	192.0369207	95.43519246	0.218994337
7.02547685	332.2216125	164.5542423	0.21605281
7.208719981	427.4218992	209.5419115	0.201195179
7.004566539	312.0738595	231.2078204	0.156796607
6.377958931	51.21923937	39.29082774	0.151368964
6.945110341	227.2444012	182.8218111	0.116463408
7.241253271	562.5241698	324.0151324	0.161739751
6.634709226	194.1445143	258.6462059	0.110725175
7.258471967	462.9715082	180.682874	0.199329474
7.093323887	341.5268085	149.1965957	0.208785311
7.021603986	610.8828338	302.524069	0.19647961
7.002738052	353.9676559	226.135045	0.157037669
7.119450168	364.7248052	351.2037087	0.147178406
7.62041808	634.7202093	257.2561519	0.172794512
6.343352013	138.0520325	159.1284553	0.087332444
7.376267588	1107.322146	372.9122421	0.207024347
7.014532238	280.5674634	232.4317656	0.157280899
7.126168072	615.2932506	225.9376778	0.225653119
7.013213381	552.1776622	233.8644949	0.214157132
6.928769261	326.5747588	194.0691318	0.176288558
6.796773839	154.1032864	100.5618153	0.172031625
7.187337282	360.4040073	171.1821494	0.222162482
7.060695016	569.2836475	347.8895221	0.156934094
6.419984907	100.8484501	83.4087256	0.124809961
6.925348347	101.4559342	61.68155112	0.167695828
6.83791521	141.4778107	118.0991124	0.159208451
7.098805166	377.0918645	154.6348774	0.198407834
6.989122037	559.9095224	202.8860872	0.238117906
7.16975204	538.412234	330.3798759	0.154695498
6.692788487	156.1775362	80.3134058	0.222273953
6.949873226	177.0075188	85.04962406	0.194156494
6.889585073	241.3128205	164.1364103	0.141730585
7.197231139	596.3305315	239.6907293	0.213058226
7.159518157	416.7541932	256.6292655	0.142179952
6.776668847	171.0364078	164.6553398	0.147622839
7.424426711	1040.00011	634.0697776	0.154310554
7.003316771	186.0520951	148.4428086	0.16381559
7.121989329	353.5213483	170.8636704	0.176569677
7.169610701	812.739506	372.0523406	0.215576007
7.311760419	903.8576676	525.9342778	0.154616997
6.468597366	108.7265823	68.97468354	0.164986723
7.274214179	402.1926463	290.8959089	0.138853374
6.804613557	107.1947853	168.8757669	0.075724394
7.257398035	826.72672	412.11296	0.184198583
7.140303997	776.5974048	395.5173297	0.208761485
6.98929599	316.4513458	242.9461698	0.123839933
6.85275165	154.5765974	103.3833718	0.176924286
7.304669033	528.0503497	273.813986	0.166618251
6.980806373	288.1171566	170.3216323	0.159687271
6.896060702	319.7233289	159.461266	0.190750622
7.319267048	424.4461833	179.4771638	0.188510691
6.772564835	162.5633383	117.0730253	0.166019368
6.963228241	256.5258765	211.7170284	0.150560784
6.916450335	93.32150313	85.7473904	0.132022703
6.874691704	212.980082	134.8664323	0.149732513
7.053389817	367.2129115	139.3155195	0.233445861
7.123506104	323.8979387	111.9190548	0.25188702
6.948165316	180.4719288	170.5152863	0.137133814
7.165256438	414.2909147	222.8213521	0.167070198
7.049371642	382.5958347	239.4692483	0.140079739
7.364621306	334.4571546	136.3587536	0.202947752
6.994512322	149.189295	131.3994778	0.113186029
7.106847801	322.9371069	158.6092243	0.187513142
7.09735885	346.4646244	190.9029905	0.194837928
6.855730405	180.4180328	115.3784153	0.164629911
7.062267321	242.948642	151.9767901	0.152194245
6.682812844	110.3619632	124.0613497	0.136380522
7.10538694	371.6356003	139.6481948	0.221428673
7.103783733	635.4958518	401.1686282	0.158582524
6.935968151	441.4684848	255.230303	0.183731001
6.985639639	566.589614	330.4820772	0.150914385
7.362126762	392.1513735	227.1741672	0.189370154
7.301556642	384.0976216	162.3326234	0.157708882
6.937551487	92.51289398	108.9465138	0.155053291
6.579722538	94.8441247	85.3764988	0.090453829
7.05953143	762.4121926	397.707655	0.178448606

log.sigma.5.0.mm.3D_gldm_DependenceNonUniformityNormalized	log.sigma.5.0.mm.3D_gldm_DependenceVariance	log.sigma.5.0.mm.3D_gldm_LargeDependenceEmphasis
0.159341435	5.656287837	18.65975495
0.117957048	7.375824302	29.87486631
0.133907824	5.092083993	22.42242503
0.136797311	8.215289425	24.63561924
0.119335723	7.029215554	29.76858108
0.114857668	13.48668503	36.12121212
0.138085629	5.07244897	21.24420457
0.116014142	7.030345937	28.49256069
0.126954092	6.895053913	25.91509019
0.14472008	5.03625181	19.50746269
0.126950693	5.90541691	25.71618037
0.129745484	6.193833833	24.86898048
0.09766534	11.70591237	42.83717193
0.122357568	8.86670857	29.70005824
0.141280953	5.612455084	21.44354839
0.156649909	3.455447321	17.65539305
0.140667043	4.893765309	21.91457286
0.097196651	13.2608629	43.11516626
0.105029428	9.471304674	37.71340524
0.148225976	5.372713271	21.01923447
0.099826608	9.890410665	39.18055556
0.134067603	4.945532755	23.50973123
0.119638443	6.000728607	27.95507813
0.118377806	8.592730092	29.825085
0.150853826	5.351973337	19.41005499
0.140237067	5.428367433	20.55255382
0.132205969	5.956052497	22.75007733
0.112988363	8.36187034	31.58435916
0.114584428	8.413775155	31.61297539
0.110635054	8.301520106	35.2969815
0.118227022	7.045410301	28.45985708
0.093834951	10.55869926	44.69840503
0.143379222	4.889716059	20.20594611
0.145330557	4.724408148	19.33957447
0.138710907	4.815522343	20.73569482
0.118028561	6.215107381	27.47215739
0.098017954	10.54447325	39.05106154
0.122983958	13.21910708	35.89633792
0.112237425	7.223078855	38.6195122
0.152313913	4.681609221	18.76836314
0.108160163	9.257325933	33.84271396
0.159113848	3.971485966	16.69019912
0.151157312	4.36159743	18.15083493
0.131259951	6.39714165	24.56913183
0.120581601	6.726793381	26.77308294
0.13129472	5.81524202	21.87650273
0.124788174	6.823307728	27.17404647
0.115784673	7.542415978	32.29047072
0.119219664	6.869244864	27.43243243
0.104643351	10.33866986	36.2943787
0.146785467	4.529789791	19.31140522
0.166095972	4.118306272	16.0762385
0.119328953	6.705217922	28.44991135
0.14146516	4.68776255	19.1884058
0.13308836	7.030957092	24.63308271
0.123750164	6.68437712	28.38564103
0.147424112	5.444110738	20.07639061
0.120518853	6.86951507	29.13765182
0.103784228	8.544335882	34.4381068
0.113922676	10.49551974	34.57739073
0.105352262	8.763088873	33.26727067
0.132404999	6.686283157	24.86516854
0.140393765	5.145828629	20.16496804
0.118569811	7.848295123	29.75482094
0.137628585	4.779580195	23.32405063
0.104141027	10.91484973	38.39720352
0.08220459	14.66645339	62.51380368
0.132276275	6.168895078	23.81696
0.132593035	7.116295032	24.09373399
0.109196462	8.340354667	34.95997239
0.118996611	7.49524624	27.90839107
0.12308866	6.917422531	27.04801865
0.126422622	6.154675124	25.90039491
0.141533125	4.699590698	20.42806552
0.135562499	7.189179898	24.7777068
0.121135125	6.497378071	26.78092399
0.107064222	8.457895184	33.65025042
0.097412843	11.60905418	41.80167015
0.124768648	6.21893722	27.05038079
0.156995687	4.264219498	16.99315947
0.162844615	5.83771831	18.47611865
0.100317915	9.543472518	37.64702613
0.126733226	5.844851582	24.92964209
0.124502387	6.12751033	27.3690205
0.137128805	6.543380333	22.99589996
0.097382046	11.16926116	43.87989556
0.135403399	5.5893436	22.44234801
0.126354713	7.517696198	26.11889132
0.123236361	6.747041939	26.86202186
0.119974638	7.033500991	28.55851852
0.084633407	15.3537652	51.6303681
0.156018304	5.027117036	18.60117548
0.122611586	7.011955819	27.58884816
0.133778329	6.010285032	23.46242424
0.130190628	5.802497837	25.39613971
0.114597128	9.509661246	30.96317943
0.136349883	5.778646029	24.4029109
0.088359975	14.24673215	47.03247373
0.113721972	6.927120174	35.91127098
0.132042292	6.200100327	24.18392795

log.sigma.5.0.mm.3D_gldm_LargeDependenceLowGrayLevelEmphasis	log.sigma.5.0.mm.3D_gldm_SmallDependenceHighGrayLevelEmphasis	log.sigma.5.0.mm.3D_gldm_LargeDependenceHighGrayLevelEmphasis
0.046903516	101.2456502	9458.080584
0.611607296	15.24165093	2174.581818
0.177645564	34.90890446	4710.071056
0.312897041	52.13803313	6195.525077
0.279819824	22.47925078	4397.461149
1.038927255	22.8887163	2172.809091
0.142295823	47.02144834	4645.643249
0.064727365	88.90718787	14151.59064
0.122499736	51.43099614	7355.6641
0.054678745	86.94374272	8458.833955
0.278034913	21.17592848	3773.69496
0.148529479	40.84688477	5696.437744
0.261689396	29.9707429	9173.137654
0.120030627	60.02273725	9558.199767
0.139277867	41.48925799	4874.963206
0.088949666	58.95813473	6109.747715
0.12749739	32.97913038	5653.436348
0.881162335	15.73594231	2985.54339
1.682759398	7.755749511	1831.378274
0.110710991	55.93812324	5651.573572
0.321631265	24.36461298	6326.529762
0.692704233	15.15861068	2347.940686
0.576045565	18.78230056	2980.780273
0.196272325	33.16055759	5559.592369
0.067842794	61.7681509	6406.958366
0.062186382	88.58092549	8975.141832
0.062369547	112.5461192	13862.59821
0.16848453	38.6914905	7134.308834
1.289339369	12.03274712	1546.051454
0.248320664	21.81687211	6074.648004
0.11148409	51.57161556	8913.620849
0.18426489	24.17432958	12799.01498
0.095846371	62.38500701	7197.40415
0.057815723	79.65800467	8418.924255
0.065244978	72.60326269	7917.594687
0.11849669	43.3446473	8098.59053
0.074408812	88.63864583	24662.60575
0.110831944	87.54200505	15252.71517
1.174452604	3.48249598	2081.109756
0.142766758	59.84989267	5120.133425
0.09501243	51.69485991	14551.54973
0.076282157	78.46994181	5315.802948
0.065726162	69.96322965	6167.25568
0.165672004	35.20137087	5124.788585
0.392206887	25.1938659	2683.453834
0.097538044	74.09555577	8143.656831
0.136874959	39.51799114	6502.071898
2.335534657	6.876217973	1129.04248
0.279339567	33.19513117	3918.797885
0.747154248	15.3939993	2852.506657
0.137905003	49.69871058	5138.945504
0.101706503	57.62714971	4045.714328
0.205884219	30.32112982	5250.218307
0.140172326	38.67660449	3580.986413
0.490945284	24.82216543	2712.494737
0.121374371	40.41490697	7859.262564
0.103268386	60.11158933	5766.036836
0.079358654	59.05782752	14421.56854
0.169387003	28.55948692	7748.612257
0.169453325	45.97309987	9303.424691
0.300937612	33.75624508	4568.549264
0.148179663	47.66721684	6381.039326
0.034283558	136.475457	13396.6464
0.126360696	58.48125938	8405.006428
0.17027385	27.92865017	3950.477215
0.12432068	57.73415797	13430.26178
0.546127222	9.745950097	7972.812117
0.069915768	82.09245801	10233.7792
0.081609625	60.43954027	8606.877412
0.269646704	22.46010338	5730.873016
0.12610253	51.53342998	8336.180908
0.076782433	74.60798959	11102.35501
0.08142408	56.37814773	10062.19658
0.110890461	43.80639877	5522.330235
0.189555701	50.35650632	5378.100607
0.113841224	39.36724799	7566.552906
0.202707369	28.15007515	6889.486227
0.848385016	18.78325968	3098.73382
0.250999792	25.45191093	4174.069127
0.077690198	117.9226891	11133.09833
0.041518902	126.5076878	11087.04827
0.126800072	44.79413415	13329.31017
0.099873235	50.13468078	7926.446926
0.113211769	44.17352364	9669.513179
0.100838176	76.37616309	8957.238622
0.546621755	17.6767385	4765.347258
0.148815818	52.9412096	7337.726625
0.097586094	66.81967923	8404.005835
0.345218414	21.93146214	3306.913934
0.204891199	30.75091144	5777.959506
1.054914062	19.5835176	3968.007669
0.159715892	51.2060129	4125.337112
0.076264522	65.81473323	12053.50473
0.136811959	39.50188907	5085.346364
0.165161632	30.99298934	5443.506434
0.061654262	122.4744698	17916.6578
0.186824406	39.11609116	6185.397586
0.166974055	56.95392511	20471.07354
0.479803147	8.206255482	3669.26259
0.149940294	41.51271777	5199.836682

log.sigma.5.0.mm.3D_gldm_SmallDependenceLowGrayLevelEmphasis	log.sigma.5.0.mm.3D_gldm_LowGrayLevelEmphasis	log.sigma.5.0.mm.3D_gldzm_DistanceZoneVariabilityNormalized
0.001630981	0.004056878	0.993630638
0.003860428	0.02116978	1
0.003134029	0.009272685	1
0.003171135	0.01698085	1
0.002056113	0.010492619	1
0.007479799	0.029325724	1
0.001531123	0.006782903	0.99669149
0.000556787	0.002775738	1
0.001313362	0.005322974	1
0.001837571	0.003964074	1
0.002388855	0.013468492	1
0.001646879	0.006783906	1
0.001387145	0.008862727	1
0.001113454	0.005627589	1
0.00242741	0.008593974	1
0.001178738	0.00519264	1
0.002184457	0.009775764	1
0.005391054	0.021903889	1
0.005885011	0.044647347	1
0.001322149	0.00564937	1
0.001134607	0.009153089	1
0.004973403	0.026577008	1
0.003378208	0.020243839	1
0.00203353	0.007594239	1
0.002053656	0.004804244	1
0.001415652	0.004294683	1
0.001023244	0.00327356	1
0.001436003	0.005994927	1
0.005945767	0.033363825	1
0.001466779	0.00759146	1
0.000941553	0.004523985	1
0.001880548	0.00743274	1
0.001650749	0.006303765	1
0.001525057	0.004230219	1
0.001049644	0.003676427	1
0.00104196	0.005344082	1
0.000547643	0.003006866	1
0.000667537	0.003621523	1
0.006121639	0.038850806	1
0.001799586	0.008560493	1
0.00114868	0.004745138	1
0.001448886	0.0051018	1
0.001396687	0.004412568	0.9978022
0.00206709	0.009013972	1
0.003090369	0.014193486	1
0.001360483	0.005751045	0.997126443
0.001092941	0.005701198	1
0.007694276	0.062496237	1
0.003009624	0.011729268	1
0.005884475	0.020251334	1
0.001903977	0.009399543	1
0.002434948	0.007514512	1
0.001844923	0.008519047	1
0.00341776	0.009364943	1
0.004125938	0.01832546	1
0.001027896	0.005511192	1
0.001742242	0.006358171	0.998062017
0.000964803	0.003884987	1
0.00262815	0.00695168	1
0.001010431	0.005438531	0.997389038
0.002552478	0.009495203	1
0.001332901	0.006768656	1
0.000673822	0.002066527	0.995798338
0.000714889	0.004419143	0.998481398
0.002741882	0.008783568	1
0.000750231	0.003889182	0.996587041
0.001709935	0.010592558	1
0.000853675	0.003503937	0.993916406
0.001596724	0.004828979	1
0.001801446	0.008423019	1
0.002034652	0.006606756	1
0.000868182	0.003347177	0.997503125
0.001093387	0.004703945	1
0.002429489	0.008657619	1
0.001989956	0.00872078	1
0.002122404	0.006305151	1
0.001947173	0.00755249	1
0.002441397	0.02003441	1
0.00202809	0.010177115	1
0.001168963	0.005796031	1
0.001584384	0.003882843	1
0.00127413	0.005278948	1
0.001724826	0.005529649	1
0.00090184	0.005728812	1
0.001086923	0.006065528	0.99667775
0.002042329	0.012558008	1
0.001554996	0.008027158	1
0.001092601	0.004406451	1
0.002736048	0.014277012	1
0.003992809	0.012668669	1
0.002220635	0.015676896	1
0.002201079	0.009909658	0.993836731
0.000919928	0.003857005	1
0.001673742	0.007142995	1
0.001489563	0.008360911	1
0.000821624	0.002603446	1
0.001888742	0.009138022	1
0.001145546	0.007654246	1
0.003174504	0.016448814	1
0.00149955	0.006077723	0.998225379

log.sigma.5.0.mm.3D_gldzm_LowIntensityEmphasis	log.sigma.5.0.mm.3D_gldzm_LargeDistanceEmphasis	log.sigma.5.0.mm.3D_gldzm_HighIntensitySmallDistanceEmphasis
0.006518132	1.009584665	423.2739617
0.026868413	1	110.4333333
0.016826212	1	192.013245
0.016648607	1	230.7345455
0.014778333	1	163.33687
0.032223026	1	112.6728395
0.0081851	1.004971002	252.5468103
0.004064402	1	548.9458288
0.007839232	1	289.4647577
0.007468114	1	405.6099291
0.016754306	1	136.9897959
0.009772062	1	242.6935123
0.01481116	1	252.6434109
0.008580397	1	352.7294833
0.012468432	1	214.3333333
0.006658936	1	325.9460727
0.01588996	1	193.7541667
0.035546675	1	112.0357143
0.05501485	1	67.67021277
0.007134007	1	305.297989
0.011148949	1	189.98
0.035560861	1	104.4248705
0.02371194	1	138.6069364
0.011678142	1	198.805293
0.008527944	1	293.4518072
0.006406438	1	418.2660099
0.005309722	1	562.1850829
0.009241459	1	251.434322
0.039466816	1	85.62068966
0.012590809	1	192.4341085
0.006620773	1	318.7703524
0.020896557	1	202.1308017
0.009022028	1	305.687915
0.00720588	1	381.8517179
0.00530223	1	373.4803719
0.00798732	1	277.7945736
0.005288727	1	601.1372213
0.004749145	1	500.6767068
0.088695993	1	40.36363636
0.009758076	1	295.7959528
0.008827017	1	318.845815
0.006284702	1	353.0939279
0.006431545	1.00330033	335.2475248
0.012654124	1	198.4980469
0.016959239	1	156.1732283
0.007336889	1.004316547	330.3971223
0.007550031	1	258.6121212
0.07393346	1	56
0.016858224	1	207.637931
0.033882017	1	102.0769231
0.011328162	1	253.9715719
0.009911009	1	249.3803109
0.012723884	1	198.8013605
0.015157853	1	179.7714286
0.021566197	1	132.730897
0.00860757	1	290.8044164
0.008396894	1.002909796	284.7005335
0.007590453	1	404.1299435
0.017416658	1	187.3207547
0.007478838	1.003921569	295.2498366
0.014593463	1	214.326284
0.008782505	1	274.9186691
0.003042296	1.006315789	640.9026316
0.005068077	1.002279635	393.5503419
0.015876941	1	177.262069
0.006525711	1.005128205	419.4482906
0.024581707	1	132.2169811
0.004802255	1.009153318	449.1880244
0.007946745	1	287.746927
0.014490846	1	183.3925
0.011761056	1	302.9325843
0.005343453	1.00375	456.3625
0.008382187	1	352.2904762
0.014264658	1	228.4296875
0.010417609	1	274.7356322
0.012135675	1	237.7471698
0.013579671	1	184.2971576
0.021406456	1	157.8783784
0.013897838	1	172.3611111
0.006670705	1	498.6
0.006186121	1	507.4104235
0.011175826	1	327.6438849
0.010386818	1	298.9353234
0.008247578	1	310.4425532
0.007089937	1.004991681	373.0203827
0.020585184	1	158.7878788
0.010073119	1	281.0056926
0.006243892	1	352.5050676
0.018705546	1	137.9446494
0.028201064	1	202.0393258
0.015708785	1	144.265
0.011029342	1.00927357	236.0823029
0.006347679	1	413.0730453
0.010148461	1	217.0898204
0.012298237	1	203.648834
0.004522839	1	656.292887
0.012299482	1	251.0573123
0.012708675	1	351.1208791
0.036600111	1	94.7721519
0.008331113	1.002664298	239.8696714

log.sigma.5.0.mm.3D_gldzm_LowIntensityLargeDistanceEmphasis	log.sigma.5.0.mm.3D_gldzm_HighIntensityEmphasis	log.sigma.5.0.mm.3D_gldzm_DistanceZoneVariability	log.sigma.5.0.mm.3D_gldzm_ZonePercentage
0.006557356	424.0047923	622.0127796	0.295004713
0.026868413	110.4333333	150	0.160427807
0.016826212	192.013245	302	0.196870926
0.016648607	230.7345455	275	0.2814739
0.014778333	163.33687	377	0.159206081
0.032223026	112.6728395	162	0.245454545
0.008241197	252.6760563	1203.006628	0.213590515
0.004064402	548.9458288	923	0.180696946
0.007839232	289.4647577	908	0.199736032
0.007468114	405.6099291	282	0.263059701
0.016754306	136.9897959	294	0.194960212
0.009772062	242.6935123	447	0.193926247
0.01481116	252.6434109	258	0.138189609
0.008580397	352.7294833	329	0.191613279
0.012468432	214.3333333	465	0.234375
0.006658936	325.9460727	853	0.194926874
0.01588996	193.7541667	240	0.201005025
0.035546675	112.0357143	196	0.158961882
0.05501485	67.67021277	188	0.144838213
0.007134007	305.297989	1094	0.210425082
0.011148949	189.98	400	0.132275132
0.035560861	104.4248705	193	0.178869323
0.02371194	138.6069364	173	0.168945313
0.011678142	198.805293	529	0.199848886
0.008527944	293.4518072	332	0.260801257
0.006406438	418.2660099	609	0.257070494
0.005309722	562.1850829	724	0.223940612
0.009241459	251.434322	472	0.170890659
0.039466816	85.62068966	87	0.194630872
0.012590809	192.4341085	258	0.125608569
0.006620773	318.7703524	823	0.172971837
0.020896557	202.1308017	237	0.114548091
0.009022028	305.687915	753	0.233199133
0.00720588	381.8517179	553	0.235319149
0.00530223	373.4803719	968	0.219800182
0.00798732	277.7945736	516	0.172057352
0.005288727	601.1372213	583	0.156678312
0.004749145	500.6767068	996	0.192985855
0.088695993	40.36363636	99	0.080487805
0.009758076	295.7959528	1779	0.244704264
0.008827017	318.845815	454	0.175019275
0.006284702	353.0939279	1054	0.27256271
0.006448383	335.4092409	907.0022002	0.248836573
0.012654124	198.4980469	512	0.205787781
0.016959239	156.1732283	254	0.198748044
0.00735375	330.6733813	693.0028777	0.253187614
0.007550031	258.6121212	825	0.180841736
0.07393346	56	115	0.132032147
0.016858224	207.637931	174	0.204465335
0.033882017	102.0769231	247	0.182692308
0.011328162	253.9715719	598	0.232775399
0.009911009	249.3803109	965	0.286265203
0.012723884	198.8013605	735	0.162898936
0.015157853	179.7714286	280	0.253623188
0.021566197	132.730897	301	0.226315789
0.00860757	290.8044164	317	0.162564103
0.008406962	284.9107662	1029.00194	0.254882571
0.007590453	404.1299435	531	0.153556969
0.017416658	187.3207547	265	0.160800971
0.007493341	295.6640523	1526.005229	0.167597765
0.014593463	214.326284	331	0.187429219
0.008782505	274.9186691	541	0.202621723
0.003060925	641.7347368	1419.012632	0.246156504
0.005078209	393.6785714	1314.00152	0.172635445
0.015876941	177.262069	145	0.183544304
0.006529898	421.0188034	583.0034188	0.151475919
0.024581707	132.2169811	106	0.081288344
0.004814517	450.9260107	1303.024409	0.20976
0.007946745	287.746927	1383	0.23612771
0.014490846	183.3925	400	0.138026225
0.011761056	302.9325843	267	0.205542725
0.005351201	456.81625	798.0025	0.186480186
0.008382187	352.2904762	420	0.184291356
0.014264658	228.4296875	512	0.22664896
0.010417609	274.7356322	696	0.222293197
0.012135675	237.7471698	265	0.197466468
0.013579671	184.2971576	387	0.161519199
0.021406456	157.8783784	148	0.154488518
0.013897838	172.3611111	288	0.168717047
0.006670705	498.6	640	0.273621206
0.006186121	507.4104235	614	0.308697838
0.011175826	327.6438849	278	0.154530295
0.010386818	298.9353234	603	0.18446008
0.008247578	310.4425532	470	0.152945005
0.007103765	373.4708819	599.0033278	0.246412464
0.020585184	158.7878788	165	0.10770235
0.010073119	281.0056926	527	0.220964361
0.006243892	352.5050676	592	0.215900802
0.018705546	137.9446494	271	0.18510929
0.028201064	202.0393258	356	0.175802469
0.015708785	144.265	200	0.153374233
0.011100897	236.5517774	643.0123648	0.271620487
0.006347679	413.0730453	972	0.187536176
0.010148461	217.0898204	668	0.202424242
0.012298237	203.648834	729	0.167509191
0.004522839	656.292887	717	0.209526593
0.012299482	251.0573123	506	0.179623713
0.012708675	351.1208791	182	0.17382999
0.036600111	94.7721519	79	0.094724221
0.008364006	239.9236234	1124.001776	0.195012123

log.sigma.5.0.mm.3D_gldzm_IntensityVariabilityNormalized	log.sigma.5.0.mm.3D_gldzm_LowIntensitySmallDistanceEmphasis	log.sigma.5.0.mm.3D_gldzm_IntensityVariability
0.046667823	0.006508326	29.21405751
0.076	0.026868413	11.4
0.058352704	0.016826212	17.62251656
0.050076033	0.016648607	13.77090909
0.067185444	0.014778333	25.32891247
0.074302698	0.032223026	12.03703704
0.057799401	0.008171076	69.76387738
0.04972128	0.004064402	45.89274106
0.054593045	0.007839232	49.57048458
0.060484885	0.007468114	17.05673759
0.073487899	0.016754306	21.60544218
0.062014224	0.009772062	27.72035794
0.049185746	0.01481116	12.68992248
0.047218706	0.008580397	15.53495441
0.054891895	0.012468432	25.52473118
0.054005654	0.006658936	46.06682298
0.064722222	0.01588996	15.53333333
0.068356935	0.035546675	13.39795918
0.089463558	0.05501485	16.81914894
0.050855756	0.007134007	55.63619744
0.0742625	0.011148949	29.705
0.076324197	0.035560861	14.73056995
0.069598049	0.02371194	12.04046243
0.062589113	0.011678142	33.10964083
0.060168384	0.008527944	19.97590361
0.049209854	0.006406438	29.96880131
0.045248161	0.005309722	32.75966851
0.063110457	0.009241459	29.78813559
0.086272955	0.039466816	7.505747126
0.069737396	0.012590809	17.99224806
0.054630763	0.006620773	44.96111786
0.073367872	0.020896557	17.38818565
0.045613033	0.009022028	34.34661355
0.048778813	0.00720588	26.97468354
0.063022932	0.00530223	61.00619835
0.066064239	0.00798732	34.08914729
0.044741077	0.005288727	26.08404803
0.043465025	0.004749145	43.29116466
0.109478625	0.088695993	10.83838384
0.04591921	0.009758076	81.69027544
0.058753323	0.008827017	26.67400881
0.052304585	0.006284702	55.12903226
0.05210213	0.006427335	47.36083608
0.061325073	0.012654124	31.3984375
0.068386137	0.016959239	17.37007874
0.054843952	0.007332673	38.11654676
0.060084481	0.007550031	49.56969697
0.106086957	0.07393346	12.2
0.058924561	0.016858224	10.25287356
0.086495435	0.033882017	21.36437247
0.055519513	0.011328162	33.2006689
0.054608714	0.009911009	52.69740933
0.05668564	0.012723884	41.66394558
0.062984694	0.015157853	17.63571429
0.066544519	0.021566197	20.02990033
0.06746012	0.00860757	21.38485804
0.05179588	0.008394377	53.40155189
0.050010462	0.007590453	26.55555556
0.071014596	0.017416658	18.81886792
0.055640993	0.007475213	85.13071895
0.057328794	0.014593463	18.97583082
0.05967931	0.008782505	32.28650647
0.053080702	0.003037639	75.64
0.053738879	0.005065545	70.72036474
0.072247325	0.015876941	10.47586207
0.049140186	0.006524665	28.74700855
0.078675685	0.024581707	8.339622642
0.04693781	0.00479919	61.53546911
0.051442237	0.007946745	71.14461316
0.072875	0.014490846	29.15
0.057919174	0.011761056	15.46441948
0.0486125	0.005341516	38.89
0.05314059	0.008382187	22.31904762
0.053817749	0.014264658	27.5546875
0.050349287	0.010417609	35.04310345
0.068422926	0.012135675	18.13207547
0.068285159	0.013579671	26.42635659
0.065467495	0.021406456	9.689189189
0.070577739	0.013897838	20.32638889
0.046191406	0.006670705	29.5625
0.046159641	0.006186121	28.34201954
0.051058434	0.011175826	14.1942446
0.048252381	0.010386818	29.09618574
0.061647804	0.008247578	28.97446809
0.048740175	0.00708648	29.29284526
0.072910927	0.020585184	12.03030303
0.050916541	0.010073119	26.83301708
0.060337153	0.006243892	35.71959459
0.075393854	0.018705546	20.43173432
0.053828431	0.028201064	19.16292135
0.0861	0.015708785	17.22
0.053278835	0.011011453	34.47140649
0.05466223	0.006347679	53.13168724
0.062031625	0.010148461	41.43712575
0.062447195	0.012298237	45.52400549
0.046484169	0.004522839	33.32914923
0.049235264	0.012299482	24.91304348
0.052650646	0.012708675	9.582417582
0.082839289	0.036600111	6.544303797
0.059363849	0.00832289	66.84369449

log.sigma.5.0.mm.3D_gldzm_HighIntensityLargeDistanceEmphasis	log.sigma.5.0.mm.3D_gldzm_SmallDistanceEmphasis	log.sigma.5.0.mm.3D_glcm_SumVariance	log.sigma.5.0.mm.3D_glcm_Homogeneity1
426.928115	0.997603834	1449.192469	0.400693067
110.4333333	1	221.5502747	0.506501443
192.013245	1	593.7513872	0.463408992
230.7345455	1	725.1401478	0.438498484
163.33687	1	429.3272931	0.491544461
112.6728395	1	229.9733128	0.480462954
253.1930406	0.998757249	686.4304755	0.443516309
548.9458288	1	1695.842164	0.477976168
289.4647577	1	923.2355552	0.454699491
405.6099291	1	1384.254391	0.428853712
136.9897959	1	389.7385416	0.486967226
242.6935123	1	716.5544309	0.468658237
252.6434109	1	698.9289607	0.522673984
352.7294833	1	1111.348997	0.465547286
214.3333333	1	643.0113446	0.440658482
325.9460727	1	1034.41429	0.416988546
193.7541667	1	680.8738061	0.461210469
112.0357143	1	246.7372029	0.517511868
67.67021277	1	138.2703007	0.527697948
305.297989	1	873.644079	0.434678228
189.98	1	504.0411496	0.526135784
104.4248705	1	267.5161255	0.478128572
138.6069364	1	314.7498876	0.498313377
198.805293	1	591.0625281	0.488765334
293.4518072	1	1023.763066	0.419112133
418.2660099	1	1313.401607	0.434072605
562.1850829	1	1951.32194	0.436557763
251.434322	1	730.297965	0.491303824
85.62068966	1	173.8709768	0.50445016
192.4341085	1	521.7797251	0.527705162
318.7703524	1	1023.624602	0.469891306
202.1308017	1	817.1104623	0.538899358
305.687915	1	1018.16291	0.430493784
381.8517179	1	1284.851621	0.421695136
373.4803719	1	1217.540935	0.446630465
277.7945736	1	904.936372	0.483893787
601.1372213	1	2137.985572	0.503550708
500.6767068	1	1599.971071	0.458519773
40.36363636	1	106.1472201	0.553896989
295.7959528	1	822.1107661	0.420466918
318.845815	1	1271.220336	0.481399632
353.0939279	1	986.2166387	0.40913954
336.0561056	0.999174917	1076.060665	0.412508242
198.4980469	1	588.3619025	0.460008031
156.1732283	1	331.7279598	0.484060923
331.7784173	0.998920863	1134.257067	0.44083623
258.6121212	1	754.9515159	0.476969958
56	1	107.5681456	0.518220039
207.637931	1	449.8127516	0.490830602
102.0769231	1	243.8545783	0.509441066
253.9715719	1	786.7914232	0.432437934
249.3803109	1	720.4701854	0.407404638
198.8013605	1	542.1837674	0.490282118
179.7714286	1	554.258624	0.428856072
132.730897	1	350.8138255	0.455840125
290.8044164	1	843.3321243	0.488885849
285.7516974	0.999272551	877.5877857	0.423545316
404.1299435	1	1510.866256	0.4763785
187.3207547	1	674.7552324	0.515720224
297.320915	0.999019608	901.6858329	0.482646682
214.326284	1	480.3742202	0.506449352
274.9186691	1	806.2693547	0.457614293
645.0631579	0.998421053	2196.918036	0.432271633
394.1914894	0.999430091	975.1927416	0.476004872
177.262069	1	494.2969176	0.468098963
427.3008547	0.998717949	1225.516608	0.502496943
132.2169811	1	374.4927976	0.590670829
457.8779558	0.99771167	1358.656005	0.44699039
287.746927	1	1105.284053	0.460607491
183.3925	1	513.7396782	0.5185682
302.9325843	1	971.9329541	0.469539133
458.63125	0.9990625	1390.336526	0.467702976
352.2904762	1	1213.835332	0.465048613
228.4296875	1	723.1009908	0.437524332
274.7356322	1	689.2165114	0.442928668
237.7471698	1	780.7674004	0.482085448
184.2971576	1	618.0219144	0.514666774
157.8783784	1	281.6921784	0.526328675
172.3611111	1	445.5124597	0.492870462
498.6	1	1801.707645	0.404710679
507.4104235	1	1753.298962	0.403823567
327.6438849	1	1091.109945	0.515119176
298.9353234	1	990.5258506	0.464528878
310.4425532	1	1053.077536	0.481531445
375.2728785	0.99875208	1161.345527	0.434018391
158.7878788	1	364.4384012	0.543403772
281.0056926	1	938.4300693	0.440081229
352.5050676	1	1089.51527	0.458532768
137.9446494	1	357.9220701	0.484488391
202.0393258	1	593.9696562	0.486000419
144.265	1	315.7610058	0.54502924
238.4296754	0.997681607	713.9599791	0.42332538
413.0730453	1	1339.170031	0.470896929
217.0898204	1	649.4198366	0.458985081
203.648834	1	593.6248337	0.477484549
656.292887	1	2105.984873	0.468378437
251.0573123	1	731.4627698	0.470202121
351.1208791	1	1327.985985	0.515499512
94.7721519	1	253.4855123	0.546737602
240.1394316	0.999333925	696.9130082	0.45602247

log.sigma.5.0.mm.3D_glcm_Homogeneity2	log.sigma.5.0.mm.3D_glcm_ClusterShade	log.sigma.5.0.mm.3D_glcm_MaximumProbability	log.sigma.5.0.mm.3D_glcm_Idmn	log.sigma.5.0.mm.3D_glcm_SumVariance2
0.319900835	-461.4092186	0.026039921	0.988748646	105.6793106
0.448025632	119.3675253	0.064178163	0.982603052	38.97825399
0.395190902	194.5623619	0.021836457	0.988210779	77.48465443
0.364920477	-394.6926212	0.032185647	0.985502963	100.8516071
0.428142137	89.30240944	0.042924745	0.988616692	48.45264768
0.414184587	160.0572433	0.058521573	0.982444779	57.47866011
0.369579128	177.0083783	0.017809154	0.992657917	72.48315608
0.413160399	183.5869884	0.032200691	0.994162262	73.23338411
0.384510901	8.394076092	0.023068211	0.990647086	74.94402647
0.350870554	-245.0545908	0.020518546	0.989787405	63.0871604
0.422207395	-4.34523076	0.026936306	0.988418979	46.34440876
0.399679261	-15.74766881	0.026470173	0.990725653	69.78443945
0.467746762	231.0803584	0.052552781	0.991907442	65.6658902
0.398107371	27.74051849	0.036975439	0.991875506	70.12114013
0.366013224	121.4720236	0.031079031	0.990399112	68.45770525
0.336068555	-350.9275187	0.017251186	0.989458299	81.87606278
0.391179454	-386.3417076	0.032295432	0.985359054	50.81517238
0.460741991	252.2682083	0.06326705	0.98368353	51.83814332
0.473017654	90.54505064	0.053909095	0.981737917	37.20953331
0.358405663	2.01637266	0.019624678	0.99182369	84.15201972
0.471319116	31.95121661	0.049090603	0.991259996	40.78760076
0.410493108	25.86265778	0.026650351	0.984505935	47.12368093
0.437109747	138.2768163	0.039561546	0.987429041	61.85564183
0.425415888	159.9604653	0.038517979	0.990946884	48.63588591
0.340168598	2.315332533	0.026636492	0.988542486	47.15666087
0.358301584	-436.7865728	0.032504519	0.991462284	74.54672713
0.363597357	-942.2148033	0.023679403	0.993825478	102.3679178
0.427412122	81.3495027	0.042726333	0.991465826	51.23723222
0.443369391	139.2936801	0.051888769	0.98106926	43.78785984
0.472937658	71.91540015	0.043299928	0.992724527	39.73724918
0.403273277	7.061921314	0.0271043	0.992259213	63.92010619
0.48872118	-287.0658135	0.058742586	0.990152644	32.48063792
0.354782589	-483.5838693	0.020790183	0.990933518	104.3201083
0.343351384	-366.6640888	0.02225268	0.991734823	81.15171053
0.373079224	-111.9215378	0.020195331	0.991922143	60.6341017
0.419353841	-102.6238803	0.025374778	0.990669888	50.06836926
0.446331295	-546.5355663	0.054728529	0.994634597	63.60462669
0.388131797	-337.2007441	0.043236016	0.993050572	128.1959649
0.505796328	3.728864839	0.048532075	0.984528928	15.31963534
0.342073251	11.59639933	0.014399464	0.990531893	108.3288886
0.41869788	-562.7938527	0.048145582	0.991232545	55.02475495
0.327189961	311.754921	0.018178538	0.992881532	94.91786247
0.333288459	158.125936	0.020770529	0.988251399	71.83666253
0.38961924	-56.79506838	0.032573029	0.992181489	50.93031545
0.419818709	303.0934587	0.03678486	0.987581739	56.08902889
0.368648441	-323.3502925	0.019547157	0.990772795	75.13075373
0.410560933	92.79867454	0.026110523	0.991584424	52.66317666
0.462067468	79.16586315	0.049269051	0.976273185	34.05104994
0.428023921	301.3168807	0.037030709	0.990405448	66.7308218
0.449160871	81.84794094	0.05736164	0.988161326	39.40829172
0.355199493	-63.5758951	0.017378906	0.98805766	77.70622037
0.325955516	105.3672673	0.016650241	0.986949602	79.5640573
0.426745637	150.0128475	0.031576161	0.991475596	59.5405545
0.35264894	8.279732164	0.022794451	0.981700318	45.17867134
0.385833365	129.7447842	0.031790841	0.983782953	69.39271271
0.424463609	3.632652117	0.041149817	0.993005836	45.25462792
0.34422567	-134.6756591	0.017268951	0.990061961	78.67987761
0.410487331	-636.6424974	0.031073726	0.992900148	72.17347215
0.460343545	-19.36026962	0.044866391	0.989549575	27.46459742
0.417005819	96.35047653	0.033736989	0.992905535	67.95640483
0.448282612	364.276105	0.049846486	0.991471192	55.72074498
0.387342379	-49.86920887	0.024959675	0.990118016	73.80587573
0.35685678	-245.9252422	0.019115685	0.994143568	77.58587189
0.410225809	381.6130136	0.028919199	0.993496617	75.19879066
0.396427208	57.36464899	0.035278779	0.987944687	32.9747231
0.442112957	78.18879092	0.045723064	0.994833397	68.63498951
0.550419249	30.91890871	0.091586419	0.992581472	22.69069214
0.374774701	-59.85655775	0.027156422	0.993723806	79.86318613
0.390686313	-228.3132093	0.025759663	0.991989775	57.40383094
0.462500697	35.06902494	0.037416727	0.989920097	40.63099453
0.403728645	-332.1863576	0.043341024	0.991167527	59.57340132
0.400687339	126.0332476	0.025917986	0.993258567	77.00602403
0.395627753	-341.0279108	0.030398527	0.991914294	59.40737232
0.363166384	-161.6807103	0.023457383	0.987249308	62.31359205
0.369173143	268.3555689	0.031999268	0.990237525	100.4649454
0.418110627	-213.6768818	0.034783856	0.988581774	43.81660098
0.457961489	-47.58383938	0.037046287	0.990293649	33.52908
0.470273843	410.9710048	0.068600184	0.990773218	62.06460646
0.42920979	36.89821505	0.035669122	0.989462671	48.14880379
0.323684874	-1676.82959	0.019269021	0.991529911	133.8966532
0.323982573	-753.4938059	0.027174511	0.98972116	120.0454021
0.458973114	-219.6832521	0.051801895	0.994842818	40.93204453
0.395856209	-138.2805132	0.0281895	0.991945898	71.14899409
0.416181186	-417.0613876	0.027206462	0.993507834	59.43158831
0.35835597	-346.2438324	0.020949319	0.992334279	103.4050017
0.492551156	169.9444016	0.059835192	0.990891336	49.77711696
0.367659416	-490.1274665	0.027118524	0.989195018	84.28934183
0.389125849	38.15211564	0.027803474	0.991652273	57.07826838
0.418739756	76.48016913	0.039651542	0.988015427	50.03813917
0.42203541	-77.7805593	0.035618585	0.989900126	59.14042558
0.495692454	189.8831292	0.090846772	0.985103712	60.53318145
0.345473592	82.01394406	0.019726191	0.988537184	90.8531253
0.403350926	-266.4335661	0.035964105	0.993133172	55.84818679
0.388330145	28.39331616	0.028354296	0.989644836	51.07528297
0.410085089	-44.88973856	0.02339911	0.991926969	50.57345028
0.402645941	-130.6335091	0.041895369	0.993880709	98.43837985
0.400624766	-156.6128292	0.025988505	0.992658324	96.30506749
0.462134177	-1168.033101	0.077780982	0.990450602	72.91358826
0.498010069	0.363916434	0.040841808	0.989374521	27.20543107
0.385733643	108.3021818	0.02267782	0.988385256	54.88608958

log.sigma.5.0.mm.3D_glcm_Contrast	log.sigma.5.0.mm.3D_glcm_DifferenceEntropy	log.sigma.5.0.mm.3D_glcm_InverseVariance	log.sigma.5.0.mm.3D_glcm_Entropy	log.sigma.5.0.mm.3D_glcm_Dissimilarity
14.40094593	3.020707228	0.311568334	7.991313973	2.82200412
5.485336479	2.339466423	0.4272412	6.299473649	1.688148808
7.754546609	2.594911938	0.391128009	7.337843549	2.043981077
10.37423125	2.796685231	0.349914555	7.533782218	2.375274351
6.392312738	2.480699434	0.403354791	6.952658744	1.819605827
8.406559136	2.607551048	0.362761882	6.730258249	2.062289543
8.758138331	2.695867801	0.3671851	7.553626637	2.202477392
7.804640318	2.616527539	0.39428458	7.326348319	1.988629886
9.388240832	2.715644782	0.369618849	7.578572833	2.210816477
9.564769225	2.730650051	0.346370876	7.226381693	2.336012343
5.878134247	2.419239647	0.404518692	6.854750846	1.80230698
7.025035442	2.579972263	0.385143265	7.306875538	1.972018131
6.20691251	2.426635169	0.413911345	6.76434907	1.703763371
8.628472055	2.691091439	0.377659615	7.247750789	2.122429334
8.981189243	2.721480779	0.36010061	7.357467513	2.242234544
9.856806996	2.718617031	0.337744208	7.616806028	2.409678753
7.550946844	2.583394073	0.383955857	6.863070886	2.036278387
6.455819978	2.45336027	0.402070076	6.562728289	1.754429502
4.436829611	2.24028856	0.43171031	6.324040535	1.536714103
9.210493655	2.734094425	0.356768474	7.714568716	2.276815833
4.826485801	2.313605877	0.428497616	6.58803306	1.573665868
5.972362565	2.430983707	0.399394092	6.833695674	1.833613384
5.311959485	2.362037515	0.422940035	6.829959054	1.703223534
6.896752051	2.539806227	0.396812254	6.987088493	1.885578764
10.78995711	2.799864008	0.338061879	7.223798988	2.472690659
10.22585769	2.800556307	0.346706998	7.476771004	2.368035244
11.2328396	2.836673772	0.359525596	7.699619576	2.408011658
5.976386576	2.476192885	0.399605815	6.938050294	1.804761168
5.204071062	2.334897048	0.408580091	6.290528042	1.695289989
4.342855394	2.258643904	0.436139824	6.560854224	1.517115511
8.224025722	2.64378943	0.386002613	7.349869747	2.061135428
5.549771481	2.351925294	0.431832162	6.155463421	1.588609881
10.25321223	2.786096193	0.355501425	7.785054449	2.372699262
10.44077381	2.804131608	0.341064327	7.597762649	2.441361597
8.02115324	2.644520666	0.37367055	7.365276811	2.132934142
6.595560565	2.503658498	0.402621662	6.998764837	1.868809136
8.524591161	2.564699826	0.409621108	6.879291521	1.919088721
10.94720836	2.815551598	0.340200831	7.910092939	2.332046327
2.744676947	1.975534297	0.470862198	5.662212051	1.267804709
10.70909476	2.839823883	0.343076288	8.040333305	2.454958492
8.268418052	2.632775912	0.397472059	6.966668762	2.015734457
11.14239537	2.853266716	0.329104472	7.877291689	2.533056771
11.81941889	2.877728226	0.336050192	7.696003173	2.572893666
7.767250593	2.631251986	0.378730881	7.16358285	2.064489353
6.38068607	2.48400631	0.408852115	6.825934893	1.844885528
10.4609721	2.784991815	0.362446209	7.564962703	2.350317501
6.877242577	2.538667275	0.394440052	7.131068183	1.921836289
4.408414023	2.229525243	0.440064081	6.170714837	1.555485836
6.236170364	2.468220557	0.40441275	6.916633708	1.828076033
5.500748576	2.36664367	0.402433808	6.489234561	1.704131197
9.124353795	2.726227485	0.353299528	7.600731098	2.281837507
11.53071269	2.886715124	0.328792614	7.81694245	2.576559162
5.98953537	2.462588182	0.407558614	7.135096951	1.7966018
10.50550332	2.779573813	0.353454106	7.170123628	2.390702205
9.235507465	2.699054164	0.368967664	7.265522409	2.200133316
5.643138374	2.421050665	0.407625163	6.826361717	1.773772594
9.887712485	2.789726533	0.335011982	7.719870129	2.400365913
7.569018026	2.564132537	0.396198422	7.163057803	1.962496018
5.88036411	2.415160395	0.430041793	6.450858825	1.687065821
7.498277348	2.618741789	0.376075561	7.387233041	1.977434459
6.528388008	2.480603729	0.413621206	6.810934009	1.782706614
8.078090595	2.652526502	0.378879573	7.398191145	2.09954874
10.61193019	2.819914307	0.352647774	7.702539158	2.387176802
7.756169875	2.61250331	0.387438537	7.353749817	2.004236982
6.721491469	2.450553819	0.374548761	6.50703272	1.940384455
6.503361842	2.506289585	0.401282837	7.089305853	1.805208397
3.079701116	2.060792271	0.448491444	5.815279955	1.217807756
9.32253982	2.739917153	0.367707987	7.608681887	2.233489996
7.966514806	2.648910324	0.378364797	7.302606264	2.079599716
5.147293096	2.338634107	0.429068977	6.643995545	1.620471493
8.328864768	2.605701293	0.396002922	6.965719883	2.053704187
8.568320887	2.652617996	0.388111089	7.464683759	2.077782076
7.542926094	2.604688879	0.381704348	7.137677776	2.025013057
9.817764888	2.766341063	0.358951031	7.442668022	2.313478416
9.777816584	2.769513489	0.351428655	7.741970467	2.303919757
6.913562344	2.527233393	0.402336482	6.822834718	1.910798349
5.924032093	2.41519146	0.419966303	6.604730203	1.700416447
5.079809408	2.355488672	0.408267022	6.52955627	1.619193082
5.338644748	2.386640674	0.413790181	6.838217432	1.72503575
13.37002565	2.945566883	0.323438544	7.898917546	2.70134117
14.68131299	3.028208698	0.313705088	8.014018637	2.820620068
5.783733203	2.412954672	0.424749306	6.567465195	1.688243846
8.0624062	2.607695968	0.386540533	7.348673814	2.052421588
6.006910612	2.443539364	0.409062647	7.046186399	1.825444876
10.24756625	2.800727367	0.345717193	7.816956145	2.37467088
4.230647388	2.231754565	0.438987952	6.433334145	1.458016538
10.17712704	2.789520819	0.363723978	7.584804095	2.335169555
8.908348906	2.698014104	0.376334119	7.27999834	2.15313935
6.10327753	2.46684562	0.397132462	6.876592098	1.834185061
6.622897269	2.509787022	0.401522314	7.060885702	1.86813019
5.843927609	2.335965067	0.413004009	6.105247759	1.627191859
10.7976726	2.843712044	0.339011252	7.768161626	2.459284469
7.256731486	2.578570982	0.390850546	7.149261648	1.97601974
7.909774728	2.618732711	0.378510377	7.180893639	2.073691001
6.078292743	2.472318485	0.398255829	7.083979985	1.851825813
10.1391076	2.758572941	0.374962863	7.521249995	2.206075481
6.341925539	2.503656284	0.387541177	7.499507518	1.909815291
9.155380571	2.593635267	0.400408518	6.561104123	1.962677715
3.195785985	2.045345052	0.468959352	6.094208486	1.337974122
8.97821159	2.690938687	0.375121843	7.328747793	2.161589765

log.sigma.5.0.mm.3D_glcm_DifferenceVariance	log.sigma.5.0.mm.3D_glcm_Idn	log.sigma.5.0.mm.3D_glcm_Idm	log.sigma.5.0.mm.3D_glcm_Correlation	log.sigma.5.0.mm.3D_glcm_Autocorrelation
6.126568784	0.9292562	0.319900835	0.761569753	463.5808602
2.421140774	0.915775199	0.448025632	0.75423663	88.93366503
3.385343469	0.928568121	0.395190902	0.81687084	210.7121089
4.498682885	0.921257196	0.364920477	0.814228238	250.7009812
2.971478544	0.930864413	0.428142137	0.766569739	158.2469059
3.693126313	0.916956291	0.414184587	0.746176005	92.02826964
3.72991599	0.941774518	0.369579128	0.785179291	239.1571115
3.760712193	0.949985384	0.413160399	0.806698701	529.8473722
4.218910517	0.936926768	0.384510901	0.778692959	309.8535493
3.811502328	0.931156828	0.350870554	0.737696746	439.07867
2.415323279	0.928252056	0.422207395	0.774899632	145.6475936
3.056469989	0.935175587	0.399679261	0.816853388	247.8591645
3.169070005	0.944032638	0.467746762	0.826492355	239.9893737
3.973405084	0.940877692	0.398107371	0.780293911	362.1554317
3.789454784	0.933753601	0.366013224	0.768958278	224.5312151
3.594552916	0.92899962	0.336068555	0.786461921	342.3201113
3.195057895	0.920200454	0.391179454	0.73945377	232.2965731
3.133025237	0.92178142	0.460741991	0.775973378	97.53385548
1.878463764	0.913344668	0.473017654	0.788522823	61.06472737
3.861444696	0.938283054	0.358405663	0.803222114	296.2884195
2.251884848	0.939307123	0.471319116	0.786372681	180.0895565
2.446354139	0.91700007	0.410493108	0.775093671	106.3186013
2.271987891	0.925849075	0.437109747	0.839163964	122.3089138
3.212594399	0.938153956	0.425415888	0.751283709	207.3417428
4.314464612	0.927656291	0.340168598	0.628582764	333.58051
4.411904647	0.937927201	0.358301584	0.75644428	420.555623
5.247852251	0.948186156	0.363597357	0.802945155	604.6405031
2.627111698	0.938164304	0.427412122	0.791033809	249.3229141
2.114337036	0.910409791	0.443369391	0.790461975	73.25990831
1.967845258	0.94327711	0.472937658	0.803128058	185.7922577
3.796972149	0.942457294	0.403273277	0.772764803	337.5234755
2.92983513	0.939542103	0.48872118	0.709152444	267.4922834
4.396556358	0.93612346	0.354782589	0.821377064	339.1861338
4.209466918	0.937609215	0.343351384	0.772443895	413.8607765
3.308944536	0.938394399	0.373079224	0.766969879	393.0415763
2.959593839	0.936361338	0.419353841	0.766371064	300.639698
4.63088235	0.95555898	0.446331295	0.763589548	646.6720296
5.161033767	0.946381919	0.388131797	0.84347756	509.9427392
1.078925026	0.915993402	0.505796328	0.695828001	48.8310975
4.503944142	0.933987932	0.342073251	0.819699562	282.3944375
4.074079557	0.940530818	0.41869788	0.738251864	403.2989874
4.469688112	0.941426355	0.327189961	0.79066899	329.6217433
4.851510139	0.927314589	0.333288459	0.719586758	352.9523418
3.379877636	0.940365438	0.38961924	0.734696303	206.8590351
2.881937079	0.927010647	0.419818709	0.795445844	126.3849891
4.632479772	0.936921114	0.368648441	0.756767305	370.2792941
3.054093757	0.938835147	0.410560933	0.768920849	257.4417638
1.84952388	0.900746552	0.462067468	0.771807676	49.2767592
2.763271908	0.935346014	0.428023921	0.829626664	165.1099414
2.352135834	0.929227679	0.449160871	0.751710185	97.41448392
3.740863027	0.925980507	0.355199493	0.788271371	269.5331453
4.688055626	0.922586042	0.325955516	0.748003435	249.3823641
2.660155155	0.938460613	0.426745637	0.815899281	194.4864233
4.509205816	0.911887849	0.35264894	0.623496141	194.8958964
4.126051409	0.918390229	0.385833365	0.766648831	133.2555441
2.380491324	0.942914911	0.424463609	0.777027933	281.9595562
3.91091174	0.931373899	0.34422567	0.776872315	296.6490886
3.530764193	0.944982058	0.410487331	0.810417436	476.0048483
2.970454313	0.935799956	0.460343545	0.647101274	227.7897497
3.456614347	0.944530604	0.417005819	0.801440689	302.8147281
3.243169609	0.94149279	0.448282612	0.787863321	172.8376787
3.545154375	0.933736415	0.387342379	0.802895954	274.3245219
4.72884923	0.948421048	0.35685678	0.760439889	671.4366649
3.579991021	0.946861454	0.410225809	0.812412009	324.1244725
2.746633695	0.926223955	0.396427208	0.659269336	174.6322356
3.147742199	0.953067618	0.442112957	0.827039235	395.3916567
1.549724858	0.945695053	0.550419249	0.760565718	136.506553
4.182486304	0.946845645	0.374774701	0.790491731	435.7750721
3.514211523	0.940037281	0.390686313	0.754657695	359.9089731
2.388470467	0.935210985	0.462500697	0.774344542	182.8495431
3.873911216	0.939330834	0.403728645	0.756006858	319.1053026
4.120756122	0.946631951	0.400687339	0.799685165	444.7071951
3.301041805	0.93968692	0.395627753	0.773507753	390.6329219
4.273016068	0.925463614	0.363166384	0.728357989	248.4546344
4.228407176	0.934298316	0.369173143	0.823430935	241.3042514
3.097778552	0.930387077	0.418110627	0.727595058	262.1632976
2.890712319	0.937679209	0.457961489	0.694259583	212.8751028
2.371575742	0.937752218	0.470273843	0.847591089	109.8197049
2.276751274	0.930890145	0.42920979	0.800137583	163.3686198
5.718769545	0.938243394	0.323684874	0.819012551	563.7816478
6.399158702	0.932816914	0.323982573	0.782851383	551.1156226
2.847373423	0.953470726	0.458973114	0.749847081	350.5905559
3.658897789	0.940929474	0.395856209	0.796589064	328.5359524
2.508199379	0.944976576	0.416181186	0.817818386	344.6242264
4.389411208	0.94086846	0.35835597	0.818777244	381.5760182
2.042990927	0.938617277	0.492551156	0.84082832	136.8939192
4.538709229	0.931650261	0.367659416	0.784374586	313.714931
4.109229892	0.94010399	0.389125849	0.731115262	355.2819368
2.617248966	0.927150042	0.418739756	0.782547883	135.6245544
3.012636099	0.934168173	0.42203541	0.797798628	209.5855066
2.817012163	0.927238492	0.495692454	0.823020731	118.7756455
4.544841948	0.92809989	0.345473592	0.787719734	248.2973084
3.21554523	0.944385022	0.403350926	0.76906023	426.0370896
3.399364572	0.932319097	0.388330145	0.732058584	225.4985726
2.53549465	0.938608772	0.410085089	0.784930119	209.4011001
5.100290698	0.950298658	0.402645941	0.813235731	646.9589621
2.571562431	0.940720579	0.400624766	0.876088669	254.8956882
5.020540121	0.942899815	0.462134177	0.779925287	416.1606028
1.31110259	0.930679803	0.498010069	0.788377415	100.3330938
4.108220105	0.930107619	0.385733643	0.718185805	239.9765543

log.sigma.5.0.mm.3D_glcm_SumEntropy	log.sigma.5.0.mm.3D_glcm_AverageIntensity	log.sigma.5.0.mm.3D_glcm_Energy	log.sigma.5.0.mm.3D_glcm_SumSquares	log.sigma.5.0.mm.3D_glcm_ClusterProminence
5.334680264	21.08567125	0.006254624	29.59790407	38137.29983
4.439880363	8.975283194	0.019927037	11.11589762	3292.431077
5.084027905	13.89968745	0.008541171	21.30980026	14130.74822
5.219873179	15.11013011	0.00863518	27.957586	29203.17214
4.793275626	12.1541244	0.012233218	13.7112401	6452.514603
4.728130726	8.929977898	0.015662255	16.47130481	7054.991614
5.103592474	14.98726162	0.007553229	20.63996785	16369.33571
5.039063086	22.69196202	0.011207636	20.01600502	19551.53071
5.136403608	17.19900979	0.007807133	21.48690257	15829.53506
4.917361971	20.71593934	0.008649631	18.30949271	14818.61249
4.753667389	11.64076811	0.011656048	13.05563575	5614.551329
5.042622305	15.25207857	0.009184008	19.41089986	12699.57362
4.843785466	15.02992888	0.018164587	18.3525084	15915.64854
4.97624819	18.66494673	0.011484718	19.80710998	17416.09257
4.989985473	14.50766827	0.010212879	19.72353059	17991.80252
5.154556675	18.07243381	0.007402899	23.3144158	23256.48705
4.664484128	14.88157647	0.01287056	14.59152981	10533.97871
4.607111319	9.283321591	0.01860558	14.57349083	6445.746316
4.491226742	7.269958683	0.017855961	10.41159073	2991.953439
5.222232885	16.72413863	0.007016624	23.70118896	20134.06508
4.637785475	13.08037491	0.016376428	11.40352164	4564.145058
4.754664075	9.798480517	0.011431767	13.27401087	5399.05907
4.899532604	10.39865486	0.012802816	16.79190033	9094.899513
4.774927495	14.0387647	0.012619594	13.99594546	7929.150036
4.777430284	18.07156366	0.009672402	14.82656036	7407.994515
5.025495933	20.17455903	0.009541115	21.05481456	25121.90194
5.24412199	24.1971051	0.008204714	28.40172282	54717.0786
4.796381056	15.43288359	0.01359477	14.39208192	8680.250538
4.547415915	7.974697414	0.017907945	12.24798273	4418.450604
4.648668943	13.30180685	0.016125534	11.02002614	4625.398142
4.998629921	18.05307668	0.009509274	18.30668648	12425.02444
4.285037726	16.1479263	0.025530754	9.50760235	7377.479455
5.304636921	17.83727624	0.007129486	28.89312801	33735.34072
5.114669887	19.97802769	0.00816025	22.70973712	26047.84473
4.968265456	19.56200421	0.008538104	17.30363408	12914.02176
4.807192681	17.02738963	0.011079309	14.22651855	8013.481218
4.769776186	25.20175328	0.017755612	18.00196065	33267.35966
5.483098544	21.99114943	0.007504664	34.54514184	49119.55346
3.988595278	6.758736508	0.025559117	4.516078071	661.5515944
5.407577662	16.12750696	0.005502256	30.15767984	33241.55457
4.704501596	19.86914198	0.015318082	15.99909924	18889.01135
5.283926905	17.664944	0.00627395	26.6064177	33179.99967
5.077347846	18.47411858	0.007369047	21.18914781	15820.54607
4.822338221	14.02124905	0.010809534	14.87395356	8531.913357
4.747920236	10.67334655	0.013524913	15.61742874	8577.395348
5.091747964	18.90824154	0.007851586	21.50987071	22310.339
4.867815318	15.71011878	0.010971522	15.18080576	8429.971098
4.367562722	6.46883223	0.0191316	9.614865991	2364.91764
4.921409935	12.24562981	0.012787348	18.24174804	13880.44556
4.563345442	9.430421313	0.016745532	11.22726007	4166.867144
5.145331206	15.91212673	0.00707403	22.05945867	16257.43268
5.171717432	15.28340672	0.006165857	23.22809163	17501.48833
4.945563095	13.45768955	0.011130064	16.41952935	10386.83567
4.73042934	13.6463559	0.009355829	13.92104366	5160.481156
4.970386137	10.87114468	0.009176261	19.65705504	10567.12158
4.737954616	16.51443025	0.013331418	12.93077555	6246.992642
5.168970256	16.77307413	0.006637159	22.51988427	18607.06142
4.958298612	21.50263355	0.011721541	19.76021937	29079.82563
4.383853268	14.91279095	0.017982645	8.336240382	2637.777684
5.049238479	17.00203438	0.009798568	19.09985085	16141.9065
4.733590812	12.67669729	0.015842822	15.68284862	10167.37189
5.054598297	16.09557623	0.008659356	20.87005074	14574.27526
5.137849036	25.66346956	0.007367352	22.36735557	25206.71382
5.057964738	17.57767731	0.010340302	20.9056857	18630.39671
4.45019974	12.96246655	0.015307313	9.924053643	3183.60441
4.967055986	19.53375516	0.013937405	18.67178993	18712.34945
4.187420173	11.47141129	0.031440507	6.442598314	2109.761043
5.136809109	20.51195431	0.008737459	22.02499566	26152.20342
4.91502298	18.72005329	0.009766956	16.57806722	12970.61958
4.629257433	13.18973746	0.014660896	11.44457191	4008.936614
4.79787769	17.55417291	0.013724479	17.3324009	17266.37396
5.117046787	20.7277882	0.009347339	21.22207927	18816.9048
4.890550959	19.49223914	0.011161255	16.9598924	15836.37142
4.975468927	15.35273987	0.008778184	18.20791838	13018.36604
5.30850484	14.84237226	0.007300036	28.15544368	26842.36165
4.661707726	15.90314762	0.01330749	12.68254083	7213.12106
4.52802554	14.35122203	0.015119592	9.863278023	3757.494816
4.732771334	9.775165671	0.02073745	16.78610397	12076.03781
4.778417902	12.35467129	0.012424643	13.37186213	5915.155144
5.363773631	23.16186788	0.006847927	36.25375192	77590.85192
5.402357642	22.99168179	0.006287685	32.99681098	52478.53966
4.569529827	18.53699892	0.018581891	11.76196086	10730.32421
5.049475099	17.74462181	0.010499199	20.12171351	18569.05396
4.879716784	18.24685511	0.011628084	16.62566565	17113.5659
5.331635701	18.98833475	0.006831504	28.33051416	36579.59808
4.667399668	11.20279364	0.018750955	13.50194109	5875.817989
5.136782493	17.23564804	0.008651217	24.02628311	24258.73318
4.921929456	18.60640667	0.009699533	16.69663736	9767.994513
4.782424877	11.16297488	0.012626384	14.03535417	6467.10192
4.906965801	14.01498828	0.012485005	16.44083071	11504.22721
4.528516302	10.25108396	0.026680139	16.59427727	6286.940109
5.2568714	15.1583446	0.006667049	25.94528589	23694.77965
4.86503328	20.40126225	0.011732074	15.73843781	15069.98158
4.845017085	14.66528789	0.010770144	14.91820614	8281.148287
4.858954801	14.08361386	0.010546247	14.20686265	7582.295815
5.189703089	25.06573648	0.010473985	27.34581361	40997.28319
5.287532422	15.26089525	0.008229364	25.87359602	26337.3815
4.584566454	20.08130681	0.022374904	20.6077851	37859.70758
4.382179197	9.712314013	0.020059771	7.600304264	2096.643206
4.894124451	15.12763795	0.00886344	16.11321677	7881.198308

log.sigma.5.0.mm.3D_glcm_SumAverage	log.sigma.5.0.mm.3D_glcm_Imc2	log.sigma.5.0.mm.3D_glcm_Imc1	log.sigma.5.0.mm.3D_glcm_DifferenceAverage	log.sigma.5.0.mm.3D_glcm_Id
41.98816602	0.903981363	-0.200534958	2.82200412	0.400693067
17.95056639	0.894465989	-0.238371234	1.688148808	0.506501443
27.7993749	0.924677702	-0.246290538	2.043981077	0.463408992
30.20060836	0.941522241	-0.259544378	2.375274351	0.438498484
24.3082488	0.892398585	-0.214885282	1.819605827	0.491544461
17.8599558	0.918251269	-0.257648165	2.062289543	0.480462954
29.88027149	0.889748847	-0.198369703	2.202477392	0.443516309
45.31988345	0.910416438	-0.222023845	1.988629886	0.477976168
34.26008533	0.893072148	-0.206770851	2.210816477	0.454699491
41.2638462	0.899191842	-0.213531889	2.336012343	0.428853712
23.28153623	0.893843691	-0.227568282	1.80230698	0.486967226
30.47220137	0.91679011	-0.22935304	1.972018131	0.468658237
30.00740998	0.936706894	-0.281145688	1.703763371	0.522673984
37.24335227	0.913396259	-0.228116221	2.122429334	0.465547286
28.95817185	0.896169456	-0.207319538	2.242234544	0.440658482
36.01679612	0.904745173	-0.210895503	2.409678753	0.416988546
29.76315293	0.866237818	-0.195964199	2.036278387	0.461210469
18.56664318	0.906802057	-0.246188083	1.754429502	0.517511868
14.53991737	0.891061241	-0.238652273	1.536714103	0.527697948
33.31874128	0.904244567	-0.207610634	2.276815833	0.434678228
26.16074982	0.897753612	-0.234460497	1.573665868	0.526135784
19.59696103	0.904820514	-0.232419296	1.833613384	0.478128572
20.79730972	0.940101167	-0.283444893	1.703223534	0.498313377
28.06386908	0.879448928	-0.203169149	1.885578764	0.488765334
36.02513795	0.839993947	-0.169404787	2.472690659	0.419112133
40.22199254	0.883394993	-0.194595428	2.368035244	0.434072605
48.24264502	0.918871088	-0.223339082	2.408011658	0.436557763
30.85421286	0.892156328	-0.21386111	1.804761168	0.491303824
15.94939483	0.932650362	-0.286864354	1.695289989	0.50445016
26.60361369	0.908831825	-0.244244901	1.517115511	0.527705162
35.97716138	0.890051898	-0.205586175	2.061135428	0.469891306
32.2958526	0.842434048	-0.194425573	1.588609881	0.538899358
35.53263161	0.921243496	-0.227596334	2.372699262	0.430493784
39.80716577	0.897803557	-0.203438679	2.441361597	0.421695136
38.9807714	0.874701224	-0.189352175	2.132934142	0.446630465
34.04461622	0.88607386	-0.209970882	1.868809136	0.483893787
50.31475869	0.897980532	-0.227893028	1.919088721	0.503550708
43.8449313	0.949482494	-0.266269308	2.332046327	0.458519773
13.51747302	0.809461634	-0.184661915	1.267804709	0.553896989
32.12386093	0.908432526	-0.205596951	2.454958492	0.420466918
39.57788124	0.859264153	-0.184087787	2.015734457	0.481399632
35.13754284	0.898426449	-0.200154075	2.533056771	0.40913954
36.76648041	0.851399482	-0.167144966	2.572893666	0.412508242
28.00470916	0.865853935	-0.185480513	2.064489353	0.460008031
21.34669311	0.905088964	-0.230817495	1.844885528	0.484060923
37.63503555	0.889915217	-0.201351693	2.350317501	0.44083623
31.36771413	0.879871294	-0.200521158	1.921836289	0.476969958
12.93766446	0.882812097	-0.233919585	1.555485836	0.518220039
24.49125963	0.941928444	-0.28200312	1.828076033	0.490830602
18.86084263	0.892336177	-0.240435421	1.704131197	0.509441066
31.76894551	0.900416121	-0.207566054	2.281837507	0.432437934
30.48705423	0.86662452	-0.171839758	2.576559162	0.407404638
26.91134171	0.906870472	-0.226822036	1.7966018	0.490282118
27.2927118	0.839418229	-0.165345201	2.390702205	0.428856072
21.74228936	0.90841678	-0.226208562	2.200133316	0.455840125
32.98805011	0.892750132	-0.220089429	1.773772594	0.488885849
33.43243659	0.883250145	-0.189317674	2.400365913	0.423545316
42.88781217	0.909000191	-0.230815337	1.962496018	0.4763785
29.8255819	0.835286025	-0.176587985	1.687065821	0.515720224
33.92253721	0.898422517	-0.211582388	1.977434459	0.482646682
25.34040513	0.906491473	-0.234213525	1.782706614	0.506449352
32.11582173	0.909779323	-0.21962867	2.09954874	0.457614293
51.1736486	0.878644205	-0.183907539	2.387176802	0.432271633
35.0574112	0.904096746	-0.220603839	2.004236982	0.476004872
25.9249331	0.870092751	-0.213066449	1.940384455	0.468098963
38.97924847	0.926907611	-0.251578336	1.805208397	0.502496943
22.94282257	0.883064298	-0.240003997	1.217807756	0.590670829
40.89588707	0.893212771	-0.199738867	2.233489996	0.44699039
37.28419741	0.861818065	-0.180713376	2.079599716	0.460607491
26.37947493	0.885545046	-0.222073401	1.620471493	0.5185682
34.99981072	0.909246627	-0.234221144	2.053704187	0.469539133
41.35588706	0.913680508	-0.224057777	2.077782076	0.467702976
38.86696101	0.892810619	-0.211315204	2.025013057	0.465048613
30.68026306	0.867723428	-0.181722766	2.313478416	0.437524332
29.57101445	0.925206407	-0.23382592	2.303919757	0.442928668
31.80629524	0.871448959	-0.202888832	1.910798349	0.482085448
28.70244407	0.856660275	-0.19911904	1.700416447	0.514666774
19.55033134	0.942556401	-0.296179835	1.619193082	0.526328675
24.70934258	0.907144248	-0.23250535	1.72503575	0.492870462
46.19842092	0.92405444	-0.22741687	2.70134117	0.404710679
45.81570814	0.923031652	-0.221251068	2.820620068	0.403823567
36.97566734	0.884088652	-0.217784022	1.688243846	0.515119176
35.36901706	0.909347263	-0.225625348	2.052421588	0.464528878
36.39984049	0.901056035	-0.226286514	1.825444876	0.481531445
37.85357732	0.924833399	-0.231946371	2.37467088	0.434018391
22.40558728	0.933166953	-0.283578763	1.458016538	0.543403772
34.36119766	0.90108982	-0.208285665	2.335169555	0.440081229
37.05204043	0.881286699	-0.195046994	2.15313935	0.458532768
22.32594976	0.907296749	-0.232548834	1.834185061	0.484488391
28.02997657	0.912514821	-0.235978906	1.86813019	0.486000419
20.50216791	0.940129233	-0.315322518	1.627191859	0.54502924
30.21540641	0.910742095	-0.211658031	2.459284469	0.42332538
40.68841143	0.87346325	-0.195924658	1.97601974	0.470896929
29.30475762	0.860326509	-0.185478566	2.073691001	0.458985081
28.16181576	0.883747761	-0.205114944	1.851825813	0.477484549
49.99419553	0.928396364	-0.241832091	2.206075481	0.468378437
30.48911227	0.947494646	-0.27408922	1.909815291	0.470202121
40.00960283	0.918438806	-0.257447283	1.962677715	0.515499512
19.42462803	0.898482048	-0.255438943	1.337974122	0.546737602
30.23185663	0.848828683	-0.173278672	2.161589765	0.45602247

log.sigma.5.0.mm.3D_glcm_ClusterTendency	log.sigma.5.0.mm.3D_firstorder_InterquartileRange	log.sigma.5.0.mm.3D_firstorder_Skewness	log.sigma.5.0.mm.3D_firstorder_Uniformity
105.6793106	163.2644656	-0.493503673	0.056881767
38.97825399	125.055994	0.495227102	0.098442621
77.48465443	175.7539845	0.194286206	0.060534023
100.8516071	159.2627674	-0.394181737	0.059370642
48.45264768	121.355021	0.270228252	0.079170043
57.47866011	162.0061445	0.455273458	0.079889807
72.48315608	153.4240494	0.320240393	0.063423862
73.23338411	133.0410414	0.156915528	0.071568452
74.94402647	154.5486975	0.087832579	0.061904617
63.0871604	154.702301	-0.466649218	0.066988402
46.34440876	137.3734412	0.05943152	0.076575681
69.78443945	154.9876328	0.025903015	0.066975593
65.6658902	99.10978413	0.378450945	0.089019703
70.12114013	132.5885124	-0.069218592	0.072867882
68.45770525	118.1708493	0.199336053	0.073899665
81.87606278	159.4869595	-0.361102713	0.059629724
50.81517238	103.0876703	-1.090671239	0.089129343
51.83814332	128.9493294	0.68771079	0.091217525
37.20953331	120.9110012	0.437077701	0.094368247
84.15201972	163.3976707	0.074832107	0.058465791
40.78760076	118.0827999	0.129359864	0.087751909
47.12368093	133.7092285	0.137289064	0.075976538
61.85564183	145.7075653	0.316569937	0.072454453
48.63588591	116.1272717	0.389337298	0.080689104
47.15666087	121.0741043	-0.19816737	0.074968729
74.54672713	125.7380028	-0.644063966	0.069461478
102.3679178	136.8054276	-0.88299405	0.064813459
51.23723222	112.3358612	0.149593293	0.08371029
43.78785984	133.0784588	0.546039438	0.087898943
39.73724918	104.3232117	0.217930572	0.089007698
63.92010619	145.1519146	0.030910507	0.068099019
32.48063792	71.38843346	-1.635176302	0.125010249
104.3201083	160.5144186	-0.369440431	0.055956294
81.15171053	141.4694967	-0.521839716	0.063487913
60.6341017	139.7220631	-0.160190812	0.068693022
50.06836926	134.3289261	-0.304431033	0.075403483
63.60462669	91.45059204	-1.150150719	0.094384227
128.1959649	206.0783358	-0.228059736	0.049846183
15.31963534	74.29942417	0.03766496	0.129372728
108.3288886	180.88238	0.062328627	0.051294669
55.02475495	100.9912119	-1.275267835	0.08960361
94.91786247	158.7224917	0.276528734	0.058427121
71.83666253	148.1284943	0.216187439	0.064019845
50.93031545	114.3015203	-0.143641805	0.078002063
56.08902889	140.4740028	0.670817435	0.078686866
75.13075373	157.233017	-0.352663581	0.062361439
52.66317666	123.8756847	0.199099361	0.076258115
34.05104994	124.747633	0.420424503	0.095762027
66.7308218	140.7200346	0.479373482	0.072481259
39.40829172	134.8326378	0.360564017	0.087351415
77.70622037	166.0062485	-0.083588283	0.060192634
79.5640573	155.471591	0.161666229	0.060185727
59.5405545	128.5270658	0.284210068	0.07322249
45.17867134	140.538208	-0.07066601	0.07274765
69.39271271	176.3619366	0.266634405	0.063947086
45.25462792	107.5157499	-0.086546866	0.084172518
78.67987761	163.2070694	-0.146634148	0.059256052
72.17347215	120.284523	-1.065510062	0.074213206
27.46459742	95.41027975	-0.372324823	0.099912221
67.95640483	142.9445686	0.225574021	0.069456652
55.72074498	129.5006905	0.779646116	0.084055951
73.80587573	164.9079819	-0.022919661	0.063993884
77.58587189	146.5364456	-0.255096949	0.064268844
75.19879066	141.2366199	0.561021163	0.068993084
32.9747231	119.4964886	0.188958162	0.087309726
68.63498951	134.6373339	0.070072768	0.075322607
22.69069214	69.15207744	0.202019066	0.129505956
79.86318613	133.329145	-0.160184732	0.065938074
57.40383094	137.0229568	-0.513547516	0.067528996
40.63099453	130.5709772	0.105840708	0.083832357
59.57340132	124.9737968	-0.564098057	0.079586891
77.00602403	150.6419334	0.0752961	0.063826104
59.40737232	120.1456299	-0.700732604	0.074735249
62.31359205	128.2778277	-0.422007036	0.070589317
100.4649454	168.2677002	0.351949305	0.057322633
43.81660098	105.3528681	-0.770159274	0.087237724
33.52908	110.7693615	-0.358998252	0.0883627
62.06460646	138.488698	0.861782712	0.089506671
48.14880379	130.1039066	0.130613349	0.079007869
133.8966532	163.1263781	-1.08927019	0.059562001
120.0454021	164.7426758	-0.691185634	0.056269007
40.93204453	92.47131062	-0.757754057	0.094783372
71.14899409	125.9694214	-0.19595818	0.068161931
59.43158831	116.4285812	-0.774849994	0.077926862
103.4050017	161.3992863	-0.306506792	0.055907648
49.77711696	135.1773996	0.480667046	0.085769894
84.28934183	131.7820778	-0.592160766	0.066502819
57.07826838	146.7453537	0.069430754	0.069621805
50.03813917	133.1152229	0.213647371	0.078810393
59.14042558	124.8474274	-0.223356348	0.075050267
60.53318145	187.169487	0.459991335	0.095139072
90.8531253	154.4441566	0.167399069	0.058626446
55.84818679	114.6559982	-0.678188909	0.077400854
51.07528297	122.3623486	0.028770643	0.077342516
50.57345028	122.8469105	-0.171536221	0.075937977
98.43837985	150.445775	-0.17926379	0.066386373
96.30506749	160.9803419	-0.156280618	0.057626064
72.91358826	78.61157811	-1.75495735	0.104055887
27.20543107	91.54181194	-0.029636372	0.102369903
54.88608958	152.5716038	0.193019579	0.068879054

log.sigma.5.0.mm.3D_firstorder_MeanAbsoluteDeviation	log.sigma.5.0.mm.3D_firstorder_Energy	log.sigma.5.0.mm.3D_firstorder_RobustMeanAbsoluteDeviation	log.sigma.5.0.mm.3D_firstorder_Median
104.212625	48208831.66	67.91299309	75.32590866
69.27130513	17567864.23	51.46366221	86.57810974
95.58381471	58748843.78	69.88832294	147.0574417
101.2252415	20672089.34	68.70622336	66.21881104
73.90787187	39118887.23	51.59609789	77.32764435
86.48345855	14801146.83	67.10359356	83.9004631
89.96349098	170820282.5	63.89520931	125.9404449
86.93633382	150689338	57.25789185	113.1200981
92.18376228	141048491.9	64.90219926	125.399128
86.20344354	36422530.4	61.7648053	150.2852325
75.33208143	27063328.59	55.65176163	95.93068314
87.56931368	72980679.24	63.12192851	131.0709534
75.63937588	32857980.22	45.14504667	68.30427551
86.70442865	39186681.64	56.097871	85.70357513
81.17890387	40920276.57	51.43985839	97.38632584
96.6994399	142402289.6	66.55994098	138.1604614
71.52357299	18963031.48	45.15406698	100.3677101
77.73650424	21214869.95	55.93901579	61.90759659
66.94090958	19154722.15	50.57300488	72.85160446
96.93972907	195802906.2	67.36266868	146.2817078
68.11760201	60766019.78	48.06419162	101.9746513
75.16525029	20499847.02	55.44814955	97.52207184
84.4164024	25433634.62	61.30266055	100.7687378
72.92811267	52624131.03	49.42615257	95.75847626
76.21538973	33428119.37	51.31424868	131.0805511
87.37639253	66169932.36	54.49012491	127.2166367
94.31747912	95221539.62	58.09019322	118.3764954
72.55754819	49292612.9	48.14239702	84.44165802
71.4937083	8755323.212	53.29741063	93.9968338
64.82224408	43429125.23	43.90268749	113.4377823
85.72255637	137528857.2	60.55312114	122.4457397
52.71533572	15295441.24	30.48256518	50.09015656
104.9544489	78234200.13	70.48567844	86.56443024
94.11176928	63685824.17	60.64144157	119.4598923
82.59578568	139738301.6	58.22894048	141.0346832
76.13017731	80969714	54.66276156	133.1264954
70.64129964	57417459.11	39.27267185	62.9210434
116.9579697	171967435	82.06035246	108.5243301
42.93514209	11110485.12	30.69973087	80.20954132
109.4914425	190293230.5	76.25515064	83.1504631
73.97410179	34451688.23	44.26841746	71.84618759
98.00085097	84450213.54	65.43517329	71.80916595
91.3433012	120827973.5	61.9532095	127.9806137
74.28747666	41038586.48	49.39514598	84.51738358
81.82585438	43018404.23	58.88120204	130.0278931
92.58738133	100927805.6	64.49357616	152.8532867
75.27979886	113214679.1	51.49595593	118.9344902
66.12334237	13776484.72	50.7968647	80.99636841
84.9772137	17602490.21	58.71623183	84.02480316
70.27483694	29953563.01	52.94849342	118.2707558
94.66245552	95369037.88	67.46655676	150.7050171
93.88698573	90032709.2	64.93424985	108.6694565
79.35250785	88730546.52	54.05901284	87.88471603
78.96464476	42517877.92	57.57195838	165.6226349
93.5358517	48214896.67	71.76167657	149.8500977
69.1909746	34748935.94	46.63140458	93.68249512
95.40229511	120625939.4	66.59690618	126.7784653
82.05091343	77605495.67	50.64212467	104.8119965
58.38091396	14469688.46	39.20803061	54.36913109
85.56620727	167715626.2	59.05827461	74.25245667
78.96853384	35850448.1	54.09722553	78.68780518
91.71584487	82777838.11	67.18588541	123.7708511
89.74402659	203108201.5	60.83707052	143.5452576
90.3201323	181378414.8	61.55464771	81.80539703
66.95081943	19728074.82	48.53368263	131.8704758
84.54596751	86160304.92	56.6739033	86.04930878
47.15415591	8090905.451	30.28091611	40.26819229
89.85224425	125646763.2	57.36441232	74.21603012
86.20082191	146304631.4	57.34761096	117.973587
70.62701058	59438673.17	52.52248883	105.3666458
78.61654647	26012030.17	51.38994275	89.01876068
91.94924871	159612048.1	62.72641223	142.133522
79.00631037	57332947.76	50.70208758	125.5474014
82.50046628	29900798.11	55.03086071	54.04545593
102.8131788	72787296.11	71.2146664	63.59296036
67.64124794	20034586.99	44.66823464	93.91366577
65.66558471	48113624.68	45.48201314	118.2031555
83.37263633	20883167.33	58.76778497	72.72561264
74.33491032	38585229.03	53.8328984	109.1364059
111.0611095	56350197.61	69.82423367	71.09803772
107.5039316	48135082.05	68.90193199	75.83676147
65.51200873	19309152.66	39.42780803	53.14927292
86.49620353	89551285.59	55.45162375	121.8152847
76.2140661	55482113.63	49.8327754	98.14971161
103.2041776	72053278.61	68.94015953	116.0083542
76.94149696	34104819.11	56.27637485	93.63201141
90.94251593	37693284.71	58.45200134	52.72166061
83.44010631	88377515.44	59.39217364	137.0428696
75.03363035	32005695.34	53.88763412	103.6120949
79.25423906	46389785.95	52.32073297	105.7876892
90.50561151	31731502.59	74.84671391	82.65011978
98.33392859	75758080.13	67.72734131	123.288147
76.04429036	81191767.98	49.00116055	76.01515961
74.742722	82390690.02	50.90919977	120.4622269
74.33652008	55701681.52	51.37884744	65.61020279
98.0503563	97394314.44	64.86056413	89.21548462
99.44550741	69420704.53	67.07736743	96.8019104
74.86318673	13541770.12	38.11533545	32.05021667
55.3028673	9332430.103	38.33863606	77.94147491
83.18985405	167527222.1	61.03790277	129.570961

log.sigma.5.0.mm.3D_firstorder_TotalEnergy	log.sigma.5.0.mm.3D_firstorder_Maximum	log.sigma.5.0.mm.3D_firstorder_RootMeanSquared	log.sigma.5.0.mm.3D_firstorder_90Percentile	log.sigma.5.0.mm.3D_firstorder_Minimum
1301638455	409.8231506	150.7268467	227.0269913	-435.0421448
474332334.2	313.5896301	137.0735554	238.4551575	-78.85006714
1586218782	448.4247742	195.698268	323.7727997	-163.6744385
558146412.3	348.5135193	145.4604426	232.5004456	-290.3502808
1056209955	359.911499	128.5293754	220.9859207	-184.9482269
399630964.4	413.3010864	149.7530636	252.719458	-78.15958405
4612147628	623.9254761	173.862958	283.7952881	-202.6932373
4068612125	470.7797546	171.7575427	288.8197235	-417.9616089
3808309281	475.2122803	176.1446706	290.6478424	-256.4872437
983408320.7	381.2938538	184.3264524	287.9522339	-334.1004333
730709871.9	372.1412354	133.9645639	220.186499	-172.694809
1970478340	436.7635193	177.9379102	287.6408203	-202.9585266
887165466	383.1476746	132.6625292	231.1386993	-265.0482483
1058040404	431.5717773	151.072041	253.8530914	-344.8326111
1104847467	486.18573	143.6145515	218.6434906	-227.3699646
3844861819	434.9825134	180.3930616	287.4785461	-294.2440796
512001849.9	261.3518372	126.023553	188.0163589	-262.173584
572801488.7	333.4135132	131.1712474	242.352002	-113.1967773
517177498.2	298.4107056	121.4788249	213.3936859	-74.59235382
5286678467	569.8655396	194.066091	311.9947693	-226.4828949
1640682534	354.8451538	141.7553641	236.3405472	-198.1535034
553495869.5	348.7361145	137.8366114	231.0506866	-120.3358307
686708134.6	373.2736511	157.5992824	271.7878845	-102.2191086
1420851538	448.2885132	140.9988265	237.4104034	-217.905777
902559222.9	433.8612366	162.0472892	249.9428955	-295.0223389
1786588174	460.1531677	167.1274608	262.2771851	-366.7952576
2570981570	553.6195068	171.6187551	262.5699402	-468.3652344
1330900548	365.8105469	133.5915789	228.3398438	-254.8555298
236393726.7	322.043396	139.9530379	239.5481659	-62.34758759
1172586381	378.9101563	145.4086764	237.7818329	-175.1823425
3713279143	492.9070129	170.014005	281.4508759	-298.6500244
412976913.6	212.3261261	85.98065707	113.9790375	-349.565918
2112323404	460.9305725	155.6554391	244.5353607	-326.7384033
1719517253	479.0179443	164.6218415	250.2406204	-358.9237061
3772934144	445.7700195	178.1287725	283.1348877	-302.6788635
2186182278	354.9596558	164.3134331	261.3613708	-270.5637512
1550271396	415.3492737	124.2201788	179.4290161	-548.2271729
4643120745	544.857666	182.5392145	298.970459	-416.2590332
299983098.2	243.3869476	95.04164735	148.3787308	-72.86873627
5137917224	518.3686523	161.7873137	267.7451965	-284.3407593
930195582.2	324.8529968	115.244516	156.0114349	-413.8152771
2280155766	617.0720215	147.7791928	234.0232788	-326.0203552
3262355284	465.8435059	181.8690967	297.0839111	-275.132782
1108041835	505.4355469	128.4313385	208.9407318	-238.2658234
1161496914	445.9264526	183.4685891	306.8004578	-82.68296051
2725050750	509.0961914	191.7495003	294.4381592	-291.9636841
3056796335	433.875946	157.5337929	253.8349518	-235.7228241
371965087.3	272.4507141	125.7650969	215.2352753	-41.81592941
475267235.7	414.7692871	143.8209906	243.1473999	-183.8179626
808746201.3	422.3338013	148.845565	231.5303284	-77.51244354
2574964023	444.3875122	192.67335	305.4131592	-220.1144257
2430883148	473.2513428	163.4258615	273.0516968	-245.7054596
2395724756	406.4135742	140.2335837	240.096492	-201.8507538
1147982704	413.1446533	196.2461989	302.5484406	-149.3611603
1301802210	462.9403687	190.3990599	306.2244263	-95.06072235
938221270.4	379.5827332	133.4914497	221.7596497	-298.775238
3256900365	485.703125	172.6875752	281.1504272	-263.3457031
2095348383	351.0944824	149.8075647	231.0647781	-407.1913757
390681588.4	268.5452881	93.70245868	149.8708984	-291.6601563
4528321906	474.9469299	135.5423966	216.6845764	-320.5216675
967962098.6	450.3730774	142.4793563	248.6345215	-179.7867584
2235001629	444.8616333	176.076505	288.2326447	-237.4148254
5483921440	563.8768921	187.3104277	293.9632385	-466.7939148
4897217201	513.2178345	154.2516581	275.5305664	-317.1400757
532658020	392.428894	158.0260947	233.8507645	-151.9791565
2326328233	495.247406	149.3645308	250.2492447	-367.711792
218454447.2	260.2237854	78.76980446	129.6172333	-215.2537079
3392462607	512.5562134	141.7867487	232.3078171	-407.2012939
3950225048	432.2017517	158.0488809	248.9405365	-311.9757996
1604844175	334.9815369	143.213963	233.605014	-184.8466339
702324814.6	416.3461304	141.508507	218.0962891	-300.7116089
4309525299	501.1512451	192.8875466	316.8317719	-327.7770996
1547989590	378.5046387	158.6097838	246.0598846	-335.6247864
807321549.1	325.2407837	115.049113	170.7467255	-322.1875305
1965256995	483.4590149	152.4706527	267.4984436	-263.2792053
540933848.6	289.1794128	122.1838875	191.8548508	-289.723999
1299067866	360.0663147	141.7067805	218.4752274	-204.1210632
563845517.9	432.7475891	147.6438732	261.5313751	-103.2822571
1041801184	363.4201355	150.3466587	248.9406799	-163.4874725
1521455336	440.5978088	155.2146146	193.0343964	-516.5167236
1299647215	409.8231506	155.5655636	227.8322083	-475.6388855
521347121.7	411.0293579	103.6014959	147.8124878	-393.8466797
2417884711	460.3628235	165.5116058	266.9670471	-292.5394592
1498017068	395.017395	134.3678037	203.2774323	-332.2674561
1945438522	545.3114014	171.8782699	272.3316284	-332.891571
920830115.9	371.9564209	149.2033219	261.6114929	-130.8283691
1017718687	352.9217529	125.7152029	177.3137299	-364.7984924
2386192917	484.9552002	179.530057	286.2408905	-286.8311768
864153774.3	377.5484009	147.8574102	242.0152161	-133.8815918
1252524221	393.3499756	151.3556615	244.9574829	-223.6217804
856750570.1	335.0726318	155.9935075	271.6394409	-103.7059555
2045468164	518.8598633	178.3378808	292.9064911	-221.2043762
2192177735	363.2440491	125.159954	194.4458069	-421.3239441
2224548630	442.7088013	158.0091003	251.6425842	-213.9458008
1503945401	386.0390625	113.1331057	180.2582901	-274.9584351
2629646490	480.2825928	168.7045516	289.1020355	-498.1465149
1874359022	448.2355957	156.9824436	255.3905029	-257.4044189
365627793.3	250.9203339	113.7272081	117.1732803	-468.9764404
251975612.8	271.0614319	105.7826269	171.364444	-134.475296
4523234998	446.0716248	170.3351604	272.6492279	-207.6784058

log.sigma.5.0.mm.3D_firstorder_Entropy	log.sigma.5.0.mm.3D_firstorder_StandardDeviation	log.sigma.5.0.mm.3D_firstorder_Range	log.sigma.5.0.mm.3D_firstorder_Variance	log.sigma.5.0.mm.3D_firstorder_10Percentile
4.439350947	136.505965	844.8652954	18633.87849	-119.6540024
3.596264774	82.80663161	392.4396973	6856.938238	15.52327785
4.213143985	116.3346667	612.0992126	13533.75467	15.86667099
4.324148267	130.3010929	638.8638	16978.37481	-121.8496765
3.897050287	92.33186867	544.859726	8525.173973	-21.01173515
3.875731187	100.3886794	491.4606705	10077.88695	-0.80240891
4.189615619	111.8485068	826.6187134	12510.08848	-4.250810146
4.145723761	113.6614919	888.7413635	12918.93474	10.13525229
4.232468368	114.9896915	731.6995239	13222.62915	-8.516824245
4.079191632	108.1771967	715.3942871	11702.30589	20.36866684
3.880692725	91.43468461	544.8360443	8360.301549	-14.75652084
4.115705276	107.6624923	639.7220459	11591.21224	14.6922266
3.934381669	103.5496032	648.1959229	10722.52033	-22.99997711
4.131463472	114.1409333	776.4043884	13028.15266	-14.54085903
4.103043709	108.4971911	713.5556946	11771.64048	-46.04283104
4.296292938	121.8261576	729.226593	14841.61268	-23.54590607
3.792779258	94.78310258	523.5254211	8983.836535	-49.04039116
3.74723904	94.0990202	446.6102905	8854.625602	-5.787828159
3.583431714	79.48365	373.0030594	6317.650618	-0.595531535
4.321103916	121.7827815	796.3484344	14831.04587	1.646044064
3.75267135	84.89814632	552.9986572	7207.695249	15.92873697
3.874196255	90.64914271	469.0719452	8217.267074	-8.401659203
3.982694869	102.166913	475.4927597	10438.07811	0.480649859
3.911138653	93.35019728	666.1942902	8714.259333	-3.121600056
3.997001866	98.13778193	728.8835754	9631.024242	2.982137346
4.205123574	118.4161574	826.9484253	14022.38634	-29.02367859
4.305178015	130.5092351	1021.984741	17032.66045	-27.58838272
3.887744916	93.74122609	620.6660767	8787.417469	-8.835349655
3.676135084	85.82884846	384.3909836	7366.591227	12.38452873
3.7431201	82.82180507	554.0924988	6859.451395	19.83157635
4.116651687	107.1263751	791.5570374	11476.06023	10.93408041
3.401553512	76.41080404	561.8920441	5838.610974	-42.26051407
4.424354692	135.282519	787.6689758	18301.35996	-123.1056137
4.289474982	123.5956321	837.9416504	15275.88027	-57.3472065
4.082099852	103.7753591	748.4488831	10769.32515	19.76748829
3.920854649	94.58484641	625.523407	8946.29317	24.09964256
3.887846588	107.025046	963.5764465	11454.36048	-26.56380844
4.558654538	146.440939	961.1166992	21444.94861	-76.54616547
3.135988579	53.12286048	316.2556839	2822.038306	8.131388569
4.496736152	137.3579315	802.7094116	18867.20135	-94.19281235
3.892842187	102.568276	738.6682739	10520.25124	-75.44986572
4.37390044	127.1216615	943.0923767	16159.91682	-80.91473694
4.228616348	116.335604	740.9762878	13533.97276	2.255735397
3.96541839	96.62258079	743.7013702	9335.923119	-40.25599365
3.896903792	99.58372595	528.6094131	9916.918475	43.01569176
4.24629588	117.3079223	801.0598755	13761.14863	8.070970535
3.966895812	95.59856606	669.5987701	9139.085833	12.27465296
3.513343803	77.41940407	314.2666435	5993.764127	7.373445034
4.04883844	105.9694244	598.5872498	11229.5189	-24.72575188
3.718534448	84.3668599	499.8462448	7117.76705	23.35789261
4.250438735	116.8518429	664.5019379	13654.35319	7.420923138
4.27149117	118.2417843	718.9568024	13981.11956	-36.748703
4.030663189	101.0003486	608.264328	10201.07041	-22.60415039
3.962126748	96.26267027	562.5058136	9266.501688	52.00074806
4.103339104	110.3802885	558.001091	12183.8081	19.37001171
3.843637337	89.14033192	678.3579712	7945.998776	-8.749777317
4.291471448	119.2659058	749.0488281	14224.35628	-23.7794693
4.08804513	113.5645428	758.2858582	12896.90538	-24.63840122
3.605422847	75.58302473	560.2054443	5712.793627	-32.6956871
4.128145898	108.3314307	795.4685974	11735.69888	-40.76005402
3.890049341	99.04639275	630.1598358	9810.187917	-3.444142818
4.164926015	112.0876456	682.2764587	12563.6403	3.501272225
4.235674653	116.1869294	1030.670807	13499.40256	11.77567978
4.157870265	114.9198549	830.3579102	13206.57305	-23.65712662
3.71483158	82.07768102	544.4080505	6736.745722	30.12110958
4.084947971	109.2800819	862.959198	11942.13631	-8.240289593
3.299417241	62.87202372	475.4774933	3952.891367	-22.56784039
4.262240115	120.2210503	919.7575073	14453.10094	-64.5055336
4.159893196	111.8493298	744.1775513	12510.27258	-30.48868713
3.762608796	85.15377554	519.8281708	7251.165488	15.64764252
3.999221575	105.0737752	717.0577393	11040.49824	-12.6552948
4.241299737	117.7007655	828.9283447	13853.4702	21.18409443
4.046733983	105.1987636	714.129425	11066.77987	-7.03179493
4.100987727	106.2445281	647.4283142	11287.89974	-95.57867279
4.368353789	129.5237131	746.7382202	16776.39226	-74.51146698
3.788338675	88.30519582	578.9034119	7797.807609	-31.14398098
3.748256875	83.06516279	564.1873779	6899.821269	14.33146572
3.844593279	101.8933279	536.0298462	10382.25027	6.617065096
3.869864925	90.81736429	526.907608	8247.793656	12.18769798
4.433189263	148.8562583	957.1145325	22158.18564	-161.7320404
4.477900313	142.875337	885.4620361	20413.36192	-125.235585
3.798138721	92.23407224	804.8760376	8507.124083	-51.27557526
4.199793413	114.1930182	752.9022827	13040.0454	-26.76487694
3.983474989	100.8427542	727.2848511	10169.26106	-30.40940933
4.430762835	133.0144925	878.2029724	17692.85521	-67.25500793
3.793137019	92.81501562	502.78479	8614.627124	17.7764822
4.226603731	119.6049103	717.7202454	14305.33456	-135.7113983
4.076459717	103.8853601	771.786377	10792.16805	21.99071522
3.885903381	92.29077277	511.4299927	8517.586738	5.136643791
4.036714839	103.0704693	616.971756	10623.52163	-9.679225922
3.652350116	101.3942813	438.7785873	10280.80027	9.495256042
4.334303778	124.1379123	740.0642395	15410.22127	-28.63263321
4.009889308	101.8521676	784.5679932	10373.86405	-50.58912201
3.9646273	95.67430924	656.6546021	9153.573448	9.97834177
3.947829097	93.75493123	660.9974976	8789.98713	-61.2788208
4.277191322	129.2127478	978.4291077	16695.9342	-16.55539112
4.359192736	127.2634183	705.6400146	16195.97763	-82.0817276
3.791012408	112.9935428	719.8967743	12767.54072	-120.489595
3.504651984	69.27051905	405.5367279	4798.40481	-6.716440392
4.038965268	101.0734225	653.7500305	10215.83673	14.02027092

log.sigma.5.0.mm.3D_firstorder_Kurtosis	log.sigma.5.0.mm.3D_firstorder_Mean	log.sigma.5.0.mm.3D_glrlm_ShortRunLowGrayLevelEmphasis	log.sigma.5.0.mm.3D_glrlm_GrayLevelVariance
3.508826522	63.91168764	0.004007037	30.6955908
2.306224739	109.2347077	0.019161477	11.31393728
2.432959205	157.3659984	0.008827972	21.71677514
2.890488234	64.65574654	0.015656911	28.27808194
2.786232633	89.4126746	0.009588052	13.98889088
2.273418231	111.121974	0.026155164	16.60268844
3.162925939	133.1098783	0.006249737	20.38462333
3.905747728	128.7700227	0.002607232	21.82632291
2.880391946	133.4328139	0.004970443	21.62318354
3.699505661	149.2445481	0.003898113	19.02060258
2.533412816	97.90915595	0.012604653	13.44129407
2.744509675	141.6710544	0.006343297	18.89720485
3.993156161	82.92663225	0.008380334	18.65325505
3.731734304	98.96771658	0.005342536	22.06210923
3.845737925	94.09303333	0.008194742	19.61561849
3.109833686	133.0415123	0.00483917	23.87159152
4.253039672	83.05479742	0.009562396	14.93967795
2.552271332	91.38528628	0.019741039	15.01007602
2.238485834	91.86650248	0.038882641	10.20612003
2.879608277	151.0979874	0.005256532	24.28235542
2.897406965	113.5204299	0.008175368	11.96781068
2.450767641	103.8347936	0.023346702	13.23094865
2.445960153	119.997732	0.018017256	16.9248292
3.345217701	105.6712342	0.007110365	14.5835633
3.54963282	128.9507646	0.004712739	15.98148447
4.378379646	117.9372792	0.004162715	23.52055664
5.447090168	111.4465641	0.003102549	28.47212979
3.367289073	95.18031566	0.005523674	14.66966545
2.407995047	110.5452921	0.0287555	11.7702432
3.076261962	119.516659	0.006874397	11.37031962
3.090506507	132.0178082	0.004222592	19.00677042
7.519516114	39.42159834	0.007424403	10.35921645
2.98242754	76.9886729	0.006028921	29.96722791
3.70646159	108.7403809	0.004126816	25.21571777
3.331117665	144.7775343	0.003476717	17.68331349
3.229005191	134.3600057	0.005040891	14.59599352
8.306029343	63.05784912	0.002897645	20.44886499
3.027579197	108.9753009	0.00337083	35.45212863
2.757495388	78.80911384	0.035743386	4.654995334
2.807143901	85.48645226	0.007984751	30.68758881
5.689429988	52.54566804	0.00467038	18.14188878
3.628788953	75.357634	0.004803204	26.40181628
3.113788318	139.7941185	0.004230031	22.32539735
3.464872608	84.60901604	0.008598362	15.41671135
2.718459744	154.0902486	0.012805151	16.26474354
3.503336155	151.6796698	0.005409633	22.81642748
3.163216965	125.2110621	0.005294022	15.27089372
2.105832128	99.11153049	0.053810679	9.656154454
3.101872004	97.23661055	0.01093916	18.78921573
2.752516588	122.6264049	0.018317497	11.60876029
2.834535462	153.1948649	0.00890788	22.26526724
2.829858702	112.8135305	0.007172162	22.53129635
3.002187547	97.28508414	0.007914454	16.92900596
2.688861191	171.0148207	0.009048843	15.22856225
2.242052814	155.1386281	0.016572716	19.40094851
3.425705528	99.36784376	0.005205138	13.22803123
2.922094764	124.8865179	0.006042746	23.40256515
5.480834787	97.70056838	0.003724595	21.85861878
3.993253433	55.38372628	0.006769476	9.861405811
3.576511381	81.46190769	0.0049676	19.57106324
3.303494338	102.4215752	0.008670513	16.50575064
2.734594592	135.7913669	0.006273078	20.4159455
4.156350001	146.921046	0.001972031	22.28247594
3.254395112	102.8931532	0.004005743	22.02920426
3.00165259	135.0388866	0.008356393	11.16617924
3.881502019	101.8215436	0.003631554	20.47408395
4.347623924	47.45303707	0.009494609	7.113387068
3.967545289	75.1690174	0.003288879	24.14855422
3.529941229	111.6654655	0.004700707	20.94292916
2.593947567	115.1480512	0.007715128	11.9125203
4.532313039	94.78480534	0.006385909	18.85684843
3.417666031	152.8140551	0.003149143	23.17417766
4.397639951	118.7025006	0.00457528	18.74110209
3.419310262	44.1406691	0.008471135	18.83764494
2.822719057	80.44195221	0.008092531	27.42599946
4.034334884	84.44580964	0.006129132	13.04561335
3.517839251	114.8084943	0.007136635	11.55376049
3.221987784	106.8478499	0.017320774	17.50036274
2.608948241	119.8178789	0.009345117	13.47953072
4.535951811	43.97034154	0.005424723	36.31612587
3.986045846	61.54090224	0.0038485	33.61855274
6.081156138	47.18205036	0.005122011	14.99723794
3.497417812	119.808373	0.005353759	21.98926982
4.669419881	88.80003162	0.005391814	16.93347572
3.344890766	108.8544186	0.005738056	29.26400826
2.467229635	116.8203927	0.011027326	14.21588129
3.499251703	38.71663324	0.007405582	23.96516965
3.119271805	146.4201944	0.004160271	17.85536299
2.640498003	115.5172153	0.013144545	14.00653539
3.424141858	110.8377852	0.012611884	18.03406062
1.777953446	118.5460843	0.012306819	16.35502287
2.989109436	128.0397534	0.00928961	25.15333872
4.75796941	72.74029167	0.003690452	17.50848042
3.20827728	125.7509535	0.006763356	15.19486349
3.049112269	63.31755274	0.007871296	14.47202057
4.54997625	108.4679286	0.002498878	28.26129545
2.855513681	91.9103365	0.008490024	26.44017322
6.811987623	12.89717571	0.007547476	22.73648864
2.899872007	79.94722848	0.015226847	7.86899516
2.641163848	137.1066379	0.005593957	16.53437383

log.sigma.5.0.mm.3D_glrlm_LowGrayLevelRunEmphasis	log.sigma.5.0.mm.3D_glrlm_GrayLevelNonUniformityNormalized	log.sigma.5.0.mm.3D_glrlm_RunVariance	log.sigma.5.0.mm.3D_glrlm_GrayLevelNonUniformity
0.004210792	0.055085615	0.149047496	105.2103125
0.021295014	0.092729503	0.250622456	74.2662313
0.009506834	0.060349074	0.197315504	81.30278415
0.017133103	0.05653084	0.181833058	48.77671148
0.010648197	0.076962734	0.229345621	155.8603311
0.029307665	0.07442853	0.242599858	42.06880453
0.006807915	0.063034954	0.190203821	314.8654661
0.002847572	0.068198864	0.226313354	299.8049757
0.00541982	0.061079435	0.208322169	241.762102
0.004118406	0.066633959	0.149419627	63.73470877
0.013777954	0.075985563	0.197852335	99.3805071
0.0069071	0.065869899	0.198919222	132.4574583
0.009312423	0.081654488	0.337621127	125.8472646
0.005814319	0.068694295	0.213947633	101.817953
0.008811436	0.07112787	0.174612082	124.9838085
0.005223654	0.05941011	0.185456752	232.4691884
0.010198246	0.086688727	0.164090444	91.07508639
0.022268644	0.083441463	0.306960402	85.26697195
0.044657545	0.091003056	0.271714518	98.52792603
0.005695468	0.057506359	0.182508316	265.0536226
0.009260045	0.084221941	0.31976422	211.4946132
0.026245229	0.075837687	0.189528261	71.39399118
0.020230041	0.070470076	0.227423684	61.95978694
0.007771649	0.078101844	0.233705087	178.0963572
0.004970278	0.07345944	0.17099111	83.68279441
0.004429296	0.06668318	0.171757273	140.4899299
0.003346166	0.062683592	0.196172985	178.5944473
0.006111736	0.07995442	0.234192425	188.4407881
0.032710239	0.085664177	0.243646577	32.66238462
0.007719264	0.086458534	0.267632921	148.9947781
0.004622624	0.066516076	0.23777317	272.3586126
0.008099035	0.117097984	0.374902124	197.485895
0.006460164	0.054739369	0.170926804	156.895813
0.004371966	0.062358384	0.176382093	130.6618561
0.003744268	0.06797601	0.176365827	264.9751195
0.005487588	0.074705219	0.245133003	192.9030702
0.003180181	0.087305762	0.308190037	271.2900778
0.003697448	0.048265046	0.30130183	213.1171509
0.040297789	0.12934735	0.291202634	130.8690665
0.008628794	0.050710232	0.153904377	329.6268248
0.005040492	0.083909132	0.257649522	184.7287518
0.005151558	0.05785116	0.134048825	201.6301637
0.004504544	0.06270721	0.169719487	205.2966852
0.009270176	0.075888212	0.194000982	165.1441378
0.014198574	0.075624501	0.212675578	83.76399177
0.005854734	0.061395841	0.180646729	149.059959
0.005799607	0.07432839	0.223876172	293.3124843
0.061499406	0.092582434	0.264516685	68.33523159
0.011985216	0.069823471	0.208370149	51.36356975
0.020472204	0.084532149	0.327714686	96.33727513
0.009572983	0.059623178	0.165299687	136.4253926
0.007627519	0.05975684	0.132769155	182.3865535
0.008702296	0.071126675	0.222348881	275.761539
0.009629552	0.07190853	0.158885792	70.8329747
0.018257433	0.063167197	0.19482832	73.69230728
0.005682906	0.081364198	0.225602905	136.3772373
0.006479104	0.058415814	0.170633154	210.6427455
0.004044276	0.071628378	0.250985082	212.3137917
0.007348945	0.095831387	0.250019611	133.1657153
0.005514915	0.067380844	0.295909114	522.8763478
0.009634724	0.078991594	0.259995283	118.4005388
0.006856462	0.062874607	0.195760162	146.8157498
0.002111574	0.063207254	0.161844952	325.4749238
0.004448878	0.066321392	0.234676288	434.1547908
0.009031661	0.085833966	0.236497598	59.26978879
0.004016061	0.070263927	0.29600129	227.2720571
0.011101094	0.118913357	0.4257277	119.9311907
0.00356945	0.063563148	0.197779604	348.4396871
0.00500827	0.065551933	0.20362764	337.9886855
0.008599747	0.081955341	0.278263238	199.5641605
0.006888263	0.075222077	0.233975173	84.6057914
0.003426627	0.061805449	0.222535418	229.6940582
0.004910343	0.072432159	0.229991421	143.3122502
0.00900234	0.068975301	0.175558628	138.0703857
0.008823839	0.055604054	0.196553689	152.7558805
0.006599584	0.084943073	0.200078553	98.65508662
0.007830672	0.086826679	0.270537398	175.9128375
0.019984821	0.081186321	0.317254066	64.46646867
0.010305666	0.077419413	0.217098452	114.049236
0.005849197	0.058065781	0.149575241	122.4565734
0.004035682	0.054419925	0.147535787	97.64357313
0.005600223	0.08907762	0.284998941	133.8923751
0.005743117	0.065606809	0.254005445	186.9483661
0.005902993	0.076170366	0.210533028	201.6175359
0.006204302	0.054796332	0.184826279	117.9803827
0.012655501	0.080237866	0.333538416	100.6875361
0.008114651	0.064212563	0.180008478	134.9442164
0.004503243	0.068037522	0.209926148	162.7971785
0.014465106	0.07663868	0.223853694	97.17465646
0.013462993	0.071960107	0.236356472	125.4110488
0.014916359	0.083902	0.386606366	88.36080022
0.009990597	0.057651691	0.15326987	123.1504992
0.004000353	0.074772349	0.211277962	334.8609562
0.007302729	0.075376232	0.194455875	218.3788213
0.008579787	0.075023432	0.194571831	283.509902
0.002700337	0.062236604	0.25865901	183.3586705
0.009289059	0.056345273	0.193575084	138.5391397
0.008291996	0.093361852	0.334277618	80.12654117
0.01703097	0.101404904	0.272816012	70.34864371
0.006111751	0.068457417	0.210377021	346.0433387

log.sigma.5.0.mm.3D_glrlm_LongRunEmphasis	log.sigma.5.0.mm.3D_glrlm_ShortRunHighGrayLevelEmphasis	log.sigma.5.0.mm.3D_glrlm_RunLengthNonUniformity	log.sigma.5.0.mm.3D_glrlm_ShortRunEmphasis
1.389530107	432.4610556	1580.977405	0.926535721
1.627978395	83.10975055	614.3658174	0.895221909
1.504672714	192.9734829	1078.231434	0.912004492
1.473464143	231.5866858	692.6688427	0.914217107
1.606859192	143.9848471	1533.9809	0.891436406
1.628085474	91.95993958	432.6597013	0.894039301
1.478147585	222.2927365	4040.438306	0.916718701
1.584173956	485.0447445	3387.658976	0.898431497
1.539564585	277.638271	3116.085355	0.905398748
1.413745533	401.0387204	778.7373125	0.918984876
1.539740668	128.1118584	1014.870122	0.899582796
1.519530611	227.0086936	1580.026614	0.906157825
1.832816025	210.5338659	1113.892231	0.871881111
1.563833352	329.4916221	1142.513515	0.898585116
1.461352525	202.2539061	1417.995803	0.915207511
1.454096991	314.4773371	3246.084487	0.924549305
1.463561985	207.8755892	830.6056778	0.908017563
1.775434729	91.703263	740.7901453	0.874085116
1.733809627	55.84380513	788.0299044	0.872610449
1.462874071	275.9842293	3740.80787	0.918202248
1.786902562	163.0811558	1842.901123	0.877834569
1.511590948	97.2254611	737.9846076	0.904414957
1.596406233	112.4661523	672.2663572	0.894454946
1.59241353	183.3017102	1760.083715	0.898548539
1.435515467	300.290077	944.0109043	0.924377476
1.445273827	394.9081457	1713.252984	0.918958072
1.491117623	547.0844094	2286.071887	0.914274467
1.617473989	224.3276781	1783.049717	0.890900948
1.641966622	70.38727895	289.7056272	0.889440263
1.70438181	166.9848505	1264.604195	0.878432315
1.597228821	302.732624	3167.914818	0.899409535
1.914108368	225.07442	1190.709707	0.862333317
1.449299585	307.5882157	2327.776535	0.917751059
1.442245599	382.6289124	1723.056622	0.922835138
1.459231112	356.9072303	3144.113653	0.916135857
1.602591848	269.1969476	2011.534808	0.901727154
1.772866628	566.7323272	2288.504075	0.877949726
1.680199861	471.6908263	3436.624887	0.900921939
1.791224712	40.9204703	718.2112358	0.863367445
1.409890252	262.5972499	5316.108726	0.921844449
1.662720023	348.1279528	1651.534057	0.887248117
1.369906797	309.9374462	2881.333285	0.926000112
1.427912956	323.1958603	2709.857354	0.925065054
1.511199277	187.880134	1716.70801	0.907019237
1.554546058	121.5010187	859.3489976	0.901015242
1.465804917	333.8473706	1957.117883	0.915963289
1.571373346	231.748167	3070.257225	0.901267847
1.680500207	48.32121999	553.4192276	0.885640274
1.560616884	159.3254966	567.8560167	0.897950306
1.775654883	91.85325435	864.0621651	0.88665996
1.431371236	244.562515	1861.575082	0.919792146
1.356111717	230.2224351	2549.756323	0.930352384
1.585239163	176.6574737	2967.69932	0.895595153
1.424173907	177.6472776	803.9185511	0.920009523
1.504334566	124.2109817	927.1854763	0.909726482
1.590501901	255.1643469	1282.837949	0.895068104
1.435841814	272.0053996	2947.646517	0.921351698
1.626750731	424.8340409	2276.348987	0.895927973
1.670194213	198.1861931	1025.775735	0.881101821
1.691625329	271.4307843	5924.637074	0.894284578
1.662135137	162.1789297	1127.314062	0.888793795
1.511230652	250.5591684	1843.788556	0.907709306
1.430697707	610.4442716	4179.829086	0.918914715
1.601265687	306.1855749	5025.95452	0.896642312
1.592073717	161.240825	552.6379971	0.906264037
1.73352289	363.2046689	2392.247284	0.8824675
2.133047188	113.6263049	642.4954795	0.821493003
1.505254137	406.125884	4359.541648	0.910675154
1.503269752	309.6295935	4148.317687	0.91485906
1.713141937	164.3516076	1809.197602	0.881920198
1.58980914	286.9539535	878.4538686	0.901677628
1.564810484	408.4923182	2899.790885	0.90289865
1.570433857	349.5477874	1548.472593	0.903882274
1.459164849	226.9495601	1614.369352	0.915621715
1.505015634	225.6216278	2187.023894	0.910523471
1.552614493	233.5694604	895.6388909	0.896718087
1.684541132	185.6939955	1523.703708	0.887362063
1.80308603	106.4275797	574.0250338	0.871915115
1.570221324	149.4678509	1137.126118	0.898480142
1.387747932	525.0754667	1756.060222	0.928426799
1.382795559	512.9437245	1492.079455	0.928272373
1.738578172	307.4911905	1099.329213	0.876700461
1.595372572	290.4671502	2268.490124	0.909078076
1.568653198	303.6238857	2025.219279	0.895050636
1.481118952	349.4189166	1731.512416	0.914074728
1.83980461	126.3736839	891.5976352	0.86636098
1.479527085	282.8031982	1675.856618	0.911316545
1.527269899	325.6159243	1895.201033	0.909395099
1.574481678	125.8086802	990.7829329	0.901462557
1.602161557	190.6177902	1346.540735	0.898266495
1.950472391	114.5981479	738.0022838	0.85767826
1.40250213	221.4305445	1758.257953	0.92415556
1.559147868	387.630871	3455.613819	0.898741815
1.499515908	206.1865811	2313.634459	0.911804751
1.529953056	189.1700062	2931.335942	0.900583693
1.618132025	589.7641929	2293.892141	0.9021991
1.518344271	231.5163843	1928.896836	0.904608315
1.847149718	357.6854896	607.3825956	0.864555727
1.741304519	88.1838105	500.9564991	0.870509823
1.524051917	221.7762657	4028.355813	0.910860891

log.sigma.5.0.mm.3D_glrlm_LongRunHighGrayLevelEmphasis	log.sigma.5.0.mm.3D_glrlm_RunPercentage	log.sigma.5.0.mm.3D_glrlm_LongRunLowGrayLevelEmphasis	log.sigma.5.0.mm.3D_glrlm_RunEntropy
665.7998482	0.899767998	0.005232832	4.946288559
138.9881596	0.856026327	0.034166265	4.260392977
320.8616639	0.878347207	0.013308006	4.775131236
378.0499696	0.882607669	0.024070057	4.872106554
253.0499765	0.855054574	0.016374761	4.580386016
143.6733979	0.855477855	0.0475644	4.521461505
348.914194	0.883791841	0.009908191	4.758599264
832.9101255	0.860279501	0.004201124	4.840992028
465.4528376	0.870723206	0.007915022	4.854035805
619.6909262	0.892149828	0.005236792	4.595819028
222.1201859	0.867271985	0.019895118	4.498148031
373.2376022	0.872250959	0.009978308	4.72403118
424.6968308	0.82386387	0.014703277	4.757116674
554.5811148	0.862909368	0.00825614	4.805354009
326.6381174	0.885429591	0.011893844	4.674501271
496.8350284	0.893527633	0.007399472	4.804512515
348.7595807	0.879783533	0.013246941	4.383941074
161.3995232	0.828311186	0.037970698	4.534591268
101.7201632	0.834064241	0.076968344	4.327867504
427.5491481	0.886324293	0.008121835	4.883630376
315.931406	0.830306268	0.015904718	4.527868314
158.6664573	0.87274542	0.040400717	4.453868564
189.534837	0.858248197	0.032033412	4.620734941
317.1390566	0.861236233	0.011624812	4.578398446
470.7370764	0.894253429	0.00648786	4.508558864
624.1489164	0.888885281	0.005853107	4.769180691
899.2681056	0.880844179	0.004645827	4.90151507
396.0084804	0.853116471	0.009367129	4.597220602
113.7536916	0.85320943	0.057052626	4.286562531
313.7513193	0.83862632	0.01259479	4.49257961
529.2753615	0.8604779	0.006941226	4.788255566
524.318747	0.813733874	0.012111724	4.282801731
496.8407058	0.887938633	0.008699672	4.976428681
609.0644616	0.891423895	0.00570327	4.819949443
567.3554258	0.884999651	0.005185134	4.652438157
477.0955281	0.861133198	0.008151898	4.56764647
1139.162225	0.833109379	0.004774871	4.707950371
832.5937869	0.855305322	0.005801962	5.211366077
89.60163747	0.822951845	0.065240781	3.915999679
397.7724214	0.894106444	0.011811142	5.032909599
679.987583	0.847755175	0.007038274	4.640024887
452.5083499	0.901294981	0.006854802	4.878786729
496.5779671	0.89563899	0.006055223	4.751530005
315.1978102	0.874505318	0.0129658	4.580595703
191.6927372	0.866257373	0.022089814	4.529716136
540.7814686	0.884321143	0.008045518	4.817299282
397.1246592	0.864954642	0.008684703	4.625945851
81.07785229	0.84712532	0.107262638	4.195077754
259.0631741	0.864051342	0.01762248	4.679120733
168.4287626	0.842512517	0.035262101	4.417412931
382.5086746	0.890588975	0.012890483	4.78781562
336.0512888	0.905460603	0.009889763	4.751430475
306.8852634	0.859110747	0.012982426	4.71504079
274.180251	0.892001115	0.01268225	4.48290923
195.6583507	0.877096588	0.027915568	4.664766408
449.5255649	0.859289941	0.008295626	4.515334965
422.3847103	0.891337834	0.008760113	4.835218284
787.9607339	0.856964897	0.005832325	4.776625607
379.0185715	0.842746453	0.010597956	4.357404159
494.4068432	0.849718143	0.008912025	4.845799958
275.0358591	0.848070389	0.015455319	4.617166634
409.8281261	0.874474215	0.009936163	4.76620072
953.378568	0.889405637	0.00284238	4.79824724
515.0181894	0.858444586	0.00697547	4.85087211
279.4315674	0.872833496	0.013278003	4.289801904
675.3603284	0.836832251	0.006389568	4.869347383
284.4561067	0.772416234	0.021133061	4.327352818
664.761814	0.876886154	0.005067278	4.883974637
521.5365917	0.879985816	0.00675922	4.762552365
306.9315848	0.840022297	0.013977646	4.502867199
497.4563333	0.864688814	0.009650142	4.650594496
687.9638742	0.865895643	0.00502693	4.897284544
610.7680784	0.867553245	0.006794165	4.684806207
371.4144858	0.88592638	0.011665415	4.667136184
358.0635001	0.877158932	0.012790059	4.960854788
416.7484925	0.865241316	0.009030883	4.436668833
352.4691	0.845415436	0.011932991	4.468466567
184.4941066	0.827123816	0.035936804	4.634472872
254.8029349	0.862917399	0.015613128	4.511009173
818.5192037	0.901239846	0.007720467	4.933033418
784.1082729	0.901728739	0.004956495	4.975635385
613.6455192	0.834566212	0.008231493	4.595994344
509.1246384	0.870437913	0.008142307	4.828784968
540.499156	0.861198027	0.008457579	4.657782966
571.7568744	0.882454978	0.008492686	5.000337748
241.1762318	0.818487648	0.022863981	4.604155182
467.6487614	0.88056765	0.011430859	4.820737319
533.2028111	0.872580374	0.006461822	4.687014607
212.4808413	0.865962589	0.021842592	4.509019092
336.1724023	0.860018993	0.018009044	4.706028678
212.3369849	0.806807456	0.033115363	4.486019572
330.9919288	0.896757734	0.013672325	4.845526917
678.7692562	0.863993232	0.005609159	4.684234287
336.4197493	0.877785548	0.010267817	4.565698252
324.1157144	0.86821267	0.012176901	4.588304188
1020.18931	0.860315605	0.0039263	4.958885806
388.7964739	0.87247754	0.013391781	4.97179897
787.90249	0.818235251	0.0119863	4.651142128
178.0433724	0.831396421	0.026973211	4.252772624
360.0963003	0.875356372	0.009195444	4.639491955

log.sigma.5.0.mm.3D_glrlm_HighGrayLevelRunEmphasis	log.sigma.5.0.mm.3D_glrlm_RunLengthNonUniformityNormalized	log.sigma.5.0.mm.3D_glszm_GrayLevelVariance
469.5387188	0.826397077	37.75044402
91.68213659	0.764074364	13.73888889
211.8224349	0.797269752	23.70128503
254.0372491	0.801033037	32.07833388
160.8378228	0.755186981	17.42072343
100.1710594	0.761980426	15.00476299
241.3338802	0.806737651	25.3586034
537.5065655	0.76908415	33.41478222
305.851387	0.783914129	26.58210425
437.057307	0.811416338	25.49821438
143.0358838	0.77235482	14.56385071
249.8820789	0.784143263	21.36964801
239.575412	0.719851436	33.1901929
364.7517008	0.769206853	36.56000961
221.5039043	0.803995578	27.90807723
340.8411752	0.825308992	27.42221715
231.2053626	0.78820828	20.06373264
102.1730874	0.72286599	17.37075698
63.11449067	0.722190693	9.489022182
299.4764433	0.809584262	30.35672724
184.1864401	0.730579877	14.549975
107.2517525	0.781044791	14.12564096
124.6792123	0.761881833	16.58745698
203.5607947	0.769554623	20.38842057
326.2319386	0.82493934	22.5915953
430.5646628	0.811230977	34.38917604
599.7835121	0.800994701	44.43402979
250.4358795	0.754338672	20.35567635
77.28131109	0.753747269	11.50244418
189.0600525	0.730835487	17.33351361
335.7266615	0.771408588	26.08186568
264.1089952	0.702376283	19.93775926
336.4917727	0.808971489	36.64950292
416.3242794	0.819990825	34.8010948
389.6462544	0.804847967	20.94871316
299.2155901	0.776470476	19.05862028
645.4403871	0.732382803	46.42565073
519.5615019	0.77558465	42.99168562
48.11167895	0.704471211	7.446178961
284.3720677	0.81629364	35.84150673
396.280159	0.747802618	28.04566361
333.960631	0.825048076	32.80619327
349.4255787	0.824906828	28.71628659
207.5313925	0.786412528	21.95077133
132.6620332	0.773965846	17.6014012
365.7405393	0.804422236	27.56277625
256.4024342	0.775312027	21.37744867
53.16307686	0.745431948	7.848468809
174.8738682	0.76887837	24.45570749
102.2418259	0.750538294	13.16805717
266.1457063	0.812057972	25.3956024
247.6398336	0.834180174	25.83907863
196.6172938	0.763405105	23.73425887
193.2645507	0.813552692	19.56816327
135.6947077	0.792188289	18.29065904
284.8107809	0.762760859	18.50236344
295.272848	0.815714405	28.5071155
476.3547295	0.764823266	34.92565993
225.6754873	0.735673626	17.2397864
302.4246168	0.761375015	26.7469264
179.1367813	0.750203235	24.36960232
275.169168	0.787539341	23.33567946
665.0416819	0.810320148	30.5869454
337.1675471	0.76563865	28.18901514
177.7265542	0.791321155	17.10868014
407.9524674	0.738132403	35.38320111
137.5049413	0.632838692	14.71956212
445.1090883	0.793568379	36.45728655
340.5477555	0.802700411	29.14890084
185.1850549	0.738836358	16.01359375
317.8944466	0.77740683	28.76348385
450.5220768	0.778250148	34.46237344
387.5956847	0.780316153	30.34435374
249.3040698	0.804023879	26.70092773
246.0301164	0.793800973	30.9989348
262.497167	0.767056265	19.26530438
209.5679854	0.748650986	17.84048768
117.8615036	0.719721337	20.60865595
165.7258249	0.769336032	16.11111111
570.2318736	0.830988982	44.51053711
555.7349324	0.830220008	40.87856635
351.3325915	0.728290394	34.10099115
319.8665709	0.792562923	32.64488503
340.5802195	0.7625078	24.0409416
382.868467	0.801371953	34.56789987
142.7453245	0.708380058	17.39415978
311.7821554	0.795239379	30.56155461
356.7320509	0.790955116	22.70228897
138.8271671	0.777023293	15.31558666
212.0278499	0.76985227	28.18473046
128.5202998	0.695480378	11.439375
239.3984165	0.821360262	27.93460723
432.1287421	0.769485797	28.0333653
226.1952725	0.796497751	20.58650364
210.5685866	0.773274669	20.34175007
649.9249087	0.777091175	42.74620542
256.0135751	0.781783316	31.18585277
417.1285667	0.705369494	35.76572878
101.6826821	0.717544236	11.93911232
242.5797278	0.794588592	21.64005628

log.sigma.5.0.mm.3D_glszm_SmallAreaHighGrayLevelEmphasis	log.sigma.5.0.mm.3D_glszm_GrayLevelNonUniformityNormalized	log.sigma.5.0.mm.3D_glszm_SizeZoneNonUniformityNormalized
216.088553	0.046667823	0.288024783
54.56758286	0.076	0.239466667
101.1867304	0.058352704	0.318999167
120.2201184	0.050076033	0.296476033
75.34462711	0.067185444	0.243518212
63.79754938	0.074302698	0.332037799
133.1796506	0.057799401	0.280982449
292.8207175	0.04972128	0.291612329
147.1050413	0.054593045	0.266095306
205.0466693	0.060484885	0.297319048
61.18242916	0.073487899	0.223888195
118.677755	0.062014224	0.258191573
114.6251983	0.049185746	0.222733009
172.561971	0.047218706	0.266322373
106.0999425	0.054891895	0.263822407
160.6131798	0.054005654	0.269308104
81.24875878	0.064722222	0.201666667
55.12228049	0.068356935	0.293263224
25.25520755	0.089463558	0.186962426
153.5428291	0.050855756	0.26239184
100.23429	0.0742625	0.2764625
38.1411108	0.076324197	0.208515665
62.26250214	0.069598049	0.239600388
95.22044638	0.062589113	0.261452039
136.7524211	0.060168384	0.283985339
219.9861506	0.049209854	0.290688927
308.3475408	0.045248161	0.312788071
131.5541519	0.063110457	0.283341712
32.08951532	0.086272955	0.195402299
78.67253597	0.069737396	0.1909741
169.2668086	0.054630763	0.292503348
100.5458052	0.073367872	0.259840837
163.1943438	0.045613033	0.275380109
203.9512491	0.048778813	0.295867682
200.8420813	0.063022932	0.29889736
137.8078108	0.066064239	0.269582657
331.8586357	0.044741077	0.314117256
261.7254012	0.043465025	0.272858502
14.87346538	0.109478625	0.18375676
152.3004615	0.04591921	0.257285279
162.9002292	0.058753323	0.289361331
192.2452509	0.052304585	0.274913675
168.7120837	0.05210213	0.286317851
98.82346078	0.061325073	0.241210938
76.18228438	0.068386137	0.26002852
190.6107862	0.054843952	0.338655349
119.1360006	0.060084481	0.234124518
22.04941219	0.106086957	0.210132325
101.7576911	0.058924561	0.242964725
46.51909222	0.086495435	0.281089675
124.413672	0.055519513	0.258425521
129.2914092	0.054608714	0.279830331
108.2542971	0.05668564	0.291902448
94.84616581	0.062984694	0.325102041
63.98451849	0.066544519	0.271862341
131.3535868	0.06746012	0.205345859
145.7411972	0.05179588	0.272509528
185.3373783	0.050010462	0.244774987
95.15780754	0.071014596	0.291705233
154.3308364	0.055640993	0.265335555
117.672099	0.057328794	0.287812269
130.2579784	0.05967931	0.242766015
354.9576186	0.053080702	0.314704586
198.5469168	0.053738879	0.241017729
88.04456021	0.072247325	0.280570749
210.0978927	0.049140186	0.250148294
43.7750157	0.078675685	0.158597366
234.9740952	0.04693781	0.26740873
151.3698001	0.051442237	0.312781116
78.56683914	0.072875	0.2084875
142.6660287	0.057919174	0.271416348
230.2835149	0.0486125	0.271878125
162.1163607	0.05314059	0.240090703
113.9484452	0.053817749	0.258880615
141.2953613	0.050349287	0.252857048
114.9414495	0.068422926	0.246934852
96.47347787	0.068285159	0.297852025
69.89135181	0.065467495	0.215850986
82.30367752	0.070577739	0.22610436
268.2924503	0.046191406	0.296391602
265.8849665	0.046159641	0.295870513
159.4343965	0.051058434	0.263211014
147.9437681	0.048252381	0.265029193
139.5457035	0.061647804	0.219846084
194.832086	0.048740175	0.26284811
82.39405769	0.072910927	0.274490358
136.6045789	0.050916541	0.257355192
189.4081613	0.060337153	0.309212929
65.05371813	0.075393854	0.256055882
92.46487763	0.053828431	0.228837899
74.7292279	0.0861	0.2761
118.0852927	0.053278835	0.261272452
195.2508773	0.05466223	0.230403987
114.2318685	0.062031625	0.287505827
98.98684418	0.062447195	0.229065879
376.8139026	0.046484169	0.333990807
116.3062951	0.049235264	0.217024168
202.9031213	0.052650646	0.32007004
26.53709417	0.082839289	0.130267585
120.4185637	0.059363849	0.284685569

log.sigma.5.0.mm.3D_glszm_SizeZoneNonUniformity	log.sigma.5.0.mm.3D_glszm_GrayLevelNonUniformity	log.sigma.5.0.mm.3D_glszm_LargeAreaEmphasis	log.sigma.5.0.mm.3D_glszm_ZoneVariance
180.3035144	29.21405751	58.99361022	47.50302647
35.92	11.4	339.3933333	300.5388889
96.33774834	17.62251656	146.9271523	121.1261348
81.53090909	13.77090909	52.67272727	40.0508562
91.80636605	25.32891247	348.4615385	309.0084782
53.79012346	12.03703704	109.8888889	93.29080933
339.1458161	69.76387738	166.1350456	144.2152715
269.1581798	45.89274106	687.3369447	656.7103738
241.6145374	49.57048458	191.1982379	166.1321149
83.84397163	17.05673759	61.78014184	47.32935969
65.82312925	21.60544218	115.3877551	89.07853209
115.4116331	27.72035794	212.901566	186.311047
57.46511628	12.68992248	602.3682171	550.0022384
87.62006079	15.53495441	278.8358663	251.5995233
122.6774194	25.52473118	149.5096774	131.305233
229.7198124	46.06682298	205.1864009	178.8681778
48.4	15.53333333	97.55	72.799375
57.47959184	13.39795918	460.9540816	421.3797116
35.14893617	16.81914894	365.4574468	317.7887053
287.0566728	55.63619744	141.2422303	118.6580158
110.585	29.705	851.595	794.4414
40.24352332	14.73056995	153.3626943	122.1070633
41.45086705	12.04046243	221.6878613	186.6524107
138.3081285	33.10964083	205.4272212	180.3893997
94.28313253	19.97590361	65.82831325	51.12617034
177.0295567	29.96880131	184.2545156	169.1225433
226.4585635	32.75966851	189.7223757	169.7819568
133.7372881	29.78813559	518.6525424	484.410209
17	7.505747126	99.25287356	72.85453825
49.27131783	17.99224806	393.1860465	329.8046992
240.7302552	44.96111786	367.8979344	334.4746438
61.58227848	17.38818565	1345.734177	1269.522014
207.3612218	34.34661355	134.2138114	115.8253185
163.6148282	26.97468354	132.9367089	114.8780513
289.3326446	61.00619835	128.6012397	107.9024998
139.1046512	34.08914729	382.6763566	348.8968324
183.1303602	26.08404803	1254.204117	1213.467756
271.7670683	43.29116466	391.0953815	364.2450878
18.19191919	10.83838384	920.040404	765.6786042
457.7105115	81.69027544	99.77740304	83.07738556
131.3700441	26.67400881	337.7444934	305.0986241
289.7590133	55.12903226	49.95920304	36.49851924
260.2629263	47.36083608	129.2024202	113.0524555
123.5	31.3984375	197.0859375	173.4724121
66.04724409	17.37007874	193.1259843	167.8100316
235.3654676	38.11654676	117.9266187	102.3269603
193.1527273	49.56969697	467.5345455	436.9569969
24.16521739	12.2	383.1565217	325.7923629
42.27586207	10.25287356	138.1666667	114.2466971
69.4291498	21.36437247	267.0445344	237.0833156
154.5384615	33.2006689	80.19565217	61.74015112
270.0362694	52.69740933	45.5388601	33.33596499
214.5482993	41.66394558	468.4761905	430.7916257
91.02857143	17.63571429	74.00714286	58.46102041
81.83056478	20.02990033	111.3421927	91.81812563
65.09463722	21.38485804	328.5930599	290.7530974
280.957323	53.40155189	77.97381183	62.58093759
129.9755179	26.55555556	423.5329567	381.1236731
77.30188679	18.81886792	444.3698113	405.6954931
405.9633987	85.13071895	452.7614379	417.1603268
95.26586103	18.97583082	295.8610272	267.395095
131.336414	32.28650647	176.7245841	152.3673488
448.4540351	75.64	163.3396491	146.8360992
317.1793313	70.72036474	363.3791793	329.8255069
40.68275862	10.47586207	154.1655172	124.4818074
146.3367521	28.74700855	721.0632479	677.4806808
16.81132075	8.339622642	1046.150943	894.8145247
350.5728452	61.53546911	215.9344012	193.206745
432.5762834	71.14461316	160.890094	142.9548996
83.395	29.15	546.8	494.309975
72.46816479	15.46441948	204.8277154	181.1578504
217.5025	38.89	371.8725	343.1160938
100.8380952	22.31904762	201.9547619	172.5112188
132.546875	27.5546875	109.0917969	89.62508774
175.9885057	35.04310345	190.3002874	170.0632163
65.43773585	18.13207547	121.0716981	95.42607334
115.2687339	26.42635659	405.6020672	367.2709306
31.94594595	9.689189189	337.0405405	295.1411614
65.11805556	20.32638889	213.2534722	178.1231554
189.690625	29.5625	101.3390625	87.98232178
181.6644951	28.34201954	56.21009772	45.71629672
73.17266187	14.1942446	468.0179856	426.1412582
159.8126036	29.09618574	392.1608624	362.771158
103.3276596	28.97446809	257.0489362	214.2995971
157.9717138	29.29284526	82.078203	65.60892135
45.29090909	12.03030303	812.4727273	726.2643159
135.626186	26.83301708	185.8690702	165.3878637
183.0540541	35.71959459	240.7432432	219.2900726
69.39114391	20.43173432	175.2398524	146.0559361
81.46629213	19.16292135	293.9466292	261.5909844
55.22	17.22	546.46	503.9496
169.0432767	34.47140649	47.94435858	34.39012539
223.9526749	53.13168724	352.3734568	323.9399852
192.0538922	41.43712575	205.6856287	181.2808455
166.9890261	45.52400549	239.7201646	204.0813938
239.4714086	33.32914923	449.4030683	426.6247479
109.8142292	24.91304348	140.2905138	109.2968684
58.25274725	9.582417582	385.8846154	352.7905144
10.29113924	6.544303797	425.2911392	313.841692
320.5559503	66.84369449	245.0373002	218.742082

log.sigma.5.0.mm.3D_glszm_ZonePercentage	log.sigma.5.0.mm.3D_glszm_LargeAreaLowGrayLevelEmphasis	log.sigma.5.0.mm.3D_glszm_LargeAreaHighGrayLevelEmphasis	log.sigma.5.0.mm.3D_glszm_HighGrayLevelZoneEmphasis
0.295004713	0.138660244	28967.6853	424.0047923
0.160427807	6.829669405	20112.14667	110.4333333
0.196870926	1.129185439	28135.84437	192.013245
0.2814739	0.475357711	13450.21091	230.7345455
0.159206081	3.18346284	43765.95225	163.33687
0.245454545	3.318501274	5895.493827	112.6728395
0.213590515	1.039923344	32797.91881	252.6760563
0.180696946	1.601796334	302914.3954	548.9458288
0.199736032	0.821780851	52401.02533	289.4647577
0.263059701	0.17374773	25302.67376	405.6099291
0.194960212	1.12054077	16419.81633	136.9897959
0.193926247	1.208502616	43236.7047	242.6935123
0.138189609	3.205381894	122076.0233	252.6434109
0.191613279	1.003876888	83921.81459	352.7294833
0.234375	0.753665327	33638.2129	214.3333333
0.194926874	0.813022222	63744.7456	325.9460727
0.201005025	0.471604925	25627.10417	193.7541667
0.158961882	9.914093447	26373.22959	112.0357143
0.144838213	15.86045717	12314.76064	67.67021277
0.210425082	0.671622582	35844.36837	305.297989
0.132275132	6.350849782	124868.45	189.98
0.178869323	3.140653798	11960.64249	104.4248705
0.168945313	3.7950323	18802.04046	138.6069364
0.199848886	1.33075472	34858.07372	198.805293
0.260801257	0.22506771	21441.62048	293.4518072
0.257070494	0.464572633	78388.53366	418.2660099
0.223940612	0.363082121	112943.489	562.1850829
0.170890659	2.710828475	105244.0233	251.434322
0.194630872	3.85008241	4455.505747	85.62068966
0.125608569	2.675320549	64367.62403	192.4341085
0.172971837	1.38468716	107287.7983	318.7703524
0.114548091	4.916167998	380855.7722	202.1308017
0.233199133	0.444730864	51019.8672	305.687915
0.235319149	0.344105638	57375.82278	381.8517179
0.219800182	0.394509214	46593.84607	373.4803719
0.172057352	1.625034652	101480.4438	277.7945736
0.156678312	2.04863707	783543.8113	601.1372213
0.192985855	1.005730865	173083.9508	500.6767068
0.080487805	25.02325798	42404.09091	40.36363636
0.244704264	0.541013396	25236.53064	295.7959528
0.175019275	0.835962701	145237.0661	318.845815
0.27256271	0.207782575	15363.73624	353.0939279
0.248836573	0.473874143	38679.79868	335.4092409
0.205787781	1.135140219	38781.85938	198.4980469
0.198748044	3.005685876	15098.26772	156.1732283
0.253187614	0.420645013	40351.86475	330.6733813
0.180841736	2.27854079	101657.7915	258.6121212
0.132032147	23.31246149	10872.67826	56
0.204465335	1.417647725	17082.48851	207.637931
0.182692308	5.503239356	18514.33603	102.0769231
0.232775399	0.449466059	21040.53344	253.9715719
0.286265203	0.267433961	10514.93575	249.3803109
0.162898936	3.376551909	73853.04354	198.8013605
0.253623188	0.552323244	12290.15357	179.7714286
0.226315789	2.144003096	10196.11296	132.730897
0.162564103	1.340812334	84896.98423	290.8044164
0.254882571	0.351347267	21679.80116	284.9107662
0.153556969	0.962176232	201118.162	404.1299435
0.160800971	2.173216229	95471.49057	187.3207547
0.167597765	1.886881097	123976.5281	295.6640523
0.187429219	2.677817942	35620.29003	214.326284
0.202621723	0.944479351	39331.56932	274.9186691
0.246156504	0.287484138	96981.48491	641.7347368
0.172635445	1.566557881	90790.53799	393.6785714
0.183544304	1.130905025	24124.31034	177.262069
0.151475919	2.286321737	236767.4855	421.0188034
0.081288344	9.463306186	122648.5	132.2169811
0.20976	0.553442198	90340.6087	450.9260107
0.23612771	0.513770486	55603.90094	287.746927
0.138026225	4.095839056	81896.92	183.3925
0.205542725	0.809971627	57038.7603	302.9325843
0.186480186	1.037056592	140345.2413	456.81625
0.184291356	0.58947188	76029.27619	352.2904762
0.22664896	0.479816595	30135.5625	228.4296875
0.222293197	1.256591014	37255.81466	274.7356322
0.197466468	0.491945638	33419.87925	237.7471698
0.161519199	2.13402206	85832.15762	184.2971576
0.154488518	6.440512023	21296.42568	157.8783784
0.168717047	1.938945911	27994.18056	172.3611111
0.273621206	0.211564095	69915.87344	498.6
0.308697838	0.113906826	32715.77687	507.4104235
0.154530295	1.377366797	166403.6871	327.6438849
0.18446008	1.429043125	114854.3234	298.9353234
0.152945005	0.823277215	94572.74894	310.4425532
0.246412464	0.283409004	31721.59567	373.4708819
0.10770235	9.834128142	80659.51515	158.7878788
0.220964361	0.694595608	60395.11195	281.0056926
0.215900802	0.889108513	69707.81419	352.5050676
0.18510929	2.005136412	20419.80074	137.9446494
0.175802469	1.829917365	53656.6573	202.0393258
0.153374233	11.49452813	35570.97	144.265
0.271620487	0.337375084	11033.2102	236.5517774
0.187536176	0.857604638	151622.7829	413.0730453
0.202424242	1.112505508	42207.46257	217.0898204
0.167509191	1.278800828	54314.46365	203.648834
0.209526593	0.877484528	237896.7545	656.292887
0.179623713	0.866752725	34916.18775	251.0573123
0.17382999	0.985028339	171051.1758	351.1208791
0.094724221	5.252299189	41186.41772	94.7721519
0.195012123	1.460071717	47083.46625	239.9236234

log.sigma.5.0.mm.3D_glszm_SmallAreaEmphasis	log.sigma.5.0.mm.3D_glszm_LowGrayLevelZoneEmphasis	log.sigma.5.0.mm.3D_glszm_ZoneEntropy	log.sigma.5.0.mm.3D_glszm_SmallAreaLowGrayLevelEmphasis
0.547612144	0.006518132	6.672521531	0.004527595
0.500339575	0.026868413	5.927501254	0.015998937
0.586097676	0.016826212	6.179056663	0.013282625
0.559485917	0.016648607	6.272356748	0.00737871
0.50672737	0.014778333	6.399727568	0.00902293
0.596998214	0.032223026	5.565604067	0.024320928
0.546572226	0.0081851	6.79815949	0.00499724
0.558437982	0.004064402	6.856413292	0.001943054
0.530486066	0.007839232	6.826653559	0.004773612
0.562680803	0.007468114	6.256565931	0.006206178
0.480733807	0.016754306	6.212677856	0.006974215
0.521809576	0.009772062	6.486557426	0.006128665
0.470581664	0.01481116	6.557963892	0.005829864
0.528465339	0.008580397	6.622511742	0.003690064
0.521302976	0.012468432	6.541559324	0.007748736
0.534222795	0.006658936	6.760947151	0.004033851
0.452326383	0.01588996	6.457471541	0.006296771
0.558834869	0.035546675	5.833521678	0.027515628
0.426794634	0.05501485	5.94105612	0.024615106
0.527545563	0.007134007	6.968162648	0.004346012
0.542620905	0.011148949	6.199082549	0.00458431
0.462827349	0.035560861	6.111630899	0.020330593
0.495365959	0.02371194	5.893180972	0.013081906
0.523051068	0.011678142	6.54208326	0.007739976
0.541004579	0.008527944	6.196927909	0.006775263
0.552503021	0.006406438	6.666398386	0.004270828
0.578401095	0.005309722	6.803127276	0.003659616
0.549161837	0.009241459	6.406866712	0.006382405
0.438602078	0.039466816	5.566154129	0.021147253
0.440161732	0.012590809	6.424845714	0.008273067
0.560438667	0.006620773	6.717629695	0.003588552
0.517818478	0.020896557	6.005754487	0.012613744
0.53879696	0.009022028	6.836065814	0.005049176
0.560843096	0.00720588	6.683773324	0.005221467
0.566695443	0.00530223	6.576724354	0.003661015
0.5344139	0.00798732	6.43287349	0.003769475
0.581013386	0.005288727	6.798963462	0.002240202
0.534850511	0.004749145	6.997409617	0.001983091
0.438002842	0.088695993	5.528409969	0.050039493
0.519471076	0.009758076	7.208394205	0.004531715
0.555904016	0.008827017	6.459809575	0.00432905
0.539287189	0.006284702	6.91401857	0.003981381
0.549738125	0.006431545	6.746027628	0.004348103
0.500107802	0.012654124	6.634647186	0.006236233
0.522859422	0.016959239	6.073297463	0.011299324
0.601848163	0.007336889	6.402758193	0.003897435
0.489717336	0.007550031	6.762086318	0.003720868
0.460155022	0.07393346	5.517272065	0.030730936
0.495794806	0.016858224	6.168209482	0.011024037
0.545807221	0.033882017	5.786678847	0.027636336
0.519725296	0.011328162	6.708502641	0.004636316
0.54319279	0.009911009	6.699565882	0.006542295
0.559924538	0.012723884	6.660606025	0.007940264
0.590437058	0.015157853	5.964000134	0.011088
0.53723345	0.021566197	6.175186929	0.012976011
0.459560308	0.00860757	6.533511176	0.003656486
0.53315859	0.008396894	6.829773752	0.004904316
0.504778861	0.007590453	6.864040695	0.004565503
0.555581831	0.017416658	5.990678961	0.014300319
0.529680782	0.007478838	6.970989805	0.003971067
0.55525486	0.014593463	6.382274656	0.011136784
0.50361573	0.008782505	6.671313963	0.004057008
0.579884999	0.003042296	6.748219193	0.002192424
0.502172004	0.005068077	7.087646857	0.002315625
0.543956619	0.015876941	5.81052047	0.012256992
0.512652289	0.006525711	6.886336178	0.003125689
0.399119478	0.024581707	6.009628327	0.015042881
0.532674746	0.004802255	7.147820156	0.002874478
0.578399499	0.007946745	6.717580837	0.005293617
0.458408288	0.014490846	6.557315475	0.009499698
0.532313847	0.011761056	6.325144578	0.008039601
0.533034923	0.005343453	6.882386519	0.003523548
0.497311672	0.008382187	6.695459536	0.003814839
0.520688405	0.014264658	6.69983656	0.007703215
0.509405498	0.010417609	6.847672528	0.006105735
0.506332304	0.012135675	6.287000315	0.008850437
0.565977967	0.013579671	6.232418675	0.009051993
0.468626973	0.021406456	6.073964389	0.008163596
0.488122842	0.013897838	6.376791562	0.008143822
0.560583546	0.006670705	6.788263638	0.002610974
0.556954843	0.006186121	6.693691067	0.004240432
0.521124432	0.011175826	6.487638057	0.005958211
0.528701307	0.010386818	6.773031218	0.007388178
0.471728853	0.008247578	6.744953941	0.00310408
0.521893452	0.007089937	6.779333365	0.002513981
0.542351524	0.020585184	5.788500299	0.013355485
0.516534082	0.010073119	6.708837716	0.00453245
0.576762856	0.006243892	6.45481982	0.003539999
0.51849971	0.018705546	6.037480221	0.009276178
0.485090564	0.028201064	6.663032788	0.017321496
0.536710899	0.015708785	5.70228558	0.010769502
0.520671544	0.011029342	6.745346579	0.005256267
0.481406254	0.006347679	6.996593326	0.003383398
0.552389322	0.010148461	6.508631685	0.005561284
0.484555396	0.012298237	6.799788994	0.004959887
0.59684247	0.004522839	6.681674293	0.00317502
0.47683414	0.012299482	6.954308976	0.006938962
0.585853085	0.012708675	6.145249787	0.003681566
0.346005871	0.036600111	5.637588464	0.023692485
0.549945715	0.008331113	6.687405447	0.005717873

log.sigma.5.0.mm.3D_ngtdm_Coarseness	log.sigma.5.0.mm.3D_ngtdm_Complexity	log.sigma.5.0.mm.3D_ngtdm_Strength	log.sigma.5.0.mm.3D_ngtdm_Busyness	log.sigma.5.0.mm.3D_ngtdm_Contrast
0.006997273	0.335932061	3.236743086	0.169185912	3.39E-05
0.012222377	0.121999438	1.252145195	0.665255269	8.85E-05
0.010305099	0.182127643	2.056722469	0.34654972	5.59E-05
0.016651863	0.305919865	3.810873501	0.161167643	0.00010344
0.006081866	0.091334638	1.094455978	0.59975195	2.34E-05
0.017798256	0.32505525	3.030016022	0.355003875	0.000140724
0.002491854	0.110879025	1.218622842	0.687949262	7.74E-06
0.002933765	0.130529137	1.832558065	0.333805804	7.23E-06
0.003304564	0.105918324	1.150627898	0.542494671	1.21E-05
0.012636474	0.412529683	4.407228198	0.128208923	5.67E-05
0.009386013	0.129860584	1.553419303	0.441735767	3.85E-05
0.006170168	0.147010608	1.674883903	0.390830774	2.60E-05
0.00837068	0.141398444	2.49889213	0.263250328	2.39E-05
0.008937785	0.261729323	3.285678103	0.167952811	2.98E-05
0.006569968	0.233559126	2.311287698	0.304395218	2.73E-05
0.003220045	0.10965	1.107091712	0.546040272	1.61E-05
0.010375819	0.191388341	2.1738262	0.248657594	5.81E-05
0.011199289	0.11162361	1.382021427	0.674841094	6.83E-05
0.010227544	0.055601258	0.794235932	1.392374971	6.42E-05
0.002958262	0.100664804	1.085033253	0.602858919	1.15E-05
0.00476008	0.067608333	0.94574092	0.642903139	1.38E-05
0.013139111	0.127161379	1.503605568	0.522359842	7.31E-05
0.015312691	0.138608843	2.019922685	0.436602342	8.32E-05
0.004984381	0.127189799	1.380208786	0.479040827	1.68E-05
0.008599642	0.352065455	2.391061096	0.215349338	5.11E-05
0.005496006	0.277871355	2.68959587	0.211144594	2.28E-05
0.004481652	0.28685922	3.541105512	0.17033708	1.30E-05
0.005010689	0.106543078	1.350432505	0.4425684	1.62E-05
0.031121444	0.192843787	2.619881814	0.431039151	0.000202945
0.006775232	0.097803433	1.3863982	0.437889316	1.91E-05
0.003066467	0.107554431	1.214931696	0.492795728	9.12E-06
0.005977343	0.100866921	1.805615717	0.326839861	1.54E-05
0.005221581	0.162966023	2.022811623	0.29944638	2.09E-05
0.005909346	0.288527896	2.937380416	0.198329833	2.31E-05
0.002946309	0.120901703	1.239875534	0.486313719	1.04E-05
0.004629851	0.10950753	1.316902914	0.444503667	1.59E-05
0.00381649	0.191882691	3.502683564	0.183007188	6.79E-06
0.003616965	0.148701707	2.060787862	0.29262633	1.09E-05
0.00975828	0.039774574	0.547466958	1.198619234	3.49E-05
0.002370237	0.073972338	0.885560464	0.811972454	9.86E-06
0.005798518	0.153825345	2.456768771	0.217029369	1.53E-05
0.004094892	0.198419949	2.408256662	0.309811367	1.19E-05
0.003644513	0.152410222	1.285226584	0.457096617	1.80E-05
0.005404782	0.171328245	1.81031298	0.373748922	1.72E-05
0.010282698	0.168488623	1.743782387	0.509796899	6.04E-05
0.004865736	0.22701492	2.029948379	0.286999376	2.09E-05
0.00305241	0.072361053	0.834267041	0.705120741	1.00E-05
0.015044271	0.060182084	0.808111652	1.709050467	0.000125111
0.016839861	0.312545901	3.840803376	0.202456888	7.63E-05
0.009060602	0.143759621	1.479891682	0.558640941	4.30E-05
0.005521919	0.143861507	1.381261809	0.454199749	3.15E-05
0.004032326	0.1377555	1.143119009	0.575320707	2.28E-05
0.003285961	0.063356423	0.796962324	0.848511808	1.17E-05
0.010953542	0.259808123	1.81032497	0.311566631	7.48E-05
0.010007379	0.201203561	1.760762134	0.509007786	7.45E-05
0.007573094	0.132209533	1.955160625	0.281269003	2.07E-05
0.003573246	0.125788209	1.217715164	0.509888518	1.58E-05
0.004679563	0.136699726	2.380940203	0.24506715	1.22E-05
0.008244537	0.123272206	1.511109973	0.315207264	2.43E-05
0.001682596	0.050811502	0.675658087	0.956242713	4.50E-06
0.008259641	0.165500489	2.169833092	0.356234886	2.85E-05
0.00553282	0.14195006	1.569910516	0.402169209	2.34E-05
0.002293302	0.188754601	1.983972589	0.306884874	5.84E-06
0.002178411	0.068530883	0.992501105	0.662589034	5.23E-06
0.014417577	0.27442061	2.278280919	0.248626188	7.70E-05
0.004612401	0.128916513	2.374838335	0.2496291	7.89E-06
0.011512288	0.085699032	1.88539741	0.304760338	1.71E-05
0.002590726	0.112735473	1.53530092	0.389969934	6.03E-06
0.002532036	0.079188929	0.961198481	0.595266469	8.44E-06
0.00490959	0.067368337	0.862400367	0.701523761	1.67E-05
0.011214877	0.311791991	4.093470057	0.138368854	3.41E-05
0.003552762	0.146672337	1.812987269	0.33213459	1.03E-05
0.00621505	0.193093501	2.538408298	0.226795148	1.99E-05
0.006366786	0.152711912	1.598441896	0.339623497	2.77E-05
0.005369425	0.142845061	1.83611228	0.428273364	2.17E-05
0.009904748	0.197771103	2.452354702	0.220198174	3.54E-05
0.005686779	0.103123858	1.19598147	0.43773537	1.73E-05
0.016492058	0.196312037	3.56463447	0.299988465	6.12E-05
0.008415395	0.111142336	1.442606181	0.458496508	3.27E-05
0.006650198	0.350060792	4.501516159	0.128163216	2.62E-05
0.007607823	0.404333366	4.250491945	0.130683076	3.49E-05
0.008731059	0.231758993	4.377766872	0.133553291	1.28E-05
0.004673164	0.133660911	1.743429482	0.345038057	1.54E-05
0.00513397	0.118636088	2.051579804	0.283213756	1.13E-05
0.006554499	0.273171789	3.229052899	0.19406384	2.32E-05
0.010404422	0.098864712	1.622051108	0.464126571	3.67E-05
0.006243784	0.181101231	2.09446145	0.269436354	2.69E-05
0.004902832	0.201762385	2.002961517	0.293031739	1.58E-05
0.008840058	0.142655882	1.493394852	0.484488452	4.42E-05
0.007543432	0.124821023	1.641328847	0.360453345	2.97E-05
0.010216912	0.085326587	1.179377294	0.798615414	8.92E-05
0.005808796	0.201754843	1.874952	0.394837318	3.22E-05
0.003078888	0.087161539	1.471083731	0.385401684	6.38E-06
0.003941102	0.107261007	1.049082115	0.569593094	1.50E-05
0.003520817	0.062988579	0.879669337	0.684365689	9.74E-06
0.00447255	0.260605466	3.416314743	0.176537962	1.23E-05
0.006610539	0.115237723	1.97858395	0.343939018	2.26E-05
0.014148623	0.364382708	6.854901789	0.083283586	4.34E-05
0.018387583	0.09733751	1.845510551	0.32925387	4.81E-05
0.002299245	0.066053902	0.576476891	1.051874871	1.03E-05

log.sigma.4.5.mm.3D_gldm_GrayLevelVariance	log.sigma.4.5.mm.3D_gldm_HighGrayLevelEmphasis	log.sigma.4.5.mm.3D_gldm_GrayLevelNonUniformityNormalized	log.sigma.4.5.mm.3D_gldm_DependenceEntropy
31.01917971	511.5419416	0.056268383	7.043106199
10.37372988	67.64705882	0.100840173	6.440963566
19.55063625	190.4713168	0.063249525	7.031906583
27.00138393	278.0470829	0.060571235	6.910762862
13.93043437	201.9028716	0.078912527	6.886692695
17.44486685	110.4212121	0.081432507	6.667216394
19.46435966	251.0684835	0.065168158	7.100829298
20.26741549	513.8326155	0.072656692	7.134456134
20.72958189	357.4109107	0.063371269	7.203800878
19.2066153	542.3442164	0.067414792	6.803280503
12.87886005	134.3461538	0.078086633	6.833802384
18.61216802	265.0681128	0.067915547	6.959188884
16.24304635	224.4863417	0.094398841	6.999942046
21.08435125	350.4740827	0.07266843	7.067744356
19.36145388	245.2847782	0.073062829	6.898048682
23.03141011	283.8066728	0.059761112	7.016462841
15.08387555	288.80067	0.085626345	6.550298508
13.62423197	112.5888078	0.097836004	6.778226043
9.832092042	71.1972265	0.096110883	6.642897282
22.91205505	273.1042508	0.059716864	7.158399022
11.56946796	168.7218915	0.089820239	6.965946562
12.50250421	120.7859129	0.078869409	6.567265544
15.6111908	131.4257813	0.077047348	6.797031991
13.61391309	192.7408387	0.081510614	6.966525344
16.27212719	347.8381775	0.073155741	6.743647748
23.31477162	462.5259603	0.067864945	6.953140898
26.50545617	584.6223322	0.06676882	7.110339497
14.41960957	237.7067343	0.082331015	6.915864116
11.54270328	83.46979866	0.095646342	6.213780678
10.7511958	201.5783836	0.092991659	6.86315917
18.00984254	388.4550231	0.069754954	7.173463867
10.25944266	334.6249396	0.121676263	6.631710077
28.14374416	326.8693094	0.056910791	7.160481436
23.93296967	363.0412766	0.063710276	7.024880123
17.05521418	370.8521798	0.069668623	6.999088643
14.60854185	349.2947649	0.076359231	6.964617389
18.09729203	584.4375168	0.095321548	7.041441981
31.42298154	532.0083317	0.053771709	7.49035582
4.741952541	74.59349593	0.129068676	6.294595993
28.86425646	271.186795	0.052570739	7.245395347
17.22635431	434.5416345	0.089470155	7.001421697
26.64659523	323.7119214	0.058055842	7.07379391
21.27116008	329.8201478	0.063265522	7.006857056
15.80448597	229.1663987	0.076663483	6.907684413
15.6395336	118.7621283	0.080287323	6.737125445
21.3818343	380.957377	0.064416906	7.084704376
15.29841604	272.9846559	0.076054673	7.044013941
9.505753051	60.92996556	0.098580224	6.523012288
17.53732182	165.7414806	0.071448396	6.827790034
12.15827472	93.65162722	0.085041665	6.828841312
21.15534608	280.3164656	0.062168463	6.987584276
22.47065725	264.5864729	0.060201215	6.942052582
15.6239472	212.7861259	0.075323566	7.035046671
15.15489048	201.9574275	0.072595043	6.659433553
19.11762338	142.1774436	0.064669569	6.875293326
13.38407784	379.0369231	0.084083629	6.754666534
22.31786408	313.3386897	0.059969289	7.062998556
20.28641015	459.4543088	0.075780556	7.009267157
10.21224314	255.6820388	0.095212496	6.66493691
18.93113429	357.8596779	0.06933462	7.375539813
15.98268669	190.1449604	0.0851519	6.922216637
19.19212165	255.5453184	0.065833158	7.005537993
21.13765085	634.9941268	0.065769597	7.090847915
21.07979812	350.8288076	0.069998588	7.23291239
11.70150457	165.5556962	0.082855312	6.582941817
18.60663341	463.1372346	0.07832347	7.129209522
6.733476396	158.2461656	0.127255919	6.781772119
22.71331441	430.97312	0.068110797	7.212017513
19.62941623	401.3891071	0.069204231	7.101834601
11.10897559	195.0024155	0.086132318	6.950277704
17.61263979	338.4334103	0.08032649	6.886221395
21.99463864	463.0044289	0.065271977	7.206964856
18.03745475	450.6555507	0.076157703	6.92993929
20.15054356	281.561753	0.067392032	6.891955454
26.06851137	262.5659534	0.059039222	7.213529275
13.24291435	286.6005961	0.087736346	6.743688261
10.68446489	198.234975	0.090027968	6.949860033
16.08859031	123.940501	0.090975458	6.773391281
12.64953743	173.7773872	0.082743135	6.795903076
34.36083833	568.9213339	0.060345415	7.003370006
33.36612454	554.899447	0.055962141	7.050093976
14.01916697	308.3924402	0.096243018	6.895800141
19.74295291	375.3117161	0.06996741	7.05645911
15.09517714	294.8978197	0.081435159	6.902968264
27.00868876	365.197622	0.057751406	7.192818056
12.6181394	144.808094	0.093105141	6.835124476
23.78617126	346.1429769	0.066958146	7.081299602
17.84543644	374.3967907	0.070163664	7.066974957
13.90505045	152.0157104	0.079425334	6.787979017
16.69306606	255.4375309	0.076619296	7.047996188
15.28898105	108.8619632	0.102900702	6.608175855
23.57091424	229.1838791	0.059838306	7.034179322
17.46644265	505.7289215	0.076493303	7.021031273
15.2929854	272.4287879	0.076658219	6.91745517
14.58171778	233.400046	0.07542657	6.926513032
25.95784497	672.3386908	0.067179364	7.277530824
23.43186816	240.1182109	0.061295656	7.142181462
19.78574524	422.0429799	0.098801415	6.926759828
7.743008471	113.2853717	0.103968624	6.543273763
16.26852538	222.1070315	0.070631052	7.033182984

log.sigma.4.5.mm.3D_gldm_DependenceNonUniformity	log.sigma.4.5.mm.3D_gldm_GrayLevelNonUniformity	log.sigma.4.5.mm.3D_gldm_SmallDependenceEmphasis
359.8152686	119.401508	0.280341447
114.7283422	94.2855615	0.144398239
204.8435463	97.02477184	0.182641804
141.4462641	59.17809621	0.234978045
313.5371622	186.8648649	0.166472579
86.83333333	53.74545455	0.213129909
799.8182623	368.2652628	0.19427698
629.4761159	371.1303837	0.174805346
612.0127585	288.0857897	0.187003264
159.1007463	72.26865672	0.210091016
195.2002653	117.7546419	0.171986163
320.7596529	156.5453362	0.191132834
185.7123728	176.2426352	0.119821991
211.2317997	124.7716948	0.196685363
281.0241935	144.9566532	0.216126631
689.5255941	261.5146252	0.203305234
175.4288107	102.2378559	0.182427023
127.6926196	120.6317924	0.150134167
146.5947612	124.751926	0.125704927
763.6031929	310.4679746	0.206032233
312.1025132	271.6164021	0.131159629
154.7924004	85.10009268	0.165253443
127.7675781	78.89648438	0.166533072
329.5976577	215.7585946	0.172998097
190.5459544	93.12725844	0.23189644
358.8387505	160.7720557	0.238338153
457.2938447	215.8635942	0.212061661
344.4989138	227.3982621	0.165709287
56.37360179	42.75391499	0.153421057
231.9834469	191.0048685	0.134548641
582.4955864	331.8940731	0.175394608
204.7873369	251.7481875	0.121964761
494.8693094	183.7649427	0.221032076
349.6391489	149.7191489	0.218861348
616.2098093	306.8206176	0.205850841
362.8279426	229.0013338	0.167327797
383.7438861	354.6914808	0.150089645
668.2637086	277.5157915	0.18450171
141.6520325	158.7544715	0.096160812
1163.275378	382.189271	0.22343115
288.4980725	232.0855821	0.168211564
647.9025084	224.5019395	0.254171064
562.7267999	231.1089515	0.217261721
337.1615756	190.738746	0.196266674
163.3661972	102.6071987	0.179766739
380.775592	176.824408	0.226943674
583.4309513	346.9614204	0.178704691
95.74856487	85.86337543	0.122763833
108.2103408	60.80258519	0.181966574
148.535503	114.9763314	0.167448101
391.2467886	159.7107824	0.211955047
584.0584396	202.9382972	0.243432471
572.4867021	339.8599291	0.171544034
162.2427536	80.14492754	0.227130692
188.2165414	86.01052632	0.202173994
261.1548718	163.9630769	0.154148073
638.1278121	242.5757726	0.236876556
441.0728745	262.0491614	0.157280769
194.2584951	156.9101942	0.166898556
1080.69219	632.9557454	0.173685483
196.4552661	150.3782559	0.167923721
364.9490637	175.7745318	0.188133853
834.5373985	380.7401969	0.227743592
937.7191394	533.5992391	0.171879229
104.3594937	65.4556962	0.182065165
438.383739	302.4852408	0.144515552
109.6518405	165.9417178	0.088503315
835.08704	425.69248	0.195142652
783.0676114	405.3291788	0.226036235
318.7819186	249.6114562	0.135624318
156.0592764	104.3441109	0.200698685
549.4456876	280.0167832	0.180198268
298.8183414	173.563405	0.176919251
342.3085436	152.2386012	0.216729457
456.9163207	184.8518045	0.205868598
166.4679583	117.7421759	0.172303243
261.7712855	215.7070117	0.151947766
98.15031315	87.15448852	0.142623914
221.2062097	141.2425308	0.150414927
375.0820864	141.1479265	0.249618981
343.2684766	111.3086978	0.287123159
194.7365203	173.1411895	0.158107696
427.694402	228.7234628	0.179921177
403.2434103	250.2502441	0.14939884
357.399344	140.8556786	0.214292223
154.5587467	142.6370757	0.113832201
333.0654088	159.6951782	0.214841689
354.7476295	192.3887673	0.208875042
192.5532787	116.2786885	0.177269
239.6834568	155.1540741	0.173356595
112.8773006	134.1825153	0.132559005
377.1738035	142.5348447	0.23792226
676.2378931	396.4647887	0.186567953
452.5630303	252.9721212	0.197359955
605.1645221	328.2564338	0.172077043
403.9748685	229.8877849	0.19635788
412.9943202	172.6698616	0.171214503
98.49188157	103.4450812	0.145324483
94.88489209	86.70983213	0.095688343
758.7485279	407.8236924	0.186547558

log.sigma.4.5.mm.3D_gldm_DependenceNonUniformityNormalized	log.sigma.4.5.mm.3D_gldm_DependenceVariance	log.sigma.4.5.mm.3D_gldm_LargeDependenceEmphasis
0.169564217	5.423373109	16.82752121
0.122704109	6.475868341	28.09090909
0.133535558	5.003595172	22.22555411
0.144776115	5.75842955	20.12384852
0.132405896	6.119966673	24.79054054
0.131565657	12.37572084	32.27272727
0.141535704	5.030024268	20.72429658
0.123233382	6.25585643	25.54150352
0.134626652	5.750886524	22.76242851
0.148414875	5.160388589	19.68843284
0.129443147	5.827420864	24.45225464
0.139158201	5.229797714	21.32364425
0.099471008	10.63423272	41.08784146
0.123023762	8.793175511	28.25684333
0.141645259	5.529721644	20.5766129
0.157569834	3.69260095	17.2321755
0.146925302	4.010552483	19.38693467
0.103562546	10.79878891	37.46390916
0.112938953	8.7126645	34.19260401
0.146875013	4.820493873	19.52010002
0.103208503	8.584923784	36.23082011
0.143459129	4.889262618	21.9823911
0.124773026	6.747421265	26.54296875
0.124517438	7.570378196	27.52058935
0.149682604	4.637681893	18.10133543
0.151472668	4.704860748	17.88644998
0.141445668	4.962009757	19.93721002
0.124728064	7.09984317	27.20347574
0.12611544	8.690669589	30.2393736
0.112942282	7.974624761	32.52872444
0.122424461	6.943814104	26.66204288
0.098978897	9.48023494	39.53649106
0.153257761	4.575878385	18.08702385
0.148782617	4.853525758	18.86978723
0.139920483	5.060823865	20.47411444
0.120982975	6.560041966	26.88062688
0.103129236	10.19153304	36.88551465
0.129483377	14.01986656	35.73319124
0.115164254	7.049311918	35.52845528
0.160010368	3.850250715	16.47180193
0.111217453	8.877447452	31.70624518
0.16754655	3.656089945	14.86914921
0.154045114	3.9671948	17.26882015
0.135515103	5.745912858	22.31028939
0.127829575	6.131609689	24.58528951
0.138716063	5.350456037	20.24808743
0.127889292	6.694158	25.35686103
0.109929466	8.450052133	34.98851894
0.127156687	5.769428653	24.23619271
0.109863538	9.250628042	32.66420118
0.152295363	4.234576373	17.98715453
0.173259697	3.888062814	15.20646692
0.126880918	5.862520866	24.80585106
0.146959016	4.311197096	18.22463768
0.141516197	5.705672452	21.44210526
0.133925575	5.072551216	23.99282051
0.157757185	4.457124714	17.12509271
0.127551439	5.689590127	25.46096009
0.1178753	6.684311375	27.65048544
0.118380128	10.67684108	32.82320079
0.111243073	7.57514214	29.95243488
0.136685043	5.544247219	22.25917603
0.144159164	5.021974921	19.22767317
0.123011825	6.84429539	26.57523285
0.132100625	4.991123217	22.86582278
0.113512102	9.472393645	33.6965303
0.084088835	15.05417027	58.6303681
0.133613926	6.255624294	23.26432
0.133697731	6.748188376	22.8453133
0.110000662	8.43886005	33.97308489
0.120138011	7.147321828	26.23325635
0.128075918	6.013446134	24.31748252
0.131118184	5.705488096	23.5792014
0.151531006	4.291499586	17.96414343
0.145933031	5.757294815	20.94762057
0.124044678	5.95980375	25.19672131
0.109253458	8.278919931	32.47579299
0.102453354	9.615556941	36.81210856
0.129587703	6.067375489	26.03104862
0.16036002	3.792838171	15.60538692
0.172583447	5.591679324	16.80693816
0.108247093	9.350895022	33.69927738
0.130833405	5.086005423	22.73936984
0.131221416	5.428425294	24.87959649
0.146535196	5.069192184	19.51168512
0.10088691	9.10035006	39.66710183
0.139650067	5.840737664	21.2591195
0.129375503	7.389368502	24.92414296
0.131525464	7.201646436	25.77868852
0.118362201	7.302448773	27.99259259
0.086562347	14.21078136	49.62423313
0.158343326	4.701061206	17.39882452
0.130472293	5.946944345	23.54871696
0.137140312	5.900055096	22.33939394
0.139054348	4.926407864	21.83226103
0.11805227	8.754948641	28.96551724
0.146607852	5.843253768	22.59886404
0.094070565	11.65587037	41.82712512
0.113770854	6.909994307	34.8705036
0.131407781	6.120220128	23.77069622

log.sigma.4.5.mm.3D_gldm_LargeDependenceLowGrayLevelEmphasis	log.sigma.4.5.mm.3D_gldm_SmallDependenceHighGrayLevelEmphasis	log.sigma.4.5.mm.3D_gldm_LargeDependenceHighGrayLevelEmphasis
0.04065258	127.8042908	9144.796418
0.875483582	10.8780204	1522.742246
0.188557054	32.38042595	4388.03455
0.195577722	60.5604254	5376.812692
0.161033798	33.24010883	4714.021115
0.720702207	29.96902923	2273.330303
0.126907319	50.60894369	4644.556008
0.06059132	91.43604403	12207.34789
0.084167738	63.10981321	7739.159261
0.043034293	108.8372094	10517.97854
0.319835562	21.52571522	3179.708223
0.118040868	49.17106291	5304.835575
0.278779178	27.67897002	8131.478307
0.103575344	65.9177021	8954.015143
0.120730463	48.20122132	5219.237399
0.109863533	56.77387913	4710.106033
0.07945686	47.3064962	6055.383585
0.573883835	19.38955484	3180.931062
1.005668374	8.703749102	1981.543914
0.113109389	59.0108251	4769.857473
0.31777994	23.90882233	5300.484458
0.412377657	19.44839531	2434.700649
0.379575606	22.4684448	3063.339844
0.191288192	31.72150787	4817.518323
0.061271588	71.86079887	6214.307934
0.048435765	108.1020394	8287.92233
0.045288249	120.239242	11644.3786
0.151816622	39.51980781	5943.152788
0.911651918	14.65244301	1569.742729
0.203229503	27.83649856	6067.706913
0.086014923	66.39290751	9654.763976
0.120124384	34.11480638	14022.60851
0.097015171	65.09474048	6306.887272
0.072461845	72.23057782	7009.706809
0.069428374	73.9340051	7406.090599
0.096616092	55.61260113	8932.47049
0.071013223	84.01347539	20844.80462
0.096427362	98.85188585	15598.49506
0.553392966	6.483143058	2843.925203
0.154947909	61.1799361	4290.566162
0.079515171	64.85521988	14591.88319
0.070115522	88.03489087	4661.358159
0.069345247	66.63242635	5702.448125
0.124342681	42.95704569	5284.616559
0.398923875	24.29257392	2260.841941
0.075142083	80.59461912	7547.852823
0.118377046	49.49994375	6444.395441
1.368795716	8.389183834	1616.799082
0.303030735	32.73217592	3511.844888
0.764635544	16.734681	2219.258136
0.121113553	56.09761541	4950.65979
0.088058792	63.8536976	3914.697419
0.157046931	36.32146838	5059.326463
0.129674327	43.50230412	3455.290761
0.343043659	28.43975966	2582.606015
0.072428529	57.57317302	8927.909744
0.074834576	70.40560405	5270.330037
0.069372597	63.12959371	12068.31087
0.118008395	37.71395626	7111.887136
0.119589532	61.24750939	10568.53007
0.231026105	35.99781068	4688.249151
0.143550247	48.15238633	5298.794007
0.037884832	141.0153135	11743.39005
0.105890892	69.00544337	8013.917093
0.167927723	29.87590764	3994.672152
0.089327139	68.50036182	13855.52149
0.438666544	13.02979604	8683.945552
0.068057361	85.47135381	9737.55968
0.063588191	78.83410103	9407.112173
0.24209184	26.17974869	5869.956867
0.110946374	65.16057203	8587.785219
0.066673033	84.81486327	10297.86597
0.062497793	72.45901784	10643.00483
0.09243996	55.46955611	5352.226649
0.137410635	59.67774458	5010.606196
0.094411784	43.13505659	7778.865872
0.203243446	28.03104375	6191.520451
0.588499646	23.19081165	3237.72547
0.214639362	27.52441378	4144.814294
0.039724838	126.055504	9904.381787
0.039774439	143.7506479	9910.835093
0.14017768	47.72338331	10553.87215
0.074140351	63.11664569	8343.964515
0.134840198	39.8809827	7575.906606
0.088204909	77.07817439	7180.326773
0.421876447	18.6782562	4775.003916
0.094508568	66.51516338	7705.715304
0.083263703	77.94149655	8523.595186
0.293520748	27.4582911	3550.224727
0.14489466	44.41473077	6798.44
1.073624499	17.99562108	3384.881902
0.154140907	52.7959743	3839.944584
0.051536716	88.0812878	12052.15416
0.103997753	51.57891089	5808.934242
0.123152373	39.12449582	5217.155101
0.053111403	129.5112486	17939.49006
0.16666499	41.08416064	5127.758963
0.168567727	55.63830598	18415.00191
0.388714087	9.637544549	4129.311751
0.162184396	41.72928186	4575.352269

log.sigma.4.5.mm.3D_gldm_SmallDependenceLowGrayLevelEmphasis	log.sigma.4.5.mm.3D_gldm_LowGrayLevelEmphasis	log.sigma.4.5.mm.3D_gldzm_DistanceZoneVariabilityNormalized
0.001564777	0.004204379	0.997222228
0.006401899	0.038489402	1
0.003711203	0.01098177	1
0.002812108	0.010495565	1
0.001860615	0.007683389	1
0.004010066	0.023948869	1
0.001366957	0.006398328	0.996779396
0.000623811	0.00289434	1
0.001163661	0.004235457	1
0.001562692	0.00328914	1
0.003052345	0.016545574	1
0.001576056	0.006714937	0.996047446
0.00173135	0.010840044	1
0.001837504	0.00524639	1
0.002603761	0.009078255	1
0.001674785	0.006840492	1
0.002375299	0.006449152	1
0.003523109	0.016015991	1
0.006414659	0.032604611	1
0.001545624	0.006375554	1
0.001438847	0.009891307	1
0.003588769	0.017058319	1
0.003545953	0.016821785	1
0.00253467	0.008385956	1
0.00200275	0.004568815	1
0.001503319	0.004009757	1
0.001070031	0.003251877	1
0.001924054	0.006938567	1
0.005193223	0.024643829	1
0.001644989	0.007097197	1
0.000952208	0.003584691	1
0.001492163	0.004717818	1
0.002206345	0.00769626	0.997630335
0.002454512	0.006515523	1
0.001068391	0.003976869	1
0.0010865	0.004243932	1
0.000796488	0.002742048	1
0.000754246	0.002989439	1
0.003479814	0.020213001	1
0.002081042	0.009296424	1
0.001141959	0.003878537	1
0.001791688	0.005567682	1
0.001578677	0.005248148	0.9978355
0.002360361	0.007750596	1
0.004345941	0.017289222	1
0.001424508	0.004678643	1
0.001209974	0.005391387	1
0.004923996	0.039019579	1
0.002630477	0.014962826	1
0.005748491	0.022797051	1
0.001918588	0.00884383	1
0.002012817	0.006434947	1
0.001584707	0.007542088	1
0.002894255	0.008463015	1
0.003881066	0.016044128	1
0.001218189	0.0037954	1
0.001852887	0.005568623	1
0.001062624	0.004033493	1
0.002138208	0.005859091	1
0.000883867	0.003941155	0.997759106
0.002066263	0.008184473	1
0.001680682	0.007058065	1
0.000678816	0.002255671	0.996015952
0.00078158	0.004348995	0.99726963
0.003280794	0.009891187	1
0.000813737	0.003066375	1
0.001761189	0.009269569	1
0.001060247	0.003724198	0.998589563
0.001300807	0.003685511	1
0.001492216	0.007954902	1
0.001495184	0.006610967	1
0.000855118	0.003196441	1
0.001102582	0.003998223	1
0.002295696	0.00769807	1
0.001925351	0.007392528	1
0.002162967	0.005832275	1
0.001997936	0.007670925	1
0.002259718	0.018044941	1
0.002013531	0.009166226	1
0.001666365	0.004230901	1
0.00173722	0.004224169	1
0.001958206	0.006879196	1
0.001140766	0.003987678	1
0.001255755	0.006105754	1
0.001300923	0.006191669	0.996779396
0.002005436	0.011134157	1
0.001637312	0.006687465	1
0.001303998	0.003868343	1
0.002305091	0.01390041	1
0.002211407	0.007270944	1
0.002318581	0.017338896	1
0.002427827	0.010376511	1
0.001012175	0.002976528	1
0.001451736	0.005751952	1
0.001710502	0.007255657	1
0.000801407	0.002371651	1
0.001872503	0.008733701	1
0.001107922	0.008192569	1
0.002347572	0.015604537	1
0.00215023	0.007247203	1

log.sigma.4.5.mm.3D_gldzm_LowIntensityEmphasis	log.sigma.4.5.mm.3D_gldzm_LargeDistanceEmphasis	log.sigma.4.5.mm.3D_gldzm_HighIntensitySmallDistanceEmphasis
0.006035212	1.004172462	460.1947149
0.052946865	1	80.20666667
0.020201758	1	178.381877
0.013711326	1	261.5036232
0.011395289	1	199.9726027
0.022094104	1	144.7988166
0.007612889	1.00483871	266.7147177
0.004196119	1	536.1629328
0.006273485	1	338.1317671
0.006568033	1	526.9562044
0.021748802	1	127.2741935
0.009342015	1.005940594	256.4257426
0.020605522	1	234.1255061
0.009557102	1	344.1085271
0.013547872	1	227.7188119
0.008461628	1	282.889115
0.012194844	1	259.5513308
0.022814804	1	135.5812808
0.052732599	1	71.40101523
0.007696193	1	293.0803213
0.012629235	1	186.0552885
0.02208722	1	123.0358974
0.021552551	1	139.915
0.014329457	1	186.094162
0.008166828	1	317.754491
0.006241002	1	459.1577381
0.005131361	1	575.5579897
0.011545035	1	246.5380228
0.030273844	1	107.4235294
0.012169854	1	205.7337662
0.005434507	1	380.3289617
0.012943398	1	267.6882129
0.010862949	1.003558719	294.3454923
0.011752246	1	329.4440735
0.005208241	1	364.7518939
0.006898932	1	335.9649446
0.005814199	1	556.8493151
0.00418301	1	536.0413534
0.041989291	1	65.90163934
0.010198361	1	280.5842405
0.007129256	1	376.578629
0.006915622	1	344.8336106
0.007700956	1.003250271	312.8605092
0.012049783	1	218.9791304
0.023385035	1	143.0187266
0.006486902	1	358.7129121
0.007228812	1	283.0181237
0.046536308	1	70.79831933
0.01727012	1	189.4130435
0.031843305	1	105.9924528
0.010718695	1	263.6949686
0.00803266	1	267.4671385
0.01033679	1	215.3261391
0.012480834	1	198.9094077
0.019180283	1	145.8167702
0.007523622	1	379.6816817
0.007846657	1	301.260177
0.007558911	1	391.5
0.012088423	1	225.2601881
0.005291813	1.003365115	353.1975603
0.011776055	1	225.0825959
0.009169029	1	261.933913
0.003033573	1.005988024	626.5598802
0.005310431	1.004101162	412.2879357
0.017074251	1	174.6582278
0.005570064	1	480.3958665
0.022176807	1	148.9661017
0.005531718	1.002117149	441.9677135
0.005748044	1	348.189899
0.011795597	1	196.7396313
0.009867832	1	324.7525424
0.004940485	1	479.2488263
0.007089422	1	412.6517094
0.011411414	1	259.2301184
0.009149736	1	293.7116883
0.011951538	1	250.4781022
0.013268452	1	185.4554455
0.02034384	1	176.2721519
0.013227719	1	185.9766667
0.006758868	1	505.4188406
0.006038634	1	503.6309696
0.014034512	1	302.0243902
0.006477951	1	352.6042945
0.008740856	1	264.9063745
0.007136016	1.00483871	355.4435484
0.019098945	1	166.2882353
0.009203395	1	307.2852459
0.005952056	1	383.9543307
0.016197148	1	155.4290657
0.013007339	1	255.9974684
0.017022369	1	137.2989691
0.011428836	1	223.8523878
0.00517864	1	474.5307018
0.008224075	1	262.0125698
0.010896258	1	225.7842227
0.004085828	1	664.3614776
0.011119155	1	246.8500882
0.012209314	1	373.2857143
0.031145852	1	104.1022727
0.011390812	1	229.6553191

log.sigma.4.5.mm.3D_gldzm_LowIntensityLargeDistanceEmphasis	log.sigma.4.5.mm.3D_gldzm_HighIntensityEmphasis	log.sigma.4.5.mm.3D_gldzm_DistanceZoneVariability	log.sigma.4.5.mm.3D_gldzm_ZonePercentage
0.0060565	460.3991655	717.0027816	0.338831291
0.052946865	80.20666667	150	0.160427807
0.020201758	178.381877	309	0.201434159
0.013711326	261.5036232	276	0.282497441
0.011395289	199.9726027	438	0.184966216
0.022094104	144.7988166	169	0.256060606
0.007652208	266.9185484	1236.006452	0.219430189
0.004196119	536.1629328	982	0.192247455
0.006273485	338.1317671	979	0.215354158
0.006568033	526.9562044	274	0.255597015
0.021748802	127.2741935	310	0.205570292
0.009401421	256.5742574	503.0039604	0.219088937
0.020605522	234.1255061	247	0.132297804
0.009557102	344.1085271	387	0.225393128
0.013547872	227.7188119	505	0.25453629
0.008461628	282.889115	983	0.224634369
0.012194844	259.5513308	263	0.220268007
0.022814804	135.5812808	203	0.164639092
0.052732599	71.40101523	197	0.151771957
0.007696193	293.0803213	1245	0.239469129
0.012629235	186.0552885	416	0.137566138
0.02208722	123.0358974	195	0.180722892
0.021552551	139.915	200	0.1953125
0.014329457	186.094162	531	0.200604458
0.008166828	317.754491	334	0.262372349
0.006241002	459.1577381	672	0.283663993
0.005131361	575.5579897	776	0.240024745
0.011545035	246.5380228	526	0.190441709
0.030273844	107.4235294	85	0.1901566
0.012169854	205.7337662	308	0.149951315
0.005434507	380.3289617	915	0.192307692
0.012943398	267.6882129	263	0.127114548
0.010935575	294.3890866	841.0023725	0.261071539
0.011752246	329.4440735	599	0.254893617
0.005208241	364.7518939	1056	0.239782016
0.006898932	335.9649446	542	0.180726909
0.005814199	556.8493151	584	0.156947057
0.00418301	536.0413534	1064	0.206161597
0.041989291	65.90163934	122	0.099186992
0.010198361	280.5842405	1929	0.265337001
0.007129256	376.578629	496	0.191210486
0.006915622	344.8336106	1202	0.310835273
0.007715402	313.0433369	921.0021668	0.252669039
0.012049783	218.9791304	575	0.231109325
0.023385035	143.0187266	267	0.208920188
0.006486902	358.7129121	728	0.265209472
0.007228812	283.0181237	938	0.205611574
0.046536308	70.79831933	119	0.136624569
0.01727012	189.4130435	184	0.216216216
0.031843305	105.9924528	265	0.196005917
0.010718695	263.6949686	636	0.247567147
0.00803266	267.4671385	989	0.293384752
0.01033679	215.3261391	834	0.184840426
0.012480834	198.9094077	287	0.259963768
0.019180283	145.8167702	322	0.242105263
0.007523622	379.6816817	333	0.170769231
0.007846657	301.260177	1130	0.279357231
0.007558911	391.5	590	0.170618855
0.012088423	225.2601881	319	0.193567961
0.005303915	353.5984296	1779.004487	0.195311644
0.011776055	225.0825959	339	0.19195923
0.009169029	261.933913	575	0.215355805
0.00304642	627.2934132	1497.011976	0.259630333
0.005327554	412.5345181	1459.005468	0.191919192
0.017074251	174.6582278	158	0.2
0.005570064	480.3958665	629	0.16286898
0.022176807	148.9661017	118	0.090490798
0.005534622	442.3535639	1415.001411	0.22672
0.005748044	348.189899	1485	0.253542769
0.011795597	196.7396313	434	0.149758454
0.009867832	324.7525424	295	0.227097768
0.004940485	479.2488263	852	0.198601399
0.007089422	412.6517094	468	0.205353225
0.011411414	259.2301184	591	0.261620186
0.009149736	293.7116883	770	0.245927819
0.011951538	250.4781022	274	0.204172876
0.013268452	185.4554455	404	0.168614357
0.02034384	176.2721519	158	0.164926931
0.013227719	185.9766667	300	0.175746924
0.006758868	505.4188406	690	0.294997862
0.006038634	503.6309696	691	0.347410759
0.014034512	302.0243902	328	0.182323513
0.006477951	352.6042945	652	0.199449373
0.008740856	264.9063745	502	0.163358282
0.007148112	355.9274194	618.0032258	0.254202542
0.019098945	166.2882353	170	0.110966057
0.009203395	307.2852459	610	0.255765199
0.005952056	383.9543307	635	0.231582786
0.016197148	155.4290657	289	0.197404372
0.013007339	255.9974684	395	0.195061728
0.017022369	137.2989691	194	0.148773006
0.011428836	223.8523878	691	0.290092359
0.00517864	474.5307018	1140	0.219949836
0.008224075	262.0125698	716	0.216969697
0.010896258	225.7842227	862	0.198069853
0.004085828	664.3614776	758	0.22150789
0.011119155	246.8500882	567	0.201277955
0.012209314	373.2857143	175	0.167144222
0.031145852	104.1022727	88	0.105515588
0.011390812	229.6553191	1175	0.203498441

log.sigma.4.5.mm.3D_gldzm_IntensityVariabilityNormalized	log.sigma.4.5.mm.3D_gldzm_LowIntensitySmallDistanceEmphasis	log.sigma.4.5.mm.3D_gldzm_IntensityVariability
0.045076901	0.00602989	32.41029207
0.077511111	0.052946865	11.62666667
0.059289283	0.020201758	18.32038835
0.049884478	0.013711326	13.76811594
0.064156294	0.011395289	28.10045662
0.068309933	0.022094104	11.5443787
0.057420656	0.007603059	71.2016129
0.047863581	0.004196119	47.00203666
0.055295005	0.006273485	54.13381001
0.060738452	0.006568033	16.64233577
0.071425598	0.021748802	22.14193548
0.058037447	0.009327164	29.30891089
0.049648413	0.020605522	12.26315789
0.046478243	0.009557102	17.9870801
0.053755514	0.013547872	27.14653465
0.054502328	0.008461628	53.5757884
0.062932817	0.012194844	16.5513308
0.066466063	0.022814804	13.49261084
0.087170502	0.052732599	17.17258883
0.050492734	0.007696193	62.86345382
0.070127589	0.012629235	29.17307692
0.074135437	0.02208722	14.45641026
0.06445	0.021552551	12.89
0.062785279	0.014329457	33.33898305
0.055882247	0.008166828	18.66467066
0.048327664	0.006241002	32.47619048
0.045292406	0.005131361	35.14690722
0.062224407	0.011545035	32.73003802
0.083460208	0.030273844	7.094117647
0.065398887	0.012169854	20.14285714
0.054340231	0.005434507	49.72131148
0.064118319	0.012943398	16.86311787
0.046029756	0.010844792	38.80308422
0.04947868	0.011752246	29.63772955
0.063143868	0.005208241	66.67992424
0.060797102	0.006898932	32.95202952
0.046397073	0.005814199	27.09589041
0.043598776	0.00418301	46.38909774
0.092985757	0.041989291	11.3442623
0.047222001	0.010198361	91.09123898
0.055207791	0.007129256	27.38306452
0.049383861	0.006915622	59.359401
0.053388252	0.007697345	49.27735645
0.059133459	0.012049783	34.00173913
0.066503949	0.023385035	17.75655431
0.052926126	0.006486902	38.53021978
0.057519288	0.007228812	53.95309168
0.089188617	0.046536308	10.61344538
0.060077977	0.01727012	11.05434783
0.081238875	0.031843305	21.52830189
0.054551837	0.010718695	34.69496855
0.053524053	0.00803266	52.93528817
0.0569645	0.01033679	47.50839329
0.062098605	0.012480834	17.82229965
0.062574746	0.019180283	20.14906832
0.060952845	0.007523622	20.2972973
0.051925758	0.007846657	58.67610619
0.047296754	0.007558911	27.90508475
0.066125529	0.012088423	21.09404389
0.055017963	0.005288788	98.09702748
0.055298857	0.011776055	18.74631268
0.060464272	0.009169029	34.76695652
0.053390226	0.003030362	80.24550898
0.052010191	0.00530615	76.09090909
0.066095177	0.017074251	10.44303797
0.047919705	0.005570064	30.14149444
0.08302212	0.022176807	9.796610169
0.0465459	0.005530992	65.95553987
0.050922241	0.005748044	75.61952862
0.06944297	0.011795597	30.13824885
0.06102844	0.009867832	18.00338983
0.049323327	0.004940485	42.02347418
0.05249653	0.007089422	24.56837607
0.052688237	0.011411414	31.13874788
0.051303761	0.009149736	39.5038961
0.063588897	0.011951538	17.42335766
0.06615773	0.013268452	26.72772277
0.058564333	0.02034384	9.253164557
0.068888889	0.013227719	20.66666667
0.044780508	0.006758868	30.89855072
0.044812254	0.006038634	30.96526773
0.052907496	0.014034512	17.35365854
0.051766156	0.006477951	33.75153374
0.061768861	0.008740856	31.00796813
0.047455775	0.007132991	29.42258065
0.071487889	0.019098945	12.15294118
0.050432679	0.009203395	30.76393443
0.057389795	0.005952056	36.44251969
0.073670095	0.016197148	21.29065744
0.052318539	0.013007339	20.66582278
0.083005633	0.017022369	16.10309278
0.055736249	0.011428836	38.51374819
0.053214835	0.00517864	60.66491228
0.057765831	0.008224075	41.3603352
0.06076087	0.010896258	52.37587007
0.04747948	0.004085828	35.98944591
0.052819848	0.011119155	29.94885362
0.05835102	0.012209314	10.21142857
0.080836777	0.031145852	7.113636364
0.059463649	0.011390812	69.86978723

log.sigma.4.5.mm.3D_gldzm_HighIntensityLargeDistanceEmphasis	log.sigma.4.5.mm.3D_gldzm_SmallDistanceEmphasis	log.sigma.4.5.mm.3D_glcm_SumVariance	log.sigma.4.5.mm.3D_glcm_Homogeneity1
461.216968	0.998956885	1577.783538	0.382730757
80.20666667	1	154.0987514	0.502324902
178.381877	1	519.6755945	0.460840607
261.5036232	1	807.8570785	0.41829015
199.9726027	1	563.2300794	0.46702912
144.7988166	1	267.917332	0.467113311
267.733871	0.998790323	719.0985835	0.43548229
536.1629328	1	1616.715895	0.462255485
338.1317671	1	1094.738836	0.440901235
526.9562044	1	1724.407912	0.427447823
127.2741935	1	356.5520159	0.477189695
257.1683168	0.998514851	765.3353765	0.450197785
234.1255061	1	656.6046751	0.518842113
344.1085271	1	1070.293086	0.451579748
227.7188119	1	717.1199535	0.426406157
282.889115	1	831.4498844	0.406714126
259.5513308	1	861.3636125	0.441515961
135.5812808	1	291.0445937	0.505650842
71.40101523	1	163.490657	0.512538785
293.0803213	1	784.5280334	0.425612721
186.0552885	1	457.7567168	0.517975879
123.0358974	1	308.6904783	0.468299254
139.915	1	338.4807222	0.483360253
186.094162	1	554.5991838	0.478445328
317.754491	1	1087.090534	0.407299665
459.1577381	1	1402.749776	0.416754839
575.5579897	1	1879.48802	0.421680625
246.5380228	1	688.1365511	0.470424884
107.4235294	1	198.6732224	0.496420261
205.7337662	1	566.4371903	0.512423875
380.3289617	1	1196.423864	0.454671558
267.6882129	1	1050.681577	0.516608884
294.5634638	0.99911032	970.0268379	0.413198637
329.4440735	1	1098.144861	0.410393853
364.7518939	1	1147.734098	0.436042674
335.9649446	1	1083.005066	0.46894906
556.8493151	1	1910.127992	0.492669515
536.0413534	1	1668.093235	0.4476411
65.90163934	1	181.9514187	0.538929723
280.5842405	1	776.9443652	0.407196587
376.578629	1	1365.730957	0.465607674
344.8336106	1	952.3379311	0.390308384
313.7746479	0.999187432	1004.082071	0.405021171
218.9791304	1	654.6603056	0.442283525
143.0187266	1	294.9389442	0.47249018
358.7129121	1	1170.802567	0.432009236
283.0181237	1	808.7403129	0.458973502
70.79831933	1	131.9631163	0.524819624
189.4130435	1	427.7924706	0.473058121
105.9924528	1	221.406164	0.496769522
263.6949686	1	830.9512231	0.419640922
267.4671385	1	772.8132604	0.393736557
215.3261391	1	599.4859454	0.470368235
198.9094077	1	576.8894303	0.420027581
145.8167702	1	371.4940889	0.44166231
379.6816817	1	1162.473654	0.466124959
301.260177	1	934.0569413	0.407772292
391.5	1	1438.589994	0.458741228
225.2601881	1	769.7744207	0.483521438
355.2019069	0.999158721	1088.689864	0.464361195
225.0825959	1	528.0960975	0.491604563
261.933913	1	734.2422082	0.447621565
630.2275449	0.998502994	2071.59846	0.42370724
413.5208476	0.99897471	1032.954505	0.458235947
174.6582278	1	448.5772298	0.459304591
480.3958665	1	1433.361269	0.486358029
148.9661017	1	446.7120603	0.575537017
443.8969654	0.999470713	1310.243798	0.437582086
348.189899	1	1291.58082	0.453734028
196.7396313	1	549.5401611	0.511365622
324.7525424	1	1045.652361	0.456604425
479.2488263	1	1434.52169	0.454233376
412.6517094	1	1416.218661	0.448664386
259.2301184	1	824.0115205	0.412688535
293.7116883	1	744.7292998	0.424446235
250.4781022	1	848.6967538	0.469943804
185.4554455	1	573.2159161	0.507696749
176.2721519	1	315.9114366	0.513155759
185.9766667	1	471.4278186	0.484382541
505.4188406	1	1773.107046	0.393695307
503.6309696	1	1727.447258	0.386070261
302.0243902	1	937.1963281	0.492079787
352.6042945	1	1177.164585	0.449775913
264.9063745	1	883.3218272	0.475105208
357.8629032	0.998790323	1095.281319	0.417920603
166.2882353	1	384.9180228	0.536734616
307.2852459	1	1047.054428	0.421948084
383.9543307	1	1145.475039	0.447562038
155.4290657	1	398.5012851	0.471103804
255.9974684	1	746.0842875	0.474191214
137.2989691	1	274.5819148	0.5431942
223.8523878	1	669.881342	0.415542907
474.5307018	1	1573.750777	0.448507427
262.0125698	1	802.6677264	0.445131308
225.7842227	1	665.4623077	0.455208593
664.3614776	1	2202.150255	0.456475822
246.8500882	1	675.4049115	0.456206932
373.2857143	1	1329.054491	0.507158404
104.1022727	1	287.857832	0.534822623
229.6553191	1	627.0562275	0.451810041

log.sigma.4.5.mm.3D_glcm_Homogeneity2	log.sigma.4.5.mm.3D_glcm_ClusterShade	log.sigma.4.5.mm.3D_glcm_MaximumProbability	log.sigma.4.5.mm.3D_glcm_Idmn	log.sigma.4.5.mm.3D_glcm_SumVariance2
0.300500107	-549.4173158	0.022947019	0.986898659	108.9788324
0.443228288	120.0483289	0.057506494	0.982037663	35.79359165
0.391806072	138.2921898	0.021518883	0.987815908	68.39308313
0.340592799	-423.4958804	0.027334062	0.984117213	97.9232721
0.398864711	56.21386969	0.035310533	0.988409574	48.04989481
0.399830619	223.5013122	0.067298491	0.9835866	61.18039142
0.359567088	166.2448775	0.01954243	0.992020988	68.45938374
0.393456758	167.8714655	0.028391156	0.993506728	68.83725797
0.368185023	-2.395411434	0.020964838	0.990227735	70.5077679
0.349613644	-270.3238051	0.022934667	0.990755975	65.25692556
0.410024314	0.31439474	0.026274356	0.987461723	44.12143379
0.377842761	-22.96840857	0.025200195	0.990818685	67.7511302
0.463219185	217.7905441	0.060318682	0.991392776	60.81705092
0.381804424	-53.13326318	0.039039776	0.989676403	69.31663565
0.349181669	32.23124656	0.029682681	0.988384982	67.84125762
0.324058331	-202.7673223	0.019492718	0.988062529	78.56270699
0.366493538	-386.3457508	0.02659728	0.986019149	51.62256548
0.447659136	263.754059	0.067059753	0.983996007	48.24670818
0.453406095	83.7552671	0.061219125	0.982264592	34.90438726
0.34757638	41.50669379	0.019351495	0.991186065	79.5763991
0.461398819	64.07149717	0.046125978	0.991384264	39.79443066
0.398408735	21.78602792	0.027640076	0.985297433	43.45976881
0.418512633	142.9181123	0.042371955	0.987236274	55.94594227
0.412525712	141.973928	0.035365307	0.990754652	45.97611675
0.326017063	-26.47111823	0.024533197	0.9867773	48.37057293
0.338524661	-372.166265	0.026459794	0.990994056	76.07824606
0.345701957	-760.9633326	0.023022137	0.992552402	96.50616013
0.40246788	69.03576522	0.037509734	0.989694101	50.52785211
0.434397315	154.6132133	0.060913989	0.981835678	41.91886196
0.45419498	53.24438474	0.045746434	0.991510958	37.89372224
0.384729574	11.0076546	0.025595373	0.991778374	59.88408343
0.46145564	-328.9691484	0.055137746	0.990937646	34.65741755
0.334327725	-425.5351756	0.017783484	0.989901018	98.51436089
0.329835112	-362.0267412	0.021780212	0.989488886	76.9180242
0.359363408	-112.1817594	0.020790474	0.990434656	58.86332603
0.400884388	-122.9919516	0.028379406	0.990548636	49.79718915
0.432573753	-449.4167108	0.054562063	0.993710177	60.793648
0.37522654	-256.4833381	0.048971178	0.992586294	115.029509
0.487262719	-8.232457407	0.046642902	0.986424518	15.26414475
0.326274397	15.10142805	0.01323341	0.988821627	102.2202491
0.39970856	-506.8420082	0.045314669	0.990938109	54.95834685
0.305892675	366.59747	0.017302508	0.991780221	95.01211994
0.324201148	70.4775688	0.017883915	0.987277896	69.55534857
0.368424414	-93.97065789	0.028312691	0.991964979	51.84355059
0.405379121	292.213059	0.036750089	0.987842269	54.13701777
0.358576383	-297.4155389	0.020794086	0.990325081	71.53572956
0.388365158	82.13881044	0.025632038	0.99115646	52.60249213
0.469129764	91.52099554	0.056702006	0.982239395	33.25108227
0.404389529	254.4658368	0.032686866	0.988487301	63.39393994
0.434010122	134.3134142	0.053708411	0.988109429	41.25278035
0.340098174	-89.73799065	0.016673231	0.987480238	72.74695093
0.310121847	81.4289726	0.016879193	0.987439951	77.26304422
0.40221072	124.2994258	0.029918616	0.990831197	56.01445782
0.341505205	46.16172853	0.02333901	0.981945284	46.18613882
0.368245073	125.2632429	0.027417743	0.983777261	66.51178597
0.396827411	-19.32028629	0.038452505	0.992997423	43.33678944
0.326545158	-142.9340815	0.015747311	0.988754256	74.32128284
0.389329445	-564.989865	0.031203184	0.991653426	68.25551512
0.419517213	-29.20729309	0.040240189	0.988852668	29.79092152
0.394930593	65.53951747	0.033992449	0.992266333	65.74236499
0.430179821	363.1255622	0.052553073	0.991365975	55.09606517
0.374935425	-18.1177808	0.025266352	0.990089922	68.0882111
0.346915996	-198.7781443	0.020655816	0.993595124	75.10000688
0.388652746	362.4787212	0.026680135	0.993034585	70.83610257
0.38562118	63.18891665	0.030581369	0.986724087	35.61717401
0.422724863	50.95543868	0.046314928	0.99424084	65.7922515
0.532713854	14.84842017	0.085131672	0.99180017	22.86382722
0.363643094	-25.21165544	0.029495627	0.992804193	76.19896278
0.382556513	-208.0000255	0.025687995	0.992543154	56.42936931
0.452981197	35.82426154	0.038053366	0.990187069	37.81939989
0.387630173	-357.4441867	0.03610227	0.990051534	57.75605901
0.384315496	139.5095628	0.028398596	0.992493438	74.68064434
0.375915098	-347.6950413	0.03051405	0.991016554	57.31981785
0.333672243	-215.4451799	0.022019582	0.985009556	66.90321656
0.346930868	260.4311014	0.02854017	0.989494142	93.43649033
0.402960175	-237.4759145	0.036643391	0.988579816	45.75385397
0.448953514	-11.37552492	0.033018244	0.990605372	32.21945284
0.454410417	377.5226389	0.06095189	0.990849103	58.64117236
0.418149009	51.14883258	0.040831674	0.989415913	44.13440452
0.312130609	-1519.145769	0.02005251	0.990221663	127.4210522
0.304698466	-817.0391479	0.023788137	0.987498873	119.7697375
0.430432267	-199.521842	0.046278652	0.993537645	41.65459756
0.37755317	-76.19440434	0.028258205	0.991337202	64.34972688
0.408233083	-326.3053684	0.028465358	0.992232494	55.15702495
0.339304799	-244.7773402	0.020122673	0.991374674	95.28522592
0.484801635	179.7801324	0.055272953	0.99165781	44.98986905
0.34658654	-534.2970326	0.025057023	0.986643977	83.19821259
0.376039021	51.21530758	0.033020384	0.991289967	56.83304343
0.401851736	51.28335042	0.036463866	0.987714738	49.37732198
0.407506019	-51.31386739	0.033435896	0.990720043	57.57040944
0.493607732	218.4335184	0.088508693	0.985004407	54.48461283
0.336663886	97.56384692	0.020134501	0.986766738	84.89279889
0.376667489	-277.8256793	0.026220308	0.992707205	58.92528459
0.371965956	-25.28074629	0.02945079	0.989447258	51.60096316
0.383229314	-54.57019952	0.023846557	0.991370301	50.66578391
0.387850807	-197.9215383	0.042994554	0.993159665	93.341553
0.384285229	-69.49028511	0.028700737	0.991614704	85.07313936
0.451573004	-1111.987055	0.061667681	0.989169953	71.42465122
0.481977578	-13.89554633	0.041447548	0.987446221	27.05729483
0.380633807	135.8551643	0.026481341	0.987623956	53.41944044

log.sigma.4.5.mm.3D_glcm_Contrast	log.sigma.4.5.mm.3D_glcm_DifferenceEntropy	log.sigma.4.5.mm.3D_glcm_InverseVariance	log.sigma.4.5.mm.3D_glcm_Entropy	log.sigma.4.5.mm.3D_glcm_Dissimilarity
17.88676923	3.161340098	0.296661031	8.11872222	3.129639637
5.688599537	2.373022958	0.427346433	6.337888183	1.717536213
8.026924374	2.624511083	0.385000215	7.310570507	2.078521296
12.36930758	2.911149008	0.331640972	7.612702832	2.593807536
7.68973984	2.600514776	0.391154358	7.092938169	2.011665448
9.361299065	2.680066479	0.365443997	6.834701468	2.182226762
9.543049065	2.757619145	0.353799713	7.572860127	2.301268787
8.704799095	2.689508728	0.379241378	7.364902395	2.117707799
10.47963071	2.786171768	0.361976203	7.613450247	2.339696508
10.48707289	2.792302444	0.34159761	7.260016575	2.403902613
6.376188775	2.484490009	0.394059869	6.914213065	1.885269782
8.011754211	2.657874641	0.37668802	7.376103883	2.120082149
6.638145169	2.464566311	0.41017393	6.703294885	1.749826283
10.42135894	2.803311145	0.362923121	7.341562983	2.308816595
10.95489696	2.850028545	0.337950055	7.483172104	2.458464724
11.2194587	2.818257426	0.322481521	7.702692154	2.5630853
9.284973947	2.720742074	0.354942569	7.04760265	2.256137382
7.045093707	2.497886564	0.409054858	6.558841841	1.828224958
4.876558894	2.311673344	0.417322891	6.390288977	1.629886218
9.94332782	2.792563264	0.346830946	7.735279479	2.375625995
5.180037642	2.359481809	0.424335404	6.629788012	1.630949947
6.246284225	2.464414263	0.394801303	6.82696349	1.892006548
5.943252793	2.442186595	0.410821294	6.861270554	1.812703667
7.583694194	2.600940085	0.385678143	7.030253174	1.979336069
12.58948766	2.891876422	0.32129882	7.319774645	2.647380453
12.13540567	2.905247822	0.335716893	7.609056034	2.571803098
13.01059063	2.926384174	0.344116007	7.752508147	2.58654644
7.274267071	2.597958711	0.384721181	7.075321218	1.991070722
5.615925281	2.395673679	0.407988608	6.283684395	1.760370613
5.097267361	2.357171143	0.423637472	6.629118162	1.639636597
9.312464889	2.724328383	0.373222816	7.386998157	2.2008824
7.019737282	2.48743129	0.414887321	6.334724011	1.780224146
12.16185179	2.905923382	0.335545657	7.889468533	2.590793948
11.9093549	2.89480482	0.324363038	7.634140968	2.601541585
8.945218451	2.725541025	0.352503411	7.417377458	2.258391999
7.773975316	2.60222228	0.385259404	7.066068668	2.020016004
9.576494928	2.638117829	0.39595914	6.933219714	2.030641998
12.30912009	2.896196636	0.326509893	7.896166632	2.477491357
3.195163155	2.070728751	0.461508807	5.761528108	1.362587908
11.9709567	2.911656083	0.33079091	8.084788982	2.601571425
10.36638516	2.768423226	0.37519319	7.111219144	2.23510031
13.60070719	2.983373896	0.312344871	7.997949824	2.790519403
12.8595835	2.933216268	0.324204284	7.75461145	2.680820294
9.630554997	2.766406232	0.357992063	7.306266249	2.279864403
6.80336544	2.529028342	0.399580468	6.867291816	1.925050782
11.68208231	2.858675639	0.355505778	7.601419148	2.469327584
8.309743397	2.664493188	0.371736433	7.253841516	2.11149758
4.294619196	2.22237062	0.433004482	6.162016382	1.528291467
6.943644135	2.55040976	0.380749918	7.018471039	1.953218742
6.075655591	2.43229363	0.398285956	6.599449455	1.792619583
10.30757395	2.81108299	0.340117391	7.651461169	2.429140703
13.49475148	2.993029359	0.31145699	7.905593776	2.779921287
6.957913135	2.561549722	0.391822331	7.203947175	1.951821498
11.2501561	2.82906548	0.339393788	7.226644468	2.488946285
10.04189256	2.765212937	0.356555719	7.350968447	2.316596047
6.941313956	2.546664794	0.39095921	6.933775884	1.96352315
11.2439661	2.868147962	0.329769882	7.770427563	2.563385838
8.95943653	2.672574415	0.382196395	7.24328198	2.134218188
7.412081125	2.571491745	0.399045249	6.690254618	1.928918998
9.252422814	2.756435759	0.352791554	7.516681032	2.193943184
7.658499385	2.571497659	0.401488097	6.883632201	1.91993084
8.679331404	2.70263348	0.367990096	7.416853424	2.18954183
11.63539454	2.884348707	0.343867213	7.73281602	2.498306419
8.819539564	2.690401769	0.375748725	7.401466768	2.150610062
7.426621295	2.539363476	0.360153166	6.696857079	2.04434365
7.707070467	2.602180692	0.389665613	7.155359988	1.955367258
3.804483787	2.153228066	0.446078814	5.914566482	1.3257819
10.75299011	2.829121255	0.354176824	7.654788257	2.376189039
8.917938477	2.717919468	0.368677428	7.352099216	2.179899986
5.467594396	2.386684226	0.418806435	6.657054104	1.67584321
9.445962481	2.687193356	0.380224706	7.007057481	2.187906075
9.58657369	2.725089279	0.377130445	7.504309999	2.202676277
9.010701971	2.705414055	0.366886101	7.167340503	2.196683951
12.50499126	2.931436216	0.329251982	7.653973493	2.624113422
11.25324904	2.861071612	0.336373734	7.816037457	2.484305193
8.136905544	2.629688338	0.384075492	6.925571227	2.055943809
6.214850778	2.448505975	0.412820717	6.63282353	1.748405387
5.483021492	2.40144347	0.405995006	6.585129912	1.694373224
5.866001221	2.450190992	0.399971604	6.845627226	1.805436984
15.52878265	3.045640252	0.315671811	7.962732634	2.893785393
18.03088251	3.159937575	0.299972017	8.117119904	3.114301277
7.294149009	2.564051808	0.400384187	6.732569531	1.899734421
9.273967946	2.693234485	0.369485903	7.356220037	2.201913927
6.297894943	2.474443847	0.404965157	7.036811268	1.871009584
11.5808298	2.873381894	0.335198652	7.839228438	2.532732276
4.230270237	2.229345328	0.443916445	6.361907011	1.474019713
12.73774282	2.929857701	0.342821995	7.688191925	2.594703556
9.901924699	2.771748181	0.365885054	7.343849392	2.272230896
6.830359351	2.546870497	0.380174937	6.953055641	1.953001754
7.615381268	2.600468987	0.386520489	7.129384601	1.995737202
5.897050169	2.353380716	0.415183372	6.062067322	1.632360392
11.71892278	2.902120871	0.332194632	7.793959904	2.562130756
9.241791606	2.735067195	0.36855476	7.354086843	2.221965947
9.29727791	2.73281141	0.362005752	7.292674805	2.24459864
7.51499025	2.60822588	0.378977609	7.231185608	2.058542519
11.38907996	2.834895119	0.359888673	7.568488114	2.339958812
7.276248746	2.591292423	0.378732832	7.49867318	2.042010094
9.792558319	2.644253908	0.392147978	6.667192335	2.035241243
3.80953047	2.171476257	0.445668604	6.183690462	1.446701528
9.622006067	2.737529889	0.368150617	7.335327558	2.225546914

log.sigma.4.5.mm.3D_glcm_DifferenceVariance	log.sigma.4.5.mm.3D_glcm_Idn	log.sigma.4.5.mm.3D_glcm_Idm	log.sigma.4.5.mm.3D_glcm_Correlation	log.sigma.4.5.mm.3D_glcm_Autocorrelation
7.739449632	0.924504141	0.300500107	0.719871586	499.6851386
2.565385931	0.914527956	0.443228288	0.724996967	65.99941125
3.531761001	0.927519636	0.391806072	0.789172912	187.479014
5.397625142	0.917714924	0.340592799	0.776509278	274.8809692
3.532805522	0.929691736	0.398864711	0.723433064	199.0161692
4.159360129	0.919313343	0.399830619	0.734557587	104.4865639
4.058066677	0.939417311	0.359567088	0.75603114	248.3876876
4.120869473	0.946969338	0.393456758	0.774533492	506.4400213
4.720907085	0.93548328	0.368185023	0.742415887	359.0218853
4.461225275	0.935334049	0.349613644	0.723410622	534.4548257
2.626297116	0.925293744	0.410024314	0.747040097	134.9448534
3.429456406	0.935030436	0.377842761	0.788242761	262.0626494
3.444093774	0.942760519	0.463219185	0.802104458	226.3120363
4.92157252	0.934584639	0.381804424	0.737932986	349.903519
4.713853257	0.928244716	0.349181669	0.72300157	246.3863516
4.159176784	0.925066508	0.324058331	0.751663308	282.1663624
3.960670527	0.921948985	0.366493538	0.69331619	285.5291368
3.490283902	0.922450577	0.447659136	0.7407818	111.5826166
2.032871303	0.913494103	0.453406095	0.755764315	69.96629425
4.131824634	0.935839276	0.34757638	0.778245927	269.1196782
2.430612031	0.939663577	0.461398819	0.766841341	165.7024218
2.522342551	0.918246322	0.398408735	0.747927594	119.3407153
2.537511602	0.92485877	0.418512633	0.804938678	129.5260471
3.539603984	0.937463253	0.412525712	0.716383223	195.8706627
5.155249552	0.923316689	0.326017063	0.587431282	351.6104825
5.28400658	0.936530291	0.338524661	0.7217498	446.2066682
6.09340044	0.943523404	0.345701957	0.763078243	583.2722393
3.200021885	0.932505038	0.40246788	0.747793841	236.378105
2.330828891	0.912159353	0.434397315	0.766025764	81.27819503
2.317344971	0.939208785	0.45419498	0.763048418	198.6430048
4.273317866	0.9405864	0.384729574	0.731747959	386.2435764
3.729785886	0.942006921	0.46145564	0.664303965	334.3970887
5.205040252	0.932703897	0.334327725	0.780346311	324.293059
4.843965443	0.930440121	0.329835112	0.732253531	359.1156897
3.678451901	0.933196478	0.359363408	0.736703829	372.3921348
3.535727916	0.936194832	0.400884388	0.7297308	351.5349998
5.223682592	0.952122034	0.432573753	0.727997479	582.8294324
5.787500423	0.944639937	0.37522654	0.807721822	527.7210142
1.273280965	0.921067638	0.487262719	0.653433321	74.37604398
5.006954577	0.928536017	0.326274397	0.789788387	268.1794182
5.199763913	0.940231613	0.39970856	0.682833188	430.2388208
5.511599139	0.937556942	0.305892675	0.750736183	318.8926411
5.275069753	0.924654922	0.324201148	0.690741125	331.7714386
4.284661967	0.94008319	0.368424414	0.686157538	226.4599602
2.994844496	0.926963456	0.405379121	0.776015115	114.3330507
5.309448742	0.935887965	0.358576383	0.719751432	379.9168664
3.705524809	0.937452773	0.388365158	0.726962229	272.9436906
1.82381015	0.913498213	0.469129764	0.771624248	58.3730964
2.996870556	0.928723764	0.404389529	0.802808906	157.9466384
2.625049998	0.928936459	0.434010122	0.738933189	89.91145735
4.232147845	0.924228333	0.340098174	0.750337243	281.9628822
5.538432487	0.924140578	0.310121847	0.704138274	264.465116
3.039522753	0.935826279	0.40221072	0.777642743	211.3362275
4.747868082	0.911926974	0.341505205	0.609101789	201.6872087
4.434636063	0.917539509	0.368245073	0.738175851	139.8514329
2.97037597	0.942926376	0.396827411	0.722354131	373.2043288
4.42862463	0.927225196	0.326545158	0.737458124	312.4328958
4.20395176	0.940680535	0.389329445	0.768517078	454.4751701
3.608014404	0.932600493	0.419517213	0.600740356	256.0055613
4.277992527	0.94234475	0.394930593	0.753238061	356.6300398
3.853317267	0.941400042	0.430179821	0.753503665	187.2763695
3.753223522	0.933248769	0.374935425	0.773744341	252.319255
5.213430509	0.946248439	0.346915996	0.732552243	635.6887002
4.009142378	0.94476291	0.388652746	0.777755671	339.949725
3.04746135	0.922743518	0.38562118	0.652513328	161.3681666
3.757696111	0.950835932	0.422724863	0.790979352	453.6997934
1.985747242	0.944099512	0.532713854	0.714169671	158.3842586
4.940272293	0.943873037	0.363643094	0.752132761	420.9345795
4.026040099	0.942522922	0.382556513	0.725268889	412.6288589
2.542099923	0.935782937	0.452981197	0.74597972	193.2488404
4.384061627	0.935807992	0.387630173	0.721290891	339.7261633
4.595188671	0.943720198	0.384315496	0.772383153	456.4526526
4.016851297	0.937082907	0.375915098	0.726601059	446.690936
5.384818069	0.919322517	0.333672243	0.68568325	278.2894015
4.827214916	0.931619464	0.346930868	0.785486193	257.4452767
3.72225872	0.930920078	0.402960175	0.698499021	282.0558238
3.026909923	0.938334319	0.448953514	0.670591219	199.3031511
2.518141082	0.937491098	0.454410417	0.827850808	120.9319382
2.518876075	0.930841542	0.418149009	0.764870652	170.6543347
6.784124155	0.934416918	0.312130609	0.783391482	554.6127089
7.966394741	0.926752083	0.304698466	0.739648747	542.8193217
3.58277269	0.94818917	0.430432267	0.699880695	306.2460227
4.201949782	0.938845288	0.37755317	0.748573436	380.9750056
2.63486952	0.940101311	0.408233083	0.795629255	294.7791987
4.920548837	0.937299221	0.339304799	0.78218444	361.2035775
1.99812321	0.940398009	0.484801635	0.824916951	142.5379278
5.763882524	0.925142086	0.34658654	0.734230266	344.5477246
4.591921611	0.938844267	0.376039021	0.703700476	370.9166928
2.887354978	0.925869844	0.401851736	0.756602301	148.2580094
3.503541451	0.936915893	0.407506019	0.76590579	254.7923064
2.906646377	0.92704883	0.493607732	0.802971814	105.0548815
4.959468727	0.923220467	0.336663886	0.757226284	234.2905839
4.145164543	0.943012941	0.376667489	0.728275102	492.8997765
4.047654686	0.931886432	0.371965956	0.694360234	270.6234553
3.145322915	0.936684399	0.383229314	0.741163671	230.6279166
5.727176148	0.947569898	0.387850807	0.78243681	672.6517732
2.974076812	0.937055538	0.384285229	0.842117201	236.8505568
5.356330349	0.939226989	0.451573004	0.761854998	417.0145681
1.617796072	0.925904163	0.481977578	0.751578062	111.0045723
4.475168387	0.928379193	0.380633807	0.693915994	218.4889683

log.sigma.4.5.mm.3D_glcm_SumEntropy	log.sigma.4.5.mm.3D_glcm_AverageIntensity	log.sigma.4.5.mm.3D_glcm_Energy	log.sigma.4.5.mm.3D_glcm_SumSquares	log.sigma.4.5.mm.3D_glcm_ClusterProminence
5.35166625	21.92996393	0.005837632	31.07016546	41186.0161
4.417616491	7.646549732	0.019586289	10.3705478	3151.692557
5.016340957	13.12731583	0.00866827	19.10500188	11424.00838
5.199034777	15.95389988	0.00785928	27.99166352	27945.7571
4.792703033	13.74485311	0.011311822	13.93490866	6721.089449
4.755918591	9.56646276	0.015855494	17.63542262	8525.905141
5.06416276	15.33601946	0.007640063	19.83505183	15076.55859
4.992315679	22.20578474	0.010705129	19.11052169	18056.61937
5.091763568	18.62304793	0.007679976	20.45338401	15298.13235
4.908026121	22.88101716	0.008713827	18.57704879	17609.5966
4.731286123	11.20232347	0.011380075	12.62440564	5433.43955
5.02882129	15.74966677	0.008810636	19.25082745	12887.38988
4.764478879	14.61014496	0.019387114	17.22767837	14830.84894
4.977699455	18.36552931	0.010857478	20.1519633	18413.57887
4.9932635	15.26934945	0.009496661	20.08125924	17742.53122
5.139418144	16.34812305	0.006992652	22.94383394	19121.61612
4.70762391	16.58107153	0.011163051	15.22688486	11412.32783
4.54583934	10.06346156	0.019485573	13.82295047	6219.992896
4.467572395	7.902310242	0.017412467	9.945236537	2780.087367
5.180095168	15.92899703	0.006927518	22.80658061	18033.48505
4.619702162	12.53177384	0.01664051	11.24361708	4572.947672
4.694632197	10.48924376	0.011550669	12.42651326	4710.436142
4.827446305	10.81626292	0.01322914	15.47229877	8206.115186
4.743869368	13.65614705	0.01213167	13.51637455	7314.043356
4.794313117	18.58710019	0.009074164	15.60575377	7776.417228
5.059645796	20.80908512	0.008555198	21.70580474	26399.94205
5.204631711	23.78107475	0.008119816	27.30924287	48592.45344
4.786863892	15.02313662	0.012340078	14.51970321	8730.846326
4.474560946	8.496431033	0.019226089	11.88369681	4094.71538
4.610299659	13.80000129	0.016249096	10.7477474	4616.016052
4.944856303	19.41811998	0.009558685	17.34214509	12189.67002
4.318262357	18.10577345	0.022952012	10.49070165	8881.758129
5.275696651	17.46529298	0.00651889	27.87045186	30804.60653
5.076833703	18.60607306	0.00798427	22.3172784	23515.26428
4.944836203	19.04539333	0.008261945	17.28249893	12013.87314
4.790336044	18.52095143	0.010863903	14.74176932	8717.986234
4.746553366	23.91377085	0.017317266	17.43185896	28589.93375
5.403737125	22.4883355	0.008137364	31.65838642	41422.97249
3.984253486	8.447117246	0.024548362	4.614826977	684.9482145
5.36901293	15.72800565	0.005306829	28.98270435	29700.9074
4.738949586	20.57392638	0.014019018	16.07902095	18280.56949
5.276556296	17.39869468	0.005789869	27.35546543	34630.09665
5.072627202	17.91446256	0.006858756	20.97577976	14724.6764
4.835251541	14.73079077	0.009859507	15.62640773	9622.537237
4.730645869	10.12256854	0.013462452	15.2350958	8278.085887
5.052405964	19.18126591	0.007815264	20.78970319	21444.18855
4.866909642	16.2238728	0.01028609	15.59606341	8964.924773
4.366602826	7.149711728	0.020278121	9.386425367	2447.494181
4.896883735	11.99196219	0.011180902	17.58439602	12096.56267
4.59094633	9.006317702	0.015570308	11.83210899	4982.309888
5.105595305	16.35857241	0.006953871	21.20225571	15323.67089
5.156128962	15.84126536	0.005860513	23.2473634	16638.21768
4.906161908	14.11773825	0.010643765	15.89401275	9587.397792
4.744611866	13.89059354	0.009139111	14.35907373	5525.608802
4.962861647	11.21153511	0.008569527	19.13841963	9887.584252
4.70948462	19.13543651	0.012601462	12.68876243	6585.923007
5.126839398	17.28918325	0.00644894	21.75033779	16847.60976
4.917632751	21.04031715	0.01140182	19.13921123	26431.18134
4.446141536	15.82429794	0.015137143	9.30075066	3214.299318
5.03047345	18.56499859	0.009100306	18.77743107	15442.94417
4.740309844	13.26348333	0.015460701	15.95621703	10419.82928
5.010376326	15.43347581	0.008587393	19.45890306	12677.31212
5.110349929	24.97185777	0.007363501	21.98834363	23942.97026
5.006648501	18.0916936	0.010084052	20.02128931	17966.93893
4.524171171	12.42082796	0.013007969	10.76094883	3761.793818
4.932565113	20.99254392	0.013474279	18.16950463	18644.24053
4.201220757	12.39398811	0.029840195	6.667077753	2296.696531
5.099076945	20.19063788	0.008883982	21.41184993	24223.62592
4.893236036	20.08648802	0.009688148	16.19318304	13183.47799
4.593528134	13.6070337	0.014614773	10.82174857	3785.420209
4.771738227	18.17473677	0.012693153	17.18388023	17115.03523
5.084902971	21.03427316	0.009225473	20.84468352	18647.18055
4.831599859	20.9267595	0.011169783	16.49968738	16663.76639
5.023236544	16.29791805	0.007562778	20.15369213	15142.74305
5.262812742	15.44602359	0.006966787	26.65960399	25250.4701
4.68841242	16.51215306	0.012951533	13.48247235	8318.104863
4.511771136	13.88480385	0.015003787	9.608575905	3412.446794
4.710746972	10.37421183	0.019964943	16.03104846	11573.26607
4.713457304	12.69118775	0.012855126	12.50010144	5348.489982
5.331019416	23.02190986	0.00665404	35.12071355	69418.09806
5.396300515	22.84885346	0.00593303	33.53144728	53213.14972
4.579924555	17.30620773	0.016867242	12.45676925	10783.35854
4.966661865	19.23748151	0.010422398	18.55111882	16313.94248
4.841702919	16.8261241	0.011836604	15.53389723	13830.96757
5.271103421	18.51870717	0.006748254	26.68837131	31877.96675
4.571399052	11.50400163	0.019944729	12.30503482	5307.438929
5.116956323	18.16127162	0.00823442	24.39247241	25458.70481
4.909707447	19.0468313	0.00974704	16.79103642	10100.69503
4.777917016	11.7301455	0.012165875	14.05192033	6684.425474
4.893282343	15.58882175	0.011790435	16.58661654	11229.3127
4.442348807	9.637944785	0.028296254	15.09541575	5577.41193
5.207828998	14.72814578	0.006597084	24.54090788	20675.49207
4.918851212	21.98160943	0.010042877	16.65552788	16168.48235
4.846585655	16.15858047	0.010159799	15.52708947	8873.644209
4.85910923	14.83626945	0.009668425	14.66289106	7928.730975
5.153181916	25.61387645	0.009991299	26.34983509	37585.91366
5.19245559	14.76004603	0.008447347	23.30221586	21121.30612
4.617658986	20.09731902	0.019185827	20.40869152	36206.89271
4.363295293	10.25624575	0.019233059	7.716706326	2218.575767
4.861680124	14.41541852	0.009237056	15.88146475	7647.499828

log.sigma.4.5.mm.3D_glcm_SumAverage	log.sigma.4.5.mm.3D_glcm_Imc2	log.sigma.4.5.mm.3D_glcm_Imc1	log.sigma.4.5.mm.3D_glcm_DifferenceAverage	log.sigma.4.5.mm.3D_glcm_Id
43.6761736	0.890280981	-0.183658918	3.129639637	0.382730757
15.29309946	0.87807875	-0.220342805	1.717536213	0.502324902
26.25463166	0.911789	-0.228678964	2.078521296	0.460840607
31.83909463	0.928458017	-0.235529038	2.593807536	0.41829015
27.48970622	0.869093214	-0.188573181	2.011665448	0.46702912
19.13292552	0.912313014	-0.246413577	2.182226762	0.467113311
30.57074014	0.870451465	-0.181264103	2.301268787	0.43548229
44.3346827	0.889377156	-0.199102292	2.117707799	0.462255485
37.09407357	0.870692771	-0.185543023	2.339696508	0.440901235
45.63903449	0.897838719	-0.208862044	2.403902613	0.427447823
22.40464694	0.875539814	-0.206098743	1.885269782	0.477189695
31.43870805	0.901987886	-0.209950755	2.120082149	0.450197785
29.17231286	0.927295904	-0.266832959	1.749826283	0.518842113
36.61471301	0.89837864	-0.208171006	2.308816595	0.451579748
30.47295334	0.875316988	-0.185211582	2.458464724	0.426406157
32.57721539	0.8836037	-0.189091356	2.5630853	0.406714126
33.16214306	0.85122745	-0.177510219	2.256137382	0.441515961
20.12692311	0.887748986	-0.22397054	1.828224958	0.505650842
15.80462048	0.870916602	-0.215388087	1.629886218	0.512538785
31.72996869	0.886073846	-0.18954792	2.375625995	0.425612721
25.06354769	0.884276481	-0.218443317	1.630949947	0.517975879
20.97848753	0.885332365	-0.21117843	1.892006548	0.468299254
21.63252585	0.92221642	-0.253953945	1.812703667	0.483360253
27.29561941	0.859836571	-0.184654791	1.979336069	0.478445328
37.02050891	0.834445698	-0.163958045	2.647380453	0.407299665
41.48188093	0.872748574	-0.181755081	2.571803098	0.416754839
47.42924025	0.899551398	-0.200592235	2.58654644	0.421680625
30.03698778	0.865843974	-0.186832702	1.991070722	0.470424884
16.99286207	0.911482956	-0.255102369	1.760370613	0.496420261
27.60000257	0.883745814	-0.215374564	1.639636597	0.512423875
38.65703992	0.862215061	-0.180553414	2.2008824	0.454671558
36.19305616	0.822704427	-0.174045246	1.780224146	0.516608884
34.79538499	0.896749525	-0.197633012	2.590793948	0.413198637
37.03188613	0.877964223	-0.184693155	2.601541585	0.410393853
37.94196207	0.853341104	-0.170400025	2.258391999	0.436042674
36.93326821	0.868218017	-0.191066726	2.020016004	0.46894906
47.75006028	0.880449241	-0.207841277	2.030641998	0.492669515
44.81130168	0.935047094	-0.242838485	2.477491357	0.4476411
16.89423449	0.780989698	-0.163572787	1.362587908	0.538929723
31.34420023	0.887058414	-0.184260066	2.601571425	0.407196587
40.94310264	0.843362586	-0.167896754	2.23510031	0.465607674
34.55589744	0.875170422	-0.176537692	2.790519403	0.390308384
35.64226863	0.834878146	-0.155100239	2.680820294	0.405021171
29.3873719	0.837506217	-0.161797325	2.279864403	0.442283525
20.24513708	0.894080934	-0.21732526	1.925050782	0.47249018
38.20700375	0.874721662	-0.184770538	2.469327584	0.432009236
32.36404184	0.854004844	-0.175986653	2.11149758	0.458973502
14.29942346	0.885017845	-0.235734915	1.528291467	0.524819624
23.98392439	0.929625737	-0.257262548	1.953218742	0.473058121
18.0126354	0.886206727	-0.228348478	1.792619583	0.496769522
32.63706759	0.87652843	-0.18286967	2.429140703	0.419640922
31.52856839	0.841079052	-0.152062231	2.779921287	0.393736557
28.21561745	0.882073637	-0.19893434	1.951821498	0.470368235
27.78118708	0.836878442	-0.162017955	2.488946285	0.420027581
22.42307022	0.891744619	-0.204996451	2.316596047	0.44166231
38.16280049	0.8617852	-0.188134408	1.96352315	0.466124959
34.44694246	0.85606299	-0.167009389	2.563385838	0.407772292
41.93513292	0.881942476	-0.200331677	2.134218188	0.458741228
31.64859588	0.806093126	-0.152397203	1.928918998	0.483521438
37.01327331	0.865906445	-0.180299983	2.193943184	0.464361195
26.48852668	0.894610844	-0.218328875	1.91993084	0.491604563
30.81819093	0.891215886	-0.200329891	2.18954183	0.447621565
49.7922055	0.859670573	-0.167835955	2.498306419	0.42370724
36.02431853	0.878159275	-0.194467356	2.150610062	0.458235947
24.84165591	0.860722619	-0.197658781	2.04434365	0.459304591
41.91265274	0.907494495	-0.224680156	1.955367258	0.486358029
24.78797623	0.869835184	-0.22426152	1.3257819	0.575537017
40.22710837	0.870592938	-0.178696792	2.376189039	0.437582086
40.03667584	0.844241655	-0.166703651	2.179899986	0.453734028
27.21406739	0.870705989	-0.205505527	1.67584321	0.511365622
36.20015635	0.892903476	-0.214790379	2.187906075	0.456604425
41.95999552	0.896459268	-0.203965062	2.202676277	0.454233376
41.69447663	0.867898716	-0.188256071	2.196683951	0.448664386
32.53790968	0.843153248	-0.158788178	2.624113422	0.412688535
30.78192647	0.902998696	-0.205718986	2.484305193	0.424446235
33.02281836	0.860968493	-0.190458187	2.055943809	0.469943804
27.76960769	0.842806836	-0.186001453	1.748405387	0.507696749
20.74842366	0.935899225	-0.281522023	1.694373224	0.513155759
25.38237549	0.885271755	-0.209105882	1.805436984	0.484382541
45.89441328	0.908870115	-0.20734687	2.893785393	0.393695307
45.49197824	0.907898611	-0.200317262	3.114301277	0.386070261
34.50518282	0.85618937	-0.186706891	1.899734421	0.492079787
38.32433715	0.883763779	-0.199291571	2.201913927	0.449775913
33.61743572	0.886805912	-0.211989533	1.871009584	0.475105208
36.89060198	0.907703822	-0.210205473	2.532732276	0.417920603
23.00800326	0.919097252	-0.264436073	1.474019713	0.536734616
36.16184553	0.875333017	-0.183114401	2.594703556	0.421948084
37.90290584	0.863383855	-0.177790628	2.272230896	0.447562038
23.46029099	0.893914182	-0.214780182	1.953001754	0.471103804
31.13009975	0.899016365	-0.21740721	1.995737202	0.474191214
19.27588957	0.926215968	-0.291726005	1.632360392	0.5431942
29.39159202	0.891571474	-0.191622547	2.562130756	0.415542907
43.83951421	0.852109002	-0.17269836	2.221965947	0.448507427
32.25123904	0.839065014	-0.166360454	2.24459864	0.445131308
29.65379717	0.855303955	-0.177054632	2.058542519	0.455208593
51.07382144	0.914738485	-0.222279081	2.339958812	0.456475822
29.48865538	0.927662351	-0.24170092	2.042010094	0.456206932
40.078939	0.913601608	-0.247071244	2.035241243	0.507158404
20.5124915	0.87880747	-0.228414763	1.446701528	0.534822623
28.81197214	0.832510258	-0.16166154	2.225546914	0.451810041

log.sigma.4.5.mm.3D_glcm_ClusterTendency	log.sigma.4.5.mm.3D_firstorder_InterquartileRange	log.sigma.4.5.mm.3D_firstorder_Skewness	log.sigma.4.5.mm.3D_firstorder_Uniformity
108.9788324	160.580369	-0.566039833	0.056268383
35.79359165	109.600462	0.559214097	0.100840173
68.39308313	161.8659744	0.134596405	0.063249525
97.9232721	151.2414916	-0.434358972	0.060571235
48.04989481	120.6650524	0.164542136	0.078912527
61.18039142	160.0324721	0.525314014	0.081432507
68.45938374	151.1892128	0.329255753	0.065168158
68.83725797	132.562294	0.135830942	0.072656692
70.5077679	150.1422997	0.03904928	0.063371269
65.25692556	152.1788273	-0.581868112	0.067414792
44.12143379	131.6102748	0.073266453	0.078086633
67.7511302	148.5509071	0.01234169	0.067915547
60.81705092	90.78926373	0.378322093	0.094398841
69.31663565	130.164566	-0.18817897	0.07266843
67.84125762	119.2576847	0.037453599	0.073062829
78.56270699	163.5550089	-0.220344091	0.059761112
51.62256548	110.397243	-1.063834685	0.085626345
48.24670818	115.4984722	0.782719444	0.097836004
34.90438726	115.0197434	0.438509951	0.096110883
79.5763991	157.0981216	0.118990442	0.059716864
39.79443066	111.269824	0.236336362	0.089820239
43.45976881	127.7826214	0.12830025	0.078869409
55.94594227	128.7111416	0.351998663	0.077047348
45.97611675	113.3953075	0.380185414	0.081510614
48.37057293	126.2144394	-0.258009887	0.073155741
76.07824606	128.7808075	-0.571470892	0.067864945
96.50616013	128.8762169	-0.780303184	0.06676882
50.52785211	110.906096	0.105731309	0.082331015
41.91886196	125.3994484	0.600023293	0.095646342
37.89372224	98.63033772	0.149947971	0.092991659
59.88408343	140.6890841	-0.009584468	0.069754954
34.65741755	73.11765099	-1.651476331	0.121676263
98.51436089	156.0751688	-0.36359755	0.056910791
76.9180242	141.4211674	-0.506400375	0.063710276
58.86332603	139.4611206	-0.177783003	0.069668623
49.79718915	132.9233685	-0.332744854	0.076359231
60.793648	90.34583664	-1.034035432	0.095321548
115.029509	187.6700921	-0.191953693	0.053771709
15.26414475	74.00901508	-0.145962065	0.129068676
102.2202491	176.7437444	0.061547632	0.052570739
54.95834685	98.06580877	-1.193962603	0.089470155
95.01211994	157.0211325	0.315668389	0.058055842
69.55534857	147.9740868	0.091146567	0.063265522
51.84355059	116.7860665	-0.173351014	0.076663483
54.13701777	132.1037083	0.665605166	0.080287323
71.53572956	147.2708893	-0.364821368	0.064416906
52.60249213	123.0870113	0.182086325	0.076054673
33.25108227	116.7626762	0.48209149	0.098580224
63.39393994	142.7746449	0.398921858	0.071448396
41.25278035	131.6652822	0.53466261	0.085041665
72.74695093	156.0416183	-0.16132928	0.062168463
77.26304422	155.3185272	0.171674459	0.060201215
56.01445782	120.769094	0.23127854	0.075323566
46.18613882	139.2104378	0.061433075	0.072595043
66.51178597	172.2571125	0.261104594	0.064669569
43.33678944	104.6177578	-0.156437904	0.084083629
74.32128284	161.8712578	-0.179584854	0.059969289
68.25551512	117.4973984	-1.011256969	0.075780556
29.79092152	97.50589037	-0.385347398	0.095212496
65.74236499	140.8643129	0.133440616	0.06933462
55.09606517	122.4134603	0.81950658	0.0851519
68.0882111	159.7318735	0.003307201	0.065833158
75.10000688	143.9709587	-0.213254746	0.065769597
70.83610257	138.4584155	0.553897275	0.069998588
35.61717401	121.3601151	0.204699367	0.082855312
65.7922515	126.8593311	0.018643484	0.07832347
22.86382722	71.25671434	0.058286768	0.127255919
76.19896278	127.6148049	-0.110211786	0.068110797
56.42936931	132.0978127	-0.529094547	0.069204231
37.81939989	121.6538143	0.094445287	0.086132318
57.75605901	126.2059669	-0.636747697	0.08032649
74.68064434	146.6783619	0.082515493	0.065271977
57.31981785	119.6695957	-0.819763361	0.076157703
66.90321656	138.0367565	-0.442932619	0.067392032
93.43649033	159.59126	0.367763679	0.059039222
45.75385397	101.6583643	-0.838012692	0.087736346
32.21945284	105.6860323	-0.188522395	0.090027968
58.64117236	133.7643752	0.846360573	0.090975458
44.13440452	121.3715649	0.173747524	0.082743135
127.4210522	157.9417849	-1.065329605	0.060345415
119.7697375	161.347682	-0.739574253	0.055962141
41.65459756	88.84459209	-0.609039143	0.096243018
64.34972688	124.3802109	-0.142035434	0.06996741
55.15702495	111.9382877	-0.731929552	0.081435159
95.28522592	157.4668255	-0.234096205	0.057751406
44.98986905	119.4719658	0.550954912	0.093105141
83.19821259	130.8655796	-0.665073768	0.066958146
56.83304343	145.6196461	0.076317429	0.070163664
49.37732198	131.518569	0.158292353	0.079425334
57.57040944	120.6688423	-0.209598204	0.076619296
54.48461283	166.6619415	0.588238953	0.102900702
84.89279889	152.0445213	0.157963302	0.059838306
58.92528459	115.1280189	-0.700641903	0.076493303
51.60096316	122.1703291	-0.088380753	0.076658219
50.66578391	124.1930784	-0.208899441	0.07542657
93.341553	144.2008481	-0.263987598	0.067179364
85.07313936	147.2914133	-0.085124698	0.061295656
71.42465122	85.28593588	-1.749481809	0.098801415
27.05729483	90.06054211	-0.167247685	0.103968624
53.41944044	147.8957663	0.26293777	0.070631052

log.sigma.4.5.mm.3D_firstorder_MeanAbsoluteDeviation	log.sigma.4.5.mm.3D_firstorder_Energy	log.sigma.4.5.mm.3D_firstorder_RobustMeanAbsoluteDeviation	log.sigma.4.5.mm.3D_firstorder_Median
105.5428104	48767177.92	68.34002184	70.19613647
65.5573537	15730872.38	46.46240284	81.01057434
90.42046297	48405946.3	65.67698713	132.6580124
100.3059487	19812986.27	66.89547086	63.71099472
73.90139762	35844034.21	50.85088187	70.04881287
89.10523799	14215983.87	67.65350667	68.18789291
88.16675554	147410291.9	62.13357509	110.0950012
85.54296023	135526556.4	56.09510119	103.7903595
90.3069128	125721057.5	62.83309939	114.1452255
86.33742692	32123960.4	61.65809732	136.324585
72.89446321	23791153.58	52.82637739	84.08535767
86.58065078	65874897.3	61.31919522	121.0841675
72.11833542	28911398.71	41.71367149	59.88602448
86.03953601	36714245.05	55.04318141	79.60371399
81.92137091	39614955.03	51.34496384	91.42054367
96.45073326	121991871.6	67.71050382	117.1964226
73.30026753	18236218.55	46.68700552	93.74375534
74.20253133	19528054.83	51.41009363	59.52434158
64.69761786	16953255.3	47.64995644	66.49534607
94.93509877	166132300.1	65.79652856	126.932579
67.33358236	52639812.17	46.71773418	89.48368454
72.89568135	18586260.55	53.80098963	91.92081451
79.70703979	21616308.65	55.87538968	87.88956451
71.71025844	47465344.17	48.51097661	87.33153534
78.56706817	30676770.05	52.92046672	120.1132813
88.48653522	63936855.76	55.12834607	118.5716782
92.59349416	87947074.42	56.30264809	108.2985764
73.03637997	45255446.3	48.20484239	75.96199799
70.99146477	7620503.98	53.35437304	78.14413452
62.67398716	37584440.83	41.20129917	102.4341469
83.98986374	120181650.6	58.56516907	109.656208
54.3105196	16098915.98	31.01598264	47.25163651
102.6883529	72216005.18	68.77046536	78.9026947
93.95059944	57944869.95	61.36030823	107.3306465
81.96174014	124625732.2	57.49354946	128.9341202
75.80463301	70336855.04	53.68759214	115.6123734
70.39156688	54222746.32	39.18143538	55.95608139
110.9147397	149273132.5	76.64370619	92.30272675
43.25635361	9874855.7	30.81442955	72.62896347
106.7120483	173435527.2	74.16307733	75.42248154
74.11780571	33889520.79	43.40833172	65.08407974
99.06246667	82165719.36	66.11152788	64.7329483
90.54537104	106968615.3	61.76699046	119.9812622
75.76391542	39547986.17	50.18501097	80.33008575
79.76702877	37962377.69	56.40334482	118.1422882
89.52185001	87832591.86	61.02152103	138.0064545
76.06131094	102312371.8	51.40337628	106.2842865
64.75103847	12539326.44	48.60929044	72.28634644
83.72966739	16439467.51	58.27104414	80.2101059
71.78768375	27550245.59	53.08572918	103.198204
91.55732178	84278076.38	64.03030014	139.7325897
93.93802846	81960767.22	65.13438745	96.98390198
76.65684618	79198610.94	51.52732271	81.52791214
79.7073749	36684811.3	57.83036831	146.9443359
92.17252835	41818914.77	70.14610937	132.6341705
69.16510306	32200563.92	45.32861648	88.18725204
94.13639244	108787900.9	65.73433343	115.4592819
81.18860999	69455374.95	49.68420647	93.86485672
60.25796865	15138491.68	39.82716162	53.32075882
85.59534005	156073556.3	58.7955576	64.80257416
78.07986731	32699794.03	52.00735795	69.40255356
88.82250587	71394881.24	64.45647111	110.052269
88.51519964	176908708.7	59.78975618	126.2178497
89.26064903	164023276.6	60.11094074	71.64244843
69.02089117	17619534.24	49.69198494	118.6849899
81.71396886	75634797.41	53.44532881	75.57509613
47.89518572	8100220.684	30.46890913	38.43460846
87.95992789	116822448.2	55.2888522	66.27958679
84.43557572	131288278.8	55.51541351	107.1440048
67.98888075	49937020.56	49.70940688	92.12366104
78.10909463	23677699.71	51.31110651	79.62141418
91.04855089	139813940.6	61.74540341	126.7074509
78.60924058	51799477	50.10564097	112.5056992
87.29016859	32358780.03	58.30383901	51.69393158
99.75672344	66959623.47	67.69991971	58.17284393
67.96847616	18846189.87	43.57700069	84.16029739
64.17454423	42135945.73	44.18931621	105.6879463
80.54924774	18777596.09	55.06000122	66.93082047
71.5287418	32296825.46	50.58723898	94.13785553
109.4500283	54206178	68.36118483	67.27352905
108.2223101	48232750.63	69.01582947	70.75809479
65.58539588	18721259.74	38.7521095	47.86157608
84.19014992	79088931.09	54.02958974	110.9176025
72.86863455	48884451.77	47.11234935	89.84285736
100.3381097	63973329.17	66.79454236	101.9940796
71.56604294	27275350.58	50.48792187	76.63046265
91.17748537	38311832.85	57.60939125	50.43408585
84.33318712	79944055.67	59.40641955	122.7614632
74.52023396	29229905.25	52.44951799	95.60486603
77.4014188	40726282.28	50.5590598	94.59480286
85.80590492	26688291.66	68.8427012	64.62216568
96.33469473	69852845.76	66.48335162	115.8087425
77.40606289	77991566.52	49.34691112	70.40546417
75.38982711	73846568.11	50.60839376	109.345623
75.24923723	53758839.18	51.88838114	61.93739128
96.07123895	88470104.84	63.41453817	81.7780838
94.00446689	59437205.85	63.03994635	84.63600922
74.15918294	13101319.58	39.13835396	32.10798264
54.41046562	7956917.173	37.24039376	69.30648041
82.33231588	143272220.2	60.01626495	111.8166046

log.sigma.4.5.mm.3D_firstorder_TotalEnergy	log.sigma.4.5.mm.3D_firstorder_Maximum	log.sigma.4.5.mm.3D_firstorder_RootMeanSquared	log.sigma.4.5.mm.3D_firstorder_90Percentile	log.sigma.4.5.mm.3D_firstorder_Minimum
1316713804	416.0115662	151.5971799	228.7772903	-458.0352783
424733554.3	329.2341919	129.7091456	227.1416382	-64.86199951
1306960550	430.0438232	177.6383283	292.2236664	-169.150177
534950629.4	334.8364868	142.4058031	223.5243774	-303.4861145
967788923.7	368.4595947	123.0318608	211.0528259	-230.6526642
383831564.5	428.6340332	146.7629705	253.3037064	-113.4404526
3980077882	597.3411255	161.5106814	264.9924927	-233.2823029
3659217022	466.7347412	162.8871248	276.2775238	-420.2236938
3394468553	468.9552917	166.2988696	276.7495117	-304.1222229
867346930.9	376.3677673	173.1080038	278.9042999	-401.0393677
642361146.6	356.9253235	125.6050439	209.7067245	-173.7235107
1778622227	453.0729065	169.0536339	273.5094055	-237.7575989
780607765.2	377.469635	124.4406848	215.5734222	-272.1108093
991284616.5	406.7745972	146.2285433	246.2309235	-325.5881958
1069603786	468.5184021	141.305397	221.3176697	-270.7718811
3293780533	450.5516968	166.9655185	270.7868805	-261.5701294
492377900.8	288.5065002	123.5848488	183.5731628	-301.645874
527257480.4	341.3527832	125.8484742	233.633432	-125.9608688
457737893.1	298.1114197	114.2849927	201.9175201	-90.24947357
4485572102	566.7073975	178.7586764	291.3918945	-228.9749451
1421274929	392.4867859	131.9368989	222.0647339	-193.2441864
501829035	339.2621155	131.2457619	221.5985168	-125.7929153
583640333.5	359.4197998	145.2916942	255.6799271	-125.1184311
1281564293	455.9589233	133.9094871	224.4186554	-224.0565491
828272791.4	419.1125183	155.2353459	244.2262268	-321.3527527
1726295106	494.2580872	164.2831824	255.8561218	-375.8087463
2374571009	548.7109375	164.9331093	254.7702087	-468.0666199
1221897050	356.5928955	128.0040141	219.6855606	-266.6333008
205753607.5	308.6750183	130.568401	228.2177368	-79.76260376
1014779902	372.042572	135.2707279	219.633725	-217.6803131
3244904567	471.2364197	158.9303586	263.6710693	-344.1262817
434670731.4	256.4736938	88.21005067	115.983963	-376.878479
1949832140	485.4312134	149.5487226	233.2751953	-348.0844421
1564511489	473.8337708	157.0267221	243.5644714	-343.4317932
3364894769	415.9898071	168.2209984	272.033255	-320.7966309
1899095086	368.5115356	153.1451474	245.9940491	-303.0427551
1464014151	417.1428833	120.7149115	173.5709991	-508.9277954
4030374576	546.7634277	170.0685049	277.4971008	-430.8673401
266621103.9	228.9889679	89.60099312	140.8022522	-104.9578934
4682759233	497.302063	154.4549485	253.2678009	-290.4920349
915017061.4	363.3654175	114.3003942	152.071312	-427.5804749
2218474423	629.5033569	145.7666741	227.2131104	-335.3760376
2888152614	453.4786682	171.1210163	279.5172729	-286.3877563
1067795627	550.2243652	126.0773288	200.8833115	-257.107605
1024984198	466.0767517	172.3499953	294.7707581	-98.68871307
2371479980	512.2658081	178.8778947	278.417218	-307.3454895
2762434039	455.1712341	149.7567601	245.2132797	-274.1192017
338561813.8	279.4656067	119.9852959	209.8562775	-57.25897217
443865622.7	392.9531555	138.988572	229.0434418	-199.7854767
743856630.9	445.7004089	142.7494329	230.4090988	-88.99720001
2275508062	438.0302429	181.123689	285.4100586	-248.2158356
2212940715	510.7387085	155.9278287	263.6655884	-254.2705078
2138362495	420.0804749	132.4872961	225.8518005	-231.1714935
990489905.1	419.0244751	182.2882216	284.5174713	-153.8675842
1129110699	459.0579834	177.321158	289.5754608	-121.209465
869415225.9	399.7754822	128.5033451	215.2673157	-358.6741333
2937273323	467.6795044	163.9951617	265.7182861	-285.4328918
1875295124	358.2970276	141.7230322	218.7659592	-408.9160156
408739275.4	287.7437439	95.84350766	152.6060852	-300.4606628
4213986020	467.6376343	130.7534207	210.518512	-366.6465149
882894438.8	496.7828979	136.074629	237.7023621	-201.7462463
1927661793	457.9127808	163.5226464	271.7167633	-238.7700958
4776535134	552.8416138	174.81264	279.0305298	-463.645813
4428628468	506.0218506	146.6863769	262.1727966	-347.8343201
475727424.4	391.6705627	149.3425857	226.4681183	-171.0013885
2042139530	467.5111694	139.9441309	234.8980545	-404.4831238
218705958.5	272.8798828	78.8151361	128.1249031	-241.1620483
3154206103	515.4616699	136.7171961	223.4485062	-404.8582153
3544783528	454.6617737	149.7184648	235.5296783	-351.4198303
1348299555	330.0339355	131.2689837	216.4615082	-217.8219757
639297892	397.7933655	135.0097642	211.767868	-328.9636841
3774976398	477.0186768	180.5288402	298.0392517	-352.3033142
1398585879	371.1397705	150.7615261	236.4432831	-385.5265503
873687060.8	345.8043213	119.6845123	173.0749298	-338.0764771
1807909834	494.8391113	146.2395923	251.9821167	-276.4386292
508847126.5	306.2490234	118.5046858	185.9146088	-309.0765686
1137670535	377.3969116	132.6120431	208.8383255	-200.3316498
506995094.3	431.5308533	140.0029678	244.3919571	-137.1973877
872014287.3	362.394043	137.5508117	230.2817444	-185.9494781
1463566806	429.1136475	152.2331671	186.5188446	-506.4660034
1302284267	416.0115662	155.7233089	228.50177	-479.1218262
505474013	430.7936401	102.0121622	140.2341675	-370.8121033
2135401139	449.3048096	155.5429719	247.450827	-329.2810974
1319880198	372.2258606	126.1258466	192.0099182	-313.4000244
1727279888	540.6500854	161.9547074	259.2636353	-338.109375
736434465.5	356.4274902	133.4307073	240.8391891	-161.8862915
1034419487	348.87323	126.742501	177.3196045	-396.3821106
2158489503	496.3628235	170.7494695	275.3013306	-311.4160461
789207441.8	382.2113037	141.3003265	235.8006744	-169.6658478
1099609622	408.1817017	141.8158818	231.7321472	-250.9870605
720583874.7	338.3457336	143.0611105	256.7253204	-106.9486008
1886026835	493.9201965	171.2462907	287.7753815	-219.7585754
2105772296	383.0232849	122.6685459	188.0968414	-452.6373596
1993857339	437.2352905	149.591938	241.0222824	-274.4963989
1451488658	402.8052979	111.1425861	174.4458527	-294.029541
2388692831	456.9559937	160.7897161	276.1768677	-504.0130005
1604804558	432.9137268	145.2565647	233.5240662	-251.3134613
353735628.6	237.6450653	111.862412	118.3364029	-467.524231
214836763.7	246.7391357	97.67634347	157.4253983	-165.2120361
3868349944	436.6948547	157.5225036	258.0964325	-207.0066833

log.sigma.4.5.mm.3D_firstorder_Entropy	log.sigma.4.5.mm.3D_firstorder_StandardDeviation	log.sigma.4.5.mm.3D_firstorder_Range	log.sigma.4.5.mm.3D_firstorder_Variance	log.sigma.4.5.mm.3D_firstorder_10Percentile
4.458550998	139.023429	874.0468445	19327.51382	-125.7783966
3.583314012	80.70780337	394.0961914	6513.749526	11.73737946
4.154462835	110.425244	599.1940002	12193.73452	1.3730178
4.309049532	129.7927399	638.3226013	16846.15533	-132.7078522
3.919854316	93.25990625	599.1122589	8697.410113	-32.54700127
3.917388334	104.360097	542.0744858	10891.02984	-13.23243265
4.165115593	110.2671383	830.6234283	12158.8418	-15.81667709
4.127574382	112.3215098	886.9584351	12616.12156	2.147080302
4.213440652	113.7251817	773.0775146	12933.41695	-20.29279423
4.078475514	108.9600124	777.407135	11872.28429	6.629126692
3.869401506	89.72254864	530.6488342	8050.135734	-21.58185024
4.118943527	107.9765026	690.8305054	11658.92512	3.48105979
3.869908363	100.5856159	649.5804443	10117.46614	-23.55511818
4.140838983	114.3773919	732.362793	13082.18778	-19.94028702
4.123445339	109.9864019	739.2902832	12097.0086	-51.30708885
4.280925099	119.7992431	712.1218262	14351.85865	-33.36553574
3.852763577	97.09646458	590.1523743	9427.723434	-54.74915123
3.699248429	92.0134548	467.313652	8466.475865	-10.55614128
3.579745339	78.05394313	388.3608932	6092.418038	-7.720550251
4.29500881	119.3843196	795.6823425	14252.61578	-11.53001595
3.752954943	84.73104303	585.7309723	7179.349652	6.092117786
3.828940992	88.12462918	465.0550308	7765.950269	-11.44972305
3.946976377	98.66437002	484.5382309	9734.657912	-7.942579889
3.895425865	92.08770024	680.0154724	8480.144535	-10.16736374
4.036995466	100.9197839	740.465271	10184.80278	-11.19503765
4.238447907	120.4616043	870.0668335	14510.99811	-28.30756912
4.283832956	128.8266625	1016.777557	16596.30896	-32.95368042
3.912813151	94.69509758	623.2261963	8967.161506	-15.55701418
3.605533102	84.69667827	388.4376221	7173.527311	4.878196049
3.724911723	81.65212955	589.7228851	6667.07026	11.5633462
4.098121882	105.8820571	815.3627014	11211.01001	0.129545012
3.463382809	79.76880914	633.3521729	6363.062912	-44.92123795
4.403153915	132.2684419	833.5156555	17494.94072	-125.0822311
4.274247957	122.6165741	817.265564	15034.82425	-70.83306427
4.064746639	103.1438146	736.786438	10638.64649	9.095128727
3.925827532	95.3663413	671.5542908	9094.739053	11.81769829
3.882730701	106.1440788	926.0706787	11266.56547	-33.79180145
4.485436542	139.9996288	977.6307678	19599.89607	-80.3549881
3.160939256	53.80523248	333.9468613	2895.003042	2.384846663
4.464193316	134.0673024	787.7940979	17974.04158	-98.36945419
3.914803729	103.622602	790.9458923	10737.64365	-83.36530991
4.387061207	128.7764732	964.8793945	16583.38006	-92.08606262
4.227494773	115.1467268	739.8664246	13258.76868	-10.52970505
3.997982023	99.15941549	807.3319702	9832.589681	-50.74059448
3.897356843	98.4900437	564.7654648	9700.288707	31.50392265
4.218982078	115.2933237	819.6112976	13292.55048	-1.72444582
3.989530038	97.30730879	729.2904358	9468.712344	0.408361289
3.52003215	76.97058011	336.7245789	5924.470203	3.662859678
4.043561129	104.3994017	592.7386322	10899.23508	-28.28562355
3.757685533	86.92048951	534.6976089	7555.171496	12.48379946
4.227123649	114.7130981	686.2460785	13159.09489	1.763445163
4.274858164	118.3703194	765.0092163	14011.53251	-48.00474167
4.003537384	98.53765939	651.2519684	9709.670319	-29.12307606
3.974948298	97.30818281	572.8920593	9468.882442	34.82438087
4.101929848	109.1248338	580.2674484	11908.22936	5.428563404
3.872542828	90.97931217	758.4496155	8277.235243	-17.83026581
4.274760683	117.8117432	753.1123962	13879.60684	-35.42006836
4.07516754	112.413075	767.2130432	12636.69943	-38.59388924
3.68334891	79.09803664	588.2044067	6256.4994	-39.14132042
4.13519185	108.6501467	834.2841492	11804.85437	-50.45595856
3.898974625	99.67036409	698.5291443	9934.181477	-14.27500439
4.134034321	109.2057728	696.6828766	11925.90082	-7.621807528
4.209757282	114.7187773	1016.487427	13160.39786	-0.316624367
4.151702204	114.5722367	853.8561707	13126.79743	-33.44700699
3.78063702	84.97826826	562.6719513	7221.306076	14.29432297
4.051138122	107.507608	871.9942932	11557.88579	-14.05453939
3.332108447	64.57010535	514.0419312	4169.298505	-25.93628807
4.242482883	118.7800249	920.3198853	14108.69432	-70.86984024
4.14207054	110.490147	806.081604	12208.07258	-38.29023132
3.73899852	83.15594639	547.8559113	6914.91142	3.948801851
3.97885111	104.7062309	726.7570496	10963.39479	-19.76610413
4.223563563	116.8975493	829.321991	13665.03703	11.07128096
4.029198973	105.7962269	756.6663208	11192.84164	-8.990043449
4.169074812	112.1899231	683.8807983	12586.57885	-106.2221375
4.352645979	127.3535654	771.2777405	16218.93062	-80.5779953
3.817356039	90.81392315	615.325592	8247.168637	-38.2295208
3.73142542	81.26396031	577.7285614	6603.831246	8.060821772
3.84676496	100.3623049	568.728241	10072.59225	-2.751912427
3.835940775	88.80017229	548.3435211	7885.470598	2.244172573
4.418113852	146.7117216	935.5796509	21524.32925	-164.2798645
4.484338371	144.4020259	895.1333923	20851.94508	-131.4132141
3.806215804	93.16590743	801.6057434	8679.886307	-63.95999603
4.158326915	110.9520802	778.585907	12310.36411	-25.92279396
3.928946643	96.87317148	685.625885	9384.411353	-37.79592667
4.392335531	129.7670001	878.7594604	16839.4743	-72.54081726
3.712851187	88.47365179	518.3137817	7827.587061	8.352425671
4.233961882	121.4481373	745.2553406	14749.65006	-135.6933838
4.086965153	105.3928359	807.7788696	11107.64987	10.92617321
3.898419614	93.00742184	551.8771515	8650.380517	-5.659340906
4.021928631	101.619949	659.1687622	10326.61403	-16.42061539
3.586779205	97.50794183	445.2943344	9507.798719	3.613143277
4.300902958	121.3649867	713.678772	14729.45999	-30.61685066
4.040921971	104.1264607	835.6606445	10842.31981	-65.18497314
3.989907727	97.53913343	711.7316895	9513.88255	-4.413369751
3.969414586	95.18177883	696.8348389	9059.571022	-69.78728256
4.259996103	127.1897119	960.9689941	16177.22282	-26.61294765
4.283970358	120.8139724	684.2271881	14596.01592	-86.33413849
3.803161868	110.9724388	705.1692963	12314.88217	-115.3249573
3.496203941	68.84122937	411.9511719	4739.114861	-17.2193819
4.022047416	100.4957883	643.7015381	10099.40347	4.160091352

log.sigma.4.5.mm.3D_firstorder_Kurtosis	log.sigma.4.5.mm.3D_firstorder_Mean	log.sigma.4.5.mm.3D_glrlm_ShortRunLowGrayLevelEmphasis	log.sigma.4.5.mm.3D_glrlm_GrayLevelVariance
3.62686797	60.44990597	0.004167587	32.19496662
2.60728112	101.5416807	0.035763251	10.77153563
2.550161995	139.1461144	0.010564598	19.53013754
2.956921116	58.59400511	0.009831469	27.62003598
2.959245329	80.24605074	0.007192696	14.42465541
2.418910575	103.1907925	0.021590674	17.97324308
3.290238339	118.0121113	0.00593616	19.82107291
4.031393607	117.9664947	0.00273213	21.3009844
3.075228901	121.3338249	0.003987703	21.15372261
4.292475167	134.5142992	0.003263814	19.62388835
2.674465117	87.9004625	0.015502352	13.13727104
2.977285793	130.0776923	0.006347946	19.00131006
4.370930799	73.26675855	0.010414058	17.77156757
3.969631211	91.10762367	0.005119952	22.48688901
3.852771405	88.7141851	0.008823213	20.12743016
2.887789306	116.2997236	0.006406382	23.18290141
4.404387317	76.45581358	0.006341529	15.61259998
2.828272787	85.85663983	0.014375589	14.34580264
2.36668425	83.47838952	0.029733933	9.967082881
2.957314389	133.0490458	0.005953067	23.49633642
3.053713851	101.1335535	0.008993782	12.06697827
2.46930406	97.25995957	0.015345528	12.44021981
2.659864267	106.6537318	0.015589108	16.11984838
3.472987677	97.21937149	0.007947422	14.22081678
3.576617422	117.9542701	0.004479481	16.8659767
4.530366588	111.704816	0.003926216	24.29771189
5.355768169	102.9884536	0.003157214	27.76898108
3.436257479	86.12703477	0.006566626	14.98122361
2.40081087	99.37092143	0.021339226	11.56846076
3.430329833	107.8475756	0.006546689	11.31877816
3.318346378	118.5236216	0.00334374	18.62762047
7.74332328	37.65567855	0.004638176	11.36138133
3.068900272	69.78452342	0.007416414	28.79670264
3.650937719	98.09468495	0.006475259	24.67287892
3.358237364	132.8896453	0.003755818	17.37691093
3.582278511	119.827781	0.003995124	15.00604619
7.672804125	57.49368988	0.002650016	20.04238393
3.181090149	96.55775616	0.002787164	32.73697284
2.939451383	71.64729531	0.018820424	4.910148397
2.852971025	76.69608546	0.008545996	29.27761799
5.613443459	48.23832973	0.003781926	18.52915252
3.735461851	68.29746138	0.005306116	27.14670706
3.12621313	126.5844917	0.005062503	21.63717086
3.766145576	77.86464636	0.00747127	16.37561289
2.819677609	141.4363185	0.015931916	16.12233124
3.73789563	136.7653127	0.004459769	22.07392055
3.360447355	113.8348578	0.00504411	15.93400258
2.278322601	92.04347355	0.034403187	9.703882812
3.035033613	91.75286416	0.013888517	17.97203899
3.029928911	113.235282	0.020506876	12.41740333
3.081135401	140.1666717	0.008405272	21.65205529
2.847644596	101.498548	0.006100671	22.73637423
3.169872991	88.56191795	0.007056118	16.16677321
2.674951435	154.1431585	0.008082016	15.43094714
2.268586905	139.7661035	0.014784295	19.15816299
3.886277804	90.75171878	0.003621924	13.88586513
2.957000222	114.0824537	0.005360454	22.84276827
5.404866271	86.30595823	0.003908891	21.40158711
4.121825005	54.12465761	0.005673261	10.87358669
3.561626772	72.74340289	0.003638012	19.67557081
3.547766822	92.63974947	0.007497001	16.84128421
2.787293246	121.7117705	0.006540744	19.50063577
4.208634731	131.9055012	0.002130348	21.69832564
3.413490356	91.59746574	0.004004613	21.8709904
2.978564988	122.8083947	0.009519255	12.0698903
4.237391002	89.59059091	0.002868506	19.87688659
4.722688859	45.19432678	0.008458113	7.450112474
4.017507415	67.69710035	0.003544427	23.73799925
3.716438947	101.0324014	0.003558804	20.57479064
2.837079215	101.5708357	0.007326459	11.44302436
4.676689148	85.23052052	0.00641018	18.55269341
3.563630749	137.570437	0.003013797	22.82368324
4.901673556	107.4066856	0.003905995	19.00598598
3.39031428	41.68697201	0.007474784	20.75164958
3.028283014	71.88245776	0.006918585	26.52487641
4.335410899	76.13272569	0.005738889	13.87882401
3.477609781	104.7956236	0.007229182	11.19386838
3.435631671	97.61269761	0.016170493	17.07634776
2.800969865	105.0464431	0.008479861	12.95552671
4.470336838	40.62767414	0.004181641	35.2981492
4.019377287	58.29068413	0.004192792	34.59845353
6.014335866	41.55231555	0.006720496	15.26988641
3.677459555	109.0103298	0.003796093	20.55480604
4.506893094	80.76705905	0.005626594	15.57663399
3.407231716	96.9012536	0.00590776	27.68892342
2.716485476	99.8807618	0.009928494	13.05709867
3.727339542	36.24929665	0.006413905	24.71210927
3.204443083	134.3418456	0.003666226	18.47331092
2.806128153	106.3738772	0.012953864	14.23888265
3.64819154	98.91981754	0.00700499	17.68034875
1.969455934	104.6837266	0.013813016	15.44392917
2.960426344	120.8132116	0.009717705	23.96136888
4.775469267	64.84791713	0.002877134	18.35331298
3.432024674	113.4189815	0.005466028	15.87333991
3.148897275	57.38556806	0.006880607	14.93647434
4.586512833	98.36722002	0.002277619	27.29498779
2.909018525	80.64399341	0.008145351	24.02812776
6.917401195	14.08250863	0.007936997	21.53934469
3.114283625	69.29324074	0.014726981	7.992343772
2.705637248	121.3010126	0.006777213	16.50898912

log.sigma.4.5.mm.3D_glrlm_LowGrayLevelRunEmphasis	log.sigma.4.5.mm.3D_glrlm_GrayLevelNonUniformityNormalized	log.sigma.4.5.mm.3D_glrlm_RunVariance	log.sigma.4.5.mm.3D_glrlm_GrayLevelNonUniformity
0.004356155	0.054212237	0.131520088	104.5592956
0.039348734	0.095584606	0.217479687	76.87011887
0.011330695	0.063161304	0.197455235	85.14544433
0.010630056	0.058258513	0.149970268	50.8367057
0.007837729	0.076414358	0.186689409	157.8887812
0.023986025	0.07440373	0.216034181	42.60766263
0.006440007	0.06444832	0.181753086	322.7785872
0.002963761	0.069500678	0.213844836	308.8149877
0.004315754	0.062564271	0.179456623	250.2401118
0.003433931	0.066892938	0.157281106	63.96526395
0.016912486	0.077080367	0.189304842	101.4209961
0.006849723	0.06663145	0.168675901	135.8363565
0.011481694	0.086044163	0.335651615	132.9776915
0.005481041	0.068385017	0.202383239	102.0581105
0.009392987	0.070122694	0.164235256	123.7796307
0.006895764	0.059310263	0.172549628	232.9191525
0.006721266	0.084001894	0.15103374	89.03317599
0.016183577	0.089521653	0.261196284	92.76713936
0.033226753	0.092146909	0.244384177	101.010425
0.006432276	0.058634285	0.163218083	271.6555437
0.010066785	0.085648011	0.307512502	216.716488
0.016967453	0.078659215	0.177421975	74.63800357
0.017085717	0.073585462	0.217205627	65.40456909
0.00862047	0.078898734	0.220106572	181.0729671
0.004718497	0.0718861	0.177736432	82.19103263
0.004142746	0.06550803	0.152661024	139.5407845
0.003357146	0.064614107	0.178935211	185.9649791
0.007139643	0.078949098	0.202017486	188.893427
0.024152353	0.090962306	0.217307165	34.96320265
0.007262263	0.089489623	0.247804795	155.956172
0.003647263	0.067933573	0.226291344	280.5145335
0.005051801	0.11425819	0.302729881	195.9068302
0.007894272	0.055732911	0.15478197	161.4243323
0.006800151	0.062469326	0.169177861	131.3321823
0.004043711	0.068806269	0.169840959	268.9348356
0.00434354	0.075063792	0.236989893	194.7456304
0.002889228	0.087736637	0.279093123	274.7922523
0.003043984	0.051180734	0.295957832	227.0746567
0.020985888	0.126982282	0.260289174	130.0968035
0.009271575	0.052004242	0.134989025	340.9629812
0.004082253	0.083485075	0.24481928	185.3732337
0.005640072	0.057440482	0.117776332	202.0590738
0.005376163	0.062462987	0.153979438	205.0053738
0.008001723	0.074553512	0.172159837	163.6166888
0.017464676	0.076970286	0.19213318	85.9600954
0.004780633	0.063262218	0.169976588	154.5780501
0.005488564	0.073879331	0.206154597	294.0570921
0.039058142	0.094116366	0.275096962	68.9276432
0.015160818	0.069758608	0.189896556	51.84863604
0.022915087	0.082018888	0.262827574	94.55769065
0.008998808	0.061294521	0.153377282	141.0719911
0.006497921	0.059569452	0.123062215	182.5487332
0.007691679	0.073270103	0.195589391	288.007066
0.008632072	0.071695447	0.151663378	70.85563651
0.016098524	0.063735957	0.172146656	75.10325851
0.003913419	0.081431203	0.198688333	138.3663978
0.005689276	0.059142794	0.146301712	215.7104491
0.004202839	0.073072155	0.217787001	219.2934742
0.006117586	0.09160552	0.208343584	130.2333286
0.003999034	0.067092686	0.275731304	525.4066949
0.00829555	0.079860492	0.23211303	120.9338031
0.0071256	0.064541965	0.176671924	151.8880407
0.002287783	0.064571074	0.154801353	334.0357625
0.004399837	0.06746782	0.209822891	446.3354184
0.010228256	0.081725072	0.209604496	56.60743893
0.003150989	0.07310572	0.261493276	239.9630664
0.009734533	0.116856585	0.409862482	119.7467907
0.003819119	0.065275097	0.181728764	359.0487555
0.003798773	0.066998761	0.181291672	347.0583427
0.008125472	0.084037901	0.255443934	205.5915026
0.006887937	0.076778299	0.209717602	86.8939921
0.003264987	0.063321874	0.200592246	237.5025232
0.004167955	0.073733393	0.204930844	147.2937371
0.007933321	0.065979966	0.144284112	133.6111427
0.007479419	0.057577602	0.165506214	160.2289214
0.006129151	0.085102979	0.193936855	99.34199994
0.007922314	0.087939399	0.263028804	179.0158716
0.018275337	0.082514738	0.29115882	66.34163452
0.009308718	0.080475335	0.202042119	119.0719941
0.00438308	0.058772275	0.134201497	124.6386775
0.004377989	0.053881988	0.132623611	97.52860315
0.007278504	0.090373506	0.246228862	138.1161357
0.004089055	0.067915198	0.231192618	194.8926074
0.006190564	0.07955345	0.188540261	212.4403324
0.006333072	0.056610457	0.16737506	123.2330809
0.011283259	0.087190109	0.29934945	110.3903875
0.006876379	0.06457894	0.168488118	136.7610151
0.003951989	0.067813006	0.199577593	163.1677896
0.014121657	0.077012941	0.212632622	98.38993012
0.007569035	0.073426158	0.212738528	128.4860507
0.016650587	0.089781053	0.355152029	94.92045173
0.010450547	0.058889527	0.139282899	126.457122
0.00308364	0.074074561	0.181841801	336.7791417
0.005881207	0.074368697	0.183941144	216.6368424
0.007433265	0.074397472	0.170227696	285.0456016
0.00245491	0.063333309	0.245301681	187.7376461
0.008857596	0.059518108	0.17953153	147.7532481
0.0087573	0.09089915	0.290516173	78.8221165
0.016320387	0.102212831	0.261816924	71.20099553
0.007352235	0.069510451	0.205795365	351.9967298

log.sigma.4.5.mm.3D_glrlm_LongRunEmphasis	log.sigma.4.5.mm.3D_glrlm_ShortRunHighGrayLevelEmphasis	log.sigma.4.5.mm.3D_glrlm_RunLengthNonUniformity	log.sigma.4.5.mm.3D_glrlm_ShortRunEmphasis
1.347030672	471.6730077	1621.805264	0.932875342
1.581860803	62.78774655	615.8156374	0.895074503
1.502559945	172.4742168	1078.305766	0.911919559
1.411540353	255.3808494	709.0169786	0.918979885
1.507385065	184.0730284	1616.548497	0.904415449
1.559032483	106.5989016	447.6095849	0.902948041
1.462091721	233.8377809	4061.484703	0.917975029
1.541785816	470.6219034	3489.507744	0.906013203
1.480631424	326.7670405	3188.452237	0.910842226
1.423174204	498.6699948	781.1736582	0.920087675
1.513058012	121.6010848	1030.448263	0.903803114
1.453353334	243.7477853	1632.785018	0.913807423
1.819799839	201.7967835	1126.710445	0.875249658
1.533736942	320.2595468	1163.421017	0.903152363
1.438326079	223.1393932	1432.600773	0.917830231
1.428728956	263.9431086	3275.147933	0.927053384
1.425219909	260.0541974	849.7291784	0.914229349
1.690556056	104.6495113	763.2460359	0.879968796
1.663087851	64.60315786	812.7879361	0.881245856
1.428378468	254.9895409	3778.097558	0.92053685
1.752761879	152.89066	1871.23627	0.881256787
1.477840947	110.8022958	752.7752382	0.909579605
1.556336149	121.2977712	695.4975706	0.903899784
1.560315682	176.1281092	1789.736461	0.902895376
1.436887328	321.8715087	956.5081922	0.927646001
1.397082567	427.8723558	1763.235385	0.926101983
1.449096868	537.3156851	2346.245831	0.920340726
1.543489019	216.3426311	1851.121338	0.899794437
1.584107006	80.01202443	295.1403923	0.895128082
1.652446271	180.8908969	1300.988057	0.885427668
1.563783358	354.7484326	3244.01677	0.905212355
1.783326637	285.4710971	1236.028884	0.871179849
1.404524451	298.867566	2389.842764	0.924608226
1.425450726	333.5036911	1738.127414	0.925090209
1.445748316	340.9614857	3167.584676	0.917860344
1.58055828	317.2552595	2032.635	0.904414781
1.716543884	517.9973509	2326.287262	0.882162341
1.660789165	492.6864345	3481.791488	0.904510753
1.722157144	63.62039501	741.224838	0.870687774
1.369356826	252.556347	5430.149665	0.926744012
1.627602571	381.8349834	1688.944272	0.892846258
1.330295202	303.4658328	2952.604262	0.932024313
1.403641238	304.4218548	2719.509107	0.925626613
1.464617121	207.439527	1751.281668	0.911995848
1.513244219	111.8106622	874.9620708	0.904737807
1.438858909	350.2898636	1989.897919	0.919906903
1.528633552	249.9194255	3137.64509	0.906850331
1.71371276	56.31435482	541.6432188	0.8803423
1.511439552	153.2048424	584.0083737	0.90521831
1.668309602	87.68726595	884.9833695	0.892042661
1.404048876	258.6491818	1889.327707	0.923367852
1.336518496	247.3766071	2573.351953	0.932483531
1.520015132	193.4973647	3074.863972	0.904324519
1.407685213	186.952302	811.1566709	0.922304526
1.453142436	132.3268873	950.0691324	0.916039521
1.524104867	343.2873963	1327.149434	0.903629168
1.382144988	290.5082923	3031.536617	0.927946898
1.556641861	410.4338826	2338.423143	0.902441556
1.56182547	227.7257665	1089.041197	0.896145574
1.645572739	325.4381881	6064.627612	0.899821706
1.605816945	176.2655168	1155.62315	0.894370408
1.470786785	235.0407791	1877.382729	0.912027252
1.411592154	587.2249449	4234.203206	0.922225927
1.546772088	325.3966262	5163.197218	0.903033304
1.541254888	149.2923719	553.1402461	0.908086707
1.657545367	420.5620342	2481.793438	0.89086905
2.06556644	133.6187709	672.646079	0.833210513
1.479519291	394.2960398	4382.288129	0.911586648
1.466755305	363.7819138	4173.763089	0.915928183
1.670977824	175.7038767	1822.530931	0.884304419
1.544628095	306.7632018	891.7832143	0.905134964
1.516869165	425.6131117	2966.170628	0.908457387
1.519128585	408.4241065	1586.668745	0.909798008
1.394793013	256.9154405	1659.140021	0.922407761
1.438857405	244.4828145	2255.926452	0.917931262
1.528906611	253.7887818	909.704927	0.901344035
1.665322023	177.2046761	1543.610019	0.890652748
1.740033161	116.9987042	595.8708074	0.880304969
1.541615945	158.5553857	1145.570132	0.900099995
1.357070606	520.9742489	1777.488448	0.931340752
1.345436198	512.5897601	1527.692545	0.934250367
1.645399396	272.9702153	1145.93586	0.887482063
1.550021112	343.0369211	2306.419828	0.912816982
1.521994888	263.4108384	2067.936521	0.899819569
1.435429462	335.4564699	1783.531782	0.921101762
1.778739017	131.2066996	909.4808836	0.87029864
1.447041912	313.8642985	1714.224564	0.917104435
1.502934879	345.3977895	1922.443802	0.912693173
1.537402644	139.1247663	1008.398738	0.906552691
1.565946715	230.7940797	1349.43284	0.897922539
1.90394825	103.494442	740.0456956	0.858086643
1.375139881	212.7487054	1780.647706	0.927099025
1.488951189	457.0354599	3595.104519	0.908365937
1.473992337	250.1870349	2343.752222	0.915047781
1.467768902	211.2811683	3040.904476	0.909772877
1.587714671	615.3011166	2326.802474	0.905564051
1.476092603	220.7549884	1980.90958	0.91168194
1.771221457	360.5008375	622.201691	0.868861605
1.71727484	97.32225848	504.9936492	0.872027851
1.513962889	206.0997225	4042.414094	0.911712614

log.sigma.4.5.mm.3D_glrlm_LongRunHighGrayLevelEmphasis	log.sigma.4.5.mm.3D_glrlm_RunPercentage	log.sigma.4.5.mm.3D_glrlm_LongRunLowGrayLevelEmphasis	log.sigma.4.5.mm.3D_glrlm_RunEntropy
696.85445	0.908576814	0.005268484	4.935516922
102.5503955	0.859646236	0.058331881	4.246750581
288.961698	0.878848661	0.015503247	4.712642034
392.641188	0.892685615	0.014315663	4.827510791
301.543615	0.87227131	0.01116736	4.547489687
160.0770918	0.866899767	0.036793158	4.530776563
360.4921914	0.886092319	0.00922056	4.729450408
780.1410708	0.869556051	0.004272862	4.785321362
526.6015024	0.879826728	0.006064803	4.803471659
771.7673879	0.891862801	0.004327545	4.593598339
202.4645503	0.872475005	0.023897207	4.47471263
382.1822493	0.884164859	0.009385659	4.688575116
396.5986017	0.826212352	0.017516283	4.695248683
528.6446973	0.868778281	0.007455286	4.791708365
355.9676482	0.88926799	0.012165669	4.681930676
404.0737283	0.896937843	0.009506087	4.780485139
417.979639	0.887643345	0.008521865	4.410910455
179.2609284	0.839852767	0.026688566	4.45256744
113.8096601	0.8443167	0.052647232	4.2960136
383.2830546	0.890999748	0.008932932	4.846473948
286.1051808	0.836233211	0.016784427	4.517031893
175.4851548	0.879446781	0.0253462	4.389145806
198.7638988	0.86733774	0.025306254	4.565723326
295.781018	0.866670541	0.012466216	4.545645952
498.5563853	0.897335186	0.006170092	4.531812464
646.7497069	0.898821314	0.005245465	4.758681301
846.3149304	0.889623831	0.004404552	4.847846666
361.4700788	0.866011252	0.010193162	4.571786256
122.5227445	0.85992084	0.04055373	4.220047095
328.5368256	0.847876564	0.011273496	4.450708904
600.9302819	0.86767226	0.00543029	4.743221714
607.3621574	0.827601591	0.007488612	4.297873436
465.2881726	0.8970865	0.010207184	4.921121295
520.9390445	0.894468085	0.008493766	4.796557056
534.5053576	0.887462447	0.005569495	4.62619103
545.4786554	0.865083233	0.006435046	4.565395528
996.7332283	0.839745312	0.004297459	4.681593016
850.4535639	0.859240159	0.004792637	5.133768781
130.5886433	0.833208255	0.032724851	3.924613133
369.3230836	0.901819913	0.012663005	4.9705399
718.0677848	0.854694265	0.00573142	4.639808861
428.0924542	0.909669591	0.007224565	4.859965641
463.7706682	0.898186949	0.007026568	4.738821914
338.0614968	0.88192555	0.010707122	4.589179237
171.6331391	0.873239437	0.025716078	4.508356584
547.1290411	0.890009808	0.006425438	4.768493916
411.9562658	0.872306343	0.007977188	4.62517289
99.3784317	0.840325002	0.066639073	4.237274105
244.4221119	0.873180873	0.022134094	4.630339153
147.3954294	0.852355485	0.037579676	4.431083892
393.9611162	0.895828967	0.01193626	4.750789348
352.4250185	0.909066016	0.008434292	4.745011786
321.4043984	0.871061784	0.011047515	4.641445357
282.0049281	0.894997213	0.011492298	4.480363551
201.8825197	0.885887796	0.023158355	4.640610468
575.7317232	0.871163708	0.005482397	4.500621048
432.7133159	0.901568888	0.007371749	4.77860565
721.6762893	0.867442274	0.005807826	4.730755138
400.2924309	0.862350635	0.008508687	4.360311411
575.167327	0.85746185	0.0063315	4.823798956
293.7806504	0.856520603	0.012849643	4.598971986
371.7671599	0.881215788	0.010186573	4.713405664
892.4973726	0.893498279	0.003095194	4.755577458
528.0170394	0.867617231	0.006575314	4.81201533
256.2212736	0.875851996	0.014322897	4.347810563
748.4961894	0.849161455	0.004866023	4.794366234
320.9782603	0.784568193	0.017822802	4.312896263
634.58855	0.87984	0.005248937	4.858117272
593.6082348	0.884149144	0.005112495	4.734423829
318.756686	0.844109996	0.012908588	4.470607709
519.7897706	0.870432877	0.009404291	4.607748981
691.39456	0.873910705	0.004668824	4.849414859
685.7055685	0.875856482	0.005600719	4.64337596
397.068843	0.896244083	0.010110062	4.69722043
371.6616216	0.888558583	0.010372807	4.905025575
445.504082	0.869769575	0.008164299	4.447376956
327.6600789	0.849268011	0.012083748	4.445124693
199.6263306	0.837883411	0.030392252	4.606761891
263.5807579	0.866612591	0.013755633	4.473748012
786.2284622	0.906271582	0.005331511	4.90309684
754.9437608	0.909656959	0.005271869	4.954987445
508.8248776	0.848676615	0.010159675	4.552287207
579.7819048	0.876861896	0.005865135	4.760510063
452.2629891	0.868832762	0.009035013	4.573255215
527.0067836	0.892295077	0.008415377	4.922681324
246.8134135	0.825818437	0.019438594	4.514293715
506.2198832	0.88743751	0.009091476	4.802522412
554.2790809	0.8774056	0.005600398	4.687524751
230.1021288	0.872688104	0.020690363	4.500892171
395.7081069	0.86351377	0.010594148	4.688634691
186.1418763	0.809579991	0.035070111	4.436921417
313.9987211	0.901407996	0.013973663	4.795032795
756.6400902	0.877098206	0.004158997	4.66575068
399.1275545	0.882517483	0.00811738	4.578796934
344.0639118	0.880320277	0.010166167	4.565445168
1051.998231	0.865553208	0.003522598	4.919463166
351.1556974	0.881024549	0.012492441	4.869838362
757.1327925	0.827198589	0.012637931	4.621203407
196.875572	0.835085778	0.024753158	4.239294877
328.3039508	0.876875117	0.01070196	4.624710575

log.sigma.4.5.mm.3D_glrlm_HighGrayLevelRunEmphasis	log.sigma.4.5.mm.3D_glrlm_RunLengthNonUniformityNormalized	log.sigma.4.5.mm.3D_glszm_GrayLevelVariance
508.2182813	0.839817744	41.54508367
69.1195784	0.763162501	13.51222222
189.7000166	0.796976235	22.81882259
277.9929949	0.810691798	33.12144245
202.8598455	0.780718635	19.26044077
114.8437499	0.779222081	18.00322118
253.2653906	0.809068674	25.09096709
517.3384825	0.784082132	35.53370029
358.2484856	0.794410146	26.83818827
542.2213414	0.814071958	26.76461186
134.5059148	0.780177159	15.39239334
266.0819524	0.79951865	23.84563866
228.0777222	0.726297453	33.45017948
353.0741162	0.7781613	37.36090913
244.0755779	0.809145084	28.48977943
284.3484292	0.830245012	26.75888476
286.3583732	0.800071799	23.35066287
116.2540158	0.734068412	18.23795773
72.49906198	0.737365002	10.29147878
275.4194815	0.813903048	30.43208722
171.5660401	0.736709574	16.86773646
121.5063394	0.791105875	14.44880999
133.1355309	0.780505983	18.364375
194.1771356	0.777965859	21.03699448
347.5914191	0.832326175	25.69253111
462.2596943	0.826001053	36.70843963
584.3621001	0.813740902	44.00595009
239.3713532	0.771654838	21.59505342
86.88147213	0.763905377	11.84415225
203.1451466	0.743899208	19.60621521
390.5205851	0.783255089	27.34083908
330.801532	0.717864241	25.60774335
324.7077993	0.822820358	36.18269216
361.6429136	0.824682505	33.36282229
371.2834997	0.808530149	20.95849116
350.4723696	0.781447106	22.43977138
586.1764403	0.739575127	44.26110668
540.098902	0.782647897	42.23275765
73.76289768	0.718085625	9.774522978
271.9703081	0.826652966	34.16423039
430.6669786	0.758585041	30.2135105
324.7731559	0.837874593	34.87444387
329.3528851	0.825764209	27.34910538
228.1965262	0.796118604	24.09753043
121.5555381	0.781484798	18.95083393
380.9976976	0.812634226	29.20065323
274.6015561	0.786173697	23.69826128
62.67524309	0.735493476	10.37709201
167.8386568	0.783113529	23.22731569
96.44377807	0.760968575	14.26665717
280.2095436	0.819482034	26.8448044
265.0056832	0.838628207	26.98199507
213.4955443	0.780497858	23.46710258
202.514888	0.81831773	20.35649334
143.6292335	0.804519737	20.0565661
379.5752616	0.779067365	23.34520106
313.2599577	0.829536061	28.80571384
456.8927044	0.777043597	36.33722781
254.9259088	0.764108724	19.30619786
360.5456363	0.772381349	26.92777774
194.1480132	0.761359235	26.01463614
256.8759652	0.796049379	23.75508204
636.1402292	0.817256322	29.95769827
356.2495117	0.77836492	29.84144042
164.9774607	0.792312523	19.19836565
468.6295232	0.754492055	36.85485579
159.5863093	0.652213703	16.91094513
431.9439524	0.795181744	36.65948367
399.1282655	0.804259319	29.88585473
197.2010961	0.74206533	16.8460203
338.949156	0.784358696	26.34155702
466.3867221	0.789204512	34.19730984
449.8129097	0.792254195	31.98492859
279.843623	0.817677024	28.6117195
264.7607087	0.80872276	31.01916006
283.7916372	0.775825074	21.8504582
198.7676791	0.754975759	18.12457112
129.1295297	0.736617413	24.10899696
175.1996334	0.772150852	17.06332222
563.4357021	0.836863667	46.0310628
551.3851036	0.84295734	43.21489232
307.9099973	0.747907762	32.58417609
375.6844222	0.800188561	30.25861907
293.6837515	0.771872236	21.68308836
364.4792852	0.8162194	35.11309834
148.2213297	0.715914392	17.87851211
343.9580969	0.807313235	32.16048374
376.9351281	0.797772736	25.46334181
152.9762259	0.786105979	16.61337867
256.5116728	0.768619195	29.17099183
115.5250463	0.69579495	12.18205973
229.4570514	0.827632405	26.55357177
504.2846045	0.788749197	29.50577024
273.0865059	0.802884566	23.18921655
232.7651462	0.791545023	21.44681607
676.7571437	0.78365106	40.68004261
241.2801095	0.795780103	28.83210312
418.204794	0.714086748	31.98648163
112.3924243	0.720227189	12.76949897
224.5030083	0.79618538	21.97655844

log.sigma.4.5.mm.3D_glszm_SmallAreaHighGrayLevelEmphasis	log.sigma.4.5.mm.3D_glszm_GrayLevelNonUniformityNormalized	log.sigma.4.5.mm.3D_glszm_SizeZoneNonUniformityNormalized
255.6553401	0.045076901	0.329543234
36.00121476	0.077511111	0.223733333
95.20224452	0.059289283	0.297786994
139.4575507	0.049884478	0.300514598
98.56141395	0.064156294	0.252548946
82.17940554	0.068309933	0.289240573
139.0745883	0.057420656	0.274704735
283.5850035	0.047863581	0.288004861
168.3793349	0.055295005	0.267170332
261.8655841	0.060738452	0.268954126
58.4654586	0.071425598	0.241165453
129.804888	0.058037447	0.265201451
105.0152282	0.049648413	0.220803488
178.5733193	0.046478243	0.30228552
118.2389318	0.053755514	0.294986766
149.9852759	0.054502328	0.282531417
121.8895804	0.062932817	0.229047695
67.88160177	0.066466063	0.27375088
23.66896444	0.087170502	0.170913963
157.8137836	0.050492734	0.284398639
98.59682203	0.070127589	0.270663831
55.27261303	0.074135437	0.239289941
63.0778832	0.06445	0.2358
88.73557718	0.062785279	0.258223655
166.0384112	0.055882247	0.327315429
258.8188665	0.048327664	0.311020762
320.3130803	0.045292406	0.310726432
121.5264332	0.062224407	0.260376758
38.37740579	0.083460208	0.20083045
96.30824362	0.065398887	0.212641255
202.6306021	0.054340231	0.295474932
131.6993038	0.064118319	0.263080282
153.3427492	0.046029756	0.285166095
172.1639598	0.04947868	0.294288477
193.7048136	0.063143868	0.292156293
175.3212687	0.060797102	0.294930625
308.7425287	0.046397073	0.313186104
287.6860193	0.043598776	0.285956315
26.12607471	0.092985757	0.160171997
146.8558724	0.047222001	0.273309808
196.4429872	0.055207791	0.279168835
200.9585409	0.049383861	0.295508595
159.463134	0.053388252	0.284463846
116.1968966	0.059133459	0.27424121
71.38160945	0.066503949	0.267292289
197.0679985	0.052926126	0.323421084
145.9222741	0.057519288	0.268886303
31.19232437	0.089188617	0.215450886
98.64655637	0.060077977	0.272566163
49.93667937	0.081238875	0.271057316
137.9957534	0.054551837	0.279968158
139.0476313	0.053524053	0.276275635
116.9781176	0.0569645	0.292425053
110.2643111	0.062098605	0.336765045
71.05647453	0.062574746	0.263184291
181.306094	0.060952845	0.234044856
160.9444473	0.051925758	0.295686428
193.2273435	0.047296754	0.266940534
112.3138221	0.066125529	0.278780672
187.7175103	0.055017963	0.280369313
120.6830763	0.055298857	0.292522689
132.9236088	0.060464272	0.266758412
358.9907981	0.053390226	0.328239949
225.8062711	0.052010191	0.271106991
95.39685252	0.066095177	0.315975004
224.3232901	0.047919705	0.230185951
59.08441034	0.08302212	0.204682562
238.9784103	0.0465459	0.278965122
199.1883862	0.050922241	0.351058509
91.46553818	0.06944297	0.239429591
189.1158712	0.06102844	0.335041655
259.5643748	0.049323327	0.294972889
194.9491864	0.05249653	0.256446782
131.8360758	0.052688237	0.273559111
157.7538997	0.051303761	0.260532974
119.5719358	0.063588897	0.252357611
91.13551207	0.06615773	0.274764729
87.48233727	0.058564333	0.234257331
85.13105239	0.068888889	0.210888889
279.0366953	0.044780508	0.312127704
284.5450353	0.044812254	0.33367401
157.5649092	0.052907496	0.265894557
182.5859469	0.051766156	0.295969927
117.4338396	0.061768861	0.227805908
193.4777777	0.047455775	0.278782518
86.51917805	0.071487889	0.264290657
161.0913968	0.050432679	0.288153722
214.3725808	0.057389795	0.323206646
82.41554562	0.073670095	0.264328732
139.4949078	0.052318539	0.282961064
67.81756402	0.083005633	0.245190775
118.0313424	0.055736249	0.282982988
239.4295699	0.053214835	0.263702678
143.248572	0.057765831	0.30794919
111.5662524	0.06076087	0.234747875
375.2289652	0.04747948	0.322731671
107.8325489	0.052819848	0.211817512
200.9017888	0.05835102	0.273795918
30.70293678	0.080836777	0.139204545
121.9334241	0.059463649	0.29947234

log.sigma.4.5.mm.3D_glszm_SizeZoneNonUniformity	log.sigma.4.5.mm.3D_glszm_GrayLevelNonUniformity	log.sigma.4.5.mm.3D_glszm_LargeAreaEmphasis	log.sigma.4.5.mm.3D_glszm_ZoneVariance
236.9415855	32.41029207	46.20305981	37.49276251
33.56	11.62666667	296.5933333	257.7388889
92.01618123	18.32038835	123.0614887	98.41620846
82.94202899	13.76811594	64.45289855	51.92232462
110.6164384	28.10045662	231.1872146	201.9581326
48.8816568	11.5443787	101.5739645	86.32239768
340.633871	71.2016129	166.6798387	145.9112376
282.8207739	47.00203666	516.6659878	489.6090401
261.5597549	54.13381001	124.7354443	103.173224
73.69343066	16.64233577	78.61313869	63.30619639
74.76129032	22.14193548	109.5354839	85.8719667
133.9267327	29.30891089	114.1564356	93.32308597
54.53846154	12.26315789	583.2672065	526.1331935
116.9844961	17.9870801	222.5917313	202.9074909
148.9683168	27.14653465	97.04950495	81.61472013
277.7283825	53.5757884	143.0824008	123.264959
60.23954373	16.5513308	74.01520913	53.40429961
55.57142857	13.49261084	350.1330049	313.2408454
33.67005076	17.17258883	318.9746193	275.5619057
354.0763052	62.86345382	102.7927711	85.35460009
112.5961538	29.17307692	924.3076923	871.4659763
46.66153846	14.45641026	146.0358974	115.4181197
47.16	12.89	179.49	153.2756
137.1167608	33.33898305	181.5536723	156.7041045
109.3233533	18.66467066	80.71556886	66.18897235
209.0059524	32.47619048	124.6532738	112.2255505
241.1237113	35.14690722	169.6920103	152.3344786
136.9581749	32.73003802	459.3536122	431.7811303
17.07058824	7.094117647	137.9411765	110.2859516
65.49350649	20.14285714	333.7467532	289.2734441
270.3595628	49.72131148	329.8819672	302.8419672
69.19011407	16.86311787	1150.840304	1088.951872
240.3950178	38.80308422	111.9655991	97.29388207
176.278798	29.63772955	113.933222	98.54168188
308.5170455	66.67992424	103.9640152	86.57132404
159.8523985	32.95202952	305.6586716	275.042255
182.9006849	27.09589041	1156.741438	1116.144466
304.2575188	46.38909774	300.487782	276.9598117
19.54098361	11.3442623	644.5737705	542.9277076
527.214619	91.09123898	61.1881804	46.98438636
138.4677419	27.38306452	341.608871	314.257658
355.2013311	59.359401	33.40682196	23.05684439
262.56013	49.27735645	88.77681473	73.11305754
157.6886957	34.00173913	162.8591304	144.1365777
71.3670412	17.75655431	188.2172285	165.3064849
235.4505495	38.53021978	109.3997253	95.18226777
252.2153518	53.95309168	307.3027719	283.6487559
25.63865546	10.61344538	376.2941176	322.7215592
50.15217391	11.05434783	122.9076087	101.5169837
71.83018868	21.52830189	234.5132075	208.4839587
178.0597484	34.69496855	79.07389937	62.75788883
273.2366026	52.93528817	41.1718908	29.55406335
243.882494	47.50839329	406.4244604	377.155582
96.65156794	17.82229965	85.71428571	70.91726256
84.74534161	20.14906832	84.35403727	67.29354577
77.93693694	20.2972973	315.3693694	281.0783216
334.1256637	58.67610619	61.89823009	49.08436448
157.4949153	27.90508475	409.1322034	374.7806837
88.93103448	21.09404389	251.1912226	224.5021767
499.8984857	98.09702748	316.8923163	290.6776866
99.16519174	18.74631268	330.0648968	302.9266366
153.386087	34.76695652	139.7947826	118.2328922
493.344644	80.24550898	143.6001331	128.7650788
396.6295284	76.09090909	318.2235133	291.0739288
49.92405063	10.44303797	121.9493671	96.94936709
144.7869634	30.14149444	608.1176471	570.4192184
24.15254237	9.796610169	1355.237288	1233.116059
395.293578	65.95553987	206.2879323	186.8334206
521.3218855	75.61952862	197.0821549	181.526169
103.9124424	30.13824885	450.9953917	406.4074625
98.83728814	18.00338983	184.4915254	165.1016834
251.3169014	42.02347418	258.7183099	233.3649573
120.017094	24.56837607	155.758547	132.0449768
161.6734349	31.13874788	78.21150592	63.60126088
200.6103896	39.5038961	109.5831169	93.04885984
69.1459854	17.42335766	119.3722628	95.38371783
111.0049505	26.72772277	397.3069307	362.1338104
37.01265823	9.253164557	335.1392405	298.3757411
63.26666667	20.66666667	164.9966667	132.6205667
215.3681159	30.89855072	68.96666667	57.47554925
230.568741	30.96526773	46.20984081	37.92444097
87.21341463	17.35365854	309.527439	279.4448896
192.9723926	33.75153374	327.7315951	302.5933677
114.3585657	31.00796813	233.252988	195.7800551
172.8451613	29.42258065	73.08870968	57.61336889
44.92941176	12.15294118	907.3294118	826.1175087
175.7737705	30.76393443	140.1754098	124.8885918
205.2362205	36.44251969	279.319685	260.673609
76.39100346	21.29065744	191.1487889	165.4870272
111.7696203	20.66582278	182.4329114	156.1510655
47.56701031	16.10309278	607.5360825	562.3555107
195.5412446	38.51374819	41.82633864	29.94330246
300.6210526	60.66491228	265.304386	244.6338035
220.4916201	41.3603352	217.6927374	196.4504229
202.3526682	52.37587007	160.8584687	135.3688557
244.6306069	35.98944591	368.055409	347.6745915
120.1005291	29.94885362	123.9171076	99.23356009
47.91428571	10.21142857	345.2171429	309.4225633
12.25	7.113636364	408.1136364	318.294938
351.88	69.86978723	234.6382979	210.4904837

log.sigma.4.5.mm.3D_glszm_ZonePercentage	log.sigma.4.5.mm.3D_glszm_LargeAreaLowGrayLevelEmphasis	log.sigma.4.5.mm.3D_glszm_LargeAreaHighGrayLevelEmphasis	log.sigma.4.5.mm.3D_glszm_HighGrayLevelZoneEmphasis
0.338831291	0.103628536	24255.57441	460.3991655
0.160427807	8.597415853	13322.25333	80.20666667
0.201434159	0.980119569	22812.35275	178.381877
0.282497441	0.3694461	16915.46377	261.5036232
0.184966216	1.444496427	40840.49315	199.9726027
0.256060606	2.219992876	6316.662722	144.7988166
0.219430189	0.976650141	33111.18548	266.9185484
0.192247455	1.225304885	225162.2434	536.1629328
0.215354158	0.437032617	41217.78958	338.1317671
0.255597015	0.175821342	39113.58759	526.9562044
0.205570292	1.194978736	13939.96774	127.2741935
0.219088937	0.56645617	29011.24356	256.5742574
0.132297804	3.230871379	114533.2308	234.1255061
0.225393128	0.795924164	65900.78036	344.1085271
0.25453629	0.462044794	24316.92079	227.7188119
0.224634369	0.796126295	33860.31536	282.889115
0.220268007	0.274588339	23765.60456	259.5513308
0.164639092	5.383389294	26103.31034	135.5812808
0.151771957	9.481960097	14281.5736	71.40101523
0.239469129	0.5526052	23173.7245	293.0803213
0.137566138	7.473617495	123084.0264	186.0552885
0.180722892	2.315589788	13612.85128	123.0358974
0.1953125	2.253341028	18097.92	139.915
0.200604458	1.20247775	30464.61582	186.094162
0.262372349	0.265500815	26724.08383	317.754491
0.283663993	0.295228139	56266.98512	459.1577381
0.240024745	0.327136792	96057.06186	575.5579897
0.190441709	2.508817193	88541.4981	246.5380228
0.1901566	3.994322417	6427.447059	107.4235294
0.149951315	1.999716787	60179.92208	205.7337662
0.192307692	1.052603989	110702.2809	380.3289617
0.127114548	3.353293709	402777.1141	267.6882129
0.261071539	0.405808022	39266.95255	294.3890866
0.254893617	0.358318419	42083.27379	329.4440735
0.239782016	0.342273091	35614.12405	364.7518939
0.180726909	1.110283667	91546.87638	335.9649446
0.156947057	2.096497252	648251.5068	556.8493151
0.206161597	0.756395819	130310.8712	536.0413534
0.099186992	10.22478318	45584.47541	65.90163934
0.265337001	0.381769753	15433.17574	280.5842405
0.191210486	0.759832785	160161.006	376.578629
0.310835273	0.14479818	10280.54908	344.8336106
0.252669039	0.342269475	26484.91549	313.0433369
0.231109325	0.817510073	36244.55652	218.9791304
0.208920188	2.999310103	14158.33708	143.0187266
0.265209472	0.357369134	38280.52198	358.7129121
0.205611574	1.444020567	69308.0064	283.0181237
0.136624569	14.81480956	13626.33613	70.79831933
0.216216216	1.341669675	14993.91304	189.4130435
0.196005917	5.601435674	12575.36226	105.9924528
0.247567147	0.412677331	21341.78145	263.6949686
0.293384752	0.216109172	10267.09302	267.4671385
0.184840426	2.474989371	72032.34892	215.3261391
0.259963768	0.621765086	13968.54704	198.9094077
0.242105263	1.320485121	9028.074534	145.8167702
0.170769231	0.930638826	110566.9309	379.6816817
0.279357231	0.246718714	18722.05575	301.260177
0.170618855	0.932575431	190672.1644	391.5
0.193567961	1.080230229	61240.0627	225.2601881
0.195311644	1.133941441	97936.02636	353.5984296
0.19195923	2.492833643	46715.74041	225.0825959
0.215355805	0.778860273	30118.61565	261.933913
0.259630333	0.264811962	81881.89953	627.2934132
0.191919192	1.254052334	85947.85509	412.5345181
0.2	0.992540452	17747.08228	174.6582278
0.16286898	1.561915936	243052.283	480.3958665
0.090490798	9.788972492	194873.9576	148.9661017
0.22672	0.54102276	83431.28299	442.3535639
0.253542769	0.534661557	77033.6862	348.189899
0.149758454	3.050918905	73762.85945	196.7396313
0.227097768	0.652558872	57695.89492	324.7525424
0.198601399	0.688465005	103219.4894	479.2488263
0.205353225	0.394235143	66765.61538	412.6517094
0.261620186	0.308670605	24495.45516	259.2301184
0.245927819	0.664061945	23038.17403	293.7116883
0.204172876	0.434429561	35886.32847	250.4781022
0.168614357	2.419614874	72011.19059	185.4554455
0.164926931	4.938761955	25848.37342	176.2721519
0.175746924	1.325023529	24170.08667	185.9766667
0.294997862	0.129982288	45729.28261	505.4188406
0.347410759	0.098824162	26365.41679	503.6309696
0.182323513	1.052977276	97793.5061	302.0243902
0.199449373	0.985701786	115482.2117	352.6042945
0.163358282	0.88561718	74174.11753	264.9063745
0.254202542	0.269374154	26589.9629	355.9274194
0.110966057	9.28627698	101008.3529	166.2882353
0.255765199	0.449180756	50011.52295	307.2852459
0.231582786	0.964942865	84402.24252	383.9543307
0.197404372	1.761672336	25219.52595	155.4290657
0.195061728	0.896176035	41484.26076	255.9974684
0.148773006	13.71214855	32131.57732	137.2989691
0.290092359	0.308123734	8915.001447	223.8523878
0.219949836	0.56750014	127871.4307	474.5307018
0.216969697	0.951054643	53535.53771	262.0125698
0.198069853	0.732173355	40514.16821	225.7842227
0.22150789	0.660961448	212101.5976	664.3614776
0.201277955	0.779485714	27687.22222	246.8500882
0.167144222	0.949159135	151241.6057	373.2857143
0.105515588	4.160791478	46567.54545	104.1022727
0.203498441	1.599371609	39452.30298	229.6553191

log.sigma.4.5.mm.3D_glszm_SmallAreaEmphasis	log.sigma.4.5.mm.3D_glszm_LowGrayLevelZoneEmphasis	log.sigma.4.5.mm.3D_glszm_ZoneEntropy	log.sigma.4.5.mm.3D_glszm_SmallAreaLowGrayLevelEmphasis
0.593468329	0.006035212	6.654337468	0.003562544
0.48128507	0.052946865	5.885630087	0.022797346
0.563359259	0.020201758	6.251392713	0.015154318
0.56598001	0.013711326	6.330731493	0.00696318
0.516312055	0.011395289	6.519782933	0.007235557
0.545812384	0.022094104	5.714848985	0.00834217
0.540612918	0.007612889	6.817339703	0.004024091
0.553081196	0.004196119	6.904508989	0.002091085
0.530640155	0.006273485	6.846603686	0.00405298
0.524861984	0.006568033	6.265870481	0.005415319
0.503251644	0.021748802	6.251088241	0.0088344
0.528073658	0.009342015	6.578335081	0.004784974
0.47483653	0.020605522	6.545522417	0.007624854
0.565726841	0.009557102	6.533054956	0.006594024
0.557236141	0.013547872	6.483116311	0.007630905
0.549526138	0.008461628	6.798341753	0.005139616
0.490117631	0.012194844	6.516177749	0.008786544
0.539603777	0.022814804	6.013135771	0.016409765
0.394973738	0.052732599	6.059976679	0.029246602
0.548588992	0.007696193	6.904782639	0.004574265
0.535838331	0.012629235	6.302740625	0.005590727
0.503109132	0.02208722	6.037022158	0.014007879
0.493342124	0.021552551	6.184685122	0.012379875
0.521821503	0.014329457	6.563577347	0.010085655
0.592012865	0.008166828	6.206981471	0.006649039
0.57475326	0.006241002	6.6848811	0.004297015
0.575779594	0.005131361	6.841056748	0.00348452
0.517859819	0.011545035	6.467339916	0.007810386
0.414963412	0.030273844	5.368173366	0.020467235
0.468343712	0.012169854	6.553326824	0.00815669
0.560502906	0.005434507	6.701957873	0.003829122
0.52493383	0.012943398	6.304491862	0.009627483
0.547805393	0.010862949	6.808756416	0.006182325
0.556180181	0.011752246	6.651074774	0.007686292
0.556911004	0.005208241	6.552032025	0.003273011
0.560423326	0.006898932	6.430048058	0.004602934
0.579899805	0.005814199	6.78057227	0.004023004
0.550143894	0.00418301	7.026754159	0.002661883
0.395187585	0.041989291	5.963415465	0.022429505
0.53759112	0.010198361	7.0951758	0.005287252
0.541426321	0.007129256	6.640887095	0.004571951
0.559649571	0.006915622	6.886524934	0.004427894
0.550049858	0.007700956	6.759053964	0.004615215
0.537979238	0.012049783	6.582665155	0.007816803
0.530553461	0.023385035	6.168145605	0.015950549
0.585028908	0.006486902	6.446923714	0.004186926
0.531399841	0.007228812	6.751075187	0.003976677
0.457075314	0.046536308	5.729949096	0.01915218
0.536683029	0.01727012	6.1197035	0.007571555
0.534986044	0.031843305	5.984138347	0.023662842
0.543442485	0.010718695	6.635942711	0.004573385
0.539166063	0.00803266	6.787015103	0.005189314
0.55946307	0.01033679	6.642907951	0.005521486
0.601268788	0.012480834	5.969683941	0.009151858
0.523016192	0.019180283	6.229984352	0.011490646
0.497538491	0.007523622	6.591277256	0.005876285
0.560003055	0.007846657	6.770874418	0.005128331
0.532645481	0.007558911	6.866086692	0.004545826
0.542323336	0.012088423	6.22721782	0.009492883
0.544478361	0.005291813	6.902670305	0.00319637
0.555037115	0.011776055	6.35450651	0.008461793
0.531633414	0.009169029	6.627281788	0.005620571
0.592501213	0.003033573	6.694208884	0.002044012
0.535982801	0.005310431	7.005148733	0.002375287
0.58029085	0.017074251	5.798902193	0.013579988
0.48447481	0.005570064	7.052904999	0.003870795
0.452114447	0.022176807	5.8486323	0.014504024
0.541453233	0.005531718	7.023881143	0.003550315
0.614992529	0.005748044	6.605690713	0.004174415
0.500146075	0.011795597	6.437684876	0.006154225
0.600013818	0.009867832	6.05245266	0.00421217
0.560325128	0.004940485	6.801320045	0.003248314
0.516493399	0.007089422	6.747292098	0.003808085
0.536598213	0.011411414	6.649886742	0.00651567
0.519820078	0.009149736	6.867525287	0.005689448
0.512908891	0.011951538	6.326960663	0.008858175
0.536143372	0.013268452	6.278130868	0.00901509
0.495733198	0.02034384	6.161782945	0.007046413
0.464739798	0.013227719	6.410997967	0.008138061
0.577057921	0.006758868	6.79429537	0.004575578
0.596612715	0.006038634	6.642403454	0.004016775
0.528811371	0.014034512	6.578964003	0.008310234
0.561307792	0.006477951	6.596542869	0.00446911
0.485393958	0.008740856	6.712095852	0.00519111
0.541216728	0.007136016	6.776112578	0.002740581
0.527685884	0.019098945	5.898238361	0.013078846
0.550704418	0.009203395	6.661702179	0.00447618
0.589152184	0.005952056	6.438477494	0.004626709
0.529389391	0.016197148	6.127948951	0.006356556
0.549875622	0.013007339	6.580982609	0.008982981
0.505609479	0.017022369	5.822799883	0.010794024
0.545559812	0.011428836	6.628535266	0.005673801
0.526292179	0.00517864	6.909891494	0.003718074
0.573340768	0.008224075	6.517216578	0.004650844
0.492018282	0.010896258	6.867291324	0.005901895
0.588825794	0.004085828	6.727451596	0.003025691
0.463456632	0.011119155	6.948385712	0.006005948
0.538343354	0.012209314	6.096323554	0.003333279
0.34124941	0.031145852	5.74029284	0.010921947
0.56583165	0.011390812	6.622819146	0.008426511

log.sigma.4.5.mm.3D_ngtdm_Coarseness	log.sigma.4.5.mm.3D_ngtdm_Complexity	log.sigma.4.5.mm.3D_ngtdm_Strength	log.sigma.4.5.mm.3D_ngtdm_Busyness	log.sigma.4.5.mm.3D_ngtdm_Contrast
0.006318896	0.408226423	3.12310526	0.172459944	3.80E-05
0.011621172	0.12799503	1.25774141	0.798814545	8.59E-05
0.009506814	0.191276666	1.908854073	0.364304959	5.19E-05
0.014817241	0.382727695	3.713744774	0.159304335	0.00010672
0.00556409	0.12422776	1.213468557	0.487395762	2.27E-05
0.017445167	0.414487431	3.640234815	0.284371826	0.000134024
0.00231817	0.117265008	1.114470934	0.710921638	8.02E-06
0.002734745	0.138465946	1.654654645	0.361706231	7.62E-06
0.003038247	0.124338185	1.180226162	0.501721322	1.20E-05
0.011779003	0.492144178	4.724961594	0.116602184	5.85E-05
0.008781742	0.139255354	1.435077254	0.476893787	4.01E-05
0.00568187	0.187186404	1.815869493	0.354439872	2.46E-05
0.007810177	0.149702461	2.304286586	0.2791367	2.40E-05
0.008400413	0.285181031	3.026348205	0.180506477	3.33E-05
0.006169003	0.257176178	2.032392447	0.307947918	3.15E-05
0.003010843	0.116803165	0.963622192	0.65019909	1.69E-05
0.009766741	0.280430159	2.693863186	0.200129036	5.21E-05
0.01003899	0.135416016	1.421524946	0.574811495	6.26E-05
0.009491412	0.070431117	0.833251664	1.113460372	5.89E-05
0.00275756	0.107754332	1.014346414	0.669763859	1.18E-05
0.004665664	0.075396061	0.992738004	0.632542077	1.32E-05
0.012364724	0.153324553	1.599845083	0.440675805	6.47E-05
0.013526384	0.173036459	2.004592197	0.393119963	7.77E-05
0.004714361	0.148224735	1.440831172	0.480844913	1.60E-05
0.008287576	0.439068613	2.634083507	0.194226213	5.03E-05
0.005143326	0.34384439	2.80824139	0.20292041	2.32E-05
0.004024641	0.313651876	3.143306891	0.191601833	1.40E-05
0.004733462	0.115733597	1.197027392	0.482724018	1.84E-05
0.025949835	0.254745364	2.71508782	0.378783157	0.000190493
0.006238741	0.11492026	1.451210699	0.413405796	1.84E-05
0.002816229	0.12667236	1.247212563	0.463787771	9.07E-06
0.005610714	0.157837301	2.307713671	0.25280446	1.39E-05
0.004631849	0.184056778	1.793438939	0.339107876	2.26E-05
0.00555086	0.270337395	2.296060253	0.248902262	2.75E-05
0.002754972	0.12169216	1.05937549	0.558391566	1.18E-05
0.004308079	0.140416502	1.509799978	0.387842971	1.50E-05
0.00364009	0.191802383	2.908779247	0.208993177	7.62E-06
0.003216935	0.161980335	1.862168002	0.310319311	1.07E-05
0.009538009	0.058284656	0.741724881	0.751029239	2.84E-05
0.002168684	0.074711327	0.746229108	0.941725525	1.09E-05
0.005481307	0.206009915	2.665781491	0.193893991	1.44E-05
0.003700912	0.240253287	2.33230868	0.335792496	1.31E-05
0.003456056	0.150404214	1.103388673	0.522154217	2.00E-05
0.00507459	0.204309051	1.831551141	0.36102251	1.89E-05
0.009625472	0.201073693	1.897761713	0.502257235	5.78E-05
0.004492578	0.26215282	2.015520272	0.285460535	2.04E-05
0.002826223	0.101181533	0.984198446	0.616849222	9.33E-06
0.01481494	0.08668703	1.130074219	0.921389207	9.14E-05
0.015587091	0.311872332	3.127635658	0.241325255	8.83E-05
0.00909464	0.170948868	1.693330108	0.564646457	4.38E-05
0.005145634	0.16702195	1.398182304	0.426920031	3.02E-05
0.003756522	0.191711194	1.357249896	0.495560898	2.04E-05
0.002910436	0.077121944	0.783352579	0.82613212	1.15E-05
0.010422525	0.272261952	1.716847702	0.329748421	7.99E-05
0.009396104	0.238963296	1.810079833	0.469006448	7.17E-05
0.006952012	0.192595737	2.461598512	0.219491511	1.93E-05
0.003320021	0.136901127	1.126035014	0.526696893	1.67E-05
0.004190029	0.148991312	2.053039056	0.278331947	1.34E-05
0.007620091	0.169872477	1.638612095	0.285402962	2.60E-05
0.001536846	0.065409895	0.714889351	0.858414317	4.47E-06
0.007782122	0.20992745	2.361099445	0.311582319	2.70E-05
0.005094401	0.151405675	1.449340246	0.441409422	2.37E-05
0.002103347	0.186149317	1.666300121	0.366273593	6.77E-06
0.001998781	0.079456746	0.98240625	0.661515959	5.35E-06
0.014036544	0.293483663	2.226700144	0.277731133	8.84E-05
0.004159474	0.152825604	2.391890649	0.241226994	7.94E-06
0.011163003	0.10363838	2.006272757	0.270238666	1.78E-05
0.002392741	0.121910635	1.40059283	0.424835011	6.44E-06
0.002362592	0.10437789	1.139853709	0.502543513	7.30E-06
0.004629834	0.078164984	0.907414537	0.647178103	1.53E-05
0.010057199	0.347941503	3.817274559	0.14815702	3.71E-05
0.003259132	0.159046453	1.705131594	0.352326379	1.10E-05
0.005732369	0.23492666	2.707177705	0.21498283	2.07E-05
0.005907077	0.187733559	1.58212127	0.334432878	3.26E-05
0.00466228	0.173750364	1.734946185	0.432925858	2.23E-05
0.009050229	0.253484515	2.620162743	0.204680352	3.53E-05
0.00525367	0.119128302	1.210370891	0.456120539	1.62E-05
0.015654586	0.229458025	3.611424983	0.26073969	5.78E-05
0.007707033	0.132622372	1.476137205	0.428797714	3.05E-05
0.006013218	0.387670951	4.003247926	0.142442972	2.82E-05
0.006913216	0.453674536	3.763259042	0.144428851	4.04E-05
0.007302922	0.270105065	3.658878599	0.171458861	1.55E-05
0.0041303	0.151164966	1.597337959	0.354975028	1.61E-05
0.004704305	0.10731149	1.579792082	0.363085083	1.28E-05
0.005871437	0.301960468	2.896456401	0.21906209	2.42E-05
0.009591876	0.114611583	1.736135556	0.408445428	3.12E-05
0.005614152	0.204750794	1.92802941	0.285027518	3.13E-05
0.004631731	0.203177762	1.802066661	0.317905984	1.85E-05
0.008306361	0.169665449	1.549916608	0.442635751	4.41E-05
0.006990397	0.17628538	2.027245009	0.285301444	2.46E-05
0.009358994	0.089287325	1.121970303	0.867501901	8.59E-05
0.005349323	0.201398285	1.571547993	0.459689331	3.55E-05
0.002803851	0.119590931	1.642523279	0.344468852	6.35E-06
0.003705959	0.137662218	1.175831825	0.485849789	1.47E-05
0.003183552	0.091332629	1.017314749	0.599058136	9.15E-06
0.004109951	0.286915585	3.217360743	0.185746783	1.30E-05
0.0057186	0.130750963	1.738851799	0.394987448	2.27E-05
0.013472359	0.376175911	6.202014094	0.092217541	4.80E-05
0.016596094	0.109119478	1.715351108	0.337448219	5.41E-05
0.002152366	0.069945345	0.535258839	1.174893233	1.08E-05

log.sigma.4.0.mm.3D_gldm_GrayLevelVariance	log.sigma.4.0.mm.3D_gldm_HighGrayLevelEmphasis	log.sigma.4.0.mm.3D_gldm_GrayLevelNonUniformityNormalized	log.sigma.4.0.mm.3D_gldm_DependenceEntropy
31.85396137	548.4198869	0.056351441	6.984901564
10.11613257	76.93582888	0.104425062	6.427202864
17.38174775	193.2985658	0.067532284	6.974119708
26.67870751	300.8065507	0.060837334	6.814558944
14.41313159	248.6013514	0.078528037	6.835347122
18.85490358	145.3151515	0.077956841	6.628430919
18.74059634	260.999823	0.067665324	7.05134942
19.46496306	490.6812843	0.074191895	6.951646426
20.04334114	411.4929608	0.064721015	7.094946263
19.17763474	606.8330224	0.069412731	6.749955607
12.34016985	146.2732095	0.080923844	6.743983145
18.80490944	313.3479393	0.068896533	6.902234321
15.06041015	271.4033208	0.102355374	6.983054753
21.5743385	373.5136867	0.072708456	6.953670258
20.23563483	300.8150202	0.073349396	6.834697539
22.03950745	291.5139397	0.061532449	6.972313744
15.79871229	312.6825796	0.082423564	6.558753349
13.26020776	106.2035685	0.09791362	6.693879241
9.466367008	80.88058552	0.097720566	6.621711979
21.69169985	248.4029621	0.062413537	7.044320584
11.33001071	155.2347884	0.093372727	6.947837379
11.85454214	112.4485635	0.081071703	6.455763394
14.54403305	139.1367188	0.083070755	6.707561748
13.4388127	209.0687571	0.082065233	6.91615299
17.10586728	366.7635507	0.07243252	6.721235752
24.19397483	492.3710426	0.066334697	6.886846387
25.9062458	564.281163	0.068104604	7.005522174
14.81400325	288.3559015	0.08098687	6.812000591
11.28634846	92.24608501	0.096687336	6.257078849
10.6065833	243.6222006	0.095940284	6.752930847
17.52154686	443.3652795	0.071911098	7.109456066
11.05245773	367.7675205	0.120044777	6.590483198
26.77232605	386.5847011	0.058376871	7.094270248
23.43181983	420.7978723	0.064539611	6.975266088
16.95986557	350.0472298	0.069950136	6.888345792
14.88176864	401.1700567	0.078885359	6.867853305
17.4675513	570.6589626	0.097331682	6.962540234
28.37182976	548.5140477	0.059801161	7.377762509
5.003985062	86.16504065	0.127391103	6.25857429
26.98043748	257.4437414	0.054530291	7.1374102
17.54804427	426.3546646	0.090320525	6.840951047
26.93387164	347.7856219	0.057879832	6.990480021
20.65022393	342.7018889	0.063419444	6.893628258
16.81688294	281.7029743	0.075738787	6.804925384
15.37525623	149.1956182	0.082258811	6.725508744
20.79838541	354.9621129	0.066447424	6.994777432
15.88220169	322.8461201	0.076752545	6.943583883
9.491216531	70.90929966	0.099181299	6.531808653
17.02370198	183.1034078	0.070564664	6.764497118
12.78295523	104.6279586	0.087069124	6.721749824
20.70149631	293.1634877	0.064333087	6.959183063
22.47745102	313.4678137	0.060447439	6.880876106
14.90102511	229.1872784	0.078186204	6.926520155
15.57734638	182.1603261	0.072511355	6.582159489
18.63860083	148.4443609	0.065924586	6.752567539
13.93299145	443.8041026	0.084789481	6.679624653
21.57658969	329.8850433	0.061484016	6.99867474
19.64768764	437.0451128	0.077913563	6.917088773
11.29393675	253.3531553	0.092814032	6.599366505
18.96470832	460.6136488	0.069259409	7.279558396
15.94475137	205.7944507	0.087407287	6.843046954
17.87426405	298.2423221	0.06947804	6.924428266
20.41935634	554.8524788	0.067737936	7.002020492
20.50735152	407.4451004	0.071272205	7.086159923
12.0951386	177.1037975	0.081954815	6.509392474
17.80863999	482.5113931	0.083627913	7.03897051
7.265130415	180.1150307	0.125585739	6.608728481
21.75839601	457.96	0.071054592	7.092532352
19.10421345	462.7182858	0.071112555	7.010452293
10.57217788	206.0990338	0.090006387	6.859136623
17.26365458	358.361047	0.07969712	6.806942301
21.18372841	433.7944056	0.068190892	7.101856043
18.20323803	513.7393594	0.077262858	6.843128296
22.34163636	312.1398849	0.063998004	6.817729887
24.93899345	249.9964867	0.060225778	7.060203499
13.95177083	308.6803279	0.090862449	6.690595645
10.28392967	160.009182	0.091802629	6.845113649
15.67370806	158.6983299	0.088061855	6.733855146
12.22501441	183.2108963	0.085294399	6.768857935
32.82697155	559.7640017	0.06148855	6.962189508
33.69524239	547.0628457	0.056379721	6.987010073
14.57240126	268.282935	0.09531977	6.82655306
18.71421619	392.9455491	0.071604076	6.950921124
13.82334154	281.0761471	0.085302439	6.796780211
25.45546143	344.0172202	0.059545069	7.088785247
11.15370614	150.1227154	0.10172968	6.720160573
24.23326539	378.0675052	0.068050227	7.020991838
18.05247757	353.1480671	0.071194978	6.970881831
14.02221205	191.0539617	0.0807308	6.777230742
16.06055175	269.774321	0.079308154	7.002305007
13.91386578	96.52300613	0.109170932	6.6157249
22.48763431	217.0327456	0.060958166	6.961769724
18.10687532	491.2008489	0.075720805	6.978486112
15.82579054	357.1906061	0.077604775	6.886044848
15.0559656	256.0402114	0.074799957	6.837126795
24.92122584	598.987142	0.068183456	7.203766929
20.9718776	196.2687256	0.065135873	7.035707127
18.44525816	421.1623687	0.09792202	6.795919439
7.830312728	125.08753	0.106004405	6.470154394
15.60993724	230.9785244	0.074282803	6.935253813

log.sigma.4.0.mm.3D_gldm_DependenceNonUniformity	log.sigma.4.0.mm.3D_gldm_GrayLevelNonUniformity	log.sigma.4.0.mm.3D_gldm_SmallDependenceEmphasis
379.7455231	119.5777568	0.29250446
117.173262	97.63743316	0.153199694
201.7640156	103.5945241	0.189565655
161.5854657	59.43807574	0.245866559
325.2753378	185.9543919	0.197362235
87.53636364	51.45151515	0.228476553
803.8529464	382.3767475	0.208477551
685.9440094	378.9722005	0.192011057
656.6726793	294.2217334	0.200561541
159.7985075	74.41044776	0.239755626
204.1458886	122.0331565	0.192124989
334.4724512	158.8065076	0.207421393
177.5302625	191.0974826	0.137748123
232.1846243	124.8404193	0.206728878
294.4858871	145.5252016	0.240168007
693.7637112	269.2659963	0.213014479
185.4137353	98.41373534	0.218984689
137.6601784	120.7274939	0.155003225
155.4468413	126.8412943	0.151951766
788.3702635	324.4879785	0.220846728
313.1593915	282.359127	0.13832663
166.5088044	87.47636701	0.174745199
133.8320313	85.06445313	0.167509002
339.0083113	217.2266717	0.198008856
193.6677141	92.20659859	0.237169667
381.6863656	157.1468974	0.263031008
478.848438	220.1821837	0.228790671
371.3041274	223.685735	0.183842593
56.73154362	43.21923937	0.176028634
246.8383642	197.0613437	0.147998467
600.8507776	342.1530055	0.195603438
214.3281779	248.3726438	0.141131584
511.5624032	188.4989161	0.233549058
355.5421277	151.6680851	0.245518634
654.2920073	308.0603996	0.226941652
380.9693231	236.5771924	0.177343449
396.2174147	362.1711905	0.167027831
654.7795001	308.6337919	0.197038475
146.6	156.6910569	0.111712669
1203.195873	396.4352132	0.23500235
308.5281419	234.2914418	0.192471846
685.155159	223.8213085	0.272831402
593.5239529	231.6712291	0.231397471
356.4766881	188.4381029	0.211892807
166.1158059	105.1267606	0.19844906
395.1253188	182.3981785	0.245671342
610.9408154	350.1451118	0.1965268
101.5487945	86.3869116	0.143002368
125.853114	60.05052879	0.203604253
155.3343195	117.7174556	0.172217801
395.1697158	165.2717011	0.230340092
596.0412341	203.768318	0.265047806
595.5070922	352.7761525	0.19012327
173.0652174	80.05253623	0.230628794
204.8195489	87.67969925	0.208147134
270.1579487	165.3394872	0.175773246
643.4123609	248.702843	0.242142543
463.5471371	269.4251012	0.165644856
214.5533981	152.9575243	0.194934555
1155.412531	632.2691423	0.193512739
204.3340883	154.3612684	0.18662228
369.9400749	185.506367	0.205728542
861.0922439	392.134911	0.234260435
1036.815296	543.3080152	0.183654533
114.9493671	64.7443038	0.187111375
440.8415329	322.9709995	0.165760127
125.6027607	163.7638037	0.098757572
868.01952	444.0912	0.205821152
818.49189	416.5062319	0.231539934
332.7453416	260.8385093	0.142670689
166.899923	103.5265589	0.189328505
566.560373	292.5389277	0.193396501
310.0495832	176.0820535	0.199923717
387.9136786	144.5714918	0.255152925
495.3564356	188.5669115	0.238465166
172.8062593	121.9374069	0.184054929
276.0959933	219.9590985	0.160358974
111.8893528	84.36325678	0.151306246
227.9748096	145.5975395	0.175773863
385.5224455	143.8217187	0.25986275
356.8139769	112.139266	0.303387298
206.4941634	171.4802668	0.20454738
460.210156	234.0737229	0.189622332
412.6752359	262.1343964	0.154429925
382.300533	145.2304223	0.229084884
161.2127937	155.8498695	0.10910091
347.7102725	162.2997904	0.236608091
364.9854121	195.2166302	0.221864511
197.7650273	118.1898907	0.19381557
244.4034568	160.5990123	0.181438652
118.4493865	142.3588957	0.13911146
394.0411419	145.202351	0.258409061
712.2263168	392.46093	0.209246961
455.6345455	256.0957576	0.217984945
656.6557904	325.5294118	0.192027968
418.9696084	233.3237873	0.220170813
424.7337593	183.4877529	0.179175745
105.9321872	102.5243553	0.150060116
98.24220624	88.40767386	0.112438503
757.9670939	428.908902	0.193508189

log.sigma.4.0.mm.3D_gldm_DependenceNonUniformityNormalized	log.sigma.4.0.mm.3D_gldm_DependenceVariance	log.sigma.4.0.mm.3D_gldm_LargeDependenceEmphasis
0.17895642	4.542482551	14.88407163
0.125318997	7.619178129	28.70053476
0.131528041	5.421005662	22.70795306
0.165389422	4.08569462	15.86386899
0.137362896	5.438503669	21.64864865
0.132630854	7.125858586	23.66363636
0.142249681	5.815648316	21.23075562
0.134288177	5.144766236	21.80931872
0.144450655	5.269812286	20.51517818
0.149065772	4.955015037	18.40858209
0.135375258	5.65371599	22.31830239
0.145107354	4.61632234	19.2681128
0.095088518	12.031864	42.56829138
0.135226922	6.823690194	23.5730926
0.148430387	4.919578401	18.36895161
0.158538325	4.185719472	17.37111517
0.155287886	4.399675654	17.77051926
0.111646536	8.903678182	32.71776156
0.119758738	6.648668925	28.10169492
0.151638827	4.696928009	18.43065974
0.103558	10.08558516	37.73148148
0.154317706	3.589940571	18.84986098
0.130695343	6.932479858	25.97070313
0.128072653	6.977174265	24.94106536
0.152134889	4.397544259	17.44933229
0.161117081	4.036572365	15.59772056
0.148112724	4.532195771	18.1419734
0.134433066	5.897003452	23.14265025
0.126916205	6.327983224	25.45637584
0.120174471	6.653244578	28.57059396
0.126282215	6.457610474	24.48886087
0.103590226	8.455344272	34.82696955
0.158427502	4.246321252	16.84453391
0.151294522	5.590819375	19.01446809
0.148567667	4.375636755	17.99046322
0.127032118	6.690214288	25.66888963
0.106481434	9.140902311	33.24079549
0.126870665	15.06041176	36.94380934
0.119186992	6.346909908	31.67317073
0.165501496	3.385790676	15.1562586
0.118939145	6.775728224	26.13030069
0.177180026	3.179470698	13.27204551
0.16247576	3.361311093	15.37010676
0.143278412	4.830261784	19.56993569
0.129981069	6.197312898	23.56494523
0.14394365	5.016730535	18.75664845
0.133919512	6.0064298	22.75142481
0.116588742	6.995545983	30.14810563
0.147888501	4.540280944	19.04465335
0.114892248	8.312103918	30.21893491
0.153822388	4.495673101	17.68820553
0.176814368	3.409004209	13.81637496
0.131982955	5.452237689	22.46054965
0.156761972	4.010698908	16.69565217
0.153999661	4.468899316	18.38796992
0.138542538	4.674341617	21.69948718
0.159063624	4.110566021	16.34932015
0.134050647	4.957156204	22.85656449
0.130190169	5.636698499	22.85315534
0.126565071	9.777118468	29.31043926
0.115704467	8.034659973	28.90600227
0.138554335	5.417828838	21.17902622
0.148746285	5.055823822	18.72499568
0.136011452	5.798205955	22.84861603
0.145505528	4.149072264	19.72658228
0.114148507	9.401011757	32.21387882
0.096321136	10.92597341	45.23466258
0.138883123	6.172101018	22.2336
0.139745926	5.780961297	20.64760116
0.114818958	7.967900568	31.63354037
0.12848339	5.89104783	23.69438029
0.132065355	6.229101994	23.51841492
0.136046329	5.211260414	21.33435717
0.171719203	3.469370367	14.36609119
0.158210296	4.280228184	16.76045992
0.128767704	7.152791505	25.72876304
0.115232051	7.732509803	30.04590985
0.116794732	8.027549566	30.80375783
0.133552905	6.133872414	24.02870533
0.164823619	3.493725462	14.6400171
0.179393654	4.706255951	14.92760181
0.114782748	7.786852742	27.80489161
0.140780103	4.945548255	20.9382074
0.134290672	4.970097939	23.74975594
0.156744786	4.399119407	17.29602296
0.105230283	8.73741044	38.34464752
0.145790471	5.866603378	20.0591195
0.133109195	7.170029809	23.78264041
0.135085401	6.263183433	23.44262295
0.120693065	7.002326231	26.67209877
0.090835419	14.95752569	48.32055215
0.165424493	4.098581511	15.54240134
0.137415843	5.623587614	21.40362724
0.138071074	6.576321763	22.37333333
0.150885981	4.195785575	18.84329044
0.122434135	7.868060724	26.10111046
0.150775207	4.874369809	20.40362087
0.101176874	10.02654786	36.97325692
0.11779641	6.167583918	31.33573141
0.131272444	5.975787811	23.23553862

log.sigma.4.0.mm.3D_gldm_LargeDependenceLowGrayLevelEmphasis	log.sigma.4.0.mm.3D_gldm_SmallDependenceHighGrayLevelEmphasis	log.sigma.4.0.mm.3D_gldm_LargeDependenceHighGrayLevelEmphasis
0.032296295	144.1795139	8536.749764
0.651139411	13.399632	1835.847059
0.184917034	34.86914462	4239.308996
0.102296212	75.72912783	4636.710338
0.108069644	47.67336158	5157.944257
0.317809724	40.23049131	2535.066667
0.124852595	57.09011014	4763.25783
0.054426324	98.02295045	9865.301879
0.062505309	79.7819266	8012.093489
0.03638808	141.2768997	10650.10354
0.229252636	27.07878659	3097.740053
0.081806915	65.88009722	5717.000868
0.196338344	38.95922316	10243.31976
0.085412475	76.8372102	8002.960396
0.077800585	66.34631197	5608.217238
0.093279115	63.06110995	4658.066956
0.065065766	61.3740787	6074.292295
0.519682545	19.17736236	2657.227088
0.587706552	12.64856979	2073.956086
0.126570374	59.77504453	3999.588575
0.365013499	23.26872176	4965.380952
0.338142555	19.31546057	2009.901761
0.329027016	23.22898283	3110.941406
0.155209938	41.45932902	4752.690215
0.057381119	81.55263294	6098.312647
0.043343901	129.9172903	7475.758126
0.042430916	129.3733217	9984.643675
0.099855906	52.18090018	6181.888125
0.591559304	19.24668897	1674.344519
0.137063918	36.19059069	6678.244401
0.066242666	85.58553334	10251.55359
0.097072087	44.76494809	13395.96472
0.05859675	86.24865908	6811.038712
0.056992714	98.87396011	7741.341702
0.080531944	79.08530654	5976.015895
0.078565095	69.85933788	9436.821941
0.064859797	93.10480437	18396.14673
0.091298467	110.3440715	16728.91785
0.425674845	8.482538608	2929.130894
0.138742807	61.7240887	3736.098762
0.06732962	72.06199654	11807.56284
0.056381532	98.24358812	4483.095681
0.061094661	75.53233176	5178.460443
0.084202168	56.267266	5672.895096
0.259864567	34.61096831	2813.394366
0.087902786	84.59716552	6292.079053
0.088223231	65.75574788	6738.995616
0.844623482	11.40265999	1682.734788
0.16231494	37.7010162	3265.0047
0.576591035	20.50291847	2267.431213
0.121676915	63.74490573	5085.207863
0.059818551	82.79569318	4272.694156
0.127265576	43.45547684	4916.559176
0.135998718	41.75466835	2780.859601
0.268435118	32.60788223	2306.489474
0.057534607	77.23595405	9551.375385
0.066813828	76.81200569	5330.897157
0.065257645	65.41263814	10242.4413
0.101763196	46.41593198	5762.98665
0.077813654	87.76450241	12332.26399
0.194820768	44.44180925	4959.352775
0.094716751	62.69077762	5905.810112
0.098967712	128.6383041	9777.503714
0.073976096	83.66323951	8214.108881
0.141238224	33.15635746	3458.387342
0.082044047	84.38128307	13897.17478
0.319321565	16.22373261	7813.579755
0.061797136	95.2740741	9840.21152
0.049162694	97.13283697	9778.030903
0.20368509	29.92553913	5882.951346
0.089415398	63.27774766	8349.177059
0.070978748	84.47658287	9191.259207
0.046644243	93.78285984	10808.92146
0.061238568	73.54100252	4788.042497
0.122844841	65.92784531	3837.412009
0.089093866	48.87831599	8347.887481
0.254949035	24.87982228	4429.022538
0.316386291	29.17946579	3776.875783
0.177714275	34.74934215	3987.531927
0.034845108	130.9782853	9061.548525
0.035524767	149.6138606	8562.200101
0.155353618	55.08587687	7483.287938
0.068047523	74.92629162	7692.902723
0.110950708	40.4276348	6869.127563
0.077463525	76.89022488	5884.774908
0.363351209	18.2697902	4912.276762
0.06788763	81.67840567	7830.363522
0.086142154	80.31852444	7456.902261
0.169244762	36.29807077	4180.605874
0.127582788	46.44877941	6879.767407
1.103847362	16.47176043	2893.78681
0.127638514	54.03465053	3282.116709
0.049349508	94.64835871	10552.77561
0.072575154	74.25145028	7707.911818
0.091473242	47.41885682	4956.995175
0.055687656	129.6444748	14462.96084
0.228096863	36.60382632	3728.189208
0.112262124	53.32971338	16290.06781
0.303767423	11.68554408	4158.763789
0.144999818	45.67028478	4652.082439

log.sigma.4.0.mm.3D_gldm_SmallDependenceLowGrayLevelEmphasis	log.sigma.4.0.mm.3D_gldm_LowGrayLevelEmphasis	log.sigma.4.0.mm.3D_gldzm_DistanceZoneVariabilityNormalized
0.001832034	0.003681553	1
0.005843571	0.02993874	1
0.003010612	0.009534274	1
0.002274257	0.008712315	1
0.001860908	0.006050938	1
0.00393386	0.014719595	1
0.001501033	0.006320985	0.995545679
0.00072208	0.003001639	0.998158381
0.001027516	0.003466106	1
0.001572236	0.003003368	1
0.002880482	0.013028306	1
0.001514528	0.005197933	0.992857235
0.001509122	0.005927658	1
0.001888327	0.005237314	1
0.002541554	0.006962381	1
0.001543009	0.00594366	1
0.002458915	0.005925224	1
0.004658579	0.018256178	1
0.005719785	0.024726685	1
0.00194993	0.007431439	1
0.001774797	0.011438667	1
0.004472985	0.020984454	1
0.00362603	0.014855757	1
0.002542006	0.007619845	1
0.001922627	0.004437963	1
0.001282288	0.00415486	1
0.001074462	0.003085919	1
0.001383506	0.005037816	1
0.005413771	0.02075974	1
0.001437618	0.005637915	1
0.000843535	0.003017246	1
0.001460133	0.004200254	1
0.001550606	0.00465268	0.997780247
0.001624419	0.004220025	1
0.001024822	0.00478209	1
0.001042232	0.003599288	1
0.000795457	0.002569555	1
0.000815892	0.002749499	1
0.003815213	0.018841191	1
0.00236219	0.009440147	1
0.00133541	0.003897526	1
0.001526001	0.004758484	1
0.001505498	0.004715421	0.998015875
0.001891265	0.005775565	1
0.003150146	0.011703314	1
0.001446367	0.005903897	1
0.001092242	0.004474135	1
0.004596738	0.029427607	1
0.003037064	0.01173751	1
0.00418711	0.018324168	1
0.002443611	0.009296595	1
0.001665286	0.00497329	1
0.001647365	0.006744154	0.997950822
0.003420763	0.009794716	1
0.004142067	0.015580451	1
0.000811359	0.003680354	1
0.001757898	0.005044476	0.996604424
0.001166268	0.004048188	1
0.002306322	0.006250901	1
0.000718794	0.002816436	0.999004975
0.002014967	0.007425634	1
0.001543149	0.005184123	1
0.000610586	0.003408108	0.998714653
0.000644388	0.003442059	0.993842423
0.002972006	0.009090351	1
0.000720147	0.003152373	1
0.001335182	0.010147186	1
0.000728747	0.003384345	0.997345725
0.001018555	0.003006258	1
0.00129	0.00738059	1
0.001786042	0.005480325	1
0.000917198	0.003679609	1
0.001313679	0.003120327	1
0.002323088	0.00636974	1
0.002141816	0.008200277	1
0.002261012	0.005548961	1
0.002394389	0.010912876	1
0.002680111	0.011410056	1
0.002127337	0.008494877	1
0.00152722	0.003720029	1
0.00208995	0.004272416	1
0.002373413	0.008167117	1
0.001078102	0.003727472	1
0.001393193	0.005658022	1
0.001516336	0.006085175	1
0.001833891	0.010137547	1
0.001890159	0.00540965	0.997005995
0.001465863	0.00419959	1
0.002537161	0.008991988	1
0.002927496	0.007491546	1
0.002923917	0.019593815	1
0.003430475	0.011844432	1
0.001108548	0.003110894	0.995279334
0.001364349	0.003996095	1
0.001595544	0.006002544	1
0.000922748	0.002802763	1
0.002968956	0.01372836	1
0.001292073	0.006205292	1
0.002596575	0.014135276	1
0.00205301	0.006911421	1

log.sigma.4.0.mm.3D_gldzm_LowIntensityEmphasis	log.sigma.4.0.mm.3D_gldzm_LargeDistanceEmphasis	log.sigma.4.0.mm.3D_gldzm_HighIntensitySmallDistanceEmphasis
0.005920148	1	501.3930481
0.045536011	1	89.72392638
0.015399947	1	190.5227964
0.010943715	1	303.7058824
0.009263611	1	243.0855019
0.017349614	1	181.2044199
0.007731546	1.006696429	282.5126488
0.004243488	1.002764977	521.7354839
0.004983343	1	400.9888164
0.005880878	1	599.0693069
0.016952116	1	143.1857143
0.007398715	1.010752688	318.2392473
0.011871354	1	281.55
0.009075968	1	379.1743341
0.011261259	1	278.675485
0.00743673	1	302.7235849
0.010226961	1	282.3597561
0.029082044	1	130.8847926
0.037721134	1	87.29487179
0.009152384	1	273.2593423
0.014308795	1	174.5491071
0.028143646	1	117.8657407
0.021402347	1	145.7562189
0.012317904	1	214.0650407
0.007632611	1	358.1574344
0.005662785	1	499.3278912
0.004575671	1	570.1892523
0.007325205	1	288.6971714
0.027929586	1	112.6770833
0.009398262	1	248.2910663
0.00426109	1	445.1019802
0.010127093	1	312.6207951
0.006632873	1.003333333	366.2233333
0.006592942	1	400.8718704
0.00519793	1	352.5516637
0.006132431	1	395.45053
0.004805927	1	555.539039
0.004097885	1	561.6010545
0.039335592	1	75.38815789
0.010954383	1	264.1763547
0.007001043	1	368.0566372
0.005602627	1	361.173947
0.006350858	1.002979146	331.4083913
0.008943109	1	262.2516129
0.015389814	1	179.0241379
0.00678932	1	350.2373096
0.00586404	1	341.4250239
0.036243256	1	85.3381295
0.017019593	1	192.3701923
0.023072955	1	126.2391304
0.011774967	1	276.4741379
0.006118789	1	314.4009132
0.009173434	1.003076923	233.7325641
0.014366038	1	190.4713805
0.020182761	1	160.9306358
0.005729357	1	443.9309463
0.007083029	1.005102041	319.1943027
0.007445801	1	390.4514107
0.011527048	1	239.172043
0.003708237	1.00149328	452.9432554
0.010385532	1	246.7640751
0.007301786	1	308.6410658
0.003141387	1.00192926	558.1789389
0.003899668	1.009264978	464.6772699
0.014913228	1	183.2674419
0.00496668	1	516.1772853
0.0193189	1	162.8857143
0.004071277	1.003986711	464.7450166
0.004339606	1	420.5073482
0.01003209	1	212.8402626
0.009822668	1	339.7735192
0.005425838	1	444.0541712
0.006120175	1	477.7721281
0.009220668	1	290.6193182
0.009452177	1	277.1303855
0.01157062	1	266.9726027
0.01578721	1	156.9555035
0.017457192	1	204.0764706
0.012237213	1	200.1652174
0.005715534	1	500.8185654
0.006675826	1	501.1145975
0.013242067	1	267.2720764
0.005777723	1	395.3475073
0.009253253	1	260.9254302
0.007511184	1	337.6065089
0.018595618	1	172.55
0.008289735	1.004497751	343.618066
0.006160028	1	375.615495
0.013195233	1	192.6850153
0.015490807	1	258.1639723
0.020467134	1	122.9609756
0.01501357	1	214.9155496
0.005148574	1.007097792	455.876183
0.006115288	1	342.8300733
0.008504181	1	244.469223
0.004307946	1	593.566706
0.017579947	1	209.8595318
0.012167584	1	345.4043716
0.027725114	1	104.7837838
0.010546367	1	244.5904685

log.sigma.4.0.mm.3D_gldzm_LowIntensityLargeDistanceEmphasis	log.sigma.4.0.mm.3D_gldzm_HighIntensityEmphasis	log.sigma.4.0.mm.3D_gldzm_DistanceZoneVariability	log.sigma.4.0.mm.3D_gldzm_ZonePercentage
0.005920148	501.3930481	748	0.352497644
0.045536011	89.72392638	163	0.174331551
0.015399947	190.5227964	329	0.214471969
0.010943715	303.7058824	289	0.29580348
0.009263611	243.0855019	538	0.227195946
0.017349614	181.2044199	181	0.274242424
0.007759516	282.9970238	1338.013393	0.237834012
0.004249201	522.0700461	1083.001843	0.212411903
0.004983343	400.9888164	1073	0.236031676
0.005880878	599.0693069	303	0.282649254
0.016952116	143.1857143	350	0.232095491
0.00748758	318.5645161	554.0143369	0.24208243
0.011871354	281.55	280	0.149973219
0.009075968	379.1743341	413	0.240535818
0.011261259	278.675485	567	0.28578629
0.00743673	302.7235849	1060	0.242230347
0.010226961	282.3597561	328	0.274706868
0.029082044	130.8847926	217	0.175993512
0.037721134	87.29487179	234	0.18027735
0.009152384	273.2593423	1338	0.257357184
0.014308795	174.5491071	448	0.148148148
0.028143646	117.8657407	216	0.200185357
0.021402347	145.7562189	201	0.196289063
0.012317904	214.0650407	615	0.232338496
0.007632611	358.1574344	343	0.269442262
0.005662785	499.3278912	735	0.310257493
0.004575671	570.1892523	856	0.264769564
0.007325205	288.6971714	601	0.217595945
0.027929586	112.6770833	96	0.214765101
0.009398262	248.2910663	347	0.168938656
0.00426109	445.1019802	1010	0.212274065
0.010127093	312.6207951	327	0.158047366
0.006725465	366.2533333	898.0022222	0.278724063
0.006592942	400.8718704	679	0.28893617
0.00519793	352.5516637	1142	0.259309718
0.006132431	395.45053	566	0.188729577
0.004805927	555.539039	666	0.178984144
0.004097885	561.6010545	1138	0.220499903
0.039335592	75.38815789	152	0.123577236
0.010954383	264.1763547	2030	0.279229711
0.007001043	368.0566372	565	0.217810332
0.005602627	361.173947	1282	0.331523145
0.006361166	331.6236346	1005.001986	0.275663838
0.008943109	262.2516129	620	0.249196141
0.015389814	179.0241379	290	0.226917058
0.00678932	350.2373096	788	0.287067395
0.00586404	341.4250239	1047	0.229504603
0.036243256	85.3381295	139	0.159586682
0.017019593	192.3701923	208	0.244418331
0.023072955	126.2391304	276	0.204142012
0.011774967	276.4741379	696	0.270922538
0.006118789	314.4009132	1095	0.324829427
0.009184081	233.9548718	973.0020513	0.216090426
0.014366038	190.4713805	297	0.269021739
0.020182761	160.9306358	346	0.260150376
0.005729357	443.9309463	391	0.200512821
0.007097162	319.6547619	1172.006803	0.290729295
0.007445801	390.4514107	638	0.184499711
0.011527048	239.172043	372	0.225728155
0.003715856	453.0164261	2007.000996	0.220067915
0.010385532	246.7640751	373	0.211211778
0.007301786	308.6410658	638	0.238951311
0.003148063	558.318328	1553.001286	0.268612887
0.003927758	465.4694256	1609.030883	0.212383576
0.014913228	183.2674419	172	0.217721519
0.00496668	516.1772853	722	0.186949767
0.0193189	162.8857143	140	0.107361963
0.004082951	465.0863787	1501.005316	0.2408
0.004339606	420.5073482	1565	0.267201639
0.01003209	212.8402626	457	0.157694962
0.009822668	339.7735192	287	0.220939184
0.005425838	444.0541712	923	0.215151515
0.006120175	477.7721281	531	0.232996928
0.009220668	290.6193182	704	0.31164232
0.009452177	277.1303855	882	0.281699138
0.01157062	266.9726027	292	0.217585693
0.01578721	156.9555035	427	0.178213689
0.017457192	204.0764706	170	0.177453027
0.012237213	200.1652174	345	0.202108963
0.005715534	500.8185654	711	0.303976058
0.006675826	501.1145975	733	0.368526898
0.013242067	267.2720764	419	0.232907171
0.005777723	395.3475073	682	0.208626491
0.009253253	260.9254302	523	0.170191995
0.007511184	337.6065089	676	0.277162772
0.018595618	172.55	160	0.104438642
0.008296931	344.3208396	665.0029985	0.27966457
0.006160028	375.615495	697	0.254194019
0.013195233	192.6850153	327	0.223360656
0.015490807	258.1639723	433	0.21382716
0.020467134	122.9609756	205	0.157208589
0.01501357	214.9155496	746	0.3131822
0.00517436	456.3706625	1262.014196	0.244645958
0.006115288	342.8300733	818	0.247878788
0.008504181	244.469223	991	0.227711397
0.004307946	593.566706	847	0.247516072
0.017579947	209.8595318	598	0.21228257
0.012167584	345.4043716	183	0.1747851
0.027725114	104.7837838	111	0.133093525
0.010546367	244.5904685	1238	0.214409422

log.sigma.4.0.mm.3D_gldzm_IntensityVariabilityNormalized	log.sigma.4.0.mm.3D_gldzm_LowIntensitySmallDistanceEmphasis	log.sigma.4.0.mm.3D_gldzm_IntensityVariability
0.043788784	0.005920148	32.7540107
0.073280891	0.045536011	11.94478528
0.059007215	0.015399947	19.41337386
0.051352354	0.010943715	14.84083045
0.06133829	0.009263611	33
0.061383963	0.017349614	11.11049724
0.055939759	0.007724553	75.18303571
0.047602625	0.00424206	51.64884793
0.054268589	0.004983343	58.23019571
0.058262262	0.005880878	17.65346535
0.068783673	0.016952116	24.07428571
0.057392634	0.007376499	32.02508961
0.052933673	0.011871354	14.82142857
0.04632143	0.009075968	19.13075061
0.051345458	0.011261259	29.11287478
0.054047704	0.00743673	57.29056604
0.064396193	0.010226961	21.12195122
0.063008346	0.029082044	13.67281106
0.084228212	0.037721134	19.70940171
0.050112722	0.009152384	67.05082212
0.067891024	0.014308795	30.41517857
0.073045267	0.028143646	15.77777778
0.063736046	0.021402347	12.81094527
0.061347082	0.012317904	37.72845528
0.054492601	0.007632611	18.6909621
0.047967051	0.005662785	35.25578231
0.046338654	0.004575671	39.66588785
0.060301605	0.007325205	36.24126456
0.080078125	0.027929586	7.6875
0.063990233	0.009398262	22.20461095
0.054098618	0.00426109	54.63960396
0.063752583	0.010127093	20.8470948
0.046617284	0.006609725	41.95555556
0.050431523	0.006592942	34.24300442
0.060150411	0.00519793	68.69176883
0.056761852	0.006132431	32.12720848
0.047678309	0.004805927	31.75375375
0.045581463	0.004097885	51.87170475
0.096520083	0.039335592	14.67105263
0.048854376	0.010954383	99.17438424
0.051953951	0.007001043	29.3539823
0.050235956	0.005602627	64.4024961
0.054507228	0.006348281	54.88877855
0.057122789	0.008943109	35.41612903
0.06216409	0.015389814	18.02758621
0.052590894	0.00678932	41.44162437
0.055203889	0.00586404	57.79847182
0.083898349	0.036243256	11.6618705
0.058848003	0.017019593	12.24038462
0.073802773	0.023072955	20.36956522
0.055683545	0.011774967	38.75574713
0.052877129	0.006118789	57.90045662
0.058219592	0.009170773	56.76410256
0.061048192	0.014366038	18.13131313
0.060509873	0.020182761	20.93641618
0.058745037	0.005729357	22.96930946
0.051322655	0.007079496	60.35544218
0.048491072	0.007445801	30.93730408
0.06019482	0.011527048	22.39247312
0.054241478	0.003706333	108.9711299
0.054474624	0.010385532	20.31903485
0.059909985	0.007301786	38.22257053
0.054649145	0.003139718	84.97942122
0.050942693	0.003892645	82.47621989
0.066590049	0.014913228	11.45348837
0.049443298	0.00496668	35.69806094
0.07744898	0.0193189	10.84285714
0.047762387	0.004068359	71.88239203
0.052216926	0.004339606	81.71948882
0.070744892	0.01003209	32.33041575
0.057533781	0.009822668	16.51219512
0.050129764	0.005425838	46.26977248
0.052961225	0.006120175	28.12241055
0.051257425	0.009220668	36.08522727
0.052010222	0.009452177	45.87301587
0.06004879	0.01157062	17.53424658
0.067751153	0.01578721	28.92974239
0.056401384	0.017457192	9.588235294
0.064499055	0.012237213	22.25217391
0.045740929	0.005715534	32.52180028
0.044011696	0.006675826	32.26057299
0.053992629	0.013242067	22.62291169
0.049651276	0.005777723	33.86217009
0.060399446	0.009253253	31.58891013
0.049381149	0.007511184	33.3816568
0.073984375	0.018595618	11.8375
0.049290947	0.008287936	32.87706147
0.055793532	0.006160028	38.88809182
0.063827399	0.013195233	20.87155963
0.053256458	0.015490807	23.06004619
0.078548483	0.020467134	16.10243902
0.053637272	0.01501357	40.01340483
0.052755774	0.005142127	66.89432177
0.056739857	0.006115288	46.41320293
0.060226193	0.008504181	59.68415742
0.047706399	0.004307946	40.40731995
0.052398743	0.017579947	31.33444816
0.054435785	0.012167584	9.961748634
0.08465222	0.027725114	9.396396396
0.059593748	0.010546367	73.77705977

log.sigma.4.0.mm.3D_gldzm_HighIntensityLargeDistanceEmphasis	log.sigma.4.0.mm.3D_gldzm_SmallDistanceEmphasis	log.sigma.4.0.mm.3D_glcm_SumVariance	log.sigma.4.0.mm.3D_glcm_Homogeneity1
501.3930481	1	1696.311792	0.365581598
89.72392638	1	178.6263377	0.494766352
190.5227964	1	527.2690192	0.457613043
303.7058824	1	886.5215511	0.394465496
243.0855019	1	717.3906004	0.442402855
181.2044199	1	367.0991896	0.442253847
284.9345238	0.998325893	746.3012584	0.426406607
523.4082949	0.999308756	1521.685423	0.442441424
400.9888164	1	1266.313554	0.423026071
599.0693069	1	1948.790066	0.420424075
143.1857143	1	392.2117723	0.462689193
319.8655914	0.997311828	921.4751659	0.42935698
281.55	1	814.1499304	0.514821902
379.1743341	1	1140.838251	0.430649081
278.675485	1	900.3215777	0.405791619
302.7235849	1	848.2483968	0.400507934
282.3597561	1	941.1216218	0.420565869
130.8847926	1	269.0582679	0.490050703
87.29487179	1	189.7860597	0.491085837
273.2593423	1	694.7557776	0.417673484
174.5491071	1	411.2746967	0.514690884
117.8657407	1	279.5274517	0.452755169
145.7562189	1	360.1985733	0.470848086
214.0650407	1	604.3226661	0.463578922
358.1574344	1	1141.470495	0.400045463
499.3278912	1	1488.148354	0.397844629
570.1892523	1	1797.972051	0.405966143
288.6971714	1	858.9501859	0.445607235
112.6770833	1	222.172871	0.480276853
248.2910663	1	705.8912732	0.493463025
445.1019802	1	1373.268092	0.4430451
312.6207951	1	1165.891689	0.492563285
366.3733333	0.999166667	1158.768507	0.398867408
400.8718704	1	1283.239095	0.402590656
352.5516637	1	1061.607883	0.42253081
395.45053	1	1247.207917	0.453781527
555.539039	1	1857.649983	0.477505619
561.6010545	1	1723.428424	0.442614304
75.38815789	1	216.3596673	0.517234078
264.1763547	1	728.3372549	0.395521702
368.0566372	1	1334.752055	0.446524295
361.173947	1	1024.630631	0.372424845
332.4846077	0.999255214	1041.419565	0.391621408
262.2516129	1	828.1615246	0.418389568
179.0241379	1	384.5155774	0.4603346
350.2373096	1	1078.69334	0.416261746
341.4250239	1	969.3913993	0.440820138
85.3381295	1	157.8563415	0.510737181
192.3701923	1	477.6095442	0.441629091
126.2391304	1	250.5802383	0.487362087
276.4741379	1	864.9554163	0.408013308
314.4009132	1	926.3164941	0.378501153
234.8441026	0.999230769	654.0279916	0.45187524
190.4713805	1	503.3140154	0.408829799
160.9306358	1	386.259673	0.425817987
443.9309463	1	1371.540794	0.448677919
321.4965986	0.99872449	983.8334506	0.39770519
390.4514107	1	1358.473055	0.443142736
239.172043	1	754.4837764	0.453449982
453.309109	0.99962668	1427.850563	0.444021794
246.7640751	1	578.1779095	0.477651636
308.6410658	1	883.1434173	0.432216508
558.8758842	0.999517685	1756.240747	0.417882986
468.6380482	0.997683755	1213.977068	0.44133528
183.2674419	1	480.0263867	0.446746219
516.1772853	1	1495.30253	0.473705629
162.8857143	1	518.1867766	0.542337436
466.4518272	0.999003322	1406.683311	0.428057759
420.5073482	1	1489.365808	0.442209655
212.8402626	1	583.8329175	0.499708461
339.7735192	1	1111.149262	0.440257234
444.0541712	1	1329.979203	0.444688456
477.7721281	1	1621.253539	0.43478774
290.6193182	1	926.3341969	0.381625461
277.1303855	1	700.0944234	0.402283061
266.9726027	1	923.209034	0.457989966
156.9555035	1	439.4138984	0.497348048
204.0764706	1	423.4151712	0.489986125
200.1652174	1	497.6456869	0.468347148
500.8185654	1	1737.2635	0.380629535
501.1145975	1	1693.568004	0.370886702
267.2720764	1	790.1882153	0.466486225
395.3475073	1	1226.863042	0.433118079
260.9254302	1	833.8282939	0.467496152
337.6065089	1	1019.903577	0.400186851
172.55	1	402.6076577	0.530563921
347.131934	0.998875562	1147.207941	0.407012066
375.615495	1	1063.855551	0.437830241
192.6850153	1	517.7997909	0.458048391
258.1639723	1	787.921079	0.467367334
122.9609756	1	234.6611726	0.535826751
214.9155496	1	625.3833751	0.401389975
458.3485804	0.998225552	1514.994393	0.429212173
342.8300733	1	1089.484284	0.429902861
244.469223	1	741.7671684	0.43170354
593.566706	1	1922.667202	0.443094935
209.8595318	1	528.0767239	0.444134964
345.4043716	1	1326.664639	0.493383297
104.7837838	1	323.3230607	0.517904123
244.5904685	1	652.5848495	0.448081249

log.sigma.4.0.mm.3D_glcm_Homogeneity2	log.sigma.4.0.mm.3D_glcm_ClusterShade	log.sigma.4.0.mm.3D_glcm_MaximumProbability	log.sigma.4.0.mm.3D_glcm_Idmn	log.sigma.4.0.mm.3D_glcm_SumVariance2
0.282134236	-619.3522803	0.020652921	0.984707535	109.4351351
0.433403858	117.1872354	0.07143873	0.982588989	33.70767393
0.387290081	100.8446922	0.025357312	0.988145463	59.52104569
0.313574279	-414.5415724	0.022021656	0.984105474	91.88549422
0.368466507	21.83381648	0.03277668	0.987607179	48.92153193
0.36937096	284.8513942	0.051965816	0.982563971	64.75264521
0.348617531	151.3437575	0.022678017	0.992518776	62.68585195
0.369404813	146.566446	0.027923728	0.993047858	63.32691224
0.346233192	-5.559503562	0.019497579	0.989967979	65.80141778
0.34188478	-320.8483191	0.025532451	0.99095328	65.0332494
0.392559598	3.68290522	0.029176042	0.986087343	41.04549652
0.352644372	-40.36678855	0.023885959	0.990751171	64.45791143
0.458781791	184.1922134	0.062845944	0.991687934	53.79269513
0.357173219	-119.1617953	0.035985715	0.988058683	67.98514754
0.326478835	-72.64086397	0.02819463	0.986927343	65.74813022
0.318109707	-103.2490908	0.021713986	0.987292764	72.24834281
0.342320019	-370.3206678	0.02376567	0.985322283	52.66560236
0.428309265	262.7401698	0.061317901	0.983732175	45.59663531
0.427088849	76.58010764	0.04540304	0.982278322	32.46614923
0.338386614	79.6704792	0.021865743	0.989967546	73.98461596
0.4568689	84.99999804	0.057866107	0.991520314	37.96200981
0.378953257	24.36799259	0.024366546	0.983826813	39.9865879
0.402145227	133.5582668	0.050869244	0.986642837	50.16569878
0.395086396	123.4342325	0.031931967	0.990240064	43.88923933
0.318799811	-32.96672097	0.023232873	0.984939354	49.33448209
0.316780728	-291.5049821	0.022796832	0.990260915	77.87929653
0.327778922	-546.8982502	0.023843769	0.990803739	91.53273832
0.37297616	35.42352932	0.034243401	0.98935824	50.03372121
0.413997298	152.9875706	0.0558144	0.980207145	39.89206279
0.430706024	39.92860947	0.042642072	0.991346702	36.4544587
0.370925774	9.741569071	0.026969519	0.991121573	57.8702014
0.432461355	-343.2355042	0.058168174	0.990029759	35.38829604
0.317918567	-318.9654565	0.018476041	0.989287045	90.09078215
0.32203179	-320.0240885	0.02114149	0.988400912	74.77472742
0.343730131	-101.1947736	0.019057668	0.988475	57.43959511
0.382385678	-109.4991862	0.037821665	0.990982397	47.02036363
0.414760386	-369.1435354	0.056265868	0.993155422	56.99810248
0.369588774	-188.7760401	0.054558271	0.992469129	101.5873919
0.458780888	-21.70935084	0.044826795	0.983166581	15.5006629
0.312517119	23.13419779	0.013515302	0.987556419	93.14269271
0.377360207	-466.7024667	0.040651763	0.989708857	54.98901831
0.285846721	387.9533941	0.016429742	0.989984225	92.83094464
0.308677105	-6.905221083	0.016076057	0.985903526	65.54771315
0.340594224	-115.3604323	0.028116538	0.991445009	51.05440782
0.389710874	271.7881184	0.034690152	0.988570096	51.41530822
0.339415726	-258.3372497	0.021217088	0.988532435	66.10104773
0.367037385	71.21780729	0.027393849	0.990754678	50.94044868
0.45254182	106.5989846	0.04932725	0.983440015	32.38082364
0.365843079	189.1778768	0.028097977	0.9873107	59.46327543
0.423679866	185.1176015	0.052567719	0.988744588	42.46678628
0.326435245	-117.1125398	0.017548486	0.986407325	67.91765531
0.293314205	78.15203059	0.017435138	0.987728642	72.15170229
0.379584123	90.74095102	0.027792526	0.989946757	51.73196887
0.329122815	88.40174017	0.023712363	0.981770386	47.34113721
0.348982427	125.5697043	0.023793245	0.982228881	63.01833509
0.375743297	-21.59535214	0.035333141	0.992780278	43.37480238
0.314813021	-141.9961654	0.016512768	0.9871185	69.16693373
0.371212471	-477.2231843	0.03438932	0.990090435	63.28900762
0.382454657	-36.96013252	0.037970892	0.987017148	32.45578032
0.371772809	34.99068742	0.031997249	0.991932996	64.7963355
0.413188063	356.3255296	0.056363458	0.991673584	53.50110147
0.355988178	-35.30169005	0.027186897	0.990227322	60.63327349
0.340054556	-108.3580136	0.023297832	0.992053135	71.44759483
0.368244883	344.6218326	0.026848484	0.992535551	67.19717751
0.371782296	67.43055352	0.028684858	0.986729718	36.46661506
0.407310157	15.18288692	0.047365462	0.9932137	60.41232954
0.492636696	0.785185661	0.079559612	0.990183087	23.48921289
0.352974357	-3.146639539	0.032174644	0.99247686	73.19656618
0.369261467	-194.5049242	0.024652546	0.99285014	56.15765569
0.438518951	34.04066044	0.040142864	0.990059009	34.51326251
0.366447894	-334.7959089	0.034190229	0.988467176	54.9281567
0.37261532	125.7445555	0.030894058	0.991238854	70.30410025
0.360076603	-359.4628483	0.027749701	0.99037702	57.58001234
0.298085202	-286.3620557	0.018977746	0.982972233	70.47938991
0.32126242	231.6655804	0.02191299	0.987727716	86.14349177
0.388052602	-279.7467958	0.048582484	0.987312421	47.73531692
0.436072042	21.51961475	0.036171328	0.989388231	30.79200332
0.426294706	356.9745478	0.050158945	0.990871281	56.23448293
0.399091316	55.54596728	0.036110846	0.9886676	40.62297158
0.297184762	-1280.240413	0.018887008	0.98732653	117.9504708
0.288592761	-873.3267609	0.021564453	0.984884446	116.3914795
0.400273257	-152.2039725	0.041418531	0.992094596	42.49651368
0.357505293	-20.72636165	0.026637131	0.990907134	59.46823612
0.398294033	-251.7947295	0.03084269	0.991581206	49.09123636
0.317736287	-164.9719501	0.019816629	0.990103862	86.50245474
0.476201777	163.4587477	0.055659076	0.991431358	39.2461041
0.33039562	-554.6974844	0.027699383	0.985513972	79.50638717
0.364480295	55.25121274	0.032551518	0.990400321	56.18503697
0.386035693	41.86909454	0.035780186	0.988173614	47.80868219
0.399700561	-64.33155199	0.033681104	0.990426846	52.74389801
0.484436356	230.5909814	0.096530841	0.984545432	47.99695001
0.319673871	92.40381357	0.018025703	0.984450575	79.95757049
0.353897813	-278.007796	0.024335921	0.990964694	58.87925727
0.35445114	-80.20281673	0.031974917	0.98895094	49.90396212
0.355031883	-75.81115228	0.020990222	0.989941527	50.68199055
0.372366728	-200.2160454	0.033935548	0.991843311	87.9570257
0.369825961	-2.070241692	0.025860559	0.989002837	74.00166428
0.435068725	-944.6609886	0.055629809	0.988525258	65.44822685
0.461603475	-29.38383832	0.043113951	0.98649372	26.76930538
0.376565807	145.2619805	0.033150514	0.986975925	49.99383216

log.sigma.4.0.mm.3D_glcm_Contrast	log.sigma.4.0.mm.3D_glcm_DifferenceEntropy	log.sigma.4.0.mm.3D_glcm_InverseVariance	log.sigma.4.0.mm.3D_glcm_Entropy	log.sigma.4.0.mm.3D_glcm_Dissimilarity
22.28131823	3.305452499	0.280596016	8.22738741	3.471176767
6.149720468	2.447082402	0.414112112	6.401661414	1.791842295
8.445152447	2.66620082	0.3750013	7.277413983	2.126601535
15.34386364	3.042971604	0.319939668	7.723067607	2.880157839
9.612793038	2.750456076	0.362842453	7.260966128	2.259872297
10.83135761	2.787405389	0.346374381	7.00665131	2.388895811
10.57966216	2.830659601	0.340686041	7.570912332	2.420225486
9.862444167	2.769826611	0.366623865	7.408438849	2.277565703
12.14314497	2.896290948	0.339418471	7.677945311	2.539393619
11.57232369	2.861876788	0.336221117	7.31580457	2.506904352
7.101678655	2.562015044	0.384403174	6.942821119	2.001984647
9.820737973	2.792603909	0.351442139	7.465490408	2.353892841
7.392452358	2.528538612	0.403138527	6.64882149	1.827138529
13.03966502	2.929001741	0.346923326	7.427823891	2.556900044
14.13773544	3.00630006	0.321895832	7.6004368	2.767053935
12.82545618	2.919290269	0.317128768	7.741009003	2.704022747
11.40234154	2.850965665	0.339381928	7.216621108	2.497841887
7.935381481	2.574715229	0.398118415	6.646726454	1.946969765
5.4727028	2.393175423	0.408226212	6.473055043	1.755663399
10.6804152	2.844892465	0.339073808	7.72901617	2.469675449
5.533284055	2.408779656	0.414274031	6.635114601	1.677892202
6.884828101	2.535332214	0.379447528	6.870104622	2.010674641
6.82719512	2.545279639	0.386640513	6.87699625	1.946517068
8.601873653	2.684362685	0.377000394	7.095804216	2.116628902
14.54539473	2.984466992	0.316492679	7.391368621	2.801291881
14.69548128	3.027279084	0.319886866	7.746812613	2.820873532
15.43320194	3.035540287	0.329512543	7.84647937	2.812144711
9.374960053	2.755449746	0.363075036	7.2332827	2.251856769
6.131283952	2.45724929	0.395225589	6.354808325	1.864064268
6.102274114	2.470740246	0.406035081	6.71393329	1.796321771
10.72020764	2.816788319	0.35939173	7.45072125	2.352083993
8.972625743	2.636944227	0.396476939	6.500234995	2.010037042
14.50605	3.024014246	0.317998964	7.955169602	2.817488085
14.05428336	3.004136935	0.314392681	7.708268626	2.786184264
10.16098854	2.808389249	0.342763244	7.489791513	2.405498611
9.094370737	2.701508837	0.36905016	7.095336224	2.178668602
11.04026884	2.723808196	0.387965926	6.979483863	2.180375084
13.81426038	2.973201449	0.315484774	7.82715454	2.600765318
4.014744072	2.217606245	0.432305283	5.897368414	1.522066884
13.38417285	2.989223603	0.316326759	8.099108465	2.75764734
12.59919508	2.880877027	0.362208211	7.207665232	2.45424023
16.68230785	3.119475925	0.29143921	8.114947664	3.085514863
14.35455457	3.000392164	0.314444643	7.792886027	2.836820927
12.18818101	2.912534218	0.338665699	7.409417139	2.558870482
7.522730123	2.600703236	0.380532119	6.919642537	2.039185327
13.14427153	2.943588558	0.340297008	7.623952704	2.634442059
9.886520251	2.780144586	0.357199087	7.340630924	2.305525644
4.521970785	2.256212041	0.436608817	6.267323397	1.589022669
8.311773333	2.665446541	0.359124757	7.186994069	2.185078794
6.839823607	2.510612303	0.39597956	6.643999284	1.889465538
12.08563598	2.920883477	0.321255884	7.676125813	2.614326652
15.76519248	3.094881774	0.300344079	7.955555219	3.000770767
8.21924925	2.675684284	0.371577977	7.268786733	2.132521456
12.31385098	2.885554448	0.334769672	7.285831626	2.614523419
11.03404287	2.841848482	0.343158991	7.390973809	2.457299925
8.650569174	2.682567119	0.369871496	7.027571777	2.166667706
12.97784674	2.966023607	0.313234502	7.81428064	2.735860494
10.74363507	2.786069812	0.369727178	7.300321987	2.321103092
9.401721018	2.72948917	0.3707392	6.906322963	2.1984675
11.46273647	2.88817669	0.340292067	7.647765353	2.43259002
9.010455839	2.668096283	0.386930983	6.954533328	2.069548999
9.756365961	2.786299941	0.350183111	7.410837586	2.342151538
12.53416843	2.940126889	0.334489583	7.737721721	2.59080837
10.02218211	2.773536919	0.363199697	7.458985372	2.304264169
8.0815159	2.596904722	0.362896775	6.796608447	2.138695311
9.159569166	2.702214976	0.37397774	7.158483679	2.109291068
5.097997333	2.306990198	0.43778953	6.111189994	1.531565616
12.50289502	2.922008106	0.343501172	7.70367861	2.534979182
10.13491033	2.793264979	0.361051441	7.415071555	2.311038959
6.02983272	2.457236974	0.408753053	6.681711514	1.766711602
11.04708723	2.801009748	0.35149291	7.12212781	2.374986273
10.62589217	2.802029055	0.36308952	7.519526068	2.322005606
11.00899806	2.823353678	0.353719343	7.278098499	2.39297239
16.40592553	3.106568639	0.304423837	7.856542738	3.023806566
13.24360366	2.965547116	0.323567494	7.894357671	2.714067594
9.861054262	2.745812623	0.362562853	6.993481636	2.230761597
6.504059503	2.482947367	0.407962362	6.65106367	1.806610501
6.415977965	2.498672663	0.395542285	6.76027899	1.85144767
6.863742902	2.551196413	0.388525807	6.893317419	1.950437482
18.34792932	3.162323654	0.296495712	8.02654277	3.135618689
22.0825741	3.292593809	0.287027457	8.183506207	3.420756894
9.545957896	2.727457097	0.38149079	6.905235468	2.166224789
11.0092423	2.808124529	0.348925605	7.415771168	2.402653261
6.844193501	2.535545409	0.391109816	7.021819343	1.950558246
13.35659435	2.969788977	0.314531726	7.878964152	2.735393818
4.324224753	2.256895454	0.433242383	6.292559744	1.510220009
15.84459731	3.058598674	0.330431458	7.748602944	2.857228459
10.95178742	2.843368334	0.35444818	7.391529402	2.393047401
7.746751969	2.63095342	0.366371385	7.031459872	2.083305157
8.475585544	2.657253387	0.382102738	7.120981273	2.080667797
6.083624664	2.394575165	0.412708545	6.07851803	1.669962504
12.93113791	2.968755668	0.319159726	7.833557768	2.710237184
11.54953182	2.878850416	0.347004695	7.497614017	2.470853867
11.13656098	2.856836357	0.345592034	7.362779724	2.450267598
9.428954486	2.756802688	0.355334939	7.382865111	2.306616462
13.03005284	2.915722867	0.351851479	7.621710617	2.49536068
8.360025487	2.684347714	0.364482546	7.500694698	2.18091422
10.39169657	2.698693691	0.389405819	6.748557992	2.123638553
4.623688102	2.290496704	0.434341472	6.269668477	1.581851613
10.1733926	2.775888401	0.365543582	7.30042746	2.279677898

log.sigma.4.0.mm.3D_glcm_DifferenceVariance	log.sigma.4.0.mm.3D_glcm_Idn	log.sigma.4.0.mm.3D_glcm_Idm	log.sigma.4.0.mm.3D_glcm_Correlation	log.sigma.4.0.mm.3D_glcm_Autocorrelation
9.827218132	0.919454374	0.282134236	0.664371031	532.1333203
2.801903251	0.915548153	0.433403858	0.687974727	74.37811324
3.766940633	0.928588498	0.387290081	0.750851395	189.1860374
6.777041386	0.917783775	0.313574279	0.715006382	297.3120453
4.375469777	0.927140968	0.368466507	0.670859807	244.8353612
4.70274711	0.915531052	0.36937096	0.7115079	136.4083772
4.531332137	0.941261974	0.348617531	0.712090318	255.5295942
4.562687634	0.944638888	0.369404813	0.729399059	478.2883126
5.394338212	0.934139973	0.346233192	0.689786409	407.0714677
5.079954681	0.936379555	0.34188478	0.697021121	596.8398769
2.918739913	0.921153448	0.392559598	0.703584714	145.613087
4.175572125	0.934637779	0.352644372	0.735663334	307.3879965
3.925944927	0.944306033	0.458781791	0.757491419	271.4352507
6.289899519	0.930522686	0.357173219	0.677682312	369.1910414
6.232699237	0.924938479	0.326478835	0.647249965	299.3913325
5.021437051	0.92390878	0.318109707	0.700828443	286.2008842
4.909356995	0.920261624	0.342320019	0.64260614	308.815551
3.964911149	0.921390913	0.428309265	0.696438304	104.2417689
2.224942705	0.912086363	0.427088849	0.711386955	78.81001677
4.411105309	0.931694011	0.338386614	0.747648265	241.3633642
2.64009459	0.940385361	0.4568689	0.741944281	150.9266235
2.711613248	0.913572217	0.378953257	0.70447842	109.4565704
2.926995381	0.923201197	0.402145227	0.757117301	135.5954143
3.990263533	0.935607209	0.395086396	0.671720213	210.2820995
6.254438156	0.91972455	0.318799811	0.54484101	366.7755592
6.465741998	0.934354391	0.316780728	0.678600258	470.3119138
7.248472877	0.937796565	0.327778922	0.712311312	559.375887
4.17149528	0.931711737	0.37297616	0.684159006	286.2369313
2.484479091	0.907434582	0.413997298	0.734619246	88.77657167
2.766148684	0.938577014	0.430706024	0.713372159	239.4155873
4.979692366	0.938657611	0.370925774	0.68783868	435.6994033
4.781499112	0.939399345	0.432461355	0.597184296	366.5679339
6.297010789	0.931155038	0.317918567	0.722883609	378.3188343
5.954166476	0.928234345	0.32203179	0.683744887	411.6915313
4.210454408	0.927194909	0.343730131	0.699085459	346.8821274
4.179294236	0.937686618	0.382385678	0.675473543	397.2894067
6.035266479	0.950128978	0.414760386	0.676004282	567.1123653
6.661408556	0.944747582	0.369588774	0.761735114	541.1674232
1.624659609	0.913167805	0.458780888	0.588134244	85.25113977
5.566880502	0.924732383	0.312517119	0.748043846	252.5796958
6.366316	0.936812496	0.377360207	0.627435113	420.9040812
6.80095855	0.931713961	0.285846721	0.697412921	339.0960446
5.853224615	0.920787006	0.308677105	0.644663597	341.7844606
5.461226659	0.938419451	0.340594224	0.614794631	276.6968042
3.256443759	0.928490087	0.389710874	0.743332842	142.8021094
5.953591642	0.930204993	0.339415726	0.669019902	352.0184674
4.41138847	0.936127174	0.367037385	0.674139768	319.0321795
1.869112216	0.915070653	0.45254182	0.754067211	67.63692662
3.399135494	0.923723816	0.365843079	0.75425076	173.2116205
3.038662637	0.931144927	0.423679866	0.716681658	99.32547895
5.068274785	0.921737083	0.326435245	0.696119249	290.8535857
6.512312361	0.925029289	0.293314205	0.643580985	308.6908529
3.556135006	0.932711746	0.379584123	0.724200941	226.8419638
5.129256654	0.911201121	0.329122815	0.587572199	179.1581228
4.775383287	0.912991003	0.348982427	0.700973794	144.2423899
3.826753896	0.942716951	0.375743297	0.665667287	431.6091555
5.222230098	0.92303648	0.314813021	0.684520638	325.8741877
5.147655654	0.936163776	0.371212471	0.71061535	430.3216169
4.458326878	0.926766435	0.382454657	0.549483911	251.8548507
5.362616929	0.941405875	0.371772809	0.699973511	452.9619768
4.605957302	0.942666345	0.413188063	0.709417161	201.8162034
4.133115254	0.933089243	0.355988178	0.723246676	295.2067852
5.656455441	0.940625129	0.340054556	0.701512331	546.362365
4.510032372	0.942631666	0.368244883	0.738658741	391.0052696
3.318015672	0.92250919	0.371782296	0.634023486	171.1708102
4.563165641	0.947435168	0.407310157	0.737937967	469.4184412
2.675738503	0.938998015	0.492636696	0.6425938	179.5677878
5.893558738	0.94330102	0.352974357	0.707828277	447.5540886
4.640166355	0.943907543	0.369261467	0.693431109	468.4905042
2.797979442	0.935144432	0.438518951	0.700264585	202.7858683
5.101924527	0.930923015	0.366447894	0.669033752	357.9712697
5.092110169	0.939433761	0.37261532	0.737054433	425.5136919
5.093417444	0.935956658	0.360076603	0.677831946	503.9608154
6.961229361	0.91395827	0.298085202	0.623450783	307.687187
5.612225899	0.925943792	0.32126242	0.733621954	243.0275912
4.678670334	0.928429884	0.388052602	0.658702235	303.2949677
3.124146692	0.934041363	0.436072042	0.644253738	158.4920984
2.87440876	0.936866212	0.426294706	0.793955276	155.1478007
2.965634012	0.928703049	0.399091316	0.71003645	177.84581
8.125084481	0.926315265	0.297184762	0.731526965	543.1167737
9.960561722	0.920657442	0.288592761	0.682932583	531.5346982
4.729940214	0.943274537	0.400273257	0.631269984	263.198187
4.970756914	0.937357311	0.357505293	0.688365413	393.8853487
2.882414465	0.937819042	0.398294033	0.755259747	279.1427606
5.602317585	0.932757691	0.317736287	0.731375545	337.9046158
1.985796269	0.938955928	0.476201777	0.797904497	147.1179385
7.387219687	0.923275986	0.33039562	0.667250738	371.7982644
5.088028169	0.935942439	0.364480295	0.673698172	346.8198045
3.271665208	0.9270606	0.386035693	0.720203121	184.7789966
4.009939934	0.936682365	0.399700561	0.722362556	266.1643698
3.029825761	0.925461653	0.484436356	0.771900799	91.7619918
5.390798029	0.916731298	0.319673871	0.721062343	220.0896737
5.271426718	0.937385265	0.353897813	0.671570259	475.6772276
4.923162359	0.930660954	0.35445114	0.634620494	352.9786244
3.956925631	0.93199861	0.355031883	0.685714944	252.7929496
6.596290675	0.943205482	0.372366728	0.741544355	593.4121158
3.463113152	0.928917032	0.369825961	0.796659183	190.6359878
5.598340243	0.936731444	0.435068725	0.729164685	416.2889231
2.018204358	0.92391146	0.461603475	0.703760591	121.9600005
4.794205908	0.926920444	0.376565807	0.660814713	225.2539784

log.sigma.4.0.mm.3D_glcm_SumEntropy	log.sigma.4.0.mm.3D_glcm_AverageIntensity	log.sigma.4.0.mm.3D_glcm_Energy	log.sigma.4.0.mm.3D_glcm_SumSquares	log.sigma.4.0.mm.3D_glcm_ClusterProminence
5.346010729	22.71512494	0.005436015	31.8788152	42511.24156
4.392835309	8.21488735	0.020385133	9.964348599	3212.637003
4.937316804	13.28442525	0.009073507	17.05811183	9358.485834
5.168449784	16.7574952	0.006977437	27.44041403	25053.89161
4.805311166	15.34549284	0.010193909	14.80113187	7432.006828
4.786200334	11.08654163	0.013298193	18.89600071	10197.60075
4.99894093	15.64658857	0.007940804	18.60982608	13495.12482
4.935613988	21.62115015	0.010175471	17.9165723	15783.61232
5.033193057	19.93127206	0.007328395	19.35805597	14167.63498
4.90840418	24.19039283	0.008692985	18.79546759	19444.97757
4.681341761	11.70969265	0.011475193	12.03679379	4983.343073
5.00220913	17.20586456	0.00847771	18.93370289	12995.9661
4.664321637	16.15394233	0.02131146	15.68276664	12789.26255
4.952643129	18.92926415	0.01005727	20.33028784	19925.16321
4.963171186	16.99661724	0.008967837	20.32379719	17688.03071
5.088567783	16.54528817	0.00709678	21.77287542	15722.94638
4.747738559	17.29463801	0.009841856	16.17361118	11851.36239
4.52699254	9.737228067	0.017941128	13.3830042	6222.434305
4.435645866	8.488452471	0.015968299	9.484713008	2564.548606
5.120615073	15.06512152	0.007120011	21.60627912	15938.31012
4.580022871	11.95051164	0.017677765	10.87382347	4620.407506
4.641105022	10.0585484	0.011040947	11.717854	4065.368128
4.731839042	11.1684473	0.013962883	14.24822347	7293.560852
4.714003864	14.20528997	0.011573953	13.27379542	6867.37513
4.799357341	19.02158021	0.008764419	16.30478416	8519.51016
5.084689746	21.40704444	0.007620531	22.64096841	27942.20072
5.181907944	23.33467943	0.007870998	26.65520136	40921.0966
4.789521595	16.64915948	0.011005253	15.15793268	8608.552097
4.425111225	8.962554408	0.018372607	11.50583668	3806.379774
4.578371968	15.22914423	0.015566101	10.68805325	4682.172428
4.909730773	20.66555099	0.009420406	16.92869714	12083.79594
4.322658349	19.00709836	0.021385659	11.23694781	9664.427006
5.226353257	19.05302887	0.006360861	25.94830063	26727.8874
5.062440605	19.99187088	0.007561741	22.03635887	21761.60084
4.920943286	18.36073453	0.007911213	17.2608502	11237.05191
4.741830547	19.77985323	0.01147012	14.18224461	9155.648149
4.709234007	23.62164439	0.017146329	16.84008029	23422.08906
5.300912716	22.88386083	0.0093174	28.6633481	34109.60164
3.98032631	9.076133217	0.0225044	4.878851744	764.0288551
5.302072999	15.31158781	0.005239049	27.10815628	24866.24138
4.738398694	20.35187519	0.013457627	16.60621156	17634.542
5.255032509	18.03873951	0.005346556	27.45763718	33614.04127
5.037146945	18.25420569	0.006557898	20.32569968	13422.00666
4.802445832	16.44871673	0.009446633	16.02186066	10988.46114
4.712147736	11.48042368	0.013040128	14.73450959	7920.22909
4.990687895	18.51758258	0.007853605	20.01907003	19413.36128
4.837685963	17.63539745	0.010063777	15.49824791	9318.048218
4.377055036	7.78851488	0.019069711	9.225698606	2646.642991
4.882943691	12.66494356	0.009573862	16.94376219	10537.47542
4.591299176	9.508592576	0.015971546	12.32665247	5831.354161
5.04884722	16.67934081	0.006959874	20.37973615	14552.36351
5.101553378	17.29502123	0.00578866	22.28782408	15908.77281
4.849681248	14.71262483	0.010324637	15.22744901	8565.787058
4.754156952	13.05356429	0.008944349	14.91374705	6080.334904
4.933941494	11.4546608	0.00832218	18.51309449	9211.815146
4.686242707	20.62868457	0.012156258	12.77467352	7285.458207
5.073922987	17.7333832	0.006385145	20.86935371	15012.77449
4.861466154	20.51511386	0.011345821	18.36592241	22701.28772
4.504027493	15.69235125	0.01330989	10.51985454	3992.741861
5.015115561	21.04246314	0.008382825	18.62863791	14730.57088
4.712373175	13.84644068	0.015611128	16.03542513	10723.75426
4.935521442	16.8815447	0.008739587	18.0653845	10519.47386
5.068163171	23.10876833	0.007600745	20.89244323	21440.79109
4.953840556	19.48132464	0.009755942	19.14211891	17060.43497
4.557695466	12.80759133	0.011995874	11.13703274	4006.834326
4.856704319	21.41244488	0.014065954	17.0780169	17317.20756
4.213636367	13.2273914	0.026253569	7.146802555	2672.411208
5.07061276	20.87846299	0.008974276	21.13532091	22161.32351
4.896952422	21.44745896	0.009380509	16.08443379	13264.27211
4.538407126	13.98768189	0.014794754	10.13577381	3507.469122
4.756712394	18.72120042	0.011220482	16.85927937	15950.71025
5.034366181	20.31517425	0.009437674	20.1338681	17404.52643
4.833176542	22.25962213	0.010218169	16.74689338	17548.26293
5.048005059	17.22751007	0.006564742	22.15233384	16874.47508
5.208944053	15.05320768	0.006360452	25.39564989	22734.94321
4.694561707	17.15249217	0.01336429	14.51315488	9883.125394
4.477523677	12.34530787	0.015135793	9.324015705	3168.386244
4.728995284	11.94900209	0.016363714	15.75180474	11699.27098
4.653429081	13.0150421	0.0127225	11.87167862	4905.848078
5.288738839	22.85397717	0.006353179	33.35515615	57525.13421
5.362102374	22.66284837	0.005685233	33.52111758	51791.17919
4.591101317	16.01437601	0.01526422	13.27527649	10603.21286
4.910965245	19.62372363	0.009814589	17.55852192	14576.95206
4.763836434	16.39962569	0.012149141	14.11172263	10513.46752
5.204609698	17.96787575	0.006557012	24.9901506	26928.88518
4.46558964	11.76352966	0.021311326	10.89258221	4511.29615
5.054367017	18.97345334	0.00837328	23.95086391	25541.48083
4.890043076	18.41863811	0.0098026	16.96341934	10090.56515
4.760542587	13.21904096	0.011781933	13.88885854	6919.420243
4.829854403	15.99284017	0.011982355	15.53309271	10330.28715
4.36938593	9.014742521	0.029431969	13.52014367	4987.199422
5.165146455	14.28946788	0.006283176	23.65273184	18155.14717
4.914968008	21.61689981	0.009176012	17.14842912	15917.38666
4.813508737	18.62394104	0.009949136	15.57781526	9371.906021
4.855150659	15.59794249	0.008713292	15.28452803	8086.782208
5.111374317	24.04015754	0.009615217	25.16020909	33483.88469
5.089938889	13.20710937	0.008438884	20.73077177	16760.27575
4.612740765	20.12127841	0.017741997	19.06877415	30047.11836
4.359099535	10.78985025	0.018517253	7.848248371	2339.210139
4.798297597	14.68544284	0.010061944	15.18975456	6958.145266

log.sigma.4.0.mm.3D_glcm_SumAverage	log.sigma.4.0.mm.3D_glcm_Imc2	log.sigma.4.0.mm.3D_glcm_Imc1	log.sigma.4.0.mm.3D_glcm_DifferenceAverage	log.sigma.4.0.mm.3D_glcm_Id
45.18127129	0.871396236	-0.164623095	3.471176767	0.365581598
16.4297747	0.860199751	-0.201016863	1.791842295	0.494766352
26.56126372	0.891800501	-0.206890498	2.126601535	0.457613043
33.35427084	0.908462624	-0.207025155	2.880157839	0.394465496
30.65973943	0.841694302	-0.16349872	2.259872297	0.442402855
22.17308325	0.909141459	-0.232318931	2.388895811	0.442253847
31.14404674	0.841866029	-0.159903187	2.420225486	0.426406607
43.12344261	0.859072694	-0.172024479	2.277565703	0.442441424
39.68062179	0.834181425	-0.157295802	2.539393619	0.423026071
48.30925726	0.890352442	-0.199786187	2.506904352	0.420424075
23.4193853	0.85143666	-0.182806372	2.001984647	0.462689193
34.27525915	0.87463583	-0.181832101	2.353892841	0.42935698
32.23823942	0.907305808	-0.240677057	1.827138529	0.514821902
37.70585673	0.876883626	-0.186506143	2.556900044	0.430649081
33.85132374	0.843374053	-0.158651543	2.767053935	0.405791619
32.94442798	0.84918394	-0.163277654	2.704022747	0.400507934
34.55380839	0.833346752	-0.159799067	2.497841887	0.420565869
19.47445613	0.873548904	-0.206302455	1.946969765	0.490050703
16.97690494	0.842648918	-0.186799943	1.755663399	0.491085837
30.0350867	0.862893575	-0.170791849	2.469675449	0.417673484
23.90102327	0.869276318	-0.203238124	1.677892202	0.514690884
20.11709681	0.856589012	-0.184231481	2.010674641	0.452755169
22.3368946	0.8969679	-0.220746341	1.946517068	0.470848086
28.38671222	0.831281441	-0.161958763	2.116628902	0.463578922
37.84427106	0.821281659	-0.153944116	2.801291881	0.400045463
42.63639218	0.856668159	-0.166785415	2.820873532	0.397844629
46.49033932	0.873344949	-0.175689156	2.812144711	0.405966143
33.23036899	0.832324879	-0.158188209	2.251856769	0.445607235
17.92510882	0.894111055	-0.229655308	1.864064268	0.480276853
30.45158967	0.852746027	-0.185988332	1.796321771	0.493463025
41.17776722	0.834710317	-0.159508166	2.352083993	0.4430451
37.94528982	0.785094289	-0.145988414	2.010037042	0.492563285
37.91582581	0.865735692	-0.169582007	2.817488085	0.398867408
39.82412119	0.85856053	-0.167378185	2.786184264	0.402590656
36.6085642	0.827455354	-0.151353087	2.405498611	0.42253081
39.3850671	0.840409908	-0.168756377	2.178668602	0.453781527
47.14300122	0.857403714	-0.185782048	2.180375084	0.477505619
45.57210505	0.915287828	-0.218741309	2.600765318	0.442614304
18.15226643	0.733527616	-0.133589731	1.522066884	0.517234078
30.50496186	0.856058689	-0.160319927	2.75764734	0.395521702
40.51176818	0.82088999	-0.15157739	2.45424023	0.446524295
35.77985886	0.842190785	-0.151221178	3.085514863	0.372424845
36.27566243	0.8091771	-0.139378529	2.836820927	0.391621408
32.67896369	0.795182553	-0.13477686	2.558870482	0.418389568
22.96084736	0.875863517	-0.198186271	2.039185327	0.4603346
36.81142386	0.845108704	-0.160002405	2.634442059	0.416261746
35.14291677	0.818319538	-0.150489308	2.305525644	0.440820138
15.57702976	0.871814769	-0.218889917	1.589022669	0.510737181
25.32988713	0.907946191	-0.222196514	2.185078794	0.441629091
19.01718515	0.876925641	-0.215546395	1.889465538	0.487362087
33.2777863	0.84682438	-0.159907702	2.614326652	0.408013308
34.32670795	0.807118133	-0.130975796	3.000770767	0.378501153
29.38873054	0.842173592	-0.165874602	2.132521456	0.45187524
26.10712859	0.832834335	-0.157055882	2.614523419	0.408829799
22.9093216	0.876053527	-0.187804284	2.457299925	0.425817987
41.13004141	0.839212308	-0.168388458	2.166667706	0.448677919
35.31653554	0.820178178	-0.143080426	2.735860494	0.39770519
40.84971461	0.850696825	-0.171646886	2.321103092	0.443142736
31.37442667	0.779846531	-0.133492766	2.1984675	0.453449982
41.93419711	0.834883575	-0.154156633	2.43259002	0.444021794
27.61794762	0.878352219	-0.199829267	2.069548999	0.477651636
33.61355568	0.860954157	-0.174187063	2.342151538	0.432216508
46.11404098	0.838142873	-0.152772217	2.59080837	0.417882986
38.81759249	0.847797111	-0.170405981	2.304264169	0.44133528
25.61518266	0.852290617	-0.186773506	2.138695311	0.446746219
42.73627178	0.876095162	-0.193434187	2.109291068	0.473705629
26.45478279	0.835886776	-0.188903283	1.531565616	0.542337436
41.58676487	0.847020247	-0.160521165	2.534979182	0.428057759
42.75375173	0.827070392	-0.153194745	2.311038959	0.442209655
27.97536378	0.840132988	-0.179371062	1.766711602	0.499708461
37.25452459	0.868806898	-0.187909518	2.374986273	0.440257234
40.52547086	0.875770315	-0.184477922	2.322005606	0.444688456
44.37618974	0.847689933	-0.168278929	2.39297239	0.43478774
34.30208243	0.807420419	-0.133699118	3.023806566	0.381625461
29.98582025	0.870764915	-0.174849216	2.714067594	0.402283061
34.28139381	0.850304327	-0.178234785	2.230761597	0.457989966
24.69061574	0.822024035	-0.169434579	1.806610501	0.497348048
23.88943488	0.921631513	-0.253168337	1.85144767	0.489986125
26.03008421	0.85477439	-0.180957134	1.950437482	0.468347148
45.52637468	0.8844224	-0.181471358	3.135618689	0.380629535
45.07564748	0.886422793	-0.177467518	3.420756894	0.370886702
31.9347686	0.828528746	-0.161042796	2.166224789	0.466486225
39.07720116	0.852839304	-0.172772265	2.402653261	0.433118079
32.77510217	0.857227328	-0.186006445	1.950558246	0.467496152
35.75378923	0.878931842	-0.181884753	2.735393818	0.400186851
23.52705933	0.901287758	-0.243481056	1.510220009	0.530563921
37.72914938	0.843996401	-0.158747685	2.857228459	0.407012066
36.63239636	0.847485039	-0.164317659	2.393047401	0.437830241
26.43808192	0.875568582	-0.1948423	2.083305157	0.458048391
31.94202344	0.878532235	-0.197870535	2.080667797	0.467367334
18.02948504	0.905811363	-0.26170874	1.669962504	0.535826751
28.51714256	0.869805803	-0.171692983	2.710237184	0.401389975
43.07394097	0.816059492	-0.145491433	2.470853867	0.429212173
37.05555778	0.801383677	-0.141025743	2.450267598	0.429902861
31.14341181	0.815766429	-0.147565761	2.306616462	0.43170354
47.9437721	0.896160922	-0.200290668	2.49536068	0.443094935
26.39848354	0.89899199	-0.206780881	2.18091422	0.444134964
40.12491425	0.898851387	-0.226731848	2.123638553	0.493383297
21.57970051	0.8580504	-0.204852456	1.581851613	0.517904123
29.34573843	0.808135979	-0.147073051	2.279677898	0.448081249

log.sigma.4.0.mm.3D_glcm_ClusterTendency	log.sigma.4.0.mm.3D_firstorder_InterquartileRange	log.sigma.4.0.mm.3D_firstorder_Skewness	log.sigma.4.0.mm.3D_firstorder_Uniformity
109.4351351	158.2417586	-0.659853532	0.056351441
33.70767393	93.69926453	0.599047114	0.104425062
59.52104569	145.5308285	0.102816069	0.067532284
91.88549422	150.1585808	-0.431987083	0.060837334
48.92153193	116.7189212	0.020953828	0.078528037
64.75264521	156.1236472	0.567223415	0.077956841
62.68585195	143.0435591	0.329404952	0.067665324
63.32691224	127.6200609	0.113070057	0.074191895
65.80141778	147.6906176	-0.04642437	0.064721015
65.0332494	144.2588263	-0.630755068	0.069412731
41.04549652	121.4469779	0.061250244	0.080923844
64.45791143	143.8411636	-0.023099204	0.068896533
53.79269513	84.09231949	0.348613206	0.102355374
67.98514754	126.8170757	-0.320752657	0.072708456
65.74813022	117.9763746	-0.147744069	0.073349396
72.24834281	158.5847378	-0.103671982	0.061532449
52.66560236	121.4415307	-1.00431658	0.082423564
45.59663531	101.2644615	0.832563122	0.09791362
32.46614923	107.68366	0.426949565	0.097720566
73.98461596	150.7153873	0.149879219	0.062413537
37.96200981	103.7186699	0.337320129	0.093372727
39.9865879	124.2780371	0.113369731	0.081071703
50.16569878	115.6674509	0.354539679	0.083070755
43.88923933	112.6856709	0.353452201	0.082065233
49.33448209	130.0146484	-0.283014543	0.07243252
77.87929653	130.5396156	-0.482360496	0.066334697
91.53273832	124.7742615	-0.619185766	0.068104604
50.03372121	112.6674824	0.018807079	0.08098687
39.89206279	122.4130507	0.612583099	0.096687336
36.4544587	90.94446373	0.056843471	0.095940284
57.8702014	135.0244884	-0.05373328	0.071911098
35.38829604	73.51083469	-1.549218661	0.120044777
90.09078215	155.7641392	-0.356236621	0.058376871
74.77472742	139.8249631	-0.522490231	0.064539611
57.43959511	139.042201	-0.187208351	0.069950136
47.02036363	130.3819485	-0.326638339	0.078885359
56.99810248	89.57393646	-0.894978622	0.097331682
101.5873919	169.6426249	-0.16165593	0.059801161
15.5006629	76.58013535	-0.326388846	0.127391103
93.14269271	170.0343881	0.065557307	0.054530291
54.98901831	94.51958323	-1.093460665	0.090320525
92.83094464	160.625926	0.329333904	0.057879832
65.54771315	146.747364	-0.040988472	0.063419444
51.05440782	116.134738	-0.23156762	0.075738787
51.41530822	125.4310369	0.631219918	0.082258811
66.10104773	140.4261475	-0.326162934	0.066447424
50.94044868	122.1071711	0.173419434	0.076752545
32.38082364	106.300211	0.55421095	0.099181299
59.46327543	134.3938317	0.257410447	0.070564664
42.46678628	127.8071938	0.662843897	0.087069124
67.91765531	148.0716248	-0.257750591	0.064333087
72.15170229	155.3704	0.159844794	0.060447439
51.73196887	113.0185103	0.160820068	0.078186204
47.34113721	142.2181263	0.192310296	0.072511355
63.01833509	166.1862402	0.260137154	0.065924586
43.37480238	102.4829998	-0.244729773	0.084789481
69.16693373	154.2755756	-0.218587946	0.061484016
63.28900762	111.7466393	-0.949843996	0.077913563
32.45578032	96.58105314	-0.402871019	0.092814032
64.7963355	138.0841079	0.018344416	0.069259409
53.50110147	111.791883	0.85612867	0.087407287
60.63327349	148.8683023	-0.000375222	0.06947804
71.44759483	139.2033157	-0.178031242	0.067737936
67.19717751	133.4686112	0.523537477	0.071272205
36.46661506	122.9602203	0.217514768	0.081954815
60.41232954	117.2900681	-0.05996085	0.083627913
23.48921289	70.74671674	-0.172205825	0.125585739
73.19656618	122.8771768	-0.073489207	0.071054592
56.15765569	126.8711319	-0.53050848	0.071112555
34.51326251	112.4455557	0.074331833	0.090006387
54.9281567	122.2662907	-0.659699429	0.07969712
70.30410025	143.1327171	0.076122505	0.068190892
57.58001234	117.6443386	-0.902075204	0.077262858
70.47938991	146.7547321	-0.474345188	0.063998004
86.14349177	154.2906828	0.364696673	0.060225778
47.73531692	99.07986403	-0.954584063	0.090862449
30.79200332	101.6773224	-0.007945778	0.091802629
56.23448293	124.0072489	0.778604812	0.088061855
40.62297158	114.5916862	0.183357864	0.085294399
117.9504708	151.3108482	-1.00766472	0.06148855
116.3914795	158.2554216	-0.797665054	0.056379721
42.49651368	88.85510635	-0.357468769	0.09531977
59.46823612	123.4493484	-0.101748086	0.071604076
49.09123636	106.0711975	-0.670221329	0.085302439
86.50245474	149.4565787	-0.187693989	0.059545069
39.2461041	100.132153	0.583020272	0.10172968
79.50638717	130.2706566	-0.743535931	0.068050227
56.18503697	144.9101858	0.085125345	0.071194978
47.80868219	118.9774857	0.080839779	0.0807308
52.74389801	114.1175461	-0.25218278	0.079308154
47.99695001	149.2501011	0.723053265	0.109170932
79.95757049	154.4522262	0.146137852	0.060958166
58.87925727	115.9613953	-0.708352137	0.075720805
49.90396212	117.2462072	-0.201952902	0.077604775
50.68199055	123.715481	-0.265933074	0.074799957
87.9570257	140.0560155	-0.344183217	0.068183456
74.00166428	141.1880141	-0.015014502	0.065135873
65.44822685	86.76497984	-1.684995738	0.09792202
26.76930538	87.31970358	-0.305990326	0.106004405
49.99383216	139.1370821	0.323348951	0.074282803

log.sigma.4.0.mm.3D_firstorder_MeanAbsoluteDeviation	log.sigma.4.0.mm.3D_firstorder_Energy	log.sigma.4.0.mm.3D_firstorder_RobustMeanAbsoluteDeviation	log.sigma.4.0.mm.3D_firstorder_Median
106.1164174	48569498.86	68.05180777	66.693367
62.53183316	13821609.06	42.05476947	74.2872467
84.71253375	38318460.66	60.99871065	114.5536308
99.62333371	18805230.97	66.24951763	60.10847092
73.70467078	32821809.65	49.65436398	62.69450951
90.73458246	13573299.07	66.8249857	59.98174477
85.59591776	124208168.1	59.64740736	92.19535828
83.63191832	118557288	54.62213464	92.41284561
87.9451382	108906890.2	60.77685786	102.9433441
85.59435035	27796412.85	60.58594472	118.8845749
70.07447588	20479427.42	49.63909323	72.94762802
85.5300288	58207999.67	59.35111336	106.501152
68.27294414	24897470.24	38.33658782	50.04101944
85.97039424	34404817.05	54.15504257	72.70811462
82.58637183	37924432.8	51.16205487	83.93916702
94.34248485	101959982	66.44733574	93.29624176
75.72834452	17358396.87	49.07881746	85.28951645
70.90143501	17651110.69	47.09870871	54.45637512
62.51121547	14661087.16	44.86545213	57.46900368
92.06804041	137851049.5	63.36341893	106.2615433
65.82758392	44369483.53	44.74125679	74.42020798
70.27474821	16427693.09	51.25832847	82.34785461
74.65877517	17940251.95	50.15187603	75.98729324
70.60596499	42137016.36	47.49811178	80.66429901
79.73230916	27660044.83	53.32784968	105.6236267
90.12720631	61057151.58	56.48763352	106.6999817
91.28870285	79809006.9	55.05649933	94.74626923
73.52469886	41295721.11	48.15953002	66.55028534
69.26222344	6502002.361	51.11590356	66.02072144
61.01694969	31699658.95	39.09962139	91.96313477
81.9881729	102971025.3	56.17899185	94.56645584
56.05190238	16707561.17	31.29362025	43.96787262
100.1427638	66121162.13	66.83086405	71.97377014
92.73850356	51661236.61	60.96267276	92.97687912
81.66912497	108772262.7	57.14032169	114.1975708
75.40158428	60011570.17	52.55539833	97.85362244
69.4611868	49973571.18	38.7002169	48.17054749
103.8558622	126227827.3	70.18187552	72.6662674
44.33193432	8664797.633	31.45491063	64.80516052
102.9992782	154860861.7	71.43203102	66.95727921
74.03961177	33007347.13	42.54884868	58.84628868
99.60232646	79035779.43	66.46067278	57.26777649
89.56219956	92022228.59	61.56380139	107.6319733
77.5428256	38023176.62	50.75826016	74.04178619
77.57532251	32724516.65	53.48950451	106.623291
87.13404003	74725075.53	58.32899965	119.6176987
76.7856617	91231026.15	50.94759214	91.53916931
62.53601602	11125223.02	44.97998084	64.77310181
82.12393988	15113336.96	57.20534172	74.93086243
73.00632531	24873454.19	52.90536546	84.54655838
89.33043019	73326143.36	61.39380623	125.4899902
93.44625999	73969492.2	64.52000817	86.75111389
74.04153066	69450585.2	48.94641361	73.20948029
80.07674803	30802985.05	57.73936185	124.173275
90.29856796	35236820.04	67.8508247	109.0267334
69.47531442	29578976.45	44.43562543	79.0812645
91.96521916	95974887.14	63.72366822	103.2328491
79.66604901	60903751.74	48.2106516	79.68107605
63.15129724	15948785.95	40.76644688	52.3899765
85.15932873	143746214.9	58.0993931	56.19917297
76.25992011	29341460.05	49.00377965	60.72860909
84.93682785	59741491.62	60.62205235	95.05817032
87.09163362	150469908.7	58.87596389	107.1974564
87.34403053	145266615.4	58.03188022	60.82342529
70.47583476	15242821.07	50.46727735	100.6992531
78.27240566	65221474.23	49.69009546	61.50121498
48.48592871	8062298.467	29.98085241	36.19703484
85.43412425	106959407	52.83518336	58.16459656
82.75858662	116015231.6	53.85515233	94.11963654
64.89348874	40983510.42	46.16886412	79.34410095
77.42158385	21092476.37	50.8277397	72.66755676
89.2382494	118622892.3	60.18733645	108.665226
78.14902007	46119205.89	49.25687148	99.18209076
92.19403275	34764254.04	61.95035459	51.77412796
96.89402834	60846178.69	65.17033327	50.18737793
68.26596267	17534094.05	42.48558487	76.85171127
62.36440469	36065305.32	42.4902073	89.46255493
76.83874457	16470442.73	50.59756989	62.85056114
69.04214106	26635835.43	47.57346509	79.74568176
107.3954531	50954895.65	67.20763247	64.43479156
107.9939137	47443306.21	68.2017742	66.99060822
66.84103923	18512258.61	39.04910199	40.93896866
81.81774128	68202325.97	52.85841711	94.22792816
69.50933231	42110308.56	44.60413632	77.97807312
96.94883228	55618752.05	64.4394918	85.89881134
66.08963795	21077387.05	44.81588122	62.45489311
91.150006	38503468	56.61826157	45.38856888
84.62595731	70373492.32	59.3062797	106.7635765
73.11922181	25955722.82	50.01502661	86.82023239
75.07274289	35070936.64	48.39201983	81.28843689
79.94809742	21824532.63	61.47782905	49.70737457
93.85549774	62857943.67	64.78079664	107.2714272
78.77682432	74917825.18	49.81999077	63.29397964
75.64943274	65224857.53	49.86781635	95.3069725
76.16293195	51803447.53	52.29852919	56.58407402
93.60922223	78565057.35	61.385323	73.54354095
88.76989159	50102214.76	59.36955355	70.03523254
72.6177616	12248593.49	39.07679752	32.86075974
54.32709951	6816483.843	36.78812018	59.77352142
80.11423224	119210594.1	57.45906389	90.98228836

log.sigma.4.0.mm.3D_firstorder_TotalEnergy	log.sigma.4.0.mm.3D_firstorder_Maximum	log.sigma.4.0.mm.3D_firstorder_RootMeanSquared	log.sigma.4.0.mm.3D_firstorder_90Percentile	log.sigma.4.0.mm.3D_firstorder_Minimum
1311376469	410.1985474	151.2896163	224.221344	-480.947876
373183444.7	333.8732605	121.5831797	213.5257996	-97.88179779
1034598438	441.1864319	158.0488526	255.8039642	-181.3616028
507741236.2	384.617157	138.7369217	210.0567871	-332.2601929
886188860.5	372.8264771	117.730886	196.201329	-275.9184265
366479074.9	424.4033508	143.407129	246.6844086	-153.0299225
3353620537	635.8903198	148.2560499	243.4741058	-272.0112305
3201046777	475.122406	152.3486759	257.8117493	-402.9624939
2940486034	458.3239746	154.7793301	253.5675125	-360.0286865
750503146.9	389.7858276	161.026363	258.4187439	-450.3471375
552944540.4	338.3232422	116.5354974	195.2610535	-180.5363464
1571615991	478.8366089	158.9116996	258.6727661	-280.5814819
672231696.6	390.5040283	115.4796482	187.4031708	-308.360199
928930060.3	406.089447	141.5547603	231.0381287	-366.35672
1023959686	456.9047852	138.2575043	210.2331787	-323.7289429
2752919515	459.5160522	152.6427629	251.5236053	-281.4782104
468676715.4	316.1824951	120.5737153	174.9471771	-329.8569641
476579988.7	357.3186646	119.6477348	224.0866547	-136.1021423
395849353.4	297.5730591	106.2785796	191.2196182	-115.7214737
3721978336	546.1828613	162.8340144	270.6687988	-226.6319275
1197976055	418.2451477	121.1298818	205.6300125	-191.4495697
443547713.3	326.946106	123.3893192	206.2401733	-133.5727539
484386802.7	353.5113525	132.3622956	237.6842743	-158.4345856
1137699442	451.6445007	126.1696562	210.3268616	-228.4324799
746821210.5	394.4172668	147.4050078	232.8719116	-337.9307556
1648543093	520.0943604	160.5409171	251.2822906	-416.7640686
2154843186	510.5341187	157.1169721	246.2479065	-452.1837769
1114984470	378.1634521	122.2758498	207.757753	-316.6571655
175554063.7	289.0769653	120.606246	215.491394	-103.9006882
855890791.7	373.0443726	124.2301688	200.4425415	-255.8841858
2780217684	437.0366821	147.1110515	243.4192505	-381.9634399
451104151.7	298.1008911	89.8620423	114.7329651	-404.4260559
1785271377	490.037262	143.098874	218.1082672	-380.659729
1394853389	442.6716309	148.2683545	231.5755844	-378.7546692
2936851093	396.437561	157.1576139	256.6908508	-320.3406677
1620312395	377.0250549	141.4585696	231.6301941	-350.2435303
1349286422	441.3812561	115.8885043	165.1322632	-515.8336182
3408151338	557.5665283	156.3905921	258.2715454	-452.344696
233949536.1	217.8465118	83.93182305	132.7953323	-134.566864
4181243267	499.0743103	145.9498424	238.1368484	-290.4708252
891198372.6	385.3691711	112.8029155	150.0845108	-431.0986328
2133966045	604.8060303	142.9633724	217.386145	-373.3594971
2484600172	437.6236267	158.7163024	262.2507751	-322.3312378
1026625769	582.5211182	123.6229237	188.7463043	-308.8621826
883561949.7	472.3600159	160.0188679	274.3193237	-129.4632721
2017577039	490.2358093	164.9916657	258.8193848	-312.9631042
2463237706	459.1976318	141.4144014	233.2635284	-315.3553467
300381021.6	293.5083923	113.0173931	197.8265839	-76.95709991
408060097.9	386.1662598	133.2647849	224.1982574	-210.3020935
671583263.1	460.3225403	135.6374774	225.0943863	-116.2204285
1979805871	439.0877075	168.9457883	264.9385498	-270.4767456
1997176289	529.5490112	148.1313369	248.2723083	-304.7286682
1875165800	423.6277161	124.0661797	210.0783997	-268.2043762
831680596.5	440.5223999	167.0366863	270.2838684	-153.4204865
951394141.2	437.0424805	162.7693142	269.9616394	-146.4749603
798632364.2	408.6347961	123.1613002	201.7399063	-418.0651855
2591321953	431.4976501	154.0350458	249.0946777	-303.0556641
1644401297	365.0538025	132.7117967	200.1620117	-410.6147766
430617220.7	314.4972534	98.37510549	155.2745499	-307.1989136
3881147803	469.4796753	125.4834947	201.8152405	-425.9606323
792219421.5	527.5634155	128.8978017	226.5980377	-225.4554291
1613020274	460.0970764	149.5830555	251.2311157	-288.070282
4062687536	509.3615112	161.2215358	262.5464417	-449.315033
3922198615	490.7695618	138.0447673	245.3158508	-396.9794006
411556169	375.7944336	138.9054002	215.8953369	-183.0977478
1760979804	427.7581482	129.9538561	217.8955627	-431.9265442
217682058.6	272.9920654	78.6304281	126.4797432	-266.480896
2887903990	541.133606	130.8185963	206.875148	-449.9157104
3132411254	475.4516907	140.7407606	219.4359467	-403.2250671
1106554781	326.6097107	118.9201332	197.5945084	-248.7792511
569496862.1	369.6006165	127.4263403	199.4043701	-352.6619568
3202818091	473.2957458	166.28597	277.1996002	-363.722168
1245218559	373.2679443	142.2553956	222.8453247	-425.1409607
938634859.2	355.4464417	124.0533058	172.7803894	-351.3468018
1642846825	493.6896667	139.4039631	232.8233185	-293.6687927
473420539.3	307.6212463	114.3050474	170.614743	-325.0202637
973763243.6	379.4894104	122.6878059	195.1917038	-199.2651672
444701953.7	426.0873718	131.1202843	227.8428223	-177.1287842
719167556.6	351.1849365	124.9155202	211.1175232	-210.2200317
1375782183	398.4905701	147.5971078	178.6631439	-515.8092651
1280969268	400.8248596	154.4436588	223.5167633	-480.947876
499830982.4	489.8387451	101.4411403	136.4450745	-342.0258484
1841462801	450.4766235	144.4415475	229.1563843	-365.0439148
1136978331	352.3834229	117.0611892	180.2835175	-314.5193176
1501706305	527.2054443	151.0096566	240.8327423	-347.5447998
569089450.5	325.8725586	117.2948658	215.4780991	-196.707901
1039593636	357.973053	127.0590876	172.0467926	-412.330658
1900084293	491.176178	160.2030672	261.1151703	-317.5735474
700804516.1	382.8454285	133.1514884	223.5171646	-207.5666504
946915289.4	415.3361816	131.6015998	214.5603973	-284.0620728
589262381	337.2714539	129.3700304	236.7215347	-110.7445526
1697164479	473.762146	162.4460689	274.0821228	-215.7198029
2022781280	398.7724609	120.2269906	179.7576569	-474.5790405
1761071153	422.6564026	140.588436	227.3551636	-326.2123108
1398693083	405.1838989	109.1025467	168.0780716	-300.7850037
2121256549	457.3601685	151.5216578	259.0353363	-485.7687988
1352759798	422.9434204	133.3629024	212.2352203	-238.9549561
330712024.3	240.8067017	108.1607699	117.5425583	-459.7459717
184045063.8	236.8329315	90.40598441	142.1419083	-199.9896088
3218686040	419.7624207	143.6875158	243.804509	-246.4768372

log.sigma.4.0.mm.3D_firstorder_Entropy	log.sigma.4.0.mm.3D_firstorder_StandardDeviation	log.sigma.4.0.mm.3D_firstorder_Range	log.sigma.4.0.mm.3D_firstorder_Variance	log.sigma.4.0.mm.3D_firstorder_10Percentile
4.463013975	140.7416259	891.1464233	19808.20527	-137.5057419
3.588758435	79.53148478	431.7550583	6325.257072	2.716003275
4.085273811	104.0524834	622.5480347	10826.91931	-13.78241301
4.312252202	128.8667453	716.8773499	16606.63803	-142.156012
3.946843293	94.65590016	648.7449036	8959.739435	-44.8611599
3.98549193	108.613078	577.4332733	11796.80072	-24.57772408
4.132279778	107.9371656	907.9015503	11650.43172	-26.30408669
4.102184782	110.2201208	878.0848999	12148.47504	-7.874757957
4.190843921	111.6617387	818.3526611	12468.3439	-30.62014961
4.07453211	109.0532701	840.1329651	11892.61571	-3.063761854
3.841235177	87.44489365	518.8595886	7646.609426	-29.62474709
4.123191785	108.1399473	759.4180908	11694.24821	-9.270345879
3.787498375	96.90639189	698.8642273	9390.848788	-25.26337395
4.148450544	115.7625101	772.446167	13400.95874	-32.11434784
4.147734458	111.8963048	780.633728	12520.78303	-62.78513947
4.249188322	116.9515605	740.9942627	13677.66751	-47.31859398
3.900797846	99.23076169	646.0394592	9846.744065	-58.75288048
3.711375533	90.43207219	493.4208069	8177.959681	-17.48143044
3.573328499	76.52467494	413.2945328	5856.025874	-16.35796146
4.250752599	116.3428847	772.8147888	13535.66682	-25.48570404
3.732742614	83.90181343	609.6947174	7039.514297	-4.413815975
3.79575013	85.75556557	460.5188599	7354.017026	-16.06524124
3.894921829	95.28141739	511.9459381	9078.548501	-17.30628834
3.886770615	91.11640151	680.0769806	8302.198624	-18.02210503
4.052680461	103.2033054	732.3480225	10650.92224	-18.84795876
4.271302007	122.7802876	936.858429	15074.99902	-38.90023117
4.274868357	126.9940923	962.7178955	16127.49948	-46.39231415
3.938849286	95.92381252	694.8206177	9201.377809	-30.98833618
3.599343796	83.15583092	392.9776535	6914.892216	-1.461732388
3.707809957	80.9225706	628.9285583	6548.462432	0.790535253
4.073749356	104.6372658	819.0001221	10948.95739	-14.48566866
3.512667633	82.97627318	702.526947	6885.061911	-52.82031784
4.375439731	129.1444688	870.696991	16678.29383	-122.8892517
4.254219143	120.8580082	821.4263	14606.65814	-80.95786133
4.062355798	102.8148118	716.7782288	10570.88553	-4.312397718
3.927928228	96.01289394	727.2685852	9218.475802	2.158456421
3.862812544	104.199677	957.2148743	10857.57268	-40.71900558
4.383687232	132.8497332	1009.911224	17649.05162	-78.45439148
3.184016187	55.55807139	352.4133759	3086.699297	-6.319317818
4.414995234	129.636525	789.5451355	16805.62863	-102.8053703
3.921732905	104.3324782	816.467804	10885.26601	-87.64760056
4.391092596	129.6383643	978.1655273	16806.1055	-103.6900116
4.21579736	113.3669271	759.9548645	12852.06017	-25.50624504
4.029887408	102.3361352	891.3833008	10472.68457	-63.48598099
3.894296648	97.49579065	601.823288	9505.429194	17.25290966
4.195216662	113.4225824	803.1989136	12864.6822	-13.36142731
4.006904232	99.35971719	774.5529785	9872.353401	-11.80450239
3.553756085	76.16293819	370.4654922	5800.793154	-1.384528637
4.058404199	102.8593163	596.4683533	10580.03895	-35.60832977
3.770798093	89.21440621	576.5429688	7959.210276	3.400050044
4.19941611	113.513123	709.5644531	12885.2291	-10.2016573
4.277761387	118.3998629	834.2776794	14018.52753	-63.83289719
3.968678049	96.14138522	691.8320923	9243.165952	-36.32942848
3.99301794	97.98399579	593.9428864	9600.863432	16.747686
4.092870753	107.4276684	583.5174408	11540.70393	-5.389084721
3.885230974	93.03432436	826.6999817	8655.385508	-25.77818108
4.248782255	115.8916674	734.5533142	13430.87856	-48.48448257
4.053998222	110.6996744	775.6685791	12254.41791	-51.26248474
3.749278981	83.96123652	621.696167	7049.489237	-47.28828239
4.139415514	108.6398122	895.4403076	11802.6088	-59.96903305
3.899173566	99.59991909	753.0188446	9920.143883	-26.03366852
4.082315259	105.5214887	748.1673584	11134.78457	-18.14212551
4.177042189	112.6591319	958.6765442	12692.08001	-11.28377094
4.13587303	113.0416743	887.7489624	12778.42012	-42.32058563
3.808623217	86.94506247	558.8921814	7559.443888	0.019685586
3.993217403	105.1827193	859.6846924	11063.40445	-25.36701527
3.376815764	66.96574949	539.4729614	4484.411605	-30.72326508
4.20417325	116.4767654	991.0493164	13566.83688	-78.5532486
4.118900651	109.0116758	878.6767578	11883.54545	-43.70596466
3.708914792	80.9861181	575.3889618	6558.751325	-5.127304316
3.981016765	103.5457774	722.2625732	10721.72802	-29.88196182
4.184049109	114.8577562	837.0179138	13192.30415	-1.158819151
4.021100886	106.5900805	798.408905	11361.44525	-19.30686722
4.237985464	118.0617067	706.7932434	13938.56659	-123.7115738
4.328471701	124.5870667	787.3584595	15521.9372	-88.49820709
3.821415861	92.82228264	632.64151	8615.976155	-48.1764328
3.708849238	79.71184584	578.7545776	6353.978367	0.963908315
3.881649167	98.55939049	603.216156	9713.953454	-13.08305569
3.81516307	87.16746279	561.4049683	7598.16657	-12.35316067
4.3914855	143.0495323	914.2998352	20463.16869	-168.1340912
4.473843419	144.7909732	881.7727356	20964.42592	-138.9074768
3.83232318	95.01379792	831.8645935	9027.621795	-76.97842255
4.121617649	107.8380788	815.5205383	11629.05123	-31.35201607
3.869452663	92.67465343	666.9027405	8588.591389	-45.91926956
4.355140612	125.759832	874.7502441	15815.53534	-79.93009644
3.623800768	83.51718714	522.5804596	6975.120548	-3.682181263
4.231239548	122.7514076	770.3037109	15067.90807	-145.3818604
4.086796995	105.9661498	808.7497253	11228.82491	-0.332607855
3.911110608	93.11484584	590.4120789	8670.374516	-13.06889019
3.989234463	99.89095636	699.3982544	9978.203163	-25.61620674
3.551586725	92.87520423	448.0160065	8625.803561	-2.974405456
4.2707081	118.3746543	689.4819489	14012.55877	-35.23796654
4.063056206	106.2148041	873.3515015	11281.5846	-76.69443207
3.996773094	99.22613718	748.8687134	9845.8263	-18.51342144
3.987757822	96.63978495	705.9689026	9339.248035	-78.93466415
4.235466452	124.4527303	943.1289673	15488.48209	-38.32453842
4.206375359	114.3426734	661.8983765	13074.24696	-83.74700623
3.795845904	107.1805281	700.5526733	11487.6656	-109.4420212
3.495080286	69.45788921	436.8225403	4824.398373	-28.28244095
3.979489599	98.65295515	666.2392578	9732.40556	-7.097352505

log.sigma.4.0.mm.3D_firstorder_Kurtosis	log.sigma.4.0.mm.3D_firstorder_Mean	log.sigma.4.0.mm.3D_glrlm_ShortRunLowGrayLevelEmphasis	log.sigma.4.0.mm.3D_glrlm_GrayLevelVariance
3.803796444	55.50083541	0.003673771	33.00380893
2.974391214	91.96310408	0.028207879	10.61878734
2.756722756	118.9643665	0.00902418	17.64130463
2.978332847	51.39353466	0.008387658	27.0650449
3.227787058	70.0058717	0.005746777	14.96401531
2.614755709	93.64189196	0.013679222	19.39701039
3.444686877	101.6337769	0.005887192	19.21344387
4.119124064	105.1743505	0.002841648	20.48288291
3.278768316	107.1834742	0.00327496	20.56034805
4.771332968	118.4773138	0.002987958	19.78072689
2.832536407	77.03189429	0.012337234	12.69441538
3.247942687	116.4417454	0.0049527	19.34734906
4.812046715	62.80684955	0.005601888	16.61708522
4.322449186	81.46650496	0.005109014	22.7535728
3.96466509	81.20563079	0.006898034	21.15388138
2.851929178	98.09253574	0.00559648	22.39653986
4.357772262	68.4928957	0.005854498	16.3669963
3.15382495	78.34296884	0.016893955	14.08129556
2.52773197	73.7503262	0.023018202	9.617070076
3.071671505	113.9265088	0.006947082	22.35945582
3.367438375	87.36666389	0.010492814	11.99692855
2.527705627	88.71813263	0.019655354	11.95290194
2.93126638	91.87616011	0.013831928	15.10806522
3.580070372	87.2730401	0.007257632	14.07500039
3.790259504	105.2488198	0.004345323	17.80709269
4.662327123	103.4330075	0.004061761	25.18892578
5.029826776	92.5107748	0.002987824	27.02365428
3.564309051	75.82879168	0.004740985	15.45539455
2.467769871	87.35544839	0.018514039	11.34773873
3.82132563	94.25854021	0.00525695	11.18526515
3.588818537	103.4055322	0.002824623	18.21213545
7.643873605	34.49818453	0.004114754	12.19667797
3.159407433	61.63597907	0.004488004	27.39978071
3.678169636	85.88857202	0.004129641	24.31623989
3.338028137	118.8597075	0.004417978	17.29730684
3.976163812	103.8847972	0.003411055	15.50537356
7.163759668	50.72053585	0.002455097	19.19663313
3.395378431	82.51645706	0.002572755	29.87929269
3.265590034	62.91145861	0.017936508	5.193364719
2.876419149	67.0501892	0.008778343	27.29589969
5.507058911	42.88626501	0.003808588	18.80938422
3.768823078	60.269564	0.004532167	27.39814797
3.161387734	111.0801714	0.004504953	21.01846237
4.108766116	69.35375035	0.005563905	17.50182709
2.943758432	126.8881747	0.010801375	15.99720722
3.881327231	119.8230676	0.005595328	21.52896419
3.602967197	100.6264355	0.004202939	16.63552155
2.581163318	83.49932926	0.026621494	9.773268861
2.975956462	84.73171742	0.011298549	17.44906034
3.302667429	102.1680724	0.016423536	13.21686277
3.367282914	125.1297339	0.008884409	21.235248
2.988998628	89.01890497	0.004747972	22.81198233
3.339491703	78.41715999	0.006346933	15.45704909
2.746143517	135.2789383	0.009406877	15.89973995
2.352107883	122.2830557	0.01458435	18.77536489
4.39591842	80.70514459	0.003536372	14.57810714
3.053891359	101.4687971	0.004846569	22.12646869
5.276135134	73.19838156	0.003909366	20.66062302
4.204549351	51.26570142	0.006080287	11.99026024
3.585009494	62.79728217	0.002609453	19.74476851
3.945781927	81.81992062	0.006878522	16.94230544
2.937318025	106.020309	0.004889671	18.31238888
4.166796989	115.3269422	0.002850657	20.89434965
3.571027198	79.2334378	0.003167214	21.31120837
2.942502302	108.3294343	0.00871347	12.47742272
4.677476186	76.31906889	0.002977301	19.10191809
5.314499992	41.21083132	0.009494339	7.977516038
4.142099114	59.55391039	0.003173005	22.82977015
3.904483716	89.01919029	0.002884244	19.95485019
3.15077301	87.08183938	0.006832672	10.9689205
4.755131656	74.26805616	0.005301839	18.12965624
3.713997505	120.2444164	0.003499804	22.00789339
5.346617379	94.2080269	0.003064173	19.22401798
3.307085059	38.08747952	0.006235542	22.91233664
3.192178983	62.54220759	0.007719523	25.34464841
4.707917212	66.70582955	0.005493734	14.82923254
3.500249235	93.26478084	0.010322205	10.8302259
3.680624662	86.47875745	0.010541353	16.5405108
3.031146731	89.47469262	0.007948253	12.6898589
4.279558639	36.35570842	0.003666798	33.70129124
4.065018666	53.74400272	0.004268211	34.83598959
5.612661757	35.53425329	0.007837748	15.71117523
3.868037786	96.09531425	0.003533817	19.40487229
4.263405281	71.51734494	0.005317239	14.26440421
3.433979473	83.59653721	0.005841045	26.15853336
3.005788848	82.35875786	0.00911501	11.61963448
3.912826005	32.80401913	0.005311731	25.35730252
3.232268613	120.1507296	0.003991749	18.83695457
3.07479279	95.1784868	0.008548277	14.520319
3.976949842	85.67833971	0.007393122	17.05059301
2.256959421	90.05998676	0.016077641	14.40268361
2.959728874	111.2482203	0.011494954	22.89709896
4.710610212	56.32889721	0.003008985	18.98389007
3.734268607	99.59559249	0.003822315	16.57492936
3.214203194	50.63711748	0.00570026	15.41097326
4.576316139	86.43107485	0.00268929	26.22050034
2.996295028	68.63976094	0.01290379	21.47236439
6.779495535	14.52881774	0.00622013	20.09602488
3.404557255	57.86919426	0.013503327	8.091434348
2.850069156	104.4686395	0.00649908	15.99067276

log.sigma.4.0.mm.3D_glrlm_LowGrayLevelRunEmphasis	log.sigma.4.0.mm.3D_glrlm_GrayLevelNonUniformityNormalized	log.sigma.4.0.mm.3D_glrlm_RunVariance	log.sigma.4.0.mm.3D_glrlm_GrayLevelNonUniformity
0.003817037	0.054507741	0.118144471	105.8369028
0.030829515	0.097079275	0.211668219	78.28796309
0.00974352	0.066901989	0.192152418	90.17818331
0.008895487	0.059619379	0.122370792	52.81278958
0.006185622	0.076109992	0.175121826	159.2959742
0.014887166	0.072677229	0.17106448	42.34817329
0.006375973	0.066419902	0.175721339	333.1800715
0.003063729	0.070986006	0.184174237	319.7285805
0.003525614	0.063736583	0.159844511	257.3904422
0.003132412	0.068149402	0.143849019	65.58129
0.01332236	0.079242248	0.176370738	105.3355584
0.005306541	0.067274853	0.154768852	138.2454752
0.006195886	0.092154891	0.344404493	142.3997001
0.005442395	0.069279624	0.171702509	104.83432
0.007247566	0.070249843	0.159444897	125.1379245
0.006002294	0.060458048	0.158619354	237.9260011
0.00616398	0.080680928	0.130805333	86.49658648
0.018684682	0.090500308	0.230632372	94.99797457
0.025291342	0.094924316	0.207500508	106.0100125
0.007490423	0.060933559	0.148665148	283.8777625
0.011696426	0.08769043	0.296975779	221.9443718
0.021278961	0.080590533	0.151662409	77.22785897
0.015114736	0.078496683	0.197206225	70.04321942
0.007829298	0.079585217	0.191008952	184.4995203
0.004578439	0.070899575	0.16204965	81.25539485
0.004273688	0.064174505	0.128595422	138.0236978
0.003165998	0.065726898	0.166575859	190.6512347
0.00513724	0.077783981	0.174548355	188.8220861
0.020641342	0.092811307	0.181526493	36.13364113
0.005780232	0.092386368	0.209430614	162.9895034
0.003065967	0.06984244	0.20430657	290.9171609
0.004460223	0.111670794	0.275543185	194.5924892
0.004762226	0.057178367	0.138457514	166.5100819
0.004356493	0.062977415	0.160166405	132.8668189
0.004790606	0.068991942	0.14786611	272.419647
0.003687707	0.076234233	0.220250702	199.1470129
0.002676929	0.089787245	0.259016141	284.535634
0.002805128	0.055504317	0.302356365	246.1236917
0.019687293	0.125242414	0.233703789	130.1779001
0.009454327	0.054038159	0.125307045	356.1320441
0.004077829	0.084778739	0.213514808	191.3888849
0.004803166	0.057246607	0.10552133	202.8367359
0.004788733	0.062725578	0.136483525	207.4751403
0.005937875	0.073472195	0.150004908	162.855621
0.01181268	0.078734294	0.171889949	88.43536324
0.005995173	0.065019369	0.151212499	159.9196185
0.004549256	0.073973527	0.177406288	297.4322914
0.02970626	0.094980793	0.228371003	70.64728991
0.012061854	0.069183159	0.147412187	52.53639456
0.018335687	0.082833837	0.2521435	96.21376054
0.009464563	0.063270539	0.141169303	146.0939746
0.005031249	0.059792682	0.108174041	184.3025564
0.006874781	0.075801997	0.170764809	300.9774356
0.009993268	0.071290011	0.137606398	70.97702761
0.01572298	0.064956111	0.150135274	77.3177042
0.003798163	0.081590337	0.174869429	140.0163429
0.005144208	0.06049155	0.132772325	221.1964397
0.004196779	0.075039629	0.189767269	227.3170922
0.006493508	0.088964379	0.179562403	128.9043989
0.002850495	0.067024405	0.23811275	531.5663405
0.007546513	0.080805299	0.22587736	123.2637347
0.005275049	0.067664094	0.167159494	160.0661656
0.003246407	0.066239541	0.14916213	343.7292685
0.003463737	0.068684796	0.178846365	460.6737762
0.009335311	0.080561197	0.169325808	56.47243766
0.003250587	0.077473555	0.250248955	255.9945514
0.010680003	0.115883665	0.314962911	123.0044442
0.003436576	0.067590748	0.169155009	373.6898103
0.003079836	0.069008516	0.166232451	359.9018761
0.007534719	0.087382032	0.242405715	215.6373533
0.005691092	0.076685871	0.180035045	87.33767753
0.003768918	0.065666737	0.193247666	247.6035271
0.003249157	0.074910053	0.180757902	150.9908434
0.006545817	0.062592152	0.116708904	128.9033033
0.008274229	0.059107338	0.133962536	167.0571477
0.005842256	0.086097434	0.183950454	100.8671384
0.011272674	0.089000582	0.239170899	182.7981813
0.011639671	0.082094033	0.233343285	67.29251045
0.008649544	0.082712777	0.179820101	123.6833363
0.003838979	0.059917915	0.120762843	127.5816802
0.004432514	0.054505949	0.11762013	99.28467323
0.008486981	0.089660569	0.207644238	139.8309389
0.003800547	0.070004054	0.195119293	202.6035077
0.005791455	0.083124298	0.180597945	222.7398888
0.006219212	0.058367876	0.149272235	128.1810631
0.010303518	0.095101444	0.292257122	120.9024924
0.005614167	0.065200385	0.158744125	139.0637774
0.00428732	0.068210773	0.193225263	164.9479752
0.009218061	0.07798483	0.186953741	100.3991013
0.007887788	0.075837228	0.211858308	133.3832146
0.019076788	0.094210318	0.338033681	100.4031293
0.012132478	0.060117614	0.125395322	130.0903455
0.003211627	0.073377353	0.163421489	336.8397698
0.004093872	0.07463757	0.176451382	218.1996406
0.006122384	0.073827779	0.146870329	286.3795689
0.002891066	0.064512732	0.219393195	193.1440332
0.013957027	0.063545249	0.156987916	158.7690513
0.006701687	0.090926382	0.265456659	79.95022476
0.014809806	0.103858894	0.226629455	73.25063517
0.007030507	0.072189459	0.199888969	366.3718218

log.sigma.4.0.mm.3D_glrlm_LongRunEmphasis	log.sigma.4.0.mm.3D_glrlm_ShortRunHighGrayLevelEmphasis	log.sigma.4.0.mm.3D_glrlm_RunLengthNonUniformity	log.sigma.4.0.mm.3D_glrlm_ShortRunEmphasis
1.316605108	509.3928735	1650.702336	0.93719882
1.568969704	71.01993572	618.1798893	0.895506479
1.497106442	177.1533063	1076.103981	0.911667194
1.343403832	279.994304	738.4571585	0.929495813
1.462042775	228.4672534	1679.522395	0.914323901
1.466131569	138.3764203	464.8167752	0.911591684
1.451938157	244.1570043	4071.157727	0.918466209
1.476414407	453.7873695	3608.462384	0.913800853
1.434333504	378.8711926	3265.358675	0.916813212
1.392290135	562.759297	792.4615759	0.923966669
1.471383984	133.8728218	1060.788703	0.911737297
1.419014829	289.7755601	1669.197813	0.919128011
1.829223514	244.1504523	1127.283788	0.875840576
1.464415173	343.9816048	1202.390229	0.910944676
1.410425071	276.8257304	1475.292838	0.925750872
1.406608149	272.880255	3279.192868	0.927412447
1.375698546	283.836205	875.3872067	0.921376006
1.621138892	99.14771635	786.1892232	0.887394204
1.569710772	73.21882605	853.2285931	0.893728681
1.398603052	233.5121719	3824.484783	0.923459042
1.739778779	141.2088417	1867.673794	0.880928064
1.42485156	103.4900275	770.8713762	0.915338872
1.52510048	128.5711577	698.9093036	0.904592045
1.503363596	192.1769772	1829.126986	0.907627993
1.41249148	341.4351143	958.4603731	0.927863645
1.34748877	460.1344146	1805.379441	0.931830749
1.416972517	523.7816406	2398.083654	0.925794534
1.475698682	264.8432757	1921.393456	0.909044051
1.514767226	87.24327778	302.2857888	0.900012186
1.5774603	218.6112539	1337.376579	0.891547893
1.517713487	406.5063317	3313.510336	0.910337667
1.70799723	319.1993385	1288.721178	0.881250956
1.372755634	356.328882	2415.714714	0.927242329
1.407689141	390.06904	1751.969936	0.926966822
1.396499778	325.2139243	3245.575758	0.923807766
1.545450935	368.2045008	2067.074565	0.908432341
1.662446413	509.5692008	2396.651133	0.889467841
1.668742875	509.1561509	3474.255014	0.904349876
1.648368093	74.47052497	766.9618266	0.880178079
1.346200985	240.6240953	5500.304974	0.929873856
1.548351104	380.1932732	1769.092296	0.904633716
1.298845564	327.1913519	3008.087848	0.936979693
1.366060066	318.9221438	2771.430947	0.930069944
1.41529814	256.7943483	1792.95856	0.917509173
1.476967981	140.3983793	885.4501853	0.907320744
1.4023613	329.3299314	2021.599219	0.92361041
1.471815002	298.28778	3212.897879	0.91251795
1.619873597	65.48246778	561.9847687	0.888526473
1.412376882	169.412239	616.1001165	0.917985969
1.632071954	98.86877712	897.5352126	0.896069512
1.383749163	271.0288596	1903.186687	0.92483346
1.307351524	293.6313631	2607.854086	0.935385563
1.467580545	209.6007388	3145.561462	0.909657662
1.375630227	170.2525749	827.4522743	0.927029667
1.40386792	139.2227915	974.4807294	0.922026727
1.473691832	404.0972596	1360.660745	0.90966182
1.361324712	306.0170114	3039.096739	0.928119368
1.501691434	393.891002	2391.884041	0.90761592
1.480762925	230.0175233	1146.899724	0.909281458
1.572525129	421.2477482	6251.662018	0.906794051
1.577864363	191.3719678	1179.41012	0.899924933
1.447882037	275.2565086	1902.104918	0.915154691
1.397222501	515.66196	4263.048751	0.924010471
1.478212725	379.1332293	5338.674111	0.910906139
1.459110312	162.1575138	567.3751495	0.914706074
1.628283336	440.3071273	2528.635149	0.895551491
1.843843811	155.3228605	737.1060562	0.856934994
1.453106538	420.8407123	4436.16222	0.914428512
1.434321368	422.367165	4244.970459	0.919776806
1.631775291	186.4069381	1860.94275	0.889717768
1.493235673	324.3990064	897.9564196	0.906523083
1.49486695	400.6384818	3009.756351	0.912186154
1.469094925	469.4449006	1623.066648	0.915091957
1.323404732	288.662366	1736.141838	0.93398367
1.366173025	234.8497705	2346.982028	0.927712001
1.506196138	275.0251994	916.4956262	0.903777678
1.61600046	144.9212866	1572.407805	0.894713234
1.616405851	147.8931248	623.2435302	0.891587981
1.491183006	168.731545	1175.641277	0.906312871
1.331978511	514.6270943	1791.553997	0.933124753
1.314504599	508.39609	1551.56418	0.937833076
1.54742545	241.7272885	1208.799125	0.900654279
1.488317039	363.1542716	2351.968646	0.917213738
1.50381256	251.6914983	2084.954184	0.902025504
1.393093957	318.9394581	1825.511413	0.927063405
1.757237293	135.7277934	919.8547698	0.873649649
1.41874333	345.4067697	1744.622105	0.921630681
1.483233091	328.6260382	1950.44008	0.916456812
1.490621155	175.0976725	1025.174994	0.910614291
1.551935	244.5547448	1372.672639	0.902327284
1.853602819	92.34308183	752.4032682	0.863314874
1.342533587	202.3739955	1815.282707	0.931599304
1.443703426	447.2526985	3681.698576	0.914215974
1.456579799	328.3032669	2361.852721	0.917075047
1.411318621	233.8475488	3140.052097	0.917709518
1.535590113	550.6971243	2381.858153	0.910746584
1.433992681	181.4614616	2007.204503	0.914842664
1.703416211	363.2561638	645.1307647	0.878009952
1.640680843	107.8870648	519.162627	0.878958052
1.50162719	214.9719602	4059.166521	0.912851271

log.sigma.4.0.mm.3D_glrlm_LongRunHighGrayLevelEmphasis	log.sigma.4.0.mm.3D_glrlm_RunPercentage	log.sigma.4.0.mm.3D_glrlm_LongRunLowGrayLevelEmphasis	log.sigma.4.0.mm.3D_glrlm_RunEntropy
727.9304872	0.914775611	0.004516677	4.9134189
116.4518161	0.861867544	0.044473011	4.249944277
287.6117108	0.878547789	0.013701622	4.658373387
403.3089912	0.906464058	0.011211259	4.775421566
361.5712187	0.883608368	0.008483975	4.531496435
202.9814567	0.882051282	0.021117436	4.551620122
370.6906222	0.887453548	0.008998882	4.69728682
714.1457792	0.881452925	0.004265245	4.714034846
586.9388204	0.888287252	0.004824047	4.753455765
840.8139673	0.89738806	0.003873931	4.58164576
213.7860051	0.881452765	0.018350225	4.416842584
441.0459137	0.89123978	0.007098724	4.67110614
482.1598724	0.825923942	0.01002277	4.622782682
538.6897281	0.881053716	0.007168489	4.744013536
426.8026468	0.89741005	0.009044324	4.674495855
405.6340251	0.898801153	0.008163401	4.754423955
437.1141501	0.897822446	0.00758185	4.421066876
163.6238615	0.850770479	0.028474957	4.426369811
124.5170551	0.860317648	0.037383185	4.233706312
340.6629498	0.895926731	0.010200648	4.78741812
260.2402678	0.836233211	0.019213448	4.509658041
158.6366886	0.888215584	0.029062714	4.333403877
206.8306099	0.870643029	0.021895281	4.513094836
309.0661868	0.875446805	0.010937761	4.507851426
515.5995853	0.899510544	0.005901531	4.549818915
661.4739	0.907685814	0.005288207	4.760411005
795.8924839	0.896571415	0.004165141	4.811235805
420.7070259	0.878738929	0.007184321	4.552661986
132.1377214	0.870246085	0.031836257	4.183339691
382.1420531	0.85840012	0.008526704	4.399605948
667.3366413	0.875141462	0.004449024	4.694839379
636.1525447	0.840948805	0.00645999	4.300689471
535.0099808	0.901946304	0.006090482	4.880707275
591.7782482	0.897545008	0.005577571	4.768406271
485.685139	0.896545099	0.006599991	4.590986588
610.8369682	0.870905687	0.005326057	4.556834589
943.7063927	0.849647531	0.003974565	4.620999935
880.7755018	0.858539639	0.004416751	5.047285488
144.1154871	0.844903064	0.028838182	3.911495724
344.8795932	0.906507248	0.012572957	4.90137773
670.2201856	0.869254493	0.005528521	4.588102345
449.7449114	0.916273796	0.006073313	4.837260911
467.9636829	0.905178041	0.006254456	4.700308282
401.1933266	0.890798912	0.007769819	4.589526394
211.8557877	0.878175033	0.017032453	4.497151893
493.1714704	0.895894634	0.008020764	4.724928416
468.5511459	0.881074428	0.006390243	4.613348092
109.5797061	0.853395743	0.047054803	4.239840979
256.075546	0.891982283	0.015750412	4.576988942
160.2430761	0.858443332	0.029818054	4.434286198
405.8046914	0.898763362	0.012519979	4.715561807
409.4752927	0.914382858	0.006364126	4.728728617
333.693704	0.879841789	0.009569044	4.576953633
247.557072	0.901477146	0.012993036	4.479514011
204.1924175	0.89496819	0.021454023	4.600311817
653.4836757	0.879763314	0.005112375	4.486608244
449.1470402	0.903907959	0.006623061	4.748357828
660.8919619	0.875739645	0.005670055	4.68174311
375.0560307	0.87887416	0.008646748	4.362621414
712.2568797	0.868474936	0.004345911	4.787919547
312.309702	0.862749368	0.011408405	4.578578614
427.6751608	0.88576779	0.007263855	4.652304212
769.7773346	0.896262142	0.005021535	4.713554691
589.806753	0.879645607	0.005019662	4.750372158
257.3254279	0.886660175	0.012643193	4.348042284
767.9212201	0.854758395	0.004859725	4.719078796
329.5492036	0.813178386	0.017063766	4.254880114
662.1528754	0.88432	0.004758788	4.805840561
667.5258292	0.8901643	0.004124005	4.687947301
329.4641521	0.851356373	0.011711128	4.416791774
534.6181693	0.876176941	0.0076188	4.589335866
636.5901981	0.878536848	0.005267619	4.795554556
755.4004963	0.884024707	0.004252192	4.607992756
417.1961739	0.911499302	0.007990924	4.706005741
336.9136025	0.902587033	0.010988701	4.827393405
470.3773212	0.872692881	0.007632598	4.453326999
254.5321395	0.856780532	0.016607301	4.398749145
244.0754659	0.854906054	0.017835353	4.573345122
268.9874618	0.875760443	0.012269526	4.423542416
757.3772373	0.910053606	0.004667421	4.864511089
725.4248832	0.915496771	0.005214516	4.918701952
415.4711516	0.866378757	0.011658435	4.509196247
579.2558724	0.884650681	0.005339217	4.694923138
425.4629538	0.871786528	0.008154638	4.504867859
481.1968645	0.900337465	0.0080584	4.860590803
255.0664232	0.829182567	0.017357041	4.415696449
540.4397275	0.893565554	0.007128713	4.780674349
513.1307724	0.881697806	0.006006503	4.676343347
281.6134844	0.879045818	0.012803754	4.498550664
415.7599628	0.867882241	0.010565712	4.640827789
161.3481336	0.816304861	0.037675687	4.392581665
290.9070897	0.908351095	0.015137094	4.739752093
712.1697814	0.885676546	0.004239571	4.652976326
518.0218749	0.885594406	0.005567159	4.579104602
363.338625	0.891261312	0.008160106	4.541866395
907.6433079	0.874230095	0.004042094	4.864433525
278.5610887	0.886895497	0.019007694	4.774316959
728.5987607	0.838806847	0.009144544	4.566344878
208.0088333	0.845508209	0.021410619	4.20285206
338.6598116	0.87867363	0.010058898	4.58211633

log.sigma.4.0.mm.3D_glrlm_HighGrayLevelRunEmphasis	log.sigma.4.0.mm.3D_glrlm_RunLengthNonUniformityNormalized	log.sigma.4.0.mm.3D_glszm_GrayLevelVariance
545.7273168	0.84915961	43.84493408
78.32468911	0.763999314	15.63724641
193.9048661	0.796014682	23.17829658
301.0815873	0.832369603	31.39878593
249.3294381	0.800906544	21.75074971
149.0521925	0.795767799	21.41247215
263.8788417	0.809843884	26.04375709
494.1727227	0.799873105	36.12154516
412.5707384	0.806362002	27.77539001
608.2014417	0.821509684	29.70355847
146.6484024	0.795737182	16.52676735
314.5105534	0.81081001	25.71305931
275.7225202	0.727067739	31.91
376.0617574	0.793420075	38.13270876
299.8058008	0.82591392	31.79213597
293.1136938	0.830328422	27.69578053
310.0460353	0.814963913	23.03193783
109.3264803	0.747217172	19.90779163
81.55459057	0.760776373	11.2177113
250.9680791	0.819722831	31.19876899
158.239407	0.735783924	18.52224171
112.8651854	0.802598285	15.35011574
140.9293573	0.781541272	20.78384198
210.6670607	0.787241723	21.69039593
367.5683791	0.832451028	27.61747231
493.1763019	0.837898832	38.27643297
565.2562796	0.825376964	42.04871605
290.0830101	0.789842246	22.20920208
94.96179628	0.773262679	12.46864149
244.5070337	0.755601786	20.16791104
445.2630148	0.793503199	27.68876091
364.7113373	0.736725379	26.73164436
385.169671	0.827820605	35.74093333
420.8630917	0.828473143	33.40725011
351.2248543	0.820529553	21.89788401
403.7917061	0.789518397	26.28115284
572.0533429	0.753341683	41.14846603
557.5473559	0.781983626	41.05557803
85.32144163	0.734439786	9.372532895
258.1841944	0.833185337	32.20694897
422.8366892	0.78180012	32.22668651
348.597522	0.848131565	33.65429954
342.8217082	0.835176936	26.4674922
280.6794683	0.807243789	26.97559834
152.3644883	0.786557914	21.21856124
356.0929594	0.820383078	29.56375293
325.2042028	0.797436658	26.03184977
72.58464111	0.751509879	11.79825061
183.9665366	0.808968127	22.99498428
107.9652357	0.767507408	17.2031611
293.1184066	0.822689203	26.59799016
313.7023282	0.844892643	28.25047518
229.9699299	0.790829161	23.27336857
183.1217472	0.82866419	21.90880749
149.963367	0.8169773	21.47228441
444.0403246	0.791119842	25.2000314
329.8315729	0.829579995	29.49714239
435.3140972	0.787485374	35.97340828
253.2014728	0.79008439	22.32266736
463.2800791	0.786274176	28.11074555
209.8450633	0.772010313	27.20000863
299.7433569	0.802436878	23.72994811
556.7812058	0.820627723	28.37128069
412.3688293	0.794071577	30.36435534
177.2893429	0.80470449	18.77190373
488.0078609	0.763714277	36.55515228
180.8154702	0.691743798	16.82122449
459.25106	0.800998869	35.23363583
460.8420175	0.812365997	29.15024549
208.1431196	0.751935157	16.34980297
358.207713	0.78579402	29.30175187
437.2114885	0.796796585	33.41007291
513.3736663	0.803105621	33.76135707
310.4074125	0.842020967	29.77846559
251.8019067	0.82888357	30.58342075
306.3083857	0.779973845	24.16790205
161.202122	0.762527756	17.08472048
162.8842574	0.75704229	27.18647059
184.86569	0.784353355	18.73603025
555.0450666	0.840331571	43.18357101
544.2956134	0.850730461	44.97455932
268.2927964	0.773412055	30.22967516
394.762851	0.809154745	32.394727
279.9583822	0.77593355	22.36175323
343.8510321	0.828480819	33.83667414
153.078155	0.721698047	18.169375
376.2496746	0.816393201	34.62416468
356.5126635	0.805627723	26.88532324
191.8621631	0.793915578	20.08809584
270.5643162	0.777791442	28.14787001
102.3426968	0.703604431	13.68199881
217.177081	0.8372156	27.19616507
490.1097093	0.80034848	29.88953269
357.7456775	0.806677163	25.09291103
255.3259952	0.807688153	21.52304138
602.5061257	0.794132494	39.17827627
197.4387059	0.801643367	28.01565978
417.0421337	0.730691808	31.35949118
123.8734416	0.7324276	12.03473744
233.6464879	0.798203735	22.22407356

log.sigma.4.0.mm.3D_glszm_SmallAreaHighGrayLevelEmphasis	log.sigma.4.0.mm.3D_glszm_GrayLevelNonUniformityNormalized	log.sigma.4.0.mm.3D_glszm_SizeZoneNonUniformityNormalized
280.4400416	0.043788784	0.339615088
42.748785	0.073280891	0.211562347
97.07398738	0.059007215	0.294777395
178.6863054	0.051352354	0.301445146
127.2864956	0.06133829	0.287178176
107.1259586	0.061383963	0.315283416
153.4370785	0.055939759	0.295580667
292.9871468	0.047602625	0.309518571
201.3420972	0.054268589	0.265907486
339.0885233	0.058262262	0.32736442
70.9556937	0.068783673	0.263085714
173.8671902	0.057392634	0.286359374
146.0456259	0.052933673	0.270765306
203.6161803	0.04632143	0.293564481
154.5502844	0.051345458	0.320511744
157.5943366	0.054047704	0.276443574
136.6957434	0.064396193	0.255707168
63.69060947	0.063008346	0.252351929
40.22602921	0.084228212	0.232851194
153.5897562	0.050112722	0.287535107
90.89219729	0.067891024	0.274832589
53.22634703	0.073045267	0.240483539
63.79510223	0.063736046	0.243979109
113.5030396	0.061347082	0.291834226
195.8448129	0.054492601	0.334834975
294.163054	0.047967051	0.333111204
325.4992779	0.046338654	0.314274172
138.9195287	0.060301605	0.258047458
57.01413754	0.080078125	0.249348958
121.3179104	0.063990233	0.237831059
255.0654035	0.054098618	0.333039898
150.1550623	0.063752583	0.26074311
201.9095352	0.046617284	0.295935802
230.4288846	0.050431523	0.323483161
201.2701052	0.060150411	0.320166789
216.1390891	0.056761852	0.307070884
323.4666867	0.047678309	0.328477126
308.5331239	0.045581463	0.297605332
28.59297534	0.096520083	0.178064404
146.1406833	0.048854376	0.288004562
205.5232715	0.051953951	0.321945336
210.8787159	0.050235956	0.313438684
172.0397174	0.054507228	0.287251405
142.8455462	0.057122789	0.291758585
100.6383033	0.06216409	0.291058264
201.4527614	0.052590894	0.338967765
180.4192387	0.055203889	0.278241649
40.71876859	0.083898349	0.241861187
95.26129174	0.058848003	0.259245562
61.07050811	0.073802773	0.2613159
150.5045811	0.055683545	0.301525961
174.700345	0.052877129	0.298414962
124.2825835	0.058219592	0.289069822
99.91833519	0.061048192	0.300615583
81.39073316	0.060509873	0.254736209
228.1558928	0.058745037	0.261556374
172.9318765	0.051322655	0.295402089
189.0866602	0.048491072	0.259465807
126.6831076	0.06019482	0.294629437
247.5076884	0.054241478	0.293534495
143.879339	0.054474624	0.31252291
169.4181022	0.059909985	0.291029962
318.4614032	0.054649145	0.32423941
245.0142084	0.050942693	0.258433954
94.93016713	0.066590049	0.265481341
276.6370588	0.049443298	0.277184798
59.38094722	0.07744898	0.183469388
252.6086181	0.047762387	0.284278098
236.6744627	0.052216926	0.330317141
100.6821674	0.070744892	0.236204147
179.9601754	0.057533781	0.298255412
242.767693	0.050129764	0.305395168
242.3882394	0.052961225	0.290937399
156.1960465	0.051257425	0.300527021
162.461843	0.052010222	0.297620333
125.8881956	0.06004879	0.26001595
79.4346372	0.067751153	0.276099798
103.4468513	0.056401384	0.229273356
102.0196221	0.064499055	0.244494854
287.32025	0.045740929	0.329527359
286.5881207	0.044011696	0.349783822
164.3907863	0.053992629	0.346255717
216.9945466	0.049651276	0.291419062
126.824328	0.060399446	0.240819072
178.776246	0.049381149	0.278955569
83.42326744	0.073984375	0.236328125
192.1768231	0.049290947	0.317115056
212.5798688	0.055793532	0.328785593
101.3150967	0.063827399	0.285825174
130.1660692	0.053256458	0.273514713
57.0511532	0.078548483	0.24297442
115.925639	0.053637272	0.303064063
240.8093687	0.052755774	0.295799789
190.7909412	0.056739857	0.319653756
122.4167808	0.060226193	0.244640717
355.0313628	0.047706399	0.356949801
97.73481756	0.052398743	0.220142951
173.9755288	0.054435785	0.276060796
35.54639516	0.08465222	0.173119065
130.6929733	0.059593748	0.30721681

log.sigma.4.0.mm.3D_glszm_SizeZoneNonUniformity	log.sigma.4.0.mm.3D_glszm_GrayLevelNonUniformity	log.sigma.4.0.mm.3D_glszm_LargeAreaEmphasis	log.sigma.4.0.mm.3D_glszm_ZoneVariance
254.0320856	32.7540107	41.33957219	33.29157968
34.48466258	11.94478528	249.6503067	216.7463585
96.98176292	19.41337386	110.8510638	89.11109469
87.11764706	14.84083045	48.64013841	37.21152764
154.5018587	33	156.3791822	137.0060944
57.06629834	11.11049724	100.2762431	86.97994567
397.2604167	75.18303571	193.2462798	175.5675085
335.8276498	51.64884793	487.3456221	465.1819202
285.3187325	58.23019571	111.6067102	93.65691822
99.19141914	17.65346535	65.39273927	52.87562222
92.08	24.07428571	105.48	86.91621224
159.7885305	32.02508961	107.109319	90.045609
75.81428571	14.82142857	652.8107143	608.3503954
121.2421308	19.13075061	157.7602906	140.4764406
181.7301587	29.11287478	94.16931217	81.92548423
293.0301887	57.29056604	125.1188679	108.0759915
83.87195122	21.12195122	45.75609756	32.50472189
54.76036866	13.67281106	284.0599078	251.7744696
54.48717949	19.70940171	269.965812	239.1965081
384.7219731	67.05082212	88.33557549	73.23729782
123.125	30.41517857	968.2946429	922.7321429
51.94444444	15.77777778	122.4398148	97.48608968
49.039801	12.81094527	199.5621891	173.60798
179.4780488	37.72845528	155.2569106	136.7319347
114.8483965	18.6909621	64.49271137	50.71844215
244.8367347	35.25578231	90.6952381	80.30667592
269.0186916	39.66588785	125.2978972	111.0331566
155.0865225	36.24126456	293.6173045	272.4970861
23.9375	7.6875	101.5520833	79.87141927
82.52737752	22.20461095	233.2910663	198.2528549
336.370297	54.63960396	385.0574257	362.8649309
85.26299694	20.8470948	858.8776758	818.8439993
266.3422222	41.95555556	73.39	60.51785062
219.6450663	34.24300442	75.39911635	63.42078904
365.6304729	68.69176883	87.97197898	73.10021746
173.8021201	32.12720848	331.5106007	303.435581
218.7657658	31.75375375	1041.656156	1010.440614
338.6748682	51.87170475	350.7214411	330.1538612
27.06578947	14.67105263	360.2631579	294.7809903
584.6492611	99.17438424	59.52019704	46.69462496
181.899115	29.3539823	378.2300885	357.1514261
401.8283931	64.4024961	31.97893916	22.8803869
289.2621648	54.88877855	61.65938431	48.49985356
180.8903226	35.41612903	128.983871	112.8804787
84.40689655	18.02758621	129.2689655	109.8482283
267.106599	41.44162437	83.41751269	71.28272276
291.3190067	57.79847182	179.3543457	160.3690574
33.61870504	11.6618705	289.6906475	250.4255473
53.92307692	12.24038462	79.11057692	62.37146357
72.12318841	20.36956522	209.0942029	185.0984037
209.862069	38.75574713	65.59913793	51.97497812
326.7643836	57.90045662	30.33150685	20.85410563
281.8430769	56.76410256	324.2605128	302.8449767
89.28282828	18.13131313	82.2020202	68.38465463
88.13872832	20.93641618	70.90751445	56.13171172
102.2685422	22.96930946	232.056266	207.1839797
347.3928571	60.35544218	52.30527211	40.47424612
165.539185	30.93730408	335.2413793	305.8642997
109.6021505	22.39247312	148.2741935	128.6483408
589.7108014	108.9711299	233.4449975	212.796591
116.5710456	20.31903485	247.077748	224.6614581
185.677116	38.22257053	131.7335423	114.2197109
504.192283	84.97942122	96.53569132	82.67623122
418.4045707	82.47621989	245.7720815	223.602467
45.6627907	11.45348837	98.23255814	77.13669551
200.1274238	35.69806094	690.7977839	662.185657
25.68571429	10.84285714	954.5428571	867.7869388
427.8385382	71.88239203	182.5408638	165.2949173
516.9463259	81.71948882	131.4319489	117.4257024
107.9452954	32.33041575	393.6323851	353.4195807
85.59930314	16.51219512	170.5191638	150.0332892
281.87974	46.26977248	260.7757313	239.1728747
154.4877589	28.12241055	114.9435028	96.52307943
211.5710227	36.08522727	67.83664773	57.54020653
262.5011338	45.87301587	88.91043084	76.30873581
75.92465753	17.53424658	121.2534247	100.131216
117.8946136	28.92974239	269.9016393	238.4156113
38.97647059	9.588235294	270.6588235	238.9022837
84.35072464	22.25217391	197.1507246	172.6697417
234.2939522	32.52180028	60.46554149	49.64319979
256.3915416	32.26057299	39.7994543	32.43633873
145.0811456	22.62291169	218.5417661	200.1071422
198.7478006	33.86217009	217.755132	194.7798372
125.9483748	31.58891013	267.6347992	233.1107488
188.5739645	33.3816568	61.60502959	48.58745098
37.8125	11.8375	1033.725	942.044375
211.5157421	32.87706147	133.2218891	120.4361717
229.1635581	38.88809182	255.6212339	240.1448553
93.4648318	20.87155963	98.04281346	77.99870942
118.4318707	23.06004619	174.6120092	152.7407261
49.8097561	16.10243902	628.6926829	588.2306722
226.0857909	40.01340483	34.71581769	24.52038037
375.0741325	66.89432177	176.7563091	160.0483305
261.4767726	46.41320293	201.4987775	185.2237672
242.4389506	59.68415742	113.2068618	93.92138123
302.3364817	40.40731995	306.1723731	289.8496283
131.6454849	31.33444816	148.0016722	125.8109557
50.51912568	9.961748634	338.3224044	305.5889994
19.21621622	9.396396396	291.0990991	234.6462138
380.3344103	73.77705977	206.6090468	184.856392

log.sigma.4.0.mm.3D_glszm_ZonePercentage	log.sigma.4.0.mm.3D_glszm_LargeAreaLowGrayLevelEmphasis	log.sigma.4.0.mm.3D_glszm_LargeAreaHighGrayLevelEmphasis	log.sigma.4.0.mm.3D_glszm_HighGrayLevelZoneEmphasis
0.352497644	0.084268941	23234.18583	501.3930481
0.174331551	5.202996994	13999.8589	89.72392638
0.214471969	0.821534461	20089.55015	190.5227964
0.29580348	0.239476879	14450.91349	303.7058824
0.227195946	0.763917043	34506.84015	243.0855019
0.274242424	1.314014991	8994.895028	181.2044199
0.237834012	1.08901114	37914.95685	282.9970238
0.212411903	1.214177885	200527.1668	522.0700461
0.236031676	0.327881352	41698.73719	400.9888164
0.282649254	0.127920103	36673.93069	599.0693069
0.232095491	0.937650232	14449.32571	143.1857143
0.24208243	0.435039022	30294.20968	318.5645161
0.149973219	2.816354378	156754.1286	281.55
0.240535818	0.524531515	51340.08959	379.1743341
0.28578629	0.347222393	28257.24339	278.675485
0.242230347	0.639505215	28809.55377	302.7235849
0.274706868	0.165941441	15145.16159	282.3597561
0.175993512	4.328021482	21081.15668	130.8847926
0.18027735	6.033954522	14545.00427	87.29487179
0.257357184	0.536786377	17565.59417	273.2593423
0.148148148	8.601154487	115832.4688	174.5491071
0.200185357	1.866885076	10634.90741	117.8657407
0.196289063	2.126666216	22105.56219	145.7562189
0.232338496	0.906396811	28745.3252	214.0650407
0.269442262	0.209973387	21836.06414	358.1574344
0.310257493	0.213659637	42148.4585	499.3278912
0.264769564	0.260161823	65842.19977	570.1892523
0.217595945	1.272255716	70745.63894	288.6971714
0.214765101	2.299993726	5802.1875	112.6770833
0.168938656	1.05249946	55211.55043	248.2910663
0.212274065	1.054867778	145678.8693	445.1019802
0.158047366	2.296594504	326144.8777	312.6207951
0.278724063	0.207906591	30947.32667	366.2533333
0.28893617	0.212189527	30084.99411	400.8718704
0.259309718	0.320283891	28038.46935	352.5516637
0.188729577	1.016307989	114239.3869	395.45053
0.178984144	1.924031862	569870.2252	555.539039
0.220499903	0.819748168	157180.862	561.6010545
0.123577236	4.550515237	32268.23026	75.38815789
0.279229711	0.369905545	14092.95468	264.1763547
0.217810332	0.839757884	175635.0053	368.0566372
0.331523145	0.122308974	10636.24259	361.173947
0.275663838	0.229427359	19373.76564	331.6236346
0.249196141	0.4874054	37239.05	262.2516129
0.226917058	1.361057256	14254.73103	179.0241379
0.287067395	0.338106831	24985.48604	350.2373096
0.229504603	0.694727199	49027.95989	341.4250239
0.159586682	8.132410828	13005.61151	85.3381295
0.244418331	0.644794275	11994.63942	192.3701923
0.204142012	4.059027608	12783.00362	126.2391304
0.270922538	0.316336467	20240.44684	276.4741379
0.324829427	0.124674894	9181.651142	314.4009132
0.216090426	1.758084849	63339.15385	233.9548718
0.269021739	0.690110912	11574.13805	190.4713805
0.260150376	1.03058854	7127.367052	160.9306358
0.200512821	0.56081566	100005.1816	443.9309463
0.290729295	0.196846102	16604.07738	319.6547619
0.184499711	0.812565246	146624.884	390.4514107
0.225728155	0.645074126	35997.24731	239.172043
0.220067915	0.614155692	95023.10602	453.0164261
0.211211778	1.652962898	39031.74531	246.7640751
0.238951311	0.602578515	32208.3558	308.6410658
0.268612887	0.234650973	48210.42251	558.318328
0.212383576	0.768504417	82783.18592	465.4694256
0.217721519	0.756011378	14799.51163	183.2674419
0.186949767	1.707346557	284390.6053	516.1772853
0.107361963	5.999127652	159116.3571	162.8857143
0.2408	0.444273908	79348.5515	465.0863787
0.267201639	0.312073839	58075.50607	420.5073482
0.157694962	2.484942554	67579.16193	212.8402626
0.220939184	0.554362211	56883.06272	339.7735192
0.215151515	0.768350239	93907.30011	444.0541712
0.232996928	0.252493953	55499.2806	477.7721281
0.31164232	0.224920686	23752.75852	290.6193182
0.281699138	0.527000661	18734.43197	277.1303855
0.217585693	0.413686209	38114.36644	266.9726027
0.178213689	2.194378908	37709.84075	156.9555035
0.177453027	2.656318534	30203.73529	204.0764706
0.202108963	1.415407021	29738.49565	200.1652174
0.303976058	0.115233573	38586.57947	500.8185654
0.368526898	0.084351035	22340.2292	501.1145975
0.232907171	0.880827214	59425.1957	267.2720764
0.208626491	0.650736063	77552.82258	395.3475073
0.170191995	1.028130797	76892.76291	260.9254302
0.277162772	0.236552696	20446.59172	337.6065089
0.104438642	9.502936349	122032.95	172.55
0.27966457	0.37560595	51627.9955	344.3208396
0.254194019	0.948383505	71610.5911	375.615495
0.223360656	0.691422996	16243.68502	192.6850153
0.21382716	0.776814515	43064.32102	258.1639723
0.157208589	14.39045397	31110.45854	122.9609756
0.3131822	0.268790467	6954.310992	214.9155496
0.244645958	0.389803545	83538.60095	456.3706625
0.247878788	0.634737089	66228.93032	342.8300733
0.227711397	0.469106457	30561.89203	244.469223
0.247516072	0.627897186	155755.9717	593.566706
0.21228257	1.134651684	26443.66054	209.8595318
0.1747851	0.834386346	150558.4973	345.4043716
0.133093525	2.542322263	37933	104.7837838
0.214409422	1.322062336	35995.28756	244.5904685

log.sigma.4.0.mm.3D_glszm_SmallAreaEmphasis	log.sigma.4.0.mm.3D_glszm_LowGrayLevelZoneEmphasis	log.sigma.4.0.mm.3D_glszm_ZoneEntropy	log.sigma.4.0.mm.3D_glszm_SmallAreaLowGrayLevelEmphasis
0.600509699	0.005920148	6.636794123	0.004526464
0.465181532	0.045536011	6.102515369	0.020016419
0.561164591	0.015399947	6.282931457	0.011425805
0.565245983	0.010943715	6.293165386	0.004924708
0.551984839	0.009263611	6.47858153	0.006501223
0.575740813	0.017349614	5.842486791	0.010659364
0.561091808	0.007731546	6.755385964	0.004394622
0.574521305	0.004243488	6.824631618	0.002331732
0.527540641	0.004983343	6.875632782	0.003344613
0.590909249	0.005880878	6.185834718	0.005087526
0.520462169	0.016952116	6.250995892	0.007964653
0.548572183	0.007398715	6.491428006	0.004709492
0.533345872	0.011871354	6.370535573	0.007780094
0.558271694	0.009075968	6.625897322	0.006370671
0.584187493	0.011261259	6.524387166	0.007041569
0.539331289	0.00743673	6.802880972	0.00448594
0.512259296	0.010226961	6.366662192	0.007463413
0.514751596	0.029082044	6.224263562	0.020526451
0.486291482	0.037721134	5.901058008	0.023443605
0.552488518	0.009152384	6.892569667	0.005422688
0.541977887	0.014308795	6.397060095	0.006976023
0.503956051	0.028143646	6.060701108	0.014500966
0.494863102	0.021402347	6.079915445	0.01362841
0.556377305	0.012317904	6.517474345	0.008997321
0.599046651	0.007632611	6.223548351	0.006151865
0.597298041	0.005662785	6.649146534	0.002937987
0.578106031	0.004575671	6.793195418	0.003237091
0.513182322	0.007325205	6.545990071	0.004796369
0.510393897	0.027929586	5.577565267	0.020677802
0.49854606	0.009398262	6.533262279	0.006522914
0.598848161	0.00426109	6.591911602	0.003081498
0.521020076	0.010127093	6.448309929	0.007816854
0.557890916	0.006632873	6.79330422	0.004306844
0.586749057	0.006592942	6.556205174	0.00457421
0.586403113	0.00519793	6.520465308	0.002690568
0.573592563	0.006132431	6.52602191	0.004327869
0.593588001	0.004805927	6.705959628	0.003619464
0.563107855	0.004097885	6.953673843	0.002902164
0.421309341	0.039335592	6.020150061	0.020366775
0.55372392	0.010954383	7.005690373	0.006033846
0.587110248	0.007001043	6.531374855	0.005010553
0.576853024	0.005602627	6.752978559	0.003469715
0.551044792	0.006350858	6.71369953	0.00423655
0.554985131	0.008943109	6.545521569	0.005995261
0.5572848	0.015389814	6.210905672	0.010698042
0.603738102	0.00678932	6.530995852	0.003559293
0.538874035	0.00586404	6.760790181	0.003285821
0.505121257	0.036243256	5.767699059	0.016289553
0.517826147	0.017019593	6.14992557	0.008324495
0.523980441	0.023072955	6.105208182	0.015759617
0.566248914	0.011774967	6.552780964	0.006394857
0.562489933	0.006118789	6.702807537	0.003954307
0.551922221	0.009173434	6.637246986	0.005314296
0.563809755	0.014366038	6.152141094	0.010147756
0.511338934	0.020182761	6.365801358	0.011927742
0.527390548	0.005729357	6.57947802	0.002501738
0.559636666	0.007083029	6.78369028	0.004793786
0.518852588	0.007445801	6.881258772	0.004803418
0.559954447	0.011527048	6.308935932	0.008580968
0.558024185	0.003708237	6.895198812	0.002419357
0.579533979	0.010385532	6.401670949	0.007505123
0.557173378	0.007301786	6.517891241	0.005058335
0.589956138	0.003141387	6.718330421	0.001595126
0.519123811	0.003899668	7.022361468	0.001824279
0.531037536	0.014913228	6.060566811	0.01105235
0.541015952	0.00496668	6.832411757	0.002627892
0.422610756	0.0193189	6.144237818	0.006530635
0.54803139	0.004071277	6.980057517	0.00198188
0.595077524	0.004339606	6.675085444	0.003053981
0.498179795	0.01003209	6.50948149	0.004549912
0.559299518	0.009822668	6.233415394	0.006609077
0.570686649	0.005425838	6.764925035	0.002952289
0.55503896	0.006120175	6.612287226	0.004899584
0.559254319	0.009220668	6.555039004	0.005842527
0.560499327	0.009452177	6.680383893	0.005217848
0.520774243	0.01157062	6.373825334	0.008858625
0.542765505	0.01578721	6.389257806	0.008567129
0.488619948	0.017457192	6.261305478	0.010967886
0.504308987	0.012237213	6.415897912	0.007585509
0.5935585	0.005715534	6.717025848	0.004108578
0.610407496	0.006675826	6.579421714	0.004924006
0.610557887	0.013242067	6.38018861	0.007927689
0.556381769	0.005777723	6.733077091	0.003935008
0.503185938	0.009253253	6.70118246	0.005982741
0.540686657	0.007511184	6.765327221	0.003450948
0.496257903	0.018595618	6.015896983	0.012587796
0.579380146	0.008289735	6.592604711	0.005269947
0.593643203	0.006160028	6.454302325	0.004786111
0.55268771	0.013195233	6.235774847	0.008513578
0.536678824	0.015490807	6.569206125	0.01174731
0.503589643	0.020467134	5.919448726	0.012999401
0.567229806	0.01501357	6.603979173	0.007436025
0.56096313	0.005148574	6.803725483	0.003706976
0.585317875	0.006115288	6.506477585	0.004479574
0.504922249	0.008504181	6.830145618	0.0050392
0.619932455	0.004307946	6.576325847	0.003017501
0.473648894	0.017579947	6.924820027	0.008660126
0.531434585	0.012167584	6.061037651	0.004274095
0.403265547	0.027725114	5.844632786	0.011409069
0.573103597	0.010546367	6.590994353	0.007591833

log.sigma.4.0.mm.3D_ngtdm_Coarseness	log.sigma.4.0.mm.3D_ngtdm_Complexity	log.sigma.4.0.mm.3D_ngtdm_Strength	log.sigma.4.0.mm.3D_ngtdm_Busyness	log.sigma.4.0.mm.3D_ngtdm_Contrast
0.005684687	0.497632292	2.978727474	0.175953649	4.22E-05
0.011166888	0.14942159	1.351294683	0.665672998	7.82E-05
0.0085544	0.225688869	1.935271797	0.33903858	4.50E-05
0.012841629	0.568557837	4.014628261	0.143321319	9.86E-05
0.004999248	0.169409508	1.318683025	0.421923245	2.30E-05
0.016738481	0.489854911	3.467534285	0.242718471	0.000148374
0.002138515	0.135721238	1.135469577	0.705516201	7.84E-06
0.002512724	0.149655573	1.473869218	0.397779509	8.01E-06
0.002760296	0.15813466	1.264701885	0.453198184	1.13E-05
0.010954781	0.653623073	5.567579361	0.097842923	5.16E-05
0.007903771	0.152787563	1.281273732	0.494052039	4.24E-05
0.005152204	0.246804397	1.944773624	0.315761777	2.45E-05
0.007041043	0.193509809	2.55656263	0.23903928	2.08E-05
0.007797681	0.339636812	2.962261516	0.177760462	3.57E-05
0.005507794	0.338958881	2.087772523	0.278523167	3.35E-05
0.002825364	0.136961917	0.955348058	0.641933847	1.64E-05
0.009268598	0.360069669	2.868286086	0.183326505	5.17E-05
0.009560826	0.163062096	1.5229885	0.559243326	5.93E-05
0.00840229	0.091780361	0.84152662	0.97405798	5.64E-05
0.002494827	0.116663181	0.935658606	0.757190978	1.21E-05
0.004449792	0.086604451	1.062043944	0.637497347	1.25E-05
0.010952678	0.170993669	1.437710886	0.49387743	6.76E-05
0.011702602	0.216405958	1.97503228	0.370538428	7.34E-05
0.004361708	0.174728495	1.442533858	0.465675742	1.60E-05
0.007930651	0.471322232	2.544435643	0.2005453	5.66E-05
0.004782854	0.426138068	2.883790547	0.197907001	2.41E-05
0.003645179	0.330502447	2.62400831	0.223519951	1.61E-05
0.004391351	0.153845203	1.284268862	0.416086305	1.84E-05
0.023974745	0.28107441	2.444680039	0.375596683	0.000203828
0.005574179	0.140153062	1.466760592	0.38369602	1.87E-05
0.002570902	0.129805912	1.077857339	0.489461992	1.03E-05
0.005356826	0.206435556	2.385796404	0.231997076	1.49E-05
0.004110806	0.238895465	1.852094835	0.312047434	2.13E-05
0.005115958	0.321659019	2.335023781	0.238148498	2.77E-05
0.002582199	0.122273196	0.891215497	0.648483855	1.37E-05
0.003971361	0.187752003	1.756629253	0.328645672	1.36E-05
0.003405604	0.206231932	2.500076564	0.225247353	8.07E-06
0.002847104	0.190687463	1.864012191	0.313177594	9.94E-06
0.008731624	0.065715127	0.686666323	0.763011917	3.40E-05
0.001936089	0.083471323	0.674179351	1.047319596	1.11E-05
0.004869715	0.246353566	2.438393809	0.212451315	1.57E-05
0.00328537	0.273393144	1.991388484	0.365202171	1.52E-05
0.003183653	0.175769894	1.096212533	0.513534939	1.97E-05
0.004569914	0.30873459	2.247302292	0.298909249	1.79E-05
0.008965201	0.256474264	2.123936797	0.380534276	5.13E-05
0.004072333	0.250317782	1.591208133	0.357192885	2.46E-05
0.002600151	0.129818807	1.061653813	0.55358317	9.48E-06
0.014391425	0.103851809	1.230536882	0.742202402	8.45E-05
0.014004105	0.387880611	3.021507066	0.229958433	8.95E-05
0.008583949	0.201099883	1.789920164	0.52908842	4.53E-05
0.004600191	0.201176135	1.364742826	0.424123381	3.05E-05
0.003456422	0.228179257	1.368084803	0.463667726	2.12E-05
0.002569478	0.094145978	0.76299748	0.817521466	1.14E-05
0.009753785	0.354180838	1.93686619	0.318793043	7.43E-05
0.008654797	0.254245744	1.645704538	0.495297516	7.55E-05
0.006427931	0.212986453	2.240740927	0.218576741	2.26E-05
0.003041773	0.150324367	1.034422411	0.547507441	1.77E-05
0.003779011	0.162406657	1.762125275	0.31360199	1.45E-05
0.006919761	0.225790833	1.703163142	0.283449139	2.80E-05
0.001381895	0.085273201	0.746258602	0.759599711	4.54E-06
0.007078326	0.276628622	2.761751785	0.288269089	2.58E-05
0.004595942	0.180552776	1.469611487	0.405433471	2.23E-05
0.001926223	0.174390444	1.249580196	0.458396168	7.84E-06
0.001826542	0.093856421	0.96862112	0.62755172	5.40E-06
0.013308074	0.341143442	2.327812028	0.262716898	8.82E-05
0.003637426	0.171343975	2.183610555	0.263624228	8.44E-06
0.010001743	0.134533286	1.96442601	0.265141034	2.08E-05
0.002159668	0.142505212	1.363899556	0.431600085	6.42E-06
0.00216073	0.123226853	1.17098434	0.483674281	7.29E-06
0.00422585	0.092613079	0.922740333	0.613548532	1.44E-05
0.009134482	0.395470806	3.496106432	0.158273469	4.08E-05
0.002995379	0.15931381	1.449844562	0.407278488	1.21E-05
0.005226974	0.301561095	2.911769371	0.199351984	2.05E-05
0.005434997	0.256045003	1.630832539	0.315851872	3.69E-05
0.004021104	0.201244174	1.50586854	0.500827792	2.45E-05
0.008153617	0.301042467	2.640292917	0.204260108	3.82E-05
0.004894792	0.115253711	1.009885173	0.586494252	1.80E-05
0.014906674	0.297852752	3.870801287	0.201208877	5.24E-05
0.006944124	0.163207172	1.453714056	0.419076425	3.04E-05
0.005233514	0.397645102	3.17208142	0.177660858	3.41E-05
0.006201526	0.516515225	3.283666433	0.160782109	4.64E-05
0.006579816	0.298097804	2.933398821	0.216325703	2.03E-05
0.00369957	0.195003073	1.637576917	0.345225145	1.50E-05
0.00422482	0.116792764	1.412133496	0.404838873	1.27E-05
0.005155261	0.341384742	2.564557393	0.253113676	2.55E-05
0.008863632	0.119570486	1.637193571	0.401228254	2.85E-05
0.005104792	0.268536299	1.990656809	0.267442129	3.17E-05
0.004318118	0.230151975	1.744962888	0.333062116	1.90E-05
0.007826284	0.219472274	1.779626848	0.350921024	4.05E-05
0.006425668	0.204017399	2.064598228	0.278141691	2.36E-05
0.00879035	0.106423644	1.13870698	0.832087086	7.38E-05
0.004864666	0.203104247	1.296032979	0.545815545	3.99E-05
0.002504391	0.135787135	1.398710412	0.393425594	7.61E-06
0.003446512	0.178137826	1.354003666	0.405941729	1.46E-05
0.00283837	0.105415114	0.896553145	0.642190824	1.09E-05
0.003764506	0.289754508	2.601373052	0.220257974	1.44E-05
0.004925663	0.124876781	1.269985763	0.556789202	2.63E-05
0.012594195	0.410203465	5.662887841	0.098805238	4.84E-05
0.015108764	0.138410327	1.777061856	0.308581919	5.44E-05
0.002005102	0.07456786	0.502430266	1.190618809	1.10E-05

log.sigma.3.5.mm.3D_gldm_GrayLevelVariance	log.sigma.3.5.mm.3D_gldm_HighGrayLevelEmphasis	log.sigma.3.5.mm.3D_gldm_GrayLevelNonUniformityNormalized	log.sigma.3.5.mm.3D_gldm_DependenceEntropy
32.19070822	582.4491046	0.057743437	6.918520382
10.16305185	104.4502674	0.10606766	6.353299661
15.34952379	195.0912647	0.072789904	6.86326156
26.24942354	322.7799386	0.059710077	6.796336523
14.90969383	266.7242399	0.079261351	6.835197277
20.43837236	159.9287879	0.074040404	6.573821252
17.63546544	337.2357105	0.072241291	6.941031609
18.49524032	507.5215348	0.076581317	6.875307753
19.11133631	466.5743511	0.066588032	6.96779124
18.69770463	573.0102612	0.071417632	6.727012395
11.52899655	158.1830239	0.085353798	6.642129937
18.90564528	362.835141	0.070409418	6.850686908
13.75518929	288.6363149	0.113517012	6.808455755
22.39887059	476.3593477	0.073466914	6.826274547
20.95082332	395.4435484	0.073961145	6.694579349
20.53683775	332.3304388	0.065847472	6.905127629
16.20614519	300.8375209	0.079701972	6.474265556
12.62531006	117.5506894	0.099931658	6.660710729
9.035724037	109.7457627	0.101426635	6.454130042
20.35361033	285.1996538	0.066021433	6.997485179
10.88228815	164.6537698	0.099638518	6.80361258
11.22582524	124.2863763	0.083816839	6.451386363
13.73217773	147.0625	0.087305069	6.626070651
13.14596233	224.2678504	0.08339198	6.771739198
17.31990233	346.6582875	0.075670969	6.706507901
25.00015983	564.7729	0.065363235	6.848724851
25.05675149	496.482524	0.069142654	6.981376418
15.15146126	341.8682114	0.082170304	6.775516627
10.71969731	100.7785235	0.098238818	6.109929343
10.34654233	255.9995131	0.099213637	6.680343472
17.11945616	457.3430013	0.075042481	7.010181401
11.72164856	361.6955051	0.115454472	6.529314636
25.47623988	410.1049861	0.060371032	7.016639143
22.44356288	398.6714894	0.067659574	6.936197822
16.98756064	327.4641235	0.070449641	6.819570036
14.79495633	413.7819273	0.083118403	6.804603728
16.54767926	652.0591239	0.099755515	6.888414436
24.94141359	610.626429	0.069645686	7.270829641
5.528101659	118.1813008	0.124671822	6.192948774
24.62590397	273.0676754	0.057216879	7.063577688
17.47131317	376.9055513	0.091337937	6.802007359
26.92116521	371.1114559	0.05753276	6.875418601
19.83378252	390.3514919	0.064872487	6.847116627
17.88780358	374.2596463	0.075621827	6.75790463
15.03871709	157.6150235	0.082789031	6.653241669
19.82476581	364.228051	0.068868385	6.904379971
16.40379896	374.7183253	0.078957059	6.835259957
9.082111302	80.0206659	0.105650762	6.446148757
16.73293464	199.920094	0.072147097	6.535273767
13.2394524	114.6050296	0.090068187	6.716367589
20.48863254	338.3382639	0.065982238	6.807507106
22.5346385	329.7353901	0.061291359	6.856799628
14.22718908	244.4835993	0.081274305	6.793358814
15.18477933	138.0652174	0.074265517	6.552330864
17.85293911	177.4857143	0.068161004	6.687001882
14.40169915	510.5030769	0.087397765	6.607290572
20.72840703	309.3166873	0.063931023	6.897526329
18.79412213	374.0043378	0.081293459	6.870870227
12.87606042	249.2293689	0.089570176	6.504173579
18.88680419	484.588783	0.069846579	7.19645105
15.61400411	220.4150623	0.092607437	6.828911791
16.50620713	342.5318352	0.074258581	6.780537058
19.42943994	521.2544481	0.070349079	6.925235533
19.48049935	466.550833	0.07327547	6.998134593
12.23179138	160.6088608	0.081595898	6.491524099
16.80761257	500.8190057	0.089658602	6.889742755
7.928021731	230.1986196	0.128427397	6.518935537
20.60159747	528.06192	0.074425651	6.99155504
18.4603337	481.8453133	0.072780618	6.924445136
10.07540433	216.7681159	0.09506592	6.68895429
16.50259423	340.1424172	0.08044383	6.641859087
19.83501638	442.0944056	0.072522753	7.005182653
18.4716132	488.8609039	0.079408476	6.798057075
24.28039453	342.3634351	0.062339007	6.784052641
23.66537344	267.1788566	0.060927185	6.936395581
14.21249786	295.1870343	0.092186185	6.57270958
9.954932365	171.5822204	0.096442039	6.657466041
14.78182191	168.9770355	0.090406684	6.675692077
11.92536333	192.5577036	0.087184683	6.677342136
30.5223296	504.1941	0.062799116	6.831453606
33.20284693	535.5801911	0.058302312	6.920858456
15.28713407	230.9638688	0.089932304	6.796544943
17.73197031	407.821658	0.074400163	6.899473839
12.44808944	235.9179954	0.089789416	6.724249233
23.57057525	357.0410004	0.061692757	6.979074367
9.80707824	181.6018277	0.110793072	6.61740263
24.23165153	371.8469602	0.069325493	6.949639429
17.68095091	295.0091174	0.073539575	6.953726308
13.73892127	203.8736339	0.08427954	6.687791642
15.24613931	316.5471605	0.08474635	6.840398729
12.19934887	84.55368098	0.119687182	6.472975811
21.24550082	230.2069689	0.063192598	6.865711624
18.69601934	476.7204322	0.075112618	6.874114523
16.02613407	372.6636364	0.080048669	6.833279368
15.40341656	247.1886489	0.074862153	6.725642154
23.61757926	483.1072472	0.07052981	7.137395351
18.7078485	207.8899539	0.068690537	6.950939626
16.86564332	419.2378223	0.099839538	6.704659939
8.109736671	160.8129496	0.106668622	6.438793184
14.67523182	270.0493592	0.080838353	6.841553453

log.sigma.3.5.mm.3D_gldm_DependenceNonUniformity	log.sigma.3.5.mm.3D_gldm_GrayLevelNonUniformity	log.sigma.3.5.mm.3D_gldm_SmallDependenceEmphasis
385.1328935	122.531574	0.322512662
124.1657754	99.17326203	0.162357438
203.0247718	111.6597132	0.191858077
169.9580348	58.33674514	0.272656426
331.6993243	187.6908784	0.21241635
102.9727273	48.86666667	0.248982789
810.453548	408.2355335	0.221574109
707.9592796	391.1773688	0.206833338
690.7589089	302.7091949	0.22777434
161.7873134	76.55970149	0.254453557
207.433687	128.7135279	0.21472885
343.0425163	162.2937093	0.242604101
174.5597215	211.9362614	0.150777351
245.1910309	126.1426907	0.237203389
319.2469758	146.7389113	0.259511027
676.7157221	288.1485375	0.227410406
203.2328308	95.1641541	0.244605664
140.7437145	123.215734	0.173292294
165.6394453	131.651772	0.172651786
792.0417388	343.2454318	0.22684406
319.042328	301.3068783	0.145105982
168.3697868	90.43836886	0.200502374
138.4550781	89.40039063	0.190300045
363.6407253	220.738572	0.217101709
195.325216	96.32914375	0.257294281
398.2663571	154.8455044	0.287558339
484.9795855	223.5381998	0.251607475
374.2860246	226.9543809	0.223111734
64.35123043	43.91275168	0.207257364
252.6290166	203.7848101	0.166664459
629.3690626	357.0521227	0.207711902
223.8351861	238.8753021	0.166092144
516.2195726	194.9380613	0.256058226
356.8382979	159	0.252882726
691.8551317	310.260218	0.241760376
379.0526842	249.2720907	0.192318735
400.3501747	371.1902714	0.179371473
617.5967836	359.4413873	0.210853892
149.795122	153.3463415	0.139310288
1228.369188	415.9667125	0.247525234
317.7062452	236.9306091	0.203972454
744.873804	222.4791828	0.305171617
596.1590474	236.9791952	0.257618178
374.9244373	188.1471061	0.243789521
173.8591549	105.8043818	0.206928897
405.2783242	189.0437158	0.254716045
651.5633494	360.2021043	0.20866554
104.2560276	92.02181401	0.138413136
143.3266745	61.39717979	0.237537484
153.5473373	121.7721893	0.188011062
428.7220708	169.508369	0.255462999
609.5843963	206.6131712	0.280724157
636.5820035	366.7096631	0.212977652
174.5235507	81.98913043	0.243909549
212.9819549	90.65413534	0.24097602
274.6615385	170.425641	0.197288634
669.4123609	258.6009889	0.259064549
459.761712	281.112782	0.196956682
242.0291262	147.6116505	0.217966487
1210.319093	637.6294227	0.214350039
206.8776897	163.5447339	0.197707623
393.7962547	198.270412	0.215842332
895.0621869	407.2508205	0.257026736
1065.498754	558.5789059	0.202103441
119.4734177	64.46075949	0.187627894
450.4179182	346.2615225	0.181114901
129.4279141	167.4693252	0.126254332
888.74208	465.16032	0.223621465
855.1205395	426.2760799	0.24779779
354.6038647	275.5010352	0.163546799
191.0862202	104.4965358	0.206812992
564.7752914	311.1226107	0.206636333
324.1978938	180.9719175	0.211616283
399.8809208	140.8238158	0.286446183
531.3302459	190.763015	0.264586249
183.2250373	123.7138599	0.205478602
293.9874791	231.0751252	0.168098797
117.4363257	86.60960334	0.170164629
237.4335091	148.8242531	0.196338958
418.2150492	146.8871313	0.283378789
360.4067371	115.9632981	0.330427369
228.0150083	161.7882157	0.218986813
457.4686448	243.2141328	0.211482947
414.1096648	275.9228767	0.164639168
394.2915129	150.4686347	0.261433533
164.6083551	169.7349869	0.114460465
346.6226415	165.3412998	0.253279481
367.4405543	201.6455142	0.241765379
203.226776	123.3852459	0.206008106
252.7382716	171.611358	0.184351798
119.7254601	156.0720859	0.152425899
398.9084803	150.5247691	0.280061285
775.9697087	389.3087015	0.230960141
457.4006061	264.1606061	0.230043536
712.6861213	325.8000919	0.220650931
433.5762712	241.3530099	0.240332843
415.5608804	193.5012425	0.218440384
115.4278892	104.5319962	0.170681976
99.58992806	88.9616307	0.157205
749.6390717	466.7606512	0.205527629

log.sigma.3.5.mm.3D_gldm_DependenceNonUniformityNormalized	log.sigma.3.5.mm.3D_gldm_DependenceVariance	log.sigma.3.5.mm.3D_gldm_LargeDependenceEmphasis
0.181495237	4.704350367	14.44863336
0.132797621	6.231954016	25.04064171
0.132349916	5.660002142	22.70925684
0.173959094	3.932312167	14.58648925
0.140075728	5.463588272	20.80827703
0.156019284	5.620881543	18.71212121
0.143417722	5.935437438	20.82976464
0.138598136	5.317456487	20.92521535
0.151948726	4.786865805	18.24637044
0.150921001	5.51400298	18.7630597
0.137555495	6.623326344	22.65517241
0.148825387	4.784231958	18.09327549
0.093497441	14.03377639	45.08141403
0.142801998	5.692047967	20.09842749
0.160910774	4.157436915	15.87701613
0.154642532	4.736246027	18.03473492
0.170211751	3.60796164	15
0.114147376	7.623320303	29.16707218
0.127611283	6.669260044	25.67026194
0.152345016	4.930839712	18.48836315
0.105503415	9.030306052	34.89748677
0.156042435	3.617469235	17.49119555
0.135210037	5.817001343	22.671875
0.137378438	5.857953826	21.51227805
0.153436933	4.89902248	17.53417125
0.168115811	3.757717927	14.29759392
0.150009151	5.330690914	18.53727188
0.135512681	5.841640533	21.49022448
0.143962484	4.630251891	19.38478747
0.122993679	6.703840513	26.68743914
0.132275969	6.369722391	23.10088272
0.108185204	7.979890959	31.20492992
0.159869796	3.914413557	15.68256426
0.151846084	7.49388067	21.47829787
0.157096987	4.352052176	16.87375114
0.126393026	6.804204936	25.18339446
0.107592092	9.074121391	32.21795216
0.119666108	16.82722584	40.15869018
0.121784652	6.285613061	28.10894309
0.168964125	3.580172592	14.85942228
0.12247735	6.936896069	25.32690825
0.192623171	2.758475852	11.44168606
0.163197111	3.439050317	14.76348207
0.150693102	4.399514454	17.31672026
0.136040027	5.206220106	21.05633803
0.147642377	5.434990063	18.80473588
0.142824057	6.066782549	21.52433143
0.119696932	8.006129381	31.2250287
0.168421474	3.746653208	15.39952996
0.113570516	8.440023108	29.71449704
0.166882861	3.80766152	15.11599844
0.180831918	3.261360333	13.06526253
0.141086437	4.781295387	19.6427305
0.158082926	3.932852867	16.10688406
0.160136808	4.85391147	17.42706767
0.140852071	4.780433925	20.41333333
0.165491313	3.853098868	15.16563659
0.132955961	5.780612496	22.68131868
0.146862334	4.831450596	19.1407767
0.132579592	9.336978197	27.1367072
0.117144785	9.010602946	29.44960362
0.147489234	5.023227146	19.37228464
0.154614301	5.57176919	18.48281223
0.139774204	5.114100573	20.64843238
0.151232174	4.216414036	19.06329114
0.116628151	8.525476693	29.57793889
0.099254535	10.61136098	40.59202454
0.142198733	5.413691494	20.12736
0.145999751	5.345945186	18.98053611
0.122361582	6.994807109	27.45341615
0.147102556	5.100857473	19.85142417
0.131649252	6.017588255	22.6997669
0.142254451	5.456246735	20.67617376
0.177016787	3.569456961	13.61266047
0.169699855	3.57931814	14.42446503
0.136531324	5.834519735	21.89418778
0.122699282	6.667689053	26.71535893
0.122584891	7.729921854	28.12108559
0.13909403	5.690749246	21.87404804
0.178800791	3.318758944	13.21205643
0.181199968	4.924372637	14.69431875
0.126745419	7.522578939	25.065592
0.139941464	5.427511199	20.77118385
0.134757457	5.066228022	22.91018549
0.161661137	4.083235371	15.66666667
0.107446707	8.738516521	37.42950392
0.145334441	5.819326064	19.64150943
0.134004579	7.265193194	23.12545587
0.138816104	6.120201783	22.15300546
0.124809023	6.424958147	25.13432099
0.091814003	13.14749097	44.12730061
0.167467876	4.378391392	15.37783375
0.149714395	4.962669816	18.61257959
0.138606244	6.359972452	21.71212121
0.163760598	3.655531728	16.01838235
0.126702592	8.478585124	25.84862653
0.147518949	5.027136702	19.29605964
0.110246313	9.451106495	32.61413563
0.119412384	6.25931485	27.61390887
0.129830113	6.569151467	23.77138899

log.sigma.3.5.mm.3D_gldm_LargeDependenceLowGrayLevelEmphasis	log.sigma.3.5.mm.3D_gldm_SmallDependenceHighGrayLevelEmphasis	log.sigma.3.5.mm.3D_gldm_LargeDependenceHighGrayLevelEmphasis
0.029452284	170.8504063	8718.584826
0.357144955	19.28242251	2258.2
0.176044758	39.28208036	3841.181226
0.070868468	83.80860847	4701.928352
0.095535896	53.71504802	5438.274071
0.226986254	47.35134026	2288.274242
0.081015832	79.83674147	6163.938595
0.048967509	109.7824991	9746.582224
0.046943969	101.9589271	8164.1652
0.040382615	142.5058109	10160.22854
0.199767816	32.11179043	3390.994695
0.061665637	85.78011045	6186.95141
0.18823266	46.48592846	11491.65185
0.056381644	112.9259341	8915.942341
0.048519094	93.5158858	6435.694556
0.074162279	78.67213511	5376.974406
0.06187211	66.16318644	4827.551089
0.363336662	22.07536261	2956.772101
0.34354864	19.21821531	2429.605547
0.092219961	69.78725982	4707.630506
0.287581591	25.64766373	4940.862765
0.233808551	25.57547299	2052.453197
0.257883608	29.21113557	2898.351563
0.121816974	49.70944862	4436.561012
0.067167916	91.28318625	5518.939513
0.030137004	162.5429764	7886.797383
0.04683562	126.4220964	8821.868852
0.073484869	75.36652856	7027.502534
0.356696104	25.16069415	1559.431767
0.116689454	42.53883879	6743.194255
0.059207486	94.53966415	9955.509878
0.092099985	53.62990061	11664.1943
0.04976211	101.2373033	6679.503561
0.072275828	92.80952136	7939.502128
0.070849588	81.32071125	5238.485922
0.07608759	79.58956944	9441.529176
0.053266999	114.4790232	20461.7893
0.082211744	134.1111678	20905.06937
0.262403439	15.48766068	3574.46748
0.088356112	70.00031124	3859.110867
0.079370382	69.45371145	9941.407479
0.043725932	118.0322833	4131.910008
0.047895018	97.6801479	5551.038598
0.051705594	85.84251289	6820.474277
0.199202604	36.41890116	2945.752739
0.069136143	90.76296166	6327.503097
0.069337868	82.07180712	7353.606532
0.645937885	12.20938095	2008.034443
0.11693407	50.04269914	2922.763807
0.47593455	25.56216822	2455.744083
0.061958179	83.14795865	4992.941612
0.052279931	92.60524423	4300.875111
0.098347748	51.79885516	4669.306294
0.21955608	35.77990292	1876.842391
0.178328728	43.59789546	2602.619549
0.043349701	100.4940145	10330.93282
0.067785432	77.27858586	4625.633127
0.092278176	68.92826527	8510.624349
0.092184244	50.98075314	4768.678398
0.067300898	102.0836806	12048.06759
0.172998021	47.24682069	5584.937146
0.068729264	74.06718873	6273.059925
0.044085571	132.5934521	8981.639316
0.053963674	102.8043232	8797.513971
0.164926698	31.39313734	2886.236709
0.070283425	95.14121789	13533.43889
0.2114322	26.69251288	9293.365798
0.044019996	119.1163736	10409.42704
0.042216589	108.8659822	9396.210347
0.158212694	36.03213412	5427.47274
0.07696766	65.70657761	6573.622017
0.063496353	94.31341461	9113.102797
0.051933875	97.84834363	9800.468627
0.048638124	88.33562736	5037.716246
0.085016665	75.03054811	3568.552859
0.087437305	51.3924758	6847.580477
0.196786935	28.78533553	4178.15985
0.265134819	32.67477018	3957.390397
0.145673478	39.4729033	4005.966022
0.034094752	129.6964804	7268.943138
0.037923042	161.1946472	8088.715435
0.143808372	51.59663956	5697.834352
0.065146235	86.58198057	7763.556745
0.125015387	37.30296503	5589.453303
0.058330427	92.09320029	5551.97991
0.261883139	21.95095581	6086.037859
0.066853389	83.11819175	7617.813417
0.107435521	74.38177959	5914.907002
0.145839284	40.04401143	4207.708333
0.093982429	54.64715916	7665.04
1.05566026	17.42419949	2379.185583
0.105582464	62.42164629	3341.40932
0.044374267	105.0719511	8913.527301
0.067548895	83.94135756	7801.341818
0.085010285	52.11721759	4129.193704
0.070809468	115.185246	11442.63501
0.139509636	46.20225558	3912.336528
0.085002348	60.92441358	14232.51767
0.191626964	22.22183979	4667.7506
0.115920074	58.68942784	5563.318497

log.sigma.3.5.mm.3D_gldm_SmallDependenceLowGrayLevelEmphasis	log.sigma.3.5.mm.3D_gldm_LowGrayLevelEmphasis	log.sigma.3.5.mm.3D_gldzm_DistanceZoneVariabilityNormalized
0.001809249	0.003435309	0.997524756
0.003526008	0.017722037	1
0.002583987	0.008838973	1
0.004151836	0.007758325	1
0.002015408	0.00590511	1
0.00392508	0.014880722	1
0.001132841	0.004084753	1
0.000770895	0.002607366	1
0.000932165	0.002937507	1
0.001615707	0.003150066	1
0.003133899	0.01086858	1
0.00184274	0.004447119	0.996884743
0.001542605	0.005351068	1
0.001928309	0.004918787	1
0.001499248	0.004612248	1
0.001429807	0.004652179	1
0.002732072	0.006417708	1
0.004076317	0.015325469	1
0.003546407	0.014390412	1
0.001604016	0.005627384	1
0.001950153	0.009574147	1
0.004106468	0.015006791	1
0.003417756	0.013566798	1
0.002074389	0.006682952	1
0.003073841	0.006106879	1
0.001378678	0.003008097	1
0.001216938	0.003386031	1
0.001387766	0.004148189	1
0.003898108	0.019827222	1
0.001639472	0.005486163	1
0.000888556	0.002930654	1
0.001659874	0.005091968	1
0.001386956	0.004339209	0.99596369
0.002077111	0.00541066	1
0.001235382	0.004981565	0.995133849
0.00088433	0.003714878	1
0.000671774	0.002079133	1
0.000668235	0.002256237	0.993506562
0.002630306	0.013183577	1
0.001863514	0.006680104	0.999080037
0.001423843	0.005079137	1
0.001514102	0.004223085	0.998608212
0.001314896	0.003773672	1
0.001497705	0.004004548	1
0.003191336	0.011025097	1
0.001665583	0.004758873	1
0.001134854	0.003676334	1
0.004669556	0.025657846	1
0.003412912	0.010161308	1
0.004207036	0.017280867	0.993421124
0.002291512	0.006233987	1
0.001710317	0.004863051	0.99825175
0.001791582	0.005959146	0.998178508
0.006123294	0.017970441	1
0.003225972	0.011494689	1
0.001155717	0.002900942	1
0.002548125	0.006412181	1
0.001551969	0.006408887	1
0.003548111	0.007914302	1
0.000768076	0.002693221	0.997357997
0.002117316	0.006878195	1
0.001426738	0.004239112	1
0.000813917	0.002864251	0.996549752
0.000707461	0.002787932	0.992152588
0.003113004	0.010334536	1
0.000829559	0.002893494	0.994818688
0.001417814	0.00811416	1
0.000696586	0.00268458	0.997549023
0.001041765	0.002829363	1
0.001476853	0.006695473	1
0.001617665	0.006159745	1
0.001065585	0.003331646	0.993896293
0.001258378	0.003864414	1
0.00305197	0.006111884	0.997435902
0.002071485	0.006695609	0.995884791
0.002968683	0.007692856	1
0.002412327	0.008655094	1
0.002479223	0.012214714	1
0.002164022	0.008639795	1
0.001836625	0.004031291	1
0.002142867	0.004180107	1
0.002736749	0.009072865	1
0.001094282	0.003632674	0.99743261
0.001438083	0.007069514	1
0.001646744	0.004785913	0.997333338
0.001514348	0.00764464	1
0.00282504	0.006072749	1
0.001556907	0.005530608	1
0.002748842	0.009143464	1
0.002250977	0.00565228	1
0.004424769	0.022828445	1
0.003779032	0.009640124	0.997527816
0.001025711	0.003081595	0.994386009
0.001477535	0.003978157	1
0.002219311	0.006760333	0.998254801
0.001213537	0.003735908	0.997860965
0.002803161	0.009266499	1
0.002105213	0.004633154	1
0.003264306	0.009773502	1
0.00136917	0.005106963	1

log.sigma.3.5.mm.3D_gldzm_LowIntensityEmphasis	log.sigma.3.5.mm.3D_gldzm_LargeDistanceEmphasis	log.sigma.3.5.mm.3D_gldzm_HighIntensitySmallDistanceEmphasis
0.005309564	1.003717472	537.3162949
0.025156553	1	122.7103825
0.013720173	1	206.7065868
0.013759186	1	311.4565217
0.009197104	1	255.5856164
0.017584824	1	192.77
0.005065114	1	364.3819683
0.003674483	1	536.4413502
0.004031844	1	449.3610197
0.00569413	1	569.4294479
0.015060554	1	150.537234
0.007225234	1.004680187	357.9177067
0.010134114	1	312.5341615
0.008707706	1	473.6901709
0.006527388	1	363.6233553
0.006236292	1	351.1077058
0.010787949	1	271.2840909
0.024486686	1	132.7639485
0.020079571	1	113.556391
0.007139002	1	310.8826494
0.014932843	1	184.2055085
0.020801716	1	131.296748
0.017720433	1	156.3198198
0.009388449	1	232.320059
0.011188707	1	359.403183
0.0045709	1	570.6683292
0.004868313	1	506.3428875
0.006036247	1	340.4311544
0.022641015	1	123.8108108
0.009619919	1	254.7963918
0.004183908	1	461.9068716
0.01123763	1	314.0208333
0.005779938	1.006066734	394.8384732
0.008654222	1	372.494964
0.005849528	1.007317073	337.4794715
0.00538379	1	421.5820433
0.003683923	1	640.264177
0.003136285	1.009771987	634.7609935
0.022479521	1	110.0680628
0.007622538	1.00138058	284.8370916
0.008066286	1	337.1734861
0.004863164	1.002089136	384.4569986
0.005014774	1	384.261302
0.005971582	1	347.9705882
0.014937351	1	181.616129
0.006569865	1	363.1089351
0.005212949	1	398.5373537
0.039661121	1	95.46206897
0.014076642	1	209.9836066
0.023249586	1.00990099	142.0470297
0.009563762	1	327.006502
0.006055254	1.002624672	331.7410324
0.008376735	1.002734731	244.8653145
0.025446917	1	153.1884984
0.014627408	1	184.0564103
0.005462863	1	506.4977477
0.009732552	1	302.2165605
0.009942018	1	349.3465982
0.015684923	1	236.8830549
0.003548938	1.003968254	476.1452822
0.010287077	1	251.2348485
0.006330626	1	346.1464873
0.003502017	1.005184332	521.4742224
0.003442493	1.01181767	516.7056837
0.015637199	1	170.6722222
0.004706424	1.007792208	531.3837662
0.015403518	1	207.4594595
0.00339263	1.003680982	533.7920245
0.004092032	1	441.9946333
0.009882018	1	223.8364662
0.009667337	1	326
0.005186709	1.009183673	463.8563776
0.006499123	1	467.7635727
0.009958613	1.003851091	313.3636072
0.008022693	1.006185567	284.6095361
0.0150668	1	251.0993976
0.013891252	1	178.2795699
0.017436655	1	198.8497409
0.012275678	1	202.5583756
0.006201922	1	461.3757881
0.00617103	1	497.4813385
0.013587702	1	238.1453362
0.005322436	1.003856041	412.1905527
0.010052181	1	225.6054889
0.006321959	1.00400534	349.2633511
0.013947972	1	192.1213873
0.010622821	1	331.2334254
0.007017203	1	311.4018568
0.014139736	1	199.3169399
0.011738215	1	301.0046296
0.027959304	1	118.4954545
0.01349208	1.003712871	228.0284653
0.004368974	1.008444757	456.1972203
0.00617811	1	367.8937143
0.009993589	1.002620087	235.1364629
0.005436072	1.003211991	482.1255353
0.01267879	1	214.3564626
0.011201461	1	355.7725118
0.019795504	1	142.1052632
0.006581519	1	293.8825301

log.sigma.3.5.mm.3D_gldzm_LowIntensityLargeDistanceEmphasis	log.sigma.3.5.mm.3D_gldzm_HighIntensityEmphasis	log.sigma.3.5.mm.3D_gldzm_DistanceZoneVariability	log.sigma.3.5.mm.3D_gldzm_ZonePercentage
0.005314664	537.9938042	805.0024783	0.380301602
0.025156553	122.7103825	183	0.195721925
0.013720173	206.7065868	334	0.217731421
0.013759186	311.4565217	322	0.329580348
0.009197104	255.5856164	584	0.246621622
0.017584824	192.77	200	0.303030303
0.005065114	364.3819683	1453	0.257122633
0.003674483	536.4413502	1185	0.231989037
0.004031844	449.3610197	1216	0.267487901
0.00569413	569.4294479	326	0.304104478
0.015060554	150.537234	376	0.24933687
0.007263914	358.0592824	639.0031201	0.278091106
0.010134114	312.5341615	322	0.172469202
0.008707706	473.6901709	468	0.272568433
0.006527388	363.6233553	608	0.306451613
0.006236292	351.1077058	1142	0.260968921
0.010787949	271.2840909	352	0.29480737
0.024486686	132.7639485	233	0.188969992
0.020079571	113.556391	266	0.204930663
0.007139002	310.8826494	1389	0.267166763
0.014932843	184.2055085	472	0.156084656
0.020801716	131.296748	246	0.227988879
0.017720433	156.3198198	222	0.216796875
0.009388449	232.320059	678	0.256139025
0.011188707	359.403183	377	0.296150825
0.0045709	570.6683292	802	0.338539468
0.004868313	506.3428875	942	0.291370244
0.006036247	340.4311544	719	0.26031861
0.022641015	123.8108108	111	0.248322148
0.009619919	254.7963918	388	0.188899708
0.004183908	461.9068716	1106	0.232450609
0.01123763	314.0208333	384	0.185596907
0.005985533	394.8847321	985.008089	0.306286776
0.008654222	372.494964	695	0.295744681
0.005875564	338.0130081	1224.014634	0.279291553
0.00538379	421.5820433	646	0.215405135
0.003683923	640.264177	723	0.194302607
0.0031514	636.3526059	1220.026059	0.237938384
0.022479521	110.0680628	191	0.155284553
0.007632126	284.8867925	2171.00092	0.298899587
0.008066286	337.1734861	611	0.235543562
0.004872449	384.5745125	1434.001393	0.371347298
0.005014774	384.261302	1106	0.302764851
0.005971582	347.9705882	714	0.286977492
0.014937351	181.616129	310	0.24256651
0.006569865	363.1089351	817	0.297632058
0.005212949	398.5373537	1111	0.243533538
0.039661121	95.46206897	145	0.166475316
0.014076642	209.9836066	244	0.286721504
0.023451647	142.1683168	301.0066007	0.224112426
0.009563762	327.006502	769	0.299338264
0.006061816	332.0034996	1141.00175	0.339068526
0.008392917	244.9808569	1095.001823	0.243129433
0.025446917	153.1884984	313	0.283514493
0.014627408	184.0564103	390	0.293233083
0.005462863	506.4977477	444	0.227692308
0.009732552	302.2165605	1256	0.310506799
0.009942018	349.3465982	779	0.225274725
0.015684923	236.8830549	419	0.254247573
0.003564648	476.4290123	2262.007937	0.24843904
0.010287077	251.2348485	396	0.224235561
0.006330626	346.1464873	669	0.250561798
0.003576346	522.0725806	1730.010369	0.299879081
0.003484701	517.6291503	1763.055149	0.233110324
0.015637199	170.6722222	180	0.227848101
0.004729085	532.312987	766.0103896	0.19937856
0.015403518	207.4594595	185	0.141871166
0.003399028	534.4472393	1626.004908	0.2608
0.004092032	441.9946333	1677	0.286324057
0.009882018	223.8364662	532	0.183574879
0.009667337	326	308	0.237105466
0.005236059	464.2857143	974.0183673	0.228438228
0.006499123	467.7635727	571	0.250548486
0.00999044	313.4801027	777.0025674	0.344842851
0.008054872	284.9443299	966.0082474	0.309805174
0.0150668	251.0993976	332	0.247391952
0.013891252	178.2795699	465	0.194073456
0.017436655	198.8497409	193	0.201461378
0.012275678	202.5583756	394	0.230814294
0.006201922	461.3757881	793	0.339033775
0.00617103	497.4813385	777	0.390648567
0.013587702	238.1453362	461	0.256253474
0.005333117	412.5385604	776.0025707	0.23799327
0.010052181	225.6054889	583	0.189716889
0.006329531	349.7930574	747.0026702	0.307093071
0.013947972	192.1213873	173	0.112924282
0.010622821	331.2334254	724	0.303563941
0.007017203	311.4018568	754	0.274981765
0.014139736	199.3169399	366	0.25
0.011738215	301.0046296	432	0.213333333
0.027959304	118.4954545	220	0.168711656
0.013529209	228.1212871	806.0024752	0.339210747
0.004413363	456.8289937	1413.022519	0.274165541
0.00617811	367.8937143	875	0.265151515
0.010098393	235.1528384	1143.001747	0.263097426
0.005442143	482.5503212	932.0021413	0.272939801
0.01267879	214.3564626	735	0.260915868
0.011201461	355.7725118	211	0.201528176
0.019795504	142.1052632	152	0.182254197
0.006581519	293.8825301	1328	0.229996536

log.sigma.3.5.mm.3D_gldzm_IntensityVariabilityNormalized	log.sigma.3.5.mm.3D_gldzm_LowIntensitySmallDistanceEmphasis	log.sigma.3.5.mm.3D_gldzm_IntensityVariability
0.042307934	0.00530829	34.1425031
0.068649407	0.025156553	12.56284153
0.057280648	0.013720173	19.13173653
0.050518884	0.013759186	16.26708075
0.061116063	0.009197104	35.69178082
0.05415	0.017584824	10.83
0.05559137	0.005065114	80.77426015
0.048340544	0.003674483	57.2835443
0.056172632	0.004031844	68.30592105
0.054650156	0.00569413	17.81595092
0.071115324	0.015060554	26.7393617
0.053784429	0.007215565	34.47581903
0.052582848	0.010134114	16.93167702
0.045894514	0.008707706	21.47863248
0.049217668	0.006527388	29.92434211
0.053056211	0.006236292	60.59019264
0.058690599	0.010787949	20.65909091
0.061614692	0.024486686	14.35622318
0.083328622	0.020079571	22.16541353
0.050873338	0.007139002	70.66306695
0.062491023	0.014932843	29.49576271
0.07098949	0.020801716	17.46341463
0.064158753	0.017720433	14.24324324
0.062055673	0.009388449	42.07374631
0.054520893	0.011188707	20.55437666
0.047512142	0.0045709	38.10473815
0.045568673	0.004868313	42.92569002
0.057789659	0.006036247	41.55076495
0.081892704	0.022641015	9.09009009
0.062945053	0.009619919	24.42268041
0.054543849	0.004183908	60.32549729
0.060492622	0.01123763	23.22916667
0.048373361	0.00572854	47.84125379
0.049295585	0.008654222	34.26043165
0.058951682	0.005843019	72.51056911
0.057630189	0.00538379	37.22910217
0.048126276	0.003683923	34.79529737
0.046910312	0.003132506	57.60586319
0.086264083	0.022479521	16.47643979
0.049261012	0.007620141	107.0441786
0.051577597	0.008066286	31.51391162
0.049568982	0.004860843	71.1810585
0.054738415	0.005014774	60.54068716
0.054782697	0.005971582	39.11484594
0.060541103	0.014937351	18.76774194
0.052430827	0.006569865	42.83598531
0.05295138	0.005212949	58.8289829
0.073388823	0.039661121	10.64137931
0.058855146	0.014076642	14.36065574
0.064427235	0.023199071	19.52145215
0.05390785	0.009563762	41.45513654
0.051636918	0.006053614	59.02099738
0.058451449	0.00837269	64.12123974
0.060396656	0.025446917	18.90415335
0.061236029	0.014627408	23.88205128
0.056783134	0.005462863	25.21171171
0.051950637	0.009732552	65.25
0.050080664	0.009942018	39.01283697
0.057900103	0.015684923	24.2601432
0.05365619	0.00354501	121.6922399
0.05396133	0.010287077	21.36868687
0.057603321	0.006330626	38.53662182
0.056002994	0.003483435	97.22119816
0.050190184	0.003431941	89.18795723
0.066790123	0.015637199	12.02222222
0.048649013	0.004700759	37.45974026
0.072958364	0.015403518	13.4972973
0.048738003	0.00339103	79.44294479
0.052279445	0.004092032	87.6726297
0.065570411	0.009882018	34.88345865
0.060233598	0.009667337	18.55194805
0.049562682	0.005174371	48.57142857
0.051738892	0.006499123	29.54290718
0.048264702	0.009950656	37.59820282
0.052600701	0.008014648	51.02268041
0.06287197	0.0150668	20.87349398
0.066953405	0.013891252	31.13333333
0.055008188	0.017436655	10.61658031
0.062743178	0.012275678	24.72081218
0.047978131	0.006201922	38.04665826
0.042567112	0.00617103	33.07464607
0.054418152	0.013587702	25.0867679
0.052051599	0.005319765	40.49614396
0.064588733	0.010052181	37.65523156
0.049862656	0.006320067	37.34712951
0.082562064	0.013947972	14.28323699
0.048392143	0.010622821	35.0359116
0.056716082	0.007017203	42.76392573
0.061169339	0.014139736	22.38797814
0.054023062	0.011738215	23.33796296
0.073264463	0.027959304	16.11818182
0.055012989	0.013482798	44.45049505
0.051456463	0.004357876	73.11963406
0.055370449	0.00617811	48.44914286
0.058728857	0.009967388	67.24454148
0.047700709	0.005434554	44.55246253
0.054160766	0.01267879	39.80816327
0.055097594	0.011201461	11.62559242
0.077562327	0.019795504	11.78947368
0.058933399	0.006581519	78.26355422

log.sigma.3.5.mm.3D_gldzm_HighIntensityLargeDistanceEmphasis	log.sigma.3.5.mm.3D_gldzm_SmallDistanceEmphasis	log.sigma.3.5.mm.3D_glcm_SumVariance	log.sigma.3.5.mm.3D_glcm_Homogeneity1
540.7038414	0.999070632	1815.611792	0.352004791
122.7103825	1	258.7397794	0.477448535
206.7065868	1	533.3473201	0.454305626
311.4565217	1	950.2325161	0.377621027
255.5856164	1	776.9341359	0.425583076
192.77	1	403.0953132	0.411702275
364.3819683	1	988.9371426	0.418613195
536.4413502	1	1577.462024	0.430151742
449.3610197	1	1443.00217	0.406457906
569.4294479	1	1819.457519	0.41252538
150.537234	1	429.2429761	0.452286644
358.625585	0.998829953	1073.638497	0.41096587
312.5341615	1	875.0545353	0.51250244
473.6901709	1	1470.95047	0.408426516
363.6233553	1	1196.095703	0.382523648
351.1077058	1	976.382145	0.399085978
271.2840909	1	898.0024828	0.400998237
132.7639485	1	301.6159325	0.475927318
113.556391	1	276.1782202	0.474396119
310.8826494	1	809.8026054	0.409385477
184.2055085	1	439.9628286	0.505654291
131.296748	1	312.2981463	0.437943556
156.3198198	1	382.7449053	0.451706702
232.320059	1	652.2201008	0.442900973
359.403183	1	1058.057718	0.398025357
570.6683292	1	1737.091528	0.383746808
506.3428875	1	1538.645588	0.394619854
340.4311544	1	1036.473878	0.425984209
123.8108108	1	244.1498194	0.454453379
254.7963918	1	746.8251557	0.47672585
461.9068716	1	1403.239503	0.42887202
314.0208333	1	1145.550354	0.464971189
395.0697674	0.998483316	1231.196375	0.385025378
372.494964	1	1207.813069	0.400089593
340.1471545	0.998170732	973.5098242	0.406316093
421.5820433	1	1281.341689	0.44567911
640.264177	1	2155.735328	0.465519502
642.7190554	0.997557003	1942.827757	0.445625381
110.0680628	1	316.3234059	0.493050236
285.085596	0.999654855	778.5174709	0.383590035
337.1734861	1	1168.678554	0.433057138
385.0445682	0.999477716	1093.106077	0.351219734
384.261302	1	1187.838717	0.380201149
347.9705882	1	1120.545453	0.39846814
181.616129	1	406.3589077	0.442574558
363.1089351	1	1102.367308	0.406591748
398.5373537	1	1134.939412	0.422710753
95.46206897	1	182.2556505	0.504422016
209.9836066	1	525.577827	0.413322856
142.6534653	0.997524752	276.7972828	0.482399403
327.006502	1	1018.108805	0.387060923
333.0533683	0.999343832	973.1528319	0.364065187
245.4430264	0.999316317	706.3431795	0.428823472
153.1884984	1	354.9420654	0.401898223
184.0564103	1	474.4410442	0.410092864
506.4977477	1	1592.179707	0.432436329
302.2165605	1	905.9570713	0.387452246
349.3465982	1	1145.836047	0.430050681
236.8830549	1	732.8621398	0.425202727
477.563933	0.999007937	1507.414988	0.427538748
251.2348485	1	624.5225509	0.468866119
346.1464873	1	1028.506606	0.41981345
524.4660138	0.998703917	1618.950738	0.407231086
521.3230163	0.997045582	1405.380473	0.4267749
170.6722222	1	420.6175243	0.436431299
536.0298701	0.998051948	1557.118945	0.460595738
207.4594595	1	686.5626011	0.517613599
537.0680982	0.999079755	1663.678773	0.416789487
441.9946333	1	1545.935488	0.427101407
223.8364662	1	617.4737443	0.481887542
326	1	1045.66478	0.420465066
466.0030612	0.997704082	1345.348858	0.435534233
467.7635727	1	1529.107363	0.422778651
313.9460847	0.999037227	1023.425354	0.359763324
286.2835052	0.998453608	753.1263736	0.3802011
251.0993976	1	868.9035986	0.441496249
178.2795699	1	472.84892	0.484435419
198.8497409	1	452.2575876	0.473535907
202.5583756	1	524.3378016	0.450759618
461.3757881	1	1540.869248	0.364373017
497.4813385	1	1650.420874	0.359167562
238.1453362	1	659.1991945	0.439637378
413.9305913	0.99903599	1270.749796	0.420484477
225.6054889	1	676.5087921	0.459288816
351.9118825	0.998998665	1054.758863	0.383180737
192.1213873	1	506.5649948	0.524973135
331.2334254	1	1124.854711	0.395415703
311.4018568	1	865.9381287	0.431516782
199.3169399	1	555.1908877	0.448011743
301.0046296	1	949.499519	0.457205031
118.4954545	1	197.5366981	0.527037735
228.4925743	0.999071782	661.8941393	0.389164596
459.3560873	0.997888811	1460.393182	0.408606021
367.8937143	1	1133.550813	0.423921541
235.2183406	0.999344978	704.3695074	0.409020555
484.2494647	0.999197002	1480.459287	0.430194971
214.3564626	1	562.9157229	0.428703429
355.7725118	1	1322.323769	0.472883058
142.1052632	1	437.1215217	0.491053493
293.8825301	1	784.5658152	0.444063463

log.sigma.3.5.mm.3D_glcm_Homogeneity2	log.sigma.3.5.mm.3D_glcm_ClusterShade	log.sigma.3.5.mm.3D_glcm_MaximumProbability	log.sigma.3.5.mm.3D_glcm_Idmn	log.sigma.3.5.mm.3D_glcm_SumVariance2
0.267634945	-723.9216156	0.021118125	0.982295009	107.1107932
0.412449186	116.9032641	0.055902496	0.983608624	32.33998083
0.384287644	90.19700901	0.026402164	0.988325682	50.85476105
0.2945999	-344.2663554	0.018981846	0.982730467	84.18581928
0.349014731	-35.16529042	0.028559922	0.985737541	48.80455798
0.332550192	332.630498	0.039599884	0.981730613	67.23269343
0.339962572	158.0887205	0.028908292	0.992934246	55.78069785
0.354462746	108.0898729	0.032269127	0.991640285	57.04359431
0.327240993	-38.70297652	0.018655436	0.989505233	61.17710195
0.331835172	-273.1792372	0.025902959	0.990561606	61.39055424
0.379786846	-4.350773707	0.033376341	0.984339624	36.80238789
0.331374485	-34.92240445	0.025782089	0.98979971	60.55291129
0.455997358	135.8207754	0.078478381	0.991415875	46.77942124
0.330994229	-162.2125146	0.029961854	0.987283456	68.70877753
0.300409096	-113.0164365	0.026276901	0.987270096	63.04886967
0.318137712	-28.88990522	0.025528491	0.987375575	63.33716898
0.320382118	-322.7701437	0.024764424	0.983595232	51.89696759
0.410933822	234.3897074	0.050878278	0.983825484	41.95789462
0.407005962	64.91013336	0.049117695	0.983507339	29.52697762
0.328781129	71.41980434	0.021800069	0.98943916	65.16362696
0.446196722	94.90535034	0.063976436	0.991462979	35.30041262
0.360590295	27.24339123	0.024500389	0.9819281	36.15977855
0.379524387	106.2420694	0.043714734	0.986182706	44.41576675
0.369470182	103.6186178	0.030107271	0.988121335	41.81266966
0.317618906	-25.30751828	0.026998532	0.985437748	48.17602873
0.300823379	-258.5946316	0.021440366	0.989945727	79.43961265
0.31452847	-305.7181446	0.026299564	0.988658239	83.04362012
0.350297037	-24.34908884	0.035341279	0.98893689	46.93969085
0.382540623	129.8909882	0.042990861	0.982207671	36.3538577
0.41036232	13.72285286	0.045727461	0.990364055	34.16393004
0.35477578	11.73202142	0.031880961	0.990308594	54.02554423
0.398707859	-309.6099029	0.053837162	0.989326566	34.12385671
0.302823765	-250.7842223	0.019786627	0.987101284	82.07708958
0.320194092	-323.7497739	0.029733579	0.985300645	67.95240915
0.324542468	-91.20226152	0.020371135	0.98808693	53.98676486
0.372715005	-86.65362933	0.036505443	0.990484914	44.74834547
0.400286637	-287.0602372	0.050683926	0.99314389	51.7497339
0.374377228	-161.602426	0.063795879	0.992392362	84.64356387
0.42914147	-33.79240373	0.043390944	0.982915847	16.29285289
0.298621582	7.878260398	0.013532094	0.986727202	80.7726315
0.361406939	-399.2579401	0.044656466	0.987662127	50.62380977
0.262575201	364.3098027	0.01383806	0.987141385	88.82731795
0.29516755	-30.26585689	0.018369001	0.986035264	59.03491122
0.319014815	-125.3509776	0.02447648	0.990446936	53.20709089
0.367525275	229.3099846	0.037868044	0.987883824	48.1974395
0.327649431	-185.871709	0.022452305	0.987194398	60.04807716
0.345819809	71.90290789	0.034204712	0.989656521	48.25170434
0.443065503	106.5752224	0.067608523	0.985638024	29.61408765
0.332852766	91.93696425	0.026091173	0.984067768	55.36349828
0.418596747	214.0989448	0.061267898	0.988405274	42.76716016
0.302651499	-165.4265626	0.01686866	0.984735394	62.72904786
0.276694723	53.13789092	0.017876779	0.985567523	68.94210655
0.35158431	46.05962208	0.026774478	0.989291045	46.96855121
0.320385477	112.1989303	0.024588288	0.979591123	45.50550894
0.329918482	112.4910839	0.026878625	0.98021087	57.79034377
0.357143718	-47.30647951	0.040814867	0.99230844	44.32709421
0.303122017	-132.3274188	0.018032895	0.983394584	65.14294168
0.355499806	-411.0857655	0.034952887	0.987579844	57.91739843
0.349101802	-50.99538809	0.034709836	0.984482194	36.23465751
0.353027923	-29.84237778	0.034493813	0.990535597	62.33010391
0.402642549	330.2181186	0.057363943	0.991001321	49.82995946
0.341152188	-34.07688246	0.029760855	0.98980932	53.15931085
0.327836435	-66.66123216	0.027836101	0.99071147	64.64570497
0.350693392	301.3522607	0.024267038	0.992383417	62.5491277
0.358793288	70.79760274	0.028930261	0.982880773	36.06813747
0.392521041	-39.44848138	0.048664741	0.991917425	54.06043534
0.462069402	-24.41095129	0.07845921	0.989033896	23.90518828
0.340687652	-31.53657921	0.030035322	0.992431971	67.95190056
0.351078903	-189.3374712	0.024535227	0.99162222	53.90944933
0.417238219	26.89416972	0.045550804	0.988730693	31.21127415
0.343468106	-279.7127953	0.030295245	0.987121183	51.52726154
0.361958376	115.6918545	0.036788461	0.990941699	62.68172281
0.346527111	-367.9218987	0.031316616	0.988943608	55.93313092
0.274001312	-335.1555511	0.020645645	0.979602018	71.14699958
0.295375538	173.5165637	0.017314398	0.98605788	76.70023341
0.369700434	-327.2883027	0.042015347	0.98395391	46.6761332
0.420162687	50.73049622	0.038681945	0.9887167	29.38855615
0.406147294	270.6306329	0.051901478	0.989369506	50.59811604
0.377620903	39.20840083	0.035140817	0.986414037	37.13697184
0.279246882	-987.4102418	0.018080105	0.985165065	104.0341539
0.276135779	-898.3970704	0.023035113	0.980953926	108.7261643
0.368186847	-89.78947321	0.037344814	0.99010374	42.91019574
0.343017645	-1.034757725	0.030036108	0.989181229	53.99163006
0.388235735	-177.0591013	0.034592334	0.98941389	42.11603875
0.298769961	-99.76373786	0.021703398	0.98901886	75.32103454
0.46889796	134.6144215	0.064484173	0.991132441	33.55363527
0.317769469	-576.3132481	0.027581512	0.98256433	75.5479762
0.357310969	37.6300567	0.035641583	0.988487303	55.11369061
0.375430687	10.80998702	0.037162884	0.987508328	44.68376304
0.387624168	-98.02904186	0.038359983	0.989816741	47.52156269
0.473401492	217.2683216	0.088074876	0.984212273	40.28260147
0.305129897	72.48704774	0.017575763	0.982419694	72.74895342
0.330666286	-276.831718	0.022852982	0.989205361	58.42037606
0.348448676	-102.98769	0.031313197	0.98826919	48.59517236
0.328514404	-90.9143672	0.019969863	0.986646679	49.0749084
0.357251174	-174.6160281	0.037642064	0.989434967	76.15561808
0.351476525	30.85080746	0.024834063	0.98707662	62.93206229
0.409954008	-726.5546834	0.054180755	0.986969327	57.17884081
0.428553449	-40.04698022	0.04150925	0.985952559	26.41641065
0.372431079	139.328872	0.040005219	0.987752793	45.60643518

log.sigma.3.5.mm.3D_glcm_Contrast	log.sigma.3.5.mm.3D_glcm_DifferenceEntropy	log.sigma.3.5.mm.3D_glcm_InverseVariance	log.sigma.3.5.mm.3D_glcm_Entropy	log.sigma.3.5.mm.3D_glcm_Dissimilarity
27.56246098	3.445199387	0.26225502	8.282451449	3.821980651
7.069716728	2.547667816	0.401751985	6.502465737	1.934436931
8.971100915	2.703471667	0.37618635	7.205255774	2.178877198
19.16463369	3.181698261	0.300388782	7.822808065	3.186214035
11.98350312	2.904326167	0.33929	7.389074292	2.514514773
13.32522407	2.935935333	0.319533302	7.226066258	2.694320315
11.67018194	2.895239725	0.334631665	7.524009005	2.529862203
11.29904914	2.867528246	0.347469895	7.436163277	2.441329234
14.27329244	3.005065846	0.325557179	7.742098832	2.754912111
12.7977998	2.943033197	0.317751952	7.346948509	2.634139913
8.037973137	2.655449784	0.367458822	6.952698652	2.129625545
12.29046104	2.939996437	0.328739752	7.55765362	2.618707432
8.211393062	2.587260589	0.394110052	6.554808704	1.898154225
16.85217769	3.070266648	0.326089941	7.559457333	2.865197994
18.41713069	3.171525745	0.301896677	7.684219697	3.13581189
14.52740019	3.007409256	0.3180835	7.711155716	2.812724035
13.81606055	2.973374507	0.328478771	7.351433337	2.742952046
8.624806508	2.643673884	0.388007218	6.698675274	2.053385706
6.330161847	2.49893371	0.396592136	6.537643806	1.892846889
11.98824402	2.927220901	0.325143497	7.712575819	2.608390571
6.038523051	2.459801674	0.411382141	6.60489266	1.747592209
7.712428106	2.620586723	0.362384072	6.918033544	2.144672802
8.426432056	2.684614638	0.368996438	6.932646123	2.153170186
9.825232552	2.780501704	0.357260798	7.168555096	2.294250058
16.11515494	3.044681279	0.318154306	7.376438223	2.889183119
17.72612196	3.147793917	0.302376434	7.851099788	3.072885036
18.32883715	3.14972284	0.308914364	7.895859389	3.042457425
11.94076281	2.90055568	0.345412936	7.331442319	2.510091878
6.822674846	2.52664041	0.389248745	6.443985388	2.003932842
7.377756884	2.584025363	0.38917153	6.766184282	1.963197972
12.46438994	2.911905693	0.349766713	7.469164114	2.528345653
11.00042491	2.775369899	0.375255315	6.638300548	2.254373728
17.6615489	3.155490105	0.301854113	8.019109094	3.083389168
16.08525337	3.092509217	0.308293991	7.66606621	2.920771496
12.05381196	2.91967173	0.325009962	7.562480357	2.609308319
10.28471355	2.778512349	0.357154535	7.082919634	2.297062266
12.80519177	2.83061889	0.370924884	7.030635838	2.348819911
15.36754323	3.03655569	0.310683642	7.672548395	2.689293063
5.26830645	2.390376479	0.410344398	6.060487909	1.726219128
15.23188691	3.077708702	0.299553921	8.092371809	2.943002843
14.43381758	2.958360935	0.35251839	7.197328214	2.614607116
20.56030759	3.262277998	0.271654472	8.233078297	3.436929444
16.16806135	3.078829637	0.301599898	7.783814257	2.99844036
16.00068786	3.084130672	0.320284849	7.579102696	2.900532567
8.597172148	2.705089622	0.356336237	7.008127061	2.212090484
14.77311024	3.030960187	0.322875876	7.635313059	2.790415931
11.81275418	2.898937104	0.340336033	7.37966902	2.521781605
4.903424385	2.335650592	0.414032744	6.276137562	1.66008151
10.56537851	2.822529349	0.338985675	7.299146267	2.476163304
7.691114506	2.591609872	0.391640483	6.703570344	1.976578725
14.69334438	3.039714608	0.308521527	7.727743937	2.871441994
18.71969033	3.213018342	0.278940854	8.023987474	3.260363993
10.05934876	2.810780955	0.346088755	7.336690177	2.374217271
12.79345455	2.916314416	0.325706896	7.279499631	2.681275859
12.33966349	2.924631318	0.325929733	7.44457688	2.624526473
10.96939853	2.832923453	0.350129544	7.138526282	2.407029375
14.8789955	3.061341198	0.299931545	7.854931358	2.910386413
12.84602145	2.901152356	0.350604315	7.353504486	2.517351814
12.24532379	2.901286003	0.34499305	7.121081354	2.520178689
14.31563852	3.026988169	0.32237443	7.756720654	2.692458402
10.43596285	2.75758932	0.372371658	6.950099737	2.205982098
10.85729349	2.860072615	0.338007174	7.41304423	2.47684735
13.98645101	3.017659874	0.322965361	7.727569476	2.740930165
11.41346744	2.861469687	0.348939292	7.510518631	2.461919243
8.868556728	2.682757716	0.346285248	6.894746592	2.25164528
11.03387937	2.808000764	0.364624048	7.154299737	2.285711769
6.904186726	2.475243782	0.414827687	6.236746841	1.755219055
14.57174827	3.010802619	0.336872581	7.725738416	2.709117944
11.96308923	2.899338367	0.342527739	7.485036248	2.502746255
6.872505585	2.539940769	0.401881603	6.701753451	1.896429724
12.38935254	2.87891601	0.34459253	7.202839031	2.546343502
11.67829276	2.864271774	0.355680479	7.468996637	2.430382968
13.5871809	2.95394424	0.336419885	7.3415288	2.616468561
21.23035879	3.287181071	0.277303153	7.995956058	3.438873294
16.08285056	3.103236369	0.298287815	7.98130715	3.011534649
11.86600061	2.845192505	0.359112389	7.027000267	2.417425297
6.906771081	2.535450277	0.400946981	6.655042789	1.887741328
7.537329288	2.601222342	0.380731619	6.819759192	2.001431546
8.303669857	2.679510944	0.366858112	6.973295687	2.141379619
21.68677419	3.275994542	0.283123884	8.096147339	3.407258791
26.84901484	3.420562791	0.269595696	8.2097318	3.737636222
12.79488607	2.920251412	0.350065138	7.14509909	2.507195705
13.2106004	2.935183637	0.330549154	7.449274139	2.615173599
7.48265096	2.601538548	0.379919948	6.994645272	2.040459901
15.72736489	3.077923926	0.302237642	7.889102927	2.969939138
4.463621037	2.288790165	0.426767326	6.226001136	1.545819097
19.37407946	3.189110148	0.312266656	7.799301267	3.127917866
11.68989805	2.889863863	0.348831173	7.399426738	2.474935823
8.885024497	2.716158055	0.363268466	7.044711128	2.212171122
9.723518638	2.732125307	0.373320221	7.097233613	2.201798533
6.205708557	2.432178165	0.410229552	6.038869874	1.707206052
14.69269178	3.064716312	0.297602695	7.870245085	2.903332873
14.73520138	3.034421168	0.328901282	7.649215344	2.771921491
12.67119453	2.936033573	0.340459726	7.389143514	2.578982931
11.85802676	2.907793745	0.333093265	7.512100218	2.583143204
15.4300776	3.026750752	0.334478705	7.650386808	2.702546909
9.906882808	2.797934534	0.346518311	7.517305475	2.367393128
11.87239108	2.792718968	0.375660429	6.819913713	2.298287822
5.951618559	2.452217976	0.411831784	6.419450059	1.797132809
11.00612177	2.82919184	0.361421659	7.242043399	2.357162509

log.sigma.3.5.mm.3D_glcm_DifferenceVariance	log.sigma.3.5.mm.3D_glcm_Idn	log.sigma.3.5.mm.3D_glcm_Idm	log.sigma.3.5.mm.3D_glcm_Correlation	log.sigma.3.5.mm.3D_glcm_Autocorrelation
12.51345854	0.914699894	0.267634945	0.593968464	563.7867994
3.205173243	0.917489704	0.412449186	0.635012332	100.6387887
4.066273653	0.929521118	0.384287644	0.699124082	189.9610902
8.674143552	0.915366549	0.2945999	0.630689353	314.7169122
5.510479608	0.922610648	0.349014731	0.605292332	261.6964819
5.634221828	0.912161831	0.332550192	0.664878949	147.5550312
5.083109178	0.94306516	0.339962572	0.654559376	324.9563417
5.218355759	0.939589206	0.354462746	0.667705351	492.2919071
6.370903736	0.932694814	0.327240993	0.62441616	456.0139363
5.677730525	0.935168907	0.331835172	0.653705874	559.6714175
3.344346027	0.916821604	0.379786846	0.639062446	156.1024866
5.303143352	0.931981194	0.331374485	0.662416388	350.2081505
4.488280241	0.944261622	0.455997358	0.700210698	287.1968385
8.3774376	0.929215111	0.330994229	0.607216566	462.1239009
8.287262587	0.926356227	0.300409096	0.550922931	383.310686
6.176667087	0.925803382	0.318137712	0.630140029	322.1480698
6.028704285	0.916255638	0.320382118	0.577992842	295.8601296
4.253990511	0.920698712	0.410933822	0.64945852	114.5951161
2.614692061	0.914703376	0.407005962	0.645626274	106.7069685
5.013732156	0.930276795	0.328781129	0.688955765	274.3661083
2.90636119	0.940329935	0.446196722	0.703313171	158.9711794
2.998621169	0.908471096	0.360590295	0.645719715	119.4577539
3.675039603	0.922340752	0.379524387	0.677492078	141.6291791
4.420449339	0.928466525	0.369470182	0.61844649	223.9140426
7.375702555	0.922379157	0.317618906	0.49885684	341.854563
7.98090784	0.933897886	0.300823379	0.63065247	540.1326615
8.752349993	0.931902959	0.31452847	0.639357258	484.326654
5.487685825	0.931263317	0.350297037	0.594047723	336.6121292
2.664017933	0.909963933	0.382540623	0.683628512	95.85664363
3.392898934	0.935786578	0.41036232	0.645171235	250.338434
5.861750002	0.936286117	0.35477578	0.626183552	442.5108782
5.750002664	0.936595138	0.398707859	0.515104839	359.7902835
7.852759366	0.925606215	0.302823765	0.646500962	397.720434
7.233306869	0.921500727	0.320194092	0.617636811	388.2259115
5.077674622	0.926296948	0.324542468	0.634424605	320.3494718
4.835161645	0.936601572	0.372715005	0.624810503	405.3995598
7.034912419	0.950122599	0.400286637	0.604717155	647.7712661
7.774933403	0.945602494	0.374377228	0.69397462	599.2596763
2.207318609	0.913291718	0.42914147	0.511044828	116.3419745
6.339413682	0.922427618	0.298621582	0.682309826	266.2589436
7.380906739	0.931488963	0.361406939	0.557270039	371.6484559
8.307975784	0.923137856	0.262575201	0.627049522	357.795896
6.718817065	0.921323476	0.29516755	0.575788337	382.2688846
7.359499785	0.935904484	0.319014815	0.53857863	360.9551165
3.594082279	0.925574268	0.367525275	0.694573846	149.1877778
6.761625717	0.926607392	0.327649431	0.605395308	357.3064121
5.280987439	0.932688872	0.345819809	0.605952966	365.1063576
2.040682924	0.920309729	0.443065503	0.713247553	75.75914012
4.277252206	0.914820062	0.332852766	0.678459838	187.4165119
3.595764763	0.931086049	0.418596747	0.687357572	107.4612644
6.258876877	0.917583055	0.302651499	0.619174229	334.0794845
7.812752304	0.919431734	0.276694723	0.575720332	321.2096088
4.292520805	0.930294186	0.35158431	0.645297622	241.0813677
5.250015088	0.905902756	0.320385477	0.560896913	132.5685064
5.246388856	0.907724662	0.329918482	0.645401723	171.1560579
5.025263138	0.941782103	0.357143718	0.602138455	493.1955287
6.138402724	0.914056591	0.303122017	0.628037004	301.9921315
6.29509296	0.929617006	0.355499806	0.637764231	368.2039915
5.748498231	0.920125694	0.349101802	0.493066245	245.6456845
6.862468317	0.937477757	0.353027923	0.627216291	474.3588716
5.449491483	0.941062565	0.402642549	0.651035676	214.5889309
4.587498881	0.931598065	0.341152188	0.660824681	336.0878578
6.314212461	0.936073645	0.327836435	0.643785417	505.7636141
5.148477639	0.941982045	0.350693392	0.689346805	444.3732716
3.613300129	0.91252561	0.358793288	0.601204525	152.8326128
5.644657356	0.943633305	0.392521041	0.663104128	484.3774788
3.72552385	0.936464279	0.462069402	0.550499679	228.4531108
7.040883782	0.943701359	0.340687652	0.646688787	518.6690162
5.522742526	0.939773949	0.351078903	0.636362302	483.3309037
3.171712082	0.930868425	0.417238219	0.635716276	211.6982459
5.588495838	0.926362489	0.343468106	0.616882063	338.5400339
5.633379601	0.938541865	0.361958376	0.685750234	427.7083688
6.534638394	0.932719164	0.346527111	0.607748931	476.6783387
9.054904068	0.906638095	0.274001312	0.541986295	334.5372496
6.738175612	0.920917769	0.295375538	0.653321815	257.6663132
5.796261895	0.920906002	0.369700434	0.595707711	286.3461267
3.243551401	0.93124061	0.420162687	0.611611652	168.0508881
3.393241367	0.932369171	0.406147294	0.739610571	163.6107879
3.617652381	0.92264969	0.377620903	0.633254691	184.9877912
9.662124823	0.920791669	0.279246882	0.656106577	485.4423937
12.43182329	0.912570777	0.276135779	0.606320901	516.748431
6.354580283	0.937045938	0.368186847	0.539626393	224.2869319
6.075941459	0.932566768	0.343017645	0.608523095	404.6864652
3.167110295	0.930852524	0.388235735	0.697545281	231.3643125
6.620825167	0.929393405	0.298769961	0.654534372	346.1326268
2.019826296	0.937596575	0.46889796	0.761343788	177.6847548
9.247712088	0.917282583	0.317769469	0.592084706	363.5270408
5.434701969	0.93024791	0.357310969	0.649191856	288.6044484
3.852018339	0.925856258	0.375430687	0.666496117	195.3498389
4.737923594	0.935541529	0.387624168	0.659641105	311.850699
3.099108484	0.92379005	0.473401492	0.728047898	79.0040487
6.065530531	0.911544966	0.305129897	0.663175193	230.1272892
6.860756676	0.932468128	0.330666286	0.596790451	459.2378531
5.821381613	0.929668677	0.348448676	0.584509537	364.5255263
5.014427413	0.922599339	0.328514404	0.609928933	240.7519223
7.910279102	0.936191465	0.357251174	0.662546109	466.7164307
4.151245404	0.923636714	0.351476525	0.727641765	200.0933653
6.308016443	0.93202579	0.409954008	0.66017099	414.0627163
2.610247549	0.922386765	0.428553449	0.630068311	156.5040859
5.279945293	0.929408202	0.372431079	0.610572132	263.0078518

log.sigma.3.5.mm.3D_glcm_SumEntropy	log.sigma.3.5.mm.3D_glcm_AverageIntensity	log.sigma.3.5.mm.3D_glcm_Energy	log.sigma.3.5.mm.3D_glcm_SumSquares	log.sigma.3.5.mm.3D_glcm_ClusterProminence
5.309292878	23.4638813	0.005403063	32.55049966	42609.36866
4.377613288	9.711549597	0.019246359	9.85242439	3461.060703
4.82929372	13.40617336	0.009912271	15.11657226	7670.586829
5.123851484	17.37962107	0.006286841	26.21940159	21500.61322
4.796132082	15.92551424	0.00955877	15.53038762	8051.782926
4.831638239	11.5805279	0.011059525	20.1865198	12227.19896
4.888356073	17.80532315	0.009031052	16.76198066	11622.3468
4.864246496	21.98307303	0.010173056	16.70632721	13145.39767
4.98332177	21.17302334	0.006984593	18.32155417	12755.62857
4.869222513	23.44365873	0.008584753	18.20050234	18065.45386
4.595993139	12.20263401	0.011917855	11.21009026	4312.942358
4.948229008	18.46088912	0.00831651	18.31078515	12739.3439
4.540199065	16.69788552	0.02480727	14.12039307	10808.93827
4.940187545	21.2961236	0.009268324	20.7287811	22853.03278
4.921767548	19.43966283	0.008580632	19.83956148	17547.04715
4.9940823	17.69783457	0.007880828	19.7784147	12978.38174
4.755604446	16.95552232	0.008979654	16.71718421	11087.22572
4.502472843	10.30751954	0.017210139	12.64567528	5875.532088
4.385668715	10.04507399	0.01618547	8.964284867	2320.085256
5.027378943	16.22882813	0.007395983	19.71535477	13214.50073
4.513313469	12.31635736	0.018967142	10.36543676	4607.860599
4.581404922	10.5991384	0.010822785	10.96805166	3435.319682
4.639615388	11.51573644	0.013678611	13.2105497	6362.086902
4.681865843	14.71075571	0.011007988	13.11806055	6335.540151
4.764758405	18.38214765	0.009256259	16.32005204	9168.540776
5.097902303	22.99120045	0.006999539	23.86945613	29513.51201
5.121074558	21.73845165	0.007871187	25.00534287	31645.82561
4.757140419	18.18496764	0.010696879	14.95776089	8544.803924
4.397041233	9.405720974	0.016303661	10.79413314	3313.12221
4.522397408	15.61886124	0.015452373	10.50256607	4549.636353
4.843242382	20.89615655	0.00991184	16.15446593	11527.98936
4.292432141	18.91033486	0.019405433	11.48787777	9175.75015
5.171371911	19.64984926	0.006166355	24.55060574	22627.71568
4.981519702	19.4760874	0.008389255	21.02406117	19178.16451
4.882479261	17.69217365	0.007686972	16.91338259	9908.177491
4.673423795	20.00213652	0.012509649	13.79466161	9352.286395
4.649502884	25.33214402	0.016955553	16.10869569	18362.58049
5.140209611	24.23116789	0.011765671	24.91846467	26788.01979
3.994024482	10.65756833	0.020579167	5.390289836	991.6188589
5.199946552	15.90062246	0.005251101	24.50454343	19145.58031
4.646961855	19.18151032	0.014050656	16.21139792	15583.53194
5.227349904	18.63494908	0.004806847	27.20661423	30523.97912
4.953775206	19.45081882	0.006772777	18.69204988	11680.45856
4.834659446	18.87536097	0.008454542	16.84859018	12261.62816
4.678675721	11.80290822	0.01228419	14.2415974	7359.695723
4.916421363	18.72878189	0.008050994	18.84268823	16355.25005
4.770802534	18.98125398	0.010522801	15.09126549	9635.42178
4.32827183	8.341088752	0.020251265	8.629378008	2605.597676
4.864701725	13.27390527	0.008927027	16.4822192	9031.717336
4.570255023	9.933824202	0.016915779	12.61456867	6391.526005
4.98364744	18.04264951	0.006797758	19.84756042	13832.18375
5.067134504	17.72659644	0.005654177	22.16257841	15878.57624
4.775058141	15.26411144	0.00991137	14.62930407	7320.265945
4.71537067	11.15278017	0.009142147	14.57474087	5994.413489
4.869979301	12.63941691	0.008244653	17.53250182	8176.10791
4.695901698	22.0889647	0.011972695	13.26040846	7947.365032
5.028310819	17.05544634	0.006380047	20.35479896	13652.73019
4.801826222	18.99450186	0.011346237	17.85686246	18790.47848
4.567443794	15.49300633	0.011736477	12.25828613	5248.047332
4.990693082	21.58083429	0.007982175	18.62533379	13751.4924
4.64455301	14.3613986	0.016545086	15.54619897	10656.76863
4.853453957	18.11192836	0.009100947	16.29190498	8886.696163
4.986907215	22.2675743	0.007890747	19.25264924	17352.37446
4.904612624	20.85884526	0.009332257	18.12140351	15306.89309
4.558933819	12.08293227	0.011256653	11.23417355	3967.733571
4.756340469	21.81677023	0.014569298	15.84210021	15640.24924
4.204851965	14.97326584	0.025438542	7.70234375	3265.821772
5.012288113	22.5709677	0.009130231	20.54744769	19485.27284
4.86345451	21.82469791	0.009037758	15.85566842	12597.82358
4.465436822	14.3388648	0.015188213	9.520944934	3207.224039
4.733535153	18.2241533	0.010419581	16.4098623	13591.45119
4.926603203	20.44636737	0.010352794	18.29399062	15003.62006
4.796342478	21.66176062	0.01022426	16.95616496	17904.06612
5.033164606	18.08474205	0.006233976	23.50979965	17553.6659
5.137440205	15.66117715	0.005790644	23.773803	19004.91534
4.650948562	16.66366762	0.012968361	14.65506717	10701.28888
4.431112685	12.74426578	0.015757922	9.073831808	3077.455768
4.684904991	12.36585094	0.015776205	14.60450563	10171.92523
4.594341542	13.33304091	0.012163782	11.36016042	4523.010378
5.215977034	21.67650447	0.006046291	30.6533734	42673.21454
5.290167776	22.41661257	0.005810473	32.73803364	47210.40957
4.620291688	14.79057373	0.0128365	14.34059409	9683.117497
4.841726915	19.96636644	0.009817672	16.66557301	12658.2605
4.659497053	14.92321451	0.012640781	12.41086784	7390.176195
5.104018805	18.341778	0.006569774	22.55887744	20815.7403
4.359473461	13.05393104	0.022860946	9.504314076	3656.83745
4.996318325	18.82774986	0.008255363	23.85449637	24319.38868
4.856442585	16.75244517	0.010107647	17.18691092	9534.694475
4.71135959	13.65349144	0.012100936	13.41609311	6789.002782
4.746772971	17.43443581	0.012577129	14.60795289	9479.936759
4.251313733	8.394437837	0.030926596	11.62207751	4142.224545
5.095556897	14.71919992	0.006197629	22.33623032	15264.21565
4.903964678	21.25669002	0.008439525	17.77892231	15420.66709
4.773020931	18.95117457	0.010315428	15.5093651	9836.091503
4.828185221	15.23007341	0.008122763	15.41757613	7831.63524
5.024501051	21.34148659	0.009680845	22.28177234	25911.47262
4.977374248	13.67863753	0.008455382	18.37809114	12684.01472
4.567926511	20.13974593	0.016887756	17.3767555	22029.6937
4.341987024	12.30386636	0.017364128	8.092007302	2505.774248
4.713287492	15.98786755	0.011609647	14.47570755	6193.495364

log.sigma.3.5.mm.3D_glcm_SumAverage	log.sigma.3.5.mm.3D_glcm_Imc2	log.sigma.3.5.mm.3D_glcm_Imc1	log.sigma.3.5.mm.3D_glcm_DifferenceAverage	log.sigma.3.5.mm.3D_glcm_Id
46.64298573	0.850105468	-0.147781766	3.821980651	0.352004791
19.42309919	0.830773712	-0.174282854	1.934436931	0.477448535
26.79270987	0.869263327	-0.187276473	2.178877198	0.454305626
34.54931863	0.88480994	-0.181575788	3.186214035	0.377621027
31.77972548	0.805983511	-0.138035491	2.514514773	0.425583076
23.15654862	0.896106825	-0.212465864	2.694320315	0.411702275
35.43535667	0.799113338	-0.135227794	2.529862203	0.418613195
43.85618992	0.816682148	-0.143925977	2.441329234	0.430151742
42.155508	0.794136074	-0.131307761	2.754912111	0.406457906
46.79727446	0.871579435	-0.181007632	2.634139913	0.41252538
24.40526802	0.807307708	-0.152668785	2.129625545	0.452286644
36.77575938	0.832214604	-0.149544632	2.618707432	0.41096587
33.31946301	0.88807472	-0.220165822	1.898154225	0.51250244
42.38582316	0.856068993	-0.164584656	2.865197994	0.408426516
38.5821435	0.805769218	-0.133132842	3.13581189	0.382523648
35.20991214	0.810459808	-0.139628118	2.812724035	0.399085978
33.84288588	0.816089945	-0.144488617	2.742952046	0.400998237
20.61503909	0.852687025	-0.185882719	2.053385706	0.475927318
20.09014797	0.805064164	-0.157244752	1.892846889	0.474396119
32.31492633	0.819327858	-0.142403846	2.608390571	0.409385477
24.6291986	0.845251034	-0.183425882	1.747592209	0.505654291
21.1982768	0.813554961	-0.152858625	2.144672802	0.437943556
23.03147288	0.856679882	-0.181869233	2.153170186	0.451706702
29.38769747	0.793431289	-0.137403739	2.294250058	0.442900973
36.54105298	0.815739438	-0.148126519	2.889183119	0.398025357
45.80988728	0.842464222	-0.154439004	3.072885036	0.383746808
43.27298604	0.840028711	-0.15045241	3.042457425	0.394619854
36.21363894	0.787064077	-0.129956656	2.510091878	0.425984209
18.81144195	0.871558937	-0.203273531	2.003932842	0.454453379
31.21790005	0.816191724	-0.159470275	1.963197972	0.47672585
41.57454838	0.802164753	-0.137669833	2.528345653	0.42887202
37.63013411	0.74904763	-0.123408347	2.254373728	0.464971189
39.06913495	0.825363716	-0.14128239	3.083389168	0.385025378
38.74246377	0.828281867	-0.147658896	2.920771496	0.400089593
35.20508537	0.787861725	-0.127326059	2.609308319	0.406316093
39.83815799	0.813621774	-0.15118391	2.297062266	0.44567911
50.51852891	0.824759399	-0.159572604	2.348819911	0.465519502
48.24640445	0.884960423	-0.191258508	2.689293063	0.445625381
21.31513666	0.693058698	-0.11115598	1.726219128	0.493050236
31.61466982	0.805133552	-0.130043143	2.943002843	0.383590035
38.08393634	0.788558795	-0.133099917	2.614607116	0.433057138
36.91733576	0.798377551	-0.124516963	3.436929444	0.351219734
38.55110568	0.772069773	-0.119613584	2.99844036	0.380201149
37.50471028	0.768732171	-0.117548988	2.900532567	0.39846814
23.60172382	0.850636451	-0.174959084	2.212090484	0.442574558
37.20099499	0.808651791	-0.1366149	2.790415931	0.406591748
37.73512083	0.768251503	-0.122630193	2.521781605	0.422710753
16.6821775	0.85431622	-0.20113788	1.66008151	0.504422016
26.54781054	0.88169639	-0.191026767	2.476163304	0.413322856
19.8676484	0.863392093	-0.199006963	1.976578725	0.482399403
35.89096573	0.804930945	-0.132551877	2.871441994	0.387060923
35.13621164	0.770727741	-0.112486906	3.260363993	0.364065187
30.45128315	0.782322434	-0.129528941	2.374217271	0.428823472
22.30556035	0.820915566	-0.148993228	2.681275859	0.401898223
25.27883382	0.838740522	-0.158823484	2.624526473	0.410092864
44.03846378	0.81661195	-0.150443137	2.407029375	0.432436329
34.02453653	0.782734224	-0.122061616	2.910386413	0.387452246
37.78504595	0.808706189	-0.143012586	2.517351814	0.430050681
30.96093114	0.759337122	-0.119297431	2.520178689	0.425202727
43.00443543	0.794055877	-0.128964425	2.692458402	0.427538748
28.61700288	0.856262979	-0.180425743	2.205982098	0.468866119
36.08322152	0.815355466	-0.143696058	2.47684735	0.41981345
44.41121243	0.796343102	-0.128637021	2.740930165	0.407231086
41.54898544	0.814585966	-0.146430858	2.461919243	0.4267749
24.16586454	0.825242847	-0.164222979	2.25164528	0.436431299
43.52541122	0.832211534	-0.159752826	2.285711769	0.460595738
29.94653169	0.792035159	-0.155350706	1.755219055	0.517613599
44.95825527	0.81257129	-0.139032541	2.709117944	0.416789487
43.48938708	0.792530578	-0.131980552	2.502746255	0.427101407
28.6777296	0.793586568	-0.148941581	1.896429724	0.481887542
36.26228054	0.84246012	-0.165462639	2.546343502	0.420465066
40.74027518	0.843619374	-0.160396883	2.430382968	0.435534233
43.1779929	0.821372261	-0.148211762	2.616468561	0.422778651
35.89157749	0.768928252	-0.112225187	3.438873294	0.359763324
31.1447481	0.820136279	-0.139031654	3.011534649	0.3802011
33.32431873	0.823799046	-0.15856099	2.417425297	0.441496249
25.48853156	0.791542514	-0.14899749	1.887741328	0.484435419
24.72500699	0.898409146	-0.222690928	2.001431546	0.473535907
26.66608181	0.805000796	-0.146168843	2.141379619	0.450759618
43.11925057	0.841542561	-0.148414902	3.407258791	0.364373017
44.55444637	0.855944994	-0.15286356	3.737636222	0.359167562
29.44513105	0.795088688	-0.135331949	2.507195705	0.439637378
39.72312098	0.811554532	-0.145014173	2.615173599	0.420484477
29.84507726	0.812139602	-0.155917758	2.040459901	0.459288816
36.39848901	0.835295072	-0.149807396	2.969939138	0.383180737
26.10786209	0.874673478	-0.216984782	1.545819097	0.524973135
37.38842488	0.809554582	-0.136908568	3.127917866	0.395415703
33.32964376	0.833757095	-0.153904945	2.474935823	0.431516782
27.30448063	0.850176773	-0.173627354	2.212171122	0.448011743
34.77838523	0.847169991	-0.172258628	2.201798533	0.457205031
16.78887567	0.87221185	-0.224228123	1.707206052	0.527037735
29.36611273	0.831857019	-0.144639329	2.903332873	0.389164596
42.34677741	0.768111544	-0.117732301	2.771921491	0.408606021
37.71134079	0.77526431	-0.125814994	2.578982931	0.423921541
30.42676343	0.758299745	-0.116234831	2.583143204	0.409020555
42.49738214	0.860522311	-0.167749517	2.702546909	0.430194971
27.33720401	0.855498182	-0.168237231	2.367393128	0.428703429
40.1356856	0.871930412	-0.197460925	2.298287822	0.472883058
24.60773272	0.818650618	-0.169095295	1.797132809	0.491053493
31.89693133	0.776008918	-0.129769642	2.357162509	0.444063463

log.sigma.3.5.mm.3D_glcm_ClusterTendency	log.sigma.3.5.mm.3D_firstorder_InterquartileRange	log.sigma.3.5.mm.3D_firstorder_Skewness	log.sigma.3.5.mm.3D_firstorder_Uniformity
107.1107932	150.7092056	-0.778216	0.057743437
32.33998083	85.26478767	0.603331127	0.10606766
50.85476105	135.633378	0.119626096	0.072789904
84.18581928	153.5757275	-0.423014706	0.059710077
48.80455798	110.4779788	-0.156979381	0.079261351
67.23269343	153.748577	0.569469247	0.074040404
55.78069785	132.5896263	0.308418853	0.072241291
57.04359431	122.6233325	0.084024887	0.076581317
61.17710195	141.6602507	-0.166720601	0.066588032
61.39055424	144.2932653	-0.615892243	0.071417632
36.80238789	110.3173883	-0.001822754	0.085353798
60.55291129	134.3665161	-0.112628153	0.070409418
46.77942124	76.79242802	0.262322752	0.113517012
68.70877753	122.2653847	-0.461559245	0.073466914
63.04886967	116.2381449	-0.324722825	0.073961145
63.33716898	146.2398627	-0.035278049	0.065847472
51.89696759	125.1335608	-0.897766789	0.079701972
41.95789462	89.70730686	0.820507419	0.099931658
29.52697762	95.73659825	0.400589462	0.101426635
65.16362696	141.1139956	0.145715491	0.066021433
35.30041262	94.51609612	0.388718247	0.099638518
36.15977855	118.189436	0.095586294	0.083816839
44.41576675	104.7983007	0.310236982	0.087305069
41.81266966	110.9125061	0.295162851	0.08339198
48.17602873	126.2635345	-0.28481364	0.075670969
79.43961265	135.0034981	-0.385823963	0.065363235
83.04362012	121.8922958	-0.428741161	0.069142654
46.93969085	108.9405787	-0.122594882	0.082170304
36.3538577	116.5133963	0.568536531	0.098238818
34.16393004	89.76205444	-0.081766353	0.099213637
54.02554423	124.7453976	-0.097815444	0.075042481
34.12385671	73.82159853	-1.359848745	0.115454472
82.07708958	150.6504784	-0.353725861	0.060371032
67.95240915	134.6420593	-0.581603582	0.067659574
53.98676486	137.0635738	-0.185220795	0.070449641
44.74834547	121.661355	-0.280021199	0.083118403
51.7497339	85.38944912	-0.749523028	0.099755515
84.64356387	143.2955382	-0.146358233	0.069645686
16.29285289	79.33232212	-0.479243663	0.124671822
80.7726315	162.3435388	0.07030501	0.057216879
50.62380977	91.49470481	-1.000446093	0.091337937
88.82731795	160.0840569	0.306653165	0.05753276
59.03491122	144.5416145	-0.163568325	0.064872487
53.20709089	120.3502983	-0.326688476	0.075621827
48.1974395	114.1175585	0.548861137	0.082789031
60.04807716	134.0311165	-0.261906984	0.068868385
48.25170434	116.2154522	0.154622123	0.078957059
29.61408765	95.61676407	0.602391067	0.105650762
55.36349828	130.5147157	0.046117088	0.072147097
42.76716016	124.2519627	0.71449605	0.090068187
62.72904786	142.3739777	-0.369331196	0.065982238
68.94210655	152.0754472	0.108608408	0.061291359
46.96855121	106.5734813	0.075094363	0.081274305
45.50550894	137.0360994	0.314424399	0.074265517
57.79034377	158.54949	0.250795513	0.068161004
44.32709421	99.43228769	-0.35218686	0.087397765
65.14294168	146.5083389	-0.266542921	0.063931023
57.91739843	106.6753769	-0.903070482	0.081293459
36.23465751	97.08795536	-0.428899344	0.089570176
62.33010391	135.6412401	-0.12037485	0.069846579
49.82995946	99.1415236	0.852856793	0.092607437
53.15931085	133.2703218	-0.055675749	0.074258581
64.64570497	135.9954491	-0.159690104	0.070349079
62.5491277	126.0206819	0.449123592	0.07327547
36.06813747	123.819665	0.221330713	0.081595898
54.06043534	105.4298553	-0.16297233	0.089658602
23.90518828	66.70198327	-0.475617304	0.128427397
67.95190056	113.1226569	-0.07308214	0.074425651
53.90944933	124.5337315	-0.523965262	0.072780618
31.21127415	103.4946623	0.029685736	0.09506592
51.52726154	118.1618309	-0.617360508	0.08044383
62.68172281	135.6344109	0.043301318	0.072522753
55.93313092	113.0229635	-0.93174779	0.079408476
71.14699958	151.6367302	-0.50762468	0.062339007
76.70023341	150.3056402	0.326446951	0.060927185
46.6761332	95.0449872	-1.129857534	0.092186185
29.38855615	94.52509022	0.148127805	0.096442039
50.59811604	106.5100036	0.647986282	0.090406684
37.13697184	104.3003674	0.130507517	0.087184683
104.0341539	148.1895218	-0.920670399	0.062799116
108.7261643	149.9979897	-0.862829952	0.058302312
42.91019574	92.68058586	-0.114133939	0.089932304
53.99163006	122.1915951	-0.091258891	0.074400163
42.11603875	103.1214495	-0.600373116	0.089789416
75.32103454	145.269802	-0.190035687	0.061692757
33.55363527	85.52216005	0.559121991	0.110793072
75.5479762	127.6255512	-0.807792513	0.069325493
55.11369061	141.1153803	0.076650871	0.073539575
44.68376304	111.2997284	-0.046577981	0.08427954
47.52156269	104.6548786	-0.3682568	0.08474635
40.28260147	122.6119907	0.853210217	0.119687182
72.74895342	145.4498334	0.116376859	0.063192598
58.42037606	117.2542686	-0.697844087	0.075112618
48.59517236	112.6968212	-0.317838747	0.080048669
49.0749084	123.7522476	-0.342621994	0.074862153
76.15561808	134.4269679	-0.422630672	0.07052981
62.93206229	130.9211006	0.025877159	0.068690537
57.17884081	88.31778765	-1.543428767	0.099839538
26.41641065	84.77245426	-0.447942464	0.106668622
45.60643518	123.9706073	0.368891318	0.080838353

log.sigma.3.5.mm.3D_firstorder_MeanAbsoluteDeviation	log.sigma.3.5.mm.3D_firstorder_Energy	log.sigma.3.5.mm.3D_firstorder_RobustMeanAbsoluteDeviation	log.sigma.3.5.mm.3D_firstorder_Median
105.6514977	47488559.62	66.95475426	64.05979347
60.62936777	11938744.59	38.98170526	66.77225494
78.30400697	29239633.6	55.24051711	92.17315674
99.00157255	17659754.24	66.71632305	53.17659378
73.3049371	30145888.36	47.9533809	56.30527306
91.06042243	12858654.87	63.71548852	54.54954338
81.88107757	102249284.2	55.78304774	72.2898407
80.89958596	100403538.6	52.35419487	77.45866394
85.41036269	91909672.24	58.84234414	89.09300232
83.73464004	23461213.76	58.26870262	99.66584396
66.991418	17339108.74	46.2917905	61.12357903
83.89268136	50467561.74	56.6371863	92.70291901
63.80379926	21083350.51	34.96335938	39.07463074
85.49266902	32259164.08	52.46114958	63.74199677
83.18761051	35960602.67	50.73410037	74.16321564
89.54370424	83117815.91	61.80082223	69.95300674
77.60356498	16230661.22	51.39425007	76.3254776
67.87044421	15567238.85	43.20461418	49.14884567
59.98506868	12410465.5	41.70970063	50.92430305
87.9616965	112055207.6	59.38455678	85.59870148
63.16075575	36340400.78	41.55703136	59.40207672
67.48239572	14091932.47	48.23348759	72.35636902
70.89845322	14688111.65	46.02799077	63.9420681
69.89184109	36779417.11	46.9073899	69.11595917
79.25765388	24398655.79	52.27361815	87.36894989
91.82686498	57421652.76	57.98431788	93.71837616
90.32067512	71199509.31	54.5403005	80.46969604
73.36736368	37507541.93	47.28952135	57.621521
66.32020753	5440767.236	47.69389769	55.55714417
59.69671074	26086043.85	37.66950147	78.71772385
79.1816714	86663086.77	52.75972279	78.94381332
57.61911235	16988616.64	31.78984311	39.03234863
97.25327439	60228560.72	64.51304692	62.70568466
89.78906506	45155018.47	58.62635219	76.45421219
81.22882266	93129918.74	56.49657705	100.0624352
74.00308063	50185802.32	50.27712208	78.21938324
67.67050068	45048919.37	37.50385903	41.0646553
95.25694335	103816451.4	62.15196263	55.3491478
46.20657669	7619646.753	32.44071636	57.38124084
98.44016672	134775655.5	68.28295872	56.10678291
72.85867975	31514890.98	41.13743676	48.96446991
99.69234422	74878544.5	66.83072702	50.35873413
87.63071045	77012372.87	60.43304955	92.814888
79.018615	36563021.05	50.57378117	66.72071075
75.72355818	27577025.74	50.99229814	92.67350006
84.62322323	62094022.76	55.62122274	100.1032028
76.88064761	80135623.12	49.91209216	75.64875412
59.46424055	9575184.291	40.7779186	55.52248764
80.50707991	13785101.3	55.00011868	69.42002869
73.26223736	21885696.21	51.99366728	69.48435211
87.75316536	62940845.05	59.43433908	108.8952713
92.46260321	66447722.92	63.09216595	74.71858215
71.81936195	60254582.75	46.7186959	63.15680885
78.80800746	25006332.71	55.95843977	103.4848442
87.41130921	28852892.72	64.626597	87.72160721
69.43113222	26897542.64	43.17418103	68.14887619
89.07511317	82868502.52	60.75510407	89.09174347
77.02475459	52441224.16	45.7956774	65.23049545
66.52194979	16710762.59	42.09779624	48.80064201
84.53511182	131667044.8	57.41494048	46.78379059
73.38834957	25872446.38	44.93751935	51.2157135
80.1007934	48605124.05	55.77785547	79.09741592
84.99838529	125138299.9	57.41365334	87.44882965
84.06715814	125690411.8	54.87418729	49.75107574
70.27947633	12681436.84	49.83693481	86.60357666
74.13877937	55372257.47	45.4161804	49.54264069
48.87885012	8021634.661	28.812266	33.71596527
82.27845153	96060674.32	50.06955674	50.9044838
80.85635148	100952204.9	52.15873943	80.67102814
61.85294182	33038142.53	42.65097911	65.1553421
75.73835263	18344500.98	49.5062041	60.19252777
85.5994927	96973954.42	56.97203243	89.13317871
77.58295299	40606617.62	48.08344798	83.32965088
96.02258434	36738375.17	64.3948082	50.1427803
94.46973611	54809261.04	63.68868511	43.35200882
68.1610435	16107606.87	41.46487001	67.98366165
60.30288727	30205395.65	40.10466279	75.23604965
73.03765017	14028531.93	46.63862129	55.21418381
66.84525433	21879746.8	44.78993252	65.13065338
104.3497516	46779721.46	65.89801156	58.36322403
106.5676353	45648317.78	66.60192985	64.46915436
69.8386955	18728930.84	41.43889733	34.94905472
79.40182128	57423186.44	51.69369262	78.34178925
66.46234075	35387612.97	42.67233821	67.95719147
93.26532225	47511985.6	62.01884475	71.85746765
59.89106542	15708845.23	39.04016903	48.53153229
90.508241	37868222.56	55.56103576	41.43390274
83.61265578	59934411.1	58.28288519	89.42153168
70.74153998	22366011.15	46.6773107	75.12208176
72.13974376	29656943.22	45.34497267	68.81984711
72.81726097	17313104.96	52.69346522	40.3957119
90.59134111	54938213.31	62.04960962	92.93805313
79.72513971	71787828.46	50.07168493	54.82818222
74.96100657	56616159.53	48.28304876	81.67711639
77.00790408	49854646.96	52.55011253	52.03662109
90.25121564	68548687.69	57.97202059	61.83439827
83.58027345	41742325.94	55.67605985	58.68760681
69.90069481	11166425.13	37.85192709	29.42769241
54.4756368	5954402.452	36.09138781	49.43175888
76.08745979	96198796.1	52.87781335	71.48194504

log.sigma.3.5.mm.3D_firstorder_TotalEnergy	log.sigma.3.5.mm.3D_firstorder_Maximum	log.sigma.3.5.mm.3D_firstorder_RootMeanSquared	log.sigma.3.5.mm.3D_firstorder_90Percentile	log.sigma.3.5.mm.3D_firstorder_Minimum
1282191110	410.0094299	149.5966295	216.4841293	-503.5231018
322346104	341.9428101	112.9987203	196.5440857	-141.4835815
789470107.2	445.6513672	138.0617212	220.5761368	-202.4212341
476813364.6	409.8947449	134.4451209	199.5477936	-360.1641235
813938985.8	352.4078369	112.8296379	185.3945267	-312.1375122
347183681.6	436.5918884	139.5808383	246.9076431	-195.380722
2760730674	643.647583	134.5139992	223.3204193	-336.388855
2710895542	444.2791443	140.2003395	235.6431168	-447.4979248
2481561150	455.5281677	142.188965	230.5516052	-414.1246338
633452771.5	411.4424744	147.9373536	239.8444977	-469.0576172
468155935.9	315.0963745	107.2291132	180.8773636	-204.8897705
1362624167	479.4977112	147.9689856	244.7137085	-340.2223511
569250463.9	388.5639343	106.2668129	168.4257385	-339.3166809
870997430.3	402.508667	137.0696826	215.0454102	-436.5730286
970936272.1	514.2729492	134.6302483	204.7392975	-382.8020325
2244181029	451.9908142	137.8187811	231.5539017	-329.0904541
438227853	336.1147461	116.5912459	167.0660263	-338.2470093
420315448.9	358.431488	112.3632406	207.7801147	-159.52211
335082568.6	283.0530396	97.78149875	174.1219666	-160.3407593
3025490606	509.7258301	146.8101606	246.7065613	-276.7123413
981190821.1	419.5334167	109.6235755	187.3390442	-207.9090424
380482176.8	303.955719	114.2811375	194.0171234	-152.2032166
396579014.7	380.5227051	119.7658509	213.3061554	-198.9122467
993044261.9	416.9578857	117.8760164	194.8193939	-251.2406158
658763706.2	438.311615	138.4422816	221.4049316	-335.2404175
1550384624	528.9837646	155.6880625	241.0820404	-460.7980652
1922386751	523.8648071	148.400597	229.6509979	-437.6412659
1012703632	404.9270935	116.5326076	191.6310806	-374.5477905
146900715.4	301.5163269	110.3256035	202.9676544	-136.2372131
704323184	362.6066895	112.6948034	178.5186813	-282.675415
2339903343	429.712738	134.9599343	222.5661377	-404.9838867
458692649.4	331.8594971	90.6147221	115.1312347	-421.7218628
1626171140	460.8030396	136.5737455	206.2159698	-401.4533386
1219185499	391.1362305	138.6178253	213.9475449	-398.1331177
2514507806	443.262085	145.4189341	237.2590698	-311.4228516
1355016663	384.8213196	129.3606541	214.5011108	-388.4504089
1216320823	459.2010803	110.0303144	154.5361328	-554.6192627
2803044188	551.343811	141.8293656	235.8460541	-503.9164124
205730462.3	224.1850281	78.70727259	121.4906044	-180.7875366
3638942697	486.714325	136.1565445	219.2511673	-303.368866
850902056.5	380.8664551	110.2231755	142.5052429	-419.3068542
2021720701	565.7232056	139.1526915	208.3591248	-397.7322998
2079334068	433.1340027	145.1962555	235.7141937	-353.1847229
987201568.3	582.7076416	121.2260203	179.7214081	-394.4003296
744579695.1	454.2940979	146.8954302	259.1204681	-152.37677
1676538614	470.1631775	150.4020389	238.2002594	-337.0348206
2163661824	439.579834	132.5363996	216.6980377	-355.5513916
258529975.9	308.7401123	104.8490484	184.5527496	-106.684433
372197735.2	369.0986633	127.2741487	211.5262299	-225.7619781
590913797.6	459.9061279	127.2306768	215.5206238	-148.5761108
1699402816	423.959259	156.5251875	247.2674622	-322.0590515
1794088519	516.1886597	140.3979204	232.2022552	-338.9747009
1626873734	440.4127197	115.5607884	193.3533279	-292.4355164
675170983.1	445.1685791	150.5013744	252.7135269	-147.225708
779028103.5	396.7676086	147.288507	249.864064	-182.2866516
726233651.2	432.9271851	117.4462073	192.3995895	-469.8951111
2237449568	396.355011	143.1315856	229.4782837	-313.8769226
1415913052	373.8796692	123.1470226	181.4358536	-394.3968506
451190589.9	341.044342	100.6976953	157.2452454	-311.0436707
3555010210	457.5775757	120.095557	191.2809509	-473.2821655
698556052.3	534.6196899	121.0384624	209.6853104	-260.0330505
1312338349	446.2684326	134.9228161	229.1203247	-343.4910278
3378734096	486.5759277	147.025732	243.6539368	-437.8670044
3393641117	483.4244385	128.406827	226.6746735	-448.2114868
342398794.8	344.3640747	126.6982703	204.3100174	-185.3887177
1495050952	402.2637329	119.7402002	199.4747116	-457.7776489
216584135.9	268.8856812	78.43188333	119.8793549	-314.1813965
2593638207	553.8381958	123.974626	190.6649414	-487.2800598
2725709531	468.5256958	131.2865671	201.0212341	-445.2181091
892029848.3	314.5148621	106.7723056	178.6321732	-273.5240173
495301526.4	365.1972961	118.8360959	185.5925629	-360.4774475
2618296769	474.0854187	150.3484332	249.7421494	-388.0160522
1096378676	379.9125061	133.4830881	206.0942383	-444.5875549
991936129.7	350.1369324	127.5269159	178.9642761	-381.7398682
1479850048	494.0630188	132.3077974	214.5696259	-302.7182617
434905385.4	290.774353	109.5567783	155.8870941	-338.83078
815545682.5	357.0722961	112.2790831	183.9925537	-210.8187866
378770362.2	413.8905334	121.0105843	204.0270035	-218.5994415
590753163.5	345.6480713	113.215108	191.7981232	-234.519928
1263052479	406.9779053	141.4209352	173.4501556	-499.8795166
1232504580	396.1825256	151.4938481	215.161969	-483.3044434
505681132.6	534.5755005	102.03306	135.2167938	-321.0767517
1550426034	441.3543091	132.5367094	213.1026276	-389.1038513
955465550.2	331.4649048	107.3110278	164.6668213	-295.7111511
1282823611	529.0391846	139.5711607	220.3841705	-350.3725891
424138821.1	278.12854	101.2611249	185.8087769	-236.2680969
1022442009	359.5686951	126.0065929	166.3059153	-414.3453979
1618229100	455.0037231	147.8442355	244.5028702	-294.260376
603882301.1	379.1362305	123.6014968	210.8217575	-248.571991
800737466.8	397.6210938	121.0181972	195.4808899	-328.2514954
467453833.8	336.1780396	115.2255223	220.3985504	-117.2792892
1483331759	448.9022827	151.8680397	256.2057343	-242.9221191
1938271368	402.4675293	117.6887103	171.6412933	-473.2010193
1528636307	401.8978271	130.9824873	212.360025	-360.8925781
1346075468	382.615509	107.0307033	160.9647797	-311.2259521
1850814568	450.5172424	141.5336054	235.6811127	-431.9875793
1127042800	395.6610107	121.7292368	191.8709595	-263.0683289
301493478.6	229.4817505	103.2722736	115.3558853	-450.3970032
160768866.2	229.104126	84.49598384	129.7712265	-238.5861511
2597367495	413.3182983	129.0762765	225.4056427	-283.677063

log.sigma.3.5.mm.3D_firstorder_Entropy	log.sigma.3.5.mm.3D_firstorder_StandardDeviation	log.sigma.3.5.mm.3D_firstorder_Range	log.sigma.3.5.mm.3D_firstorder_Variance	log.sigma.3.5.mm.3D_firstorder_10Percentile
4.448701722	141.342744	913.5325317	19977.77128	-142.131308
3.60192867	79.24583806	483.4263916	6279.90285	-9.665810585
4.002384765	97.6793139	648.0726013	9541.248365	-23.8947897
4.323989491	127.3481712	770.0588684	16217.55671	-149.9190094
3.961319198	96.19035578	664.5453491	9252.584545	-56.13325233
4.07576547	112.5991322	631.9726105	12678.56458	-44.80485649
4.079521807	104.7030666	980.036438	10962.73216	-33.62533188
4.066331526	107.1321733	891.7770691	11477.30256	-17.71442032
4.156342595	109.0606729	869.6528015	11894.23037	-40.41276169
4.051663608	107.858278	880.5000916	11633.40813	-11.6814414
3.791799545	84.93192816	519.986145	7213.43242	-36.70404739
4.120699608	108.2309287	819.7200623	11713.93392	-20.96783447
3.694202895	92.72581104	727.8806152	8598.076033	-28.04284363
4.163758083	117.7255541	839.0816956	13859.30609	-50.89553757
4.151422537	114.0138105	897.0749817	12999.14899	-74.69878387
4.193053991	112.8601034	781.0812683	12737.40293	-53.79929733
3.943666009	100.3868669	674.3617554	10077.52305	-67.97870178
3.701911411	88.81219637	517.953598	7887.606225	-23.23692017
3.55747177	74.88564204	443.3937988	5607.859384	-25.58052864
4.197124835	112.5660425	786.4381714	12671.11393	-38.3911644
3.685808926	82.12136035	627.4424591	6743.917826	-14.78851147
3.764910887	83.45946739	456.1589355	6965.482696	-24.19632835
3.852994144	92.43671968	579.4349518	8544.547144	-26.89244022
3.872042588	90.17400227	668.1985016	8131.350685	-28.09239578
4.039368584	104.2079501	773.5520325	10859.29686	-23.30758896
4.292358068	124.7346725	989.7818298	15558.73853	-46.64670792
4.262726342	124.8426175	961.506073	15585.67914	-61.8958046
3.951312874	97.10153572	779.474884	9428.708239	-44.36298676
3.591432235	81.17572092	437.75354	6589.497667	-12.14028435
3.681798093	80.18619702	645.2821045	6429.826192	-9.93055191
4.049770883	103.1237612	834.6966248	10634.51012	-27.58876953
3.579328033	85.49240957	753.5813599	7308.952094	-61.4633461
4.340826323	126.0078581	862.2563782	15877.98031	-128.0601074
4.204313372	118.1604417	789.2693481	13961.88997	-88.15756226
4.067343416	102.7144822	754.6849365	10550.26485	-20.34927368
3.899971613	95.90773016	773.2717285	9198.292704	-9.421392059
3.838252239	101.328761	1013.820343	10267.5178	-47.28913498
4.246837392	124.7944367	1055.260223	15573.65143	-75.09049225
3.237764857	58.35000947	404.9725647	3404.723605	-19.29519043
4.34949437	123.8257553	790.0831909	15332.81768	-102.8566254
3.917038219	103.9807053	800.1733093	10811.98707	-92.9009491
4.390039157	129.3419561	963.4555054	16729.34161	-113.1404205
4.18518406	110.9130145	786.3187256	12301.69678	-40.8869751
4.056801451	105.7148359	977.1079712	11175.62653	-79.47033997
3.901793081	96.74185571	606.6708679	9358.986647	4.215472078
4.157350659	111.2451838	807.197998	12375.49093	-27.59273872
4.006925734	101.0454675	795.1312256	10210.18651	-24.80172501
3.535477507	74.84296432	415.4245453	5601.469308	-8.04920578
4.048532696	102.1007743	594.8606415	10424.56811	-51.47443008
3.782888128	90.45368243	608.4822388	8181.868664	-5.286004591
4.18614037	113.0734964	746.0183105	12785.61559	-24.50069542
4.279678601	118.2993643	855.1633606	13994.7396	-77.16677094
3.930517331	94.12408141	732.8482361	8859.342701	-44.77539215
3.970799124	97.4753969	592.3942871	9501.453001	1.177976441
4.067106027	105.1240862	579.0542603	11051.0735	-17.91672459
3.891378648	94.78999579	902.8222961	8985.143302	-40.46688957
4.212273736	113.4904983	710.2319336	12880.09321	-56.81418915
4.020742462	108.1834927	768.2765198	11703.6681	-61.31566277
3.823798241	89.2468923	652.0880127	7965.007785	-58.89805031
4.132807024	108.2908012	930.8597412	11726.89763	-72.1974411
3.865935032	98.67036258	794.6527405	9735.840451	-34.69363403
4.022420389	101.29645	789.7594604	10260.97079	-28.2765934
4.13250493	109.9610769	924.4429321	12091.43842	-24.16074829
4.104328549	110.111982	931.6359253	12124.64857	-51.94100952
3.817162653	87.19226684	529.7527924	7602.491397	-20.27738571
3.924440418	102.1761598	860.0413818	10439.96763	-34.85318794
3.394156139	69.91512907	583.0670776	4888.125273	-39.48727913
4.155419632	113.1225425	1041.118256	12796.70961	-86.81429062
4.095764398	107.1639248	913.7438049	11484.10677	-58.03996811
3.66782875	78.78758246	588.0388794	6207.483151	-14.87008333
3.966553198	101.2960572	725.6747437	10260.89119	-45.12471008
4.125025472	111.2015803	862.1014709	12365.79146	-13.09674549
4.013743492	107.1786382	824.500061	11487.26049	-35.67886963
4.289351436	123.0892951	731.8768005	15150.97456	-138.3013885
4.304266921	121.3929752	796.7812805	14736.25442	-97.03456879
3.804946527	93.94465484	629.6051331	8825.598173	-60.23834724
3.673591936	78.42577458	567.8910828	6150.602119	-11.36496115
3.859579096	96.05284786	632.489975	9226.149582	-23.94384918
3.80007405	86.07458923	580.1679993	7408.83491	-26.13959846
4.356455723	137.9189421	906.8574219	19021.6346	-176.1725281
4.443669214	143.7312553	879.486969	20658.67376	-144.7560638
3.893763919	97.72312871	855.6522522	9549.809885	-87.3577652
4.073660373	104.6955745	830.4581604	10961.16331	-36.96408997
3.804012288	88.18248176	627.1760559	7776.15009	-53.12801895
4.30168489	121.1440226	879.4117737	14675.8742	-89.30335999
3.533135639	77.72104817	514.396637	6040.561328	-14.49165955
4.214713655	122.7679665	773.914093	15071.97361	-143.7227203
4.061922483	105.0209583	749.2640991	11029.40169	-11.36251154
3.884866578	92.37837661	627.7082214	8533.764465	-23.96807156
3.929534384	97.63854476	725.8725891	9533.285423	-37.47844009
3.47068302	87.40698483	453.4573288	7639.980997	-9.933507347
4.229207863	115.0293047	691.8244019	13231.74094	-41.38082199
4.082760957	107.726672	875.6685486	11605.03587	-86.34072266
3.986784625	100.0326798	762.7904053	10006.53702	-32.34179077
3.99731091	97.92584267	693.8414612	9589.470662	-88.33401413
4.201437375	121.1899983	882.5048218	14687.01569	-51.2043293
4.135187758	107.9425605	658.7293396	11651.59636	-85.86853638
3.77098847	102.3140621	679.8787537	10468.16731	-105.9483307
3.512105171	70.81433746	467.6902771	5014.67039	-41.72285042
3.916955545	95.37674756	696.9953613	9096.723975	-17.53726368

log.sigma.3.5.mm.3D_firstorder_Kurtosis	log.sigma.3.5.mm.3D_firstorder_Mean	log.sigma.3.5.mm.3D_glrlm_ShortRunLowGrayLevelEmphasis	log.sigma.3.5.mm.3D_glrlm_GrayLevelVariance
4.045371589	49.00388032	0.003428687	33.47998754
3.411920036	80.55313738	0.016665035	10.74722828
3.055505567	97.5694137	0.008343572	15.91278905
3.012104015	43.10375638	0.007738942	26.65899947
3.598597717	58.97408443	0.005666733	15.50646055
2.862683361	82.48785266	0.014135279	21.1499144
3.668443463	84.44692897	0.003816089	18.25575172
4.217164772	90.43689865	0.002455974	19.46257817
3.504009392	91.23306089	0.002791107	19.64615619
4.955672337	101.2524196	0.003115228	19.35865277
3.027280364	65.45724021	0.010280817	11.97679765
3.580350012	100.9003805	0.004317067	19.65015033
5.367776502	51.90914646	0.005070684	15.49321439
4.890585397	70.20535453	0.004905597	23.75918662
4.255725057	71.59717018	0.00452621	21.90839566
3.081036702	79.09875788	0.004409753	21.14713405
4.117675516	59.29583089	0.006319698	16.74844756
3.490921995	68.83234431	0.014423489	13.34781402
2.750625775	62.87576731	0.013281238	9.320700974
3.246825418	94.24494317	0.005303507	21.07370121
3.808160337	72.61825166	0.008870057	11.63422516
2.6609337	78.06853197	0.014094175	11.39340017
3.279000417	76.15321328	0.012743931	14.30089072
3.645118556	75.91708997	0.006329883	13.72335016
4.283453053	91.14257228	0.0060949	18.10703551
4.706840086	93.1667015	0.002956321	26.04304137
4.62045918	80.23127848	0.003277904	26.29985869
3.856182401	64.42934422	0.003948083	15.90492114
2.638164995	74.71439698	0.018462736	10.88185265
4.248640761	79.18517871	0.005205995	10.95871666
3.9317226	87.06132169	0.002766498	17.90595282
7.253881463	30.03457619	0.005076063	12.98996233
3.272337723	52.67264615	0.004199935	26.12193204
3.862241127	72.47766211	0.005370956	23.50950593
3.354840787	102.9388242	0.004711213	17.46400677
4.343350769	86.80948179	0.003523349	15.53860002
6.915278494	42.88533878	0.00197458	18.18596526
3.723740794	67.39374993	0.002097879	26.8597344
3.780618246	52.82150276	0.012599081	5.777044325
2.890034253	56.61966906	0.006311492	25.08488219
5.43233025	36.56721672	0.0049766	18.77662769
3.686897837	51.32377562	0.004039071	27.35706652
3.263012964	93.70301932	0.003607592	20.29377466
4.406662473	59.33061163	0.003878212	18.61128183
3.097537755	110.5408556	0.010385497	15.5476693
3.972608435	101.2189824	0.004586206	20.66232659
3.92401119	85.76544008	0.003476866	17.25134384
3.042246345	73.42924243	0.024003086	9.597282066
3.030225673	75.98776762	0.009833587	17.26349088
3.51844277	89.47500466	0.0158819	13.88271511
3.706294213	108.2336303	0.0061215	21.15561702
3.236623289	75.60976435	0.004676831	22.95884741
3.493015495	67.04441156	0.005651386	14.77145353
2.916141367	114.6700078	0.017406379	15.57798314
2.512872497	103.1640965	0.010907692	18.03767646
4.87488353	69.3431201	0.002822564	15.22451498
3.211013542	87.21558119	0.006279487	21.31124131
5.220163068	58.8346928	0.006195517	19.86429395
4.271762018	46.63708886	0.007869866	13.61622107
3.699902664	51.92345492	0.002518792	19.73529541
4.433028927	70.10327338	0.006459103	16.77449997
3.25828421	89.12460673	0.004045331	17.09682377
4.08092534	97.59675944	0.002726857	20.07414245
3.757148659	66.05804007	0.002590149	20.30150944
2.937305165	91.92366563	0.009810918	12.5741694
5.229364252	62.43194635	0.002730435	18.16593487
6.289667126	35.54483155	0.00778406	8.740160295
4.335970505	50.72473045	0.002549335	21.66202358
4.0677822	75.84230954	0.002726532	19.322475
3.55170685	72.0613772	0.006248854	10.60600053
4.710626553	62.1379634	0.006040006	17.28156747
3.919995785	101.1872518	0.003166764	20.81725687
5.639896634	79.56427789	0.003791942	19.56019351
3.251979147	33.34875895	0.006099583	25.03862398
3.269079984	52.62222759	0.006355223	24.10056744
5.222616384	56.36567668	0.007725908	15.01692186
3.616482654	80.34917789	0.008113576	10.50233501
3.948902705	73.60307014	0.011482365	15.72164914
3.314773923	73.54471959	0.008233024	12.4371839
4.019648	31.27692943	0.003980719	31.28031967
4.134339307	47.87183149	0.004150585	34.38091065
5.067676213	29.34170136	0.008846742	16.34901362
4.075946911	81.27001918	0.003447963	18.46006063
4.003449338	61.1515052	0.006674925	12.88949702
3.419154673	69.31258685	0.004610892	24.25524334
3.375566257	64.90958399	0.006908009	10.31515693
4.052567152	28.38464092	0.006075033	25.4519684
3.230234538	104.0601571	0.005240933	18.42938732
3.491815787	82.11921551	0.008852536	14.38485799
4.456977357	71.49908135	0.005568457	16.24098186
2.655516451	75.07955768	0.019620867	12.94110897
3.014403786	99.15725153	0.00941014	21.68911489
4.579928703	47.38772702	0.002964299	19.57926508
4.079720399	84.55693325	0.003828451	16.85785858
3.256408261	43.19838873	0.006491653	15.77205692
4.592386764	73.10776813	0.003587233	24.98920638
3.141798936	56.2708692	0.008851722	19.30890721
6.275027195	14.03549746	0.004670223	18.34855355
3.76304732	46.09664734	0.009411964	8.494681213
3.121714389	86.97103646	0.004737049	15.28547661

log.sigma.3.5.mm.3D_glrlm_LowGrayLevelRunEmphasis	log.sigma.3.5.mm.3D_glrlm_GrayLevelNonUniformityNormalized	log.sigma.3.5.mm.3D_glrlm_RunVariance	log.sigma.3.5.mm.3D_glrlm_GrayLevelNonUniformity
0.003555809	0.055522804	0.113183125	108.2437609
0.018140814	0.099405944	0.193458197	81.07509389
0.009015259	0.071431737	0.193225129	96.4275548
0.008049538	0.058640651	0.11282265	52.30616906
0.006061989	0.076902483	0.163187953	161.7092428
0.015122322	0.069908504	0.132315529	41.5071354
0.004119526	0.070045962	0.17215556	352.4359936
0.002646252	0.073093445	0.174877747	331.1316548
0.002984939	0.065566236	0.143237277	267.4870531
0.003273264	0.069685804	0.137785093	67.13555977
0.011097436	0.082714522	0.160648965	110.3402504
0.004571842	0.068270965	0.142326306	141.4056605
0.005605941	0.099302106	0.336911634	153.1334807
0.005153026	0.069865518	0.149397009	107.1251005
0.004753608	0.070948112	0.139583403	127.6956521
0.004717286	0.063758556	0.15338468	250.7645806
0.006629481	0.078077684	0.111704932	84.7120187
0.015765893	0.093867121	0.222438568	99.60953205
0.01457084	0.096785989	0.186618446	109.4342884
0.005695507	0.064075788	0.141430153	298.8121501
0.009817448	0.092734079	0.284218614	236.5973063
0.015185017	0.083042238	0.137489846	80.21458473
0.01380723	0.083236461	0.175799005	75.11257959
0.006812976	0.080787857	0.165462612	189.6088137
0.006362181	0.073550207	0.143414908	84.46055847
0.00309406	0.063483359	0.114474497	137.4182381
0.003473841	0.065976554	0.159368855	191.8187676
0.004241931	0.078371022	0.161927797	191.906669
0.020001706	0.095090057	0.147175561	37.8795834
0.005657487	0.095037123	0.196896025	169.2464365
0.002984184	0.072314149	0.187063204	303.2658055
0.00543273	0.106846919	0.232626327	188.7866915
0.004438549	0.058959788	0.132277835	172.5760584
0.005635215	0.065063673	0.174511932	136.8302353
0.00504051	0.069145476	0.134410701	274.8077099
0.003807752	0.079413455	0.206527694	208.15082
0.002154119	0.091717714	0.243743056	291.8318284
0.002299012	0.062235491	0.314128114	274.1831854
0.013706083	0.121920238	0.2047274	128.7927625
0.006734988	0.05651241	0.119787379	373.5836557
0.005315465	0.085480632	0.197877661	193.9059609
0.004256048	0.056994106	0.092020004	203.8898706
0.003828774	0.064044734	0.124770096	212.7079967
0.004110483	0.073324132	0.133753351	164.271414
0.011223073	0.079386191	0.153934171	89.87940463
0.004874407	0.067003017	0.14266781	165.1654181
0.003739863	0.07531982	0.168137664	304.9964278
0.026437116	0.099305874	0.214534508	73.80503261
0.010385203	0.070350003	0.127325275	54.32935203
0.017529193	0.084002081	0.223862919	97.854518
0.006422267	0.064672059	0.125535088	151.0595555
0.004931982	0.060549435	0.102492431	187.3762057
0.006071278	0.078854738	0.15011148	316.78505
0.018404774	0.072705965	0.139264367	72.61842441
0.0116488	0.066998573	0.138011647	80.38563733
0.003003172	0.083156317	0.16029213	143.8095609
0.006595095	0.062702333	0.12479356	230.6122322
0.006642253	0.077373535	0.179418052	235.6417015
0.008272639	0.085869346	0.153308966	126.4288916
0.002731469	0.067293076	0.213382065	538.4488884
0.007041924	0.084929829	0.216848035	129.8143929
0.004326015	0.071681707	0.152469011	170.9130226
0.002909684	0.068082318	0.139592502	354.8739937
0.002811724	0.070757961	0.160152131	478.4172206
0.010545064	0.080219892	0.149981938	56.44723223
0.002973321	0.082484734	0.229312342	274.8691998
0.00858635	0.117577371	0.299761434	127.0936147
0.002735914	0.070834082	0.153832627	394.5334558
0.002898154	0.070524965	0.149860427	370.3952954
0.006839371	0.091274358	0.206186072	228.7371505
0.006399361	0.077673471	0.153778346	89.92319963
0.003408461	0.06918202	0.18016806	261.716703
0.00401754	0.076425515	0.168846885	154.8339172
0.006333107	0.060476436	0.110380347	125.2403559
0.006762145	0.059952427	0.110511525	171.1557673
0.008128327	0.08804677	0.160124235	104.5556763
0.008859086	0.092727164	0.198399315	192.5489768
0.012538573	0.084022093	0.208193095	69.67287851
0.008860945	0.084394815	0.157360338	127.3461379
0.004151146	0.061208265	0.10583698	131.3754498
0.004315519	0.055979741	0.114084999	102.2795675
0.009456855	0.084956969	0.172237842	134.1423401
0.003702019	0.072415403	0.186247874	210.3677044
0.007244225	0.086920863	0.169425641	234.0369517
0.004882368	0.060399833	0.128698101	133.6993176
0.007785899	0.103022334	0.281522616	131.5464208
0.006362906	0.066122857	0.151455341	141.3043251
0.00563517	0.070044702	0.177413441	170.0649091
0.009451634	0.081124199	0.169695221	105.0805057
0.005933477	0.080626309	0.195174952	142.5213569
0.022698602	0.103765644	0.297140338	111.623104
0.009891928	0.062178942	0.118958022	134.9335575
0.003155238	0.072730108	0.139675611	337.9443372
0.004087652	0.076498223	0.166779792	224.180686
0.006899669	0.073691685	0.126421491	289.6840143
0.00385196	0.066262299	0.194917492	199.2411576
0.009469006	0.066847557	0.149119136	168.2229042
0.004969721	0.09255893	0.227016941	82.77869366
0.010204607	0.10364587	0.195641615	74.43295918
0.005153584	0.076989964	0.19575723	390.9183873

log.sigma.3.5.mm.3D_glrlm_LongRunEmphasis	log.sigma.3.5.mm.3D_glrlm_ShortRunHighGrayLevelEmphasis	log.sigma.3.5.mm.3D_glrlm_RunLengthNonUniformity	log.sigma.3.5.mm.3D_glrlm_ShortRunEmphasis
1.301577217	543.6348719	1671.073399	0.940488583
1.520170495	96.69224903	636.4122435	0.9029488
1.496972222	181.5392927	1081.263912	0.912354074
1.315923002	301.060231	753.5596207	0.934697913
1.436894364	244.7283515	1696.504833	0.916655507
1.372489777	152.7727993	487.5864292	0.923842452
1.441137875	315.6394081	4105.980473	0.920602798
1.45145427	471.2385474	3662.965872	0.917640956
1.39024994	431.9031189	3353.237557	0.923668453
1.381198627	531.6184782	792.1862391	0.924010127
1.444697706	144.7713924	1065.133902	0.912505367
1.386865119	337.494425	1705.90586	0.924562802
1.825729582	259.5603773	1115.795224	0.873182998
1.409830677	441.3362836	1245.98901	0.919600023
1.362483566	366.449444	1515.763962	0.932700664
1.401775583	311.8924437	3261.309201	0.926044023
1.325951674	276.8095306	904.4548627	0.929793552
1.583201831	108.0932475	811.265957	0.895490376
1.512723972	101.0259263	879.1734569	0.901899746
1.387877466	267.3382483	3821.83208	0.92315169
1.704283784	150.2638554	1911.364735	0.886736054
1.390081521	115.2681221	786.9879644	0.920422953
1.472238065	136.6744858	718.7956513	0.911404453
1.445719097	207.6384482	1888.893466	0.915228327
1.381090997	325.1866931	956.1981441	0.927974719
1.316652784	529.6420513	1833.034669	0.93550254
1.402870565	462.2281082	2409.391233	0.926975608
1.440580641	314.2563181	1966.804519	0.91480225
1.415185129	95.36006128	322.1837578	0.916869494
1.539029871	230.2301589	1373.190948	0.898310607
1.480650877	421.1674526	3367.669108	0.914461537
1.619503463	318.2459939	1335.290364	0.889981538
1.353610727	379.8668793	2451.145199	0.931066921
1.42886229	370.1447895	1741.133852	0.926237537
1.36607199	305.6091367	3296.728612	0.927629206
1.524160681	380.9444229	2076.649242	0.908901463
1.631463037	583.305539	2416.351278	0.891731364
1.698028672	564.2559257	3412.868604	0.900289226
1.571111166	103.8033269	800.2989345	0.891201445
1.332794356	256.0903794	5543.510544	0.931860082
1.517469983	338.2031074	1786.727998	0.906918602
1.262236792	351.5024095	3089.98673	0.943689025
1.341937977	365.170554	2796.385665	0.932383229
1.372234976	343.127875	1846.028637	0.924892063
1.436668904	146.4234215	902.7764423	0.912077003
1.38691148	339.0099592	2026.625267	0.92420936
1.443699004	348.0553468	3275.629309	0.917461948
1.600026199	73.75160458	557.593987	0.886896171
1.348077131	187.3579484	646.9387953	0.930861539
1.586135165	108.6830255	897.3968028	0.896987959
1.338845092	316.0235795	1966.471746	0.933325806
1.291601863	309.3930329	2638.020289	0.938369012
1.416123994	224.9786989	3246.536524	0.917461312
1.369559321	130.9078761	837.1348452	0.930227156
1.372084693	167.1475489	996.5923275	0.927715779
1.43814954	467.4689882	1388.509554	0.914853659
1.339224799	288.4080337	3090.425344	0.93243108
1.476400926	339.8667414	2425.778764	0.911320536
1.413093009	229.0100637	1198.744424	0.920342363
1.523187725	445.2502171	6385.421456	0.911825279
1.562496851	203.8567971	1184.003578	0.90065522
1.412040274	317.3506536	1943.952805	0.920695795
1.376085253	486.1953888	4310.12716	0.926654966
1.436988208	433.5413259	5440.18961	0.915425106
1.425312726	148.2872794	570.4363047	0.915760956
1.582540806	457.8089539	2575.086139	0.899863792
1.775382455	199.7002261	777.5198712	0.870758158
1.418337463	486.5497496	4519.261199	0.918860937
1.399487127	441.7572152	4318.406664	0.9238108
1.551838652	197.4375058	1929.370995	0.898204439
1.422648113	312.6693745	940.1279196	0.918240872
1.472600606	409.0290604	3021.767634	0.912903855
1.44284032	449.190135	1639.72083	0.917548329
1.303479706	317.3342843	1762.552463	0.937812017
1.315666341	251.784934	2400.200394	0.933214157
1.448088788	265.2190284	948.784769	0.9120554
1.540388015	156.7302313	1605.604093	0.899330265
1.555192809	156.7902138	642.6152393	0.899975043
1.442637385	176.8378512	1199.940702	0.911296541
1.296434469	467.5582324	1829.379036	0.938378556
1.303109487	500.3114117	1566.087415	0.940428464
1.476793813	210.2096725	1244.592658	0.907438967
1.467735839	378.4557881	2368.959243	0.919223363
1.478408989	212.0286879	2108.425235	0.904886142
1.35039125	332.5110025	1860.005683	0.931538135
1.735946715	162.8076288	926.2817743	0.874806964
1.405316504	339.4425649	1753.760018	0.922871525
1.457241392	275.7206682	1963.635836	0.917572154
1.457374659	187.3635618	1041.407466	0.914174735
1.520628016	287.1058826	1385.330033	0.904537546
1.777410366	81.1035656	769.2570948	0.869267827
1.327961912	215.6769954	1827.042425	0.933374355
1.388147186	438.3281605	3797.100296	0.921893541
1.440494772	343.0730131	2369.846703	0.917559504
1.355864835	227.6454684	3253.805298	0.926817613
1.497169625	445.2393494	2396.690738	0.911866475
1.407878314	191.9561418	2048.705146	0.920059703
1.609964356	367.6755718	674.6097557	0.889887988
1.560080766	141.4507903	545.2475412	0.891060448
1.49618938	251.7230618	4058.840129	0.912788073

log.sigma.3.5.mm.3D_glrlm_LongRunHighGrayLevelEmphasis	log.sigma.3.5.mm.3D_glrlm_RunPercentage	log.sigma.3.5.mm.3D_glrlm_LongRunLowGrayLevelEmphasis	log.sigma.3.5.mm.3D_glrlm_RunEntropy
763.3869337	0.918400638	0.004175456	4.887996819
154.1967538	0.871657754	0.025788972	4.234935777
285.8224981	0.879650988	0.012746503	4.580792586
425.6171554	0.912920243	0.009536248	4.75876032
382.2935675	0.887798857	0.008084776	4.531320184
211.7206755	0.899300699	0.019801898	4.571994063
476.7633922	0.890026272	0.005793077	4.641135766
726.2806548	0.886512861	0.003686858	4.659329025
647.0966892	0.897356933	0.003967626	4.685598057
786.3530295	0.898464409	0.004061843	4.561031541
227.2793212	0.884462355	0.015091514	4.364749855
499.5725701	0.898147839	0.005866488	4.647592192
511.4396825	0.82415228	0.009034528	4.558577179
665.0774979	0.892477936	0.006356692	4.723779981
542.6545546	0.906792804	0.005902597	4.642851192
459.8233942	0.898203488	0.00633765	4.713028303
403.0606671	0.908645793	0.008019388	4.418533175
180.7415938	0.859941356	0.023041824	4.380865394
161.9788227	0.870807159	0.021367234	4.186073119
389.4914049	0.896844068	0.007631741	4.737953791
271.4989707	0.842846968	0.01561333	4.446190607
171.2668922	0.89520211	0.020388726	4.285555813
211.5915942	0.880558894	0.019317785	4.438214011
319.4638983	0.886286362	0.00934822	4.451979372
472.802557	0.901746329	0.007833578	4.537514637
741.7003239	0.91359548	0.003756125	4.757927379
691.6218921	0.898689	0.0045065	4.795375956
489.6240134	0.886314265	0.005744111	4.538898396
137.712032	0.890724488	0.02763203	4.115348229
394.0914873	0.866526852	0.008003029	4.345777789
671.0952777	0.881139457	0.004202137	4.654181495
591.7757892	0.853106294	0.007344503	4.326124458
558.3454593	0.906520237	0.005615847	4.828384667
566.3260677	0.894631751	0.007122636	4.732938982
444.2907937	0.90236149	0.006635496	4.578262593
619.3593549	0.873573242	0.00540991	4.527228792
1058.765601	0.853430633	0.003195223	4.584793176
1000.263265	0.852682098	0.003702895	4.958048714
188.5027374	0.858786742	0.019377384	3.918930656
361.5661999	0.909290022	0.008752982	4.83041222
577.9883593	0.873524702	0.007046842	4.569814061
466.7172403	0.925125818	0.005251596	4.798783914
522.3722891	0.909031565	0.004926137	4.659691876
518.147348	0.900228791	0.005238338	4.581112095
221.8657093	0.885337667	0.01538235	4.47555031
500.202677	0.897828219	0.006312013	4.685539295
533.3053312	0.88724581	0.005150322	4.593029915
123.3065698	0.853130796	0.039012086	4.235527902
267.4277298	0.907168038	0.013163295	4.514734703
171.5635752	0.861060537	0.026669615	4.453779075
452.2789332	0.909123574	0.007865845	4.6641256
425.8336043	0.918033909	0.006117512	4.719368963
344.9971234	0.890190262	0.008166228	4.50041941
184.5917958	0.904264214	0.023445759	4.445823191
239.3320327	0.902082128	0.015352681	4.546845798
733.181205	0.886390533	0.003925897	4.47573683
414.1825618	0.909099553	0.008134927	4.693323345
553.7914344	0.880344352	0.008767063	4.636434674
352.3958931	0.8929705	0.010292088	4.380234373
726.6911271	0.876193365	0.004011083	4.755029902
334.2280933	0.864578796	0.010322844	4.5513699
480.1625284	0.892768655	0.005772771	4.573890822
711.3773312	0.90026177	0.00382351	4.658814944
661.1386847	0.886870705	0.00394347	4.693204473
226.8039748	0.890262902	0.01421491	4.347862499
777.8276163	0.861988607	0.004362415	4.629196675
408.9670091	0.827866918	0.012966208	4.224409115
746.6849689	0.890929231	0.003675638	4.734836685
678.6726224	0.896442127	0.003790158	4.641763529
331.1538599	0.864495408	0.010036543	4.337674828
482.8773319	0.89074436	0.008145015	4.516928553
640.5883713	0.881369912	0.004714732	4.735162823
703.098066	0.888412597	0.005169957	4.585082788
451.2041744	0.916573024	0.007441483	4.737322154
348.3445509	0.911800113	0.008639464	4.767871779
433.1866236	0.884328786	0.01006229	4.390578036
260.0152154	0.866251445	0.012813844	4.334584967
253.6951633	0.864782399	0.018045508	4.522156842
275.6185501	0.88373665	0.011911409	4.388073703
661.6377107	0.917486105	0.004942071	4.799507959
702.2346195	0.918242642	0.005102004	4.882803998
340.4832951	0.877367768	0.01239896	4.524503751
590.8174533	0.887803845	0.005135972	4.638035747
351.12676	0.87599189	0.009987786	4.428659246
484.0434026	0.907559845	0.006202362	4.780109413
308.7594293	0.832446274	0.012867566	4.320091061
527.2236171	0.895468473	0.007799113	4.759926124
420.4953571	0.885288672	0.00777928	4.641339928
294.7542455	0.884457755	0.012527983	4.459877552
479.098254	0.872098765	0.007877747	4.571704857
136.3841025	0.824386503	0.040842702	4.303845935
304.4140235	0.91090228	0.012177171	4.692760565
663.4785169	0.896362368	0.004106062	4.633637162
534.3662148	0.887762238	0.005454266	4.568571545
337.3871374	0.903227517	0.008816872	4.505829985
713.7135423	0.878163917	0.005261276	4.822649464
291.7263588	0.893176047	0.012521977	4.679605892
682.7042723	0.853353905	0.006607803	4.491513756
253.711892	0.860726803	0.014170665	4.172139234
394.3467808	0.878940076	0.007519625	4.531376222

log.sigma.3.5.mm.3D_glrlm_HighGrayLevelRunEmphasis	log.sigma.3.5.mm.3D_glrlm_RunLengthNonUniformityNormalized	log.sigma.3.5.mm.3D_glszm_GrayLevelVariance
580.0151926	0.856476594	45.8357986
105.9931607	0.778031614	18.29102093
197.3468183	0.798093141	24.86055434
322.407208	0.843639133	33.64423633
266.9056042	0.805459369	22.29137737
163.0315794	0.820193326	27.1331
340.5420876	0.814388125	26.53581052
511.0678743	0.807626554	35.32384483
467.1842166	0.820279288	26.51079291
574.5308106	0.82088686	29.36606572
158.5061716	0.796765308	15.95255206
364.1389324	0.822367027	28.98496645
293.5899808	0.721645593	32.33567378
478.5225646	0.811535392	41.88532307
393.9728398	0.840426142	33.37642562
334.8590655	0.826788096	29.59328811
299.0795556	0.832489657	25.18643466
119.3829497	0.762937912	21.1125274
111.0886009	0.775641655	12.04697835
287.7024036	0.818605828	30.60346516
167.5047129	0.747033113	20.93991938
124.8409351	0.813162327	15.16546037
148.7666202	0.795114308	20.10358331
225.8205051	0.802948939	20.61326694
348.8789551	0.830485748	29.74473893
565.5549662	0.845518391	39.94623168
497.8505056	0.827621066	41.0752397
342.8415051	0.801667109	24.98588868
102.6347336	0.806667901	12.51781511
256.1334819	0.76871498	21.28963625
459.2972152	0.801564367	28.58204386
359.4107966	0.753241026	28.90353733
408.8798622	0.835935229	34.34565662
399.3966601	0.826606847	33.82905647
328.6208154	0.828367762	23.8301778
417.1103905	0.790490705	26.02782544
653.3886989	0.757022834	39.23453262
620.5398775	0.773689594	39.88733766
117.2166868	0.754847122	10.79970396
274.0522382	0.83731586	31.34015907
374.6441924	0.786097398	32.15383544
371.8706193	0.863001454	34.6813354
391.0454265	0.839676191	26.72312538
372.4164331	0.822635909	28.34229574
159.2977865	0.795873909	21.63443288
365.8260074	0.82108367	29.6107052
377.4538489	0.807577148	28.72818335
81.7774399	0.747222628	15.57545779
200.7171925	0.836001321	23.28050255
118.3251399	0.767281526	19.85785707
338.5187873	0.840811415	29.32061465
329.7272686	0.851443256	29.6148292
244.9195803	0.806893979	23.00616997
139.6697243	0.835781878	21.34234299
179.075807	0.829012213	21.00315582
510.7927797	0.801685726	27.93285346
309.3433432	0.838889256	28.54592225
373.3865264	0.794826971	34.56885411
249.0435534	0.812991841	24.37763512
487.148934	0.796322858	28.49455716
223.8809536	0.773415395	28.12480232
343.811832	0.813960359	25.26004723
523.3068709	0.826145104	26.90754077
470.459879	0.802966951	31.77030644
161.3905015	0.806553446	17.60033951
505.5361617	0.771686942	36.99038792
229.8183221	0.716753295	18.35979547
528.9053845	0.810062563	34.58188904
479.8553092	0.82076945	29.37426596
218.54152	0.768114019	18.62545579
340.4108334	0.809741831	26.53023275
445.6131026	0.797704401	35.45768846
489.399348	0.807745976	34.67175601
340.1582176	0.850249631	33.229294
268.6264288	0.839863628	29.60369859
292.7683798	0.796716817	22.92563326
173.1302082	0.770984749	18.0529726
172.0576043	0.772816956	28.09390856
193.3996394	0.79392673	19.82836455
500.8240958	0.851480777	40.27226568
533.6035676	0.856413748	45.38537986
231.313738	0.786729892	28.74371944
410.1901378	0.812746804	30.18024762
235.0191673	0.781334088	18.99568389
356.8365378	0.837865807	31.96366138
184.0734096	0.723754393	16.13739183
369.6572853	0.819050774	35.57395035
298.2664526	0.807706961	24.96643366
204.5563116	0.801429114	21.39341276
317.1251072	0.781612573	28.44898834
89.43636383	0.713516075	15.84171488
230.723164	0.840694223	26.25627389
476.0194416	0.816002274	31.26780706
373.4240757	0.807424958	26.67893029
246.2951399	0.826446552	22.37519956
486.2129015	0.795952424	38.39695262
208.3122068	0.812549398	26.42407145
415.9838247	0.752482891	31.77251185
159.692573	0.755701453	13.76021468
273.4064857	0.798015485	23.17101312

log.sigma.3.5.mm.3D_glszm_SmallAreaHighGrayLevelEmphasis	log.sigma.3.5.mm.3D_glszm_GrayLevelNonUniformityNormalized	log.sigma.3.5.mm.3D_glszm_SizeZoneNonUniformityNormalized
327.8476396	0.042307934	0.38666163
56.81836771	0.068649407	0.219086864
112.9121686	0.057280648	0.294596436
173.4986238	0.050518884	0.316577293
134.6877329	0.061116063	0.293834444
113.7291239	0.05415	0.30375
208.4993746	0.05559137	0.303409563
309.8076185	0.048340544	0.316313269
243.6298899	0.056172632	0.300686569
328.6269096	0.054650156	0.333396063
82.00903795	0.071115324	0.306515957
203.5668738	0.053784429	0.332142396
166.4216257	0.052582848	0.281181282
282.1969467	0.045894514	0.335397034
203.0069098	0.049217668	0.331804017
196.4625857	0.053056211	0.306170083
138.6623195	0.058690599	0.282589747
71.61288677	0.061614692	0.299968686
53.03797531	0.083328622	0.242240941
174.9428491	0.050873338	0.292580136
94.65444601	0.062491023	0.27398736
68.7957937	0.07098949	0.272522969
81.39330716	0.064158753	0.276316857
127.7821389	0.062055673	0.298996702
216.621251	0.054520893	0.349759725
352.1714022	0.047512142	0.363051225
306.4603631	0.045568673	0.347354637
196.9922221	0.057789659	0.328204642
68.59088096	0.081892704	0.278629981
133.2959982	0.062945053	0.261079817
264.4459582	0.054543849	0.326818374
172.9177306	0.060492622	0.304077148
230.7742573	0.048373361	0.32746971
206.8875697	0.049295585	0.33251074
198.4215749	0.058951682	0.319132791
232.1295275	0.057630189	0.312607233
380.4111887	0.048126276	0.343235214
376.8655172	0.046910312	0.335319208
51.90341325	0.086264083	0.226446643
158.1763761	0.049261012	0.288388707
191.5779384	0.051577597	0.327549214
236.3571462	0.049568982	0.334135753
218.497688	0.054738415	0.327745423
202.3805748	0.054782697	0.323192022
102.6943278	0.060541103	0.291467222
212.3607069	0.052430827	0.348783276
218.4916219	0.05295138	0.287170841
37.94237922	0.073388823	0.197431629
115.6545835	0.058855146	0.281913464
76.64126073	0.064427235	0.29281443
184.1780696	0.05390785	0.319748851
193.1908468	0.051636918	0.319866294
137.5261246	0.058451449	0.307234697
85.34671544	0.060396656	0.319703171
100.2931034	0.061236029	0.297094017
274.9630033	0.056783134	0.278000974
168.1592142	0.051950637	0.314343381
189.2440008	0.050080664	0.30026811
124.9161534	0.057900103	0.302749472
268.1680899	0.05365619	0.309689834
141.6339117	0.05396133	0.323092031
186.8033005	0.057603321	0.287690393
304.6346069	0.056002994	0.337225122
287.7531083	0.050190184	0.285277489
83.16337565	0.066790123	0.231975309
302.5596605	0.048649013	0.309033564
97.6750809	0.072958364	0.23926954
305.2038911	0.048738003	0.307176032
254.9735869	0.052279445	0.343849884
113.755539	0.065570411	0.266797162
164.8910971	0.060233598	0.288033395
268.3871464	0.049562682	0.327580175
243.3999353	0.051738892	0.290138357
176.1401129	0.048264702	0.333341023
171.6121986	0.052600701	0.32864704
121.5592864	0.06287197	0.276255625
86.05094912	0.066953405	0.267975488
99.94478962	0.055008188	0.241805149
109.1275649	0.062743178	0.268043495
263.7664009	0.047978131	0.335586126
306.2975367	0.042567112	0.396947968
141.1826639	0.054418152	0.326993568
235.4852892	0.052051599	0.322962444
108.4585926	0.064588733	0.226888778
208.4844499	0.049862656	0.33164112
93.16931149	0.082562064	0.236793745
186.109684	0.048392143	0.33839779
190.8892901	0.056716082	0.360478861
98.93949597	0.061169339	0.269058497
153.0886653	0.054023062	0.28495799
66.80356077	0.073264463	0.303099174
129.677278	0.055012989	0.331735247
251.4230962	0.051456463	0.304155373
212.3627007	0.055370449	0.327503673
121.6398795	0.058728857	0.26715814
301.6624829	0.047700709	0.382082544
116.9420898	0.054160766	0.282421213
173.7988423	0.055097594	0.277195032
66.72714347	0.077562327	0.252510388
166.3364525	0.058933399	0.320404358

log.sigma.3.5.mm.3D_glszm_SizeZoneNonUniformity	log.sigma.3.5.mm.3D_glszm_GrayLevelNonUniformity	log.sigma.3.5.mm.3D_glszm_LargeAreaEmphasis	log.sigma.3.5.mm.3D_glszm_ZoneVariance
312.0359356	34.1425031	38.63692689	31.722699
40.09289617	12.56284153	196.6502732	170.5454328
98.39520958	19.13173653	123.3353293	102.2413855
101.9378882	16.26708075	43.86024845	34.6541125
171.5993151	35.69178082	100.5308219	84.08946331
60.75	10.83	68.58	57.69
440.854095	80.77426015	139.9621473	124.8363113
374.8312236	57.2835443	384.0472574	365.4664288
365.6348684	68.30592105	85.56085526	71.58457139
108.6871166	17.81595092	48.16564417	37.35244081
115.25	26.7393617	77.93617021	61.85095066
212.9032761	34.47581903	73.38065523	60.44984314
90.54037267	16.93167702	665.6801242	632.0617357
156.965812	21.47863248	115.8568376	102.3967191
201.7368421	29.92434211	72.07236842	61.42416898
349.6462347	60.59019264	147.5201401	132.8368825
99.47159091	20.65909091	34.34090909	22.83493673
69.89270386	14.35622318	322.3218884	294.3182597
64.43609023	22.16541353	249.1578947	225.34643
406.3938085	70.66306695	103.287977	89.27807348
129.3220339	29.49576271	980.1271186	939.080293
67.04065041	17.46341463	77.66260163	58.42403662
61.34234234	14.24324324	174.6216216	153.3454265
202.719764	42.07374631	111.0722714	95.83004194
131.8594164	20.55437666	59.13793103	47.73611297
291.1670823	38.10473815	61.93640898	53.21108855
327.2080679	42.92569002	155.5552017	143.7761696
235.9791377	41.55076495	279.2100139	264.4533031
30.92792793	9.09009009	91.55855856	75.34161188
101.2989691	24.42268041	202.6391753	174.614704
361.4611212	60.32549729	392.3707052	373.8635946
116.765625	23.22916667	574.4348958	545.4041273
323.867543	47.84125379	61.5520728	50.89240902
231.094964	34.26043165	91.13956835	79.70641271
392.5333333	72.51056911	73.05853659	60.23864366
201.9442724	37.22910217	331.0665635	309.5145477
248.1590595	34.79529737	602.1479945	575.6603862
411.771987	57.60586319	413.9242671	396.2610021
43.2513089	16.47643979	250.3350785	208.8641759
626.6686608	107.0441786	50.89093419	39.69786034
200.1325696	31.51391162	197.4369885	179.4127252
479.8189415	71.1810585	21.48259053	14.23089662
362.4864376	60.54068716	41.02983725	30.12073304
230.7591036	39.11484594	78.43417367	66.29177946
90.35483871	18.76774194	105.0774194	88.08174818
284.9559364	42.83598531	90.68176255	79.39314955
319.0468047	58.8289829	235.229523	218.3685573
28.62758621	10.64137931	211.3172414	175.2344352
68.78688525	14.36065574	46.20081967	34.03673408
88.72277228	19.52145215	216.9768977	197.0670414
245.8868661	41.45513654	58.7373212	47.57702994
365.6071741	59.02099738	28.49256343	19.79445045
337.0364631	64.12123974	237.4749316	220.5578702
100.0670927	18.90415335	76.87539936	64.43456604
115.8666667	23.88205128	60.11794872	48.48809993
123.4324324	25.21171171	126.8873874	107.598673
394.8152866	65.25	42.94824841	32.57636149
233.9088575	39.01283697	228.8883184	209.1833808
126.8520286	24.2601432	110.2959427	94.82608324
702.3765432	121.6922399	188.0912698	171.88958
127.9444444	21.36868687	343.6717172	323.783721
192.4648729	38.53662182	147.6143498	131.6860182
585.4228111	97.22119816	107.7897465	96.66967305
506.9380979	89.18795723	154.3066967	135.90419
41.75555556	12.02222222	70.62222222	51.35987654
237.9558442	37.45974026	677.3662338	652.2101467
44.26486486	13.4972973	734.9621622	685.2787144
500.6969325	79.44294479	206.7128834	192.0105988
576.6362552	87.6726297	97.66785927	85.46998022
141.9360902	34.88345865	452.5413534	422.8675307
88.71428571	18.55194805	150.1850649	132.3974848
321.0285714	48.57142857	339.9979592	320.8350062
165.6690018	29.54290718	133.9737303	118.0437062
259.6726573	37.59820282	60.76893453	52.35967906
318.7876289	51.02268041	59.74329897	49.32437985
91.71686747	20.87349398	79.21686747	62.87773988
124.6086022	31.13333333	299.6301075	273.0799121
46.66839378	10.61658031	218.3626943	193.7240731
105.6091371	24.72081218	133.0329949	114.2625486
266.1197982	38.04665826	52.24337957	43.54348341
308.4285714	33.07464607	38.55212355	31.99931426
150.7440347	25.0867679	186.7180043	171.4893869
251.2647815	40.49614396	239.3303342	221.6752153
132.2761578	37.65523156	201.1509434	173.3673758
248.3991989	37.34712951	46.75967957	36.15591773
40.96531792	14.28323699	1017.387283	938.9675566
245	35.0359116	118.1616022	107.3098558
271.801061	42.76392573	181.3448276	168.1199333
98.47540984	22.38797814	84.54644809	68.54644809
123.1018519	23.33796296	281.4699074	259.4972512
66.68181818	16.11818182	678.1	642.967438
268.0420792	44.45049505	29.72524752	21.03442677
432.2047854	73.11963406	147.6826179	134.3788622
286.5657143	48.44914286	165.4514286	151.2277551
305.8960699	67.24454148	144.660262	130.2136237
356.8650964	44.55246253	240.5867238	227.1632086
207.5795918	39.80816327	76.81496599	62.1257365
58.48815166	11.62559242	268.9810427	244.358752
38.38157895	11.78947368	180.1184211	150.0129848
425.496988	78.26355422	254.8885542	235.9843931

log.sigma.3.5.mm.3D_glszm_ZonePercentage	log.sigma.3.5.mm.3D_glszm_LargeAreaLowGrayLevelEmphasis	log.sigma.3.5.mm.3D_glszm_LargeAreaHighGrayLevelEmphasis	log.sigma.3.5.mm.3D_glszm_HighGrayLevelZoneEmphasis
0.380301602	0.074626374	22620.41016	537.9938042
0.195721925	2.610899898	16355.01093	122.7103825
0.217731421	0.872017585	21172.75449	206.7065868
0.329580348	0.180618412	14237.30435	311.4565217
0.246621622	0.444994361	24904.77226	255.5856164
0.303030303	0.764572924	7415.625	192.77
0.257122633	0.554977146	37564.56091	364.3819683
0.231989037	0.888480356	169302.1857	536.4413502
0.267487901	0.210948005	37738.93339	449.3610197
0.304104478	0.101366692	25503.84356	569.4294479
0.24933687	0.599970478	12094.32447	150.537234
0.278091106	0.247041756	24010.43838	358.0592824
0.172469202	2.662661782	169698.9441	312.5341615
0.272568433	0.297885978	49373.21154	473.6901709
0.306451613	0.200821808	28290.06908	363.6233553
0.260968921	0.585020413	39904.80035	351.1077058
0.29480737	0.129800097	11224.40909	271.2840909
0.188969992	3.97269741	28167.74249	132.7639485
0.204930663	3.433834819	19787.02256	113.556391
0.267166763	0.487452047	24136.07127	310.8826494
0.156084656	7.62802352	130435.2352	184.2055085
0.227988879	0.928054046	8264.784553	131.296748
0.216796875	1.712863688	20791.11712	156.3198198
0.256139025	0.614573685	21582.22566	232.320059
0.296150825	0.213020572	18606.61008	359.403183
0.338539468	0.124420477	32869.82668	570.6683292
0.291370244	0.360732377	70479.60297	506.3428875
0.26031861	0.95835734	83318.75661	340.4311544
0.248322148	1.731306738	5900.198198	123.8108108
0.188899708	0.847917617	50808.74485	254.7963918
0.232450609	1.014062023	155996.4087	461.9068716
0.185596907	1.577951518	213784.1771	314.0208333
0.306286776	0.165269455	26720.13448	394.8847321
0.295744681	0.281284224	32828.58129	372.494964
0.279291553	0.286116739	21067.78049	338.0130081
0.215405135	0.994673104	114414.8947	421.5820433
0.194302607	0.979337195	374796.9129	640.264177
0.237938384	0.806805332	217562.6539	636.3526059
0.155284553	2.288228954	30589.82723	110.0680628
0.298899587	0.258672962	12817.32628	284.8867925
0.235543562	0.529273673	78680.18494	337.1734861
0.371347298	0.074420865	7823.449861	384.5745125
0.302764851	0.1288303	14981.62568	384.261302
0.286977492	0.22216918	29815.2381	347.9705882
0.24256651	0.981928344	12729.83226	181.616129
0.297632058	0.325053804	27936.75398	363.1089351
0.243533538	0.766076198	74379.18542	398.5373537
0.166475316	4.771506154	11207.58621	95.46206897
0.286721504	0.334784316	8151.967213	209.9836066
0.224112426	3.409424232	15330.13861	142.1683168
0.299338264	0.220844837	18480.57607	327.006502
0.339068526	0.107679188	9233.914261	332.0034996
0.243129433	1.182103235	49935.86509	244.9808569
0.283514493	1.033917358	7259.661342	153.1884984
0.293233083	0.604800326	7706.861538	184.0564103
0.227692308	0.26256001	63546.77703	506.4977477
0.310506799	0.176133391	12790.34395	302.2165605
0.225274725	0.690234992	83099.07702	349.3465982
0.254247573	0.502422982	26186.26253	236.8830549
0.24843904	0.471151392	79199.97751	476.4290123
0.224235561	2.055520551	59385.56313	251.2348485
0.250561798	0.520010432	44498.14649	346.1464873
0.299879081	0.25286832	48405.88134	522.0725806
0.233110324	0.407499812	61243.24986	517.6291503
0.227848101	0.619855112	9639.494444	170.6722222
0.19937856	1.574806762	295255.0922	532.312987
0.141871166	3.432343232	163336.6324	207.4594595
0.2608	0.41717411	105302.8276	534.4472393
0.286324057	0.223041195	44858.84914	441.9946333
0.183574879	2.674633257	80172.40226	223.8364662
0.237105466	0.522483143	47183.31494	326
0.228438228	0.978616458	121818.352	464.2857143
0.250548486	0.321694402	59482.87916	467.7635727
0.344842851	0.182858788	22686.95635	313.4801027
0.309805174	0.303549183	14102.28969	284.9443299
0.247391952	0.302024233	23985.30422	251.0993976
0.194073456	2.180025081	44237.73978	178.2795699
0.201461378	1.936485638	27023.9171	198.8497409
0.230814294	0.837337494	22929.79188	202.5583756
0.339033775	0.105431691	30692.01513	461.3757881
0.390648567	0.086191387	20626.04762	497.4813385
0.256253474	0.890613515	42388.39046	238.1453362
0.23799327	0.70336168	85053.51285	412.5385604
0.189716889	0.919160734	48755.65352	225.6054889
0.307093071	0.159661709	16492.58611	349.7930574
0.112924282	7.240493172	151274.6936	192.1213873
0.303563941	0.340606044	44745.54006	331.2334254
0.274981765	0.838256284	41674.33289	311.4018568
0.25	0.529082845	15292.81694	199.3169399
0.213333333	1.015309104	81343.81481	301.0046296
0.168711656	16.25531053	31024.06364	118.4954545
0.339210747	0.194737241	6393.97896	228.1212871
0.274165541	0.330592705	68754.05489	456.8289937
0.265151515	0.503062895	56338.32457	367.8937143
0.263097426	0.599980293	37496.05328	235.1528384
0.272939801	0.623437134	97432.23876	482.5503212
0.260915868	0.489688681	15187.39592	214.3564626
0.201528176	0.649964974	116802.8436	355.7725118
0.182254197	1.142015569	30849.93421	142.1052632
0.229996536	1.291462685	52917.24623	293.8825301

log.sigma.3.5.mm.3D_glszm_SmallAreaEmphasis	log.sigma.3.5.mm.3D_glszm_LowGrayLevelZoneEmphasis	log.sigma.3.5.mm.3D_glszm_ZoneEntropy	log.sigma.3.5.mm.3D_glszm_SmallAreaLowGrayLevelEmphasis
0.645113151	0.005309564	6.571628724	0.004206976
0.455507965	0.025156553	6.179683825	0.010803176
0.556873228	0.013720173	6.321392154	0.009267153
0.57876486	0.013759186	6.337995704	0.011323203
0.560510356	0.009197104	6.441865747	0.006625946
0.565476857	0.017584824	6.092601814	0.008742438
0.5700617	0.005065114	6.774666057	0.003305023
0.580108141	0.003674483	6.79825566	0.002600038
0.564474109	0.004031844	6.684159472	0.00271155
0.59877172	0.00569413	6.23793234	0.00483582
0.572407329	0.015060554	6.082713849	0.009526966
0.597433714	0.007225234	6.490299401	0.005658727
0.541724213	0.010134114	6.405136548	0.007188281
0.600093371	0.008707706	6.548241757	0.005676919
0.593313423	0.006527388	6.466223694	0.003587663
0.569771549	0.006236292	6.708359363	0.004218151
0.547300443	0.010787949	6.418179502	0.007852222
0.566817633	0.024486686	6.059123893	0.016370724
0.500410147	0.020079571	6.050800124	0.012943483
0.555718357	0.007139002	6.856203891	0.004459499
0.53925665	0.014932843	6.386393304	0.00845317
0.537883687	0.020801716	6.10847521	0.013494322
0.540806168	0.017720433	6.095944832	0.011358885
0.564720447	0.009388449	6.469388173	0.006371854
0.61310068	0.011188707	6.235590816	0.009168031
0.625191883	0.0045709	6.577440974	0.003517007
0.608329559	0.004868313	6.661301307	0.003385114
0.593128455	0.006036247	6.4188415	0.004357151
0.536385825	0.022641015	5.407312124	0.009245134
0.521301219	0.009619919	6.503620561	0.006891906
0.591628089	0.004183908	6.605238968	0.003017285
0.566250072	0.01123763	6.29626407	0.006977662
0.590755054	0.005779938	6.68359223	0.003411899
0.596287585	0.008654222	6.572579325	0.005573035
0.584448714	0.005849528	6.555336839	0.00300719
0.578384022	0.00538379	6.478903644	0.002793781
0.607464236	0.003683923	6.630152606	0.002872158
0.598944881	0.003136285	6.769802836	0.002227884
0.474775768	0.022479521	6.016656677	0.010058104
0.552321428	0.007622538	6.940145943	0.004444836
0.589169742	0.008066286	6.478217816	0.004313229
0.598186897	0.004863164	6.707303756	0.003183061
0.594034843	0.005014774	6.621762526	0.003488833
0.587867639	0.005971582	6.508336314	0.004284074
0.557772175	0.014937351	6.213838336	0.010069811
0.611943381	0.006569865	6.436626682	0.004453091
0.547367571	0.005212949	6.749206919	0.003647983
0.440121042	0.039661121	5.990629973	0.016388108
0.53811302	0.014076642	6.148554758	0.009057381
0.558699296	0.023249586	6.213213218	0.014122458
0.583954023	0.009563762	6.559384704	0.006015723
0.584078475	0.006055254	6.670583601	0.003927392
0.572716899	0.008376735	6.583066314	0.0056804
0.58353914	0.025446917	6.107473069	0.01646976
0.560230545	0.014627408	6.211318858	0.007933227
0.543235302	0.005462863	6.553535663	0.004351853
0.578683453	0.009732552	6.700761285	0.006731821
0.566311092	0.009942018	6.745556092	0.004768207
0.566554451	0.015684923	6.35817768	0.012225737
0.57443933	0.003548938	6.821133376	0.002360257
0.588299987	0.010287077	6.334993173	0.007656988
0.551676187	0.006330626	6.564689825	0.004569668
0.601327909	0.003502017	6.566920212	0.001933471
0.549087095	0.003442493	6.984003013	0.002223015
0.489391438	0.015637199	6.112531258	0.010653385
0.574657598	0.004706424	6.768637624	0.003303113
0.492019431	0.015403518	5.995374819	0.006422054
0.571964733	0.00339263	6.885889439	0.0018426
0.607941348	0.004092032	6.642462662	0.002984089
0.527839872	0.009882018	6.45916182	0.005490989
0.550073236	0.009667337	6.224725955	0.004343439
0.5934243	0.005186709	6.745933079	0.003737296
0.552800031	0.006499123	6.651988974	0.003823548
0.596808442	0.009958613	6.524215372	0.007712906
0.593314717	0.008022693	6.60663506	0.005020296
0.537965387	0.0150668	6.305909854	0.009685168
0.528547485	0.013891252	6.377851877	0.009790249
0.498413249	0.017436655	6.297956248	0.00816879
0.532949661	0.012275678	6.442892015	0.006380842
0.598172358	0.006201922	6.616509548	0.004632391
0.653833159	0.00617103	6.500180997	0.004818819
0.588863142	0.013587702	6.330412251	0.007925126
0.586262987	0.005322436	6.57033378	0.003614494
0.485707838	0.010052181	6.721622828	0.004612638
0.596071604	0.006321959	6.569700303	0.00417848
0.500470101	0.013947972	5.970671151	0.009995489
0.601120622	0.010622821	6.536220565	0.008111958
0.623235997	0.007017203	6.363041669	0.004134185
0.532896015	0.014139736	6.352648311	0.008173897
0.548030108	0.011738215	6.488988774	0.009028313
0.569305519	0.027959304	5.843328757	0.020351668
0.595442972	0.01349208	6.439270973	0.009052937
0.568352518	0.004368974	6.78193911	0.002916583
0.593156625	0.00617811	6.525141334	0.004641566
0.526344639	0.009993589	6.716947707	0.0065653
0.642082422	0.005436072	6.520724272	0.003622429
0.546750691	0.01267879	6.689085975	0.008131289
0.536957194	0.011201461	6.202327506	0.009305291
0.515897305	0.019795504	5.886145873	0.015038503
0.586139114	0.006581519	6.552394955	0.004519522

log.sigma.3.5.mm.3D_ngtdm_Coarseness	log.sigma.3.5.mm.3D_ngtdm_Complexity	log.sigma.3.5.mm.3D_ngtdm_Strength	log.sigma.3.5.mm.3D_ngtdm_Busyness	log.sigma.3.5.mm.3D_ngtdm_Contrast
0.00498656	0.607419763	2.801732799	0.183300044	4.64E-05
0.01034898	0.208069799	1.529604963	0.479941523	7.05E-05
0.007816434	0.264852545	1.975042693	0.32138934	3.88E-05
0.011697222	0.661025696	3.633213364	0.15273957	0.000111479
0.004363509	0.210322328	1.259684553	0.432766048	2.57E-05
0.015407071	0.606931453	3.43729412	0.244366649	0.000170511
0.001933712	0.155697534	1.081767387	0.628766848	7.61E-06
0.002274074	0.144120301	1.125559454	0.463839936	9.46E-06
0.00244362	0.180184085	1.149710639	0.467721253	1.21E-05
0.010007836	0.645380558	4.591454422	0.114721653	5.82E-05
0.00688523	0.171639572	1.13768188	0.514579761	4.43E-05
0.004599588	0.324793444	2.009993432	0.292059394	2.50E-05
0.006169264	0.228361034	2.517287212	0.241592419	1.94E-05
0.00694189	0.48149343	3.409701982	0.152205977	3.55E-05
0.00495259	0.455229751	2.2138081	0.242261892	3.55E-05
0.002600199	0.171172593	1.028414625	0.563204731	1.46E-05
0.008769438	0.39879775	2.498187026	0.201322898	6.01E-05
0.008960211	0.1928426	1.569491965	0.484707194	5.49E-05
0.00761931	0.132767541	0.975677159	0.682978285	4.77E-05
0.002228865	0.136137289	0.909671998	0.718938853	1.15E-05
0.004061466	0.100841984	1.101505185	0.615866077	1.18E-05
0.009808466	0.188906183	1.274743971	0.508045097	7.09E-05
0.010153903	0.279446727	1.925089489	0.355804904	7.29E-05
0.003939874	0.177594213	1.163121495	0.519677823	1.87E-05
0.007686942	0.519317789	2.574417324	0.198908355	5.61E-05
0.0044401	0.560229409	3.189260865	0.179464246	2.37E-05
0.003342763	0.324900974	1.965489828	0.281690489	1.95E-05
0.00412855	0.214165337	1.49851696	0.340145023	1.72E-05
0.02167661	0.355178409	2.631325376	0.317309438	0.000189709
0.005116704	0.18361859	1.615067098	0.354064888	1.72E-05
0.002329043	0.162557076	1.127928717	0.472065107	9.81E-06
0.004934057	0.245626854	2.043976752	0.247500596	1.71E-05
0.003618675	0.288906989	1.733492268	0.32238904	2.17E-05
0.004656083	0.308476828	1.852821215	0.29072636	3.29E-05
0.00240356	0.139776835	0.833848471	0.677402731	1.41E-05
0.003610142	0.2008517	1.575621425	0.349996515	1.48E-05
0.003115422	0.240453576	2.360745605	0.221226003	8.08E-06
0.002501912	0.20620567	1.69282853	0.316465402	9.40E-06
0.007799105	0.102747892	0.858798399	0.597116469	3.42E-05
0.001706786	0.101066828	0.64606319	1.040486989	1.05E-05
0.00434998	0.253699796	1.943143672	0.264994964	1.87E-05
0.002864776	0.284039185	1.504076949	0.442591964	1.98E-05
0.002894996	0.207769565	1.08678258	0.486506438	1.93E-05
0.00403999	0.355883661	1.983205807	0.293435234	2.22E-05
0.008184861	0.285325742	1.945626795	0.395056015	5.54E-05
0.003739683	0.276188851	1.469595447	0.378143794	2.56E-05
0.002370283	0.155397985	1.060825749	0.531497444	1.03E-05
0.012929904	0.149697714	1.516507156	0.540497014	6.90E-05
0.012481571	0.43575958	2.651801902	0.231998067	0.000100228
0.008019515	0.260308673	2.083208019	0.460363354	4.28E-05
0.00417674	0.24262166	1.378443254	0.399939793	3.13E-05
0.003079333	0.289668139	1.362939826	0.451116728	2.14E-05
0.002222788	0.127324302	0.795791402	0.791652818	1.08E-05
0.008935351	0.345786039	1.690187934	0.436377518	8.35E-05
0.007637126	0.282263434	1.416358574	0.491824833	8.15E-05
0.005751475	0.325876094	2.782826158	0.183795469	2.04E-05
0.002738166	0.142357089	0.794083149	0.688261931	2.16E-05
0.003447854	0.162480335	1.350971471	0.384816583	1.66E-05
0.006342858	0.274925617	1.603892136	0.30182402	3.43E-05
0.001244363	0.110192002	0.75766176	0.74680909	4.64E-06
0.00636064	0.319402496	2.61325276	0.284109088	2.60E-05
0.004068929	0.218589402	1.474289989	0.383435934	2.12E-05
0.001753148	0.19171602	1.139104796	0.51129671	8.15E-06
0.001621443	0.111769396	0.951179342	0.616769931	5.40E-06
0.012430811	0.322665772	1.824408887	0.327850053	0.000107316
0.003145958	0.182366824	1.811939278	0.303308439	9.52E-06
0.008657289	0.184450423	2.082220244	0.252249993	2.49E-05
0.001907696	0.171478182	1.339250656	0.42939938	6.45E-06
0.001956284	0.152476785	1.191233435	0.477068006	6.97E-06
0.003812026	0.10137632	0.855115002	0.639223342	1.50E-05
0.008119578	0.443348421	2.991252287	0.181386167	4.40E-05
0.002715575	0.183599048	1.405427311	0.409244195	1.16E-05
0.004772481	0.345119248	2.576528227	0.212873321	2.21E-05
0.004788894	0.324756293	1.530066139	0.329741566	4.46E-05
0.003454932	0.254182401	1.377256181	0.516673267	2.55E-05
0.007281234	0.306985191	2.177929465	0.244672598	4.70E-05
0.004557025	0.120681976	0.956088976	0.599923787	1.83E-05
0.013138329	0.332325047	3.330073491	0.210660745	5.59E-05
0.006218337	0.18487327	1.280025664	0.4443071	3.38E-05
0.00462383	0.448342076	2.610946761	0.209781437	3.61E-05
0.005395708	0.555238637	2.711432323	0.191414455	5.53E-05
0.006088717	0.339267079	2.342919484	0.271480826	2.73E-05
0.003285519	0.222325878	1.469885789	0.37464743	1.60E-05
0.003812204	0.107708623	1.032128788	0.535415177	1.45E-05
0.004527628	0.344289141	1.992474515	0.303908326	2.99E-05
0.008082089	0.102968962	1.25660636	0.441778876	3.20E-05
0.00457296	0.305873759	1.730267718	0.297059294	3.62E-05
0.003982705	0.226682458	1.442182861	0.411399386	2.13E-05
0.006924369	0.263777562	1.780589596	0.345357576	4.04E-05
0.005762109	0.240944952	2.124939862	0.261586255	2.26E-05
0.007707922	0.128762574	1.144749178	0.841021854	6.19E-05
0.004386414	0.224488134	1.156828275	0.564236494	4.15E-05
0.002183136	0.167052635	1.223493961	0.441818691	8.78E-06
0.003219595	0.203607861	1.35724262	0.399433587	1.50E-05
0.00247391	0.113345649	0.700591571	0.791783774	1.41E-05
0.003426569	0.276219744	1.907956013	0.286672996	1.69E-05
0.004287431	0.143486862	1.080037489	0.594007802	2.67E-05
0.011051417	0.469019163	4.877796691	0.112399665	5.07E-05
0.013159564	0.208780715	2.056433639	0.258533873	5.35E-05
0.001845098	0.093531044	0.570173932	0.990755469	9.44E-06

log.sigma.3.0.mm.3D_gldm_GrayLevelVariance	log.sigma.3.0.mm.3D_gldm_HighGrayLevelEmphasis	log.sigma.3.0.mm.3D_gldm_GrayLevelNonUniformityNormalized	log.sigma.3.0.mm.3D_gldm_DependenceEntropy
31.75323637	567.0584354	0.060222737	6.77465911
10.04803569	135.7037433	0.109542738	6.252371193
13.45240052	171.2053455	0.081682642	6.804111159
24.96264859	343.8976459	0.062612032	6.756242845
15.32117404	284.0291385	0.081443814	6.745555784
21.36318641	198.5757576	0.07161157	6.471749488
16.28810294	423.6549283	0.079041483	6.849134733
17.04307104	521.6294049	0.082455607	6.751943671
18.2172205	522.9736032	0.069215715	6.909335931
18.00020101	495.3330224	0.072578456	6.596186456
11.02223156	170.5384615	0.09026395	6.487156646
18.8824566	412.9006508	0.075258257	6.750507418
12.55898389	306.2581682	0.129473111	6.680300271
22.82977481	589.7245195	0.075631706	6.776277045
21.57431402	458.4138105	0.075690707	6.614743495
18.52490478	339.2671389	0.07455679	6.894778665
16.11330873	255.3777219	0.07804657	6.470278453
11.9996106	128.6326034	0.105455607	6.493339863
8.668291386	142.03698	0.105745238	6.467990081
18.85813017	360.8128486	0.071625734	6.894122522
10.21265054	200.8151455	0.110205438	6.704824441
10.57665037	134.0769231	0.088178479	6.342497854
13.15647793	179.5009766	0.092496872	6.501625468
12.74889208	210.1582924	0.0846879	6.680484256
17.16974272	399.3676355	0.081022308	6.659761222
25.31908903	589.7475728	0.064641232	6.73796175
23.97869309	391.2350758	0.070213424	6.946292736
15.3747084	399.0778421	0.085071213	6.667658655
9.977128157	130.4854586	0.098068656	5.977742438
10.10146327	236.1689387	0.103375369	6.490251592
16.44038858	428.6635141	0.081488649	6.853941281
12.16350551	353.618173	0.115311039	6.475569133
24.24540474	394.0065036	0.063198075	6.959042412
21.11259846	375.753617	0.072297691	6.81332258
17.06918711	303.7654405	0.071862567	6.735497101
14.39699751	385.6048683	0.090612509	6.715955784
15.43748263	634.3117442	0.10474835	6.778779862
21.55787119	577.7444294	0.084288469	7.153844404
6.332291625	154.9739837	0.121032454	6.107230067
21.9260195	288.5001376	0.060616579	6.92552365
16.73444263	329.2906708	0.095516966	6.669451569
26.13483469	356.2236876	0.057825129	6.772892116
18.67573678	361.4678347	0.067955876	6.798862314
19.07434787	522.073955	0.076126177	6.710245073
14.97552416	189.2237872	0.084340507	6.504928661
18.86561226	409.6659381	0.072362202	6.771071762
16.59568753	388.6238492	0.081793905	6.773329088
8.597581728	108.1159587	0.112689665	6.387512857
17.01050951	216.2150411	0.073712961	6.417293136
13.08652916	145.966716	0.094985295	6.648232208
20.44549765	424.5196575	0.068369294	6.731213553
22.38722783	310.268763	0.062767822	6.685720837
13.85677889	229.7096631	0.083361725	6.657897102
14.56300869	140.6775362	0.0784056	6.457148135
16.78403584	236.4699248	0.07093674	6.550077581
14.69040789	580.105641	0.088958317	6.506699613
19.62738194	356.9421508	0.067016705	6.803970157
17.6005785	428.0555234	0.087295083	6.749400802
14.11822767	273.4884709	0.088073805	6.451096482
18.63257345	507.9828021	0.071265849	7.112099685
15.12964945	265.1670442	0.100420168	6.754175091
15.27899676	390.506367	0.08005639	6.72541683
18.36859746	488.3047158	0.074517253	6.824559105
18.08459582	528.8042765	0.076698178	6.911454219
11.62360038	143.1177215	0.085374139	6.382175955
15.74253834	475.4241326	0.095014671	6.763391882
8.668483477	319.7147239	0.130276356	6.460373329
19.07727862	509.80256	0.078894899	6.893086752
17.71696435	500.6057709	0.075217387	6.809158663
9.518257993	228.0641822	0.103227709	6.579299198
15.49623474	320.7097768	0.082248973	6.56467801
18.05175673	448.425641	0.078861993	6.832295347
18.5207233	505.4791575	0.081421245	6.706788027
25.67864982	371.9614874	0.062510276	6.76051793
22.39681548	284.8968381	0.061858517	6.811791647
14.35201648	349.8532042	0.095885315	6.497442622
9.706431107	182.6464942	0.101834304	6.588423292
13.74074054	205.9885177	0.096218636	6.488885711
11.72781843	202.5301699	0.092513716	6.551333893
27.95084484	491.3689611	0.064172193	6.779358349
32.00456407	521.5062846	0.06113539	6.73666221
16.49335419	196.9244024	0.085320391	6.739988375
16.37414065	381.0801468	0.080113053	6.834485711
11.1604969	221.301985	0.093011373	6.569158439
21.72490994	300.1590816	0.065030281	6.797799211
8.177635593	215.9928198	0.125112483	6.507091295
23.56622602	401.1962264	0.0720789	6.917359438
16.88521319	240.3997082	0.076984648	6.805789831
13.17851384	244.1188525	0.0893549	6.681935291
14.46929822	367.1298765	0.090885414	6.750254051
10.57471995	108.5452454	0.127513503	6.390980661
19.94140228	272.1003359	0.065611735	6.740085479
18.91139278	420.3017557	0.075360688	6.77016216
15.88871065	386.4978788	0.083002755	6.745164477
15.72163217	301.8382353	0.075175988	6.666151259
22.23965738	544.5932203	0.075810899	6.998214394
16.56852401	219.5374512	0.075068216	6.779697565
15.49091825	339.5940783	0.104816691	6.568477676
8.605552968	201.1294964	0.107997056	6.334480857
13.27722256	277.9733287	0.092509283	6.74938421

log.sigma.3.0.mm.3D_gldm_DependenceNonUniformity	log.sigma.3.0.mm.3D_gldm_GrayLevelNonUniformity	log.sigma.3.0.mm.3D_gldm_SmallDependenceEmphasis
400.8124411	127.7926484	0.343125706
123.3294118	102.4224599	0.186823622
190.8956975	125.3011734	0.206356888
170.037871	61.17195496	0.29678685
342.8462838	192.8589527	0.240958763
108.5333333	47.26363636	0.268536315
814.8701115	446.6634224	0.23473443
704.968285	421.183242	0.219145586
694.1033876	314.6546414	0.256964559
173.1865672	77.80410448	0.271622632
227.3395225	136.1180371	0.242174294
355.6872017	173.470282	0.2662205
174.033744	241.7262989	0.15677206
260.1228888	129.8596389	0.247213522
324.9858871	150.1703629	0.294663462
618.6115174	326.2605119	0.24051339
207.5293132	93.18760469	0.255312425
144.0851582	130.026764	0.178883398
167.1571649	137.257319	0.189028621
793.8559338	372.3821889	0.246322415
313.9265873	333.2612434	0.153539336
176.9647822	95.14457831	0.214462864
140.8691406	94.71679688	0.233266301
386.6955043	224.1688704	0.229695575
183.683425	103.1413983	0.249911546
428.9392149	153.1350781	0.30412112
489.0167028	227	0.272097908
393.0724113	234.9666908	0.232988498
77.2147651	43.83668904	0.20453468
272.7497566	212.3330088	0.191833929
628.0937369	387.7229929	0.231710267
240.4557757	238.5785404	0.185358276
524.1619697	204.0665841	0.278142166
362.6834043	169.8995745	0.252405112
714.2048138	316.482743	0.265848368
375.9263088	271.7469156	0.212969634
419.7411986	389.7686106	0.196211329
575.8649487	435.0127882	0.21211416
167.8650407	148.8699187	0.17221764
1270.444292	440.6825309	0.262738315
327.3708558	247.77101	0.221208808
797.8973364	223.609775	0.33979421
597.9756365	248.2428141	0.260068245
393.7532154	189.4019293	0.2725648
190.4600939	107.7871674	0.221193614
433.856102	198.6342441	0.279878868
656.1613327	373.1437966	0.23950028
108.8094145	98.15269805	0.172430372
154.9388954	62.72972973	0.259519066
155.8032544	128.4201183	0.189083665
440.3079019	175.6407162	0.25998442
673.4556511	211.5903293	0.317492165
692.4259752	376.1281028	0.243071196
180.6630435	86.55978261	0.259544054
222.6586466	94.34586466	0.260037243
287.6748718	173.4687179	0.218843993
679.8484549	271.0825711	0.279109003
463.8629265	301.8663968	0.220612636
255.2184466	145.1456311	0.246508962
1267.954321	650.5859349	0.240533181
208.4575311	177.3420159	0.218705972
391.4089888	213.7505618	0.249091172
897.3485922	431.3803766	0.27339403
1092.393415	584.6702086	0.222909478
122.1949367	67.44556962	0.206493667
465.744174	366.9466598	0.207074072
133.5521472	169.8803681	0.169678036
906.94848	493.09312	0.235356776
900.3143247	440.5482329	0.273301808
349.5279503	299.1538992	0.194545474
197.0138568	106.8414165	0.229516233
584.4368298	338.3179487	0.220716889
338.7955244	185.5590171	0.233415805
413.5037627	141.2107127	0.312314645
584.2117534	193.6790163	0.303694462
187.8897168	128.6780924	0.214583278
298.8196995	243.9949917	0.197623377
125.0334029	92.17745303	0.188924075
249.0831869	157.9209139	0.221093365
431.761864	150.0987602	0.309219126
375.2282554	121.5982906	0.337654455
247.7871039	153.4913841	0.264958893
454.8244111	261.8895687	0.224002452
437.7881549	285.8239505	0.181282519
419.0926609	158.6088561	0.277684352
158.8537859	191.6723238	0.120523292
358.9563941	171.9081761	0.264648164
391.9540481	211.0919037	0.232820517
198.1188525	130.8155738	0.217948204
254.4291358	184.042963	0.207362542
124.9723926	166.2776074	0.164076984
431.0344249	156.2871537	0.29712699
827.4831179	390.5944434	0.256929269
475.969697	273.9090909	0.248566326
731.4140625	327.1659007	0.266103866
452.7884278	259.4248977	0.254706854
426.7302094	211.4671636	0.230909872
125.6456543	109.7430755	0.198128403
107.7721823	90.06954436	0.187006192
707.3207482	534.1485972	0.207365889

log.sigma.3.0.mm.3D_gldm_DependenceNonUniformityNormalized	log.sigma.3.0.mm.3D_gldm_DependenceVariance	log.sigma.3.0.mm.3D_gldm_LargeDependenceEmphasis
0.188884279	3.749962024	12.54194156
0.131903114	5.413622351	22.64278075
0.124443088	7.025127106	25.2125163
0.17404081	4.312545769	14.7011259
0.144783059	5.235082633	19.12077703
0.164444444	3.778751148	14.93030303
0.144199276	7.075843806	22.02211998
0.138012585	5.795170442	21.20555991
0.152684423	4.794876986	17.42190937
0.161554634	3.892208732	15.16977612
0.150755652	5.516720726	19.07161804
0.15431115	4.718521746	17.00347072
0.093215717	15.39518586	46.67005892
0.151498479	5.039122632	18.14967967
0.163803371	4.565043534	15.63810484
0.141364606	6.747299546	21.71160878
0.173810145	3.33416384	14.04690117
0.116857387	7.05426534	27.4541768
0.128780558	7.140735658	25.80739599
0.152693967	4.982064515	17.99557607
0.103811702	11.18731585	37.79298942
0.164008139	3.277585998	15.76552363
0.13756752	6.49779892	21.98632813
0.146088215	5.617940148	19.94446543
0.144291771	5.159236274	18.90102121
0.181063409	3.127695018	12.44449135
0.151257873	5.889475884	18.84101454
0.142314414	5.47661996	19.95076032
0.172739967	3.220885946	15.51006711
0.13278956	5.542643655	22.54722493
0.132007931	6.683321433	22.78436318
0.116218355	7.447539818	27.80425326
0.162329504	4.173462066	15.42056364
0.154333364	7.986755274	22.04765957
0.162171847	4.290685497	15.8760218
0.125350553	8.350877435	26.66722241
0.112803332	9.329385337	30.77317925
0.11158011	18.38664561	44.34508816
0.136475643	4.644288453	21.86341463
0.174751622	3.584296794	14.20275103
0.126203106	6.769994097	23.99537394
0.206334972	2.482681431	10.0804241
0.163694398	3.766519297	15.12044895
0.158260939	4.327549731	16.00482315
0.149029807	4.58540462	18.46948357
0.158053225	5.284201977	17.23387978
0.143831945	5.736748555	19.99473915
0.124924701	7.759981335	27.73708381
0.182066857	3.165240037	13.37367803
0.115239094	8.335606246	29.20266272
0.171392722	3.778851083	14.76488906
0.19977919	2.626753696	10.79946603
0.153463204	4.612357394	17.4304078
0.163644061	3.987788148	15.47644928
0.167412516	3.961894963	15.19699248
0.147525575	4.398696647	18.35179487
0.168071311	3.829542248	14.54808405
0.134141968	5.744681364	21.51417004
0.154865562	3.947166026	16.37621359
0.138893014	8.900597287	25.09661518
0.118039372	10.07219417	30.30690827
0.146595127	5.845774804	19.72209738
0.155009258	5.526062334	18.01848333
0.143302298	5.263064964	19.81254099
0.154677135	4.311193719	18.17974684
0.120596627	7.805407748	26.74210254
0.102417291	10.28255674	35.80674847
0.145111757	5.431742054	19.54048
0.153715951	4.814776766	17.03551306
0.120610059	6.970652519	26.1573499
0.151665787	4.240063862	17.43725943
0.136232361	5.776771263	21.32867133
0.14865973	5.544760419	19.50460728
0.183047261	3.885649081	13.40283311
0.186589509	3.392235423	12.63142766
0.140007241	6.433538927	22.33830104
0.124716068	7.630345233	26.54340568
0.130515034	5.954402221	23.58037578
0.14591868	5.884444252	20.56766257
0.184592503	3.314090991	12.50491663
0.188651712	3.768282113	12.64203117
0.137736022	7.312868832	22.34519177
0.139132582	7.648466864	23.70480269
0.14246279	4.37445079	20.29384966
0.171829709	3.540558718	13.97088971
0.103690461	9.275818228	37.56266319
0.150505826	6.812784304	20.44234801
0.142944584	6.355703031	21.26622903
0.135327085	6.28961562	22.30054645
0.125644018	6.531915501	24.2217284
0.095837724	11.04521011	39.15797546
0.180954838	3.53324084	13.16876574
0.159653312	4.450769221	16.47559329
0.144233242	5.774014325	19.82969697
0.168063893	3.702202292	14.62316176
0.132316899	8.918599388	25.47983635
0.151483922	4.369717859	17.59637913
0.1200054	7.127513093	26.45558739
0.12922324	6.117431235	24.05275779
0.122500996	8.684672605	27.77797021

log.sigma.3.0.mm.3D_gldm_LargeDependenceLowGrayLevelEmphasis	log.sigma.3.0.mm.3D_gldm_SmallDependenceHighGrayLevelEmphasis	log.sigma.3.0.mm.3D_gldm_LargeDependenceHighGrayLevelEmphasis
0.029153218	183.4579453	7276.079171
0.225553571	28.1936052	2721.147594
0.213020853	37.48298558	3706.107562
0.059785547	96.07393324	5066.558854
0.082945277	65.27705016	5368.542652
0.12426308	55.11433265	2701.357576
0.062816872	103.0936343	8340.222615
0.046965951	118.536097	10231.24608
0.037743699	130.0797621	8899.690717
0.035630574	129.804014	7379.969216
0.138617401	38.45588208	3173.942971
0.048541558	107.1544387	6765.849892
0.177005607	48.68766638	12896.28066
0.036680156	141.0451536	10220.61328
0.038057284	122.6727059	7176.217238
0.08283895	82.91093184	6591.069241
0.073654653	59.66956343	3768.732831
0.289226237	24.16636352	3112.334144
0.233325334	26.46513162	3300.306626
0.061473183	91.89643728	6050.27813
0.235308926	31.80446123	6622.987103
0.165937148	28.58577482	2044.83874
0.16858121	44.40998524	3498.663086
0.122834022	49.44202805	3865.685304
0.057616816	99.77841562	6981.329929
0.025031508	178.8668445	7149.795694
0.064912793	108.9229631	6900.505722
0.056325214	90.58020394	7681.851195
0.175390915	28.72142089	1738.991051
0.106532984	43.87561772	5264.391918
0.062565949	99.85910211	9037.122951
0.086946882	59.8718194	10013.94877
0.057119062	106.9405396	6112.342211
0.0860689	89.80704828	7610.113617
0.068940615	82.81309085	4516.419391
0.086103485	84.43208819	9136.743248
0.051399406	120.3622698	19119.3233
0.092085165	126.0366289	22400.94303
0.155407383	24.19822654	3591.291057
0.069610375	78.44383999	3927.767813
0.087433931	66.11366841	8172.104857
0.039273915	123.925276	3498.030773
0.050977856	91.16494559	5341.173282
0.032488389	132.4404448	8684.573151
0.130619546	42.94040414	3138.707355
0.051707655	114.7720957	6531.214208
0.062025298	97.02502904	7188.224463
0.343770381	20.11145409	2659.032147
0.082797331	52.50911614	2920.854289
0.300636168	31.40558906	3482.997781
0.043071314	108.0450152	6168.585053
0.051280352	98.20795497	3336.799169
0.093143194	55.0328698	3903.007979
0.198618522	38.66769184	1819.380435
0.090640346	63.94316221	3179.549624
0.033489889	122.9890699	10703.60513
0.049973947	97.06518263	5051.550309
0.057551899	89.43902719	9214.125506
0.069153677	63.94282342	4476.949636
0.058087004	121.2341908	11792.92047
0.137731734	62.15210583	7141.256512
0.058313038	96.29209922	7330.017978
0.043742295	132.5422431	8219.732942
0.042911108	122.7703245	9893.278893
0.183404525	30.98694178	2356.31519
0.064610899	101.7739612	11797.27965
0.121522058	48.95080917	11431.05215
0.046507436	118.8745554	9796.77152
0.036032899	126.9983764	8796.466792
0.133530256	44.34853494	5581.52795
0.066607724	69.83415294	5599.670516
0.056988078	99.1493413	8704.459207
0.044399287	116.0160837	9540.43484
0.051527902	105.1418964	5267.181496
0.063867422	90.20626641	3356.255509
0.072961197	65.12694417	8245.693741
0.177874621	38.21356282	4394.244157
0.148166621	42.39212735	4295.126305
0.126373861	46.43602853	3999.261277
0.031094934	141.391322	6574.287302
0.035014081	169.0711353	6703.892408
0.1504222	53.30456089	4384.171762
0.084514506	85.87610845	8086.996329
0.11788833	38.61519514	4560.587374
0.067884208	80.13549923	4274.533825
0.200952351	27.22312464	7529.860966
0.059801156	97.63391848	8469.180294
0.124493889	57.07689113	4452.270605
0.114715573	53.73893948	5141.695355
0.074584753	71.03591324	8679.055802
0.562038451	21.69026133	3155.617331
0.066413584	78.94592757	3447.009656
0.0446742	101.726045	6989.773876
0.058649358	92.55469387	7381.043939
0.056475617	76.429339	4599.65671
0.054637681	135.3789899	12909.49854
0.105029587	52.240511	3855.181044
0.08691103	59.54985651	9363.231137
0.12565041	32.28872075	5099
0.127078308	60.92857133	6608.312781

log.sigma.3.0.mm.3D_gldm_SmallDependenceLowGrayLevelEmphasis	log.sigma.3.0.mm.3D_gldm_LowGrayLevelEmphasis	log.sigma.3.0.mm.3D_gldzm_DistanceZoneVariabilityNormalized
0.001981056	0.003863226	0.997647062
0.00423309	0.01390057	1
0.003510984	0.010463457	1
0.003186109	0.006413912	1
0.002664169	0.00687515	1
0.003723549	0.011946509	1
0.000937932	0.003042623	1
0.000761639	0.002470766	0.998437501
0.000903718	0.002543207	1
0.001941001	0.003671585	1
0.00363435	0.009839935	1
0.001778684	0.003934828	0.997142863
0.001520611	0.004928236	1
0.002051643	0.003623925	1
0.001812901	0.00361645	1
0.001447149	0.004687805	1
0.003190027	0.008666272	1
0.004944338	0.014253644	1
0.0028326	0.010119742	1
0.001299512	0.003936888	0.998685076
0.001678872	0.006968317	1
0.004898908	0.013694269	1
0.003635723	0.010389352	1
0.00248552	0.007812499	1
0.003080006	0.005269337	1
0.001271287	0.002740522	1
0.002235389	0.005377774	1
0.001276518	0.003523795	1
0.005023263	0.013275414	1
0.003261691	0.00717293	1
0.001319261	0.003436267	1
0.00124614	0.004856517	0.995454569
0.001629764	0.004949754	0.996212135
0.00277221	0.007636197	1
0.001901251	0.005509816	0.995614056
0.001059115	0.00397885	1
0.000725795	0.002102481	1
0.000711646	0.002339385	0.995219151
0.003554118	0.010889912	1
0.001632844	0.005467543	1
0.001944296	0.005668383	1
0.001770983	0.004416883	0.99492711
0.001697767	0.004344467	1
0.001191137	0.002769657	1
0.003620713	0.009216527	1
0.001436197	0.003658895	1
0.001163091	0.00380255	1
0.004633762	0.016888561	1
0.004553348	0.009941839	1
0.00412986	0.012292043	0.993442694
0.001629086	0.004051558	1
0.002025875	0.005890501	0.998472117
0.002275035	0.006722989	1
0.006703622	0.017043713	1
0.002738635	0.006976539	1
0.001069425	0.002527742	1
0.001735602	0.004358433	0.998502995
0.001346383	0.003736494	1
0.002617512	0.006224945	1
0.000814797	0.002587838	0.996857824
0.001895869	0.005516541	1
0.001385443	0.003621293	1
0.001026303	0.002853771	0.993510074
0.000687858	0.002372418	0.993957759
0.003767373	0.012173609	1
0.001126974	0.003145256	0.995642722
0.001935288	0.005151374	1
0.000704966	0.002874154	0.997653962
0.001028386	0.00267376	1
0.002199442	0.006354005	1
0.002165531	0.005807929	1
0.001141964	0.003158812	0.998106062
0.001371221	0.003268749	1
0.002725149	0.006558706	0.997638728
0.002155719	0.005876115	0.994704362
0.001936322	0.00576074	1
0.002200793	0.00774158	1
0.002881183	0.008323065	1
0.002944066	0.008872483	0.995545679
0.0016839	0.003663633	1
0.001961521	0.004442677	1
0.004642361	0.012096258	1
0.001315987	0.004604588	0.997624706
0.001542229	0.007198749	1
0.002420982	0.00645208	0.997515532
0.001385203	0.006115602	1
0.00217326	0.004748151	1
0.003125049	0.008007087	1
0.00307709	0.007378749	1
0.002117058	0.004691524	1
0.003073142	0.014384497	1
0.002595002	0.006397765	0.995327128
0.001432998	0.003640077	0.9949875
0.001507768	0.003827055	1
0.001790941	0.004964757	0.998552823
0.001216506	0.002857749	0.995955527
0.002420477	0.007812876	1
0.002621291	0.00580197	1
0.003023012	0.007717478	1
0.00129943	0.004820745	0.99850075

log.sigma.3.0.mm.3D_gldzm_LowIntensityEmphasis	log.sigma.3.0.mm.3D_gldzm_LargeDistanceEmphasis	log.sigma.3.0.mm.3D_gldzm_HighIntensitySmallDistanceEmphasis
0.005718237	1.003533569	533.2653121
0.023723705	1	155.352657
0.016687761	1	184.0733696
0.01013072	1	328.26
0.011413346	1	273.4545455
0.01577888	1	214.6201923
0.003871302	1	446.2627119
0.003383017	1.002345582	545.289484
0.003412482	1	508.1696882
0.006464375	1	488.5843023
0.014845215	1	160.713948
0.006409065	1.004291845	404.9685265
0.009855654	1	316.310559
0.007514153	1	576.3426854
0.00580584	1	424.4164223
0.006339179	1	350.5389718
0.01350644	1	238.2050562
0.027493923	1	136.8252033
0.014427669	1	145.2307692
0.005123725	1.001973684	376.0177632
0.011167386	1	213.7803922
0.022393583	1	134.7715356
0.015027221	1	192.0145455
0.011227906	1	215.1908078
0.011214927	1	409.9505495
0.003968675	1	595.2188235
0.008317969	1	403.9703557
0.005262179	1	394.5443709
0.022446449	1	142.6203704
0.015936532	1	231.6772727
0.005481656	1	437.0746988
0.008200388	1.006833713	316.5267654
0.006385998	1.0056926	382.7459677
0.012087678	1	360.5550787
0.007333106	1.006593407	313.089011
0.005615655	1	399.6619916
0.003600285	1	617.4189526
0.003273116	1.007188498	596.9876198
0.021753892	1	137.3539095
0.006219424	1	298.2145661
0.008778639	1	297.5619335
0.005105447	1.007628735	363.2603306
0.006319337	1	355.6044643
0.004162301	1	485.6457286
0.015145083	1	204.7401813
0.004958326	1	418.5422773
0.005107973	1	409.9928344
0.027633175	1	121.0163934
0.016411718	1	206.010989
0.020402948	1.009868421	170.4572368
0.006042978	1	420.4040404
0.006780009	1.002293578	311.1095183
0.009331475	1	228.7179487
0.025908003	1	155.1891892
0.009934831	1	247.5434783
0.004547221	1	560.8487903
0.006022947	1.002247191	351.8878277
0.006074845	1	407.6862069
0.010408171	1	263.3135593
0.003365744	1.004720692	504.0108183
0.008311463	1	288.1183036
0.005311937	1	389.1105332
0.003710463	1.009766685	488.2788931
0.002988202	1.009090909	557.4412879
0.017644905	1	155.8410256
0.005317516	1.006550218	498.6091703
0.011434705	1	287.9755102
0.003393697	1.003523194	504.9729888
0.00368486	1	467.6630197
0.011046464	1	231.4333868
0.009522351	1	309.4845938
0.004983718	1.002843602	459.6170616
0.005640215	1	498.4510433
0.009368091	1.003546099	338.0591017
0.007106016	1.007964602	296.4904867
0.010025465	1	303.5654762
0.010896016	1	198.6097561
0.014891646	1	227.8238095
0.013793514	1.006696429	210.9866071
0.005171478	1	461.1349481
0.006005063	1	498.5126904
0.018864794	1	198.6124314
0.006529878	1.003567182	387.68044
0.009710113	1	212.3143297
0.008817377	1.003731343	288.5314055
0.011484283	1	224.0247525
0.007708732	1	372.2260184
0.012837331	1	249.7214854
0.013166759	1	245.8376963
0.009601443	1	348.7142857
0.01826565	1	140.5021834
0.008371439	1.007025761	269.7450234
0.005351257	1.007537688	398.4558731
0.005905094	1	376.4526882
0.006702283	1.002172339	285.3606083
0.004628446	1.006079027	532.164387
0.010700532	1	225.161039
0.01282945	1	295.9446809
0.015421709	1	175.0502793
0.006248872	1.002250563	302.2865716

log.sigma.3.0.mm.3D_gldzm_LowIntensityLargeDistanceEmphasis	log.sigma.3.0.mm.3D_gldzm_HighIntensityEmphasis	log.sigma.3.0.mm.3D_gldzm_DistanceZoneVariability	log.sigma.3.0.mm.3D_gldzm_ZonePercentage
0.005733942	533.4640754	847.0023557	0.400094251
0.023723705	155.352657	207	0.221390374
0.016687761	184.0733696	368	0.239895698
0.01013072	328.26	350	0.358239509
0.011413346	273.4545455	660	0.278716216
0.01577888	214.6201923	208	0.315151515
0.003871302	446.2627119	1534	0.271456379
0.00338677	545.6559812	1277.001564	0.250391543
0.003412482	508.1696882	1379	0.303343599
0.006464375	488.5843023	344	0.320895522
0.014845215	160.713948	423	0.280503979
0.006438869	405.1230329	697.0028612	0.303253796
0.009855654	316.310559	322	0.172469202
0.007514153	576.3426854	499	0.29062318
0.00580584	424.4164223	682	0.34375
0.006339179	350.5389718	1206	0.27559415
0.01350644	238.2050562	356	0.298157454
0.027493923	136.8252033	246	0.199513382
0.014427669	145.2307692	286	0.220338983
0.005143461	376.0671053	1518.001316	0.292363916
0.011167386	213.7803922	510	0.168650794
0.022393583	134.7715356	267	0.247451344
0.015027221	192.0145455	275	0.268554688
0.011227906	215.1908078	718	0.271250472
0.011214927	409.9505495	364	0.285938727
0.003968675	595.2188235	850	0.358801182
0.008317969	403.9703557	1012	0.313021961
0.005262179	394.5443709	755	0.273352643
0.022446449	142.6203704	108	0.241610738
0.015936532	231.6772727	440	0.214216164
0.005481656	437.0746988	1245	0.261664565
0.00823076	316.9111617	437.0045558	0.212179797
0.006730717	382.8235294	1050.00759	0.326416847
0.012087678	360.5550787	699	0.297446809
0.007360176	313.5186813	1359.013187	0.309945504
0.005615655	399.6619916	713	0.237745915
0.003600285	617.4189526	802	0.215533459
0.003285024	598.0838658	1246.014377	0.242588646
0.021753892	137.3539095	243	0.197560976
0.006219424	298.2145661	2293	0.315405777
0.008778639	297.5619335	662	0.255204318
0.005136668	363.7552448	1565.020343	0.406775278
0.006319337	355.6044643	1120	0.306597317
0.004162301	485.6457286	796	0.319935691
0.015145083	204.7401813	331	0.258998435
0.004958326	418.5422773	887	0.323132969
0.005107973	409.9928344	1256	0.275317843
0.027633175	121.0163934	183	0.21010333
0.016411718	206.010989	273	0.32079906
0.020557142	170.6151316	302.0065789	0.224852071
0.006042978	420.4040404	792	0.308291164
0.006787945	311.2752294	1306.001529	0.388015426
0.009331475	228.7179487	1287	0.285239362
0.025908003	155.1891892	333	0.301630435
0.009934831	247.5434783	414	0.311278195
0.004547221	560.8487903	496	0.254358974
0.006041519	351.9558052	1333.001498	0.330037083
0.006074845	407.6862069	870	0.251590515
0.010408171	263.3135593	472	0.286407767
0.00338134	504.4834776	2534.012589	0.278453281
0.008311463	288.1183036	448	0.253680634
0.005311937	389.1105332	769	0.288014981
0.004008318	488.9918611	1831.039067	0.318362411
0.003025414	558.05	1968.036364	0.25974026
0.017644905	155.8410256	195	0.246835443
0.005350369	498.9923581	912.0087336	0.237182807
0.011434705	287.9755102	245	0.187883436
0.003400707	505.4468585	1699.004698	0.27248
0.00368486	467.6630197	1828	0.312105173
0.011046464	231.4333868	623	0.214975845
0.009522351	309.4845938	357	0.27482679
0.005003465	459.7194313	1053.001896	0.245920746
0.005640215	498.4510433	623	0.273365511
0.009392717	338.1867612	844.0023641	0.374501992
0.007143017	297.0672566	1124.015929	0.360907058
0.010025465	303.5654762	336	0.250372578
0.010896016	198.6097561	533	0.22245409
0.014891646	227.8238095	210	0.219206681
0.013840017	211.2276786	446.0044643	0.26244874
0.005171478	461.1349481	867	0.370671227
0.006005063	498.5126904	788	0.396178984
0.018864794	198.6124314	547	0.30405781
0.006542222	387.9381688	839.0023781	0.257265219
0.009710113	212.3143297	649	0.211194273
0.008824431	289.0248756	802.0024876	0.329643296
0.011484283	224.0247525	202	0.131853786
0.007708732	372.2260184	761	0.319077568
0.012837331	249.7214854	754	0.274981765
0.013166759	245.8376963	382	0.260928962
0.009601443	348.7142857	483	0.238518519
0.01826565	140.5021834	229	0.175613497
0.008409253	270.2025761	850.0093677	0.35852225
0.005405634	399.0640704	1584.020101	0.307158017
0.005905094	376.4526882	930	0.281818182
0.006762626	285.3801593	1379.001448	0.317325368
0.004643573	532.8125633	983.0081054	0.28842782
0.010700532	225.161039	770	0.273340433
0.01282945	295.9446809	235	0.224450812
0.015421709	175.0502793	179	0.214628297
0.006264501	302.3675919	1331.0015	0.230862487

log.sigma.3.0.mm.3D_gldzm_IntensityVariabilityNormalized	log.sigma.3.0.mm.3D_gldzm_LowIntensitySmallDistanceEmphasis	log.sigma.3.0.mm.3D_gldzm_IntensityVariability
0.043891449	0.005714311	37.26383981
0.0664893	0.023723705	13.76328502
0.058276229	0.016687761	21.44565217
0.051493878	0.01013072	18.02285714
0.057203857	0.011413346	37.75454545
0.054086538	0.01577888	11.25
0.054944084	0.003871302	84.28422425
0.049894213	0.003382079	63.81469898
0.054842633	0.003412482	75.6279913
0.0542692	0.006464375	18.66860465
0.070603424	0.014845215	29.86524823
0.051004808	0.006401614	35.65236052
0.054492496	0.009855654	17.54658385
0.046911458	0.007514153	23.40881764
0.047884005	0.00580584	32.6568915
0.053546529	0.006339179	64.57711443
0.059020326	0.01350644	21.01123596
0.062793311	0.027493923	15.44715447
0.078243435	0.014427669	22.37762238
0.052423823	0.00511879	79.68421053
0.062775855	0.011167386	32.01568627
0.070712172	0.022393583	18.88014981
0.060495868	0.015027221	16.63636364
0.061777919	0.011227906	44.35654596
0.054009178	0.011214927	19.65934066
0.045948789	0.003968675	39.05647059
0.046337234	0.008317969	46.89328063
0.053038025	0.005262179	40.04370861
0.082475995	0.022446449	8.907407407
0.061115702	0.015936532	26.89090909
0.053421719	0.005481656	66.51004016
0.062406276	0.008192795	27.39635535
0.047396923	0.006299818	49.95635674
0.05034169	0.012087678	35.1888412
0.055921856	0.007326339	76.33333333
0.056838635	0.005615655	40.5259467
0.049601682	0.003600285	39.78054863
0.047263675	0.003270139	59.17412141
0.084421413	0.021753892	20.51440329
0.051900502	0.006219424	119.00785
0.052404597	0.008778639	34.6918429
0.050558758	0.005097642	79.52892562
0.054139031	0.006319337	60.63571429
0.053035277	0.004162301	42.2160804
0.05563111	0.015145083	18.41389728
0.051914857	0.004958326	46.04847802
0.051662846	0.005107973	64.88853503
0.069545224	0.027633175	12.72677596
0.056689342	0.016411718	15.47619048
0.064599204	0.0203644	19.63815789
0.050804765	0.006042978	40.23737374
0.052730083	0.006778025	68.97094801
0.060112813	0.009331475	77.36519037
0.060592124	0.025908003	20.17717718
0.05875283	0.009934831	24.3236715
0.056207726	0.004547221	27.87903226
0.05178078	0.006018304	69.12734082
0.051885322	0.006074845	45.14022989
0.056251795	0.010408171	26.55084746
0.053591867	0.003361844	136.2305271
0.052236129	0.008311463	23.40178571
0.059082354	0.005311937	45.4343303
0.054790177	0.003635999	100.9782963
0.051298847	0.002978899	101.5717172
0.065825115	0.017644905	12.83589744
0.05143876	0.005309303	47.11790393
0.065589338	0.011434705	16.06938776
0.050129835	0.003391945	85.37110981
0.053404613	0.00368486	97.62363239
0.063146531	0.011046464	39.34028892
0.058776452	0.009522351	20.98319328
0.050041104	0.004978781	52.79336493
0.050114781	0.005640215	31.22150883
0.046001487	0.009361934	38.91725768
0.051659488	0.007096766	58.37522124
0.06079932	0.010025465	20.42857143
0.064236912	0.010896016	34.23827392
0.053197279	0.014891646	11.17142857
0.062340561	0.013781889	27.92857143
0.049010961	0.005171478	42.49250288
0.045186039	0.006005063	35.60659898
0.053564565	0.018864794	29.29981718
0.052973288	0.006526793	44.55053508
0.065842674	0.009710113	42.73189522
0.053371204	0.008815614	42.91044776
0.07562984	0.011484283	15.27722772
0.047753406	0.007708732	36.34034166
0.055727543	0.012837331	42.01856764
0.06184041	0.013166759	23.62303665
0.055214777	0.009601443	26.66873706
0.077019889	0.01826565	17.63755459
0.056705187	0.008361986	48.42622951
0.051179263	0.005337662	81.47738693
0.054861834	0.005905094	51.02150538
0.05975636	0.006687197	82.52353367
0.046662335	0.004624664	46.05572442
0.055260584	0.010700532	42.55064935
0.056767768	0.01282945	13.34042553
0.073874099	0.015421709	13.22346369
0.058245056	0.006244965	77.64066017

log.sigma.3.0.mm.3D_gldzm_HighIntensityLargeDistanceEmphasis	log.sigma.3.0.mm.3D_gldzm_SmallDistanceEmphasis	log.sigma.3.0.mm.3D_glcm_SumVariance	log.sigma.3.0.mm.3D_glcm_Homogeneity1
534.2591284	0.999116608	1752.791121	0.337312736
155.352657	1	354.272343	0.46273078
184.0733696	1	453.0894506	0.455920267
328.26	1	1021.686376	0.368842364
273.4545455	1	831.5008215	0.408598346
214.6201923	1	521.581607	0.383888539
446.2627119	1	1284.084863	0.416218564
547.1219703	0.999413604	1622.22025	0.422239051
508.1696882	1	1631.372291	0.392408827
488.5843023	1	1533.550652	0.393606973
160.713948	1	467.9334625	0.429927008
405.7410587	0.998927039	1233.961416	0.394341245
316.310559	1	938.4583642	0.508241509
576.3426854	1	1877.286194	0.395166364
424.4164223	1	1405.441561	0.371409001
350.5389718	1	1000.251911	0.407742597
238.2050562	1	730.9367715	0.391227339
136.8252033	1	334.1467532	0.466203969
145.2307692	1	377.879677	0.46341599
376.2644737	0.999506579	1053.466845	0.399800967
213.7803922	1	559.4065025	0.503185527
134.7715356	1	340.0708712	0.422656618
192.0145455	1	485.1807524	0.435907969
215.1908078	1	598.1865458	0.428368631
409.9505495	1	1217.494407	0.402161089
595.2188235	1	1814.188605	0.367604011
403.9703557	1	1159.375311	0.382811355
394.5443709	1	1218.822068	0.4100546
142.6203704	1	332.5844195	0.430220661
231.6772727	1	675.6203048	0.456546295
437.0746988	1	1301.110041	0.418728916
318.4487472	0.998291572	1110.793392	0.445485718
383.1337761	0.99857685	1172.259182	0.372717531
360.5550787	1	1130.923682	0.398308984
315.2373626	0.998351648	874.7229059	0.393705786
399.6619916	1	1179.696903	0.438512568
617.4189526	1	2088.255329	0.452883714
602.4688498	0.998202875	1828.932116	0.455355022
137.3539095	1	433.5978995	0.458377012
298.2145661	1	829.9504938	0.37463426
297.5619335	1	1008.008891	0.425620871
365.7349015	0.998092816	1038.115983	0.33031161
355.6044643	1	1091.344455	0.378421006
485.6457286	1	1631.336933	0.382074142
204.7401813	1	501.5748457	0.422493545
418.5422773	1	1245.448636	0.393503398
409.9928344	1	1168.298708	0.409323351
121.0163934	1	265.2149533	0.483336186
206.010989	1	573.4542589	0.3899534
171.2467105	0.997532895	371.4421318	0.475445357
420.4040404	1	1286.13925	0.372097384
311.9380734	0.999426606	899.6186219	0.345094071
228.7179487	1	652.1499112	0.409068899
155.1891892	1	358.4755261	0.396541246
247.5434783	1	671.2529644	0.394074615
560.8487903	1	1840.643364	0.417479527
352.2277154	0.999438202	1066.569159	0.373600696
407.6862069	1	1314.217985	0.418550301
263.3135593	1	810.8684942	0.403114956
506.3741149	0.998819827	1586.755303	0.410441623
288.1183036	1	769.854906	0.458839587
389.1105332	1	1189.066262	0.408450956
491.843733	0.997558329	1499.410258	0.397079888
560.4848485	0.997727273	1632.791094	0.415470059
155.8410256	1	362.145254	0.430280006
500.5251092	0.998362445	1464.422314	0.445068504
287.9755102	1	1001.936952	0.486245724
507.3423371	0.999119201	1594.302795	0.408211255
467.6630197	1	1603.450672	0.41100399
231.4333868	1	656.1382666	0.471060903
309.4845938	1	969.5478364	0.407157699
460.12891	0.9992891	1358.960202	0.424337983
498.4510433	1	1575.846805	0.408729972
338.6973995	0.999113475	1106.142054	0.342355745
299.3743363	0.99800885	810.6600251	0.358591787
303.5654762	1	1060.649773	0.432218076
198.6097561	1	505.4974077	0.47346056
227.8238095	1	573.3544457	0.455036336
212.1919643	0.998325893	555.7491238	0.433736254
461.1349481	1	1495.071955	0.350481118
498.5126904	1	1593.060892	0.344513124
198.6124314	1	534.4901234	0.415554614
388.9690844	0.999108205	1174.716161	0.417909242
212.3143297	1	627.7119964	0.444441482
290.9987562	0.999067164	872.6037738	0.370419485
224.0247525	1	625.745374	0.520897652
372.2260184	1	1221.367911	0.386533445
249.7214854	1	667.8627496	0.42868295
245.8376963	1	687.5048791	0.442412218
348.7142857	1	1123.589372	0.445761599
140.5021834	1	269.403963	0.515692884
272.0327869	0.99824356	794.8624245	0.371192911
401.4968593	0.998115578	1259.79014	0.384753314
376.4526882	1	1175.855208	0.413593385
285.4583635	0.999456915	889.1841882	0.386508355
535.4052685	0.998480243	1695.312282	0.421397361
225.161039	1	600.1948277	0.414087641
295.9446809	1	1049.211164	0.453647323
175.0502793	1	568.8543304	0.465661232
302.6916729	0.999437359	817.2599993	0.447918015

log.sigma.3.0.mm.3D_glcm_Homogeneity2	log.sigma.3.0.mm.3D_glcm_ClusterShade	log.sigma.3.0.mm.3D_glcm_MaximumProbability	log.sigma.3.0.mm.3D_glcm_Idmn	log.sigma.3.0.mm.3D_glcm_SumVariance2
0.252683069	-758.7547487	0.021819447	0.978628696	98.17933459
0.394574608	103.8929924	0.053004609	0.983955859	29.90848442
0.385657524	79.45051266	0.036209959	0.986638722	41.03584269
0.286593665	-295.5236973	0.022347926	0.979738054	74.09906919
0.330011329	-88.14382385	0.032826393	0.982721693	47.17372648
0.299870937	336.87955	0.027828737	0.981462887	67.61127191
0.337720151	112.9750592	0.037984696	0.992415559	50.2314444
0.345939326	77.6125446	0.040086485	0.991362382	50.15845393
0.311429654	-80.38845876	0.018903659	0.988622247	56.29886571
0.310498938	-189.3209337	0.022346842	0.988051626	54.06295017
0.353345471	-18.98718764	0.036202663	0.981147661	32.83998623
0.313061514	-60.50353679	0.02878999	0.987109034	55.75916572
0.450627602	82.09775001	0.103482404	0.990193756	39.96412054
0.316498442	-274.1112491	0.034042688	0.986476295	70.16921967
0.290213004	-181.4090454	0.028535693	0.985948504	63.46139079
0.329903534	-18.98030042	0.035300101	0.986211162	54.14404401
0.309730972	-240.6856166	0.023753545	0.979703243	48.413817
0.399528163	194.4420783	0.05055794	0.982070894	38.00955849
0.392474855	53.63859336	0.057798403	0.984013013	26.33735582
0.318030648	64.50740666	0.026606861	0.988549141	55.50331376
0.442504062	84.81379082	0.074141022	0.991171692	31.57766172
0.342566114	20.2354601	0.026648474	0.979234934	31.91990132
0.361284786	73.98297401	0.04408309	0.985224205	39.07795056
0.351831005	76.96956455	0.030258136	0.986535967	38.44548246
0.322958569	16.91880556	0.036980557	0.98706696	44.79622159
0.283065255	-198.6757162	0.018468186	0.98861911	76.87593545
0.301690948	-127.4822192	0.033649467	0.98621838	69.45531299
0.33245013	-55.90708259	0.037612523	0.987578201	44.3082681
0.351355715	92.93937552	0.03540014	0.981460334	31.59839769
0.386812798	-16.82325025	0.042039842	0.987524374	30.97693345
0.34358066	-10.29959289	0.037866825	0.98872069	48.14554792
0.376586757	-263.7002159	0.05378744	0.98690868	33.36576463
0.289817299	-200.1621752	0.019542867	0.983696846	71.46492223
0.319779941	-336.3625764	0.036313497	0.98240726	61.00818237
0.31106339	-65.58668995	0.024242509	0.987911943	50.41290663
0.365398867	-58.16357196	0.05031321	0.989719773	40.75374556
0.38598749	-202.4669503	0.051008568	0.992342462	45.37388399
0.387640821	-106.066675	0.084297456	0.990795184	70.93936658
0.386851284	-48.58239466	0.042072049	0.982672639	17.47014912
0.289152411	6.697212512	0.014115784	0.984189864	68.33059172
0.353503609	-342.6727918	0.045638042	0.985149705	48.1193802
0.239701264	273.4301521	0.013058951	0.984476918	80.23184851
0.293877152	-84.68692148	0.01915622	0.983651414	53.91498209
0.301129956	-207.5426865	0.023646387	0.988802762	57.14129062
0.344643279	175.6335316	0.036626031	0.986111359	44.95767297
0.312940098	-108.343203	0.025439091	0.986235442	54.36099827
0.331297222	66.18150181	0.035962978	0.989015631	45.42889923
0.417433986	89.40636446	0.056298744	0.986136119	26.00593651
0.307039614	-13.26506998	0.023879594	0.978226191	51.84128343
0.409755235	202.2612911	0.056415223	0.988075964	41.22402949
0.285879237	-143.1774355	0.025561105	0.984331987	55.99004587
0.256248112	13.48935755	0.014694575	0.983644865	65.3540761
0.329012782	22.02045247	0.025815158	0.98698542	42.67533607
0.313817453	126.8573039	0.026752779	0.978583322	42.18826798
0.311845892	106.754651	0.025106869	0.981887246	52.56077383
0.340394099	-86.41545591	0.035808691	0.991563969	43.46916216
0.288025513	-133.6937391	0.018341941	0.982814557	55.91874224
0.343593985	-298.651318	0.03947107	0.987931714	51.22756341
0.323942004	-73.1451206	0.028586648	0.982077858	38.47904714
0.334454467	-98.46980089	0.036434632	0.988042217	58.2936049
0.391572861	264.7940868	0.063553455	0.990479232	44.16454122
0.328756863	-43.68565208	0.034640452	0.988597767	44.9657244
0.317056929	-54.96207788	0.031740186	0.988051314	56.81085358
0.33781611	187.9434412	0.025434806	0.991424911	56.7465917
0.352315096	49.50779311	0.031623255	0.981814762	32.7482617
0.37558888	-73.98774428	0.048368682	0.990699653	47.08388299
0.424096745	-64.25874629	0.068798326	0.989296428	24.35142074
0.331250351	-31.9185469	0.029967396	0.99078984	59.41215662
0.332509085	-180.7252941	0.025238685	0.990911015	50.70875568
0.404815267	14.04576944	0.048669281	0.987963595	27.42722676
0.327290239	-207.0439979	0.031951411	0.984390177	46.94518636
0.348537067	77.62507116	0.034552948	0.990773933	54.97721999
0.331199028	-324.5839005	0.032628241	0.987338713	52.81387926
0.255998051	-324.7633989	0.025267752	0.977113513	69.4049063
0.271348403	107.132877	0.017759505	0.983030206	66.882742
0.358879983	-362.7649343	0.045936493	0.982064801	44.82160022
0.406390151	62.55531289	0.045528905	0.988472522	27.56834848
0.385556949	181.5823487	0.048281288	0.987900105	44.65319547
0.357795952	19.56853783	0.043501529	0.985894063	33.73865881
0.263831634	-698.7377912	0.01898086	0.983365303	89.76677312
0.260800597	-845.6273759	0.024025605	0.978456644	98.70626657
0.340597821	-52.47405133	0.036075616	0.986115649	44.0735901
0.34138694	-9.831373017	0.035763427	0.987480174	46.81864585
0.370605178	-125.7567908	0.035115026	0.985993645	35.514965
0.285418056	-143.3926332	0.020639408	0.984656481	66.1827936
0.464107063	90.85959359	0.070738091	0.991292839	26.75215674
0.309328371	-509.3897141	0.037021746	0.979367156	67.92962008
0.354193072	49.74297498	0.041558236	0.984336529	51.33067736
0.369345738	-27.06598388	0.036518223	0.98738296	40.52685392
0.375288564	-120.7724693	0.03976099	0.988787671	40.70177103
0.459647963	188.9539849	0.087426679	0.98654858	33.23090623
0.285340663	25.73480003	0.018135355	0.980837531	63.58344682
0.303078145	-231.092009	0.022395624	0.985887918	53.44125749
0.336898831	-118.2690816	0.032176706	0.986676804	45.86263119
0.302491085	-117.481434	0.020100938	0.98298447	46.81247863
0.348117438	-198.2107514	0.048853726	0.98802414	69.29485815
0.334626977	16.22514608	0.029176806	0.98565545	52.05369995
0.388671627	-511.1411825	0.048459967	0.983754648	49.84712715
0.398485305	-61.27657658	0.045967889	0.98458431	26.27954146
0.376886429	119.1111577	0.048416777	0.98876709	39.34497913

log.sigma.3.0.mm.3D_glcm_Contrast	log.sigma.3.0.mm.3D_glcm_DifferenceEntropy	log.sigma.3.0.mm.3D_glcm_InverseVariance	log.sigma.3.0.mm.3D_glcm_Entropy	log.sigma.3.0.mm.3D_glcm_Dissimilarity
33.89646936	3.578089128	0.251244527	8.29728857	4.201638096
8.372318278	2.662262607	0.38532292	6.523585182	2.095018135
9.624473024	2.755142664	0.362311386	7.053124712	2.231199279
22.85855772	3.28685529	0.294948121	7.808744976	3.434525695
14.72877484	3.040092704	0.322654103	7.461766227	2.775683986
16.82595039	3.094577646	0.289679224	7.394217726	3.056542994
13.21885258	2.982727315	0.322184619	7.503581256	2.655058803
13.03167535	2.965831429	0.335457962	7.404829205	2.602135732
17.32125806	3.137851975	0.306277694	7.80183893	3.016916073
14.59141256	3.02892032	0.310325044	7.384446166	2.835396894
9.804461162	2.788903982	0.350400173	6.967978041	2.354691409
15.77045579	3.096143371	0.309991416	7.57082473	2.92140419
9.519566549	2.664999035	0.381204399	6.436022533	2.002091973
21.43892807	3.199123554	0.310813548	7.639703031	3.159613009
23.95376476	3.335034289	0.288391627	7.748643515	3.50662842
16.12434555	3.074449308	0.319701715	7.596160552	2.862804831
16.1891906	3.080578221	0.315324617	7.426314819	2.943023777
9.608754347	2.724216892	0.378023173	6.699246116	2.169973305
7.502160705	2.622793356	0.368552758	6.58331868	2.045309447
13.89186064	3.022213394	0.312573671	7.659424505	2.780270262
6.764719632	2.533648899	0.395189175	6.523968529	1.823151473
8.914436016	2.724665267	0.347197531	6.936672444	2.310809288
10.66214135	2.829338105	0.350348702	6.937442571	2.384396036
11.20311992	2.872241237	0.339593164	7.213009119	2.460538196
17.16590244	3.074929447	0.318530766	7.266138514	2.913265993
21.20966987	3.26060064	0.289937603	7.939723369	3.346234215
21.432547	3.255283891	0.296186738	7.867900358	3.275686036
15.31192003	3.052355834	0.326900936	7.391942071	2.793876689
7.875172356	2.627217546	0.361172884	6.540152011	2.187655042
8.999263592	2.703973493	0.378662775	6.800225528	2.15824225
14.66520241	3.013317823	0.338967075	7.421402104	2.711310987
13.64991271	2.919331911	0.360843424	6.775007899	2.511296714
21.53028933	3.275853116	0.290757607	8.010776583	3.354243409
18.48300427	3.169232942	0.308080079	7.621809192	3.055589095
14.77587309	3.049684996	0.311352563	7.632302866	2.848659788
11.87521045	2.87332889	0.34934987	7.042078662	2.442432276
15.04720935	2.932496009	0.361925847	7.033176496	2.533760874
17.20832065	3.079379422	0.31320418	7.482784996	2.743892147
7.457502636	2.602640705	0.37946415	6.299879918	2.037996129
17.25115817	3.161732406	0.291195523	8.059411165	3.114692801
16.60490375	3.044737327	0.343220353	7.243291081	2.779322937
24.99184972	3.401732753	0.248675004	8.322963087	3.819637149
18.11659791	3.156977885	0.294873575	7.780045659	3.118431709
20.89925784	3.249068337	0.299200986	7.709043369	3.262033162
10.68572085	2.846165689	0.340366619	7.108034078	2.45706388
16.94569971	3.12343025	0.312160506	7.63385816	2.984280774
14.18335942	3.014214808	0.331329761	7.401065998	2.733208273
5.83097464	2.458249384	0.400181028	6.314551532	1.818584875
14.87415516	3.043447687	0.311605394	7.411154993	2.881162251
8.586188732	2.672894701	0.376468408	6.717932477	2.077788353
18.42825322	3.180516683	0.288276247	7.724456485	3.164958913
22.64224803	3.335628937	0.265698628	8.108493664	3.582366245
12.34120886	2.945659077	0.329303706	7.403312921	2.625375225
13.48088966	2.963375105	0.318598547	7.25108263	2.750495433
14.17182359	3.023157626	0.313927585	7.468412698	2.826645479
13.44490978	2.956657891	0.337476324	7.203934457	2.632302967
17.686061	3.175872216	0.287839538	7.835768276	3.150539063
15.93401657	3.025996703	0.341789999	7.357450029	2.751674424
15.34059192	3.051497231	0.325575109	7.271926498	2.824264888
18.36737807	3.181271164	0.305915303	7.844866965	3.013344712
12.46189249	2.874453035	0.357849221	6.938374393	2.39248099
13.03689586	2.976324797	0.325620698	7.372013271	2.677311855
16.33465868	3.122669535	0.310502123	7.712857339	2.946930846
13.62710832	2.980813721	0.331701115	7.56970451	2.664845562
9.450608162	2.735894701	0.346321129	6.877874667	2.319826177
13.55049809	2.923415873	0.35855928	7.163764653	2.501360433
9.86582637	2.689026149	0.38534457	6.382123078	2.081623736
17.11516881	3.104975142	0.325761996	7.697443997	2.895090485
14.4969628	3.018691814	0.326389969	7.537498578	2.735262517
8.006243656	2.636703619	0.392827508	6.6732034	2.027773001
14.1956144	2.980949883	0.324455726	7.284183549	2.731499902
13.34600726	2.945196034	0.342662138	7.427197133	2.581325089
16.70730414	3.07767834	0.325367178	7.412080766	2.860700002
27.26089136	3.454611681	0.256700085	8.079704066	3.87243773
19.78793989	3.242304074	0.280078218	8.041079509	3.346741721
14.61420231	2.963070361	0.341726269	7.034646644	2.621821531
7.645842296	2.614673631	0.386043017	6.658298172	1.9957395
9.358753859	2.722176513	0.375642083	6.839683535	2.201465969
10.17703813	2.805939563	0.350384243	6.98510577	2.351431165
25.92104724	3.394799975	0.265806732	8.138040201	3.703332861
32.52157027	3.541350477	0.258802171	8.212704114	4.083621358
17.33735723	3.109663897	0.322636928	7.346677707	2.896993492
15.4598491	3.038925838	0.320889007	7.403910128	2.776950376
8.559475437	2.686595879	0.369273365	6.984797685	2.181937618
18.91746679	3.201200393	0.289852565	7.903380867	3.230471575
4.785794061	2.337930084	0.421942645	6.122372619	1.592842277
23.33121949	3.304630839	0.302834841	7.807863025	3.39244203
12.30024456	2.922928317	0.345582433	7.359279005	2.528226363
10.46830515	2.817944193	0.35158475	7.017961289	2.3515455
11.58255065	2.823703262	0.370048503	7.041435663	2.360649434
6.370513421	2.471569194	0.410623176	6.031224861	1.754575738
17.23877778	3.170215323	0.284282229	7.894093486	3.161443206
18.49363662	3.183869373	0.306217808	7.726842247	3.110207439
14.57071491	3.012255787	0.335227547	7.386731056	2.730546714
15.37470745	3.077752811	0.305892801	7.633902963	2.92387867
18.70705581	3.132404839	0.324658508	7.646446849	2.920881474
11.90596702	2.921310279	0.328585532	7.48428013	2.580560316
14.06504618	2.894788353	0.376152043	6.860486786	2.499628306
7.999281328	2.629189145	0.392024247	6.522384495	2.055512311
11.47017747	2.868049153	0.351654873	7.09973766	2.388470068

log.sigma.3.0.mm.3D_glcm_DifferenceVariance	log.sigma.3.0.mm.3D_glcm_Idn	log.sigma.3.0.mm.3D_glcm_Idm	log.sigma.3.0.mm.3D_glcm_Correlation	log.sigma.3.0.mm.3D_glcm_Autocorrelation
15.76680857	0.907769424	0.252683069	0.490461849	542.9007971
3.880535422	0.918679192	0.394574608	0.554364768	130.0406255
4.490264837	0.925822791	0.385657524	0.618173364	163.7201151
10.66728422	0.91010613	0.286593665	0.531155953	333.472505
6.860833619	0.915820727	0.330011329	0.524021528	276.5069546
7.025781858	0.910636036	0.299870937	0.595594787	184.0152716
5.993549728	0.941990419	0.337720151	0.58325245	407.8678295
6.143984921	0.939158673	0.345939326	0.58535795	502.509436
7.907101971	0.930568489	0.311429654	0.532831382	507.2807867
6.377810558	0.927085863	0.310498938	0.573624994	477.5897091
4.105749564	0.90926877	0.353345471	0.536659318	166.5462891
7.081528031	0.925338676	0.313061514	0.559237087	393.8313757
5.390956816	0.941822727	0.450627602	0.61388362	302.7530769
11.15095767	0.928603282	0.316498442	0.533594793	575.1820698
11.32199512	0.924529401	0.290213004	0.455830284	441.4499187
7.591971425	0.925238943	0.329903534	0.544595308	326.6422074
7.272765542	0.908473097	0.309730972	0.49664797	246.1351958
4.763431151	0.916871673	0.399528163	0.584612578	124.3242745
3.20684576	0.916502113	0.392474855	0.553935258	137.6255715
5.988398036	0.928291978	0.318030648	0.598656768	343.1230489
3.364640574	0.940258546	0.442504062	0.642278136	193.4774369
3.471205079	0.902407273	0.342566114	0.559808281	127.2908397
4.858000089	0.921130871	0.361284786	0.567087305	171.6916834
5.001857645	0.92387197	0.351831005	0.547274375	206.7351471
8.364094775	0.928038267	0.322958569	0.447291488	385.2075983
9.686723459	0.930291622	0.283065255	0.561828903	560.6196557
10.37585166	0.925770211	0.301690948	0.529939708	372.7378152
7.341956102	0.928661765	0.33245013	0.486159519	386.6791879
2.965337102	0.906701535	0.351355715	0.59854233	123.6776609
4.211541715	0.92788929	0.386812798	0.549418262	227.9762728
7.112807762	0.93239309	0.34358066	0.534806107	411.0102021
7.158922888	0.930347575	0.376586757	0.422794957	349.4386521
9.959694231	0.918268447	0.289817299	0.538741227	378.0739951
8.860088423	0.91662488	0.319779941	0.53527789	363.941622
6.48490285	0.926815105	0.31106339	0.545163878	290.154641
5.74635025	0.935012603	0.365398867	0.547335444	374.2146479
8.401179158	0.947798781	0.38598749	0.503747854	626.5312545
9.368299916	0.942720722	0.387640821	0.610257451	563.0009975
3.218786451	0.91313272	0.386851284	0.401606893	151.6778095
7.318246612	0.916403747	0.289152411	0.596474265	279.7083695
8.643401094	0.925979495	0.353503609	0.488156684	324.8142946
9.932106652	0.915577171	0.239701264	0.529099857	339.7811731
7.977759285	0.91673575	0.293877152	0.503729643	352.963066
9.990717083	0.932126386	0.301129956	0.466095983	504.8287215
4.52589666	0.921133105	0.344643279	0.612222353	177.7595617
7.826968877	0.924198723	0.312940098	0.523644192	396.3223918
6.545036406	0.931543271	0.331297222	0.523623935	372.5716395
2.44289714	0.921188403	0.417433986	0.629208835	102.2918134
6.38293103	0.903502643	0.307039614	0.553105153	200.5028523
4.120520095	0.930512208	0.409755235	0.645339988	136.8099813
8.212085017	0.917719518	0.285879237	0.504757392	408.3729441
9.499721675	0.914691516	0.256248112	0.487902217	298.1209528
5.316123499	0.923868611	0.329012782	0.548404182	223.512919
5.623649834	0.90386035	0.313817453	0.514301509	133.081899
5.987906089	0.910984398	0.311845892	0.569947174	229.7475178
6.358038903	0.939966158	0.340394099	0.525903336	561.05434
7.488885677	0.913260506	0.288025513	0.52020488	346.2497056
8.150575836	0.931881817	0.343593985	0.527210931	413.8143929
7.195101443	0.914615446	0.323942004	0.428284287	268.1137626
9.06285996	0.93113222	0.334454467	0.521976974	494.7134756
6.617527844	0.939994365	0.391572861	0.557025498	255.8377755
5.734128921	0.928882391	0.328756863	0.550826334	379.3455644
7.485104329	0.928594894	0.317056929	0.552823755	469.4152212
6.31847941	0.93925314	0.33781611	0.609485638	507.0216145
3.904907785	0.910308622	0.352315096	0.546683202	134.0023315
7.124153086	0.940545827	0.37558888	0.556130289	455.6219586
5.415546295	0.937592276	0.424096745	0.424711952	317.8757676
8.541549047	0.93915929	0.331250351	0.55249262	496.3590139
6.823384638	0.937878845	0.332509085	0.5549685	497.8790774
3.799083398	0.929410255	0.404815267	0.543480092	221.4324355
6.401620221	0.919359559	0.327290239	0.540454385	315.5071119
6.544645136	0.938353068	0.348537067	0.609504737	429.4100611
8.319043862	0.929286728	0.331199028	0.518616604	488.2633722
11.86950359	0.902288492	0.255998051	0.438930295	356.5882253
8.285277799	0.913305151	0.271348403	0.543183722	272.8199452
7.496348084	0.918375706	0.358879983	0.509771	339.8213667
3.575559661	0.930276535	0.406390151	0.557366352	176.9432051
4.353227106	0.929038014	0.385556949	0.653543071	199.225518
4.536290676	0.921863787	0.357795952	0.534977384	193.0382298
11.78771368	0.916930671	0.263831634	0.553587151	470.1627807
15.37802169	0.908109743	0.260800597	0.508026232	497.507809
8.74420849	0.926843136	0.340597821	0.434770696	186.1005307
7.455110247	0.929228239	0.34138694	0.506092503	374.7166824
3.638163731	0.921309892	0.370605178	0.610608684	215.3254599
8.177147914	0.918318877	0.285418056	0.555052609	290.928587
2.193542544	0.938462846	0.464107063	0.692398796	211.216844
11.45456956	0.911687297	0.309328371	0.489905873	388.8707105
5.795778691	0.919940485	0.354193072	0.609954874	229.0017544
4.803508519	0.927034093	0.369345738	0.586488492	233.300374
5.869095715	0.933645097	0.375288564	0.556401485	359.059154
3.160350657	0.928139442	0.459647963	0.671296373	101.9586326
7.033860459	0.907547467	0.285340663	0.572617399	268.27391
8.615847113	0.923403839	0.303078145	0.486738076	400.0196243
6.927499924	0.926259141	0.336898831	0.515604854	375.0393609
6.628899828	0.913926532	0.302491085	0.504982144	293.5889574
9.937618106	0.933550289	0.348117438	0.574270454	525.4339583
5.089106187	0.920344685	0.334626977	0.627204799	209.6581712
7.566663445	0.924706317	0.388671627	0.564096893	335.5012855
3.653191051	0.919943451	0.398485305	0.530496407	195.0752238
5.616999898	0.932777238	0.376886429	0.548239144	270.5975891

log.sigma.3.0.mm.3D_glcm_SumEntropy	log.sigma.3.0.mm.3D_glcm_AverageIntensity	log.sigma.3.0.mm.3D_glcm_Energy	log.sigma.3.0.mm.3D_glcm_SumSquares	log.sigma.3.0.mm.3D_glcm_ClusterProminence
5.22859212	23.12304552	0.005419548	31.56426662	38940.44703
4.319857615	11.16451596	0.019057327	9.570200675	3553.003372
4.670317858	12.48679802	0.011934129	12.71913497	5622.911559
5.031462276	18.04554533	0.006674568	24.49517916	17453.00011
4.759723251	16.42535197	0.009527391	15.85224054	8428.110663
4.870788471	13.12611549	0.009140215	21.64468895	13669.66427
4.804267007	20.02386973	0.009875582	15.56802379	9743.984562
4.768129568	22.27692326	0.011088164	15.29508838	10564.54051
4.923751318	22.40520042	0.006865284	17.64626334	11278.72157
4.789558306	21.71183784	0.008427972	16.78341762	13989.72265
4.501518513	12.67996407	0.012034099	10.66111185	3674.421104
4.858244839	19.68783464	0.008867475	17.70884197	12163.54896
4.384427089	17.22203775	0.029173024	12.71648758	9066.372046
4.944949114	23.81481428	0.00905007	22.29623657	26488.86003
4.915589234	20.92974497	0.008521948	20.93215957	18514.47687
4.857917086	17.91187362	0.009727068	17.81952798	10989.95032
4.734765559	15.43845665	0.008490173	16.25239975	9155.243116
4.445025805	10.82602018	0.01793218	11.90457821	5334.121566
4.308522607	11.52885647	0.016468853	8.459879131	2072.008394
4.890569084	18.32997281	0.008279299	17.40945013	10858.3034
4.394871764	13.68720037	0.021917492	9.630077251	4287.383771
4.494638582	11.02428461	0.01107325	10.20858433	2893.58542
4.537293807	12.82992014	0.014374624	12.45808674	5675.807652
4.620097295	14.15081485	0.01066827	12.56255635	5430.256474
4.655401373	19.5887034	0.010919036	15.14847248	9391.562205
5.081370256	23.49727561	0.006449918	24.07927387	27310.11017
4.971835517	19.154704	0.008416743	22.26543761	22084.94816
4.68674323	19.5880812	0.010806135	14.6609536	8635.848491
4.353230443	10.85091556	0.014416084	9.868392511	2744.831055
4.44185563	14.9175802	0.015105837	10.01908859	4264.407757
4.734400441	20.20189203	0.0112578	15.26407122	10471.91995
4.297955364	18.66800532	0.018548418	11.98395253	8201.645736
5.062601005	19.29100144	0.006479314	22.86115789	18489.95653
4.88364321	18.9164833	0.009093192	20.07399718	16858.30753
4.829911641	16.86310843	0.007699512	16.56230142	8953.072773
4.565759223	19.27246879	0.014465812	13.2009301	8531.054887
4.559158321	24.97272118	0.017225145	15.00028675	13801.96417
4.957252854	23.55799972	0.016161682	21.74164642	20465.62297
4.02829631	12.21365217	0.018072782	6.231912938	1347.171723
5.079017612	16.43212595	0.005394145	21.87038534	13795.96042
4.623382126	17.91184621	0.013652313	16.38793228	13466.43943
5.159643931	18.27183752	0.004450533	26.07102881	24195.05316
4.886112394	18.70324221	0.006875507	18.15715533	10243.80848
4.85550832	22.37193769	0.008076503	18.80991403	14334.38591
4.645951967	13.00899176	0.011876722	13.96056788	7016.719651
4.83065087	19.79380447	0.008418423	17.61367924	13435.04909
4.688055893	19.24005643	0.011205432	14.65359808	9473.990719
4.256284338	9.861141336	0.020199406	7.959227788	2456.543039
4.820393241	13.82912901	0.008488083	16.67885965	8318.587606
4.512307169	11.34492878	0.017516217	12.52299368	6119.919811
4.875643262	20.1454775	0.007331852	18.30906191	12917.306
5.024712854	17.10986741	0.005365072	22.19943758	15339.84571
4.701264893	14.72522362	0.00960614	14.07568767	6476.358109
4.657046556	11.22046081	0.009638031	13.91728941	5694.78238
4.80103797	14.8586937	0.008056887	16.97467057	7517.918407
4.661845752	23.58773846	0.011710088	13.70926738	8260.207621
4.907553154	18.46531028	0.006700019	18.67257779	11121.3781
4.710089101	20.25609405	0.011915578	16.2782315	14607.39781
4.601206526	16.2276615	0.010701196	13.74503465	6184.478278
4.937834495	22.10913676	0.00778593	18.47002268	12646.46828
4.551609524	15.81813426	0.018141763	14.50638355	9657.458106
4.717023032	19.37482529	0.010010861	14.47370302	7632.456783
4.880663234	21.52025881	0.008512124	17.77263414	13539.98785
4.853328329	22.35034249	0.00911376	17.34379282	13020.5228
4.493658223	11.32023214	0.011823627	10.54971747	3420.336593
4.648429586	21.22879443	0.014775684	14.56515633	13160.86281
4.188260635	17.75860266	0.023579365	8.731103084	4105.891336
4.90334843	22.13860844	0.009469106	18.77751221	15855.05715
4.814167279	22.19481144	0.008937627	15.60181013	11485.97722
4.358762192	14.71619869	0.016641482	8.858367605	2861.42419
4.686879365	17.58267772	0.010152008	15.62062948	10912.19478
4.828287691	20.55634891	0.010755601	16.56523356	12401.66119
4.756854753	21.98805836	0.010061101	16.76339796	16323.39895
5.006653196	18.812457	0.006258798	24.07112975	17149.49683
5.041498184	16.27670263	0.005518854	22.21814267	14981.64883
4.584658129	18.25020606	0.013150139	15.01271498	11279.63252
4.36513085	13.11292854	0.01664711	8.803547695	2932.636632
4.603979566	13.81230008	0.015714544	13.72258707	8463.290371
4.512938242	13.6817791	0.012795009	10.99854279	4241.243433
5.137010784	21.44785601	0.005831778	28.04965046	29672.61861
5.204722272	22.11665581	0.005847239	31.33962735	40455.90555
4.643960918	13.43983419	0.011464243	15.68301629	9344.681
4.724083466	19.2831259	0.010843291	15.49703352	10342.04947
4.549854631	14.44188098	0.012492191	11.01861011	5027.096019
5.016189843	16.83180203	0.006475115	21.59175111	15797.47599
4.211780597	14.3429318	0.02580468	7.8844877	2697.024132
4.908033036	19.6048684	0.008856529	22.71500548	20497.69539
4.783514993	14.81913654	0.010938721	16.08703275	8825.729391
4.616551776	15.03390337	0.012645754	12.85431044	6401.889519
4.604528504	18.84370455	0.013463658	13.22560361	8337.082192
4.150634577	9.758388702	0.031894662	9.900354914	3429.441237
5.000950271	16.07920117	0.006036053	20.7405999	12170.58862
4.829115722	19.93571317	0.00809353	17.4888662	13078.7872
4.710227353	19.26786733	0.01066378	15.19495172	9498.714271
4.783501481	16.94508814	0.007596772	15.82921083	7368.88166
4.965483927	22.73912926	0.010614797	21.28266588	21778.53828
4.84504816	14.14044819	0.008998667	16.16832912	9206.941061
4.52921474	18.11980453	0.016358867	16.26334711	14638.72639
4.31136442	13.80225735	0.016983712	8.569705698	2766.550317
4.582077489	16.30874614	0.014446086	13.14191879	5151.404938

log.sigma.3.0.mm.3D_glcm_SumAverage	log.sigma.3.0.mm.3D_glcm_Imc2	log.sigma.3.0.mm.3D_glcm_Imc1	log.sigma.3.0.mm.3D_glcm_DifferenceAverage	log.sigma.3.0.mm.3D_glcm_Id
45.90520627	0.808458298	-0.12353657	4.201638096	0.337312736
22.32903193	0.805162608	-0.155173677	2.095018135	0.46273078
24.96790494	0.833831166	-0.163486664	2.231199279	0.455920267
35.81199226	0.864054023	-0.164807844	3.434525695	0.368842364
32.76523402	0.760129504	-0.114236561	2.775683986	0.408598346
26.17538152	0.891218783	-0.199222368	3.056542994	0.383888539
39.93000601	0.751207857	-0.111992699	2.655058803	0.416218564
44.41684009	0.766117883	-0.117940979	2.602135732	0.422239051
44.61046672	0.746732711	-0.106894915	3.016916073	0.392408827
43.2528026	0.839320352	-0.1557204	2.835396894	0.393606973
25.35992814	0.75057856	-0.120504892	2.354691409	0.429927008
39.18203007	0.781196642	-0.121409884	2.92140419	0.394341245
34.35900544	0.849922587	-0.187493531	2.002091973	0.508241509
47.45421777	0.845903604	-0.155770276	3.159613009	0.395166364
41.54832756	0.786619127	-0.121097676	3.50662842	0.371409001
35.61612649	0.765374697	-0.117098229	2.862804831	0.407742597
30.85944723	0.783379227	-0.125728483	2.943023777	0.391227339
21.65204036	0.824203396	-0.164939596	2.169973305	0.466203969
23.05771295	0.759896302	-0.129857223	2.045309447	0.46341599
36.48070724	0.755462422	-0.111650473	2.780270262	0.399800967
27.36906681	0.809234493	-0.15963488	1.823151473	0.503185527
22.04856922	0.769628452	-0.127291911	2.310809288	0.422656618
25.65728309	0.818488669	-0.153496504	2.384396036	0.435907969
28.27838216	0.751068877	-0.115533309	2.460538196	0.428368631
38.89883409	0.801085744	-0.13823157	2.913265993	0.402161089
46.75992064	0.817359832	-0.137803666	3.346234215	0.367604011
37.9855157	0.789542003	-0.121967515	3.275686036	0.382811355
38.95765672	0.739916348	-0.106826097	2.793876689	0.4100546
21.70183112	0.835108494	-0.171103592	2.187655042	0.430220661
29.83148213	0.761586906	-0.127063565	2.15824225	0.456546295
40.13131374	0.748024854	-0.111295025	2.711310987	0.418728916
37.1218253	0.717475289	-0.106031764	2.511296714	0.445485718
38.24008296	0.776247053	-0.115156461	3.354243409	0.372717531
37.59260323	0.791576422	-0.127063121	3.055589095	0.398308984
33.53976013	0.736569597	-0.10340853	2.848659788	0.393705786
38.31355586	0.772847466	-0.12821953	2.442432276	0.438512568
49.75727711	0.778871654	-0.130721157	2.533760874	0.452883714
46.88540077	0.850376358	-0.167389639	2.743892147	0.455355022
24.42730435	0.639101435	-0.085459821	2.037996129	0.458377012
32.67637833	0.740181642	-0.101477868	3.114692801	0.37463426
35.60516297	0.759782111	-0.117298591	2.779322937	0.425620871
36.10909571	0.738008991	-0.096587074	3.819637149	0.33031161
37.09484977	0.733416418	-0.102205769	3.118431709	0.378421006
44.53155898	0.75715514	-0.109575294	3.262033162	0.382074142
26.01278194	0.810309365	-0.146797921	2.45706388	0.422493545
39.34245283	0.772303248	-0.11742739	2.984280774	0.393503398
38.19681697	0.72009779	-0.10109395	2.733208273	0.409323351
19.72228267	0.817657524	-0.168741291	1.818584875	0.483336186
27.65825803	0.845738592	-0.159833792	2.881162251	0.3899534
22.68301744	0.842147829	-0.180464877	2.077788353	0.475445357
39.94815576	0.753220248	-0.107689895	3.164958913	0.372097384
33.90730726	0.723711871	-0.092641991	3.582366245	0.345094071
29.38711436	0.712464237	-0.098279758	2.625375225	0.409068899
22.44092162	0.788843989	-0.13180846	2.750495433	0.396541246
29.67180702	0.812623378	-0.140724426	2.826645479	0.394074615
47.05463194	0.782844648	-0.129984388	2.632302967	0.417479527
36.69794094	0.715992441	-0.093914355	3.150539063	0.373600696
40.24837579	0.765207887	-0.118182189	2.751674424	0.418550301
32.39285403	0.734279028	-0.10559505	2.824264888	0.403114956
44.03282208	0.739340828	-0.102653803	3.013344712	0.410441623
31.48993977	0.819563301	-0.15305301	2.39248099	0.458839587
38.54105369	0.754773086	-0.113359746	2.677311855	0.408450956
42.86178926	0.738209619	-0.10232658	2.946930846	0.397079888
44.55254848	0.769338133	-0.121773333	2.664845562	0.415470059
22.64046427	0.798050718	-0.146746005	2.319826177	0.430280006
42.2958744	0.781551648	-0.128828793	2.501360433	0.445068504
35.45428003	0.756827166	-0.129995645	2.081623736	0.486245724
44.08060398	0.758981499	-0.112483586	2.895090485	0.408211255
44.21838711	0.743984042	-0.108537017	2.735262517	0.41100399
29.43239738	0.733706792	-0.118956777	2.027773001	0.471060903
35.06049507	0.813134635	-0.143791156	2.731499902	0.407157699
40.93846192	0.804488434	-0.135854371	2.581325089	0.424337983
43.78260651	0.788842341	-0.127723279	2.860700002	0.408729972
37.20474713	0.729892377	-0.094499702	3.87243773	0.342355745
32.31321306	0.756200335	-0.106780873	3.346741721	0.358591787
36.455863	0.795098686	-0.138595295	2.621821531	0.432218076
26.22585708	0.74756885	-0.125608738	1.9957395	0.47346056
27.59648732	0.8668226	-0.190967502	2.201465969	0.455036336
27.35998343	0.753759859	-0.118961801	2.351431165	0.433736254
42.62279076	0.795225936	-0.120939396	3.703332861	0.350481118
43.8614892	0.822013734	-0.131259783	4.083621358	0.344513124
26.78907798	0.765649092	-0.117456009	2.896993492	0.415554614
38.30772305	0.761489077	-0.119788354	2.776950376	0.417909242
28.88376197	0.748385508	-0.122674877	2.181937618	0.444441482
33.41228236	0.778895171	-0.118991933	3.230471575	0.370419485
28.68586361	0.829637445	-0.1827145	1.592842277	0.520897652
38.86969268	0.771985808	-0.116289872	3.39244203	0.386533445
29.61124409	0.807601228	-0.139235342	2.528226363	0.42868295
30.05133329	0.812567625	-0.149186701	2.3515455	0.442412218
37.5110499	0.795582435	-0.14005969	2.360649434	0.445761599
19.5167774	0.837689936	-0.193120507	1.754575738	0.515692884
32.04176367	0.774861244	-0.11459568	3.161443206	0.371192911
39.56155037	0.699396731	-0.088905212	3.110207439	0.384753314
38.32529665	0.74189321	-0.110512512	2.730546714	0.413593385
33.80704985	0.685793223	-0.086610377	2.92387867	0.386508355
45.28841316	0.830664727	-0.147601731	2.920881474	0.421397361
28.25696991	0.791115873	-0.129632375	2.580560316	0.414087641
36.14084214	0.844175197	-0.170556594	2.499628306	0.453647323
27.60451471	0.788178275	-0.146197387	2.055512311	0.465661232
32.47308293	0.733186627	-0.111255678	2.388470068	0.447918015

log.sigma.3.0.mm.3D_glcm_ClusterTendency	log.sigma.3.0.mm.3D_firstorder_InterquartileRange	log.sigma.3.0.mm.3D_firstorder_Skewness	log.sigma.3.0.mm.3D_firstorder_Uniformity
98.17933459	146.7920866	-0.913243089	0.060222737
29.90848442	85.42516518	0.52509398	0.109542738
41.03584269	117.7910242	0.149252612	0.081682642
74.09906919	155.609787	-0.474603674	0.062612032
47.17372648	109.4847803	-0.369467159	0.081443814
67.61127191	141.534787	0.512910747	0.07161157
50.2314444	118.8615019	0.2406016	0.079041483
50.15845393	110.0706787	0.016722351	0.082455607
56.29886571	134.7197056	-0.323229963	0.069215715
54.06295017	132.3462615	-0.581224267	0.072578456
32.83998623	100.0095775	-0.139461448	0.09026395
55.75916572	123.7678165	-0.306386232	0.075258257
39.96412054	67.36379814	0.073753204	0.129473111
70.16921967	119.2831483	-0.624847595	0.075631706
63.46139079	111.6344974	-0.49586897	0.075690707
54.14404401	123.016408	-0.054954428	0.07455679
48.413817	125.7432852	-0.738913218	0.07804657
38.00955849	80.29811096	0.710253706	0.105455607
26.33735582	89.89285171	0.34198176	0.105745238
55.50331376	126.0147376	0.065375817	0.071625734
31.57766172	85.58330464	0.319782497	0.110205438
31.91990132	108.2508087	0.041871618	0.088178479
39.07795056	102.3203253	0.172030552	0.092496872
38.44548246	109.722868	0.186496574	0.0846879
44.79622159	116.4205647	-0.330899422	0.081022308
76.87593545	137.8655319	-0.295138383	0.064641232
69.45531299	123.7299604	-0.267298381	0.070213424
44.3082681	104.7502774	-0.335519316	0.085071213
31.59839769	108.585346	0.458208762	0.098068656
30.97693345	84.64782286	-0.298671886	0.103375369
48.14554792	112.7820821	-0.176318666	0.081488649
33.36576463	69.82456183	-1.163550364	0.115311039
71.46492223	139.2131977	-0.369009286	0.063198075
61.00818237	124.6921617	-0.684415599	0.072297691
50.41290663	130.7877269	-0.190065367	0.071862567
40.75374556	107.8968487	-0.212811703	0.090612509
45.37388399	79.09515131	-0.649759638	0.10474835
70.93936658	116.6817664	-0.168480293	0.084288469
17.47014912	81.2544083	-0.681622604	0.121032454
68.33059172	153.6839857	0.061086513	0.060616579
48.1193802	86.77168304	-0.932033353	0.095516966
80.23184851	159.8643284	0.24486247	0.057825129
53.91498209	138.4236012	-0.280510891	0.067955876
57.14129062	116.252104	-0.48698308	0.076126177
44.95767297	110.190073	0.371135422	0.084340507
54.36099827	125.7813721	-0.213492349	0.072362202
45.42889923	109.4148369	0.078452474	0.081793905
26.00593651	85.71301651	0.528725436	0.112689665
51.84128343	121.9068077	-0.227741083	0.073712961
41.22402949	119.7466118	0.642107879	0.094985295
55.99004587	135.8635387	-0.50150054	0.068369294
65.3540761	146.2205958	0.005620759	0.062767822
42.67533607	104.8120249	-0.036407163	0.083361725
42.18826798	128.9385853	0.401470354	0.0784056
52.56077383	146.4911618	0.209463835	0.07093674
43.46916216	96.3323226	-0.489478248	0.088958317
55.91874224	137.2782669	-0.334740025	0.067016705
51.22756341	97.29222858	-0.896898898	0.087295083
38.47904714	99.76639676	-0.495488738	0.088073805
58.2936049	131.0819302	-0.282918335	0.071265849
44.16454122	85.00959253	0.725808998	0.100420168
44.9657244	118.3351293	-0.213324194	0.08005639
56.81085358	127.0776596	-0.18260687	0.074517253
56.7465917	115.1858611	0.294051059	0.076698178
32.7482617	113.2664323	0.181533584	0.085374139
47.08388299	94.39972705	-0.302002346	0.095014671
24.35142074	65.14671216	-0.846711735	0.130276356
59.41215662	106.4047632	-0.137013944	0.078894899
50.70875568	118.8077221	-0.52674982	0.075217387
27.42722676	90.6710124	-0.082835887	0.103227709
46.94518636	113.0951927	-0.533564966	0.082248973
54.97721999	120.3583608	-0.058029473	0.078861993
52.81387926	110.9036617	-0.904143755	0.081421245
69.4049063	148.6203861	-0.550935996	0.062510276
66.882742	150.8557262	0.23275208	0.061858517
44.82160022	93.89180183	-1.322527467	0.095885315
27.56834848	88.82417393	0.215690892	0.101834304
44.65319547	95.6452167	0.416712427	0.096218636
33.73865881	95.86052847	-0.015092886	0.092513716
89.76677312	143.7753563	-0.824750034	0.064172193
98.70626657	141.8891239	-0.93480842	0.06113539
44.0735901	98.8848505	-0.03450472	0.085320391
46.81864585	117.1352625	-0.127273954	0.080113053
35.514965	97.98526192	-0.538509331	0.093011373
66.1827936	137.8666539	-0.262241368	0.065030281
26.75215674	70.86911607	0.424879547	0.125112483
67.92962008	122.5210533	-0.835709486	0.0720789
51.33067736	131.0921736	0.010955591	0.076984648
40.52685392	101.7508512	-0.275872641	0.0893549
40.70177103	95.8112793	-0.562757344	0.090885414
33.23090623	91.27370906	0.948593395	0.127513503
63.58344682	134.8411484	0.04167535	0.065611735
53.44125749	115.9686995	-0.68096051	0.075360688
45.86263119	106.5220604	-0.460020482	0.083002755
46.81247863	125.0278602	-0.444236609	0.075175988
69.29485815	120.2114501	-0.532847148	0.075810899
52.05369995	122.4440384	-0.003493481	0.075068216
49.84712715	79.77847338	-1.362673963	0.104816691
26.27954146	84.31397796	-0.632242191	0.107997056
39.34497913	107.7627637	0.374103233	0.092509283

log.sigma.3.0.mm.3D_firstorder_MeanAbsoluteDeviation	log.sigma.3.0.mm.3D_firstorder_Energy	log.sigma.3.0.mm.3D_firstorder_RobustMeanAbsoluteDeviation	log.sigma.3.0.mm.3D_firstorder_Median
103.9195743	45548121.39	64.57486715	56.41321373
58.88620268	10148274.48	36.17092746	55.74443436
71.55297293	21830638.77	48.47183431	68.73027039
97.2999506	16351939.39	65.88098052	51.36367416
72.7135028	27855290.2	46.17411935	49.2592926
90.18940236	12056180.35	60.18228571	49.95099449
77.10665589	82716934.09	50.88234699	54.79553986
76.69604435	82163732.86	48.44794672	61.69963837
82.41970641	76287776.14	55.83036272	75.7438736
80.34096302	19253186.94	54.26631845	81.73307037
64.17519903	14651679.59	43.20301214	50.12731552
81.97456093	43234542.35	53.58918846	74.77468872
58.87000403	17746309.86	31.01887185	30.73645973
84.57271988	30160929.83	50.48942447	53.41445541
83.46597786	33845014.75	49.8218533	65.46077728
82.09527806	66289597.77	54.00605189	49.83741951
78.0259144	14895522.61	51.92288393	65.59177399
63.9221104	13271053.66	38.77347926	41.5622406
57.45186134	10361576.71	38.75727824	40.74809456
82.95603375	90074223.03	54.47449466	65.62826538
59.16892163	29039175.66	37.06808133	45.25657082
64.58632045	11734529.71	44.82369958	60.2468605
67.96664501	12086806.13	42.99994828	50.6027298
68.80617877	31564023.75	45.9500672	56.87274933
76.33817278	21037000.31	48.61911829	68.48974609
92.64134399	52836423.51	58.50691276	79.98931885
89.25034157	62577965.39	54.45065152	63.46143341
71.76243844	33933048.8	44.61222517	48.34454727
63.23282014	4499923.119	44.36505344	46.57966232
58.04515471	21143983.1	35.94180562	64.01677322
75.5172616	71983599.81	48.47662045	61.96547699
58.59653295	16853676.95	32.04020262	33.08096695
94.03978184	54673622.58	61.74066874	53.52703476
85.34768969	38981386.44	54.30498468	61.68148613
80.45149731	79017866.93	55.02672147	82.71992493
70.98966335	41056999.26	46.2349201	57.70758057
65.05244033	40296163.63	35.15446335	33.352108
85.16082145	83205723.62	52.57267323	37.42066574
48.28236995	6926960.038	33.16523846	46.30999374
92.71280862	114123093.9	64.22010998	44.66346741
70.59755321	29164541.66	39.25400695	41.81946945
99.32139004	69678986.87	67.14628304	41.12604523
84.20920711	63165131.42	57.38845889	74.33369446
80.09417238	35179001.68	49.94028518	57.79608727
73.83116102	22873296.77	48.19646021	78.62030029
81.6362209	50460907.94	52.7554818	78.76181793
75.64793191	69138115.64	47.86085486	59.17946434
55.64014243	8023264.948	35.59982024	51.11087036
79.56903467	12691680.38	52.3334207	69.31025696
71.89252003	18658842.67	50.18364422	52.52007484
86.27928942	53561982.03	57.56612127	87.96469879
91.20474156	59848916.19	60.93718449	62.89406967
70.21290255	52387512.06	45.22024407	52.48421288
75.6029824	19520251.72	52.35468654	80.40045166
83.23852595	23054655.51	60.12646745	68.35902786
68.79851537	24071829.58	41.94691357	57.12143135
85.62080361	70297609.57	57.23452113	73.19095612
73.37759523	44557503.66	42.51310078	49.36827469
68.88475937	17165092.21	42.40263823	44.60989189
83.39808249	120836891.6	56.21416057	36.5581131
69.8097248	22543076.58	40.62458265	41.83430099
75.06344095	39130919.36	50.49246971	63.30675316
82.00139924	102559555.7	54.67346791	68.4588089
79.49202154	106681072.2	50.53914553	42.2296524
67.83986763	10075202.05	47.17296022	68.33010864
69.62825706	46659632.03	41.19345973	38.38228989
49.80990112	8076681.599	28.16468877	29.69340706
78.62364015	84825955.86	47.24196043	43.86502647
78.69969496	86888341.1	50.37461325	68.24833679
58.34361975	26502521.74	38.33631678	49.52166557
72.78941095	15625993.21	46.85553444	48.85412979
80.00483851	76476161.85	51.73777997	68.3246727
76.80197551	35549106.97	46.93954483	66.14527893
97.87308254	37855018.89	64.61806179	46.34937668
92.27944686	49271523.64	62.66758795	36.91590881
67.6417613	14700899.23	40.3464139	60.12298775
58.39950756	24898879.54	37.5362511	59.76226997
68.28169655	11613030.74	41.90501232	45.24749947
64.64221464	18170293.84	41.86537257	51.3262825
100.497772	42139749.44	64.52428066	51.84906006
103.7962306	42959598.64	63.81189095	56.29119873
73.22622948	19401276.55	44.10653122	29.80688095
76.3829268	47268236.14	49.58355173	58.12091064
63.39683949	29021419.37	41.10302572	56.651474
89.3092719	40307096	59.12501556	60.2491188
52.90356412	11327535.38	32.61923711	35.78318214
88.74118203	36195618.16	53.52717419	37.67953491
80.35724319	49294759.69	54.89108263	72.03277588
67.85296777	18789376.22	43.43524937	63.11332893
68.04628055	24725415.17	41.34117537	55.67512894
64.42624659	13327571.64	42.7882753	32.29890442
87.05778593	46557650.58	59.06155036	80.28959274
79.95207761	68509673.92	49.87931988	45.74972916
73.23025166	48230892.51	46.05120744	66.63674545
77.52562689	48060030.13	52.37012118	45.52788734
85.68312733	59401763.23	52.89827333	51.94076347
78.13196825	34651457.24	51.27219334	49.01515579
67.44945484	10260627.29	36.43808189	26.23974991
54.86742078	5353799.493	35.35922004	43.18123436
70.26482553	75477208.98	46.40881532	52.22208405

log.sigma.3.0.mm.3D_firstorder_TotalEnergy	log.sigma.3.0.mm.3D_firstorder_Maximum	log.sigma.3.0.mm.3D_firstorder_RootMeanSquared	log.sigma.3.0.mm.3D_firstorder_90Percentile	log.sigma.3.0.mm.3D_firstorder_Minimum
1229799278	404.5023193	146.5084066	202.3849045	-500.2509766
274003410.9	341.6274719	104.1814259	176.0366241	-196.1173248
589427246.8	415.8430786	119.2945331	194.7475586	-215.2949677
441502363.6	383.3768005	129.3711243	184.4826813	-389.0366211
752092835.4	336.2234802	108.4583504	170.4152008	-342.2017212
325516869.5	451.8030701	135.1552438	241.2072296	-244.4421844
2233357220	579.2668457	120.9858446	202.4430389	-402.2584839
2218420787	467.6286316	126.8278519	212.6474564	-471.0213928
2059769956	455.1578674	129.5426455	207.17202	-461.5364075
519836047.3	392.5494385	134.0151575	217.9110168	-443.2217102
395595348.9	291.9234924	98.5696094	164.8836456	-242.6888275
1167332644	433.8617859	136.9556547	224.8693054	-383.374176
479150366.2	367.5284119	97.49489393	146.3517883	-358.3380127
814345105.3	413.1419678	132.5370243	202.8105835	-522.1710205
913815398.3	541.2356567	130.6100272	193.3346695	-430.3246765
1789819140	425.822113	123.0790216	205.5016632	-363.89151
402179110.6	348.5778503	111.6929365	160.4895218	-317.910614
358318448.9	349.9364624	103.745953	190.5691345	-187.340332
279762571.2	295.4604492	89.34609527	156.4045151	-206.8579559
2432004022	459.8383789	131.6255969	221.4924866	-350.3973083
784057742.8	390.0675049	97.99439782	166.7976654	-250.1364288
316832302.3	286.4005127	104.2850664	173.8077606	-190.6666107
326343765.6	386.9621582	108.6440132	188.0357361	-237.6629333
852228641.1	416.3435974	109.199138	181.4377625	-273.4483948
567999008.3	458.951355	128.5516646	207.4706055	-390.0658264
1426583435	534.8945313	149.3427609	231.7289185	-476.2721558
1689605066	544.2665405	139.1258616	212.6329987	-389.1066284
916192317.7	407.9126282	110.8407878	175.2595673	-423.3310242
121497924.2	294.1412659	100.3341522	182.1260864	-186.4705353
570887543.7	341.0658569	101.4596084	155.7649704	-291.2584839
1943557195	435.9596252	122.9998443	199.1908539	-416.3695068
455049277.7	334.6472778	90.25413063	111.2646088	-420.9969788
1476187810	436.5235901	130.1232503	193.4872833	-412.9362183
1052497434	362.0513	128.7937266	196.5709885	-396.7625427
2133482407	509.919342	133.948852	218.1596542	-318.0326233
1108538980	414.7380676	117.0052556	196.3057709	-391.976593
1087996418	486.3632202	104.0643595	140.22052	-573.9535522
2246554538	521.0042114	126.9725002	207.5151672	-514.5535278
187027921	231.9655914	75.04448566	111.7212654	-243.5847015
3081323535	444.2929077	125.2909098	197.6589706	-328.1545105
787442624.9	393.1696472	106.0333767	133.9532852	-386.4071655
1881332645	554.1220703	134.2344005	195.4121429	-387.2310486
1705458548	414.766571	131.4964008	213.4054413	-368.3927612
949833045.3	526.3787842	118.9095039	167.3001663	-475.5657349
617579012.8	440.4916077	133.7823916	240.3540176	-218.9632416
1362444514	426.4898376	135.5833486	214.0698975	-376.4992371
1866729122	469.5866394	123.106541	200.8253555	-389.4930115
216628153.6	305.3111877	95.97684941	159.9003448	-160.7104492
342675370.2	344.6506042	122.1222461	191.0677338	-254.0369873
503788752.1	439.7142334	117.4773133	199.8547073	-189.354599
1446173515	411.0914612	144.3930425	228.4403137	-387.7217102
1615920737	548.0421753	133.2443396	208.7513885	-337.8738403
1414462825	440.6134949	107.7529928	175.5904099	-298.9541931
527046796.4	421.238678	132.9713782	225.9889999	-172.1426849
622475698.8	389.391571	131.6598931	223.4416626	-265.6196594
649939398.7	431.6274414	111.1059314	178.5914688	-501.5198364
1898035458	376.8646545	131.8290176	209.5799072	-352.8514404
1203052599	405.2653503	113.5136089	158.7529663	-428.0719604
463457489.7	347.2671204	102.0573922	150.6201233	-347.2292786
3262596074	445.1760864	115.0504096	177.1837677	-484.6817627
608663067.8	525.2113647	112.9825165	191.6041641	-308.1778564
1056534823	411.0186157	121.0610385	201.9529083	-395.8535461
2769108005	438.5091858	133.1025239	221.6480042	-442.9143677
2880388948	450.2845459	118.2989053	203.1084534	-492.1632385
272030455.2	327.1645813	112.9310423	183.6806656	-177.6009064
1259810065	432.0230408	109.9169123	172.6931763	-463.1314087
218070403.2	310.413208	78.70053515	108.383181	-380.5997009
2290300808	536.65448	116.4995834	171.6271835	-497.8594971
2345985210	495.8709412	121.7988368	185.2572113	-465.3862305
715568086.9	301.3717651	95.63005579	159.8196381	-287.3802185
421901816.7	341.2979126	109.6779287	169.8922913	-356.7473145
2064856370	495.3954773	133.5163329	222.8559357	-401.024292
959825888.1	396.3827515	124.8941754	184.5929718	-468.839325
1022085510	380.3687439	129.450466	178.3525635	-424.9251709
1330331138	466.3070679	125.4458973	196.4701538	-326.3911438
396924279.2	264.2732849	104.6636057	145.1000778	-397.1747131
672269747.5	355.1312866	101.940438	168.8022995	-246.3983459
313551830.1	378.278595	110.1006881	175.7967697	-256.1995239
490597933.7	366.5587158	103.1725619	175.8167267	-256.0227966
1137773235	446.0644226	134.2242122	164.166571	-482.2111816
1159909163	404.5023193	146.9645929	200.6963745	-482.6848755
523834466.9	536.8029785	103.848343	131.9404236	-289.6785583
1276242376	437.3031006	120.2478273	193.4257721	-396.1162415
783578323	298.1611633	97.18025725	147.3388977	-288.2420654
1088291592	495.4663391	128.5537811	200.2966217	-339.9137878
305843455.1	261.0871887	85.98809566	155.9007324	-281.6944275
977281690.3	334.5190735	123.1923691	158.4612915	-437.5036621
1330958512	396.6116028	134.0808183	228.6887985	-274.7489014
507313158	381.3692932	113.2884521	188.7677658	-292.7407837
667586209.7	364.118927	110.4992379	172.3639069	-388.2623291
359844434.4	325.5603027	101.0966389	197.1925522	-174.6983643
1257056566	416.289917	139.8056264	235.9568405	-285.2705994
1849761196	403.5219116	114.9702156	162.5025635	-429.3480225
1302234098	377.0262451	120.8942595	194.9426254	-376.2444763
1297620814	331.555481	105.0866569	151.7712067	-366.4605408
1603847607	441.6829834	131.752743	208.4969955	-476.4846191
935589345.5	394.6156921	110.9091375	168.6671661	-299.329895
277036936.9	253.0313721	98.99508103	108.5482529	-411.6335449
144552586.3	230.0640106	80.12130711	114.7256676	-276.5813904
2037884642	432.2870789	114.3324543	201.6578308	-315.0462952

log.sigma.3.0.mm.3D_firstorder_Entropy	log.sigma.3.0.mm.3D_firstorder_StandardDeviation	log.sigma.3.0.mm.3D_firstorder_Range	log.sigma.3.0.mm.3D_firstorder_Variance	log.sigma.3.0.mm.3D_firstorder_10Percentile
4.406254779	140.6483424	904.7532959	19781.95622	-147.3185898
3.584917013	79.265433	537.7447968	6283.008869	-16.16106453
3.89085173	91.90402481	631.1380463	8446.349776	-33.33346024
4.287318436	124.8256229	772.4134216	15581.43613	-147.9847717
3.95775432	97.48642881	678.4252014	9503.603802	-67.53399811
4.133390012	115.6726397	696.2452545	13380.15957	-53.32676506
4.001470688	100.6682559	981.5253296	10134.09775	-42.26044464
3.996358317	102.9605267	938.6500244	10600.87006	-30.70667286
4.118346954	106.335588	916.6942749	11307.25727	-56.50777435
4.044613667	105.128411	835.7711487	11051.98281	-26.97059498
3.740453334	82.83549696	534.6123199	6861.719556	-48.22619362
4.082109809	108.3177381	817.2359619	11732.73239	-34.60000076
3.563504796	88.436716	725.8664246	7821.052737	-32.68708191
4.156171877	119.3053875	935.3129883	14233.77548	-65.79515076
4.138005565	115.9107101	971.5603333	13435.29272	-89.74009781
4.089637981	107.3491343	789.713623	11523.83664	-64.70521545
3.955242983	100.3039891	666.4884644	10060.89022	-87.75565338
3.667451367	86.29760017	537.2767944	7447.275795	-31.19517326
3.53983337	73.2216347	502.3184052	5361.407789	-36.67224197
4.126485807	108.3587575	810.2356873	11741.62032	-53.67528229
3.601809116	79.33209434	640.2039337	6293.581192	-23.62376308
3.73053826	81.17625493	477.0671234	6589.584364	-36.03981476
3.81378778	90.45018879	624.6250916	8181.236651	-32.78712692
3.855663198	88.91497717	689.7919922	7905.873164	-39.993293
3.990926612	103.5290362	849.0171814	10718.26134	-32.03763657
4.305661588	125.3797103	1011.166687	15720.07175	-55.76544418
4.240271597	122.1442087	933.3731689	14919.20772	-78.06571045
3.939169143	97.77734462	831.2436523	9560.409121	-56.75859909
3.589488647	79.11278012	480.6118011	6258.831978	-26.57719574
3.643599287	79.22996688	632.3243408	6277.387651	-25.93178596
3.987129717	101.0797996	852.3291321	10217.12588	-39.19957657
3.60961215	86.85391152	755.6442566	7543.601947	-69.49884644
4.295234917	122.7569585	849.4598083	15069.27087	-132.7646118
4.136143992	114.7454913	758.8138428	13166.52776	-88.879879
4.066250992	102.9086776	827.9519653	10590.19592	-38.76738548
3.848504095	94.39779528	806.7146606	8910.943753	-22.43250923
3.782101869	98.22132462	1060.316772	9647.428609	-54.7874794
4.080208298	115.85274	1035.557739	13421.85736	-76.43930817
3.315469618	62.28951864	475.550293	3879.984133	-35.09231148
4.266198887	116.6887348	772.4474182	13616.26084	-105.080703
3.87088251	101.8450815	779.5768127	10372.42062	-98.48843307
4.376596684	127.6061066	941.3531189	16283.31843	-122.9404495
4.133364383	107.8779887	783.1593323	11637.66045	-54.85066986
4.078096218	108.6680773	1001.944519	11808.75102	-94.82935715
3.905688664	96.48731554	659.4548492	9309.80206	-13.00028019
4.116564843	108.237558	802.9890747	11715.36895	-41.0157959
3.994871999	101.5040607	859.0796509	10303.07433	-39.02904625
3.505184508	73.28616366	466.021637	5370.861784	-18.34608269
4.042737247	103.1227545	598.6875916	10634.30249	-67.83564758
3.759035698	89.95922194	629.0688324	8092.661612	-16.08050709
4.163118588	113.0221046	798.8131714	12773.99613	-40.96107788
4.270435281	118.1320828	885.9160156	13955.18899	-91.00354004
3.906998403	92.75616443	739.567688	8603.70604	-55.89290924
3.927574489	95.23314726	593.3813629	9069.352337	-15.58076334
4.037448592	102.10911	655.0112305	10426.27034	-34.60336266
3.884806539	95.4151523	933.1472778	9104.051289	-55.76055374
4.167290104	110.607526	729.716095	12234.02482	-67.07127838
3.954646442	104.6330595	833.3373108	10948.07714	-74.70137024
3.870549674	93.79181429	694.4963989	8796.904427	-71.12127991
4.112575626	107.6527148	929.8578491	11589.107	-82.23647919
3.818703454	97.0640624	833.3892212	9421.43221	-42.84324074
3.956995277	97.5564851	806.8721619	9517.267785	-38.83308067
4.081131749	106.8587994	881.4235535	11418.80302	-37.63133392
4.054867964	106.0013646	942.4477844	11236.2893	-61.52934036
3.787249901	85.09951884	504.7654877	7241.928106	-33.62661476
3.864634849	98.61553653	895.1544495	9725.024045	-44.91811104
3.415320967	73.34630788	691.0129089	5379.680879	-49.85346336
4.085106587	108.9209305	1034.513977	11863.7691	-89.93259964
4.062465213	104.8721847	961.2571716	10998.17513	-72.60827484
3.606325603	76.77270366	588.7519836	5894.048027	-24.06633492
3.942968094	97.96209376	698.0452271	9596.571814	-63.19691544
4.037612333	106.045544	896.4197693	11245.6574	-23.98648033
4.007930548	107.1397669	865.2220764	11478.92965	-49.4653656
4.305715262	126.4623509	805.2939148	15992.7262	-152.2676666
4.273535125	118.0567265	792.6982117	13937.39067	-107.0314178
3.775103874	94.27152215	661.447998	8887.119889	-70.49351196
3.638724644	77.34723609	601.5296326	5982.594931	-23.95425606
3.802832851	92.66847518	634.4781189	8587.446292	-31.69892235
3.765582486	85.45073884	622.5815125	7301.828769	-41.58102722
4.316149457	131.7531302	928.2756042	17358.88731	-168.8648132
4.393673638	141.2061274	887.1871948	19939.17041	-149.2898712
3.956049052	101.2157187	826.4815369	10244.62172	-112.3602463
3.994454225	101.1409864	833.419342	10229.49912	-46.74955673
3.736183038	83.36570197	586.4032288	6949.840265	-61.4981308
4.227716688	116.3604057	835.380127	13539.74401	-99.65309906
3.406841543	71.09777976	542.7816162	5054.894287	-23.3306284
4.185773851	120.997487	772.0227356	14640.39187	-136.933374
4.012295321	102.3781184	671.3605042	10481.27913	-25.58142395
3.827874598	90.84982184	674.1100769	8253.690128	-30.10702381
3.862951684	94.6416344	752.3812561	8957.038961	-45.02339172
3.421133975	81.19043488	500.258667	6591.886717	-15.6399087
4.182908791	111.3019487	701.5605164	12388.12378	-53.00782356
4.085915736	108.3830335	832.8699341	11746.88194	-94.10063934
3.960061448	99.41804317	753.2707214	9883.947307	-43.26518631
4.000912878	98.96808522	698.0160217	9794.681892	-99.2752861
4.142757181	117.7386581	918.1676025	13862.39162	-62.48953781
4.038469491	101.8000312	693.9455872	10363.24635	-88.04410095
3.720901211	98.20075751	664.664917	9643.388775	-114.7360611
3.529669364	72.28598697	506.645401	5225.263913	-51.16858025
3.807839598	90.8774765	747.333374	8258.715736	-25.59985886

log.sigma.3.0.mm.3D_firstorder_Kurtosis	log.sigma.3.0.mm.3D_firstorder_Mean	log.sigma.3.0.mm.3D_glrlm_ShortRunLowGrayLevelEmphasis	log.sigma.3.0.mm.3D_glrlm_GrayLevelVariance
4.318189758	41.02142089	0.003850134	32.87778008
3.99398957	67.60740068	0.013435989	10.69528799
3.366903246	76.05810846	0.009986373	14.18918004
3.136583037	33.99193563	0.0063638	25.56480669
4.019331162	47.53535489	0.006779036	16.07870953
3.164812526	69.90551023	0.01160593	22.02878602
4.008516241	67.10794924	0.002852475	17.11027271
4.412765446	74.05696418	0.002326238	18.08176881
3.788797018	73.98675372	0.002428371	18.89066702
4.776452439	83.11485808	0.003651749	18.72286289
3.308329683	53.42516579	0.009538119	11.49680087
4.062544202	83.81001705	0.003853377	19.72922133
6.070336246	41.0390254	0.00469867	14.26074795
5.663985199	57.72596746	0.003664992	24.15432884
4.630631914	60.19706383	0.003578426	22.73990895
3.628221445	60.20472505	0.004471126	19.45393075
3.754130179	49.13676678	0.00855972	16.64008937
3.836295508	57.58425969	0.013745408	12.79029625
3.127457053	51.19879834	0.009430567	9.048711855
3.520251012	74.7240084	0.003737794	19.62780612
4.280497759	57.52669651	0.00641788	11.0803116
2.924135995	65.46595083	0.013228448	10.85548933
3.750500904	60.18542151	0.010039585	13.91692346
3.659298921	63.39225956	0.007496118	13.32043166
5.133247347	76.20544039	0.005324309	18.19929166
4.596178468	81.1368504	0.002686653	26.28610577
4.289792661	66.60929109	0.005302999	25.25747927
4.390521358	52.20412926	0.003381865	16.31684864
2.942827448	61.70988674	0.012728496	10.21234555
4.767822855	63.37716064	0.007093816	10.77600178
4.456738479	70.08449062	0.003305034	17.44510908
6.639349313	24.53988894	0.004741947	13.25109391
3.449088188	43.16004395	0.004760671	25.03248126
4.24252494	58.49184773	0.007657225	22.30511757
3.512001543	85.74438189	0.00530711	17.65649054
4.612016552	69.13238081	0.003780197	15.34634834
6.965639083	34.37967883	0.002004817	17.05008652
4.232675585	51.96304895	0.002171094	23.72513745
4.559894146	41.85320412	0.010651564	6.652106084
2.92163201	45.62402056	0.005184597	22.378968
5.380434562	29.50688648	0.005550046	17.97919522
3.477455935	41.66000307	0.004253183	26.57142251
3.493441172	75.19071075	0.004210271	19.27007223
4.626471949	48.27752175	0.002702856	19.97087442
3.378431031	92.67106475	0.008924796	15.67897035
4.069992415	81.65461084	0.003521375	19.7169716
4.342499147	69.65734783	0.003632327	17.56189835
3.705848586	61.97333168	0.016193785	9.165154698
3.302317039	65.41819695	0.009871063	17.55625166
3.601812636	75.55301135	0.011596348	13.77902448
4.154424514	89.86297682	0.003959511	21.1627848
3.487409865	61.63493377	0.005685003	22.82637164
3.656560461	54.83613242	0.006457419	14.40483673
3.203800972	92.80105111	0.016515022	15.04340472
2.805292405	83.11472261	0.00670248	17.19865096
5.236747909	56.92518512	0.002469291	15.55753766
3.409803083	71.72771475	0.004218787	20.31460378
5.361759033	44.01434159	0.003623406	18.74575002
4.447937351	40.23439905	0.006120364	14.88342357
3.933773181	40.58928103	0.002439417	19.56716516
4.819703642	57.82401592	0.005225844	16.52450243
3.870494803	71.68338201	0.003477626	15.98044179
4.044586323	79.35665601	0.002727168	19.14360485
4.014032966	52.51991722	0.002224619	18.92682474
3.029687026	74.23942487	0.011599217	12.0215998
5.820496702	48.54589132	0.003022904	17.08553193
7.673817582	28.53232121	0.005040535	9.642946761
4.531562178	41.33260021	0.002708359	20.11110586
4.233085035	61.94175909	0.002578529	18.52725254
4.143033862	57.0180633	0.006061816	10.19100812
4.514467229	49.32216775	0.0057064	16.15602528
4.300391734	81.12307782	0.003013292	19.1072195
5.741226756	64.18430804	0.003205131	19.67436499
3.296639712	27.65315432	0.006498932	26.51780907
3.248620885	42.41794995	0.005633376	22.93176953
5.881488095	45.46812593	0.005716545	15.27850041
3.854513628	66.40224367	0.007241908	10.37414717
4.296660705	59.45347118	0.008024664	14.700353
3.745004168	57.81650941	0.008635118	12.30437848
3.835816163	25.63692316	0.003603897	28.67690122
4.248930837	40.73599344	0.004395107	33.01669555
4.556166614	23.23481511	0.012017599	17.65054633
4.332639418	65.03876413	0.004396789	17.30875726
3.772748533	49.94158722	0.00678876	11.56821253
3.422936897	54.64732945	0.006260877	22.21369191
3.958828817	48.36381196	0.005607057	8.824446005
4.142927309	23.15098149	0.00470323	24.86674937
3.334687584	86.58167644	0.007794407	17.63909215
4.157263613	67.68000626	0.007215324	13.88210987
5.177028886	57.03545044	0.004636878	15.49781811
3.185193252	60.23822445	0.012673391	11.37128522
3.127679565	84.60194673	0.00619639	20.37712315
4.436054385	38.35711843	0.00353567	19.79348957
4.442584634	68.78571556	0.003698823	16.79996668
3.337778364	35.3344529	0.004793072	16.14506828
4.858980927	59.13031084	0.002778025	23.79225918
3.348383485	44.01806952	0.007503243	17.20137335
5.620144717	12.51548216	0.00582558	16.66091425
4.259259656	34.55661934	0.007544429	9.13754975
3.586753467	69.37718915	0.004450608	14.11136281

log.sigma.3.0.mm.3D_glrlm_LowGrayLevelRunEmphasis	log.sigma.3.0.mm.3D_glrlm_GrayLevelNonUniformityNormalized	log.sigma.3.0.mm.3D_glrlm_RunVariance	log.sigma.3.0.mm.3D_glrlm_GrayLevelNonUniformity
0.003984357	0.058105628	0.10598904	114.0067754
0.014397659	0.103276588	0.179781784	84.91843319
0.010767285	0.078541682	0.198751417	105.4184256
0.006634089	0.061011725	0.10820781	54.53012978
0.007147543	0.078148671	0.142338817	165.6888175
0.012239641	0.069349668	0.112595238	41.65771334
0.003074801	0.075360096	0.170014233	379.0689107
0.002507058	0.077628472	0.164714312	352.0374627
0.002586723	0.067771402	0.133142313	277.8504374
0.003810818	0.070913941	0.112246572	69.13243589
0.010118772	0.087013113	0.139433638	117.7079168
0.004055306	0.072508634	0.129874757	151.0689865
0.005177744	0.111182139	0.358492427	171.2586223
0.003812315	0.071719114	0.138829031	110.7809154
0.003737862	0.072044063	0.124566156	130.188564
0.00478914	0.070388943	0.163054882	274.2115459
0.008950444	0.076439644	0.106953719	83.29434949
0.014829977	0.098077559	0.204707662	104.6547146
0.010297846	0.100383122	0.17826282	113.6880072
0.003994833	0.06894804	0.135168878	322.5636388
0.007130803	0.100408928	0.279894145	255.3398346
0.014032029	0.086701124	0.124075523	84.4391187
0.010710817	0.087027516	0.159710076	79.09734771
0.007999872	0.081972094	0.153712965	193.8046997
0.005549144	0.077569172	0.151313068	88.52192823
0.002805113	0.06295576	0.09980898	137.3728607
0.005574153	0.066487496	0.147945888	193.5957025
0.003610371	0.080531752	0.147037055	198.4883772
0.013536245	0.09575532	0.123120573	38.68848207
0.007520085	0.099050014	0.167590367	179.1002004
0.00353343	0.077172952	0.176826139	324.7758863
0.005090877	0.106309802	0.213724557	190.4261589
0.005042314	0.061429999	0.122761859	180.4147073
0.008013572	0.068640479	0.164893653	144.2952302
0.005618174	0.070054969	0.123438297	280.0229873
0.004081727	0.084282156	0.202499629	221.0117259
0.002175406	0.096014283	0.232851576	307.8816699
0.002393579	0.071987605	0.329647069	313.5248941
0.011368462	0.117699013	0.155410969	127.2539111
0.005512575	0.05976117	0.11270754	396.7435136
0.005911931	0.088886163	0.183088177	202.8531205
0.004454349	0.057243259	0.079457732	206.4048552
0.004443037	0.066618595	0.120279862	221.230989
0.002845107	0.073516683	0.124452395	165.9574373
0.009477321	0.08045325	0.142259346	92.09409213
0.003730594	0.069889854	0.128665185	173.7546397
0.003881749	0.077605589	0.152908883	316.3576673
0.017519417	0.106109362	0.188899431	80.09776047
0.01027697	0.07189157	0.109890453	56.02466817
0.012647158	0.088066614	0.204144051	102.7592335
0.004158399	0.066757242	0.115723169	156.2440973
0.005959399	0.062010341	0.085651821	194.087796
0.006865483	0.080692231	0.130314054	327.98328
0.017439917	0.076193667	0.124915169	76.413361
0.007096489	0.069251793	0.120918456	83.78122669
0.002613488	0.084688727	0.139713348	147.8157059
0.004446014	0.065434565	0.115441394	241.5757236
0.003862993	0.082301088	0.164571796	252.1769801
0.006437193	0.084716386	0.127483378	126.0967185
0.002628372	0.068353357	0.192750509	551.5715129
0.005673262	0.090432745	0.212940373	138.3896482
0.003704886	0.076711486	0.146546953	183.4104951
0.002901562	0.071388498	0.134424984	373.0757782
0.002399289	0.073904872	0.151011445	502.4517317
0.0124185	0.083097895	0.140195924	58.78403914
0.003254268	0.087564993	0.197527546	294.817037
0.005460335	0.118847403	0.241231398	130.9353934
0.002913548	0.075075496	0.149559761	419.5945161
0.00273154	0.072817745	0.13283102	385.6312987
0.006552525	0.097885034	0.193693875	246.864192
0.006018618	0.079444152	0.1319992	92.78093033
0.003229914	0.074770067	0.170277972	284.6111839
0.003385486	0.07782579	0.152260019	158.7650422
0.006762955	0.060322553	0.103107932	125.3585082
0.005939639	0.060747196	0.097831694	175.2929394
0.00604038	0.090762577	0.155664675	107.8482324
0.007893908	0.096396632	0.187740332	201.3323188
0.00861028	0.089976177	0.178089331	75.65904352
0.009174403	0.088496615	0.14759394	134.6438064
0.003757667	0.062282489	0.096349842	134.3268492
0.004563953	0.058929807	0.107547055	108.3219394
0.012663437	0.080086395	0.15838599	128.1795082
0.004719791	0.076236915	0.18689591	220.5905739
0.007339171	0.090238596	0.154332856	245.45018
0.00658977	0.063687236	0.112542502	142.029103
0.006275853	0.11507737	0.269515998	147.181468
0.004944069	0.067924032	0.150858624	145.297499
0.008273749	0.073019591	0.160486874	178.2320385
0.007671773	0.085599522	0.161023802	110.9016988
0.004923833	0.086052425	0.177852315	152.8651872
0.014429699	0.111918142	0.274099224	121.8974766
0.006511252	0.064532689	0.103692816	141.2807228
0.003733981	0.072772674	0.125766935	341.4396084
0.003930435	0.079011202	0.150228006	233.2265214
0.005062383	0.073637996	0.110738207	292.0774672
0.002958346	0.070303927	0.191565721	212.3020698
0.007988832	0.072586223	0.141232505	183.7854108
0.006168893	0.098159641	0.189631942	89.44206802
0.008063413	0.103619338	0.170859713	75.69945611
0.004872017	0.085502042	0.208338468	429.9750319

log.sigma.3.0.mm.3D_glrlm_LongRunEmphasis	log.sigma.3.0.mm.3D_glrlm_ShortRunHighGrayLevelEmphasis	log.sigma.3.0.mm.3D_glrlm_RunLengthNonUniformity	log.sigma.3.0.mm.3D_glrlm_ShortRunEmphasis
1.278708605	533.4917493	1704.561743	0.94563397
1.482906608	125.9481491	650.3920578	0.908673797
1.517877064	159.1275483	1062.292739	0.907960748
1.307607228	320.9024504	756.275762	0.935360361
1.393969865	261.4582939	1727.420448	0.920911532
1.324535195	187.1974163	503.0268069	0.931056883
1.438205126	395.3715919	4096.637972	0.920230115
1.43809473	483.8970279	3664.147791	0.917469685
1.367051943	484.8430413	3392.533096	0.92644765
1.324613877	461.3463365	814.7433407	0.93074111
1.387101582	157.2000502	1107.275754	0.922423411
1.357551983	384.4321071	1731.000061	0.928457143
1.850069331	274.1423876	1118.667676	0.874673062
1.379627453	547.6424178	1273.326616	0.92522849
1.335489014	426.4060481	1525.179216	0.933988556
1.433050089	316.3414263	3166.310201	0.919089538
1.309977072	236.4255036	916.0490383	0.933184822
1.549826331	118.2400676	822.9993582	0.898801401
1.49781463	130.0171155	881.6774386	0.902852713
1.37330479	336.9770968	3856.438539	0.925404309
1.706511946	181.912998	1886.316721	0.883571076
1.355615432	124.5244239	803.2990935	0.925468968
1.435781622	167.1239897	731.6302529	0.915794229
1.413556326	195.3922993	1928.372808	0.920630516
1.403871334	371.9698894	935.8558376	0.922617167
1.280895272	556.0465721	1871.523199	0.940863654
1.386612298	365.4763125	2411.604571	0.926985102
1.407077484	368.0533252	1999.608453	0.919130385
1.353419618	123.1782007	334.1680664	0.926160615
1.465954535	214.6807213	1430.730133	0.908969454
1.461024851	396.5308774	3389.089969	0.915817458
1.559505905	316.0334953	1386.278828	0.900076274
1.335258511	367.0346335	2466.698086	0.93251177
1.419713022	349.2027117	1732.665052	0.924747428
1.340279115	284.922306	3343.95998	0.93112709
1.517492519	356.2919318	2075.128868	0.908877546
1.596000065	570.5536721	2471.635292	0.897840625
1.748072413	528.5637374	3299.22683	0.891861383
1.456050659	138.7638491	847.7907619	0.905489882
1.315089014	270.9602326	5606.76228	0.934701515
1.485767681	296.8314874	1812.689988	0.910289061
1.230816665	338.9051382	3153.935426	0.948770209
1.335913067	337.0714855	2784.88248	0.931473555
1.343835298	482.4647389	1888.005644	0.9307081
1.396209369	176.7275451	934.100754	0.921046089
1.351939514	383.5892622	2073.09792	0.929632512
1.410499862	361.7558588	3328.41576	0.92138373
1.527546077	99.06455016	581.0921478	0.897926186
1.305909686	201.9533182	662.5205793	0.93715264
1.556712198	135.5942055	895.2660873	0.896297251
1.323549302	396.2851112	1969.604842	0.933330162
1.247414849	293.6002309	2718.824654	0.945846966
1.365440717	212.8588471	3350.317671	0.925438691
1.342181363	133.8481761	841.505791	0.931688069
1.333471229	223.2852642	1015.511935	0.932199962
1.392233951	532.6504777	1419.653544	0.920290771
1.320012209	334.1644355	3118.611408	0.934607876
1.444545265	390.8553511	2462.408568	0.915088694
1.358767379	252.8695363	1231.479321	0.926590719
1.478617921	469.172925	6518.16449	0.916937257
1.555166272	244.2987249	1186.494672	0.901381231
1.398589094	361.6941961	1955.422319	0.922133602
1.364694969	456.1803996	4338.417882	0.928205176
1.413088588	490.3155003	5519.821554	0.919135449
1.397542891	133.164442	577.978109	0.91999587
1.521220892	435.6725344	2633.165963	0.904673659
1.653885268	280.900373	814.0609134	0.882091311
1.404741072	470.2682173	4560.314752	0.921247761
1.360271151	461.8151911	4406.524061	0.928780094
1.520845707	207.9289757	1963.669555	0.902798867
1.375088901	295.1641902	956.8292131	0.922580651
1.447078628	416.2220693	3071.836468	0.916869454
1.40679816	467.7160393	1669.720632	0.922202539
1.28789781	346.5021449	1778.893046	0.939951388
1.277338315	270.2857805	2476.257977	0.941097958
1.438401779	315.0224916	950.0112383	0.912961471
1.511834486	167.8640671	1629.331862	0.903582376
1.4855063	190.9192335	663.81674	0.907977015
1.412434978	186.6311604	1230.032127	0.917430472
1.27581832	458.1364078	1850.671495	0.940955693
1.281902699	490.9013399	1595.579448	0.945266214
1.426724908	180.7475216	1296.295718	0.918197557
1.476311278	353.7982148	2340.946889	0.916583087
1.436738819	201.1113919	2170.157976	0.912076511
1.313872034	279.9865815	1891.687961	0.935881149
1.718951471	192.3317067	928.596678	0.875054294
1.401268589	367.9242189	1760.969251	0.924109195
1.426278597	224.6718309	1981.476115	0.919343641
1.445859842	224.3794836	1036.257906	0.912816773
1.486749651	333.668315	1400.088355	0.907281561
1.718629846	101.8885546	793.4999433	0.876684987
1.290461329	256.2096733	1871.043211	0.939290302
1.349553698	388.9314364	3900.661753	0.928706362
1.404028556	357.5574833	2414.055193	0.922202166
1.317327154	279.7892405	3331.389124	0.93271081
1.48215081	501.6803024	2424.5935	0.914869865
1.384511405	203.0652094	2081.297542	0.923979895
1.522083096	302.1582237	707.1429012	0.901055015
1.483041592	179.5398441	570.9058191	0.903769487
1.533877817	258.1805057	3946.472451	0.905941107

log.sigma.3.0.mm.3D_glrlm_LongRunHighGrayLevelEmphasis	log.sigma.3.0.mm.3D_glrlm_RunPercentage	log.sigma.3.0.mm.3D_glrlm_LongRunLowGrayLevelEmphasis	log.sigma.3.0.mm.3D_glrlm_RunEntropy
728.5012675	0.924418183	0.004635409	4.818903632
196.5425685	0.878815302	0.019331052	4.19093478
253.6701733	0.874435864	0.015075353	4.502386458
451.529611	0.914494922	0.007866477	4.724694926
395.7215799	0.895140333	0.008919167	4.506260803
259.8585811	0.91002331	0.015116824	4.57987428
599.4029469	0.889767638	0.004295472	4.575915673
741.0325854	0.887476658	0.00346437	4.593300099
713.5511014	0.901790247	0.003374545	4.634789931
655.3329391	0.909299656	0.004550987	4.515446906
236.4329902	0.896857784	0.012982102	4.271108972
558.5754677	0.903654263	0.00505899	4.595494519
552.5440374	0.823369453	0.008379083	4.442275453
808.9093545	0.899198065	0.004554198	4.689826356
613.8921333	0.910476117	0.004537966	4.622146134
478.9423225	0.889677964	0.006453726	4.660102353
336.8483357	0.912575699	0.01069323	4.412658722
194.5876478	0.864745149	0.020633198	4.3406969
209.6345274	0.872288728	0.014730827	4.170567273
490.8584812	0.899714442	0.005264929	4.661750817
332.7674772	0.840074278	0.01146607	4.388192012
181.3245784	0.902545092	0.017747372	4.223330689
252.9882574	0.887094351	0.014121658	4.38529063
293.667509	0.89288309	0.010570004	4.410738915
554.491786	0.895884948	0.00671859	4.528004923
753.6377862	0.92106374	0.003362568	4.736333574
537.0888644	0.900045207	0.006947651	4.773265343
559.2841655	0.892135019	0.004752217	4.511161212
173.4448714	0.903631045	0.017357695	4.06196003
345.9254662	0.879859187	0.009687865	4.261295863
619.1969216	0.884130372	0.004776024	4.591092198
555.2719912	0.864929174	0.00693285	4.305334502
526.6670745	0.909474236	0.006352944	4.776895951
528.9663917	0.894238953	0.009812561	4.676730211
404.1348383	0.907549081	0.007115691	4.560893681
573.2664851	0.873855388	0.00576484	4.491851076
1009.697795	0.860355984	0.003163125	4.507032435
977.9359374	0.842502198	0.00392944	4.857653217
228.0363222	0.878861789	0.014728812	3.925929913
377.5029342	0.913130886	0.007079815	4.732911094
493.4164966	0.878832809	0.007777757	4.504906914
437.1611564	0.932446142	0.005350695	4.755810304
482.1314185	0.908863105	0.005571405	4.617648359
706.502963	0.90703067	0.003543531	4.577767221
259.7940971	0.89514867	0.012271745	4.445819027
548.5672697	0.905506515	0.004780745	4.617282344
541.6995437	0.893231713	0.005165786	4.564373362
161.544016	0.8665548	0.024236526	4.162596275
283.0542542	0.915574437	0.012190008	4.478530503
219.1311712	0.86276741	0.018251351	4.436542154
561.1658722	0.910980028	0.005114269	4.638737962
387.2718919	0.928507861	0.007185383	4.666465746
312.8121062	0.900760366	0.008844643	4.436436483
184.7169738	0.908096433	0.021849705	4.40060518
311.3840632	0.909543088	0.009011644	4.503229151
808.9215868	0.894792899	0.003320078	4.440060779
470.1631283	0.912541599	0.005545384	4.638455963
619.5690748	0.885727633	0.005073899	4.556106123
372.1377656	0.902865945	0.007959385	4.388843526
741.2328726	0.88367586	0.003726839	4.709052423
402.7935561	0.865449952	0.008176959	4.512815872
543.1015729	0.895188706	0.004865695	4.504926621
661.3444048	0.902520696	0.003778245	4.604995075
741.1870737	0.89175471	0.003286177	4.62705365
197.5970865	0.895228822	0.016408353	4.303838153
712.9104134	0.871091105	0.00451029	4.539616181
529.8634302	0.844148183	0.007721067	4.194942543
714.6386024	0.893993846	0.003924451	4.654575761
685.1319834	0.90400704	0.003497246	4.579335875
342.9725323	0.869989913	0.009176176	4.268202532
441.9080172	0.898738675	0.007482951	4.464691409
639.52506	0.88678501	0.004398216	4.631785435
708.5864333	0.89475816	0.004307888	4.561341195
483.6046492	0.919807948	0.007945616	4.742659807
361.3023418	0.921553694	0.007347978	4.700298285
509.2280468	0.885073942	0.007606193	4.36721692
271.2724189	0.871195582	0.011322953	4.295630319
299.5565919	0.876987313	0.011652374	4.425659128
284.4910763	0.89108197	0.011795768	4.329434954
632.8611464	0.921860098	0.004455987	4.744327821
670.7782556	0.923889082	0.00538247	4.806045994
281.1108099	0.889340232	0.015791559	4.53695771
553.3903873	0.884344777	0.006455971	4.585950279
319.022832	0.885003379	0.00996941	4.324452217
396.641743	0.914246066	0.008127143	4.681342226
365.6974169	0.83390239	0.010087837	4.208846421
566.7624118	0.896468312	0.006149045	4.732031593
336.2626813	0.889945576	0.010749287	4.579137611
350.4960302	0.884562842	0.009952161	4.413210445
544.6848546	0.876695157	0.00640586	4.493337347
174.9251185	0.834532798	0.024559133	4.226125322
350.0846148	0.919072531	0.007983753	4.614223904
569.0852127	0.905089123	0.004687271	4.600449911
540.1952499	0.894265734	0.005120934	4.521215775
400.1386245	0.911358173	0.006312702	4.478539352
798.6805224	0.881940386	0.003977115	4.754964716
303.5413414	0.89858278	0.010374351	4.567009583
522.7457811	0.869370362	0.007918341	4.380291902
302.0204708	0.875576462	0.010602751	4.142943008
414.9805089	0.870400469	0.007265763	4.472466934

log.sigma.3.0.mm.3D_glrlm_HighGrayLevelRunEmphasis	log.sigma.3.0.mm.3D_glrlm_RunLengthNonUniformityNormalized	log.sigma.3.0.mm.3D_glszm_GrayLevelVariance
565.4487312	0.868085251	44.84060094
137.3736348	0.789231207	19.57950011
173.52954	0.788872165	23.10071332
343.3685492	0.845067838	32.91122449
284.0006282	0.813775387	24.86676768
199.9746904	0.835825641	29.63403754
427.1665219	0.813222163	27.61483259
524.9860576	0.807100319	33.24780465
523.2540071	0.826000657	27.1643165
495.571163	0.834675746	28.95557565
170.502648	0.817251558	16.37332126
413.5974985	0.830001928	31.84294342
310.4505755	0.72440389	32.1472937
591.1349863	0.823398024	41.42643604
457.4470512	0.842696029	36.29175876
342.0912764	0.81117275	29.63601286
254.2731706	0.839777214	24.76608856
130.3098432	0.769661469	21.21899993
143.1750399	0.777086745	13.06117659
362.7018528	0.823458993	29.56847472
203.8399886	0.740312572	21.6046905
134.3172192	0.823465472	16.14925164
181.2439931	0.803777829	22.5414876
211.3799485	0.814085042	20.94175441
401.5963408	0.818460404	33.76107958
590.4636943	0.856827062	41.03183391
393.0616833	0.82735317	38.6770337
399.8825905	0.810298334	29.00035963
131.8694717	0.825423356	14.38571674
236.201006	0.789661455	22.48049587
431.19118	0.804101934	30.16965146
352.2036313	0.772320904	25.34970242
393.7536863	0.838744998	34.96917859
376.9897584	0.823102392	33.35407828
305.0318039	0.83566614	25.71304915
389.4336554	0.790136072	26.94999498
635.0410428	0.768737719	38.18249887
586.7425075	0.75680802	37.96015257
154.0067584	0.782329811	12.52156683
289.284921	0.843439792	28.24086085
327.5268146	0.792886968	30.91572731
356.7758641	0.874162001	32.82988075
361.7257557	0.836990421	27.89749283
520.0070552	0.835203173	30.92760915
190.79051	0.814677488	25.85965809
411.4501912	0.832727284	30.04795563
390.9667938	0.815421216	31.01485798
109.3932175	0.767805319	15.70169309
215.8364863	0.849116255	24.79797126
148.9661226	0.764945044	21.9195702
424.6326552	0.840518122	32.53289843
310.303346	0.867843843	29.3536716
229.9228113	0.823370719	22.00192348
142.3727578	0.837639382	22.07441676
238.2055303	0.838158952	23.62912203
579.420487	0.812445781	29.15585572
357.3154147	0.843512842	28.95278991
427.5485882	0.802295177	33.06350773
273.1952864	0.826141893	26.46667624
510.4309608	0.80649952	29.11654542
268.5542844	0.774545685	30.32947525
391.5505653	0.816772482	24.79214896
490.161721	0.829479461	27.59306952
531.3973357	0.810505105	30.51262397
144.0526048	0.813996215	18.53070348
479.0025337	0.781032875	34.72159546
319.0909694	0.73719286	22.48506456
510.2309514	0.814859054	32.90146607
498.7323235	0.830920984	28.36159977
229.3790221	0.777162228	20.36799105
320.2898869	0.817762572	25.1324216
451.6609994	0.805939372	35.16414815
506.4379229	0.817157733	36.30456884
370.0583307	0.855024848	36.01881193
286.1597663	0.857380235	29.90389224
347.2338898	0.797910377	24.99348073
184.4357144	0.77878174	18.50630612
208.4223317	0.78801223	29.01380952
203.0691635	0.806913462	20.44252232
488.8325491	0.857194643	36.30912252
520.3253	0.867319537	42.24574197
196.8795966	0.808820143	29.99247349
384.0568256	0.80682422	29.23889091
220.8647977	0.796048228	17.69248411
299.5104109	0.846643396	28.54775872
218.0827006	0.724278426	17.49085874
399.4266921	0.821688379	36.48420278
242.7512494	0.810930637	25.51653779
245.0698833	0.797981715	22.40563581
367.5004651	0.786444328	28.80069785
112.7945819	0.727084603	16.40842089
272.4875738	0.853535176	24.55017715
419.4722751	0.830403634	31.39789873
387.2658616	0.816920588	27.80749798
300.7863147	0.838911034	21.86453372
546.9605551	0.801973406	40.30539465
219.6530793	0.820650162	25.87465003
337.2354561	0.774281568	28.51780896
199.7312604	0.779437837	16.20579882
282.0112514	0.783738484	23.60536421

log.sigma.3.0.mm.3D_glszm_SmallAreaHighGrayLevelEmphasis	log.sigma.3.0.mm.3D_glszm_GrayLevelNonUniformityNormalized	log.sigma.3.0.mm.3D_glszm_SizeZoneNonUniformityNormalized
347.7782298	0.043891449	0.410872071
82.05445194	0.0664893	0.275642372
104.6776275	0.058276229	0.316236413
193.2607713	0.051493878	0.356587755
157.5962096	0.057203857	0.337511478
122.8952215	0.054086538	0.344535873
258.9835109	0.054944084	0.324631261
318.2817375	0.049894213	0.325104335
296.5877377	0.054842633	0.334652545
285.7903976	0.0542692	0.349394943
87.93953335	0.070603424	0.319640751
245.8897431	0.051004808	0.364102816
173.1823284	0.054492496	0.314359014
324.8378387	0.046911458	0.324572994
253.3779666	0.047884005	0.384207222
206.352405	0.053546529	0.346802472
127.7811553	0.059020326	0.310440601
73.38788402	0.062793311	0.299424945
74.7189219	0.078243435	0.287740232
218.0260989	0.052423823	0.316882791
111.4160316	0.062775855	0.290957324
70.40601735	0.070712172	0.272903253
115.7283939	0.060495868	0.337335537
122.5505304	0.061777919	0.305972176
244.1750877	0.054009178	0.362033571
367.6583596	0.045948789	0.365846367
254.3964011	0.046337234	0.37417785
221.4638874	0.053038025	0.320461383
67.6931771	0.082475995	0.234910837
126.569685	0.061115702	0.301869835
263.4096002	0.053421719	0.356249093
175.4175679	0.062406276	0.311823828
235.3219294	0.047396923	0.36172852
201.8963581	0.05034169	0.32654661
190.204381	0.055921856	0.344137181
237.2617927	0.056838635	0.339668627
368.4545297	0.049601682	0.353778894
351.6902685	0.047263675	0.339377762
67.35210324	0.084421413	0.254280343
173.42177	0.051900502	0.305399223
176.1486534	0.052404597	0.351174232
232.1136446	0.050558758	0.369018429
202.4312362	0.054139031	0.32723852
290.7185248	0.053035277	0.356354638
108.4253735	0.05563111	0.300572284
258.7901422	0.051914857	0.377850424
246.3244123	0.051662846	0.338781492
60.21956999	0.069545224	0.246678014
105.9734494	0.056689342	0.288250211
94.01317641	0.064599204	0.2904259
236.10147	0.050804765	0.311157662
186.6142093	0.052730083	0.340776356
130.3377242	0.060112813	0.318471911
87.04595724	0.060592124	0.331349367
142.7873778	0.05875283	0.318443838
308.8709444	0.056207726	0.295988879
207.7496755	0.05178078	0.346709871
233.391803	0.051885322	0.33062756
153.9303971	0.056251795	0.346407282
299.1469742	0.053591867	0.338384379
172.0356201	0.052236129	0.338239397
233.0971837	0.059082354	0.345716069
298.1741852	0.054790177	0.362542317
317.4985584	0.051298847	0.305911132
81.64347561	0.065825115	0.272583826
290.9540672	0.05143876	0.330585801
156.0322837	0.065589338	0.317950854
296.1867283	0.050129835	0.327643628
288.0790545	0.053404613	0.380550062
133.778459	0.063146531	0.336498948
166.1889065	0.058776452	0.295937983
264.4070632	0.050041104	0.342180095
281.3173144	0.050114781	0.3168663
199.9784253	0.046001487	0.364706112
183.6266129	0.051659488	0.349844154
160.6351083	0.06079932	0.302543934
115.0798174	0.064236912	0.325225546
125.5149589	0.053197279	0.284353741
120.024788	0.062340561	0.292390784
276.778756	0.049010961	0.35717298
324.2426594	0.045186039	0.400087609
133.5480488	0.053564565	0.393661287
219.7987712	0.052973288	0.322580417
105.7964132	0.065842674	0.248601024
169.5991232	0.053371204	0.335805426
103.2732533	0.07562984	0.225075973
214.7997582	0.047753406	0.342406855
138.2769441	0.055727543	0.311607765
138.1676865	0.06184041	0.294070886
190.4079749	0.055214777	0.325351817
80.23505832	0.077019889	0.346046033
158.1657668	0.056705187	0.345265975
224.6862545	0.051179263	0.323787594
224.5277489	0.054861834	0.360927275
164.8922091	0.05975636	0.321610499
337.5484099	0.046662335	0.389151164
131.0681813	0.055260584	0.301001855
173.4362047	0.056767768	0.342399276
85.29458053	0.073874099	0.283542961
175.1863519	0.058245056	0.339142738

log.sigma.3.0.mm.3D_glszm_SizeZoneNonUniformity	log.sigma.3.0.mm.3D_glszm_GrayLevelNonUniformity	log.sigma.3.0.mm.3D_glszm_LargeAreaEmphasis	log.sigma.3.0.mm.3D_glszm_ZoneVariance
348.8303887	37.26383981	49.9057715	43.65871579
57.05797101	13.76328502	241.47343	221.0709702
116.375	21.44565217	110.1195652	92.74335421
124.8057143	18.02285714	30.42	22.62792653
222.7575758	37.75454545	103.8424242	90.96955005
71.66346154	11.25	49.5	39.43158284
497.9843546	84.28422425	124.9471969	111.3765704
415.8084441	63.81469898	386.7568413	370.8068413
461.4858593	75.6279913	67.25888325	56.39136619
120.1918605	18.66860465	46.62790698	36.91671174
135.2080378	29.86524823	74.37825059	61.66894131
254.5078684	35.65236052	71.84120172	60.9672473
101.2236025	17.54658385	981.2391304	947.6207419
161.9619238	23.40881764	115.3807615	103.5410942
262.0293255	32.6568915	70.23460411	61.77179419
418.2437811	64.57711443	302.0464345	288.8802477
110.5168539	21.01123596	44.11235955	32.86349577
73.65853659	15.44715447	274.1910569	249.068957
82.29370629	22.37762238	221.4195804	200.8219473
481.6618421	79.68421053	76.18881579	64.48971563
148.3882353	32.01568627	747.8039216	712.6459977
72.86516854	18.88014981	66.21348315	49.88219781
92.76727273	16.63636364	110.2690909	96.40362314
219.6880223	44.35654596	111.862117	98.27087973
131.7802198	19.65934066	90.07967033	77.84889355
310.9694118	39.05647059	37.70470588	29.937009
378.6679842	46.89328063	119.5662055	109.3603273
241.9483444	40.04370861	250.8291391	237.4461401
25.37037037	8.907407407	79.28703704	62.1566358
132.8227273	26.89090909	190.4681818	168.6762603
443.5301205	66.51004016	373.5309237	358.9256341
136.8906606	27.39635535	424.3895216	402.1773029
381.2618596	49.95635674	76.4487666	67.06332162
228.2560801	35.1888412	74.71244635	63.40976789
469.7472527	76.33333333	79.6014652	69.19197842
242.1837307	40.5259467	367.3674614	349.675586
283.7306733	39.78054863	724.1483791	702.6220188
424.9009585	59.17412141	492.9400958	475.9475261
61.79012346	20.51440329	166.7325103	141.1114159
700.2804187	119.00785	34.48495421	24.43276366
232.4773414	34.6918429	281.3927492	266.0386634
580.4659886	79.52892562	17.3941513	11.35061829
366.5071429	60.63571429	48.98839286	38.3503117
283.6582915	42.2160804	65.79145729	56.02190601
99.48942598	18.41389728	108.1812689	93.27373792
335.1533258	46.04847802	114.6685457	105.0913699
425.5095541	64.88853503	210.3232484	197.1306214
45.1420765	12.72677596	136.4535519	113.8001135
78.69230769	15.47619048	33.3003663	23.58332998
88.28947368	19.63815789	286.3552632	266.5761773
246.4368687	40.23737374	55.63762626	45.11612176
445.735474	68.97094801	18.66743119	12.02538308
409.8733489	77.36519037	91.02719503	78.73636853
110.3393393	20.17717718	71.33333333	60.34201769
131.8357488	24.3236715	49.41062802	39.09008378
146.8104839	27.87903226	174.2258065	158.7694947
462.8576779	69.12734082	41.98277154	32.8020985
287.645977	45.14022989	236.583908	220.7855674
163.5042373	26.55084746	97.31355932	85.12280954
860.1730921	136.2305271	182.5786782	169.6814816
151.53125	23.40178571	274.4553571	258.9162747
265.8556567	45.4343303	123.976593	111.92152
668.165491	100.9782963	112.9837222	103.1173742
605.7040404	101.5717172	111.9146465	97.09214646
53.15384615	12.83589744	85.17948718	68.76660092
302.8165939	47.11790393	380.7445415	362.9685599
77.89795918	16.06938776	476.7591837	448.4307205
557.9770992	85.37110981	205.994128	192.5252711
695.6455142	97.62363239	93.89989059	83.63396611
209.6388443	39.34028892	401.8523274	380.2141504
105.6498599	20.98319328	73.45938375	60.21957018
361	52.79336493	277.0919431	260.556735
197.4077047	31.22150883	112.9422151	99.56047603
308.5413712	38.91725768	44.90189125	37.77185504
395.3238938	58.37522124	37.0840708	29.40675777
101.6547619	20.42857143	77.93452381	61.98210743
173.3452158	34.23827392	195.7223265	175.5145183
59.71428571	11.17142857	165.3714286	144.5604535
130.9910714	27.92857143	107.8102679	93.29212671
309.6689735	42.49250288	25.28373702	18.00556613
315.2690355	35.60659898	52.14593909	45.77479934
215.3327239	29.29981718	145.0877514	134.2712285
271.2901308	44.55053508	233.0380499	217.9289759
161.3420647	42.73189522	209.6533128	187.2333067
269.9875622	42.91044776	58.3221393	49.11951901
45.46534653	15.27722772	914.6534653	857.1340065
260.5716163	36.34034166	122.0906702	112.2685
234.9522546	42.01856764	190.8275862	177.6026919
112.3350785	23.62303665	60.30890052	45.62114525
157.1449275	26.66873706	261.5258799	243.9484331
79.24454148	17.63755459	711.5982533	679.1729372
294.8571429	48.42622951	31.53161593	23.75182774
515.4698492	81.47738693	75.8951005	65.29582231
335.6623656	51.02150538	243.7806452	231.1895942
444.1440985	82.52353367	44.1115134	34.18057206
384.0921986	46.05572442	270.3748734	258.3542856
231.7714286	42.55064935	88.14155844	74.75736381
80.46382979	13.34042553	210.1234043	190.2735355
50.75418994	13.22346369	135.5642458	113.8559346
452.0772693	77.64066017	401.5828957	382.8202853

log.sigma.3.0.mm.3D_glszm_ZonePercentage	log.sigma.3.0.mm.3D_glszm_LargeAreaLowGrayLevelEmphasis	log.sigma.3.0.mm.3D_glszm_LargeAreaHighGrayLevelEmphasis	log.sigma.3.0.mm.3D_glszm_HighGrayLevelZoneEmphasis
0.400094251	0.100309601	27570.3086	533.4640754
0.221390374	2.260640181	27053.9372	155.352657
0.239895698	0.931107098	15130.20924	184.0733696
0.358239509	0.117460529	10555.67429	328.26
0.278716216	0.405454981	28801.17424	273.4545455
0.315151515	0.398664817	7654.355769	214.6201923
0.271456379	0.364225573	44422.9987	446.2627119
0.250391543	0.86949642	174217.2611	545.6559812
0.303343599	0.141804255	33829.38651	508.1696882
0.320895522	0.109557518	21771.96221	488.5843023
0.280503979	0.507925979	12103.49409	160.713948
0.303253796	0.206591608	26816.40629	405.1230329
0.172469202	3.642365062	267348.1553	316.310559
0.29062318	0.222718115	62961.05611	576.3426854
0.34375	0.166176385	31128.84311	424.4164223
0.27559415	1.115033172	84341.82007	350.5389718
0.298157454	0.213525949	11452.58146	238.2050562
0.199513382	2.842607292	28338.69106	136.8252033
0.220338983	2.017856038	25636.25175	145.2307692
0.292363916	0.254049867	24468.59868	376.0671053
0.168650794	4.417555798	130254.3863	213.7803922
0.247451344	0.642651574	8229.906367	134.7715356
0.268554688	0.830378983	16169.61091	192.0145455
0.271250472	0.655485949	20527.35794	215.1908078
0.285938727	0.274329649	31628.28846	409.9505495
0.358801182	0.075726651	20384.95059	595.2188235
0.313021961	0.367397914	42183.39229	403.9703557
0.273352643	0.712741073	89790.90728	394.5443709
0.241610738	0.887071088	8051.546296	142.6203704
0.214216164	0.86241701	43917.36591	231.6772727
0.261664565	1.029917269	138221.6972	437.0746988
0.212179797	1.219164225	151354.8383	316.9111617
0.326416847	0.219088648	30230.12239	382.8235294
0.297446809	0.264258856	25201.2289	360.5550787
0.309945504	0.336651795	20591	313.5186813
0.237745915	1.185199728	116678.1865	399.6619916
0.215533459	1.208307235	437312.9227	617.4189526
0.242588646	0.987781177	249702.0439	598.0838658
0.197560976	1.061281116	28092.54321	137.3539095
0.315405777	0.158045392	9466.560837	298.2145661
0.255204318	0.871256296	95329.6858	297.5619335
0.406775278	0.063167296	6034.656707	363.7552448
0.306597317	0.163663736	16374.86964	355.6044643
0.319935691	0.127141031	35761.53015	485.6457286
0.258998435	0.732377579	17285.49245	204.7401813
0.323132969	0.340566682	40373.28861	418.5422773
0.275317843	0.654245432	69630.74682	409.9928344
0.21010333	1.701059152	12091.31694	121.0163934
0.32079906	0.194937875	7092.692308	206.010989
0.224852071	3.109697008	28104.03618	170.6151316
0.308291164	0.158723336	21567.16035	420.4040404
0.388015426	0.082311998	5655.133028	311.2752294
0.285239362	0.475609028	18898.68531	228.7179487
0.301630435	0.860161323	7081.123123	155.1891892
0.311278195	0.293693866	9717.350242	247.5434783
0.254358974	0.313731386	98612.80645	560.8487903
0.330037083	0.138326098	14245.3161	351.9558052
0.251590515	0.588319834	97918.10115	407.6862069
0.286407767	0.379974215	26568.17161	263.3135593
0.278453281	0.428404501	80998.86192	504.4834776
0.253680634	1.247017072	61684.80134	288.1183036
0.288014981	0.369777356	43208.50715	389.1105332
0.318362411	0.277309239	47626.76723	488.9918611
0.25974026	0.244802275	53155.99444	558.05
0.246835443	0.818467503	10118.81538	155.8410256
0.237182807	0.919633324	159857.1223	498.9923581
0.187883436	1.511982255	153181.6735	287.9755102
0.27248	0.426816044	102521.7663	505.4468585
0.312105173	0.20372586	44899.34683	467.6630197
0.214975845	2.073598815	79856.95024	231.4333868
0.27482679	0.261775073	22971.94398	309.4845938
0.245920746	0.748866297	105024.3412	459.7194313
0.273365511	0.249394478	53535.11236	498.4510433
0.374501992	0.136499813	17577.86879	338.1867612
0.360907058	0.170147671	9446.631858	297.0672566
0.250372578	0.239242221	28670.96726	303.5654762
0.22245409	1.305892004	30830.65854	198.6097561
0.219206681	1.012574479	28697.95714	227.8238095
0.26244874	0.635380498	19758.22991	211.2276786
0.370671227	0.058772834	13623.94464	461.1349481
0.396178984	0.119973532	26222.09137	498.5126904
0.30405781	0.811139456	28226.12249	198.6124314
0.257265219	0.742752726	76226.94293	387.9381688
0.211194273	0.983046729	47704.47612	212.3143297
0.329643296	0.227415025	17631.33333	289.0248756
0.131853786	4.799571031	178181.0446	224.0247525
0.319077568	0.315135725	50089.27595	372.2260184
0.274981765	1.111122366	34671.08753	249.7214854
0.260928962	0.302673162	13683.96859	245.8376963
0.238518519	0.780585966	90179.92133	348.7142857
0.175613497	10.15819323	51988.24891	140.5021834
0.35852225	0.149225881	8032.802108	270.2025761
0.307158017	0.197368422	30963.79397	399.0640704
0.281818182	0.713749383	85155.88495	376.4526882
0.317325368	0.155920952	14025.39464	285.3801593
0.28842782	0.581228061	128384.2128	532.8125633
0.273340433	0.45246986	19296.45584	225.161039
0.224450812	0.629844988	74262.02128	295.9446809
0.214628297	0.666800195	29181.44134	175.0502793
0.230862487	1.828571713	90125.57089	302.3675919

log.sigma.3.0.mm.3D_glszm_SmallAreaEmphasis	log.sigma.3.0.mm.3D_glszm_LowGrayLevelZoneEmphasis	log.sigma.3.0.mm.3D_glszm_ZoneEntropy	log.sigma.3.0.mm.3D_glszm_SmallAreaLowGrayLevelEmphasis
0.665942694	0.005718237	6.410028539	0.004327761
0.533783859	0.023723705	5.997975708	0.014794552
0.581437991	0.016687761	6.240160901	0.01207831
0.614614432	0.01013072	6.190058041	0.007808378
0.601989624	0.011413346	6.3829336	0.007763098
0.607078917	0.01577888	5.962623721	0.008470017
0.590263621	0.003871302	6.686436445	0.002708308
0.587857798	0.003383017	6.673907528	0.002413204
0.598906672	0.003412482	6.563569065	0.002414867
0.611323819	0.006464375	6.191530587	0.005445142
0.582232573	0.014845215	6.03608342	0.010428906
0.626904317	0.006409065	6.453172104	0.005097151
0.579059691	0.009855654	6.318176175	0.007292359
0.588668489	0.007514153	6.594652352	0.006458089
0.642444221	0.00580584	6.354423168	0.004691326
0.610031121	0.006339179	6.519558022	0.004008473
0.574625043	0.01350644	6.269702296	0.008175461
0.563356127	0.027493923	5.926772008	0.020707268
0.554334482	0.014427669	5.982367264	0.01005942
0.581675408	0.005123725	6.723101411	0.003519618
0.553761256	0.011167386	6.346200943	0.00761249
0.537985986	0.022393583	6.126362314	0.015957482
0.600884653	0.015027221	6.063485254	0.010705231
0.570798271	0.011227906	6.446463183	0.007081204
0.623734132	0.011214927	6.27089635	0.010029237
0.627821534	0.003968675	6.621822661	0.003055584
0.63499673	0.008317969	6.584815577	0.005966977
0.583809653	0.005262179	6.53988926	0.003840244
0.48455741	0.022446449	5.679526711	0.017316713
0.567170043	0.015936532	6.386712066	0.013537379
0.619200435	0.005481656	6.507724639	0.004282956
0.570636548	0.008200388	6.214720425	0.00401426
0.623220398	0.006385998	6.562720692	0.003949004
0.589503276	0.012087678	6.55492429	0.006915538
0.6084409	0.007333106	6.531565476	0.004795458
0.604756082	0.005615655	6.421286522	0.003172057
0.615865622	0.003600285	6.530574908	0.002831249
0.602097724	0.003273116	6.691276863	0.002363555
0.51588024	0.021753892	6.063445337	0.014215355
0.570957733	0.006219424	6.827762549	0.003757388
0.613990328	0.008778639	6.381312044	0.006043688
0.63021694	0.005105447	6.536975121	0.00351179
0.592465468	0.006319337	6.59123547	0.00457714
0.618355563	0.004162301	6.451596776	0.003181546
0.565366668	0.015145083	6.307911329	0.01180274
0.637600728	0.004958326	6.340964227	0.00370251
0.60154608	0.005107973	6.580549068	0.003186392
0.503609128	0.027633175	6.074733839	0.016228586
0.547078937	0.016411718	6.201393245	0.012026364
0.553927685	0.020402948	6.193516511	0.015517641
0.575564276	0.006042978	6.636667693	0.004287702
0.6039183	0.006780009	6.560200503	0.00393134
0.581883227	0.009331475	6.488406552	0.006273008
0.593008945	0.025908003	6.065828084	0.018819149
0.582515241	0.009934831	6.253878902	0.007386622
0.560867434	0.004547221	6.548827529	0.003621053
0.609594141	0.006022947	6.553169055	0.00436393
0.596115828	0.006074845	6.616464861	0.004393277
0.608838988	0.010408171	6.244316552	0.007757868
0.601356654	0.003365744	6.678910101	0.002290789
0.602187717	0.008311463	6.349823422	0.006222429
0.609657738	0.005311937	6.360204635	0.004018479
0.624338641	0.003710463	6.504714974	0.002603186
0.570488842	0.002988202	6.846191737	0.002021135
0.530988767	0.017644905	5.989410812	0.011844606
0.594200477	0.005317516	6.655440395	0.003990623
0.582770138	0.011434705	5.988203145	0.008989078
0.592581479	0.003393697	6.758182094	0.001811908
0.641161297	0.00368486	6.49363529	0.002727179
0.600886631	0.011046464	6.25339047	0.008581491
0.559716791	0.009522351	6.297909657	0.006290129
0.606377889	0.004983718	6.65820343	0.003893243
0.577698465	0.005640215	6.577269473	0.004275467
0.623964113	0.009368091	6.455963348	0.0058762
0.612217741	0.007106016	6.524642052	0.004739299
0.568669471	0.010025465	6.259581495	0.005843276
0.591873658	0.010896016	6.224537606	0.007746891
0.547981555	0.014891646	6.244533375	0.010819441
0.555582429	0.013793514	6.323924538	0.008582962
0.619340511	0.005171478	6.536037653	0.003862934
0.656763241	0.006005063	6.369266983	0.004098162
0.651224538	0.018864794	6.174948009	0.012437935
0.584855242	0.006529878	6.549624586	0.003921769
0.508794497	0.009710113	6.527377683	0.004631594
0.599082029	0.008817377	6.481734497	0.005876749
0.461255616	0.011484283	6.080476829	0.008025027
0.60409336	0.007708732	6.551835525	0.005942982
0.573795465	0.012837331	6.474599827	0.009384784
0.560349189	0.013166759	6.350712628	0.010162973
0.590256818	0.009601443	6.374493035	0.007909997
0.610899131	0.01826565	5.672801667	0.013624485
0.606106679	0.008371439	6.35853773	0.005986666
0.587272422	0.005351257	6.702485503	0.003859133
0.623941552	0.005905094	6.418150378	0.004526309
0.5857522	0.006702283	6.499420001	0.004473613
0.648529546	0.004628446	6.552790892	0.003666556
0.565861387	0.010700532	6.581668102	0.006697813
0.606321957	0.01282945	6.11552546	0.010317913
0.546657747	0.015421709	5.932904661	0.01198988
0.604020776	0.006248872	6.463681316	0.004327403

log.sigma.3.0.mm.3D_ngtdm_Coarseness	log.sigma.3.0.mm.3D_ngtdm_Complexity	log.sigma.3.0.mm.3D_ngtdm_Strength	log.sigma.3.0.mm.3D_ngtdm_Busyness	log.sigma.3.0.mm.3D_ngtdm_Contrast
0.004280934	0.70064243	2.381858685	0.210749167	5.29E-05
0.009532241	0.284794497	1.729468998	0.370353645	6.38E-05
0.006887068	0.264589761	1.631606353	0.388078834	3.96E-05
0.010427348	0.849229516	3.608487663	0.14346425	0.00010479
0.003829046	0.240343645	1.119997785	0.468545804	3.04E-05
0.014034037	0.814215351	3.647799112	0.202524074	0.00017515
0.001714529	0.172618568	0.985554742	0.604700165	7.74E-06
0.002013888	0.169023483	1.047326216	0.477125704	9.14E-06
0.002123821	0.222209544	1.063783968	0.470861543	1.26E-05
0.00912029	0.698731488	3.969515687	0.133825627	6.24E-05
0.005871675	0.199798241	0.99318583	0.552980328	4.88E-05
0.004005868	0.394626715	1.929781877	0.289569475	2.69E-05
0.005401634	0.23216883	2.087639914	0.269013574	2.09E-05
0.006046198	0.677379933	3.81512896	0.136860657	3.55E-05
0.004449694	0.605859458	2.321410755	0.225908952	3.76E-05
0.00233352	0.183518887	0.942239299	0.583254028	1.41E-05
0.008110134	0.40984205	1.926763283	0.255323689	7.31E-05
0.00819624	0.204606975	1.388170594	0.476379218	5.71E-05
0.006846439	0.169290733	0.994633653	0.577651706	4.67E-05
0.001963726	0.160235006	0.890972173	0.654539339	1.10E-05
0.003554362	0.117827821	1.101181012	0.572016665	1.11E-05
0.008564947	0.212336233	1.117010614	0.527085036	7.57E-05
0.009273988	0.377782805	2.074660699	0.28795653	6.67E-05
0.003540216	0.198305901	1.03975722	0.588267979	2.03E-05
0.007310117	0.67212883	3.218023394	0.163363501	4.78E-05
0.00415141	0.614579103	2.870120099	0.189308049	2.66E-05
0.003033094	0.351365984	1.614336784	0.363366606	2.24E-05
0.003580441	0.26345915	1.428999408	0.339344982	1.89E-05
0.019471132	0.465816314	2.642868129	0.257158597	0.00017909
0.004550732	0.18723643	1.283594074	0.4297098	2.05E-05
0.002044084	0.190715002	1.067149319	0.514795449	9.91E-06
0.004591322	0.304915973	1.927392364	0.259657052	1.93E-05
0.003169482	0.308559532	1.396861501	0.384774054	2.50E-05
0.004157275	0.316558075	1.531305828	0.338404804	3.63E-05
0.002217283	0.177354962	0.872488343	0.677075923	1.38E-05
0.003301571	0.245085266	1.587671254	0.351323549	1.39E-05
0.002725467	0.272726305	2.052338926	0.253312763	8.58E-06
0.002162351	0.210024892	1.428554469	0.367743554	9.23E-06
0.006733151	0.179032106	1.076155367	0.470800748	3.48E-05
0.001479051	0.099100356	0.480255745	1.245487715	1.19E-05
0.003876941	0.266465875	1.548778788	0.328515771	2.14E-05
0.002481608	0.332136322	1.25769394	0.516484831	2.25E-05
0.002635853	0.227433068	0.982520325	0.53554065	1.98E-05
0.003604224	0.487958199	2.092467267	0.245727327	2.33E-05
0.007374845	0.342209977	1.842456459	0.357043503	5.76E-05
0.003365345	0.324304377	1.40381673	0.368727606	2.54E-05
0.002170529	0.182889166	1.012726322	0.538357756	1.10E-05
0.011328761	0.211228945	1.681998275	0.396509354	5.97E-05
0.010897872	0.510671075	2.267989944	0.24355208	0.000121261
0.007286972	0.297379953	1.992839982	0.392299774	4.21E-05
0.00376612	0.344546638	1.615612443	0.328462812	2.89E-05
0.00270562	0.31086128	1.113965956	0.545305594	2.59E-05
0.001961532	0.125317617	0.586695757	1.015905784	1.40E-05
0.008125587	0.36900847	1.549831958	0.457083312	8.59E-05
0.006803988	0.420316574	1.696468288	0.354475878	6.72E-05
0.005005383	0.377053631	2.519784126	0.1964178	2.40E-05
0.002433168	0.188966036	0.840794529	0.618292108	2.00E-05
0.003031289	0.216447358	1.429405615	0.363198046	1.55E-05
0.005727323	0.338431344	1.573226397	0.305719175	4.01E-05
0.00110904	0.128566002	0.661118334	0.805421356	5.33E-06
0.005496003	0.387784728	2.613473612	0.274581364	2.65E-05
0.003537824	0.280595109	1.493799732	0.35539566	1.95E-05
0.001581318	0.188007781	0.900778317	0.620374121	9.48E-06
0.001405858	0.134423468	0.899818423	0.620398045	5.38E-06
0.011817377	0.341582566	1.698806363	0.354450098	0.000108589
0.002758269	0.231571443	1.742445724	0.320336154	9.13E-06
0.007146221	0.345614137	2.796366321	0.19435126	2.28E-05
0.001643651	0.183475316	1.079912776	0.51321787	7.13E-06
0.001730336	0.172533137	1.051798815	0.522528793	7.57E-06
0.003377396	0.122048397	0.843877681	0.628547279	1.45E-05
0.007312754	0.465037374	2.400376972	0.215573138	5.01E-05
0.00241119	0.2134014	1.330093048	0.419257443	1.09E-05
0.004278926	0.412430335	2.405713602	0.222959933	2.39E-05
0.004205804	0.409111739	1.414068209	0.342344689	5.20E-05
0.002981932	0.298940041	1.147751799	0.562619871	2.83E-05
0.006452575	0.390159026	2.308005177	0.235159667	4.97E-05
0.004090692	0.1438255	0.975383659	0.592738026	1.78E-05
0.011519152	0.401765414	3.177400338	0.191175302	5.40E-05
0.005529019	0.229380836	1.260057306	0.438366759	3.48E-05
0.00411679	0.46045253	1.958138919	0.258088487	4.27E-05
0.004591265	0.683465963	2.393031546	0.212027175	5.85E-05
0.005466693	0.387698559	1.839750027	0.355303038	3.85E-05
0.00299527	0.242108859	1.28844682	0.410861286	1.62E-05
0.003336815	0.104105398	0.750630281	0.701176588	1.73E-05
0.003912148	0.371833415	1.540006987	0.387966907	3.36E-05
0.00677223	0.11330166	1.173950765	0.434244553	2.86E-05
0.004114481	0.352509949	1.553166181	0.313601292	4.04E-05
0.003646624	0.178052916	0.920537396	0.617476582	2.94E-05
0.006334943	0.339433502	1.992333512	0.29309006	3.61E-05
0.005097648	0.292504724	2.158602947	0.25218003	2.23E-05
0.007203645	0.155733998	1.228293866	0.659706382	5.11E-05
0.003907998	0.277120582	1.125894084	0.522072119	4.05E-05
0.001907762	0.182624019	0.929615832	0.561542227	1.11E-05
0.002943002	0.223021969	1.257746217	0.422216876	1.63E-05
0.002149331	0.145715245	0.665877678	0.773500218	1.59E-05
0.003013412	0.32960827	1.874628765	0.285139233	1.68E-05
0.003594393	0.18200865	1.011909341	0.603289852	2.50E-05
0.00927409	0.483250855	3.26422176	0.15465898	5.97E-05
0.011428816	0.276182237	2.004160273	0.243800669	6.17E-05
0.00164517	0.106833777	0.599454774	0.975528675	8.34E-06

log.sigma.2.5.mm.3D_gldm_GrayLevelVariance	log.sigma.2.5.mm.3D_gldm_HighGrayLevelEmphasis	log.sigma.2.5.mm.3D_gldm_GrayLevelNonUniformityNormalized	log.sigma.2.5.mm.3D_gldm_DependenceEntropy
31.38384378	549.9868049	0.062691822	6.703397399
10.18881409	197.0737968	0.116762847	6.28381197
12.40565096	150.0013038	0.093492314	6.666778735
23.83230054	365.7226203	0.068154032	6.606619322
15.82187043	337.7576014	0.084749789	6.610843623
22.50189164	303.1151515	0.071354454	6.362019662
15.13838844	436.1033445	0.086103342	6.704215973
15.70288396	447.0700861	0.091381217	6.670849841
17.65114935	535.2186538	0.073759094	6.775022802
16.78490477	343.6119403	0.078239864	6.411089231
10.94632693	183.6909814	0.092981552	6.297250934
19.3326412	509.5180043	0.078513276	6.606925289
11.68328729	291.7032673	0.148178165	6.432061892
23.12714168	713.987187	0.079720456	6.690559
22.45913172	438.2983871	0.077770856	6.524117697
16.46285184	347.6928702	0.088541789	6.718783118
16.26155414	241.9907873	0.076131635	6.386883172
11.10439528	187.8240065	0.120350144	6.370515068
8.483396882	179.472265	0.107036783	6.304322014
17.95064988	408.0644355	0.077466441	6.737499831
9.429403288	241.0347222	0.122560521	6.595411215
10.37205012	194.6923077	0.09247484	6.260016129
13.10247803	278.9453125	0.094091415	6.360661645
12.46755057	222.5039668	0.086695718	6.551694188
16.88215268	498.7415554	0.090088483	6.490235149
25.1306119	475.4157873	0.065297663	6.668112031
22.92133733	446.8252397	0.071700754	6.825065896
15.56010334	418.9706734	0.090346069	6.567369589
10.01652578	190.3087248	0.096897537	5.993742968
10.08990106	313.1786758	0.109556104	6.33945328
15.91956416	528.1740227	0.090733052	6.699227897
12.37302059	309.79797	0.117339183	6.417568504
22.99586331	416.9489006	0.067151102	6.890201348
20.21488981	473.6148936	0.078205885	6.731368722
17.69750912	350.4037239	0.073772316	6.657730061
13.63185772	288.1257086	0.101645864	6.554261133
15.23357996	617.1109917	0.111080504	6.623241824
18.38429067	458.6277853	0.103360834	6.9783983
7.67281975	224.4804878	0.115912486	6.004503883
19.25782192	271.4514443	0.065247848	6.779813008
15.4881559	284.1877409	0.102627854	6.549535085
25.32439627	273.8249289	0.057424292	6.694621267
17.87575591	370.2419929	0.073521503	6.686085616
20.28026424	594.6993569	0.079126754	6.594408375
15.41631706	286.5571205	0.088001842	6.393367849
17.84985584	417.5584699	0.077450971	6.628781426
16.4508489	401.5377028	0.085703218	6.636630377
8.630669843	164.3214696	0.118304969	6.188604901
18.65325234	262.7250294	0.074494512	6.426813626
12.71454464	180.5051775	0.098557692	6.468528041
20.85367606	519.7473725	0.071364857	6.615196248
22.67888382	325.7342035	0.063898798	6.620884189
13.96240443	215.4614362	0.084852431	6.505651549
13.71783436	191.1576087	0.084626392	6.379810545
15.8600424	340.9165414	0.0739974	6.457862365
14.49775148	510.0738462	0.092002104	6.501963306
18.84068604	369.7475896	0.07106327	6.616650876
16.36772661	444.7547715	0.094062402	6.627108065
15.36911909	297.5194175	0.089153372	6.39752723
18.67547043	488.6992004	0.072923495	6.954864486
14.84565897	315.002265	0.109070411	6.575900016
15.29510317	402.559176	0.086748306	6.56725465
17.60902911	416.1869062	0.078576452	6.682595754
16.89417709	549.1335432	0.081326665	6.777012155
10.63526518	126.1025316	0.093690114	6.257383239
14.84804577	495.1864319	0.102426651	6.641851018
9.94571152	423.5253067	0.128517963	6.297481638
17.89085494	449.04784	0.083591168	6.779595622
17.26999556	434.4072051	0.078022504	6.661611316
9.254973326	305.0403727	0.114973273	6.393951051
14.47039323	301.8498845	0.085952894	6.463094818
16.39899419	414.1736597	0.089368239	6.686808781
18.36301804	568.5423431	0.084011236	6.703797734
26.44524553	363.2709163	0.063863576	6.670891086
21.5121647	271.0229958	0.062384062	6.719685813
14.36098667	409.5469449	0.100410669	6.315010897
9.718266532	252.5108514	0.105743504	6.439029739
12.97389307	217.302714	0.107528733	6.358807665
11.78992803	274.9630931	0.096339584	6.398928694
25.75599392	568.4527576	0.065102567	6.648927286
30.88873874	550.97637	0.064145409	6.705357699
18.17833884	249.475264	0.084344616	6.652442488
15.36241869	392.6059957	0.087281068	6.746808861
10.07661514	207.3726001	0.096373323	6.456923379
20.64969495	349.1225912	0.069217736	6.741130231
6.835428611	257.6194517	0.148272877	6.361263622
22.49799964	390.9287212	0.075427572	6.817345964
15.9364763	348.8326039	0.083267762	6.65608306
13.09400428	323.6864754	0.094730732	6.501631055
13.63077433	464.7264198	0.101980796	6.595367806
9.126180888	187.3404908	0.14581373	6.156574909
18.95074449	315.7388749	0.067784835	6.571510468
19.12591257	406.8427552	0.077179066	6.662096787
15.44318641	325.950303	0.087600184	6.653444152
16.31135026	362.0730699	0.075754977	6.549575435
21.42518952	565.4924021	0.083950551	6.839089115
15.18801626	265.3038694	0.080960984	6.601663901
15.42967263	452.3075454	0.110970627	6.357580555
8.755481945	217.4436451	0.11417916	6.234752646
12.05719259	287.6608937	0.108325498	6.62365083

log.sigma.2.5.mm.3D_gldm_DependenceNonUniformity	log.sigma.2.5.mm.3D_gldm_GrayLevelNonUniformity	log.sigma.2.5.mm.3D_gldm_SmallDependenceEmphasis
414.8228087	133.0320452	0.369037288
112.428877	109.173262	0.204991784
187.9243807	143.4172099	0.211353584
163.0429887	66.58648925	0.288321466
364.1342905	200.6875	0.272184386
123.169697	47.09393939	0.297448433
823.3029552	486.5699876	0.256844998
686.8003132	466.7752545	0.246061934
720.8073031	335.3088429	0.280435396
189.1996269	83.87313433	0.296770997
236.6591512	140.2161804	0.256245107
374.1843818	180.973102	0.299558003
175.3760043	276.6486342	0.177562588
265.7874199	136.8800233	0.282319105
332.0514113	154.297379	0.304555161
593.0685558	387.4588665	0.248059604
216.0251256	90.90117253	0.291163075
138.3657745	148.3917275	0.195399361
182.2989214	138.9337442	0.218973427
808.0044239	402.7480285	0.270619308
310.797619	370.6230159	0.168858896
175.0611677	99.78035218	0.25261558
147.0546875	96.34960938	0.249658722
403.2017378	229.4835663	0.244842137
188.6433621	114.6826394	0.262721928
447.883073	154.6901646	0.320676726
514.9554593	231.808537	0.295109483
396.0550326	249.5358436	0.264065997
77.56375839	43.31319911	0.270821024
283.677702	225.0282376	0.21738904
630.06095	431.7078604	0.250309132
249.2754954	242.7747704	0.221858769
528.3440694	216.8309074	0.298080351
351.1608511	183.7838298	0.275182316
741.6180745	324.8932788	0.292230681
368.3071024	304.8359453	0.233823315
436.4560602	413.3305563	0.221566502
533.4138733	533.4452625	0.216324287
180.9837398	142.5723577	0.213209147
1302.498487	474.3518569	0.278001871
335.6260601	266.2166538	0.23489976
834.0871477	222.0597362	0.35637921
605.2419929	268.5740487	0.278255862
413.3697749	196.8673633	0.305241437
196.5336463	112.4663537	0.260045489
450.0302368	212.6029144	0.302119315
672.086804	390.9780798	0.261332604
114.0470723	103.043628	0.201740937
154.8542891	63.39482961	0.317161273
170.0192308	133.25	0.219129339
455.984819	183.3363176	0.301054724
695.2153664	215.4028478	0.352044871
735.733156	382.8541667	0.275139081
180.4456522	93.42753623	0.2645926
233.606015	98.41654135	0.286088629
281.985641	179.4041026	0.247073854
712.3651422	287.4509271	0.301635367
477.4089069	325.2677848	0.232480263
264.5946602	146.9247573	0.274061806
1363.321832	665.7185891	0.27386973
211.0249151	192.6183465	0.246782934
403.8389513	231.6179775	0.271735264
929.1115909	454.879081	0.293150934
1130.914994	619.953168	0.248155043
121.8455696	74.01518987	0.222851639
477.6949767	395.5717245	0.224153263
150.357362	167.5874233	0.211098276
919.70976	522.4448	0.258265699
956.5847704	456.9778043	0.288690671
346.9006211	333.1925466	0.203108479
198.2902232	111.6528099	0.25413234
565.5202797	383.3897436	0.233632012
329.879333	191.461606	0.264362817
435.9048251	144.2678176	0.35592254
607.9805174	195.324497	0.319303655
193.3338301	134.7511177	0.240325868
316.484975	253.3614357	0.220071107
125.2985386	103.0125261	0.21371316
258.3872291	164.4516696	0.257039207
454.1534844	152.2749038	0.330119582
385.6405229	127.5852187	0.371868021
262.2984992	151.7359644	0.285893276
431.8179872	285.321811	0.246646922
452.6654735	296.1552229	0.201137755
413.3936039	168.8220582	0.300074529
147.0221932	227.154047	0.140221052
369.9157233	179.8947589	0.295288831
393.5594457	228.3202042	0.260201201
211.8114754	138.6857923	0.240160329
248.9980247	206.5111111	0.213038428
126.8650307	190.1411043	0.172078337
463.6994123	161.4634761	0.326332893
868.5917422	400.0191009	0.292266453
476.0175758	289.0806061	0.258614418
778.5358456	329.6856618	0.297743207
463.3512566	287.2787843	0.266693519
453.8817891	228.0670927	0.256277705
132.7574021	116.1862464	0.23952972
110.5779376	95.22541966	0.210862414
660.088327	625.4714236	0.220491442

log.sigma.2.5.mm.3D_gldm_DependenceNonUniformityNormalized	log.sigma.2.5.mm.3D_gldm_DependenceVariance	log.sigma.2.5.mm.3D_gldm_LargeDependenceEmphasis
0.195486715	4.249875413	12.80867107
0.120244788	7.191253968	26.1486631
0.122506115	8.275269468	27.14732725
0.166881258	3.731892902	14.30603889
0.153772927	5.713259422	18.39864865
0.186620753	2.805913682	11.87878788
0.145691551	7.434761744	21.87099628
0.134455817	6.967102443	22.58104933
0.15855858	5.012446654	16.85173779
0.176492189	3.918213271	14.0130597
0.156935777	4.223177536	16.35278515
0.162335957	4.59467742	15.67592191
0.093934657	15.13889105	44.99250134
0.154797565	5.361178716	17.66627839
0.167364623	4.065264048	14.52318548
0.135527549	7.219593161	22.81992687
0.180925566	3.108137	12.69011725
0.112218795	7.973277449	29.0081103
0.14044601	5.85052742	21.22804314
0.155415354	4.851504985	17.01615695
0.10277699	13.10019666	39.90410053
0.1622439	3.749759715	15.41334569
0.143608093	5.258239746	19.02929688
0.152324041	4.771126518	17.78503967
0.14818803	5.804387333	19.17596229
0.189059972	3.043445119	11.74208527
0.159280996	5.24629141	16.93875657
0.143394291	5.264818754	18.75887038
0.173520712	3.064606699	13.31319911
0.138109884	5.13716604	20.35248296
0.132421385	7.477509969	23.48759983
0.120481148	7.744281916	26.17738038
0.163624673	4.873893019	16.05048002
0.149430149	6.935844273	20.54723404
0.168396475	4.6396631	15.50090827
0.122809971	8.976165113	27.32677559
0.117295367	8.768106755	28.24321419
0.103354752	18.99151323	46.97829878
0.147141252	4.147044748	18.23089431
0.179160727	3.486363549	13.53535076
0.129385528	6.676942639	22.99306091
0.215693599	2.308233774	9.365658133
0.165683546	4.271708698	15.37612921
0.166145408	4.227259334	14.82636656
0.153782196	4.57518472	16.9514867
0.163945441	5.20096297	16.44116576
0.147322842	5.334404775	18.58132398
0.130938085	5.443734256	22.29047072
0.181967437	3.301202291	12.47121034
0.125753869	7.447093502	25.10946746
0.177495064	3.560744094	13.28960685
0.206234164	2.722838041	10.28329872
0.163061426	3.862883375	14.96808511
0.163447149	4.17805805	15.78985507
0.17564362	3.574528803	13.67067669
0.144608021	4.769699934	18.38769231
0.176110048	3.371874569	13.07021014
0.138059256	5.86307139	20.90167727
0.160555012	4.358740221	15.87257282
0.149339668	7.919919093	22.00974915
0.119493157	9.781730921	29.10985277
0.151250544	5.612290816	18.48614232
0.160496043	5.646561773	17.51684229
0.148355633	5.075227591	18.37767283
0.154234898	4.640179458	18.13417722
0.123691087	8.754670519	27.17659244
0.115304725	10.04166275	30.88957055
0.147153562	5.868404838	19.35904
0.163323335	4.289776175	15.35956975
0.119703458	7.073476341	25.95307108
0.152648363	4.613941796	17.31254811
0.131822909	6.323334257	22.20792541
0.144747404	6.060666855	19.78543221
0.192963623	3.771588968	12.27401505
0.194180938	3.202165302	11.83423826
0.144063957	5.650962485	19.82265276
0.132088888	6.318190306	22.70784641
0.130791794	6.742073126	23.69102296
0.151369203	5.182489552	18.12478032
0.194165662	2.651420117	10.84437794
0.193886638	4.371661534	13.02513826
0.14580239	6.828270044	20.43079489
0.132094826	9.237154725	26.07249924
0.147304092	4.213196427	18.73641393
0.169493073	4.245706262	14.75563756
0.095967489	11.01969473	40.65535248
0.155100932	6.725088406	19.49559748
0.143530068	7.184410747	21.64770241
0.14467997	5.421099092	19.46311475
0.122961988	6.764699528	24.68790123
0.097289134	11.29663329	38.61809816
0.194668099	2.90123449	11.24853065
0.167584747	4.250755216	14.97723326
0.14424775	5.851502663	19.64484848
0.178891509	3.22868458	12.78860294
0.13540364	8.871223469	24.75920514
0.161122396	4.07047228	15.8029819
0.126797901	6.802221109	23.7086915
0.132587455	6.413775454	23.03357314
0.114320805	10.31296068	31.01593349

log.sigma.2.5.mm.3D_gldm_LargeDependenceLowGrayLevelEmphasis	log.sigma.2.5.mm.3D_gldm_SmallDependenceHighGrayLevelEmphasis	log.sigma.2.5.mm.3D_gldm_LargeDependenceHighGrayLevelEmphasis
0.031447642	185.0288921	7257.150801
0.157556967	41.21483089	4761.392513
0.275234209	34.14378554	3477.182529
0.046196405	95.96319141	5627.595701
0.062412423	83.71328854	6332.4375
0.051481778	95.07241127	3311.569697
0.057674567	112.9466792	8788.302601
0.057016504	108.6900509	9466.986296
0.03429132	141.8138795	8945.971843
0.054389163	96.63916028	4800.550373
0.108096374	44.72230479	2994.94695
0.034742303	147.8571821	7746.515835
0.178192886	49.47142757	12118.77772
0.027590875	193.0519912	12444.18171
0.038469922	125.3828155	6314.729839
0.079509376	86.74338737	7306.482633
0.073030395	68.51719389	3134.590452
0.18444157	36.48128123	4982.8897
0.135996781	39.03070983	3659.205701
0.048154394	110.3685865	6616.97538
0.192158507	40.53500762	8756.579034
0.091016764	44.36663376	2997.897127
0.079200589	66.85987291	5085.402344
0.101353971	53.79820934	3707.962221
0.049082605	128.8956782	9062.516889
0.0314213	154.5352951	5392.996201
0.045343453	130.2260144	7195.978348
0.049577646	107.1985074	7691.685373
0.088249508	53.33652729	2379.302013
0.069120149	62.32274213	6412.699611
0.04909109	128.8079734	11816.12484
0.089793456	61.78178492	8253.17158
0.046596278	120.201349	6591.641375
0.05100068	123.6589708	9256.580851
0.053624432	101.4302325	5129.201181
0.125355107	68.44985512	6963.31077
0.047991122	130.4309335	17161.82102
0.120184798	103.2003831	19202.49564
0.096318789	43.044448	4349.85122
0.065521881	75.71440784	3528.487758
0.098037953	61.82998029	6610.281033
0.051217637	98.69203067	2505.94647
0.049143988	98.09226987	5564.196003
0.026144655	167.3956719	9136.108521
0.070761914	74.15468239	4561.205008
0.046289557	126.0625943	6446.728597
0.054101107	103.5423794	7078.058965
0.153145522	32.09019819	3607.657865
0.061621492	82.61817797	3309.831962
0.187870613	40.35867847	3880.031805
0.0296881	149.7895735	6803.566757
0.040128075	111.0298393	3437.469594
0.093127057	56.30757392	3205.528369
0.110771169	54.83636451	2640.778986
0.048606985	96.98318727	4365.006767
0.038324485	119.466028	9420.907692
0.040760732	105.414515	4824.786403
0.053525193	98.09369451	9253.251012
0.059197015	74.4005541	4810.985437
0.052604516	132.5334773	10035.82988
0.105498385	77.73363475	8436.231031
0.055107446	104.093963	7162.4603
0.050872052	118.4016456	6813.59924
0.036702237	136.5320562	9846.22865
0.199022439	27.38982877	2133.558228
0.060514977	110.7451366	12836.2522
0.075870152	80.59616885	13121.24463
0.047132352	112.7632487	8694.73328
0.038083798	114.1721217	6917.154004
0.093694597	59.37272801	7538.0245
0.071272852	72.62154498	5204.830639
0.062817254	94.53756774	8532.148951
0.038387401	147.030835	10911.32953
0.044355031	118.8654652	4668.64409
0.060620043	88.33850274	3077.59917
0.050041301	87.77136206	8528.084948
0.101932125	56.76920511	5402.035893
0.138185735	46.1603515	4741.442589
0.073281857	67.39747654	4948.146456
0.022095893	180.6081675	6438.03805
0.028802518	190.9021812	7327.483157
0.096898661	67.66736262	5155.367426
0.078926775	96.10895284	9247.792903
0.103327918	40.03034826	4012.667751
0.049663543	97.99359972	5288.208282
0.170764534	33.93723117	10056.90535
0.055838159	107.184072	7888.807128
0.074549638	88.44598054	6830.240336
0.069746413	74.9634175	6185.760246
0.057680798	93.20458516	11338.40741
0.255300969	35.25685489	6156.141104
0.045967782	100.333046	3456.805206
0.042166494	115.0171329	6113.979549
0.070447927	79.86015068	6229.929697
0.03934186	102.029471	4779.627757
0.052782625	148.5996397	13143.15868
0.069733314	65.81575313	4222.876464
0.054032557	94.01969208	11186.42407
0.109723639	40.71808738	5331.645084
0.130671571	64.49323542	7939.42778

log.sigma.2.5.mm.3D_gldm_SmallDependenceLowGrayLevelEmphasis	log.sigma.2.5.mm.3D_gldm_LowGrayLevelEmphasis	log.sigma.2.5.mm.3D_gldzm_DistanceZoneVariabilityNormalized
0.002894359	0.005074906	0.991179886
0.00403147	0.008994631	1
0.005514498	0.016403406	1
0.00329257	0.005934742	1
0.002348974	0.005541635	1
0.003479961	0.006538739	1
0.001051696	0.002983999	0.99529691
0.001111633	0.003007198	1
0.001021026	0.002510192	0.998652292
0.002245243	0.006180325	1
0.00368924	0.009453679	1
0.001412452	0.003027987	0.997452233
0.002536883	0.006147621	1
0.001962068	0.003050419	1
0.002200451	0.004171436	1
0.002118542	0.004982947	1
0.003247985	0.008057427	1
0.003809899	0.008996226	1
0.002788102	0.007915961	1
0.001344597	0.003462224	1
0.001502398	0.005549437	1
0.003511455	0.008039893	0.99378888
0.002810233	0.006025161	1
0.002257667	0.007277149	1
0.001647064	0.004438957	1
0.001643073	0.003629546	1
0.001471853	0.003425155	1
0.001471427	0.003532117	1
0.004249897	0.008841029	1
0.001718372	0.004470986	1
0.000960392	0.002532454	1
0.002191484	0.005288687	1
0.001744829	0.003972814	0.994642896
0.001636739	0.003580326	1
0.001896244	0.004477429	0.992110577
0.00218493	0.006342234	1
0.000837074	0.002182387	1
0.001359935	0.003460647	1
0.003060368	0.008777285	1
0.002076965	0.005763029	0.998349836
0.002894443	0.0073651	1
0.003083976	0.006814318	0.99880024
0.001796813	0.004246996	1
0.00117682	0.002479226	1
0.002861824	0.005922721	1
0.001477863	0.003475455	1
0.001266069	0.00372187	1
0.004853297	0.01069081	1
0.005729492	0.00957607	1
0.004701944	0.01029543	0.994047672
0.001504727	0.003126953	1
0.002666637	0.00557567	0.998596492
0.004314828	0.009769688	1
0.003891464	0.008933158	1
0.001980123	0.004450659	1
0.00127064	0.002866694	1
0.001890664	0.004159875	0.995783575
0.001102352	0.003584521	1
0.003882864	0.006692238	1
0.000978003	0.002781503	0.995853508
0.001913123	0.004651839	1
0.002123632	0.004749924	1
0.001270686	0.003588752	0.986889124
0.000733544	0.002276314	0.989936214
0.005761528	0.015522428	1
0.001224587	0.003016251	0.985773088
0.001820361	0.003860303	1
0.001119405	0.003126182	0.995675696
0.001357208	0.0032992	1
0.001529436	0.004373696	1
0.002183161	0.006349093	1
0.002057952	0.004136632	0.994594634
0.001195133	0.002661081	1
0.004190749	0.007203736	1
0.002552183	0.006463224	0.998329157
0.002249234	0.004315195	1
0.001711204	0.005242541	1
0.004289109	0.010372683	1
0.002337557	0.005547783	0.996031762
0.001406397	0.002861615	1
0.002633738	0.004161619	1
0.003558261	0.00789418	1
0.001777003	0.003996156	1
0.001935929	0.006976086	1
0.002104337	0.004868425	0.995288601
0.001453085	0.005056345	1
0.002322659	0.004679923	1
0.001679578	0.004203073	1
0.002161442	0.005223922	1
0.001215593	0.003198547	1
0.001930158	0.007201982	1
0.002282723	0.005137473	0.995671016
0.001514917	0.00374707	0.994372583
0.002467036	0.00544768	1
0.001610508	0.003913756	0.998687665
0.001350516	0.00332229	0.99611652
0.002097124	0.005744774	1
0.001889619	0.003740678	1
0.003105775	0.007415872	1
0.001408411	0.004938107	0.998592541

log.sigma.2.5.mm.3D_gldzm_LowIntensityEmphasis	log.sigma.2.5.mm.3D_gldzm_LargeDistanceEmphasis	log.sigma.2.5.mm.3D_gldzm_HighIntensitySmallDistanceEmphasis
0.007832842	1.013289037	503.942691
0.019241461	1	203.8846154
0.0286468	1	165.2884097
0.010515099	1	331.8925373
0.008853761	1	311.9744624
0.010821998	1	327.1403509
0.003944753	1.007071302	443.8535651
0.00439448	1	448.8411681
0.003478104	1.002022927	511.5084289
0.008446135	1	332.5333333
0.014542424	1	174.6099773
0.004573086	1.003826531	497.6772959
0.013985936	1	288.3252033
0.006348302	1	689.9418182
0.006854995	1	417.4719424
0.008271314	1	356.5329536
0.011275771	1	237.0746988
0.018529757	1	192.6455224
0.012397227	1	178.6238245
0.004809082	1	413.3248762
0.008874141	1	244.2206406
0.013011928	1.009345794	179.9602804
0.010700792	1	273.1909722
0.009834692	1	223.6888889
0.007063252	1	506.6266319
0.004961146	1	486.0324748
0.004784805	1	446.5004525
0.005380862	1	411.5095694
0.013974176	1	202.7191781
0.007752261	1	288.7791411
0.003729971	1	522.0648567
0.009745851	1	280.8387716
0.005709417	1.008057296	403.7594002
0.005706362	1	454.096817
0.006270518	1.011881188	349.4929043
0.009876024	1	299.9047014
0.00367493	1	594.7569677
0.006034378	1	482.8047244
0.015025641	1	199.0068493
0.007244645	1.002477291	275.8693229
0.013078547	1	261.7610619
0.008323749	1.00180072	278.0858343
0.006199933	1	357.6624791
0.003692453	1	552.1851429
0.010234466	1	293.167979
0.004723939	1	422.7167019
0.005080064	1	402.7785924
0.022728409	1	165.3826531
0.016637738	1	257.9535604
0.020101396	1.008955224	189.8395522
0.004769166	1	505.8090395
0.007424648	1.002106742	315.0407303
0.015766369	1	207.3764124
0.01392281	1	210.5015015
0.006434408	1	346.2377778
0.004824981	1	486.4172662
0.005992398	1.006338028	353.9163732
0.005257329	1	424.2945652
0.013029168	1	277.302682
0.003569363	1.006232687	483.8148373
0.007372385	1	319.743487
0.00794976	1	388.433414
0.004520359	1.019796954	410.1003807
0.002895349	1.015172414	554.458046
0.025048932	1	126.4444444
0.005221347	1.021494371	500.2436029
0.008452167	1	383.5641892
0.004350851	1.006500542	437.1382719
0.004656625	1	397.6254527
0.00729973	1	297.6273292
0.009489267	1	288.1145833
0.008291358	1.008130081	415.7215447
0.004274771	1	557.4404591
0.011420712	1	337.018143
0.00798472	1.002508361	277.951714
0.008630253	1	368.0454545
0.00761854	1	262.4382609
0.021529683	1	219.1497797
0.008781373	1.005964215	265.2470179
0.00399799	1	548.547046
0.006667907	1	515.2698225
0.012433111	1	235.6908784
0.00678434	1	394.7785016
0.009921226	1	198.2320675
0.006962347	1.007083825	327.5067887
0.010001544	1	242.2669492
0.007380773	1	367.2935561
0.006231654	1	347.841133
0.008825915	1	315.9875622
0.005667273	1	439.14375
0.010744006	1	212.8498024
0.006692545	1.006507592	312.1415401
0.005129646	1.008465011	394.287105
0.009309313	1	310.8606811
0.005230838	1.001969796	345.4793171
0.005340146	1.005836576	561.6678016
0.008061695	1	258.5221445
0.007617837	1	390.0875912
0.014163617	1	189.7772277
0.006497722	1.002112676	300.1558099

log.sigma.2.5.mm.3D_gldzm_LowIntensityLargeDistanceEmphasis	log.sigma.2.5.mm.3D_gldzm_HighIntensityEmphasis	log.sigma.2.5.mm.3D_gldzm_DistanceZoneVariability	log.sigma.2.5.mm.3D_gldzm_ZonePercentage
0.007869926	506.0099668	895.0354374	0.425541942
0.019241461	203.8846154	208	0.222459893
0.0286468	165.2884097	371	0.241851369
0.010515099	331.8925373	335	0.342886387
0.008853761	311.9744624	744	0.314189189
0.010821998	327.1403509	228	0.345454545
0.003975508	444.3653506	1689.018857	0.300300832
0.00439448	448.8411681	1404	0.27486296
0.003481096	511.8503034	1481.001349	0.326220853
0.008446135	332.5333333	375	0.349813433
0.014542424	174.6099773	441	0.292440318
0.004611352	497.7729592	782.002551	0.340130152
0.013985936	288.3252033	369	0.197643278
0.006348302	689.9418182	550	0.32032615
0.006854995	417.4719424	695	0.350302419
0.008271314	356.5329536	1229	0.280850091
0.011275771	237.0746988	415	0.347571189
0.018529757	192.6455224	268	0.217356042
0.012397227	178.6238245	319	0.245762712
0.004809082	413.3248762	1616	0.310829006
0.008874141	244.2206406	562	0.185846561
0.013067228	180.3551402	319.0062305	0.297497683
0.010700792	273.1909722	288	0.28125
0.009834692	223.6888889	765	0.289006422
0.007063252	506.6266319	383	0.300864101
0.004961146	486.0324748	893	0.376952301
0.004784805	446.5004525	1105	0.341787813
0.005380862	411.5095694	836	0.302679218
0.013974176	202.7191781	146	0.326621924
0.007752261	288.7791411	489	0.238072055
0.003729971	522.0648567	1326	0.278688525
0.009745851	280.8387716	521	0.25181247
0.006040242	404.1575649	1111.016115	0.345927532
0.005706362	454.096817	754	0.320851064
0.006312872	350.5062706	1503.047525	0.34400545
0.009876024	299.9047014	787	0.262420807
0.00367493	594.7569677	897	0.24106423
0.006034378	482.8047244	1270	0.246076342
0.015025641	199.0068493	292	0.237398374
0.007270173	275.9318745	2418.003303	0.333149931
0.013078547	261.7610619	678	0.261372398
0.008332936	278.1740696	1664.0012	0.430824929
0.006199933	357.6624791	1194	0.32685464
0.003692453	552.1851429	875	0.351688103
0.010234466	293.167979	381	0.298122066
0.004723939	422.7167019	946	0.344626594
0.005080064	402.7785924	1364	0.29899167
0.022728409	165.3826531	196	0.225028703
0.016637738	257.9535604	323	0.379553467
0.020211954	190.0208955	333.0059701	0.247781065
0.004769166	505.8090395	885	0.34449202
0.007429001	315.2956461	1422.001404	0.42242658
0.015766369	207.3764124	1416	0.313829787
0.01392281	210.5015015	333	0.301630435
0.006434408	346.2377778	450	0.338345865
0.004824981	486.4172662	556	0.285128205
0.006044041	354.1408451	1414.012676	0.35105068
0.005257329	424.2945652	920	0.26604974
0.013029168	277.302682	522	0.316747573
0.003589635	484.4664127	2876.024931	0.316354475
0.007372385	319.743487	499	0.282559456
0.00794976	388.433414	826	0.309363296
0.004776096	411.5162437	1944.171574	0.34030057
0.002946675	555.8735632	2153.111264	0.28532074
0.025048932	126.4444444	216	0.273417722
0.005319143	501.9416581	963.1003071	0.252977732
0.008452167	383.5641892	296	0.226993865
0.004372854	437.6229686	1838.017335	0.29536
0.004656625	397.6254527	1933	0.33003244
0.00729973	297.6273292	644	0.222222222
0.009489267	288.1145833	384	0.295612009
0.00835659	416.0542005	1101.01626	0.258041958
0.004274771	557.4404591	697	0.305835893
0.011420712	337.018143	937	0.414785303
0.00800545	278.027592	1194.001672	0.381986586
0.008630253	368.0454545	374	0.278688525
0.00761854	262.4382609	575	0.239983306
0.021529683	219.1497797	227	0.236951983
0.008816664	265.499006	501.0039761	0.29466901
0.00399799	548.547046	914	0.390765284
0.006667907	515.2698225	845	0.424836601
0.012433111	235.6908784	592	0.329071707
0.00678434	394.7785016	921	0.281737534
0.009921226	198.2320675	711	0.231369997
0.00698131	328.1841795	843.0094451	0.347273473
0.010001544	242.2669492	236	0.154046997
0.007380773	367.2935561	838	0.351362683
0.006231654	347.841133	812	0.296134209
0.008825915	315.9875622	402	0.274590164
0.005667273	439.14375	480	0.237037037
0.010744006	212.8498024	253	0.194018405
0.006731051	312.4164859	918.0086768	0.387069689
0.005172272	394.7471783	1762.028217	0.341886938
0.009309313	310.8606811	969	0.293636364
0.005261616	345.5108339	1521.001313	0.349954044
0.005358328	562.1420233	1024.007782	0.300409117
0.008061695	258.5221445	858	0.30457934
0.007617837	390.0875912	274	0.261700096
0.014163617	189.7772277	202	0.242206235
0.006515182	300.2197183	1418.001408	0.245930031

log.sigma.2.5.mm.3D_gldzm_IntensityVariabilityNormalized	log.sigma.2.5.mm.3D_gldzm_LowIntensitySmallDistanceEmphasis	log.sigma.2.5.mm.3D_gldzm_IntensityVariability
0.042917113	0.007823572	38.75415282
0.066845414	0.019241461	13.90384615
0.059538946	0.0286468	22.08894879
0.048875028	0.010515099	16.37313433
0.055934935	0.008853761	41.6155914
0.048399508	0.010821998	11.03508772
0.054460903	0.003937064	92.42015321
0.051048693	0.00439448	71.67236467
0.053719634	0.003477356	79.66621713
0.055004444	0.008446135	20.62666667
0.067281637	0.014542424	29.67120181
0.050665738	0.00456352	39.72193878
0.058276599	0.013985936	21.50406504
0.046201653	0.006348302	25.41090909
0.047891931	0.006854995	33.28489209
0.053058014	0.008271314	65.20829943
0.058104224	0.011275771	24.11325301
0.059562263	0.018529757	15.96268657
0.073279547	0.012397227	23.37617555
0.051199025	0.004809082	82.73762376
0.06629222	0.008874141	37.25622776
0.067303306	0.012998103	21.60436137
0.058617863	0.010700792	16.88194444
0.061677133	0.009834692	47.18300654
0.054121304	0.007063252	20.72845953
0.045950274	0.004961146	41.03359462
0.046702565	0.004784805	51.60633484
0.053421396	0.005380862	44.66028708
0.0722462	0.013974176	10.54794521
0.063403047	0.007752261	31.00408998
0.053501271	0.003729971	70.94268477
0.059545168	0.009745851	31.02303263
0.047240138	0.005626711	52.76723366
0.050422504	0.005706362	38.01856764
0.054384646	0.00625993	82.39273927
0.057534362	0.009876024	45.27954257
0.049033754	0.00367493	43.98327759
0.048322897	0.006034378	61.37007874
0.077500469	0.015025641	22.63013699
0.052362019	0.007238262	126.8208092
0.052762332	0.013078547	35.77286136
0.050007278	0.008321452	83.31212485
0.053555045	0.006199933	63.94472362
0.049801143	0.003692453	43.576
0.052803439	0.010234466	20.11811024
0.052138972	0.004723939	49.32346723
0.051528195	0.005080064	70.28445748
0.069918784	0.022728409	13.70408163
0.053590085	0.016637738	17.30959752
0.063969704	0.020073757	21.42985075
0.051802483	0.004769166	45.84519774
0.049998619	0.00742356	71.19803371
0.058119075	0.015766369	82.29661017
0.057724391	0.01392281	19.22222222
0.057925926	0.006434408	26.06666667
0.055399565	0.004824981	30.80215827
0.05062686	0.005979487	71.89014085
0.053586957	0.005257329	49.3
0.05221591	0.013029168	27.25670498
0.051665215	0.003564295	149.2091413
0.052847177	0.007372385	26.37074148
0.055511259	0.00794976	45.85230024
0.053880286	0.004456425	106.1441624
0.052175981	0.002882518	113.4827586
0.069401578	0.025048932	14.99074074
0.05167051	0.005196899	50.48208802
0.062819576	0.008452167	18.59459459
0.051260727	0.00434535	94.62730228
0.053596036	0.004656625	103.6011381
0.061798349	0.00729973	39.79813665
0.060180664	0.009489267	23.109375
0.05027953	0.008275051	55.65943993
0.050556906	0.004274771	35.23816356
0.044415008	0.011420712	41.61686233
0.05086772	0.007979537	60.83779264
0.058651949	0.008630253	21.93582888
0.064692628	0.00761854	37.19826087
0.05598789	0.021529683	12.7092511
0.063962152	0.00877255	32.17296223
0.049799137	0.00399799	45.51641138
0.044167921	0.006667907	37.32189349
0.051023786	0.012433111	30.20608108
0.053408171	0.00678434	49.18892508
0.069502553	0.009921226	49.41631505
0.051221827	0.006957606	43.38488784
0.077779374	0.010001544	18.3559322
0.04791497	0.007380773	40.15274463
0.054438958	0.006231654	44.2044335
0.062436573	0.008825915	25.09950249
0.056163194	0.005667273	26.95833333
0.078348357	0.010744006	19.82213439
0.056679105	0.006682918	52.25813449
0.050265861	0.00511899	89.07110609
0.055434677	0.009309313	53.71620227
0.056691251	0.005223144	86.34077479
0.046435222	0.005335601	47.73540856
0.05694655	0.008061695	48.86013986
0.055250679	0.007617837	15.13868613
0.078374669	0.014163617	15.83168317
0.058467566	0.006493357	83.02394366

log.sigma.2.5.mm.3D_gldzm_HighIntensityLargeDistanceEmphasis	log.sigma.2.5.mm.3D_gldzm_SmallDistanceEmphasis	log.sigma.2.5.mm.3D_glcm_SumVariance	log.sigma.2.5.mm.3D_glcm_Homogeneity1
514.2790698	0.996677741	1689.877905	0.329493339
203.8846154	1	553.7386704	0.464095194
165.2884097	1	384.6486512	0.453685645
331.8925373	1	1098.832822	0.363139876
311.9744624	1	1010.116208	0.390012895
327.1403509	1	851.3984698	0.357038386
446.4124926	0.998232174	1325.388945	0.405759459
448.8411681	1	1356.320197	0.414202193
513.2178018	0.999494268	1669.834955	0.377762749
332.5333333	1	1027.986743	0.381344718
174.6099773	1	509.2425098	0.409984768
498.1556122	0.999043367	1549.686886	0.377353975
288.3252033	1	890.6368998	0.502818705
689.9418182	1	2320.960302	0.380186753
417.4719424	1	1321.586948	0.358754288
356.5329536	1	1036.3713	0.413215991
237.0746988	1	681.9890664	0.371194235
192.6455224	1	527.5519329	0.465769977
178.6238245	1	500.7481475	0.438194915
413.3248762	1	1202.503681	0.385297226
244.2206406	1	698.3481884	0.498386662
181.9345794	0.997663551	534.1162896	0.405255769
273.1909722	1	823.9616512	0.416828522
223.6888889	1	634.5522463	0.414946249
506.6266319	1	1555.418155	0.40353391
486.0324748	1	1391.580434	0.351922357
446.5004525	1	1342.760379	0.368303538
411.5095694	1	1282.053038	0.395755808
202.7191781	1	521.5321852	0.397600094
288.7791411	1	943.1286407	0.436670172
522.0648567	1	1648.295917	0.417023969
280.8387716	1	944.1906028	0.437864132
405.7502238	0.997985676	1244.215381	0.366113648
454.096817	1	1448.151996	0.392807654
354.559736	0.997029703	1009.502991	0.374291821
299.9047014	1	851.300154	0.440759911
594.7569677	1	2023.612977	0.438943963
482.8047244	1	1407.952763	0.466095631
199.0068493	1	663.8212519	0.425536265
276.1820809	0.999380677	776.4375764	0.364117019
261.7610619	1	852.421266	0.418942233
278.5270108	0.99954982	776.0818316	0.313638651
357.6624791	1	1120.633478	0.371381189
552.1851429	1	1887.404158	0.364815985
293.167979	1	821.9662973	0.403582057
422.7167019	1	1265.100543	0.383589077
402.7785924	1	1206.410423	0.398230954
165.3826531	1	443.0236563	0.454389262
257.9535604	1	726.8969566	0.364580209
190.7462687	0.997761194	478.5800808	0.453987065
505.8090395	1	1589.973014	0.357601722
316.315309	0.999473315	939.8068589	0.32432095
207.3764124	1	597.5829884	0.38898701
210.5015015	1	519.4902794	0.395374848
346.2377778	1	1030.235659	0.375809915
486.4172662	1	1587.381917	0.405540095
355.0387324	0.998415493	1104.842898	0.360141627
424.2945652	1	1373.941829	0.4092975
277.302682	1	889.2945549	0.384794919
487.0727147	0.998441828	1514.963263	0.387336426
319.743487	1	934.3979612	0.447866096
388.433414	1	1229.083903	0.392352733
417.1796954	0.995050761	1253.715707	0.384236391
561.5356322	0.996206897	1705.792456	0.398002901
126.4444444	1	309.1662398	0.427790595
508.7338792	0.994626407	1537.931796	0.433991051
383.5641892	1	1353.11753	0.443286391
439.5617551	0.998374865	1374.691692	0.393907688
397.6254527	1	1352.246828	0.391246521
297.6273292	1	928.2573142	0.461281141
288.1145833	1	908.6645595	0.394515946
417.3848238	0.99796748	1240.953521	0.420831754
557.4404591	1	1800.396703	0.402646369
337.018143	1	1077.655801	0.330301202
278.3311037	0.99937291	760.8420928	0.340933209
368.0454545	1	1275.526624	0.40623607
262.4382609	1	740.2367717	0.453965109
219.1497797	1	609.5843092	0.442603507
266.5069583	0.998508946	803.5706295	0.411407165
548.547046	1	1774.266699	0.336819571
515.2698225	1	1696.899312	0.338897413
235.6908784	1	707.1118821	0.392764044
394.7785016	1	1210.764909	0.417276205
198.2320675	1	582.1812145	0.428707206
330.8937426	0.998229044	1038.355174	0.358861444
242.2669492	1	775.9623685	0.523327662
367.2935561	1	1175.228064	0.371111226
347.841133	1	1039.34969	0.413263574
315.9875622	1	960.1107031	0.422693206
439.14375	1	1454.308715	0.438634952
212.8498024	1	527.5275519	0.508929779
313.516269	0.998373102	941.3844848	0.348122945
396.5874718	0.997883747	1206.046983	0.364476485
310.8606811	1	969.7710722	0.409945654
345.6369009	0.999507551	1099.518655	0.358138051
564.0389105	0.998540856	1769.56024	0.413155895
258.5221445	1	757.3049018	0.394261812
390.0875912	1	1422.230914	0.424427285
189.7772277	1	626.1481738	0.448936
300.4753521	0.999471831	854.821252	0.454607299

log.sigma.2.5.mm.3D_glcm_Homogeneity2	log.sigma.2.5.mm.3D_glcm_ClusterShade	log.sigma.2.5.mm.3D_glcm_MaximumProbability	log.sigma.2.5.mm.3D_glcm_Idmn	log.sigma.2.5.mm.3D_glcm_SumVariance2
0.246781854	-749.7890662	0.020776659	0.971056173	86.50622762
0.396566108	68.79826848	0.069414413	0.984897452	27.71780127
0.383627524	60.76655242	0.044865527	0.982204527	33.85693666
0.281675329	-303.1639532	0.028736526	0.974527605	63.83526871
0.309709797	-137.7474902	0.040675829	0.979408544	43.59787724
0.271091411	252.2168798	0.02619317	0.979897778	62.87050028
0.327347195	64.2020818	0.045847696	0.989996925	42.17192652
0.337871116	27.25783496	0.040515062	0.988944258	41.17411069
0.296700008	-128.3187259	0.021722233	0.984860512	49.33445777
0.297615501	-166.4495844	0.025940298	0.980084325	46.91037206
0.330190834	-42.8104962	0.032851692	0.979788963	29.53574075
0.296658238	-130.4867873	0.028392881	0.981977825	53.2203012
0.445525176	21.07021306	0.12540045	0.986983693	33.97964296
0.300157919	-389.6126848	0.04077463	0.984636533	65.61300713
0.278091417	-249.3193493	0.027704977	0.980979325	58.93967529
0.338745683	-45.14043132	0.048143372	0.98471163	44.32356045
0.286748849	-159.9678629	0.023817496	0.975199718	44.60457777
0.400447104	125.1963973	0.062046056	0.985098753	32.80785757
0.36326156	39.22970406	0.045073459	0.986590033	23.37036829
0.303254637	35.96873811	0.030395171	0.986947932	47.87498777
0.437990945	48.28366034	0.092806919	0.989390065	26.66347028
0.322907756	2.311523268	0.02771831	0.977910325	28.28149282
0.341050575	10.27028291	0.040669576	0.98302931	35.57674819
0.337807382	40.54392382	0.03155574	0.982776731	34.50887625
0.326261241	-4.910267092	0.043097666	0.986720461	44.1599282
0.265966369	-60.83060093	0.01920626	0.984251635	68.6581432
0.287226148	-37.12894835	0.029936837	0.983671895	63.07515465
0.317273824	-113.9102541	0.032666425	0.984296461	41.2761037
0.312250501	61.60382024	0.035223922	0.978602823	27.97437054
0.363821364	-56.86987813	0.04286048	0.986043743	28.54521701
0.343235116	-59.94257093	0.047211645	0.988143966	45.70067198
0.369162812	-205.9409207	0.055668045	0.981092435	32.55588885
0.285164893	-145.785034	0.021574766	0.980028831	62.62989563
0.31582593	-299.4220672	0.036090906	0.981330112	55.17906623
0.290257259	-35.22414616	0.030152034	0.987135314	45.52282757
0.370197228	-37.40241591	0.063429177	0.986826331	37.71348758
0.371409885	-167.8568927	0.057290717	0.990914923	40.59343284
0.402567885	-76.4893356	0.102810577	0.98755934	53.07521928
0.349101482	-75.44259786	0.036066275	0.980083198	19.10460155
0.277780149	-4.985029796	0.015496545	0.980751744	55.7564326
0.346002609	-249.7801618	0.047974724	0.982578165	42.52289777
0.222408384	69.92719515	0.011440889	0.976762136	70.28332481
0.28731367	-106.5824734	0.025426771	0.980031972	47.40897608
0.283284359	-294.276966	0.026923635	0.985112315	56.55198413
0.324770323	74.78391376	0.03786656	0.983603403	41.94558316
0.302925719	-60.21400712	0.03094837	0.985473925	47.47554004
0.31984269	26.44080328	0.036035339	0.987072307	42.70529083
0.382724172	46.18127617	0.052957039	0.983812947	23.18006816
0.279851726	-115.2759933	0.023801072	0.97223261	51.93840059
0.384059087	157.4012424	0.061633445	0.987115038	37.2644102
0.271344073	-159.4903227	0.024713874	0.982022398	55.60967527
0.234492512	-30.90406603	0.016267267	0.981452727	58.8719107
0.307119718	-11.21951289	0.026285845	0.982249804	38.13099221
0.313575324	111.3825731	0.029253187	0.978360067	36.76913962
0.291013079	47.4636573	0.022838196	0.980994736	44.75605424
0.327797316	-105.672744	0.03965461	0.987716439	38.73035881
0.274327871	-128.8987056	0.020330958	0.980303185	48.67905257
0.334147903	-250.8223648	0.039060461	0.986623535	45.18962746
0.303488308	-119.0007898	0.032781484	0.979772627	38.4930307
0.309818704	-142.8948546	0.03661726	0.984270085	53.08784918
0.379999835	167.319932	0.067881405	0.987659214	38.42408349
0.312584847	-90.85941585	0.041330403	0.984456483	39.89996581
0.303772189	-64.1425751	0.034078726	0.983922875	48.13562583
0.318425435	66.94531518	0.027602265	0.989208205	48.15770984
0.351333644	23.30757838	0.04304855	0.980161939	27.7883789
0.363715492	-108.0468214	0.059053714	0.98815087	40.1752498
0.374382272	-101.022436	0.061163443	0.987362417	23.45006881
0.3157792	-52.18883745	0.031598683	0.986795882	49.58017799
0.311174451	-158.9579851	0.024120094	0.988697953	45.41715795
0.393736045	-3.665769301	0.055892073	0.987120852	24.6120539
0.313791306	-138.1316585	0.036181544	0.982725441	40.45609147
0.345504194	15.24319609	0.039704756	0.989568197	45.08974711
0.325890026	-266.6235268	0.037637945	0.987337407	49.52034809
0.245376369	-345.8883841	0.022348421	0.970050154	66.55037384
0.253127456	38.76292328	0.01636681	0.979558139	57.84310903
0.329174965	-353.9177704	0.040396775	0.980980266	38.70280002
0.383686475	65.45815631	0.046961101	0.988036806	26.18860352
0.371849073	69.80677273	0.057532271	0.984131575	37.26181041
0.331903486	-13.88283986	0.04385822	0.985150092	31.13871419
0.250406693	-511.8366671	0.017343981	0.982111091	77.51790394
0.257008651	-771.8288093	0.022871058	0.972627501	86.77707248
0.317603655	-79.90412445	0.038427413	0.97940813	44.52804778
0.342437356	-23.81942855	0.049302077	0.985245251	40.07901869
0.35215863	-82.64685066	0.032125136	0.981796751	29.31358128
0.274099956	-154.9604963	0.023933332	0.979438922	55.11922604
0.46825799	38.52354934	0.097908987	0.990842897	20.46437842
0.293081594	-391.8111231	0.036752722	0.977223227	59.73349672
0.336710879	-20.2233035	0.04483337	0.984140988	43.66122378
0.34699093	-94.44328051	0.03713245	0.984897889	36.33210894
0.367998113	-118.6859011	0.056622133	0.987108705	34.53524284
0.451547289	145.6898604	0.106147818	0.988489291	26.2442789
0.258981658	-25.36855744	0.017542393	0.978277899	54.08798287
0.28132013	-191.2254227	0.023701597	0.98354462	49.99684336
0.333460616	-140.5940202	0.038747965	0.981615768	43.23693521
0.27183497	-143.6984562	0.024414837	0.979630487	41.62452677
0.34135705	-246.7880694	0.054834406	0.985459107	61.87211025
0.312908299	-21.50487147	0.028458917	0.983962277	43.22244722
0.35557357	-339.5058282	0.052330512	0.983385178	42.83593961
0.37891769	-87.12680635	0.046991566	0.980016493	24.21330666
0.386490425	90.47390821	0.067961056	0.988033668	33.15696688

log.sigma.2.5.mm.3D_glcm_Contrast	log.sigma.2.5.mm.3D_glcm_DifferenceEntropy	log.sigma.2.5.mm.3D_glcm_InverseVariance	log.sigma.2.5.mm.3D_glcm_Entropy	log.sigma.2.5.mm.3D_glcm_Dissimilarity
42.84265244	3.718994018	0.245242852	8.294147017	4.629852204
10.26387317	2.777280232	0.366134975	6.474130026	2.234915565
11.2375446	2.84844018	0.349599567	6.933584866	2.359761851
27.81236508	3.401434709	0.286121618	7.717960348	3.707930337
19.16447088	3.206178373	0.301635718	7.504050413	3.134693977
22.32892016	3.292757524	0.272065887	7.523728193	3.515550278
15.98777595	3.103271879	0.314345647	7.45413409	2.879556295
16.0475907	3.091595314	0.322715444	7.319279311	2.829072725
22.25970912	3.305841862	0.290668072	7.817299994	3.37949404
17.08009477	3.130252182	0.302775106	7.364160779	3.048868504
12.64879968	2.953412594	0.327017098	7.039879558	2.643162313
22.78513207	3.306480752	0.296923037	7.643985082	3.386627132
11.40864657	2.734533832	0.381757475	6.277921126	2.124005211
27.41535333	3.348664507	0.292494813	7.667498234	3.519317264
31.60381096	3.49764548	0.275735416	7.77253168	3.944743975
18.25567806	3.125898138	0.330549192	7.399219159	2.93237995
20.14568485	3.236276379	0.289640169	7.561837549	3.284940737
10.93938637	2.808046436	0.372940032	6.573745757	2.276163456
9.616017873	2.775644077	0.353773097	6.673676288	2.301667686
17.96473537	3.179867442	0.301253192	7.640148405	3.093825107
8.258891118	2.63897628	0.386688977	6.440508326	1.957733719
11.58375584	2.907168657	0.322451058	6.992792883	2.596953357
14.51485024	2.996282916	0.339638709	6.990687718	2.705844958
13.58603882	2.99427759	0.332446159	7.242531724	2.678896991
20.06605307	3.145999154	0.320696479	7.189112639	3.03133058
25.82094047	3.394231347	0.270899107	7.979005899	3.681460691
27.05569709	3.411265196	0.281810364	7.969133419	3.654214746
19.83802896	3.198467213	0.313616728	7.386957115	3.107265015
11.12322963	2.858144689	0.31657859	6.748883357	2.594814823
11.80508618	2.868522702	0.358768415	6.828749779	2.433499879
18.40261624	3.135081553	0.328252303	7.393775243	2.9284753
16.67907871	3.03295089	0.352215558	6.875769097	2.724335268
27.00165904	3.409741798	0.285612111	8.026009975	3.66880586
24.01107972	3.302685313	0.303224747	7.617110528	3.351923999
19.79352869	3.234656321	0.292268851	7.680615576	3.237561974
13.69821604	2.951801676	0.349534167	6.971383566	2.551372561
19.80400571	3.0688862	0.355010345	7.043901279	2.829713638
19.66882782	3.103361048	0.322235263	7.200890376	2.799536365
11.62608327	2.876839788	0.346849546	6.543363393	2.477959174
20.01174838	3.265602012	0.278091606	8.017329122	3.335659154
18.58354684	3.118163793	0.333902745	7.211747437	2.921490709
30.92738194	3.558938104	0.226198079	8.411246531	4.248750494
21.22820357	3.263982467	0.288352778	7.745317647	3.325587991
28.37654932	3.432334098	0.281483207	7.786299101	3.725092136
14.84360907	3.042561598	0.325915409	7.178490893	2.809631289
20.19918431	3.230336519	0.304481542	7.5995334	3.214393057
17.90549497	3.156203364	0.316934911	7.456916683	3.009366186
8.386330252	2.687721291	0.369000568	6.370006466	2.140385782
22.94482495	3.305968893	0.278661313	7.544627339	3.475026151
10.06200015	2.776456778	0.365074199	6.752171589	2.26080029
24.25726727	3.350026594	0.274034095	7.828887701	3.545755077
28.80911721	3.507214649	0.24176716	8.168917918	4.050125122
16.0783534	3.116971838	0.31097229	7.479242932	2.964001164
14.85419396	3.037327958	0.315822172	7.177816739	2.84583978
17.25166307	3.151690592	0.294925229	7.519568914	3.108354278
17.00481681	3.10679934	0.320727695	7.226399733	2.91388427
21.94256035	3.311819425	0.278489211	7.83289413	3.453466907
19.93488547	3.157286144	0.328343398	7.353384266	3.017527812
20.16943472	3.22026753	0.301905782	7.363910984	3.193777864
24.63273516	3.368191751	0.288220502	7.945701326	3.466939613
15.49357483	3.00463011	0.34553271	6.877239327	2.629825766
18.36136944	3.169320482	0.313263784	7.37748896	3.055320325
20.00492233	3.248047855	0.300037536	7.678169329	3.223055663
17.37587028	3.141985958	0.311262557	7.593787236	2.980808019
10.39168412	2.804378347	0.350361861	6.781263154	2.400979555
17.58783999	3.071347571	0.341230065	7.146108026	2.785263665
15.17247349	2.956871596	0.358175898	6.514688538	2.585806603
21.63959378	3.255125799	0.306983718	7.687717042	3.220254859
19.20207413	3.194537743	0.307971477	7.602711037	3.10797178
10.12285423	2.767128167	0.377088335	6.627308578	2.217072115
16.86711249	3.109199106	0.309610684	7.294895597	2.974689892
16.19184699	3.036554484	0.336113903	7.274941343	2.746573718
20.82758238	3.201938351	0.315087881	7.452094383	3.114774837
34.63866926	3.602357061	0.251740332	8.151314861	4.291863011
25.58210927	3.424719278	0.256325725	8.120209809	3.78867117
19.34684073	3.124789437	0.322375325	7.005161894	2.994819733
9.298903002	2.734833737	0.374495493	6.697487985	2.192098034
12.62151431	2.895975976	0.355600253	6.764150752	2.473777252
13.4387144	2.978919018	0.325276728	7.03831735	2.670882832
32.83685572	3.534601809	0.256714116	8.175575173	4.094365266
40.49649462	3.662424025	0.25508622	8.194487569	4.447825122
25.10563119	3.329836751	0.305929172	7.502872839	3.429498966
18.57852669	3.136477926	0.315819491	7.329144237	2.952461899
10.40935896	2.818577281	0.350797713	6.997460713	2.389627326
24.53117552	3.36594886	0.274890956	7.874796656	3.596772696
5.547011349	2.411534876	0.419053889	5.94509919	1.651081022
29.25715849	3.462306574	0.281131146	7.869191717	3.797249056
15.45164367	3.064299083	0.327063438	7.338355401	2.796786002
13.76733539	2.96481917	0.339010839	6.98989834	2.629608721
14.47084794	2.935432436	0.359619314	6.887709479	2.55936789
7.06224246	2.544332302	0.402467878	5.930796003	1.836055611
21.10781281	3.308843488	0.260517757	7.910452165	3.513952315
24.35214223	3.368325166	0.283155934	7.837236379	3.546518015
17.08712914	3.101938036	0.325711209	7.357959757	2.891153073
21.35854029	3.292051477	0.282680012	7.715544334	3.421955218
24.44274511	3.261091145	0.322206763	7.566580374	3.233665153
15.42526596	3.087247292	0.310843652	7.489239946	2.905626195
21.24814582	3.147525053	0.343864569	6.923172445	3.035311999
10.71861652	2.797055118	0.36967132	6.524789334	2.3161965
12.29605039	2.904570711	0.354065025	6.907027476	2.422301993

log.sigma.2.5.mm.3D_glcm_DifferenceVariance	log.sigma.2.5.mm.3D_glcm_Idn	log.sigma.2.5.mm.3D_glcm_Idm	log.sigma.2.5.mm.3D_glcm_Correlation	log.sigma.2.5.mm.3D_glcm_Autocorrelation
20.92647971	0.89608637	0.246781854	0.342185048	520.5115949
5.183501069	0.923692329	0.396566108	0.450678817	188.900074
5.510649334	0.917039842	0.383627524	0.498319448	140.4878255
13.66531426	0.902199353	0.281675329	0.397100802	352.7361792
9.158430444	0.90963854	0.309709797	0.390347113	325.8536009
9.49329203	0.907470485	0.271091411	0.472113178	281.4619523
7.532765222	0.934964042	0.327347195	0.450731203	416.2054212
7.935245685	0.933206692	0.337871116	0.438272233	423.9608799
10.5476072	0.921780455	0.296700008	0.382127504	514.6221483
7.620023787	0.908303275	0.297615501	0.464022266	331.7574493
5.511040108	0.907295628	0.330190834	0.396214876	177.366042
11.12881602	0.915926456	0.296658238	0.403504366	480.654998
6.787683331	0.935415142	0.445525176	0.494756541	285.8160127
14.69546759	0.925319934	0.300157919	0.412909	695.2204071
15.6898761	0.915172396	0.278091417	0.307160834	414.3959082
9.424778981	0.924367223	0.338745683	0.419812875	333.4473101
9.112939316	0.899593138	0.286748849	0.374672234	230.1862293
5.651754002	0.925344537	0.400447104	0.48603573	181.799168
4.221763571	0.923203306	0.36326156	0.413245842	173.5344346
8.210603715	0.925449879	0.303254637	0.454710077	382.5964044
4.3524512	0.936762033	0.437990945	0.520833923	231.8513261
4.739080738	0.900990519	0.322907756	0.415643382	185.0610707
7.069866615	0.918022469	0.341050575	0.416198914	270.2027915
6.262142048	0.915680189	0.337807382	0.432992546	215.9679689
10.64556446	0.929620733	0.326261241	0.377680923	478.6967495
11.93359142	0.919245988	0.265966369	0.44656245	438.574361
13.37132326	0.920407245	0.287226148	0.401915218	423.3504058
10.01135413	0.922192771	0.317273824	0.35196925	401.966918
4.293580788	0.900530571	0.312250501	0.427279862	180.1707903
5.755860238	0.925215587	0.363821364	0.415374091	303.6261701
9.657361582	0.933249367	0.343235116	0.426881836	506.6125596
9.081318159	0.918954249	0.369162812	0.32471587	301.6954249
13.20862127	0.912340471	0.285164893	0.400602712	395.8014198
12.50968076	0.917471578	0.31582593	0.395121522	451.1224159
9.124395534	0.926031156	0.290257259	0.393804117	326.5281301
7.052085257	0.929145673	0.370197228	0.464302315	278.1771466
11.60151955	0.945025872	0.371409885	0.346307905	605.3270346
11.5769314	0.937077888	0.402567885	0.461453176	438.7086259
5.391011936	0.909468587	0.349101482	0.243730968	218.7427378
8.679214316	0.909014642	0.277780149	0.471465392	261.4065016
9.829205369	0.920759418	0.346002609	0.39241623	278.2056019
12.42627357	0.898579249	0.222408384	0.39354106	260.1733276
9.830660471	0.909953413	0.28731367	0.388601387	358.8071283
14.2216902	0.924182525	0.283284359	0.333948656	573.6470342
6.817738636	0.91731646	0.324770323	0.473691422	271.2809881
9.674195582	0.923309553	0.302925719	0.401569005	399.1599697
8.692593719	0.927662114	0.31984269	0.408916417	381.4502802
3.730515809	0.916759195	0.382724172	0.463875841	155.859254
10.59941142	0.895065824	0.279851726	0.387527016	243.9295765
4.820860737	0.927508989	0.384059087	0.562613824	168.5235579
11.48347804	0.914251352	0.271344073	0.392838274	492.7601403
12.07907076	0.909463204	0.234492512	0.347001044	307.0876934
7.160357785	0.91314051	0.307119718	0.403147373	205.2576668
6.566462873	0.904794042	0.313575324	0.421917027	181.1443953
7.394436018	0.909234891	0.291013079	0.438828949	332.6446338
8.347568523	0.929765161	0.327797316	0.391676507	487.8871502
9.766740183	0.909056347	0.274327871	0.379792732	354.3253693
10.63680488	0.930026825	0.334147903	0.390233157	427.9304204
9.791547929	0.910945196	0.303488308	0.312388618	289.2240965
12.37765942	0.922362752	0.309818704	0.36812275	471.3542053
8.466565468	0.933374666	0.379999835	0.421684901	301.1796409
8.884522569	0.920886424	0.312584847	0.372216672	387.3122194
9.453466037	0.919116586	0.303772189	0.412669399	396.3196884
8.287457441	0.933029009	0.318425435	0.466074015	524.3550025
4.487386009	0.907972941	0.351333644	0.448916947	116.0368838
9.663057948	0.935009197	0.363715492	0.395210091	472.8008342
8.349056405	0.932638767	0.374382272	0.222888091	413.5788702
11.08177983	0.928885806	0.3157792	0.393523588	430.6082568
9.334103457	0.932176874	0.311174451	0.40692489	424.3695915
5.12039618	0.929092264	0.393736045	0.411556376	297.8194338
7.704135566	0.915780165	0.313791306	0.416107381	295.9693691
8.519016996	0.936836823	0.345504194	0.472620467	391.9764054
10.93442816	0.931126356	0.325890026	0.406551228	548.7713274
15.83086409	0.891407615	0.245376369	0.31864747	345.6926616
10.89634621	0.906181309	0.253127456	0.386074747	255.3836736
10.08259395	0.916575972	0.329174965	0.340352126	396.7370478
4.418049	0.929281253	0.383686475	0.467220035	244.7454626
6.351061327	0.922095777	0.371849073	0.494650554	207.4327028
6.195129497	0.920818757	0.331903486	0.39577344	264.1483016
15.66217968	0.915558744	0.250406693	0.406827881	545.5047835
20.2582538	0.899818559	0.257008651	0.367880267	522.6141857
13.09159397	0.913756617	0.317603655	0.280957782	235.7691661
9.588345942	0.925920477	0.342437356	0.3706591	381.6497576
4.542423183	0.911846879	0.35215863	0.474938889	199.8437328
11.27926178	0.908683651	0.274099956	0.385891139	335.595172
2.770572211	0.939271917	0.46825799	0.570078078	252.0214362
14.4405892	0.90780183	0.293081594	0.34564692	373.0908434
7.516908709	0.920498527	0.336710879	0.474720218	334.9680829
6.71270603	0.92241237	0.34699093	0.447257569	310.1586338
7.77756789	0.931216129	0.367998113	0.411488829	448.1688449
3.60710067	0.933542957	0.451547289	0.566575484	178.8436399
8.549286651	0.901579398	0.258981658	0.438473657	308.7887826
11.56145456	0.918373845	0.28132013	0.347459662	382.5671014
8.553290175	0.916148377	0.333460616	0.430153249	314.1899246
9.424850418	0.907166505	0.27183497	0.324895801	351.0358343
13.73711088	0.929633808	0.34135705	0.433336711	542.4941522
6.820730031	0.917058084	0.312908299	0.473524819	253.8951615
11.76040024	0.924992416	0.35557357	0.34466438	438.3865196
5.223289167	0.911922174	0.37891769	0.383927557	209.9033343
6.313990968	0.932284983	0.386490425	0.457851579	278.8363808

log.sigma.2.5.mm.3D_glcm_SumEntropy	log.sigma.2.5.mm.3D_glcm_AverageIntensity	log.sigma.2.5.mm.3D_glcm_Energy	log.sigma.2.5.mm.3D_glcm_SumSquares	log.sigma.2.5.mm.3D_glcm_ClusterProminence
5.106264878	22.78571571	0.005516761	30.60608043	32978.19628
4.233410952	13.58394212	0.021223512	9.495418608	3640.254032
4.494095929	11.61123165	0.014707734	11.27362031	4344.77155
4.908695084	18.72104918	0.007910767	23.02093187	14742.75062
4.673874954	17.94785036	0.010033761	15.97920823	8297.923345
4.862895152	16.55327836	0.008190244	21.78416837	13783.88145
4.657891563	20.34385119	0.010950772	14.01445785	7717.547093
4.609346228	20.55819181	0.012587377	13.56543139	7877.13093
4.815631764	22.67209417	0.007216889	16.85944305	9612.175697
4.694343749	18.07101046	0.009086362	16.39879954	10030.5017
4.414519259	13.15817025	0.011909196	10.54613511	3326.607856
4.814848973	21.89169963	0.008729559	17.91132791	12046.72833
4.207968752	16.77116138	0.035111256	11.68947379	7592.429477
4.88008376	26.31174374	0.009419527	22.6710488	27413.96284
4.842516371	20.39273595	0.008525071	21.30138305	17167.65944
4.665652743	18.20159256	0.012825188	15.80730232	9077.145181
4.691852272	14.97946958	0.007840188	16.29780954	7377.068184
4.314679401	13.27957709	0.021900979	10.96158581	4547.273878
4.235153699	13.04469917	0.015296139	8.286769886	1995.467349
4.755907799	19.510144	0.009024737	15.88265	9205.244571
4.232450834	15.07745413	0.025504685	8.773191255	3517.477121
4.407838796	13.4491731	0.011147445	9.966312165	2602.487327
4.475553605	16.30395323	0.013340728	12.78674354	5281.253153
4.537720544	14.52581837	0.010780041	12.13485313	4578.412993
4.607197195	21.84691942	0.01226586	15.238045	10430.61575
4.996438775	20.85174817	0.006266174	22.69364034	21955.43701
4.93809591	20.53231577	0.007821985	21.81629898	16678.45207
4.605292828	20.05967571	0.011285656	14.72621215	8670.225513
4.313543992	13.26474198	0.012507542	9.774400043	2522.336729
4.366658431	17.32561443	0.015308073	10.25738988	4056.974573
4.679273314	22.45426477	0.012805396	15.19297265	10349.32584
4.316346019	17.28616189	0.018881928	12.49781139	6824.754701
4.964955359	19.8737857	0.006761526	21.69690279	14870.25609
4.788300201	21.19712486	0.00980874	19.01975526	14954.62813
4.734202374	18.07093031	0.008266349	15.96471024	8329.228661
4.471595145	16.56869123	0.01728274	13.24681537	7326.011731
4.463988695	24.61934495	0.018179704	14.83374412	11407.53799
4.681391487	20.86298759	0.022288797	17.49336122	14212.77847
4.06202859	14.72658528	0.015884246	7.682671206	1960.899614
4.933073276	15.98327085	0.005641084	19.49184304	9395.403877
4.53956761	16.5797698	0.014735146	15.59845608	10208.15966
5.077123945	16.04492632	0.004085141	25.89784062	16555.30509
4.777186356	18.95408986	0.00762586	17.35439265	9054.375626
4.818319497	23.94114795	0.008294519	20.34181212	14918.70585
4.59813342	16.28711743	0.011948261	14.43041458	6815.006104
4.720326614	19.94648642	0.009387786	16.39978768	10345.59843
4.62947809	19.51375084	0.011164383	14.6198708	8877.291086
4.180371209	12.33516139	0.01924916	7.891599604	2334.291989
4.788702327	15.41023684	0.008073704	19.00833623	9441.772301
4.425818431	12.72070932	0.017818678	11.91597258	5118.622542
4.870928217	22.1782997	0.007014632	18.85581981	13418.14652
4.935682436	17.54141534	0.005337805	21.68648001	13490.27877
4.613095412	14.14800258	0.009423298	13.72354082	5721.936082
4.536760384	13.25366819	0.010628493	12.90583339	5047.139858
4.705254804	18.13699762	0.007836327	16.00579521	6278.789866
4.576583815	22.07183411	0.011690533	13.1485195	7015.948117
4.791506092	18.79140168	0.007138233	17.70908549	9168.267469
4.614471331	20.66861598	0.012509866	15.45549375	11473.33538
4.574221086	16.94345749	0.010231594	15.02998509	7006.66691
4.858164989	21.67905193	0.007588598	18.4962174	11426.57788
4.44411534	17.26690194	0.020029473	13.64262726	8081.860875
4.601345733	19.69464757	0.011104727	14.2984141	7512.203643
4.738983601	19.87955666	0.009366114	16.67804343	10301.36338
4.746023035	22.81792814	0.009254466	15.88540255	10278.61627
4.361639732	10.56710141	0.013847051	9.545015756	2647.582523
4.526598839	21.71889192	0.016075526	13.65819503	10257.74211
4.106900281	20.45752225	0.021074499	9.125567169	4712.791657
4.762099329	20.72831267	0.009772311	17.03438638	11875.24342
4.731101772	20.58211715	0.008788884	15.21856857	9586.588628
4.243337451	17.16912307	0.019027333	8.829766294	2779.680674
4.597721148	17.11374056	0.010571561	14.78787387	7585.822348
4.648768426	19.73307614	0.012854246	14.65309869	9725.418337
4.700973872	23.35658557	0.010722634	17.05282742	14303.16017
4.956212776	18.59712767	0.00615657	25.3186877	16263.90583
4.938473347	15.86681907	0.005224317	21.32781164	11496.26899
4.424459422	19.94182569	0.0138967	14.47333421	10380.37586
4.295664234	15.51821926	0.016953747	8.989188452	2955.398674
4.451050704	14.20060002	0.017845552	12.65938354	6686.290379
4.439205197	16.14430947	0.012775132	11.34400723	4143.197356
5.04004779	23.26597027	0.005720874	26.74520913	21625.84606
5.086152964	22.80932909	0.006073154	30.18568656	33071.29203
4.650983865	15.30557568	0.010937972	17.7741627	8951.915736
4.580447819	19.54809739	0.01271952	14.46343214	8331.170956
4.423578209	13.96797126	0.012353014	9.930735062	3269.308773
4.862295299	18.31317799	0.007154428	19.92249513	11890.16993
4.028139842	15.75716906	0.033107297	6.502847443	1906.015693
4.835751843	19.3265597	0.00860699	21.79830247	15779.20934
4.661970174	18.20800517	0.011532089	15.21319972	7247.217997
4.50713506	17.49380536	0.012972176	12.81670218	6326.908337
4.422581336	21.18245662	0.01665352	11.7718228	7150.706218
3.995945926	13.19210036	0.037036877	8.326630339	2685.014475
4.884899187	17.45205388	0.005868594	19.43569496	9492.821518
4.788653367	19.58678551	0.007732902	17.78458644	11074.71635
4.640740063	17.60162513	0.011414194	15.44498297	8746.306437
4.675279481	18.78993968	0.007563248	15.9286159	6466.710638
4.855064383	23.2182762	0.012213022	20.59724858	19125.72882
4.706650504	15.74885757	0.00930573	14.9405974	6758.445617
4.476670386	20.94928272	0.016362815	15.25593775	9735.031768
4.207851172	14.37106757	0.017661288	8.732980795	2704.728108
4.41819596	16.61467123	0.019128384	11.77271041	4471.446471

log.sigma.2.5.mm.3D_glcm_SumAverage	log.sigma.2.5.mm.3D_glcm_Imc2	log.sigma.2.5.mm.3D_glcm_Imc1	log.sigma.2.5.mm.3D_glcm_DifferenceAverage	log.sigma.2.5.mm.3D_glcm_Id
45.14815629	0.764096843	-0.102061852	4.629852204	0.329493339
27.16788424	0.785227917	-0.143485483	2.234915565	0.464095194
23.22246329	0.767785445	-0.128705471	2.359761851	0.453685645
37.07851802	0.83955563	-0.148220249	3.707930337	0.363139876
35.76244881	0.703514266	-0.090106037	3.134693977	0.390012895
32.94255141	0.879987775	-0.184488011	3.515550278	0.357038386
40.47953993	0.678866977	-0.084155209	2.879556295	0.405759459
40.87388053	0.686580506	-0.08676249	2.829072725	0.414202193
45.07080961	0.667825634	-0.077290772	3.37949404	0.377762749
36.01580078	0.802628175	-0.133902016	3.048868504	0.381344718
26.3163405	0.682371671	-0.090241531	2.643162313	0.409984768
43.49828542	0.735097548	-0.098235699	3.386627132	0.377353975
33.4763786	0.797822329	-0.154489755	2.124005211	0.502818705
52.36998301	0.816738848	-0.136735461	3.519317264	0.380186753
40.37604335	0.7534833	-0.103985917	3.944743975	0.358754288
36.16199192	0.70967127	-0.094377053	2.93237995	0.413215991
29.93788961	0.74834112	-0.106507424	3.284940737	0.371194235
26.55629205	0.781496514	-0.141184258	2.276163456	0.465769977
26.08402397	0.698818461	-0.09992201	2.301667686	0.438194915
38.73530818	0.690623262	-0.085748491	3.093825107	0.385297226
30.14902926	0.748876336	-0.12737215	1.957733719	0.498386662
26.8983462	0.706483887	-0.097244394	2.596953357	0.405255769
32.55311076	0.786769287	-0.132728091	2.705844958	0.416828522
29.03299278	0.698966549	-0.093050876	2.678896991	0.414946249
43.48122841	0.798349048	-0.134734502	3.03133058	0.40353391
41.36571996	0.784237704	-0.117221646	3.681460691	0.351922357
40.71061777	0.751657514	-0.102472145	3.654214746	0.368303538
39.82971349	0.691887937	-0.087110704	3.107265015	0.395755808
26.52948397	0.781604793	-0.132957959	2.594814823	0.397600094
34.60853519	0.701914777	-0.098944545	2.433499879	0.436670172
44.71137715	0.713698483	-0.096317658	2.9284753	0.417023969
34.50937957	0.698483913	-0.096225592	2.724335268	0.437864132
39.33865957	0.729668453	-0.094672114	3.66880586	0.366113648
42.11034702	0.763835249	-0.11116147	3.351923999	0.392807654
35.78154259	0.673317556	-0.079941693	3.237561974	0.374291821
32.9944714	0.741915058	-0.113503185	2.551372561	0.440759911
48.99499518	0.736496884	-0.108335044	2.829713638	0.438943963
41.48982049	0.799361999	-0.13751323	2.799536365	0.466095631
29.45317056	0.583751839	-0.064851133	2.477959174	0.425536265
31.77846845	0.650277724	-0.071974666	3.335659154	0.364117019
32.99802267	0.720324927	-0.100074652	2.921490709	0.418942233
31.64377778	0.659400349	-0.069343604	4.248750494	0.313638651
37.53691601	0.670353152	-0.079149758	3.325587991	0.371381189
47.60668025	0.720695229	-0.092689609	3.725092136	0.364815985
32.52587951	0.77795206	-0.126444324	2.809631289	0.403582057
39.61450657	0.731559657	-0.098847326	3.214393057	0.383589077
38.74220255	0.686077174	-0.085556069	3.009366186	0.398230954
24.67032278	0.758287123	-0.130466583	2.140385782	0.454389262
30.76814614	0.834270453	-0.148181427	3.475026151	0.364580209
25.43175755	0.812013145	-0.156984416	2.26080029	0.453987065
44.04124437	0.725539553	-0.093375381	3.545755077	0.357601722
34.61544865	0.655384586	-0.069501316	4.050125122	0.32432095
28.26472134	0.621275104	-0.06815617	2.964001164	0.38898701
26.50733638	0.761120287	-0.117226735	2.84583978	0.395374848
36.09602371	0.760628976	-0.113025975	3.108354278	0.375809915
43.92921365	0.733878129	-0.105283771	2.91388427	0.405540095
37.2899756	0.639414403	-0.069037943	3.453466907	0.360141627
41.06629309	0.710359212	-0.094326223	3.017527812	0.4092975
33.74242662	0.707437357	-0.092386039	3.193777864	0.384794919
43.09211421	0.664253702	-0.0748607	3.466939613	0.387336426
34.37670462	0.782593301	-0.131431688	2.629825766	0.447866096
39.08547638	0.677936597	-0.083667258	3.055320325	0.392352733
39.46007445	0.667736344	-0.078080606	3.223055663	0.384236391
45.45996408	0.687744136	-0.088443793	2.980808019	0.398002901
21.13420282	0.766058982	-0.130070822	2.400979555	0.427790595
43.22738664	0.721222187	-0.100850185	2.785263665	0.433991051
40.57129813	0.695254962	-0.09712009	2.585806603	0.443286391
41.16391135	0.680539142	-0.081940573	3.220254859	0.393907688
40.88076404	0.667157321	-0.079260847	3.10797178	0.391246521
34.30369752	0.67413089	-0.094312489	2.217072115	0.461281141
34.06258372	0.768013266	-0.118924839	2.974689892	0.394515946
39.22962475	0.74742841	-0.109312921	2.746573718	0.420831754
46.54414825	0.765141172	-0.114733528	3.114774837	0.402646369
36.75385918	0.698030447	-0.081786338	4.291863011	0.330301202
31.45226876	0.676924785	-0.077100717	3.78867117	0.340933209
39.59239078	0.739625335	-0.110597431	2.994819733	0.40623607
31.01666915	0.701875684	-0.10334809	2.192098034	0.453965109
28.37352639	0.818108527	-0.155165985	2.473777252	0.442603507
32.23161595	0.701352764	-0.095593836	2.670882832	0.411407165
46.23047675	0.76274944	-0.104288345	4.094365266	0.336819571
45.21236786	0.786048196	-0.112446493	4.447825122	0.338897413
30.39149954	0.757455402	-0.108773242	3.429498966	0.392764044
38.79537696	0.726566727	-0.103342268	2.952461899	0.417276205
27.93594252	0.653339178	-0.085130481	2.389627326	0.428707206
36.21820185	0.711672238	-0.09111711	3.596772696	0.358861444
31.51433813	0.769583866	-0.147622806	1.651081022	0.523327662
38.23428619	0.730006054	-0.095509198	3.797249056	0.371111226
36.21515514	0.744500837	-0.108344065	2.796786002	0.413263574
34.90003418	0.761309268	-0.121044223	2.629608721	0.422693206
42.10202727	0.749871126	-0.117231492	2.55936789	0.438634952
26.38420072	0.772684734	-0.151922179	1.836055611	0.508929779
34.67137581	0.698600808	-0.084166598	3.513952315	0.348122945
38.78929075	0.630511581	-0.065855821	3.546518015	0.364476485
35.079431	0.712962023	-0.097812848	2.891153073	0.409945654
37.20047052	0.583157518	-0.056419176	3.421955218	0.358138051
46.17846206	0.791974983	-0.126453037	3.233665153	0.413155895
31.42855875	0.706632553	-0.092467558	2.905626195	0.394261812
41.6162998	0.797807678	-0.136608436	3.035311999	0.424427285
28.74213514	0.736231976	-0.118436842	2.3161965	0.448936
33.08279233	0.683826513	-0.093246242	2.422301993	0.454607299

log.sigma.2.5.mm.3D_glcm_ClusterTendency	log.sigma.2.5.mm.3D_firstorder_InterquartileRange	log.sigma.2.5.mm.3D_firstorder_Skewness	log.sigma.2.5.mm.3D_firstorder_Uniformity
86.50622762	139.7161674	-1.037423398	0.062691822
27.71780127	71.70474291	0.222949625	0.116762847
33.85693666	100.6151563	0.089753795	0.093492314
63.83526871	145.6444626	-0.669390903	0.068154032
43.59787724	100.3748705	-0.623135217	0.084749789
62.87050028	130.2043223	0.343866786	0.071354454
42.17192652	104.4331316	0.088694543	0.086103342
41.17411069	97.5773375	-0.185019705	0.091381217
49.33445777	121.7656912	-0.534721149	0.073759094
46.91037206	111.2407105	-0.632286823	0.078239864
29.53574075	93.73021364	-0.348190859	0.092981552
53.2203012	114.1557369	-0.674816469	0.078513276
33.97964296	58.42560339	-0.292747587	0.148178165
65.61300713	109.2682745	-0.839946318	0.079720456
58.93967529	106.0770164	-0.734793548	0.077770856
44.32356045	98.67883539	-0.243733304	0.088541789
44.60457777	122.5975189	-0.556209961	0.076131635
32.80785757	67.32895947	0.375796449	0.120350144
23.37036829	82.45935899	0.194839818	0.107036783
47.87498777	112.382609	-0.158658264	0.077466441
26.66347028	71.11539912	0.090607236	0.122560521
28.28149282	97.735834	-0.159095376	0.09247484
35.57674819	91.18834347	-0.21114055	0.094091415
34.50887625	105.2944596	0.005044654	0.086695718
44.1599282	99.80226517	-0.568746288	0.090088483
68.6581432	139.1273973	-0.233277807	0.065297663
63.07515465	119.6805656	-0.21618553	0.071700754
41.2761037	95.53653729	-0.651360904	0.090346069
27.97437054	95.02318636	0.259035977	0.096897537
28.54521701	77.63643026	-0.634137316	0.109556104
45.70067198	99.04257548	-0.394416229	0.090733052
32.55588885	67.27470684	-1.091995644	0.117339183
62.62989563	128.1288776	-0.418289755	0.067151102
55.17906623	112.6984912	-0.836859419	0.078205885
45.52282757	122.7843704	-0.254924198	0.073772316
37.71348758	93.43847346	-0.218672464	0.101645864
40.59343284	72.83915186	-0.712769222	0.111080504
53.07521928	88.25277376	-0.294589592	0.103360834
19.10460155	85.33614802	-1.033582411	0.115912486
55.7564326	142.8773966	-0.001662662	0.065247848
42.52289777	78.97894597	-0.885554634	0.102627854
70.28332481	163.153471	0.143102986	0.057424292
47.40897608	121.7754693	-0.431319556	0.073521503
56.55198413	107.33476	-0.761220016	0.079126754
41.94558316	99.6914854	-0.040944264	0.088001842
47.47554004	114.44098	-0.221679963	0.077450971
42.70529083	101.6915562	-0.139854068	0.085703218
23.18006816	71.34725761	0.075848562	0.118304969
51.93840059	118.3269405	-0.533397873	0.074494512
37.2644102	113.8276246	0.382046251	0.098557692
55.60967527	128.2257061	-0.677412267	0.071364857
58.8719107	139.7434464	-0.143467251	0.063898798
38.13099221	102.126685	-0.215358315	0.084852431
36.76913962	110.7742414	0.345621916	0.084626392
44.75605424	130.5121017	0.054816539	0.0739974
38.73035881	91.8741076	-0.694415112	0.092002104
48.67905257	124.872243	-0.456606459	0.07106327
45.18962746	88.96515882	-0.947130146	0.094062402
38.4930307	95.2437768	-0.663753309	0.089153372
53.08784918	127.0349083	-0.458481842	0.072923495
38.42408349	73.89975893	0.349338384	0.109070411
39.89996581	106.0909027	-0.5836776	0.086748306
48.13562583	115.9581947	-0.313067226	0.078576452
48.15770984	106.1573868	-0.005334273	0.081326665
27.7883789	99.64272761	0.00785041	0.093690114
40.1752498	84.42494017	-0.529403459	0.102426651
23.45006881	63.65621471	-1.262308552	0.128517963
49.58017799	101.9202514	-0.294059585	0.083591168
45.41715795	112.1290578	-0.58978845	0.078022504
24.6120539	75.8259629	-0.38273815	0.114973273
40.45609147	100.4673319	-0.506269487	0.085952894
45.08974711	102.8515551	-0.332240961	0.089368239
49.52034809	104.0845246	-0.815851188	0.084011236
66.55037384	140.1269207	-0.636811654	0.063863576
57.84310903	147.5119991	0.066935666	0.062384062
38.70280002	87.30278599	-1.414766354	0.100410669
26.18860352	82.47312403	0.112632863	0.105743504
37.26181041	79.54119015	-0.045039096	0.107528733
31.13871419	90.96906053	-0.304686549	0.096339584
77.51790394	145.2887535	-0.74119502	0.065102567
86.77707248	134.2125454	-1.017494394	0.064145409
44.52804778	101.3440638	-0.254768491	0.084344616
40.07901869	106.6801013	-0.233281496	0.087281068
29.31358128	92.40460944	-0.502581998	0.096373323
55.11922604	128.2114525	-0.42084888	0.069217736
20.46437842	54.89583313	0.000341919	0.148272877
59.73349672	121.224102	-0.798622388	0.075427572
43.66122378	114.1409287	-0.20209269	0.083267762
36.33210894	93.76502514	-0.696635201	0.094730732
34.53524284	84.12756538	-0.851890587	0.101980796
26.2442789	64.50943351	0.89956396	0.14581373
54.08798287	134.9692848	-0.10115552	0.067784835
49.99684336	111.8206987	-0.669497309	0.077179066
43.23693521	98.31574273	-0.653145801	0.087600184
41.62452677	124.1600614	-0.559451112	0.075754977
61.87211025	105.8243942	-0.754351443	0.083950551
43.22244722	108.8071251	-0.159022661	0.080960984
42.83593961	75.77783775	-1.277686565	0.110970627
24.21330666	82.06316853	-0.932523395	0.11417916
33.15696688	85.36366141	0.253375503	0.108325498

log.sigma.2.5.mm.3D_firstorder_MeanAbsoluteDeviation	log.sigma.2.5.mm.3D_firstorder_Energy	log.sigma.2.5.mm.3D_firstorder_RobustMeanAbsoluteDeviation	log.sigma.2.5.mm.3D_firstorder_Median
102.3515508	43728070.16	62.34144095	48.28226471
56.13199186	8636729.063	32.07217681	46.15458679
66.04351357	16547357.41	42.39947155	47.54277039
93.83990362	15086013.53	61.55529541	43.46133804
72.25894754	26380406.78	44.31112814	41.78599167
88.62542606	11316969.13	56.03200656	45.16466522
72.41336879	67732667.89	45.8863277	40.14797592
71.09007499	66223895.76	42.63061133	48.47934914
79.37048354	64239556.82	51.83242016	61.47342491
76.31954923	15739766.8	48.8734309	67.72161484
62.41157984	12779069.44	40.64707598	42.26807022
79.44247809	37698279.88	49.26829619	62.33815765
54.11459467	15295161.2	26.86890317	24.01608658
83.03410292	28302452.85	47.92229131	44.78622437
83.99216254	32337775.37	48.95880612	56.68537712
73.2493854	52862522.17	44.6397107	35.2675724
77.94977352	13795623.03	51.8002224	50.90634537
58.33524775	11004046.79	32.71418758	34.74972153
55.02329283	8832131.582	35.70019075	34.35497284
78.27445431	74565190.99	49.03872477	51.6880188
54.8246152	23326089.96	32.41378397	33.71543121
62.07427627	9757783.017	41.54546414	51.23950195
66.00169951	10418841.31	40.47474504	39.53241348
67.51315839	27242443.33	44.44581341	45.60248947
72.69822593	18212137.87	44.48706009	53.08450699
92.5349161	48081966.4	58.02030825	66.29187775
87.57989568	55178766.4	53.52037109	49.63311005
69.74060423	31005371.04	41.35185742	40.62771225
62.21505298	3864308.506	42.48638642	36.6873436
56.57808888	17616190	33.95015075	51.97973251
71.04386025	60548801.59	42.99940965	45.6414299
59.18521614	16565869.3	31.6269265	28.35523796
90.1113465	50060609.24	57.48165499	43.98698044
80.7700442	34276896.62	49.50354858	47.64193344
80.04265098	69012575.52	52.79434486	65.77647018
66.39048671	33339898.14	40.44216127	40.47684097
63.06324931	37648092.47	32.68507356	27.57959175
74.60848624	66301633.17	42.5277957	25.09867287
52.39192822	7004342.507	35.08532546	41.14739037
86.55686036	95913457.95	59.48504535	34.8949852
67.56552925	26240359.2	37.01339263	33.26490402
98.69129014	64641262.28	67.58566886	33.88993073
79.99024551	52285876.92	52.55090314	55.10607529
80.95337678	34687209.19	48.49102244	50.65154266
72.0411523	19234348.44	44.78757039	65.09188461
78.20619037	40970458.48	49.44587388	58.85808563
73.31078321	59531623.45	44.68469433	46.0112896
52.39649992	6808461.06	31.14768737	47.67842484
80.67436291	12305145.71	50.60052058	61.32702255
69.4446906	15740431.36	47.12738702	41.57457161
84.67169604	46248875.93	54.46008045	71.06140137
91.03232254	55350280.14	59.84178686	52.47187424
69.70707134	47322708.62	44.14013942	43.72288895
71.21518915	14882235.32	47.28955685	58.61055756
77.78536062	18354894.01	53.3470027	54.1082325
67.52683486	21254672.48	40.61651396	46.05484962
82.31215438	60051756.55	53.5724868	59.76319885
69.65416275	38199503.6	39.36411935	36.12022781
69.87367535	17499179.03	41.33157261	38.43456078
82.38669799	114144291.1	54.54614559	25.85389328
66.03734983	20010209.22	35.99279471	35.55097771
71.54637862	33248450.22	45.6564474	49.71209717
78.51250209	85502572.3	50.60818817	54.07439804
75.08567319	91941235.63	46.06886738	36.10784912
63.33698916	7756175.428	42.37392094	49.5347538
65.6368586	40361263.14	37.2421381	30.91582966
51.74766787	8565072.746	28.04876454	26.2734251
75.44963062	75934387.46	44.47230278	36.96652222
76.82924553	76352922.8	48.63126504	55.60459518
54.53573717	21914755.78	33.3530346	38.02120018
70.22498436	13415231.12	44.34051735	39.76203537
73.00791581	59924861.27	44.86231957	51.83843231
75.91115721	31566187.17	45.47451022	50.21728897
98.20555475	38375038.71	63.372509	40.67620087
91.00684075	45433715.31	62.11698413	29.74857521
66.9736404	13717751.47	38.82197258	50.84355545
57.31516608	20893273.42	35.77756337	47.54950905
63.08974229	9676487.986	36.47303268	36.77176666
62.62402557	15736275.24	38.80168466	42.56177902
96.42006918	38401042.5	62.44556198	40.97473526
101.171656	40511395.45	61.09869458	48.25589371
76.97204368	20888115.29	46.08139638	29.15288162
72.0392066	38902382.59	45.06523003	39.79319763
60.6177902	23731543.14	39.81884043	44.92855835
85.81161929	35314816.3	55.63397606	48.04896164
45.30395534	8135027.113	25.85407864	26.93464661
87.0292636	34252851.79	52.45593296	34.32313156
75.5005076	40148318.01	49.35426355	58.28509903
64.90365759	15980289.68	39.84394896	52.55295753
63.34888056	20832992.5	36.66413776	43.32660294
55.6449051	10114225.73	32.91748235	27.16533279
84.6999281	39118031.94	57.32432917	64.22073746
80.00495749	66189102.01	49.11541587	39.48067856
70.67382539	40997333.86	43.13769487	52.65520287
78.44078532	47506792.46	52.38560273	39.73142624
80.66582318	52818205.91	46.92212396	42.48080254
73.47477741	29538335.93	47.04698786	39.21905899
67.14740865	10232030.35	35.2288429	24.74102211
54.49633632	5038892.565	33.74084226	33.99078178
63.45315394	59078013.06	38.80547522	37.42430878

log.sigma.2.5.mm.3D_firstorder_TotalEnergy	log.sigma.2.5.mm.3D_firstorder_Maximum	log.sigma.2.5.mm.3D_firstorder_RootMeanSquared	log.sigma.2.5.mm.3D_firstorder_90Percentile	log.sigma.2.5.mm.3D_firstorder_Minimum
1180657894	373.5844421	143.5514111	185.021965	-516.0214233
233191684.7	338.7020569	96.1100587	153.4232605	-273.8578491
446778650.1	359.1340637	103.8607953	175.9214066	-221.5218964
407322365.4	347.5782471	124.262465	159.3542999	-410.8236694
712270983	309.794342	105.5479724	156.8385208	-393.8573914
305558166.6	425.1471252	130.9462597	220.9885468	-331.400238
1828782033	506.8499451	109.4804156	177.705307	-440.951355
1788045186	451.6871643	113.8628155	182.0884628	-433.0189514
1734468034	422.4206238	118.8739113	181.476799	-487.5892639
424973703.6	315.4377441	121.1718543	198.0696259	-366.5860291
345034875	319.4610596	92.05533109	147.2476578	-260.9544067
1017853557	371.7673645	127.8866768	203.1191925	-453.9032288
412969352.4	321.2275696	90.51173091	125.5483307	-358.9414368
764166227	379.3240356	128.3887262	180.5112946	-590.2734985
873119935	509.6199341	127.6686412	174.3744293	-439.2289734
1427288099	411.3769226	109.909513	178.4764481	-389.7349548
372481821.8	335.1217346	107.4901072	157.4580414	-301.5023499
297109263.3	356.7386475	94.47016543	166.8420898	-259.4111633
238467552.7	362.1419983	82.4888823	136.9426559	-254.4466705
2013260157	459.5774231	119.7589994	195.1922546	-420.81427
629804428.9	337.193634	87.82741079	146.5268967	-320.8718567
263460141.5	273.2526245	95.09657068	156.4896667	-270.7527771
281308715.3	341.4963379	100.8694687	164.5035507	-336.3103638
735545969.9	369.2081604	101.4485998	164.8764832	-285.6412354
491727722.5	429.0148315	119.6096619	187.5628601	-452.1858826
1298213093	514.5396729	142.4651304	216.8351532	-432.1140137
1489826693	515.2955322	130.6420965	200.1828705	-428.7444763
837145018.1	380.848999	105.9513828	157.6104721	-448.9613647
104336329.7	270.5462952	92.97841401	163.3085144	-250.9556274
475637130.1	312.5007629	92.60954986	139.4185699	-368.5004272
1634817643	426.7869873	112.8081708	175.8030533	-485.2089844
447278471	298.5300293	89.48018429	106.8348511	-386.0875549
1351636450	421.553894	124.5128124	177.4511108	-442.9216919
925476208.7	358.4846497	120.7721551	178.4738464	-453.9718628
1863339539	564.5778809	125.1815897	199.7211563	-362.4199524
900177249.8	403.1270447	105.437209	173.359259	-347.883606
1016498497	538.9558716	100.5869531	124.3405151	-563.3544312
1790144096	470.3956299	113.3431253	177.5363464	-462.1835632
189117247.7	232.5752869	75.46249005	104.5933784	-323.6138
2589663365	423.0977783	114.8609936	175.7435547	-348.1533203
708489698.5	379.9340515	100.5772836	123.2003319	-372.8882141
1745314082	502.155365	129.2908612	186.3298065	-334.1978455
1411718677	370.8486328	119.637509	190.6307526	-395.9202271
936554648.1	486.1633301	118.075418	154.6328934	-534.0371704
519327407.8	397.5338745	122.6798716	209.6029953	-324.0115967
1106202379	458.0420227	122.1699054	192.1622375	-402.4703369
1607353833	471.0814819	114.2342205	179.0895279	-414.568634
183828448.6	290.4873047	88.41285227	137.6219025	-240.6110535
332238934.1	339.6279907	120.2482045	179.3122253	-317.7467651
424991646.8	412.2767334	107.8996334	188.3786713	-245.9372406
1248719650	390.9613953	134.174053	211.8154449	-462.7088013
1494457564	558.687561	128.1387654	189.0104828	-356.917572
1277713133	401.4483948	102.4118595	158.0839142	-298.4493103
401820353.6	373.3904724	116.1046322	190.2720886	-230.9395752
495582138.2	349.3935242	117.4762622	196.5751953	-355.6855774
573876157.1	382.2213745	104.4022607	154.8747314	-490.5853271
1621397427	394.1190796	121.8438448	189.8855042	-384.8700562
1031386597	433.4786072	105.1033032	138.2710464	-460.2807007
472477833.9	359.2970276	103.0457854	143.4059418	-373.1654358
3081895861	438.0702209	111.8189717	163.6988556	-476.817749
540275649.1	461.2628479	106.4462742	176.2885513	-350.7471924
897708155.8	377.0643616	111.5912321	180.4086517	-423.1698608
2308569452	411.3517761	121.5312082	198.520105	-421.5983887
2482413362	427.3353882	109.8227231	177.7627747	-514.460083
209416736.6	333.2313538	99.08553669	162.6463242	-189.6196747
1089754105	415.3847046	102.2295008	146.4589722	-485.4915771
231256964.1	343.3833923	81.04509437	100.1012901	-450.6020813
2050228461	490.9254456	110.2247794	149.2539886	-458.7935791
2061528916	548.4414063	114.1761044	164.6435333	-440.7539063
591698406.1	286.9292297	86.9599191	140.6773392	-363.3673096
362211240.2	361.3929749	101.6235832	151.5356842	-361.4795837
1617971254	508.0858765	118.1884052	191.766095	-417.237915
852287053.6	433.015686	117.6898207	168.0360992	-501.4733887
1036126045	364.5708008	130.336573	169.8890381	-420.3433838
1226710314	489.429657	120.461313	182.0255127	-330.7688599
370379289.8	277.8071899	101.1032712	131.5407333	-434.9671021
564118382.3	349.7685852	93.38128309	151.5682068	-317.4500122
261265175.6	361.4955139	100.5023291	155.5025497	-288.986969
424879431.4	371.4830017	96.01392217	151.9369934	-348.2633972
1036828148	462.7065735	128.1316434	158.0060242	-525.7952271
1093807677	373.5844421	142.7155219	184.8744049	-515.8444824
563979112.9	455.4678345	107.7541601	130.3202972	-330.7885437
1050364330	414.5370483	109.0889195	171.1745728	-403.2492981
640751664.9	267.9807739	87.87831265	133.0978943	-286.21521
953500040	422.8328247	120.3296284	178.8508789	-383.0986633
219645732.1	239.7555542	72.87022655	122.4828484	-333.9161682
924826998.5	381.0682373	119.8406556	147.1265076	-448.8538818
1084004586	361.2105103	121.0040629	204.0735992	-360.1446228
431467821.3	347.896759	104.4772627	163.8046585	-359.3144226
562490797.4	320.5814209	101.4292735	148.163559	-455.8621826
273084094.6	307.0581055	88.06990446	169.6888687	-266.7584839
1056186862	381.5383911	128.1497077	210.6472183	-340.9394836
1787105754	468.6484375	113.0062959	152.153009	-431.9480286
1106928014	333.2362976	111.4604616	175.5150513	-358.8439636
1282683396	331.2208557	104.4800595	142.4363632	-404.8965454
1426091560	448.889801	124.2372473	190.0847198	-523.1585083
797535070	384.0314636	102.3999133	150.6077759	-334.6769104
276264819.5	338.9741211	98.85703236	105.2576706	-488.1948547
136050099.2	212.2168732	77.72925837	100.7891731	-302.4055481
1595106353	415.6176147	101.1520178	177.5048721	-347.3977356

log.sigma.2.5.mm.3D_firstorder_Entropy	log.sigma.2.5.mm.3D_firstorder_StandardDeviation	log.sigma.2.5.mm.3D_firstorder_Range	log.sigma.2.5.mm.3D_firstorder_Variance	log.sigma.2.5.mm.3D_firstorder_10Percentile
4.362386472	139.9684288	889.6058655	19591.16105	-155.8503525
3.555818813	79.66920327	612.559906	6347.18195	-25.36296959
3.777632713	87.61693547	580.6559601	7676.727382	-39.48995667
4.214869263	121.8086077	758.4019165	14837.33692	-146.5387451
3.944910594	99.15586227	703.6517334	9831.885023	-86.77052002
4.183583458	118.2217042	756.5473633	13976.37134	-71.52948303
3.925715605	97.14571197	947.8013	9437.289355	-55.24744034
3.899744183	98.73270776	884.7061157	9748.147582	-43.16517334
4.068087357	104.7264519	910.0098877	10967.62973	-72.88232422
3.988593016	102.5243837	682.0237732	10511.24924	-50.69693527
3.726830103	82.24936866	580.4154663	6764.958646	-58.18443642
4.0533659	109.6248287	825.6705933	12017.60307	-50.35383682
3.42336757	85.12925107	680.1690063	7246.989388	-38.03101807
4.130224544	120.3399479	969.5975342	14481.70305	-90.5277832
4.123129555	118.4318488	948.8489075	14026.10282	-107.5797775
3.944025025	101.3683125	801.1118774	10275.53478	-71.34590912
3.994006042	100.3670592	636.6240845	10073.54657	-97.69004974
3.567396862	82.940428	616.1498108	6879.114597	-37.93358154
3.53921265	72.56571567	616.5886688	5265.783091	-47.42794075
4.065976739	105.6675649	880.3916931	11165.63428	-65.39575195
3.508205366	76.57141982	658.0654907	5863.182334	-32.45267296
3.706383956	79.95209723	544.0054016	6392.337852	-42.6084549
3.794128411	90.34968739	677.8067017	8163.066011	-47.85297165
3.833952906	88.13946944	654.8493958	7768.566074	-55.61287003
3.906047011	102.6725028	881.2007141	10541.64283	-36.64203873
4.299277881	125.237354	946.6536865	15684.39484	-70.99240112
4.214952451	119.6573953	944.0400085	14317.89225	-99.37605896
3.896815248	98.26920194	829.8103638	9656.836051	-66.96338959
3.632694893	79.17392398	521.5019226	6268.510238	-45.71184921
3.600517425	79.25012093	681.0011902	6280.581667	-38.86722603
3.90853246	99.41164445	911.9959717	9882.675052	-46.9776886
3.616237185	87.5557596	684.6175842	7666.011039	-86.51976471
4.242821408	119.8915847	864.4755859	14373.99207	-131.9158508
4.076006474	112.1451083	812.4565125	12576.52532	-89.31441193
4.073486555	104.8256586	926.9978333	10988.4187	-60.31430359
3.75996158	91.92781846	751.0106506	8450.723807	-35.59605865
3.737550332	97.21029498	1102.310303	9449.841451	-67.59947968
3.888994612	107.0644232	932.5791931	11462.79072	-75.25479889
3.401217018	68.92268699	556.1890869	4750.336782	-53.46022835
4.173720729	109.5531648	771.2510986	12001.89591	-107.9149345
3.798459639	98.11337334	752.8222656	9626.234028	-98.55321426
4.3616508	125.3474106	836.3532104	15711.97335	-132.9382294
4.074101071	105.3861855	766.7688599	11106.2481	-69.89710693
4.077085936	112.1421152	1020.2005	12575.85399	-111.3634972
3.908999886	97.95370442	721.5454712	9594.92821	-26.67945747
4.060393788	105.1501087	860.5123596	11056.54537	-61.4397934
3.966760883	101.157459	885.650116	10232.83151	-55.73162651
3.475107643	73.22314574	531.0983582	5361.629073	-35.42815781
4.07851653	107.7814742	657.3747559	11616.84618	-90.46903992
3.738374543	88.77964889	658.213974	7881.826057	-29.66585484
4.145559995	113.9726173	853.6701965	12989.75749	-55.02754211
4.269195256	118.9445467	915.6051331	14147.80519	-108.3068008
3.901118124	93.1972276	699.8977051	8685.723232	-69.60686035
3.87488848	92.21531012	604.3300476	8503.66342	-31.92903347
4.011814631	99.10035889	705.0791016	9820.881132	-53.28083229
3.857984562	94.73518275	872.8067017	8974.75485	-62.1486763
4.118766629	108.3531544	778.9891357	11740.40606	-86.88928833
3.872639308	100.7694928	893.7593079	10154.49067	-83.75116272
3.893067786	97.84374248	732.4624634	9573.397942	-80.72300034
4.098172432	107.8773206	914.88797	11637.51631	-93.38900909
3.76935677	96.18407147	812.0100403	9251.375605	-57.45586777
3.905263599	97.30219803	800.2342224	9467.717742	-52.14711876
4.037649648	104.796534	832.9501648	10982.31355	-55.62894211
4.000089675	102.5422235	941.7954712	10514.9076	-73.79022064
3.715590585	81.5400588	522.8510284	6648.781189	-45.63411217
3.795457635	95.89676409	900.8762817	9196.189363	-56.07691803
3.444641265	78.32629016	793.9854736	6135.00773	-58.43315544
4.020789999	105.568911	949.7190247	11144.79497	-96.92002869
4.030693021	103.5520782	989.1953125	10723.03291	-84.7110611
3.532559544	75.79493872	650.2965393	5744.872735	-33.99304619
3.896985837	94.82407321	722.8725586	8991.60486	-83.13425293
3.920330651	101.0650651	925.3237915	10214.14738	-35.43358421
3.998093777	106.9918113	934.4890747	11447.24768	-67.37496948
4.303918931	128.571664	784.9141846	16530.67279	-165.1198547
4.250507723	116.0307828	820.1985168	13463.14255	-114.1171188
3.747300288	95.01055146	712.774292	9027.004889	-78.70310669
3.620474114	77.47469994	667.2185974	6002.329131	-34.21729469
3.715180761	89.97859651	650.4824829	8096.14783	-45.43897781
3.748361609	85.87360481	719.7463989	7374.276003	-57.87937546
4.281504578	126.5833645	988.5018005	16023.34818	-159.9470398
4.342152741	138.9366903	889.4289246	19303.40392	-158.184317
4.005538111	106.3215603	786.2563782	11304.27419	-129.4596405
3.91880716	97.86421127	817.7863464	9577.403848	-56.98536682
3.669706686	79.0512394	554.1959839	6249.098451	-67.86227112
4.173438065	113.3302789	805.931488	12843.75211	-111.1897186
3.253800825	64.6651178	573.6717224	4181.57746	-29.59920597
4.146294077	118.5635488	829.9221191	14057.31511	-146.4228912
3.947116561	99.63326054	721.3551331	9926.786605	-34.18015175
3.76626712	90.20072733	707.2111816	8136.171211	-36.55571098
3.751132334	91.73893448	776.4436035	8416.032099	-51.17634277
3.310536955	75.02878658	573.8165894	5629.318815	-23.77582378
4.141462406	108.5271597	722.4778748	11778.14439	-63.43581047
4.077914007	109.0323435	900.5964661	11888.05192	-112.0922501
3.901639002	98.02325282	692.0802612	9608.558094	-54.90319633
4.010066186	100.8007713	736.1174011	10160.7955	-112.0103653
4.054776088	115.5849997	972.0483093	13359.89216	-71.94442825
3.964337994	97.12770835	718.708374	9433.79173	-96.61456299
3.684079938	98.33954316	827.1689758	9670.66575	-131.377533
3.485384316	73.95910811	514.6224213	5469.949672	-63.48984642
3.682914785	86.44324653	763.0153503	7472.43487	-32.80231628

log.sigma.2.5.mm.3D_firstorder_Kurtosis	log.sigma.2.5.mm.3D_firstorder_Mean	log.sigma.2.5.mm.3D_glrlm_ShortRunLowGrayLevelEmphasis	log.sigma.2.5.mm.3D_glrlm_GrayLevelVariance
4.501858638	31.87234799	0.005129976	32.77715838
4.982634443	33.75836151	0.008859424	11.10711787
3.734983172	55.77039918	0.016155868	13.28149937
3.516986672	24.57281625	0.005978305	24.66041646
4.493385242	36.17304882	0.005494671	16.74866007
3.61047129	56.30765124	0.006490943	23.4378839
4.480597558	50.48437429	0.00282707	16.11856231
4.83153977	56.71501715	0.002866307	16.8654318
4.200153755	56.24390682	0.002421077	18.50916759
4.48323397	64.58613659	0.006082891	17.52355685
3.75291866	41.34277852	0.009237494	11.44042952
4.98284712	65.8589329	0.002963978	20.39326087
6.959377937	30.74709816	0.006090935	13.34909102
6.47496064	44.74328975	0.003113355	24.68334813
4.913420007	47.67786826	0.004149346	23.68581022
4.538342479	42.48018674	0.004882796	17.62467204
3.401946844	38.47826096	0.00786046	16.78604552
4.446525628	45.22717722	0.00877905	12.1095687
3.921967702	39.22540773	0.007570898	8.935678863
4.037683507	56.3611892	0.003328003	18.88775721
4.857121658	43.01711	0.005145869	10.39577952
3.527013127	51.48805592	0.00785478	10.80802102
4.543189108	44.85068232	0.00593187	13.91280783
3.727800947	50.23198519	0.006994558	13.02417238
6.487711143	61.35819745	0.004401923	18.08786663
4.33799606	67.91110762	0.003549973	26.09711132
4.047103595	52.43534235	0.003319212	24.0776981
5.245265656	39.60883077	0.003430481	16.60201885
3.347334088	48.74910495	0.008656101	10.30108616
5.570087728	47.91604177	0.004320297	10.77169791
5.480197387	53.31986818	0.002419896	17.11614509
5.99402295	18.45785314	0.00522006	13.50971535
3.765899781	33.60726691	0.00387867	23.99676997
4.86100977	44.82619919	0.003516618	21.48192405
3.870863387	68.42522715	0.004350451	18.4770641
4.892335299	51.63604595	0.006111779	14.72384969
7.379274789	25.84363932	0.002094457	16.88889708
5.182421022	37.20044804	0.003345778	20.76927222
5.707141495	30.72866125	0.008671511	8.096993347
3.014810859	34.51306925	0.005536703	19.80669945
5.278129495	22.12591128	0.007325834	16.70244788
3.177127226	31.68838018	0.006633067	25.76873212
3.960179777	56.62936932	0.004132406	18.62744596
4.921983213	36.95876546	0.002433321	21.33246163
4.243091011	73.86083319	0.005823768	16.25476007
4.192659218	62.20080722	0.003356089	18.76601974
4.807502716	53.07189109	0.003582626	17.48177406
4.659528001	49.55000881	0.010581336	9.251753284
3.795355478	53.31776908	0.009687958	19.3904579
3.634039168	61.32295518	0.010082353	13.42151884
4.805575264	70.80197038	0.00307359	21.71159047
3.64375793	47.66275289	0.005485658	23.30207284
3.907867933	42.45545589	0.009651132	14.56593476
3.669266036	70.5451784	0.008659975	14.35008408
3.321148186	63.08558517	0.004323404	16.38183049
5.467027975	43.87570163	0.002797224	15.42235144
3.665246677	55.72716078	0.00406243	19.607658
5.70489737	29.86994585	0.003467548	17.49953681
4.954675592	32.32701559	0.006752379	16.30746808
4.212581227	29.42730241	0.002647289	19.7278424
5.031951271	45.60080806	0.004470995	16.34571461
5.059225965	54.63410414	0.004710645	16.20114944
4.190080228	61.54283889	0.003448042	18.50082835
4.416992928	39.32076951	0.00215503	17.746861
3.298587787	56.29531412	0.015134983	11.13230004
6.232701339	35.42148313	0.002924532	16.23667665
8.912075971	20.81584951	0.003840539	11.04940198
4.716523651	31.69711374	0.003009672	18.94441694
4.536609156	48.09521717	0.003207219	18.06792927
5.261751704	42.62809864	0.004152499	10.06740884
4.277051511	36.5478838	0.006230642	15.18685751
5.192537872	61.27276514	0.004088288	17.59342447
5.587281106	49.02699473	0.002600996	19.62934476
3.42418471	21.37637662	0.007272195	27.47615827
3.199247915	32.36951293	0.006259336	22.10812457
6.462052014	34.56684179	0.004331902	15.38062459
4.290336178	52.13189906	0.004954261	10.39639138
4.981158002	44.77242811	0.010400978	14.08651466
4.657400788	42.94644626	0.005404184	12.454074
3.977315182	19.85874782	0.002814082	26.44158896
4.409472492	32.62386036	0.004201037	32.19625263
4.268342098	17.51241965	0.007831938	19.38260986
4.745731135	48.19739103	0.003890325	16.52397257
3.584225146	38.3861874	0.006650954	10.45860574
3.611639366	40.44091198	0.004776442	21.46622791
5.161286064	33.5930418	0.004714506	7.590406438
4.101311011	17.44900037	0.004651515	23.80288116
3.8951185	68.66728933	0.004037515	16.89764335
5.324200973	52.71932474	0.005126594	13.80714966
6.258586298	43.26506016	0.003099955	14.692049
3.958010767	46.11929376	0.006549225	10.05110416
3.322972463	68.14839099	0.004996323	19.37326583
4.356104941	29.70473024	0.00363832	20.05038813
4.80770036	53.05540885	0.00537736	16.38307597
3.539505337	27.48249123	0.003794222	16.83926741
5.561403296	45.55218371	0.003278802	23.06502817
3.638690859	32.43378676	0.005548937	15.81857147
5.655160286	10.10183631	0.003738156	16.68180447
5.055359748	23.91417854	0.007319222	9.294428
4.418321831	52.52899989	0.004637627	13.09867784

log.sigma.2.5.mm.3D_glrlm_LowGrayLevelRunEmphasis	log.sigma.2.5.mm.3D_glrlm_GrayLevelNonUniformityNormalized	log.sigma.2.5.mm.3D_glrlm_RunVariance	log.sigma.2.5.mm.3D_glrlm_GrayLevelNonUniformity
0.005275029	0.060154133	0.097988754	118.2177051
0.009445239	0.107620107	0.183412794	87.71383691
0.017228931	0.087624692	0.208173947	117.2255297
0.006191745	0.065674545	0.109316384	58.62781693
0.005760001	0.080139604	0.134428887	171.1393506
0.006738184	0.068918526	0.093375083	41.97370753
0.00303142	0.080623797	0.159717615	406.6928943
0.00307398	0.084827048	0.16445426	384.2389093
0.002562032	0.071390966	0.124505894	294.125669
0.006358711	0.07557986	0.101803109	74.25077248
0.009737175	0.089607579	0.122299189	122.2599878
0.003111095	0.074995216	0.115379625	157.4025958
0.006596513	0.124861272	0.318590997	193.5037515
0.003219412	0.074478753	0.130391574	115.6082235
0.00431905	0.073902826	0.109356811	134.0624675
0.005172743	0.081658153	0.169074618	316.9628896
0.008238846	0.074237783	0.095313207	81.49672825
0.009434315	0.10912772	0.202693813	116.0791117
0.008114047	0.102371323	0.152085184	117.9609076
0.003530975	0.073827056	0.125914369	347.1513784
0.005702451	0.109016085	0.28329583	276.9438218
0.008268206	0.090080327	0.117290226	88.18395479
0.006251392	0.08902026	0.141197672	81.67281254
0.007437585	0.0838134	0.138113994	199.6461904
0.004624905	0.084453439	0.136203231	96.56810723
0.003704248	0.063379472	0.092527065	138.8915539
0.003502873	0.067840865	0.127473471	198.9814566
0.003635588	0.085447987	0.137046908	211.7963949
0.009069784	0.094450716	0.102867686	38.64844437
0.004609136	0.104302229	0.147389434	190.3960599
0.002594205	0.083963236	0.170072836	353.540368
0.005550828	0.107310426	0.194500599	194.0271088
0.004076566	0.064740358	0.126191061	190.2413567
0.003707021	0.073500381	0.149352192	154.9009931
0.00457789	0.070989444	0.115631576	285.0702654
0.006566607	0.092223791	0.193438105	241.746696
0.002258226	0.100944307	0.202131734	326.6391873
0.003641451	0.085594778	0.32844599	369.451467
0.009144977	0.111774952	0.133685829	122.9527735
0.005844102	0.064055813	0.107055074	426.8587714
0.007737339	0.094747748	0.168162412	217.2191859
0.006902999	0.056808221	0.073061054	205.6846643
0.004350779	0.070974099	0.112763899	236.0542271
0.002548163	0.075561235	0.113434762	171.7313002
0.006123902	0.083298929	0.124411147	96.18987323
0.003543886	0.073755371	0.121483557	184.1809244
0.003807841	0.080853239	0.140047601	331.4796084
0.011215094	0.112264374	0.152189271	86.12118714
0.009979745	0.07236211	0.09414006	56.78801298
0.010739813	0.090801326	0.180681614	107.6697907
0.003209115	0.069016236	0.102415196	162.8657132
0.005691073	0.062646725	0.079615738	196.9754536
0.010097466	0.081942866	0.112224149	336.5902266
0.009144702	0.081424575	0.118039474	81.58254497
0.004542284	0.072118351	0.10557708	87.8909404
0.002960152	0.087391201	0.133348087	152.8032912
0.004253902	0.06890564	0.101812475	256.0920143
0.003695613	0.088486015	0.154346799	272.1987909
0.007006284	0.085174549	0.117751504	127.4622575
0.00282896	0.069467408	0.165102295	567.1667388
0.004814462	0.096923836	0.202592975	148.9552053
0.004939453	0.082085428	0.136218378	197.4210565
0.003654531	0.074389244	0.128679621	390.2291815
0.002308363	0.078040081	0.136621068	534.4048046
0.01601765	0.089679181	0.135501122	63.57518147
0.003132585	0.092968296	0.192715566	313.7549606
0.004088306	0.117876079	0.208144677	132.6763986
0.003200319	0.079074685	0.143981781	443.4975887
0.003377348	0.075348153	0.115027773	401.8567973
0.004495367	0.107022969	0.186424966	270.3869521
0.006563745	0.082021928	0.133132169	96.07127999
0.004320599	0.083480264	0.163700612	317.181179
0.002748575	0.07945993	0.147612165	162.3228779
0.007481607	0.061398153	0.092307064	128.4917616
0.006558982	0.061216675	0.091197756	177.3932702
0.004533082	0.094625258	0.145414475	113.5393516
0.005346578	0.099780714	0.164067358	211.1123712
0.010959178	0.09870112	0.17392065	83.26601419
0.005713405	0.091433613	0.1295525	140.5067362
0.002925845	0.063547507	0.086037373	138.0139646
0.004322487	0.061450408	0.099341188	113.1236026
0.008228724	0.078664618	0.14212972	126.9227907
0.004143219	0.081112901	0.194243949	233.7435788
0.007128894	0.093572962	0.139545759	256.4641411
0.005000997	0.06681162	0.117322481	148.932563
0.005242291	0.131868521	0.294921675	167.721345
0.004871474	0.07061053	0.136895725	151.8134423
0.004304229	0.077955477	0.154369119	190.7511527
0.005414967	0.091122716	0.149760668	119.3485627
0.003320813	0.095153467	0.181423828	168.8062103
0.0073369	0.126006395	0.264304313	137.8046605
0.005221293	0.066695283	0.089562981	147.3329381
0.00383052	0.074010008	0.110845152	350.0619248
0.005662158	0.083011663	0.143899018	245.3941652
0.003985458	0.073931734	0.096810338	295.8844913
0.003466977	0.077073626	0.179438204	233.5669197
0.005871878	0.078081678	0.11977917	199.4540534
0.003944027	0.102826265	0.171012647	94.84378952
0.007762858	0.108889312	0.162235972	80.10752566
0.005050874	0.097181033	0.218077777	484.8351459

log.sigma.2.5.mm.3D_glrlm_LongRunEmphasis	log.sigma.2.5.mm.3D_glrlm_ShortRunHighGrayLevelEmphasis	log.sigma.2.5.mm.3D_glrlm_RunLengthNonUniformity	log.sigma.2.5.mm.3D_glrlm_ShortRunEmphasis
1.266112398	516.5918432	1706.838024	0.945802115
1.508442306	180.1618495	634.6184294	0.902558848
1.535248153	139.2467862	1048.805911	0.905230139
1.310781725	337.7955892	753.9153368	0.934798267
1.367824106	311.2369916	1765.889735	0.926828611
1.2708102	288.0210357	523.0514525	0.941193628
1.419384177	405.7087619	4116.748146	0.921313409
1.439557725	413.0206649	3649.505468	0.916643715
1.345447503	496.9576883	3437.858688	0.930094925
1.294965373	321.1086639	832.5319007	0.936351942
1.348874346	169.8327986	1131.411398	0.927383756
1.323630259	476.2434769	1763.070006	0.93303907
1.788345731	260.0218824	1127.76105	0.876268903
1.358246363	662.5863658	1288.767585	0.928323726
1.30894234	409.3817806	1535.858214	0.935723439
1.446305068	322.431818	3134.820833	0.917060953
1.280216047	226.4209549	934.5394131	0.938148204
1.553523221	171.0575197	813.6713096	0.896189345
1.424417657	164.790441	924.1746751	0.914874057
1.35090954	380.8623678	3903.237967	0.928330177
1.712865327	216.2919287	1883.193968	0.883029327
1.33536122	180.5241838	815.740947	0.929632522
1.391036634	258.1702216	750.7254779	0.922322881
1.377373713	207.2133315	1963.87071	0.925262884
1.381128898	463.4464646	937.6778501	0.923146689
1.262960604	450.2774386	1893.421512	0.943723036
1.345879755	417.9865968	2455.576194	0.931428131
1.382661079	386.9546444	2027.805386	0.922532246
1.299332217	179.207744	345.9114889	0.935017572
1.418551766	285.482092	1465.612045	0.915044662
1.451946997	486.2298655	3386.392495	0.915592803
1.511605042	278.7851412	1421.827984	0.90665316
1.338153735	388.889506	2472.859599	0.933130918
1.396474775	439.2602315	1733.155662	0.924451986
1.32070731	328.9588335	3386.349738	0.934378299
1.507404909	266.4695637	2065.308108	0.907742947
1.53484947	557.6921086	2522.776386	0.903066282
1.771295386	416.3232778	3211.22981	0.885013062
1.387697054	203.9616379	891.2907571	0.918838627
1.299936382	255.2813023	5662.632923	0.937269498
1.455992737	258.1766492	1831.931856	0.913015948
1.215172589	261.0695736	3189.090903	0.951351973
1.322878257	345.1364755	2789.702634	0.931985616
1.315056906	552.4307508	1921.523524	0.935242605
1.354555805	267.4700134	955.0724315	0.926535275
1.333199699	391.9067036	2099.700547	0.932976415
1.381027583	373.4049268	3372.369487	0.924800329
1.445666075	149.2356903	604.0086503	0.907430273
1.27306774	246.5024362	672.4345763	0.940512974
1.489196465	167.3993897	938.099277	0.90812376
1.289211966	487.774033	2012.064824	0.938876087
1.230659509	307.9855655	2753.636139	0.949095317
1.320732275	200.5534124	3442.230939	0.932153009
1.336415818	180.7514661	835.3890353	0.929581986
1.298988672	321.2314338	1034.311647	0.936849934
1.38033367	468.910714	1425.960303	0.921342296
1.288708894	346.586663	3169.109822	0.938752939
1.423027126	407.4212506	2484.823974	0.917269323
1.333644899	275.3934073	1249.418309	0.930384006
1.419839273	454.3472714	6710.134037	0.923931918
1.531111553	288.8619694	1199.663305	0.90424684
1.372159906	373.6331229	1985.081906	0.926020644
1.349240677	389.7147261	4385.393362	0.930978833
1.379822212	508.660466	5620.753825	0.923608934
1.383251597	116.9106113	580.4432514	0.92200803
1.508028607	452.1835845	2650.881919	0.906595794
1.555615814	378.7355374	866.5156345	0.898709349
1.389069323	413.9167215	4601.76946	0.923669545
1.323816247	402.064634	4478.987544	0.932697958
1.508336938	277.4507676	1968.929161	0.903525608
1.367770412	279.2754028	969.0376435	0.926546289
1.443971543	382.3830271	3045.985599	0.914678458
1.397234634	525.9216101	1674.126752	0.923072861
1.259113405	340.6639797	1814.059527	0.945159902
1.260769419	256.9302312	2505.455466	0.94393689
1.402085539	372.4752076	976.3894505	0.920118368
1.451054509	232.4344276	1685.341032	0.912155699
1.468519782	200.1239266	668.6366091	0.910343598
1.366532144	253.8966884	1263.278144	0.924484662
1.248082521	534.8983877	1885.05172	0.945664987
1.269203249	518.1148824	1597.276667	0.945382504
1.389345809	229.1359783	1321.110774	0.9226928
1.493701745	363.1490072	2313.42712	0.913786375
1.400424969	189.0475173	2212.843049	0.917160413
1.318808185	325.1117012	1891.554549	0.936400371
1.76056738	226.7192328	918.8688722	0.873485627
1.37336956	359.3150119	1779.902156	0.926585451
1.413272244	324.879682	1994.508359	0.921008676
1.405604716	298.1062687	1070.209756	0.921454201
1.492274755	421.1136459	1395.995544	0.906769784
1.698432455	170.9785808	800.4408317	0.878759025
1.255011657	298.8463579	1917.284177	0.945266253
1.313789166	379.2499238	3984.343156	0.934080611
1.393623285	300.8207483	2417.264913	0.922270472
1.281216153	338.149492	3410.704163	0.938623665
1.459581193	521.777447	2444.247377	0.916759598
1.33909468	246.1709492	2127.532364	0.929444282
1.467894861	406.3476554	730.6064357	0.909726952
1.454339063	194.7322412	582.8186691	0.909713074
1.564569519	264.0848703	3861.110283	0.900702936

log.sigma.2.5.mm.3D_glrlm_LongRunHighGrayLevelEmphasis	log.sigma.2.5.mm.3D_glrlm_RunPercentage	log.sigma.2.5.mm.3D_glrlm_LongRunLowGrayLevelEmphasis	log.sigma.2.5.mm.3D_glrlm_RunEntropy
700.7149882	0.925940695	0.005945852	4.777051527
293.1045921	0.870999589	0.012483567	4.215101724
224.0354662	0.871376993	0.022897426	4.421044316
485.1858455	0.913392646	0.007194685	4.662171081
464.2300781	0.901474792	0.007049424	4.47301394
381.443675	0.922610723	0.007867398	4.58726847
611.9147764	0.89232675	0.004119438	4.500785151
637.6740493	0.886482742	0.00416733	4.5099071
720.8268247	0.906122035	0.003257173	4.574429223
445.4039811	0.916260046	0.007588977	4.437835265
248.1384142	0.904509284	0.012087967	4.229399316
672.8730124	0.910428834	0.003816977	4.544435506
512.3248974	0.828313625	0.009723816	4.296944016
968.8413046	0.903543748	0.003750137	4.654259023
574.3497373	0.914081886	0.00512116	4.596868931
496.6984339	0.886549009	0.00674453	4.545538437
310.7356071	0.919404716	0.009894407	4.423493631
287.5142222	0.862062512	0.012898759	4.27142112
254.4697457	0.887637786	0.010807516	4.11565505
548.2196957	0.904315919	0.004514904	4.589808778
404.7665402	0.839336589	0.009100828	4.308094058
260.84453	0.907107721	0.010210472	4.192320904
386.3021255	0.895733173	0.007849469	4.336412799
304.0616154	0.899712301	0.009626075	4.361245298
684.0410136	0.897818599	0.005678182	4.450216756
598.2219605	0.925025165	0.004412789	4.717102986
597.601843	0.906945205	0.004426018	4.720323804
578.2522468	0.897175959	0.0046408	4.453133824
245.1812718	0.915332989	0.010990054	4.059504351
445.9742599	0.888435323	0.006018114	4.190296688
761.7207145	0.884566883	0.003526317	4.522584991
470.9989619	0.873331598	0.007326374	4.28324768
556.9993741	0.90987922	0.00506448	4.727014489
658.4512871	0.896563011	0.00468542	4.622048938
460.248235	0.911706141	0.005678501	4.55529362
425.3078481	0.87370149	0.008955591	4.415824522
945.5077213	0.868728423	0.003160205	4.438057511
789.0083229	0.834990237	0.005606126	4.728779513
314.7615984	0.894121326	0.011313662	3.956456008
351.383024	0.916537932	0.007304347	4.631031319
415.476479	0.883103019	0.009777618	4.423153224
331.985714	0.936285333	0.008085252	4.724223234
489.6872711	0.910295016	0.005383161	4.560305505
787.3192817	0.913245115	0.003098977	4.55710218
385.4522011	0.903153967	0.00756207	4.420886465
552.4761153	0.909513801	0.004472961	4.551036636
550.869711	0.898475702	0.004928941	4.521052847
237.1575618	0.880597015	0.0142727	4.082496332
335.0787954	0.921992226	0.011290779	4.494408939
262.5993513	0.876820665	0.01426571	4.358043669
669.9030039	0.918495673	0.003844619	4.594825731
402.3321639	0.93270657	0.006601497	4.653270038
284.7897921	0.910290507	0.012139963	4.393752575
250.9372144	0.907399666	0.011460237	4.361040165
440.4256824	0.916252169	0.005571387	4.453853915
705.6033735	0.8965286	0.00374216	4.411291044
477.3004127	0.918684035	0.005144908	4.56855361
633.9456522	0.889309071	0.004818981	4.46356572
398.8444113	0.907953697	0.008207063	4.3923416
686.5798913	0.894090683	0.003830531	4.657953472
475.0692668	0.869370154	0.006713421	4.45811549
550.4162954	0.900460962	0.00606063	4.441674544
557.5385564	0.905948948	0.00467955	4.55150058
756.3759108	0.898182625	0.003062642	4.549251151
172.8612309	0.897176241	0.020312497	4.232541078
740.3482565	0.873381668	0.004258761	4.468396503
660.9025198	0.862848042	0.005438725	4.15243446
624.2442326	0.897193846	0.004163636	4.580191676
579.1083266	0.910495003	0.004185777	4.523484592
456.6293848	0.871343632	0.006304794	4.20091253
413.6643037	0.901403446	0.008148913	4.411776556
591.3012816	0.885171239	0.005536467	4.533095626
791.9448193	0.895973268	0.003508208	4.552322472
460.9097579	0.926311847	0.008430114	4.717971012
340.0782485	0.92546004	0.007924174	4.66147564
580.1256697	0.893671902	0.005535765	4.307263021
362.8811427	0.882753307	0.007360352	4.234609041
314.8794586	0.880118837	0.013770117	4.337212601
376.107652	0.900094633	0.007195265	4.279276165
713.4565753	0.928371757	0.003426034	4.683963434
702.663891	0.925320029	0.004901652	4.758582095
348.139707	0.896609227	0.010145421	4.555294848
577.5120331	0.880650399	0.00559208	4.536053049
292.0558115	0.891887156	0.009406183	4.233160757
463.2458113	0.913772984	0.006095813	4.638202619
449.854273	0.829082145	0.008410234	4.095597333
540.7125317	0.901015965	0.005963927	4.680541003
486.8987073	0.892189867	0.00569584	4.515319487
454.424589	0.894335855	0.006874147	4.313069713
693.6240266	0.875631529	0.004469088	4.393525399
306.2886372	0.837423313	0.011898631	4.124278458
395.6254765	0.927339663	0.006254738	4.539255581
535.3785993	0.912494991	0.004727246	4.566266895
453.3818986	0.895617716	0.007075371	4.459407942
466.2007125	0.919541855	0.004853118	4.457998002
817.3376274	0.88515488	0.004470202	4.662229519
356.0589478	0.906720188	0.007389046	4.464543502
670.9990687	0.88031739	0.00499835	4.305862879
320.7482246	0.8816639	0.009951879	4.078695731
440.4710085	0.86345954	0.007461842	4.394007166

log.sigma.2.5.mm.3D_glrlm_HighGrayLevelRunEmphasis	log.sigma.2.5.mm.3D_glrlm_RunLengthNonUniformityNormalized	log.sigma.2.5.mm.3D_glszm_GrayLevelVariance
547.8941826	0.868041896	47.25334157
198.5635689	0.777132084	24.99399038
152.2121687	0.782318091	23.35105092
363.2588605	0.843704335	34.81901537
336.7026682	0.826348635	26.93659491
304.7466491	0.857842657	38.14035088
438.6011206	0.81518233	27.743608
449.1484978	0.805108065	32.29167529
534.7615887	0.83351978	28.73998188
343.2850829	0.846751984	27.44888889
183.4163999	0.827930417	18.21822183
510.0636395	0.839491874	34.53518716
294.6441418	0.726719602	29.89126108
713.9802371	0.829709813	45.33588099
437.8933035	0.845826938	39.54715387
349.9650188	0.806505976	30.86115247
241.6176622	0.850492796	24.4069444
189.5190609	0.763968499	24.51752896
179.8632839	0.801103761	15.60136005
409.310382	0.829405364	31.5529356
243.407832	0.739558238	20.92558352
194.3856207	0.832361101	17.05699673
279.5354052	0.817228582	25.67504581
223.3583436	0.823307292	20.30027767
500.625868	0.818943555	34.50067831
476.3730061	0.863252608	39.65447571
448.0330911	0.836502078	37.50931062
419.2904724	0.8172	30.139128
191.1027367	0.844207746	15.62924564
312.508743	0.80172461	21.73408442
529.909214	0.803356515	31.87323492
308.5603608	0.78518265	26.12697419
416.9311359	0.840300821	35.6688117
474.5722749	0.821617163	34.39040766
351.3730665	0.842628595	28.19120304
291.2966164	0.78708633	27.42402994
617.4569279	0.77826469	39.06964004
465.6273632	0.743372418	38.15942712
223.0914616	0.809316632	15.27439482
271.9819642	0.848895865	27.52460159
283.4247009	0.798017817	31.42000157
274.1598186	0.880162202	31.87627704
370.289518	0.837629766	28.42540522
592.4409256	0.84467452	34.21956441
287.7246441	0.826017928	29.63834639
419.056144	0.83994476	29.95637132
402.8594908	0.822001967	31.28405543
164.4238671	0.786068264	19.04672532
262.3951681	0.85599172	29.22305399
182.7241693	0.788879794	22.41502339
519.6663519	0.852141747	34.50211114
325.05357	0.875120966	31.87936685
215.3092687	0.837425106	23.61694387
193.0183464	0.832465242	24.21589157
342.0311448	0.847897339	23.9156
509.4400363	0.814618153	29.25749185
369.4391094	0.852010909	30.19154731
444.4122079	0.806613995	32.89995156
296.6590204	0.833935671	30.41551064
490.6799688	0.820919674	30.67905295
317.5793043	0.78002974	30.94785965
403.1419377	0.824728221	28.44481119
417.6314399	0.835433517	28.17164807
549.966018	0.819683081	29.70379051
126.565316	0.817013782	17.9375
497.3086439	0.784654891	33.35432239
422.4498884	0.768952783	24.99925813
448.5906666	0.819701514	31.96932843
432.5716158	0.838942863	28.26199408
306.2404027	0.778340996	21.77855793
301.5763937	0.826203217	23.88278537
416.4858357	0.800962967	35.89227132
569.2909153	0.818666729	35.47858521
361.6792226	0.866350622	39.64891471
271.6951279	0.863795251	29.75434489
406.9466151	0.812587981	28.55990306
253.8387338	0.795692115	20.05397656
218.7818768	0.791924368	30.11756487
274.7908584	0.821228156	21.56801537
566.8205587	0.867347514	35.62862164
549.3578633	0.86715894	45.17558069
248.8348071	0.817842803	31.51960544
395.5051339	0.80083465	29.97264928
206.6824981	0.806030746	16.34264848
348.0303903	0.847478851	30.40779806
258.7523089	0.721181866	16.37623887
389.2053248	0.826645404	35.8624068
351.1054851	0.814379789	27.77493236
323.6629434	0.815642988	23.67964283
464.5930933	0.78537943	28.65926649
191.4553713	0.730998715	17.40277149
315.9798045	0.866888395	25.18384066
406.3908628	0.841706857	31.90057911
326.1239887	0.816821799	27.89898196
361.0604698	0.851538066	24.55290449
567.7976515	0.805750531	41.78734349
265.0350708	0.831872234	24.48207193
449.2400027	0.790938069	29.91922852
215.5809621	0.790878541	15.72014999
290.9076398	0.773094491	24.56618925

log.sigma.2.5.mm.3D_glszm_SmallAreaHighGrayLevelEmphasis	log.sigma.2.5.mm.3D_glszm_GrayLevelNonUniformityNormalized	log.sigma.2.5.mm.3D_glszm_SizeZoneNonUniformityNormalized
332.7969066	0.042917113	0.442159701
125.5394572	0.066845414	0.371995192
96.29935369	0.059538946	0.325128414
199.9248396	0.048875028	0.360106928
183.177589	0.055934935	0.366440484
200.9408506	0.048399508	0.360495537
265.9135713	0.054460903	0.349019327
277.2352649	0.051048693	0.381995682
307.9039303	0.053719634	0.367496495
198.1640152	0.055004444	0.366172444
105.4165258	0.067281637	0.354548773
321.233911	0.050665738	0.411986542
160.5683493	0.058276599	0.353574078
435.2235429	0.046201653	0.39772562
263.1354404	0.047891931	0.407061746
217.9846276	0.053058014	0.37423044
142.3237613	0.058104224	0.350477573
109.1475161	0.059562263	0.350328581
105.18091	0.073279547	0.330706263
253.0189429	0.051199025	0.365177127
138.2404152	0.06629222	0.327668089
96.1649053	0.067303306	0.325938219
162.6481002	0.058617863	0.367139275
126.2660676	0.061677133	0.3210748
302.8124855	0.054121304	0.372795506
308.4452059	0.045950274	0.380429344
282.9291737	0.046702565	0.392146762
255.8630042	0.053421396	0.385061583
115.6473491	0.0722462	0.321261025
164.4242927	0.063403047	0.344783603
324.359575	0.053501271	0.388887751
165.6523622	0.059545168	0.366020609
256.2508304	0.047240138	0.390005041
273.7115605	0.050422504	0.37129298
215.4921139	0.054384646	0.367581827
183.1644608	0.057534362	0.380921551
369.6499468	0.049033754	0.391155207
299.3647526	0.048322897	0.372804266
108.8228671	0.077500469	0.308641396
159.7576623	0.052362019	0.322184953
161.99828	0.052762332	0.381757903
176.2108601	0.050007278	0.374232046
209.8816477	0.053555045	0.350904438
348.3589645	0.049801143	0.403303184
173.4300428	0.052803439	0.359084052
271.9624029	0.052138972	0.401268946
245.6196667	0.051528195	0.371218213
92.01158215	0.069918784	0.325124948
164.9086591	0.053590085	0.37112404
111.1622362	0.063969704	0.356489196
311.5556451	0.051802483	0.383640716
200.6902334	0.049998619	0.385326584
125.3320851	0.058119075	0.370685906
130.4632123	0.057724391	0.35816898
204.3256111	0.057925926	0.348819753
293.0791788	0.055399565	0.364473889
214.2894094	0.05062686	0.376121801
247.4777791	0.053586957	0.342696125
162.1016373	0.05221591	0.365937083
301.8831486	0.051665215	0.372045756
201.5051907	0.052847177	0.397922097
238.5294514	0.055511259	0.384817874
252.2066057	0.053880286	0.384160375
333.2201589	0.052175981	0.342995904
64.40886207	0.069401578	0.279278121
299.9856145	0.05167051	0.35433287
235.5038016	0.062819576	0.39848886
270.918968	0.051260727	0.366581018
245.251661	0.053596036	0.390060562
171.1623223	0.061798349	0.355961383
168.6956833	0.060180664	0.344848633
248.2724348	0.05027953	0.375539904
346.2810333	0.050556906	0.371193206
219.8797099	0.044415008	0.428581191
173.4261912	0.05086772	0.364881265
207.3042567	0.058651949	0.334839429
160.4701652	0.064692628	0.362431758
125.6526185	0.05598789	0.338391973
158.3377745	0.063962152	0.35954057
346.1530169	0.049799137	0.381040848
348.661451	0.044167921	0.453859459
154.7466955	0.051023786	0.413354867
240.202411	0.053408171	0.371346115
106.4913024	0.069502553	0.28167178
204.4334848	0.051221827	0.384710813
117.8485355	0.077779374	0.273089629
226.209873	0.04791497	0.381818855
207.288763	0.054438958	0.373653207
186.872049	0.062436573	0.345337987
259.7579936	0.056163194	0.356467014
116.9904634	0.078348357	0.325501101
189.9516836	0.056679105	0.368373008
245.016663	0.050265861	0.371974507
189.2752728	0.055434677	0.374436212
208.9349039	0.056691251	0.362567142
365.1609966	0.046435222	0.410874881
147.8688405	0.05694655	0.323993023
248.3041762	0.055250679	0.421040013
107.1607974	0.078374669	0.31384178
182.7974033	0.058467566	0.378170998

log.sigma.2.5.mm.3D_glszm_SizeZoneNonUniformity	log.sigma.2.5.mm.3D_glszm_GrayLevelNonUniformity	log.sigma.2.5.mm.3D_glszm_LargeAreaEmphasis	log.sigma.2.5.mm.3D_glszm_ZoneVariance
399.2702104	38.75415282	30.6910299	25.16879014
77.375	13.90384615	302.8221154	282.6153615
120.6226415	22.08894879	146.7708895	129.6745592
120.6358209	16.37313433	53.50746269	45.00196926
272.6317204	41.6155914	109.9193548	99.78916638
82.19298246	11.03508772	50.55263158	42.17313019
592.2857985	92.42015321	242.4213318	231.332471
536.3219373	71.67236467	372.8062678	359.5699386
544.9973028	79.66621713	66.27376939	56.87704344
137.3146667	20.62666667	47.26933333	39.09735822
156.3560091	29.67120181	89.95011338	78.25712537
322.997449	39.72193878	72.30229592	63.65839592
130.4688347	21.50406504	1066.626016	1041.026256
218.7490909	25.41090909	153.7109091	143.9651603
282.9079137	33.28489209	83.14532374	74.9961472
459.9292107	65.20829943	356.4621644	343.784161
145.4481928	24.11325301	30.18313253	21.90537959
93.8880597	15.96268657	345.3171642	324.150298
105.4952978	23.37617555	181.3103448	164.7538644
590.1262376	82.73762376	99.41769802	89.06730307
184.1494662	37.25622776	1211.44484	1182.492015
104.6261682	21.60436137	49.06853583	37.76972273
105.7361111	16.88194444	128.6458333	116.003858
245.6222222	47.18300654	90.27581699	78.3033124
142.7806789	20.72845953	134.2898172	123.2424381
339.7234043	41.03359462	57.34042553	50.302783
433.3221719	51.60633484	125.9683258	117.4080678
321.9114833	44.66028708	222.8373206	211.9220428
46.90410959	10.54794521	38.67808219	29.30441922
168.599182	31.00408998	144.8261759	127.1827401
515.6651584	70.94268477	528.1809955	515.3055629
190.696737	31.02303263	477.4606526	461.69015
435.6356312	52.76723366	84.96060877	76.60400629
279.9549072	38.01856764	96.31830239	86.60441571
556.8864686	82.39273927	67.43234323	58.98209718
299.7852605	45.27954257	381.3354511	366.814219
350.8662207	43.98327759	646.212932	629.0047713
473.4614173	61.37007874	623.3173228	606.8030188
90.12328767	22.63013699	113.3356164	95.5919028
780.3319571	126.8208092	36.55078448	27.54087258
258.8318584	35.77286136	433.3569322	418.7189722
623.4705882	83.31212485	13.82172869	8.434091404
418.9798995	63.94472362	69.11306533	59.75274544
352.8902857	43.576	93.75314286	85.66805682
136.8110236	20.11811024	79.09186352	67.84032901
379.6004228	49.32346723	106.9143763	98.49456374
506.3416422	70.28445748	278	266.8138195
63.7244898	13.70408163	221.9234694	202.1754217
119.873065	17.30959752	25.18575851	18.24424657
119.4238806	21.42985075	241.7850746	225.4972243
339.5220339	45.84519774	80.28135593	71.85496377
548.7050562	71.19803371	18.10463483	12.50064258
524.8912429	82.29661017	139.5127119	129.3593077
119.2702703	19.22222222	87.27927928	76.28796364
156.9688889	26.06666667	41.06222222	32.32691358
202.647482	30.80215827	160.9892086	148.6887972
534.0929577	71.89014085	55.96126761	47.84679379
315.2804348	49.3	251.0826087	236.9548157
191.0191571	27.25670498	80.55172414	70.58451872
1074.468144	149.2091413	159.591759	149.599768
198.5631263	26.37074148	364.3406814	351.8156072
317.8595642	45.85230024	211.8232446	201.3745405
756.7959391	106.1441624	107.4654822	98.83023757
746.016092	113.4827586	93.45057471	81.1667583
60.32407407	14.99074074	80.0462963	66.66966735
346.1832139	50.48208802	512.1944729	496.5689193
117.9527027	18.59459459	457.3918919	437.9842951
676.708559	94.62730228	179.4940412	168.0310848
753.9870667	103.6011381	141.2576306	132.0766993
229.2391304	39.79813665	523.0869565	502.8369565
132.421875	23.109375	85.1171875	73.67376709
415.7226739	55.65943993	384.2023487	369.1840982
258.7216643	35.23816356	132.6700143	121.9788971
401.5805763	41.61686233	43.42902882	37.61665845
436.3979933	60.83779264	39.80351171	32.95014807
125.2299465	21.93582888	79.39037433	66.51494181
208.3982609	37.19826087	262.8521739	245.4886473
76.81497797	12.7092511	235.5066079	217.695977
180.8489066	32.17296223	119.333996	107.817216
348.2713348	45.51641138	28.1214442	21.57254883
383.5112426	37.32189349	31.90887574	26.36828402
244.7060811	30.20608108	137.3766892	128.1420717
342.009772	49.18892508	332.4636265	319.8653661
200.2686357	49.41631505	161.0168776	142.3364885
325.850059	43.38488784	50.80637544	42.51442343
64.44915254	18.3559322	1098.338983	1056.199081
319.9642005	40.15274463	130.3997613	122.2996921
303.4064039	44.2044335	249.7561576	238.35306
138.8258706	25.09950249	110.3432836	97.08064157
171.1041667	26.95833333	225.84375	208.0458984
82.35177866	19.82213439	746.2371542	719.6718899
339.6399132	52.25813449	20.51843818	13.84389307
659.1388262	89.07110609	68.34819413	59.79289926
362.8286894	53.71620227	160.4396285	148.8417027
552.1897571	86.34077479	45.66250821	37.49709876
422.3793774	47.73540856	257.0758755	245.9950075
277.986014	48.86013986	76.55594406	65.77643161
115.3649635	15.13868613	182.3686131	167.7672891
63.3960396	15.83168317	109.7821782	92.73590824
537.0028169	83.02394366	583.9394366	567.4054771

log.sigma.2.5.mm.3D_glszm_ZonePercentage	log.sigma.2.5.mm.3D_glszm_LargeAreaLowGrayLevelEmphasis	log.sigma.2.5.mm.3D_glszm_LargeAreaHighGrayLevelEmphasis	log.sigma.2.5.mm.3D_glszm_HighGrayLevelZoneEmphasis
0.425541942	0.067552354	17045.86711	506.0099668
0.222459893	1.732819575	54396.45192	203.8846154
0.241851369	1.398106288	17448.8248	165.2884097
0.342886387	0.154220987	21023.65075	331.8925373
0.314189189	0.348568608	36509.61559	311.9744624
0.345454545	0.216820064	12906.07895	327.1403509
0.300300832	0.654556078	90960.46317	444.3653506
0.27486296	0.932144129	150815.2991	448.8411681
0.326220853	0.131757423	34818.38975	511.8503034
0.349813433	0.164901438	15474.21333	332.5333333
0.292440318	0.583616922	14964.17234	174.6099773
0.340130152	0.156915162	34731.74617	497.7729592
0.197643278	4.049698142	283876.4661	288.3252033
0.32032615	0.232411922	104800.3418	689.9418182
0.350302419	0.210737675	34417.83885	417.4719424
0.280850091	1.179671697	109863.1587	356.5329536
0.347571189	0.155112886	7250.084337	237.0746988
0.217356042	2.130935837	57483.78731	192.6455224
0.245762712	1.174793155	29070.07837	178.6238245
0.310829006	0.275142425	37145.79146	413.3248762
0.185846561	5.784938695	256760.2046	244.2206406
0.297497683	0.286944939	9224.109034	180.3551402
0.28125	0.535119567	32274.78472	273.1909722
0.289006422	0.492550613	17915.31242	223.6888889
0.300864101	0.312407902	60944.96606	506.6266319
0.376952301	0.140377102	25015.71333	486.0324748
0.341787813	0.323691219	50728.64977	446.5004525
0.302679218	0.590781937	85510.8433	411.5095694
0.326621924	0.265628936	6213.945205	202.7191781
0.238072055	0.470279184	45868.29652	288.7791411
0.278688525	1.117045238	251883.5098	522.0648567
0.25181247	1.555417066	148599.858	280.8387716
0.345927532	0.216996967	35302.5103	404.1575649
0.320851064	0.230168638	42156.16844	454.096817
0.34400545	0.230784471	20955.61848	350.5062706
0.262420807	1.619386446	91953.44473	299.9047014
0.24106423	1.072061571	391820.631	594.7569677
0.246076342	1.521502005	258972.9937	482.8047244
0.237398374	0.504290869	28277.11986	199.0068493
0.333149931	0.167012571	9363.117258	275.9318745
0.261372398	1.527325209	126490.9838	261.7610619
0.430824929	0.07304577	3633.630852	278.1740696
0.32685464	0.216568657	23420.43719	357.6624791
0.351688103	0.158740995	56841.13371	552.1851429
0.298122066	0.329698681	20071.39108	293.167979
0.344626594	0.30094453	39141.86469	422.7167019
0.29899167	0.796954539	98663.47581	402.7785924
0.225028703	1.506524189	33833.76531	165.3826531
0.379553467	0.122917475	6413.390093	257.9535604
0.247781065	1.86096877	32988.92836	190.0208955
0.34449202	0.179439927	37532.57966	505.8090395
0.42242658	0.068183407	5848.432584	315.2956461
0.313829787	0.731767723	28538.12006	207.3764124
0.301630435	0.615671876	13278.31231	210.5015015
0.338345865	0.146399778	12473.75333	346.2377778
0.285128205	0.326822906	80847.05755	486.4172662
0.35105068	0.169225935	19663.80493	354.1408451
0.26604974	0.600844514	107507.0109	424.2945652
0.316747573	0.293843987	23424.24904	277.302682
0.316354475	0.387961359	67868.70118	484.4664127
0.282559456	1.312044539	102454.6573	319.743487
0.309363296	0.59468257	77481.4322	388.433414
0.34030057	0.297604367	40237.20609	411.5162437
0.28532074	0.185540533	48617.97379	555.8735632
0.273417722	0.848327641	8517.476852	126.4444444
0.252977732	1.142825748	231426.7369	501.9416581
0.226993865	1.073001838	197617.0169	383.5641892
0.29536	0.418764757	78666.6831	437.6229686
0.33003244	0.339043012	60431.27522	397.6254527
0.222222222	1.890275502	146399.9674	297.6273292
0.295612009	0.315695182	24996.57031	288.1145833
0.258041958	1.067082313	140469.598	416.0542005
0.305835893	0.251638129	71596.69727	557.4404591
0.414785303	0.136037943	15975.15582	337.018143
0.381986586	0.18016224	10052.59615	278.027592
0.278688525	0.196783446	33855.86631	368.0454545
0.239983306	1.231666724	57364.68522	262.4382609
0.236951983	1.273456895	45552.96035	219.1497797
0.29466901	0.475277742	30974.13917	265.499006
0.390765284	0.052750854	17052.88293	548.547046
0.424836601	0.066244926	17640.4426	515.2698225
0.329071707	0.578742273	34307.19932	235.6908784
0.281737534	0.980803643	114478.4593	394.7785016
0.231369997	0.807007286	34072.95921	198.2320675
0.347273473	0.156316697	18136.57615	328.1841795
0.154046997	4.604092251	264864.3432	242.2669492
0.351362683	0.339393221	52046.13246	367.2935561
0.296134209	0.882956084	72246.18842	347.841133
0.274590164	0.387101523	33171.70896	315.9875622
0.237037037	0.517780049	100810.2771	439.14375
0.194018405	4.902243535	115163.6759	212.8498024
0.387069689	0.083094792	6085.616052	312.4164859
0.341886938	0.181504031	27130.50564	394.7471783
0.293636364	0.56102256	47587.41383	310.8606811
0.349954044	0.12848154	17504.99278	345.5108339
0.300409117	0.523141619	129445.9874	562.1420233
0.30457934	0.296971961	21101.88462	258.5221445
0.261700096	0.40589793	84187.84672	390.0875912
0.242206235	0.503913874	25298.30693	189.7772277
0.245930031	2.451771293	141000.4958	300.2197183

log.sigma.2.5.mm.3D_glszm_SmallAreaEmphasis	log.sigma.2.5.mm.3D_glszm_LowGrayLevelZoneEmphasis	log.sigma.2.5.mm.3D_glszm_ZoneEntropy	log.sigma.2.5.mm.3D_glszm_SmallAreaLowGrayLevelEmphasis
0.691362172	0.007832842	6.342622119	0.006094514
0.633206294	0.019241461	5.816146229	0.016263116
0.590190565	0.0286468	6.218901191	0.018081935
0.620562577	0.010515099	6.216040276	0.008666806
0.628071283	0.008853761	6.316518567	0.006223342
0.620808147	0.010821998	6.081527802	0.008991199
0.611730868	0.003944753	6.493358437	0.002869948
0.642115353	0.00439448	6.464738992	0.003398691
0.628801604	0.003478104	6.454686043	0.002633399
0.626429114	0.008446135	6.056865744	0.004894756
0.617595393	0.014542424	6.023156467	0.010257791
0.666220605	0.004573086	6.279838081	0.003631333
0.616148328	0.013985936	6.156849629	0.011408082
0.655261942	0.006348302	6.355688372	0.005774367
0.662228965	0.006854995	6.286209647	0.005645477
0.634468033	0.008271314	6.394188209	0.006460892
0.612321662	0.011275771	6.135468845	0.007683356
0.614328445	0.018529757	5.999922662	0.015639668
0.59565893	0.012397227	5.919177092	0.009314465
0.626961958	0.004809082	6.558548589	0.0035952
0.592895947	0.008874141	6.1892531	0.006440197
0.590421694	0.013011928	6.010079	0.010164329
0.628383353	0.010700792	5.959917407	0.008792031
0.585392586	0.009834692	6.34465198	0.005835565
0.632858272	0.007063252	6.193617096	0.004293675
0.639562054	0.004961146	6.517966927	0.003713591
0.65020952	0.004784805	6.478425962	0.003701588
0.64329558	0.005380862	6.280651216	0.004163775
0.583866137	0.013974176	5.602830662	0.011500364
0.60966312	0.007752261	6.236331809	0.006098614
0.647888655	0.003729971	6.387928761	0.00290644
0.626998038	0.009745851	6.120364211	0.007487651
0.648010231	0.005709417	6.465727097	0.004327773
0.632526031	0.005706362	6.371470247	0.004461689
0.628682962	0.006270518	6.433075041	0.004661926
0.641260257	0.009876024	6.229512065	0.006963096
0.650090269	0.00367493	6.473818043	0.002988429
0.633431794	0.006034378	6.541842084	0.004815103
0.575439567	0.015025641	5.954258714	0.009551041
0.5866316	0.007244645	6.708092243	0.004910442
0.641511872	0.013078547	6.294721682	0.00913268
0.634269075	0.008323749	6.487221984	0.006018949
0.614488403	0.006199933	6.482334356	0.00465047
0.659158729	0.003692453	6.299190526	0.002929879
0.620456976	0.010234466	6.198159559	0.008532757
0.658341233	0.004723939	6.28423749	0.003648627
0.633025141	0.005080064	6.508071801	0.003332941
0.590692933	0.022728409	5.823929772	0.019052127
0.631850512	0.016637738	6.104971756	0.013800527
0.618604412	0.020101396	6.038799213	0.017041286
0.643276358	0.004769166	6.387695793	0.003806127
0.644206042	0.007424648	6.446208157	0.005410436
0.631822184	0.015766369	6.303370748	0.011597773
0.61998983	0.01392281	6.122557362	0.011190845
0.61126837	0.006434408	6.159099547	0.005041712
0.626335691	0.004824981	6.229125053	0.003935824
0.636570117	0.005992398	6.443426509	0.004640582
0.605565588	0.005257329	6.503152053	0.003095404
0.626807322	0.013029168	6.274732888	0.011264332
0.632839466	0.003569363	6.562675076	0.002490704
0.654829606	0.007372385	6.136947852	0.005955675
0.644699296	0.00794976	6.286997458	0.00587153
0.643198704	0.004520359	6.35397575	0.00295355
0.607162532	0.002895349	6.675410059	0.00201353
0.53912961	0.025048932	5.974541957	0.017238515
0.617662077	0.005221347	6.538376177	0.004184217
0.656465939	0.008452167	5.890284635	0.007240189
0.628268547	0.004350851	6.563568487	0.003062102
0.648709879	0.004656625	6.412973761	0.003438825
0.617840024	0.00729973	6.161154239	0.005813674
0.609441497	0.009489267	6.19447655	0.005655816
0.636643119	0.008291358	6.495999208	0.007247415
0.632022447	0.004274771	6.437562501	0.003416739
0.679360135	0.011420712	6.267613359	0.009120637
0.623684109	0.00798472	6.418973765	0.005389557
0.597885075	0.008630253	6.217824993	0.007265359
0.62520415	0.00761854	6.147645127	0.005777839
0.603112517	0.021529683	6.124810609	0.015162617
0.621875696	0.008781373	6.089140857	0.006798563
0.640449569	0.00399799	6.42817134	0.003122912
0.700683782	0.006667907	6.292125662	0.005754859
0.668195924	0.012433111	6.146943962	0.009324222
0.632565289	0.00678434	6.384739764	0.005583868
0.543486171	0.009921226	6.352292794	0.006076521
0.644699841	0.006962347	6.379568673	0.005077383
0.531747575	0.010001544	6.025027347	0.007816826
0.640011833	0.007380773	6.377667754	0.005862467
0.634293389	0.006231654	6.28101737	0.004823205
0.608987329	0.008825915	6.165822821	0.006652486
0.619494633	0.005667273	6.287423855	0.004365099
0.588634509	0.010744006	5.754852822	0.00816977
0.630193592	0.006692545	6.324182486	0.004969217
0.632757324	0.005129646	6.513739956	0.003679712
0.635675556	0.009309313	6.386873202	0.007277687
0.624663813	0.005230838	6.393602831	0.003822126
0.666276703	0.005340146	6.456030329	0.003778023
0.58621108	0.008061695	6.434848825	0.005541835
0.674589699	0.007617837	5.774023469	0.006558075
0.578493392	0.014163617	5.756451492	0.010925651
0.639024974	0.006497722	6.30861438	0.004339682

log.sigma.2.5.mm.3D_ngtdm_Coarseness	log.sigma.2.5.mm.3D_ngtdm_Complexity	log.sigma.2.5.mm.3D_ngtdm_Strength	log.sigma.2.5.mm.3D_ngtdm_Busyness	log.sigma.2.5.mm.3D_ngtdm_Contrast
0.003756165	0.728157079	1.84023402	0.25906693	6.82E-05
0.008720073	0.377827392	1.989169247	0.276572578	6.03E-05
0.005840116	0.244406613	1.163105996	0.528468988	4.80E-05
0.008756533	0.915315888	2.961604315	0.16584296	0.000123684
0.003346063	0.309405749	1.101595724	0.454069014	3.46E-05
0.012507179	1.293401558	4.30690478	0.139076967	0.000165594
0.001505809	0.186296132	0.827985069	0.681916351	8.62E-06
0.00173052	0.204358785	0.99774053	0.552957132	8.99E-06
0.001820168	0.276337231	0.990986129	0.502630362	1.35E-05
0.007867638	0.612272981	2.47394224	0.21005383	8.30E-05
0.005259006	0.258917783	0.965905114	0.533465442	5.18E-05
0.003424682	0.413743417	1.508771881	0.325228169	3.75E-05
0.004564706	0.242780888	1.728348623	0.32689653	2.35E-05
0.005091085	0.870036126	3.624551205	0.140027476	4.08E-05
0.003825431	0.702748667	1.969928504	0.264946268	4.62E-05
0.001967566	0.200520931	0.848595857	0.636943106	1.37E-05
0.007214034	0.521079191	1.664585661	0.2852551	8.22E-05
0.006756395	0.286498017	1.567722646	0.372684235	4.99E-05
0.006085036	0.304871446	1.434949489	0.411765483	3.69E-05
0.001704563	0.198559117	0.852696735	0.648998589	1.14E-05
0.003069655	0.125277642	0.88763871	0.610412823	1.27E-05
0.007279586	0.319957601	1.290555342	0.408203367	7.22E-05
0.007941901	0.590335116	2.382688757	0.215402321	6.34E-05
0.003166676	0.207578383	0.820378758	0.662418149	2.45E-05
0.006535009	0.797871101	3.502264504	0.150788454	4.73E-05
0.003715078	0.619578244	2.159766731	0.254546959	3.35E-05
0.002776882	0.430318976	1.460063008	0.352741791	2.34E-05
0.003054418	0.325924157	1.35481888	0.361503661	2.10E-05
0.016427348	0.589024015	2.213083133	0.244480884	0.000228653
0.003890581	0.243100573	1.276090674	0.400413325	2.24E-05
0.001777114	0.228827763	1.01758521	0.494355223	1.03E-05
0.004208155	0.278437758	1.29180923	0.349645421	2.82E-05
0.002795092	0.355587508	1.216315932	0.412722266	2.74E-05
0.003616277	0.456716257	1.749792848	0.289715568	3.32E-05
0.001942529	0.240087181	0.893112285	0.645046649	1.51E-05
0.002974228	0.201324008	0.995414298	0.530929703	1.91E-05
0.002352912	0.346875081	1.873146158	0.284858358	9.60E-06
0.001863916	0.199327409	1.117616836	0.48770764	9.47E-06
0.005534414	0.29342363	1.173772566	0.41902328	4.54E-05
0.001254514	0.114811097	0.401725614	1.421696722	1.20E-05
0.003343124	0.261465606	1.144568074	0.441287923	2.56E-05
0.002137412	0.351359131	0.90702275	0.747968124	2.91E-05
0.002328129	0.236972433	0.829961138	0.603193906	2.27E-05
0.003085336	0.536475194	1.627893109	0.277534801	3.15E-05
0.006474764	0.502279555	2.110331822	0.261306767	5.60E-05
0.002995202	0.41565072	1.413714425	0.363474384	2.40E-05
0.001904562	0.236210893	1.013144924	0.542332934	1.12E-05
0.008686925	0.297307491	1.579559903	0.359658633	6.85E-05
0.009170574	0.750694399	2.258908137	0.22569074	0.000143764
0.006703576	0.35314798	1.90204789	0.347547851	4.19E-05
0.003367525	0.460247701	1.727655163	0.295791136	3.05E-05
0.002289509	0.400520369	1.007565749	0.58881545	2.99E-05
0.001708654	0.147544839	0.485896359	1.185509816	1.68E-05
0.007362547	0.437954161	1.507439551	0.369373976	8.19E-05
0.006272892	0.473138741	1.621097135	0.300867035	7.06E-05
0.004390441	0.417110514	2.086448868	0.235032569	2.72E-05
0.002105621	0.220769472	0.736924401	0.675688991	2.23E-05
0.002578588	0.268312726	1.272655259	0.390904709	1.58E-05
0.004920006	0.46236133	1.590521038	0.305783507	4.51E-05
0.000969397	0.128309965	0.455155745	1.049121649	7.57E-06
0.004661367	0.406859859	1.931461119	0.299905662	3.26E-05
0.002949717	0.334522651	1.247679584	0.404430694	2.36E-05
0.001439141	0.182322026	0.661347121	0.785522805	1.15E-05
0.001166334	0.132890018	0.624747205	0.801553034	7.04E-06
0.010538219	0.418729709	1.698163099	0.357361868	9.84E-05
0.002379391	0.243363284	1.303578486	0.383161226	1.14E-05
0.005938961	0.46392871	2.58889117	0.203852064	3.36E-05
0.001386499	0.204966054	0.834638833	0.655745059	8.37E-06
0.001484652	0.202295958	0.880154801	0.64372167	8.71E-06
0.002909398	0.168024117	0.992141029	0.536595502	1.37E-05
0.006397896	0.503777007	1.906187908	0.26227301	5.83E-05
0.002082067	0.210777338	1.064786008	0.522269474	1.24E-05
0.00383281	0.504468226	2.188523547	0.22423155	2.61E-05
0.003571702	0.482558969	1.196240181	0.403165048	6.44E-05
0.00256499	0.338664127	0.889405391	0.68881067	3.48E-05
0.005648338	0.56927999	2.534103314	0.210889214	4.71E-05
0.003607013	0.17959637	0.981901592	0.52653975	1.88E-05
0.008726192	0.495192955	2.535490792	0.229863051	6.32E-05
0.004665459	0.326185964	1.350037453	0.381740945	3.54E-05
0.003571915	0.666895123	2.019901811	0.241615567	3.97E-05
0.004018089	0.762475554	2.012885801	0.240171807	6.98E-05
0.004594299	0.540267526	1.645513891	0.34148467	4.68E-05
0.002808581	0.287257799	1.259530619	0.398475346	1.60E-05
0.002854308	0.112112284	0.567120501	0.883647239	1.99E-05
0.003297858	0.438873913	1.279816115	0.408376179	3.82E-05
0.005661796	0.137801166	1.25975119	0.403043242	2.34E-05
0.003577976	0.441253724	1.381569329	0.342743242	4.25E-05
0.003160247	0.243153942	1.027219868	0.489985068	2.72E-05
0.005534925	0.42818134	2.045019536	0.264965367	3.84E-05
0.004325346	0.322465978	1.921454762	0.264987376	2.56E-05
0.006082064	0.170841778	1.124441391	0.526209484	4.81E-05
0.003361225	0.326387017	0.976071109	0.544549481	4.47E-05
0.001624121	0.221338111	0.777217259	0.666787162	1.37E-05
0.002568619	0.200434805	0.853507487	0.595176994	2.19E-05
0.001822343	0.191852883	0.609757357	0.784465206	1.88E-05
0.002596009	0.375259036	1.624426975	0.313006807	1.86E-05
0.00297916	0.235256002	0.939702384	0.59215585	2.53E-05
0.007626989	0.565557125	2.516271874	0.171480547	8.01E-05
0.009419694	0.337570501	1.743855914	0.275218862	7.60E-05
0.001452363	0.111197964	0.554997299	1.023962321	8.01E-06

log.sigma.2.0.mm.3D_gldm_GrayLevelVariance	log.sigma.2.0.mm.3D_gldm_HighGrayLevelEmphasis	log.sigma.2.0.mm.3D_gldm_GrayLevelNonUniformityNormalized	log.sigma.2.0.mm.3D_gldm_DependenceEntropy
34.58713393	731.3779453	0.06263186	6.545563979
11.80591267	304.0513369	0.12292602	6.03839886
12.87385792	238.8135593	0.104757186	6.394084731
24.58302472	470.3244626	0.074791861	6.481231872
18.0958556	399.1532939	0.087806808	6.413427445
25.68056933	474.8636364	0.073494031	6.262795237
16.09148382	496.7336755	0.087558039	6.587120708
16.40991606	461.2333594	0.102265383	6.477923871
19.48341319	506.3622965	0.07586215	6.606706587
18.82392724	394.2145522	0.081861564	6.283717495
12.17613928	258.9244032	0.09213461	6.196816342
23.08599263	572.0885033	0.078357433	6.478797298
12.40303596	427.2790573	0.155191986	6.237776077
25.31908704	798.042516	0.078954536	6.571855496
26.69023051	507.1925403	0.075808078	6.471651329
16.25660074	526.5457038	0.100092452	6.561822198
18.1976332	329.2738693	0.07144741	6.256192249
11.22947413	328.6934307	0.130636346	6.153246084
9.863537836	319.7103236	0.104058395	6.192258083
20.25288017	509.2335064	0.080957799	6.522638778
9.986110127	323.536045	0.127991938	6.410426329
12.02316187	300.9286376	0.093067501	6.055986669
15.46776962	532.9414063	0.092908859	6.289041928
14.17224994	338.0090669	0.085682104	6.459528654
19.30151697	615.3440691	0.094524072	6.34312966
27.9030737	455.6192486	0.062919613	6.634204015
24.50230471	467.9610269	0.070228732	6.657127882
17.08538608	483.4076756	0.091320554	6.356417349
12.75726319	267.6845638	0.085576726	5.842879485
12.05163981	447.6699124	0.110127815	6.187254941
17.43339904	594.8062211	0.0979601	6.544575108
13.94290781	374.6491058	0.113346903	6.316555112
24.10875044	444.5809848	0.070332628	6.801382814
22.09674405	542.1502128	0.081757175	6.661327222
21.23410763	488.5163488	0.072551293	6.603143053
14.28703733	410.9329777	0.106213353	6.435390617
17.88724889	814.9744692	0.110675906	6.499990779
17.38170908	718.3024608	0.118287744	6.777307476
11.49325732	424.3097561	0.099132791	5.999583132
19.03665549	324.0554333	0.067513873	6.608625687
15.16065498	307.5917502	0.1024406	6.414752539
26.62848291	328.8955262	0.055272181	6.5252015
19.21576774	463.1524774	0.077674403	6.52142213
24.71684778	579.3685691	0.07615364	6.413216941
18.44074699	490.2276995	0.086694047	6.333185257
18.8071883	352.504918	0.078433316	6.516913442
18.38180086	459.0872424	0.084901175	6.521151267
10.76904096	299.3008037	0.116678376	6.041216869
23.70039809	352.3278496	0.073425748	6.235546823
14.21746219	318.1420118	0.101734008	6.259839588
24.18288208	629.2203192	0.071774872	6.481041499
25.80906032	461.7775141	0.061316175	6.452270089
16.51697286	367.4148936	0.08132146	6.33591386
14.4448637	285.7690217	0.088781243	6.314213359
16.59227825	469.2578947	0.080633162	6.299551604
15.29848284	366.3707692	0.092978304	6.262054369
20.40725729	465.9772559	0.071430828	6.521312735
16.84298503	509.8935801	0.095269149	6.446679026
18.49787327	478.7669903	0.086147375	6.315025106
21.40758424	473.7047869	0.071073909	6.78471566
16.66647471	372.6489241	0.108833137	6.482255025
19.22398813	751.6865169	0.085520627	6.400748701
19.61677553	563.4353083	0.080759696	6.544306238
18.65528899	529.6536797	0.082706046	6.539102851
10.98077872	156.9316456	0.097638199	6.068172438
16.1893806	476.4707405	0.103072307	6.466449959
13.38158133	498.833589	0.117200732	6.18896483
19.42198275	567.0344	0.08470697	6.602983478
19.64216239	544.7147004	0.077305279	6.481529351
10.35058471	438.7177364	0.118093152	6.268207854
15.39193588	356.830639	0.088380296	6.386477441
17.24332839	705.9081585	0.098358409	6.472869157
20.18947981	416.8266784	0.083363545	6.626291653
29.54723823	475.1328021	0.061537922	6.580484042
23.81985566	327.3650591	0.060624425	6.566259179
16.39073518	395.5081967	0.096341737	6.192679572
11.7545875	337.2287145	0.103716615	6.252596788
14.59305007	365.1189979	0.106973034	6.165769661
13.46159873	483.7949619	0.096072583	6.186907282
27.10717482	560.4031637	0.062770601	6.587466314
33.60380484	734.5087984	0.063704068	6.526809258
22.41411617	383.3507504	0.08109162	6.590227069
16.52975208	490.5408382	0.094364864	6.585002309
10.31807639	253.5785877	0.09668656	6.342878656
22.87784584	407.3690037	0.070808666	6.570022061
6.263869479	307.4177546	0.164294869	6.218386291
23.87617825	383.9844864	0.072644278	6.731694011
17.44744089	440.4832239	0.086176296	6.550932977
15.28867641	457.9829235	0.096473842	6.338065834
14.41114074	532.0241975	0.107603841	6.394800384
8.555889853	363.3466258	0.156402904	6.058639958
20.70897689	402.6964736	0.065851781	6.391121374
21.37474324	396.902759	0.074806701	6.526056008
16.25882195	341.6457576	0.090509826	6.504887368
19.16044427	431.5434283	0.071964251	6.487112558
23.87999924	691.7746932	0.090792005	6.659976982
15.9353968	284.4554491	0.083535243	6.445603893
19.35702407	683.686724	0.098425574	6.272117949
10.11046127	237.6354916	0.113905998	6.135641551
12.37425071	412.5197437	0.122815701	6.392598052

log.sigma.2.0.mm.3D_gldm_DependenceNonUniformity	log.sigma.2.0.mm.3D_gldm_GrayLevelNonUniformity	log.sigma.2.0.mm.3D_gldm_SmallDependenceEmphasis
463.6182846	132.9048068	0.402830161
122.4331551	114.9358289	0.252565153
203.166884	160.6975228	0.253928418
160.6601842	73.0716479	0.317693531
398.2060811	207.9265203	0.324591741
138.2060606	48.50606061	0.392548545
895.4855778	494.7904796	0.307979635
697.0191856	522.371574	0.28048998
799.5961285	344.8693357	0.336689478
195.738806	87.75559701	0.34611395
259.6246684	138.938992	0.311523245
411.2264642	180.6138829	0.343122007
186.0433851	289.7434387	0.205316562
287.6534653	135.5649388	0.310923669
364.1774194	150.4032258	0.350970014
603.5772395	438.0045704	0.2770883
257.2512563	85.30820771	0.371561584
145.9748581	161.0746148	0.221232469
197.3297381	135.0677966	0.271717599
917.3027505	420.8995961	0.330637945
334.9332011	387.047619	0.215592441
198.3901761	100.4198332	0.323398776
160.9160156	95.13867188	0.293190326
432.535323	226.8005289	0.317082373
195.153967	120.3291438	0.301443301
480.6479527	149.056564	0.373205366
585.4918033	227.0494896	0.347458047
452.9319334	252.2273715	0.31539519
97.17897092	38.25279642	0.351330516
308.7088608	226.2025316	0.269841959
664.1214796	466.0941572	0.295527181
272.3765104	234.5147414	0.2603889
546.5961598	227.104057	0.318076587
354.9582979	192.1293617	0.312421817
797.5690282	319.5158946	0.345416266
394.6512171	318.5338446	0.260989571
480.7592045	411.825047	0.26632393
536.5636505	610.4830459	0.237628708
214.4195122	121.9333333	0.293425796
1416.444292	490.8258597	0.336109706
365.5304549	265.7309175	0.264227544
950.8924231	213.7375226	0.426731562
658.3566931	283.7445935	0.326967334
481.1913183	189.4702572	0.352529446
208.1909233	110.7949922	0.306601184
479.5632058	215.2994536	0.341039623
741.2889084	387.3191583	0.308068356
130.5729047	101.6268657	0.261766006
181.7990599	62.4853114	0.397759032
190.9497041	137.5443787	0.267769514
502.6800311	184.3896458	0.354823777
812.7063186	206.6968259	0.418224743
841.2371454	366.9224291	0.346338353
181.2264493	98.01449275	0.297777878
235.3353383	107.2421053	0.32369347
326.7179487	181.3076923	0.286879191
788.1851669	288.9377009	0.351659889
519.086177	329.4407172	0.286745049
295.9332524	141.9708738	0.335798645
1583.058933	648.8337167	0.330429212
223.4337486	192.1993205	0.288563646
457.7325843	228.3400749	0.333933232
972.4544826	467.5178787	0.332411783
1290.499672	630.4681884	0.310635614
132.0177215	77.13417722	0.277462149
534.5484205	398.0652512	0.281637859
178.0046012	152.8297546	0.271424342
1014.84736	529.41856	0.307130946
1119.90729	452.777019	0.365192181
373.3795721	342.2339545	0.239301127
205.4526559	114.8060046	0.29790019
597.183683	421.9575758	0.269468925
353.2887231	189.98552	0.30213846
481.9437804	139.0141656	0.378866517
684.1031619	189.8150751	0.381048432
226.561848	129.290611	0.314619655
364.4966611	248.5050083	0.272509916
136.263048	102.480167	0.259668382
288.4997071	163.9958992	0.303328632
485.5412569	146.8204361	0.380240477
434.7958773	126.7073906	0.409897266
298.4652585	145.8838243	0.334560841
462.7191802	308.4787397	0.292466897
477.5733811	297.1178002	0.262171722
458.6924969	172.702337	0.347600578
143.3733681	251.6997389	0.163061742
405.1417191	173.2566038	0.337919468
411.3391685	236.2954048	0.298040013
231.5737705	141.2377049	0.307245752
276.4103704	217.8977778	0.25428868
134.4861963	203.9493865	0.21527715
542.3845508	156.8589421	0.386720056
977.5000965	387.7231333	0.341582511
502.4393939	298.6824242	0.291941321
867.7674632	313.1884191	0.361314635
492.3436587	310.6902396	0.299623328
492.6308129	235.3187788	0.309712319
164.8032474	103.0515759	0.313657211
120.6235012	94.99760192	0.244799954
688.7440249	709.1378594	0.248913797

log.sigma.2.0.mm.3D_gldm_DependenceNonUniformityNormalized	log.sigma.2.0.mm.3D_gldm_DependenceVariance	log.sigma.2.0.mm.3D_gldm_LargeDependenceEmphasis
0.218481755	2.966174567	9.905749293
0.130944551	6.547417427	22.26631016
0.132442558	8.33001297	24.82398957
0.164442358	4.524549804	15.24564995
0.168161352	5.320147005	16.14780405
0.209403122	2.872277319	10.0969697
0.158464976	7.202016446	19.6841267
0.136456379	7.186869032	22.05794832
0.175890041	4.608402469	14.45358557
0.18259217	3.679100997	12.69029851
0.172164899	3.707909716	13.63793103
0.178406275	3.675630361	12.91670282
0.099648305	12.45777578	38.16979111
0.167532595	4.339019616	14.82877111
0.183557167	3.634786965	12.53225806
0.137928985	7.316818679	22.16819013
0.215453313	2.411342026	9.380234506
0.11838999	7.619831493	26.46796431
0.152025992	4.434851769	16.63636364
0.176438306	4.306545775	14.06751298
0.110758334	10.97990678	32.90608466
0.183864853	3.074945823	12.00834106
0.157144547	4.259765625	15.65039063
0.163405864	4.645136899	15.48507745
0.153302409	5.309835245	17.38177533
0.202890651	2.913068959	10.48079358
0.181098609	4.154618763	13.45777915
0.163986942	4.405019817	15.08906589
0.21740262	2.073219925	9.053691275
0.150296427	4.79720952	17.35929893
0.139579966	7.838200161	22.54392602
0.131646453	6.10865825	21.40889319
0.169277225	4.852837512	15.41684732
0.151046084	6.674878407	19.53702128
0.181101051	4.223691657	13.55086285
0.13159427	7.576777899	23.50483494
0.129201614	7.1418886	23.11824778
0.103965055	17.70754366	44.43770587
0.174324807	2.886334854	12.62276423
0.194834153	3.023809176	11.29160935
0.140913822	6.154497449	20.30531997
0.245899256	1.740314647	7.254978019
0.180223568	4.093904496	13.59676978
0.193404871	3.335288484	11.64951768
0.162903696	4.417353014	15.26917058
0.174704264	4.587873298	14.48342441
0.162492089	4.768905052	15.81192459
0.149911486	4.765054881	17.5717566
0.213629918	2.468996867	9.317273796
0.141234988	5.970781835	20.02218935
0.19567148	3.113942762	11.20397042
0.241087606	1.978354825	7.767724711
0.186444403	3.087676165	11.6179078
0.164154392	4.429111531	15.34782609
0.176943863	3.655539601	13.13082707
0.167547666	3.895700197	14.53333333
0.194854182	3.519548222	11.8815822
0.150111676	5.175792397	17.61885483
0.179571148	3.536842245	12.70509709
0.173409895	6.10412344	16.92288312
0.126519676	8.775938868	25.96602492
0.171435425	5.088627979	15.51161049
0.167983155	5.396242199	16.20124374
0.169290263	4.14701406	14.44536272
0.16711104	3.541137638	14.42278481
0.138412331	6.94523516	21.46090109
0.136506596	6.553850352	21.25613497
0.162375578	5.10584064	16.29888
0.191208347	3.571753157	11.97353594
0.128840432	6.3212277	22.47619048
0.158162168	5.351529364	17.16628176
0.139203656	6.078065214	20.26759907
0.155019185	6.097220535	18.41026766
0.21334386	3.116267996	10.43426295
0.218493504	2.61904604	9.533375918
0.16882403	4.401740401	14.81222057
0.152127154	4.788830717	17.17946578
0.142237002	5.11249951	18.75156576
0.169009787	3.66124257	13.84358524
0.207584975	2.786697158	10.09448482
0.21860024	3.079342619	10.04374057
0.165906203	6.222522487	17.483602
0.141547623	9.280454778	24.27623126
0.155409496	4.375381182	16.5196876
0.188065804	3.63402755	12.37515375
0.093585749	12.30850984	41.66057441
0.169870742	5.921948587	16.81886792
0.150014285	6.01470866	18.71553611
0.158178805	5.318192168	16.99180328
0.136498948	6.955711385	22.10222222
0.103133586	11.31274229	35.28067485
0.227701323	2.272953251	8.711167086
0.188597356	3.261571124	11.83156473
0.152254362	5.524726905	17.91939394
0.199395097	2.954417298	10.77435662
0.143875996	8.010937582	22.27469316
0.174877818	4.009141214	13.95278665
0.157405203	4.706049668	16.09264565
0.144632495	5.574360655	19.55155875
0.11928369	9.403584071	28.30966401

log.sigma.2.0.mm.3D_gldm_LargeDependenceLowGrayLevelEmphasis	log.sigma.2.0.mm.3D_gldm_SmallDependenceHighGrayLevelEmphasis	log.sigma.2.0.mm.3D_gldm_LargeDependenceHighGrayLevelEmphasis
0.015481962	274.9057772	7454.374175
0.081815976	74.7009215	6528
0.129307525	64.48477098	5230.113429
0.034820235	127.8642345	7723.073695
0.046014932	118.1714735	6631.228463
0.025878156	184.1426712	4619.806061
0.043755351	152.0808907	9278.903911
0.052440255	125.170046	9758.216915
0.032202844	159.63713	7357.611307
0.036235043	127.2509576	5071.216418
0.059672321	77.45662906	3556.282493
0.027353644	186.0869069	7361.601735
0.095263971	83.54403792	15727.01982
0.020710763	241.2227434	11871.33663
0.030291558	167.3200375	6460.233871
0.045324559	141.5541732	11413.87386
0.034635624	123.1193606	3046.526801
0.088921317	68.20896936	8389.36983
0.054039753	81.86280315	5460.881356
0.030880054	165.1296282	7021.072706
0.110538077	69.96037658	10112.72652
0.043320777	93.17830341	3681.134384
0.031674312	148.8570857	8377.273438
0.052073605	106.9132972	4994.519078
0.031688581	182.8058	10301.36606
0.029413492	170.0781684	4582.101309
0.035977346	160.3480377	6135.053511
0.034616111	144.0412435	7267.01593
0.041105953	91.73754567	2362.977629
0.040734952	112.9199339	7883.220058
0.041586652	169.4529702	12925.67255
0.059732599	89.56864197	8199.699855
0.040586653	139.0683883	6761.745432
0.043002355	160.4258362	10351.2234
0.031449235	164.9169572	6403.839691
0.063670771	108.0982822	9141.285762
0.02939541	209.2054113	18794.53641
0.066428773	173.5673874	30274.77756
0.030721157	115.8878453	5648.968293
0.042567889	108.2819174	3571.500825
0.074306606	76.45927258	6300.745952
0.030111328	140.9984465	2361.506336
0.03486068	145.628035	6219.139338
0.022763706	193.8975149	6929.793006
0.035963678	144.0102508	7227.607981
0.048225262	119.1300132	4897.244444
0.039037392	138.6970353	7053.70057
0.061492861	73.12656018	5398.092997
0.033469956	133.348239	3353.519389
0.073617549	84.99138971	5848.968935
0.020431522	216.8558969	6939.499805
0.020915677	193.7480479	3582.704835
0.034735887	121.4023136	4341.345745
0.064193778	86.18255265	4162.111413
0.030815786	145.077541	6107.224812
0.045804966	97.98475655	5376.275385
0.028966123	159.2914112	5538.200247
0.036514959	139.5531104	9035.806246
0.028259904	150.6410835	6266.375
0.04160938	156.045756	7597.683974
0.076535037	102.6830341	9312.996602
0.02205926	236.3907615	11512.42959
0.031525298	178.8488184	8823.953878
0.029558386	159.8866751	7676.779221
0.120087231	42.63685461	2181.063291
0.048878775	130.8042298	10008.21699
0.04498013	125.9238527	10671.35199
0.030306688	166.7510901	9343.44624
0.023229804	186.1418401	6769.182858
0.054461164	100.469139	9654.518288
0.052273135	97.79593507	6207.113934
0.030791479	184.273385	13853.38415
0.054307471	121.2736968	7422.917069
0.025584391	167.0881857	5208.729969
0.037918973	122.6240837	3104.07793
0.042953949	116.3331971	5983.433681
0.056127167	91.13635913	5548.471619
0.056972476	88.92443464	6790.438413
0.029997385	138.4557389	6848.041008
0.022415527	212.8192446	5752.987174
0.015564406	283.7748523	7539.24183
0.052203217	118.4243708	6803.132852
0.055781842	141.633616	11152.3469
0.070331518	64.09380281	4308.410999
0.036076466	135.6452254	5109.092661
0.139817034	46.14398632	12757.82507
0.050572361	125.4369014	6581.363941
0.050896312	127.6340105	7774.246171
0.041334376	135.4650859	7657.696038
0.043598806	126.3764599	11776.58519
0.106194425	78.63664857	12011.06595
0.026416175	156.7523916	3416.096138
0.034803974	128.1837091	4780.28497
0.061071164	92.23711302	6041.352424
0.027987255	149.8868703	4778.497932
0.035942782	199.6616341	14839.03653
0.056428677	87.49465571	4013.724885
0.024580947	199.5934999	11392.04776
0.084758484	51.92246498	4922.285372
0.075529368	101.4473141	10954.2655

log.sigma.2.0.mm.3D_gldm_SmallDependenceLowGrayLevelEmphasis	log.sigma.2.0.mm.3D_gldm_LowGrayLevelEmphasis	log.sigma.2.0.mm.3D_gldzm_DistanceZoneVariabilityNormalized
0.001554348	0.002509152	0.993896293
0.002871269	0.005872282	1
0.00358038	0.007679708	1
0.002863129	0.004553388	1
0.002226169	0.004743271	1
0.002954403	0.004439957	1
0.001048315	0.002616768	0.993933322
0.001402165	0.003133759	0.99873177
0.001317109	0.003074561	0.997701152
0.00300603	0.00488922	1
0.003037693	0.006400067	1
0.002092903	0.003681891	0.990950413
0.001539434	0.00356334	1
0.00167296	0.002652298	1
0.002431183	0.004385669	1
0.001169423	0.002713095	1
0.002677027	0.00503206	1
0.002034005	0.004876535	1
0.002012711	0.004426285	1
0.001669765	0.003199498	0.994918732
0.001384549	0.004069473	1
0.002867851	0.005367209	1
0.001851604	0.003268919	1
0.001947875	0.004266742	0.997910138
0.002195317	0.00338471	1
0.002226943	0.003998597	1
0.001853453	0.003992298	1
0.001803384	0.003496712	1
0.004127277	0.006881933	1
0.001933861	0.003665301	1
0.001382177	0.00273596	1
0.002151383	0.004322032	1
0.001910631	0.003899618	0.996598649
0.001555322	0.003665953	1
0.001420601	0.002990274	0.988584851
0.001394737	0.003450866	0.997671714
0.000804177	0.001744906	1
0.000758301	0.001892764	1
0.002183858	0.003907807	1
0.001940425	0.004577348	0.997224155
0.002750028	0.005737781	1
0.002872311	0.005304833	0.995884791
0.00156633	0.003654874	1
0.00197969	0.003317227	0.998002
0.002535003	0.004110648	1
0.00268841	0.004976886	1
0.001710966	0.003509423	0.99749844
0.003163149	0.005803109	1
0.00474532	0.007016489	1
0.002554714	0.005123676	0.995049535
0.001583677	0.002759558	0.998076925
0.001953019	0.003600734	0.998790079
0.00194829	0.003962741	1
0.002910603	0.006053122	1
0.00176442	0.003338652	1
0.003193513	0.005705715	1
0.00158997	0.003316326	0.99400485
0.001356362	0.002873217	1
0.001966474	0.003482209	1
0.001558727	0.003334641	0.996567516
0.002399175	0.004510978	1
0.001066369	0.001996264	0.998013905
0.001157747	0.002448974	0.991743261
0.0012895	0.002776666	0.988126583
0.00588097	0.012646787	1
0.001585584	0.003257183	0.988235706
0.001930213	0.003542601	1
0.001046684	0.002402962	0.990804794
0.001480481	0.002761584	1
0.001191259	0.003068698	1
0.002413122	0.004703563	1
0.000849441	0.001936454	0.993695887
0.001936649	0.00469443	1
0.002544982	0.004161219	1
0.0031561	0.005821608	0.991453149
0.00332319	0.005617092	1
0.001715934	0.004063331	1
0.003957867	0.006192646	1
0.001603627	0.003135297	0.996539803
0.001809635	0.00338216	1
0.001570232	0.002497587	1
0.004193115	0.00646267	1
0.001305344	0.002994997	1
0.001935395	0.005321882	1
0.002845079	0.004930812	0.997959186
0.001570964	0.00446562	1
0.002492774	0.004848047	1
0.001556894	0.003902044	1
0.002625677	0.004302226	1
0.00145417	0.002949916	1
0.001496815	0.003791471	0.98692867
0.002149619	0.004030869	0.998163454
0.002009464	0.004179207	0.989279064
0.002885938	0.005588791	1
0.001604669	0.003414408	0.998893806
0.001260223	0.002475609	0.994755281
0.00327975	0.006370872	1
0.001702274	0.002749954	1
0.004228851	0.007672352	1
0.001162299	0.003167271	0.993678951

log.sigma.2.0.mm.3D_gldzm_LowIntensityEmphasis	log.sigma.2.0.mm.3D_gldzm_LargeDistanceEmphasis	log.sigma.2.0.mm.3D_gldzm_HighIntensitySmallDistanceEmphasis
0.00360815	1.009183673	689.7191327
0.011085262	1	305.7509579
0.01357049	1	259.5273973
0.008475404	1	405.2142857
0.00707483	1	368.6270023
0.007013663	1	479.3024055
0.003349207	1.009127789	497.1251268
0.00484999	1.001903553	453.9811231
0.004049978	1.003452244	479.7695627
0.00801645	1	373.4125874
0.009520678	1	248.4238532
0.006012442	1.013636364	548.9744318
0.007293902	1	412.5676329
0.005019193	1	780.5709516
0.006838005	1	482.5809285
0.004139847	1	514.3975994
0.006753416	1	331.9712644
0.009264879	1	312.755102
0.007014215	1	303.1909548
0.004805721	1.007641365	504.7361182
0.006271482	1	328.6216968
0.008432396	1	291.4245524
0.005986036	1	509.4592145
0.005908368	1.003138075	339.3933054
0.006638941	1	615.578341
0.005582364	1	461.6183057
0.005519895	1	465.8603175
0.005592832	1	463.3205791
0.0103345	1	267.5699482
0.006852197	1	419.7656766
0.004527101	1	581.8817481
0.008043991	1	344.8456261
0.005796502	1.005110733	439.9118399
0.005371675	1	520.8552473
0.003977372	1.017221584	482.7092423
0.005239755	1.003496503	420.8371212
0.002936839	1	790.4156456
0.003046716	1	737.4959469
0.006851235	1	396.2650602
0.005650011	1.004169562	324.3858582
0.010221757	1	288.6284953
0.006435953	1.006185567	330.6015464
0.004913733	1	451.3158273
0.005307576	1.003	553.93325
0.007660533	1	481.8382688
0.00751741	1	352.163074
0.005351701	1.003757044	454.6593613
0.011101037	1	284.7251908
0.0111268	1	339.193299
0.008962069	1.007444169	324.9503722
0.004197451	1.002887392	620.1571222
0.004488409	1.001815981	461.3694007
0.005439559	1	353.0981282
0.009519666	1	290.8010753
0.0051202	1	453.2865854
0.010659462	1	346.1156894
0.00440738	1.009019844	453.7784125
0.004586347	1	490.2578558
0.005552901	1	452.9454254
0.00458187	1.005157593	473.7227794
0.007958747	1	361.4794275
0.003015752	1.002982107	719.3014414
0.003343794	1.012436665	547.3513358
0.004017981	1.017917133	517.4059351
0.020556618	1	156.6733871
0.005443473	1.017751479	467.3590448
0.007019355	1	467.1898396
0.003324718	1.013856813	543.0243649
0.003880762	1	514.9200659
0.004870114	1	427.2065789
0.007628937	1	334.7628635
0.003029348	1.009486166	692.4843874
0.006890848	1	407.212548
0.006245254	1	444.92
0.007867675	1.012875536	323.7784335
0.010164888	1	370.2484211
0.006013529	1	341.4671233
0.01427238	1	349.6131387
0.005036951	1.005199307	459.7192374
0.00462945	1	558.6860465
0.003589838	1	700.1569892
0.011640579	1	360.1007194
0.00441129	1	489.3956563
0.007300831	1	245.2879291
0.007689094	1.003064351	391.8743616
0.009266277	1	280.1971326
0.007079262	1	370.4707135
0.005559591	1	434.997819
0.007889195	1	445.9921415
0.005418614	1	501.8519164
0.006645199	1.019736842	374.3782895
0.005249937	1.002757353	405.7058824
0.005767377	1.016168545	377.9118079
0.009611601	1	321.179702
0.004376026	1.00166021	416.5170172
0.004055537	1.007887818	675.6932515
0.010011002	1	281.7942913
0.005195551	1	636.2231638
0.016020244	1	212.5367965
0.004568157	1.009511731	418.4324667

log.sigma.2.0.mm.3D_gldzm_LowIntensityLargeDistanceEmphasis	log.sigma.2.0.mm.3D_gldzm_HighIntensityEmphasis	log.sigma.2.0.mm.3D_gldzm_DistanceZoneVariability	log.sigma.2.0.mm.3D_gldzm_ZonePercentage
0.003633661	691.2642857	974.0183673	0.461828464
0.011085262	305.7509579	261	0.279144385
0.01357049	259.5273973	438	0.285528031
0.008475404	405.2142857	350	0.358239509
0.00707483	368.6270023	874	0.369087838
0.007013663	479.3024055	291	0.440909091
0.003385603	497.7636917	1960.036511	0.348964785
0.004861253	454.0615482	1574.001269	0.30853563
0.004056812	480.2882624	1734.004603	0.382314122
0.00801645	373.4125874	429	0.400186567
0.009520678	248.4238532	545	0.361405836
0.006192966	549.7636364	872.0363636	0.381778742
0.007293902	412.5676329	414	0.221746117
0.005019193	780.5709516	599	0.348864298
0.006838005	482.5809285	797	0.40171371
0.004139847	514.3975994	1333	0.304616088
0.006753416	331.9712644	522	0.43718593
0.009264879	312.755102	294	0.238442822
0.007014215	303.1909548	398	0.306625578
0.004927612	505.1456954	1953.025471	0.37757261
0.006271482	328.6216968	719	0.23776455
0.008432396	291.4245524	391	0.362372567
0.005986036	509.4592145	331	0.323242188
0.005924379	339.5470711	954.0020921	0.361163581
0.006638941	615.578341	434	0.340926944
0.005582364	461.6183057	1027	0.433516252
0.005519895	465.8603175	1260	0.3897309
0.005592832	463.3205791	967	0.350108617
0.0103345	267.5699482	193	0.431767338
0.006852197	419.7656766	606	0.29503408
0.004527101	581.8817481	1556	0.327028163
0.008043991	344.8456261	583	0.281778637
0.006111979	439.9693356	1170.006814	0.363580056
0.005371675	520.8552473	829	0.352765957
0.004018821	484.7801378	1722.114811	0.3955495
0.005268652	420.9428904	856.002331	0.286095365
0.002936839	790.4156456	1061	0.285138404
0.003046716	737.4959469	1357	0.26293354
0.006851235	396.2650602	415	0.337398374
0.005687188	324.5813065	2870.011119	0.395873453
0.010221757	288.6284953	751	0.289514264
0.006475764	331.1489691	1932.016495	0.50168089
0.004913733	451.3158273	1390	0.380509171
0.005315887	554.204	998.002	0.40192926
0.007660533	481.8382688	439	0.343505477
0.00751741	352.163074	1067	0.38870674
0.005366377	454.8998121	1593.005009	0.350065761
0.011101037	284.7251908	262	0.300803674
0.0111268	339.193299	388	0.455934195
0.00900005	325.3151365	401.0049628	0.298076923
0.00420545	620.4177093	1037.001925	0.404437524
0.0044909	461.7003632	1650.001211	0.490062296
0.005439559	353.0981282	1763	0.390735816
0.009519666	290.8010753	372	0.336956522
0.0051202	453.2865854	492	0.369924812
0.010659462	346.1156894	631	0.323589744
0.004459606	454.3926639	1653.030066	0.411124845
0.004586347	490.2578558	1082	0.312897629
0.005552901	452.9454254	623	0.378033981
0.00459794	474.2269341	3478.02063	0.382298171
0.007958747	361.4794275	559	0.316534541
0.003020524	719.7673956	1004.001988	0.376779026
0.00340416	548.528328	2153.07462	0.375021593
0.004106384	518.6360582	2647.191116	0.351436442
0.020556618	156.6733871	248	0.313924051
0.006292445	468.1065089	1169.08284	0.30631797
0.007019355	467.1898396	374	0.286809816
0.003356291	544.7394919	2145.092379	0.3464
0.003880762	514.9200659	2427	0.41437596
0.004870114	427.2065789	760	0.262249827
0.007628937	334.7628635	447	0.344110855
0.003054648	693.4798419	1257.025296	0.294871795
0.006890848	407.212548	781	0.342694164
0.006245254	444.92	1000	0.442673749
0.007954369	324.4206009	1386.051502	0.446502715
0.010164888	370.2484211	475	0.353949329
0.006013529	341.4671233	730	0.304674457
0.01427238	349.6131387	274	0.286012526
0.005057261	460.0519931	575.0034662	0.338019918
0.00462945	558.6860465	1032	0.441214194
0.003589838	700.1569892	930	0.467571644
0.011640579	360.1007194	695	0.386325737
0.00441129	489.3956563	1059	0.323952279
0.007300831	245.2879291	903	0.293849658
0.007698551	392.1225741	977.0020429	0.401394014
0.009266277	280.1971326	279	0.182114883
0.007079262	370.4707135	939	0.393710692
0.005559591	434.997819	917	0.334427425
0.007889195	445.9921415	509	0.347677596
0.005418614	501.8519164	574	0.28345679
0.006722296	375.6414474	300.0263158	0.233128834
0.005255634	406.0395221	1086.001838	0.456759026
0.005879258	379.2229299	2019.118569	0.393787382
0.009611601	321.179702	1074	0.325454545
0.004383405	416.610404	1805.001107	0.415211397
0.004077813	676.4425942	1135.015776	0.333430742
0.010011002	281.7942913	1016	0.360667377
0.005195551	636.2231638	354	0.338108883
0.016020244	212.5367965	231	0.276978417
0.004605631	419.1686747	1567.031706	0.273120887

log.sigma.2.0.mm.3D_gldzm_IntensityVariabilityNormalized	log.sigma.2.0.mm.3D_gldzm_LowIntensitySmallDistanceEmphasis	log.sigma.2.0.mm.3D_gldzm_IntensityVariability
0.041720117	0.003601773	40.88571429
0.062433024	0.011085262	16.29501916
0.056764871	0.01357049	24.8630137
0.04757551	0.008475404	16.65142857
0.052249318	0.00707483	45.66590389
0.046019768	0.007013663	13.39175258
0.052686392	0.003340109	103.8975659
0.050486034	0.004847174	79.56598985
0.049840365	0.00404827	86.62255466
0.051700436	0.00801645	22.17948718
0.064617456	0.009520678	35.21651376
0.048396178	0.005967311	42.58863636
0.057667624	0.007293902	23.87439614
0.044657066	0.005019193	26.74958264
0.045721959	0.006838005	36.44040151
0.055181275	0.004139847	73.55663916
0.054175658	0.006753416	28.27969349
0.061640983	0.009264879	18.12244898
0.070149744	0.007014215	27.91959799
0.047734074	0.004775249	93.70198675
0.061561704	0.006271482	44.26286509
0.062041719	0.008432396	24.25831202
0.055101724	0.005986036	18.23867069
0.057023687	0.005904366	54.51464435
0.051317718	0.006638941	22.2718894
0.045187911	0.005582364	46.40798442
0.044543966	0.005519895	56.12539683
0.051249667	0.005592832	49.55842813
0.067142742	0.0103345	12.95854922
0.060337222	0.006852197	36.56435644
0.052503453	0.004527101	81.69537275
0.055138589	0.008043991	32.1457976
0.046180881	0.005717632	54.21635434
0.047492801	0.005371675	39.37153197
0.048410908	0.00396701	84.33180253
0.056158682	0.005232531	48.18414918
0.04957978	0.002936839	52.60414703
0.048745852	0.003046716	66.14812085
0.066709247	0.006851235	27.68433735
0.051821322	0.005640717	149.1417651
0.056203801	0.010221757	42.20905459
0.047268041	0.006426001	91.7
0.050517054	0.004913733	70.21870504
0.046182	0.005305499	46.182
0.053543724	0.007660533	23.50569476
0.050682088	0.00751741	54.07778819
0.051205513	0.005348032	81.77520351
0.063924014	0.011101037	16.7480916
0.047401424	0.0111268	18.39175258
0.057022702	0.008952574	22.98014888
0.046710532	0.004195452	48.53224254
0.04731443	0.004487786	78.16343826
0.054687824	0.005439559	96.41463415
0.056625043	0.009519666	21.06451613
0.053803953	0.0051202	26.47154472
0.055171149	0.010659462	34.81299525
0.050251865	0.004394323	83.56885147
0.051515131	0.004586347	55.73937153
0.051918306	0.005552901	32.34510433
0.048472673	0.004577853	169.1696275
0.049542212	0.007958747	27.6940966
0.05237956	0.003014559	52.69383698
0.050374891	0.003328702	109.3638876
0.049395705	0.00399588	132.3310937
0.069719043	0.020556618	17.29032258
0.050749238	0.00523123	60.03634827
0.056392805	0.007019355	21.09090909
0.049852311	0.003316825	107.930254
0.051633279	0.003880762	125.3139679
0.062583102	0.004870114	47.56315789
0.056959396	0.007628937	25.46085011
0.051508382	0.003023022	65.15810277
0.04797192	0.006890848	37.46606914
0.04383	0.006245254	43.83
0.047856022	0.007846001	66.90271817
0.05454626	0.010164888	25.90947368
0.059324451	0.006013529	43.30684932
0.054584688	0.01427238	14.95620438
0.061157184	0.005031873	35.28769497
0.046840259	0.00462945	48.33914729
0.041953983	0.003589838	39.0172043
0.045391025	0.011640579	31.54676259
0.050814057	0.00441129	53.81208687
0.065926425	0.007300831	59.53156146
0.04818763	0.007686729	47.17568948
0.080638738	0.009266277	22.49820789
0.047040957	0.007079262	44.171459
0.052450442	0.005559591	48.09705562
0.059560524	0.007889195	30.31630648
0.056167976	0.005418614	32.24041812
0.07217365	0.006625925	21.94078947
0.052241025	0.005248513	56.83823529
0.048894046	0.005739407	99.79274865
0.054667804	0.009611601	58.7132216
0.052439799	0.004374181	94.7587161
0.044815924	0.004049968	51.13496933
0.054699609	0.010011002	55.57480315
0.050480386	0.005195551	17.8700565
0.065759637	0.016020244	15.19047619
0.056697638	0.004558789	89.41217502

log.sigma.2.0.mm.3D_gldzm_HighIntensityLargeDistanceEmphasis	log.sigma.2.0.mm.3D_gldzm_SmallDistanceEmphasis	log.sigma.2.0.mm.3D_glcm_SumVariance	log.sigma.2.0.mm.3D_glcm_Homogeneity1
697.444898	0.997704082	2351.427288	0.29647428
305.7509579	1	920.6813037	0.432269432
259.5273973	1	682.7877558	0.429526302
405.2142857	1	1435.333933	0.35191472
368.6270023	1	1204.460582	0.353871073
479.3024055	1	1386.545318	0.317806064
500.3179513	0.997718053	1540.457708	0.381199016
454.3832487	0.999524112	1403.58083	0.400696126
482.363061	0.999136939	1560.486572	0.34891237
373.4125874	1	1185.499916	0.353504862
248.4238532	1	762.4221275	0.379132407
552.9204545	0.996590909	1757.305816	0.347994929
412.5676329	1	1346.138467	0.472297271
780.5709516	1	2615.764944	0.350115136
482.5809285	1	1544.641565	0.336083831
514.3975994	1	1651.411436	0.403495583
331.9712644	1	984.5708081	0.322812157
312.755102	1	1016.796698	0.443367921
303.1909548	1	981.8058065	0.39203903
506.7840041	0.998089659	1539.970127	0.353262832
328.6216968	1	988.8013168	0.46095194
291.4245524	1	895.0962454	0.364256734
509.4592145	1	1680.651075	0.37832577
340.1621339	0.999215481	1031.829986	0.37669805
615.578341	1	1967.471108	0.384236788
461.6183057	1	1305.748128	0.321976467
465.8603175	1	1398.987735	0.336385656
463.3205791	1	1495.981681	0.366468354
267.5699482	1	770.5542568	0.338050088
419.7656766	1	1387.04154	0.389905128
581.8817481	1	1903.802253	0.396253745
344.8456261	1	1167.107928	0.405732316
440.1993186	0.998722317	1328.782333	0.349982871
520.8552473	1	1673.930884	0.377246673
493.0637199	0.995694604	1454.342979	0.346899282
421.3659674	0.999125874	1255.019928	0.415007213
790.4156456	1	2711.846601	0.404127516
737.4959469	1	2362.218827	0.454144639
396.2650602	1	1355.726705	0.357850026
325.3630994	0.998957609	947.9089234	0.330136227
288.6284953	1	933.371958	0.394558648
333.3386598	0.998453608	938.3363403	0.280304992
451.3158273	1	1408.302644	0.348684418
555.287	0.99925	1811.844019	0.327579514
481.8382688	1	1483.166496	0.369440606
352.163074	1	1036.111607	0.357365215
455.8616155	0.999060739	1391.373231	0.367812758
284.7251908	1	897.4304582	0.406998487
339.193299	1	1019.631518	0.312904885
326.7741935	0.998138958	937.1412124	0.407424679
621.4600577	0.999278152	1975.800325	0.330851208
463.0242131	0.999546005	1381.826207	0.290662076
353.0981282	1	1102.687668	0.336592721
290.8010753	1	844.9997383	0.372963811
453.2865854	1	1444.810296	0.353571219
346.1156894	1	1108.795034	0.374056574
456.8496693	0.997745039	1409.28029	0.331225071
490.2578558	1	1603.634939	0.382538377
452.9454254	1	1479.462078	0.350427603
476.243553	0.998710602	1451.041717	0.346327654
361.4794275	1	1114.060587	0.417741704
721.6312127	0.999254473	2472.089181	0.359525044
553.2362966	0.996890834	1750.254466	0.365588702
523.556551	0.995520717	1625.183729	0.358789874
156.6733871	1	405.7383984	0.39915824
471.0963652	0.99556213	1467.290111	0.398713938
467.1898396	1	1599.211276	0.398902695
551.6	0.996535797	1798.609129	0.364641837
514.9200659	1	1732.848159	0.352762511
427.2065789	1	1394.432816	0.421800301
334.7628635	1	1095.805319	0.369436193
697.4616601	0.997628458	2296.889842	0.40355707
407.212548	1	1245.888566	0.374928692
444.92	1	1418.511839	0.302010135
326.9892704	0.996781116	940.5874704	0.304364427
370.2484211	1	1213.058318	0.364817661
341.4671233	1	1022.441404	0.398362575
349.6131387	1	1112.153579	0.395329637
461.3830156	0.998700173	1510.903662	0.368327284
558.6860465	1	1747.025536	0.310496512
700.1569892	1	2360.992885	0.304678509
360.1007194	1	1135.10961	0.354396496
489.3956563	1	1537.664691	0.401613229
245.2879291	1	737.4400754	0.397291841
393.1154239	0.999233912	1205.223178	0.331563184
280.1971326	1	959.8914841	0.512644678
370.4707135	1	1134.112574	0.341041123
434.997819	1	1323.585671	0.382341693
445.9921415	1	1394.877809	0.381896357
501.8519164	1	1682.46913	0.412126929
380.6940789	0.995065789	1143.445169	0.482247075
407.3740809	0.999310662	1200.228005	0.306843772
384.4674179	0.995957864	1161.004702	0.3316277
321.179702	1	1019.773252	0.388962479
416.9839513	0.999584947	1296.215451	0.321993253
679.4399649	0.998028046	2225.077498	0.388484468
281.7942913	1	822.843068	0.363879006
636.2231638	1	2250.20535	0.374487751
212.5367965	1	692.8635373	0.418254472
422.1135067	0.997622067	1271.101564	0.434056816

log.sigma.2.0.mm.3D_glcm_Homogeneity2	log.sigma.2.0.mm.3D_glcm_ClusterShade	log.sigma.2.0.mm.3D_glcm_MaximumProbability	log.sigma.2.0.mm.3D_glcm_Idmn	log.sigma.2.0.mm.3D_glcm_SumVariance2
0.211768686	-773.8914897	0.01938054	0.969975727	83.89035526
0.361281118	17.60840271	0.07391566	0.983531015	28.1807912
0.357684084	38.95715121	0.067131868	0.982419039	30.84872467
0.273228411	-345.4931052	0.039391746	0.969962872	59.11064268
0.271075404	-160.0857647	0.040259518	0.976238269	40.15139448
0.230503937	184.1566122	0.02448381	0.977010701	62.81796421
0.301457085	15.0684299	0.045341665	0.987054685	38.87865909
0.326213976	-48.80396334	0.049922708	0.984006793	37.64209583
0.266827357	-171.3797342	0.023023865	0.980146341	47.08003489
0.269529232	-185.27651	0.031334376	0.975928242	41.37065225
0.296068288	-68.11721781	0.030434241	0.978168667	29.0690028
0.266323518	-305.723287	0.024002172	0.977331401	57.12867376
0.411273296	-45.5967959	0.116973352	0.986068313	29.32891813
0.267997672	-456.6004224	0.034901335	0.981217777	59.7143377
0.255307989	-373.4074311	0.02396827	0.974440016	61.88450616
0.330138746	-101.1314829	0.048689236	0.983332803	40.79671083
0.232957499	-92.42998795	0.018395848	0.969942697	42.98758034
0.375653631	15.45265483	0.068378761	0.984104235	29.05933597
0.309890044	11.74573652	0.03455921	0.987168478	22.24005818
0.270211058	-50.38346575	0.029519399	0.982055045	49.43825401
0.395372841	13.97828161	0.081499098	0.986477984	24.60142703
0.277716023	-45.11957785	0.030826009	0.977975009	28.28900508
0.298413262	-77.45859687	0.035800283	0.980453457	35.19943623
0.294561664	-9.034861105	0.028265415	0.978328499	33.79604944
0.306714083	-97.3046534	0.046912344	0.983680161	45.94422844
0.234589627	-6.960534422	0.018824837	0.977329932	65.80595149
0.253426086	-49.33075642	0.024654536	0.975056316	57.44048576
0.285402127	-189.5851226	0.0307501	0.979046585	40.96738205
0.245834699	22.80298483	0.025593066	0.97178242	29.97564749
0.310303431	-97.5627826	0.044720115	0.982972004	27.85690419
0.320934241	-158.7681492	0.048948382	0.987526027	43.75659952
0.332860318	-193.9199373	0.047801093	0.974573509	31.66079498
0.269151042	-117.282681	0.025299903	0.980221054	61.73380281
0.301348126	-331.8560032	0.040842799	0.976068174	54.26028843
0.26339558	-56.1201789	0.027660384	0.982958233	51.53874741
0.341731777	-33.82492465	0.057075641	0.985716914	33.23437324
0.331952028	-179.2637858	0.054203641	0.988193838	40.75635544
0.391593392	-137.9413886	0.103513935	0.990109993	42.41102003
0.271428132	-136.5174753	0.027105338	0.975006342	24.16006299
0.241414942	-31.27955026	0.014956588	0.976989998	45.11920802
0.318127717	-181.6181434	0.044614232	0.978100917	36.71720498
0.188842293	31.14848245	0.011036076	0.970869504	61.8128747
0.264450267	-109.8959743	0.028399099	0.978676534	46.04489051
0.244211636	-390.2800661	0.027196476	0.976593739	59.5748991
0.288910346	-80.06162112	0.031511961	0.979469097	42.87277635
0.273508792	-42.92646538	0.03513795	0.982528943	40.83004535
0.286747322	-31.97968578	0.034206526	0.982588124	42.90517955
0.328797314	-35.71902393	0.044705114	0.98376774	24.17345058
0.224525824	-238.0574301	0.023997086	0.967727949	52.56075346
0.331007516	70.73770433	0.051691241	0.983591307	35.16177222
0.244738012	-230.1766561	0.023405712	0.980131838	59.15478318
0.200936226	-57.47912283	0.013363268	0.977205556	63.31923539
0.248341538	-66.23826578	0.024973739	0.978303746	36.12123342
0.289060812	48.93774296	0.034162906	0.979924476	33.93709168
0.267769078	8.710647353	0.02606051	0.980502671	40.07024942
0.291359911	-163.5407908	0.033495613	0.979196563	34.2570178
0.244683081	-133.7645316	0.023269823	0.9742246	46.20917428
0.304947337	-211.8816893	0.035175499	0.982646753	41.54617081
0.26763515	-164.2237723	0.025815344	0.979751396	41.25899832
0.2660528	-166.4330654	0.030602876	0.97629521	52.34247128
0.347565133	44.04305971	0.069325971	0.982901617	34.61768402
0.278702436	-231.9283202	0.033864939	0.981791937	45.91197959
0.285855834	-108.8231665	0.038111842	0.980065633	48.91616684
0.275854057	-41.29789226	0.02575701	0.982135651	44.54525678
0.319329725	-11.75929126	0.040226314	0.977252345	24.90641141
0.324272237	-154.5384164	0.047114193	0.982330644	37.44650386
0.324634184	-174.9309942	0.048502691	0.980983063	29.23846337
0.284957727	-134.2019265	0.034523566	0.983810632	47.99964956
0.269203042	-206.9302694	0.024278212	0.984720262	47.17162451
0.34717937	-45.11302842	0.062141962	0.985244752	21.59235972
0.287117539	-124.3314766	0.040944033	0.977100861	35.36067391
0.328746998	-111.8057766	0.044390062	0.988209666	42.03327166
0.29739668	-166.0335475	0.035164973	0.980158964	44.7079302
0.217118659	-309.5454704	0.018812525	0.966663987	68.22613566
0.215454966	-31.50606582	0.017655691	0.971630912	51.94838802
0.28316397	-317.1742345	0.041177012	0.976023589	37.47819709
0.317684992	41.21755123	0.039170384	0.987988407	24.91394315
0.317803822	-87.61046889	0.048295444	0.980415665	32.70906045
0.283709251	-71.46541599	0.032081846	0.98266595	30.78307217
0.224339706	-399.1735582	0.015720655	0.977574524	69.91054811
0.220277665	-752.2479402	0.020597454	0.97151298	82.49963784
0.276158789	-160.4877336	0.031952004	0.971486728	45.78102642
0.328070809	-41.20367731	0.050675016	0.982064097	38.73614051
0.315882641	-58.76358691	0.032626088	0.977934171	25.70918479
0.248072663	-165.2661197	0.023592897	0.974306238	53.1083414
0.455658838	-14.21459841	0.114556376	0.989299619	15.78644425
0.260260651	-279.1644927	0.030229377	0.97441481	54.59376809
0.303259921	-55.25675438	0.048242039	0.979904075	38.82779478
0.301581138	-163.5952551	0.038661824	0.980802532	35.52421415
0.338923738	-161.6103342	0.053995398	0.983663667	33.85304328
0.419460492	85.31284758	0.110010183	0.989908313	21.43383275
0.214945144	-26.6351114	0.016475451	0.975919838	46.77541072
0.246228947	-159.3840164	0.019920089	0.978516615	49.27230901
0.310613916	-161.6653891	0.04152153	0.975375225	39.96381526
0.234154679	-122.4119738	0.017590696	0.977208014	43.73157446
0.315725433	-365.4773352	0.052369792	0.981153078	59.74322901
0.280477371	-73.5976674	0.027708738	0.978878808	38.23124554
0.297722137	-350.2138272	0.038355114	0.980900917	46.71263794
0.34389553	-119.8669048	0.048640908	0.972959052	24.19930292
0.36429403	59.18442424	0.068141056	0.98746421	28.12062956

log.sigma.2.0.mm.3D_glcm_Contrast	log.sigma.2.0.mm.3D_glcm_DifferenceEntropy	log.sigma.2.0.mm.3D_glcm_InverseVariance	log.sigma.2.0.mm.3D_glcm_Entropy	log.sigma.2.0.mm.3D_glcm_Dissimilarity
60.80092146	3.958002522	0.214867384	8.428765679	5.533255319
16.55584303	3.034102024	0.344681464	6.566025828	2.733518215
16.30997119	3.062058039	0.332468137	6.896311483	2.777444199
40.52962983	3.627141963	0.267138786	7.723338031	4.360337682
29.02997032	3.476750332	0.273415787	7.553086769	3.843378776
36.39332961	3.611674644	0.23703271	7.657265447	4.455173341
23.21972521	3.342200358	0.288505681	7.591590174	3.418126702
24.02400657	3.30618228	0.312214699	7.305334127	3.313474731
33.17145277	3.564801002	0.26519522	7.953979005	4.08149204
27.63767743	3.418606702	0.278901698	7.402682806	3.755512052
18.90105916	3.201751489	0.298515621	7.20036781	3.168812462
39.1708048	3.609389094	0.26753208	7.811947645	4.260236634
18.23378911	2.969879807	0.363883213	6.334140131	2.601749735
39.42924579	3.585423965	0.267260655	7.757070245	4.207985967
48.95372705	3.732382115	0.257112315	7.919906193	4.749645785
25.45473028	3.305405157	0.318078099	7.371339039	3.333900403
30.5662572	3.523777084	0.243828519	7.83474884	4.11755223
15.76419022	2.99877784	0.356079256	6.583680263	2.654892883
15.73615951	3.10040013	0.304906013	6.909790585	2.93243782
29.8424909	3.494118796	0.272095243	7.848754213	3.873976971
13.27725692	2.914347414	0.361348444	6.606911238	2.437408238
18.82698337	3.22448873	0.2832048	7.208173079	3.267312941
25.43496565	3.32429297	0.298183371	7.204258908	3.467137868
21.71718836	3.291804092	0.290964515	7.481316914	3.336799669
29.63279172	3.356248258	0.301671588	7.249431888	3.569468137
38.28345927	3.662966637	0.235361057	8.192198529	4.464004577
38.68728977	3.651737821	0.256058624	8.136000693	4.3748841
29.06658497	3.421061537	0.290797981	7.529826413	3.695909935
20.95367315	3.27614166	0.250439663	7.151112277	3.55799121
20.37328094	3.221521181	0.308741208	7.015878771	3.168450466
27.58474262	3.353200858	0.306166531	7.426096914	3.453489139
25.04299933	3.274259962	0.323541053	7.062527921	3.299662814
38.67911572	3.629181208	0.260805811	8.140567662	4.28701846
35.78697394	3.527318186	0.285445831	7.720784345	3.962769698
32.77425773	3.559505469	0.260686666	8.005330776	4.055815441
20.30937501	3.172064786	0.329010607	7.049410355	3.020376513
32.66722899	3.356411981	0.320497687	7.239732968	3.545964576
25.82705526	3.20117415	0.333886482	7.020449162	3.081586095
24.02946301	3.333816516	0.276332632	7.074121495	3.523595514
29.15270903	3.525350444	0.244148285	8.109679172	4.03049259
23.79767027	3.289008564	0.309396633	7.282222717	3.325120903
44.03185226	3.809420429	0.197850264	8.573276854	5.104481266
30.79013346	3.508400682	0.265438731	7.859064111	3.937543754
46.55046385	3.747170987	0.246758608	8.046941478	4.721867293
28.33842807	3.396865526	0.290458847	7.35334666	3.660890606
27.64749208	3.427600269	0.277594373	7.631850804	3.720096877
27.61470338	3.429660277	0.28392329	7.672546036	3.66948196
15.94628602	3.057701439	0.325143163	6.64856791	2.834186792
40.61690508	3.668954331	0.234160863	7.719018386	4.607682619
17.10048483	3.125513204	0.317329875	6.976524253	2.943709119
37.17897814	3.626222085	0.248918491	8.020773999	4.308614958
43.88676829	3.795791512	0.209300913	8.465702445	4.991722757
27.41934082	3.477447247	0.253372366	7.744314414	3.889391994
21.18299062	3.272070791	0.284609036	7.271378188	3.333558295
24.558108	3.382191611	0.268470244	7.52633501	3.638540674
22.43274481	3.267262752	0.300432997	7.265211987	3.334409243
33.35451838	3.591801369	0.24663142	8.008298257	4.206338073
27.89877994	3.375364571	0.304773257	7.495382875	3.548972853
32.45871019	3.513054888	0.273812116	7.563610668	3.985031556
38.36659976	3.672353454	0.253269687	8.224719005	4.35750781
23.41220774	3.232218267	0.331139692	6.90127341	3.172094574
34.26113794	3.525216507	0.277069266	7.690243548	3.972459361
30.18289368	3.493071662	0.278892162	7.821351041	3.834134867
28.45132227	3.456310151	0.27586362	7.789251226	3.756454802
14.32627687	3.019746618	0.32623205	6.818157794	2.80681018
27.35521963	3.328252671	0.314426431	7.311576715	3.416859828
26.61672207	3.300557049	0.318411593	6.912866481	3.38300239
32.73149899	3.515517685	0.280554491	7.860834525	3.914300453
30.67957944	3.491987818	0.273615413	7.860371021	3.881438149
15.63059605	3.016559285	0.344465168	6.67839665	2.70741543
24.46655615	3.3600215	0.28018447	7.38185886	3.539595036
25.02058536	3.262397032	0.321164326	7.298531181	3.255598175
30.30044906	3.452920309	0.291892774	7.608772899	3.755147443
52.21377255	3.880438661	0.21791853	8.411348128	5.243049109
38.6314234	3.705531553	0.224651473	8.286715795	4.663764816
28.17876059	3.39109933	0.291232791	7.214668345	3.667895206
15.67817091	3.070681464	0.321121624	6.921616143	2.855132875
22.55901341	3.242099924	0.309809968	6.949754943	3.239339898
21.98591044	3.286998795	0.284555021	7.225787923	3.375513425
48.49430654	3.800993338	0.227864696	8.36098009	4.936661351
57.78978869	3.905371942	0.223489162	8.328786924	5.328155681
40.78954397	3.6312475	0.273136301	7.711031229	4.327129142
27.48055957	3.349027046	0.307114793	7.404834234	3.455991594
15.22317214	3.071033382	0.313523038	7.148392547	2.859765332
39.56550748	3.660022092	0.254093997	8.047627997	4.458727761
7.860486728	2.570530839	0.398514641	5.841082027	1.856868316
41.39466404	3.699706465	0.247202438	8.035716098	4.530834408
24.52776161	3.32913669	0.300984679	7.388364499	3.418883505
24.94036858	3.307427507	0.295695177	7.176746718	3.417770697
22.46503041	3.205787776	0.325978829	7.030947951	3.125056686
9.662765949	2.732589955	0.377810272	6.008986257	2.124588677
32.19541936	3.593731172	0.223170301	8.046473433	4.345216597
36.06731895	3.630941938	0.249871144	8.058212308	4.306803197
23.837996	3.299648673	0.302542646	7.443860706	3.348698228
34.36848932	3.62041114	0.239725247	8.055876911	4.316707448
37.97852849	3.506909362	0.300194331	7.643648173	3.927527234
23.72038649	3.362602621	0.280504124	7.619912858	3.537187535
36.35553171	3.469776355	0.298547174	7.292435721	3.928524134
16.61121681	3.051994215	0.33652687	6.690755511	2.815667223
16.60744582	3.060310336	0.347043912	6.793006488	2.745908615

log.sigma.2.0.mm.3D_glcm_DifferenceVariance	log.sigma.2.0.mm.3D_glcm_Idn	log.sigma.2.0.mm.3D_glcm_Idm	log.sigma.2.0.mm.3D_glcm_Correlation	log.sigma.2.0.mm.3D_glcm_Autocorrelation
29.73746042	0.893875938	0.211768686	0.166366661	699.5246488
9.013022599	0.923118188	0.361281118	0.254223791	293.2082915
8.425180705	0.919379673	0.357684084	0.304314117	226.8121516
21.10804039	0.897416475	0.273228411	0.193146867	444.218491
14.05837909	0.903918484	0.271075404	0.167803553	376.9735858
16.04555688	0.902470942	0.230503937	0.267875133	431.4874694
11.38625484	0.927900071	0.301457085	0.252834908	473.5132068
12.94992684	0.924227362	0.326213976	0.221976785	433.3183333
16.26294007	0.912403226	0.266827357	0.176855434	479.913856
13.35647221	0.903261407	0.269529232	0.201024586	372.3372134
8.730116044	0.905600421	0.296068288	0.208061887	249.9735664
20.79021635	0.910177598	0.266323518	0.190858947	534.6590175
11.34762861	0.935483651	0.411273296	0.238531109	410.5416806
21.40275643	0.918338607	0.267997672	0.209001328	770.2548135
26.02407389	0.905930322	0.255307989	0.122325655	473.0643287
14.18528825	0.92424802	0.330138746	0.233684258	503.0288576
13.35973306	0.888440818	0.232957499	0.169224969	315.9242987
8.628980629	0.925198592	0.375653631	0.287946296	321.1433604
7.054667834	0.924984202	0.309890044	0.170697917	310.8691479
14.64629613	0.91613221	0.270211058	0.248832827	475.2643104
7.257710242	0.9304657	0.395372841	0.293584808	312.8044345
8.064825296	0.902274196	0.277716023	0.200065457	288.3700783
13.27748168	0.914868923	0.298413262	0.160815478	509.4287
10.4428802	0.907418066	0.294561664	0.218401929	329.0246445
16.66771044	0.924878193	0.306714083	0.219792618	590.4003278
18.01231365	0.90498569	0.234589627	0.259630402	410.5280638
19.23926127	0.90312539	0.253426086	0.198617563	435.276266
15.24530869	0.912419465	0.285402127	0.173202702	459.3482212
8.205622152	0.887315531	0.245834699	0.174761099	251.9410122
10.20669712	0.91869636	0.310303431	0.161282524	425.7881432
15.51815709	0.934060931	0.320934241	0.228539195	573.11506
13.99018203	0.908010425	0.332860318	0.121984633	362.7769615
19.95239516	0.914896636	0.269151042	0.233528157	416.5652812
19.86482357	0.91040684	0.301348126	0.207387266	510.846384
16.12203409	0.918145835	0.26339558	0.221928788	451.4829891
11.07166123	0.928449401	0.341731777	0.242543876	389.6201954
19.92485625	0.939281088	0.331952028	0.113295682	789.1798339
16.15272522	0.945674948	0.391593392	0.247296082	695.0279813
11.48422711	0.900043728	0.271428132	0.009956638	413.8430492
12.72571138	0.901218162	0.241414942	0.21785093	307.0536176
12.53796868	0.911510701	0.318127717	0.216423028	298.8811995
17.60748686	0.886931424	0.188842293	0.175535526	303.6998928
15.00740666	0.908951357	0.264450267	0.204554555	437.2007384
23.98355117	0.907593493	0.244211636	0.127075795	548.5192463
14.74697001	0.913055887	0.288910346	0.209648681	456.2428548
13.65184084	0.91718012	0.273508792	0.193351729	329.6725177
14.00906956	0.91844855	0.286747322	0.217659037	431.5710441
7.813225487	0.919577495	0.328797314	0.204828563	286.5941296
18.96856804	0.887938737	0.224525824	0.136368281	324.1432856
8.296823686	0.919239222	0.331007516	0.334850263	301.3422837
18.41918358	0.91202546	0.244738012	0.227795135	597.5891736
18.61288972	0.900744535	0.200936226	0.185478707	431.3660115
12.14682508	0.90426428	0.248341538	0.140354601	348.4702797
9.941989238	0.909750639	0.289060812	0.22966081	274.5853812
11.15753836	0.90979976	0.267769078	0.238856029	446.8044594
11.1808789	0.910345628	0.291359911	0.211422583	349.6071532
15.4333252	0.898357222	0.244683081	0.165269981	437.2012673
15.14219092	0.921534361	0.304947337	0.19891628	489.0551522
16.3910831	0.912512784	0.26763515	0.1239014	454.1662271
19.15491324	0.905632085	0.2660528	0.157386286	449.4638927
13.24325481	0.924071321	0.347565133	0.19377391	348.5273439
18.30655484	0.918791372	0.278702436	0.150060629	729.7005268
15.32218219	0.913494102	0.285855834	0.237752256	533.5815892
14.14959643	0.916571839	0.275854057	0.219293745	497.9396569
6.319549742	0.902567858	0.319329725	0.263845044	143.8571989
15.52277522	0.922853394	0.324272237	0.16093613	449.5829969
15.01983519	0.91972271	0.324634184	0.053887029	481.6483006
17.23297925	0.922915003	0.284957727	0.190937509	545.7627947
15.40986884	0.922946289	0.269203042	0.213116261	528.256902
8.225286281	0.925452573	0.34717937	0.162114287	424.5409523
11.66836236	0.905449731	0.287117539	0.188369267	345.9403697
14.2925744	0.936234499	0.328746998	0.254835583	681.3809262
15.99160038	0.915533519	0.29739668	0.193669787	389.2793315
24.34329129	0.88665422	0.217118659	0.138062315	439.7529181
16.51680834	0.890979355	0.215454966	0.150463177	303.5275413
14.40439246	0.905979989	0.28316397	0.150607688	376.2882959
7.440482163	0.928925603	0.317684992	0.226804372	323.0640508
11.87116193	0.915619868	0.317803822	0.19039429	348.7093215
10.48004418	0.91573722	0.283709251	0.170566018	461.9358608
23.68873702	0.907035596	0.224339706	0.184458789	532.9603748
28.96785762	0.897479047	0.220277665	0.18281477	702.3490407
21.77075106	0.90075442	0.276158789	0.066022035	355.4075983
15.3083781	0.921996018	0.328070809	0.174861105	470.3165861
6.902595824	0.904593302	0.315882641	0.255949809	243.0700093
19.30674382	0.901436733	0.248072663	0.151269859	379.126604
4.3549722	0.937930008	0.455658838	0.334224983	300.8192027
20.48110504	0.902590004	0.260260651	0.142842953	357.8914805
12.72694823	0.913679968	0.303259921	0.226958202	410.8840362
13.09176969	0.9160025	0.301581138	0.176976936	429.5000713
12.53417065	0.924813084	0.338923738	0.206425547	509.1725474
5.099838718	0.93801822	0.419460492	0.370887837	353.0305833
13.14176504	0.896543139	0.214945144	0.189119884	378.5510976
17.32174808	0.908003105	0.246228947	0.158692993	366.6155059
12.42994621	0.905662318	0.310613916	0.251145211	325.5914374
15.50629377	0.90290214	0.234154679	0.125690904	404.6286614
22.29949705	0.922658565	0.315725433	0.223628138	663.7060462
11.05787302	0.907609169	0.280477371	0.235214414	269.5225963
20.67513201	0.920550061	0.297722137	0.129135694	665.451845
8.534949145	0.900781526	0.34389553	0.185415897	227.2985073
8.980749811	0.932422907	0.36429403	0.258159334	392.5423593

log.sigma.2.0.mm.3D_glcm_SumEntropy	log.sigma.2.0.mm.3D_glcm_AverageIntensity	log.sigma.2.0.mm.3D_glcm_Energy	log.sigma.2.0.mm.3D_glcm_SumSquares	log.sigma.2.0.mm.3D_glcm_ClusterProminence
5.059696854	26.71974483	0.005162555	34.74193725	31927.9195
4.201190454	17.08860517	0.021483165	11.52185065	4484.59497
4.345062353	14.96525733	0.018378484	12.06223359	4398.614785
4.834560906	21.2172964	0.008620827	23.54145024	14851.17262
4.565981757	19.60800414	0.010597759	16.59186613	8348.783913
4.841820884	20.87019816	0.007585116	23.10064359	16975.97883
4.590047259	21.77469329	0.010777837	14.90292743	7301.191398
4.510015747	20.90127087	0.014749043	14.28483888	7539.98432
4.752156833	22.01586367	0.007106532	18.66802209	9829.006129
4.588611323	19.39421462	0.009950793	16.98304259	8253.779676
4.379108936	15.7503281	0.010962594	12.16192305	3703.763586
4.817442141	23.20857717	0.007976641	22.14339519	16393.76099
4.098623593	20.32699081	0.033086919	11.11649728	7205.249513
4.766463899	27.91477937	0.009007653	23.78067247	27423.67512
4.844524333	21.91936639	0.007737465	25.70172255	19765.56297
4.552819933	22.47414341	0.014637929	15.64241752	9064.028765
4.688389648	17.86899821	0.006296993	18.97612152	6422.396683
4.226145814	17.88709631	0.023096847	11.49767273	4329.563315
4.193683767	17.69457041	0.013126284	9.569208236	2336.524466
4.76820378	21.86066274	0.008574297	18.52291604	11188.47613
4.16024479	17.6662655	0.023933646	9.737648168	3470.455587
4.381567088	16.94624066	0.010315183	12.02787016	3097.638219
4.468652725	22.67974481	0.011720352	14.04048152	6603.855285
4.519366432	18.18101445	0.009460028	14.27120996	4796.106641
4.592873804	24.36337394	0.012973691	18.09273595	12758.46208
4.967651561	20.34338506	0.005561011	24.83899429	19419.41464
4.873902644	20.99756585	0.0069197	22.85508835	14321.08075
4.581065316	21.55035734	0.010221694	16.27882519	9663.00788
4.389195042	15.82054664	0.009439277	12.91756917	3137.080262
4.311293011	20.78747882	0.01386693	11.23104982	4786.856082
4.582046426	24.02440116	0.013941168	17.23161012	11680.14274
4.310123809	19.11306956	0.016245969	14.27744715	6179.782652
4.940742324	20.4986226	0.006891584	23.91736951	15036.55611
4.753872404	22.67190575	0.010507802	21.08255371	15880.99745
4.820590502	21.32879277	0.007219876	20.02317715	11303.34539
4.35924659	19.80678657	0.017105927	12.73924881	6450.990906
4.430029794	28.270496	0.016266232	17.20459908	13022.99297
4.404837544	26.49452832	0.025621417	16.30064733	12318.05435
4.194035166	20.53508175	0.010988947	11.86131269	3718.371717
4.77088184	17.62776949	0.005511805	18.67683814	7079.80649
4.444840456	17.32423117	0.013800426	15.39366258	7498.522533
4.991856186	17.65901311	0.003642962	26.18189694	12615.51569
4.726944848	21.028047	0.007783026	17.97542561	9797.467558
4.841546966	23.59149042	0.007169856	24.95024664	17607.5942
4.59778785	21.47162653	0.010643932	16.28537145	9064.120419
4.583274809	18.30137916	0.009780935	16.52571504	8258.680554
4.642515015	20.86198947	0.009938779	16.49419575	9524.194551
4.185435505	16.89195157	0.016417812	10.20716948	3206.585921
4.743769508	18.18615494	0.00748238	23.17283912	12150.46202
4.423623105	17.3285185	0.015681877	13.55556495	4912.468371
4.886464114	24.50445064	0.006604047	23.10992587	16987.10349
4.992796552	20.91956657	0.004323628	25.67503542	14793.20797
4.559404087	18.84096334	0.008035697	15.50110305	5869.910643
4.468875874	16.56419888	0.010657047	14.23868943	5032.870242
4.611244563	21.23591418	0.008617919	15.20972828	6567.672626
4.456808656	18.76376181	0.011279983	14.20708395	6472.692232
4.744802083	21.06672146	0.006560982	18.86551178	8912.974567
4.551324765	22.18695571	0.01177663	16.25590643	9766.978103
4.595258963	21.44295099	0.00902914	17.05037224	9141.331865
4.836969129	21.33024179	0.006620069	21.43107981	11813.75434
4.332291785	18.80895351	0.020398836	13.7930702	7450.344334
4.661700763	27.12947436	0.009597936	19.3841897	11820.29287
4.748027611	23.15205939	0.009589172	18.55215363	10833.7117
4.691037862	22.38052558	0.00846139	17.16810073	10055.4134
4.251133716	11.88255189	0.013987937	9.808172068	2450.93279
4.474322816	21.31280037	0.014244252	15.01516602	9098.23035
4.240301007	22.10526814	0.016667219	12.76815444	6890.176764
4.719164646	23.45622021	0.009341923	19.45134874	11522.30253
4.731035584	23.06245745	0.007875246	18.4449399	10881.12533
4.084334846	20.74631816	0.019679051	8.969221703	3039.821213
4.487898542	18.71216509	0.010814415	15.1716293	6017.619663
4.557922489	26.14731679	0.014262821	16.27701653	10405.78422
4.619221773	19.85665077	0.010247461	17.88949327	11709.43747
5.002988641	21.18198279	0.00491129	28.27814841	16783.40692
4.842254472	17.63098371	0.004936597	22.56066645	10239.85598
4.389521531	19.55626581	0.012672113	16.01522868	10106.99112
4.235536613	18.07241836	0.014836871	10.00713765	3393.499911
4.35655099	18.75503021	0.015114826	13.76789506	6514.572793
4.410597774	21.60799124	0.011070661	12.26191681	5035.82938
4.987024796	23.21366324	0.004945493	28.80650941	19283.52003
5.036570363	26.74927978	0.005610835	33.69822528	31256.22851
4.633250425	19.12403753	0.009795018	20.72871652	10709.6899
4.527654499	21.78734249	0.013347271	15.70364892	8501.193385
4.334348191	15.50612867	0.011424215	10.23308923	2479.633956
4.825211279	19.67003881	0.006716265	21.84250916	11915.52379
3.847486531	17.28774247	0.037681452	5.922135087	1501.672475
4.805254297	19.1202436	0.007380055	22.96473607	12846.36901
4.519874196	20.41438894	0.012463219	15.08652165	7719.949424
4.451443726	20.85509253	0.011874332	13.94356659	7718.43773
4.400214372	22.64498353	0.015644434	12.96534763	7426.062979
3.924563823	18.7525143	0.035753896	7.956347003	2309.36895
4.762371021	19.66901262	0.005478203	18.99782371	8521.814449
4.778886866	19.30911799	0.006689028	20.20580982	10691.40599
4.561989686	18.01669181	0.011564614	16.22863589	8168.68337
4.723786396	20.33399303	0.005939038	18.3966485	7296.112926
4.77995176	25.86806533	0.01278387	23.38724617	20739.91542
4.601639777	16.39219535	0.008888855	15.77781625	5952.076516
4.550983638	25.90385865	0.012358602	19.84221199	12270.91302
4.168192741	15.01332375	0.016427554	10.20262993	3133.212018
4.223720006	19.90188187	0.022321713	10.59354592	4478.612656

log.sigma.2.0.mm.3D_glcm_SumAverage	log.sigma.2.0.mm.3D_glcm_Imc2	log.sigma.2.0.mm.3D_glcm_Imc1	log.sigma.2.0.mm.3D_glcm_DifferenceAverage	log.sigma.2.0.mm.3D_glcm_Id
52.67820047	0.759112957	-0.097227559	5.533255319	0.29647428
34.07530304	0.774390097	-0.132032719	2.733518215	0.432269432
29.8775558	0.744355046	-0.115037676	2.777444199	0.429526302
41.93103456	0.833508077	-0.142274229	4.360337682	0.35191472
38.68779309	0.660515873	-0.073042305	3.843378776	0.353871073
41.22405108	0.874361952	-0.172932439	4.455173341	0.317806064
43.34001578	0.604343595	-0.060051479	3.418126702	0.381199016
41.46832941	0.620223307	-0.065443974	3.313474731	0.400696126
43.65455087	0.603113932	-0.056442108	4.08149204	0.34891237
38.4132142	0.75647125	-0.109372001	3.755512052	0.353504862
31.45930161	0.655632965	-0.07623796	3.168812462	0.379132407
46.05006978	0.716653527	-0.088525328	4.260236634	0.347994929
40.38632793	0.751514672	-0.123231001	2.601749735	0.472297271
55.32362936	0.787287441	-0.118154781	4.207985967	0.350115136
43.35088575	0.756801078	-0.102029207	4.749645785	0.336083831
44.68498481	0.665638022	-0.077537991	3.333900403	0.403495583
35.37322629	0.714541365	-0.08738071	4.11755223	0.322812157
35.65383976	0.743200581	-0.119954992	2.654892883	0.443367921
35.17048798	0.634631106	-0.072659448	2.93243782	0.39203903
43.37536109	0.62001708	-0.061859393	3.873976971	0.353262832
35.21155774	0.671087978	-0.090087525	2.437408238	0.46095194
33.8226847	0.653536902	-0.074673333	3.267312941	0.364256734
45.03254823	0.768741782	-0.116776803	3.467137868	0.37832577
36.1106644	0.637284822	-0.068427957	3.336799669	0.37669805
48.42770839	0.796628509	-0.131223224	3.569468137	0.384236788
40.1777632	0.745596323	-0.095776224	4.464004577	0.321976467
41.50097643	0.686561555	-0.076112564	4.3748841	0.336385656
42.72554696	0.656000937	-0.072470614	3.695909935	0.366468354
31.60253234	0.788507621	-0.127952402	3.55799121	0.338050088
41.17830673	0.632467475	-0.070064736	3.168450466	0.389905128
47.71009316	0.664770339	-0.078097882	3.453489139	0.396253745
38.00635306	0.670577961	-0.081527384	3.299662814	0.405732316
40.53591497	0.717168631	-0.086826121	4.28701846	0.349982871
44.99872272	0.744330539	-0.099552613	3.962769698	0.377246673
42.27413233	0.647758023	-0.067405873	4.055815441	0.346899282
39.31300279	0.687403824	-0.08835973	3.020376513	0.415007213
56.1125794	0.712358649	-0.093115458	3.545964576	0.404127516
52.56915583	0.746515561	-0.111927346	3.081586095	0.454144639
40.68459236	0.623625245	-0.067374578	3.523595514	0.357850026
34.81725383	0.506317265	-0.038017962	4.03049259	0.330136227
34.38886847	0.678231129	-0.082282738	3.325120903	0.394558648
34.59787958	0.57251015	-0.046151763	5.104481266	0.280304992
41.63547056	0.635817356	-0.064803651	3.937543754	0.348684418
46.7015186	0.70265772	-0.081450735	4.721867293	0.327579514
42.54842565	0.756564006	-0.109561295	3.660890606	0.369440606
36.13108097	0.6801797	-0.079239049	3.720096877	0.357365215
41.36373654	0.632715343	-0.065915541	3.66948196	0.367812758
33.73623768	0.703082096	-0.099021851	2.834186792	0.406998487
35.84132429	0.821361122	-0.136527448	4.607682619	0.312904885
34.45531105	0.739758125	-0.11325293	2.943709119	0.407424679
48.66556026	0.702350728	-0.082376914	4.308614958	0.330851208
41.3034524	0.632052572	-0.059588022	4.991722757	0.290662076
37.21731332	0.528249431	-0.042422175	3.889391994	0.336592721
32.94774688	0.737801924	-0.103195137	3.333558295	0.372963811
42.0901385	0.735134245	-0.098971773	3.638540674	0.353571219
37.23670224	0.654996293	-0.07590405	3.334409243	0.374056574
41.66451322	0.587648722	-0.052168358	4.206338073	0.331225071
44.07444231	0.656154273	-0.073274573	3.548972853	0.382538377
42.51871525	0.710708195	-0.088433088	3.985031556	0.350427603
42.23574364	0.585317551	-0.050824851	4.35750781	0.346327654
37.18704173	0.755886924	-0.115678499	3.172094574	0.417741704
53.91768778	0.674325459	-0.076823003	3.972459361	0.359525044
45.99497995	0.623428618	-0.06307099	3.834134867	0.365588702
44.44821854	0.57999064	-0.054405262	3.756454802	0.358789874
23.76510378	0.722516623	-0.106014524	2.80681018	0.39915824
42.28753251	0.652762907	-0.073794062	3.416859828	0.398713938
43.86302723	0.727228852	-0.102250232	3.38300239	0.398902695
46.5593294	0.613019471	-0.060062521	3.914300453	0.364641837
45.78774632	0.59299457	-0.055616488	3.881438149	0.352762511
41.1361093	0.590620796	-0.064065222	2.70741543	0.421800301
37.05193772	0.716310899	-0.093924267	3.539595036	0.369436193
52.04315914	0.682621597	-0.084761635	3.255598175	0.40355707
39.27704997	0.731522902	-0.096039407	3.755147443	0.374928692
41.74909444	0.700214783	-0.077021972	5.243049109	0.302010135
34.65219604	0.611016399	-0.056626079	4.663764816	0.304364427
38.67588361	0.724053117	-0.098473987	3.667895206	0.364817661
35.81892651	0.610485137	-0.067359511	2.855132875	0.398362575
37.2111783	0.758364983	-0.11672135	3.239339898	0.395329637
42.88276378	0.662926049	-0.076753091	3.375513425	0.368327284
45.93889988	0.737874631	-0.091048479	4.936661351	0.310496512
52.76996277	0.781986071	-0.107815809	5.328155681	0.304678509
37.63813933	0.751203542	-0.10149888	4.327129142	0.354396496
43.24346686	0.705070504	-0.09019206	3.455991594	0.401613229
31.01225735	0.547820949	-0.052018266	2.859765332	0.397291841
38.76781143	0.694798596	-0.080168591	4.458727761	0.331563184
34.57372596	0.690593459	-0.11056324	1.856868316	0.512644678
37.66096496	0.703567619	-0.08191562	4.530834408	0.341041123
40.36272274	0.684025485	-0.083638064	3.418883505	0.382341693
41.3205058	0.715335546	-0.095213862	3.417770697	0.381896357
45.00310917	0.704766937	-0.095376396	3.125056686	0.412126929
37.42043291	0.715377624	-0.118184776	2.124588677	0.482247075
38.72425704	0.632291014	-0.062067817	4.345216597	0.306843772
38.12145173	0.573516531	-0.049134195	4.306803197	0.3316277
35.86365861	0.651384457	-0.074713328	3.348698228	0.388962479
40.11417287	0.557050303	-0.045730945	4.316707448	0.321993253
51.31251242	0.744083429	-0.102524797	3.927527234	0.388484468
32.61221766	0.609253406	-0.060406495	3.537187535	0.363879006
51.49195872	0.819423031	-0.14229044	3.928524134	0.374487751
30.02664751	0.699976568	-0.09757451	2.815667223	0.418254472
39.47955264	0.612828036	-0.068554171	2.745908615	0.434056816

log.sigma.2.0.mm.3D_glcm_ClusterTendency	log.sigma.2.0.mm.3D_firstorder_InterquartileRange	log.sigma.2.0.mm.3D_firstorder_Skewness	log.sigma.2.0.mm.3D_firstorder_Uniformity
83.89035526	136.3279023	-1.022779771	0.06263186
28.1807912	69.5623343	-0.360425562	0.12292602
30.84872467	80.51277468	-0.059670527	0.104757186
59.11064268	128.4657745	-1.024858752	0.074791861
40.15139448	101.0285287	-0.858611421	0.087806808
62.81796421	112.128674	-0.020077225	0.073494031
38.87865909	97.71685982	-0.112901903	0.087558039
37.64209583	81.42282736	-0.59714989	0.102265383
47.08003489	115.5366855	-0.729287458	0.07586215
41.37065225	105.1066973	-0.879322847	0.081861564
29.0690028	94.70581484	-0.564209618	0.09213461
57.12867376	106.123323	-1.164466853	0.078357433
29.32891813	51.30833262	-0.791635397	0.155191986
59.7143377	104.433795	-1.006060322	0.078954536
61.88450616	107.9488824	-1.002567222	0.075808078
40.79671083	80.17145371	-0.655310084	0.100092452
42.98758034	126.8752704	-0.374485691	0.07144741
29.05933597	62.24699926	-0.409938273	0.130636346
22.24005818	85.05400777	-0.140358714	0.104058395
49.43825401	101.5283146	-0.528260202	0.080957799
24.60142703	62.56522149	-0.11669022	0.127991938
28.28900508	95.2129941	-0.588217033	0.093067501
35.19943623	92.37878156	-0.823948187	0.092908859
33.79604944	103.2746515	-0.221121416	0.085682104
45.94422844	88.61268997	-0.922147194	0.094524072
65.80595149	139.6205988	-0.209791837	0.062919613
57.44048576	117.9580517	-0.33070478	0.070228732
40.96738205	91.17012477	-1.028622235	0.091320554
29.97564749	108.1916971	-0.039864917	0.085576726
27.85690419	76.37242901	-1.03371087	0.110127815
43.75659952	87.50054167	-0.767526045	0.0979601
31.66079498	71.48263168	-1.223203344	0.113346903
61.73380281	119.6520405	-0.458638617	0.070332628
54.26028843	105.8234929	-0.970998294	0.081757175
51.53874741	119.7024332	-0.394112373	0.072551293
33.23437324	78.22099721	-0.432508329	0.106213353
40.75635544	71.93600845	-0.984377401	0.110675906
42.41102003	69.90312338	-0.628378915	0.118287744
24.16006299	95.77993584	-1.324005593	0.099132791
45.11920802	135.7777834	-0.136812409	0.067513873
36.71720498	81.37037492	-0.813458707	0.1024406
61.8128747	173.3264008	0.033646375	0.055272181
46.04489051	111.9103031	-0.618109748	0.077674403
59.5748991	111.6477356	-1.016438416	0.07615364
42.87277635	97.50202394	-0.851458511	0.086694047
40.83004535	106.5166435	-0.249813733	0.078433316
42.90517955	99.21392393	-0.428957669	0.084901175
24.17345058	74.74661374	-0.75955829	0.116678376
52.56075346	115.5718322	-0.725468044	0.073425748
35.16177222	92.16200507	-0.033899547	0.101734008
59.15478318	122.4184339	-0.799160976	0.071774872
63.31923539	145.4872303	-0.284813018	0.061316175
36.12123342	107.2485747	-0.451961354	0.08132146
33.93709168	101.639941	-0.009092199	0.088781243
40.07024942	105.9446619	-0.329210725	0.080633162
34.2570178	91.12479579	-0.986647992	0.092978304
46.20917428	123.4959373	-0.631809392	0.071430828
41.54617081	85.02074718	-0.987427669	0.095269149
41.25899832	98.52131796	-0.873142391	0.086147375
52.34247128	128.9169121	-0.544300299	0.071073909
34.61768402	71.77904786	-0.234137554	0.108833137
45.91197959	97.32590598	-1.063121853	0.085520627
48.91616684	107.0062239	-0.570207285	0.080759696
44.54525678	104.3939342	-0.418792413	0.082706046
24.90641141	85.91940773	-0.303159544	0.097638199
37.44650386	78.46168208	-0.84708889	0.103072307
29.23846337	69.45911407	-1.464467637	0.117200732
47.99964956	100.2508526	-0.534936318	0.08470697
47.17162451	112.113863	-0.727325301	0.077305279
21.59235972	72.19501334	-0.920842599	0.118093152
35.36067391	93.65458536	-0.673288071	0.088380296
42.03327166	85.37251085	-0.775060284	0.098358409
44.7079302	99.4317162	-0.638620563	0.083363545
68.22613566	148.2910137	-0.723062683	0.061537922
51.94838802	148.2880039	-0.153576503	0.060624425
37.47819709	85.82820022	-1.224993034	0.096341737
24.91394315	81.59211302	-0.078706839	0.103716615
32.70906045	75.33009148	-0.814481589	0.106973034
30.78307217	91.11631918	-0.740398486	0.096072583
69.91054811	152.96245	-0.617616792	0.062770601
82.49963784	132.1028938	-1.013117998	0.063704068
45.78102642	101.4880161	-0.669791715	0.08109162
38.73614051	87.69015598	-0.421147604	0.094364864
25.70918479	91.43479919	-0.525036959	0.09668656
53.1083414	124.3814249	-0.636612244	0.070808666
15.78644425	46.91928311	-0.984541238	0.164294869
54.59376809	123.54422	-0.673247727	0.072644278
38.82779478	105.1271127	-0.580159028	0.086176296
35.52421415	89.82847043	-1.190689946	0.096473842
33.85304328	77.06096965	-1.200991513	0.107603841
21.43383275	50.52889243	0.435421868	0.156402904
46.77541072	140.1661305	-0.245246296	0.065851781
49.27230901	113.6206226	-0.625062594	0.074806701
39.96381526	92.18257464	-0.849379073	0.090509826
43.73157446	131.4912977	-0.569090943	0.071964251
59.74322901	92.5260663	-1.081414453	0.090792005
38.23124554	103.8363132	-0.427200198	0.083535243
46.71263794	83.395998	-1.311738114	0.098425574
24.19930292	75.38643599	-1.246356538	0.113905998
28.12062956	65.53709668	-0.097443837	0.122815701

log.sigma.2.0.mm.3D_firstorder_MeanAbsoluteDeviation	log.sigma.2.0.mm.3D_firstorder_Energy	log.sigma.2.0.mm.3D_firstorder_RobustMeanAbsoluteDeviation	log.sigma.2.0.mm.3D_firstorder_Median
106.2942681	46908717.91	62.69122234	43.34813499
56.46076447	8352505.852	29.83733366	38.32665634
63.66493362	14460566.33	37.76807074	29.21935368
92.45450486	15267693.61	57.10140028	39.37636948
75.96108068	28320622.97	44.40326548	38.80828285
89.57070675	11747080.75	52.20641711	37.77222252
71.83558397	63895848.12	43.12724472	31.97363472
68.2104075	60293472.83	37.04062666	38.12592697
80.62163462	62196620.45	49.82340855	48.6916256
78.06676431	15022131.79	46.53707527	55.78208351
64.27875133	12681027.19	40.63617813	32.3696804
82.49605853	38500798.69	47.24263639	56.01924515
52.56906346	15308674.77	24.17724047	20.07188606
85.27585713	28988102.7	47.20562038	39.35081482
89.19936422	35432276	49.53621196	47.52552605
68.09001004	47343679.5	37.75982829	27.94479084
82.19983632	14503703.07	54.13516017	36.92318344
54.74066113	9912103.146	28.22084893	30.08559799
57.49029331	8970624.352	36.04256362	29.8139143
79.04447517	74001875.86	46.16981736	43.43123627
53.14070803	21393274.82	29.04588538	26.19895363
64.40205675	9629149.251	40.48577444	44.27323151
68.26696959	10768195.01	39.66756453	33.74994087
69.4437499	26958172.61	43.7232205	34.30535889
72.97213918	18238522.93	40.82666607	44.68996048
96.47261674	48276543.84	59.40777769	54.36984634
89.58803927	54255190	54.00442988	41.65710068
70.82234884	31496766.3	40.04418762	33.48308372
68.75603351	4175036.563	45.60865504	38.47058868
59.40183275	17693473.76	33.81356414	42.45627785
69.62637738	58665025.08	38.51946409	35.25136757
62.85501796	18190927.11	33.2260833	24.24621964
89.32200445	50607748.91	54.21319168	34.20747757
81.28693115	34734142.75	46.85847475	38.41852379
84.25369148	70516123.93	52.40103327	53.11214447
64.09066242	30325398.27	35.6448317	30.68908119
66.50976563	42683637.08	32.91663833	23.6017189
66.96005269	58701249.19	34.28143991	17.88962936
62.51418398	9254155.992	40.57255562	33.94823456
84.8020289	90446682.04	57.07775761	26.55885887
67.07681737	24945060.78	36.97712134	25.55360126
102.5508387	66068890.97	71.29510567	27.08517265
79.02826471	49294497.41	48.19355236	42.88116455
87.64385713	40052400.59	50.35889016	42.20925903
74.12681365	18565497.72	43.25261455	54.41036415
78.72728269	37589488.01	48.06852585	44.07117081
75.11371642	58581697.01	43.66582539	37.28591156
56.33674294	7021684.198	32.17169905	44.88129044
88.72888615	14013593.65	53.43550897	55.02572632
68.51398164	15108877.64	42.06583057	33.99014282
86.80321662	45468796.09	52.41627674	54.04975891
96.27184264	58139436.85	62.70024431	42.5905571
74.7459433	50743550.37	46.2385229	37.74530602
69.49211261	12557919.52	43.01868936	41.57166481
75.10709586	16374368.63	47.15326244	45.15870094
68.32418914	20344137.41	39.76704937	38.53363419
84.02697435	57911872.17	53.03085672	48.4139595
69.88990202	37125849.15	38.74412284	26.36332798
75.09973783	19892964.2	43.16695654	35.22909927
85.76817929	124822906.1	55.13832796	15.76961327
66.93531909	20410279.97	34.0967635	32.89053726
75.48509218	35996358.45	44.15481138	42.13403702
79.17953659	82592472.79	47.87528946	43.81999207
76.53306817	94135306.15	44.97783289	32.25117874
61.43208378	6604524.086	38.95525766	41.73044586
66.25269651	41012520.05	35.20394743	26.7486496
58.89057478	10960821.11	30.66547676	21.87951851
77.43488462	78748851.21	44.50998093	32.18913269
80.18374472	78877104.49	49.08434198	46.5160141
54.72317744	21215383.17	31.27323084	30.87769794
71.00653984	13179447.75	43.14358347	32.90533066
69.66312969	54041422.17	38.88626365	39.87161827
78.19796475	31487064.25	44.78119965	39.0798378
102.9296783	42094163.7	65.64101965	36.27468872
94.53600854	48202925.23	63.52197958	28.36506844
70.0641736	14452253.46	39.63543472	38.35389519
60.29419874	21050293.25	35.36428627	37.46187019
63.50543849	9571152.953	34.46494507	33.92841339
65.39378478	15904117.06	39.29473839	37.26826859
97.91121721	40052848.31	63.16066697	31.77416611
104.3445828	42988796.26	61.18091047	43.78559113
83.92407381	25358589.73	48.45730952	27.80975723
69.65068735	36853223.3	39.22026353	29.69802475
61.06551343	21928680.27	39.76281224	34.87512589
87.68367568	36478368.31	54.11416562	38.34881592
40.72707636	6614195.908	21.68191107	22.39048672
89.87320289	35816693.31	54.81877876	27.70437431
74.85030917	37115446.83	45.95080053	45.67763519
66.58254797	16108819.46	38.39769674	42.59773636
62.96710975	20086068.96	34.23017093	37.47917557
49.41038443	8403633.148	26.04371862	22.86856461
87.49291411	36967447.57	58.35250305	51.36606216
84.21321012	71561814.19	51.07154342	35.04364395
70.58723905	38219990.28	41.07621834	42.29588127
83.9423098	53623289.87	55.32089617	35.67720985
80.27773689	54694470.32	43.05438416	33.76245308
73.73065167	29303840.5	45.63862153	32.50619125
74.71479041	12665556.67	39.24382507	24.82598114
56.6558145	5395184.353	33.55220615	28.28917217
58.8578088	52330503.67	31.71940836	29.20246983

log.sigma.2.0.mm.3D_firstorder_TotalEnergy	log.sigma.2.0.mm.3D_firstorder_Maximum	log.sigma.2.0.mm.3D_firstorder_RootMeanSquared	log.sigma.2.0.mm.3D_firstorder_90Percentile	log.sigma.2.0.mm.3D_firstorder_Minimum
1266535384	420.7990112	148.680527	184.3471161	-603.2866211
225517658	369.5023499	94.51540266	126.0351761	-371.4686584
390435290.9	376.7020569	97.09122287	154.94561	-321.0200195
412227727.5	337.9416199	125.0084689	145.6776611	-489.5667114
764656820.1	356.1592102	109.3605164	139.2595856	-447.706665
317171180.2	460.0400391	133.4114208	212.2285049	-458.6706543
1725187899	524.3804321	106.3343709	159.1684418	-478.7298279
1627923766	448.0793762	108.6449903	160.1794815	-453.6217041
1679308752	464.1495361	116.9684347	164.6295929	-494.2208252
405597558.3	370.3818054	118.3772894	174.471727	-406.5899353
342387734.1	338.1616516	91.70152247	133.2217896	-339.6825867
1039521565	447.7078552	129.2407306	179.1243225	-506.2695313
413334218.8	372.7804871	90.55170652	108.9857254	-455.2427673
782678772.9	424.1105347	129.9345803	171.9083862	-628.6890869
956671451.9	514.0289917	133.6376073	169.221138	-489.5161743
1278279346	386.2249756	104.0141315	149.6697464	-522.6132202
391599983	329.0481873	110.2141325	157.4504547	-382.8464661
267626784.9	328.2364197	89.66054369	132.0055084	-395.4940796
242206857.5	443.7049561	83.13310263	116.5203079	-387.1933289
1998050648	459.2680969	119.3057721	173.8626801	-492.7899475
577618420.1	340.9581299	84.11002105	123.6151207	-383.2209778
259987029.8	324.1816101	94.46767717	140.3183502	-374.6051636
290741265.2	314.9998474	102.54665	145.7554031	-506.9171448
727870660.4	346.6098938	100.9179115	153.7339569	-385.4235535
492440119.1	439.0002441	119.6962736	180.3369019	-540.0652466
1303466684	516.6212769	142.7531023	208.9986115	-441.5789185
1464890130	446.0139771	129.5441459	190.3888458	-462.8243713
850412690.2	367.2948303	106.7876789	143.0708191	-493.2597656
112725987.2	278.2957764	96.64433009	150.2465118	-344.3725586
477723791.5	327.2139893	92.81247062	121.3031128	-458.131134
1583955677	559.473999	111.039478	158.5914825	-544.8522949
491155032	265.9990234	93.76638471	106.8466232	-435.2268982
1366409221	552.3768921	125.1913975	167.4255188	-469.1513672
937821854.4	368.0952759	121.5750233	165.9832596	-517.9677734
1903935346	571.9942017	126.537882	189.2710999	-460.4274902
818785753.4	425.1529236	100.5576288	152.9045349	-428.7988892
1152458201	559.6037598	107.1028122	119.7290573	-664.5374756
1584933728	580.5288696	106.6489888	148.2854004	-619.2839355
249862211.8	236.1231384	86.73928786	107.9278702	-459.0945435
2442060415	443.6293335	111.5396082	159.0309357	-383.3331909
673516641	372.6430359	98.06347916	117.6396072	-391.6446228
1783860056	506.7790833	130.7107849	183.0949402	-392.6195068
1330951430	410.0500488	116.1647562	171.4350525	-467.3510437
1081414816	482.0860901	126.8788073	151.7280029	-541.6867065
501268438.6	388.4246216	120.5279782	184.6571762	-474.8524475
1014916176	549.5076904	117.0205219	180.087439	-393.2063599
1581705819	468.9138184	113.3191566	161.1960327	-467.5445557
189585473.3	352.4931335	89.78660885	115.7255707	-358.5480347
378367028.6	409.0917358	128.324618	173.473999	-381.6766357
407939696.3	379.548645	105.7128439	189.047937	-352.707428
1227657494	451.3481445	133.0376839	195.3528107	-534.8790283
1569764795	550.744812	131.327608	185.5452576	-457.5033875
1370075860	411.3849487	106.0488295	147.5323303	-405.3531799
339063827.2	406.0187988	106.6533033	162.8910065	-335.9253235
442107953	352.5338745	110.9574484	176.6126633	-467.0833435
549291710.2	337.6738586	102.1415243	135.8910568	-408.5422058
1563620548	366.5584106	119.6532611	174.2464844	-451.8594666
1002397927	418.8126221	103.6157329	125.1002106	-516.1085815
537110033.5	443.1963196	109.8679818	134.9060837	-499.0020142
3370218465	429.0426025	116.9325825	160.8307861	-490.6062317
551077559.3	446.4075317	107.5051158	153.0330811	-419.4920044
971901678.3	387.1003113	116.111077	167.7920441	-623.5772705
2229996765	396.328949	119.4451334	186.2251709	-508.0003052
2541653266	409.789978	111.125393	145.3555237	-513.6020508
178322150.3	326.4549561	91.43389454	149.8337845	-236.9606171
1107338041	404.5033569	103.0509721	131.4496262	-494.3942871
295942169.9	326.6804504	91.68171933	103.5120277	-505.0982971
2126218983	509.4769897	112.2489029	138.0483749	-536.2751465
2129681821	543.2871094	116.0480555	156.8431396	-522.6522827
572815345.6	281.5973511	85.56107834	125.1905594	-458.5342712
355845089.3	328.7114563	100.7265666	143.7008728	-406.0599365
1459118399	477.549469	112.2366611	174.1878326	-596.1109009
850150734.8	465.2875977	117.5422291	161.7711334	-442.3901672
1136542420	417.3367004	136.5063415	167.8951599	-496.8492737
1301478981	467.1349182	124.0781083	175.1968536	-379.4489746
390210843.5	339.3804016	103.7747096	126.6673431	-443.7966614
568357917.9	461.6610413	93.73152184	135.7153473	-380.6152344
258421129.7	361.3120422	99.95381477	136.0526962	-406.362915
429411160.5	349.8580017	96.52460236	127.0809708	-478.1042786
1081426904	526.8566895	130.8583958	155.7468079	-527.269104
1160697499	420.7990112	147.0145269	184.6094482	-603.2866211
684681922.6	401.5533447	118.7263091	133.8296021	-439.0339661
995037029.1	401.4414063	106.1769581	149.5828461	-496.6311646
592074367.3	267.2674255	84.47437117	121.6886459	-341.7389526
984915944.4	469.6672363	122.2958731	164.1570374	-443.3758545
178583289.5	230.6509705	65.7066226	92.42597733	-390.9495239
967050719.4	487.4481201	122.5458342	144.2330231	-431.598114
1002117064	357.6930237	116.343901	184.6614182	-439.0662231
434938125.4	361.6665649	104.8965775	150.1503906	-466.757782
542323861.9	327.4123535	99.59440948	128.6022919	-501.5137024
226898095	302.2282715	80.2776678	142.2557465	-421.5263367
998121084.3	425.368927	124.5772828	196.4589157	-409.4134216
1932168983	517.6610107	117.5033008	147.501535	-446.0913086
1031939738	323.7414856	107.6188424	159.8531342	-377.5785828
1447828826	425.0645752	111.0023784	142.8772873	-459.6361694
1476750699	429.4558716	126.4246348	171.3298813	-578.8122559
791203693.4	405.5907288	101.9926433	133.1375061	-373.6712952
341970030	389.0484314	109.9863527	109.488884	-612.0802002
145669977.5	222.3055267	80.43037975	95.79697571	-334.3161011
1412923599	418.7635498	95.20046454	152.4752502	-430.9025269

log.sigma.2.0.mm.3D_firstorder_Entropy	log.sigma.2.0.mm.3D_firstorder_StandardDeviation	log.sigma.2.0.mm.3D_firstorder_Range	log.sigma.2.0.mm.3D_firstorder_Variance	log.sigma.2.0.mm.3D_firstorder_10Percentile
4.413924297	146.9835081	1024.085632	21604.15164	-184.7759064
3.571171246	85.57682418	740.9710083	7323.392837	-43.64656219
3.739436167	89.30717624	697.7220764	7975.771727	-54.85762863
4.160944778	124.0314647	827.5083313	15383.80424	-151.5912323
3.977814537	106.3442183	803.8658752	11309.09276	-106.1923264
4.231117666	126.4046549	918.7106934	15978.13679	-104.4804443
3.953662993	100.1658759	1003.11026	10033.2027	-79.27910614
3.840388631	101.0891517	901.7010803	10219.01659	-64.40208817
4.093998574	110.1919962	958.3703613	12142.27602	-97.3693161
4.016209092	108.789877	776.9717407	11835.23734	-85.57181778
3.776305453	86.69521986	677.8442383	7516.061146	-72.3728096
4.10535068	119.8211364	953.9773865	14357.10472	-74.96689911
3.398450675	87.9094853	828.0232544	7728.077606	-45.3142662
4.171009494	125.8404328	1052.799622	15835.81454	-112.8814987
4.18792498	128.9181863	1003.545166	16619.89877	-122.5132431
3.855083824	100.411534	908.8381958	10082.47615	-81.79285049
4.092779526	106.5591555	711.8946533	11354.85362	-118.6655396
3.51700086	83.40652423	723.7304993	6956.648284	-46.06263428
3.620561287	78.38050707	830.8982849	6143.503889	-67.74461746
4.087402518	112.2502035	952.0580444	12600.10818	-88.68990021
3.506953198	78.49072108	724.1791077	6160.793295	-48.67212448
3.767825264	86.78443584	698.7867737	7531.538305	-67.21899567
3.86446844	97.70210792	821.9169922	9545.701891	-75.73118057
3.89878965	93.72718086	732.0334473	8784.784433	-72.0620697
3.925684603	109.6873879	979.0654907	12031.32307	-58.75283813
4.367586893	131.8313481	958.2001953	17379.50434	-97.95409546
4.250585777	123.5335043	908.8383484	15260.52668	-119.1488968
3.909910847	103.1952822	860.5545959	10649.26626	-80.28557434
3.830982442	89.45789517	622.668335	8002.715008	-68.76737213
3.651825719	86.26236857	785.3451233	7441.196231	-63.31716499
3.887432711	104.3420983	1104.326294	10887.27348	-64.04822083
3.670125733	92.94018069	701.2259216	8637.877187	-105.6613434
4.242179586	122.6794619	1021.528259	15050.25038	-130.7633728
4.089054035	117.1262024	886.0630493	13718.54729	-100.7685928
4.168668901	115.0166971	1032.421692	13228.84062	-88.60471497
3.760976977	94.11989116	853.9518127	8858.553912	-56.06045609
3.789106524	105.5524665	1224.141235	11141.32319	-83.82523346
3.764439903	103.8550357	1199.812805	10785.86844	-76.81665802
3.654077379	84.31053103	695.2176819	7108.265643	-76.06635284
4.159063019	108.9196364	826.9625244	11863.48719	-118.4218826
3.791934142	96.90445464	764.2876587	9390.47333	-112.1094223
4.40268978	128.8315141	899.3985901	16597.55903	-149.221579
4.082387516	109.3033636	877.4010925	11947.22529	-88.6580719
4.17281161	124.100252	1023.772797	15400.87255	-128.3795044
3.956024242	107.0553417	863.2770691	11460.84618	-46.12942047
4.074333173	108.3063426	942.7140503	11730.26385	-87.74477997
4.015525965	106.9719731	936.458374	11443.00303	-81.12947159
3.552518577	81.80870566	711.0411682	6692.664322	-57.73179245
4.172998469	121.6460837	790.7683716	14797.76968	-122.897583
3.775239022	94.09631528	732.256073	8854.116548	-49.26668358
4.20860885	122.2421198	986.2271729	14943.13585	-80.77485504
4.348459603	126.6964918	1008.248199	16052.00102	-133.8718719
3.998629973	101.4563157	816.7381287	10293.38399	-95.42070923
3.885005138	94.52540367	741.9441223	8935.05194	-56.1076767
3.996943425	101.5644096	819.617218	10315.32929	-78.02925873
3.855243296	97.32638398	746.2160645	9472.425019	-79.05037079
4.14930164	112.647912	818.4178772	12689.55207	-111.6629456
3.882097924	102.0983585	934.9212036	10424.07481	-100.7065796
3.980040677	107.2839546	942.1983337	11509.84693	-96.50723495
4.17595552	115.3407589	919.6488342	13303.49067	-115.0615997
3.806489495	101.8780763	865.8995361	10379.14244	-72.58164215
3.998513598	109.2938345	1010.677582	11945.14225	-78.8638649
4.067894435	110.4556849	904.3292542	12200.45832	-85.28384857
4.032672179	107.6900525	923.3920288	11597.14741	-94.98615875
3.696652389	82.50349326	563.4155731	6806.8264	-63.86446342
3.815294639	100.242274	898.897644	10048.51349	-77.51758041
3.618818225	90.71406469	831.7787476	8229.041532	-73.67938919
4.042115439	109.9771713	1045.752136	12094.97821	-112.8763878
4.086561186	110.492959	1065.939392	12208.694	-102.3205704
3.54723758	80.22099987	740.1316223	6435.40882	-48.9084465
3.912776308	97.60955761	734.7713928	9527.625736	-100.3667038
3.869509828	103.4679661	1073.66037	10705.62	-56.48821259
4.050992937	112.0968039	907.6777649	12565.69344	-98.63355713
4.375523524	135.6624865	914.1859741	18404.31024	-178.8269318
4.314478309	121.8884076	846.5838928	14856.7839	-138.0943604
3.840097726	100.8766727	783.177063	10176.10309	-98.85354919
3.703956581	85.33674498	842.2762756	7282.360043	-55.38337135
3.748373855	95.16790972	767.6749573	9056.93104	-74.28450317
3.791545581	91.84509261	827.9622803	8435.521037	-76.78957214
4.334736254	130.0397827	1054.125793	16910.34508	-154.6939972
4.397203635	144.9955838	1024.085632	21023.71932	-175.2858337
4.11722533	118.0599522	840.5873108	13938.15231	-146.0095093
3.905458325	101.2759487	898.0725708	10256.81778	-81.08644562
3.680830277	79.93653384	609.0063782	6389.849442	-81.37299194
4.203397759	119.1187277	913.0430908	14189.27129	-135.6363342
3.139068678	62.16154837	621.6004944	3864.058096	-40.48335609
4.192697517	121.9719631	919.0462341	14877.15979	-165.8819855
3.962812615	104.1211767	796.7592468	10841.21944	-56.90349159
3.80038803	97.56793732	828.4243469	9519.502394	-55.98239975
3.730559866	94.57063923	828.9260559	8943.605805	-68.04270325
3.282777464	72.9166524	723.7546082	5316.838198	-35.09694939
4.202990292	113.610545	834.7823486	12907.35593	-85.16072159
4.138331396	115.4241114	963.7523193	13322.7255	-132.2806793
3.896743398	100.4836579	701.3200684	10096.96551	-70.9969574
4.116961618	109.1319153	884.7007446	11909.77493	-126.8495346
4.045191372	121.8403161	1008.268127	14845.06264	-93.32289047
3.963242412	99.54865047	779.2620239	9909.933811	-108.5863541
3.883899008	109.7432941	1001.128632	12043.59061	-139.7340851
3.531912065	79.05661491	556.6216278	6249.94836	-77.89482651
3.612557324	87.47351303	849.6660767	7651.615482	-44.5945118

log.sigma.2.0.mm.3D_firstorder_Kurtosis	log.sigma.2.0.mm.3D_firstorder_Mean	log.sigma.2.0.mm.3D_glrlm_ShortRunLowGrayLevelEmphasis	log.sigma.2.0.mm.3D_glrlm_GrayLevelVariance
4.490228351	22.39972029	0.002507057	35.89832796
6.650123788	40.12192046	0.005844971	12.93562618
4.737801558	38.09112537	0.0075848	13.99814859
4.531289039	15.59849574	0.004632466	25.89825625
5.051854462	25.50744547	0.004716401	19.21046473
4.471000172	42.66697093	0.004468202	26.71752754
4.936794133	35.69027494	0.002505852	17.21098772
5.69921942	39.80850823	0.003040785	17.84280203
4.802669419	39.23440686	0.003002641	20.54407546
4.822249609	46.66846147	0.004908449	19.81166858
4.402320525	29.88491387	0.006305205	12.79419928
6.474707788	48.43616148	0.003689808	24.3288904
8.617801698	21.71483242	0.003485346	14.22438456
6.857453932	32.36017042	0.00269021	26.92505851
5.301511303	35.2010129	0.004401935	28.04770515
6.012090068	27.13785924	0.002631288	17.57700731
3.351692351	28.14784873	0.004952944	18.77408578
6.250943603	32.89931323	0.00477705	12.31952426
5.298873945	27.70575505	0.004320648	10.4462321
5.15326595	40.41978573	0.003159916	21.42782496
5.87103921	30.22750975	0.003846447	11.1362109
4.592431149	37.3176061	0.005316495	12.61008561
6.164064443	31.1466458	0.003267543	16.41108056
4.375797885	37.41176859	0.004140137	14.97366067
7.844021854	47.91528808	0.003438952	20.81132152
4.219385185	54.76261389	0.003953953	28.90005435
4.103619866	39.00203917	0.003930017	25.69925335
6.391655764	27.46528906	0.003464667	18.20888504
3.676862922	36.57063756	0.00685526	13.10578374
6.789145365	34.2484813	0.003645783	12.91749256
7.230019648	37.98015529	0.002703071	18.96616988
6.021438164	12.42005291	0.004285712	15.14721159
4.343949205	24.95266772	0.003845355	25.35993666
5.664381044	32.58740533	0.003633997	23.70676107
4.449305294	52.75409904	0.002914466	22.29890164
5.691340154	35.4017343	0.003321294	15.53791908
8.565522212	18.15734553	0.001700741	19.64264712
7.41970211	24.25156454	0.001803645	19.97617217
6.823688204	20.38230645	0.003900022	12.08514058
3.307647055	24.03324785	0.00443422	19.65363927
5.098136359	15.03238552	0.005681702	16.25326429
2.996066351	22.08506853	0.005211158	27.12183299
4.826644063	39.33224251	0.003579266	20.16925907
5.335743307	26.40755951	0.003324286	25.93846973
6.437587052	55.37280328	0.00414861	19.58259369
4.447845802	44.31183474	0.004930068	19.81068297
5.268927132	37.39289013	0.003449049	19.4903881
6.542864642	36.99960551	0.005809046	11.57535135
4.17204887	40.85875543	0.007103156	24.67286745
4.342531815	48.17767957	0.005031731	15.22608284
5.539828275	52.49656643	0.002743095	25.29883805
3.785974889	34.56789845	0.003545005	26.50546638
4.246611568	30.87021637	0.00387876	17.25133025
4.585489439	49.39509252	0.005962963	15.228532
4.333458547	44.67914584	0.00329513	17.38749272
5.82102661	30.9913853	0.005748193	16.19749704
4.071626249	40.34043647	0.003249841	21.27671796
6.092593246	17.66763424	0.002812121	18.05806206
5.791410062	23.6881088	0.00346581	19.54050749
4.406481664	19.22857704	0.003261106	22.6121203
5.712930069	34.32502715	0.0044756	18.35208035
6.747056985	39.19999926	0.001967191	20.45643406
4.749355834	45.46076946	0.002377132	20.81554223
5.254335059	27.41724923	0.00271582	19.69058622
3.774092095	39.41231623	0.012496023	11.50693184
6.688783846	23.89538385	0.003199649	17.60052381
8.664103443	13.2851845	0.00355188	14.64956942
5.174605888	22.46859986	0.002331769	20.60939186
5.164328021	35.47474013	0.002724241	20.55261911
7.179436452	29.75381162	0.002947959	11.25936986
4.505348351	24.86393954	0.004661104	16.3331681
6.96017596	43.49078182	0.001873742	18.65548046
5.141781816	35.36215754	0.004636153	21.54101156
3.669614807	15.1549013	0.004167981	30.59748858
3.303230731	23.20760762	0.00575766	24.51990616
6.162259563	24.35338323	0.00568754	17.42438945
5.340967649	38.77161517	0.003920472	12.56665729
6.571582621	30.55869838	0.006346247	15.82899177
6.324619992	29.68969221	0.003093397	14.2159477
4.556764974	14.61419447	0.003349805	28.04713458
4.584956879	24.28068787	0.002496963	34.952918
4.687202534	12.56121683	0.00661322	23.9451279
5.725806064	31.88618281	0.002902448	17.95578167
3.710668928	27.31428093	0.00511266	10.80987091
4.153763778	27.69493241	0.004932662	23.89526293
8.098102192	21.29089376	0.004279181	7.106564173
3.964668483	11.84574533	0.004811529	25.19387285
5.10026134	51.91034436	0.003813384	18.61943814
7.062130953	38.51998945	0.004342304	16.34253607
7.497748558	31.23204436	0.002913427	15.65510448
5.785530647	33.58073481	0.003609035	9.57883022
3.720342487	51.10913297	0.003969364	21.27517462
4.421042639	22.00682164	0.004109623	22.28649376
5.225226462	38.53374784	0.005573562	17.2781516
3.864877223	20.29169961	0.003336718	19.82493172
6.750639946	33.73611791	0.002455923	25.9340582
4.200653607	22.1938162	0.006324371	16.74092807
6.638043608	7.308020284	0.002779563	20.72726315
5.897419481	14.8019467	0.007729901	10.83540252
5.984714313	37.57010736	0.00299515	13.67095101

log.sigma.2.0.mm.3D_glrlm_LowGrayLevelRunEmphasis	log.sigma.2.0.mm.3D_glrlm_GrayLevelNonUniformityNormalized	log.sigma.2.0.mm.3D_glrlm_RunVariance	log.sigma.2.0.mm.3D_glrlm_GrayLevelNonUniformity
0.002576827	0.060044141	0.077511081	119.4178763
0.006158567	0.111790207	0.161964365	92.6639935
0.008021131	0.095042876	0.211487629	128.8207442
0.004778618	0.069922697	0.114311617	62.36623922
0.004913614	0.081887011	0.117666064	176.866709
0.004584039	0.070304531	0.078654442	43.39473636
0.002661386	0.081403939	0.140784608	415.3034569
0.003231408	0.093080664	0.154802047	423.381065
0.003145004	0.07276642	0.105005173	303.6404441
0.005069551	0.077894751	0.091959111	77.08914239
0.006581954	0.088626387	0.102839632	122.6064732
0.003815879	0.075104297	0.098286818	159.5542191
0.003767911	0.132224612	0.265425514	208.3957663
0.002777075	0.074122414	0.108650227	116.3535953
0.004545464	0.072219329	0.095191211	132.3941028
0.002796867	0.091329082	0.155400333	355.9363157
0.005127517	0.069582203	0.072459041	77.84484327
0.005102108	0.117677999	0.184104227	126.4939091
0.004555039	0.099700912	0.123384509	117.0116362
0.00329221	0.076517019	0.103410701	365.3509926
0.004190715	0.113874159	0.232406898	295.7240642
0.005520494	0.089550705	0.091895795	89.24375039
0.003402469	0.088111817	0.113920602	81.99517367
0.00435961	0.082043253	0.11357237	198.0627605
0.003562074	0.087352577	0.12309186	100.6515157
0.004090937	0.060957881	0.082850564	134.6900311
0.004096866	0.066653432	0.097883443	198.5318388
0.003614209	0.086508261	0.110536384	218.1190019
0.007061631	0.083689044	0.07015126	35.04816373
0.003815172	0.103622328	0.126767607	192.0498964
0.002848569	0.089223719	0.159383424	378.3827731
0.004519204	0.104550458	0.153146171	192.1295799
0.004017954	0.066913706	0.115205423	197.4041912
0.003811487	0.075455053	0.135862824	159.7121118
0.003050116	0.069193471	0.100380059	280.6887414
0.00354977	0.09660229	0.16798582	256.46438
0.00180898	0.1007947	0.161011784	331.9793609
0.001972126	0.097636944	0.295452465	423.6320906
0.004046865	0.096062142	0.097842389	108.5296147
0.00463985	0.066049114	0.089233232	445.5605429
0.005981259	0.094826674	0.14286701	219.9472152
0.005372355	0.054618874	0.05784375	200.2612981
0.003741603	0.074096364	0.100522549	249.0357693
0.003428251	0.072540795	0.088632009	167.430734
0.004293656	0.082184698	0.115401442	95.77859065
0.005131839	0.074358035	0.105292326	187.2975569
0.003613406	0.079928846	0.117141109	332.1176868
0.006067036	0.110138774	0.128265906	86.43226913
0.007261144	0.070629021	0.076971781	56.38170562
0.005310992	0.093129572	0.147434134	112.6171938
0.002836284	0.068982303	0.087535705	164.649947
0.003654455	0.059966769	0.061696871	191.2154467
0.004043374	0.078104117	0.090070243	326.3946208
0.006240382	0.084534024	0.112770138	85.07513366
0.003432035	0.077084957	0.100022132	94.33758694
0.005952706	0.088514242	0.108546731	157.6040875
0.003384841	0.068784687	0.0888753	258.0319884
0.002961937	0.089375305	0.128284535	279.0765809
0.003594024	0.082315611	0.094873594	125.085662
0.003419356	0.06732816	0.123782903	560.6332455
0.004735982	0.097356528	0.174054368	151.1984059
0.002054544	0.08032835	0.113222099	196.1366561
0.002501255	0.07534078	0.117098963	397.8694801
0.002845631	0.078902714	0.110084509	550.4307349
0.013058965	0.093556824	0.111669358	67.37354035
0.003378831	0.09473944	0.153699761	326.4100554
0.003722442	0.108339223	0.147149601	125.9104403
0.002456916	0.079657723	0.120858414	453.0399479
0.002828893	0.074412258	0.090407339	404.0565422
0.003156819	0.109682653	0.162501157	281.0130862
0.004877098	0.08274203	0.121200666	97.43520813
0.001990165	0.090713582	0.151416948	347.8754195
0.004863906	0.077950392	0.131020105	160.5392442
0.004286255	0.059239111	0.079077198	125.0574141
0.005946418	0.059255885	0.074436351	173.9104442
0.005874432	0.091005856	0.107422255	111.6902237
0.004155273	0.09809774	0.125269143	212.2832683
0.006576322	0.098789159	0.13474871	84.8750705
0.003229015	0.091939774	0.103314106	143.7285511
0.003462144	0.06099524	0.077732209	133.3363295
0.002565952	0.060964838	0.078365273	113.6283427
0.006815529	0.075446224	0.117294884	123.4657374
0.003085176	0.086594867	0.165695707	251.9487867
0.005427333	0.092965529	0.122901958	258.399926
0.005098383	0.067822616	0.091824841	153.0002481
0.004700062	0.144630118	0.300435085	184.2339342
0.005018393	0.068246685	0.117864812	148.4009104
0.004022027	0.080366588	0.133038669	198.6595007
0.00450564	0.091405756	0.121408161	121.431992
0.003076613	0.099397621	0.150835825	178.9476028
0.003938869	0.134677053	0.229980682	149.4149472
0.004099281	0.064686959	0.071023632	144.9824672
0.004275705	0.072021227	0.090842574	345.634396
0.00582212	0.085036392	0.130494964	253.4634774
0.003473896	0.069937727	0.081899001	283.3517937
0.002579643	0.081903635	0.161104437	250.440997
0.006580318	0.079918943	0.103496561	206.519124
0.002879548	0.091945362	0.120401499	87.49706969
0.008075225	0.1077653	0.13788153	80.43180441
0.003245791	0.109151807	0.19290617	549.3131708

log.sigma.2.0.mm.3D_glrlm_LongRunEmphasis	log.sigma.2.0.mm.3D_glrlm_ShortRunHighGrayLevelEmphasis	log.sigma.2.0.mm.3D_glrlm_RunLengthNonUniformity	log.sigma.2.0.mm.3D_glrlm_ShortRunEmphasis
1.217098238	693.8866694	1761.512983	0.953514367
1.440066359	279.4957248	665.1144666	0.914937114
1.505215096	223.067011	1092.335975	0.916304446
1.317861504	433.379391	753.184495	0.934897602
1.321775975	370.8778321	1825.556897	0.935361266
1.223892195	452.89402	543.9471349	0.951426582
1.3706127	464.1580715	4250.994968	0.929600042
1.41914937	425.7018249	3685.609245	0.918936669
1.293882754	473.5810976	3556.498768	0.938611161
1.267397567	369.5429668	849.1697551	0.941332905
1.29351421	242.1911435	1176.612905	0.937692974
1.277309315	537.6923633	1822.888818	0.941304046
1.676990132	380.7868787	1173.82627	0.885820756
1.308039243	745.2963198	1326.283593	0.935257381
1.269599676	476.1337836	1580.046183	0.942746189
1.419930239	484.7490655	3160.363475	0.919159866
1.212696965	313.8929227	986.8688331	0.952058391
1.504202821	297.948983	837.4034119	0.903451323
1.348617293	294.9456128	971.2699324	0.927338492
1.290592542	478.7904858	4073.672841	0.939092197
1.594907178	292.9029774	1992.04894	0.89709105
1.265440152	282.6688018	857.1921104	0.942330807
1.326847191	494.6251467	776.8294895	0.930719309
1.31856062	317.139868	2032.731489	0.933864292
1.347578672	573.2516054	956.1070158	0.928069973
1.233781924	434.0784472	1939.081991	0.949895071
1.277996608	441.0474426	2553.36959	0.940881873
1.312397354	451.0094735	2125.974341	0.934565338
1.210589565	254.8679575	368.6592389	0.951248855
1.357715694	412.9145437	1530.00338	0.92624747
1.421883745	549.2720714	3451.170413	0.92046266
1.424158482	340.9053759	1478.32401	0.916028834
1.316319028	416.2889699	2497.787034	0.935857723
1.371367943	502.3591037	1748.395707	0.926381028
1.280647168	460.6341504	3479.378679	0.941140004
1.447400447	379.2386281	2133.009512	0.915580131
1.441696405	744.2737433	2636.351665	0.914111831
1.720473104	644.1652496	3235.123451	0.886123277
1.28498697	394.767404	960.5042303	0.937691867
1.252155538	306.9891076	5857.210943	0.945864288
1.397974068	281.9465172	1886.1787	0.920145719
1.1707909	316.2003242	3306.508398	0.960664514
1.284244662	435.2710584	2875.582275	0.940008636
1.252134649	545.5659783	2003.542211	0.945793305
1.320453129	458.8556824	983.0919794	0.93438307
1.295241686	331.8688594	2147.116663	0.93853746
1.324571504	429.5280468	3494.369727	0.933488761
1.363979543	275.3384303	646.2330922	0.924907917
1.216083285	334.9616032	707.9958368	0.953743167
1.400469081	296.4578449	986.7617875	0.921495928
1.246710707	596.2034489	2078.822613	0.947213649
1.179816894	443.0383215	2865.067113	0.959249
1.256749288	345.8924076	3614.284182	0.944543644
1.319381776	267.9341302	846.5334557	0.933300415
1.283303193	440.6704422	1046.882556	0.939930691
1.309824923	341.1761252	1500.318531	0.934288747
1.253620274	439.8922728	3253.482345	0.945327179
1.357479817	471.721839	2585.356084	0.927305813
1.27237526	448.2823374	1302.560816	0.941057549
1.328027735	445.383737	7052.438909	0.936025811
1.471583779	340.1889098	1229.307655	0.909811839
1.31083793	702.9817374	2068.87837	0.936487581
1.320695976	527.7536351	4458.86212	0.93509359
1.306225383	495.5444958	5917.622017	0.936729528
1.316996025	147.0785437	606.2650154	0.933730581
1.412604132	439.3756865	2800.594164	0.920356622
1.409568266	456.5026329	940.7546433	0.918414184
1.331037039	527.3268408	4777.465183	0.932938885
1.255719032	511.9596873	4710.989038	0.945453286
1.445206808	401.2106078	2045.647319	0.913125008
1.341462133	330.244912	980.4765294	0.929420353
1.406378347	652.6623077	3130.419281	0.921752138
1.358288295	387.9038525	1711.153244	0.92861901
1.225403534	448.7564226	1856.457964	0.950875431
1.213770716	311.9057859	2595.868055	0.952953844
1.305929478	368.0295098	1037.436023	0.935268101
1.353561504	314.1691786	1790.04016	0.92693505
1.382290833	336.2497069	699.9120496	0.921014847
1.297345866	450.9272838	1323.91387	0.93621372
1.224068307	532.0687312	1922.076088	0.950735501
1.218386177	697.49235	1651.028001	0.953553219
1.328610104	355.440989	1371.679519	0.932103148
1.433881967	454.3514561	2366.044367	0.91992205
1.347807424	234.2159972	2305.090615	0.927860258
1.263530642	383.0750934	1946.28489	0.943133846
1.765728547	267.9761309	922.7119555	0.874354465
1.324902934	357.2991542	1837.070073	0.934536824
1.36532352	410.4567107	2043.152084	0.926754943
1.340485304	426.1299122	1109.598115	0.930326625
1.419939725	486.1592858	1449.402852	0.916241422
1.617660622	327.1779253	830.6641583	0.888324961
1.20187337	385.4943461	1997.61312	0.955835652
1.258607165	373.3934031	4150.799604	0.944394121
1.358871906	316.6057109	2472.901665	0.928033469
1.236761509	407.5516717	3535.075603	0.947889675
1.417082485	640.2611354	2502.97594	0.922693663
1.294087421	266.2966763	2196.9116	0.937632145
1.333528189	632.8719781	797.2508264	0.932033371
1.390750182	216.0003546	606.5277299	0.919891292
1.51364654	377.2699651	3935.400412	0.905047466

log.sigma.2.0.mm.3D_glrlm_LongRunHighGrayLevelEmphasis	log.sigma.2.0.mm.3D_glrlm_RunPercentage	log.sigma.2.0.mm.3D_glrlm_LongRunLowGrayLevelEmphasis	log.sigma.2.0.mm.3D_glrlm_RunEntropy
894.1761024	0.937142029	0.002904136	4.780183012
435.0574851	0.885972851	0.007767167	4.175684953
350.8613604	0.88225855	0.010616533	4.345486121
628.5120012	0.912526573	0.0054802	4.627986959
530.8413263	0.911902287	0.005860203	4.468150925
579.4343949	0.935081585	0.005115725	4.591005988
676.2628076	0.902576807	0.003478351	4.4895147
651.0510966	0.89014216	0.004213726	4.446337627
657.2749006	0.917780636	0.00381264	4.558218627
501.1440936	0.923005166	0.005801111	4.441858088
335.8089142	0.917261783	0.007863003	4.230913618
731.690077	0.921541799	0.004396066	4.550860423
711.5408471	0.843434552	0.005439029	4.221107434
1045.112633	0.913892747	0.003187684	4.649301589
646.3428202	0.923736042	0.00520213	4.618785021
746.1483427	0.890240473	0.003649451	4.456674664
398.7167391	0.936928231	0.005894417	4.449040524
492.1674408	0.871482937	0.006790554	4.187755103
433.3581983	0.904112836	0.005674278	4.129396404
656.1794099	0.918297898	0.003918336	4.556972292
511.0442201	0.858363858	0.006176495	4.245703461
382.0086034	0.92350467	0.006452916	4.193520738
708.727192	0.908653846	0.004038683	4.357505268
443.2781007	0.911830519	0.005413848	4.384580211
825.7149621	0.904828086	0.004157947	4.449717221
560.1113292	0.932655778	0.004726866	4.749993512
596.8494731	0.921149682	0.004859387	4.695928956
634.9961772	0.912744388	0.004321614	4.401510679
323.7390369	0.936843917	0.007951498	4.165124578
609.9864781	0.902142162	0.004633679	4.19183701
842.122262	0.890969056	0.003614796	4.485538539
536.6332227	0.888017251	0.005689706	4.278233422
584.6988108	0.913452605	0.0048523	4.719585889
742.6394791	0.900523732	0.004671281	4.629110664
623.9899487	0.920998393	0.003692723	4.616256124
589.7070698	0.884961654	0.004751097	4.379310041
1175.998108	0.884729084	0.002362731	4.425625849
1219.530087	0.83961069	0.003040312	4.60677355
549.4481989	0.918449031	0.004715262	4.101793614
404.9630324	0.927870067	0.005587977	4.57038917
431.404752	0.893778542	0.007441335	4.369677332
384.806481	0.948141075	0.006064947	4.712668329
594.9602087	0.919897239	0.004490503	4.534360646
728.3904931	0.92755998	0.003904917	4.59356971
645.4777856	0.911761165	0.005013248	4.435862685
454.912791	0.917472327	0.006092291	4.535164143
606.4469304	0.91064985	0.004405922	4.516389175
410.1116892	0.900821337	0.007312356	4.085506896
430.2889013	0.93781072	0.007978049	4.523292085
440.9432484	0.894287665	0.006729634	4.339753892
783.8923929	0.929065485	0.003268308	4.617840423
544.7983684	0.94591881	0.004130207	4.673671967
463.1858107	0.926128614	0.004799299	4.424768545
374.8719868	0.911371237	0.007559929	4.361539369
603.0417657	0.920069404	0.004073337	4.429787743
481.1489303	0.913017751	0.006923895	4.337894583
584.9841985	0.927241609	0.004001857	4.565204599
693.4522157	0.902789518	0.003694617	4.422634282
612.1158847	0.922003361	0.004182115	4.423059972
624.9742512	0.911954296	0.004224556	4.665975483
545.4158038	0.878996428	0.006098832	4.456754559
985.4013751	0.91428983	0.002483299	4.48009353
742.2206164	0.912034761	0.003112925	4.563106878
693.275342	0.91503446	0.003466241	4.514567205
205.9940726	0.911587147	0.015708614	4.160016575
671.2356482	0.891925268	0.004313521	4.411346153
705.0371163	0.890986314	0.004572697	4.213921493
756.6008865	0.909784615	0.003072283	4.551772927
687.8701026	0.926977581	0.0033167	4.510062216
632.4125001	0.883872166	0.004225623	4.169935468
480.7486446	0.906259253	0.005908554	4.415328639
988.7834151	0.893580778	0.002595404	4.457381873
564.5126497	0.903500186	0.005961058	4.578226745
586.3537639	0.934416181	0.004820872	4.751577554
397.3790782	0.937326487	0.006786168	4.675307675
518.8942199	0.914364324	0.006758566	4.318055481
454.0411402	0.903075639	0.005290347	4.236914566
505.1346466	0.896418821	0.007679492	4.306277834
630.099998	0.915731603	0.003858986	4.260013666
687.1831304	0.934488769	0.003959917	4.71148731
898.2891211	0.936960978	0.002890336	4.764683948
511.6301319	0.909394108	0.007773954	4.620149823
697.046455	0.889521613	0.004071524	4.500654052
343.3389059	0.904428146	0.006924621	4.196411393
516.2420016	0.924748478	0.005854415	4.627831468
543.2790211	0.830086363	0.007255594	3.98568643
510.8328411	0.911498145	0.006017542	4.679161663
597.5722647	0.90139146	0.005037563	4.499552338
613.3064513	0.907051282	0.005300224	4.307547181
756.6276105	0.888774929	0.003892382	4.32978571
579.7480697	0.850165172	0.005770898	4.058975744
482.8535726	0.940870632	0.004695651	4.544402256
501.1778336	0.92586711	0.00504482	4.565099586
464.4695474	0.903053613	0.007046735	4.425238574
535.7952997	0.930907098	0.004090074	4.51586219
975.2016011	0.893202356	0.003253694	4.634479465
368.6887185	0.917178668	0.007767858	4.426675771
917.470864	0.908676806	0.003368558	4.387653344
334.2213171	0.89466888	0.009738222	4.083361764
617.489157	0.871226453	0.004601724	4.307346272

log.sigma.2.0.mm.3D_glrlm_HighGrayLevelRunEmphasis	log.sigma.2.0.mm.3D_glrlm_RunLengthNonUniformityNormalized	log.sigma.2.0.mm.3D_glszm_GrayLevelVariance
729.3724601	0.885376125	51.01937838
304.9365069	0.801610683	29.37625695
241.5439769	0.805144906	27.55641563
466.5297391	0.843869619	39.53062041
397.6874786	0.844853903	32.06824668
475.5441636	0.880705781	41.75952103
498.292611	0.832687508	30.04630136
462.4448472	0.809808842	35.76042566
505.3764457	0.851755938	33.88284651
393.361328	0.857515207	32.92836922
258.5558629	0.849791591	21.2166316
571.6129328	0.857600362	42.79747417
428.8328847	0.744051118	35.70137576
797.427292	0.844419262	50.21807074
506.0287163	0.861163724	45.68176458
527.0573249	0.810345031	32.90297593
329.5196934	0.88167068	27.06079256
329.3784726	0.778186812	26.75408395
318.7910126	0.826968031	19.29385622
509.6036879	0.852743759	37.21584774
325.2482327	0.766071918	24.19442859
300.3916558	0.859784496	22.07853167
532.1534093	0.834236736	31.15835014
338.9349445	0.841363691	25.00423115
616.6934457	0.828973552	42.33999979
456.4194821	0.877078002	42.46544657
468.4317954	0.856733332	41.30410431
483.0044064	0.842731594	34.5693811
267.8252915	0.879784618	18.32274692
446.6585323	0.824883338	25.94623621
595.9928024	0.813200962	36.7372787
373.2889001	0.803643498	31.68312596
444.7267951	0.84592605	40.30997855
542.2962416	0.825396126	40.93347166
489.0801592	0.857277626	35.76355121
412.8249894	0.802893063	31.54764563
814.5474454	0.799780629	43.59647373
723.4075006	0.745078749	43.59863238
422.4588236	0.84951283	22.42516476
324.3524459	0.867731698	28.43762885
306.95629	0.812384426	30.23678327
329.0319538	0.901544664	34.33290759
463.0891054	0.854931391	32.48046219
577.9713587	0.867663277	43.353596
490.8006609	0.842838831	36.81089243
353.1457846	0.851789137	32.06503181
459.7020331	0.840525971	34.38020098
298.4314422	0.822387439	24.0629334
351.6560791	0.886223617	37.88454538
320.1217799	0.815188683	29.09943414
629.3678328	0.870704783	42.10548012
461.8144198	0.898265302	36.74659632
366.7417947	0.864594346	27.95361385
286.5603244	0.840347573	27.17522257
468.9944188	0.854719086	28.44075534
365.7297536	0.842050919	29.77197666
465.6113304	0.866722368	32.08151379
509.2545489	0.827323568	35.92193292
477.4253368	0.856791342	34.96282422
475.0195502	0.846463111	35.43848113
373.4715547	0.79091946	36.38369053
751.2398449	0.847003386	35.57573051
564.0366326	0.844021204	34.46513259
529.2343292	0.847671608	35.06298905
157.2591444	0.8411354	19.46239269
477.0411103	0.812471771	36.60509943
497.900325	0.808715836	33.45909949
566.0749916	0.839435799	35.60664658
542.9262214	0.867043332	32.31502145
439.1613018	0.797697177	24.72907029
355.9804725	0.831855821	26.7485849
707.2172137	0.815758399	39.00997422
417.3693572	0.830250579	38.18893995
473.319832	0.87905511	43.3184
327.3330255	0.883989842	33.56929069
394.4038866	0.844501395	31.3784554
338.2016147	0.826455594	25.16333458
365.0675876	0.81389666	35.00394267
482.6278727	0.846328152	25.93996318
559.9440263	0.878835967	40.9471749
732.8413482	0.885515593	50.72930975
382.2497825	0.83761796	40.12841157
492.5955699	0.812423927	34.12337258
252.9271472	0.828466903	18.29015868
406.695474	0.861964655	36.11236164
306.9701504	0.722930753	16.68765817
383.0589615	0.843718016	37.43736624
441.9000236	0.826062807	32.595094
458.0770031	0.834234409	29.80007797
531.3090105	0.804222557	32.53328619
366.1930592	0.748303017	20.98830289
403.0802857	0.890758699	29.61532815
396.0760262	0.864461321	34.65766054
341.4131108	0.829008106	29.29170351
430.6411175	0.872172927	28.75803224
693.1702315	0.818040515	48.72272658
284.1987992	0.849562774	27.77469833
681.1056934	0.837335589	39.84261387
236.0644286	0.811657341	21.59464778
415.0191993	0.781365696	28.87445038

log.sigma.2.0.mm.3D_glszm_SmallAreaHighGrayLevelEmphasis	log.sigma.2.0.mm.3D_glszm_GrayLevelNonUniformityNormalized	log.sigma.2.0.mm.3D_glszm_SizeZoneNonUniformityNormalized
470.1894431	0.041720117	0.464446064
193.230426	0.062433024	0.418886981
166.4955367	0.056764871	0.393465524
259.919076	0.04757551	0.439134694
239.3078747	0.052249318	0.436544675
336.9064614	0.046019768	0.48540995
330.5015018	0.052686392	0.423757144
303.4387098	0.050486034	0.45140222
317.3070513	0.049840365	0.439832036
244.057885	0.051700436	0.433169783
159.7269336	0.064617456	0.397936201
374.3880577	0.048396178	0.465358988
259.0999213	0.057667624	0.411689888
517.0605136	0.044657066	0.423357237
322.3008186	0.045721959	0.445615538
344.0827363	0.055181275	0.436138104
219.8728112	0.054175658	0.407730362
194.6718986	0.061640983	0.410708501
188.2717336	0.070149744	0.385053408
335.0391633	0.047734074	0.427640592
206.2674361	0.061561704	0.388151137
191.8497317	0.062041719	0.425605536
338.6406104	0.055101724	0.432699592
227.6618767	0.057023687	0.430172353
409.8725733	0.051317718	0.434368961
311.7749461	0.045187911	0.436367067
321.3647565	0.044543966	0.457459058
308.8417964	0.051249667	0.450229871
161.2638594	0.067142742	0.368573653
268.9606963	0.060337222	0.411266869
396.9514524	0.052503453	0.465068464
231.9603306	0.055138589	0.452609528
289.6269756	0.046180881	0.413046444
347.4056158	0.047492801	0.448257598
324.6908504	0.048410908	0.439565618
279.0903899	0.056158682	0.427002679
549.2196877	0.04957978	0.470261281
494.720274	0.048745852	0.429371109
246.2147829	0.066709247	0.380148062
207.6051475	0.051821322	0.388147655
188.6059871	0.056203801	0.418203159
229.984314	0.047268041	0.445201934
285.8043199	0.050517054	0.400344703
370.3102701	0.046182	0.43942
312.2401652	0.053543724	0.432599457
241.1034621	0.050682088	0.448590193
298.5317301	0.051205513	0.420710953
169.9986385	0.063924014	0.373113455
233.830711	0.047401424	0.46862047
207.0283611	0.057022702	0.416177675
411.6090419	0.046710532	0.43460294
320.9233291	0.04731443	0.443798404
239.08943	0.054687824	0.447258498
192.3509376	0.056625043	0.406044051
292.8564335	0.053803953	0.415113358
212.9905354	0.055171149	0.39391854
298.9493749	0.050251865	0.414151663
328.3010025	0.051515131	0.44102282
301.5997629	0.051918306	0.443793172
314.5954492	0.048472673	0.417091978
254.7964065	0.049542212	0.500283217
478.649205	0.05237956	0.449434605
364.9564859	0.050374891	0.450401963
338.7322956	0.049395705	0.412721204
96.28210193	0.069719043	0.385373309
321.354137	0.050749238	0.459691359
319.4059715	0.056392805	0.473662387
360.798296	0.049852311	0.425554779
349.6859135	0.051633279	0.458546034
267.6586168	0.062583102	0.402132964
206.4187193	0.056959396	0.400537513
454.2044504	0.051508382	0.430773797
264.9179562	0.04797192	0.423753322
291.0097247	0.04383	0.42963
215.102632	0.047856022	0.421361192
244.8346478	0.05454626	0.431986704
216.3645168	0.059324451	0.399958716
217.7072934	0.054584688	0.411369812
298.9057784	0.061157184	0.418158226
387.701264	0.046840259	0.441629484
486.6100055	0.041953983	0.47895248
236.6065739	0.045391025	0.443569173
332.6565996	0.050814057	0.450082525
152.6105021	0.065926425	0.370441092
258.976167	0.04818763	0.424613513
157.6095534	0.080638738	0.330327205
251.15488	0.047040957	0.432938537
284.7079593	0.052450442	0.430307686
292.8034341	0.059560524	0.426982295
312.3730897	0.056167976	0.398098799
242.9002222	0.07217365	0.425164474
271.4387401	0.052241025	0.414278763
245.4582835	0.048894046	0.417424906
203.8352231	0.054667804	0.422689332
278.5285446	0.052439799	0.430546951
454.2227746	0.044815924	0.452782551
182.6025692	0.054699609	0.38873884
447.2212745	0.050480386	0.485285199
122.4139503	0.065759637	0.34791327
271.4307759	0.056697638	0.433089565

log.sigma.2.0.mm.3D_glszm_SizeZoneNonUniformity	log.sigma.2.0.mm.3D_glszm_GrayLevelNonUniformity	log.sigma.2.0.mm.3D_glszm_LargeAreaEmphasis	log.sigma.2.0.mm.3D_glszm_ZoneVariance
455.1571429	40.88571429	29.26938776	24.58083715
109.3295019	16.29501916	251.0842912	238.2508771
172.3378995	24.8630137	279.7214612	267.4554742
153.6971429	16.65142857	80.40857143	72.61649796
381.5400458	45.66590389	83.81006865	76.46931701
141.2542955	13.39175258	37.21649485	32.07248379
835.6490872	103.8975659	246.744929	238.5331587
711.4098985	79.56598985	630.3997462	619.8949084
764.4280783	86.62255466	67.87226697	61.03064115
185.8298368	22.17948718	29.77622378	23.53204992
216.8752294	35.21651376	70.41100917	62.75487249
409.5159091	42.58863636	68.56704545	61.70621772
170.4396135	23.87439614	875.3792271	855.0421772
253.590985	26.74958264	71.44073456	63.22423293
355.1555834	36.44040151	27.42032622	21.22353745
581.372093	73.55663916	606.3465866	595.5696749
212.835249	28.27969349	14.83908046	9.607081517
120.7482993	18.12244898	475.8129252	458.2243163
153.2512563	27.91959799	68.0201005	57.3839802
839.4584819	93.70198675	79.7050433	72.69050589
279.0806676	44.26286509	1039.702364	1022.013262
166.4117647	24.25831202	55.79283887	48.17749753
143.223565	18.23867069	123.5951662	114.0244613
411.2447699	54.51464435	60.08682008	52.42040909
188.516129	22.2718894	142.9101382	134.306595
448.1489776	46.40798442	36.20155794	30.88060725
576.3984127	56.12539683	91.16587302	84.58216868
435.3722854	49.55842813	96.94519131	88.78699033
71.13471503	12.95854922	12.61658031	7.252436307
249.2277228	36.56435644	252.3234323	240.835136
723.6465296	81.69537275	431.2583548	421.9079655
263.8713551	32.1457976	434.9451115	422.3505262
484.9165247	54.21635434	104.3739353	96.80909266
371.6055489	39.37153197	127.0784077	119.0426532
765.7233065	84.33180253	52.35820896	45.9667748
366.3682984	48.18414918	503.9219114	491.7045237
498.9472196	52.60414703	746.1800189	733.8804873
582.6565954	66.14812085	813.7472366	799.2825834
157.7614458	27.68433735	34.38072289	25.59628393
1117.088951	149.1417651	26.04100069	19.66002277
314.0705726	42.20905459	350.4793609	338.5488217
863.6917526	91.7	9.227319588	5.254078808
556.4791367	70.21870504	70.68561151	63.778925
439.42	46.182	61.972	55.781856
189.9111617	23.50569476	111.1298405	102.6549779
478.6457357	54.07778819	79.50421743	72.88577404
671.8753914	81.77520351	255.5679399	247.4077413
97.75572519	16.7480916	103.2633588	92.2115407
181.8247423	18.39175258	21.21391753	16.40335716
167.719603	22.98014888	264.1538462	252.8989034
451.5524543	48.53224254	44.43984601	38.3262447
733.1549637	78.16343826	14.71004843	10.54617596
788.5167328	96.41463415	51.85479297	45.30490974
151.0483871	21.06451613	86.1344086	77.32691641
204.2357724	26.47154472	45.91056911	38.60299755
248.562599	34.81299525	81.45800317	71.90784632
688.7342153	83.56885147	38.40709561	32.49076338
477.1866913	55.73937153	308.6377079	298.4237173
276.4831461	32.34510433	49.21348315	42.21605703
1455.651003	169.1696275	118.2449857	111.4027889
279.6583184	27.6940966	267.9248658	257.9442398
452.1312127	52.69383698	108.7634195	101.7193025
977.8226624	109.3638876	191.8908337	184.7805415
1105.680105	132.3310937	59.24636058	51.14969108
95.57258065	17.29032258	102.8467742	92.6994667
543.8148774	60.03634827	415.1310228	404.47353
177.1497326	21.09090909	221.1283422	208.9717464
921.326097	107.930254	208.4184758	200.0846535
1112.891224	125.3139679	54.17676143	48.35290185
305.6210526	47.56315789	582.3921053	567.8519321
179.0402685	25.46085011	75.99328859	67.54821855
544.9288538	65.15810277	501.0608696	489.5599244
330.9513444	37.46606914	183.7298335	175.2147957
429.63	43.83	31.199	26.095919
589.0629471	66.90271817	28.30114449	23.28521073
205.1936842	25.90947368	53.03157895	45.04946704
291.969863	43.30684932	102.4712329	91.69844999
112.7153285	14.95620438	154.4014599	142.176994
241.2772964	35.28769497	93.74870017	84.9965368
455.7616279	48.33914729	18.03972868	12.90282942
445.4258065	39.0172043	30.17526882	25.60118973
308.2805755	31.54676259	66.5942446	59.89396822
476.6373938	53.81208687	374.5410765	365.0122829
334.5083056	59.53156146	127.8527132	116.2716183
415.6966292	47.17568948	35.6618999	29.45523616
92.16129032	22.49820789	810.4229391	780.2714251
406.5292865	44.171459	94.5228967	88.0716213
394.5921483	48.09705562	224.8069793	215.8657706
217.3339882	30.31630648	58.29862475	50.02593783
228.5087108	32.24041812	197.412892	184.966993
129.25	21.94078947	681.2763158	662.8767313
450.7352941	56.83823529	12.76286765	7.969665739
851.9642332	99.79274865	42.30132288	35.85256
453.9683426	58.7132216	193.2458101	183.8047814
777.9983398	94.7587161	26.67515219	20.87470511
516.6248904	51.13496933	358.8080631	349.8133209
394.9586614	55.57480315	51.65846457	43.97094422
171.7909605	17.8700565	123.8615819	115.1140237
80.36796537	15.19047619	113.6796537	100.6447405
682.9822448	89.41217502	880.7266963	867.3209754

log.sigma.2.0.mm.3D_glszm_ZonePercentage	log.sigma.2.0.mm.3D_glszm_LargeAreaLowGrayLevelEmphasis	log.sigma.2.0.mm.3D_glszm_LargeAreaHighGrayLevelEmphasis	log.sigma.2.0.mm.3D_glszm_HighGrayLevelZoneEmphasis
0.461828464	0.043447165	21537.52245	691.2642857
0.279144385	0.901877873	71227.69349	305.7509579
0.285528031	1.402926657	57113.41096	259.5273973
0.358239509	0.171183129	40030.92286	405.2142857
0.369087838	0.218972351	33845.754	368.6270023
0.440909091	0.091608575	16355.7732	479.3024055
0.348964785	0.549656236	111682.8996	497.7636917
0.30853563	1.469220539	272102.9619	454.0615482
0.382314122	0.140481161	34283.39758	480.2882624
0.400186567	0.083434218	11748.29837	373.4125874
0.361405836	0.289371872	17967.20734	248.4238532
0.381778742	0.130660722	38310.55682	549.7636364
0.221746117	2.17675606	353900.6184	412.5676329
0.348864298	0.096170256	56057.00668	780.5709516
0.40171371	0.063817706	13796.13049	482.5809285
0.304616088	1.181507709	312952.3646	514.3975994
0.43718593	0.054612147	4782.189655	331.9712644
0.238442822	1.561782158	146786.3163	312.755102
0.306625578	0.223152868	21554.64322	303.1909548
0.37757261	0.169682853	38637.25471	505.1456954
0.23776455	3.427932015	317238.1822	328.6216968
0.362372567	0.193397741	16892.27621	291.4245524
0.323242188	0.248514796	63048.82779	509.4592145
0.361163581	0.19777578	19098.5251	339.5470711
0.340926944	0.253233306	82672.40323	615.578341
0.433516252	0.097122736	14682.57254	461.6183057
0.3897309	0.222908279	39122.24603	465.8603175
0.350108617	0.215524943	45093.35057	463.3205791
0.431767338	0.060406268	3197.829016	267.5699482
0.29503408	0.570508838	113037.0495	419.7656766
0.327028163	0.785848306	238652.1234	581.8817481
0.281778637	1.149610136	166527.7033	344.8456261
0.363580056	0.241492343	46979.41567	439.9693356
0.352765957	0.256462863	65553.04825	520.8552473
0.3955495	0.121806972	23466.11596	484.7801378
0.286095365	1.342647892	190632.1189	420.9428904
0.285138404	0.928175551	602374.5259	790.4156456
0.26293354	1.20092988	553609.3272	737.4959469
0.337398374	0.081879523	15642.33976	396.2650602
0.395873453	0.092882251	8203.395066	324.5813065
0.289514264	1.140132206	109926.5979	288.6284953
0.50168089	0.037534739	3009.379897	331.1489691
0.380509171	0.167353531	31303.17266	451.3158273
0.40192926	0.112480702	35935.637	554.204
0.343505477	0.254948944	50168.73576	481.8382688
0.38870674	0.255857956	25698.59044	352.163074
0.350065761	0.60154166	109992.7652	454.8998121
0.300803674	0.361184338	30540.64504	284.7251908
0.455934195	0.071753658	7555.873711	339.193299
0.298076923	0.959662878	73998.85856	325.3151365
0.404437524	0.080250966	26021.0154	620.4177093
0.490062296	0.036619887	6850.831719	461.7003632
0.390735816	0.148068564	18964.33012	353.0981282
0.336956522	0.347445833	22325.88978	290.8010753
0.369924812	0.105796677	21030.00203	453.2865854
0.323589744	0.238304542	29647.62758	346.1156894
0.411124845	0.088969857	17616.6819	454.3926639
0.312897629	0.613879927	156807.8189	490.2578558
0.378033981	0.108767351	23392.78331	452.9454254
0.382298171	0.289410572	49651.84785	474.2269341
0.316534541	0.78630072	92656.46154	361.4794275
0.376779026	0.153875404	78215.57555	719.7673956
0.375021593	0.372745037	99827.24136	548.528328
0.351436442	0.116699808	31381.891	518.6360582
0.313924051	0.76859284	14734.46774	156.6733871
0.30631797	0.914550153	190135.2527	468.1065089
0.286809816	0.448822374	110952.9866	467.1898396
0.3464	0.368222758	119224.8522	544.7394919
0.41437596	0.103077049	29527.26329	514.9200659
0.262249827	1.367309606	249618.8342	427.2065789
0.344110855	0.221734217	27214.87472	334.7628635
0.294871795	0.75600912	333732.4538	693.4798419
0.342694164	0.490102608	70990.45327	407.212548
0.442673749	0.069672972	15597.883	444.92
0.446502715	0.10137582	8971.165951	324.4206009
0.353949329	0.144795873	21301.6	370.2484211
0.304674457	0.337218413	31896.14247	341.4671233
0.286012526	0.453908893	54466.18978	349.6131387
0.338019918	0.195022383	46206.86655	460.0519931
0.441214194	0.037485243	10496.62694	558.6860465
0.467571644	0.044541252	22167.36129	700.1569892
0.386325737	0.184447979	25999.0705	360.1007194
0.323952279	0.813984107	173881.3352	489.3956563
0.293849658	0.514617423	32755.90144	245.2879291
0.401394014	0.098414441	14418.34014	392.1225741
0.182114883	2.757103179	239976.5878	280.1971326
0.393710692	0.260695797	35911.99681	370.4707135
0.334427425	0.581977936	88622.29553	434.997819
0.347677596	0.13918703	26044.50098	445.9921415
0.28345679	0.388684184	101942.7404	501.8519164
0.233128834	2.034382894	229549.1579	375.6414474
0.456759026	0.039004038	4933.289522	406.0395221
0.393787382	0.116267914	16583.31455	379.2229299
0.325454545	0.617194283	62203.46089	321.179702
0.415211397	0.065948655	11780.35141	416.610404
0.333430742	0.556077794	234001.9781	676.4425942
0.360667377	0.191254852	15118.31004	281.7942913
0.338108883	0.180839592	87171.57627	636.2231638
0.276978417	0.482026574	28014.15584	212.5367965
0.273120887	2.302534141	338556.7933	419.1686747

log.sigma.2.0.mm.3D_glszm_SmallAreaEmphasis	log.sigma.2.0.mm.3D_glszm_LowGrayLevelZoneEmphasis	log.sigma.2.0.mm.3D_glszm_ZoneEntropy	log.sigma.2.0.mm.3D_glszm_SmallAreaLowGrayLevelEmphasis
0.708644839	0.00360815	6.315796951	0.003085712
0.670917012	0.011085262	5.759346828	0.009230188
0.650005119	0.01357049	5.988567323	0.011120079
0.688834372	0.008475404	6.053487667	0.007446767
0.68616064	0.00707483	6.09649976	0.005177967
0.72412695	0.007013663	5.922324663	0.006286172
0.676883727	0.003349207	6.308093038	0.002524965
0.698544025	0.00484999	6.181527025	0.004018808
0.689837071	0.004049978	6.303227177	0.002874055
0.684522835	0.00801645	6.065471397	0.006972217
0.655549878	0.009520678	5.909334527	0.007301758
0.709241879	0.006012442	6.219134927	0.004976214
0.66729416	0.007293902	6.055312089	0.006240345
0.676331037	0.005019193	6.377996402	0.004470791
0.694052548	0.006838005	6.281798707	0.005419957
0.686909062	0.004139847	6.217237362	0.003332946
0.663152438	0.006753416	6.027816214	0.00541151
0.665131786	0.009264879	5.828060461	0.007430489
0.644968448	0.007014215	5.881356534	0.005743967
0.679506756	0.004805721	6.370965934	0.003951357
0.645434464	0.006271482	6.078697089	0.004882506
0.678179718	0.008432396	5.819696274	0.007083908
0.684033521	0.005986036	5.960566961	0.005266227
0.681810038	0.005908368	6.086237661	0.004706636
0.685267329	0.006638941	6.089875937	0.006100193
0.687100549	0.005582364	6.362440211	0.004628547
0.703245478	0.005519895	6.306898731	0.004062215
0.697705009	0.005592832	6.158747195	0.004611114
0.623050864	0.0103345	5.628104079	0.008723362
0.666973687	0.006852197	6.057579654	0.005943655
0.708932699	0.004527101	6.158992311	0.003824373
0.698779274	0.008043991	5.949257126	0.006912135
0.668277414	0.005796502	6.457274378	0.004597113
0.696357417	0.005371675	6.255373973	0.003674247
0.689351986	0.003977372	6.303637439	0.003130627
0.679302333	0.005239755	6.125352265	0.004199655
0.712815606	0.002936839	6.22583873	0.002528397
0.681604002	0.003046716	6.414228689	0.002548314
0.640632614	0.006851235	5.954452825	0.005888652
0.647005311	0.005650011	6.441188182	0.004063588
0.672617191	0.010221757	6.178426236	0.00842864
0.693974069	0.006435953	6.284996877	0.005041438
0.657452935	0.004913733	6.347563245	0.003421929
0.687943725	0.005307576	6.261616452	0.004513211
0.683803581	0.007660533	6.033398876	0.006885924
0.695982568	0.00751741	6.149651114	0.006249151
0.674426451	0.005351701	6.333094675	0.004332026
0.630703331	0.011101037	5.89217757	0.009619171
0.711742864	0.0111268	6.027114106	0.009750561
0.667329371	0.008962069	5.886271415	0.007770698
0.685332114	0.004197451	6.330693977	0.003557126
0.692554571	0.004488409	6.289150384	0.003512857
0.695651448	0.005439559	6.148415201	0.004376028
0.662417911	0.009519666	5.989655007	0.007559175
0.669700159	0.0051202	6.042548567	0.004278935
0.650946279	0.010659462	6.146961604	0.008924914
0.668236686	0.00440738	6.293752211	0.00333681
0.690685278	0.004586347	6.24777379	0.003836283
0.692758606	0.005552901	6.14126122	0.00473687
0.671292798	0.00458187	6.439056927	0.003528639
0.734744596	0.007958747	5.988208743	0.007032915
0.696848681	0.003015752	6.105150189	0.002560432
0.697616221	0.003343794	6.205926979	0.002717792
0.667890143	0.004017981	6.467710635	0.003178453
0.644526868	0.020556618	5.717915284	0.016102502
0.705006677	0.005443473	6.212556599	0.004658967
0.714955317	0.007019355	5.851687785	0.006175347
0.678232313	0.003324718	6.392797753	0.002592691
0.704203839	0.003880762	6.2192021	0.003199188
0.659412214	0.004870114	6.086550588	0.003895292
0.65703202	0.007628937	6.020410204	0.006284303
0.682491896	0.003029348	6.292553777	0.002536536
0.676439711	0.006890848	6.262484898	0.004666335
0.680116926	0.006245254	6.321791182	0.005263381
0.674782248	0.007867675	6.308476475	0.00624616
0.683257722	0.010164888	6.012402725	0.008591173
0.657471675	0.006013529	6.117455404	0.004862855
0.66628114	0.01427238	5.918132875	0.013077643
0.672439161	0.005036951	5.956651556	0.004229553
0.690500033	0.00462945	6.307716279	0.003635196
0.719562323	0.003589838	6.254786463	0.003096344
0.691778614	0.011640579	6.238713251	0.010175082
0.69774284	0.00441129	6.180871667	0.003509413
0.63247456	0.007300831	6.121673483	0.005313162
0.677250599	0.007689094	6.302704405	0.006419645
0.589471055	0.009266277	5.796205584	0.007487633
0.683586258	0.007079262	6.244802387	0.005639168
0.682199639	0.005559591	6.196521603	0.003832037
0.678994167	0.007889195	5.986505829	0.007028816
0.654907939	0.005418614	6.189032633	0.004626575
0.677485431	0.006645199	5.546886449	0.005709769
0.668805102	0.005249937	6.230305931	0.004145056
0.671576481	0.005767377	6.397138813	0.004406403
0.675946319	0.009611601	6.180224316	0.007917848
0.682155465	0.004376026	6.2501485	0.003300584
0.699686602	0.004055537	6.353468717	0.003392028
0.646142331	0.010011002	6.247551455	0.008054897
0.723843505	0.005195551	5.923744929	0.004710488
0.610491073	0.016020244	5.806827084	0.013917835
0.683643033	0.004568157	6.120477884	0.003634384

log.sigma.2.0.mm.3D_ngtdm_Coarseness	log.sigma.2.0.mm.3D_ngtdm_Complexity	log.sigma.2.0.mm.3D_ngtdm_Strength	log.sigma.2.0.mm.3D_ngtdm_Busyness	log.sigma.2.0.mm.3D_ngtdm_Contrast
0.003300357	1.229346425	2.023558124	0.212810179	7.41E-05
0.007185376	0.785670374	2.885607683	0.190589581	5.76E-05
0.005176738	0.413097123	1.445008818	0.351658356	4.45E-05
0.00730341	1.308181466	2.929917891	0.151996273	0.000138582
0.002733465	0.478696806	1.140719091	0.438803044	4.14E-05
0.009958792	2.195100276	4.670683654	0.111685194	0.000195833
0.001296764	0.285291092	0.835256709	0.634004092	9.30E-06
0.001434256	0.245665844	0.82080517	0.639226363	1.16E-05
0.001530676	0.383858862	0.882631978	0.56471317	1.71E-05
0.006315448	1.036867929	2.532075375	0.193907219	9.38E-05
0.004686601	0.451116874	1.227209276	0.390419827	5.14E-05
0.002813036	0.777185399	1.802663173	0.288218524	4.18E-05
0.00397087	0.427327942	2.184136474	0.245457584	2.24E-05
0.004338663	1.286577542	3.493157192	0.140574332	4.92E-05
0.003426032	0.974923213	1.841074186	0.257324144	6.26E-05
0.00163338	0.294501273	0.978875078	0.527440226	1.36E-05
0.006097846	0.658520557	1.353262153	0.312275683	0.000119915
0.005441549	0.496990914	1.997536349	0.263295969	4.52E-05
0.005128686	0.517578674	1.644331133	0.306963228	4.45E-05
0.001353789	0.32380651	0.86697111	0.617297152	1.38E-05
0.002452528	0.190287529	0.867195249	0.586760808	1.51E-05
0.005859493	0.629800717	1.592006162	0.305406749	8.06E-05
0.00697498	0.996117361	2.949310049	0.151967009	7.91E-05
0.002805158	0.303983312	0.842321336	0.544094488	3.06E-05
0.005776825	1.283552271	4.174907768	0.12594317	5.04E-05
0.003151088	0.787330522	1.68043327	0.314763544	4.83E-05
0.002373745	0.47998212	1.043828053	0.442707602	3.56E-05
0.002562046	0.467624106	1.345140899	0.36642306	2.59E-05
0.013683955	1.090253675	2.24709683	0.201410687	0.000313573
0.003152589	0.434306196	1.469048139	0.341286416	2.69E-05
0.001514469	0.390455616	1.285433471	0.436034667	1.02E-05
0.003574346	0.385080377	1.236836504	0.360940273	3.76E-05
0.002400589	0.528478847	1.260562794	0.407767631	3.01E-05
0.003238491	0.580795485	1.560476076	0.289112372	4.25E-05
0.001684794	0.432120546	1.051221817	0.502561496	1.70E-05
0.002553325	0.323960558	1.14578021	0.423105795	1.90E-05
0.001979909	0.586710012	2.112749561	0.255253498	1.17E-05
0.001628323	0.35994386	1.652293286	0.33154653	7.08E-06
0.004875354	0.786282663	2.142979361	0.248568778	5.56E-05
0.001035517	0.178777568	0.402664101	1.312419533	1.25E-05
0.002910005	0.340991442	1.021231849	0.465112829	2.83E-05
0.001809375	0.470569321	0.759648391	0.775712849	3.69E-05
0.001960137	0.401437009	0.982110955	0.500313367	2.24E-05
0.002655258	0.788136281	1.489673751	0.308167931	4.60E-05
0.005469449	0.961498296	2.868057102	0.182566635	6.34E-05
0.00262856	0.476355576	1.102686939	0.471359084	3.31E-05
0.001629478	0.318266728	0.887872065	0.571612617	1.53E-05
0.006813196	0.787044442	2.637466942	0.218012154	6.69E-05
0.007532726	1.485587184	2.689533237	0.183131919	0.000176631
0.005283939	0.556022886	1.924143571	0.289186321	5.40E-05
0.002797889	0.808946863	1.937394518	0.258058593	3.35E-05
0.002005466	0.644521005	1.085340608	0.48393557	3.51E-05
0.001497335	0.290575977	0.640966677	0.780290336	1.75E-05
0.006389545	0.799262014	2.0647309	0.24765613	7.22E-05
0.005299589	0.706461001	1.781578056	0.240293352	7.23E-05
0.003666609	0.463253855	1.440771901	0.343710827	3.83E-05
0.0017661	0.343951095	0.766573035	0.616801682	2.56E-05
0.00217355	0.400440891	1.243078268	0.386053686	1.74E-05
0.004111319	0.898364898	2.126513628	0.225120216	4.87E-05
0.000844132	0.192118108	0.416847078	1.101860683	9.65E-06
0.003950969	0.612561023	1.940568351	0.281894684	3.89E-05
0.002537751	0.621682446	1.567791445	0.2817547	3.01E-05
0.001246073	0.258897525	0.664812763	0.712455737	1.44E-05
0.000927767	0.205743494	0.581653194	0.903450795	8.86E-06
0.008663335	0.567679591	1.564882775	0.346010159	0.000114438
0.001924737	0.346932393	1.124174921	0.446250127	1.46E-05
0.005367911	0.834929847	2.767955663	0.172463408	4.47E-05
0.00114625	0.329444071	0.861882138	0.611873822	9.71E-06
0.001237266	0.331924115	0.911981734	0.60237923	1.07E-05
0.002450426	0.267029639	1.135699886	0.454018715	1.56E-05
0.005451299	0.768337218	1.947877687	0.240529703	6.34E-05
0.001743249	0.417142125	1.575569479	0.34653967	1.06E-05
0.0033738	0.610293843	1.585846302	0.302193094	3.67E-05
0.003043503	0.807984475	1.317820035	0.349363888	7.29E-05
0.002208821	0.489330796	0.804176292	0.656003741	4.43E-05
0.004964277	0.796225618	2.112913424	0.230094371	6.14E-05
0.002969026	0.325168907	1.171955515	0.455014208	2.27E-05
0.00694745	0.886889933	2.765130377	0.186559812	7.96E-05
0.003776132	0.650382766	1.991523465	0.262781221	3.45E-05
0.003113826	1.055405136	2.034869576	0.246258219	4.39E-05
0.003536265	1.279938543	2.207900833	0.197409342	7.50E-05
0.004066315	0.798716697	1.623304444	0.277761566	6.29E-05
0.002405549	0.425419316	1.303362143	0.363543441	1.82E-05
0.002369288	0.157797285	0.501619572	0.902113932	2.42E-05
0.002831359	0.675464458	1.27217664	0.382577164	4.59E-05
0.004642223	0.229376796	1.609674047	0.351719602	1.97E-05
0.003058884	0.6892619	1.319150639	0.362102721	4.87E-05
0.002630307	0.386285229	1.092437654	0.441800926	3.14E-05
0.004717001	0.681348556	2.123450303	0.22523733	4.79E-05
0.003467131	0.577957957	2.231288682	0.24576077	2.58E-05
0.005219019	0.26657303	1.414586446	0.33172166	3.96E-05
0.00289003	0.633867257	1.250790455	0.410203969	4.29E-05
0.00135553	0.341079358	0.731977293	0.7303868	1.69E-05
0.002219759	0.242971084	0.720942762	0.663551719	2.83E-05
0.001573364	0.365058301	0.757092839	0.651386993	2.00E-05
0.002127314	0.589222093	1.729010849	0.297682844	2.18E-05
0.002481632	0.371129411	0.942030111	0.579424546	2.78E-05
0.006517393	1.349920877	3.590908774	0.119603451	8.42E-05
0.007906012	0.533673654	1.761497427	0.27342445	9.55E-05
0.001205099	0.171116693	0.691062106	0.834874587	7.54E-06

log.sigma.1.5.mm.3D_gldm_GrayLevelVariance	log.sigma.1.5.mm.3D_gldm_HighGrayLevelEmphasis	log.sigma.1.5.mm.3D_gldm_GrayLevelNonUniformityNormalized	log.sigma.1.5.mm.3D_gldm_DependenceEntropy
34.98911542	999.0301602	0.061420636	6.453184193
12.79112814	357.4791444	0.117099145	5.886175673
12.48998366	316.9856584	0.117030915	6.304892632
23.87287343	640.7113613	0.077888676	6.370395125
19.14843161	385.3179899	0.087372383	6.337044455
28.27157025	587.8090909	0.070091827	6.129842021
16.4275481	475.1270572	0.087679541	6.401821094
17.63041755	668.5295615	0.106649235	6.285238505
20.17008658	524.6201056	0.078032853	6.419933752
21.94156462	584.454291	0.077329653	6.175190158
12.56398193	310.5842175	0.092055457	6.131165042
26.55027898	744.8832972	0.075612857	6.359503691
13.76929898	592.6523835	0.152572705	6.088392284
24.82087617	666.5888177	0.078909762	6.371737521
28.94208685	728.0700605	0.074624211	6.328322621
15.75617122	597.0002285	0.105984968	6.29724136
19.41920659	283.1959799	0.070044527	6.200336556
11.50928541	467.8669911	0.12958523	6.077466726
12.09418603	504.298151	0.099188986	6.00987749
22.49079067	675.9180612	0.078418953	6.338007131
9.539742794	380.4970238	0.130442571	6.216097159
15.33285806	356.1612604	0.082847967	6.008756367
17.18587875	707.5820313	0.091213226	6.146551781
15.53522681	521.7257272	0.086808184	6.292351015
20.82571926	639.5962294	0.09160157	6.173983685
29.47990801	764.3727311	0.064264906	6.515093354
25.02974095	627.8818435	0.07006494	6.462273394
17.58694114	651.4341057	0.092646609	6.209282713
16.2677457	322.0872483	0.075987568	5.789724341
13.95450135	610.6314508	0.104091193	6.096116801
18.52061659	719.579235	0.101135743	6.391560075
14.71359462	533.8221363	0.1089318	6.191214843
23.44932849	608.3880458	0.074457718	6.600168672
23.19896587	568.3519149	0.084058307	6.422739709
23.57991991	507.4600363	0.073453371	6.453543759
14.98088737	471.6915639	0.108441282	6.212156317
20.07497457	1042.25262	0.101398897	6.301509454
16.18618436	1044.829297	0.133145725	6.521440409
14.78699517	589.4219512	0.092507106	5.923327819
18.85684511	346.1283356	0.070443514	6.452831689
13.83398808	332.6781033	0.105744292	6.228911743
26.44916769	316.6814068	0.055770252	6.423305681
19.20293765	569.1330413	0.080595926	6.3330334
28.02018111	662.3392283	0.070097238	6.370538317
20.708881	750.0469484	0.084095601	6.247051713
19.56798232	445.5956284	0.079591375	6.288879658
20.23253028	570.105217	0.084728773	6.334603247
13.28480006	392.4385763	0.109684291	5.866058522
26.8465357	412.1774383	0.068540364	6.149968882
15.15578717	538.387574	0.097964663	6.146684251
25.50418992	695.8283379	0.07291158	6.292968726
26.53745116	574.0086028	0.060385839	6.34659948
18.85735124	431.1489362	0.076412565	6.185459058
14.58072671	364.8197464	0.091275467	6.056245198
18.804444	533.1390977	0.085560518	6.164206666
15.45678396	426.2569231	0.093948718	6.131633344
20.89165467	485.1156984	0.071986505	6.340899831
16.56130034	685.2877386	0.096406987	6.323795881
20.00891342	650.9696602	0.08419517	6.158278447
22.99885317	695.7161792	0.070123282	6.570647243
17.72594073	566.9195923	0.109531493	6.196993448
23.34007996	1017.077154	0.08189847	6.303978848
21.76643375	791.352047	0.081634175	6.443664483
20.52319691	706.4298832	0.083104875	6.410929727
10.40660151	220.1063291	0.100865246	5.923370981
17.16591597	646.5235629	0.103959599	6.314097773
16.24310992	535.3420245	0.106177547	6.059942129
20.74456105	649.12512	0.082071245	6.398771969
21.52849148	831.4396449	0.076563568	6.320486468
11.18787629	420.9499655	0.115139019	6.122649699
16.41304942	344.2209392	0.091067269	6.236271409
18.27011639	728.6230769	0.104601692	6.312194899
20.61849294	398.2632734	0.085105224	6.465583424
30.11267899	553.4466578	0.059213836	6.495114011
24.3031106	389.8198659	0.062596034	6.481144908
16.67374806	381.2503726	0.089608676	6.131313548
14.05536635	436.0525876	0.100650848	6.112720969
16.40881207	466.7964509	0.102854329	5.985158637
14.54196357	509.3503222	0.09197491	6.073518543
26.6933626	646.8289867	0.065044076	6.422201854
33.75770559	1003.223731	0.063269804	6.414653819
23.95239898	500.1089494	0.081095946	6.418166168
16.92149255	603.4086877	0.101754844	6.391500344
10.29701369	341.2456883	0.098924966	6.152411216
23.41709352	471.7486675	0.072192157	6.464812763
5.580711573	328.1723238	0.178929572	5.98316605
23.47462873	414.5522013	0.072577825	6.521918655
18.64932998	457.3088986	0.085874111	6.349008467
16.89751812	616.9132514	0.0976552	6.116839754
14.81470849	655.1920988	0.106625453	6.164061541
8.499755354	556.5766871	0.150090331	5.926900477
22.10016437	499.2346767	0.065100625	6.282435487
22.250215	467.2216863	0.073022793	6.322492432
15.60838788	396.6387879	0.094675115	6.281026107
20.59067496	506.5236673	0.068644382	6.325264444
25.30204745	1014.167446	0.094536811	6.464453068
16.57383685	342.8782393	0.084343006	6.278835856
22.44782336	682.30468	0.089038678	6.161816099
10.28652186	259.9568345	0.114044017	5.966535854
12.47776549	563.6782127	0.126774659	6.153148601

log.sigma.1.5.mm.3D_gldm_DependenceNonUniformity	log.sigma.1.5.mm.3D_gldm_GrayLevelNonUniformity	log.sigma.1.5.mm.3D_gldm_SmallDependenceEmphasis
517.1460886	130.33459	0.452832533
140.715508	109.4877005	0.288665255
200.6205997	179.5254237	0.283681609
181.7451382	76.09723644	0.365559828
422.9738176	206.8978041	0.354640808
158.5090909	46.26060606	0.438233863
989.4137321	495.4770837	0.343198283
752.3848865	544.7642913	0.314071142
878.8706555	354.7373515	0.372221232
222.4981343	82.89738806	0.370963024
271.2718833	138.8196286	0.325072002
461.1475054	174.2876356	0.382126272
208.1258704	284.8532405	0.234974948
322.2055911	135.4880606	0.355232807
413.4717742	148.0544355	0.38850638
660.2189214	463.7902194	0.304792139
274.6247906	83.63316583	0.390175054
162.2181671	159.7785888	0.260290261
228.9953775	128.7473035	0.335667116
1044.037892	407.7001346	0.380188705
371.7109788	394.4583333	0.257525344
234.9592215	89.39295644	0.374692471
184.4316406	93.40234375	0.348846294
476.5005667	229.7812618	0.343750816
226.8727416	116.6087981	0.35237424
529.9953567	152.2435627	0.414168996
668.2112589	226.5199505	0.399048033
492.2469225	255.8899348	0.336877581
115.9888143	33.96644295	0.428111484
346.8510224	213.8033106	0.328091761
714.2374947	481.2038672	0.315712415
312.2179797	225.3798937	0.302215806
581.6918551	240.4239703	0.354997027
409.146383	197.5370213	0.339020561
887.5526794	323.4886467	0.385936564
445.012004	325.2154051	0.307164332
579.0392368	377.3052943	0.313528446
569.0930052	687.1650843	0.261729617
242.900813	113.7837398	0.348133297
1521.580743	512.1243466	0.370382254
397.767926	274.3006939	0.290842617
1018.191104	215.6635635	0.457606108
712.9775527	294.4169176	0.359936076
556.772508	174.4019293	0.415893369
238.943662	107.4741784	0.356257236
537.2302368	218.4783242	0.383285757
848.4739149	386.5326611	0.370215368
152.7428243	95.53501722	0.328549709
213.2068155	58.32784959	0.451859736
225.6715976	132.4482249	0.333860565
556.0221876	187.3098482	0.399942855
861.2919015	203.5606645	0.44253111
979.5	344.7734929	0.387870078
203.6666667	100.7681159	0.348287975
256.9984962	113.7954887	0.354114038
348.2605128	183.2	0.332557897
857.9537701	291.1854141	0.387874852
564.5083864	333.3753615	0.320375224
324.9563107	138.7536408	0.372951469
1868.592069	640.1554387	0.393592434
259.6840317	193.4326161	0.318517375
528.4868914	218.6689139	0.385553659
1069.493004	472.5802384	0.365988277
1410.514102	633.5084612	0.350782045
139.4556962	79.6835443	0.320483071
588.736406	401.4919731	0.315629218
212.0966258	138.4555215	0.326933892
1163.6864	512.94528	0.3633003
1272.136418	448.4328154	0.407168831
427.7556936	333.6728778	0.275142003
228.0777521	118.2963818	0.342148547
641.3184149	448.7412587	0.314566765
378.515577	193.9548047	0.341944397
540.8769367	133.7640549	0.439734874
712.0009582	195.9881827	0.419590765
258.3159463	120.2548435	0.352954511
418.9315526	241.1594324	0.337807851
162.0250522	98.53444676	0.336482906
326.0626831	157.0011716	0.346935724
521.7533134	152.1380932	0.414120692
484.1131222	125.84364	0.458342809
335.0755976	145.8916064	0.382579334
492.5466504	332.6365861	0.310965814
527.1060853	303.9964204	0.289121176
479.7052071	176.0766708	0.374509653
141.7167102	274.1201044	0.175212648
468.1027254	173.0981132	0.374902664
476.9467542	235.4668125	0.351638174
258.5887978	142.9672131	0.336950561
315.3397531	215.9165432	0.312505862
153.0230061	195.7177914	0.246521402
590.0016793	155.0696893	0.43315066
1119.809956	378.4771368	0.38973004
552.890303	312.4278788	0.33053373
990.1571691	298.7403493	0.406131008
550.5254237	323.5049679	0.34502772
537.9236777	237.5942492	0.3618262
189.6475645	93.2234957	0.351671389
129.4892086	95.11270983	0.286860026
757.0114306	731.9968826	0.2774172

log.sigma.1.5.mm.3D_gldm_DependenceNonUniformityNormalized	log.sigma.1.5.mm.3D_gldm_DependenceVariance	log.sigma.1.5.mm.3D_gldm_LargeDependenceEmphasis
0.243706922	2.632829094	8.550424128
0.15049787	5.115623552	17.54973262
0.13078266	10.3416858	27.47718383
0.186023683	4.010030078	12.92425793
0.1786207	4.707820917	14.39442568
0.240165289	1.948861341	7.636363636
0.175086486	5.724874718	16.09644311
0.147295397	6.335686678	19.39624119
0.193328345	3.895693027	12.28860537
0.20755423	2.623158693	9.951492537
0.179888517	3.320251321	12.4801061
0.200063994	3.585478329	11.50585683
0.111476096	9.479825653	30.14408141
0.187656139	3.606787312	12.2813046
0.208403112	2.937365862	10.24697581
0.150872697	6.109519391	18.76919561
0.230004012	2.332455129	8.763819095
0.131563801	6.965847941	22.63665856
0.176421708	3.933734725	13.46841294
0.200815136	3.110211949	10.80419311
0.122920297	9.260207581	27.26653439
0.217756461	2.309609437	9.135310473
0.180109024	4.246444702	13.69726563
0.180015326	3.727925163	12.90102002
0.178218964	4.432165052	13.96779262
0.22372113	2.697303231	9.281975517
0.206684584	3.403669187	10.96659449
0.178221188	3.592671515	12.85083273
0.259482806	1.387865411	6.503355705
0.168866126	4.086611822	14.21226874
0.150112967	6.79757762	19.76544767
0.150902842	5.752258068	18.21121315
0.18014613	4.559735388	14.04552493
0.174104844	5.274177275	15.55234043
0.201533306	3.655159087	11.57266122
0.148386797	6.071520784	18.91797266
0.155613877	5.00133058	16.64848159
0.110267972	14.15013117	37.20538655
0.197480336	2.59837134	10.42926829
0.209295838	3.050455585	10.51004127
0.153341529	4.835808155	16.79876638
0.263302587	1.762135751	6.817946729
0.195175897	3.274092693	11.43087873
0.223783162	2.868412754	9.54340836
0.186966872	3.235687119	11.74491393
0.19571229	3.472729022	11.55737705
0.185987268	4.177922725	13.12801403
0.175364896	3.76429431	13.39494834
0.250536798	2.244711068	7.81786134
0.166916862	4.810966003	15.32544379
0.216435262	2.61375768	9.429739198
0.255500416	1.786582487	7.065262533
0.217087766	2.238096164	8.927748227
0.184480676	3.144166142	11.8423913
0.193231952	3.318573125	11.62857143
0.178595135	3.49874451	12.75282051
0.212102292	2.575577534	9.571569839
0.163247075	4.78496816	15.67611336
0.197182227	3.130419926	11.03033981
0.204687487	4.381189105	12.46960237
0.147046451	7.237031688	20.77576444
0.197935165	3.691829595	11.78127341
0.184745725	4.479338334	13.65849024
0.18503399	3.826694069	12.70589007
0.176526198	3.457516424	13.01265823
0.152443399	5.603610958	17.78663905
0.162650787	5.562676427	16.80368098
0.186189824	4.155943322	13.06784
0.21719932	2.847271618	9.709578282
0.147603759	5.284733623	18.19806763
0.175579486	4.835278633	14.75365666
0.149491472	5.863983352	18.48717949
0.16608845	5.452684824	16.31461167
0.239432022	2.37244206	8.272244356
0.227403692	2.711452561	9.166719898
0.192485802	3.388207649	11.76453055
0.174846224	3.734678136	13.25792988
0.169128447	4.053360123	14.16283925
0.191015046	2.889372785	11.13942589
0.223066829	2.498691171	9.011115861
0.243395235	2.709054147	8.667169432
0.186256586	4.532160879	13.6459144
0.150671964	7.63862385	20.89232181
0.171528176	3.710991113	14.00813537
0.196681102	3.524465175	11.60926609
0.09250438	12.20150454	41.32767624
0.196269487	4.27645109	12.72662474
0.173941194	4.730891697	14.72866521
0.176631692	4.389202425	14.1079235
0.155723335	5.551525926	17.47703704
0.117348931	8.819684124	27.94018405
0.247691721	2.157412754	7.787573468
0.2160544	2.561501598	9.429480996
0.167542516	4.472924151	14.82181818
0.227517732	2.210447529	8.488051471
0.160878265	7.255214907	19.45821157
0.190956222	3.633784383	12.09265176
0.181134255	3.821908049	12.94842407
0.15526284	5.717509446	18.04316547
0.131106933	7.595895699	23.40526498

log.sigma.1.5.mm.3D_gldm_LargeDependenceLowGrayLevelEmphasis	log.sigma.1.5.mm.3D_gldm_SmallDependenceHighGrayLevelEmphasis	log.sigma.1.5.mm.3D_gldm_LargeDependenceHighGrayLevelEmphasis
0.009703687	445.2432577	8559.768143
0.053712884	102.7612018	6103.8
0.096411737	89.30283593	8137.9309
0.021838511	215.4852576	8537.839304
0.045839005	123.7081785	5745.694679
0.016336647	263.9679802	4314.030303
0.036642116	163.4828708	7419.875597
0.030520057	205.2396684	12702.05305
0.025480937	187.9241949	6460.591069
0.01915407	206.7235631	5914.094216
0.043531333	96.05716749	3948.869363
0.017479934	270.1964915	8589.290239
0.053952723	138.8062748	17524.96626
0.020773634	233.7794596	8184.711124
0.015756793	276.2185987	7422.649698
0.03285096	175.0255236	11144.94081
0.038567141	112.5783183	2488.892797
0.051488391	115.5436504	10453.85239
0.027577963	164.2496105	6954.353621
0.017553057	254.5844172	7217.319485
0.075551819	98.42152587	10074.44213
0.02889423	129.4022507	3297.458758
0.020987255	239.3184313	9672.117188
0.0269436	180.8454587	6540.451077
0.025593866	227.5754923	8557.123331
0.013867963	321.7616395	6879.894892
0.019315558	250.3670669	6751.270028
0.020939867	211.2859377	8374.812093
0.025830245	138.9641884	2053.116331
0.023988602	192.4433458	8873.309153
0.02919638	224.5777308	13909.11097
0.035211606	150.9603214	9860.057999
0.02512474	214.8749584	8400.165067
0.03050332	187.0112112	8651.41234
0.025735288	193.6971232	5713.160763
0.043199847	143.3999929	8698.937312
0.016664619	320.5753999	17335.4781
0.037050043	275.4189258	37868.48208
0.018638796	193.6323889	6384.417073
0.035400754	128.4566921	3560.128886
0.05420253	93.44700239	5635.27101
0.03024622	150.366994	2103.308766
0.021736762	197.7727158	6524.549411
0.016256312	266.9675303	6422.976688
0.017396132	261.0489344	8739.99687
0.029217392	168.3689366	5024.555191
0.025062004	211.7900707	7331.350723
0.03669197	121.5962006	5340.877153
0.025160826	177.9339617	3268.041128
0.031071068	180.6515976	7998.681953
0.015573726	276.7133935	6426.768782
0.014051167	253.9396883	4081.450311
0.022611352	160.5432201	3906.764184
0.036712406	126.176155	4184.674819
0.024260346	182.8032807	6106.256391
0.032563361	134.9563657	5489.997436
0.022257134	180.78008	4670.109518
0.023815668	215.7224709	10781.72672
0.017875301	233.6685313	7347.174757
0.019508669	274.9320867	8427.272538
0.039097421	175.1804879	11495.08834
0.012302018	378.1094066	11996.10262
0.018502002	284.83722	10582.67905
0.018797798	240.8529425	9050.291355
0.066333408	68.51989591	2860.724051
0.028773805	198.4149813	11459.59606
0.033014839	167.7413011	9060.087423
0.021041948	225.6371353	8623.2032
0.012208744	328.3071916	8219.381253
0.045788521	111.8601387	7596.479296
0.047545142	111.3830792	5143.6097
0.027107232	226.9076863	13124.34242
0.047870672	136.3166357	6257.236507
0.017555204	236.7108287	4714.740151
0.028589825	163.056195	3531.960396
0.034954675	128.7621249	4521.225037
0.032795836	149.7420104	5646.189482
0.033836236	146.7699602	6714.458246
0.023834764	172.7677026	5683.991213
0.015825413	271.879784	5820.347584
0.009837081	453.7299602	8664.384113
0.029762242	180.8432521	6937.2607
0.037507755	185.3774692	12204.33191
0.043141753	95.96684666	4846.913114
0.028556019	172.7968939	5456.794998
0.127733585	51.71776603	13698.27676
0.035546709	150.8819088	5258.350943
0.036401864	159.1289355	6453.055069
0.024717388	201.6526612	8592.455601
0.027958925	195.1043316	11439.74123
0.052968844	140.1765359	15088.46933
0.018291165	216.1309931	3774.943325
0.022219999	176.2689946	4456.211846
0.041433415	129.3116493	5821.192121
0.018469795	201.8330768	4367.649127
0.020524342	347.5349086	19209.41467
0.039483352	120.782156	4177.227547
0.021413442	228.3590176	9019.649475
0.07308201	68.54155845	4832.23741
0.043741742	153.9138326	12783.00554

log.sigma.1.5.mm.3D_gldm_SmallDependenceLowGrayLevelEmphasis	log.sigma.1.5.mm.3D_gldm_LowGrayLevelEmphasis	log.sigma.1.5.mm.3D_gldzm_DistanceZoneVariabilityNormalized
0.001136601	0.001735984	0.996330288
0.002991287	0.005097726	1
0.002505658	0.004953943	1
0.002171255	0.003204887	1
0.004216532	0.006953361	1
0.002877289	0.003933424	1
0.001332685	0.002840411	0.989071368
0.0009032	0.001973325	0.997691867
0.00179275	0.003087425	0.998937301
0.002207675	0.003376397	1
0.002743597	0.005148562	1
0.00193267	0.002834749	0.995901657
0.001798601	0.003169266	1
0.001885962	0.002995049	1
0.001511961	0.0024273	1
0.001171783	0.002385712	1
0.004471754	0.007212814	1
0.001705014	0.003453084	1
0.001635475	0.002997342	1
0.001423965	0.002404957	0.998176847
0.001251469	0.003327243	1
0.003200913	0.005096212	1
0.001651547	0.00261728	1
0.001353152	0.002703074	0.996078447
0.002724565	0.00382486	1
0.001184297	0.002044755	1
0.001395438	0.002443227	1
0.001266318	0.002319841	1
0.004205165	0.006298021	1
0.001290552	0.002431682	1
0.000994998	0.00200807	1
0.001448956	0.002803577	0.990977629
0.001280827	0.002443085	0.998457981
0.001979436	0.003294918	1
0.001805296	0.003180664	0.994791702
0.001498474	0.003110737	1
0.000721584	0.001410139	1
0.000537402	0.001265366	0.997206709
0.00181204	0.002933926	1
0.002093245	0.004301864	0.996782505
0.002274718	0.004685477	1
0.004250567	0.006659148	0.998067635
0.001365017	0.00260945	1
0.001684969	0.002711848	1
0.001720334	0.00260682	1
0.001758348	0.00332376	1
0.001273082	0.00251513	0.998931624
0.0027469	0.004598296	1
0.004995821	0.006642989	1
0.001636297	0.002946334	1
0.001707567	0.002649863	0.9982684
0.001541311	0.002655231	0.998847263
0.001901787	0.003440118	1
0.002854869	0.004767601	1
0.002009558	0.00330929	1
0.002747308	0.004390003	1
0.001903541	0.00330985	0.996657391
0.000944469	0.001996742	1
0.001502301	0.002497214	1
0.000918378	0.001873842	0.997045069
0.001536222	0.002797169	1
0.000941505	0.001555227	1
0.000849268	0.001703692	0.991521985
0.000901844	0.001877084	0.98326759
0.004241707	0.007726577	1
0.001041752	0.002171263	0.992372347
0.001921423	0.003234161	1
0.001223638	0.002256978	0.992063618
0.000880761	0.001628299	1
0.001761139	0.003588382	1
0.004044071	0.006207334	1
0.000979599	0.001980667	0.991780962
0.00290978	0.005010873	1
0.002198345	0.003391823	1
0.0025697	0.004484626	0.994691478
0.002754173	0.00472169	1
0.00161114	0.003232272	1
0.004037951	0.005494521	1
0.001880704	0.003248359	0.996960493
0.001469018	0.002517418	1
0.001167619	0.001754703	1
0.002368806	0.003751822	1
0.00142839	0.002647496	1
0.001538782	0.003801669	1
0.002441292	0.004058374	0.998082456
0.001605564	0.004279584	1
0.00288325	0.004675924	1
0.002029349	0.003643766	1
0.001604017	0.002726447	1
0.001227145	0.002322227	1
0.001279398	0.00267976	0.988024387
0.001899821	0.003180709	0.994979111
0.001772299	0.003229135	0.993013186
0.002645853	0.004559523	1
0.001517056	0.002824818	0.999009901
0.000839348	0.00152456	0.995356062
0.002765202	0.004939574	1
0.002772276	0.003774009	1
0.003404307	0.006572267	1
0.000892657	0.002220421	0.997660822

log.sigma.1.5.mm.3D_gldzm_LowIntensityEmphasis	log.sigma.1.5.mm.3D_gldzm_LargeDistanceEmphasis	log.sigma.1.5.mm.3D_gldzm_HighIntensitySmallDistanceEmphasis
0.002361126	1.005514706	983.6410846
0.009683171	1	361.8255034
0.008298911	1	323.4772727
0.005522221	1	596.7443609
0.011903985	1	352.9235737
0.006059593	1	603.2676923
0.003738896	1.016483516	477.1964286
0.002798245	1.003466205	659.9718371
0.004591342	1.001594896	507.9080276
0.005509198	1	563.6371681
0.008062337	1	296.1727273
0.004760445	1.006160164	714.2209959
0.007583763	1	593.3088889
0.005040914	1	658.2681481
0.003655616	1	715.5684695
0.003710155	1	578.6445825
0.010625849	1	287.4677716
0.006528049	1	447.4866469
0.004620351	1	489.5505155
0.003555555	1.002737226	672.9402372
0.004820376	1	382.1197098
0.007773375	1	348.6575053
0.004411497	1	689.074813
0.003760443	1.00589391	527.0994597
0.00722134	1	649.6369168
0.00269722	1	781.8397493
0.003327359	1	630.8044229
0.003591587	1	631.5411765
0.008896835	1	326.2863436
0.003809377	1	587.258597
0.003056459	1	716.409957
0.004686515	1.013595166	499.1412387
0.00343491	1.002314815	606.9955633
0.005513191	1	558.1762065
0.004456671	1.007832898	506.8793734
0.00472409	1	472.740519
0.002241015	1	1026.1668
0.001999824	1.004195804	1058.188811
0.004767095	1	561.0121704
0.00546076	1.004834032	348.0182082
0.007561932	1	321.9260143
0.008690218	1.002901354	324.7768375
0.003639691	1	553.5712366
0.003847732	1	643.5607477
0.004469629	1	741.1237721
0.004458627	1	443.7674419
0.0033587	1.001603421	572.9985302
0.008038104	1	373.3022508
0.010044592	1	400.9483146
0.004706971	1	543.7659138
0.004000639	1.002599653	695.1553293
0.003313746	1.001730104	571.6326413
0.004694364	1	417.1461303
0.007787957	1	369.6043165
0.005270642	1	522.8579439
0.007740273	1	409.8036212
0.004629298	1.005022321	470.664202
0.002866446	1	673.4740741
0.003779228	1	628.6868687
0.002267259	1.004438964	698.7334772
0.004612659	1	556.4486134
0.002306169	1	988.0918635
0.002240555	1.012771392	784.9650915
0.002499327	1.025312184	685.1731353
0.012417819	1	217.8450704
0.003243916	1.011485452	630.5457504
0.005588645	1	514.8329764
0.003235427	1.011952191	623.7824701
0.0020799	1	809.8237918
0.006229868	1	410.7649824
0.011014501	1	328.4071856
0.003019465	1.012379642	727.8739684
0.008311265	1	400.8495887
0.004706422	1	536.6
0.005874125	1.007984032	389.0883234
0.007392918	1	366.2761194
0.004628934	1	446.3183908
0.01132829	1	442.6282421
0.005144509	1.00456621	498.2104262
0.003374066	1	652.6696833
0.002397445	1	991.8149951
0.005906917	1	478.575
0.004386921	1	598.7037702
0.00517786	1	331.0957129
0.006150128	1.002879079	462.2351248
0.009229826	1	292.4768683
0.007235268	1	403.4274432
0.005546743	1	459.2836946
0.004561729	1	600.7940075
0.003792348	1	626.8833819
0.005083076	1.018072289	575.1792169
0.004125335	1.007550336	499.2160235
0.004366284	1.01051709	453.0173094
0.007686939	1	392.3498322
0.003579597	1.001485884	498.4052749
0.002331767	1.006982157	1013.115981
0.007393527	1	333.2768412
0.007265376	1	657.9755501
0.011471188	1	239.3880597
0.003133598	1.003512881	564.8871487

log.sigma.1.5.mm.3D_gldzm_LowIntensityLargeDistanceEmphasis	log.sigma.1.5.mm.3D_gldzm_HighIntensityEmphasis	log.sigma.1.5.mm.3D_gldzm_DistanceZoneVariability	log.sigma.1.5.mm.3D_gldzm_ZonePercentage
0.002375021	984.9549632	1084.007353	0.512723845
0.009683171	361.8255034	298	0.318716578
0.008298911	323.4772727	484	0.315514993
0.005522221	596.7443609	399	0.40839304
0.011903985	352.9235737	929	0.392314189
0.006059593	603.2676923	325	0.492424242
0.003796394	478.8159341	2160.131868	0.386480269
0.002807076	660.4471404	1727.004622	0.338880188
0.0045929	508.3163211	1879.001063	0.413770348
0.005509198	563.6371681	452	0.421641791
0.008062337	296.1727273	550	0.364721485
0.004884029	714.3110883	970.0082136	0.422559653
0.007583763	593.3088889	450	0.241028388
0.005040914	658.2681481	675	0.393127548
0.003655616	715.5684695	869	0.438004032
0.003710155	578.6445825	1449	0.331124314
0.010625849	287.4677716	543	0.454773869
0.006528049	447.4866469	337	0.273317113
0.004620351	489.5505155	485	0.373651772
0.003644515	673.082573	2188.00365	0.421619542
0.004820376	382.1197098	827	0.273478836
0.007773375	348.6575053	473	0.43836886
0.004411497	689.074813	401	0.391601563
0.003778983	527.8634578	1014.007859	0.384586324
0.00722134	649.6369168	493	0.387274156
0.00269722	781.8397493	1117	0.471506965
0.003327359	630.8044229	1447	0.447571915
0.003591587	631.5411765	1020	0.36929761
0.008896835	326.2863436	227	0.507829978
0.003809377	587.258597	727	0.353943525
0.003056459	716.409957	1627	0.341950399
0.004715124	500.9380665	656.0271903	0.319961334
0.00345404	607.0655864	1294.001543	0.401362651
0.005513191	558.1762065	891	0.379148936
0.004494914	507.4715405	1905.02611	0.434831971
0.00472409	472.740519	1002	0.33411137
0.002241015	1026.1668	1247	0.335124966
0.00200863	1058.830769	1426.005594	0.277078086
0.004767095	561.0121704	493	0.400813008
0.005535331	348.3744763	3093.016113	0.426822558
0.007561932	321.9260143	838	0.3230532
0.008694685	325.2736944	2064.003868	0.534781484
0.003639691	553.5712366	1488	0.407336436
0.003847732	643.5607477	1177	0.47307074
0.004469629	741.1237721	509	0.39827856
0.004458627	443.7674419	1161	0.42295082
0.003364248	573.1143773	1869.001069	0.410127137
0.008038104	373.3022508	311	0.35706085
0.010044592	400.9483146	445	0.522914219
0.004706971	543.7659138	487	0.360207101
0.004006534	695.4419411	1152.001733	0.449202024
0.003315668	572.0219146	1732.001153	0.514387422
0.004694364	417.1461303	1964	0.435283688
0.007787957	369.6043165	417	0.377717391
0.005270642	522.8579439	535	0.402255639
0.007740273	409.8036212	718	0.368205128
0.00464383	471.1668527	1786.010045	0.443016069
0.002866446	673.4740741	1215	0.351359167
0.003779228	628.6868687	693	0.420509709
0.002276515	699.329963	4043.017756	0.444188849
0.004612659	556.4486134	613	0.347112118
0.002306169	988.0918635	1143	0.428089888
0.002261984	787.1260111	2329.085143	0.405769563
0.002572703	688.483969	2913.42187	0.388692116
0.012417819	217.8450704	284	0.359493671
0.005695449	631.9257274	1296.038285	0.338166753
0.005588645	514.8329764	467	0.358128834
0.003263969	625.2047809	2490.079681	0.4016
0.0020799	809.8237918	2690	0.459279495
0.006229868	410.7649824	851	0.293650794
0.011014501	328.4071856	501	0.385681293
0.003046179	729.4786795	1442.049519	0.338927739
0.008311265	400.8495887	851	0.37340939
0.004706422	536.6	1135	0.502434706
0.005929298	389.4271457	1495.021291	0.480038326
0.007392918	366.2761194	536	0.399403875
0.004628934	446.3183908	870	0.363105175
0.01132829	442.6282421	347	0.362212944
0.005151815	498.9238965	655.0030441	0.384885764
0.003374066	652.6696833	1105	0.472424113
0.002397445	991.8149951	1027	0.516339869
0.005906917	478.575	760	0.422456921
0.004386921	598.7037702	1114	0.340776996
0.00517786	331.0957129	1003	0.326391149
0.006161375	462.4193858	1040.001919	0.427224272
0.009229826	292.4768683	281	0.183420366
0.007235268	403.4274432	1013	0.424737945
0.005546743	459.2836946	1061	0.386943837
0.004561729	600.7940075	534	0.364754098
0.003792348	626.8833819	686	0.338765432
0.005128597	576.9909639	328.0240964	0.254601227
0.004142071	500.2944631	1186.015101	0.500419815
0.004404895	454.130149	2266.056091	0.440285549
0.007686939	392.3498322	1192	0.361212121
0.003581635	498.6760773	2017.000991	0.463924632
0.00234312	1014.327386	1283.013964	0.376680304
0.007393527	333.2768412	1127	0.400070998
0.007265376	657.9755501	409	0.390639924
0.011471188	239.3880597	268	0.321342926
0.003141428	565.3495316	1704.004684	0.295808798

log.sigma.1.5.mm.3D_gldzm_IntensityVariabilityNormalized	log.sigma.1.5.mm.3D_gldzm_LowIntensitySmallDistanceEmphasis	log.sigma.1.5.mm.3D_gldzm_IntensityVariability
0.041399154	0.002357652	45.04227941
0.065582631	0.009683171	19.54362416
0.056118093	0.008298911	27.16115702
0.047229603	0.005522221	18.84461153
0.051576924	0.011903985	47.91496233
0.045604734	0.006059593	14.82153846
0.052747924	0.003724522	115.2014652
0.050140487	0.002796037	86.79318313
0.049037622	0.004590953	92.23976608
0.047762158	0.005509198	21.58849558
0.061897521	0.008062337	34.04363636
0.045556122	0.004729549	44.37166324
0.055525926	0.007583763	24.98666667
0.045249931	0.005040914	30.5437037
0.043064989	0.003655616	37.42347526
0.054644192	0.003710155	79.17943409
0.051154998	0.010625849	27.7771639
0.061293135	0.006528049	20.65578635
0.062608141	0.004620351	30.36494845
0.047023599	0.003533315	103.0757299
0.062665862	0.004820376	51.82466747
0.058208815	0.007773375	27.53276956
0.055839205	0.004411497	22.3915212
0.053074135	0.003755808	54.02946955
0.049311044	0.00722134	24.31034483
0.043607822	0.00269722	48.70993733
0.045011269	0.003327359	65.13130615
0.051043829	0.003591587	52.06470588
0.059519882	0.008896835	13.51101322
0.057251352	0.003809377	41.62173315
0.050474684	0.003056459	82.122311
0.0582324	0.004679362	38.54984894
0.044705456	0.003430127	57.9382716
0.046451546	0.005513191	41.38832772
0.046037263	0.004447111	88.1613577
0.057212919	0.00472409	57.32734531
0.04989103	0.002241015	62.21411387
0.050481686	0.001997622	72.18881119
0.061316854	0.004767095	30.22920892
0.051305334	0.005442117	159.2004512
0.057538405	0.007561932	48.21718377
0.046272013	0.008689101	95.69052224
0.051401354	0.003639691	76.48521505
0.044180841	0.003847732	52.00084962
0.051408633	0.004469629	26.16699411
0.050043734	0.004458627	58.10077519
0.049059872	0.003357313	91.79102084
0.061796301	0.008038104	19.21864952
0.047059715	0.010044592	20.94157303
0.054319915	0.004706971	26.45379877
0.045521718	0.003999166	52.53206239
0.045848749	0.003313265	79.5017301
0.050222747	0.004694364	98.63747454
0.057410187	0.007787957	23.94004796
0.051718054	0.005270642	27.66915888
0.056005152	0.007740273	40.21169916
0.048803835	0.004625665	87.45647321
0.054304053	0.002866446	65.97942387
0.049397292	0.003779228	34.23232323
0.046888878	0.002264945	190.134402
0.051571577	0.004612659	31.61337684
0.047433157	0.002306169	54.21609799
0.047189995	0.002235197	110.8492976
0.048350286	0.002480983	143.2618967
0.068761159	0.012417819	19.52816901
0.052598561	0.002631032	68.69372129
0.05626602	0.005588645	26.27623126
0.048565578	0.003228291	121.8996016
0.050826827	0.0020799	136.7241636
0.065491486	0.006229868	55.73325499
0.054880259	0.011014501	27.49500998
0.051217625	0.003012786	74.47042641
0.047656659	0.008311265	40.55581669
0.043842496	0.004706422	49.76123348
0.046184411	0.005860332	69.41516966
0.055232234	0.007392918	29.60447761
0.05643546	0.004628934	49.09885057
0.054937754	0.01132829	19.06340058
0.058860324	0.005142682	38.67123288
0.04573944	0.003374066	50.54208145
0.042199465	0.002397445	43.33885102
0.045003463	0.005906917	34.20263158
0.052030788	0.004386921	57.96229803
0.065473569	0.00517786	65.66999003
0.046899695	0.006147317	48.86948177
0.085801852	0.009229826	24.11032028
0.046521577	0.007235268	47.12635735
0.049140951	0.005546743	52.13854854
0.054545582	0.004561729	29.12734082
0.058436536	0.003792348	40.08746356
0.071527072	0.005071696	23.74698795
0.04994989	0.004121151	59.54026846
0.047382979	0.004356631	108.1279579
0.054748378	0.007686939	65.26006711
0.051150769	0.003579088	103.2734027
0.045076168	0.002328928	58.10318076
0.052600169	0.007393527	59.28039042
0.050448048	0.007265376	20.63325183
0.068918467	0.011471188	18.47014925
0.058604912	0.00313164	100.0971897

log.sigma.1.5.mm.3D_gldzm_HighIntensityLargeDistanceEmphasis	log.sigma.1.5.mm.3D_gldzm_SmallDistanceEmphasis	log.sigma.1.5.mm.3D_glcm_SumVariance	log.sigma.1.5.mm.3D_glcm_Homogeneity1
990.2104779	0.998621324	3305.375197	0.276086466
361.8255034	1	1102.505032	0.394961978
323.4772727	1	962.9099992	0.41716822
596.7443609	1	2044.691012	0.337012179
352.9235737	1	1166.218857	0.335960606
603.2676923	1	1795.426664	0.289442075
485.293956	0.995879121	1465.610174	0.356680206
662.3483536	0.999133449	2166.472536	0.38270044
509.9494949	0.999601276	1632.660712	0.328862358
563.6371681	1	1824.12472	0.321053657
296.1727273	1	946.260056	0.359568854
714.6714579	0.998459959	2390.383996	0.315506873
593.3088889	1	1947.51525	0.446968663
658.2681481	1	2138.553734	0.328787968
715.5684695	1	2321.983221	0.309236001
578.6445825	1	1935.13783	0.383309752
287.4677716	1	821.8091787	0.304003552
447.4866469	1	1483.589234	0.411948198
489.5505155	1	1612.926718	0.358986119
673.6519161	0.999315693	2158.890049	0.318058136
382.1197098	1	1194.887123	0.429910919
348.6575053	1	1083.351858	0.321368379
689.074813	1	2334.432086	0.354452697
530.9194499	0.998526523	1644.879241	0.351414573
649.6369168	1	2045.705315	0.352409283
781.8397493	1	2400.095613	0.299092
630.8044229	1	1984.838061	0.313071943
631.5411765	1	2118.202149	0.350230594
326.2863436	1	955.4351784	0.292133133
587.258597	1	1967.501739	0.368109895
716.409957	1	2337.841039	0.371463686
508.1253776	0.996601208	1705.950558	0.378907574
607.345679	0.999421296	1938.051247	0.332166386
558.1762065	1	1776.880196	0.349033538
509.8402089	0.998041775	1521.931709	0.321682305
472.740519	1	1476.88141	0.390129828
1026.1668	1	3564.847141	0.365361713
1061.398601	0.998951049	3591.998644	0.441087256
561.0121704	1	1905.24828	0.332672176
349.7995488	0.998791492	1019.72214	0.309110743
321.9260143	1	1023.865209	0.37468787
327.2611219	0.999274662	903.1857871	0.264345732
553.5712366	1	1798.226959	0.333904343
643.5607477	1	2108.96072	0.296111431
741.1237721	1	2430.576067	0.342990865
443.7674419	1	1355.230191	0.33822724
573.5777659	0.999599145	1797.51895	0.341555878
373.3022508	1	1204.308407	0.357068861
400.9483146	1	1194.475142	0.282916449
543.7659138	1	1672.48494	0.36909706
696.5883882	0.999350087	2215.382244	0.301700791
573.5790081	0.999567474	1790.651772	0.274614388
417.1461303	1	1294.844824	0.305863852
369.6043165	1	1111.186942	0.347261882
522.8579439	1	1662.998718	0.336903597
409.8036212	1	1302.490363	0.346138692
473.1774554	0.99874442	1471.643392	0.313105309
673.4740741	1	2267.695075	0.360175659
628.6868687	1	2115.955639	0.325729551
701.7159063	0.998890259	2277.399686	0.309207235
556.4486134	1	1797.603636	0.394300965
988.0918635	1	3428.328024	0.324135573
795.7696892	0.996807152	2589.548526	0.335942609
701.7273034	0.993671954	2277.820752	0.327299535
217.8450704	1	622.2086256	0.379865144
637.4456355	0.997128637	2096.746262	0.37552877
514.8329764	1	1717.708122	0.366615475
630.8940239	0.997011952	2108.426052	0.330594208
809.8237918	1	2740.28413	0.320269959
410.7649824	1	1332.739312	0.392991294
328.4071856	1	1047.732944	0.348864873
735.8975241	0.996905089	2385.355403	0.378320394
400.8495887	1	1180.216231	0.355469606
536.6	1	1717.558955	0.278679024
390.7824351	0.998003992	1135.450443	0.28628373
366.2761194	1	1147.064196	0.335883307
446.3183908	1	1345.050498	0.366875925
442.6282421	1	1440.00762	0.35584724
501.7777778	0.998858447	1603.001055	0.3434035
652.6696833	1	2077.416015	0.290711189
991.8149951	1	3318.661015	0.283776112
478.575	1	1532.872823	0.335780256
598.7037702	1	1962.640937	0.385914126
331.0957129	1	1052.747396	0.369765633
463.1564299	0.99928023	1429.343094	0.317402769
292.4768683	1	1048.029979	0.510299829
403.4274432	1	1237.017551	0.318541681
459.2836946	1	1373.347359	0.351325787
600.7940075	1	1962.289733	0.360870478
626.8833819	1	2135.439979	0.384932558
584.2379518	0.995481928	1823.569691	0.451602419
504.6082215	0.998112416	1521.669541	0.290205269
458.5815074	0.997370727	1404.805472	0.308119815
392.3498322	1	1201.125194	0.360257894
499.7592868	0.999628529	1561.459783	0.298721236
1019.173002	0.998254461	3382.837576	0.362931056
333.2768412	1	1027.750865	0.333587459
657.9755501	1	2232.820652	0.339592612
239.3880597	1	772.9652937	0.39904781
567.1990632	0.99912178	1821.001207	0.418951716

log.sigma.1.5.mm.3D_glcm_Homogeneity2	log.sigma.1.5.mm.3D_glcm_ClusterShade	log.sigma.1.5.mm.3D_glcm_MaximumProbability	log.sigma.1.5.mm.3D_glcm_Idmn	log.sigma.1.5.mm.3D_glcm_SumVariance2
0.191376438	-457.1604305	0.016181708	0.972978013	75.13600384
0.317214954	-28.22630234	0.053569385	0.980562114	25.51982588
0.345362493	-4.552593297	0.086099228	0.981290181	25.65923587
0.257294074	-352.4515438	0.030480851	0.96892527	53.95661861
0.252000857	-177.0583062	0.038881382	0.969281704	38.19664344
0.199867157	-40.49673669	0.018673868	0.976324129	64.05077267
0.275120454	-14.03005029	0.042342918	0.983802674	34.83255971
0.306951594	-108.5781564	0.052560743	0.985077645	36.79950517
0.24499791	-181.1034816	0.023396269	0.979100967	44.79358227
0.234409514	-217.1992417	0.021790406	0.975178538	47.13553917
0.273865809	-77.79406653	0.027909474	0.976496295	27.04130503
0.23030576	-412.2012153	0.023902945	0.975017037	56.59880689
0.383853397	-92.94269483	0.098794946	0.985787077	30.97103081
0.245158109	-335.9265935	0.027338662	0.976438579	55.76244581
0.225457177	-334.1322565	0.025061148	0.979507247	57.76743286
0.306856235	-142.5308137	0.051865605	0.985005099	36.24592672
0.21327277	-42.9440984	0.017572785	0.965532425	41.63750665
0.338103759	-53.79616755	0.065942151	0.984068701	26.18068949
0.273970754	-12.18988492	0.039498798	0.98567246	25.48819522
0.230901224	-173.5905622	0.022539757	0.982349903	46.6416107
0.358889274	-4.494216246	0.074397129	0.985282343	19.46461248
0.231273763	-91.84668971	0.021897051	0.970787861	30.41810327
0.272060117	-126.5639108	0.03588244	0.982698887	34.97747971
0.267318846	-29.38487623	0.028033976	0.978301178	33.16373563
0.271100523	-135.3550796	0.038534518	0.980079864	44.46559823
0.21140583	-19.11801393	0.022644151	0.980549638	59.50117613
0.228188327	-124.4922183	0.023529335	0.975749469	55.47898513
0.267087684	-224.7679393	0.029808346	0.980476644	40.29063181
0.198347763	-41.75373213	0.018993909	0.966532204	34.18676691
0.286534366	-144.1450751	0.037735689	0.982529647	31.18419628
0.29286402	-146.9742854	0.048552961	0.987970954	37.75160024
0.302429614	-173.9983949	0.047679787	0.978071286	31.64035275
0.249428458	-115.4380371	0.026256696	0.981668407	52.89958316
0.270172896	-293.1190393	0.044334079	0.978236955	53.25871431
0.237260694	-93.33872349	0.026276235	0.980253895	52.07700508
0.313431204	-58.39029367	0.056022372	0.987205286	32.58824409
0.286576951	-236.7310413	0.039908018	0.987634663	41.90054039
0.377698109	-153.5639014	0.096421722	0.990182604	37.05934026
0.24478671	-178.1614825	0.027622664	0.974097979	31.01649345
0.219415479	-36.33388698	0.017650129	0.974508712	38.25944731
0.295469392	-107.0055676	0.041888221	0.976890977	29.36198583
0.17332683	-13.45859025	0.01005239	0.963633628	54.37031459
0.249697334	-152.5778865	0.026042165	0.979977456	43.25774435
0.210504297	-393.7364198	0.019462667	0.97017932	61.14969223
0.258863839	-250.8702558	0.026897065	0.979696596	45.09518423
0.254097399	-20.59543651	0.029349021	0.981769372	40.62256341
0.258995138	-60.34726277	0.03172038	0.981674445	43.66498769
0.271002312	-84.08450887	0.034167085	0.98009527	24.64787386
0.194687285	-210.4034958	0.020506826	0.963178681	55.61400127
0.288677723	-17.37385919	0.040457974	0.979962937	32.95829502
0.212678475	-197.9616248	0.024936039	0.981957036	51.33596601
0.184471693	-105.3448907	0.012575832	0.974232921	58.80609525
0.215885032	-70.71978789	0.017973795	0.976482434	38.35840662
0.260405206	5.624670776	0.031616943	0.979478398	28.41419089
0.252703961	-25.06319228	0.031325314	0.977579415	42.27561051
0.259541096	-139.8663732	0.028404317	0.976619303	30.49940045
0.226260612	-134.2558633	0.020242375	0.9757977	44.16084694
0.278989107	-185.2428213	0.037370335	0.984357479	36.17231562
0.239885442	-208.4730752	0.025111152	0.981063712	41.72340317
0.225303954	-189.8354633	0.024769509	0.978193857	49.96843335
0.321984207	-42.71305565	0.066413504	0.982249667	37.30086907
0.240699178	-289.7431165	0.027340944	0.98464804	50.28148548
0.253080075	-140.2955592	0.030505391	0.982317079	44.38928129
0.240908608	-135.7378031	0.03118909	0.985230703	40.26748597
0.297014935	-39.03700455	0.038570314	0.973207541	21.14049371
0.297893223	-187.7934431	0.044719141	0.983909108	36.65965883
0.288505863	-180.9405459	0.047134114	0.9771168	33.18803384
0.246565464	-174.0656652	0.026582264	0.982097776	43.78428325
0.233805243	-177.3819409	0.022400453	0.985851726	45.5518998
0.313243777	-70.9470933	0.056306164	0.982423372	20.91581281
0.265871842	-132.4766078	0.036460359	0.972711607	33.69017613
0.300397837	-132.7244725	0.045649947	0.986241978	37.23851211
0.27599545	-83.97751179	0.038903718	0.976208361	40.19870439
0.191991071	-289.0280128	0.015047571	0.972621693	66.62097517
0.196756409	-46.12617841	0.016619279	0.969107603	47.34586699
0.250643817	-188.1056951	0.028013468	0.976306787	34.82173806
0.283854906	38.58455235	0.033252096	0.986676574	28.93316822
0.27336633	-155.8416277	0.043105799	0.975082901	33.08788351
0.256409555	-101.6659569	0.025147147	0.980171514	30.87913306
0.203443405	-266.0738268	0.017631019	0.977108759	57.67382843
0.199225019	-431.8951033	0.017526879	0.974373897	72.35681165
0.257013185	-207.2251216	0.02928964	0.974585083	50.19239392
0.311339907	-98.29878307	0.048092395	0.98344833	36.59765012
0.284107251	-46.7881156	0.030699514	0.97740812	22.94922513
0.234156712	-171.0873188	0.023200873	0.971462035	50.25734195
0.453174022	-42.12381294	0.131492911	0.987842625	11.96553318
0.235528626	-179.8554389	0.028312672	0.972891418	49.45886084
0.268454503	-73.05412162	0.032751348	0.974078398	36.74086018
0.279955922	-202.6373139	0.037902809	0.978253622	37.34744394
0.307551438	-174.8741718	0.050093018	0.98167624	31.89452247
0.38323092	30.27137096	0.090292191	0.990185924	18.91192001
0.19966432	-49.87820959	0.015445243	0.97442384	48.06875542
0.221177589	-129.2627537	0.017910308	0.977075005	48.12615969
0.277460926	-118.8538736	0.035576196	0.977864949	32.58838442
0.2091977	-106.0057798	0.015437385	0.977776325	45.36610004
0.28811736	-348.8464352	0.055867732	0.983090055	56.88063959
0.246351861	-95.87504388	0.026996378	0.974305645	33.17267749
0.258423992	-312.2217703	0.03692169	0.97395462	49.3220037
0.32189323	-100.2479189	0.053294858	0.97066037	21.85986896
0.347616998	2.440156887	0.070724745	0.985927083	26.83766139

log.sigma.1.5.mm.3D_glcm_Contrast	log.sigma.1.5.mm.3D_glcm_DifferenceEntropy	log.sigma.1.5.mm.3D_glcm_InverseVariance	log.sigma.1.5.mm.3D_glcm_Entropy	log.sigma.1.5.mm.3D_glcm_Dissimilarity
72.88562122	4.108846896	0.195247378	8.558162666	6.161183354
22.46046364	3.242393663	0.310911504	6.727162656	3.228229243
21.2355621	3.234118874	0.312554912	6.85978963	3.134188604
47.36764215	3.72941321	0.259843435	7.757312054	4.701167197
36.29480992	3.635078625	0.251027703	7.650547914	4.302404236
50.70687955	3.828295348	0.205158904	7.786591959	5.218213663
29.40978762	3.508299964	0.270794255	7.677902072	3.873607143
31.61972532	3.466238191	0.296138621	7.370009359	3.749418803
40.68081384	3.709275232	0.246332737	8.016838172	4.53079028
42.50934124	3.708821239	0.233614514	7.708235702	4.632431349
23.59227265	3.356980048	0.272998771	7.286756412	3.533970413
52.9000916	3.834358786	0.229073971	7.998404318	5.017669659
25.79573511	3.155411565	0.35476022	6.46689396	3.052704727
47.77798612	3.745764518	0.245102552	7.881007145	4.711426182
59.18942404	3.881910068	0.237819674	8.027053608	5.281325864
30.58948625	3.428086555	0.301477683	7.380361073	3.662614447
37.97365079	3.687084211	0.216221369	7.947591266	4.615672761
21.65969453	3.196611685	0.324910368	6.683502975	3.09643882
23.2777039	3.352997003	0.273824003	7.166380033	3.541868714
40.86489039	3.710262678	0.238158589	8.003623523	4.562680418
16.45924767	3.062873796	0.34404373	6.631778433	2.758434163
29.46312758	3.520026855	0.242860716	7.509128609	4.090302076
32.27722514	3.492444857	0.275104843	7.323013008	3.927209071
29.70927372	3.497670283	0.265262698	7.609851162	3.878958951
38.22692863	3.56270248	0.271648741	7.432280381	4.1533935
49.00831971	3.83939303	0.218451715	8.22949676	5.065242698
48.10261632	3.812049632	0.234111731	8.257251038	4.916385625
35.22557512	3.542938226	0.273131093	7.589992494	4.044857112
33.52548777	3.589954732	0.21036025	7.450116737	4.533029308
27.93063708	3.413462321	0.285020909	7.225120934	3.654465533
34.04827196	3.498397309	0.288632153	7.408413046	3.859677213
32.39869387	3.452438717	0.300202097	7.215365942	3.779506392
44.55400892	3.745646956	0.243112574	8.116755045	4.644607644
45.58435041	3.698762843	0.26526765	7.844430874	4.521447098
43.9600069	3.766601867	0.237259872	8.15077682	4.712123938
27.01478915	3.350025892	0.306784714	7.174221437	3.458468274
43.12561904	3.555855891	0.289801194	7.494807652	4.135167233
31.1498517	3.308339077	0.327699715	6.987452193	3.362949968
34.65041364	3.554943892	0.249872362	7.391938504	4.155195411
36.46964068	3.690349355	0.220405937	8.137747204	4.534192758
26.66534078	3.386558419	0.297411332	7.285622144	3.581205067
53.1241188	3.953785344	0.178057533	8.633380262	5.638129558
37.1558067	3.639520641	0.2556098	7.883504442	4.320848219
60.79529839	3.958261096	0.215123092	8.285965662	5.493823611
39.02168691	3.578461416	0.265243801	7.508532693	4.217269546
35.36981228	3.596574151	0.261270631	7.799886147	4.198701596
37.26095269	3.63073676	0.262261316	7.845281092	4.267971119
25.55279129	3.366423388	0.273763564	6.883955894	3.605770704
55.46753688	3.912406854	0.202102928	7.952316237	5.481750009
25.58429488	3.388446133	0.288433778	7.202857207	3.591568532
45.44999343	3.778913557	0.223900429	8.02724408	4.862112379
52.51159292	3.929483257	0.189747376	8.55023967	5.478916731
37.27959082	3.694547483	0.22390968	8.008031812	4.570022322
26.19771086	3.395320655	0.272962268	7.250001873	3.706043903
34.0995253	3.583657014	0.25752571	7.580622253	4.191716873
28.7435023	3.455738411	0.26727531	7.348920644	3.822846013
40.92087254	3.735880775	0.233276072	8.113731817	4.660959466
33.23695968	3.517978958	0.278367825	7.552379538	3.929299691
40.33408929	3.680628305	0.242835596	7.700164115	4.49532487
48.64886873	3.859839008	0.226133069	8.388060433	5.038611465
31.75291743	3.423653859	0.309486381	7.104182935	3.683479286
49.35651643	3.771226788	0.247481644	7.947638792	4.782593232
39.16867274	3.673909243	0.255488951	7.890492184	4.399527101
38.48834919	3.6628942	0.245599627	7.846486203	4.386387865
17.16867445	3.147355578	0.30557248	6.842856776	3.081301718
34.82632584	3.492060997	0.291731808	7.409357854	3.860781947
36.29487079	3.514149525	0.291437031	7.189506305	3.987693613
41.52296193	3.704875936	0.249552421	7.986066076	4.511680634
41.11226787	3.71310983	0.240218937	8.052295398	4.563628744
20.17447079	3.18352961	0.318009368	6.814845047	3.088661432
31.78879126	3.525207448	0.266263187	7.443254838	4.006484495
32.22029378	3.436328765	0.299185365	7.312376018	3.713349615
36.85379269	3.595449897	0.272880153	7.659843377	4.17148859
61.92893454	4.011706802	0.198594035	8.553385661	5.771967695
47.35101346	3.863614274	0.198096553	8.348650182	5.197722696
34.4071377	3.575905235	0.257803209	7.457144813	4.182998116
23.93699761	3.349217819	0.288659641	7.221824794	3.492663519
33.42265949	3.498340022	0.272590498	7.151256803	3.955920044
28.49457916	3.465668923	0.261245453	7.417406489	3.846933698
56.27059623	3.925553148	0.210810019	8.335082407	5.395583538
69.13925239	4.059939989	0.203075397	8.440060547	5.941458172
51.10011644	3.782456489	0.258768404	7.869655316	4.858745001
34.13768721	3.477569355	0.29747034	7.39397718	3.811832514
19.62909647	3.246783832	0.284235234	7.244043429	3.262000781
49.37605349	3.818897598	0.235381993	8.126905417	4.971685057
9.170902247	2.620850119	0.393483291	5.677642549	1.938980963
48.96617201	3.817548139	0.235119524	8.130196964	4.957882458
32.46768758	3.526634549	0.272452907	7.552986636	3.972302819
33.95448048	3.509800708	0.277136463	7.338812102	3.952028871
28.4572143	3.368371641	0.304053932	7.134200597	3.540634678
12.83687295	2.904983342	0.35748941	6.178560538	2.447083102
42.79485106	3.79358295	0.203726258	8.242790995	4.959908423
44.77527451	3.788090294	0.229557432	8.214506805	4.840121842
28.76578816	3.449170133	0.278791251	7.427989486	3.752075344
43.0392993	3.784294298	0.215364931	8.260355791	4.859490145
49.43644437	3.704136341	0.276916567	7.756376272	4.526652467
31.27849095	3.550428488	0.250939809	7.6694189	4.082294574
48.81357516	3.701286943	0.26154293	7.571761037	4.642298669
19.64911397	3.183082653	0.316889981	6.747969114	3.098097438
21.12773451	3.198981269	0.332625997	6.864458622	3.048860684

log.sigma.1.5.mm.3D_glcm_DifferenceVariance	log.sigma.1.5.mm.3D_glcm_Idn	log.sigma.1.5.mm.3D_glcm_Idm	log.sigma.1.5.mm.3D_glcm_Correlation	log.sigma.1.5.mm.3D_glcm_Autocorrelation
34.70362185	0.897341399	0.191376438	0.021204705	957.6596864
11.99652145	0.915864245	0.317214954	0.062562982	342.5140125
11.30078259	0.918139816	0.345362493	0.094794754	304.0941421
25.07314386	0.895966572	0.257294074	0.068382344	610.8272205
17.66906767	0.891816671	0.252000857	0.030845226	363.6689505
23.08198275	0.901688411	0.199867157	0.11859756	542.8469186
14.32942052	0.919532273	0.275120454	0.086045298	449.5422893
17.52226737	0.92764991	0.306951594	0.077181119	641.6060558
20.04090258	0.91006078	0.24499791	0.050374625	497.0496501
20.91357437	0.9024632	0.234409514	0.054978794	549.6974252
11.04452237	0.902392156	0.273865809	0.067051282	300.9946469
27.60210862	0.904701126	0.23030576	0.039332078	705.8433777
16.4076818	0.935905601	0.383853397	0.093512783	574.5233004
25.41757345	0.908054584	0.245158109	0.080313435	636.761012
31.11272228	0.913974584	0.225457177	-0.004631046	685.2086715
17.11682176	0.927752399	0.306856235	0.085958669	577.9779205
16.53314069	0.880543509	0.21327277	0.046219146	266.6816562
12.02096985	0.925263463	0.338103759	0.094544254	449.3197933
10.69420288	0.921663308	0.273970754	0.045797067	487.3598735
19.9644876	0.91604355	0.230901224	0.070102637	643.0369262
8.809300479	0.926524738	0.358889274	0.081752499	367.1997683
12.65985808	0.888653677	0.231273763	0.019443083	340.0824674
16.80002838	0.919157436	0.272060117	0.038178653	687.1681882
14.59051355	0.907843562	0.267318846	0.057948854	498.8817371
20.81190974	0.915863602	0.271100523	0.078962218	609.7449295
23.17938827	0.911217924	0.21140583	0.097011823	711.4033204
23.79529831	0.903711854	0.228188327	0.074101168	596.5465892
18.78414365	0.915677958	0.267087684	0.068573163	629.4424923
12.9383186	0.877442626	0.198347763	0.011032184	303.5722009
14.52279388	0.918901072	0.286534366	0.056283174	585.6550821
19.09527949	0.934751804	0.29286402	0.056137451	686.9524611
18.0356841	0.91378381	0.302429614	-0.006984556	511.8041333
22.7926012	0.916949789	0.249428458	0.090070345	583.4599918
25.04390855	0.913473409	0.270172896	0.080436298	536.8790912
21.66636674	0.91226031	0.237260694	0.085918998	467.5997401
14.99515781	0.932482367	0.313431204	0.095278398	450.1061932
25.94616429	0.936847096	0.286576951	-0.010972495	1017.932504
19.75294425	0.946224248	0.377698109	0.09005791	1023.312156
17.33160194	0.900095417	0.24478671	-0.051911994	567.1910752
15.84238421	0.895900638	0.219415479	0.029044489	323.9347368
13.73922635	0.907870825	0.295469392	0.052243503	322.1436137
21.17719995	0.874330749	0.17332683	0.017852439	290.0144463
18.36284385	0.911824491	0.249697334	0.078377343	543.2609515
30.48770817	0.894673285	0.210504297	0.007421207	628.2887639
21.10648072	0.914630404	0.258863839	0.077054426	715.426871
17.6500195	0.915747189	0.254097399	0.068940043	418.7821583
18.98038093	0.916516587	0.258995138	0.080081275	542.6953819
12.49232331	0.911095371	0.271002312	-0.011961798	370.6202189
25.17795875	0.879129115	0.194687285	0.008418733	371.5078268
12.61879867	0.911610231	0.288677723	0.12131007	506.16748
21.73924111	0.914278043	0.212678475	0.066135852	658.2726693
22.29905682	0.89466981	0.184471693	0.061238558	543.4583735
16.33127496	0.899927242	0.215885032	0.018443704	401.7337257
12.40945811	0.908666103	0.260405206	0.04357824	347.1096212
16.47137353	0.905958928	0.252703961	0.106014303	505.4331311
14.0618347	0.904247249	0.259541096	0.036140138	401.177806
19.0997553	0.901234303	0.226260612	0.04144852	452.2675583
17.7300764	0.924191805	0.278989107	0.044744775	668.7673486
20.04329963	0.914125567	0.239885442	0.021026029	628.5418892
23.1859089	0.906753959	0.225303954	0.018961304	675.9661922
18.12408407	0.923145273	0.321984207	0.080708082	538.665424
26.35321399	0.924703822	0.240699178	0.015405157	987.3620011
19.74374304	0.917470552	0.253080075	0.06711358	760.0037305
19.16953665	0.923291034	0.240908608	0.027517789	673.1202439
7.602374096	0.89454539	0.297014935	0.100810008	206.1096219
19.83964786	0.926108135	0.297893223	0.02873236	621.213067
20.30379021	0.911872542	0.288505863	-0.039874479	514.113403
21.09410894	0.917328203	0.246565464	0.030437327	627.5709571
20.20133472	0.924512584	0.233805243	0.05495685	802.517005
10.61060951	0.918669854	0.313243777	0.021306784	405.7444406
15.5765346	0.898131289	0.265871842	0.034614767	329.5204985
18.3655774	0.93116493	0.300397837	0.075751273	700.3508725
19.34744797	0.907606171	0.27599545	0.046002208	367.5494706
28.45559123	0.895107917	0.191991071	0.041036406	521.5542093
20.14653493	0.885880852	0.196756409	0.005274691	356.6136251
16.72573034	0.904110503	0.250643817	0.013118571	356.9050232
11.70014173	0.926396892	0.283854906	0.094001006	413.5443143
17.64757627	0.905066288	0.27336633	0.00232865	438.5035121
13.64495743	0.910259504	0.256409555	0.04301417	485.9422543
26.95461816	0.90483303	0.203443405	0.017598693	620.47236
33.61672681	0.900660563	0.199225019	0.028529066	961.1227538
27.31133902	0.905999942	0.257013185	-0.003363655	466.7649269
19.48452195	0.925582849	0.311339907	0.039448012	584.5269376
8.905548785	0.902952071	0.284107251	0.079622084	331.1428376
24.39820528	0.896852078	0.234156712	0.014112751	439.3090845
5.360485675	0.93600502	0.453174022	0.133706023	321.506324
24.18440497	0.899175095	0.235528626	0.009945174	384.6708863
16.63240332	0.901865546	0.268454503	0.065454891	422.4320399
18.2652055	0.911869165	0.279955922	0.048497432	585.196351
15.80381279	0.920102524	0.307551438	0.060832368	631.224545
6.819440155	0.938692602	0.38323092	0.189267416	539.4158898
18.13753759	0.894750044	0.19966432	0.060270224	467.4504054
21.2807362	0.904411277	0.221177589	0.038995712	433.5381397
14.58684162	0.908638229	0.277460926	0.0653973	373.3435017
19.33426158	0.903400175	0.2091977	0.028871384	477.7304404
28.80866723	0.925778907	0.28811736	0.073027964	975.5179882
14.55409245	0.898288856	0.246351861	0.034473017	324.663094
27.08754117	0.906251595	0.258423992	0.009405307	659.1354075
9.964755722	0.896042522	0.32189323	0.053858634	248.6081012
11.79287046	0.929759611	0.347616998	0.117915784	543.2447557

log.sigma.1.5.mm.3D_glcm_SumEntropy	log.sigma.1.5.mm.3D_glcm_AverageIntensity	log.sigma.1.5.mm.3D_glcm_Energy	log.sigma.1.5.mm.3D_glcm_SumSquares	log.sigma.1.5.mm.3D_glcm_ClusterProminence
5.038759461	31.36681348	0.004651376	35.43426852	24418.94121
4.155119236	18.63006752	0.017581354	11.9795435	4229.929324
4.198503863	17.55514295	0.020837679	12.01745595	3499.358862
4.745532855	24.8799202	0.008272545	23.48974387	14025.7765
4.529088345	19.32194662	0.00986185	18.10080585	7792.791631
4.844667301	23.45879099	0.006397971	27.17255884	19376.06365
4.515106198	21.31318912	0.010106016	15.23435176	6087.431298
4.460270535	25.46533053	0.015102187	16.54714256	8006.76771
4.694887228	22.47616281	0.007133296	20.0484091	9335.764274
4.687563487	23.63446422	0.00756735	20.80935173	10002.43925
4.329804727	17.41878669	0.01007044	13.0123942	3459.483993
4.791331743	26.86948429	0.006893133	25.63543564	17717.30898
4.106064694	24.07893784	0.029609162	13.55366715	8626.839968
4.751451806	25.41554361	0.007942164	24.99282176	20482.18475
4.782742973	26.59393107	0.007452419	27.15009505	18906.8662
4.447208866	24.16996356	0.014403806	16.21855279	7945.046433
4.673337322	16.49024202	0.005777847	20.73585384	6318.86355
4.164723478	21.33126714	0.019945739	10.91231045	4392.887194
4.284699161	22.18094112	0.011599906	11.54939511	3104.904994
4.700098214	25.61933714	0.007390147	21.01803712	11972.17876
4.001081461	19.28831229	0.022074351	8.932745951	2489.677817
4.420798155	18.60197408	0.008018587	15.24115508	3987.339571
4.449403295	26.33154529	0.010926739	16.5200767	7569.489513
4.486361385	22.48884664	0.00908417	14.72104658	5241.806046
4.587560841	24.84265437	0.01125885	19.9187933	12085.25887
4.865355849	27.0107497	0.005969336	26.11669333	17828.33352
4.84849803	24.64860398	0.006317382	25.34368881	13509.5198
4.542740274	25.22499835	0.009959138	18.00670764	9932.573931
4.485042488	17.5828841	0.007329956	17.51664563	4108.75985
4.363346827	24.29383588	0.012293989	13.81650226	6198.318219
4.424888178	26.4829523	0.014080566	16.66168116	10545.19463
4.336226813	22.79869561	0.014252019	14.78203557	5911.547071
4.805003855	24.40037	0.007008382	24.0162942	13360.50242
4.741853679	23.37045653	0.009913393	23.53988499	15195.84324
4.815302905	21.83192599	0.006896438	22.99527069	11979.64408
4.36173762	21.31459025	0.016031141	14.23177195	6407.686773
4.465292677	32.1817456	0.012399173	20.3139948	14627.85718
4.308972997	32.1697283	0.026207152	16.45645148	10495.76468
4.377278556	23.99993048	0.009288566	15.02315318	5688.605207
4.643206126	18.28827176	0.005643955	18.31927493	5662.173139
4.323397818	18.11367397	0.01341788	14.09626889	4705.938224
4.906927646	17.40003254	0.003462009	26.94650256	9680.619157
4.657970303	23.44859053	0.007959842	19.00801239	9234.580745
4.875101762	25.38746687	0.005757607	29.06362081	18533.95579
4.596629944	26.88662947	0.009199704	19.95559366	12134.47493
4.606253626	20.62178436	0.00859231	18.16869317	8028.888848
4.64374424	23.45741957	0.009238963	19.52190317	10224.69925
4.168518731	19.47967624	0.013639579	11.81898529	3901.152172
4.800025847	19.62677848	0.006003238	26.4099673	13669.7925
4.422764031	22.61502151	0.012695204	13.51378741	5205.324223
4.736965986	26.00381407	0.006941915	23.12525523	14783.89354
4.941330404	23.57461245	0.004022288	27.16627917	12803.60107
4.626054962	20.30831961	0.006399261	17.53600269	6241.652157
4.326426054	18.81972989	0.010725469	13.3361124	4501.786455
4.614055087	22.63965557	0.00910074	17.83145989	8850.162329
4.371890131	20.2843424	0.010550961	13.89888954	5242.193678
4.712951833	21.4729787	0.006190501	19.93368881	8364.276212
4.453693521	26.04261186	0.011182363	16.97779515	7822.64042
4.583862185	25.27441992	0.008173579	19.86715011	9958.243245
4.79041285	26.35467003	0.005752749	24.30429361	11337.76628
4.402456478	23.35647016	0.017525674	16.24246249	8669.374099
4.716298859	31.70366843	0.007906258	23.79439882	15715.97067
4.639279422	27.85120393	0.009334579	20.1277118	10716.74186
4.569157559	26.27308919	0.008614049	18.68972465	10025.99435
4.126879508	14.32153298	0.013569347	9.57729204	2030.853161
4.441724549	25.08310042	0.013525228	17.0759754	8857.270523
4.339535091	22.88259088	0.013834717	16.03496062	7674.574179
4.641848729	25.32778178	0.008078002	20.75363728	10579.7311
4.707482242	28.58714036	0.006853194	20.22742814	10937.34748
4.05779499	20.34343116	0.017574518	9.821558262	3133.69051
4.435252375	18.34725417	0.010420557	16.46758993	5871.829234
4.423439352	26.67089932	0.014686101	16.60843314	10017.46939
4.535286901	19.41767388	0.010312275	18.3449939	9236.31992
4.992048484	23.17458351	0.004186356	31.10979478	16420.40737
4.781938303	19.25144256	0.004768711	22.65265986	8787.26201
4.428125526	19.15212817	0.009701816	16.46387446	7018.295527
4.331707575	20.43748823	0.012147484	12.49875619	5192.236694
4.376096116	21.16641825	0.012640703	15.30406127	7093.0738
4.411148504	22.1900628	0.009470711	13.70111505	5143.064404
4.863095401	25.29338982	0.005118416	28.06243696	15235.01182
5.00165081	31.40216474	0.005097113	33.77370304	23695.61422
4.714386038	21.87657458	0.00863675	23.80886897	12055.40361
4.441790265	24.3508403	0.014035128	17.22528796	8432.593185
4.258440734	18.24419754	0.010627871	10.93897978	2088.603144
4.772778058	21.23343576	0.006552931	23.25022648	11027.89958
3.634255017	17.9115057	0.041333329	5.288452431	1161.501718
4.758813402	19.91997342	0.006772739	23.26948295	10412.60419
4.494408061	20.81072509	0.010578409	16.23034945	7482.874742
4.472433984	24.33004863	0.011502705	16.42089788	8631.85986
4.349702421	25.22087913	0.014431977	14.06887588	6782.225693
3.903814873	23.25634445	0.029358028	7.484486133	2272.616677
4.792594609	21.8268694	0.004790799	21.36922162	8642.816036
4.769758436	21.05070427	0.005989134	21.93876085	9943.169952
4.410646672	19.5261259	0.011606078	14.62996048	5782.511476
4.750422705	22.06436219	0.005110909	21.15317419	8003.407995
4.735699034	31.49630353	0.01236353	24.99175972	20250.18047
4.473593781	18.2641478	0.008769226	16.02307088	5101.44579
4.614491798	25.90380475	0.010030752	23.36327382	13872.47812
4.093177599	15.74971844	0.016657775	10.37724573	2555.52321
4.196292429	23.37409215	0.021529732	11.21468746	4531.753457

log.sigma.1.5.mm.3D_glcm_SumAverage	log.sigma.1.5.mm.3D_glcm_Imc2	log.sigma.1.5.mm.3D_glcm_Imc1	log.sigma.1.5.mm.3D_glcm_DifferenceAverage	log.sigma.1.5.mm.3D_glcm_Id
61.8738978	0.75533912	-0.0937939	6.161183354	0.276086466
36.97230741	0.727549163	-0.106091491	3.228229243	0.394961978
34.81262803	0.705409693	-0.096167137	3.134188604	0.41716822
49.3627133	0.817351152	-0.132147828	4.701167197	0.337012179
38.11493739	0.625472861	-0.06289541	4.302404236	0.335960606
46.45388886	0.870815264	-0.167833495	5.218213663	0.289442075
42.34057729	0.538030925	-0.043815303	3.873607143	0.356680206
50.608445	0.577184526	-0.053914799	3.749418803	0.38270044
44.54285592	0.562222454	-0.046330506	4.53079028	0.328862358
46.84162398	0.769578586	-0.10953772	4.632431349	0.321053657
34.64817713	0.628671704	-0.066594106	3.533970413	0.359568854
53.10029086	0.6806739	-0.07486854	5.017669659	0.315506873
47.88433679	0.72876287	-0.111450026	3.052704727	0.446968663
50.38892668	0.772583469	-0.109104212	4.711426182	0.328787968
52.366375	0.750137553	-0.096786177	5.281325864	0.309236001
48.02335286	0.614207397	-0.062472401	3.662614447	0.383309752
32.60451034	0.708617751	-0.084050717	4.615672761	0.304003552
42.34059657	0.708073707	-0.098777086	3.09643882	0.411948198
44.12730057	0.648019121	-0.0732251	3.541868714	0.358986119
50.65917732	0.542657997	-0.043153161	4.562680418	0.318058136
38.28546099	0.582680746	-0.061633745	2.758434163	0.429910919
36.86918795	0.669485191	-0.076161626	4.090302076	0.321368379
52.40188677	0.749037405	-0.10684688	3.927209071	0.354452697
44.63234011	0.615610676	-0.060461552	3.878958951	0.351414573
49.32256076	0.770810228	-0.115001973	4.1533935	0.352409283
53.24369539	0.715008424	-0.083442305	5.065242698	0.299092
48.77293048	0.663806964	-0.068181078	4.916385625	0.313071943
50.12672708	0.637797868	-0.067158595	4.044857112	0.350230594
34.83681628	0.826078558	-0.143696179	4.533029308	0.292133133
48.3669064	0.631592764	-0.067956172	3.654465533	0.368109895
52.38398522	0.620064634	-0.062975256	3.859677213	0.371463686
45.25450542	0.665907437	-0.077423198	3.779506392	0.378907574
48.22312148	0.671705071	-0.071982396	4.644607644	0.332166386
46.25827278	0.731772064	-0.093132605	4.521447098	0.349033538
43.15368851	0.610595418	-0.05592099	4.712123938	0.321682305
42.36537785	0.646378474	-0.073140416	3.458468274	0.390129828
63.81967419	0.6870598	-0.081727762	4.135167233	0.365361713
63.93223962	0.693964155	-0.090639033	3.362949968	0.441087256
47.6696174	0.678289314	-0.079341036	4.155195411	0.332672176
35.97144351	0.441680702	-0.026398096	4.534192758	0.309110743
35.8590466	0.615443017	-0.063465612	3.581205067	0.37468787
34.04129198	0.538370551	-0.038957411	5.638129558	0.264345732
46.55013728	0.601439368	-0.055593252	4.320848219	0.333904343
50.12769345	0.706136755	-0.080018441	5.493823611	0.296111431
53.43770454	0.740607703	-0.101111795	4.217269546	0.342990865
40.86353652	0.662234296	-0.071328844	4.198701596	0.33822724
46.52266471	0.597898832	-0.05546449	4.267971119	0.341555878
38.51465807	0.703698756	-0.093574703	3.605770704	0.357068861
38.54685121	0.838187614	-0.140837813	5.481750009	0.282916449
44.91320397	0.708720483	-0.09386118	3.591568532	0.36909706
51.25609189	0.661577453	-0.069040928	4.862112379	0.301700791
46.55644852	0.609629852	-0.053168806	5.478916731	0.274614388
40.07271636	0.502112318	-0.035330721	4.570022322	0.305863852
37.23179853	0.707289171	-0.091362026	3.706043903	0.347261882
44.87130167	0.740094652	-0.099343903	4.191716873	0.336903597
40.03675491	0.616057888	-0.062205632	3.822846013	0.346138692
42.4949184	0.562154812	-0.045409569	4.660959466	0.313105309
51.69263516	0.617207921	-0.061583327	3.929299691	0.360175659
50.12742766	0.693683217	-0.081595536	4.49532487	0.325729551
51.9858992	0.525920487	-0.038012315	5.038611465	0.309207235
46.35828669	0.746200682	-0.108489963	3.683479286	0.394300965
62.83710004	0.683375496	-0.076249252	4.782593232	0.324135573
55.08871795	0.557058475	-0.046462963	4.399527101	0.335942609
51.87189901	0.512866156	-0.038578727	4.386387865	0.327299535
28.64306596	0.672688505	-0.085262604	3.081301718	0.379865144
49.82984669	0.623687573	-0.064815355	3.860781947	0.37552877
45.3823144	0.747551106	-0.106701791	3.987693613	0.366615475
50.08011673	0.550731658	-0.04457528	4.511680634	0.330594208
56.61819774	0.556187604	-0.044651703	4.563628744	0.320269959
40.27685545	0.553208173	-0.052258014	3.088661432	0.392991294
36.27902535	0.701711429	-0.087202852	4.006484495	0.348864873
52.88076572	0.616853232	-0.063923196	3.713349615	0.378320394
38.29926775	0.710146108	-0.086901652	4.17148859	0.355469606
45.62363498	0.702634766	-0.076623081	5.771967695	0.278679024
37.76832796	0.584846317	-0.048721381	5.197722696	0.28628373
37.7781061	0.717597253	-0.091648025	4.182998116	0.335883307
40.6099703	0.612202319	-0.063005137	3.492663519	0.366875925
41.88476356	0.758504006	-0.111287992	3.955920044	0.35584724
44.06103656	0.629603273	-0.065344735	3.846933698	0.3434035
49.80431838	0.708185038	-0.080221595	5.395583538	0.290711189
61.97789895	0.767278841	-0.099716657	5.941458172	0.283776112
43.21991922	0.757422006	-0.102186265	4.858745001	0.335780256
48.32839027	0.675068166	-0.07954654	3.811832514	0.385914126
36.34873903	0.491748456	-0.037748929	3.262000781	0.369765633
41.90873757	0.689198116	-0.076256067	4.971685057	0.317402769
35.82216275	0.591284632	-0.076898033	1.938980963	0.510299829
39.21964111	0.695006912	-0.0775392	4.957882458	0.318541681
41.05363246	0.652552059	-0.070648647	3.972302819	0.351325787
48.34641447	0.714520663	-0.092392512	3.952028871	0.360870478
50.21403113	0.668573692	-0.080385033	3.540634678	0.384932558
46.38480846	0.651000387	-0.086926554	2.447083102	0.451602419
43.17971736	0.634237407	-0.060213565	4.959908423	0.290205269
41.60281844	0.542016233	-0.041294884	4.840121842	0.308119815
38.59438877	0.596813274	-0.057851657	3.752075344	0.360257894
43.68742153	0.547346362	-0.042096251	4.859490145	0.298721236
62.40645008	0.702838233	-0.084563521	4.526652467	0.362931056
36.01041628	0.563274891	-0.048749693	4.082294574	0.333587459
51.34217703	0.82771316	-0.142018174	4.642298669	0.339592612
31.49943688	0.69289581	-0.092719922	3.098097438	0.39904781
46.55376215	0.540886883	-0.049983503	3.048860684	0.418951716

log.sigma.1.5.mm.3D_glcm_ClusterTendency	log.sigma.1.5.mm.3D_firstorder_InterquartileRange	log.sigma.1.5.mm.3D_firstorder_Skewness	log.sigma.1.5.mm.3D_firstorder_Uniformity
75.13600384	140.9640589	-0.814135088	0.061420636
25.51982588	71.49405527	-0.599961329	0.117099145
25.65923587	72.53081822	-0.25139496	0.117030915
53.95661861	109.6042137	-1.197441184	0.077888676
38.19664344	101.2994933	-0.927620422	0.087372383
64.05077267	117.7305655	-0.271473698	0.070091827
34.83255971	96.67033863	-0.235933188	0.087679541
36.79950517	75.36653173	-0.750662341	0.106649235
44.79358227	109.4998808	-0.779526604	0.078032853
47.13553917	109.618331	-1.015136018	0.077329653
27.04130503	96.50826311	-0.656704391	0.092055457
56.59880689	112.4820938	-1.272363878	0.075612857
30.97103081	51.87283993	-1.01017854	0.152572705
55.76244581	107.5714512	-0.871429729	0.078909762
57.76743286	111.7325654	-0.983710439	0.074624211
36.24592672	74.98581219	-0.916476731	0.105984968
41.63750665	125.6827478	-0.219496836	0.070044527
26.18068949	64.66355467	-1.011239606	0.12958523
25.48819522	87.33540821	-0.405422952	0.099188986
46.6416107	105.4680405	-0.755390346	0.078418953
19.46461248	63.34898949	-0.141112535	0.130442571
30.41810327	109.7649279	-0.749823422	0.082847967
34.97747971	91.94055557	-0.905787788	0.091213226
33.16373563	97.62512875	-0.404567535	0.086808184
44.46559823	91.1565547	-0.842859767	0.09160157
59.50117613	129.5042782	-0.183298589	0.064264906
55.47898513	118.4400749	-0.445997144	0.07006494
40.29063181	90.46709728	-1.140195639	0.092646609
34.18676691	115.5646524	-0.271278863	0.075987568
31.18419628	81.85846305	-1.16714155	0.104091193
37.75160024	80.64457417	-0.876197059	0.101135743
31.64035275	76.31864166	-1.294816777	0.1089318
52.89958316	112.7238464	-0.323731569	0.074457718
53.25871431	103.1742101	-0.901480625	0.084058307
52.07700508	114.9279115	-0.480774441	0.073453371
32.58824409	76.14125299	-0.565887484	0.108441282
41.90054039	81.45929146	-1.109267233	0.101398897
37.05934026	58.32483673	-0.887935232	0.133145725
31.01649345	95.93734312	-1.381036038	0.092507106
38.25944731	126.5160904	-0.205072026	0.070443514
29.36198583	77.18800592	-0.709807365	0.105744292
54.37031459	166.7480774	0.010922799	0.055770252
43.25774435	104.7558975	-0.750375703	0.080595926
61.14969223	123.9553709	-0.938646971	0.070097238
45.09518423	100.54159	-1.27620485	0.084095601
40.62256341	106.1348381	-0.171604254	0.079591375
43.66498769	98.43802071	-0.379628006	0.084728773
24.64787386	78.12172127	-0.95395317	0.109684291
55.61400127	127.4702187	-0.672663032	0.068540364
32.95829502	83.2476896	-0.519600655	0.097964663
51.33596601	115.7994919	-0.732619899	0.07291158
58.80609525	148.7157536	-0.309396238	0.060385839
38.35840662	114.366107	-0.515065614	0.076412565
28.41419089	94.05246925	-0.256243008	0.091275467
42.27561051	98.30350447	-0.443110118	0.085560518
30.49940045	90.8217411	-1.099365217	0.093948718
44.16084694	121.3483543	-0.662646197	0.071986505
36.17231562	86.04455996	-0.949836587	0.096406987
41.72340317	102.2015619	-0.853020126	0.08419517
49.96843335	127.2326736	-0.45350638	0.070123282
37.30086907	74.66048789	-0.518602106	0.109531493
50.28148548	99.69873857	-1.081911899	0.08189847
44.38928129	100.5559921	-0.595278386	0.081634175
40.26748597	103.3616743	-0.639237171	0.083104875
21.14049371	85.63081002	-0.567709619	0.100865246
36.65965883	77.45111895	-0.962400781	0.103959599
33.18803384	78.15207911	-1.23706249	0.106177547
43.78428325	104.0514832	-0.654272978	0.082071245
45.5518998	111.944397	-0.768347769	0.076563568
20.91581281	71.32353306	-1.164150006	0.115139019
33.69017613	91.30661583	-0.828417134	0.091067269
37.23851211	77.13441563	-0.830933444	0.104601692
40.19870439	95.52236652	-0.469746878	0.085105224
66.62097517	150.4347973	-0.61716982	0.059213836
47.34586699	143.9490623	-0.271728367	0.062596034
34.82173806	97.40850163	-0.917714004	0.089608676
28.93316822	84.45422482	-0.084419086	0.100650848
33.08788351	81.25343752	-1.183698584	0.102854329
30.87913306	94.27164078	-0.926276167	0.09197491
57.67382843	138.7537785	-0.49486703	0.065044076
72.35681165	134.9968109	-0.827959927	0.063269804
50.19239392	101.686842	-0.817976827	0.081095946
36.59765012	77.24198723	-0.656294982	0.101754844
22.94922513	89.83341789	-0.56997679	0.098924966
50.25734195	118.9007702	-0.710808794	0.072192157
11.96553318	43.87701643	-1.761684988	0.178929572
49.45886084	121.9553299	-0.597329117	0.072577825
36.74086018	98.09313178	-0.659517389	0.085874111
37.34744394	85.27305818	-1.290318775	0.0976552
31.89452247	77.57470608	-1.293056847	0.106625453
18.91192001	51.08076167	-0.132637297	0.150090331
48.06875542	138.5842323	-0.322094901	0.065100625
48.12615969	118.7656498	-0.562284857	0.073022793
32.58838442	86.57824278	-0.848312127	0.094675115
45.36610004	134.4883261	-0.436529408	0.068644382
56.88063959	86.69634795	-1.167253766	0.094536811
33.17267749	100.4248943	-0.535318969	0.084343006
49.3220037	97.14559555	-1.169030483	0.089038678
21.85986896	76.76366234	-1.131117104	0.114044017
26.83766139	62.4881103	-0.408948349	0.126774659

log.sigma.1.5.mm.3D_firstorder_MeanAbsoluteDeviation	log.sigma.1.5.mm.3D_firstorder_Energy	log.sigma.1.5.mm.3D_firstorder_RobustMeanAbsoluteDeviation	log.sigma.1.5.mm.3D_firstorder_Median
107.1047159	46689511.13	64.29765387	31.50684357
58.41996606	8065066.977	31.41317414	27.1101284
60.11229845	12740485.22	33.49832408	17.14630318
86.8333183	14588955.06	50.26975668	27.61798096
77.59644099	28702842.65	44.92927431	31.33901024
92.19154943	12099334.09	52.79115278	29.32581615
71.85959035	60894573.85	42.62341876	23.19658089
68.21949539	59280133.15	34.97801924	27.95349979
80.6244822	59985677.97	48.30253704	34.16837883
83.40383709	15575330.03	49.2176927	43.59416008
65.30732399	12313538.86	41.29650409	24.94867134
87.75281142	40532968.76	49.47151986	44.44513702
53.36544069	16229130.98	23.64750604	16.38395882
84.93828652	27333798.21	47.25857577	31.37478638
91.79522239	36787668.4	50.42052022	35.09003067
65.31607441	43831850.61	34.9139795	20.73966408
84.09235073	14763391.85	55.25535716	25.22230911
54.47578697	9373334.434	28.38884795	23.45432091
62.65853582	10093272.53	38.47917686	25.4389019
82.17572978	76520880.36	47.40801055	32.35398483
51.62177602	19108198.11	27.86880561	19.03166866
73.0166953	10957784.88	46.31247629	33.25180435
71.39169252	11232976.73	40.82181751	27.14177608
70.74038083	27241211.46	42.98721376	24.90590668
75.69877673	18038367.55	41.25882083	32.93999863
97.23631356	47248085.01	58.15850519	42.62537003
89.32040176	52385961	53.27783701	30.18917465
70.82343051	30951102.34	39.4777923	23.85918713
77.76691512	4828518.41	51.17554047	34.14068222
63.66067698	18856233.09	35.80541429	33.08270645
69.38659033	57552211.68	36.493957	26.11614418
64.13162466	19036278.53	33.94597582	18.64861298
85.5089821	48190047.84	50.03301795	23.17507744
81.59548505	35057005.67	45.53220807	26.79785156
86.28631921	70850416.9	51.09619957	41.64064026
63.81263194	29252094.07	34.13386489	22.9672451
70.55898189	46816300.47	35.9793313	18.45117378
61.04162676	52947395.13	28.71293207	10.65299606
69.06750982	11573074.31	42.67928148	25.55558777
82.43539333	86852394.09	53.98952015	18.7654686
64.36096099	22529052.35	35.57855196	17.80230999
101.3283018	64278002.23	69.81246171	15.55314159
77.81946192	45696978.16	46.40679576	30.39393044
93.38359633	44070214.92	54.21135284	30.85545349
76.71172388	18258891.2	43.64278432	43.59672737
79.08519783	35824453.49	47.04578757	30.84522057
77.30657242	60044906.21	43.72784037	27.04119778
61.36165618	7699216.365	34.20355109	35.36376953
94.36090666	14920922.64	56.42259493	42.91184235
68.02447794	14394153.95	39.60854148	30.77870941
87.7774628	43898852.48	50.68430574	41.64873505
97.52491715	57501688.38	63.38566241	31.72823334
80.00969097	54839778.34	49.52082138	30.3183651
67.64578673	11042678.97	40.51802975	31.91171265
75.10568581	16679437.89	43.22805738	32.11251831
68.56776518	19464868.46	39.70789781	28.4940834
84.25570583	55430790.98	52.43209316	35.29548645
69.21528224	35907753.4	38.33192262	18.33411503
77.70264904	20958925.15	44.59918358	26.57748795
87.43068518	131769883.9	54.50674738	9.416220665
67.7101518	20520425.98	33.95355759	24.60770988
81.35543857	40495509.21	45.64252986	32.51232147
81.0554673	83741682.15	46.56362407	31.69756508
78.45705084	99743722.59	44.82744731	26.31801414
59.17539669	5541457.011	37.1897063	28.57452297
67.73169756	42063676.24	35.55055831	21.17718124
65.77954251	13192575.82	34.42701332	17.47809029
79.67462392	82090038.02	45.78594868	24.6012373
82.16996682	81888567.86	49.03957998	33.64958954
55.87546083	21020346.8	31.62510767	21.89014435
71.28149777	13496448.32	41.52924149	26.48146057
68.6629821	52336619.46	35.60228189	28.09859276
78.42465633	30473273.91	44.11436085	26.69198418
103.3916654	42519587.72	66.08163154	27.90428543
93.70171806	48058556.45	61.31358107	22.80469513
72.69963524	14248677.59	42.4686176	27.99029732
64.07876356	22520512.24	36.50633581	28.47938442
66.90618055	10053188.8	36.00612305	27.51577377
67.80159136	16032939.57	41.07774158	27.44684601
94.77506539	39190023.32	59.12136551	21.47129059
104.3118575	42421655.6	62.00246783	31.64349365
84.45915823	26703332.01	47.21566669	22.34378052
67.94511773	35562751.38	36.10289395	21.1096344
60.00823794	20470709.59	38.63595548	24.79286194
87.11640182	36166430.43	52.32834867	27.31686211
37.49553714	5428050.368	19.9054004	16.16175652
88.08492192	34930637.42	53.49729905	20.86450195
75.11235613	35340264.97	43.56157081	35.34900475
68.59589393	16361420.36	38.19283814	31.99996853
64.19928892	19552731.38	34.99565107	28.37468529
46.96304561	7574498.845	23.62675049	19.40757561
89.30388229	35405154.97	58.70487042	36.31629562
85.98536994	73124387.49	52.3470634	27.34523773
68.47921461	34167249.7	39.06135576	30.90004539
86.53082576	56661411.5	56.99551225	25.20189381
80.31659956	55805000.07	41.15423612	25.32611465
73.70675168	29476891.8	44.73080111	22.76369858
79.76465042	14625788.29	42.7713936	19.74440956
56.60007943	5325814.245	33.07577717	18.92910862
57.03457081	48311613.2	29.19276022	21.94919872

log.sigma.1.5.mm.3D_firstorder_TotalEnergy	log.sigma.1.5.mm.3D_firstorder_Maximum	log.sigma.1.5.mm.3D_firstorder_RootMeanSquared	log.sigma.1.5.mm.3D_firstorder_90Percentile	log.sigma.1.5.mm.3D_firstorder_Minimum
1260616801	468.5677185	148.3327244	179.145488	-726.3479614
217756808.4	356.8818665	92.87486249	117.4350937	-405.6069641
343993101	378.6378784	91.13397313	135.628537	-378.4751282
393901786.7	277.6601563	122.1982008	139.2142151	-590.5610352
774976751.7	336.2980957	110.0960166	134.4264465	-448.4664917
326682020.5	529.7116699	135.3969142	172.8774643	-543.9641724
1644153494	509.7266541	103.8070009	143.8770142	-485.1216736
1600563595	499.6993408	107.7281349	135.843959	-578.3897705
1619613305	515.8327026	114.8706478	148.1566925	-510.1991577
420533910.8	420.6257935	120.5372381	155.8674973	-527.9746704
332465549.3	336.8539124	90.36302728	119.7319489	-396.0690002
1094390156	436.9432678	132.6076999	167.2093689	-615.2279663
438186536.5	423.1036987	93.2342483	93.43104248	-561.0818481
738012551.5	429.7047424	126.1725395	146.6186493	-578.149292
993267046.8	629.1907349	136.1696426	166.2915588	-618.4591064
1183459966	480.1795044	100.0820625	122.1803856	-572.1240234
398611579.9	381.0253296	111.1964468	147.0560638	-370.8829346
253080029.7	364.3120728	87.18976718	105.5291885	-491.1274414
272518358.4	436.3651428	88.18173791	111.0952477	-505.7911377
2066063770	539.7545776	121.3193487	157.5719818	-599.7666626
515921348.9	343.460022	79.49118555	101.1288506	-439.669342
295860191.9	312.3215637	100.7745025	136.3105835	-407.1356201
303290371.6	394.0777588	104.7363539	134.7717941	-604.3341064
735512709.5	347.8603821	101.4463061	143.9771912	-507.6459961
487035923.9	448.9316711	119.0376691	162.4288513	-564.3134766
1275698295	556.6469116	141.224347	195.3129089	-616.7860718
1414420947	465.6698303	127.2930244	174.2519409	-553.0133057
835679763.1	375.5736389	105.8586189	126.2964417	-580.00354
130369997.1	345.6487732	103.9329332	146.8023499	-395.9282227
509118293.4	369.9594116	95.81362012	115.4095833	-562.6936646
1553909715	628.5916138	109.9812851	139.2916962	-602.2737427
513979520.4	333.4610291	95.92035557	102.6493195	-532.3000488
1301131292	597.5241699	122.1643972	151.816571	-557.0263062
946539153	515.2243042	122.138752	157.8228745	-525.2719116
1912961256	605.5480347	126.8374639	181.8006271	-488.052124
789806539.9	575.0136108	98.76208453	128.5179657	-483.028717
1264040113	601.2675171	112.1679315	113.1629715	-762.7156982
1429579668	569.7335205	101.2873876	124.7093048	-754.2522583
312473006.4	267.3569336	97.00001807	110.0701912	-550.2449951
2345014640	457.1125183	109.3008883	144.8301605	-421.5401611
608284413.3	359.23349	93.19368318	108.2042114	-414.4934998
1735506060	492.4440308	128.9270659	174.9722168	-397.6248779
1233818410	459.0801086	111.8456003	148.2628235	-535.100647
1189895803	429.9773254	133.0906041	154.8661591	-585.2130127
492990062.5	389.2586975	119.5285837	157.861969	-602.8538208
967260244.1	567.0192261	114.24011	161.3169281	-457.3985291
1621212468	519.710083	114.7256283	146.9713089	-527.2659912
207878841.9	385.8098755	94.01868762	109.5038376	-425.5615234
402864911.3	447.4065552	132.4137421	165.4450836	-429.2575684
388642156.5	324.6626587	103.1821863	160.8983185	-512.3709717
1185269017	584.4699097	130.7207503	178.7987457	-591.8245239
1552545586	501.6094971	130.6053364	171.8848267	-547.7300415
1480674015	450.0793457	110.2461164	140.7251221	-450.9631042
298152332.1	402.480957	100.0121323	136.8241547	-413.0023193
450344823.1	396.0574951	111.9862971	146.9679642	-514.0030518
525551448.4	339.2141113	99.90987852	125.9531227	-463.0403748
1496631356	474.5036316	117.0620904	156.468985	-485.041748
969509341.8	462.1520081	101.9017453	115.8690407	-612.0535889
565890978.9	494.5033264	112.7731989	127.0201096	-584.253418
3557786866	499.045166	120.1424484	155.2084167	-601.8453369
554051501.4	449.6740112	107.7948063	132.3679581	-541.480835
1093378749	586.130127	123.1537957	157.0160431	-735.3656616
2261025418	450.9007568	120.2732552	175.2148712	-639.1016846
2693080510	591.2211304	114.3878314	130.0735107	-615.5220337
149619339.3	263.6398926	83.7526271	118.5461159	-303.526886
1135719258	486.3401489	104.3632219	118.0825401	-586.6571045
356199547.2	357.9675598	100.5833286	103.324836	-531.8720703
2216431026	546.4417725	114.6054365	132.5579544	-597.2807617
2210991332	600.3140259	118.242615	147.1309692	-667.3056641
567549363.6	311.3051453	85.16688201	110.0447449	-473.6442261
364404104.7	367.1134644	101.9307389	128.5417114	-420.6064758
1413088725	501.4476929	110.4521534	147.7413773	-601.0206909
822778395.7	458.5201721	115.6344915	149.6139984	-449.1599121
1148028868	564.7175293	137.194407	163.5603821	-531.2008667
1297581024	466.3558655	123.8921606	164.3235474	-445.4282532
384714294.8	430.7283325	103.0412271	118.9037575	-440.2995605
608053830.5	543.6373901	96.94953388	119.9598694	-466.8915405
271436097.7	344.0234375	102.4399045	117.3428108	-478.6594543
432889368.5	359.7433472	96.91473606	122.0585968	-506.9868774
1058130630	570.0599365	129.4412356	153.8497009	-575.5256958
1145384701	468.5677185	146.0415438	178.3981445	-726.3479614
720989964.3	507.3128357	121.8336193	135.9826447	-515.1669922
960194287.4	486.4680176	104.3014189	130.6462952	-573.4862061
552709158.9	268.0395508	81.61785342	108.0043243	-417.3042297
976493621.5	474.9399109	121.7718554	156.5031464	-491.2894287
146557359.9	207.9217682	59.52406082	71.58325729	-404.3529663
943127210.4	511.7877197	121.0205337	136.0028564	-450.0190125
954187154.1	347.8744202	113.5275264	168.4999329	-456.5094604
441758349.6	347.865448	105.7158152	133.7972763	-561.7033691
527923747.2	315.9159241	98.26326788	117.1050507	-575.8621826
204511468.8	303.0091248	76.21459675	107.8373367	-548.3112793
955939184.1	450.2951355	121.9164677	179.8340424	-484.008728
1974358462	541.3806763	118.7792329	142.9000732	-477.5492249
922515742	385.5720215	101.7531918	138.9607697	-449.8111572
1529858110	512.1204224	114.1035749	140.4692581	-521.2532349
1506735002	509.3007507	127.7016633	164.7233047	-725.3708496
795876078.5	391.3773804	102.2933544	123.4020325	-418.8023071
394896283.8	374.7602539	118.1915153	121.8373245	-615.9189453
143796984.6	212.9295807	79.91162868	93.69394379	-355.9720459
1304413556	374.0271912	91.47183257	126.7522583	-542.4287109

log.sigma.1.5.mm.3D_firstorder_Entropy	log.sigma.1.5.mm.3D_firstorder_StandardDeviation	log.sigma.1.5.mm.3D_firstorder_Range	log.sigma.1.5.mm.3D_firstorder_Variance	log.sigma.1.5.mm.3D_firstorder_10Percentile
4.454624097	147.6800519	1194.91568	21809.39774	-189.4517258
3.61042157	88.88247273	762.4888306	7900.093958	-57.72800217
3.660549458	88.06373941	757.1130066	7755.222199	-68.33862686
4.134856112	121.8972199	868.2211914	14858.93222	-151.2677429
3.9976676	108.9549012	784.7645874	11871.17049	-124.5995583
4.269620391	132.2606876	1073.675842	17492.88949	-107.0710052
3.958445828	101.2436014	994.8483276	10250.26683	-97.68112183
3.83709226	104.7269168	1078.089111	10967.7271	-83.56523666
4.089528476	112.3498113	1026.03186	12622.4801	-113.0824547
4.100281791	116.7141487	948.6004639	13622.19251	-110.8346756
3.786556638	88.31581394	732.9229126	7799.682992	-88.84310074
4.187463698	128.5857405	1052.171234	16534.29266	-104.485878
3.420544614	92.16550894	984.1855469	8494.481038	-54.56278229
4.157550168	124.4255149	1007.854034	15481.70877	-121.2231812
4.243743289	134.1673763	1247.649841	18000.88487	-142.1515396
3.798439055	98.90168732	1052.303528	9781.543755	-90.30568695
4.135855801	109.6589541	751.9082642	12025.08621	-125.5701065
3.507662368	84.45318957	855.4395142	7132.341229	-57.32144775
3.732984512	86.47750479	942.1562805	7478.358835	-91.80355911
4.13960349	118.3830294	1139.52124	14014.54165	-108.8209595
3.470220777	77.12929312	783.129364	5948.927858	-61.97075233
3.927246194	97.81009877	719.4571838	9566.815421	-96.13405457
3.912562156	102.9080129	998.4118652	10590.05912	-101.5106468
3.929180999	98.3435805	855.5063782	9671.459825	-83.88870544
3.981212763	113.936213	1013.245148	12981.46062	-83.50265503
4.383314539	135.4426377	1173.432983	18344.70811	-120.2657379
4.266269478	124.5178114	1018.683136	15504.68537	-123.0568893
3.906848789	104.5211738	955.577179	10924.67578	-94.15443954
3.999514387	100.8866311	741.5769958	10178.11233	-97.8875351
3.736340926	93.10115337	932.6530762	8667.82476	-82.01280975
3.88377887	107.2385101	1230.865356	11500.09805	-81.67458267
3.713876383	95.64069536	865.7610779	9147.142609	-104.1864899
4.197732645	120.9483467	1154.550476	14628.50258	-129.5848511
4.101448272	120.2154384	1040.496216	14451.75163	-114.9562935
4.207229116	121.157251	1093.600159	14679.07946	-107.3823257
3.764693455	96.36619179	1058.042328	9286.44292	-74.13674316
3.88363445	111.5612745	1363.983215	12445.91796	-92.11877441
3.63000431	100.2978557	1323.985779	10059.65986	-70.41710663
3.807666145	96.28923186	817.6019287	9271.616173	-92.83496628
4.136543502	108.2952516	878.6526794	11727.86151	-124.7976974
3.746355945	92.76380011	773.7269897	8605.12261	-111.2856178
4.399766267	128.2209611	890.0689087	16440.61488	-158.661554
4.058115342	109.2877635	994.1807556	11943.81525	-104.7658264
4.281061718	132.017885	1015.190338	17428.72195	-144.3852798
4.009989854	113.5045423	992.1125183	12883.28112	-73.2036705
4.087856872	110.5996244	1024.417755	12232.27692	-105.8845016
4.057591004	112.2441729	1046.976074	12598.75434	-104.1307556
3.660577318	90.85469001	811.3713989	8254.574696	-75.65418243
4.264232896	129.4010111	876.6641235	16744.62166	-148.6431122
3.833403747	97.36702045	837.0336304	9480.336672	-72.48345566
4.227191576	125.9301508	1176.294434	15858.40288	-104.2970001
4.371155394	128.6299445	1049.339539	16545.66262	-143.9832764
4.08835311	108.3760365	901.04245	11745.36529	-120.5024689
3.862187827	95.24030621	815.4832764	9070.715928	-80.45902023
4.008671521	107.9852128	910.0605469	11660.80618	-94.67145157
3.843014386	98.04833871	802.2544861	9613.476725	-93.39643784
4.157874946	114.126415	959.5453796	13024.83861	-122.666803
3.871782612	101.507678	1074.205597	10303.80869	-104.2836945
4.023711139	111.7046068	1078.756744	12477.91918	-110.8215401
4.227849571	119.6433339	1100.890503	14314.52735	-128.4418274
3.816594557	105.2117571	991.1548462	11069.51383	-86.09582901
4.106606816	120.4656137	1321.495789	14511.96408	-107.1412674
4.111168851	116.2322747	1090.002441	13509.94169	-105.236319
4.063198867	113.0954329	1206.743164	12790.57695	-110.449942
3.651294426	80.23186061	567.1667786	6437.151457	-73.90356445
3.828876279	103.3400636	1072.997253	10679.16875	-94.28529129
3.769075156	100.3301316	889.8396301	10066.1353	-94.30341034
4.08281934	113.7130371	1143.722534	12930.65481	-125.4742226
4.129857323	115.8099664	1267.61969	13411.94832	-115.4535248
3.581684485	83.10325068	784.9493713	6906.150274	-63.03623924
3.906174103	100.7818035	787.7199402	10156.97193	-109.5165756
3.855211861	106.6633435	1102.468384	11377.06885	-72.91686401
4.056929519	113.250268	907.6800842	12825.62321	-115.1707855
4.41571675	136.8642585	1095.918396	18731.82526	-182.0614594
4.309704036	122.9454847	911.7841187	15115.5922	-145.7737122
3.907352486	101.9567232	871.0278931	10395.1734	-109.4412056
3.786357837	93.35290103	1010.528931	8714.764131	-74.57373428
3.80520075	100.8341052	822.6828918	10167.51677	-85.70589981
3.842964243	95.12941205	866.7302246	9049.605036	-90.18947754
4.320845416	129.0571988	1145.585632	16655.76057	-152.7258942
4.419573909	145.1431604	1194.91568	21066.53701	-178.4458374
4.131027014	121.5513273	1022.479828	14774.72518	-151.0295135
3.849915305	102.7263417	1059.954224	10552.70128	-90.18002319
3.670007346	79.78214755	685.3437805	6365.191068	-86.72110901
4.199284112	120.6140506	966.2293396	14547.74921	-135.7827332
2.991132428	58.33355722	612.2747345	3402.803898	-44.88019142
4.202189132	120.814812	961.8067322	14596.21881	-155.9372498
3.997650813	107.6282482	804.3838806	11583.8398	-77.89777222
3.845518798	102.6860156	909.5688171	10544.41781	-82.79699249
3.738096311	96.1397457	891.7781067	9242.850704	-85.8271225
3.30611529	72.73418916	851.3204041	5290.262273	-48.97793884
4.243369404	117.1821895	934.3038635	13731.66554	-111.0451141
4.173175879	117.8496326	1018.929901	13888.5359	-142.2613831
3.860345171	98.52061242	835.3831787	9706.311072	-86.61681824
4.179651996	113.2841111	1033.373657	12833.28982	-135.4551483
4.038992653	125.523293	1234.6716	15756.09708	-100.4753067
3.971310253	101.3261614	810.1796875	10266.99098	-114.2245026
4.017195633	118.0886865	990.6791992	13944.93788	-132.6259918
3.551989608	79.5163278	568.9016266	6322.846386	-91.24517288
3.584940243	88.14077047	916.4559021	7768.795419	-58.64483681

log.sigma.1.5.mm.3D_firstorder_Kurtosis	log.sigma.1.5.mm.3D_firstorder_Mean	log.sigma.1.5.mm.3D_glrlm_ShortRunLowGrayLevelEmphasis	log.sigma.1.5.mm.3D_glrlm_GrayLevelVariance
4.527984769	13.89961877	0.00173085	36.25261888
7.164040061	26.93781959	0.005115982	13.87707542
5.630567674	23.45589176	0.004866118	13.81933848
5.598507918	8.571350256	0.003249019	25.2282308
5.17971882	15.8101987	0.007079515	20.2789001
5.472552162	28.97300274	0.00397457	29.41377338
5.128302728	22.92654821	0.002754326	17.50238944
6.517523747	25.25121669	0.001905745	19.19366932
5.24065487	23.93294006	0.003063902	21.25598861
5.49859888	30.11699289	0.003399393	22.99308068
4.923116104	19.12573415	0.005094856	13.20243418
6.839184788	32.41156284	0.002868255	28.0204598
10.5215938	14.07636383	0.003215101	15.58423747
6.390882416	20.92369381	0.003016107	26.24258486
5.65517464	23.26556909	0.002423149	30.41160863
7.364840226	15.32564804	0.002332801	17.05585918
3.679189269	18.42725101	0.007212616	20.10479834
8.452393403	21.67289258	0.003417561	12.63325692
5.734014981	17.25282777	0.002962536	12.83472592
6.138848256	26.53003443	0.002387832	23.68891228
6.275690968	19.23332324	0.003158696	10.5797665
4.677478582	24.26283033	0.0050965	15.97630583
6.781501275	19.48447355	0.002632164	18.25206765
5.261541099	24.89765454	0.002640357	16.43219411
7.371652239	34.47471586	0.003911575	22.23762324
4.644327718	39.99510049	0.002018474	30.64864093
4.597932169	26.43536849	0.002410804	26.27706099
7.117400256	16.77412919	0.002291346	18.68944822
3.760586658	24.97883669	0.00630239	16.68002486
7.4976996	22.6368072	0.002399431	14.87780763
8.026934124	24.40870806	0.001962865	20.13926331
6.765027574	7.319289843	0.00276386	15.88607374
5.196053789	17.19410814	0.002398961	24.85691473
6.10534859	21.58988478	0.003305046	24.7858933
4.803496803	37.5321568	0.003145142	24.81278175
6.753272771	21.62189678	0.003038003	16.24385731
9.449200924	11.65018806	0.001386327	21.69589188
9.625073405	14.12356258	0.001211037	18.50973204
7.482628009	11.7212343	0.002938669	15.53557445
3.721351617	14.79265628	0.00420291	19.59143108
5.180146656	8.940915784	0.00461137	14.78845077
3.110937164	13.47491896	0.006630689	27.05347945
5.367743688	23.78283127	0.002567122	20.14530604
5.32908483	16.86377591	0.002706893	29.26189398
8.012973645	37.46733503	0.002628167	21.93883032
4.814633664	28.60989009	0.00325743	20.56105418
5.767901567	23.7321608	0.002459636	21.47540338
7.442076396	24.18551067	0.004610834	14.16824934
4.187121158	28.08518197	0.006741909	27.76896368
5.623790436	34.15006438	0.002913333	16.17767074
5.720594686	35.06439327	0.002651743	26.70229715
3.952024459	22.62943385	0.00262113	27.32666289
4.327993254	20.21981463	0.003391815	19.63415466
5.588736373	30.52393607	0.004748317	15.41689392
5.732438112	29.66689335	0.003306136	19.8341124
6.182845277	19.19653879	0.004415153	16.40300917
4.467147989	26.0517636	0.003275109	21.75550889
6.502395169	8.953045151	0.001944805	17.70147587
6.190841655	15.48790523	0.002486412	21.14069075
4.529477811	10.93986057	0.001818729	24.1459014
6.754929	23.45647943	0.002769363	19.37901304
7.453087214	25.59088331	0.001546019	24.70044306
5.251430786	30.91462815	0.001658529	23.03848618
6.142025898	17.14639954	0.001828747	21.70055433
4.445045329	24.02813121	0.007664654	10.95659371
7.319179672	14.57783734	0.002117487	18.54434894
7.688593414	7.132369814	0.003238915	17.56173268
5.594826659	14.27414702	0.002224091	21.95049747
5.960107315	23.86142674	0.001597748	22.47261917
8.065862583	18.63457853	0.003529298	12.08589514
5.332396968	15.26117986	0.006298693	17.44786677
7.954515108	28.68116716	0.001935108	19.80961699
5.077220959	23.36048854	0.005025017	22.03493745
3.958184819	9.512100857	0.003388565	31.07104099
3.568828383	15.28644075	0.004435425	25.15932468
5.307587594	14.91043526	0.004708204	17.50985424
6.469278671	26.16195691	0.003158007	14.98061107
7.62560844	18.06702152	0.005663514	17.58846505
6.674791544	18.51650692	0.003230483	15.28913024
4.857991038	9.963579642	0.002492277	27.74388189
4.774100457	16.17391457	0.001751194	35.01827123
5.312748572	8.28888552	0.003768721	25.44209943
6.958754257	18.0578155	0.00261935	18.37513803
4.305204581	17.21287099	0.003671706	10.82442945
4.571310212	16.75218032	0.004059853	24.54872145
11.16309901	11.8452488	0.00413481	6.398023281
4.367326306	7.053422013	0.004683709	24.63374238
5.462961289	36.12006999	0.00360755	19.87213907
7.726927173	25.12798779	0.00271868	18.03534891
7.847930003	20.31795048	0.002292132	15.96046045
8.342072944	22.76845374	0.002621113	9.51744208
3.979543091	33.64460657	0.003145845	22.78947308
4.543586345	14.83139495	0.003184768	23.2092914
5.558017865	25.44407514	0.004557703	16.60140747
4.103110376	13.65049387	0.002774747	21.26595618
7.791473304	23.48654352	0.001505106	27.4558563
4.650546367	14.0335088	0.004900476	17.41396057
6.887272201	4.929137675	0.003879311	23.84620703
5.595969305	7.938640496	0.006548394	11.04035926
7.144003768	24.46018675	0.002113411	13.74569585

log.sigma.1.5.mm.3D_glrlm_LowGrayLevelRunEmphasis	log.sigma.1.5.mm.3D_glrlm_GrayLevelNonUniformityNormalized	log.sigma.1.5.mm.3D_glrlm_RunVariance	log.sigma.1.5.mm.3D_glrlm_GrayLevelNonUniformity
0.001774989	0.058963987	0.064061679	118.2335757
0.005330216	0.108254367	0.12747109	91.40956918
0.005165036	0.102678566	0.195175665	138.6570057
0.00333965	0.073585096	0.094055933	66.41771989
0.007280647	0.081412993	0.104815267	177.1575676
0.004049978	0.067947556	0.061008499	42.46010934
0.002895564	0.081850146	0.11432645	423.1007125
0.002020759	0.096731353	0.136844293	444.5048295
0.003173241	0.074564501	0.089248532	314.2655332
0.003488581	0.074500176	0.075663264	74.62623674
0.005296625	0.088780755	0.093172889	123.4622767
0.002941918	0.072063121	0.084995104	154.5207321
0.003393805	0.133655686	0.200837551	215.5968408
0.003106909	0.074647801	0.092129704	118.5990502
0.002495551	0.070895487	0.076966586	131.4620675
0.002458599	0.097098826	0.131747579	383.1965718
0.007401086	0.068224445	0.068748443	76.65306309
0.003608961	0.118334779	0.154893571	129.345669
0.003084866	0.093758811	0.095713349	111.932246
0.002467327	0.074726894	0.082246289	362.0182649
0.003409757	0.117598098	0.188341625	311.3342277
0.005237661	0.080449919	0.06964844	81.42901937
0.002718494	0.085959239	0.100793591	81.00312543
0.002759333	0.082779485	0.098352886	202.0423922
0.00401288	0.085415635	0.10033152	100.0196053
0.002083364	0.061793412	0.072134175	137.5805188
0.002496422	0.066596026	0.081899568	200.8206942
0.002383653	0.088044295	0.097681849	224.0862748
0.006436412	0.07463912	0.05166593	31.74554662
0.002503611	0.098399915	0.106264083	185.179219
0.002069785	0.092146072	0.139315183	394.6477509
0.002901908	0.100840925	0.126754254	188.379471
0.00250311	0.070475934	0.103194079	209.3899551
0.003424783	0.077476722	0.109936686	166.6476603
0.00325728	0.069685779	0.08605449	285.5135598
0.003205106	0.098874881	0.134576279	267.1075456
0.001454146	0.09419475	0.122580656	318.0722357
0.001313428	0.112997786	0.253921553	498.2027113
0.003026926	0.089116129	0.081569114	102.0380302
0.004369805	0.068336014	0.082540915	463.7615105
0.004839975	0.098583004	0.124253948	231.5891736
0.006783217	0.05495832	0.054517027	202.3206864
0.002666975	0.076977494	0.087587201	261.1499764
0.0027817	0.06718435	0.072730464	156.9877222
0.00270528	0.080490318	0.08789452	95.29289157
0.003388688	0.075557793	0.087716016	192.7285193
0.002567214	0.079531046	0.097221298	335.069398
0.004771998	0.103989284	0.099354612	83.25217517
0.006854767	0.066216478	0.058665763	53.40463732
0.003040193	0.091243292	0.109433755	112.7301075
0.002723181	0.069886191	0.073772935	168.4246158
0.002693829	0.05900695	0.055981577	188.9876976
0.003504592	0.07387813	0.070538844	313.0064448
0.004916869	0.087252463	0.08667779	89.11764951
0.003413601	0.081282008	0.088259753	100.2863216
0.004558966	0.089559041	0.094948341	160.9420319
0.003382208	0.06956389	0.074431458	263.597988
0.002044719	0.090329953	0.11310443	284.7595672
0.002568463	0.080302362	0.081554704	123.1366777
0.001898875	0.066686062	0.092197707	565.6432064
0.002908873	0.098273568	0.141402112	155.7322086
0.001598122	0.077364363	0.089026601	191.9302688
0.001735337	0.07629043	0.100296185	407.2279029
0.00191153	0.07909675	0.097292284	557.1099467
0.007967772	0.096481976	0.098999962	70.09268891
0.002232049	0.095931181	0.12557602	335.0518031
0.00336724	0.098337584	0.117825387	116.6430789
0.002313489	0.077458684	0.096943818	447.1986988
0.00165489	0.073706793	0.075265419	404.8401585
0.003713063	0.107810367	0.133065639	281.298571
0.006489874	0.085340208	0.106225641	101.713326
0.002039652	0.095519009	0.132622055	369.6080162
0.005215815	0.079007863	0.117659683	164.181177
0.003471917	0.057505141	0.064013619	122.7675671
0.00457349	0.060664285	0.068856286	178.6872095
0.004867025	0.085856286	0.088685909	106.8343024
0.003303181	0.095612497	0.100470504	210.7327427
0.005800232	0.095827103	0.105702494	84.12870737
0.00334171	0.08857227	0.086414834	140.3160433
0.002568949	0.062852873	0.070102988	138.2428561
0.001795333	0.060658294	0.06457303	113.9697521
0.00389091	0.076101959	0.097375617	126.2897718
0.002749657	0.093468173	0.142925418	274.5888443
0.003869294	0.095013515	0.105351175	267.186587
0.004186314	0.06891272	0.087552535	156.1789758
0.004525723	0.156983285	0.289267747	200.0564603
0.004832028	0.068896127	0.094045624	152.2956155
0.003756469	0.080476709	0.10573095	202.3261105
0.00282019	0.092237746	0.10356989	124.0571028
0.002401941	0.09907422	0.12374164	181.730171
0.00280176	0.13366234	0.184985755	151.7181786
0.003233793	0.06362989	0.062844474	143.5695788
0.003291275	0.070395484	0.075418207	342.1332201
0.004731586	0.089046509	0.108551951	268.8215282
0.002867607	0.066938889	0.067318305	274.4560342
0.001577121	0.085043346	0.134876891	263.1843632
0.005077882	0.080793234	0.090156285	210.9123823
0.00396676	0.083366693	0.097337425	80.51509659
0.006843915	0.106304951	0.12547552	80.12103505
0.002268979	0.11375531	0.164349761	581.8025064

log.sigma.1.5.mm.3D_glrlm_LongRunEmphasis	log.sigma.1.5.mm.3D_glrlm_ShortRunHighGrayLevelEmphasis	log.sigma.1.5.mm.3D_glrlm_RunLengthNonUniformity	log.sigma.1.5.mm.3D_glrlm_ShortRunEmphasis
1.184441537	956.9572972	1799.036415	0.958739641
1.355588845	332.6801989	698.4541103	0.927220237
1.494808063	292.4142074	1077.818346	0.912855811
1.267404987	600.5116479	777.2332773	0.942809786
1.290488295	359.5698336	1859.629647	0.939852973
1.177743696	566.3181761	562.6913766	0.959866554
1.310823501	446.6280117	4389.837042	0.937353833
1.373768068	620.0012504	3786.172419	0.925788945
1.253717876	495.05191	3654.627583	0.945468645
1.221835817	553.3656129	878.5114616	0.950022164
1.270597428	291.1062624	1191.389092	0.940852815
1.241014363	704.3212064	1869.478268	0.947778258
1.543855837	534.3181445	1238.548882	0.898303999
1.261218124	628.8467349	1372.553477	0.944102832
1.223031353	691.7359262	1628.585743	0.950481937
1.363130703	552.9379743	3261.528064	0.926940927
1.198797072	270.8270142	1000.253228	0.95571168
1.43009563	428.0363988	876.219213	0.914938926
1.278591208	471.82873	1016.983519	0.938872333
1.234440462	641.7737277	4236.494588	0.948912523
1.496050787	347.3882579	2090.020309	0.908850832
1.207134487	338.6468586	893.8075864	0.952555355
1.282382762	664.8735797	806.9162328	0.940669109
1.275825401	492.8499923	2096.029263	0.941749399
1.28364303	603.7984485	1000.21272	0.939759663
1.205138266	732.3643399	1979.815167	0.955157254
1.231928827	597.3416201	2644.580277	0.949917934
1.276161568	612.2470755	2182.480545	0.941294917
1.156741104	310.5553665	385.417289	0.962640764
1.298787762	569.8276753	1595.265583	0.936900168
1.37501988	668.2828698	3537.157177	0.926605112
1.35539355	492.6909478	1544.910441	0.927109225
1.28600441	573.1596145	2546.696919	0.940831203
1.30483889	533.221844	1825.454034	0.937193358
1.24221809	482.0820176	3577.36637	0.94823642
1.368741695	438.115509	2228.649354	0.926201329
1.339070541	969.9508538	2821.534513	0.931102767
1.628398668	941.3709056	3368.290664	0.896092665
1.236191987	555.7379164	998.0303668	0.947676003
1.231132491	329.5144887	5961.011201	0.95046822
1.345645663	308.4578601	1953.718179	0.929271703
1.158310686	306.1386047	3348.863299	0.964000134
1.248130084	537.9244484	2950.66978	0.946642417
1.207232087	630.6332975	2074.470501	0.954587572
1.254581894	708.0972833	1022.599902	0.944005949
1.247039835	422.773922	2221.283861	0.94714393
1.270705237	538.8488882	3632.240759	0.943290495
1.283803978	367.0479462	682.0510466	0.938848661
1.172899318	394.8265643	726.9193732	0.960350387
1.30857323	505.1531995	1043.598068	0.935246419
1.210623258	664.5033167	2134.482188	0.953754235
1.164166326	552.1014906	2900.280472	0.962304282
1.20502973	410.3200833	3754.171947	0.954013878
1.25680135	344.81661	879.268048	0.942556287
1.251469115	504.3641803	1070.644157	0.94571993
1.273797351	399.8896803	1538.315984	0.940509185
1.214635984	461.1251162	3343.568043	0.952341082
1.317749776	639.2261938	2651.879069	0.933756889
1.237543261	614.3827266	1334.386045	0.947072213
1.251289446	661.0986032	7407.118846	0.948167513
1.386290685	524.5659443	1299.14466	0.923717858
1.248017154	962.276569	2162.616377	0.947611936
1.277716247	746.8886119	4592.540278	0.942409268
1.269997116	665.2502597	6077.43313	0.943526979
1.282794	206.7137985	620.3662572	0.939503001
1.349683971	599.8860535	2898.036426	0.928524887
1.327451619	498.0755479	994.547896	0.932607796
1.269861808	610.3484668	4978.25627	0.943372376
1.213196066	790.8870801	4864.187863	0.953624616
1.368174496	389.5940788	2149.323592	0.925632225
1.295815304	321.9465871	1015.109972	0.938461661
1.363242201	677.7614188	3201.509371	0.927393404
1.321783104	373.8140435	1752.825931	0.934811044
1.184131971	529.1328763	1917.037543	0.958979604
1.19931794	372.7213435	2620.297302	0.955411764
1.253085049	359.5098155	1078.462862	0.945250402
1.2833925	411.1701028	1884.494627	0.940123164
1.298174091	435.7511041	744.4138249	0.936932102
1.248195374	481.2849451	1374.063108	0.94577027
1.2015581	618.2783877	1955.907415	0.955286507
1.185760577	961.2672813	1685.056949	0.958547021
1.273659105	469.3032385	1426.666902	0.942170971
1.383692511	559.9662697	2420.438822	0.925475329
1.300679931	318.594583	2378.868809	0.93596356
1.247418682	446.4079523	1974.790666	0.947145516
1.748876833	284.8015807	922.036041	0.873816781
1.260436515	391.864678	1921.375148	0.945983811
1.295925001	430.7140547	2138.740604	0.938186166
1.290041624	579.3959522	1148.305216	0.93936748
1.34442959	607.9151839	1527.031287	0.929624398
1.506821115	505.814532	881.8597025	0.903026142
1.17781184	480.3912625	2034.782174	0.960687029
1.213195514	444.7409941	4305.383563	0.953783601
1.304728561	371.4705881	2556.435252	0.936282344
1.194346509	483.6679724	3657.247306	0.956534594
1.359530675	945.9395552	2580.117188	0.930158072
1.255612599	323.2342257	2258.854031	0.944788411
1.274440434	640.239534	829.7492764	0.941829265
1.352644793	239.4052224	624.0591832	0.927457036
1.441124188	518.5561251	4107.131389	0.915728122

log.sigma.1.5.mm.3D_glrlm_LongRunHighGrayLevelEmphasis	log.sigma.1.5.mm.3D_glrlm_RunPercentage	log.sigma.1.5.mm.3D_glrlm_LongRunLowGrayLevelEmphasis	log.sigma.1.5.mm.3D_glrlm_RunEntropy
1184.153898	0.944899587	0.001971813	4.785559165
482.5357091	0.902838338	0.006378167	4.140599778
467.709067	0.879249824	0.006868373	4.301325193
816.4576444	0.923628061	0.003762164	4.562137925
500.8482083	0.918756497	0.008211019	4.458337524
689.7878061	0.946736597	0.004382327	4.578005392
620.5238279	0.914596464	0.003594902	4.444704557
916.2002154	0.899463888	0.0026002	4.40953134
658.8138462	0.927036448	0.003679985	4.515962879
716.0192854	0.93434271	0.003884611	4.479213152
395.9276008	0.922056723	0.006216544	4.222967413
926.0685515	0.930218588	0.00328092	4.595153868
911.6615809	0.863623254	0.004345658	4.161683846
841.246662	0.925182564	0.003527294	4.587651807
890.7276428	0.934475806	0.002819988	4.631765432
814.0786822	0.901613697	0.003082726	4.354205314
339.2925893	0.940922562	0.008235207	4.477729189
668.5649805	0.886206251	0.004572611	4.121522504
647.0015892	0.919698945	0.00364031	4.183512154
833.7148099	0.931776821	0.002831453	4.547249512
566.4906575	0.875254375	0.004773369	4.138608295
430.7729032	0.937976759	0.005850452	4.285229189
908.2190898	0.920222356	0.003128145	4.357768501
663.4838913	0.921972625	0.003321826	4.375489171
817.0252587	0.919693033	0.004488822	4.438855194
918.3598341	0.93976686	0.002379145	4.738549244
772.1012862	0.932689334	0.002892122	4.661754035
832.085866	0.921433744	0.002818415	4.362055351
371.8366933	0.951471347	0.007000129	4.271054764
796.0989824	0.916073702	0.002995709	4.212828508
986.6960244	0.899957966	0.002614606	4.448816913
726.1500479	0.902702904	0.003575624	4.256270412
781.1154198	0.920003812	0.003004438	4.654177365
740.5396823	0.915155483	0.004001508	4.583215065
628.8488337	0.930238245	0.003779911	4.619589058
643.3917277	0.900607895	0.004036415	4.322536598
1396.377082	0.907200298	0.001786356	4.41569109
1691.402408	0.853785045	0.001933165	4.423690996
732.257677	0.93083177	0.003424501	4.206153343
425.2611517	0.933414453	0.005141599	4.526620509
448.5767043	0.905432655	0.005945323	4.276761086
365.861176	0.951980267	0.007444939	4.69245375
711.4799499	0.928615048	0.003127869	4.474750809
801.6445585	0.939092258	0.003119564	4.644625792
940.8787147	0.926267004	0.003055139	4.429984077
554.6400414	0.929101864	0.003998429	4.498920602
722.925489	0.923397295	0.003076842	4.506337945
505.4525994	0.919102711	0.005516927	4.113473665
484.793185	0.947663382	0.007340018	4.565783876
702.4814131	0.913746017	0.003643104	4.314046354
841.0610525	0.938048328	0.003047458	4.598447199
668.646538	0.950094699	0.00300863	4.677089981
520.7652358	0.938983224	0.004006261	4.454724013
457.0735407	0.925027871	0.005671242	4.287984934
666.8638848	0.927588201	0.003910701	4.420872641
544.2929638	0.921459566	0.005219781	4.290979401
590.1834909	0.936731007	0.003862311	4.533432418
903.9609422	0.911531788	0.002521656	4.375285202
808.3057212	0.930358476	0.002935772	4.43112858
867.77129	0.929076401	0.002287758	4.644501877
783.8660885	0.896942242	0.003616043	4.390285451
1270.970983	0.929069432	0.001844785	4.525820566
1009.334758	0.921934172	0.00210455	4.567241125
899.1097585	0.923853924	0.002304775	4.509891243
282.4847777	0.919571568	0.009370058	4.090568073
872.9260678	0.904214636	0.002790253	4.374374265
712.191007	0.909509202	0.003988113	4.276887837
826.8642016	0.923643077	0.002737879	4.53071915
1011.346085	0.937707674	0.001916439	4.505799962
575.7206992	0.900249509	0.00462036	4.133970844
447.564076	0.91733286	0.007397234	4.36923284
990.0296992	0.90181101	0.002563021	4.414735559
524.1916437	0.911702164	0.006142764	4.552026643
657.5779216	0.945040351	0.003841413	4.742755259
467.171071	0.940741469	0.005183694	4.659193592
478.9151271	0.927146624	0.005591753	4.32132847
557.8819418	0.91977013	0.003988854	4.248987976
608.0129698	0.916171511	0.006445516	4.275953067
636.2473482	0.927988824	0.003849408	4.256753718
776.7470277	0.940309797	0.002912009	4.673143568
1189.761193	0.944579804	0.001992767	4.752496892
639.1863309	0.922350024	0.004465102	4.579761109
831.6979754	0.89843989	0.003420642	4.415838092
444.9054227	0.915041678	0.004791793	4.14518039
589.0264614	0.929100829	0.004777246	4.608158139
576.5222613	0.830889737	0.006832938	3.835121408
522.961412	0.926656991	0.005534216	4.621541876
590.0258946	0.916849015	0.004466466	4.470180727
795.6017603	0.918558218	0.003305655	4.307164791
881.9512537	0.905641026	0.002939371	4.262540532
833.4916983	0.870280793	0.003746688	3.998290809
586.9280115	0.947200155	0.003629526	4.558454815
568.0191924	0.937666038	0.003779389	4.548436188
517.1820902	0.914731935	0.005558102	4.344778427
606.1975884	0.942095588	0.003280438	4.5262662
1373.909288	0.904104662	0.001947691	4.587308989
431.3389936	0.926599492	0.005892976	4.394474876
872.6281688	0.922268753	0.004377059	4.45541549
353.8615342	0.903431101	0.008262627	4.072950412
808.408446	0.885534625	0.003083463	4.220845873

log.sigma.1.5.mm.3D_glrlm_HighGrayLevelRunEmphasis	log.sigma.1.5.mm.3D_glrlm_RunLengthNonUniformityNormalized	log.sigma.1.5.mm.3D_glszm_GrayLevelVariance
998.5573654	0.897070596	52.18027462
358.2476039	0.826896325	31.02508896
318.9881082	0.797694798	28.47535602
638.6308428	0.860865199	41.84080502
383.7698161	0.854388537	33.55705001
589.0202285	0.90002811	47.75905325
475.9400734	0.848986723	30.86568651
669.2533458	0.823786489	40.0889806
524.0736618	0.866822415	36.47293099
583.4069098	0.876777481	39.71342705
309.9455797	0.856331873	23.20225124
743.9562	0.871744837	49.31725584
593.8792897	0.767518726	43.56271111
666.319036	0.863701036	47.40946173
727.9105636	0.878018713	52.25407562
596.766949	0.826227936	34.715314
283.2973903	0.890091742	30.93711018
467.909379	0.801240977	29.3630304
503.3200157	0.851645974	23.88081199
676.1679887	0.874356839	42.02324957
381.4932359	0.788995802	24.14598884
355.7918689	0.88271177	26.10198052
707.165425	0.856045435	32.51406397
522.6398003	0.858452752	29.80207831
641.2531349	0.853932659	43.15887743
765.6980753	0.888964048	46.44440562
628.4425272	0.876819504	42.93463348
650.9278019	0.857306861	36.65951173
322.3945587	0.90596882	23.34235091
609.283507	0.847489528	29.27231997
720.5538654	0.825683882	41.46424749
532.542492	0.826634333	32.43138754
608.8692393	0.856743293	43.11303477
568.7230423	0.848484913	45.46633563
508.026998	0.873005896	41.31750956
472.4839955	0.824693653	33.41557205
1041.950476	0.835307265	46.93357145
1048.171187	0.763815615	44.29690254
587.7540809	0.871473407	27.71137507
346.4574724	0.878106862	29.78131338
332.2659267	0.83125279	28.52754313
317.1669437	0.909511348	35.68151514
568.6355188	0.869444448	33.37569192
661.3872251	0.887595166	46.03449866
750.0754479	0.863355721	41.83822048
446.0486149	0.870591284	34.48073737
570.7497781	0.861966211	38.19733529
391.634347	0.851718065	28.48959378
411.5791964	0.900986516	39.69208938
539.3479011	0.844197268	31.53841354
696.3576455	0.885549864	45.14774622
573.8377545	0.905370039	39.08844748
430.5672868	0.885963649	32.33518273
365.4075738	0.860292738	29.79001547
533.2514618	0.867408954	34.65446415
425.6620622	0.85562425	31.50653898
484.6222314	0.882172018	34.49213238
684.8837308	0.840874428	33.98779996
649.7182008	0.870021691	38.33010792
696.6591128	0.873088315	37.95870748
567.541061	0.819570439	39.68372591
1016.214133	0.871630172	43.72616306
792.0413016	0.860141429	39.29328405
705.6430747	0.862544459	39.4812609
220.0560281	0.853477465	19.4717318
646.2955355	0.829524338	37.51444564
534.639401	0.838293812	36.18852854
647.9391391	0.862082274	38.36863288
830.2583825	0.885368407	35.57765094
420.9450028	0.823402901	27.09309984
343.5676468	0.851357567	30.58639607
729.8866427	0.827203372	40.90354172
399.1817818	0.843255659	40.20672714
552.477758	0.897769569	43.5252584
389.9851763	0.889354511	36.32228911
380.7296481	0.866314238	29.80006683
436.7952206	0.854802388	30.58035275
465.8509546	0.847716182	35.68191746
509.0998149	0.867195293	27.89516482
647.0435406	0.889078198	42.64674024
1003.039038	0.89669379	50.93254665
499.0227345	0.85945399	43.79762985
604.4328559	0.823488799	37.09035646
340.7716377	0.845578387	19.1489758
471.5077577	0.870858774	38.85770481
327.0039167	0.721971968	16.51501374
414.3666386	0.868611333	38.93036722
458.3465909	0.850511598	36.03354295
616.8876906	0.853383055	35.88615354
654.5499408	0.832107976	31.55183639
558.5797395	0.776656123	24.88252105
499.6943119	0.901676787	32.65383809
466.6922215	0.885718976	37.89505358
396.7244064	0.846454333	30.68005875
506.0111709	0.891861223	31.33262829
1015.854918	0.833505963	52.79822043
342.4597631	0.865076248	29.67636516
680.9092067	0.858761329	43.56599973
258.9517428	0.827522911	21.05435509
565.1653566	0.802800596	30.86819259

log.sigma.1.5.mm.3D_glszm_SmallAreaHighGrayLevelEmphasis	log.sigma.1.5.mm.3D_glszm_GrayLevelNonUniformityNormalized	log.sigma.1.5.mm.3D_glszm_SizeZoneNonUniformityNormalized
726.8120733	0.041399154	0.512612524
240.3142836	0.065582631	0.434372326
216.7466093	0.056118093	0.457516563
408.9068062	0.047229603	0.48112135
240.9442769	0.051576924	0.482059369
445.5565661	0.045604734	0.501671006
332.834202	0.052747924	0.453024578
475.8739565	0.050140487	0.506046835
357.4455778	0.049037622	0.479993703
376.6474562	0.047762158	0.439404025
196.0629074	0.061897521	0.427008264
502.1506054	0.045556122	0.490778727
424.6777711	0.055525926	0.489461728
468.8277714	0.045249931	0.474133333
502.167726	0.043064989	0.473238157
398.6936762	0.054644192	0.465406999
196.9699612	0.051154998	0.424335846
302.7905038	0.061293135	0.462881596
337.8978024	0.062608141	0.451129769
481.9768224	0.047023599	0.485243703
271.6477831	0.062665862	0.472018294
228.9486235	0.058208815	0.419181242
476.8036243	0.055839205	0.461234694
368.1224397	0.053074135	0.454973541
469.0792363	0.049311044	0.491822637
560.4945182	0.043607822	0.477768899
457.1792876	0.045011269	0.493371172
438.344592	0.051043829	0.469603998
221.7523981	0.059519882	0.430495449
416.4952473	0.057251352	0.481182679
513.1665449	0.050474684	0.491515525
350.7963851	0.0582324	0.4853917
427.7422614	0.044705456	0.465511164
379.9044399	0.046451546	0.455437276
357.4533376	0.046037263	0.477689534
333.602719	0.057212919	0.482061824
741.6447135	0.04989103	0.496840211
777.5098118	0.050481686	0.508684043
364.5900553	0.061316854	0.4168501
235.2125872	0.051305334	0.425886958
215.4495723	0.057538405	0.430947078
237.2542351	0.046272013	0.468509273
375.7732776	0.051401354	0.447731494
458.6719005	0.044180841	0.48460474
515.1917727	0.051408633	0.46942462
320.2263923	0.050043734	0.503254271
417.2364204	0.049059872	0.493958964
255.5174048	0.061796301	0.468285067
279.9101278	0.047059715	0.483842949
396.7284079	0.054319915	0.500508077
496.5648824	0.045521718	0.481466018
406.1494096	0.045848749	0.462938795
287.9894391	0.050222747	0.465207752
260.793032	0.057410187	0.488869336
348.7445236	0.051718054	0.440076863
277.620381	0.056005152	0.454931293
322.2594699	0.048803835	0.45900555
472.8930418	0.054304053	0.464928449
436.8981736	0.049397292	0.463932252
502.615793	0.046888878	0.48002609
397.3857696	0.051571577	0.500054555
707.5505819	0.047433157	0.498022502
558.3768897	0.047189995	0.487173604
481.8638064	0.048350286	0.466577382
144.7074486	0.068761159	0.438677842
456.4306718	0.052598561	0.502212664
359.5302848	0.05626602	0.474948301
441.0161239	0.048565578	0.483861526
576.8137523	0.050826827	0.484699493
276.0388596	0.065491486	0.446054341
222.3490402	0.054880259	0.453651579
525.7343614	0.051217625	0.501928939
290.3526809	0.047656659	0.497846592
388.5917621	0.043842496	0.492489278
278.2465839	0.046184411	0.477237142
247.3074407	0.055232234	0.442964747
325.7469011	0.05643546	0.496105166
310.6905164	0.054937754	0.504837678
345.6832856	0.058860324	0.456567721
475.8705561	0.04573944	0.480466002
741.0707478	0.042199465	0.52581374
346.374034	0.045003463	0.518594183
423.3775202	0.052030788	0.475445529
210.4815698	0.065473569	0.383031365
322.217384	0.046899695	0.459097926
185.6607839	0.085801852	0.413102671
281.4662775	0.046521577	0.467645193
326.6542759	0.049140951	0.492526123
424.041447	0.054545582	0.481378614
440.5612013	0.058436536	0.48425826
415.5767439	0.071527072	0.493921469
352.8265031	0.04994989	0.465070549
314.7447726	0.047382979	0.46174996
279.4366209	0.054748378	0.479936152
348.4936817	0.051150769	0.460545325
743.4080668	0.045076168	0.511354957
236.9587948	0.052600169	0.475273771
456.0666812	0.050448048	0.473604295
151.6392623	0.068918467	0.407496102
394.1352532	0.058604912	0.485001015

log.sigma.1.5.mm.3D_glszm_SizeZoneNonUniformity	log.sigma.1.5.mm.3D_glszm_GrayLevelNonUniformity	log.sigma.1.5.mm.3D_glszm_LargeAreaEmphasis	log.sigma.1.5.mm.3D_glszm_ZoneVariance
557.7224265	45.04227941	18.89522059	15.09128663
129.442953	19.54362416	139.1845638	129.3401311
221.4380165	27.16115702	285	274.9547674
191.9674185	18.84461153	43.36591479	37.37016727
447.8331539	47.91496233	179.3347686	172.8374828
163.0430769	14.82153846	13.50153846	9.377514793
989.4056777	115.2014652	108.7394689	102.0445496
875.9670711	86.79318313	614.8561525	606.1483686
902.8681552	92.23976608	62.5645933	56.72367312
198.6106195	21.58849558	21.16814159	15.54326885
234.8545455	34.04363636	49.87272727	42.35516033
478.0184805	44.37166324	35.51848049	29.91801732
220.2577778	24.98666667	789.34	772.126721
320.04	30.5437037	59.7762963	53.30586776
411.2439586	37.42347526	24.52704258	19.31457001
674.3747412	79.17943409	353.3761215	344.2556381
230.4143646	27.7771639	14.92449355	10.08935421
155.9910979	20.65578635	269.421365	256.0348863
218.7979381	30.36494845	58.26391753	51.10139654
1063.654197	103.0757299	66.90374088	61.27827447
390.3591294	51.82466747	627.1100363	613.7393852
198.2727273	27.53276956	18.74630021	13.54250008
184.9551122	22.3915212	70.67331671	64.15236224
463.1630648	54.02946955	57.92436149	51.16333984
242.4685598	24.31034483	65.42596349	58.75846434
533.6678603	48.70993733	20.22112802	15.72308324
713.9080857	65.13130615	56.1292329	51.13723554
478.9960784	52.06470588	138.954902	131.6224875
97.72246696	13.51101322	7.792951542	3.915348639
349.8198074	41.62173315	68.47042641	60.48805269
799.6957591	82.122311	452.1745544	443.6224347
321.3293051	38.54984894	232.6661631	222.8981777
603.3024691	57.9382716	93.41898148	87.21134771
405.7946128	41.38832772	111.2390572	104.282725
914.7754569	88.1613577	67.48616188	62.19736286
483.0259481	57.32734531	523.1447106	514.1865779
619.5597434	62.21411387	287.0048115	278.1007853
727.4181818	72.18881119	827.734965	814.7094278
205.5070994	30.22920892	28.77079108	22.54612033
1321.527232	159.2004512	23.08217854	17.59302637
361.1336516	48.21718377	268.2816229	258.6997169
968.877176	95.69052224	7.996615087	4.500004443
666.2244624	76.48521505	58.06653226	52.03963925
570.3797791	52.00084962	28.47408666	24.00573005
238.9371316	26.16699411	37.57563851	31.27149424
584.2782084	58.10077519	65.28251507	59.69240631
924.1972207	91.79102084	136.2565473	130.311395
145.6366559	19.21864952	66.38906752	58.54546582
215.3101124	20.94157303	8.856179775	5.199060725
243.7474333	26.45379877	140.8459959	133.1388166
555.6117851	52.53206239	31.87781629	26.92198412
802.7358708	79.5017301	10.01441753	6.235048338
913.6680244	98.63747454	32.9592668	27.68143902
203.8585132	23.94004796	41.08872902	34.079568
235.4411215	27.66915888	37.44672897	31.26662591
326.6406685	40.21169916	77.32033426	69.94434401
822.5379464	87.45647321	30.22377232	25.1285746
564.8880658	65.97942387	232.8131687	224.7129374
321.5050505	34.23232323	21.6046176	15.94941791
1946.505795	190.134402	86.87225647	81.80392866
306.5334421	31.61337684	167.954323	159.6546602
569.23972	54.21609799	66.30446194	60.84776214
1144.370796	110.8492976	97.12345679	91.0499284
1382.468782	143.2618967	50.96692541	44.34798401
124.584507	19.52816901	79.87323944	72.1354394
655.8897397	68.69372129	369.9188361	361.1742716
221.8008565	26.27623126	85.99571734	78.19882708
1214.49243	121.8996016	90.05577689	83.85547848
1303.841636	136.7241636	28.69702602	23.95628875
379.5922444	55.73325499	551.8119859	540.2151999
227.2794411	27.49500998	62.94411178	56.22142541
729.8046768	74.47042641	618.5515818	609.8462412
423.6674501	40.55581669	160.6333725	153.46155
558.9753304	49.76123348	19.53568282	15.57435541
717.2874251	69.41516966	22.9427811	18.60319636
237.4291045	29.60447761	34.42910448	28.16043384
431.6114943	49.09885057	97.25747126	89.67282864
175.1786744	19.06340058	77.70028818	70.07823335
299.9649924	38.67123288	39.62100457	32.87049895
530.9149321	50.54208145	25.03438914	20.5537921
540.0107108	43.33885102	19.46153846	15.71069725
394.1315789	34.20263158	70.46710526	64.86391794
529.6463196	57.96229803	385.6364452	377.0253288
384.1804586	65.66999003	74.39780658	65.0108836
478.3800384	48.86948177	37.1756238	31.69678954
116.0818505	24.11032028	1092.989324	1063.265485
473.7245805	47.12635735	64.57551826	59.03235237
522.5702168	52.13854854	159.3817154	152.7028278
257.0561798	29.12734082	110.7003745	103.1841518
332.2011662	40.08746356	175.3921283	166.6784439
163.9819277	23.74698795	530.7710843	515.3441719
554.364094	59.54026846	12.34228188	8.348990473
1053.713409	108.1279579	39.15907099	34.0004795
572.0838926	65.26006711	199.2348993	191.5705486
929.8410104	103.2734027	24.15255077	19.50627336
659.13654	58.10318076	251.2909232	244.2431134
535.6335404	59.28039042	54.12866016	47.88087824
193.7041565	20.63325183	87.07334963	80.52025036
109.2089552	18.47014925	127.7462687	118.0620962
828.381733	100.0971897	1039.76815	1028.33995

log.sigma.1.5.mm.3D_glszm_ZonePercentage	log.sigma.1.5.mm.3D_glszm_LargeAreaLowGrayLevelEmphasis	log.sigma.1.5.mm.3D_glszm_LargeAreaHighGrayLevelEmphasis	log.sigma.1.5.mm.3D_glszm_HighGrayLevelZoneEmphasis
0.512723845	0.020796569	18763.30331	984.9549632
0.318716578	0.418201375	47426.43289	361.8255034
0.315514993	0.989633917	82894.23967	323.4772727
0.40839304	0.069915385	28860.52882	596.7443609
0.392314189	0.467250946	71367.29925	352.9235737
0.492424242	0.029709251	7436.344615	603.2676923
0.386480269	0.246897192	48596.39423	478.8159341
0.338880188	0.943956614	401984.4547	660.4471404
0.413770348	0.12221657	33149.84848	508.3163211
0.421641791	0.040461349	12543.85177	563.6371681
0.364721485	0.170623397	15367.06727	296.1727273
0.422559653	0.052195494	26271.72279	714.3110883
0.241028388	1.371544344	457180.5556	593.3088889
0.393127548	0.09378289	40153.85037	658.2681481
0.438004032	0.036791086	17759.12198	715.5684695
0.331124314	0.604681612	207760.7005	578.6445825
0.454773869	0.065380453	4157.653775	287.4677716
0.273317113	0.607300342	121070.2493	447.4866469
0.373651772	0.116093105	30135.0866	489.5505155
0.421619542	0.105188672	43751.35493	673.082573
0.273478836	1.699196106	232919.0484	382.1197098
0.43836886	0.057799401	6870.53277	348.6575053
0.391601563	0.103600727	49955.26683	689.074813
0.384586324	0.119018148	28992.70923	527.8634578
0.387274156	0.113760135	39956.68966	649.6369168
0.471506965	0.029772678	14828.36437	781.8397493
0.447571915	0.096358144	33668.09261	630.8044229
0.36929761	0.222790822	87851.90882	631.5411765
0.507829978	0.032374639	2466.938326	326.2863436
0.353943525	0.11196456	43057.89409	587.258597
0.341950399	0.656236976	313137.9201	716.409957
0.319961334	0.436981978	125220.6405	500.9380665
0.401362651	0.154808981	57554.93904	607.0655864
0.379148936	0.207832079	61164.10213	558.1762065
0.434831971	0.145278636	32264.00836	507.4715405
0.33411137	1.139703748	241581.504	472.740519
0.335124966	0.273035065	303888.9575	1026.1668
0.277078086	0.812639692	845407.4098	1058.830769
0.400813008	0.049509028	18028.41988	561.0121704
0.426822558	0.074971317	7817.645827	348.3744763
0.3230532	0.789940382	92382.21599	321.9260143
0.534781484	0.03598544	2460.744197	325.2736944
0.407336436	0.106936943	32512.94825	553.5712366
0.47307074	0.046474919	18784.87511	643.5607477
0.39827856	0.054708524	27657.89391	741.1237721
0.42295082	0.156845681	27950.33247	443.7674419
0.410127137	0.252026299	74620.64351	573.1143773
0.35706085	0.175143624	26321.40193	373.3022508
0.522914219	0.031016779	3646.321348	400.9483146
0.360207101	0.27674611	72858.25462	543.7659138
0.449202024	0.050864575	21350.94021	695.4419411
0.514387422	0.019740081	5814.00519	572.0219146
0.435283688	0.080945954	14110.67413	417.1461303
0.377717391	0.127918946	13993.40288	369.6043165
0.402255639	0.077134657	19284.2785	522.8579439
0.368205128	0.19343734	32082.51393	409.8036212
0.443016069	0.068041796	14323.50167	471.1668527
0.351359167	0.341500263	159958.3235	673.4740741
0.420509709	0.035074465	14406.38384	628.6868687
0.444188849	0.135401748	56546.64636	699.329963
0.347112118	0.307146178	93083.4323	556.4486134
0.428089888	0.067106454	67073.11986	988.0918635
0.405769563	0.127971324	74697.28097	787.1260111
0.388692116	0.073138748	36461.79177	688.483969
0.359493671	0.390573768	17016.98592	217.8450704
0.338166753	0.585512475	235059.268	631.9257274
0.358128834	0.162677837	46837.41756	514.8329764
0.4016	0.141960861	58195.23347	625.2047809
0.459279495	0.035636723	23954.39033	809.8237918
0.293650794	1.33589293	229574.9365	410.7649824
0.385681293	0.188209351	22342.32136	328.4071856
0.338927739	0.8814075	435876.368	729.4786795
0.37340939	0.446377115	59241.99295	400.8495887
0.502434706	0.038048731	11317.58238	536.6
0.480038326	0.066509006	8797.760479	389.4271457
0.399403875	0.09570345	13458.35821	366.2761194
0.363105175	0.232610926	41426.86437	446.3183908
0.362212944	0.172503215	37203.68012	442.6282421
0.384885764	0.081140178	20474.29985	498.9238965
0.472424113	0.040595805	16620.12579	652.6696833
0.516339869	0.021402999	19301.01071	991.8149951
0.422456921	0.145356992	35549.03421	478.575
0.340776996	0.649819852	230654.5485	598.7037702
0.326391149	0.218825313	26052.5005	331.0957129
0.427224272	0.085794211	17445.28503	462.4193858
0.183420366	3.334096781	360530.2989	292.4768683
0.424737945	0.167071618	26213.31392	403.4274432
0.386943837	0.369841285	69797.66352	459.2836946
0.364754098	0.183917695	68030.28277	600.7940075
0.338765432	0.276887274	112500.6851	626.8833819
0.254601227	0.996907965	284335.3855	576.9909639
0.500419815	0.029149195	5914.168624	500.2944631
0.440285549	0.086223096	18616.07011	454.130149
0.361212121	0.523385974	77195.8255	392.3498322
0.463924632	0.049342731	12631.24468	498.6760773
0.376680304	0.260299903	244400.5531	1014.327386
0.400070998	0.159501099	19377.00621	333.2768412
0.390639924	0.130616164	61199.75306	657.9755501
0.321342926	0.493232958	34064.23881	239.3880597
0.295808798	1.948159837	556341.4496	565.3495316

log.sigma.1.5.mm.3D_glszm_SmallAreaEmphasis	log.sigma.1.5.mm.3D_glszm_LowGrayLevelZoneEmphasis	log.sigma.1.5.mm.3D_glszm_ZoneEntropy	log.sigma.1.5.mm.3D_glszm_SmallAreaLowGrayLevelEmphasis
0.743996471	0.002361126	6.198190984	0.002061986
0.684606638	0.009683171	5.697284838	0.008733193
0.702788487	0.008298911	5.83588344	0.007274118
0.720039273	0.005522221	6.047559719	0.005024288
0.721921159	0.011903985	6.026125743	0.009825052
0.735730332	0.006059593	5.987887719	0.005554573
0.700095894	0.003738896	6.165578375	0.003008168
0.73934595	0.002798245	6.056930225	0.002370097
0.720305252	0.004591342	6.162029349	0.00395428
0.689347952	0.005509198	6.100989925	0.004870501
0.679201758	0.008062337	5.912772163	0.006741082
0.728000663	0.004760445	6.206043328	0.004310306
0.726749692	0.007583763	5.93079128	0.007009998
0.715749999	0.005040914	6.231866872	0.004455124
0.71523173	0.003655616	6.269886619	0.003192097
0.70946074	0.003710155	6.11763968	0.00316458
0.676822504	0.010625849	6.036212675	0.008998004
0.706774202	0.006528049	5.775946683	0.005622125
0.698485286	0.004620351	5.802523356	0.003957382
0.724234071	0.003555555	6.246942393	0.003092317
0.714087497	0.004820376	5.831283597	0.003919617
0.671865189	0.007773375	5.919912453	0.006734893
0.705509142	0.004411497	5.828067386	0.003937126
0.70147755	0.003760443	6.092374229	0.003122471
0.728787536	0.00722134	5.974597311	0.006726337
0.718665278	0.00269722	6.298089118	0.002273902
0.730256992	0.003327359	6.202004632	0.002831113
0.712539457	0.003591587	6.117819793	0.003109807
0.681867691	0.008896835	5.699213983	0.007687804
0.720746346	0.003809377	5.894718385	0.003283524
0.728686698	0.003056459	6.151670861	0.002617526
0.723997699	0.004686515	5.896666974	0.004084998
0.709598269	0.00343491	6.31507761	0.002867505
0.70180207	0.005513191	6.263723249	0.004823096
0.718728063	0.004456671	6.245564698	0.003764018
0.721745084	0.00472409	5.957878563	0.004001047
0.73227356	0.002241015	6.176306806	0.001944532
0.74088925	0.001999824	6.13813757	0.001737102
0.671354095	0.004767095	5.920431536	0.004173968
0.678689524	0.00546076	6.309080624	0.004220338
0.682133906	0.007561932	6.039248853	0.006256674
0.71190366	0.008690218	6.195086131	0.00729255
0.695587212	0.003639691	6.184661742	0.002972912
0.723390076	0.003847732	6.187923181	0.00327737
0.712180963	0.004469629	6.0509472	0.004068582
0.737194422	0.004458627	5.988639802	0.003722512
0.730599704	0.0033587	6.140975575	0.002751996
0.710002394	0.008038104	5.681345742	0.007089225
0.722935185	0.010044592	5.961082189	0.009119119
0.73497531	0.004706971	5.790593122	0.004163446
0.721485537	0.004000639	6.224714501	0.003533978
0.707552582	0.003313746	6.240377292	0.002685681
0.709351396	0.004694364	6.135556172	0.003906807
0.72563786	0.007787957	5.832924218	0.0069806
0.689316699	0.005270642	6.070321352	0.004605804
0.701321701	0.007740273	6.007717424	0.006955205
0.704490243	0.004629298	6.187351408	0.003900801
0.709078292	0.002866446	6.086955221	0.002366557
0.707998783	0.003779228	6.111308946	0.003283262
0.720466661	0.002267259	6.276396878	0.001803276
0.734780505	0.004612659	5.983910869	0.004080649
0.733337692	0.002306169	6.114027474	0.002031515
0.725659333	0.002240555	6.193212024	0.001853656
0.710238986	0.002499327	6.346875945	0.002023434
0.688715	0.012417819	5.596731509	0.010732346
0.736364122	0.003243916	6.05362226	0.002743742
0.716402726	0.005588645	5.894294121	0.004974286
0.72312769	0.003235427	6.2297188	0.002744375
0.723809639	0.0020799	6.179175278	0.00170874
0.694221126	0.006229868	5.879658279	0.005369768
0.700314113	0.011014501	5.943753476	0.009837039
0.736315539	0.003019465	6.086965709	0.002604156
0.733342214	0.008311265	6.093827353	0.00715879
0.729582537	0.004706422	6.203408855	0.004059463
0.718388772	0.005874125	6.18456429	0.004816143
0.692129878	0.007392918	6.000777696	0.006288328
0.731637844	0.004628934	5.927390015	0.00394393
0.735843828	0.01132829	5.796130225	0.010718642
0.70251415	0.005144509	5.913481411	0.004463891
0.720333377	0.003374066	6.190593505	0.002821751
0.753251472	0.002397445	6.138711937	0.002114424
0.748012502	0.005906917	6.060238604	0.005232879
0.716966693	0.004386921	6.077159379	0.003842707
0.642630639	0.00517786	6.072737211	0.003913498
0.70470849	0.006150128	6.231453844	0.005234568
0.666885512	0.009229826	5.467793911	0.007722619
0.710992195	0.007235268	6.18775771	0.006282043
0.729426682	0.005546743	6.038653386	0.004779017
0.721073405	0.004561729	5.958669313	0.00405136
0.722927404	0.003792348	5.857322744	0.003296803
0.729765256	0.005083076	5.407752486	0.004583084
0.709086522	0.004125335	6.091582538	0.003440926
0.706660917	0.004366284	6.25013061	0.003589916
0.720183989	0.007686939	5.984286083	0.006737718
0.705837606	0.003579597	6.153402953	0.002886608
0.742963648	0.002331767	6.193558898	0.002043121
0.716449564	0.007393527	6.062861489	0.006233156
0.715436134	0.007265376	5.966212294	0.006796448
0.663060502	0.011471188	5.629938588	0.009485171
0.724171549	0.003133598	5.923209193	0.002628751

log.sigma.1.5.mm.3D_ngtdm_Coarseness	log.sigma.1.5.mm.3D_ngtdm_Complexity	log.sigma.1.5.mm.3D_ngtdm_Strength	log.sigma.1.5.mm.3D_ngtdm_Busyness	log.sigma.1.5.mm.3D_ngtdm_Contrast
0.003162684	1.707892215	2.27591835	0.170093559	7.06E-05
0.006850667	0.967990731	2.678352763	0.184764664	7.43E-05
0.004881092	0.615930153	1.855186561	0.257325689	3.64E-05
0.006940356	1.766520668	3.540928871	0.124479493	0.000134007
0.002627811	0.53073381	0.979497316	0.465980264	5.01E-05
0.009385808	2.977769171	4.890818542	0.097625054	0.000229414
0.001229165	0.344989344	0.775317393	0.658213154	1.04E-05
0.001333225	0.357707157	0.9306365	0.494129275	1.19E-05
0.001470817	0.429433914	0.806050893	0.575128966	1.99E-05
0.00589211	1.461534636	2.682210671	0.160117137	0.000118671
0.00438667	0.59368348	1.351157189	0.341158175	5.17E-05
0.002672129	1.144266094	1.987109412	0.234888394	4.74E-05
0.003671207	0.654820976	2.576518775	0.19541145	2.37E-05
0.003963008	1.412374964	2.776062026	0.167965563	5.52E-05
0.003385919	1.475754925	2.353130945	0.185231749	6.06E-05
0.001539395	0.432846085	1.226412406	0.432391767	1.13E-05
0.005551878	0.94649401	1.499883221	0.314786833	0.000119484
0.005269793	0.76498033	2.655585347	0.186012164	4.02E-05
0.004924351	0.8222941	1.915126823	0.212924046	5.06E-05
0.001265604	0.510966211	1.039840028	0.487527597	1.46E-05
0.002254183	0.23498338	0.816557418	0.551815754	1.54E-05
0.005477161	0.914313904	1.626470167	0.282980754	0.000112236
0.006346024	1.269820475	2.742632124	0.140522994	9.86E-05
0.002603778	0.489972869	1.119111166	0.386790455	3.05E-05
0.005444554	1.518824712	3.659928939	0.133025891	6.43E-05
0.002871511	1.156187581	1.925188214	0.228818456	4.94E-05
0.002214685	0.737128374	1.292407952	0.334796186	3.22E-05
0.002459975	0.64226942	1.597109857	0.292416403	2.53E-05
0.012978012	1.798677024	2.658682504	0.163323046	0.000388403
0.002973236	0.646443275	1.714745118	0.278271623	3.12E-05
0.00143979	0.566500882	1.522286759	0.359373043	9.97E-06
0.003385067	0.585683332	1.520761602	0.269849726	3.47E-05
0.002247635	0.726185164	1.472502604	0.316667491	2.67E-05
0.003086395	0.973945207	2.020051443	0.22949668	3.56E-05
0.001587098	0.582839125	1.085410604	0.474946967	1.93E-05
0.002320678	0.498683178	1.430926919	0.369644854	1.87E-05
0.001874978	0.792409611	2.235614431	0.22288937	1.37E-05
0.001435303	0.454283588	1.874928055	0.288151433	6.70E-06
0.00487574	1.045296764	2.317457748	0.200376646	7.42E-05
0.000946574	0.218772939	0.378018307	1.300550296	1.35E-05
0.002710563	0.379459081	0.94509498	0.475860215	2.85E-05
0.001686702	0.525406853	0.661550279	0.84575726	4.17E-05
0.001787333	0.555557961	1.117127867	0.43485137	2.17E-05
0.002562418	1.130907728	1.648029483	0.268426803	5.05E-05
0.005088906	1.561458501	3.831101862	0.130863305	6.41E-05
0.002536556	0.68486949	1.311528251	0.369102523	3.12E-05
0.001523629	0.490725245	1.030784185	0.463720404	1.56E-05
0.00662664	1.220579426	2.947683337	0.173848443	8.38E-05
0.007505547	1.827024996	2.584740342	0.168805815	0.000220745
0.004762925	0.805214265	2.073269914	0.221801716	6.21E-05
0.002591888	0.936047252	1.694157741	0.266472577	4.11E-05
0.001876947	0.799442959	1.078682119	0.432107561	3.69E-05
0.001398577	0.413398774	0.690565954	0.698833252	2.02E-05
0.006029092	1.09269565	2.421617109	0.196238958	6.66E-05
0.004872703	1.150971084	2.427504447	0.192118638	7.05E-05
0.00333075	0.591558576	1.43955936	0.333129081	4.21E-05
0.001669431	0.4417263	0.777216831	0.586842934	2.66E-05
0.002049096	0.496063893	1.263027264	0.340154773	1.84E-05
0.003927064	1.171810589	2.226669321	0.189666687	5.44E-05
0.000800551	0.309353949	0.545769138	0.784414535	9.00E-06
0.003868863	0.85743729	2.272456869	0.206265569	4.02E-05
0.002487633	1.077060296	2.170197824	0.19947841	3.09E-05
0.001177224	0.446177799	0.913202425	0.498654485	1.32E-05
0.000848543	0.373005333	0.792268291	0.657158554	8.03E-06
0.0078827	0.703814129	1.625621278	0.287649742	0.000111043
0.001802165	0.457614534	1.184972715	0.381601719	1.63E-05
0.005419915	1.131140818	2.733852615	0.152347299	5.44E-05
0.001087371	0.399967199	0.79304988	0.588554342	1.16E-05
0.001157475	0.524029397	1.136394753	0.436679187	1.06E-05
0.002312709	0.322771719	1.002485787	0.472304165	1.84E-05
0.005028235	0.864459397	1.709262564	0.263482196	7.78E-05
0.001607653	0.545827003	1.58965081	0.337392694	1.14E-05
0.003221726	0.726493854	1.464947496	0.31425249	4.06E-05
0.002929779	1.245057435	1.638357516	0.280983053	6.47E-05
0.002081633	0.623006165	0.838694668	0.570166188	4.54E-05
0.004843024	0.910902394	1.824772565	0.24732465	7.17E-05
0.002687736	0.584243677	1.549679547	0.348381975	2.33E-05
0.006603189	1.384652285	3.26465919	0.146185117	8.40E-05
0.003590677	0.811023661	1.884141163	0.261024298	4.11E-05
0.003008482	1.21377517	1.898406165	0.22974891	4.63E-05
0.003341388	1.707888067	2.36196579	0.164384416	7.43E-05
0.003828896	1.250999473	2.128928658	0.212601787	5.82E-05
0.002181926	0.538179103	1.345036527	0.330048892	1.90E-05
0.002175193	0.220835426	0.572645427	0.743159062	2.36E-05
0.002707471	0.808743762	1.237974463	0.355548015	5.04E-05
0.004404736	0.281373507	1.746780253	0.325166774	1.75E-05
0.002976536	0.846498832	1.365730216	0.332709261	4.76E-05
0.002480307	0.477998936	1.045847328	0.442079552	3.70E-05
0.004466218	0.98227703	2.491402224	0.182294589	5.27E-05
0.003294759	0.644965883	1.871301352	0.242861888	3.29E-05
0.004918962	0.402987654	1.756750689	0.234741663	3.55E-05
0.0027363	0.845152452	1.31301294	0.357711677	4.81E-05
0.00127958	0.436418087	0.731460528	0.670314725	1.83E-05
0.002047309	0.322827543	0.798945126	0.59545545	2.64E-05
0.001479866	0.5379053	0.881009883	0.561691904	2.03E-05
0.002026126	0.947138377	2.280007144	0.209978371	1.95E-05
0.00237768	0.448160232	0.897737769	0.525326308	3.15E-05
0.006568906	1.725048753	3.362078241	0.114401419	0.000104089
0.007899316	0.596339358	1.539829979	0.264810183	0.000110668
0.001122843	0.209804223	0.701371059	0.726113645	8.17E-06

log.sigma.1.0.mm.3D_gldm_GrayLevelVariance	log.sigma.1.0.mm.3D_gldm_HighGrayLevelEmphasis	log.sigma.1.0.mm.3D_gldm_GrayLevelNonUniformityNormalized	log.sigma.1.0.mm.3D_gldm_DependenceEntropy
13.43527681	487.1437323	0.09411968	6.162415142
5.103411593	150.0032086	0.173503389	5.674248196
4.612799151	124.4967405	0.18652737	5.938758014
9.169479398	254.0583419	0.122972691	6.057282874
7.60935146	197.8116554	0.131484261	5.996226212
11.76440771	270.0969697	0.106818182	5.783405858
6.521651275	230.1911166	0.137093326	6.064894751
7.440470182	296.57361	0.158862309	5.997447378
8.031896076	294.9564452	0.12270085	6.035674167
9.358623162	268.0522388	0.11223442	5.886631286
5.077405033	145.5344828	0.143287084	5.774734178
11.38735278	340.7193059	0.110365564	6.038250801
5.885056871	287.4847349	0.214386596	5.747992789
9.556571732	329.6476412	0.123987098	6.065104313
11.64039737	370.6512097	0.113065817	6.075781085
6.278341185	255.3496344	0.162901496	5.955725237
7.916076755	125.8040201	0.109117615	5.906929043
4.799625598	227.8815896	0.18696511	5.746082523
5.534033039	225.1810478	0.141916805	5.738553015
9.433273173	299.1661858	0.117590526	6.013464011
3.602675838	169.3257275	0.202802186	5.835648491
7.246789969	150.291937	0.118498661	5.81377737
7.023921967	291.8554688	0.132390976	5.861121874
6.571116213	231.2765395	0.132881541	5.958525181
8.275269372	271.0054988	0.134083994	5.898409683
11.86221956	384.4385817	0.103310828	6.130252244
9.981796906	266.9328797	0.110390461	6.14817606
7.03367217	289.9967415	0.144823641	5.960564987
7.303644981	150.7136465	0.115305116	5.662811764
6.082469762	260.1387537	0.152577107	5.781930155
7.405505093	330.4150904	0.156920465	6.038191238
5.933445479	250.3441276	0.167183592	5.892424779
8.929021514	260.0371632	0.123861728	6.189798147
9.38466727	232.1561702	0.127907288	6.048029387
9.590956605	213.1196639	0.114071119	6.085952567
6.241156744	228.5781927	0.163672319	5.885058548
8.205447542	480.5229777	0.150907337	6.010321598
6.254647849	437.341988	0.203896347	6.037146936
6.699375372	286.2414634	0.139056117	5.733428512
7.509077912	145.9969739	0.113188529	6.098447731
5.345977224	141.3191981	0.165941033	5.918794364
10.29858947	172.9451771	0.090634157	6.038123224
7.379627228	261.1245552	0.12790248	6.007256548
11.51755759	294.6712219	0.103493231	6.046994375
8.660274637	341.5086072	0.128330407	5.899480711
8.017171011	178.4979964	0.124289568	5.982129928
8.429343455	232.1488382	0.129902088	6.003602556
5.78663953	148.6865672	0.160860012	5.551587062
11.15577029	181.226792	0.105742743	5.79648657
6.271086447	301.5414201	0.143591786	5.896964519
10.22944225	305.6839237	0.111986934	5.986886572
10.37665973	233.6446158	0.095766929	6.035057257
7.999191282	200.9578901	0.114942109	5.945918774
5.773943729	151.321558	0.144351567	5.76508001
8.546263214	237.2834586	0.132229069	5.832117683
6.223064037	171.3389744	0.142453649	5.812361223
8.384387018	233.5698393	0.114060026	6.051109142
6.580030263	284.142568	0.147723772	5.962693704
8.122160062	290.3743932	0.129846268	5.858281669
9.377726234	288.0617811	0.109658484	6.264848755
7.296165843	295.1613817	0.164414273	5.89271149
9.916326642	410.4108614	0.120760286	6.071754975
9.233299996	375.1798238	0.123033507	6.103693147
8.561855266	291.8280205	0.128077757	6.070629799
3.810094536	102.8	0.164204454	5.506402184
7.022604023	288.6747799	0.154570647	5.970149572
6.897817358	220.2952454	0.157860194	5.882743836
8.57859031	324.58752	0.124935885	6.091299194
8.907480718	408.0201468	0.118435746	6.071540326
4.598411793	196.4658385	0.172043879	5.74742386
6.881471565	170.2055427	0.141364738	5.856160499
7.582061823	266.3944056	0.160687456	5.995600712
8.199809736	232.0035103	0.132648843	6.156082199
11.83570082	227.5055334	0.093485289	6.139116756
9.486848375	173.483871	0.099477199	6.076030304
6.572762143	169.4679583	0.135650685	5.833984093
6.014907288	202.3188648	0.153081025	5.848463633
6.80350613	197.7797495	0.147900768	5.683264314
5.95688246	197.71529	0.140402952	5.829146142
10.13530592	226.1209919	0.106058742	6.090008325
12.95055384	488.8878834	0.097446211	6.141069125
9.055317929	195.7065036	0.129279097	6.045637456
6.795289809	289.8149281	0.158979376	6.010720992
4.041160697	143.6700293	0.156866609	5.765667375
8.983477625	228.6404264	0.117859452	6.127794164
1.978086291	116.9099217	0.287391181	5.578459755
9.126262411	223.0582809	0.116570811	6.165269218
7.604803258	210.6309263	0.132640754	6.067706241
6.920539207	262.4398907	0.146771141	5.791118647
5.999530316	258.8330864	0.161901905	5.888452366
3.567531122	258.7093558	0.217567936	5.648074451
9.010639727	237.995382	0.102157872	5.929036644
8.950627969	199.2128111	0.111649123	6.038360807
5.996269605	202.2678788	0.14878641	5.949052323
8.383494869	226.5836397	0.105686491	6.030039592
10.20197191	538.4763296	0.144732694	6.15826152
6.796961851	170.1955982	0.131508594	5.950724825
9.290274026	284.9073543	0.134623051	5.981564495
3.993905595	117.1330935	0.179065381	5.688559844
5.080498212	293.2802217	0.18760382	5.812939034

log.sigma.1.0.mm.3D_gldm_DependenceNonUniformity	log.sigma.1.0.mm.3D_gldm_GrayLevelNonUniformity	log.sigma.1.0.mm.3D_gldm_SmallDependenceEmphasis
377.6390198	199.7219604	0.335718933
102.6064171	162.2256684	0.21756171
144.0430248	286.1329857	0.19330415
134.1668373	120.1443193	0.264708978
316.6064189	311.3547297	0.260902454
120.8060606	70.5	0.347050526
711.4611573	774.7143868	0.250108283
564.6401723	811.4686766	0.24455519
644.2349318	557.7980642	0.268780209
178.1026119	120.3152985	0.305379905
205.8103448	216.0769231	0.244650011
369.3392625	254.3926247	0.310122839
163.1360471	400.259775	0.176115357
233.1782178	212.8858474	0.265614237
295.5504032	224.3225806	0.305073001
466.8240402	712.856947	0.221397458
197.961474	130.2864322	0.291081009
124.0624493	230.5279805	0.187129964
174.6579353	184.2080123	0.244160742
788.6522408	611.3531448	0.29364739
264.0350529	613.2738095	0.182198101
176.3772011	127.8600556	0.295191272
138.3652344	135.5683594	0.262757691
360.5413676	351.7374386	0.250847707
172.6653574	170.6889238	0.271245771
399.9598987	244.7433516	0.321521638
490.3448809	356.89236	0.290762499
344.7856626	400.0028965	0.23997257
81.65771812	51.54138702	0.335038307
264.4771178	313.3933788	0.249112837
527.7440101	746.6275746	0.237731392
220.5137748	345.9028516	0.216086658
436.2245277	399.94952	0.270784125
312.6731915	300.5821277	0.267428465
665.4959128	502.3692098	0.292942715
325.5535178	490.8532844	0.222236868
430.1281913	561.5262026	0.239095018
431.0015501	1052.309049	0.178693151
173.4910569	171.0390244	0.251132362
1113.12132	822.8806052	0.278481328
280.0346955	430.4510409	0.207888558
762.2795449	350.482286	0.330230362
514.482617	467.227758	0.254580962
419.1848875	257.4911576	0.313797238
180.8888889	164.0062598	0.283267452
391.3249545	341.1748634	0.284908739
621.9522139	592.6133275	0.2730875
113.2663605	140.10907	0.237058825
157.1621622	89.98707403	0.335206785
172.5650888	194.1360947	0.23288443
408.0276372	287.6944336	0.295414653
629.0391575	322.8303174	0.331682402
716.9117908	518.6187943	0.292596973
144.6322464	159.3641304	0.225433665
189.2255639	175.8646617	0.269607507
260.0964103	277.7846154	0.244865772
618.0037083	461.3728059	0.282335957
406.9016773	510.8288028	0.225324185
243.4393204	213.9866505	0.298290332
1360.714536	1001.072297	0.282420522
199.7542469	290.3556059	0.25053487
381.058427	322.4299625	0.28159914
804.5605459	712.2409743	0.282730622
1043.867637	976.3367441	0.262412943
101.3265823	129.721519	0.206301574
447.0683584	596.9518384	0.236025159
149.9294479	205.8496933	0.237606917
861.5328	780.84928	0.275032404
914.8804849	693.6781629	0.310031319
317.1180124	498.5831608	0.193288914
167.3294842	183.6327945	0.24854061
472.4713287	689.3491841	0.236179045
275.4019307	302.3067135	0.251233816
403.9092519	211.1832669	0.319373898
548.2194187	311.4631108	0.319098672
183.9642325	182.0432191	0.259204984
297.5342237	366.7821369	0.237435909
127.1920668	141.6889353	0.253004031
234.370826	239.6678383	0.252149154
379.0547242	248.071398	0.305432184
350.0809452	193.8205128	0.333597085
236.3496387	232.5730962	0.272291291
357.6534108	519.7035791	0.228940178
389.7334852	482.0510901	0.200747299
344.2710127	287.4592046	0.277581937
103.654047	440.2832898	0.117750208
337.9928721	278.0213836	0.291080333
352.6535376	363.7009482	0.249883407
196.1188525	214.8729508	0.260189808
229.9283951	327.851358	0.233492139
114.7837423	283.708589	0.176734832
436.5759866	243.3400504	0.329211393
815.8736253	578.6774069	0.283452154
402.4951515	490.9951515	0.244089766
728.4692096	459.9476103	0.29009567
396.5634132	495.2752776	0.250035228
397.770323	370.4597089	0.255190699
138.4861509	140.9503343	0.269181211
92.76019185	149.3405276	0.196275155
564.5538621	1083.224454	0.200860962

log.sigma.1.0.mm.3D_gldm_DependenceNonUniformityNormalized	log.sigma.1.0.mm.3D_gldm_DependenceVariance	log.sigma.1.0.mm.3D_gldm_LargeDependenceEmphasis
0.177963723	3.998294426	13.48539114
0.109739484	8.303022677	29.30588235
0.093900277	15.21879722	44.91916558
0.13732532	6.690285994	21.44626407
0.133702035	7.285575694	22.77618243
0.183039486	3.475528007	12.38484848
0.125900046	9.409006714	27.14546098
0.110540363	10.64672257	31.96045419
0.141714679	5.75580259	19.52045755
0.166140496	4.055938823	14.58208955
0.136479009	5.293448206	20.02519894
0.160233953	4.991757803	16.270282
0.087378708	13.49512421	45.44456347
0.135805602	6.757301425	21.6721025
0.148966937	5.528533713	18.06955645
0.106678254	10.31643747	32.68829982
0.165796879	3.913728329	14.62646566
0.100618369	10.53136213	35.79156529
0.134559272	5.521321174	20.46687211
0.151693064	5.357844683	17.66243508
0.087313179	14.42891393	46.33796296
0.163463578	4.045143574	14.93697868
0.135122299	5.810970306	20.48046875
0.136207543	6.200198127	21.15111447
0.135636573	6.633419087	21.45561665
0.168830688	4.926930286	15.54368932
0.151668692	5.815518687	18.43086916
0.124831884	7.518367353	24.72194062
0.182679459	3.135744636	11.9753915
0.128761985	6.499512197	22.50730282
0.110917194	12.29570621	34.41614124
0.106579881	9.59843355	31.85548574
0.135095859	8.564591887	24.36946423
0.133052422	7.478414486	23.08595745
0.151111697	5.857712129	18.4373297
0.108554024	9.717988913	31.40146716
0.115594784	8.184195095	27.37570546
0.083511248	15.36904273	50.41057935
0.14104964	4.834690991	18.66341463
0.153111598	5.124678192	17.4605227
0.107954779	10.13268328	32.51426369
0.197124268	2.607342844	10.67106284
0.140838384	6.154400393	20.48973446
0.168482672	4.138705012	14.46221865
0.141540602	5.336879563	18.96557121
0.142559182	5.738414405	19.21493625
0.136333234	7.299374914	22.33581762
0.130041746	5.918483182	21.90929966
0.184679391	3.743631947	12.82843713
0.1276369	7.566198225	24.6612426
0.158827418	4.60286274	15.9793694
0.186603132	3.038189608	11.71017502
0.158890025	4.247220179	15.49468085
0.13100747	5.385751418	21.29891304
0.14227486	5.573608457	19.26917293
0.133382774	6.006086522	21.33230769
0.152782128	5.328951276	17.72039555
0.117669658	8.05549867	27.02255639
0.147718034	5.389785501	18.18082524
0.149054062	6.652442605	20.01347355
0.113111125	12.15454495	33.75651189
0.142718512	6.404264894	20.19700375
0.13898092	7.544560494	22.2281914
0.136936592	6.690587006	21.48930867
0.128261497	5.644505688	22.90379747
0.115760839	9.158952263	28.7410668
0.11497657	9.473869924	29.36656442
0.137845248	6.677865882	21.18848
0.156202917	5.400873455	17.16049172
0.109426505	8.042550466	29.84610076
0.128814076	6.885340236	23.10161663
0.110133177	10.27107742	31.57715618
0.120843322	8.863616349	27.22290478
0.178800023	3.668394838	13.13103143
0.175094033	3.91548828	13.66240818
0.13708214	5.885616814	20.50968703
0.124179559	7.071660196	24.2754591
0.132768337	6.104998671	21.50939457
0.137299839	5.477009962	19.94551845
0.162058454	4.707628204	15.77982044
0.176008519	4.079561773	13.71794872
0.131378343	7.098828606	22.71428571
0.10940759	10.75920213	32.57601713
0.126825085	6.229784009	23.93654409
0.141152527	6.395217056	20.46617466
0.0676593	18.89078084	72.20365535
0.141716089	7.283794154	21.47631027
0.128611793	8.477649721	25.43690737
0.133960965	7.280539879	22.80327869
0.113544886	9.478634598	29.70419753
0.088024342	13.58914524	45.34202454
0.183281271	3.554741304	12.65071369
0.157413395	4.443978666	16.03916651
0.121968228	7.959853444	25.73757576
0.167387226	3.811805382	14.38924632
0.115886445	11.08213912	31.57218001
0.141203523	5.76975584	19.88888889
0.132269485	7.832781887	23.76408787
0.111223252	11.00344473	33.73141487
0.097775175	11.50984952	37.45029442

log.sigma.1.0.mm.3D_gldm_LargeDependenceLowGrayLevelEmphasis	log.sigma.1.0.mm.3D_gldm_SmallDependenceHighGrayLevelEmphasis	log.sigma.1.0.mm.3D_gldm_LargeDependenceHighGrayLevelEmphasis
0.029148967	159.1160159	6590.619698
0.205481458	29.79722041	4322.315508
0.386460372	23.89763179	5383.709257
0.09019216	63.47466751	5492.230297
0.118164027	46.90372623	4627.514358
0.052133769	95.23116456	3214.530303
0.123391654	56.79657053	6132.692267
0.111811176	70.80511482	9311.287001
0.068095383	76.23525969	5804.041355
0.058863719	76.96178408	3876.011194
0.144964269	34.35286433	2949.334218
0.050495897	100.5386014	5532.019957
0.162750504	48.91257711	12920.70809
0.068913619	84.57592775	7149.170064
0.051350117	109.3077283	6647.609879
0.13154115	54.03787747	8341.037477
0.134997439	37.25903694	1825.157454
0.161215461	39.85399949	8125.970803
0.092660386	53.48176495	4680.455316
0.062675557	86.83334381	5228.92614
0.282230069	31.17916313	7724.726521
0.109348628	42.27381615	2261.333642
0.073009393	74.0378118	5968.001953
0.097468648	57.88338046	4763.630147
0.086601581	74.94598567	5603.475255
0.04390451	127.3304904	5762.25707
0.07436882	77.17230272	4818.169502
0.087415632	66.164601	7203.738957
0.092799361	52.00840464	1736.440716
0.088080761	60.53185058	5934.163096
0.107595279	77.14068799	11217.63115
0.129101757	50.60392301	8061.665539
0.099622295	71.60941166	6177.165686
0.108274452	61.97270348	5237.834468
0.094870852	61.76157461	3815.110581
0.143672065	50.60016266	7042.465488
0.058134913	112.3770811	13121.8366
0.117986642	78.24700602	21742.91029
0.066211604	67.25883017	5509.649593
0.135261698	40.94199125	2473.610729
0.239806048	27.93944407	4642.898998
0.073091296	59.44567909	1800.430049
0.08105707	62.96474075	5386.113879
0.052503232	89.29665149	4318.471061
0.058001315	92.36880194	6477.873239
0.119002021	52.43559291	3297.638251
0.102673743	64.14024424	5072.963832
0.155706593	33.28455632	3284.385763
0.084015577	57.79901347	2322.345476
0.085413958	67.22043147	7330.723373
0.055939058	88.2413874	4790.821331
0.05522866	76.78349304	2736.768911
0.082798848	57.91468197	3113.923759
0.15151823	33.6491477	3175.214674
0.0880886	63.63045571	4445.761654
0.132797123	39.74606575	3639.23641
0.080148767	63.53403196	4140.329295
0.097181913	62.76310499	7706.491324
0.064235212	84.0511832	5348.952063
0.074372329	82.25642355	5618.056523
0.118568534	72.25428821	9790.823896
0.051192671	111.0301476	8241.272285
0.061988768	104.5823799	8172.079461
0.076073472	74.67741265	6292.115834
0.237811422	20.43782402	2343.913924
0.102370534	66.85801997	8271.545313
0.136689329	51.06715776	6540.06365
0.066934495	85.42507948	6913.52064
0.043349461	123.3634278	7040.122247
0.156931058	36.06680052	5819.188406
0.14448298	41.00912824	3935.015396
0.126600066	62.56536747	8132.826107
0.124815293	56.59210344	6164.39491
0.066992691	71.34070969	3021.269146
0.089297428	55.28946578	2348.347493
0.12611814	42.30509304	3546.140089
0.126911683	48.18347726	4795.240401
0.1150178	46.52386247	4257.343424
0.105507667	47.71564836	3960.466315
0.077782574	70.29936175	3524.259513
0.029565362	159.3867408	6706.429361
0.124455283	51.70717259	4447.384102
0.116639691	65.06899362	9290.297645
0.173145469	28.12363603	3454.44712
0.095046394	60.95395782	4658.99221
0.607968484	11.96097403	8690.052219
0.102157435	64.08841776	4791.589099
0.130782786	52.76125554	5165.66229
0.091922257	67.63446243	5882.352459
0.117252236	57.33255412	7704.034074
0.181053447	46.35744004	11492.90721
0.057521343	77.74689217	2977.184299
0.086390886	55.1043161	3221.012927
0.134444641	48.37534823	5111.66303
0.067828002	65.06286032	3273.699908
0.060565237	132.0595376	16749.82466
0.125102957	42.49188884	3369.445864
0.086239803	74.36851531	6868.334288
0.294064274	21.91815987	4020.140288
0.131807319	57.71918113	10776.14409

log.sigma.1.0.mm.3D_gldm_SmallDependenceLowGrayLevelEmphasis	log.sigma.1.0.mm.3D_gldm_LowGrayLevelEmphasis	log.sigma.1.0.mm.3D_gldzm_DistanceZoneVariabilityNormalized
0.001425939	0.00286403	0.992565159
0.004278647	0.009702815	1
0.004888556	0.012136711	1
0.003903013	0.007165826	1
0.003224792	0.007329462	1
0.003869574	0.00653796	1
0.001761125	0.00526123	0.994712529
0.001479196	0.004158461	0.998457981
0.001591478	0.004217061	0.998449613
0.002920865	0.005687671	1
0.00353455	0.009434708	1
0.002321035	0.004441205	0.992238151
0.002173013	0.005158706	1
0.001802766	0.004218462	1
0.001817531	0.003810339	1
0.001804599	0.005096227	1
0.006295716	0.013489239	1
0.002133224	0.005898585	1
0.00226078	0.005779516	1
0.002432671	0.004951645	0.996323542
0.001771394	0.006871093	1
0.006754371	0.011993187	1
0.002233853	0.004872155	1
0.002151815	0.005608937	0.994505536
0.003169532	0.006159123	1
0.001541086	0.003473357	1
0.002231179	0.005175143	1
0.001904144	0.00463467	1
0.00571766	0.010905032	1
0.002091582	0.005089531	1
0.001499426	0.003934891	1
0.001867295	0.005175928	0.991247434
0.001972272	0.005035306	1
0.004401965	0.007941118	1
0.003291755	0.007002564	0.994257047
0.001951965	0.005635782	0.99705883
0.000986372	0.002610629	1
0.000790076	0.00272724	0.993833564
0.002351803	0.005068357	1
0.003961274	0.009854108	0.998161766
0.003701986	0.010480098	1
0.00364717	0.008396091	0.997250864
0.001866365	0.004890739	1
0.002777259	0.00538547	1
0.002363674	0.004507789	1
0.003408343	0.007925106	1
0.00218605	0.005772781	0.998497371
0.006772922	0.012339304	1
0.007313165	0.012035582	1
0.001809733	0.004442378	0.993902496
0.0021453	0.004581794	1
0.002683311	0.005919187	1
0.002844906	0.006796778	0.99858357
0.004636668	0.010228459	1
0.003601859	0.006927002	1
0.005071014	0.009829792	1
0.002323895	0.005706848	0.995238122
0.001539603	0.004404736	1
0.002191336	0.004703567	1
0.001594384	0.004336448	0.997157078
0.001939811	0.004579661	1
0.001534932	0.003353832	1
0.001232955	0.003244241	0.99318577
0.001866037	0.004548341	0.980209329
0.004938957	0.013739059	1
0.001660759	0.004464206	0.987448199
0.002952474	0.006740708	1
0.001428814	0.0037702	0.991179886
0.001358922	0.003128181	1
0.0021694	0.006430453	1
0.004431252	0.009507416	1
0.0025782	0.005687767	0.996108964
0.00232691	0.005829398	1
0.002959926	0.007121408	1
0.004419956	0.009163259	0.994570176
0.003630138	0.008474493	1
0.002259398	0.006296988	1
0.005185611	0.009131848	1
0.002842044	0.006949303	1
0.003322684	0.006967567	1
0.001442232	0.002875137	1
0.004688449	0.009033269	1
0.001597097	0.004399302	0.997490594
0.002451826	0.008481381	1
0.002540641	0.006050592	1
0.003179571	0.010949926	1
0.003339384	0.006886981	1
0.00252683	0.006559044	1
0.002246258	0.005323173	1
0.00214349	0.00519859	1
0.001579588	0.004858164	1
0.002652656	0.005727098	0.997706425
0.003219808	0.007296328	0.991048774
0.00240906	0.006425467	1
0.002265376	0.00571956	0.998559079
0.000933574	0.002382867	0.99782609
0.003401502	0.008335859	1
0.002882246	0.005635312	1
0.003966915	0.011641297	1
0.001105238	0.003924307	1

log.sigma.1.0.mm.3D_gldzm_LowIntensityEmphasis	log.sigma.1.0.mm.3D_gldzm_LargeDistanceEmphasis	log.sigma.1.0.mm.3D_gldzm_HighIntensitySmallDistanceEmphasis
0.004039685	1.01119403	475.2322761
0.019451511	1	141.4759615
0.024628382	1	126.1090343
0.014046869	1	240.8391608
0.012366479	1	178.4150077
0.010583176	1	276.0555556
0.007045113	1.007952286	227.1520875
0.006033715	1.002314815	292.9645062
0.00593083	1.002327386	281.867533
0.009229957	1	255.2640449
0.014855111	1	139.1488251
0.007238966	1.011688312	325.7522727
0.012087942	1	281.7725948
0.006776312	1	319.3039832
0.005810853	1	360.7039877
0.008341109	1	245.3629929
0.02088549	1	127.1857506
0.011935974	1	210.7733333
0.009134851	1	218.452381
0.008063553	1.005524862	297.0007673
0.009899028	1	171.2918919
0.021798002	1	143.0228571
0.008223536	1	282.024055
0.008323582	1.008264463	231.5733471
0.011535449	1	280.9194444
0.004634944	1	396.193662
0.00748868	1	267.1108949
0.007860787	1	276.3453757
0.016423682	1	154.0838323
0.00839173	1	244.1837121
0.006228843	1	326.8193172
0.008856965	1.013186813	229.121978
0.007196388	1	264.3375796
0.015950864	1	232.5616642
0.010971976	1.008639309	212.5827934
0.008951068	1.004418262	228.8324742
0.004167882	1	469.6254218
0.004367578	1.009278351	438.3590206
0.009192354	1	266.3784615
0.014212041	1.00275989	147.8846596
0.019081581	1	133.2786885
0.010735411	1.004129387	179.4778045
0.007237309	1	246.5866534
0.008571092	1	284.4081395
0.008163697	1	331.056
0.011851673	1	183.7891566
0.008113199	1.002255639	235.8430451
0.028147717	1	139.9861111
0.02019751	1	175.6936937
0.007660979	1.009174312	290.0825688
0.007067112	1	302.8539604
0.007886419	1	232.040032
0.009746105	1.002126152	196.6399717
0.020550668	1	151.8346457
0.012731753	1	239.0280612
0.020581338	1	163.4329268
0.008121529	1.007159905	225.1945107
0.006888009	1	278.0188917
0.007308055	1	280.8681102
0.005602541	1.004270463	290.872331
0.00755073	1	290.8216561
0.005302252	1	396.9640644
0.004293451	1.01025641	372.7264957
0.00705993	1.029985721	283.1136364
0.024259074	1	99.43930636
0.007054334	1.018947368	281.1376316
0.012559255	1	212.4797508
0.005174552	1.013289037	311.3971484
0.004302089	1	397.6399796
0.01141935	1	187.5045208
0.017657746	1	164.7238372
0.010964515	1.005847953	268.9885478
0.009155285	1	227.4590164
0.009613194	1	224.7372986
0.013533934	1.008166969	174.5594374
0.01391578	1	162
0.009490726	1	206.3112245
0.020072988	1	185.2380952
0.011049225	1	191.2633833
0.010653532	1	228.9131545
0.004093633	1	479.770557
0.016959934	1	187.8811321
0.006902077	1.003768844	284.3128141
0.012555324	1	139.4822006
0.009072389	1	220.204918
0.028033345	1	99.36723164
0.011274806	1	217.9221477
0.010137867	1	213.6305556
0.00858703	1	259.582716
0.009204512	1	244.2328482
0.009041449	1	265.1013216
0.007771856	1.003444317	237.8705511
0.011362122	1.013487476	192.5637444
0.00982407	1	199.9010727
0.007673851	1.002162942	223.7355804
0.00364985	1.003264418	530.7181719
0.013165736	1	166.8490323
0.01028819	1	276.7973856
0.020238587	1	112.1213873
0.005520072	1	291.1341256

log.sigma.1.0.mm.3D_gldzm_LowIntensityLargeDistanceEmphasis	log.sigma.1.0.mm.3D_gldzm_HighIntensityEmphasis	log.sigma.1.0.mm.3D_gldzm_DistanceZoneVariability	log.sigma.1.0.mm.3D_gldzm_ZonePercentage
0.004081611	476.608209	798.0223881	0.378887842
0.019451511	141.4759615	208	0.222459893
0.024628382	126.1090343	321	0.209256845
0.014046869	240.8391608	286	0.292732856
0.012366479	178.4150077	653	0.275760135
0.010583176	276.0555556	252	0.381818182
0.007111341	227.5904573	1501.021206	0.267032384
0.006056863	293.0223765	1294.001543	0.253719655
0.00593523	282.1753297	1287.001552	0.283545974
0.009229957	255.2640449	356	0.332089552
0.014855111	139.1488251	383	0.25397878
0.007577204	326.0649351	764.0233766	0.334056399
0.012087942	281.7725948	343	0.183717193
0.006776312	319.3039832	477	0.277810134
0.005810853	360.7039877	652	0.328629032
0.008341109	245.3629929	989	0.226005484
0.02088549	127.1857506	393	0.329145729
0.011935974	210.7733333	225	0.182481752
0.009134851	218.452381	336	0.258859784
0.008210786	297.1356661	1623.01105	0.313329486
0.009899028	171.2918919	555	0.183531746
0.021798002	143.0228571	350	0.324374421
0.008223536	282.024055	291	0.284179688
0.008397518	232.0950413	722.0110193	0.274272762
0.011535449	280.9194444	360	0.282796544
0.004634944	396.193662	852	0.35964542
0.00748868	267.1108949	1028	0.317970925
0.007860787	276.3453757	692	0.250543085
0.016423682	154.0838323	167	0.37360179
0.00839173	244.1837121	528	0.257059396
0.006228843	326.8193172	1201	0.252416982
0.008914244	229.9956044	451.0175824	0.219913001
0.007196388	264.3375796	942	0.291731186
0.015950864	232.5616642	673	0.286382979
0.011148064	212.938085	1381.023038	0.315395095
0.008987583	228.9661267	677.0029455	0.226408803
0.004167882	469.6254218	889	0.23891427
0.004404898	439.2721649	964.0185567	0.187948072
0.009192354	266.3784615	325	0.264227642
0.014563161	147.9636615	2170.00368	0.299037139
0.019081581	133.2786885	549	0.211642251
0.010785613	179.6352374	1449.005506	0.37574347
0.007237309	246.5866534	1004	0.274842595
0.008571092	284.4081395	860	0.345659164
0.008163697	331.056	375	0.29342723
0.011851673	183.7891566	830	0.302367942
0.008131841	235.9112782	1328.001504	0.291538799
0.028147717	139.9861111	216	0.247990815
0.02019751	175.6936937	333	0.391304348
0.007696816	290.6697248	325.0061162	0.241863905
0.007067112	302.8539604	808	0.314519268
0.007886419	232.040032	1249	0.370513201
0.009779326	196.6739901	1409.001417	0.312721631
0.020550668	151.8346457	254	0.230072464
0.012731753	239.0280612	392	0.294736842
0.020581338	163.4329268	492	0.252307692
0.008303014	225.3031026	1251.01432	0.310754017
0.006888009	278.0188917	794	0.229612493
0.007308055	280.8681102	508	0.308252427
0.00562589	291.0765125	2802.011388	0.307810275
0.00755073	290.8216561	471	0.266704417
0.005302252	396.9640644	807	0.302247191
0.004375237	373.3726496	1743.041026	0.303161168
0.007282332	284.6611138	2059.4198	0.275613276
0.024259074	99.43930636	173	0.218987342
0.011397106	282.0368421	938.0757895	0.245986535
0.012559255	212.4797508	321	0.246165644
0.005250021	312.0636766	1790.070875	0.28896
0.004302089	397.6399796	1961	0.334813044
0.01141935	187.5045208	553	0.190821256
0.017657746	164.7238372	344	0.264819092
0.011008986	269.1822612	1022.007797	0.239160839
0.009155285	227.4590164	610	0.267661255
0.009613194	224.7372986	807	0.357237716
0.013665637	174.6914701	1096.016334	0.351964229
0.01391578	162	370	0.275707899
0.009490726	206.3112245	588	0.245409015
0.020072988	185.2380952	252	0.263048017
0.011049225	191.2633833	467	0.273579379
0.010653532	228.9131545	783	0.334758444
0.004093633	479.770557	754	0.379084967
0.016959934	187.8811321	530	0.294608116
0.006921306	284.4974874	794.0025126	0.243499541
0.012555324	139.4822006	618	0.201106411
0.009072389	220.204918	732	0.300123001
0.028033345	99.36723164	177	0.115535248
0.011274806	217.9221477	745	0.312368973
0.010137867	213.6305556	720	0.262582057
0.00858703	259.582716	405	0.276639344
0.009204512	244.2328482	481	0.237530864
0.009041449	265.1013216	227	0.174079755
0.007781397	238.1814007	869.0022962	0.36565911
0.011466221	193.2048812	1543.062942	0.300405171
0.00982407	199.9010727	839	0.254242424
0.007679842	223.9307859	1385.001442	0.318704044
0.003662602	530.9270947	917.0021763	0.2685564
0.013165736	166.8490323	775	0.275115371
0.01028819	276.7973856	306	0.29226361
0.020238587	112.1213873	173	0.207434053
0.005520072	291.1341256	1178	0.204018012

log.sigma.1.0.mm.3D_gldzm_IntensityVariabilityNormalized	log.sigma.1.0.mm.3D_gldzm_LowIntensitySmallDistanceEmphasis	log.sigma.1.0.mm.3D_gldzm_IntensityVariability
0.058275166	0.004029203	46.85323383
0.084781805	0.019451511	17.63461538
0.089391601	0.024628382	28.69470405
0.07015013	0.014046869	20.06293706
0.073785966	0.012366479	48.18223583
0.064531368	0.010583176	16.26190476
0.078628296	0.007028555	118.6500994
0.070825713	0.006027928	91.79012346
0.070171247	0.00592973	90.45073701
0.068283676	0.009229957	24.30898876
0.086530006	0.014855111	33.14099217
0.063606004	0.007154407	48.97662338
0.08062117	0.012087942	27.65306122
0.067565014	0.006776312	32.22851153
0.060225263	0.005810853	39.26687117
0.078529139	0.008341109	77.6653185
0.07463305	0.02088549	29.3307888
0.084760494	0.011935974	19.07111111
0.086663832	0.009134851	29.11904762
0.067783472	0.008026745	110.4192756
0.096449963	0.009899028	53.52972973
0.075297959	0.021798002	26.35428571
0.077904134	0.008223536	22.67010309
0.076949055	0.008305098	55.86501377
0.071157407	0.011535449	25.61666667
0.060710507	0.004634944	51.72535211
0.064754955	0.00748868	66.56809339
0.075311571	0.007860787	52.11560694
0.078991717	0.016423682	13.19161677
0.082895719	0.00839173	43.76893939
0.069667866	0.006228843	83.67110741
0.081289699	0.008842646	36.98681319
0.066480047	0.007196388	62.62420382
0.065001358	0.015950864	43.74591382
0.064755943	0.010927954	89.94600432
0.077370559	0.008941939	52.53460972
0.067419441	0.004167882	59.93588301
0.072464661	0.004358248	70.29072165
0.085557396	0.009192354	27.80615385
0.073576744	0.014124261	159.9558418
0.085285052	0.019081581	46.82149362
0.065637746	0.010722861	95.37164487
0.075046428	0.007237309	75.34661355
0.062368848	0.008571092	53.6372093
0.071815111	0.008163697	26.93066667
0.071400784	0.011851673	59.2626506
0.069748431	0.008108539	92.76541353
0.08590535	0.028147717	18.55555556
0.070367665	0.02019751	23.43243243
0.080473959	0.00765202	26.31498471
0.065321415	0.007067112	52.77970297
0.065950599	0.007886419	82.37229784
0.071680895	0.0097378	101.1417434
0.085560171	0.020550668	21.73228346
0.071532695	0.012731753	28.04081633
0.079887964	0.020581338	39.30487805
0.070351616	0.008076157	88.43198091
0.077590747	0.006888009	61.6070529
0.071176142	0.007308055	36.15748031
0.064325933	0.005596704	180.7558719
0.075382819	0.00755073	35.50530786
0.065300676	0.005302252	52.6976456
0.065805635	0.004273004	115.4888889
0.067009409	0.00700433	140.7867682
0.099134619	0.024259074	17.15028902
0.076740166	0.005968641	72.90315789
0.081084229	0.012559255	26.02803738
0.071348857	0.005155685	128.8560354
0.069853994	0.004302089	136.9836818
0.090458423	0.01141935	50.02350814
0.08470795	0.017657746	29.13953488
0.069297676	0.010953398	71.0994152
0.067637732	0.009155285	41.25901639
0.062269577	0.009613194	50.25154895
0.0677534	0.013501008	74.66424682
0.080073046	0.01391578	29.62702703
0.085201768	0.009490726	50.09863946
0.079868985	0.020072988	20.12698413
0.08726254	0.011049225	40.751606
0.063382315	0.010653532	49.62835249
0.059002737	0.004093633	44.48806366
0.065987896	0.016959934	34.97358491
0.076282796	0.00689727	60.72110553
0.102805794	0.012555324	63.53398058
0.066170982	0.009072389	48.43715847
0.129113601	0.028033345	22.85310734
0.067119499	0.011274806	50.00402685
0.071608796	0.010137867	51.55833333
0.079689072	0.00858703	32.27407407
0.081262616	0.009204512	39.08731809
0.107104737	0.009041449	24.31277533
0.068595027	0.007769471	59.74626866
0.06740603	0.011336097	104.9511882
0.080493408	0.00982407	67.53396901
0.070437251	0.007672353	97.6964672
0.06331455	0.003646662	58.18607182
0.077637461	0.013165736	60.16903226
0.067901235	0.01028819	20.77777778
0.104480604	0.020238587	18.07514451
0.088152634	0.005520072	103.8438031

log.sigma.1.0.mm.3D_gldzm_HighIntensityLargeDistanceEmphasis	log.sigma.1.0.mm.3D_gldzm_SmallDistanceEmphasis	log.sigma.1.0.mm.3D_glcm_SumVariance	log.sigma.1.0.mm.3D_glcm_Homogeneity1
482.1119403	0.997201493	1543.956327	0.349955103
141.4759615	1	426.1790903	0.486401952
126.1090343	1	343.9052845	0.51812533
240.8391608	1	762.2390922	0.418209309
178.4150077	1	570.6917094	0.423210052
276.0555556	1	789.2082112	0.366854727
229.3439364	0.998011928	685.7837495	0.444102815
293.253858	0.999421296	917.2639702	0.459832952
283.4065167	0.999418154	909.4671618	0.409604194
255.2640449	1	799.2287308	0.392561121
139.1488251	1	411.7069508	0.444823164
327.3155844	0.997077922	1047.420546	0.378841117
281.7725948	1	913.9020352	0.520490202
319.3039832	1	1022.138347	0.41175181
360.7039877	1	1145.400216	0.381384567
245.3629929	1	783.7304352	0.473531907
127.1857506	1	326.2411648	0.388385651
210.7733333	1	700.5172789	0.498828732
218.452381	1	683.2723305	0.436647623
297.6752609	0.998618785	906.681986	0.393870882
171.2918919	1	503.305621	0.52964296
143.0228571	1	409.7925963	0.396999866
282.024055	1	905.0464763	0.428817108
234.1818182	0.997933884	693.05935	0.43623676
280.9194444	1	815.152259	0.429126319
396.193662	1	1177.493917	0.373537454
267.1108949	1	792.0130349	0.395649903
276.3453757	1	900.062944	0.441622196
154.0838323	1	406.8430818	0.373995974
244.1837121	1	794.921128	0.445799265
326.8193172	1	1043.65573	0.458025141
233.4901099	0.996703297	772.0130395	0.4709704
264.3375796	1	780.3778806	0.420490407
232.5616642	1	677.4023506	0.427412227
214.3592513	0.997840173	596.6592599	0.405287398
229.5007364	0.998895434	689.1171146	0.474594136
469.6254218	1	1568.418835	0.444534776
442.9247423	0.997680412	1434.857832	0.513079967
266.3784615	1	892.3474675	0.420021528
148.2796688	0.999310028	393.8443039	0.401579718
133.2786885	1	398.438512	0.474030869
180.264969	0.998967653	471.0517664	0.351521594
246.5866534	1	791.2280478	0.422599236
284.4081395	1	889.8749217	0.371969512
331.056	1	1059.989235	0.417563749
183.7891566	1	503.1261838	0.424023205
236.1842105	0.99943609	683.7366626	0.425594958
139.9861111	1	416.3520645	0.448473428
175.6936937	1	491.4947083	0.368254877
293.0183486	0.997706422	929.0101454	0.450120831
302.8539604	1	928.1390389	0.383093015
232.040032	1	675.8631111	0.360262927
196.8100638	0.999468462	576.3600607	0.391670261
151.8346457	1	425.2464705	0.445377323
239.0280612	1	702.2559457	0.419589827
163.4329268	1	487.0349977	0.437976223
225.7374702	0.998210024	683.8123086	0.402618482
278.0188917	1	886.7678917	0.45098564
280.8681102	1	893.8144655	0.40734622
291.8932384	0.998932384	879.6431886	0.394480491
290.8216561	1	917.2613008	0.473843566
396.9640644	1	1290.963668	0.396919088
375.957265	0.997435897	1184.43551	0.408070094
290.8510233	0.99250357	890.6073751	0.412269919
99.43930636	1	273.9573531	0.470688826
285.6336842	0.995263158	896.2180405	0.452840429
212.4797508	1	664.8367574	0.453335518
314.7297896	0.996677741	1015.740056	0.404781342
397.6399796	1	1289.371045	0.396695613
187.5045208	1	590.8992321	0.486044458
164.7238372	1	484.5300398	0.435727132
269.957115	0.998538012	808.3305609	0.466746626
227.4590164	1	683.3930578	0.447277083
224.7372986	1	654.7172894	0.359471471
175.2196007	0.997958258	475.0349171	0.372378253
162	1	480.9785452	0.428636323
206.3112245	1	595.9623617	0.457989764
185.2380952	1	576.0418158	0.438347715
191.2633833	1	580.8815623	0.433039748
228.9131545	1	654.2644455	0.380540005
479.770557	1	1551.075573	0.358984438
187.8811321	1	558.5454869	0.424787292
285.2361809	0.999057789	906.7687238	0.47055101
139.4822006	1	408.3457408	0.459855478
220.204918	1	671.2289297	0.41037336
99.36723164	1	347.0081783	0.610850411
217.9221477	1	652.603691	0.41040514
213.6305556	1	604.8744033	0.443156865
259.582716	1	794.4478694	0.441663595
244.2328482	1	796.4452397	0.467765628
265.1013216	1	819.0913932	0.535566293
239.4247991	0.999138921	696.9965447	0.374528972
195.7694284	0.996628131	563.463397	0.392934967
199.9010727	1	592.4545409	0.456598064
224.7116078	0.999459265	661.5920135	0.381540556
531.7627856	0.999183896	1745.194223	0.443761278
166.8490323	1	478.438666	0.424561218
276.7973856	1	876.021361	0.422523046
112.1213873	1	325.08579	0.488123111
291.1341256	1	929.2949695	0.502244338

log.sigma.1.0.mm.3D_glcm_Homogeneity2	log.sigma.1.0.mm.3D_glcm_ClusterShade	log.sigma.1.0.mm.3D_glcm_MaximumProbability	log.sigma.1.0.mm.3D_glcm_Idmn	log.sigma.1.0.mm.3D_glcm_SumVariance2
0.267031479	-73.48609255	0.034631222	0.976137804	26.50950614
0.424955731	-5.868431878	0.106628779	0.981852807	9.878412488
0.461917773	-3.626472338	0.153368959	0.980684209	9.106490444
0.344007791	-74.75928416	0.064139829	0.973731383	19.02593796
0.348692536	-42.56296448	0.069297037	0.973790321	15.46603087
0.281663129	-12.74604708	0.036795614	0.977621462	24.4774659
0.372166277	-7.022290709	0.084959016	0.985358899	12.79296584
0.394223677	-20.36131938	0.109540824	0.987187259	14.5159991
0.332753117	-39.27233396	0.052149504	0.983660742	16.46822777
0.311891204	-49.98980537	0.038654873	0.977117564	18.30458551
0.372227218	-18.89503552	0.059839097	0.978177335	10.37921354
0.29729713	-86.72090303	0.041428535	0.979135014	22.31449537
0.468772698	-22.16094979	0.152580589	0.987768873	12.05214732
0.335923313	-58.38858945	0.064839122	0.98037272	19.77852207
0.302819145	-64.33802473	0.052762975	0.981928126	21.35088738
0.409504629	-29.71034211	0.110980768	0.986437744	12.98107522
0.303948223	-5.834430235	0.035304387	0.971929018	16.30176797
0.439954517	-15.59519098	0.119511604	0.987476478	10.07412475
0.362156648	-5.847751955	0.063404345	0.983523433	10.53509491
0.313160383	-38.99011986	0.051026637	0.984183727	18.9388911
0.476423788	-0.587041704	0.15222731	0.98701625	7.161007417
0.315957704	-24.69909428	0.044401536	0.96636779	13.93983856
0.354554426	-21.69826915	0.062765661	0.983914679	13.26689404
0.362855801	-11.13168042	0.060529326	0.981033792	13.24455544
0.356069512	-25.47405667	0.067100085	0.98088227	15.94175771
0.291314219	-9.731982036	0.049661014	0.982249566	21.87822276
0.316690913	-23.80116683	0.057908731	0.978570064	20.4506602
0.370739717	-44.0107108	0.074835716	0.983363926	14.51460524
0.286312666	-16.42430089	0.040720066	0.966498901	14.99441401
0.37513255	-34.47918218	0.076088128	0.983155213	12.24692652
0.391713542	-35.49540141	0.111118708	0.987323612	14.69781873
0.407818305	-39.07229065	0.104008814	0.982659362	11.97253275
0.346827447	-8.131307076	0.065710484	0.983890251	19.08949285
0.356495711	-56.68068017	0.07885827	0.981319145	19.43892542
0.328188327	-24.02374525	0.054717191	0.981443719	19.12451612
0.41088627	-10.96127332	0.109568777	0.988789762	12.26434185
0.377323675	-52.04010725	0.081069888	0.987841847	16.89506126
0.460196077	-25.2294716	0.141961367	0.98933328	12.74626029
0.342504521	-48.77380618	0.052829462	0.979970596	13.19369471
0.321184673	-10.10862864	0.038840824	0.975760459	15.37459507
0.409892634	-19.97456186	0.10805611	0.979650831	11.03808999
0.26282491	-1.970216269	0.021888908	0.971030613	21.39250776
0.347154555	-36.74420066	0.063873479	0.982644205	15.07059811
0.290130053	-75.69579493	0.038527821	0.973464579	23.37463802
0.341732984	-49.33818537	0.049744875	0.982838892	17.40852347
0.349680674	-1.327759614	0.058948512	0.983945829	15.22404509
0.352432792	-6.352338776	0.075795204	0.982552181	16.56076524
0.378613595	-23.69907177	0.077304545	0.97984089	10.76685607
0.285059197	-54.35346676	0.044829836	0.971314679	23.47575284
0.379109203	-18.85696126	0.087518433	0.984137325	12.66636172
0.301056443	-39.18170963	0.04599792	0.98292781	20.68149282
0.272975829	-14.5177273	0.027003521	0.977143129	21.05311521
0.309009487	-22.45955839	0.043335647	0.977989756	15.80106543
0.374076255	-2.911298745	0.063527063	0.982309264	11.15286006
0.344923373	-0.476908642	0.065616086	0.979968173	17.53345792
0.364915042	-34.69858591	0.066324085	0.980467905	12.37435618
0.323536878	-31.81257295	0.049859542	0.981595749	16.7791305
0.382550362	-32.38925744	0.082918492	0.985094036	13.4584849
0.329454685	-39.91560977	0.059839006	0.982747117	15.95128069
0.31620433	-28.12322471	0.045043449	0.978841015	20.005756
0.411186646	-13.0291908	0.127076621	0.98617314	13.97689506
0.319071471	-55.41606279	0.058510058	0.98488195	19.77579461
0.33171146	-26.52486708	0.07001625	0.983397106	17.84955155
0.335773617	-33.72778353	0.064087317	0.9849712	17.07362013
0.40313148	-11.3213093	0.073520897	0.975761155	7.512882696
0.385771082	-36.51606716	0.092979822	0.986761866	14.05651943
0.386101306	-39.34586072	0.100118809	0.980414192	13.09513681
0.327766143	-36.34286105	0.062288533	0.983964676	17.3458274
0.316705778	-39.53049565	0.049486149	0.987828871	17.6586208
0.423071171	-17.45673128	0.098042787	0.983101109	8.862676225
0.363994234	-30.7095379	0.078421042	0.977819506	13.85133084
0.402511697	-18.66315523	0.108988765	0.985791501	15.02821537
0.379535581	-19.8870692	0.07863411	0.981058231	15.92106595
0.27501556	-47.28531033	0.032795371	0.976165493	24.3629398
0.287203518	-18.40618553	0.033408564	0.974255807	18.61598533
0.354304436	-37.74564286	0.05946073	0.97963241	13.53090724
0.389434273	10.02113502	0.07236179	0.987195026	11.36606695
0.367325782	-41.95120908	0.076741874	0.976243447	13.22326228
0.357594542	-23.50138402	0.061777476	0.979360158	11.82087474
0.299210015	-38.74343037	0.043337036	0.977610129	21.16242297
0.277183975	-68.35697946	0.036838902	0.977412456	25.38139649
0.353600465	-46.30185856	0.06060774	0.9783241	17.57122458
0.407122171	-22.32968448	0.103631299	0.985696738	13.52705867
0.389737191	-10.10993921	0.073124417	0.978042973	8.450658968
0.335544127	-39.26020337	0.057707616	0.976519448	18.13742617
0.575640074	-9.394226949	0.235169384	0.987689985	4.01144131
0.335623752	-44.75701935	0.058999371	0.977765874	18.665881
0.372666519	-19.01906558	0.079587086	0.979923322	14.95682776
0.370556909	-45.9018064	0.0844405	0.981212988	13.88211312
0.402382319	-37.64626858	0.103046846	0.983972803	11.77583088
0.482901793	4.41081333	0.159450736	0.990979207	7.217127229
0.288865448	-19.32688778	0.03306127	0.975317239	18.10490592
0.312598598	-31.58758767	0.042052507	0.978697263	18.01303803
0.388586394	-24.90975449	0.084295277	0.983026683	12.47245433
0.297206307	-20.80630152	0.032582946	0.981560878	17.12167439
0.377513019	-77.77112027	0.10838005	0.986261988	21.51433884
0.349188741	-20.56519611	0.058240444	0.978155159	13.79557132
0.349400512	-56.71601505	0.085628544	0.97579258	18.45308895
0.425352049	-16.83291349	0.12494047	0.975753365	8.115192466
0.444313018	0.402493125	0.132272684	0.989249581	9.959465298

log.sigma.1.0.mm.3D_glcm_Contrast	log.sigma.1.0.mm.3D_glcm_DifferenceEntropy	log.sigma.1.0.mm.3D_glcm_InverseVariance	log.sigma.1.0.mm.3D_glcm_Entropy	log.sigma.1.0.mm.3D_glcm_Dissimilarity
28.81362215	3.495441921	0.270631443	7.457360461	3.888669896
8.88598176	2.681645247	0.392313481	5.67092146	2.042817454
7.773497719	2.610793973	0.383646941	5.563294376	1.875915533
17.37764828	3.111638198	0.326702042	6.625293735	2.884110805
14.33205383	3.038353703	0.331671762	6.541264163	2.701140138
20.81503023	3.247776454	0.28827394	6.84643233	3.332581921
11.45868966	2.903183946	0.346121252	6.385007804	2.419012161
13.30005282	2.937278882	0.359271127	6.251150133	2.456392386
15.91743676	3.101161502	0.324612868	6.76276948	2.84863493
17.26666113	3.131103008	0.314545354	6.731685511	2.992442718
9.646283585	2.777901561	0.365627043	6.095209616	2.262999135
22.33476484	3.293833644	0.296493143	7.004868581	3.336051524
10.71407141	2.664559356	0.402888159	5.468291174	2.004273479
18.47426435	3.15170923	0.319827021	6.746408298	2.959985166
24.25757525	3.326846225	0.306079863	6.950780493	3.42680918
11.66449311	2.829943003	0.371952926	6.101028332	2.268331947
15.22475814	3.089646669	0.298913888	6.804419402	2.916094701
8.546126996	2.625861254	0.401790618	5.595435496	1.950410514
10.21368487	2.824929264	0.354101834	6.146749462	2.348586755
17.5770522	3.159933072	0.310118628	6.891078767	3.001916905
6.173240664	2.477505523	0.410861497	5.360778636	1.691330347
13.82055864	3.025689438	0.322131956	6.572432657	2.787793599
12.78856199	2.934507407	0.347958853	6.310435416	2.541063389
12.05397443	2.915462449	0.34765187	6.421222164	2.470877974
14.49753818	2.985449088	0.34599768	6.388011081	2.634780879
21.03046232	3.272780288	0.300550244	7.056372354	3.308683683
18.68253471	3.194192601	0.311223355	7.016733856	3.069055322
13.44381185	2.940651193	0.354435102	6.347496999	2.511377735
15.00148875	3.076472361	0.288353276	6.593669669	2.991849371
11.59624993	2.862271261	0.364112016	6.137117599	2.379029324
14.14288724	2.955230004	0.356084874	6.285525463	2.494441675
12.09468883	2.844984307	0.376971108	6.018403765	2.312662539
16.89213025	3.115258231	0.327507383	6.805142773	2.853070748
17.59690542	3.095780918	0.343321746	6.67176839	2.826961059
17.08805865	3.142870877	0.320989988	6.878758414	2.936827856
10.90861817	2.792661212	0.379241362	6.011812758	2.212224211
18.39081067	3.03557238	0.3591865	6.415696804	2.725704798
12.6916662	2.786028538	0.382950036	5.785475629	2.183496171
14.10737596	2.979334632	0.332919781	6.312602305	2.639940057
14.14594549	3.056071312	0.317622349	6.851822782	2.791326939
9.986922711	2.775554606	0.376801858	6.019659133	2.183501765
20.02228555	3.292486079	0.268519131	7.367847484	3.426940002
13.79912744	3.003402441	0.336836168	6.570773624	2.646419919
23.6972202	3.346819043	0.291004035	7.191717331	3.459233047
16.06218177	3.040042309	0.335799604	6.463803039	2.763339867
13.69430314	2.985795369	0.346242413	6.558744992	2.622704284
15.01868085	3.04197984	0.340836067	6.611445735	2.705546611
10.83007648	2.816530081	0.371910841	5.914159252	2.321503079
22.04838702	3.30047185	0.288856256	6.985303221	3.410565528
10.78922494	2.839998915	0.355856611	6.155850119	2.329155426
19.00376744	3.214293298	0.302498204	6.977753595	3.145936508
19.58342109	3.269887716	0.276102813	7.245857261	3.337830696
15.03916676	3.093222488	0.308367406	6.820336827	2.89053099
10.23133169	2.796896373	0.371015581	6.089752123	2.292929958
14.97804537	3.038970539	0.337060153	6.490328668	2.734893934
11.42688813	2.86894097	0.357675324	6.228673841	2.407292971
15.68566251	3.1051238	0.316996876	6.833862542	2.874366782
12.7920473	2.909574287	0.362428481	6.316718954	2.43761352
15.83435744	3.077947608	0.326092029	6.571634334	2.834644985
19.60089515	3.256072597	0.299398315	7.157156324	3.171055139
12.75501866	2.869423302	0.369820469	6.008228438	2.346712732
21.47859159	3.25110371	0.306579805	6.886925815	3.189198112
17.35359294	3.153301487	0.319206619	6.806088988	2.944085265
16.74487284	3.118073326	0.322256289	6.755983648	2.866989278
6.848229696	2.566527595	0.396200712	5.630941245	1.943701389
13.92737709	2.935729069	0.364380079	6.284253483	2.486089342
13.89346085	2.93166537	0.360923298	6.123301215	2.490336939
17.77998606	3.169342487	0.316360432	6.854575929	2.984570677
17.71960045	3.171929336	0.307915776	6.884736199	3.009446916
8.267087706	2.643329785	0.393773999	5.742924916	1.982503388
13.07952573	2.950734594	0.349337997	6.309215889	2.550610672
13.21289891	2.888902497	0.370087364	6.171308925	2.385324474
14.21766127	2.985696808	0.352974413	6.465500312	2.573093582
23.68953486	3.374846128	0.276267767	7.356602009	3.566837664
17.77620942	3.204270387	0.290473807	7.094969733	3.165479107
12.91744717	2.954481815	0.341757921	6.359031536	2.571118467
9.968716459	2.787657531	0.372102809	6.042106533	2.23430141
13.2322728	2.90938131	0.362758613	6.140159662	2.502820286
11.05592289	2.865034821	0.348309765	6.234532395	2.410273948
20.89895126	3.277456537	0.295366594	7.131334307	3.282839969
27.2598788	3.447752811	0.279121369	7.344579942	3.74786222
17.72121088	3.11762515	0.335974415	6.606120604	2.880232152
13.28497736	2.894865318	0.366457069	6.162749016	2.386544392
7.838190725	2.658448183	0.381596978	5.945110101	2.064917892
17.76525953	3.150173996	0.324870745	6.810793697	2.961399144
3.503027501	2.072320379	0.444842807	4.369629136	1.190208497
18.13564215	3.159740158	0.324869017	6.852303436	2.973226346
12.88111034	2.942245261	0.351735022	6.417903445	2.497836373
13.13714584	2.92618022	0.354709106	6.229125329	2.494595052
11.12948538	2.81023041	0.374594767	6.006751967	2.256195698
5.514801839	2.403184335	0.416307492	5.122085279	1.608074636
16.95996852	3.17423449	0.290537097	7.021738646	3.103489751
17.12300057	3.155947988	0.312883543	6.951690176	2.997953533
10.70840203	2.821791283	0.372089307	6.192502529	2.287627344
16.76694266	3.163457948	0.295863295	7.002449607	3.046020493
20.70668759	3.157591653	0.334694565	6.588584469	2.926837186
12.76225999	2.965932863	0.340194029	6.513861245	2.580894152
18.89965784	3.13584999	0.325162057	6.55688187	2.928119088
7.851984437	2.640861409	0.385379681	5.627426098	1.980135067
8.422385769	2.640490107	0.399747532	5.670454514	1.943959316

log.sigma.1.0.mm.3D_glcm_DifferenceVariance	log.sigma.1.0.mm.3D_glcm_Idn	log.sigma.1.0.mm.3D_glcm_Idm	log.sigma.1.0.mm.3D_glcm_Correlation	log.sigma.1.0.mm.3D_glcm_Autocorrelation
13.65881414	0.902604428	0.267031479	-0.037064605	467.5885244
4.703904515	0.918367454	0.424955731	0.049451398	143.2398977
4.235844939	0.917872217	0.461917773	0.077192123	118.7097065
9.029913294	0.902326423	0.344007791	0.045446735	245.0120575
7.016449427	0.899666734	0.348692536	0.038499727	189.0042934
9.5931999	0.904306906	0.281663129	0.079965841	254.3878436
5.595153878	0.923121134	0.372166277	0.055257676	221.4610686
7.257781424	0.931872758	0.394223677	0.044415516	286.871066
7.79029313	0.919176587	0.332753117	0.017236593	286.8405013
8.261950574	0.904563681	0.311891204	0.029441626	255.9014778
4.515535771	0.905633843	0.372227218	0.035063492	140.4309185
11.17965953	0.91031596	0.29729713	0.002173912	327.2041277
6.676786634	0.938997072	0.468772698	0.059294515	280.9853474
9.69410061	0.914820813	0.335923313	0.034578894	318.9738042
12.47497435	0.917620987	0.302819145	-0.056371129	353.1996553
6.511273509	0.930732336	0.409504629	0.053480109	248.1355223
6.69611476	0.891502081	0.303948223	0.031947284	117.0525405
4.73265712	0.93285997	0.439954517	0.079223559	222.0745029
4.679885329	0.916436277	0.362156648	0.016084744	219.2120796
8.549525745	0.919894447	0.313160383	0.038439904	287.1797539
3.308290676	0.9307409	0.476423788	0.070089589	163.8284184
6.022102194	0.88199721	0.315957704	0.005433542	141.3068282
6.321221585	0.919826612	0.354554426	0.01675717	283.3900078
5.931057585	0.91342019	0.362855801	0.04832778	223.79807
7.524860665	0.915164774	0.356069512	0.045239241	258.9633847
10.04819885	0.915061009	0.291314219	0.021927358	364.178741
9.240516434	0.908802001	0.316690913	0.045295113	255.0235433
7.126149358	0.921360789	0.370739717	0.037689271	282.1862217
6.040304536	0.878992198	0.286312666	-0.001014192	140.6818848
5.930791718	0.919405085	0.37513255	0.026038974	251.2404329
7.911811984	0.933039437	0.391713542	0.020408184	321.9661595
6.735214935	0.92242013	0.407818305	-0.003436778	243.6293468
8.721276667	0.921882248	0.346827447	0.061264533	251.1700684
9.590372334	0.918584538	0.356495711	0.049567378	220.7023396
8.451465276	0.914715994	0.328188327	0.055304624	198.2482356
6.002556513	0.936026099	0.41088627	0.058697843	220.7280577
10.94710667	0.936852331	0.377323675	-0.040860193	467.0740536
7.905456578	0.943070745	0.460196077	0.006300554	426.2898486
7.132374502	0.911713214	0.342504521	-0.033183422	279.1088062
6.346330022	0.899443203	0.321184673	0.041487302	137.731147
5.206033566	0.913502334	0.409892634	0.05005765	136.2347479
8.259968431	0.88768831	0.26282491	0.033147155	162.2378808
6.779962631	0.916974349	0.347154555	0.044187333	252.6014817
11.71455052	0.899188255	0.290130053	-0.006405337	282.9039038
8.392120353	0.919545065	0.341732984	0.041926218	328.5037531
6.796590372	0.920227776	0.349680674	0.050610936	169.2476412
7.687020305	0.918422071	0.352432792	0.047796238	221.9795311
5.427002344	0.911819968	0.378613595	-0.002047195	140.9826636
10.38116881	0.893377435	0.285059197	0.031513886	167.1253835
5.347291985	0.920587389	0.379109203	0.077100641	290.0441146
9.099145125	0.916454298	0.301056443	0.043655867	293.8220105
8.410858066	0.900454418	0.272975829	0.036749349	222.2971723
6.671178177	0.903317237	0.309009487	0.024875039	191.4921379
4.965907797	0.915596058	0.374076255	0.043380346	144.5407051
7.478234782	0.912043031	0.344923373	0.074288402	227.4696713
5.620173803	0.912173226	0.364915042	0.040054106	163.175775
7.408551418	0.913229558	0.323536878	0.034066344	222.8540467
6.842366208	0.925832165	0.382550362	0.025575376	277.7821626
7.787458515	0.917153136	0.329454685	0.005007521	281.5079049
9.538680462	0.908833391	0.31620433	0.010566345	280.0088549
7.234782263	0.931028472	0.411186646	0.045527802	285.4899129
11.27007724	0.924499251	0.319071471	-0.035787526	394.092171
8.669806545	0.919376982	0.33171146	0.017908284	364.3371561
8.506788716	0.923434149	0.335773617	0.010636124	281.3909583
3.060259429	0.8993431	0.40313148	0.044956278	97.57270912
7.73102281	0.931274355	0.385771082	0.005410632	280.3733703
7.675394173	0.917248152	0.386101306	-0.028194743	213.5201474
8.859834212	0.920594538	0.327766143	-0.01049048	316.8599092
8.653126138	0.929283651	0.316705778	0.001608642	393.9709515
4.333998421	0.920214253	0.423071171	0.03363941	190.817407
6.530517539	0.908133242	0.363994234	0.030374562	162.7436835
7.512839049	0.930028936	0.402511697	0.064192356	255.8260288
7.576867026	0.917318548	0.379535581	0.055936951	221.2541412
10.94756686	0.901528891	0.27501556	0.014024729	216.1375787
7.728168182	0.895450452	0.287203518	0.022698223	162.5061763
6.271723535	0.910387775	0.354304436	0.023959289	161.5477813
4.972808186	0.928395475	0.389434273	0.064267539	194.0932603
6.944903394	0.906670167	0.367325782	0.001195681	188.5926162
5.236773925	0.908251591	0.357594542	0.034035591	190.4796208
10.09513302	0.906015164	0.299210015	0.005987794	215.2668775
13.17813977	0.905802058	0.277183975	-0.031101359	469.1409069
9.398680911	0.911890547	0.353600465	-0.003292118	185.3712579
7.568240355	0.930063547	0.407122171	0.011152823	282.7005629
3.558027377	0.904211837	0.389737191	0.038117807	138.6604165
8.950218027	0.906197946	0.335544127	0.011181957	218.9490716
2.07131475	0.936337029	0.575640074	0.068976264	114.4899866
9.267204775	0.908942776	0.335623752	0.014516798	213.6618021
6.631284649	0.913195908	0.372666519	0.073210194	198.7186867
6.904808533	0.916414502	0.370556909	0.026747391	251.8433961
6.01223156	0.923619208	0.402382319	0.029687617	250.9637667
2.91876977	0.940945298	0.482901793	0.131961897	252.526213
7.316075849	0.897046488	0.288865448	0.032815809	227.5703383
8.130310479	0.907284228	0.312598598	0.025222342	188.1973627
5.463696914	0.919334819	0.388586394	0.074993688	194.1339415
7.48016106	0.910913023	0.297206307	0.010616058	216.81445
12.11453278	0.932752507	0.377513019	0.020608306	519.0915905
6.093971744	0.906557857	0.349188741	0.039037263	161.7474502
10.28162603	0.908148676	0.349400512	-0.010324747	276.1821199
3.915442453	0.904297525	0.425352049	0.017008396	112.7902661
4.635465514	0.937378014	0.444313018	0.081572621	286.4962044

log.sigma.1.0.mm.3D_glcm_SumEntropy	log.sigma.1.0.mm.3D_glcm_AverageIntensity	log.sigma.1.0.mm.3D_glcm_Energy	log.sigma.1.0.mm.3D_glcm_SumSquares	log.sigma.1.0.mm.3D_glcm_ClusterProminence
4.319805964	21.86887591	0.01041287	13.06218819	3058.7982
3.512199664	11.95775534	0.038177591	4.691098562	616.2807245
3.462704926	10.88001674	0.050445601	4.219997041	459.7505209
4.017453287	15.6396201	0.019170935	9.10089656	1936.661542
3.911879455	13.73747124	0.021002944	7.449521176	1199.787139
4.187823916	15.94990705	0.013683927	11.56713625	3179.208093
3.798632332	14.87247543	0.024369154	6.084675588	848.0859645
3.810776696	16.97922452	0.032124263	7.137505318	1290.399742
3.981484566	16.98186945	0.017251455	8.286099186	1349.306877
4.032804041	15.99347406	0.01545224	8.926228543	1605.989667
3.652026172	11.84255141	0.023651878	5.006374281	518.6536439
4.160652456	18.22871437	0.013976844	11.32896202	2706.640016
3.474483108	16.77555828	0.056305433	5.85707371	1372.53904
4.04134408	17.91076088	0.018866955	9.746866575	2492.233263
4.099043521	19.07176265	0.01673528	10.75726648	2609.905339
3.721381634	15.74932258	0.033831312	6.222401746	1129.649241
4.00852926	10.80659076	0.013486824	7.88163153	1033.501291
3.50234144	14.88925847	0.041796869	4.655062936	733.1481039
3.669091685	14.80306509	0.023928212	5.187194945	518.7974342
4.077654609	17.0166707	0.015902477	9.312308002	1941.463753
3.30540978	12.78984491	0.053748897	3.33356202	321.6742598
3.876059989	11.88587594	0.016408096	6.9400993	820.6296532
3.798643367	16.83602444	0.022001969	6.549754807	1023.568114
3.82659649	14.94984603	0.021470959	6.324632466	888.121734
3.891973051	16.08727338	0.023001687	7.656010171	1554.829744
4.161145479	19.3163594	0.014380809	10.52143185	2561.534099
4.134191877	15.9816678	0.015417863	9.962226117	1994.258718
3.82270516	16.80022652	0.024569115	7.057008006	1391.774837
3.927002935	11.86095807	0.014553899	7.49897569	817.7245874
3.714578604	15.84541233	0.026721854	5.960794112	982.9207835
3.801717056	18.03785006	0.031262336	7.330105574	1510.368501
3.650415695	15.60960095	0.033857366	6.016805396	930.2192682
4.070508304	15.89178767	0.018789132	9.332120648	1950.162601
4.030352522	14.85478146	0.021983064	9.354497997	2216.980623
4.091950896	14.07080004	0.01673412	9.130931627	1760.239725
3.674638751	14.85630382	0.034977602	5.871839724	993.7648765
3.851745187	21.7276534	0.026980047	8.188044614	2221.338399
3.582090076	20.7771665	0.052490628	5.99002623	1318.986158
3.776263781	16.71337476	0.020948078	6.825267668	1090.047664
3.991336428	11.7227872	0.014360454	7.380135138	915.4035295
3.638964136	11.66068478	0.033785829	5.256253176	655.6650908
4.242490313	12.72380242	0.008694427	10.35369833	1485.593848
3.907257382	15.88631784	0.019605833	7.239813462	1218.280934
4.207983028	16.87047546	0.012660708	12.05134025	2795.958973
3.941801744	18.15396815	0.019791232	8.486593292	1892.583009
3.900931067	12.99725861	0.020738186	7.257412298	1226.578458
3.942282093	14.89853409	0.022031659	8.013132134	1614.512778
3.609505895	11.8742501	0.029123725	5.399233139	678.856782
4.194012809	12.91934412	0.013647826	11.4372163	2515.38924
3.76244682	17.01670707	0.026246723	5.863896663	888.0489152
4.133979454	17.23848695	0.014340103	10.23722489	2178.520161
4.20533093	14.91104018	0.010309531	10.27442955	1785.602165
3.986250565	13.83110353	0.015110154	7.710058047	1062.88188
3.676580445	12.01289393	0.024913264	5.346047937	709.5496323
3.954365553	15.07137923	0.021238525	8.241051507	1796.156438
3.742818326	12.7647143	0.023751076	5.950311078	828.5617449
4.011285068	14.92337285	0.015732435	8.148734755	1311.942655
3.771775874	16.68014955	0.026595322	6.6728643	1090.88684
3.925957003	16.8023758	0.019056926	8.058364899	1445.695454
4.141335421	16.78046966	0.013985087	10.14830596	1768.979724
3.720106213	16.9148451	0.038008662	6.837695643	1366.64746
4.071299945	20.03148231	0.016763557	9.618368249	2400.507649
4.013391201	19.25196907	0.019134799	8.919302922	1753.683745
3.988943215	16.84550636	0.018733142	8.675188456	1689.485366
3.415793102	9.869327407	0.031357773	3.590278098	269.1470906
3.785600211	16.78891349	0.029041658	7.141861301	1307.23725
3.709170881	14.61914825	0.030787833	6.747149415	1177.130999
4.009853841	17.9294696	0.017695227	8.946230744	1590.711285
4.037119319	20.01631796	0.015614536	8.178186424	1675.55394
3.491087427	13.80823819	0.035488795	4.282440983	500.798762
3.804074978	12.74949896	0.024577682	6.732714143	1013.063862
3.795119256	15.99893247	0.033511361	7.183223719	1585.798317
3.885072457	14.86181586	0.025005635	7.551219556	1454.573555
4.284993543	14.73580226	0.010348519	12.2618512	2369.263519
4.115126333	12.73951339	0.011645837	9.098048688	1366.3906
3.78782124	12.70407181	0.021686358	6.612088602	947.2654505
3.65994572	13.92138904	0.028091333	5.365959774	930.3990862
3.742277631	13.73295718	0.026132248	6.613883768	1108.88933
3.734042339	13.7944818	0.022714841	5.719199407	741.5572566
4.178005997	14.69504188	0.012768016	10.70234515	2013.770749
4.280870043	21.89361918	0.011408058	12.39049393	2948.153279
3.974149734	13.62316662	0.021321684	8.885139059	1602.243008
3.736647492	16.84117832	0.033052837	6.837720877	1248.397707
3.540454613	11.76887375	0.026598609	4.072212423	302.255856
4.031932783	14.79926429	0.017596492	9.02278563	1562.692056
2.868056501	10.694051	0.097178636	1.878617203	135.9611585
4.04720876	14.61693212	0.017440896	9.234001618	1658.644095
3.868344237	14.07817719	0.024038655	6.959484525	1239.044607
3.788880707	15.86367765	0.025593442	6.754814739	1248.288376
3.66155091	15.83671538	0.032441061	5.726329065	936.0424745
3.261899074	15.87758839	0.059410299	3.182982267	356.8370562
4.096350165	15.07587239	0.011945665	8.766218611	1281.273075
4.065935235	13.71503162	0.014420433	8.826149102	1473.769466
3.751838927	13.91730097	0.027684326	5.79521409	822.0438127
4.056809928	14.73195654	0.012268797	8.551110665	1210.911899
4.041046767	22.89323333	0.028726417	9.718077371	2957.293038
3.860119539	12.70780809	0.019973638	6.639457829	892.9782936
3.959970938	16.63583019	0.022560441	9.406990872	2020.109345
3.430688923	10.61714403	0.038986657	3.991794226	317.8052215
3.509164915	16.92008885	0.045638353	4.633131358	667.5127005

log.sigma.1.0.mm.3D_glcm_SumAverage	log.sigma.1.0.mm.3D_glcm_Imc2	log.sigma.1.0.mm.3D_glcm_Imc1	log.sigma.1.0.mm.3D_glcm_DifferenceAverage	log.sigma.1.0.mm.3D_glcm_Id
43.27419842	0.589568805	-0.055150295	3.888669896	0.349955103
23.91551067	0.583581891	-0.071649284	2.042817454	0.486401952
21.76003347	0.55508792	-0.064992567	1.875915533	0.51812533
31.2792402	0.651537924	-0.08028313	2.884110805	0.418209309
27.47494248	0.484020985	-0.040203285	2.701140138	0.423210052
31.84127609	0.721966482	-0.102597468	3.332581921	0.366854727
29.74061832	0.406989912	-0.028383414	2.419012161	0.444102815
33.85644946	0.450143192	-0.035818715	2.456392386	0.459832952
33.8645333	0.421640038	-0.028635149	2.84863493	0.409604194
31.97750402	0.614694674	-0.068472703	2.992442718	0.392561121
23.68510281	0.484345195	-0.04313854	2.262999135	0.444823164
36.17773138	0.54123957	-0.048215886	3.336051524	0.378841117
33.50523944	0.597886746	-0.078956199	2.004273479	0.520490202
35.70134947	0.607977582	-0.066086257	2.959985166	0.41175181
37.62577235	0.604604988	-0.062818929	3.42680918	0.381384567
31.48368961	0.47127803	-0.04050544	2.268331947	0.473531907
21.61318153	0.54219474	-0.050174325	2.916094701	0.388385651
29.77851694	0.554945125	-0.06501876	1.950410514	0.498828732
29.60613018	0.499722681	-0.045891336	2.348586755	0.436647623
33.87259031	0.413562412	-0.026898554	3.001916905	0.393870882
25.57968982	0.46158309	-0.04484324	1.691330347	0.52964296
23.77175187	0.531137659	-0.04949586	2.787793599	0.396999866
33.66122945	0.590375923	-0.066424354	2.541063389	0.428817108
29.89969206	0.472979773	-0.038850501	2.470877974	0.43623676
32.1621219	0.60915327	-0.070678954	2.634780879	0.429126319
38.15509345	0.56247999	-0.05277429	3.308683683	0.373537454
31.9111745	0.50752794	-0.041659688	3.069055322	0.395649903
33.58080748	0.483079034	-0.04141027	2.511377735	0.441622196
23.72191613	0.670478053	-0.08725765	2.991849371	0.373995974
31.69082467	0.488801567	-0.043774447	2.379029324	0.445799265
35.87900302	0.47788961	-0.040587077	2.494441675	0.458025141
31.21920191	0.522608892	-0.051727058	2.312662539	0.4709704
31.66188162	0.524294005	-0.0463194	2.853070748	0.420490407
29.68103267	0.570841324	-0.057632116	2.826961059	0.427412227
28.12377468	0.453861568	-0.033329797	2.936827856	0.405287398
29.6908788	0.507027145	-0.048953733	2.212224211	0.474594136
43.24106556	0.568280119	-0.058804751	2.725704798	0.444534776
41.29287794	0.561556405	-0.063104259	2.183496171	0.513079967
33.42674952	0.526388253	-0.050216383	2.639940057	0.420021528
23.4455744	0.336187218	-0.017560583	2.791326939	0.401579718
23.32136956	0.477957194	-0.042570309	2.183501765	0.474030869
25.44760484	0.386385955	-0.021753651	3.426940002	0.351521594
31.76677732	0.435562525	-0.031903528	2.646419919	0.422599236
33.64421075	0.544666189	-0.047628783	3.459233047	0.371969512
36.23061808	0.60741279	-0.068964191	2.763339867	0.417563749
25.98932237	0.514789116	-0.046344133	2.622704284	0.424023205
29.77197101	0.459248453	-0.035651524	2.705546611	0.425594958
23.7485002	0.542194506	-0.057553511	2.321503079	0.448473428
25.82762571	0.69232403	-0.089694905	3.410565528	0.368254877
34.03341414	0.548195873	-0.058206347	2.329155426	0.450120831
34.25788974	0.504929465	-0.041030067	3.145936508	0.383093015
29.7944785	0.438187213	-0.029158194	3.337830696	0.360262927
27.66220705	0.366533919	-0.021131211	2.89053099	0.391670261
24.02578786	0.549917382	-0.057676358	2.292929958	0.445377323
30.12102833	0.598285872	-0.066719257	2.734893934	0.419589827
25.5294286	0.454545876	-0.036713167	2.407292971	0.437976223
29.83821978	0.428099938	-0.029265734	2.874366782	0.402618482
33.32356636	0.484085182	-0.041740125	2.43761352	0.45098564
33.55458465	0.540048944	-0.051093566	2.834644985	0.40734622
33.4608398	0.430728866	-0.028283751	3.171055139	0.394480491
33.77471614	0.592108325	-0.069761945	2.346712732	0.473843566
39.72486723	0.557790821	-0.052062259	3.189198112	0.396919088
38.16863165	0.440416582	-0.031233364	2.944085265	0.408070094
33.544497	0.402561021	-0.026244215	2.866989278	0.412269919
19.73865481	0.487023057	-0.047485404	1.943701389	0.470688826
33.4867592	0.499746397	-0.044889644	2.486089342	0.452840429
29.2382965	0.583512679	-0.066155254	2.490336939	0.453335518
35.60720015	0.419124758	-0.027959931	2.984570677	0.404781342
39.69814666	0.416034733	-0.02691721	3.009446916	0.396695613
27.61647638	0.416192781	-0.033239472	1.982503388	0.486044458
25.49899792	0.556908192	-0.057816454	2.550610672	0.435727132
31.96069159	0.495747495	-0.044955406	2.385324474	0.466746626
29.7204737	0.570686765	-0.059377515	2.573093582	0.447277083
29.39176035	0.538067491	-0.045366421	3.566837664	0.359471471
25.47902678	0.445279237	-0.030885204	3.165479107	0.372378253
25.40814362	0.557313321	-0.057174566	2.571118467	0.428636323
27.83832393	0.469616911	-0.040777745	2.23430141	0.457989764
27.46591437	0.599013998	-0.070132488	2.502820286	0.438347715
27.58896361	0.475528926	-0.040467315	2.410273948	0.433039748
29.339412	0.523358183	-0.043968471	3.282839969	0.380540005
43.34095332	0.607770946	-0.060325918	3.74786222	0.358984438
27.23294924	0.587425254	-0.062145617	2.880232152	0.424787292
33.62375999	0.534352112	-0.053466125	2.386544392	0.47055101
23.5377475	0.353960132	-0.022614159	2.064917892	0.459855478
29.58753447	0.531021487	-0.048045799	2.961399144	0.41037336
21.38810201	0.433079285	-0.050534002	1.190208497	0.610850411
29.22523626	0.559087898	-0.053703867	2.973226346	0.41040514
28.15635438	0.51416195	-0.047658963	2.497836373	0.443156865
31.7273553	0.553822556	-0.057471339	2.494595052	0.441663595
31.67343075	0.511211988	-0.049851243	2.256195698	0.467765628
31.75517678	0.500508182	-0.057205067	1.608074636	0.535566293
30.15174479	0.458764255	-0.033226254	3.103489751	0.374528972
27.42075177	0.415795852	-0.027013728	2.997953533	0.392934967
27.83460195	0.453773069	-0.037097848	2.287627344	0.456598064
29.44319783	0.39460283	-0.023893766	3.046020493	0.381540556
45.55814445	0.5790997	-0.060454603	2.926837186	0.443761278
25.41561618	0.436077079	-0.03193533	2.580894152	0.424561218
33.24412204	0.667910764	-0.086783885	2.928119088	0.422523046
21.23428807	0.530294378	-0.057225653	1.980135067	0.488123111
33.82960913	0.420543809	-0.035113433	1.943959316	0.502244338

log.sigma.1.0.mm.3D_glcm_ClusterTendency	log.sigma.1.0.mm.3D_firstorder_InterquartileRange	log.sigma.1.0.mm.3D_firstorder_Skewness	log.sigma.1.0.mm.3D_firstorder_Uniformity
26.50950614	90.58059454	-0.677860269	0.09411968
9.878412488	46.49548244	-0.529066824	0.173503389
9.106490444	41.29746795	-0.390558915	0.18652737
19.02593796	66.70136833	-1.152367038	0.122972691
15.46603087	65.29584932	-0.893681785	0.131484261
24.4774659	81.0048542	-0.286937799	0.106818182
12.79296584	61.62198639	-0.281977327	0.137093326
14.5159991	50.94427967	-0.658507926	0.158862309
16.46822777	69.27736092	-0.737918459	0.12270085
18.30458551	76.71027422	-0.918155299	0.11223442
10.37921354	62.4005065	-0.647617622	0.143287084
22.31449537	78.2862978	-1.088714038	0.110365564
12.05214732	35.50232887	-1.015424682	0.214386596
19.77852207	67.46342659	-0.691022615	0.123987098
21.35088738	71.57671928	-0.837450262	0.113065817
12.98107522	48.72861528	-0.914733663	0.162901496
16.30176797	81.85209703	-0.173611197	0.109117615
10.07412475	42.52455616	-1.022897035	0.18696511
10.53509491	59.44325447	-0.423561972	0.141916805
18.9388911	70.87602425	-0.738940409	0.117590526
7.161007417	38.79879045	-0.137968408	0.202802186
13.93983856	73.24586296	-0.660613205	0.118498661
13.26689404	62.53048372	-0.725833602	0.132390976
13.24455544	64.39832592	-0.458601969	0.132881541
15.94175771	61.32740116	-0.677447517	0.134083994
21.87822276	80.32187271	-0.150606457	0.103310828
20.4506602	75.7329216	-0.441484469	0.110390461
14.51460524	57.28065252	-1.033612466	0.144823641
14.99441401	77.92748356	-0.343563322	0.115305116
12.24692652	54.93813205	-1.084807787	0.152577107
14.69781873	51.99019384	-0.815608133	0.156920465
11.97253275	47.25492859	-1.196820571	0.167183592
19.08949285	66.06396103	-0.152069592	0.123861728
19.43892542	64.02222681	-0.770040354	0.127907288
19.12451612	74.12895441	-0.48347657	0.114071119
12.26434185	48.77240086	-0.474474292	0.163672319
16.89506126	53.2085247	-1.050555535	0.150907337
12.74626029	34.65862274	-0.859970395	0.203896347
13.19369471	63.00997829	-1.338063142	0.139056117
15.37459507	77.39374876	-0.198242406	0.113188529
11.03808999	47.6603415	-0.636538743	0.165941033
21.39250776	100.836422	0.021219101	0.090634157
15.07059811	67.32978439	-0.763745538	0.12790248
23.37463802	81.91329145	-0.789835226	0.103493231
17.40852347	64.94287014	-1.062218238	0.128330407
15.22404509	65.28742409	-0.086701277	0.124289568
16.56076524	63.58452511	-0.256682752	0.129902088
10.76685607	51.11168957	-0.818367131	0.160860012
23.47575284	81.32547092	-0.60031138	0.105742743
12.66636172	57.11981201	-0.759049994	0.143591786
20.68149282	75.11499786	-0.630247148	0.111986934
21.05311521	93.06958199	-0.251728456	0.095766929
15.80106543	75.34866047	-0.444651308	0.114942109
11.15286006	59.25620651	-0.253467691	0.144351567
17.53345792	62.28410387	-0.292223776	0.132229069
12.37435618	61.26698589	-1.013839622	0.142453649
16.7791305	76.14567757	-0.589142124	0.114060026
13.4584849	55.84579039	-0.889797777	0.147723772
15.95128069	66.93495321	-0.740763999	0.129846268
20.005756	78.75377083	-0.359634939	0.109658484
13.97689506	47.80670309	-0.530699097	0.164414273
19.77579461	70.48724031	-0.925274889	0.120760286
17.84955155	67.1875515	-0.447475025	0.123033507
17.07362013	66.15616131	-0.654872764	0.128077757
7.512882696	52.85439897	-0.71963276	0.164204454
14.05651943	51.80305004	-0.888082025	0.154570647
13.09513681	52.86344814	-1.007740179	0.157860194
17.3458274	66.51460123	-0.605853348	0.124935885
17.6586208	72.55718613	-0.715571564	0.118435746
8.862676225	47.20087504	-1.092594938	0.172043879
13.85133084	57.15009499	-0.797759941	0.141364738
15.02821537	49.65773964	-0.64965558	0.160687456
15.92106595	61.10286045	-0.416464981	0.132648843
24.3629398	95.97359276	-0.479494037	0.093485289
18.61598533	88.15844154	-0.284907162	0.099477199
13.53090724	63.44874334	-0.777440358	0.135650685
11.36606695	55.13888717	0.018216308	0.153081025
13.22326228	55.84947062	-1.169657234	0.147900768
11.82087474	60.19151211	-0.855515946	0.140402952
21.16242297	82.46245956	-0.457546405	0.106058742
25.38139649	86.18840981	-0.699345196	0.097446211
17.57122458	62.05255699	-0.78267097	0.129279097
13.52705867	48.59697723	-0.755697169	0.158979376
8.450658968	56.30848503	-0.550550459	0.156866609
18.13742617	71.31765175	-0.645660557	0.117859452
4.01144131	25.67845976	-1.851103987	0.287391181
18.665881	73.12261581	-0.607838735	0.116570811
14.95682776	62.21227551	-0.513022441	0.132640754
13.88211312	57.39825916	-1.18680109	0.146771141
11.77583088	50.13351631	-1.157914158	0.161901905
7.217127229	36.48025012	-0.231086575	0.217567936
18.10490592	87.67400503	-0.349153519	0.102157872
18.01303803	77.00953484	-0.515402776	0.111649123
12.47245433	54.37428117	-0.724006821	0.14878641
17.12167439	87.19657516	-0.343860976	0.105686491
21.51433884	55.01491666	-1.052069715	0.144732694
13.79557132	63.9632473	-0.477632329	0.131508594
18.45308895	62.85665226	-0.985232558	0.134623051
8.115192466	47.81302261	-0.852963041	0.179065381
9.959465298	42.22573519	-0.47007445	0.18760382

log.sigma.1.0.mm.3D_firstorder_MeanAbsoluteDeviation	log.sigma.1.0.mm.3D_firstorder_Energy	log.sigma.1.0.mm.3D_firstorder_RobustMeanAbsoluteDeviation	log.sigma.1.0.mm.3D_firstorder_Median
66.28207711	17790484.64	40.19231302	12.99256849
37.56855855	3102016.179	20.7201142	13.70211029
36.19111988	4607277.546	19.92748964	7.581020594
53.1448634	5614996.747	30.76450088	13.13407421
48.91421962	11177530.01	28.92525202	15.27101851
59.30824006	4928494.595	34.19558576	14.22535753
45.15115184	23508589.93	26.91485339	11.36641121
44.28683355	24306026.31	22.94915818	13.21196651
50.41385078	23239815.92	30.10820371	16.94943428
55.27136567	6453860.332	33.51723689	22.95978832
41.27237582	4802244.446	26.33774182	12.91162491
58.25524639	16874759.53	33.68485659	23.74970627
35.09199022	6845956.868	15.84895545	8.153153419
52.82205758	10300012.39	29.83622744	15.24710846
58.53829627	14547514.5	32.84723466	17.83259964
41.02238782	17046762.54	22.25490944	10.02537489
52.75289451	5859109.734	34.40478053	13.05777884
35.3804755	3778221.106	19.09233807	12.14226246
42.39696266	4480858.054	26.16521548	12.96213818
53.20095919	31148447.11	31.20748824	16.07486153
31.75742406	6946373.774	17.39175383	9.224973202
49.87188726	4963106.846	31.78163041	16.74107742
46.69761521	4539639.745	27.64947251	13.407691
45.57857281	11063834.33	27.71307851	11.72421741
48.98032777	6913994.92	27.67881819	15.6707058
60.78111572	18419197.99	35.82266842	20.55641747
56.26005739	20594788.08	33.56199085	14.673666
44.63096559	12177066.52	24.92929459	11.56316328
51.21048085	2087466.141	33.35358298	17.27333069
41.82803004	7881202.182	23.87569225	17.47236156
44.01895218	22419188.71	23.34209792	12.55656195
40.44688217	7614343.501	21.61369171	9.188030243
51.6558253	18110360.04	29.67768872	10.40223598
51.8055036	13866763.42	28.96093734	13.89665937
54.92823771	27879809.17	32.40483321	21.37578773
41.14187622	11905181.6	22.11570427	11.1193924
45.52021498	18954869.26	23.73097922	9.003048897
37.23762427	20106111.94	17.07806062	5.072403908
45.39818937	5062978.787	27.517877	14.50809479
51.39328561	34224408.24	33.1877511	8.370133877
39.56369101	8549970.598	22.05898705	8.683179379
62.58722594	24888397.75	42.52974293	6.388998985
48.40141365	17099108.88	29.21263137	15.82720375
60.23457794	17896103.11	35.85595626	14.78806782
49.83639322	7298205.897	28.43214148	23.91561413
50.13461912	14204528.21	29.66852566	14.55271816
49.67908569	24392278.62	28.22327608	12.93341732
40.49109782	3210469.646	22.98144874	15.47907925
60.00852238	5989878.936	35.89509824	21.26153755
43.4855633	5573453.149	25.84547052	17.0042057
55.99100376	17121114.88	32.84576697	20.37081337
60.94478953	22101411.65	39.82040485	16.34927559
52.18058119	22762071.56	32.64548707	15.0471406
42.40261057	4163569.946	25.47530805	15.84453106
49.56623043	7402831.937	27.51497804	16.46425343
43.6640809	7650862.888	25.64628934	13.87776947
52.77147302	21611885.21	32.58485484	17.5509758
43.41494401	14045999.71	24.35640658	8.106189728
49.49666198	8369068.023	28.70602631	13.92612028
55.29233952	53204459.98	33.96381427	4.958934784
43.09088846	8153618.95	22.07346358	12.17825937
53.61845901	16923669.23	30.4580149	16.74026585
52.65078579	34600547.35	30.1618656	15.28552628
50.3305648	41028615.95	28.86756344	12.99908543
35.73141879	1929540.219	22.73500148	14.2615509
43.54644543	16997561.54	23.26365083	10.5799036
43.40989542	5593055.292	23.05319034	9.390732765
51.11351347	33499204.23	29.6791451	11.80643559
52.38995647	33158272.21	31.19085255	16.53112793
36.1433925	8421096.767	20.77967053	10.46588278
45.28048819	5572155.367	26.06135658	12.32857609
43.99105911	21036675.57	22.60268059	14.11694145
49.06238468	11831604.89	27.5348364	13.15626621
64.60458685	16674373.94	41.19645502	14.19503975
58.1082811	18495854.67	37.81423129	11.39513683
45.98134544	5506960.185	27.60586405	13.8337636
41.68767111	9335467.975	23.87419275	13.84476233
43.93747325	4117230.372	24.46168264	14.81878376
43.4988911	6359962.678	26.65338356	14.24556923
57.75092249	14843889.42	35.55931325	10.51817322
64.39069792	16068061.34	38.72886324	13.19628334
52.1437041	10198671.9	29.34322325	11.62078857
42.31184113	13829691.73	22.43058506	8.787262917
37.1180823	7779005.489	23.83269902	11.77324486
53.5161772	13779981.93	31.90456309	12.36277103
21.94616679	1855743.93	11.74128034	7.364732504
53.88200283	13465279.14	32.18637327	9.491560936
47.92507693	13831352.14	27.71026021	18.8314991
44.04845435	6446894.058	24.96521303	15.68967533
40.98875484	7678351.902	22.76245542	14.08389664
30.7706465	3036816.728	16.32024092	9.497834206
57.00126651	14003774.08	37.39986998	17.83796024
54.56074531	29039911.35	33.62298307	13.86793327
42.32845583	12718557.48	24.21463793	15.0095253
55.0748675	22772426.93	36.4187959	12.54909897
51.01292318	22146932.46	26.33421035	12.62462378
46.50491539	11893055.23	27.95674047	11.41642094
51.1852803	6025880.594	27.63267374	10.26098537
35.29539106	2039272.583	20.77595124	8.867621422
36.47633411	18837994.42	19.14587393	10.53753424

log.sigma.1.0.mm.3D_firstorder_TotalEnergy	log.sigma.1.0.mm.3D_firstorder_Maximum	log.sigma.1.0.mm.3D_firstorder_RootMeanSquared	log.sigma.1.0.mm.3D_firstorder_90Percentile	log.sigma.1.0.mm.3D_firstorder_Minimum
480343085.2	286.7011108	91.56325008	111.2729904	-502.5111694
83754436.83	244.7350769	57.59916981	73.40239563	-258.6973877
124396493.7	217.3698425	54.803653	77.21884079	-248.290863
151604912.2	206.3257599	75.81017037	89.24919128	-373.0960083
301793310.1	209.3660889	68.70400797	82.90274582	-307.492981
133069354.1	337.3200989	86.4142121	107.7741501	-362.4914856
634731928.2	323.7756042	64.49865178	86.62988281	-345.0723572
656262710.4	360.7756042	68.98132587	85.03427277	-383.7451172
627475029.8	342.7807312	71.49927304	87.41932297	-384.3026428
174254229	258.8742676	77.59118572	96.69889221	-365.1428528
129660600	219.2437897	56.43148263	72.72056274	-257.5276184
455618507.3	325.9449768	85.56247361	108.1085281	-405.2306213
184840835.4	276.450592	60.55429055	59.68759842	-383.9767761
278100334.5	292.0558777	77.45219424	92.39791412	-404.6757507
392782891.4	411.6424255	85.62953101	102.6303329	-442.1347046
460262588.5	324.6344604	62.41404059	72.64895248	-360.4248657
158195962.8	280.2818298	70.05088922	89.10872421	-245.9830322
102011969.9	260.2957458	55.35567446	67.17674561	-325.2833252
120983167.5	242.4565735	58.75478575	71.44372101	-333.1778259
841008071.9	391.24823	77.40308872	97.37016907	-386.5374756
187552091.9	209.0856934	47.92787576	58.93619232	-275.275238
134003884.8	196.1129761	67.82129687	87.99305878	-271.9329529
122570273.1	273.0772095	66.58259486	82.79594116	-386.4660034
298723527	229.9455414	64.6510915	87.76724701	-345.7321472
186677862.8	261.8060913	73.69708772	98.84937286	-354.8457947
497318345.8	363.2203979	88.1764942	118.4071259	-425.3013306
556059278.3	317.4775391	79.81339992	106.0300537	-364.350708
328780796	252.7750854	66.39868861	75.94398346	-385.3608398
56361585.79	218.4671631	68.33700771	91.66105042	-253.4367828
212792458.9	238.0174255	61.9435391	74.17604752	-366.4304199
605318095.2	372.5895081	68.6432326	85.16087036	-408.5704956
205587274.5	230.4121857	60.66469063	66.3172226	-355.2524719
488979721	398.8340454	74.89098102	89.79989929	-373.2358398
374402612.2	352.949585	76.81634195	95.78590622	-342.2538452
752754847.6	385.4857788	79.56484906	111.2049606	-318.9483948
321439903.1	399.734314	63.00569105	77.13538361	-325.6660767
511781470	389.0434265	71.37244286	74.5022583	-514.4187622
542865022.4	316.9378662	62.41617039	73.84025574	-486.7036133
136700427.2	208.3832092	64.15795287	73.34961319	-384.0026855
924059022.6	289.9842224	68.61210893	89.70534515	-269.7449341
230849206.2	233.3043671	57.41129257	63.87603951	-272.6494751
671986739.4	324.6063232	80.2253062	106.5393265	-291.3910828
461675939.8	297.624115	68.41666536	86.91148071	-374.9436951
483194783.9	282.9548035	84.81136414	97.42412796	-389.7089844
197051559.2	299.0422974	75.56881792	94.70806808	-400.5531921
383522261.7	398.0515442	71.9353287	94.36531525	-285.8537598
658591522.7	346.2671204	73.1220815	89.5425415	-338.8779602
86682680.45	251.0748901	60.71209321	69.10263062	-269.0621948
161726731.3	326.6192322	83.89657709	109.0891495	-284.4044495
150483235	205.456955	64.20573642	91.23187027	-381.8583374
462270101.6	390.5713501	81.63642322	108.8169037	-387.916687
596738114.5	326.2858276	80.97121024	104.5143509	-347.3426208
614575932	293.002655	71.02665248	88.27369232	-307.1376343
112416388.5	276.742218	61.41131482	78.79454727	-259.478241
199876462.3	286.7480164	74.60589153	92.8872757	-347.8613586
206573298	262.895752	62.63800308	75.17013626	-293.3444519
583520900.7	342.3215332	73.09489778	96.11053314	-333.709198
379241992.3	293.8471985	63.73292242	68.75937576	-391.4679565
225964836.6	309.9490356	71.26231806	83.10242691	-379.0370178
1436520420	314.2078857	76.34180363	96.5987854	-385.936554
220147711.6	314.3204651	67.94849721	79.96979523	-379.7272949
456939069.2	422.8525085	79.61440131	100.0281815	-460.710144
934214778.4	324.1835022	77.31071854	105.9625366	-429.177124
1107772631	387.6860352	73.36357284	85.73569641	-384.4518433
52097585.9	162.875824	49.42120975	63.00412178	-201.4844055
458934161.7	361.6758728	66.34178866	75.15856781	-384.6629639
151012492.9	260.7280884	65.49162413	69.94693985	-340.7894897
904478514.3	358.2336731	73.21115132	86.67287292	-420.3146362
895273349.6	437.8847656	75.24165407	91.95401459	-460.0982361
227369612.7	187.6569672	53.90575565	66.86496506	-321.1386719
150448194.9	266.9620972	65.49483209	78.77129669	-286.7373657
567990240.4	342.8551636	70.02609499	89.78336258	-367.4375
319453331.9	297.4234314	72.05260159	90.30759735	-331.9836731
450208096.4	381.9251709	85.91453679	103.7230728	-342.3549805
499388076	301.5239868	76.85916612	99.64801788	-297.2276917
148687925	275.0829773	64.05893445	72.24326019	-281.9018555
252057635.3	342.9224243	62.42012584	74.0908699	-324.3373108
111165220.1	218.659668	65.55711444	74.45777359	-311.1514893
171718992.3	213.2333679	61.03943753	80.82612152	-313.9656982
400785014.4	355.4069519	79.66338283	94.97935333	-348.9838867
433837656.2	286.7011108	89.88026597	109.4510941	-502.5111694
275364141.4	338.7140198	75.29328205	85.01956024	-321.7772522
373401676.7	312.3144836	65.04273421	79.10450592	-395.5055237
210033148.2	167.5216827	50.31306316	65.27076416	-267.2184448
372059512.1	287.5323792	75.16547804	90.83570251	-332.3526611
50105086.11	130.7450409	34.8040386	40.86743698	-238.6472015
363562536.8	307.9350281	75.13866745	83.57688141	-341.5506897
373446507.9	258.1136169	71.02293151	101.8381844	-303.2186279
174066139.6	231.350235	66.35974804	81.1328598	-368.5909119
207315501.4	241.5537109	61.57742051	74.00897522	-374.9681702
81994051.66	217.5070801	48.25813076	63.20628548	-369.9934998
378101900.2	250.6427002	76.67462654	107.120623	-334.8939209
784077606.5	348.1105042	74.85262561	90.37943726	-308.148468
343401051.9	256.5763855	62.08146524	83.4436409	-309.6954651
614855527.2	363.7046204	72.33695366	87.81816864	-335.2077637
597967176.4	359.9611206	80.44828459	101.4780373	-529.5641479
321112491.3	265.0306396	64.97604822	79.42763672	-286.2237854
162698776	239.8834686	75.86420655	78.08294373	-375.1214294
55060359.74	154.4839325	49.44866998	60.02380295	-235.9374084
508625849.3	253.6916351	57.11878255	75.43101196	-377.8802185

log.sigma.1.0.mm.3D_firstorder_Entropy	log.sigma.1.0.mm.3D_firstorder_StandardDeviation	log.sigma.1.0.mm.3D_firstorder_Range	log.sigma.1.0.mm.3D_firstorder_Variance	log.sigma.1.0.mm.3D_firstorder_10Percentile
3.813859576	91.3239446	789.2122803	8340.062857	-113.455011
3.015951665	56.04811646	503.4324646	3141.391359	-39.84200058
2.958801966	53.66080495	465.6607056	2879.481988	-43.92416
3.466368001	75.71082798	579.4217682	5732.129473	-93.32169647
3.369253391	68.30882817	516.8590698	4666.096005	-80.85931473
3.644626748	85.23417983	699.8115845	7264.865411	-78.32839661
3.301899027	63.53238805	668.8479614	4036.364331	-63.84212875
3.240369751	67.85616201	744.5207214	4604.458723	-59.13802719
3.436395296	70.64743709	727.083374	4991.060367	-73.06376648
3.535553267	76.20379328	624.0171204	5807.01811	-79.38818436
3.144947846	55.70087594	476.7714081	3102.587581	-58.50039253
3.622437453	84.01103321	731.1755981	7057.853702	-75.447435
2.87612604	60.15961936	660.4273682	3619.179802	-37.3290451
3.497878769	76.79818979	696.7316284	5897.961955	-76.75453491
3.630756098	84.87458345	853.7771301	7203.694915	-89.63718109
3.14896713	62.02246998	685.0593262	3846.786783	-58.54527092
3.492596262	69.48245443	526.2648621	4827.811474	-81.43564911
2.931340567	54.28151425	585.579071	2946.482789	-40.82146225
3.196812456	58.19542945	575.6343994	3386.708009	-64.63770065
3.527555634	76.3041604	777.7857056	5822.324895	-71.80838928
2.804883962	47.03101684	484.3609314	2211.916545	-41.79279823
3.408265636	66.75939345	468.045929	4456.816613	-72.64850922
3.33224817	65.9280746	659.5432129	4346.511021	-70.48215179
3.313890222	63.50381974	575.6776886	4032.735122	-60.33885345
3.380415962	71.54927034	616.651886	5119.298086	-56.17851257
3.719623066	85.80609013	788.5217285	7362.685103	-79.80186005
3.608931384	78.71858009	681.8282471	6196.614852	-80.73483429
3.261957449	65.93594312	638.1359253	4347.548596	-60.99712639
3.432565179	67.17596643	471.9039459	4512.610465	-68.70044861
3.167807394	60.91297576	604.4478455	3710.390616	-56.3747303
3.239776081	67.6296191	781.1600037	4573.76538	-56.01969032
3.090454347	60.57011931	585.6646576	3668.739353	-63.67379379
3.485193218	74.38992648	772.0698853	5533.861161	-78.86239777
3.470212039	76.08155684	695.2034302	5788.403291	-75.35995865
3.559649518	77.21847164	704.4341736	5962.692362	-72.35907211
3.151819681	62.16344772	725.4003906	3864.294233	-53.58054428
3.279289509	71.14632394	903.4621887	5061.79941	-61.67182159
2.967300088	62.06996655	803.6414795	3852.680747	-44.79546738
3.236889372	63.94297117	592.3858948	4088.703562	-63.23675537
3.469466428	68.26259235	559.7291565	4659.781515	-78.38322983
3.088562972	57.2630031	505.9538422	3279.051524	-67.80233536
3.721899828	79.9832272	615.997406	6397.316633	-97.58077698
3.374102631	67.56058999	672.5678101	4564.433319	-67.75792084
3.682823159	84.43191563	672.6637878	7128.748376	-94.28598557
3.403406844	73.23938939	699.5954895	5364.008158	-53.05249443
3.444805646	70.56249214	683.905304	4979.065298	-68.01618652
3.433426886	72.22207297	685.1450806	5216.027824	-70.89167404
3.095008087	59.63977178	520.137085	3556.902378	-52.39938354
3.651928541	82.7589772	611.0236816	6849.048307	-99.24051666
3.237187323	61.91967034	587.3152924	3834.045575	-49.99206581
3.591158458	79.78933386	778.4880371	6366.337798	-71.88231659
3.699699584	80.2037807	673.6284485	6432.646439	-92.68198395
3.484628114	70.34313588	600.1402893	4948.156766	-79.2978508
3.196173281	59.73207666	536.220459	3567.920982	-51.67994957
3.411322832	73.02485103	634.609375	5332.628868	-64.66672592
3.214864742	61.99468962	556.2402039	3843.341542	-59.84840355
3.504968419	72.00474872	676.0307312	5184.683838	-78.71424255
3.231475605	63.6146575	685.315155	4046.824649	-65.99769058
3.399646888	70.84607665	688.9860535	5019.166577	-74.48594894
3.586227479	76.18317117	700.1444397	5803.875569	-86.0252594
3.203715872	66.93589637	694.04776	4480.414223	-56.45191574
3.521203377	78.61342966	883.5626526	6180.071323	-72.74610596
3.50935045	75.72598313	753.3606262	5734.424521	-71.52068481
3.439806062	72.91462314	772.1378784	5316.542268	-73.71302948
2.937729726	48.19879883	364.3602295	2323.124209	-47.47893181
3.225247545	65.97296029	746.3388367	4352.431489	-65.51503448
3.190628227	65.42035101	601.5175781	4279.822327	-65.5955574
3.463012735	72.88727257	778.5483093	5312.554503	-80.39896011
3.499405732	74.31332141	897.9830017	5522.469739	-73.30511932
2.976682255	53.17107087	508.795639	2827.162777	-45.33169365
3.280734544	65.08875129	553.6994629	4236.545545	-69.84899139
3.2305512	68.5149772	710.2926636	4694.302101	-48.63740463
3.404406507	71.12966123	629.4071045	5059.428707	-69.21365967
3.761927966	85.79640729	724.2801514	7361.023504	-113.5420349
3.639632858	76.48016017	598.7516785	5849.2149	-90.00891113
3.28002484	63.69906533	556.9848328	4057.570924	-69.85703812
3.16936146	61.08488932	667.2597351	3731.363703	-51.01777077
3.222263959	65.04493309	529.8111572	4230.84332	-59.19242897
3.22174719	60.38756866	527.1990662	3646.658449	-58.74025192
3.629987932	79.506092	704.3908386	6321.218666	-90.0966217
3.777479153	89.53011324	789.2122803	8015.641177	-106.7697983
3.442167161	75.18364912	660.491272	5652.581094	-91.05029449
3.200719496	64.54377049	707.8200073	4165.898309	-59.57079773
3.004045545	49.65673897	434.7401276	2465.791725	-54.33590012
3.506879659	74.76928404	619.8850403	5590.445835	-87.58596039
2.275218026	34.40342635	369.3922424	1183.595745	-27.97238903
3.525168717	75.06922358	649.4857178	5635.388329	-92.51452332
3.372572374	68.58963378	561.3322449	4704.537863	-55.2482235
3.236176725	65.26712878	599.9411469	4259.798099	-56.27899971
3.128346813	60.80667649	616.5218811	3697.451905	-57.84521713
2.726806705	46.83105316	587.5005798	2193.14754	-31.80939369
3.598431936	74.97919007	585.5366211	5621.878943	-76.45846939
3.542506174	74.49597416	656.2589722	5549.650167	-88.36997223
3.198631779	60.79875763	566.2718506	3696.488929	-55.61681061
3.54287117	72.02200447	698.912384	5187.169128	-86.83342896
3.414634934	79.5363432	889.5252686	6326.029889	-64.63843689
3.333102929	64.59428225	551.254425	4172.4213	-71.01827698
3.422617948	75.82040863	615.0048981	5748.734364	-82.29184418
2.910349362	49.32562868	390.4213409	2433.017644	-58.28137856
2.977541225	55.86056952	631.5718536	3120.403227	-41.05020256

log.sigma.1.0.mm.3D_firstorder_Kurtosis	log.sigma.1.0.mm.3D_firstorder_Mean	log.sigma.1.0.mm.3D_glrlm_ShortRunLowGrayLevelEmphasis	log.sigma.1.0.mm.3D_glrlm_GrayLevelVariance
4.721983116	6.615580654	0.002801495	14.21196055
6.718124042	13.27678438	0.009435787	5.724658203
5.958341108	11.13366042	0.011786474	5.347905078
5.931750002	3.879749729	0.007197698	9.950061248
5.0727449	7.358308663	0.007158918	8.281981332
5.796553447	14.23202871	0.006512792	12.46883855
5.251085964	11.12257839	0.004950621	7.184812467
6.751842128	12.40824706	0.003909416	8.399001323
5.440732816	11.00389373	0.004030903	8.676327618
5.434286873	14.60732662	0.005601462	9.960944916
5.063534629	9.051223742	0.009093413	5.467699011
6.443151017	16.21983933	0.004378194	12.23059715
11.01226171	6.902340351	0.00503163	6.961709532
6.0252615	10.04392542	0.00408611	10.38166025
5.708454314	11.3455571	0.003713838	12.58370408
7.85665108	6.980378236	0.004838758	7.081222904
4.013802607	8.90593095	0.013277974	8.366286381
8.869760699	10.85209221	0.005637695	5.488352072
5.347395636	8.08806779	0.005557613	6.006956167
6.394488753	12.99666299	0.004856255	10.17550223
6.133054868	9.228473857	0.006228706	4.197958923
4.416443212	11.95456798	0.011983272	7.693763473
6.294214794	9.312943585	0.004753358	7.638111816
5.528093698	12.12553134	0.005362151	7.131133328
6.557253597	17.66246451	0.006097892	9.038214054
5.047716348	20.30785629	0.003358155	12.65983239
4.795584353	13.17436737	0.005007993	10.71408767
7.282476184	7.82542358	0.004474293	7.756554631
3.743179066	12.54337107	0.010736904	7.664313199
7.39828201	11.25217401	0.00490651	6.665866687
7.899398789	11.752787	0.003748933	8.393534652
7.068763391	3.386050209	0.004929043	6.667779815
5.853251081	8.648576623	0.004814625	9.786853068
6.20125381	10.59939142	0.007977708	10.29923626
4.914695353	19.18001157	0.006838061	10.32376511
7.224984968	10.26756404	0.005326823	7.030207431
9.351526181	5.676811627	0.002481734	9.184693161
10.02487828	6.564874626	0.002491462	7.450497729
7.705336878	5.247795164	0.004973516	7.23218472
3.942262926	6.916644913	0.009515549	7.989034267
5.45245459	4.123710803	0.010152237	5.982719611
3.278031283	6.227609665	0.008148458	10.69741506
5.434755643	10.78919737	0.004686678	7.968003393
5.226103712	8.013682754	0.005325357	12.25323596
7.848807702	18.61821914	0.004451089	9.425739357
5.089758469	13.98664428	0.007655972	8.666218143
5.940726539	11.43726273	0.005534043	9.21459968
7.023135209	11.3602766	0.012436096	6.36655206
4.355835234	13.76907186	0.012139432	11.76739087
6.464157948	16.98031256	0.004274223	6.865784113
5.774787809	17.26753595	0.004463316	10.98338654
4.031045641	11.12162077	0.005752514	10.86039605
4.27499463	9.82998458	0.006576715	8.506692872
5.871221168	14.26284005	0.01002856	6.292903967
6.201681529	15.27776757	0.006862399	9.295686935
6.117566113	8.95421063	0.009780615	6.789042943
4.673440424	12.57697274	0.005504792	8.973828361
6.733625146	3.880818308	0.004172825	7.301533411
6.135139734	7.690994579	0.004577762	8.796273392
4.601840441	4.918883209	0.004128419	10.08538698
7.291668664	11.68691788	0.004400193	8.28023866
7.286566731	12.58497407	0.003260894	10.78400741
5.453647168	15.57313969	0.003079914	10.05915682
6.429491161	8.103798631	0.004386649	9.335764165
4.912710032	10.92390792	0.01323903	4.15390683
7.453225043	6.985802246	0.00425058	7.840469475
7.162283737	3.054587415	0.006597004	7.710127043
5.575042779	6.878820696	0.003589715	9.314808442
6.455961968	11.7829015	0.003018072	9.532881402
7.760025505	8.86948222	0.00606902	5.151183607
5.709386055	7.281997409	0.009383258	7.522417532
7.906143886	14.46899715	0.005556328	8.559035566
5.368941174	11.49559434	0.005585518	9.06612885
3.992734302	4.503790432	0.006958393	12.42615794
3.759309584	7.623419017	0.008994984	9.984848072
5.128782592	6.780572189	0.008244997	7.11233315
7.202826845	12.84166683	0.005974571	6.625926791
7.389794591	8.178748855	0.009190213	7.470994074
6.042645526	8.896880647	0.00671949	6.433093296
4.85765532	5.003588489	0.006832867	10.7925136
5.008690863	7.925972128	0.002814356	13.72806299
5.556653357	4.061677992	0.009027099	9.920011877
7.451678816	8.041079826	0.004166969	7.665365548
4.588365766	8.100160451	0.007966202	4.378322159
4.714115915	7.707350664	0.005848946	9.708650639
11.58376313	5.26548749	0.010166124	2.452107676
4.939558301	3.229708563	0.006771723	9.883551119
5.347970416	18.43146595	0.006300299	8.341867146
7.493308215	11.99241682	0.005152106	7.579195109
7.670052618	9.712199118	0.005006183	6.724005305
8.948063616	11.64901901	0.004525107	4.171863857
4.042308425	16.03494346	0.005569578	9.493563178
4.606536241	7.298314473	0.007118572	9.540807928
5.664306299	12.5546564	0.006116185	6.587945317
4.197289271	6.742828593	0.005506111	8.844349692
7.915745772	12.07876664	0.002268473	11.47688371
4.90314698	7.033174426	0.008070518	7.32750236
6.482080894	2.577493142	0.005588305	10.19238549
5.092707714	3.486161019	0.011135232	4.459443735
7.428321628	11.92275527	0.003593124	5.814929388

log.sigma.1.0.mm.3D_glrlm_LowGrayLevelRunEmphasis	log.sigma.1.0.mm.3D_glrlm_GrayLevelNonUniformityNormalized	log.sigma.1.0.mm.3D_glrlm_RunVariance	log.sigma.1.0.mm.3D_glrlm_GrayLevelNonUniformity
0.002928617	0.089210666	0.101244042	174.1840814
0.010150314	0.156985534	0.201079319	126.5832473
0.012870402	0.157766989	0.320238685	200.7990617
0.007537798	0.113533594	0.148289331	98.83239221
0.007600573	0.120367715	0.155177214	252.9097014
0.006746267	0.101459671	0.09156633	61.85808939
0.005361237	0.124071226	0.188186241	614.7048271
0.004266701	0.138407002	0.215168227	608.7906416
0.004302678	0.114826883	0.139052028	467.6500989
0.005855895	0.10721477	0.107778751	105.0216885
0.009698403	0.135805067	0.143639243	182.4597357
0.004583026	0.103707019	0.117989765	217.3886967
0.005507795	0.182468896	0.300700551	279.885872
0.004347174	0.113749957	0.151420892	173.8548872
0.003919954	0.104664376	0.127047116	187.4087109
0.00527265	0.143623598	0.217797458	538.554573
0.013892686	0.104777787	0.10645891	114.1760338
0.006157169	0.166487249	0.242387686	173.5662208
0.005936601	0.13268884	0.143528429	153.2634361
0.005107793	0.109779208	0.126853459	515.7521991
0.007036223	0.174274726	0.313343018	432.9632866
0.012468392	0.112993303	0.105520577	111.1472485
0.005039103	0.122966919	0.147807277	112.2384928
0.005739137	0.123972053	0.14915273	292.036267
0.006429709	0.12349582	0.141836841	140.0003564
0.003537623	0.097000636	0.114731466	209.850042
0.00530137	0.102388758	0.132137008	298.5694512
0.004789818	0.132830079	0.173174463	322.5355882
0.011180343	0.111096844	0.08614404	45.89282161
0.005246653	0.140937471	0.155014198	256.1435672
0.004070644	0.13631919	0.233629882	556.4375961
0.00536302	0.148759561	0.215757873	263.8393704
0.005158869	0.113195451	0.165968363	323.7783524
0.008368321	0.115626871	0.157638579	240.9392406
0.007210365	0.106250481	0.129202774	422.1286573
0.005807865	0.144352327	0.212905859	372.1443266
0.002685494	0.13630108	0.190339889	441.344386
0.002818417	0.170691799	0.335255189	714.5715182
0.005250475	0.130903277	0.136879628	144.1964257
0.010077154	0.107729477	0.12881389	707.6168398
0.010969392	0.147214559	0.218177623	327.1945066
0.008515278	0.088369539	0.08334566	317.5653497
0.0050046	0.119215957	0.14425317	388.8915319
0.005553303	0.097984969	0.108510004	223.0545659
0.004684812	0.120369503	0.132070685	137.9209164
0.008128195	0.115461567	0.13788657	284.4343805
0.005914285	0.118220739	0.156512037	479.7045438
0.01304344	0.148390807	0.155239256	114.3570858
0.012512499	0.099654725	0.097807386	78.25897857
0.004584094	0.131169762	0.169992405	155.9957342
0.004697317	0.10515726	0.117156687	245.5212829
0.006019021	0.092515405	0.088260231	288.5473588
0.006942184	0.109086289	0.114189192	447.6810462
0.010646676	0.135235088	0.151013014	132.1731289
0.007212526	0.121912926	0.137040803	145.3164612
0.010300974	0.132906146	0.14851842	230.1365626
0.005834857	0.107420263	0.125328289	392.4614814
0.004523456	0.13434673	0.18119107	404.6860223
0.004836893	0.120683474	0.131218378	179.2156051
0.00440713	0.102448874	0.143330579	839.832789
0.004758475	0.141301926	0.218443416	214.6208859
0.003453812	0.111637528	0.14455971	266.9933124
0.003301958	0.111736024	0.154010802	576.4804147
0.004673866	0.118060147	0.153999063	801.5848584
0.014202711	0.154728975	0.162420234	107.4288453
0.004601212	0.138239039	0.192676558	463.6564889
0.007058263	0.140391275	0.202081705	158.7409546
0.0038447	0.115025763	0.146584773	641.3362554
0.003192129	0.111667702	0.123037812	592.9930518
0.006628608	0.156869276	0.204238671	390.5227869
0.009936311	0.129128401	0.159099484	148.2366086
0.005964463	0.140852633	0.218103108	520.3877084
0.006013025	0.119922643	0.183406749	238.8177608
0.007279831	0.089437809	0.096918185	185.9153749
0.009396997	0.095553266	0.101488621	274.7661409
0.008755828	0.127135195	0.144085874	152.1149659
0.006447847	0.141590488	0.167974398	298.2407643
0.009633988	0.13616791	0.147572839	115.8001989
0.00715183	0.131803855	0.141256826	200.7715867
0.007166884	0.100326361	0.113341706	213.6712881
0.00294251	0.092334888	0.102383078	168.8106083
0.009492754	0.118376817	0.156317548	188.8373261
0.004537916	0.140446638	0.21942987	394.4592465
0.008650263	0.14719914	0.167429788	396.5902983
0.006217951	0.109437412	0.142628024	238.7270834
0.011750209	0.24043149	0.493595226	279.5468089
0.00714377	0.108129487	0.150689157	230.5143487
0.006752551	0.120729709	0.17515962	291.3969888
0.00549064	0.134611223	0.158450699	174.8425481
0.005398558	0.14465643	0.198511171	253.6276352
0.00504928	0.187109503	0.301254852	200.5584652
0.005831142	0.098148301	0.096904105	215.6818229
0.007491311	0.10578854	0.119754729	497.6163435
0.006597732	0.135977715	0.176177323	393.2870924
0.005814772	0.101755466	0.108036805	404.5084492
0.002457564	0.124521232	0.21178024	368.4602103
0.008577539	0.123140192	0.139286294	310.1535853
0.005898987	0.120904357	0.168018796	112.0559915
0.012107112	0.158728749	0.221179796	113.2634722
0.004001135	0.16276341	0.256862976	792.0769586

log.sigma.1.0.mm.3D_glrlm_LongRunEmphasis	log.sigma.1.0.mm.3D_glrlm_ShortRunHighGrayLevelEmphasis	log.sigma.1.0.mm.3D_glrlm_RunLengthNonUniformity	log.sigma.1.0.mm.3D_glrlm_ShortRunEmphasis
1.2836252	457.3957092	1669.701584	0.940187896
1.548069603	134.5494112	615.6923438	0.896301353
1.783873884	109.9619223	920.4366225	0.874680439
1.410980357	232.51721	704.2766029	0.918369527
1.426866876	179.7136741	1685.867042	0.915641715
1.264660038	255.6138286	523.870575	0.942093106
1.49134907	209.9464157	3918.966883	0.909906466
1.565406037	266.9529909	3370.414675	0.897713032
1.386439784	271.2443032	3324.081228	0.922091164
1.306507819	250.2752768	826.3465721	0.934963905
1.405202747	133.0255396	1083.200097	0.917342652
1.328281029	317.1864584	1756.7446	0.932424399
1.787853718	249.9342331	1085.852168	0.866719656
1.415289883	302.2772918	1235.117554	0.918247653
1.356857444	343.3555171	1478.619846	0.926634764
1.583170441	227.8523272	2841.133617	0.893233456
1.308016433	117.5538424	915.8942819	0.933641019
1.644776174	201.3965986	772.7484967	0.884711581
1.407685482	205.6425835	928.4533348	0.916322737
1.35248108	277.9316969	3893.937443	0.928149965
1.799604114	147.7551352	1766.613214	0.868338769
1.310034467	139.7290672	823.4810554	0.932102837
1.408603782	267.7444123	738.4976232	0.918609858
1.413776799	212.8951272	1899.530842	0.917413788
1.404375798	249.9139258	911.6162828	0.916593581
1.314713238	361.6837423	1830.899114	0.93611288
1.362392814	248.0728014	2411.40833	0.927281391
1.468872846	263.2874742	1922.112876	0.91030321
1.257967129	142.5440665	354.3438072	0.941426244
1.434663855	236.4275612	1447.477574	0.912693705
1.595284447	297.0335595	3125.378217	0.897147231
1.579036191	222.5885242	1344.274543	0.893423609
1.443198612	239.1182326	2299.201898	0.915910295
1.431253342	213.23592	1671.913965	0.915548951
1.358689665	198.2068405	3283.853481	0.927130571
1.568253533	205.5036439	1963.829808	0.895471025
1.512956131	434.103572	2519.311504	0.903607121
1.859168862	377.9431987	2913.975448	0.860293421
1.384982102	262.1501874	897.3761244	0.921437717
1.355291834	135.9510962	5443.158004	0.92803699
1.583561827	125.744385	1683.978097	0.893148
1.241967285	164.2505412	3122.652189	0.946482296
1.400311738	239.4433786	2646.040467	0.919671199
1.303735763	275.1877275	1928.422061	0.936625197
1.377089552	314.2067333	933.0629501	0.921502279
1.381017351	165.7919327	2016.788869	0.923389429
1.421694965	213.9466927	3279.971971	0.918439854
1.434242215	135.2811904	614.0481269	0.912853526
1.274122407	170.4924677	675.4593459	0.942290854
1.465723237	274.4341355	940.2692756	0.909447331
1.328605555	285.3099477	1953.539119	0.931880141
1.256862731	220.3092454	2686.582939	0.943223894
1.323804679	187.1363348	3433.297547	0.931863802
1.429928547	138.3068777	777.6324232	0.912152017
1.383081801	219.6117313	971.5107064	0.921768699
1.417862254	156.6392312	1386.709011	0.915029951
1.352584577	216.1925603	3020.530409	0.927161122
1.500547903	256.3160454	2339.420627	0.903005847
1.364523627	268.1419036	1224.727969	0.92612648
1.384551803	267.3348077	6743.542342	0.925129002
1.573311979	265.5366715	1162.769821	0.897216933
1.3918657	378.4647593	1954.852383	0.922821584
1.41418007	345.9411637	4187.816786	0.919994473
1.416336595	267.8260503	5497.060604	0.918972838
1.460485846	93.20503682	546.2774295	0.907686475
1.520369111	260.3108072	2597.843318	0.901898729
1.533779785	198.0815848	876.085605	0.901936124
1.404430935	297.7691752	4517.578357	0.919394171
1.340530372	379.1037928	4431.383415	0.930735846
1.561249874	175.4378025	1883.979418	0.893005627
1.44208196	155.0487939	914.1559442	0.912352887
1.568842234	240.7532162	2831.59376	0.897749085
1.49501812	210.8980393	1561.101493	0.906405884
1.278520642	213.420371	1775.053742	0.939776764
1.287723853	163.0582189	2450.882695	0.938912964
1.404233874	154.7965489	965.5758638	0.917746327
1.463722331	184.6683091	1662.192563	0.909150789
1.41866385	180.4670042	680.6143926	0.914562865
1.398799055	181.2420377	1231.010304	0.918385439
1.320311094	211.3407961	1787.285739	0.932959596
1.287327853	458.8378467	1560.38417	0.939384314
1.430445114	178.8232102	1279.724522	0.915336974
1.578025654	260.1302525	2144.176645	0.895937136
1.470105333	130.0054089	2111.313642	0.906416248
1.395250441	210.3502005	1776.094299	0.92098112
2.257953933	93.67168973	723.0212878	0.814224773
1.405862616	205.4107851	1741.995721	0.921980007
1.467468901	193.139531	1920.79562	0.912182002
1.431433661	240.8592694	1043.797806	0.915985729
1.535901859	232.315643	1352.231763	0.899892618
1.784525789	226.0989867	761.7669541	0.86826503
1.272729207	224.3648093	1886.96249	0.941880475
1.334461382	185.0876143	3927.101324	0.931042657
1.479734556	184.0190116	2274.006384	0.907665034
1.307322504	211.644512	3350.925149	0.93476325
1.551924472	486.2266289	2288.193385	0.901023454
1.391837867	156.4960319	2043.193186	0.919874306
1.446428664	259.6582456	741.1225837	0.914329432
1.592454972	103.9727892	538.9822381	0.891559503
1.667236646	260.3857037	3600.376471	0.883995991

log.sigma.1.0.mm.3D_glrlm_LongRunHighGrayLevelEmphasis	log.sigma.1.0.mm.3D_glrlm_RunPercentage	log.sigma.1.0.mm.3D_glrlm_LongRunLowGrayLevelEmphasis	log.sigma.1.0.mm.3D_glrlm_RunEntropy
625.9197736	0.91999565	0.003530568	4.266896091
231.5286324	0.862196627	0.013948496	3.72778624
219.7964672	0.828853676	0.019609414	3.814720114
359.4815657	0.890717266	0.009238767	4.043332808
284.2113065	0.8870842	0.009811922	3.976776633
339.9703905	0.923659674	0.00781099	4.068428287
342.1403725	0.876482039	0.007596911	3.957640252
462.7431802	0.860896934	0.00623824	3.983760801
409.8912137	0.895766354	0.005647135	4.007391075
350.4956729	0.913676808	0.007054308	4.010074465
205.1180563	0.890838604	0.012643076	3.722543117
453.2335983	0.909294177	0.005573546	4.129468358
512.6626695	0.821062173	0.008310815	3.798789754
466.8302654	0.889924287	0.0056643	4.09145076
502.9143482	0.902256514	0.004918283	4.17364311
404.4955898	0.85654268	0.007625214	3.900773467
164.3842742	0.912575699	0.016755842	3.966903306
374.9869641	0.845155655	0.009047665	3.724558737
318.002528	0.889771246	0.007773244	3.781079957
404.2387808	0.90354654	0.006336117	4.067229544
303.4897404	0.821199634	0.011913818	3.700242967
197.4079246	0.911527768	0.014665434	3.890666583
411.3316744	0.891150841	0.006473351	3.909307548
325.7337511	0.889744558	0.00762624	3.90843966
378.4625677	0.890386126	0.008030531	3.970448468
502.9927813	0.91314089	0.004416124	4.2080883
362.7565412	0.901853482	0.006753013	4.149961519
426.7742423	0.878933883	0.006434183	3.912167531
188.7141971	0.924109448	0.013129715	3.844266554
374.5198108	0.884577934	0.006935582	3.786188456
525.8551734	0.857567821	0.005924523	3.992159275
396.8034462	0.857010075	0.007706368	3.837224771
373.6081979	0.885556376	0.006965974	4.103669286
331.055337	0.886513912	0.010353442	4.085805739
288.587095	0.90204709	0.009031941	4.102712779
357.0900159	0.859363378	0.008407222	3.893322546
727.1107185	0.869968784	0.003767182	3.980113435
810.0316564	0.81078503	0.00482477	3.947800714
398.8070937	0.895434647	0.006598816	3.799184539
197.0457488	0.903375304	0.01283202	4.000788546
224.5272415	0.856503173	0.01535118	3.834783007
214.0417871	0.9292435	0.010164868	4.12859515
366.6419373	0.892838342	0.006580588	3.954830093
385.0412392	0.91488375	0.006639098	4.163475904
470.7878874	0.896472854	0.005807707	3.964288396
244.9165501	0.89726776	0.010472224	4.007802683
328.8676971	0.889319799	0.007861859	4.037919701
213.8182377	0.884659543	0.016057163	3.707412273
231.3054205	0.922534575	0.014245121	4.078979604
441.7922136	0.87943787	0.006171278	3.864990663
405.3039526	0.908734317	0.005825061	4.103356426
293.6967449	0.925199096	0.007221311	4.128539244
266.221818	0.909472177	0.00866424	3.990226046
215.8945825	0.885033445	0.013662495	3.802565542
326.8374952	0.896124928	0.008922703	3.97813826
243.1266349	0.887889546	0.012845956	3.814876892
316.273721	0.903071218	0.00742435	4.038894234
426.7865719	0.870956978	0.006324118	3.916333667
397.4204977	0.900905527	0.006107833	3.947086097
397.2915541	0.897874062	0.005834816	4.149286759
462.738312	0.859700322	0.006758291	3.942422269
571.3406378	0.895620859	0.004431733	4.096546627
529.1301994	0.8910799	0.004449716	4.107920439
414.0276177	0.890503436	0.006138604	4.041774422
150.2151296	0.878675755	0.018978357	3.560378975
438.864854	0.868262757	0.006451381	3.929648177
338.8521414	0.866918358	0.009542564	3.888962505
456.6965447	0.891950769	0.005119309	4.056727702
547.810703	0.906568078	0.004045478	4.024081552
306.5600942	0.858868185	0.009561371	3.708282289
245.7695523	0.883579085	0.012693265	3.898230382
415.1309357	0.860928815	0.008222955	3.975254313
345.5841141	0.873662538	0.008269918	4.07305229
291.2361146	0.920148466	0.008747406	4.210938995
223.1616225	0.918384394	0.011268743	4.097743885
238.9966902	0.891379113	0.011240611	3.858930429
294.8784466	0.878932837	0.008859933	3.81584641
281.0841356	0.887506022	0.011801182	3.823455072
277.0412204	0.89216349	0.009251299	3.797624818
298.026186	0.910481139	0.008741716	4.128984548
629.682189	0.919054801	0.003549366	4.235756812
280.3668105	0.886475392	0.011828826	4.05577066
456.282506	0.858813563	0.006593868	3.945766809
211.4963595	0.876617688	0.01206524	3.649889453
319.3736007	0.894187403	0.008045094	4.079888199
266.9471125	0.757632055	0.022389115	3.417696212
313.8879526	0.893565554	0.009062947	4.099684157
307.3126123	0.880070695	0.009153363	4.00984769
374.8280296	0.88692728	0.007218121	3.842425981
398.1135246	0.865489079	0.007497284	3.836762421
458.9239607	0.821731949	0.008171281	3.620424408
302.5462929	0.922463347	0.0070544	4.036076429
266.3087039	0.907493433	0.009287844	4.059447272
298.5413304	0.876293706	0.00909371	3.85598042
296.4597232	0.913373162	0.007260808	4.026477972
833.5223504	0.864361822	0.003509915	4.144427824
236.8532109	0.893940635	0.011040527	3.908349945
413.2592004	0.884946	0.007506706	4.035599346
187.2839273	0.855100535	0.017356256	3.637737811
486.9701779	0.842570142	0.006343907	3.797974071

log.sigma.1.0.mm.3D_glrlm_HighGrayLevelRunEmphasis	log.sigma.1.0.mm.3D_glrlm_RunLengthNonUniformityNormalized	log.sigma.1.0.mm.3D_glszm_GrayLevelVariance
486.802711	0.854913026	25.04570896
150.1205919	0.763262015	15.0216115
125.2003155	0.722608843	13.72261527
253.5859762	0.808495094	20.2231894
196.9634121	0.802262704	17.05109414
270.7906659	0.858961619	23.34649786
230.5350602	0.790857405	15.65551774
297.057031	0.766208082	20.64504684
294.524131	0.81588227	18.67352865
267.8718122	0.843333708	19.69328368
145.2536478	0.805803733	12.04233446
340.4162564	0.837886491	25.11002867
287.972865	0.707619216	22.02721655
329.4275274	0.807909503	22.48237368
370.6028054	0.825511801	25.84766786
255.2188319	0.757410771	18.08221682
125.8718006	0.840263435	15.35378021
227.76391	0.740998724	16.75938765
224.7531699	0.803525577	14.03047052
299.3241255	0.828664729	21.9709079
169.782692	0.710697524	11.98655953
150.0730837	0.836866345	14.49204898
291.6720886	0.808716352	16.59165574
231.7314245	0.806054469	15.72958169
271.9166879	0.803931008	20.90381944
385.5137522	0.846136534	23.91203658
267.2748804	0.826812515	20.73084377
289.5934955	0.791442605	19.00257902
151.1058747	0.857398702	13.51228083
259.5456807	0.796141811	15.93342157
330.8177269	0.765507413	20.72957936
249.6995885	0.757693958	16.38613694
260.6380126	0.803402097	21.48075874
232.59597	0.802161399	23.28008584
213.5034926	0.82644218	21.08866176
229.0943619	0.761665125	18.52388833
480.4688994	0.777885925	24.83779629
438.437623	0.695890339	22.3547072
285.2629502	0.814429566	16.34938698
146.2909802	0.828432435	15.52316052
141.0281844	0.757359421	14.47265935
173.2795662	0.868762985	17.62946918
260.6982934	0.810756307	16.629366
294.2149533	0.847007709	23.36024202
341.234375	0.814180449	22.23395556
179.1043082	0.818507814	18.55826535
232.6046919	0.808232298	20.06436769
148.4251755	0.796487013	16.4264189
181.0561602	0.859842559	19.78134892
301.6542611	0.789964271	16.24567704
305.9768978	0.836615376	23.86474365
233.6088675	0.861255039	19.00625833
200.8792603	0.836437822	16.81756735
151.5010954	0.795090126	16.21303243
237.8260368	0.814859608	20.78773818
171.2557035	0.800664153	16.44194511
233.3663409	0.826486401	17.45709025
283.9775657	0.776453749	18.98729927
289.8898532	0.824479878	19.74032411
288.6010404	0.822445844	19.74175618
295.6605613	0.765463007	20.00606741
410.2765466	0.817299387	23.47371589
375.6831557	0.811587397	21.02445467
291.5975809	0.809384659	21.62242906
102.7456155	0.786141496	10.33880183
288.6545798	0.774384778	19.56273573
219.9188872	0.774708111	18.91450976
324.2013295	0.810084184	19.75971353
407.6330802	0.834311806	18.37378914
196.4796542	0.756562628	15.24482929
170.0599135	0.795756499	16.5781419
267.4069471	0.766306188	22.60656175
232.4142113	0.783634887	20.19343187
227.2931804	0.853762684	21.41867703
173.5865086	0.852083552	17.64645044
169.0255606	0.806631911	15.39371804
202.7826734	0.788925048	16.78074818
197.5393242	0.799984941	18.29528849
197.5112889	0.807901721	13.75198199
226.3468627	0.838997139	20.67682832
488.6656782	0.853175991	24.56512921
195.5200147	0.801908082	21.17892844
290.139475	0.763040968	18.92214843
143.5412489	0.783298596	10.52375865
228.5009949	0.81373016	19.5878796
115.9300901	0.619906873	8.418142935
222.9135129	0.816469916	19.50939868
211.2755305	0.795604551	18.40756173
262.7386294	0.803302426	17.53058375
258.4981374	0.770822426	17.04460129
259.6670592	0.710418321	13.31293058
238.1235894	0.858506506	17.19320733
198.9877456	0.834776417	19.47995276
202.5429558	0.785979362	15.11609683
226.4873698	0.842759462	16.67044224
539.1746959	0.773138203	26.70715555
170.1715693	0.810962446	15.57490281
284.4078939	0.799508762	22.2593447
116.8484954	0.754743729	10.37976544
294.0005259	0.739664768	16.27262979

log.sigma.1.0.mm.3D_glszm_SmallAreaHighGrayLevelEmphasis	log.sigma.1.0.mm.3D_glszm_GrayLevelNonUniformityNormalized	log.sigma.1.0.mm.3D_glszm_SizeZoneNonUniformityNormalized
322.8147169	0.058275166	0.441347368
92.68776026	0.084781805	0.482063609
78.608585	0.089391601	0.392940674
154.9903927	0.07015013	0.410215658
118.6747113	0.073785966	0.44509145
196.1480829	0.064531368	0.474741749
153.8604865	0.078628296	0.437278691
209.6751775	0.070825713	0.503931851
194.6390038	0.070171247	0.453219069
167.4541091	0.068283676	0.440853428
95.69672071	0.086530006	0.441812269
224.0911971	0.063606004	0.467798954
183.1389773	0.08062117	0.431410382
220.2586713	0.067565014	0.461150007
256.0670941	0.060225263	0.490557605
169.823481	0.078529139	0.474020085
84.42821698	0.07463305	0.39380637
143.0534902	0.084760494	0.464296296
147.6914461	0.086663832	0.427118764
208.2670977	0.067783472	0.466689729
120.5629346	0.096449963	0.462160539
95.35352847	0.075297959	0.430677551
190.8169812	0.077904134	0.441244199
151.466822	0.076949055	0.405641691
199.8777347	0.071157407	0.48191358
274.7609243	0.060710507	0.439677974
180.9179205	0.064754955	0.435748459
187.0551588	0.075311571	0.451355709
107.5472252	0.078991717	0.439133709
166.333775	0.082895719	0.466382576
223.4638395	0.069667866	0.450989011
161.4014614	0.081289699	0.472324598
184.172497	0.066480047	0.445062004
160.8607519	0.065001358	0.451702143
146.1508895	0.064755943	0.455158577
161.0944148	0.077370559	0.475669192
345.0892162	0.067419441	0.513410374
296.7393526	0.072464661	0.43240727
176.668721	0.085557396	0.431081657
100.2684736	0.073576744	0.431512675
90.64082574	0.085285052	0.442589772
120.0864889	0.065637746	0.396694501
159.123412	0.075046428	0.40824352
193.2499567	0.062368848	0.441730665
238.0607174	0.071815111	0.517539556
133.1273505	0.071400784	0.480275802
165.4088867	0.069748431	0.457718356
93.53396124	0.08590535	0.44058642
111.7793974	0.070367665	0.409301193
190.2554504	0.080473959	0.429079109
205.3822561	0.065321415	0.453987354
157.1840302	0.065950599	0.431140108
137.1215299	0.071680895	0.451647755
100.1517913	0.085560171	0.429443859
158.2184738	0.071532695	0.431031341
109.2939988	0.079887964	0.450550268
147.9037481	0.070351616	0.416010516
192.7301146	0.077590747	0.453996916
209.3795534	0.071176142	0.527985306
197.9271864	0.064325933	0.425613911
201.9845563	0.075382819	0.472685392
271.4915514	0.065300676	0.459773451
259.7504719	0.065805635	0.466866016
196.561349	0.067009409	0.45364835
61.65801913	0.099134619	0.374386047
198.7092675	0.076740166	0.465473684
151.0864646	0.081084229	0.466658903
217.8636049	0.071348857	0.47657188
282.9311498	0.069853994	0.478153415
125.2519817	0.090458423	0.449035182
111.6338905	0.08470795	0.438057734
193.8397349	0.069297676	0.513012551
153.9279362	0.067637732	0.453942489
149.903847	0.062269577	0.421573008
118.9989601	0.0677534	0.438149084
112.4151659	0.080073046	0.446983199
142.5454875	0.085201768	0.461908233
124.8942558	0.079868985	0.466805241
123.703576	0.08726254	0.414275823
160.2155942	0.063382315	0.440237877
324.5198946	0.059002737	0.437521547
133.7728624	0.065987896	0.474097544
193.7878117	0.076282796	0.442788692
90.09899294	0.102805794	0.395377091
149.8412954	0.066170982	0.44936397
62.21840722	0.129113601	0.41281241
157.8576302	0.067119499	0.479745957
145.1589266	0.071608796	0.441720679
180.2668215	0.079689072	0.453180918
171.6449334	0.081262616	0.481053419
187.7958566	0.107104737	0.48153467
160.5513008	0.068595027	0.43349753
132.4327682	0.06740603	0.439240771
137.6738198	0.080493408	0.462686864
148.04031	0.070437251	0.407733465
363.595945	0.06331455	0.452638708
109.7966551	0.077637461	0.412621852
188.9918176	0.067901235	0.445256098
68.76462854	0.104480604	0.370643857
200.3242642	0.088152634	0.471291735

log.sigma.1.0.mm.3D_glszm_SizeZoneNonUniformity	log.sigma.1.0.mm.3D_glszm_GrayLevelNonUniformity	log.sigma.1.0.mm.3D_glszm_LargeAreaEmphasis	log.sigma.1.0.mm.3D_glszm_ZoneVariance
354.8432836	46.85323383	83.14427861	76.17835573
100.2692308	17.63461538	500.1201923	479.9134384
126.1339564	28.69470405	1063.551402	1040.714318
117.3216783	20.06293706	157.3181818	145.6485525
290.6447167	48.18223583	643.8070444	630.6567028
119.6349206	16.26190476	70.96825397	64.10884354
659.8535454	118.6500994	1226.481776	1212.457769
653.095679	91.79012346	1956.861111	1941.326808
584.1993794	90.45073701	399.5655547	387.1274838
156.9438202	24.30898876	66.91573034	57.84818836
169.2140992	33.14099217	293.6814621	278.1788409
360.2051948	48.97662338	148.9181818	139.9571007
147.9737609	27.65306122	1878.848397	1849.220529
219.9685535	32.22851153	396.5932914	383.6363101
319.8435583	39.26687117	223.3803681	214.1208551
468.8058645	77.6653185	2133.003033	2113.425316
154.7659033	29.3307888	55.99491094	46.76444652
104.4666667	19.07111111	1007.124444	977.0940444
143.5119048	29.11904762	402.047619	387.1241142
760.2375691	110.4192756	386.9386126	376.7527582
256.4990991	53.52972973	2518.518919	2488.831147
150.7371429	26.35428571	96.71142857	87.20742041
128.4020619	22.67010309	233.0309278	220.6482682
294.4958678	55.86501377	357.6267218	344.3333656
173.4888889	25.61666667	213.7638889	201.2598071
374.6056338	51.72535211	206.6866197	198.9553481
447.9494163	66.56809339	407.25	397.3593421
312.3381503	52.11560694	993.8757225	977.9450115
73.33532934	13.19161677	33.31137725	26.14693965
246.25	43.76893939	614.2083333	599.0750545
541.6378018	83.67110741	1579.142381	1563.447326
214.9076923	36.98681319	941.2505495	920.5730419
419.2484076	62.62420382	466.7165605	454.9666574
303.9955423	43.74591382	478.6389302	466.4460699
632.2152628	89.94600432	256.9388049	246.8859334
322.9793814	52.53460972	1471.170839	1451.662811
456.4218223	59.93588301	1585.668166	1568.148904
419.4350515	70.29072165	4475.708247	4447.399266
140.1015385	27.80615385	301.6123077	287.2889941
938.1085557	159.9558418	414.8160074	403.6332284
242.9817851	46.82149362	1391.82878	1369.503585
576.3971094	95.37164487	24.8678596	17.78486166
409.876494	75.34661355	434.9312749	421.692984
379.8883721	53.6372093	239.5511628	231.1815792
194.0773333	26.93066667	239.3653333	227.7508693
398.6289157	59.2626506	299.5746988	288.6369357
608.7654135	92.76541353	908.0135338	896.248118
95.16666667	18.55555556	380.4768519	364.2165424
136.2972973	23.43243243	55.61861862	49.08775442
140.3088685	26.31498471	470.9724771	453.877919
366.8217822	52.77970297	199.6076733	189.4987363
538.4939952	82.37229784	46.9071257	39.62274511
637.2749823	101.1417434	385.8355776	375.610087
109.0787402	21.73228346	342.0393701	323.1476843
168.9642857	28.04081633	263.0765306	251.565051
221.6707317	39.30487805	526.4593496	510.750694
522.9252188	88.43198091	220.6833731	210.3279822
360.4735516	61.6070529	1337.027708	1318.060257
268.2165354	36.15748031	311.3385827	300.8144336
1195.975089	180.7558719	300.466548	289.9121426
222.6348195	35.50530786	682.6199575	668.5614381
371.0371747	52.6976456	407.0656753	396.119171
819.3498575	115.4888889	891.9048433	881.024243
953.1151832	140.7867682	926.8391242	913.6747647
64.76878613	17.15028902	304.150289	283.2976043
442.2	72.90315789	1436.644211	1420.117846
149.7975078	26.02803738	536.9158879	520.4135635
860.6888151	128.8560354	1023.573643	1011.597292
937.6588475	136.9836818	343.5119837	334.5913591
248.3164557	50.02350814	2037.699819	2010.236913
150.6918605	29.13953488	324.0319767	309.7725713
526.3508772	71.0994152	1997.469786	1979.986628
276.904918	41.25901639	536.5032787	522.5450927
340.2094176	50.25154895	138.4349442	130.5991073
482.8402904	74.66424682	111.2041742	103.1317692
165.3837838	29.62702703	296.9567568	283.8014317
271.6020408	50.09863946	898.462585	881.8583461
117.6349206	20.12698413	296.5555556	282.1034895
193.4668094	40.751606	304.8201285	291.4593033
344.706258	49.62835249	161.0383142	152.1147794
329.8912467	44.48806366	93.71750663	86.75882649
251.2716981	34.97358491	361.5566038	350.0350623
352.459799	60.72110553	1231.94598	1215.080306
244.3430421	63.53398058	1413.156958	1388.431282
328.9344262	48.43715847	198.9494536	187.847448
73.06779661	22.85310734	3343.819209	3268.903827
357.4107383	50.00402685	371.0053691	360.7567767
318.0388889	51.55833333	697.4666667	682.9632639
183.5382716	32.27407407	415.4024691	402.335583
231.3866944	39.08731809	894.1101871	876.3862622
109.30837	24.31277533	1425.568282	1392.569078
377.576349	59.74626866	58.33065442	50.8515912
683.8978805	104.9511882	492.8278741	481.746715
388.1942789	67.53396901	923.2467223	907.7762363
565.5263158	97.6964672	97.07137707	87.22617009
415.9749728	58.18607182	1118.668118	1104.802827
319.7819355	60.16903226	212.1870968	198.9750443
136.248366	20.77777778	260.5196078	248.812476
64.12138728	18.07514451	502.3583815	479.1181797
555.1816638	103.8438031	3583.185059	3559.160083

log.sigma.1.0.mm.3D_glszm_ZonePercentage	log.sigma.1.0.mm.3D_glszm_LargeAreaLowGrayLevelEmphasis	log.sigma.1.0.mm.3D_glszm_LargeAreaHighGrayLevelEmphasis	log.sigma.1.0.mm.3D_glszm_HighGrayLevelZoneEmphasis
0.378887842	0.17921157	39362.51368	476.608209
0.222459893	3.470845185	73002.39904	141.4759615
0.209256845	9.15876826	124824.1121	126.1090343
0.292732856	0.638088196	39912.84266	240.8391608
0.275760135	3.186761786	131349.5161	178.4150077
0.381818182	0.286273819	18265.68254	276.0555556
0.267032384	5.591356207	270264.6733	227.5904573
0.253719655	6.85699018	559877.8997	293.0223765
0.283545974	1.339462054	120163.4228	282.1753297
0.332089552	0.25769615	18115.50562	255.2640449
0.25397878	2.025492149	43436.56919	139.1488251
0.334056399	0.449306826	50244.48052	326.0649351
0.183717193	6.715182784	528201.102	281.7725948
0.277810134	1.193061443	132981.4319	319.3039832
0.328629032	0.622758374	80940.2362	360.7039877
0.226005484	8.433994632	542203.5854	245.3629929
0.329145729	0.488737364	6967.725191	127.1857506
0.182481752	4.41629418	231540.3644	210.7733333
0.258859784	1.753196705	93176.53869	218.452381
0.313329486	1.329805452	113597.6654	297.1356661
0.183531746	15.29314364	417219.9477	171.2918919
0.324374421	0.655032505	14934.26571	143.0228571
0.284179688	0.810828628	67708.35739	282.024055
0.274272762	1.638406511	79038.69008	232.0950413
0.282796544	0.857920318	54279.85556	280.9194444
0.35964542	0.563289087	76555.60681	396.193662
0.317970925	1.597811833	104608.8891	267.1108949
0.250543085	3.413312947	291270.8454	276.3453757
0.37360179	0.242790586	4959.51497	154.0838323
0.257059396	2.375327986	159638.9299	244.1837121
0.252416982	4.945689541	505777.9359	326.8193172
0.219913001	3.73180516	238758.8791	229.9956044
0.291731186	1.838259122	119252.0106	264.3375796
0.286382979	2.155755473	107384.8247	232.5616642
0.315395095	1.284038686	52162.51908	212.938085
0.226408803	6.712798884	324128.1325	228.9661267
0.23891427	3.328056735	758651.6513	469.6254218
0.187948072	10.44170229	1922917.367	439.2721649
0.264227642	0.98528437	93471.25231	266.3784615
0.299037139	2.896535589	60360.18583	147.9636615
0.211642251	10.04183974	194963.6029	133.2786885
0.37574347	0.166127121	4176.64212	179.6352374
0.274842595	1.680369442	113457.7291	246.5866534
0.345659164	0.808519507	72033.52326	284.4081395
0.29342723	0.711574429	81749.584	331.056
0.302367942	1.788360775	51034.43976	183.7891566
0.291538799	4.071502757	203557.6398	235.9112782
0.247990815	2.566555996	57464.89815	139.9861111
0.391304348	0.336690442	9902.006006	175.6936937
0.241863905	1.625881454	137459.2294	290.6697248
0.314519268	0.694089426	58172.22525	302.8539604
0.370513201	0.213056271	10876.06565	232.040032
0.312721631	1.954735788	76915.04181	196.6739901
0.230072464	2.345718848	50883.55512	151.8346457
0.294736842	1.169736936	59971.02806	239.0280612
0.252307692	3.174891416	88589.0874	163.4329268
0.310754017	0.962775568	51241.92363	225.3031026
0.229612493	4.700953051	382735.9408	278.0188917
0.308252427	1.067507647	91552.97835	280.8681102
0.307810275	1.107494697	82399.95302	291.0765125
0.266704417	2.381234447	196453.1359	290.8216561
0.302247191	1.019589206	163521.8971	396.9640644
0.303161168	2.464773056	323711.9339	373.3726496
0.275613276	3.134751759	275441.7796	284.6611138
0.218987342	3.159888901	30004.56647	99.43930636
0.245986535	5.038097527	411277.5411	282.0368421
0.246165644	2.414612595	120394.4206	212.4797508
0.28896	3.084055672	341262.3666	312.0636766
0.334813044	0.843344493	140849.9602	397.6399796
0.190821256	10.63798446	393997.7143	187.5045208
0.264819092	1.933851232	55501.88081	164.7238372
0.239160839	7.74829357	517452.2768	269.1822612
0.267661255	2.443591351	119119.3311	227.4590164
0.357237716	0.617008437	32079.42379	224.7372986
0.351964229	0.695399555	18481.76225	174.6914701
0.275707899	1.668669193	53747.08108	162
0.245409015	4.567945115	178436.2925	206.3112245
0.263048017	1.522873016	58685.5119	185.2380952
0.273579379	1.522317725	62012.56959	191.2633833
0.334758444	0.718234322	36976.65262	228.9131545
0.379084967	0.201353717	44411.1565	479.770557
0.294608116	1.842934147	72278.22642	187.8811321
0.243499541	4.324643349	352152.5716	284.4974874
0.201106411	9.638162203	209459.6084	139.4822006
0.300123001	0.891128257	45096.52459	220.204918
0.115535248	28.62993839	393297.9209	99.36723164
0.312368973	1.675379402	83291.55436	217.9221477
0.262582057	3.56948059	137786.7111	213.6305556
0.276639344	1.613590754	107826.1778	259.582716
0.237530864	3.464983893	232017.5946	244.2328482
0.174079755	5.747630164	355420.8767	265.1013216
0.36565911	0.259087483	13609.98278	238.1814007
0.300405171	2.455789035	100538.6076	193.2048812
0.254242424	4.827547699	178181.3611	199.9010727
0.318704044	0.43536453	22333.32949	223.9307859
0.2685564	2.118197888	592187.1926	530.9270947
0.275115371	1.259802579	36576.14065	166.8490323
0.29226361	0.908878486	75563.9281	276.7973856
0.207434053	4.243251134	60274.21387	112.1213873
0.204018012	12.56510419	1024092.463	291.1341256

log.sigma.1.0.mm.3D_glszm_SmallAreaEmphasis	log.sigma.1.0.mm.3D_glszm_LowGrayLevelZoneEmphasis	log.sigma.1.0.mm.3D_glszm_ZoneEntropy	log.sigma.1.0.mm.3D_glszm_SmallAreaLowGrayLevelEmphasis
0.690997797	0.004039685	5.97462844	0.00333384
0.72097623	0.019451511	5.095665412	0.017430508
0.649249495	0.024628382	5.40280153	0.020695331
0.665548033	0.014046869	5.64795231	0.012182489
0.693638914	0.012366479	5.61677029	0.010173841
0.715595185	0.010583176	5.456195358	0.009336628
0.687579985	0.007045113	5.695667904	0.005395454
0.737532834	0.006033715	5.6016636	0.004999787
0.70009098	0.00593083	5.765691207	0.004687082
0.690381593	0.009229957	5.619623414	0.007865624
0.690680049	0.014855111	5.415800451	0.011560969
0.710792138	0.007238966	5.806318185	0.006268819
0.680984401	0.012087942	5.487059123	0.010775508
0.70591823	0.006776312	5.67232698	0.005644694
0.727943176	0.005810853	5.762270269	0.00493868
0.715851375	0.008341109	5.572085732	0.006795815
0.652242717	0.02088549	5.676286187	0.016494157
0.708002881	0.011935974	5.175008437	0.010142241
0.678811493	0.009134851	5.398230146	0.00743891
0.710337126	0.008063553	5.796196583	0.006932435
0.706482918	0.009899028	5.232358998	0.007797376
0.682425915	0.021798002	5.557990123	0.019033916
0.690125945	0.008223536	5.450804753	0.006993815
0.662016161	0.008323582	5.737125472	0.006656721
0.721199149	0.011535449	5.476749542	0.010180944
0.688764796	0.004634944	5.89600608	0.00369361
0.686528069	0.00748868	5.879199211	0.006032827
0.697248096	0.007860787	5.576017969	0.006628553
0.688796879	0.016423682	5.234702049	0.01365602
0.709793792	0.00839173	5.429944576	0.007060672
0.698437523	0.006228843	5.761420533	0.005133618
0.714336365	0.008856965	5.41480465	0.007287194
0.693650026	0.007196388	5.834889652	0.00574023
0.6987578	0.015950864	5.791866759	0.014168445
0.701595404	0.010971976	5.827085633	0.009199187
0.716910183	0.008951068	5.532908651	0.007308175
0.743958943	0.004167882	5.68048062	0.003568647
0.683395218	0.004367578	5.801191309	0.003560295
0.682224472	0.009192354	5.423723911	0.007880244
0.68323491	0.014212041	5.823225375	0.01111279
0.691882072	0.019081581	5.45167034	0.014513728
0.654861907	0.010735411	6.024719783	0.008063638
0.664014479	0.007237309	5.800462468	0.005691074
0.690860937	0.008571092	5.845643984	0.00715965
0.74607056	0.008163697	5.444085246	0.007389113
0.720467039	0.011851673	5.551762039	0.009840353
0.703224192	0.008113199	5.745162895	0.006249741
0.687562167	0.028147717	5.163300704	0.025212046
0.663444026	0.02019751	5.62110822	0.017177397
0.681010391	0.007660979	5.482842629	0.006504314
0.700589126	0.007067112	5.79710016	0.005987175
0.682691878	0.007886419	5.882562705	0.006189544
0.69872392	0.009746105	5.713230018	0.007710156
0.680612168	0.020550668	5.387247251	0.017896997
0.680300608	0.012731753	5.615537707	0.011132947
0.698074863	0.020581338	5.479423822	0.018300808
0.670867232	0.008121529	5.874137188	0.006305689
0.700672746	0.006888009	5.649141132	0.005627292
0.75208536	0.007308055	5.449633738	0.006316588
0.678543429	0.005602541	6.035471819	0.004236961
0.714806152	0.00755073	5.525262023	0.006473625
0.704839739	0.005302252	5.797666935	0.004493652
0.710564145	0.004293451	5.80139712	0.003432997
0.700477487	0.00705993	5.909066164	0.005822483
0.635003331	0.024259074	5.184414164	0.018786555
0.709370509	0.007054334	5.635463204	0.005792328
0.710068283	0.012559255	5.404523154	0.010624627
0.717530301	0.005174552	5.736334482	0.00416288
0.71887254	0.004302089	5.746630376	0.003513869
0.696858375	0.01141935	5.368216973	0.009654932
0.687569186	0.017657746	5.432459349	0.014838979
0.743839764	0.010964515	5.58447179	0.00973853
0.700557871	0.009155285	5.677009445	0.007542222
0.675092347	0.009613194	5.909523131	0.006787018
0.688388044	0.013533934	5.793553464	0.010949688
0.694941287	0.01391578	5.462745919	0.01142334
0.705808782	0.009490726	5.404483302	0.007775423
0.709884478	0.020072988	5.399382426	0.018345248
0.669274847	0.011049225	5.520466622	0.008923666
0.690129826	0.010653532	5.863709089	0.008693848
0.687928203	0.004093633	5.948867082	0.003385278
0.713877009	0.016959934	5.576505958	0.0144985
0.691926882	0.006902077	5.687912151	0.005621049
0.653832257	0.012555324	5.440221577	0.009451764
0.696921955	0.009072389	5.813783861	0.007294451
0.667531757	0.028033345	4.869498677	0.024130941
0.71979611	0.011274806	5.691190015	0.00955553
0.69130972	0.010137867	5.717510173	0.008116872
0.700059662	0.00858703	5.485698622	0.007079594
0.720427767	0.009204512	5.465072852	0.007945088
0.720757207	0.009041449	4.909885909	0.007904902
0.684519783	0.007771856	5.73084995	0.006249293
0.689349959	0.011362122	5.85656845	0.009188664
0.70735544	0.00982407	5.527252723	0.00808936
0.664146948	0.007673851	5.936432663	0.005875852
0.699691205	0.00364985	5.898776125	0.003021894
0.668045781	0.013165736	5.707119243	0.010517525
0.693639871	0.01028819	5.654776209	0.008970368
0.632177517	0.020238587	5.207493934	0.01578408
0.713939701	0.005520072	5.411178087	0.004458147

log.sigma.1.0.mm.3D_ngtdm_Coarseness	log.sigma.1.0.mm.3D_ngtdm_Complexity	log.sigma.1.0.mm.3D_ngtdm_Strength	log.sigma.1.0.mm.3D_ngtdm_Busyness	log.sigma.1.0.mm.3D_ngtdm_Contrast
0.00323206	0.464690923	0.991773636	0.372086786	4.23E-05
0.007026039	0.282785853	1.129900732	0.415928734	4.52E-05
0.004693305	0.138434808	0.615384064	0.726478647	2.51E-05
0.007010181	0.480581712	1.36210989	0.29362793	7.88E-05
0.002766383	0.170737163	0.493279358	0.876250159	2.78E-05
0.009829029	0.973614883	2.435233099	0.198146949	0.000141661
0.001228098	0.095698918	0.322287574	1.440404852	6.49E-06
0.001363237	0.113955535	0.440859405	1.068902189	7.57E-06
0.00148805	0.167599362	0.496594267	0.926832172	8.94E-06
0.005912269	0.551759122	1.391363487	0.309612715	6.52E-05
0.004314803	0.187886094	0.640001076	0.711036876	2.95E-05
0.002796471	0.402839122	1.018385668	0.457346201	2.73E-05
0.00360931	0.268447911	1.542333649	0.366849144	1.24E-05
0.00401507	0.350197084	1.007631163	0.385966074	3.67E-05
0.003467515	0.445787195	1.089882753	0.373128799	3.55E-05
0.001571118	0.123163446	0.520103706	0.974676363	6.88E-06
0.005584248	0.324210784	0.79273907	0.636227511	6.63E-05
0.005323646	0.273121424	1.409800715	0.358291452	2.22E-05
0.005063771	0.260364765	0.850704276	0.462610663	3.58E-05
0.001287643	0.173147211	0.5064799	0.995462182	8.40E-06
0.002247954	0.068151097	0.354321622	1.249046424	9.06E-06
0.005734109	0.286195206	0.685362295	0.626130435	8.29E-05
0.006391103	0.445146068	1.338079402	0.292676353	5.33E-05
0.002588353	0.177312664	0.595098458	0.765624259	1.66E-05
0.005367748	0.409142062	1.349862138	0.32760136	4.25E-05
0.002880099	0.37859492	0.971407273	0.439136957	2.72E-05
0.002219472	0.213792567	0.563639759	0.765014949	1.97E-05
0.00251831	0.197439023	0.741556414	0.61245168	1.43E-05
0.01390478	0.704863872	1.554037268	0.265688271	0.000205722
0.003189707	0.237199211	0.93082337	0.525804319	1.72E-05
0.001479185	0.151362357	0.599379597	0.810079319	6.51E-06
0.003464534	0.205574575	0.819225535	0.511638159	1.75E-05
0.00224592	0.244265149	0.781387546	0.639840038	1.29E-05
0.003121248	0.266516951	0.813693147	0.555348635	2.34E-05
0.001583072	0.164782632	0.446622861	1.120961458	1.25E-05
0.002336573	0.167900249	0.715787797	0.732971489	1.08E-05
0.001896258	0.270110651	1.1404572	0.449389006	7.51E-06
0.001345142	0.132808035	0.79216769	0.713628373	4.01E-06
0.004952041	0.388722571	1.314635911	0.366533931	4.15E-05
0.00093344	0.062293561	0.159565333	3.023867207	8.35E-06
0.002743624	0.114252699	0.42715234	1.040174735	1.58E-05
0.001681296	0.16703409	0.324941496	1.530003868	2.20E-05
0.001801856	0.162350272	0.507753117	0.92997525	1.23E-05
0.002649429	0.33268679	0.727651747	0.584851357	3.11E-05
0.005007484	0.502907003	1.737732567	0.279793952	3.85E-05
0.002537489	0.195550162	0.567552002	0.878748401	1.98E-05
0.001528767	0.14632683	0.446509571	1.078667298	9.93E-06
0.007225273	0.375335558	1.270767589	0.38375785	5.50E-05
0.008059247	0.597643539	1.328220664	0.329167511	0.000124894
0.004938106	0.283499061	1.073624978	0.386579053	3.51E-05
0.002589028	0.348113693	0.967622979	0.521779745	2.06E-05
0.001864957	0.244707118	0.509306079	0.958144999	2.01E-05
0.001423364	0.1163391	0.281788198	1.56965433	1.38E-05
0.005881017	0.321996126	1.081163808	0.456375231	3.95E-05
0.004841905	0.400013829	1.166599313	0.3939948	4.41E-05
0.003398479	0.157996057	0.555648746	0.839902422	2.95E-05
0.001679703	0.14318409	0.391747815	1.163488035	1.46E-05
0.002046311	0.154742676	0.596016711	0.757529174	1.04E-05
0.004053529	0.391573204	1.1368366	0.374599976	2.90E-05
0.000792012	0.089133778	0.233681501	1.852148886	5.66E-06
0.003998682	0.293986869	1.173304152	0.382957357	2.27E-05
0.002612007	0.407123303	1.243923045	0.383653686	1.57E-05
0.001199731	0.147608616	0.447141013	0.998734023	7.84E-06
0.000875668	0.12162402	0.381159059	1.392140622	4.68E-06
0.007724493	0.201229813	0.753478953	0.614453435	5.87E-05
0.001827395	0.175321404	0.684815857	0.728395932	8.14E-06
0.005451306	0.370933571	1.275050439	0.337217682	3.26E-05
0.001116894	0.124682232	0.373309321	1.187301638	7.12E-06
0.001149313	0.176534477	0.577996434	0.87174099	6.03E-06
0.002324537	0.103515642	0.477602739	1.007545176	1.13E-05
0.005144349	0.298583555	0.89726757	0.496818724	4.37E-05
0.001649989	0.148551298	0.597032915	0.848592988	7.76E-06
0.003201739	0.203794225	0.649254703	0.634190973	2.55E-05
0.002980019	0.381935484	0.76765798	0.613055126	3.58E-05
0.002072431	0.198024236	0.414539167	1.16621574	2.39E-05
0.004799233	0.290513209	0.874820735	0.533279001	4.00E-05
0.002687831	0.2093561	0.808175616	0.682969217	1.32E-05
0.006744385	0.39322091	1.288776164	0.343601299	5.74E-05
0.003768633	0.22239505	0.745407462	0.620774514	2.70E-05
0.002987392	0.30564394	0.73047422	0.620403902	2.89E-05
0.00340324	0.485553398	1.080991347	0.34844771	4.25E-05
0.003806456	0.369419923	0.971457494	0.4910645	3.09E-05
0.002186034	0.188019476	0.751815978	0.635615899	9.81E-06
0.002167578	0.071348921	0.284844436	1.589989859	1.28E-05
0.002813809	0.234474503	0.584465056	0.699944029	2.70E-05
0.004218804	0.06575936	0.613887773	0.95678955	1.13E-05
0.00295599	0.279092111	0.728902276	0.607976847	2.38E-05
0.002504337	0.169246095	0.535078227	0.841985156	1.93E-05
0.004653336	0.307970324	1.124853915	0.385390811	3.11E-05
0.003444864	0.23143339	0.975273549	0.483002647	1.66E-05
0.004908986	0.138692496	0.870229644	0.489836322	2.19E-05
0.002755397	0.258308385	0.602876498	0.73665756	2.88E-05
0.001289738	0.131876581	0.330471261	1.485357438	1.08E-05
0.002070626	0.119145396	0.453470602	1.04186279	1.21E-05
0.001480894	0.1688955	0.424521605	1.224481499	1.22E-05
0.00206275	0.309237306	1.119660788	0.400778168	1.10E-05
0.002424234	0.146952563	0.449038593	1.013668092	1.78E-05
0.0068422	0.540485461	1.609582846	0.250548455	6.35E-05
0.008287285	0.189235825	0.723432257	0.526752634	5.86E-05
0.001152698	0.074088836	0.369055015	1.332161723	4.44E-06

log.sigma.0.5.mm.3D_gldm_GrayLevelVariance	log.sigma.0.5.mm.3D_gldm_HighGrayLevelEmphasis	log.sigma.0.5.mm.3D_gldm_GrayLevelNonUniformityNormalized	log.sigma.0.5.mm.3D_gldm_DependenceEntropy
0.939564066	44.1196984	0.323752066	5.272284415
0.428750036	13.89411765	0.466139724	4.717259783
0.409419945	13.80769231	0.459785072	4.856445642
0.675168591	21.39303992	0.392362097	5.236968845
0.560205898	21.82601351	0.426441803	5.07005479
0.862525253	22.33030303	0.352203857	4.962164034
0.512744098	22.11360821	0.416562469	5.029399229
0.580741037	22.44753328	0.434379042	5.032209634
0.590335988	22.02727673	0.412565574	5.072299724
0.668853726	22.19776119	0.389793105	5.017497507
0.411585162	13.35344828	0.457789227	4.828115726
0.811237666	33.09631236	0.379240263	5.165780684
0.509531975	21.90573112	0.450448391	4.891082089
0.700149826	32.39895166	0.395473475	5.07778553
0.827519348	32.58165323	0.369482066	5.192368905
0.50445461	21.84437843	0.436374846	5.015165111
0.565549692	13.43886097	0.384871033	5.104334407
0.429239441	22.13138686	0.47688104	4.721694134
0.46128333	21.71494607	0.434946736	4.848164438
0.681320897	22.34256588	0.38944182	5.119508731
0.349548847	13.66335979	0.480511289	4.807980656
0.549518528	13.74142725	0.407370124	4.994026716
0.547118187	21.92871094	0.412614822	5.010078101
0.531020355	22.06384586	0.407543935	5.062111951
0.618078418	22.60408484	0.404359934	4.968926103
0.824008435	33.51498523	0.361095307	5.336578228
0.717813294	22.42251779	0.367936153	5.299068589
0.557378349	21.80340333	0.42345953	5.078845709
0.537253077	13.8344519	0.403410257	4.908059693
0.475272795	22.08373905	0.471794177	4.815789868
0.578324182	32.68705338	0.433131713	5.038224127
0.483749034	21.37602707	0.450435565	4.870527457
0.663533607	21.9108083	0.382599873	5.388768419
0.699076324	22.20893617	0.390570213	5.130160447
0.678816879	22.89963669	0.387806663	5.188986585
0.517014174	22.07335779	0.430372311	4.92773295
0.639294933	44.01289976	0.410541115	5.081489498
0.535567289	31.94419686	0.419468018	5.14303377
0.51154802	21.41056911	0.439459317	4.924679135
0.560427242	13.31623109	0.385520832	5.253531365
0.462191678	13.26060139	0.437242638	4.960885591
0.735099279	13.38013964	0.331374352	5.33559391
0.545461848	21.99151382	0.421538863	5.067440488
0.796843498	21.85048232	0.36461575	5.166240189
0.644916622	33.09389671	0.431223229	4.920383379
0.605213121	14.07103825	0.394879247	5.112250855
0.638779533	22.16089434	0.399367975	5.110676995
0.471493104	13.83467279	0.48406954	4.727470388
0.771484712	14.1786134	0.370254943	4.942317699
0.479853975	22.65754438	0.446707267	4.879054194
0.737186998	22.7493188	0.380204223	5.091536743
0.730086422	22.01690893	0.35234508	5.25338572
0.595334231	21.85904255	0.391825404	5.061954519
0.464319471	13.96195652	0.45119854	4.916857431
0.65393691	22.5887218	0.408724066	4.936424697
0.492498356	13.58205128	0.437432742	4.875651758
0.613031333	22.23955501	0.392176091	5.158716209
0.529439116	21.39039907	0.421678668	4.978775162
0.599160721	21.81614078	0.417096481	5.005309815
0.698708586	21.36937233	0.354670494	5.299496317
0.576362178	22.39467724	0.433928784	5.022409199
0.72598241	32.71835206	0.386382752	5.141554079
0.685510947	33.06080497	0.381105468	5.203230781
0.643510807	21.92824347	0.402081899	5.177102072
0.341530203	13.53037975	0.493510655	4.596144379
0.561669949	21.80942517	0.42724822	5.041090717
0.566597821	21.38650307	0.429246726	4.871922858
0.644729062	31.93488	0.392003686	5.187155637
0.647359939	32.45603551	0.400665086	5.230258125
0.403197203	21.6973775	0.467405474	4.756549868
0.531000041	13.54426482	0.423927093	4.89106198
0.587645199	22.5988345	0.430146978	5.065829835
0.622597419	22.12549364	0.400717889	5.148908231
0.825865002	21.71226206	0.334601195	5.323659721
0.68475505	13.48291281	0.353155987	5.254827019
0.511750929	13.39567809	0.433370128	4.882928707
0.471554392	22.2090985	0.451308316	4.904244651
0.544414468	21.81210856	0.441150448	4.786464369
0.47720455	21.76567077	0.433713964	4.990303076
0.7227072	21.5754596	0.365524013	5.379437869
0.913931601	44.23931624	0.331905672	5.275441654
0.687445715	21.50416898	0.389901313	5.164170896
0.561955188	21.84765983	0.418044178	5.126427033
0.344525749	13.40123658	0.479363898	4.947814721
0.673875611	21.70807708	0.381825774	5.254258728
0.273866394	13.6116188	0.547035735	4.649747049
0.658031284	21.31572327	0.379243261	5.200812949
0.571119831	22.88657914	0.412998014	5.077570181
0.527658336	22.20355191	0.454737003	4.922771178
0.495732724	22.1308642	0.466263314	4.919334622
0.37100039	22.23236196	0.469946178	4.734930834
0.639235915	22.40134341	0.368571028	5.136272104
0.656071592	21.69766544	0.382455347	5.130248412
0.488989807	22.26393939	0.439951148	4.978901629
0.609978118	21.49563419	0.379654406	5.225873794
0.742692196	45.24430158	0.392252182	5.137876726
0.521238452	13.39652112	0.411561657	5.095753605
0.689021893	21.64565425	0.394507799	5.025675891
0.370450402	13.19784173	0.475544744	4.693157882
0.444244855	22.27000346	0.45014067	4.914419445

log.sigma.0.5.mm.3D_gldm_DependenceNonUniformity	log.sigma.0.5.mm.3D_gldm_GrayLevelNonUniformity	log.sigma.0.5.mm.3D_gldm_SmallDependenceEmphasis
149.2591894	687.001885	0.093379989
63.09197861	435.8406417	0.053426136
98.83441982	705.3102999	0.048328559
61.18628454	383.3377687	0.060559956
139.160473	1009.814189	0.064846258
50.59090909	232.4545455	0.08116068
373.7573881	2353.994514	0.055941632
294.6898982	2218.808144	0.061259939
275.1007479	1875.523097	0.068818145
70.71455224	417.858209	0.083940702
101.9708223	690.3461538	0.051410595
144.4308026	874.1488069	0.081371855
126.5179432	840.9871452	0.044409789
107.6790914	679.0279557	0.063702689
127.0524194	733.0524194	0.082926854
274.5690128	1909.576325	0.052697754
86.51758794	459.5360134	0.062583548
80.03325223	587.9943228	0.04529734
92.04930663	564.5608629	0.059966927
337.8459319	2024.708021	0.072999784
210.2592593	1453.066138	0.031422679
71.53012048	439.5523633	0.066834454
67.6484375	422.5175781	0.063310562
187.6173026	1078.768795	0.061477532
83.717989	514.7501964	0.065335163
149.3343183	855.4347826	0.087300808
200.915249	1189.537581	0.067694904
172.5611875	1169.595221	0.056763844
32.74049217	180.3243848	0.068047494
128.5316456	969.0652386	0.058737825
278.7822615	2060.840689	0.058895687
135.8912518	931.9511841	0.046668774
192.6308455	1235.414989	0.065231412
147.2961702	917.84	0.068704476
268.1257947	1707.900545	0.075348577
203.3134378	1290.686562	0.050299709
252.3136254	1527.623488	0.057222084
339.8761868	2164.874443	0.04266113
78.70569106	540.5349593	0.061497673
480.7568088	2802.736451	0.063515767
168.5512722	1134.207402	0.044485707
279.4370313	1281.424619	0.079578702
227.2879825	1539.881467	0.059500091
161.3432476	907.1639871	0.08094743
77.52425665	551.1032864	0.076181138
178.424408	1083.943534	0.068782257
287.7308198	1821.916703	0.066324711
53.62686567	421.6245695	0.065883041
59.30082256	315.0869565	0.08194748
87.29142012	603.9482249	0.060574105
167.3425457	976.7446477	0.073050344
231.7878968	1187.755265	0.084052629
298.6702128	1767.916223	0.070912322
69.98550725	498.1231884	0.053319771
86.62556391	543.6030075	0.072131454
131.5825641	852.9938462	0.052185491
249.7470952	1586.352287	0.064954708
243.2903412	1458.164835	0.052222451
104.663835	687.375	0.078885028
650.2974039	3237.786943	0.068900459
106.3782559	766.3182333	0.061709412
167.247191	1031.641948	0.070661057
361.2442563	2206.219554	0.0705872
478.7754165	3065.070314	0.070350934
58.21012658	389.8734177	0.046892649
252.1165199	1650.032626	0.054076842
86.00306748	559.7377301	0.057352551
394.46144	2450.02304	0.064898908
357.7625064	2346.695407	0.086661774
207.0904072	1354.541063	0.041516673
84.69822941	550.6812933	0.055571669
255.1431235	1845.330536	0.059159082
143.546731	913.2360685	0.06275101
155.3116423	755.8640992	0.076768879
215.6962632	1105.731396	0.07470955
89.29955291	581.5827124	0.057530967
159.3823038	1081.334725	0.058183826
67.03340292	422.6221294	0.061965451
114.3848858	740.3497364	0.056893504
142.9572467	854.960667	0.077695821
140.5455003	660.1603821	0.096275433
113.7426348	701.4324625	0.070216647
204.2132151	1366.586418	0.057329351
203.1705174	1473.085259	0.040505987
150.2439524	931.2730627	0.068941526
96.19321149	838.0587467	0.02455657
154.2201258	904.4951782	0.06570689
166.2363239	1132.440554	0.063015099
95.07786885	665.7349727	0.072441039
117.982716	944.1832099	0.062317948
90.86042945	612.809816	0.034628073
165.6003359	877.9361881	0.080454161
346.1811692	1982.266062	0.068124889
204.4290909	1451.838788	0.057692143
287.7012868	1652.255974	0.068635444
212.7971946	1342.286967	0.069024755
186.9481718	1159.369187	0.056233625
68.47086915	413.0496657	0.067619653
57.00479616	396.6043165	0.039756633
370.2868029	2599.112227	0.047719375

log.sigma.0.5.mm.3D_gldm_DependenceNonUniformityNormalized	log.sigma.0.5.mm.3D_gldm_DependenceVariance	log.sigma.0.5.mm.3D_gldm_LargeDependenceEmphasis
0.070338921	15.7467774	69.86145146
0.067478052	16.83433212	102.0459893
0.064429218	18.0657619	109.7457627
0.062626699	19.26363054	98.50460594
0.058767092	21.57809005	107.25
0.076652893	13.21651974	69.64848485
0.066140044	17.12248993	98.52433198
0.057691836	22.13255145	111.7364918
0.060514903	20.36002237	101.0514738
0.065965067	17.35052002	83.9738806
0.067619909	16.77977401	102.6246684
0.062659784	19.6147927	87.91193059
0.067765369	16.62676446	109.8366363
0.062713507	18.82495813	97.32847991
0.064038518	18.93367809	87.27923387
0.06274429	18.57081087	108.7166362
0.072460291	14.37498772	83.09547739
0.064909369	17.9235027	116.6139497
0.070916261	15.94070764	97.8027735
0.064982868	17.71707093	91.27928448
0.069530178	16.05483119	118.1289683
0.066292975	17.56953586	87.09638554
0.066062927	17.23611069	95.27539063
0.070879223	15.20220523	89.25840574
0.065764328	17.44758656	92.56323645
0.063036859	19.54652798	86.14563107
0.062145144	19.4231543	93.24064337
0.062476896	19.00457067	104.577118
0.073244949	14.85510663	76.901566
0.062576264	19.53570159	109.3330088
0.058592321	21.28651429	112.4211854
0.065679677	17.34813553	110.5746738
0.059656502	21.22872109	102.89904
0.062679221	18.77781802	95.32170213
0.060882333	20.58109546	93.55086285
0.067793744	16.32540134	102.7552518
0.067808015	16.4720385	100.6468691
0.065854716	17.09711964	108.4086417
0.063988367	18.60901844	105.0536585
0.06612886	17.25765708	92.77303989
0.06497736	17.6685079	109.692367
0.072261968	14.89545115	71.1939488
0.062219541	19.18956875	101.4757733
0.064848572	18.29665223	83.87540193
0.060660608	21.01470657	102.9186228
0.064999784	17.54192003	90.41566485
0.063071201	18.71191423	95.92985533
0.061569306	20.67959417	112.9586682
0.069683693	15.83628854	75.85781434
0.06456466	18.08239382	102.3091716
0.065139177	17.63409614	87.82444531
0.068759388	16.25558798	77.23079205
0.066194639	16.9494673	87.7748227
0.06339267	19.0675508	111.0815217
0.065132003	17.6947278	91.60300752
0.067478238	16.82667613	101.014359
0.061742174	19.91216026	94.51990111
0.070355796	15.70443705	102.4129555
0.063509609	18.93666786	97.35800971
0.071234243	14.74846011	78.8413846
0.060236838	20.58866676	105.0079275
0.062639397	19.05737267	92.25917603
0.062401841	19.74954161	91.40317844
0.062806692	19.22616159	100.4293585
0.073683705	15.02479731	107.6227848
0.065281336	17.74697654	104.6970482
0.065953273	17.50157844	102.7239264
0.06311383	18.65800151	94.98688
0.061082893	21.03695413	89.17483353
0.071459768	15.1426471	108.3071084
0.06520264	17.77096019	100.7505774
0.059473922	20.92505387	111.2149184
0.062986718	18.62482275	99.08863537
0.068752387	16.49904675	76.7521027
0.068890534	16.00515446	78.58735228
0.066542141	16.93104804	95.71982116
0.06652016	17.17914805	102.4716194
0.069972237	15.98649326	97.55114823
0.067009306	17.12701242	98.31693029
0.06111896	20.68524331	91.94655836
0.070661388	15.65354295	68.42584213
0.063225478	18.84667197	93.85825459
0.062469628	18.76767667	101.0501682
0.066114714	17.52635368	121.9410999
0.061600637	20.18032649	94.19393194
0.062789303	19.15431968	148.6266319
0.064662527	17.74019382	90.83731656
0.060625939	20.24054966	100.5200584
0.064943899	18.17953656	100.0177596
0.05826307	22.20528188	117.5303704
0.069678243	15.5329143	109.7300613
0.069521552	16.08113961	76.89756507
0.066791659	16.63469857	87.77869959
0.061948209	19.08188099	105.3690909
0.066107832	17.26453017	88.953125
0.062185036	19.08038405	95.24196376
0.066364278	16.81292835	95.00070998
0.065397201	17.74106033	93.63228271
0.068351075	16.65910437	110.4748201
0.064130032	17.91190302	109.5857291

log.sigma.0.5.mm.3D_gldm_LargeDependenceLowGrayLevelEmphasis	log.sigma.0.5.mm.3D_gldm_SmallDependenceHighGrayLevelEmphasis	log.sigma.0.5.mm.3D_gldm_LargeDependenceHighGrayLevelEmphasis
1.555107348	3.889739988	3230.836004
7.049910873	0.631695321	1544.163636
7.784120672	0.606459107	1628.722947
4.537735548	1.180300372	2261.038895
4.652812845	1.190954373	2553.516892
3.210402404	1.875945115	1590.539394
4.412123037	1.209532554	2285.52504
4.777736148	1.2000814	2684.646829
4.434119472	1.382740023	2384.256049
3.697804176	1.682674545	1979.06903
7.421690429	0.680952497	1503.531167
2.599337728	2.405246618	3046.295445
4.88532469	0.855307728	2562.573112
2.916013872	1.933400076	3341.295865
2.615269239	2.467746916	2998.672379
4.82684374	1.015056478	2539.021709
6.602897822	0.967492589	1144.663317
5.023590596	0.764771844	2778.38605
4.391436332	1.244217134	2258.802773
4.075195081	1.513595765	2130.435276
8.498006871	0.42735331	1736.326058
6.26520415	0.821511538	1289.683967
4.259442656	1.313738812	2214.963867
4.088462933	1.289108955	2036.468833
4.047946639	1.388924055	2192.02121
2.594360306	2.90956588	2946.562685
4.167983438	1.435815638	2181.471698
4.659694247	1.171217695	2440.568067
5.652854835	0.939695574	1119.973154
4.678415387	1.063480419	2620.703992
3.311275816	1.638275182	3891.733291
4.913036412	0.87521649	2583.639923
4.74576473	1.487060061	2342.013007
4.23559198	1.419555645	2241.161277
4.042345752	1.641253667	2240.277475
4.576350267	1.014213229	2396.94965
2.23805674	2.227383042	4621.726955
3.368612564	1.26597746	3596.740167
4.670055887	1.145939244	2446.773171
7.422603699	0.899380704	1265.986933
8.059048231	0.586819134	1599.40478
6.028985179	1.227279126	940.4497026
4.462628284	1.18875117	2392.168355
3.763614157	1.695251918	1963.503215
2.99499538	2.249325924	3595.247261
6.569961518	1.020867586	1333.075774
4.258003788	1.509499088	2247.580009
7.679972892	0.89037361	1725.409874
5.450890935	1.135609214	1153.410106
4.410058132	1.22430991	2440.45784
3.853671104	1.561692479	2081.924095
3.572298015	1.913660348	1760.068526
3.937057701	1.475417529	2039.303635
7.878112419	0.628437869	1650.230072
3.994925886	1.489602448	2172.468421
7.325717379	0.673883174	1483.021026
4.164039282	1.312104648	2227.357973
4.693386652	1.048488803	2339.801041
4.323499071	1.607546716	2272.846481
3.902040854	1.540448779	1697.105378
4.525172675	1.294179266	2509.738958
2.750752336	2.098853617	3173.740824
2.707037259	2.140236191	3162.226809
4.499609104	1.432479449	2334.374524
7.627405767	0.568489595	1595.046835
4.67120596	1.059335838	2442.591403
4.576530104	1.077230452	2393.141104
2.883868752	1.875481925	3219.3752
2.659588345	2.705046498	3064.328325
4.82829413	0.749058935	2514.549689
7.228390856	0.685316362	1493.337952
4.789097798	1.168183274	2664.015152
4.402365543	1.299064678	2322.92409
3.581109634	1.642780253	1755.811421
6.188228953	1.040983914	1100.395081
7.008267304	0.704290137	1412.577496
4.538780773	1.335933957	2391.295075
4.306347833	1.167848052	2287.864301
4.391758034	1.129468329	2283.927358
4.318984183	1.751637826	2065.038478
1.519400461	3.984508991	3168.110608
4.237639749	1.475034096	2173.823235
4.544115631	1.171873038	2339.918018
9.024066511	0.507989461	1752.445818
4.233337029	1.411373415	2191.280033
10.24360748	0.210037859	2242.244778
4.180693488	1.353187266	2076.495597
4.327688838	1.385349288	2408.539387
4.36762795	1.507230072	2363.916667
5.017276115	1.2094386	2822.860741
4.820839398	0.628765905	2576.786043
3.48211997	1.699154431	1776.026868
4.000153754	1.495922261	2020.103222
4.597247044	1.234944958	2496.103333
4.180936538	1.498359173	1991.974494
2.052497469	2.8495932	4500.351257
7.159763046	0.774842745	1360.405396
4.193111758	1.174702393	2186.354346
7.898036904	0.46811785	1631.711031
4.778549701	0.887152202	2591.455317

log.sigma.0.5.mm.3D_gldm_SmallDependenceLowGrayLevelEmphasis	log.sigma.0.5.mm.3D_gldm_LowGrayLevelEmphasis	log.sigma.0.5.mm.3D_gldzm_DistanceZoneVariabilityNormalized
0.00365589	0.025613836	0.988571806
0.010928376	0.087824718	1
0.008905069	0.085613864	1
0.006345432	0.058585087	1
0.005905445	0.053080065	1
0.010389457	0.057902426	1
0.004208681	0.0507137	0.992337278
0.006794315	0.052425666	1
0.006584455	0.053047229	1
0.009114064	0.054663629	1
0.009727403	0.090276488	1
0.005101883	0.03543853	1
0.005478084	0.05353978	1
0.003989197	0.035158366	1
0.004990315	0.035722035	1
0.005475438	0.053021958	1
0.009106774	0.092763354	1
0.005774129	0.051196255	1
0.005141051	0.05152818	1
0.009342416	0.055726445	0.994100347
0.00403436	0.08310718	1
0.013398277	0.094101277	1
0.005749113	0.052577964	1
0.005655721	0.05178002	1
0.007107958	0.052890858	1
0.004418545	0.034053656	1
0.007388443	0.054135622	1
0.005558014	0.05420073	1
0.012019026	0.090314442	1
0.006641443	0.052289482	1
0.003627614	0.033998407	1
0.004308135	0.053539875	1
0.006112958	0.05389238	1
0.007872658	0.056593153	1
0.006852435	0.05181213	1
0.004939687	0.051805267	1
0.002479009	0.024799535	1
0.002764327	0.034529263	1
0.006854784	0.054533304	1
0.011070686	0.095063121	0.9950125
0.008434692	0.092450099	1
0.013735264	0.100150739	1
0.005755704	0.052595981	1
0.008876491	0.059824416	1
0.005048908	0.034455687	1
0.01225667	0.092257814	1
0.005448863	0.053056392	1
0.013899648	0.092364779	1
0.018710245	0.103034818	1
0.005279949	0.049735975	1
0.008415858	0.054800444	1
0.007040546	0.054242031	1
0.005965652	0.052838504	1
0.012289091	0.087740036	1
0.00730463	0.053517697	1
0.010798935	0.094791738	1
0.00563435	0.052248782	1
0.005598249	0.054172109	1
0.008106013	0.054596561	1
0.006780934	0.05621241	1
0.005917561	0.052405253	1
0.005216989	0.035716694	1
0.00368683	0.033767816	0.98870093
0.008316129	0.055406283	0.987654797
0.007145955	0.084598453	1
0.006282346	0.054628796	0.977780583
0.007359819	0.055982947	1
0.003360964	0.034817598	0.988920011
0.004010308	0.03458248	1
0.003910492	0.051008262	1
0.011745959	0.097167265	1
0.00705871	0.053461323	0.990654412
0.005751866	0.052708137	1
0.007076457	0.057344885	1
0.013923004	0.100512214	0.990521542
0.012625154	0.096100969	1
0.00435888	0.0500848	1
0.008575577	0.054938648	1
0.004841075	0.051919461	1
0.006907367	0.056525853	1
0.003793534	0.02551665	1
0.007588514	0.056775872	1
0.006816131	0.054143364	1
0.006227551	0.085251925	1
0.006364541	0.054899447	1
0.005050525	0.082851755	1
0.00690528	0.05639733	1
0.004983055	0.050078718	1
0.007196428	0.052645785	1
0.006993711	0.052499435	1
0.003107672	0.04901041	1
0.007423338	0.052338973	0.987730529
0.006040107	0.054400807	0.98726167
0.004553779	0.050655461	1
0.005593103	0.053938039	0.992307807
0.002727854	0.024397156	1
0.010115021	0.094169822	1
0.010113884	0.058990437	1
0.006428375	0.088203437	1
0.004256268	0.049876414	1

log.sigma.0.5.mm.3D_gldzm_LowIntensityEmphasis	log.sigma.0.5.mm.3D_gldzm_LargeDistanceEmphasis	log.sigma.0.5.mm.3D_gldzm_HighIntensitySmallDistanceEmphasis
0.042193326	1.017241379	41.64367816
0.253708333	1	11.425
0.20739418	1	13.19047619
0.12430035	1	18.98181818
0.102560631	1	18.125
0.136801724	1	23.42592593
0.084668247	1.011538462	21.82788462
0.125160701	1	19.57664234
0.102873538	1	20.67465753
0.117895025	1	20.08235294
0.217144608	1	13.97058824
0.071042811	1	28.30681818
0.150813492	1	19.86363636
0.068539981	1	30.54736842
0.064251973	1	29.76923077
0.125634663	1	19.09625668
0.154598765	1	16.87301587
0.149703737	1	15.17391304
0.093032372	1	21.40298507
0.143412602	1.00887574	20.77588757
0.160189594	1	14.12698413
0.231827257	1	13.03125
0.09693695	1	21.40677966
0.102374783	1	21.47794118
0.117172097	1	21.73684211
0.053514028	1	33.95876289
0.124032348	1	21.40106952
0.118311144	1	20.55714286
0.189017857	1	14.71428571
0.132133273	1	17.87628866
0.0692265	1	27.32340426
0.114100456	1	17.75641026
0.101628544	1	24.14736842
0.134371407	1	20.66901408
0.100346285	1	21.9760274
0.114671295	1	19.79674797
0.04936638	1	37.11931818
0.076419111	1	29.92814371
0.127726591	1	18.11764706
0.201295139	1.0075	14.778125
0.230872585	1	13.51086957
0.188950642	1	16.02491103
0.107183852	1	20.29473684
0.136022968	1	20.14516129
0.071228823	1	30.07865169
0.214608175	1	15.60377358
0.095247756	1	23.09363296
0.244323899	1	13.8490566
0.251700275	1	14.35714286
0.098164862	1	19.73239437
0.128019396	1	21.68786127
0.090068668	1	23.5095057
0.092848486	1	21.13357401
0.242309671	1	13.35185185
0.113750693	1	21.04444444
0.249819902	1	13.25274725
0.096683147	1	20.5
0.124827324	1	20.1
0.113356493	1	20.50833333
0.112755461	1	23.02729045
0.110751295	1	21.55670103
0.08354653	1	29.03636364
0.057436584	1.017045455	30.23082386
0.132062248	1.01863354	20.68995859
0.171227599	1	12.5483871
0.139446212	1.033707865	18.8511236
0.148524445	1	18.33333333
0.05774294	1.016713092	28.11977716
0.049951453	1	31.57234043
0.109662085	1	17.92783505
0.265281924	1	11.71641791
0.145077449	1.014084507	18.96830986
0.100805	1	21.35483871
0.106563422	1	21.67320261
0.207332631	1.014285714	14.73809524
0.252504085	1	13.20588235
0.07911785	1	24.171875
0.154991497	1	18.76923077
0.095484356	1	20.05952381
0.097128121	1	23.74853801
0.041592148	1	41.54748603
0.122824752	1	20.29059829
0.137183718	1	20.58125
0.181965352	1	13.08602151
0.100205722	1	20.88050314
0.321578283	1	5.545454545
0.118457244	1	20.5971223
0.089401115	1	22.11888112
0.109664757	1	21.16842105
0.123875714	1	20.01851852
0.111696783	1	16.03125
0.101181693	1.018518519	21.49382716
0.098955926	1.019230769	22.16746795
0.086160751	1	22.26282051
0.089715661	1.011583012	22.40540541
0.044155681	1	39.86956522
0.216946565	1	14.30534351
0.184771777	1	16.68965517
0.188900463	1	12.45833333
0.105580864	1	17.68246445

log.sigma.0.5.mm.3D_gldzm_LowIntensityLargeDistanceEmphasis	log.sigma.0.5.mm.3D_gldzm_HighIntensityEmphasis	log.sigma.0.5.mm.3D_gldzm_DistanceZoneVariability	log.sigma.0.5.mm.3D_gldzm_ZonePercentage
0.043270913	41.71264368	172.0114943	0.081998115
0.253708333	11.425	40	0.042780749
0.20739418	13.19047619	63	0.0410691
0.12430035	18.98181818	55	0.05629478
0.102560631	18.125	128	0.054054054
0.136801724	23.42592593	54	0.081818182
0.085950299	21.85384615	258.0076923	0.046009556
0.125160701	19.57664234	274	0.053641347
0.102873538	20.67465753	292	0.064232292
0.117895025	20.08235294	85	0.079291045
0.217144608	13.97058824	68	0.045092838
0.071042811	28.30681818	176	0.076355748
0.150813492	19.86363636	66	0.03535083
0.068539981	30.54736842	95	0.055329062
0.064251973	29.76923077	156	0.078629032
0.125634663	19.09625668	187	0.04273309
0.154598765	16.87301587	63	0.052763819
0.149703737	15.17391304	46	0.03730738
0.093032372	21.40298507	67	0.051617874
0.152288342	20.77810651	336.0059172	0.065012502
0.160189594	14.12698413	63	0.020833333
0.231827257	13.03125	64	0.05931418
0.09693695	21.40677966	59	0.057617188
0.102374783	21.47794118	136	0.05137892
0.117172097	21.73684211	76	0.059701493
0.053514028	33.95876289	194	0.081891093
0.124032348	21.40106952	187	0.057841015
0.118311144	20.55714286	140	0.050687907
0.189017857	14.71428571	28	0.062639821
0.132133273	17.87628866	97	0.047224927
0.0692265	27.32340426	235	0.0493905
0.114100456	17.75641026	78	0.037699372
0.101628544	24.14736842	190	0.058841747
0.134371407	20.66901408	142	0.060425532
0.100346285	21.9760274	292	0.066303361
0.114671295	19.79674797	123	0.041013671
0.04936638	37.11931818	176	0.047299113
0.076419111	29.92814371	167	0.03235807
0.127726591	18.11764706	68	0.055284553
0.208795139	14.78	398.005	0.055020633
0.230872585	13.51086957	92	0.035466461
0.188950642	16.02491103	281	0.072666149
0.107183852	20.29473684	190	0.052012045
0.136022968	20.14516129	186	0.074758842
0.071228823	30.07865169	89	0.069640063
0.214608175	15.60377358	159	0.057923497
0.095247756	23.09363296	267	0.058526962
0.244323899	13.8490566	53	0.060849598
0.251700275	14.35714286	70	0.082256169
0.098164862	19.73239437	71	0.052514793
0.128019396	21.68786127	173	0.067341378
0.090068668	23.5095057	263	0.078018392
0.092848486	21.13357401	277	0.061391844
0.242309671	13.35185185	54	0.048913043
0.113750693	21.04444444	90	0.067669173
0.249819902	13.25274725	91	0.046666667
0.096683147	20.5	234	0.057849197
0.124827324	20.1	150	0.043377675
0.113356493	20.50833333	120	0.072815534
0.112755461	23.02729045	513	0.056194545
0.110751295	21.55670103	97	0.054926387
0.08354653	29.03636364	165	0.061797753
0.058916224	30.28409091	348.0227273	0.060804975
0.134477707	20.76604555	477.0372671	0.063360882
0.171227599	12.5483871	31	0.039240506
0.156644102	19.06179775	174.0449438	0.046090109
0.148524445	18.33333333	66	0.050613497
0.058787508	28.18662953	355.0222841	0.05744
0.049951453	31.57234043	470	0.08024586
0.109662085	17.92783505	97	0.03347136
0.265281924	11.71641791	67	0.051578137
0.146642394	19	211.0093897	0.04965035
0.100805	21.35483871	124	0.054409829
0.106563422	21.67320261	153	0.067729084
0.21090406	14.75238095	208.0095238	0.067071223
0.252504085	13.20588235	68	0.050670641
0.07911785	24.171875	128	0.053422371
0.154991497	18.76923077	52	0.054279749
0.095484356	20.05952381	84	0.049209139
0.097128121	23.74853801	171	0.073108166
0.041592148	41.54748603	179	0.089994972
0.122824752	20.29059829	117	0.065036131
0.137183718	20.58125	160	0.048944631
0.181965352	13.08602151	93	0.030263586
0.100205722	20.88050314	159	0.065190652
0.321578283	5.545454545	22	0.014360313
0.118457244	20.5971223	139	0.058280922
0.089401115	22.11888112	143	0.052151714
0.109664757	21.16842105	95	0.06489071
0.123875714	20.01851852	108	0.053333333
0.111696783	16.03125	32	0.024539877
0.101696096	21.66049383	160.0123457	0.068010076
0.099419252	22.37179487	308.025641	0.060196797
0.086160751	22.26282051	156	0.047272727
0.090037412	22.50965251	257.007722	0.059512868
0.044155681	39.86956522	207	0.060490941
0.216946565	14.30534351	131	0.046503372
0.184771777	16.68965517	58	0.055396371
0.188900463	12.45833333	24	0.028776978
0.105580864	17.68246445	211	0.036543124

log.sigma.0.5.mm.3D_gldzm_IntensityVariabilityNormalized	log.sigma.0.5.mm.3D_gldzm_LowIntensitySmallDistanceEmphasis	log.sigma.0.5.mm.3D_gldzm_IntensityVariability
0.220306513	0.04192393	38.33333333
0.27	0.253708333	10.8
0.310657596	0.20739418	19.57142857
0.257520661	0.12430035	14.16363636
0.303955078	0.102560631	38.90625
0.211248285	0.136801724	11.40740741
0.320473373	0.084347735	83.32307692
0.248548138	0.125160701	68.10218978
0.295505723	0.102873538	86.28767123
0.250103806	0.117895025	21.25882353
0.306228374	0.217144608	20.82352941
0.20745093	0.071042811	36.51136364
0.203397612	0.150813492	13.42424242
0.242548476	0.068539981	23.04210526
0.245397765	0.064251973	38.28205128
0.235265521	0.125634663	43.99465241
0.294532628	0.154598765	18.55555556
0.280718336	0.149703737	12.91304348
0.345956783	0.093032372	23.17910448
0.243828998	0.141193667	82.41420118
0.310657596	0.160189594	19.57142857
0.346679688	0.231827257	22.1875
0.295604711	0.09693695	17.44067797
0.257893599	0.102374783	35.07352941
0.246191136	0.117172097	18.71052632
0.189658837	0.053514028	36.79381443
0.227086848	0.124032348	42.46524064
0.262857143	0.118311144	36.8
0.323979592	0.189017857	9.071428571
0.311085131	0.132133273	30.17525773
0.239909461	0.0692265	56.3787234
0.319526627	0.114100456	24.92307692
0.243933518	0.101628544	46.34736842
0.212854592	0.134371407	30.22535211
0.253049353	0.100346285	73.89041096
0.255998414	0.114671295	31.48780488
0.197766012	0.04936638	34.80681818
0.213596759	0.076419111	35.67065868
0.312283737	0.127726591	21.23529412
0.3266375	0.199420139	130.655
0.315926276	0.230872585	29.06521739
0.264041742	0.188950642	74.19572954
0.261218837	0.107183852	49.63157895
0.232281189	0.136022968	43.20430108
0.242772377	0.071228823	21.60674157
0.228353309	0.214608175	36.3081761
0.236319769	0.095247756	63.09737828
0.263082948	0.244323899	13.94339623
0.229387755	0.251700275	16.05714286
0.302122595	0.098164862	21.45070423
0.242607504	0.128019396	41.97109827
0.271176394	0.090068668	71.31939163
0.320830455	0.092848486	88.8700361
0.27914952	0.242309671	15.07407407
0.249382716	0.113750693	22.44444444
0.35032001	0.249819902	31.87912088
0.298013003	0.096683147	69.73504274
0.286933333	0.124827324	43.04
0.266388889	0.113356493	31.96666667
0.240586847	0.112755461	123.4210526
0.229036029	0.110751295	22.21649485
0.231955923	0.08354653	38.27272727
0.232889979	0.057066674	81.97727273
0.26630917	0.131458383	128.6273292
0.329864724	0.171227599	10.22580645
0.249779068	0.135146739	44.46067416
0.272268136	0.148524445	17.96969697
0.269155267	0.057481797	96.62674095
0.207487551	0.049951453	97.51914894
0.322350941	0.109662085	31.26804124
0.294274894	0.265281924	19.71641791
0.240141947	0.144686213	51.15023474
0.249219563	0.100805	30.90322581
0.230039728	0.106563422	35.19607843
0.294013605	0.206439774	61.74285714
0.334775087	0.252504085	22.76470588
0.274902344	0.07911785	35.1875
0.24704142	0.154991497	12.84615385
0.286848073	0.095484356	24.0952381
0.278342054	0.097128121	47.59649123
0.212758653	0.041592148	38.08379888
0.221710863	0.122824752	25.94017094
0.22421875	0.137183718	35.875
0.378887733	0.181965352	35.23655914
0.254538982	0.100205722	40.47169811
0.495867769	0.321578283	10.90909091
0.244656074	0.118457244	34.00719424
0.258154433	0.089401115	36.91608392
0.250304709	0.109664757	23.77894737
0.274348422	0.123875714	29.62962963
0.37890625	0.111696783	12.125
0.28494132	0.101053092	46.16049383
0.27681213	0.098840094	86.36538462
0.299556213	0.086160751	46.73076923
0.332091054	0.089635224	86.01158301
0.202408458	0.044155681	41.89855072
0.288736088	0.216946565	37.82442748
0.253269917	0.184771777	14.68965517
0.395833333	0.188900463	9.5
0.29157027	0.105580864	61.52132701

log.sigma.0.5.mm.3D_gldzm_HighIntensityLargeDistanceEmphasis	log.sigma.0.5.mm.3D_gldzm_SmallDistanceEmphasis	log.sigma.0.5.mm.3D_glcm_SumVariance	log.sigma.0.5.mm.3D_glcm_Homogeneity1
41.98850575	0.995689655	116.1530476	0.636183187
11.425	1	30.45638386	0.725637783
13.19047619	1	30.26808771	0.731743804
18.98181818	1	48.91528965	0.691486366
18.125	1	51.85237694	0.701897802
23.42592593	1	47.95310269	0.652146826
21.95769231	0.997115385	53.19870536	0.703129434
19.57664234	1	53.57800352	0.710156287
20.67465753	1	52.01135393	0.695133368
20.08235294	1	51.04513996	0.680574715
13.97058824	1	29.19539917	0.726445589
28.30681818	1	82.50373073	0.670213501
19.86363636	1	53.843685	0.718386495
30.54736842	1	82.63651241	0.686664183
29.76923077	1	81.20694083	0.666960875
19.09625668	1	53.14581428	0.714049955
16.87301587	1	26.24161457	0.682806735
15.17391304	1	55.72156188	0.72970899
21.40298507	1	53.79838509	0.709930434
20.78698225	0.997781065	50.77776724	0.681963273
14.12698413	1	30.94536562	0.739171753
13.03125	1	27.82752037	0.685918405
21.40677966	1	52.65285027	0.698188505
21.47794118	1	52.8873912	0.700479835
21.73684211	1	52.97617546	0.693385123
33.95876289	1	82.49681109	0.675455776
21.40106952	1	50.56880379	0.676280138
20.55714286	1	52.05370359	0.70358598
14.71428571	1	27.54084159	0.676833932
17.87628866	1	54.80514442	0.722196503
27.32340426	1	86.6546588	0.711006104
17.75641026	1	52.41153564	0.712455146
24.14736842	1	49.7792364	0.695943217
20.66901408	1	50.50134609	0.687652716
21.9760274	1	51.9875112	0.686561534
19.79674797	1	53.68653886	0.71149028
37.11931818	1	123.1281738	0.694584278
29.92814371	1	84.41059044	0.714130302
18.11764706	1	52.34757068	0.708624265
14.7875	0.998125	26.05817686	0.689264873
13.51086957	1	28.05363416	0.714421584
16.02491103	1	24.14494845	0.648429806
20.29473684	1	52.65668558	0.703114412
20.14516129	1	48.59636355	0.661074874
30.07865169	1	86.1398362	0.701800692
15.60377358	1	28.19584641	0.694480512
23.09363296	1	51.66691795	0.691996085
13.8490566	1	30.00934443	0.725110409
14.35714286	1	25.98315982	0.661206648
19.73239437	1	55.31490341	0.716922641
21.68786127	1	51.04698821	0.673932004
23.5095057	1	49.12774503	0.660866284
21.13357401	1	50.97740862	0.680372766
13.35185185	1	29.9310889	0.722363342
21.04444444	1	52.65806047	0.691397972
13.25274725	1	28.12618753	0.709757964
20.5	1	51.83696879	0.688727078
20.1	1	51.32532733	0.703609344
20.50833333	1	51.59284108	0.695089958
23.02729045	1	47.75554458	0.661247792
21.55670103	1	54.18532371	0.713195214
29.03636364	1	83.60922342	0.678980853
30.49715909	0.995738636	84.51053671	0.685059339
21.07039337	0.995341615	50.4830391	0.693264242
12.5483871	1	31.05397322	0.740404836
19.90449438	0.991573034	52.05564844	0.70401872
18.33333333	1	51.82236415	0.697177444
28.454039	0.995821727	81.99337708	0.684630206
31.57234043	1	84.49679712	0.694102003
17.92783505	1	55.36586907	0.729423502
11.71641791	1	27.8097144	0.703075191
19.12676056	0.996478873	53.82975709	0.712139534
21.35483871	1	51.56878235	0.698136447
21.67320261	1	47.25274347	0.647572075
14.80952381	0.996428571	25.20882625	0.660628626
13.20588235	1	27.61616256	0.70268457
24.171875	1	55.12695585	0.721968106
18.76923077	1	52.57206076	0.703039622
20.05952381	1	53.35047559	0.709574523
23.74853801	1	48.00399083	0.672826747
41.54748603	1	116.9756691	0.643103828
20.29059829	1	49.02843717	0.683345194
20.58125	1	52.38332587	0.705003441
13.08602151	1	30.18700564	0.742107518
20.88050314	1	49.58303363	0.678899205
5.545454545	1	33.91387724	0.776104279
20.5971223	1	48.69807216	0.677643091
22.11888112	1	54.14862161	0.702474749
21.16842105	1	53.58060893	0.713915352
20.01851852	1	54.72891768	0.724579568
16.03125	1	57.02195375	0.732382034
22.32716049	0.99537037	51.62336052	0.673217642
23.18910256	0.995192308	49.31859322	0.676024847
22.26282051	1	54.16443497	0.715072993
22.92664093	0.997104247	49.34790813	0.67949907
39.86956522	1	123.2786987	0.686118523
14.30534351	1	26.99524496	0.701062405
16.68965517	1	50.06003647	0.683741914
12.45833333	1	29.70662316	0.732582015
17.68246445	1	55.90615563	0.723285025

log.sigma.0.5.mm.3D_glcm_Homogeneity2	log.sigma.0.5.mm.3D_glcm_ClusterShade	log.sigma.0.5.mm.3D_glcm_MaximumProbability	log.sigma.0.5.mm.3D_glcm_Idmn	log.sigma.0.5.mm.3D_glcm_SumVariance2
0.607201363	-1.323446986	0.240661285	0.979306314	1.86234212
0.716058858	-0.353236223	0.374333537	0.979404707	0.848673612
0.723649454	-0.249418879	0.367132667	0.980958817	0.819052919
0.671835528	-1.311456952	0.310440964	0.976600665	1.402422787
0.685230514	-0.915319751	0.350608201	0.979972025	1.156339604
0.628077985	-0.264541766	0.245252269	0.978123144	1.73767989
0.690423181	-0.257896296	0.312116309	0.986222703	1.0139094
0.695190724	-0.619107995	0.367898403	0.984795718	1.123570315
0.677468073	-0.752143095	0.332839541	0.983418302	1.182692211
0.661393984	-0.830931544	0.306978691	0.976746712	1.312540567
0.716692939	-0.465929596	0.350835816	0.979480353	0.840924142
0.64746434	-1.519658725	0.309889019	0.982413325	1.595632594
0.707471608	-0.605429484	0.345842445	0.982362357	1.007083156
0.668147034	-1.220119421	0.318092782	0.981429548	1.434225138
0.642911852	-1.31038443	0.301933392	0.985071792	1.616961384
0.702089256	-0.654790792	0.337438302	0.986066199	1.029963562
0.665408091	-0.150550524	0.254512466	0.972377007	1.169830298
0.720681123	-0.504707979	0.379267317	0.984509333	0.888202344
0.69863821	-0.224643018	0.319485364	0.983326109	0.898431267
0.663745118	-0.690919259	0.296855651	0.982164809	1.350410675
0.733285623	-0.188128245	0.36452196	0.982749797	0.70591604
0.668961338	-0.509560263	0.308070764	0.962536767	1.066035858
0.683753049	-0.471813321	0.31187006	0.980775435	1.048154043
0.686739535	-0.276923464	0.293979449	0.981131623	1.071021943
0.677697874	-0.628216919	0.321862072	0.979086701	1.197952363
0.653514953	-0.377635694	0.299397986	0.983736548	1.626077343
0.655791776	-0.624967606	0.286736133	0.980627154	1.438392055
0.689184968	-0.840308262	0.328355133	0.980142134	1.138402124
0.658292107	-0.310241766	0.282790738	0.971920461	1.114318381
0.709730323	-0.76915853	0.387228331	0.982622581	0.955955802
0.697115618	-0.881751862	0.358744443	0.987701398	1.141082126
0.699182942	-0.775343442	0.345065991	0.981641167	0.992297794
0.677685791	-0.276881316	0.297555012	0.986062265	1.384730914
0.669339095	-1.251280863	0.314882571	0.981376961	1.437062574
0.667367297	-0.659824021	0.32349685	0.982172031	1.340868122
0.700171362	-0.421727972	0.330240207	0.989060853	1.003167873
0.679484252	-1.010663086	0.296639241	0.988123796	1.25461533
0.70234424	-0.55993493	0.309616495	0.988539544	1.0939984
0.694184971	-0.750203488	0.337179111	0.980352898	1.008066598
0.67269785	-0.226007836	0.254759157	0.973157693	1.14209147
0.702517022	-0.480695366	0.332696258	0.977504738	0.943539738
0.62284681	-0.037158674	0.192536909	0.973364173	1.521011585
0.687890031	-0.709020028	0.336971767	0.980491082	1.107314762
0.636868297	-1.399377904	0.282037241	0.971493749	1.579320358
0.685421512	-0.994143636	0.365292429	0.986198154	1.267147721
0.679331804	-0.174125503	0.307488777	0.984257025	1.155529689
0.675001793	-0.377447503	0.314046956	0.983325263	1.246599741
0.712464172	-0.564863367	0.401990216	0.977302013	0.893386081
0.636313559	-0.961982688	0.301285854	0.972385392	1.637451472
0.70482551	-0.460300715	0.363135861	0.983137081	0.964797903
0.653632305	-0.775517585	0.298165519	0.980814051	1.471636968
0.637496678	-0.356580395	0.251506432	0.980178936	1.451887236
0.662448285	-0.474453864	0.29020221	0.97868835	1.178295861
0.711427122	-0.158194974	0.358439993	0.978248498	0.920964405
0.674206107	-0.366195978	0.32739922	0.978385811	1.30650241
0.69762842	-0.623352008	0.333356043	0.97668011	0.986369926
0.670418986	-0.589483522	0.313638851	0.983146591	1.212661894
0.690249572	-0.690548755	0.306526791	0.98094147	1.07094414
0.678236922	-0.79322713	0.323416713	0.983418939	1.170181914
0.639108356	-0.437748398	0.212847752	0.979919524	1.443053324
0.699641517	-0.473519376	0.36291514	0.98510532	1.084987133
0.658631674	-1.160626862	0.30887321	0.986700708	1.422886297
0.666359197	-0.628881749	0.311876385	0.985683135	1.361403307
0.675885295	-0.684171305	0.312662149	0.982803856	1.271892625
0.734686116	-0.321791962	0.379682673	0.976022786	0.683100676
0.68981829	-0.797093734	0.3284286	0.984307321	1.111033823
0.682182595	-0.931522613	0.319655209	0.97906903	1.087244622
0.665735989	-0.772099496	0.300851329	0.985592712	1.315175103
0.676697696	-0.814793019	0.328928901	0.98882302	1.260872925
0.721594733	-0.409344686	0.350667631	0.980555641	0.785274495
0.688427218	-0.618318295	0.330747645	0.974657396	1.060375988
0.698209435	-0.697168229	0.360766848	0.984723753	1.155481505
0.681905199	-0.533367392	0.32174455	0.983731502	1.224545825
0.620862113	-0.94421346	0.244627104	0.977082523	1.671118135
0.638932614	-0.35033272	0.236292667	0.975685541	1.346376056
0.687683092	-0.759244041	0.334990795	0.974452992	1.055005993
0.71154881	-0.015933692	0.351080048	0.987676425	0.902259261
0.688838096	-0.904104989	0.335642513	0.980002901	1.067069442
0.697280674	-0.452740639	0.32370658	0.982543539	0.951568651
0.650145117	-0.717957075	0.266312769	0.979478908	1.493425263
0.61586506	-1.269238047	0.248802343	0.980146288	1.799855412
0.662675128	-0.920926529	0.304532109	0.980779364	1.34459645
0.690773198	-0.6123077	0.320489726	0.984410621	1.105439157
0.735809196	-0.265228689	0.355836589	0.975516469	0.721758038
0.657955319	-0.871815111	0.298496047	0.975571453	1.368358728
0.773083166	-0.366274929	0.450931252	0.980516429	0.564751059
0.657441224	-0.856556006	0.280921889	0.981179729	1.340705513
0.687919807	-0.516315858	0.342361596	0.980773383	1.132629202
0.700533061	-0.867946473	0.363001545	0.980982966	1.068615005
0.711984418	-0.874312533	0.394416942	0.98225824	0.988873571
0.726762428	-0.085550995	0.35595533	0.987011467	0.744462046
0.653669925	-0.415725678	0.267841066	0.977384023	1.282081908
0.656261405	-0.611231538	0.282718094	0.98174439	1.315992403
0.70304273	-0.594907136	0.356860047	0.98274034	1.010738181
0.660176788	-0.388351719	0.264652904	0.982427203	1.243645743
0.667402591	-1.382452733	0.321564318	0.987199636	1.493428202
0.686456571	-0.404319405	0.301332698	0.975011702	1.058090464
0.664348776	-1.04850622	0.306937061	0.975231976	1.36002373
0.72407161	-0.424493157	0.365756282	0.973354861	0.760541377
0.714287145	-0.20596688	0.353617486	0.984613363	0.856737795

log.sigma.0.5.mm.3D_glcm_Contrast	log.sigma.0.5.mm.3D_glcm_DifferenceEntropy	log.sigma.0.5.mm.3D_glcm_InverseVariance	log.sigma.0.5.mm.3D_glcm_Entropy	log.sigma.0.5.mm.3D_glcm_Dissimilarity
1.827658741	1.798459537	0.474387183	3.825799898	0.951264434
0.802063614	1.343279414	0.468612691	2.666778317	0.606653006
0.733129592	1.295402032	0.469461937	2.643086868	0.582626884
1.271783524	1.60428046	0.44806399	3.285034014	0.756136942
1.061755491	1.511559399	0.455942647	3.034069426	0.70078588
1.529516183	1.682168363	0.487697134	3.553626262	0.870808926
0.928050549	1.419808519	0.487573794	2.975303024	0.670146286
1.042565417	1.49525362	0.45227545	2.986392488	0.6801057
1.137220332	1.5463624	0.459969746	3.145222779	0.725538404
1.254468994	1.584551374	0.474689892	3.267113329	0.77134038
0.797129049	1.345499369	0.465814482	2.680756805	0.604912862
1.548114576	1.699659371	0.459824654	3.440619454	0.839075917
0.94440639	1.409567285	0.468838505	2.802246852	0.64290641
1.302640832	1.600113259	0.465799778	3.300169662	0.765358904
1.596834189	1.721514669	0.454959292	3.51340206	0.855580177
0.951273587	1.428840369	0.469746629	2.899453209	0.653570751
1.095117851	1.510076155	0.488862151	3.227183575	0.739660695
0.812811023	1.343918485	0.463911776	2.653465573	0.600267173
0.868153964	1.378878695	0.486126564	2.8325151	0.646542903
1.235327996	1.574159082	0.479858277	3.279216658	0.763257715
0.656544657	1.231478148	0.473192912	2.482226935	0.553897097
1.06396094	1.495782694	0.486805764	3.078315839	0.728693465
1.017350393	1.468243576	0.481839873	3.004781641	0.695603926
0.9992491	1.456271746	0.48292835	3.057365659	0.687389677
1.125776092	1.51288142	0.478780814	3.130558691	0.722306299
1.417904062	1.660040347	0.45938522	3.515033662	0.80931639
1.349473504	1.627159351	0.470676616	3.4336912	0.795202702
1.065334721	1.491575979	0.469248262	3.038187293	0.693686827
1.107543179	1.511627846	0.49233154	3.170055647	0.753761678
0.91911818	1.428253876	0.450216669	2.763119105	0.635947755
1.061885589	1.488699145	0.458025969	2.983672972	0.678801617
0.974821476	1.45252539	0.462440209	2.858539898	0.662607624
1.204521685	1.574150763	0.45172295	3.318105288	0.735014696
1.306048919	1.600073321	0.465016144	3.316199309	0.764301991
1.230094476	1.586788512	0.463100544	3.299554723	0.757269382
0.932218871	1.409793734	0.480540221	2.889673275	0.653572881
1.279068309	1.550792314	0.476286623	3.135016121	0.739484054
0.985815988	1.4356802	0.469843056	2.979824501	0.657824947
1.055470693	1.491201675	0.458935295	2.913927785	0.683581482
1.064896309	1.502272797	0.48276455	3.199201679	0.722319361
0.87994402	1.398334206	0.471805708	2.84669283	0.642240868
1.426181438	1.660490911	0.487532651	3.636049218	0.865131625
1.036228713	1.490736815	0.465109574	3.042005246	0.691722912
1.583690251	1.716205322	0.466605249	3.533710463	0.863111548
1.201968588	1.55193572	0.453795312	3.06042998	0.720408943
1.077768896	1.496798349	0.482132294	3.14045531	0.712467471
1.145808611	1.540788724	0.47114192	3.185901846	0.73086989
0.895801924	1.418377084	0.443571741	2.672350197	0.628212222
1.515252542	1.700471347	0.463471744	3.548071262	0.854362534
0.881797579	1.402223585	0.465186109	2.838597651	0.638442253
1.334530673	1.620394327	0.477530644	3.389306512	0.796305906
1.365169225	1.639450729	0.480561472	3.516050635	0.829617697
1.128257055	1.529824756	0.488880889	3.205683498	0.749730188
0.853058748	1.380229484	0.463493303	2.78555524	0.622802783
1.162531528	1.545491065	0.470358811	3.167751852	0.734635021
0.919366148	1.414836098	0.47839096	2.870191903	0.656681967
1.152838418	1.552002479	0.467865647	3.221982837	0.740199127
1.014780878	1.462496146	0.478660977	3.005947462	0.684070388
1.139158452	1.537793912	0.465952866	3.092769122	0.724543944
1.392995378	1.644437022	0.487990039	3.533731674	0.830624103
1.024177356	1.472337379	0.457115644	2.925681927	0.669374166
1.43012748	1.645780139	0.464374023	3.37100031	0.799102145
1.23571665	1.582016408	0.469828882	3.309496788	0.759192722
1.193088322	1.561146831	0.46424894	3.196190878	0.735827757
0.645558835	1.219844988	0.47227224	2.397910791	0.549782946
1.086666205	1.495259445	0.46932049	3.011395222	0.695037655
1.134472924	1.514885123	0.475837787	2.994455783	0.71577042
1.2424675	1.586846646	0.468949182	3.28584988	0.761664149
1.176782485	1.556759012	0.462804252	3.207341057	0.732270161
0.754223493	1.303088953	0.475468594	2.590759918	0.589419515
1.00660929	1.468748149	0.470414291	2.975485926	0.686531295
1.055623363	1.487376133	0.455495259	2.994137397	0.676356227
1.118878815	1.526107514	0.465062308	3.162627962	0.714765773
1.599103168	1.72777708	0.474913669	3.689271404	0.895203788
1.298759627	1.602356329	0.495719173	3.445361852	0.816899911
1.015456314	1.47672194	0.468292549	2.972831751	0.689194091
0.826827362	1.364964713	0.46911472	2.742713677	0.618236387
1.075003953	1.488002494	0.471506552	2.92271124	0.695828428
0.915618686	1.41537851	0.477729795	2.868595208	0.656515551
1.435079167	1.67079789	0.458570686	3.507048077	0.818329065
1.753065481	1.771183257	0.475669535	3.75680314	0.924478658
1.337982513	1.629374311	0.45499827	3.295329866	0.78228529
1.07635167	1.493422414	0.467305584	3.032132074	0.692249143
0.665232673	1.243899382	0.463743308	2.50424916	0.551090486
1.323182332	1.623193294	0.462590347	3.359200961	0.788414694
0.520436025	1.119676313	0.422782794	2.041592752	0.46493406
1.303663352	1.611113785	0.471498215	3.356924578	0.785625635
1.019582468	1.476757607	0.471975114	3.054602304	0.68899104
1.021530919	1.469014346	0.457011792	2.904263943	0.667359026
0.946586741	1.437658933	0.44342706	2.800813851	0.636165618
0.663313531	1.217658499	0.489030352	2.515482327	0.565715317
1.203112811	1.564631272	0.48897587	3.340352874	0.776648814
1.257562025	1.592563612	0.479726995	3.337886628	0.780433266
0.907452371	1.413802089	0.468587741	2.900971587	0.645468015
1.204426189	1.571920877	0.477673955	3.313655209	0.765506181
1.370896833	1.620518112	0.463316387	3.325602442	0.776346362
0.985857707	1.462171165	0.475647176	3.039149822	0.686538686
1.369257122	1.622684871	0.462936476	3.290051982	0.78234719
0.727677959	1.296379402	0.46484059	2.537467779	0.581123372
0.801118257	1.33569338	0.477853826	2.709365731	0.609309908

log.sigma.0.5.mm.3D_glcm_DifferenceVariance	log.sigma.0.5.mm.3D_glcm_Idn	log.sigma.0.5.mm.3D_glcm_Idm	log.sigma.0.5.mm.3D_glcm_Correlation	log.sigma.0.5.mm.3D_glcm_Autocorrelation
0.921761353	0.912130809	0.607201363	0.009552219	43.12252749
0.433372152	0.916647882	0.716058858	0.02679919	13.37219697
0.392448692	0.919355687	0.723649454	0.05444034	13.32904868
0.698038871	0.912053909	0.671835528	0.049123403	20.72801465
0.569141653	0.917140673	0.685230514	0.043025393	21.15033924
0.76463256	0.909972613	0.628077985	0.061371308	21.0690405
0.478226084	0.928282949	0.690423181	0.044424792	21.41339095
0.579411741	0.928191487	0.695190724	0.037537803	21.62360862
0.610198588	0.923678978	0.677468073	0.019568447	21.34629375
0.656398969	0.909782926	0.661393984	0.022806834	21.35434849
0.430552041	0.916864633	0.716692939	0.02629893	12.94240375
0.842672173	0.921954058	0.64746434	0.015315973	31.89734821
0.529504049	0.923443648	0.707471608	0.032492409	21.2757557
0.716213507	0.920375799	0.668147034	0.047785741	31.52937768
0.862901532	0.927385583	0.642911852	0.007370596	31.5481492
0.523484912	0.930457017	0.702089256	0.039798318	21.27645253
0.545923383	0.90035671	0.665408091	0.031393184	12.79121367
0.451511483	0.92772123	0.720681123	0.04403543	21.60804045
0.448459811	0.92212901	0.69863821	0.017599115	21.22286133
0.651836407	0.920033698	0.663745118	0.04440029	21.366735
0.349443545	0.922656774	0.733285623	0.035476006	13.19362295
0.531316951	0.886122901	0.668961338	0.001721899	13.06379173
0.532643361	0.917246721	0.683753049	0.014058457	21.2602969
0.525149891	0.918127475	0.686739535	0.035657539	21.47710504
0.601590015	0.914852248	0.677697874	0.029697638	21.70999209
0.76107461	0.92416631	0.653514953	0.065531079	32.13203388
0.71539932	0.917310743	0.655791776	0.032056397	21.50271234
0.58364355	0.918036904	0.689184968	0.03273055	21.19458034
0.53869969	0.898380422	0.658292107	0.00245505	13.10275725
0.514247524	0.924189094	0.709730323	0.018851789	21.42401116
0.600674818	0.935326809	0.697115618	0.035674917	31.91632071
0.535228641	0.921214729	0.699182942	0.009615472	20.82569612
0.662969829	0.930466305	0.677685791	0.068649755	21.1426222
0.721061985	0.920541118	0.669339095	0.047533941	21.34606741
0.656014263	0.920755494	0.667367297	0.042534461	21.78077484
0.50418075	0.937011054	0.700171362	0.036972281	21.41938529
0.731500573	0.936431199	0.679484252	-0.00990695	43.23008285
0.552034777	0.936960524	0.70234424	0.052237777	31.28579274
0.587643754	0.919298258	0.694184971	-0.022621902	20.9036774
0.542644036	0.90259749	0.67269785	0.034889726	12.69866289
0.466648494	0.912297364	0.702517022	0.035083725	12.82887957
0.676524138	0.899215306	0.62284681	0.032270519	12.63188023
0.556796514	0.918019159	0.687890031	0.033350867	21.37893098
0.837408033	0.901102569	0.636868297	-0.001186234	20.95356952
0.680536332	0.93196055	0.685421512	0.026915276	32.04079189
0.568827213	0.924628272	0.679331804	0.03342743	13.36549568
0.610948492	0.923095791	0.675001793	0.041552615	21.39353549
0.499895614	0.914771145	0.712464172	-0.001023273	13.2049159
0.783014251	0.901586068	0.636313559	0.038230213	13.22664679
0.47307669	0.923419576	0.70482551	0.043756468	21.8356297
0.70002499	0.917030125	0.653632305	0.048833333	21.7058578
0.675132845	0.913414328	0.637496678	0.03118642	21.17139325
0.565251491	0.911264751	0.662448285	0.021921174	21.13514842
0.464402131	0.914913656	0.711427122	0.038566502	13.41288207
0.620971921	0.913659557	0.674206107	0.056876017	21.8109638
0.487252778	0.910598434	0.69762842	0.035265607	12.94704172
0.604065507	0.922083349	0.670418986	0.025617625	21.44743041
0.546389057	0.918747585	0.690249572	0.027072654	20.84546901
0.613562657	0.923797722	0.678236922	0.01401524	21.11672366
0.702748497	0.913501697	0.639108356	0.017631998	20.69281313
0.575179872	0.929270414	0.699641517	0.02880341	21.63130569
0.789332501	0.931686404	0.658631674	-0.001062184	31.8364859
0.658297273	0.928114476	0.666359197	0.048330112	32.11753545
0.650708408	0.922914596	0.675885295	0.031819981	21.04873275
0.342263508	0.910602292	0.734686116	0.027895192	13.08237522
0.602305249	0.926751948	0.68981829	0.01135747	21.0939805
0.620943559	0.915803466	0.682182595	-0.020261396	20.8602653
0.661533864	0.927911881	0.665735989	0.028277375	31.20649223
0.640095101	0.936581243	0.676697696	0.034399741	31.80382818
0.406487953	0.918575362	0.721594733	0.019797598	21.22113447
0.532709788	0.907295371	0.688427218	0.026958045	12.96752702
0.597338866	0.928718123	0.698209435	0.045082742	21.75916875
0.606642181	0.92476088	0.681905199	0.04472564	21.30178234
0.796425113	0.907777053	0.620862113	0.021853349	20.85541485
0.629782214	0.904183843	0.638932614	0.017773368	12.7091967
0.538335014	0.907020262	0.687683092	0.019652573	12.82012633
0.444217193	0.933531647	0.71154881	0.043148628	21.54080317
0.589051077	0.917848039	0.688838096	-0.00246375	21.07472576
0.483850206	0.921346127	0.697280674	0.019758427	21.18818563
0.764262667	0.915372331	0.650145117	0.019508432	20.78498681
0.897376611	0.914402541	0.61586506	0.013142838	43.21583239
0.724052692	0.918776853	0.662675128	0.003224813	20.68863614
0.596096569	0.927006397	0.690773198	0.014309533	21.24222485
0.360410522	0.910768644	0.735809196	0.040951802	12.98440559
0.699000765	0.908306383	0.657955319	0.01746954	20.98252264
0.301399407	0.923783964	0.773083166	0.041112517	13.31232223
0.684624082	0.918046571	0.657441224	0.014245401	20.65372516
0.544085974	0.918179496	0.687919807	0.051899098	21.95464224
0.575462093	0.921085236	0.700533061	0.022312803	21.42747663
0.540005094	0.924461623	0.711984418	0.022723353	21.5003232
0.341984039	0.930603294	0.726762428	0.05729767	21.68394462
0.59872889	0.908506641	0.653669925	0.031950795	21.64449392
0.648170097	0.918238207	0.656261405	0.02263949	20.86821165
0.490037162	0.922748825	0.70304273	0.053586204	21.62591657
0.617957525	0.919535855	0.660176788	0.016098677	20.78198626
0.766945626	0.933523213	0.667402591	0.042422326	44.13977094
0.514046202	0.906996567	0.686456571	0.035459861	12.77720442
0.754688433	0.909507856	0.664348776	-0.002380163	20.95561993
0.388870123	0.906542927	0.72407161	0.022600794	12.84288948
0.429208354	0.926359195	0.714287145	0.032684505	21.70315708

log.sigma.0.5.mm.3D_glcm_SumEntropy	log.sigma.0.5.mm.3D_glcm_AverageIntensity	log.sigma.0.5.mm.3D_glcm_Energy	log.sigma.0.5.mm.3D_glcm_SumSquares	log.sigma.0.5.mm.3D_glcm_ClusterProminence
2.441599232	6.566112018	0.116769426	0.922500215	14.28657459
1.869255276	3.655183807	0.228016333	0.412684307	3.13580005
1.869467362	3.647937294	0.225132131	0.388045628	2.497213102
2.20554352	4.54919686	0.164363998	0.668551578	8.882098836
2.072756	4.596361778	0.192863743	0.554523774	5.773410919
2.371097972	4.584396145	0.135929384	0.816799018	14.48522115
2.02641476	4.62513186	0.186661328	0.485489987	4.231467294
2.05337506	4.647933745	0.20643972	0.541533933	6.174055076
2.108549129	4.618968014	0.181180693	0.579978136	6.155710709
2.18733361	4.619496477	0.16351618	0.64175239	7.11808364
1.867280476	3.596019365	0.218944202	0.409513298	2.788974929
2.298657502	5.646706993	0.15666381	0.785936792	12.52531498
1.952940598	4.610862715	0.21475694	0.487872386	5.941006737
2.213158198	5.612168325	0.171168545	0.684216493	11.50157437
2.311364621	5.616319536	0.151032427	0.803448893	12.68968854
2.001968885	4.6105036	0.202280372	0.495309287	5.221127852
2.141285862	3.573860838	0.151945506	0.566237037	4.633941227
1.888010313	4.646402685	0.23201952	0.425253342	3.937700395
1.938967591	4.606000704	0.198414286	0.441646308	3.030336931
2.208240416	4.619294928	0.164415465	0.646434668	8.831736712
1.762284161	3.630596409	0.237080728	0.340615174	1.815186385
2.055732101	3.614288661	0.174152715	0.5324992	4.24175535
2.036635891	4.610048206	0.1837704	0.516376109	4.805761309
2.066556129	4.632389206	0.176279895	0.517567761	4.75218463
2.119437545	4.657448482	0.179568646	0.580932114	6.854756859
2.334992796	5.663830572	0.150270595	0.760995351	12.56756773
2.260233333	4.634700669	0.151224701	0.69696639	9.042316909
2.068108182	4.601767715	0.189530588	0.550934211	6.463246947
2.098624225	3.619526144	0.159064172	0.55546539	4.096085002
1.917134825	4.627609715	0.227885918	0.468768496	4.807784129
2.048071032	5.647691243	0.204599901	0.550741929	7.509112987
1.955401785	4.563037577	0.206442423	0.491779818	4.553531784
2.229900103	4.593199123	0.16037426	0.64731315	8.751425817
2.228807934	4.616627054	0.167788666	0.685777873	10.4800036
2.211483251	4.664006764	0.167074102	0.64274065	7.586720348
1.994140026	4.626180513	0.200658514	0.483846686	4.859321223
2.111274317	6.575421661	0.181217165	0.63342091	9.912100818
2.054196756	5.59094847	0.189634081	0.519953597	6.059004347
1.981573023	4.573347059	0.199259218	0.515884323	4.590626798
2.130142077	3.560807102	0.155745834	0.551746945	4.493645308
1.952441621	3.579518935	0.200892609	0.45587094	3.462779224
2.345302818	3.550796963	0.112806	0.736798256	7.187809602
2.063881202	4.621805032	0.189039011	0.535885869	5.513678983
2.298534419	4.577619193	0.145853276	0.790752652	11.76922597
2.105651496	5.65900587	0.201415624	0.617279077	9.13969321
2.106620764	3.653196077	0.175881073	0.558324646	5.975302246
2.144535464	4.62258302	0.176034471	0.598102088	7.564711008
1.872203684	3.633928589	0.238794578	0.447297001	3.653998739
2.331340613	3.63262398	0.145993744	0.788176003	11.01390502
1.969121444	4.670606988	0.210140491	0.461648871	4.142762598
2.269634985	4.655267454	0.157123546	0.70154191	9.972803119
2.293086725	4.598873902	0.133598333	0.704264115	7.990488619
2.135119342	4.595922236	0.163288012	0.576638229	5.339827537
1.934079445	3.660035833	0.214586664	0.443505788	3.8594024
2.166760791	4.666295014	0.180967814	0.617258485	8.484786028
1.982295391	3.595864072	0.200055765	0.476434019	4.08209019
2.144054279	4.629516408	0.168312937	0.591375078	5.968589405
2.039285991	4.564142878	0.186276587	0.521431255	5.062479062
2.08812056	4.594442105	0.184003795	0.577335092	6.690556307
2.289854324	4.547555713	0.130235172	0.709012176	8.208564625
2.011679306	4.649304448	0.211263888	0.527291122	6.394488896
2.21959469	5.642536463	0.16184614	0.713253444	11.0088443
2.210391574	5.664448753	0.166058927	0.649279989	8.725463684
2.156495649	4.585737466	0.17494313	0.616245237	7.849959971
1.720408687	3.615631986	0.247835044	0.332164878	1.675678367
2.046835118	4.592154176	0.19340064	0.549425007	6.363583081
2.014446585	4.568594029	0.193423952	0.555429387	6.372552111
2.187264425	5.58464811	0.164835868	0.639410651	7.906759951
2.151933102	5.637621641	0.177395056	0.609413853	7.919232472
1.823786261	4.60579224	0.226561301	0.384874497	2.628130106
2.026622851	3.599172208	0.19250887	0.51674632	4.899107936
2.066344178	4.661990525	0.204323103	0.552776217	7.401289537
2.129790036	4.612512852	0.178782361	0.58585616	6.784917666
2.378358626	4.564797413	0.121696354	0.817555326	10.37027696
2.241937671	3.563315321	0.134767324	0.661283921	6.587452085
2.00483165	3.579127339	0.194381273	0.517615577	4.762726926
1.914623475	4.639171166	0.216247255	0.432271656	4.125451847
2.005323895	4.59093414	0.201111024	0.535518349	5.991685229
1.965527417	4.602081922	0.197501752	0.466796834	3.824761688
2.295289361	4.55744797	0.140665437	0.732126108	8.959950919
2.414046887	6.572982101	0.123048626	0.888230223	13.88012413
2.191392227	4.548291478	0.164388966	0.670644741	7.835160023
2.055467946	4.608134774	0.189247156	0.545447707	6.111461654
1.774819921	3.601418424	0.231445382	0.346747678	1.710736517
2.215317346	4.579431077	0.157297914	0.672885265	7.696742066
1.519419754	3.647083736	0.300304173	0.271296771	1.179892236
2.205624392	4.543613935	0.153114246	0.661092216	7.324651824
2.083916393	4.682531426	0.189946991	0.538052918	5.850119466
2.00905446	4.627704489	0.21035062	0.522536481	5.836405993
1.940832251	4.635698872	0.227795295	0.483865078	4.94899988
1.807078224	4.654404689	0.231219827	0.351943894	2.081205733
2.20543096	4.650228022	0.146294595	0.62129868	6.078713385
2.204830142	4.566571628	0.154929753	0.643388607	7.007318016
2.004583561	4.647581477	0.203982401	0.479547638	4.36946695
2.179620642	4.55764819	0.149869876	0.612017983	5.844553201
2.247319915	6.641455111	0.16997562	0.716081259	12.23240562
2.05121887	3.571991012	0.178094026	0.510987043	4.251448495
2.177618753	4.577977676	0.168392871	0.682320213	9.188850979
1.785109651	3.582546491	0.230598059	0.372054834	1.946164218
1.894905605	4.65716464	0.219245571	0.414464013	3.242582123

log.sigma.0.5.mm.3D_glcm_SumAverage	log.sigma.0.5.mm.3D_glcm_Imc2	log.sigma.0.5.mm.3D_glcm_Imc1	log.sigma.0.5.mm.3D_glcm_DifferenceAverage	log.sigma.0.5.mm.3D_glcm_Id
13.13222404	0.269671663	-0.020261059	0.951264434	0.636183187
7.310367614	0.243160325	-0.025087654	0.606653006	0.725637783
7.295874587	0.202782549	-0.016854337	0.582626884	0.731743804
9.098393721	0.286131446	-0.026530574	0.756136942	0.691486366
9.192723556	0.204041729	-0.01460933	0.70078588	0.701897802
9.168792289	0.356806442	-0.040439211	0.870808926	0.652146826
9.25026372	0.17949899	-0.011474394	0.670146286	0.703129434
9.29586749	0.22074473	-0.017702403	0.6801057	0.710156287
9.237936029	0.175708185	-0.010548363	0.725538404	0.695133368
9.238992954	0.259542709	-0.022543854	0.77134038	0.680574715
7.19203873	0.164262666	-0.010523431	0.604912862	0.726445589
11.29341399	0.232090374	-0.017347526	0.839075917	0.670213501
9.22172543	0.286259567	-0.032557342	0.64290641	0.718386495
11.22433665	0.29772906	-0.028373136	0.765358904	0.686664183
11.23263907	0.279700171	-0.023589199	0.855580177	0.666960875
9.221007201	0.198115179	-0.013916458	0.653570751	0.714049955
7.147721677	0.217112945	-0.016536802	0.739660695	0.682806735
9.292805369	0.24432702	-0.024459971	0.600267173	0.72970899
9.212001408	0.198115617	-0.014851776	0.646542903	0.709930434
9.238589855	0.201385184	-0.013316918	0.763257715	0.681963273
7.261192817	0.179500823	-0.013911563	0.553897097	0.739171753
7.228577321	0.186834448	-0.012173297	0.728693465	0.685918405
9.220096411	0.253560682	-0.023239236	0.695603926	0.698188505
9.264778412	0.222718105	-0.016865236	0.687389677	0.700479835
9.314896964	0.268091224	-0.025833381	0.722306299	0.693385123
11.32766114	0.281810746	-0.024897981	0.80931639	0.675455776
9.269401339	0.263053151	-0.021363184	0.795202702	0.676280138
9.20353543	0.222627995	-0.01790807	0.693686827	0.70358598
7.239052288	0.241793683	-0.019411147	0.753761678	0.676833932
9.255219431	0.198630973	-0.015886815	0.635947755	0.722196503
11.29538249	0.237287048	-0.020361453	0.678801617	0.711006104
9.126075155	0.218958456	-0.017391286	0.662607624	0.712455146
9.186398245	0.259513934	-0.022527556	0.735014696	0.695943217
9.233254108	0.255598205	-0.02092614	0.764301991	0.687652716
9.328013529	0.21726897	-0.015107282	0.757269382	0.686561534
9.252361026	0.247202725	-0.022986656	0.653572881	0.71149028
13.15084332	0.300768445	-0.030579427	0.739484054	0.694584278
11.18189694	0.266610437	-0.024989884	0.657824947	0.714130302
9.146694119	0.22075809	-0.017418043	0.683581482	0.708624265
7.121614204	0.154056109	-0.007922451	0.722319361	0.689264873
7.159037869	0.208223639	-0.016365801	0.642240868	0.714421584
7.101593925	0.159196534	-0.007407344	0.865131625	0.648429806
9.243610064	0.198093018	-0.014095209	0.691722912	0.703114412
9.155238386	0.248854265	-0.018761606	0.863111548	0.661074874
11.31801174	0.286367373	-0.029582724	0.720408943	0.701800692
7.306392154	0.248486385	-0.021421489	0.712467471	0.694480512
9.245166041	0.22238498	-0.016755546	0.73086989	0.691996085
7.267857178	0.216388347	-0.019029952	0.628212222	0.725110409
7.26524796	0.311470872	-0.028811633	0.854362534	0.661206648
9.341213977	0.227744693	-0.020007174	0.638442253	0.716922641
9.310534908	0.217624258	-0.014689677	0.796305906	0.673932004
9.197747804	0.177104828	-0.01011448	0.829617697	0.660866284
9.191844473	0.164857983	-0.009325662	0.749730188	0.680372766
7.320071666	0.234453143	-0.021089721	0.622802783	0.722363342
9.332590028	0.271615072	-0.025546042	0.734635021	0.691397972
7.191728145	0.185741625	-0.012940806	0.656681967	0.709757964
9.259032817	0.200514588	-0.013062357	0.740199127	0.688727078
9.128285756	0.216123386	-0.016271334	0.684070388	0.703609344
9.188884209	0.219799127	-0.016384398	0.724543944	0.695089958
9.095111426	0.201469936	-0.01190064	0.830624103	0.661247792
9.298608896	0.273226615	-0.026802513	0.669374166	0.713195214
11.28507293	0.270626943	-0.023186464	0.799102145	0.678980853
11.32889751	0.244569427	-0.019098463	0.759192722	0.685059339
9.171474931	0.210750562	-0.01572243	0.735827757	0.693264242
7.231263971	0.185115805	-0.014958764	0.549782946	0.740404836
9.184308352	0.247316238	-0.02179247	0.695037655	0.70401872
9.137188058	0.249566282	-0.02170936	0.71577042	0.697177444
11.16929622	0.209557644	-0.014387216	0.761664149	0.684630206
11.27524328	0.203634665	-0.013645564	0.732270161	0.694102003
9.211584479	0.160925469	-0.010668406	0.589419515	0.729423502
7.198344416	0.228957765	-0.019494602	0.686531295	0.703075191
9.323981051	0.248685145	-0.021859123	0.676356227	0.712139534
9.225025703	0.278537437	-0.026357029	0.714765773	0.698136447
9.129594827	0.247112983	-0.017566259	0.895203788	0.647572075
7.126630643	0.193006231	-0.011761703	0.816899911	0.660628626
7.158254677	0.248592152	-0.022327439	0.689194091	0.70268457
9.278342332	0.203415902	-0.016898779	0.618236387	0.721968106
9.181868279	0.249865325	-0.023015255	0.695828428	0.703039622
9.204163844	0.186792593	-0.01363607	0.656515551	0.709574523
9.114895939	0.243382182	-0.017622244	0.818329065	0.672826747
13.1459642	0.277025837	-0.021663143	0.924478658	0.643103828
9.096582956	0.264527904	-0.022023931	0.78228529	0.683345194
9.216269548	0.237740867	-0.019468049	0.692249143	0.705003441
7.202836848	0.156437804	-0.010961698	0.551090486	0.742107518
9.158862153	0.241090244	-0.01931187	0.788414694	0.678899205
7.294167473	0.140474738	-0.01654266	0.46493406	0.776104279
9.08722787	0.250082042	-0.0204575	0.785625635	0.677643091
9.365062852	0.239598629	-0.020875635	0.68899104	0.702474749
9.255408978	0.228069813	-0.018791876	0.667359026	0.713915352
9.271397743	0.223923542	-0.019655046	0.636165618	0.724579568
9.308809377	0.221965888	-0.022450399	0.565715317	0.732382034
9.300456043	0.177303878	-0.009836206	0.776648814	0.673217642
9.133143256	0.196905362	-0.012276424	0.780433266	0.676024847
9.295162954	0.201907005	-0.01551473	0.645468015	0.715072993
9.11529638	0.188582956	-0.0111643	0.765506181	0.67949907
13.28291022	0.261056634	-0.022051297	0.776346362	0.686118523
7.143982023	0.187686717	-0.012112098	0.686538686	0.701062405
9.155955351	0.31038268	-0.03137668	0.78234719	0.683741914
7.165092983	0.173718283	-0.012729639	0.581123372	0.732582015
9.31432928	0.215886996	-0.019157409	0.609309908	0.723285025

log.sigma.0.5.mm.3D_glcm_ClusterTendency	log.sigma.0.5.mm.3D_firstorder_InterquartileRange	log.sigma.0.5.mm.3D_firstorder_Skewness	log.sigma.0.5.mm.3D_firstorder_Uniformity
1.86234212	22.69390106	-0.673269429	0.323752066
0.848673612	11.63540828	-0.521274044	0.466139724
0.819052919	10.37441692	-0.403702183	0.459785072
1.402422787	16.72363234	-1.146675842	0.392362097
1.156339604	16.33584416	-0.892959209	0.426441803
1.73767989	20.47964323	-0.285794601	0.352203857
1.0139094	15.51062727	-0.285837703	0.416562469
1.123570315	12.84458745	-0.649035207	0.434379042
1.182692211	17.36667478	-0.735880878	0.412565574
1.312540567	19.34512854	-0.907274498	0.389793105
0.840924142	15.61498821	-0.6473245	0.457789227
1.595632594	19.58512211	-1.069409386	0.379240263
1.007083156	9.022771955	-1.017975325	0.450448391
1.434225138	17.01929903	-0.683338238	0.395473475
1.616961384	18.06755555	-0.828362695	0.369482066
1.029963562	12.40125233	-0.909227185	0.436374846
1.169830298	20.38285375	-0.175555617	0.384871033
0.888202344	10.75493813	-1.007476022	0.47688104
0.898431267	15.0482626	-0.41736614	0.434946736
1.350410675	17.87628579	-0.735653772	0.38944182
0.70591604	9.708653629	-0.140536308	0.480511289
1.066035858	18.36064029	-0.651278195	0.407370124
1.048154043	15.77226114	-0.709444735	0.412614822
1.071021943	16.19801474	-0.460467182	0.407543935
1.197952363	15.4264822	-0.668329096	0.404359934
1.626077343	20.29998541	-0.15207688	0.361095307
1.438392055	19.01500082	-0.440411748	0.367936153
1.138402124	14.35930419	-1.024540777	0.42345953
1.114318381	19.68024087	-0.346999762	0.403410257
0.955955802	13.75258857	-1.080350599	0.471794177
1.141082126	12.98555201	-0.811882499	0.433131713
0.992297794	11.90496182	-1.187829791	0.450435565
1.384730914	16.55331373	-0.146760116	0.382599873
1.437062574	16.10346437	-0.762308571	0.390570213
1.340868122	18.56297553	-0.485003663	0.387806663
1.003167873	12.27083457	-0.463555137	0.430372311
1.25461533	13.33630657	-1.044378403	0.410541115
1.0939984	8.692008257	-0.849524733	0.419468018
1.008066598	15.92619061	-1.333311072	0.439459317
1.14209147	19.45520604	-0.198064353	0.385520832
0.943539738	12.01970088	-0.635269569	0.437242638
1.521011585	25.13153315	0.020683591	0.331374352
1.107314762	16.82226563	-0.766343819	0.421538863
1.579320358	20.6668843	-0.781243962	0.36461575
1.267147721	16.29309106	-1.030355327	0.431223229
1.155529689	16.50215673	-0.082000431	0.394879247
1.246599741	16.0586158	-0.251795508	0.399367975
0.893386081	12.81502187	-0.809493041	0.48406954
1.637451472	20.38176894	-0.601950272	0.370254943
0.964797903	14.55508298	-0.772074711	0.446707267
1.471636968	19.06412697	-0.625264183	0.380204223
1.451887236	23.41193008	-0.244560529	0.35234508
1.178295861	18.78363764	-0.438447932	0.391825404
0.920964405	14.90717071	-0.248247748	0.45119854
1.30650241	15.71125251	-0.280490675	0.408724066
0.986369926	15.19704545	-1.00541827	0.437432742
1.212661894	19.09102964	-0.583532114	0.392176091
1.07094414	14.14665639	-0.888687367	0.421678668
1.170181914	16.82579267	-0.735064554	0.417096481
1.443053324	19.74970436	-0.355106096	0.354670494
1.084987133	12.02555853	-0.52765306	0.433928784
1.422886297	17.83734882	-0.914866833	0.386382752
1.361403307	16.94438457	-0.434651381	0.381105468
1.271892625	16.53453851	-0.65764249	0.402081899
0.683100676	13.22119999	-0.735656977	0.493510655
1.111033823	13.01029927	-0.880372357	0.42724822
1.087244622	13.20446992	-0.9972786	0.429246726
1.315175103	16.73949182	-0.600901437	0.392003686
1.260872925	18.21904325	-0.71268988	0.400665086
0.785274495	11.94819009	-1.082863974	0.467405474
1.060375988	14.52343321	-0.793919672	0.423927093
1.155481505	12.51001692	-0.634198581	0.430146978
1.224545825	15.33271492	-0.417472594	0.400717889
1.671118135	23.75675058	-0.471669263	0.334601195
1.346376056	22.0322957	-0.285607002	0.353155987
1.055005993	15.8987143	-0.775517031	0.433370128
0.902259261	13.88636088	0.022870456	0.451308316
1.067069442	14.08321261	-1.167268388	0.441150448
0.951568651	15.27279162	-0.846950598	0.433713964
1.493425263	20.68809748	-0.457477505	0.365524013
1.799855412	21.69249821	-0.694842709	0.331905672
1.34459645	15.60317445	-0.779736704	0.389901313
1.105439157	12.23327446	-0.761417897	0.418044178
0.721758038	14.04398775	-0.549220911	0.479363898
1.368358728	17.85437083	-0.639472032	0.381825774
0.564751059	6.405395687	-1.836601279	0.547035735
1.340705513	18.27730894	-0.612246556	0.379243261
1.132629202	15.54565912	-0.500914252	0.412998014
1.068615005	14.43029028	-1.182235696	0.454737003
0.988873571	12.86592913	-1.150165173	0.466263314
0.744462046	9.090936601	-0.222706891	0.469946178
1.282081908	21.76960909	-0.352048693	0.368571028
1.315992403	19.25467753	-0.515422853	0.382455347
1.010738181	13.65899241	-0.71224323	0.439951148
1.243645743	21.86777568	-0.340629842	0.379654406
1.493428202	13.95165914	-1.040327006	0.392252182
1.058090464	16.08524942	-0.471268515	0.411561657
1.36002373	15.59750271	-0.973169629	0.394507799
0.760541377	11.98038745	-0.83015681	0.475544744
0.856737795	10.66185546	-0.468112919	0.45014067

log.sigma.0.5.mm.3D_firstorder_MeanAbsoluteDeviation	log.sigma.0.5.mm.3D_firstorder_Energy	log.sigma.0.5.mm.3D_firstorder_RobustMeanAbsoluteDeviation	log.sigma.0.5.mm.3D_firstorder_Median
16.57687694	1113201.798	10.05593579	3.175710082
9.425525158	194715.8525	5.200812064	3.444401264
9.038534633	287649.9167	4.975077625	1.977944314
13.32222189	352816.6108	7.718936768	3.066188335
12.26553499	701785.8741	7.26843776	3.827770114
14.89289037	310406.701	8.596175712	3.474011421
11.31300827	1475840.446	6.744389632	2.86079216
11.1334502	1534470.208	5.776263714	3.312461376
12.63052739	1459133.441	7.541880701	4.246758938
13.89787833	406662.76	8.454417503	5.606221676
10.35871139	302152.2568	6.61548947	3.222209454
14.6475177	1064917.136	8.486334817	5.827358246
8.82830332	432596.6821	3.994358466	2.045005798
13.23540757	645690.1513	7.487566744	3.82461381
14.67584901	913387.3991	8.243868136	4.394723654
10.29194386	1072180.707	5.587449247	2.52539134
13.22150099	368337.0026	8.620653987	3.152316213
8.900471321	238528.234	4.815468327	2.995011806
10.67630812	283963.3521	6.589884323	3.227053046
13.35414925	1960279.078	7.841378983	4.0353899
7.945391459	434043.5493	4.354700647	2.313488722
12.56738528	315270.3754	8.007113271	4.177198887
11.73519275	285675.0685	6.974005061	3.337430716
11.45176037	697388.749	6.969032455	2.971572399
12.28723056	432925.8352	6.964363509	4.001272678
15.22826387	1156363.097	8.965864431	5.13501358
14.11395601	1296101.975	8.417631112	3.658699512
11.19690167	766304.8128	6.257817074	2.949423313
12.87029979	131839.7099	8.380171153	4.391053677
10.50865298	497249.429	6.002427664	4.327906132
11.03975593	1407625.149	5.85972115	3.122099042
10.1426315	478613.4172	5.421694321	2.310272932
12.91960613	1133072.703	7.425817353	2.534808874
12.99352347	871072.193	7.26766936	3.51651895
13.77113002	1751040.113	8.124948278	5.367922783
10.33943388	751740.1095	5.557966521	2.816029072
11.41984199	1190666.012	5.960282927	2.238063335
9.333654678	1262859.471	4.278486539	1.272457123
11.40362179	319843.9963	6.907643401	3.69966197
12.8857614	2153348.051	8.315737494	2.084411263
9.917283926	537809.3488	5.525630378	2.171794057
15.68051291	1563603.373	10.64854034	1.624345541
12.1154579	1070546.937	7.315442196	3.941797256
15.11196979	1124715.644	9.013467421	3.702225685
12.51284347	459342.8987	7.147059803	5.99668479
12.58312245	895216.8892	7.442642209	3.670768261
12.46805792	1534856.875	7.087342829	3.222666979
10.18132891	202041.8543	5.800952251	3.884773016
15.04900314	376844.3365	9.001848322	5.349025249
10.89772636	349768.6817	6.480522189	4.24118495
14.04560782	1075535.174	8.250785988	5.146748543
15.26739736	1386097.57	9.981319013	4.134631157
13.10557204	1435097.402	8.203338782	3.827631831
10.62543175	261247.8307	6.387551731	3.905232906
12.49572635	471061.3237	6.927892139	4.163564205
10.9613007	481585.969	6.44818837	3.469530821
13.22822573	1359396.349	8.1602455	4.447813034
10.88296591	883171.8417	6.107376756	2.029376745
12.41875051	526048.7655	7.216092338	3.438904524
13.86360718	3345492.448	8.512324854	1.219269156
10.81371335	512895.3851	5.546509298	3.029772878
13.46898978	1065222.553	7.664521798	4.191136837
13.2326316	2185120.663	7.579721794	3.798702478
12.6256095	2580982.115	7.24580738	3.273042917
8.912385753	120044.2903	5.669461585	3.638978958
10.93449204	1069898.187	5.85322189	2.646607161
10.90298117	352231.7409	5.799256508	2.301304102
12.83612173	2110067.243	7.460545646	2.952932835
13.14045443	2085867.005	7.827306897	4.079095364
9.078916979	530256.6817	5.226639873	2.605609179
11.36459725	351673.5325	6.532801758	3.044665337
11.04625577	1325231.032	5.677817038	3.54144001
12.29184608	743159.8529	6.897594939	3.36799407
16.19624631	1047776.106	10.32953533	3.53521657
14.54448093	1159559.529	9.459166005	2.838468552
11.51674412	345202.1564	6.920364596	3.453483105
10.45592292	587462.6073	5.987263431	3.488809109
11.03701891	259048.3226	6.157845764	3.66916573
10.91005292	399875.3365	6.681614717	3.509372711
14.45689516	929256.9942	8.906081452	2.597470999
16.10186049	1004927.725	9.691268096	3.187271357
13.04944772	638203.2996	7.354535898	2.921532631
10.60156201	867988.5691	5.622987007	2.204926252
9.295798786	487372.5122	5.972315547	2.949286461
13.38125291	862302.6131	7.970332038	3.091123343
5.474426338	115436.8779	2.928495587	1.834332883
13.48951323	845449.132	8.046663521	2.364152193
12.0243422	870015.8429	6.9515203	4.675748587
11.04142881	404217.8894	6.262206603	3.898447156
10.28623509	482517.8582	5.721553232	3.504956722
7.748225201	191836.9825	4.121587681	2.353660583
14.30112154	881315.7135	9.381762313	4.450805426
13.68847097	1826561.855	8.441208472	3.433962345
10.60902915	797901.2971	6.073613216	3.796742678
13.8191328	1432939.129	9.141051915	3.166134953
12.79597476	1391743.132	6.612363746	3.120098829
11.66824642	749300.8105	7.012871119	2.889277697
12.84532944	379049.6302	6.944874768	2.493265867
8.849947795	127950.2138	5.216593172	2.210402489
9.156565092	1185800.691	4.812289555	2.614223719

log.sigma.0.5.mm.3D_firstorder_TotalEnergy	log.sigma.0.5.mm.3D_firstorder_Maximum	log.sigma.0.5.mm.3D_firstorder_RootMeanSquared	log.sigma.0.5.mm.3D_firstorder_90Percentile	log.sigma.0.5.mm.3D_firstorder_Minimum
30056448.54	71.21192169	22.90415425	27.66503906	-126.1284027
5257328.019	61.47484589	14.4309476	18.22762146	-64.75788879
7766547.751	53.53659439	13.69365703	19.22487106	-62.71178436
9526048.493	51.89054489	19.00322148	22.26964149	-93.17070007
18948218.6	52.80057526	17.2151758	20.78422813	-77.50046539
8380980.928	84.64702606	21.68670522	27.11957436	-91.25188446
39847692.03	81.1064682	16.16058398	21.61105347	-86.96133423
41430695.6	91.19960785	17.33220376	21.45147133	-96.26242828
39396602.92	86.36907959	17.91565909	21.86183167	-97.18520355
10979894.52	64.47081757	19.47689887	24.41676445	-91.81764984
8158110.934	54.57297897	14.15507747	18.26872845	-65.15393829
28752762.67	83.35797119	21.49425728	27.28873978	-101.633812
11680110.42	69.22296906	15.22191993	15.03793507	-96.33330536
17433634.09	73.41452026	19.39219339	22.80784149	-101.8091202
24661459.78	103.2125168	21.45639097	25.71142063	-111.6016083
28948879.09	81.6696167	15.65291898	18.22094631	-90.6147995
9945099.07	70.72168732	17.56388203	22.26720581	-61.89440155
6440262.319	65.79846191	13.90875823	16.86645813	-81.66195679
7667010.506	60.16286469	14.79087275	18.07525921	-83.30603027
52927535.11	98.64559174	19.41775618	24.55108681	-97.18328857
11719175.83	52.76083374	11.98052251	14.83007774	-68.70906067
8512300.135	49.88861847	17.09349462	22.15891876	-68.4101181
7713226.85	68.88970947	16.70268119	20.56890736	-96.71522522
18829496.22	58.24201202	16.23156883	22.08504906	-86.88170624
11688997.55	65.11818695	18.44134318	24.81435394	-88.9907608
31221803.61	90.9934845	22.09350306	29.49613876	-106.6801834
34994753.32	79.97556305	20.02242769	26.6349781	-91.10819244
20690229.95	63.79180527	16.65669907	18.99286518	-96.68186951
3559672.167	54.37399292	17.17391692	23.01607895	-63.4323616
13425734.58	59.76424789	15.55918795	18.56336727	-91.90028381
38005879.01	92.4642334	17.20011259	21.33545532	-102.8789291
12922562.27	57.96892929	15.20940377	16.51673889	-89.29679871
30592962.99	100.1205444	18.73246225	22.39166145	-93.64574432
23518949.21	88.72647095	19.25276651	23.99543514	-85.97541046
47278083.05	96.31455994	19.93996503	27.84428196	-80.19637299
20296982.96	100.605957	15.83235899	19.21962166	-81.92415619
32147982.33	97.2461853	17.88813828	18.86519814	-129.0684357
34097205.72	78.64164734	15.64265919	18.54159164	-121.7357178
8635787.901	52.90376663	16.12562462	18.45862904	-96.88195038
58140397.38	72.53829193	17.21035824	22.39338112	-68.05292511
14520852.42	58.71775818	14.39889576	16.05367527	-68.63224792
42217291.07	81.6436615	20.10833969	26.73468781	-73.85913086
28904767.3	74.82006073	17.11898536	21.74350204	-94.42045593
30367322.4	71.30202484	21.26161158	24.38161469	-98.12716675
12402258.26	76.72809601	18.95846087	23.64873867	-100.3254547
24170856.01	100.1112213	18.05896928	23.63467293	-71.35826111
41441135.64	86.51296234	18.34240598	22.47903042	-85.21465302
5455130.067	63.05482101	15.23041003	17.40083122	-67.18429565
10174797.08	82.45761108	21.04341482	27.24007607	-72.22444916
9443754.406	51.69695282	16.08429813	22.76833096	-96.54263306
29039449.69	98.25940704	20.46116025	27.18245354	-97.2879715
37424634.4	82.24510193	20.27764543	26.15246964	-86.81916809
38747629.85	73.49217224	17.83430311	22.12635307	-77.22131348
7053691.428	69.80615997	15.38302726	19.69667587	-64.94390106
12718655.74	72.89405823	18.81970899	23.52604446	-87.71576691
13002821.16	66.58460999	15.71518894	18.96185818	-73.34158325
36703701.42	86.6055603	18.33216623	23.92831612	-84.48606873
23845639.73	73.45413971	15.98122671	17.43885212	-98.0463028
14203316.67	77.59403992	17.86629083	20.80973988	-95.1552124
90328296.08	79.23059845	19.14337132	24.26672058	-96.32692719
13848175.4	79.23547363	17.04194056	20.34614372	-95.79438019
28761008.92	106.0586777	19.97397701	25.14772511	-115.0535431
58998257.91	82.08063507	19.42835083	26.70493279	-107.4170914
69686517.11	96.53473663	18.40049633	21.46698456	-97.21296692
3241195.838	40.19482422	12.32699468	15.79338808	-50.42861938
28887251.04	90.93824768	16.64428298	18.89735222	-96.30687714
9510257.003	65.96592712	16.43521724	17.63395634	-85.23960876
56971815.56	89.76519775	18.3741873	21.69969006	-105.6837921
56318409.14	111.0572128	18.87146844	23.02609367	-116.2353745
14316930.41	46.44808197	13.52676311	16.84233036	-80.64229584
9495185.379	67.66475677	16.4537641	19.87465286	-72.36495972
35781237.86	87.53183746	17.57588298	22.47351246	-92.15107727
20065316.03	75.30047607	18.05797126	22.58817368	-84.27285004
28289954.86	95.79428864	21.5365498	26.21704216	-86.09126282
31308107.29	76.20359039	19.24442728	24.75824928	-74.94553375
9320458.222	68.688797	16.03837974	18.02558136	-70.44567108
15861490.4	86.84640503	15.65837564	18.58323479	-81.51600647
6994304.709	54.99953079	16.44400642	18.91767292	-78.12824249
10796634.09	52.78248596	15.30543047	20.24172974	-78.34790039
25089938.84	89.06395721	19.93208857	23.6935009	-87.20498657
27133048.58	71.21192169	22.47760435	27.02226601	-126.1284027
17231489.09	84.56942749	18.83492698	21.16298752	-80.27064514
23435691.37	77.90683746	16.29481907	19.63425941	-99.72615051
13159057.83	41.95920181	12.59358088	16.3486187	-66.87797546
23282170.55	72.80583191	18.80286167	22.56227455	-83.84356689
3116795.704	33.05236816	8.680463266	10.15008678	-59.13832092
22827126.56	76.93357086	18.82779884	21.06083221	-86.47075653
23490427.76	65.49730682	17.81270417	25.46974487	-76.4311676
10913883.01	58.45092773	16.61641104	20.56743298	-92.49519348
13027982.17	61.05373383	15.43633453	18.44801521	-93.88408661
5179598.527	55.12905502	12.12906634	15.74611969	-92.87042999
23795524.27	62.70555496	19.23511903	26.86258831	-84.52487183
49317170.09	87.18520355	18.77269341	22.6582695	-78.06622314
21543335.02	64.30461121	15.5495425	20.82429657	-78.03275299
38689356.48	91.75500488	18.14552039	22.1323431	-84.15639496
37577064.55	91.17636871	20.16691839	25.52927647	-133.8673859
20231121.88	67.46447754	16.30927583	20.04127197	-72.23345184
10234340.02	59.84417725	19.02719188	19.5831089	-93.26378632
3454655.773	38.99674225	12.38618273	14.95482626	-59.06682587
32016618.65	63.91834259	14.33070182	18.73131294	-95.44705963

log.sigma.0.5.mm.3D_firstorder_Entropy	log.sigma.0.5.mm.3D_firstorder_StandardDeviation	log.sigma.0.5.mm.3D_firstorder_Range	log.sigma.0.5.mm.3D_firstorder_Variance	log.sigma.0.5.mm.3D_firstorder_10Percentile
1.948691337	22.8442639	197.3403244	521.8603932	-28.14769745
1.386408968	14.04476983	126.2327347	197.2555596	-9.896975517
1.37714106	13.40749129	116.2483788	179.7608228	-10.9218462
1.670660468	18.97809522	145.061245	360.168098	-23.31659698
1.548818227	17.11698353	130.3010406	292.9911251	-20.31572037
1.863242617	21.39432587	175.8989105	457.7171794	-19.98110676
1.540731725	15.91972602	168.0678024	253.4376764	-16.01037788
1.566102223	17.05027495	187.4620361	290.7118759	-14.95180311
1.602248573	17.70453005	183.5542831	313.4503843	-18.34679413
1.68605793	19.13102291	156.2884674	365.9960377	-20.20610123
1.364694043	13.97416126	119.7269173	195.2771829	-14.5119606
1.778803921	21.10714048	184.9917831	445.511379	-19.04126511
1.458566689	15.12368185	165.5562744	228.7257527	-9.467154312
1.698053654	19.22976342	175.2236404	369.7838012	-19.35645714
1.809857376	21.26869556	214.8141251	452.3574107	-22.5695158
1.483159781	15.55482735	172.2844162	241.9526539	-14.64861441
1.624625267	17.42295682	132.6160889	303.5594243	-20.46282673
1.35894188	13.63972226	147.4604187	186.0420234	-10.1810379
1.45767914	14.65244245	143.468895	214.6940698	-16.37528133
1.697555024	19.14470807	195.8288803	366.5198472	-18.06199036
1.273056325	11.75701405	121.4698944	138.2273794	-10.45927029
1.573075171	16.82895258	118.2987366	283.213645	-18.40148621
1.568224311	16.53951691	165.6049347	273.5556195	-17.4150528
1.563593536	15.94724995	145.1237183	254.3147809	-15.15070629
1.636594117	17.90706326	154.1089478	320.6629147	-14.19186916
1.843940186	21.50751324	197.6736679	462.5731257	-20.01692581
1.764066518	19.75026288	171.0837555	390.0728838	-20.35596008
1.549592444	16.54154566	160.4736748	273.622733	-15.29694738
1.57866034	16.88516372	117.8063545	285.1087538	-17.62195206
1.409078734	15.30291802	151.6645317	234.1793001	-14.05324383
1.554475667	16.94808975	195.3431625	287.2377462	-14.06658449
1.433345266	15.18575307	147.265728	230.6070962	-15.98312378
1.701336454	18.60780962	193.7662888	346.2505789	-19.76037941
1.703104374	19.07020275	174.7018814	363.6726328	-18.81372108
1.710955373	19.35716074	176.5109329	374.6996718	-18.21748981
1.51413075	15.6219096	182.5301132	244.0440597	-13.4269722
1.608518078	17.83179859	226.314621	317.9730408	-15.67119122
1.536018988	15.55586502	200.3773651	241.9849364	-11.43946362
1.470394162	16.07230467	149.785717	258.3189774	-16.09994831
1.619398387	17.12352428	140.591217	293.2150838	-19.7539856
1.447527671	14.36186115	127.3500061	206.2630558	-16.88312454
1.823897786	20.04820297	155.5027924	401.9304423	-24.31350441
1.553323618	16.90700664	169.2405167	285.8468736	-16.87905884
1.800412615	21.16755608	169.4291916	448.0654305	-23.59464111
1.604121532	18.37862891	177.0535507	337.7740006	-13.2955883
1.647515234	17.71663788	171.4694824	313.8792578	-17.01223259
1.658848388	18.1183014	171.7276154	328.2728456	-17.76332874
1.385075524	14.96640757	130.2391167	223.9933556	-13.3424778
1.79119494	20.76271602	154.6820602	431.0903763	-25.01732445
1.470708356	15.52042385	148.2395859	240.8835564	-12.65717669
1.751577521	20.00173508	195.5473785	400.0694064	-17.85104675
1.797777299	20.08568924	169.06427	403.4349123	-23.11148834
1.64049055	17.66462214	150.7134857	312.0388755	-20.10371552
1.44132793	14.96550781	134.750061	223.966424	-12.95360403
1.653224948	18.42345181	160.6098251	339.4235767	-16.15709572
1.475551617	15.55530929	139.9261932	241.967647	-15.30387545
1.655146056	18.06099291	171.091629	326.1994648	-19.65551491
1.52576906	15.95128905	171.5004425	254.4436223	-16.46689968
1.591703239	17.76183619	172.7492523	315.4828249	-18.56233673
1.768050185	19.10375858	175.5575256	364.953592	-21.65998154
1.557860381	16.790187	175.0298538	281.9103794	-14.04379225
1.723494845	19.72428692	221.1122208	389.0474945	-18.40667763
1.724586035	19.03366064	189.4977264	362.2802374	-17.94252663
1.651674254	18.28912377	193.7477036	334.4920484	-18.53043289
1.234778805	12.023691	90.6234436	144.5691454	-12.12512083
1.546483792	16.55174254	187.2451248	273.9601811	-16.38478413
1.527924467	16.41744003	151.2055359	269.532337	-16.27210083
1.669577778	18.29349256	195.4489899	334.6518702	-20.16812801
1.660068419	18.64116343	227.2925873	347.492974	-18.29382973
1.336256663	13.34399775	127.0903778	178.0622761	-11.32678928
1.530971273	16.35246726	140.0297165	267.4031855	-17.65544701
1.571801621	17.19769992	179.6829147	295.7608824	-12.15730066
1.649564342	17.82834253	159.5733261	317.8497973	-17.35789757
1.872676785	21.50722182	181.8855515	462.5605903	-28.23663635
1.761347639	19.14953397	151.1491241	366.7046514	-22.62043762
1.498534396	15.94933612	139.1344681	254.3813228	-17.41153526
1.443876241	15.32776628	168.3624115	234.9404191	-12.85732841
1.500153787	16.31672453	133.1277733	266.2354994	-15.03508825
1.466492043	15.14242871	131.1303864	229.2931472	-14.76862659
1.764733262	19.89299727	176.2689438	395.7313403	-22.44397163
1.922105211	22.39037085	197.3403244	501.3287066	-26.67306213
1.692492912	18.80767097	164.8400726	353.7284875	-22.45407181
1.562619352	16.16999172	177.632988	261.4686323	-14.81903229
1.255560896	12.430662	108.8371773	154.5213578	-13.60895157
1.704201843	18.70443943	156.6493988	349.8560544	-22.04667015
1.030445218	8.579878599	92.19068909	73.61431677	-6.88290267
1.695423875	18.81053033	163.4043274	353.8360513	-23.10598183
1.598528237	17.20592142	141.9284744	296.043732	-13.93260603
1.474332572	16.34596422	150.9461212	267.1905462	-14.14177065
1.437588127	15.24567034	154.9378204	232.4304642	-14.59191303
1.314072764	11.77201986	147.999485	138.5804517	-8.021440458
1.704898975	18.81656208	147.2304268	354.0630084	-19.16284122
1.696266813	18.68424356	165.2514267	349.1009575	-22.07815704
1.484615717	15.22951108	142.3373642	231.9380079	-13.93884888
1.665863707	18.06740386	175.9113998	326.4310822	-21.84235058
1.72313162	19.93907275	225.0437546	397.5666219	-16.17988892
1.547431906	16.21296414	139.6979294	262.8602063	-17.96709747
1.681476095	19.01614419	153.1079636	361.6137399	-20.61347122
1.278172621	12.3551871	98.06356812	152.6506484	-14.75668716
1.424416933	14.01794501	159.3654022	196.5027824	-10.34317055

log.sigma.0.5.mm.3D_firstorder_Kurtosis	log.sigma.0.5.mm.3D_firstorder_Mean	log.sigma.0.5.mm.3D_glrlm_ShortRunLowGrayLevelEmphasis	log.sigma.0.5.mm.3D_glrlm_GrayLevelVariance
4.734636859	1.655260868	0.022098559	1.106909559
6.682745951	3.316125619	0.075920021	0.524805206
5.975681784	2.784855497	0.071077295	0.501429807
5.932854983	0.976897456	0.051191491	0.815432194
5.068989639	1.836069924	0.045170608	0.690142228
5.813185812	3.549084951	0.051594802	1.040704391
5.263348091	2.779711876	0.041262476	0.624499605
6.759325938	3.113424386	0.044739427	0.744175849
5.45193194	2.742345031	0.045129595	0.733679934
5.410132017	3.654251205	0.04804472	0.816810141
5.063707848	2.255888998	0.076598524	0.490143724
6.410555601	4.060999485	0.030711502	1.012487758
11.01098206	1.726584364	0.044494405	0.634861365
6.008982429	2.504668275	0.029450113	0.867834361
5.714707613	2.831837305	0.030695822	1.018676077
7.870302683	1.749633917	0.044120363	0.623161928
4.034088909	2.220479135	0.078136417	0.663088397
8.860729197	2.722412928	0.041944162	0.538969529
5.303604338	2.018872723	0.041841632	0.557160182
6.4018042	3.24490493	0.048922042	0.84555239
6.121628659	2.30337586	0.065309921	0.41385796
4.401327431	2.995649053	0.083680058	0.659250058
6.250332247	2.328935298	0.044089275	0.665865575
5.531834803	3.024738946	0.042628947	0.636207261
6.496898967	4.406838281	0.045243542	0.766936923
5.063945681	5.054676228	0.028727196	1.016271851
4.801208277	3.290095247	0.046690478	0.881351776
7.289816643	1.955221421	0.045897548	0.688033405
3.740808814	3.136027538	0.078618827	0.633150303
7.388547428	2.812299686	0.044350057	0.595816739
7.877508164	2.933620116	0.027863426	0.739671895
7.086639565	0.847860096	0.044174441	0.585172111
5.880472891	2.157443644	0.044918292	0.820044065
6.203483157	2.645068147	0.049855306	0.866581562
4.92310626	4.785659168	0.044479935	0.844420274
7.249544813	2.572844998	0.042443131	0.636009067
9.315323332	1.418608566	0.020059829	0.797982344
9.994440141	1.645554629	0.027376434	0.65657042
7.719887353	1.310264077	0.04632335	0.628501651
3.950485247	1.72665774	0.080675799	0.667341967
5.472626221	1.032057864	0.078211845	0.551147879
3.286330493	1.553989375	0.086910718	0.850563917
5.441999909	2.685663066	0.044200172	0.668780251
5.219317483	1.997672821	0.054213173	0.967948695
7.814264045	4.652874146	0.029069407	0.833568414
5.102921236	3.499587615	0.080851466	0.737439952
5.937088003	2.858498092	0.044904373	0.790820877
6.97925537	2.823479051	0.083025816	0.601953218
4.383962916	3.425628542	0.095679153	0.922025457
6.536048372	4.22150327	0.040805204	0.599225144
5.779370064	4.311574246	0.048280703	0.914349943
4.036199576	2.783521503	0.046601126	0.867894765
4.272308383	2.454280335	0.044749503	0.716003617
5.875411952	3.559649383	0.074251783	0.581227759
6.216069591	3.84159729	0.046260718	0.816953244
6.103119074	2.235959868	0.082904516	0.601834226
4.691538211	3.141473206	0.044212757	0.749482767
6.747312083	0.97774481	0.044695484	0.640038154
6.137324049	1.929124945	0.046956598	0.74600995
4.605159351	1.230883243	0.047670836	0.823020139
7.325564218	2.918451392	0.044511062	0.728999133
7.253843337	3.148374665	0.030736488	0.902882359
5.465275411	3.896226182	0.028276736	0.841516139
6.43618931	2.021439264	0.047935601	0.803479756
4.947890739	2.717655697	0.06909907	0.403301421
7.439021543	1.752705042	0.046220642	0.694784036
7.135476997	0.764217709	0.048404761	0.698841448
5.565787354	1.720142041	0.028853051	0.783374984
6.492164776	2.939276638	0.029419537	0.788934539
7.719748684	2.21608756	0.040693987	0.484991829
5.728043948	1.822955693	0.086734458	0.649379157
7.893141268	3.626400429	0.046011791	0.752427633
5.401481447	2.870632096	0.044224608	0.767049339
3.986634151	1.123560047	0.050155001	0.970819786
3.772773055	1.908750862	0.089175983	0.805444066
5.130491634	1.687691308	0.084393244	0.607849987
7.256546262	3.200673136	0.040360615	0.584472076
7.361185256	2.042020504	0.047557065	0.674422034
5.987413361	2.227791421	0.042716661	0.573775384
4.851008009	1.247723753	0.048707356	0.879762343
5.021102905	1.978380867	0.022092616	1.076501489
5.557052017	1.012910174	0.049572015	0.840300637
7.47283256	2.013081262	0.045966091	0.690903379
4.591865746	2.019138797	0.067203802	0.405906488
4.718822695	1.921341315	0.047128161	0.819410853
11.50505786	1.317621244	0.064827492	0.324149199
4.97704379	0.806199676	0.048549128	0.789149339
5.34292527	4.609631004	0.041715213	0.711881102
7.472761415	2.985727633	0.044583141	0.661793769
7.659143477	2.418668977	0.044361311	0.63203173
8.925062825	2.921266619	0.038384779	0.444295913
4.047479419	3.990838986	0.044895227	0.758683479
4.60521352	1.820181435	0.046262007	0.7899803
5.669217412	3.138513017	0.041467384	0.601795753
4.202272745	1.681911986	0.044942208	0.728699332
7.899487262	3.022908412	0.020219512	0.930165454
4.925825521	1.769822509	0.081042486	0.621750872
6.435246823	0.648298458	0.052699585	0.845275892
5.049145968	0.875713537	0.072714263	0.430217359
7.447331408	2.977621929	0.040282507	0.545769772

log.sigma.0.5.mm.3D_glrlm_LowGrayLevelRunEmphasis	log.sigma.0.5.mm.3D_glrlm_GrayLevelNonUniformityNormalized	log.sigma.0.5.mm.3D_glrlm_RunVariance	log.sigma.0.5.mm.3D_glrlm_GrayLevelNonUniformity
0.026493433	0.284621012	0.471768875	456.5862973
0.094936596	0.408550191	0.730387921	261.3570729
0.091379528	0.406251937	0.850213628	418.211523
0.063130042	0.340845868	0.681764706	232.1283574
0.056685257	0.361502854	0.764256679	584.7844147
0.061348311	0.311521288	0.456474386	154.2137408
0.05272677	0.368910769	0.69929306	1442.608624
0.056296	0.361746188	0.801734467	1246.893598
0.056342289	0.352824147	0.732623511	1112.901524
0.058117268	0.337402975	0.555142363	262.2132241
0.096879918	0.41405684	0.700813809	426.3605412
0.03738693	0.31965477	0.620100649	531.4900446
0.057241523	0.397732574	0.755854304	495.7520604
0.036951787	0.337281113	0.677917722	404.4205306
0.037573801	0.311937748	0.595055387	446.328225
0.056403478	0.385831377	0.789614784	1137.795793
0.096874868	0.350580448	0.559297832	301.4110739
0.054717032	0.415520645	0.786542104	336.5806531
0.053844221	0.390179938	0.641682635	349.942351
0.059655145	0.337658686	0.638562286	1244.849957
0.087299881	0.434945051	0.900286571	855.1698396
0.101252969	0.357586105	0.573407194	277.1904663
0.055295422	0.363805655	0.666848297	260.4239512
0.053788633	0.368068917	0.623753184	689.9806756
0.056105883	0.348622907	0.638848283	313.0882241
0.035276283	0.307491937	0.624466785	528.4079024
0.057361719	0.318641225	0.660979415	729.8888624
0.057800218	0.36985991	0.737577013	697.7458658
0.095204841	0.362339176	0.484267036	119.2382921
0.056139928	0.403655618	0.805667753	559.7696421
0.035810439	0.366702893	0.836016198	1172.433497
0.057082162	0.39599856	0.773825789	546.9186021
0.056719676	0.33528818	0.737934428	748.6550461
0.060937715	0.334885474	0.683261506	552.8591111
0.05467444	0.327833511	0.670038992	1026.064556
0.054509908	0.379706956	0.70969715	775.8371187
0.025776677	0.36449301	0.710298127	930.616634
0.035894714	0.376823339	0.765887515	1305.357699
0.058482014	0.387406259	0.702607592	324.7973791
0.100320197	0.349923863	0.644900753	1792.599864
0.099684128	0.389392684	0.776494366	676.8252605
0.104418011	0.303966479	0.469715072	882.0020835
0.055721017	0.365448636	0.750626368	922.1296663
0.06448361	0.314358176	0.580466901	568.9504152
0.036513238	0.357689166	0.724528494	315.9323387
0.098718455	0.347644111	0.655966604	678.3329732
0.055951802	0.346197674	0.695184382	1107.106614
0.102669389	0.408412588	0.786053348	238.3962462
0.11220085	0.315463179	0.513116361	199.2029836
0.052193206	0.384562042	0.727580381	356.6882286
0.058478575	0.325062068	0.611511939	598.5227834
0.056504116	0.313742209	0.530431257	781.0450873
0.055328974	0.34496671	0.608943302	1113.073668
0.09458255	0.394326675	0.765939917	291.9297733
0.056943997	0.349535062	0.63218314	329.2799183
0.103108487	0.384673356	0.669884658	514.6769423
0.05492322	0.338922129	0.670454631	969.4160549
0.057285098	0.377415309	0.712706687	888.3223727
0.058302245	0.361139528	0.650600807	415.7627797
0.05830979	0.323901288	0.57143539	2161.217592
0.055892053	0.370430026	0.720181267	448.612998
0.037784513	0.330826047	0.646830426	627.0182389
0.035092247	0.328347352	0.633688791	1355.903691
0.059418614	0.347618679	0.717579796	1834.782553
0.08989285	0.445433113	0.749102564	235.4946794
0.058488779	0.373169136	0.706954066	980.3091559
0.060367497	0.37698994	0.699193496	336.0973614
0.036292953	0.342437376	0.663143746	1504.20947
0.0360219	0.34800517	0.662064179	1470.78415
0.053480111	0.421809724	0.760186296	816.0748348
0.106707976	0.370641424	0.671212824	331.6725541
0.057726784	0.363009644	0.822702798	1048.784096
0.055603077	0.346111715	0.7011023	547.3745339
0.06028308	0.295693105	0.530932678	494.6095191
0.106869235	0.318071765	0.530080151	731.4951392
0.104044437	0.382594297	0.659940119	358.2016732
0.052151397	0.399037953	0.723237873	653.7305683
0.059083036	0.38643181	0.680719357	256.059189
0.054442432	0.389364538	0.654643414	460.3098719
0.059649087	0.321920858	0.630763135	537.7588807
0.026385595	0.292657793	0.463595755	442.1211457
0.060808746	0.337718985	0.650183562	429.0150457
0.057600758	0.369906984	0.719764929	834.5715296
0.08999006	0.438609108	0.879989097	870.5643096
0.058155311	0.330093841	0.646404557	570.3362903
0.090042514	0.490225466	1.152016855	451.8685275
0.059834686	0.335205449	0.644774597	568.1388897
0.052481401	0.351660695	0.719221497	669.5132831
0.056045281	0.392879556	0.707028393	397.8101301
0.056637993	0.391421227	0.81338985	526.097561
0.050898467	0.423492764	0.757449163	367.4591683
0.054417749	0.330606902	0.536236291	581.9905135
0.057165609	0.335601114	0.609664753	1242.801997
0.053143243	0.379512071	0.750208842	853.7852707
0.056134808	0.339975869	0.596243225	1055.528119
0.025386316	0.332525297	0.671452937	800.2625028
0.100589526	0.369637606	0.671581852	727.7650312
0.064129315	0.342763032	0.644799077	252.6673032
0.094463428	0.429686014	0.726302422	238.8591759
0.052266896	0.39737224	0.800486284	1538.264263

log.sigma.0.5.mm.3D_glrlm_LongRunEmphasis	log.sigma.0.5.mm.3D_glrlm_ShortRunHighGrayLevelEmphasis	log.sigma.0.5.mm.3D_glrlm_RunLengthNonUniformity	log.sigma.0.5.mm.3D_glrlm_ShortRunEmphasis
2.234392727	35.1286586	984.2236956	0.810398939
2.890430011	9.793715996	333.3433196	0.744329112
3.11877596	9.662971299	521.6742301	0.731982017
2.770121052	15.51746407	367.2371957	0.758449145
2.92897165	15.52145351	855.6571695	0.75270735
2.253483128	17.82052724	296.7752949	0.799375197
2.805447688	16.32554447	2084.192721	0.75577405
3.010072388	16.03058085	1786.49437	0.745851697
2.836079739	16.12741125	1701.996159	0.760246767
2.469921186	16.86449433	444.1696452	0.782662708
2.879190931	9.368992678	531.9687953	0.74096742
2.559331097	25.37373677	953.119168	0.784431798
3.018339403	15.34466556	621.8731903	0.729077682
2.746449421	23.97424914	650.5570472	0.762750962
2.534389936	24.82309587	815.7058528	0.782131292
3.017460989	15.50425321	1510.136346	0.739276556
2.509240674	10.35437208	484.4072399	0.775332894
3.125185349	15.05544278	393.5364113	0.718856866
2.753587294	15.67980374	468.5466399	0.746037627
2.641874067	16.9337567	2048.3658	0.772122912
3.290326134	9.361976229	951.1857503	0.716502638
2.528889332	10.29733266	435.9514326	0.775901485
2.725665917	16.23122001	388.6659269	0.762546019
2.641830048	16.66836131	1028.232086	0.765451015
2.669438382	16.87576647	491.1705919	0.763698671
2.544935833	26.37193626	1002.45378	0.790771695
2.670850443	17.03761453	1280.158426	0.774233711
2.895017309	15.74460179	990.2537981	0.750082261
2.346253818	10.7030204	191.4265011	0.78801619
3.034924847	15.42159868	711.4710231	0.740019583
3.071415081	23.3950286	1646.374124	0.742594863
3.034397351	14.66496841	689.5804202	0.729494248
2.856191	16.33115376	1198.240049	0.75725987
2.727670734	16.51144475	904.4755057	0.766183164
2.666501957	17.46754557	1762.616614	0.77759863
2.879298836	15.90258695	1055.474025	0.742890143
2.853539956	32.24133185	1344.42216	0.750930886
3.001592792	22.99048053	1753.840732	0.735255558
2.880883294	15.03902971	433.0553048	0.742372277
2.674831759	10.13102275	2806.28978	0.766125773
3.031704328	9.149592167	876.9698914	0.732649864
2.261179547	10.88074441	1748.083004	0.802673016
2.88760463	15.91214456	1342.823887	0.753441847
2.482558985	16.73135661	1051.023115	0.789332631
2.838534245	24.17724253	470.1669347	0.754747703
2.65919535	10.695386	1088.186937	0.772342983
2.744955221	16.60089754	1753.62352	0.767162103
3.047586848	9.456345608	295.4134064	0.734224872
2.359585499	10.93542888	372.8859638	0.793752202
2.876071292	16.45751257	486.2796573	0.748121893
2.569355917	17.45036574	1044.015049	0.780468616
2.376663447	17.35499337	1473.586689	0.796335766
2.573823355	16.51806414	1815.459242	0.777254761
3.029814216	9.880658037	373.6909078	0.732223458
2.637844855	16.9601629	519.5361513	0.768244709
2.814264284	9.684214435	694.8244068	0.744213833
2.693295191	16.690949	1589.2775	0.771593301
2.886709616	15.26035141	1210.262797	0.740684556
2.718808819	15.94482964	619.4718117	0.758799213
2.455216835	16.9307135	3896.469818	0.7914364
2.867079245	16.18831166	638.0805773	0.751171152
2.644077301	24.67856059	1057.363783	0.773882301
2.610287198	25.23885707	2337.73944	0.780016094
2.822662702	16.10116441	2828.921496	0.757774048
3.026421561	9.186297927	263.6508718	0.724899181
2.885451538	15.56615496	1354.466751	0.742400943
2.848171341	15.18846145	466.259253	0.748791969
2.702567107	23.70979018	2407.528716	0.766712752
2.599405827	24.99317704	2458.574447	0.790059315
3.021549424	15.09849898	962.7320578	0.727380096
2.809595402	9.589660288	469.676173	0.747148719
3.043548599	16.2338621	1494.30677	0.744646754
2.793367025	16.30709144	850.2723287	0.759165882
2.370609766	17.04025636	996.9733959	0.798825421
2.399075448	10.57844901	1349.219495	0.792483763
2.738784896	9.610187495	501.0451186	0.755930807
2.882311915	16.16210403	854.2042746	0.746034147
2.788566891	15.71882803	351.018821	0.752970037
2.763281045	15.76106702	624.4045019	0.751013307
2.604130213	16.56457039	945.2536654	0.779641446
2.210510063	35.36822311	934.8726478	0.813580496
2.678284908	15.84998779	699.2916645	0.767422467
2.842531924	15.95619703	1199.38909	0.754475992
3.309905195	9.042394222	945.7009437	0.709224089
2.667730134	16.17701015	959.6751197	0.770972411
4.048564304	8.111413884	398.5822414	0.662821364
2.664282789	15.88147284	943.7454016	0.770990713
2.807686513	17.10239169	1034.313153	0.763633041
2.827301038	16.04683676	534.2443298	0.75060609
3.114708492	15.24012602	673.5771165	0.730216847
3.03121538	15.64468032	432.0008982	0.727863434
2.381808792	17.68122622	1045.780038	0.797708892
2.581321796	16.39849517	2076.413641	0.775435872
2.920904894	16.08962751	1175.934684	0.74772334
2.579829019	16.33695413	1730.23451	0.772581199
2.707981333	34.01765202	1328.132084	0.76925949
2.737890304	9.891565731	1064.911947	0.760976872
2.681636011	15.77647105	399.5499155	0.761428236
3.013764247	8.795774619	274.8620932	0.721713984
3.039668824	15.85280018	1975.890664	0.738965725

log.sigma.0.5.mm.3D_glrlm_LongRunHighGrayLevelEmphasis	log.sigma.0.5.mm.3D_glrlm_RunPercentage	log.sigma.0.5.mm.3D_glrlm_LongRunLowGrayLevelEmphasis	log.sigma.0.5.mm.3D_glrlm_RunEntropy
100.161225	0.755528166	0.054506598	3.06426893
41.68921053	0.68342246	0.229339191	2.74629689
44.51188597	0.670193561	0.245208395	2.792281675
61.09982573	0.696086922	0.147044231	3.009349837
66.46882269	0.682464917	0.142620545	2.939084779
50.72139532	0.7495338	0.120234434	3.005263066
63.2677543	0.691450118	0.135106146	2.902579893
69.71212621	0.674387085	0.144052105	3.01238475
64.43142887	0.692967613	0.138564555	2.964060161
56.09764908	0.724526406	0.124276303	2.919080048
39.95441234	0.682105693	0.237236752	2.732898292
86.34322776	0.720607375	0.084263187	3.046103338
67.9395612	0.667133616	0.149047225	2.912428394
91.10950602	0.697683796	0.090367719	3.046599441
84.37599001	0.720494727	0.084401173	3.075535497
67.97118344	0.673287864	0.147755336	2.909699685
33.99339138	0.71962376	0.219605922	2.866880314
71.57305946	0.656372824	0.147871151	2.842349104
61.37154352	0.690470546	0.133919899	2.805827065
60.08915812	0.708583012	0.134395285	3.010841581
46.58158617	0.6498779	0.255617973	2.767678044
35.79014959	0.717758608	0.214871725	2.819352259
61.30441384	0.698692909	0.133890014	2.890054458
59.08796518	0.70747726	0.130015866	2.86044413
61.53776199	0.705118134	0.13063282	2.939081617
86.04533988	0.724583563	0.08237592	3.09565034
60.93601681	0.708011135	0.133612288	3.069055253
65.00671902	0.68267142	0.144258939	2.950941157
33.01375388	0.735501635	0.197035599	2.752800902
69.63999926	0.673994457	0.144961704	2.832023366
103.1790736	0.671290458	0.097694666	3.006551828
67.59190704	0.66710042	0.149654724	2.880485378
63.67947035	0.690878338	0.14360824	3.076841042
62.14939187	0.701963993	0.139617283	3.030148125
62.24955199	0.709914064	0.128113741	3.012797699
65.09738129	0.680893631	0.139754783	2.917082259
127.9758201	0.685589068	0.067410957	3.012989043
97.5767187	0.670934375	0.098747292	2.983018159
63.98035976	0.680863039	0.143952488	2.863991674
35.95361704	0.704232356	0.236819969	2.93130329
41.96787362	0.669503588	0.25391705	2.874695885
30.10554658	0.749875674	0.21327683	2.952723002
65.52393483	0.689570216	0.1404973	2.917792676
55.67022651	0.726997279	0.13340796	3.037495249
96.16094949	0.690381606	0.090853584	2.978707315
38.08474271	0.710130307	0.223077495	2.935824235
62.31362937	0.700485617	0.135149963	2.997482078
44.12324404	0.668992317	0.246376061	2.823048136
34.35512118	0.740486306	0.21407148	2.950849292
66.70074153	0.6854802	0.134311677	2.847203466
59.41637513	0.716231997	0.128848558	3.035972183
52.93779742	0.73812838	0.121601812	2.984138543
57.63161211	0.714777687	0.127527585	2.912234006
43.47831373	0.669523411	0.24186831	2.8693788
60.7585816	0.707807981	0.129960647	2.955169429
39.47752511	0.68556213	0.239386406	2.851956639
61.33836666	0.706247029	0.131229523	2.958914385
63.5293733	0.680317658	0.14551014	2.930238145
61.01422477	0.697862211	0.136265869	2.9347954
52.4460103	0.730545936	0.131540312	2.987655547
65.97520128	0.685077097	0.138382278	2.955350557
88.32409527	0.709392106	0.087611723	3.030337682
87.91458606	0.712890495	0.083592664	3.011990707
63.48415302	0.691873783	0.142692682	3.032128554
42.56069166	0.66835443	0.237766302	2.639296675
64.80346283	0.679819145	0.144544162	2.96630915
63.12988245	0.683400189	0.144944261	2.923229188
88.47077972	0.702436923	0.088960221	2.998375308
86.36730529	0.720978185	0.08466013	2.922646359
67.55726498	0.66722408	0.145267794	2.774620768
39.56399705	0.688103275	0.241355179	2.888287493
70.73080211	0.672996235	0.147352425	3.022958837
63.39772588	0.693455294	0.136928569	3.010885964
52.48827442	0.739912146	0.126055167	3.047665377
32.77902713	0.734196497	0.22070406	2.95568724
38.18226844	0.69706523	0.236303501	2.823398603
65.38520853	0.683254142	0.136857988	2.840777581
62.75296176	0.69110326	0.139584072	2.866034052
61.76439007	0.691902122	0.134961336	2.812028595
57.00734696	0.71378301	0.136837434	3.048446048
99.29776439	0.759059442	0.053755607	3.028071933
59.43695258	0.705349126	0.138536894	3.012198019
63.65851055	0.689578088	0.1420736	2.939724883
45.88623079	0.645447946	0.263898119	2.767500084
59.50153544	0.70757246	0.135423476	3.005885255
58.25659445	0.600823459	0.304978404	2.670684289
58.40163906	0.709627479	0.138487339	2.98746438
65.51982711	0.693850642	0.131978475	2.95008743
64.54641085	0.690521227	0.137196967	2.846467359
71.58513232	0.66294397	0.148968931	2.912120245
68.99301496	0.665172251	0.141712502	2.744308987
53.94223332	0.738519667	0.118001608	2.878807242
57.28860786	0.714050372	0.131053729	2.980487866
66.79266728	0.681188811	0.138887746	2.881219604
56.29280805	0.712811086	0.13190389	2.946676647
124.8508668	0.702805377	0.062656038	3.064760826
37.70576777	0.698369788	0.235390643	2.877591283
59.88069922	0.70340166	0.141524894	2.991339832
41.79455847	0.665928795	0.243672147	2.68714715
69.59846348	0.670205963	0.142723401	2.860625545

log.sigma.0.5.mm.3D_glrlm_HighGrayLevelRunEmphasis	log.sigma.0.5.mm.3D_glrlm_RunLengthNonUniformityNormalized	log.sigma.0.5.mm.3D_glszm_GrayLevelVariance
43.68396637	0.612491394	3.897212313
13.50514182	0.520064439	2.274375
13.48306783	0.504170546	2.394557823
20.89928123	0.537531932	2.689586777
21.17449501	0.527772072	2.374023438
22.28051447	0.597379257	4.163580247
21.82672463	0.532119937	2.763668639
21.92708092	0.517875272	3.224359316
21.57132375	0.538269013	2.902080597
21.84639782	0.570668727	3.030311419
12.97350334	0.514668931	2.530276817
32.67411628	0.57261409	3.646565083
21.45045025	0.498023378	3.497933884
31.86632887	0.541672136	3.725872576
32.11239924	0.569419512	3.470742932
21.36826688	0.510674525	2.967657068
13.38692546	0.56133727	2.481229529
21.51330532	0.484866944	2.463137996
21.33152982	0.521343723	2.66830029
22.10201547	0.554812094	3.548238857
13.3314546	0.482381803	2.153187201
13.47431777	0.561283834	2.46875
21.56587343	0.542050678	2.726802643
21.89310826	0.546945746	2.848561851
22.32094109	0.545187837	3.110803324
33.39504682	0.582298515	3.538420661
22.18242776	0.557783973	3.553833395
21.35229148	0.524210758	3.036938776
13.69127125	0.58011654	2.464285714
21.42858118	0.512191428	2.690402806
32.04017532	0.514184183	3.250810321
20.70225949	0.498573132	2.66617357
21.70005484	0.53488217	3.564432133
21.84498189	0.546903162	3.347004563
22.63806769	0.562547724	3.208141771
21.70629337	0.515682287	3.073302928
43.42553996	0.525542082	3.99144499
31.56905901	0.505349248	3.77747499
20.80056482	0.515210649	2.815743945
13.25582174	0.546706937	2.704375
12.84475958	0.502944396	2.66387051
13.4631616	0.600963296	2.822937906
21.51487637	0.530183134	2.743047091
21.48248276	0.579928579	3.405624928
32.52305074	0.53163609	3.712157556
13.93641946	0.556251995	3.065938847
21.8498305	0.547720991	3.362734784
13.33107756	0.504791334	2.821644713
13.94868197	0.589056691	3.086734694
22.29489276	0.523035271	2.468954573
22.52247261	0.566492508	3.588492766
21.88118315	0.590860266	3.070855441
21.51786539	0.562101272	2.801574372
13.70046388	0.502648132	2.608024691
22.30088041	0.550871889	3.029135802
13.27000817	0.51813813	2.672382562
21.89522312	0.554488483	2.780937249
20.9636576	0.513212618	3.29
21.37857885	0.537115339	3.077708333
21.37437665	0.583038079	3.429841661
21.9419445	0.525893607	3.428419598
32.25883757	0.557276206	3.915004591
32.6587899	0.565557939	3.2559078
21.55084884	0.534845558	3.51689964
13.0943972	0.496034047	1.933402706
21.34740779	0.514549328	3.285727812
20.79746968	0.522497936	3.288337925
31.37927656	0.547129152	2.907302085
31.98414285	0.580842461	2.664558624
21.20262569	0.496662011	2.499946859
13.15971726	0.522808779	2.536422366
22.15383871	0.516597797	3.336242809
21.75803734	0.536853308	3.223725286
21.48128382	0.594767163	3.289845786
13.39573733	0.585108596	2.734603175
13.02173017	0.533866022	2.738754325
21.90235841	0.520179563	2.78125
21.30877031	0.52906286	3.378698225
21.3460945	0.52679049	2.399518141
21.38233903	0.565161868	3.208029821
43.81094205	0.617629006	3.714678069
21.03708452	0.549075741	3.319015268
21.46564073	0.530434621	3.410585938
13.05692296	0.474371485	2.188923575
21.30301447	0.553706317	2.911435465
12.92658436	0.427635044	0.785123967
20.91479719	0.554278928	3.125925159
22.60126001	0.542480499	2.771186855
21.76401593	0.526687903	2.993905817
21.463089	0.499422501	2.964677641
21.83885923	0.496836811	1.733398438
22.26318741	0.592994962	2.936175888
21.38402792	0.559831784	3.203114727
21.85059191	0.521631387	2.414653189
21.29566054	0.555963548	2.794681057
44.66918843	0.550879118	3.927512894
13.16489009	0.539769135	2.714177495
21.15719237	0.541285148	3.329369798
12.67446982	0.492087542	2.164930556
21.82254356	0.50945556	2.394735069

log.sigma.0.5.mm.3D_glszm_SmallAreaHighGrayLevelEmphasis	log.sigma.0.5.mm.3D_glszm_GrayLevelNonUniformityNormalized	log.sigma.0.5.mm.3D_glszm_SizeZoneNonUniformityNormalized
26.8164105	0.220306513	0.4115471
8.059475609	0.27	0.47875
6.917544441	0.310657596	0.332829428
11.00710637	0.257520661	0.292561983
11.75792525	0.303955078	0.4296875
12.66351433	0.211248285	0.302469136
13.79745754	0.320473373	0.374585799
11.62071946	0.248548138	0.378576376
11.89378379	0.295505723	0.360034716
12.40356242	0.250103806	0.399861592
9.061743977	0.306228374	0.377595156
16.84607034	0.20745093	0.356404959
10.70596707	0.203397612	0.355371901
18.46691807	0.242548476	0.374626039
17.8579983	0.245397765	0.371055227
12.0126472	0.235265521	0.418799508
11.42452955	0.294532628	0.389770723
9.460913932	0.280718336	0.427221172
14.44571592	0.345956783	0.438627757
12.96875552	0.243828998	0.416249431
8.117745497	0.310657596	0.314184933
6.463349801	0.346679688	0.315429688
12.82325425	0.295604711	0.354208561
13.27756672	0.257893599	0.414900519
12.29122452	0.246191136	0.336565097
20.90047402	0.189658837	0.377245191
12.66476264	0.227086848	0.364923218
12.03478375	0.262857143	0.332959184
7.98782791	0.323979592	0.303571429
11.79997386	0.311085131	0.484110958
16.89340067	0.239909461	0.421892259
12.07717561	0.319526627	0.392504931
14.77718526	0.243933518	0.359612188
12.91552349	0.212854592	0.38176949
13.64981679	0.253049353	0.38794802
11.99730567	0.255998414	0.373521052
24.82688771	0.197766012	0.439243285
15.9273155	0.213596759	0.308257736
12.29762183	0.312283737	0.41133218
9.287506843	0.3266375	0.36705
9.190127864	0.315926276	0.356332703
9.990199274	0.264041742	0.332708552
11.69736243	0.261218837	0.339445983
13.16621851	0.232281189	0.376517516
18.70308212	0.242772377	0.427092539
10.3154967	0.228353309	0.419010324
14.79893982	0.236319769	0.367279664
10.01311218	0.263082948	0.428266287
8.002995562	0.229387755	0.305306122
12.6100013	0.302122595	0.427097798
12.46315112	0.242607504	0.351598784
15.1260458	0.271176394	0.382295537
13.64287767	0.320830455	0.407056002
5.717815981	0.27914952	0.344993141
12.20424556	0.249382716	0.371604938
7.091996712	0.35032001	0.304673349
10.73179604	0.298013003	0.31214844
13.92113939	0.286933333	0.4448
14.21580196	0.266388889	0.459583333
14.53231812	0.240586847	0.383263226
12.84318692	0.229036029	0.367201615
18.28093152	0.231955923	0.389237833
18.50772283	0.232889979	0.381618027
13.83457972	0.26630917	0.442991311
8.86329438	0.329864724	0.448491155
12.1210385	0.249779068	0.388713546
11.74112456	0.272268136	0.389348026
16.95897921	0.269155267	0.352162072
19.67977868	0.207487551	0.374622001
9.514000248	0.322350941	0.353385057
7.076016572	0.294274894	0.308977501
12.42424846	0.240141947	0.42652472
12.80135052	0.249219563	0.378902185
12.9270844	0.230039728	0.339655688
8.638783994	0.294013605	0.34675737
7.43949655	0.334775087	0.366782007
14.86396061	0.274902344	0.364990234
12.54031749	0.24704142	0.452662722
12.35828791	0.286848073	0.386621315
14.64613837	0.278342054	0.359597825
25.01127078	0.212758653	0.369495334
13.42275439	0.221710863	0.349989042
12.11176547	0.22421875	0.345234375
9.175370093	0.378887733	0.465140479
11.10041225	0.254538982	0.293857047
3.633840885	0.495867769	0.446280992
13.02293409	0.244656074	0.356555044
13.66212322	0.258154433	0.383441733
15.0439595	0.250304709	0.486537396
13.46835628	0.274348422	0.470336077
7.164442951	0.37890625	0.322265625
13.56389797	0.28494132	0.434156379
14.43496756	0.27681213	0.374486358
13.84155051	0.299556213	0.401216305
14.4145328	0.332091054	0.391124163
25.12948428	0.202408458	0.387663656
8.934294341	0.288736088	0.346891207
10.4132999	0.253269917	0.476813317
8.31041098	0.395833333	0.416666667
10.78942827	0.29157027	0.416949305

log.sigma.0.5.mm.3D_glszm_SizeZoneNonUniformity	log.sigma.0.5.mm.3D_glszm_GrayLevelNonUniformity	log.sigma.0.5.mm.3D_glszm_LargeAreaEmphasis	log.sigma.0.5.mm.3D_glszm_ZoneVariance
71.6091954	38.33333333	7687.977011	7539.249174
19.15	10.8	10054.725	9508.334375
20.96825397	19.57142857	16620.4127	16027.52885
16.09090909	14.16363636	6318.890909	6003.344132
55	38.90625	18033.57813	17691.32813
16.33333333	11.40740741	2744.074074	2594.691358
97.39230769	83.32307692	49846.35	49373.95649
103.729927	68.10218978	40273.20438	39925.6677
105.130137	86.28767123	28199.18493	27956.80695
33.98823529	21.25882353	4965.529412	4806.472803
25.67647059	20.82352941	14737.70588	14245.91003
62.72727273	36.51136364	10822.38068	10650.85999
23.45454545	13.42424242	23544.04545	22743.84137
35.58947368	23.04210526	11975.8	11649.14194
57.88461538	38.28205128	8420.75641	8259.010191
78.31550802	43.99465241	43657.06952	43109.45946
24.55555556	18.55555556	8416.253968	8057.061224
19.65217391	12.91304348	15619.41304	14900.93998
29.3880597	23.17910448	10817.97015	10442.65182
140.6923077	82.41420118	30113.72485	29877.12949
19.79365079	19.57142857	68970.60317	66666.60317
20.1875	22.1875	7205.390625	6921.1521
20.89830508	17.44067797	7157.966102	6856.737719
56.42647059	35.07352941	20390.66912	20011.85159
25.57894737	18.71052632	8323.118421	8042.555921
73.18556701	36.79381443	9486.860825	9337.743995
68.24064171	42.46524064	19574.65775	19275.75618
46.61428571	36.8	22404.07143	22014.8549
8.5	9.071428571	2815.821429	2560.96301
46.95876289	30.17525773	20317.42268	19869.03114
99.14468085	56.3787234	40656.05106	40246.11781
30.61538462	24.92307692	24499.75641	23796.14678
68.32631579	46.34736842	20226.05789	19937.23681
54.21126761	30.22535211	14566.50704	14292.62785
113.2808219	73.89041096	23894.17808	23666.70557
45.94308943	31.48780488	30798.57724	30204.08976
77.30681818	34.80681818	31644.85795	31197.87187
51.47904192	35.67065868	65450.05389	64494.98483
27.97058824	21.23529412	9695.588235	9368.403979
146.82	130.655	49195.76	48865.42938
32.7826087	29.06521739	31585.84783	30790.85302
93.4911032	74.19572954	16004.48043	15815.09973
64.49473684	49.63157895	28366.45789	27996.80668
70.03225806	43.20430108	11612.69892	11433.77234
38.01123596	21.60674157	7719.595506	7513.398813
66.62264151	36.3081761	17963.45283	17665.40192
98.06367041	63.09737828	30179.75281	29887.81655
22.69811321	13.94339623	6805.528302	6535.453186
21.37142857	16.05714286	3474.242857	3326.446735
30.32394366	21.45070423	11217.29577	10854.68836
60.8265896	41.97109827	13910.01734	13689.50342
100.5437262	71.31939163	14276.01141	14111.72334
112.7545126	88.8700361	27922.51264	27657.18735
18.62962963	15.07407407	9899.555556	9481.580247
33.44444444	22.44444444	7802.777778	7584.395062
27.72527473	31.87912088	18067.01099	17607.82732
73.04273504	69.73504274	24564.67094	24265.85392
66.72	43.04	33250.37333	32718.91716
55.15	31.96666667	9229.516667	9040.912222
196.6140351	123.4210526	54558.35283	54241.67935
35.6185567	22.21649485	13686.30928	13354.84409
64.22424242	38.27272727	16020.65455	15758.80331
134.3295455	81.97727273	34355.35511	34084.88345
213.9648033	128.6273292	47193.81988	46944.72914
13.90322581	10.22580645	9704.193548	9054.765869
69.19101124	44.46067416	35240.7191	34769.97538
25.6969697	17.96969697	10975.0303	10584.6685
126.4261838	96.62674095	41164.08357	40860.99389
176.0723404	97.51914894	24871.34681	24716.05279
34.27835052	31.26804124	40110.06186	39217.46923
20.70149254	19.71641791	10337.74627	9961.84941
90.84976526	51.15023474	36129.53991	35723.88627
46.98387097	30.90322581	16263.3629	15925.57408
51.96732026	35.19607843	10340.83007	10122.83353
72.81904762	61.74285714	15433.8619	15211.568
24.94117647	22.76470588	11332.82353	10943.3417
46.71875	35.1875	19506.95313	19156.56152
23.53846154	12.84615385	7716.807692	7377.397929
32.47619048	24.0952381	14648.32143	14235.36097
61.49122807	47.59649123	11043.26901	10856.1714
66.1396648	38.08379888	6479.73743	6356.266846
40.94871795	25.94017094	10251.61538	10015.19191
55.2375	35.875	27088.11875	26670.68277
43.25806452	35.23655914	48305.19355	47213.35299
46.72327044	40.47169811	13388.77358	13153.46956
9.818181818	10.90909091	58293.81818	53444.59504
49.56115108	34.00719424	14747.10072	14452.69437
54.83216783	36.91608392	20592.68531	20225.01139
46.22105263	23.77894737	10010.4	9772.915679
50.7962963	29.62962963	17475.13889	17123.57639
10.3125	12.125	24657	22996.4375
70.33333333	46.16049383	12203.88889	11987.68999
116.8397436	86.36538462	31765.75962	31489.79511
62.58974359	46.73076923	29635.88462	29188.39941
101.3011583	86.01158301	26851.15058	26568.80679
80.24637681	41.89855072	21146.57971	20873.29249
45.44274809	37.82442748	24212.31298	23749.89884
27.65517241	14.68965517	7341.327586	7015.462842
10	9.5	13730.08333	12522.52083
87.97630332	61.52132701	69649.40284	68900.56351

log.sigma.0.5.mm.3D_glszm_ZonePercentage	log.sigma.0.5.mm.3D_glszm_LargeAreaLowGrayLevelEmphasis	log.sigma.0.5.mm.3D_glszm_LargeAreaHighGrayLevelEmphasis	log.sigma.0.5.mm.3D_glszm_HighGrayLevelZoneEmphasis
0.081998115	172.8935773	348869.8161	41.71264368
0.042780749	722.3019583	147561.525	11.425
0.0410691	1207.550617	241704.8095	13.19047619
0.05629478	286.3544115	144986.7091	18.98181818
0.054054054	795.5741861	421398.0625	18.125
0.081818182	128.1333975	61413.33333	23.42592593
0.046009556	2283.721846	1130420.5	21.85384615
0.053641347	1762.38567	946520.7007	19.57664234
0.064232292	1263.795517	650787.0342	20.67465753
0.079291045	221.4857382	115142.8471	20.08235294
0.045092838	1098.15337	210384.5147	13.97058824
0.076355748	323.2121633	369561.767	28.30681818
0.03535083	1065.15143	539514.7879	19.86363636
0.055329062	365.2905588	402155.2526	30.54736842
0.078629032	250.4217655	288514.9103	29.76923077
0.04273309	1995.802546	991895.2995	19.09625668
0.052763819	663.9553351	115213.2381	16.87301587
0.03730738	691.6911711	363878.6087	15.17391304
0.051617874	500.5762513	243375.1642	21.40298507
0.065012502	1366.92137	688114.7604	20.77810651
0.020833333	5098.502037	990294.6984	14.12698413
0.05931418	532.0552995	103664.7656	13.03125
0.057617188	326.825242	162888.3898	21.40677966
0.05137892	947.3866403	457261.5809	21.47794118
0.059701493	371.4182972	192887.0921	21.73684211
0.081891093	284.7150836	322804.0464	33.95876289
0.057841015	893.7683329	445499.8717	21.40106952
0.050687907	1014.622237	512974.8071	20.55714286
0.062639821	210.9453175	40139.92857	14.71428571
0.047224927	891.0207649	476737.7113	17.87628866
0.0493905	1222.736074	1379832.838	27.32340426
0.037699372	1106.73253	562063.4744	17.75641026
0.058841747	942.127696	452639.2632	24.14736842
0.060425532	655.5987242	335285.1338	20.66901408
0.066303361	1053.012767	558748.2466	21.9760274
0.041013671	1396.90426	704279.3171	19.79674797
0.047299113	713.0707883	1432393.318	37.11931818
0.03235807	2054.2762	2144012.323	29.92814371
0.055284553	443.9576122	220020.1324	18.11764706
0.055020633	3961.033651	659734.9875	14.78
0.035466461	2379.013889	447378.5652	13.51086957
0.072666149	1336.647407	208279.669	16.02491103
0.052012045	1267.484705	656226.9	20.29473684
0.074758842	529.5879658	264550.8065	20.14516129
0.069640063	229.181563	264747.3483	30.07865169
0.057923497	1345.26232	255699.9937	15.60377358
0.058526962	1371.98044	688779.2734	23.09363296
0.060849598	474.0724581	101941.1698	13.8490566
0.082256169	242.2508392	52353.37143	14.35714286
0.052514793	491.5253636	263464.8592	19.73239437
0.067341378	620.7891827	322261.3815	21.68786127
0.078018392	668.5787383	318103.3802	23.5095057
0.061391844	1282.24194	632124.0722	21.13357401
0.048913043	719.7015432	143939.2222	13.35185185
0.067669173	347.1933016	181193.4111	21.04444444
0.046666667	1335.659463	259489.0659	13.25274725
0.057849197	1095.70738	569108.047	20.5
0.043377675	1545.517477	745340.0933	20.1
0.072815534	418.5395959	211084.475	20.50833333
0.056194545	2691.59379	1161162.926	23.02729045
0.054926387	607.7266666	318196.6495	21.55670103
0.061797753	487.50583	538865.8667	29.03636364
0.060804975	1045.078187	1155511.986	30.28409091
0.063360882	2144.877468	1077183.501	20.76604555
0.039240506	702.0136918	141560.9355	12.5483871
0.046090109	1593.411094	807799.6854	19.06179775
0.050613497	504.1536918	248492.0303	18.33333333
0.05744	1275.204328	1363578.292	28.18662953
0.08024586	754.0737063	838655.117	31.57234043
0.03347136	1827.855238	913459.0619	17.92783505
0.051578137	764.4368201	148542.806	11.71641791
0.04965035	1588.830187	846325.3192	19
0.054409829	732.8709588	373940.629	21.35483871
0.067729084	479.4705489	232905.0065	21.67320261
0.067071223	1221.091933	210313.5667	14.75238095
0.050670641	836.869906	163139.5147	13.20588235
0.053422371	874.6354553	449988.8359	24.171875
0.054279749	347.6072992	177470.0769	18.76923077
0.049209139	674.6429678	330848.2619	20.05952381
0.073108166	523.6527145	243571.2924	23.74853801
0.089994972	144.2181184	296665.2626	41.54748603
0.065036131	467.1917811	233675.2137	20.29059829
0.048944631	1244.827018	612864.275	20.58125
0.030263586	3636.035606	684106.3011	13.08602151
0.065190652	608.6068163	305741.4465	20.88050314
0.014360313	4142.561162	860881.6818	5.545454545
0.058280922	683.8349089	331464.5324	20.5971223
0.052151714	907.1601528	481818.9161	22.11888112
0.06489071	440.6408197	234257.4947	21.16842105
0.053333333	762.6456658	411551.213	20.01851852
0.024539877	1112.84898	566111.9688	16.03125
0.068010076	567.5268881	273615.5432	21.66049383
0.060196797	1457.963813	719480.7147	22.37179487
0.047272727	1305.878232	692989.8654	22.26282051
0.059512868	1268.386138	593802.7297	22.50965251
0.060490941	461.8499933	983196.1739	39.86956522
0.046503372	1855.226283	338414.458	14.30534351
0.055396371	337.3986806	166515.6724	16.68965517
0.028776978	1024.7675	195864.2083	12.45833333
0.036543124	3131.048175	1603561.18	17.68246445

log.sigma.0.5.mm.3D_glszm_SmallAreaEmphasis	log.sigma.0.5.mm.3D_glszm_LowGrayLevelZoneEmphasis	log.sigma.0.5.mm.3D_glszm_ZoneEntropy	log.sigma.0.5.mm.3D_glszm_SmallAreaLowGrayLevelEmphasis
0.666208156	0.042193326	4.276393288	0.031922748
0.713698426	0.253708333	3.45015896	0.194745809
0.59313587	0.20739418	3.721515893	0.15952521
0.551502796	0.12430035	4.082121315	0.06726396
0.678726339	0.102560631	3.821508059	0.074393176
0.555664754	0.136801724	4.15714804	0.099819889
0.635625674	0.084668247	4.052163996	0.057303874
0.638924073	0.125160701	4.218186311	0.095829353
0.619772867	0.102873538	4.065357237	0.074578049
0.654353949	0.117895025	3.853731881	0.090597322
0.630348239	0.217144608	3.534207264	0.156903673
0.613985821	0.071042811	4.352811573	0.048304959
0.611129874	0.150813492	4.107974143	0.111150616
0.634185361	0.068539981	4.081941926	0.052298205
0.627938433	0.064251973	4.253837878	0.04639455
0.671935448	0.125634663	4.161709868	0.091531497
0.645092922	0.154598765	3.646298274	0.1094465
0.672810645	0.149703737	3.311635739	0.123474784
0.687410847	0.093032372	3.329072645	0.071450996
0.67094989	0.143412602	4.2339996	0.115273092
0.564486257	0.160189594	3.992399507	0.099845844
0.576150961	0.231827257	3.69553081	0.161393166
0.614605623	0.09693695	3.757927008	0.069126404
0.669302049	0.102374783	3.957755735	0.081017162
0.598666282	0.117172097	4.054726938	0.088108738
0.637340812	0.053514028	4.530380101	0.038415021
0.627184187	0.124032348	4.368981607	0.094866591
0.591368142	0.118311144	4.17203026	0.073381875
0.561882683	0.189017857	3.54952346	0.13481353
0.720917448	0.132133273	3.46289523	0.109152726
0.673595931	0.0692265	4.202019103	0.053494977
0.647716471	0.114100456	3.594125343	0.072012576
0.620125386	0.101628544	4.305444418	0.074124493
0.64034418	0.134371407	4.484967871	0.092961515
0.647112511	0.100346285	4.265513254	0.074403354
0.628148378	0.114671295	4.111088832	0.083962837
0.688372699	0.04936638	4.272237838	0.038183019
0.574814859	0.076419111	4.657612349	0.057034382
0.663309845	0.127726591	3.439120159	0.092583354
0.629069428	0.201295139	4.012841432	0.140818535
0.619328787	0.230872585	3.84063224	0.159515662
0.596262256	0.188950642	4.268522879	0.13416057
0.601983719	0.107183852	4.297985183	0.077124363
0.635311941	0.136022968	4.198327709	0.082751717
0.677448687	0.071228823	3.90920151	0.057260483
0.672021113	0.214608175	4.080912081	0.152932864
0.625720875	0.095247756	4.371366145	0.059827246
0.678559121	0.244323899	3.540105591	0.173287372
0.565829022	0.251700275	4.256519891	0.169616256
0.674999522	0.098164862	3.585605525	0.074013907
0.61422944	0.128019396	4.324334843	0.094138184
0.641920947	0.090068668	4.213258877	0.063288578
0.662937595	0.092848486	3.860616013	0.067116299
0.606373667	0.242309671	3.552693794	0.207197392
0.632062941	0.113750693	3.95890008	0.079088842
0.562035207	0.249819902	3.800124585	0.152784525
0.577184505	0.096683147	4.33152768	0.064496086
0.692973761	0.124827324	3.76371249	0.096373341
0.704039594	0.113356493	3.713419044	0.086672494
0.643384294	0.112755461	4.420566755	0.08519977
0.621927063	0.110751295	4.122053609	0.074894276
0.648005772	0.08354653	4.303646916	0.065123121
0.640693862	0.057436584	4.407700655	0.041162249
0.691817901	0.132062248	4.047944968	0.103697921
0.692065805	0.171227599	2.99436123	0.126661065
0.647026782	0.139446212	4.129103837	0.099400979
0.647206311	0.148524445	3.594464178	0.11135859
0.614081678	0.05774294	4.31133579	0.037860731
0.63602825	0.049951453	4.574028733	0.032118427
0.615328288	0.109662085	3.710849091	0.079415249
0.564066455	0.265281924	3.819873992	0.148954827
0.677910561	0.145077449	4.137749562	0.107107824
0.63916267	0.100805	4.100007349	0.073984672
0.602481115	0.106563422	4.429551772	0.068147703
0.608850299	0.207332631	4.219246844	0.145331226
0.617910876	0.252504085	3.432637831	0.184210944
0.62414968	0.07911785	3.990491632	0.055169178
0.69705857	0.154991497	3.587888996	0.130852752
0.640214812	0.095484356	3.770850991	0.065731574
0.622079104	0.097128121	4.165603465	0.063294494
0.629208382	0.041592148	4.411655702	0.030531307
0.612742883	0.122824752	4.221008234	0.084180984
0.608529935	0.137183718	4.364309422	0.105054984
0.707743258	0.181965352	3.204429222	0.139565452
0.555374412	0.100205722	4.408926338	0.064475327
0.68970984	0.321578283	2.642423962	0.233273384
0.619175315	0.118457244	4.285111752	0.084004564
0.642446185	0.089401115	4.228348191	0.063525316
0.72297406	0.109664757	3.582977183	0.086646251
0.711951866	0.123875714	3.588213865	0.10343943
0.572035178	0.111696783	3.177549678	0.082529991
0.682352333	0.101181693	3.807123646	0.082196883
0.635523358	0.098955926	4.216957617	0.068996858
0.656445244	0.086160751	3.959512264	0.063576341
0.650268197	0.089715661	3.912217427	0.063213426
0.645520887	0.044155681	4.474063539	0.032019924
0.610292967	0.216946565	4.103416277	0.144334437
0.715449756	0.184771777	3.59105819	0.147878126
0.662671044	0.188900463	2.855388542	0.139070436
0.671008965	0.105580864	3.84642052	0.080149923

log.sigma.0.5.mm.3D_ngtdm_Coarseness	log.sigma.0.5.mm.3D_ngtdm_Complexity	log.sigma.0.5.mm.3D_ngtdm_Strength	log.sigma.0.5.mm.3D_ngtdm_Busyness	log.sigma.0.5.mm.3D_ngtdm_Contrast
0.002752067	0.014164046	0.110959663	4.457800069	9.47E-06
0.005232716	0.009174515	0.078109513	6.351158673	1.69E-05
0.003405053	0.005268115	0.050509832	9.839404174	9.24E-06
0.005583603	0.013647623	0.112176768	4.132265118	2.09E-05
0.002194278	0.005487302	0.048597482	10.07365577	6.76E-06
0.008244213	0.028216637	0.209498303	2.447901095	3.31E-05
0.000924823	0.002998962	0.027928886	19.95466237	1.90E-06
0.001030928	0.00330339	0.030638286	17.67208598	2.43E-06
0.001200501	0.003810057	0.033635934	15.48900199	2.84E-06
0.00530732	0.012498623	0.103327219	4.318506539	1.91E-05
0.003234337	0.005595629	0.049040792	10.49869494	9.85E-06
0.002445568	0.010789992	0.089349364	5.495365681	6.79E-06
0.002597125	0.006660663	0.063342508	8.365589179	7.54E-06
0.003213891	0.010931382	0.104821899	4.884556127	9.36E-06
0.002830992	0.013632974	0.110817915	4.824818345	8.31E-06
0.001164763	0.003760914	0.036889844	15.64042898	2.35E-06
0.004616344	0.007600026	0.057436325	8.135575843	1.93E-05
0.003967946	0.009666652	0.102685379	5.291895921	8.91E-06
0.003830506	0.009824393	0.090457271	5.734813603	9.75E-06
0.001045877	0.003406923	0.028563771	18.14864254	3.00E-06
0.001591344	0.002712126	0.025539438	20.6363844	3.94E-06
0.004813814	0.005804093	0.043543801	9.983368153	3.20E-05
0.004977848	0.013079077	0.109023558	4.498586652	1.58E-05
0.002028076	0.004875937	0.044122047	11.0916597	5.66E-06
0.004349759	0.010218484	0.090717982	5.169938921	1.43E-05
0.00253631	0.010088783	0.090807507	5.420723165	6.47E-06
0.001795648	0.005624824	0.04494543	10.7944082	5.23E-06
0.001889731	0.00460066	0.041873659	11.76533133	5.73E-06
0.011808958	0.020703813	0.150940248	3.041836943	4.95E-05
0.002408738	0.005966543	0.059294977	8.794282182	6.20E-06
0.001115933	0.00462696	0.048709048	11.5354814	2.02E-06
0.002291265	0.006208627	0.055567767	9.500404953	6.52E-06
0.001785998	0.00692018	0.068195055	9.197967938	3.49E-06
0.002434745	0.007408713	0.064162738	7.795763095	6.73E-06
0.001352905	0.003971137	0.034556564	13.94001543	3.46E-06
0.001707632	0.007347886	0.074972222	9.147812837	2.80E-06
0.001348396	0.008099547	0.084268135	7.281663371	2.45E-06
0.001012517	0.004175564	0.045688662	12.9126746	1.67E-06
0.003946542	0.010572948	0.091784705	5.558304849	1.19E-05
0.00074208	0.001244126	0.009407385	50.8622941	3.14E-06
0.001911663	0.003384642	0.027490073	18.22464262	6.77E-06
0.001490514	0.003776476	0.025751904	21.83550102	6.28E-06
0.001469003	0.003492232	0.032034761	15.09005145	4.21E-06
0.002207886	0.005759543	0.040319948	10.84822131	1.09E-05
0.004276902	0.017302965	0.179972602	3.016865015	8.55E-06
0.002024886	0.006770036	0.061332231	11.81707987	4.90E-06
0.001175891	0.003843586	0.032781993	16.10305149	3.16E-06
0.005676506	0.009576302	0.083766958	5.831538856	2.03E-05
0.006984323	0.016335079	0.118776881	4.289688133	2.92E-05
0.003980547	0.009117445	0.092719049	5.37730086	9.41E-06
0.002229581	0.006955868	0.057250473	8.57151308	6.72E-06
0.001665186	0.005639285	0.041401188	11.95449475	5.31E-06
0.001158962	0.003053021	0.024053286	19.82976185	4.03E-06
0.004760718	0.007440248	0.06739123	7.166185777	1.54E-05
0.004080333	0.00977796	0.085581299	5.567671913	1.47E-05
0.002611202	0.004457924	0.036885083	13.19823407	9.62E-06
0.001388681	0.004386139	0.037234819	13.56561989	3.38E-06
0.001461211	0.003714754	0.033013383	15.33083844	4.34E-06
0.003138338	0.010634472	0.091214262	5.936362568	8.10E-06
0.000607582	0.002056835	0.015436722	33.21130811	1.85E-06
0.002932007	0.009538827	0.088083615	6.239177336	7.02E-06
0.002110222	0.011329106	0.100320621	5.498973718	4.06E-06
0.000984168	0.004100091	0.037642294	13.57618967	2.13E-06
0.000688209	0.002298278	0.019606935	27.45818046	1.91E-06
0.006064665	0.00702319	0.069172807	7.004880564	2.17E-05
0.001306711	0.004471508	0.039556284	14.09668976	3.17E-06
0.003657549	0.010277898	0.08275007	6.096493731	1.31E-05
0.000851068	0.003791369	0.034313216	15.74553193	1.84E-06
0.00096036	0.004806426	0.049314705	11.99357188	1.52E-06
0.001637399	0.003267766	0.034866228	16.12470814	5.03E-06
0.003917943	0.006889639	0.053198857	8.987083639	1.64E-05
0.001249733	0.003873326	0.036837053	14.5782208	2.91E-06
0.002392156	0.007648712	0.066603295	7.876134465	6.06E-06
0.002576226	0.008799186	0.059466908	7.909368374	9.46E-06
0.001745645	0.004640359	0.031830459	17.70029966	7.11E-06
0.003728535	0.006673736	0.051575141	9.366755139	1.51E-05
0.002100196	0.006689305	0.069036741	8.580497013	3.91E-06
0.004987743	0.013674037	0.117092152	4.379978659	1.66E-05
0.002904268	0.00751174	0.068806546	7.53996897	7.70E-06
0.002492443	0.007716354	0.061857685	7.941412596	7.29E-06
0.002932047	0.014808132	0.120909557	4.140414843	9.63E-06
0.002891721	0.010370357	0.078695733	6.648294194	9.16E-06
0.00157491	0.00526405	0.046861144	11.7836932	3.74E-06
0.001590143	0.001800578	0.017729534	27.31260459	5.60E-06
0.002270513	0.005621581	0.043883563	10.30196774	8.63E-06
0.003104805	0.003399449	0.039448584	12.79771535	7.98E-06
0.002257734	0.007743157	0.060262223	8.612733156	6.48E-06
0.00204099	0.004708727	0.043651307	10.8613811	5.91E-06
0.003613791	0.008319569	0.08326374	5.945678687	9.77E-06
0.002578057	0.005963485	0.060872574	8.252178549	6.51E-06
0.003755014	0.007111708	0.077971178	6.959019599	9.80E-06
0.002398444	0.005792942	0.046529629	9.768114844	8.20E-06
0.001043127	0.003524261	0.028034275	18.4841051	2.94E-06
0.001664188	0.003695568	0.037840389	13.00499059	3.90E-06
0.001249978	0.004153974	0.033842559	15.50230495	3.19E-06
0.001641131	0.008871835	0.093204155	6.003051067	3.13E-06
0.001828255	0.003150755	0.024768166	19.70651903	7.31E-06
0.005063813	0.012890478	0.100768583	4.568298892	2.09E-05
0.005560272	0.00700785	0.060969324	7.857955715	2.37E-05
0.000867181	0.00215127	0.021297057	24.7129266	2.04E-06

original_gldm_GrayLevelVariance	original_gldm_HighGrayLevelEmphasis	original_gldm_GrayLevelNonUniformityNormalized	original_gldm_DependenceEntropy	original_gldm_DependenceNonUniformity
121.7524336	408.6253534	0.04681	6.850597267	546.7587182
23.99117618	69.36256684	0.108165518	6.020501472	146.5743316
56.11858457	163.2829205	0.060610516	7.03034161	198.2607562
113.4390406	422.5076766	0.058167955	6.733420168	183.0368475
52.95846774	150.8999155	0.065581629	6.538002622	472.691723
39.63591368	176.2424242	0.070211203	6.166308837	152.7818182
71.21682057	298.3986905	0.043421483	7.268532481	1020.994337
43.23511126	220.5920125	0.090450575	6.632538646	726.9099452
86.28860909	304.4062912	0.050601961	6.956258848	949.983722
65.02968019	191.886194	0.056918648	6.453975169	241.2817164
60.41592497	237.7931034	0.053476947	6.64190434	321.3992042
49.22541189	293.9544469	0.06387604	6.572468557	460.1288503
29.55558858	71.60042849	0.147796031	6.171479229	204.5018747
78.09204403	215.5154339	0.070025362	6.595699329	322.5864881
67.75656461	254.0020161	0.072488172	6.59304252	377.3205645
93.64739441	243.6930987	0.087106024	6.715644908	629.8048446
71.43193354	470.8291457	0.044054724	6.709867311	308.7236181
29.56361587	79.46472019	0.10058285	6.282072418	170.9059205
33.39716727	225.9822804	0.066302074	6.417678087	251.6918336
54.10924231	299.650125	0.059437688	6.780891107	1036.474899
32.14395845	88.17096561	0.098598995	6.616425116	379.3187831
37.08095489	187.9221501	0.059290989	6.297234846	258.5625579
49.41156006	137.7421875	0.076330185	6.401276577	189.4160156
85.55255274	321.3090291	0.038604266	7.203702309	541.8832641
40.58867567	121.1932443	0.097414486	6.238516506	203.5326002
75.75571086	396.088645	0.038000514	7.084963375	559.056564
90.07357929	432.7185277	0.050977255	6.906952697	695.3680792
54.4953415	219.5648081	0.06191428	6.738696429	530.7313541
31.72641873	258.8791946	0.052054712	6.123834079	134.4004474
29.48963952	107.5374878	0.068745147	6.506817814	381.5228822
52.72992585	233.5979403	0.079940759	6.758333963	719.1424968
58.40749203	133.7757371	0.091042924	6.619529719	299.4311261
132.1938046	398.401982	0.048399353	7.073509216	631.1876742
92.86866981	242.4259574	0.067864554	6.947523773	370.946383
53.90691065	270.6730245	0.055756636	6.828289127	896.1035422
43.46705187	147.0260087	0.103652979	6.437940035	421.5448483
80.10454706	188.8500403	0.087343701	6.611780822	571.9959688
232.0364851	486.5814765	0.083575897	7.142947228	523.8320093
61.96089365	308.4764228	0.037453896	6.903677107	293.5235772
116.9930138	555.4950481	0.028801447	7.445046145	1666.122421
73.44158663	257.2875867	0.068514673	6.767327282	459.3222822
105.2288183	733.2580812	0.026634966	7.141453032	1231.190328
69.46706624	231.285245	0.052796868	6.934284266	729.0355872
92.6071547	365.6402733	0.047622479	6.784619746	613.818328
36.3594482	122.9600939	0.070923367	6.391033596	248.0985915
77.15725349	308.9176685	0.056867097	6.787036462	556.091439
59.63776703	311.9057431	0.055381637	6.945583512	854.2078036
28.38291893	219.8128588	0.060011257	6.435764603	180.6073479
55.07633102	223.893067	0.045927857	6.568848026	212.3160987
23.11085429	135.1856509	0.112114203	6.08344247	206.3106509
52.62005154	206.9443363	0.068183227	6.491309557	536.1693266
93.40456883	549.6597449	0.030710491	7.081483705	1000.076239
50.90210458	353.4168883	0.046624682	6.795044288	1063.229167
59.21824459	167.5742754	0.071717142	6.491833345	211.057971
47.0000701	144.9654135	0.067441913	6.662717434	225.9413534
36.42853938	160.5738462	0.068708481	6.526685682	378.1384615
83.90647466	261.2472188	0.045243116	6.967509448	895.0247219
86.86626296	353.1844997	0.061873608	6.972468882	604.9456333
54.46509413	241.6134709	0.060809955	6.518679655	369.538835
146.4176342	864.4216234	0.029423338	7.534585377	2076.021799
41.52000862	181.0702152	0.085171139	6.643095324	251.8301246
56.00254794	182.1146067	0.063907195	6.75354072	504.7250936
60.00429439	205.5256521	0.067804239	6.77776421	1035.536535
71.0508421	361.1133412	0.041048386	7.314024591	1442.33963
26.23784009	201.1468354	0.077067778	6.253801987	151.5189873
44.73502009	141.178405	0.080810891	6.731499648	578.5758674
42.1004907	183.7177914	0.082238699	6.442849212	218.9064417
93.70446543	345.43696	0.047252122	7.212313901	1195.06752
133.8056431	550.5663309	0.038085352	7.156080982	1455.054635
32.07470241	134.5117322	0.080100219	6.576816385	449.3809524
65.04898361	155.4819092	0.081823467	6.512465092	213.886836
45.75913133	220.7568765	0.105832396	6.385997045	608.7226107
62.29638489	307.9425186	0.06695411	6.713184292	401.4695042
109.4168292	476.0571049	0.037726816	6.915437582	614.1394422
92.68263002	404.0907058	0.032960695	7.094535951	823.3839029
53.98405354	268.3599106	0.048401856	6.629953769	329.7317437
42.58349109	221.6807179	0.059778958	6.797699973	441.0584307
26.2226847	113.736952	0.102056738	6.131407155	148.085595
51.43241465	167.1177504	0.060394688	6.592441303	357.056239
152.053829	626.1351005	0.029287025	7.276264581	595.231723
126.0563481	404.6726998	0.049138285	6.790531219	499.2182001
71.64345024	257.8999444	0.068751369	6.7285568	328.5252918
112.6944985	271.1355154	0.081114703	6.92010883	436.7308045
60.55129838	312.0312398	0.04148124	7.045567212	653.5629678
84.7627764	360.6441164	0.05367658	6.924281948	505.5887659
12.61771972	58.6573107	0.159370335	6.167678775	148.6096606
125.1363995	427.2255765	0.042152868	7.094024969	506.4683438
42.14240592	144.2983224	0.073926085	6.61867231	465.2582057
44.11120396	194.4583333	0.060185807	6.643710133	295.7855191
46.3787169	130.4562963	0.075327298	6.546587299	341.8098765
18.2301972	61.49309816	0.152448577	6.045845338	145.5015337
91.82017313	409.3240974	0.035297265	6.93451386	646.6414777
67.49327654	409.9039167	0.051401849	6.826425266	1210.212039
57.79914564	231.9751515	0.063636915	6.803413928	590.3418182
71.19068096	383.7539063	0.036846332	6.988760951	1136.703585
72.77807819	206.9979544	0.081737074	6.820713797	508.7895967
98.14247389	383.8448704	0.03681424	7.242355518	604.6450124
101.841709	255.9856734	0.072437829	6.650817927	186.4937918
37.34310968	161.5827338	0.056990379	6.622517037	155.5899281
37.55598269	123.2066159	0.100602548	6.641038043	708.8330447

original_gldm_GrayLevelNonUniformity	original_gldm_SmallDependenceEmphasis	original_gldm_DependenceNonUniformityNormalized	original_gldm_DependenceVariance	original_gldm_LargeDependenceEmphasis
99.33081998	0.487531601	0.257661978	2.856897046	8.524976437
101.1347594	0.295745511	0.156763991	4.135727073	15.46417112
92.97653194	0.28326146	0.1292443	10.83935277	28.57366362
56.83009212	0.38972913	0.187345801	4.693542051	13.81678608
155.2972973	0.395464912	0.199616437	4.127065119	12.30067568
46.33939394	0.415606509	0.231487603	2.030018365	8.06969697
245.3748009	0.370138156	0.180674984	5.456865282	15.32631393
462.0215348	0.305897307	0.142308133	6.449256743	20.17462803
230.0365156	0.408119394	0.208971342	3.161963283	10.47074351
61.01679104	0.425215615	0.225076228	2.393235548	8.746268657
80.64323607	0.408488622	0.213129446	2.710479564	9.655172414
147.2342733	0.393478134	0.199622061	3.339604839	11.16746204
275.9351901	0.226073571	0.109535016	8.283560582	29.75415104
120.2335469	0.370014135	0.187877978	3.875066865	12.57018055
143.8165323	0.393567876	0.190181736	4.370935224	13.17842742
381.1759598	0.311066506	0.143922496	6.663869361	20.25731261
52.60134003	0.450291271	0.258562494	1.46362185	6.494137353
124.0186537	0.275738804	0.13860983	5.598752606	19.77858881
86.06009245	0.360139111	0.193907422	2.69169114	10.64252696
309.0165416	0.377088355	0.199360435	3.00110379	10.72071552
298.1633598	0.254600863	0.125436106	7.426134137	24.25132275
63.97497683	0.433773221	0.239631657	2.030435279	7.78776645
78.16210938	0.367176952	0.184976578	3.49937439	12.19726563
102.185493	0.396724155	0.204716005	3.514644245	11.19342652
124.008641	0.31562042	0.159884211	4.231589808	15.1948154
90.02321655	0.444202577	0.235988419	2.382360378	8.374419586
164.8094649	0.414923529	0.215084466	3.06038993	10.07887411
171.0072411	0.37386019	0.192154726	3.367668487	11.59015206
23.26845638	0.513650185	0.300672142	1.432197749	5.604026846
141.2025316	0.342334579	0.185746291	3.515878486	12.31256086
380.3581337	0.322566987	0.151143862	6.666141192	19.45901639
188.3678105	0.312698586	0.144722632	6.515652708	19.96955051
156.2815113	0.392740907	0.195474659	4.10545516	12.43821617
159.4817021	0.336759187	0.157849525	6.147423087	18.07489362
245.5522252	0.404780286	0.203474919	3.868143567	11.76521344
310.8552851	0.30116729	0.140561803	6.137391801	19.96765589
325.0059124	0.313976432	0.153721034	5.215308481	17.18382155
431.3352064	0.238262708	0.101498161	14.43901572	40.15714009
46.06829268	0.38529924	0.238637055	1.715145747	7.687804878
209.3865199	0.418633921	0.229177775	3.228491152	9.866024759
177.7270625	0.352566885	0.177071042	4.32268642	13.89591365
102.997414	0.538688868	0.318383845	1.220368967	5.021205069
192.8669587	0.390383859	0.199571746	3.44846025	11.31699973
118.4847267	0.456126464	0.246711547	2.282906504	7.92926045
90.6400626	0.369431234	0.194130353	3.282593842	11.40375587
156.1001821	0.403821528	0.202583402	3.458868949	11.20327869
252.6510302	0.371794691	0.187244148	4.219689711	13.10170978
52.26980482	0.36666525	0.207356312	2.45922116	9.732491389
39.08460635	0.454290798	0.249490128	2.542841007	8.294947121
151.5784024	0.285488539	0.152596635	4.351438588	16.4260355
175.1627092	0.386143085	0.208707406	2.774961396	10.00350331
103.5250667	0.514640013	0.296670495	1.54382913	5.812815188
210.3705674	0.429064009	0.235644762	2.194342078	8.140070922
79.17572464	0.376047386	0.191175698	4.117123503	12.73007246
89.69774436	0.356156476	0.169880717	4.748790774	15.04661654
133.9815385	0.361827364	0.19391716	2.921781986	10.92923077
183.0084054	0.423866222	0.221266928	2.833839088	9.524598269
213.9589358	0.341837849	0.1749409	4.027812558	13.63100058
100.2148058	0.420767532	0.22423473	2.414052927	8.822815534
268.6056523	0.438052347	0.227409552	4.570419045	11.81476613
150.412231	0.316539258	0.142599165	6.497934433	20.38052095
170.6322097	0.38335958	0.189035616	4.099852712	12.83220974
392.5187424	0.380335199	0.178880037	5.076293723	14.93539471
312.9118457	0.361876413	0.189208924	4.950469478	14.06099961
60.8835443	0.341615821	0.191796187	3.195808364	11.6278481
312.0916624	0.304202114	0.149812498	6.473542016	19.31227343
107.2392638	0.323975812	0.167873038	4.249171967	14.52453988
295.32576	0.373716581	0.191210803	4.141785702	12.77504
223.065904	0.45694534	0.248430021	2.223784151	7.796995049
232.1304348	0.301623525	0.155065891	4.242506076	15.84195997
106.2886836	0.347359382	0.164654993	5.103667711	15.95458045
454.020979	0.299924941	0.141893382	5.995557729	19.58741259
152.588416	0.353254724	0.176160379	3.962577984	13.42650285
85.22487826	0.487067101	0.27186341	1.941189646	6.853917663
103.1999361	0.475059693	0.262977931	1.874539447	6.93165123
64.95529061	0.413608436	0.245701746	1.952250017	7.709388972
143.230384	0.338901437	0.184081148	3.365816985	12.21619366
97.77035491	0.313608941	0.154577865	4.571606644	16.19415449
103.0937317	0.398107439	0.209171786	2.79256063	9.944346807
68.50235143	0.470496017	0.254481284	2.633801877	8.229585293
97.73604827	0.48012086	0.250989543	2.984003775	8.883358472
123.6837132	0.380846989	0.182615504	4.590059143	13.95386326
265.1639645	0.290613699	0.133597677	7.650227987	23.12358519
127.4718516	0.357322069	0.21267913	2.454935754	9.650178978
130.9171792	0.412175354	0.207293467	4.392742911	12.33825338
244.1553525	0.213015399	0.097003695	11.77043098	38.13315927
100.5345912	0.414617714	0.2123557	3.360398015	10.68553459
202.7053246	0.347138174	0.169678412	5.178538349	15.62873815
88.11202186	0.387115871	0.202039289	3.855950835	11.81420765
152.5377778	0.322985296	0.168795001	4.316390794	14.63555556
198.7929448	0.251938565	0.111580931	9.40535963	30.2392638
84.07808564	0.473450831	0.271469974	1.962337176	6.909319899
266.4157824	0.436603509	0.233496438	2.366755636	8.436233841
210.0018182	0.359359357	0.17889146	4.454616713	13.94848485
160.355239	0.457308905	0.261191081	1.795594106	6.909926471
279.7042665	0.334936782	0.148681939	7.392871306	20.92460549
103.7057153	0.40429012	0.214641467	3.282051301	10.42917998
75.84240688	0.362966434	0.178122055	4.070524873	13.45272206
47.52997602	0.360040923	0.186558667	3.859784115	12.65947242
580.8791133	0.273507473	0.12276291	7.630944181	25.21163838

original_gldm_LargeDependenceLowGrayLevelEmphasis	original_gldm_SmallDependenceHighGrayLevelEmphasis	original_gldm_LargeDependenceHighGrayLevelEmphasis	original_gldm_SmallDependenceLowGrayLevelEmphasis
0.09581059	274.2694875	1618.874175	0.003640212
1.011198943	43.98116192	423.7775401	0.014838261
7.391026173	93.94623074	1152.453716	0.004944834
0.109406952	204.825273	6519.977482	0.003255714
0.701181109	102.4263832	589.0865709	0.010554474
0.127033094	112.3499852	813.6909091	0.008197105
0.291905514	164.6427285	1906.987967	0.002954142
0.217736226	116.0804162	2283.927565	0.00194011
0.169572666	154.2195924	3064.881874	0.004701361
0.329411152	127.8511767	743.9682836	0.00936522
0.169303049	132.7277968	1380.082228	0.004657581
0.088242094	178.1402008	1826.912364	0.002184512
1.858692644	40.39499868	777.0637386	0.009100875
0.302329197	137.4816925	1082.355271	0.005548768
0.175017879	163.3027254	1457.704637	0.003691032
0.406585461	116.7731785	4202.785878	0.003603177
0.028169757	239.0786053	2793.420436	0.002957904
1.43024874	55.57562415	523.0170316	0.014025018
0.101784551	118.5555322	1524.24114	0.003178196
0.08780509	161.2526849	1953.247355	0.002161405
1.577877514	46.79832866	763.9566799	0.007310501
0.12454711	121.8641533	814.6580167	0.005461264
0.489379513	91.17177886	654.9941406	0.010803704
0.220884264	165.0904303	2358.975066	0.004393353
0.489809889	78.87388183	733.8955224	0.007077503
0.071002783	215.4388668	2508.010131	0.003163807
0.068403935	238.3411387	2562.839777	0.00225225
0.175467207	128.241582	1288.713251	0.004306608
0.04600754	151.3831814	1265.017897	0.010903682
0.616371099	61.82147671	610.7263875	0.011827211
0.245654895	133.8471738	2005.739596	0.002235119
1.148384054	76.64036062	867.8666022	0.012402172
0.157823228	181.7246992	5922.04088	0.004506279
0.483755268	128.6177971	1820.629362	0.004980786
0.137056139	155.4788736	1681.992734	0.002932576
0.42137829	83.61767227	1369.990997	0.003945429
0.485810791	114.6800684	1235.298307	0.005927225
0.836359473	115.2213231	34089.89634	0.003859045
0.121232799	150.6499889	1696.850407	0.004249374
0.075248609	242.4701784	6838.39326	0.002559627
0.208283245	137.749596	1700.495759	0.002949123
0.047387009	406.4666277	3424.768813	0.002819163
0.28381588	141.0541877	1215.120996	0.006338757
0.100295881	236.5026181	1476.422428	0.003474909
0.465516088	78.84576853	589.3106416	0.008513479
0.130090463	191.1959725	1761.87286	0.002793807
0.118345189	166.8074447	2204.769399	0.002177391
0.087172316	107.9343311	1541.167623	0.004481602
0.190530499	134.9885456	1137.350176	0.008879027
0.241121155	71.29280281	1333.664941	0.003573918
0.151239055	126.4165363	1069.070455	0.004599067
0.039483384	334.9444471	2300.874221	0.002346708
0.053575714	197.15003	1909.92797	0.002226535
0.396847556	114.8152278	764.3985507	0.008944258
0.608754937	91.37570615	801.1458647	0.008439825
0.203446537	91.40438985	979.1902564	0.005403006
0.270056494	154.6304971	1580.510012	0.007096499
0.104748869	176.5003826	3163.618276	0.002412508
0.10275249	152.9939899	1182.994539	0.004211733
0.030885613	342.5701055	15366.47705	0.001847388
0.30775021	107.28693	1724.788222	0.003261617
0.32300322	116.3871037	967.1895131	0.007442766
0.301221591	130.5632657	1420.192088	0.003911081
0.330605023	181.3857552	2558.270366	0.00210223
0.104014369	94.74241495	1692.41519	0.003643775
0.79735676	77.95515927	1111.165458	0.005259844
0.204726738	100.8078123	1405.157975	0.004581021
0.18883181	175.9235807	2677.72784	0.00270978
0.051193473	341.1033097	2478.595015	0.002525849
0.362359205	75.89835399	1086.790545	0.004612882
0.767553439	88.07535892	1963.771363	0.009500524
0.198251887	116.1719102	2295.763869	0.002007389
0.113090046	168.8269764	2196.776218	0.002178048
0.058774824	293.0742021	2153.188579	0.002693972
0.080164955	234.6689052	1972.840626	0.004130564
0.093115833	149.7382713	1296.792101	0.005409393
0.142537024	105.1679863	1838.978715	0.003269181
0.356300349	64.09295815	957.9665971	0.005895969
0.53134982	97.24266696	1079.069713	0.009820826
0.063345525	319.233006	5989.079094	0.003124358
0.100633161	257.7986111	2077.751634	0.003728826
0.215310103	137.3946642	2456.702613	0.005601841
0.480978832	110.9362631	8034.438666	0.004525802
0.106698474	132.0647228	2548.325415	0.002750051
0.108366148	199.9979302	3145.655187	0.002590088
1.571196827	27.9221267	1092.490209	0.005265772
0.147985784	250.4221474	2100.165618	0.002688974
0.535329304	93.46373186	832.4128373	0.005954346
0.227379663	109.2512601	1200.866803	0.00621679
0.657310504	76.83957855	739.6967901	0.010437359
1.418238152	36.34263207	798.2829755	0.008396785
0.07720536	218.0750943	2835.474391	0.003639101
0.043285583	230.472052	2460.949257	0.002041947
0.224737565	127.7451568	1547.098788	0.004006897
0.058249009	203.8309424	2040.786535	0.003181257
0.4638897	124.7488949	1614.44623	0.003913043
0.123363212	185.7049036	4576.561945	0.003964444
0.300661138	144.3818325	1874.982808	0.006416653
0.382114132	89.9260748	1072.781775	0.006272895
0.738945982	75.67876754	1220.784725	0.003800786

original_gldm_LowGrayLevelEmphasis	original_gldzm_DistanceZoneVariabilityNormalized	original_gldzm_LowIntensityEmphasis	original_gldzm_LargeDistanceEmphasis	original_gldzm_HighIntensitySmallDistanceEmphasis
0.00882798	0.993168313	0.007540928	1.010282776	548.9515853
0.061700525	1	0.052433364	1	146.0430464
0.089754834	1	0.019139562	1	327.1149897
0.008107239	1	0.007942404	1	533.4389671
0.035966804	1	0.026507058	1	254.0354746
0.017037058	1	0.019074081	1	258.9903537
0.011792173	0.992350304	0.007781004	1.011518771	443.1321459
0.00855235	0.998789347	0.006092457	1.001817081	388.5427014
0.01362494	0.999040307	0.011799714	1.00144023	370.4672348
0.028908928	1	0.022346212	1	291.8245614
0.013630962	1	0.011244806	1	323.4379458
0.00654059	0.992055737	0.005508804	1.011964108	437.4376869
0.053690983	1	0.039434273	1	190.1902552
0.019053304	1	0.014879171	1	358.4520167
0.010990109	1	0.009297049	1	410.2972973
0.01626819	1	0.011007926	1	395.2338377
0.005288449	1	0.006262568	1	519.7694754
0.063551366	1	0.051619394	1	199.9183099
0.008648982	1	0.008484825	1	321.9906542
0.006900178	0.996373539	0.005725793	1.005449591	422.979337
0.047970206	1	0.026987638	1	191.5819277
0.013739608	1	0.012417016	1	268.411215
0.035249691	1	0.028999218	1	246.2184466
0.013846918	0.998323555	0.010988279	1.002516779	414.6602349
0.030076813	1	0.024932587	1	242.1723356
0.007875429	1	0.007497496	1	473.4682081
0.005791489	1	0.00538695	1	574.9041005
0.013533154	1	0.012181309	1	338.0242424
0.014587614	1	0.019594553	1	288.2765152
0.040049982	1	0.035532934	1	174.8606658
0.009460908	1	0.006639938	1	415.4760479
0.048137259	0.997187066	0.03816061	1.004225352	252.9609155
0.012356651	0.995807146	0.011525002	1.006302521	456.3609944
0.021480003	1	0.014825422	1	395.5965116
0.008529187	0.995005026	0.00705599	1.007511267	379.9111167
0.017644674	0.997923159	0.01277927	1.003118503	281.8742204
0.023431857	1	0.018101863	1	372.0917874
0.023548555	0.998430142	0.015264019	1.002356638	501.9632757
0.012013637	1	0.010999176	1	377.2041522
0.0068087	0.997740116	0.00611848	1.003393665	575.8315187
0.011213854	1	0.008171017	1	395.8631476
0.006972042	0.996686007	0.005504721	1.004979253	753.8742739
0.019514396	0.998760075	0.01597563	1.001861042	354.2575993
0.009259769	0.998445999	0.00805294	1.002332815	504.8413686
0.032598758	1	0.024509061	1	206.693609
0.008804704	1	0.006848083	1	463.2686688
0.006954472	0.997905761	0.005690255	1.003144654	447.6700734
0.009664218	1	0.011602421	1	284.3709677
0.019519399	1	0.019194047	1	285.4263736
0.013930532	1	0.01267883	1	249.0074074
0.013108646	0.998231655	0.011837909	1.002654867	315.7772124
0.005722779	0.997994989	0.004929953	1.003010537	638.3569995
0.005559956	0.999091735	0.005085098	1.001363017	452.9669468
0.028080606	1	0.02457278	1	294.544086
0.030416973	1	0.023752072	1	251.575188
0.01666124	1	0.015373414	1	245.1889862
0.021020322	0.994842737	0.016440052	1.007755946	358.0473113
0.007216423	1	0.00694446	1	522.8498113
0.010568414	1	0.010026243	1	350.2297818
0.003951645	0.996935879	0.004226552	1.004603244	765.1516879
0.012653568	1	0.010103633	1	335.3553719
0.021976037	1	0.019550785	1	297.0722997
0.015092289	0.989211936	0.010225925	1.016270338	338.0671673
0.010058174	0.983806747	0.005885554	1.024489796	487.1672684
0.00925445	1	0.010239744	1	272.3878205
0.025293828	0.99223011	0.017539992	1.011700468	256.0113105
0.012943571	1	0.01375004	1	305.9594883
0.009944271	0.992467151	0.007242489	1.011342155	465.6904537
0.005728948	1	0.005419373	1	728.4085526
0.019422363	1	0.015496771	1	248.2732068
0.040626177	1	0.0279653	1	253.5357873
0.008463717	0.991130996	0.00643432	1.013363029	397.8962509
0.007021986	1	0.005926907	1	479.1939121
0.006307718	1	0.005474432	1	589.7782194
0.010180944	0.993003002	0.009068978	1.010532475	484.2636044
0.011552429	1	0.012473519	1	348.0286145
0.010284569	1	0.009468043	1	304.7805405
0.020121722	1	0.018673318	1	202.809816
0.034322027	0.997385625	0.025864941	1.003926702	242.332788
0.00720071	1	0.006745532	1	677.7995227
0.009155021	1	0.007864473	1	527.3355019
0.014760662	1	0.01441374	1	370.9291339
0.019434869	0.998027615	0.014508894	1.0029615	401.9447187
0.008827204	1	0.007596636	1	368.9809306
0.007905675	0.998275863	0.006728692	1.002588438	474.6145384
0.034450371	1	0.023802297	1	134.1101449
0.009199252	1	0.006345972	1	605.2193375
0.024027775	1	0.016286099	1	264.0507177
0.015246585	1	0.015234593	1	276.1513261
0.037315351	1	0.032450956	1	238.1589041
0.042633747	0.994152097	0.033196262	1.008797654	146.1678886
0.009196339	0.998472117	0.007652577	1.002293578	455.9298547
0.004851327	0.991463098	0.004633873	1.012860483	519.8042673
0.013043123	1	0.011200097	1	357.489842
0.007343962	0.996542793	0.006875736	1.005194805	443.4648268
0.016593198	0.995172997	0.011485962	1.007258065	375.5993952
0.01121859	1	0.009730724	1	453.2803667
0.020948107	1	0.018064557	1	397.8931116
0.022009932	1	0.017955919	1	240.9193084
0.023358051	0.997555016	0.013417759	1.003671971	282.6813035

original_gldzm_LowIntensityLargeDistanceEmphasis	original_gldzm_HighIntensityEmphasis	original_gldzm_DistanceZoneVariability	original_gldzm_ZonePercentage	original_gldzm_IntensityVariabilityNormalized
0.007572563	550.4490146	1159.027421	0.549952875	0.030219056
0.052433364	146.0430464	302	0.322994652	0.05515109
0.019139562	327.1149897	487	0.317470665	0.038609599
0.007942404	533.4389671	426	0.436028659	0.033040182
0.026507058	254.0354746	1043	0.440456081	0.039995441
0.019074081	258.9903537	311	0.471212121	0.044612856
0.007844966	444.6523038	2326.069113	0.414793842	0.032961217
0.006095612	388.804361	1649.001211	0.323218481	0.043108429
0.011857323	370.4762362	2081.00096	0.458205015	0.034877361
0.022346212	291.8245614	513	0.478544776	0.03908895
0.011244806	323.4379458	701	0.464854111	0.037800086
0.00553081	440.1505484	995.0319043	0.435140998	0.04022131
0.039434273	190.1902552	431	0.230851634	0.0585645
0.014879171	358.4520167	719	0.41875364	0.039413031
0.009297049	410.2972973	851	0.428931452	0.037275563
0.011007926	395.2338377	1454	0.33226691	0.039121789
0.006262568	519.7694754	629	0.52680067	0.036181791
0.051619394	199.9183099	355	0.287915653	0.04718905
0.008484825	321.9906542	535	0.412172573	0.044150581
0.005744262	424.0658492	2194.014532	0.423542989	0.037767664
0.026987638	191.5819277	830	0.274470899	0.050837567
0.012417016	268.411215	535	0.495829472	0.043857105
0.028999218	246.2184466	412	0.40234375	0.043418324
0.010993479	414.9647651	1190.001678	0.450321118	0.031621605
0.024932587	242.1723356	441	0.346425766	0.048698845
0.007497496	473.4682081	1211	0.511186154	0.032904404
0.00538695	574.9041005	1512	0.46767708	0.034211318
0.012181309	338.0242424	1155	0.418175235	0.03805251
0.019594553	288.2765152	264	0.590604027	0.045884986
0.035532934	174.8606658	811	0.394839338	0.049685201
0.006639938	415.4760479	1670	0.35098781	0.039477213
0.038246841	253.0126761	708.0028169	0.343160947	0.042110692
0.011612846	457.6561625	1422.012605	0.44224218	0.032067729
0.014825422	395.5965116	860	0.365957447	0.035408329
0.007084596	380.565348	1987.025038	0.453451408	0.040009188
0.012783248	282.485447	960.002079	0.320773591	0.047164388
0.018101863	372.0917874	1242	0.333781242	0.04066606
0.01526542	502.9536528	1271.001571	0.246657624	0.035638363
0.010999176	377.2041522	578	0.469918699	0.034392548
0.006125771	576.4027149	3528.00905	0.486382393	0.028004616
0.008171017	395.8631476	1023	0.394371627	0.039311859
0.005545846	754.1493776	2402.013278	0.623222136	0.02752673
0.015983901	354.3622829	1610.001241	0.441281139	0.035653812
0.008054556	505.6835148	1284.001555	0.516881029	0.031717334
0.024509061	206.693609	532	0.41627543	0.046427158
0.006848083	463.2686688	1232	0.448816029	0.034720758
0.005699758	448.0041929	1904.004193	0.418237615	0.036525564
0.011602421	284.3709677	372	0.427095293	0.045872355
0.019194047	285.4263736	455	0.5346651	0.038473614
0.01267883	249.0074074	405	0.299556213	0.055217193
0.011849709	315.9265487	1128.00177	0.439859868	0.041444122
0.005002604	638.4119418	1989.004014	0.591219223	0.028398189
0.005091156	453.0436165	2199.000909	0.487810284	0.036943061
0.02457278	294.544086	465	0.421195652	0.039574517
0.023752072	251.575188	532	0.4	0.041233252
0.015373414	245.1889862	799	0.40974359	0.04534924
0.016534705	358.8531541	1924.025853	0.478121137	0.033743847
0.00694446	522.8498113	1325	0.383169462	0.034338199
0.010026243	350.2297818	779	0.472694175	0.040285676
0.00423364	765.9848751	4548.021482	0.499726147	0.024962805
0.010103633	335.3553719	605	0.342582106	0.042289461
0.019550785	297.0722997	1148	0.429962547	0.039084789
0.010344011	340.1009595	2371.14101	0.41406115	0.038041746
0.005941895	491.3152276	3133.42449	0.417814509	0.033912398
0.010239744	272.3878205	312	0.394936709	0.05138478
0.017810857	258.375975	1272.039002	0.331952356	0.046884621
0.01375004	305.9594883	469	0.359662577	0.043421334
0.007272825	467.6018904	2625.075614	0.4232	0.034361369
0.005419373	728.4085526	3040	0.51903705	0.028088643
0.015496771	248.2732068	948	0.327122153	0.0458794
0.0279653	253.5357873	489	0.376443418	0.041455999
0.006496702	398.9569414	1335.053452	0.313986014	0.048054766
0.005926907	479.1939121	887	0.389205792	0.03751673
0.005474432	589.7782194	1258	0.556883577	0.0299059
0.009551707	484.8964307	1697.04213	0.545832003	0.029081916
0.012473519	348.0286145	664	0.494783905	0.03764153
0.009468043	304.7805405	925	0.3860601	0.042594595
0.018673318	202.809816	326	0.340292276	0.055854567
0.025882393	242.5536649	762.0026178	0.447568834	0.041634687
0.006745532	677.7995227	1257	0.537409149	0.025515031
0.007864473	527.3355019	1076	0.540975365	0.031202927
0.01441374	370.9291339	762	0.423568649	0.036872852
0.014555168	401.9921027	1011.001974	0.309880697	0.039781946
0.007596636	368.9809306	1311	0.426618939	0.037778674
0.006732243	475.0862813	1157.001726	0.475194752	0.033112208
0.023802297	134.1101449	345	0.225195822	0.070413779
0.006345972	605.2193375	1117	0.468343816	0.027682379
0.016286099	264.0507177	1045	0.38110868	0.043331426
0.015234593	276.1513261	641	0.43784153	0.041143299
0.032450956	238.1589041	730	0.360493827	0.043918184
0.033304875	146.3460411	339.0058651	0.261503067	0.072625794
0.007656247	456.2882263	1306.001529	0.549118388	0.031620281
0.004682162	521.1742011	2544.09431	0.495080069	0.03568428
0.011200097	357.489842	1329	0.402727273	0.038412085
0.00690779	444.3229437	2302.013853	0.530790441	0.033380559
0.0115177	376.1080645	1234.014516	0.362361192	0.039146722
0.009730724	453.2803667	1309	0.464678736	0.03165661
0.018064557	397.8931116	421	0.402101242	0.037333348
0.017955919	240.9193084	347	0.416067146	0.042446993
0.013431289	282.9736842	1630.004896	0.282992726	0.044764783

original_gldzm_LowIntensitySmallDistanceEmphasis	original_gldzm_IntensityVariability	original_gldzm_HighIntensityLargeDistanceEmphasis	original_gldzm_SmallDistanceEmphasis	original_glcm_SumVariance
0.007533019	35.26563839	556.4387318	0.997429306	1178.427772
0.052433364	16.65562914	146.0430464	1	131.6877553
0.019139562	18.80287474	327.1149897	1	398.4743274
0.007942404	14.07511737	533.4389671	1	1266.825313
0.026507058	41.71524449	254.0354746	1	345.026917
0.019074081	13.87459807	258.9903537	1	436.4000221
0.007765014	77.26109215	450.7329352	0.997120307	816.5995878
0.006091669	71.17201696	389.8509994	0.99954573	582.339046
0.011785312	72.64954393	370.512242	0.999639942	795.664849
0.022346212	20.05263158	291.8245614	1	442.8009819
0.011244806	26.4978602	323.4379458	1	568.2780906
0.005503303	40.34197408	451.001994	0.997008973	791.8986336
0.039434273	25.2412993	190.1902552	1	162.9607538
0.014879171	28.3379694	358.4520167	1	569.6982802
0.009297049	31.72150411	410.2972973	1	660.4093309
0.011007926	56.88308116	395.2338377	1	648.9919664
0.006262568	22.75834658	519.7694754	1	1400.386338
0.051619394	16.75211268	199.9183099	1	164.2550405
0.008484825	23.62056075	321.9906542	1	571.8129889
0.005721176	83.164396	428.4118983	0.998637602	811.8915049
0.026987638	42.19518072	191.5819277	1	199.5760235
0.012417016	23.4635514	268.411215	1	442.3873631
0.028999218	17.88834951	246.2184466	1	314.5086259
0.010986979	37.69295302	416.1828859	0.999370805	804.6029312
0.024932587	21.47619048	242.1723356	1	260.6304341
0.007497496	39.84723369	473.4682081	1	1134.864357
0.00538695	51.72751323	574.9041005	1	1249.655755
0.012181309	43.95064935	338.0242424	1	547.1007627
0.019594553	12.11363636	288.2765152	1	689.8770896
0.035532934	40.2946979	174.8606658	1	243.4496158
0.006639938	65.92694611	415.4760479	1	616.6276744
0.038139052	29.89859155	253.2197183	0.998943662	374.076632
0.01150304	45.79271709	462.8368347	0.99842437	1128.1298
0.014825422	30.45116279	395.5965116	1	653.1591326
0.007048839	79.89834752	383.1822734	0.998122183	736.8329901
0.012778276	45.37214137	284.9303534	0.999220374	362.0419345
0.018101863	50.50724638	372.0917874	1	511.7546303
0.015263668	45.36763551	506.915161	0.999410841	1521.941861
0.010999176	19.87889273	377.2041522	1	797.2773213
0.006116657	99.02432127	578.6875	0.999151584	1602.507587
0.008171017	40.21603128	395.8631476	1	711.9863501
0.005494439	66.33941909	755.2497925	0.998755187	2165.110494
0.015973562	57.47394541	354.7810174	0.999534739	560.9257771
0.008052536	40.78849145	509.0520995	0.999416796	996.8221804
0.024509061	24.69924812	206.693609	1	274.0841783
0.006848083	42.77597403	463.2686688	1	782.3342425
0.005687879	69.69077568	449.3406709	0.999213836	827.1605992
0.011602421	17.06451613	284.3709677	1	572.0144032
0.019194047	17.50549451	285.4263736	1	598.1587805
0.01267883	22.36296296	249.0074074	1	328.0987355
0.011834959	46.83185841	316.5238938	0.999336283	527.6902502
0.00491179	56.59759157	638.631711	0.999247366	1549.245395
0.005083583	81.31167651	453.3502953	0.999659246	967.3975875
0.02457278	18.40215054	294.544086	1	352.6208079
0.023752072	21.93609023	251.575188	1	310.5386689
0.015373414	36.23404255	245.1889862	1	413.9219577
0.016416388	65.26059979	362.0765253	0.998061013	655.2849883
0.00694446	45.49811321	522.8498113	1	1015.7132
0.010026243	31.38254172	350.2297818	1	616.3749472
0.00422478	113.8803157	769.3176238	0.998849189	2557.047234
0.010103633	25.58512397	335.3553719	1	449.7529066
0.019550785	44.86933798	297.0722997	1	438.882529
0.010196403	91.18606592	348.2361285	0.995932416	498.663536
0.005871468	108.010989	507.9070644	0.993877551	1048.475696
0.010239744	16.03205128	272.3878205	1	503.2288967
0.017472276	60.10608424	267.8346334	0.997074883	354.6023575
0.01375004	20.36460554	305.9594883	1	491.1880387
0.007234905	90.88582231	475.2476371	0.997164461	929.7918803
0.005419373	85.38947368	728.4085526	1	1215.683355
0.015496771	43.49367089	248.2732068	1	310.9302793
0.0279653	20.27198364	253.5357873	1	386.7246737
0.006418724	64.72976986	403.199703	0.996659243	569.3010856
0.005926907	33.27733935	479.1939121	1	871.8337577
0.005474432	37.62162162	589.7782194	1	1370.870345
0.008948296	49.70099473	487.4277355	0.997366881	1130.091398
0.012473519	24.9939759	348.0286145	1	718.1672984
0.009468043	39.4	304.7805405	1	529.6024382
0.018673318	18.20858896	202.809816	1	279.3304619
0.025860578	31.80890052	243.4371728	0.999018325	363.3629985
0.006745532	32.07239459	677.7995227	1	1887.54239
0.007864473	33.57434944	527.3355019	1	1155.489773
0.01441374	28.09711286	370.9291339	1	694.1129294
0.014497326	40.29911155	402.1816387	0.999259625	674.2030455
0.007596636	49.52784134	368.9809306	1	873.896173
0.006727804	38.37704918	476.9732528	0.99935289	983.7289644
0.023802297	24.29275362	134.1101449	1	116.6908829
0.006345972	30.92121755	605.2193375	1	1242.896029
0.016286099	45.28133971	264.0507177	1	323.4423601
0.015234593	26.37285491	276.1513261	1	484.7263091
0.032450956	32.06027397	238.1589041	1	308.8742612
0.033169109	24.76539589	147.058651	0.997800587	106.4105699
0.007651659	41.35932722	457.7217125	0.999426606	1064.634741
0.0046218	91.56586126	526.6539361	0.996784879	1191.520319
0.011200097	51.0496614	357.489842	1	596.2231392
0.006867722	77.10909091	447.7554113	0.998701299	1067.899977
0.011478028	48.54193548	378.1427419	0.998185484	540.2866343
0.009730724	41.43850267	453.2803667	1	1043.685256
0.018064557	15.71733967	397.8931116	1	769.2101112
0.017955919	14.72910663	240.9193084	1	393.0071201
0.013414376	73.14565483	284.1432069	0.999082007	256.1450898

original_glcm_Homogeneity1	original_glcm_Homogeneity2	original_glcm_ClusterShade	original_glcm_MaximumProbability	original_glcm_Idmn	original_glcm_SumVariance2	original_glcm_Contrast	original_glcm_DifferenceEntropy
0.254436909	0.175193555	9689.113906	0.014702619	0.963015825	365.1718144	137.8090859	4.478065218
0.387250016	0.311858388	753.287003	0.042080225	0.969473806	55.90510956	33.9495971	3.393467891
0.402100881	0.334267271	2082.837572	0.047267272	0.979904426	165.9537803	42.81414915	3.552900715
0.303760872	0.229814752	7478.792548	0.025220248	0.96315329	359.704905	96.64969319	4.173708611
0.308123418	0.228399074	2066.306381	0.022380769	0.976121114	134.0589728	67.20368305	3.999142676
0.28996075	0.205536995	980.0912112	0.019852307	0.962598713	88.55218817	67.90133892	3.975812974
0.330304804	0.251458193	2407.530329	0.020439612	0.975306824	222.4793716	54.54424688	3.866792303
0.373873669	0.301193032	2624.854644	0.035983775	0.981629685	112.9919411	50.08629959	3.66566158
0.29538391	0.214467472	5252.254149	0.014748157	0.969148015	248.77638	75.26268977	4.081867636
0.29637509	0.214710854	3676.705742	0.017737836	0.966221433	172.4869528	69.50180994	4.009653857
0.312407009	0.228394018	2347.681897	0.016519183	0.964803108	161.9322854	50.31744228	3.817540055
0.304038693	0.222284916	1787.876829	0.017965106	0.969951442	116.680487	78.44385095	4.047690359
0.45280107	0.394071774	2314.841682	0.06328075	0.979172578	84.2464801	39.62412548	3.19627055
0.309097537	0.229392643	6716.237969	0.024010668	0.9744658	225.4900046	84.5393821	4.058101583
0.310607807	0.23613213	3338.717731	0.027762327	0.956843666	168.7241176	103.7577146	4.161507339
0.373961618	0.303846611	10479.03003	0.037297519	0.976275194	290.4318588	61.69032602	3.733248126
0.262000432	0.172238032	3938.51893	0.009310335	0.97365846	226.794664	71.07816245	4.073580684
0.397129913	0.326435481	1225.897134	0.038311503	0.975152725	75.77165387	38.55972403	3.440285722
0.328799808	0.244086824	738.961916	0.019271363	0.972477001	75.36397761	38.53672668	3.650179227
0.310691218	0.226708171	2351.689559	0.017359926	0.98071375	149.9771703	58.01095686	3.889832442
0.418539428	0.349604854	2289.96571	0.039526153	0.982309607	97.80247519	27.65018745	3.289895038
0.2929132	0.204721544	768.4245438	0.01565284	0.967428397	85.88171714	48.09424354	3.829832644
0.334955512	0.253979931	2417.377786	0.027638614	0.970706379	131.6749697	53.2171695	3.751746301
0.312951638	0.230709203	3648.6939	0.012539216	0.969652015	242.4404502	56.40358066	3.897913042
0.358661305	0.28403225	1995.881345	0.037145366	0.975820367	91.56486971	62.49054806	3.732267117
0.275860959	0.191629143	2716.463677	0.009182155	0.968134537	220.40252	86.27475776	4.166383998
0.286641258	0.204079362	7707.928331	0.015426872	0.974973033	273.6476246	86.4638258	4.145192434
0.313755544	0.2312254	2685.946774	0.017417938	0.972866735	139.9191345	64.60069427	3.929929064
0.264273736	0.170969955	63.77067133	0.013619152	0.966281465	73.27017897	46.1058116	3.796348528
0.340396675	0.257926879	913.4165862	0.019258061	0.974518406	82.02025289	42.78071094	3.670546078
0.358744953	0.284653363	2890.606464	0.03573547	0.979973349	145.0251959	59.88532843	3.787871859
0.36770434	0.297050921	5243.761463	0.04430357	0.968284989	198.8609611	62.00980119	3.762669344
0.303135205	0.225209922	10769.16515	0.014815846	0.96302432	419.2069886	90.29196828	4.141349905
0.350772775	0.28023586	8222.941265	0.03497987	0.965449454	286.1207397	82.5786363	3.941770801
0.306156368	0.224677083	2838.847125	0.014590655	0.973423342	154.5107994	64.20044974	3.990529316
0.38799075	0.316347869	3625.202216	0.043743825	0.978971844	121.9712467	45.60608633	3.548011388
0.354833165	0.279572403	10733.67106	0.034859163	0.979283375	250.1007519	74.85741299	3.819053337
0.452656404	0.397848547	26500.08377	0.047059465	0.977518405	835.8857724	67.64099958	3.442909859
0.285955674	0.195288358	918.8648663	0.008837398	0.974271374	174.5031258	51.44606619	3.854138486
0.279815288	0.195937893	3621.202019	0.00769484	0.967747993	366.7510254	71.89206453	4.117029138
0.332506562	0.254439197	5653.94935	0.023781855	0.971979701	226.0951116	64.14733536	3.868162486
0.220276008	0.134585856	-399.0566292	0.00377389	0.963587435	309.643535	103.1201747	4.392422971
0.304143106	0.223192136	3429.804588	0.015390409	0.97378691	180.1515162	72.1426442	4.034210507
0.262059045	0.177639061	5821.918511	0.012219834	0.969973235	258.9620687	101.3510195	4.277376547
0.330366979	0.248407668	1145.964592	0.022042129	0.965851656	95.63300935	54.46909109	3.768271868
0.310411106	0.229732668	4621.445637	0.018630642	0.973885881	202.3031313	72.35087496	3.987999622
0.321627982	0.240566657	2727.663896	0.018754919	0.976754509	162.8417284	58.35929607	3.891688329
0.321706155	0.232987205	375.8595292	0.0173427	0.970879238	71.78486258	36.5831943	3.609437
0.265919152	0.181938115	1492.514487	0.015799254	0.955109436	147.2350408	86.04216795	4.18378979
0.384735816	0.310153579	766.938311	0.040944743	0.984396891	51.3195489	35.70124897	3.446791011
0.298474586	0.214533282	3112.754135	0.018865658	0.97456229	138.0547213	70.83649511	4.01108451
0.233119387	0.146831242	2031.797442	0.007004655	0.975192796	262.0820378	94.68631553	4.329712395
0.27860647	0.190933569	1211.873287	0.0094594	0.969701665	129.9510155	60.58610308	4.003454201
0.328340702	0.247421495	2863.371263	0.028526317	0.971323557	134.7933411	60.14406768	3.821086184
0.341630301	0.265387612	1533.930421	0.026859727	0.966272255	107.250305	62.09448636	3.847446054
0.324363721	0.240391681	1474.063644	0.018146899	0.975683105	102.8928129	49.41787154	3.759259656
0.286124123	0.203452899	4270.053781	0.011048954	0.967574442	234.3751884	75.93556257	4.115895231
0.329379487	0.247929066	9780.321116	0.020518545	0.981913823	277.8085523	62.20762225	3.837643871
0.28629047	0.200899898	2365.948073	0.013811226	0.97173559	130.6023236	71.9838037	4.045861813
0.27568563	0.195823428	1004.55443	0.018698718	0.971593799	466.6858012	90.58749752	4.263576123
0.377845944	0.306504173	1938.612528	0.034415833	0.981378375	102.6333632	50.95352024	3.664907104
0.316725541	0.236820808	2900.625999	0.018790822	0.972770417	146.9080329	73.80625207	3.999510535
0.33452678	0.259958844	3557.686463	0.022123578	0.970266321	158.3161706	70.78836316	3.954399658
0.31571608	0.232976189	1997.186158	0.014760397	0.981410178	223.7287779	55.72954447	3.901735928
0.341651065	0.257817682	577.1029567	0.023085754	0.980117693	55.07117377	37.08975077	3.55355641
0.368127461	0.294389256	3249.806947	0.031640667	0.974904099	131.3738171	56.34179723	3.711657799
0.339699223	0.259576194	2902.122439	0.024546914	0.981273774	118.3365389	55.68364327	3.756213748
0.30829628	0.22808862	6580.789896	0.012891146	0.976139816	267.6743278	77.01891887	4.051934396
0.279432209	0.192847172	7732.658386	0.01018462	0.989443139	271.6219803	69.63117631	4.058082198
0.374986264	0.297560162	1374.786017	0.02507866	0.98496296	83.0766216	35.31864072	3.487151258
0.343359217	0.268694488	5116.39754	0.035821374	0.964160668	190.1437158	66.84659576	3.8671517
0.376561789	0.302954913	3318.525008	0.043746345	0.983296349	110.8434803	52.10773981	3.622648276
0.330499886	0.252419322	4252.058488	0.022921177	0.974845284	185.1689074	64.32948361	3.882403615
0.238393813	0.155767131	5039.723948	0.009702252	0.957597064	312.4777991	120.0146915	4.440363185
0.249211094	0.163611461	2625.521148	0.007162628	0.971239004	270.7658511	86.78723861	4.256421924
0.283739998	0.196689756	1967.821305	0.011508948	0.966624725	154.19459	66.35975022	3.988049753
0.348828327	0.264949733	1511.780989	0.018694708	0.981796564	104.1289977	34.09108144	3.534282863
0.368332643	0.291878428	1135.739773	0.036842518	0.977057701	69.19100666	45.31604609	3.582491967
0.311656889	0.226195948	2189.08815	0.017945112	0.981767389	127.2345596	50.29288183	3.815505538
0.249633224	0.16839211	8588.082364	0.008594232	0.963351636	495.2290504	116.4360571	4.410557183
0.266170375	0.186783492	12482.65008	0.016070847	0.966089656	388.7740155	130.8676699	4.405840442
0.312929475	0.238285991	4407.709347	0.026378236	0.961247021	195.8625714	86.22036046	4.110416225
0.389938254	0.322725883	11682.00967	0.035345343	0.983883855	323.9156719	61.18254185	3.67459879
0.304816842	0.21731796	2401.529913	0.010217015	0.975716656	195.1305404	43.12446379	3.764865351
0.293297969	0.215429909	4683.897982	0.02080606	0.960605587	232.3722966	91.1023804	4.189652919
0.482382139	0.423332603	352.8763015	0.099396215	0.985780896	28.96904656	16.5382152	2.924661889
0.28423197	0.206014136	7829.400254	0.015846045	0.964196804	398.385961	101.3204779	4.250658326
0.351257662	0.274141032	1633.45769	0.028190987	0.96963669	108.0116835	52.58886218	3.748586213
0.329638919	0.247123005	1416.51086	0.022397657	0.972250363	122.6843928	49.77784968	3.741888492
0.355245164	0.27786315	3145.845625	0.025735973	0.978063312	134.603676	49.66696374	3.668730017
0.459371218	0.396323579	536.5562452	0.079140679	0.986906479	33.56884758	20.5536744	3.018148918
0.254937646	0.16908514	3012.368746	0.008071884	0.962215642	253.8613492	80.76793072	4.20735663
0.277002297	0.192059485	4346.582372	0.012493035	0.974306478	199.7595075	78.4570739	4.13222347
0.336112239	0.257266237	3275.363847	0.025292748	0.97307691	169.1194868	55.10340259	3.815350923
0.259429417	0.171520309	2065.514565	0.006462126	0.964240907	207.1848363	73.03709929	4.136275282
0.363913405	0.294689102	7031.590436	0.032685069	0.975084558	210.794256	80.87710255	3.879622812
0.298113396	0.213587772	5528.280144	0.009124592	0.974223706	302.9384383	58.55346912	3.951083403
0.321116534	0.244957997	11543.34705	0.024838629	0.970310885	342.7237064	88.62838711	4.018125625
0.338520916	0.254789436	837.3514903	0.018063648	0.967350885	106.5215385	39.27853445	3.621555621
0.415244664	0.349338543	1550.53343	0.045637632	0.987709584	82.39691161	41.1461126	3.443375592

original_glcm_InverseVariance	original_glcm_Entropy	original_glcm_Dissimilarity	original_glcm_DifferenceVariance	original_glcm_Idn	original_glcm_Idm	original_glcm_Correlation	original_glcm_Autocorrelation
0.177119561	9.101884971	8.137868322	70.7442583	0.886199776	0.175193555	0.459979009	351.2872465
0.309179268	6.879354059	3.724987765	19.98380751	0.902158912	0.311858388	0.246234222	48.54801074
0.290401223	7.787593047	4.10618631	25.48706351	0.921746988	0.334267271	0.594506934	136.7956266
0.224240869	8.242296038	6.674648093	51.29108249	0.889321811	0.229814752	0.581956795	384.0438593
0.229157703	8.29925414	5.665606808	34.71474821	0.907102332	0.228399074	0.338727884	114.2021761
0.212939806	7.827186089	5.796555306	33.86945009	0.883328894	0.205536995	0.137023718	144.636621
0.243974414	8.974798991	5.049118374	28.80947092	0.906386767	0.251458193	0.610108418	268.2384056
0.290053811	7.680851253	4.453645682	30.13404579	0.924839423	0.301193032	0.389380954	192.1288878
0.218690574	8.910205779	6.036159072	38.39211265	0.895039217	0.214467472	0.540256154	253.1678335
0.223587149	8.194961184	5.837790354	35.16768604	0.889769863	0.214710854	0.430901945	143.833632
0.234427093	8.300064717	5.036763226	24.61583873	0.886052243	0.228394018	0.527687012	190.3763355
0.221041759	8.297939252	5.925967964	43.05725463	0.899588655	0.222284916	0.202277208	252.0008789
0.366636794	6.373552197	3.426766817	27.64401823	0.930120408	0.394071774	0.367426307	54.10254201
0.226707958	8.206808441	6.007837148	48.00513039	0.90855867	0.229392643	0.458906145	177.2821575
0.233067621	8.214137822	6.682605193	58.59251128	0.884947529	0.23613213	0.247296055	204.0128289
0.291994793	7.790712087	4.820412871	38.15736793	0.917687647	0.303846611	0.652028013	201.5039936
0.175984584	8.844636726	6.16147413	32.62091666	0.89680506	0.172238032	0.525324444	436.5926125
0.317482415	7.106743904	3.856854369	23.54862548	0.91417323	0.326435481	0.32402382	58.71605115
0.250205243	7.936449662	4.417729881	18.89103293	0.897347466	0.244086824	0.322979537	194.4231897
0.235088947	8.578056832	5.217548314	30.55453373	0.916214612	0.226708171	0.447601919	263.5279899
0.330186767	7.184092633	3.325364851	16.44255265	0.925336262	0.349604854	0.559261455	72.2488266
0.214733293	8.132169809	5.122037557	21.69258042	0.885494056	0.204721544	0.285121788	152.7246183
0.255582982	7.685942244	4.835952918	29.59431168	0.90112708	0.253979931	0.426142744	105.1933073
0.231686813	8.953697539	5.237700125	28.64696202	0.89499131	0.230709203	0.624933764	263.3010381
0.279714468	7.344735408	4.846316112	38.76912929	0.915543291	0.28403225	0.191215237	85.6644553
0.194660868	9.191271779	6.462599369	43.87170675	0.892611289	0.191629143	0.444319289	357.2786982
0.209916798	8.86926701	6.339456059	45.78141266	0.906123365	0.204079362	0.526746012	386.3115953
0.232792133	8.402776544	5.390907496	35.27208773	0.903474775	0.2312254	0.370943033	180.2923239
0.184195891	8.014065511	5.322834706	17.69555271	0.876698485	0.170969955	0.230094581	229.4448007
0.257458149	7.964809887	4.444371067	22.83920151	0.904921364	0.257926879	0.318408403	88.11701202
0.27266556	8.011706003	4.877216908	35.88980944	0.921520528	0.284653363	0.421954609	200.9305831
0.284122012	7.735808815	4.930087707	37.36033202	0.903893973	0.297050921	0.531973505	118.2354011
0.222569794	8.935037631	6.374245457	48.95078816	0.889887462	0.225209922	0.649654889	344.4576371
0.26676838	8.219934487	5.679249983	49.94334141	0.9004862	0.28023586	0.555908756	201.9349731
0.224238758	8.663009688	5.568576553	32.9708448	0.901660918	0.224677083	0.418439632	240.3526308
0.305121659	7.29309996	4.15062699	28.20968029	0.921491718	0.316347869	0.460525788	121.0380028
0.2724035	7.882997969	5.16451513	47.85223247	0.923901325	0.279572403	0.544842699	156.712946
0.340324425	7.473665055	4.305892336	48.77973524	0.931438605	0.397848547	0.851550764	438.4665277
0.198624737	8.825023443	5.237722615	23.78434002	0.897421075	0.195288358	0.545505944	264.7595826
0.194939285	9.773496578	6.114044563	34.11291223	0.88857656	0.195937893	0.673300929	499.4940723
0.256480475	8.265221451	5.234116379	36.2066041	0.904998504	0.254439197	0.561059858	228.0844445
0.14162276	10.05227445	7.785488795	41.49133512	0.875282568	0.134585856	0.506761207	656.7593422
0.223743565	8.752437805	5.805405959	37.91343412	0.903914721	0.223192136	0.436048743	183.2455161
0.183880866	8.962559793	7.021924563	51.56176729	0.895582123	0.177639061	0.444566328	307.9760867
0.253546035	7.845034567	4.896484777	30.22246589	0.893403123	0.248407668	0.280771789	94.43619686
0.235116424	8.476841428	5.695722418	39.54921662	0.906044048	0.229732668	0.479478978	249.912089
0.239273001	8.618122921	5.187915349	31.23859866	0.909513874	0.240566657	0.475754808	268.7230469
0.237453499	7.933350874	4.357995478	17.43286692	0.892923502	0.232987205	0.328000889	195.7780771
0.178395738	8.548026932	6.723949658	40.14599747	0.870134039	0.181938115	0.270260348	192.9223407
0.307760165	6.99945839	3.807373803	21.14602553	0.928846293	0.310153579	0.183304688	115.2942221
0.217797729	8.296349652	5.72200091	37.86586439	0.905045132	0.214533282	0.32788813	171.7256288
0.152357975	9.751528428	7.34158439	40.17163087	0.89682877	0.146831242	0.476182194	479.5195921
0.198428005	8.939403797	5.701048783	27.89499096	0.890108937	0.190933569	0.369772808	312.4902662
0.248844649	7.78452181	5.109710022	33.77190055	0.902886287	0.247421495	0.384718835	116.4102246
0.253494236	7.950158919	5.170408594	34.97690512	0.896277427	0.265387612	0.271303277	104.7700071
0.245878248	8.114833426	4.792914168	26.15149143	0.906743887	0.240391681	0.357433371	142.3612778
0.204863889	9.045461548	6.155512618	37.6282155	0.890831816	0.203452899	0.514082879	210.9759926
0.25030102	8.458819253	5.090734467	35.95501703	0.922976533	0.247929066	0.638451375	318.9391848
0.206239412	8.357464371	5.922011363	36.6425433	0.898156795	0.200899898	0.296112265	200.2516013
0.190884004	9.942087055	6.774055399	44.19282185	0.896441124	0.195823428	0.678266637	768.4680406
0.286615668	7.559859371	4.484323548	30.67284171	0.924443707	0.306504173	0.340736261	151.449691
0.231316396	8.370362795	5.67478937	41.3278622	0.905083201	0.236820808	0.337580644	143.0924429
0.255901705	8.344348571	5.522455697	40.07005056	0.902611665	0.259958844	0.388227837	162.2790722
0.225238091	9.152803258	5.176514133	28.65916251	0.916467003	0.232976189	0.603674134	339.8409423
0.262768159	7.446431463	4.226282558	18.82447064	0.913972569	0.257817682	0.193185155	171.4216522
0.281360662	7.930615231	4.638403496	34.53550952	0.913751893	0.294389256	0.405881972	118.5929473
0.258863264	7.720874191	4.830944929	32.12749823	0.921383653	0.259576194	0.366263628	162.4240784
0.226392094	9.049658381	5.855280396	42.37748679	0.909938431	0.22808862	0.556740398	294.6967414
0.199976193	9.217785247	5.90979638	34.27689109	0.934429678	0.192847172	0.585629373	382.5223676
0.295092313	7.676902043	3.875348664	20.18802613	0.928704343	0.297560162	0.40425505	111.8454594
0.257305166	7.746772158	5.308741133	37.82795067	0.894558022	0.268694488	0.488495642	122.0629017
0.295022486	7.337339235	4.37941295	32.76662307	0.93012978	0.302954913	0.365510573	184.7311573
0.254159471	8.222770598	5.27778096	36.13943104	0.909190609	0.252419322	0.489813644	278.2385211
0.160556248	9.380750047	7.974311138	55.50325168	0.873271156	0.155767131	0.454445802	417.6186685
0.168624982	9.582837194	6.920083766	38.27952999	0.891463463	0.163611461	0.520106724	356.3808829
0.204812578	8.591812732	5.753234266	32.58449867	0.888284685	0.196689756	0.405122975	234.9573732
0.267445044	8.103146713	4.028632149	17.75407039	0.917430962	0.264949733	0.505193831	183.1775675
0.283325728	7.164590923	4.291885847	26.5957989	0.91500551	0.291878428	0.217503369	97.06796866
0.23040971	8.240152326	4.970432605	25.37171856	0.916300701	0.226195948	0.432124291	124.9573655
0.170904938	9.645302595	7.709350148	55.87933745	0.883003019	0.16839211	0.624317361	567.6521937
0.188954301	8.903987912	7.756629047	69.87202961	0.892846851	0.186783492	0.503659379	343.7369918
0.230429528	8.2998182	6.271629085	46.40104898	0.887152969	0.238285991	0.394511907	218.4591442
0.301999417	7.748927714	4.664420996	39.14789348	0.93333917	0.322725883	0.682933612	208.0977679
0.22308303	8.956371633	4.826419006	19.32804408	0.900170766	0.21731796	0.642687683	289.6673606
0.212660385	8.788108265	6.583718568	47.19645078	0.883880891	0.215429909	0.441929026	307.1107447
0.367540696	6.02831012	2.489327004	10.25359924	0.934489575	0.423332603	0.2699487	47.41330443
0.207399009	9.110628147	6.927709135	52.45004615	0.889063426	0.206014136	0.600002336	379.9637152
0.266778407	7.918940241	4.732690652	30.05530279	0.901442012	0.274141032	0.348752691	111.0868002
0.252829499	8.134287197	4.758264163	26.84937702	0.901802411	0.247123005	0.425009708	164.9296128
0.278639868	7.816317962	4.520680638	28.91874889	0.916107658	0.27786315	0.464960521	104.4546082
0.36375387	6.087049784	2.729919889	13.0627443	0.937562352	0.396323579	0.237543798	42.16895341
0.172833344	9.287145876	6.669899855	35.910843	0.877047802	0.16908514	0.521152004	337.2130805
0.200444054	9.057775225	6.243577718	39.14135685	0.901880685	0.192059485	0.441781778	373.0513181
0.254469599	8.333973083	4.964435336	30.11234535	0.904783021	0.257266237	0.51276137	197.5649586
0.17825118	9.439689541	6.310324542	32.81979064	0.880268453	0.171520309	0.483249128	341.5135954
0.278517203	7.960247546	5.422401227	51.09710049	0.917048764	0.294689102	0.451484808	168.4991617
0.214482394	9.168825705	5.466390285	28.3252071	0.900543371	0.213587772	0.678370495	335.0044867
0.241827213	7.983434325	6.052378225	50.90309764	0.904300092	0.244957997	0.596458731	232.5309627
0.251632985	8.003783851	4.375894291	19.77542279	0.891149788	0.254789436	0.463663113	139.1781634
0.326033493	7.293008855	3.835897353	26.3113865	0.940824383	0.349338543	0.337210491	90.44240358

original_glcm_SumEntropy	original_glcm_AverageIntensity	original_glcm_Energy	original_glcm_SumSquares	original_glcm_ClusterProminence	original_glcm_SumAverage	original_glcm_Imc2	original_glcm_Imc1
5.801484078	17.85159112	0.003327278	134.5699107	601717.9607	34.31298348	0.893829959	-0.159743868
4.418526235	6.64832289	0.015059836	24.12158137	20585.42829	13.12177879	0.749961848	-0.113562942
5.344386338	10.5699527	0.009547792	56.58654176	122553.3489	20.58884872	0.939140831	-0.243225407
5.56338368	18.41389949	0.006145702	119.3018657	392993.6105	35.66870816	0.942996756	-0.23245378
5.223904032	10.23222492	0.005979216	54.51683055	86625.43783	19.73903259	0.756405825	-0.096866981
4.969672106	12.13447308	0.006337807	42.68496483	35724.47035	23.61636548	0.866597671	-0.161695845
5.710260639	15.35009142	0.004218939	72.83252567	138424.9916	30.07412166	0.857260252	-0.138235945
4.89872187	13.61427401	0.011000801	44.41401161	119913.7206	26.56187822	0.69087736	-0.080277461
5.582860018	14.94848373	0.003844119	84.22723186	257958.4657	28.96416976	0.791681949	-0.104559114
5.292974543	11.23923797	0.005319609	65.64925937	177257.4312	21.73051773	0.889810026	-0.172963817
5.33595562	13.01063931	0.005030118	56.06313028	97386.67792	25.4828651	0.851191517	-0.143963231
5.156190243	16.02625929	0.005494709	53.15678853	74438.92167	31.14029795	0.738015295	-0.089511469
4.234342958	6.70006249	0.025886782	34.13262878	98438.83367	13.10494185	0.720038751	-0.109383522
5.284012738	12.42498077	0.006342485	84.53417827	363857.6456	23.83163652	0.862952696	-0.151415919
5.232073721	14.24676351	0.007002489	74.81921386	146324.1749	27.40430721	0.800997731	-0.115935077
5.090894141	12.31658539	0.011207817	92.63812126	580175.9652	24.02341923	0.797541305	-0.121235257
5.625444345	20.36359904	0.00296517	76.47981265	210237.9937	39.8767954	0.917806705	-0.189025767
4.652987939	7.152040637	0.013460061	30.83718604	41300.60239	14.05340451	0.776565752	-0.122349004
4.93763069	13.7667982	0.006339639	29.72244505	26357.31386	27.21719261	0.78536617	-0.113832266
5.290758011	15.89918013	0.005373033	54.65513876	97130.91246	31.01619784	0.721091161	-0.081952385
4.705508653	7.502650076	0.014333381	33.44564777	96219.24077	14.78770781	0.768409367	-0.118184238
5.059051398	12.18148192	0.005064583	35.54042708	28826.11523	23.93588089	0.80260547	-0.118525391
4.980337679	9.452596345	0.008682713	49.95343644	93574.92618	18.498438	0.8630275	-0.162703822
5.738989831	15.08999008	0.003122221	78.03157982	201744.0789	29.43633523	0.880664179	-0.15395425
4.705752428	9.167673613	0.011788219	43.48067589	87858.50478	17.7066963	0.78297274	-0.119868395
5.747117266	18.5038232	0.002483491	79.97431821	176452.8253	35.97889079	0.869611049	-0.142764626
5.610881059	18.9638728	0.004058963	95.40948491	452719.5792	36.85044629	0.849848771	-0.13359975
5.234703028	13.08776503	0.00530027	55.5011864	120589.982	25.4123939	0.77281581	-0.10222476
5.012265376	15.15537349	0.004668502	30.89874691	17220.10695	29.84134124	0.901009205	-0.188849732
4.993223469	9.029242809	0.006354521	33.79101722	35181.34449	17.69724858	0.749739792	-0.098710978
5.090037291	13.77131214	0.009544401	55.89992198	120801.2427	26.80502063	0.74164822	-0.09441925
5.096070392	9.490341435	0.01140884	71.46784624	247517.5154	18.32976531	0.836724502	-0.143412972
5.761522759	16.71450053	0.003761373	131.9071536	610324.476	32.38340316	0.869899524	-0.146585581
5.422832567	12.68171426	0.007789657	98.53469114	433722.2411	24.57342049	0.872658079	-0.159096557
5.383207956	15.19813343	0.004412687	58.27785892	137632.5551	29.5132609	0.747179465	-0.089377078
4.699458984	10.34391322	0.013983406	45.89048749	178130.3342	20.19275163	0.729699318	-0.098671397
5.075647889	11.01911653	0.009711859	88.59880401	717432.4312	21.24814645	0.803331518	-0.121798067
5.204540725	15.9428481	0.014563122	228.5684292	1736301.272	31.36520182	0.92131097	-0.223794988
5.638312891	15.57816797	0.002772984	58.01279111	76803.60943	30.59236507	0.896268255	-0.168882357
6.115779406	21.01881506	0.001682078	110.7951695	329014.541	41.26616823	0.85004145	-0.125158034
5.350348367	14.01906666	0.006950999	76.92483582	279375.0784	27.38734478	0.840227033	-0.138406422
6.12391441	25.24693378	0.001146022	102.8694361	230614.6713	49.1955696	0.824294317	-0.108605265
5.486434981	12.94388208	0.004179935	67.83203751	173366.0779	24.99579052	0.791364124	-0.106016731
5.612831844	17.00613226	0.003540458	96.29696851	313649.5157	32.77461622	0.850491616	-0.132506716
4.988451646	9.396259389	0.007278934	41.06365863	39015.39358	18.34174611	0.791745527	-0.11809342
5.406818976	15.23916927	0.0052344	74.04431668	224510.6898	29.48638733	0.829092129	-0.127199922
5.377078366	15.94369355	0.005062972	58.64264187	125384.4565	31.14668476	0.766513749	-0.097608042
4.982161899	13.85789537	0.005552528	28.85695029	15899.36728	27.34686339	0.835659422	-0.139708389
5.421224566	13.8174333	0.003608541	62.06155678	80760.79313	26.65015543	0.912670176	-0.188273693
4.471717943	10.7849039	0.014399425	24.43667696	26358.10202	21.10762581	0.720621121	-0.097835985
5.154327596	12.88570317	0.005586304	57.04751617	147229.0754	24.89123259	0.756272669	-0.095823296
5.964362862	21.54248271	0.001576864	91.80499343	186708.2874	41.83865159	0.82685879	-0.111959504
5.421351146	17.60523683	0.003139968	49.98440063	57534.1286	34.35809641	0.688595956	-0.069426275
5.014979745	10.1337778	0.008029169	52.91760694	123107.1981	19.76942166	0.850689781	-0.151868226
5.079724155	9.976586904	0.006879537	46.61313638	60933.11771	19.33418681	0.832150197	-0.137286108
5.079172949	11.63802467	0.005995578	41.36110387	58900.74858	22.71445653	0.775794143	-0.107002022
5.666120028	13.53650315	0.003213492	82.56962728	226493.8503	26.17532226	0.79994101	-0.106731466
5.395863314	16.66783057	0.00605655	89.2809172	583414.4452	32.5548601	0.850912643	-0.14075447
5.206508998	14.08490589	0.004715503	55.48400094	107267.6071	27.24632412	0.813725622	-0.119742727
6.219944624	26.54286175	0.002172757	138.5063246	426257.6701	51.93840796	0.868431178	-0.133914207
4.908463818	12.08131961	0.01076766	41.99363273	79953.83326	23.53719587	0.812899014	-0.132189893
5.257491516	11.5273138	0.005545793	60.13883594	134367.9425	22.33952327	0.774222183	-0.102876829
5.249461061	12.25314448	0.006594295	62.33746857	173599.3508	23.69592455	0.711650909	-0.080551201
5.797745461	17.59820295	0.003175065	73.04280867	173688.016	34.51594931	0.849724166	-0.133081397
4.670013144	13.19894301	0.008202477	25.54858854	19962.04725	25.8391726	0.797475204	-0.126564677
5.042802707	10.24068521	0.008956726	50.95932323	157497.2174	19.98133563	0.758263136	-0.102554822
4.919694758	12.4579223	0.008218805	47.77968961	140177.9032	24.22795493	0.826377073	-0.136548984
5.702960844	16.17723329	0.003698767	90.29715731	380878.9285	31.4334814	0.819244878	-0.115829112
5.717866524	18.69354451	0.002812438	88.52119248	514770.7626	36.42923773	0.814393908	-0.113453541
4.895897387	10.19452918	0.008599121	32.25361013	54698.22317	19.98947814	0.747543875	-0.100981676
5.083879988	9.869349271	0.009348301	69.28578535	247927.2821	19.09601555	0.845655189	-0.149666275
4.668644665	13.36313989	0.013921601	44.53578195	168984.7303	26.07900474	0.662526889	-0.074964988
5.293470385	16.16370406	0.006722695	66.98746353	205329.8156	31.49587906	0.838068615	-0.136033537
5.912209155	19.96157054	0.002303512	114.3336447	304993.2353	38.44162144	0.86153422	-0.133942866
5.92229514	18.18001578	0.001869415	92.67198481	190432.4291	35.22786285	0.823929971	-0.112553069
5.44139305	14.96119039	0.003772144	58.46349203	93927.28869	29.1861039	0.869699102	-0.151577042
5.115423275	13.06594964	0.005867395	36.38793545	69169.8589	25.73924783	0.794935967	-0.116866359
4.593107716	9.758471593	0.012218889	31.74295122	39792.16627	19.08761991	0.781576742	-0.12326553
5.197704245	10.50371999	0.00534277	46.41776512	94229.22705	20.56159389	0.801200995	-0.117009645
6.182148161	22.4308223	0.001942043	156.0098336	686703.6197	43.48716586	0.922374393	-0.179838982
5.732606705	17.36828537	0.003884725	138.0766004	815457.0107	33.41851767	0.903989236	-0.171899701
5.322542268	14.34040513	0.006596439	75.94015933	214474.2605	27.64373895	0.851162303	-0.14200564
5.15298884	12.268062	0.010645681	100.4153237	649259.8326	23.8482303	0.851376406	-0.152691112
5.675177744	16.17494146	0.002899164	61.38148257	136976.8888	31.72476744	0.849633498	-0.135764617
5.561346517	17.02080388	0.004489846	85.45600875	237803.2688	32.96790304	0.834494642	-0.126161052
3.945936083	6.674373474	0.032742976	11.77181098	8636.712717	13.31105235	0.625333615	-0.082567155
5.908453023	18.06663738	0.003493646	131.1746747	477914.3153	34.96281686	0.898895439	-0.165777014
5.043769532	10.09712831	0.008248481	43.74064041	60699.05391	19.71945991	0.749795255	-0.098655562
5.197351864	12.37740664	0.005991312	46.13718379	54576.88866	24.22002296	0.854667003	-0.14881554
5.04449122	9.34086058	0.008249783	49.98973754	145048.3235	18.23704419	0.818136249	-0.132418123
3.941943529	6.261850699	0.029548257	13.98189395	15782.88801	12.47480892	0.622846906	-0.078442873
5.815861217	17.69836941	0.002277341	86.79871126	177844.5685	34.2797343	0.859311723	-0.134014191
5.533458977	19.01210654	0.003465989	73.49800297	232785.5011	37.02532668	0.737666025	-0.083147101
5.338644005	13.29901332	0.006580246	60.18110625	147381.4167	26.00019368	0.803033073	-0.117148205
5.760621537	18.00518408	0.002037501	72.09618369	129584.9011	35.09670302	0.760451644	-0.088176646
5.1737433	12.09676006	0.009452212	79.42752552	403767.9432	23.32246025	0.804662661	-0.121295044
5.88424923	16.92770395	0.002615386	92.96331041	331653.0423	33.09427565	0.895092036	-0.161943632
5.349874877	13.46098624	0.007312726	114.9611958	663025.2775	25.98835252	0.93089045	-0.221484192
5.19837631	11.21104036	0.005619978	38.4524313	34558.86466	22.12344246	0.889687549	-0.179046278
4.721643514	9.182883576	0.014324991	33.93805137	58542.15432	17.90251316	0.682489301	-0.082647495

original_glcm_DifferenceAverage	original_glcm_Id	original_glcm_ClusterTendency	original_firstorder_InterquartileRange	original_firstorder_Skewness	original_firstorder_Uniformity	original_firstorder_MeanAbsoluteDeviation
8.137868322	0.254436909	365.1718144	282.75	1.526851485	0.04681	210.8438934
3.724987765	0.387250016	55.90510956	99	2.162032039	0.108165518	84.32701421
4.10618631	0.402100881	165.9537803	174	1.419445782	0.060610516	135.0133395
6.674648093	0.303760872	359.704905	316	1.129094038	0.058167955	218.5640415
5.665606808	0.308123418	134.0589728	190.25	1.621247003	0.065581629	137.0990037
5.796555306	0.28996075	88.55218817	150	1.743586285	0.070211203	114.168191
5.049118374	0.330304804	222.4793716	304	0.938463314	0.043421483	172.7941543
4.453645682	0.373873669	112.9919411	124	2.350133623	0.090450575	112.0791985
6.036159072	0.29538391	248.77638	266.75	1.189782217	0.050601961	185.1923702
5.837790354	0.29637509	172.4869528	205	1.581540523	0.056918648	151.0161332
5.036763226	0.312407009	161.9322854	237	1.078681645	0.053476947	156.1766371
5.925967964	0.304038693	116.680487	176	1.727719419	0.06387604	129.0879207
3.426766817	0.45280107	84.2464801	63	3.313255525	0.147796031	79.31348838
6.007837148	0.309097537	225.4900046	167	2.131797221	0.070025362	153.3924688
6.682605193	0.310607807	168.7241176	164	1.741034566	0.072488172	150.7299022
4.820412871	0.373961618	290.4318588	143.25	1.968230821	0.087106024	171.947169
6.16147413	0.262000432	226.794664	204	1.088091822	0.044054724	157.4414311
3.856854369	0.397129913	75.77165387	102	2.326281722	0.10058285	91.51356354
4.417729881	0.328799808	75.36397761	159	1.34116049	0.066302074	109.6224439
5.217548314	0.310691218	149.9771703	204.5	1.409787203	0.059437688	142.2582289
3.325364851	0.418539428	97.80247519	113	2.388054873	0.098598995	96.26161541
5.122037557	0.2929132	85.88171714	181	1.199133235	0.059290989	117.5628929
4.835952918	0.334955512	131.6749697	159.25	1.830336306	0.076330185	128.4922562
5.237700125	0.312951638	242.4404502	310	0.90099388	0.038604266	186.9400467
4.846316112	0.358661305	91.56486971	113	2.63828302	0.097414486	104.9579094
6.462599369	0.275860959	220.40252	294	0.956541461	0.038000514	173.0033397
6.339456059	0.286641258	273.6476246	218	1.648046985	0.050977255	173.8375043
5.390907496	0.313755544	139.9191345	176	1.723711749	0.06191428	134.55908
5.322834706	0.264273736	73.27017897	190	0.360919171	0.052054712	112.4053071
4.444371067	0.340396675	82.02025289	154	1.549916144	0.068745147	101.6033303
4.877216908	0.358744953	145.0251959	162	1.917025439	0.079940759	131.8910136
4.930087707	0.36770434	198.8609611	139	2.157206936	0.091042924	132.7233219
6.374245457	0.303135205	419.2069886	312	1.177801453	0.048399353	231.9206151
5.679249983	0.350772775	286.1207397	183	1.767422809	0.067864554	176.020113
5.568576553	0.306156368	154.5107994	201	1.50841081	0.055756636	138.7704494
4.15062699	0.38799075	121.9712467	101	2.624571693	0.103652979	106.9028843
5.16451513	0.354833165	250.1007519	125	2.796358939	0.087343701	140.7442177
4.305892336	0.452656404	835.8857724	542	1.032564299	0.083575897	329.2361353
5.237722615	0.285955674	174.5031258	292.75	0.494836263	0.037453896	162.1852231
6.114044563	0.279815288	366.7510254	406	0.478432487	0.028801447	227.2723222
5.234116379	0.332506562	226.0951116	184	1.785987499	0.068514673	156.6888178
7.785488795	0.220276008	309.643535	405	-0.093396797	0.026634966	214.9952359
5.805405959	0.304143106	180.1515162	217	1.535678924	0.052796868	157.289024
7.021924563	0.262059045	258.9620687	247	1.416661203	0.047622479	184.3345489
4.896484777	0.330366979	95.63300935	156.75	1.639847749	0.070923367	112.8896677
5.695722418	0.310411106	202.3031313	207	1.560484583	0.056867097	164.7110753
5.187915349	0.321627982	162.8417284	230	1.381267816	0.055381637	149.8178897
4.357995478	0.321706155	71.78486258	174.5	0.906157888	0.060011257	105.2672713
6.723949658	0.265919152	147.2350408	251.5	0.973368728	0.045927857	147.7310581
3.807373803	0.384735816	51.3195489	86	2.72681883	0.112114203	76.9912293
5.72200091	0.298474586	138.0547213	159	2.043397225	0.068183227	127.6523895
7.34158439	0.233119387	262.0820378	360	0.540135918	0.030710491	199.0909547
5.701048783	0.27860647	129.9510155	223	0.933504612	0.046624682	141.2169672
5.109710022	0.328340702	134.7933411	172.5	1.921148403	0.071717142	137.83312
5.170408594	0.341630301	107.250305	169.75	1.680728864	0.067441913	125.7548917
4.792914168	0.324363721	102.8928129	156.75	1.778535373	0.068708481	109.5466351
6.155512618	0.286124123	234.3751884	261	1.242350939	0.045243116	178.5574328
5.090734467	0.329379487	277.8085523	174	2.063524713	0.061873608	161.0409331
5.922011363	0.28629047	130.6023236	166.25	1.792217603	0.060809955	132.163642
6.774055399	0.27568563	466.6858012	564	-0.004087425	0.029423338	261.873313
4.484323548	0.377845944	102.6333632	132.75	2.182841667	0.085171139	112.301551
5.67478937	0.316725541	146.9080329	173	1.818581911	0.063907195	135.2145324
5.522455697	0.33452678	158.3161706	169	1.771918901	0.067804239	141.8202801
5.176514133	0.31571608	223.7287779	235.5	0.838123294	0.041048386	160.737544
4.226282558	0.341651065	55.07117377	126.5	1.631390254	0.077067778	92.71362282
4.638403496	0.368127461	131.3738171	132	2.453489731	0.080810891	111.4920951
4.830944929	0.339699223	118.3365389	123.25	2.420332516	0.082238699	107.6293119
5.855280396	0.30829628	267.6743278	252.5	1.45059865	0.047252122	184.1007533
5.90979638	0.279432209	271.6219803	308	1.405925223	0.038085352	220.527945
3.875348664	0.374986264	83.0766216	121.75	2.122124493	0.080100219	97.13799884
5.308741133	0.343359217	190.1437158	162	1.885364065	0.081823467	145.7738747
4.37941295	0.376561789	110.8434803	104	2.889811786	0.105832396	106.9825491
5.27778096	0.330499886	185.1689074	175.5	1.760462014	0.06695411	145.0432422
7.974311138	0.238393813	312.4777991	309	1.038865818	0.037726816	209.5265883
6.920083766	0.249211094	270.7658511	353.5	0.666990739	0.032960695	199.9450669
5.753234266	0.283739998	154.19459	234.75	1.095330868	0.048401856	144.8756377
4.028632149	0.348828327	104.1289977	194	1.524565736	0.059778958	123.6792439
4.291885847	0.368332643	69.19100666	101	2.535430927	0.102056738	85.26604879
4.970432605	0.311656889	127.2345596	186.5	1.527400977	0.060394688	134.6810827
7.709350148	0.249633224	495.2290504	466	0.71180745	0.029288487	257.6808051
7.756629047	0.266170375	388.7740155	270	1.645523226	0.049138285	211.2795832
6.271629085	0.312929475	195.8625714	187	1.605453539	0.068751369	158.3633857
4.664420996	0.389938254	323.9156719	166	1.72381949	0.081114703	195.9783451
4.826419006	0.304816842	195.1305404	275	0.852780145	0.04148124	157.333861
6.583718568	0.293297969	232.3722966	252.5	1.296931555	0.05367658	180.9730098
2.489327004	0.482382139	28.96904656	64	2.649652091	0.159370335	58.8949035
6.927709135	0.28423197	398.385961	361	1.067325026	0.042152868	228.0590216
4.732690652	0.351257662	108.0116835	158.75	1.747317094	0.073926085	119.1275315
4.758264163	0.329638919	122.6843928	195	1.243058725	0.060185807	129.9907246
4.520680638	0.355245164	134.603676	147	2.191609011	0.075327298	117.8898743
2.729919889	0.459371218	33.56884758	66	3.136662183	0.152448577	66.13567856
6.669899855	0.254937646	253.8613492	361	0.657597754	0.035297265	201.7337081
6.243577718	0.277002297	199.7595075	201	1.539758831	0.051401849	151.6452908
4.964435336	0.336112239	169.1194868	185	1.572945242	0.063636915	142.1625069
6.310324542	0.259429417	207.1848363	282	0.713450758	0.036846332	169.5234174
5.422401227	0.363913405	210.794256	142	2.371757267	0.081737074	143.3808024
5.466390285	0.298113396	302.9384383	299	0.978498475	0.03681424	197.415106
6.052378225	0.321116534	342.7237064	164	1.961961682	0.072437829	178.6754278
4.375894291	0.338520916	106.5215385	186.5	1.035519356	0.056990379	119.6317162
3.835897353	0.415244664	82.39691161	103	2.452019348	0.100602548	101.7425193

original_firstorder_Energy	original_firstorder_RobustMeanAbsoluteDeviation	original_firstorder_Median	original_firstorder_TotalEnergy	original_firstorder_Maximum	original_firstorder_RootMeanSquared
1029934195	130.8587368	-748.5	27808223265	329	696.6779239
497612463	45.47283911	-761	13435536501	-130	729.5243949
926069537	82.49312194	-789	25003877499	55	776.9787109
336872933	156.139727	-641	9095569191	215	587.1996351
1051090436	86.18193785	-705	28379441772	356	666.2376831
359243497	67.14269135	-766	9699574419	-43	737.7725454
2722472841	125.6752239	-718	73506766707	74	694.0953331
2676475732	59.2333714	-760	72264844764	190	723.8627205
1589741462	123.8849194	-636	42923019474	241	591.3553564
470909054	92.51719997	-697	12714544458	140	662.7826446
441493194	107.5592986	-573	11920316238	39	541.0798185
962328800	78.29278165	-679	25982877600	156	646.1394784
1158694179	30.49562462	-815	31284742833	31	787.7932469
913016275	79.43630704	-773	24651439425	335	729.2125124
979096185	84.36174592	-751	26435596995	96	702.492748
2440785638	83.70720627	-804	65901212226	158	746.8375798
256864450	95.59644658	-461	6935340150	301	463.820391
645971755	46.71577929	-753	17441237385	16	723.8110787
480609062	69.63830696	-632	12976444674	-67	608.4972662
2346928852	92.32743356	-710	63367079004	259	671.8774315
1546265239	51.77636031	-748	41749161453	27	715.0741902
389210226	77.84249658	-621	10508676102	1	600.5945664
502183260	74.38255988	-744	13558948020	63	700.2951805
954285048	130.9880494	-617	25765696296	66	600.4295931
874223586	53.67091502	-865	23604036822	106	828.6994638
742268823	121.0016552	-558	20041258221	244	559.7551261
1345574673	102.266568	-678	36330516171	314	645.1356622
1061599299	79.28129759	-650	28663181073	204	619.966859
137146812	78.57020455	-543	3702963924	-40	553.9098611
819568130	65.57310568	-650	22128339510	122	631.6729962
2336195215	75.82479843	-743	63077270805	253	700.7165034
1077267526	67.04857971	-767	29086223202	86	721.575101
1090105856	159.9914634	-630	29432858112	214	581.0323509
1003526339	95.92000878	-705	27095211153	199	653.4772258
1579630705	87.60083939	-627	42650029035	209	598.8997452
1806217550	50.5395721	-813	48767873850	68	776.0626743
2581029753	60.7612813	-877	69687803331	318	832.8497706
2694891583	263.3425773	-831	72762072741	213	722.6095875
319651592	119.4680023	-495	8630592984	176	509.7836238
2273982023	172.2316487	-545.5	61397514621	92	559.2761611
1298568213	88.52603701	-756.5	35061341751	98	707.5341363
759456688	162.7784375	-357	20505330576	259	443.1639447
1678113235	97.99274713	-713	45309057345	274	677.7753894
1039157429	116.2830791	-683	28057250583	346	646.2722158
620149573	69.98708007	-728	16744038471	-5	696.5989213
1318715008	97.55837697	-737	35605305216	190	693.1134077
1900143488	98.5953698	-680	51303874176	171	645.3800827
321174719	73.04702877	-618	8671717413	-103	607.2417161
211672165	104.0703812	-500	5715148455	219	498.7318407
632464230	38.85361537	-705	17076534210	179	683.9583223
1443332636	72.31431432	-785	38969981172	187	749.5509583
1053100245	146.8080748	-545	28433706615	436	558.9275034
1401479389	96.65716303	-571	37839943503	77	557.3253688
665530930	75.96017643	-821	17969335110	27	776.4251331
672845200	74.26825924	-745.5	18166820400	26	711.2655247
828960339	66.98326929	-676	22381929153	129	652.0029648
1458728770	117.3494825	-629	39385676790	232	600.5207402
1671717504	82.67413103	-733	45136372608	300	695.2947505
690991279	76.40103546	-674.5	18656764533	204	647.526698
2071696559	214.429615	-379	55935807093	290	476.37775
955082276	61.75126879	-772	25787221452	221	735.4024006
1525530222	77.58361217	-792	41189315994	190	755.8833466
2749178677	82.00990031	-730	74227824279	164	689.1277025
3431839892	104.5413954	-671	92659677084	247	670.966055
354600276	55.45469928	-685.5	9574207452	27	669.9709761
2533511998	58.86476514	-843	68404823946	48	809.9446628
701595502	55.43642682	-763.5	18943078554	253	733.5075735
2740863566	114.4397946	-700	74003316282	307	662.2221459
1968953270	141.1990854	-597	53161738290	964	579.8025118
1226632065	53.46279874	-672	33119065755	253	650.5908725
560204996	79.34088018	-705	15125534892	110	656.7028684
2423488088	49.94607035	-788	65434178376	246	751.6087002
1115420466	85.21681454	-743	30116352582	113	699.5957166
630920586	144.5112158	-547	17034855822	284	528.4808241
1117323558	148.0731938	-600	30167736066	322	597.3762386
446517006	98.43495833	-596	12055959162	122	576.8232327
864978871	81.21091037	-623	23354429517	115	600.8406989
635010447	45.64660482	-841.5	17145282069	-10	814.1560989
819101688	82.65448273	-728	22115745576	313	692.7111049
736396076	193.7866565	-541	19882694052	275	561.1004563
986421624	126.6150355	-757	26633383848	372	704.2289923
776440644	92.58713656	-705	20963897388	118	656.959427
1604542426	106.4298018	-763	43322645502	485	700.5967884
1102523932	112.1754841	-607	29768146164	49	598.9805669
999944021	117.3516034	-689	26998488567	99	640.297703
916352931	30.73339376	-796	24741529137	-147	773.3961468
852548665	162.3597681	-639	23018813955	251	597.8818885
1359804097	70.52241117	-739	36714710619	11	704.2136639
578547489	87.36987234	-659	15620782203	53	628.6355948
937584539	65.79808947	-712	25314782553	216	680.4444949
846349725	30.96440223	-831	22851442575	-34	805.630933
714751566	152.6403125	-561	19298292282	148	547.7806401
1788591524	91.86167177	-611	48291971148	284	587.441981
1441441840	86.49172913	-697	38918929680	92	660.9088875
875999515	119.8139952	-433	23651986905	205	448.6498114
1824630722	69.97961568	-775	49265029494	302	730.2095169
1074172329	134.1761726	-639	29002652883	148	617.509377
583551233	88.83257937	-802	15755883291	231	746.5624712
236007973	79.93984117	-546	6372215271	7	531.9616349
3224592405	49.47482393	-781	87063994935	423	747.3069541

original_firstorder_90Percentile	original_firstorder_Minimum	original_firstorder_Entropy	original_firstorder_StandardDeviation	original_firstorder_Range	original_firstorder_Variance	original_firstorder_10Percentile
-197	-1034	4.92575119	275.9316066	1363	76138.25153	-873.9
-574.4	-867	3.704223243	122.1045102	737	14909.51141	-816
-513.3	-1000	4.469157532	186.9461869	1055	34948.87679	-962
-13.4	-933	4.661246217	266.2656979	1148	70897.42188	-749.4
-377.7	-861	4.383525213	182.0141496	1217	33129.15066	-798.3
-519.9	-980	4.272871428	157.3741082	937	24766.60994	-861
-349	-1012	4.841428897	211.0247715	1086	44531.45417	-879
-497	-1005	4.030833293	164.1595977	1195	26948.37352	-829
-118	-894	4.741842405	232.0330699	1135	53839.34555	-753
-347.2	-890	4.561883247	201.2938544	1030	40519.21583	-812
-163.4	-819	4.558496148	194.2636133	858	37738.35144	-691.3
-382.4	-982	4.394941157	175.3332309	1138	30741.74187	-777
-664.2	-923	3.359154235	135.9152558	954	18472.95676	-859
-412.2	-961	4.400375184	220.8457403	1296	48772.84103	-861
-342.5	-993	4.367279664	205.6633909	1089	42297.43035	-832
-310.5	-977	4.180948175	241.7384297	1135	58437.46839	-873
-77	-887	4.85450138	211.0866834	1188	44557.58793	-630
-536.2	-875	3.809396588	136.0792274	891	18517.55613	-818
-397.4	-924	4.281259665	144.3872279	857	20847.67159	-729.3
-368.8	-1006	4.523904118	183.8208433	1265	33790.10245	-811
-531	-859	3.850355846	141.3233401	886	19972.28646	-810
-362.4	-851	4.393381493	151.8381575	852	23054.82607	-735
-423.6	-895	4.231188057	175.4639667	958	30787.60362	-820.7
-172.6	-917	4.929719676	231.1505219	983	53430.56379	-801.4
-637	-1024	3.932023418	158.9708399	1130	25271.72794	-931
-225	-947	4.96745179	217.3739326	1191	47251.42659	-753
-258.2	-1028	4.777823987	236.8751191	1342	56109.82206	-801
-339.1	-900	4.466871023	184.5579609	1104	34061.64092	-751.9
-348.8	-891	4.479463327	141.1145142	851	19913.30611	-703.4
-436	-824	4.188273881	135.703684	946	18415.48985	-752
-415.4	-988	4.229841048	181.4579839	1241	32926.99992	-819
-440.8	-896	4.084967055	190.727728	982	36377.06621	-833
54.2	-883	4.811412433	287.1890616	1097	82477.55708	-752
-207.7	-883	4.4754595	240.7224147	1082	57947.28092	-791
-319.3	-915	4.551564273	183.5329522	1124	33684.34455	-743
-576.8	-986	3.872150055	164.5915356	1054	27090.37361	-873
-572	-1035	4.151612556	223.656306	1353	50022.14321	-948
49	-995	4.201717415	380.8722592	1208	145063.6778	-903
-179.9	-845	4.881504138	196.842467	1021	38746.9568	-712
-42	-976	5.229163032	270.3135353	1068	73069.40735	-817
-352.3	-990	4.410059573	213.9362217	1088	45768.70696	-842
-15.6	-962	5.33219476	256.2861349	1221	65682.58297	-702.8
-345	-943	4.674276476	208.1681927	1217	43333.99646	-832.8
-220	-980	4.852006491	240.4732948	1326	57827.40553	-814
-456	-886	4.208348421	151.1377119	881	22842.60795	-818
-306.8	-1008	4.62355217	219.6067512	1198	48227.12516	-844
-339	-980	4.589856149	192.8264688	1151	37182.04709	-794
-414	-912	4.318350942	133.2579251	809	17757.67461	-738
-213	-774	4.699958202	185.4469284	993	34390.56325	-663
-552	-920	3.699436119	119.7754968	1099	14346.16964	-763
-496.8	-1006	4.367261836	181.0170816	1193	32767.18382	-877
-151	-1018	5.207387176	241.4438589	1454	58295.137	-785
-254	-941	4.691206249	178.2709235	1018	31780.52217	-720
-504.3	-992	4.329083597	192.2321494	1019	36953.19927	-904
-451.5	-905	4.313498756	171.3495879	931	29360.68128	-839
-441	-896	4.261508403	150.7034241	1025	22711.52203	-772
-179.4	-861	4.808831427	228.8350755	1093	52365.49179	-774
-369	-1027	4.573946375	232.9713999	1327	54275.67315	-832
-361.7	-944	4.476333659	184.2810592	1148	33959.5088	-787
34	-1010	5.303699858	302.3340587	1300	91405.88303	-779
-517	-997	4.09483817	161.1641228	1218	25973.87449	-843
-465.9	-999	4.436685954	187.0779267	1189	34998.15067	-893
-369.6	-929	4.389834317	193.4377087	1093	37418.14715	-822
-338.2	-1030	4.919203074	210.6477237	1277	44372.46351	-915
-484.9	-959	4.071420728	128.7885277	986	16586.48486	-780
-616.1	-1011	4.139445031	166.9970934	1059	27888.0292	-922
-534.3	-995	4.115556786	161.8661461	1248	26200.64926	-842
-226.9	-1000	4.84177225	241.9387808	1307	58534.37364	-832
2.4	-1000	5.125481637	289.0207442	1964	83532.99055	-766
-465.7	-855	4.081467855	141.396906	1108	19993.08504	-759
-304.8	-839	4.1963234	201.3844805	949	40555.70897	-776
-555.9	-1037	3.869003765	168.8449282	1283	28508.60979	-846
-391.8	-1050	4.42269943	197.0586878	1163	38832.12642	-833
-9	-922	5.041996311	261.3597845	1206	68308.93697	-720
-173	-975	5.104080929	240.6311223	1297	57903.33702	-820
-284.1	-882	4.678938634	183.3274869	1004	33608.96745	-735
-360	-900	4.412516207	163.0885266	1015	26597.86752	-735
-662.2	-1005	3.789817545	128.3462438	995	16472.7583	-904
-416.6	-921	4.457600628	178.9992569	1234	32040.73396	-831
17	-991	5.324219914	308.3049533	1266	95051.94424	-800
-197	-1034	4.887230462	280.9060993	1406	78908.23663	-876
-285.6	-941	4.459135216	211.3041685	1059	44649.45164	-787.2
-87	-950	4.253565817	265.2665259	1435	70366.32975	-835
-286	-949	4.820195656	194.3301597	998	37764.21097	-778
-207.8	-994	4.713968648	229.9407217	1093	52872.73552	-798
-666.2	-910	3.198674614	88.58488363	763	7847.281607	-835
-45	-933	4.969735716	279.4633347	1184	78099.75545	-790
-455	-908	4.228591566	162.0450573	919	26258.60061	-822
-353.3	-896	4.434901984	165.6623758	949	27444.02276	-767
-456.4	-868	4.21193509	169.7569734	1084	28817.43	-796
-699.3	-928	3.300303985	106.7753909	894	11400.9841	-872
-95	-917	5.040240898	239.5196415	1065	57369.65867	-751.9
-254	-982	4.716704317	205.0838653	1266	42059.39181	-735
-358.9	-945	4.471789229	190.0001274	1037	36100.0484	-793
-66.1	-805	4.962095884	210.782684	1010	44429.33988	-635.9
-433	-966	4.230718803	213.2212413	1268	45463.29773	-847
-148	-951	5.032702153	247.6753298	1099	61343.06897	-826
-282.2	-993	4.426829167	252.4995147	1224	63756.00491	-888
-283.3	-753	4.419407987	152.4833771	760	23251.18029	-671
-551.3	-950	3.883606548	153.1402976	1373	23451.95076	-844

original_firstorder_Kurtosis	original_firstorder_Mean	original_glrlm_ShortRunLowGrayLevelEmphasis	original_glrlm_GrayLevelVariance	original_glrlm_LowGrayLevelRunEmphasis	original_glrlm_GrayLevelNonUniformityNormalized
4.688848284	-639.704524	0.008270016	124.002302	0.008707098	0.0446864
7.993503794	-719.2331551	0.056832742	25.50189883	0.06128874	0.101801023
5.478453834	-754.1531943	0.055654854	57.73268528	0.073470004	0.05655397
2.926126998	-523.3602866	0.00762487	111.6355543	0.008099647	0.054561359
5.46221561	-640.8927365	0.032120744	54.59520319	0.034787653	0.062232844
6.430656795	-720.7924242	0.016468584	41.03014522	0.017128027	0.06798012
3.254660382	-661.2388958	0.010258645	72.26314917	0.011286745	0.041176324
9.292460808	-705.0027408	0.007537118	45.96992556	0.008335175	0.08399585
3.348670509	-543.9318082	0.012634502	85.53886599	0.013439503	0.048991564
5.152013624	-631.4757463	0.026779119	66.40919107	0.02840413	0.054912983
3.119907341	-505.0039788	0.012593564	61.11030548	0.013401227	0.05168253
6.156819504	-621.8958785	0.006063043	51.07886549	0.006454291	0.061148417
15.28216354	-775.9801821	0.046158034	32.65132153	0.052546137	0.136145964
7.373539625	-694.9662202	0.017395695	81.13042395	0.018699606	0.066107114
5.305970254	-671.7132056	0.01010404	70.65029935	0.010826043	0.067882768
5.824351525	-706.6320841	0.014312321	94.6955208	0.015841741	0.080742121
3.920732442	-413.0033501	0.005188459	71.98692289	0.005342719	0.043457822
9.140545196	-710.9042985	0.056951003	31.92751852	0.062608445	0.094433563
4.739693198	-591.1186441	0.008112917	34.48809637	0.008602982	0.064056161
4.752763607	-646.2423543	0.006404161	55.59822013	0.006815669	0.056926699
9.641915876	-700.9699074	0.040700365	34.33362926	0.046111566	0.091649737
4.216338485	-581.0843373	0.012998302	38.03756744	0.0136308	0.057696524
6.159546868	-677.9570313	0.032643779	51.5650261	0.034866719	0.072079482
2.870831715	-554.1526256	0.01260266	85.50743222	0.013515232	0.037732569
11.39177475	-813.3087196	0.027617087	43.17237638	0.029816329	0.091237126
3.708862124	-515.8239764	0.007491849	76.25943078	0.007842265	0.037488028
5.436946544	-600.0751624	0.005429752	91.65573118	0.005738919	0.049112239
5.997234534	-591.85916	0.012631141	56.53505005	0.013425912	0.059305712
3.234620027	-535.6331096	0.014679594	32.24872586	0.014899188	0.051542373
6.184121529	-616.9240506	0.036827159	30.48548134	0.039481948	0.066682179
6.696507361	-676.8135771	0.008308806	55.72590145	0.009177212	0.073394034
7.327533844	-695.9120348	0.04295527	61.50515362	0.047202962	0.083315861
3.026607316	-505.0950759	0.011597046	129.3642863	0.012300956	0.046781685
5.203242899	-607.5238298	0.0191222	96.02787881	0.020965664	0.062721698
5.267835518	-570.0846957	0.007800882	55.06464921	0.008354177	0.053895686
10.51038504	-758.408136	0.01565879	46.25452477	0.017280293	0.09611987
11.18646595	-802.2571889	0.021067731	84.63243958	0.022998522	0.081393567
2.26692868	-614.0854486	0.020642724	215.1289937	0.02346106	0.078085045
2.499070548	-470.2471545	0.011243909	62.29214708	0.011860664	0.037164732
2.159600941	-489.6125172	0.006402753	113.9090097	0.00674956	0.02859076
5.555991556	-674.4151889	0.010099623	75.65271402	0.010938303	0.064644573
2.166624912	-361.5407292	0.006604576	104.6121481	0.006870108	0.026706933
5.144236028	-645.0158774	0.017936336	71.20050994	0.01917063	0.050843784
4.435543052	-599.8669614	0.008654229	94.58276036	0.009120289	0.045993628
5.924411142	-680.0054773	0.02979339	37.68905369	0.031976057	0.06803968
4.764379126	-657.4032787	0.00806104	79.1663943	0.008628805	0.054405556
4.579745241	-615.9004822	0.006345151	61.31415315	0.006822271	0.05265863
3.871095177	-592.4397245	0.009324035	29.24493178	0.009741592	0.058668847
3.677415449	-462.9717979	0.018500594	55.75732572	0.019380622	0.04487702
13.3411577	-673.3890533	0.012808588	24.84708547	0.013857833	0.105767633
7.770108827	-727.3647334	0.01227273	54.48343846	0.01299257	0.065490531
2.610576929	-504.0881044	0.005454557	93.88679656	0.005669717	0.030469385
3.34800256	-528.0445479	0.005253411	51.82146582	0.005514717	0.045709019
6.456340965	-752.2518116	0.026078902	61.95182325	0.027821933	0.067402741
5.543373642	-690.3172932	0.027240662	49.08104443	0.029681473	0.063971419
6.916087803	-634.3471795	0.015578949	37.85543456	0.016544592	0.066484843
3.761087535	-555.2113721	0.019382701	84.45840842	0.020619846	0.043960048
7.122583449	-655.1023713	0.006727205	89.20290767	0.007185617	0.058842094
6.506235646	-620.7506068	0.01000619	56.29364193	0.010512007	0.058995242
1.752671976	-368.1438274	0.00385348	143.0248724	0.00400842	0.02780193
8.480609493	-717.5254813	0.011253046	44.50519673	0.012400302	0.078905681
6.383062365	-732.3670412	0.020345871	58.35262026	0.021742204	0.060906838
5.83457104	-661.4218345	0.013488151	62.11109682	0.014680644	0.064046243
3.908439409	-637.0423718	0.008334092	70.95434616	0.009326386	0.040385926
6.40692596	-657.4759494	0.008817849	27.15426372	0.009296793	0.07454062
10.15382968	-792.5416882	0.021634917	47.00968545	0.024189846	0.076887781
10.33982151	-715.4248466	0.012062018	44.50236075	0.012902405	0.078426554
4.476377378	-616.44448	0.008905052	94.24363796	0.009650073	0.045782348
4.555364479	-502.6310398	0.005443403	136.0860121	0.005693131	0.037018446
8.679682767	-635.0396825	0.017550418	33.99003988	0.019113441	0.076776726
5.876546759	-625.0623557	0.036570366	65.47112302	0.039805837	0.076437209
12.5805618	-732.3981352	0.007540596	48.94990656	0.008300851	0.097626129
5.835446738	-671.2689776	0.006438644	64.69001952	0.006921724	0.063267169
3.102853292	-459.3289066	0.00593596	109.9562161	0.00621687	0.03700607
2.539064126	-546.7678058	0.009676154	93.04915782	0.010102837	0.032469869
3.907300334	-546.9150522	0.011102538	54.85570905	0.011558704	0.047389147
5.904780555	-578.283389	0.009559968	43.57074019	0.010199646	0.058031302
11.27911483	-803.9759916	0.018449042	27.96236922	0.01996415	0.096213944
5.212537696	-669.1845343	0.030865514	52.12243895	0.033295735	0.058487194
2.47082411	-468.8088927	0.006856153	148.5382399	0.007166439	0.028739252
5.117048852	-645.7787833	0.00858066	127.2108109	0.009040076	0.046873471
4.896115908	-622.0500278	0.013774249	72.54320621	0.014689758	0.064426699
4.639351982	-648.436219	0.017338688	109.815854	0.019159826	0.076125852
3.22196508	-566.5805402	0.008194731	60.02033276	0.008707881	0.041029208
3.751277821	-597.5854859	0.007366614	85.40603774	0.007829879	0.050710262
12.29502315	-768.3061358	0.028625635	14.18192293	0.033352089	0.141822508
3.00323505	-528.5480084	0.008298732	126.7415968	0.008924015	0.04008787
5.958586537	-685.3161926	0.021254702	44.1013099	0.023272841	0.070067842
4.177761135	-606.4146175	0.014207435	45.14846857	0.015092944	0.057919785
8.494091535	-658.9288889	0.034074052	48.48653657	0.036799277	0.071483436
15.49020449	-798.523773	0.037224537	20.35027557	0.042010577	0.137098283
2.332167497	-492.6397985	0.008675148	90.92821134	0.009083646	0.034914983
5.380805813	-550.4804167	0.004621424	68.78890935	0.004835581	0.049883009
5.136405377	-633.0090909	0.011916542	59.59216033	0.0128346	0.059994786
2.809165492	-396.0521599	0.006998514	71.41196142	0.007298478	0.036497627
8.595364175	-698.3857393	0.014504685	76.97799614	0.016073024	0.075533867
3.113055484	-565.6631168	0.010536975	96.24054858	0.01113893	0.036287387
6.018192617	-702.5663801	0.019448059	104.3277041	0.020802623	0.068346495
3.601679836	-509.6390887	0.019934826	38.3445885	0.021531682	0.055623859
9.777610446	-731.4476966	0.019990243	40.9691473	0.022609584	0.093024578

original_glrlm_RunVariance	original_glrlm_GrayLevelNonUniformity	original_glrlm_LongRunEmphasis	original_glrlm_ShortRunHighGrayLevelEmphasis	original_glrlm_RunLengthNonUniformity	original_glrlm_ShortRunEmphasis
0.061014327	89.79558495	1.176747446	412.4545379	1809.386879	0.960127781
0.116957921	86.55263249	1.329118095	71.54525172	712.0318138	0.931915592
0.188398919	76.06451457	1.495455332	173.1876655	1061.362442	0.908431945
0.09969409	49.1813264	1.277292754	402.5592378	776.3516675	0.942511
0.088673848	136.8567743	1.249631385	155.5785761	1913.696394	0.946817279
0.064802138	42.35561103	1.187649513	176.9243898	557.6699828	0.957809533
0.108473452	213.5595105	1.297321983	302.404837	4428.029341	0.939348723
0.141380199	384.512323	1.388536381	222.6606001	3742.712592	0.922755769
0.078901009	208.1309252	1.225037214	294.1963151	3738.479379	0.951049745
0.068347089	55.42839218	1.196973574	194.7546703	899.7592358	0.956180123
0.07288637	73.04334158	1.212520668	236.9564747	1247.893902	0.952465801
0.085450978	131.2147974	1.240339749	294.9292636	1875.658228	0.948603152
0.19748706	218.7400914	1.552392661	75.29881445	1218.330647	0.893365034
0.091132843	105.0290554	1.261103684	220.276293	1371.211464	0.943630841
0.09469826	124.5149973	1.266234123	258.7633703	1583.236736	0.943707766
0.140958011	316.9021579	1.38674743	239.2635354	3214.785932	0.923257136
0.051612575	49.40916243	1.155003157	457.7559676	1032.473087	0.963409036
0.142802785	104.0734016	1.396657115	83.62221624	896.7801647	0.920816249
0.08202269	77.34077013	1.239328457	224.1057525	1050.226985	0.946736412
0.081327481	275.7424316	1.23408867	298.0335315	4232.596133	0.948609028
0.16742473	244.1808482	1.460178313	91.60261034	2112.944252	0.910492741
0.060906195	58.90735491	1.178522989	188.7009313	917.4907153	0.959300839
0.092216656	68.28441556	1.26177118	141.2918428	817.7784842	0.943756811
0.084851158	93.08029392	1.237441731	316.6184305	2161.401241	0.949406925
0.112057903	105.8382675	1.317997817	125.035476	974.2876387	0.933171956
0.064830932	83.8658493	1.187088072	388.7628239	2005.110469	0.958167845
0.076190301	148.7193728	1.216937382	430.4505058	2674.864002	0.952628593
0.089169545	152.0479242	1.250539806	221.2201265	2229.247031	0.946601791
0.042911445	22.11531021	1.128536682	253.9314175	395.8742445	0.969476801
0.092534123	126.621624	1.264127733	108.8245068	1635.743535	0.942933683
0.134705594	314.6866657	1.368376425	238.5495553	3543.626037	0.926766907
0.140974641	154.7515334	1.384664508	138.8510583	1524.153544	0.924001827
0.091989376	140.1089755	1.255838401	376.7721175	2604.009761	0.946333918
0.122622298	133.5083403	1.343199501	246.3599036	1771.45084	0.929554434
0.084946001	220.8497755	1.240893858	271.2178231	3577.73175	0.94815484
0.13882799	258.1728873	1.387475395	149.837358	2189.274689	0.921578395
0.124575019	274.2735414	1.345642647	194.765369	2802.34299	0.929394001
0.266509411	339.9852119	1.677086851	382.3378135	3248.500539	0.886474434
0.061182544	43.17466599	1.182894913	303.4722084	1039.098251	0.957541561
0.073461088	195.2515774	1.207545739	525.4770576	6068.662754	0.954874148
0.105388911	154.2226703	1.289781643	259.3917152	2044.140988	0.940706256
0.039453398	99.50086286	1.117039038	716.0019553	3464.105144	0.972372194
0.08271633	172.8574479	1.238727095	233.913871	2964.637975	0.947599305
0.061899367	108.381599	1.177562225	368.1650141	2125.266474	0.960551509
0.084642126	80.78159495	1.244930441	125.4040257	1031.41441	0.946132777
0.0835106	139.1153496	1.237055846	311.529222	2235.523892	0.948653532
0.095290496	221.9674509	1.268174412	311.8930458	3635.116045	0.943258672
0.07785873	47.7707048	1.223572437	215.9750329	716.6503633	0.951077366
0.062879815	36.14100717	1.181160683	222.943562	724.5479085	0.959493499
0.121911045	129.3992109	1.344537549	134.954877	1014.622421	0.928321119
0.075962103	157.3289767	1.220238165	208.434226	2112.031436	0.950979543
0.045888351	98.50090317	1.133532727	543.9101264	2982.012026	0.969316062
0.063470919	194.8356286	1.18438242	349.9866406	3820.463561	0.958358141
0.091653665	68.89526482	1.260673912	172.5806282	884.2723702	0.944308177
0.103042583	77.87168445	1.299109871	149.2314522	1028.335792	0.935319971
0.082240278	120.5341504	1.24038856	160.8031357	1575.857617	0.946464454
0.071772655	166.9751116	1.206544972	260.3431467	3368.285935	0.954196279
0.09877645	187.0726826	1.283299341	348.6680906	2711.831653	0.939129583
0.069247058	91.5080901	1.199128728	242.6550069	1381.88311	0.955904492
0.084850282	237.3246577	1.229544407	792.16634	7547.566688	0.952861139
0.144338665	124.7937085	1.395396331	185.1187549	1292.350459	0.922077709
0.092170957	150.4077318	1.262228223	185.9978078	2130.323548	0.943577725
0.10479907	340.299623	1.293166861	209.2140114	4529.51098	0.938852133
0.102858183	283.9572959	1.279926579	360.2050377	6061.47461	0.94290661
0.088142804	54.57842049	1.25364937	196.9904516	633.8371158	0.944684859
0.131362946	267.4807039	1.365683538	144.2428123	2868.538329	0.925937847
0.107533743	93.61364193	1.302832454	184.7608965	1011.352458	0.936466934
0.092912986	264.8338677	1.261548758	342.2599799	5002.432537	0.944405436
0.060309229	205.4831648	1.174477973	550.4202015	5007.66345	0.960731478
0.116332129	201.9380395	1.332016141	136.0684896	2191.020646	0.930021887
0.1139681	90.55950649	1.318511025	153.1992791	998.041803	0.934186986
0.137478416	375.6348771	1.382742768	222.3120436	3142.988978	0.922447348
0.100326379	132.6917839	1.282744493	309.1580187	1794.519885	0.94027709
0.053792493	79.68839091	1.154863223	472.8947315	1964.440046	0.965039881
0.054330531	96.78502054	1.158350034	399.5755232	2711.029188	0.963873944
0.063765437	60.17690406	1.181776952	266.8311793	1143.691647	0.959911971
0.092326029	128.4829333	1.264734969	217.9705367	1907.405593	0.942846829
0.116316293	83.65910568	1.333088033	114.9332841	724.4494972	0.92988236
0.075530579	93.41484898	1.218808783	166.4257137	1405.90731	0.951326586
0.059175427	63.69196809	1.173658216	600.2523414	1996.978432	0.960334627
0.063074227	88.11412229	1.183240716	404.0384951	1686.167759	0.958642925
0.098587046	106.742666	1.279327828	254.798109	1417.677102	0.940399289
0.157016579	220.8278246	1.429937373	249.7322855	2330.657939	0.915824434
0.07450544	117.92843	1.218519538	302.2283452	2526.173445	0.950759786
0.086662031	115.0463802	1.243935093	355.9134181	1980.186038	0.947922567
0.263053332	182.9702001	1.689462924	60.29915957	952.2016611	0.881538875
0.081229129	89.34657744	1.228033527	430.7327748	1962.981589	0.951311653
0.112351205	175.643116	1.309990252	148.7275659	2124.046637	0.936510989
0.087416233	78.86545014	1.245377845	194.6588145	1189.531418	0.948023893
0.106868141	132.4729752	1.303508421	133.7264142	1569.520065	0.936149741
0.197623391	154.3270162	1.54407085	64.0210413	861.2587527	0.896568429
0.053975389	79.25244775	1.155664355	398.2843902	2067.780998	0.964564807
0.066764258	244.0065479	1.190107139	404.8897688	4381.194314	0.958024161
0.101728508	182.1689228	1.284634187	233.9541053	2598.636892	0.940341971
0.053493217	151.1298179	1.158482729	376.8890883	3758.236131	0.963175624
0.146255638	231.9302007	1.391074618	213.8547289	2524.123073	0.92457033
0.078278898	95.64492068	1.221485033	367.1143134	2322.602855	0.951704151
0.095772279	65.89982903	1.277099902	256.0563908	824.3169468	0.939950104
0.09244508	42.8885911	1.264476751	162.2223515	664.3634063	0.942882154
0.172013752	471.304571	1.473722001	128.6306674	3988.383484	0.907979769

original_glrlm_LongRunHighGrayLevelEmphasis	original_glrlm_RunPercentage	original_glrlm_LongRunLowGrayLevelEmphasis	original_glrlm_RunEntropy	original_glrlm_HighGrayLevelRunEmphasis
455.1486826	0.946893352	0.010650001	5.24488763	420.4486548
82.55093369	0.909008638	0.083009627	4.206366646	73.51691531
203.8995471	0.876491826	0.178400911	5.052418371	178.6610899
537.2125958	0.922289583	0.010360424	5.075215241	424.6919612
171.243004	0.928501819	0.047811582	4.790369429	158.4891326
198.9970194	0.943939394	0.020019278	4.595230198	181.0051812
354.2077517	0.917632005	0.016573782	5.286215337	311.8916613
278.3409362	0.895970122	0.012408642	4.609981285	232.2104538
368.1597029	0.934481708	0.017065703	5.112912485	307.0210248
215.5869801	0.94151837	0.035675889	4.891231543	198.4943222
274.6651104	0.937104673	0.01703066	4.911047806	243.9839517
342.6117261	0.930852661	0.008321958	4.79517548	303.5667566
93.95836193	0.860244737	0.086387135	4.101319614	78.55821565
249.0603324	0.925048161	0.024856739	4.830586904	225.5889999
295.3201546	0.924317618	0.014317718	4.805800913	265.4296179
321.6946602	0.896656588	0.023555539	4.752511607	251.8986445
538.2842382	0.952196882	0.005994503	5.124947373	473.3040629
96.12331755	0.893630295	0.090966051	4.385929625	85.78382666
266.5939704	0.930010667	0.01083137	4.672382857	232.0069433
351.353247	0.931599272	0.008707039	4.926476778	307.8555919
111.4262879	0.880698006	0.075010678	4.48754421	95.1278424
210.6966143	0.946175233	0.016440164	4.70533151	192.8785807
158.5054852	0.925030048	0.045407118	4.655642341	144.473835
381.0443919	0.931882247	0.017944116	5.297263049	328.3011529
143.8389706	0.911112454	0.040181863	4.433013187	128.2833657
457.6018876	0.944312758	0.009430844	5.279283659	401.6027642
501.6813063	0.936567608	0.007171337	5.145113086	443.6183342
256.2014528	0.928173564	0.017169358	4.881302093	227.5689392
288.6146837	0.959903631	0.015813281	4.695497957	260.5884347
125.7938544	0.924387686	0.051993208	4.608113955	111.9677377
287.1980449	0.900895658	0.013655761	4.787494213	247.0317568
162.1674027	0.897386326	0.068985059	4.65707876	143.1189022
504.2965281	0.927436453	0.015636578	5.209168393	397.8341592
294.9944669	0.905630115	0.0300708	4.99536551	255.2198178
315.9426386	0.930377978	0.011005762	4.946432629	279.4044259
183.3147905	0.895426681	0.025346194	4.457752627	155.7164371
227.2689323	0.905401774	0.032596237	4.692752672	200.4104169
938.4918738	0.843411384	0.039185402	4.969183833	452.3347094
354.9703941	0.944465291	0.014543175	5.181878117	313.1885221
681.0326185	0.939371495	0.008359788	5.561109519	552.1673231
306.2081318	0.919459107	0.015141868	4.865661743	267.8707362
815.0086953	0.96347795	0.008004494	5.547063946	735.0544853
266.7326893	0.930573396	0.025022247	5.0703216	240.0601654
408.8457139	0.947069008	0.011195427	5.165220922	375.6148056
141.1156324	0.928855182	0.041987834	4.608994121	128.3308675
357.8457613	0.931427771	0.011306665	5.015712234	319.8348581
370.4985137	0.9238357	0.009147708	5.024795744	322.5772126
259.6793145	0.934734611	0.011659968	4.67313447	223.9336583
253.5060765	0.94621712	0.023384437	4.989992339	228.730352
168.7551777	0.904813382	0.018889156	4.227912202	140.8480295
237.3013046	0.935054047	0.016262542	4.751541037	213.8250026
608.9964004	0.958994135	0.006593694	5.451044086	556.1828368
403.7198243	0.944677441	0.006692177	5.017721576	360.1010771
191.7946979	0.925585284	0.035978871	4.752974511	176.1396092
170.1293713	0.915037594	0.041276842	4.778054109	153.0753175
187.5673334	0.929625247	0.020951255	4.664682698	165.8010422
301.8626762	0.938974993	0.026260376	5.160025167	267.6488638
429.2881468	0.919273035	0.009337253	5.034330205	363.4303646
274.4584382	0.9410941	0.012793304	4.820893585	248.5316436
1116.41706	0.934941059	0.004698955	5.670152425	844.4878502
226.1712664	0.895156373	0.018274622	4.666281171	192.0958418
210.8924575	0.924805532	0.028288378	4.869621362	190.5310508
243.0381646	0.917695364	0.020480881	4.859926911	215.306255
431.4417541	0.922370559	0.014930049	5.32711299	373.2454301
242.7459638	0.92677702	0.01152326	4.467302168	205.2972326
173.2148061	0.900649325	0.037336279	4.672442743	149.3862426
221.509362	0.915172251	0.016998943	4.588917509	191.3664598
414.2403154	0.925452308	0.013242432	5.253198557	354.9885487
619.0164309	0.947662889	0.006808098	5.442728287	563.2032326
164.1222957	0.907469342	0.026593925	4.592370593	141.0770129
194.5839818	0.911766448	0.055429432	4.681211075	160.138775
278.4601218	0.896664874	0.012099507	4.454183134	232.046842
367.5819404	0.920140412	0.009253183	4.874724076	319.7284066
531.5694518	0.95321279	0.007492318	5.313263979	484.0961651
455.3649912	0.951969142	0.011947558	5.382574251	410.0029887
304.6112251	0.946176774	0.01364846	4.982481061	273.8672838
268.1505799	0.924040067	0.013222092	4.831751734	227.0362602
139.1531803	0.907339008	0.027178504	4.29854809	119.2214381
193.8552794	0.935604524	0.043916666	4.816856685	171.5222226
737.5450377	0.947479199	0.00852279	5.611639601	625.3685852
457.6602883	0.945043895	0.011081883	5.212470119	414.2076185
312.1902983	0.920767948	0.019097547	4.900357138	265.3245721
393.0419091	0.887168506	0.028410904	4.843126138	271.282298
372.4278896	0.935292498	0.011035488	5.170686756	315.3743774
429.6328141	0.930046993	0.01000551	5.10689294	369.0349749
84.72778876	0.840982125	0.06119411	4.014439984	64.03751984
490.1058114	0.934365425	0.011957689	5.340912115	441.9036465
169.8929712	0.914127812	0.033264846	4.705741217	152.4798129
227.8842515	0.929960067	0.019377475	4.81981225	200.6431714
153.9708973	0.914909782	0.049830338	4.687250055	137.4254443
81.59427915	0.862907032	0.067454238	4.028498378	66.93175503
471.8052572	0.952916102	0.010857718	5.303559752	411.0457397
471.5648218	0.943706496	0.005810183	5.056049919	417.1376514
275.5423101	0.91995338	0.017236454	4.923453683	241.5186726
435.3472696	0.951481193	0.008614381	5.246631065	388.0199695
253.4885649	0.896978825	0.024429074	4.80320511	220.8006106
471.0307178	0.935638022	0.013841954	5.376680236	384.6349382
305.4887004	0.920652413	0.027088671	4.869262667	265.2730738
190.9797037	0.924368198	0.028874038	4.808808826	167.4552959
156.9614991	0.877194852	0.036277842	4.549884252	133.403794

original_glrlm_RunLengthNonUniformityNormalized	original_glszm_GrayLevelVariance	original_glszm_SmallAreaHighGrayLevelEmphasis	original_glszm_GrayLevelNonUniformityNormalized
0.900310917	139.8918282	463.0055072	0.030219056
0.837086581	43.57770493	126.1701574	0.05515109
0.788160691	73.01698789	267.212604	0.038609599
0.860786312	109.1369933	421.4253752	0.033040182
0.869956339	67.76401137	215.9451355	0.039995441
0.894888207	59.00892257	219.0129606	0.044612856
0.853434599	76.82768855	351.8299929	0.032961217
0.817348472	67.84986578	322.1773724	0.043108429
0.879578699	81.75365399	298.8918106	0.034877361
0.891207921	77.05639342	247.2858674	0.03908895
0.882639461	64.87521596	253.4444651	0.037800086
0.873872312	68.77192152	374.3895116	0.04022131
0.757617416	63.35353492	160.8247267	0.0585645
0.862753555	108.5130677	302.3452071	0.039413031
0.862940654	94.99488678	352.2278128	0.037275563
0.818626652	108.6140609	309.2111568	0.039121789
0.907885017	83.29832854	394.0673296	0.036181791
0.813366444	57.06531244	183.7570216	0.04718905
0.869494212	47.174824	254.2099616	0.044150581
0.873672975	68.82381243	336.159045	0.037767664
0.792286778	50.94518943	152.0781816	0.050837567
0.898394019	48.15232771	226.4703717	0.043857105
0.862926156	72.89994344	210.724182	0.043418324
0.875803321	84.71342226	322.6440072	0.031621605
0.839538127	71.10254472	211.8730151	0.048698845
0.895915524	84.03334558	375.7493085	0.032904404
0.883078047	103.3651734	452.9065557	0.034211318
0.869316052	78.07492813	277.2119431	0.03805251
0.92244895	39.37808483	228.5304015	0.045884986
0.860997351	41.71581567	140.7704416	0.049685201
0.826132266	79.53324572	346.3182634	0.039477213
0.820246189	80.81174569	201.9003994	0.042110692
0.869032229	116.6228663	360.0005933	0.032067729
0.831877044	109.0031084	312.1077416	0.035408329
0.872882101	64.0746558	308.4114538	0.040009188
0.814765632	72.23544699	239.4439337	0.047164388
0.831381543	131.6170972	314.8875544	0.04066606
0.745178796	165.006227	389.8771014	0.035638363
0.894146853	67.00551957	271.1935765	0.034392548
0.888278334	97.51583582	419.645907	0.028004616
0.856504553	89.56182581	308.2987391	0.039311859
0.929589126	98.36441246	572.7151781	0.02752673
0.871582468	85.37786545	291.4517971	0.035653812
0.901596352	113.1445069	419.3646838	0.031717334
0.868306593	50.96009879	174.5395319	0.046427158
0.873971831	94.29820519	391.2323265	0.034720758
0.862016819	74.3479985	352.5509585	0.036525564
0.879705717	41.6144641	217.8798696	0.045872355
0.89916902	61.74312281	228.4363876	0.038473614
0.828984344	48.9770218	215.0317219	0.055217193
0.878997899	76.17010024	261.6480669	0.041444122
0.92227786	98.46172441	511.4383221	0.028398189
0.896136398	61.84518169	358.1705693	0.036943061
0.864611179	88.60615562	254.868133	0.039574517
0.8442733	65.85772231	210.5622406	0.041233252
0.868853946	55.92853081	200.5755379	0.04534924
0.886556052	88.96975689	293.5699577	0.033743847
0.852606683	118.854981	406.8391179	0.034338199
0.890653077	77.83149458	297.0583786	0.040285676
0.88401827	119.619455	578.3859131	0.024962805
0.816465221	69.96165289	289.9273898	0.042289461
0.862416442	79.76669241	249.732419	0.039084789
0.852205297	76.27229385	292.508645	0.038041746
0.861447971	79.5629606	376.4413395	0.033912398
0.865149254	39.72755383	206.5544761	0.05138478
0.824163413	71.78293228	211.2788676	0.046884621
0.84691925	76.20111747	251.7387188	0.043421334
0.864447874	96.27117599	363.055393	0.034361369
0.901889109	158.6756228	597.7952554	0.028088643
0.832634957	55.8892839	210.897345	0.0458794
0.842040915	75.7034723	215.3350426	0.041455999
0.816560368	79.98847889	333.0869133	0.048054766
0.855253518	83.73562507	387.8220039	0.03751673
0.912056309	115.4181297	477.3001508	0.0299059
0.909271737	94.98992735	384.4630049	0.029081916
0.900232895	65.59629255	266.6229374	0.03764153
0.860990949	53.94343316	233.9453686	0.042594595
0.832652644	48.31864203	171.2478922	0.055854567
0.879912092	61.89050979	196.2158963	0.041634687
0.900831592	128.7417935	521.8392926	0.025515031
0.896824024	134.9936594	440.4127047	0.031202927
0.855372554	81.66135532	292.2701009	0.036872852
0.802930219	115.2364942	314.3082542	0.039781946
0.878535937	57.16621488	253.4923302	0.037778674
0.872471386	92.05511431	378.5786391	0.033112208
0.736439062	27.0326402	110.9021228	0.070413779
0.880315919	128.5183776	479.3755594	0.027682379
0.847039853	60.83136925	227.2745005	0.043331426
0.873017093	56.73250406	223.5709748	0.041143299
0.846397984	69.58603115	192.7107416	0.043918184
0.764364285	40.63628624	129.8284514	0.072625794
0.9107623	84.78323175	349.1137972	0.031620281
0.895498728	83.1970624	416.1143138	0.03568428
0.855468015	71.32259527	282.8587691	0.038412085
0.907397006	73.3519979	333.5915506	0.033380559
0.821667831	106.8305515	316.7332263	0.039146722
0.880835771	91.37988224	348.4918457	0.03165661
0.854535817	127.0131177	327.6402723	0.037333348
0.861087805	48.05365047	192.4627094	0.042446993
0.786803888	68.765201	249.3094052	0.044764783

original_glszm_SizeZoneNonUniformityNormalized	original_glszm_SizeZoneNonUniformity	original_glszm_GrayLevelNonUniformity	original_glszm_LargeAreaEmphasis	original_glszm_ZoneVariance	original_glszm_ZonePercentage
0.549571221	641.3496144	35.26563839	12.93230506	9.625953363	0.549952875
0.442195518	133.5430464	16.65562914	83.76490066	74.17952064	0.322994652
0.445526186	216.9712526	18.80287474	99.63449692	89.71264373	0.317470665
0.52335295	222.9483568	14.07511737	45.10093897	39.84113051	0.436028659
0.506278905	528.0488974	41.71524449	33.65675935	28.50216161	0.440456081
0.476742383	148.266881	13.87459807	13.7170418	9.21336628	0.471212121
0.489535769	1147.471843	77.26109215	135.6966724	129.8845413	0.414793842
0.525661631	867.8673531	71.17201696	271.9951545	262.4230456	0.323218481
0.505400115	1052.74844	72.64954393	29.7205953	24.95759813	0.458205015
0.505139283	259.1364522	20.05263158	21.33333333	16.96661841	0.478544776
0.48803116	342.1098431	26.4978602	17.7746077	13.14689225	0.464854111
0.512457642	513.995015	40.34197408	27.22532403	21.9440343	0.435140998
0.519479331	223.8955916	25.2412993	533.2227378	514.4583632	0.230851634
0.476556259	342.6439499	28.3379694	30.37413074	24.67140074	0.41875364
0.543541089	462.5534665	31.72150411	36.75910693	31.32379823	0.428931452
0.521329009	758.0123796	56.88308116	258.213205	249.1553406	0.33226691
0.467653757	294.154213	22.75834658	8.041335453	4.437977864	0.52680067
0.503431859	178.7183099	16.75211268	146.4197183	134.3563261	0.287915653
0.445701808	238.4504673	23.62056075	20.55700935	14.67071709	0.412172573
0.473379827	1042.38238	83.164396	44.51271571	38.93822745	0.423542989
0.451955291	375.1228916	42.19518072	280.460241	267.1860705	0.274470899
0.482693685	258.2411215	23.4635514	12.33084112	8.263268408	0.495829472
0.507752851	209.1941748	17.88834951	80.22815534	74.05075879	0.40234375
0.482192525	574.7734899	37.69295302	27.35654362	22.42531235	0.450321118
0.47478674	209.3809524	21.47619048	93.36734694	85.03476432	0.346425766
0.496701377	601.5053675	39.84723369	10.89925681	7.072403327	0.511186154
0.501936886	758.9285714	51.72751323	28.32208995	23.75007305	0.46767708
0.484459437	559.5506494	43.95064935	29.30909091	23.59057439	0.418175235
0.512941919	135.4166667	12.11363636	5.291666667	2.424801997	0.590604027
0.423500238	343.458693	40.2946979	28.10850801	21.69406177	0.394839338
0.498440245	832.3952096	65.92694611	200.142515	192.0251339	0.35098781
0.497385439	353.143662	29.89859155	195.3732394	186.8813509	0.343160947
0.501268154	715.8109244	45.79271709	21.45868347	16.34563776	0.44224218
0.511930773	440.2604651	30.45116279	90.71860465	83.25173067	0.365957447
0.509624477	1017.72008	79.89834752	31.9569354	27.0935522	0.453451408
0.515006851	495.4365904	45.37214137	295.1403326	285.4217532	0.320773591
0.512213432	636.1690821	50.50724638	160.9460548	151.9701931	0.333781242
0.531124713	676.1217596	45.36763551	697.5655931	681.1290338	0.246657624
0.381760276	220.6574394	19.87889273	8.761245675	4.232743861	0.469918699
0.4612403	1630.945701	99.02432127	19.85463801	15.62752062	0.486382393
0.474619432	485.5356794	40.21603128	59.24731183	52.81764194	0.394371627
0.52699678	1270.062241	66.33941909	4.404564315	1.829937673	0.623222136
0.487156654	785.2965261	57.47394541	23.75868486	18.62334407	0.441281139
0.512301396	658.8195956	40.78849145	12.12286159	8.37987031	0.516881029
0.471613715	250.8984962	24.69924812	34.40977444	28.63894228	0.41627543
0.520781066	641.6022727	42.77597403	32.98133117	28.01697101	0.448816029
0.4811101	917.9580713	69.69077568	64.50733753	58.7905267	0.418237615
0.422028558	156.9946237	17.06451613	14.68548387	9.203339982	0.427095293
0.476923077	217	17.50549451	8.076923077	4.578790001	0.5346651
0.483261698	195.7209877	22.36296296	212.9333333	201.789276	0.299556213
0.461029055	520.9628319	46.83185841	19.82389381	14.65531287	0.439859868
0.523104574	1042.547416	56.59759157	6.258404415	3.397502901	0.591219223
0.48596039	1069.598819	81.31167651	15.61744662	11.41503975	0.487810284
0.485850387	225.9204301	18.40215054	27.35053763	21.71374263	0.421195652
0.496798858	264.2969925	21.93609023	34.68421053	28.43421053	0.4
0.455686943	364.0938673	36.23404255	28.50813517	22.55184751	0.40974359
0.504927338	976.5294726	65.26059979	17.42244054	13.04798394	0.478121137
0.459622642	609	45.49811321	43.23622642	36.42511129	0.383169462
0.499737163	389.2952503	31.38254172	16.7830552	12.30757645	0.472694175
0.50565159	2306.782552	113.8803157	55.34480491	51.34041967	0.499726147
0.528075951	319.4859504	25.58512397	112.1256198	103.6050106	0.342582106
0.502343115	576.6898955	44.86933798	42.29790941	36.88863832	0.429962547
0.544173646	1304.38423	91.18606592	79.51647893	73.68376024	0.41406115
0.440509847	1403.023862	108.010989	40.98869702	35.2603019	0.417814509
0.411283695	128.3205128	16.03205128	27.87179487	21.46051118	0.394936709
0.468155013	600.174727	60.10608424	195.3447738	186.269735	0.331952356
0.458267602	214.9275053	20.36460554	45.29637527	37.56584122	0.359662577
0.470183711	1243.635917	90.88582231	36.12627599	30.5427482	0.4232
0.509796139	1549.780263	85.38947368	13.59703947	9.885079532	0.51903705
0.474547793	449.871308	43.49367089	59.45991561	50.11489879	0.327122153
0.523780847	256.1288344	20.27198364	66.6400818	59.5833992	0.376443418
0.527142998	710.0616184	64.72976986	493.4936897	483.3503868	0.313986014
0.499207518	442.7970688	33.27733935	96.77226607	90.17078456	0.389205792
0.515993034	649.1192369	37.62162162	8.90063593	5.676070604	0.556883577
0.507067701	866.578701	49.70099473	8.805734348	5.449270222	0.545832003
0.42592811	282.8162651	24.9939759	10.38554217	6.30076027	0.494783905
0.423597955	391.8281081	39.4	25.96540541	19.25590533	0.3860601
0.493131093	160.7607362	18.20858896	76.79141104	68.15574542	0.340292276
0.4866266	371.7827225	31.80890052	20.7447644	15.75269832	0.447568834
0.515559328	648.0580748	32.07239459	11.34367542	7.881174571	0.537409149
0.548491245	590.1765799	33.57434944	13.72211896	10.30511861	0.540975365
0.519257927	395.6745407	28.09711286	42.98293963	37.40912676	0.423568649
0.512922335	519.5903258	40.29911155	302.3247779	291.9109367	0.309880697
0.387180351	507.5934401	49.52784134	16.90694127	11.41254805	0.426618939
0.488760728	566.4736842	38.37704918	16.87748059	12.44897977	0.475194752
0.467372401	161.2434783	24.29275362	723.5884058	703.8696576	0.225195822
0.502888941	561.7269472	30.92121755	32.33213966	27.77313096	0.468343816
0.495134269	517.415311	45.28133971	76.97033493	70.08536068	0.38110868
0.478286901	306.5819033	26.37285491	25.49453978	20.27819734	0.43784153
0.447082004	326.369863	32.06027397	69.45342466	61.75850066	0.360493827
0.53676009	183.0351906	24.76539589	560.6627566	546.0394217	0.261503067
0.48776057	637.9908257	41.35932722	7.818042813	4.501634262	0.549118388
0.502434559	1289.247077	91.56586126	14.29579111	10.21589492	0.495080069
0.481584903	640.0263356	51.0496614	47.33634312	41.1707066	0.402727273
0.475988831	1099.534199	77.10909091	8.418181818	4.868791065	0.530790441
0.538015088	667.1387097	48.54193548	180.3435484	172.7277289	0.362361192
0.467802678	612.3537051	41.43850267	19.35905271	14.7278435	0.464678736
0.504618006	212.4441805	15.71733967	31.33254157	25.14769156	0.402101242
0.436387645	151.426513	14.72910663	18.25360231	12.47699092	0.416067146
0.552660044	903.0465116	73.14565483	380.9510404	368.4642893	0.282992726

original_glszm_LargeAreaLowGrayLevelEmphasis	original_glszm_LargeAreaHighGrayLevelEmphasis	original_glszm_HighGrayLevelZoneEmphasis	original_glszm_SmallAreaEmphasis	original_glszm_LowGrayLevelZoneEmphasis
0.149594244	2052.326478	550.4490146	0.769291676	0.007540928
6.502876703	1592.741722	146.0430464	0.691182478	0.052433364
19.75465802	3282.398357	327.1149897	0.694071682	0.019139562
0.45320332	9772.220657	533.4389671	0.750828087	0.007942404
2.014766807	1185.444871	254.0354746	0.739241564	0.026507058
0.214329599	1301.03537	258.9903537	0.717463183	0.019074081
2.777410111	8548.718003	444.6523038	0.727331855	0.007781004
3.053117005	25543.87765	388.804361	0.75182364	0.006092457
0.416309417	6876.281805	370.4762362	0.738399638	0.011799714
0.737342544	1382.865497	291.8245614	0.738201533	0.022346212
0.336872193	2017.971469	323.4379458	0.726126274	0.011244806
0.228525764	3962.545364	440.1505484	0.74283456	0.005508804
31.12004842	11156.44548	190.1902552	0.747528874	0.039434273
0.71584815	2133.421419	358.4520167	0.717280911	0.014879171
0.481004601	3547.379553	410.2972973	0.764125152	0.009297049
5.746218792	16670.8879	395.2338377	0.749579147	0.011007926
0.033581634	3268.54372	519.7694754	0.710978941	0.006262568
10.30185737	3157.047887	199.9183099	0.736295242	0.051619394
0.209188348	2618.801869	321.9906542	0.693733044	0.008484825
0.384765711	6164.680745	424.0658492	0.715413005	0.005725793
16.98763808	6029.579518	191.5819277	0.699128428	0.026987638
0.221368482	1121.880374	268.411215	0.72212144	0.012417016
3.425827747	2487.11165	246.2184466	0.740314098	0.028999218
0.648422779	3661.32802	414.9647651	0.721932092	0.010988279
2.773648957	3697.829932	242.1723356	0.715351425	0.024932587
0.09958116	2922.982659	473.4682081	0.732376177	0.007497496
0.211389698	5192.644841	574.9041005	0.736118498	0.00538695
0.41825319	2912.434632	338.0242424	0.723253872	0.012181309
0.050011789	1144.034091	288.2765152	0.743974795	0.019594553
1.418504714	1179.617756	174.8606658	0.676364236	0.035532934
2.654049232	16236.1982	415.4760479	0.733666444	0.006639938
11.37546547	4304.574648	253.0126761	0.732374258	0.03816061
0.297526597	7951.781513	457.6561625	0.734938349	0.011525002
2.446555153	5446.59186	395.5965116	0.74280057	0.014825422
0.400122863	3573.190786	380.565348	0.741681507	0.00705599
6.261876711	15036.10915	282.485447	0.745151612	0.01277927
4.453444058	7267.322866	372.0917874	0.742397066	0.018101863
21.03619601	292180.8853	502.9536528	0.755347254	0.015264019
0.135144965	1949.539792	377.2041522	0.640714742	0.010999176
0.236680283	10878.70843	576.4027149	0.706281705	0.00611848
0.966407446	4834.514174	395.8631476	0.71620579	0.008171017
0.038610541	2947.351452	754.1493776	0.754353096	0.005504721
0.564079674	2081.788462	354.3622829	0.725434193	0.01597563
0.132012555	2014.403577	505.6835148	0.743655485	0.00805294
1.364520717	1385.118421	206.693609	0.713349771	0.024509061
0.397814382	3959.301136	463.2686688	0.749390451	0.006848083
0.674393653	7502.749476	448.0041929	0.721233065	0.005690255
0.129935317	2319.497312	284.3709677	0.675571925	0.011602421
0.186645299	1063.628571	285.4263736	0.717662218	0.019194047
2.855338811	16606.62963	249.0074074	0.722084364	0.01267883
0.315276696	1850.80531	315.9265487	0.706091549	0.011837909
0.042082231	2291.758655	638.4119418	0.751570035	0.004929953
0.113103338	3122.558383	453.0436165	0.724792766	0.005085098
0.815980689	1497.384946	294.544086	0.724337953	0.02457278
1.361418206	1638.672932	251.575188	0.731389173	0.023752072
0.521126979	2214.769712	245.1889862	0.701880731	0.015373414
0.584860881	2249.477249	358.8531541	0.738283616	0.016440052
0.339809872	7127.474717	522.8498113	0.704931952	0.00694446
0.178931153	2205.947368	350.2297818	0.734253213	0.010026243
0.062298953	92865.39961	765.9848751	0.739187153	0.004226552
1.687877188	8441.74876	335.3553719	0.753501543	0.010103633
1.155208314	2395.857143	297.0722997	0.73634576	0.019550785
1.639556918	5284.631623	340.1009595	0.765115104	0.010225925
1.158467437	4943.146939	491.3152276	0.690342901	0.005885554
0.269264092	3532.224359	272.3878205	0.666243003	0.010239744
5.212310552	8698.930577	258.375975	0.71135108	0.017539992
0.629876991	4031.910448	305.9594883	0.703539135	0.01375004
0.577852446	4691.349338	467.6018904	0.71295023	0.007242489
0.099491336	3535.815461	728.4085526	0.741938581	0.005419373
1.338681086	3810.041139	248.2732068	0.715045272	0.015496771
3.291442793	3340.693252	253.5357873	0.750729261	0.0279653
5.115239731	48770.08686	398.9569414	0.753360265	0.00643432
0.897183685	11761.73732	479.1939121	0.734272486	0.005926907
0.082583289	2258.886328	589.7782194	0.746456568	0.005474432
0.102739129	2128.803979	484.8964307	0.74006643	0.009068978
0.125411957	1626.712349	348.0286145	0.675431321	0.012473519
0.321720704	3337.730811	304.7805405	0.676415024	0.009468043
1.752921049	3886.01227	202.809816	0.729005291	0.018673318
0.780179417	1484.382199	242.5536649	0.724762422	0.025864941
0.100303149	6395.384248	677.7995227	0.746119558	0.006745532
0.160577465	2313.451673	527.3355019	0.768449309	0.007864473
0.607267873	4383.47769	370.9291339	0.74817712	0.01441374
7.622177719	24517.04837	401.9921027	0.743191005	0.014508894
0.213707442	3399.852021	368.9809306	0.646031911	0.007596636
0.15271397	3301.85591	475.0862813	0.726754605	0.006728692
27.1499603	20412.11884	134.1101449	0.710303578	0.023802297
0.556217577	3576.170994	605.2193375	0.736983801	0.006345972
2.454181465	3282.562679	264.0507177	0.731122201	0.016286099
0.538663258	2172.198128	276.1513261	0.719010519	0.015234593
3.014536635	2396.394521	238.1589041	0.69489649	0.032450956
26.72172796	12652.99413	146.3460411	0.759003598	0.033196262
0.083255835	3498.411315	456.2882263	0.72600047	0.007652577
0.073767223	3701.162899	521.1742011	0.736549172	0.004633873
0.714615343	4220.299473	357.489842	0.721419779	0.011200097
0.073092694	2203.320779	444.3229437	0.717350942	0.006875736
4.289756227	9010.079839	376.1080645	0.760588571	0.011485962
0.277963452	6198.356761	453.2803667	0.711313448	0.009730724
0.732016086	2815.332542	397.8931116	0.736986785	0.018064557
0.477803658	1604.161383	240.9193084	0.686497729	0.017955919
11.42807682	14473.36781	282.9736842	0.769804818	0.013417759

original_glszm_ZoneEntropy	original_glszm_SmallAreaLowGrayLevelEmphasis	original_ngtdm_Coarseness	original_ngtdm_Complexity	original_ngtdm_Strength	original_ngtdm_Busyness	original_ngtdm_Contrast
6.527622549	0.005505194	0.002668042	3.374878714	5.084641804	0.546056415	0.000204761
5.948516907	0.030561819	0.005456199	1.281425221	4.208307135	0.958973393	0.000158052
6.395635545	0.006886483	0.005707856	1.381174539	6.756578079	0.446753136	0.000114244
6.32899421	0.006404447	0.005642692	3.853991153	7.418634267	0.269976099	0.000494913
6.268448349	0.018482154	0.002225267	1.373066649	3.009674145	1.387306998	0.000105064
6.028087575	0.014992208	0.007624877	3.860480841	6.528779439	0.299014694	0.000338994
6.652550617	0.005345433	0.001587285	0.469296429	1.308018063	1.358136412	4.56E-05
6.283471084	0.0043698	0.001092282	0.648123944	2.210853125	1.197934138	2.38E-05
6.530763965	0.00820387	0.001400038	0.82936722	1.719134879	1.298054116	7.31E-05
6.181815031	0.014778757	0.005661775	2.980659186	6.260287836	0.513979891	0.000279324
6.261410729	0.008046899	0.004727041	1.035298776	2.489323094	0.7924831	0.000238303
6.311260929	0.003940208	0.002309372	1.455265124	2.647145363	0.658467956	0.000101471
5.815728063	0.027254215	0.002475845	1.036407268	5.725079663	1.401365475	5.30E-05
6.419444236	0.010002958	0.003238768	2.855341262	7.375833923	0.560150415	0.000142742
6.311003899	0.006921413	0.002243849	2.003551616	2.953627179	0.804094215	0.00016664
6.390905451	0.007849805	0.00125153	0.708390796	2.366874933	1.605521659	7.40E-05
6.487661353	0.005006969	0.00661512	2.929402818	5.763770443	0.168959709	0.000215059
5.885654518	0.034660463	0.004411207	1.298169448	5.027510058	0.966993692	0.000114339
6.242636745	0.006298241	0.00508459	1.152808973	2.520650979	0.441578878	0.000144644
6.564732636	0.003846465	0.001247435	0.794268012	1.985993507	0.941914478	2.98E-05
6.192119995	0.01617818	0.002116466	0.556478732	2.999385585	1.693392603	3.63E-05
6.078949135	0.008899788	0.00565124	1.721625475	3.158711885	0.472733875	0.000205349
6.070197956	0.02044419	0.005489278	2.457249731	6.40117938	0.605376299	0.000223536
6.594585639	0.007651729	0.003389251	0.809776816	2.15240506	0.953830818	0.000142711
6.084874105	0.01383826	0.003827682	2.47671351	7.423858073	0.632341156	0.00012507
6.586551857	0.0050506	0.003283442	1.680620215	2.959583965	0.430751831	0.000120821
6.621418567	0.004003769	0.002003115	1.702913433	3.495180912	0.50704538	7.89E-05
6.509465455	0.007818086	0.002395251	1.106908078	2.819610109	0.782089032	7.31E-05
5.825755594	0.017513035	0.014883072	3.291453023	5.068391271	0.151808629	0.000520737
6.266529484	0.022487147	0.003113323	1.017709221	3.043469489	0.965397834	6.41E-05
6.454764825	0.004679949	0.001184652	0.829829022	2.527077171	1.140485483	3.17E-05
6.220825339	0.027800543	0.002559573	1.209740994	4.147354482	1.42221905	0.000111799
6.638281315	0.00829433	0.002029804	1.077461032	2.420200605	0.847152841	0.000164561
6.459361156	0.009396103	0.002511459	1.404398234	3.569667711	0.987918669	0.000157588
6.362034854	0.00523518	0.001433849	0.772335021	1.588806161	1.094804487	4.65E-05
6.115320616	0.008817164	0.001757898	0.869357816	3.340644512	1.215724945	4.84E-05
6.510719409	0.013227269	0.001440887	1.481440744	5.655824659	1.200050733	4.86E-05
6.527896808	0.01052163	0.001244418	0.667007788	3.467155395	0.786351404	0.000107591
6.623177916	0.007054085	0.00728834	1.551528799	3.755243749	0.338028382	0.000240651
6.897713098	0.004209573	0.001335421	0.376996726	0.8762671	1.393088852	6.60E-05
6.460725862	0.005508533	0.002430442	1.03849176	3.182135533	0.920498369	0.000107175
6.633398629	0.003532415	0.002097403	1.14410904	1.569160808	0.472457024	0.000114442
6.582044749	0.011225816	0.001800112	1.202240028	2.922500032	1.020488031	6.24E-05
6.601535789	0.005361518	0.002477601	2.450872599	4.222570915	0.523335082	0.000121932
6.062395282	0.014558395	0.004695935	1.399961926	3.350622398	0.794854613	0.000168937
6.42950718	0.004857756	0.002319715	1.327256074	2.904488907	0.737228011	0.000107459
6.555092321	0.004068483	0.001532874	0.683419546	1.666232101	0.991482289	4.63E-05
6.210768843	0.009064152	0.00855187	1.463388183	3.367094506	0.265616537	0.000179256
6.149578039	0.014399038	0.007324293	3.13529952	4.477482072	0.380176206	0.000407087
5.908837954	0.008560496	0.003702267	1.510747172	5.451435028	0.49375301	6.22E-05
6.462631659	0.008212086	0.002135838	1.757568332	4.092293444	0.707282107	6.67E-05
6.64202193	0.003162468	0.002326065	1.574908762	2.264010251	0.498555026	0.000101867
6.496536846	0.00370834	0.001543349	0.626088251	1.023022372	0.998293501	5.12E-05
6.263083639	0.016254488	0.005308548	2.708775067	6.831048773	0.505789468	0.000228392
6.232816644	0.016432252	0.004289306	1.85694692	4.012447053	0.756631088	0.000186817
6.338210824	0.010112623	0.003263484	1.288501207	3.759729874	0.63177317	7.01E-05
6.56868996	0.011585182	0.0017078	0.90131771	1.913480599	1.460920902	8.44E-05
6.796669208	0.004974939	0.002128332	1.260948923	4.395046595	0.507141895	5.69E-05
6.30660575	0.007265154	0.003673405	2.299049351	4.784254079	0.42737503	0.000124388
6.920660453	0.00314554	0.001032327	0.506523382	1.045136257	0.692384725	4.92E-05
6.160977968	0.007449427	0.003247183	1.75068206	5.628101339	0.498288596	7.47E-05
6.387204212	0.013946544	0.002132542	1.600497394	3.602194424	0.9170935	7.67E-05
6.310891343	0.006946992	0.000966669	0.624508393	1.413319383	2.099623312	4.01E-05
6.85216447	0.003580185	0.001145076	0.52188741	1.556494947	0.981905391	2.38E-05
6.063651864	0.007536578	0.007980374	1.758238949	4.407480849	0.28111789	0.000199585
6.360241902	0.010965988	0.001522679	0.75597521	2.493908727	1.475881035	3.98E-05
6.291721401	0.010513792	0.004457794	2.633394648	8.773587487	0.341836031	9.82E-05
6.755556448	0.004707126	0.001125718	0.757605832	1.866840986	1.35246918	4.38E-05
6.91150451	0.004101095	0.001470436	1.716160524	5.52300187	0.455183717	3.41E-05
6.330259674	0.010117458	0.002312334	0.729101938	3.000379461	0.952677125	3.75E-05
6.109334821	0.017729846	0.003667541	1.954170165	4.631807681	0.957426064	0.000257902
6.20226078	0.004700143	0.001157867	0.89720044	3.315640445	0.999634204	2.65E-05
6.396089124	0.004440756	0.002634389	1.427068706	3.578876633	0.544556947	9.74E-05
6.585569691	0.004033082	0.002729995	2.073794836	2.763977013	0.662959332	0.000230583
6.641693135	0.006112272	0.002435016	1.130933193	1.73848371	1.014773325	0.000146555
6.430019395	0.009391113	0.005469592	1.784429085	3.907241773	0.3900975	0.000184851
6.520555324	0.00645773	0.003357205	0.818724457	2.874010521	0.563245026	6.16E-05
5.78824849	0.013625595	0.005013723	2.160096898	6.611482051	0.483936509	0.000120012
6.25749727	0.01618717	0.00377405	1.266550772	3.265049431	0.864004135	0.000154019
6.754671652	0.004854898	0.003482874	2.154815127	3.71906263	0.466703728	0.000234243
6.470071982	0.005709307	0.002844102	3.685595508	6.054656057	0.492369513	0.000211233
6.324274462	0.011212151	0.002842395	1.82930453	3.434928815	0.681262054	0.000181312
6.40964602	0.010880542	0.001731082	1.009194606	3.71304336	0.997318178	0.000110796
6.69389843	0.004804564	0.003019538	0.619335394	1.903035071	0.754844336	7.44E-05
6.542761527	0.004168626	0.002416745	1.350492753	2.261519966	0.831234539	0.000168197
5.595496164	0.015844328	0.003454036	0.5349599	3.363339398	1.13447551	3.67E-05
6.680779383	0.004363864	0.002937379	1.719479341	3.451163204	0.782727545	0.000213259
6.240677984	0.011475719	0.002060903	0.815037117	2.0610459	1.406396215	7.37E-05
6.240834973	0.012069811	0.004786304	1.358647302	3.651618993	0.517489925	0.000135245
6.330469485	0.021365692	0.00306922	1.512895442	5.537784	0.816931512	7.32E-05
5.461836499	0.022981434	0.003931541	1.040721179	5.864422983	0.889237222	5.33E-05
6.535272415	0.005317242	0.003171209	1.294488587	2.19638907	0.65466216	0.000184762
6.59171751	0.003410104	0.001266033	0.885006439	1.757384134	0.825639356	4.09E-05
6.449741494	0.007713853	0.002060935	0.752540432	2.122225193	0.984330962	6.68E-05
6.605616686	0.004938967	0.001780816	0.646515035	1.091553498	1.134724906	7.82E-05
6.371616956	0.008045557	0.001493355	1.38321193	4.158160591	1.109770457	6.00E-05
6.692896406	0.006694985	0.003221162	0.991914229	2.789017278	0.626564802	0.000122544
6.319100224	0.012186276	0.005150848	4.222148508	11.22484802	0.369655269	0.00031846
6.172055496	0.011064376	0.009428443	1.300913422	3.714619674	0.434330526	0.000274061
6.165940868	0.008828892	0.000967717	0.502406703	2.235560836	2.188564682	1.94E-05

wavelet.LHL_gldm_GrayLevelVariance	wavelet.LHL_gldm_HighGrayLevelEmphasis	wavelet.LHL_gldm_GrayLevelNonUniformityNormalized	wavelet.LHL_gldm_DependenceEntropy	wavelet.LHL_gldm_DependenceNonUniformity
92.37157542	1652.844958	0.031427858	6.904066144	737.1847314
40.33631388	1455.544385	0.056247534	6.471907656	230.0780749
31.07438861	566.9335072	0.059005013	6.614269526	332.2972621
48.0056761	2540.539406	0.052556811	6.531564881	240.9856704
38.26189405	711.9142736	0.052439409	6.626362235	583.4603041
51.82564509	676.2954545	0.042548209	6.331489912	223.5424242
41.71385649	1461.740577	0.050845028	6.739846821	1433.342948
30.21697363	1583.962608	0.071274795	6.514741672	1059.060689
31.48334777	1137.609107	0.06667426	6.597724533	963.4148702
42.34109768	1156.854478	0.055989293	6.339571553	298.3283582
23.6033335	544.6717507	0.068031507	6.306098307	343.7665782
55.5784522	1734.373536	0.047818521	6.648954507	629.3722343
34.51936819	1565.478843	0.092575385	6.551889555	288.1130155
38.42293499	1117.910891	0.057070529	6.581270498	392.7414094
79.17278449	2261.626008	0.043749695	6.773865614	567.8629032
37.81726647	1909.427559	0.051278538	6.712760406	1081.275594
64.84203612	1763.262144	0.039808198	6.64516745	367.6582915
28.35488121	955.7623682	0.076779481	6.317074547	236.8588808
19.27490497	343.6910632	0.07174247	6.26880651	283.8844376
59.02072724	2622.351991	0.046514967	6.746228935	1442.810348
15.4002775	604.875	0.0979035	6.49525105	435.2453704
34.26524749	785.3503244	0.056004728	6.307403478	288.5755329
71.78768063	729.0791016	0.037723541	6.692889682	314.0839844
28.74941416	640.2417832	0.058380452	6.400429574	684.4250094
61.55340139	1775.500393	0.051562792	6.570797426	341.0974077
39.31869916	1000.371043	0.052383565	6.602416354	609.8180667
62.50632297	1571.803588	0.040804172	6.827127215	937.8270956
36.50932944	1585.966329	0.059219174	6.570766438	645.2925416
43.8768624	1631.908277	0.051273967	6.214490896	134.4899329
42.51965695	1391.096884	0.05187304	6.612934502	521.9717624
31.23570757	823.1057167	0.072674663	6.685999554	877.2501051
38.78583785	1170.527791	0.050714581	6.620893841	514.2556791
55.1326818	1849.831836	0.041785975	6.83017369	916.3118613
47.96267651	875.7612766	0.043070349	6.644695318	695.1182979
32.33564896	1031.090827	0.055974525	6.525036811	1110.72525
27.07625605	873.306102	0.075127521	6.468298804	583.3734578
39.33821355	1630.900027	0.066795004	6.564325379	807.4450417
48.37787332	1690.896532	0.05241024	7.002029021	1113.500291
39.16502677	1122.217073	0.051014608	6.368880562	366.702439
52.76077281	2475.255433	0.043330565	6.815881997	2032.057772
28.70077336	753.8546646	0.066818095	6.547655768	540.1781033
67.23172242	1230.734937	0.037976649	6.683507097	1311.782002
41.713852	1492.045442	0.056617794	6.655110357	861.57268
61.80944353	1510.150322	0.040291793	6.669567703	795.8786174
42.53451084	713.741784	0.050612141	6.437599186	366.85759
33.61794022	1027.073224	0.05599278	6.577574895	668.1832423
38.6803215	2533.268961	0.054694625	6.7016325	1060.3516
65.96424923	2999.971297	0.057981311	6.547227145	202.4626866
56.92383744	2888.014101	0.046250972	6.460191294	248.2714454
28.38139421	342.5517751	0.079302852	6.273777476	270.9615385
70.43910226	5259.316855	0.042636241	6.79091506	743.7637213
76.62822594	1278.851676	0.034637402	6.831410454	1135.231979
37.58510751	877.7240691	0.056090575	6.512294193	1177.001773
23.47991165	1294.686594	0.069365679	6.305354364	241.6974638
23.70719034	362.4609023	0.073398157	6.282194895	291.4526316
61.4377712	2469.227179	0.049826167	6.663847868	512.2605128
46.43798833	1255.603214	0.046408375	6.691446695	1117.625464
40.90778786	1159.672932	0.0504688	6.618899499	916.5465587
41.15514503	912.2287621	0.05437011	6.513031463	421.618932
37.60974094	1225.26487	0.059150797	6.770456045	2040.59426
49.278487	1249.630238	0.043147332	6.533202698	553.3499434
61.13185779	2239.853558	0.049868283	6.647323446	746.552809
38.45002207	912.664018	0.05481041	6.65124696	1424.843496
60.520061	1817.419782	0.040326205	6.78950274	2322.149154
25.16618491	559.5367089	0.071209742	6.23799195	182.6177215
28.47719068	1062.544278	0.073494654	6.628383139	727.6431901
37.51347435	796.9723926	0.055615802	6.48118501	323.7592025
64.49110845	1683.43968	0.03781079	6.860497199	1922.46464
30.52114774	779.5344033	0.060080108	6.520904557	1461.599283
29.42665642	696.573844	0.061369755	6.49496808	668.6825397
30.37738866	531.56505	0.068025917	6.37117168	280.7167052
85.98263561	1718.519347	0.036550443	7.110658182	1167.193939
66.20488844	3332.78631	0.049471865	6.892181395	541.1342694
66.03608565	1235.379371	0.038157929	6.640690648	770.4484285
37.12904093	743.5918237	0.052244067	6.607872883	803.0836793
49.47987245	1244.155738	0.045040323	6.524252746	408.8122206
47.10621278	1376.189482	0.049991499	6.628203554	649.3973289
42.65481322	1531.100209	0.057005941	6.42404176	245.4509395
26.22917385	722.2677211	0.064681801	6.384961764	396.9765671
53.68229956	1355.846088	0.042837943	6.609531375	715.6695169
94.23345949	1476.39819	0.031405258	6.90390785	697.4605329
45.59105253	1162.503057	0.057618633	6.601299182	431.5169539
19.67044759	1097.313246	0.095325715	6.385925279	539.4661976
12.33280509	243.6241458	0.087696299	6.336197812	532.8893589
43.0018446	889.4563346	0.045206188	6.549967122	717.0631406
15.30120314	695.4144909	0.100565653	6.30161079	233.3407311
36.03518567	1110.033124	0.059891267	6.594104659	566.2167715
30.68874761	785.1345733	0.070143448	6.567382975	545.8942378
23.77492423	784.4644809	0.068897623	6.351706512	314.1803279
46.20684749	1732.718519	0.057320287	6.680267014	458.862716
37.0941205	1242.099693	0.076585965	6.5095308	240.0460123
49.36041089	1518.161209	0.042973082	6.573738973	736.1494542
45.9702606	1041.924947	0.048767641	6.680373349	1417.523635
30.68360882	1009.235758	0.059983655	6.503082798	780.5078788
33.97559982	886.3821232	0.052745568	6.592898552	1103.35386
49.38853077	1337.195792	0.04779163	6.690855753	943.0455874
47.97227014	1180.98651	0.048652452	6.742167799	712.2822151
52.52053395	1446.369628	0.054271585	6.564852281	255.7946514
16.72510768	309.118705	0.085189402	6.258026677	149.323741
50.18829631	1941.835643	0.048258506	6.863594157	1416.20194

wavelet.LHL_gldm_GrayLevelNonUniformity	wavelet.LHL_gldm_SmallDependenceEmphasis	wavelet.LHL_gldm_DependenceNonUniformityNormalized	wavelet.LHL_gldm_DependenceVariance
66.68991517	0.576035502	0.34740091	1.10275992
52.59144385	0.421757208	0.246072807	1.801165604
90.5136897	0.395836995	0.216621422	2.735791422
51.34800409	0.440296456	0.246658823	1.861573614
124.1765203	0.431758375	0.246393709	1.849775583
28.08181818	0.548384654	0.338700643	0.955472911
287.3252522	0.45720801	0.253644125	2.281995494
364.0716523	0.391367067	0.207333729	3.50022076
303.1011879	0.397746662	0.21192584	3.016940387
60.02052239	0.484973991	0.278291379	1.574177851
102.5915119	0.404707607	0.227961922	2.134613626
110.221692	0.495676832	0.273046522	2.087994316
172.8382432	0.33260297	0.154318701	6.872384771
97.99009901	0.415327403	0.228736989	2.255779252
86.79939516	0.508346937	0.286221221	1.707157258
224.3948812	0.447595425	0.247092229	2.312071788
47.53098827	0.516795596	0.307921517	1.197517459
94.66909976	0.356009124	0.19209966	2.999769123
93.12172573	0.378917604	0.21870912	2.264132801
241.8313137	0.496381119	0.277516897	1.612475745
296.0601852	0.27991091	0.143930347	6.055884494
60.42910102	0.473526583	0.267447204	1.545633593
38.62890625	0.52062283	0.306722641	1.12852478
154.5330563	0.457856566	0.258566305	1.937688545
65.63943441	0.483045574	0.267947689	1.875404883
124.0966653	0.458368257	0.257415815	2.019080707
131.9198886	0.515667406	0.290079522	1.768338399
163.5633599	0.425746719	0.233632347	2.451659299
22.91946309	0.487324136	0.300872333	1.048811615
106.5472249	0.447356268	0.254124519	1.860969026
345.7860446	0.357790997	0.184373709	4.326250022
104.9284679	0.435296391	0.248552769	1.772939438
134.9269124	0.500090922	0.283775739	1.868672541
101.2153191	0.492636795	0.29579502	1.298578542
246.5118074	0.449602577	0.252208277	1.855028168
225.3074358	0.359759996	0.19452266	3.192288282
248.5442085	0.404838075	0.216996786	2.737265436
270.4892463	0.402610888	0.215752817	4.551802583
62.74796748	0.492018392	0.298132064	1.438062
315.013205	0.488691322	0.279512761	1.734199713
173.3261372	0.376790211	0.208241366	2.976585549
146.8557021	0.559428839	0.339224722	1.000525556
206.8248015	0.424123818	0.235853457	2.160079926
100.2459807	0.537461514	0.319886904	1.229668324
64.68231612	0.49318084	0.287056017	1.400902721
153.7001821	0.431232183	0.243418303	2.03535967
249.5168786	0.420241458	0.232431302	2.468704839
50.50172216	0.418920675	0.232448549	2.108175013
39.35957697	0.501017554	0.291740829	1.546023825
107.2174556	0.369620141	0.200415339	2.995612461
109.5325029	0.507016196	0.289514878	1.535669852
116.7626817	0.552983642	0.336764159	1.009826516
253.0806738	0.460537554	0.260860322	1.777227433
76.57971014	0.374563791	0.218928862	2.326556264
97.61954887	0.384749023	0.219137317	2.476671378
97.16102564	0.466826431	0.262697699	1.796338199
187.7218789	0.485891327	0.276298013	1.587001853
174.5211105	0.459332949	0.265051058	1.675801596
89.60194175	0.461528471	0.255836731	2.047717445
539.9876219	0.41497318	0.223528783	3.415150074
76.198188	0.53068599	0.313335189	1.203669668
133.1483146	0.504285862	0.279607794	1.935041311
317.2974607	0.450092877	0.246129469	2.2806443
307.406664	0.518472543	0.304624053	1.270465361
56.2556962	0.402367476	0.231161673	2.35866688
283.8363542	0.365565044	0.188410976	4.29856754
72.52300613	0.447336695	0.248281597	2.070794441
236.31744	0.522639091	0.307594342	1.30688
351.8891924	0.447814084	0.249547428	2.173454353
177.8495514	0.389125405	0.230739317	1.864597131
88.3656659	0.382687619	0.216102159	2.206189282
156.8013986	0.480704668	0.272073179	1.84926837
112.74638	0.458622998	0.237443734	3.234131734
86.19876051	0.560830259	0.341057295	0.997843068
163.5761737	0.452336605	0.256494308	2.037645816
60.44411326	0.521277349	0.304629076	1.311395453
119.7796327	0.482649508	0.271033944	1.786406671
54.61169102	0.452343665	0.256211837	1.991705929
110.4118336	0.393440453	0.232558036	2.015351516
100.1979478	0.516304459	0.305972431	1.238563306
62.46505782	0.578423469	0.35065889	1.101679145
103.65592	0.44580781	0.239864899	2.330440511
311.6197614	0.32950744	0.165024839	5.333346684
269.4907257	0.303450107	0.17341014	3.667406272
110.2578926	0.500747316	0.293998828	1.325575363
154.0665796	0.295905929	0.152311182	5.484698921
142.8406709	0.435156907	0.237407451	2.762066198
192.3333333	0.389260946	0.199086155	3.443524056
100.8661202	0.399556194	0.214604049	2.529181224
116.0735802	0.428982757	0.226598872	2.973087273
99.86809816	0.362663719	0.184084365	3.990797546
102.3618808	0.517663192	0.30904679	1.161707489
252.7626857	0.479301103	0.273494817	1.627389577
197.9460606	0.418217839	0.236517539	2.064703765
229.5487132	0.428098629	0.253528001	1.777324954
163.5429573	0.488021155	0.275583164	2.071205979
137.0539581	0.455840521	0.252851337	2.12684839
56.82234957	0.449889819	0.244311988	2.133695308
71.04796163	0.347198871	0.179045253	4.549551726
278.6446138	0.443621458	0.245272244	2.137765786

wavelet.LHL_gldm_LargeDependenceEmphasis	wavelet.LHL_gldm_LargeDependenceLowGrayLevelEmphasis	wavelet.LHL_gldm_SmallDependenceHighGrayLevelEmphasis	wavelet.LHL_gldm_LargeDependenceHighGrayLevelEmphasis
4.501413761	0.004126016	923.3797623	7650.975024
7.382887701	0.006408523	602.3257471	10932.85989
9.610169492	0.020160961	223.9047027	5381.683181
7.360286592	0.004031424	1095.177595	18692.74821
7.382601351	0.012741434	305.5715071	5175.164274
4.406060606	0.011668388	371.3917692	2934.786364
7.777207574	0.006027097	673.5853863	11065.76358
11.08966327	0.007492297	616.4487019	17371.96222
10.15310163	0.009828886	449.0969256	11456.67818
6.201492537	0.00689018	550.4959119	7300.229478
8.377984085	0.018478637	223.3623324	4584.304377
7.027765727	0.00825352	864.6307523	11881.28373
19.44884842	0.013998829	524.2009043	29790.45367
8.472335469	0.008951413	475.5566143	9249.740827
6.222782258	0.00373735	1151.220846	13893.87802
7.98857404	0.004620334	852.9121522	15099.89008
5.150753769	0.010140322	927.3302181	8779.788107
11.22871046	0.013671921	328.8579818	10683.27088
8.996918336	0.033602505	129.0246609	3151.791217
6.242546644	0.002681829	1294.144563	16379.07444
19.57804233	0.034755389	167.0197217	11566.52315
6.366079703	0.010220309	359.4683249	5090.52734
5.05078125	0.016511381	373.4806573	3753.311523
7.149603325	0.013306482	287.6574519	4556.420098
6.83503535	0.005096753	855.6059239	11883.39827
7.317433516	0.011568328	459.8227683	7176.110173
6.248376121	0.00466179	799.4746955	9940.192082
8.606082549	0.006124405	672.188761	13432.26539
5.129753915	0.005612672	802.462523	8346.422819
7.168451801	0.006386285	606.2756156	10139.70545
13.44010088	0.018756334	295.3752583	10848.11749
7.206863219	0.007022143	497.6907823	8652.318995
6.48405079	0.004127413	930.6579534	11863.37721
5.540425532	0.008033925	426.831183	4939.32383
7.18982743	0.007605907	455.5031859	7523.143506
11.32210737	0.014151337	314.22699	9782.689897
9.573501747	0.006425462	662.2079017	15535.90836
12.2408448	0.008005814	685.6484205	20057.98276
5.749593496	0.006560164	543.2551817	6480.039837
6.400275103	0.002882152	1212.154364	15761.79574
10.3924441	0.015229084	278.8809773	7855.234002
4.439358676	0.004719242	691.3093353	5432.176881
8.121270189	0.006092586	617.4501449	12260.68382
5.026527331	0.004155605	798.8428594	7724.733923
5.791862285	0.023838947	352.4526367	4165.328638
7.716939891	0.008487387	440.5492172	7899.740619
8.680403332	0.003779321	1059.153281	21712.97918
8.150401837	0.003981406	1245.228248	24397.07348
5.925969448	0.003275607	1417.07802	17466.36898
10.73816568	0.041552	143.9678353	3277.952663
5.913195796	0.001541482	2663.964562	31161.51849
4.490358944	0.004798322	705.0432402	5773.640759
6.865691489	0.009770061	406.1086839	6023.469193
9.141304348	0.0081392	468.2666763	11949.5
9.260150376	0.032851091	141.6996725	3232.1
6.846153846	0.003551155	1135.083064	17042.2241
6.245982695	0.00573618	606.2206478	7846.903585
6.654713707	0.006587457	526.0607217	7869.6631
7.370145631	0.009894709	421.5962289	6514.225121
10.33881038	0.01026791	519.4490784	12130.98291
5.06002265	0.005127771	659.4962623	6409.532843
6.67340824	0.003600883	1129.585665	14834.77491
7.947659354	0.010141103	406.5975092	7161.950596
5.293060475	0.003382387	927.62249	9783.967073
8.678481013	0.019262358	225.539384	4828.086076
13.20559296	0.013344803	384.6480189	13866.24858
7.613496933	0.012564813	352.4071259	6139.266871
5.30688	0.0037998	863.8145691	9082.83936
7.727334813	0.011364514	341.2868386	6071.758067
8.026915114	0.013367259	271.2324949	5580.356453
8.964588145	0.021491105	213.3033014	4525.95766
6.722144522	0.005017751	813.4251566	11327.20513
9.59763054	0.003432958	1524.459207	31650.39754
4.420097388	0.004715569	680.8895866	5541.922089
7.374321303	0.011861255	343.0555548	5312.998403
5.347242921	0.005698078	638.8427994	6861.964978
6.635225376	0.005615379	644.0445669	9378.492487
7.342379958	0.00631834	687.4402737	11153.1858
8.176332748	0.013245909	279.6123579	6022.26655
5.236853356	0.004736557	703.3559929	7104.248824
4.471091001	0.008479677	831.2788164	6758.851684
8.190661479	0.008335879	515.0320114	9528.419678
16.286632	0.015774615	369.2185015	17495.00673
13.5961601	0.062858207	73.17041692	3301.294175
5.556785568	0.00770302	446.5108871	4933.915539
17.8537859	0.027312182	202.286707	12275.53068
8.925366876	0.008993472	485.326674	9881.718658
11.34573304	0.016646618	320.1982096	8454.033917
9.366120219	0.013468154	312.1907227	7275.838798
9.574814815	0.006549163	749.7358007	16050.06272
12.99079755	0.012810196	465.9916136	15479.30061
5.088161209	0.004042557	764.6026083	8025.943745
6.366390122	0.007502398	499.2285839	6668.813236
7.976969697	0.00872041	420.7400101	8098.372121
7.165441176	0.009310055	378.6843432	6373.727711
6.959672706	0.006016026	648.5736513	9110.081239
7.567980121	0.007564199	538.0416375	8893.447994
7.785100287	0.007022143	643.9601696	11203.52722
14.13429257	0.054340652	111.9020605	4214.142686
7.772428126	0.004498742	872.3810208	14853.62816

wavelet.LHL_gldm_SmallDependenceLowGrayLevelEmphasis	wavelet.LHL_gldm_LowGrayLevelEmphasis	wavelet.LHL_gldzm_DistanceZoneVariabilityNormalized	wavelet.LHL_gldzm_LowIntensityEmphasis
0.001054611	0.001438777	1	0.001743154
0.001467413	0.001889118	1	0.003068092
0.002052346	0.003325684	1	0.004794752
0.001240489	0.001466024	1	0.002453617
0.001501689	0.002480082	1	0.003274546
0.004760412	0.005611924	1	0.007755
0.00064545	0.001046299	0.993261533	0.001328302
0.000510351	0.000916954	0.998232436	0.001238723
0.000743427	0.001327464	0.999025341	0.001776184
0.001541037	0.00204019	1	0.002877866
0.001796165	0.003202383	1	0.004264993
0.000639896	0.001672774	0.995395266	0.001638916
0.000965547	0.001494527	1	0.002809609
0.001146232	0.001731958	1	0.002546456
0.000915475	0.001181016	1	0.001653126
0.000503842	0.00080766	1	0.001052488
0.000946506	0.002505303	1	0.002356417
0.001966052	0.002656845	1	0.004992357
0.003938571	0.006493976	1	0.009835156
0.0004262	0.00062624	0.997971605	0.000801583
0.001314189	0.002571664	0.995731076	0.004450508
0.002440201	0.003145553	0.996598649	0.004664112
0.003487415	0.005138316	1	0.00647289
0.00205259	0.002983762	1	0.004144748
0.001283546	0.00159674	1	0.002393065
0.000914941	0.002143339	1	0.002279635
0.000796215	0.001138457	1	0.001444904
0.000709829	0.001092251	1	0.001540192
0.002582652	0.002923305	1	0.004611684
0.001414719	0.001834779	1	0.002847053
0.001285917	0.00224696	1	0.003451236
0.001004426	0.001523638	1	0.002117124
0.000651743	0.000947259	0.998929336	0.001209666
0.001446569	0.00215574	1	0.002739234
0.00081737	0.001389334	0.989556424	0.001720228
0.000988458	0.001787014	0.996763763	0.002583101
0.000644798	0.001027805	1	0.001483006
0.000542552	0.000925519	0.993437315	0.001250402
0.001540133	0.002038953	1	0.002836971
0.000391123	0.000609579	0.999033116	0.000749363
0.001292373	0.00220285	1	0.00321006
0.001116866	0.001545855	0.995194261	0.001871502
0.00071023	0.001135814	1	0.001561434
0.00093556	0.001284233	1	0.001610097
0.002631728	0.005371293	1	0.005375505
0.000943574	0.001550767	1	0.002039674
0.000411299	0.000647163	0.999087175	0.00090888
0.001359263	0.00155628	1	0.002833519
0.001388099	0.001565391	1	0.00241217
0.004000991	0.006431983	0.996509609	0.010059364
0.00049699	0.000593271	1	0.000876941
0.001035106	0.001495609	0.999076639	0.001769842
0.001384869	0.002180711	0.996692857	0.002843866
0.001256893	0.001751664	1	0.002995637
0.004107681	0.006257327	1	0.009792461
0.000890968	0.001120132	1	0.001700127
0.00080002	0.001254659	0.993857013	0.001554483
0.000948034	0.001467391	1	0.001913132
0.001532707	0.002202353	1	0.00309091
0.000612845	0.001309423	0.998155409	0.001530825
0.00119185	0.00161699	0.996350377	0.002060368
0.00074044	0.000974575	1	0.001350149
0.001267802	0.00193896	0.98870093	0.002625346
0.000572198	0.00086355	0.981419093	0.001038662
0.002462199	0.003863759	1	0.005623415
0.000754277	0.001394068	0.992495416	0.00196117
0.002193959	0.003142818	1	0.004526467
0.000682483	0.001010499	0.993139923	0.001230571
0.001460468	0.002247323	1	0.003048747
0.001328915	0.00234504	1	0.003190464
0.002811073	0.004215833	1	0.006708184
0.00078836	0.001153866	0.994970694	0.001523963
0.000602998	0.000771857	1	0.001211893
0.00121409	0.00163107	0.995912824	0.002007341
0.001259322	0.002139996	0.995198107	0.002631775
0.001550142	0.001985984	1	0.002714902
0.00103991	0.001438317	1	0.001981636
0.001597797	0.001981566	1	0.003137865
0.002018213	0.002937176	1	0.00464899
0.000981172	0.001379781	1	0.001759266
0.00152069	0.002652069	1	0.002807237
0.001326321	0.001847238	1	0.002750128
0.000667438	0.001312593	0.996686007	0.001922319
0.002467804	0.005856376	1	0.007948439
0.001290289	0.001949855	0.995774667	0.002417265
0.001241823	0.002310212	1	0.003994397
0.001004775	0.001560317	1	0.002150123
0.001225636	0.00211118	1	0.002993062
0.001405961	0.002234766	1	0.003270794
0.000934688	0.001283386	1	0.001983091
0.001981754	0.002530978	0.992495416	0.004966382
0.000863878	0.001217244	0.997202803	0.001549077
0.001234348	0.001832729	0.988182552	0.002412476
0.000855378	0.001490731	1	0.001929053
0.000933574	0.001677564	0.997259028	0.002073609
0.00081241	0.001228576	0.998952331	0.001564809
0.001064807	0.001586631	1	0.002163198
0.00151683	0.001953047	1	0.00304614
0.003694271	0.006443069	1	0.010051288
0.000475453	0.000789713	0.997986579	0.001006913

wavelet.LHL_gldzm_LargeDistanceEmphasis	wavelet.LHL_gldzm_HighIntensitySmallDistanceEmphasis	wavelet.LHL_gldzm_LowIntensityLargeDistanceEmphasis	wavelet.LHL_gldzm_HighIntensityEmphasis
1	1615.737258	0.001743154	1615.737258
1	1434.047312	0.003068092	1434.047312
1	567.2320675	0.004794752	567.2320675
1	2524.150198	0.002453617	2524.150198
1	712.4507161	0.003274546	712.4507161
1	680.3	0.007755	680.3
1.010141988	1474.357843	0.001335694	1478.140297
1.002653693	1582.025763	0.001240492	1583.074303
1.001462701	1132.558386	0.001777706	1132.9098
1	1141.573554	0.002877866	1141.573554
1	549.2920228	0.004264993	549.2920228
1.006923077	1742.757308	0.001642292	1746.316923
1	1581.008889	0.002809609	1581.008889
1	1141.296703	0.002546456	1141.296703
1	2267.848616	0.001653126	2267.848616
1	1911.679858	0.001052488	1911.679858
1	1790.67872	0.002356417	1790.67872
1	934.4649299	0.004992357	934.4649299
1	339.6265271	0.009835156	339.6265271
1.003045685	2609.836464	0.000802327	2613.256514
1.006417112	598.1604278	0.00446967	598.7540107
1.005110733	768.1601363	0.004676889	768.6712095
1	718.6335505	0.00647289	718.6335505
1	631.6889368	0.004144748	631.6889368
1	1784.536827	0.002393065	1784.536827
1	1003.282072	0.002279635	1003.282072
1	1554.934623	0.001444904	1554.934623
1	1584.064397	0.001540192	1584.064397
1	1641.492188	0.004611684	1641.492188
1	1358.399247	0.002847053	1358.399247
1	828.91	0.003451236	828.91
1	1147.693916	0.002117124	1147.693916
1.001606856	1857.145287	0.001210622	1857.820568
1	867.5599112	0.002739234	867.5599112
1.015748031	1015.861111	0.001755131	1018.8014
1.004862237	875.3219206	0.002589554	876.3338736
1	1635.293294	0.001483006	1635.293294
1.009876543	1703.825	0.001258235	1707.146914
1	1107.668085	0.002836971	1107.668085
1.001451028	2478.028053	0.000750097	2478.858767
1	742.69375	0.00321006	742.69375
1.007226014	1232.353473	0.001880655	1234.405058
1	1462.980545	0.001561434	1462.980545
1	1490.77892	0.001610097	1490.77892
1	713	0.005375505	713
1	1025.018827	0.002039674	1025.018827
1.001369863	2525.77226	0.000909333	2526.808219
1	2988.912941	0.002833519	2988.912941
1	2845.742515	0.00241217	2845.742515
1.005244755	384.4248252	0.010072476	384.9493007
1	5252.830779	0.000876941	5252.830779
1.001385681	1276.061894	0.001772705	1276.229561
1.004968944	881.3098344	0.002850486	882.3175983
1	1263.394191	0.002995637	1263.394191
1	370.8771044	0.009792461	370.8771044
1	2443.50047	0.001700127	2443.50047
1.009242958	1244.172095	0.001561807	1247.319982
1	1145.902517	0.001913132	1145.902517
1	919.8294664	0.00309091	919.8294664
1.002769444	1251.701304	0.001534596	1252.597046
1.005484461	1243.133455	0.002067806	1244.230347
1	2243.020888	0.001350149	2243.020888
1.017045455	904.5326705	0.002648351	908.6233289
1.028135224	1785.434188	0.001064244	1796.313195
1	561.1552632	0.005623415	561.1552632
1.011299435	1054.353578	0.002012347	1056.286252
1	789.7510981	0.004526467	789.7510981
1.010325655	1656.729415	0.001236715	1661.966905
1	767.8153693	0.003048747	767.8153693
1	696.9563253	0.003190464	696.9563253
1	556.4652778	0.006708184	556.4652778
1.007563025	1704.88687	0.001529633	1707.660924
1	3331.287307	0.001211893	3331.287307
1.006143345	1220.147611	0.002013483	1221.972696
1.007220217	757.0415162	0.002645507	758.2743682
1	1226.993781	0.002714902	1226.993781
1	1340.078137	0.001981636	1340.078137
1	1523.550495	0.003137865	1523.550495
1	708.3014049	0.00464899	708.3014049
1	1355.675695	0.001759266	1355.675695
1	1446.875285	0.002807237	1446.875285
1	1156.705947	0.002750128	1156.705947
1.004979253	1119.556224	0.001930118	1120.550207
1	241.4124294	0.007948439	241.4124294
1.006351447	890.4124912	0.002427544	891.4446013
1	687.1227364	0.003994397	687.1227364
1	1114.580537	0.002150123	1114.580537
1	821.215604	0.002993062	821.215604
1	785.6146927	0.003270794	785.6146927
1	1755.645519	0.001983091	1755.645519
1.011299435	1281.601224	0.004975179	1285.693032
1.004201681	1485.453957	0.001553081	1486.565826
1.017832168	1035.726399	0.003483364	1040.590909
1	1007.150685	0.001929053	1007.150685
1.004117109	884.1108188	0.002078483	885.2557182
1.001572327	1332.05097	0.001565521	1332.919287
1	1180.708614	0.002163198	1180.708614
1	1436.729282	0.00304614	1436.729282
1	318.9131737	0.010051288	318.9131737
1.003023178	1960.04392	0.001009705	1960.963722

wavelet.LHL_gldzm_DistanceZoneVariability	wavelet.LHL_gldzm_ZonePercentage	wavelet.LHL_gldzm_IntensityVariabilityNormalized	wavelet.LHL_gldzm_LowIntensitySmallDistanceEmphasis	wavelet.LHL_gldzm_IntensityVariability
1393	0.656456173	0.028212543	0.001743154	39.30007179
465	0.497326203	0.041359695	0.003068092	19.23225806
711	0.463494133	0.043865636	0.004794752	31.18846695
506	0.517911975	0.039916262	0.002453617	20.19762846
1187	0.501266892	0.039875256	0.003274546	47.33192923
420	0.636363636	0.035770975	0.007755	15.02380952
2938.067613	0.523447177	0.037974053	0.001326454	112.3272481
2257.003538	0.442638998	0.043387666	0.001238281	98.09951349
2049.000975	0.45116586	0.042279979	0.001775803	86.71623598
605	0.564365672	0.040601052	0.002877866	24.56363636
702	0.465517241	0.049910309	0.004264993	35.03703704
1294.013846	0.563991323	0.034880473	0.001638072	45.34461538
675	0.361542582	0.041224691	0.002809609	27.82666667
819	0.476994758	0.038751508	0.002546456	31.73748474
1156	0.58266129	0.030514781	0.001653126	35.27508651
2249	0.513939671	0.039575516	0.001052488	89.0053357
719	0.602177554	0.033079865	0.002356417	23.78442281
499	0.404703974	0.045746804	0.004992357	22.82765531
573	0.441448382	0.053772283	0.009835156	30.81151832
2949.006091	0.568378534	0.034308079	0.000801398	101.3803723
931.0085561	0.309193122	0.052166204	0.004445717	48.77540107
585.0034072	0.544022243	0.043431069	0.004660918	25.49403748
614	0.599609375	0.032557375	0.00647289	19.99022801
1392	0.525878353	0.04503464	0.004144748	62.68821839
706	0.554595444	0.036983685	0.002393065	26.11048159
1255	0.529759392	0.039568261	0.002279635	49.65816733
1912	0.591401175	0.032595827	0.001444904	62.32322176
1351	0.489138306	0.041365855	0.001540192	55.88527017
256	0.572706935	0.042785645	0.004611684	10.953125
1062	0.517039922	0.039491277	0.002847053	41.93973635
1900	0.399327449	0.04100831	0.003451236	77.91578947
1052	0.508458192	0.039766731	0.002117124	41.83460076
1865.001071	0.578197584	0.034683817	0.001209427	64.75468666
1352	0.575319149	0.036820314	0.002739234	49.78106509
2262.125984	0.519073569	0.042412295	0.001711502	96.95450569
1230.006483	0.41147049	0.044553428	0.002581488	54.97893031
1715	0.460897608	0.041554454	0.001483006	71.26588921
2414.052675	0.470838985	0.034540128	0.001248444	83.93251029
705	0.573170732	0.04213269	0.002836971	29.7035461
4131.001935	0.568775791	0.035330509	0.00074918	146.0916566
1120	0.431765613	0.042640306	0.00321006	47.75714286
2479.028904	0.644168606	0.032368474	0.001869214	80.62986752
1799	0.492471941	0.038792165	0.001561434	69.78710395
1556	0.625401929	0.034024524	0.001610097	52.94215938
737	0.576682316	0.040851006	0.005375505	30.10719132
1381	0.503096539	0.04216267	0.002039674	58.22664736
2188.000913	0.480052609	0.038860741	0.000908767	85.10502283
425	0.487944891	0.038937024	0.002833519	16.54823529
501	0.588719154	0.037724949	0.00241217	18.9001996
570.0034965	0.423076923	0.046909384	0.010056086	26.83216783
1501	0.584274037	0.033176639	0.000876941	49.79813458
2163.000924	0.642242658	0.030747617	0.001769127	66.56859122
2407.013251	0.535239362	0.041472851	0.002842211	100.1569358
482	0.436594203	0.050464007	0.002995637	24.32365145
594	0.446616541	0.049252344	0.009792461	29.25589226
1063	0.545128205	0.036378874	0.001700127	38.67074318
2258.043134	0.561681088	0.037145795	0.001552652	84.39524648
1867	0.539907461	0.03993156	0.001913132	74.55222282
862	0.523058252	0.038102723	0.00309091	32.84454756
4325.007385	0.474641253	0.039603377	0.001529882	171.6014309
1090.007313	0.619479049	0.037562373	0.002058508	41.09323583
1532	0.573782772	0.035453408	0.001350149	54.31462141
2958.193182	0.516842287	0.039079926	0.002619595	116.927139
4499.806543	0.601469238	0.033785244	0.001032266	154.9053435
380	0.481012658	0.048711911	0.005623415	18.51052632
1581.045198	0.41248058	0.044406851	0.001948376	70.74011299
683	0.523773006	0.040800533	0.004526467	27.86676428
3751.089489	0.60432	0.032504262	0.001229035	122.7685994
3006	0.51323203	0.045000042	0.003048747	135.2701264
1328	0.458247067	0.045298665	0.003190464	60.15662651
576	0.443418014	0.045096692	0.006708184	25.97569444
2368.030252	0.554778555	0.028574959	0.001522546	68.00840336
1166	0.511627907	0.030414341	0.001211893	35.46312178
1459.012287	0.648517043	0.032803178	0.002005806	48.05665529
1654.019254	0.530820824	0.039889307	0.002628342	66.29602888
804	0.599105812	0.038619094	0.002714902	31.04975124
1331	0.555509182	0.038848789	0.001981636	51.70773854
505	0.527139875	0.042211548	0.003137865	21.31683168
783	0.458699473	0.047397686	0.00464899	37.11238825
1403	0.599828987	0.034889599	0.001759266	48.95010691
1315	0.661136249	0.028208591	0.002807237	37.09429658
908	0.504724847	0.038017427	0.002750128	34.51982379
1201.006639	0.368614255	0.050432327	0.001920369	60.77095436
1062	0.345590628	0.060949919	0.007948439	64.72881356
1411.012703	0.58097581	0.038127108	0.002414695	54.0261115
497	0.324412533	0.057127473	0.003994397	28.39235412
1192	0.499790356	0.041929418	0.002150123	49.97986577
1192	0.434719183	0.041597225	0.002993062	49.58389262
667	0.455601093	0.047623115	0.003270794	31.76461769
993	0.49037037	0.036563092	0.001983091	36.30715005
527.0150659	0.407208589	0.039973613	0.004964182	21.2259887
1424.005602	0.599496222	0.037220771	0.001548077	53.1512605
2826.202098	0.551803975	0.037805761	0.002144755	108.1244755
1606	0.486666667	0.043929288	0.001929053	70.55043587
2180.008234	0.502297794	0.041924867	0.00207239	91.64775846
1906.001048	0.557568673	0.035105415	0.001564631	66.98113208
1486	0.527511537	0.035766753	0.002163198	53.14939435
543	0.518624642	0.037181744	0.00304614	20.18968692
334	0.400479616	0.05656352	0.010051288	18.89221557
2971.006046	0.515587115	0.03561884	0.001006215	106.0372859

wavelet.LHL_gldzm_HighIntensityLargeDistanceEmphasis	wavelet.LHL_gldzm_SmallDistanceEmphasis	wavelet.LHL_glcm_SumVariance	wavelet.LHL_glcm_Homogeneity1	wavelet.LHL_glcm_Homogeneity2
1615.737258	1	5664.52207	0.201056166	0.116516331
1434.047312	1	4993.905037	0.281860147	0.191743052
567.2320675	1	1827.833002	0.297685316	0.20952149
2524.150198	1	9067.891753	0.26936732	0.181207792
712.4507161	1	2224.387561	0.272404232	0.180931325
680.3	1	2166.028263	0.227436122	0.138573742
1493.270115	0.997464503	5027.294837	0.264433894	0.176002247
1587.268465	0.999336577	5477.643664	0.308143444	0.221021747
1134.315456	0.999634325	3858.463801	0.301676283	0.214751856
1141.573554	1	3836.412226	0.251835335	0.162548321
549.2920228	1	1710.730305	0.30562251	0.21629344
1760.555385	0.998269231	5952.502738	0.247352945	0.16101461
1581.008889	1	5464.180447	0.361989748	0.285210364
1141.296703	1	3796.130729	0.281890733	0.193437679
2267.848616	1	7853.500997	0.233457432	0.149466997
1911.679858	1	6688.171044	0.272609095	0.183227121
1790.67872	1	6178.934072	0.230388064	0.141127745
934.4649299	1	3152.707179	0.321925655	0.236708928
339.6265271	1	1042.967254	0.310562625	0.221229988
2626.936717	0.999238579	9290.425404	0.241402259	0.153784435
601.1283422	0.998395722	1990.070866	0.391954823	0.3142145
770.7155026	0.998722317	2551.852934	0.262020157	0.169812883
718.6335505	1	2340.653025	0.236138527	0.146841058
631.6889368	1	2074.985775	0.277411089	0.186814739
1784.536827	1	6070.4567	0.256640831	0.171077545
1003.282072	1	3300.001469	0.271277493	0.182521562
1554.934623	1	5374.011823	0.232959429	0.145944684
1584.064397	1	5468.882991	0.281472405	0.192350139
1641.492188	1	5738.361038	0.242120605	0.149555827
1358.399247	1	4812.771094	0.268675125	0.177490613
828.91	1	2669.356684	0.326141366	0.243321339
1147.693916	1	3962.459685	0.264516812	0.174243292
1860.521693	0.999598286	6399.581143	0.242223662	0.153420977
867.5599112	1	2839.500662	0.23949408	0.149918321
1030.562555	0.996062992	3449.341687	0.273518237	0.18436635
880.3816856	0.998784441	2884.94174	0.318820972	0.233943107
1635.293294	1	5679.877168	0.288455273	0.200920232
1720.434568	0.997530864	5859.525999	0.291978888	0.208802467
1107.668085	1	3800.831409	0.243030013	0.152189019
2482.18162	0.999637243	8766.670877	0.24143256	0.151826765
742.69375	1	2460.895043	0.3046679	0.217708025
1242.611401	0.998193497	4049.854765	0.207264049	0.121300956
1462.980545	1	5157.061051	0.276752439	0.190064984
1490.77892	1	5108.434521	0.217879372	0.130624428
713	1	2226.509945	0.244612398	0.155822526
1025.018827	1	3420.890311	0.281656525	0.19136963
2530.952055	0.999657534	9086.807762	0.282078512	0.193239239
2988.912941	1	10871.60212	0.289746123	0.201388706
2845.742515	1	10355.81751	0.238755092	0.149538924
387.0472028	0.998688811	976.7183061	0.324255188	0.236189647
5252.830779	1	19263.67744	0.230286693	0.142226167
1276.900231	0.99965358	4181.804553	0.208476159	0.121218578
886.3486542	0.998757764	2855.841703	0.261662394	0.171261987
1263.394191	1	4505.707699	0.308072676	0.217166708
370.8771044	1	1055.748001	0.311242874	0.221201357
2443.50047	1	8669.648032	0.258089884	0.168823148
1259.911532	0.997689261	4229.651398	0.249425614	0.159960897
1145.902517	1	3875.586471	0.252852827	0.162975873
919.8294664	1	2965.893331	0.266741696	0.179817548
1256.180014	0.999307639	4151.729152	0.288497194	0.203857149
1248.617916	0.998628885	4161.565147	0.232770969	0.142410232
2243.020888	1	7830.02654	0.243603173	0.157315941
924.9859626	0.995738636	2963.103592	0.279085049	0.191260257
1839.829226	0.992966194	6245.695138	0.226320411	0.138030157
561.1552632	1	1757.623419	0.296852143	0.210164567
1064.016949	0.997175141	3585.681038	0.323674888	0.239738892
789.7510981	1	2564.852946	0.257598976	0.168880125
1682.916865	0.997418586	5808.334813	0.22440277	0.13650078
767.8153693	1	2575.902754	0.29038545	0.201460764
696.9563253	1	2247.646141	0.291146273	0.199903866
556.4652778	1	1663.287553	0.301678093	0.213252057
1718.757143	0.998109244	5914.254245	0.244389865	0.157742661
3331.287307	1	12127.1058	0.274385803	0.190991663
1229.273038	0.998464164	4091.268505	0.206157868	0.121003911
763.2057762	0.998194946	2321.001775	0.263509706	0.172979649
1226.993781	1	4237.176554	0.238513719	0.146859058
1340.078137	1	4751.707168	0.259332912	0.170244053
1523.550495	1	5268.939212	0.26806736	0.180492159
708.3014049	1	2366.240211	0.295251168	0.20382055
1355.675695	1	4619.448348	0.23059184	0.141450097
1446.875285	1	4989.962079	0.202757036	0.118600402
1156.705947	1	3873.229749	0.274246929	0.188935243
1124.526141	0.998755187	3778.379629	0.364974981	0.285202456
241.4124294	1	716.1167007	0.369164695	0.283726832
895.5730416	0.998412138	2832.788367	0.244974057	0.154130633
687.1227364	1	2309.673613	0.378535269	0.29912736
1114.580537	1	3718.32802	0.277265762	0.189296143
821.215604	1	2504.036674	0.31784648	0.231810995
785.6146927	1	2621.913079	0.313310813	0.224037572
1755.645519	1	6032.945162	0.287406333	0.20218936
1302.060264	0.997175141	4295.914586	0.329584553	0.246484988
1491.013305	0.99894958	5221.190083	0.232365112	0.143082982
1060.048951	0.995541958	3448.890841	0.249417199	0.160247311
1007.150685	1	3365.464884	0.285994343	0.196119586
889.8353156	0.998970723	2902.457782	0.273849785	0.182327515
1336.392558	0.999606918	4546.079563	0.251100845	0.164127531
1180.708614	1	3969.091492	0.261243618	0.173812583
1436.729282	1	4938.592801	0.257229457	0.170244987
318.9131737	1	935.2740164	0.345912841	0.259169363
1964.642929	0.999244206	6757.01302	0.269001717	0.180652313

wavelet.LHL_glcm_ClusterShade	wavelet.LHL_glcm_MaximumProbability	wavelet.LHL_glcm_Idmn	wavelet.LHL_glcm_SumVariance2	wavelet.LHL_glcm_Contrast	wavelet.LHL_glcm_DifferenceEntropy
-1279.439167	0.005383348	0.975414132	247.3318627	132.8351356	4.538256668
-510.5392124	0.014942893	0.988937012	103.5453797	58.04359345	3.876518105
-116.6668874	0.019086488	0.979810641	71.80223151	51.9770554	3.836516127
-599.7015938	0.014381426	0.987516299	109.4894512	83.8666464	4.051357769
15.56742949	0.013211868	0.978611895	92.4159379	53.88441775	3.900090665
95.57235868	0.010908833	0.963066234	113.2929785	86.11090338	4.180474332
176.159433	0.012172053	0.985307509	96.10602514	70.99029707	4.087948282
-278.4225934	0.02274062	0.987747501	61.41099929	52.95418727	3.809232483
-340.2467856	0.018236472	0.984023152	74.76794706	55.45462257	3.844398782
-548.3043993	0.018888926	0.977790912	90.51200102	77.50420719	4.097161023
-157.4571081	0.017288105	0.978936863	58.14269413	43.27129874	3.730300314
-601.7819936	0.011174239	0.979515719	119.56102	104.0283215	4.285077517
-660.6624602	0.053058978	0.988512872	71.27914253	59.67546313	3.641943895
113.1523409	0.016055126	0.982617907	91.00229051	62.57418451	3.948872612
-1710.546888	0.009925136	0.978421264	178.24191	146.7430716	4.456723098
-235.388964	0.011309795	0.987833339	91.81500094	61.67982662	3.993395025
-168.8007346	0.008629925	0.981852043	158.7987585	88.72319084	4.213855195
-588.0029132	0.02421531	0.97708637	64.68348808	58.07004676	3.747996313
-159.2809139	0.018067613	0.966643141	40.92450845	37.4162828	3.606473446
-556.4933613	0.009456707	0.98600045	124.8075743	111.1189207	4.313340275
-105.8191999	0.042267838	0.989790132	37.35920412	25.76509853	3.275031681
-215.2707657	0.015309713	0.976003955	71.00653959	65.9553686	3.952771766
-467.63575	0.009262472	0.975830136	176.6248148	88.32238292	4.176250054
-182.1584945	0.011334482	0.974727708	67.25465032	52.03616409	3.894519208
-1337.17953	0.013650447	0.977886384	130.6804583	123.9565924	4.266102155
-422.5054642	0.011560599	0.97950416	94.75987775	61.00202714	3.988136486
-705.2434315	0.007145557	0.981381211	153.0635914	102.7976063	4.339914454
-464.4494008	0.017682549	0.985762148	81.24452115	63.9031254	3.949130885
-279.4279454	0.015282844	0.983041288	91.74880426	71.48074512	3.979112222
-380.8555894	0.012006536	0.984935697	104.6451196	65.74715984	3.986934368
-347.2651879	0.027101535	0.982026444	72.17226252	55.09397305	3.801605231
-464.8570671	0.010416085	0.980315963	98.13462731	59.98956413	3.989973676
-276.8375509	0.00790495	0.984936338	139.2747065	82.5219838	4.177893307
-348.5300311	0.007109553	0.974088467	122.8964937	77.40883093	4.17517175
-230.7475015	0.010746113	0.979241083	74.62192352	59.21053292	3.966988282
-100.9167798	0.023526634	0.984380223	59.34780739	52.43750654	3.786564452
-542.1027511	0.017225174	0.988147154	82.74894143	77.57413103	3.99568715
-304.9191625	0.018967165	0.98484681	125.0558946	74.45448306	4.067988614
-619.7658329	0.010178237	0.977365439	86.92018227	69.63315534	4.085335209
-405.7143499	0.008543434	0.99083133	130.8723384	81.38501843	4.191020512
-334.8751741	0.019364184	0.977661858	67.36273939	51.13135341	3.809364452
-185.1281906	0.006280884	0.983156204	151.543336	125.4167908	4.464708837
-728.5667758	0.012860041	0.981151106	91.22490055	79.42572199	4.070069229
-1005.276424	0.007448567	0.974236067	144.5117571	111.4695152	4.393903267
-139.5408792	0.009999923	0.972001944	90.26868958	91.42811467	4.198992442
-215.9880503	0.011988024	0.98365348	81.0350704	55.99181202	3.911162573
-269.7470128	0.016223339	0.990512227	85.54728137	61.54169975	3.949855351
-551.4011401	0.015948643	0.988324316	119.0437052	102.1733261	3.912319837
-712.7513632	0.012544857	0.987046727	114.9065489	97.51202526	4.170287636
763.9928578	0.024499852	0.98166714	61.50688826	39.66130482	3.613839002
-1811.586945	0.008777304	0.99165867	170.0382581	122.7709719	4.369598487
-215.3032455	0.00561729	0.980890866	195.4312825	109.7349341	4.400230697
-301.5398126	0.0104886	0.981787687	81.93963195	69.71033434	4.050596322
-270.1193985	0.018172824	0.985201615	51.09781276	38.50154362	3.625616185
142.8387719	0.02249393	0.974299533	49.6216089	37.47979963	3.638193848
-2418.157761	0.011472624	0.988151477	171.3682402	87.51930795	4.121402609
-314.0950387	0.007319462	0.982743786	113.4899472	76.52281235	4.149492922
-506.9956447	0.011100913	0.979607302	94.68807443	72.74188316	4.11523808
-163.8566704	0.013348952	0.975458347	92.43370524	74.43054656	4.09544243
-112.0854661	0.018908075	0.986589891	97.54104282	66.80309376	4.022727732
-409.3353762	0.008520397	0.977428222	108.1074853	80.5385286	4.17808538
-886.7858136	0.011184758	0.98258897	130.5094547	123.510164	4.335112137
-312.6821109	0.012906298	0.977296901	91.76169143	63.50704607	4.00283449
-741.8702268	0.005844652	0.981194757	145.1521955	101.0503598	4.328365029
-164.7187047	0.019650216	0.973988933	57.77839696	54.76190865	3.814019428
-414.1763495	0.027021359	0.986181038	64.53401327	53.07304686	3.777027771
-361.6129533	0.012801587	0.975011075	83.48689158	76.0294634	4.124033322
-461.4413338	0.004949208	0.981431115	161.6311883	101.751201	4.349685235
-144.1555539	0.013422818	0.984524633	70.1031738	50.48811312	3.853698287
-80.26664784	0.015758082	0.981916032	71.17375837	47.79610217	3.794273989
418.9947653	0.018094418	0.983647206	68.33122979	50.61672023	3.784118287
-1467.1993	0.007830466	0.980422033	235.6306497	103.8389752	4.301353968
-891.2120214	0.01866258	0.986960115	136.7448592	105.7271852	4.217993547
-686.8745929	0.006306866	0.970395205	148.765338	123.3552132	4.479731465
17.35287318	0.011589907	0.978979637	83.77554793	62.24692091	4.021792543
-885.4181689	0.010540786	0.978175883	116.917065	76.01260043	4.09961242
-966.2696486	0.009781446	0.980330431	107.327601	79.53051516	4.112809775
-924.6536727	0.014637374	0.978569233	99.24782567	82.66019004	4.06193532
-323.9788634	0.016304309	0.978035322	58.44268906	47.98993428	3.7566823
-455.869787	0.008130839	0.980324963	134.799882	87.68956692	4.251964807
-1665.187	0.006608758	0.973668249	261.6815478	135.418447	4.542573315
-563.1631657	0.014871553	0.980692731	97.32696278	86.79507361	4.107052256
-152.1549495	0.040343042	0.988226431	46.25132222	36.38836671	3.513240099
-3.593997571	0.024177733	0.980594083	31.9040015	18.98098248	3.213326811
-213.8361719	0.007089299	0.977106732	109.5678931	67.76550867	4.073915078
-105.9334226	0.05243143	0.983141474	33.67825669	30.61056901	3.375034667
-260.9251544	0.016328253	0.983166821	84.62881148	68.75375722	4.032158632
30.3234647	0.022478666	0.984744662	67.69963915	46.56383393	3.757648569
-159.9245126	0.019978177	0.980279147	51.35699857	38.35152512	3.646881092
-107.949484	0.016062457	0.985951619	105.1078597	76.11940461	4.015546119
436.4302113	0.026693478	0.986601767	82.99727154	60.69520921	3.757068582
-415.5630351	0.008446698	0.982911725	121.5301338	82.48483295	4.212062735
-558.6400222	0.010980034	0.979716914	110.8206616	80.85236395	4.174961596
-253.0932798	0.012391014	0.984883184	74.42214788	53.04464047	3.881038028
-204.7005056	0.009454261	0.979609832	83.7423253	53.75081878	3.912835191
-80.10782372	0.013567345	0.982599304	118.6953371	84.81679699	4.210960934
-390.3924852	0.010067145	0.979605402	104.9904527	81.74371889	4.131841307
-978.100315	0.015864359	0.977589639	115.8784201	102.9940011	4.208061676
7.607526473	0.038497858	0.979719468	34.98904313	29.00466578	3.446859072
-282.2588235	0.011981528	0.987100149	123.0393731	77.09305175	4.088341147

wavelet.LHL_glcm_InverseVariance	wavelet.LHL_glcm_Entropy	wavelet.LHL_glcm_Dissimilarity	wavelet.LHL_glcm_DifferenceVariance	wavelet.LHL_glcm_Idn	wavelet.LHL_glcm_Idm	wavelet.LHL_glcm_Correlation
0.121295902	9.756691951	8.763253079	53.97055252	0.895947338	0.116516331	0.310116164
0.201225183	8.272985999	5.437644429	27.61369706	0.932016631	0.191743052	0.286073142
0.209062689	8.341555164	5.149248826	24.81160694	0.910158283	0.20952149	0.167134506
0.192173757	8.418293774	6.22301062	43.89566361	0.931104539	0.181207792	0.133033349
0.187514686	8.649935648	5.490591246	22.63003064	0.904029217	0.180931325	0.267578166
0.152389195	8.560823262	7.078847162	34.19812489	0.87426344	0.138573742	0.144781774
0.181746323	8.994672569	6.130776794	32.41704275	0.921272819	0.176002247	0.152217545
0.227024264	8.200536654	4.998739915	27.13138587	0.931329823	0.221021747	0.083766011
0.221671596	8.406604307	5.173550893	27.32255152	0.921464672	0.214751856	0.165353661
0.169485294	8.367980621	6.455702901	34.31601541	0.903858619	0.162548321	0.085805682
0.222750596	8.100010253	4.754177304	20.293111	0.907583582	0.21629344	0.146721442
0.164640968	8.98389027	7.176283512	50.65835259	0.91059564	0.16101461	0.079865923
0.282919311	7.47168389	4.567466188	37.79590117	0.941840869	0.285210364	0.107553078
0.200538218	8.514367514	5.612522475	30.14682165	0.91659769	0.193437679	0.189989031
0.156378431	9.175505001	8.292840194	74.10363209	0.910902758	0.149466997	0.114373886
0.18936232	8.908757389	5.73203361	28.12191375	0.927574229	0.183227121	0.201166614
0.148829128	8.99193238	7.059032464	37.16891941	0.910386991	0.141127745	0.28239217
0.240223593	7.821864419	4.950192538	32.26120331	0.912220455	0.236708928	0.075013282
0.23339579	7.792555946	4.487726213	16.24696798	0.88387249	0.221229988	0.065476412
0.161250246	9.252654785	7.324077123	55.35223222	0.926001516	0.153784435	0.060996948
0.309155424	7.333739486	3.340286149	14.15696183	0.939340125	0.3142145	0.194724625
0.17635468	8.275852574	5.881905073	29.80371231	0.900712136	0.169812883	0.046493555
0.160019838	8.95306701	6.918795312	37.89531497	0.899435732	0.146841058	0.339400139
0.195735315	8.533590948	5.376906673	22.62475695	0.896654018	0.186814739	0.132725842
0.179116982	8.603369735	7.331501682	67.21013429	0.912839297	0.171077545	0.045249025
0.188321941	8.746687689	5.763164374	27.08974102	0.906870297	0.182521562	0.220934808
0.150985888	9.413628999	7.427871782	46.19091778	0.911485894	0.145944684	0.203572227
0.201038416	8.489393885	5.608986482	31.35978629	0.924740514	0.192350139	0.126317955
0.154797888	8.005034497	6.323997674	29.79085382	0.912787355	0.149555827	0.1481574
0.181556494	8.72468998	5.807203656	31.24324473	0.920946942	0.177490613	0.229987711
0.241782813	8.250069988	4.939568622	29.68615353	0.920518997	0.243321339	0.14187339
0.180829734	8.784599979	5.81064596	25.46230423	0.907361596	0.174243292	0.246359142
0.159366436	9.305960327	6.72283402	35.96848529	0.918479225	0.153420977	0.264091192
0.157620368	9.173298066	6.678066419	32.00853521	0.893800998	0.149918321	0.233322702
0.192818818	8.753272983	5.671149706	26.05335992	0.906682238	0.18436635	0.124948187
0.235853748	8.056001624	4.914813913	27.43789057	0.924151454	0.233943107	0.061463715
0.208334136	8.410897505	5.812927437	42.55311986	0.934815879	0.200920232	0.037212277
0.208311189	9.032034701	5.944090972	38.11413643	0.924398625	0.208802467	0.26066307
0.159334349	8.630865397	6.372691795	28.34841501	0.899284762	0.152189019	0.109225658
0.156489636	9.392671758	6.700578554	35.30474079	0.935281965	0.151826765	0.241540574
0.224261918	8.3003392	5.038343226	24.6607782	0.907259882	0.217708025	0.149169959
0.127826572	9.612154768	8.393865553	51.85110019	0.913502946	0.121300956	0.099279189
0.19765004	8.744663912	6.116525384	40.45481421	0.916024244	0.190064984	0.075971553
0.135441128	9.362631497	7.862679642	47.97120312	0.895352924	0.130624428	0.139676754
0.164749662	8.682079442	6.860822085	43.19009793	0.894667949	0.155822526	0.008641854
0.197371397	8.616847835	5.410222779	26.25208132	0.91756909	0.19136963	0.189865255
0.200195366	8.709836869	5.556810383	29.90901582	0.937115176	0.193239239	0.166069115
0.21534469	8.040078792	6.004899682	64.3170441	0.94039073	0.201388706	0.081959577
0.153243945	8.542321273	6.996111629	46.7778678	0.926107686	0.149538924	0.068284291
0.239638391	7.585146004	4.41075356	19.51697734	0.915598849	0.236189647	0.206891768
0.148360761	9.284478004	7.679191012	62.50482908	0.941737842	0.142226167	0.165216926
0.125204278	9.711462507	8.036092982	42.45303576	0.906461105	0.121218578	0.287544343
0.176790384	8.768226138	6.064965577	31.55889803	0.912960981	0.171261987	0.088609087
0.223253533	7.865624097	4.528143113	17.3462777	0.920481712	0.217166708	0.146218008
0.229420037	7.792653417	4.486566826	16.85302863	0.897432815	0.221201357	0.142734129
0.176473843	8.837701516	6.454462573	44.32376075	0.93164764	0.168823148	0.312108129
0.166062415	9.156915089	6.476186127	33.51432987	0.913760196	0.159960897	0.201292431
0.170848783	8.962458838	6.309078132	32.17079339	0.906909296	0.162975873	0.139628606
0.187832096	8.635552112	6.233949575	34.51757938	0.901169638	0.179817548	0.116696596
0.206738374	8.933580664	5.763906627	32.62938452	0.926899045	0.203857149	0.189155309
0.151411515	8.981822597	6.80861772	32.92842599	0.900007681	0.142410232	0.148722405
0.16485668	8.998652041	7.541024896	63.72645213	0.919639595	0.157315941	0.041269264
0.198741564	8.877347978	5.729333012	29.8959621	0.904930456	0.191260257	0.188142356
0.145392419	9.601857415	7.430296599	44.0246564	0.910130768	0.138030157	0.187037786
0.216917229	7.823169825	5.267998274	25.29238815	0.89978936	0.210164567	0.028045495
0.244616227	8.143164658	4.864919882	28.52716939	0.929511567	0.239738892	0.113842484
0.170250971	8.548159106	6.374172122	34.73736997	0.898894144	0.168880125	0.054322822
0.143061757	9.686634376	7.525559347	43.4711677	0.910074145	0.13650078	0.235233908
0.210394303	8.594235523	5.177463317	23.00900252	0.919297366	0.201460764	0.164705012
0.208825862	8.397138821	5.014270038	22.10026945	0.913320735	0.199903866	0.202704006
0.221114628	8.038263301	5.012170763	24.66059421	0.919598077	0.213252057	0.148788633
0.161194963	9.649732336	7.240748035	48.72503397	0.911953422	0.157742661	0.391215373
0.195650935	8.841482711	6.831824411	57.05770881	0.931862221	0.190991663	0.121539627
0.129882287	9.460723657	8.443744298	49.62279003	0.886911967	0.121003911	0.106884648
0.176977532	8.809624031	5.883112854	27.03581622	0.904984383	0.172979649	0.152979726
0.155752013	8.8056955	6.523483537	31.94973896	0.902371092	0.146859058	0.219844444
0.178134828	8.847181287	6.333467529	38.45768787	0.91144933	0.170244053	0.151331381
0.189201169	8.31581187	6.299303192	40.95738462	0.909912244	0.180492159	0.106194112
0.207892279	8.133122496	4.966968694	22.22879938	0.906054497	0.20382055	0.113789502
0.149826725	9.221091106	7.054644121	37.08898449	0.906780393	0.141450097	0.214759239
0.123067128	9.716767979	8.802785498	55.62776326	0.893153597	0.118600402	0.327760883
0.195134358	8.552328331	6.379881144	43.73579762	0.915406513	0.188935243	0.062404511
0.281257506	7.578435992	3.96785935	20.14957576	0.935383896	0.285202456	0.127357704
0.288495907	7.483179542	3.236393516	8.260856611	0.908879744	0.283726832	0.250902012
0.165390735	9.057936291	6.278630808	27.44362311	0.898980622	0.154130633	0.243757412
0.290795248	7.212528696	3.65021436	16.6182111	0.92331771	0.29912736	0.053901552
0.194234637	8.577433213	5.875470785	33.51012383	0.918239679	0.189296143	0.113432354
0.231851873	8.119762052	4.78356028	23.01145856	0.922942054	0.231810995	0.17768617
0.229891207	7.949119641	4.50377984	17.53948997	0.909788711	0.224037572	0.144377617
0.207489398	8.565264505	5.921299755	39.57938758	0.927616276	0.20218936	0.160728669
0.245750421	7.81472134	4.944577054	35.29574486	0.933176419	0.246484988	0.150065387
0.149419886	9.16635055	6.910026534	33.75873358	0.911724022	0.143082982	0.198464027
0.167211461	9.152899178	6.561739129	36.81919163	0.908147185	0.160247311	0.166836948
0.205210699	8.540883953	5.280873898	24.62480113	0.920479711	0.196119586	0.175707044
0.189753315	8.799175857	5.443462755	23.60308188	0.906509665	0.182327515	0.224937709
0.171551453	9.156274945	6.724341928	38.59807747	0.914692947	0.164127531	0.170636508
0.17627801	8.955412965	6.467725003	37.7498601	0.909785746	0.173812583	0.132844888
0.169189939	8.498816275	6.970989578	51.79544401	0.90871861	0.170244987	0.057236652
0.253311997	7.323494421	3.886546685	13.41746118	0.909512732	0.259169363	0.089324401
0.186930099	9.117197621	6.139535811	38.28236953	0.928287146	0.180652313	0.232280976

wavelet.LHL_glcm_Autocorrelation	wavelet.LHL_glcm_SumEntropy	wavelet.LHL_glcm_AverageIntensity	wavelet.LHL_glcm_Energy	wavelet.LHL_glcm_SumSquares	wavelet.LHL_glcm_ClusterProminence	wavelet.LHL_glcm_SumAverage
1609.758569	5.925005197	40.55646378	0.001507879	92.7684311	236075.537	79.52451598
1421.397961	5.169352398	37.96950442	0.004601103	38.96677771	70993.9384	75.09971424
555.5340518	5.024264768	23.80554361	0.004752217	31.32738961	23089.87826	46.92812334
2498.757671	5.197902451	50.46128018	0.004232886	46.95373287	85759.45943	99.84649655
668.755614	5.173052452	26.07439142	0.003877283	35.53740299	41489.62089	51.34548931
647.0096917	5.29858209	25.81503582	0.003286356	49.06650089	44795.21671	50.60417771
1429.272346	5.222806208	38.23018589	0.003407509	41.27876165	44785.35595	75.44505243
1539.083993	4.81340103	39.59060856	0.0067036	27.45877358	30606.24234	78.4083458
1109.851482	4.972055701	33.64064091	0.005719888	31.7808105	38924.17605	66.48364803
1101.28595	5.069674146	33.64943322	0.00464684	41.11341882	47319.21977	66.27270285
521.8830284	4.874946654	22.99932307	0.005544441	24.94447409	14557.3581	45.52623964
1673.005619	5.335856691	41.45747821	0.003102051	53.25084614	85537.48993	81.70938925
1525.817349	4.612615224	39.40054343	0.014025595	31.19077218	65190.75956	78.04884149
1096.178012	5.13246141	33.44423376	0.004607439	37.77034818	41841.42244	66.00203428
2178.326272	5.56784981	47.33246253	0.002800758	78.70377487	200904.5583	93.17605137
1876.646339	5.2487319	43.63637826	0.003252131	37.67872279	34401.78223	86.4664129
1744.76495	5.530825808	42.17830216	0.002554952	61.2178124	132737.2178	83.11949189
913.2563814	4.815221705	30.49566291	0.007386182	28.17337643	31894.605	60.38501177
329.675285	4.610543551	18.40045432	0.006477202	19.838044	8170.134224	36.26541126
2559.4318	5.376825454	51.21111461	0.00281247	56.70835473	99246.53835	101.1137837
595.0762191	4.480055396	24.52149778	0.012614477	15.76434636	9607.407945	48.66913909
750.8237304	4.948415096	27.82655485	0.004498352	33.04531553	28869.02215	54.7560664
701.396386	5.609126671	26.62591119	0.002615902	65.18948788	125321.8179	52.12666831
624.375263	5.014931901	25.23790329	0.003933015	29.44128739	18284.83248	49.82202226
1696.68414	5.270737209	41.75656776	0.003886201	60.63197687	135841.9475	82.34036887
964.9678393	5.24074722	31.33709208	0.003477218	38.27485768	39269.7304	61.85520209
1526.748455	5.569131085	39.54930226	0.002169572	61.83188506	108235.6593	77.82488044
1542.463528	5.03740718	39.67327421	0.004756884	34.91944706	44936.27252	78.43783836
1613.084166	5.056821209	40.64861881	0.004847201	39.23912115	44255.4136	80.19941933
1374.308382	5.265154723	37.39873945	0.003465082	41.32740437	64386.13569	73.88028348
784.7768304	4.912769262	28.27458664	0.006991183	30.5085552	37315.11645	55.8750203
1145.852053	5.255275127	34.14077141	0.003308208	38.53224851	37842.00667	67.41815361
1806.208918	5.542306865	42.9027244	0.002286727	54.77352038	78709.98364	84.66422719
840.2403509	5.459052504	29.28938644	0.002319925	48.43217447	50853.38734	57.57914251
1002.149549	5.099479156	31.97816635	0.003580181	32.63975412	22508.75911	63.19141727
841.2920919	4.794461903	29.29204883	0.006992912	27.07538665	25264.57618	57.95033889
1592.626029	4.969238901	40.39185713	0.005661646	38.96523435	60405.59383	79.78296178
1657.871154	5.39640758	41.05128658	0.003963971	48.47075508	81146.45735	81.12251496
1095.197218	5.115628153	33.51243164	0.003310886	37.69430186	42867.15979	66.05642216
2433.818126	5.487371271	49.80645301	0.002280679	51.88774122	90546.46922	98.41629449
729.1297392	4.930718682	27.29096145	0.005784854	28.81996591	24191.53279	53.85421331
1163.068442	5.57957533	34.7622961	0.001809624	67.31226257	101543.4124	68.01562386
1459.025894	5.142857068	38.65312148	0.004031932	40.96465077	54273.08923	76.3170184
1451.655267	5.528968972	38.62345825	0.002107187	61.65225542	91253.41665	75.98390527
660.1769449	5.179841568	26.23917901	0.003284101	44.1188156	35632.96222	51.39901604
995.9527161	5.127204789	31.86351782	0.003891369	33.77312965	32053.04187	62.91856142
2505.995869	5.124893056	50.49094492	0.004125573	35.59222754	45390.4361	99.9997508
2961.77052	5.072562302	54.93869449	0.005506868	51.44573443	156073.3544	108.765572
2836.335612	5.235469294	53.85827883	0.003531647	51.70888919	117504.1265	106.4325556
309.2710102	4.606432201	17.71469998	0.008930593	25.60788528	33283.78006	34.85773843
5176.995168	5.558655603	72.62258364	0.002451233	70.70973879	237382.0497	143.738179
1208.690158	5.775677196	35.16630782	0.001622971	75.06551943	146856.1505	68.91313014
837.1931736	5.095211632	29.328267	0.00370772	36.9863017	38938.33706	57.76260829
1281.014458	4.751755095	36.04526297	0.006228634	21.68438779	13633.85983	71.49437496
334.0352156	4.667048554	18.47810408	0.007380345	21.42943475	13610.58698	36.38577453
2402.693978	5.420084588	49.34946554	0.003330547	62.00743687	273651.4903	97.60585627
1218.20275	5.382948088	35.28326664	0.002585442	46.49301979	59486.72767	69.5401114
1118.486213	5.234157046	33.86928322	0.00313367	40.25878586	39145.64986	66.7230224
868.0079185	5.166385799	29.84338824	0.003894446	40.36543536	42669.61036	58.77070738
1194.628484	5.231695579	34.9188399	0.004189255	40.59470259	52104.05277	68.90387487
1196.698339	5.320470099	35.03554301	0.002591804	45.20236668	49576.795	68.9870488
2167.649033	5.33152333	47.21955333	0.003277905	60.81832944	137897.9864	93.07818192
871.6320285	5.222270139	29.80926429	0.003685678	37.05027025	38570.81758	58.80697663
1761.259418	5.565826612	42.4752344	0.001931564	59.69207348	95192.41553	83.67156499
529.8150928	4.773121563	23.28580784	0.006331265	27.17183086	18331.53631	46.00195194
1031.849193	4.816540292	32.46108695	0.007470094	28.26345727	32268.67959	64.15555048
755.2412806	5.082423765	27.93033882	0.003852538	38.37284853	38086.5661	54.89537132
1647.153391	5.656150614	41.06446055	0.001752986	64.26778102	108529.3979	80.8004522
763.6432203	5.03247598	27.91070965	0.004241663	29.27578079	23809.94801	55.09020017
672.8677261	5.000837097	26.19787627	0.004776755	29.18198597	26956.2696	51.65304996
505.9378296	4.85191758	22.74218584	0.0060199	30.28095589	36072.82853	44.78843601
1682.033992	5.860788949	41.20933412	0.002026557	81.94147067	230656.2006	81.21640567
3305.358572	5.349697772	58.03584207	0.004356434	58.90872652	116704.6132	114.8491633
1174.176262	5.557569224	34.90768324	0.001912374	65.83624474	84029.20757	68.34652324
693.2707946	5.156229891	26.6768418	0.003534872	35.98548542	29449.00309	52.45476588
1218.654795	5.336317414	35.28354628	0.002983316	46.55258685	62157.49996	69.52442373
1354.84513	5.278452638	37.22520375	0.003178185	44.20504017	62681.86207	73.42680333
1487.029557	5.115752511	38.97783435	0.004531052	43.0766097	68039.91053	77.01584402
702.1495018	4.858052427	26.75447069	0.005299238	26.02963058	18124.33648	52.89732835
1324.191349	5.487265983	36.79830266	0.002344625	54.66213413	76555.92178	72.45444027
1426.913391	5.945697093	38.15634157	0.001565642	96.94566878	267834.1492	74.70574898
1109.872925	5.102878916	33.76880798	0.00440874	44.25472838	64540.86717	66.55022828
1080.295943	4.569722589	33.05512947	0.011618205	20.48752172	17274.64854	65.66062302
238.2038162	4.500199338	15.38329508	0.008776838	13.11063442	3805.764852	30.65736126
838.8492286	5.379620214	29.25219601	0.00250467	43.30734335	41166.43961	57.5634012
679.1483143	4.384444759	26.18179222	0.013199224	15.82614634	7718.406317	52.09131484
1072.033701	5.082391451	33.16752697	0.004576348	37.10959653	40658.73767	65.36242107
740.3197793	4.86367305	27.42829603	0.006841516	27.65819305	29890.47177	54.22281934
772.0474673	4.754009333	28.00486874	0.006191862	22.46320236	14300.40453	55.45414644
1696.587637	5.210723715	41.58212907	0.004511767	44.33436753	71081.12911	82.20282779
1221.388473	4.829809777	35.2402072	0.008346841	34.98971338	56323.99662	69.7364026
1485.906515	5.4295815	39.00768702	0.002344015	49.4667473	56192.33349	76.84110792
1003.371534	5.339027964	32.07145764	0.002845537	46.48170112	62673.56276	63.11488492
979.1516629	5.044073635	31.5998803	0.004310233	31.17946353	28322.95452	62.41162497
856.3073023	5.177215343	29.51827756	0.003364901	33.36163222	30065.49445	58.26861878
1302.049922	5.394400207	36.51115134	0.002947279	49.97850549	58609.0123	71.93229698
1143.236969	5.289574413	34.22714077	0.003159887	45.18587236	53000.46095	67.45122613
1395.962104	5.193508204	37.89442826	0.004212386	52.5633407	86815.18562	74.63873022
297.8235916	4.424336381	17.44501502	0.010232207	15.88327156	7166.925858	34.42804427
1897.376177	5.404626335	43.93401126	0.003108016	48.43636849	82006.15433	86.85361734

wavelet.LHL_glcm_Imc2	wavelet.LHL_glcm_Imc1	wavelet.LHL_glcm_DifferenceAverage	wavelet.LHL_glcm_Id	wavelet.LHL_glcm_ClusterTendency	wavelet.LHL_firstorder_InterquartileRange	wavelet.LHL_firstorder_Skewness
0.892369999	-0.151252351	8.763253079	0.201056166	247.3318627	287.8878215	-0.352931314
0.893439714	-0.176358677	5.437644429	0.281860147	103.5453797	152.1982741	-0.609271312
0.807969839	-0.119982711	5.149248826	0.297685316	71.80223151	152.0014237	-0.093685376
0.902340171	-0.182719958	6.22301062	0.26936732	109.4894512	161.8022707	-1.009840432
0.778458318	-0.104406513	5.490591246	0.272404232	92.4159379	168.0602264	-0.159229913
0.934971641	-0.216569255	7.078847162	0.227436122	113.2929785	218.477035	-0.170026788
0.693456967	-0.071684945	6.130776794	0.264433894	96.10602514	169.6918252	-0.020058353
0.673307571	-0.073415177	4.998739915	0.308143444	61.41099929	120.677845	-0.656279615
0.71799917	-0.085426874	5.173550893	0.301676283	74.76794706	125.7344546	-0.530876364
0.878185024	-0.162616452	6.455702901	0.251835335	90.51200102	155.8932895	-0.66346237
0.769172649	-0.105719807	4.754177304	0.30562251	58.14269413	129.4717635	-0.410016175
0.833699874	-0.124795108	7.176283512	0.247352945	119.56102	186.7321874	-0.711211537
0.852258994	-0.159773223	4.567466188	0.361989748	71.27914253	87.84441707	-1.382489297
0.829494778	-0.129939348	5.612522475	0.281890733	91.00229051	147.5031978	-0.166529335
0.899376094	-0.166770762	8.292840194	0.233457432	178.24191	193.0960216	-0.763972016
0.68167672	-0.069127048	5.73203361	0.272609095	91.81500094	172.6340514	-0.39705994
0.905724703	-0.175393525	7.059032464	0.230388064	158.7987585	233.1970654	-0.359006242
0.824265254	-0.136289021	4.950192538	0.321925655	64.68348808	111.4435408	-1.508186445
0.736745989	-0.098082564	4.487726213	0.310562625	40.92450845	128.137086	-0.509781368
0.761207373	-0.092489096	7.324077123	0.241402259	124.8075743	183.8831985	-0.632143047
0.702168856	-0.091729338	3.340286149	0.391954823	37.35920412	84.92721089	-0.46373538
0.852990372	-0.146878122	5.881905073	0.262020157	71.00653959	161.0063081	-0.626925608
0.932382261	-0.204268853	6.918795312	0.236138527	176.6248148	235.2181403	-0.143884365
0.700368981	-0.076688597	5.376906673	0.277411089	67.25465032	154.3668279	-0.436958854
0.912449268	-0.189169254	7.331501682	0.256640831	130.6804583	158.8585651	-1.032186152
0.772553858	-0.100231461	5.763164374	0.271277493	94.75987775	167.4704116	-0.503088638
0.795269159	-0.101674978	7.427871782	0.232959429	153.0635914	224.7845544	-0.435573285
0.766571505	-0.101154135	5.608986482	0.281472405	81.24452115	146.9815451	-0.685816898
0.942473143	-0.240904252	6.323997674	0.242120605	91.74880426	184.6693919	-0.509772863
0.820919487	-0.122029795	5.807203656	0.268675125	104.6451196	171.2493507	-0.52088068
0.732985008	-0.091737925	4.939568622	0.326141366	72.17226252	112.6911073	-0.603080702
0.774863172	-0.099814946	5.81064596	0.264516812	98.13462731	174.013397	-0.539514657
0.776309345	-0.09608031	6.72283402	0.242223662	139.2747065	212.6322187	-0.1986484
0.778618287	-0.096734922	6.678066419	0.23949408	122.8964937	215.0325866	-0.355803873
0.653110952	-0.064000636	5.671149706	0.273518237	74.62192352	158.1338382	-0.490362349
0.750981159	-0.099601324	4.914813913	0.318820972	59.34780739	112.4441916	-0.489588297
0.767544865	-0.10237417	5.812927437	0.288455273	82.74894143	125.2029259	-0.686590872
0.764856885	-0.094646358	5.944090972	0.291978888	125.0558946	163.1982692	-0.431160535
0.824618395	-0.124306087	6.372691795	0.243030013	86.92018227	178.3496765	-0.764095968
0.671608897	-0.063686994	6.700578554	0.24143256	130.8723384	206.6312146	-0.3052313
0.741899168	-0.094731591	5.038343226	0.3046679	67.36273939	128.830647	-0.611815945
0.777097927	-0.095001992	8.393865553	0.207264049	151.543336	240.8538736	-0.106954889
0.76558127	-0.098651909	6.116525384	0.276752439	91.22490055	149.0200504	-0.924894347
0.813898943	-0.109990425	7.862679642	0.217879372	144.5117571	225.6381595	-0.6220055
0.851738302	-0.138479957	6.860822085	0.244612398	90.26868958	187.8703967	-0.30916746
0.746828161	-0.091238456	5.410222779	0.281656525	81.0350704	159.545988	-0.313924836
0.707515071	-0.079103607	5.556810383	0.282078512	85.54728137	159.730087	-0.615411679
0.933533084	-0.227277819	6.004899682	0.289746123	119.0437052	143.3888884	-1.306292625
0.914865046	-0.193941863	6.996111629	0.238755092	114.9065489	198.3434264	-0.999055206
0.826875258	-0.142128556	4.41075356	0.324255188	61.50688826	105.8598238	1.146042892
0.854720828	-0.132452136	7.679191012	0.230286693	170.0382581	201.5225992	-0.853995559
0.81534522	-0.108391845	8.036092982	0.208476159	195.4312825	264.389633	-0.102797973
0.700188819	-0.076729336	6.064965577	0.261662394	81.93963195	156.8465593	-0.379986777
0.77490162	-0.11336374	4.528143113	0.308072676	51.09781276	131.7302438	-1.013689806
0.783767677	-0.11644049	4.486566826	0.311242874	49.6216089	116.3769355	0.128888214
0.884143165	-0.158898871	6.454462573	0.258089884	171.3682402	177.9380761	-0.93007073
0.727477295	-0.080063117	6.476186127	0.249425614	113.4899472	193.1046814	-0.332405344
0.705062576	-0.074253221	6.309078132	0.252852827	94.68807443	179.8768296	-0.634610664
0.835058067	-0.129774074	6.233949575	0.266741696	92.43370524	159.0840093	-0.409039743
0.665536211	-0.066956613	5.763906627	0.288497194	97.54104282	147.2714241	-0.122801368
0.811824559	-0.113175786	6.80861772	0.232770969	108.1074853	219.6006796	-0.515626098
0.846806695	-0.13260949	7.541024896	0.243603173	130.5094547	175.2927	-0.772100768
0.668963894	-0.06593786	5.729333012	0.279085049	91.76169143	157.1503422	-0.553231925
0.679806783	-0.064391588	7.430296599	0.226320411	145.1521955	223.5520771	-0.494312987
0.879201578	-0.174676468	5.267998274	0.296852143	57.77839696	119.4810804	-0.535989899
0.734920495	-0.092138488	4.864919882	0.323674888	64.53401327	116.3225805	-0.693564905
0.835263777	-0.130467778	6.374172122	0.257598976	83.48689158	163.5359837	-0.575315006
0.715656542	-0.072750385	7.525559347	0.22440277	161.6311883	241.7776264	-0.301653152
0.635866287	-0.061199385	5.177463317	0.29038545	70.1031738	145.2395758	-0.331543952
0.730337902	-0.088075388	5.014270038	0.291146273	71.17375837	143.9935248	-0.297895215
0.834718502	-0.139723362	5.012170763	0.301678093	68.33122979	123.6767587	0.60782147
0.843587516	-0.123795928	7.240748035	0.244389865	235.6306497	242.6746608	-0.516488401
0.875434238	-0.15496055	6.831824411	0.274385803	136.7448592	169.2802588	-0.678879399
0.828950316	-0.116334111	8.443744298	0.206157868	148.765338	241.6996934	-0.489643184
0.706789604	-0.076210522	5.883112854	0.263509706	83.77554793	164.8177691	-0.011463088
0.860552738	-0.142835213	6.523483537	0.238513719	116.917065	204.7405391	-0.714172251
0.800751163	-0.110545278	6.333467529	0.259332912	107.327601	178.9229865	-0.977675257
0.900316463	-0.182504749	6.299303192	0.26806736	99.24782567	150.5596062	-1.163536016
0.764189219	-0.104125439	4.966968694	0.295251168	58.44268906	142.0665854	-0.718473046
0.810062802	-0.109861038	7.054644121	0.23059184	134.799882	221.8474853	-0.310231282
0.910269032	-0.166975746	8.802785498	0.202757036	261.6815478	286.6754559	-0.373065931
0.861123217	-0.148451573	6.379881144	0.274246929	97.32696278	141.2903996	-0.568302071
0.705801744	-0.08874399	3.96785935	0.364974981	46.25132222	88.62134016	-0.504954635
0.568989353	-0.055768934	3.236393516	0.369164695	31.9040015	102.4212723	-0.147807051
0.759347717	-0.091523698	6.278630808	0.244974057	109.5678931	207.3215864	-0.289114763
0.756685294	-0.116015928	3.65021436	0.378535269	33.67825669	83.29944376	-0.766350542
0.781283955	-0.104092475	5.875470785	0.277265762	84.62881148	143.4760787	-0.389384021
0.760082413	-0.102986701	4.78356028	0.31784648	67.69963915	119.5206329	-0.027544836
0.762088634	-0.105080397	4.50377984	0.313310813	51.35699857	125.3716958	-0.585747634
0.844642282	-0.139936534	5.921299755	0.287406333	105.1078597	149.4714873	-0.424345402
0.880030513	-0.175363221	4.944577054	0.329584553	82.99727154	105.0277762	-0.181215623
0.784608405	-0.099562924	6.910026534	0.232365112	121.5301338	221.2276204	-0.449701835
0.701636661	-0.072376646	6.561739129	0.249417199	110.8206616	181.1105425	-0.495333526
0.705136787	-0.078102212	5.280873898	0.285994343	74.42214788	146.4469615	-0.443264626
0.663814042	-0.064957816	5.443462755	0.273849785	83.7423253	172.69334	-0.39274057
0.767044253	-0.09367573	6.724341928	0.251100845	118.6953371	181.7031756	-0.263839641
0.786379097	-0.106092892	6.467725003	0.261243618	104.9904527	176.1713291	-0.483700008
0.915039664	-0.194279328	6.970989578	0.257229457	115.8784201	163.3486602	-0.81784851
0.794406085	-0.129178979	3.886546685	0.345912841	34.98904313	101.9671888	-0.121428721
0.745389873	-0.087100919	6.139535811	0.269001717	123.0393731	182.8795828	-0.368971716

wavelet.LHL_firstorder_Uniformity	wavelet.LHL_firstorder_MeanAbsoluteDeviation	wavelet.LHL_firstorder_Energy	wavelet.LHL_firstorder_RobustMeanAbsoluteDeviation	wavelet.LHL_firstorder_Median
0.031427858	184.8140197	127600185.5	122.4536351	61.21079319
0.056247534	112.4597652	24372270.93	67.0747823	40.96814495
0.059005013	102.1546023	30038127.01	63.89820445	15.33578084
0.052556811	119.4718273	30608488.5	70.98834573	39.2215972
0.052439409	114.3637294	59582939.22	71.76601206	38.70007386
0.042548209	138.0097044	22216850.34	91.70752513	37.74999525
0.050845028	118.5590286	152044279.7	74.19687163	25.47161404
0.071274795	92.31412324	98948622.37	51.77170704	25.35830728
0.06667426	97.01645631	90780639.62	55.47328046	21.4817442
0.055989293	116.5045182	28712804.56	69.9027572	40.35593731
0.068031507	89.33666862	22283356.4	55.84622333	14.60315011
0.047818521	131.8012856	83036177.73	78.7475551	39.80236564
0.092575385	86.46327781	40584563.04	40.61697177	16.59212639
0.057070529	110.5522913	43259032.37	66.09411651	28.98871365
0.043749695	152.1993081	99744990.39	85.64614401	38.34768209
0.051278538	114.8459303	104682885.6	74.15819815	20.20451876
0.039808198	150.257268	53864606.8	97.60341587	61.82838234
0.076779481	87.39559424	22424549.39	49.00936052	28.98683029
0.07174247	82.72926146	15690966.28	53.90272061	22.48024361
0.046514967	135.0995248	195725396.7	80.04334456	35.35698929
0.0979035	66.49091797	29967033.25	37.63768145	18.56625438
0.056004728	108.0506278	23492444.78	69.19602589	32.38156277
0.037723541	159.7218395	48752532.49	101.7376825	59.49145811
0.058380452	100.9832489	50046245.77	65.4076845	33.88112238
0.051562792	130.4306106	49551256.27	71.72909482	29.76707901
0.052383565	115.442879	61537487.41	72.30296109	40.99728245
0.040804172	147.9037144	126561452	94.56418228	27.7223967
0.059219174	105.8602389	63900792.15	62.60751882	26.2015726
0.051273967	122.6508673	12812321.15	77.8657774	37.41935971
0.05187304	117.6610448	56320011.65	72.98977873	38.2480268
0.072674663	93.32102744	93664755.83	51.09252391	19.15104891
0.050714581	117.4498724	50098133.08	76.45205788	15.78726103
0.041785975	140.2099369	112532600.6	91.28017173	25.46028871
0.043070349	134.0007889	72452792.83	89.9526433	44.22167953
0.055974525	106.5374166	92396997.63	67.89190585	37.31735306
0.075127521	87.84128125	51232404.38	49.25785388	19.86457233
0.066795004	101.9833489	91897912.65	55.12120927	17.99330248
0.05241024	122.6283816	159135091.3	72.41508475	22.73000961
0.051014608	116.8527787	30245208.65	75.7137087	22.23412575
0.043330565	135.692698	241860120.2	87.96107482	21.79843276
0.066818095	95.46137158	46635887.18	56.46621929	20.60489935
0.037976649	155.7098698	163287272.2	101.8729272	25.52664406
0.056617794	113.3460413	95769868.06	66.37159105	28.96591326
0.040291793	148.0458111	96246124.56	96.30615077	28.58907361
0.050612141	121.0127183	35415405.27	77.58609291	41.98936804
0.05599278	107.145715	59404055.65	67.63054296	29.30762947
0.054694625	111.551295	115854994.2	69.0515863	35.20436667
0.057981311	124.332487	37392635.11	63.31979658	46.70203394
0.046250972	133.5346288	31786589.23	83.38421008	55.87621395
0.079302852	88.13665208	25209663.27	46.41808555	19.59644112
0.042636241	147.6905627	113562017.9	89.04777541	29.84475009
0.034637402	167.8586253	164215965	111.6110993	29.86987024
0.056090575	110.036164	106546091.6	66.91160724	18.34686851
0.069365679	87.61646792	17008833.62	54.76460465	33.30252243
0.073398157	87.02802994	20367637.04	50.85048903	16.24027531
0.049826167	131.9084336	75048781.16	75.18295195	25.8775775
0.046408375	127.7833514	121234122.7	82.38460834	38.59965624
0.0504688	119.3659772	90345752.71	76.13741387	37.71872222
0.05437011	114.6701371	43342056.76	68.32712669	27.37321479
0.059150797	108.6549731	219616781.4	64.80670976	14.42337304
0.043147332	134.9732671	55667764.72	90.82712331	38.61602369
0.049868283	132.227654	104124947.8	74.6752108	39.41257016
0.05481041	112.3123526	142969734	68.75476984	31.61562846
0.040326205	146.3840869	288900101	94.70181506	22.73987399
0.071209742	89.39136818	12576617.86	53.29556772	25.8897993
0.073494654	90.10429999	69551919.63	50.73254454	18.63369801
0.055615802	111.3551307	30697472.26	69.11677211	9.978390955
0.03781079	153.3469294	253829558.9	101.7050391	24.8691534
0.060080108	100.2776929	113995693.4	62.17900219	24.69272084
0.061369755	98.70600639	56233860.32	61.27679847	38.22427082
0.068025917	94.98421428	25294661.83	54.78556796	16.33325709
0.036550443	170.4898009	252873444.8	106.4646914	69.7755011
0.049471865	139.7053877	94553421.31	78.79568762	22.88204317
0.038157929	155.9112033	93954889.89	102.4150295	35.07995341
0.052244067	113.7288998	72449948.58	72.28566601	2.840360316
0.045040323	132.5388264	42309053.75	85.86566614	41.57773803
0.049991499	124.6481772	71972112.7	77.21740884	42.93132691
0.057005941	113.3051225	26290576.69	66.60235095	35.09702696
0.064681801	94.56012848	28857031.45	59.65009588	34.26618703
0.042837943	138.4528308	78693969.96	90.58311962	27.06496769
0.031405258	185.9712361	120321781.2	122.6416544	56.88606419
0.057618633	115.4545214	51198635.86	64.62128794	8.681832408
0.095325715	72.42775185	40213582.72	38.67393284	7.921710725
0.087696299	66.44696569	24434973.46	43.20971032	20.22381594
0.045206188	127.053477	65657594.25	85.80530974	20.21270263
0.100565653	66.03570763	14895214.01	36.95215927	16.2594322
0.059891267	105.4556001	53845391.28	61.94898004	7.0994302
0.070143448	94.58089808	53999200.73	52.72561347	20.1761662
0.068897623	88.45326262	22811727.95	54.56736585	29.00979246
0.057320287	115.3422021	61482687.39	65.10831359	38.40671344
0.076585965	96.27404852	31472167.12	48.67277348	27.67980944
0.042973082	135.1265223	74273730.51	91.13386971	24.62597614
0.048767641	123.9059958	152334722.5	76.96967045	35.26442549
0.059983655	101.2097725	64437771.02	63.03985035	27.48193169
0.052745568	110.4999758	93324252	72.17647479	24.70394983
0.04779163	128.8192124	109905365.8	79.63444411	31.97281857
0.048652452	125.9415031	86720786.65	77.19723307	37.67488293
0.054271585	125.4342624	34377375.85	71.36781038	15.39964087
0.085189402	73.24755345	8883676.804	44.72602853	18.29914732
0.048258506	126.929022	184320164	77.31079385	30.58712425

wavelet.LHL_firstorder_TotalEnergy	wavelet.LHL_firstorder_Maximum	wavelet.LHL_firstorder_RootMeanSquared	wavelet.LHL_firstorder_90Percentile	wavelet.LHL_firstorder_Minimum	wavelet.LHL_firstorder_Entropy
3445205007	823.6020414	245.2183484	344.8666315	-901.9407629	5.270861915
658051315.2	837.6442558	161.4515405	195.5130384	-891.7542389	4.544101185
811029429.2	632.9561656	139.9341599	185.4004389	-539.5641031	4.452562096
826429189.6	740.8387774	177.0001605	230.9750148	-1182.129083	4.631200162
1608739359	587.8902785	158.6244439	220.4006011	-575.228458	4.611664618
599854959.1	536.7095295	183.4717811	271.7523007	-572.3334873	4.833013046
4105195552	762.0039015	164.0296617	222.9905634	-881.3769631	4.67343408
2671612804	613.4058398	139.1808331	166.863066	-933.0721874	4.309104337
2451077270	576.1579018	141.3129307	176.0058235	-775.6171583	4.382821229
775245723.2	593.6204688	163.6591966	194.3489949	-787.3029408	4.582903562
601650622.7	509.1508378	121.5597036	150.1411057	-525.0443448	4.239214491
2241976799	698.1923756	189.8008749	253.52909	-956.637867	4.798607616
1095783202	705.1203298	147.4376086	162.8273414	-948.4349105	4.114371086
1167993874	638.3909152	158.7278935	225.9893303	-752.3769253	4.561512859
2693114741	779.513997	224.2201881	277.0832727	-1107.201953	5.001667163
2826437911	669.3321559	154.6675428	204.7687086	-1043.959254	4.614378155
1454344384	701.5774492	212.3975895	324.1736817	-939.462681	4.954951614
605462833.6	420.2522439	134.8591208	172.7943539	-703.7792025	4.162855052
423656089.7	332.6309084	109.9480417	134.5851774	-418.3896332	4.108492294
5284585712	890.6237378	194.0276762	241.8956487	-1214.506644	4.857715599
809109897.8	604.4060289	99.54764295	125.9961053	-554.9050444	3.843007702
634296009.1	558.5912692	147.5548147	183.9140781	-630.0274959	4.488071955
1316318377	830.6741908	218.196918	309.2497233	-572.5510986	5.047108787
1351248636	483.1279628	137.501926	187.1233356	-564.8207323	4.417472717
1337883919	722.7625151	197.2936616	228.7885035	-990.4630163	4.753251855
1661512160	561.3977577	161.1711667	223.7677038	-724.2597643	4.615801699
3417159205	823.5687929	197.8553837	228.882858	-943.1684302	4.957665172
1725321388	628.4018359	152.1042272	186.278849	-939.655509	4.501657548
345932671	582.9418545	169.3012429	221.7552617	-938.5396959	4.602799576
1520640314	678.8747357	165.5888722	209.4521433	-869.1795439	4.644766949
2528948407	601.0982557	140.3058838	164.0877941	-660.2277095	4.312500531
1352649593	499.9043777	155.6075001	184.2224262	-823.2786007	4.618708447
3038380216	735.9968948	186.683165	249.1996705	-1002.25549	4.892450658
1956225407	602.4457012	175.5875157	246.9964484	-662.2169256	4.801412725
2494718936	514.9061433	144.8455891	194.609861	-726.6927531	4.498303261
1383274918	655.5996805	130.7025727	150.6583066	-688.894938	4.224244707
2481243642	927.5043946	157.1531131	160.4734675	-950.9006459	4.42292001
4296647466	679.8151768	175.5965785	231.1764376	-966.4589171	4.715147523
816620633.5	500.0113071	156.8107156	191.0882809	-786.1097183	4.601263503
6530223245	1087.4786	182.3958391	232.4967297	-1191.350156	4.853353147
1259168954	472.9200518	134.0834376	161.4202567	-631.4453946	4.345804255
4408756348	1231.449094	205.4892376	248.6677027	-808.6686087	5.031745667
2585786438	591.2987414	161.9159411	187.8792879	-918.3027324	4.582698414
2598645363	610.4328912	196.682825	235.2742078	-911.5175737	4.945380134
956215942.3	707.101912	166.467969	211.5384002	-594.9371334	4.639154582
1603909503	629.9161741	147.1081974	187.8275758	-746.4655592	4.514536148
3128084844	733.7532258	159.3601535	220.017945	-1181.258205	4.576071833
1009601148	841.3782391	207.1972363	231.2524711	-1284.869724	4.6273038
858237909.3	762.2972227	193.2667684	239.7125544	-1264.962638	4.763411748
680660908.2	696.2725103	136.5510917	165.4383713	-398.3069636	4.171125737
3066174484	1127.523121	210.2492709	231.9254865	-1766.377088	4.960630355
4433831054	992.3075752	220.7132024	293.8538523	-822.2799438	5.14323412
2876744472	755.9877986	153.668275	184.7208831	-690.6532167	4.559016935
459238507.8	351.489203	124.1231332	169.7639072	-833.9049656	4.198215982
549926200.2	468.8048325	123.7497993	174.9378009	-418.1378506	4.200029399
2026317091	844.6026251	196.1799033	203.834509	-1197.177067	4.784087597
3273321312	766.9956409	173.1223641	228.8742235	-801.3928325	4.759481019
2439335323	617.4190488	161.6372213	205.5286063	-776.8059007	4.649908572
1170235532	577.1652547	162.1721053	205.8384563	-694.6743986	4.607145706
5929653098	857.9669024	155.1033427	207.4029208	-818.74937	4.541897928
1503029647	577.8855367	177.5442215	236.6726126	-815.487507	4.801014855
2811373592	838.6762999	197.4793858	229.7557163	-1112.885578	4.805597025
3860182819	544.6749346	157.1521329	211.1197877	-698.4674341	4.586444533
7800302727	719.1138861	194.6753154	237.1101502	-1000.559673	4.954101936
339568682.3	521.2680942	126.1735687	145.5874545	-543.6650019	4.215879844
1877901830	687.6409104	134.1987362	160.3908737	-762.1407897	4.267352915
828831751	616.8809905	153.4307868	167.4319211	-659.0468824	4.541085898
6853398090	788.5503048	201.5260019	260.2954978	-955.4361564	5.019652493
3077883723	704.8233089	139.5104085	181.8606391	-640.2556232	4.436063102
1518314229	615.5872687	139.2995644	184.5625838	-579.5803726	4.399945801
682955869.4	781.495942	139.5435808	168.0973323	-516.6971856	4.327536084
6827583011	781.328203	242.7856234	359.4013968	-912.2405631	5.155404946
2552942375	748.7963652	203.6884772	245.2829066	-1391.457941	4.862305744
2536782027	688.073968	203.9396072	252.2759947	-801.8605571	5.014120031
1956148612	641.4248044	152.1169142	191.132536	-633.0987309	4.604239353
1142344451	554.1402479	177.5580629	229.4226745	-807.7626315	4.771306827
1943247043	621.100157	173.3160245	208.6238186	-860.4811725	4.690011951
709845570.6	503.6156054	165.6598645	204.6988748	-914.0183289	4.547710515
779139849.1	473.5707704	130.0196719	167.2870749	-620.0976979	4.29931621
2124737189	717.7452329	183.4237705	223.9129117	-858.5878478	4.865062807
3248688092	823.6020414	245.9544783	327.2277826	-873.1195801	5.28114066
1382363168	727.6920879	168.6994292	163.3789762	-816.8377485	4.596796714
1085766733	506.7187472	110.9120987	126.8201694	-793.9869914	3.949725481
659744283.5	395.3878068	89.17120916	120.5799273	-326.2358173	3.825609226
1772755045	587.8300516	164.0727974	214.0964007	-675.5169042	4.732493713
402170778.3	360.3656888	98.60387695	124.6496078	-607.3796369	3.812266756
1453825565	708.6855861	150.2553958	173.4688312	-788.9150137	4.500287614
1457978420	669.2697716	140.3330424	178.7340742	-635.3705667	4.336345658
615916654.5	378.0135791	124.8270058	178.9894743	-637.9748551	4.234878698
1660032559	757.38785	174.2464374	228.8538323	-964.6210008	4.60970038
849748512.3	736.8833666	155.3547472	182.0482056	-810.7045122	4.299013118
2005390724	741.6198222	176.5821256	234.3279631	-915.6035859	4.806443202
4113037508	737.9777953	171.4386935	218.2464253	-747.2776145	4.728420193
1739819817	674.2627963	139.7376015	171.075785	-741.4624874	4.431336571
2519754804	505.3892893	146.4376718	189.5460459	-682.8629387	4.550007858
2967444877	812.9147163	179.2129734	257.5731626	-848.9402381	4.781706698
2341461240	693.5768952	175.4559825	225.3874867	-778.2856037	4.746377572
928189148	634.758918	181.202014	177.5639065	-903.9881772	4.672902195
239859273.7	480.9025456	103.207997	128.1368808	-376.9629093	3.96133932
4976644427	776.8683729	178.6685177	226.2601001	-1027.420158	4.776328187

wavelet.LHL_firstorder_StandardDeviation	wavelet.LHL_firstorder_Range	wavelet.LHL_firstorder_Variance	wavelet.LHL_firstorder_10Percentile	wavelet.LHL_firstorder_Kurtosis	wavelet.LHL_firstorder_Mean
240.085867	1725.542804	57641.22352	-258.6193021	3.588229716	49.9080642
159.0540608	1729.398495	25298.19425	-165.9678594	6.792074528	27.72013126
138.9873805	1172.520269	19317.49194	-143.516165	4.446560153	16.25045122
173.2981521	1922.96786	30032.24951	-137.5323468	9.06789663	36.01121071
154.4023662	1163.118737	23840.09068	-146.8592803	4.215648339	36.35414031
179.642103	1109.043017	32271.28518	-191.709073	3.279959147	37.29087385
161.3538446	1643.380865	26035.06318	-164.9519906	4.812254565	29.50706236
137.2773037	1546.478027	18845.05812	-117.1263941	7.728229124	22.94005636
140.0130373	1351.77506	19603.65063	-133.7830829	6.014911179	19.1231213
162.1612334	1380.92341	26296.26562	-183.1213417	5.391815285	22.09223836
121.2995378	1034.195183	14713.57788	-133.8163464	4.676850157	7.948815013
186.1994042	1654.830243	34670.21812	-174.3660468	6.14772038	36.79883154
146.6103717	1653.55524	21494.60108	-93.32080977	11.70799365	15.59638927
155.0727876	1390.767841	24047.56945	-141.7799993	5.266910275	33.8670155
222.1207365	1886.71595	49337.6216	-219.6716084	6.198459927	30.61161777
153.5028512	1713.29141	23563.12532	-171.5544542	4.863162498	18.94527619
201.2697087	1641.04013	40509.49563	-162.5072111	5.288213274	67.84718402
132.7322436	1124.031446	17617.84849	-98.12962997	8.696056741	23.85652917
109.2308078	751.0205416	11931.36938	-125.0237954	4.190879948	12.53804159
191.9883783	2105.130382	36859.5374	-190.9505826	5.986322205	28.05711553
97.62902858	1159.311073	9531.427221	-83.54032536	6.825450822	19.4500899
145.8279929	1188.618765	21265.80351	-146.3429085	5.221779988	22.50821699
211.4774913	1403.225289	44722.72933	-209.2617575	3.64902001	53.73235224
134.0253078	1047.948695	17962.78314	-133.9972385	4.30103636	30.7245264
195.9603202	1713.225531	38400.4471	-168.7211328	7.293394032	22.89851087
156.7001198	1285.657522	24554.92756	-146.5771809	4.661951178	37.69903715
197.6610611	1766.737223	39069.89507	-245.3189812	4.537017821	8.766858153
150.5516509	1568.057345	22665.79959	-142.2739982	6.377147737	21.67709237
165.8877393	1521.48155	27518.74204	-158.6689104	5.97912376	33.82556439
162.7928601	1548.05428	26501.5153	-164.1611387	5.766919143	30.30114371
139.3935654	1261.325965	19430.56606	-129.8504964	6.57279494	15.97419693
155.5699017	1323.182978	24201.99433	-202.6147589	4.318666483	3.420492125
185.425927	1738.252384	34382.77441	-214.745031	4.288753426	21.62937064
172.6982744	1264.662627	29824.69399	-187.0089357	3.438982759	31.72194319
142.1883642	1241.598896	20217.53091	-150.9152816	4.266584715	27.61727329
129.8627312	1344.494619	16864.32895	-126.2343947	6.970769418	14.79302425
156.8472284	1878.40504	24601.05305	-141.4125647	9.086417031	9.800403375
173.7002795	1646.274094	30171.78708	-168.1542211	5.444121079	25.73657484
156.4717128	1286.121025	24483.3969	-178.6240838	5.22906597	10.30551456
181.5006699	2278.828755	32942.49318	-202.839022	5.324936003	18.04851626
133.6517467	1104.365446	17862.78941	-153.0026145	4.983318025	10.75075902
204.9084476	2040.117702	41987.4719	-246.6602283	4.272081654	15.43874622
161.2620845	1509.601474	26005.45988	-172.2553856	6.234045159	14.53657802
196.1975204	1521.950465	38493.46701	-225.2962237	4.381781349	13.80820966
162.7316997	1302.039045	26481.6061	-162.9208673	4.820335465	35.07105105
144.8647345	1376.381733	20985.7913	-147.284955	4.811604436	25.59356237
155.243603	1915.01143	24100.57626	-140.1591594	6.742405228	35.98725144
202.9310921	2126.247963	41181.02813	-114.094101	11.68022625	41.82901616
188.4702817	2027.259861	35521.0471	-163.569519	8.921192852	42.79014671
133.0883535	1094.579474	17712.50983	-92.78891226	6.959740867	30.55635463
209.8260715	2893.900209	44026.9803	-231.3490432	8.554933715	13.33325232
218.7692928	1814.587519	47860.00348	-242.7722289	3.692954894	29.22865403
153.1760215	1446.641015	23462.89357	-160.3805154	5.420443829	12.29004348
120.7792796	1185.394169	14587.63439	-115.3736037	7.042811029	28.61673997
121.6752254	886.9426831	14804.86047	-108.6502484	4.636516453	22.56440434
195.6704595	2041.779692	38286.92871	-187.4368852	8.113868736	14.12889709
170.2089013	1568.388473	28971.07009	-174.3318301	4.499655872	31.62724878
159.8699573	1394.22495	25558.40325	-175.2510034	4.582474467	23.83669528
160.1786672	1271.839653	25657.20543	-158.6943337	5.009715447	25.34928586
153.2316392	1676.716272	23479.93525	-143.7051975	5.464680692	24.0231489
175.2239957	1393.373044	30703.44866	-185.5208447	4.204482105	28.60947255
195.2631281	1951.561878	38127.68918	-175.4338831	7.40952646	29.50285801
154.8512848	1243.142369	23978.9204	-160.0916416	4.761965367	26.7931425
194.3962616	1719.67356	37789.90651	-232.605711	4.462594708	10.41978468
125.2610282	1064.933096	15690.32519	-139.2854823	5.350638376	15.14741749
133.1890333	1449.7817	17739.3186	-118.9679101	6.716142755	16.4311345
153.4231061	1275.927873	23538.64948	-183.4602703	5.350345192	1.535203622
200.6792597	1743.986461	40272.16529	-231.6980119	4.120455398	18.45437994
137.8220904	1345.078932	18994.92861	-139.0199783	5.092515964	21.63851816
135.3108989	1195.167641	18309.03937	-122.7858726	5.092328491	33.09575909
137.8710487	1298.193128	19008.42607	-126.1455572	6.90133967	21.54030848
231.6700831	1693.568766	53671.02739	-203.8458872	4.180951604	72.62115081
203.036813	2140.254307	41223.94742	-247.1142533	5.99714949	16.28030513
203.1882845	1489.934525	41285.47894	-247.65697	3.756720885	17.48955249
152.1022602	1274.523535	23135.09756	-184.9663295	3.98423091	2.1114062
175.5576452	1361.902879	30820.4868	-192.2200288	4.496418851	26.57778933
171.6835065	1481.581329	29475.22639	-171.1858349	5.808322919	23.73221392
163.4316828	1417.633934	26709.91493	-153.8668833	7.281698612	27.07906511
128.1167286	1093.668468	16413.89614	-122.6412343	5.197245551	22.16345974
182.8394353	1576.333081	33430.25909	-208.9795356	4.329049323	14.62943939
242.3833523	1696.721621	58749.68945	-271.0486362	3.664092893	41.76021985
168.6840869	1544.529836	28454.32118	-196.3664427	6.009874515	-2.275135901
110.6110296	1300.705739	12234.79987	-98.91165456	7.871323742	8.166625545
87.41332683	721.6236241	7641.089708	-91.37524541	3.822065401	17.61859347
163.4103552	1263.346956	26702.94419	-184.8855745	3.582035784	14.72883744
97.56144058	967.7453256	9518.234689	-85.40106514	7.299531738	14.29999515
150.094832	1497.6006	22528.45861	-157.0281536	5.779892107	6.944448734
138.2528364	1304.640338	19113.84678	-120.6633811	5.898962631	24.07313837
121.8896525	1015.988434	14857.08738	-108.606287	5.371997706	26.92014114
169.7855824	1722.008851	28827.14399	-140.3370837	6.77113817	39.17495305
152.4284104	1547.587879	23234.4203	-107.9261373	8.381702657	30.01128432
175.3891593	1657.223408	30761.35721	-200.6454773	4.11431692	20.49121407
169.3710195	1485.25541	28686.54224	-174.049942	4.864159325	26.54587356
138.3861738	1415.725284	19150.7331	-141.537091	5.212920616	19.38721699
145.3849461	1188.252228	21136.78257	-158.1714426	4.091074191	17.52738319
175.7877503	1661.854954	30901.33315	-178.490894	4.35610542	34.87057049
173.0269404	1471.862499	29938.3221	-184.8805392	4.838743805	29.09432402
181.1568732	1538.747095	32817.8127	-214.7762413	6.215286699	-4.044400115
102.0756538	857.8654549	10419.4391	-109.9354437	5.285367355	15.24636203
176.9281579	1804.288531	31303.57306	-180.8800715	5.684463548	24.87702042

wavelet.LHL_glrlm_ShortRunLowGrayLevelEmphasis	wavelet.LHL_glrlm_GrayLevelVariance	wavelet.LHL_glrlm_LowGrayLevelRunEmphasis	wavelet.LHL_glrlm_GrayLevelNonUniformityNormalized	wavelet.LHL_glrlm_RunVariance
0.001439776	93.54014401	0.001459705	0.031145559	0.03601029
0.001925847	41.56602885	0.001954256	0.055033091	0.059568469
0.003318642	32.23521323	0.003414082	0.057131211	0.072653714
0.001508863	49.5944038	0.001524532	0.051285243	0.059683889
0.002460819	39.32016833	0.002525816	0.051241515	0.059292785
0.005696468	52.73525782	0.005741846	0.041927093	0.035616298
0.00103509	43.02481994	0.001063268	0.049456377	0.061587189
0.000902919	31.87367981	0.000935917	0.067765686	0.082960874
0.001308659	32.8762689	0.0013542	0.063986135	0.076838271
0.002058827	43.46417495	0.002090969	0.054517606	0.048881346
0.003169007	24.34749118	0.003265651	0.066324832	0.06392591
0.001633077	57.40314682	0.001685022	0.046403846	0.052876105
0.001528949	37.71566624	0.001581046	0.083745395	0.130256362
0.001735705	39.85943265	0.001778263	0.055085784	0.066780037
0.001195306	81.67730961	0.001211825	0.042436799	0.049410044
0.000800506	39.01032762	0.000822349	0.050015295	0.061194899
0.002444427	66.10847296	0.002512724	0.039241634	0.041999536
0.002722497	29.99994383	0.002780692	0.07339835	0.087272878
0.006514073	19.89640092	0.006682788	0.070219941	0.072265733
0.000623967	60.79772839	0.000637069	0.045406022	0.050343465
0.002535579	16.63684	0.002668883	0.091287286	0.138809751
0.003187806	35.28449707	0.003233768	0.054910428	0.052041277
0.005131432	72.82874721	0.005219577	0.037283219	0.040622741
0.002988875	29.613435	0.003051275	0.057147485	0.057469999
0.001624563	63.75071827	0.001646271	0.050025112	0.051644616
0.00209632	40.47980016	0.002163697	0.051165827	0.057825934
0.001136845	64.10361196	0.001158938	0.039966876	0.04808822
0.001089941	37.96673802	0.001118345	0.057416029	0.066125143
0.002996393	44.7203824	0.003016242	0.050673165	0.040971623
0.001864413	43.81948432	0.001892519	0.050616892	0.058616945
0.002241465	33.07792543	0.002320413	0.068577999	0.100772166
0.001522926	39.79921016	0.001557565	0.049784271	0.058531343
0.000944775	56.38588263	0.000964082	0.041095987	0.050339875
0.002149665	48.79293581	0.002190778	0.042555645	0.045131533
0.001370513	33.28119307	0.001409245	0.054699436	0.057532306
0.001765787	28.53579658	0.001831341	0.071807906	0.085035623
0.001024579	41.28809132	0.001054069	0.064208825	0.07424188
0.000915582	50.74908349	0.000947119	0.049757949	0.089658127
0.002055474	40.07018247	0.002085514	0.050227612	0.046297537
0.000604076	54.08881842	0.000618322	0.042561244	0.049729015
0.00218927	30.00269311	0.0022595	0.064018384	0.080632948
0.001542912	68.38262294	0.001566435	0.03751969	0.035170762
0.001131239	43.22392277	0.001161014	0.054871815	0.064500947
0.001285243	63.00284539	0.001305354	0.039758936	0.03938011
0.005297445	43.53382318	0.005407346	0.049799504	0.045204271
0.001537358	34.70414341	0.001579257	0.054690857	0.061185794
0.000644435	40.15433775	0.00066185	0.053016526	0.068295936
0.001614966	69.05505536	0.001629448	0.056289599	0.064543275
0.001607898	58.45057516	0.001619119	0.045397972	0.046106432
0.00644234	29.91190377	0.006635403	0.075595741	0.081622824
0.000604754	72.4047566	0.000610853	0.041759552	0.047463811
0.001488722	77.58918627	0.00151346	0.034322651	0.035566254
0.002172917	38.73506968	0.002222663	0.05478045	0.054900515
0.0017785	24.36360562	0.001815626	0.067488783	0.070142488
0.006298043	24.58831862	0.006452292	0.070877612	0.071702211
0.001140236	63.45997688	0.001155492	0.048499782	0.053414208
0.001243914	47.59235262	0.001272962	0.045625086	0.04956697
0.001461136	41.92622906	0.001494432	0.049483183	0.052735841
0.002208226	42.6385759	0.002254708	0.052769865	0.056849359
0.001279433	39.1868344	0.001327914	0.056518883	0.078159772
0.00162052	50.15465917	0.00164542	0.042702648	0.039730003
0.000983054	63.26953274	0.000998818	0.048373542	0.051824921
0.001933427	39.7851116	0.001980963	0.053164927	0.061002078
0.000857102	61.61118744	0.000874429	0.039789425	0.04243098
0.003880153	26.18624726	0.003973471	0.069050544	0.068856404
0.001371699	30.10431737	0.00142789	0.069503782	0.096093626
0.003172311	38.80575985	0.003232331	0.054033587	0.060445213
0.001005174	65.60117064	0.001024474	0.037362643	0.041766231
0.002241687	31.47387473	0.002295318	0.058603079	0.061045663
0.002320839	30.3532648	0.002390201	0.060058575	0.064987057
0.004245556	31.62517748	0.004348718	0.065851182	0.070955059
0.001152732	87.91046319	0.001176771	0.035714383	0.05237665
0.000787327	69.08278157	0.000800707	0.046786321	0.07188409
0.001632357	67.10647495	0.001655161	0.037711319	0.035204689
0.002111483	38.22646294	0.002170897	0.050942139	0.057463313
0.002005393	50.39279672	0.002030984	0.044465287	0.043801158
0.001445532	48.46140074	0.001471815	0.048959076	0.052085982
0.002024445	44.09641718	0.002050834	0.055367397	0.056868822
0.002963144	27.12761381	0.003026764	0.063210628	0.066018892
0.00137929	54.86360582	0.001402839	0.042164138	0.041121386
0.002622535	95.4400427	0.002672643	0.031112076	0.035464847
0.001863857	47.44253809	0.001901438	0.055575421	0.064852133
0.001284822	21.09204268	0.001348131	0.088605231	0.114609431
0.005676523	12.89821124	0.005967209	0.085006272	0.102457054
0.001938565	43.84265897	0.001978139	0.044640158	0.044076137
0.002291551	16.44635617	0.00239901	0.093317738	0.132653131
0.001556382	37.47546548	0.00159709	0.057841454	0.067350708
0.002089684	32.32474783	0.002163358	0.066655018	0.085893862
0.002230421	24.74806958	0.002293489	0.066581835	0.074309631
0.001298943	48.34984358	0.001326801	0.054956228	0.071110265
0.002621761	39.52905949	0.002671382	0.071914096	0.094517842
0.001217241	50.14106153	0.001237346	0.042561702	0.041260459
0.001830561	47.16234544	0.001868045	0.047815991	0.050059823
0.001472005	31.74300183	0.001516103	0.058529316	0.062734555
0.001651286	34.80691118	0.001699806	0.051864117	0.05750366
0.001221387	50.86166845	0.001249363	0.046324639	0.053835255
0.001586994	49.55115403	0.001622613	0.047372441	0.060411815
0.001990122	54.3375866	0.002020345	0.052447441	0.064865602
0.006441796	17.68032115	0.006674817	0.080425678	0.101339326
0.000780855	51.86187526	0.000802949	0.047026239	0.059927596

wavelet.LHL_glrlm_GrayLevelNonUniformity	wavelet.LHL_glrlm_LongRunEmphasis	wavelet.LHL_glrlm_ShortRunHighGrayLevelEmphasis	wavelet.LHL_glrlm_RunLengthNonUniformity	wavelet.LHL_glrlm_ShortRunEmphasis
63.94636	1.104435442	1609.065814	1926.617657	0.975837867
48.76090103	1.174072437	1395.923018	799.0772949	0.960530559
82.17967053	1.211481524	540.5066098	1271.087128	0.952626484
47.5188254	1.173338485	2439.757261	837.1357329	0.961048175
115.0341222	1.172909432	684.7295777	2026.004842	0.960788949
26.76146948	1.105470343	659.9288335	597.8458632	0.975125877
265.0370081	1.174109469	1408.574246	4847.473577	0.961734018
322.8147911	1.234222224	1505.574143	4179.196275	0.949808297
272.2121804	1.220235267	1082.775896	3750.579081	0.951717511
55.86132784	1.144587696	1116.23704	938.1902962	0.966369075
94.25391895	1.190707649	520.6012494	1265.312275	0.955777941
101.9404	1.154813338	1675.452094	2002.268772	0.964582147
141.1394854	1.363090803	1455.085343	1395.076477	0.926842441
89.15876416	1.193518302	1071.589771	1444.057337	0.956444999
80.56349993	1.143181345	2189.749069	1744.428909	0.967528159
207.238483	1.177364071	1834.228015	3731.644227	0.960079982
45.07485862	1.123104182	1716.721514	1065.404678	0.971378098
84.00809159	1.250777155	903.3918096	994.9864211	0.945825579
85.55301415	1.208348119	327.1095214	1079.77555	0.953758651
225.6131048	1.145991106	2534.570823	4554.700109	0.96675154
247.6735186	1.383969619	559.4795313	2221.302095	0.922938254
56.52214805	1.151673083	756.5424061	940.4081251	0.965417193
36.73805585	1.120979159	707.130041	913.8749421	0.971374199
143.8173214	1.164733504	616.2812706	2284.583187	0.963090778
60.67884144	1.153888095	1713.40842	1103.342389	0.96391439
115.1475037	1.166696519	964.1133164	2040.516818	0.962606245
123.6691131	1.140382903	1519.27175	2843.491394	0.967782496
149.5619623	1.191538795	1519.46037	2328.945211	0.957168351
21.76249241	1.124830073	1583.273947	396.6108071	0.969804365
98.75872868	1.167720586	1336.095446	1767.560516	0.962353969
301.0302549	1.27846496	777.1716543	3782.103309	0.942570238
97.73320338	1.16999508	1122.572878	1774.456197	0.961489926
126.8440862	1.145581532	1790.218015	2831.21551	0.967023228
95.9329041	1.132356918	847.8010055	2079.59334	0.969322664
228.7785945	1.167636628	990.3792284	3787.266079	0.962007184
200.043624	1.245957234	826.9373662	2420.683242	0.946056213
224.1088696	1.212516643	1555.789842	3092.659063	0.953530438
239.3545107	1.242898128	1610.49011	4224.155884	0.950104698
59.22387951	1.134801334	1086.523209	1086.263178	0.968897901
295.6220437	1.145796198	2392.535252	6357.121105	0.966331841
155.0793051	1.22918491	715.7561101	2128.767327	0.950406618
140.3183473	1.104732032	1200.517088	3502.581791	0.975076109
189.3561184	1.186826433	1427.219817	3094.965167	0.958192758
95.31569343	1.117041744	1466.325729	2229.555054	0.97238922
60.96591397	1.135727438	690.5002596	1124.922191	0.967835756
142.1434145	1.177322912	986.1534575	2340.053695	0.96002036
227.9906497	1.194644671	2426.359008	3844.815525	0.957191945
46.28673875	1.189158815	2871.539256	736.2610873	0.957305792
37.01095861	1.136919153	2790.951532	750.1536752	0.968059668
95.21177529	1.236707158	329.3416049	1100.240177	0.947862914
102.7775751	1.137618078	5093.62866	2266.451746	0.968600707
111.8481835	1.105936898	1246.066108	3049.133159	0.974781742
235.2384114	1.159829819	845.9692197	3904.488889	0.963643746
69.89266652	1.207442431	1231.453484	915.3525567	0.952837569
88.46143763	1.208565639	346.7534423	1105.179968	0.953496933
90.04220291	1.157227358	2377.251013	1687.829019	0.963756946
176.3329599	1.145422259	1212.853382	3537.421296	0.96636615
163.0109083	1.155293022	1116.195118	2997.92175	0.964096372
82.596782	1.16645588	879.2784877	1416.684279	0.961911092
483.6362072	1.217380233	1173.058672	7582.427119	0.953733667
72.61738923	1.118699893	1213.438633	1579.083816	0.97182165
123.3178427	1.149633658	2164.69646	2333.073703	0.966138968
291.43474	1.176861469	876.5610727	4937.862896	0.960159485
291.5813387	1.124880648	1761.926913	6786.495368	0.970840483
51.38475042	1.199244178	535.3569238	664.9602345	0.956220422
247.9628615	1.26956294	1002.770604	3076.35418	0.943145619
66.79616418	1.174389497	764.9854189	1116.474765	0.961000959
224.5350786	1.123793649	1631.949588	5566.347422	0.970891469
325.331488	1.174876386	748.126061	5014.066999	0.961111824
164.1316374	1.190599434	666.6261597	2441.262377	0.956826929
80.286822	1.207820432	509.4919615	1079.442868	0.953347624
146.1064164	1.152724782	1659.226652	3732.167757	0.965053661
100.3906339	1.201031853	3190.094041	1916.461748	0.956743656
82.40625929	1.104313287	1203.346829	2047.444691	0.975288311
151.4672493	1.16691461	717.3403199	2690.702537	0.961994597
57.35757219	1.126835308	1205.688663	1195.520181	0.9709907
111.8888188	1.152345367	1324.509205	2085.708556	0.965054426
50.36922693	1.167321263	1472.779955	823.160552	0.961732158
101.7544869	1.192011749	689.7435927	1437.846801	0.956752133
94.83128983	1.122895894	1316.327641	2081.861075	0.97076598
59.89317832	1.103201582	1438.094985	1807.156615	0.976008308
94.52316369	1.184995657	1114.646658	1528.783108	0.95912303
263.9647199	1.32092343	1027.307258	2505.180348	0.93343199
239.6202008	1.292344146	228.3482064	2395.622547	0.937611161
104.4519383	1.130944852	862.1421336	2157.321635	0.969196452
129.1645806	1.363054606	646.0650563	1150.830054	0.928168134
130.0978287	1.192866795	1063.209867	2012.31154	0.957450706
170.0461256	1.242373492	749.0125543	2228.897478	0.948195656
91.43061961	1.212314255	748.1649397	1215.480744	0.95334683
104.5791625	1.205241794	1658.392996	1690.027928	0.95447024
86.58160865	1.269967305	1176.198183	1035.505948	0.942088269
97.55450939	1.121582536	1470.662089	2126.488165	0.971540476
236.6143582	1.14753882	1005.967432	4522.178941	0.965780847
182.5168146	1.18322293	966.8315522	2795.434938	0.958371064
214.2443033	1.167846213	852.2393375	3735.383981	0.961727245
151.1481176	1.154491733	1291.833647	2978.869456	0.965276085
126.6252386	1.173017476	1135.886183	2419.916301	0.961682244
52.01792146	1.181994198	1389.603854	895.4921044	0.96070056
61.68696231	1.286805931	291.7977871	655.4731416	0.939499967
257.1923939	1.175315949	1867.03738	4926.145919	0.960119858

wavelet.LHL_glrlm_LongRunHighGrayLevelEmphasis	wavelet.LHL_glrlm_RunPercentage	wavelet.LHL_glrlm_LongRunLowGrayLevelEmphasis	wavelet.LHL_glrlm_RunEntropy	wavelet.LHL_glrlm_HighGrayLevelRunEmphasis
1830.345959	0.967556007	0.001544201	5.468124149	1650.585983
1712.171721	0.947593583	0.002078663	4.842608361	1454.204299
686.2202864	0.937619095	0.003836464	4.808418965	567.1928074
2981.784102	0.94827179	0.001594498	4.927776664	2539.649823
834.0657823	0.947992464	0.002810219	4.919738668	712.3472895
746.9395885	0.967016317	0.005933411	5.017928821	676.5770739
1712.34485	0.948300505	0.001191351	4.989790089	1463.40657
1953.240183	0.932504066	0.001089461	4.713400521	1584.650423
1387.813884	0.935716945	0.001560862	4.76666522	1137.688933
1326.504902	0.955726177	0.002227843	4.847609761	1155.827161
648.6294411	0.942358702	0.003677685	4.574776835	544.6664579
1999.308844	0.952978475	0.001900895	5.085190227	1735.891843
2127.356681	0.902064192	0.001835787	4.670395968	1568.045479
1330.780314	0.942565297	0.001967108	4.897241695	1119.349271
2583.621751	0.956730769	0.001283908	5.267663296	2262.520434
2246.384526	0.946825341	0.000919376	4.935832328	1910.078917
1975.428581	0.961989434	0.002791156	5.178606202	1765.553272
1196.046083	0.928130264	0.003049967	4.571463056	955.4320226
416.1437923	0.938663032	0.007431492	4.455664609	343.2981703
3005.79244	0.95570154	0.000694565	5.137360925	2622.012695
834.7795638	0.897028897	0.003333165	4.409992765	605.67515
906.5354456	0.954017252	0.003434609	4.758324241	784.3818515
818.6742494	0.962289663	0.005590405	5.262195333	728.4403927
745.8971414	0.950655314	0.00332937	4.716190149	640.0701518
2045.893727	0.952806816	0.001738808	5.038921844	1776.68967
1165.66502	0.949930188	0.002451309	4.917373536	1001.042209
1794.762432	0.957053463	0.001254708	5.22422779	1570.704853
1887.796337	0.943045731	0.001245079	4.843164561	1586.822317
1834.915332	0.960764068	0.003098157	4.815712125	1632.271386
1627.65207	0.949853944	0.002017462	4.943666911	1389.568866
1049.893569	0.922349403	0.002707203	4.765866978	823.9670938
1373.375844	0.948804699	0.001709722	4.921567905	1168.821698
2117.325559	0.955832956	0.001049173	5.162762862	1850.647255
993.1304667	0.959247136	0.002366638	5.049860955	875.1113671
1206.13739	0.949626214	0.001579283	4.805649556	1030.157066
1087.108182	0.928796778	0.002129025	4.640822489	873.7554252
1976.519015	0.937899241	0.001188644	4.799422131	1631.310979
2094.553361	0.931811068	0.001100756	5.114630177	1693.418523
1274.50279	0.958599124	0.002215013	4.849481241	1121.747406
2835.192383	0.955380383	0.000679974	5.133785567	2475.636929
927.5796913	0.933722792	0.00258157	4.733389555	753.4297444
1358.959658	0.967138111	0.001665072	5.244062911	1231.013589
1773.864088	0.944555581	0.001293281	4.918174094	1490.614181
1689.063846	0.96351719	0.001390682	5.17277153	1508.969077
810.8012907	0.957866859	0.006145457	4.892427393	713.5973025
1209.101607	0.946784363	0.001764248	4.835568755	1027.149505
3023.529287	0.942602772	0.000741117	4.921071521	2534.263042
3567.602213	0.943919456	0.001693609	4.949143641	2999.768746
3289.60746	0.957968001	0.001667093	5.004164466	2885.177962
417.5460049	0.931440601	0.00750894	4.566162793	345.2967332
5984.962317	0.957990239	0.000637491	5.22110986	5258.850172
1414.798572	0.96670698	0.00161696	5.352353118	1278.562676
1017.82524	0.951701446	0.002438504	4.858397151	877.8132154
1565.736423	0.938057414	0.001978859	4.549895442	1293.444761
436.324045	0.938288028	0.007140634	4.55677395	363.1676419
2860.354171	0.952031558	0.001221565	5.075634807	2467.799152
1438.586019	0.955443568	0.001398501	5.036327511	1255.325688
1342.203034	0.952640477	0.001637944	4.938788621	1158.583404
1061.544737	0.949729276	0.002457456	4.907280488	913.3012072
1484.912174	0.937258272	0.00155037	4.921049248	1228.003114
1399.401206	0.962932311	0.001749833	5.030353815	1249.034369
2573.626002	0.954739268	0.001068519	5.088142042	2240.318803
1073.472162	0.946901949	0.002191707	4.913101509	912.8497287
2047.179421	0.961321507	0.000948576	5.201575985	1815.996408
670.4579877	0.941772152	0.004392343	4.547338608	559.7422902
1347.604219	0.923674461	0.001693606	4.711141654	1062.977826
937.1250065	0.947911751	0.00349578	4.846856695	796.4433384
1894.824894	0.961538462	0.001106377	5.263664252	1682.003812
916.8913607	0.947820491	0.002536498	4.755942415	778.9321969
829.1584633	0.942984552	0.002694886	4.737286387	696.6490233
638.4928239	0.938473382	0.004805067	4.684642346	533.1764566
1979.498021	0.953559261	0.001281126	5.439722547	1719.022959
3999.051352	0.941438553	0.000863176	5.211475373	3333.857884
1365.980623	0.967310246	0.001751009	5.220632344	1234.521096
865.0936651	0.949610594	0.00243052	4.910173921	744.7812645
1405.359842	0.961194543	0.002141703	5.002846525	1242.715915
1590.14557	0.953769102	0.001586299	4.970984609	1374.106514
1786.71219	0.949494138	0.002165975	4.839377239	1531.287584
862.7981684	0.942994908	0.003307304	4.633618642	721.4963495
1522.528542	0.961554905	0.00150149	5.106599022	1355.892884
1632.555171	0.967861701	0.002877955	5.477143657	1474.688423
1378.375467	0.945354257	0.00207015	4.92383924	1162.273034
1444.960216	0.9111702	0.001652806	4.457879356	1099.166536
314.9252234	0.917269519	0.007315546	4.28679127	243.5810876
1005.62013	0.959346517	0.002146201	4.98357328	889.5312372
947.0428008	0.903193412	0.002939509	4.336862216	695.7775273
1323.440634	0.942976939	0.001781005	4.841203894	1110.297518
969.293782	0.930342815	0.002506075	4.746091044	787.844975
950.188378	0.937894073	0.002580807	4.591955022	784.6942102
2081.976547	0.939639126	0.001452595	4.966435483	1735.465445
1568.800929	0.923076923	0.002902935	4.741065584	1245.752443
1707.599451	0.962248918	0.001323175	5.039484302	1515.715978
1196.287255	0.954733671	0.002028182	5.012635093	1041.716587
1194.738813	0.944941725	0.00170925	4.763391373	1008.998475
1035.463838	0.949183399	0.001911182	4.858482268	886.2514545
1542.211637	0.953423549	0.001373543	5.069778573	1337.849492
1384.907362	0.948745255	0.001782617	5.054224746	1181.099073
1709.697834	0.947028139	0.002158547	4.976320896	1446.401769
395.9337473	0.919387567	0.007763966	4.40405241	309.9386232
2278.744232	0.947163678	0.000899886	5.100086564	1943.407096

wavelet.LHL_glrlm_RunLengthNonUniformityNormalized	wavelet.LHL_glszm_GrayLevelVariance	wavelet.LHL_glszm_SmallAreaHighGrayLevelEmphasis	wavelet.LHL_glszm_GrayLevelNonUniformityNormalized
0.938237393	107.7559421	1237.201736	0.028212543
0.901490588	61.0659267	972.9132031	0.041359695
0.883116397	47.57163006	390.4541003	0.043865636
0.902903691	71.74755113	1751.871703	0.039916262
0.902085193	54.07650843	496.4319532	0.039875256
0.936474268	65.26	508.7040466	0.035770975
0.904340573	60.61016057	1081.949359	0.037974053
0.87686494	55.30517881	1123.207721	0.043387666
0.881139788	52.22832448	806.966079	0.042279979
0.915266442	59.2179332	823.9625284	0.040601052
0.890029285	35.44966559	389.9270789	0.049910309
0.911114774	80.42130178	1323.034281	0.034880473
0.826946666	79.58990398	1169.261704	0.041224691
0.891779812	61.38389382	822.3764234	0.038751508
0.918389215	114.5193267	1711.445292	0.030514781
0.900323253	54.60258074	1380.699364	0.039575516
0.927289171	83.92015258	1328.228791	0.033079865
0.868323322	56.45321103	628.4272513	0.045746804
0.885724431	29.66991646	229.8638852	0.053772283
0.916285934	85.60007444	1952.445363	0.034308079
0.818034964	34.14525551	402.4162783	0.052166204
0.913123598	50.75989424	551.486417	0.043431069
0.927184455	87.51113805	532.5781177	0.032557375
0.907522136	41.91794191	452.3370896	0.04503464
0.909273413	93.64981061	1316.884901	0.036983685
0.906416208	56.75149537	725.7016732	0.039568261
0.918702294	83.81896954	1172.202363	0.032595827
0.893672076	58.21078993	1126.171448	0.041365855
0.923229621	58.32269287	1173.04675	0.042785645
0.905637877	61.85681708	958.6578107	0.039491277
0.860931561	58.20473435	590.1477787	0.04100831
0.903610791	55.17777473	799.7636951	0.039766731
0.916979971	72.29412492	1384.308516	0.034683817
0.922291976	60.73941684	624.5992265	0.036820314
0.905032913	46.5402553	725.543696	0.042412295
0.868307174	50.21398044	604.0312413	0.044553428
0.885507231	70.17974347	1174.121513	0.041554454
0.87776161	77.94491372	1192.517482	0.034540128
0.921150113	52.75454152	790.4463974	0.04213269
0.915032851	71.50293355	1819.041048	0.035330509
0.878251418	48.32419563	508.1983997	0.042640306
0.936287939	84.10335788	944.551088	0.032368474
0.896258581	63.996011	1021.039222	0.038792165
0.929775795	78.44909827	1109.689513	0.034024524
0.918619729	57.09801553	520.8963257	0.040851006
0.900124799	50.23185877	717.3809721	0.04216267
0.893726606	61.30029315	1793.322438	0.038860741
0.894481116	117.4661536	2083.123535	0.038937024
0.919577427	78.47853993	2053.044984	0.037724949
0.872634716	52.77955829	282.2020917	0.046909384
0.920658299	99.19790271	3923.535354	0.033176639
0.935495932	90.42136787	961.6499757	0.030747617
0.908854074	55.57056424	637.6358557	0.041472851
0.883447291	38.39419087	820.13121	0.050464007
0.885149884	37.53803183	252.6013118	0.049252344
0.90891334	90.93108041	1752.98745	0.036378874
0.91508631	63.71165951	915.3142561	0.037145795
0.909720337	55.80636597	811.7499552	0.03993156
0.904681498	63.89366579	676.2795709	0.038102723
0.88583003	58.41846916	903.4895042	0.039603377
0.92830345	62.22834875	925.0571132	0.037562373
0.914859849	91.80507741	1705.644256	0.035453408
0.900522918	57.68841141	656.6865126	0.039079926
0.925897336	77.16637625	1325.489779	0.033785244
0.892448999	40.23470222	379.0113011	0.048711911
0.861821908	51.47982877	743.778052	0.044406851
0.902752815	56.07892148	562.3293071	0.040800533
0.92601647	80.53069507	1228.024439	0.032504262
0.902891048	44.24832272	549.544736	0.045000042
0.892830822	45.0154685	467.0572689	0.045298665
0.884829102	51.77657215	390.4083783	0.045096692
0.912055792	112.1283898	1234.422166	0.028574959
0.892832514	104.2483553	2537.073114	0.030414341
0.936776613	81.14714254	919.5870609	0.032803178
0.904717317	52.60806938	542.9817643	0.039889307
0.926490257	62.6054847	915.4462582	0.038619094
0.91221091	66.37657975	973.6481325	0.038848789
0.904381065	63.09994706	1082.968803	0.042211548
0.892621897	41.43509344	476.6399477	0.047397686
0.925535812	71.70751099	1009.991953	0.034889599
0.938619488	109.6522118	1108.42565	0.028208591
0.898365774	73.58565444	851.5968969	0.038017427
0.840364149	40.18695684	794.731146	0.050432327
0.849331599	21.69182617	156.9056672	0.060949919
0.921828363	56.0020798	655.2766306	0.038127108
0.830117099	32.29832111	472.8505576	0.057127473
0.894355545	56.36653754	807.5723549	0.041929418
0.873234722	55.01276899	610.4463705	0.041597225
0.884734248	38.85869509	555.9978823	0.047623115
0.887519609	77.20446347	1275.875625	0.036563092
0.859518341	74.10246098	927.9266681	0.039973613
0.927587129	61.87340034	1086.519629	0.037220771
0.913666059	64.00655582	765.2795312	0.037805761
0.896183034	47.23132742	702.4406753	0.043929288
0.904067895	47.62196783	610.4641658	0.041924867
0.912741714	69.76510027	987.310875	0.035105415
0.904669029	70.50953312	854.4755096	0.035766753
0.902320401	78.2027275	1038.339981	0.037181744
0.853920346	29.20015956	225.80358	0.05656352
0.900404555	74.74367689	1416.016301	0.03561884

wavelet.LHL_glszm_SizeZoneNonUniformityNormalized	wavelet.LHL_glszm_SizeZoneNonUniformity	wavelet.LHL_glszm_GrayLevelNonUniformity	wavelet.LHL_glszm_LargeAreaEmphasis	wavelet.LHL_glszm_ZoneVariance
0.563139253	784.4529792	39.30007179	4.028715004	1.70817785
0.436346398	202.9010753	19.23225806	8.694623656	4.651497283
0.443152708	315.0815752	31.18846695	15.27707454	10.62216209
0.465403303	235.4940711	20.19762846	9.148221344	5.420116702
0.460476419	546.5855097	47.33192923	10.63352991	6.653723396
0.521303855	218.947619	15.02380952	3.995238095	1.52585034
[truncated: 1,155,878 more chars]
