# Supplementary material for: Peritumoral radiomics features predict distant metastasis in locally advanced NSCLC
Source: PLoS One. 2018 Nov 2;13(11):e0206108. doi: 10.1371/journal.pone.0206108 (PMC6214508; doi:10.1371/journal.pone.0206108)
Supplement: S6 File — (PDF) [file pone.0206108.s006.pdf]

X	general_info_BoundingBox
97	(6, 5, 6, 18, 14, 9)
98	(5, 6, 6, 7, 6, 4)
99	(6, 6, 6, 8, 12, 11)
100	(6, 6, 6, 6, 6, 5)
102	(6, 6, 5, 13, 14, 12)
103	(6, 5, 6, 18, 11, 6)
104	(6, 6, 6, 35, 34, 39)
105	(6, 6, 6, 21, 33, 31)
106	(6, 6, 6, 23, 29, 24)
109	(7, 5, 6, 13, 13, 11)
110	(5, 6, 6, 37, 34, 25)
111	(6, 6, 6, 17, 21, 26)
113	(6, 6, 6, 12, 12, 7)
115	(5, 6, 5, 9, 11, 8)
116	(7, 5, 6, 15, 12, 13)
118	(6, 5, 6, 26, 40, 28)
119	(6, 6, 5, 10, 10, 11)
120	(6, 6, 6, 6, 7, 8)
121	(6, 5, 6, 11, 15, 9)
122	(6, 6, 5, 33, 32, 39)
123	(6, 6, 6, 12, 15, 19)
124	(6, 6, 5, 7, 12, 6)
125	(6, 6, 5, 9, 8, 9)
127	(6, 5, 6, 27, 29, 16)
128	(6, 6, 6, 10, 18, 10)
129	(6, 6, 6, 15, 16, 21)
130	(6, 5, 6, 18, 22, 17)
131	(6, 6, 5, 16, 17, 20)
132	(6, 6, 6, 5, 3, 3)
133	(6, 6, 6, 16, 17, 10)
134	(5, 5, 6, 23, 23, 22)
137	(5, 6, 6, 8, 10, 12)
210	(6, 6, 5, 13, 24, 23)
310	(6, 5, 5, 21, 25, 18)
410	(5, 6, 6, 26, 24, 33)
510	(6, 6, 6, 16, 20, 17)
610	(6, 8, 6, 20, 19, 17)
710	(6, 6, 6, 29, 32, 40)
810	(5, 6, 6, 12, 8, 7)
910	(5, 47, 9, 30, 46, 39)
1010	(6, 6, 6, 13, 11, 13)
1110	(6, 5, 6, 31, 30, 27)
1210	(5, 5, 6, 25, 25, 10)
138	(6, 6, 6, 11, 17, 13)
142	(6, 6, 6, 14, 14, 14)
151	(5, 5, 5, 18, 27, 13)
161	(6, 5, 5, 29, 29, 26)
171	(6, 5, 6, 10, 7, 6)
181	(6, 6, 5, 10, 7, 4)
191	(6, 6, 6, 15, 6, 15)
201	(6, 6, 6, 21, 23, 23)
221	(6, 6, 5, 21, 20, 22)
231	(6, 6, 6, 31, 39, 25)
241	(5, 5, 6, 14, 18, 15)
251	(6, 6, 6, 7, 13, 8)
261	(6, 5, 6, 12, 13, 10)
271	(6, 6, 6, 31, 33, 21)
281	(6, 6, 5, 12, 15, 16)
291	(6, 7, 6, 11, 8, 11)
301	(6, 5, 5, 45, 41, 42)
311	(6, 6, 5, 11, 13, 13)
321	(6, 6, 5, 15, 22, 16)
331	(6, 5, 5, 29, 41, 38)
341	(6, 6, 5, 33, 50, 29)
351	(6, 6, 6, 7, 10, 11)
361	(6, 6, 6, 21, 20, 21)
371	(5, 6, 6, 7, 9, 6)
381	(6, 6, 6, 22, 31, 38)
391	(6, 6, 5, 29, 26, 30)
401	(6, 6, 6, 21, 17, 14)
411	(6, 5, 5, 11, 11, 5)
421	(6, 6, 5, 25, 25, 30)
431	(5, 6, 6, 12, 14, 12)
481	(6, 6, 6, 14, 13, 18)
491	(6, 6, 6, 22, 29, 31)
501	(5, 6, 6, 16, 8, 11)
541	(5, 6, 6, 16, 16, 17)
551	(5, 6, 6, 20, 16, 10)
561	(6, 6, 5, 19, 10, 13)
601	(5, 6, 5, 16, 17, 16)
611	(6, 5, 6, 18, 14, 9)
621	(6, 5, 6, 9, 10, 11)
641	(5, 6, 6, 21, 21, 25)
661	(6, 5, 5, 16, 17, 19)
681	(5, 6, 6, 14, 19, 22)
691	(6, 6, 5, 8, 8, 9)
701	(5, 8, 6, 18, 16, 8)
711	(6, 6, 5, 23, 26, 21)
721	(6, 6, 6, 17, 14, 12)
751	(6, 5, 6, 14, 14, 10)
771	(6, 6, 5, 9, 10, 12)
781	(6, 6, 6, 20, 22, 14)
791	(6, 6, 6, 29, 22, 27)
801	(6, 5, 6, 23, 26, 18)
811	(7, 5, 6, 28, 29, 21)
821	(6, 6, 5, 19, 17, 20)
841	(6, 6, 5, 19, 15, 17)
861	(5, 6, 5, 7, 6, 4)
871	(5, 5, 6, 6, 6, 4)
901	(6, 5, 6, 32, 38, 30)

general_info_GeneralSettings

{'distances': [1], 'verbose': True, 'additionalInfo': True, 'enableCExtensions': True, 'force2D': False, 'interpolator': 3, 'resampledPixelSpacing': [3, 3, 3], 'label': 1, 'normalizeScale': 1, 'normalize': False, 'force2Ddimension': 0, 'removeOutliers': None, 'minimumROISi
{'distances': [1], 'verbose': True, 'additionalInfo': True, 'enableCExtensions': True, 'force2D': False, 'interpolator': 3, 'resampledPixelSpacing': [3, 3, 3], 'label': 1, 'normalizeScale': 1, 'normalize': False, 'force2Ddimension': 0, 'removeOutliers': None, 'minimumROISi
{'distances': [1], 'verbose': True, 'additionalInfo': True, 'enableCExtensions': True, 'force2D': False, 'interpolator': 3, 'resampledPixelSpacing': [3, 3, 3], 'label': 1, 'normalizeScale': 1, 'normalize': False, 'force2Ddimension': 0, 'removeOutliers': None, 'minimumROISi
{'distances': [1], 'verbose': True, 'additionalInfo': True, 'enableCExtensions': True, 'force2D': False, 'interpolator': 3, 'resampledPixelSpacing': [3, 3, 3], 'label': 1, 'normalizeScale': 1, 'normalize': False, 'force2Ddimension': 0, 'removeOutliers': None, 'minimumROISi
{'distances': [1], 'verbose': True, 'additionalInfo': True, 'enableCExtensions': True, 'force2D': False, 'interpolator': 3, 'resampledPixelSpacing': [3, 3, 3], 'label': 1, 'normalizeScale': 1, 'normalize': False, 'force2Ddimension': 0, 'removeOutliers': None, 'minimumROISi
{'distances': [1], 'verbose': True, 'additionalInfo': True, 'enableCExtensions': True, 'force2D': False, 'interpolator': 3, 'resampledPixelSpacing': [3, 3, 3], 'label': 1, 'normalizeScale': 1, 'normalize': False, 'force2Ddimension': 0, 'removeOutliers': None, 'minimumROISi
{'distances': [1], 'verbose': True, 'additionalInfo': True, 'enableCExtensions': True, 'force2D': False, 'interpolator': 3, 'resampledPixelSpacing': [3, 3, 3], 'label': 1, 'normalizeScale': 1, 'normalize': False, 'force2Ddimension': 0, 'removeOutliers': None, 'minimumROISi
{'distances': [1], 'verbose': True, 'additionalInfo': True, 'enableCExtensions': True, 'force2D': False, 'interpolator': 3, 'resampledPixelSpacing': [3, 3, 3], 'label': 1, 'normalizeScale': 1, 'normalize': False, 'force2Ddimension': 0, 'removeOutliers': None, 'minimumROISi
{'distances': [1], 'verbose': True, 'additionalInfo': True, 'enableCExtensions': True, 'force2D': False, 'interpolator': 3, 'resampledPixelSpacing': [3, 3, 3], 'label': 1, 'normalizeScale': 1, 'normalize': False, 'force2Ddimension': 0, 'removeOutliers': None, 'minimumROISi
{'distances': [1], 'verbose': True, 'additionalInfo': True, 'enableCExtensions': True, 'force2D': False, 'interpolator': 3, 'resampledPixelSpacing': [3, 3, 3], 'label': 1, 'normalizeScale': 1, 'normalize': False, 'force2Ddimension': 0, 'removeOutliers': None, 'minimumROISi
{'distances': [1], 'verbose': True, 'additionalInfo': True, 'enableCExtensions': True, 'force2D': False, 'interpolator': 3, 'resampledPixelSpacing': [3, 3, 3], 'label': 1, 'normalizeScale': 1, 'normalize': False, 'force2Ddimension': 0, 'removeOutliers': None, 'minimumROISi
{'distances': [1], 'verbose': True, 'additionalInfo': True, 'enableCExtensions': True, 'force2D': False, 'interpolator': 3, 'resampledPixelSpacing': [3, 3, 3], 'label': 1, 'normalizeScale': 1, 'normalize': False, 'force2Ddimension': 0, 'removeOutliers': None, 'minimumROISi
{'distances': [1], 'verbose': True, 'additionalInfo': True, 'enableCExtensions': True, 'force2D': False, 'interpolator': 3, 'resampledPixelSpacing': [3, 3, 3], 'label': 1, 'normalizeScale': 1, 'normalize': False, 'force2Ddimension': 0, 'removeOutliers': None, 'minimumROISi
{'distances': [1], 'verbose': True, 'additionalInfo': True, 'enableCExtensions': True, 'force2D': False, 'interpolator': 3, 'resampledPixelSpacing': [3, 3, 3], 'label': 1, 'normalizeScale': 1, 'normalize': False, 'force2Ddimension': 0, 'removeOutliers': None, 'minimumROISi
{'distances': [1], 'verbose': True, 'additionalInfo': True, 'enableCExtensions': True, 'force2D': False, 'interpolator': 3, 'resampledPixelSpacing': [3, 3, 3], 'label': 1, 'normalizeScale': 1, 'normalize': False, 'force2Ddimension': 0, 'removeOutliers': None, 'minimumROISi
{'distances': [1], 'verbose': True, 'additionalInfo': True, 'enableCExtensions': True, 'force2D': False, 'interpolator': 3, 'resampledPixelSpacing': [3, 3, 3], 'label': 1, 'normalizeScale': 1, 'normalize': False, 'force2Ddimension': 0, 'removeOutliers': None, 'minimumROISi
{'distances': [1], 'verbose': True, 'additionalInfo': True, 'enableCExtensions': True, 'force2D': False, 'interpolator': 3, 'resampledPixelSpacing': [3, 3, 3], 'label': 1, 'normalizeScale': 1, 'normalize': False, 'force2Ddimension': 0, 'removeOutliers': None, 'minimumROISi
{'distances': [1], 'verbose': True, 'additionalInfo': True, 'enableCExtensions': True, 'force2D': False, 'interpolator': 3, 'resampledPixelSpacing': [3, 3, 3], 'label': 1, 'normalizeScale': 1, 'normalize': False, 'force2Ddimension': 0, 'removeOutliers': None, 'minimumROISi
{'distances': [1], 'verbose': True, 'additionalInfo': True, 'enableCExtensions': True, 'force2D': False, 'interpolator': 3, 'resampledPixelSpacing': [3, 3, 3], 'label': 1, 'normalizeScale': 1, 'normalize': False, 'force2Ddimension': 0, 'removeOutliers': None, 'minimumROISi
{'distances': [1], 'verbose': True, 'additionalInfo': True, 'enableCExtensions': True, 'force2D': False, 'interpolator': 3, 'resampledPixelSpacing': [3, 3, 3], 'label': 1, 'normalizeScale': 1, 'normalize': False, 'force2Ddimension': 0, 'removeOutliers': None, 'minimumROISi
{'distances': [1], 'verbose': True, 'additionalInfo': True, 'enableCExtensions': True, 'force2D': False, 'interpolator': 3, 'resampledPixelSpacing': [3, 3, 3], 'label': 1, 'normalizeScale': 1, 'normalize': False, 'force2Ddimension': 0, 'removeOutliers': None, 'minimumROISi
{'distances': [1], 'verbose': True, 'additionalInfo': True, 'enableCExtensions': True, 'force2D': False, 'interpolator': 3, 'resampledPixelSpacing': [3, 3, 3], 'label': 1, 'normalizeScale': 1, 'normalize': False, 'force2Ddimension': 0, 'removeOutliers': None, 'minimumROISi
{'distances': [1], 'verbose': True, 'additionalInfo': True, 'enableCExtensions': True, 'force2D': False, 'interpolator': 3, 'resampledPixelSpacing': [3, 3, 3], 'label': 1, 'normalizeScale': 1, 'normalize': False, 'force2Ddimension': 0, 'removeOutliers': None, 'minimumROISi
{'distances': [1], 'verbose': True, 'additionalInfo': True, 'enableCExtensions': True, 'force2D': False, 'interpolator': 3, 'resampledPixelSpacing': [3, 3, 3], 'label': 1, 'normalizeScale': 1, 'normalize': False, 'force2Ddimension': 0, 'removeOutliers': None, 'minimumROISi
{'distances': [1], 'verbose': True, 'additionalInfo': True, 'enableCExtensions': True, 'force2D': False, 'interpolator': 3, 'resampledPixelSpacing': [3, 3, 3], 'label': 1, 'normalizeScale': 1, 'normalize': False, 'force2Ddimension': 0, 'removeOutliers': None, 'minimumROISi
{'distances': [1], 'verbose': True, 'additionalInfo': True, 'enableCExtensions': True, 'force2D': False, 'interpolator': 3, 'resampledPixelSpacing': [3, 3, 3], 'label': 1, 'normalizeScale': 1, 'normalize': False, 'force2Ddimension': 0, 'removeOutliers': None, 'minimumROISi
{'distances': [1], 'verbose': True, 'additionalInfo': True, 'enableCExtensions': True, 'force2D': False, 'interpolator': 3, 'resampledPixelSpacing': [3, 3, 3], 'label': 1, 'normalizeScale': 1, 'normalize': False, 'force2Ddimension': 0, 'removeOutliers': None, 'minimumROISi
{'distances': [1], 'verbose': True, 'additionalInfo': True, 'enableCExtensions': True, 'force2D': False, 'interpolator': 3, 'resampledPixelSpacing': [3, 3, 3], 'label': 1, 'normalizeScale': 1, 'normalize': False, 'force2Ddimension': 0, 'removeOutliers': None, 'minimumROISi
{'distances': [1], 'verbose': True, 'additionalInfo': True, 'enableCExtensions': True, 'force2D': False, 'interpolator': 3, 'resampledPixelSpacing': [3, 3, 3], 'label': 1, 'normalizeScale': 1, 'normalize': False, 'force2Ddimension': 0, 'removeOutliers': None, 'minimumROISi
{'distances': [1], 'verbose': True, 'additionalInfo': True, 'enableCExtensions': True, 'force2D': False, 'interpolator': 3, 'resampledPixelSpacing': [3, 3, 3], 'label': 1, 'normalizeScale': 1, 'normalize': False, 'force2Ddimension': 0, 'removeOutliers': None, 'minimumROISi
{'distances': [1], 'verbose': True, 'additionalInfo': True, 'enableCExtensions': True, 'force2D': False, 'interpolator': 3, 'resampledPixelSpacing': [3, 3, 3], 'label': 1, 'normalizeScale': 1, 'normalize': False, 'force2Ddimension': 0, 'removeOutliers': None, 'minimumROISi
{'distances': [1], 'verbose': True, 'additionalInfo': True, 'enableCExtensions': True, 'force2D': False, 'interpolator': 3, 'resampledPixelSpacing': [3, 3, 3], 'label': 1, 'normalizeScale': 1, 'normalize': False, 'force2Ddimension': 0, 'removeOutliers': None, 'minimumROISi
{'distances': [1], 'verbose': True, 'additionalInfo': True, 'enableCExtensions': True, 'force2D': False, 'interpolator': 3, 'resampledPixelSpacing': [3, 3, 3], 'label': 1, 'normalizeScale': 1, 'normalize': False, 'force2Ddimension': 0, 'removeOutliers': None, 'minimumROISi
{'distances': [1], 'verbose': True, 'additionalInfo': True, 'enableCExtensions': True, 'force2D': False, 'interpolator': 3, 'resampledPixelSpacing': [3, 3, 3], 'label': 1, 'normalizeScale': 1, 'normalize': False, 'force2Ddimension': 0, 'removeOutliers': None, 'minimumROISi
{'distances': [1], 'verbose': True, 'additionalInfo': True, 'enableCExtensions': True, 'force2D': False, 'interpolator': 3, 'resampledPixelSpacing': [3, 3, 3], 'label': 1, 'normalizeScale': 1, 'normalize': False, 'force2Ddimension': 0, 'removeOutliers': None, 'minimumROISi
{'distances': [1], 'verbose': True, 'additionalInfo': True, 'enableCExtensions': True, 'force2D': False, 'interpolator': 3, 'resampledPixelSpacing': [3, 3, 3], 'label': 1, 'normalizeScale': 1, 'normalize': False, 'force2Ddimension': 0, 'removeOutliers': None, 'minimumROISi
{'distances': [1], 'verbose': True, 'additionalInfo': True, 'enableCExtensions': True, 'force2D': False, 'interpolator': 3, 'resampledPixelSpacing': [3, 3, 3], 'label': 1, 'normalizeScale': 1, 'normalize': False, 'force2Ddimension': 0, 'removeOutliers': None, 'minimumROISi
{'distances': [1], 'verbose': True, 'additionalInfo': True, 'enableCExtensions': True, 'force2D': False, 'interpolator': 3, 'resampledPixelSpacing': [3, 3, 3], 'label': 1, 'normalizeScale': 1, 'normalize': False, 'force2Ddimension': 0, 'removeOutliers': None, 'minimumROISi
{'distances': [1], 'verbose': True, 'additionalInfo': True, 'enableCExtensions': True, 'force2D': False, 'interpolator': 3, 'resampledPixelSpacing': [3, 3, 3], 'label': 1, 'normalizeScale': 1, 'normalize': False, 'force2Ddimension': 0, 'removeOutliers': None, 'minimumROISi
{'distances': [1], 'verbose': True, 'additionalInfo': True, 'enableCExtensions': True, 'force2D': False, 'interpolator': 3, 'resampledPixelSpacing': [3, 3, 3], 'label': 1, 'normalizeScale': 1, 'normalize': False, 'force2Ddimension': 0, 'removeOutliers': None, 'minimumROISi
{'distances': [1], 'verbose': True, 'additionalInfo': True, 'enableCExtensions': True, 'force2D': False, 'interpolator': 3, 'resampledPixelSpacing': [3, 3, 3], 'label': 1, 'normalizeScale': 1, 'normalize': False, 'force2Ddimension': 0, 'removeOutliers': None, 'minimumROISi
{'distances': [1], 'verbose': True, 'additionalInfo': True, 'enableCExtensions': True, 'force2D': False, 'interpolator': 3, 'resampledPixelSpacing': [3, 3, 3], 'label': 1, 'normalizeScale': 1, 'normalize': False, 'force2Ddimension': 0, 'removeOutliers': None, 'minimumROISi
{'distances': [1], 'verbose': True, 'additionalInfo': True, 'enableCExtensions': True, 'force2D': False, 'interpolator': 3, 'resampledPixelSpacing': [3, 3, 3], 'label': 1, 'normalizeScale': 1, 'normalize': False, 'force2Ddimension': 0, 'removeOutliers': None, 'minimumROISi
{'distances': [1], 'verbose': True, 'additionalInfo': True, 'enableCExtensions': True, 'force2D': False, 'interpolator': 3, 'resampledPixelSpacing': [3, 3, 3], 'label': 1, 'normalizeScale': 1, 'normalize': False, 'force2Ddimension': 0, 'removeOutliers': None, 'minimumROISi
{'distances': [1], 'verbose': True, 'additionalInfo': True, 'enableCExtensions': True, 'force2D': False, 'interpolator': 3, 'resampledPixelSpacing': [3, 3, 3], 'label': 1, 'normalizeScale': 1, 'normalize': False, 'force2Ddimension': 0, 'removeOutliers': None, 'minimumROISi
{'distances': [1], 'verbose': True, 'additionalInfo': True, 'enableCExtensions': True, 'force2D': False, 'interpolator': 3, 'resampledPixelSpacing': [3, 3, 3], 'label': 1, 'normalizeScale': 1, 'normalize': False, 'force2Ddimension': 0, 'removeOutliers': None, 'minimumROISi
{'distances': [1], 'verbose': True, 'additionalInfo': True, 'enableCExtensions': True, 'force2D': False, 'interpolator': 3, 'resampledPixelSpacing': [3, 3, 3], 'label': 1, 'normalizeScale': 1, 'normalize': False, 'force2Ddimension': 0, 'removeOutliers': None, 'minimumROISi
{'distances': [1], 'verbose': True, 'additionalInfo': True, 'enableCExtensions': True, 'force2D': False, 'interpolator': 3, 'resampledPixelSpacing': [3, 3, 3], 'label': 1, 'normalizeScale': 1, 'normalize': False, 'force2Ddimension': 0, 'removeOutliers': None, 'minimumROISi
{'distances': [1], 'verbose': True, 'additionalInfo': True, 'enableCExtensions': True, 'force2D': False, 'interpolator': 3, 'resampledPixelSpacing': [3, 3, 3], 'label': 1, 'normalizeScale': 1, 'normalize': False, 'force2Ddimension': 0, 'removeOutliers': None, 'minimumROISi
{'distances': [1], 'verbose': True, 'additionalInfo': True, 'enableCExtensions': True, 'force2D': False, 'interpolator': 3, 'resampledPixelSpacing': [3, 3, 3], 'label': 1, 'normalizeScale': 1, 'normalize': False, 'force2Ddimension': 0, 'removeOutliers': None, 'minimumROISi
{'distances': [1], 'verbose': True, 'additionalInfo': True, 'enableCExtensions': True, 'force2D': False, 'interpolator': 3, 'resampledPixelSpacing': [3, 3, 3], 'label': 1, 'normalizeScale': 1, 'normalize': False, 'force2Ddimension': 0, 'removeOutliers': None, 'minimumROISi
{'distances': [1], 'verbose': True, 'additionalInfo': True, 'enableCExtensions': True, 'force2D': False, 'interpolator': 3, 'resampledPixelSpacing': [3, 3, 3], 'label': 1, 'normalizeScale': 1, 'normalize': False, 'force2Ddimension': 0, 'removeOutliers': None, 'minimumROISi
{'distances': [1], 'verbose': True, 'additionalInfo': True, 'enableCExtensions': True, 'force2D': False, 'interpolator': 3, 'resampledPixelSpacing': [3, 3, 3], 'label': 1, 'normalizeScale': 1, 'normalize': False, 'force2Ddimension': 0, 'removeOutliers': None, 'minimumROISi
{'distances': [1], 'verbose': True, 'additionalInfo': True, 'enableCExtensions': True, 'force2D': False, 'interpolator': 3, 'resampledPixelSpacing': [3, 3, 3], 'label': 1, 'normalizeScale': 1, 'normalize': False, 'force2Ddimension': 0, 'removeOutliers': None, 'minimumROISi
{'distances': [1], 'verbose': True, 'additionalInfo': True, 'enableCExtensions': True, 'force2D': False, 'interpolator': 3, 'resampledPixelSpacing': [3, 3, 3], 'label': 1, 'normalizeScale': 1, 'normalize': False, 'force2Ddimension': 0, 'removeOutliers': None, 'minimumROISi
{'distances': [1], 'verbose': True, 'additionalInfo': True, 'enableCExtensions': True, 'force2D': False, 'interpolator': 3, 'resampledPixelSpacing': [3, 3, 3], 'label': 1, 'normalizeScale': 1, 'normalize': False, 'force2Ddimension': 0, 'removeOutliers': None, 'minimumROISi
{'distances': [1], 'verbose': True, 'additionalInfo': True, 'enableCExtensions': True, 'force2D': False, 'interpolator': 3, 'resampledPixelSpacing': [3, 3, 3], 'label': 1, 'normalizeScale': 1, 'normalize': False, 'force2Ddimension': 0, 'removeOutliers': None, 'minimumROISi
{'distances': [1], 'verbose': True, 'additionalInfo': True, 'enableCExtensions': True, 'force2D': False, 'interpolator': 3, 'resampledPixelSpacing': [3, 3, 3], 'label': 1, 'normalizeScale': 1, 'normalize': False, 'force2Ddimension': 0, 'removeOutliers': None, 'minimumROISi
{'distances': [1], 'verbose': True, 'additionalInfo': True, 'enableCExtensions': True, 'force2D': False, 'interpolator': 3, 'resampledPixelSpacing': [3, 3, 3], 'label': 1, 'normalizeScale': 1, 'normalize': False, 'force2Ddimension': 0, 'removeOutliers': None, 'minimumROISi
{'distances': [1], 'verbose': True, 'additionalInfo': True, 'enableCExtensions': True, 'force2D': False, 'interpolator': 3, 'resampledPixelSpacing': [3, 3, 3], 'label': 1, 'normalizeScale': 1, 'normalize': False, 'force2Ddimension': 0, 'removeOutliers': None, 'minimumROISi
{'distances': [1], 'verbose': True, 'additionalInfo': True, 'enableCExtensions': True, 'force2D': False, 'interpolator': 3, 'resampledPixelSpacing': [3, 3, 3], 'label': 1, 'normalizeScale': 1, 'normalize': False, 'force2Ddimension': 0, 'removeOutliers': None, 'minimumROISi
{'distances': [1], 'verbose': True, 'additionalInfo': True, 'enableCExtensions': True, 'force2D': False, 'interpolator': 3, 'resampledPixelSpacing': [3, 3, 3], 'label': 1, 'normalizeScale': 1, 'normalize': False, 'force2Ddimension': 0, 'removeOutliers': None, 'minimumROISi
{'distances': [1], 'verbose': True, 'additionalInfo': True, 'enableCExtensions': True, 'force2D': False, 'interpolator': 3, 'resampledPixelSpacing': [3, 3, 3], 'label': 1, 'normalizeScale': 1, 'normalize': False, 'force2Ddimension': 0, 'removeOutliers': None, 'minimumROISi
{'distances': [1], 'verbose': True, 'additionalInfo': True, 'enableCExtensions': True, 'force2D': False, 'interpolator': 3, 'resampledPixelSpacing': [3, 3, 3], 'label': 1, 'normalizeScale': 1, 'normalize': False, 'force2Ddimension': 0, 'removeOutliers': None, 'minimumROISi
{'distances': [1], 'verbose': True, 'additionalInfo': True, 'enableCExtensions': True, 'force2D': False, 'interpolator': 3, 'resampledPixelSpacing': [3, 3, 3], 'label': 1, 'normalizeScale': 1, 'normalize': False, 'force2Ddimension': 0, 'removeOutliers': None, 'minimumROISi
{'distances': [1], 'verbose': True, 'additionalInfo': True, 'enableCExtensions': True, 'force2D': False, 'interpolator': 3, 'resampledPixelSpacing': [3, 3, 3], 'label': 1, 'normalizeScale': 1, 'normalize': False, 'force2Ddimension': 0, 'removeOutliers': None, 'minimumROISi
{'distances': [1], 'verbose': True, 'additionalInfo': True, 'enableCExtensions': True, 'force2D': False, 'interpolator': 3, 'resampledPixelSpacing': [3, 3, 3], 'label': 1, 'normalizeScale': 1, 'normalize': False, 'force2Ddimension': 0, 'removeOutliers': None, 'minimumROISi
{'distances': [1], 'verbose': True, 'additionalInfo': True, 'enableCExtensions': True, 'force2D': False, 'interpolator': 3, 'resampledPixelSpacing': [3, 3, 3], 'label': 1, 'normalizeScale': 1, 'normalize': False, 'force2Ddimension': 0, 'removeOutliers': None, 'minimumROISi
{'distances': [1], 'verbose': True, 'additionalInfo': True, 'enableCExtensions': True, 'force2D': False, 'interpolator': 3, 'resampledPixelSpacing': [3, 3, 3], 'label': 1, 'normalizeScale': 1, 'normalize': False, 'force2Ddimension': 0, 'removeOutliers': None, 'minimumROISi
{'distances': [1], 'verbose': True, 'additionalInfo': True, 'enableCExtensions': True, 'force2D': False, 'interpolator': 3, 'resampledPixelSpacing': [3, 3, 3], 'label': 1, 'normalizeScale': 1, 'normalize': False, 'force2Ddimension': 0, 'removeOutliers': None, 'minimumROISi
{'distances': [1], 'verbose': True, 'additionalInfo': True, 'enableCExtensions': True, 'force2D': False, 'interpolator': 3, 'resampledPixelSpacing': [3, 3, 3], 'label': 1, 'normalizeScale': 1, 'normalize': False, 'force2Ddimension': 0, 'removeOutliers': None, 'minimumROISi
{'distances': [1], 'verbose': True, 'additionalInfo': True, 'enableCExtensions': True, 'force2D': False, 'interpolator': 3, 'resampledPixelSpacing': [3, 3, 3], 'label': 1, 'normalizeScale': 1, 'normalize': False, 'force2Ddimension': 0, 'removeOutliers': None, 'minimumROISi
{'distances': [1], 'verbose': True, 'additionalInfo': True, 'enableCExtensions': True, 'force2D': False, 'interpolator': 3, 'resampledPixelSpacing': [3, 3, 3], 'label': 1, 'normalizeScale': 1, 'normalize': False, 'force2Ddimension': 0, 'removeOutliers': None, 'minimumROISi
{'distances': [1], 'verbose': True, 'additionalInfo': True, 'enableCExtensions': True, 'force2D': False, 'interpolator': 3, 'resampledPixelSpacing': [3, 3, 3], 'label': 1, 'normalizeScale': 1, 'normalize': False, 'force2Ddimension': 0, 'removeOutliers': None, 'minimumROISi
{'distances': [1], 'verbose': True, 'additionalInfo': True, 'enableCExtensions': True, 'force2D': False, 'interpolator': 3, 'resampledPixelSpacing': [3, 3, 3], 'label': 1, 'normalizeScale': 1, 'normalize': False, 'force2Ddimension': 0, 'removeOutliers': None, 'minimumROISi
{'distances': [1], 'verbose': True, 'additionalInfo': True, 'enableCExtensions': True, 'force2D': False, 'interpolator': 3, 'resampledPixelSpacing': [3, 3, 3], 'label': 1, 'normalizeScale': 1, 'normalize': False, 'force2Ddimension': 0, 'removeOutliers': None, 'minimumROISi
{'distances': [1], 'verbose': True, 'additionalInfo': True, 'enableCExtensions': True, 'force2D': False, 'interpolator': 3, 'resampledPixelSpacing': [3, 3, 3], 'label': 1, 'normalizeScale': 1, 'normalize': False, 'force2Ddimension': 0, 'removeOutliers': None, 'minimumROISi
{'distances': [1], 'verbose': True, 'additionalInfo': True, 'enableCExtensions': True, 'force2D': False, 'interpolator': 3, 'resampledPixelSpacing': [3, 3, 3], 'label': 1, 'normalizeScale': 1, 'normalize': False, 'force2Ddimension': 0, 'removeOutliers': None, 'minimumROISi
{'distances': [1], 'verbose': True, 'additionalInfo': True, 'enableCExtensions': True, 'force2D': False, 'interpolator': 3, 'resampledPixelSpacing': [3, 3, 3], 'label': 1, 'normalizeScale': 1, 'normalize': False, 'force2Ddimension': 0, 'removeOutliers': None, 'minimumROISi
{'distances': [1], 'verbose': True, 'additionalInfo': True, 'enableCExtensions': True, 'force2D': False, 'interpolator': 3, 'resampledPixelSpacing': [3, 3, 3], 'label': 1, 'normalizeScale': 1, 'normalize': False, 'force2Ddimension': 0, 'removeOutliers': None, 'minimumROISi
{'distances': [1], 'verbose': True, 'additionalInfo': True, 'enableCExtensions': True, 'force2D': False, 'interpolator': 3, 'resampledPixelSpacing': [3, 3, 3], 'label': 1, 'normalizeScale': 1, 'normalize': False, 'force2Ddimension': 0, 'removeOutliers': None, 'minimumROISi
{'distances': [1], 'verbose': True, 'additionalInfo': True, 'enableCExtensions': True, 'force2D': False, 'interpolator': 3, 'resampledPixelSpacing': [3, 3, 3], 'label': 1, 'normalizeScale': 1, 'normalize': False, 'force2Ddimension': 0, 'removeOutliers': None, 'minimumROISi
{'distances': [1], 'verbose': True, 'additionalInfo': True, 'enableCExtensions': True, 'force2D': False, 'interpolator': 3, 'resampledPixelSpacing': [3, 3, 3], 'label': 1, 'normalizeScale': 1, 'normalize': False, 'force2Ddimension': 0, 'removeOutliers': None, 'minimumROISi
{'distances': [1], 'verbose': True, 'additionalInfo': True, 'enableCExtensions': True, 'force2D': False, 'interpolator': 3, 'resampledPixelSpacing': [3, 3, 3], 'label': 1, 'normalizeScale': 1, 'normalize': False, 'force2Ddimension': 0, 'removeOutliers': None, 'minimumROISi
{'distances': [1], 'verbose': True, 'additionalInfo': True, 'enableCExtensions': True, 'force2D': False, 'interpolator': 3, 'resampledPixelSpacing': [3, 3, 3], 'label': 1, 'normalizeScale': 1, 'normalize': False, 'force2Ddimension': 0, 'removeOutliers': None, 'minimumROISi
{'distances': [1], 'verbose': True, 'additionalInfo': True, 'enableCExtensions': True, 'force2D': False, 'interpolator': 3, 'resampledPixelSpacing': [3, 3, 3], 'label': 1, 'normalizeScale': 1, 'normalize': False, 'force2Ddimension': 0, 'removeOutliers': None, 'minimumROISi
{'distances': [1], 'verbose': True, 'additionalInfo': True, 'enableCExtensions': True, 'force2D': False, 'interpolator': 3, 'resampledPixelSpacing': [3, 3, 3], 'label': 1, 'normalizeScale': 1, 'normalize': False, 'force2Ddimension': 0, 'removeOutliers': None, 'minimumROISi
{'distances': [1], 'verbose': True, 'additionalInfo': True, 'enableCExtensions': True, 'force2D': False, 'interpolator': 3, 'resampledPixelSpacing': [3, 3, 3], 'label': 1, 'normalizeScale': 1, 'normalize': False, 'force2Ddimension': 0, 'removeOutliers': None, 'minimumROISi
{'distances': [1], 'verbose': True, 'additionalInfo': True, 'enableCExtensions': True, 'force2D': False, 'interpolator': 3, 'resampledPixelSpacing': [3, 3, 3], 'label': 1, 'normalizeScale': 1, 'normalize': False, 'force2Ddimension': 0, 'removeOutliers': None, 'minimumROISi
{'distances': [1], 'verbose': True, 'additionalInfo': True, 'enableCExtensions': True, 'force2D': False, 'interpolator': 3, 'resampledPixelSpacing': [3, 3, 3], 'label': 1, 'normalizeScale': 1, 'normalize': False, 'force2Ddimension': 0, 'removeOutliers': None, 'minimumROISi
{'distances': [1], 'verbose': True, 'additionalInfo': True, 'enableCExtensions': True, 'force2D': False, 'interpolator': 3, 'resampledPixelSpacing': [3, 3, 3], 'label': 1, 'normalizeScale': 1, 'normalize': False, 'force2Ddimension': 0, 'removeOutliers': None, 'minimumROISi
{'distances': [1], 'verbose': True, 'additionalInfo': True, 'enableCExtensions': True, 'force2D': False, 'interpolator': 3, 'resampledPixelSpacing': [3, 3, 3], 'label': 1, 'normalizeScale': 1, 'normalize': False, 'force2Ddimension': 0, 'removeOutliers': None, 'minimumROISi
{'distances': [1], 'verbose': True, 'additionalInfo': True, 'enableCExtensions': True, 'force2D': False, 'interpolator': 3, 'resampledPixelSpacing': [3, 3, 3], 'label': 1, 'normalizeScale': 1, 'normalize': False, 'force2Ddimension': 0, 'removeOutliers': None, 'minimumROISi
{'distances': [1], 'verbose': True, 'additionalInfo': True, 'enableCExtensions': True, 'force2D': False, 'interpolator': 3, 'resampledPixelSpacing': [3, 3, 3], 'label': 1, 'normalizeScale': 1, 'normalize': False, 'force2Ddimension': 0, 'removeOutliers': None, 'minimumROISi
{'distances': [1], 'verbose': True, 'additionalInfo': True, 'enableCExtensions': True, 'force2D': False, 'interpolator': 3, 'resampledPixelSpacing': [3, 3, 3], 'label': 1, 'normalizeScale': 1, 'normalize': False, 'force2Ddimension': 0, 'removeOutliers': None, 'minimumROISi
{'distances': [1], 'verbose': True, 'additionalInfo': True, 'enableCExtensions': True, 'force2D': False, 'interpolator': 3, 'resampledPixelSpacing': [3, 3, 3], 'label': 1, 'normalizeScale': 1, 'normalize': False, 'force2Ddimension': 0, 'removeOutliers': None, 'minimumROISi

general_info_ImageHash	general_info_ImageSpacing	general_info_InputImages	general_info_MaskHash	general_info_Version	general_info_VolumeNum
ed65c08c80974cb4fd975d41e064408072eb9fc5	(1.0, 1.0, 1.0)	{'LoG': {}, 'Original': {}, 'Wavelet': {}}	4abe8c16bc87a898311af27e6e3e8d2eb9350abd	1.1.1-beta1	1
65ad1ca0547df3f3064fed30066db1a82774efcf	(1.0, 1.0, 1.0)	{'LoG': {}, 'Original': {}, 'Wavelet': {}}	d05eee13494ab313d99b2ca141adc2d16b7d12da	1.1.1-beta1	1
7da59d8ecc6ae1e315f9ebbade22cbdcbf329bff	(1.0, 1.0, 1.0)	{'LoG': {}, 'Original': {}, 'Wavelet': {}}	3a8f8417d38bfe40218238acb9a641469630a354	1.1.1-beta1	1
1ad8fc837aa7f23c48e4dbce3b8ae95447fca758	(1.0, 1.0, 1.0)	{'LoG': {}, 'Original': {}, 'Wavelet': {}}	90dc3e1ba8c816a038eb15b893f008440ca069dc	1.1.1-beta1	1
314fd58bf855390c8419fda7f6ac787be3a080af	(1.0, 1.0, 1.0)	{'LoG': {}, 'Original': {}, 'Wavelet': {}}	89452b1626ef35fd38d062ee6b18dae1f4d8dee0	1.1.1-beta1	1
f7303295530dfe4c40be14bae21df46c9dac8f12	(1.0, 1.0, 1.0)	{'LoG': {}, 'Original': {}, 'Wavelet': {}}	f294c3b7364c1ba780094b4c98dd5d76b5fd1259	1.1.1-beta1	2
aa1d92a0b4bfd52bb01fba1ce0891d056f607af6	(1.0, 1.0, 1.0)	{'LoG': {}, 'Original': {}, 'Wavelet': {}}	c1ac05591c239f5a22ed8d8405acd7cb32e387c2	1.1.1-beta1	1
ed7f9b7a46fb4f2c715cd36f3f6ca2aba60dbe72	(1.0, 1.0, 1.0)	{'LoG': {}, 'Original': {}, 'Wavelet': {}}	ed120901fbc5516cded38e6c5c6bbbcb0f2fa934	1.1.1-beta1	1
a2885c5a291b9e749465e2a0ec811843c1356957	(1.0, 1.0, 1.0)	{'LoG': {}, 'Original': {}, 'Wavelet': {}}	4e18b32143ca1050b051aa36d374dec9aaba749e	1.1.1-beta1	1
45186d25cd3d063f3db17aa6ad67e7f04c255dfd	(1.0, 1.0, 1.0)	{'LoG': {}, 'Original': {}, 'Wavelet': {}}	5c0691d8f0296f9961acf1b53b384cacd0440057	1.1.1-beta1	1
73bb392ea6f27824996321b54d01efa59d755779	(1.0, 1.0, 1.0)	{'LoG': {}, 'Original': {}, 'Wavelet': {}}	0492968d65182737fd84c8188941fff120422996	1.1.1-beta1	1
df2a327f0fd4e4a0a788f198a38a51886ce4e7fe	(1.0, 1.0, 1.0)	{'LoG': {}, 'Original': {}, 'Wavelet': {}}	240bac4abe7d463f8271f55b1198912143aa02eb	1.1.1-beta1	1
cb57eb7e4f3b9b6a9d2032f40065cf95023e077a	(1.0, 1.0, 1.0)	{'LoG': {}, 'Original': {}, 'Wavelet': {}}	8ff7f4339d7d558e7fd6c6d07899b622eb6e86c1	1.1.1-beta1	1
8ef38ffe29df9cac94a667a31964ea2ca652e5bb	(1.0, 1.0, 1.0)	{'LoG': {}, 'Original': {}, 'Wavelet': {}}	913bd647f1e3c6e1ab09f89ac335ecb049b33b0b	1.1.1-beta1	1
89c8f005ffea423f2786070d8f173966baa33df8	(1.0, 1.0, 1.0)	{'LoG': {}, 'Original': {}, 'Wavelet': {}}	3fc45f6cd08ab1486926b71599658f8363f2ca36	1.1.1-beta1	1
019cb604b3fbc1d0b89d44e8dcdeebed40cab556	(1.0, 1.0, 1.0)	{'LoG': {}, 'Original': {}, 'Wavelet': {}}	12a71d649dc4315d3b6177b9c7405d5b9bfafc77	1.1.1-beta1	1
e48cf6ca33cd38279eacb0fa088efc1e13829fcb	(1.0, 1.0, 1.0)	{'LoG': {}, 'Original': {}, 'Wavelet': {}}	590cdeeb908f0c51ebba3a5ed94a547a91e3185c	1.1.1-beta1	1
16dd0656efdc9e17c85f904f727385322aedba74	(1.0, 1.0, 1.0)	{'LoG': {}, 'Original': {}, 'Wavelet': {}}	26ad771e496e74c38789499ec4290bb41899e372	1.1.1-beta1	1
16010807008a65eb9589d163a02dca4259c592eb	(1.0, 1.0, 1.0)	{'LoG': {}, 'Original': {}, 'Wavelet': {}}	721d55423d6364ec819310df2ad1b9f7ffe16f33	1.1.1-beta1	1
7f89bd9f0d38399c5078bdaf8c23dbb400da36d5	(1.0, 1.0, 1.0)	{'LoG': {}, 'Original': {}, 'Wavelet': {}}	24727a87d836e18bc49ad2de773306168395783d	1.1.1-beta1	1
e4c2a9d419938e931e307ed2fef3aa78ae25b852	(1.0, 1.0, 1.0)	{'LoG': {}, 'Original': {}, 'Wavelet': {}}	3bae02090b41b5b42ade1d34d6e2f28bb47b2062	1.1.1-beta1	1
b72f76ef35094b561f82869dbd9ce66688dfdc0d	(1.0, 1.0, 1.0)	{'LoG': {}, 'Original': {}, 'Wavelet': {}}	8382c87798d7c5377b42183dc60caaca53bdcb9b	1.1.1-beta1	1
598b8f00d15ac8a578c0f506c2fa81a09ca45dae	(1.0, 1.0, 1.0)	{'LoG': {}, 'Original': {}, 'Wavelet': {}}	ee319ae99510c91d8578b092990a97e77050307d	1.1.1-beta1	1
585288372551a5a7b62426ba406e35cc42ba2307	(1.0, 1.0, 1.0)	{'LoG': {}, 'Original': {}, 'Wavelet': {}}	eeca9d4cecf21180be4534f50d1c4d7c54549f8a	1.1.1-beta1	1
88dfd708f1296121cc8bc50d5a588774b97fcaee	(1.0, 1.0, 1.0)	{'LoG': {}, 'Original': {}, 'Wavelet': {}}	0d75ddac8125c5677f1825721fdc833ebc422363	1.1.1-beta1	1
0899aa8ed9ab2538553f22b4ca145c56d5b05c5d	(1.0, 1.0, 1.0)	{'LoG': {}, 'Original': {}, 'Wavelet': {}}	7d6d3a1d9abef77236f19844248f03e06301b415	1.1.1-beta1	1
75992e6c089e7148cd27cfcb442e09ecc06d0933	(1.0, 1.0, 1.0)	{'LoG': {}, 'Original': {}, 'Wavelet': {}}	6b138657d355e3e90848e4d85a2e2e586422cc0a	1.1.1-beta1	1
4618b426281c2e2b2187e4f17e263612b29add3d	(1.0, 1.0, 1.0)	{'LoG': {}, 'Original': {}, 'Wavelet': {}}	d8340bf2de91758dfba83788709e005f9b7d115f	1.1.1-beta1	1
8fe9f25f5324cac50cf02b6ee52d869af931a01a	(1.0, 1.0, 1.0)	{'LoG': {}, 'Original': {}, 'Wavelet': {}}	7d692433fb23eab5577569435da6eaf5d43ac71e	1.1.1-beta1	1
c1d572ada6df0c9ad45d058195f67c5e18373f83	(1.0, 1.0, 1.0)	{'LoG': {}, 'Original': {}, 'Wavelet': {}}	acd063947635800043dbe5a31d0c3d666c143824	1.1.1-beta1	1
10d556d912f25c09216bafa5a66952f7d81fe841	(1.0, 1.0, 1.0)	{'LoG': {}, 'Original': {}, 'Wavelet': {}}	5ce22805c506753062ad4b563df2b0b9e5dd3f4d	1.1.1-beta1	1
eb139341e5bdcb3228c5d66ca8cb59b543f7c121	(1.0, 1.0, 1.0)	{'LoG': {}, 'Original': {}, 'Wavelet': {}}	0bed8eda2dd56e150a607e361d2f7ca989558000	1.1.1-beta1	1
0f8bb6a679f462279fdc7030b67755129892e2a4	(1.0, 1.0, 1.0)	{'LoG': {}, 'Original': {}, 'Wavelet': {}}	49dba026a5935b80ad48b421de2836ca0f5cb6cc	1.1.1-beta1	1
488004ba1d23d972f102431a393957dd51c2b9bb	(1.0, 1.0, 1.0)	{'LoG': {}, 'Original': {}, 'Wavelet': {}}	2dd1fe2bb35511caab5599bc6e121471837cf36f	1.1.1-beta1	1
e8f2c5199d3012336ad8ba7b56d5e4f689a00ae8	(1.0, 1.0, 1.0)	{'LoG': {}, 'Original': {}, 'Wavelet': {}}	10f4462bfc58a9c4f712eff232ca129fa4c5d1db	1.1.1-beta1	2
a910d2fc98f00540b1f7953fcd1ae06a9ad06bc2	(1.0, 1.0, 1.0)	{'LoG': {}, 'Original': {}, 'Wavelet': {}}	da469960bd72b43637f816ade18cc2f4901d93cb	1.1.1-beta1	1
41230a7cf76b04c1c8be28f95e81055d7454f454	(1.0, 1.0, 1.0)	{'LoG': {}, 'Original': {}, 'Wavelet': {}}	545e14b362741d8496b6d46cd6d7282de2d5bac3	1.1.1-beta1	1
c9833ed94573172487d3a2392d8edbd9ce169831	(1.0, 1.0, 1.0)	{'LoG': {}, 'Original': {}, 'Wavelet': {}}	e4054de6c5618281be850ba246bc917cabe035c5	1.1.1-beta1	1
b3585b13296efcf884530d0066f687afb24148d1	(1.0, 1.0, 1.0)	{'LoG': {}, 'Original': {}, 'Wavelet': {}}	859e3d32529d8b49f28b4cb275979100e4cf6757	1.1.1-beta1	1
6baca2b585341fcdca50a5175b832a8667da594c	(1.0, 1.0, 1.0)	{'LoG': {}, 'Original': {}, 'Wavelet': {}}	fe62ddf39c39f5302a44e191a5ba987c764d2093	1.1.1-beta1	1
0040252eb47789872b18d46df6e5ebd5f36ed66a	(1.0, 1.0, 1.0)	{'LoG': {}, 'Original': {}, 'Wavelet': {}}	3476c15d8f3699b264bd52bb1cdd95e9eafc6426	1.1.1-beta1	1
4c13260d78c556a515754d7d4d7105016427b20b	(1.0, 1.0, 1.0)	{'LoG': {}, 'Original': {}, 'Wavelet': {}}	03cdad8fe97b4a80f101eb5cc486f2d172e0a571	1.1.1-beta1	1
0a3248d12a3ad904aff856fe2235bba407b54a68	(1.0, 1.0, 1.0)	{'LoG': {}, 'Original': {}, 'Wavelet': {}}	d759559eca8672a658ca44af5234d19269ef03c6	1.1.1-beta1	1
300ec0022da2f22a510eeaf86521db72d2680cfe	(1.0, 1.0, 1.0)	{'LoG': {}, 'Original': {}, 'Wavelet': {}}	0a65ea2be0fc6c355fe3f33e856faf2f827eb9c8	1.1.1-beta1	1
fa63779c19a9655e95a0cbbf52490f5fd7bb8592	(1.0, 1.0, 1.0)	{'LoG': {}, 'Original': {}, 'Wavelet': {}}	78fb1dbde1c91b855b420a18c28ba4b12ff9f1f4	1.1.1-beta1	1
26420ffcbcae814458ecb352f9922a16cdcf7531	(1.0, 1.0, 1.0)	{'LoG': {}, 'Original': {}, 'Wavelet': {}}	72125e20fff412968789946803fa5bf7c08136e0	1.1.1-beta1	1
afd835f657fc4009b364a8267baa496797ba8a7a	(1.0, 1.0, 1.0)	{'LoG': {}, 'Original': {}, 'Wavelet': {}}	7626d55449e6ac03388a70f43d00ae252cadba7c	1.1.1-beta1	1
3ad47ff43e11e3e7c5d94798ba0a1dcc8d191316	(1.0, 1.0, 1.0)	{'LoG': {}, 'Original': {}, 'Wavelet': {}}	d206df915f9d83c748a5f9a7f490c74f9b876752	1.1.1-beta1	1
be465182b41363fb0a24c0f39e83be527145cef5	(1.0, 1.0, 1.0)	{'LoG': {}, 'Original': {}, 'Wavelet': {}}	2631cfc049d8fb8a81ac1795733983753d4c1a6f	1.1.1-beta1	1
5ccee5534ebee2035cdc08fb25c930f46d416e76	(1.0, 1.0, 1.0)	{'LoG': {}, 'Original': {}, 'Wavelet': {}}	b81cbffc44a17a9fdcde8fdc38050425dd414883	1.1.1-beta1	2
6b086e9648536757c76fe9546dc229c2078bc31e	(1.0, 1.0, 1.0)	{'LoG': {}, 'Original': {}, 'Wavelet': {}}	69e430aedf320f99025c1ecb0f2603bd28cdf121	1.1.1-beta1	1
deb3739483bf9882150472db24506ccdffc88835	(1.0, 1.0, 1.0)	{'LoG': {}, 'Original': {}, 'Wavelet': {}}	8ada44ad84ea34ede6e8d8adfd2c4171b9d5b2d3	1.1.1-beta1	1
ae2fb26d74deda60fcd9b5512d2d8c4cdc02f09b	(1.0, 1.0, 1.0)	{'LoG': {}, 'Original': {}, 'Wavelet': {}}	a61662d0fd084d4fd715b4b6c10501b5b1db88e5	1.1.1-beta1	1
e4c1f1b50a2622af66579a0cc705ed95224707ba	(1.0, 1.0, 1.0)	{'LoG': {}, 'Original': {}, 'Wavelet': {}}	44522da0d508650be294d7d2da02985f94840457	1.1.1-beta1	1
81301f5e7f57e66819369a50fb8580038cd4307f	(1.0, 1.0, 1.0)	{'LoG': {}, 'Original': {}, 'Wavelet': {}}	539536388d4cc330519b873ae1e07432e42deee0	1.1.1-beta1	1
0988a7b4d40284e7aa00cdda8fdfd6e8170db508	(1.0, 1.0, 1.0)	{'LoG': {}, 'Original': {}, 'Wavelet': {}}	3e2228e681b6605c36dd7894d4f305b2fb78d70d	1.1.1-beta1	1
bba9fd993814f7c2d8700f76c15be963fa6a13f9	(1.0, 1.0, 1.0)	{'LoG': {}, 'Original': {}, 'Wavelet': {}}	95314ccfe67c6a731029525fa67368edf0f4e0c2	1.1.1-beta1	1
d31fc0b625e1403fbcef8e23f7659a794f8d2a92	(1.0, 1.0, 1.0)	{'LoG': {}, 'Original': {}, 'Wavelet': {}}	1a4ada51c3a7a0130a3e31cff8eca4c7a931293a	1.1.1-beta1	1
ad39cbbd4fab07012ddc60e4825c63227300facc	(1.0, 1.0, 1.0)	{'LoG': {}, 'Original': {}, 'Wavelet': {}}	0ab9ba0a59c0c6aa3e1a915e177badb67c65554d	1.1.1-beta1	1
fbb72fe0721ecb66f8f14a631d06f617d8749d63	(1.0, 1.0, 1.0)	{'LoG': {}, 'Original': {}, 'Wavelet': {}}	f63ebfabc0bdad63fc052fae5874b5fcc1ec82b4	1.1.1-beta1	1
1af4acb389de9db01f9f800373b5b5ee9f832914	(1.0, 1.0, 1.0)	{'LoG': {}, 'Original': {}, 'Wavelet': {}}	0a3ac3b15a7db0e0b4aaece1e772f94023a52677	1.1.1-beta1	1
934ce4d2a7a6080c14f4281770c6b6b8e7762379	(1.0, 1.0, 1.0)	{'LoG': {}, 'Original': {}, 'Wavelet': {}}	9c7b0f9542120a5d1c63b414ce684a6a4d051e7f	1.1.1-beta1	1
69b1efb62fc712b7b2c2e9f857250ca3efbaa6a1	(1.0, 1.0, 1.0)	{'LoG': {}, 'Original': {}, 'Wavelet': {}}	e1cb3aaa1aabecd163e8fa4b10d63f7c1e6c26e8	1.1.1-beta1	1
6f2d3c3db98af76d85136544b1253c8130449a21	(1.0, 1.0, 1.0)	{'LoG': {}, 'Original': {}, 'Wavelet': {}}	a7fa7ddbf56852c3dc68f50beb2946a492b038f8	1.1.1-beta1	2
cf2cdbe1128946803077cd3d274cbc70fc76011f	(1.0, 1.0, 1.0)	{'LoG': {}, 'Original': {}, 'Wavelet': {}}	04002b87fa65c33fb6db7928af26ad8ef51b19aa	1.1.1-beta1	1
ef087ab4b8bf4d7fd7bef824258b7d926e5e1fe4	(1.0, 1.0, 1.0)	{'LoG': {}, 'Original': {}, 'Wavelet': {}}	4bb3d7ca1fd126cfd229fc730dc790e9460c2909	1.1.1-beta1	1
054fba93d28e56e01c378e31bf15e0717dfbf45e	(1.0, 1.0, 1.0)	{'LoG': {}, 'Original': {}, 'Wavelet': {}}	a23bfd0d12c13749d5c3677115627535329f08ce	1.1.1-beta1	1
60e228850daf01015d767e6d5ac6f1d54dc80fbd	(1.0, 1.0, 1.0)	{'LoG': {}, 'Original': {}, 'Wavelet': {}}	aa1cd104dc22479f98d1f5f2d99768008a3479f7	1.1.1-beta1	1
050ceb7940625f4865e6256707da469e33ecd8d7	(1.0, 1.0, 1.0)	{'LoG': {}, 'Original': {}, 'Wavelet': {}}	400772ff43d63c595be5f6f8ef3ec03b8a11d355	1.1.1-beta1	1
f218a4e109f512b25c86d77cc7e1ccf678b55635	(1.0, 1.0, 1.0)	{'LoG': {}, 'Original': {}, 'Wavelet': {}}	3b5e3602dfa58615d178009d3c4bbda3b1b278a2	1.1.1-beta1	1
8bf3c8a5059fea0ae370db32a372863938187979	(1.0, 1.0, 1.0)	{'LoG': {}, 'Original': {}, 'Wavelet': {}}	4eb1581db5b7ed1119d121887e41852eadb10263	1.1.1-beta1	1
a4207feed93d67307a4d9c541fc4036fe9faf03f	(1.0, 1.0, 1.0)	{'LoG': {}, 'Original': {}, 'Wavelet': {}}	f9c177657849d34c35fdb80a94caeee0a3fc2b68	1.1.1-beta1	1
9a847ecddf2df6803b4253a70439f6719bd63f43	(1.0, 1.0, 1.0)	{'LoG': {}, 'Original': {}, 'Wavelet': {}}	d1b7ed26bb355d5b2fb351fff8007b44e15f7715	1.1.1-beta1	1
2b60599e673e3162bc243e1fa284da7c7222285a	(1.0, 1.0, 1.0)	{'LoG': {}, 'Original': {}, 'Wavelet': {}}	54f204f937643b1bd1f3916027f29aabf218e1ec	1.1.1-beta1	1
6871c8e2428a7f0df42ad9c0b267ce065b97fddd	(1.0, 1.0, 1.0)	{'LoG': {}, 'Original': {}, 'Wavelet': {}}	36772e785b999225a59e21bf9e0bcadb4e7d57ae	1.1.1-beta1	1
78520834edfc54938eb50245deb0b7bda563bc0d	(1.0, 1.0, 1.0)	{'LoG': {}, 'Original': {}, 'Wavelet': {}}	4ba85bcae710213d15a71a5d42a1ca2aafa658cf	1.1.1-beta1	1
19d2879a142774e357d38ac0e8fbb7492a584776	(1.0, 1.0, 1.0)	{'LoG': {}, 'Original': {}, 'Wavelet': {}}	f612ce80c75e02ae0279d46efbb01bdeced693a1	1.1.1-beta1	1
c5454410a0192ace0171bad6421cfd2e11cd6772	(1.0, 1.0, 1.0)	{'LoG': {}, 'Original': {}, 'Wavelet': {}}	75fdf53e234f7811f59d089f0b61aae1b84d46bb	1.1.1-beta1	2
e2e99cdda7f3e61d56e946a0b0bcc16d6fed45a6	(1.0, 1.0, 1.0)	{'LoG': {}, 'Original': {}, 'Wavelet': {}}	f4efd2c635601a5129e0bc8bd05d5f88f35c4cdb	1.1.1-beta1	1
d6df8d2df93ef59b1bba950be2acb73a3f2f84c7	(1.0, 1.0, 1.0)	{'LoG': {}, 'Original': {}, 'Wavelet': {}}	a527ffcf95e309da3632da83c1a057c4995b991e	1.1.1-beta1	1
ed65c08c80974cb4fd975d41e064408072eb9fc5	(1.0, 1.0, 1.0)	{'LoG': {}, 'Original': {}, 'Wavelet': {}}	4abe8c16bc87a898311af27e6e3e8d2eb9350abd	1.1.1-beta1	1
b54fcec088350813b092acb7a2f155e1fbdb8574	(1.0, 1.0, 1.0)	{'LoG': {}, 'Original': {}, 'Wavelet': {}}	3ffaa3ae6b5f33edfb8788f6b5f85efed9ce3d6c	1.1.1-beta1	2
f7bab373d06471ab6d846c8ae3aa0b8840feb195	(1.0, 1.0, 1.0)	{'LoG': {}, 'Original': {}, 'Wavelet': {}}	5d67b205d4304530e5504ca5db4d96b7dbc1fa1c	1.1.1-beta1	1
8ea1a010ff3e496743303208f45e2b6a46e9638a	(1.0, 1.0, 1.0)	{'LoG': {}, 'Original': {}, 'Wavelet': {}}	6f4b19bfb1b98803960ff5fead6a2ba43e5ce39f	1.1.1-beta1	1
6dff4408189ff68146aa5b8501b7c858b599c1a0	(1.0, 1.0, 1.0)	{'LoG': {}, 'Original': {}, 'Wavelet': {}}	38a6e0b09762103771f6baf8b4b286a608ea33c6	1.1.1-beta1	1
c88b281b945d81f98496f84645bb20b68ffee8f9	(1.0, 1.0, 1.0)	{'LoG': {}, 'Original': {}, 'Wavelet': {}}	d723f06a337f25cbb0a0e859f86803285935721c	1.1.1-beta1	1
9e3af65703fe371a72314f30f8610cee46c07806	(1.0, 1.0, 1.0)	{'LoG': {}, 'Original': {}, 'Wavelet': {}}	48f393cb69683f67dc82a1aad858447a3959e912	1.1.1-beta1	1
6dc8bb58620b346a123fda450f59eb23c3f875b2	(1.0, 1.0, 1.0)	{'LoG': {}, 'Original': {}, 'Wavelet': {}}	8d9f955505794172ad3ae3b21de02213ff46b21f	1.1.1-beta1	1
8a6ee45d54882e971c721576805518b0223edd45	(1.0, 1.0, 1.0)	{'LoG': {}, 'Original': {}, 'Wavelet': {}}	29db10ab660dd156cfdc56d449ed24cf07b7bf4f	1.1.1-beta1	1
62ec0bfda20ae2f050345ff4ab504a7f5d0ee9d6	(1.0, 1.0, 1.0)	{'LoG': {}, 'Original': {}, 'Wavelet': {}}	5f004fbfe00397005b5d0f81ee59953a72f71ffd	1.1.1-beta1	1
0f7b3f928363b4c91b7257db151003acc51dc16d	(1.0, 1.0, 1.0)	{'LoG': {}, 'Original': {}, 'Wavelet': {}}	85ba5240e3300fe62f037894084f3e0f0f39d4b7	1.1.1-beta1	1
21632cf06e539ea4939f3b3dd4814f74dfb8d133	(1.0, 1.0, 1.0)	{'LoG': {}, 'Original': {}, 'Wavelet': {}}	0e33f078d00e9d4de67495b6c8e2ab26a7da4f94	1.1.1-beta1	1
285ebc6336a666cb2c34c76f74d2f91004ce6428	(1.0, 1.0, 1.0)	{'LoG': {}, 'Original': {}, 'Wavelet': {}}	8fe9406e3eecac70a9110e2c178602f6bb51aaab	1.1.1-beta1	1
8652292d9b0a653fb4d837fd11c908a3093e7e67	(1.0, 1.0, 1.0)	{'LoG': {}, 'Original': {}, 'Wavelet': {}}	8b1ffcee09c55ebd1e8eb80e899624a0e0c22d43	1.1.1-beta1	1
38de3f3af0ee7c556478b9c3d7d7f92423576c6b	(1.0, 1.0, 1.0)	{'LoG': {}, 'Original': {}, 'Wavelet': {}}	a6f1f39e0da247744ba09a7f6b4577a8002cc749	1.1.1-beta1	1
5a8ac6e079824e746b392f693cae4de3bf2660e8	(1.0, 1.0, 1.0)	{'LoG': {}, 'Original': {}, 'Wavelet': {}}	a59a37790a3d2a30c9b65fb4bacfb6fa4cb4e489	1.1.1-beta1	1
e7e5d7043754b07480d07b9ad918e2a543ea5b0d	(1.0, 1.0, 1.0)	{'LoG': {}, 'Original': {}, 'Wavelet': {}}	b88dd6742bf3c0ea34274ba6d913f3eacc3f9447	1.1.1-beta1	1
53184def7583c0f3c45375d7b7c7a6817f6be21a	(1.0, 1.0, 1.0)	{'LoG': {}, 'Original': {}, 'Wavelet': {}}	69b3db8561c16c2b909e33df26a9d5887f59bb5e	1.1.1-beta1	1
d76dda1554fb0894e0ccc0d972f7c221c9e3eb53	(1.0, 1.0, 1.0)	{'LoG': {}, 'Original': {}, 'Wavelet': {}}	a415e1b4f4dee3f1cde66fe885256e5d7e63e7b6	1.1.1-beta1	1
014c1dd75c7dc2418eda6e673725cd7aa7f1ef8e	(1.0, 1.0, 1.0)	{'LoG': {}, 'Original': {}, 'Wavelet': {}}	35b1c0f4bc27838967e48542d5f4f51a07686671	1.1.1-beta1	1

general_info_VoxelNum	original_shape_Maximum3DDiameter	original_shape_Compactness2	original_shape_Maximum2DDiameterSlice	original_shape_Sphericity	original_shape_Compactness1	original_shape_Elongation
666	55.39855594	0.203155458	46.86149806	0.587863052	0.023911847	1.338463196
113	22.04540769	0.656290853	21.21320344	0.869024693	0.042978084	1.226394715
483	42.10700654	0.500291818	30.59411708	0.793854907	0.037524125	1.288988604
92	18.49324201	0.685254631	17.49285568	0.881625195	0.043916211	1.074879219
746	42.63801121	0.465236886	40.24922359	0.774862625	0.036185614	1.05525927
103	57.78408085	0.275054204	56.36488268	0.650338446	0.02782326	4.551966222
16306	136.0955547	0.336214281	107.3312629	0.695353084	0.030761461	1.240431289
5046	104.6565813	0.184721785	87.46427842	0.569516144	0.022801212	1.12026905
4229	103.2714869	0.305010675	79.25906888	0.673139403	0.029299243	1.690403365
364	47.52893855	0.289930962	35.11409973	0.661858065	0.028565786	1.786082055
5464	113.8815174	0.101198135	109.4897255	0.466005279	0.016876607	1.128616945
3073	79.3158244	0.35294472	61.18823416	0.706700768	0.031517533	1.230711154
331	43.05810028	0.438129085	38.18376618	0.75951093	0.035115586	1.583140358
190	39.57271787	0.373318801	24.73863375	0.720045522	0.032414461	1.923936832
920	44.59820624	0.382026409	42.42640687	0.725600871	0.032790314	1.01949709
8527	138.8452376	0.264283436	101.2027668	0.641736362	0.027273059	1.770558111
436	33.9411255	0.544435142	30	0.816548622	0.039144608	1.061823893
111	24.91987159	0.570931876	16.15549442	0.82958603	0.040085843	1.685496365
567	47.33920151	0.429404577	46.95742753	0.754435686	0.034764198	1.588059546
11927	144.0624864	0.284680075	87.46427842	0.657838102	0.028305929	1.805334305
1452	57.23635209	0.512419541	42.95346319	0.800218451	0.037976219	1.377547935
158	33.27160952	0.479520497	30.59411708	0.78271272	0.036736896	1.232235221
281	28.93095228	0.574224706	25.63201124	0.831177844	0.040201274	1.139160403
4804	92.0271699	0.33531537	89.49860334	0.694732827	0.030720311	1.243197821
726	52.04805472	0.419602461	51.0881591	0.748650885	0.034365122	1.517604541
2025	66.34003316	0.431418228	40.24922359	0.755613132	0.034845614	1.695292644
939	88.07950954	0.24224244	48.83646179	0.623376	0.026111029	2.239472233
1759	63.35613625	0.34022951	48.37354649	0.698110216	0.0309446	1.358151648
28	13.74772708	0.690786593	13.41640786	0.883991251	0.04409312	1.653948734
1151	48.92851929	0.519727104	45.89117562	0.804004455	0.038246047	1.05455872
3511	87.15503428	0.369573906	69.26037828	0.717629748	0.032251471	1.45778333
255	37.58989226	0.316753767	24.73863375	0.681669605	0.029857935	1.80965461
1279	72.86974681	0.156296379	67.68308504	0.538661958	0.020973617	1.323537225
3149	76.4852927	0.358826701	63.78087488	0.710604986	0.031779075	1.066714338
3477	115.3342967	0.133410178	65.8634952	0.51097108	0.019377304	2.192808916
1128	75.05997602	0.223232911	47.4341649	0.606623748	0.025065593	1.313154441
390	76.13146524	0.091482804	54.74486277	0.450588208	0.016046067	2.053614652
11738	129.3135724	0.194749273	88.28363382	0.579640357	0.023411908	1.703208264
197	33.54101966	0.441534531	30.59411708	0.761473671	0.035251793	1.420932327
12114	153.2644773	0.15961266	115.8015544	0.542445085	0.021194957	1.519805868
631	42.74342055	0.347089224	36.24913792	0.702770803	0.031254996	1.269022056
7237	122.8169369	0.128224865	109.8590005	0.504263365	0.018996999	1.328471088
1527	78.34538914	0.175424719	75.71657678	0.559796609	0.022220011	1.077729318
707	57.93962375	0.343549007	39.45883931	0.700373275	0.031095191	2.08155448
1088	54.41507144	0.466242131	45.79301257	0.775420309	0.036224686	1.27333352
1955	80.33056703	0.365976808	75.95393341	0.7152939	0.032094134	1.474317247
6969	91.43850392	0.331169765	88.23264702	0.691857882	0.030529818	1.065788094
219	27.82085549	0.666048701	25.63201124	0.87331046	0.043296408	1.271536708
114	27.82085549	0.436910649	27.16615541	0.75880621	0.035066724	1.330761878
220	58.01723882	0.454120486	24.73863375	0.768641268	0.03575069	2.779945058
1906	88.84255737	0.227313749	56.04462508	0.610297937	0.025293663	1.270976623
2159	70.80254233	0.198381556	62.42595614	0.58322182	0.023629227	1.127161592
10102	119.6244122	0.306409235	103.3150521	0.674166679	0.029366339	1.216333208
847	54.74486277	0.173270375	53.41348144	0.557495593	0.022083151	1.173184196
287	38.06573262	0.491281623	25.63201124	0.789060263	0.037184688	1.519185272
663	39.34463115	0.550198821	37.10795063	0.819419985	0.039351266	1.046008631
5287	110.6752005	0.211693654	89.49860334	0.595985847	0.024409158	1.058690226
1545	49.56813493	0.598461587	44.59820624	0.842711188	0.041040913	1.051316132
272	38.06573262	0.362491665	31.32091953	0.713016109	0.031940954	1.481133867
23513	155.0161282	0.217547707	135.5654823	0.601429653	0.024744355	1.245775137
797	46.28174586	0.381896423	43.26661531	0.725518566	0.032784735	1.127722268
1603	75.59761901	0.315981139	46.95742753	0.681114909	0.029821498	1.871627178
4572	166.6553329	0.04817452	89.49860334	0.363864035	0.011644144	1.873116622
8520	159.7904878	0.142314683	148.946299	0.522095444	0.020013532	1.905616616
321	35.36947837	0.546424094	24.73863375	0.817541763	0.039216045	1.433581721
2185	66.61080993	0.211841851	60.74537019	0.596124889	0.0244177	1.11290236
119	24.18677324	0.60848158	24.18677324	0.84738833	0.041383059	1.200539151
4128	114.3940558	0.132350816	90.44888059	0.509615006	0.019300217	1.5798919
10023	96.51424765	0.494711236	90.24965374	0.790892137	0.037314254	1.091618421
1527	63.63961031	0.38018975	63.5688603	0.724436184	0.032711396	1.370531282
198	38.88444419	0.47775501	34.98571137	0.781750948	0.036669206	1.424493883
7099	100.5783277	0.383784209	73.54590403	0.72671206	0.032865665	1.23483728
749	41.78516483	0.536655803	40.24922359	0.812640776	0.038863937	1.264443351
1073	57.07889277	0.270877292	40.80441153	0.647029675	0.027611193	1.200884119
3216	103.4021276	0.094780799	67.41661516	0.455939048	0.01633274	1.711673447
604	46.95742753	0.408124119	45.89117562	0.741761156	0.033891829	1.292886512
1482	56.36488268	0.399292038	53.41348144	0.736371351	0.033523102	1.217090408
115	71.18286311	0.377255486	20.1246118	0.722567662	0.03258492	5.963488544
651	56.92099788	0.275634828	56.92099788	0.650795734	0.027852612	1.501808874
1596	50.64582905	0.401207144	48.09365863	0.737546748	0.033603399	1.113100985
666	55.39855594	0.203155458	46.86149806	0.587863052	0.023911847	1.338463196
369	34.07345007	0.511363514	27.65863337	0.799668359	0.037937066	1.228308878
3418	81.3879598	0.209363715	68.41052551	0.593791266	0.02427446	1.341594833
1175	57.62811814	0.285482698	48.46648326	0.658455755	0.028345804	1.223984154
1805	71.11961755	0.262938876	49.65883607	0.640646219	0.027203594	1.281906643
176	25.45584412	0.500210848	22.84731932	0.793812077	0.037521089	1.074201903
725	58.403767	0.220522402	51.6139516	0.604158521	0.024912954	1.410075876
4066	76.01315676	0.480820282	75.95393341	0.783419288	0.036786652	1.125010718
1175	60.37383539	0.449990344	51	0.766303951	0.035587745	1.446414021
800	50.019996	0.445635371	45	0.763823856	0.035415119	1.107542611
276	39.45883931	0.281363806	27.65863337	0.655273709	0.028140577	1.405057393
1430	81.22191822	0.219803467	69.97142274	0.603501258	0.024872311	1.195799479
3422	92.80624979	0.140195287	83.67795409	0.519490732	0.019863949	1.43795523
3854	79.93747557	0.429255487	76.4852927	0.754348362	0.034758162	1.284973048
2067	108.5817664	0.077130174	71.30918594	0.425671693	0.014733673	2.210558781
1184	64.41273166	0.188305144	55.80322571	0.573175204	0.023021307	1.383304687
1465	63.5688603	0.254392574	54.08326913	0.633628655	0.026757842	1.199199952
87	21.84032967	0.661386012	21.21320344	0.871267809	0.043144593	1.326679133
71	20.1246118	0.68068429	17.49285568	0.879660807	0.043769516	1.362612921
9604	121.6018092	0.227810604	109.4897255	0.610742269	0.025321291	1.343667174

original_shape_SurfaceVolumeRatio	original_shape_Volume	original_shape_SphericalDisproportion	original_shape_Flatness	original_shape_SurfaceArea	original_shape_Maximum2DDiameterColumn
0.313999263	17982	1.701076461	1.36505848	5646.334743	51.0881591
0.383681864	3051	1.150715288	1.308745543	1170.613368	18.97366596
0.258805018	13041	1.259676034	1.257055259	3375.076241	32.44996148
0.405026029	2484	1.134268854	1.120562334	1006.084657	17.49285568
0.229381584	20142	1.290551341	1.048877496	4620.203862	35.11409973
0.528783261	2781	1.537660899	1.633983718	1470.54625	17.49285568
0.091420096	440262	1.438118307	1.315486377	40248.79419	115.8015544
0.16502171	136242	1.755876478	1.640183667	22482.88775	92.4175308
0.148085304	114183	1.485576383	1.183082747	16908.82427	68.41052551
0.341112428	9828	1.510897959	1.713466127	3352.452946	31.32091953
0.19639713	147528	2.145898435	1.728820566	28974.07572	108.6646217
0.156894042	82971	1.415026055	1.186076592	13017.6556	77.88452991
0.306821774	8937	1.316636746	1.088219719	2742.066195	28.3019434
0.389420116	5130	1.388801081	1.516609771	1997.725197	24
0.228420879	24840	1.378168136	1.18535974	5673.974638	42.95346319
0.122953473	230229	1.558272304	1.417661373	28307.45514	89.49860334
0.260346648	11772	1.224666815	1.312241042	3064.800737	32.44996148
0.404321758	2997	1.205420491	1.145491236	1211.75231	21.84032967
0.258154457	15309	1.325494033	1.205205563	3952.086578	29.54657341
0.107250693	322029	1.520130861	1.19452557	34537.83333	124.7798061
0.177895694	39204	1.249658763	1.176811678	6974.222797	56.04462508
0.380955969	4266	1.277607958	1.359974012	1625.158162	20.1246118
0.296096635	7587	1.203111954	1.269648143	2246.485169	28.3019434
0.137513093	129708	1.439402259	1.615150674	17836.54825	78
0.239573064	19602	1.335736083	1.272853317	4696.111201	32.44996148
0.168624897	54675	1.323428562	1.134886995	9219.566237	63.07138812
0.264073159	25353	1.604168272	1.408816784	6695.046809	42.63801121
0.191286181	47493	1.432438571	1.131145234	9084.754592	58.24946352
0.600521959	756	1.131232916	1.187478432	453.994601	13.41640786
0.191313657	31077	1.243774203	1.522524319	5945.454517	46.95742753
0.14779242	94797	1.393476236	1.275990047	14010.27802	66.61080993
0.372914165	6885	1.466986342	1.508063678	2567.514026	35.11409973
0.275691292	34533	1.85645187	1.858055622	9520.447399	48.83646179
0.154766537	85023	1.407251594	1.266019594	13158.71529	64.20280368
0.208240398	93879	1.957057921	1.27897261	19549.40034	99.72462083
0.255274301	30456	1.64846827	1.420985088	7774.634107	46.57252409
0.489660245	10530	2.219321283	3.590684445	5156.122378	50.28916384
0.122369416	316926	1.725207688	1.198392731	38782.04943	125.3554945
0.363819442	5319	1.313243042	1.557107939	1935.15561	33.54101966
0.129393135	327078	1.843504583	1.236179125	42321.64777	111.3642672
0.267427485	17037	1.422939023	1.152141829	4556.162059	41.67733197
0.165266918	195399	1.983090722	1.301051887	32292.99051	88.63972022
0.25006501	41229	1.78636309	3.072878	10309.93028	72.12489168
0.258360926	19089	1.427810048	1.136134483	4931.851718	38.41874542
0.202123154	29376	1.289623173	1.260932164	5937.569769	49.2036584
0.180230973	52785	1.39802674	1.063963302	9513.491915	46.86149806
0.121980148	188163	1.445383547	1.023452629	22952.15054	85.32877592
0.306228897	5913	1.145068158	1.166025382	1810.731466	27.16615541
0.438123999	3078	1.317859536	1.854613624	1348.545668	24.18677324
0.347401438	5940	1.300997021	1.254408068	2063.564541	57.31491952
0.213032895	51462	1.638543963	1.805068501	10963.09884	64.89992296
0.213851134	58293	1.714613489	1.272183522	12466.02418	69.7782201
0.110609679	272754	1.483312705	1.407756233	30169.23236	88.23264702
0.305604887	22869	1.793736153	2.071614668	6988.878153	47.4341649
0.309712498	7749	1.267330325	1.192357114	2399.962148	25.8069758
0.225606604	17901	1.22037541	1.246015301	4038.58382	34.20526275
0.155259132	142749	1.677892194	1.688625267	22163.0858	95.85927185
0.165465688	41715	1.186646165	1.291772029	6902.401161	47.4341649
0.348931807	7344	1.402492857	1.432120187	2562.555193	34.98571137
0.093556798	634851	1.662704849	1.655477141	59394.62707	126.6056871
0.239641244	21519	1.37832448	1.209315159	5156.839933	39
0.202223354	43281	1.468181047	1.062476782	8752.428984	45.69463864
0.266924434	123444	2.748279309	1.556965437	32950.21985	100.8464179
0.151170286	230040	1.915358602	1.347855383	34775.21258	91.43850392
0.287972652	8667	1.223179103	1.416525516	2495.858971	31.32091953
0.208389165	58995	1.677500838	1.279639321	12293.9188	63.5688603
0.386750963	3213	1.180096497	1.316158202	1242.630845	17.49285568
0.197185102	111456	1.96226561	1.29232035	21977.46274	105.3850084
0.094532186	270621	1.264394919	1.182902728	25582.39475	95.34149149
0.193233783	41229	1.380383837	1.092451891	7966.835639	53.16013544
0.353784979	5346	1.279179772	1.399421374	1891.334497	27.65863337
0.115416556	191673	1.37606083	1.052026459	22122.23751	90.24965374
0.218425659	20223	1.230556022	1.086553554	4417.222094	36.12478374
0.243354153	28971	1.545524167	1.19999703	7050.213179	45.89117562
0.239524937	86832	2.193275624	1.379580271	20798.42935	81.05553652
0.257090761	16308	1.348142851	1.730009493	4192.636133	46.57252409
0.192006789	40014	1.358010465	1.311896421	7682.959662	51.26402247
0.458759498	3105	1.383953438	1.358275267	1424.44824	20.1246118
0.285797117	17577	1.536580447	1.373517601	5023.455917	53.66563146
0.187023288	43092	1.35584626	1.128429938	8059.207534	46.57252409
0.313999263	17982	1.701076461	1.36505848	5646.334743	51.0881591
0.28104606	9963	1.250518403	1.104303146	2800.061899	32.31098884
0.180220855	92286	1.684093482	1.334283544	16631.86181	80.49844719
0.232001246	31725	1.518704929	1.23237944	7360.239516	54.74486277
0.20665799	48735	1.560923908	1.616502782	10071.47712	68.54195795
0.362360579	4752	1.259743998	1.183069526	1721.937472	24.73863375
0.297006506	19575	1.655194731	1.521833025	5813.902354	45.89117562
0.12891778	109782	1.27645568	1.122072003	14152.85174	68.47627326
0.199349821	31725	1.304965214	1.167783593	6324.373059	54.08326913
0.2273385	21600	1.309202368	1.135480524	4910.511592	40.80441153
0.377836375	7452	1.526079845	1.29578421	2815.636666	36.24913792
0.237086055	38610	1.656997375	1.999309812	9153.892598	58.94064811
0.205916776	92394	1.924962156	1.24149695	19025.47463	86.53323061
0.136297225	104058	1.325647473	1.164061014	14182.81667	65.520989
0.297286102	55809	2.349228329	1.467932177	16591.24007	83.19254774
0.265842803	31968	1.744667237	1.195887867	8498.462734	54.33231083
0.22400043	39555	1.578211452	1.713409695	8860.337005	57.31491952
0.417546438	2349	1.147752722	1.207325755	980.816584	18.24828759
0.44254915	1917	1.136801813	1.239001655	848.3667209	16.15549442
0.124171189	259308	1.637351876	1.261561284	32198.58277	108.1665383

original_shape_Maximum2DDiameterRow	log.sigma.5.0.mm.3D_gldm_GrayLevelVariance	log.sigma.5.0.mm.3D_gldm_HighGrayLevelEmphasis	log.sigma.5.0.mm.3D_gldm_GrayLevelNonUniformityNormalized
39	19.14053693	148.7447447	0.067563059
17.49285568	27.86670843	155.1769912	0.064296343
38.18376618	22.63725251	166.1407867	0.058515404
15.29705854	23.77173913	134.0217391	0.064272212
36.49657518	16.81074578	77.7386059	0.079738947
16.97056275	16.58308983	122.8932039	0.075878971
129.7998459	6.587297002	108.6671777	0.194830134
97.9489663	11.15321244	139.3596908	0.087199351
89.49860334	7.558476443	91.98628517	0.107402798
46.86149806	11.0500166	91.21153846	0.086840357
106.0235823	3.278473307	150.2230966	0.19719215
78.74642849	8.674402004	95.81386267	0.110810817
27.16615541	32.81315432	162.6767372	0.049972162
32.44996148	18.79127424	95.58421053	0.065263158
41.67733197	9.825973535	76.21304348	0.089829868
130.6981255	9.706619234	111.1094171	0.123971897
31.32091953	7.328402491	41.64220183	0.106114805
22.84731932	22.53810567	113.045045	0.067770473
37.58989226	7.267962512	59.86243386	0.10401289
114.629839	9.42716923	117.8021296	0.115770809
55.31726674	16.01608307	91.86914601	0.075239624
30.59411708	24.20834001	140.9303797	0.060807563
26.83281573	15.15348083	93.80071174	0.072301516
80.04998438	5.017289401	97.63988343	0.197944954
48.83646179	15.01635627	190.5606061	0.072733344
63.28506933	12.15061509	104.8518519	0.089304191
51.6139516	11.02096241	115.6091587	0.090340368
59.77457654	8.438854057	94.5412166	0.095116127
8.485281374	4.489795918	32.42857143	0.145408163
48.83646179	10.70298407	102.9530843	0.085012768
80.72174428	10.04703596	105.6086585	0.0885012
37.58989226	17.65084198	138.3921569	0.067404844
66.61080993	13.23762273	109.1673182	0.081870426
75	9.27560827	113.2197523	0.101056544
96.04686356	8.716621963	85.38107564	0.097124627
72.24956747	17.01503241	138.1515957	0.06938943
59.54829972	21.32760684	175.9153846	0.060315582
126.4634335	9.136715466	144.5523087	0.139710365
24.18677324	13.16385375	80.37055838	0.07681208
152.7939789	6.760864408	292.3189698	0.142694346
37.58989226	10.7273088	73.61648177	0.089910865
108.2081328	2.867542509	80.19911566	0.19798063
76.89603371	18.8629502	143.0825147	0.066833238
57.62811814	14.63035837	83.14285714	0.082622952
40.02499219	10.21088006	97.51654412	0.094669118
72.99315036	11.62102132	110.1329923	0.083970408
84.95881355	7.497906982	118.0388865	0.11453842
21.63330765	12.56950439	68.64840183	0.083880653
20.1246118	21.55278547	90.12280702	0.068944291
19.20937271	16.62353306	131.7954545	0.072355372
85.27602242	11.72253853	141.9171039	0.091413981
66.40783086	15.88067529	187.5525706	0.079329051
118.6802427	4.489690565	104.9415957	0.219545804
51	7.528798774	111.8724911	0.101235139
37.58989226	32.98680329	184.9547038	0.052119122
36.12478374	17.19810997	88.66817496	0.079725822
93.04837452	8.345126669	130.902213	0.102578604
48.37354649	9.94845718	72.69126214	0.087264691
29.69848481	13.7324827	116.3970588	0.075719074
148.946299	4.280161293	158.431506	0.205912702
42.42640687	12.96340889	110.7176913	0.079235338
69.97142274	24.13655618	219.4036182	0.058592183
129.4179277	18.78821619	215.4133858	0.067104035
150.029997	10.38909057	195.9497653	0.103683545
32.44996148	9.643365262	70.78504673	0.08946924
63.78087488	23.04850525	216.812357	0.06192691
24.18677324	16.71972318	106.4117647	0.075630252
113.5649594	9.972966748	167.4304748	0.096168021
91.0933587	7.078946339	102.3511923	0.134976086
51.6139516	15.9036569	128.6221349	0.071350058
21.84032967	11.55575962	66.52525253	0.085399449
89.49860334	11.48471909	206.5471193	0.129633955
40.80441153	9.178119112	57.78638184	0.097630842
55.80322571	12.35498628	108.4790308	0.088448219
98.27003612	8.811615798	164.4636194	0.113017391
33.54101966	9.06353888	68.74172185	0.099419982
53.66563146	49.22039189	212.7219973	0.050190227
16.15549442	30.25890359	230.173913	0.064650284
37.94733192	21.53001055	140.109063	0.060190986
50.28916384	7.866936687	110.5031328	0.108553809
39	19.14053693	148.7447447	0.067563059
33.54101966	25.259979	124.9891599	0.056088013
78	11.18939359	208.329725	0.116080633
56.92099788	7.243483205	94.5506383	0.106210593
65.520989	11.57511069	124.900277	0.087280177
24.73863375	36.84216813	287.5965909	0.050426136
45.09988914	14.90333603	125.6027586	0.071611891
75.23961722	8.677106881	107.4707329	0.144298611
40.02499219	32.9655091	169.666383	0.067564328
42.95346319	19.481775	107.185	0.067515625
36.24913792	30.75278303	320.7391304	0.062303088
64.89992296	9.004960634	144.1496503	0.099948164
73.05477397	9.672127117	110.2632963	0.092388406
70.03570518	9.136401865	104.0648677	0.099423994
84.85281374	7.22318302	61.99177552	0.103928413
61.84658438	20.32654995	150.3327703	0.065632989
55.07267925	14.26963156	90.72764505	0.078184254
16.15549442	16.05311138	109.9425287	0.074646585
16.15549442	10.81650466	77.54929577	0.089466376
118.6802427	12.90137452	223.8887963	0.092870443

log.sigma.5.0.mm.3D_gldm_DependenceEntropy	log.sigma.5.0.mm.3D_gldm_DependenceNonUniformity	log.sigma.5.0.mm.3D_gldm_GrayLevelNonUniformity	log.sigma.5.0.mm.3D_gldm_SmallDependenceEmphasis
6.535099836	113.6666667	44.996997	0.270840867
5.502534246	28.32743363	7.265486726	0.410737463
6.441130352	86.54451346	28.26293996	0.26636109
5.083306592	28.65217391	5.913043478	0.503227657
6.344749429	102.6595174	59.48525469	0.253663118
5.287594679	30.12621359	7.815533981	0.39197411
6.758653617	818.8134429	3176.900159	0.055565133
7.185557166	440.1482362	440.0079271	0.120654947
7.025140733	292.0621896	454.2064318	0.075161846
6.095025376	55.0989011	31.60989011	0.241367271
6.898459709	272.1669107	1077.457906	0.053969387
7.131772095	187.6742597	340.5216401	0.082919045
5.954231793	81.98489426	16.5407855	0.406667786
5.556319427	45.98947368	12.4	0.413070175
6.679909489	102.2217391	82.64347826	0.164327858
7.22207665	456.6289434	1057.108362	0.082576731
5.978763518	55.18807339	46.26605505	0.154172274
5.315019475	28.22522523	7.522522523	0.438128128
6.231723231	67.74779541	58.97530864	0.116848091
7.55234152	643.3786367	1380.798441	0.083954038
6.724119481	182.2754821	109.2479339	0.188128161
5.759941055	33.01265823	9.607594937	0.319404448
5.89757624	56.06761566	20.31672598	0.263810951
6.883363052	216.970025	950.9275604	0.049104668
6.546791276	107.2837466	52.80440771	0.184734667
7.019604731	192.5298765	180.8409877	0.154966714
6.676573879	111.2087327	84.82960596	0.210077627
6.920369834	162.760091	167.3092666	0.107045902
3.940759833	6.285714286	4.071428571	0.277103175
6.844568786	122.2302346	97.84969592	0.149447053
6.980970005	318.1187696	310.7277129	0.117268874
5.874784394	55.54901961	17.18823529	0.333983905
6.75867826	156.4542611	104.7122752	0.18467759
7.205380959	217.5138139	318.2270562	0.088785844
6.855618448	341.1616336	337.7023296	0.140131443
6.952294311	137.2996454	78.2712766	0.22106238
5.933614995	103.8512821	23.52307692	0.421150997
7.279635704	561.4184699	1639.920259	0.070108795
5.57539575	42.10659898	15.1319797	0.254132826
7.242226826	608.1609708	1728.599307	0.066425306
6.384816968	81.27892235	56.73375594	0.207375768
6.938549253	313.1636037	1432.785823	0.049701473
7.006983574	196.1211526	102.0543549	0.24381013
6.465635047	88.4427157	58.41442716	0.237042952
6.940422992	90.23897059	103	0.104222818
6.930291845	197.9370844	164.1621483	0.160487022
7.381358835	365.5538815	798.2182523	0.073066262
5.785255999	35.29223744	18.36986301	0.178063373
5.323125681	30.80701754	7.859649123	0.330820466
5.899142247	45.09090909	15.91818182	0.306890036
7.010886619	165.3378804	174.2350472	0.091233276
7.007306288	238.3765632	171.271422	0.184081715
6.833661615	403.6107701	2217.851713	0.040275881
6.501865676	100.4096812	85.74616293	0.149911756
6.090763722	63.41114983	14.95818815	0.39903395
6.33186322	89.86877828	52.85822021	0.230150773
7.339968541	337.7017212	542.3330811	0.084365753
6.646108736	173.436246	134.8239482	0.152257424
6.014996843	47.08823529	20.59558824	0.282759687
6.960101425	932.9335687	4841.625356	0.040764657
6.561268182	104.0840652	63.15056462	0.2191602
7.063315983	207.1110418	93.92326887	0.227604569
7.085936143	619.5787402	306.79965	0.227814559
7.403731864	537.6685446	883.3838028	0.115100405
5.905393596	54.85046729	28.71962617	0.199968097
6.954202251	333.1098398	135.3102975	0.222580293
5.182375487	34.32773109	9	0.376139122
7.131912232	338.497093	396.9815891	0.148187888
7.198171594	487.376534	1352.86531	0.052403697
6.740600175	207.8120498	108.951539	0.204473195
5.620659903	39.24242424	16.90909091	0.258074253
7.206271017	372.2232709	920.2714467	0.082404165
6.306462045	93.98531375	73.12550067	0.205686906
6.897988549	107.1323392	94.90493942	0.160524424
7.23055259	216.4291045	363.4639303	0.113739004
6.280208229	78.67218543	60.04966887	0.152998411
7.014631712	262.9581646	74.38191633	0.243222386
5.520241112	34.5826087	7.434782609	0.453352657
6.428021116	120.9907834	39.1843318	0.276213044
6.930237611	126.2819549	173.2518797	0.093817307
6.535099836	113.6666667	44.996997	0.270840867
6.199169409	73.63414634	20.69647696	0.316589313
7.229081935	216.842598	396.7636044	0.098311588
6.723485981	106.4791489	124.7974468	0.108389238
6.987582135	176.0559557	157.5407202	0.138374913
5.557633364	66.47727273	8.875	0.457741477
6.776155439	95.86344828	51.91862069	0.225403867
6.990478707	217.9862273	586.7181505	0.073876414
6.951122018	163.0153191	79.38808511	0.213608349
6.56382166	107.005	54.0125	0.227699093
6.010586885	64.12318841	17.19565217	0.404648218
6.968295596	126.1328671	142.9258741	0.108604333
7.265666175	281.0917592	316.1531268	0.139820615
7.11724602	289.4291645	383.1800727	0.10063518
6.655854664	220.5549105	214.820029	0.149318512
6.589274702	200.9949324	77.70945946	0.308326938
6.609502478	192.2491468	114.5399317	0.221143024
4.995663002	25.20689655	6.494252874	0.43307152
4.69131226	24.01408451	6.352112676	0.344483568
7.567804246	629.2207414	891.9277384	0.1138655

log.sigma.5.0.mm.3D_gldm_DependenceNonUniformityNormalized	log.sigma.5.0.mm.3D_gldm_DependenceVariance	log.sigma.5.0.mm.3D_gldm_LargeDependenceEmphasis
0.170670671	5.008485963	16.38138138
0.250685253	1.42376067	6.840707965
0.179181187	2.48455778	12.45341615
0.311436673	1.021266541	4.934782609
0.137613294	6.026759338	20.80697051
0.292487511	0.978980111	6.184466019
0.050215469	75.59454869	269.6965534
0.087227157	18.36383795	57.21482362
0.069061762	29.95402607	95.09789548
0.151370607	5.075745683	18.34615385
0.049810928	36.99694098	142.4725476
0.061072001	37.21775742	112.8958672
0.247688502	2.59853415	8.63141994
0.242049861	1.687977839	7.347368421
0.111110586	8.162660681	31.01521739
0.053550949	50.5497159	145.5910637
0.12657815	5.116446427	25.5733945
0.254281308	1.516597679	6.711711712
0.119484648	5.670508167	31.38095238
0.05394304	44.37929351	131.450239
0.125534079	6.200821134	24.50413223
0.208940875	2.099983977	9.784810127
0.199528881	2.127911247	11.21352313
0.045164451	55.98314893	197.0407993
0.147773756	6.332073553	22.84297521
0.095076482	14.67777863	45.25185185
0.118433155	7.471508561	26.67625133
0.092529898	12.42496948	48.77373508
0.224489796	1.780612245	10.35714286
0.106194817	8.423544366	33.63249348
0.090606314	14.87151639	51.76217602
0.217839293	2.312433679	9.698039216
0.122325458	6.10537699	25.02345582
0.069073933	32.76136879	96.09749127
0.098119538	12.3943664	42.68075928
0.121719544	8.868593129	27.76950355
0.266285339	1.257172913	6.256410256
0.047829142	52.91088284	165.0131198
0.213739081	1.67868278	10.19796954
0.050203151	51.17775364	160.6853228
0.128809703	8.222914851	26.46434231
0.043272572	48.46005507	184.1502004
0.128435594	7.218455489	23.74132286
0.125095779	6.820079664	23.84299859
0.08294023	17.2373351	60.59375
0.10124659	12.06549512	39.28132992
0.052454281	42.33961211	135.4126847
0.161151769	3.004607911	16.85388128
0.270236996	1.530624808	7.736842105
0.204958678	2.197438017	10.19090909
0.086746002	27.49657513	75.89611752
0.110410636	8.28118794	30.23946271
0.039953551	59.99277142	240.9701049
0.118547439	6.458979466	28.28453365
0.220944773	2.177396836	8.700348432
0.135548685	5.094112642	20.24886878
0.063873978	31.29660152	99.56289011
0.11225647	7.045791309	30.14174757
0.173118512	2.988538062	12.98529412
0.039677352	59.50338218	241.3511249
0.130594812	5.496408269	21.66750314
0.129202147	7.25765204	24.09170306
0.13551591	8.415202775	25.16929134
0.063106637	43.69407503	113.0133803
0.17087373	2.614629128	15.24922118
0.152453016	4.563231938	18.11304348
0.288468329	0.940611539	5.974789916
0.082000265	15.87603073	52.82994186
0.048625814	48.88982556	165.6259603
0.136091716	6.404743433	22.8637852
0.198194062	3.258034894	13
0.052433198	58.82391991	163.7832089
0.12548106	5.987393962	23.64085447
0.099843746	12.05783577	39.59832246
0.067297607	33.15235042	92.66666667
0.130251963	6.136627779	25.6192053
0.177434659	2.919239602	13.57624831
0.300718336	0.961512287	5.208695652
0.185853738	3.62802353	13.53456221
0.079124032	20.25779047	68.69799499
0.170670671	5.008485963	16.38138138
0.199550532	3.339208731	11.92140921
0.063441369	44.60398893	116.9286132
0.090620552	12.41786582	48.44851064
0.097537926	10.18375672	39.48753463
0.377711777	0.569085744	4.170454545
0.132225446	5.904810464	21.87172414
0.053611959	54.24605301	155.7073291
0.138736442	6.265854957	22.08255319
0.13375625	6.405	22.405
0.232330393	1.775624869	7.739130435
0.088204802	16.16900778	54.26293706
0.082142536	16.2112627	53.69199299
0.075098382	26.66666837	78.47379346
0.106702908	10.29593008	36.13497823
0.169759233	3.915220964	14.08614865
0.131228087	6.501227038	22.95767918
0.289734443	1.007002246	5.528735632
0.338226542	0.674866098	5.816901408
0.065516529	35.86479113	97.51770096

log.sigma.5.0.mm.3D_gldm_LargeDependenceLowGrayLevelEmphasis	log.sigma.5.0.mm.3D_gldm_SmallDependenceHighGrayLevelEmphasis	log.sigma.5.0.mm.3D_gldm_LargeDependenceHighGrayLevelEmphasis
0.292397918	49.26581042	1613.477477
0.271481869	82.78910275	723.920354
0.25659802	59.80692379	1754.935818
0.32951406	89.91995471	456.6521739
1.285055897	34.01347461	711.6072386
0.372288319	62.48914509	645.2621359
2.375426064	6.152804309	35610.99871
0.469199809	19.32567791	9912.655767
1.131442811	8.204778694	11598.95531
0.513936052	31.11029804	1104.118132
0.922901035	7.726939953	22957.35706
1.337714219	9.953611877	13661.93231
2.800354918	93.26934066	696.6344411
0.855400772	54.38400731	398.8894737
0.80281877	17.15712584	2342.2
1.418060523	9.208769286	20605.50815
3.573123606	11.20064623	731.5963303
0.313918082	66.99510761	522.9009009
1.683007728	9.575456784	1743.746032
1.314569193	12.21462359	16576.93905
0.934959841	28.29920239	1565.885675
0.369546101	50.97754747	1012.487342
1.760955334	33.27129909	846.7117438
1.973418262	4.984249708	22712.53955
0.152882419	39.15424327	5238.053719
0.692773135	24.10419581	4234.421235
0.812962277	32.14146392	2758.923323
0.690625942	11.38313708	5512.775441
0.733217936	13.19138889	232.5
0.633302619	20.91082668	2869.486533
0.659613749	15.12360439	7006.100256
0.281213124	57.58677158	957.6392157
0.854417691	29.22727673	2516.935887
0.896037267	10.56915215	13164.67799
0.805020161	16.45811635	3973.503595
0.673221428	38.24124106	4467.321809
0.104033658	90.38684473	893.6282051
1.115833843	9.319030499	29622.08852
0.533296721	26.70270446	689.5482234
0.522924588	20.7824751	51032.68483
0.836578282	24.79115972	1332.884311
2.440973716	5.024615527	14796.61282
0.585705611	47.25956445	2941.907662
0.922276818	33.09725872	1189.598303
0.895600971	12.52857394	6906.549632
0.571881569	25.9997886	4567.349361
1.13964825	8.627664425	19429.25886
2.487762804	14.4105372	1086
0.928385146	37.11262884	469.3157895
0.200986799	46.62401317	1150.504545
0.545479392	11.2996746	14732.36516
0.274972041	48.10316819	4787.041223
2.193529273	4.721929667	28853.95961
0.353238549	17.27997345	2946.146399
0.426087494	94.99577098	1103.867596
0.852448545	35.16319418	970.4690799
0.783372826	12.58039284	15357.99054
1.050125242	15.5676507	2200.733981
0.362811672	42.56394038	1085.852941
1.467846294	7.020730403	41238.26802
0.422479403	34.62469325	1905.006274
0.226628557	67.38886537	4594.73612
0.191861373	61.04836513	5152.240595
0.550818946	25.62030044	26619.28286
0.911955905	19.6309161	824.0498442
0.173327294	58.74665205	3103.918078
0.391866687	52.20941643	528.9663866
0.378610877	33.320279	9300.323886
1.659335382	6.191889284	20378.29233
0.445704685	37.82738914	2222.495088
1.356868779	25.16981053	479.0808081
0.707258698	14.99555408	43311.81124
1.299874451	20.16420445	876.4726302
0.691652168	24.4936984	3880.492078
0.54061554	20.09000648	18048.49036
0.930951178	13.704213	1300.456954
0.308100504	68.32373604	2098.221323
0.11120832	100.0694155	1190.034783
0.498380119	50.80755664	1225.479263
0.699854799	12.21134105	8941.181078
0.292397918	49.26581042	1613.477477
1.67353361	58.9341086	692.0677507
0.519965236	22.12216467	30544.86454
0.611652951	11.89833839	5274.436596
0.521407994	21.70256372	5096.607756
0.052918815	138.0804924	1161.380682
0.542712943	34.10867647	2559.666207
1.513820022	9.755305663	20891.6183
0.378154063	50.54436481	2466.224681
0.917986416	41.60129207	1258.0025
0.056600616	144.104428	2298.913043
0.437903539	18.43001946	8515.258741
0.826225171	21.13259622	5905.868206
0.994638458	14.03211832	9770.832901
1.23921803	12.76755681	1674.025641
0.232504797	64.36396005	1474.75
0.770820468	31.29280722	1312.866894
0.406140078	58.88644955	519.2068966
0.255658653	32.82443271	391.3802817
0.450039634	29.3711188	24121.55539

log.sigma.5.0.mm.3D_gldm_SmallDependenceLowGrayLevelEmphasis	log.sigma.5.0.mm.3D_gldm_LowGrayLevelEmphasis	log.sigma.5.0.mm.3D_gldzm_DistanceZoneVariabilityNormalized
0.004493366	0.015733267	0.972227733
0.009818669	0.039885311	0.839950754
0.00351324	0.020393401	0.873200981
0.012349684	0.047927817	0.964297521
0.006070123	0.0426824	0.926371825
0.008157192	0.046538675	1
0.001174051	0.016370004	0.942515886
0.001514276	0.012084509	0.957766161
0.001680312	0.018556986	0.962278107
0.005459388	0.028427665	0.942906574
0.000677462	0.007698114	0.962278107
0.001625705	0.022062671	0.983123585
0.004792495	0.058661528	0.915826114
0.009104846	0.058273554	0.957010023
0.003482537	0.029206801	0.973688889
0.001479188	0.018487829	0.928997982
0.005042149	0.092068836	1
0.015801828	0.039240186	0.966111772
0.003310752	0.040644368	0.967222222
0.001305431	0.01515272	0.9543866
0.002723495	0.030800001	0.884702575
0.010396365	0.030486294	0.938523558
0.005338934	0.059080063	0.97727573
0.000885135	0.01621653	0.978380961
0.003160936	0.009815569	0.958352196
0.00257928	0.017912188	0.772401305
0.002539198	0.023876228	0.990338392
0.002477618	0.019978098	0.953243214
0.042766891	0.084222248	1
0.002624247	0.018504599	0.954826423
0.001628441	0.01759937	0.935696172
0.007846316	0.022526252	0.963648834
0.002840507	0.02759211	0.98455692
0.001600969	0.014776233	0.959201389
0.002531716	0.02392446	0.963727829
0.002945777	0.027278265	0.985294925
0.006373069	0.015831281	1
0.001023144	0.012252819	0.90511665
0.007417574	0.044137905	0.967222222
0.000396748	0.00396772	0.958121281
0.00552495	0.037270074	0.986395196
0.000925646	0.015275513	0.981133787
0.003629414	0.023710084	0.912425027
0.005347179	0.035456226	0.989418289
0.002095428	0.02365621	0.963315573
0.002352433	0.017163952	0.939269465
0.001168936	0.012984773	0.986637576
0.006077803	0.07918509	1
0.009910149	0.067072929	0.961553249
0.008922918	0.023207676	0.931122449
0.001815759	0.012614774	0.973688889
0.001787638	0.010132565	0.940829641
0.000620718	0.013117463	0.965572607
0.003313821	0.013863723	0.886134068
0.00548457	0.028449874	0.926577705
0.0046091	0.03211541	0.943230396
0.001125096	0.011465506	0.985149346
0.003282763	0.03037872	0.878950014
0.006646619	0.025557052	1
0.000408082	0.007564674	0.959222063
0.004001541	0.018954036	0.930120098
0.001719719	0.009914756	0.936345781
0.001881625	0.009319646	0.915475034
0.000921794	0.007638062	0.945140657
0.004687357	0.046483814	0.972227733
0.002025614	0.009501969	0.849809016
0.008990414	0.050848434	0.964923469
0.001270941	0.009598469	0.977815787
0.000935914	0.015654318	0.985623458
0.002274284	0.017759442	0.905252898
0.007667147	0.051713628	0.969238281
0.000821314	0.007440567	0.905782212
0.007293162	0.05073995	0.921480114
0.003188624	0.019428503	0.977903311
0.001346862	0.009476275	0.984085905
0.00394048	0.031546862	0.895061728
0.003598557	0.018280339	0.491445692
0.011279348	0.028806581	0.969704142
0.003665129	0.025390048	0.91090925
0.001937746	0.014320641	0.971122596
0.004493366	0.015733267	0.972227733
0.00476552	0.051569252	0.971836735
0.000970242	0.007055121	0.947406866
0.002168516	0.019275257	0.967750605
0.002188773	0.015348787	0.955578512
0.010217224	0.015306583	0.837662974
0.003255347	0.024660843	1
0.001319329	0.016996097	0.939166376
0.003093456	0.016034981	0.524404071
0.003107233	0.026807257	0.96117353
0.006782955	0.011381785	0.886134068
0.001664125	0.011756616	1
0.001731415	0.016849249	0.970839721
0.001536074	0.018721603	0.946307813
0.00416498	0.034251919	0.993630638
0.003715981	0.015845951	0.937744182
0.003924965	0.029182888	0.966490186
0.012088109	0.053979141	1
0.02096211	0.046456946	0.937565036
0.000720892	0.006517957	0.920859464

log.sigma.5.0.mm.3D_gldzm_LowIntensityEmphasis	log.sigma.5.0.mm.3D_gldzm_LargeDistanceEmphasis	log.sigma.5.0.mm.3D_gldzm_HighIntensitySmallDistanceEmphasis
0.01627748	1.042253521	187.5762911
0.034152919	1.263157895	189.6052632
0.016970961	1.204081633	222.2636054
0.036742972	1.054545455	165.0045455
0.02571556	1.184834123	139.0014481
0.035004451	1	144.7843137
0.023582534	1.312958435	119.8809996
0.013795902	1.064748201	167.8390288
0.024864807	1.057692308	126.4423077
0.028872555	1.088235294	127.8161765
0.013452096	1.057692308	143.6067308
0.02087492	1.025531915	132.8776596
0.017028768	1.132075472	220.5062893
0.028394115	1.065934066	129.1923077
0.026990813	1.04	109.1283333
0.017204614	1.151414309	120.8240895
0.038024533	1	80.8852459
0.035758154	1.051724138	144.5387931
0.037980544	1.05	86.27083333
0.016265844	1.120575221	161.771336
0.017432115	1.295373665	156.9208432
0.032996941	1.095238095	161.5436508
0.028036312	1.034482759	123.8045977
0.022040568	1.032786885	111.2677596
0.01752499	1.063829787	211.7109929
0.016902413	1.643712575	147.5913382
0.016684724	1.014563107	154.9805825
0.031097503	1.071856287	108.8188623
0.12210641	1	43.72727273
0.018421754	1.069364162	144.9248555
0.016025565	1.192307692	136.9068605
0.023611905	1.055555556	166.125
0.017503075	1.023346304	159.1673152
0.019187131	1.0625	125.7666667
0.020946415	1.079918033	125.6640625
0.016367584	1.022222222	173.9490741
0.015467853	1	205.0597015
0.0173769	1.199417758	137.2921317
0.037856429	1.05	99.41666667
0.006367401	1.071225071	319.5445552
0.035626933	1.020547945	122.5753425
0.020367875	1.028571429	110.052381
0.015978976	1.137681159	191.7349034
0.027158649	1.015957447	139.0598404
0.029148872	1.056074766	125.8738318
0.01553589	1.094043887	170.2194357
0.017366396	1.020179372	124.4624439
0.051508761	1	78.35555556
0.044810934	1.058823529	107.7696078
0.029213789	1.107142857	146.6458333
0.024096552	1.04	119.21
0.009807468	1.091549296	268.4712441
0.017565964	1.148255814	129.2275
0.022369018	1.181818182	115.6212121
0.018203022	1.114503817	236.6698473
0.022191468	1.087719298	155.6169591
0.014439845	1.02244389	158.7973815
0.02075288	1.360169492	104.819209
0.028495184	1	149.0795455
0.011456026	1.148771022	184.9333491
0.018435748	1.134020619	160.7157789
0.009075839	1.111111111	302.7070707
0.008959582	1.132653061	271.5182823
0.009184706	1.089627392	228.4391015
0.033987134	1.042253521	97.80633803
0.01004758	1.290018832	255.2332601
0.037451676	1.053571429	129.2366071
0.010200327	1.033653846	229.9282853
0.020353132	1.03373494	134.3323963
0.01333685	1.16374269	194.5994964
0.036892693	1.046875	96.12890625
0.01084372	1.194706994	182.1599979
0.041751421	1.122807018	98.41520468
0.020785998	1.033519553	158.6438547
0.014189587	1.024064171	178.334893
0.031542934	1.166666667	93.7662037
0.015558987	2.633093525	146.8968658
0.029516195	1.046153846	224.65
0.015914138	1.140186916	184.3890187
0.022562511	1.080291971	140.2027575
0.01627748	1.042253521	187.5762911
0.019216797	1.042857143	181.9517857
0.010392596	1.081081081	231.8280781
0.02762054	1.049180328	110.0020492
0.016446585	1.068181818	159.8664773
0.019486438	1.267326733	292.5173267
0.018452346	1	149.9562842
0.018049976	1.178988327	147.451497
0.014175726	2.411552347	132.4901976
0.015156928	1.108374384	184.3586481
0.015664309	1.181818182	350.2575758
0.019452158	1	177.7189542
0.013854259	1.044397463	159.3530655
0.019031689	1.153846154	152.9701129
0.034516002	1.009584665	89.39057508
0.013463288	1.120689655	209.6774083
0.018857598	1.051136364	148.7535511
0.041582262	1	127.2826087
0.059801478	1.096774194	87.62096774
0.006919425	1.142056075	267.4009605

log.sigma.5.0.mm.3D_gldzm_LowIntensityLargeDistanceEmphasis	log.sigma.5.0.mm.3D_gldzm_HighIntensityEmphasis	log.sigma.5.0.mm.3D_gldzm_DistanceZoneVariability	log.sigma.5.0.mm.3D_gldzm_ZonePercentage
0.018078906	188.685446	207.084507	0.31981982
0.096930697	190.8421053	47.87719298	0.504424779
0.042278154	224.4217687	128.3605442	0.304347826
0.091288427	165.0181818	53.03636364	0.597826087
0.0598394	140.3933649	195.464455	0.282841823
0.035004451	144.7843137	51	0.495145631
0.031786147	121.0207824	770.9779951	0.050165583
0.015063372	169.0017986	532.5179856	0.110186286
0.031831055	126.6730769	250.1923077	0.061480255
0.041331705	128.0294118	96.17647059	0.28021978
0.014943905	144.8961538	250.1923077	0.047584187
0.025484849	132.9191489	231.0340426	0.076472502
0.044577248	221.3836478	145.6163522	0.480362538
0.0333758	129.4725275	87.08791209	0.478947368
0.027550227	109.8533333	146.0533333	0.163043478
0.027632966	121.9317804	558.327787	0.070481998
0.038024533	80.8852459	61	0.139908257
0.087482292	144.5517241	56.03448276	0.522522523
0.087980544	86.28333333	58.03333333	0.105820106
0.029501624	163.5154867	862.7654867	0.075794416
0.035055389	160.5587189	248.6014235	0.193526171
0.083592179	161.7460317	59.12698413	0.398734177
0.028994167	124.1149425	85.02298851	0.309608541
0.023229093	111.7431694	179.0437158	0.038093256
0.018624293	212.8865248	135.1276596	0.194214876
0.021653539	168.5688623	257.9820359	0.164938272
0.020325501	154.9951456	204.0097087	0.219382322
0.033962592	109.7305389	159.1916168	0.094940307
0.12210641	43.72727273	11	0.392857143
0.038597305	145.4277457	165.1849711	0.150304083
0.019686788	139.4587912	340.5934066	0.103674167
0.051956576	166.4722222	104.0740741	0.423529412
0.018556901	159.3190661	253.0311284	0.200938233
0.021723483	127.4041667	230.2083333	0.076214671
0.032770149	126.1577869	470.2991803	0.140350877
0.017775763	174.1518519	266.0296296	0.239361702
0.015467853	205.0597015	201	0.515384615
0.024096815	141.5604076	621.8151383	0.058527858
0.038637679	100.2166667	58.03333333	0.304568528
0.006556322	326.531339	672.6011396	0.05794948
0.040763919	122.5958904	144.0136986	0.231378764
0.021725018	110.2095238	309.0571429	0.043526323
0.027431431	193.3961353	377.7439614	0.271119843
0.027355654	139.3829787	186.0106383	0.265912306
0.030842559	126.3925234	103.0747664	0.098345588
0.019182145	172.1285266	299.6269592	0.163171355
0.018029175	124.6793722	440.0403587	0.063997704
0.051508761	78.35555556	45	0.205479452
0.103634463	107.7843137	49.03921569	0.447368421
0.033866567	147.3333333	78.21428571	0.381818182
0.024751628	119.86	146.0533333	0.078698846
0.019188493	269.6103286	400.7934272	0.197313571
0.022225531	130.1918605	332.1569767	0.034052663
0.024746608	119.3712121	116.969697	0.155844156
0.048441377	237.6946565	121.3816794	0.456445993
0.050943905	155.8538012	161.2923977	0.257918552
0.016401607	158.8778055	395.0448878	0.075846416
0.037997894	109.3771186	207.4322034	0.152750809
0.028495184	149.0795455	88	0.323529412
0.012951493	187.2121604	741.4786546	0.032875431
0.039343179	161.7783505	180.443299	0.243412798
0.018971388	304.9924242	370.7929293	0.247036806
0.019058581	274.0705782	1076.598639	0.257217848
0.011848154	230.3736153	938.5246727	0.116549296
0.035677275	98.07042254	69.02816901	0.221183801
0.015471487	272.2542373	451.2485876	0.243020595
0.091023104	129.25	54.03571429	0.470588235
0.011332693	230.3910256	610.1570513	0.151162791
0.021463189	134.6963855	409.0337349	0.041404769
0.031325678	196.2777778	309.5964912	0.223968566
0.083767693	96.140625	62.03125	0.323232323
0.02013749	186.5330813	479.1587902	0.074517538
0.068820822	100.1345029	157.5730994	0.228304406
0.037662162	159.2513966	175.0446927	0.166821994
0.014409379	179.5962567	368.0481283	0.116293532
0.080154045	94.2037037	96.66666667	0.178807947
0.037560746	297.4292566	204.9328537	0.281376518
0.0346444	224.7538462	63.03076923	0.565217391
0.033930847	186.1448598	194.9345794	0.328725038
0.023079418	142.0218978	133.0437956	0.085839599
0.018078906	188.685446	207.084507	0.31981982
0.019988936	182.5571429	136.0571429	0.379403794
0.012043782	234.0870871	315.4864865	0.097425395
0.02880544	110.5245902	118.0655738	0.103829787
0.031830801	160.375	252.2727273	0.146260388
0.061250899	295.8514851	84.6039604	0.573863636
0.018452346	149.9562842	183	0.252413793
0.022501717	149.1167315	241.3657588	0.063207083
0.0183763	250.5559567	145.2599278	0.235744681
0.034349616	185.3448276	195.1182266	0.25375
0.048133778	351.8257576	116.969697	0.47826087
0.019452158	177.7189542	153	0.106993007
0.023797881	159.5433404	459.2071882	0.138223261
0.02348346	154.5137363	344.456044	0.094447327
0.035580965	89.41214058	311.0063898	0.151427189
0.027406169	210.8669951	380.7241379	0.342905405
0.038207668	148.9772727	340.2045455	0.240273038
0.041582262	127.2826087	46	0.528735632
0.156575672	87.64516129	29.06451613	0.436619718
0.008850002	271.9766355	985.3196262	0.111411912

log.sigma.5.0.mm.3D_gldzm_IntensityVariabilityNormalized	log.sigma.5.0.mm.3D_gldzm_LowIntensitySmallDistanceEmphasis	log.sigma.5.0.mm.3D_gldzm_IntensityVariability
0.061165113	0.015827124	13.02816901
0.056940597	0.018458475	3.245614035
0.06076172	0.010644163	8.931972789
0.060495868	0.023106609	3.327272727
0.070371285	0.017409739	14.84834123
0.07266436	0.035004451	3.705882353
0.076909512	0.022233019	62.91198044
0.066889654	0.013479034	37.19064748
0.081745562	0.023123245	21.25384615
0.075163399	0.025757767	7.666666667
0.103816568	0.013079144	26.99230769
0.078279765	0.019722437	18.39574468
0.05525889	0.010141648	8.786163522
0.072817293	0.027148694	6.626373626
0.083022222	0.02685096	12.45333333
0.086276062	0.014798648	51.85191348
0.118516528	0.038024533	7.229508197
0.065992866	0.02282712	3.827586207
0.102222222	0.025480544	6.133333333
0.065723138	0.013130092	59.41371681
0.069008751	0.013291309	19.39145907
0.060720585	0.020348132	3.825396825
0.078874356	0.027796849	6.862068966
0.093971155	0.021743437	17.19672131
0.066646547	0.017250164	9.397163121
0.066836387	0.015969135	22.32335329
0.0759261	0.01577453	15.6407767
0.088242676	0.030381231	14.73652695
0.123966942	0.12210641	1.363636364
0.082027465	0.013377866	14.19075145
0.084379906	0.01523999	30.71428571
0.067558299	0.016525737	7.296296296
0.068025254	0.017239618	17.48249027
0.088888889	0.018553043	21.33333333
0.082513101	0.018024584	40.26639344
0.069876543	0.016015539	18.86666667
0.062151927	0.015467853	12.49253731
0.078850518	0.015812106	54.17030568
0.083888889	0.037661116	5.033333333
0.068972654	0.006323441	48.41880342
0.083223869	0.034342686	12.15068493
0.101879567	0.020028589	32.09206349
0.058927863	0.013115862	24.39613527
0.069714803	0.027109397	13.10638298
0.077998078	0.02872545	8.345794393
0.071215888	0.014624326	22.71786834
0.084759396	0.017200701	37.80269058
0.088395062	0.051508761	3.977777778
0.07112649	0.030105051	3.62745098
0.065759637	0.028050595	5.523809524
0.076177778	0.023932782	11.42666667
0.063666821	0.007462212	27.12206573
0.092651433	0.016559593	31.87209302
0.099862259	0.02177462	13.18181818
0.057514131	0.010643434	7.534351145
0.067131767	0.015003359	11.47953216
0.077592801	0.013949404	31.11471322
0.095554438	0.016843785	22.55084746
0.071797521	0.028495184	6.318181818
0.080422875	0.011194874	62.16688228
0.075087682	0.013298381	14.56701031
0.053795531	0.006621437	21.3030303
0.059409563	0.006434833	69.86564626
0.066113347	0.008524552	65.65055388
0.095020829	0.033564599	6.746478873
0.057493767	0.008736433	30.52919021
0.078443878	0.024058819	4.392857143
0.063871425	0.009917235	39.85576923
0.076150385	0.020097745	31.60240964
0.069782155	0.008856219	23.86549708
0.084960938	0.025173943	5.4375
0.077619077	0.008582419	41.06049149
0.083205089	0.034984071	14.22807018
0.064948035	0.016566957	11.62569832
0.067831508	0.014134638	25.36898396
0.100308642	0.019390156	10.83333333
0.043412752	0.010360985	18.10311751
0.054201183	0.028234144	3.523076923
0.06450345	0.011409961	13.80373832
0.074857478	0.022474663	10.25547445
0.061165113	0.015827124	13.02816901
0.06377551	0.019023762	8.928571429
0.068491915	0.009979799	22.80780781
0.08559527	0.027324315	10.44262295
0.068583563	0.012600532	18.10606061
0.050093128	0.009045323	5.059405941
0.069306339	0.018452346	12.68306011
0.071507517	0.017112582	18.37743191
0.05263981	0.013371034	14.58122744
0.068310321	0.010458726	13.86699507
0.051882461	0.007546942	6.848484848
0.080951771	0.019452158	12.38562092
0.078090011	0.011368353	36.93657505
0.073360705	0.018048372	26.7032967
0.088058467	0.034249762	27.56230032
0.05992623	0.010012767	24.33004926
0.077188791	0.014020081	27.17045455
0.072778828	0.041582262	3.347826087
0.086368366	0.03560793	2.677419355
0.062326841	0.006469724	66.68971963

log.sigma.5.0.mm.3D_gldzm_HighIntensityLargeDistanceEmphasis	log.sigma.5.0.mm.3D_gldzm_SmallDistanceEmphasis	log.sigma.5.0.mm.3D_glcm_SumVariance	log.sigma.5.0.mm.3D_glcm_Homogeneity1
193.1220657	0.98943662	351.295603	0.391810841
195.7894737	0.934210526	284.7142923	0.287704209
233.0544218	0.948979592	348.9816532	0.355021353
165.0727273	0.986363636	239.576167	0.27934786
150.8056872	0.969589258	127.6950187	0.410518217
144.7843137	1	245.6082644	0.345943346
147.5525672	0.975496779	299.5550967	0.732489037
173.6528777	0.98381295	358.5309067	0.521582558
127.5961538	0.985576923	221.9804729	0.589320809
128.8823529	0.977941176	177.662215	0.420020099
150.0538462	0.985576923	435.048778	0.685714752
133.0851064	0.993617021	229.4325151	0.61116939
224.8930818	0.966981132	299.7509623	0.300333383
130.5934066	0.983516484	154.5916771	0.317828011
112.7533333	0.99	160.0438847	0.476705917
128.344426	0.971390275	280.7626602	0.620481016
80.8852459	1	63.03556149	0.47565015
144.6034483	0.987068966	203.146948	0.317203894
86.33333333	0.9875	112.4342274	0.506218847
174.8440265	0.981576942	295.0806046	0.611992931
187.4911032	0.951982009	179.3276871	0.432281593
162.5555556	0.976190476	260.650037	0.334708142
125.3563218	0.99137931	174.5563361	0.380031897
113.6448087	0.991803279	260.6730962	0.705391123
217.5886525	0.984042553	487.5770429	0.424076844
315.9341317	0.899160013	241.6092936	0.48346815
155.0533981	0.996359223	261.4744837	0.453826342
113.3772455	0.982035928	224.3817316	0.532040534
43.72727273	1	70.75913685	0.45181701
147.4393064	0.98265896	226.628466	0.4857492
166.0164835	0.97348138	256.4655306	0.516092426
167.8611111	0.986111111	275.2577954	0.334510572
159.92607	0.994163424	232.4456271	0.46007506
133.9541667	0.984375	285.0901688	0.585085158
132.0286885	0.985783811	185.9330961	0.506102452
174.962963	0.994444444	310.6024538	0.445418997
205.0597015	1	356.4342084	0.32947089
163.3755459	0.961466925	400.05077	0.650746492
103.4166667	0.9875	149.1857905	0.389954557
357.542735	0.983776512	909.6519203	0.655797623
122.6780822	0.994863014	132.823161	0.445087118
110.8380952	0.992857143	203.4881408	0.715829516
200.0410628	0.96557971	321.0376683	0.41106234
140.6755319	0.996010638	155.0665996	0.438670048
128.4672897	0.985981308	220.5429995	0.551665826
179.7648903	0.976489028	247.1666403	0.476644753
125.5470852	0.994955157	308.784022	0.633434369
78.35555556	1	136.7311135	0.432874056
107.8431373	0.985294118	164.9351642	0.355894491
150.0833333	0.973214286	282.3416841	0.35867907
122.46	0.99	376.7917493	0.562660948
274.1666667	0.977112676	458.4234464	0.459007605
147.1976744	0.985763081	288.6384967	0.738314299
134.3712121	0.954545455	277.5933321	0.49263045
241.7938931	0.971374046	384.4670262	0.308070378
156.8011696	0.978070175	161.9614901	0.414933933
159.1995012	0.994389027	341.2995893	0.599824358
143.0889831	0.947622411	150.7640063	0.456139397
149.0795455	1	234.0216649	0.395101386
211.3880983	0.983117364	464.9610255	0.733540724
167.4948454	0.972222222	224.4249371	0.428123187
315.75	0.975028058	540.6975801	0.415376563
284.2797619	0.966836735	531.3974485	0.424372219
238.9889225	0.978712096	545.9971698	0.57955617
99.12676056	0.98943662	128.6238129	0.416320983
351.0433145	0.937957732	560.7469995	0.394212384
129.3035714	0.986607143	186.8052008	0.309219145
232.2419872	0.991586538	439.6393308	0.526454409
137.4481928	0.994243641	262.7431327	0.659150354
205.5380117	0.96231319	271.1983741	0.417719823
96.1875	0.98828125	102.473298	0.386073567
211.1247637	0.961825247	605.8831133	0.632557779
107.0116959	0.969298246	96.76557279	0.435238095
161.6815642	0.991620112	251.5626323	0.50065968
184.6417112	0.993983957	450.0132753	0.589850027
95.9537037	0.958333333	129.1507784	0.456988801
1298.553957	0.721372902	635.4325312	0.343661422
225.1692308	0.988461538	519.5484953	0.292012826
193.1682243	0.964953271	278.2735424	0.368428543
155.6569343	0.988037307	274.8701432	0.569184407
193.1220657	0.98943662	351.295603	0.391810841
184.9785714	0.989285714	225.1407568	0.344173969
243.1231231	0.97972973	596.7753573	0.593347381
112.6147541	0.987704918	223.3093255	0.542393704
162.4090909	0.982954545	292.0318906	0.500177865
309.1881188	0.933168317	645.7641418	0.26363472
149.9562842	1	283.7563513	0.446610851
166.0116732	0.974843277	273.5285773	0.637043617
955.7436823	0.747643402	489.515612	0.391463338
194.1280788	0.983853311	200.9129979	0.418543635
358.0984848	0.954545455	798.7391045	0.305113097
177.7189542	1	368.9182651	0.541763878
160.3044397	0.988900634	252.166426	0.53178963
170.8296703	0.978437118	245.7408033	0.549967663
89.49840256	0.997603834	119.4452156	0.510595433
219.0517241	0.97530104	317.7819635	0.369813388
149.8721591	0.987215909	169.8087572	0.422925857
127.2826087	1	204.6969497	0.3109041
87.74193548	0.975806452	134.6925105	0.338805953
292.8130841	0.968639668	621.2947617	0.559591277

log.sigma.5.0.mm.3D_glcm_Homogeneity2	log.sigma.5.0.mm.3D_glcm_ClusterShade	log.sigma.5.0.mm.3D_glcm_MaximumProbability	log.sigma.5.0.mm.3D_glcm_Idmn	log.sigma.5.0.mm.3D_glcm_SumVariance2
0.305539668	261.6105999	0.029353158	0.982880267	59.51761716
0.192391126	251.5535982	0.028812035	0.954485766	71.22433129
0.265441724	19.40414999	0.017902195	0.969250686	65.44504835
0.184210011	125.373592	0.026628662	0.948191212	58.73995243
0.330406711	299.4952413	0.03372506	0.981134716	41.22031634
0.254366814	-8.578730119	0.037245419	0.966231052	43.51185367
0.708280007	-140.1090723	0.296417361	0.995591707	23.26290435
0.464015645	-77.54378424	0.052413292	0.990767478	38.04714804
0.546840525	-40.74039097	0.105010537	0.991838395	26.16401322
0.337894402	44.41937641	0.035151844	0.979019979	26.96398427
0.663857845	-46.43635405	0.206337679	0.995483021	11.79034477
0.571782905	-77.08653199	0.104114031	0.994448788	30.14177421
0.204617466	134.6664519	0.017307254	0.957412058	91.66281424
0.221558525	109.3674048	0.027143436	0.942944018	46.83051885
0.410038082	37.96746845	0.027138079	0.984877805	30.18379218
0.580032179	-136.1677065	0.180150277	0.990765086	34.99141585
0.407075379	26.45099608	0.036740357	0.979960257	20.78693464
0.221592719	352.4311368	0.030227235	0.963544413	59.1733486
0.445394762	-15.64221979	0.037727341	0.980012693	24.4320483
0.572337637	-2.311494685	0.111832223	0.995844293	31.78724972
0.356088614	243.0411809	0.023515826	0.983697606	45.73453309
0.240360313	149.6415931	0.02608006	0.955944539	67.20825253
0.291794495	-8.023712196	0.023640002	0.973403873	46.5002666
0.681777433	-91.87714888	0.290709028	0.995114369	18.08312616
0.344990209	-9.16724748	0.025961065	0.983161393	47.13581868
0.41967509	136.4350564	0.047438402	0.985595638	36.1982085
0.382023032	-56.53489828	0.030388091	0.987278653	33.64387727
0.476850863	-8.371067906	0.04531887	0.990111819	29.40850775
0.36995058	15.79666927	0.139217929	0.949727553	9.544743318
0.420538219	35.32376102	0.028056472	0.988217496	33.61109111
0.457814671	-14.18627354	0.048824209	0.99185923	34.96808378
0.243445251	42.63202588	0.024554902	0.956980065	47.46658469
0.390422971	4.874439691	0.032951897	0.987067162	42.16241985
0.5402916	-43.66736308	0.122663237	0.990897989	32.39273521
0.445859158	-2.930503697	0.033597084	0.98955899	27.07374521
0.374086312	-121.6826543	0.026293389	0.98062668	56.72295897
0.236684455	102.9895619	0.016608686	0.97194645	59.50829602
0.616013494	-188.8996542	0.182364244	0.994297692	32.9827399
0.300374542	62.58417814	0.02563863	0.970054392	39.89010128
0.624327744	-68.9176689	0.209661895	0.99751411	21.57748387
0.371406722	64.24850682	0.042347963	0.977626392	25.21567795
0.697367102	-7.744110445	0.234746858	0.995475686	8.45618053
0.333799669	-26.63519754	0.022256306	0.978205739	57.07942456
0.362947969	212.6074153	0.032869045	0.98745946	39.11403584
0.501817379	-75.83248482	0.065224726	0.990462699	35.08094755
0.410449391	16.43598423	0.029303943	0.985539524	34.77479536
0.598639197	-87.18058752	0.130305813	0.993018565	26.22785882
0.353649301	-25.46544872	0.03275044	0.966730849	43.54936113
0.261865316	494.8399195	0.035816103	0.970295736	66.03521768
0.266503925	-22.52151209	0.024870398	0.962916034	47.72436826
0.511347346	-115.1913645	0.120286347	0.990734738	42.61985569
0.390574669	61.76791198	0.031370859	0.99153417	46.28031044
0.719132237	-76.3365255	0.316412132	0.99725553	15.71867525
0.429013791	21.23290244	0.038119031	0.983395528	24.59650923
0.214356541	130.2430932	0.016607348	0.97109506	100.3866123
0.335699665	310.9926311	0.034727511	0.978031195	44.9783931
0.559927218	-55.79042611	0.105815163	0.994586216	28.13342799
0.382863532	49.50901088	0.024001534	0.977018494	31.76489125
0.308549632	-8.019812552	0.027278105	0.975533625	41.6923209
0.714210528	-61.82910091	0.281950041	0.998023607	14.02797641
0.350905154	46.40099436	0.025189746	0.982807933	36.25491423
0.33628291	196.144773	0.020917271	0.98886178	76.1270263
0.349522591	11.34012158	0.022246305	0.985353404	59.33336074
0.531394825	-102.2656438	0.122109737	0.993808969	34.26926118
0.33343176	4.476121194	0.028351747	0.970517139	28.19195467
0.309996669	333.791293	0.017587027	0.987766266	79.86257558
0.213197709	6.744256155	0.027736028	0.935728527	44.23951571
0.472416948	-19.38495468	0.037142466	0.992018961	28.56480176
0.627828702	-76.6173621	0.148202718	0.995825113	23.86493052
0.337699863	111.1113411	0.020431153	0.981647801	44.57216208
0.297458608	71.42635502	0.030808813	0.964738598	28.98325431
0.593010863	-268.1865974	0.181248508	0.993366043	42.79397721
0.358767861	44.54728745	0.03436822	0.975043375	21.22653613
0.439172769	63.9531995	0.057867422	0.990663453	39.24098641
0.545764674	-124.5601231	0.102127229	0.994869154	30.04346033
0.383922594	97.57584531	0.03920821	0.978316581	26.60164733
0.256795553	3719.393338	0.012049932	0.986067668	208.0093252
0.195410169	-90.09635502	0.03119588	0.953591769	99.26578775
0.2784877	156.5655099	0.019316609	0.979929606	65.00543093
0.523940124	-46.44649289	0.068795679	0.993582506	25.21579417
0.305539668	261.6105999	0.029353158	0.982880267	59.51761716
0.252195045	176.059868	0.018935659	0.97136014	70.26768919
0.545333885	-152.2410384	0.18174411	0.994970812	39.70162143
0.490954376	-47.81662068	0.055189275	0.986695478	24.31469866
0.44040816	-82.58114637	0.036684306	0.986951688	37.88335723
0.165620874	-15.58002943	0.019199011	0.9656822	111.6496252
0.372873013	-47.13978219	0.022677745	0.981388702	50.58077139
0.600069743	-118.768777	0.228489574	0.992634905	29.78204353
0.313023087	2154.158544	0.027367847	0.985512958	138.0623115
0.34040371	314.6239356	0.02628873	0.981787045	53.99332546
0.21092523	-516.4214048	0.02580258	0.971941605	104.9360208
0.48905819	-70.64001753	0.061495607	0.991759941	27.98737204
0.479066244	-24.00154371	0.034705852	0.990749164	30.90205919
0.498428355	-34.4966719	0.088782201	0.992823057	29.49347108
0.450674126	21.01658371	0.046496449	0.987836342	20.79629643
0.283897629	250.7921655	0.020198715	0.980164836	53.60871298
0.345326435	174.1223669	0.029226087	0.984251583	38.48423406
0.213391787	-14.22487482	0.031128594	0.939191962	42.56262548
0.242415578	11.54633451	0.039455734	0.947575605	27.66640889
0.509610552	-68.27732122	0.108918318	0.993727101	44.25354702

log.sigma.5.0.mm.3D_glcm_Contrast	log.sigma.5.0.mm.3D_glcm_DifferenceEntropy	log.sigma.5.0.mm.3D_glcm_InverseVariance	log.sigma.5.0.mm.3D_glcm_Entropy	log.sigma.5.0.mm.3D_glcm_Dissimilarity
12.20100764	2.920651832	0.298809648	7.30649593	2.725163967
31.91721419	3.406561561	0.180559594	6.587444054	4.592333893
17.76316589	3.091915351	0.26317103	7.562856574	3.308830057
31.20085196	3.347921222	0.202706615	6.368985223	4.566134983
11.65097412	2.887366355	0.330673555	6.869145863	2.579494316
18.02815204	2.989682381	0.259288479	6.021795137	3.360443056
2.234305843	1.771152292	0.306629107	4.679265696	0.807944133
5.093396028	2.383078897	0.408179599	6.605474028	1.627270429
3.422044764	2.107913065	0.427996761	5.881627547	1.260799277
8.931993555	2.670783144	0.328811425	6.465430249	2.347400681
1.684750188	1.702744683	0.432479279	4.785181157	0.822671875
3.02852072	2.064784723	0.415241345	5.830747221	1.170912104
27.58116274	3.364521761	0.204320152	7.616200541	4.219984158
21.52026758	3.190000187	0.223523832	6.83678049	3.75285071
6.51169024	2.481897118	0.401313368	6.567400167	1.881944214
3.922714479	2.153599741	0.369131003	5.723863755	1.254801539
5.476282296	2.366913191	0.401153404	6.063345027	1.806784586
21.28853396	3.143051362	0.233676142	6.402654949	3.728186266
4.183966459	2.165657539	0.431189285	6.039067458	1.572822404
3.594524418	2.145515857	0.400471815	6.034772173	1.228925315
9.147952044	2.729894856	0.35786423	7.085109661	2.292527179
19.72773865	3.120772283	0.221946276	6.769096962	3.58585735
11.50551967	2.778542866	0.315199873	6.899813792	2.697602315
1.63897036	1.711909563	0.379760383	4.74039332	0.787677369
10.34573476	2.726903453	0.335206995	7.001793909	2.421173212
8.142589373	2.639003914	0.38060652	6.801201349	2.024028345
8.385864668	2.655670827	0.369496038	6.829308836	2.140682759
3.69955386	2.181130036	0.435617666	6.278658099	1.446616424
5.7035194	2.001876534	0.336574409	3.959337493	1.930405773
5.446255109	2.396815564	0.412562172	6.654529721	1.758686993
4.856967858	2.32620112	0.418084043	6.498220126	1.615723279
21.3533768	3.217142466	0.239186681	7.117929611	3.671355232
7.886444244	2.608101507	0.38509507	6.967977109	2.0653457
3.43837178	2.138652731	0.416102265	6.071449323	1.283957597
5.284668111	2.399153766	0.414597828	6.489505711	1.685106977
10.17404154	2.788335138	0.361429602	7.280912002	2.321790625
20.61787633	3.158501552	0.254167531	7.345950553	3.594934928
3.146325936	2.045435251	0.369705779	5.536476703	1.094033441
9.355071029	2.607881743	0.317195585	6.567211482	2.488512922
2.600203687	1.943247019	0.398982174	5.465968142	1.006985199
8.734432088	2.718626208	0.356586937	6.59728111	2.227076614
1.350419588	1.60156095	0.405458091	4.536166294	0.71899923
14.96550277	2.924280918	0.32069761	7.465133451	2.80506167
8.899248237	2.705551808	0.35608345	6.804649686	2.245034465
4.010653569	2.18671839	0.433627905	6.177440943	1.419341677
7.437648303	2.601870444	0.38391082	6.831444339	1.987806998
2.628714812	1.983217399	0.415395745	5.724926584	1.068872228
7.145494915	2.477409915	0.360456824	6.360072197	2.127709932
14.20224241	2.876124188	0.266378417	6.226608154	3.040439637
14.87737919	2.986841746	0.269755055	6.934291618	3.075925356
3.463058533	2.136226267	0.408090939	6.144470361	1.354689672
9.025701418	2.710868922	0.379960559	7.111917558	2.163932011
1.48952481	1.620969975	0.35044751	4.439798247	0.698063636
5.10104316	2.298863648	0.415900711	6.303352539	1.710791593
26.2973739	3.364872403	0.21656658	7.600735107	4.11028059
11.5938763	2.864327636	0.335260861	6.936017127	2.545194994
3.232645745	2.075074762	0.432261748	6.007100451	1.208157342
7.181851578	2.599370778	0.366863771	6.72167152	2.046389081
10.49449924	2.777612146	0.313783562	6.856766497	2.571287133
1.582874345	1.638767109	0.357444221	4.570927688	0.716046144
9.671496446	2.764545473	0.348732099	6.983436178	2.355969776
11.92339758	2.90356045	0.330829206	7.631658099	2.572458313
13.1443626	2.9539166	0.336478442	7.675268985	2.62179154
5.048817811	2.356976194	0.369976861	6.253507233	1.470853686
8.209365131	2.608533662	0.344162922	6.584143818	2.281998397
12.24328137	2.932746137	0.313986575	7.80579323	2.703414624
21.78836963	3.161629494	0.22934136	6.464268871	3.823026681
5.666870282	2.400342931	0.422692271	6.478438618	1.641481916
2.272065729	1.893483938	0.399340419	5.382502827	0.967644707
10.35613419	2.82241033	0.334956232	7.254326218	2.453876268
11.21686965	2.788140148	0.299419952	6.416320874	2.677761726
3.662759382	2.154253326	0.356396196	5.6939754	1.203968876
8.835882315	2.719415994	0.354910154	6.5667033	2.258365908
5.609469143	2.428180905	0.409244445	6.600260993	1.733616206
4.459415391	2.264998835	0.39284718	6.079142642	1.376842405
6.712853466	2.503522463	0.374674536	6.378316614	1.99904146
26.04131007	3.389424691	0.261875315	8.417016883	3.780669554
30.43629602	3.370505503	0.194956408	6.525858617	4.415771774
14.42320593	3.008481221	0.279087312	7.526982439	2.99453827
3.213907579	2.082226281	0.452834546	5.899588682	1.280041236
12.20100764	2.920651832	0.298809648	7.30649593	2.725163967
17.81206121	3.06311671	0.25415607	7.378899058	3.375362973
4.04823343	2.233729911	0.35931503	5.977188483	1.351703693
3.589086957	2.156611915	0.446852651	6.093775159	1.401963979
6.114637761	2.46024688	0.415702666	6.676961097	1.775846893
33.57935665	3.447359405	0.183503571	7.216683965	4.791365706
7.970327719	2.635548533	0.371300119	7.097212021	2.135112893
3.419581975	2.102092254	0.374428583	5.53405885	1.155361771
20.04692895	3.196011674	0.304160155	7.831585575	3.197724857
11.20036114	2.867016276	0.33550008	7.165641919	2.521875302
27.32275946	3.366369112	0.212111979	7.419086003	4.177744078
4.140355357	2.233905364	0.426663136	6.205004944	1.466939073
5.153689101	2.360781731	0.428436424	6.557668371	1.585684926
4.663135151	2.315419553	0.409852582	6.292644014	1.49971273
4.592464666	2.307036806	0.419975563	6.220680382	1.608236753
16.6545203	3.119926581	0.293915174	7.568454832	3.117449207
10.40799386	2.812070828	0.343538595	7.047801403	2.438832424
20.55358664	3.065741854	0.236084427	6.172355107	3.704759873
15.31327566	2.848324637	0.258717137	5.804441402	3.20287827
5.470042368	2.402896622	0.386604817	6.626885912	1.561561406

log.sigma.5.0.mm.3D_glcm_DifferenceVariance	log.sigma.5.0.mm.3D_glcm_Idn	log.sigma.5.0.mm.3D_glcm_Idm	log.sigma.5.0.mm.3D_glcm_Correlation	log.sigma.5.0.mm.3D_glcm_Autocorrelation
4.542643379	0.910077474	0.305539668	0.661285621	132.3221279
10.02408251	0.854834906	0.192391126	0.388316941	102.3039907
6.15825297	0.88235651	0.265441724	0.573959303	130.6062047
9.64971303	0.845253775	0.184210011	0.315777954	87.41705818
4.853551504	0.908831604	0.330406711	0.556177302	54.94880957
6.28416832	0.876024807	0.254366814	0.409071749	91.16392105
1.558982024	0.96718031	0.708280007	0.824160622	107.5533985
2.37080194	0.937407003	0.464015645	0.763647273	134.5750667
1.751486131	0.944169583	0.546840525	0.767941117	88.93215373
3.130513993	0.900770241	0.337894402	0.503781945	73.22258976
0.985172348	0.960753831	0.663857845	0.750546765	148.6154227
1.619910472	0.954059458	0.571782905	0.816794241	91.56510803
8.912429679	0.860301824	0.204617466	0.538365616	111.3971822
6.845636819	0.839785046	0.221558525	0.374018253	62.01003132
2.822596365	0.919094287	0.410038082	0.645134447	68.39003486
2.203492302	0.945334493	0.580032179	0.798053751	106.984609
2.097024635	0.904412584	0.407075379	0.580848523	31.87014238
6.84887089	0.868945376	0.221592719	0.469923805	77.19306194
1.5770416	0.904956784	0.445394762	0.707004587	51.24216973
2.042240543	0.961362756	0.572337637	0.795826802	113.0562398
3.731175598	0.91449142	0.356088614	0.66334541	75.12883593
6.27187018	0.858017502	0.240360313	0.540267906	98.41311772
3.944797865	0.887948106	0.291794495	0.602935133	72.46502866
1.001632257	0.960610782	0.681777433	0.833285819	96.43584857
4.010595824	0.913726698	0.344990209	0.641119322	174.3039972
3.85865247	0.92449342	0.41967509	0.63259934	96.13341006
3.583541849	0.925497937	0.382023032	0.603426648	102.4869576
1.550541204	0.932625747	0.476850863	0.776003538	91.04399406
1.548119364	0.849491566	0.36995058	0.248500565	28.84412894
2.273741776	0.926616465	0.420538219	0.720584416	91.99009573
2.106300079	0.939917655	0.457814671	0.756219694	101.7583596
7.182093983	0.861622028	0.243445251	0.387389341	103.98586
3.471662289	0.925242341	0.390422971	0.685300012	94.00408979
1.728005219	0.940449755	0.5402916	0.807547365	110.4866084
2.383662796	0.932598645	0.445859158	0.672905975	77.67267241
4.617865715	0.911151504	0.374086312	0.696560535	119.6927267
6.792225736	0.885757936	0.236684455	0.493169525	131.1905964
1.902712226	0.957491161	0.616013494	0.825325224	143.0982093
2.808763525	0.879266449	0.300374542	0.619318884	63.45665022
1.560156992	0.970799282	0.624327744	0.783666982	291.3427148
3.658921031	0.902019123	0.371406722	0.481578093	57.8377783
0.819914674	0.961765468	0.697367102	0.722221796	77.68732044
5.977153002	0.906513376	0.333799669	0.591063534	122.281837
3.702187858	0.924647508	0.362947969	0.628135613	66.14601838
1.914607202	0.93744283	0.501817379	0.793964831	89.4249099
3.353486183	0.92222054	0.410449391	0.645670179	98.29050923
1.459718849	0.949934011	0.598639197	0.817086877	116.5691328
2.344496517	0.876389485	0.353649301	0.715771892	58.81473236
4.645018154	0.880651502	0.261865316	0.644639168	64.6233519
5.107240941	0.869664149	0.266503925	0.522558137	107.8024128
1.505296285	0.936913575	0.511347346	0.848898838	139.7973573
4.229756694	0.939865985	0.390574669	0.671835982	166.0231464
0.989426053	0.972135257	0.719132237	0.826535798	103.5155406
1.929742756	0.913809743	0.429013791	0.65765703	106.8545926
8.760301954	0.883011826	0.214356541	0.586944869	139.4091541
4.914994044	0.903207696	0.335699665	0.587863412	67.60393426
1.714032621	0.954503251	0.559927218	0.792780155	127.9884834
2.893803067	0.899399822	0.382863532	0.63155967	65.08511275
3.652925829	0.892484686	0.308549632	0.598431492	93.01548383
1.054789483	0.976232646	0.714210528	0.796124794	156.5978275
3.926977366	0.912476361	0.350905154	0.579607867	90.22625118
5.095242514	0.929401644	0.33628291	0.728657249	193.4834295
5.964595441	0.922311431	0.349522591	0.638548832	189.7261542
2.839183204	0.952968815	0.531394825	0.74252324	189.9286692
2.811267878	0.88261499	0.33343176	0.548251133	56.62000836
4.726636839	0.923557437	0.309996669	0.734817746	200.0143288
6.715934291	0.829380864	0.213197709	0.345765935	72.58726299
2.901840455	0.943912285	0.472416948	0.667468629	158.1762796
1.30846739	0.961689956	0.627828702	0.825352719	100.4056713
4.187515239	0.909202408	0.337699863	0.622424854	106.4010205
3.728979016	0.872500414	0.297458608	0.444597515	45.6380624
2.175718879	0.953518608	0.593010863	0.841899624	204.9204857
3.607321027	0.895948999	0.358767861	0.410452671	44.54390392
2.52139239	0.936023141	0.439172769	0.749462941	100.3186463
2.502354694	0.957105716	0.545764674	0.741996641	160.2021646
2.516878435	0.901104	0.383922594	0.595158193	56.86354625
11.08196254	0.922120958	0.256795553	0.78159553	218.1059435
10.19800998	0.855329256	0.195410169	0.530703164	176.301712
5.150360141	0.902207541	0.2784877	0.636871464	108.2820492
1.530862788	0.947560118	0.523940124	0.772304269	105.8882014
4.542643379	0.910077474	0.305539668	0.661285621	132.3221279
5.717414091	0.883870877	0.252195045	0.587870064	88.58930763
2.127108816	0.956246966	0.545333885	0.81442394	204.2480444
1.571614329	0.924020604	0.490954376	0.741509184	89.75255782
2.851222588	0.926806757	0.44040816	0.722527918	113.0580455
9.877167377	0.869565362	0.165620874	0.537186261	218.0667521
3.308376226	0.909344223	0.372873013	0.727396511	111.2839129
2.053209959	0.951461932	0.600069743	0.793497696	103.3576185
9.2482648	0.923732386	0.313023087	0.750419863	173.8541455
4.676543538	0.91067701	0.34040371	0.654773402	81.77718645
9.198190026	0.884787996	0.21092523	0.591930597	266.4295799
1.892668991	0.940633732	0.48905819	0.738121934	136.1728748
2.584712175	0.939201094	0.479066244	0.714103216	100.2757119
2.355700894	0.946385314	0.498428355	0.726768636	97.40199452
1.91221593	0.926044122	0.450674126	0.636519701	54.01499177
6.622410651	0.905786952	0.283897629	0.525088985	120.2471002
4.230311475	0.916140197	0.345326435	0.573539504	71.32657966
6.234628146	0.833483121	0.213391787	0.356855745	77.77631817
4.771675477	0.844138881	0.242415578	0.291705093	54.04819634
2.970453467	0.951728952	0.509610552	0.779152585	215.3321877

log.sigma.5.0.mm.3D_glcm_SumEntropy	log.sigma.5.0.mm.3D_glcm_AverageIntensity	log.sigma.5.0.mm.3D_glcm_Energy	log.sigma.5.0.mm.3D_glcm_SumSquares	log.sigma.5.0.mm.3D_glcm_ClusterProminence
4.87260733	10.97873667	0.008704044	17.98643838	11523.213
4.61962909	9.610368057	0.011589157	25.78538637	14888.72871
4.949994137	10.89234566	0.006568886	20.80205356	10468.97414
4.503406189	8.968771497	0.013181805	22.4852011	9361.835545
4.492648503	6.895009176	0.012595279	13.21782262	7368.742298
4.198822053	9.198231481	0.017409554	15.38500143	5536.740218
3.606342762	10.1141327	0.121240503	6.374302548	2108.693408
4.577991378	11.23990239	0.016427666	10.78513602	3535.066564
4.254684137	9.123892516	0.028206075	7.396514497	1562.350315
4.302219648	8.287844079	0.014557369	8.973994457	2248.611607
3.600222582	12.0867195	0.074997552	3.368773739	645.3711111
4.298991003	9.207870756	0.034440391	8.292573732	2430.67115
5.107472792	9.759806766	0.005997895	29.81099425	19092.22954
4.544112762	7.459973679	0.010243235	17.08769661	5327.438117
4.412452245	7.903849745	0.013497942	9.173870605	2288.689864
4.244553745	9.960721564	0.050420833	9.728532582	2871.86829
4.090577217	5.294663071	0.018150856	6.565804234	1219.995454
4.462328101	8.225363765	0.013265616	20.11547064	12271.35422
4.21017309	6.794981998	0.018795385	7.15400369	1182.924631
4.365756165	10.29680677	0.036003415	8.863115026	5158.556215
4.687306732	8.122448017	0.009992241	13.72062128	7591.930333
4.698654456	9.295580482	0.010567283	21.7339978	10233.10164
4.64814884	7.981031523	0.010031264	14.50144657	4886.668672
3.641930814	9.608515473	0.103484305	4.93052413	1272.7252
4.711978874	12.84909485	0.010493505	14.37038836	5465.995204
4.548477844	9.44020784	0.014724738	11.08519947	5057.793385
4.51947967	9.806658403	0.012572721	10.50743548	3712.5678
4.434320932	9.198632054	0.017126838	8.277015402	2069.87767
2.75492501	5.27514694	0.074048724	3.812065679	232.7526282
4.540436945	9.216443253	0.013276918	9.764336554	2981.836224
4.53196973	9.707156895	0.015391661	9.95626291	2797.030811
4.650916354	9.87045339	0.008654746	17.20499037	6424.81064
4.6917936	9.24278265	0.011375876	12.51221602	5369.684428
4.425840174	10.16103631	0.029631788	8.957776747	2530.866434
4.393211614	8.498417361	0.015958196	8.089603329	2079.686642
4.856363346	10.39458245	0.008847982	16.72425013	7295.447082
4.811515544	11.01852044	0.007134177	20.03154309	9472.564021
4.133875468	11.64637635	0.058415746	9.032266458	3520.604477
4.489597408	7.469337015	0.01218047	12.31129308	3444.205388
4.057915287	16.94399388	0.060743821	6.127275324	1866.867753
4.28467728	7.328619367	0.014896914	8.487527511	2221.254829
3.46007634	8.712656524	0.087741851	2.45165003	298.4277433
4.895485827	10.57043567	0.00820203	18.01123183	9012.748154
4.54073792	7.6555182	0.012914453	12.05067464	6128.818309
4.454488991	9.036249347	0.021976324	9.772900281	2694.665694
4.552767992	9.562968149	0.012272883	10.55311092	3249.774074
4.230638022	10.51986664	0.037383012	7.214143407	1864.958006
4.448957819	7.049850493	0.014517236	12.67371401	3380.835596
4.428437704	7.182034658	0.015311851	20.05936502	14913.26243
4.641938804	9.977470432	0.009716635	15.65043686	5315.497817
4.523942824	11.40204328	0.026910454	11.52072856	3981.028655
4.73514791	12.5373073	0.011719055	14.07167434	9654.297009
3.475557312	9.997893503	0.124745418	4.302050015	1037.404237
4.292546978	10.09782071	0.016595187	7.424388099	1595.764734
5.135042559	11.03725246	0.006061798	32.47261574	22931.07907
4.579728913	7.696918075	0.011683319	14.14306735	8190.203456
4.372931548	11.03454828	0.027914961	7.841518433	2142.219909
4.445805742	7.677161265	0.011467494	9.736685707	2243.319675
4.59410145	9.229393723	0.010548351	13.04670503	4379.269219
3.542781934	12.38930256	0.110490836	3.908082479	1106.922236
4.566565891	9.141069752	0.010466294	11.48160267	3576.555793
5.086750657	13.35005773	0.007076797	22.37484515	15586.75808
4.969675093	13.36474487	0.00776299	18.35901087	10893.31866
4.406297606	13.51785825	0.030058028	9.890273553	4097.648521
4.348273826	7.183574023	0.012754732	9.10032995	1982.105206
5.134611583	13.55626431	0.006438063	23.33250278	20464.00868
4.429449318	8.181547961	0.012735237	16.50697134	4477.010934
4.419383168	12.34830713	0.017708102	8.578188482	3125.109259
4.038708252	9.747141388	0.054461413	6.534249062	1600.350288
4.72916977	9.891335212	0.008831621	13.73207407	5858.043466
4.264110933	6.414834058	0.013918983	10.05003099	2456.085734
4.20889548	13.96913578	0.056471002	11.61418415	5309.248042
4.184082613	6.437574618	0.014349425	7.515604612	1530.414115
4.602820111	9.586872219	0.016624005	11.21261389	5659.843384
4.310675974	12.4029216	0.032454019	8.645183456	3179.8694
4.280127917	7.202667221	0.016507695	8.3286252	2510.683697
5.60258658	13.28170406	0.004166269	60.46004393	195127.2834
4.729280177	12.59896379	0.012090727	32.42552094	21665.46204
4.955376771	9.777836615	0.006820831	19.86433945	10770.53905
4.238469592	10.01932943	0.025402188	7.107425438	1793.493807
4.87260733	10.97873667	0.008704044	17.98643838	11523.213
4.930632614	8.681990274	0.007146885	22.0199376	11633.81488
4.350053454	13.98014459	0.045247858	10.99386778	4598.229012
4.286136858	9.196134907	0.02075765	6.975946405	1637.533787
4.563137324	10.25235922	0.014713718	10.99949875	3632.381742
5.070313446	14.16952797	0.007537006	37.30214579	29751.29562
4.7930306	10.03079439	0.009404308	14.63777478	6048.627592
4.061706792	9.836918138	0.066036799	8.300406377	2413.867449
5.282481358	12.10696333	0.007353334	40.71688882	93582.79729
4.740372629	8.429085226	0.009705347	16.29842165	9633.653918
5.094588583	15.83903693	0.00756183	34.32509257	29397.6415
4.358336622	11.41078372	0.020925814	8.031931848	2409.069951
4.499031804	9.686845017	0.015829187	9.013937072	2822.713576
4.393828106	9.549543392	0.022252207	8.539151558	2560.692044
4.204514815	7.068255931	0.018549329	6.347190274	1198.300756
4.818231666	10.54488427	0.007470151	17.75581219	9990.261079
4.578399116	8.018442205	0.010921365	12.22305698	5653.594887
4.270599886	8.498064519	0.015246097	15.77905303	4186.307093
3.931419981	7.134729178	0.019795824	10.74492114	1816.51772
4.658388278	14.34487624	0.024367055	12.49438492	7065.980324

log.sigma.5.0.mm.3D_glcm_SumAverage	log.sigma.5.0.mm.3D_glcm_Imc2	log.sigma.5.0.mm.3D_glcm_Imc1	log.sigma.5.0.mm.3D_glcm_DifferenceAverage	log.sigma.5.0.mm.3D_glcm_Id
21.95274045	0.879125777	-0.189482537	2.725163967	0.391810841
19.22073611	0.980016676	-0.395215125	4.592333893	0.287704209
21.78469132	0.884701089	-0.191206061	3.308830057	0.355021353
17.93754299	0.983229234	-0.424737679	4.566134983	0.27934786
13.79001835	0.78411125	-0.135794411	2.579494316	0.410518217
18.39646296	0.977685566	-0.421445913	3.360443056	0.345943346
20.22826539	0.925145126	-0.350982015	0.807944133	0.732489037
22.47980478	0.873145595	-0.20542192	1.627270429	0.521582558
18.24778503	0.893937597	-0.255738	1.260799277	0.589320809
16.57568816	0.850064676	-0.184449414	2.347400681	0.420020099
24.173439	0.854817094	-0.251721006	0.822671875	0.685714752
18.41574151	0.920037272	-0.286254487	1.170912104	0.61116939
19.51961353	0.934717615	-0.241393265	4.219984158	0.300333383
14.91994736	0.928089557	-0.255490409	3.75285071	0.317828011
15.80769949	0.816255586	-0.167458629	1.881944214	0.476705917
19.92144313	0.918799909	-0.298472031	1.254801539	0.620481016
10.58932614	0.798929292	-0.161973379	1.806784586	0.47565015
16.45072753	0.975433477	-0.385870059	3.728186266	0.317203894
13.589964	0.855224798	-0.206424288	1.572822404	0.506218847
20.59196492	0.905448874	-0.258568436	1.228925315	0.611992931
16.24489603	0.810868319	-0.150450057	2.292527179	0.432281593
18.59116096	0.966233978	-0.337146524	3.58585735	0.334708142
15.96206305	0.902942919	-0.222754133	2.697602315	0.380031897
19.21703095	0.904649244	-0.31318854	0.787677369	0.705391123
25.69818971	0.870075389	-0.203532831	2.421173212	0.424076844
18.88041568	0.814646389	-0.16042077	2.024028345	0.48346815
19.61331681	0.807029046	-0.154427862	2.140682759	0.453826342
18.39726411	0.880012831	-0.221747941	1.446616424	0.532040534
10.55029388	0.970066025	-0.556955027	1.930405773	0.45181701
18.43288651	0.855259823	-0.188690773	1.758686993	0.4857492
19.41431379	0.868026748	-0.210332013	1.615723279	0.516092426
19.74090678	0.915423572	-0.229624579	3.671355232	0.334510572
18.4855653	0.851026387	-0.179290379	2.0653457	0.46007506
20.32207261	0.91534822	-0.273669498	1.283957597	0.585085158
16.99683472	0.808780625	-0.159581847	1.685106977	0.506102452
20.7891649	0.864882487	-0.179566183	2.321790625	0.445418997
22.03704089	0.917672056	-0.228035554	3.594934928	0.32947089
23.29275269	0.918997329	-0.297661802	1.094033441	0.650746492
14.93867403	0.91113706	-0.243338225	2.488512922	0.389954557
33.85840427	0.907243562	-0.281979931	1.006985199	0.655797623
14.65723873	0.746882377	-0.124066842	2.227076614	0.445087118
17.42531305	0.850891426	-0.25918798	0.71899923	0.715829516
21.14087133	0.815676024	-0.172799391	2.80506167	0.41106234
15.30732884	0.830396132	-0.16611804	2.245034465	0.438670048
18.07249869	0.914754843	-0.267197818	1.419341677	0.551665826
19.1259363	0.806211305	-0.154990085	1.987806998	0.476644753
21.03973328	0.915228438	-0.2824052	1.068872228	0.633434369
14.09970099	0.915644645	-0.258294075	2.127709932	0.432874056
14.36406932	0.977396982	-0.401298968	3.040439637	0.355894491
19.95494086	0.914967911	-0.235522895	3.075925356	0.35867907
22.80408656	0.932548758	-0.2979172	1.354689672	0.562660948
25.03514597	0.83644346	-0.163881265	2.163932011	0.459007605
19.99578701	0.906538337	-0.332794532	0.698063636	0.738314299
20.19564141	0.814792782	-0.180459841	1.710791593	0.49263045
21.98349965	0.945152818	-0.258156498	4.11028059	0.308070378
15.39383615	0.823224337	-0.157936442	2.545194994	0.414933933
22.06909656	0.908675725	-0.267689756	1.208157342	0.599824358
15.35432253	0.765353789	-0.130473509	2.046389081	0.456139397
18.45878745	0.896179344	-0.218297518	2.571287133	0.395101386
24.77792392	0.899074609	-0.316385814	0.716046144	0.733540724
18.2821395	0.791541318	-0.140119288	2.355969776	0.428123187
26.63958174	0.882005652	-0.187417421	2.572458313	0.415376563
26.69450406	0.797321398	-0.137012932	2.62179154	0.424372219
27.02763692	0.888644007	-0.22957221	1.470853686	0.57955617
14.36714805	0.826869785	-0.168058263	2.281998397	0.416320983
27.06196692	0.861292309	-0.167596105	2.703414624	0.394212384
16.36309592	0.955703208	-0.32149664	3.823026681	0.309219145
24.69414378	0.830936635	-0.176273886	1.641481916	0.526454409
19.49428278	0.914889262	-0.298512693	0.967644707	0.659150354
19.78267042	0.787203875	-0.133339962	2.453876268	0.417719823
12.82966812	0.874800778	-0.207528005	2.677761726	0.386073567
27.93827156	0.924544818	-0.2971935	1.203968876	0.632557779
12.87514924	0.661157613	-0.092287388	2.258365908	0.435238095
19.17374444	0.88905875	-0.220606494	1.733616206	0.50065968
24.80355855	0.885125336	-0.233396425	1.376842405	0.589850027
14.40533444	0.799691988	-0.16176671	1.99904146	0.456988801
26.27528066	0.929120854	-0.217216139	3.780669554	0.343661422
25.19792757	0.989338882	-0.462130313	4.415771774	0.292012826
19.55513842	0.865496172	-0.174616235	2.99453827	0.368428543
20.03865887	0.886696932	-0.241388512	1.280041236	0.569184407
21.95274045	0.879125777	-0.189482537	2.725163967	0.391810841
17.36398055	0.905775799	-0.218473935	3.375362973	0.344173969
27.95231388	0.918448644	-0.283094105	1.351703693	0.593347381
18.39226981	0.860967228	-0.209923083	1.401963979	0.542393704
20.50471845	0.863481577	-0.196300779	1.775846893	0.500177865
28.16713112	0.983105729	-0.381543978	4.791365706	0.26363472
20.06158877	0.8830546	-0.199866171	2.135112893	0.446610851
19.67383628	0.906913102	-0.278460202	1.155361771	0.637043617
24.02813849	0.917853159	-0.218884707	3.197724857	0.391463338
16.85817045	0.844447928	-0.168405447	2.521875302	0.418543635
31.42741172	0.966747467	-0.30921465	4.177744078	0.305113097
22.82156745	0.868853365	-0.220108376	1.466939073	0.541763878
19.37369003	0.852669344	-0.187900691	1.585684926	0.53178963
19.09908678	0.868457969	-0.208961825	1.49971273	0.549967663
14.13651186	0.76805208	-0.147485156	1.608236753	0.510595433
21.07095328	0.775461545	-0.121702841	3.117449207	0.369813388
16.03688441	0.753996125	-0.124358055	2.438832424	0.422925857
16.99612904	0.973569141	-0.391026031	3.704759873	0.3109041
14.26945836	0.966990856	-0.384187581	3.20287827	0.338805953
28.67996112	0.902675824	-0.234664731	1.561561406	0.559591277

log.sigma.5.0.mm.3D_glcm_ClusterTendency	log.sigma.5.0.mm.3D_firstorder_InterquartileRange	log.sigma.5.0.mm.3D_firstorder_Skewness	log.sigma.5.0.mm.3D_firstorder_Uniformity
59.51761716	150.2282829	0.522047463	0.067563059
71.22433129	161.566391	0.22362969	0.064296343
65.44504835	169.9618607	-0.002249532	0.058515404
58.73995243	160.5901909	0.088818024	0.064272212
41.22031634	138.5008392	0.916295197	0.079738947
43.51185367	130.835115	0.028358844	0.075878971
23.26290435	72.31289423	-1.109552664	0.194830134
38.04714804	126.0927944	-0.229713496	0.087199351
26.16401322	110.2222137	-0.209995853	0.107402798
26.96398427	114.4091578	0.343259266	0.086840357
11.79034477	45.72976303	-1.091396049	0.19719215
30.14177421	106.4338019	-0.331869024	0.110810817
91.66281424	220.9994278	0.025814784	0.049972162
46.83051885	175.0477295	0.12847862	0.065263158
30.18379218	117.6409969	0.350319106	0.089829868
34.99141585	117.1726587	-0.65599968	0.123971897
20.78693464	99.54706001	0.353659673	0.106114805
59.1733486	159.6466293	0.537748726	0.067770473
24.4320483	107.7887535	-0.23755483	0.10401289
31.78724972	79.72791004	0.139159242	0.115770809
45.73453309	133.9115028	0.743799798	0.075239624
67.20825253	177.4519596	0.049753855	0.060807563
46.5002666	147.1897049	-0.145406335	0.072301516
18.08312616	67.7693367	-1.114419085	0.197944954
47.13581868	149.3694935	-0.110979557	0.072733344
36.1982085	103.9305668	0.725564496	0.089304191
33.64387727	101.1512527	-0.0005384	0.090340368
29.40850775	108.5840511	-0.069885493	0.095116127
9.544743318	72.18400574	0.301660747	0.145408163
33.61109111	115.8329277	0.214733326	0.085012768
34.96808378	121.0591831	0.011404298	0.0885012
47.46658469	138.4655762	-0.036133381	0.067404844
42.16241985	116.4386139	0.170370216	0.081870426
32.39273521	112.826484	-0.212417946	0.101056544
27.07374521	99.85442352	0.162647366	0.097124627
56.72295897	158.5609932	-0.340250811	0.06938943
59.50829602	172.8898735	-0.074175749	0.060315582
32.9827399	100.3575058	-0.963308306	0.139710365
39.89010128	135.5845108	0.14290171	0.07681208
21.57748387	76.45756206	-0.595800794	0.142694346
25.21567795	108.7364883	0.502752929	0.089910865
8.45618053	45.06517458	-0.14526056	0.19798063
57.07942456	139.8111572	0.121639326	0.066833238
39.11403584	122.4513321	0.8635188	0.082622952
35.08094755	118.1161928	-0.332507141	0.094669118
34.77479536	120.0179977	0.23307954	0.083970408
26.22785882	95.07776064	-0.579227465	0.11453842
43.54936113	151.7033615	-0.209551849	0.083880653
66.03521768	157.8473711	0.632960337	0.068944291
47.72436826	152.350049	-0.13769778	0.072355372
42.61985569	144.6529188	-0.410593581	0.091413981
46.28031044	117.2578096	0.472265132	0.079329051
15.71867525	59.48080707	-1.029353248	0.219545804
24.59650923	95.98298264	0.00058618	0.101235139
100.3866123	207.7215805	0.06736983	0.052119122
44.9783931	118.9430923	0.933012163	0.079725822
28.13342799	102.9275062	-0.254843261	0.102578604
31.76489125	123.7983551	0.226330664	0.087264691
41.6923209	125.4949532	-0.070500807	0.075719074
14.02797641	50.51714528	-1.024531207	0.205912702
36.25491423	128.5979156	0.295558384	0.079235338
76.1270263	187.0095472	0.344449373	0.058592183
59.33336074	136.933063	0.041658048	0.067104035
34.26926118	116.3934867	-0.311263099	0.103683545
28.19195467	111.3677368	0.007571998	0.08946924
79.86257558	153.4560394	0.437830119	0.06192691
44.23951571	151.7788086	-0.22939853	0.075630252
28.56480176	99.21221733	0.208611791	0.096168021
23.86493052	93.37747818	-0.463041685	0.134976086
44.57216208	137.9127979	0.378278998	0.071350058
28.98325431	109.8268127	0.390502968	0.085399449
42.79397721	119.9293414	-0.965473992	0.129633955
21.22653613	101.4931488	0.648195081	0.097630842
39.24098641	105.3609085	0.519284303	0.088448219
30.04346033	95.57799655	-0.58376086	0.113017391
26.60164733	101.7957611	0.541363324	0.099419982
208.0093252	198.8492537	1.349148768	0.050190227
99.26578775	206.0737381	-0.41579258	0.064650284
65.00543093	173.3627887	0.154878335	0.060190986
25.21579417	99.83121681	-0.153210378	0.108553809
59.51761716	150.2282829	0.522047463	0.067563059
70.26768919	192.435318	0.2065125	0.056088013
39.70162143	126.1644425	-0.513174796	0.116080633
24.31469866	93.89264679	-0.364450989	0.106210593
37.88335723	111.9048309	-0.21445701	0.087280177
111.6496252	207.2373295	-0.235659677	0.050426136
50.58077139	140.2517776	-0.163756933	0.071611891
29.78204353	103.5139127	-0.560311107	0.144298611
138.0623115	128.0542473	1.503299834	0.067564328
53.99332546	164.4621849	0.626466081	0.067515625
104.9360208	170.2940025	-0.594324022	0.062303088
27.98737204	103.8860188	-0.400934197	0.099948164
30.90205919	102.6741333	0.090207423	0.092388406
29.49347108	108.4485497	0.022780387	0.099423994
20.79629643	95.25334835	0.289606574	0.103928413
53.60871298	152.1676579	0.544059595	0.065632989
38.48423406	132.5174904	0.60789549	0.078184254
42.56262548	151.9163971	-0.239013459	0.074646585
27.66640889	120.057148	-0.197935521	0.089466376
44.25354702	106.8276702	-0.151811626	0.092870443

log.sigma.5.0.mm.3D_firstorder_MeanAbsoluteDeviation	log.sigma.5.0.mm.3D_firstorder_Energy	log.sigma.5.0.mm.3D_firstorder_RobustMeanAbsoluteDeviation	log.sigma.5.0.mm.3D_firstorder_Median
87.90493142	23459702.14	62.37832649	-166.0083542
104.8430284	9354782.375	71.66753835	-251.620636
96.92508011	23947414.26	69.78277386	-188.0699158
98.81190644	7127230.08	67.56608772	-248.0916443
81.29820006	28573751.12	56.91743368	-188.8031464
78.97608944	1695470.753	53.08761914	-77.53528595
49.74156188	86008933.52	32.62597724	-9.055666447
68.61916907	90058630.91	50.59169139	-97.99324036
57.10606324	48880331.04	43.44822268	-76.58130646
66.47806759	5323345.181	46.91809966	-93.8712616
33.03293375	11425434.95	20.23862048	-0.260039464
59.85176012	25444288.93	43.62672809	-36.63316345
119.8447227	27953070.25	90.59609216	-250.1364441
91.79536298	21561089.09	70.20886993	-320.7453156
64.23336584	28718576.17	47.54777115	-161.9824524
65.85091961	82781523.2	49.62279359	-30.98145676
55.41867642	5650310.92	41.23992146	-91.35277176
95.09387405	13192442.85	66.23618548	-335.7677002
56.72234552	8912933.317	43.58010496	-97.08777618
56.71565086	78460319.05	35.97007081	-13.78229809
79.15715553	44855621.04	54.67631128	-158.6851273
103.3002231	6810585.266	78.72981744	-177.1115265
80.53293809	10464211.97	60.03276456	-154.3072052
44.1403127	25365525.02	29.8424948	-25.35903454
80.04258063	24728436.02	59.3877675	-159.0269089
67.00837607	25720675.85	44.87852395	-77.44608307
63.51017689	37399131.35	42.70171052	-179.4014435
59.76655868	28923732.02	44.26624084	-104.6224823
42.07248608	82378.46076	30.60318707	15.97856236
66.19191816	24827959.64	47.39561211	-126.1945114
65.73209486	70859444.74	49.50031429	-120.1732483
85.04991662	23872592.54	59.71466137	-288.3094788
70.63021235	28183085.65	48.47147933	-111.4984818
63.58674032	28602050.49	47.19976281	-45.2984848
58.48636938	49250836.56	41.04906348	-92.9070282
85.41689039	41712580.75	63.04782539	-153.5905685
95.50490072	29934910.56	70.84579497	-246.6172943
61.38405579	91849673.42	43.39858384	-15.68591118
74.63619974	6453258.482	56.25652937	-162.4885254
50.36597138	69957595.34	33.46047889	-23.1435976
64.60407312	33109271.99	44.96075174	-223.7974243
31.16768584	14853398.17	19.8075783	-11.22514725
85.29299257	56499321.82	58.0849063	-157.7313538
74.55990669	29471404.32	50.45201109	-197.0389404
66.63937706	13742382.77	49.72846143	-63.59066582
68.54256656	39599865.76	49.0176324	-115.8479462
56.10146782	49243678.04	40.71769842	-33.79049301
77.28105264	5202637.8	61.29712301	-119.8222809
93.92869633	2775607.277	66.61951437	-117.288208
84.88372069	5394747.023	62.92476723	-123.9693871
72.89031506	33695252.27	56.78656328	-93.21080399
75.14595983	36874755.3	49.35583623	-91.50331116
41.22301847	41569189.11	26.94025791	-14.9911952
55.28750163	6715132.925	39.46706099	-58.02698135
117.6390419	17945275.99	86.37300777	-190.5148621
80.73107275	27654383.33	54.71382084	-198.2578583
58.56889887	46818310.81	42.26044011	-53.69013214
66.31100254	51364630.56	50.48358247	-167.8922424
74.64343577	17239925.3	52.6555102	-233.7217789
38.15976376	78393158.45	23.26261644	-8.713603973
72.09691091	22123704.64	51.3893774	-139.3642883
101.0982655	44691383.68	74.41654463	-121.1048813
84.83912081	87728344.18	57.75520014	-82.96275711
64.77591963	98682596.15	46.81236887	-58.5575943
63.34353629	6343766.653	45.67801815	-114.6709747
94.180562	60023277.36	64.11753156	-127.1134491
83.64920782	13830738.55	61.74771927	-320.9831238
60.44027854	65865955.32	40.80383003	-98.43053436
54.2557586	62615605.85	39.28905683	-22.35063934
80.36410906	37753032.93	57.79891918	-126.6893768
67.34638988	9455137.879	47.32523457	-205.9889297
70.40248198	79180999.3	52.07331512	-29.35167313
59.38814065	38202597.03	41.05256481	-222.8809357
66.57181848	17324514.19	44.14957222	-92.10083771
58.41994409	25833655.7	40.17742998	-35.63352013
59.65144295	8725567.99	41.51276213	-101.9071579
130.5469801	48319332.57	82.47800766	-78.01925278
110.4538431	9887492.122	78.39895013	-246.9716644
95.06587691	14065574.75	70.1010789	-89.96580505
56.51965487	18979500.52	41.06335481	-73.36302185
87.90493142	23459702.14	62.37832649	-166.0083542
104.7738639	18760182.41	79.24223599	-193.8307037
68.37620185	49294293.22	50.45137858	-68.92451096
53.46208783	24663959.65	38.05122228	-121.7626343
67.99572298	29604920.7	48.05172515	-85.56521606
125.6585069	14252874.86	91.91792337	-226.9439468
79.68967638	17905783.79	58.5414325	-120.0405197
60.31066671	38324734.17	43.83974678	-39.6007061
100.5302612	26589890.82	57.02920081	-67.32537842
90.08999347	28679817.3	65.3673461	-168.8253021
106.9488337	8737144.821	69.77452354	-92.0176239
60.6362675	21423736.47	43.24655835	-85.44434357
61.20955786	46407151.74	42.34045433	-85.36132431
60.70435661	40182145.35	43.49289132	-63.87589264
54.37593578	21517303.19	39.11113884	-80.9462738
90.27785949	52327624.52	63.50527685	-193.2110672
76.24755348	33774426.09	54.24342324	-134.2093964
83.90244149	7187387.694	61.79932321	-263.6098938
66.69763204	2262160.988	49.04896985	-143.8875732
70.08054002	100471096	47.44115437	-33.9371109

log.sigma.5.0.mm.3D_firstorder_TotalEnergy	log.sigma.5.0.mm.3D_firstorder_Maximum	log.sigma.5.0.mm.3D_firstorder_RootMeanSquared	log.sigma.5.0.mm.3D_firstorder_90Percentile	log.sigma.5.0.mm.3D_firstorder_Minimum
633411957.9	216.1522675	187.6826524	-10.02361679	-420.9569092
252579124.1	80.82699585	287.7250158	-87.82322083	-520.5327759
646580185.1	81.48881531	222.6669437	-27.74422302	-474.4062195
192435212.2	60.44514465	278.3341376	-89.49266205	-497.5015259
771491280.3	225.0426941	195.7105399	-20.57829285	-335.5439758
45777710.33	207.125	128.2999653	43.94074936	-318.2434082
2322241205	254.525528	72.62699284	13.0038991	-267.1408386
2431583035	180.3048401	133.594644	-11.27864933	-361.8019409
1319768938	197.8565674	107.5098428	-2.880333614	-287.9196167
143730319.9	179.1820068	120.9321077	25.92121296	-291.686615
308486743.6	161.2899628	45.7278756	36.27685661	-278.4250793
686995801.2	286.4219971	90.9942352	20.0167984	-266.3098755
754732896.8	65.67544556	290.6034468	-69.82165527	-523.1911011
582149405.5	-75.52719116	336.8670603	-163.5249664	-522.992981
775401556.6	148.5324707	176.6800602	-58.54300613	-337.4138794
2235101126	193.9187927	98.53002095	14.48403778	-286.4217529
152558394.8	173.3341827	113.8394866	-8.686633348	-216.9026337
356195957	19.39143181	344.7474967	-181.2579346	-542.5065918
240649199.5	51.50348282	125.3772671	-23.62727089	-264.1995239
2118428614	437.0835571	81.10720307	49.44360962	-270.7582397
1211101768	203.861908	175.7620579	-6.558108234	-338.6795959
183885802.2	73.20293427	207.6173645	14.7640542	-415.4789124
282533723.3	101.4570236	192.97458	-48.99419785	-373.5419006
684869175.6	158.1128235	72.66418863	2.022114754	-264.8327332
667667772.4	102.0162811	184.5567843	-26.19975758	-461.8377991
694458248	273.0530396	112.7012348	41.26077652	-275.6813965
1009776546	179.4410858	199.5712426	-87.13735352	-421.9980469
780940764.5	141.1744537	128.231359	-12.64837837	-311.1028442
2224218.441	128.4398041	54.24101662	85.15150604	-79.8600769
670354910.2	167.6848907	146.8699184	-12.85194778	-337.5427856
1913205008	244.3247986	142.06381	-15.02479267	-341.5107422
644559998.7	-32.03004456	305.9706031	-154.6883392	-528.4467773
760943312.5	233.6728363	148.4427523	-11.79314899	-348.4143677
772255363.1	168.3900452	95.30424695	29.80481339	-280.3225708
1329772587	227.3656464	119.0157562	-8.323934555	-292.5664978
1126239680	113.6680832	192.2998655	-38.85853653	-424.0984192
808242585	78.6348877	277.0490587	-111.3168213	-529.9581909
2479941182	246.9133759	88.45894503	21.82310123	-319.1433411
174237979	65.11903381	180.9907657	-27.75687523	-330.2142639
1888855074	336.9917297	75.99301089	25.29468651	-434.4802551
893950343.6	66.57752228	229.0657384	-102.7222061	-390.6433105
401041750.6	196.9937744	45.30369505	27.01131134	-208.837738
1525481689	181.3314209	192.3543885	-23.09629593	-418.7773743
795727916.7	261.4697266	204.1694263	-48.84434433	-360.8157043
371044334.9	176.5852814	112.3871279	11.68847752	-290.1862183
1069196376	188.1637573	142.3224712	-15.52179813	-326.5217285
1329579307	163.4122009	84.06012072	26.73618507	-279.0428162
140471220.6	33.20191956	154.1309086	-21.02431812	-297.5169373
74941396.49	215.2636108	156.0366375	59.62437325	-297.3517456
145658169.6	99.00260162	156.5936697	4.273439217	-351.0481262
909771811.3	86.11316681	132.9605822	-3.146421671	-354.6809692
995618393.2	398.5427856	130.6887645	33.61904907	-377.5374756
1122368106	295.0504761	64.14784842	5.682744646	-253.6403046
181308589	107.9874039	89.04009235	34.08979034	-276.4128113
484522451.7	217.8442688	250.0541822	-15.94887962	-484.2258606
746668349.8	171.4792328	204.2326747	-21.93644485	-354.0671997
1264094392	258.0866089	94.10294498	21.5150135	-304.3233032
1386845025	72.88040161	182.3340768	-54.87124863	-329.7922058
465477983.1	23.0907402	251.7579757	-114.4675797	-456.9482727
2116615278	350.6159668	57.74110082	15.26576443	-305.3679504
597340025.4	192.5275879	166.6095016	-24.26126251	-352.0671997
1206667359	345.4195862	166.9725729	43.65460205	-445.4375
2368665293	296.0866394	138.5213946	51.17312775	-409.7101135
2664430096	279.1499329	107.6218581	5.082147074	-392.4777222
171281699.6	89.17188263	140.5792052	-18.88148117	-291.976593
1620628489	316.9167175	165.7425941	44.54923401	-425.9949036
373429940.9	-143.2901001	340.9174295	-196.8266479	-549.6143799
1778380794	235.6636658	126.3166657	-12.97629423	-393.8682251
1690621358	287.5838013	79.03918043	15.52189426	-257.6572266
1019331889	196.4349213	157.2375995	11.3311739	-359.7573242
255288722.7	42.03538513	218.5251052	-85.70092545	-354.0934753
2137886981	173.2041473	105.6116678	16.76829834	-381.3304749
1031470120	70.22636414	225.8424283	-114.8890366	-362.0899353
467761883.2	263.9457397	127.0663841	15.06516953	-308.0046692
697508703.8	355.1282043	89.62618557	23.23092175	-349.3179321
235590335.7	135.5071869	120.1927811	9.973527718	-252.2151489
1304621979	676.9649658	180.5661597	167.2131714	-326.7720337
266962287.3	-10.82260323	293.2203819	-75.24113007	-597.5040283
379770518.3	275.4693909	146.9901548	64.15231323	-347.3285522
512446513.9	218.3289185	109.0500693	-10.86296797	-303.1217041
633411957.9	216.1522675	187.6826524	-10.02361679	-420.9569092
506524925.2	154.5982208	225.4786082	-14.64929886	-421.2261963
1330945917	260.4077148	120.0915065	-4.581291533	-401.2053528
665926910.5	33.56315231	144.8813444	-48.28525696	-326.7814331
799332858.8	171.6008759	128.0688023	-2.216809177	-326.0055542
384827621.2	112.688446	284.5737927	-51.69772339	-606.8635254
483456162.2	124.6865311	157.1548052	-1.103748846	-372.6173096
1034767822	209.7023621	97.08583908	0.986592174	-276.7015076
717927052.2	563.8496094	150.4316932	132.0344574	-307.9831238
774355067	219.2360229	189.3403592	-1.790792057	-359.0789185
235902910.2	200.9022827	177.9222353	55.96580505	-515.2194824
578440884.7	150.7364655	122.3994849	-12.29524698	-362.2970276
1252993097	243.4665375	116.4534755	6.910118961	-321.8004456
1084917925	321.2398071	102.1082199	12.86686192	-295.1506653
580967186.2	205.6572113	102.0290121	12.5440176	-243.5462341
1412845862	228.322998	210.2275644	-19.71484032	-430.9214172
911909504.3	290.025177	151.8361477	8.05173111	-310.2946167
194059467.7	-84.51973724	287.4259064	-136.0414886	-497.5193787
61078346.68	30.5221138	178.4976817	-57.38502502	-342.9240112
2712719593	313.9454956	102.280889	42.76612511	-385.1832886

log.sigma.5.0.mm.3D_firstorder_Entropy	log.sigma.5.0.mm.3D_firstorder_StandardDeviation	log.sigma.5.0.mm.3D_firstorder_Range	log.sigma.5.0.mm.3D_firstorder_Variance	log.sigma.5.0.mm.3D_firstorder_10Percentile
4.100958053	109.1785456	637.1091766	11919.95482	-277.1206207
4.229293338	132.0452729	601.3597717	17435.95409	-431.8963623
4.240009777	118.7333637	555.8950348	14097.61165	-351.189447
4.141965734	123.0710966	557.9466705	15146.49481	-433.2657043
3.894430047	101.6865768	560.5866699	10340.15991	-280.9627533
3.911198713	100.3733688	525.3684082	10074.81316	-206.3650635
2.926853923	63.24495827	521.6663666	3999.924746	-133.8865051
3.725517429	83.18763028	542.106781	6920.181832	-220.0480423
3.409117948	68.32138029	485.7761841	4667.811005	-178.4770416
3.729982525	82.58951469	470.8686218	6821.027937	-185.264035
2.744595178	44.75064806	439.7150421	2002.620502	-69.69374771
3.469486649	72.96851478	552.7318726	5324.404149	-161.9954498
4.42864175	143.137401	588.8665466	20488.31557	-452.0215149
4.025844826	109.2308551	447.4657898	11931.37971	-469.1997742
3.639166846	78.10928149	485.9463501	6101.059856	-257.846228
3.414152767	77.70765506	480.3405457	6038.479655	-181.0317902
3.38597985	67.70881995	390.2368164	4584.484299	-183.2178421
4.087668827	118.9897909	561.8980236	14158.57033	-475.9930725
3.379504536	66.97171414	315.7030067	4485.210494	-201.1780762
3.529920977	76.35450142	707.8417969	5830.009887	-130.1323639
3.945453046	99.71491263	542.5415039	9943.0638	-260.6670807
4.153880291	123.1184824	488.6818466	15158.16071	-333.8336823
3.924056599	97.02032681	474.9989243	9412.943814	-303.1943054
2.872186109	55.62886247	422.9455566	3094.57034	-131.9341034
3.939641496	97.16043698	563.8540802	9440.150514	-281.1820068
3.76400674	86.79670327	548.734436	7533.667698	-174.6758453
3.737384706	81.85976256	601.4391327	6701.020726	-288.1928345
3.557017893	72.4092928	452.277298	5243.105684	-199.2093475
2.927740976	50.94348387	208.299881	2595.238548	-38.35612984
3.737733837	81.44822537	505.2276764	6633.813416	-225.5988007
3.654987909	78.95282969	585.8355408	6233.549316	-223.1325531
4.064969686	105.2374124	496.4167328	11074.91297	-422.7324951
3.8672932	90.49519759	582.087204	8189.380787	-237.230722
3.56360166	76.0147577	448.712616	5778.243388	-165.1908081
3.585325348	73.33594669	519.9321442	5378.161078	-190.6694641
4.016530053	103.2761079	537.7665024	10665.95446	-310.8455444
4.204909487	114.9062006	608.5930786	13203.43493	-409.6736633
3.299654598	75.18887346	566.0567169	5653.366693	-162.9789551
3.814464431	88.94296373	395.3332977	7910.850796	-276.4037659
3.276300706	64.88636204	771.4719849	4210.239979	-127.6036903
3.700593705	81.31291784	457.2208328	6611.790608	-313.15802
2.725576757	41.82068199	405.8315125	1748.969442	-74.83081055
4.129317179	108.3027611	600.1087952	11729.48807	-301.3830688
3.856638879	95.27952441	622.2854309	9078.187771	-286.3887085
3.617398516	79.78335595	466.7714996	6365.383886	-196.6980133
3.774201927	84.93644028	514.6854858	7214.198888	-227.9356171
3.390114992	67.99341186	442.4550171	4623.104056	-150.9442688
3.665815071	89.01318922	330.7188568	7923.347855	-252.4032288
4.028924885	115.511912	512.6153564	13343.00181	-239.694368
3.965032717	101.846722	450.0507278	10372.75477	-252.4996185
3.652558876	85.4019074	440.794136	7293.485788	-224.7695999
3.969835486	99.36206668	776.0802612	9872.820295	-191.8128357
2.738099038	52.93559716	548.6907806	2802.177447	-119.1533173
3.481192227	68.31715234	384.4002151	4667.233303	-145.8881775
4.432044213	143.532774	702.0701294	20601.65721	-410.4044922
3.914678725	102.9027884	525.5464325	10588.98387	-288.5062378
3.526900901	71.82618197	562.4099121	5159.000416	-161.1173584
3.62260433	78.63833657	402.6726074	6183.987978	-265.8102661
3.898346941	92.36070295	480.0390129	8530.499449	-361.5913208
2.816032772	51.27455541	655.9839172	2629.080033	-98.99788361
3.845072301	89.9574234	544.5947876	8092.338025	-258.4183594
4.284559213	122.7771968	790.8570862	15074.24007	-260.2075623
4.150499185	107.9753022	705.7967529	11658.66589	-226.0623642
3.606518412	80.31322244	671.627655	6450.213698	-175.6045624
3.642109177	77.3592137	381.1484756	5984.447945	-224.7457581
4.270182248	119.8131769	742.9116211	14355.19735	-252.0662781
3.889907266	102.1729966	406.3242798	10439.32123	-473.4258484
3.663493048	78.59222163	629.5318909	6176.7373	-193.157373
3.263899337	65.90776676	545.2410278	4343.833719	-140.840094
3.995615345	99.2607642	556.1922455	9852.69931	-246.3872955
3.72187378	84.20908455	396.1288605	7091.169921	-311.7107788
3.390442377	84.52920922	554.5346222	7145.187212	-198.3284363
3.578560456	75.22523063	432.3162994	5658.835324	-301.9641602
3.793782129	87.44934173	571.9504089	7647.387369	-201.9506927
3.486874517	73.85556697	704.4461365	5454.644772	-155.6934357
3.555481407	74.54320699	387.7223358	5556.689708	-184.6001465
4.614203018	175.2875843	1003.737	30725.73721	-224.7010574
4.210532744	137.0882228	586.6814251	18793.18083	-457.6590027
4.201813203	115.6676466	622.7979431	13379.00447	-243.4846497
3.440890408	69.45369406	521.4506226	4823.815619	-180.5911179
4.100958053	109.1785456	637.1091766	11919.95482	-277.1206207
4.26259251	125.0571601	575.8244171	15639.2933	-353.4530945
3.533745678	83.56749364	661.6130676	6983.525994	-207.8098892
3.44035796	66.18638219	360.3445854	4380.637188	-220.4018524
3.748486551	84.66699859	497.6064301	7168.500651	-222.7128265
4.484327477	151.7290653	719.5519714	23021.70927	-466.3477478
3.956502253	96.27536853	497.3038406	9268.946586	-252.7432007
3.296755516	73.5202058	486.4038696	5405.22066	-179.313736
4.271658814	143.4813082	871.8327332	20586.88579	-193.7762054
4.066520158	110.4670342	578.3149414	12202.96565	-288.6899292
4.349368254	138.3323658	716.1217651	19135.84344	-320.1313934
3.577483745	74.74477354	513.033493	5586.781171	-198.8013489
3.670707993	77.53422212	565.266983	6011.5556	-187.9063995
3.579039316	75.50972202	616.3904724	5701.718119	-169.4725418
3.450308034	66.83431541	449.2034454	4466.825716	-160.822226
4.153835027	112.3630027	659.2444153	12625.44437	-308.904007
3.882370273	94.3791639	600.3197937	8907.426578	-232.2896729
3.881107172	100.8193146	412.9996414	10164.53421	-413.5566895
3.645430748	81.51856782	373.446125	6645.276899	-272.0122375
3.802566569	89.65922433	699.1287842	8038.776507	-173.0050797

log.sigma.5.0.mm.3D_firstorder_Kurtosis	log.sigma.5.0.mm.3D_firstorder_Mean	log.sigma.5.0.mm.3D_glrlm_ShortRunLowGrayLevelEmphasis	log.sigma.5.0.mm.3D_glrlm_GrayLevelVariance
3.053507733	-152.6591732	0.014439707	19.45927648
2.703527097	-255.6359338	0.037812668	28.1535794
2.381510796	-188.3692018	0.018917526	22.95603572
2.611018524	-249.6465449	0.04481516	23.89316051
3.45842704	-167.2197821	0.036039421	17.25560369
3.07008272	-79.91287714	0.043129048	16.66987779
4.131912891	-35.70371609	0.017859685	8.248960342
2.664118494	-104.5339518	0.011153162	11.37057979
2.592101439	-83.00936865	0.016826667	7.42925455
2.957285636	-88.33768582	0.026121945	11.36362631
5.033953223	-9.403090198	0.006456486	3.909777617
3.080462003	-54.36493991	0.021047413	9.40065513
2.133622055	-252.9071919	0.040639418	32.09419248
2.085750285	-318.6660267	0.050911844	18.65974731
3.027329004	-158.4764456	0.026205415	10.02868716
2.518499232	-60.57792811	0.018143116	10.3473085
3.139414575	-91.51472241	0.073196543	7.381161397
3.067649286	-323.5618429	0.037051801	22.69242216
2.130834284	-105.9917384	0.033324435	7.128943923
5.208903871	-27.35632473	0.013880347	11.40628472
3.294176755	-144.7385132	0.026015969	16.46198168
2.016224813	-167.1729922	0.028097641	24.12296611
2.361894506	-166.8120041	0.046711617	15.01017426
3.956785051	-46.74948096	0.015512474	5.990715157
2.516331517	-156.9109815	0.009485494	14.852207
3.928766942	-71.88811184	0.015914105	13.1727059
3.79987172	-182.0100551	0.019469233	11.35190596
2.532000001	-105.8308827	0.018352498	8.391991089
2.433685508	18.62389154	0.082021093	4.616434117
2.769968896	-122.2168544	0.016177133	11.03087996
2.58024274	-118.104093	0.015837454	9.814675345
2.62890035	-287.3031448	0.020806909	17.650178
3.496016379	-117.6684746	0.023286812	13.64729315
2.469040645	-57.48613832	0.013829579	9.839740227
3.147582813	-93.7368079	0.02141809	9.043746747
2.419669142	-162.2136979	0.024041948	17.15051444
2.426343792	-252.0966997	0.015174464	21.35137706
3.388117149	-46.60062514	0.012169832	10.07553011
2.184790003	-157.6286981	0.040639515	13.14190737
4.49642868	-39.55626025	0.003454316	8.365880767
3.072653949	-214.1478972	0.034103998	11.34146446
4.597752419	-17.41996967	0.012206746	3.832705055
3.003800181	-158.9676781	0.020824121	19.45723332
3.881028978	-180.5739928	0.030985106	15.27198324
2.445573652	-79.1548017	0.021807717	10.73029188
2.986081877	-114.1993298	0.015292801	12.05754145
2.756404453	-49.4267118	0.012015545	8.076691359
1.864600157	-125.8292062	0.062889137	12.26837028
2.870350182	-104.902004	0.059381074	21.45430009
2.321623515	-118.9488235	0.022298812	16.8242062
2.219863051	-101.9069704	0.012006988	11.43021913
4.852424693	-84.89247835	0.009161847	16.83126375
4.588402306	-36.23215437	0.012064778	5.864471197
2.61558767	-57.10433208	0.012757843	7.640370737
2.409990119	-204.757019	0.025247195	32.66534672
3.533751479	-176.4142895	0.027487154	17.80663964
2.978320003	-60.79772888	0.010459884	9.015921881
2.25465841	-164.5044911	0.025888799	9.757837328
2.597063986	-234.2041393	0.02370365	13.81050975
5.838410495	-26.55098286	0.006446301	6.118668194
3.025713641	-140.2368996	0.017125415	13.32351342
2.853427396	-113.1618312	0.009011812	24.64286161
3.116990799	-86.77275426	0.008615185	19.55404571
3.821855394	-71.63972808	0.007209098	11.41955266
2.513911162	-117.3800025	0.040471348	9.66684035
3.279712599	-114.5225311	0.008758309	23.3781235
2.228584333	-325.2466333	0.047113501	16.77804895
4.230684935	-98.88964925	0.008724871	10.88538951
3.057364006	-43.62749505	0.014428862	8.046636502
2.856173251	-121.9465595	0.015592331	16.28166835
2.698742354	-201.6483367	0.043514873	11.43842223
2.992108304	-63.31379922	0.007367612	11.99538064
3.458576816	-212.9459253	0.044427916	9.573493739
4.055512918	-92.1871933	0.017119426	13.29824919
3.972940183	-50.77606097	0.009087507	9.763430017
3.006190831	-94.2847544	0.02768243	9.12364386
5.267144523	-43.34052179	0.01649466	50.02505309
2.413405475	-259.2007167	0.028249343	30.56756351
2.442869	-90.70336897	0.022202424	21.40451376
3.351758306	-84.07200483	0.013010977	8.291530025
3.053507733	-152.6591732	0.014439707	19.45927648
2.21600941	-187.6201201	0.040115605	24.92509457
3.238564959	-86.24641405	0.006701607	11.78308052
2.792014841	-128.87966	0.018052199	7.543616487
2.807262636	-96.0891121	0.013791649	11.96325258
2.446945777	-240.7499413	0.01518024	36.68693935
2.451008822	-124.2122628	0.021872083	15.02758492
3.000419017	-63.40693565	0.01620931	9.724147392
5.960792484	-45.19743938	0.014367152	34.27126484
2.900250406	-153.7751799	0.022300682	19.76191959
3.106465608	-111.8949435	0.011312982	31.26226682
3.16887984	-96.92704847	0.010988491	9.5850758
3.377282915	-86.88990945	0.014496266	10.05312185
3.51697947	-68.73405598	0.016717906	9.644906141
2.803853821	-77.09146246	0.029766872	7.523611378
3.020053696	-177.6800058	0.01463224	20.80040055
3.24214777	-118.9402757	0.025585339	14.70036595
2.262458253	-269.1637372	0.049982006	16.12095687
2.457265283	-158.795924	0.045063747	10.87627629
3.634859255	-49.21995282	0.005993347	14.66709249

log.sigma.5.0.mm.3D_glrlm_LowGrayLevelRunEmphasis	log.sigma.5.0.mm.3D_glrlm_GrayLevelNonUniformityNormalized	log.sigma.5.0.mm.3D_glrlm_RunVariance	log.sigma.5.0.mm.3D_glrlm_GrayLevelNonUniformity
0.01558821	0.066294168	0.132040325	40.13275109
0.039631068	0.063466936	0.052259891	6.805801911
0.020290628	0.057989547	0.120135529	25.68936244
0.046964197	0.063661447	0.037476997	5.635534478
0.040765272	0.076516269	0.156179052	50.85031967
0.045640607	0.075937559	0.057273857	7.436850866
0.021882877	0.111102481	5.915875058	913.2487789
0.012634597	0.083830138	0.513750322	337.9733712
0.019953961	0.103479519	0.858592805	318.6536501
0.028375484	0.085166883	0.151342628	27.85411372
0.008206587	0.163299501	1.385570396	575.2803093
0.024670439	0.095937535	1.018892703	207.7537979
0.049477831	0.050106271	0.064743507	15.65657157
0.055376937	0.065258133	0.058141396	11.74267995
0.029365471	0.089096247	0.236866807	70.03995689
0.021376821	0.090794052	1.760206936	515.2469077
0.086321417	0.105945535	0.195318863	39.92703103
0.038866584	0.066797695	0.049001999	7.049819619
0.039234799	0.104360421	0.239418404	49.90714986
0.016654534	0.092743587	1.37711827	752.7351419
0.029682325	0.073889459	0.197137504	93.76219762
0.030094586	0.060226116	0.069666344	8.865886967
0.053891244	0.072492886	0.094031161	18.78767595
0.019320493	0.129197401	2.654933084	362.2889832
0.010149369	0.07280751	0.208384875	46.63673355
0.01811369	0.084326716	0.352769449	141.147756
0.022807231	0.088854185	0.193685377	72.59152524
0.020830908	0.095452286	0.385555487	135.4162106
0.085907544	0.143620735	0.083895199	3.731611722
0.018395638	0.083767442	0.264150847	81.53003296
0.018143881	0.088925633	0.422630094	251.2577202
0.022213797	0.067389043	0.079789154	16.04687164
0.026650182	0.080303952	0.208667212	89.47984167
0.016030777	0.088199773	0.879520514	203.5326269
0.024307687	0.095653816	0.338308275	275.0527932
0.02696964	0.068373626	0.21358707	67.19902318
0.015788003	0.060370756	0.052861107	22.42471528
0.014508041	0.100756404	1.890913768	748.1375351
0.043568906	0.076747333	0.092645272	14.00309876
0.004266374	0.103667108	2.040557786	799.4372533
0.037536096	0.085520846	0.191699767	47.18778884
0.016224098	0.154010254	2.302306862	659.481801
0.023174177	0.065418707	0.220244615	88.13706057
0.034765294	0.079305126	0.16971476	49.34073611
0.02507917	0.087488161	0.472771208	74.84273787
0.017306699	0.082587191	0.306238708	135.2799211
0.01446488	0.09878744	1.451950508	459.8024468
0.073519124	0.083756681	0.138453601	16.4176993
0.06445547	0.068819724	0.059997658	7.396479271
0.023437775	0.071843298	0.07816202	14.6947241
0.013639194	0.08230029	0.639289028	121.0162907
0.010202353	0.07646095	0.236871314	141.7225384
0.015763119	0.134605647	3.77161084	711.3489123
0.014098514	0.100380613	0.212940566	73.00474575
0.027378605	0.052064112	0.062968385	14.0488251
0.030981502	0.076402659	0.15643862	45.02341501
0.012330481	0.093735428	1.014121245	357.4451406
0.029645963	0.087941303	0.233675747	115.9497867
0.025455875	0.075725605	0.109822789	18.89088583
0.008591475	0.130425454	3.941566107	1599.399466
0.018850467	0.077939825	0.17450379	54.90769928
0.009902397	0.057665	0.194877596	81.41350178
0.009393607	0.065171349	0.204951039	262.5879892
0.008359142	0.08965812	1.160415421	549.1356228
0.044966015	0.08931744	0.136314892	25.84156107
0.009492069	0.061092146	0.147297548	119.7250535
0.049825408	0.075232784	0.049000083	8.524056585
0.009937136	0.092659423	0.428213806	307.9764239
0.017807469	0.103291441	1.974250738	645.8247085
0.017400759	0.070056079	0.181561342	94.3996394
0.048911739	0.085679798	0.097530361	15.57963533
0.008668335	0.091110786	2.03163886	417.7985333
0.049903885	0.094679355	0.170970843	62.1884451
0.019501061	0.083007552	0.288571539	74.54460382
0.010396542	0.098384101	0.74899935	234.9658359
0.031128163	0.097691802	0.230527689	51.29274035
0.017944313	0.049763216	0.119764724	67.33167658
0.029018277	0.063560797	0.040676481	7.01193023
0.024466079	0.060146066	0.112551866	35.92281775
0.015042845	0.102605993	0.569276732	126.2845088
0.01558821	0.066294168	0.132040325	40.13275109
0.046416769	0.056265712	0.091780383	19.21399089
0.007774916	0.089409999	1.269890467	217.9435655
0.020326459	0.102770988	0.360905519	97.53008953
0.015565033	0.084662907	0.356162917	126.9066509
0.015470934	0.050431576	0.034450448	8.569852231
0.024341051	0.071214968	0.170676947	45.68982677
0.019434323	0.098207441	1.819120445	260.3928514
0.015852988	0.065095483	0.183040959	67.76823961
0.025435029	0.066026005	0.162989747	46.72908102
0.011627243	0.060832086	0.064776893	15.86233076
0.01237826	0.094036702	0.432884371	107.7727766
0.016929747	0.090987075	0.420922207	250.0750292
0.019634663	0.093857966	0.68452502	275.5890231
0.033983982	0.101117848	0.286890273	176.2176904
0.015731429	0.064243398	0.113113272	69.6663147
0.028548558	0.075830788	0.186637236	98.05946038
0.052757281	0.074305242	0.047086429	6.183271814
0.046693255	0.089446892	0.048519592	6.041659156
0.006951634	0.078193378	0.953568788	553.5470888

log.sigma.5.0.mm.3D_glrlm_LongRunEmphasis	log.sigma.5.0.mm.3D_glrlm_ShortRunHighGrayLevelEmphasis	log.sigma.5.0.mm.3D_glrlm_RunLengthNonUniformity	log.sigma.5.0.mm.3D_glrlm_ShortRunEmphasis
1.347165984	144.8824749	511.2871511	0.934332052
1.165033874	153.5326811	96.59330135	0.959563359
1.318634376	160.2471728	381.3220836	0.939892826
1.119039157	133.489997	81.99337599	0.970240211
1.420635044	78.9882024	539.1944633	0.919082961
1.167721262	120.5070779	89.08873248	0.96272675
9.993869596	62.84180526	4122.926076	0.73300694
2.100953047	115.4341061	2860.147431	0.864819888
2.79818955	68.49298474	1953.912403	0.818118905
1.400117377	89.187429	271.2786551	0.925830608
3.884984663	107.5949344	1873.180635	0.751973413
3.078811558	70.78418996	1329.687408	0.807970532
1.189049704	163.0277945	280.0751186	0.957637227
1.175111002	95.47340171	161.7813668	0.95921762
1.623166778	69.60052467	600.7931059	0.893319222
4.144545437	75.79029346	3427.680147	0.796429053
1.543579643	40.1988221	290.3486431	0.897635635
1.156429539	112.049893	95.26809356	0.960892615
1.676580926	53.84905319	354.0286336	0.877154281
3.5717646	92.95342671	4860.540189	0.800178892
1.512147198	89.3169516	999.2660729	0.907429404
1.226068562	136.9602788	127.9166353	0.944717229
1.279730581	90.63529	223.3353729	0.939864726
5.729689717	62.94381849	1436.022564	0.739785209
1.517581526	172.3735883	521.6997175	0.915839038
1.835322344	96.82680479	1223.555955	0.877037325
1.524223381	107.6882549	637.0213646	0.902415398
1.94320122	78.9050797	998.091563	0.861156614
1.281452991	31.77761193	22.69438746	0.93747151
1.67204706	95.83936003	730.4286395	0.888154493
1.990448151	87.27126577	2008.530372	0.864208838
1.231808649	135.4835707	209.3550723	0.94917115
1.536951409	101.7966906	877.3602534	0.906053872
2.779585773	87.28238166	1501.342274	0.829361813
1.813604018	75.80279848	2103.389967	0.877566965
1.538199791	124.3529175	776.4253437	0.90807533
1.15673551	172.6730311	338.2227437	0.963882889
4.467277604	94.24050706	4151.377713	0.772825808
1.26290915	77.55370017	158.0137311	0.944042883
4.565141211	217.829403	4385.070555	0.779026583
1.505683108	71.84296488	435.2838353	0.907987433
5.258965688	59.8415893	2138.790927	0.729670348
1.529076887	136.2055516	1101.077197	0.917533479
1.467886551	82.48312549	492.8069748	0.909272916
2.135676554	79.94532552	583.3828248	0.846067698
1.742273872	99.60631735	1222.533748	0.885596753
3.738196578	83.6918754	2720.831438	0.790179907
1.401255474	63.98221111	159.7667113	0.917647199
1.188980514	88.67330964	95.6332883	0.954464273
1.23852696	126.8922991	177.6184783	0.945123559
2.36569159	108.7694968	1010.561426	0.848569851
1.601503063	175.8535786	1425.439261	0.897747269
7.598349312	62.70824507	2378.071046	0.694488384
1.581701621	101.1408796	554.2877259	0.892878661
1.195838341	182.1605699	238.6929985	0.953010995
1.424757938	88.73335846	474.9987848	0.917107241
2.993248286	101.4563248	2416.378025	0.819015766
1.615959531	65.90751791	1000.666473	0.892095906
1.304132475	113.3269423	212.5255415	0.937654097
7.779057642	102.0349861	5568.840915	0.697249236
1.461288776	106.2115798	567.5711272	0.915522197
1.489554411	208.2879024	1134.175392	0.914889044
1.50019477	201.9454923	3255.539471	0.916519663
3.12653996	152.4626234	3959.979586	0.829077466
1.377535232	68.2671071	240.6079488	0.926347203
1.392977439	206.7517725	1612.705471	0.924690196
1.154559692	104.3246475	102.803961	0.962144206
1.99095006	146.1668184	2349.145728	0.863863878
4.618371532	70.50332962	3426.167605	0.765347528
1.470634196	123.9306323	1081.808783	0.914698972
1.289741082	65.87901605	154.772634	0.936646925
4.492956672	137.8382117	2727.052416	0.796827459
1.476606622	56.60914542	516.3461251	0.906740854
1.728534691	99.90028439	665.0853792	0.882667342
2.593676634	128.3473404	1543.605486	0.828340918
1.586584995	65.22356755	412.7868547	0.902968797
1.322394397	206.6608131	1146.051673	0.93594317
1.128818683	222.6654587	101.4145606	0.967795329
1.303615954	137.2925819	509.7984903	0.938919636
2.277998536	90.63611068	827.9744438	0.843529584
1.347165984	144.8824749	511.2871511	0.934332052
1.264530964	125.5163671	296.0524159	0.943549863
3.336714615	157.2787321	1581.349818	0.825502943
1.907402552	78.91473148	662.8087679	0.859283017
1.825453324	111.4114159	1112.699379	0.882400533
1.108177517	280.5386653	158.3082132	0.972955621
1.451828416	117.2893337	515.8247237	0.915539418
4.209568805	76.02468976	1569.813544	0.795817967
1.464887127	165.3486101	842.3566452	0.917688505
1.44396973	107.2140192	567.3633951	0.914610692
1.18748526	310.1954088	233.9128418	0.958159271
2.020968058	122.9623573	806.7199402	0.859804278
1.984917498	97.42052208	1932.314567	0.862467686
2.433684734	85.14986304	1972.83661	0.843484268
1.705078098	58.4970107	1307.466705	0.887321007
1.307449558	147.5645611	923.6570733	0.938149446
1.479409218	89.24022515	1041.325535	0.915186331
1.142259195	108.018235	76.38489946	0.966655203
1.155562547	75.53364115	61.0556016	0.961109363
2.817208638	182.6386891	4664.351384	0.836443684

log.sigma.5.0.mm.3D_glrlm_LongRunHighGrayLevelEmphasis	log.sigma.5.0.mm.3D_glrlm_RunPercentage	log.sigma.5.0.mm.3D_glrlm_LongRunLowGrayLevelEmphasis	log.sigma.5.0.mm.3D_glrlm_RunEntropy
190.1812173	0.908754909	0.02147578	4.532890853
175.4721134	0.94894486	0.046927494	4.435953747
213.9043046	0.917025004	0.026854079	4.615857796
146.0250407	0.962374582	0.055560345	4.278399518
98.76663267	0.890595999	0.065200227	4.41669283
141.6201727	0.950709485	0.055802068	4.091949034
1286.801452	0.502613479	0.101746089	4.941557561
314.1773756	0.79872862	0.022652285	4.602634411
290.121853	0.728031722	0.043879969	4.481655016
121.4558439	0.898351648	0.039429115	4.182815814
604.7203336	0.64345647	0.027378451	4.280500008
328.6317293	0.703872437	0.053639183	4.679118661
184.1318086	0.943992563	0.08984479	4.655988168
107.4666957	0.946963563	0.074545724	4.251433868
122.520948	0.854598662	0.046367024	4.302252149
527.754786	0.663503261	0.056403741	4.767914574
60.17966231	0.86450247	0.155874484	3.980708203
126.8134933	0.950796951	0.046125713	4.285065983
98.88780356	0.84344051	0.071361264	4.057937455
435.4695812	0.679569948	0.044141691	4.907650468
129.1803641	0.873913965	0.048842217	4.540328187
168.7295318	0.931840312	0.038103458	4.42849097
117.0659829	0.922529428	0.08685586	4.256714895
624.4921764	0.581662717	0.068942321	4.632657119
297.855408	0.882178428	0.013812174	4.454824154
184.7640477	0.825792972	0.03156581	4.568527036
171.9473525	0.869910707	0.037674649	4.339022475
191.8437209	0.806577164	0.035523294	4.380578673
39.53647945	0.925824176	0.104935462	3.109889275
165.188019	0.845351868	0.030851261	4.4380744
224.5384247	0.804855071	0.032255247	4.476467322
165.5794851	0.933936652	0.02823536	4.344348259
164.183935	0.871173393	0.043792489	4.47498363
340.6466376	0.73236925	0.034660333	4.645743975
156.0276249	0.826795867	0.041415754	4.368058013
217.4028697	0.871249318	0.04196078	4.604874943
199.9570064	0.952465483	0.018368726	4.439094935
734.8028371	0.631237139	0.040156585	4.834655117
99.93038416	0.926200703	0.056688552	4.142625725
1404.696434	0.635977445	0.0160617	4.810850915
103.1085559	0.874192369	0.054762742	4.291379573
423.1354852	0.590438027	0.072963602	4.468926411
210.0242991	0.882121807	0.037422041	4.676917799
112.0365683	0.879773692	0.053161431	4.42665948
218.6770704	0.785209276	0.044149	4.538610702
190.8236149	0.837812316	0.028891204	4.505108267
492.1634067	0.667406206	0.039004666	4.721926206
96.1663406	0.895328416	0.122230531	4.100024366
104.4648208	0.942645074	0.0847965	4.245685045
160.9578907	0.92972028	0.028158827	4.281819875
379.5619491	0.770885463	0.024879544	4.573387382
288.5716216	0.858232088	0.015840931	4.651863086
876.1361496	0.52104686	0.07728715	4.794533279
174.7495421	0.858777586	0.021314606	4.124857123
214.0559263	0.940230501	0.036193029	4.694237757
115.3016096	0.888734192	0.048061939	4.446903129
418.8308618	0.72067917	0.029478841	4.679850896
115.5293846	0.853622106	0.051001689	4.279264238
146.2983978	0.916855204	0.033176246	4.261470832
1300.010036	0.519804102	0.050342219	4.901755706
154.9323552	0.883795	0.027735426	4.393293897
316.8539335	0.880656461	0.014592213	4.845600249
317.1785671	0.881031698	0.013562295	4.740477254
672.3545508	0.718237631	0.019432514	4.767876304
93.71929607	0.901749341	0.067281066	4.072240322
291.9467156	0.896884351	0.013207964	4.779787689
120.8108418	0.952165482	0.060689037	4.080842773
335.8888485	0.80465489	0.017587757	4.554013118
527.3681869	0.622905778	0.056699128	4.808274401
179.2980559	0.882424059	0.02675336	4.558448587
81.51772594	0.918414918	0.072179283	4.057504467
1088.573829	0.644424458	0.024656633	4.822732353
79.0980286	0.876861456	0.076111909	4.166345516
180.869916	0.836619112	0.032794064	4.543883645
457.8011761	0.741747991	0.020556537	4.608237326
103.800527	0.868695874	0.050508268	4.128281108
268.8833951	0.912903561	0.024726596	5.047273301
259.9340754	0.959197324	0.032094013	4.385939426
173.9930377	0.917405175	0.03490452	4.586237875
263.9812416	0.770773087	0.029359881	4.414029687
190.1812173	0.908754909	0.02147578	4.532890853
148.0401229	0.92578695	0.077463229	4.57783055
780.6144995	0.711369672	0.019042431	4.721664122
187.4169878	0.807594108	0.03303982	4.287989272
230.685453	0.830257831	0.026835166	4.504260686
317.8852808	0.965472028	0.016633708	4.625128642
179.39887	0.884774536	0.035813952	4.485421616
518.1938674	0.651046199	0.052884047	4.729967326
231.0349256	0.885499182	0.023711484	4.825650474
140.878371	0.884615385	0.042186602	4.597801643
377.8836259	0.944537347	0.012943812	4.611000664
297.3094654	0.801075847	0.021489753	4.46090482
217.4323454	0.8029942	0.032263211	4.540826512
269.6492432	0.761626282	0.039625659	4.569326449
98.93135845	0.84295337	0.058211783	4.175878393
188.027727	0.915800416	0.020804566	4.5815108
123.7728728	0.882436335	0.044478775	4.446707029
123.9943018	0.956675508	0.063908946	4.036863423
88.46987678	0.951245937	0.053211288	3.817695786
657.635616	0.736463973	0.015907278	4.94000793

log.sigma.5.0.mm.3D_glrlm_HighGrayLevelRunEmphasis	log.sigma.5.0.mm.3D_glrlm_RunLengthNonUniformityNormalized	log.sigma.5.0.mm.3D_glszm_GrayLevelVariance
152.4890462	0.843483775	22.239106
157.825893	0.899576713	31.28531856
168.5238655	0.857546668	24.20889444
135.9970058	0.925046005	26.20694215
82.37747642	0.810380235	17.92924687
124.0665602	0.908007367	18.11149558
92.47765732	0.499412608	13.77931295
135.3175096	0.706573576	17.52591416
86.32090993	0.627996699	13.41436391
94.03917845	0.826496646	15.2949827
146.0832258	0.526567076	8.635428994
90.18019927	0.610682942	15.1735627
166.9493961	0.895319232	26.08741743
97.81660252	0.898393984	16.31904359
77.04808039	0.760345696	13.02773333
100.7603644	0.596021623	10.82404534
43.35517392	0.767634853	8.216608439
115.0026131	0.901996002	24.30915577
60.64116573	0.732183184	8.549722222
116.3059349	0.597406255	20.04597071
95.55955602	0.786231928	18.34672813
143.0741644	0.866440786	23.02998236
95.2278418	0.857528628	14.22671423
89.38179604	0.508675243	9.876616202
188.7476852	0.808229849	21.42729239
107.8946644	0.728755675	18.76298899
117.5788953	0.777179967	15.50299274
92.59234083	0.699910178	12.2296963
33.27297517	0.864036525	6.628099174
105.6724339	0.748890877	13.2442113
102.4564965	0.7062619	12.89629121
140.7775297	0.876767044	20.01740398
110.8741443	0.784714472	18.5690018
108.0253566	0.646738948	11.21373264
85.69143894	0.729341816	12.74401623
137.1612392	0.788427737	17.531262
177.6899669	0.909728923	21.26165194
130.3563226	0.556293428	14.16959165
81.29704129	0.864350769	12.79416667
283.475401	0.565501902	19.48309876
76.68893598	0.787765177	14.35039407
80.84006772	0.495141023	9.4461678
146.1536918	0.811387186	23.26381012
87.32015835	0.790377421	18.6169081
95.40093835	0.675593964	16.82836929
111.1764059	0.744359894	17.01282417
110.0894949	0.58196195	11.49076495
69.11083098	0.809977051	11.47950617
91.59503068	0.888127761	21.03883122
133.0763113	0.866827142	19.96598639
132.872674	0.680646221	14.04448889
192.1352763	0.767623274	25.78137054
95.75653349	0.447571693	11.98945376
112.6755586	0.758580733	9.915231864
188.373543	0.883730643	28.21875182
93.17996595	0.805517704	19.74269006
126.1569285	0.628805407	14.51847936
73.35001615	0.75651685	9.461846452
118.8761511	0.850498174	17.09077996
150.7306767	0.450948596	15.84949684
113.6296283	0.803916753	16.16561271
223.9444294	0.802090646	29.31503801
218.0880938	0.806307736	23.72606828
187.2923318	0.645011445	20.34259149
72.40565202	0.827529529	10.34477286
220.7198438	0.822094528	27.51738006
107.4989349	0.90588451	16.73852041
167.9989326	0.704389542	21.44029421
95.27409165	0.544821245	14.331694
132.4840009	0.801516743	17.64126569
68.59199143	0.848553688	12.01733398
184.0180382	0.59346742	14.02546446
60.34986039	0.784722501	13.13251941
111.0313359	0.738765306	22.40061172
158.2344086	0.643614193	18.22474906
70.67798651	0.780550586	10.17592593
217.4389486	0.846174507	61.43482336
230.119182	0.918647039	33.46366864
143.5890964	0.852642735	20.16859551
108.2255695	0.66944722	16.48377644
152.4890462	0.843483775	22.239106
129.4796271	0.864223215	20.54061224
195.7915561	0.642609803	19.76803831
92.78132535	0.695840639	12.1572158
125.295104	0.739159921	17.01066058
288.0079884	0.931099103	35.71610626
126.9319309	0.803058176	16.00412076
98.88714729	0.59123037	16.01517056
175.8279058	0.807969557	41.40379778
112.81837	0.80081484	19.15081657
322.3472858	0.896400653	37.83809688
143.1548871	0.698864524	16.77739331
111.5185543	0.701251993	14.42489798
101.5752609	0.668996204	16.45123626
64.23953967	0.747845248	10.63942676
154.1901738	0.851095609	23.19049237
94.61393503	0.803241157	15.02295325
110.885776	0.916569645	17.0326087
78.12088827	0.902960817	13.46305931
219.8648239	0.657221494	20.65043323

log.sigma.5.0.mm.3D_glszm_SmallAreaHighGrayLevelEmphasis	log.sigma.5.0.mm.3D_glszm_GrayLevelNonUniformityNormalized	log.sigma.5.0.mm.3D_glszm_SizeZoneNonUniformityNormalized
114.1111442	0.061165113	0.331658181
141.8131756	0.056940597	0.40166205
156.99252	0.06076172	0.345643019
138.9046667	0.060495868	0.475702479
99.94745963	0.070371285	0.391073875
108.04378	0.07266436	0.377931565
82.34673318	0.076909512	0.355518559
115.2909468	0.066889654	0.359971016
79.59113849	0.081745562	0.314970414
84.93832134	0.075163399	0.321030373
79.53718751	0.103816568	0.274023669
86.56280467	0.078279765	0.32095971
171.3053831	0.05525889	0.416241446
99.55450078	0.072817293	0.437990581
74.7615688	0.083022222	0.347822222
83.5087723	0.086276062	0.377183341
58.56979269	0.118516528	0.373824241
115.2980854	0.065992866	0.440546968
58.12734433	0.102222222	0.315555556
101.2296577	0.065723138	0.341691793
109.0199397	0.069008751	0.336355923
95.00555778	0.060720585	0.332325523
83.16438134	0.078874356	0.307702471
75.87631294	0.093971155	0.365164681
129.5380846	0.066646547	0.293999296
105.615454	0.066836387	0.324500699
112.3547276	0.0759261	0.398199642
73.80893138	0.088242676	0.337301445
28.1540404	0.123966942	0.272727273
91.43114282	0.082027465	0.313842761
100.1882149	0.084379906	0.390366502
109.0757141	0.067558299	0.326646091
110.9246007	0.068025254	0.325682448
83.65775423	0.088888889	0.346770833
83.69790654	0.082513101	0.351619188
126.219465	0.069876543	0.382112483
150.2059752	0.062151927	0.396698102
94.43463107	0.078850518	0.359080787
63.22319935	0.083888889	0.258333333
212.8484834	0.068972654	0.354027159
82.80518593	0.083223869	0.313098142
63.15529268	0.101879567	0.277802973
138.5467265	0.058927863	0.38689351
103.1926744	0.069714803	0.39141014
86.37489665	0.077998078	0.323084986
119.6007622	0.071215888	0.36409823
79.77936826	0.084759396	0.355728046
41.73864456	0.088395062	0.20691358
64.31151961	0.07112649	0.285659362
91.24200083	0.065759637	0.31292517
65.01525955	0.076177778	0.253688889
181.7047423	0.063666821	0.350999581
83.20482925	0.092651433	0.300804489
62.20867323	0.099862259	0.271235078
181.5432558	0.057514131	0.460287862
110.1598954	0.067131767	0.360145002
104.9606882	0.077592801	0.341658323
72.7173676	0.095554438	0.329431198
100.911899	0.071797521	0.35356405
117.0891792	0.080422875	0.315527782
110.6881494	0.075087682	0.357955149
209.0442953	0.053795531	0.378048158
174.5334926	0.059409563	0.333917581
149.5532951	0.066113347	0.33371871
60.16935634	0.095020829	0.271176354
168.7190127	0.057493767	0.325225829
92.39662657	0.078443878	0.34375
167.319802	0.063871425	0.389017299
79.70234706	0.076150385	0.287234722
122.9152052	0.069782155	0.313207483
59.67180124	0.084960938	0.269042969
119.2726012	0.077619077	0.332499527
71.21764944	0.083205089	0.354673233
103.8353188	0.064948035	0.33272994
117.2630751	0.067831508	0.331965455
47.30493241	0.100308642	0.224108368
175.5303936	0.043412752	0.297172334
141.1470043	0.054201183	0.40591716
116.8513404	0.06450345	0.318237401
85.93069718	0.074857478	0.313442378
114.1111442	0.061165113	0.331658181
127.4048355	0.06377551	0.342857143
142.5983213	0.068491915	0.2769887
71.30061012	0.08559527	0.287019618
99.65949043	0.068583563	0.29884068
194.5688944	0.050093128	0.399862759
99.41075307	0.069306339	0.350771895
101.0023852	0.071507517	0.340610759
152.77219	0.05263981	0.31637321
130.7877169	0.068310321	0.339173482
251.9727687	0.051882461	0.428259871
99.80246609	0.080951771	0.247639797
112.1899037	0.078090011	0.381135213
98.30637344	0.073360705	0.301986475
55.55419231	0.088058467	0.308005594
155.3913747	0.05992623	0.422626125
100.8837883	0.077188791	0.351853048
95.56995761	0.072778828	0.406427221
57.74557348	0.086368366	0.298647242
172.5572133	0.062326841	0.344943663

log.sigma.5.0.mm.3D_glszm_SizeZoneNonUniformity	log.sigma.5.0.mm.3D_glszm_GrayLevelNonUniformity	log.sigma.5.0.mm.3D_glszm_LargeAreaEmphasis	log.sigma.5.0.mm.3D_glszm_ZoneVariance
70.64319249	13.02816901	38.25352113	28.47688951
22.89473684	3.245614035	6.824561404	2.894429055
50.80952381	8.931972789	38.02040816	27.2244898
26.16363636	3.327272727	4.618181818	1.820165289
82.51658768	14.84834123	110.4170616	97.916983
19.2745098	3.705882353	5.823529412	1.744713572
290.8141809	62.91198044	56176.8533	55779.48953
200.1438849	37.19064748	2376.021583	2293.656164
81.89230769	21.25384615	5637.65	5373.087263
32.74509804	7.666666667	40.94117647	28.20607459
71.24615385	26.99230769	19312.19231	18870.54592
75.42553191	18.39574468	3400.46383	3229.466474
66.18238994	8.786163522	9.251572327	4.91784344
39.85714286	6.626373626	9.78021978	5.420842893
52.17333333	12.45333333	286.32	248.7022222
226.687188	51.85191348	11880.16805	11678.86814
22.80327869	7.229508197	252.9180328	201.8306907
25.55172414	3.827586207	6.224137931	2.561533888
18.93333333	6.133333333	402.6833333	313.3808333
308.8893805	59.41371681	14166.35951	13992.28885
94.51601423	19.39145907	280.9679715	254.2673978
20.93650794	3.825396825	13.71428571	7.424540186
26.77011494	6.862068966	26.5862069	16.15404941
66.82513661	17.19672131	22706.20765	22017.07343
41.45390071	9.397163121	130.3829787	103.871435
108.3832335	22.32335329	660.9850299	624.2265858
82.02912621	15.6407767	177.7718447	156.9941795
56.32934132	14.73652695	1209.05988	1098.117179
3	1.363636364	9.454545455	2.975206612
54.29479769	14.19075145	377.0693642	332.8045708
142.0934066	30.71428571	1927.96978	1834.932096
35.27777778	7.296296296	10.71296296	5.138117284
83.70038911	17.48249027	214.3618677	189.5947857
83.225	21.33333333	3257.320833	3085.164566
171.5901639	40.26639344	885.5922131	834.8265881
103.1703704	18.86666667	120.1037037	102.6498765
79.73631841	12.49253731	6.07960199	2.314843692
246.6885007	54.17030568	23647.88064	23355.95332
15.5	5.033333333	22.95	12.16972222
248.5270655	48.41880342	25039.73219	24741.9485
45.71232877	12.15068493	133.0753425	114.3963689
87.50793651	32.09206349	27950.9619	27423.12951
160.173913	24.39613527	119.9782609	106.3739235
73.58510638	13.10638298	106.3776596	92.23525917
34.57009346	8.345794393	846.8224299	743.4296445
116.1473354	22.71786834	563.3448276	525.7859887
158.6547085	37.80269058	9793.360987	9549.202844
9.311111111	3.977777778	53.04444444	29.36
14.56862745	3.62745098	7.294117647	2.297577855
26.28571429	5.523809524	13.73809524	6.878684807
38.05333333	11.42666667	1507.613333	1346.153956
149.5258216	27.12206573	417.0399061	391.3545207
103.4767442	31.87209302	59684.74419	58822.36584
35.8030303	13.18181818	290.8863636	249.7127525
60.29770992	7.534351145	12.9389313	8.13915273
61.58479532	11.47953216	104.2046784	89.17205294
137.0049875	31.11471322	5053.603491	4879.771432
77.74576271	22.55084746	441.0381356	398.1800309
31.11363636	6.318181818	27.38636364	17.83264463
243.9029754	62.16688228	135147.0026	134221.7568
69.44329897	14.56701031	110.5412371	93.66354023
149.7070707	21.3030303	158.770202	142.3840616
392.6870748	69.86564626	202.9183673	187.8037276
331.3826788	65.65055388	4614.116818	4540.499381
19.25352113	6.746478873	61.73239437	41.29180718
172.6949153	30.52919021	166.3483992	149.4161817
19.25	4.392857143	8.267857143	3.752232143
242.7467949	39.85576923	1484.044872	1440.281558
119.2024096	31.60240964	28741.0241	28157.7143
107.1169591	23.86549708	205.5760234	185.6405817
17.21875	5.4375	21.46875	11.89746094
175.8922495	41.06049149	9784.599244	9604.511976
60.64912281	14.22807018	177.8421053	158.6566807
59.55865922	11.62569832	337.3240223	301.3910302
124.1550802	25.36898396	2011.609626	1937.66802
24.2037037	10.83333333	168.5740741	137.2969822
123.9208633	18.10311751	61.1942446	48.56363542
26.38461538	3.523076923	4.507692308	1.377514793
68.10280374	13.80373832	31.89252336	22.6384182
42.94160584	10.25547445	1535.284672	1399.570675
70.64319249	13.02816901	38.25352113	28.47688951
48	8.928571429	17.97857143	11.03158163
92.23723724	22.80780781	2805.285285	2699.930164
35.01639344	10.44262295	874.4344262	781.6754233
78.89393939	18.10606061	530.9507576	484.2045311
40.38613861	5.059405941	4.198019802	1.161454759
64.19125683	12.68306011	86.19125683	70.49580459
87.53696498	18.37743191	7945.735409	7695.430998
87.63537906	14.58122744	135.4548736	117.461351
68.85221675	13.86699507	108.0295567	92.49896867
56.53030303	6.848484848	9.090909091	4.719008264
37.88888889	12.38562092	888.7320261	801.3767354
180.2769556	36.93657505	1238.782241	1186.441758
109.9230769	26.7032967	2819.39011	2707.286228
96.4057508	27.56230032	663.5750799	619.9644581
171.5862069	24.33004926	76.07389163	67.56934165
123.8522727	27.17045455	211.59375	194.2720735
18.69565217	3.347826087	5.891304348	2.314272212
9.258064516	2.677419355	8.161290323	2.915712799
369.0897196	66.68971963	5210.91028	5130.347073

log.sigma.5.0.mm.3D_glszm_ZonePercentage	log.sigma.5.0.mm.3D_glszm_LargeAreaLowGrayLevelEmphasis	log.sigma.5.0.mm.3D_glszm_LargeAreaHighGrayLevelEmphasis	log.sigma.5.0.mm.3D_glszm_HighGrayLevelZoneEmphasis
0.31981982	0.545830937	4082.798122	188.685446
0.504424779	0.233338359	809.7719298	190.8421053
0.304347826	0.606559447	5092.517007	224.4217687
0.597826087	0.212835912	474.4	165.0181818
0.282841823	5.814205147	3087.843602	140.3933649
0.495145631	0.276514983	651.2941176	144.7843137
0.050165583	449.6144439	7443690.637	121.0207824
0.110186286	17.57100973	375727.7752	169.0017986
0.061480255	72.20264692	558817.8231	126.6730769
0.28021978	0.954224499	2647.656863	128.0294118
0.047584187	123.2130937	3092062.758	144.8961538
0.076472502	39.72299906	371194.366	132.9191489
0.480362538	1.263775154	872.2641509	221.3836478
0.478947368	0.911030759	501.4615385	129.4725275
0.163043478	7.242621509	18200.25333	109.8533333
0.070481998	112.0025035	1619927.388	121.9317804
0.139908257	20.3892191	8054.52459	80.8852459
0.522522523	0.274069136	509.6551724	144.5517241
0.105820106	12.68960524	24518.5	86.28333333
0.075794416	129.4384929	1727928.088	163.5154867
0.193526171	8.136320691	14699.40214	160.5587189
0.398734177	0.35934042	1504.587302	161.7460317
0.309608541	1.736890594	2153.425287	124.1149425
0.038093256	215.6680602	2569997.082	111.7431694
0.194214876	0.926011355	24251.87943	212.8865248
0.164938272	9.944614809	54198.31437	168.5688623
0.219382322	2.431117048	18595.86893	154.9951456
0.094940307	16.64075189	114165.1377	109.7305389
0.392857143	0.687447448	228	43.72727273
0.150304083	6.201746502	31083.72832	145.4277457
0.103674167	24.88254775	206464.3956	139.4587912
0.423529412	0.224204096	1190.574074	166.4722222
0.200938233	3.55003388	22190.40856	159.3190661
0.076214671	34.81480461	396267.0125	127.4041667
0.140350877	14.55918443	69924.88934	126.1577869
0.239361702	1.859874207	17436.1	174.1518519
0.515384615	0.098883529	894.1940299	205.0597015
0.058527858	148.8538856	4155325.844	141.5604076
0.304568528	1.025858589	1523.116667	100.2166667
0.05794948	82.59609005	7761091.818	326.531339
0.231378764	3.603445543	6206.630137	122.5958904
0.043526323	358.840985	2268203.006	110.2095238
0.271119843	2.029669473	14636.9686	193.3961353
0.265912306	3.374049449	4920.755319	139.3829787
0.098345588	11.18019762	91538.26168	126.3925234
0.163171355	8.04918932	52926.16301	172.1285266
0.063997704	84.48684415	1299652.845	124.6793722
0.205479452	4.068470238	3746.488889	78.35555556
0.447368421	0.666680034	512.3137255	107.7843137
0.381818182	0.233529535	1696.321429	147.3333333
0.078698846	11.58676205	257098.92	119.86
0.197313571	3.171946362	62027.18779	269.6103286
0.034052663	524.4378376	7093192.983	130.1918605
0.155844156	3.506067064	27876.13636	119.3712121
0.456445993	0.432837564	1663.312977	237.6946565
0.257918552	3.070608852	4846.263158	155.8538012
0.075846416	41.57723387	706963.8853	158.8778055
0.152750809	14.64243299	23457.16102	109.3771186
0.323529412	0.553246898	2411.590909	149.0795455
0.032875431	814.2745886	22907736.98	187.2121604
0.243412798	2.033477931	8996.721649	161.7783505
0.247036806	1.46119957	25063.05051	304.9924242
0.257217848	1.260611433	39137.52211	274.0705782
0.116549296	23.52505111	999306.3484	230.3736153
0.221183801	2.420306967	3283.352113	98.07042254
0.243020595	1.364928381	24859.10923	272.2542373
0.470588235	0.365093933	744.7321429	129.25
0.151162791	9.909351904	239522.4311	230.3910256
0.041404769	287.0107205	3357638.598	134.6963855
0.223968566	3.632547199	16463	196.2777778
0.323232323	1.433455441	883.15625	96.140625
0.074517538	41.09684811	2547245.13	186.5330813
0.228304406	6.867295077	6304.19883	100.1345029
0.166821994	4.728353533	31976.68156	159.2513966
0.116293532	11.47257623	376886.1471	179.5962567
0.178807947	5.358796292	7966.703704	94.2037037
0.281376518	1.113432898	6935.976019	297.4292566
0.565217391	0.100665086	1048.215385	224.7538462
0.328725038	0.967293274	2523.67757	186.1448598
0.085839599	15.46753684	183681.4526	142.0218978
0.31981982	0.545830937	4082.798122	188.685446
0.379403794	1.476880497	1123.35	182.5571429
0.097425395	11.91785844	726307.973	234.0870871
0.103829787	10.46957256	86070.90984	110.5245902
0.146260388	5.520297399	70207.54167	160.375
0.573863636	0.053406947	1186.287129	295.8514851
0.252413793	1.410282987	9959.530055	149.9562842
0.063207083	72.72217344	1030530.187	149.1167315
0.235744681	1.799838646	14659.80505	250.5559567
0.25375	3.851715887	5146.384236	185.3448276
0.47826087	0.06525642	2690.787879	351.8257576
0.106993007	6.736257223	134486.8627	177.7189542
0.138223261	14.79113134	127120.2389	159.5433404
0.094447327	33.7125278	298452.6593	154.5137363
0.151427189	18.69893438	30133.98403	89.41214058
0.342905405	1.109798231	6620.866995	210.8669951
0.240273038	6.27343176	10091.14773	148.9772727
0.528735632	0.304381609	551.8913043	127.2826087
0.436619718	0.277083032	607.2903226	87.64516129
0.111411912	24.05977347	1227390.174	271.9766355

log.sigma.5.0.mm.3D_glszm_SmallAreaEmphasis	log.sigma.5.0.mm.3D_glszm_LowGrayLevelZoneEmphasis	log.sigma.5.0.mm.3D_glszm_ZoneEntropy	log.sigma.5.0.mm.3D_glszm_SmallAreaLowGrayLevelEmphasis
0.591987709	0.01627748	5.862537533	0.010756102
0.656575942	0.034152919	5.152979707	0.011485639
0.609935578	0.016970961	5.587803693	0.005288855
0.71585073	0.036742972	4.824465341	0.012694902
0.648385485	0.02571556	5.534266786	0.013019048
0.61754902	0.035004451	4.969007636	0.008451437
0.618270027	0.023582534	5.932901905	0.011020432
0.623702477	0.013795902	6.032984649	0.00749745
0.577136994	0.024864807	5.712795191	0.014819717
0.584198498	0.028872555	5.390384597	0.012362565
0.539250202	0.013452096	5.720438483	0.009239002
0.585860241	0.02087492	5.677054104	0.007289181
0.669626043	0.017028768	5.42848044	0.004149865
0.687539077	0.028394115	4.942743647	0.009806584
0.610575612	0.026990813	5.313674361	0.009224452
0.638098474	0.017204614	5.661076541	0.00898061
0.632816256	0.038024533	4.577535084	0.008983602
0.685401886	0.035758154	4.789460849	0.024752706
0.578075371	0.037980544	4.800815129	0.012062666
0.60437108	0.016265844	6.206369811	0.008747229
0.600610609	0.017432115	5.837993988	0.005496566
0.590409605	0.032996941	5.090386225	0.021998518
0.568713609	0.028036312	5.209607577	0.005893645
0.628007192	0.022040568	5.345017913	0.008089806
0.558767855	0.01752499	5.863159442	0.013002489
0.58315217	0.016902413	5.902674414	0.00964034
0.655633732	0.016684724	5.470823594	0.005958156
0.602842615	0.031097503	5.424155828	0.01469348
0.509318182	0.12210641	3.459431619	0.098509215
0.574993814	0.018421754	5.546416921	0.010862403
0.649057777	0.016025565	5.511422355	0.006742543
0.586338791	0.023611905	5.524001419	0.015197727
0.5852836	0.017503075	5.80023722	0.00639203
0.610856319	0.019187131	5.468453282	0.01168182
0.613572516	0.020946415	5.768425511	0.008162167
0.642552714	0.016367584	5.598933184	0.005192555
0.65254916	0.015467853	5.562191129	0.009807782
0.622329576	0.0173769	5.887125566	0.007401976
0.514510027	0.037856429	5.026408572	0.010659336
0.618516662	0.006367401	6.135816252	0.004552362
0.576198617	0.035626933	5.373439061	0.011979355
0.541632303	0.020367875	5.820361227	0.011207239
0.64647968	0.015978976	5.969744068	0.008107657
0.648902436	0.027158649	5.516507262	0.012649553
0.588142816	0.029148872	5.362597305	0.007811411
0.620674318	0.01553589	5.691957766	0.008240753
0.617552959	0.017366396	5.667236544	0.010530253
0.45625219	0.051508761	5.058302541	0.011384827
0.527953431	0.044810934	4.998611068	0.010616502
0.575549351	0.029213789	5.475725536	0.01936935
0.515932536	0.024096552	5.855210792	0.014598609
0.614427505	0.009807468	6.102112089	0.006009369
0.5616951	0.017565964	5.736588934	0.007007572
0.535692613	0.022369018	5.449337891	0.016048566
0.705330093	0.018203022	5.354097788	0.006893182
0.622847235	0.022191468	5.536098295	0.0111549
0.605162916	0.014439845	5.869851867	0.008104128
0.590884686	0.02075288	5.427141976	0.011187282
0.616285434	0.028495184	5.250855382	0.014055397
0.582373936	0.011456026	6.150831701	0.006024221
0.620246836	0.018435748	5.584135136	0.01179183
0.63716202	0.009075839	6.100415808	0.004011631
0.598006375	0.008959582	6.407184752	0.004724389
0.598292685	0.009184706	6.308551571	0.004427165
0.533528477	0.033987134	5.154380437	0.008114856
0.590409296	0.01004758	6.380731067	0.005391636
0.593456111	0.037451676	4.686445995	0.008156154
0.648182053	0.010200327	5.975070965	0.0047235
0.55101491	0.020353132	6.056576193	0.009507167
0.573916362	0.01333685	5.94318257	0.005199994
0.513399468	0.036892693	5.189464016	0.0113301
0.597547819	0.01084372	5.950064053	0.006308039
0.616125715	0.041751421	5.466449443	0.019776239
0.597732674	0.020785998	5.884318817	0.013007216
0.595870314	0.014189587	6.031018196	0.006729287
0.459548697	0.031542934	5.446855249	0.009807308
0.55984844	0.015558987	6.728972154	0.008000218
0.656358974	0.029516195	5.310603774	0.014878356
0.579921052	0.015914138	5.818825778	0.005765364
0.57799354	0.022562511	5.55769819	0.01537161
0.591987709	0.01627748	5.862537533	0.010756102
0.602304708	0.019216797	5.593828637	0.005068404
0.54229585	0.010392596	6.263048899	0.006292448
0.547706132	0.02762054	5.347208318	0.008374851
0.558053705	0.016446585	5.922431827	0.009040769
0.632288229	0.019486438	5.378681291	0.016425602
0.612841271	0.018452346	5.589226187	0.00652492
0.604098995	0.018049976	5.764680117	0.009861128
0.579760314	0.014175726	6.238971607	0.008586161
0.602836782	0.015156928	5.744137472	0.005413506
0.679781072	0.015664309	5.616851295	0.01294421
0.504786503	0.019452158	5.824457122	0.008467917
0.641752011	0.013854259	5.723864427	0.006131987
0.562902214	0.019031689	5.997113376	0.006411329
0.572579417	0.034516002	5.749643461	0.015116115
0.675782512	0.013463288	5.818163573	0.006934656
0.615055565	0.018857598	5.779517251	0.007388557
0.655742384	0.041582262	4.586183359	0.014744734
0.550853495	0.059801478	4.502583407	0.040925636
0.608067312	0.006919425	6.264276942	0.003287666

log.sigma.5.0.mm.3D_ngtdm_Coarseness	log.sigma.5.0.mm.3D_ngtdm_Complexity	log.sigma.5.0.mm.3D_ngtdm_Strength	log.sigma.5.0.mm.3D_ngtdm_Busyness	log.sigma.5.0.mm.3D_ngtdm_Contrast
0.015873174	0.66645384	3.798942745	0.232395322	0.000145153
0.062282118	5.726242909	12.92193698	0.062849	0.002331066
0.021268928	0.787108418	3.178212746	0.214602008	0.000369916
0.07292577	5.042298892	9.984052413	0.080292565	0.003591219
0.012763281	0.450819558	2.547417741	0.600819472	0.000165947
0.075694713	2.722334512	9.427284553	0.078437233	0.00191694
0.001061133	0.006970208	0.255058755	2.708159425	9.03E-07
0.002739402	0.043032552	0.514454512	1.166551426	8.61E-06
0.003428506	0.031329208	0.527425932	1.253798247	7.17E-06
0.027100235	0.5548427	3.072995697	0.241941382	0.000260873
0.002315686	0.014630702	0.423365166	1.330353121	2.08E-06
0.005170685	0.0572712	1.240110464	0.660311374	8.05E-06
0.027762594	1.459785197	4.817212256	0.188064182	0.00088608
0.036376644	1.442713932	3.55705031	0.265591964	0.001592539
0.012160745	0.1908063	1.596058064	0.53899446	7.36E-05
0.001694028	0.015176351	0.265227934	2.217934172	4.57E-06
0.022868765	0.279010917	2.374653459	0.451484934	0.000153944
0.066505588	3.521868701	10.55509312	0.102219052	0.002286948
0.02011276	0.123155998	1.265901881	0.50301654	0.000129567
0.001324797	0.025853606	0.652527642	1.738348289	1.46E-06
0.007786277	0.2132592	1.564158876	0.74338243	6.26E-05
0.052548254	1.939908893	6.865781863	0.121367573	0.001711265
0.035113857	0.751302292	4.054533164	0.187888305	0.000500181
0.003113804	0.014806841	0.47433043	1.252644782	3.45E-06
0.015249507	0.432794268	2.685723664	0.215503159	0.000118555
0.006177609	0.127776839	1.152328458	0.688209098	2.89E-05
0.01066603	0.33045864	2.252143681	0.341857471	6.43E-05
0.007747791	0.063552624	0.884400197	0.750560252	2.64E-05
0.209217503	1.479327858	6.052789127	0.192287378	0.005917124
0.010422576	0.174404029	1.606596599	0.468947364	4.96E-05
0.003823184	0.057653955	0.734761484	1.053698374	1.18E-05
0.029430492	1.479566842	3.762621539	0.177579993	0.000783728
0.009776988	0.222343559	2.136930526	0.385780466	4.51E-05
0.004836331	0.036223819	0.611243186	0.954062577	1.34E-05
0.00356175	0.049366783	0.532833164	1.441038587	1.35E-05
0.009897717	0.237470146	1.546745892	0.407186054	9.47E-05
0.02125131	1.216060938	4.104811701	0.176293586	0.000423842
0.001300336	0.013379134	0.309573642	1.957650015	2.07E-06
0.047399216	0.805298314	4.338853608	0.204859368	0.000738906
0.001375368	0.020909393	0.688775559	0.93038786	7.82E-07
0.015978198	0.325498953	1.799884071	0.458518855	0.000149725
0.001939113	0.008537613	0.259858664	2.396242187	1.42E-06
0.006964247	0.270265608	1.381514406	0.525356413	7.25E-05
0.015524706	0.453440872	3.48742749	0.363137245	0.000117075
0.014648152	0.124511806	1.997743043	0.310620527	4.07E-05
0.006139006	0.122273747	0.992565857	0.698324315	3.17E-05
0.002296488	0.014687701	0.305300601	1.728761731	4.24E-06
0.050323859	0.362826093	3.202399811	0.236306068	0.000725704
0.080996541	2.332311558	12.22021082	0.114131326	0.001741386
0.036868313	1.145837885	4.288004019	0.157014623	0.000839413
0.009156547	0.056067448	1.194855161	0.456456775	2.62E-05
0.006199656	0.255404917	2.663258607	0.305880655	1.86E-05
0.001464384	0.01066041	0.444641779	1.928362421	9.06E-07
0.013245478	0.144236945	1.296710801	0.441009333	6.68E-05
0.031391904	2.293855079	7.619967226	0.110683835	0.000798631
0.015960984	0.45187003	2.750320964	0.435353227	0.000180804
0.003032734	0.035581332	0.715160665	0.938166125	4.23E-06
0.006453832	0.095620512	0.599076975	1.382749618	6.11E-05
0.032556828	0.905811176	4.211748479	0.164655541	0.000437158
0.000633742	0.006461655	0.295885434	3.003231404	2.68E-07
0.013403775	0.362659894	2.26333288	0.340945303	0.000102802
0.008291309	0.397291526	3.051839041	0.258802763	4.60E-05
0.002676799	0.113820832	0.730916873	0.829292224	1.58E-05
0.001649729	0.036510311	0.546084036	1.135979164	2.97E-06
0.028440973	0.415653795	2.181277857	0.326767048	0.000358762
0.005901425	0.263266953	2.055902243	0.382663119	3.36E-05
0.052379134	2.13695578	4.859543546	0.143102611	0.002486795
0.003127607	0.071731741	0.801618577	0.769502917	7.84E-06
0.001676115	0.011735676	0.334988191	2.101349003	2.00E-06
0.007589381	0.195041916	1.265136593	0.595766223	6.06E-05
0.042638097	0.909056211	3.831594599	0.237167236	0.000732183
0.002077441	0.024703991	0.508141921	1.050034028	4.84E-06
0.01206621	0.261418848	1.319652655	0.714777934	0.000123932
0.011748119	0.24350281	2.79019723	0.320145852	4.52E-05
0.004345781	0.073607784	1.248268566	0.526332728	7.71E-06
0.016412063	0.228365754	1.591159822	0.52583582	0.000135406
0.009325874	0.961419836	9.108206502	0.213639457	6.53E-05
0.052527807	5.323486382	12.61265579	0.054991375	0.002551111
0.017397805	0.564084437	3.249079292	0.261686228	0.000202294
0.009225266	0.0906963	1.647459974	0.396985605	1.78E-05
0.015873174	0.66645384	3.798942745	0.232395322	0.000145153
0.027799701	1.018134825	4.755025452	0.212607699	0.000528396
0.004232588	0.083454144	1.526958426	0.414300474	7.12E-06
0.010960818	0.072650071	0.93728877	0.580193494	4.36E-05
0.007315689	0.102003975	1.050105485	0.551361853	3.27E-05
0.043025537	5.146654617	13.24853445	0.05255671	0.001412792
0.016801871	0.259505043	2.094194691	0.31889231	0.000137255
0.003741047	0.032943532	0.667955738	0.923190828	7.30E-06
0.010407121	0.767842497	7.606609643	0.219293048	6.72E-05
0.013837748	0.459363688	2.890463758	0.407615178	0.000141443
0.029796492	2.788073155	9.659184655	0.063148233	0.000631284
0.009076206	0.130558633	1.64201565	0.344695535	2.52E-05
0.003997294	0.063137533	0.790104873	0.911680632	1.09E-05
0.003713986	0.060048289	0.912083673	0.885508484	7.73E-06
0.005824593	0.06465024	0.71644387	1.229735454	2.21E-05
0.008291121	0.530494525	2.251651765	0.391177795	9.08E-05
0.007264232	0.236676465	1.509956538	0.725526266	5.91E-05
0.076911645	2.841101935	7.007973301	0.096581977	0.003066418
0.084053089	2.44211925	6.136034729	0.115333729	0.003046214
0.001594219	0.034320489	0.546012378	1.124047631	3.04E-06

log.sigma.4.5.mm.3D_gldm_GrayLevelVariance	log.sigma.4.5.mm.3D_gldm_HighGrayLevelEmphasis	log.sigma.4.5.mm.3D_gldm_GrayLevelNonUniformityNormalized	log.sigma.4.5.mm.3D_gldm_DependenceEntropy
17.90158853	159.9264264	0.070854638	6.445654192
25.13556269	150.6548673	0.068055447	5.325543095
20.00800724	149.1573499	0.06349635	6.413981214
24.73298677	132.2608696	0.060964083	5.217703636
13.77705583	65.82573727	0.085949011	6.251089457
17.05702705	109.0679612	0.071920068	5.039833232
6.057752936	111.5575862	0.208996976	6.751783568
10.73917564	146.2405866	0.090962275	7.146019677
7.21814934	96.56065264	0.115407745	6.978907573
8.930405446	90.47527473	0.100335104	5.954919486
3.263706613	205.102306	0.204211742	6.90203335
7.911071615	97.738041	0.120663698	7.08190736
29.56170535	150.8942598	0.0528564	6.044484982
17.10304709	84.51578947	0.068421053	5.69297767
9.506180293	84.875	0.090302457	6.614368922
9.207543601	115.0630937	0.142342973	7.104221144
6.948194596	42.50917431	0.110491541	6.019046441
17.48721695	78.91891892	0.077509942	5.198625921
6.73441393	50.47619048	0.107552669	6.214081771
8.691983286	141.0856879	0.12460242	7.466759232
14.06212007	80.59917355	0.079339602	6.746141836
20.64480852	112.8291139	0.063130909	5.605881306
13.56988893	97.60854093	0.076328821	6.052193606
4.601790478	100.8532473	0.215283233	6.774770039
15.26624813	205.1060606	0.073583316	6.595905659
12.17603073	127.8898765	0.089859229	6.987098572
10.43096853	126.056443	0.092524733	6.626221221
8.37982005	101.7839682	0.096483253	6.891466776
4.24872449	19.96428571	0.206632653	3.896291529
9.657162095	110.4057341	0.090435469	6.799272995
9.859322221	94.99686699	0.09007091	7.018608516
17.60445982	126.827451	0.069865436	5.81126486
11.87657358	114.5684128	0.083881624	6.762351781
8.872201562	116.9291839	0.11339514	7.116369461
8.35598922	89.50100661	0.098807241	6.832664709
16.63209141	145.0966312	0.069419295	6.920498699
21.65196581	172.1230769	0.060670611	5.835832728
8.470203848	148.7629068	0.155027059	7.1778069
12.09384421	65.59898477	0.082223196	5.824881518
6.431719431	369.7032359	0.156475156	7.1775205
9.216211533	85.7163233	0.097113982	6.350011175
2.796805616	99.32057482	0.204812777	6.878982717
18.12145494	148.6823838	0.068040926	6.95685473
12.61922301	89.59688826	0.086580147	6.367367564
9.37979749	100.5928309	0.101670631	6.962176983
11.2862098	115.7544757	0.085194367	6.916812292
7.081669697	121.8349835	0.122598385	7.304093807
13.31836284	74.85844749	0.081879027	5.888055759
19.20629424	96.30701754	0.070175439	5.422643032
17.61867769	148.4090909	0.070909091	5.795968404
11.04316407	128.4459601	0.104601474	6.869160289
14.72599571	164.8480778	0.082913045	6.913070212
4.119751422	88.06285884	0.231374469	6.774597286
9.269643955	119.9539551	0.090056021	6.478269863
27.83180566	137.8989547	0.056926756	6.079288182
14.46697106	77.17043741	0.083834392	6.361648697
7.941291345	113.2608284	0.110406063	7.236767525
10.31057237	67.91326861	0.085454907	6.710899431
11.31352725	86.97794118	0.085261678	5.990802033
4.016168323	160.2188577	0.219753005	6.879890505
12.36286325	98.61731493	0.08092612	6.570632434
21.76051921	225.966937	0.061893074	6.980608529
19.00127842	246.0465879	0.067328786	7.097279149
10.01372345	202.731338	0.1102046	7.320464075
9.606098543	75.00934579	0.089876845	5.969097989
21.77342626	221.0160183	0.064561054	6.9401631
15.09243697	96.09243697	0.077325048	5.305955054
9.807653572	201.2022771	0.096314848	7.115266783
6.404656957	105.6041105	0.146375946	7.108291529
14.64247175	135.0956123	0.074702077	6.688741883
10.1372564	57.8030303	0.092286501	5.678652725
10.83463346	159.4004789	0.143150518	7.116568815
8.225981772	66.22162884	0.101227984	6.217309173
10.72610175	113.9767008	0.095499201	6.703617862
8.3883691	166.7599502	0.119754152	7.123287688
9.396704092	74.40728477	0.094222841	6.346994417
48.05435264	209.4574899	0.051508794	6.951612089
32.12143667	257.6434783	0.057693762	5.50175679
19.75500766	138.5683564	0.062366535	6.315540401
6.978926012	116.1002506	0.118943976	6.894560473
17.90158853	159.9264264	0.070854638	6.445654192
22.29400489	113.8617886	0.060127349	6.066661597
10.55508424	188.0105325	0.129675237	7.084327219
7.119620462	84.81446809	0.108787687	6.735911668
10.23596504	108.4160665	0.092677312	6.969163324
34.69821798	264.8409091	0.051394628	5.682831852
14.41170797	153.9448276	0.074024257	6.768073994
7.849582479	91.41957698	0.157379505	6.919142711
34.28832304	194.4885106	0.068494341	6.946482791
16.44517344	93.73125	0.073234375	6.589168274
30.32140044	318.2137681	0.063405797	5.923527988
8.338739792	147.8594406	0.106832608	6.876111159
8.873522769	95.93191116	0.096297174	7.159199801
8.603928981	108.3860924	0.105566999	7.073426908
6.80131558	64.55249153	0.108916138	6.586185836
19.03080987	156.9940878	0.067607515	6.599499248
13.09440343	93.70170648	0.08093979	6.611012838
18.93565861	145.045977	0.073589642	4.987732474
12.41460028	88.91549296	0.084308669	4.797083739
12.25552013	227.4592878	0.099238638	7.509209609

log.sigma.4.5.mm.3D_gldm_DependenceNonUniformity	log.sigma.4.5.mm.3D_gldm_GrayLevelNonUniformity	log.sigma.4.5.mm.3D_gldm_SmallDependenceEmphasis
111.021021	47.18918919	0.260613861
31.05309735	7.690265487	0.436779744
84.77225673	30.66873706	0.245579323
29.7173913	5.608695652	0.374378019
104.5549598	64.11796247	0.24189697
37.0776699	7.40776699	0.557882956
737.4924568	3407.904698	0.055020278
429.0443916	458.9956401	0.122896198
282.1738	488.0593521	0.074841802
52.86813187	36.52197802	0.264022694
266.5278184	1115.812958	0.0567059
176.7826228	370.7995444	0.080993347
76.21450151	17.49546828	0.375045991
47.75789474	13	0.456997173
104.3673913	83.07826087	0.159081228
436.4427114	1213.758532	0.079120894
52.51376147	48.17431193	0.145804057
27.91891892	8.603603604	0.372202202
68.5026455	60.98236332	0.117464593
632.1915821	1486.133059	0.087288645
168.4393939	115.2011019	0.183863657
31.13924051	9.974683544	0.327007027
46.40213523	21.44839858	0.234487159
212.561199	1034.220649	0.049919099
98.8677686	53.4214876	0.194172168
192.1367901	181.9649383	0.162865906
113.0383387	86.88072417	0.208310506
158.1335986	169.7140421	0.106631156
5	5.785714286	0.278104248
117.086881	104.091225	0.148695872
296.7225862	316.2389633	0.115854346
55.21960784	17.81568627	0.356911565
152.1696638	107.2845973	0.183497642
208.1190854	357.0812956	0.091663953
335.3974691	343.5527754	0.13781965
134.5212766	78.30496454	0.223332686
105.4974359	23.66153846	0.427757457
544.5421707	1819.707616	0.071638928
34.91878173	16.19796954	0.276432866
571.2052171	1895.540036	0.066893746
76.93660856	61.27892235	0.218452658
317.7953572	1482.230068	0.052635268
200.4695481	103.8984938	0.247122783
96.53889675	61.21216407	0.209036185
84.49080882	110.6176471	0.096233678
195.1248082	166.5549872	0.154382086
354.360023	854.3881475	0.071156081
33.35616438	17.93150685	0.189548938
26.21052632	8	0.383540299
53.45454545	15.6	0.356780896
157.5991605	199.3704092	0.096813054
239.8411302	179.0092635	0.193715347
397.0958226	2337.344882	0.041949377
110.107438	76.27744982	0.165478533
58.89547038	16.33797909	0.353433843
90.8974359	55.58220211	0.217113584
325.4486476	583.7168527	0.087169738
175.4517799	132.0278317	0.165342849
46.50735294	23.19117647	0.251189185
909.2391018	5167.052397	0.043458926
103.8130489	64.49811794	0.240437101
208.0867124	99.21459763	0.243384153
613.6146107	307.8272091	0.239142566
534.2323944	938.9431925	0.118711954
49.04984424	28.85046729	0.210640902
338.9176201	141.0659039	0.240120569
31.84033613	9.201680672	0.361111111
345.9602713	397.5876938	0.157204723
467.8610197	1467.12611	0.05536751
203.5854617	114.070072	0.201320574
30.48484848	18.27272727	0.285352329
357.7040428	1016.225525	0.082538992
96.90921228	75.81975968	0.195033337
113.4547996	102.4706431	0.141255119
219.9782338	385.1293532	0.116779237
80.53311258	56.91059603	0.174877581
263.9176788	76.33603239	0.277627118
37.3826087	6.634782609	0.493297101
123.9400922	40.60061444	0.30357147
119.1666667	189.8345865	0.088702281
111.021021	47.18918919	0.260613861
70.6802168	22.18699187	0.341399854
213.2843768	443.229959	0.101100931
103.5855319	127.8255319	0.109827727
168.0880886	167.2825485	0.149359802
54.875	9.045454545	0.487099116
93.58206897	53.66758621	0.233136421
210.758485	639.9050664	0.075096484
162.7531915	80.48085106	0.224516083
105.375	58.5875	0.210122582
61.9057971	17.5	0.377967492
123.2699301	152.7706294	0.114472221
286.4365868	329.5289305	0.141510684
273.3580695	406.8552154	0.105413558
224.8829221	225.1296565	0.153933291
202.5574324	80.0472973	0.304886032
201.8423208	118.5767918	0.227695406
25.64367816	6.402298851	0.460009579
19.28169014	5.985915493	0.435250391
616.0343607	953.08788	0.119661014

log.sigma.4.5.mm.3D_gldm_DependenceNonUniformityNormalized	log.sigma.4.5.mm.3D_gldm_DependenceVariance	log.sigma.4.5.mm.3D_gldm_LargeDependenceEmphasis
0.16669823	3.425281137	14.45645646
0.274806171	1.580076748	6.592920354
0.175511919	2.857305745	13.51759834
0.323015123	0.896502836	5.717391304
0.140154102	5.10598078	19.37533512
0.359977378	0.82250919	4.048543689
0.045228288	73.8015083	267.7429167
0.085026633	24.52083527	66.98493857
0.066723528	35.43239568	107.347127
0.145242121	6.290152156	20.20879121
0.048778883	39.088359	145.5889458
0.0575277	43.00138945	126.5515783
0.230255292	2.32507918	8.873111782
0.251357341	2.35900277	7.968421053
0.113442817	8.249995274	31.31086957
0.051183618	56.03859891	161.9424182
0.120444407	5.771063042	27.32110092
0.251521792	1.44825907	7.234234234
0.120815953	5.44223286	30.65432099
0.053005079	44.96501825	133.1977027
0.116005092	6.913591209	27.32506887
0.197083801	2.584521711	10.69620253
0.165132154	3.247147326	14.9430605
0.044246711	58.66143222	202.4217319
0.136181499	10.14481403	28.77961433
0.094882365	13.47770255	43.04444444
0.120381617	6.294898273	24.77848775
0.089899715	14.46432462	52.39283684
0.178571429	4.744897959	16.5
0.101726222	8.812740932	35.72458732
0.084512272	17.661862	57.98490459
0.216547482	2.828819685	10.12941176
0.118975499	6.566761684	26.393276
0.066090532	38.08410015	108.0765322
0.09646174	13.79990155	45.06269773
0.119256451	9.00324116	28.32269504
0.270506246	1.276686391	6.184615385
0.046391393	54.99430727	172.2637587
0.177252699	2.599654719	12.66497462
0.047152486	52.76669842	167.3100545
0.121928064	7.65068402	26.10935024
0.043912582	47.24025006	176.5998342
0.131283267	6.536540052	22.27046496
0.136547237	6.323325644	22.51626591
0.077656993	22.68922672	72.90625
0.099808086	12.84352771	41.20255754
0.050848045	43.73702201	141.9570957
0.152311253	5.454098121	21.05479452
0.229916898	2.020006156	8.49122807
0.242975207	1.540826446	7.836363636
0.082685814	35.80611291	90.45435467
0.11108899	8.34781426	29.82260306
0.039308634	58.62911167	237.6673926
0.129996975	5.136919108	23.55489965
0.205210698	2.745256104	10.37979094
0.137100205	4.531006509	19.61538462
0.061556393	33.39564557	104.5294118
0.113561023	10.54308187	34.70873786
0.170982915	3.589100346	15.02941176
0.038669634	57.61637586	235.7440139
0.130254767	6.352444628	22.44291092
0.1298108	6.979177766	23.21459763
0.13421142	8.928370912	25.66097988
0.062703333	45.89154511	117.3615023
0.152803253	4.683980163	18.77570093
0.155111039	5.201299478	18.27688787
0.26756585	1.032554198	6.529411765
0.083808205	17.7488843	53.89437984
0.046678741	47.85498713	166.061758
0.133323813	6.116088105	22.93844139
0.153963881	4.561881441	16.56565657
0.050387948	61.55826062	173.8215242
0.129384796	5.066939275	22.25233645
0.105736067	9.217688428	35.34855545
0.068401192	35.25799137	94.44651741
0.133332968	5.302344195	23.2384106
0.178082104	3.173874164	13.13765182
0.325066163	0.750396975	4.443478261
0.190384166	3.382252519	12.47465438
0.074665831	25.18797464	80.09273183
0.16669823	3.425281137	14.45645646
0.191545303	3.923384817	12.76151762
0.062400344	49.57621082	126.8186074
0.0881579	14.15644654	52.32255319
0.093123595	13.34889511	44.83933518
0.311789773	0.977143595	4.931818182
0.129078716	6.154855648	22.40965517
0.051834354	58.20768607	167.2267585
0.138513354	6.376565686	21.88340426
0.13171875	5.89609375	22.4
0.224296366	2.255618568	8.985507246
0.086202748	21.65279671	62.72027972
0.083704438	15.86380695	52.44827586
0.070928404	28.78064265	84.16606124
0.108796769	10.40230507	35.5476536
0.171078913	4.168964573	14.46959459
0.137776328	6.203606798	21.59249147
0.294754921	0.904743031	5.137931034
0.271573101	1.079150962	5.845070423
0.064143519	40.25199608	105.2051229

log.sigma.4.5.mm.3D_gldm_LargeDependenceLowGrayLevelEmphasis	log.sigma.4.5.mm.3D_gldm_SmallDependenceHighGrayLevelEmphasis	log.sigma.4.5.mm.3D_gldm_LargeDependenceHighGrayLevelEmphasis
0.170082172	52.79821146	1860.599099
0.2755908	88.01935595	602.3362832
0.34368218	46.83015229	1610.503106
0.472301889	70.00588768	564.326087
1.272802432	28.76963925	655.3780161
0.20917391	78.24543689	282.8932039
2.241949584	6.210202477	35533.2366
0.450689853	19.29072538	12712.61871
1.079855012	8.091539099	13966.84772
0.432332802	34.10925754	1234.717033
0.692917658	10.96855442	31388.67954
1.317255879	9.285533331	15638.40905
0.48377201	81.00692344	702.9546828
0.82585963	51.2200359	334.5684211
0.580343519	17.67186252	2938.053261
1.436827044	8.979053458	23125.98722
2.431016658	10.49630061	865.1651376
0.740423782	44.38625626	332.5045045
2.206295945	7.969107027	1523.075838
1.002986477	14.77303784	20033.70663
1.182098886	23.25166954	1683.239669
0.808508196	53.32199615	663.3101266
0.63843781	29.52992133	1175.715302
1.891161936	4.913571381	23896.66507
0.157042537	42.09162899	7657.670799
0.476627476	29.73647608	5184.198025
0.337946978	32.61971679	2896.033014
0.595688701	11.49111316	6678.85162
3.313667604	8.487416991	169.1785714
0.508231013	20.94706115	3642.612511
0.79564951	12.46491024	7515.266876
0.829497993	55.73903515	792.0980392
0.470830086	28.13821905	3072.393276
0.916335452	10.57982744	15296.90632
0.710737614	16.32946788	4676.634167
0.381215891	37.715625	5034.194149
0.124926235	89.43871157	828.6410256
1.078622336	9.44164511	31194.25652
1.385110514	25.049702	612.9796954
0.429358178	26.02145179	66453.65255
0.521395101	27.69618438	1777.992076
1.839546985	6.285740376	17724.69684
0.43181198	49.14286683	3126.713818
0.534625959	30.13652047	1514.471004
0.890534637	11.65729891	8795.719669
0.479450751	23.83679585	5487.641944
1.11050031	8.620080809	20774.4411
2.241812115	16.46904869	1259.159817
0.434741452	49.58287554	475.9561404
0.13605107	59.48887325	1168.663636
0.703703771	10.00585019	16158.92235
0.322958706	45.17573615	4393.265864
2.611711342	3.98629587	23835.83439
0.289549777	20.01720673	2745.361275
0.813120112	65.85411336	887.5644599
0.819027227	27.85878102	967.3815988
0.93618002	10.60796492	14182.62758
1.27496098	14.5997379	3037.405825
0.582645355	29.56018524	965.9926471
1.411904533	7.436687239	40600.84804
0.498234384	34.47960439	1901.139272
0.172458826	72.37333323	4915.562695
0.142135241	72.2701418	6302.403106
0.530545429	26.64683108	28741.57923
0.928814494	21.52163155	969.3271028
0.174570749	64.80419218	3208.529062
0.464919277	44.2075747	505.2857143
0.281169429	38.89158414	11975.21148
1.555106397	6.813357171	20763.40098
0.343751651	38.41785489	2538.570399
1.688504165	24.06007423	528.1161616
1.003459604	11.06296072	35827.80335
0.686649689	19.45583934	1179.214953
0.444309756	20.23004871	3887.741845
0.533689647	20.595781	18599.76679
0.692340081	16.94577505	1341.413907
0.292537961	78.73746064	1872.202429
0.046978287	126.9106884	1067.382609
0.343612189	58.52214835	1072.494624
0.696363364	11.94604281	11177.88972
0.170082172	52.79821146	1860.599099
0.743868448	56.75493432	629.5528455
0.605420594	19.19312016	30322.8502
0.758766526	10.5868491	5282.589787
0.644725812	20.09626478	5369.78338
0.074033708	138.8798658	1155.352273
0.266009602	40.50718391	3356.391724
1.868293878	7.916702952	19269.89916
0.269806543	58.22351833	2944.44766
0.95807003	31.05235611	1173.52375
0.079156012	137.6556914	2532.192029
0.447078769	20.16454582	10572.43147
0.904218985	18.30013033	5330.949445
0.846295059	14.68543169	11079.6274
0.986284027	13.55591504	1849.717465
0.193942804	65.23234201	1716.524493
0.577574022	32.30292054	1402.851195
0.125937101	78.0956258	627.6206897
0.244760026	49.07932316	393.4225352
0.462975555	30.0017686	26467.93555

log.sigma.4.5.mm.3D_gldm_SmallDependenceLowGrayLevelEmphasis	log.sigma.4.5.mm.3D_gldm_LowGrayLevelEmphasis	log.sigma.4.5.mm.3D_gldzm_DistanceZoneVariabilityNormalized
0.004033811	0.013154371	0.959815911
0.015078641	0.029121884	0.87359954
0.003381078	0.023035868	0.917959184
0.010421003	0.058647837	0.957466919
0.006354653	0.050402284	0.951426331
0.013134674	0.041680935	1
0.001089734	0.014890708	0.923496688
0.001609169	0.011083509	0.945233882
0.001613996	0.016972698	0.992094986
0.006321081	0.021410203	0.946469152
0.00055388	0.005490705	0.965996576
0.001939455	0.019559833	0.973099174
0.007333993	0.027550559	0.921185964
0.017104491	0.058420241	0.980584391
0.00429793	0.023143342	0.958440786
0.001627682	0.016780153	0.896932286
0.004900121	0.068863381	1
0.011637739	0.062327773	1
0.003742416	0.059366252	1
0.000991031	0.010467431	0.944626333
0.003725192	0.035052083	0.922238798
0.010374533	0.037684958	0.969704142
0.006870188	0.030435808	0.929506246
0.000965592	0.014379236	0.979168975
0.003126897	0.009059374	0.963427337
0.001964582	0.01354832	0.746061467
0.002684667	0.015833833	0.980954142
0.002622113	0.018135734	0.976881776
0.04992647	0.150810461	1
0.002546576	0.015686518	0.906658135
0.002144218	0.021578277	0.918430208
0.00686532	0.032085675	1
0.003272241	0.018429189	0.984064773
0.001560406	0.014241606	0.968135849
0.002535851	0.020095951	0.983238085
0.003928274	0.020393223	0.978650173
0.006382379	0.01703223	1
0.001103948	0.010819296	0.894806049
0.009399791	0.073225473	0.907908429
0.000305646	0.003037028	0.94295125
0.004993877	0.022689148	0.98726167
0.000841384	0.011909383	0.970951557
0.002868529	0.020252537	0.956119676
0.003755096	0.027017656	0.97546391
0.002004166	0.020975893	0.959201389
0.002358601	0.015960875	0.901040583
0.001274908	0.012458089	0.973397298
0.008944054	0.050420517	0.924644368
0.015526881	0.040060107	0.931122449
0.010425085	0.02155603	0.88166205
0.001928909	0.016653071	0.953783131
0.002219704	0.014670383	0.94623332
0.000835435	0.016766736	0.989011324
0.00377536	0.013832181	0.859785969
0.005506449	0.040060743	0.983194484
0.005072367	0.038319596	0.952124657
0.001853966	0.01503608	0.985402249
0.003988668	0.039675392	0.872767892
0.005148076	0.036422984	1
0.000451546	0.00738908	0.972195564
0.00499674	0.024875107	0.945497423
0.002080461	0.008477377	0.93143685
0.001784431	0.007251688	0.930852212
0.000984603	0.007252568	0.951814635
0.00494784	0.041233763	0.926934786
0.001812461	0.009490748	0.801376364
0.008857782	0.052996821	1
0.001297404	0.00696673	0.949439508
0.00086005	0.014047364	0.969777207
0.002523514	0.014148967	0.89443012
0.011847058	0.053972673	0.907908429
0.001275874	0.012380734	0.850019786
0.005052296	0.032953967	0.885230395
0.002904112	0.016348558	0.987578125
0.001367916	0.009111859	0.98433518
0.004215861	0.029202653	0.946469152
0.003541291	0.016339079	0.477634883
0.015796797	0.019457563	0.942080642
0.004562007	0.019192786	0.926035503
0.00173865	0.012697537	0.97037707
0.004033811	0.013154371	0.959815911
0.005555476	0.034729295	0.947132944
0.000960849	0.008455185	0.912459102
0.003023092	0.024827099	0.985075471
0.002298126	0.019029809	0.935634732
0.010705399	0.016571924	0.826183432
0.00396442	0.014117377	0.967922968
0.001807962	0.023898073	0.91103293
0.00322983	0.012797879	0.483514256
0.003637457	0.030351192	0.908177263
0.006250504	0.01159924	0.894272
0.001666849	0.01111425	1
0.002149061	0.020605885	0.967295673
0.001632527	0.016410832	0.969079795
0.00505264	0.031908781	0.98235434
0.004488497	0.015540098	0.893607071
0.004401788	0.025708134	0.967323952
0.01709714	0.031174733	0.959201389
0.021031146	0.043358442	0.947406866
0.000841062	0.006167205	0.906177423

log.sigma.4.5.mm.3D_gldzm_LowIntensityEmphasis	log.sigma.4.5.mm.3D_gldzm_LargeDistanceEmphasis	log.sigma.4.5.mm.3D_gldzm_HighIntensitySmallDistanceEmphasis
0.015849228	1.061538462	205.25
0.033115044	1.203389831	187.2288136
0.018396241	1.128571429	195.3482143
0.042884841	1.065217391	164.9619565
0.029695721	1.123762376	120.4755226
0.032327353	1	129.057971
0.021704444	1.245073892	120.1666407
0.015249481	1.119089317	158.1297431
0.024336648	1.011904762	122.7113095
0.024885027	1.082568807	127.162844
0.010184526	1.051903114	193.9913495
0.02689857	1.040909091	124.8556818
0.02025618	1.123287671	208.0958904
0.042769708	1.029411765	109.1372549
0.028993001	1.098591549	114.0266041
0.022296689	1.255892256	118.2050767
0.039957205	1	79.18644068
0.041858935	1	110.64
0.039476679	1	71.734375
0.011619636	1.106203996	180.7856058
0.02175186	1.200729927	136.5791008
0.031351551	1.046153846	156.2653846
0.029736762	1.109756098	120.5426829
0.02302988	1.031578947	108.8789474
0.015574235	1.055900621	209.8524845
0.013595929	1.761363636	161.9126618
0.016358903	1.028846154	155.3269231
0.031981366	1.035087719	108.5160819
0.167047068	1	27
0.018651086	1.147239264	147.0690184
0.021527838	1.155367232	112.801946
0.025730586	1	150.8571429
0.018528348	1.024096386	155.0411647
0.020382438	1.048582996	120.8066802
0.019694083	1.035864979	124.0420769
0.020490976	1.032374101	166.3642086
0.015699991	1	202.0097087
0.017159804	1.199730094	131.9485305
0.043604672	1.14516129	86.48790323
0.004825074	1.115598886	392.0131538
0.024876132	1.051282051	124.6474359
0.017679385	1.058823529	126.5586601
0.013096184	1.067331671	199.0411471
0.024626761	1.037267081	145.2189441
0.030380636	1.0625	127.9192708
0.016514852	1.241935484	157.5207213
0.021199017	1.040449438	124.6219101
0.045887034	1.117647059	80.98529412
0.039478241	1.107142857	120.46875
0.02875069	1.189473684	154.1315789
0.030727735	1.071005917	95.43934911
0.013971992	1.094252874	237.0235632
0.02388954	1.016574586	103.5096685
0.022557512	1.227586207	120.4568966
0.021484807	1.025423729	181.0444915
0.031320965	1.073619632	132.8128834
0.025540639	1.022058824	128.1672794
0.026794554	1.300395257	89.23869126
0.028995121	1	115.3690476
0.011680643	1.042303173	182.0190952
0.024174439	1.08411215	142.7570093
0.009041778	1.106635071	295.0776066
0.007622462	1.107579462	303.1721679
0.009625497	1.090729783	228.2799214
0.034206683	1.113924051	103.4651899
0.009334984	1.431893688	240.9038044
0.037799336	1	116.2641509
0.008930157	1.096036585	249.918064
0.017175737	1.177242888	137.2882932
0.012918655	1.182662539	197.4783282
0.041463568	1.14516129	83.77016129
0.019725591	1.394736842	132.0418986
0.029720381	1.248322148	100.8818046
0.02146661	1.01875	153.6515625
0.014157809	1.023684211	176.7769737
0.029263408	1.082568807	105.4816514
0.012478755	2.692144374	147.1512886
0.027994532	1.089552239	262.2723881
0.014844143	1.115384615	187.9540598
0.02099472	1.045112782	142.4078947
0.015849228	1.061538462	205.25
0.01897374	1.114864865	165.9290541
0.011755745	1.152439024	196.2088415
0.036830579	1.060150376	94.17126149
0.017854082	1.136029412	136.5743464
0.019829942	1.288461538	282.6875
0.017966069	1.048913043	173.1127717
0.027735382	1.177606178	116.0307808
0.014135205	2.61409396	142.0897651
0.019043895	1.248677249	153.8928571
0.015551458	1.168	349.828
0.018477584	1	183.2738854
0.017741329	1.060165975	135.1674159
0.01903415	1.047120419	151.0765707
0.039013748	1.026706231	89.72551929
0.016196537	1.221411192	209.2178291
0.02093792	1.063535912	145.5986034
0.037246141	1.0625	161.4635417
0.049254811	1.081081081	104.4932432
0.007198019	1.171202809	256.8136279

log.sigma.4.5.mm.3D_gldzm_LowIntensityLargeDistanceEmphasis	log.sigma.4.5.mm.3D_gldzm_HighIntensityEmphasis	log.sigma.4.5.mm.3D_gldzm_DistanceZoneVariability	log.sigma.4.5.mm.3D_gldzm_ZonePercentage
0.016598013	206.7846154	187.1641026	0.292792793
0.10188623	187.8135593	51.54237288	0.522123894
0.044544538	196.3071429	128.5142857	0.289855072
0.108102233	164.9782609	44.04347826	0.5
0.045866444	121.8861386	192.1881188	0.27077748
0.032327353	129.057971	69	0.669902913
0.031630387	121.9889163	749.8793103	0.049797621
0.018180309	160.5954466	539.7285464	0.113158938
0.024579602	122.8571429	250.0079365	0.059588555
0.054609797	127.5137615	103.1651376	0.299450549
0.010751355	195.916955	279.1730104	0.052891654
0.02787016	125.3772727	214.0818182	0.071591279
0.054602467	208.5684932	134.4931507	0.441087613
0.050122649	109.1666667	100.0196078	0.536842105
0.029821813	116.1197183	136.0985915	0.154347826
0.038851196	120.543771	532.7777778	0.069661077
0.039957205	79.18644068	59	0.135321101
0.041858935	110.64	50	0.45045045
0.039476679	71.734375	64	0.11287478
0.014862751	184.06204	898.3396425	0.079735055
0.037440323	139.0109489	252.6934307	0.188705234
0.077505398	156.2769231	63.03076923	0.411392405
0.068085028	121.3292683	76.2195122	0.291814947
0.027135144	109.2894737	186.0421053	0.039550375
0.01649324	211.1055901	155.1118012	0.221763085
0.017547015	191.9715909	262.6136364	0.17382716
0.016815884	156.3798077	204.0384615	0.221512247
0.034106122	108.994152	167.0467836	0.097214326
0.167047068	27	10	0.357142857
0.041366126	149.3742331	147.7852761	0.141615986
0.026597548	114.9943503	325.1242938	0.100825976
0.025730586	150.8571429	112	0.439215686
0.033588589	155.0562249	245.0321285	0.194683346
0.021768974	121.3927126	239.1295547	0.078437599
0.020739566	124.3902954	466.0548523	0.136324418
0.022533093	166.6043165	272.0647482	0.246453901
0.015699991	202.0097087	206	0.528205128
0.019399833	137.0175439	663.0512821	0.063128301
0.059713131	87.32258065	56.29032258	0.314720812
0.005070838	404.1183844	677.0389972	0.059270266
0.025232257	125.4679487	154.0128205	0.247226624
0.018682672	127.4176471	330.1235294	0.046980793
0.015067527	200.032419	383.40399	0.262606418
0.024910156	146.4534161	157.0496894	0.227722772
0.031506673	128.8020833	92.08333333	0.088235294
0.02033205	161.3774194	279.3225806	0.158567775
0.023715137	125.1191011	433.1617978	0.063854212
0.046961415	84.23529412	47.15686275	0.232876712
0.106442526	120.5357143	52.14285714	0.49122807
0.035809919	157.8421053	83.75789474	0.431818182
0.031679138	96.81065089	161.1893491	0.088667366
0.015959533	238.7954023	411.6114943	0.201482168
0.024979479	103.6298343	358.0220994	0.035834488
0.024897095	126.337931	124.6689655	0.171192444
0.022003658	181.3559322	116.0169492	0.411149826
0.077333235	132.8588957	155.196319	0.245852187
0.028655426	128.2205882	402.0441176	0.077170418
0.030961774	93.83399209	220.8102767	0.163754045
0.028995121	115.3690476	84	0.308823529
0.012241809	183.1128085	827.3384254	0.036192744
0.045862307	143.1775701	202.3364486	0.268506901
0.018820051	298.0473934	393.0663507	0.263256394
0.015817639	306.0749796	1142.155664	0.268372703
0.011062949	230.4852071	965.1400394	0.119014085
0.037713481	104.7088608	73.2278481	0.246105919
0.013834893	269.5315615	482.4285714	0.275514874
0.037799336	116.2641509	53	0.445378151
0.014720936	252.0731707	622.8323171	0.158914729
0.020066096	138.3041575	443.1881838	0.045595131
0.028793065	200.6842105	288.9009288	0.211525868
0.093721633	84.38709677	56.29032258	0.313131313
0.031147862	138.0169173	452.2105263	0.074940132
0.036004611	105.4161074	131.8993289	0.198931909
0.021577557	154.44375	158.0125	0.149114632
0.014316952	177.9473684	374.0473684	0.118159204
0.066725181	105.5779817	103.1651376	0.180463576
0.029690928	295.4989384	224.9660297	0.317813765
0.075569159	262.4626866	63.11940299	0.582608696
0.032373204	189.5854701	216.6923077	0.359447005
0.021570138	143.7894737	129.0601504	0.083333333
0.016598013	206.7846154	187.1641026	0.292792793
0.026370167	166.6621622	140.1756757	0.401084011
0.013301854	199.9878049	299.2865854	0.095962551
0.037186498	95.30075188	131.0150376	0.113191489
0.022651878	138.2904412	254.4926471	0.150692521
0.063005599	285.2115385	85.92307692	0.590909091
0.035123879	173.548913	178.0978261	0.253793103
0.031260764	117.984556	235.957529	0.063698967
0.0220703	276.1610738	144.0872483	0.253617021
0.029042466	156.5502646	171.6455026	0.23625
0.048439791	352.504	111.784	0.452898551
0.018477584	183.2738854	157	0.10979021
0.023423678	135.7012448	466.2365145	0.140853302
0.022016811	151.6753927	370.1884817	0.0991178
0.040251865	90.06824926	331.0534125	0.16303822
0.031975707	212.3819951	367.2725061	0.347128378
0.030897773	146.320442	350.1712707	0.247098976
0.099746141	161.4791667	46.04166667	0.551724138
0.130335893	104.5135135	35.05405405	0.521126761
0.009809029	262.6988586	1032.136084	0.118596418

log.sigma.4.5.mm.3D_gldzm_IntensityVariabilityNormalized	log.sigma.4.5.mm.3D_gldzm_LowIntensitySmallDistanceEmphasis	log.sigma.4.5.mm.3D_gldzm_IntensityVariability
0.059040105	0.015662032	11.51282051
0.060614766	0.015922247	3.576271186
0.062040816	0.011859167	8.685714286
0.052930057	0.026580494	2.434782609
0.077982551	0.025753505	15.75247525
0.072043688	0.032327353	4.971014493
0.074328423	0.019593668	60.3546798
0.065847547	0.014572082	37.59894921
0.087962963	0.024275909	22.16666667
0.082737143	0.017453834	9.018348624
0.097831683	0.010042819	28.2733564
0.077438017	0.026655672	17.03636364
0.052917996	0.011669608	7.726027397
0.067666282	0.040931472	6.901960784
0.076869669	0.02882572	10.91549296
0.08432813	0.018427434	50.09090909
0.107153117	0.039957205	6.322033898
0.0752	0.041858935	3.76
0.114257813	0.039476679	7.3125
0.066235	0.010861154	62.98948475
0.074138207	0.017959194	20.31386861
0.068402367	0.01981309	4.446153846
0.077037478	0.020149695	6.317073171
0.10166205	0.022003565	19.31578947
0.069788974	0.015344484	11.23602484
0.064275568	0.01286674	22.625
0.079650518	0.016244658	16.56730769
0.077459731	0.031450176	13.24561404
0.14	0.167047068	1.4
0.082991456	0.012972326	13.52760736
0.080181302	0.020307811	28.38418079
0.067442602	0.025730586	7.553571429
0.06817632	0.014763288	16.97590361
0.085511974	0.020035804	21.12145749
0.079385426	0.019480552	37.62869198
0.067258423	0.019980447	18.69784173
0.063719483	0.015699991	13.12621359
0.080106578	0.016681517	59.35897436
0.083766909	0.039577557	5.193548387
0.06777958	0.004774024	48.66573816
0.076101249	0.024836562	11.87179487
0.095155709	0.017455572	32.35294118
0.059570525	0.012603348	23.88778055
0.066856989	0.024555912	10.76397516
0.082465278	0.030099127	7.916666667
0.070551509	0.015686498	21.87096774
0.08077263	0.020569987	35.94382022
0.101114956	0.045618439	5.156862745
0.066964286	0.022737169	3.75
0.061717452	0.026985883	5.863157895
0.07860369	0.030489885	13.28402367
0.064595059	0.013486459	28.09885057
0.091053387	0.023617055	32.96132597
0.093745541	0.021972616	13.59310345
0.059465671	0.021355094	7.016949153
0.068914901	0.019817898	11.23312883
0.075211457	0.024761942	30.68627451
0.089909232	0.026013617	22.74703557
0.089569161	0.028995121	7.523809524
0.077126378	0.011540352	65.63454759
0.07533409	0.018752472	16.12149533
0.054075605	0.00659721	22.81990521
0.057110159	0.005573668	70.07416463
0.065590607	0.009282216	66.50887574
0.087646211	0.033329984	6.924050633
0.056776415	0.008278009	34.17940199
0.081523674	0.037799336	4.320754717
0.06432648	0.007495695	42.19817073
0.076098042	0.016677522	34.77680525
0.067450086	0.008965341	21.78637771
0.095213319	0.028399052	5.903225806
0.083321556	0.017059649	44.32706767
0.085897032	0.028272581	12.79865772
0.066015625	0.021438874	10.5625
0.067756233	0.014118023	25.74736842
0.097214039	0.019897964	10.59633028
0.046073539	0.008532839	21.70063694
0.05368679	0.016100875	3.597014925
0.065928848	0.010461878	15.42735043
0.079654022	0.020850865	10.59398496
0.059040105	0.015662032	11.51282051
0.068480643	0.017958801	10.13513514
0.072241225	0.011392743	23.69512195
0.089829838	0.036791032	11.94736842
0.072204801	0.016758841	19.63970588
0.054733728	0.009036028	5.692307692
0.068761815	0.013676616	12.65217391
0.073746664	0.026988099	19.1003861
0.048714022	0.012528006	14.51677852
0.072310406	0.01681243	13.66666667
0.054848	0.007329374	6.856
0.077771918	0.018477584	12.21019108
0.080792342	0.016367787	38.94190871
0.073079137	0.018288484	27.91623037
0.085595541	0.038704219	28.84569733
0.057725209	0.012306487	23.72506083
0.070586978	0.018473323	25.55248619
0.075520833	0.021621141	3.625
0.084002922	0.028984541	3.108108108
0.063303531	0.006575008	72.10272169

log.sigma.4.5.mm.3D_gldzm_HighIntensityLargeDistanceEmphasis	log.sigma.4.5.mm.3D_gldzm_SmallDistanceEmphasis	log.sigma.4.5.mm.3D_glcm_SumVariance	log.sigma.4.5.mm.3D_glcm_Homogeneity1
212.9230769	0.984615385	392.3841037	0.392201385
190.1525424	0.949152542	277.0681964	0.289927898
200.1428571	0.967857143	312.4612348	0.365286431
165.0434783	0.983695652	235.9128209	0.278993115
132.3910891	0.980060506	105.5860903	0.409752868
129.057971	1	202.226405	0.314235014
140.4704433	0.968200602	311.8433063	0.732771792
176.0157618	0.978011286	388.3708556	0.526915227
123.4404762	0.99702381	242.2358321	0.599474882
128.9174312	0.979357798	181.8543436	0.428376837
203.6193772	0.987024221	625.2906066	0.684878847
127.4636364	0.989772727	241.1113492	0.620322618
210.4589041	0.969178082	275.7571834	0.312795225
109.2843137	0.992647059	132.7522279	0.323093918
130.6267606	0.983176839	191.520243	0.475693516
138.9040404	0.957605686	298.6916483	0.633232776
79.18644068	1	67.28790806	0.481275417
110.64	1	127.3003862	0.337101557
71.734375	1	91.62062987	0.506817483
199.2754995	0.978122444	372.8019488	0.613111519
159.6350365	0.968192417	155.8272995	0.441424604
156.3230769	0.988461538	194.3663179	0.345524242
124.4756098	0.972560976	190.3030946	0.398440338
110.9315789	0.992105263	275.7693038	0.707451648
216.1180124	0.986024845	543.9221295	0.429398389
418.5965909	0.885811237	316.7550381	0.479477089
160.5913462	0.992788462	303.0209846	0.452754024
110.9064327	0.99122807	251.2211906	0.533544629
27	1	35.61437641	0.476697274
158.595092	0.963190184	255.2849179	0.490122046
127.480226	0.967435656	229.6777591	0.523962668
150.8571429	1	246.8267401	0.334507658
155.1164659	0.993975904	255.1491426	0.465678636
123.7368421	0.987854251	301.9365773	0.594431598
126.242616	0.993377872	203.0338283	0.507777578
167.5647482	0.991906475	340.9680373	0.446437986
202.0097087	1	345.1909783	0.311316543
160.2145749	0.957564852	419.233457	0.656003327
90.66129032	0.963709677	114.7501644	0.403874853
468.2743733	0.977290313	1186.734782	0.652980622
132.8525641	0.994301994	175.9297884	0.449952474
132.4352941	0.988562092	267.3236252	0.706490883
203.9975062	0.983167082	344.9118924	0.409845765
151.3913043	0.99068323	180.4953419	0.438064638
132.3333333	0.984375	235.8144094	0.569571965
190.5322581	0.959341398	270.9623192	0.480585121
127.1078652	0.98988764	325.0210688	0.640959084
97.23529412	0.970588235	154.8903386	0.432192553
120.8035714	0.973214286	177.469296	0.349913812
172.6842105	0.952631579	339.5125319	0.333215598
102.295858	0.982248521	342.7509973	0.575005066
248.1816092	0.97899106	396.2128343	0.456871608
104.1104972	0.995856354	236.5640946	0.736565926
149.862069	0.943103448	300.433244	0.468781209
182.6016949	0.993644068	259.9349282	0.334299624
133.0429448	0.981595092	139.4027129	0.418903608
128.4338235	0.994485294	290.5293499	0.603108196
121.5256917	0.946859903	142.1979663	0.457601573
115.3690476	1	164.0357347	0.414561439
187.4876616	0.989424207	476.5790521	0.729985856
144.8598131	0.978971963	196.4657841	0.426707236
309.9265403	0.973341232	571.0912453	0.410890588
317.6862266	0.973105134	635.3446821	0.417247445
243.2534517	0.981186993	575.4431046	0.581131041
109.6835443	0.971518987	142.796392	0.415632464
425.6744186	0.9151555	586.7520396	0.385593104
116.2641509	1	165.6727478	0.317957839
266.25	0.980278201	558.4251061	0.518441725
160.9781182	0.987999179	276.7728441	0.661028658
216.6037152	0.957774338	296.9536215	0.418060012
86.85483871	0.963709677	87.56759047	0.402004064
192.5827068	0.937770191	454.2636845	0.641339791
130.7718121	0.952833706	128.0875156	0.433365773
157.6125	0.9953125	274.3319248	0.497594384
182.6289474	0.994078947	461.6247506	0.588838884
105.9633028	0.979357798	145.8037832	0.445348009
1256.764331	0.708259613	625.7400118	0.336076612
263.2238806	0.97761194	592.7306351	0.282347584
196.1111111	0.971153846	278.4549379	0.366733116
149.3157895	0.988721805	300.08132	0.5808865
212.9230769	0.984615385	392.3841037	0.392201385
169.8648649	0.978791291	201.8863011	0.355341819
217.054878	0.965277778	538.629396	0.60084325
105.4661654	0.993316625	200.1879958	0.548197826
149.1580882	0.974162582	249.3372354	0.506575913
295.3076923	0.927884615	574.9927121	0.266321385
175.2934783	0.987771739	372.9355942	0.448210971
127.996139	0.964178464	228.9671593	0.647398725
1020.181208	0.710547166	570.3436056	0.383952737
176.9153439	0.961346267	171.9608841	0.417689449
363.208	0.958	786.4876379	0.30956605
183.2738854	1	390.8859615	0.550959769
138.2883817	0.987263716	214.9081801	0.528635382
154.0706806	0.988219895	263.4993104	0.556883423
91.43916914	0.993323442	128.9310187	0.506784107
236.3065693	0.956897134	346.4728768	0.367547228
150.6933702	0.98718539	183.1921428	0.415841666
161.5416667	0.984375	296.9946606	0.296665944
104.5945946	0.97972973	156.3066183	0.321718403
290.5847234	0.962595113	639.9589019	0.562812537

log.sigma.4.5.mm.3D_glcm_Homogeneity2	log.sigma.4.5.mm.3D_glcm_ClusterShade	log.sigma.4.5.mm.3D_glcm_MaximumProbability	log.sigma.4.5.mm.3D_glcm_Idmn	log.sigma.4.5.mm.3D_glcm_SumVariance2
0.307357858	225.8758069	0.024659408	0.982425617	54.1907424
0.19597969	213.3164262	0.033609598	0.952983479	62.15171394
0.278028638	14.64174541	0.020420894	0.968094567	56.4876357
0.182634402	131.5309855	0.0309521	0.94033331	61.27640751
0.3291347	198.1947888	0.033213862	0.980075338	32.11313976
0.220207002	61.74659	0.032247461	0.962473419	42.22949576
0.709160644	-129.6876087	0.293810332	0.995457335	20.83849831
0.469023417	-84.54352068	0.084727885	0.989714936	36.13667467
0.557303885	-51.57244454	0.129478079	0.992807275	24.93778924
0.348263496	30.24581655	0.047269262	0.975253195	20.84803408
0.662045159	-46.27756781	0.205737662	0.995944499	11.3744784
0.580882359	-85.74087874	0.120413157	0.993819559	27.17966969
0.220341453	263.4553175	0.018348581	0.958686584	77.30852792
0.22968542	136.59034	0.028168083	0.948552432	40.06376735
0.40769512	21.70729102	0.0268095	0.986107381	29.58477981
0.593747462	-144.9428744	0.21754877	0.991495863	32.63144145
0.414556665	31.24638362	0.040393084	0.982252658	19.30982238
0.244016042	243.3971712	0.03173273	0.963100149	43.27364682
0.446803501	-10.95940169	0.036992185	0.977752586	22.55663489
0.573861273	-14.42317983	0.123292772	0.996244322	28.76142069
0.366700917	177.7736914	0.023085382	0.981328581	39.89334887
0.25210984	156.8324566	0.025073499	0.954813817	54.71524972
0.312497212	0.778780095	0.026188317	0.972827015	41.3266766
0.683674225	-81.99659485	0.30763788	0.995048419	16.23928
0.350563342	-37.00279161	0.042900113	0.981250652	48.85894348
0.415851497	156.1349933	0.042393685	0.987641096	37.53497837
0.380488931	-51.03786311	0.030241828	0.987711363	32.40124039
0.478217852	-24.20712687	0.053421609	0.989669643	28.80749365
0.398368295	18.50371568	0.168414441	0.933764554	8.868142147
0.425245285	14.70294638	0.031412085	0.987198609	29.93865359
0.46707771	-31.92735604	0.058626455	0.991180315	34.31416816
0.244565968	26.83580569	0.023455002	0.955615634	46.58518643
0.397357283	-12.87412616	0.025667906	0.986278983	37.71719877
0.550131011	-61.35880923	0.150522202	0.99078411	30.31400426
0.447533807	-11.6726141	0.046959996	0.988431495	26.01039754
0.375823564	-122.5238414	0.026461596	0.978130312	55.20657036
0.218322797	64.5188641	0.017452886	0.95955866	57.82033663
0.621911692	-177.8065048	0.198244096	0.994600785	29.90189865
0.316218461	69.19082613	0.028846002	0.968545818	35.43712457
0.620037341	-58.7245425	0.221888764	0.997692976	19.06727881
0.376907152	28.48898832	0.040437257	0.979137672	21.91235662
0.686324993	-10.40489441	0.236658886	0.995472075	8.03588736
0.332936558	-41.26231647	0.020793086	0.979723628	54.55511791
0.362991237	121.3710012	0.035725475	0.986841108	32.65602172
0.523147074	-82.24549167	0.086718594	0.990302966	32.04483102
0.415601429	-0.622466581	0.034885484	0.986520308	34.16840133
0.607581061	-89.20908596	0.142049949	0.993757336	24.21518865
0.349446546	11.5689898	0.045396096	0.969907996	45.81112369
0.255484849	374.1334628	0.032403468	0.96801571	56.44995363
0.239392123	-87.91774509	0.0216412	0.96170779	51.38094674
0.525338002	-133.3540892	0.160331767	0.988547646	39.10752251
0.388051398	28.69863375	0.029165493	0.991159739	41.86359228
0.717342456	-68.05567774	0.317233995	0.996668487	14.05963201
0.399429609	25.04290884	0.03034008	0.982411366	30.98791625
0.245797488	214.7149775	0.018977014	0.969262802	79.09572228
0.340621316	212.5941033	0.026620581	0.978852256	36.3939916
0.563118632	-65.84605715	0.127939991	0.993457869	26.22657399
0.384287961	45.04534094	0.025092114	0.976563745	33.72702821
0.3321943	-1.873383016	0.03015631	0.976177948	32.86798498
0.710395626	-57.31337364	0.284475116	0.997796794	12.80225068
0.350232023	39.75196429	0.02477754	0.980309945	33.6340448
0.331707005	142.3813151	0.020124489	0.987508977	66.66993742
0.341994647	-31.88876453	0.023165844	0.984324442	59.51555997
0.533067774	-116.1446949	0.130756018	0.993438427	32.56899582
0.330150768	-7.739948722	0.034975778	0.969325322	28.41870568
0.300615332	313.6778735	0.018919544	0.985229931	75.64531482
0.219702969	16.02532986	0.028445483	0.934361657	39.01990237
0.461152884	-49.11949955	0.041133712	0.991997701	28.08638367
0.630354639	-67.94050893	0.160682805	0.99575938	20.95139775
0.338284396	87.99308616	0.021228247	0.982618652	40.34806372
0.315957227	58.0531321	0.040741075	0.962003616	24.20346503
0.603047794	-255.6542905	0.192865642	0.991320549	39.56608024
0.355557905	29.14701507	0.037638433	0.973234647	20.87686312
0.436698864	27.24439025	0.049571771	0.991090209	32.77066477
0.544993462	-118.9347836	0.114600263	0.994278938	28.25168702
0.368786091	105.2539215	0.029201314	0.978795151	28.16212304
0.248354509	3551.449953	0.012744913	0.983684341	201.328331
0.186454627	-103.5492889	0.025078265	0.952588088	104.632848
0.278011125	164.4014448	0.01962478	0.979705117	57.16887891
0.53647693	-51.31260566	0.09680362	0.99369099	22.0581537
0.307357858	225.8758069	0.024659408	0.982425617	54.1907424
0.265700223	215.2582411	0.024287679	0.970923816	59.43436068
0.55350079	-158.9849227	0.213234895	0.994148818	36.4460654
0.497766339	-57.94045168	0.056623678	0.985199459	24.08625578
0.447819667	-71.27323158	0.043077094	0.985840119	33.02472657
0.167216387	11.40525878	0.017101182	0.958631488	104.3845155
0.375771107	-74.98471879	0.02638156	0.985055433	48.01134517
0.611821726	-113.1776413	0.252538414	0.99138992	26.64578763
0.305455008	2274.67761	0.025504862	0.983415797	141.1802415
0.33825871	226.3408844	0.026978968	0.981560702	43.23488031
0.216785576	-558.9229692	0.027416637	0.969031748	104.0804993
0.500092418	-73.00705919	0.095316873	0.992013524	25.67808052
0.475182146	-16.08598286	0.03819276	0.990564015	27.74481706
0.506842015	-45.2535659	0.101288487	0.992755723	27.57375597
0.446386008	13.47110869	0.053715503	0.987227551	19.27794447
0.281324291	152.222101	0.018595832	0.98044126	48.52420444
0.336387778	146.2602639	0.026893173	0.984766375	34.97357972
0.19994185	-68.18446332	0.030718137	0.941467377	51.91552215
0.222426559	15.08908778	0.038399947	0.945100757	30.89571549
0.513197208	-82.97865476	0.126810225	0.993310944	41.18709554

log.sigma.4.5.mm.3D_glcm_Contrast	log.sigma.4.5.mm.3D_glcm_DifferenceEntropy	log.sigma.4.5.mm.3D_glcm_InverseVariance	log.sigma.4.5.mm.3D_glcm_Entropy	log.sigma.4.5.mm.3D_glcm_Dissimilarity
12.58708666	2.939140398	0.308299146	7.267083051	2.739620805
30.54736686	3.336643784	0.200093803	6.45292457	4.484099701
17.03295332	3.085055839	0.277906305	7.382378424	3.196465506
33.41772011	3.371972671	0.173897921	6.319393303	4.728857345
11.36988935	2.872726583	0.333702244	6.784604517	2.55277458
20.19440945	3.02069441	0.274227959	6.105789751	3.613329461
2.313773981	1.774406595	0.309587333	4.585460071	0.812451794
5.221759144	2.408553362	0.392141955	6.52451215	1.632378754
3.31003253	2.099960013	0.412099645	5.726102567	1.229272801
8.689485096	2.677081494	0.337254595	6.354900578	2.284909736
1.85029844	1.742005261	0.423246902	4.776747375	0.844760766
3.097116374	2.082544973	0.39401535	5.679900866	1.164380146
26.86392303	3.368784088	0.224942589	7.502521388	4.090599225
21.51462478	3.190288185	0.245797312	6.76873974	3.698722494
6.55619282	2.493474261	0.392201933	6.555974091	1.892178598
3.97580087	2.152850017	0.349890532	5.53165219	1.23350301
5.451073586	2.370575967	0.405608888	5.999998774	1.785161537
18.11602551	3.033735034	0.261843901	6.24485589	3.395233582
4.047837687	2.137367087	0.440249172	5.968470554	1.551124044
3.70518372	2.157093544	0.399284955	5.940800074	1.236815096
8.820744993	2.714789793	0.357556083	6.987379802	2.236075552
18.48186553	3.096391598	0.233788473	6.705095813	3.426039729
10.67635747	2.746785042	0.313445448	6.805143597	2.565930407
1.662240597	1.71800338	0.373568104	4.604281023	0.78775519
10.66840717	2.773332798	0.321370491	6.988504867	2.439836281
8.888215669	2.682015212	0.382361063	6.852324338	2.086162834
8.094371079	2.629315492	0.373799139	6.789868235	2.113433432
3.875035718	2.217212419	0.426792442	6.256999106	1.466402707
6.33246028	2.081929426	0.291051046	3.698744342	1.97175996
5.385213985	2.40528916	0.406919354	6.571862651	1.745170666
4.852353813	2.333446281	0.412997781	6.410127586	1.596510181
22.23242441	3.256433552	0.244497026	7.079947984	3.714638168
7.732938578	2.598550706	0.388688721	6.884252852	2.030030398
3.489856144	2.151831063	0.399566812	5.908930599	1.271935679
5.370471105	2.412219803	0.409979929	6.439645221	1.689592967
10.6181448	2.819997184	0.360898407	7.264798089	2.349075594
26.22545861	3.300405984	0.229012172	7.412869397	4.052027861
3.240615823	2.051384719	0.361665146	5.389889926	1.093772873
8.751937705	2.592811168	0.317259624	6.445775492	2.389386083
2.886605068	1.994040092	0.387429566	5.365717109	1.047044049
8.109011415	2.671590241	0.364240348	6.525484391	2.150189851
1.517759824	1.657647277	0.407221868	4.546177007	0.760770721
15.00524447	2.928747536	0.328031006	7.467091188	2.799940517
8.663402591	2.68608738	0.366542323	6.767824465	2.220359765
3.690738107	2.151899264	0.43073241	6.020624749	1.341263564
7.577522155	2.608255841	0.38546385	6.800075633	1.982451954
2.599082597	1.970225697	0.411894436	5.600341745	1.047197718
7.328514232	2.466877228	0.328261184	6.340267477	2.167508643
15.44706002	2.931872148	0.25930687	6.273866022	3.156015838
18.8316305	3.147701477	0.253732614	7.058701349	3.468639593
3.45961047	2.147911644	0.395249759	5.93322095	1.328733952
9.475887651	2.733176983	0.378394543	7.06247121	2.196398665
1.516960765	1.62789408	0.353145317	4.35326416	0.704248716
6.057961063	2.382382012	0.396585269	6.557868533	1.885184644
22.76195283	3.298219384	0.255400353	7.389214609	3.733751196
11.20047046	2.838278943	0.345312281	6.864171796	2.483637032
3.309757746	2.092530086	0.423160966	5.90490019	1.211164921
7.337025772	2.621239027	0.360031421	6.753631909	2.061636456
9.251786887	2.709667543	0.334527808	6.653067308	2.382826804
1.646177611	1.6550597	0.361410011	4.477056632	0.729908719
10.24796185	2.806185933	0.349421274	6.999063261	2.406198426
12.63863208	2.941621607	0.330660587	7.575243402	2.637195631
15.13417483	3.044348896	0.325149116	7.752607012	2.78983624
5.37548597	2.385600396	0.362482586	6.171550466	1.497331839
8.540621039	2.634364018	0.316049424	6.549532444	2.333947177
13.98596839	3.023485106	0.302227904	7.850393707	2.868567771
19.82771976	3.108576169	0.221626937	6.447483853	3.644643346
6.118910978	2.460660488	0.404424715	6.521586375	1.714438041
2.312724052	1.897049633	0.399632896	5.256082189	0.966917019
10.6660553	2.847212443	0.332619395	7.212647072	2.476750919
10.88707084	2.772262953	0.299073683	6.194747122	2.5891431
3.672575102	2.146524262	0.347525037	5.530665668	1.186943644
8.48285113	2.695004773	0.352814743	6.570229649	2.236362898
5.816065524	2.440179754	0.417978508	6.508562312	1.752613436
4.662294165	2.2840767	0.393616002	6.014655368	1.396361177
7.368422905	2.563593053	0.359193571	6.53502782	2.096943869
27.93408648	3.437698409	0.255103623	8.414666398	3.91818396
34.00531042	3.42089803	0.198055608	6.596525437	4.638794798
14.63368488	3.015195911	0.293368855	7.470000236	2.998133654
3.161934189	2.082833495	0.436637902	5.739968028	1.250589445
12.58708666	2.939140398	0.308299146	7.267083051	2.739620805
16.72740379	3.04879221	0.274322615	7.256204256	3.224190963
4.086233803	2.234578063	0.348972743	5.801685974	1.33872754
3.530489637	2.151312026	0.444015649	6.038451404	1.382091879
6.06146998	2.462030516	0.412119583	6.574366686	1.753941391
33.3636495	3.458098512	0.173482426	7.221578162	4.769864234
8.393914372	2.666504584	0.374737418	7.058228643	2.157386715
3.309341225	2.075575477	0.366769567	5.362377451	1.119666673
23.17806132	3.284007832	0.294832187	7.896141251	3.410688877
11.35620696	2.884733575	0.324515705	7.089533024	2.541880693
28.42377738	3.414986755	0.205424434	7.420489904	4.237768327
4.017134418	2.218307075	0.425849393	6.055821362	1.428301807
5.265806466	2.373645964	0.427481045	6.491122677	1.603677344
4.718426725	2.321654403	0.406748684	6.193404975	1.487001771
4.844584991	2.340853607	0.419109846	6.209824732	1.642806232
17.63539188	3.161350003	0.287185866	7.586379826	3.191078274
10.8890801	2.838885093	0.337670113	7.066557863	2.496013309
24.45030962	3.152971278	0.22161887	6.232320607	4.049937536
18.20926051	2.97043694	0.228718326	5.868693085	3.500182249
5.85860925	2.434542453	0.37689194	6.545005586	1.589908512

log.sigma.4.5.mm.3D_glcm_DifferenceVariance	log.sigma.4.5.mm.3D_glcm_Idn	log.sigma.4.5.mm.3D_glcm_Idm	log.sigma.4.5.mm.3D_glcm_Correlation	log.sigma.4.5.mm.3D_glcm_Autocorrelation
4.887145744	0.909884074	0.307357858	0.624274317	144.9417817
9.646364635	0.852945957	0.19597969	0.348718983	100.0909425
6.270323875	0.881876485	0.278028638	0.538222107	118.4160622
10.15414657	0.835375625	0.182634402	0.304125621	84.79493006
4.733472924	0.906220174	0.3291347	0.47487534	46.98760662
6.506243947	0.867673811	0.220207002	0.34729423	76.82882605
1.632884517	0.967084662	0.709160644	0.799162678	110.5217964
2.485117593	0.934850049	0.469023417	0.747035022	143.1108677
1.723772076	0.947845289	0.557303885	0.764418919	94.77900884
3.253642632	0.894333842	0.348263496	0.412393922	73.80814528
1.115264438	0.963414741	0.662045159	0.720350287	203.1539747
1.706766387	0.952579046	0.580882359	0.794699801	94.41754519
9.398806389	0.864617373	0.220341453	0.484618252	103.2088991
7.139677218	0.848931211	0.22968542	0.308912014	53.96728411
2.842839629	0.922048202	0.40769512	0.637105519	79.05999939
2.324372441	0.948705382	0.593747462	0.781721731	111.3999846
2.142566392	0.910395966	0.414556665	0.55616549	33.46460156
6.100227766	0.869739847	0.244016042	0.412643161	51.30479149
1.494519047	0.899884315	0.446803501	0.694310336	43.43145381
2.13659218	0.963490461	0.573861273	0.770473091	136.5081413
3.684173344	0.909665947	0.366700917	0.634934757	66.80222317
6.115047636	0.857967023	0.25210984	0.490569193	76.51992292
3.814037894	0.888258702	0.312497212	0.588085715	78.03707079
1.025474274	0.960660657	0.683674225	0.813468758	100.0817732
4.269510658	0.910126668	0.350563342	0.643043328	191.1748577
4.350138628	0.930521323	0.415851497	0.618184644	120.2903474
3.427664062	0.926230372	0.380488931	0.602590427	115.7191192
1.669875614	0.931951569	0.478217852	0.762095756	99.53665133
2.019510167	0.836690786	0.398368295	0.179817129	16.00055538
2.266113341	0.923969074	0.425245285	0.694396581	100.937236
2.17437288	0.938399415	0.46707771	0.751931976	92.57094398
7.744260649	0.860714904	0.244565968	0.362136137	94.33150767
3.481787213	0.923696447	0.397357283	0.660247889	101.1567894
1.816013775	0.941169908	0.550131011	0.792661064	114.827196
2.45664918	0.929691012	0.447533807	0.657258012	83.05357087
4.959256816	0.906915948	0.375823564	0.677742524	129.2049859
8.493442896	0.865500463	0.218322797	0.391273093	126.1400201
2.000405019	0.959218437	0.621911692	0.803579759	147.5845735
2.732803624	0.877558243	0.316218461	0.604017504	50.97706456
1.762559054	0.972190703	0.620037341	0.73567172	367.2149629
3.388408747	0.904812967	0.376907152	0.457035082	72.49854483
0.924901013	0.961792499	0.686324993	0.679505692	96.84325996
6.118002298	0.909752966	0.332936558	0.57497317	129.8709376
3.596771884	0.922708479	0.362991237	0.579327821	75.03575606
1.824784731	0.937948002	0.523147074	0.792427561	94.00506553
3.520486307	0.925545107	0.415601429	0.635265521	105.8772484
1.478931411	0.953188705	0.607581061	0.805032749	120.866055
2.228794658	0.88125166	0.349446546	0.720379106	65.16465793
5.118073125	0.877050916	0.255484849	0.569951572	69.47375677
6.426583141	0.867390025	0.239392123	0.46316802	125.284176
1.587465016	0.931892521	0.525338002	0.836186055	127.7586383
4.542882617	0.939160652	0.388051398	0.629060979	145.9527165
1.008616396	0.969461968	0.717342456	0.804595817	86.94456809
2.16086519	0.910544378	0.399429609	0.672523242	115.1005979
8.375905214	0.882620898	0.245797488	0.55251205	98.67595714
4.884589411	0.905328229	0.340621316	0.527773921	59.7365429
1.787977539	0.950759219	0.563118632	0.774022268	111.2973121
2.989379171	0.898879637	0.384287961	0.64277868	62.02163937
3.406535233	0.89508904	0.3321943	0.561960403	69.07151847
1.098850549	0.97497111	0.710395626	0.770659622	158.773342
4.289375174	0.907616355	0.350232023	0.533817797	80.49170431
5.479733477	0.92585865	0.331707005	0.680566244	201.9757903
7.019004901	0.920574701	0.341994647	0.59657476	220.8544749
3.089161479	0.952333051	0.533067774	0.715856011	197.5424737
2.864425419	0.880359543	0.330150768	0.537325052	61.4765998
5.52792248	0.917274407	0.300615332	0.689203423	207.2494135
6.153475079	0.827684477	0.219702969	0.331458633	65.76515973
3.104185103	0.943571619	0.461152884	0.640766262	193.5842441
1.351962423	0.961774144	0.630354639	0.800140414	103.8648014
4.395171851	0.911876687	0.338284396	0.581532906	114.3523751
3.880947119	0.870480139	0.315957227	0.381034417	39.88090314
2.230243221	0.948247135	0.603047794	0.829444882	158.491192
3.365842455	0.891401448	0.355557905	0.421836213	55.93970808
2.673596303	0.937778923	0.436698864	0.698036686	106.8181479
2.656801441	0.955215752	0.544993462	0.716833013	162.9343416
2.761577784	0.901809434	0.368786091	0.584344557	62.74200784
11.90058098	0.916184965	0.248354509	0.760856608	214.4692699
11.64567881	0.854633009	0.186454627	0.511327665	198.445919
5.369896483	0.902244243	0.278011125	0.592455213	107.9192077
1.557519371	0.948799926	0.53647693	0.74757925	112.7432539
4.887145744	0.909884074	0.307357858	0.624274317	144.9417817
5.783199376	0.884667438	0.265700223	0.553162419	80.62006763
2.20622629	0.953736965	0.55350079	0.797398581	185.4791269
1.571521697	0.92075873	0.497766339	0.743224011	81.93922596
2.887507504	0.924606557	0.447819667	0.690098411	98.62313902
9.900214296	0.858450989	0.167216387	0.514407285	196.4770007
3.647196217	0.919202511	0.375771107	0.701594661	139.70434
2.028503358	0.948601698	0.611821726	0.778291188	88.31317571
10.89700093	0.919560896	0.305455008	0.722443086	198.5147659
4.749781666	0.910039907	0.33825871	0.582056837	71.64552834
9.735213321	0.880324235	0.216785576	0.576277745	262.3910885
1.894729492	0.942164985	0.500092418	0.724564196	141.9235456
2.641206958	0.938576666	0.475182146	0.680809744	87.54596089
2.453997306	0.946942861	0.506842015	0.707411666	102.5590008
2.05535016	0.924731356	0.446386008	0.596208581	57.12258717
7.147836032	0.906849717	0.281324291	0.467070118	129.1196119
4.431612118	0.917261739	0.336387778	0.524751805	75.71463715
7.381563559	0.836259597	0.19994185	0.36673074	106.8550766
5.629193956	0.840502143	0.222426559	0.262524324	61.18013479
3.270718563	0.951098289	0.513197208	0.749752302	219.7335239

log.sigma.4.5.mm.3D_glcm_SumEntropy	log.sigma.4.5.mm.3D_glcm_AverageIntensity	log.sigma.4.5.mm.3D_glcm_Energy	log.sigma.4.5.mm.3D_glcm_SumSquares	log.sigma.4.5.mm.3D_glcm_ClusterProminence
4.80863927	11.60473589	0.008854743	16.8174106	10247.8176
4.542327598	9.595911378	0.013191742	23.1747702	11619.31083
4.838445968	10.41734943	0.007682406	18.38014726	7958.069289
4.42848398	8.817195314	0.013796936	23.67353191	9819.584782
4.358955937	6.464713407	0.012562098	10.87075728	4842.285853
4.240594098	8.436605453	0.015957914	15.6059763	4855.383642
3.521831995	10.29028278	0.12592212	5.788068072	1890.111265
4.503084652	11.63528486	0.019579853	10.33960845	3191.426813
4.166605628	9.453537439	0.03430733	7.061955443	1433.155853
4.135177852	8.410989565	0.016958329	7.384379794	1437.308494
3.561550745	14.1694276	0.07792765	3.306194211	666.5233859
4.17761324	9.401877885	0.041389327	7.569196517	1961.563325
4.948997874	9.513479677	0.006668178	26.04311274	14253.34125
4.420591239	7.021926149	0.010873278	15.39459803	4339.528167
4.398464998	8.561617412	0.013365562	9.035243157	2050.065245
4.107944878	10.20950001	0.065560088	9.151810579	2703.729638
4.027233056	5.476561666	0.019636329	6.190223992	1070.863776
4.254805421	6.706277901	0.0149747	15.34741808	6917.759722
4.147794271	6.228785528	0.01986348	6.651118139	998.7441029
4.276619746	11.41453286	0.040216101	8.149238303	4483.909002
4.59922959	7.683058479	0.010473781	12.17852347	5601.333067
4.609474453	8.206752816	0.011048878	18.29927881	7239.501411
4.572043651	8.388164351	0.010907296	13.00075852	3688.508829
3.530661616	9.820186479	0.114604997	4.475380149	1078.226759
4.703820532	13.47673198	0.011408432	14.88183766	5674.150282
4.562666177	10.63866253	0.01455786	11.66115467	6334.631189
4.491827535	10.47089522	0.012746791	10.12390287	3367.195049
4.405451665	9.659235839	0.018070724	8.170632341	1996.929893
2.69781981	3.914183727	0.09168115	3.800150607	216.1317389
4.461415829	9.736245915	0.014360267	8.830966894	2436.427091
4.48460839	9.230483355	0.016841235	9.791630492	2542.347307
4.636314144	9.392402009	0.008953435	17.20440271	6421.62247
4.610098234	9.67765469	0.011743095	11.36253434	3956.313853
4.315418843	10.3980395	0.038865535	8.450965101	2345.358492
4.346637796	8.825583656	0.016720458	7.845217162	1788.315835
4.826614532	10.86521638	0.009034883	16.45617879	6841.782545
4.798240688	10.87098236	0.006885824	21.01144881	8492.78803
4.010465142	11.87088862	0.067857856	8.285628617	3097.143165
4.408684702	6.654220966	0.013315434	11.04726557	2856.261477
3.942949182	19.09725967	0.067616643	5.448925974	1658.612781
4.208730991	8.309163616	0.015248136	7.505342008	1639.044806
3.413196326	9.757724779	0.089331074	2.388411796	303.0659778
4.867722365	10.95478442	0.008162424	17.41589667	8405.553359
4.458832416	8.30839528	0.01307259	10.32985608	4092.853532
4.371082529	9.322761884	0.026149436	8.933892282	2334.32234
4.534794451	9.961207458	0.0128657	10.43648087	3096.96696
4.1470463	10.74522548	0.043347165	6.703567812	1717.589189
4.461607542	7.451811303	0.015515408	13.28490948	3680.741723
4.388710717	7.690964817	0.014682093	17.97425341	10697.09639
4.672490171	10.82182266	0.008845214	17.55314431	6319.391209
4.378150784	10.90158048	0.037963004	10.64178325	3504.873901
4.658018491	11.75588034	0.012123269	13.0497951	8330.472043
3.392883217	9.154698169	0.128765771	3.894148193	864.951819
4.454985328	10.43304872	0.01348037	9.261469327	2329.280929
4.947413277	9.19506571	0.007208967	25.49451581	14794.38251
4.470869506	7.309559963	0.011689241	11.89861551	5733.314097
4.292160741	10.27450326	0.033372642	7.384082933	1929.737169
4.474809333	7.444671196	0.011209179	10.26601349	2434.333511
4.442662113	7.946348693	0.012376574	10.52994297	2786.229035
3.443303347	12.48974269	0.116107589	3.618436187	1015.742847
4.518504242	8.638905628	0.010424138	10.97050166	3182.636382
4.997304775	13.76492248	0.007461435	20.25243579	12593.34464
4.969749178	14.5244138	0.007565317	19.11149329	11072.30853
4.322441123	13.81586461	0.034019092	9.550616046	3792.96145
4.33929678	7.515867523	0.013585444	9.23983168	1930.059185
5.093381395	13.8806116	0.006457378	22.81742547	18828.62137
4.363126967	7.80612104	0.013067305	14.71190553	3592.808798
4.400454112	13.71723746	0.017307825	8.588218375	2982.645442
3.926002782	9.960138687	0.060362924	5.81603045	1304.307159
4.662456976	10.34055469	0.009119528	12.75352976	4932.896277
4.1317664	6.043179849	0.016871271	8.772633969	1733.725463
4.091464467	12.22769298	0.06523802	10.80966383	4663.523906
4.184403982	7.269111309	0.014213329	7.339928562	1262.315454
4.465838823	10.00385419	0.017614005	9.646682573	4206.196197
4.245418262	12.53195355	0.035998504	8.239137251	2883.806919
4.32503903	7.585072755	0.014212133	8.882636486	3006.693124
5.561766969	13.23259972	0.004203775	59.41204504	179105.5906
4.8047202	13.43273351	0.011290617	34.65953961	23762.09421
4.851146474	9.862172858	0.007196152	17.95776169	8710.67027
4.112680094	10.39316788	0.030561126	6.305021973	1367.137209
4.80863927	11.60473589	0.008854743	16.8174106	10247.8176
4.790504217	8.358452104	0.008123803	19.04044112	8787.568959
4.228138801	13.31987207	0.056909344	10.15094969	3981.042759
4.257174582	8.76341937	0.021935663	6.904186355	1594.141441
4.463529553	9.585329867	0.016101326	9.771549137	2725.490443
5.042754861	13.39271142	0.007429755	34.90326111	25583.73336
4.760494669	11.39258079	0.010095229	14.10131489	5935.213014
3.939722817	9.081689694	0.077607668	7.488782215	1996.716915
5.285273737	13.11434495	0.007143071	42.39392527	98839.61762
4.613391779	7.97854811	0.010003543	13.64777182	6792.600464
5.084676516	15.69844523	0.007415114	34.22788457	28938.20663
4.257831691	11.68339044	0.025370824	7.423803735	2132.734165
4.421819316	9.051184684	0.01667359	8.252655883	2209.048528
4.322196193	9.840959986	0.025545998	8.073045675	2216.036356
4.159055042	7.315223939	0.019655539	6.030632366	1100.017908
4.774730221	11.03219244	0.007325204	16.77896584	8342.178918
4.521848498	8.350058272	0.010667148	11.5265438	5247.905759
4.34446966	9.996831145	0.014740147	19.09145794	6170.811487
4.03521581	7.612015702	0.01881196	12.276244	2289.999211
4.575140573	14.52874999	0.028694167	11.83911246	6210.345877

log.sigma.4.5.mm.3D_glcm_SumAverage	log.sigma.4.5.mm.3D_glcm_Imc2	log.sigma.4.5.mm.3D_glcm_Imc1	log.sigma.4.5.mm.3D_glcm_DifferenceAverage	log.sigma.4.5.mm.3D_glcm_Id
23.19755417	0.865121854	-0.177748302	2.739620805	0.392201385
19.19182276	0.979941446	-0.401954403	4.484099701	0.289927898
20.83469886	0.870385008	-0.184080062	3.196465506	0.365286431
17.63439063	0.989014495	-0.465887658	4.728857345	0.278993115
12.92942681	0.728029377	-0.110554724	2.55277458	0.409752868
16.87321091	0.978531437	-0.420617415	3.613329461	0.314235014
20.58056556	0.911429699	-0.332180939	0.812451794	0.732771792
23.27056972	0.865494149	-0.200546893	1.632378754	0.526915227
18.90707488	0.890383624	-0.256504841	1.229272801	0.599474882
16.82197913	0.785507193	-0.143869629	2.284909736	0.428376837
28.33885519	0.839598338	-0.236335129	0.844760766	0.684878847
18.80375577	0.908439995	-0.274865466	1.164380146	0.620322618
19.02695935	0.918416402	-0.223043793	4.090599225	0.312795225
14.0438523	0.918564705	-0.242935059	3.698722494	0.323093918
17.12323482	0.819559814	-0.168305314	1.892178598	0.475693516
20.41900002	0.910819232	-0.293631613	1.23350301	0.633232776
10.95312333	0.782316008	-0.15364944	1.785161537	0.481275417
13.4125558	0.955144643	-0.331453314	3.395233582	0.337101557
12.45757106	0.849787788	-0.203279002	1.551124044	0.506817483
22.82481853	0.891817401	-0.24469511	1.236815096	0.613111519
15.36611696	0.791473435	-0.140991211	2.236075552	0.441424604
16.41350563	0.953637836	-0.307924136	3.426039729	0.345524242
16.7763287	0.890471485	-0.212069568	2.565930407	0.398440338
19.64037296	0.890233144	-0.299856404	0.78775519	0.707451648
26.95346395	0.872266251	-0.203627971	2.439836281	0.429398389
21.27223649	0.816630534	-0.159212884	2.086162834	0.479477089
20.94179044	0.801257284	-0.151749157	2.113433432	0.452754024
19.31847168	0.875687227	-0.217224296	1.466402707	0.533544629
7.828367455	0.934544834	-0.458918015	1.97175996	0.476697274
19.47249183	0.834932312	-0.174837721	1.745170666	0.490122046
18.46096671	0.86861312	-0.212634143	1.596510181	0.523962668
18.78480402	0.899111238	-0.211918305	3.714638168	0.334507658
19.35530938	0.840477188	-0.172247595	2.030030398	0.465678636
20.79607901	0.908546012	-0.26923601	1.271935679	0.594431598
17.65116731	0.798913325	-0.154150856	1.689592967	0.507777578
21.73043275	0.856653404	-0.173038637	2.349075594	0.446437986
21.74196473	0.909835553	-0.218388076	4.052027861	0.311316543
23.74177723	0.904900892	-0.283009911	1.093772873	0.656003327
13.30844193	0.897251586	-0.23054784	2.389386083	0.403874853
38.11400648	0.883182565	-0.255941953	1.047044049	0.652980622
16.61832723	0.719819084	-0.113059416	2.150189851	0.449952474
19.51544956	0.822448516	-0.231259371	0.760770721	0.706490883
21.90614274	0.80404854	-0.164102822	2.799940517	0.409845765
16.61679056	0.804494195	-0.150295238	2.220359765	0.438064638
18.64552377	0.912967332	-0.270238444	1.341263564	0.569571965
19.92241492	0.816153559	-0.160267165	1.982451954	0.480585121
21.49045096	0.910621581	-0.280896946	1.047197718	0.640959084
14.90362261	0.924905897	-0.272505614	2.167508643	0.432192553
15.38192963	0.976258724	-0.396184709	3.156015838	0.349913812
21.64364532	0.917696835	-0.234002653	3.468639593	0.333215598
21.80316095	0.924534942	-0.29255289	1.328733952	0.575005066
23.48123121	0.813802821	-0.150343708	2.196398665	0.456871608
18.30939634	0.888898311	-0.311771533	0.704248716	0.736565926
20.86609744	0.834849251	-0.188049391	1.885184644	0.468781209
18.38783506	0.924915918	-0.233881364	3.733751196	0.334299624
14.61911993	0.79877022	-0.14351789	2.483637032	0.418903608
20.54900651	0.898291377	-0.257743083	1.211164921	0.603108196
14.88934239	0.781040791	-0.13734878	2.061636456	0.457601573
15.89269739	0.871336647	-0.197374224	2.382826804	0.414561439
24.97871706	0.878984382	-0.293763465	0.729908719	0.729985856
17.27781126	0.772170975	-0.128834902	2.406198426	0.426707236
27.45574302	0.860528046	-0.17103538	2.637195631	0.410890588
28.96421709	0.78241632	-0.12681077	2.78983624	0.417247445
27.62199719	0.877687985	-0.220269702	1.497331839	0.581131041
15.03173505	0.830291616	-0.170815127	2.333947177	0.415632464
27.69958398	0.830610974	-0.145983147	2.868567771	0.385593104
15.61224208	0.948324217	-0.304943429	3.644643346	0.317957839
27.42922914	0.810815728	-0.162093796	1.714438041	0.518441725
19.92027737	0.899717549	-0.282064152	0.966917019	0.661028658
20.68110939	0.762675678	-0.12095198	2.476750919	0.418060012
12.0863597	0.855510699	-0.195315587	2.5891431	0.402004064
24.45538596	0.91263627	-0.285453805	1.186943644	0.641339791
14.53822262	0.669818568	-0.093678462	2.236362898	0.433365773
20.00770837	0.85986428	-0.194602966	1.752613436	0.497594384
25.0626603	0.874324944	-0.223533747	1.396361177	0.588838884
15.17014551	0.804746875	-0.161104984	2.096943869	0.445348009
26.16149719	0.919297436	-0.204083117	3.91818396	0.336076612
26.86546701	0.991541591	-0.476569964	4.638794798	0.282347584
19.72381091	0.846779452	-0.16095192	2.998133654	0.366733116
20.78633575	0.86858174	-0.227163596	1.250589445	0.5808865
23.19755417	0.865121854	-0.177748302	2.739620805	0.392201385
16.71690421	0.883487585	-0.195977482	3.224190963	0.355341819
26.637308	0.909281284	-0.27601722	1.33872754	0.60084325
17.52683874	0.858085656	-0.208271678	1.382091879	0.548197826
19.17065973	0.84237924	-0.181717561	1.753941391	0.506575913
26.72324366	0.980212074	-0.367350108	4.769864234	0.266321385
22.78516159	0.881781029	-0.197781272	2.157386715	0.448210971
18.16337939	0.898558052	-0.273848234	1.119666673	0.647398725
25.99996522	0.911180193	-0.20801526	3.410688877	0.383952737
15.95709622	0.801888059	-0.141414666	2.541880693	0.417689449
31.20149159	0.958257302	-0.287552818	4.237768327	0.30956605
23.36678089	0.863529427	-0.218172824	1.428301807	0.550959769
18.10236937	0.830122382	-0.172443911	1.603677344	0.528635382
19.68191997	0.860768885	-0.204254362	1.487001771	0.556883423
14.63044788	0.739868841	-0.132340785	1.642806232	0.506784107
22.03565322	0.750758223	-0.109384608	3.191078274	0.367547228
16.69555047	0.723841151	-0.110468485	2.496013309	0.415841666
19.99366229	0.980327202	-0.420015728	4.049937536	0.296665944
15.2240314	0.973908837	-0.410711028	3.500182249	0.321718403
29.0447912	0.89017101	-0.222983616	1.589908512	0.562812537

log.sigma.4.5.mm.3D_glcm_ClusterTendency	log.sigma.4.5.mm.3D_firstorder_InterquartileRange	log.sigma.4.5.mm.3D_firstorder_Skewness	log.sigma.4.5.mm.3D_firstorder_Uniformity
54.1907424	142.2835789	0.508028263	0.070854638
62.15171394	148.2523651	0.259894304	0.068055447
56.4876357	164.4009628	0.029110523	0.06349635
61.27640751	173.750145	0.089950169	0.060964083
32.11313976	120.6790504	0.968027578	0.085949011
42.22949576	152.9913979	0.177232107	0.071920068
20.83849831	61.55152428	-1.198128135	0.208996976
36.13667467	125.9421554	-0.300856068	0.090962275
24.93778924	107.6569309	-0.349237884	0.115407745
20.84803408	100.048274	0.400867637	0.100335104
11.3744784	41.46012783	-1.194266284	0.204211742
27.17966969	101.1420644	-0.471593399	0.120663698
77.30852792	221.664917	0.240545254	0.0528564
40.06376735	158.4759712	0.276463751	0.068421053
29.58477981	117.2408543	0.232748521	0.090302457
32.63144145	107.0108259	-0.77535936	0.142342973
19.30982238	101.5934629	0.405169424	0.110491541
43.27364682	144.6308975	0.655480901	0.077509942
22.55663489	106.5479202	-0.206298227	0.107552669
28.76142069	72.61191177	0.105826352	0.12460242
39.89334887	127.7125702	0.718830989	0.079339602
54.71524972	178.4598789	0.1512391	0.063130909
41.3266766	138.4731674	-0.10599494	0.076328821
16.23928	62.56636369	-1.183472657	0.215283233
48.85894348	155.3907099	-0.206880817	0.073583316
37.53497837	103.6172638	0.704469679	0.089859229
32.40124039	100.5123062	0.041042287	0.092524733
28.80749365	110.6402931	-0.157869842	0.096483253
8.868142147	88.92256832	0.560629289	0.206632653
29.93865359	109.7511845	0.152397762	0.090435469
34.31416816	126.0065804	-0.088071204	0.09007091
46.58518643	138.2460098	-0.00459047	0.069865436
37.71719877	116.4082222	0.094555255	0.083881624
30.31400426	108.0791928	-0.332002979	0.11339514
26.01039754	103.2058029	0.087938349	0.098807241
55.20657036	153.1001053	-0.383852502	0.069419295
57.82033663	177.2906342	-0.12081171	0.060670611
29.90189865	90.67131877	-1.0493851	0.155027059
35.43712457	120.8658142	0.205399165	0.082223196
19.06727881	70.13309	-0.620918905	0.156475156
21.91235662	95.39477539	0.396381477	0.097113982
8.03588736	42.37931204	-0.248316771	0.204812777
54.55511791	134.5903893	0.080029997	0.068040926
32.65602172	112.7519188	0.772456263	0.086580147
32.04483102	114.241034	-0.418417609	0.101670631
34.16840133	117.8068657	0.162393376	0.085194367
24.21518865	89.43061399	-0.69030813	0.122598385
45.81112369	158.4723473	-0.085702733	0.081879027
56.44995363	152.0082235	0.582528251	0.070175439
51.38094674	146.0220928	-0.271579613	0.070909091
39.10752251	138.9605138	-0.531804273	0.104601474
41.86359228	111.7804594	0.470559055	0.082913045
14.05963201	53.24577847	-1.106612469	0.231374469
30.98791625	113.4798312	0.000970526	0.090056021
79.09572228	196.1317863	0.232993252	0.056926756
36.3939916	113.7112961	0.978279705	0.083834392
26.22657399	96.68762589	-0.388821323	0.110406063
33.72702821	124.4497681	0.188204851	0.085454907
32.86798498	115.7581367	0.02174331	0.085261678
12.80225068	45.36134231	-1.077758709	0.219753005
33.6340448	124.128212	0.30370937	0.08092612
66.66993742	172.3942099	0.331999588	0.061893074
59.51555997	136.4072361	-0.011622743	0.067328786
32.56899582	113.7924683	-0.414339103	0.1102046
28.41870568	112.3821411	-0.054061382	0.089876845
75.64531482	145.468338	0.410378286	0.064561054
39.01990237	135.685997	-0.14851527	0.077325048
28.08638367	99.94576454	-0.021822391	0.096314848
20.95139775	85.19486701	-0.491108692	0.146375946
40.34806372	133.0664005	0.37420255	0.074702077
24.20346503	113.9076614	0.394880663	0.092286501
39.56608024	113.0730598	-1.033597507	0.143150518
20.87686312	96.29829407	0.45259416	0.101227984
32.77066477	95.60705566	0.473847931	0.095499201
28.25168702	90.77249834	-0.633828126	0.119754152
28.16212304	105.5756245	0.479598632	0.094222841
201.328331	188.7185874	1.359541138	0.051508794
104.632848	215.6583939	-0.414821751	0.057693762
57.16887891	169.555881	0.245635934	0.062366535
22.0581537	95.20571756	-0.287118382	0.118943976
54.1907424	142.2835789	0.508028263	0.070854638
59.43436068	188.7517929	0.352519636	0.060127349
36.4460654	121.1246881	-0.60007546	0.129675237
24.08625578	90.87875748	-0.458782478	0.108787687
33.02472657	110.8695641	-0.235665686	0.092677312
104.3845155	214.2996407	-0.270977766	0.051394628
48.01134517	131.5925636	-0.193568159	0.074024257
26.64578763	97.91178823	-0.65199347	0.157379505
141.1802415	128.9353564	1.508826693	0.068494341
43.23488031	144.4010582	0.679721857	0.073234375
104.0804993	156.8771873	-0.65235925	0.063405797
25.67808052	103.1609478	-0.470245672	0.106832608
27.74481706	101.5770316	0.108161297	0.096297174
27.57375597	105.2299614	-0.091242641	0.105566999
19.27794447	87.5470562	0.248064482	0.108916138
48.52420444	145.007658	0.443597449	0.067607515
34.97357972	120.6800346	0.584183439	0.08093979
51.91552215	155.6655807	-0.394697686	0.073589642
30.89571549	131.4232597	-0.198020486	0.084308669
41.18709554	102.6168192	-0.223189453	0.099238638

log.sigma.4.5.mm.3D_firstorder_MeanAbsoluteDeviation	log.sigma.4.5.mm.3D_firstorder_Energy	log.sigma.4.5.mm.3D_firstorder_RobustMeanAbsoluteDeviation	log.sigma.4.5.mm.3D_firstorder_Median
84.57652714	20474072.1	59.73704987	-149.66716
100.1341869	9313644.299	68.52959313	-255.0992889
90.91018503	21445995.03	65.28032987	-182.6659698
100.4265265	7289688.273	69.55227765	-256.2872086
72.99858053	24785453.23	50.22337455	-172.8631821
83.85717858	2052472.089	59.79042607	-102.1098938
46.52297878	75922932.88	28.92396729	-6.286376476
67.8680378	80724842.82	50.5382242	-88.2865181
56.2965544	43449361.44	43.23819156	-67.11906433
58.6344436	4769146.479	40.260169	-90.6014328
32.0103868	11130752.9	18.80203034	0.959652305
57.00338122	22867927.05	41.19257812	-33.36216736
114.400315	24753226.15	88.15996994	-241.7350006
85.65071856	19933577.88	65.0225027	-310.8527985
63.59028255	24919759.98	47.43015233	-147.9365845
63.38797615	74584372.67	46.58502172	-22.46056175
54.17447414	5252320.208	40.75968003	-89.53620148
85.41026969	12307708.84	61.26753046	-332.0170288
54.49632649	7682790.377	41.8762729	-89.81802368
53.79163219	71460195.71	33.44025278	-10.68883514
74.0572745	38394928.91	51.63644724	-143.8236771
95.90801021	6737639.966	72.89196297	-179.7594681
76.15469074	9375336.164	57.23689318	-151.8044891
41.93167981	22083987.21	27.76008917	-21.02392292
80.80511255	21720519.59	60.84000198	-144.8345871
66.64218848	24811214.7	44.11627436	-73.95565033
62.25888373	32693440.93	42.06425977	-167.7502441
59.81001304	25337809.63	44.66324238	-93.4631958
44.74018836	77496.02839	34.89356564	-4.374350905
62.76839976	21346920.44	44.72389892	-114.8242722
65.84194329	61347568.14	50.54211639	-104.9774628
83.57575457	22190459.29	57.5351811	-275.0325928
67.920038	24676787.71	47.66735327	-105.1292496
61.96767696	26004036.03	45.62256071	-33.11521149
58.31342053	44492936.78	41.93329615	-84.85071564
83.75395008	38295488.28	62.21004332	-144.1379929
97.23361742	30748164.87	72.79174928	-244.0110703
58.42497997	81629958	40.11373056	-10.65297985
71.52309921	6217015.588	53.11671361	-161.3821106
48.18605642	63532073.79	31.14635441	-17.20385647
58.57168797	27330901.99	39.46008534	-197.9059753
30.16661122	14268376.63	18.7063707	-10.25041962
83.77929447	52361506.16	57.18943676	-150.8747711
68.54284567	25416815.06	46.29166012	-177.6634979
63.38650617	12179239.36	46.92227927	-55.32587433
67.43816879	36039308.86	48.48532539	-105.052063
54.12190113	44357691.96	38.50346484	-26.34144402
79.74371054	4794725.113	64.37956994	-111.3861084
89.42101387	2924847.05	64.14951912	-132.9082489
87.44135932	5934043.076	64.57102781	-128.559269
71.34650733	28977321.84	56.00261686	-76.04660797
71.58132184	33978621.95	46.48781332	-85.44542694
38.85796077	36481236.74	24.71831034	-12.03551579
62.31772337	6900148.101	45.77543926	-51.09941864
108.3827479	16478435.33	80.75688723	-191.6123352
72.95692214	23869864.64	48.63975796	-179.4641571
57.0160406	42528942.37	40.74266004	-43.98703766
67.62509727	43565991	51.6577188	-151.5192108
67.1615266	15062919.34	46.84644109	-217.2008591
36.26625309	71918151.08	21.375984	-7.395323277
69.82065282	19638262.55	49.45512607	-133.7270203
95.21984653	39455899.42	69.41300162	-109.4175491
84.92590325	87591769.88	57.48358135	-81.66209412
63.69631077	89491411.1	46.01150525	-47.89293671
63.17030428	5831064.283	46.06687408	-107.6991119
90.91186805	55864121.95	61.16414552	-121.0667572
79.5736609	12664850.68	59.19305735	-310.3725891
60.49482948	59657410.47	41.32525817	-88.97418213
50.99651089	54312162.83	36.32039022	-17.1357193
77.13383895	33407718.38	55.3179278	-117.1974945
63.3601178	8418132.241	45.15608857	-196.2181015
68.21975373	71418098.65	49.90436107	-21.05609131
56.5712217	32765294.33	39.39147102	-204.7420349
61.90112901	14709584.05	40.46096564	-82.73683167
56.68681912	23935914.93	38.68555603	-30.40880013
60.61704524	7973994.884	42.51403278	-91.59080505
129.1299001	47373622.61	81.74359847	-82.18030167
113.9167916	10182936.06	81.48809397	-247.5944061
91.58951037	13323347.3	67.46462032	-94.59076691
53.49862751	16232314.09	38.84143298	-65.99010086
84.57652714	20474072.1	59.73704987	-149.66716
99.47209373	16216448.37	75.78028227	-185.7489014
66.83314835	43951133.36	49.57027117	-59.83984375
53.53868041	21355569.27	38.40982058	-109.5266724
64.737378	26162182.92	46.47896866	-79.99804688
122.2978582	13398519.4	90.2560704	-222.0360947
76.8206257	16383742.39	55.55793534	-114.7992935
57.47344655	33859081.55	41.69344591	-33.24951172
101.5289361	27576472.34	56.24795821	-69.61785889
80.9938509	24509927.67	57.83069976	-156.0429764
106.4295943	8775911.56	68.88323724	-92.99885941
58.52188813	19535642.67	41.92614575	-81.18107986
59.01315031	40391535.03	41.26115658	-77.92312241
59.13240925	35681100.53	42.66904084	-55.10967827
52.04453054	19551923.16	36.53763921	-77.54841614
85.83079154	47791154.38	59.16020111	-177.868187
71.61930722	30766323.36	49.61691211	-123.7973938
88.76460971	6717455.747	65.05367038	-246.6704712
71.30144771	2590241.206	52.21080907	-162.95578
68.1991575	93273154.8	45.9206739	-27.31520939

log.sigma.4.5.mm.3D_firstorder_TotalEnergy	log.sigma.4.5.mm.3D_firstorder_Maximum	log.sigma.4.5.mm.3D_firstorder_RootMeanSquared	log.sigma.4.5.mm.3D_firstorder_90Percentile	log.sigma.4.5.mm.3D_firstorder_Minimum
552799946.7	224.1532898	175.3335393	-2.88005203	-403.7025757
251468396.1	70.57078552	287.0916769	-96.17166138	-500.873291
579041865.9	78.43811798	210.7169809	-26.30887604	-434.6148376
196821583.4	33.14432144	281.4884404	-85.40473328	-491.5499573
669207237.2	246.7127838	182.2758024	-28.95503712	-316.0250854
55416746.41	201.8990631	141.1627199	38.3345665	-304.9730835
2049919188	251.1347961	68.23587567	12.72524691	-268.7607727
2179570756	173.8853455	126.482366	-5.667118311	-350.1130371
1173132759	207.8687592	101.3614598	-0.997881925	-288.1335754
128766954.9	148.2835388	114.4641899	10.82521496	-283.8697205
300530328.4	168.2918701	45.13432227	37.90721359	-333.7608948
617434030.3	269.4614258	86.26450189	16.63152351	-259.2354736
668337106	78.11560822	273.4650994	-54.82083893	-475.3188477
538206602.9	-45.54760361	323.9036397	-164.7536179	-492.0080261
672833519.5	151.9736786	164.5803622	-47.07760086	-328.552063
2013778062	201.657959	93.52458486	12.37301731	-278.03125
141812645.6	178.8793793	109.7570316	-8.874893188	-211.6115417
332308138.7	22.24150467	332.9868764	-174.1002808	-485.6402588
207435340.2	56.72581863	116.4040156	-15.18397217	-248.0110626
1929425284	468.6231995	77.40455015	49.0504425	-277.4318542
1036663081	192.3247375	162.6123873	-15.28064566	-318.6483154
181916279.1	66.2230835	206.5025204	-7.192695713	-382.9447937
253134076.4	91.37765503	182.6586588	-43.33376312	-353.0577087
596267654.8	154.1124268	67.80117872	3.203712153	-263.9795227
586454028.8	83.2672348	172.9684106	-15.73822117	-452.1454468
669902796.9	302.3721619	110.6907932	41.55049286	-302.7669983
882722905.2	181.5535278	186.5939334	-70.03737793	-404.5726929
684120860	140.9864197	120.0194451	-5.641659069	-306.9224854
2092392.767	125.5840073	52.60908001	70.77702942	-60.72027206
576366851.9	146.7092438	136.1852128	-14.53618145	-328.2946472
1656384340	228.2234039	132.1853305	-7.252826214	-324.7679749
599142400.9	-11.98409748	294.9939135	-146.3833069	-501.7584229
666273268.2	215.5536041	138.9021731	-6.756315899	-326.5789185
702108972.8	171.0718536	90.87282916	24.32518311	-287.269928
1201309293	223.7391663	113.1209883	-4.108404064	-278.9672852
1033978184	80.3502655	184.254989	-29.29485836	-417.9949341
830200451.5	36.97179413	280.7871941	-109.4239838	-535.5915527
2204008866	256.1830444	83.39264215	22.30997353	-318.6888428
167859420.9	56.2960434	177.6469948	-26.64209824	-319.1605225
1715365992	353.3908997	72.41903462	23.12714825	-478.8796387
737934353.7	64.1307373	208.1192742	-94.79257965	-382.6362915
385246168.9	195.2366486	44.40255858	26.09583511	-228.0733948
1413760666	204.1042175	185.176788	-22.09700127	-423.3938293
686254006.6	236.0713501	189.6054679	-46.74827042	-363.1786194
328839462.7	155.1227264	105.8024283	7.798378038	-279.4390259
973061339.4	200.4697418	135.7734477	-9.993111801	-333.2037964
1197657683	195.567627	79.78095636	26.932481	-279.0409241
129457578	50.54566956	147.9652729	-0.759862638	-281.0324402
78970870.35	175.4808044	160.1766308	41.54220963	-302.4273376
160219163.1	101.8168106	164.2343541	-0.435073099	-376.5561218
782387689.8	61.89078522	123.3013053	-1.299955547	-345.7613525
917422792.6	415.5057068	125.4517025	30.88332443	-366.1891785
984993391.9	270.9018555	60.09399746	5.970790482	-240.8228455
186303998.7	136.3252716	90.25837307	50.59381561	-290.1938171
444917753.8	177.643692	239.6166751	-13.31976929	-438.440033
644486345.3	198.8760223	189.7440709	-39.48959427	-335.9047546
1148281444	225.6194	89.68868079	19.67188454	-293.6122437
1176281757	91.67668152	167.9227573	-34.02118073	-316.3968201
406698822.2	27.28295326	235.326114	-106.6303314	-417.4923706
1941790079	326.0936584	55.30510675	13.16573296	-300.1523743
530233089	190.2567139	156.9720646	-20.58628883	-341.3598633
1065309284	321.4525757	156.8878143	40.33158493	-445.1194458
2364977787	295.3069458	138.4135285	51.10731735	-434.5785522
2416268100	283.6724243	102.4874956	7.267377806	-392.7485352
157438735.7	88.80792999	134.778742	-9.73948288	-295.8484802
1508331293	296.09375	159.8971665	49.63310242	-443.6982422
341950968.3	-126.0942078	326.2319981	-187.6716125	-521.152832
1610750083	237.2288971	120.2160225	-5.326736498	-403.1682739
1466428396	278.9693909	73.61218072	15.86464634	-265.3992004
902008396.2	200.5685425	147.9121635	9.568622398	-357.9777527
227289570.5	38.3828392	206.1936454	-77.00818481	-330.6963501
1928288663	147.0244904	100.3010672	17.54781151	-348.4054871
884662947	40.3170929	209.1539775	-99.9981842	-355.6745605
397158769.5	293.2767639	117.0847506	12.07348537	-305.3748779
646269703.2	329.4981079	86.27142991	20.87161446	-346.7946472
215297861.9	159.2821808	114.899862	11.94418507	-260.4206543
1279087810	646.4557495	178.7904001	170.6454819	-325.6971436
274939273.5	-21.57416916	297.5689333	-77.78822021	-613.87677
359730377.2	286.2015991	143.0593278	59.12322998	-337.0049744
438272480.4	207.8856964	100.849506	-6.92744565	-317.2974854
552799946.7	224.1532898	175.3335393	-2.88005203	-403.7025757
437844105.9	161.8376007	209.6354325	-6.593364429	-381.5908813
1186680601	246.5853882	113.3963329	-2.375807047	-399.9729309
576600370.3	42.14302444	134.8145117	-39.46498184	-320.7991943
706378938.9	174.3227844	120.3922097	-1.07883358	-312.9081726
361760023.7	72.20727539	275.9129412	-51.3828125	-577.09198
442361044.4	150.6670227	150.3271943	0.232717174	-378.3255005
914195201.9	194.7468109	91.25441799	1.742459238	-259.2582703
744564753.2	542.7504272	153.1970566	135.7445007	-326.967926
661768047.1	227.4618683	175.0354524	-9.73431673	-348.5015869
236949612.1	186.9979858	178.3165192	43.77135277	-516.8590698
527462352.2	155.8363647	116.8815151	-11.59609661	-353.375
1090571446	258.9926758	108.6438573	10.05677958	-299.1309204
963389714.4	319.5191956	96.21953758	13.74446182	-298.5244751
527901925.4	202.3862	97.25781369	14.06567612	-238.9539032
1290361168	263.7770081	200.9083141	-21.75834618	-442.5758057
830690730.8	300.6341553	144.9168842	9.621994019	-305.2816162
181371305.2	-65.44165802	277.8707164	-122.0443619	-511.4342957
69936512.58	36.71542358	191.0033259	-63.06232452	-368.4101257
2518375180	317.3012085	98.5490081	44.75882454	-376.0505676

log.sigma.4.5.mm.3D_firstorder_Entropy	log.sigma.4.5.mm.3D_firstorder_StandardDeviation	log.sigma.4.5.mm.3D_firstorder_Range	log.sigma.4.5.mm.3D_firstorder_Variance	log.sigma.4.5.mm.3D_firstorder_10Percentile
4.054802528	105.7927551	627.8558655	11192.10704	-262.0678101
4.173710683	126.3008339	571.4440765	15951.90065	-441.4890503
4.132941182	111.5501169	513.0529556	12443.42858	-319.7610107
4.214682647	124.1650071	524.6942787	15416.94898	-427.5656403
3.776848511	92.71361485	562.7378693	8595.814378	-259.6720123
3.984592544	102.4930514	506.8721466	10504.82559	-233.3925323
2.844305665	60.51774842	519.8955688	3662.397874	-125.5839348
3.678971897	81.69465608	523.9983826	6674.016832	-209.77314
3.325934688	66.78958902	496.0023346	4460.849202	-172.0818878
3.570110707	74.54314635	432.1532593	5556.680668	-172.528656
2.721609655	44.5210566	502.0527649	1982.124481	-66.79444046
3.36989253	69.63618795	528.6968994	4849.198673	-155.6703979
4.346127316	135.2803669	553.4344559	18300.77767	-415.5600281
3.986285283	102.3408011	446.4604225	10473.63958	-443.3137665
3.62781045	76.83218492	480.5257416	5903.18464	-241.8094543
3.310336774	75.65487232	479.689209	5723.659706	-176.424057
3.337610631	65.60429316	390.490921	4303.923282	-173.9422379
3.872098888	105.6802828	507.8817635	11168.32217	-446.7024841
3.332342914	64.42586198	304.7368813	4150.691691	-190.0082001
3.452781208	73.29372835	746.0550537	5371.970615	-124.5942703
3.858552523	93.24793679	510.973053	8695.177716	-242.7899063
4.076035042	114.0231766	449.1678772	13001.28479	-323.4998352
3.844551211	91.55966027	444.4353638	8383.171389	-289.9836426
2.77450082	53.25001507	418.0919495	2835.564105	-124.9626495
3.930277802	97.12042878	535.4126816	9432.377687	-266.4030762
3.771414488	86.85698778	605.1391602	7544.136326	-170.6089417
3.690918991	80.1857811	586.1262207	6429.759491	-268.0804077
3.543401941	72.06442229	447.908905	5193.28096	-192.3593933
2.534185619	51.48193916	186.3042793	2650.39006	-45.7754879
3.664760873	77.40566914	475.003891	5991.637615	-210.3999481
3.615867597	78.14488951	552.9913788	6106.623756	-211.480835
4.017061312	104.6836736	489.7743254	10958.67152	-407.4959656
3.799085659	85.76156256	542.1325226	7355.045614	-223.8945923
3.480934315	74.27277787	458.3417816	5516.445533	-160.5351654
3.545287049	72.05052286	502.7064514	5191.277844	-182.141925
3.991197417	101.1225283	498.3451996	10225.76572	-300.5143311
4.198074176	116.1499778	572.5633469	13490.81735	-415.4239136
3.197395448	72.42038626	574.8718872	5244.712346	-155.8859299
3.716671821	86.10359426	375.4565659	7413.828944	-269.3371399
3.198403961	63.18462399	832.2705383	3992.296709	-122.8761566
3.6114598	75.43771971	446.7670288	5690.849555	-291.2182007
2.694346038	41.16872722	423.3100433	1694.864101	-71.57193756
4.108468683	106.1474869	627.4980469	11267.28897	-292.2371399
3.792287627	88.38349734	599.2499695	7811.642602	-267.5571472
3.541529462	76.04305969	434.5617523	5782.546926	-186.2975464
3.760295521	83.56605593	533.6735382	6983.285704	-216.6507294
3.328515952	66.19435437	474.608551	4381.692551	-143.5447235
3.685759326	90.8198995	331.5781097	8248.254145	-241.667569
4.021376959	108.4609372	477.9081421	11763.77491	-250.3371002
4.008665441	105.241554	478.3729324	11075.78469	-267.6280487
3.548107536	83.04817768	407.6521378	6896.999816	-211.1218262
3.911344748	95.76976931	781.6948853	9171.848714	-187.2730499
2.65858528	50.4432987	511.7247009	2544.526383	-111.1849304
3.625554986	75.42648163	426.5190887	5689.154131	-146.0320801
4.278029975	131.4412198	616.083725	17276.79426	-375.8190247
3.829998898	94.40293709	534.780777	8911.914531	-267.2208984
3.462348654	70.06277563	519.2316437	4908.792529	-157.5376984
3.647425524	79.95007331	408.0735016	6392.014223	-251.6319702
3.750905502	83.79780267	444.7753239	7022.071733	-333.1185333
2.735195112	49.56485523	626.2460327	2456.674874	-93.92584991
3.828597888	86.9770548	531.6165771	7565.008062	-240.2282318
4.21622119	116.582269	766.5720215	13591.42544	-248.2754364
4.156640176	108.5248486	729.885498	11777.64277	-225.7710129
3.54903931	78.92870411	676.4209595	6229.740332	-170.0729614
3.634407591	77.33828796	384.6564102	5981.210785	-216.1542664
4.225528915	116.6261197	739.7919922	13601.6518	-241.0461578
3.831902115	96.88472113	395.0586243	9386.649189	-446.3426025
3.655325854	77.9002645	640.397171	6068.45121	-186.7104553
3.173925038	62.55070839	544.3685913	3912.59112	-132.7548645
3.939069662	95.36740986	558.5462952	9094.942864	-234.1482239
3.58785421	78.32865213	369.0791893	6135.377745	-295.1219727
3.288048107	82.04257479	495.4299774	6730.984078	-191.0605164
3.527287726	70.88820047	395.9916534	5025.136966	-282.0024292
3.693699905	82.03755262	598.6516418	6730.160041	-188.0569946
3.431676439	71.82394311	676.2927551	5158.678803	-148.1566162
3.612534654	76.00676467	419.7028351	5777.028276	-180.0617828
4.581085941	173.0752609	972.1528931	29955.04592	-223.1084839
4.316792826	140.9597933	592.3026009	19869.66334	-461.1765198
4.149005378	111.018097	623.2065735	12325.01786	-231.5379944
3.329054241	65.87769985	525.1831818	4339.871338	-167.6888809
4.054802528	105.7927551	627.8558655	11192.10704	-262.0678101
4.171166894	117.9631016	543.4284821	13915.29334	-318.5593994
3.438172184	81.42509406	646.5583191	6630.045943	-200.2402191
3.403532414	66.10756824	362.9422188	4370.210578	-209.9392822
3.659876659	80.02971584	487.230957	6404.755417	-208.1225647
4.441655091	146.6374529	649.2992554	21502.54259	-456.3323822
3.94007032	94.16428464	528.9925232	8866.912502	-246.6907135
3.200788472	70.15447772	454.0050812	4921.650743	-170.5704956
4.281827171	146.1886287	869.7183533	21371.11517	-193.0107758
3.96613403	100.7353534	575.9634552	10147.61143	-263.6228455
4.28165105	137.5495331	703.8570557	18919.87406	-329.4939423
3.502321021	72.06814751	509.2113647	5193.817885	-187.6248688
3.605880129	74.30238838	558.1235962	5520.844919	-176.3946274
3.51627305	73.15053172	618.0436707	5351.00029	-162.7348969
3.412410098	64.87352833	441.3401031	4208.574678	-155.8155121
4.122304277	108.5264116	706.3528137	11777.98201	-297.4945587
3.847978077	90.51779972	605.9157715	8193.472065	-221.1105469
3.948796261	106.7121155	445.9926376	11387.4756	-413.0434631
3.737167545	87.66354394	405.1255493	7684.896935	-293.946106
3.742069218	87.40801988	693.3517761	7640.161939	-170.4091125

log.sigma.4.5.mm.3D_firstorder_Kurtosis	log.sigma.4.5.mm.3D_firstorder_Mean	log.sigma.4.5.mm.3D_glrlm_ShortRunLowGrayLevelEmphasis	log.sigma.4.5.mm.3D_glrlm_GrayLevelVariance
3.216191912	-139.8203954	0.012452503	18.3963303
2.729371457	-257.8172421	0.027479982	25.17191151
2.423030401	-178.7686143	0.02100754	20.15655298
2.454237827	-252.6238174	0.05405962	25.01667359
3.947216093	-156.9351897	0.043306094	14.23331099
2.714281081	-97.06743993	0.039788332	17.09977752
4.607535767	-31.52359203	0.016153799	8.011765052
2.634551011	-96.55968141	0.01053964	10.98078653
2.588587437	-76.24497575	0.015897693	7.08135126
3.231797676	-86.86408981	0.01994018	9.28527528
5.869422676	-7.41502302	0.004565796	4.029953139
3.064247228	-50.91527877	0.019213304	8.606691549
2.079440218	-237.6602257	0.024324082	29.41551108
2.22329411	-307.3108007	0.052345576	17.01092029
2.791527174	-145.5455633	0.021367391	9.63614583
2.726808128	-54.98352723	0.016824412	10.26988782
3.194156155	-87.99251502	0.057115989	7.056370936
3.253560769	-315.771971	0.056368076	17.56592509
2.112577507	-96.94948764	0.0486999	6.639639192
5.748479039	-24.88963175	0.009463284	10.72522775
3.396631863	-133.2201591	0.029570049	14.4000491
2.030105922	-172.1685399	0.032276682	20.52690433
2.274830654	-158.0538334	0.026693263	13.44015421
4.132957405	-41.9694619	0.013784041	5.596247905
2.476488625	-143.128241	0.008818935	15.05373256
4.20721974	-68.61716533	0.012259653	13.21285002
3.791281299	-168.4860128	0.014555721	10.74197729
2.52245568	-95.97596706	0.017165953	8.401468751
2.337919003	10.83167762	0.134722108	4.310147262
2.80132878	-112.0480905	0.014033865	10.01565127
2.416207187	-106.6130284	0.019855511	9.572916145
2.720723346	-275.7947379	0.027058274	17.25447519
3.332650023	-109.2646698	0.016553745	12.19191932
2.601115807	-52.35862438	0.013696176	9.686533377
3.037628512	-87.20711072	0.017996325	8.583543653
2.406528009	-154.0264108	0.01917722	16.74997925
2.312259539	-255.6376949	0.016117095	21.60726994
3.609304047	-41.34755639	0.010828217	9.747547016
2.249331395	-155.3854106	0.063706506	12.07713486
5.120193203	-35.38671879	0.002615509	8.248830618
3.287365183	-193.9659319	0.020837792	9.78629251
5.143796468	-16.63499648	0.009573751	3.754461351
3.038985049	-151.7338256	0.018045685	18.61559827
3.94309399	-167.7456135	0.024671273	13.30623302
2.522152422	-73.56362483	0.019677132	9.947777987
3.056479369	-107.0100153	0.014641777	11.69624876
2.995684277	-44.53435132	0.011936381	7.879107872
1.764141768	-116.8138169	0.039867053	12.74383041
2.700140138	-117.8676298	0.037305069	19.34918159
2.469676399	-126.0838546	0.021055711	17.73052295
2.315583323	-91.13842254	0.016371523	10.99858988
5.30466981	-81.03259191	0.013651958	15.74638883
4.812019909	-32.66132495	0.015937226	5.481460112
2.468403801	-49.5723691	0.012894714	9.288688162
2.356748204	-200.3480888	0.034347545	27.37718341
4.014479369	-164.5931283	0.034100538	15.03633758
2.994983812	-55.99345437	0.014406443	8.723450193
2.207494058	-147.6686771	0.034524078	9.911301833
2.696460912	-219.900678	0.032966672	11.44438155
6.471944546	-24.53527987	0.00628117	5.829787162
3.095755902	-130.672189	0.022858076	12.74635428
2.986668321	-104.9874319	0.007861207	22.34669781
3.216636221	-85.91077988	0.006824073	19.85048733
3.930891018	-65.3754267	0.006988803	11.13775898
2.437599243	-110.381604	0.036102466	9.644377654
3.292529657	-109.3867088	0.00869523	22.15074565
2.202349336	-311.5135108	0.048693903	15.0226167
4.077388574	-91.56113183	0.006426304	10.58358319
3.2201303	-38.8093034	0.01283242	7.454299404
2.971806076	-113.0622185	0.012616131	15.10267318
2.596045845	-190.7365766	0.044637781	10.07906469
3.054684638	-57.70025996	0.013180388	11.64865102
3.032056256	-196.7746156	0.030047356	8.547338815
4.366028958	-83.53848682	0.015066296	11.54463483
4.018482133	-47.79205807	0.008777458	9.353005635
3.158457227	-86.16814962	0.026257419	9.560013201
5.161426291	-44.84374244	0.014670467	48.59518185
2.399563322	-262.0641271	0.019682062	32.27305519
2.538876246	-90.22723213	0.017190821	19.73626718
3.34812812	-76.35935781	0.011668921	7.413874442
3.216191912	-139.8203954	0.012452503	18.3963303
2.271961255	-173.2966278	0.029981956	22.13635266
3.282635722	-78.92200179	0.008336856	11.22232554
2.795819255	-117.4935828	0.02380562	7.391896493
2.790528753	-89.94180758	0.017210994	10.58772627
2.310874535	-233.7207918	0.016293868	34.47865464
2.641272472	-117.1808553	0.013218675	14.65297898
3.02825465	-58.3585303	0.02464804	8.973941
5.943816862	-45.80636406	0.011802822	35.56915492
3.234073104	-143.1425798	0.025600798	16.73412286
3.152392642	-113.4764599	0.011355626	30.48957105
3.308972387	-92.01886056	0.010521523	8.905497433
3.330784528	-79.26312393	0.01784168	9.299498993
3.492583378	-62.50759251	0.015350696	9.219959779
2.96334621	-72.46038674	0.028725737	7.205632739
3.184316432	-169.0744471	0.014718448	19.63104799
3.489480555	-113.169922	0.023170156	13.61297709
2.357351506	-256.5631685	0.030737227	18.9741523
2.481695355	-169.6978891	0.041942339	12.4546607
3.690971768	-45.51642624	0.005705949	14.16774062

log.sigma.4.5.mm.3D_glrlm_LowGrayLevelRunEmphasis	log.sigma.4.5.mm.3D_glrlm_GrayLevelNonUniformityNormalized	log.sigma.4.5.mm.3D_glrlm_RunVariance	log.sigma.4.5.mm.3D_glrlm_GrayLevelNonUniformity
0.013265902	0.069392079	0.122502703	42.09855281
0.028820427	0.066859625	0.047367094	7.195737106
0.022716017	0.063101256	0.138761995	27.81667747
0.057277681	0.060284154	0.047420647	5.291343028
0.048614992	0.083046606	0.143029758	55.33913455
0.041171491	0.071835366	0.033702159	7.173214945
0.019784964	0.118109061	5.454748871	971.823515
0.011827678	0.085169636	0.626689001	338.7019118
0.018704618	0.106805871	0.987872403	321.9086431
0.021519776	0.096794441	0.157488695	31.55676025
0.005820863	0.166520575	1.409717429	585.313013
0.022403906	0.101132832	1.171704157	213.7481596
0.026364539	0.052600167	0.070153213	16.36780441
0.056239841	0.06835533	0.059405111	12.3033587
0.023618266	0.090210533	0.234457834	70.83860416
0.019829404	0.09393033	1.979464679	516.4524406
0.066332583	0.109313248	0.199905169	40.99220371
0.060458484	0.076954518	0.053813775	8.080088685
0.057386249	0.107812463	0.246145793	51.66464103
0.011387495	0.097126348	1.358971351	785.787348
0.033831299	0.078472522	0.210732237	98.51959591
0.035831008	0.06316209	0.073723765	9.270385908
0.02960006	0.076368651	0.118188368	19.46594253
0.017055484	0.136792362	2.696339565	380.9141931
0.009453673	0.072659549	0.219256003	46.03669857
0.013813898	0.085405738	0.351507678	143.5644967
0.016010254	0.091268841	0.194510429	74.83284425
0.019281392	0.09587523	0.396460818	135.2213889
0.147719914	0.195222629	0.110208547	4.968067573
0.01583422	0.088611629	0.282826396	85.59585241
0.022644502	0.089629933	0.465055358	249.8978919
0.03012381	0.070176294	0.075159902	16.72041863
0.018408721	0.082936674	0.208064199	91.98360243
0.015810157	0.091150194	0.982034645	205.9636151
0.020450697	0.097228452	0.354032521	278.3612773
0.020909421	0.068360302	0.209862855	67.03404379
0.016861606	0.060658107	0.051618853	22.54945483
0.012873392	0.106175913	2.009815278	777.1877343
0.070413794	0.082172138	0.103221083	14.83459417
0.003252832	0.10792872	2.059705308	820.7384945
0.02291019	0.092912443	0.184325374	51.20544023
0.012608541	0.158712397	2.134265217	691.8447218
0.019947779	0.067036348	0.190142763	90.68335483
0.027048306	0.083269452	0.171278802	52.02706531
0.022640379	0.091427183	0.546590767	76.2657991
0.016388437	0.083987334	0.326587731	136.8924154
0.014203977	0.101408921	1.553580488	464.9881132
0.046539502	0.082019086	0.165056824	15.91130105
0.03957047	0.069802864	0.071482866	7.487213682
0.021840411	0.070468635	0.062237993	14.60349438
0.018527301	0.08562789	0.777610859	123.2114357
0.015013358	0.07983633	0.239385723	148.3209255
0.020592778	0.143037537	3.522294315	760.1300793
0.014070433	0.089986643	0.188372847	66.55590638
0.037850461	0.056866221	0.081599144	15.21163959
0.037790175	0.081787969	0.145342982	48.20974003
0.016716058	0.095974507	1.049513547	362.7530258
0.039283098	0.086914727	0.248004674	114.0203077
0.035944184	0.084684018	0.121249582	20.92854561
0.008367999	0.139518727	3.568378601	1729.158435
0.024981686	0.079700503	0.17201528	56.17788232
0.008545508	0.060923143	0.196355807	86.28933517
0.007378622	0.065193308	0.203977096	262.7020806
0.008037692	0.092007937	1.192593664	559.8044753
0.040114593	0.089492772	0.161137502	25.66573371
0.00944741	0.063397067	0.141055183	124.5835626
0.051786879	0.077377596	0.054237888	8.731843316
0.007243255	0.093294493	0.429306143	311.0342883
0.015910045	0.109956246	1.908468304	684.9704319
0.013992531	0.073150974	0.183506012	98.38363194
0.050673428	0.091412182	0.125625258	16.38549114
0.015263967	0.094953613	2.133959412	426.2410093
0.033047874	0.098256534	0.170857114	64.69168535
0.016787114	0.090403718	0.267729686	81.70137614
0.00999872	0.101685788	0.761656648	243.1132217
0.029110874	0.093177601	0.208397564	49.31345133
0.015951414	0.050971055	0.105124299	69.27502166
0.019895775	0.057375312	0.035353908	6.364645703
0.018656002	0.062293657	0.107703772	37.41035763
0.013540161	0.109807515	0.643134269	132.1310185
0.013265902	0.069392079	0.122502703	42.09855281
0.033072077	0.059514242	0.095093067	20.29246613
0.009575411	0.092742978	1.344947747	222.606794
0.026586671	0.104805957	0.377827328	98.65706262
0.019503202	0.089175002	0.375243474	132.4493464
0.016694362	0.051654936	0.038872029	8.745101236
0.014326186	0.073278785	0.172005642	46.94321217
0.028931329	0.101786832	1.988505249	264.0315006
0.012845455	0.065847461	0.180540067	68.6746447
0.029070981	0.071824112	0.16548423	50.69106883
0.011785169	0.061909376	0.070029634	16.04596332
0.011832566	0.098432802	0.497045922	111.5573303
0.020790958	0.094261844	0.422197322	259.9816037
0.017685571	0.096137024	0.734500595	278.8440432
0.032254349	0.104502579	0.278989219	182.7092033
0.015655396	0.066001541	0.110248978	71.51136327
0.025463243	0.078611592	0.176304289	102.2617596
0.031507168	0.073530504	0.048576674	6.138609619
0.043443711	0.084396391	0.045332465	5.719442119
0.006612699	0.079667741	1.034601994	557.9716772

log.sigma.4.5.mm.3D_glrlm_LongRunEmphasis	log.sigma.4.5.mm.3D_glrlm_ShortRunHighGrayLevelEmphasis	log.sigma.4.5.mm.3D_glrlm_RunLengthNonUniformity	log.sigma.4.5.mm.3D_glrlm_ShortRunEmphasis
1.331017791	154.1338692	512.3287309	0.934713848
1.153060477	149.9031012	97.60747905	0.961734881
1.349605466	144.0731703	377.3994847	0.93787919
1.151720188	130.7329588	80.12409459	0.963138329
1.399397404	66.15866418	540.98787	0.919513271
1.10013523	109.1348014	94.13546233	0.97674856
9.522996368	64.65334254	4006.400896	0.723436051
2.256587374	118.1070903	2798.05096	0.861981759
3.01373918	69.07732341	1877.774426	0.811306706
1.415691483	88.19973791	268.8926842	0.923888639
3.917369443	148.6381614	1858.78688	0.751109731
3.337891804	69.91157786	1270.041424	0.799764072
1.203781843	150.8405149	276.7211496	0.954657541
1.175904481	84.80273531	162.3298834	0.959949143
1.618594292	75.68731523	596.8022727	0.892105769
4.504799885	77.19254614	3217.408216	0.786503762
1.562485009	40.52087923	285.6097063	0.892481157
1.172535675	78.76568175	93.76763996	0.956866081
1.67678568	45.25435428	359.2092786	0.882125488
3.56925342	110.4323873	4805.243662	0.797199636
1.554483299	77.36189834	971.5528816	0.900531022
1.236307386	112.3190508	126.6531741	0.942790241
1.344523056	93.32149112	212.7591582	0.927588389
5.818748881	64.94447245	1434.244037	0.741999031
1.555734793	180.9010652	504.6882887	0.907848205
1.823157035	116.9374038	1238.754052	0.879792814
1.515454188	116.6033714	645.2765596	0.905982897
1.972943941	82.9430843	982.0088519	0.857318532
1.341672345	19.82083016	21.22347817	0.925304525
1.712736037	100.4047403	714.8356445	0.883038408
2.070509987	75.47000269	1948.641684	0.858451418
1.225423213	124.7898265	209.2408344	0.948877942
1.546951636	104.6088778	864.3593128	0.902351468
2.964627337	87.66262723	1434.991782	0.820655845
1.842699663	77.78178974	2089.072443	0.876205455
1.53772815	128.6134761	770.9529547	0.906326395
1.154348042	169.3342215	338.9948569	0.964133796
4.658188999	96.52626865	4029.100951	0.76761947
1.298352845	63.78956158	153.1554898	0.936232173
4.657344647	273.0937164	4247.435427	0.772589767
1.501057479	81.70609618	431.0626326	0.904829093
4.97942748	73.75600588	2211.958556	0.73563559
1.483798362	140.0711163	1105.592855	0.91805029
1.457456248	86.7393865	501.4214664	0.914411153
2.287143006	80.14575171	551.2214871	0.835295727
1.780075045	101.7907529	1211.783161	0.883332246
3.915088209	84.95416775	2628.287744	0.78285908
1.471168034	69.85522625	157.2559024	0.910792486
1.208347777	96.03792761	95.63390105	0.954665636
1.191154903	142.1979178	184.2416051	0.954727935
2.57443652	94.87030877	975.5007181	0.844082901
1.597210262	153.7025438	1439.19318	0.900662519
7.300852401	52.0435401	2365.909431	0.691198284
1.51077512	109.0503475	581.1229478	0.904507517
1.235483705	136.1081837	234.3299276	0.948621185
1.412300649	75.93718035	473.5411599	0.915722672
3.058901272	85.62956058	2361.236874	0.81451244
1.643285056	59.23105909	989.613722	0.890098599
1.336192501	84.35985914	206.8048949	0.931352719
7.31790419	102.4477189	5561.83122	0.69355202
1.455458584	93.99383136	566.4987781	0.915203676
1.483900122	212.6695461	1147.315459	0.917673587
1.498380599	229.1295027	3258.46415	0.91691016
3.185229408	155.2562483	3905.120973	0.826642331
1.426777576	72.535609	236.0150656	0.921710235
1.379999759	211.0327212	1623.331817	0.926146644
1.168194301	94.15293692	101.5980941	0.95949868
1.981579456	173.588068	2378.124633	0.86740093
4.576705635	72.41525573	3353.257691	0.758994806
1.478163258	128.8544653	1074.84407	0.912929391
1.358650686	57.38762065	149.3940698	0.927346952
4.69058479	101.4038991	2600.700719	0.787287345
1.470946332	62.92077982	520.761005	0.908825263
1.68885807	102.904077	668.983274	0.883073816
2.598825321	130.5042824	1557.404396	0.831600773
1.538259949	70.44119466	420.048736	0.907001081
1.296582184	204.7247664	1155.99985	0.937595135
1.11149843	251.3063198	103.1635976	0.972125393
1.286734176	136.8081549	519.5971341	0.94347766
2.433726183	92.35451724	785.6520058	0.83205791
1.331017791	154.1338692	512.3287309	0.934713848
1.270269298	114.753226	294.5330443	0.942704117
3.465592981	137.5955934	1540.661087	0.821747982
1.952146025	69.68368149	648.6920393	0.854113046
1.87002194	94.93600312	1086.319717	0.876858664
1.120172168	259.1700664	156.655135	0.970474958
1.458653232	142.1799203	513.9179266	0.914260209
4.488123261	62.15906355	1507.558341	0.788973482
1.459152059	187.9964067	846.7600096	0.918661712
1.452791409	92.76860614	561.9246452	0.912106859
1.20760899	307.488337	229.3162259	0.952681989
2.118938981	124.5285382	791.2140732	0.857224555
1.976108284	83.9546773	1953.606146	0.865035638
2.52875207	86.52062564	1921.899823	0.838007706
1.688336321	60.33886069	1315.975361	0.88863458
1.306884547	152.8581031	919.3007763	0.936612663
1.454392337	91.25377381	1056.961106	0.918637333
1.140118831	142.5743888	77.23500031	0.96904889
1.144407682	87.6601768	61.6814508	0.963898079
2.940242806	183.4643063	4589.266478	0.834015336

log.sigma.4.5.mm.3D_glrlm_LongRunHighGrayLevelEmphasis	log.sigma.4.5.mm.3D_glrlm_RunPercentage	log.sigma.4.5.mm.3D_glrlm_LongRunLowGrayLevelEmphasis	log.sigma.4.5.mm.3D_glrlm_RunEntropy
206.7328396	0.910718411	0.017135789	4.492584641
167.9849612	0.952348536	0.034182207	4.37270774
194.1656826	0.912884217	0.031668309	4.512672029
148.4181562	0.954013378	0.070192657	4.378461308
83.64374452	0.89317385	0.074490356	4.299315989
117.0224657	0.969380134	0.046769041	4.100186488
1232.060867	0.5028352	0.092316821	4.926188823
364.9497687	0.787828897	0.0212586	4.582640997
333.9255446	0.71229787	0.041619591	4.45124541
121.0312322	0.894970414	0.029973221	4.056808114
825.0424823	0.64154184	0.019913572	4.287677587
367.1867051	0.686900799	0.050071014	4.636390738
172.5414686	0.940041831	0.035910086	4.616433229
94.55943995	0.947368421	0.073321906	4.201317064
138.5122414	0.853762542	0.036059291	4.285021665
589.368263	0.642655456	0.054447452	4.773142011
62.92283124	0.859915314	0.112892837	3.963419553
88.81999637	0.945945946	0.076820115	4.104099484
84.20899849	0.845339845	0.102505069	4.001670538
519.7893571	0.677183636	0.031776648	4.858908119
117.5124265	0.86469591	0.05746673	4.482478961
132.4127223	0.928919182	0.050272184	4.359684186
128.5799342	0.906925814	0.042255602	4.247984451
650.1845205	0.577307372	0.064215165	4.562706268
334.2328552	0.8724306	0.012859117	4.477882515
227.5028351	0.829325736	0.023393202	4.559182612
187.6629015	0.873105595	0.023230489	4.290027213
213.6878659	0.801591814	0.031657078	4.389251108
24.82948288	0.906593407	0.210433746	2.900957712
184.220894	0.838936042	0.026195035	4.390680982
216.4299895	0.794229126	0.03969104	4.466795736
150.0495359	0.93453997	0.043348124	4.30259948
175.9467692	0.867203945	0.02839165	4.422330335
378.703347	0.716686616	0.034318675	4.640958962
169.4297338	0.823410986	0.03516889	4.335766187
231.0343772	0.869408074	0.029502657	4.595813591
194.4687679	0.953254438	0.019980238	4.424783753
782.7635154	0.621957613	0.037473381	4.807723403
82.29648025	0.916438891	0.100856997	4.078061022
1797.605567	0.626827193	0.012781023	4.802050922
122.4268857	0.873217116	0.033307954	4.221411103
498.0916148	0.60101402	0.054359731	4.409501992
216.0594421	0.885899955	0.030585611	4.648354792
123.4127664	0.883690567	0.038938945	4.356176331
244.8848452	0.765907805	0.040829999	4.531181455
210.6083614	0.83364155	0.026556743	4.506280005
529.4681088	0.657284458	0.038428839	4.71545671
107.1994023	0.886547243	0.085491729	4.129082947
111.7943686	0.940620783	0.049083124	4.24465182
176.6265941	0.941958042	0.025035482	4.27915599
383.3258841	0.754136734	0.033156232	4.541311802
254.9714429	0.860227313	0.02204792	4.589785233
703.2412288	0.523826203	0.092149133	4.725584202
180.276098	0.873399328	0.020270494	4.205118613
162.9857975	0.932189761	0.054960343	4.57333088
101.6448965	0.88908226	0.054933417	4.368210031
375.7097413	0.714073708	0.037102895	4.654696227
114.9408715	0.849390092	0.065838476	4.30008114
111.6945861	0.908371041	0.049127922	4.154597744
1230.812396	0.525136667	0.04679266	4.828047397
138.2673372	0.884181064	0.035672367	4.374875671
329.366888	0.883487691	0.012262279	4.76508671
365.4550217	0.881132647	0.010388601	4.749973202
710.8580073	0.713308053	0.018615344	4.741480811
100.6533946	0.894080997	0.061453351	4.089954398
295.0146379	0.899383911	0.013114822	4.73090528
110.2083569	0.948287007	0.064202572	4.039676854
408.254361	0.807226446	0.012705534	4.525570941
533.0333725	0.620296395	0.051681669	4.754328744
191.2946285	0.880711299	0.021127033	4.51615132
73.21528701	0.905205905	0.081455081	3.983750252
885.8503229	0.63094477	0.038382228	4.801931541
92.96476253	0.879018178	0.048077602	4.112693993
189.3528633	0.841852463	0.026064586	4.436356726
464.957427	0.742561232	0.019817922	4.554583219
109.2457582	0.875573102	0.044725564	4.166696296
259.232251	0.917055953	0.022062859	5.002020258
285.4051189	0.964548495	0.020750627	4.458804112
169.1198073	0.922486116	0.025879842	4.513823954
301.8280203	0.753470214	0.027140363	4.372539449
206.7328396	0.910718411	0.017135789	4.492584641
133.8048769	0.924119241	0.047998556	4.512688978
739.6106276	0.700432102	0.022618219	4.672480083
173.7446226	0.800851064	0.042607086	4.27953707
208.3112926	0.822629448	0.033030663	4.450643434
294.091309	0.961975524	0.018308598	4.586884654
221.9173753	0.883395225	0.019973476	4.485178732
474.2562124	0.636876159	0.07171369	4.693060427
266.5596182	0.88700491	0.018388629	4.833144407
124.8797047	0.882211538	0.047366278	4.509708026
379.7581096	0.938684504	0.013555858	4.562393529
325.8594796	0.791984938	0.020689173	4.407316861
190.9295017	0.805826552	0.038579648	4.473299999
294.7968405	0.75222546	0.035032091	4.557093393
103.5270812	0.845595624	0.052023215	4.138931301
197.5385827	0.91502079	0.019929258	4.560931206
127.6479361	0.887582043	0.037842814	4.397665363
164.3288835	0.959328028	0.03465097	4.087178826
99.52162588	0.954496208	0.049449198	3.894964553
699.8666075	0.728486528	0.015642578	4.916209563

log.sigma.4.5.mm.3D_glrlm_HighGrayLevelRunEmphasis	log.sigma.4.5.mm.3D_glrlm_RunLengthNonUniformityNormalized	log.sigma.4.5.mm.3D_glszm_GrayLevelVariance
162.6696793	0.843751364	23.63350427
153.5194732	0.905265812	29.22436082
151.7480158	0.852761774	21.1230102
134.1845282	0.910134668	29.71124764
69.27164457	0.810924808	15.75534261
110.5543051	0.941598924	17.43415249
96.5320463	0.485535683	14.28439631
140.0016487	0.701398974	17.93731463
88.95519573	0.616997736	12.4404132
93.30689168	0.82241459	12.88628903
200.9353526	0.524823543	9.485805965
90.81684539	0.597634609	14.84072314
154.9317521	0.888361269	29.2653875
86.69509693	0.900731457	16.66753172
84.74802722	0.757046375	14.84809562
104.205005	0.579592373	11.84383963
44.05236676	0.758472609	9.081298477
80.77654467	0.89237245	18.48
50.85299077	0.741527681	6.982177734
139.0806516	0.592638207	20.37813315
83.57173766	0.77246277	15.50909745
116.2630112	0.861239829	19.56686391
99.14894242	0.830969806	14.1310232
92.26865663	0.512000849	9.394709141
201.2148277	0.791199367	19.94490953
130.5755939	0.734253324	20.30204029
127.4781663	0.784430984	15.02142659
98.55460463	0.692898426	13.84610649
20.78824835	0.829763199	5.84
112.21858	0.738725144	12.9289021
90.40273904	0.695308035	13.29225318
129.4037088	0.875811321	18.60714286
115.248905	0.776950104	18.38228416
110.5067017	0.632143498	12.28749857
88.80404937	0.727165254	13.07444053
142.9640677	0.78480397	17.90471508
174.1015003	0.91065552	20.67602979
134.4948923	0.548254886	13.78057882
66.98257756	0.846834203	12.02497399
358.9871584	0.555826983	20.71144117
88.23870398	0.780900544	14.54384451
99.489225	0.503431605	11.0316263
150.7778828	0.81192954	22.23247368
92.53317806	0.800980597	19.64623278
97.46897803	0.656128547	16.13270399
115.1079832	0.740673974	17.29998959
113.3564995	0.570605742	12.61613433
75.89894331	0.800290408	11.14955786
98.62880223	0.889423138	21.24872449
148.6437016	0.888093725	21.56720222
117.9237592	0.672902241	13.01775148
168.056889	0.773453408	25.97090236
80.331983	0.443127586	11.74542902
120.3114251	0.781986461	11.92171225
141.0683735	0.874613653	24.46969262
80.33692995	0.802663039	19.42986187
107.9805851	0.620899255	14.75631968
66.98336939	0.75235357	10.18019341
88.97091936	0.836038575	11.76573129
152.6294552	0.446129132	16.42680692
100.8365795	0.802655833	15.50869072
229.040493	0.808394037	29.14956987
248.0133441	0.807105719	25.19158515
192.4784942	0.640850802	20.54968508
77.1971929	0.818152218	11.67184746
224.9318369	0.825156439	26.54457456
97.18413606	0.899309019	13.22249911
200.0076904	0.711003721	20.90339642
98.73540484	0.535328918	14.20358249
138.4506702	0.797808902	18.8936729
60.07102085	0.829550529	11.31217482
139.3418447	0.578383042	12.38315832
67.92414506	0.789269999	12.40367551
115.3388915	0.738707013	20.31839844
160.368953	0.649202498	17.99144044
76.27481522	0.788633748	11.00681761
214.5344089	0.849619569	54.09575326
258.1260796	0.929292185	34.7680998
142.2537702	0.863725618	18.91469428
112.4827133	0.64957104	15.91248799
162.6696793	0.843751364	23.63350427
118.3664343	0.862006692	17.75620891
174.104555	0.636534122	17.40838786
82.76695455	0.686530257	11.29594663
107.8912016	0.728539655	15.09676417
266.0842812	0.924883617	33.50110947
154.843214	0.800881225	17.70625591
82.64266745	0.58054112	14.71501617
200.5016969	0.810395655	44.05891627
98.21006804	0.795436869	17.33523698
320.6345269	0.883813405	32.8096
145.7751338	0.693953295	16.41989533
96.29996255	0.70617869	13.69501214
104.7354006	0.659748829	16.33688769
66.44273476	0.750315181	11.29604029
160.4305878	0.847651588	24.6719946
96.9295344	0.810481055	17.0397729
146.068248	0.923299536	18.97526042
90.03246662	0.90933507	13.49159971
222.31903	0.653458885	20.33492251

log.sigma.4.5.mm.3D_glszm_SmallAreaHighGrayLevelEmphasis	log.sigma.4.5.mm.3D_glszm_GrayLevelNonUniformityNormalized	log.sigma.4.5.mm.3D_glszm_SizeZoneNonUniformityNormalized
134.9587592	0.059040105	0.345851414
148.2027873	0.060614766	0.435794312
117.5916903	0.062040816	0.300816327
118.9600317	0.052930057	0.331758034
89.25022668	0.077982551	0.369424566
109.2149919	0.072043688	0.49968494
82.51893135	0.074328423	0.345133951
110.2702878	0.065847547	0.358375174
84.29275737	0.087962963	0.352481733
93.00021443	0.082737143	0.384058581
102.0753512	0.097831683	0.271823853
84.12760185	0.077438017	0.330785124
158.0240538	0.052917996	0.395477576
86.15744173	0.067666282	0.486543637
81.7419145	0.076869669	0.350922436
81.40875486	0.08432813	0.341932229
54.35333797	0.107153117	0.311117495
85.79670835	0.0752	0.3632
45.12039689	0.114257813	0.277832031
117.883891	0.066235	0.349573917
92.32419401	0.074138207	0.369865203
108.8903293	0.068402367	0.338224852
75.28717048	0.077037478	0.274241523
67.1124498	0.10166205	0.342936288
126.6600314	0.069788974	0.280274681
127.5139997	0.064275568	0.355840005
110.6469281	0.079650518	0.382673817
71.48134801	0.077459731	0.309462741
19.40771605	0.14	0.32
99.57809885	0.082991456	0.38138432
84.23130885	0.080181302	0.391490312
103.8039305	0.067442602	0.36846301
109.7465666	0.06817632	0.349752423
82.33088171	0.085511974	0.382910718
84.38612164	0.079385426	0.333155299
120.6270566	0.067258423	0.390714766
145.4178231	0.063719483	0.402205674
88.97181982	0.080106578	0.33645127
62.51074028	0.083766909	0.361082206
253.7227979	0.06777958	0.332807008
88.9249104	0.076101249	0.354125575
75.0727065	0.095155709	0.297041522
149.4820522	0.059570525	0.423187667
103.688313	0.066856989	0.33405347
87.23964619	0.082465278	0.336588542
109.2557183	0.070551509	0.342434964
82.21178086	0.08077263	0.337679586
45.58912989	0.101114956	0.229527105
86.51833407	0.066964286	0.37627551
109.9290824	0.061717452	0.361108033
54.05140581	0.07860369	0.26326109
174.2608002	0.064595059	0.387998415
67.70825598	0.091053387	0.316489118
64.18787915	0.093745541	0.272294887
135.3685403	0.059465671	0.40405056
90.76945206	0.068914901	0.347209154
88.25332283	0.075211457	0.373426086
66.40879605	0.089909232	0.38446156
73.88771631	0.089569161	0.305555556
119.9427763	0.077126378	0.3436325
104.7469853	0.07533409	0.385841558
219.8308847	0.054075605	0.415736394
205.3710748	0.057110159	0.359175413
155.1255089	0.065590607	0.354210676
62.53899614	0.087646211	0.28889601
170.1356951	0.056776415	0.323252503
79.25753145	0.081523674	0.33001068
183.5603817	0.06432648	0.415391694
85.84824765	0.076098042	0.311933502
131.8338037	0.067450086	0.337672172
62.81601083	0.095213319	0.421436004
88.69539442	0.083321556	0.33931398
75.44696341	0.085897032	0.374712851
83.06311585	0.066015625	0.265546875
114.5031989	0.067756233	0.320263158
63.8685891	0.097214039	0.322447605
193.923731	0.046073539	0.349786559
179.2373549	0.05368679	0.456003564
131.7886324	0.065928848	0.347432245
87.24106481	0.079654022	0.295494375
134.9587592	0.059040105	0.345851414
118.997936	0.068480643	0.39490504
124.4152715	0.072241225	0.318709102
60.6607756	0.089829838	0.269150319
96.37686577	0.072204801	0.373837587
202.2388462	0.054733728	0.44637574
117.5780817	0.068761815	0.389532136
83.2057967	0.073746664	0.378512545
171.8112396	0.048714022	0.342079186
99.62907184	0.072310406	0.320455754
249.4883247	0.054848	0.398912
114.230612	0.077771918	0.288084709
94.94135678	0.080792342	0.378970748
102.2069783	0.073079137	0.34279488
55.57211702	0.085595541	0.289013727
155.1285165	0.057725209	0.396333197
102.1837328	0.070586978	0.357574555
121.7875696	0.075520833	0.436631944
81.37790556	0.084002922	0.427319211
167.4292776	0.063303531	0.351699387

log.sigma.4.5.mm.3D_glszm_SizeZoneNonUniformity	log.sigma.4.5.mm.3D_glszm_GrayLevelNonUniformity	log.sigma.4.5.mm.3D_glszm_LargeAreaEmphasis	log.sigma.4.5.mm.3D_glszm_ZoneVariance
67.44102564	11.51282051	52.86153846	41.19668639
25.71186441	3.576271186	8.186440678	4.518241885
42.11428571	8.685714286	44.39285714	32.49035714
15.26086957	2.434782609	5.956521739	1.956521739
74.62376238	15.75247525	123.1584158	109.5196549
34.47826087	4.971014493	2.913043478	0.684730099
280.2487685	60.3546798	61271.52709	60868.26926
204.6322242	37.59894921	2442.287215	2364.192387
88.82539683	22.16666667	6454.940476	6173.313476
41.86238532	9.018348624	52.49541284	41.34348961
78.55709343	28.2733564	17805.03806	17447.57951
72.77272727	17.03636364	4033.213636	3838.103533
57.73972603	7.726027397	12.65068493	7.51083693
49.62745098	6.901960784	7.549019608	4.079200308
49.83098592	10.91549296	306.1830986	264.2073001
203.1077441	50.09090909	14586.15993	14380.08762
18.3559322	6.322033898	245.5932203	190.9836254
18.16	3.76	8.86	3.9316
17.78125	7.3125	367.359375	288.8708496
332.444795	62.98948475	14654.80021	14497.5101
101.3430657	20.31386861	349.2919708	321.2097075
21.98461538	4.446153846	11.2	5.291360947
22.48780488	6.317073171	30.45121951	18.70806068
65.15789474	19.31578947	24075.10526	23435.81396
45.1242236	11.23602484	101.4161491	81.08221133
125.2556818	22.625	589.6789773	556.5837987
79.59615385	16.56730769	178.2163462	157.8363304
52.91812865	13.24561404	1224.918129	1119.105024
3.2	1.4	14.4	6.56
62.16564417	13.52760736	476.4478528	426.5851933
138.5875706	28.38418079	2071.477401	1973.109108
41.26785714	7.553571429	10.6875	5.503746811
87.08835341	16.97590361	210.6385542	184.2544475
94.57894737	21.12145749	3619.493927	3456.957236
157.9156118	37.62869198	946.0907173	892.2819927
108.618705	18.69784173	120.9280576	104.4643134
82.85436893	13.12621359	6.40776699	2.823546046
249.3103914	59.35897436	24287.50607	24036.57653
22.38709677	5.193548387	26.24193548	16.14594173
238.9554318	48.66573816	27116.88579	26832.22592
55.24358974	11.87179487	125.7115385	109.3505506
100.9941176	32.35294118	27231.13824	26778.07449
169.6982544	23.88778055	134.5610973	120.0603852
53.7826087	10.76397516	147.4223602	128.1388064
32.3125	7.916666667	1077.9375	949.4930556
106.1548387	21.87096774	553.9258065	514.1544745
150.2674157	35.94382022	10568.89663	10323.63991
11.70588235	5.156862745	47.31372549	28.87427912
21.07142857	3.75	6.714285714	2.570153061
34.30526316	5.863157895	10.08421053	4.72132964
44.49112426	13.28402367	1549.100592	1421.904905
168.7793103	28.09885057	452.5034483	427.8699115
114.5690608	32.96132597	60188.71823	59409.96908
39.48275862	13.59310345	188.7655172	154.643805
47.6779661	7.016949153	14.60169492	8.686081586
56.59509202	11.23312883	118.9386503	102.3942188
152.3578431	30.68627451	5583.973039	5416.054636
97.2687747	22.74703557	349.8221344	312.530113
25.66666667	7.523809524	29.92857143	19.44331066
292.4312573	65.63454759	131636.9436	130873.5351
82.57009346	16.12149533	91.9953271	78.12492357
175.4407583	22.81990521	146.2819905	131.8527942
440.7082315	70.07416463	194.9437653	181.0594867
359.1696252	66.50887574	5170.635108	5100.03534
22.82278481	6.924050633	62.16455696	45.65422208
194.5980066	34.17940199	132.6162791	119.4425144
17.49056604	4.320754717	8.283018868	3.241723033
272.4969512	42.19817073	1470.140244	1430.542385
142.5536105	34.77680525	28340.08534	27859.06542
109.0681115	21.78637771	212.9504644	190.6006959
26.12903226	5.903225806	36.29032258	26.09157128
180.5150376	44.32706767	10904.42669	10726.36476
55.83221477	12.79865772	239.738255	214.469078
42.4875	10.5625	436.60625	391.6324609
121.7	25.74736842	2107.973684	2036.348643
35.14678899	10.59633028	166.1651376	135.4593048
164.7494692	21.70063694	55.23142251	45.3309803
30.55223881	3.597014925	4.492537313	1.54644687
81.2991453	15.42735043	28.54273504	20.80292571
39.30075188	10.59398496	1788.571429	1644.571429
67.44102564	11.51282051	52.86153846	41.19668639
58.44594595	10.13513514	20.88513514	14.66887327
104.5365854	23.69512195	3381.219512	3272.627863
35.79699248	11.94736842	772.6390977	694.5891797
101.6838235	19.63970588	510.2242647	466.1873784
46.42307692	5.692307692	4.25	1.386094675
71.67391304	12.65217391	95.82065217	80.29533908
98.03474903	19.1003861	8778.138996	8531.685365
101.9395973	14.51677852	134.2516779	118.7047993
60.56613757	13.66666667	119.5449735	101.6283419
49.864	6.856	10.768	5.892736
45.22929936	12.21019108	947.8089172	864.848148
182.6639004	38.94190871	1237.634855	1187.230747
130.947644	27.91623037	2786.884817	2685.09679
97.39762611	28.84569733	546.3590504	508.738846
162.892944	23.72506083	52.52068127	44.22179599
129.441989	25.55248619	179.7541436	163.3762477
20.95833333	3.625	5.354166667	2.069010417
15.81081081	3.108108108	5.810810811	2.128560993
400.5856014	72.10272169	5151.799824	5080.701911

log.sigma.4.5.mm.3D_glszm_ZonePercentage	log.sigma.4.5.mm.3D_glszm_LargeAreaLowGrayLevelEmphasis	log.sigma.4.5.mm.3D_glszm_LargeAreaHighGrayLevelEmphasis	log.sigma.4.5.mm.3D_glszm_HighGrayLevelZoneEmphasis
0.292792793	0.592445013	6312.066667	206.7846154
0.522123894	0.203867397	867.7627119	187.8135593
0.289855072	0.871683672	4696.964286	196.3071429
0.5	0.361881108	642.173913	164.9782609
0.27077748	6.409904593	3377.762376	121.8861386
0.669902913	0.137536378	251.0434783	129.057971
0.049797621	482.0322428	8141695.042	121.9889163
0.113158938	16.44659114	420678.2487	160.5954466
0.059588555	73.10344027	704112.2103	122.8571429
0.299450549	0.997966951	3387.12844	127.5137615
0.052891654	83.72416502	3836959.644	195.916955
0.071591279	44.70173241	451624.3773	125.3772727
0.441087613	0.553034127	1036.013699	208.5684932
0.536842105	0.566827668	358.372549	109.1666667
0.154347826	6.191672218	22954.80986	116.1197183
0.069661077	123.9162424	2047047.591	120.543771
0.135321101	16.19878349	8308.440678	79.18644068
0.45045045	0.742206723	476.84	110.64
0.11287478	15.97197987	18839.84375	71.734375
0.079735055	106.1596392	2161109.707	184.06204
0.188705234	12.40448463	15849.62774	139.0109489
0.411392405	0.539200456	805.1538462	156.2769231
0.291814947	0.863801901	2712.463415	121.3292683
0.039550375	217.6247052	2803412.647	109.2894737
0.221763085	0.583429068	23187.77019	211.1055901
0.17382716	6.345513184	65066.76989	191.9715909
0.221512247	1.890400037	20168.35096	156.3798077
0.097214326	14.13606086	132208.4561	108.994152
0.357142857	2.418491512	214.9	27
0.141615986	6.337104361	45633.42945	149.3742331
0.100825976	28.20477716	215584.7966	114.9943503
0.439215686	0.391018792	1110.205357	150.8571429
0.194683346	2.658382865	23943.81124	155.0562249
0.078437599	33.50288101	476654.3968	121.3927126
0.136324418	13.50599144	83531.26793	124.3902954
0.246453901	1.403610017	19183.02518	166.6043165
0.528205128	0.11139964	840.3009709	202.0097087
0.063128301	143.1041966	4383683.645	137.0175439
0.314720812	1.884414237	1316.33871	87.32258065
0.059270266	70.49085917	10573149.89	404.1183844
0.247226624	2.083350729	8840.173077	125.4679487
0.046980793	277.102118	2748628.291	127.4176471
0.262606418	1.891250322	16827.07731	200.032419
0.227722772	3.103537217	8949.372671	146.4534161
0.088235294	13.07982315	119620.1563	128.8020833
0.158567775	6.596724912	61416.5871	161.3774194
0.063854212	83.09118797	1471903.613	125.1191011
0.232876712	2.982697887	3353.764706	84.23529412
0.49122807	0.310145897	454.5178571	120.5357143
0.431818182	0.149457321	1532.042105	157.8421053
0.088667366	12.09787803	253015.7515	96.81065089
0.201482168	3.884352998	59914.18621	238.7954023
0.035834488	633.8209526	5993979.738	103.6298343
0.171192444	2.243847832	20547.48276	126.337931
0.411149826	0.890008508	1224.635593	181.3559322
0.245852187	3.872959671	5156.03681	132.8588957
0.077170418	49.99476802	708640.5882	128.2205882
0.163754045	12.47257403	21441.3834	93.83399209
0.308823529	0.942536108	1933.97619	115.3690476
0.036192744	780.6667105	22571682.4	183.1128085
0.268506901	1.988829501	6586.130841	143.1775701
0.263256394	1.106914092	26625.01185	298.0473934
0.268372703	0.925801854	47199.51345	306.0749796
0.119014085	24.94853235	1169512.107	230.4852071
0.246105919	1.92628062	3565.291139	104.7088608
0.275514874	0.994380039	21116.94684	269.5315615
0.445378151	0.458252595	629.0377358	116.2641509
0.158914729	7.797073133	294947.0229	252.0731707
0.045595131	266.5157386	3397230.035	138.3041575
0.211525868	3.117501409	19402.91331	200.6842105
0.313131313	2.335730374	1366.5	84.38709677
0.074940132	59.74050025	2218904.011	138.0169173
0.198931909	6.151128547	11422.33557	105.4161074
0.149114632	5.139217916	44994.99375	154.44375
0.118159204	11.69160114	401208.7105	177.9473684
0.180463576	4.567907279	8814.275229	105.5779817
0.317813765	0.974079328	5752.025478	295.4989384
0.582608696	0.057286805	1064.522388	262.4626866
0.359447005	0.687976648	2148.777778	189.5854701
0.083333333	16.16521687	227722.3008	143.7894737
0.292792793	0.592445013	6312.066667	206.7846154
0.401084011	1.125939582	963.0743243	166.6621622
0.095962551	15.66966555	796122.0366	199.9878049
0.113191489	9.775914108	72428.54887	95.30075188
0.150692521	6.27534115	60965.54044	138.2904412
0.590909091	0.063102984	967.4807692	285.2115385
0.253793103	0.928061755	13494.01087	173.548913
0.063698967	95.99033321	971279.5367	117.984556
0.253617021	1.324446138	17458.10067	276.1610738
0.23625	4.593695827	5076.671958	156.5502646
0.452898551	0.083851922	3157.832	352.504
0.10979021	6.75483386	151056.1083	183.2738854
0.140853302	16.97974672	112226.5456	135.7012448
0.0991178	28.90971686	324221.9686	151.6753927
0.16303822	13.1921938	26455.31751	90.06824926
0.347128378	0.595395681	5961.583942	212.3819951
0.247098976	4.206891887	9943.389503	146.320442
0.551724138	0.123574397	676.2708333	161.4791667
0.521126761	0.2032615	464.5675676	104.5135135
0.118596418	22.28873978	1262888.637	262.6988586

log.sigma.4.5.mm.3D_glszm_SmallAreaEmphasis	log.sigma.4.5.mm.3D_glszm_LowGrayLevelZoneEmphasis	log.sigma.4.5.mm.3D_glszm_ZoneEntropy	log.sigma.4.5.mm.3D_glszm_SmallAreaLowGrayLevelEmphasis
0.610116814	0.015849228	5.904630971	0.010547019
0.679910258	0.033115044	5.085701524	0.026362787
0.558010426	0.018396241	5.740755811	0.005538196
0.555140399	0.042884841	5.045301086	0.01033755
0.629911073	0.029695721	5.453801736	0.012334476
0.732161836	0.032327353	4.859527218	0.013063159
0.60822287	0.021704444	5.984885191	0.010409293
0.621775191	0.015249481	6.061867309	0.00809925
0.615461069	0.024336648	5.546321798	0.014470768
0.64352552	0.024885027	5.111830256	0.016314416
0.53112239	0.010184526	5.778609444	0.007347011
0.593876553	0.02689857	5.639758835	0.012312826
0.652068701	0.02025618	5.599502622	0.012503398
0.722427085	0.042769708	5.069737848	0.025408453
0.613514491	0.028993001	5.503945338	0.017680037
0.605702959	0.022296689	5.780032213	0.012339376
0.571902385	0.039957205	4.880619058	0.00866577
0.617357855	0.041858935	4.79656563	0.012090502
0.532373344	0.039476679	4.929632915	0.008792719
0.611429226	0.011619636	6.157017095	0.006631652
0.631166423	0.02175186	5.709963932	0.010506465
0.599402787	0.031351551	4.984153298	0.020434851
0.529361047	0.029736762	5.373758901	0.01752641
0.603440849	0.02302988	5.260317202	0.011378081
0.545643203	0.015574235	5.83458231	0.011632636
0.6128689	0.013595929	5.869982833	0.006516003
0.642678319	0.016358903	5.474134918	0.006704786
0.574760938	0.031981366	5.639411978	0.01511981
0.551234568	0.167047068	3.321928095	0.11788966
0.641562144	0.018651086	5.279175173	0.012217066
0.649809836	0.021527838	5.574920421	0.009110158
0.626556428	0.025730586	5.373540802	0.010199033
0.613777412	0.018528348	5.865114499	0.011169504
0.641664891	0.020382438	5.515166596	0.010044498
0.597946438	0.019694083	5.825802024	0.010318551
0.649180652	0.020490976	5.654753257	0.010010903
0.65550918	0.015699991	5.525922191	0.009773744
0.601478354	0.017159804	6.024418173	0.009400531
0.616147188	0.043604672	4.957834415	0.012279847
0.597225465	0.004825074	6.229958181	0.003413018
0.614493761	0.024876132	5.43637942	0.014255747
0.56383418	0.017679385	5.823423694	0.011000346
0.676300777	0.013096184	5.873440979	0.00590097
0.59799555	0.024626761	5.594693395	0.007992586
0.599223947	0.030380636	5.146888413	0.010594427
0.603785647	0.016514852	5.830532375	0.008741309
0.601558898	0.021199017	5.787896091	0.011328769
0.484499465	0.045887034	4.914990234	0.028280829
0.629800195	0.039478241	4.98817094	0.027327839
0.621325744	0.02875069	5.460380972	0.020902425
0.528763714	0.030727735	5.749975808	0.010760812
0.647529638	0.013971992	5.985999787	0.004959429
0.582414687	0.02388954	5.817050183	0.009337293
0.538074265	0.022557512	5.621672065	0.017113037
0.660745664	0.021484807	5.404533757	0.006874118
0.606588675	0.031320965	5.551868032	0.010022815
0.635267159	0.025540639	5.784544657	0.014879704
0.644151355	0.026794554	5.341077078	0.011714759
0.565000377	0.028995121	5.189513108	0.008385354
0.609025319	0.011680643	6.142577879	0.006729717
0.643904952	0.024174439	5.469269716	0.012332401
0.67028276	0.009041778	6.03080898	0.005858921
0.621930765	0.007622462	6.375946222	0.004591951
0.615596086	0.009625497	6.152021194	0.004812298
0.542529865	0.034206683	5.218355356	0.00924345
0.585051062	0.009334984	6.348555867	0.004053351
0.587819706	0.037799336	4.864251822	0.008888835
0.67001423	0.008930157	5.897324421	0.00577556
0.576515497	0.017175737	5.969387264	0.008083517
0.60176269	0.012918655	5.920197429	0.007630169
0.6712078	0.041463568	4.588534464	0.029402814
0.600786492	0.019725591	5.805397785	0.007844196
0.63504196	0.029720381	5.248657969	0.011666339
0.521424515	0.02146661	5.927890051	0.012844565
0.583983587	0.014157809	6.047502742	0.006728378
0.587116292	0.029263408	5.177447891	0.012056063
0.61074146	0.012478755	6.499161556	0.007216949
0.700559701	0.027994532	5.166616616	0.026059015
0.609136281	0.014844143	5.751326845	0.008834262
0.55548634	0.02099472	5.598599828	0.014097274
0.610116814	0.015849228	5.904630971	0.010547019
0.650659013	0.01897374	5.285147475	0.00752654
0.58443506	0.011755745	5.979372839	0.005126477
0.532563625	0.036830579	5.414252901	0.009764386
0.634577299	0.017854082	5.660856147	0.008038183
0.691124466	0.019829942	5.355118535	0.016895321
0.648402703	0.017966069	5.590141786	0.012273585
0.638168515	0.027735382	5.597742172	0.009531805
0.600083168	0.014135205	6.220165182	0.009478526
0.584229121	0.019043895	5.76786462	0.008109882
0.656141311	0.015551458	5.419868609	0.01251912
0.551235627	0.018477584	5.707686377	0.008428581
0.639279611	0.017741329	5.717562451	0.008260249
0.60431117	0.01903415	5.900569726	0.007672193
0.554709433	0.039013748	5.889052663	0.019827428
0.65422886	0.016196537	5.941893411	0.00919784
0.619473411	0.02093792	5.797057136	0.010009888
0.685422099	0.037246141	4.629247855	0.027392311
0.67149001	0.049254811	4.317033095	0.035328851
0.614748013	0.007198019	6.227948675	0.004360886

log.sigma.4.5.mm.3D_ngtdm_Coarseness	log.sigma.4.5.mm.3D_ngtdm_Complexity	log.sigma.4.5.mm.3D_ngtdm_Strength	log.sigma.4.5.mm.3D_ngtdm_Busyness	log.sigma.4.5.mm.3D_ngtdm_Contrast
0.014678556	0.691941954	3.494433935	0.22400106	0.000141655
0.059514511	5.288725791	11.16197486	0.072259728	0.00229211
0.019105359	0.750924142	2.662285506	0.265583202	0.000369865
0.073562304	5.56255107	10.33609144	0.077200034	0.003568144
0.011585934	0.442301589	2.212683948	0.680897808	0.000154073
0.072796769	3.184411937	9.313406003	0.085751392	0.001941202
0.000940238	0.007335372	0.236525877	2.97855179	8.77E-07
0.002656039	0.039588686	0.441596341	1.224664174	9.28E-06
0.003189832	0.036157757	0.601333385	1.169543007	6.21E-06
0.024854833	0.506533362	2.371935147	0.25731875	0.000241155
0.002161476	0.01804059	0.5182597	1.131277045	1.96E-06
0.004724984	0.053149975	1.00644078	0.731770282	8.28E-06
0.025840737	1.570296569	4.563355879	0.218334223	0.000819386
0.036477936	1.674686488	4.052206245	0.25678505	0.001307
0.011791161	0.246655588	1.960385226	0.427101155	5.91E-05
0.001557199	0.015833425	0.259777401	2.290784497	4.47E-06
0.021444625	0.290207967	2.354353251	0.467776446	0.000148832
0.058227003	2.872388496	8.523130689	0.147138934	0.002066555
0.019470922	0.103310658	1.03826149	0.636816548	0.000140447
0.001211167	0.03092668	0.725182756	1.543544846	1.22E-06
0.0071457	0.177859439	1.168054957	0.978744585	6.72E-05
0.049731273	1.766499764	5.62203579	0.170006172	0.001660683
0.032412782	0.684844012	3.327443402	0.21401746	0.000505958
0.002911829	0.015331357	0.459109849	1.290361233	3.23E-06
0.014853807	0.399302065	2.419971334	0.223626975	0.000135171
0.005949338	0.177415637	1.53562883	0.50957095	2.35E-05
0.009977349	0.274976774	1.719979151	0.402668005	7.40E-05
0.007513764	0.073945517	0.955314762	0.650794239	2.43E-05
0.1282677	1.491166896	4.707354776	0.302345513	0.007764991
0.009666374	0.143394095	1.170090025	0.554506802	5.63E-05
0.003731757	0.053618324	0.650817538	1.180380816	1.28E-05
0.027735061	1.572684063	3.648143798	0.191688322	0.000809981
0.0095608	0.203455872	1.760148925	0.399725727	4.45E-05
0.004548255	0.036887122	0.598322313	0.93895738	1.30E-05
0.00333743	0.057709967	0.557002094	1.333655529	1.22E-05
0.009183951	0.229675013	1.285430115	0.466886709	0.000108627
0.018247353	1.404198491	3.437704748	0.201145939	0.000501401
0.001191554	0.01399195	0.302016113	2.057106399	1.98E-06
0.045742261	0.60439005	3.314306127	0.297755767	0.000868286
0.001255672	0.025317448	0.801812234	0.821473883	6.84E-07
0.015005802	0.327728944	1.650598383	0.396417668	0.000127107
0.001806469	0.010410522	0.283764187	2.11038613	1.32E-06
0.006550137	0.31247157	1.474585974	0.496031544	6.63E-05
0.014441059	0.456640587	3.108836011	0.33841366	0.000102877
0.014183646	0.107621566	1.718594872	0.328756355	4.06E-05
0.005870246	0.140020854	1.08751196	0.642945445	2.89E-05
0.002183662	0.015117616	0.308006152	1.733525498	4.05E-06
0.047406127	0.465663335	3.666639332	0.22919508	0.000692628
0.077570829	2.712763161	12.06154278	0.095306396	0.001489883
0.033027067	1.636919869	4.753538977	0.131022877	0.000834342
0.008502498	0.043819424	0.926559186	0.580328795	3.15E-05
0.00570712	0.260023035	2.448978707	0.362565471	1.95E-05
0.001320658	0.010179517	0.37593154	2.439629719	9.49E-07
0.013426265	0.168589117	1.451875096	0.420809944	7.80E-05
0.029762169	1.578685472	5.274614026	0.185945128	0.000905721
0.014598828	0.485576288	2.719690915	0.44842415	0.000155689
0.002815041	0.027880254	0.500579156	1.283812511	5.46E-06
0.006376834	0.09874322	0.603401672	1.449790761	6.50E-05
0.030625316	0.715231814	3.273740914	0.227731823	0.000439208
0.000570842	0.007350125	0.305993769	3.129212893	2.44E-07
0.012724737	0.370514642	2.051844161	0.376767963	0.000103904
0.007473257	0.392407806	2.516921269	0.285105011	4.70E-05
0.002498988	0.133587081	0.727309943	0.776164992	1.62E-05
0.001511058	0.038712552	0.510127577	1.190489044	3.03E-06
0.02766486	0.432573009	2.090856602	0.325273809	0.000368905
0.005364573	0.264604335	1.67661799	0.428127597	3.70E-05
0.052536426	1.820449137	4.216594133	0.169365565	0.002466401
0.002905936	0.084186051	0.819284815	0.702278332	7.62E-06
0.00152971	0.012200639	0.315337087	2.207175205	1.88E-06
0.006932495	0.231710314	1.320834254	0.548957538	5.36E-05
0.039216455	0.73344466	2.704692585	0.359915959	0.0008509
0.001880944	0.018561124	0.342807776	1.554309562	6.20E-06
0.011628705	0.222001123	1.014627992	0.723020764	0.000123355
0.010846227	0.258118256	2.637358982	0.315688011	4.08E-05
0.004045991	0.070044987	1.063979581	0.584361987	8.31E-06
0.016011917	0.275261662	1.757151259	0.467387427	0.000132371
0.008342689	0.931954194	7.044771783	0.263311629	7.71E-05
0.055058537	6.099510958	13.38714094	0.051582685	0.002666772
0.016222054	0.609140374	3.052374769	0.278792817	0.000195556
0.008418148	0.094548588	1.60671008	0.402777097	1.60E-05
0.014678556	0.691941954	3.494433935	0.22400106	0.000141655
0.026083877	0.945004092	4.072846403	0.272460037	0.000516472
0.00394453	0.072757167	1.209996802	0.505851982	7.98E-06
0.01060774	0.0624876	0.799119094	0.687437484	4.88E-05
0.006830092	0.094449458	0.887835882	0.653203288	3.27E-05
0.040576739	4.523414968	10.47054604	0.066261235	0.001592143
0.015458779	0.354755896	2.506850885	0.249409253	0.000116104
0.003541255	0.026542294	0.499167641	1.178699293	8.16E-06
0.009395667	0.813313738	6.469999202	0.223670301	7.74E-05
0.012352783	0.507576329	2.76554087	0.432276072	0.000126228
0.028848262	2.609014564	8.498124604	0.072649067	0.000740812
0.008549331	0.132787988	1.609787246	0.349655171	2.35E-05
0.003609321	0.069792171	0.777876682	1.053702845	1.06E-05
0.003436804	0.068973589	0.970320572	0.853331908	6.93E-06
0.005498588	0.058568727	0.572234918	1.361536129	2.45E-05
0.007735859	0.469426015	1.698127775	0.44892404	0.000104878
0.006876244	0.281119492	1.61677824	0.649545936	5.23E-05
0.069969028	4.000796441	9.048154451	0.070122225	0.003099672
0.083490661	3.011789308	6.888700307	0.099044318	0.003307567
0.001465709	0.036752545	0.508095357	1.183932837	3.08E-06

log.sigma.4.0.mm.3D_gldm_GrayLevelVariance	log.sigma.4.0.mm.3D_gldm_HighGrayLevelEmphasis	log.sigma.4.0.mm.3D_gldm_GrayLevelNonUniformityNormalized	log.sigma.4.0.mm.3D_gldm_DependenceEntropy
16.62228219	170.9264264	0.071337103	6.457732819
21.43582113	111.6017699	0.069934999	5.320858803
17.78605078	135.4389234	0.067671429	6.443793167
23.06427221	115.3913043	0.060727788	5.009603124
11.69843994	71.17024129	0.091318129	6.252814233
18.41662739	119	0.067772646	5.160689697
5.492391699	114.2803876	0.223070493	6.763627228
10.34573093	153.8182719	0.096762421	7.024041589
6.79380306	101.6869236	0.127340239	6.889146346
7.983297609	92.43131868	0.105678662	5.941182696
3.19240794	268.5889458	0.213497465	6.865812462
7.144197136	99.83729255	0.132124911	7.035152119
26.45222296	142.0634441	0.059209025	6.058476078
14.09864266	94.43684211	0.077063712	5.517761235
9.289034735	94.67065217	0.09106569	6.624521034
8.633794935	119.1511669	0.161769479	6.997378611
6.652949036	44.86009174	0.111312179	5.980448651
12.7895463	55.45045045	0.08789871	5.268836362
6.392921686	55.664903	0.10921369	6.241048769
7.932364943	142.7254968	0.135307603	7.371634729
12.0880613	71.0137741	0.084521018	6.706434172
15.78132511	73.75316456	0.074747637	5.541914372
11.69541926	86.41637011	0.081774547	5.859763678
4.129266887	104.1417569	0.231607057	6.683153683
14.71866676	168.3966942	0.079658341	6.565376799
11.94125871	131.0582716	0.092347142	7.012943716
10.03642876	118.0724175	0.091767124	6.528426862
8.23607656	90.50994883	0.098792824	6.847443511
5.320153061	25.89285714	0.137755102	3.780394654
8.741708377	98.19287576	0.095212036	6.754053152
9.547005988	104.0037026	0.094979112	6.96794943
16.43269512	119.4352941	0.07432526	5.818583908
10.66259007	101.202502	0.088829538	6.719297345
8.354963559	120.9075897	0.129834097	6.989888356
8.057748652	77.05234397	0.102075027	6.840414252
16.05310347	178.0283688	0.071566445	6.988544079
22.35203156	150.5641026	0.060815253	5.748214141
7.797112089	153.406628	0.173531516	7.051771891
10.8383107	67.43147208	0.087737381	5.697092036
6.048174476	456.7883441	0.172459279	7.060444011
8.22202074	82.35657686	0.10406092	6.15367592
2.758613125	120.6867487	0.212465939	6.834929238
17.03494274	156.9155206	0.069959674	6.932032989
11.03658505	99.56152758	0.089176931	6.428147252
8.213937304	86.05238971	0.117310837	6.847281261
10.74843937	122.3074169	0.088169753	6.923752486
6.648943672	125.6244798	0.135140368	7.185710253
12.82483685	65.60273973	0.080919914	5.966759756
15.40512465	70.86842105	0.079562942	5.37575726
21.66702479	171.6181818	0.063057851	5.777484489
10.17756706	161.1605456	0.123818416	6.784558067
13.91049027	169.229736	0.085694254	6.935680089
3.704371273	90.48257771	0.245523594	6.685623949
10.59106869	127.5985832	0.084703426	6.368016067
22.117957	119.2125436	0.061637266	5.994497531
12.08916461	67.87631976	0.089307936	6.380502261
7.524304577	139.0856819	0.122133727	7.172484946
10.42475718	79.30938511	0.085270577	6.692243845
9.385907764	63.61397059	0.093777033	5.930456649
3.722139292	162.2605793	0.235670404	6.776218163
11.64999236	126.5457967	0.086121261	6.515694263
19.89623635	235.2514036	0.06467949	6.967370457
19.15806372	249.4446632	0.067793021	7.093554616
9.623744614	209.6303991	0.118425742	7.230573962
9.68773595	81.27725857	0.088091148	5.892919769
21.37397672	228.2334096	0.065884829	6.890137016
12.35957913	91.55462185	0.0826919	5.123873861
9.901970284	210.5964147	0.096812372	7.037076273
5.664465016	108.8420633	0.159576116	7.037599537
13.65133941	121.6234447	0.077398789	6.658257557
8.936766656	49.88383838	0.094225079	5.66278868
10.02763833	218.0917031	0.15690845	7.025156583
8.047165691	62.42056075	0.100810872	6.150090267
9.590089366	119.6542404	0.102416425	6.661923308
7.804215764	169.7092662	0.129283232	7.058525033
9.518782071	79.86754967	0.093499189	6.324580169
44.95005109	205.9419703	0.054291625	6.859311588
31.07448015	233.2086957	0.056483932	5.497153964
17.86511122	138.0890937	0.066665723	6.35119269
6.176809112	121.8740602	0.127994642	6.845914907
16.62228219	170.9264264	0.071337103	6.457732819
19.25834857	104.3577236	0.06694281	6.063346138
9.794321598	195.6243417	0.145058806	6.995700346
6.941395383	93.48680851	0.111792123	6.695708351
9.219080271	113.067036	0.096701836	6.89209764
30.71484375	191.6875	0.052750517	5.628692469
13.45104209	161.1489655	0.078647325	6.588476398
7.028747566	94.5427939	0.177008286	6.80277513
34.42196541	195.0748936	0.068640652	6.938094009
13.77619375	99.4775	0.080265625	6.429815643
28.12271582	284.8188406	0.062539382	6.051497909
7.625001223	109.2398601	0.113190865	6.764536208
8.318005234	101.7323203	0.099922084	7.096150643
7.968516929	113.1826674	0.114083885	7.031686522
6.573544367	67.55249153	0.113337916	6.522969714
18.04634927	191.4890203	0.069470759	6.495968525
12.37088562	98.15767918	0.083566728	6.611375273
20.33637204	162.137931	0.068569164	5.136636134
13.57349732	104.2253521	0.086689149	4.788772606
11.60534084	176.1305706	0.10674035	7.42115906

log.sigma.4.0.mm.3D_gldm_DependenceNonUniformity	log.sigma.4.0.mm.3D_gldm_GrayLevelNonUniformity	log.sigma.4.0.mm.3D_gldm_SmallDependenceEmphasis
107.7897898	47.51051051	0.262668592
31.5840708	7.902654867	0.508316126
79.30227743	32.68530021	0.252026847
33.54347826	5.586956522	0.439311594
103.386059	68.1233244	0.24691818
33.50485437	6.980582524	0.532278857
664.6168282	3637.387465	0.056313333
425.6904479	488.2631788	0.122608923
266.4471506	538.5218728	0.076273636
55.49450549	38.46703297	0.259680201
265.5409956	1166.550146	0.058800929
168.3068663	406.0198503	0.08963009
67.69486405	19.59818731	0.361966942
44.30526316	14.64210526	0.409409998
103.5652174	83.78043478	0.154386891
424.3303624	1379.40835	0.081156298
56.77522936	48.53211009	0.147597979
25.12612613	9.756756757	0.351038385
67.83597884	61.92416226	0.127000595
613.7195439	1613.813784	0.08614152
165.6129477	122.7245179	0.174579426
32.59493671	11.81012658	0.336645592
49.95373665	22.97864769	0.189784722
209.0491257	1112.6403	0.050017472
95.67493113	57.83195592	0.192438608
182.702716	187.002963	0.165153562
124.6080937	86.16932907	0.203503773
155.7220011	173.7765776	0.110584714
7.142857143	3.857142857	0.398571429
114.0894874	109.589053	0.138630648
281.2876673	333.4716605	0.108003677
48.90588235	18.95294118	0.351467372
144.9702893	113.6129789	0.177119097
193.488409	408.8475707	0.090462237
326.4063848	354.9148691	0.136028545
130.4060284	80.72695035	0.22629454
113.4410256	23.71794872	0.428777066
532.600443	2036.912932	0.07225803
38.17766497	17.28426396	0.261605114
557.4579825	2089.171702	0.070803434
82.76228209	65.66244057	0.197421946
315.0588642	1537.616001	0.059194029
200.2966601	106.8284217	0.236999495
97.16973126	63.04809052	0.197493674
80.20772059	127.6341912	0.086330436
188.7319693	172.371867	0.148359921
348.8685608	941.7932271	0.069967791
33.47488584	17.72146119	0.166057225
26.35087719	9.070175439	0.337066277
59.80909091	13.87272727	0.369477273
143.9317943	235.9979014	0.093421086
238.5173692	185.0138953	0.193824978
393.8930905	2480.279351	0.04389557
125.4769776	71.74380165	0.181665903
57.51567944	17.68989547	0.339860027
88.41779789	59.21116139	0.205247871
309.2368073	645.7210138	0.090292772
172.6090615	131.7430421	0.172722373
41.11764706	25.50735294	0.240040361
911.5344278	5541.318207	0.042945835
100.6411543	68.63864492	0.239508894
212.1952589	103.6812227	0.237084157
618.0634296	309.9496938	0.257789779
534.3622066	1008.987324	0.126386508
54.57632399	28.27725857	0.230330114
345.3963387	143.9583524	0.27102047
31.55462185	9.840336134	0.392403056
360.5431202	399.6414729	0.163815783
452.8523396	1599.431408	0.057731973
200.78389	118.1879502	0.21127232
34.74747475	18.65656566	0.265329307
350.2632765	1113.893084	0.081808229
104.0173565	75.50734312	0.19453127
108.0624418	109.8928239	0.156488286
217.7419154	415.7748756	0.117675415
77.96688742	56.47350993	0.17664223
265.5236167	80.46018893	0.286955237
40.02608696	6.495652174	0.385688406
125.1167435	43.39938556	0.282604751
111.4799499	204.2794486	0.089576501
107.7897898	47.51051051	0.262668592
66.06233062	24.70189702	0.338980954
204.8291398	495.8110006	0.097769475
104.7668085	131.3557447	0.1017868
166.0304709	174.5468144	0.131479377
51.48863636	9.284090909	0.436041667
98.00413793	57.01931034	0.229610489
197.6920807	719.7156911	0.073103378
177.2553191	80.65276596	0.224184036
109.35	64.2125	0.241246874
61.66666667	17.26086957	0.371574423
121.8741259	161.8629371	0.112376617
285.1753361	341.9333723	0.143155425
263.4966269	439.6792942	0.10276342
228.2346396	234.2694727	0.156254781
210.7804054	82.25337838	0.317046031
197.8860068	122.425256	0.233818887
25.85057471	5.965517241	0.471264368
23.11267606	6.154929577	0.455007825
613.1880466	1025.134319	0.120963383

log.sigma.4.0.mm.3D_gldm_DependenceNonUniformityNormalized	log.sigma.4.0.mm.3D_gldm_DependenceVariance	log.sigma.4.0.mm.3D_gldm_LargeDependenceEmphasis
0.161846531	3.976787598	15.57357357
0.279505051	1.750489467	6.451327434
0.16418691	3.624894444	15.11180124
0.364603025	0.628544423	4.456521739
0.13858721	4.790101273	18.97855228
0.325289848	0.891507211	4.475728155
0.040759035	70.63622814	264.6357169
0.08436196	24.81129455	67.74157749
0.063004765	41.40873903	120.1350201
0.152457433	5.148864871	18.02197802
0.048598279	39.28617382	144.6039531
0.054769563	43.11785685	128.3660918
0.204516206	3.30853132	11.25377644
0.233185596	2.175623269	8.294736842
0.112570888	7.930415879	31.89782609
0.049763148	55.03245377	163.9994136
0.130218416	5.478831748	25.52293578
0.226361497	2.279198117	9.342342342
0.119640174	5.22197027	28.96825397
0.051456321	44.538484	135.4691876
0.114058504	9.580451396	31.79752066
0.206297068	3.827591732	12.30379747
0.177771305	2.427932777	14.66548043
0.043515638	59.58923073	207.7352206
0.131783652	17.40401764	39.8815427
0.090223563	15.88306514	47.90469136
0.132702975	5.19375857	21.94355698
0.08852871	15.06742713	52.68845935
0.255102041	2.530612245	9.142857143
0.099122057	10.05459839	38.98609904
0.080116111	22.08816122	67.96553688
0.191787774	2.982237601	11.29019608
0.113346591	7.584043926	29.45191556
0.061444398	40.8792349	116.7389648
0.093875866	18.39657442	52.99884958
0.115608181	9.179832503	29.14361702
0.290874425	1.140433925	5.779487179
0.045374037	55.37907897	176.2554098
0.193795254	2.199335206	11.56852792
0.046017664	51.48150918	167.3894667
0.13116051	5.397600468	22.25832013
0.043534457	46.90950193	172.2275805
0.131170046	6.851276456	23.06679764
0.137439507	5.203435438	21.3281471
0.073720331	27.22139922	84.69117647
0.096538092	15.5611452	46.54987212
0.05006006	45.34963157	148.1149376
0.15285336	3.301182211	19.19178082
0.231148046	1.714373653	8.456140351
0.271859504	1.307933884	6.809090909
0.075515107	43.13724484	107.3725079
0.110475854	9.242843759	31.17508106
0.038991595	57.03201873	232.8477529
0.148142831	3.941163269	19.48170012
0.200403064	2.768565844	10.71428571
0.133360178	5.0115545	21.19306184
0.058490034	38.17413093	115.0605258
0.111721075	14.06472324	39.05177994
0.15116782	4.000865052	17.08823529
0.038767253	53.09270411	225.2945605
0.126274974	7.589684655	24.74404015
0.132373836	6.780392659	22.81534623
0.135184477	9.759282796	26.21391076
0.062718569	47.45469478	119.7086854
0.170019701	2.736386487	14.45794393
0.158076128	4.64496667	16.5624714
0.265164889	1.340300826	6.680672269
0.087340872	19.21263371	54.12790698
0.045181317	46.33886067	166.4187369
0.131489122	6.043431291	22.57694826
0.175492297	3.142536476	13.52525253
0.049339805	61.35524851	177.8668827
0.138874975	4.706130649	20.61014686
0.10071057	12.92943025	40.92171482
0.067705819	36.64746193	97.24129353
0.129084251	5.317332573	23.28145695
0.179165733	3.334254873	13.18758435
0.34805293	0.677807183	5.069565217
0.192191618	3.878117324	13.61136713
0.069849593	29.20660988	88.17669173
0.161846531	3.976787598	15.57357357
0.179030706	4.106300629	13.63414634
0.059926606	52.17272958	136.6319485
0.089163241	13.94368493	51.64851064
0.09198364	12.593241	45.98947368
0.29254907	0.998450413	5.465909091
0.135178121	5.10075434	20.32689655
0.048620777	61.02741291	178.8027545
0.150855591	6.138506836	20.52680851
0.1366875	5.456475	20.3175
0.223429952	1.984877127	8.565217391
0.085226661	17.52882586	57.65174825
0.083335867	17.53073539	54.69725307
0.068369649	32.66560437	92.92734821
0.110418307	9.229155651	33.22351234
0.178023991	3.984657483	13.82432432
0.135075773	6.653298699	22.36518771
0.297133043	0.929845422	5.068965517
0.325530649	0.7462805	4.690140845
0.063847152	42.19772248	109.119117

log.sigma.4.0.mm.3D_gldm_LargeDependenceLowGrayLevelEmphasis	log.sigma.4.0.mm.3D_gldm_SmallDependenceHighGrayLevelEmphasis	log.sigma.4.0.mm.3D_gldm_LargeDependenceHighGrayLevelEmphasis
0.141892395	55.1348374	2504.821321
0.528000915	75.196588	385.7610619
0.367463446	41.25565986	1802.590062
0.794447407	62.87167874	382.673913
0.712373047	29.29105523	883.650134
0.136613133	75.60451456	380.2038835
2.150268005	6.353593934	35128.14044
0.407682793	18.61593609	13461.5547
1.063393135	7.997055037	16370.09979
0.331511741	32.16477675	1280.673077
0.528192616	14.85371042	40263.806
1.259711818	10.03590006	16036.13667
0.320588652	75.31383386	767.1873112
0.246272637	51.98150994	473.0368421
0.448703474	17.36248505	3602.459783
1.337105733	9.045232475	23751.37352
1.769286277	10.25112833	856.2912844
1.069440813	31.92770459	237.3063063
1.170553653	8.383424111	1579.051146
0.985956503	14.42479452	20499.47841
1.215194276	18.7486933	2319.972452
1.582462231	36.64936563	396.664557
0.542915088	21.3001673	1129.825623
1.840218752	5.13519506	24985.97939
0.236832359	32.22158826	9685.608815
0.48924342	30.54497751	6289.844444
0.306412433	28.08792925	2555.691161
0.685449736	9.439548079	6265.889142
1.593507293	9.367380952	134.5357143
0.576100666	17.16870084	3988.823632
0.713031756	11.98873957	9871.230134
0.536582149	55.98783748	874.9254902
0.492240753	21.86751533	3346.903831
0.919912609	10.51405182	16883.0759
0.898905908	13.1337291	5189.451539
0.211326345	42.53692833	6313.833333
0.203435778	81.10824929	646.174359
1.049783478	9.813989323	32170.0834
0.788437857	22.34693043	601.3502538
0.350431856	33.57398739	80945.90061
0.427125169	22.38663795	1700.277338
1.447544731	8.288840511	21098.36396
0.283074431	47.12138395	3599.942371
0.353012975	29.31021185	1850.526167
1.167900836	7.95822192	9091.607537
0.450841291	23.20943243	6979.88491
1.114796173	8.481270531	21932.03401
1.625936876	11.66000139	1302.415525
0.667098761	29.72131335	417.0614035
0.108184097	63.68296465	1119.963636
0.559831147	11.06192997	22916.17943
0.265102331	43.70584686	5092.607226
2.44763397	4.204163971	23655.53128
0.231152867	22.83451957	2537.145218
0.435611364	58.49478942	721.4355401
0.924564771	21.99049907	994.1025641
0.781016827	12.75128022	18912.10138
0.716599463	16.38677121	4520.526214
2.078403716	20.55694348	867.0772059
1.330338093	7.389523997	39115.13601
0.290397989	39.1108006	3035.343789
0.141124148	70.4271249	5230.044916
0.139714096	77.51754119	6701.072835
0.508140543	28.31556955	30323.04096
0.394341298	26.63510803	1068.037383
0.13722941	74.79964125	3106.443021
0.234692773	47.3126231	429.789916
0.254203289	40.98893724	13144.09641
1.481540978	7.193001915	20984.06146
0.367317256	36.32524584	2369.669286
1.131237433	18.90851152	486.1666667
0.710463554	14.81653805	47686.64418
0.756202179	15.87525105	1201.931909
0.416238151	23.62798696	5160.481827
0.537611074	20.84629061	19145.99596
0.62325238	17.8154809	1645.47351
0.241782565	77.9695427	1827.758435
0.057440909	91.60646135	1112.06087
0.243245226	52.22996458	1306.331797
0.676707985	11.53071074	13078.6297
0.141892395	55.1348374	2504.821321
0.582126765	52.69552086	681.0948509
0.611333765	18.93252977	33462.1182
0.602077119	9.491378724	6005.946383
0.560267989	18.89550295	5796.350139
0.225818745	95.90653567	833.2556818
0.197091478	42.26946819	3331.435862
1.818213095	7.549321581	20996.80251
0.245586999	55.31541306	2953.505532
0.538750537	35.98634954	1308.2675
0.094483927	120.2353385	2151.289855
0.723900845	14.21915215	7179.985315
0.693813449	18.47561113	6093.562244
0.818649869	13.77942579	12762.43176
0.781136889	14.10672025	1933.011611
0.124237431	77.97322643	2199.830236
0.467337497	33.3032878	1737.608874
0.096993536	84.93542465	769.1264368
0.123677197	53.15121283	427.915493
0.64529537	23.21373646	21264.74427

log.sigma.4.0.mm.3D_gldm_SmallDependenceLowGrayLevelEmphasis	log.sigma.4.0.mm.3D_gldm_LowGrayLevelEmphasis	log.sigma.4.0.mm.3D_gldzm_DistanceZoneVariabilityNormalized
0.004258336	0.011318196	0.980394079
0.017039955	0.043359193	0.91322314
0.005621513	0.02082942	0.879693878
0.01464972	0.069455968	0.961553249
0.006124386	0.033005615	0.922099786
0.019399535	0.030778742	0.970156107
0.00115543	0.0134967	0.903871304
0.001970285	0.010389176	0.926116379
0.001556033	0.015703669	0.944729466
0.007580464	0.020463248	0.94499822
0.000463153	0.004139154	0.946001604
0.002288168	0.017929299	0.959856221
0.008211317	0.022297619	0.957163408
0.012278268	0.028129323	1
0.004054475	0.019461182	0.929671266
0.001703857	0.015170131	0.859417874
0.004829544	0.058410931	1
0.021445519	0.080848224	1
0.004563365	0.041410097	0.970156107
0.001043886	0.010198052	0.922575516
0.004052665	0.040945685	0.875540657
0.008356866	0.068405465	1
0.007091043	0.032604326	0.903476013
0.001015771	0.013052712	0.969395152
0.003086797	0.013159449	0.891272189
0.001815644	0.013488215	0.681307129
0.003601676	0.016006903	0.989637587
0.002970852	0.023453467	0.978083697
0.060920091	0.12677378	1
0.002352687	0.018909454	0.962113741
0.001850894	0.017493761	0.893496094
0.008353321	0.029440287	0.963315573
0.00490921	0.023075056	0.958178099
0.001697393	0.013370019	0.951920033
0.003829402	0.0292979	0.953910011
0.003619063	0.011982633	0.924260204
0.006720299	0.02719918	1
0.001148372	0.009884699	0.842049104
0.007560599	0.054683366	1
0.000266877	0.00241985	0.933233624
0.004664421	0.024479697	0.970483404
0.000764599	0.009548656	0.9753125
0.002905011	0.015691462	0.954798078
0.004410994	0.019404135	0.933412604
0.003184555	0.028181326	0.975907198
0.001988609	0.015257157	0.947247078
0.001166702	0.011803845	0.985949181
0.006670049	0.076742721	0.909297052
0.014383916	0.061648529	1
0.010534763	0.021079143	0.826183432
0.002014245	0.010032146	0.940883403
0.002324239	0.012516864	0.952511943
0.001018692	0.015254546	0.968931049
0.003893324	0.01352796	0.818994502
0.006582342	0.031139862	1
0.007397949	0.04481226	0.900277008
0.001362206	0.0102584	0.950037858
0.004487296	0.028600099	0.845586547
0.006084091	0.061068036	1
0.000421455	0.007218066	0.958543945
0.003852128	0.014259656	0.953078772
0.002051925	0.00743443	0.915657439
0.001949321	0.007362017	0.938523558
0.001176987	0.006686534	0.927830731
0.005488202	0.031912775	0.975004006
0.002146187	0.009049955	0.757570946
0.014359378	0.033695576	1
0.001344486	0.0066287	0.954310728
0.000837251	0.01278044	0.945500865
0.002581914	0.017026079	0.902198476
0.019068156	0.077140166	1
0.000787214	0.006365089	0.854121501
0.007097706	0.041106868	0.876457773
0.002990116	0.014441448	0.966111772
0.001441412	0.008391391	0.958024567
0.003692254	0.029792037	0.937565036
0.0035195	0.014520255	0.491276042
0.015437376	0.0211062	0.966676242
0.005485368	0.017573819	0.898596939
0.001798238	0.011511646	0.985294925
0.004258336	0.011318196	0.980394079
0.007136369	0.029391397	0.972608025
0.000911224	0.007547575	0.906807372
0.002866499	0.020622535	1
0.002133291	0.015660485	0.974363299
0.010467862	0.034982835	0.864800347
0.003966355	0.012478517	0.978380961
0.001680549	0.018883073	0.912908962
0.00410143	0.016569468	0.503747893
0.004052265	0.022266322	0.892790231
0.006474444	0.012630166	0.9232
0.002076334	0.020010506	1
0.003011905	0.016966609	0.964991667
0.001760102	0.01464922	0.983828568
0.005179142	0.029444415	0.994318228
0.003382248	0.009980983	0.915541409
0.005319434	0.024080972	0.968061842
0.018865511	0.027825624	0.921699292
0.023093772	0.037429573	0.902695595
0.001306588	0.009793209	0.904983129

log.sigma.4.0.mm.3D_gldzm_LowIntensityEmphasis	log.sigma.4.0.mm.3D_gldzm_LargeDistanceEmphasis	log.sigma.4.0.mm.3D_gldzm_HighIntensitySmallDistanceEmphasis
0.015632258	1.02970297	209.7240099
0.033127039	1.136363636	141.3219697
0.021949439	1.192857143	165.0196429
0.046827493	1.058823529	135.5931373
0.02650174	1.146464646	118.665404
0.034112779	1.045454545	135.4242424
0.022544925	1.329369798	117.5636279
0.018414189	1.169844021	148.8373772
0.026840664	1.133603239	111.3393219
0.028263036	1.08490566	122.5896226
0.008396608	1.100346021	249.2595156
0.033419632	1.06147541	116.8565574
0.021310577	1.065693431	208.1952555
0.028785658	1	123.5806452
0.028554492	1.109489051	111.0127737
0.021476724	1.266452648	112.836499
0.044974135	1	73.34848485
0.067548169	1	84.77083333
0.042787358	1.045454545	67.39772727
0.013370526	1.136507937	178.2721634
0.026883453	1.235294118	112.4963235
0.036053062	1	102.2608696
0.037969244	1.152542373	107.7415254
0.0234502	1.046632124	107.8678756
0.020000287	1.173076923	160.8205128
0.0132833	2.033994334	149.5401912
0.01875543	1.015625	139.5690104
0.039963985	1.060773481	83.96009822
0.138634203	1	26.64285714
0.022059134	1.08974359	126.8377849
0.020890665	1.184375	115.6546875
0.02398545	1.056074766	154.6214953
0.030999692	1.064102564	125.7767094
0.02204231	1.142857143	121.3275794
0.036400426	1.12793177	100.3040156
0.01656399	1.135714286	183.1027778
0.020221164	1	180.1052632
0.017867599	1.289986996	129.5620214
0.045513495	1	82.44262295
0.003912337	1.150127226	471.5755319
0.029277061	1.082089552	114.9908789
0.014261431	1.0375	143.876875
0.014169571	1.06940874	196.3688946
0.023436806	1.103448276	147.3672414
0.048587418	1.036585366	102.5152439
0.016518987	1.097972973	160.6891892
0.019969902	1.021226415	123.6167453
0.051037201	1.142857143	68.53571429
0.050346127	1	88.51020408
0.026633481	1.288461538	156.3317308
0.025518895	1.091463415	107.6615854
0.014612277	1.07300885	226.1808628
0.027900632	1.060367454	102.3436862
0.021760985	1.301886792	119.1572327
0.023972156	1	169.5913043
0.044496348	1.157894737	110.2417763
0.016557688	1.088372093	142.7686693
0.029148338	1.306818182	93.60216751
0.035897672	1	84.64102564
0.011575467	1.063529412	181.6347059
0.016617485	1.072115385	163.5432692
0.008927452	1.132352941	296.0079657
0.008049864	1.095238095	300.2363946
0.009838294	1.117041199	225.6706721
0.031127975	1.037974684	113.0506329
0.009410727	1.570783133	238.8547733
0.036048546	1	114.245614
0.008964785	1.070175439	248.5899123
0.016504436	1.12749004	135.5739265
0.015101733	1.215909091	175.2968947
0.080405296	1	69.96774194
0.010169197	1.416826004	173.5900808
0.043475084	1.261437908	79.41684822
0.021016994	1.051724138	153.2198276
0.013300301	1.064343164	177.0529491
0.03004706	1.096774194	103.6330645
0.012161927	2.60625	149.2552807
0.03223818	1.050847458	242.9872881
0.019213695	1.160714286	178.0535714
0.021717555	1.022222222	131.6444444
0.015632258	1.02970297	209.7240099
0.021664155	1.041666667	154.1111111
0.01138817	1.16091954	187.5388729
0.040205291	1	92.04347826
0.016825158	1.038961039	149.2900433
0.026926512	1.21875	213.5260417
0.017592192	1.032786885	182.3784153
0.024697357	1.136882129	109.0095057
0.021557742	2.421221865	133.0692212
0.018967437	1.215962441	150.5589463
0.016103102	1.12	314.37
0.022874	1	130.5704698
0.02210391	1.053465347	132.419802
0.019569358	1.024456522	146.5040761
0.037364586	1.008547009	93.1460114
0.010953351	1.155092593	238.8503729
0.023876743	1.075471698	146.831162
0.036945013	1.12244898	173.0306122
0.048544347	1.153846154	113.1602564
0.011977376	1.187062937	196.5923235

log.sigma.4.0.mm.3D_gldzm_LowIntensityLargeDistanceEmphasis	log.sigma.4.0.mm.3D_gldzm_HighIntensityEmphasis	log.sigma.4.0.mm.3D_gldzm_DistanceZoneVariability	log.sigma.4.0.mm.3D_gldzm_ZonePercentage
0.016038485	210.4405941	198.039604	0.303303303
0.09278613	141.5606061	60.27272727	0.584070796
0.054726225	166.9642857	123.1571429	0.289855072
0.105651022	135.6078431	49.03921569	0.554347826
0.031938851	121.020202	182.5757576	0.26541555
0.045476415	135.469697	64.03030303	0.640776699
0.033494896	119.8406659	760.1557669	0.051576107
0.022149714	152.2842288	534.3691508	0.114347998
0.028309804	113.3319838	233.3481781	0.058406243
0.059465867	122.8867925	100.1698113	0.291208791
0.008944752	253.5051903	273.3944637	0.052891654
0.035773248	117.6557377	234.204918	0.079401237
0.048074568	208.2992701	131.1313869	0.413897281
0.028785658	123.5806452	93	0.489473684
0.030532222	112.9343066	127.3649635	0.148913043
0.038123253	116.2166934	535.4173355	0.073062038
0.044974135	73.34848485	66	0.151376147
0.067548169	84.77083333	48	0.432432432
0.04460554	67.68181818	64.03030303	0.116402116
0.016512969	181.4232804	871.8338624	0.079231995
0.044879698	115.5772059	238.1470588	0.187327824
0.036053062	102.2608696	69	0.436708861
0.095504123	108.4915254	53.30508475	0.209964413
0.039419412	108.6217617	187.0932642	0.040174854
0.024150744	163.5512821	139.0384615	0.214876033
0.017757336	193.121813	240.5014164	0.174320988
0.03438043	139.5729167	190.0104167	0.204472843
0.040448468	85.1878453	177.0331492	0.102899375
0.138634203	26.64285714	14	0.5
0.024454106	127.724359	150.0897436	0.135534318
0.024273119	119.06875	285.91875	0.091142125
0.055138098	154.6915888	103.0747664	0.419607843
0.058310945	126.0299145	224.2136752	0.182955434
0.023548405	123.1632653	233.2204082	0.077802477
0.051590048	101.2515991	447.3837953	0.134886396
0.018530992	186.0392857	258.7928571	0.24822695
0.020221164	180.1052632	209	0.535897436
0.022628193	138.4408322	647.5357607	0.065513716
0.045513495	82.44262295	61	0.30964467
0.004156055	490.0381679	733.5216285	0.064883606
0.029785797	116.9179104	130.0447761	0.212361331
0.014618078	144.955	390.125	0.055271521
0.015961777	198.1619537	371.4164524	0.254747872
0.024222261	151.0344828	135.3448276	0.205091938
0.050050833	102.7439024	80.02439024	0.075367647
0.018738624	162.6114865	280.3851351	0.15140665
0.021011017	123.8325472	418.0424528	0.060840867
0.05535207	69.85714286	38.19047619	0.191780822
0.050346127	88.51020408	49	0.429824561
0.031670763	173.0480769	85.92307692	0.472727273
0.026258563	110.8536585	154.304878	0.086044071
0.016582782	227.5597345	430.5353982	0.209356183
0.031420007	102.9448819	369.1627297	0.037715304
0.024689764	127.6289308	130.2201258	0.18772137
0.023972156	169.5913043	115	0.400696864
0.087899122	112.3684211	136.8421053	0.229260935
0.017928934	144.4976744	408.5162791	0.081331568
0.033288038	99.05681818	223.2348485	0.170873786
0.035897672	84.64102564	78	0.286764706
0.012222972	183.4964706	814.7623529	0.036150215
0.018630039	164.5673077	198.2403846	0.26097867
0.019661082	299.8259804	373.5882353	0.25452277
0.01646283	303.0204082	1241.666667	0.289370079
0.011437764	229.6385768	990.923221	0.125352113
0.03172133	113.6582278	77.02531646	0.246105919
0.014717663	278.001506	503.0271084	0.30389016
0.036048546	114.245614	57	0.478991597
0.010816497	251.2236842	652.748538	0.165697674
0.018687974	137.440239	474.6414343	0.050084805
0.02431121	177.7869318	317.5738636	0.230517354
0.080405296	69.96774194	62	0.313131313
0.012803852	183.0573614	446.7055449	0.073672348
0.055554146	84.14379085	134.0980392	0.204272363
0.021405783	155.2586207	168.1034483	0.162162162
0.013760158	180.5737265	357.3431635	0.115982587
0.040789462	104.1048387	116.2580645	0.205298013
0.028562336	287.3854167	235.8125	0.32388664
0.083085638	243	57.03389831	0.513043478
0.042640737	179.6071429	201.2857143	0.344086022
0.021871876	132.4444444	133.0148148	0.084586466
0.016038485	210.4405941	198.039604	0.303303303
0.028174571	154.2152778	140.0555556	0.390243902
0.012606962	193.0201149	315.5689655	0.101813926
0.040205291	92.04347826	115	0.09787234
0.017433924	149.9134199	225.0779221	0.127977839
0.070793434	215.1041667	83.02083333	0.545454545
0.034149569	182.7923497	179.0437158	0.252413793
0.027652891	110.9087452	240.095057	0.064682735
0.026500083	254.2090032	156.6655949	0.264680851
0.030965691	153.5117371	190.1643192	0.26625
0.047384769	315.6	115.4	0.452898551
0.022874	130.5704698	149	0.104195804
0.036418175	133.0257426	487.3207921	0.147574518
0.020189194	146.7853261	362.048913	0.09548521
0.037706466	93.1994302	349.005698	0.169811321
0.015862603	242.1134259	395.5138889	0.364864865
0.033465504	147.6118598	359.1509434	0.253242321
0.100618483	173.4285714	45.16326531	0.563218391
0.144698193	113.2564103	35.20512821	0.549295775
0.015804492	202.1381119	1035.300699	0.119117035

log.sigma.4.0.mm.3D_gldzm_IntensityVariabilityNormalized	log.sigma.4.0.mm.3D_gldzm_LowIntensitySmallDistanceEmphasis	log.sigma.4.0.mm.3D_gldzm_IntensityVariability
0.055288697	0.015530701	11.16831683
0.065197429	0.018212266	4.303030303
0.063265306	0.013755242	8.857142857
0.056516724	0.032121611	2.882352941
0.08198143	0.025181432	16.23232323
0.066115702	0.031271869	4.363636364
0.073530888	0.020226938	61.83947681
0.063079515	0.01753916	36.39688042
0.082791064	0.026540755	20.44939271
0.087219651	0.020462329	9.245283019
0.093593228	0.008271438	27.04844291
0.071049449	0.032831227	17.33606557
0.057808088	0.01461958	7.919708029
0.074806336	0.028785658	6.956989247
0.072406628	0.028060059	9.919708029
0.083088355	0.017409534	51.76404494
0.105142332	0.044974135	6.939393939
0.081597222	0.067548169	3.916666667
0.1184573	0.042332813	7.818181818
0.065735002	0.012618539	62.11957672
0.078800822	0.022526231	21.43382353
0.074144087	0.036053062	5.115942029
0.08072393	0.023585525	4.762711864
0.098767752	0.019457897	19.06217617
0.069115713	0.018962673	10.78205128
0.06226677	0.012514094	21.98016997
0.079155816	0.01484918	15.19791667
0.081590916	0.039874185	14.7679558
0.153061224	0.138634203	2.142857143
0.086374096	0.021519254	13.47435897
0.081132813	0.020069164	25.9625
0.072058695	0.016197288	7.710280374
0.067389875	0.024171878	15.76923077
0.075818409	0.021771193	18.5755102
0.079118571	0.032755717	37.10660981
0.06625	0.016095721	18.55
0.064536068	0.020221164	13.48803828
0.077186355	0.016711222	59.35630689
0.082504703	0.045513495	5.032786885
0.065448789	0.003868052	51.44274809
0.082423702	0.029198941	11.04477612
0.09145	0.014172269	36.58
0.059641425	0.013721519	23.20051414
0.06530321	0.023240442	9.468965517
0.084176086	0.048221565	6.902439024
0.067932798	0.015980761	20.10810811
0.07868681	0.019709623	33.36320755
0.10430839	0.049958484	4.380952381
0.08121616	0.050346127	3.979591837
0.058986686	0.02537416	6.134615385
0.077706722	0.025333978	12.74390244
0.060595975	0.014119651	27.38938053
0.090003513	0.02704489	34.29133858
0.085320992	0.021028791	13.56603774
0.06389414	0.023972156	7.347826087
0.073234072	0.033645655	11.13157895
0.073834505	0.01622806	31.74883721
0.084079431	0.028249548	22.1969697
0.091387245	0.035897672	7.128205128
0.073386851	0.01141359	62.37882353
0.073317308	0.016114347	15.25
0.054510285	0.006244044	22.24019608
0.055771801	0.005946622	73.78609221
0.06533792	0.009444582	69.78089888
0.091491748	0.030979637	7.227848101
0.053917477	0.008172715	35.80120482
0.095106187	0.036048546	5.421052632
0.062151602	0.008501857	42.51169591
0.079109855	0.016052082	39.71314741
0.065696023	0.012899933	23.125
0.090010406	0.080405296	5.580645161
0.08825024	0.009666347	46.15487572
0.08496732	0.040582027	13
0.067776457	0.020919796	11.79310345
0.066981003	0.013185337	24.98391421
0.083896982	0.02736146	10.40322581
0.046901042	0.008431373	22.5125
0.054294743	0.019526316	3.203389831
0.060267857	0.013356935	13.5
0.083127572	0.021678975	11.22222222
0.055288697	0.015530701	11.16831683
0.069251543	0.02003655	9.972222222
0.068370987	0.011099762	23.79310345
0.085217391	0.040205291	9.8
0.076816401	0.016672966	17.74458874
0.056206597	0.015959781	5.395833333
0.070261877	0.013452847	12.8579235
0.078344345	0.023958474	20.60456274
0.048810496	0.020510972	15.18006431
0.073332011	0.016025355	15.61971831
0.057664	0.008282686	7.208
0.082473762	0.022874	12.2885906
0.076216057	0.018525344	38.48910891
0.075629135	0.019414399	27.83152174
0.083205493	0.037279116	29.20512821
0.058213306	0.009742876	25.14814815
0.068765847	0.021530299	25.51212938
0.065389421	0.021026646	3.204081633
0.075608153	0.024505886	2.948717949
0.061282031	0.011086967	70.10664336

log.sigma.4.0.mm.3D_gldzm_HighIntensityLargeDistanceEmphasis	log.sigma.4.0.mm.3D_gldzm_SmallDistanceEmphasis	log.sigma.4.0.mm.3D_glcm_SumVariance	log.sigma.4.0.mm.3D_glcm_Homogeneity1
213.3069307	0.992574257	434.8341346	0.395701197
142.5151515	0.965909091	184.0095223	0.295013962
174.7428571	0.951785714	285.354584	0.371066366
135.6666667	0.985294118	197.8131959	0.27873873
133.6717172	0.968995511	127.0163605	0.409624993
135.6515152	0.988636364	227.5903208	0.308975247
147.1688466	0.959909985	323.8761546	0.733155026
177.4176776	0.970116022	421.1307864	0.528406398
129	0.97798583	265.591551	0.609515316
124.0754717	0.978773585	193.8409396	0.422253142
275.4705882	0.97875817	851.0495486	0.681980804
120.852459	0.984631148	253.9296615	0.624424339
208.7153285	0.983576642	262.6455084	0.329826611
123.5806452	1	169.4120536	0.339067308
120.620438	0.972627737	229.882463	0.477990952
133.3900482	0.942304263	317.6368309	0.638557225
73.34848485	1	75.34837957	0.472412712
84.77083333	1	80.93731226	0.367137083
68.81818182	0.988636364	107.8384615	0.497761812
195.9936508	0.969400353	382.5897851	0.616608011
130.8419118	0.949346405	137.6596509	0.45059644
102.2608696	1	111.9897672	0.367700182
111.4915254	0.961864407	169.309271	0.411291508
111.6373057	0.988341969	291.5375102	0.712159698
174.474359	0.956730769	439.6920302	0.447168008
533.7195467	0.850586245	334.1450445	0.48654542
139.5885417	0.99609375	288.9650048	0.444925081
94.91160221	0.990945365	221.7049404	0.532358233
26.64285714	1	44.71147369	0.411481311
134.7179487	0.98468661	226.6073063	0.49467388
134.725	0.957378472	263.6236082	0.535002558
154.9719626	0.985981308	234.9348889	0.345912074
127.042735	0.983974359	225.0185099	0.474267589
140.4612245	0.980300454	320.7962001	0.603942217
110.315565	0.98112118	171.8966709	0.514942025
200.4678571	0.970039683	455.0424121	0.44709717
180.1052632	1	287.5052483	0.294251001
181.0013004	0.934727641	440.6962323	0.660138982
82.44262295	1	124.2347917	0.406478388
593.0661578	0.973114574	1506.499376	0.65833564
130.2313433	0.987769486	177.5152287	0.446072671
149.2675	0.990625	341.2052139	0.699362996
205.3341902	0.982647815	380.7666023	0.412239204
165.7034483	0.974137931	220.7138893	0.431747404
103.6585366	0.990853659	200.6766277	0.586144224
173.6790541	0.979260511	299.4360249	0.48889692
124.6957547	0.994693396	342.2363613	0.646572626
75.14285714	0.964285714	133.3279946	0.440282189
88.51020408	1	117.6798542	0.360331116
239.9134615	0.927884615	419.214474	0.305684186
123.6219512	0.977134146	461.5585039	0.593702614
233.0752212	0.981747788	419.1442788	0.454777812
106.7611549	0.987824439	248.2584816	0.733687828
161.5157233	0.924528302	324.3842143	0.44941367
169.5913043	1	223.0647317	0.348729583
120.875	0.960526316	123.6422529	0.426737313
153.4395349	0.980490956	380.3621331	0.610766827
125.6060606	0.935921717	178.7928218	0.459515723
84.64102564	1	113.2251741	0.430073113
190.9435294	0.984117647	489.7891885	0.724997125
168.6634615	0.981971154	285.6795683	0.428156946
315.0980392	0.966911765	609.3418993	0.406172434
314.1564626	0.976190476	652.2092646	0.411244112
246.2134831	0.971780067	606.3317273	0.581397503
116.0886076	0.990506329	163.8430143	0.407066965
508.4442771	0.893438337	622.6381255	0.373090053
114.245614	1	162.666446	0.335701698
261.7587719	0.98245614	598.004588	0.510316975
149.3067729	0.978156817	291.4954951	0.663275441
197.4886364	0.960325915	265.2667281	0.416466073
69.96774194	1	76.40087154	0.398954459
272.0707457	0.93938149	662.0905181	0.647340425
111.0980392	0.949164851	127.2819696	0.428019081
163.4137931	0.987068966	300.0522586	0.50094853
194.6568365	0.983914209	476.9286934	0.590304474
105.9919355	0.975806452	163.4861324	0.442986772
1159.78125	0.721267361	609.8532107	0.333006054
243.0508475	0.987288136	523.704783	0.28817625
185.8214286	0.959821429	283.3810786	0.369623583
135.6444444	0.994444444	326.9542692	0.588605626
213.3069307	0.992574257	434.8341346	0.395701197
154.6319444	0.989583333	184.718071	0.369010081
217.4482759	0.962962963	575.2319307	0.612038329
92.04347826	1	233.218186	0.547819972
152.4069264	0.99025974	269.7827414	0.511650437
221.4166667	0.9453125	371.4835239	0.274421481
184.4480874	0.991803279	403.911009	0.444633101
118.5057034	0.965779468	245.3245295	0.658559316
910.2508039	0.725192033	569.0855876	0.374980018
172.9389671	0.956442358	196.3707317	0.410355757
320.52	0.97	687.4061322	0.312265316
130.5704698	1	276.6070451	0.550513135
135.449505	0.986633663	237.2302252	0.526977414
147.9103261	0.99388587	284.0055388	0.565520179
93.41310541	0.997863248	140.6572409	0.500302381
261.4722222	0.966370885	467.9501238	0.361559071
153.6576819	0.987121893	201.4507158	0.416127613
175.0204082	0.969387755	346.3850635	0.291790133
113.6410256	0.961538462	192.402312	0.303340264
229.888986	0.962040841	476.5018748	0.565107947

log.sigma.4.0.mm.3D_glcm_Homogeneity2	log.sigma.4.0.mm.3D_glcm_ClusterShade	log.sigma.4.0.mm.3D_glcm_MaximumProbability	log.sigma.4.0.mm.3D_glcm_Idmn	log.sigma.4.0.mm.3D_glcm_SumVariance2
0.311484014	148.4209714	0.022349481	0.982203139	48.08311199
0.200381944	184.220269	0.031428274	0.9498236	49.59960871
0.284132517	13.29032859	0.021715216	0.965890221	48.67072076
0.18244729	162.2510149	0.026313591	0.934590702	56.10850535
0.328033552	127.6967168	0.032616083	0.981963842	26.86483259
0.213146415	79.72755062	0.037016029	0.957681638	43.86845704
0.710117023	-114.1610645	0.284575265	0.995328581	18.22118137
0.471448138	-94.38811248	0.100800707	0.990477475	33.93047041
0.568119379	-61.35459015	0.160264352	0.992993592	23.04352015
0.341702535	5.319780646	0.043143329	0.97864068	18.65564329
0.65851139	-43.62741482	0.210423284	0.996318519	10.68108087
0.58670162	-85.82264405	0.131106308	0.993256921	23.91378753
0.241433297	337.8708008	0.026443369	0.959287952	63.55513499
0.247456294	115.2772241	0.030789259	0.953721235	31.30564375
0.409866735	-1.465358461	0.031688714	0.984935415	29.3162579
0.600943694	-149.4621259	0.233341939	0.991442585	29.84333945
0.402857926	28.31718612	0.042025127	0.979386964	18.51280784
0.278121046	161.5866613	0.040405724	0.963893652	28.74635742
0.434296787	-9.412551695	0.03494653	0.976667338	21.11266326
0.578029985	-24.1208805	0.137465683	0.996389077	25.47437165
0.377235846	113.9230685	0.024088648	0.980806555	34.44985309
0.28025308	144.8460813	0.040829514	0.952713053	39.27407722
0.328510174	7.800175985	0.026131937	0.972928279	35.37204479
0.688960069	-69.99192717	0.318236139	0.994539035	14.13254357
0.372499638	-65.14780687	0.077572696	0.978521787	47.48983316
0.42459237	175.0982767	0.051028607	0.988066347	38.05974572
0.370742551	-31.02764261	0.027007911	0.986867171	31.7984998
0.477272481	-40.67767002	0.054886357	0.988138153	27.5546594
0.325801723	15.08543177	0.109994167	0.912177009	12.12123686
0.429903196	-4.226907451	0.036312424	0.986061235	26.81380395
0.479654126	-52.83858952	0.074519343	0.989482759	32.80207587
0.256725408	26.55589164	0.028830531	0.95732812	42.47497072
0.407356719	-29.27692654	0.031064992	0.98541746	33.82181659
0.561352968	-75.61243347	0.170438506	0.990752319	27.55626084
0.455331631	-23.38556315	0.068656188	0.987097719	24.92337036
0.376016734	-146.6310169	0.028987055	0.977726682	52.77428007
0.199912894	75.92272087	0.018387452	0.951002934	55.79210234
0.626964469	-164.0680333	0.211560025	0.994841696	26.67416524
0.320920037	57.80910236	0.031320328	0.970750592	31.05172751
0.626415818	-51.17052065	0.234712598	0.997838977	17.98041216
0.371673299	6.356938575	0.040837093	0.980130874	20.25410552
0.677753093	-12.23958249	0.242135531	0.995841926	7.616881906
0.334821362	-49.81169692	0.021096862	0.981465589	50.85493089
0.35242318	56.57012177	0.028180062	0.984764512	28.84460962
0.542453077	-86.82168318	0.136797527	0.98748896	27.80411707
0.425347068	-23.54199141	0.052115383	0.986678053	32.65267212
0.613719569	-90.89035002	0.156897928	0.994828479	21.97293198
0.360303417	32.94785778	0.033846444	0.967953186	43.57267471
0.26975459	225.8667467	0.032414238	0.955345003	41.11817965
0.211990462	-69.56855392	0.02136392	0.953608504	66.46922273
0.547049815	-148.2536348	0.200231217	0.989845581	34.59844699
0.385323377	11.12549185	0.029790568	0.990448971	38.6913544
0.714400232	-58.87429962	0.319532746	0.996256093	12.17686429
0.375254311	26.06620118	0.025377923	0.982474073	35.46150495
0.260541286	257.8699088	0.0216912	0.966445786	58.90180289
0.349366753	129.8661399	0.029601645	0.980112255	29.90042676
0.571349322	-75.58010095	0.153408189	0.993362585	24.09456324
0.387079827	28.46486854	0.042421206	0.980660061	34.19453912
0.351357627	1.791575927	0.034931282	0.972944471	25.79297614
0.705479262	-51.43483129	0.281051884	0.997863345	11.44875816
0.351207536	23.19468097	0.03115991	0.981161254	31.16807684
0.326354654	79.18133623	0.020404139	0.986533068	58.46278208
0.335772185	-55.34241728	0.026423291	0.982424061	58.62969679
0.533938833	-122.6635949	0.130951088	0.993464874	30.51735135
0.322445978	-1.288128863	0.02867383	0.968835513	28.87128867
0.287278258	298.3992822	0.016641951	0.983804714	73.03072898
0.241335413	30.22834583	0.032636843	0.943902884	30.31212903
0.451203937	-68.20814452	0.057232718	0.991152403	28.0006211
0.633213288	-57.36881893	0.166441779	0.995299518	17.82942575
0.337003374	72.33716897	0.01937064	0.980529908	37.08795145
0.313240791	36.88218688	0.037293524	0.958981187	20.36420721
0.610533421	-235.4803162	0.198385928	0.992094046	35.49962088
0.348713311	31.57049755	0.03228074	0.966985536	23.01329814
0.439924348	6.212802851	0.055630528	0.991332705	28.58136615
0.546236841	-100.4762135	0.135302669	0.993201801	25.8870442
0.365745785	92.6249216	0.029722336	0.979876112	29.20717318
0.244914723	3156.842826	0.015740702	0.982199551	184.836835
0.192698083	-43.96140723	0.024482703	0.948913327	97.59239967
0.281492735	134.0326302	0.022435927	0.979196026	49.11376174
0.545565398	-53.14485078	0.120162148	0.993151822	19.25311616
0.311484014	148.4209714	0.022349481	0.982203139	48.08311199
0.28234059	219.3303979	0.02545184	0.970576061	48.52895598
0.566701424	-155.2743519	0.23630784	0.994009082	32.70779479
0.497682358	-65.7789197	0.056394095	0.98697996	23.4267927
0.453567713	-62.63655534	0.050023647	0.987159428	29.21618495
0.175663384	57.20821117	0.018429325	0.946970913	86.62159678
0.37233975	-85.38837156	0.028499095	0.984808978	44.04755869
0.624247995	-104.3602283	0.277708957	0.99247883	23.44143479
0.296672529	2076.44033	0.02262696	0.980351047	136.907754
0.329012909	148.815676	0.026200272	0.981671207	35.15950754
0.22012502	-462.4894964	0.023968493	0.965308863	96.16206113
0.500599598	-68.90308349	0.095328052	0.990606947	23.28519584
0.472506155	-15.25447986	0.048190608	0.990095565	25.24586631
0.51655546	-53.88344943	0.121603241	0.992803724	25.24906974
0.438577895	5.845464767	0.050928461	0.984852517	18.34362494
0.275145163	63.89529826	0.017972971	0.981602632	46.15469052
0.336947621	108.9127453	0.027335366	0.984125514	32.70440081
0.19491076	-108.0764764	0.026776047	0.942754987	56.44181945
0.20497855	12.51277353	0.040389756	0.943022703	33.28953969
0.515909099	-87.87017323	0.142389709	0.991969467	37.91695633

log.sigma.4.0.mm.3D_glcm_Contrast	log.sigma.4.0.mm.3D_glcm_DifferenceEntropy	log.sigma.4.0.mm.3D_glcm_InverseVariance	log.sigma.4.0.mm.3D_glcm_Entropy	log.sigma.4.0.mm.3D_glcm_Dissimilarity
12.8089107	2.957108484	0.307210467	7.234265895	2.736292686
27.75102162	3.262662758	0.217443784	6.394080843	4.255119592
16.81687979	3.104878735	0.276332255	7.340817561	3.149312733
30.75272173	3.327866165	0.200761947	6.347280025	4.538477453
11.14832144	2.862820781	0.331071369	6.736553858	2.531509161
23.15286439	3.100848966	0.242559009	6.111175748	3.842589637
2.391295562	1.776222032	0.312130144	4.480577825	0.816793663
5.267510856	2.41362257	0.395009157	6.432562179	1.632913341
3.221499723	2.093263562	0.39905882	5.540078747	1.200710897
9.179915347	2.723140309	0.338756535	6.353654213	2.341770839
2.01827821	1.773395089	0.421167694	4.747230521	0.868653222
3.093318953	2.074431519	0.399073279	5.532116425	1.151347145
26.68768017	3.391142109	0.239502718	7.294102159	3.980409575
19.36491453	3.124858433	0.266032048	6.602799362	3.448289686
6.472500919	2.49490387	0.386836963	6.528601044	1.883702771
4.012758744	2.145137973	0.353117358	5.350142193	1.221141741
5.657638066	2.402913619	0.396804186	6.05694018	1.833914376
14.70528582	2.927980424	0.293228751	6.046001222	2.990528615
4.247578715	2.191719067	0.426671255	5.992824819	1.602681533
3.797512977	2.162258969	0.396089129	5.815359608	1.236416248
8.251366053	2.680792702	0.360451227	6.834630795	2.156359931
15.80963009	3.001170862	0.283978605	6.466028426	3.098577818
9.589637016	2.689511625	0.335466091	6.68896165	2.412428366
1.639792408	1.708739863	0.368597666	4.470434818	0.77614514
10.32308781	2.773576999	0.32489627	6.797625739	2.356930842
9.307759231	2.698546315	0.381099208	6.823466294	2.08465242
7.985206051	2.621963504	0.374804448	6.792733562	2.128604229
4.022578501	2.238991476	0.427653403	6.200776044	1.484737611
8.965783047	2.193226013	0.311817892	4.063266412	2.393895365
5.303089265	2.406942475	0.398922683	6.459497639	1.731692321
4.863192524	2.346287848	0.401904109	6.275003279	1.574077912
21.41399991	3.243244692	0.245587657	6.925511791	3.598847473
7.574299381	2.596725191	0.386112439	6.750472919	1.990524753
3.513630021	2.151482382	0.393110473	5.697925104	1.251780095
5.476634462	2.433490934	0.398340105	6.362150171	1.685406606
10.85049286	2.842950687	0.353433678	7.215647125	2.366511811
32.59428228	3.449825591	0.200628391	7.472081937	4.509983683
3.363020434	2.054767553	0.358457107	5.223694321	1.094618157
8.078427159	2.55565639	0.341934876	6.389676822	2.306548894
3.026531657	1.99974428	0.382407363	5.23461215	1.046529468
7.692189691	2.629826239	0.370120624	6.446934522	2.116862067
1.72021458	1.712178305	0.405731558	4.535401329	0.801665116
14.69594222	2.931080848	0.323820508	7.39885275	2.769475853
8.512000707	2.686507639	0.34396514	6.76087444	2.245872842
3.428608897	2.122467525	0.423929491	5.793456263	1.27407838
7.511972923	2.604813669	0.383115475	6.716411599	1.949285801
2.591131015	1.966129863	0.404869566	5.461463587	1.034118874
6.823974548	2.417657157	0.344321558	6.38098671	2.087435933
14.75097178	2.886193112	0.284063381	6.145268198	3.044729093
28.23298554	3.413213877	0.221356493	7.2828127	4.186878523
3.431105819	2.146783932	0.380820249	5.670012831	1.28382849
10.32117072	2.783317759	0.368831223	7.045042194	2.263893116
1.553857852	1.638305137	0.357754206	4.255686814	0.713582703
6.705947632	2.429421013	0.383740747	6.698127198	2.009264993
19.78586387	3.201645737	0.273194988	7.217309289	3.438746835
10.53319306	2.801427209	0.349020256	6.776183105	2.398183736
3.362342757	2.104624903	0.410431309	5.759476151	1.20281159
7.462464243	2.635089155	0.359741095	6.747931933	2.070558928
8.544498431	2.67238937	0.348750061	6.458793986	2.258922598
1.719726501	1.668297906	0.370904255	4.364613136	0.745604167
10.71416486	2.836805444	0.338717175	6.893574315	2.438021252
13.69899386	2.997045265	0.322297597	7.56407291	2.732493219
17.13979473	3.121179423	0.31679889	7.798275749	2.942316342
5.757768448	2.411983207	0.361289898	6.063205066	1.527709864
8.708760519	2.624134529	0.337883083	6.595394573	2.361461609
16.49116234	3.128071103	0.292948162	7.916510299	3.08836754
16.67850464	2.977317837	0.267190866	6.35709194	3.303902163
6.804425322	2.528741656	0.394438411	6.555793233	1.802142805
2.357047837	1.900471026	0.398888936	5.122308395	0.965798116
11.09611312	2.874538684	0.333768071	7.186009636	2.510582801
10.48014874	2.76046238	0.319769808	6.247511515	2.539548017
3.674341287	2.134382922	0.346782638	5.367276328	1.171826582
8.268377226	2.66971671	0.353516635	6.615024313	2.236619223
6.148815511	2.472007598	0.40669973	6.402731383	1.771256771
4.815334812	2.301402809	0.386514661	5.919689719	1.408316266
7.78937513	2.602912413	0.351947144	6.600777275	2.142670127
29.14631144	3.471218061	0.24942307	8.378175123	3.999298099
34.20863891	3.419716934	0.187429293	6.597746706	4.638534442
15.05234196	3.053009198	0.289070078	7.42491506	3.018807063
3.136752859	2.086467405	0.431297634	5.60736274	1.23223353
12.8089107	2.957108484	0.307210467	7.234265895	2.736292686
15.62596364	3.026511511	0.294786379	7.083451747	3.063366219
4.203803837	2.234857623	0.340266967	5.624111216	1.32201066
3.504361398	2.148534653	0.447131626	5.971109969	1.380650462
6.029623559	2.468133489	0.40773152	6.489680948	1.739137051
32.00586092	3.436544262	0.174353371	7.215536956	4.65130969
8.567465779	2.666472944	0.382350497	6.996091112	2.173521326
3.185459022	2.047858773	0.356439366	5.133869268	1.082182236
26.32920086	3.360899443	0.292183141	7.96078663	3.617040517
11.27487821	2.877667181	0.324647315	6.980957517	2.557126853
27.92404297	3.410030241	0.211714427	7.445060997	4.187620519
3.933371645	2.20340635	0.436087694	5.937224106	1.416480673
5.534629152	2.414688933	0.416875073	6.447215576	1.640388186
4.694991811	2.319864783	0.39860443	6.072184161	1.463804897
5.220421347	2.386345701	0.414965167	6.223436614	1.696732711
18.89350326	3.209070689	0.285293556	7.612638062	3.294174971
11.41583787	2.876204226	0.334769562	7.081035135	2.530715999
26.45871923	3.237843202	0.212733422	6.272999991	4.200291291
21.28226481	3.041026972	0.232412901	5.857850251	3.791644752
6.159679492	2.456176803	0.371489851	6.450572141	1.609641988

log.sigma.4.0.mm.3D_glcm_DifferenceVariance	log.sigma.4.0.mm.3D_glcm_Idn	log.sigma.4.0.mm.3D_glcm_Idm	log.sigma.4.0.mm.3D_glcm_Correlation	log.sigma.4.0.mm.3D_glcm_Autocorrelation
5.156805875	0.910188629	0.311484014	0.579893236	157.6195919
8.915848614	0.849035511	0.200381944	0.289098422	69.77816691
6.444995306	0.879065611	0.284132517	0.488673834	109.257447
9.177983901	0.828250763	0.18244729	0.302184281	72.913634
4.639784821	0.910155565	0.328033552	0.412670474	54.89423197
7.662433055	0.861251709	0.213146415	0.304600348	84.10368523
1.704616935	0.966993041	0.710117023	0.766398936	113.3041509
2.532880326	0.937401296	0.471448138	0.730332856	152.4903492
1.712860437	0.94904506	0.568119379	0.752588751	101.1744359
3.541875737	0.901350928	0.341702535	0.340910944	77.02635831
1.242982251	0.965540768	0.65851139	0.68168165	266.221175
1.736516646	0.95115376	0.58670162	0.769831564	97.53449373
10.27041647	0.868699063	0.241433297	0.407303643	98.21980027
6.814673434	0.8578642	0.247456294	0.248786475	66.84465134
2.807235019	0.918967503	0.409866735	0.63771673	91.69935473
2.408053405	0.949329041	0.600943694	0.760768891	116.0269906
2.190078002	0.903204679	0.402857926	0.528433665	36.63060791
5.435630731	0.873417311	0.278121046	0.326306324	35.12320391
1.541577315	0.896836785	0.434296787	0.66303877	49.36272006
2.234032783	0.964618906	0.578029985	0.738789744	138.4091172
3.490368381	0.908769336	0.377235846	0.611318345	60.2894497
5.652879767	0.857844491	0.28025308	0.424184833	47.48136132
3.516271005	0.88920136	0.328510174	0.571147344	70.73248584
1.023046754	0.959204438	0.688960069	0.790649433	103.8616707
4.391293653	0.906235616	0.372499638	0.644079793	158.4794243
4.802605009	0.933171147	0.42459237	0.608801043	125.5028045
3.271571404	0.922808162	0.370742551	0.60001836	111.2276324
1.76556918	0.927935819	0.477272481	0.74399869	89.44786987
2.793385873	0.808754667	0.325801723	0.174162468	19.48090072
2.23263506	0.921048456	0.429903196	0.668901743	91.04237399
2.267165375	0.934290216	0.479654126	0.740681606	102.9207999
7.866292096	0.864804651	0.256725408	0.338023826	90.21864436
3.498736723	0.922206991	0.407356719	0.634553375	90.73194314
1.897748798	0.942244223	0.561352968	0.772404865	119.4246582
2.57983937	0.926998763	0.455331631	0.63896774	72.20926065
5.129278779	0.906427435	0.376016734	0.658458731	164.7322359
10.50627673	0.853595318	0.199912894	0.286722168	105.5105051
2.123868695	0.960792397	0.626964469	0.774662339	152.4706267
2.544399411	0.880907241	0.320920037	0.585515671	54.54899899
1.905664025	0.973687036	0.626415818	0.709778194	453.6562008
3.128285035	0.905757357	0.371673299	0.447583189	72.84195861
1.062640668	0.963645382	0.677753093	0.628162073	118.2978077
6.086556977	0.913410449	0.334821362	0.556241628	141.0112096
3.33508679	0.91566063	0.35242318	0.543515949	88.38671098
1.750251482	0.931292075	0.542453077	0.779122864	81.42819558
3.599388183	0.926845441	0.425347068	0.624062726	114.673915
1.499791739	0.957616366	0.613719569	0.787248897	125.1497218
2.104477599	0.878246675	0.360303417	0.724878347	57.78651186
5.142855079	0.858839429	0.26975459	0.471866954	48.48184573
10.14177055	0.857320832	0.211990462	0.405698891	149.3059397
1.690745769	0.93760445	0.547049815	0.817514266	162.2490952
5.086544471	0.937647569	0.385323377	0.577462526	152.3613696
1.033240453	0.967676055	0.714400232	0.772595837	89.64950252
2.257314712	0.909484659	0.375254311	0.679916244	123.1310215
7.683503483	0.879072864	0.260541286	0.495463736	86.83371304
4.673369583	0.908171509	0.349366753	0.478862864	54.2299825
1.864790137	0.951214269	0.571349322	0.752052333	138.0747368
3.081361197	0.90774807	0.387079827	0.641447202	74.72101356
3.338827979	0.890310004	0.351357627	0.504176102	50.75086038
1.149816285	0.975315197	0.705479262	0.736866992	161.2202926
4.618697286	0.910263715	0.351207536	0.489336254	109.2838299
6.02794181	0.923602521	0.326354654	0.619372367	212.7008889
8.138258367	0.917071633	0.335772185	0.549845537	225.1985653
3.381685029	0.953097717	0.533938833	0.681454735	205.2962222
2.873963741	0.879056394	0.322445978	0.53501159	68.62668398
6.702062689	0.914448684	0.287278258	0.633514154	217.4344646
5.507103034	0.840954232	0.241335413	0.29484123	65.47605383
3.476316952	0.941043315	0.451203937	0.607424027	204.9308744
1.400341765	0.960307408	0.633213288	0.765076474	107.3152913
4.67175491	0.907667939	0.337003374	0.539281599	103.632405
3.774349962	0.865568176	0.313240791	0.319754775	35.5406325
2.271690953	0.951128467	0.610533421	0.811359286	218.195171
3.152114999	0.879081147	0.348713311	0.471424137	55.92794423
2.95417031	0.939540798	0.439924348	0.645396364	113.9856045
2.781354998	0.951837632	0.546236841	0.685823132	166.5025968
2.997410935	0.904627298	0.365745785	0.578431815	68.94385719
12.48146131	0.912837097	0.244914723	0.732568773	209.2677274
11.91013855	0.850048003	0.192698083	0.48390355	176.9498841
5.7095452	0.90186393	0.281492735	0.530678057	109.1158132
1.581454631	0.947428081	0.545565398	0.717944575	120.0049076
5.156805875	0.910188629	0.311484014	0.579893236	157.6195919
5.882452959	0.885879426	0.28234059	0.505979187	74.64201368
2.376523261	0.954487111	0.566701424	0.770541213	194.7484075
1.554402399	0.925107715	0.497682358	0.738531344	92.35979097
2.920134019	0.928372733	0.453567713	0.65760749	104.6621103
9.706736489	0.842593489	0.175663384	0.459428956	132.3759461
3.760686569	0.918706403	0.37233975	0.672922692	148.8952864
1.989995864	0.95249358	0.624247995	0.759596103	92.32910859
12.56327699	0.913512795	0.296672529	0.682541324	197.3875131
4.606288489	0.90932698	0.329012909	0.512677618	79.85716432
9.657468254	0.874447192	0.22012502	0.555352914	232.5159007
1.849224868	0.93746739	0.500599598	0.705569024	105.6333902
2.794838354	0.937376928	0.472506155	0.639848032	94.31254714
2.50479036	0.947823347	0.51655546	0.685738979	108.2962839
2.254747582	0.918929118	0.438577895	0.553817507	60.86591634
7.750066756	0.909645291	0.275145163	0.420052843	166.7655409
4.79935506	0.916454332	0.336947621	0.482329333	81.66701162
8.134930896	0.838321934	0.19491076	0.367380391	122.3444035
6.483156273	0.837480526	0.20497855	0.226027328	72.11285747
3.512266752	0.947440602	0.515909099	0.718841223	169.533173

log.sigma.4.0.mm.3D_glcm_SumEntropy	log.sigma.4.0.mm.3D_glcm_AverageIntensity	log.sigma.4.0.mm.3D_glcm_Energy	log.sigma.4.0.mm.3D_glcm_SumSquares	log.sigma.4.0.mm.3D_glcm_ClusterProminence
4.731273548	12.20501289	0.00870825	15.36007332	8246.830276
4.44417302	8.013990825	0.013599588	19.33765758	7655.428032
4.744376051	10.06345056	0.007831824	16.37190014	5940.28596
4.415039056	8.155190419	0.013449703	21.71530677	8379.019502
4.270563313	7.138622155	0.012711897	9.503288508	3209.551717
4.213009715	8.878362746	0.016300205	16.75533036	4851.208822
3.430861619	10.45685559	0.129511437	5.153119234	1622.751591
4.433087862	12.0548997	0.022738787	9.799495315	2905.233655
4.044714453	9.80889389	0.043914553	6.566254968	1292.277275
4.044491987	8.639249569	0.01658564	6.95888966	1247.17734
3.510467392	16.24977489	0.081535318	3.17483977	656.4490558
4.051530575	9.608736227	0.047694839	6.751776622	1603.640027
4.757637952	9.431018733	0.008535127	22.56070379	11031.51859
4.232473952	7.990345341	0.012545789	12.66763957	3113.839848
4.384509642	9.272800705	0.013760962	8.947189704	1887.087927
3.970727332	10.46738984	0.076050179	8.464024548	2506.073796
4.022242959	5.780275606	0.018711049	6.042611476	926.4292434
4.023096364	5.620809081	0.017931502	10.86291081	3536.182573
4.125186571	6.718766415	0.01906559	6.340060495	920.3926836
4.166827395	11.53387328	0.045740425	7.343000666	3737.216229
4.502235471	7.330584667	0.011431312	10.67530478	3818.597238
4.374121961	6.445219148	0.013901188	13.77092683	4200.502551
4.462318627	8.017461305	0.01172286	11.24042045	2699.71592
3.418336556	10.03676852	0.123540416	3.943083994	895.2579534
4.624584589	12.21407605	0.015456292	14.45323024	5056.840592
4.547646943	10.8855885	0.015476793	12.00280275	7357.643348
4.48513413	10.25995326	0.012057573	9.945926463	2995.616466
4.349391233	9.141178163	0.019366704	7.894309476	1828.283384
2.950161093	4.320437497	0.065107184	5.271754978	330.5824441
4.376357646	9.255329937	0.015584669	8.029223303	1928.629186
4.397253281	9.794367249	0.020219409	9.416317099	2276.390005
4.560501522	9.216032385	0.010285159	15.97224266	5517.42235
4.521636688	9.174277381	0.012894494	10.34902899	3019.342648
4.175113306	10.64947438	0.049228645	7.767472714	2111.591891
4.290307085	8.206333803	0.018742621	7.600001205	1577.145694
4.783464453	12.41951416	0.009707947	15.90619323	6525.424391
4.7546923	9.981132054	0.006650032	22.09659615	8053.206222
3.871787279	12.10955689	0.078298889	7.509296418	2717.758114
4.320562767	6.984554395	0.01412229	9.782538668	2353.907288
3.841292373	21.23378346	0.076775938	5.122602468	1577.257903
4.157649319	8.348506522	0.015690359	6.986573802	1294.720887
3.352733313	10.80847176	0.092899554	2.334274121	320.0030698
4.81336376	11.4937141	0.008478738	16.48076248	7348.085068
4.402903682	9.126776438	0.012270223	9.339152581	2751.11554
4.211129674	8.679408813	0.036279022	7.808181491	1878.997131
4.488802963	10.41082934	0.014429193	10.04116126	2846.924314
4.040936431	10.96821099	0.051542701	6.141015749	1613.872084
4.469135162	6.970391028	0.014431806	12.59916231	3433.123793
4.193794953	6.468333365	0.016014215	13.96728786	5431.951959
4.862696032	11.82010237	0.007480911	23.67555207	11021.58637
4.193353138	12.42795772	0.053199949	9.507388202	3039.101401
4.600187801	12.07362287	0.012315084	12.52861031	7201.480912
3.28918423	9.327018447	0.134150855	3.432680536	698.4731831
4.539662586	10.76651709	0.01198999	10.54186315	2898.164189
4.743621558	8.775956809	0.008276794	19.67191669	9633.880498
4.373450844	7.027389147	0.012158616	10.10840496	3818.726531
4.181017675	11.52770326	0.041713948	6.864226498	1771.202839
4.472589329	8.248370939	0.011839572	10.41425084	2407.106121
4.279123574	6.813695933	0.01402842	8.584368644	1776.510238
3.331296281	12.60180282	0.120805642	3.303125418	910.6139471
4.459179822	10.20600757	0.011747993	10.47056043	2773.060774
4.919919438	14.23428686	0.007522538	18.44989321	10060.49676
4.950637016	14.70857813	0.007547771	19.43996873	11006.23363
4.225002249	14.11631142	0.038738436	9.133104551	3375.015264
4.330191579	7.972973352	0.012732607	9.395012297	1920.025561
5.073269702	14.31318699	0.006290603	22.98982494	18268.80411
4.253073292	7.876776574	0.014038112	11.74765842	2358.798895
4.383761234	14.13291252	0.017872796	8.74807564	2932.504915
3.799052782	10.17085801	0.066615626	5.046618397	1017.690634
4.605684418	9.855577645	0.009244419	12.04601614	4305.914632
4.015386047	5.749070568	0.015935125	7.711088986	1114.707548
3.967518554	14.49955355	0.07271403	9.793490541	3975.358945
4.245411371	7.227605741	0.013311684	7.820418841	1443.604395
4.344734122	10.41477152	0.019152354	8.766747929	3560.043694
4.15816656	12.69864464	0.040931877	7.691558344	2569.34005
4.360700636	7.973805908	0.013801929	9.249137078	3151.688112
5.487763155	13.21089716	0.004453569	55.53613925	148462.8401
4.745422233	12.68104591	0.011166755	32.95025964	20780.37515
4.754236279	10.02920038	0.007568532	16.04853202	6902.838572
3.997317634	10.76911392	0.035548012	5.597467254	1052.342741
4.731273548	12.20501289	0.00870825	15.36007332	8246.830276
4.628505332	8.146118188	0.009545176	16.03872991	6723.963994
4.103030082	13.69998246	0.067552915	9.262473262	3363.836255
4.211702299	9.347451871	0.022888115	6.732788524	1529.394295
4.376152474	9.942943843	0.017138881	8.811452128	2153.326822
4.91347276	10.88967953	0.007494563	29.65686442	17132.30758
4.696768958	11.83293323	0.01063053	13.15375612	5352.16269
3.787548343	9.341467678	0.091887498	6.656723454	1620.383888
5.268716255	13.16048437	0.006929547	42.17348539	89763.80359
4.494359647	8.595161656	0.010552567	11.60859644	4595.02853
5.041299544	14.73732348	0.007105545	31.84244771	24131.17513
4.164604085	10.03928963	0.027194999	6.804641872	1773.075744
4.349063411	9.454280477	0.017768155	7.695123866	1851.047936
4.227534121	10.15661345	0.030559106	7.486015389	1873.371039
4.117839253	7.58843196	0.020370905	5.891011571	1055.224972
4.756823789	12.69928226	0.007098947	16.85797631	7170.218692
4.484974304	8.738611931	0.010717806	11.06559866	4746.442455
4.405609716	10.71466043	0.013968484	20.72513467	7249.435806
4.012776734	8.309290137	0.019140811	13.64295113	2745.07514
4.481646672	12.7144171	0.033450003	11.0624878	5430.217722

log.sigma.4.0.mm.3D_glcm_SumAverage	log.sigma.4.0.mm.3D_glcm_Imc2	log.sigma.4.0.mm.3D_glcm_Imc1	log.sigma.4.0.mm.3D_glcm_DifferenceAverage	log.sigma.4.0.mm.3D_glcm_Id
24.39606712	0.850445786	-0.166698713	2.736292686	0.395701197
16.02798165	0.969414648	-0.362633529	4.255119592	0.295013962
20.12690112	0.842359381	-0.162993677	3.149312733	0.371066366
16.31038084	0.985067129	-0.435665653	4.538477453	0.27873873
14.27724431	0.699256822	-0.098439872	2.531509161	0.409624993
17.75672549	0.981395389	-0.431262074	3.842589637	0.308975247
20.91371117	0.894118798	-0.311659475	0.816793663	0.733155026
24.1097994	0.852682736	-0.192040501	1.632913341	0.528406398
19.61778778	0.881849187	-0.253020793	1.200710897	0.609515316
17.27849914	0.764668076	-0.131413599	2.341770839	0.422253142
32.49954978	0.818113775	-0.218125249	0.868653222	0.681980804
19.21747245	0.892336883	-0.259539004	1.151347145	0.624424339
18.86203747	0.900828663	-0.207826606	3.980409575	0.329826611
15.98069068	0.907854399	-0.236665658	3.448289686	0.339067308
18.54560141	0.820248779	-0.167782734	1.883702771	0.477990952
20.93477967	0.895579636	-0.27974178	1.221141741	0.638557225
11.56055121	0.763960579	-0.141329666	1.833914376	0.472412712
11.24161816	0.92356579	-0.277809335	2.990528615	0.367137083
13.43753283	0.829155109	-0.186125534	1.602681533	0.497761812
23.06413926	0.875209128	-0.230634609	1.236416248	0.616608011
14.66116933	0.77911051	-0.136188477	2.156359931	0.45059644
12.8904383	0.918316428	-0.252907458	3.098577818	0.367700182
16.03492261	0.871803465	-0.197451654	2.412428366	0.411291508
20.07353705	0.870520769	-0.282327506	0.77614514	0.712159698
24.42815211	0.878253041	-0.212250536	2.356930842	0.447168008
21.75434288	0.823184217	-0.162612776	2.08465242	0.48654542
20.51990652	0.801401601	-0.149652844	2.128604229	0.444925081
18.28235633	0.862125326	-0.207410237	1.484737611	0.532358233
8.640874993	0.97550988	-0.565206078	2.393895365	0.411481311
18.51065987	0.820949089	-0.16720678	1.731692321	0.49467388
19.5887345	0.863271367	-0.210949652	1.574077912	0.535002558
18.43206477	0.906061609	-0.223880061	3.598847473	0.345912074
18.34855476	0.826463243	-0.165082628	1.990524753	0.474267589
21.29894875	0.89613277	-0.260761185	1.251780095	0.603942217
16.41266761	0.790526787	-0.15083201	1.685406606	0.514942025
24.83902833	0.856748792	-0.172924511	2.366511811	0.44709717
19.96226411	0.901031634	-0.206660675	4.509983683	0.294251001
24.21911378	0.886002818	-0.265646859	1.094618157	0.660138982
13.96910879	0.887210058	-0.219992317	2.306548894	0.406478388
42.42249793	0.868280447	-0.244287195	1.046529468	0.65833564
16.69701304	0.712740693	-0.110010199	2.116862067	0.446072671
21.61694351	0.792312827	-0.206994435	0.801665116	0.699362996
22.97467408	0.806669897	-0.161590642	2.769475853	0.412239204
18.25355288	0.774801845	-0.133268814	2.245872842	0.431747404
17.35881763	0.900281963	-0.26208108	1.27407838	0.586144224
20.82165868	0.819157815	-0.162978205	1.949285801	0.48889692
21.93642198	0.898263109	-0.270278945	1.034118874	0.646572626
13.94078206	0.923836693	-0.272925655	2.087435933	0.440282189
12.93666673	0.950132299	-0.326528288	3.044729093	0.360331116
23.64020475	0.945385855	-0.269342033	4.186878523	0.305684186
24.85591544	0.908359353	-0.280296618	1.28382849	0.593702614
24.10469621	0.793017116	-0.138041921	2.263893116	0.454777812
18.65403689	0.864773649	-0.285997609	0.713582703	0.733687828
21.53303419	0.845172865	-0.191275813	2.009264993	0.44941367
17.55191362	0.908012001	-0.216995514	3.438746835	0.348729583
14.05477829	0.763122885	-0.125218842	2.398183736	0.426737313
23.05540651	0.884991023	-0.246942451	1.20281159	0.610766827
16.49674188	0.792220372	-0.142960237	2.070558928	0.459515723
13.62739187	0.827785631	-0.16682095	2.258922598	0.430073113
25.20220014	0.852897358	-0.268622089	0.745604167	0.724997125
20.41201514	0.773415197	-0.12861454	2.438021252	0.428156946
28.39009872	0.828096808	-0.149234692	2.732493219	0.406172434
29.31220421	0.768134393	-0.117767099	2.942316342	0.411244112
28.22094632	0.862198324	-0.208250305	1.527709864	0.581397503
15.9459467	0.842687784	-0.179466751	2.361461609	0.407066965
28.51555832	0.803251365	-0.128671403	3.08836754	0.373090053
15.75355315	0.926398543	-0.270210987	3.303902163	0.335701698
28.25803922	0.79249584	-0.149901764	1.802142805	0.510316975
20.34171602	0.878114967	-0.260825545	0.965798116	0.663275441
19.71115529	0.737604505	-0.109679722	2.510582801	0.416466073
11.49814114	0.825818125	-0.171803413	2.539548017	0.398954459
28.9991071	0.89778072	-0.27185702	1.171826582	0.647340425
14.45521148	0.682807831	-0.097010248	2.236619223	0.428019081
20.82067362	0.836331314	-0.177348106	1.771256771	0.50094853
25.39537407	0.859293114	-0.211559847	1.408316266	0.590304474
15.94761182	0.812686356	-0.16218984	2.142670127	0.442986772
26.10206716	0.899652301	-0.184197674	3.999298099	0.333006054
25.36209182	0.990874317	-0.468085516	4.638534442	0.28817625
20.05786595	0.81273331	-0.139873842	3.018807063	0.369623583
21.53822784	0.848832897	-0.212776333	1.23223353	0.588605626
24.39606712	0.850445786	-0.166698713	2.736292686	0.395701197
16.29223638	0.855525856	-0.176204513	3.063366219	0.369010081
27.39479736	0.893826759	-0.262705969	1.32201066	0.612038329
18.69490374	0.852911577	-0.205761061	1.380650462	0.547819972
19.88588769	0.824643687	-0.170966634	1.739137051	0.511650437
21.77935906	0.972060194	-0.336533664	4.65130969	0.274421481
23.66586647	0.871957308	-0.189950212	2.173521326	0.444633101
18.68293536	0.88507946	-0.266675645	1.082182236	0.658559316
26.05585333	0.899092511	-0.193638213	3.617040517	0.374980018
17.19032331	0.758930929	-0.120770924	2.557126853	0.410355757
29.35168731	0.959553955	-0.29040354	4.187620519	0.312265316
20.07857925	0.846906398	-0.20686166	1.416480673	0.550513135
18.90856095	0.804701228	-0.156285358	1.640388186	0.526977414
20.3132269	0.848715891	-0.197144744	1.463804897	0.565520179
15.17686392	0.713655648	-0.119312683	1.696732711	0.500302381
25.29404531	0.739002397	-0.103113689	3.294174971	0.361559071
17.4745299	0.710341592	-0.103477428	2.530715999	0.416127613
21.42932087	0.985488815	-0.444232368	4.200291291	0.291790133
16.61858027	0.977550746	-0.425013824	3.791644752	0.303340264
25.423757	0.874506781	-0.209986659	1.609641988	0.565107947

log.sigma.4.0.mm.3D_glcm_ClusterTendency	log.sigma.4.0.mm.3D_firstorder_InterquartileRange	log.sigma.4.0.mm.3D_firstorder_Skewness	log.sigma.4.0.mm.3D_firstorder_Uniformity
48.08311199	139.0560179	0.418757324	0.071337103
49.59960871	146.9870911	0.335623311	0.069934999
48.67072076	153.6301956	0.08325791	0.067671429
56.10850535	170.9058685	0.179983225	0.060727788
26.86483259	111.5522728	0.918590874	0.091318129
43.86845704	146.7326374	0.122902381	0.067772646
18.22118137	50.2323246	-1.267461822	0.223070493
33.93047041	125.0780892	-0.389522221	0.096762421
23.04352015	106.2737827	-0.502991387	0.127340239
18.65564329	87.23304558	0.250811858	0.105678662
10.68108087	38.06658077	-1.296569896	0.213497465
23.91378753	91.91262263	-0.597828983	0.132124911
63.55513499	201.7553635	0.44330116	0.059209025
31.30564375	139.8291054	0.412363151	0.077063712
29.3162579	117.7972813	0.086562793	0.09106569
29.84333945	94.86559731	-0.902985159	0.161769479
18.51280784	97.89670086	0.418554608	0.111312179
28.74635742	126.8036728	0.884926071	0.08789871
21.11266326	97.69342232	-0.19370397	0.10921369
25.47437165	66.70917749	0.052384805	0.135307603
34.44985309	122.4538975	0.64257214	0.084521018
39.27407722	165.8866138	0.337608517	0.074747637
35.37204479	126.3188858	-0.07902702	0.081774547
14.13254357	55.46156514	-1.253435781	0.231607057
47.48983316	152.6996908	-0.279994987	0.079658341
38.05974572	102.1742344	0.737621403	0.092347142
31.7984998	105.1475334	0.092207058	0.091767124
27.5546594	109.2334461	-0.270128938	0.098792824
12.12123686	94.61612129	0.526851386	0.137755102
26.81380395	106.9705601	0.057276955	0.095212036
32.80207587	127.9578753	-0.214842946	0.094979112
42.47497072	121.7593231	0.082866313	0.07432526
33.82181659	117.9860954	0.008302241	0.088829538
27.55626084	99.54667716	-0.475138994	0.129834097
24.92337036	103.8843899	-0.004285085	0.102075027
52.77428007	149.3404522	-0.453461787	0.071566445
55.79210234	189.1681747	-0.067155346	0.060815253
26.67416524	77.4128983	-1.136592012	0.173531516
31.05172751	111.6439514	0.25467705	0.087737381
17.98041216	62.41007059	-0.605189621	0.172459279
20.25410552	83.97434235	0.267378	0.10406092
7.616881906	39.78757	-0.372257617	0.212465939
50.85493089	137.2501869	0.038272734	0.069959674
28.84460962	108.6324768	0.578399893	0.089176931
27.80411707	104.7072451	-0.527463657	0.117310837
32.65267212	120.1761646	0.073195389	0.088169753
21.97293198	81.14051378	-0.801309309	0.135140368
43.57267471	160.9778357	0.036154852	0.080919914
41.11817965	140.1865664	0.570509071	0.079562942
66.46922273	163.8405132	-0.149710283	0.063057851
34.59844699	129.4678936	-0.685619589	0.123818416
38.6913544	105.7561836	0.463822179	0.085694254
12.17686429	45.4440192	-1.197529787	0.245523594
35.46150495	122.0957031	-0.014508835	0.084703426
58.90180289	168.6755905	0.4401184	0.061637266
29.90042676	110.848629	0.917845979	0.089307936
24.09456324	90.60238349	-0.546601155	0.122133727
34.19453912	128.5950012	0.121329752	0.085270577
25.79297614	103.807663	0.124817508	0.093777033
11.44875816	39.31633317	-1.123504068	0.235670404
31.16807684	121.7561378	0.271929676	0.086121261
58.46278208	161.0100155	0.289169354	0.06467949
58.62969679	134.8251872	-0.027081143	0.067793021
30.51735135	108.6252069	-0.503322018	0.118425742
28.87128867	112.8429756	-0.054608423	0.088091148
73.03072898	138.0061874	0.437978095	0.065884829
30.31212903	126.9482727	-0.024772332	0.0826919
28.0006211	104.6678128	-0.167798998	0.096812372
17.82942575	74.96852565	-0.514005368	0.159576116
37.08795145	128.8928413	0.381121614	0.077398789
20.36420721	107.8762379	0.310312124	0.094225079
35.49962088	104.2236893	-1.111093544	0.15690845
23.01329814	95.90345764	0.295980214	0.100810872
28.58136615	91.06420898	0.396212299	0.102416425
25.8870442	85.84236629	-0.629573864	0.129283232
29.20717318	104.5822558	0.400742326	0.093499189
184.836835	181.0035534	1.336908988	0.054291625
97.59239967	207.1472473	-0.363188521	0.056483932
49.11376174	151.0112705	0.296309357	0.066665723
19.25311616	88.76491499	-0.463705033	0.127994642
48.08311199	139.0560179	0.418757324	0.071337103
48.52895598	162.2243195	0.511645099	0.06694281
32.70779479	114.4327837	-0.706875767	0.145058806
23.4267927	92.94535065	-0.527270555	0.111792123
29.21618495	106.6156082	-0.277472256	0.096701836
86.62159678	219.7239799	-0.236120874	0.052750517
44.04755869	129.0298233	-0.23300182	0.078647325
23.44143479	88.84026742	-0.761562229	0.177008286
136.907754	127.7175521	1.427917251	0.068640652
35.15950754	129.3599701	0.687616392	0.080265625
96.16206113	153.9218254	-0.641632168	0.062539382
23.28519584	102.3178725	-0.487801313	0.113190865
25.24586631	97.92170715	0.094229651	0.099922084
25.24906974	100.6962374	-0.205775241	0.114083885
18.34362494	82.5733881	0.219696169	0.113337916
46.15469052	139.4693508	0.304401774	0.069470759
32.70440081	111.4517899	0.510014841	0.083566728
56.44181945	166.4772568	-0.434669715	0.068569164
33.28953969	133.3656349	-0.177059025	0.086689149
37.91695633	95.39125961	-0.291166428	0.10674035

log.sigma.4.0.mm.3D_firstorder_MeanAbsoluteDeviation	log.sigma.4.0.mm.3D_firstorder_Energy	log.sigma.4.0.mm.3D_firstorder_RobustMeanAbsoluteDeviation	log.sigma.4.0.mm.3D_firstorder_Median
81.06066586	17614676.03	57.09700251	-130.8847504
92.64354751	8636862.158	63.81996261	-263.3556213
86.22617846	18691354.2	62.27856444	-169.66539
97.59073863	6933033.706	69.23776734	-254.4674988
66.96957038	21096209.85	46.66262531	-155.9309921
87.58221935	2472191.051	63.19819514	-114.4957428
42.96955731	66366073.72	25.00975875	-4.470636845
66.84538667	71477686.98	50.1338427	-76.68587494
54.96394663	37899300.8	42.41940918	-56.06761932
55.15653116	4314795.363	37.32035336	-85.43636322
30.88111234	10829282.83	17.44784202	2.084449768
54.03005029	20355073.44	38.62023406	-27.53421402
108.4673009	21161563.29	83.74833697	-242.1421967
76.81578087	17497974.62	56.41564803	-298.1269226
63.44193131	21251556.02	47.76528038	-134.3267593
60.33143293	66266336.28	42.68208496	-15.89584351
52.79550173	4779366.539	39.80793606	-85.55139542
71.3861578	10771035.26	51.817751	-315.3710938
52.07900944	6486526.039	39.40005691	-81.59184265
50.51122936	64236346.51	30.54307909	-8.147875786
69.33655822	31888381.96	49.29347356	-129.5378952
84.01519525	6290658.105	63.88596077	-180.6924591
71.34151742	8092961.633	53.82376815	-145.5901184
39.40009701	18940403.19	25.37471775	-17.37140942
81.2298236	18528227.94	62.77546631	-128.6509323
65.07827974	23457057.02	42.62900025	-67.57011414
61.85793101	27675122.14	42.63389263	-153.6311188
59.33060082	21789370.07	44.47885666	-81.12424469
48.22515951	93299.34333	36.12854497	-14.20167208
60.15704121	18016232.42	43.30737743	-100.8462067
65.40242771	52121113.83	50.91376952	-88.36044312
79.01158059	19680579.3	52.89365318	-259.8783875
65.1360727	21185021.78	46.56227901	-94.91585541
59.44948544	23282601.54	42.84963908	-22.69070244
57.79762743	39478484.53	42.37626916	-77.03546906
82.91575745	34704818.25	61.55018327	-131.3023605
98.38311623	30635607.74	73.74437902	-244.3779984
54.86760767	71712001.97	36.00937193	-7.534134626
66.51256389	5680394.265	48.25536238	-159.1404114
45.50023585	57082291.1	28.34401922	-12.3604846
54.67612813	21900448.08	36.43225739	-171.8467102
29.3825366	13926210.67	17.69922124	-9.513074875
81.6513844	46990256.86	56.24892633	-141.9189758
65.5374037	21399684.74	45.09755447	-160.1517334
59.38238443	10419447.68	43.62383273	-46.45671844
66.05761279	32056325.25	47.86619484	-95.49419403
51.68410079	39518071.07	35.76695285	-18.15969658
78.89900065	4235393.395	63.98688489	-109.8120956
82.22585378	2931199.282	59.62949196	-148.3386154
93.13953885	6667969.134	66.27245543	-132.6173859
68.4664736	24288800.88	53.53840553	-57.79019547
69.00496854	30955425.28	44.76737636	-77.01139069
36.18205058	31523280.66	22.19495983	-9.664883614
68.15069521	7046777.09	51.47344993	-46.98600769
96.0799187	14410388.21	70.46035078	-194.6284943
67.48426104	20020486.74	46.01419869	-163.32724
55.11208791	38108244.93	38.71041178	-34.46193695
68.07140899	36199788	52.27022203	-134.894577
60.97180899	12769374.08	42.36188296	-199.3650436
34.06629951	64983758.45	19.22084769	-6.387158871
68.73397928	17226934.49	49.05528086	-121.5614777
90.10988681	34511736.58	64.91553422	-98.62651062
85.25428668	85942711.06	57.46978824	-77.916996
62.13506966	80316919.77	44.52120879	-37.76470184
63.65056052	5251998.163	46.4800705	-99.62034607
89.29854217	52293686.76	59.55311721	-114.0491333
72.07752232	10959072.5	53.22182578	-293.3016052
61.20033165	53966632.74	42.35519883	-79.6172142
47.21700328	46053794.12	32.88653395	-12.17156601
74.13250881	29041652.67	52.85074984	-105.4407501
61.63141425	7256498.305	45.72117383	-184.5146179
65.26402408	63266651.32	46.77961258	-14.5644207
56.6483448	27473349.9	40.11593263	-184.0120392
58.08099504	12436137.63	38.13405365	-71.11408997
54.61087233	21821083.22	37.08159831	-25.39852238
62.04527673	7235591.755	44.0839973	-80.03115463
125.2442523	44545861.6	79.28021372	-80.98415375
114.0839605	9860959.875	82.55968005	-240.3274078
86.52374894	12317421.08	62.93840748	-95.2917099
50.2040792	13581406.23	36.37989493	-57.47731018
81.06066586	17614676.03	57.09700251	-130.8847504
91.71280276	13486510.57	68.77791019	-180.15065
64.72556132	38381560.17	48.08656061	-47.88347626
53.33746586	18012546.24	38.4166348	-95.72893524
61.54262751	22660974.11	44.38954356	-72.82449341
115.847782	11892417.08	86.80521618	-208.2768173
73.37268224	14688214.17	51.86755935	-107.1112442
54.23078346	29247926.25	38.96195613	-27.06416035
101.8226397	27634393.23	56.20949888	-70.76800537
74.2774261	20529931.59	52.79302411	-139.3101425
102.6844611	8295861.076	67.23388648	-89.56376266
56.10040992	17165269.77	40.89006936	-76.09184265
57.17228797	34875143.63	40.1753884	-69.06999588
57.04745918	31054947.61	41.18005728	-44.91242218
49.91302341	17707842.26	33.9478919	-70.14273834
83.31900614	42798941.19	57.05107446	-164.2355804
68.48859971	27679882.71	46.64795905	-112.2428818
93.5186676	6045746.417	69.04679215	-216.985321
72.31562069	2730774.606	52.54786633	-166.5824127
65.85059224	85746209.07	43.80916119	-20.68602371

log.sigma.4.0.mm.3D_firstorder_TotalEnergy	log.sigma.4.0.mm.3D_firstorder_Maximum	log.sigma.4.0.mm.3D_firstorder_RootMeanSquared	log.sigma.4.0.mm.3D_firstorder_90Percentile	log.sigma.4.0.mm.3D_firstorder_Minimum
475596252.9	219.6908569	162.6298328	-1.445371568	-401.635437
233195278.3	51.9022522	276.4641179	-96.51100006	-451.2706299
504666563.4	90.86322021	196.7192311	-25.24920578	-423.7958069
187191910.1	19.13520241	274.5160506	-77.7504921	-455.8016052
569597665.9	253.4347534	168.1639131	-34.14991379	-300.1908264
66749158.36	179.0540924	154.9253203	26.24880219	-330.4441528
1791883990	266.0000305	63.79686584	12.25307655	-261.9309082
1929897548	189.3759918	119.0177188	-1.236379325	-352.2626038
1023281122	213.7476807	94.66659495	0.758359766	-291.2469788
116499474.8	182.8661804	108.8753113	9.195625639	-299.0757141
292390636.4	173.1877747	44.51890798	38.29273643	-394.1004333
549586982.9	248.8796692	81.38700307	13.18777294	-268.1233826
571362208.9	106.3103561	252.8482062	-32.75299072	-454.6209106
472445314.7	-26.22291374	303.4709266	-162.0837067	-479.7486877
573792012.6	138.0035858	151.9852539	-30.19865379	-325.7597351
1789191080	210.5457764	88.15528393	10.77004223	-286.9482727
129042896.5	172.1480713	104.6988532	-6.189440489	-206.9305878
290817952	19.57257462	311.5065869	-187.088562	-429.6987
175136203.1	59.84341431	106.9583152	-9.120912743	-230.2298584
1734381356	489.9452515	73.38795846	47.93389511	-298.7195129
860986313	187.3547058	148.1947893	-13.20077353	-286.54776
169847768.8	50.1603775	199.5351894	-29.15737801	-344.3106079
218509964.1	78.1384201	169.7073222	-37.34955215	-332.2230225
511390886	137.462616	62.79037987	4.310406876	-253.4677582
500262154.3	88.24333191	159.7528564	-8.350152493	-417.849762
633340539.5	344.0308228	107.6277467	30.23721428	-321.8006287
747228297.7	189.330658	171.6769452	-51.49485016	-387.5480042
588312991.8	127.2921371	111.2985273	0.705005336	-297.8821411
2519082.27	119.545929	57.72451303	83.27793808	-88.40011597
486438275.4	135.7500458	125.1106632	-10.02842236	-307.6369629
1407270073	196.7530823	121.8404318	-1.557679653	-315.5822449
531375641.1	6.553898335	277.8106232	-126.2012695	-478.9936218
571995588	216.3242493	128.7001891	-4.691549778	-314.4280701
628630241.5	174.6266785	85.98633185	20.97301445	-285.6113281
1065919082	210.5703583	106.5559956	0.568438649	-270.1746521
937030092.6	67.72743988	175.404341	-22.730583	-442.0382385
827161409	53.13142014	280.2727963	-108.1204369	-521.1390991
1936224053	282.5435181	78.16257723	22.03049831	-309.2941284
153370645.1	51.59703827	169.8072102	-26.03565292	-314.5351563
1541221860	357.8153076	68.6446845	20.26917801	-525.218811
591312098.2	84.39678955	186.2995563	-80.89227295	-358.77771
376007688	202.7806854	43.86692519	25.68041229	-254.8095551
1268736935	230.107666	175.4221344	-15.32148991	-417.552124
577791488	189.6593781	173.9778585	-44.17879639	-356.1212769
281325087.3	122.8264313	97.86060614	5.586576366	-265.3332825
865520781.8	207.4850006	128.0511512	-3.781261778	-338.9873047
1066987919	249.3842926	75.30306212	25.95542717	-279.9866943
114355621.7	63.64906311	139.0672351	13.81298485	-267.8631287
79142380.62	122.9419708	160.3504737	29.25779819	-288.5838318
180035166.6	130.2254028	174.0946599	3.320017934	-410.3618164
655797623.7	72.04199219	112.8863911	0.354791477	-352.5093994
835796482.6	415.9063721	119.7407754	27.86528511	-366.8471375
851128577.9	240.4362335	55.86142655	6.241706371	-228.6392975
190262981.4	156.2913513	91.21233249	63.91163864	-296.9789124
389080481.7	126.431076	224.0767991	-23.5319355	-410.3185425
540553142	222.2507629	173.772301	-43.42687607	-323.3219299
1028922613	216.3128204	84.89943866	18.53417206	-311.8743896
977394276.1	127.9441223	153.0695381	-17.96875	-305.3019104
344773100	29.31306076	216.670783	-110.1382126	-374.7564087
1754561478	357.9217529	52.5712606	11.75970001	-310.06427
465127231.2	177.3782959	147.0194658	-23.85396919	-353.5623169
931816887.6	308.0436401	146.7292323	37.41349716	-425.4386597
2320453199	287.5590515	137.1044067	51.41515007	-440.5011902
2168556834	303.8912964	97.09206207	8.780505657	-383.5424194
141803950.4	81.66709137	127.9115514	2.643993855	-287.4702148
1411929542	316.418335	154.7030621	53.16061859	-441.2879028
295894957.4	-111.5005493	303.4683611	-169.6544556	-479.435791
1457099084	235.2198944	114.3385858	0.680556512	-402.625061
1243452441	264.8175659	67.78503777	15.31885548	-267.6990051
784124622	215.4111176	137.908535	7.623600769	-347.2617188
195925454.2	32.77001572	191.4392367	-74.29171066	-312.9497986
1708199586	110.3403702	94.40366084	18.10775719	-375.6906433
741780447.3	8.77875042	191.5203381	-78.5810257	-347.7705688
335775716.1	321.2546692	107.657155	4.364488602	-307.6135559
589169247	282.8084106	82.372099	19.55426598	-329.1979675
195360977.4	187.551239	109.4507037	17.59934158	-264.7049866
1202738263	602.182251	173.3722478	167.6679962	-328.7406311
266245916.6	-20.50345039	292.8267016	-81.67729187	-597.8307495
332570369.2	282.3504028	137.5527888	51.36514282	-329.0068359
366697968.2	180.4825897	92.2477806	-3.267640591	-317.8158264
475596252.9	219.6908569	162.6298328	-1.445371568	-401.635437
364135785.4	166.6270905	191.1774284	3.58725853	-351.260376
1036302124	242.464447	105.968133	-0.317988271	-383.8348083
486338748.6	51.4651947	123.8136769	-29.24237061	-310.2589722
611846301	180.4410553	112.0471164	1.62628634	-303.4085693
321095261.1	33.02777481	259.9433622	-47.27469254	-513.789978
396581782.6	170.317688	142.3362419	1.688473606	-376.4408264
789694008.8	206.3126373	84.81327868	2.036008596	-256.9204102
746128617.1	510.1351624	153.3578577	150.2348846	-341.4049683
554308152.9	234.9547272	160.1949265	-9.279654408	-345.2658386
223988249.1	166.7703247	173.3708963	34.82722473	-488.1168518
463462283.9	156.7032318	109.5613306	-13.439291	-324.6588135
941628878	266.6611633	100.9527142	11.51123257	-287.9038086
838483585.6	303.4136353	89.76551882	15.19072952	-292.5661011
478111741	186.2398682	92.55770637	15.88405266	-248.2359619
1155571412	285.0954285	190.1256279	-17.34198322	-463.7037048
747356833.2	301.3464966	137.4558762	7.420376968	-308.7012939
163235153.3	-43.33333969	263.6120978	-108.6658829	-505.1582642
73730914.35	39.05835724	196.1163281	-68.72802734	-375.1503601
2315147645	318.0856934	94.48902955	46.98927345	-345.9091187

log.sigma.4.0.mm.3D_firstorder_Entropy	log.sigma.4.0.mm.3D_firstorder_StandardDeviation	log.sigma.4.0.mm.3D_firstorder_Range	log.sigma.4.0.mm.3D_firstorder_Variance	log.sigma.4.0.mm.3D_firstorder_10Percentile
4.028155216	101.6824519	621.3262939	10339.32102	-246.2518768
4.055491739	115.6811432	503.1728821	13382.12689	-413.3650208
4.045927032	105.0665027	514.6590271	11038.96999	-301.4311951
4.171455338	119.2724909	474.9368076	14225.92709	-411.0955811
3.690096638	85.06987229	553.6255798	7236.883172	-242.5567398
4.035358345	106.6410188	509.4982452	11372.3069	-260.8787109
2.757248642	57.59419523	527.9309387	3317.091324	-116.6083832
3.624730484	80.1347148	541.6385956	6421.572516	-198.5644608
3.2325724	64.99714824	504.9946594	4224.62928	-163.5070007
3.501060556	70.52864858	481.9418945	4974.290271	-164.9586517
2.684971027	44.17534499	567.288208	1951.461105	-61.83883705
3.271738315	66.40003521	517.0030518	4408.964676	-148.5046997
4.23596854	127.6339827	560.9312668	16290.43353	-364.4593811
3.861397292	93.23837453	453.525774	8693.394486	-404.1117462
3.605800206	76.04598316	463.7633209	5782.991555	-231.3539795
3.195634467	73.16198551	497.4940491	5352.676124	-170.382605
3.332838841	63.51948711	379.0786591	4034.725242	-162.7753677
3.678500834	88.68928205	449.2712746	7865.78875	-401.7892456
3.314919124	62.13123212	290.0732727	3860.290004	-177.0332458
3.366463086	69.91018786	788.6647644	4887.434367	-117.5402786
3.754397477	86.71173953	473.9024658	7518.925772	-226.8784851
3.87439776	99.71319091	394.4709854	9942.720442	-295.7654175
3.744472363	85.60501928	410.3614426	7328.219326	-266.7974548
2.675006021	50.682848	390.9303741	2568.751081	-116.3612
3.851635869	95.73116123	506.0930939	9164.455231	-251.7191696
3.744171063	86.05966871	665.8314514	7406.266578	-165.5974152
3.677507649	78.76138479	576.8786621	6203.355735	-247.0249603
3.509102185	71.19197678	425.1742783	5068.297557	-182.8256409
2.999169548	57.71334031	207.9460449	3330.82965	-63.38068047
3.591240567	73.91312875	443.3870087	5463.150602	-192.4917755
3.552816667	76.81247696	512.3353271	5900.156617	-200.8175964
3.96583948	100.5298366	485.5475202	10106.24805	-385.4278625
3.70302545	81.34853367	530.7523193	6617.58393	-207.1475861
3.373513073	72.03812219	460.2380066	5189.491048	-155.2441223
3.499973948	70.6512307	480.7450104	4991.596399	-173.6293823
3.959416133	99.98408981	509.7656784	9996.818215	-283.5968658
4.200513401	117.8968731	574.2705193	13899.6727	-416.3513855
3.086514788	69.39506624	591.8376465	4815.675218	-149.2339462
3.676263356	81.20617702	366.1321945	6594.443186	-254.6804108
3.103103021	61.18604208	883.0341187	3743.731745	-114.9489258
3.529252041	71.22131441	443.1744995	5072.475626	-266.2784424
2.663757206	40.93105567	457.5902405	1675.351318	-69.10156555
4.062918757	103.0720606	647.65979	10623.84967	-280.5543091
3.726933674	83.13216616	545.7806549	6910.95705	-250.2537018
3.406842241	71.36512751	388.1597137	5092.981424	-174.0779358
3.717178534	81.6888908	546.4723053	6673.07488	-204.8906555
3.249249967	64.07060743	529.3709869	4105.042736	-135.3017609
3.699317084	89.78147158	331.5121918	8060.712639	-224.3950623
3.813979152	98.45968913	411.5258026	9694.310383	-245.7870529
4.169754468	114.8306849	540.5872192	13186.0862	-289.3729187
3.40708711	79.81466464	424.5513916	6370.380692	-197.859993
3.870142827	92.70129347	782.7535095	8593.529811	-180.2976959
2.575012457	47.76362749	469.075531	2281.364111	-103.0952888
3.707899879	80.9629005	453.2702637	6554.991257	-144.3201538
4.177054642	117.345946	536.7496185	13770.07105	-332.7001465
3.72561146	86.66857979	545.5726929	7511.442723	-249.7139374
3.383769547	68.19277917	528.1872101	4650.255131	-153.6058716
3.657081469	80.64474465	433.2460327	6503.57484	-237.2920013
3.601973349	75.88408344	404.0694695	5758.394119	-302.4855804
2.640754847	47.62049769	667.9860229	2267.7118	-88.92125549
3.767261996	84.95187489	530.9406128	7216.821047	-223.6690277
4.161614834	111.1848866	733.4822998	12362.07902	-231.789801
4.157214207	109.0961421	728.0602417	11901.96823	-225.5515945
3.477518625	77.19367783	687.4337158	5958.863897	-162.806044
3.652035972	77.63088843	369.1373062	6026.554838	-202.8834534
4.210058238	115.3697249	757.7062378	13310.17343	-234.8444672
3.707716148	87.73510229	367.9352417	7697.448174	-416.955658
3.652486391	78.11735463	637.8449554	6102.321095	-179.0119156
3.074637151	58.69426708	532.516571	3445.016988	-122.3829636
3.893105853	91.9832729	562.6728363	8460.922493	-218.2174927
3.536059226	73.88199222	345.7198143	5458.548774	-271.1989838
3.186939253	78.94494217	486.0310135	6232.303894	-182.6371002
3.501364391	70.40299648	356.5493193	4956.581913	-266.8599426
3.608160208	77.04375552	628.8682251	5935.740264	-174.1069305
3.35906691	69.38457147	612.0063782	4814.218757	-141.8503647
3.631275124	77.33688453	452.2562256	5980.993709	-171.8960464
4.516026094	167.3876595	930.9228821	28018.62854	-218.3396179
4.28887823	139.6310097	577.3272991	19496.81888	-459.5483582
4.082680529	105.3372327	611.3572388	11095.93259	-224.0322418
3.231996285	61.92157227	498.2984161	3834.281112	-154.7962189
4.028155216	101.6824519	621.3262939	10339.32102	-246.2518768
4.059551535	109.5717058	517.8874664	12005.95871	-288.07146
3.332857528	78.61246886	626.2992554	6179.92026	-188.5935989
3.36566548	65.39096801	361.7241669	4275.978698	-197.4196991
3.593114012	75.70383599	483.8496246	5731.070784	-193.868103
4.370257819	138.0701451	546.8177528	19063.36496	-424.8422394
3.884508338	91.57832885	546.7585144	8386.590315	-228.6991394
3.065785921	66.3233606	463.2330475	4398.788161	-159.552948
4.289768723	146.3678288	851.5401306	21423.5413	-189.2424927
3.842919823	92.88046906	580.2205658	8626.781533	-241.250531
4.286641756	132.5333076	654.8871765	17565.07762	-308.0007935
3.416268379	68.64782202	481.3620453	4712.523468	-173.2942978
3.558741988	71.6606804	554.5649719	5135.253116	-164.4142807
3.438409937	70.39218083	595.9797363	4955.059122	-155.3730453
3.392456101	63.70125898	434.4758301	4057.850396	-147.6635895
4.097598934	105.5324179	748.7991333	11137.09124	-282.6384796
3.824586926	87.68843048	610.0477905	7689.260841	-213.0230072
4.038751441	110.9602976	461.8249245	12312.18765	-401.2569031
3.750670743	89.78666897	414.2087173	8061.645924	-304.7606201
3.678357283	84.88388451	663.994812	7205.273849	-164.7422455

log.sigma.4.0.mm.3D_firstorder_Kurtosis	log.sigma.4.0.mm.3D_firstorder_Mean	log.sigma.4.0.mm.3D_glrlm_ShortRunLowGrayLevelEmphasis	log.sigma.4.0.mm.3D_glrlm_GrayLevelVariance
3.317676058	-126.9217928	0.010859793	17.16867164
2.726558831	-251.0981513	0.039293905	21.37302101
2.489835946	-166.3114125	0.018970434	17.92886127
2.298590715	-247.2511576	0.061355209	23.08416881
4.183203027	-145.059362	0.029449704	12.13502
2.475883205	-112.3812616	0.030141249	18.45653903
5.210464573	-27.43991193	0.014328749	7.621418524
2.707381998	-87.99798225	0.010099069	10.69958529
2.662099019	-68.82684738	0.015196867	6.746073853
3.517359399	-82.94301142	0.019395062	8.34022444
7.137812724	-5.520150659	0.003434487	4.026301096
3.174709171	-47.0625073	0.017672602	7.869253239
2.190905264	-218.2699747	0.020548934	26.66801351
2.531865488	-288.7926744	0.026761706	14.26892153
2.518187372	-131.5922713	0.01833075	9.337202563
3.008504863	-49.18005653	0.015422667	10.04306229
3.117580854	-83.22934949	0.050066467	6.730922514
3.749690447	-298.6144085	0.073603319	12.95853498
2.180748709	-87.06199627	0.03667626	6.35568241
6.306034285	-22.32393511	0.009348713	9.958161709
3.371372338	-120.1780754	0.035852865	12.29897234
2.171712636	-172.8339416	0.057266293	15.65687641
2.231304752	-146.534487	0.029614127	11.67711231
4.446060116	-37.06589703	0.012423866	5.203411573
2.374408202	-127.8926108	0.013081583	14.41402499
4.791634458	-64.63331404	0.012234082	13.03494807
3.552196332	-152.5438225	0.014824733	10.20653954
2.532140809	-85.55153194	0.022504282	8.263469888
2.223318494	-1.135673757	0.11741056	5.29181058
2.786473213	-100.9431892	0.017264306	9.041489161
2.311740894	-94.57766223	0.016342591	9.213928896
2.855261048	-258.9835792	0.025931908	16.44304163
3.20273786	-99.73041038	0.021710612	10.92664004
2.815743406	-46.94846342	0.013095736	9.445619985
2.949068034	-79.76580592	0.027708059	8.280314571
2.491566297	-144.1175375	0.011640712	16.23803289
2.27408756	-254.2698717	0.025405851	22.40722037
3.951996021	-35.96822572	0.009928449	9.393337496
2.381463604	-149.1309675	0.048891865	10.89433109
5.987425267	-31.11849877	0.002050645	8.008458966
3.395301056	-172.1483345	0.022967286	8.62311612
5.855069766	-15.77833348	0.007747273	3.764599467
3.048116026	-141.9474395	0.014621576	17.54389839
3.595886251	-152.8310773	0.01810273	11.58300632
2.631347768	-66.9605616	0.027524894	8.835992344
3.115551716	-98.61045804	0.014309257	11.08799263
3.370361685	-39.56650641	0.011262359	7.685483956
1.76146924	-106.2025575	0.067360329	12.51223956
2.522830163	-126.5620955	0.056775492	15.46059162
2.624493783	-130.8543634	0.020750275	21.85625595
2.500062245	-79.83079981	0.010220852	10.526334
5.52408782	-75.79131541	0.011890616	14.94758352
5.152332685	-28.96782466	0.014397809	5.076163073
2.319426266	-42.00831276	0.012691345	10.53950156
2.517957599	-190.8935327	0.028440563	22.15526455
4.295553869	-150.6166321	0.040612058	12.61150988
3.12568862	-50.5733087	0.009680789	8.45887055
2.189314703	-130.102685	0.026782431	10.00687123
2.776816315	-202.9478605	0.047804365	9.44241033
7.346440968	-22.27163309	0.006090048	5.456267663
3.113258678	-119.9912592	0.013314841	12.05802056
3.073282131	-95.74648083	0.006975817	20.51665015
3.291552853	-83.04005127	0.006983566	20.04547743
4.032921679	-58.88976668	0.006495958	10.82626178
2.38412644	-101.6602683	0.030001188	9.862906163
3.438176685	-103.0672789	0.008458088	21.90536274
2.232132091	-290.5092047	0.032393679	12.38987265
4.007116404	-83.49246133	0.006201479	10.62908108
3.484112399	-33.90861775	0.01153216	6.78964301
3.124790838	-102.7513578	0.015378748	14.08332494
2.364914311	-176.6081328	0.070544944	9.051091047
3.187187049	-51.76627556	0.006318106	11.17110457
2.628589876	-178.1108026	0.037935294	8.117582856
4.669051881	-75.19523101	0.013524505	10.381037
4.085607732	-44.39531435	0.007973263	8.862862415
3.236950915	-77.44974396	0.026867923	9.771768766
4.893068623	-45.1586953	0.01319518	45.28577309
2.345436006	-257.3920323	0.021341568	31.36523061
2.71631122	-88.45810942	0.016479449	18.21089599
3.293392195	-68.3766913	0.010784841	6.591427784
3.317676058	-126.9217928	0.010859793	17.16867164
2.479920146	-156.6615793	0.025965451	19.39725711
3.314987811	-71.05860231	0.007445801	10.6181913
2.79185047	-105.1372812	0.019996996	7.12286258
2.806765333	-82.60439159	0.014108184	9.563662913
2.143948212	-220.2434712	0.033124408	30.64527483
2.915542472	-108.9633674	0.011869689	13.69230843
3.146249519	-52.86306915	0.01899669	8.140841555
5.642851993	-45.77216654	0.015896105	35.71612656
3.473742552	-130.5206227	0.019881354	14.07575201
3.104606082	-111.7693609	0.012232941	28.25369454
3.341769722	-85.38829948	0.018479043	8.02523394
3.352879606	-71.10694333	0.015471623	8.79437084
3.504382253	-55.70268617	0.013932699	8.689625688
3.17911902	-67.14967321	0.027238338	7.019657382
3.249818907	-158.1475993	0.00945095	18.64583583
3.648883255	-105.8529974	0.022304128	12.99360731
2.347907189	-239.1216228	0.02774652	20.41799174
2.495804846	-174.3558667	0.037201796	13.72152506
3.776793665	-41.50786499	0.009472034	13.6420914

log.sigma.4.0.mm.3D_glrlm_LowGrayLevelRunEmphasis	log.sigma.4.0.mm.3D_glrlm_GrayLevelNonUniformityNormalized	log.sigma.4.0.mm.3D_glrlm_RunVariance	log.sigma.4.0.mm.3D_glrlm_GrayLevelNonUniformity
0.011522842	0.070212285	0.121278736	42.43875595
0.041855406	0.069132089	0.049284676	7.462606584
0.02055923	0.067143282	0.13215164	29.44989091
0.065981196	0.060504516	0.038490592	5.36213186
0.032466902	0.088648625	0.146581959	59.09834715
0.03089093	0.067572129	0.035847328	6.721296627
0.017657646	0.128128399	4.876345515	1054.831237
0.011226743	0.087379394	0.62568933	346.9433301
0.017735217	0.110365331	1.130739794	325.6494764
0.020727496	0.102593955	0.145837911	33.63915393
0.004371795	0.172479303	1.408075739	608.5067099
0.020623701	0.107754383	1.188339737	226.6463464
0.021945453	0.057491518	0.084602362	17.70024947
0.028065474	0.076015023	0.059852052	13.62520274
0.020111771	0.090962098	0.248669847	71.14087536
0.018114474	0.102020698	1.913746235	556.356119
0.056926542	0.110456042	0.201846378	41.71381691
0.079068134	0.086451958	0.06489752	8.984921528
0.041507452	0.10938945	0.227296997	52.7689556
0.011209776	0.102859611	1.339654077	825.4908407
0.040541873	0.084323077	0.222463364	104.9321504
0.064151066	0.073549819	0.084780783	10.76809488
0.032296676	0.08164633	0.123527701	20.73479377
0.015459368	0.145893976	2.720029635	401.4019981
0.014001381	0.075493182	0.281471405	46.93885882
0.01384347	0.087782783	0.363257468	146.0424208
0.016178708	0.091156461	0.172382273	75.39165128
0.025135214	0.096904353	0.387488853	136.8242023
0.124970046	0.13433008	0.066137108	3.535163583
0.019390711	0.093432518	0.299776729	89.46615528
0.018652258	0.092289132	0.537198375	252.0525562
0.028313925	0.073854893	0.088187838	17.46867458
0.023772341	0.087651531	0.224121641	96.25826981
0.015085443	0.097040675	1.048986302	215.497212
0.030939182	0.09923553	0.407318796	280.2815731
0.012513905	0.070080393	0.224707044	68.50548698
0.026708871	0.060663705	0.048022847	22.60689317
0.011792124	0.114537941	2.080063272	830.2845025
0.053349673	0.087144729	0.091724315	15.80390577
0.002578046	0.115608549	1.913133495	876.3828206
0.024939377	0.100642324	0.158338777	55.91059954
0.010110012	0.162562648	1.970600772	716.9192446
0.015855586	0.068883521	0.20049631	92.92916278
0.019656471	0.086882538	0.165920907	54.30809429
0.03140435	0.098631155	0.647943007	80.23615756
0.015936823	0.086565318	0.35727586	139.5068016
0.013494181	0.10538851	1.611781022	477.1161674
0.075340467	0.080878662	0.148019894	15.67182523
0.060607957	0.079145149	0.066833018	8.468660934
0.021383347	0.062581259	0.056065361	13.05587866
0.011430035	0.091238553	0.954295732	127.1902022
0.012959464	0.082429311	0.234526601	152.789229
0.018512821	0.153180764	3.283748071	821.4531567
0.013737955	0.084909544	0.15641798	63.77205869
0.030511116	0.061190594	0.086596746	16.33258086
0.04471526	0.087268053	0.16012249	51.13749573
0.01132249	0.099561211	1.170327974	369.8375714
0.029480661	0.086461927	0.286367135	113.0122667
0.056357292	0.092798851	0.122862287	22.70163061
0.008134752	0.153388378	3.083803023	1933.277876
0.01447036	0.083768874	0.186564247	58.70865737
0.007543495	0.063499953	0.187169321	90.03680736
0.007520209	0.065465844	0.199037036	264.1959553
0.007433625	0.095579069	1.212157057	580.347774
0.03211757	0.08744128	0.131446194	25.4315385
0.00908039	0.064548874	0.130396327	127.7396734
0.033695172	0.0826082	0.058547231	9.336465793
0.006932149	0.092703196	0.440989462	310.5593601
0.014406691	0.11831917	1.814843813	733.4283181
0.016962825	0.076114377	0.167534793	102.5269596
0.076324616	0.093451542	0.108305959	16.91688144
0.007470813	0.100710503	2.094344342	446.6962465
0.041364127	0.099477516	0.153863453	65.92931225
0.014994401	0.097390172	0.289984398	87.28466493
0.009144799	0.106151321	0.778726573	252.6125885
0.029836703	0.092280483	0.200685099	48.81735172
0.014264963	0.053340319	0.106642042	72.55108954
0.021643869	0.056376013	0.041985525	6.210137986
0.017557433	0.065713143	0.106858366	39.29180638
0.012443259	0.115648241	0.719098536	137.2332472
0.011522842	0.070212285	0.121278736	42.43875595
0.028421156	0.065506476	0.10232491	22.23188577
0.008600172	0.097229219	1.411870892	228.2314651
0.022218892	0.108130464	0.3566408	101.9449966
0.016064978	0.092798816	0.357458774	136.7311616
0.034586654	0.052906017	0.044565401	8.911971559
0.012757702	0.077822008	0.156834291	50.13086859
0.022599094	0.10916607	2.118873222	276.183175
0.016979689	0.066022579	0.156063773	69.26983417
0.021878921	0.078749299	0.160784383	56.08289576
0.012749777	0.061554126	0.064689678	15.96650567
0.021026762	0.104575198	0.452809371	118.9318944
0.017594131	0.097227273	0.423670063	267.5448425
0.016016333	0.0994542	0.806737353	283.7809398
0.030127083	0.108114167	0.267249355	190.0726748
0.010037294	0.067978069	0.105773972	73.87081836
0.024227966	0.080934418	0.17975749	105.0751625
0.028283232	0.068336139	0.045478475	5.708773782
0.037944207	0.085745127	0.037751577	5.857668708
0.01083464	0.081863426	1.061418609	569.7492883

log.sigma.4.0.mm.3D_glrlm_LongRunEmphasis	log.sigma.4.0.mm.3D_glrlm_ShortRunHighGrayLevelEmphasis	log.sigma.4.0.mm.3D_glrlm_RunLengthNonUniformity	log.sigma.4.0.mm.3D_glrlm_ShortRunEmphasis
1.339134772	161.7015204	504.8207727	0.930323662
1.14912819	112.0867365	98.80619625	0.965307955
1.351896772	129.0047118	369.2376289	0.932440542
1.118719498	114.1742203	82.34650317	0.971362444
1.401085001	69.98004605	541.9820758	0.920436038
1.110429426	118.1426283	92.89914984	0.973292329
8.948186708	66.15812058	3836.161955	0.708502168
2.260167029	123.2263975	2789.169957	0.861276396
3.238432769	70.32755142	1804.009219	0.805059658
1.385198703	89.13799528	273.0920285	0.928205293
3.89373283	196.878513	1872.493412	0.753000963
3.37460191	70.86469377	1250.773703	0.79637534
1.242805456	142.9954785	268.8630315	0.947613021
1.186063346	93.97447075	159.7469684	0.955435501
1.642646797	82.27330131	589.5036205	0.889348929
4.468641519	78.91343821	3101.578752	0.777712785
1.540552528	42.7015237	292.7672925	0.900727304
1.207313065	56.00786368	90.95039761	0.949026435
1.628713948	49.77521563	363.3550328	0.885177054
3.585742965	110.5846251	4686.548188	0.790870294
1.589411949	65.56225292	948.1003735	0.894534831
1.254663169	74.23373065	126.5287309	0.94276699
1.355866226	81.72891259	210.9982564	0.926473204
5.913306817	66.21385116	1381.405585	0.733082631
1.666389407	142.3698323	485.9404344	0.900945915
1.86392523	117.9651567	1205.827728	0.873840879
1.470542061	108.4034079	659.5565092	0.910634864
1.953789805	72.21513099	984.8954387	0.858859954
1.206107824	25.53065414	23.36230331	0.952034298
1.757738873	86.90812199	698.0852572	0.876866415
2.210814344	79.60846163	1860.619748	0.848584092
1.25628527	117.8159641	205.2039559	0.944131737
1.590520244	89.80069339	840.1390643	0.895210441
3.103504807	88.68377889	1369.327859	0.810674939
1.936261415	64.6347873	2029.252508	0.870508544
1.561095375	156.3788818	764.8045838	0.904535527
1.145504998	148.6005082	341.2380053	0.965655064
4.780640716	99.9630052	3934.435209	0.762767428
1.275637777	65.25448326	154.743955	0.938321922
4.530040686	335.7933879	4122.395624	0.763371561
1.451912252	76.7452519	439.1268279	0.908911818
4.749625389	89.27206108	2255.413381	0.738850868
1.499725997	145.9299416	1098.818015	0.917245585
1.451056037	94.0347462	500.5545996	0.91355519
2.475172664	65.62300486	523.5893065	0.825260201
1.842785468	104.5384726	1180.711016	0.877782606
4.036009777	86.99496012	2541.31025	0.77570852
1.44925088	59.38060396	155.1252421	0.907231584
1.20901618	69.7162028	94.76817249	0.951210762
1.170226645	165.1149138	187.9654401	0.959790123
2.862733934	116.6479466	914.4390039	0.832661033
1.598014839	155.2893706	1423.379122	0.897447234
6.988326436	53.92191743	2374.795497	0.689614381
1.438929787	116.4440387	604.5643664	0.913721694
1.245438219	118.8092494	233.2219602	0.947753556
1.442367712	66.02653595	466.8721574	0.912610429
3.254493848	103.717148	2284.465001	0.809112347
1.693458299	67.03775402	995.6988827	0.893459434
1.363526458	60.96024158	200.0548934	0.921886951
6.711940468	102.7643509	5555.686293	0.687066723
1.487192854	118.1664549	560.4812273	0.91266328
1.471457797	219.7618715	1147.322594	0.917481873
1.488433389	231.6127984	3265.04665	0.917402411
3.214704841	159.3880893	3901.395631	0.827095322
1.364001619	77.25202816	245.0153002	0.929442665
1.351192656	218.1775148	1654.380381	0.931043172
1.169368065	90.57323262	102.5099348	0.962308667
1.97871729	180.2092421	2417.946002	0.871867901
4.509839459	74.3493527	3245.758636	0.74932106
1.456716247	115.384074	1073.8511	0.912344517
1.312823856	48.55479389	153.690761	0.934667766
4.708519074	141.9236328	2518.54342	0.779950043
1.435793662	58.10958207	528.5192715	0.912207056
1.731938231	106.0336529	657.7066395	0.879830452
2.630442673	133.0995441	1537.395257	0.828966993
1.527667119	74.35290656	417.3362554	0.905240089
1.295633785	201.3775611	1160.439204	0.938927167
1.13371467	226.2884181	101.0206888	0.966571332
1.294792022	134.8218427	511.7700319	0.939799264
2.557877057	94.5466001	764.9588853	0.827557983
1.339134772	161.7015204	504.8207727	0.930323662
1.288623578	105.1401783	290.2496158	0.939124676
3.616422927	139.492576	1455.609352	0.809878568
1.919426927	75.213242	648.5965299	0.854284546
1.872783459	97.04889535	1052.112249	0.867993953
1.137457362	188.2318686	154.4576957	0.96668141
1.428031707	148.3341096	517.6442426	0.915573474
4.748509877	62.51502262	1430.283678	0.778772091
1.417499528	187.162904	856.3404791	0.920803925
1.425099819	97.36057509	578.4606938	0.919812071
1.198150926	275.409467	229.3535568	0.952978253
2.067982757	91.08178437	791.3682922	0.854650831
1.982887645	87.66304697	1938.650888	0.863200999
2.659676971	87.90818026	1850.083001	0.830030802
1.661036544	62.60262044	1334.496552	0.892133707
1.294888128	184.8726238	927.113873	0.938768759
1.461283422	94.2595764	1048.488336	0.916575923
1.133616689	158.3862374	77.41333073	0.969913417
1.120587744	102.1035235	63.24628144	0.969853064
2.988633776	141.0166466	4528.501908	0.83175052

log.sigma.4.0.mm.3D_glrlm_LongRunHighGrayLevelEmphasis	log.sigma.4.0.mm.3D_glrlm_RunPercentage	log.sigma.4.0.mm.3D_glrlm_LongRunLowGrayLevelEmphasis	log.sigma.4.0.mm.3D_glrlm_RunEntropy
225.5954643	0.907484407	0.01463109	4.484845523
122.9285442	0.955071477	0.053063761	4.236649509
178.2987405	0.908106386	0.028934669	4.460694806
126.5747662	0.963210702	0.084550293	4.298270626
92.95135214	0.893586306	0.047327293	4.218787654
129.5433354	0.965646004	0.033914646	4.17311567
1157.69641	0.502755003	0.0836363	4.90138119
384.0757059	0.786456904	0.019664348	4.548616391
381.6323076	0.697200648	0.03989772	4.417101088
123.107717	0.90046492	0.027573047	3.968420718
1066.56702	0.643752112	0.015035921	4.259516061
378.7724976	0.683346266	0.046276892	4.572716934
165.1546618	0.930048803	0.028528528	4.567148302
107.464897	0.943319838	0.033483286	4.12948344
159.3284917	0.850167224	0.030184621	4.280154894
597.8734162	0.636972152	0.049239898	4.73432913
65.80390944	0.866266761	0.093033666	3.924204093
62.99682833	0.936243936	0.101141069	3.960969974
90.45749542	0.851037851	0.066676167	3.96851206
526.7175986	0.671701569	0.031032733	4.815757732
110.7294498	0.85717313	0.065436732	4.402898054
86.31506894	0.926484907	0.095099012	4.181606246
115.7707485	0.903914591	0.044601922	4.174399473
675.958206	0.57032601	0.060636641	4.514876134
306.3879584	0.856113583	0.0190623	4.4537263
241.2173399	0.820816714	0.02351808	4.563069169
172.4447459	0.881051855	0.022972873	4.24202575
190.9389113	0.802553899	0.03983371	4.355013176
30.13856971	0.93956044	0.156098305	3.184210977
172.358784	0.831584575	0.031545202	4.341212211
258.3386422	0.777950617	0.033499962	4.455240679
143.2360847	0.92760181	0.039976366	4.277966784
163.5682457	0.858603476	0.034559142	4.365267785
408.7861157	0.703471187	0.033254417	4.619744493
158.776457	0.812216544	0.049869158	4.326129139
289.2359095	0.866612111	0.017046404	4.57350413
168.4375987	0.955621302	0.032128186	4.418894805
818.2616142	0.615600876	0.035632498	4.76327831
83.91692243	0.920734088	0.072107359	4.03338542
2138.672417	0.624382469	0.010062023	4.761229672
116.8834997	0.880531513	0.03431309	4.11267008
578.1914226	0.607901702	0.041906926	4.371603626
231.9590752	0.883582691	0.023175295	4.61328883
140.3964375	0.884016973	0.027376895	4.296980737
231.62796	0.74688914	0.05602583	4.480508404
235.8654344	0.824355695	0.025406792	4.486227822
557.4236295	0.648564522	0.037693102	4.701157203
95.65134132	0.885142255	0.11451077	4.167745658
83.12410326	0.938596491	0.076470393	4.07418874
200.1832276	0.948251748	0.024010751	4.402012448
530.0555718	0.730204213	0.022114098	4.514751013
265.7739882	0.858338975	0.018600698	4.562277675
682.8302762	0.528478748	0.083466524	4.644107098
183.8482294	0.886840432	0.018904014	4.236143976
140.6468741	0.930045564	0.040125354	4.488645562
91.72734051	0.883745214	0.064343139	4.285911385
489.3483956	0.701211971	0.027788922	4.638853323
144.4024383	0.846203635	0.04531944	4.307174818
84.01067332	0.899321267	0.091811736	4.044360626
1136.898634	0.533747289	0.042472147	4.728890452
185.4752411	0.879162243	0.020492808	4.344039801
342.9976324	0.88444743	0.010546363	4.714978202
369.4040496	0.882444983	0.010420819	4.747102327
742.7906956	0.711529433	0.017411143	4.693006265
108.1975754	0.906781692	0.043047269	4.064353497
299.4067622	0.905685619	0.012086933	4.69910343
104.0340495	0.949579832	0.039301366	3.907093359
433.6487931	0.811195587	0.011885353	4.50476463
534.3171013	0.616996293	0.047454121	4.698134094
171.1177148	0.882071432	0.024763413	4.467320443
62.83964663	0.914529915	0.102525253	3.91567902
1174.004569	0.623305558	0.022973434	4.765694725
87.54028541	0.885077539	0.058432216	4.048073893
207.5563992	0.834970249	0.023202252	4.365731067
475.9065178	0.739069078	0.018769719	4.514940611
118.9080956	0.875445746	0.044499857	4.199675649
254.7109357	0.917730717	0.019360559	4.934980106
263.3914585	0.957859532	0.022853071	4.444508827
171.1271109	0.918468628	0.022755819	4.489370997
336.3083203	0.743107769	0.025439035	4.309699864
225.5954643	0.907484407	0.01463109	4.484845523
124.8669348	0.919741505	0.040145579	4.429644083
798.3478329	0.684993473	0.021334988	4.655031636
190.5386556	0.802291326	0.034604453	4.234051236
217.8868456	0.816194332	0.027513045	4.419696095
214.3716044	0.957167832	0.040585376	4.540241673
229.1717935	0.888381963	0.017063034	4.415579919
514.8446191	0.621060199	0.0636602	4.634433476
263.9029466	0.892569558	0.02232979	4.824926935
131.9748392	0.890192308	0.032855306	4.363175517
337.1430125	0.939799331	0.014847328	4.565207002
234.2316095	0.794835933	0.037164092	4.31708798
205.7152645	0.803983276	0.030920148	4.436858887
325.5645856	0.739890623	0.032348266	4.538472291
107.8916689	0.850061404	0.046469547	4.111943584
241.1617706	0.917814449	0.012745419	4.529405137
136.2894136	0.886006826	0.034947703	4.387855526
183.1354991	0.960212202	0.030488502	4.171828382
115.6276563	0.962080173	0.040913852	3.89822537
551.4666324	0.723825009	0.023608731	4.885206095

log.sigma.4.0.mm.3D_glrlm_HighGrayLevelRunEmphasis	log.sigma.4.0.mm.3D_glrlm_RunLengthNonUniformityNormalized	log.sigma.4.0.mm.3D_glszm_GrayLevelVariance
172.4912409	0.834045769	26.31450348
114.1349219	0.913862661	21.55853994
137.1532593	0.839669978	20.0352551
116.6009628	0.928029569	25.97462514
73.90942786	0.812421038	15.4912764
120.319126	0.932805663	19.41804408
100.8609693	0.464961154	14.97916952
146.7545298	0.700329377	18.89574053
92.35191138	0.606601197	12.92671573
94.46907795	0.831151192	13.6251335
264.5120272	0.527291962	10.72781696
92.6706802	0.591919695	16.17488578
147.0824388	0.872406595	26.54163781
96.61139163	0.890000274	15.01768991
93.37141704	0.75126664	15.54478129
108.2531885	0.565172971	12.35998341
46.25211499	0.77342538	9.105831038
57.39471644	0.874358807	14.97873264
55.90295277	0.746916026	7.266528926
140.6290921	0.582619557	20.36166513
72.1663018	0.760555441	14.61903925
76.54142626	0.862162702	14.60785549
87.31509388	0.827469714	14.82849756
95.48708899	0.499355936	9.861419098
161.6430031	0.777245734	18.93523997
133.0146762	0.722635125	21.5340786
118.5894889	0.794164929	14.41276042
86.57513509	0.695160046	12.7028479
26.4066532	0.884552708	5.086734694
98.56644302	0.727314481	12.43733563
97.46339339	0.677815112	13.493125
122.4274475	0.865270141	17.34195126
100.5859331	0.762624308	17.92324129
113.8576427	0.615215043	14.23783424
75.08958569	0.716433949	13.51582326
174.7815609	0.78107062	19.35274235
152.441328	0.914313182	21.75495067
139.7528959	0.540991041	14.91018177
68.55329709	0.851468753	11.7172803
446.0250702	0.541658452	21.03467164
83.49738336	0.789111243	14.06198485
120.4303589	0.507640637	11.436975
158.1235554	0.80969633	22.82078495
101.388838	0.799200913	19.33907253
81.89498886	0.639322786	14.79610351
120.0279229	0.73010721	18.04335966
117.2226163	0.559425535	13.2355765
65.48343176	0.792345937	8.868480726
72.17572619	0.882410449	15.39025406
171.8613297	0.899986766	24.63008506
147.6140424	0.652786394	13.51859012
171.2565297	0.766687893	26.25888382
83.31355539	0.441037567	12.17050034
127.4966775	0.801051278	12.64040188
122.6861198	0.872658941	21.17534972
70.37237377	0.796200877	18.58448753
132.1960971	0.612037465	14.85561925
76.53213824	0.759876158	12.01639979
64.94485794	0.816219388	9.973701512
155.0280578	0.438328687	17.60207059
128.0563105	0.798032813	16.38350592
237.415251	0.807674124	27.49509203
250.9324545	0.807801438	26.40040358
198.23821	0.641880682	20.5274797
82.32341654	0.836261701	12.38839929
231.7498211	0.835437176	28.65647908
92.99222622	0.906322829	12.12927054
207.6474164	0.719357158	21.59485611
102.55137	0.521077037	13.75216663
124.2613647	0.796166265	19.04124968
51.03226938	0.845410389	11.02497399
195.0918981	0.567114406	11.2619576
63.16907207	0.795735743	11.2813875
119.9839924	0.732675055	20.88522262
163.7506761	0.644316433	18.26749276
81.10988358	0.784193852	12.77907128
210.8453855	0.852548988	49.90447483
233.7090262	0.91602895	35.93277794
141.1894385	0.854927803	22.68678253
116.853133	0.64179416	14.84246914
172.4912409	0.834045769	26.31450348
108.5865236	0.853629345	17.61762153
180.328101	0.61593318	17.91421423
90.22784003	0.686235587	11.35621928
112.0327047	0.711522659	13.90330016
193.4031803	0.916116229	30.03081597
161.5881767	0.8025201	18.02777031
85.0014144	0.564926248	13.55263196
199.8058166	0.81461782	44.00562442
103.1542495	0.811619096	16.63272278
287.0407482	0.883585296	30.125184
107.4257093	0.690307812	13.08814918
101.3349627	0.702653637	14.84085874
108.3923647	0.645926209	15.72016039
69.04123244	0.757062112	11.58287676
194.5159067	0.852333121	25.02867263
100.8114133	0.805683351	17.98350782
162.7013169	0.925087025	22.48979592
104.8083501	0.924496847	16.8139382
171.7265929	0.649434042	21.1133185

log.sigma.4.0.mm.3D_glszm_SmallAreaHighGrayLevelEmphasis	log.sigma.4.0.mm.3D_glszm_GrayLevelNonUniformityNormalized	log.sigma.4.0.mm.3D_glszm_SizeZoneNonUniformityNormalized
140.8947565	0.055288697	0.351877267
119.1393902	0.065197429	0.535812672
104.5941422	0.063265306	0.329795918
96.66876906	0.056516724	0.387158785
92.67509975	0.08198143	0.411335578
107.4950715	0.066115702	0.488062443
82.10977665	0.073530888	0.358682051
101.5614607	0.063079515	0.351405255
83.56325472	0.082791064	0.37989477
86.40123684	0.087219651	0.367034532
138.5517708	0.093593228	0.292321692
87.68264356	0.071049449	0.399959688
156.3773755	0.057808088	0.428951995
93.50959109	0.074806336	0.434847959
81.50026951	0.072406628	0.369598807
77.57560852	0.083088355	0.339312445
45.52288836	0.105142332	0.251147842
65.19195097	0.081597222	0.353298611
43.21582418	0.1184573	0.307621671
116.0630711	0.065735002	0.355688811
75.21260248	0.078800822	0.326448962
71.85314487	0.074144087	0.343835329
65.29096316	0.08072393	0.226659006
71.38996139	0.098767752	0.330881366
100.5984426	0.069115713	0.299720579
133.5506499	0.06226677	0.382612813
99.55198078	0.079155816	0.406684028
52.58400125	0.081590916	0.304233693
13.04968254	0.153061224	0.387755102
83.77788954	0.086374096	0.331196581
85.98177218	0.081132813	0.381503906
113.2046164	0.072058695	0.402393222
89.57816046	0.067389875	0.377565929
78.56080642	0.075818409	0.339675135
68.27806007	0.079118571	0.334809353
133.0617275	0.06625	0.408367347
131.0407601	0.064536068	0.391955312
89.82862521	0.077186355	0.319806345
52.10164153	0.082504703	0.305025531
306.6866796	0.065448789	0.342498171
78.46825713	0.082423702	0.343172199
88.72066417	0.09145	0.3114
144.2864325	0.059641425	0.395338387
109.0063676	0.06530321	0.332413793
61.95335816	0.084176086	0.298631767
111.174449	0.067932798	0.341878196
85.66983152	0.07868681	0.376568619
34.90296026	0.10430839	0.218820862
54.74977779	0.08121616	0.332778009
101.5522817	0.058986686	0.336723373
55.5839876	0.077706722	0.261005354
159.1902231	0.060595975	0.362097658
69.8612352	0.090003513	0.335510227
70.54884272	0.085320992	0.306910328
124.0745388	0.06389414	0.384650284
73.92412329	0.073234072	0.333275623
101.1738769	0.073834505	0.388004327
71.01680798	0.084079431	0.386966253
55.50489572	0.091387245	0.322485207
118.2193772	0.073386851	0.323211073
119.337977	0.073317308	0.417067308
214.4585706	0.054510285	0.401348039
210.4320319	0.055771801	0.391205882
159.4688178	0.06533792	0.381813814
85.60650839	0.091491748	0.353949688
187.5974251	0.053917477	0.374886595
83.79356501	0.095106187	0.383810403
186.7457588	0.062151602	0.420189118
85.1145362	0.079109855	0.323240901
119.8228587	0.065696023	0.347995222
47.85745113	0.090010406	0.308532778
117.6131975	0.08825024	0.343638152
55.5072629	0.08496732	0.326583793
100.3495238	0.067776457	0.326859559
117.2981745	0.066981003	0.34008007
59.02730998	0.083896982	0.266649324
189.8097893	0.046901042	0.371553819
133.7494729	0.054294743	0.326055731
117.2295019	0.060267857	0.307716837
77.77919665	0.083127572	0.273964335
140.8947565	0.055288697	0.351877267
115.2967183	0.069251543	0.42814429
117.7453149	0.068370987	0.277992469
53.14797558	0.085217391	0.233119093
105.2431992	0.076816401	0.361181387
147.8616638	0.056206597	0.393663194
125.0416201	0.070261877	0.38027412
75.53569896	0.078344345	0.323396319
148.0492526	0.048810496	0.288530929
108.7472789	0.073332011	0.385395314
213.8583987	0.057664	0.377664
84.51847025	0.082473762	0.302373767
89.65691069	0.076216057	0.349350064
95.88314613	0.075629135	0.33875827
56.35543977	0.083205493	0.279746106
175.9423738	0.058213306	0.403581533
104.169208	0.068765847	0.377046084
129.1301587	0.065389421	0.43940025
80.19957265	0.075608153	0.413543721
129.5530419	0.061282031	0.354843146

log.sigma.4.0.mm.3D_glszm_SizeZoneNonUniformity	log.sigma.4.0.mm.3D_glszm_GrayLevelNonUniformity	log.sigma.4.0.mm.3D_glszm_LargeAreaEmphasis	log.sigma.4.0.mm.3D_glszm_ZoneVariance
71.07920792	11.16831683	40.98019802	30.10979316
35.36363636	4.303030303	5.560606061	2.629247016
46.17142857	8.857142857	39.75	27.8475
19.74509804	2.882352941	4.862745098	1.608612072
81.44444444	16.23232323	132.8888889	118.6935007
32.21212121	4.363636364	3.287878788	0.852387511
301.6516052	61.83947681	63855.36504	63479.43859
202.7608319	36.39688042	2426.467938	2349.98882
93.8340081	20.44939271	7741.267206	7448.1229
38.90566038	9.245283019	50.24528302	38.45318619
84.48096886	27.04844291	18905.9654	18548.50684
97.59016393	17.33606557	4149.479508	3990.864065
58.76642336	7.919708029	21.80291971	15.96558154
40.44086022	6.956989247	10.06451613	5.890623193
50.6350365	9.919708029	298.6715328	253.5758964
211.3916533	51.76404494	16069.46549	15882.13156
16.57575758	6.939393939	198.5757576	154.9357208
16.95833333	3.916666667	11.5625	6.21484375
20.3030303	7.818181818	386.0454545	312.2417355
336.1259259	62.11957672	16621.76825	16462.47447
88.79411765	21.43382353	292.5294118	264.0326557
23.72463768	5.115942029	11.62318841	6.379752153
13.37288136	4.762711864	59.40677966	36.72335536
63.86010363	19.06217617	25606.82902	24987.25759
46.75641026	10.78205128	133.1282051	111.4699211
135.0623229	21.98016997	625.5949008	592.6869648
78.08333333	15.19791667	188.2552083	164.3369954
55.06629834	14.7679558	1186.414365	1091.970331
5.428571429	2.142857143	6.428571429	2.428571429
51.66666667	13.47435897	517.4166667	462.9787558
122.08125	25.9625	2572.878125	2452.496084
43.05607477	7.710280374	12.86915888	7.189623548
88.35042735	15.76923077	246.3888889	216.5137884
83.22040816	18.5755102	4351.889796	4186.688613
157.0255864	37.10660981	1001.835821	946.8736731
114.3428571	18.55	148.3785714	132.1491837
81.91866029	13.48803828	5.598086124	2.116022985
245.9310793	59.35630689	26534.83485	26301.84579
18.60655738	5.032786885	25.29508197	14.86535877
269.2035623	51.44274809	27679.8855	27442.34916
45.98507463	11.04477612	191.261194	169.0869347
124.56	36.58	23690.9975	23363.65894
153.7866324	23.20051414	133.4678663	118.0587096
48.2	9.468965517	153.8827586	130.1087277
24.48780488	6.902439024	1499.073171	1323.02558
101.1959459	20.10810811	664.7601351	621.1376803
159.6650943	33.36320755	13062.79009	12792.63745
9.19047619	4.380952381	64.97619048	37.78741497
16.30612245	3.979591837	10.16326531	4.750520616
35.01923077	6.134615385	7.673076923	3.198224852
42.80487805	12.74390244	1955.414634	1820.344884
163.6681416	27.38938053	339.4579646	316.6425474
127.8293963	34.29133858	60368.01575	59665.00045
48.79874214	13.56603774	115.3899371	87.01253906
44.23478261	7.347826087	18.04347826	11.81519849
50.65789474	11.13157895	109.5065789	90.4809124
166.8418605	31.74883721	6080.723256	5929.547653
102.1590909	22.1969697	396.2462121	361.997116
25.15384615	7.128205128	37.74358974	25.58316897
274.7294118	62.37882353	142777.2694	142012.0636
86.75	15.25	123.9855769	109.3034162
163.75	22.24019608	148.5171569	133.0807322
517.5653817	73.78609221	193.3499622	181.4075308
407.7771536	69.78089888	5375.951311	5312.310356
27.96202532	7.227848101	63.15189873	46.64156385
248.9246988	35.80120482	116.1370482	105.3085875
21.87719298	5.421052632	8.228070175	3.869498307
287.4093567	42.51169591	1407.482456	1371.060172
162.2669323	39.71314741	28401.79084	28003.14427
122.4943182	23.125	166.2698864	147.4510508
19.12903226	5.580645161	24.51612903	14.31737773
179.7227533	46.15487572	12651.21797	12466.97498
49.96732026	13	167.9673203	144.0021359
56.87356322	11.79310345	423.0862069	385.0584291
126.849866	24.98391421	2575.651475	2501.312868
33.06451613	10.40322581	124.0645161	100.3381894
178.3458333	22.5125	59.14583333	49.61317708
19.23728814	3.203389831	5.237288136	1.438092502
68.92857143	13.5	34.00446429	25.55817522
36.98518519	11.22222222	1836.740741	1696.975802
71.07920792	11.16831683	40.98019802	30.10979316
61.65277778	9.972222222	25.20138889	18.63498264
96.74137931	23.79310345	3612.149425	3515.680902
26.80869565	9.8	899.5043478	795.1092628
83.43290043	17.74458874	725.8744589	664.8181631
37.79166667	5.395833333	5.083333333	1.722222222
69.59016393	12.8579235	99.56830601	83.87285377
85.05323194	20.60456274	9461.376426	9222.362475
89.73311897	15.18006431	95.57877814	81.30447369
82.08920188	15.61971831	104.8826291	90.77608058
47.208	7.208	9.056	4.180736
45.05369128	12.2885906	1038.724832	946.6163686
176.4217822	38.48910891	1188.154455	1142.237059
124.6630435	27.83152174	3286.891304	3177.211218
98.19088319	29.20512821	633.9287749	599.2497626
174.3472222	25.14814815	54.72685185	47.21519204
139.884097	25.51212938	185.916442	170.3235228
21.53061224	3.204081633	4.755102041	1.602665556
16.12820513	2.948717949	5.051282051	1.737015122
405.9405594	70.10664336	5864.213287	5793.735501

log.sigma.4.0.mm.3D_glszm_ZonePercentage	log.sigma.4.0.mm.3D_glszm_LargeAreaLowGrayLevelEmphasis	log.sigma.4.0.mm.3D_glszm_LargeAreaHighGrayLevelEmphasis	log.sigma.4.0.mm.3D_glszm_HighGrayLevelZoneEmphasis
0.303303303	0.365403786	6105.242574	210.4405941
0.584070796	0.349531855	391.5909091	141.5606061
0.289855072	0.741210292	4316.492857	166.9642857
0.554347826	0.424952754	454.9803922	135.6078431
0.26541555	4.698604951	4841.863636	121.020202
0.640776699	0.096316774	323.6212121	135.469697
0.051576107	492.9566693	8495052.69	119.8406659
0.114347998	14.34940563	457204.9757	152.2842288
0.058406243	74.66740173	933141.085	113.3319838
0.291208791	0.846562693	3570.669811	122.8867925
0.052891654	68.45247336	5268361.834	253.5051903
0.079401237	41.07117281	483186.9672	117.6557377
0.413897281	0.617190104	1336.89781	208.2992701
0.489473684	0.288614334	581.4301075	123.5806452
0.148913043	4.313437513	29456.9854	112.9343066
0.073062038	121.8031502	2326443.175	116.2166934
0.151376147	10.88741578	6384.348485	73.34848485
0.432432432	1.131396689	319.8541667	84.77083333
0.116402116	12.00615422	21883.93939	67.68181818
0.079231995	117.4864855	2463552.996	181.4232804
0.187327824	11.27568622	13669.83088	115.5772059
0.436708861	1.183072204	442.7971014	102.2608696
0.209964413	1.687849747	4879.169492	108.4915254
0.040174854	223.5870144	3031038.036	108.6217617
0.214876033	0.84553831	28301.02564	163.5512821
0.174320988	6.062250693	74771.71388	193.121813
0.204472843	1.980278801	22131.90625	139.5729167
0.102899375	14.54658491	120005.895	85.1878453
0.5	0.897004013	128.5	26.64285714
0.135534318	7.414562046	45882.19231	127.724359
0.091142125	28.04575312	314463.1156	119.06875
0.419607843	0.509855542	1172.504673	154.6915888
0.182955434	3.228364051	26772.34188	126.0299145
0.077802477	35.6814958	605548.8082	123.1632653
0.134886396	16.3051446	81230.73134	101.2515991
0.24822695	0.998970994	29764.33571	186.0392857
0.535897436	0.170293049	638.0287081	180.1052632
0.065513716	148.4792004	4869573.434	138.4408322
0.30964467	1.125276446	1412.262295	82.44262295
0.064883606	58.15015552	13271581.06	490.0381679
0.212361331	3.155582997	13687.41045	116.9179104
0.055271521	197.0685269	2902690.41	144.955
0.254747872	1.395326586	19189.74807	198.1619537
0.205091938	2.363021681	12686.38621	151.0344828
0.075367647	19.58309676	148615.4268	102.7439024
0.15140665	6.946597865	81228.07095	162.6114865
0.060840867	99.11206331	1851186.823	123.8325472
0.191780822	4.674924597	4217.880952	69.85714286
0.429824561	0.706725673	483.4081633	88.51020408
0.472727273	0.113203375	1353.153846	173.0480769
0.086044071	10.72749143	398820.9756	110.8536585
0.209356183	2.513571226	51170.87389	227.5597345
0.037715304	620.6276583	6096059.735	102.9448819
0.18772137	1.31398269	13675.39623	127.6289308
0.400696864	0.659659051	1146.26087	169.5913043
0.229260935	3.626000447	5063.868421	112.3684211
0.081331568	40.99448681	963121.2837	144.4976744
0.170873786	9.746716323	29233.33712	99.05681818
0.286764706	2.066020011	1901.5	84.64102564
0.036150215	834.2405514	24746885.11	183.4964706
0.26097867	1.425880929	13678.71635	164.5673077
0.25452277	0.86958602	32053.69608	299.8259804
0.289370079	0.894730419	47685.78609	303.0204082
0.125352113	24.09194791	1283739.409	229.6385768
0.246105919	1.325529392	4729.443038	113.6582278
0.30389016	0.756181549	20302.36446	278.001506
0.478991597	0.265726794	551.6666667	114.245614
0.165697674	6.922336048	306086.2807	251.2236842
0.050084805	253.865949	3469229.522	137.440239
0.230517354	2.297004167	15620.69602	177.7869318
0.313131313	1.64535599	859.6935484	69.96774194
0.073672348	49.15992002	3396021.008	183.0573614
0.204272363	4.417384145	8793.196078	84.14379085
0.162162162	4.009576111	50689.44253	155.2586207
0.115982587	13.99397986	494720.1796	180.5737265
0.205298013	2.936437235	7796.032258	104.1048387
0.32388664	1.068702532	5385.595833	287.3854167
0.513043478	0.073468037	1102.152542	243
0.344086022	0.563642592	2964.232143	179.6071429
0.084586466	14.36774226	254502.8963	132.4444444
0.303303303	0.365403786	6105.242574	210.4405941
0.390243902	1.011123175	1191.222222	154.2152778
0.101813926	15.40245759	884171.2126	193.0201149
0.09787234	9.598928767	97379.17391	92.04347826
0.127977839	7.693415221	89334.18182	149.9134199
0.545454545	0.20253871	798.7395833	215.1041667
0.252413793	0.806804775	15793.27322	182.7923497
0.064682735	87.99667845	1098447.487	110.9087452
0.264680851	0.950778942	12999.97106	254.2090032
0.26625	2.671826172	5816.112676	153.5117371
0.452898551	0.089697194	2352.832	315.6
0.104195804	10.36246197	126738.3826	130.5704698
0.147574518	13.82801998	119547.2079	133.0257426
0.09548521	30.67511672	407528.5272	146.7853261
0.169811321	12.7536739	35264.83476	93.1994302
0.364864865	0.412357778	8376.178241	242.1134259
0.253242321	3.286572662	13293.63612	147.6118598
0.563218391	0.090577963	756.3265306	173.4285714
0.549295775	0.129482288	481.6410256	113.2564103
0.119117035	32.36454533	1128110.719	202.1381119

log.sigma.4.0.mm.3D_glszm_SmallAreaEmphasis	log.sigma.4.0.mm.3D_glszm_LowGrayLevelZoneEmphasis	log.sigma.4.0.mm.3D_glszm_ZoneEntropy	log.sigma.4.0.mm.3D_glszm_SmallAreaLowGrayLevelEmphasis
0.614702116	0.015632258	5.991900607	0.011670173
0.757924826	0.033127039	4.89077343	0.024851562
0.594208793	0.021949439	5.663935186	0.014907727
0.620099401	0.046827493	4.976155244	0.018122402
0.666588378	0.02650174	5.331118874	0.015064147
0.724301347	0.034112779	4.883345779	0.028007505
0.62182779	0.022544925	5.99885578	0.01222837
0.614859878	0.018414189	6.155998513	0.011631687
0.640323258	0.026840664	5.441291976	0.012793126
0.626630409	0.028263036	5.17260208	0.021938094
0.554336654	0.008396608	5.765570514	0.006370603
0.657007588	0.033419632	5.604763851	0.017267115
0.679200673	0.021310577	5.401114843	0.016695445
0.683912713	0.028785658	5.024900613	0.021519324
0.628378613	0.028554492	5.56622576	0.018615181
0.604607717	0.021476724	5.939515757	0.013027503
0.506106887	0.044974135	4.942830113	0.009122485
0.610449174	0.067548169	4.871115365	0.034906617
0.570808197	0.042787358	4.797717226	0.014911898
0.614781864	0.013370526	6.122058792	0.007351436
0.586652349	0.026883453	5.686994776	0.008892426
0.597385243	0.036053062	4.936149776	0.009839241
0.481595115	0.037969244	5.126216092	0.023598328
0.594863594	0.0234502	5.458059547	0.013459929
0.560356549	0.020000287	5.774321288	0.008869558
0.638307693	0.0132833	5.800541869	0.006015784
0.661575364	0.01875543	5.37190887	0.012412082
0.56868738	0.039963985	5.58413115	0.014607583
0.637777778	0.138634203	3.093069208	0.112050335
0.59632466	0.022059134	5.451861271	0.008067805
0.641223396	0.020890665	5.555348468	0.009090396
0.658040845	0.02398545	5.192617075	0.016420442
0.637428217	0.030999692	5.676719926	0.018473852
0.604905376	0.02204231	5.740190912	0.012001275
0.598345788	0.036400426	5.857180075	0.014254856
0.660759506	0.01656399	5.548821567	0.011192115
0.640859767	0.020221164	5.482151125	0.007488861
0.586768572	0.017867599	6.197488871	0.010297592
0.560127725	0.045513495	5.107149945	0.012429299
0.607057838	0.003912337	6.263008168	0.002875846
0.592295909	0.029277061	5.260922025	0.01265079
0.576013585	0.014261431	5.839469245	0.008635905
0.652873088	0.014169571	5.940127255	0.006745495
0.597570828	0.023436806	5.625315277	0.014603924
0.565298658	0.048587418	5.146063359	0.020222145
0.598888306	0.016518987	5.785648399	0.006426163
0.637551745	0.019969902	5.658843517	0.011081593
0.476929061	0.051037201	4.67173022	0.009529485
0.584204944	0.050346127	4.954209273	0.020407445
0.585439889	0.026633481	5.56290354	0.018731548
0.523218561	0.025518895	5.834260963	0.016413168
0.624171613	0.014612277	6.135698191	0.005962773
0.59928358	0.027900632	5.700312977	0.014099713
0.573242739	0.021760985	5.666136686	0.016380565
0.642745382	0.023972156	5.30237747	0.009694264
0.590498526	0.044496348	5.57725893	0.019417976
0.646692767	0.016557688	5.774362522	0.010934279
0.645512826	0.029148338	5.427770662	0.014800076
0.585353912	0.035897672	5.081680226	0.01039371
0.588079405	0.011575467	6.21454141	0.005602564
0.670536494	0.016617485	5.405191964	0.011465532
0.656675165	0.008927452	5.979561642	0.00610218
0.649873464	0.008049864	6.251894136	0.004800194
0.641880687	0.009838294	6.111892065	0.006444053
0.61491346	0.031127975	4.813294155	0.009306768
0.635799019	0.009410727	6.285917653	0.004797133
0.634366323	0.036048546	4.583428945	0.024559872
0.674034951	0.008964785	5.908312071	0.005798383
0.583242432	0.016504436	5.881632764	0.007701175
0.609441817	0.015101733	5.876229731	0.00616459
0.569868678	0.080405296	4.931872472	0.043694348
0.607350392	0.010169197	5.787290141	0.006461784
0.591845751	0.043475084	5.337060543	0.018521839
0.592146894	0.021016994	5.806716931	0.013158824
0.603825625	0.013300301	6.013111277	0.008270369
0.522976124	0.03004706	5.440958405	0.008240775
0.632709361	0.012161927	6.441731064	0.007549129
0.554225374	0.03223818	5.382477371	0.028141453
0.56675281	0.019213695	5.960665712	0.012140099
0.533325361	0.021717555	5.667799556	0.01456169
0.614702116	0.015632258	5.991900607	0.011670173
0.679815431	0.021664155	5.305114068	0.013648676
0.541092381	0.01138817	6.212775563	0.004591402
0.489138682	0.040205291	5.49754312	0.013424121
0.623138732	0.016825158	5.631080922	0.010288633
0.645183015	0.026926512	5.241882324	0.011798657
0.640751928	0.017592192	5.558937542	0.012739097
0.58902156	0.024697357	5.739995653	0.012747422
0.55091633	0.021557742	6.433447226	0.010084052
0.642725179	0.018967437	5.595368782	0.009324913
0.636433561	0.016103102	5.577007666	0.012870001
0.569237537	0.022874	5.595218752	0.007970069
0.611288771	0.02210391	5.907161757	0.014369792
0.601289755	0.019569358	5.811100607	0.010268431
0.543749002	0.037364586	5.980000555	0.019121693
0.657446441	0.010953351	5.899396121	0.007260639
0.637986926	0.023876743	5.811824748	0.014246873
0.68856576	0.036945013	4.760132232	0.031367579
0.65977208	0.048544347	4.682587475	0.03797633
0.617627808	0.011977376	6.246459817	0.00611046

log.sigma.4.0.mm.3D_ngtdm_Coarseness	log.sigma.4.0.mm.3D_ngtdm_Complexity	log.sigma.4.0.mm.3D_ngtdm_Strength	log.sigma.4.0.mm.3D_ngtdm_Busyness	log.sigma.4.0.mm.3D_ngtdm_Contrast
0.014201036	0.723455743	3.248095607	0.214015108	0.000136668
0.056498788	4.120166024	8.492034265	0.112235854	0.002544884
0.0172959	0.713097501	2.210119549	0.308625766	0.000375843
0.074968372	4.312509376	8.366803756	0.109141247	0.003915734
0.010970917	0.456803779	2.045173633	0.596661773	0.000132837
0.068670809	3.540620347	8.420610808	0.098020488	0.002347229
0.000825439	0.00771684	0.220017011	3.311879167	8.39E-07
0.002518589	0.045176508	0.483188421	1.134018575	8.31E-06
0.003018729	0.033029031	0.526993807	1.237610436	6.48E-06
0.022605426	0.626924005	2.625942178	0.232692614	0.000202558
0.002002265	0.021942838	0.607713526	0.991543238	1.84E-06
0.004304404	0.048668925	0.818621389	0.819279288	8.45E-06
0.022932144	1.707558484	4.271522217	0.274460698	0.000761076
0.037830733	1.612060881	3.872594054	0.210528657	0.001012374
0.011583924	0.216014173	1.605536135	0.432455282	6.35E-05
0.001398275	0.018287725	0.27785539	2.258478703	3.89E-06
0.020219315	0.297982299	2.066706978	0.486959471	0.000150284
0.061329589	1.92888606	6.226233979	0.253273593	0.002017995
0.017703693	0.11221221	0.939159398	0.678255316	0.000143933
0.001084361	0.035152406	0.769500989	1.608135341	1.09E-06
0.006719735	0.162911159	1.027003267	1.088871186	6.27E-05
0.045809615	1.352729253	4.298942518	0.276012933	0.001533099
0.031819414	0.596454756	2.871540261	0.250712199	0.000475368
0.002617003	0.013967122	0.392225199	1.486482902	3.32E-06
0.013966852	0.354828658	2.065770718	0.260270677	0.0001442
0.005726105	0.199304812	1.692770811	0.47887768	2.17E-05
0.009861233	0.320534539	1.987973057	0.389995736	6.50E-05
0.007253752	0.067617613	0.808739421	0.755234441	2.74E-05
0.188335123	1.633815272	5.062245816	0.30973157	0.008178608
0.008992288	0.146299148	1.109689059	0.58724637	5.16E-05
0.003548374	0.053328053	0.589974924	1.126299037	1.26E-05
0.027596915	1.557175729	3.627137191	0.190173769	0.000750009
0.008681717	0.194280068	1.521408821	0.47489229	4.48E-05
0.0041085	0.037754008	0.582679138	0.965110276	1.24E-05
0.003149831	0.047792609	0.420203573	1.73277636	1.48E-05
0.00864205	0.247315624	1.290408205	0.431503367	0.000108922
0.016715034	1.449774046	2.840859393	0.261502386	0.000647475
0.001072367	0.016023978	0.324246097	2.084414778	1.73E-06
0.043934349	0.704771834	3.620537923	0.240710925	0.000679683
0.001103433	0.026028876	0.750716931	0.860981374	7.30E-07
0.013853612	0.293831423	1.429239923	0.445158122	0.000125885
0.00168617	0.01404994	0.354428065	1.768463393	1.13E-06
0.006076213	0.296815295	1.280993896	0.545311367	6.91E-05
0.01339241	0.418354823	2.450880995	0.34288838	9.94E-05
0.01314304	0.070121737	1.101205266	0.47058982	4.97E-05
0.005569765	0.142543659	1.041215009	0.629663674	2.79E-05
0.002014895	0.019732607	0.392678393	1.545786582	3.15E-06
0.050689026	0.356688458	3.237043807	0.284953288	0.000740413
0.068926117	1.734383656	6.454991075	0.180011418	0.00191973
0.032912543	2.269268877	5.310387122	0.117533789	0.00100742
0.007809831	0.053224509	1.150490258	0.471007061	2.57E-05
0.005296484	0.29491864	2.413878943	0.358388501	1.84E-05
0.001181162	0.009478852	0.295613667	2.820797956	9.92E-07
0.013469635	0.194968499	1.626183661	0.383568258	8.32E-05
0.02964945	1.548542048	4.912810054	0.212667521	0.000702846
0.013413939	0.515068882	2.761676996	0.458669231	0.000130101
0.002618866	0.030705822	0.511009433	1.116683826	4.79E-06
0.006191501	0.134407616	0.783779592	1.039454484	5.36E-05
0.029474351	0.559756311	2.422322503	0.325515442	0.000461838
0.000498689	0.008488079	0.325394735	3.367190047	2.23E-07
0.011366347	0.394037186	1.8057501	0.350165675	0.000103658
0.006699121	0.401521347	2.097321514	0.313765528	4.97E-05
0.002342224	0.143718755	0.671007568	0.818662525	1.77E-05
0.001376701	0.044928165	0.528120817	1.194836312	2.88E-06
0.028783603	0.428324107	2.098752623	0.302949652	0.000369598
0.004875502	0.316337193	1.650905989	0.427795901	3.76E-05
0.053113944	1.625854973	3.746746489	0.193343653	0.00217737
0.002715907	0.090617128	0.762428346	0.723858257	8.28E-06
0.001350753	0.012820903	0.292270446	2.398393529	1.74E-06
0.006498762	0.219685602	1.124766161	0.645232417	5.70E-05
0.036549484	0.642802807	2.177746981	0.451668593	0.000887523
0.001724351	0.022437137	0.43871192	1.331020564	5.30E-06
0.011369489	0.162663203	0.728216433	0.977883758	0.000152551
0.009895682	0.301375088	2.769774051	0.299368102	3.52E-05
0.00368141	0.078355811	1.047245597	0.58944636	7.43E-06
0.015402359	0.321879836	1.990315992	0.407790563	0.000122702
0.007335351	0.884669118	5.45410128	0.322069243	8.64E-05
0.053242037	5.425436972	11.09548845	0.06778645	0.003152941
0.014815531	0.654964936	2.792791337	0.282284303	0.000187751
0.007813127	0.087461948	1.293049443	0.437560489	1.61E-05
0.014201036	0.723455743	3.248095607	0.214015108	0.000136668
0.024645666	0.974536428	3.904344782	0.289195062	0.000446332
0.003584653	0.068984547	1.080662225	0.561301234	8.13E-06
0.010095525	0.075511266	0.915558298	0.587777617	4.22E-05
0.006427212	0.112292503	0.965055389	0.601040029	2.73E-05
0.037113741	3.180284396	6.681897475	0.11665303	0.002028645
0.014270448	0.407849572	2.604777212	0.230802711	0.000102665
0.003149727	0.028098807	0.491951878	1.249284546	7.40E-06
0.008731201	0.814635757	5.362789999	0.251504146	9.01E-05
0.011078425	0.480876655	2.240135675	0.443805206	0.000118255
0.027989998	2.569924205	7.438960435	0.077792493	0.000709122
0.007934932	0.109388892	1.225641286	0.489161155	2.63E-05
0.003310528	0.066873534	0.618318178	1.166756772	1.16E-05
0.003104433	0.064892765	0.803685079	0.946340059	7.21E-06
0.005198725	0.068759218	0.591630754	1.220290373	2.22E-05
0.00709886	0.59927122	1.866416955	0.360090291	9.05E-05
0.006583479	0.314253754	1.614054896	0.586145797	4.74E-05
0.071772585	4.603938347	9.995465992	0.063159754	0.003144638
0.073242142	3.892869059	7.397786308	0.090360484	0.003529094
0.001343961	0.033416256	0.399083956	1.594838138	3.57E-06

log.sigma.3.5.mm.3D_gldm_GrayLevelVariance	log.sigma.3.5.mm.3D_gldm_HighGrayLevelEmphasis	log.sigma.3.5.mm.3D_gldm_GrayLevelNonUniformityNormalized	log.sigma.3.5.mm.3D_gldm_DependenceEntropy
15.00834393	157.8933934	0.074980386	6.379815916
16.54585324	81.39823009	0.073850732	5.310582171
16.11906262	125.3064182	0.070217627	6.342710439
19.04950378	85.68478261	0.077268431	5.241894532
9.925100087	62.49731903	0.094926291	6.159677796
20.61834292	177.4757282	0.064756339	5.07309461
4.907927757	116.8722556	0.240299826	6.73987656
9.852891292	187.9320254	0.106831105	6.958323506
6.340265709	128.6547647	0.145692393	6.771652276
8.276619672	97.01923077	0.101165318	5.81985017
3.144858721	377.8195461	0.222276209	6.787869135
6.56931851	123.2346241	0.14678203	6.974665375
23.73143728	137.5951662	0.063900476	6.087799685
10.96180055	74.37368421	0.092022161	5.523910365
9.228486531	105.8445652	0.091999055	6.452474059
7.929227738	123.7686173	0.186364042	6.849884502
6.142638456	35.51605505	0.113942429	6.052156447
8.323999675	40.63963964	0.124259395	4.992959962
5.858502157	47.34391534	0.114949501	6.201099744
7.118110494	168.6320114	0.148617562	7.233951486
10.28178242	78.79958678	0.090628296	6.628242122
11.10915719	57.05696203	0.093734978	5.529604957
10.23844683	94.66903915	0.085472575	5.935354734
3.694222862	88.1856786	0.24643953	6.62480663
13.93811139	135.7272727	0.08780138	6.540209499
11.25952166	134.6395062	0.097803871	6.98922449
9.710842772	111.9382322	0.091637831	6.476252235
7.75501611	98.37805571	0.10601694	6.752997888
7.846938776	40.5	0.130102041	3.994680368
8.092416899	88.23457863	0.100079182	6.667973164
9.104790161	93.96923953	0.102629073	6.881366026
13.55287966	113.3960784	0.086658977	5.876616305
9.658710107	108.6137608	0.094729867	6.74276563
7.749170675	125.4258495	0.150499874	6.877267215
7.667337412	121.0834052	0.107114595	6.820623577
15.95039155	217.8271277	0.074178864	6.867417634
23.55903353	159.0128205	0.057777778	5.781705065
7.07771077	183.6338388	0.194010677	6.916050357
9.176737355	72.69035533	0.09469453	5.770289018
5.607367672	507.6795443	0.192549188	6.921341841
7.674408091	98.75435816	0.106949199	6.063810434
2.796455864	121.6417024	0.224141504	6.736535516
15.59716073	167.1434185	0.07315387	6.828488246
10.09863779	92.12022631	0.090049195	6.304966953
6.956469473	90.03768382	0.136323394	6.795408531
9.968871475	129.7677749	0.093596719	6.883433092
6.168323194	129.777156	0.153068812	7.028828257
11.97126832	71.31050228	0.088717917	5.789844594
12.06894429	65.33333333	0.094336719	5.268681462
27.41603306	202.1363636	0.060619835	5.869320385
9.083466471	147.250787	0.153621028	6.657584433
13.13907357	175.2501158	0.088172543	6.944360999
3.282539871	93.09859434	0.26237807	6.567094064
11.24281128	134.8016529	0.082442512	6.380880978
17.31646615	88.18118467	0.072272335	5.966072062
10.36548983	76.50678733	0.093280008	6.29862416
6.958512533	168.5133346	0.140410532	7.04749765
10.29364837	91.55016181	0.088303642	6.76649821
8.169928633	74.90441176	0.100210856	6.048726725
3.397802865	164.6585293	0.256402665	6.633768261
11.39839014	136.5181932	0.087594792	6.493209057
18.21268761	189.2788522	0.068700335	6.902316914
19.08234573	287.5489939	0.068653752	7.078796548
9.136030536	217.0203052	0.129368732	7.153042938
9.654254132	88.42679128	0.087722363	6.058909179
21.40725751	208.8402746	0.066348151	6.846116378
9.604406468	58.25210084	0.09031848	5.014636349
10.07599805	220.5649225	0.099019717	6.986091045
4.877188473	112.2124115	0.175085033	6.891719959
12.70847684	130.6876228	0.080817711	6.62857276
8.164575043	59.02020202	0.101061116	5.676374316
9.131874419	254.8647697	0.173697388	6.948889257
8.495432272	75.564753	0.096090738	6.136383738
8.419113911	104.9273066	0.108869837	6.583146686
7.175361435	148.1097637	0.140741991	6.978955448
9.591802443	86.79304636	0.094140608	6.453011393
40.19994728	202.1693657	0.057521568	6.834861389
28.04431002	189.8869565	0.056181474	5.507112634
16.02126942	117.625192	0.07055434	6.321964379
5.418754829	107.3452381	0.143351801	6.706819874
15.00834393	157.8933934	0.074980386	6.379815916
16.51926763	80	0.075844038	5.946161383
8.962647752	152.1772967	0.16042851	6.855740805
6.395389045	102.9531915	0.118118244	6.548450873
8.352956162	119.3313019	0.103560593	6.806184801
26.40828383	158.0056818	0.058755165	5.570096646
12.59417646	169.4827586	0.083308442	6.573303908
6.136877527	98.02508608	0.200620771	6.682355472
33.34953373	221.2144681	0.067984427	6.942186522
12.199375	109.715	0.085053125	6.255452835
24.32150546	200.076087	0.06747532	5.801404038
6.738107487	115.4223776	0.122062693	6.712306204
7.854842032	108.1472823	0.105172431	7.014487716
7.271616654	118.5210171	0.125662445	6.972564412
6.5299047	71.57135946	0.115885377	6.571328352
16.99878376	204.6934122	0.070289673	6.551068778
11.80424979	104.3447099	0.085834896	6.636827142
20.06605892	137.5517241	0.073853878	5.070231749
12.33644118	87.36619718	0.091053362	4.775819302
10.75218655	179.757601	0.117458564	7.27585253

log.sigma.3.5.mm.3D_gldm_DependenceNonUniformity	log.sigma.3.5.mm.3D_gldm_GrayLevelNonUniformity	log.sigma.3.5.mm.3D_gldm_SmallDependenceEmphasis
109.1711712	49.93693694	0.277323377
30.52212389	8.345132743	0.413130456
82.95445135	33.91511387	0.25901343
22.36956522	7.108695652	0.35797083
106.1233244	70.8150134	0.219631492
37	6.669902913	0.564439051
625.4653502	3918.328959	0.058234619
402.8818866	539.0697582	0.121424127
250.9843935	616.1331284	0.078345947
61.06593407	36.82417582	0.268235218
263.9370425	1214.517204	0.066862804
163.0123658	451.061178	0.084949037
68.78851964	21.1510574	0.388203552
36.57894737	17.48421053	0.363822141
110.2152174	84.63913043	0.149151514
418.3160549	1589.126187	0.082211684
56.26605505	49.67889908	0.151716104
20.8018018	13.79279279	0.403179866
64.65432099	65.17636684	0.130133848
604.2205919	1772.561667	0.090946827
156.6790634	131.5922865	0.167402064
26.58227848	14.81012658	0.305250791
46.23131673	24.01779359	0.165165955
197.4034138	1183.895504	0.053490509
81.43801653	63.74380165	0.178817026
176.7195062	198.0528395	0.163681684
128.8658147	86.04792332	0.19680595
151.0568505	186.4837976	0.123281169
8.857142857	3.642857143	0.421130952
113.9400521	115.1911381	0.147630803
279.7177442	360.330675	0.10444426
49.50196078	22.09803922	0.352475075
132.5903049	121.1594996	0.178774271
178.9422039	473.9241029	0.096026384
307.0638481	372.4374461	0.14182815
136.570922	83.67375887	0.23801024
121.9897436	22.53333333	0.49879416
516.6798432	2277.297325	0.073555518
33.96446701	18.65482234	0.231787213
545.1953112	2332.540862	0.077715562
85.54199683	67.48494453	0.231025852
320.1991157	1622.112063	0.067804982
196.8952194	111.7059594	0.241207095
104.8896747	63.66478076	0.2038937
71.94301471	148.3198529	0.099609109
171.631202	182.9815857	0.158696069
344.6220405	1066.736548	0.072556624
37.72146119	19.42922374	0.170670598
26.49122807	10.75438596	0.383452519
58.01818182	13.33636364	0.390570037
128.224554	292.8016789	0.092843709
235.7114405	190.3645206	0.205548431
397.6388834	2650.543259	0.046543884
127.9964581	69.82880756	0.184032803
56.05226481	20.74216028	0.319323827
93.5158371	61.84464555	0.217032762
294.4303007	742.3504823	0.089394666
158.7333333	136.4291262	0.160936425
37.83823529	27.25735294	0.244420067
945.9177051	6028.795858	0.045228532
103.3437892	69.81304893	0.242811107
212.4934498	110.1266376	0.251597665
622.7576553	313.8849519	0.263455965
522.9380282	1102.221596	0.128305636
53.8411215	28.1588785	0.22761435
350.2869565	144.9707094	0.270294736
27.47058824	10.74789916	0.345808752
366.0014535	408.7533915	0.170418007
451.5652998	1754.877282	0.058317202
207.1362148	123.4086444	0.212664959
34.19191919	20.01010101	0.231205596
333.9559093	1233.077757	0.081500241
109.5313752	71.97196262	0.188249859
108.3532153	116.8173346	0.151364359
213.64801	452.6262438	0.123217607
77.25827815	56.86092715	0.178198475
260.0931174	85.24696356	0.289588713
33.46956522	6.460869565	0.457292271
113.2918587	45.93087558	0.293683002
104.3859649	228.7894737	0.098360851
109.1711712	49.93693694	0.277323377
63.09214092	27.98644986	0.299541928
204.0877706	548.344646	0.095903756
103.3761702	138.7889362	0.101181258
162.3950139	186.9268698	0.141645474
48.70454545	10.34090909	0.467065972
93.19862069	60.39862069	0.219209133
191.5307427	815.7240531	0.075565442
177.6382979	79.88170213	0.254404129
117.615	68.0425	0.248343382
63.57971014	18.62318841	0.373094211
118.1776224	174.5496503	0.116073398
282.3331385	359.9000584	0.153784647
246.0871821	484.3030618	0.10544396
216.7706821	239.535075	0.174620113
200.3141892	83.22297297	0.332476967
188.8102389	125.7481229	0.233709152
24.31034483	6.425287356	0.432072158
19.22535211	6.464788732	0.401971831
607.1261974	1128.072053	0.127394947

log.sigma.3.5.mm.3D_gldm_DependenceNonUniformityNormalized	log.sigma.3.5.mm.3D_gldm_DependenceVariance	log.sigma.3.5.mm.3D_gldm_LargeDependenceEmphasis
0.163920677	4.018018018	15.12912913
0.270107291	1.37661524	6.469026549
0.171748346	4.049415103	15.36853002
0.243147448	1.879962193	8.239130435
0.142256467	4.293605215	18.70509383
0.359223301	0.841926666	4.067961165
0.038357988	65.5376991	253.8125843
0.079841832	28.69090691	76.69718589
0.059348402	45.56691439	132.2650745
0.167763555	3.287163386	14.01098901
0.04830473	39.19095359	141.7251098
0.053046653	43.59187316	132.9752685
0.207820301	2.924945921	10.26888218
0.192520776	3.687645429	12.18947368
0.119799149	9.786181474	33.45652174
0.049057823	51.90093436	162.5095579
0.129050585	4.487185422	24.40825688
0.18740362	4.884993101	14.65765766
0.114028785	7.075228079	31.82892416
0.050659897	43.99742756	136.1506666
0.107905691	17.27376887	44.42561983
0.168242269	4.049351066	14.46835443
0.164524259	3.037676828	17.94661922
0.041091468	57.11659275	206.4608659
0.112173576	19.74068256	47.80440771
0.087268892	17.45525719	51.52049383
0.137237289	4.774539792	21.28115016
0.085876549	17.44849602	56.03808982
0.316326531	0.780612245	5.071428571
0.098992226	14.26781381	44.12250217
0.079668967	27.41356915	77.44773569
0.194125336	4.044044598	12.71764706
0.103667166	11.70773932	37.08444097
0.056825089	41.77160245	123.8497936
0.08831287	21.70852165	59.43715847
0.121073512	11.43669521	31.19503546
0.312794214	0.937172913	4.794871795
0.044017707	54.68603587	180.4208553
0.172408462	3.221726919	14.68527919
0.045005391	49.11617862	164.8464586
0.135565764	4.3646113	19.82408875
0.044244731	45.86955573	163.7646815
0.128942514	7.149690208	23.62999345
0.148358804	4.549938081	19.36209335
0.066124094	34.25940744	99.20404412
0.087790896	18.14938259	53.10741688
0.049450716	46.90505095	152.3760941
0.172244115	2.669460603	17.55707763
0.232379194	2.973838104	9.807017544
0.263719008	1.420165289	6.836363636
0.067274163	51.92729977	130.4533054
0.109176211	10.66320481	33.01389532
0.039362392	52.31894572	222.1383884
0.151117424	4.14223128	19.34946871
0.195304059	2.384756401	10.56794425
0.141049528	4.298014646	19.26546003
0.055689484	42.34942362	125.8261774
0.102740022	18.55937244	48.84854369
0.139111159	5.075259516	19.47058824
0.040229563	47.76213761	212.0037001
0.129665984	7.633544235	24.49811794
0.132559856	9.340632758	25.9095446
0.136211211	10.58215227	27.17191601
0.061377703	47.42069028	121.5948357
0.16772935	3.101542105	15.03738318
0.160314397	3.898519655	15.29107551
0.230845279	1.934609138	8.764705882
0.088663143	21.81531473	57.27664729
0.045052908	44.20639097	164.1026639
0.135649126	7.245253629	23.81074001
0.172686461	2.893072135	14.34343434
0.047042669	60.23477067	182.1911537
0.146236816	4.641674436	20.46061415
0.100981561	13.08215983	41.60950606
0.066432839	38.93230327	101.891791
0.127911057	5.524088856	23.74172185
0.175501429	3.757489332	13.90958165
0.291039698	1.112589792	5.504347826
0.174027433	3.520864745	13.5437788
0.06540474	32.02623884	95.88596491
0.163920677	4.018018018	15.12912913
0.170981412	3.814396193	14.12737127
0.059709705	50.33252759	135.8273844
0.087979719	15.01834314	54.30723404
0.089969537	14.25046032	48.04487535
0.276730372	1.341813017	5.954545455
0.128549822	5.943202854	22.80413793
0.047105446	61.31559688	185.2769306
0.15118153	5.491834495	18.69191489
0.14701875	4.35374375	17.5125
0.230361269	1.836746482	8.15942029
0.082641694	20.47432344	65.41118881
0.082505301	20.34371607	58.46405611
0.063852408	35.6340055	101.5158277
0.104872125	10.05758751	34.48040639
0.169184281	4.035082063	14.08783784
0.128880709	7.254222647	24.0894198
0.279429251	0.99326199	5.712643678
0.270779607	1.055346161	6.070422535
0.063215972	42.0734864	109.4966681

log.sigma.3.5.mm.3D_gldm_LargeDependenceLowGrayLevelEmphasis	log.sigma.3.5.mm.3D_gldm_SmallDependenceHighGrayLevelEmphasis	log.sigma.3.5.mm.3D_gldm_LargeDependenceHighGrayLevelEmphasis
0.144820222	51.00084355	2268.184685
0.430881114	41.46142511	340.300885
0.248882051	40.74016033	1877.890269
0.920819759	42.81237849	397.1413043
0.742524775	20.9949295	962.3284182
0.050671031	107.8555124	655.1067961
2.021980582	6.817977322	33579.24782
0.360414204	22.07672	18145.57927
0.876826716	9.040157574	21828.87136
0.22443947	34.32859332	1197.892857
0.369578941	24.32920081	54894.12994
0.991698839	10.78636793	19818.89424
0.22214644	71.99155567	812.1268882
0.413861133	36.14403503	590.8157895
0.3463492	16.51519473	4723.727174
1.250162518	9.336325484	23774.79641
2.717190612	8.165564079	726.1169725
1.014287474	26.6435083	309.9189189
1.474091961	7.981931396	1608.451499
0.812668923	17.60441989	24109.82527
0.822435403	18.9334001	4438.086088
1.166947754	25.85338947	410.9303797
0.39493619	17.49080435	1663.572954
2.189720914	4.467939254	20772.03913
0.362904901	24.11480315	9361.716253
0.46002419	29.45540224	7308.637531
0.277353557	24.64959811	2521.275825
0.571235273	11.28233415	7263.786811
0.358354681	21.36359127	155.0714286
0.646134817	15.95153287	4626.688097
0.813819246	9.637969212	10431.90943
0.247051315	53.73147357	1068.407843
0.424064843	21.11700927	4933.057858
0.92500984	11.64732417	18298.62051
0.470502046	19.34829042	9014.067012
0.145020553	51.02585801	8701.185284
0.137394868	97.04360541	556.7410256
0.892850883	11.79722119	38132.64321
0.397700716	21.30496815	896.0101523
0.312405182	41.37226758	87723.28149
0.267958225	28.81900479	1887.546751
1.359488663	9.301659863	20211.64944
0.217449469	48.51259476	4212.898494
0.32656797	24.31643504	1744.091938
1.076254496	8.527200143	11175.84835
0.426601819	25.00983766	8682.376471
1.089611879	8.971461057	22831.88937
0.756420561	13.90217165	1149.283105
0.495228448	30.09561311	390.8245614
0.088283701	82.79924284	1216.881818
0.75471361	9.526623739	24827.18468
0.237911483	45.50564255	5878.137564
2.272349943	4.501463616	22819.72619
0.214285282	22.71523387	2736.662338
0.452741885	41.01287582	600.5993031
0.500427602	24.06497434	1252.355958
0.683874063	14.58383111	24527.89446
0.627648	16.54545514	6552.218123
0.477055706	23.72019185	1354.419118
1.236020467	7.653933166	37125.56484
0.230380635	41.71315602	3514.964868
0.183265692	59.16992637	5248.457891
0.108751396	89.02963928	8289.31671
0.495195651	29.03640522	31504.23157
0.313023718	22.43363476	1319.461059
0.13085661	65.57245344	2747.643021
0.972174021	28.48633806	324.7058824
0.242519408	41.88936518	14945.51066
1.392652228	7.518639445	20934.45406
0.256799961	36.93591683	3157.790439
0.515975678	18.98279755	734.7373737
0.625548049	17.32652515	55275.65953
0.458443219	16.52822481	1788.667557
0.440453365	19.5546663	4965.503262
0.648286611	19.24344828	17296.03451
0.492096254	18.90228473	1845.120861
0.221308786	73.15076746	2061.019568
0.15259412	97.06566667	909.8347826
0.287904558	47.1328256	1154.639017
0.79938576	10.90812675	12700.5094
0.144820222	51.00084355	2268.184685
0.835347788	39.50705385	546.6720867
0.784111201	13.4667823	25979.04798
0.520702069	10.05530379	6980.810213
0.440289281	19.45516221	6732.829363
0.259603733	88.547631	639.9034091
0.179884484	43.69018478	4021.802759
1.723148226	7.563508208	22107.5578
0.161680676	71.18713618	3299.459574
0.308189524	40.48582841	1557.6275
0.331387991	91.34122381	1311.423913
0.581251121	14.5986796	9104.100699
0.595521016	20.14977822	7166.466978
0.817914584	14.49476681	14261.5698
0.692957047	16.00723252	2279.432027
0.112547588	84.62467566	2603.230574
0.398589209	34.75100892	2223.240273
0.235105273	70.4510696	656.0804598
0.273068069	46.31158451	397.1690141
0.60819386	24.65301244	21515.24261

log.sigma.3.5.mm.3D_gldm_SmallDependenceLowGrayLevelEmphasis	log.sigma.3.5.mm.3D_gldm_LowGrayLevelEmphasis	log.sigma.3.5.mm.3D_gldzm_DistanceZoneVariabilityNormalized
0.004840145	0.01245156	1
0.022391027	0.063456487	0.901902497
0.006742339	0.020287837	0.91739558
0.017853581	0.057367674	0.95125
0.008505912	0.040897861	0.912771178
0.016541448	0.020800355	1
0.001137409	0.012328713	0.905202065
0.001609368	0.00771213	0.918580868
0.001151697	0.010843392	0.942304527
0.00692881	0.020308682	0.982144306
0.000393186	0.002937849	0.954964328
0.001319299	0.01198804	0.952029024
0.011774473	0.023183658	0.945987654
0.014280802	0.03509829	1
0.00378688	0.01678444	0.942906574
0.001522178	0.013491342	0.827060465
0.011625681	0.09198005	0.969704142
0.032821497	0.093540162	0.959201389
0.006780697	0.058041699	0.972978045
0.000989154	0.007745666	0.901766088
0.003775377	0.025888584	0.866319582
0.014435622	0.054250898	0.966111772
0.007260785	0.023467683	1
0.001431588	0.016771213	0.938140496
0.00467723	0.02053171	0.924168975
0.001898441	0.01293772	0.668547685
0.003578445	0.017216476	0.98989925
0.00343458	0.019206345	0.952218917
0.051503777	0.092441859	1
0.003076892	0.023158596	0.872261224
0.003185646	0.022263796	0.902989721
0.009053801	0.023204539	0.981652949
0.003932527	0.017135897	0.966676242
0.001890288	0.012330771	0.958496173
0.002051988	0.011978191	0.95955901
0.00307065	0.008555283	0.945987654
0.00707682	0.021902646	1
0.000792271	0.007164065	0.807940379
0.008435272	0.036917808	1
0.000259879	0.00216126	0.930883279
0.005423827	0.016984376	0.973860796
0.000952803	0.009562264	0.962929571
0.003064787	0.012047222	0.930438941
0.006179821	0.022017697	0.858817802
0.004462838	0.021156617	0.979594006
0.00216128	0.012694054	0.943444245
0.001278604	0.011067616	0.954984806
0.008702194	0.041519117	0.95240928
0.022710982	0.046616449	0.962278107
0.008961818	0.016260553	0.815529741
0.002339308	0.012273578	0.954299621
0.002091471	0.010216264	0.94621373
0.001004973	0.014031742	0.985748953
0.003626282	0.012629553	0.886134068
0.011731571	0.043162452	0.981310075
0.007466809	0.029165733	0.923687558
0.001066499	0.007780079	0.968688017
0.00409482	0.022343445	0.805035403
0.00912313	0.028812043	0.973688889
0.000450639	0.007018716	0.950923434
0.003873552	0.012869811	0.926035503
0.003383553	0.010741659	0.886111724
0.001596644	0.005408319	0.923090808
0.001111612	0.006194735	0.94133507
0.007126165	0.025298267	0.950648934
0.003585615	0.010185026	0.765808688
0.011907252	0.071234078	1
0.001434619	0.006209663	0.940999762
0.00080701	0.011690491	0.988072
0.003036427	0.01336784	0.909890696
0.010932055	0.042079964	0.9608
0.000666259	0.005024555	0.810673131
0.0066577	0.028657773	0.867310497
0.004342179	0.017291358	0.988304498
0.002084605	0.010019638	0.950648934
0.004385216	0.024798242	0.952029024
0.003503403	0.012719282	0.542701416
0.010425937	0.032914835	1
0.007149458	0.024536122	0.955969483
0.002201629	0.013397121	0.985508019
0.004840145	0.01245156	1
0.008869163	0.045997802	0.968757874
0.001446425	0.010918585	0.890544193
0.002860821	0.015280061	0.98333451
0.002334285	0.013476236	0.983872034
0.013310792	0.035992027	0.941230487
0.003248632	0.01137015	1
0.0015084	0.016532753	0.914151259
0.003781385	0.012220456	0.507796203
0.003836742	0.016649915	0.90898677
0.004190015	0.026738437	0.924926758
0.002374362	0.01367727	0.951607987
0.002588558	0.014540561	0.964173974
0.001705971	0.012778507	0.948753463
0.005714196	0.02584483	0.984616309
0.003075141	0.008636785	0.931902841
0.004015357	0.020759691	0.967922968
0.010774641	0.04000275	1
0.01615203	0.051958069	0.892244898
0.001421903	0.008657664	0.908365549

log.sigma.3.5.mm.3D_gldzm_LowIntensityEmphasis	log.sigma.3.5.mm.3D_gldzm_LargeDistanceEmphasis	log.sigma.3.5.mm.3D_gldzm_HighIntensitySmallDistanceEmphasis
0.017200072	1	182.7370892
0.056942212	1.155172414	98.80603448
0.026307669	1.129496403	160.1510791
0.048312905	1.075	117.35625
0.04287123	1.164772727	96.203125
0.026048161	1	185.8714286
0.021178894	1.171557562	119.8690431
0.014671865	1.144067797	172.2565443
0.018511117	1.107407407	114.8639918
0.026579034	1.027027027	125.2094595
0.006063838	1.083333333	360.7291667
0.018924213	1.073770492	132.0204918
0.028454773	1.083333333	185.5989583
0.038659097	1	96.5
0.027745005	1.088235294	108.9963235
0.019863423	1.286833856	110.3589342
0.080632525	1.046153846	59.25
0.09664848	1.0625	64.52083333
0.055689323	1.04109589	61.26027397
0.011448606	1.169811321	198.4083637
0.022369718	1.235059761	118.2942674
0.045953265	1.051724138	82
0.043841454	1	109.6041667
0.036873283	1.15	86.15369318
0.034268376	1.118421053	125.3914474
0.014017796	2.09939759	141.1307313
0.022392187	1.015228426	122.4162437
0.036254621	1.097560976	85.5903794
0.110349607	1	46.625
0.027764189	1.205714286	104.5671429
0.039606843	1.153354633	94.23083067
0.025660089	1.027777778	145.3055556
0.023014761	1.050847458	120.7902542
0.021472994	1.06360424	121.29947
0.015438315	1.082304527	137.6451189
0.013643607	1.083333333	204.2369792
0.017215505	1	188.0826087
0.011726725	1.391468005	147.4374739
0.046435339	1	94.64150943
0.003426869	1.140311804	521.0610616
0.024431768	1.039735099	122.6837748
0.015415591	1.077405858	137.2160623
0.013408443	1.108247423	197.0972938
0.031791851	1.229299363	110.5843949
0.053573739	1.030927835	87.84536082
0.015344603	1.087378641	158.3082524
0.021842106	1.08045977	121.4742018
0.051586435	1.073170732	81.00609756
0.052227684	1.057692308	79.26923077
0.02094365	1.308411215	174.9182243
0.032668833	1.127906977	90.03197674
0.012114084	1.093418259	221.7636825
0.024370909	1.0215311	101.132177
0.019218984	1.181818182	123.0075758
0.040867813	1.028301887	127.5566038
0.036871153	1.119205298	112.7152318
0.013207117	1.047727273	159.9613636
0.027698922	1.365461847	99.63241856
0.038252159	1.04	91.84
0.012094418	1.119825708	174.3223905
0.016147811	1.115384615	171.9290865
0.0138523	1.204176334	233.5625806
0.006080707	1.120178042	333.0804896
0.009290718	1.099725526	226.4357528
0.032157297	1.075949367	100.8006329
0.013292575	1.513196481	207.945259
0.047793724	1	78.33333333
0.008760572	1.104827586	242.7327969
0.015886732	1.018	141.361
0.014368129	1.184750733	179.1375041
0.045332215	1.06	81.285
0.008734669	1.558659218	192.6342393
0.042288188	1.24822695	81.46985816
0.02969041	1.017647059	130.7308824
0.018174709	1.075949367	153.6924051
0.030269704	1.073770492	108.8217213
0.011685153	2.344195519	152.8998359
0.029882105	1	207.40625
0.026848803	1.067567568	160.1024775
0.026520118	1.02189781	112.1970803
0.017200072	1	182.7370892
0.033458185	1.047619048	130.9880952
0.01883823	1.174311927	133.8478593
0.030985691	1.067226891	94.40149393
0.017087292	1.024390244	143.9796748
0.032375856	1.090909091	184.5277778
0.016475248	1	197.0595238
0.023886093	1.152985075	104.7518657
0.016224865	2.564102564	147.1798433
0.015218435	1.18957346	163.7730384
0.017476226	1.1171875	232.8476563
0.022267258	1.104938272	123.7779492
0.017774327	1.063752277	132.040427
0.017424582	1.078947368	145.6203947
0.034387452	1.023255814	93.61111111
0.009541566	1.117370892	247.9527256
0.019236079	1.048913043	149.357337
0.034775111	1	153.2553191
0.052452505	1.171428571	106.8357143
0.012352315	1.16446281	192.8849403

log.sigma.3.5.mm.3D_gldzm_LowIntensityLargeDistanceEmphasis	log.sigma.3.5.mm.3D_gldzm_HighIntensityEmphasis	log.sigma.3.5.mm.3D_gldzm_DistanceZoneVariability	log.sigma.3.5.mm.3D_gldzm_ZonePercentage
0.017200072	182.7370892	213	0.31981982
0.162459453	99.15517241	52.31034483	0.513274336
0.062209614	160.8201439	127.5179856	0.287784679
0.123312905	117.375	38.05	0.434782609
0.044718094	99.38068182	160.6477273	0.235924933
0.026048161	185.8714286	70	0.67961165
0.028007357	122.4841986	802.0090293	0.054335827
0.016703614	177.3661017	541.9627119	0.116924296
0.019338003	117.9925926	254.4222222	0.063844881
0.053606061	125.2162162	109.018018	0.304945055
0.00637807	365.5689655	332.3275862	0.063689605
0.020591059	133.1639344	232.295082	0.079401237
0.035345699	186.8541667	136.2222222	0.435045317
0.038659097	96.5	84	0.442105263
0.028858482	111.4117647	128.2352941	0.147826087
0.029540335	115.1175549	527.6645768	0.074821156
0.126786371	59.26153846	63.03076923	0.149082569
0.098384591	65.08333333	46.04166667	0.432432432
0.056100282	62.28767123	71.02739726	0.128747795
0.013515174	204.5789474	908.0784508	0.084430284
0.027571759	121.8645418	217.4462151	0.172865014
0.058884299	82.05172414	56.03448276	0.367088608
0.043841454	109.6041667	48	0.170818505
0.038804611	88.37727273	206.3909091	0.045795171
0.037496934	127.7697368	140.4736842	0.209366391
0.018513549	191.1686747	221.9578313	0.163950617
0.022544471	122.7969543	195.0101523	0.209797657
0.03737603	87.70243902	195.204878	0.116543491
0.110349607	46.625	16	0.571428571
0.033404118	108	152.6457143	0.152041703
0.042240377	96.69648562	282.6357827	0.089148391
0.026431694	145.5555556	106.0185185	0.423529412
0.024352733	121.5720339	228.1355932	0.184519156
0.022199999	122.7491166	271.254417	0.0898698
0.016824881	139.345679	466.345679	0.139775669
0.014632487	206.8229167	272.4444444	0.255319149
0.017215505	188.0826087	230	0.58974359
0.015012719	161.3287327	643.9284818	0.067899131
0.046435339	94.64150943	53	0.269035533
0.00363094	541.9610245	835.9331849	0.074129107
0.024778412	124.0596026	147.0529801	0.239302694
0.016079966	139.4267782	460.2803347	0.066049468
0.017389729	199.8324742	361.0103093	0.254092993
0.033837622	117.5541401	134.8343949	0.222065064
0.055506729	87.96907216	95.02061856	0.089154412
0.016764285	160.5242718	291.5242718	0.158056266
0.023678029	122.7126437	415.4183908	0.062419285
0.052489777	82.48780488	39.04878049	0.187214612
0.066650761	79.32692308	50.03846154	0.456140351
0.022256283	212.1308411	87.26168224	0.486363636
0.033441748	93.23837209	164.1395349	0.090241343
0.014953029	224.8089172	445.6666667	0.218156554
0.026423779	101.3421053	412.0430622	0.041377945
0.021074515	127.7030303	146.2121212	0.194805195
0.041150832	128.2641509	104.0188679	0.369337979
0.063001099	114.8211921	139.4768212	0.22775264
0.013590178	161.55	426.2227273	0.083223
0.031580085	108.0803213	200.4538153	0.161165049
0.038652159	92.84	73.02666667	0.275735294
0.013103588	176.8311547	872.9477124	0.039042232
0.018429487	174.1394231	192.6153846	0.26097867
0.023789935	238.4524362	381.9141531	0.268870867
0.011324413	338.4695846	1244.326409	0.294838145
0.010704097	229.3769442	1028.879231	0.128286385
0.034269639	101.6455696	75.10126582	0.246105919
0.018550505	243.8841642	522.2815249	0.312128146
0.047793724	78.33333333	51	0.428571429
0.01009694	247.7586207	682.2248276	0.175629845
0.016052059	141.88	494.036	0.049885264
0.020370194	182.4662757	310.2727273	0.223313687
0.046556705	82.02	48.04	0.252525253
0.011260863	208.8752328	435.3314711	0.075644457
0.044171132	89.04255319	122.2907801	0.188251001
0.030396292	130.8411765	168.0117647	0.158434296
0.019029065	158.0481013	375.5063291	0.122823383
0.039533707	109.295082	116.147541	0.201986755
0.025441055	261.6171079	266.4663951	0.331309042
0.029882105	207.40625	64	0.556521739
0.048200154	160.3018018	212.2252252	0.341013825
0.026862272	112.5474453	135.0145985	0.085839599
0.017200072	182.7370892	213	0.31981982
0.059913212	131.047619	122.0634921	0.341463415
0.020517883	138.5840979	291.2079511	0.095669982
0.031328685	95.86554622	117.0168067	0.101276596
0.017547997	144.3943089	242.0325203	0.136288089
0.071096395	184.8383838	93.18181818	0.5625
0.016475248	197.0595238	168	0.231724138
0.026520446	106.880597	244.9925373	0.065912445
0.021770741	281.1452991	178.2364672	0.298723404
0.017678947	168.0995261	191.7962085	0.26375
0.042739536	234.3359375	118.390625	0.463768116
0.023176807	126.4506173	154.1604938	0.113286713
0.019023914	133.1420765	529.3315118	0.160432496
0.020691029	146.6526316	360.5263158	0.098598858
0.03579734	93.89405685	381.0465116	0.187227866
0.011330365	251.0352113	396.9906103	0.359797297
0.020272202	150.2336957	356.1956522	0.251194539
0.034775111	153.2553191	47	0.540229885
0.159595362	106.9428571	31.22857143	0.492957746
0.014383341	198.385124	1099.122314	0.125989171

log.sigma.3.5.mm.3D_gldzm_IntensityVariabilityNormalized	log.sigma.3.5.mm.3D_gldzm_LowIntensitySmallDistanceEmphasis	log.sigma.3.5.mm.3D_gldzm_IntensityVariability
0.060018956	0.017200072	12.78403756
0.079072533	0.030562902	4.586206897
0.063609544	0.017332183	8.841726619
0.07375	0.029562905	2.95
0.079093492	0.042453356	13.92045455
0.060816327	0.026048161	4.257142857
0.071526479	0.019535758	63.3724605
0.061821316	0.014176256	36.47457627
0.086886145	0.018325392	23.45925926
0.086437789	0.019822277	9.594594595
0.09892324	0.00599252	34.42528736
0.075114217	0.018507501	18.32786885
0.054012346	0.026732041	7.777777778
0.077664399	0.038659097	6.523809524
0.075692042	0.027466636	10.29411765
0.084993269	0.017444196	54.22570533
0.097751479	0.069094063	6.353846154
0.102430556	0.096214452	4.916666667
0.109776694	0.055586584	8.01369863
0.064550135	0.010943005	65.0019861
0.078903509	0.021138375	19.80478088
0.096313912	0.042720506	5.586206897
0.076388889	0.043841454	3.666666667
0.107231405	0.036496104	23.59090909
0.070290859	0.033461237	10.68421053
0.061184497	0.01322494	20.31325301
0.077482027	0.022354116	15.26395939
0.07964307	0.03600634	16.32682927
0.1015625	0.110349607	1.625
0.081404082	0.026354207	14.24571429
0.078831059	0.038948459	24.67412141
0.070130316	0.025467188	7.574074074
0.071387532	0.022680268	16.84745763
0.07323103	0.021291242	20.72438163
0.074870023	0.015113499	36.38683128
0.059968171	0.013396387	17.27083333
0.059659735	0.017215505	13.72173913
0.07861507	0.01099128	62.65621079
0.081523674	0.046435339	4.320754717
0.063873691	0.003384856	57.35857461
0.077145739	0.024345108	11.64900662
0.08883773	0.015279394	42.46443515
0.065389521	0.012413122	25.37113402
0.071605339	0.031280408	11.24203822
0.077266447	0.053090491	7.494845361
0.071605869	0.014989683	22.12621359
0.076348263	0.02139824	33.21149425
0.089827484	0.051360599	3.682926829
0.090976331	0.048621915	4.730769231
0.054065857	0.020615492	5.785046729
0.08207139	0.032533025	14.11627907
0.060849888	0.011413563	28.66029724
0.090760285	0.023857692	37.93779904
0.086868687	0.018755101	14.33333333
0.065681737	0.040797058	6.962264151
0.069163633	0.030338667	10.44370861
0.070320248	0.013111352	30.94090909
0.086853438	0.026788277	21.62650602
0.085511111	0.038152159	6.413333333
0.073706219	0.011910881	67.66230937
0.072670118	0.015577393	15.11538462
0.05453782	0.011385732	23.50580046
0.053684324	0.004769781	72.36646884
0.063999551	0.008945698	69.95150961
0.084762057	0.031629212	6.696202532
0.05481119	0.012060698	37.38123167
0.094194541	0.047793724	4.803921569
0.061920571	0.00843903	44.89241379
0.076264	0.0158454	38.132
0.068463464	0.012935953	23.34604106
0.0928	0.045026092	4.64
0.082619838	0.008283713	44.36685289
0.084150697	0.041852476	11.86524823
0.066297578	0.029513939	11.27058824
0.071661593	0.01796112	28.30632911
0.085192153	0.027953703	10.39344262
0.049663806	0.008557554	24.38492872
0.0546875	0.029882105	3.5
0.064929795	0.021510965	14.41441441
0.083488731	0.02643458	11.4379562
0.060018956	0.017200072	12.78403756
0.075459814	0.026844429	9.507936508
0.071701783	0.018418317	23.44648318
0.081844503	0.03094758	9.739495798
0.076839183	0.016972116	18.90243902
0.064789307	0.022695721	6.414141414
0.070436508	0.016475248	11.83333333
0.082145244	0.023256296	22.01492537
0.04473178	0.015102708	15.7008547
0.074324476	0.014679371	15.68246445
0.069458008	0.011160398	8.890625
0.082837982	0.022074864	13.41975309
0.073533266	0.017478656	40.36976321
0.07365651	0.01660797	27.98947368
0.081625704	0.03403498	31.58914729
0.057814807	0.009101592	24.62910798
0.06777233	0.018977049	24.94021739
0.067451335	0.034775111	3.170212766
0.084081633	0.025666791	2.942857143
0.059725429	0.011866432	72.2677686

log.sigma.3.5.mm.3D_gldzm_HighIntensityLargeDistanceEmphasis	log.sigma.3.5.mm.3D_gldzm_SmallDistanceEmphasis	log.sigma.3.5.mm.3D_glcm_SumVariance	log.sigma.3.5.mm.3D_glcm_Homogeneity1
182.7370892	1	403.8269565	0.396154037
100.5517241	0.961206897	124.5182968	0.313687087
163.4964029	0.967625899	269.7363323	0.37524586
117.45	0.98125	137.5912319	0.31760242
115.7272727	0.965119949	114.5484854	0.412839774
185.8714286	1	393.4237729	0.292108173
134.8510158	0.962126912	336.1201144	0.729355217
202.4220339	0.967749529	545.5433865	0.538544908
133.7333333	0.977263374	362.3598562	0.620143125
125.2432432	0.993243243	210.2914544	0.407051053
390.5603448	0.982359515	1248.737285	0.676372804
137.7377049	0.981557377	336.8700157	0.630232451
191.875	0.979166667	263.1640597	0.325286502
96.5	1	132.0319102	0.371906702
121.0735294	0.977941176	274.5184496	0.476895343
134.1520376	0.928291536	340.308568	0.643187272
59.30769231	0.988461538	56.66467765	0.474069671
67.33333333	0.984375	58.89595293	0.414033337
66.39726027	0.989726027	89.51326672	0.499782353
233.2423039	0.960857332	474.7721031	0.617669803
137.2788845	0.945661797	167.1579928	0.46851193
82.25862069	0.987068966	85.70945023	0.398975547
109.6041667	1	203.1774232	0.4272917
103.4590909	0.975284091	242.6963476	0.713410357
137.2828947	0.970394737	347.3701726	0.470050863
585.9096386	0.84270415	354.3927874	0.495649502
124.319797	0.996192893	279.5253512	0.442904276
99.81463415	0.98102981	251.7766979	0.535483759
46.625	1	78.27619079	0.350247449
121.7314286	0.948571429	204.4876949	0.495934422
106.5591054	0.961661342	238.5720336	0.544134594
146.5555556	0.993055556	234.3599783	0.369710796
124.6991525	0.987288136	256.319411	0.487109824
128.5477032	0.98409894	343.3435742	0.613804812
149.9979424	0.983996342	314.1716204	0.522821637
217.1666667	0.979166667	598.1129507	0.44576771
188.0826087	1	317.1310078	0.274913833
231.7327478	0.918862401	551.0215467	0.66646345
94.64150943	1	145.7625472	0.430488194
651.9454343	0.972345954	1710.202693	0.660170141
129.5629139	0.990066225	237.6358585	0.441302142
151.1799163	0.985297536	348.2780274	0.68730106
210.7731959	0.972938144	424.7685368	0.416441261
145.433121	0.942675159	210.5210262	0.426193494
88.46391753	0.992268041	221.6000387	0.601758244
169.3883495	0.97815534	333.3151434	0.502168568
129.3931034	0.982439336	361.6525347	0.649761243
88.41463415	0.981707317	150.7829286	0.439850355
79.55769231	0.985576923	108.3584715	0.372480684
360.9813084	0.922897196	550.0817638	0.283655086
117.6918605	0.980943152	428.0090238	0.619590141
239.4055202	0.979004482	448.3201948	0.452663648
102.1818182	0.994617225	261.1398351	0.729337131
146.4848485	0.954545455	348.4986505	0.442493752
131.0943396	0.992924528	157.0685615	0.355586274
123.2450331	0.970198675	156.6055416	0.422015799
167.9045455	0.988068182	487.9630865	0.619450747
147.5301205	0.917559125	220.5519722	0.473993771
96.84	0.99	156.4600275	0.44167299
193.3594771	0.980375212	505.4419943	0.718450918
182.9807692	0.971153846	325.2756548	0.423272092
263.9721578	0.954111885	470.3086071	0.407655945
360.0259644	0.96995549	785.6996757	0.405934039
243.2561757	0.977101759	640.0338432	0.583199388
105.0253165	0.981012658	188.9480335	0.408095784
436.5351906	0.897768003	570.3688269	0.363782552
78.33333333	1	92.0774602	0.358079891
271.0496552	0.976858238	642.3991155	0.505018351
143.956	0.9955	308.1303563	0.664086484
201.8680352	0.963587488	300.7053911	0.416080827
84.96	0.985	107.8194083	0.409658465
352.9590317	0.91933968	801.2255296	0.655113804
126.4255319	0.945823483	174.3684185	0.426844572
131.2823529	0.995588235	261.8651896	0.502921777
175.4708861	0.981012658	411.6501712	0.593536687
111.1885246	0.981557377	186.3513952	0.441686845
913.3299389	0.762601833	577.5925908	0.334840093
207.40625	1	408.5817616	0.291913093
161.0990991	0.983108108	235.2008537	0.370821672
113.9489051	0.994525547	288.7496275	0.596359372
182.7370892	1	403.8269565	0.396154037
131.2857143	0.988095238	130.7169759	0.379030906
157.529052	0.956422018	438.1484055	0.616404614
109.0420168	0.992530345	270.6750787	0.553565756
146.0528455	0.993902439	297.0545796	0.512851757
186.0808081	0.977272727	292.0569594	0.285192099
197.0595238	1	440.8667119	0.448624215
117.7835821	0.965899668	263.7384173	0.666818206
1124.307692	0.735992403	652.0251294	0.365728587
192.2511848	0.963138494	234.8098017	0.401245983
240.2890625	0.970703125	436.5887728	0.318938368
142.5185185	0.980624143	306.5720924	0.564716051
138.5282332	0.986085813	262.9357962	0.524659079
150.7815789	0.980263158	307.0762073	0.575876433
95.02583979	0.994186047	155.6556474	0.498535262
267.1314554	0.973265519	527.0116824	0.360994463
153.7391304	0.987771739	227.2137391	0.422088137
153.2553191	1	285.3482862	0.301897643
107.3714286	0.957142857	152.0386019	0.319852938
224.7421488	0.963475666	495.4957545	0.565881678

log.sigma.3.5.mm.3D_glcm_Homogeneity2	log.sigma.3.5.mm.3D_glcm_ClusterShade	log.sigma.3.5.mm.3D_glcm_MaximumProbability	log.sigma.3.5.mm.3D_glcm_Idmn	log.sigma.3.5.mm.3D_glcm_SumVariance2
0.313083314	63.21677119	0.021946087	0.982438458	41.00225661
0.219282106	129.8975982	0.030868539	0.941099476	35.61461279
0.290052475	24.84964965	0.019894771	0.966375775	42.60187551
0.223480338	181.8229706	0.042073492	0.935105694	43.73563641
0.331377303	70.34541778	0.028498973	0.979964444	23.63201055
0.195633401	9.724088829	0.031430584	0.954196722	50.62138532
0.707189643	-95.42568636	0.263921254	0.995523379	15.45014234
0.483043559	-103.4433393	0.126794164	0.991870172	31.01141385
0.57960896	-68.43922024	0.194636946	0.993663323	20.68017144
0.325258233	-14.50689186	0.032425554	0.978140377	20.69348126
0.652292963	-40.27242752	0.216380174	0.996934308	9.858278481
0.593464703	-82.74220673	0.142771944	0.993729877	21.01558176
0.235082303	299.2717903	0.024215166	0.959579397	52.96083776
0.28366429	67.83923794	0.042735286	0.958322474	23.34202494
0.40925892	-26.30082434	0.045104472	0.983420435	29.00272074
0.607841587	-146.2726545	0.245507383	0.991438338	26.33309479
0.405741719	22.9334716	0.036776352	0.974040084	16.94757289
0.335733801	76.49522644	0.066986536	0.966945332	15.96139913
0.437123281	-10.26644251	0.042287809	0.975721086	18.76280261
0.579533474	-30.39247209	0.150479216	0.996681381	22.04698162
0.397712053	59.98264226	0.052492017	0.982205014	29.22960554
0.317509602	101.3012141	0.044450622	0.951454035	25.00667768
0.346779811	0.146899526	0.029766799	0.971718317	31.02020303
0.690584187	-57.15818175	0.322941445	0.994454825	12.0510287
0.401093351	-88.13018248	0.100942365	0.974980885	44.48014425
0.436073907	192.3496361	0.060931375	0.988904569	36.72071405
0.367772444	-14.59073115	0.026254848	0.985701412	30.67995225
0.48211099	-55.45315802	0.069954637	0.986501824	24.8930704
0.256242439	23.66392297	0.088193085	0.904770156	17.64186538
0.431504111	-17.57025685	0.053400746	0.982505749	24.3492183
0.490897767	-71.08884966	0.08837462	0.988445166	30.29139456
0.284857209	28.70409465	0.035968719	0.961960623	33.63528535
0.42247889	-43.58934656	0.04877425	0.985609441	30.13653089
0.573443417	-87.34926596	0.179686802	0.991468346	24.36440379
0.464788387	-34.11467676	0.086361135	0.989160652	23.30690236
0.375217435	-179.7020249	0.038081612	0.980357702	50.98764331
0.181313159	131.6929203	0.015962881	0.948715756	54.54997998
0.635115303	-144.3872346	0.225388433	0.995368018	23.2197281
0.349937094	34.77649823	0.041147325	0.971394554	25.11698717
0.629213912	-39.74124393	0.243167447	0.997816638	15.89045648
0.365842246	-1.613569957	0.035508138	0.982485487	20.07982331
0.66363155	-13.67482774	0.24997539	0.994597186	7.278520735
0.339366619	-54.96136409	0.023394335	0.982625483	45.51408965
0.346292091	28.27299892	0.027632142	0.980130273	27.00399159
0.560320718	-81.76003556	0.173099635	0.986871891	23.1631502
0.441294745	-40.90535363	0.070177487	0.987343885	30.01072018
0.617282073	-85.41357701	0.173781837	0.995600567	19.38897455
0.36032543	55.77692882	0.03772914	0.972704496	38.90576178
0.285859767	136.9573833	0.042877136	0.957018421	28.46215973
0.192835501	261.3044299	0.020057941	0.956841625	90.7362791
0.577012297	-146.6215873	0.252819168	0.990134284	29.10926139
0.382755871	5.487419463	0.031780407	0.989097193	35.61232782
0.710611551	-48.8900016	0.315019601	0.996186764	10.24431817
0.365912921	16.46298202	0.026055021	0.98325224	36.94656056
0.26589115	217.0719271	0.024939722	0.96458301	42.90892003
0.34296655	69.65026031	0.028792149	0.982455434	26.07431428
0.580877723	-81.97600717	0.183363974	0.993783161	21.26753045
0.404168386	-2.899289347	0.063546456	0.9822203	33.0909899
0.36555405	-0.840693496	0.041653916	0.971590305	21.81198162
0.699227912	-44.67381879	0.273664314	0.99789449	9.912948256
0.345477046	10.3140782	0.037412951	0.97816896	30.11137298
0.328552595	42.81439873	0.03568777	0.98280399	51.00298988
0.330852418	-72.76793326	0.031463411	0.982529792	55.55288213
0.5364559	-118.6062335	0.133271629	0.993458229	27.81171839
0.322855184	-4.603372532	0.026738926	0.96918477	29.09424995
0.277922287	335.6344261	0.016971975	0.981142813	71.66630837
0.265681174	25.14346279	0.034837959	0.940641302	21.98813402
0.444878391	-70.91072825	0.077715127	0.992646319	27.92662881
0.634963595	-45.98717386	0.171597935	0.995250297	14.6035232
0.336982232	58.47075919	0.023960083	0.982702112	33.78689211
0.326115915	20.93482153	0.043362934	0.955128643	18.82332908
0.620152421	-209.6802596	0.209058422	0.992829345	30.9245487
0.346990611	33.78570299	0.028420006	0.97068478	26.16209663
0.442553991	-14.14225764	0.059843759	0.991306966	24.00768285
0.549875364	-73.229044	0.163240067	0.993371291	23.23148599
0.363852652	83.8365223	0.030024614	0.981020191	29.64743488
0.247632886	2328.857756	0.016323019	0.977579409	154.6639566
0.195626439	-10.76667007	0.022062889	0.947762492	83.90675697
0.282796856	90.36844891	0.021456598	0.975232925	41.24412115
0.553835059	-50.91292094	0.149060831	0.990806149	16.40675938
0.313083314	63.21677119	0.021946087	0.982438458	41.00225661
0.294291328	185.6719165	0.031309062	0.966395313	38.22817804
0.57373648	-149.7030701	0.245037785	0.993434756	28.4411087
0.504645732	-61.92142005	0.062260635	0.987611464	20.83418671
0.455045316	-60.20542843	0.057408506	0.987203136	25.83912158
0.189202642	96.50454877	0.019710983	0.939774288	69.71294375
0.375674881	-94.30816485	0.03075544	0.985814345	39.66881046
0.633571138	-92.87785444	0.30014319	0.992884265	19.88854724
0.286847636	1710.330599	0.021160765	0.978172663	127.0235644
0.318247407	100.4430553	0.023675639	0.981249057	30.3061118
0.22796256	-292.150452	0.022409389	0.959717336	78.43936351
0.517471458	-58.16001854	0.096306213	0.990273687	20.38147047
0.468744724	-18.28969342	0.070073538	0.989416922	22.75823875
0.52902982	-56.95955587	0.142765951	0.991540516	22.36192323
0.436127928	1.784588362	0.057637562	0.983797343	17.71735571
0.274413664	8.513450768	0.016746501	0.981117394	43.1881537
0.34482248	82.19782214	0.026027992	0.983701037	31.28407422
0.204297948	-51.6832869	0.032066579	0.938580628	53.9214373
0.224559444	17.12838239	0.043166346	0.939702687	28.30422733
0.517425361	-90.24144066	0.157626881	0.991635407	33.75359541

log.sigma.3.5.mm.3D_glcm_Contrast	log.sigma.3.5.mm.3D_glcm_DifferenceEntropy	log.sigma.3.5.mm.3D_glcm_InverseVariance	log.sigma.3.5.mm.3D_glcm_Entropy	log.sigma.3.5.mm.3D_glcm_Dissimilarity
12.67622132	2.948185856	0.32020772	7.149946496	2.709033423
22.48184878	3.175101773	0.242196034	6.37492755	3.807389643
16.71933296	3.104291161	0.290942172	7.279025116	3.099989311
24.95744509	3.201771489	0.208380947	5.996427296	3.998922684
10.46441827	2.829216896	0.334635773	6.703575519	2.467607728
27.58658072	3.256680622	0.224186976	6.215417971	4.208333897
2.508685827	1.78405518	0.324954124	4.366668222	0.83119693
5.283647806	2.419243213	0.38528639	6.282347323	1.612044816
3.184362552	2.093241946	0.383818231	5.331569074	1.178096672
10.36848954	2.798702589	0.337608568	6.563281998	2.49436585
2.318385771	1.816826252	0.422912247	4.715077895	0.908818729
3.156860297	2.077137077	0.393620989	5.367897559	1.143809542
26.50899093	3.403568001	0.238655273	7.275031553	3.974064374
15.53433778	3.011514211	0.282965144	6.418562311	3.037649022
6.405193299	2.506610799	0.389876246	6.486712667	1.891307158
4.03144189	2.129125438	0.362019319	5.126952023	1.204427308
5.568558838	2.399888093	0.405816307	5.999884166	1.81450335
10.87329788	2.734265535	0.35112166	5.555671157	2.488608962
4.443037326	2.250584713	0.423477002	5.971671115	1.621501009
3.93619934	2.173800296	0.395142722	5.678644996	1.245939577
7.624205249	2.648756796	0.356322879	6.602136227	2.05013869
12.90115173	2.89037858	0.31936084	6.090712568	2.733793229
8.990698863	2.684889438	0.332940406	6.576460153	2.312044962
1.667696476	1.711900142	0.368104488	4.348994944	0.77661059
10.05653659	2.764111979	0.331064956	6.591098354	2.260404678
9.363232281	2.673540661	0.388916999	6.685782783	2.036692324
8.022860276	2.621454849	0.370487167	6.785662226	2.140040367
4.117948282	2.255098102	0.432250103	6.085945309	1.485271538
14.69791206	2.369233701	0.264632983	4.169915777	3.115583831
5.387059394	2.420437627	0.395672694	6.364344037	1.742350404
4.88152948	2.348562469	0.399654213	6.118569722	1.552133009
17.25836106	3.108381441	0.291912158	6.738489341	3.174746384
7.496279403	2.599454945	0.380582763	6.606808026	1.947577753
3.590584824	2.149914688	0.388542918	5.46447915	1.236001301
5.531555543	2.442301376	0.393881167	6.242941191	1.670602082
11.3952581	2.868203671	0.355670613	7.164454379	2.4038988
40.47649865	3.577490204	0.192448121	7.487774397	5.010495526
3.523677713	2.048097168	0.357866016	5.027666802	1.090258868
6.970380529	2.5062496	0.360196376	6.271349855	2.112352707
3.238088657	2.00861009	0.383587578	5.053653711	1.056927013
7.437624918	2.601151454	0.375315486	6.45032444	2.105685801
2.042969046	1.788937312	0.407114241	4.52850873	0.86603973
13.71746947	2.894180056	0.330014053	7.283971584	2.681900789
8.506861178	2.683791404	0.348956049	6.748386062	2.263056213
3.161682143	2.088463285	0.41472701	5.540327731	1.211394375
7.137553375	2.574216848	0.384959272	6.529119916	1.875714337
2.612365214	1.968251884	0.40078447	5.288411544	1.029712725
6.606694743	2.415802625	0.356075473	6.300932576	2.055392427
14.32604127	2.910935509	0.307018656	5.942298071	2.938714546
45.36607331	3.692108627	0.194554013	7.376751213	5.13192508
3.346959133	2.125140288	0.362012854	5.323153949	1.213983511
11.18167895	2.834282556	0.360106496	7.028646575	2.333039763
1.587252544	1.641362398	0.370446682	4.134653431	0.722850606
7.067334346	2.448749301	0.369714274	6.700202226	2.073982779
17.59172908	3.128012764	0.276725847	7.006590935	3.246077119
10.02940427	2.773524547	0.350698845	6.719335803	2.374188729
3.433364352	2.115498918	0.397147542	5.551458076	1.194362512
7.584444782	2.658283119	0.349706649	6.653214032	2.044697303
8.063394724	2.643387193	0.358103976	6.32181985	2.168930717
1.824843275	1.681739083	0.384920726	4.222934212	0.765837646
11.51410505	2.884744994	0.331439608	6.906345372	2.516850005
14.53335996	3.036152177	0.317567	7.476943655	2.784404246
19.39835765	3.196893589	0.310928774	7.812152343	3.103123185
6.189055162	2.435904863	0.357938963	5.956752954	1.556453463
8.604359727	2.618517464	0.334244271	6.609751186	2.347663878
19.45974089	3.23836986	0.282802669	7.986678406	3.316591669
13.62293089	2.908921542	0.282643136	6.171354016	2.98236479
7.907158759	2.615837944	0.380625885	6.568896829	1.912045526
2.389680298	1.897490092	0.40416674	4.957186473	0.963991434
11.59839786	2.900627921	0.333606421	7.126971151	2.542812934
10.1507317	2.755867086	0.322670394	6.198755306	2.47457753
3.648261869	2.113003697	0.346195233	5.178729438	1.148208513
8.235025211	2.666547692	0.350807042	6.685678886	2.241446854
6.187160223	2.475979409	0.407917563	6.266413885	1.766361322
5.06959168	2.320417471	0.379542325	5.804662222	1.423102125
8.126628052	2.642171963	0.344557197	6.656437259	2.178283942
30.17861018	3.5028172	0.248935202	8.291098609	4.04175558
32.2976157	3.371909115	0.192261411	6.587028718	4.499716522
15.50483998	3.080735233	0.285416337	7.366922625	3.042230249
3.12980876	2.093051598	0.419053201	5.419789137	1.2177675
12.67622132	2.948185856	0.32020772	7.149946496	2.709033423
15.04120457	3.023233428	0.303113967	6.892067555	2.970909521
4.281985291	2.233664203	0.351061669	5.436114807	1.31230711
3.327544618	2.12100732	0.449645645	5.816610313	1.34411033
6.018620171	2.46726394	0.408049562	6.361762598	1.733668287
31.18197494	3.434617162	0.196882614	7.112878279	4.512971803
8.693640216	2.687253843	0.368505541	6.902464662	2.175634458
3.006565311	2.015368106	0.351404697	4.907926392	1.045295687
29.56206624	3.433788259	0.28718882	8.011830386	3.816745213
11.5357595	2.891187629	0.325318729	6.942041348	2.606005834
26.10751488	3.372968187	0.229788572	7.313425787	4.025632133
3.68452356	2.17073952	0.433315537	5.742739024	1.354871883
5.930875727	2.46703302	0.401863502	6.373481651	1.692494014
4.721533223	2.315401423	0.394350382	5.907435282	1.438642029
5.623168902	2.442357843	0.405231283	6.241342657	1.744538928
19.46467783	3.232883482	0.281249916	7.607972465	3.329845284
11.81511804	2.896988967	0.33744948	7.04947782	2.529844565
26.23161757	3.259876501	0.203627348	6.245936575	4.116371913
20.32044135	3.028244907	0.233662787	5.797070434	3.674352309
6.44549023	2.471499192	0.372603477	6.317797221	1.627672106

log.sigma.3.5.mm.3D_glcm_DifferenceVariance	log.sigma.3.5.mm.3D_glcm_Idn	log.sigma.3.5.mm.3D_glcm_Idm	log.sigma.3.5.mm.3D_glcm_Correlation	log.sigma.3.5.mm.3D_glcm_Autocorrelation
5.201333404	0.911038612	0.313083314	0.527577872	147.274156
7.471095149	0.838374327	0.219282106	0.231121214	49.97484292
6.74056817	0.881041064	0.290052475	0.438966331	103.681285
7.980251929	0.832645768	0.223480338	0.28540612	52.88528627
4.293954466	0.905161868	0.331377303	0.386095692	50.66513491
9.224156224	0.855848597	0.195633401	0.291234943	135.5730171
1.800308421	0.96780932	0.707189643	0.718028089	115.9045493
2.621582895	0.942719807	0.483043559	0.707299252	188.8508723
1.73734787	0.952094356	0.57960896	0.729624909	129.5579396
3.997683207	0.900074538	0.325258233	0.332292309	82.78223655
1.472760616	0.969099867	0.652292963	0.61799589	375.0953195
1.82049125	0.953573994	0.593464703	0.737003838	121.9382404
10.26186674	0.868728853	0.235082303	0.329927927	98.28649537
5.865245286	0.866701286	0.28366429	0.212594913	54.71114194
2.73163489	0.914873522	0.40925892	0.636929806	105.8664371
2.483734049	0.950116121	0.607841587	0.730602997	121.2599866
2.185153338	0.892699854	0.405741719	0.502322496	29.20735558
4.578077873	0.881911932	0.335733801	0.190053712	27.22438866
1.698821899	0.896154879	0.437123281	0.61479993	42.41698981
2.353308223	0.96635285	0.579533474	0.694701677	164.7357013
3.333950131	0.912902786	0.397712053	0.584589493	70.24149615
5.098223735	0.859046975	0.317509602	0.319533877	38.07690943
3.457277315	0.888225628	0.346779811	0.548070442	81.91124039
1.051386378	0.959243652	0.690584187	0.754747248	88.15332378
4.638091485	0.902443179	0.401093351	0.631499439	128.7618444
5.088813473	0.936915144	0.436073907	0.595387832	131.2569316
3.256117356	0.919448068	0.367772444	0.585815872	108.0167585
1.862496837	0.924418845	0.48211099	0.714308177	98.41906582
4.185641606	0.797999915	0.256242439	0.103820907	29.76606531
2.277611488	0.912683093	0.431504111	0.6365087	83.13632865
2.365565184	0.932505259	0.490897767	0.720301168	93.9209333
6.830489171	0.873577223	0.284857209	0.328634154	90.22422036
3.603865818	0.923986381	0.42247889	0.601366769	100.2598326
2.018893075	0.945702571	0.573443417	0.74094912	124.5912708
2.690497599	0.933577593	0.464788387	0.615153612	117.4808295
5.505909809	0.912384637	0.375217435	0.632702262	207.6694067
13.16146441	0.850773901	0.181313159	0.181244321	112.6756107
2.299126075	0.963732904	0.635115303	0.734229979	183.0078559
2.397675269	0.883701237	0.349937094	0.562424626	62.14126783
2.097432008	0.974171	0.629213912	0.658003862	505.9796539
2.928781315	0.909932829	0.365842246	0.458189865	92.52361045
1.277067815	0.959211385	0.66363155	0.557549022	119.4957279
5.709331735	0.915687117	0.339366619	0.538701138	154.3818018
3.261725058	0.904056568	0.346292091	0.519892009	84.8755624
1.648167756	0.930669305	0.560320718	0.758135212	87.01193688
3.523025457	0.929475214	0.441294745	0.613208684	124.6314612
1.53223618	0.961045934	0.617282073	0.759816357	129.7231235
2.088957832	0.886398255	0.36032543	0.704748464	64.1623666
5.409246649	0.86372373	0.285859767	0.334665582	45.38164962
18.15624923	0.8657036	0.192835501	0.341431723	185.6934963
1.79769473	0.941205039	0.577012297	0.790688949	149.4507282
5.633476788	0.934279423	0.382755871	0.521021145	160.6692571
1.054422719	0.967270752	0.710611551	0.729930835	92.53493837
2.274748607	0.91089037	0.365912921	0.675548282	130.7035217
6.886876477	0.875608338	0.26589115	0.415353595	64.19717057
4.302982819	0.912000042	0.34296655	0.44517604	65.87193287
1.961383162	0.953589229	0.580877723	0.717216674	168.5407192
3.323847462	0.913157644	0.404168386	0.626168886	88.44471861
3.304619166	0.888809045	0.36555405	0.460384836	65.42508002
1.225219271	0.975505196	0.699227912	0.686498537	164.0007921
5.047787562	0.904363895	0.345477046	0.447206632	121.4544413
6.585424056	0.915429245	0.328552595	0.554529899	169.0994725
9.411416922	0.918074669	0.330852418	0.485735861	263.7552834
3.727461109	0.953891127	0.5364559	0.634264367	213.6777058
2.837790909	0.879627918	0.322855184	0.541331871	77.29629081
8.191181091	0.909301109	0.277922287	0.575013121	200.5336262
4.561552942	0.837185606	0.265681174	0.237054112	40.57017647
4.16563825	0.946754476	0.444878391	0.55718989	217.3388785
1.43899969	0.960427008	0.634963595	0.716845187	110.9212366
5.022988374	0.91327858	0.336982232	0.488506497	114.4345403
3.81156059	0.861376696	0.326115915	0.297799785	47.20897421
2.304872475	0.954104777	0.620152421	0.787372922	256.0589973
3.09420441	0.885003661	0.346990611	0.520602554	72.54352293
3.020235186	0.939751548	0.442553991	0.589583694	100.8537999
2.997634539	0.953142547	0.549875364	0.640414614	145.6034648
3.206361266	0.907525035	0.363852652	0.568921331	76.8117191
13.22266694	0.903770455	0.247632886	0.678069933	199.2647396
11.34850798	0.848675729	0.195626439	0.447596015	141.2901854
6.065683483	0.894510058	0.282796856	0.453232583	92.66347932
1.613502765	0.940454826	0.553835059	0.677377131	106.8956789
5.201333404	0.911038612	0.313083314	0.527577872	147.274156
5.984660073	0.880071058	0.294291328	0.428320036	55.3689243
2.488872789	0.953321117	0.57373648	0.73564111	152.2937754
1.479316017	0.926859535	0.504645732	0.722855077	103.420417
2.936570333	0.928598804	0.455045316	0.621206683	112.5225981
10.29158043	0.836026767	0.189202642	0.381138612	106.8642231
3.872670532	0.921706695	0.375674881	0.637674595	159.6205432
1.892893369	0.953994459	0.633571138	0.735597819	96.76811672
14.32892437	0.909622317	0.286847636	0.628074827	222.3724601
4.620912374	0.907650692	0.318247407	0.447511953	92.2717887
9.248489306	0.866462812	0.22796256	0.506473948	155.2324431
1.784894376	0.937259618	0.517471458	0.68815534	113.936222
3.018295584	0.935686128	0.468744724	0.585360546	101.7753644
2.610970261	0.944891669	0.52902982	0.650082883	114.481643
2.505726116	0.917152235	0.436127928	0.513610252	65.66544864
8.098983118	0.908897495	0.274413664	0.379378725	184.2904878
5.230272583	0.916829021	0.34482248	0.451816062	89.95971302
8.690596104	0.835332556	0.204297948	0.348590767	102.7022314
6.354739161	0.834818787	0.224559444	0.171866697	58.43159545
3.746202155	0.947047012	0.517425361	0.67684718	173.8645018

log.sigma.3.5.mm.3D_glcm_SumEntropy	log.sigma.3.5.mm.3D_glcm_AverageIntensity	log.sigma.3.5.mm.3D_glcm_Energy	log.sigma.3.5.mm.3D_glcm_SumSquares	log.sigma.3.5.mm.3D_glcm_ClusterProminence
4.633351579	11.84509737	0.009142847	13.51893264	5782.305914
4.235386119	6.828499734	0.013580011	14.52411539	3934.831441
4.648302653	9.85883859	0.008125229	14.83030212	4592.465768
4.197235973	6.938537056	0.018205533	17.17327038	5397.450935
4.231293191	6.882525092	0.012435217	8.524107204	1941.767582
4.27240292	11.38983644	0.014750975	19.55199151	6336.934085
3.321962166	10.61452853	0.132154653	4.489707041	1341.318571
4.329615607	13.50603109	0.028891666	9.073765415	2610.948927
3.893071503	11.1882764	0.057130684	5.966133497	1149.844938
4.141476721	8.954414566	0.013656501	7.7654927	1403.923691
3.439609469	19.32254207	0.085912892	3.063326853	666.4208938
3.904963003	10.83843305	0.05601516	6.043110515	1370.037388
4.650311715	9.572972097	0.008497707	19.86745717	8853.919753
4.103530922	7.262913061	0.014981549	9.719090678	1906.433767
4.353576711	10.01050818	0.014790956	8.851978509	1832.67107
3.792227375	10.75548587	0.087999229	7.59113417	2223.738603
3.967116112	5.134057167	0.018795397	5.629032932	735.5894627
3.638669915	5.09355169	0.027582597	6.708674253	1350.179865
4.052490137	6.231626489	0.019834914	5.801459983	763.1397713
4.037426696	12.66264678	0.052307206	6.549360315	2986.136519
4.36053071	8.05224216	0.014224026	9.213452698	2479.039291
4.048907239	5.916815071	0.018444815	9.476957353	2077.74797
4.360946625	8.74069792	0.012687327	10.00272547	2039.109983
3.312588667	9.249620404	0.130248603	3.429681295	721.6619123
4.519743064	10.96135852	0.020915094	13.63417021	4248.694821
4.485504198	11.16704115	0.017544565	11.76189721	7952.583382
4.460669595	10.11618022	0.011917786	9.675703131	2621.408272
4.248574258	9.65509217	0.022851179	7.252754671	1584.979684
3.010438631	5.383939261	0.059540298	8.08494436	706.8151307
4.287118706	8.85390782	0.017324388	7.434069423	1594.419995
4.284662355	9.357308937	0.025208208	8.793231011	1990.560461
4.393418677	9.280110467	0.012554341	12.7234116	3663.377148
4.413570666	9.726046236	0.015115925	9.408202574	2333.981786
3.994077084	10.92677734	0.060471273	6.988747153	1851.228607
4.209488305	10.63166598	0.02202738	7.209614476	1368.416989
4.736379765	14.06262649	0.010828761	15.59572535	6519.388318
4.704222397	10.4436395	0.006530049	23.82297852	8443.334988
3.715762179	13.34550415	0.090226687	6.695617221	2355.272875
4.197586371	7.588697425	0.016232166	8.021841925	1689.688279
3.685245023	22.43645037	0.086847475	4.71392854	1485.634458
4.157390907	9.452926091	0.015252549	6.879362057	1172.851618
3.276813362	10.8713467	0.098211204	2.330372445	337.1981252
4.729419686	12.10519582	0.009330941	14.87943202	5926.9881
4.370617552	8.957966802	0.011868607	8.877713191	2109.921513
4.025495485	9.055904928	0.047322292	6.581208084	1415.572819
4.394327321	10.9045372	0.017589952	9.287068389	2399.160985
3.907777853	11.20384029	0.062336782	5.50033494	1447.112909
4.40145071	7.488232882	0.015760834	11.37811413	2889.114923
4.000866743	6.466324875	0.019375338	10.69705025	2799.066297
4.978048662	13.38707299	0.007016228	35.95213349	24965.8878
3.945178293	11.95850688	0.077632913	8.114055131	2469.975037
4.549374122	12.4561538	0.012612479	12.00542364	5845.585739
3.17310154	9.506318096	0.138293939	2.957892679	549.7079983
4.553564928	11.099872	0.011845843	11.00347373	2973.289753
4.529292509	7.60578531	0.009670283	15.12516228	6266.525779
4.30580045	7.864959756	0.01221559	9.025929638	2525.996119
4.016206245	12.80927786	0.054095802	6.175223699	1593.850597
4.427281584	9.058902981	0.014299437	10.16885867	2193.42187
4.143651208	7.872746345	0.016059301	7.468844086	1202.622972
3.193791191	12.72810314	0.126573306	2.94891051	778.6472406
4.435028675	10.80743729	0.011690955	10.40636951	2655.0276
4.81986051	12.65936923	0.00849969	16.56179723	7870.972802
4.898872626	16.05951726	0.007756493	19.30106514	10127.14143
4.120343119	14.44049982	0.044591788	8.579925733	2880.7606
4.347713558	8.494583068	0.012346461	9.42465242	1872.864191
5.053723112	13.75629674	0.006072693	23.56106272	18532.23052
4.03616393	6.201513561	0.016193081	8.902766228	1262.936065
4.35521299	14.59227642	0.0195098	9.126070859	2963.383346
3.639417166	10.38589928	0.074103501	4.248300875	763.4754458
4.532271629	10.43487188	0.009775695	11.34632249	3696.168677
3.989967095	6.710568488	0.016694977	7.243515194	848.3482834
3.820476693	15.78726312	0.081823515	8.643202642	3281.223598
4.327325263	8.249368182	0.01212426	8.59928046	1767.959436
4.214296147	9.821035268	0.020772084	7.60199892	2479.75157
4.046030778	11.87849477	0.048269708	7.10526604	2298.004164
4.385474072	8.45131328	0.013211535	9.443515732	3178.37982
5.369192811	13.10281705	0.004861928	47.71241092	98527.15366
4.645470186	11.32225038	0.011256122	29.05109317	15503.58653
4.645245139	9.285219522	0.007954066	14.18724028	5115.130412
3.852085072	10.1771106	0.043833923	4.884142036	809.7095895
4.633351579	11.84509737	0.009142847	13.51893264	5782.305914
4.463817352	7.03804851	0.011208199	13.31734565	4826.370652
3.946144732	12.09334814	0.075017944	8.180773497	2699.598823
4.098437353	9.951779593	0.026043042	6.040432833	1232.846286
4.274624947	10.3712942	0.019617262	7.964435438	1729.277514
4.809013274	9.855956299	0.008203187	25.22372967	10891.4996
4.618408657	12.32348576	0.011622076	12.09061267	4864.064197
3.624815486	9.620053051	0.106673482	5.723778139	1277.281844
5.230642815	14.22253012	0.00673978	40.45874312	72938.72472
4.416387952	9.358183519	0.0106163	10.46046783	3304.001072
4.920371242	11.92048223	0.00759332	26.1367196	15709.05297
4.037710395	10.47626787	0.031266153	6.016498509	1295.457137
4.257842748	9.87763153	0.020083832	7.172278619	1563.902158
4.109694406	10.49142323	0.037796548	6.770864114	1537.488817
4.085590616	7.914563394	0.020808444	5.835131152	1027.209162
4.715034443	13.4158087	0.00696745	16.24427802	5935.641995
4.452167823	9.227713672	0.011001243	10.84996656	4383.768545
4.360931883	9.78513637	0.014508154	20.03826372	6509.401594
3.900312918	7.508670894	0.020388154	12.15616717	2042.014068
4.360648829	12.92672619	0.039386671	10.09232027	4485.818541

log.sigma.3.5.mm.3D_glcm_SumAverage	log.sigma.3.5.mm.3D_glcm_Imc2	log.sigma.3.5.mm.3D_glcm_Imc1	log.sigma.3.5.mm.3D_glcm_DifferenceAverage	log.sigma.3.5.mm.3D_glcm_Id
23.67966028	0.819043329	-0.147306631	2.709033423	0.396154037
13.65699947	0.949557067	-0.311733453	3.807389643	0.313687087
19.71767718	0.827093287	-0.152101353	3.099989311	0.37524586
13.87707411	0.966278641	-0.371366213	3.998922684	0.31760242
13.76505018	0.669137501	-0.087331369	2.467607728	0.412839774
22.77967287	0.985186269	-0.44739322	4.208333897	0.292108173
21.22905706	0.866160779	-0.281370941	0.83119693	0.729355217
27.01206219	0.835903548	-0.182942441	1.612044816	0.538544908
22.3765528	0.860270163	-0.237785078	1.178096672	0.620143125
17.90882913	0.759232273	-0.125029671	2.49436585	0.407051053
38.63728954	0.790740942	-0.196811644	0.908818729	0.676372804
21.67686611	0.867321033	-0.238320764	1.143809542	0.630232451
19.14594419	0.880815785	-0.187528488	3.974064374	0.325286502
14.52582612	0.861060177	-0.194166098	3.037649022	0.371906702
20.02101637	0.814478449	-0.162790857	1.891307158	0.476895343
21.51097175	0.869879104	-0.258718346	1.204427308	0.643187272
10.26811433	0.733574051	-0.12868608	1.81450335	0.474069671
10.18710338	0.864206284	-0.221587762	2.488608962	0.414033337
12.46325298	0.787296357	-0.160969763	1.621501009	0.499782353
25.31453615	0.850328069	-0.211045389	1.245939577	0.617669803
16.10448432	0.776549529	-0.137955038	2.05013869	0.46851193
11.83363014	0.863313843	-0.205165274	2.733793229	0.398975547
17.48139584	0.865394782	-0.193599399	2.312044962	0.4272917
18.49924081	0.843055781	-0.258008723	0.77661059	0.713410357
21.92271704	0.879598929	-0.217101975	2.260404678	0.470050863
22.30824672	0.830819614	-0.169161694	2.036692324	0.495649502
20.23236044	0.788196519	-0.143644465	2.140040367	0.442904276
19.31018434	0.839865343	-0.191961737	1.485271538	0.535483759
10.76787852	0.985274171	-0.617448774	3.115583831	0.350247449
17.70781564	0.793744816	-0.15287712	1.742350404	0.495934422
18.71461787	0.848834956	-0.202734597	1.552133009	0.544134594
18.56022093	0.882590347	-0.203152499	3.174746384	0.369710796
19.45209247	0.810404139	-0.157425471	1.947577753	0.487109824
21.85355468	0.872540718	-0.242381647	1.236001301	0.613804812
21.26333196	0.783433434	-0.148707793	1.670602082	0.522821637
28.12525297	0.850408884	-0.169880292	2.4038988	0.44576771
20.88149102	0.915031768	-0.227543835	5.010495526	0.274913833
26.68948595	0.862435081	-0.247250112	1.090258868	0.66646345
15.17739485	0.873388444	-0.208937478	2.112352707	0.430488194
44.84710378	0.836449785	-0.220566732	1.056927013	0.660170141
18.90585218	0.713527049	-0.10944299	2.105685801	0.441302142
21.74269339	0.748907018	-0.175980078	0.86603973	0.68730106
24.20039095	0.798364623	-0.155544109	2.681900789	0.416441261
17.9159336	0.759718139	-0.125494581	2.263056213	0.426193494
18.11180986	0.877540978	-0.245287282	1.211394375	0.601758244
21.80907441	0.82081437	-0.167887471	1.875714337	0.502168568
22.40768058	0.876899398	-0.252219998	1.029712725	0.649761243
14.97646576	0.911789626	-0.25690553	2.055392427	0.439850355
12.93264975	0.925481436	-0.289089514	2.938714546	0.372480684
26.40560005	0.96660185	-0.311884961	5.13192508	0.283655086
23.91701376	0.88450979	-0.263929282	1.213983511	0.619590141
24.86402596	0.769764678	-0.125671972	2.333039763	0.452663648
19.01263619	0.832465608	-0.256920567	0.722850606	0.729337131
22.199744	0.854368601	-0.195897261	2.073982779	0.442493752
15.21157062	0.86973982	-0.184310874	3.246077119	0.355586274
15.72991951	0.745394501	-0.117135985	2.374188729	0.422015799
25.61855573	0.860725023	-0.229491228	1.194362512	0.619450747
18.11780596	0.797382838	-0.147579051	2.044697303	0.473993771
15.74549269	0.82098319	-0.165118841	2.168930717	0.44167299
25.4541448	0.811076352	-0.234002881	0.765837646	0.718450918
21.61487459	0.75729689	-0.120495401	2.516850005	0.423272092
25.29618516	0.796450095	-0.132904592	2.784404246	0.407655945
31.91838453	0.752652207	-0.108772336	3.103123185	0.405934039
28.86316256	0.83919064	-0.191682488	1.556453463	0.583199388
16.98916614	0.835528238	-0.173613781	2.347663878	0.408095784
27.38370909	0.775057273	-0.113651774	3.316591669	0.363782552
12.40302712	0.902237856	-0.244398345	2.98236479	0.358079891
29.14308864	0.775508786	-0.139236555	1.912045526	0.505018351
20.77179856	0.850043575	-0.237765672	0.963991434	0.664086484
20.86974377	0.718716911	-0.102116829	2.542812934	0.416080827
13.42113698	0.7927068	-0.150863021	2.47457753	0.409658465
31.57452623	0.87761137	-0.255230636	1.148208513	0.655113804
16.49873636	0.723691773	-0.112117856	2.241446854	0.426844572
19.63623896	0.804530351	-0.158566961	1.766361322	0.502921777
23.75363255	0.839835175	-0.197640724	1.423102125	0.593536687
16.90262656	0.807120019	-0.156229098	2.178283942	0.441686845
25.93301778	0.868829858	-0.160103632	4.04175558	0.334840093
22.64450076	0.989494853	-0.459309426	4.499716522	0.291913093
18.57043904	0.774523133	-0.121077367	3.042230249	0.370821672
20.35422119	0.823923893	-0.198431381	1.2177675	0.596359372
23.67966028	0.819043329	-0.147306631	2.709033423	0.396154037
14.07609702	0.817763291	-0.153212713	2.970909521	0.379030906
24.18669627	0.870556994	-0.24343229	1.31230711	0.616404614
19.90355919	0.837417295	-0.197403827	1.34411033	0.553565756
20.7425884	0.804703063	-0.160750631	1.733668287	0.512851757
19.7119126	0.954736755	-0.293165859	4.512971803	0.285192099
24.64697152	0.861856079	-0.183089016	2.175634458	0.448624215
19.2401061	0.863446104	-0.251758209	1.045295687	0.666818206
28.14183027	0.889559951	-0.181152872	3.816745213	0.365728587
18.71636704	0.710920463	-0.1010695	2.606005834	0.401245983
23.84096446	0.938458227	-0.254250244	4.025632133	0.318938368
20.95253575	0.833228747	-0.200769809	1.354871883	0.564716051
19.75526306	0.77253065	-0.139536355	1.692494014	0.524659079
20.98284646	0.833584264	-0.18943161	1.438642029	0.575876433
15.82912679	0.685156737	-0.106224704	1.744538928	0.498535262
26.70989796	0.720236848	-0.095102926	3.329845284	0.360994463
18.44872706	0.70684626	-0.101412923	2.529844565	0.422088137
19.57027274	0.979567796	-0.411325426	4.116371913	0.301897643
15.01734179	0.968358906	-0.391959114	3.674352309	0.319852938
25.84839002	0.853041571	-0.194341527	1.627672106	0.565881678

log.sigma.3.5.mm.3D_glcm_ClusterTendency	log.sigma.3.5.mm.3D_firstorder_InterquartileRange	log.sigma.3.5.mm.3D_firstorder_Skewness	log.sigma.3.5.mm.3D_firstorder_Uniformity
41.00225661	133.4416199	0.263123292	0.074980386
35.61461279	132.2014771	0.428589235	0.073850732
42.60187551	152.5558014	0.153758644	0.070217627
43.73563641	169.2801208	0.36932486	0.077268431
23.63201055	109.8973293	0.769335454	0.094926291
50.62138532	156.6086502	-0.115056149	0.064756339
15.45014234	37.90236712	-1.311584746	0.240299826
31.01141385	120.6533229	-0.50810115	0.106831105
20.68017144	98.71956968	-0.675865981	0.145692393
20.69348126	94.30310535	0.053874029	0.101165318
9.858278481	35.3209033	-1.371908657	0.222276209
21.01558176	82.90849149	-0.696170348	0.14678203
52.96083776	179.9402428	0.5390749	0.063900476
23.34202494	112.0082588	0.470818068	0.092022161
29.00272074	121.0410137	-0.098448102	0.091999055
26.33309479	80.80351271	-1.03585932	0.186364042
16.94757289	92.09734917	0.3838529	0.113942429
15.96139913	86.46125793	1.055381015	0.124259395
18.76280261	91.07310486	-0.190197964	0.114949501
22.04698162	57.89844036	-0.026215054	0.148617562
29.22960554	118.2593699	0.506516037	0.090628296
25.00667768	117.9446259	0.581160267	0.093734978
31.02020303	123.6350784	-0.111605878	0.085472575
12.0510287	47.82413346	-1.323891948	0.24643953
44.48014425	158.1286612	-0.342837132	0.08780138
36.72071405	99.67195415	0.826873373	0.097803871
30.67995225	105.6742783	0.070728527	0.091637831
24.8930704	105.9373446	-0.40578908	0.10601694
17.64186538	94.19822311	0.390305053	0.130102041
24.3492183	102.357214	-0.052802382	0.100079182
30.29139456	125.5927305	-0.358671058	0.102629073
33.63528535	107.2004395	0.245847714	0.086658977
30.13653089	113.073267	-0.093789091	0.094729867
24.36440379	87.18881977	-0.635129426	0.150499874
23.30690236	105.3851795	-0.122576144	0.107114595
50.98764331	146.8920021	-0.534955047	0.074178864
54.54997998	190.4011154	0.099063153	0.057777778
23.2197281	61.00834608	-1.213398199	0.194010677
25.11698717	99.28011322	0.244472916	0.09469453
15.89045648	53.02156834	-0.538474281	0.192549188
20.07982331	82.85884094	0.194420352	0.106949199
7.278520735	36.3914113	-0.484979322	0.224141504
45.51408965	136.731945	-0.011663488	0.07315387
27.00399159	107.4330788	0.368466946	0.090049195
23.1631502	95.76970696	-0.657157071	0.136323394
30.01072018	117.9718275	-0.035216636	0.093596719
19.38897455	71.38971233	-0.900366694	0.153068812
38.90576178	151.7173533	0.146083429	0.088717917
28.46215973	117.9333115	0.594812975	0.094336719
90.7362791	171.7317114	0.39301782	0.060619835
29.10926139	116.3708168	-0.868914908	0.153621028
35.61232782	103.223547	0.456547586	0.088172543
10.24431817	37.93754409	-1.300917952	0.26237807
36.94656056	135.3645649	-0.061051663	0.082442512
42.90892003	147.8325806	0.61278198	0.072272335
26.07431428	104.9183235	0.752628194	0.093280008
21.26753045	81.13623917	-0.71815322	0.140410532
33.0909899	132.6142082	0.017078527	0.088303642
21.81198162	101.269474	0.103034166	0.100210856
9.912948256	32.83481264	-1.162400656	0.256402665
30.11137298	121.6308823	0.196984589	0.087594792
51.00298988	149.4473104	0.23250737	0.068700335
55.55288213	134.6855628	-0.001139547	0.068653752
27.81171839	100.9633995	-0.584467781	0.129368732
29.09424995	114.3806992	-0.050973769	0.087722363
71.66630837	137.6089859	0.515699322	0.066348151
21.98813402	113.2675171	0.064499708	0.09031848
27.92662881	107.5080719	-0.174444154	0.099019717
14.6035232	64.6332196	-0.52426392	0.175085033
33.78689211	127.081584	0.405698168	0.080817711
18.82332908	116.9432259	0.147536317	0.101061116
30.9245487	92.25060248	-1.207388143	0.173697388
26.16209663	104.2253952	0.189825745	0.096090738
24.00768285	90.55589676	0.285318801	0.108869837
23.23148599	81.00595146	-0.52532825	0.140741991
29.64743488	108.8332057	0.326158685	0.094140608
154.6639566	176.0662675	1.279050568	0.057521568
83.90675697	211.390419	-0.234631442	0.056181474
41.24412115	137.4417896	0.275342051	0.07055434
16.40675938	83.66559005	-0.668797111	0.143351801
41.00225661	133.4416199	0.263123292	0.074980386
38.22817804	140.7747116	0.647792118	0.075844038
28.4411087	107.6774874	-0.839594169	0.16042851
20.83418671	91.11144257	-0.595080149	0.118118244
25.83912158	101.4594116	-0.340076439	0.103560593
69.71294375	215.4247131	-0.114748975	0.058755165
39.66881046	114.7618637	-0.286442739	0.083308442
19.88854724	79.33087534	-0.893888111	0.200620771
127.0235644	124.8322788	1.276976568	0.067984427
30.3061118	124.8270226	0.649522139	0.085053125
78.43936351	139.9411836	-0.532462412	0.06747532
20.38147047	99.27395916	-0.481758924	0.122062693
22.75823875	96.65974593	0.043901611	0.105172431
22.36192323	90.5874835	-0.313586732	0.125662445
17.71735571	76.121171	0.217448733	0.115885377
43.1881537	133.9047012	0.180009009	0.070289673
31.28407422	107.5865631	0.435319205	0.085834896
53.9214373	173.3568993	-0.29379958	0.073853878
28.30422733	123.086853	-0.112472474	0.091053362
33.75359541	86.1456337	-0.361517822	0.117458564

log.sigma.3.5.mm.3D_firstorder_MeanAbsoluteDeviation	log.sigma.3.5.mm.3D_firstorder_Energy	log.sigma.3.5.mm.3D_firstorder_RobustMeanAbsoluteDeviation	log.sigma.3.5.mm.3D_firstorder_Median
77.0944558	14856495.2	54.29149622	-112.5447884
82.84050755	7418498.614	58.33694958	-243.4108429
82.68885286	15885896.62	60.35039817	-154.9006348
90.87324048	6093206.471	67.11871804	-256.1966248
62.92246089	17537442.77	44.73592774	-143.0637131
90.88588678	2871568.929	63.02862117	-120.9133453
39.07455446	57381549.43	20.80677066	-3.425783992
65.0698577	62144010.13	48.6960224	-64.76654053
52.97896135	32268899.63	40.67446785	-44.08626938
56.25314293	3989503.145	38.67873707	-77.47467041
29.65045075	10503938.21	16.20209143	2.325954914
51.20203297	17966323.89	36.03858129	-21.14865303
101.8851018	17468906.04	76.55574171	-227.2523651
66.34693969	14469376.12	46.60310598	-274.1777954
63.3733523	17728315.87	48.42548015	-113.4861069
56.4408677	57752284.19	38.00411637	-11.14448547
50.63968653	4229967.681	37.75722196	-79.7950058
55.97421799	8786825.513	37.92268083	-287.7082214
49.71175983	5365976.383	36.77875882	-70.75255585
46.99605906	56912199.34	27.45729917	-6.494796753
64.97289388	25512056.96	47.57584969	-111.0248337
67.14705468	5545218.643	49.11162495	-175.903862
66.94230225	6726850.824	50.6389142	-130.5567474
36.35595439	15953722.4	22.48320509	-13.54913044
79.89131474	15274483.39	63.06561789	-107.1194
62.4274673	21454997.17	40.87032257	-60.11847687
61.61192107	22582101.66	43.11967615	-137.0977173
57.84995365	18251276.96	43.37434542	-66.59495544
55.5310497	138552.3695	39.40119239	-26.73376083
58.04189317	14864630.19	42.39512824	-86.65880585
63.86793469	43222286.43	49.9525898	-70.32655334
70.73890822	16478708.02	45.87448979	-240.3683624
62.82790487	17803820.29	45.59098031	-80.08939362
56.03136532	20413870.52	39.0156032	-14.50542831
56.56743637	34214700.8	41.86050583	-66.90432739
82.60979769	30900398.15	61.16368285	-115.8151894
100.1883197	29395460.66	74.76339493	-247.2743149
50.55342196	62096658.96	30.91975153	-4.982363224
60.01601579	4902965.211	42.21513813	-143.7760162
42.1500064	50488466	24.97147669	-9.076364517
53.59331841	17008067.19	36.25406177	-151.5393982
28.63151497	13797440.04	16.44818001	-8.375534058
78.96739682	40514065.35	55.2702964	-132.9794769
63.79844262	17549614.81	45.02784484	-141.2586365
54.46353421	8555563.047	39.70481364	-39.90052605
64.2252877	27602802.8	47.17048806	-83.83333588
48.68920679	34703964.51	32.32624104	-11.68006992
74.99234973	3563471.128	59.96746028	-102.9943619
71.98405948	2788769.865	52.65509633	-143.857338
101.107138	7630158.591	67.27612566	-134.6297302
64.19236201	19710038.11	48.98215296	-37.63914299
66.99996364	27865554.58	43.15266986	-68.48438263
33.19621329	26728803.62	19.33882774	-7.600171566
71.15689612	6942799.541	54.62080951	-33.61221695
83.44477907	12071991.02	58.80593388	-189.8526154
62.82595603	16228588.71	43.71739619	-142.2761841
52.39595495	33367273.79	35.76314819	-24.70837212
67.98235117	29345817.01	52.97694427	-111.1881561
57.97954703	10535481.08	40.94900238	-179.2744598
31.48783936	57665986.88	16.71291106	-5.538680077
67.72302635	14913483.98	48.44348825	-107.1310043
85.99418315	29910122.65	61.48270805	-83.27822876
85.08952727	82141504.14	57.13682824	-71.05391693
60.08231003	71329764.67	42.249491	-28.24758911
64.21510405	4604084.083	47.63130509	-90.53964233
88.52502465	49087778.41	58.18711938	-105.4840317
63.29687082	8928868.72	46.39578526	-268.0852051
61.97982216	48688114.87	43.1564449	-67.48160553
42.88290181	38060101.85	28.99109221	-8.634181976
71.13800723	24743627.66	50.32439377	-94.86330414
61.04316066	6040579.286	47.2144478	-167.4578094
61.46872589	54820998.77	42.74087228	-10.11782742
59.20855249	22413733.44	42.96079429	-163.6400604
55.06975127	10461341.15	36.86792122	-62.74952316
52.23946106	19589927.59	35.28232921	-20.4783783
62.35522639	6442107.934	44.50091091	-68.64577103
119.6117541	40120521.12	76.32575078	-80.65283585
109.3722014	8892912.701	80.04054085	-233.5066376
79.82022171	11133073.3	56.53956053	-90.94048309
47.03147682	11072109.02	33.9748036	-46.7134285
77.0944558	14856495.2	54.29149622	-112.5447884
82.50537305	10811463.57	59.5545316	-155.002533
61.81174082	32616787.72	45.52044458	-33.4398098
52.17649936	14646057.94	37.82903684	-80.49349213
58.45466753	19163890.05	42.19461357	-63.35057831
108.7437135	9973500.604	83.74029047	-192.8570251
69.84859925	12834024.27	48.3731444	-97.74351501
50.40429984	24557489.91	35.55463679	-19.77652836
101.7317981	26771746.57	56.94614107	-68.97454834
69.96502763	16835322.5	49.85671192	-126.8905716
96.23708654	7359565.33	64.35193083	-86.37710953
53.1018276	14416240.64	39.45541463	-67.57956696
55.58418875	29967925.69	38.87959652	-58.89725876
54.27388174	26447955.18	38.58282425	-33.77186775
48.80070849	15972617.28	32.51571842	-61.69810486
81.38423119	37362525.1	56.06918729	-148.5688705
66.32474823	24446763.1	44.77215041	-100.4637527
94.14567331	5204234.475	70.2044597	-204.9055481
70.01231822	2625300.968	51.22887524	-172.4994812
62.72957284	77628876.63	40.71905957	-15.21745062

log.sigma.3.5.mm.3D_firstorder_TotalEnergy	log.sigma.3.5.mm.3D_firstorder_Maximum	log.sigma.3.5.mm.3D_firstorder_RootMeanSquared	log.sigma.3.5.mm.3D_firstorder_90Percentile	log.sigma.3.5.mm.3D_firstorder_Minimum
401125370.3	240.9582672	149.355448	-5.575326443	-385.6044617
200299462.6	23.69515228	256.223399	-99.68195496	-423.3191528
428919208.8	107.4107513	181.3561554	-25.84709167	-394.9673462
164516574.7	14.98816013	257.3528805	-75.59242554	-412.60672
473510954.7	237.737442	153.3252635	-26.67093945	-296.5804443
77532361.07	132.0354614	166.9709855	21.95430164	-413.2212524
1549301835	291.6859436	59.32153995	11.69542599	-259.640564
1677888274	206.1004333	110.9752199	1.610683501	-389.1806946
871260289.9	211.8343811	87.35207694	2.237810659	-314.149292
107716584.9	223.549057	104.6908471	13.32010374	-289.9454041
283606331.8	185.5031128	43.84506658	38.95114746	-453.8991394
485090745	226.5858154	76.4624716	12.28199978	-280.7019958
471660463	125.6786728	229.7306057	-14.59461689	-433.4643555
390673155.3	-23.14046669	275.9612494	-149.7374557	-441.7483215
478664528.6	121.5200424	138.8160962	-19.2340292	-336.7021484
1559311673	220.2983398	82.29746378	9.41812439	-287.5746155
114209127.4	147.5611267	98.49751639	1.535794795	-198.3436432
237244288.8	10.77296829	281.3549186	-174.7911224	-389.4041443
144881362.3	78.81691742	97.28208028	0.719745028	-221.2534332
1536629382	505.5862732	69.0775737	47.11453705	-323.0043335
688825537.9	185.4909058	132.5529598	-8.351144886	-277.8174438
149720903.3	38.47032928	187.3401199	-40.4541935	-314.3708496
181624972.3	64.3279953	154.7222379	-29.06113052	-325.8795471
430750504.8	172.3267822	57.62746506	5.148042917	-247.202774
412411051.5	96.76283264	145.0490721	-2.610411644	-359.5176697
579284923.6	372.6472473	102.9323095	23.9513649	-323.9094849
609716744.8	176.808075	155.0777113	-32.3676796	-372.3770752
492784477.9	118.4621506	101.8623539	4.952111149	-285.5896301
3740913.978	110.3495102	70.34414626	93.41036987	-127.5895462
401345015.2	116.5668869	113.6421372	-5.81840992	-298.9779663
1167001734	183.4985199	110.9528478	2.563958883	-294.7237549
444925116.5	24.06346512	254.2093318	-111.5888565	-451.5888977
480703147.8	218.9910583	117.9835145	-1.330598187	-306.2973022
551174503.9	189.140686	80.51491663	19.01986351	-298.0823669
923796921.5	214.3306732	99.19825394	4.068425179	-301.3437805
834310750.1	90.88890076	165.5112383	-16.47365627	-462.7019348
793677437.8	108.147316	274.5413922	-88.68893509	-512.8723145
1676609792	311.6038513	72.73393171	21.59854069	-325.8492432
132380060.7	45.00791931	157.7597803	-28.59863014	-312.0165405
1363188582	357.8559265	64.55833233	16.86580753	-574.1060791
459217814.2	103.021553	164.1771838	-61.11135101	-356.7067871
372530881	196.3512115	43.66364339	24.57353668	-271.4906311
1093879764	241.1565704	162.8858632	-7.24257164	-405.0621643
473839599.9	129.4654999	157.5520605	-31.87188187	-338.6655579
231000202.3	99.90713501	88.67676684	4.34029417	-252.1856384
745275675.5	223.6441498	118.823738	-0.806744337	-337.2556152
937007041.7	286.8417969	70.56743193	23.6538784	-276.2799988
96213720.44	87.8132019	127.5600159	20.64525185	-260.5770874
75296786.35	105.5194855	156.4061813	11.60581946	-277.2424316
206014282	290.8985901	186.2324866	20.93649712	-427.1816711
532171029.1	77.73077393	101.6909452	1.944610238	-343.0357056
752369973.7	378.4281006	113.6076363	29.98183632	-354.1600952
721677697.7	239.1438446	51.43823734	6.503115988	-234.343689
187455587.6	181.4533539	90.53689745	74.11142883	-289.3502808
325943757.6	138.1609802	205.0918977	-21.27877235	-364.1684875
438171895.2	237.1747437	156.4528982	-34.42621765	-315.5871887
900916392.2	214.4826355	79.44301302	16.88654785	-342.5999451
792337059.3	170.0361786	137.8189258	-7.425626087	-302.9092102
284457989.2	24.73915672	196.8079936	-104.723941	-359.9699402
1556981646	392.3838501	49.52287423	11.13778057	-304.6681519
402664067.5	156.1826324	136.7919043	-14.58968182	-352.7168884
807573311.5	292.9660034	136.5973694	39.31661682	-387.4802551
2217820612	308.7698669	134.0380851	53.96555023	-456.4082947
1925903646	341.8948975	91.4988421	11.13925905	-399.4571228
124310270.2	82.32345581	119.7620185	13.65361309	-278.8121948
1325370017	332.6165161	149.8859649	56.65888443	-416.3931274
241079455.4	-95.51448822	273.9206276	-153.6671509	-423.2970886
1314579101	369.3075256	108.6029533	5.535323095	-406.8986206
1027622750	258.6083069	61.62204516	14.07412434	-259.9634399
668077946.9	264.4229126	127.2952409	15.42929535	-326.6331787
163095640.7	24.95405388	174.6653262	-58.9061821	-319.9873962
1480166967	124.2771912	87.87693149	18.00717735	-416.3809509
605170802.8	31.72494888	172.9880856	-53.29147949	-349.3096313
282456211.1	340.7459412	98.74015883	4.888238621	-291.6157532
528928045	336.3034668	78.04739198	19.3418169	-305.1641541
173936914.2	217.7015076	103.2750774	27.71566372	-271.2632141
1083254070	521.7542114	164.5353354	176.5756607	-336.4024963
240108642.9	23.55171585	278.0821383	-75.71273193	-536.4579468
300592979	259.3361816	130.7726848	44.44900894	-316.9316406
298946943.4	129.5223236	83.29112596	-0.522826314	-298.7835388
401125370.3	240.9582672	149.355448	-5.575326443	-385.6044617
291909516.3	167.7902527	171.170556	10.66275311	-313.3313904
880653268.3	250.3016205	97.68650332	1.222251058	-343.8284912
395443564.5	59.79459763	111.645556	-19.11918488	-313.4596558
517425031.3	181.9953156	103.0393789	4.370564079	-300.7520142
269284516.3	41.90200806	238.0496105	-36.59003353	-466.6543274
346518655.4	178.8195648	133.0492482	5.527582741	-380.5318604
663052227.4	210.5960388	77.71561711	2.244033575	-272.1052246
722837157.3	488.0926819	150.945239	154.1905365	-365.3586426
454553707.4	230.4089661	145.0660302	-8.231224871	-338.1239929
198708263.9	160.9649506	163.294494	30.79080963	-424.8032227
389238497.2	148.6838074	100.4056132	-8.68184557	-305.7834473
809133993.6	262.197052	93.58114162	15.28695822	-278.5631104
714094790	267.8850708	82.84001999	17.43384857	-276.1162109
431260666.7	180.2995758	87.90585544	18.69869881	-249.4399872
1008788178	281.7714233	177.6406112	-12.38795443	-464.3354797
660062603.7	310.8100281	129.1789856	7.095619297	-317.6688232
140514330.8	-7.632575035	244.5787952	-76.88308716	-468.6438904
70883126.14	35.3205719	192.2916274	-68.980896	-367.5857239
2095979669	318.466095	89.90535807	48.9459362	-344.7114563

log.sigma.3.5.mm.3D_firstorder_Entropy	log.sigma.3.5.mm.3D_firstorder_StandardDeviation	log.sigma.3.5.mm.3D_firstorder_Range	log.sigma.3.5.mm.3D_firstorder_Variance	log.sigma.3.5.mm.3D_firstorder_10Percentile
3.956328023	96.71099942	626.5627289	9353.017409	-237.8883743
3.891804652	101.3097625	447.0143051	10263.66798	-362.3891113
3.996029594	100.2066249	502.3780975	10041.36768	-281.8347595
3.842111899	108.4435987	427.5948801	11760.01411	-370.2431549
3.608031562	78.80726711	534.3178864	6210.58535	-223.1272812
4.115327313	112.22695	545.2567139	12594.88831	-280.9968445
2.657355561	54.49268438	551.3265076	2969.452651	-106.0740395
3.548340393	78.16053195	595.2811279	6109.068755	-188.5710297
3.114793066	62.75049704	525.9836731	3937.624879	-153.8026398
3.542650101	71.37617971	513.4944611	5094.55903	-166.208876
2.651740537	43.68695786	639.4022522	1908.550287	-55.87374077
3.166480472	63.40573773	507.2878113	4020.287577	-140.9489624
4.166604814	121.4500263	559.1430283	14750.1089	-336.8553772
3.672803177	82.74077974	418.6078548	6846.036632	-365.0877625
3.582328769	75.30789332	458.2221909	5671.278796	-215.6540955
3.052464101	70.07194318	507.8729553	4910.077222	-161.8005737
3.275103398	61.25566002	345.9047699	3752.255885	-152.860817
3.316995218	71.85833769	400.1771126	5163.620695	-344.6309204
3.262965481	60.22782899	300.0703506	3627.391385	-159.9858734
3.264544392	66.20443865	828.5906067	4383.027697	-109.0762527
3.634855189	80.02889484	463.3083496	6404.624009	-205.2180588
3.596730412	82.10548203	352.8411789	6741.31018	-262.1388153
3.667488759	79.89590166	390.2075424	6383.355102	-244.0138092
2.583957207	47.88315415	419.5295563	2292.796451	-105.537574
3.755321394	92.83810721	456.2805023	8618.914151	-234.200882
3.673962196	83.83733353	696.5567322	7028.698493	-158.3488434
3.660921662	77.32168922	549.1851501	5978.643624	-229.2208557
3.436399554	69.36361502	404.0517807	4811.311088	-170.9879944
3.168986958	68.24288893	237.9390564	4657.09189	-95.05791779
3.520500683	70.72727107	415.5448532	5002.346873	-181.811615
3.472179382	74.67292322	478.2222748	5576.045462	-189.7201691
3.818026557	92.29236533	475.6523628	8517.880699	-345.4688843
3.61587972	77.43492731	525.2883606	5996.167967	-193.1430084
3.237224009	69.15661636	487.223053	4782.637586	-148.974588
3.448363928	68.841741	515.6744537	4739.185304	-164.845929
3.944125739	99.49313288	553.5908356	9898.883489	-271.2763184
4.253423337	120.8348125	621.0196304	14601.05191	-406.1760651
2.963606021	66.01486987	637.4530945	4357.963044	-139.9125748
3.591391018	74.94979493	357.0244598	5617.471761	-231.9329803
2.986250116	58.74997836	931.9620056	3451.559958	-107.3148491
3.487686416	68.91008134	459.7283401	4748.599311	-239.6549683
2.632250134	41.08154352	467.8418427	1687.693218	-65.84292755
4.000508237	98.85030084	646.2187347	9771.381975	-259.1593323
3.681789533	79.49185528	468.1310577	6318.955057	-236.9382538
3.24174328	65.82905086	352.0927734	4333.463937	-159.9337646
3.640294898	78.82684019	560.899765	6213.670735	-197.5196808
3.147551804	61.52756838	563.1217957	3785.641671	-126.168483
3.637957179	86.07993608	348.3902893	7409.755395	-203.2457703
3.597492535	87.33333626	382.7619171	7627.111622	-228.6437943
4.279262248	131.1620406	718.0802612	17203.48089	-289.494516
3.220007266	75.49482954	420.7664795	5699.469287	-183.6500015
3.832936415	90.17801022	732.5881958	8132.073528	-174.2953857
2.477137776	44.86281687	473.4875336	2012.672338	-95.32241287
3.728042078	83.68564698	470.8036346	7003.287511	-143.5515167
3.984346217	104.2639189	502.3294678	10870.96478	-300.7729553
3.651169467	79.94905128	552.7619324	6391.850801	-231.5646851
3.26686598	65.81211069	557.0825806	4331.233914	-144.2126678
3.631560986	80.0862168	472.9453888	6413.802121	-219.8521484
3.51836	71.52798413	384.7090969	5116.252514	-281.5205017
2.522804527	45.40959273	697.052002	2062.031112	-82.18998566
3.744588073	83.79565116	508.8995209	7021.711154	-211.3105316
4.090706468	106.5369323	680.4462585	11350.11794	-219.135556
4.148697343	109.0475625	765.1781616	11891.37089	-219.7366837
3.401125179	75.22466556	741.3520203	5658.750309	-155.6796539
3.642226887	77.44110941	361.1356506	5997.125426	-194.4682312
4.205176654	115.6401387	749.0096436	13372.64167	-232.4685638
3.599196054	77.22681334	327.7826004	5963.980698	-364.016925
3.637924028	78.91459798	776.2061462	6227.513774	-173.5049759
2.959625475	54.39188337	518.5717468	2958.476977	-110.8406937
3.840193709	88.90476797	591.0560913	7904.057768	-200.8483429
3.45272334	71.56086966	344.9414501	5120.958067	-248.001886
3.073302535	75.14618935	540.6581421	5646.949775	-173.6814178
3.541582537	72.37894692	381.0345802	5238.711958	-248.9253174
3.508013566	72.37476957	632.3616943	5238.107271	-164.0466675
3.276941432	66.75142434	641.4676208	4455.752651	-134.0091934
3.62645318	77.48630095	488.9647217	6004.126836	-165.4744095
4.432093796	158.5276998	858.1567078	25131.03161	-205.1701447
4.298759184	132.5171859	560.0096626	17560.80456	-431.6814575
4.019481956	99.33148072	576.2678223	9866.743062	-210.0173187
3.113009147	57.69545484	428.3058624	3328.765509	-141.7009048
3.956328023	96.71099942	626.5627289	9353.017409	-237.8883743
3.911209049	101.2788621	481.1216431	10257.40791	-258.0106262
3.219294291	74.94912265	594.1301117	5617.370985	-177.8429337
3.29232027	63.46871545	373.2542534	4028.277841	-184.5020264
3.508064421	71.5871646	482.7473297	5124.722135	-176.1069672
4.214857201	127.780357	508.5563354	16327.81963	-380.7322845
3.835184259	88.44019627	559.3514252	7821.668316	-215.3624298
2.924464159	61.98647953	482.7012634	3842.323644	-148.2986984
4.29949336	144.1258638	853.4513245	20772.26463	-189.3164307
3.767005287	87.1415705	568.532959	7593.653308	-221.6671951
4.156096176	123.5182502	585.7681732	15256.75814	-292.5966034
3.299128054	64.37556971	454.4672546	4144.213976	-160.8606766
3.503556701	69.75082302	540.7601624	4865.177313	-150.7727905
3.344336489	67.26007204	544.0012817	4523.917291	-146.9179367
3.382583258	63.26431528	429.739563	4002.373588	-140.4809753
4.060656187	102.8284178	746.1069031	10573.68351	-263.4883453
3.790556129	85.41300053	628.4788513	7295.380659	-204.7799744
3.944321545	112.0854578	461.0113153	12563.14984	-382.8693054
3.676185022	86.74030188	402.9062958	7523.87997	-289.4402466
3.59220596	81.84870583	663.1775513	6699.210646	-156.069487

log.sigma.3.5.mm.3D_firstorder_Kurtosis	log.sigma.3.5.mm.3D_firstorder_Mean	log.sigma.3.5.mm.3D_glrlm_ShortRunLowGrayLevelEmphasis	log.sigma.3.5.mm.3D_glrlm_GrayLevelVariance
3.356948929	-113.8157829	0.012045668	15.5248335
2.678712548	-235.3439233	0.060564973	16.60151915
2.550412866	-151.157823	0.019452138	16.39727675
2.238364672	-233.3891407	0.050787511	18.89337886
3.968458141	-131.5220554	0.037587565	10.19535181
2.574344727	-123.6301811	0.020915829	20.86963764
5.989878944	-23.44338821	0.012617725	7.151071902
2.914199609	-78.780903	0.00749634	10.36832246
2.859790162	-60.76808757	0.010512193	6.546861471
3.530398618	-76.58729951	0.019528091	8.614633002
8.991767148	-3.720158043	0.002453574	4.074020893
3.413518144	-42.73431861	0.011491979	7.437120199
2.424133741	-195.0026725	0.022318212	24.1383528
2.896973982	-263.2652171	0.033152479	11.25152957
2.356949844	-116.6131629	0.016134863	9.149067793
3.383723908	-43.16011264	0.013630261	9.614632504
2.914221073	-77.13303346	0.079186476	6.220198657
4.572082863	-272.02384	0.09015932	8.759685168
2.298243218	-76.39641195	0.052777616	5.895799651
6.807468372	-19.71505747	0.006962707	9.124292284
3.198792508	-105.6677016	0.023544411	10.45934618
2.61551509	-168.3894603	0.047489446	11.20007759
2.237832225	-132.4976068	0.02199308	10.22338235
5.01498402	-32.06443946	0.016772868	4.842630355
2.244930953	-111.4464857	0.021046088	13.84094893
5.682946399	-59.71902408	0.011995062	12.43734411
3.211711181	-134.4263848	0.016324797	9.878128142
2.602698054	-74.59643463	0.018816865	7.907636292
2.219305093	-17.06478897	0.091241801	7.947743112
2.759240054	-88.95048331	0.021738635	8.378841892
2.304779012	-82.06393228	0.022114471	8.9409501
3.123888506	-236.8638927	0.022226404	13.96381148
3.115345277	-89.01652498	0.016248155	9.910729098
3.131164671	-41.23122862	0.012079907	9.070457589
2.925532083	-71.42204339	0.011243913	7.931295315
2.635571952	-132.268993	0.008474739	16.23024327
2.371729578	-246.5196222	0.020695587	23.58885732
4.466100652	-30.53296215	0.006848691	8.944490696
2.572975491	-138.8188623	0.035449695	9.508358656
7.207490094	-26.76225542	0.001814643	7.65721895
3.406059946	-149.0152622	0.016176725	8.02806389
6.652464	-14.7925838	0.007934442	3.867717934
3.02927866	-129.4620502	0.011355495	15.9278533
3.0905976	-136.0282938	0.0209808	10.44743682
2.782454178	-59.41468707	0.021240559	7.586430158
3.140034085	-88.91237248	0.011988057	10.27494512
3.880962183	-34.55605269	0.010718542	7.494654045
1.85620784	-94.13714604	0.037925211	11.87507643
2.471272244	-129.7527723	0.04441203	12.14461494
3.510624788	-132.2083892	0.015966992	27.72070665
2.793591335	-68.12913504	0.013017012	9.786648502
5.405377321	-69.09863607	0.009637507	14.12149383
5.69081346	-25.16386145	0.012787454	4.596642202
2.203474076	-34.54912862	0.011867849	11.18598975
2.867641635	-176.6117825	0.040631716	17.60370168
4.232170959	-134.4829304	0.02722503	10.76739766
3.481736813	-44.49672353	0.007291154	8.128549366
2.208440074	-112.1617323	0.021283811	9.887987395
2.760203814	-183.3497582	0.027083183	8.387858002
8.598570406	-19.76066698	0.005833424	4.993296492
3.112767184	-108.1217551	0.012263064	11.84503998
3.095119702	-85.49107201	0.010328144	19.01368462
3.355584728	-77.94124305	0.005132327	20.06209219
4.201967657	-52.08922919	0.005996651	10.48521504
2.304580196	-91.35543575	0.024025827	9.764114791
3.689213314	-95.35806626	0.009712053	21.95229444
2.305838431	-262.8089221	0.06315183	9.636407786
4.278151194	-74.61291909	0.005886613	10.85249207
3.918219889	-28.96203502	0.010428538	6.024580412
3.345171476	-91.10444883	0.012491947	13.20712556
2.141775362	-159.3330415	0.039956907	8.319865072
3.458817587	-45.55661657	0.004865291	10.5903293
2.435822078	-157.1183178	0.027063517	8.380703211
4.901989125	-67.16778763	0.016684508	9.088377759
4.427727615	-40.44308031	0.009681022	8.325202767
3.300272909	-68.27601904	0.023049879	9.943200521
4.494230114	-44.05502228	0.011594856	40.1584208
2.242526937	-244.4767292	0.031877568	28.16183931
2.904136567	-85.05734547	0.02330528	16.48350732
3.249415405	-60.07200808	0.012873291	5.883167158
3.356948929	-113.8157829	0.012045668	15.5248335
2.769096999	-137.9925771	0.041080498	16.83527745
3.379471172	-62.65207056	0.011174075	9.991266615
2.801274494	-91.85016235	0.014578943	6.585372033
2.854755243	-74.11067039	0.012626236	8.742010631
1.998219319	-200.8476971	0.034168794	26.0893938
3.206289731	-99.40037289	0.010929849	12.99064459
3.391451989	-46.87636397	0.016862409	7.355269476
5.170369413	-44.85755828	0.011829073	34.82145346
3.489081908	-115.9762899	0.015190388	12.5414614
2.929027084	-106.8098012	0.023899749	24.17347366
3.231334038	-77.05240544	0.012810138	7.027865594
3.408263951	-62.38792154	0.01348025	8.438078297
3.572995312	-48.35857339	0.012151751	8.191577046
3.385811589	-61.03331741	0.02402771	7.020610259
3.177538779	-144.8533853	0.008151853	17.43383576
3.831859825	-96.91145262	0.019359813	12.4830839
2.254338901	-217.3836177	0.038153311	20.05029903
2.495137792	-171.6164037	0.050260544	12.49592165
3.922306644	-37.19896187	0.008373435	12.88986221

log.sigma.3.5.mm.3D_glrlm_LowGrayLevelRunEmphasis	log.sigma.3.5.mm.3D_glrlm_GrayLevelNonUniformityNormalized	log.sigma.3.5.mm.3D_glrlm_RunVariance	log.sigma.3.5.mm.3D_glrlm_GrayLevelNonUniformity
0.012713419	0.073528449	0.119230997	44.58131492
0.063191246	0.073780337	0.052542982	7.935210678
0.020623725	0.069472366	0.119365705	30.50528565
0.055098157	0.076594465	0.065471806	6.634878389
0.0408952	0.09328355	0.14304642	62.10596518
0.021153992	0.064149095	0.030508253	6.405381124
0.015708234	0.143879542	4.053733615	1204.347879
0.00837951	0.091012537	0.691077153	354.8301707
0.012304025	0.114581988	1.25847614	330.0722861
0.020646797	0.098932264	0.119548407	32.86778883
0.003101928	0.176964791	1.359599771	629.5957194
0.013596779	0.114658252	1.198193362	238.2620654
0.023353496	0.062047203	0.079418237	19.18926993
0.035139218	0.089034089	0.087265975	15.66963013
0.017549763	0.091289058	0.243952157	71.49760942
0.016064417	0.11577621	1.797879378	629.0419082
0.089755384	0.113553729	0.186218205	42.91297811
0.095025926	0.11675302	0.097793691	11.90297911
0.059085492	0.114078868	0.23626807	54.7740113
0.008411153	0.110311023	1.302344287	882.3451773
0.026362772	0.090449539	0.307192323	110.0547689
0.052396483	0.091299818	0.103242071	13.19379254
0.02380024	0.085609781	0.144929161	21.41095299
0.020535772	0.156522053	2.579248647	429.3459021
0.022490098	0.078643452	0.353754723	47.71892347
0.013476949	0.092464305	0.39291956	152.5358549
0.017619807	0.090898386	0.169422309	75.28511154
0.020819974	0.100962641	0.418280272	142.040726
0.093530453	0.127939216	0.040830136	3.435067155
0.024185402	0.097429872	0.320281195	92.94002215
0.024706643	0.095244023	0.622783677	256.3889957
0.02341421	0.083965424	0.092073732	19.81504873
0.017827533	0.092214973	0.268333043	99.64022237
0.014024599	0.107230312	1.073137271	233.7897612
0.012610325	0.101546193	0.456504356	283.2779879
0.009020578	0.071406192	0.226395411	69.88802009
0.021570151	0.057781675	0.041186081	21.69658267
0.008311938	0.125415381	2.075529793	897.4137742
0.037627081	0.092527217	0.107458858	16.55830185
0.002296214	0.127577813	1.793213891	968.4603376
0.017374861	0.104301825	0.147812378	58.38830613
0.010186723	0.167674952	1.735806018	755.3717686
0.012263513	0.072362907	0.203836037	97.48790509
0.022506027	0.088788068	0.146311533	55.8904541
0.024093884	0.108631202	0.735953375	86.2249689
0.013374059	0.09006358	0.417286097	142.8039515
0.012767924	0.111521541	1.588968997	501.2408638
0.041468335	0.087467288	0.146064245	17.04790475
0.046666343	0.092585004	0.067573723	9.902215188
0.016467227	0.060021417	0.05529193	12.53091077
0.014549738	0.100090484	1.223893481	133.4072053
0.010538538	0.084709085	0.238856381	156.6821033
0.016608673	0.169517933	2.903294938	924.50676
0.012822249	0.082432553	0.153647015	62.01961919
0.043096061	0.071024089	0.082785705	18.92501589
0.029492604	0.091838691	0.138612714	54.16968156
0.008600076	0.105726285	1.243620789	384.9225759
0.023487315	0.087759986	0.343798625	112.0825037
0.029221318	0.098581297	0.144410668	23.93292119
0.007831371	0.173814854	2.607373919	2239.892652
0.013196644	0.084883403	0.190501182	59.58306924
0.011068823	0.066285391	0.189562333	93.72520755
0.005525439	0.065837387	0.202299604	265.496911
0.006880689	0.100330676	1.208949053	605.8220016
0.02562471	0.087329388	0.139512342	25.37761408
0.010323862	0.065024409	0.121439378	129.1072234
0.068592089	0.09019514	0.067163156	10.06630221
0.006543686	0.092489493	0.460662049	309.2079188
0.013085795	0.12923484	1.715424355	801.7470008
0.013552807	0.079252828	0.177993713	106.7381701
0.04253341	0.099294933	0.120449804	17.85295903
0.005829564	0.109270856	2.063102305	477.296104
0.029205817	0.09600712	0.152664159	63.66015602
0.01827168	0.103616312	0.296231206	92.63120691
0.0110555	0.11135708	0.823539387	263.0069479
0.025222844	0.092314357	0.200105733	48.78036436
0.01252404	0.056586224	0.105711097	76.81177554
0.032949632	0.055974095	0.041990655	6.165932419
0.02473762	0.069324021	0.109314741	41.3758643
0.014787736	0.123777061	0.763874722	144.5100848
0.012713419	0.073528449	0.119230997	44.58131492
0.044841272	0.073736527	0.102685698	24.8966322
0.01277632	0.104271908	1.32620361	244.0982065
0.016338051	0.113198227	0.378833842	106.0853552
0.01412174	0.098009664	0.376896663	144.1905012
0.035658659	0.058876267	0.046775481	9.904376173
0.011734201	0.081819267	0.176138019	52.24542288
0.020010019	0.118105317	2.171434415	293.7873258
0.012545839	0.065367322	0.144264639	69.0444865
0.016519046	0.083617674	0.136270724	60.13743391
0.025824369	0.066949081	0.063353809	17.39893968
0.014589727	0.11235927	0.490255371	125.5093717
0.015225394	0.100196303	0.451964384	274.6996385
0.014034035	0.103542286	0.873968461	290.1362095
0.026548734	0.109728155	0.262228856	192.4995717
0.008672758	0.069145467	0.107798372	75.0331733
0.02103455	0.082867205	0.174200267	106.9187619
0.039765407	0.073574242	0.04490296	6.112814949
0.052108348	0.090318232	0.049841742	6.108178168
0.009560588	0.086082065	1.043764774	598.1850352

log.sigma.3.5.mm.3D_glrlm_LongRunEmphasis	log.sigma.3.5.mm.3D_glrlm_ShortRunHighGrayLevelEmphasis	log.sigma.3.5.mm.3D_glrlm_RunLengthNonUniformity	log.sigma.3.5.mm.3D_glrlm_ShortRunEmphasis
1.329598364	149.1889816	510.263893	0.933114252
1.160225608	80.65097599	97.74826835	0.962469052
1.335579584	118.2306402	368.575249	0.93156152
1.199506972	85.23596572	77.15944911	0.953843525
1.401347772	59.78878964	538.818115	0.918570303
1.096804368	174.351188	93.8780025	0.975798908
7.99984433	68.55779331	3760.564257	0.696048278
2.387785859	148.4292701	2677.130213	0.852439979
3.467551321	87.44112053	1709.746532	0.794917438
1.32287932	92.89740949	281.2054648	0.935695868
3.797923649	281.1634764	1909.011002	0.757505273
3.439690488	86.87933806	1200.943958	0.785871836
1.227448479	137.2457902	272.3139292	0.950734997
1.256312929	73.42260319	152.3310363	0.943642344
1.632219344	89.90696305	593.8116651	0.891751337
4.368267293	81.69918883	2986.629215	0.766734205
1.524934725	33.24478859	291.0668199	0.897851354
1.285505957	41.15775156	86.74058829	0.937960857
1.647854123	41.77678327	359.0281019	0.883875171
3.562875763	129.8609271	4604.413544	0.785980826
1.738450088	69.64662732	912.4469518	0.887706666
1.305412343	57.18850077	122.0520519	0.933881135
1.413745141	87.31663648	201.3041669	0.915220722
5.791777168	55.44567215	1343.974744	0.724251137
1.814274413	110.4830388	457.8225094	0.887385987
1.917646825	118.5099091	1184.475181	0.870384238
1.461843106	101.6219688	661.0071244	0.911450236
1.99640299	77.86423579	982.3976484	0.859380606
1.134358974	39.96475783	24.73880342	0.966410256
1.792331966	76.17285472	696.763323	0.87702497
2.346370638	69.36337249	1817.244861	0.845322018
1.264196815	111.0185916	204.1362309	0.943476051
1.680643492	93.3040517	810.7346378	0.887647899
3.206865189	89.76285468	1286.601016	0.794886727
2.021185061	99.86043742	1980.997158	0.866099027
1.560536293	188.9634184	768.36107	0.905964485
1.121876245	157.713751	349.4920698	0.971895137
4.847446413	120.5295021	3762.63327	0.752046061
1.322337461	69.35335324	148.5589563	0.928563584
4.405723729	371.6660349	4049.84962	0.757022365
1.421471719	91.41175245	448.0113439	0.913940142
4.392957177	90.43252918	2340.669545	0.745297097
1.506084867	153.9353375	1096.843952	0.916889791
1.412759884	85.5090198	508.4473714	0.916858007
2.651280701	66.31684033	494.4300997	0.813886668
1.95444609	108.1140445	1141.157504	0.870601992
4.045699236	89.83174466	2478.723856	0.76986873
1.42973588	66.05660708	157.8941386	0.913598028
1.208648781	64.41964704	94.41110364	0.951228224
1.167413103	196.2031325	188.496224	0.960635854
3.311215676	102.4247059	839.8623095	0.818192158
1.606568565	159.2293647	1417.901513	0.896807235
6.478350887	56.14584014	2390.795701	0.685844125
1.43248138	122.5293178	608.1640194	0.914779392
1.245214433	87.12664633	231.1135515	0.945109929
1.404636612	72.45659582	473.1358106	0.915099242
3.410122886	123.4272509	2170.513851	0.797576729
1.815620295	74.77360138	941.42936	0.880958163
1.405125885	70.42878273	196.7581051	0.918520285
6.075516352	104.3584749	5629.094541	0.683605439
1.487158618	125.8722551	562.9244588	0.914017377
1.479794649	174.6133009	1137.928341	0.915807091
1.493415639	265.074254	3260.420405	0.917181403
3.235690824	163.7171465	3835.353785	0.822956464
1.376348048	82.75204454	244.4577338	0.929185383
1.334459352	198.7195352	1663.771248	0.932024187
1.207363665	57.68485532	98.53840018	0.951301279
2.002416743	186.3277058	2413.498885	0.872142989
4.405943187	76.68143107	3194.448475	0.743570759
1.467943835	121.4811237	1077.748238	0.913716275
1.339229972	56.27304766	150.6884148	0.930433434
4.759148985	165.6807801	2392.719157	0.76706289
1.432717783	68.13963645	528.7370924	0.91232629
1.74591355	91.03050934	656.1280044	0.879338859
2.703089742	115.9063187	1510.101782	0.825505272
1.530266213	80.36026501	415.7951265	0.904383958
1.299101836	196.0666577	1152.220136	0.937090387
1.13345488	185.4551574	101.0007716	0.96663628
1.301603638	114.8339761	507.9146311	0.937867655
2.661966472	80.27544479	732.968497	0.817632113
1.329598364	149.1889816	510.263893	0.933114252
1.299557977	80.69182241	284.9660217	0.934675081
3.53314803	105.3083456	1422.763675	0.80304907
1.966135956	82.23733516	641.4731211	0.851351534
1.896960022	100.1903117	1049.776366	0.8680074
1.143024405	156.4737546	153.96904	0.965811526
1.471675119	154.7163194	507.7808995	0.91114685
4.902899148	63.38367208	1362.545802	0.766881428
1.386375353	211.7199282	872.776265	0.925827625
1.377080296	105.7199209	591.4054511	0.924462404
1.192094352	195.2319523	231.0610253	0.954976671
2.159899703	92.92295034	752.9675084	0.843283025
2.022720034	91.9081525	1930.366616	0.862784821
2.796953738	90.77539174	1773.580004	0.820880673
1.660409904	65.34204318	1322.531582	0.889651694
1.300565999	195.8862702	923.1397219	0.937578836
1.468627919	98.47177512	1026.741414	0.911371262
1.143398949	134.6268224	75.70175444	0.964150263
1.154167157	86.19047984	61.38048016	0.962773135
2.974215694	143.7048805	4489.103319	0.829466072

log.sigma.3.5.mm.3D_glrlm_LongRunHighGrayLevelEmphasis	log.sigma.3.5.mm.3D_glrlm_RunPercentage	log.sigma.3.5.mm.3D_glrlm_LongRunLowGrayLevelEmphasis	log.sigma.3.5.mm.3D_glrlm_RunEntropy
207.6459193	0.91025641	0.015909582	4.407336339
91.60253004	0.951667801	0.07400456	4.073055173
165.6564498	0.909061953	0.026332172	4.428326634
98.22069714	0.941471572	0.073566778	4.061042113
83.65358152	0.892452052	0.057416986	4.138704187
193.3183639	0.969380134	0.022106647	4.23908468
1030.646522	0.510718093	0.072886023	4.83156461
493.5757148	0.771974755	0.015443051	4.543921721
508.166485	0.680339051	0.030093087	4.396351485
125.9524445	0.912510566	0.025899443	3.980753712
1455.396423	0.649003266	0.010429231	4.223852152
468.7800273	0.674835415	0.033139701	4.539857288
160.6226338	0.934231931	0.028045489	4.487031808
89.18656649	0.926315789	0.043877961	4.018271118
182.6215132	0.851337793	0.02514249	4.24160486
595.9685996	0.633959098	0.044196231	4.665755061
52.34675573	0.866796048	0.146706752	3.877595949
48.26900901	0.918225918	0.116693089	3.702433503
78.74459751	0.847103514	0.091997686	3.936996295
614.8745681	0.669244313	0.024264	4.747508867
142.1559037	0.838048315	0.042673768	4.319815911
69.44217311	0.914313535	0.074812407	3.971599746
133.5741137	0.889953463	0.032214519	4.148236396
555.1299333	0.568436559	0.073528267	4.459976089
274.5542743	0.834710744	0.030357838	4.446179387
258.7917306	0.813979107	0.022658936	4.518411656
164.5026205	0.882198738	0.024049732	4.221764044
214.0072225	0.79953645	0.032686874	4.30356103
44.99851852	0.958791209	0.102685065	3.298142305
163.0292152	0.828309831	0.03776461	4.281052149
253.9910149	0.766404487	0.041844834	4.426832661
138.2424084	0.925188537	0.028783864	4.178446105
189.9952565	0.844710411	0.02663872	4.324316368
435.8659521	0.690011481	0.031818484	4.587538975
261.9706793	0.802216765	0.021661024	4.310866323
357.9454567	0.867498636	0.011999406	4.558427128
174.4302639	0.962919132	0.02520556	4.434095162
973.3975568	0.60718639	0.028047333	4.71609454
93.72875464	0.908238969	0.047144115	4.011548589
2296.205187	0.624699966	0.008819916	4.685381682
138.5251242	0.887236377	0.023258209	4.048842225
538.4427851	0.620847993	0.038645518	4.316307189
251.5753553	0.882323309	0.01745232	4.549417045
128.6578009	0.890436296	0.029886416	4.21604669
262.6634642	0.728506787	0.04426545	4.397837689
270.4960868	0.811056463	0.022119211	4.460735444
570.7148411	0.643465015	0.035632061	4.668556419
101.2026682	0.890059712	0.059903554	4.115868956
76.11619101	0.937921727	0.056183671	3.861324485
233.7160745	0.948951049	0.018519827	4.507593687
561.296031	0.697634999	0.029230378	4.480939428
279.7141697	0.856628781	0.015385771	4.533739403
641.8525215	0.537250811	0.074306337	4.534120035
194.2492732	0.888475161	0.017634154	4.246171788
105.0374999	0.928437416	0.053722765	4.333042816
103.9649794	0.889662374	0.040181507	4.192583728
618.4287326	0.687055332	0.022391447	4.612518851
182.7796559	0.826786159	0.036411058	4.349830234
103.4828264	0.892533937	0.03929368	4.013888279
1038.661411	0.545550906	0.037933616	4.589257318
202.608842	0.880513464	0.018168206	4.314983048
280.967885	0.881904122	0.014871006	4.661219763
431.1656248	0.88180564	0.007677739	4.742408067
769.3125137	0.707213796	0.01645976	4.663836829
121.7103694	0.905583513	0.033814618	4.072497039
272.4308743	0.908642845	0.01321864	4.682680029
67.42798277	0.937944409	0.091322928	3.84997963
465.1257411	0.809425313	0.011109004	4.500243683
531.2201648	0.617318629	0.043491727	4.609777448
189.6727174	0.881920306	0.019063598	4.419815248
77.43320143	0.908313908	0.055437071	3.868108454
1357.292069	0.613715908	0.019168879	4.732457658
108.7360865	0.885488343	0.039836407	4.07198383
187.4841019	0.833034626	0.027153359	4.27726625
425.2863945	0.733280712	0.022575172	4.467529054
129.4517224	0.874299542	0.036505578	4.210796744
253.1649571	0.915914045	0.016976472	4.857435343
213.0528278	0.957859532	0.037237887	4.464419331
146.6624137	0.916696207	0.031292352	4.436935432
311.6535584	0.731106613	0.02993923	4.252371266
207.6459193	0.91025641	0.015909582	4.407336339
96.27195145	0.914946842	0.06244968	4.311580174
608.379023	0.682832966	0.028878443	4.59012933
215.318293	0.797381342	0.026708477	4.178001819
236.437407	0.814873215	0.023202524	4.341301373
176.2027159	0.955856643	0.041775978	4.394398044
249.4958024	0.880530504	0.015723814	4.401822219
544.5116906	0.610238753	0.058667696	4.576233694
295.6409301	0.898723404	0.016069036	4.816337923
144.510304	0.898942308	0.023291499	4.269338118
233.9193277	0.941750279	0.033587623	4.421680455
264.8043583	0.78063475	0.026031692	4.262121684
226.5369361	0.800993571	0.026562504	4.396255325
357.1795667	0.726278392	0.029843678	4.517691629
115.965393	0.848386737	0.04056006	4.11557994
260.9816168	0.916515073	0.011120433	4.494842954
148.4160623	0.88065109	0.029727543	4.383678369
154.9835923	0.954907162	0.046213789	4.109051163
98.38726122	0.952329361	0.059581745	3.849403418
557.3024729	0.722647615	0.021221288	4.827795402

log.sigma.3.5.mm.3D_glrlm_HighGrayLevelRunEmphasis	log.sigma.3.5.mm.3D_glrlm_RunLengthNonUniformityNormalized	log.sigma.3.5.mm.3D_glszm_GrayLevelVariance
158.9730377	0.840382334	23.59827195
82.74285056	0.907219743	15.96819263
126.1463561	0.837391151	21.95341856
87.74380864	0.888036749	18.869375
63.98816529	0.808490577	14.97052557
178.1446231	0.938983493	23.85489796
105.7350025	0.448615795	15.43411304
178.9824022	0.684668356	19.79978167
116.9993732	0.589972891	11.82628258
98.24704668	0.845771615	13.54273192
373.9467276	0.533610813	11.30733915
115.1525216	0.575432469	14.67535609
141.379285	0.879568744	26.93706597
76.29221621	0.863853513	14.20918367
102.7805846	0.75609116	15.19874567
113.6371952	0.547594249	12.62573334
36.33359071	0.767792158	9.178698225
42.44111447	0.850339539	11.30555556
47.22801801	0.742712104	7.365359354
166.3459664	0.57474692	20.91858283
78.19562337	0.748559978	14.39951747
59.35707694	0.842616247	12.12752675
94.98302728	0.802833349	15.75651042
81.20948352	0.487158901	9.248161157
128.4758992	0.750720387	18.20944425
135.2351802	0.716175475	22.79460009
111.662947	0.7954758	14.82692159
93.60783768	0.696194798	12.73218322
40.97150997	0.918339728	9.109375
87.19214276	0.728217993	12.7424
86.40702579	0.672346586	14.13632884
115.7626764	0.863643412	17.11111111
106.4549194	0.748128778	15.98770109
118.037936	0.589550187	15.55711771
117.2459866	0.708461759	14.32935359
212.2338031	0.783957888	21.65508054
160.8681802	0.929506471	23.23933837
169.7333299	0.524867825	14.69699264
73.78676656	0.82940068	13.30153079
497.4779326	0.532052594	21.58262236
99.47726182	0.799140243	15.26503224
121.25216	0.51638507	12.36954885
167.4394361	0.80926619	20.83841933
92.75534462	0.805918333	17.04393687
84.67551506	0.619850799	13.86714847
126.0197596	0.716975402	16.67956976
121.7863217	0.550442136	14.45699036
71.7919762	0.803401806	12.09161214
66.6645804	0.881017648	12.52773669
203.2860014	0.902024082	32.52074417
133.1820377	0.628505949	11.92411439
176.3811239	0.765471754	24.92575313
86.85232871	0.436806089	11.63673565
134.3157092	0.803825162	11.97759412
90.39791609	0.86633096	19.78996084
77.99151048	0.801271446	18.75523003
160.0890917	0.593557774	15.68948347
87.21746084	0.735693824	12.99824196
75.81215066	0.808937042	11.84
158.1027132	0.434593909	17.90091299
137.0560896	0.800421103	17.04694434
189.8111302	0.803881845	27.56273922
288.0456676	0.807301063	27.7635815
205.1957084	0.634819173	21.38935202
88.72123535	0.835468829	11.51578273
211.3448708	0.837337327	28.41448087
59.58907051	0.881173243	9.54094579
215.8067524	0.719916375	21.93027348
106.4792082	0.512766912	14.731824
131.8508361	0.799179457	19.21132429
59.81120666	0.835374349	9.77
230.9405377	0.547242486	12.77093585
75.14525	0.795855925	12.07615311
103.9455429	0.732223118	20.09650519
143.1052097	0.63820326	17.13016504
87.96017473	0.782393141	13.83257189
206.1827329	0.84831904	40.692174
190.9746914	0.91601869	28.515625
120.3417934	0.850260483	21.12557828
101.6370129	0.625220799	13.56673238
158.9730377	0.840382334	23.59827195
83.49445635	0.843074842	16.59158478
138.9733369	0.60427037	16.77541172
99.171896	0.68189189	11.96610409
116.870743	0.711375651	13.82188182
160.2143834	0.91431679	22.08264463
169.5815748	0.793917259	18.32808957
88.17549128	0.547038435	12.76163956
225.5158225	0.825087044	49.16896778
112.3545867	0.821305241	16.14424654
202.4336769	0.888356687	22.04193115
111.9952864	0.669530721	12.80448865
106.8614044	0.70216522	15.01012273
113.4140311	0.630747009	15.97163435
72.61660566	0.751970292	11.86756939
207.0421669	0.849749149	23.98832352
106.3778399	0.794421571	18.60795723
138.6981764	0.91019408	20.76052512
88.56251199	0.906870908	15.33061224
175.5102946	0.645233363	21.86864558

log.sigma.3.5.mm.3D_glszm_SmallAreaHighGrayLevelEmphasis	log.sigma.3.5.mm.3D_glszm_GrayLevelNonUniformityNormalized	log.sigma.3.5.mm.3D_glszm_SizeZoneNonUniformityNormalized
122.99217	0.060018956	0.361326016
68.26258261	0.079072533	0.390606421
109.2763982	0.063609544	0.348894985
83.26961214	0.07375	0.3475
70.5011463	0.079093492	0.36253874
143.7194444	0.060816327	0.513469388
85.89898136	0.071526479	0.361708849
119.901075	0.061821316	0.341286986
80.67175118	0.086886145	0.365651578
89.66411209	0.086437789	0.365148933
208.6182911	0.09892324	0.313978068
88.07964404	0.075114217	0.34711771
141.6362027	0.054012346	0.471547068
70.86840944	0.077664399	0.414399093
71.64443432	0.075692042	0.309039792
78.9706088	0.084993269	0.342601783
39.48063245	0.097751479	0.324023669
58.04218866	0.102430556	0.612847222
40.46728073	0.109776694	0.260273973
139.3706437	0.064550135	0.388143965
80.58897136	0.078903509	0.33245504
58.11650401	0.096313912	0.366825208
59.06573003	0.076388889	0.255208333
56.34421623	0.107231405	0.33838843
79.83347482	0.070290859	0.283760388
136.2448433	0.061184497	0.417422703
84.59909858	0.077482027	0.361694452
59.62467601	0.07964307	0.334443783
32.20703125	0.1015625	0.3671875
72.74844529	0.081404082	0.325126531
66.91458161	0.078831059	0.3353816
109.2568122	0.070130316	0.407750343
83.26877749	0.071387532	0.389435507
83.89786553	0.07323103	0.356041404
96.58993672	0.074870023	0.362275398
149.6286921	0.059968171	0.414303627
147.2715821	0.059659735	0.468960302
105.6554244	0.07861507	0.336800958
56.99937196	0.081523674	0.303666785
353.3820551	0.063873691	0.356379681
97.25999263	0.077145739	0.460199114
87.37107961	0.08883773	0.330648973
149.024359	0.065389521	0.429296418
80.89898596	0.071605339	0.318836464
57.09684143	0.077266447	0.372303114
121.5331083	0.071605869	0.421057593
88.27177662	0.076348263	0.382523451
45.95772311	0.089827484	0.265913147
54.26866	0.090976331	0.409023669
131.7685052	0.054065857	0.364835357
46.64279782	0.08207139	0.256219578
160.274605	0.060849888	0.400480524
70.10970685	0.090760285	0.350621552
63.02909469	0.086868687	0.286538108
93.68617437	0.065681737	0.384834461
84.28270036	0.069163633	0.392833648
109.1843744	0.070320248	0.347561983
74.11790152	0.086853438	0.367784391
67.04691505	0.085511111	0.371022222
115.9407454	0.073706219	0.341668209
129.341677	0.072670118	0.43791605
176.7952387	0.05453782	0.432453529
238.7098649	0.053684324	0.401801988
161.5546475	0.063999551	0.38525207
62.24781592	0.084762057	0.327351386
158.2972881	0.05481119	0.358975241
56.19859053	0.094194541	0.361783929
178.0790261	0.061920571	0.409396433
92.26910238	0.076264	0.345216
124.2159478	0.068463464	0.364006157
57.37745883	0.0928	0.3232
134.5265408	0.082619838	0.333173815
60.96057621	0.084150697	0.35244706
86.34620184	0.066297578	0.320415225
104.5216939	0.071661593	0.348514661
63.03071296	0.085192153	0.282450954
171.1996986	0.049663806	0.368544182
148.9155208	0.0546875	0.421875
112.4768948	0.064929795	0.377201526
82.85199897	0.083488731	0.399648356
122.99217	0.060018956	0.361326016
97.72538068	0.075459814	0.390778534
87.29070836	0.071701783	0.280550646
54.78537607	0.081844503	0.230845279
101.8954274	0.076839183	0.390904885
138.1923393	0.064789307	0.456177941
144.6041324	0.070436508	0.404691043
76.05529886	0.082145244	0.37795166
182.6152479	0.04473178	0.340541067
124.8657803	0.074324476	0.409132769
164.143078	0.069458008	0.37097168
87.28932668	0.082837982	0.367398262
92.17696382	0.073533266	0.368217093
100.6982285	0.07365651	0.353434903
61.99852606	0.081625704	0.340290714
198.6043561	0.057814807	0.491249532
112.4983301	0.06777233	0.406353379
112.7414617	0.067451335	0.403349932
80.45658041	0.084081633	0.382857143
135.9822587	0.059725429	0.3894652

log.sigma.3.5.mm.3D_glszm_SizeZoneNonUniformity	log.sigma.3.5.mm.3D_glszm_GrayLevelNonUniformity	log.sigma.3.5.mm.3D_glszm_LargeAreaEmphasis	log.sigma.3.5.mm.3D_glszm_ZoneVariance
76.96244131	12.78403756	46.14084507	36.36421345
22.65517241	4.586206897	6.396551724	2.600772889
48.49640288	8.841726619	40.75539568	28.68102065
13.9	2.95	9.25	3.96
63.80681818	13.92045455	137.4659091	119.4998709
35.94285714	4.257142857	2.9	0.734897959
320.4740406	63.3724605	65629.68172	65290.97217
201.359322	36.47457627	2865.616949	2792.470968
98.72592593	23.45925926	7670.92963	7425.601221
40.53153153	9.594594595	39.67567568	28.92200308
109.2643678	34.42528736	16330.22414	16083.69804
84.69672131	18.32786885	4636.028689	4477.413246
67.90277778	7.777777778	18.4375	13.15388696
34.80952381	6.523809524	16.07142857	10.95521542
42.02941176	10.29411765	293.7058824	247.9446367
218.5799373	54.22570533	18781.02978	18602.40111
21.06153846	6.353846154	221.2307692	176.2376331
29.41666667	4.916666667	23.6875	18.33984375
19	8.01369863	323.5205479	263.1923438
390.8609732	65.0019861	17231.54419	17091.26169
83.44621514	19.80478088	380.9322709	347.4676592
21.27586207	5.586206897	21.93103448	14.51010702
12.25	3.666666667	92.52083333	58.24956597
74.44545455	23.59090909	23545.60909	23068.78231
43.13157895	10.68421053	161.3947368	138.5815443
138.5843373	20.31325301	680.5090361	643.3063852
71.25380711	15.26395939	150.4111675	127.6916695
68.56097561	16.32682927	1074.180488	1000.555717
5.875	1.625	3.875	0.8125
56.89714286	14.24571429	460.6342857	417.3754776
104.9744409	24.67412141	2792.968051	2667.1413
44.03703704	7.574074074	17.52777778	11.9529321
91.90677966	16.84745763	267.8516949	238.4808065
100.7597173	20.72438163	4439.685512	4315.870744
176.0658436	36.38683128	985.9897119	934.8054031
119.3194444	17.27083333	145.3402778	130
107.8608696	13.72173913	4.52173913	1.646502836
268.4303639	62.65621079	30122.60728	29905.70128
16.09433962	4.320754717	43.56603774	29.750089
320.0289532	57.35857461	27543.97327	27361.99377
69.49006623	11.64900662	164.8410596	147.3786237
158.0502092	42.46443515	21583.82218	21354.59751
166.5670103	25.37113402	145.435567	129.9468793
50.05732484	11.24203822	110.2738854	89.99521279
36.11340206	7.494845361	1426.824742	1301.014986
130.1067961	22.12621359	674.184466	634.1552979
166.3977011	33.21149425	14727.28046	14470.61796
10.90243902	3.682926829	78.36585366	49.83462225
21.26923077	4.730769231	11.30769231	6.50147929
39.03738318	5.785046729	8.018691589	3.791248144
44.06976744	14.11627907	2375.488372	2252.691049
188.626327	28.66029724	397.2377919	376.2259817
146.5598086	37.93779904	58948.90431	58364.83798
47.27878788	14.33333333	97.21818182	70.86707071
40.79245283	6.962264151	25.66981132	18.33899964
59.31788079	10.44370861	120.0331126	100.754616
152.9272727	30.94090909	7296.220455	7151.838383
91.57831325	21.62650602	398.0522088	359.5524266
27.82666667	6.413333333	48.58666667	35.43395556
313.6514161	67.66230937	144838.6852	144182.6446
91.08653846	15.11538462	109.5817308	94.89957008
186.387471	23.50580046	164.9118329	151.0789563
541.6290801	72.36646884	222.4332344	210.9296639
421.0805124	69.95150961	5899.965233	5839.202281
25.86075949	6.696202532	54.29113924	37.78080436
244.8211144	37.38123167	86.30058651	76.03617315
18.45098039	4.803921569	10.92156863	5.477124183
296.8124138	44.89241379	1177.988966	1145.569686
172.608	38.132	31699.614	31297.77188
124.1260997	23.34604106	217.1290323	197.0764957
16.16	4.64	47.92	32.2384
178.9143389	44.36685289	14018.527	13843.76549
49.69503546	11.86524823	163.0992908	134.881344
54.47058824	11.27058824	446.8294118	406.9910381
137.6632911	28.30632911	2610.177215	2543.888761
34.45901639	10.39344262	138.4262295	113.9156141
180.9551935	24.38492872	58.10183299	48.99151737
27	3.5	4.921875	1.693115234
83.73873874	14.41441441	41.84234234	33.24318237
54.75182482	11.4379562	2195.372263	2059.658266
76.96244131	12.78403756	46.14084507	36.36421345
49.23809524	9.507936508	45.97619048	37.39965986
91.74006116	23.44648318	4470.159021	4360.902187
27.47058824	9.739495798	922.6302521	825.1353718
96.16260163	18.90243902	677.8902439	624.052829
45.16161616	6.414141414	5.636363636	2.475869809
67.98809524	11.83333333	133.3869048	114.76357
101.2910448	22.01492537	10958.00746	10727.82875
119.5299145	15.7008547	92.67521368	81.46893288
86.32701422	15.68246445	93.76303318	79.38779452
47.484375	8.890625	9.546875	4.897460938
59.51851852	13.41975309	1050.296296	972.3775339
202.151184	40.36976321	1143.224044	1104.37187
134.3052632	27.98947368	3647.442105	3544.579806
131.6925065	31.58914729	542.9069767	514.3797849
209.2723005	24.62910798	59.38497653	51.66023055
149.5380435	24.94021739	200.4375	184.5893121
18.95744681	3.170212766	5.170212766	1.743775464
13.4	2.942857143	7.685714286	3.570612245
471.2528926	72.2677686	6464.109091	6401.110105

log.sigma.3.5.mm.3D_glszm_ZonePercentage	log.sigma.3.5.mm.3D_glszm_LargeAreaLowGrayLevelEmphasis	log.sigma.3.5.mm.3D_glszm_LargeAreaHighGrayLevelEmphasis	log.sigma.3.5.mm.3D_glszm_HighGrayLevelZoneEmphasis
0.31981982	0.386402027	6967.384977	182.7370892
0.513274336	0.448664658	357.4137931	99.15517241
0.287784679	0.635976523	4395.834532	160.8201439
0.434782609	0.730452378	499.425	117.375
0.235924933	4.695398794	5579.960227	99.38068182
0.67961165	0.043571399	492.5428571	185.8714286
0.054335827	506.046968	8675327.809	122.4841986
0.116924296	12.87982532	669298.3373	177.3661017
0.063844881	53.27343974	1197912.33	117.9925926
0.304945055	0.569588435	3457.918919	125.2162162
0.063689605	42.29774073	6337668.989	365.5689655
0.079401237	34.69181687	668201.9508	133.1639344
0.435045317	0.373453371	1394.458333	186.8541667
0.442105263	0.474611707	781.8928571	96.5
0.147826087	3.035847705	36896.59559	111.4117647
0.074821156	134.6316717	2762533.281	115.1175549
0.149082569	17.39219477	6168.6	59.26153846
0.432432432	1.658553872	495.1458333	65.08333333
0.128747795	12.08029988	15531.26027	62.28767123
0.084430284	100.9373604	3019984.651	204.5789474
0.172865014	9.830908277	23906.13147	121.8645418
0.367088608	1.526239977	676.2931034	82.05172414
0.170818505	1.640321199	8533.333333	109.6041667
0.045795171	246.0348518	2327594.882	88.37727273
0.209366391	1.204278824	30279.5	127.7697368
0.163950617	6.069984449	88123.09337	191.1686747
0.209797657	1.687284058	16896.41624	122.7969543
0.116543491	10.32932848	128856.5707	87.70243902
0.571428571	0.296981659	134.375	46.625
0.152041703	6.661134123	40579.85143	108
0.089148391	30.93509993	336028.639	96.69648562
0.423529412	0.311442615	1610.37037	145.5555556
0.184519156	2.775593351	33816.97881	121.5720339
0.0898698	32.1304974	652670.9965	122.7491166
0.139775669	8.269878541	135171.0556	139.345679
0.255319149	0.626740235	39489.53819	206.8229167
0.58974359	0.112809794	548.3521739	188.0826087
0.067899131	143.6885441	6404254.801	161.3287327
0.269035533	1.088805188	2563.962264	94.64150943
0.074129107	52.10651369	14620293.94	541.9610245
0.239302694	2.024646868	14814.06623	124.0596026
0.066049468	178.3621779	2650788.358	139.4267782
0.254092993	1.214840557	23485.54124	199.8324742
0.222065064	1.765906116	8902.006369	117.5541401
0.089154412	15.85767689	151738.6907	87.96907216
0.158056266	5.7728351	94962.88026	160.5242718
0.062419285	106.4980022	2142041.593	122.7126437
0.187214612	2.905416762	4908.073171	82.48780488
0.456140351	0.59970127	462.1923077	79.32692308
0.486363636	0.089975897	1522.018692	212.1308411
0.090241343	13.51294396	444276.8372	93.23837209
0.218156554	2.673320303	64747.14437	224.8089172
0.041377945	590.2708485	6039492.215	101.3421053
0.194805195	1.110265566	12764.85455	127.7030303
0.369337979	1.112983012	1146.490566	128.2641509
0.22775264	2.662157293	7229.019868	114.8211921
0.083223	39.45697115	1392121.464	161.55
0.161165049	6.622313143	39278.9759	108.0803213
0.275735294	0.966977092	3634.56	92.84
0.039042232	837.5257223	25329981.05	176.8311547
0.26097867	1.090975264	13633.90865	174.1394231
0.268870867	1.205733992	29259.88863	238.4524362
0.294838145	0.811774075	65978.47329	338.4695846
0.128286385	24.64055952	1476555.177	229.3769442
0.246105919	1.027077645	4732.797468	101.6455696
0.312128146	0.608661443	14425.46041	243.8841642
0.428571429	0.853183116	431.372549	78.33333333
0.175629845	4.979227257	291156.9683	247.7586207
0.049885264	273.8533409	3919663.39	141.88
0.223313687	2.451594628	23548.43988	182.4662757
0.252525253	1.809807409	2174	82.02
0.075644457	46.52663374	4298839.354	208.8752328
0.188251001	3.104114632	11487.95745	89.04255319
0.158434296	4.611903083	48365.54706	130.8411765
0.122823383	15.96871306	441598.0228	158.0481013
0.201986755	2.660609361	9732.778689	109.295082
0.331309042	0.924533681	5648.869654	261.6171079
0.556521739	0.139915123	831.453125	207.40625
0.341013825	0.78554247	3163.774775	160.3018018
0.085839599	19.22570349	270024.4599	112.5474453
0.31981982	0.386402027	6967.384977	182.7370892
0.341463415	2.157136022	1548.952381	131.047619
0.095669982	24.40833541	857122.8502	138.5840979
0.101276596	8.45405449	111787.042	95.86554622
0.136288089	5.987925548	91529.83333	144.3943089
0.5625	0.229467525	614.030303	184.8383838
0.231724138	0.979053011	21719.625	197.0595238
0.065912445	100.3036642	1281567.276	106.880597
0.298723404	0.696128463	14837.14815	281.1452991
0.26375	1.681481373	6922.843602	168.0995261
0.463768116	0.269997475	1473.429688	234.3359375
0.113286713	9.171187593	139521.3457	126.4506173
0.160432496	11.74552222	126512.8998	133.1420765
0.098598858	30.17897487	483559.9158	146.6526316
0.187227866	9.575054712	33772.63566	93.89405685
0.359797297	0.380027408	10879.223	251.0352113
0.251194539	2.63483191	18241.60598	150.2336957
0.540229885	0.18570473	664.3617021	153.2553191
0.492957746	0.281770047	542.6571429	106.9428571
0.125989171	34.21652778	1266349.383	198.385124

log.sigma.3.5.mm.3D_glszm_SmallAreaEmphasis	log.sigma.3.5.mm.3D_glszm_LowGrayLevelZoneEmphasis	log.sigma.3.5.mm.3D_glszm_ZoneEntropy	log.sigma.3.5.mm.3D_glszm_SmallAreaLowGrayLevelEmphasis
0.623040219	0.017200072	5.837700005	0.012382304
0.641525383	0.056942212	4.859426737	0.032258271
0.611556858	0.026307669	5.694902805	0.018077817
0.606937287	0.048312905	4.715311532	0.032625547
0.624686265	0.04287123	5.45070953	0.024346748
0.739781746	0.026048161	4.930480918	0.023511067
0.624188609	0.021178894	6.075140554	0.011820954
0.606638442	0.014671865	6.226483474	0.009943568
0.625442012	0.018511117	5.423836914	0.008818648
0.621911986	0.026579034	5.131385765	0.01797118
0.575680936	0.006063838	5.653981988	0.004793184
0.606268824	0.018924213	5.690739696	0.008619905
0.713130993	0.028454773	5.511750743	0.0242927
0.667950055	0.038659097	4.9696989	0.02821257
0.573564432	0.027745005	5.659138581	0.017800697
0.608095898	0.019863423	5.947607365	0.01069842
0.589226471	0.080632525	5.014278776	0.047052279
0.806899294	0.09664848	4.103846169	0.059104873
0.523122872	0.055689323	4.979351573	0.022810144
0.645677006	0.011448606	6.061607538	0.007829603
0.589835159	0.022369718	5.634101133	0.012305202
0.623184542	0.045953265	4.579001522	0.031694815
0.511345284	0.043841454	5.162782032	0.03446526
0.598466526	0.036873283	5.239468573	0.01496152
0.532029749	0.034268376	5.703464771	0.013103833
0.671227946	0.014017796	5.814471772	0.006775543
0.623668106	0.022392187	5.546518411	0.010735146
0.599649532	0.036254621	5.645196335	0.018786145
0.595920139	0.110349607	3.875	0.080367906
0.590702526	0.027764189	5.461635946	0.009816757
0.597479489	0.039606843	5.712367394	0.019201149
0.657341061	0.025660089	5.229108165	0.017684195
0.646550996	0.023014761	5.657225778	0.015454557
0.619827358	0.021472994	5.823062757	0.013851051
0.625673684	0.015438315	5.929717814	0.009527887
0.66833282	0.013643607	5.712777089	0.009718976
0.711113158	0.017215505	5.47953376	0.00833339
0.602369846	0.011726725	6.075239026	0.00674879
0.559311337	0.046435339	4.952082427	0.018145766
0.620211151	0.003426869	6.264353632	0.002524758
0.702658318	0.024431768	5.218797427	0.018303795
0.592658707	0.015415591	5.76951525	0.009885722
0.680668381	0.013408443	5.813512426	0.008754089
0.584516764	0.031791851	5.693390986	0.020749915
0.630994974	0.053573739	5.063492033	0.03758628
0.674166351	0.015344603	5.483534225	0.008744321
0.642901739	0.021842106	5.681256248	0.012144687
0.512162208	0.051586435	4.802554748	0.03274581
0.660509393	0.052227684	4.728721929	0.043518197
0.620869034	0.02094365	5.540108693	0.016351383
0.521336051	0.032668833	5.791114651	0.017739879
0.657054327	0.012114084	5.990268216	0.00618182
0.61190387	0.024370909	5.596295397	0.013770488
0.552408023	0.019218984	5.676289133	0.014372997
0.64311804	0.040867813	5.399918983	0.021857388
0.650097421	0.036871153	5.478882617	0.024107854
0.612766216	0.013207117	6.037602705	0.008368508
0.630279137	0.027698922	5.579586056	0.016225237
0.628153856	0.038252159	4.976639984	0.026818092
0.603589062	0.012094418	6.096862103	0.006003453
0.687354668	0.016147811	5.485124363	0.011715524
0.68365038	0.0138523	5.93513132	0.009881285
0.658986473	0.006080707	6.296103631	0.004110742
0.644849409	0.009290718	6.12346752	0.005947667
0.591339309	0.032157297	5.134114571	0.021094423
0.621176982	0.013292575	6.319555247	0.009419819
0.605702292	0.047793724	4.483617685	0.015409176
0.665041317	0.008760572	5.986918215	0.006095878
0.606865274	0.015886732	5.81907308	0.00784247
0.625550282	0.014368129	5.882236797	0.009155848
0.584725121	0.045332215	4.74146788	0.028169877
0.597054811	0.008734669	5.891182344	0.005685062
0.61425834	0.042288188	5.3669972	0.024132406
0.583479281	0.02969041	5.828517302	0.020990446
0.612238168	0.018174709	5.942637258	0.012585916
0.537896768	0.030269704	5.566332618	0.012223027
0.629788138	0.011685153	6.353222657	0.007912999
0.672282986	0.029882105	5.140319531	0.011936391
0.63519573	0.026848803	5.771137417	0.01500263
0.65524007	0.026520118	5.226995702	0.017504343
0.623040219	0.017200072	5.837700005	0.012382304
0.648137736	0.033458185	5.171043983	0.016450915
0.545962731	0.01883823	6.189632544	0.00799833
0.489040679	0.030985691	5.672129058	0.01937458
0.649399069	0.017087292	5.553471106	0.011137576
0.699196042	0.032375856	5.054572945	0.018423949
0.660974779	0.016475248	5.38490184	0.010508153
0.637927753	0.023886093	5.468019707	0.010178457
0.601722191	0.016224865	6.433034464	0.009285445
0.663149119	0.015218435	5.508518698	0.010269911
0.629103546	0.017476226	5.28278345	0.00473869
0.629296742	0.022267258	5.468729631	0.01484866
0.630314085	0.017774327	5.893635595	0.010932652
0.615073611	0.017424582	5.800045012	0.010104377
0.603757127	0.034387452	5.763154831	0.022123111
0.72810184	0.009541566	5.656649044	0.007009086
0.662866559	0.019236079	5.71958323	0.009180786
0.656722632	0.034775111	4.799061724	0.012261437
0.637688508	0.052452505	4.414438731	0.021781895
0.648572634	0.012352315	6.17462428	0.007301848

log.sigma.3.5.mm.3D_ngtdm_Coarseness	log.sigma.3.5.mm.3D_ngtdm_Complexity	log.sigma.3.5.mm.3D_ngtdm_Strength	log.sigma.3.5.mm.3D_ngtdm_Busyness	log.sigma.3.5.mm.3D_ngtdm_Contrast
0.0131614	0.697192702	2.817370001	0.237418914	0.000138489
0.053624138	2.401751618	4.947730101	0.215907784	0.002851221
0.016344295	0.727773688	2.105009161	0.325343438	0.000348519
0.061323786	3.361516248	6.071749661	0.184757824	0.004246951
0.010416248	0.417739068	1.751837056	0.67604519	0.000123682
0.06337406	4.464114809	8.619739145	0.073795435	0.002685771
0.000693162	0.009250892	0.232423153	3.651040804	7.35E-07
0.002353309	0.055587397	0.593066508	0.934917616	6.74E-06
0.0027121	0.031752097	0.483862178	1.258663737	6.86E-06
0.021149882	0.607367525	2.252439328	0.264980718	0.000251617
0.001876574	0.029174468	0.760290512	0.786407281	1.61E-06
0.003845252	0.055375814	0.857620874	0.74963168	7.31E-06
0.021461751	1.807534905	3.942479891	0.254031923	0.000716572
0.038113012	1.181827649	3.228436642	0.245092137	0.00089736
0.011210344	0.193856886	1.343911244	0.443394837	7.08E-05
0.001214967	0.018874882	0.264919739	2.462222307	3.68E-06
0.019213255	0.228263881	1.430740502	0.736918469	0.000185685
0.056199829	1.538881967	5.065836696	0.242521485	0.001337519
0.016453688	0.119829137	0.891548532	0.75090121	0.000137431
0.000969151	0.041593375	0.841271197	1.496289418	9.11E-07
0.006342306	0.163109259	0.954402693	0.935184613	5.21E-05
0.041120119	1.010550528	3.066050707	0.405658927	0.001296638
0.030329846	0.606277128	2.689143094	0.231875099	0.000413155
0.002400822	0.014524913	0.361706617	1.724003338	3.07E-06
0.013359637	0.276072201	1.583154556	0.336946883	0.000167076
0.005490989	0.21667361	1.901799678	0.448956092	1.88E-05
0.009684744	0.286908981	1.650121666	0.445220779	6.99E-05
0.006756934	0.061011237	0.668744626	0.803552155	3.00E-05
0.168187962	3.293310497	7.310780238	0.121341403	0.009818071
0.008344904	0.115637677	0.79895747	0.767121783	6.13E-05
0.003337128	0.049394752	0.509394359	1.275597265	1.34E-05
0.027934578	1.270823482	3.301315379	0.188948323	0.000625504
0.008049318	0.202454214	1.414244098	0.465186028	4.16E-05
0.003570253	0.045007221	0.623412239	0.952272695	1.09E-05
0.002953632	0.067675304	0.546043755	1.119336197	1.08E-05
0.008180678	0.324366723	1.568661545	0.339306151	9.42E-05
0.016223213	1.761399155	2.846489384	0.264398357	0.000704508
0.000957859	0.018513246	0.351141921	1.986568802	1.50E-06
0.045600999	0.566373048	3.037873739	0.243796882	0.000619234
0.000954745	0.029467459	0.748181056	0.892356227	6.79E-07
0.012964776	0.383756629	1.799347244	0.347963391	9.44E-05
0.00151913	0.013981444	0.280506283	2.07636447	1.42E-06
0.005705324	0.332110264	1.363104772	0.499102529	5.84E-05
0.013208364	0.332521334	1.741268679	0.421569585	0.000110976
0.011545646	0.063188774	0.941799506	0.534962178	4.79E-05
0.005306665	0.144108222	1.015148576	0.607988677	2.57E-05
0.001772554	0.023091053	0.447896872	1.576281636	2.74E-06
0.047057451	0.445222796	3.581036836	0.251072815	0.000611901
0.059322106	1.674873574	5.077411291	0.193409012	0.001751629
0.031063754	3.617432658	7.612529095	0.105277219	0.001144101
0.006740654	0.053020908	1.061067564	0.524573465	2.28E-05
0.00496083	0.288409342	1.970434068	0.386453005	1.98E-05
0.001023601	0.009983316	0.275033547	3.163852261	9.25E-07
0.013067375	0.204474026	1.604418215	0.388131239	9.17E-05
0.026367843	1.315530348	3.884273118	0.293825453	0.00065651
0.012542063	0.469286106	2.209183303	0.451242166	0.000123595
0.00233502	0.035460667	0.554643395	1.022219612	4.20E-06
0.005964723	0.13555432	0.750992069	0.933981379	5.41E-05
0.029293471	0.541599705	2.234393118	0.274909566	0.000389175
0.000423847	0.009115705	0.306349172	3.88298541	2.17E-07
0.010469896	0.420413549	1.637355786	0.355558834	0.000108022
0.006073962	0.365773235	1.601088215	0.411857611	5.61E-05
0.002232068	0.179531948	0.741774575	0.711926984	1.66E-05
0.001230139	0.047920441	0.498491599	1.288439067	2.90E-06
0.0283411	0.436745893	2.108656167	0.296360534	0.000367846
0.004427397	0.326871206	1.384413346	0.524525806	4.50E-05
0.054514002	1.174509782	2.943257559	0.27354955	0.001852715
0.00254202	0.134552163	1.078184941	0.611066034	6.83E-06
0.001177518	0.014906805	0.29867171	2.474557859	1.44E-06
0.006035043	0.289117757	1.340030102	0.546767678	4.70E-05
0.032085195	0.665797932	1.920558998	0.383105946	0.00081913
0.001571918	0.02345508	0.448702643	1.3191418	4.90E-06
0.011177966	0.194015501	0.861064576	0.814454801	0.000139952
0.009275228	0.264509456	2.198411381	0.369750199	3.75E-05
0.003410507	0.083411652	1.011511278	0.668925621	7.17E-06
0.015089633	0.33971625	2.031776587	0.391474473	0.000126909
0.00647502	0.779243684	3.687986727	0.407990819	9.93E-05
0.05655463	4.914860772	9.832815979	0.071862882	0.002845358
0.013524718	0.627488406	2.277498859	0.345602188	0.000195791
0.007030187	0.07024156	0.905476664	0.575462197	1.81E-05
0.0131614	0.697192702	2.817370001	0.237418914	0.000138489
0.021780365	0.834315701	2.9775633	0.409065703	0.000481934
0.003310469	0.067013568	0.964886583	0.700377252	8.30E-06
0.00992548	0.076305797	0.954078728	0.547275162	3.81E-05
0.00588371	0.11767026	0.911056523	0.610360138	2.55E-05
0.032014384	2.826493763	4.905555237	0.161236766	0.002200099
0.013141869	0.466949853	2.762207108	0.219970143	9.13E-05
0.002820334	0.032215666	0.540332971	1.22718551	5.82E-06
0.008065157	0.880315827	4.489450403	0.24814079	9.73E-05
0.010143168	0.511897182	2.123873631	0.425997191	0.00010883
0.02500282	1.964091989	5.106860314	0.122075008	0.000791682
0.007389054	0.09774016	1.069812637	0.527460567	2.53E-05
0.003015569	0.079739874	0.635306509	1.118531967	1.07E-05
0.00286733	0.059490044	0.646033757	1.027451265	7.35E-06
0.004849516	0.073079418	0.538736155	1.245639986	2.37E-05
0.00670628	0.586889741	1.63439664	0.381388704	9.56E-05
0.006237879	0.325579315	1.522739842	0.57609489	4.68E-05
0.066952678	3.639562699	7.621939016	0.089553773	0.003939432
0.075523004	3.376728622	6.257908677	0.107392153	0.003607396
0.001226234	0.035523252	0.371181001	1.680725061	3.50E-06

log.sigma.3.0.mm.3D_gldm_GrayLevelVariance	log.sigma.3.0.mm.3D_gldm_HighGrayLevelEmphasis	log.sigma.3.0.mm.3D_gldm_GrayLevelNonUniformityNormalized	log.sigma.3.0.mm.3D_gldm_DependenceEntropy
13.10579949	145.5285285	0.081090099	6.36703048
11.69629572	61.44247788	0.09092333	4.963713203
15.06411361	98.80745342	0.074341268	6.333727271
13.93431002	78.2826087	0.085066163	5.180639401
8.823545055	87.2386059	0.099371806	6.255790138
22.71901216	257.1359223	0.067207088	5.320788969
4.362363373	141.996443	0.261026248	6.63972006
9.179858461	226.5114943	0.118342796	6.910600966
5.735917382	135.9300071	0.164088383	6.657261968
9.197107837	85.80769231	0.09509721	5.835050184
3.041619283	420.1310395	0.229291446	6.710901709
5.989559614	105.885454	0.167385993	6.789482958
21.93959529	138.0060423	0.064265569	5.946546075
8.690415512	60.37894737	0.098060942	5.61951456
8.86850189	118.4347826	0.10055293	6.330547101
7.060483281	128.1817755	0.213794594	6.679846089
5.583552521	38.01605505	0.118592711	6.022261618
6.313286259	42.85585586	0.123772421	5.054860237
5.646470019	53.05467372	0.118850101	6.325373464
6.300247399	197.0629664	0.166033773	7.053979507
8.546370448	70.6976584	0.103959391	6.608217595
6.863203012	33.60126582	0.118811088	5.508085398
8.980863971	86.55871886	0.09436304	5.956423552
3.286935594	111.4356786	0.256183267	6.528392175
12.77496414	127.9380165	0.10796166	6.503892352
10.30106533	139.16	0.108208139	6.918973339
9.113789963	107.5015974	0.093370806	6.41873133
7.088143458	127.5758954	0.115628841	6.643463739
9.891581633	46.32142857	0.114795918	3.923251797
7.357423492	79.02519548	0.109821022	6.607465078
8.274992514	103.119624	0.117646691	6.866645832
10.66365244	93.86666667	0.096255286	5.945181015
8.779043929	97.24784988	0.102178023	6.624519059
6.963966543	129.8294697	0.179644503	6.698483495
7.095051098	151.3851021	0.115165198	6.757826066
15.74991198	205.4468085	0.078039334	6.88848233
25.14451019	175.3641026	0.059552926	5.683086277
6.295921334	162.7738967	0.219348198	6.785485832
7.518256075	63.35025381	0.104795279	5.82512262
5.065997896	660.2510319	0.217905763	6.714014211
7.457556114	97.57210777	0.104864615	6.185097175
2.863547066	122.753489	0.239864598	6.583872948
14.06628643	156.3582187	0.077638954	6.784878494
9.445652587	105.4413013	0.092381899	6.291359378
5.74101157	77.30514706	0.160701976	6.585846249
9.021167051	138.5150895	0.104168078	6.722429749
5.548739811	133.6259148	0.176827762	6.87751615
10.50232481	63.47945205	0.091136548	5.997526848
9.556478917	50.56140351	0.098491844	5.121849224
36.09247934	188.7272727	0.056115702	5.791558707
7.859723092	133.909234	0.17634267	6.514280355
12.54353728	157.7577582	0.090820313	6.881678679
2.870225629	115.8813106	0.281962411	6.424127617
11.04240398	119.1582054	0.083142252	6.469532013
14.62239435	65.89198606	0.078731076	5.934750155
8.873887831	70.60180995	0.098899149	6.166491536
6.288945384	174.6355211	0.164921442	6.885252057
9.808646328	104.6472492	0.095561211	6.670140153
8.338005515	90.46691176	0.09939987	5.886610578
3.064410736	142.5811679	0.27695708	6.446104493
11.28096422	148.2032622	0.087843529	6.428345295
16.88950264	199.7716781	0.072721181	6.849659163
18.70129082	264.6874453	0.070753432	7.0684885
8.675912848	255.3870892	0.142171995	7.072138168
9.504197358	78.58566978	0.089294553	6.092943501
21.9601661	219.5524027	0.066181841	6.840180021
8.103241297	61.18487395	0.098933691	5.193618368
10.2801343	203.6167636	0.105287533	6.944895378
4.106021898	95.30918887	0.197423916	6.700416069
11.94105061	99.83628029	0.083748583	6.603317489
8.096852362	70.57070707	0.099377615	5.674627995
8.058525828	294.9073109	0.194597939	6.835475923
9.051481192	111.8477971	0.093862578	6.158491853
7.454102172	90.78564772	0.117564137	6.595053313
6.678571926	152.25	0.153579893	6.878432777
9.383700276	92.96688742	0.095774308	6.336262714
34.74306751	175.2503374	0.061462699	6.751381984
23.64688091	158.4086957	0.060567108	5.21788546
14.19506797	120.3072197	0.075934224	6.306620782
4.592811524	93.43170426	0.162095401	6.53413803
13.10579949	145.5285285	0.081090099	6.36703048
14.24413745	74.86449864	0.084642445	6.196519643
7.970806124	159.6828555	0.185925645	6.708640983
5.851475962	113.4987234	0.127427071	6.472987375
7.373886327	105.7407202	0.116241895	6.746216817
22.49609375	153.3125	0.063081095	5.647752878
11.43648038	179.72	0.088369084	6.492803839
5.235670463	122.8829316	0.225115767	6.552356477
31.58536895	221.6731915	0.066477863	6.936794346
11.18636094	102.07875	0.087509375	6.344429658
20.24952741	153	0.070127074	5.808724513
5.760680718	102.0475524	0.133738569	6.649014922
7.576984503	115.2767387	0.110860861	6.90588181
6.541640735	103.8357551	0.139770675	6.895716778
6.501496435	93.87469763	0.116584267	6.558971554
16.11954152	193.7922297	0.071908955	6.509607571
11.14037904	93.00273038	0.089354564	6.661074527
19.09261461	136.0804598	0.073061171	5.055951651
10.00912517	75.14084507	0.091450109	4.839612612
9.861287883	183.5662224	0.131203445	7.171271086

log.sigma.3.0.mm.3D_gldm_DependenceNonUniformity	log.sigma.3.0.mm.3D_gldm_GrayLevelNonUniformity	log.sigma.3.0.mm.3D_gldm_SmallDependenceEmphasis
102.2762763	54.00600601	0.27005462
28.23893805	10.27433628	0.392858899
75.82401656	35.9068323	0.277062729
19.69565217	7.826086957	0.421442389
93.1152815	74.13136729	0.224640532
29.77669903	6.922330097	0.517834412
632.6706734	4256.294002	0.060638468
386.4970273	597.1577487	0.116597782
243.8257271	693.9297706	0.07510441
66.56593407	34.61538462	0.287421044
268.1006589	1252.848463	0.070627943
163.6319557	514.3771559	0.089688164
75.51963746	21.27190332	0.418768912
38.6	18.63157895	0.295512889
104.6913043	92.50869565	0.16004084
411.5896564	1823.026504	0.080695412
56.81651376	51.70642202	0.154464713
20.67567568	13.73873874	0.286586433
62.3968254	67.38800705	0.147061955
604.9783684	1980.284816	0.088728771
140.2520661	150.9490358	0.154852235
23.43037975	18.7721519	0.26611696
39.07829181	26.51601423	0.213741479
204.3676103	1230.704413	0.052201809
71.05785124	78.38016529	0.15682429
168.1822222	219.1214815	0.14202193
124.3120341	87.67518637	0.193537387
147.4161455	203.3911313	0.114929784
10	3.214285714	0.488095238
112.6924414	126.4039965	0.140623293
251.821988	413.0575335	0.110611502
41.58823529	24.54509804	0.288024567
131.5942142	130.6856919	0.177839244
170.0308034	565.7005399	0.09554924
292.6554501	400.4293932	0.13936876
128.1294326	88.02836879	0.231110989
128.5025641	23.22564103	0.542646346
504.826887	2574.70915	0.073472881
29.80203046	20.64467005	0.22007205
549.0090804	2639.710418	0.081119647
81.95721078	66.16957211	0.209878226
329.0207268	1735.900097	0.078395064
195.0144073	118.5546824	0.225718834
98.7708628	65.31400283	0.19931582
71	174.84375	0.091940535
171.5984655	203.6485934	0.14920399
339.3555747	1232.31267	0.068926881
32.6803653	19.95890411	0.161486401
24.07017544	11.22807018	0.404444755
71.61818182	12.34545455	0.409352273
124.9391396	336.1091291	0.091182805
233.3723946	196.081056	0.212024124
409.2783607	2848.38428	0.051844994
122.716647	70.4214876	0.181780864
53.06968641	22.59581882	0.332569104
91.63348416	65.57013575	0.217205446
281.7925099	871.9396633	0.092405559
151.0349515	147.6420712	0.153347961
42.54411765	27.03676471	0.274891196
1017.783907	6512.091822	0.047845302
102.4730238	70.01129235	0.227296575
205.4266999	116.5720524	0.254676437
614.9908136	323.4846894	0.266139653
498.6610329	1211.305399	0.134804448
49.14330218	28.6635514	0.222058333
355.6462243	144.6073227	0.289281997
24.88235294	11.77310924	0.322832218
356.251938	434.626938	0.175383073
461.5331737	1978.779906	0.060193819
198.1879502	127.8840864	0.236003145
32.82828283	19.67676768	0.214667173
324.0877588	1381.450768	0.082233283
104.6368491	70.30307076	0.175698875
97.47903075	126.1463187	0.160583089
206.3942786	493.9129353	0.122999392
80.74834437	57.84768212	0.204606429
256.7233468	91.0877193	0.282629476
33.34782609	6.965217391	0.480707729
114.9569892	49.43317972	0.289556326
99.79197995	258.7042607	0.096346179
102.2762763	54.00600601	0.27005462
55.84552846	31.23306233	0.301540569
184.3774137	635.4938561	0.092560881
104.0331915	149.7268085	0.100790495
146.6864266	209.8166205	0.142308757
46.11363636	11.10227273	0.423510053
94.15586207	64.06758621	0.213266502
187.7343827	915.3207083	0.07115823
179.8340426	78.11148936	0.274152655
111.5175	70.0075	0.259724006
63.24637681	19.35507246	0.390288105
112.8881119	191.2461538	0.107944326
283.9105786	379.3658679	0.16862335
231.5059678	538.6761806	0.108325269
222.6081277	240.9796807	0.17876702
197.9594595	85.1402027	0.325826272
175.2648464	130.9044369	0.218957769
19.71264368	6.356321839	0.399508301
17.67605634	6.492957746	0.348423318
601.3096626	1260.077884	0.128255492

log.sigma.3.0.mm.3D_gldm_DependenceNonUniformityNormalized	log.sigma.3.0.mm.3D_gldm_DependenceVariance	log.sigma.3.0.mm.3D_gldm_LargeDependenceEmphasis
0.153567982	4.613090568	16.97897898
0.249902107	1.729657765	7.566371681
0.156985542	5.723810381	18.09730849
0.214083176	2.915406427	10.06521739
0.124819412	10.096716	28.95978552
0.289094165	1.590536337	6.029126214
0.03879987	58.37074317	236.2870109
0.076594734	32.28665127	85.64090369
0.057655646	40.08905842	126.268385
0.182873445	2.585979954	11.85164835
0.049066738	36.67114337	134.9073939
0.053248277	38.7073567	125.1132444
0.228156004	2.449119668	8.782477341
0.203157895	2.831135734	11.70526316
0.113794896	17.62046786	46.15
0.048268987	48.34901252	162.1926821
0.130313105	4.823373453	25.1146789
0.186267348	2.330654979	11.99099099
0.110047311	7.893172084	32.57671958
0.050723432	42.37339897	135.6115536
0.096592332	29.41644848	66.95316804
0.148293543	4.118570742	16.86075949
0.139068654	5.069641975	20.75800712
0.042541135	49.24062917	188.337219
0.097875828	24.87050065	61.76859504
0.083052949	20.16430081	59.39209877
0.132387683	5.410085503	22.74014909
0.083806791	19.51936035	60.94201251
0.357142857	0.551020408	4
0.09790829	18.87521371	52.02519548
0.071723722	33.88598652	92.59099972
0.163091119	4.302745098	15.8627451
0.102888361	12.88312862	39.60046912
0.053995174	40.71155597	128.4779295
0.084168953	24.28807723	65.72878919
0.113589923	11.34475127	32.49822695
0.329493754	1.061275477	4.661538462
0.043007913	52.35686258	182.4544215
0.151279342	6.48086784	21.3248731
0.045320215	44.17215172	158.9382533
0.129884645	5.008406147	21.84310618
0.04546369	42.78189078	152.8245129
0.12771081	6.707277733	23.78847413
0.139704191	7.567001234	24.38048091
0.065257353	35.15246202	104.8933824
0.087774151	23.3374889	63.19846547
0.048695017	44.68277754	152.1496628
0.149225412	4.521590459	21
0.21114189	2.843028624	10.33333333
0.32553719	0.888099174	5.336363636
0.06555044	44.70423107	121.6390346
0.108092818	11.2942129	34.03195924
0.040514587	48.00138669	210.8606217
0.144883881	4.03961478	19.7107438
0.184911799	3.316636113	12.19163763
0.138210383	7.12965655	24.02262443
0.053299132	40.16875772	125.6132022
0.09775725	23.97574638	59.76634304
0.156412197	4.544117647	16.79411765
0.043286008	40.86142539	193.5031259
0.12857343	10.78838933	29.14805521
0.128151404	10.02676049	27.50155958
0.134512426	11.64306854	29.06255468
0.05852829	46.99639175	124.3248826
0.153094399	3.902233092	17.25545171
0.162767151	4.106226665	15.1006865
0.209095403	2.912929878	10.93277311
0.086301342	28.37265991	68.18362403
0.046047408	39.56066909	156.4305098
0.129789096	9.312969903	26.76424361
0.165799408	3.490358127	16.06060606
0.045652593	53.67923859	174.0911396
0.139702068	7.815765034	26.44726302
0.090847186	18.62913902	52.35135135
0.064177325	39.07213002	105.2394279
0.133689312	5.702129293	22.15562914
0.173227629	3.675182714	14
0.289981096	1.065860113	5.313043478
0.176585237	3.535154471	13.51920123
0.062526303	30.17505072	96.09398496
0.153567982	4.613090568	16.97897898
0.151342896	5.900323881	18.52303523
0.05394307	45.45349766	135.9204213
0.088538886	17.47277067	58.8587234
0.081266718	20.19924157	60.08144044
0.262009298	2.325284091	7.965909091
0.129870155	6.607935315	24.04
0.046171762	57.96540336	185.3787506
0.153050249	6.419121775	19.53276596
0.139396875	5.87549375	20.145
0.229153539	2.291587902	8.760869565
0.078942736	27.28289696	77.54405594
0.082966271	24.48051987	63.80829924
0.060069011	34.45546274	103.017644
0.10769624	10.58020793	34.49879052
0.167195489	3.958226808	14.1722973
0.119634707	9.440330813	28.87849829
0.226582111	1.859162373	8.08045977
0.24895854	1.319182702	7.394366197
0.062610336	41.63266759	110.724698

log.sigma.3.0.mm.3D_gldm_LargeDependenceLowGrayLevelEmphasis	log.sigma.3.0.mm.3D_gldm_SmallDependenceHighGrayLevelEmphasis	log.sigma.3.0.mm.3D_gldm_LargeDependenceHighGrayLevelEmphasis
0.158668645	44.54337065	2535.546547
0.476571983	31.25342183	281.159292
0.413105976	35.87240537	2000.910973
0.378203415	44.96749693	423.2608696
0.42115424	24.82005703	3089.83378
0.03942194	136.7681553	1545.330097
1.558830176	8.6653489	36804.0785
0.329340668	24.35952784	23696.86465
0.802530647	8.586749873	21210.98132
0.284216649	28.35702112	952.0934066
0.320002081	28.63356568	57336.12701
1.081735013	9.524103654	15816.3124
0.161505798	78.39381315	761.9818731
0.380064833	23.79054652	632.4210526
0.340359902	16.02744762	7825.697826
1.192733692	9.63054249	23877.10965
1.479995385	7.888908869	960.4701835
0.513133169	17.73215093	405.5945946
1.004457713	8.816199602	1822.733686
0.684848136	19.32138767	27774.56561
1.106432895	15.23230122	6565.358815
1.941776105	15.33014014	352.1329114
0.409371795	20.83989084	2049.967972
1.577957562	5.311682412	23209.39446
0.457491915	19.28414328	11169.02893
0.47391785	25.68596156	9010.788642
0.273982587	21.94856202	2885.951012
0.426461076	13.33623314	9803.293917
1.09704581	31.71428571	120.4642857
0.714459822	12.79265259	5249.673328
0.790861961	10.57428957	13110.05383
0.3178419	37.35487	1160.290196
0.468429079	19.29649593	4878.312744
0.923669653	11.79325687	19192.28866
0.396097395	23.06185621	12177.09462
0.175000517	47.46870653	8505.739362
0.085813164	114.275094	552.025641
1.036141421	10.46478529	33549.9971
0.610034245	15.83951039	1255.563452
0.233870197	55.26417707	108595.3853
0.280502716	23.54160124	2235.822504
1.251736571	10.54385291	19032.99834
0.242574225	41.34891491	3991.310413
0.266874294	22.75644867	3179.528996
1.3013084	6.983093253	10073.51838
0.41642229	22.65089423	11393.70486
1.069239366	8.62182483	22862.43177
0.812695799	12.46339442	1175.031963
0.706021812	27.97827909	305.4385965
0.103634783	87.06561995	822.3727273
0.782227182	8.483469821	20225.92602
0.252332568	42.09907604	5577.141732
1.731209519	6.032337119	26348.59364
0.242993383	20.58153129	2558.303424
0.829434536	31.51464759	511.2508711
0.512626122	21.05471885	2077.781297
0.660865169	14.78652352	24900.45924
0.53467523	17.33032422	9093.040777
0.257478339	30.57302871	1645.0625
1.310464218	6.870773725	29218.00812
0.21228944	40.65191582	5085.528231
0.164890543	63.70282243	5949.094198
0.12449955	81.98376791	8219.187008
0.433780907	34.61699069	36859.3439
0.436987774	20.20617384	1302.672897
0.112972646	75.50648534	2895.623799
0.415364942	27.06608593	431.302521
0.305782419	39.84154981	16596.04845
1.572279686	6.71738597	16706.05767
0.391998466	32.12601395	2948.188605
0.357980368	15.50687787	1191.318182
0.524503687	20.0594507	59253.68939
0.252648073	19.93103995	4038.032043
0.604384863	17.05457599	5807.17521
0.651799174	20.60929453	17958.26679
0.333769236	22.45511083	2080.354305
0.2418255	61.60884882	1683.362348
0.122098407	89.10756763	639.4173913
0.216515046	44.27076593	1383.867896
0.916559979	8.304003814	10990.22744
0.158668645	44.54337065	2535.546547
0.884780696	34.85743345	742.1924119
0.75273074	12.73608872	26306.93739
0.480042233	10.76499135	8380.874043
0.549517636	16.55536868	7800.770083
0.171053851	81.75450584	794.9034091
0.153020751	41.37917862	4886.197241
1.352345752	8.558548988	26666.9695
0.158360195	71.62090728	3608.24
0.313304814	36.9745915	1993.24625
0.30124526	73.19269121	1030.971014
0.699991349	11.3230352	9857.518881
0.545548369	22.64679222	8501.946523
0.965394015	12.46282881	12394.80566
0.448836005	21.0838643	3220.138849
0.103999021	77.06251867	2710.748311
0.499857291	27.93389281	2651.095563
0.229153941	71.02846743	799.5287356
0.254212841	32.18315336	401.1408451
0.601595636	25.12108393	21801.5304

log.sigma.3.0.mm.3D_gldm_SmallDependenceLowGrayLevelEmphasis	log.sigma.3.0.mm.3D_gldm_LowGrayLevelEmphasis	log.sigma.3.0.mm.3D_gldzm_DistanceZoneVariabilityNormalized
0.005653694	0.013782669	0.989950005
0.025086314	0.063940965	0.92737629
0.010913516	0.034877624	0.906268598
0.021185831	0.040300583	1
0.00583241	0.019961869	0.915061728
0.013394646	0.01589706	1
0.000804483	0.008880646	0.88812117
0.001271671	0.005948441	0.914875088
0.001257705	0.009843778	0.91955597
0.010434197	0.032017812	0.98425296
0.000384509	0.00264754	0.899346784
0.003069413	0.015367581	0.955079931
0.012053768	0.022325015	0.938334212
0.018034854	0.049873709	1
0.005483717	0.015781776	0.9608
0.001463888	0.012112117	0.854570637
0.012072549	0.068255125	0.970156107
0.03709667	0.068686206	0.945987654
0.011578178	0.042728308	0.975004006
0.000704429	0.006120643	0.897234035
0.00452115	0.028615036	0.874488889
0.021747804	0.110477342	1
0.006590725	0.027926036	1
0.001168109	0.01126202	0.963520233
0.00392491	0.01844924	0.953871908
0.00177411	0.012233824	0.614461416
0.004468705	0.017814198	0.939756772
0.002416757	0.011473484	0.960455544
0.026993727	0.141301763	1
0.005357786	0.026876429	0.930323704
0.002772692	0.017053244	0.888688752
0.009438529	0.024565452	0.977530992
0.004659141	0.01982142	0.982380247
0.001645013	0.011525003	0.938628248
0.001568134	0.008749893	0.987377586
0.002711677	0.010226887	0.958207216
0.009021321	0.015812537	1
0.000919461	0.008256309	0.770689859
0.009827382	0.043745284	1
0.000217141	0.001649154	0.922919903
0.005435892	0.016883448	0.95695029
0.001173171	0.009587822	0.957666262
0.003016588	0.012897273	0.966583473
0.005470086	0.016686308	0.935555556
0.004019484	0.025371951	0.978949751
0.002454925	0.011085772	0.940453587
0.001351335	0.010341381	0.986270679
0.011955674	0.050895378	0.915061728
0.028945566	0.06666129	1
0.010635627	0.021669279	0.7731569
0.003475678	0.012983913	0.935208015
0.003414406	0.011331707	0.944708837
0.000866734	0.010226724	0.970227036
0.004073808	0.015349949	0.950648934
0.021210796	0.074534813	0.897735573
0.010745188	0.034487415	0.972794293
0.001019467	0.007479503	0.950951307
0.003424829	0.017251695	0.853644019
0.008125278	0.021779348	0.929506246
0.000663188	0.008290794	0.936447965
0.00389414	0.011540778	0.960607055
0.002578949	0.008611641	0.91322864
0.001852322	0.006052866	0.929522783
0.001076902	0.005044448	0.92889649
0.008408504	0.033074651	0.95125
0.00281686	0.0084091	0.739523871
0.016782775	0.041861415	1
0.001609256	0.007008475	0.925652614
0.001204303	0.013965628	0.952222643
0.005872432	0.02175352	0.934421115
0.012513261	0.03165286	0.961553249
0.00058164	0.004114594	0.787870756
0.004285525	0.014917206	0.888504801
0.004755687	0.021229972	0.988439697
0.001566589	0.008828391	0.95183661
0.00546739	0.020744593	0.943310778
0.00408657	0.01465214	0.575428881
0.016479767	0.025821389	1
0.007216123	0.021507438	0.946942149
0.003513575	0.016000148	1
0.005653694	0.013782669	0.989950005
0.010500256	0.039184456	0.884497041
0.00156123	0.009161722	0.915572849
0.002313073	0.012757148	0.98333451
0.003220957	0.01543651	0.94779877
0.009794391	0.020835878	0.977780583
0.00356324	0.009971597	0.987578125
0.001283109	0.010926585	0.929241853
0.004270203	0.012200327	0.537176386
0.004662944	0.017888204	0.891556211
0.011121215	0.026702845	0.926035503
0.002217269	0.015306398	0.987097318
0.002902395	0.013124069	0.958083751
0.002538939	0.015322938	0.942362445
0.00314032	0.015730046	0.985342501
0.003491103	0.009264338	0.953729701
0.00690572	0.027575707	0.965626567
0.010423445	0.037005861	0.95240928
0.023034059	0.04176403	0.889273356
0.001659596	0.008160692	0.866902283

log.sigma.3.0.mm.3D_gldzm_LowIntensityEmphasis	log.sigma.3.0.mm.3D_gldzm_LargeDistanceEmphasis	log.sigma.3.0.mm.3D_gldzm_HighIntensitySmallDistanceEmphasis
0.021246155	1.015151515	162.4507576
0.066736839	1.113207547	79.29245283
0.042384206	1.147887324	128.7676056
0.046563032	1	106.0444444
0.02575579	1.133333333	108.5833333
0.02270933	1	263.1111111
0.014898837	1.240860215	141.0684364
0.011762854	1.180530973	194.8610251
0.019970478	1.125954198	107.0591603
0.040549784	1.023809524	95.38095238
0.00566003	1.240331492	394.8691682
0.039706581	1.068965517	105.3764368
0.027534631	1.095541401	182.5700637
0.064817977	1	74.78571429
0.036107965	1.06	92.95666667
0.020059332	1.236842105	114.7883127
0.081478818	1.045454545	54.53030303
0.122901364	1.083333333	57.33333333
0.083624408	1.037974684	59.53164557
0.008308101	1.221991701	219.9830247
0.029692205	1.275555556	101.92
0.101461678	1	56.9787234
0.037900265	1	98.359375
0.025887932	1.078703704	101.537037
0.03211899	1.070866142	115.7283465
0.015654949	2.302816901	130.9289662
0.027220068	1.093264249	107.0738342
0.023336571	1.085427136	105.2889447
0.090775616	1	57.94117647
0.046025049	1.108433735	89.37198795
0.029959102	1.177464789	89.58380282
0.032408633	1.034090909	124.7755682
0.028024019	1.026666667	108.9433333
0.020217011	1.095070423	120.334507
0.011978556	1.040169133	164.8175358
0.014216877	1.06405694	195.3994662
0.016069553	1	203.9430894
0.014040322	1.48574969	126.5620956
0.060784163	1	68.59183673
0.002730993	1.158613445	659.7138553
0.026633879	1.102189781	108.6222628
0.016254815	1.091561939	134.969629
0.015164759	1.050991501	180.2514164
0.027581591	1.1	110.6983333
0.054254108	1.085106383	76.57565012
0.017567413	1.092150171	150.8728669
0.023154024	1.020737327	123.2085253
0.084830471	1.133333333	74.15555556
0.070252062	1	68.17307692
0.025126688	1.391304348	159.4173913
0.04152415	1.100558659	79.64944134
0.01676608	1.085365854	194.8739837
0.018301726	1.056034483	118.9520474
0.023213769	1.075949367	110.3338608
0.071139659	1.162162162	89.68693694
0.05613593	1.04137931	95.79482759
0.012926396	1.075471698	155.1850105
0.024211411	1.238493724	106.3661088
0.031267057	1.109756098	98.99085366
0.016792293	1.185524975	144.8777602
0.017159522	1.060301508	175.9949749
0.010424606	1.158371041	242.0828934
0.007005344	1.109729334	302.3615582
0.008283438	1.114836547	249.5908308
0.044967444	1.075	87.98125
0.009794313	1.611961057	215.4572419
0.050412231	1	82.45652174
0.009899115	1.122503329	220.4670439
0.02329399	1.123595506	120.3483796
0.025647406	1.114583333	138.5191696
0.054214766	1.058823529	69.78921569
0.007480967	1.538033395	218.0732471
0.025889154	1.177777778	102.6111111
0.033727339	1.01744186	108.193314
0.013544599	1.074074074	162.5240741
0.028423731	1.087591241	110.1514599
0.014041552	2.154320988	139.5679012
0.030906719	1	176.8550725
0.02700977	1.081818182	149.975
0.0414737	1	83.36111111
0.021246155	1.015151515	162.4507576
0.034891992	1.184615385	113.8
0.01813616	1.132492114	130.7799685
0.025788545	1.025210084	97.90756303
0.023509416	1.08045977	117.0057471
0.02263056	1.033707865	185.508427
0.016990426	1.01875	195.6515625
0.019524507	1.130081301	122.9452349
0.01683486	2.31420765	150.3224423
0.017594588	1.193832599	139.3195056
0.027437007	1.115384615	178.2211538
0.026195221	1.019480519	104.612013
0.017938504	1.064250412	134.8134267
0.02540921	1.118518519	119.1555556
0.018514041	1.034398034	116.5608108
0.011047146	1.071090047	226.8815166
0.037580881	1.052478134	129.5889213
0.036508541	1.073170732	170.8109756
0.053945756	1.176470588	89.68382353
0.013733438	1.25545675	190.4048774

log.sigma.3.0.mm.3D_gldzm_LowIntensityLargeDistanceEmphasis	log.sigma.3.0.mm.3D_gldzm_HighIntensityEmphasis	log.sigma.3.0.mm.3D_gldzm_DistanceZoneVariability	log.sigma.3.0.mm.3D_gldzm_ZonePercentage
0.02155537	162.6363636	196.010101	0.297297297
0.179944386	79.32075472	49.1509434	0.469026549
0.049591426	131.2605634	128.6901408	0.293995859
0.046563032	106.0444444	45	0.489130435
0.026883373	112.65	164.7111111	0.241286863
0.02270933	263.1111111	63	0.611650485
0.018268971	145.444086	825.9526882	0.057034221
0.012643057	203.9026549	516.9044248	0.111969877
0.021204247	110.3396947	240.9236641	0.06195318
0.040921808	95.76190476	124.015873	0.346153846
0.006509421	407.7513812	325.5635359	0.06625183
0.040774491	106.5402299	249.2758621	0.08493329
0.031240148	184.8917197	147.3184713	0.474320242
0.064817977	74.78571429	70	0.368421053
0.036562144	95.18666667	144.12	0.163043478
0.023715049	119.4241486	552.0526316	0.075759353
0.082741444	54.93939394	64.03030303	0.151376147
0.123734697	59.41666667	34.05555556	0.324324324
0.083888121	60.89873418	77.02531646	0.139329806
0.009866423	227.56639	864.93361	0.080825019
0.034251862	105.5688889	196.76	0.154958678
0.101461678	56.9787234	47	0.297468354
0.037900265	98.359375	64	0.227758007
0.026675612	103.0740741	208.1203704	0.044962531
0.032842064	117.511811	121.1417323	0.174931129
0.020738142	189.9894366	174.5070423	0.140246914
0.031685178	109.8290155	181.373057	0.205537806
0.023845275	108.1859296	191.1306533	0.113132462
0.090775616	57.94117647	17	0.607142857
0.04774999	91.42771084	154.4337349	0.144222415
0.032143491	93.37183099	315.484507	0.101110795
0.033772269	124.9886364	86.02272727	0.345098039
0.028666498	109.2266667	221.0355556	0.175918686
0.021196913	122.7323944	266.5704225	0.090187361
0.012249233	165.7991543	467.0295983	0.136036813
0.014695077	198.3594306	269.2562278	0.249113475
0.016069553	203.9430894	246	0.630769231
0.018407646	143.4399009	621.9467162	0.068751065
0.060784163	68.59183673	49	0.248730964
0.002923483	687.302521	878.6197479	0.078586759
0.027186105	111.8175182	131.1021898	0.217115689
0.016825152	137.8402154	533.4201077	0.076965593
0.015761566	181.9830028	341.203966	0.231172233
0.028256154	114.7533333	140.3333333	0.212164074
0.055583895	77.18085106	92.0212766	0.086397059
0.019032704	153.5733788	275.552901	0.149872123
0.023449557	123.6059908	428.0414747	0.062275793
0.08772399	75.82222222	41.17777778	0.205479452
0.070252062	68.17307692	52	0.456140351
0.027971566	209.8695652	88.91304348	0.522727273
0.042254383	83.45810056	167.4022346	0.093913956
0.019246615	198.6056911	464.796748	0.227883279
0.019006198	119.9439655	450.1853448	0.045931499
0.023893877	112.5506329	150.2025316	0.186540732
0.099879226	92.61261261	99.64864865	0.386759582
0.076969263	96.54482759	141.0551724	0.218702866
0.013592933	157.6457023	453.6037736	0.090221298
0.02662845	112.5355649	204.0209205	0.154692557
0.031924264	103.6463415	76.2195122	0.301470588
0.018339242	147.9745158	918.6554536	0.041721601
0.018762416	177.0050251	191.160804	0.249686324
0.011724158	246.9660633	403.6470588	0.275733001
0.011426018	308.1536211	1270.657644	0.298993876
0.009077317	255.1475272	1108.173512	0.140023474
0.046498056	88.9	76.1	0.249221184
0.012891501	262.1808067	531.7176634	0.329061785
0.050412231	82.45652174	46	0.386554622
0.010555792	227.4167776	695.1651132	0.181928295
0.025743406	121.588015	508.4868914	0.053277462
0.029264055	140.3463542	358.8177083	0.251473477
0.055415246	70.50980392	49.03921569	0.257575758
0.009566083	240.8886827	424.6623377	0.075926187
0.027056944	110	119.9481481	0.18024032
0.033942671	108.5465116	170.0116279	0.160298229
0.013792639	168.4222222	385.4938272	0.125932836
0.031982126	112.3576642	129.2335766	0.226821192
0.027743732	221.7510288	279.6584362	0.327935223
0.030906719	176.8550725	69	0.6
0.038108262	150.7318182	208.3272727	0.337941628
0.0414737	83.36111111	144	0.090225564
0.02155537	162.6363636	196.010101	0.297297297
0.056490995	114.7692308	114.9846154	0.352303523
0.019207928	135.1167192	290.2365931	0.092744295
0.025917168	99.14285714	117.0168067	0.101276596
0.025490383	118.091954	247.3754789	0.144598338
0.056338425	185.5168539	87.02247191	0.505681818
0.017101373	196.44375	158.0125	0.220689655
0.020964109	125.6260163	228.5934959	0.060501722
0.022095781	264.0464481	196.6065574	0.311489362
0.020252888	143.9823789	202.3832599	0.28375
0.074877356	179.2769231	120.3846154	0.471014493
0.026310491	105.4350649	152.012987	0.107692308
0.020634361	136.0032949	581.5568369	0.177381648
0.028032705	120.6592593	381.6567901	0.105085625
0.018745344	117.4520885	401.034398	0.196903725
0.011819036	229.7322275	402.4739336	0.356418919
0.039568831	130.5422741	331.2099125	0.234129693
0.044638622	170.9756098	39.04878049	0.471264368
0.164239874	89.79411765	30.23529412	0.478873239
0.016023579	200.3080032	1072.358124	0.1288005

log.sigma.3.0.mm.3D_gldzm_IntensityVariabilityNormalized	log.sigma.3.0.mm.3D_gldzm_LowIntensitySmallDistanceEmphasis	log.sigma.3.0.mm.3D_gldzm_IntensityVariability
0.060146924	0.021168852	11.90909091
0.092203631	0.038434952	4.886792453
0.063281095	0.0405824	8.985915493
0.082469136	0.046563032	3.711111111
0.08154321	0.025473894	14.67777778
0.059208869	0.02270933	3.73015873
0.067919991	0.01415251	63.1655914
0.063813924	0.011572668	36.05486726
0.084231688	0.019662035	22.06870229
0.082388511	0.040456778	10.38095238
0.085467477	0.0055066	30.93922652
0.071534475	0.039439603	18.67049808
0.054647247	0.026608252	8.579617834
0.074693878	0.064817977	5.228571429
0.078222222	0.035994421	11.73333333
0.0779649	0.019145403	50.36532508
0.101010101	0.081163161	6.666666667
0.094135802	0.122693031	3.388888889
0.101426054	0.08355848	8.012658228
0.065260154	0.007982284	62.91078838
0.082824691	0.028673389	18.63555556
0.096423721	0.101461678	4.531914894
0.093261719	0.037900265	5.96875
0.088263032	0.025726734	19.06481481
0.072478145	0.031938221	9.204724409
0.058892085	0.01479619	16.72535211
0.074391259	0.02610379	14.35751295
0.077599051	0.02323421	15.44221106
0.100346021	0.090775616	1.705882353
0.07736972	0.045593814	12.84337349
0.083896052	0.029413005	29.78309859
0.068956612	0.032067724	6.068181818
0.06982716	0.027863399	15.71111111
0.073571712	0.019972035	20.8943662
0.074201378	0.011930503	35.09725159
0.060827497	0.014097326	17.09252669
0.056249587	0.016069553	13.83739837
0.079037357	0.013069092	63.78314746
0.082882132	0.060784163	4.06122449
0.061473854	0.002690988	58.52310924
0.076242741	0.026531868	10.44525547
0.084786736	0.016146015	47.22621185
0.064642201	0.015015557	22.81869688
0.068088889	0.027412951	10.21333333
0.086238117	0.054106354	8.106382979
0.068224441	0.01720109	19.98976109
0.072203699	0.023080141	31.33640553
0.082469136	0.084107092	3.711111111
0.090976331	0.070252062	4.730769231
0.052703214	0.024415469	6.060869565
0.081988702	0.041341591	14.67597765
0.055472933	0.016145946	27.29268293
0.088352779	0.018142238	40.99568966
0.080035251	0.023043742	12.64556962
0.07199091	0.063954767	7.990990991
0.073388823	0.050927596	10.64137931
0.069068119	0.012759762	32.94549266
0.078447506	0.023607151	18.74895397
0.076145152	0.031102755	6.243902439
0.072853119	0.016548312	71.46890928
0.063205475	0.016758799	12.57788945
0.053684404	0.010123291	23.72850679
0.051919287	0.005900175	70.97366496
0.062884991	0.008087548	75.0217938
0.078125	0.044584791	6.25
0.051030929	0.009118606	36.69123783
0.103969754	0.050412231	4.782608696
0.060381099	0.009739512	45.34620506
0.074878312	0.022826132	39.98501873
0.067586263	0.024772179	25.953125
0.095732411	0.053914646	4.882352941
0.081092245	0.007082385	43.70871985
0.080384088	0.025597207	10.85185185
0.071119524	0.033673506	12.23255814
0.071970736	0.013482589	29.14814815
0.082316586	0.027534133	11.27737226
0.054200749	0.010916411	26.34156379
0.061961773	0.030906719	4.275362319
0.066115702	0.024235146	14.54545455
0.092785494	0.0414737	13.36111111
0.060146924	0.021168852	11.90909091
0.069467456	0.029492241	9.030769231
0.074107614	0.017868218	23.49211356
0.088058753	0.025756389	10.4789916
0.074793382	0.023014174	19.5210728
0.070824391	0.014203594	6.303370787
0.068125	0.016962689	10.9
0.075979906	0.019191332	18.69105691
0.04658246	0.015732212	17.04918033
0.075239186	0.016954986	17.07929515
0.066508876	0.01557692	8.646153846
0.090487435	0.026166404	13.93506494
0.069461445	0.01726454	42.1630972
0.075092212	0.024801562	30.41234568
0.079982372	0.018475174	32.55282555
0.056995575	0.010854173	24.0521327
0.066460403	0.037083893	22.79591837
0.069601428	0.034476021	2.853658537
0.096885813	0.026372227	3.294117647
0.058524908	0.013197927	72.39531124

log.sigma.3.0.mm.3D_gldzm_HighIntensityLargeDistanceEmphasis	log.sigma.3.0.mm.3D_gldzm_SmallDistanceEmphasis	log.sigma.3.0.mm.3D_glcm_SumVariance	log.sigma.3.0.mm.3D_glcm_Homogeneity1
163.3787879	0.996212121	377.3054448	0.403849289
79.43396226	0.971698113	94.72100912	0.345506811
141.2323944	0.963028169	208.0580941	0.384636571
106.0444444	1	136.695018	0.358413777
128.9166667	0.966666667	196.9867386	0.432608031
263.1111111	1	646.274924	0.297530617
172.3602151	0.954756571	427.2101649	0.72187606
256.5433628	0.965744838	691.9381199	0.550974259
123.4618321	0.96851145	396.4317426	0.623043159
97.28571429	0.994047619	181.4753681	0.387926994
483.4668508	0.958333333	1408.233207	0.666069604
111.1954023	0.982758621	287.685674	0.628452602
194.1783439	0.97611465	279.3988736	0.314596429
74.78571429	1	112.4968978	0.392744067
104.1066667	0.985	327.3681747	0.490408701
137.9674923	0.940789474	364.0045097	0.650490634
56.57575758	0.988636364	67.00138413	0.480037942
67.75	0.979166667	76.67337377	0.413857784
66.36708861	0.990506329	107.7097734	0.494563204
271.0072614	0.958410846	580.7455219	0.616867155
130.2088889	0.948549383	153.7366996	0.498716085
56.9787234	1	47.00686033	0.428274541
98.359375	1	192.2802456	0.434585446
112.1851852	0.985468107	324.967998	0.70526424
124.6456693	0.982283465	334.9962207	0.500981659
637.2147887	0.81196205	379.3294543	0.510871891
120.8497409	0.976683938	275.5264254	0.446210251
124.798995	0.984226689	354.4073564	0.541204998
57.94117647	1	87.64581648	0.306691578
99.65060241	0.972891566	185.0463085	0.503844586
108.5239437	0.955633803	275.0469298	0.560804209
125.8409091	0.991477273	199.3735717	0.407786342
110.36	0.993333333	231.5015737	0.494534406
132.3239437	0.976232394	367.625758	0.624924651
174.2452431	0.994655861	423.1694468	0.53371453
210.1992883	0.983985765	573.6939163	0.450083387
203.9430894	1	377.295484	0.260675684
229.6431227	0.900592042	486.9565188	0.674119607
68.59183673	1	130.1421813	0.458720182
838.5840336	0.969136613	2303.282107	0.660875051
131.8978102	0.982562855	243.552864	0.445902494
153.5834829	0.983093956	356.4639724	0.674570619
188.9093484	0.987252125	404.8508149	0.423068236
130.9733333	0.975	265.2222464	0.433183385
82.62765957	0.990543735	191.3622001	0.612567513
164.3754266	0.976962457	374.0912227	0.516717124
125.1958525	0.994815668	382.3853833	0.652995787
82.48888889	0.966666667	133.6818788	0.452885457
68.17307692	1	81.30731745	0.394154909
411.6782609	0.902173913	530.1967579	0.261291147
98.69273743	0.974860335	392.3976635	0.622145948
213.5325203	0.978658537	402.7476232	0.453488649
125.2909483	0.988386015	344.1769527	0.723772708
121.4177215	0.981012658	301.3311281	0.444923805
104.3153153	0.959459459	114.3231414	0.359489409
99.54482759	0.989655172	151.0604998	0.432709695
167.4884696	0.981132075	519.4766848	0.623858019
137.2133891	0.940376569	268.3379891	0.491039124
122.2682927	0.972560976	216.4051718	0.429412733
172.8634047	0.974158455	435.6955466	0.70897504
181.0452261	0.984924623	373.2003325	0.426407529
271.1040724	0.965434892	515.9773033	0.409363672
331.3218727	0.972567666	721.6742046	0.408642538
278.7191953	0.972222222	783.441292	0.58635746
92.575	0.98125	168.1104298	0.422841017
528.8998609	0.884194869	615.4823448	0.352056499
82.45652174	1	113.7806771	0.378856175
257.1331558	0.970853677	596.0094213	0.508753661
130.5168539	0.980610175	258.6426616	0.662947213
148.8125	0.974247685	217.5331398	0.420154823
73.39215686	0.985294118	150.8217181	0.417988964
394.5769944	0.908752046	956.6464494	0.656562389
139.5555556	0.955555556	298.0456857	0.436422479
109.9593023	0.995639535	225.8639534	0.518683972
192.0148148	0.981481481	433.2731056	0.598163975
121.1824818	0.97810219	208.8297663	0.437209703
676.8312757	0.785793896	487.8499383	0.337368795
176.8550725	1	333.6252973	0.303042872
153.7590909	0.979545455	255.0558724	0.371338363
83.36111111	1	252.4719746	0.603016297
163.3787879	0.996212121	377.3054448	0.403849289
118.6461538	0.953846154	126.0247755	0.389874393
152.4637224	0.966876972	476.8861585	0.627668898
104.0840336	0.993697479	314.6326781	0.559376878
122.4367816	0.979885057	264.625694	0.52739526
185.5505618	0.991573034	294.9914083	0.302670914
199.6125	0.9953125	488.5570167	0.452779704
139.402439	0.971996387	355.0662946	0.673923472
893.0081967	0.756640862	648.196425	0.359247516
166.4713656	0.956436613	222.7980431	0.405742271
183.5	0.971153846	318.2674741	0.323869052
108.7272727	0.99512987	273.2374145	0.577168532
140.7627677	0.983937397	291.9680571	0.523500147
130.6740741	0.977314815	266.5209871	0.583063035
122.5896806	0.994130494	226.7282271	0.493547191
241.1350711	0.982227488	505.6105559	0.364203037
134.3556851	0.986880466	203.3131304	0.436814198
171.6341463	0.981707317	293.1205295	0.322379054
90.23529412	0.955882353	127.6380549	0.347287508
249.9232013	0.945595302	516.7936239	0.567780163

log.sigma.3.0.mm.3D_glcm_Homogeneity2	log.sigma.3.0.mm.3D_glcm_ClusterShade	log.sigma.3.0.mm.3D_glcm_MaximumProbability	log.sigma.3.0.mm.3D_glcm_Idmn	log.sigma.3.0.mm.3D_glcm_SumVariance2
0.320634703	-1.418937905	0.026777178	0.981860135	33.6750858
0.253774299	74.8815915	0.041023333	0.938501366	23.52055125
0.300561787	15.48050033	0.030981322	0.964083037	38.64307597
0.272027957	134.6341502	0.048952113	0.946109465	28.98210098
0.353800711	34.08194772	0.03533922	0.979631893	21.7321873
0.20412914	-92.70554672	0.039481672	0.957952626	57.77336892
0.700558688	-75.31173632	0.243258287	0.995972388	12.73129545
0.498103178	-105.7502626	0.147513098	0.99310719	27.23245998
0.585111922	-68.28939851	0.198187129	0.993260442	17.70605683
0.301792704	-12.75638859	0.029649645	0.977205606	23.70255708
0.640491413	-32.7892912	0.193129806	0.996889554	8.720923169
0.593010499	-72.74741308	0.161844202	0.992395648	17.73665218
0.221452966	217.8406786	0.023521317	0.959111349	46.25462496
0.308756221	15.10380648	0.03663352	0.959436927	18.62478924
0.423924429	-51.8887916	0.087053253	0.983858804	26.70348015
0.617933885	-132.3779181	0.256913362	0.992297384	22.13698361
0.413478537	14.38788404	0.036146428	0.975431352	15.46060769
0.333009015	18.31446554	0.054771377	0.962520648	12.12769463
0.430494864	-12.45511525	0.050402027	0.973361531	17.20418325
0.578635306	-33.63129033	0.165157804	0.996686816	18.59038744
0.431980264	17.13895025	0.103266518	0.982179856	24.12000961
0.348881921	41.06883538	0.05222005	0.952913748	14.14847921
0.35582913	-14.55966708	0.035440533	0.970099846	27.27106134
0.683563656	-45.07521043	0.302042332	0.995903965	10.02520135
0.438482089	-104.6353977	0.140085447	0.976366401	39.53650978
0.454244282	185.60616	0.081077096	0.989662233	33.442969
0.372079065	-24.45416075	0.02711164	0.984578839	28.20765445
0.488582333	-60.41975668	0.088685605	0.987743043	21.47515995
0.202319813	36.53368689	0.07442228	0.909080779	21.88730576
0.440644711	-26.1097189	0.086563075	0.982557448	21.48644685
0.51026585	-79.94047968	0.11704207	0.989625102	26.18137991
0.32773961	23.05402584	0.040465722	0.96767402	25.13260831
0.432377233	-52.43613366	0.061636554	0.985948664	26.39986265
0.588059802	-89.65545269	0.194550314	0.991422314	20.45308603
0.47820121	-43.85779451	0.10017497	0.990223432	20.68269284
0.380414004	-209.3060434	0.046214501	0.981086152	48.12313678
0.167131347	206.2120157	0.019684138	0.950646444	54.25161623
0.645460632	-117.3592342	0.237606745	0.995262242	19.46169278
0.383713219	8.025274792	0.055665807	0.972413587	19.57556393
0.632156926	-25.95402561	0.247115593	0.998103935	13.05059937
0.370022223	-4.88657462	0.031656253	0.980994065	20.20425534
0.649170725	-13.87086172	0.251917146	0.995140796	6.946473069
0.34609084	-57.0639342	0.026864641	0.982980147	39.66646289
0.353960967	3.382932186	0.03035082	0.980083028	25.67288244
0.573198226	-68.72667991	0.200931081	0.986160891	18.27251299
0.458029836	-51.40376001	0.097370111	0.989100759	26.33592528
0.621520509	-73.37613144	0.191225193	0.995627545	16.2609884
0.377690177	46.93083436	0.044967474	0.977226552	32.6025349
0.315009474	72.31656905	0.05084071	0.956177452	19.79174522
0.171743908	1162.609988	0.01672272	0.953979907	121.9982985
0.58336138	-128.8121135	0.253913974	0.988995856	23.27022614
0.384911677	16.15437836	0.038915698	0.988140066	32.47871958
0.705491256	-38.81112428	0.311488908	0.996410136	8.334321497
0.368516843	-10.75185222	0.026081882	0.981721638	34.80511081
0.268717133	164.506727	0.029154104	0.962569202	34.59964258
0.355254003	28.75496151	0.030178345	0.98225012	22.72444968
0.587327821	-79.99976763	0.196455364	0.994173317	18.0440741
0.425204844	-32.5111911	0.080317472	0.985372079	30.32969273
0.351351503	-16.52529041	0.034568012	0.973399449	22.28989224
0.690312517	-36.70108568	0.259406385	0.997762295	8.320766641
0.350016854	-8.827259511	0.039821924	0.976505833	28.57543414
0.332050185	7.367352937	0.040760449	0.981246216	44.29384799
0.335008713	-50.08867822	0.039908827	0.981044864	52.19599926
0.541531732	-113.5816211	0.136372906	0.993970368	24.56685404
0.342188736	-13.1434879	0.031847546	0.970569925	27.84588499
0.265990956	331.9608953	0.017072819	0.979696769	68.72669436
0.292103991	17.55877989	0.043762665	0.937652638	18.47736567
0.449420597	-56.24191052	0.104914409	0.993548244	26.89400184
0.635255935	-34.96323212	0.17282486	0.995226177	11.50424755
0.342557112	48.8020549	0.03194745	0.982042581	30.58374901
0.336888708	8.222762331	0.040288129	0.955143918	19.34120821
0.623739845	-175.3845378	0.213571789	0.994418874	25.50798968
0.357957514	13.05494199	0.038832644	0.976111625	27.84954707
0.460472834	-31.79489761	0.081319831	0.991107877	20.4050716
0.555529528	-35.66079929	0.181185162	0.994449904	20.79061626
0.361243258	64.99689319	0.032361736	0.981675139	28.20426677
0.251512489	1666.609612	0.018801468	0.974250184	128.4321084
0.209899001	63.09295076	0.025522699	0.952621132	65.49226234
0.284345303	35.00622236	0.021902327	0.970307444	34.42460503
0.563222448	-44.51476107	0.16920031	0.987509616	13.42783667
0.320634703	-1.418937905	0.026777178	0.981860135	33.6750858
0.305023768	130.7789131	0.042270606	0.963820298	30.39141747
0.589014856	-133.5916011	0.256670279	0.992929621	23.74994312
0.511536251	-58.69197342	0.085941058	0.98786749	18.18208769
0.471127109	-56.63568889	0.089403531	0.984949337	21.8262298
0.210101787	133.2210577	0.029574807	0.941566553	52.31419051
0.380823691	-106.4087592	0.029098574	0.986303174	34.56594878
0.642870595	-78.40823952	0.311620695	0.993943795	16.1971153
0.280784843	1250.997365	0.024193762	0.975201869	112.5688569
0.323614495	64.10184954	0.024731017	0.977556605	27.13022942
0.233017683	-117.9853658	0.020517413	0.958717477	61.64182971
0.531692476	-46.08943814	0.133231073	0.989698096	16.82784817
0.467090675	-24.16524556	0.091313861	0.98746582	20.52232456
0.53879352	-53.15749888	0.152717896	0.989942118	19.1192995
0.429993271	2.210681511	0.053177515	0.984245558	16.85205404
0.277250902	-14.74888555	0.017886562	0.977635686	39.91921738
0.363302172	61.04626317	0.030872212	0.985904257	29.19611975
0.228314988	12.35037659	0.034300295	0.949889004	47.35030838
0.256376552	19.90497988	0.040298895	0.943786569	21.52051336
0.51994578	-86.28897593	0.170217696	0.991827223	29.23089902

log.sigma.3.0.mm.3D_glcm_Contrast	log.sigma.3.0.mm.3D_glcm_DifferenceEntropy	log.sigma.3.0.mm.3D_glcm_InverseVariance	log.sigma.3.0.mm.3D_glcm_Entropy	log.sigma.3.0.mm.3D_glcm_Dissimilarity
12.1884386	2.923778782	0.313929164	7.017632679	2.640672664
16.63586472	2.995696847	0.281838822	6.07829822	3.231504111
16.58129792	3.095519336	0.294499758	7.130236706	3.026475415
18.36872966	3.017840427	0.283898621	5.904987615	3.310931823
9.705213624	2.799291041	0.327185564	6.544789284	2.353273172
31.95561334	3.386050631	0.196702216	6.171790339	4.495977027
2.663919345	1.79587881	0.344982669	4.233876742	0.854148647
5.206332192	2.404471717	0.383076744	6.09299659	1.569798003
3.095176139	2.068767381	0.399781315	5.130860561	1.155702156
11.88746136	2.87752419	0.316088838	6.714119549	2.691408028
2.729278882	1.876834002	0.425798729	4.693271243	0.969487472
3.211037021	2.068198452	0.407984252	5.167358133	1.143920982
26.82137892	3.414251684	0.230106115	7.312941287	4.037691077
11.92028266	2.853409436	0.326872629	6.258071251	2.66591966
6.198146948	2.51203035	0.373285074	6.273191589	1.848241301
3.972427306	2.095697498	0.371150865	4.871902798	1.172887831
5.260055598	2.369074194	0.416031684	5.950247049	1.757896728
9.617914161	2.677390813	0.346692347	5.678725532	2.3972217
4.924160347	2.332735765	0.411235094	6.014917902	1.692779778
4.166696439	2.198169166	0.391941832	5.524905678	1.270362085
6.913369478	2.603353154	0.341618339	6.28111853	1.905136413
9.313656293	2.722763366	0.334077287	5.834329882	2.339562272
8.482672384	2.665319734	0.342962982	6.441544506	2.237215288
1.690935882	1.709640126	0.393174122	4.256010348	0.789638416
9.550572848	2.726574898	0.331706808	6.262683852	2.120761193
9.426405548	2.648374168	0.38873712	6.471906926	1.975185494
7.940819987	2.626342969	0.371241172	6.707709573	2.128212029
4.176465719	2.270707171	0.425062882	5.944171814	1.479508757
19.22536432	2.415972841	0.204405608	4.239274344	3.637559629
5.363755098	2.431107473	0.388172675	6.186786453	1.726074459
4.815287418	2.34179884	0.388155713	5.876092786	1.506323709
13.26111053	2.933368228	0.320800602	6.461916679	2.702509674
7.333246389	2.582538119	0.388982472	6.426148813	1.905965395
3.637980296	2.126695759	0.393354169	5.165360285	1.206265009
5.441870557	2.42781272	0.394404535	6.070823062	1.629580653
11.98964765	2.887750323	0.350851812	7.070890268	2.426073149
48.40043057	3.699426405	0.177618879	7.450274088	5.461163291
3.639197698	2.018045172	0.364089359	4.791651288	1.069845927
5.874300084	2.424815062	0.374292244	5.996111698	1.909237741
3.602267267	2.011007321	0.395434775	4.824492381	1.076596209
7.320627232	2.60190796	0.36542518	6.437470302	2.085351254
2.464130903	1.866535962	0.411189074	4.499669948	0.938634079
12.45873802	2.843128258	0.334435898	7.091800342	2.56264053
8.509263896	2.703986352	0.338655008	6.659578204	2.252610877
2.907859827	2.046381382	0.414340414	5.252119451	1.157456585
6.651030203	2.536544039	0.382105751	6.271945271	1.791202252
2.597353261	1.959037139	0.402059181	5.055165449	1.019905444
6.207945044	2.418921363	0.372808397	6.271673458	1.971743409
13.17649496	2.827204775	0.348915153	5.848865206	2.741006326
69.10178411	3.893487318	0.182048984	7.489570967	6.081097814
3.371875604	2.10815373	0.386971676	5.11092586	1.196779826
12.34268371	2.872922293	0.361348877	6.962397944	2.394391057
1.64779448	1.648875705	0.384116363	3.992007311	0.73758233
7.00270974	2.432751395	0.368376447	6.618067754	2.058942746
17.02280607	3.120618114	0.268301743	6.862469608	3.192005691
9.290783515	2.734613722	0.352115607	6.548194556	2.28417661
3.502431186	2.109765796	0.402820014	5.306331501	1.18689121
7.496660586	2.651387475	0.350642385	6.449735102	1.983332866
8.489857036	2.662504677	0.361683783	6.374399416	2.234797842
1.95419142	1.692133107	0.406772966	4.076723514	0.791985527
12.54245585	2.943151693	0.324759776	6.864298562	2.585055098
16.03457152	3.093030483	0.316775703	7.403367959	2.87192168
21.30821683	3.246224478	0.308402242	7.768527417	3.1914331
6.910634167	2.46098966	0.359051592	5.834357001	1.597562857
8.26677671	2.607036256	0.35225443	6.511518572	2.25428249
24.0084037	3.373330255	0.268884952	8.070317723	3.630339975
12.50827019	2.831339006	0.306417383	6.079610231	2.810239061
9.30823013	2.69246298	0.364420963	6.522071813	2.003740928
2.411652087	1.885484222	0.417846066	4.75285862	0.961870118
12.14958676	2.92593199	0.335315172	7.053885443	2.558389993
10.16556642	2.785825409	0.325368721	6.190821686	2.451396324
3.641060969	2.090572527	0.361371132	4.978021296	1.133980263
8.372107503	2.686176542	0.340689538	6.615423312	2.230009804
5.866625135	2.455613538	0.3984509	6.045742768	1.699141935
5.597532984	2.344284794	0.373335716	5.669853032	1.447890961
8.693525373	2.679040569	0.362437107	6.649054304	2.222878716
31.32961282	3.523683073	0.253879584	8.182528122	4.074456587
28.91392325	3.348300669	0.227265651	6.527019369	4.230650192
15.89935994	3.10257764	0.289416518	7.250303562	3.063828731
2.991319455	2.068124123	0.428611086	5.193820743	1.18550645
12.1884386	2.923778782	0.313929164	7.017632679	2.640672664
14.83306982	3.038491003	0.288055807	6.75377253	2.918028599
4.281589779	2.202415664	0.360715803	5.15178403	1.275247717
3.257047969	2.115448119	0.447879845	5.640798377	1.322029344
5.863009606	2.46504146	0.393235798	6.14068601	1.687162056
30.50203651	3.441369384	0.211974299	6.920526712	4.361814943
8.405059603	2.660658903	0.373584533	6.763507431	2.132990812
2.803137093	1.970464112	0.358399307	4.661061386	1.003251165
34.12271996	3.525501591	0.278122143	8.081110651	4.069251367
11.77050088	2.911559959	0.321360692	6.886315423	2.610589559
24.63651692	3.334810739	0.237445653	7.27520675	3.904730167
3.515097182	2.151300692	0.425083014	5.537748766	1.309295946
6.491136665	2.525608362	0.389882167	6.297593527	1.753083412
4.756722919	2.298124091	0.401344653	5.731421498	1.414107953
6.10208638	2.493862534	0.397901146	6.247406425	1.804247054
19.12274789	3.21973952	0.280333703	7.554646588	3.282352176
11.85913229	2.885490327	0.346264027	6.94545238	2.461331665
25.79679953	3.217820853	0.211975258	6.161083932	3.971396422
16.77872735	2.98906275	0.269360463	5.803571469	3.279784226
6.781979448	2.490460276	0.369871415	6.158457614	1.648008981

log.sigma.3.0.mm.3D_glcm_DifferenceVariance	log.sigma.3.0.mm.3D_glcm_Idn	log.sigma.3.0.mm.3D_glcm_Idm	log.sigma.3.0.mm.3D_glcm_Correlation	log.sigma.3.0.mm.3D_glcm_Autocorrelation
5.114268186	0.910099003	0.320634703	0.466999663	138.0926284
5.92431169	0.836839263	0.253774299	0.174230183	40.18539965
7.11281479	0.879336879	0.300561787	0.401382358	82.63356529
6.813847582	0.850849502	0.272027957	0.236675574	53.99861641
4.098253261	0.905537352	0.353800711	0.381813693	78.73819488
11.27311695	0.862718232	0.20412914	0.281515504	211.1108842
1.919347483	0.969392425	0.700558688	0.649855574	141.0897729
2.686000971	0.947990558	0.498103178	0.676528896	230.1414802
1.708769198	0.950951307	0.585111922	0.697246921	138.1375611
4.485941503	0.897308693	0.301792704	0.330215392	73.43411776
1.769280122	0.969359508	0.640491413	0.521851701	417.0992286
1.878834944	0.949503133	0.593010499	0.690609523	105.3431928
10.15909319	0.866499609	0.221452966	0.261102107	103.6375318
4.632776814	0.867928386	0.308756221	0.223222201	48.37863065
2.695681028	0.916754974	0.423924429	0.621333679	121.5516947
2.516765334	0.9534153	0.617933885	0.689476337	126.3201259
2.105549883	0.895580408	0.413478537	0.489728778	33.3539272
3.784491452	0.87221009	0.333009015	0.115098363	34.54381406
1.952331641	0.892624638	0.430494864	0.552753423	48.81403595
2.525851808	0.966694158	0.578635306	0.630736611	193.9342176
3.217631282	0.915138711	0.431980264	0.55288873	64.84557453
3.740829848	0.860094261	0.348881921	0.205524407	23.53955588
3.329684599	0.885758808	0.35582913	0.522470205	77.93713972
1.056001307	0.964193689	0.683563656	0.707994007	111.9000744
4.815035033	0.908608808	0.438482089	0.609336668	123.3224505
5.423825732	0.940883698	0.454244282	0.561868287	137.8625452
3.223516369	0.916704853	0.372079065	0.559039136	106.2355831
1.941816548	0.928533281	0.488582333	0.671876039	129.1790638
5.023486159	0.797527139	0.202319813	0.065741502	31.37204278
2.313255161	0.913586303	0.440644711	0.598546776	75.87686454
2.453589255	0.937365383	0.51026585	0.685486092	104.191421
5.797218406	0.885359502	0.32773961	0.31428559	78.71426441
3.613405285	0.925584281	0.432377233	0.563883327	91.34604854
2.145216217	0.947206811	0.588059802	0.694683914	129.6159503
2.742650914	0.937746558	0.47820121	0.581279028	149.6215155
5.998683468	0.915196746	0.380414004	0.597126746	199.2239332
15.98834074	0.853578374	0.167131347	0.097842147	129.5689385
2.464323112	0.964563464	0.645460632	0.681542478	162.1749524
2.151505708	0.88730473	0.383713219	0.534343988	56.34812327
2.42043324	0.976685571	0.632156926	0.56374219	661.473522
2.899283526	0.906712346	0.370022223	0.466690406	94.45259601
1.566898849	0.96164829	0.649170725	0.471510604	120.8829636
5.223012115	0.916166413	0.34609084	0.518802456	147.4272008
3.324400556	0.904603282	0.353960967	0.500535474	102.3158943
1.525769638	0.929348446	0.573198226	0.722999602	75.78111464
3.366258496	0.934905323	0.458029836	0.593014773	136.0143048
1.538649497	0.961428738	0.621520509	0.720699855	134.0092965
2.110384489	0.896414276	0.377690177	0.675752876	58.4037679
5.475320012	0.866029848	0.315009474	0.203125866	35.5614888
30.90901467	0.865783142	0.171743908	0.289527865	173.1675488
1.876498939	0.9391868	0.58336138	0.742652841	136.7381471
6.509119336	0.933100913	0.384911677	0.448414907	145.6826501
1.094849941	0.96809349	0.705491256	0.667098573	115.6577193
2.243612296	0.907505476	0.368516843	0.65973535	115.3844732
6.708908211	0.87231754	0.268717133	0.337597138	48.66673173
4.004592959	0.91158403	0.355254003	0.419972147	63.6968405
2.054553811	0.95571861	0.587327821	0.667983262	175.3818254
3.492680538	0.922569784	0.425204844	0.602069359	103.2020504
3.432000052	0.891405124	0.351351503	0.447618131	85.11448462
1.315199203	0.974717065	0.690312517	0.616218824	142.1724391
5.736778967	0.902647847	0.350016854	0.3894828	135.6189185
7.608645918	0.913589996	0.332050185	0.466036785	181.6219754
10.79865271	0.916539539	0.335008713	0.422756572	243.6081728
4.321160806	0.956907826	0.541531732	0.558495793	253.1834316
2.985212056	0.884303917	0.342188736	0.539217138	70.0023779
10.54015706	0.907472481	0.265990956	0.485131589	212.9041156
4.516869576	0.83655901	0.292103991	0.194036428	48.19182933
5.206310598	0.951564327	0.449420597	0.484685795	202.3662699
1.468772636	0.960538186	0.635255935	0.650812702	94.19550845
5.5078363	0.913192828	0.342557112	0.430418841	86.73234061
4.005798684	0.862987147	0.336888708	0.306514347	62.01535072
2.334401259	0.959575959	0.623739845	0.747778904	297.2562004
3.277676481	0.896656194	0.357957514	0.536029444	112.7870473
2.945302631	0.939892124	0.460472834	0.552596467	88.36325629
3.45925134	0.958205415	0.555529528	0.573681625	150.5965807
3.614016878	0.910022841	0.361243258	0.527436112	84.01246744
14.14649135	0.898562934	0.251512489	0.612780773	170.4877841
10.42707027	0.856233339	0.209899001	0.391208164	118.5096957
6.365686229	0.886107552	0.284345303	0.367494386	98.5483429
1.558301459	0.931855965	0.563222448	0.632877155	94.20564646
5.114268186	0.910099003	0.320634703	0.466999663	138.0926284
6.135422125	0.877219427	0.305023768	0.337152066	53.64617853
2.593099146	0.953089333	0.589014856	0.690588217	161.3282473
1.471820178	0.928017133	0.511536251	0.693712994	115.8574402
2.955237814	0.924247736	0.471127109	0.574524927	101.0394829
11.0397358	0.841774939	0.210101787	0.261238932	107.0329228
3.757000083	0.923078132	0.380823691	0.604423578	172.9736065
1.779084337	0.957579182	0.642870595	0.70221229	122.7111506
16.93497719	0.904897341	0.280784843	0.541338649	220.4583578
4.850794368	0.900646955	0.323614495	0.393776738	87.87613377
8.873494058	0.865242698	0.233017683	0.435213961	117.2679839
1.742757263	0.936317231	0.531692476	0.647902251	102.250433
3.373968161	0.931225394	0.467090675	0.517307257	109.9257341
2.723407868	0.941446104	0.53879352	0.599547561	100.6074515
2.785514292	0.91872788	0.429993271	0.462457606	88.53958869
8.114616771	0.901997533	0.277250902	0.350667163	177.2733589
5.649730719	0.924346755	0.363302172	0.422196936	81.68776082
9.563898345	0.853401818	0.228314988	0.294601711	105.2614092
5.64028354	0.842579206	0.256376552	0.130921533	51.22033094
4.022235993	0.948301956	0.51994578	0.619935962	178.4164274

log.sigma.3.0.mm.3D_glcm_SumEntropy	log.sigma.3.0.mm.3D_glcm_AverageIntensity	log.sigma.3.0.mm.3D_glcm_Energy	log.sigma.3.0.mm.3D_glcm_SumSquares	log.sigma.3.0.mm.3D_glcm_ClusterProminence
4.504761909	11.51986007	0.010289791	11.4658811	3688.485896
3.964873994	6.199235447	0.017524862	10.03910399	1750.687185
4.548713372	8.780964396	0.009406495	13.80609347	3681.962302
3.954275234	7.163440816	0.019950966	11.83770766	2871.57297
4.167195405	8.702038458	0.013915461	7.85935023	1292.871292
4.353871309	14.32133036	0.01569026	22.65865207	8771.740568
3.184758532	11.77165476	0.136125334	3.848803698	1072.309135
4.194225913	14.98956981	0.035977823	8.131824566	2281.332546
3.733342523	11.5964306	0.064844404	5.200308243	968.5793269
4.230571777	8.394262387	0.012118399	8.897504612	1635.371692
3.362572174	20.40571144	0.085469514	2.900175154	609.1572963
3.740689006	10.08511012	0.065126849	5.2369223	1114.117418
4.608358394	9.938052838	0.008043152	18.26900097	7392.886232
3.981727582	6.833349101	0.016197744	7.636267976	1077.59405
4.241331318	10.78961918	0.020600206	8.225406774	1678.40503
3.581362142	11.03513162	0.10153727	6.527352728	1860.029691
3.921968812	5.549788949	0.019514294	5.180165822	573.7162521
3.615847118	5.823285537	0.02381657	5.436402198	547.6124593
4.013755731	6.763143225	0.020369186	5.532085901	686.96197
3.882229764	13.8100168	0.060308674	5.791091417	2142.829887
4.177417437	7.780872063	0.022367774	7.758344771	1503.165071
3.719222424	4.723993925	0.022048651	5.865533876	749.3761798
4.271005494	8.557775947	0.014210891	8.93843343	1608.335059
3.212235449	10.47923095	0.126117962	2.929034307	595.1794866
4.336028351	10.76194706	0.031783	12.27177066	3313.397927
4.368345335	11.50250272	0.021801174	11.04955615	7624.152456
4.392712886	10.05720503	0.012321974	9.03711861	2162.754118
4.101849563	11.17359584	0.02742792	6.412906417	1264.201084
2.996719634	5.532356347	0.056059863	10.27816752	1146.691389
4.164133798	8.475897661	0.021824286	6.712550486	1239.264876
4.110233062	9.941764395	0.034435737	7.749166832	1606.976826
4.207124215	8.702877042	0.015256362	9.598429711	2143.994663
4.289056627	9.304487456	0.018142192	8.43327726	1781.722615
3.765191067	11.19859241	0.075171767	6.022766582	1538.999684
4.08895145	12.07499808	0.026839416	6.531140848	1159.878743
4.65805537	13.79007065	0.012555037	15.02819611	6311.524056
4.682237606	11.36974573	0.00694791	26.35138223	9211.841113
3.535543154	12.57974136	0.10304217	5.793176625	1969.802587
4.036535908	7.273803241	0.01999697	6.362466003	1090.641094
3.490085545	25.69245705	0.098306661	4.270329411	1234.509446
4.159220618	9.551145469	0.014603106	6.881220643	1098.23786
3.192129286	10.94356341	0.103373827	2.352650993	370.7182431
4.610524485	11.85754204	0.010538537	13.03522411	4538.904728
4.325285255	9.900298691	0.012500853	8.545536584	1690.919204
3.807479839	8.481593173	0.060228809	5.295093205	985.9669643
4.25169025	11.44933142	0.023576768	8.246738871	1874.126541
3.721388666	11.42761212	0.075618997	4.714585415	1193.692564
4.341809009	7.196723402	0.016487252	9.702619987	2268.729795
3.807142647	5.82146587	0.021481735	8.242060044	1429.139512
5.091904792	12.93543369	0.006322351	51.31907754	61714.62614
3.745425198	11.47862258	0.084242576	6.660525437	1871.512724
4.476975929	11.88219921	0.013613017	11.52063243	4908.461572
3.026974258	10.67639555	0.14449801	2.495528994	414.839586
4.50337461	10.41192822	0.012658225	10.45195514	2618.212544
4.378997614	6.652877974	0.010732202	12.90561216	4623.009864
4.207975352	7.767471583	0.013562777	8.003808298	1648.748997
3.818242452	13.10501481	0.065941939	5.386626322	1384.697263
4.321009563	9.873565058	0.018647648	9.456588329	1882.006998
4.142319152	9.036350156	0.015046302	7.694937319	1224.442594
3.040468802	11.8575776	0.131418393	2.583034007	633.1929824
4.380681296	11.47198605	0.012599948	10.2794725	2432.907618
4.705992402	13.2301863	0.009576866	15.34269552	6051.206859
4.843638105	15.45801446	0.00863485	18.97483923	9193.247302
3.997443273	15.80235676	0.051559237	7.992522254	2457.233953
4.294630509	8.06804088	0.013606425	9.028165426	1654.361043
5.023957633	14.31977649	0.005735105	24.15649998	18076.69108
3.908127656	6.832153813	0.017518031	7.746408964	865.990138
4.284771493	14.12251397	0.024053638	9.334220186	3175.875444
3.454844276	9.587570267	0.083514145	3.47897491	550.3750278
4.451879487	9.062154499	0.010608958	10.68333394	3174.546974
3.991802223	7.727380273	0.016411587	7.376693657	840.2348601
3.649265433	17.08177017	0.09052459	7.287262663	2560.048013
4.340451699	10.38750771	0.012877725	9.055413644	1781.132015
4.07637869	9.204563885	0.024970735	6.567924183	1670.124868
3.92289853	12.1239788	0.055954266	6.717930515	2254.918097
4.352174323	8.895411383	0.013475873	9.224448037	2903.090032
5.225852931	12.2201633	0.005484655	41.54707451	61956.03117
4.542797889	10.45286637	0.011934781	23.6015464	9756.773294
4.528939144	9.690647251	0.008683553	12.58099124	3636.035518
3.680694142	9.57041849	0.053057758	4.104789031	595.8063684
4.504761909	11.51986007	0.010289791	11.4658811	3688.485896
4.327942364	7.052295065	0.012559006	11.30612182	3274.390144
3.730234095	12.50821373	0.087487513	7.007883225	2056.529853
3.960961585	10.58860524	0.031076172	5.359783914	986.5270043
4.121140806	9.851086359	0.025350008	6.922309851	1282.975888
4.57820153	10.07603431	0.00977269	20.70405676	6934.847261
4.49573098	12.9003807	0.012847377	10.7427521	4004.155992
3.442427756	10.92521982	0.118769127	4.750063099	969.1779123
5.20114988	14.35277122	0.006518764	37.96655558	53533.15788
4.346550374	9.167007109	0.010939284	9.725182576	2374.146837
4.766724765	10.39193406	0.007736382	21.56958666	9354.364132
3.880858288	9.945408786	0.038726528	5.085736338	856.7069389
4.156527877	10.3158514	0.023780356	6.753365306	1343.651799
3.970715436	9.849596906	0.044899523	5.969005605	1202.17353
4.045423216	9.265465619	0.020625578	5.738535105	966.4319785
4.657174784	13.13469268	0.007239313	14.97384527	4913.617608
4.395818398	8.798222199	0.012196555	10.34305115	4008.128727
4.311445531	9.992297604	0.015539832	18.28677698	5569.608023
3.845573843	7.070962572	0.019754281	9.574810178	1265.728054
4.210515264	13.14861998	0.046475721	9.052810114	3617.142755

log.sigma.3.0.mm.3D_glcm_SumAverage	log.sigma.3.0.mm.3D_glcm_Imc2	log.sigma.3.0.mm.3D_glcm_Imc1	log.sigma.3.0.mm.3D_glcm_DifferenceAverage	log.sigma.3.0.mm.3D_glcm_Id
23.03972014	0.78102902	-0.128587253	2.640672664	0.403849289
12.39847089	0.908016427	-0.2547514	3.231504111	0.345506811
17.56192879	0.818414608	-0.148970926	3.026475415	0.384636571
14.32688163	0.953334966	-0.341512707	3.310931823	0.358413777
17.40407692	0.698947153	-0.100564996	2.353273172	0.432608031
28.60700415	0.987755804	-0.466406708	4.495977027	0.297530617
23.54330952	0.823307868	-0.242795819	0.854148647	0.72187606
29.97513978	0.811615311	-0.171220116	1.569798003	0.550974259
23.1928612	0.826457686	-0.214594219	1.155702156	0.623043159
16.78852477	0.763275011	-0.123754539	2.691408028	0.387926994
40.77258988	0.748182081	-0.16774992	0.969487472	0.666069604
20.17022024	0.831131882	-0.211546943	1.143920982	0.628452602
19.87610568	0.869102312	-0.176775122	4.037691077	0.314596429
13.6666982	0.845511174	-0.184505536	2.66591966	0.392744067
21.57923835	0.79532475	-0.156560271	1.848241301	0.490408701
22.07026324	0.834550465	-0.233714797	1.172887831	0.650490634
11.0995779	0.719771378	-0.121874943	1.757896728	0.480037942
11.64657107	0.840328581	-0.196461977	2.3972217	0.413857784
13.52628645	0.754287834	-0.139831074	1.692779778	0.494563204
27.59176473	0.810149219	-0.182982967	1.270362085	0.616867155
15.56174413	0.757904768	-0.133843487	1.905136413	0.498716085
9.44798785	0.781006511	-0.151638646	2.339562272	0.428274541
17.11555189	0.830163408	-0.170417494	2.237215288	0.434585446
20.95846191	0.807564596	-0.228684263	0.789638416	0.70526424
21.52389412	0.865110704	-0.213451955	2.120761193	0.500981659
22.9655154	0.816182962	-0.163631855	1.975185494	0.510871891
20.11441006	0.763003852	-0.133086286	2.128212029	0.446210251
22.34719168	0.802550478	-0.169371177	1.479508757	0.541204998
11.06471269	0.985379338	-0.608947495	3.637559629	0.306691578
16.95179532	0.763261041	-0.139297123	1.726074459	0.503844586
19.88352879	0.821309891	-0.188789768	1.506323709	0.560804209
17.40575408	0.867230212	-0.196853515	2.702509674	0.407786342
18.60897491	0.796074673	-0.15208324	1.905965395	0.494534406
22.39718482	0.831894039	-0.215286377	1.206265009	0.624924651
24.14999616	0.765367186	-0.142408753	1.629580653	0.53371453
27.58014129	0.840571165	-0.164724601	2.426073149	0.450083387
22.61468939	0.921923894	-0.237119459	5.461163291	0.260675684
25.15691215	0.827667691	-0.223794892	1.069845927	0.674119607
14.54760648	0.859430177	-0.20343175	1.909237741	0.458720182
51.3462996	0.789737821	-0.190501442	1.076596209	0.660875051
19.10229094	0.714674235	-0.109684073	2.085351254	0.445902494
21.88712682	0.69511129	-0.144785004	0.938634079	0.674570619
23.71472387	0.786825405	-0.149942436	2.56264053	0.423068236
19.80059738	0.736718146	-0.116958175	2.252610877	0.433183385
16.96318635	0.841424533	-0.220929177	1.157456585	0.612567513
22.89866284	0.807219625	-0.164062041	1.791202252	0.516717124
22.85522425	0.842476303	-0.226849957	1.019905444	0.652995787
14.3934468	0.895685795	-0.240739319	1.971743409	0.452885457
11.64293174	0.910023394	-0.266737054	2.741006326	0.394154909
25.28822421	0.973974725	-0.32936422	6.081097814	0.261291147
22.95724517	0.839724357	-0.227010607	1.196779826	0.622145948
23.71867676	0.754742549	-0.118816524	2.394391057	0.453488649
21.3527911	0.784903243	-0.220688599	0.73758233	0.723772708
20.82385644	0.853991447	-0.198216693	2.058942746	0.444923805
13.30575595	0.833891348	-0.16024616	3.192005691	0.359489409
15.53494317	0.727100842	-0.111404975	2.28417661	0.432709695
26.21002962	0.819227101	-0.202015519	1.18689121	0.623858019
19.74713012	0.789044452	-0.146863788	1.983332866	0.491039124
18.07270031	0.808631183	-0.155808674	2.234797842	0.429412733
23.71331755	0.755013098	-0.195455263	0.791985527	0.70897504
22.94397209	0.765697758	-0.124258461	2.585055098	0.426407529
26.4235211	0.768421665	-0.119517063	2.87192168	0.409363672
30.71591534	0.747906158	-0.105296039	3.1914331	0.408642538
31.54470361	0.817095283	-0.177463298	1.597562857	0.58635746
16.13608176	0.836632126	-0.177226198	2.25428249	0.422841017
28.40466031	0.739665739	-0.097170081	3.630339975	0.352056499
13.66430763	0.879596447	-0.220593148	2.810239061	0.378856175
28.13994264	0.761521077	-0.131321581	2.003740928	0.508753661
19.17514053	0.803997279	-0.20509197	0.961870118	0.662947213
18.124309	0.703530032	-0.096441813	2.558389993	0.420154823
15.45476055	0.784369166	-0.147108305	2.451396324	0.417988964
34.16354034	0.844652408	-0.229579866	1.133980263	0.656562389
20.77501542	0.753812387	-0.127755256	2.230009804	0.436422479
18.40912777	0.784913256	-0.150671508	1.699141935	0.518683972
24.23167795	0.820338211	-0.185310374	1.447890961	0.598163975
17.79082277	0.792044483	-0.145364879	2.222878716	0.437209703
24.18259732	0.83038709	-0.137271306	4.074456587	0.337368795
20.90573275	0.984108984	-0.423657433	4.230650192	0.303042872
19.3812945	0.745858536	-0.108358144	3.063828731	0.371338363
19.14083698	0.778220588	-0.172585223	1.18550645	0.603016297
23.03972014	0.78102902	-0.128587253	2.640672664	0.403849289
14.10459013	0.772984501	-0.128785547	2.918028599	0.389874393
25.01642747	0.832252188	-0.217995219	1.275247717	0.627668898
21.17721048	0.805803132	-0.179669434	1.322029344	0.559376878
19.70217272	0.773995756	-0.147289707	1.687162056	0.52739526
20.15206861	0.943763219	-0.278170829	4.361814943	0.302670914
25.80076141	0.841765069	-0.172714695	2.132990812	0.452779704
21.85043965	0.831300811	-0.230997601	1.003251165	0.673923472
28.34289627	0.867355541	-0.161849256	4.069251367	0.359247516
18.33401422	0.695263704	-0.095002624	2.610589559	0.405742271
20.78386812	0.919799072	-0.228384562	3.904730167	0.323869052
19.89081757	0.800919428	-0.183970453	1.309295946	0.577168532
20.63170279	0.735638662	-0.122325069	1.753083412	0.523500147
19.69919381	0.807300849	-0.174995489	1.414107953	0.583063035
18.53093124	0.653935092	-0.093040185	1.804247054	0.493547191
26.23428773	0.705596966	-0.090435592	3.282352176	0.364203037
17.58968751	0.708778876	-0.102390374	2.461331665	0.436814198
19.98459521	0.978757738	-0.413983973	3.971396422	0.322379054
14.14192514	0.961814386	-0.370396643	3.279784226	0.347287508
26.29075075	0.824474542	-0.176057213	1.648008981	0.567780163

log.sigma.3.0.mm.3D_glcm_ClusterTendency	log.sigma.3.0.mm.3D_firstorder_InterquartileRange	log.sigma.3.0.mm.3D_firstorder_Skewness	log.sigma.3.0.mm.3D_firstorder_Uniformity
33.6750858	123.1934509	0.053937413	0.081090099
23.52055125	134.454422	0.489149036	0.09092333
38.64307597	149.8657913	0.207679995	0.074341268
28.98210098	130.410305	0.585563179	0.085066163
21.7321873	105.3616428	0.539785336	0.099371806
57.77336892	152.1528969	-0.322563276	0.067207088
12.73129545	26.56176203	-1.314585053	0.261026248
27.23245998	112.9681344	-0.649804464	0.118342796
17.70605683	88.90851927	-0.862768242	0.164088383
23.70255708	98.40797877	0.047337791	0.09509721
8.720923169	32.94973564	-1.381702153	0.229291446
17.73665218	74.4712435	-0.793483818	0.167385993
46.25462496	159.2599602	0.5216607	0.064265569
18.62478924	94.32572174	0.338217175	0.098060942
26.70348015	120.1721115	-0.329420093	0.10055293
22.13698361	59.8379041	-1.169575571	0.213794594
15.46060769	86.22091675	0.316476257	0.118592711
12.12769463	77.51013184	0.698691128	0.123772421
17.20418325	82.75862694	-0.149310373	0.118850101
18.59038744	49.1099844	-0.124609754	0.166033773
24.12000961	114.7290821	0.309036953	0.103959391
14.14847921	85.87899208	0.756296033	0.118811088
27.27106134	123.7183647	-0.214796884	0.09436304
10.02520135	39.56753387	-1.388958892	0.256183267
39.53650978	155.7813134	-0.410041423	0.10796166
33.442969	97.5883255	0.973306977	0.108208139
28.20765445	103.6147995	-0.03953173	0.093370806
21.47515995	97.50853682	-0.563232001	0.115628841
21.88730576	107.4219754	0.306336292	0.114795918
21.48644685	101.6382341	-0.163263815	0.109821022
26.18137991	118.0564208	-0.507860263	0.117646691
25.13260831	91.9429245	0.466502892	0.096255286
26.39986265	109.3538971	-0.206987775	0.102178023
20.45308603	72.61503744	-0.805414235	0.179644503
20.68269284	102.2163477	-0.258792431	0.115165198
48.12313678	147.4827023	-0.596284117	0.078039334
54.25161623	179.6031189	0.340936545	0.059552926
19.46169278	42.56103051	-1.248289169	0.219348198
19.57556393	93.95671082	0.106935702	0.104795279
13.05059937	42.84242111	-0.413476242	0.217905763
20.20425534	92.16520691	0.174876802	0.104864615
6.946473069	32.6519804	-0.511268135	0.239864598
39.66646289	128.6256695	-0.082176237	0.077638954
25.67288244	110.30019	0.203795086	0.092381899
18.27251299	82.28729987	-0.807070851	0.160701976
26.33592528	114.9486332	-0.175503953	0.104168078
16.2609884	61.29312086	-0.981896514	0.176827762
32.6025349	135.3019109	0.250409248	0.091136548
19.79174522	102.5288143	0.564203693	0.098491844
121.9982985	172.4999971	1.104375961	0.056115702
23.27022614	100.3239165	-1.074745745	0.17634267
32.47871958	102.3415627	0.50064715	0.090820313
8.334321497	29.63574734	-1.401303794	0.281962411
34.80511081	137.4414425	-0.134177093	0.083142252
34.59964258	129.0941238	0.705561227	0.078731076
22.72444968	103.6500168	0.508713546	0.098899149
18.0440741	69.55983893	-0.868842103	0.164921442
30.32969273	134.72645	-0.121676318	0.095561211
22.28989224	104.1177998	-0.022721634	0.09939987
8.320766641	26.12972331	-1.171041033	0.27695708
28.57543414	120.0806389	0.105056415	0.087843529
44.29384799	140.3789387	0.193405044	0.072721181
52.19599926	132.5803519	0.068082232	0.070753432
24.56685404	91.03473321	-0.655516427	0.142171995
27.84588499	115.9995155	-0.097964316	0.089294553
68.72669436	135.8038483	0.601533166	0.066181841
18.47736567	113.6867218	0.151480494	0.098933691
26.89400184	102.6059718	-0.032204525	0.105287533
11.50424755	54.66966939	-0.499976015	0.197423916
30.58374901	119.0291252	0.446442872	0.083748583
19.34120821	119.5437889	0.017453979	0.099377615
25.50798968	78.57476377	-1.322362424	0.194597939
27.84954707	117.274025	0.082378107	0.093862578
20.4050716	85.67592812	0.130071033	0.117564137
20.79061626	74.95911145	-0.260171008	0.153579893
28.20426677	103.0874195	0.327804201	0.095774308
128.4321084	168.4731436	1.182133405	0.061462699
65.49226234	185.6283951	-0.013610527	0.060567108
34.42460503	126.099102	0.203125476	0.075934224
13.42783667	74.58851242	-0.868102497	0.162095401
33.6750858	123.1934509	0.053937413	0.081090099
30.39141747	119.9927597	0.719239444	0.084642445
23.74994312	93.11230636	-0.996026125	0.185925645
18.18208769	84.81980133	-0.679316602	0.127427071
21.8262298	92.73060894	-0.426951361	0.116241895
52.31419051	200.5248241	0.068536041	0.063081095
34.56594878	112.2080612	-0.349462385	0.088369084
16.1971153	69.04495841	-1.053563821	0.225115767
112.5688569	130.7785563	1.101613751	0.066477863
27.13022942	118.8824482	0.55508383	0.087509375
61.64182971	135.523026	-0.312715718	0.070127074
16.82784817	90.10800552	-0.520923591	0.133738569
20.52232456	92.02721035	-0.027427952	0.110860861
19.1192995	81.78618766	-0.385708353	0.139770675
16.85205404	75.35636044	0.26561796	0.116584267
39.91921738	136.056798	0.120608523	0.071908955
29.19611975	104.5127258	0.440860855	0.089354564
47.35030838	147.6835556	0.023860274	0.073061171
21.52051336	108.7432098	0.00719709	0.091450109
29.23089902	75.4286384	-0.431044232	0.131203445

log.sigma.3.0.mm.3D_firstorder_MeanAbsoluteDeviation	log.sigma.3.0.mm.3D_firstorder_Energy	log.sigma.3.0.mm.3D_firstorder_RobustMeanAbsoluteDeviation	log.sigma.3.0.mm.3D_firstorder_Median
72.42626308	12174033.38	50.75375999	-93.70762634
70.35102547	5878697.368	50.45893411	-218.6560974
80.47206475	13156940.16	59.56174855	-135.7139893
77.65035967	4934668.848	55.75114846	-226.282692
60.14112407	14169097.19	44.04136336	-123.8492126
94.90247191	3116409.747	65.4894171	-111.0630341
34.92282953	48982766.7	16.56946564	-2.769171238
62.27271756	52592258.66	45.98154925	-49.16261864
50.06781937	26612349.65	37.68053075	-29.80328751
60.34039215	3761017.724	42.65660411	-66.30292892
28.12229758	10103087.07	14.90818529	1.825404644
48.32134804	15715912.77	33.09697344	-14.07670498
96.21904264	13889780.84	70.37934997	-185.1061859
59.33567869	11230541.71	41.68104675	-236.6435547
62.90617401	14385647.42	48.67912518	-95.05734634
51.51169806	48955038.49	32.16378087	-8.327529907
48.55061602	3613561.009	36.04879574	-73.04541397
48.39603694	6657597.623	33.16020323	-240.2352905
47.62717952	4346940.134	34.0812762	-59.54584885
43.17069136	49743563.77	24.0050204	-5.869174957
60.2948379	19471318.84	45.38064651	-92.76174927
51.40671528	4630197.282	34.85472893	-169.4549255
62.57111268	5363240.83	47.74912958	-115.1153717
32.8797168	13137173.87	19.2616508	-10.25850439
77.36741817	12167753.05	62.00272754	-78.56332016
58.99627806	18838618.63	39.03653363	-51.03718948
60.499532	17658013.98	42.92684062	-115.9038391
54.76707652	14718295.88	40.45316279	-50.83140182
64.9760667	212769.8752	44.94369955	-44.93682098
55.76680265	11911704.39	41.30619705	-72.47969055
60.93312199	34680150.88	47.5790494	-51.07286453
61.41016449	12934096.24	39.08866705	-213.37854
60.26024041	14575467.73	43.81574635	-67.49967194
51.57784309	17385498.47	33.97373129	-10.76146126
54.71415497	28720470.96	40.811093	-54.1646843
81.68697246	26854391.02	59.94821624	-100.2179222
102.6038123	26904828.56	75.22000605	-237.4621506
45.45507564	52737427.82	25.13728033	-3.060092568
54.22527754	3991947.588	37.50998014	-128.4238892
38.13979613	43754590.59	20.99952367	-7.092069149
54.08279333	12786139.63	37.94194514	-126.9934998
27.77725725	13828919.5	14.94346582	-7.500597954
74.90353514	33187867.77	53.17475479	-116.3012238
62.98897482	13961392.52	45.93189052	-120.8727341
49.04908518	6715474.61	35.26858617	-30.83646202
61.4465101	22787818.03	45.94464455	-69.9561615
44.91189113	29815313.88	28.29162116	-7.446573734
69.41593906	2849255.898	54.22661538	-90.61875916
63.7122112	2517256.555	46.31071495	-138.9892044
110.3508629	8580546.41	70.80001051	-143.267662
58.44435628	15342202.1	42.63460691	-24.15207005
65.54155388	24755840.58	42.34224901	-61.63486481
29.82667526	22148848.67	16.20154169	-6.029387712
70.43636641	6440740.966	54.27888245	-21.89476776
77.1083448	9772515.758	55.016405	-174.5935364
58.92469161	12594011.23	42.05987215	-121.1789703
48.5464922	28172998.73	31.58894269	-16.0225296
66.79606234	23095743.18	52.84206532	-88.5821991
58.88732918	8462845.106	42.63523074	-158.7264709
28.56047225	50193498.1	14.10919654	-4.859352112
67.21314028	12726407.58	47.96914636	-93.53312683
82.32388754	25711475.44	58.12277088	-65.58939362
83.89002549	76103329.41	55.94534477	-63.98399353
57.38568637	62742183.32	39.07154934	-20.36945534
63.96011202	3894027.562	48.28269031	-76.42199707
88.76559113	46005713.53	57.83589806	-95.29740143
59.17384866	6870465.717	43.90514492	-237.2424164
61.85579049	43567208.21	42.5910696	-54.11899185
38.09177939	30623015.39	24.7082378	-6.494909763
68.78903388	20649313.61	48.65613765	-83.05160522
60.7054065	4827723.359	47.48468203	-146.8050232
56.61180382	46268193.11	37.43459904	-7.178065777
61.90499337	17624326.23	46.19929409	-139.7720795
52.25026546	8712583.874	35.78386522	-52.71075439
49.44984293	17377457.18	32.98106307	-16.38345146
60.58877291	5588188.774	42.91041784	-56.68782616
112.9392578	34664438.15	72.99246056	-76.80815125
100.9649692	7387769.006	75.25685495	-228.8512726
74.46735873	9836888.571	51.92195958	-82.02101898
43.32472856	8759419.556	30.89056704	-36.55499268
72.42626308	12174033.38	50.75375999	-93.70762634
75.41261919	8396692.859	52.97353975	-131.2961426
57.84368131	26762919.93	41.76787151	-19.56265354
49.20395623	11331378.5	35.84056889	-65.52119446
55.12900523	15723610.31	39.80158983	-50.66371918
101.6796641	7938654.917	78.57891106	-180.4917755
66.25737635	10820195.7	45.45901212	-85.56742859
45.77356604	19865820.1	31.26797051	-13.47536421
100.4206155	25221389.11	57.84391117	-64.04507446
67.69567595	13496039.96	49.09007639	-109.9748306
88.80322339	6166185.35	61.52641488	-83.55078125
49.41989781	11495478.64	36.93236235	-56.18913269
54.26400672	25676678.22	37.57915482	-47.13921356
50.82195023	22053571.89	35.22575078	-21.92159176
48.81254408	14308994.64	32.46888081	-53.89635086
79.99346404	31666465.26	55.70024463	-134.8641739
64.12350161	21028543.87	43.22561438	-85.22528839
90.42515292	4275229.305	65.54583078	-195.1065826
63.95956187	2268828.084	46.71383419	-169.9367218
58.68774444	68835060.4	36.65268507	-11.32679319

log.sigma.3.0.mm.3D_firstorder_TotalEnergy	log.sigma.3.0.mm.3D_firstorder_Maximum	log.sigma.3.0.mm.3D_firstorder_RootMeanSquared	log.sigma.3.0.mm.3D_firstorder_90Percentile	log.sigma.3.0.mm.3D_firstorder_Minimum
328698901.3	243.1236877	135.2010703	3.781780005	-357.4068604
158724828.9	-2.976895332	228.0874186	-89.60764618	-369.6670227
355237384.3	141.3535309	165.0455747	-19.74215469	-339.526886
133236058.9	12.07845306	231.5981538	-72.98442383	-383.4881897
382565624.1	195.1797028	137.8166458	-16.55572987	-300.3152161
84143063.16	147.0975647	173.9436843	20.18140488	-475.940979
1322534701	307.42453	54.80850258	10.79553843	-288.0222473
1419990984	229.3438568	102.0909601	4.31172657	-410.8394775
718533440.4	197.8387451	79.32731735	3.634177923	-321.2480469
101547478.5	270.3670349	101.6487397	23.58795033	-255.1530457
272783351	205.7817383	43.0003215	39.18423615	-498.859375
424329644.8	207.0297699	71.51358085	11.49583549	-271.3475952
375024082.7	150.9735718	204.84893	2.149630308	-408.4385681
303224626.2	-18.95220566	243.1216039	-134.0799301	-386.3990173
388412480.4	122.9517899	125.0462846	-8.438683701	-325.7211914
1321786039	229.1065826	75.77057215	8.891801453	-281.4154968
97566147.23	139.0457611	91.03836534	4.137654305	-184.860611
179755135.8	-2.407288551	244.9047916	-148.1017151	-367.3735962
117367383.6	98.22946167	87.55890065	12.05845261	-207.9788666
1343076222	523.6108398	64.58071343	45.08885269	-346.7044373
525725608.7	184.4727936	115.801551	-3.724614954	-256.6010742
125015326.6	49.96675873	171.1871668	-54.42648697	-268.1457214
144807502.4	56.29956818	138.1530539	-23.06150246	-321.4708862
354703694.5	213.9628906	52.29371246	6.122618294	-255.7044983
328529332.4	119.1886215	129.4603817	0.309248462	-346.4103088
508642703	394.616394	96.45217232	15.39907761	-309.2719116
476766377.6	195.7680359	137.1317859	-12.84290695	-341.2640686
397393988.9	120.4594193	91.4736155	8.481998062	-307.7144775
5744786.629	132.7887268	87.17180801	76.08780136	-172.4711761
321616018.4	139.6165314	101.7300579	-0.881251693	-274.9610596
936364073.8	202.6113892	99.38596854	5.46980238	-297.4577942
349220598.4	43.6912384	225.2153326	-102.4625	-411.4074097
393537628.7	236.4526672	106.7519896	1.774416304	-277.980072
469408458.7	195.9918213	74.30315302	18.05683365	-291.5168457
775452715.9	210.5760498	90.88525567	7.944026184	-339.7965698
725068557.5	128.850235	154.2954446	-9.448442745	-449.5092468
726430371	176.8332977	262.6532693	-59.04212456	-514.9993896
1423910551	342.4449768	67.02895048	20.77262325	-316.8926697
107782584.9	45.84478378	142.3506001	-31.26948967	-296.8454895
1181373946	418.8399048	60.09910819	13.56923227	-632.045166
345225770	120.2199936	142.3492036	-36.24240494	-348.2197571
373380826.6	250.0689392	43.71342527	22.45969467	-271.0766296
896072429.9	228.1685791	147.4246675	1.500197959	-381.7731018
376957598.1	129.0974426	140.5253455	-16.42257042	-325.6582947
181317814.5	94.28794861	78.56405808	5.471851301	-237.7939301
615271086.7	226.7481995	107.9637574	1.005316448	-339.8196411
805013474.7	289.7966919	65.40854081	18.30178223	-286.3483276
76929909.25	139.3628693	114.062706	29.38520966	-244.5775299
67965926.98	119.3890762	148.5974355	-6.334539318	-264.500885
231674753.1	448.7546082	197.4904648	37.97931786	-400.9511719
414239456.7	77.27097321	89.71858212	3.292961717	-310.6000977
668407695.6	401.2001038	107.0810245	33.75341949	-347.0613098
598018914	247.1455383	46.82436493	7.121833992	-257.9286804
173900006.1	182.8893127	87.20195295	77.97657471	-254.3122406
263857925.5	160.8868256	184.5279858	-29.33538666	-321.9476318
340038303.2	235.4453735	137.8241345	-21.03696556	-293.1637878
760670965.6	238.131485	72.99815483	14.29686108	-341.7434387
623585065.8	202.5894623	122.2648808	-0.273526692	-300.1256409
228496817.9	49.78826141	176.3899122	-76.74991608	-371.6252747
1355224449	421.1386414	46.20295046	11.63838425	-299.9978638
343613004.7	161.4191284	126.364113	-6.256667709	-364.1619873
694209836.8	291.7023926	126.6475341	46.31666565	-396.5757751
2054789894	337.4228821	129.0175283	55.25772324	-432.4773254
1694038950	375.8915405	85.81436956	13.27498169	-411.5544434
105138744.2	100.1575623	110.1404875	19.50271797	-253.4840546
1242154265	386.8673706	145.1042607	62.93539581	-402.762207
185502574.4	-76.34511566	240.2810979	-129.0038422	-390.1222839
1176314622	505.0709534	102.733012	8.982640839	-388.9284363
826821415.6	284.2286987	55.27453671	12.84390335	-244.9476776
557531467.4	306.1538391	116.287569	22.8189312	-297.5657654
130348530.7	15.20639801	156.1487791	-41.33485413	-316.9187012
1249241214	154.487854	80.73143678	18.01046562	-432.8624573
475856808.2	69.64276886	153.3964654	-24.99725151	-357.5752869
235239764.6	339.7563477	90.11013182	6.592018223	-255.5419922
469191344	429.5846558	73.50808241	19.1404314	-303.7067871
150881096.9	244.8030548	96.18715178	32.05699043	-252.8359528
935939830.1	490.5134583	152.9389066	179.6216812	-320.4775085
199469763.2	63.37963486	253.4590097	-59.17010803	-475.1358337
265595991.4	215.4597931	122.9244805	37.81433487	-310.1199341
236504328	89.69545746	74.08345374	0.978956848	-257.1353455
328698901.3	243.1236877	135.2010703	3.781780005	-357.4068604
226710707.2	162.4513092	150.8484843	19.65032463	-279.1252441
722598838	242.329895	88.48725404	2.972109938	-330.6518555
305947219.5	66.05194092	98.20247646	-10.18353596	-311.8230896
424537478.3	163.5186462	93.33349584	6.550933361	-279.6020508
214343682.8	63.60150146	212.3817173	-26.20147133	-432.0189819
292145283.8	195.9406433	122.1654937	11.80432034	-392.7038879
536377142.6	200.2547302	69.89877457	2.542939782	-276.4963989
680977506	480.5535278	146.5094263	153.7299622	-366.5782471
364393079	216.8404236	129.8847564	2.563694167	-307.8742371
166487004.5	194.3951874	149.4699009	25.84626579	-368.3518066
310377923.2	128.7450562	89.65933442	-3.533846092	-280.7796631
693270312.1	245.910141	86.62224131	19.73140526	-289.3323364
595446441	249.4016876	75.64559002	18.8557497	-270.6842651
386342855.4	194.8306885	83.20210658	22.99713402	-258.4934998
854994562.1	240.8501282	163.539981	-0.742093045	-434.3688049
567770684.6	394.645813	119.8079916	7.856733608	-294.1908875
115431191.2	57.76701355	221.6767167	-36.09424362	-432.2871094
61258358.26	26.68653297	178.7605245	-64.26997375	-345.4093018
1858546631	328.5497131	84.66009924	48.92629089	-336.9028931

log.sigma.3.0.mm.3D_firstorder_Entropy	log.sigma.3.0.mm.3D_firstorder_StandardDeviation	log.sigma.3.0.mm.3D_firstorder_Range	log.sigma.3.0.mm.3D_firstorder_Variance	log.sigma.3.0.mm.3D_firstorder_10Percentile
3.856920285	90.86578324	600.5305481	8256.590563	-221.9280167
3.606301514	85.71293944	366.6901274	7346.707987	-313.009375
3.916468902	96.96948934	480.8804169	9403.081862	-262.7688538
3.75240569	93.62399486	395.5666428	8765.452413	-313.2206573
3.534525871	73.96244889	495.4949188	5470.443846	-205.9671402
4.118403363	117.2169699	623.0385437	13739.81804	-280.9129395
2.545986602	51.21287589	595.4467773	2622.758657	-94.17765808
3.457600942	75.36146496	640.1833344	5679.3504	-176.8516235
2.996212439	59.80076464	519.086792	3576.131452	-143.2829773
3.593114091	75.5447187	525.5200806	5707.004523	-167.4467346
2.614050416	42.9518195	704.6411133	1844.858799	-50.04756126
3.043248399	60.63208832	478.3773651	3676.250135	-130.5018616
4.164278509	116.8676044	559.4121399	13658.03696	-306.3502197
3.554423282	73.76234902	367.4468117	5440.884133	-321.0901398
3.492109164	74.22475816	448.6729813	5509.314724	-199.4562653
2.891869018	66.16544868	510.5220795	4377.866599	-151.9965454
3.224303742	58.61732565	323.9063721	3435.990866	-144.8914261
3.239930215	62.34843145	364.9663076	3887.326904	-316.1291504
3.256275355	58.51233869	306.2083282	3423.693778	-148.0178467
3.142730433	62.26981311	870.3152771	3877.529624	-100.7152252
3.462718819	73.06631889	441.0738678	5338.686956	-185.0091248
3.295507752	65.44458779	318.1124802	4282.994071	-234.111348
3.531277043	74.67634569	377.7704544	5576.556605	-220.6769409
2.511209875	44.78218869	469.6673889	2005.444423	-92.26099548
3.579896894	88.81638446	465.5989304	7888.350149	-213.09758
3.552623449	80.14850486	703.8883057	6423.782831	-146.3584869
3.604145514	75.42312538	537.0321045	5688.647843	-210.8088989
3.346140504	66.14727735	428.1738968	4375.462301	-158.1960541
3.311844101	79.70001978	305.259903	6352.093153	-130.2311531
3.412784605	67.45336637	414.5775909	4549.956635	-164.4690247
3.346048788	71.38783542	500.0691833	5096.223046	-173.9915924
3.6678288	81.53350105	455.0986481	6647.711794	-306.714917
3.522409379	73.87043821	514.4327393	5456.841641	-180.9382996
3.055461525	65.44136742	487.508667	4282.57257	-141.2602753
3.370194021	66.25971673	550.3726196	4390.350061	-153.0904022
3.914573765	99.05938031	578.3594818	9812.760828	-260.7414978
4.272852023	124.8675907	691.8326874	15591.91522	-380.7126801
2.816231719	62.13349061	659.3376465	3860.570655	-130.0301605
3.458022689	68.5162036	342.6902733	4694.470156	-213.6764496
2.835274613	55.79652916	1050.885071	3113.252666	-98.06648483
3.460310071	68.12184242	468.4397507	4640.585415	-208.6517334
2.58597987	41.541225	521.1455688	1725.673374	-62.52710037
3.905941771	93.14895163	609.9416809	8676.72719	-229.3255615
3.617444892	76.85741146	454.7557373	5907.061696	-214.933493
3.060201218	59.69037528	332.0818787	3562.940901	-143.6833679
3.518873926	74.76255823	566.5678406	5589.440113	-180.4989288
3.013729228	58.36003276	576.1450195	3405.893423	-115.3105362
3.605089643	80.39799651	383.9403992	6463.837842	-177.9745514
3.497835817	77.67523762	383.8899612	6033.44254	-218.5575287
4.388396591	149.7812205	849.70578	22434.41402	-301.0765778
3.062447971	69.92960357	387.8710709	4890.149455	-166.9035492
3.788937651	88.04703988	748.2614136	7752.281232	-164.3541534
2.368305925	41.71418433	505.0742188	1740.073174	-85.04075699
3.71068677	82.81771518	437.2015533	6858.773947	-141.0563965
3.828224801	95.38987044	482.8344574	9099.227383	-270.0035461
3.536338877	73.96526376	528.6091614	5470.860243	-206.5367859
3.115806016	62.45420509	579.8749237	3900.527733	-133.7582581
3.547631333	77.88778118	502.7151031	6066.506457	-199.0503815
3.515839431	71.68940241	421.4135361	5139.370417	-259.2091766
2.40168412	42.95428135	721.1365051	1845.070286	-74.36044159
3.73976115	83.33926497	525.5811157	6945.433085	-202.0256592
4.032833223	102.5952897	688.2781677	10525.79347	-207.9990173
4.121463368	108.0217658	769.9002075	11668.70189	-212.8617188
3.320568738	73.13870235	787.4459839	5349.269781	-148.3198807
3.6156948	76.04989628	353.6416168	5783.586725	-177.2821503
4.216491246	116.8029215	789.6295776	13642.92246	-224.1501984
3.465301037	71.01698269	313.7771683	5043.41183	-311.1923401
3.607420333	79.65340891	893.9993896	6344.665551	-166.3395142
2.810656359	49.78385051	529.1763763	2478.431771	-98.29571838
3.791183253	86.03684603	603.7196045	7402.338875	-185.0064026
3.44979094	70.94991698	332.1250992	5033.89072	-229.3186447
2.947659102	70.61277223	587.3503113	4986.163602	-157.6292816
3.555274793	74.58353307	427.2180557	5562.703406	-224.9519714
3.391670351	67.82242143	595.2983398	4599.880849	-150.7934082
3.191023373	64.2115626	733.2914429	4123.124772	-124.885788
3.605173025	76.14952548	497.6390076	5798.75023	-153.9314514
4.339634224	147.2511955	810.9909668	21682.91458	-188.0642883
4.175525513	120.2871089	538.5154686	14468.98857	-384.1281189
3.923719826	93.67945647	525.5797272	8775.840565	-198.1253662
2.959723303	53.29607981	346.8308029	2840.472123	-129.800621
3.856920285	90.86578324	600.5305481	8256.590563	-221.9280167
3.787978983	94.30416285	441.5765533	8893.275131	-226.0618103
3.041943967	70.31168629	572.9817505	4943.73323	-166.301622
3.190103571	60.01806804	377.8750305	3602.168491	-169.3773621
3.379549072	67.44131576	443.120697	4548.331071	-163.5078918
4.112349532	118.6780244	495.6204834	14084.47348	-320.8310089
3.759687	84.38823945	588.6445313	7121.374958	-198.5674469
2.768373663	57.04843115	476.7511292	3254.523497	-133.6928329
4.306926186	140.1318972	847.1317749	19636.9486	-189.0867462
3.71605809	83.3047568	524.7146606	6939.682505	-199.9737167
4.06594279	112.1731336	562.746994	12582.81191	-276.8765869
3.165542725	59.37796429	409.5247192	3525.742643	-147.3207352
3.46139142	68.43972078	535.2424774	4683.99538	-141.7963348
3.241266005	63.81459435	520.0859528	4072.302453	-134.1970383
3.373429373	63.31801171	453.3241882	4009.170607	-133.6063416
4.02416269	100.2994038	675.2189331	10059.9704	-252.8240891
3.736340081	83.07040666	688.8367004	6900.692463	-193.3431458
3.952616239	110.2925421	490.0541229	12164.44484	-339.5101746
3.619415159	78.45664512	372.0958347	6155.445163	-262.3624878
3.493740813	78.12528479	665.4526062	6103.560124	-148.4232071

log.sigma.3.0.mm.3D_firstorder_Kurtosis	log.sigma.3.0.mm.3D_firstorder_Mean	log.sigma.3.0.mm.3D_glrlm_ShortRunLowGrayLevelEmphasis	log.sigma.3.0.mm.3D_glrlm_GrayLevelVariance
3.36091615	-100.1136296	0.013482029	13.6042651
2.531453206	-211.3697294	0.061297054	11.78928658
2.578557609	-133.5550818	0.033550465	15.39619364
2.498366955	-211.8307165	0.03880342	14.19581928
3.326195793	-116.2883658	0.018808244	8.919961544
2.885086817	-128.5168754	0.016235957	23.2552222
7.045588019	-19.52468432	0.008245992	6.590022584
3.210235266	-68.87099347	0.005746578	9.88818966
3.215547458	-52.12189393	0.009425253	6.203347727
3.4859796	-68.01074732	0.030896214	9.465926984
11.49701227	-2.041776225	0.002212346	4.005354039
3.846224483	-37.92020716	0.015610204	7.031008376
2.588728338	-168.2410388	0.021795186	22.44982305
2.912996484	-231.6618875	0.048921011	9.017623702
2.443945557	-100.6342812	0.015962466	8.741438361
3.883462573	-36.92306874	0.012087708	8.937388863
2.741432076	-69.65624953	0.062745187	5.670162204
4.11007929	-236.8354493	0.06854244	6.630614108
2.51874655	-65.1372958	0.040454734	5.803327729
7.116298865	-17.12130028	0.005320955	8.201009662
2.909537197	-89.84048223	0.026734555	8.653822964
3.244021374	-158.1836022	0.100216752	7.14294332
2.258694196	-116.2312767	0.026750033	8.911441074
6.02293778	-27.00348013	0.01031456	4.42190931
2.191972306	-94.18938519	0.018892878	12.87166792
6.924806742	-53.65853813	0.011462673	11.5344638
2.962679676	-114.5271971	0.017132819	9.189418417
2.818121936	-63.18195971	0.011122296	7.41961349
2.446077033	-35.31049362	0.128458231	9.897338391
2.753530579	-76.15148091	0.026410234	7.657814307
2.426781318	-69.14728988	0.017070191	8.379301434
3.612470785	-209.938644	0.023516104	11.15740655
3.089255929	-77.06585262	0.019056613	9.051778556
3.578100058	-35.19070869	0.011279393	8.465194416
3.00244069	-62.2075529	0.008195027	7.424837137
2.793167142	-118.2976052	0.010087504	16.05694488
2.633334768	-231.073202	0.015303726	25.18059311
5.254491475	-25.14576599	0.007908394	8.319142035
2.784118923	-124.7766933	0.04181055	7.906402054
9.002658088	-22.33047558	0.001362593	7.065207101
3.231246989	-124.9908411	0.016154157	7.711523341
7.647279863	-13.60845968	0.008123232	4.00230738
2.99878494	-114.268567	0.012076418	14.28599852
2.738765894	-117.6448513	0.016114925	9.600936589
2.997243357	-51.08199606	0.025774898	6.388969095
3.227255187	-77.88923411	0.010676966	9.300545511
4.491921291	-29.53614374	0.009864409	6.979760297
2.104308504	-80.91021606	0.04896077	10.67982082
2.709107988	-126.6797352	0.063117917	9.713886781
5.311680677	-128.7170139	0.021170273	36.4108213
3.237975803	-56.20742409	0.013832157	8.92437794
5.284636284	-60.94312567	0.010954635	13.64647064
6.522169411	-21.27082454	0.008787111	4.114520104
2.15740216	-27.30213637	0.014569885	11.06388303
3.240041595	-157.9599638	0.070797922	14.9392649
3.820254389	-116.2954505	0.033337054	9.075236003
4.116849087	-37.79157148	0.00704138	7.598622788
2.278969508	-94.24539579	0.016848061	9.550802494
2.740329378	-161.1646075	0.021050301	8.574504754
10.29512755	-17.01888201	0.006980509	4.475680574
3.13087561	-94.98660946	0.011187236	11.76364686
3.183448845	-74.25499603	0.008219288	17.81186709
3.443357614	-70.5465855	0.005776175	19.82501433
4.573122908	-44.88692729	0.004845988	10.28268875
2.215157043	-79.67019681	0.031575847	9.648437545
3.917882601	-86.09485471	0.007979788	22.69981031
2.265489675	-229.5464968	0.040153088	8.268355581
5.161847182	-64.87993684	0.006737081	11.31280998
4.675508489	-24.01754852	0.012619428	5.209286747
3.634844123	-78.23336768	0.020673129	12.46422108
2.061519646	-139.0990672	0.030922033	8.197304443
3.950472939	-39.13312257	0.003855735	9.762207143
2.327094409	-134.0439189	0.014400173	8.765072914
4.865582979	-59.32920872	0.02073354	8.044484166
5.559639172	-35.78146737	0.008261989	7.944917986
3.555877367	-58.76408714	0.019847142	9.765374767
4.048656702	-41.32063129	0.013429751	35.02591743
2.17900377	-223.0974698	0.025406991	23.66409034
2.982419278	-79.59012091	0.020781172	14.64933328
3.376318157	-51.45761357	0.015802734	5.062122222
3.36091615	-100.1136296	0.013482029	13.6042651
2.955685808	-117.7369529	0.0347799	14.7144496
3.509787726	-53.72393226	0.009101924	9.149930998
2.874626046	-77.72745905	0.012137119	6.007450141
2.948820921	-64.5198448	0.014935699	7.892023774
2.013462573	-176.1292717	0.02006806	22.38178094
3.446041135	-88.33477741	0.009731103	11.80955869
3.784058274	-40.38954306	0.010622466	6.516575918
4.701538477	-42.75585812	0.011878807	33.0971318
3.268040861	-99.65122904	0.016669321	11.51588771
2.693781365	-98.7848134	0.0249087	20.1123061
3.071660101	-67.1792647	0.014777456	6.092218132
3.522440044	-53.09818556	0.012501042	8.369064139
3.76299892	-40.61961149	0.014833738	7.555089634
3.602952751	-53.97610519	0.014444813	6.969924361
3.028598861	-129.1718042	0.008903941	16.61223179
4.32618362	-86.33227901	0.026580028	11.87986196
2.343267283	-192.2917624	0.035845076	19.32631555
2.526520588	-160.6234104	0.041000603	10.12480248
4.16394118	-32.61552206	0.007876593	12.06293298

log.sigma.3.0.mm.3D_glrlm_LowGrayLevelRunEmphasis	log.sigma.3.0.mm.3D_glrlm_GrayLevelNonUniformityNormalized	log.sigma.3.0.mm.3D_glrlm_RunVariance	log.sigma.3.0.mm.3D_glrlm_GrayLevelNonUniformity
0.014208128	0.079107609	0.13090413	47.5918293
0.063996855	0.088993766	0.058607936	9.509030838
0.035664814	0.072910254	0.138441111	31.81315442
0.040616916	0.082321305	0.072896672	7.089892837
0.020425128	0.098679662	0.191297833	64.14184227
0.016397225	0.065948561	0.049128692	6.507164891
0.010597769	0.165709828	3.268874083	1427.652369
0.006478529	0.095662427	0.723601993	366.0649739
0.011100633	0.12475666	1.147423198	360.2529359
0.032517212	0.093574192	0.092837841	31.38687761
0.002786296	0.185168697	1.234063284	667.0193204
0.017952185	0.127250312	1.057837525	266.8496875
0.022591888	0.062443933	0.069098211	19.46716629
0.05103403	0.096175296	0.099852661	16.8854059
0.017118151	0.095963876	0.310401683	73.54923575
0.014361893	0.134549889	1.718240447	724.5365298
0.069252642	0.118033236	0.187487676	44.52032848
0.07112419	0.120418628	0.100190786	12.28817858
0.04441671	0.116194432	0.24970623	55.81231124
0.006533049	0.120678981	1.25351587	962.2483077
0.030066085	0.098937427	0.469883048	115.3230468
0.109463343	0.114666629	0.122791776	16.3293911
0.02871369	0.094105126	0.151137272	23.43323668
0.012964634	0.173266373	2.093553388	488.7897322
0.020465861	0.087460276	0.451043373	51.19231065
0.012924743	0.09968241	0.440940745	161.0867012
0.018415305	0.093221741	0.175749433	76.86096638
0.012385445	0.105830163	0.434524581	147.313098
0.136114179	0.114183929	0.03273951	3.092072258
0.028931553	0.10317748	0.369451745	97.0900253
0.019188961	0.101287354	0.748138031	264.7516484
0.024994366	0.093081	0.115145108	21.54431706
0.020838953	0.09818146	0.285981824	105.4595792
0.013189314	0.125020643	1.092480378	268.0489141
0.009238719	0.10604431	0.496196526	291.6572834
0.010794238	0.074040184	0.248853441	71.97388399
0.01575468	0.059168216	0.039041465	22.28108518
0.009657032	0.142871469	2.031573615	1010.117075
0.044885767	0.101687846	0.147131258	17.83691948
0.001743683	0.147160436	1.599217365	1120.92229
0.017358414	0.103304059	0.171656924	57.40525739
0.010273271	0.177874525	1.513112996	819.7248351
0.013102723	0.076590629	0.213442456	102.8822123
0.017261023	0.091634226	0.164744152	57.05454346
0.02922952	0.122703684	0.790360876	95.94510461
0.011897915	0.096190173	0.462012404	149.6812882
0.011845261	0.124418063	1.543782721	556.4291024
0.052485917	0.088848543	0.165546946	17.16724465
0.066547792	0.096667426	0.079726605	10.28612742
0.021796093	0.055825157	0.045466578	11.75728799
0.01540226	0.116763882	1.057108356	156.4584817
0.011847144	0.08662762	0.247625223	159.9554089
0.011608923	0.189293569	2.544866476	1052.12376
0.015679013	0.082857159	0.165791679	62.1937992
0.074847312	0.077306914	0.091862953	20.50015201
0.035733514	0.098221251	0.161651674	57.32464348
0.008315278	0.119850215	1.179269418	434.035836
0.018535335	0.09150675	0.418266174	114.2749337
0.022323953	0.097577737	0.120642515	23.9893972
0.009281188	0.199035401	2.10734796	2647.073981
0.011991076	0.085203435	0.197920004	59.33744593
0.008848309	0.069217286	0.195705952	97.40241685
0.006215602	0.067043582	0.210336752	269.1817154
0.00559496	0.106176453	1.193100939	635.2116811
0.033773806	0.088548989	0.14863566	25.52841372
0.008489312	0.064573129	0.116729512	128.5351512
0.042195019	0.097310659	0.08120862	10.76450854
0.007481954	0.093121448	0.538447355	306.0881669
0.015742244	0.147186633	1.545267318	921.2581387
0.022319091	0.081992539	0.189277298	109.9043623
0.032621578	0.098234135	0.123890808	17.54909654
0.004686337	0.125106358	1.809376785	549.285829
0.015482077	0.094245825	0.193510199	61.56124632
0.022810353	0.110373775	0.343626783	96.56273043
0.00959276	0.117746946	0.839369267	275.2029979
0.02134412	0.093549039	0.19519197	49.90657915
0.014485398	0.059942325	0.105209445	81.26986949
0.026059283	0.060564605	0.040687072	6.680913103
0.021888638	0.074491736	0.10573602	44.47007145
0.017983266	0.136205091	0.769599727	157.9985975
0.014208128	0.079107609	0.13090413	47.5918293
0.038363879	0.081087893	0.119777622	26.9846693
0.010586712	0.119626509	1.260394271	276.1704119
0.013651583	0.119760125	0.401277192	111.3260555
0.016627358	0.105686981	0.435301232	151.8671558
0.020872864	0.062510681	0.063952678	10.42027947
0.010387582	0.086947936	0.178750746	55.34833724
0.012818939	0.132770103	2.060267769	327.2773223
0.012566116	0.063590139	0.1399152	67.1994058
0.018010677	0.086099839	0.149754778	61.52481047
0.026377985	0.06977526	0.065623988	18.11428934
0.016684424	0.119006897	0.6102069	130.3789127
0.013953581	0.101428372	0.494264251	276.8682708
0.017067729	0.111518194	0.87344288	309.8920068
0.016025929	0.111322064	0.248603828	195.6824001
0.009400079	0.070617314	0.111624499	76.55244156
0.028660347	0.085942237	0.199522732	109.4079997
0.037300942	0.071778325	0.062689151	5.88641293
0.042374504	0.091327383	0.060699375	6.118896892
0.009012932	0.092747811	1.02198986	641.2206216

log.sigma.3.0.mm.3D_glrlm_LongRunEmphasis	log.sigma.3.0.mm.3D_glrlm_ShortRunHighGrayLevelEmphasis	log.sigma.3.0.mm.3D_glrlm_RunLengthNonUniformity	log.sigma.3.0.mm.3D_glrlm_ShortRunEmphasis
1.360895082	135.2211247	498.8441135	0.927217335
1.178707588	61.08593756	95.72559839	0.95777308
1.372610837	92.37065893	364.1276467	0.928944686
1.220234342	78.45990495	75.79085731	0.949574094
1.511673524	78.60695024	509.2511395	0.905584559
1.143186296	249.2374569	90.75406297	0.96755694
6.991791805	86.30441378	3782.84682	0.688010125
2.482939463	175.9787362	2549.813588	0.841468807
3.347026409	92.88496051	1675.337448	0.786832045
1.273590408	81.48615623	286.9756083	0.93970466
3.607701349	317.0529703	1951.704398	0.761315779
3.250919407	74.31268671	1206.43283	0.784465582
1.198535131	137.3215234	278.2411309	0.956264427
1.276161283	58.21228479	152.1503991	0.943937692
1.761229453	95.51846607	568.7709109	0.883154367
4.327890008	84.83030265	2856.952547	0.754259761
1.531319596	34.56624008	289.8412488	0.897111272
1.288416908	41.59884306	87.20674358	0.938620187
1.658741289	46.75038651	360.476782	0.885838539
3.528089476	150.8158895	4513.23175	0.779784748
2.029249107	58.95594934	839.4141141	0.871350833
1.35795903	33.28244297	116.9765553	0.923826671
1.431954662	78.20436389	199.265898	0.912605315
5.118491023	72.24848187	1371.066616	0.721095584
2.018367893	99.17234821	419.7665018	0.868464035
2.028488964	119.0328426	1126.51655	0.859023414
1.480268639	95.74983209	654.2068382	0.908930566
2.045311984	100.4987941	953.1324528	0.852035961
1.106587771	46.34640227	25.37425378	0.973353057
1.882669784	65.6764407	679.6226068	0.871960252
2.579041283	73.8285888	1695.606999	0.830477667
1.337405845	90.70591858	193.3423233	0.928894929
1.714702205	82.45455829	800.0817983	0.884832113
3.301931263	91.49470478	1216.122131	0.780838119
2.106470046	123.0608753	1913.935343	0.858172614
1.60221906	177.7433358	758.0010121	0.903199997
1.113797164	174.5222485	352.333675	0.974020541
4.872390219	106.3452648	3587.846616	0.739258531
1.416243446	58.9310903	141.3685508	0.915538318
4.19859199	481.2011271	3963.563698	0.748011523
1.464491937	88.78057788	441.8105007	0.91133807
4.045911362	91.7683614	2425.36352	0.750993012
1.527210614	142.8917215	1083.928407	0.913486531
1.458374952	94.0260045	493.5578475	0.909942122
2.772059013	55.87340808	476.7271098	0.805778781
2.059381633	110.9680156	1090.328466	0.860051558
4.03096765	93.12718186	2419.266221	0.762765131
1.465453304	58.79671272	154.7292101	0.91045035
1.230571167	50.44729273	93.3468669	0.949432479
1.13794714	185.0197102	193.3954011	0.967155208
3.118316196	93.68475791	821.8566333	0.809059883
1.619910018	142.6328515	1414.193476	0.896588708
5.980577671	71.85616751	2435.168649	0.68543003
1.454830728	107.6012294	608.3687456	0.914776972
1.266079258	65.0015762	228.3929167	0.942183978
1.454993807	63.85105153	461.4016287	0.909338763
3.373795254	126.6534857	2112.329263	0.789185248
1.957926025	82.71012769	898.5134963	0.871231132
1.348752213	83.9362374	203.4324765	0.926745971
5.349147543	91.08744269	5841.925868	0.684797179
1.514406781	133.6118983	552.1746988	0.909412839
1.499051077	182.9756216	1124.753138	0.912686912
1.512322587	242.7682794	3223.602114	0.914553009
3.261845608	192.1063597	3712.112266	0.814355888
1.403658928	73.20269197	237.8335369	0.922901993
1.323294374	209.2115495	1675.752847	0.933863356
1.241261288	60.3616587	96.22981881	0.945991241
2.132291175	169.3146752	2344.905375	0.867273343
4.183878279	65.18714029	3181.22795	0.738719077
1.490948313	91.95656071	1067.312818	0.911698139
1.356924119	65.05648136	147.3882481	0.925378347
4.482990022	193.988192	2330.921589	0.756006482
1.513866495	97.07369079	513.3190319	0.90609654
1.856211495	75.50790786	620.7552914	0.866607318
2.75792736	119.1151324	1464.892277	0.818284175
1.500431204	85.3592787	428.2121984	0.912422078
1.30169694	170.3203217	1146.618798	0.935533679
1.128685997	156.1353641	101.404388	0.967828501
1.296860499	115.6298221	508.1109855	0.938012547
2.692069799	68.85951673	719.3855527	0.81288026
1.360895082	135.2211247	498.8441135	0.927217335
1.351296256	74.85062805	273.3545799	0.924430132
3.534747274	108.8038693	1334.45184	0.785209237
2.012893663	89.62797582	629.5696227	0.847311445
2.030645798	85.42012365	992.3379944	0.855223583
1.179920131	151.7474223	150.3944516	0.960525291
1.484965038	161.5166021	505.0644538	0.909277212
4.849350448	79.7518401	1307.073223	0.755098942
1.379542901	211.0107148	872.2480582	0.925796496
1.405679242	96.35419736	582.5424682	0.921230117
1.19845581	149.5455845	230.6516792	0.954086855
2.350580697	79.9704864	735.6382126	0.840944514
2.078482378	96.86162588	1923.179341	0.863287199
2.82926574	79.12731996	1725.736477	0.814086504
1.640092024	84.71384877	1328.245921	0.890355316
1.306935003	183.6145909	922.0184847	0.937537913
1.529057728	85.79964239	992.4876237	0.903446103
1.19080078	133.9035535	73.14792703	0.955402252
1.185906798	73.78020088	59.82087622	0.956134444
2.972153473	146.9644691	4400.854507	0.824084333

log.sigma.3.0.mm.3D_glrlm_LongRunHighGrayLevelEmphasis	log.sigma.3.0.mm.3D_glrlm_RunPercentage	log.sigma.3.0.mm.3D_glrlm_LongRunLowGrayLevelEmphasis	log.sigma.3.0.mm.3D_glrlm_RunEntropy
197.9584682	0.903210903	0.017716533	4.337500574
69.84016544	0.945541184	0.075119486	3.847785051
136.1009281	0.903169294	0.045373204	4.374700097
90.37440611	0.935618729	0.048482304	4.01088998
133.3888518	0.871416787	0.028968944	4.111878263
293.8055182	0.957430919	0.017087293	4.291543168
1063.517579	0.525441319	0.050795376	4.713160629
612.3262354	0.757523095	0.012539759	4.52393568
507.258573	0.681412227	0.027045918	4.340266826
108.0644872	0.921386306	0.039561566	3.988736275
1526.165125	0.657253069	0.008981838	4.16927594
376.1803242	0.680943203	0.038959234	4.446750088
158.366552	0.941668603	0.026152897	4.46199149
75.57990927	0.92388664	0.060643892	3.906448495
229.1089	0.833026756	0.023757251	4.199227907
600.6730564	0.62808635	0.040513098	4.583282747
57.93421742	0.865208186	0.101562581	3.842627271
53.39482983	0.918918919	0.082929335	3.609120448
88.90919093	0.847374847	0.065859658	3.937362853
708.1863433	0.667077284	0.019620925	4.657697374
158.1895423	0.802818394	0.051031881	4.255514511
42.52507635	0.901168452	0.151839921	3.750645695
126.0936417	0.886120996	0.037940763	4.035834314
605.3742801	0.584849164	0.048443377	4.352670974
290.7701221	0.804831532	0.02985587	4.422394304
287.691117	0.797568851	0.022131333	4.467746263
163.1060666	0.878348489	0.024727948	4.172962643
281.3654356	0.790921415	0.020456358	4.267559332
49.81952851	0.967032967	0.166737971	3.411408357
158.1005842	0.817015304	0.043566492	4.213711328
305.998543	0.74377232	0.034772213	4.407702702
120.8812402	0.907692308	0.031806132	4.109077824
175.8206686	0.839658387	0.030724942	4.255514039
459.7725295	0.67813958	0.030837294	4.50657795
340.7974577	0.790867459	0.016515828	4.285404427
347.662679	0.861565739	0.014578027	4.559365441
190.4190928	0.965483235	0.017739959	4.451038747
857.1374583	0.599768012	0.032428381	4.641036839
88.79344893	0.890277235	0.058632712	3.94218983
2823.5338	0.626427147	0.006485432	4.565228781
143.2617883	0.880653419	0.023522815	4.035871557
500.0041851	0.634995376	0.035511481	4.246055079
238.9698033	0.879502292	0.019005927	4.471723871
157.3660405	0.88075291	0.022904812	4.171410818
235.2876025	0.717265271	0.05503391	4.281370665
310.28535	0.7956325	0.019788765	4.393398463
575.7904009	0.639745245	0.034014652	4.601944862
91.40534329	0.88233228	0.07083601	4.112775061
58.94688495	0.933198381	0.082293543	3.789339286
211.6015241	0.957342657	0.024374728	4.575853834
467.1136361	0.70110582	0.030003154	4.375389846
255.482912	0.855061104	0.016838851	4.499909763
725.6147676	0.547629563	0.05333976	4.402180082
175.87367	0.886204704	0.021515231	4.243905756
79.30260642	0.923880997	0.093801871	4.186397452
104.1822003	0.880380555	0.046922871	4.100276729
627.6668792	0.68293783	0.021394075	4.5288414
229.8937336	0.808364451	0.029073029	4.335605885
122.6177146	0.903846154	0.027979984	3.980407769
788.7918109	0.563275962	0.039270886	4.407489601
229.0432762	0.873660844	0.016190474	4.33256348
301.2189186	0.877681271	0.012191222	4.631749022
403.1727376	0.877935931	0.0086118	4.735097879
900.2367478	0.70024377	0.013770309	4.646442192
110.0409317	0.897915169	0.044252294	4.074571467
284.4151666	0.910931174	0.010910817	4.688734371
72.78956532	0.929541047	0.051191469	3.771931138
461.5382204	0.795784884	0.013002988	4.522200178
424.3719661	0.622667864	0.049256001	4.477890689
149.1805254	0.877789532	0.030677841	4.381485581
96.28618999	0.902097902	0.040762463	3.883299011
1444.400907	0.616414013	0.01545804	4.657627358
178.7436389	0.872445312	0.020995444	4.111659277
177.2320317	0.815112194	0.034283855	4.21954936
441.6372102	0.725602755	0.021025715	4.432205648
139.1730919	0.882450331	0.029232346	4.164994243
218.6391313	0.914875947	0.019463404	4.780655842
175.5674896	0.959197324	0.028668453	4.321829426
152.2724979	0.91693253	0.02690218	4.347879837
274.0819284	0.726190476	0.035366796	4.136418346
197.9584682	0.903210903	0.017716533	4.337500574
93.69602684	0.901813634	0.054556937	4.263022104
625.2802977	0.67263807	0.025617578	4.504999522
244.6157001	0.791031097	0.022813473	4.102494836
229.1730551	0.795567867	0.027173039	4.301442341
176.593203	0.947115385	0.024431451	4.33004174
270.918228	0.877877984	0.013722127	4.336001438
656.615374	0.604317227	0.042088709	4.48402481
296.6824406	0.899181669	0.015876159	4.824019811
140.1931798	0.893173077	0.024827786	4.239250571
179.0263533	0.940635452	0.032653115	4.346999588
260.3766178	0.765788058	0.030404026	4.169881993
250.6614722	0.79726206	0.02398837	4.377586737
313.3882714	0.719991218	0.035657529	4.470556878
152.575797	0.850359123	0.024839894	4.098506435
250.8379101	0.915540541	0.011758945	4.466634226
140.0894116	0.868889472	0.039054291	4.377703147
157.0552595	0.942528736	0.043388745	4.179225463
86.62529863	0.943661972	0.048308109	3.822125929
563.3704254	0.718763015	0.02025366	4.774349813

log.sigma.3.0.mm.3D_glrlm_HighGrayLevelRunEmphasis	log.sigma.3.0.mm.3D_glrlm_RunLengthNonUniformityNormalized	log.sigma.3.0.mm.3D_glszm_GrayLevelVariance
145.6837224	0.827720188	22.72808897
62.72454318	0.895084794	12.26628693
99.02387667	0.83233204	20.41822059
80.68523761	0.87809148	14.73580247
86.87901497	0.782528295	14.30972222
257.3491461	0.918912607	28.97253716
132.4530594	0.438588372	17.31629206
215.6403114	0.664979687	19.95224685
125.2143586	0.577107413	11.63319445
86.32294379	0.854444147	13.61501638
418.0084476	0.538900793	13.47660328
99.21075588	0.573174372	17.05297926
141.1741572	0.891900818	27.12531949
61.08414669	0.864975102	13.72265306
112.2098799	0.740211236	14.18666667
119.2296986	0.528916286	14.09173863
38.25520308	0.766140248	8.451790634
43.67207011	0.853151069	12.34182099
52.8517623	0.745964823	9.022272072
194.7299562	0.565139876	19.83576815
68.20554987	0.718937277	12.63928889
34.94506769	0.820167443	9.456767768
85.97880567	0.79778855	11.34545898
105.0364992	0.482968498	12.11899863
119.4151882	0.714589978	17.19660239
138.4780228	0.695297001	24.90313182
106.1790318	0.790527991	14.84227765
121.8341056	0.682955647	13.83252948
47.04102752	0.934520979	10.57439446
76.64013562	0.719786109	13.54176949
94.27747323	0.64653094	12.67577068
95.95030998	0.832483248	17.34801136
94.71679368	0.742818135	16.03982222
122.610485	0.567039165	15.85756794
146.2907128	0.694158383	14.34788516
200.0689668	0.778689504	21.30846874
177.5653461	0.934668587	25.94844339
151.7732577	0.506581426	15.27617854
63.89017864	0.803662467	13.10453978
650.3502159	0.519070641	22.6161288
97.46790373	0.794063897	14.28387234
122.3015354	0.523952987	13.97970018
156.3196443	0.80191593	20.34023225
104.1724229	0.791256066	16.74333333
72.44201972	0.606725724	11.29481666
132.4378536	0.698048841	18.04563827
126.7599459	0.54001106	15.18703413
64.09992023	0.795860205	15.32839506
52.01699561	0.876373185	10.47152367
190.1913049	0.917472358	41.32143667
123.0240174	0.612183967	12.39037483
158.3167731	0.765033594	27.69789808
109.9669236	0.436545198	12.12438689
118.2486391	0.805127239	12.93001923
67.65357016	0.860201031	17.5015015
70.28236939	0.789577014	18.00856124
166.3711621	0.580653554	16.20030853
98.39057922	0.718115391	14.3680958
90.43551173	0.826194751	13.85916121
137.5251047	0.437023893	18.02381227
147.1908858	0.791412651	20.44079695
199.9138173	0.797968617	27.43021744
264.7724039	0.801829994	28.94345608
242.9464015	0.620264174	21.618917
78.92406213	0.820672492	13.21
222.1011019	0.841360994	31.93500477
62.64431266	0.868686999	9.465500945
197.9494796	0.711537586	23.28079206
91.00283705	0.506292613	15.65997559
100.233567	0.795248951	18.48545329
70.40854969	0.823668585	10.82660515
272.343866	0.53043463	13.76868454
108.9573112	0.783952812	13.23270233
88.48800034	0.708324136	17.41062737
147.9049588	0.625832448	17.64807804
93.25218576	0.798729426	14.82401833
179.1409635	0.844940954	35.47908093
160.0217892	0.918625856	24.74396135
121.9663388	0.850523951	20.25311983
88.1538963	0.617641697	12.28684414
145.6837224	0.827720188	22.72808897
78.21366131	0.820715502	18.1252071
147.139412	0.575240501	15.56974395
108.9072499	0.674718921	10.87762164
102.1086421	0.688545053	14.6918865
155.8504267	0.901696788	20.58199722
178.4485445	0.791090247	19.71996094
112.3990071	0.529619085	13.92002115
225.2688623	0.824571774	45.475268
103.4695295	0.814526184	15.00483223
155.0734339	0.887120683	20.51692308
97.57030491	0.666337598	11.44581717
113.1400689	0.703152011	15.90769414
99.69873735	0.619265054	14.59468983
94.54251904	0.753419167	12.52805631
194.9152233	0.849711931	24.40098493
94.0246258	0.77837956	19.89395575
138.3745205	0.890691374	21.06246282
76.28212004	0.891716895	11.41955017
180.1720058	0.636009539	22.5805228

log.sigma.3.0.mm.3D_glszm_SmallAreaHighGrayLevelEmphasis	log.sigma.3.0.mm.3D_glszm_GrayLevelNonUniformityNormalized	log.sigma.3.0.mm.3D_glszm_SizeZoneNonUniformityNormalized
117.2761391	0.060146924	0.407560453
56.50167391	0.092203631	0.425418298
99.2980275	0.063281095	0.423626265
82.93153422	0.082469136	0.503209877
80.71745337	0.08154321	0.410185185
199.1919111	0.059208869	0.517258755
104.9875097	0.067919991	0.38559371
129.1154049	0.063813924	0.317077297
72.91187753	0.084231688	0.332061069
62.41031507	0.082388511	0.331443689
239.174103	0.085467477	0.340786301
75.54973833	0.071534475	0.361606553
147.4832917	0.054647247	0.490121303
51.85075152	0.074693878	0.31755102
60.57751685	0.078222222	0.337333333
81.14208351	0.0779649	0.337887836
36.69074019	0.101010101	0.343434343
46.25800392	0.094135802	0.408950617
41.50104723	0.101426054	0.350745073
156.8140752	0.065260154	0.400847523
74.23418931	0.082824691	0.370666667
44.21117176	0.096423721	0.407876867
66.64817323	0.093261719	0.370117188
61.56275792	0.088263032	0.289909122
73.26638151	0.072478145	0.271994544
133.7332995	0.058892085	0.377777227
75.40079472	0.074391259	0.354774625
68.87279379	0.077599051	0.285018055
47.74836601	0.100346021	0.418685121
60.24175395	0.07736972	0.308172449
68.24481556	0.083896052	0.354540766
90.08430313	0.068956612	0.366477273
83.3057555	0.06982716	0.437471605
85.90783812	0.073571712	0.360369966
118.3404873	0.074201378	0.373474158
147.4817872	0.060827497	0.430528995
165.1199162	0.056249587	0.52006081
96.45925552	0.079037357	0.372058921
42.8292035	0.082882132	0.307788421
461.533381	0.061473854	0.385855077
82.1212872	0.076242741	0.419468272
88.52654328	0.084786736	0.364600692
137.3928923	0.064642201	0.444133249
70.94867414	0.068088889	0.318044444
48.76219319	0.086238117	0.318017202
110.8021293	0.068224441	0.39673147
78.20096577	0.072203699	0.313926819
37.69332022	0.082469136	0.191111111
54.21167531	0.090976331	0.519970414
133.4158726	0.052703214	0.356219282
38.87955719	0.081988702	0.219999376
145.4028902	0.055472933	0.405016855
88.01743552	0.088352779	0.417350996
63.92791255	0.080035251	0.299471239
70.22837894	0.07199091	0.408164922
78.00758883	0.073388823	0.44627824
101.5444276	0.069068119	0.329905199
81.1669771	0.078447506	0.361355719
82.2323478	0.076145152	0.421475312
100.9277104	0.072853119	0.36818512
127.4325106	0.063205475	0.383348905
189.2753288	0.053684404	0.436835855
217.4996666	0.051919287	0.404416679
181.1101512	0.062884991	0.403641949
58.38226666	0.078125	0.320625
180.6907862	0.051030929	0.391923182
56.50021074	0.103969754	0.348771267
166.914678	0.060381099	0.420256347
83.41873704	0.074878312	0.368570186
103.7779577	0.067586263	0.413764106
36.51746533	0.095732411	0.257208766
160.6152117	0.081092245	0.37592119
71.7625748	0.080384088	0.307983539
77.68919284	0.071119524	0.380543537
113.5211238	0.071970736	0.34961134
72.03742162	0.082316586	0.337737759
142.7264752	0.054200749	0.35582313
131.9478824	0.061961773	0.439193447
104.3010062	0.066115702	0.362272727
57.46366868	0.092785494	0.350887346
117.2761391	0.060146924	0.407560453
84.80269963	0.069467456	0.405680473
84.44715362	0.074107614	0.306351939
60.111052	0.088058753	0.23946049
83.13092144	0.074793382	0.368682198
140.0394102	0.070824391	0.422295165
139.2687187	0.068125	0.4225
89.90121689	0.075979906	0.369257717
174.7395788	0.04658246	0.386888232
107.5451805	0.075239186	0.415047837
131.2927655	0.066508876	0.403313609
67.85569874	0.090487435	0.306038118
95.77367216	0.069461445	0.38811613
82.98276771	0.075092212	0.371790886
78.98502254	0.079982372	0.331502152
182.3640345	0.056995575	0.474023495
94.74679155	0.066460403	0.395591973
132.0088119	0.069601428	0.449137418
54.98558007	0.096885813	0.326989619
135.0750482	0.058524908	0.385981548

log.sigma.3.0.mm.3D_glszm_SizeZoneNonUniformity	log.sigma.3.0.mm.3D_glszm_GrayLevelNonUniformity	log.sigma.3.0.mm.3D_glszm_LargeAreaEmphasis	log.sigma.3.0.mm.3D_glszm_ZoneVariance
80.6969697	11.90909091	59.98989899	48.6758494
22.54716981	4.886792453	12.09433962	7.548593806
60.15492958	8.985915493	47.40140845	35.831829
22.64444444	3.711111111	10.08888889	5.909135802
73.83333333	14.67777778	129.6111111	112.4346914
32.58730159	3.73015873	4.428571429	1.755605946
358.6021505	63.1655914	69193.40215	68885.98437
179.1486726	36.05486726	3563.578761	3483.816474
87	22.06870229	9230.805344	8970.266316
41.76190476	10.38095238	24.49206349	16.14638448
123.3646409	30.93922652	16703.66298	16475.83648
94.37931034	18.67049808	5143.605364	5004.979551
76.94904459	8.579617834	16.94904459	12.50419895
22.22857143	5.228571429	15.45714286	8.089795918
50.6	11.73333333	315.92	278.3022222
218.2755418	50.36532508	21758.53096	21584.29913
22.66666667	6.666666667	228.4848485	184.8448118
14.72222222	3.388888889	29.08333333	19.57638889
27.70886076	8.012658228	307.7088608	256.1964429
386.4170124	62.91078838	20913.3973	20760.32086
83.4	18.63555556	526.5777778	484.9322667
19.17021277	4.531914894	41.95744681	30.65640561
23.6875	5.96875	65.640625	46.36303711
62.62037037	19.06481481	25182.17593	24687.52538
34.54330709	9.204724409	304.6614173	271.98264
107.2887324	16.72535211	1007.517606	956.6766887
68.47150259	14.35751295	165.4663212	141.7953234
56.71859296	15.44221106	1344.125628	1265.994243
7.117647059	1.705882353	3.529411765	0.816608997
51.15662651	12.84337349	516.560241	468.4835607
125.8619718	29.78309859	2803.602817	2705.787931
32.25	6.068181818	32.73863636	24.34181302
98.43111111	15.71111111	358.7777778	326.4648691
102.3450704	20.8943662	5537.419014	5414.474645
176.653277	35.09725159	1137.553911	1083.517425
120.9786477	17.09252669	171.1459075	155.0318258
127.9349593	13.83739837	4.235772358	1.722387468
300.2515489	63.78314746	34738.95415	34527.39046
15.08163265	4.06122449	47.32653061	31.16284881
367.3340336	58.52310924	30066.41176	29904.49149
57.46715328	10.44525547	157.1605839	135.9468272
203.0825853	47.22621185	19986.01975	19817.20641
156.7790368	22.81869688	170.8300283	152.1176641
47.70666667	10.21333333	140.3666667	118.1511556
29.89361702	8.106382979	1687	1553.031689
116.2423208	19.98976109	796.4129693	751.8926487
136.2442396	31.33640553	17164.57373	16906.7271
8.6	3.711111111	63.75555556	40.07111111
27.03846154	4.730769231	11.65384615	6.847633136
40.96521739	6.060869565	5.495652174	1.835916824
39.37988827	14.67597765	2568.759777	2455.378921
199.2682927	27.29268293	382.5630081	363.306609
193.6508621	40.99568966	57627.62069	57153.62023
47.3164557	12.64556962	118.943038	90.20529563
45.30630631	7.990990991	21.68468468	14.99943186
64.71034483	10.64137931	125.4689655	104.5619976
157.3647799	32.94549266	7845.503145	7722.651249
86.36401674	18.74895397	473.3849372	431.5960155
34.56097561	6.243902439	39.58536585	28.58239143
361.1896024	71.46890928	148655.2773	148080.7931
76.28643216	12.57788945	121.3517588	105.3115325
193.081448	23.72850679	165.2873303	152.1344004
552.8376006	70.97366496	230.3087052	219.1226898
481.5448449	75.0217938	6156.50964	6105.506337
25.65	6.25	53.6125	37.51234375
281.7927677	36.69123783	92.11265647	82.87748205
16.04347826	4.782608696	15.36956522	8.677221172
315.6125166	45.34620506	1349.541944	1319.328551
196.8164794	39.98501873	33713.78839	33361.48814
158.8854167	25.953125	176.2526042	160.4395549
13.11764706	4.882352941	39.17647059	24.10380623
202.6215213	43.70871985	16099.77922	15926.31224
41.57777778	10.85185185	165.8296296	135.0476818
65.45348837	12.23255814	501.9360465	463.0187601
141.5925926	29.14814815	2842.57284	2779.517476
46.27007299	11.27737226	135.3138686	115.8767116
172.9300412	26.34156379	57.72427984	48.42554489
30.30434783	4.275362319	3.782608696	1.004830918
79.7	14.54545455	39.54090909	30.78469008
50.52777778	13.36111111	2437.652778	2314.8125
80.6969697	11.90909091	59.98989899	48.6758494
52.73846154	9.030769231	40.11538462	32.05852071
97.11356467	23.49211356	5461.425868	5345.167133
28.49579832	10.4789916	1053.302521	955.8076407
96.22605364	19.5210728	796.5708812	748.7438529
37.58426966	6.303370787	8.764044944	4.853427598
67.6	10.9	159.16875	138.6365234
90.83739837	18.69105691	13817.77236	13544.58252
141.6010929	17.04918033	91.88797814	81.58142226
94.21585903	17.07929515	77.71806167	65.2978711
52.43076923	8.646153846	8.630769231	4.123313609
47.12987013	13.93506494	1157.584416	1071.359926
235.5864909	42.1630972	1050.378913	1018.59681
150.5753086	30.41234568	3887.812346	3797.25715
134.9213759	32.55282555	495.1375921	469.3451696
200.0379147	24.0521327	69.30331754	61.43143685
135.6880466	22.79591837	257.7638484	239.5212369
18.41463415	2.853658537	8.951219512	4.448542534
11.11764706	3.294117647	6.323529412	1.962802768
477.4591754	72.39531124	7253.691188	7193.412346

log.sigma.3.0.mm.3D_glszm_ZonePercentage	log.sigma.3.0.mm.3D_glszm_LargeAreaLowGrayLevelEmphasis	log.sigma.3.0.mm.3D_glszm_LargeAreaHighGrayLevelEmphasis	log.sigma.3.0.mm.3D_glszm_HighGrayLevelZoneEmphasis
0.297297297	0.51277976	8829.424242	162.6363636
0.469026549	0.728457115	415.2264151	79.32075472
0.293995859	1.201947839	4157.239437	131.2605634
0.489130435	0.403066779	436.6222222	106.0444444
0.241286863	2.318969487	9062.688889	112.65
0.611650485	0.039091513	1136.904762	263.1111111
0.057034221	450.0125357	10749593.7	145.444086
0.111969877	13.4318718	973401.6159	203.9026549
0.06195318	57.81880664	1529812.26	110.3396947
0.346153846	0.466022454	2091.333333	95.76190476
0.06625183	39.31926602	7125296.718	407.7513812
0.08493329	43.45985508	639480.9732	106.5402299
0.474320242	0.307391547	1312.343949	184.8917197
0.368421053	0.492206537	864.8857143	74.78571429
0.163043478	2.524327215	48199.66667	95.18666667
0.075759353	151.3416089	3220871.409	119.4241486
0.151376147	13.86377041	6857.045455	54.93939394
0.324324324	1.196768627	975.1944444	59.41666667
0.139329806	8.278904226	16647.82278	60.89873418
0.080825019	104.1159337	4266838.575	227.56639
0.154958678	12.04391414	37141.05333	105.5688889
0.297468354	3.953950135	804.5319149	56.9787234
0.227758007	1.224499851	5801.296875	98.359375
0.044962531	209.5356351	3072456.403	103.0740741
0.174931129	2.417492309	51713.0315	117.511811
0.140246914	7.907814837	142166.581	189.9894366
0.205537806	1.783429107	18722.75648	109.8290155
0.113132462	9.478522553	204392.8844	108.1859296
0.607142857	0.658189831	126.2941176	57.94117647
0.144222415	7.498698547	44883.36145	91.42771084
0.101110795	23.25951989	384390.8535	93.37183099
0.345098039	0.544481823	2603.25	124.9886364
0.175918686	3.915854006	42164.87111	109.2266667
0.090187361	37.68351869	835727.7711	122.7323944
0.136036813	6.859782802	201760.6638	165.7991543
0.249113475	0.802336989	43634.59786	198.3594306
0.630769231	0.075143541	529.1707317	203.9430894
0.068751065	190.7156737	6417837.66	143.4399009
0.248730964	1.184232258	2943.877551	68.59183673
0.078586759	44.04908494	20565082.69	687.302521
0.217115689	1.890691815	14972.0146	111.8175182
0.076965593	163.2324281	2475724.44	137.8402154
0.231172233	1.454992352	27734.52975	181.9830028
0.212164074	1.538743037	15504.56667	114.7533333
0.086397059	19.8854845	158192.4468	77.18085106
0.149872123	5.477768037	130787.2389	153.5733788
0.062275793	118.6528573	2549434.509	123.6059908
0.205479452	2.382958141	3373.688889	75.82222222
0.456140351	0.710044555	379.7692308	68.17307692
0.522727273	0.097366251	900.1391304	209.8695652
0.093913956	15.92483759	426842.1061	83.45810056
0.227883279	2.661641339	59028.63211	198.6056911
0.045931499	468.6923498	7193570.942	119.9439655
0.186540732	1.380027607	14886.32278	112.5506329
0.386759582	1.298965851	845.2882883	92.61261261
0.218702866	2.596940677	8339.731034	96.54482759
0.090221298	40.31542711	1551217.065	157.6457023
0.154692557	4.829773391	63924.21757	112.5355649
0.301470588	0.525001652	3932.134146	103.6463415
0.041721601	992.3325833	22490026.2	147.9745158
0.249686324	0.96010814	18200.43216	177.0050251
0.275733001	0.901754832	35345.97285	246.9660633
0.298993876	0.874614113	65549.45647	308.1536211
0.140023474	21.41701924	1805553.041	255.1475272
0.249221184	1.083421872	4448.075	88.9
0.329061785	0.524976107	18232.66759	262.1808067
0.386554622	0.585714157	621.8913043	82.45652174
0.181928295	6.120296916	310063.739	227.4167776
0.053277462	338.1784964	3523917.944	121.588015
0.251473477	2.613439063	15715.66146	140.3463542
0.257575758	0.930275749	2696.529412	70.50980392
0.075926187	47.27299739	5540219.868	240.8886827
0.18024032	1.690371577	20632.54815	110
0.160298229	5.743790161	49691.97093	108.5465116
0.125932836	17.05076697	484064.5975	168.4222222
0.226821192	2.05301489	10989.32847	112.3576642
0.327935223	0.959456599	5435.619342	221.7510288
0.6	0.086422515	512.2173913	176.8550725
0.337941628	0.557817813	3714.872727	150.7318182
0.090225564	23.86288961	263485.0347	83.36111111
0.297297297	0.51277976	8829.424242	162.6363636
0.352303523	1.55051968	1554.1	114.7692308
0.092744295	28.63768514	1069416.946	135.1167192
0.101276596	8.39281813	142033.7395	99.14285714
0.144598338	7.026026836	99251.1954	118.091954
0.505681818	0.187516099	894.8876404	185.5168539
0.220689655	1.030917407	28302.01875	196.44375
0.060501722	98.73368858	1978610.041	125.6260163
0.311489362	0.642360126	15830.5847	264.0464481
0.28375	1.323438879	6087.819383	143.9823789
0.471014493	0.252996347	1110.292308	179.2769231
0.107692308	10.64557326	141421.7662	105.4350649
0.177381648	9.018707409	131658.6606	136.0032949
0.105085625	35.37319149	457313.8691	120.6592593
0.196903725	5.790977719	44870.086	117.4520885
0.356418919	0.450475169	12294.82464	229.7322275
0.234129693	3.744820573	20857.75219	130.5422741
0.471264368	0.236131989	938.0243902	170.9756098
0.478873239	0.229349871	375.4411765	89.79411765
0.1288005	37.47674117	1436032.456	200.3080032

log.sigma.3.0.mm.3D_glszm_SmallAreaEmphasis	log.sigma.3.0.mm.3D_glszm_LowGrayLevelZoneEmphasis	log.sigma.3.0.mm.3D_glszm_ZoneEntropy	log.sigma.3.0.mm.3D_glszm_SmallAreaLowGrayLevelEmphasis
0.662885534	0.021246155	5.643587436	0.015996943
0.662241369	0.066736839	4.494258606	0.042622945
0.67545106	0.042384206	5.471734684	0.028293203
0.734449205	0.046563032	4.374860596	0.039432728
0.665060796	0.02575579	5.371739108	0.019489847
0.746752401	0.02270933	4.951419757	0.021345185
0.644478016	0.014898837	5.994606652	0.008194643
0.5844362	0.011762854	6.363138466	0.008366484
0.597864828	0.019970478	5.773627198	0.012365147
0.593587025	0.040549784	5.394334731	0.023270001
0.597923288	0.00566003	5.707376779	0.00460568
0.625035127	0.039706581	5.783341707	0.027265335
0.727414688	0.027534631	5.425817086	0.022805901
0.568837089	0.064817977	5.332505338	0.0370119
0.602528246	0.036107965	5.592962524	0.027716843
0.602963824	0.020059332	6.005764105	0.011182849
0.605249789	0.081478818	5.023423241	0.049911697
0.660357559	0.122901364	4.340083252	0.103635505
0.613576295	0.083624408	5.022851009	0.066277214
0.657883464	0.008308101	6.020310625	0.005612318
0.630312926	0.029692205	5.497386357	0.018484543
0.660649048	0.101461678	4.519925224	0.049597958
0.62589831	0.037900265	4.831704689	0.018379449
0.551384139	0.025887932	5.747444681	0.016331921
0.533547432	0.03211899	5.692798644	0.013897372
0.638459199	0.015654949	5.966547855	0.007357178
0.618634303	0.027220068	5.58994714	0.01525542
0.548519728	0.023336571	5.761024477	0.015144629
0.66380719	0.090775616	3.969815782	0.025642854
0.570982581	0.046025049	5.630139407	0.024447396
0.618884899	0.029959102	5.74883785	0.016698004
0.623782294	0.032408633	5.344509642	0.02283107
0.686733646	0.028024019	5.503512195	0.019142011
0.623166322	0.020217011	5.804319045	0.010844122
0.635321771	0.011978556	5.850177958	0.007742361
0.681688332	0.014216877	5.675099426	0.007538019
0.749416556	0.016069553	5.469834373	0.012893373
0.634073758	0.014040322	5.989279428	0.00787316
0.564213579	0.060784163	4.915431017	0.02598495
0.645418414	0.002730993	6.170653069	0.002105016
0.672001698	0.026633879	5.255859591	0.020434086
0.625589405	0.016254815	5.766989011	0.010961704
0.691912986	0.015164759	5.697710342	0.009234587
0.581378673	0.027581591	5.663656362	0.020546084
0.582014282	0.054254108	5.193438088	0.029530149
0.653903572	0.017567413	5.721856283	0.012365061
0.577845711	0.023154024	5.972296746	0.01431614
0.411025638	0.084830471	5.011638864	0.037556948
0.740951142	0.070252062	4.358623612	0.056123759
0.599247585	0.025126688	5.63311459	0.017231037
0.477935397	0.04152415	6.010883186	0.029334225
0.661367557	0.01676608	6.071180378	0.011650985
0.671519468	0.018301726	5.479714302	0.012235275
0.567175805	0.023213769	5.654941528	0.015796874
0.662171748	0.071139659	5.263438229	0.036036319
0.690328031	0.05613593	5.350720233	0.039014164
0.595603219	0.012926396	6.116199789	0.00682068
0.624443595	0.024211411	5.6648395	0.014603375
0.666727758	0.031267057	5.019154371	0.022207099
0.630321025	0.016792293	6.042616662	0.009869791
0.642902373	0.017159522	5.648477793	0.012681298
0.687344661	0.010424606	5.924211476	0.007285073
0.660315787	0.007005344	6.317444911	0.004750296
0.659320295	0.008283438	6.072090817	0.005903603
0.581388263	0.044967444	5.284506614	0.022601573
0.649884601	0.009794313	6.285793665	0.006705247
0.606315905	0.050412231	4.544876086	0.035649866
0.673890486	0.009899115	6.011053921	0.006557171
0.627224441	0.02329399	5.76863651	0.012916024
0.668730034	0.025647406	5.721654107	0.017482317
0.498834698	0.054214766	5.01537093	0.04018343
0.637577017	0.007480967	5.887389177	0.005312033
0.575419666	0.025889154	5.530607814	0.01800272
0.640502986	0.033727339	5.538594333	0.02025149
0.613370641	0.013544599	5.961845113	0.008765956
0.595607656	0.028423731	5.424022412	0.017345992
0.617102432	0.014041552	6.223335658	0.009424631
0.685362319	0.030906719	4.97785761	0.025606711
0.62386029	0.02700977	5.750383332	0.016389925
0.614794357	0.0414737	5.318610653	0.030150574
0.662885534	0.021246155	5.643587436	0.015996943
0.661478677	0.034891992	5.387591443	0.023706239
0.570446592	0.01813616	6.016828626	0.011723479
0.503413658	0.025788545	5.678825668	0.015471085
0.629961215	0.023509416	5.64780625	0.015494618
0.672134987	0.02263056	4.97761641	0.016330983
0.675173394	0.016990426	5.495060769	0.013358771
0.630885201	0.019524507	5.726784067	0.013199659
0.642689595	0.01683486	6.208139957	0.010750494
0.669374017	0.017594588	5.469385104	0.011966765
0.657924477	0.027437007	5.344452572	0.020378563
0.57399034	0.026195221	5.454449951	0.011504788
0.647596399	0.017938504	5.868235119	0.011880504
0.632460248	0.02540921	5.72617816	0.016445981
0.595822015	0.018514041	5.832574361	0.010888063
0.715646075	0.011047146	5.69170642	0.008005772
0.652339294	0.037580881	5.71242659	0.020314642
0.688867941	0.036508541	4.485004748	0.013692273
0.557908497	0.053945756	4.4548224	0.043199011
0.645509504	0.013733438	6.231833727	0.009561884

log.sigma.3.0.mm.3D_ngtdm_Coarseness	log.sigma.3.0.mm.3D_ngtdm_Complexity	log.sigma.3.0.mm.3D_ngtdm_Strength	log.sigma.3.0.mm.3D_ngtdm_Busyness	log.sigma.3.0.mm.3D_ngtdm_Contrast
0.012551128	0.583591842	2.226646641	0.275881043	0.000143605
0.04920201	1.502665356	3.029363622	0.402498095	0.002717604
0.015192163	0.676221284	1.854802511	0.403973414	0.00036744
0.064642477	2.809284721	5.781804807	0.175531917	0.002768464
0.009887785	0.39280168	1.45669735	0.543297381	0.000107714
0.052239596	5.756639547	9.168776996	0.063920264	0.003027255
0.000573754	0.011942445	0.264557668	3.579970463	5.99E-07
0.002252988	0.061130408	0.667037649	0.812507865	5.75E-06
0.002415335	0.034716975	0.506970689	1.227238416	5.63E-06
0.020060114	0.583596062	2.044542913	0.341413788	0.000335946
0.001703451	0.037435344	0.86052077	0.73536216	1.42E-06
0.003279938	0.046555087	0.625538094	1.046974394	8.43E-06
0.020667648	1.866271622	3.661474864	0.252083492	0.000692792
0.035348847	0.962564672	2.719239217	0.281885801	0.000747372
0.010551707	0.193566801	1.364200595	0.402619577	6.67E-05
0.001048781	0.019512708	0.260230579	2.711804736	3.34E-06
0.019398439	0.220961639	1.385831769	0.674915485	0.000163634
0.054556616	1.083493527	3.207432013	0.270412504	0.001313497
0.015713081	0.123684194	0.814532026	0.741200091	0.000141305
0.00084691	0.03532752	0.613688925	1.810459012	1.10E-06
0.005856858	0.144942342	0.838752498	0.994649919	4.62E-05
0.042039691	0.691651176	2.307369611	0.488097794	0.000961498
0.028155874	0.470417522	2.072257204	0.296076764	0.000457787
0.002104125	0.022005654	0.530660635	1.409538731	2.07E-06
0.011357112	0.285880097	1.537737494	0.366843608	0.000151698
0.005000261	0.218222109	1.888604923	0.461103439	1.71E-05
0.009398858	0.211954029	1.138013753	0.600323026	9.08E-05
0.006159822	0.075354693	0.7686893	0.663920573	2.54E-05
0.168209384	4.581520042	9.352383473	0.128242838	0.012120991
0.007675698	0.120400013	0.786026543	0.800009569	5.59E-05
0.002984226	0.05117508	0.490061413	1.276979427	1.25E-05
0.02779534	1.055242941	3.038481546	0.21274	0.000496966
0.007364724	0.193558598	1.232330508	0.547171615	4.28E-05
0.003041797	0.046591311	0.601351837	1.042043631	9.97E-06
0.002774311	0.076228977	0.591499559	0.955856172	9.22E-06
0.007804014	0.360614812	1.582458333	0.32803112	8.88E-05
0.015130915	2.557684653	3.539156558	0.229070775	0.000655266
0.000837137	0.020766054	0.371161805	2.25546248	1.27E-06
0.041925359	0.478309092	2.511337799	0.276141678	0.000564167
0.000813296	0.030700667	0.715938805	0.936503508	6.98E-07
0.012810828	0.303644798	1.39529142	0.429097184	0.000111733
0.00130543	0.021353879	0.371458023	1.981200913	1.22E-06
0.005388136	0.305574603	1.230903162	0.55669132	5.58E-05
0.012225206	0.307327532	1.462408142	0.434560686	0.000116319
0.010222998	0.055447743	0.755526648	0.662401373	4.53E-05
0.004807651	0.162201948	1.125998142	0.570729296	2.12E-05
0.001542838	0.019475377	0.346754969	1.97236403	3.09E-06
0.047669998	0.449182415	3.618996188	0.265369946	0.000532219
0.057350673	1.292498783	3.648373612	0.287084189	0.001827304
0.030179983	5.350420718	10.38067478	0.116603748	0.001511685
0.005798661	0.049827793	0.859122287	0.653519104	2.32E-05
0.004571869	0.298873631	1.760442766	0.459549425	2.19E-05
0.000886057	0.010542886	0.255528476	3.229190422	8.60E-07
0.013263039	0.17835957	1.419871755	0.429691982	9.94E-05
0.023864984	1.033411493	2.87036694	0.493475081	0.000767739
0.011656274	0.430604304	1.919977621	0.501306525	0.00011453
0.00198739	0.03408933	0.495897766	1.202735261	4.29E-06
0.005534641	0.156996952	0.850493628	0.789061164	4.67E-05
0.030027562	0.55270431	2.361116826	0.23939808	0.000391078
0.00035477	0.010128743	0.297426193	4.930304516	2.12E-07
0.010006305	0.448532587	1.528874261	0.34926149	0.000114106
0.005774586	0.393988447	1.452282477	0.401516483	5.57E-05
0.002120572	0.190859231	0.696740579	0.773434859	1.73E-05
0.001141368	0.055501312	0.54379622	1.205405431	2.71E-06
0.028064766	0.431466932	2.118895529	0.300363157	0.000357413
0.003981616	0.470201614	1.612029216	0.480645459	4.33E-05
0.049662789	1.04919449	2.367335892	0.316116126	0.001787478
0.002403639	0.162707061	1.298595758	0.638121154	6.97E-06
0.000993741	0.016102626	0.295320404	3.084919488	1.27E-06
0.005659409	0.293446495	1.291826238	0.702543733	5.04E-05
0.031503899	0.690046362	1.921720075	0.337210879	0.000810508
0.001365411	0.024145205	0.419405418	1.379814294	4.44E-06
0.010925373	0.261586905	1.189887794	0.521735252	0.000116059
0.008633442	0.222734293	1.7560394	0.463401002	3.98E-05
0.003180737	0.101289707	1.222114386	0.662477469	6.47E-06
0.014369277	0.445405682	2.390350511	0.319836005	0.000106533
0.005678139	0.756349925	2.858479548	0.531622215	0.000105073
0.053801481	4.199227768	7.788870564	0.096894271	0.002877049
0.012295843	0.598450025	1.780062215	0.372461968	0.000200289
0.006248821	0.048939057	0.579338258	0.856959374	2.25E-05
0.012551128	0.583591842	2.226646641	0.275881043	0.000143605
0.019447729	0.783915158	2.391448306	0.472317483	0.00047775
0.002775545	0.068632221	0.876210095	0.782147782	7.57E-06
0.00918364	0.069603418	0.832198183	0.593513607	4.02E-05
0.005341497	0.095810858	0.674386987	0.784591116	2.79E-05
0.02991517	2.69149996	4.016341084	0.206620172	0.002160109
0.012697519	0.471424366	2.729630945	0.210601169	8.26E-05
0.00248939	0.035763675	0.594160115	1.123367641	4.47E-06
0.007635116	0.925385903	3.556073241	0.269062724	0.000111386
0.009523613	0.469997569	1.620750844	0.486904495	0.0001136
0.022905492	1.690299026	3.826075878	0.175659105	0.000813851
0.007167347	0.078303633	0.825389854	0.651408342	2.67E-05
0.002844504	0.075218836	0.501111328	1.195687201	1.21E-05
0.002586884	0.050024068	0.476214286	1.34103278	8.23E-06
0.004476238	0.088882339	0.547987682	1.063519484	2.28E-05
0.006502837	0.547582849	1.371619748	0.421160409	9.99E-05
0.006056164	0.256208536	1.208695583	0.756658739	5.78E-05
0.070590204	4.191746541	8.426474356	0.083681805	0.003401058
0.079462409	2.70864111	5.337451568	0.129794341	0.00315366
0.001096913	0.041455038	0.383670683	1.718302666	3.17E-06

log.sigma.2.5.mm.3D_gldm_GrayLevelVariance	log.sigma.2.5.mm.3D_gldm_HighGrayLevelEmphasis	log.sigma.2.5.mm.3D_gldm_GrayLevelNonUniformityNormalized	log.sigma.2.5.mm.3D_gldm_DependenceEntropy
11.46580815	157.7087087	0.086717348	6.253677415
8.836244029	48.20353982	0.100634349	5.441872715
14.40734025	113.0455487	0.077847648	6.276587674
10.42296786	60.95652174	0.104442344	5.212375103
8.244523069	80.6769437	0.101010573	6.279081789
22.63644076	261.5339806	0.068715242	5.187280717
3.827318615	169.5617564	0.284920273	6.466004333
8.297951082	208.9137931	0.137475848	6.787803103
5.167392148	168.1960274	0.186767985	6.414830686
10.74384132	77.31868132	0.087368675	5.958469133
2.911295838	464.2095534	0.243393802	6.557440782
5.545400356	154.1425317	0.190772873	6.58174105
20.71875941	119.5740181	0.066036272	6.036613018
8.455096953	66.61578947	0.099889197	5.562839186
8.55020794	155.9086957	0.111323251	6.280467183
6.169675753	156.6613111	0.242717849	6.446252518
5.034593048	41.58715596	0.124810622	6.120756302
6.601574548	51.64864865	0.12263615	5.130216345
5.197807701	59.41622575	0.130477248	6.259161464
5.627539107	228.1267712	0.185690171	6.830409157
6.942480306	64.03236915	0.117946937	6.447275039
5.141523794	38.09493671	0.1334722	5.25662646
7.976089462	81.22775801	0.105051861	6.115102791
2.867087369	115.5830558	0.274802829	6.305148406
11.55698988	165.7134986	0.137528554	6.420872514
9.348122298	144.7802469	0.122204542	6.668791011
8.629484837	103.8945687	0.096249267	6.363328672
6.222858419	161.0477544	0.130792633	6.658700379
12.17346939	67.35714286	0.119897959	3.878783493
6.622537272	70.3032146	0.119108455	6.550775892
7.271697422	112.3648533	0.140237692	6.73720064
8.852041522	78.03529412	0.107481738	5.766950252
8.140331487	88.07427678	0.115035019	6.511328922
6.045935737	158.371229	0.208543877	6.535863319
6.471292471	160.3111878	0.131612489	6.616963698
15.64049532	222.9698582	0.085279287	6.772899761
26.97639053	200.6076923	0.057343853	5.824733686
5.506227972	167.085023	0.249405207	6.579770711
6.576000412	73.28426396	0.113968409	5.748051935
4.560365047	832.388311	0.243942834	6.462130996
7.58995984	97.31378764	0.103910227	6.246518116
2.968613478	124.2454056	0.252235811	6.396617499
11.92051735	147.2534381	0.084395314	6.740336776
8.936454809	120.9009901	0.094394507	6.237213523
4.76371158	82.18106618	0.176945353	6.485572073
7.873963148	199.9693095	0.119681321	6.596985375
4.881010035	137.3241498	0.203815326	6.66598562
8.80682221	55.28310502	0.102062092	5.770524533
8.249923053	54.8245614	0.105878732	5.353570199
42.66402893	157.6409091	0.057107438	5.907722117
6.426582703	143.8179433	0.206932545	6.295244069
12.18985082	167.1398796	0.095264585	6.807366681
2.503693577	118.9116017	0.303849027	6.202275777
9.921740597	85.18299882	0.091129328	6.416308754
13.8606029	95.93031359	0.083854363	5.943539632
7.548362146	82.21568627	0.103803953	6.293891624
5.442751632	155.9330433	0.192652401	6.647729941
9.002736042	117.4860841	0.110937673	6.615205463
8.89570718	90.55882353	0.094912413	6.00688942
2.751582381	169.9143878	0.298723109	6.17229253
11.47153142	137.9987453	0.09510728	6.492246315
16.21133799	185.5196507	0.078104101	6.771478646
18.61023062	245.4446632	0.073523153	6.985708672
8.331186149	369.7996479	0.159617195	6.8830935
8.746401918	85.97819315	0.098339496	6.149705034
22.87809142	204.3935927	0.064696783	6.808779071
9.051761881	69.86554622	0.11220959	5.255400327
10.43791402	162.1131298	0.113273973	6.862347464
3.435991861	98.26080016	0.227053981	6.452374032
11.43032231	130.5789129	0.088684834	6.574245506
8.377844098	69.58080808	0.102285481	5.680479917
6.975397653	237.8492745	0.213835335	6.676981527
9.216892662	132.9639519	0.097402678	6.237065263
6.607162679	95.59552656	0.127248597	6.480813281
6.426521679	112.0954602	0.166677496	6.738307137
9.056738191	81.89900662	0.101229113	6.360332409
29.25814588	150.9419703	0.064685356	6.683051705
18.00952741	94.07826087	0.072060491	5.26472739
12.88067749	148.4546851	0.079921944	6.293478486
3.888733425	81.12155388	0.181648514	6.320559329
11.46580815	157.7087087	0.086717348	6.253677415
12.93587738	71.25745257	0.085773459	5.987760685
6.804090296	121.2214745	0.213843963	6.370968397
4.978155545	104.5021277	0.143947125	6.37182987
6.554267079	93.52409972	0.134781961	6.643316468
20.61454029	112.8181818	0.065276343	5.609381527
10.17755244	165.7558621	0.095020214	6.431574194
4.302497236	106.9065421	0.246045391	6.318221868
29.71257655	196.9029787	0.066340244	6.886558811
10.78824375	95.935	0.088659375	6.376805908
17.16645663	137.8478261	0.070074564	5.842809687
4.793914617	110.006993	0.151954619	6.448499677
7.490056946	123.4015196	0.119455334	6.731065734
5.959679576	109.5407369	0.158493767	6.738973797
6.638433943	100.0174165	0.118772219	6.542630566
15.87258035	160.5219595	0.072372626	6.520928852
10.61076728	121.5283276	0.093996203	6.632659472
19.51803409	138.2528736	0.078874356	5.095146013
7.144217417	53.42253521	0.107319976	4.774412746
8.873807266	187.9608496	0.148431967	6.927869861

log.sigma.2.5.mm.3D_gldm_DependenceNonUniformity	log.sigma.2.5.mm.3D_gldm_GrayLevelNonUniformity	log.sigma.2.5.mm.3D_gldm_SmallDependenceEmphasis
106.3213213	57.75375375	0.286258372
21.99115044	11.37168142	0.346328437
73	37.60041408	0.267902548
17.60869565	9.608695652	0.322314974
90.39410188	75.3538874	0.229347102
35.87378641	7.077669903	0.51881068
686.3804734	4645.909972	0.062580365
359.085216	693.7031312	0.114017971
238.8481911	789.8418066	0.080124631
67.92307692	31.8021978	0.309846363
272.1943631	1329.903734	0.081528897
165.14904	586.2450374	0.092195693
74.0694864	21.85800604	0.389462706
37.07368421	18.97894737	0.34559133
96.77391304	102.4173913	0.153554805
422.8318283	2069.655096	0.080582414
48.32110092	54.41743119	0.142073737
21.7027027	13.61261261	0.344613287
58.11816578	73.98059965	0.163233269
632.1089126	2214.726671	0.099073175
140.3471074	171.2589532	0.138957313
26.6835443	21.08860759	0.259245897
34.06049822	29.51957295	0.206672063
220.9608659	1320.152789	0.057953828
62.5261708	99.84573003	0.181485355
163.2182716	247.4641975	0.140043943
123.5218317	90.37806177	0.197223631
134.8419557	230.064241	0.117046283
10.5	3.357142857	0.463730159
106.2441355	137.0938315	0.145891209
232.5004272	492.3745372	0.103530072
40.44313725	27.40784314	0.280654894
127.9741986	147.1297889	0.184052857
165.6351223	656.7046681	0.090339822
279.5539258	457.6166235	0.144414269
130.8475177	96.19503546	0.223874352
130.5846154	22.36410256	0.556421609
515.4862839	2927.518317	0.07313743
27.95431472	22.45177665	0.198524058
581.5070167	2955.123493	0.084069833
79.43740095	65.56735341	0.217526537
354.6725162	1825.430565	0.094883978
182.3686968	128.8716437	0.215058257
93.59405941	66.73691655	0.213729729
66.87132353	192.5165441	0.094985631
158.9375959	233.9769821	0.145575714
340.2879897	1420.389008	0.076880695
35.3196347	22.35159817	0.156363667
20.92982456	12.07017544	0.351292133
59.90909091	12.56363636	0.402535354
111.956978	394.4134313	0.105223001
226.2691061	205.676239	0.224411174
446.6844189	3069.482875	0.05492359
111.0306966	77.18654073	0.183672688
55.11149826	24.06620209	0.343120112
80.75263952	68.82202112	0.192669048
282.3943635	1018.553244	0.094482115
134.5288026	171.3987055	0.158474901
40.97794118	25.81617647	0.25394031
1160.889253	7023.876451	0.053735458
100.9372647	75.80050188	0.237323151
200.5246413	125.2008734	0.256074336
606.2751531	336.1478565	0.277182363
501.2561033	1359.938498	0.140616371
42.50778816	31.56697819	0.19917466
365.0723112	141.3624714	0.311683318
23.90756303	13.35294118	0.320235773
331.6787791	467.5949612	0.189007802
484.9648808	2275.762047	0.063897953
190.6254093	135.421742	0.234753826
34.25252525	20.25252525	0.212669736
331.5169742	1518.017045	0.082428389
93.09078772	72.95460614	0.176782991
91.00372787	136.5377446	0.161125618
207.5982587	536.0348259	0.129753924
74.68874172	61.14238411	0.207607261
260.1592443	95.86369771	0.302143661
31.10434783	8.286956522	0.488325939
109.4485407	52.02918587	0.340568361
97.19799499	289.9110276	0.1011627
106.3213213	57.75375375	0.286258372
62.79403794	31.6504065	0.26814013
191.0713868	730.9186659	0.099742314
94.12340426	169.1378723	0.108380587
138.5047091	243.2814404	0.138213222
43.53409091	11.48863636	0.435112663
91.41655172	68.88965517	0.186349535
189.7747172	1000.420561	0.069837244
177.0493617	77.94978723	0.273246271
106.4625	70.9275	0.263096284
62.66666667	19.34057971	0.360717602
106.7104895	217.2951049	0.097059133
292.9795441	408.7761543	0.179735313
228.8858329	610.8349766	0.115558169
222.5529753	245.5021771	0.190965498
190.8462838	85.68918919	0.306172305
167.1802048	137.7044369	0.21421453
21.7816092	6.862068966	0.418157531
14.66197183	7.61971831	0.342852113
601.9900042	1425.540608	0.134671984

log.sigma.2.5.mm.3D_gldm_DependenceNonUniformityNormalized	log.sigma.2.5.mm.3D_gldm_DependenceVariance	log.sigma.2.5.mm.3D_gldm_LargeDependenceEmphasis
0.159641624	4.325298271	15.73873874
0.194611951	2.634818702	10.80530973
0.151138716	9.18810574	23.71635611
0.191398866	2.709829868	11.19565217
0.121171718	15.44609679	37.33243968
0.348289188	0.662079367	4.029126214
0.042093737	46.46980733	201.2346376
0.07116235	31.25449464	88.21720174
0.056478645	34.60652105	117.7831639
0.18660186	2.862365656	11.76373626
0.049815952	34.25046946	128.0538067
0.053741959	32.36482029	115.2382037
0.223774883	2.312994587	8.86102719
0.195124654	2.388919668	10.76842105
0.105189036	20.41763233	52.53695652
0.049587408	40.24848818	148.789844
0.110828213	7.97496002	33.43577982
0.195519844	2.291859427	10.24324324
0.102501174	10.79656225	37.62786596
0.052998148	37.48328269	123.1423661
0.096657787	28.76629936	68.87741047
0.168883192	2.811248197	13.81012658
0.121211737	10.89031294	31.28469751
0.045995184	37.63075438	159.815154
0.086124202	34.42949404	81.78512397
0.080601616	20.9912089	62.47753086
0.131546147	8.658128819	27.47284345
0.076658303	21.62457479	67.67765776
0.375	0.780612245	4.5
0.092305939	18.41039975	53.65334492
0.066220572	36.45394914	102.6183424
0.158600538	4.222775855	16.05098039
0.100058013	16.25113199	45.26270524
0.052599277	36.10024444	125.4204509
0.080400899	26.50490371	71.22203049
0.115999572	17.66703134	41.7535461
0.334832347	1.082287968	4.605128205
0.043916024	47.05489462	173.5091157
0.141900075	6.470818625	22.75634518
0.048002891	36.76420282	143.9073799
0.125891285	6.784366123	24.8637084
0.049008224	36.06600082	131.8959514
0.119429402	7.863165917	26.85723641
0.132381979	7.488309469	24.58132956
0.061462614	30.5576307	99.38051471
0.081298003	23.14535776	65.76521739
0.048828812	38.96077418	142.0463481
0.161276871	4.141531661	20.17808219
0.183594952	3.543859649	12.54385965
0.27231405	1.325619835	6.490909091
0.058739233	36.691168	112.2151102
0.104802736	14.38720815	39.08244558
0.044217424	39.94454999	187.6216591
0.131087009	5.887174541	23.94214876
0.192026126	2.879141425	11.42508711
0.121798853	7.262859756	26.9668175
0.053412968	35.4250742	118.3153017
0.087073659	29.86001571	72.35016181
0.150654196	3.756001298	16.26470588
0.04937223	31.97975308	163.748437
0.126646505	25.02368197	49.89836888
0.125093351	11.77802771	30.7654398
0.132606114	12.39034398	30.23228346
0.058832876	43.16867239	118.7239437
0.132423016	5.191215147	21.92211838
0.167081149	4.425885667	14.98169336
0.200903891	3.034955159	11.53781513
0.080348541	27.26788669	69.43604651
0.048385202	33.81154903	146.4153447
0.124836548	12.41264915	32.2259332
0.172992552	3.532190593	15.60606061
0.046699109	45.31962724	157.8756163
0.124286766	12.54100082	35.37383178
0.084812421	21.49253602	60.65703635
0.064551697	36.23592818	99.59079602
0.123656857	7.501820096	26.31788079
0.175546049	3.362733367	13.08097166
0.27047259	1.874480151	6.791304348
0.168123718	4.745680166	15.1812596
0.060900999	27.78341059	93.66165414
0.159641624	4.325298271	15.73873874
0.170173545	3.780421707	14.94579946
0.055901517	38.06302503	123.4821533
0.080105025	21.10079421	66.63744681
0.076733911	28.85125314	76.74072022
0.247352789	2.120738636	7.761363636
0.126091795	7.638742449	26.88965517
0.046673565	46.13906427	164.2990654
0.150680308	6.130132368	19.33021277
0.133078125	8.05999375	24.04
0.22705314	3.515385423	11.05797101
0.07462272	29.22581838	83.77902098
0.085616465	22.23487312	59.11689071
0.059389163	32.30760539	100.6808511
0.107669557	11.7807119	35.49153362
0.16118774	5.186895088	16.48648649
0.114116181	10.73106781	32.13037543
0.250363324	2.033557934	7.804597701
0.206506646	1.976988693	9.366197183
0.062681175	37.08925026	103.5718451

log.sigma.2.5.mm.3D_gldm_LargeDependenceLowGrayLevelEmphasis	log.sigma.2.5.mm.3D_gldm_SmallDependenceHighGrayLevelEmphasis	log.sigma.2.5.mm.3D_gldm_LargeDependenceHighGrayLevelEmphasis
0.114824636	44.50854389	2690.366366
0.64252511	24.40937496	339.1769912
0.274466678	36.88770805	3497.795031
0.394371611	23.19004541	521.5652174
0.491997029	21.70816247	4110.757373
0.033234828	139.3884844	1035.631068
1.133081275	10.71379619	36400.67153
0.371151167	21.25798012	22043.41914
0.617536113	11.19901478	23330.73753
0.309929221	27.57683163	930.3296703
0.277149287	37.71207615	59531.85121
0.686653674	13.87472916	20234.23039
0.226049661	59.54091658	777.9848943
0.32183374	25.46668719	661.4789474
0.275473034	19.14198556	11043.54348
0.895427304	11.75709189	25874.11012
1.174151532	7.248718081	1594.761468
0.406845511	22.81849783	423.4144144
0.757996914	10.98604903	2439.22575
0.536024048	24.4282327	28914.76683
1.097237031	11.5333774	5987.161846
0.667215696	13.49097191	439.3987342
0.447782869	18.30513796	3398.715302
1.313107386	6.186682735	19923.33118
0.414755743	27.70193352	17771.96143
0.429337074	25.82016551	10038.72198
0.282494635	19.64100925	3819.372737
0.380972346	16.92686497	12941.51507
0.158682385	28.59777778	288.7857143
0.822903793	10.90273761	4767.25543
0.792291898	10.47933788	14957.33552
0.312954549	29.72721605	1160.403922
0.532140061	17.60677883	5151.971071
0.738754763	13.19408352	22180.09432
0.393516096	24.00121384	13769.24015
0.179553107	49.60627985	12167.02128
0.054138714	125.8961211	720.3923077
0.970594092	11.06483226	31945.97018
0.347107444	13.2694776	1887.081218
0.169462159	71.99521341	122643.2699
0.290255925	22.16252532	2848.442155
1.069687962	13.44138457	16560.96808
0.218267187	35.34678508	4608.112639
0.211319916	26.67295605	3706.439887
1.127460262	7.271954422	9771.932904
0.291026798	30.37135353	16050.66803
0.978394315	9.638599847	21420.53609
0.723015153	11.04117254	1126.552511
0.597013762	25.89715654	531.0701754
0.257241521	78.10467551	690.6954545
0.686427796	11.3773026	19016.25236
0.250396246	47.26193905	6976.976841
1.523471839	6.526819911	23560.10087
0.419798525	14.03992794	2342.214876
0.236333234	43.90881398	853.8606272
0.399522271	18.34309729	2592.242836
0.707993816	13.5692256	20473.10346
0.520836607	17.90081955	11892.57087
0.258030517	22.56228795	1554.602941
0.934417362	9.335431907	29110.97916
0.33223876	38.52206352	8786.129235
0.181168006	57.1017146	6314.857143
0.137300779	81.01155283	7887.646325
0.292032351	52.19071588	49037.04096
0.390065623	19.02628899	1988.102804
0.113774686	75.63746455	2772.615103
0.346990406	28.32144601	610.7731092
0.408367132	35.92948432	13177.31953
1.428534929	7.487349397	15676.82969
0.266413879	38.58187837	4802.306483
0.340336516	14.55189564	1315.025253
0.596833884	16.56479255	42812.76771
0.241742014	21.63185846	6562.59012
0.581759909	16.40240965	7185.384902
0.864533531	16.70090493	12300.15267
0.464440464	22.74353838	2169.455298
0.240728616	56.21163166	1428.778677
0.368875584	63.1609826	377.8695652
0.147510257	60.33760732	2052.089094
1.022776784	6.763447846	9211.483709
0.114824636	44.50854389	2690.366366
0.568740125	28.9785748	785.4254743
0.908738304	9.658797235	17994.78087
0.563041785	9.789208162	8679.557447
0.760497951	14.04673497	8901.700277
0.306926617	65.24787129	533.2045455
0.204485896	32.99361795	5239.358621
1.404365326	6.944582221	20075.67585
0.179509093	66.29814768	3386.40766
0.337217351	34.36885063	2558.195
0.242605744	60.77763299	993.0507246
0.669428063	10.05536162	11262.73846
0.463794174	25.28982069	8258.468147
0.892893891	13.91686121	12324.9014
0.401572476	23.28502482	3614.522496
0.140018916	58.88204002	2949.165541
0.306331105	33.73175675	4246.19727
0.114867538	61.97206128	946.2988506
0.456291418	25.56947574	351.3380282
0.544280402	26.88316942	20617.19284

log.sigma.2.5.mm.3D_gldm_SmallDependenceLowGrayLevelEmphasis	log.sigma.2.5.mm.3D_gldm_LowGrayLevelEmphasis	log.sigma.2.5.mm.3D_gldzm_DistanceZoneVariabilityNormalized
0.006053643	0.011982162	0.972482423
0.031251916	0.067198643	0.957466919
0.007782218	0.022015149	0.920665874
0.034131457	0.055926645	1
0.008811527	0.024028847	0.933082309
0.012735406	0.015580747	1
0.000692966	0.006971481	0.902022049
0.001374566	0.00642332	0.872877158
0.001134576	0.007431917	0.928209459
0.015248991	0.042746768	0.94035503
0.000393714	0.002409779	0.870117473
0.002004531	0.009156012	0.978130407
0.008959627	0.028453786	0.922706184
0.01948347	0.038577735	0.972978045
0.003548561	0.010160458	0.973514707
0.001100424	0.008515591	0.841432606
0.013579316	0.052312791	0.967750605
0.016798562	0.052495699	0.955578512
0.008936009	0.031555329	0.875555556
0.000659699	0.005061628	0.831249392
0.004440793	0.030182192	0.92015893
0.019033281	0.055893792	1
0.008207176	0.027176831	0.938523558
0.001236704	0.010521646	0.938539893
0.003683419	0.010742818	0.947406866
0.001833978	0.009780358	0.56721277
0.005490108	0.018723751	0.948489684
0.001519178	0.00876326	0.942113518
0.056803673	0.071649127	1
0.008591031	0.032700214	0.928288371
0.002447747	0.013911354	0.900606945
0.012654911	0.029310766	1
0.00715213	0.025357974	1
0.001356937	0.008295692	0.929309091
0.001634896	0.008028263	0.959699623
0.002989415	0.008613754	0.964550561
0.007489693	0.012132153	1
0.000892556	0.007604096	0.783052128
0.012976734	0.028467561	1
0.000190437	0.001311706	0.918912203
0.006298659	0.017242653	0.959748546
0.001323218	0.009501777	0.889402636
0.004350736	0.013509968	0.970436373
0.004366576	0.013395243	0.952690542
0.004229319	0.020071487	0.942878688
0.001606904	0.00671707	0.955599858
0.001387275	0.009610436	0.971087691
0.01281674	0.053633686	0.953514739
0.021242108	0.054029019	1
0.011686461	0.03136315	0.808673469
0.002548238	0.010317277	0.990338392
0.002714828	0.009460739	0.917748671
0.000973316	0.009786546	0.95183661
0.01168353	0.03034253	0.9753125
0.010454193	0.024099934	0.899443966
0.005798896	0.021713344	0.895061728
0.001411255	0.008321015	0.944529704
0.003508216	0.013964893	0.84063996
0.010450207	0.024328621	0.950032873
0.000545649	0.006542338	0.863618973
0.004394688	0.012927788	0.961919125
0.003491201	0.009681506	0.921524468
0.002253414	0.006756357	0.911212757
0.000659789	0.003183103	0.9323375
0.006381117	0.027535758	0.971020761
0.004620178	0.010584083	0.733604571
0.012859771	0.041241798	0.957466919
0.00284566	0.010873563	0.936809442
0.001303218	0.012658633	0.961392333
0.004231152	0.013130708	0.943642659
0.010957933	0.03269615	0.924644368
0.000801766	0.005232331	0.811883382
0.003835692	0.011978537	0.908109394
0.004760682	0.016927995	0.96532872
0.002818624	0.014069605	0.936432366
0.006538162	0.026616258	0.943310778
0.005930147	0.019168355	0.548296845
0.015195038	0.050746391	1
0.00546827	0.012359121	0.960645796
0.004419438	0.018208404	0.987180021
0.006053643	0.011982162	0.972482423
0.009806477	0.039793799	0.8828125
0.002883472	0.014349741	0.899209348
0.002954974	0.013498084	0.954223633
0.003616187	0.017740873	0.924934518
0.013926751	0.046870553	0.95654321
0.002264214	0.012650582	0.959476813
0.001465831	0.012747378	0.960202593
0.004763708	0.015564001	0.557582568
0.00845122	0.021983872	0.892466913
0.008563939	0.021067513	0.967222222
0.002301148	0.01243502	1
0.003325277	0.012213447	0.962861607
0.002413157	0.013298803	0.940559363
0.003269907	0.014345487	0.972670397
0.004675681	0.012782997	0.942637746
0.003972415	0.014016458	0.975613474
0.017818186	0.025783352	1
0.019936357	0.062732219	1
0.001727443	0.00773012	0.858517252

log.sigma.2.5.mm.3D_gldzm_LowIntensityEmphasis	log.sigma.2.5.mm.3D_gldzm_LargeDistanceEmphasis	log.sigma.2.5.mm.3D_gldzm_HighIntensitySmallDistanceEmphasis
0.020222295	1.041860465	152.0662791
0.089209887	1.065217391	65.32608696
0.030972683	1.124137931	129.6637931
0.092552178	1	77.91428571
0.040362406	1.132183908	91.43263091
0.021534461	1	264.90625
0.012258199	1.174897119	166.697045
0.013089194	1.268206039	163.2285006
0.016217607	1.128378378	128.6351351
0.05705942	1.092307692	83.45
0.004983945	1.300947867	446.0460111
0.023816637	1.051470588	144.1148897
0.028086452	1.120805369	147.0587248
0.05664576	1.04109589	71.2739726
0.023407242	1.040268456	115.6191275
0.014259304	1.260465116	137.6593023
0.10054524	1.049180328	52.54098361
0.059057167	1.068181818	62.77840909
0.059536634	1.2	62.25
0.006945176	1.509571559	234.0962445
0.032725729	1.150259067	88.195308
0.074327219	1	52.53488372
0.041088337	1.095238095	83.59126984
0.025096519	1.177865613	97.81225296
0.020342575	1.081081081	143.6452703
0.013870498	2.586956522	119.8925624
0.032774189	1.079365079	92.93518519
0.016752754	1.148514851	129.6757426
0.100521406	1	65.52941176
0.068756689	1.141975309	72.45061728
0.027130924	1.244648318	93.45211094
0.044546803	1	101.0853659
0.043987391	1	92.74273859
0.015952187	1.189090909	139.7312374
0.011997406	1.071868583	162.0430071
0.01350791	1.054151625	209.4684116
0.013080696	1	223.3183673
0.013334696	1.578947368	129.9033094
0.06121951	1	69.86363636
0.002302586	1.165496489	823.6771565
0.029146324	1.061643836	97.52910959
0.015115117	1.275297619	135.1033135
0.022490341	1.05988024	158.2044245
0.020999948	1.072727273	116.1787879
0.051017992	1.252427184	69.45091694
0.011775209	1.113207547	205.9106132
0.021300649	1.054393305	122.3740121
0.091130777	1.071428571	69.83333333
0.06234564	1	70.17391304
0.028278674	1.321428571	134.0892857
0.026167477	1.014563107	99.04975728
0.012829301	1.138401559	201.6629846
0.019524752	1.074074074	120.4855967
0.067376128	1.0375	72.2625
0.029181867	1.159292035	118.5884956
0.032232249	1.166666667	92.80357143
0.016285035	1.129817444	136.7150665
0.023220814	1.261904762	103.0555556
0.040782036	1.076923077	84.90064103
0.011316067	1.892416226	161.9294426
0.018987979	1.058252427	159.2354369
0.013840821	1.133786848	217.3381204
0.008167906	1.139684814	282.0248926
0.004817324	1.119166667	361.4404398
0.041359741	1.044117647	92.73897059
0.014464084	1.625	199.9953673
0.047902584	1.065217391	84.82608696
0.017337712	1.110275689	179.6189084
0.022292176	1.128113879	124.4334964
0.0179278	1.1	166.1243421
0.049732207	1.117647059	66.45588235
0.010591737	1.527075812	175.9607401
0.021936396	1.144827586	108.65
0.031524659	1.052941176	100.5132353
0.025255676	1.098591549	123.3849765
0.036656981	1.087591241	105.5729927
0.020792158	2.273833671	122.5897707
0.039326307	1	119.4626866
0.015644737	1.060240964	173.6556225
0.047175532	1.019354839	63.89032258
0.020222295	1.041860465	152.0662791
0.037938392	1.1875	104.4441964
0.033705729	1.206128134	86.35238703
0.02896979	1.0703125	82.20898438
0.028696353	1.13618677	97.76761781
0.043219889	1.066666667	144.3444444
0.017353058	1.062068966	168.1758621
0.025655968	1.08097166	98.30566802
0.019587714	2.214092141	138.4687782
0.031552606	1.171052632	126.1787281
0.024073738	1.05	163.4104167
0.025651302	1	100.9347826
0.019274523	1.056782334	140.2527603
0.022256154	1.091981132	121.9864387
0.017630169	1.041570439	120.3314088
0.015934775	1.100737101	183.2407862
0.019492231	1.037037037	159.0756173
0.037211377	1	149.0444444
0.069764097	1	70.43333333
0.013734733	1.302521008	188.7282542

log.sigma.2.5.mm.3D_gldzm_LowIntensityLargeDistanceEmphasis	log.sigma.2.5.mm.3D_gldzm_HighIntensityEmphasis	log.sigma.2.5.mm.3D_gldzm_DistanceZoneVariability	log.sigma.2.5.mm.3D_gldzm_ZonePercentage
0.020505704	153.6744186	209.0837209	0.322822823
0.105514235	65.39130435	44.04347826	0.407079646
0.032642682	132.3793103	133.4965517	0.300207039
0.092552178	77.91428571	35	0.380434783
0.041690357	94.35632184	162.3563218	0.233243968
0.021534461	264.90625	64	0.621359223
0.013734897	172.2582305	876.7654321	0.05960996
0.014069607	178.5186501	491.4298401	0.111573524
0.017039918	133.2939189	274.75	0.069992906
0.060494844	85.58461538	122.2461538	0.357142857
0.006061358	465.4597156	367.1895735	0.077232796
0.024188631	145.3161765	266.0514706	0.088512854
0.03052833	150.1946309	137.4832215	0.450151057
0.057787313	71.64383562	71.02739726	0.384210526
0.023629105	117.4563758	145.0536913	0.161956522
0.016954033	145	542.724031	0.075642078
0.101037043	53.7704918	59.03278689	0.139908257
0.059620653	64.84090909	42.04545455	0.396396396
0.062153197	67.63333333	78.8	0.158730159
0.009254912	251.1257976	911.8805834	0.091976188
0.035365955	90.69948187	177.5906736	0.13292011
0.074327219	52.53488372	43	0.272151899
0.041846297	86.79365079	59.12698413	0.224199288
0.026601673	102.3833992	237.4505929	0.052664446
0.020959504	146.6756757	140.2162162	0.203856749
0.020478705	191.8405797	156.5507246	0.136296296
0.033399435	95.53439153	179.2645503	0.201277955
0.017379381	135.2079208	190.3069307	0.114837976
0.100521406	65.52941176	17	0.607142857
0.070995292	74.83950617	150.382716	0.140747176
0.029267682	98.01529052	294.4984709	0.093135859
0.044546803	101.0853659	82	0.321568627
0.043987391	92.74273859	241	0.18842846
0.016988592	144.8509091	255.56	0.087329311
0.012582471	164.2936345	467.3737166	0.140063273
0.01381369	212.3140794	267.1805054	0.245567376
0.013080696	223.3183673	245	0.628205128
0.017466044	147.4760766	654.6315789	0.071221673
0.06121951	69.86363636	44	0.223350254
0.002465162	859.3570712	916.1554664	0.082301469
0.029525815	100.1643836	140.1232877	0.231378764
0.016847381	143.516369	597.6785714	0.092856156
0.023108687	159.7095808	324.1257485	0.218729535
0.021440866	119.2787879	157.1939394	0.233380481
0.055333675	70.9223301	97.11650485	0.094669118
0.012295026	209.7320755	253.2339623	0.135549872
0.021703216	123.9121339	464.1799163	0.068589468
0.092246849	70.97619048	40.04761905	0.191780822
0.06234564	70.17391304	46	0.403508772
0.029210522	191.3839286	90.57142857	0.509090909
0.026287833	99.49029126	204.0097087	0.108079748
0.014103176	209.7719298	470.8050682	0.237610005
0.020468439	122.3559671	462.5925926	0.048109285
0.068084461	72.9	156.05	0.188902007
0.030328041	124.3893805	101.6371681	0.393728223
0.0341399	97.02380952	112.7777778	0.190045249
0.017374611	139.5720081	465.653144	0.093247588
0.025594585	110.7103175	211.8412698	0.163106796
0.041149208	88.94871795	74.1025641	0.286764706
0.016554506	173.0537919	979.3439153	0.048228639
0.020140142	161.4854369	198.1553398	0.25846926
0.014920109	222.2131519	406.3922902	0.27510917
0.009485427	290.9663324	1272.053009	0.305336833
0.005286835	369.9058333	1118.805	0.14084507
0.042260101	93.27941176	66.02941176	0.211838006
0.018072802	244.9210526	557.5394737	0.347826087
0.048554758	86.45652174	44.04347826	0.386554622
0.017909065	186.2869674	747.5739348	0.193313953
0.02377099	126.0231317	540.3024911	0.056071037
0.019182431	168.5789474	358.5842105	0.248853962
0.051046659	69.11764706	47.15686275	0.257575758
0.012847685	194.6732852	449.7833935	0.07803916
0.022781792	115.1103448	131.6758621	0.193591455
0.032650735	101.1705882	164.1058824	0.158434296
0.02578711	129.42723	398.9201878	0.132462687
0.043530473	106.7445255	129.2335766	0.226821192
0.039904943	191.8965517	270.3103448	0.33265857
0.039326307	119.4626866	67	0.582608696
0.016924566	175.7309237	239.2008032	0.382488479
0.04730994	64.58709677	153.0129032	0.097117794
0.020505704	153.6744186	209.0837209	0.322822823
0.050890189	106.6339286	98.875	0.303523035
0.035749523	90.37325905	322.816156	0.105032183
0.029536508	84.6640625	122.140625	0.10893617
0.030801652	100.2607004	237.7081712	0.142382271
0.047386556	144.6111111	86.08888889	0.511363636
0.017627891	171.8689655	139.1241379	0.2
0.02643253	100.1052632	237.1700405	0.060747664
0.024565204	239.6937669	205.7479675	0.314042553
0.034277411	130.4254386	203.4824561	0.285
0.02594488	164.0166667	116.0666667	0.434782609
0.025651302	100.9347826	138	0.096503497
0.02009217	142.1041009	610.4542587	0.185271771
0.023602205	124.8679245	398.7971698	0.110015568
0.017954557	121.8729792	421.1662818	0.209482342
0.016769589	187.0884521	383.6535627	0.34375
0.020677511	159.7376543	316.0987654	0.22116041
0.037211377	149.0444444	45	0.517241379
0.069764097	70.43333333	30	0.422535211
0.015639934	202.7998472	1123.799083	0.136297376

log.sigma.2.5.mm.3D_gldzm_IntensityVariabilityNormalized	log.sigma.2.5.mm.3D_gldzm_LowIntensitySmallDistanceEmphasis	log.sigma.2.5.mm.3D_gldzm_IntensityVariability
0.065094646	0.020151443	13.99534884
0.080340265	0.085133801	3.695652174
0.05902497	0.030555184	8.55862069
0.07755102	0.092552178	2.714285714
0.074118113	0.040068204	12.89655172
0.062011719	0.021534461	3.96875
0.065498569	0.011915814	63.66460905
0.065119933	0.012884001	36.6625222
0.084276844	0.016028713	24.94594595
0.073964497	0.056200564	9.615384615
0.084903753	0.004772127	35.82938389
0.071637111	0.023752007	19.48529412
0.057970362	0.027475982	8.637583893
0.082004128	0.056360372	5.98630137
0.078059547	0.023351776	11.63087248
0.078002524	0.013585622	50.31162791
0.094329481	0.100422289	5.754098361
0.104338843	0.058916296	4.590909091
0.087901235	0.058882493	7.911111111
0.06468873	0.006573661	70.96353692
0.085559344	0.032113252	16.51295337
0.104380746	0.074327219	4.488372093
0.092970522	0.040898847	5.857142857
0.082722742	0.02491944	20.92885375
0.070032871	0.020188343	10.36486486
0.060202689	0.01287528	16.61594203
0.077517427	0.032617877	14.65079365
0.082246839	0.016650485	16.61386139
0.107266436	0.100521406	1.823529412
0.075826856	0.068281714	12.28395062
0.080754519	0.026729035	26.40672783
0.0743605	0.044546803	6.097560976
0.071055939	0.043987391	17.12448133
0.073586777	0.015777112	20.23636364
0.073563577	0.011860052	35.82546201
0.059625435	0.013431465	16.51624549
0.052794669	0.013080696	12.93469388
0.077527987	0.012619895	64.81339713
0.087809917	0.06121951	3.863636364
0.060821381	0.002269707	60.63891675
0.076562207	0.029051452	11.17808219
0.082168545	0.014835063	55.2172619
0.065330417	0.022352727	21.82035928
0.07922865	0.020889718	13.07272727
0.090771986	0.050585878	9.349514563
0.070501958	0.011692425	18.68301887
0.070061799	0.021213762	33.48953975
0.078231293	0.090851759	3.285714286
0.09073724	0.06234564	4.173913043
0.053252551	0.028045712	5.964285714
0.082241493	0.026137388	16.94174757
0.055633452	0.012518327	28.53996101
0.089230978	0.01928883	43.36625514
0.078828125	0.067199044	12.6125
0.069151852	0.028895324	7.814159292
0.081506677	0.031755336	10.26984127
0.072977054	0.016079408	35.97768763
0.077160494	0.022627372	19.44444444
0.079552926	0.040690243	6.205128205
0.077721166	0.010945183	88.13580247
0.060231879	0.018699939	12.40776699
0.051424047	0.013579718	22.67800454
0.049237075	0.007838525	68.73495702
0.057533333	0.004706702	69.04
0.081314879	0.041134651	5.529411765
0.048556094	0.013702472	36.90263158
0.085066163	0.04773954	3.913043478
0.05502478	0.017205752	43.90977444
0.074017553	0.022108585	41.59786477
0.062132964	0.017627138	23.61052632
0.093425606	0.049403594	4.764705882
0.068983044	0.010216395	38.2166065
0.080047562	0.021725047	11.60689655
0.075709343	0.03124314	12.87058824
0.066664463	0.025122817	28.39906103
0.075816506	0.034938608	10.38686131
0.05370522	0.016765619	26.47667343
0.068389396	0.039326307	4.582089552
0.063369946	0.01532478	15.77911647
0.097273673	0.047141929	15.07741935
0.065094646	0.020151443	13.99534884
0.071269133	0.034700443	7.982142857
0.084581901	0.033285105	30.36490251
0.090332031	0.02882811	11.5625
0.076897455	0.028195611	19.76264591
0.069382716	0.042178222	6.244444444
0.069678954	0.017284349	10.10344828
0.081283089	0.025493067	20.07692308
0.045159774	0.018623801	16.66395664
0.069713758	0.030871405	15.89473684
0.07125	0.023605953	8.55
0.085591262	0.025651302	11.8115942
0.064230911	0.019070112	40.72239748
0.070943841	0.021919642	30.08018868
0.074900394	0.017549072	32.43187067
0.055134652	0.015734497	22.43980344
0.060890108	0.019195911	19.72839506
0.067654321	0.037211377	3.044444444
0.106666667	0.069764097	3.2
0.057082045	0.013347315	74.72039725

log.sigma.2.5.mm.3D_gldzm_HighIntensityLargeDistanceEmphasis	log.sigma.2.5.mm.3D_gldzm_SmallDistanceEmphasis	log.sigma.2.5.mm.3D_glcm_SumVariance	log.sigma.2.5.mm.3D_glcm_Homogeneity1
160.1069767	0.989534884	433.3159677	0.403617112
65.65217391	0.983695652	79.66001241	0.381601251
143.2413793	0.968965517	264.4406441	0.399781105
77.91428571	1	111.9435179	0.381634435
110.3678161	0.973339719	190.8211686	0.448283547
264.90625	1	684.7302248	0.274462619
197.744856	0.960848194	531.449147	0.704105573
262.1563055	0.947811822	645.9479571	0.561444306
155.3074324	0.971659159	520.6787543	0.622518368
94.12307692	0.976923077	160.2956908	0.374344232
576.0687204	0.945826751	1582.916956	0.646346913
152.4742647	0.99121732	462.1596546	0.626073825
162.738255	0.969798658	241.9327548	0.31732518
73.12328767	0.989726027	147.664659	0.386918219
124.8053691	0.989932886	467.8204261	0.501963739
174.3627907	0.934883721	472.4815742	0.64925138
58.68852459	0.987704918	81.90333663	0.502019061
73.09090909	0.982954545	113.7707623	0.390249202
89.16666667	0.95	130.3705771	0.501530655
382.7602552	0.928372954	699.5388565	0.60541922
103.5025907	0.968192286	142.7377375	0.513887476
52.53488372	1	72.6383794	0.436238923
99.6031746	0.976190476	190.9749757	0.462307273
128.6363636	0.975454545	345.7390206	0.689870271
158.7972973	0.97972973	475.9885781	0.530358592
745.4528986	0.781400966	410.0098651	0.517623418
105.9312169	0.98015873	272.9370285	0.44811041
171.5940594	0.976794554	477.7812455	0.553478737
65.52941176	1	142.5791091	0.30240855
86.61728395	0.971364883	166.6723419	0.509177017
129.8379205	0.959437649	314.0673179	0.575758746
101.0853659	1	174.6522472	0.424298706
92.74273859	1	212.5290553	0.501393855
181.3381818	0.971035354	476.760278	0.631977208
175.6324435	0.984314397	465.6188281	0.543978946
223.6967509	0.986462094	650.2663722	0.459714506
223.3183673	1	480.7407033	0.255993146
253.6136364	0.905940657	511.233414	0.672926508
69.86363636	1	171.4895374	0.471220278
1047.114343	0.967611167	2959.38996	0.648943634
110.7054795	0.984589041	251.8695424	0.450753815
192.5833333	0.954434937	366.5266689	0.653731946
168.3383234	0.98835662	393.1621829	0.439505814
131.6787879	0.981818182	329.4994741	0.436579373
85.91262136	0.974986516	214.9739142	0.614976288
234.5698113	0.982311321	599.8739718	0.528163042
131.6359833	0.988726174	404.64456	0.650449513
75.54761905	0.982142857	115.5948857	0.449666795
70.17391304	1	103.0010857	0.405166571
420.5625	0.919642857	460.3873201	0.26485396
101.2524272	0.996359223	438.1484411	0.62413974
244.711501	0.967565519	444.9862178	0.456417038
129.8374486	0.981481481	361.8575694	0.71120631
75.45	0.990625	199.4432442	0.458408168
147.5929204	0.960176991	211.4022058	0.362746729
113.9047619	0.958333333	197.3773808	0.451288617
157.3509128	0.977758057	466.6385572	0.623582666
141.3293651	0.93452381	320.5018572	0.511094193
105.1410256	0.980769231	231.6020981	0.427166271
335.3941799	0.939055979	541.140625	0.689803695
170.4854369	0.985436893	354.0237405	0.445235641
244.6258503	0.969072814	482.0260894	0.41430245
326.7320917	0.965078797	671.3559075	0.408096286
410.0975	0.973478009	1194.466223	0.576011318
95.44117647	0.988970588	198.1952513	0.445128796
483.8710526	0.881076389	571.5427732	0.341048793
92.97826087	0.983695652	154.0021238	0.373232752
215.8107769	0.975215817	461.2107237	0.517853267
138.044484	0.983911625	275.5409984	0.660760749
181.0289474	0.977923977	319.4762712	0.428130623
79.76470588	0.970588235	158.5894291	0.41663149
327.2797834	0.919581328	762.956499	0.652102282
140.9517241	0.963793103	379.3051997	0.453371601
103.8	0.986764706	251.6547024	0.536047209
153.5962441	0.975352113	305.6698144	0.594158964
111.4306569	0.97810219	181.9047596	0.449097805
576.0588235	0.766128578	406.5320743	0.333694887
119.4626866	1	176.7663221	0.324166317
184.0321285	0.984939759	355.6928613	0.374477089
67.37419355	0.99516129	221.0171486	0.605818007
160.1069767	0.989534884	433.3159677	0.403617112
115.3928571	0.953125	125.772052	0.383208701
111.7465181	0.959397245	356.6168273	0.626100162
94.484375	0.982421875	294.6278728	0.569878196
113.155642	0.970276697	236.3405254	0.545626431
145.6777778	0.983333333	200.8348614	0.306957717
186.6413793	0.984482759	458.024759	0.464030411
109.8947368	0.984255511	310.3830542	0.669214834
775.8075881	0.771943692	565.2190947	0.353377832
147.4122807	0.957236842	213.3466516	0.414282282
166.4416667	0.9875	292.3110903	0.333507019
100.9347826	1	311.2259972	0.586659775
149.5094637	0.985804416	324.6171445	0.514375738
136.3938679	0.977004717	291.7037037	0.585521239
128.039261	0.98960739	250.9074718	0.489676827
206.017199	0.977545728	414.7521383	0.371962256
162.3858025	0.990740741	301.5967403	0.44503699
149.0444444	1	315.5545455	0.321157677
70.43333333	1	86.27276592	0.387217842
274.4568373	0.941399287	542.2075882	0.56548349

log.sigma.2.5.mm.3D_glcm_Homogeneity2	log.sigma.2.5.mm.3D_glcm_ClusterShade	log.sigma.2.5.mm.3D_glcm_MaximumProbability	log.sigma.2.5.mm.3D_glcm_Idmn	log.sigma.2.5.mm.3D_glcm_SumVariance2
0.320954231	-36.51819906	0.028024683	0.980852027	27.75830622
0.294137501	39.25329338	0.043726618	0.951590017	17.48688134
0.319455245	-13.94165776	0.048146309	0.967542544	34.8116377
0.296003175	56.46677641	0.055138429	0.945971036	19.42654024
0.372381863	5.85445536	0.066594089	0.977063728	20.32101883
0.174661511	-117.5737468	0.034375977	0.960123336	57.94482534
0.68403624	-53.9031597	0.230924445	0.996003839	10.04129314
0.511854199	-101.3533858	0.159603176	0.99281139	22.39925458
0.587054481	-64.31896947	0.199740332	0.993295445	14.53861646
0.287138667	7.200760183	0.027096177	0.973999779	26.59854403
0.620238158	-23.82346283	0.178651348	0.996709754	7.006601337
0.592270765	-68.64908104	0.169753057	0.993500431	14.65105468
0.224656474	146.9024038	0.022944669	0.955775868	41.56900626
0.298454324	-17.81808938	0.035061741	0.962181118	18.6590828
0.438461189	-73.73970456	0.111163402	0.986571285	23.51004285
0.619580909	-111.6028252	0.249057372	0.992983029	17.68975133
0.439946288	2.806175307	0.043933882	0.981952209	13.46447122
0.305427351	7.957841967	0.053524161	0.950509809	14.87542511
0.439767881	-9.215706055	0.067698245	0.978351781	14.57364857
0.567130015	-32.52829013	0.167091742	0.99647152	15.02679092
0.452332787	-8.939440328	0.125678068	0.982365023	18.90335282
0.361139045	16.07397327	0.06113373	0.965955793	10.62031323
0.39032281	-32.47490256	0.059925998	0.968886768	23.76193491
0.669218437	-32.88047416	0.274320115	0.996424105	7.896072608
0.473489879	-124.2345038	0.195647272	0.982255197	32.98248122
0.462432085	174.2068544	0.103311657	0.990446794	29.22642544
0.372235593	-32.55494222	0.033378029	0.984390067	25.12407735
0.503892735	-58.99444265	0.105225314	0.990035401	17.2322129
0.205858132	59.70038125	0.082274844	0.908556488	25.57922973
0.446770095	-33.82502666	0.095628258	0.980549295	18.17903495
0.528207893	-78.36478073	0.144316107	0.990716013	21.17750444
0.346621865	15.72993628	0.042778107	0.9703555	19.40226637
0.440604685	-62.15454513	0.081559917	0.985384728	22.51569163
0.598606422	-80.58065798	0.189995655	0.992891869	15.99043699
0.491318429	-48.23158579	0.120552535	0.991145437	17.65514332
0.392039081	-231.4346854	0.087017034	0.982808953	43.92426214
0.164246536	238.5145456	0.017000513	0.953811967	53.98536495
0.645984786	-87.85104457	0.239485011	0.996395	15.64169778
0.401077928	-12.80557535	0.057474659	0.975769482	15.59972612
0.620700761	-11.31020751	0.235572358	0.998279932	10.69422609
0.376829813	-10.47094575	0.033136877	0.98032901	19.77900315
0.62672316	-10.72866072	0.23477842	0.994869797	6.537297481
0.365404215	-59.92067796	0.029687425	0.981563805	31.88301317
0.358844547	-14.30666874	0.039970162	0.980232288	23.36275374
0.577984522	-54.91353287	0.193162362	0.986687203	14.0494995
0.472975446	-53.23525857	0.115446341	0.991240306	21.589538
0.620886318	-57.63542194	0.199077776	0.995545135	12.93157147
0.373725345	37.35361052	0.046698885	0.978864385	25.31576075
0.324048971	20.57203006	0.052569141	0.957981502	15.59992484
0.177639396	2545.3731	0.018101652	0.951432538	151.8614163
0.588398361	-100.4261615	0.250199378	0.991196962	16.95960687
0.389307892	33.26666758	0.051702966	0.986422743	29.26691709
0.693705978	-28.99460635	0.299891902	0.99649216	6.4780027
0.38516475	-29.70001059	0.038319378	0.978531495	29.15938586
0.275373909	123.8356303	0.031011224	0.970582535	31.90344678
0.378223403	1.916361647	0.034845869	0.98295987	19.57549819
0.589095772	-66.39606341	0.200633486	0.99454939	14.16944479
0.449861126	-56.05906986	0.106409722	0.987063235	25.98725321
0.34921106	-34.41030823	0.038993421	0.977364912	23.83679062
0.671163302	-27.84005211	0.23541522	0.99779151	6.742523595
0.371260091	-22.36967121	0.09606688	0.97627126	26.76221033
0.339636453	-2.236415492	0.0514172	0.981314973	38.43147519
0.336412645	-23.99932392	0.048283652	0.979427804	47.4345306
0.531011464	-94.44153088	0.138387995	0.994066558	20.65683146
0.368590915	-27.20517169	0.037087145	0.97300093	24.32933349
0.255682029	310.729311	0.016703351	0.97499446	65.44133143
0.286152825	63.61914108	0.053913556	0.95357983	22.2405971
0.462574163	-32.16129048	0.116925578	0.992992581	25.05504071
0.634687529	-24.03768691	0.177467294	0.995516346	8.641867336
0.353256099	38.95605584	0.045012547	0.98312117	27.28875852
0.337047049	3.270738593	0.040820931	0.954310638	20.34315291
0.621557244	-137.1434859	0.204927706	0.995162586	19.99796136
0.379207894	-20.32340799	0.065484932	0.980433496	26.53486218
0.481771753	-41.89543411	0.096233061	0.989934101	17.10265557
0.552808696	4.726405856	0.182035409	0.994192513	18.20873706
0.377153999	62.80896157	0.045345966	0.981611786	24.9498832
0.24734188	1079.208544	0.019065395	0.968594303	101.6405226
0.232273045	98.41416811	0.036727806	0.949780475	44.22872934
0.289021022	4.141362913	0.02686047	0.971644726	28.86451322
0.567739888	-36.48012357	0.1773637	0.986474337	10.66771312
0.320954231	-36.51819906	0.028024683	0.980852027	27.75830622
0.297785553	81.17475436	0.031156524	0.959496975	25.85452867
0.590221471	-109.3915662	0.247752766	0.990572853	18.50458656
0.523635803	-47.13256932	0.126904251	0.98830433	14.14439758
0.492340507	-52.89544053	0.134393472	0.983495103	18.03294147
0.215406669	131.2191682	0.024348005	0.93552513	42.14803009
0.394521368	-94.87310968	0.037097654	0.98595545	28.40978826
0.640989858	-60.92598812	0.295210175	0.992367729	12.3651791
0.275169094	853.3005731	0.022781112	0.968428722	97.9807598
0.335016501	28.54340177	0.028783272	0.974444891	24.47614392
0.244635226	7.27252675	0.025643664	0.958073584	46.45001582
0.542632117	-38.6457796	0.159784143	0.990027057	12.98841699
0.457477252	-25.53404481	0.094734346	0.985637759	18.13862362
0.543734111	-45.13177242	0.164372644	0.989487363	15.87124194
0.426132981	4.677704657	0.059995247	0.986629614	15.81131641
0.286843092	-4.936842277	0.01851805	0.97524234	37.66300727
0.373171505	51.29937289	0.041736815	0.988633372	26.73373698
0.230972742	74.86930817	0.043535466	0.953359765	40.89154172
0.30504579	6.594866831	0.059083372	0.938235471	14.31739104
0.51927671	-76.52491308	0.172185273	0.990678361	23.96629437

log.sigma.2.5.mm.3D_glcm_Contrast	log.sigma.2.5.mm.3D_glcm_DifferenceEntropy	log.sigma.2.5.mm.3D_glcm_InverseVariance	log.sigma.2.5.mm.3D_glcm_Entropy	log.sigma.2.5.mm.3D_glcm_Dissimilarity
11.93725743	2.897458195	0.327084286	6.877507866	2.602859985
12.93424603	2.851680989	0.30962195	5.954145074	2.779150148
16.56924135	3.087529289	0.303120814	6.933478022	2.949630149
14.52283974	2.894284832	0.297674428	5.719559769	2.927188675
9.075939967	2.760491501	0.331821611	6.413009124	2.24542413
32.27285401	3.316814449	0.17677343	6.134097023	4.627479925
2.883977136	1.806158045	0.38927101	4.082361737	0.896157255
5.05493686	2.373273504	0.391150632	5.811553128	1.522086742
3.088920942	2.045942492	0.420743757	4.874521275	1.146548395
15.16726598	3.023206417	0.296925563	6.862376702	2.976264581
3.128534142	1.910812456	0.457050212	4.530625994	1.037226343
3.341914639	2.055644456	0.425602323	4.930918596	1.148101284
26.97735318	3.428135446	0.230858681	7.325530239	4.028703296
10.85252792	2.800653474	0.312344307	6.253262812	2.61736993
6.265027426	2.518722234	0.372464135	6.040113034	1.822292659
3.963468064	2.058679291	0.398703755	4.59056306	1.157579097
4.984616408	2.348272659	0.415278025	5.818412312	1.664675684
11.36312345	2.784963828	0.330707542	5.785039843	2.633466428
5.318086915	2.390205476	0.409983932	5.876910232	1.706012645
4.710608839	2.244340169	0.406207543	5.372541491	1.34115278
6.138661253	2.525434031	0.366785293	6.027966382	1.779418669
7.467441223	2.553871427	0.386691554	5.658637944	2.12077907
7.854296316	2.643070267	0.354432781	6.190361916	2.091489178
1.786890983	1.713634803	0.431209828	4.111104833	0.822820209
9.522371129	2.688862849	0.325219164	5.850262247	2.016291351
10.0588282	2.637558899	0.390475101	6.211178624	1.965114043
8.063273976	2.635506153	0.352191794	6.593099962	2.139955594
4.175771316	2.263742008	0.425325052	5.738342445	1.442907123
26.20388205	2.57054583	0.201255619	4.224909107	4.141407451
5.348102104	2.430405848	0.384694227	6.000129413	1.70976209
4.72206688	2.320405408	0.383927019	5.57182936	1.456671495
11.13182106	2.785755774	0.349332085	6.252543571	2.433441203
7.690277543	2.607564696	0.382615504	6.204959834	1.913890446
3.654102791	2.088038658	0.409410205	4.861297698	1.173214481
5.361761861	2.410224164	0.398847756	5.81538892	1.588914609
12.80073602	2.91726408	0.344260049	6.89863237	2.441656632
54.67954623	3.782835699	0.175079744	7.529688584	5.767756157
3.896766255	2.002111778	0.380943118	4.542355083	1.072712455
5.922742215	2.449365659	0.388606894	5.838040357	1.867723461
4.079634148	2.032737611	0.419093599	4.634778345	1.129102615
7.63145914	2.630376503	0.366414103	6.370492351	2.097575818
3.126309843	1.955166413	0.429733007	4.482664723	1.044874975
10.65155654	2.758795533	0.350295516	6.836463591	2.363950589
8.459652429	2.698023233	0.345717415	6.540059659	2.232217986
2.799280756	2.016348812	0.430796326	5.04297209	1.132213387
6.224367706	2.483926174	0.39507959	5.941786884	1.712897528
2.65889255	1.94758279	0.42140581	4.797592588	1.023377671
6.505570048	2.479992912	0.369898827	6.143293019	2.004266942
12.60092752	2.840912811	0.323691245	5.798202313	2.665393678
90.02656264	3.963327226	0.175932414	7.399518062	6.618646004
3.344020275	2.083755409	0.408017144	4.820834786	1.178279458
14.42550091	2.943001823	0.353618287	6.870230501	2.504589147
1.775406189	1.65837486	0.413909229	3.842600283	0.769114432
6.676679851	2.424401716	0.373998183	6.427148169	1.984577582
17.49112532	3.108627688	0.291452249	6.809310281	3.177448664
8.071778174	2.665004193	0.367732679	6.403752282	2.125215617
3.563522451	2.091996869	0.420169997	5.02537414	1.180983178
7.244651781	2.617522762	0.354268584	6.1148677	1.89532689
8.895843572	2.684039082	0.359472407	6.398477169	2.280013584
2.223959814	1.716578446	0.442965413	3.94184178	0.846633075
14.02230778	3.008812182	0.297587704	6.664455843	2.628338761
18.55784168	3.159463161	0.316877814	7.26427376	2.98294095
24.95517771	3.318485247	0.309343375	7.712348728	3.368494007
8.120760447	2.525412063	0.365855913	5.706334353	1.705780348
7.583050143	2.582018043	0.356874823	6.305078039	2.116187976
30.31675102	3.524240596	0.256157088	8.15719662	4.011584499
15.7136171	3.025386249	0.286008584	5.953793318	3.047608611
10.85563666	2.715829369	0.372591704	6.348412178	2.046435839
2.491900513	1.871238536	0.434968595	4.498086707	0.963837037
13.37466528	2.977105283	0.333606257	6.926955012	2.606068772
10.39775475	2.781766598	0.335993061	6.134980469	2.471243695
3.690851852	2.065563894	0.390389366	4.804803827	1.129697629
8.406722177	2.69791168	0.342293658	6.453368231	2.179231086
5.67811858	2.42858937	0.39872789	5.785752552	1.631947711
6.808487685	2.374931765	0.385629176	5.533931004	1.514263806
9.888372917	2.726882783	0.366998035	6.509843211	2.242850579
32.44997176	3.545012296	0.253574471	8.118334375	4.132836047
23.32526969	3.223306793	0.245768059	6.306299567	3.799441488
16.60473559	3.133256594	0.292916347	7.182742041	3.089649812
2.827856582	2.035011081	0.439590139	4.948238089	1.157477045
11.93725743	2.897458195	0.327084286	6.877507866	2.602859985
15.18591908	3.056946928	0.29516531	6.739012276	2.952535844
4.031925745	2.155280216	0.39044948	4.838496346	1.243413073
3.1424052	2.099491625	0.442095013	5.39356046	1.283423749
5.833043744	2.458445502	0.380390139	5.864110534	1.640370039
31.81779261	3.485828505	0.222382141	6.919106569	4.354876158
7.983662645	2.626927262	0.38001577	6.544442719	2.053916624
2.6271626	1.92651303	0.393891455	4.427228515	0.983340925
40.0596596	3.623154866	0.26701829	8.076364546	4.357110256
12.47556515	2.952832564	0.32437025	6.817525333	2.621254103
25.20778426	3.386871073	0.235947423	7.225198401	3.893941724
3.389200659	2.136226689	0.418933318	5.292515481	1.276300093
7.545917782	2.599259125	0.389849205	6.222797708	1.862583566
5.050964974	2.29337927	0.414287642	5.529955823	1.418404346
6.91556359	2.56882474	0.392734214	6.235769891	1.886748849
20.10527349	3.241881668	0.282756941	7.49331396	3.283762847
12.49168029	2.884206676	0.347034458	6.799751114	2.447226186
29.0312003	3.281044053	0.238992259	6.030431658	4.099337812
12.48730366	2.779332815	0.327992061	5.574894974	2.769409037
7.290703406	2.50219027	0.383716919	5.952457687	1.679012574

log.sigma.2.5.mm.3D_glcm_DifferenceVariance	log.sigma.2.5.mm.3D_glcm_Idn	log.sigma.2.5.mm.3D_glcm_Idm	log.sigma.2.5.mm.3D_glcm_Correlation	log.sigma.2.5.mm.3D_glcm_Autocorrelation
5.086899836	0.908111427	0.320954231	0.395375789	154.0245841
5.039647561	0.856093355	0.294137501	0.149941648	35.51047098
7.635194648	0.887331149	0.319455245	0.354038014	100.5616454
5.740357946	0.850788345	0.296003175	0.153102169	46.18505022
3.983009792	0.901570652	0.372381863	0.380431024	76.45702198
10.2715382	0.862478111	0.174661511	0.276410418	221.8193119
2.067912908	0.969061105	0.68403624	0.547825926	168.7356786
2.68904683	0.947832659	0.511854199	0.627873846	214.0829942
1.729407762	0.951348449	0.587054481	0.642550219	171.9687244
6.104087154	0.892925385	0.287138667	0.272157352	65.78444556
2.035125407	0.96836914	0.620238158	0.382907258	460.1588238
2.002177908	0.953566873	0.592270765	0.623816381	155.0424509
10.46734073	0.862384333	0.224656474	0.206895576	91.28597607
3.918411717	0.868637807	0.298454324	0.260175852	60.80698026
2.87400848	0.924958385	0.438461189	0.576457235	162.6222912
2.559464422	0.955807627	0.619580909	0.624865994	155.536461
2.174134411	0.911618873	0.439946288	0.456699985	38.72813985
4.323074903	0.853973018	0.305427351	0.133422825	47.85033087
2.3159338	0.904920027	0.439767881	0.462935993	56.06365322
2.88873274	0.965917405	0.567130015	0.518894676	225.3985469
2.921745483	0.916378123	0.452332787	0.507713963	60.46777424
2.934777426	0.877567976	0.361139045	0.168833037	33.50489332
3.383779292	0.887136147	0.39032281	0.500393815	76.72892067
1.100023202	0.965819419	0.669218437	0.625975042	116.3460544
5.258285542	0.923619915	0.473489879	0.546567402	163.5468249
6.115454086	0.944916893	0.462432085	0.489987698	145.4896537
3.286668258	0.916341725	0.372235593	0.509857249	104.6843836
2.05372925	0.936619246	0.503892735	0.606672784	164.2208557
8.145181785	0.801987537	0.205858132	-0.018776325	47.30452116
2.357937398	0.909931829	0.446770095	0.542282964	68.73082541
2.52479269	0.941941795	0.528207893	0.62946614	114.4093837
5.109678275	0.890348569	0.346621865	0.276383667	70.17501999
3.95436857	0.925684686	0.440604685	0.487662916	83.87756012
2.24696866	0.952921978	0.598606422	0.62332659	158.9586737
2.803839988	0.941595307	0.491318429	0.531007445	160.3376106
6.757451301	0.920906092	0.392039081	0.541230615	220.2682151
19.01076451	0.858942236	0.164246536	0.030541923	159.8817006
2.721168547	0.96967812	0.645984786	0.59650878	166.5758867
2.396404787	0.896331212	0.401077928	0.443371448	69.45868305
2.784844128	0.978038299	0.620700761	0.444420744	830.9343105
3.16175107	0.906611275	0.376829813	0.440755624	96.73211704
2.018579634	0.960997389	0.62672316	0.348038632	122.5485316
4.592350567	0.913151374	0.365404215	0.488951376	142.5218048
3.372278636	0.905490145	0.358844547	0.465689945	121.8749138
1.483033394	0.930706988	0.577984522	0.664766426	81.9427343
3.230319081	0.941834954	0.472975446	0.546665493	200.9014346
1.59621047	0.961348286	0.620886318	0.653885583	138.1231973
2.337984874	0.90037797	0.373725345	0.588028472	51.59542447
5.383947972	0.869244001	0.324048971	0.098121775	43.67430413
44.90169259	0.867912814	0.177639396	0.268930085	143.362011
1.906032811	0.945698439	0.588398361	0.665109366	147.5248386
8.05250112	0.93098235	0.389307892	0.339450547	157.044532
1.176562723	0.968179838	0.693705978	0.565741766	118.9159675
2.254956194	0.901788356	0.38516475	0.620765416	81.43727857
7.296315253	0.887089601	0.275373909	0.290442023	82.33467053
3.507486803	0.91360195	0.378223403	0.415109163	78.94818809
2.136705355	0.957597698	0.589095772	0.588670043	156.877119
3.595982696	0.92886902	0.449861126	0.561534387	118.0136228
3.602527963	0.899516467	0.34921106	0.455058319	90.19006498
1.49705978	0.974685099	0.671163302	0.499871423	169.8960401
7.001236722	0.90603719	0.371260091	0.310509626	128.0615439
9.512508809	0.916637673	0.339636453	0.345681572	169.2301723
13.3406788	0.915522935	0.336412645	0.312307041	226.6332219
5.177087883	0.95784321	0.531011464	0.433997739	366.190178
2.972991674	0.890894745	0.368590915	0.521726707	79.70038892
13.94041386	0.899849668	0.255682029	0.36932641	197.6017799
6.267825028	0.860255114	0.286152825	0.178384132	61.08281686
6.586406498	0.95230949	0.462574163	0.394315459	160.6137911
1.549488141	0.962094009	0.634687529	0.548699474	97.26711277
6.504713086	0.918068056	0.353256099	0.340148491	118.4519223
4.143114586	0.862271553	0.337047049	0.31856624	64.59201046
2.397102017	0.962448997	0.621557244	0.684806513	240.3568339
3.546270091	0.907950318	0.379207894	0.515855076	137.2810996
2.987161786	0.937887424	0.481771753	0.499830639	95.04535486
4.477170105	0.959223254	0.552808696	0.452662982	110.3114524
4.759619011	0.91399293	0.377153999	0.430258316	74.17817488
14.88761201	0.889063441	0.24734188	0.520568712	144.3346814
8.550478918	0.852578941	0.232273045	0.312758279	68.1311655
6.933183276	0.889904421	0.289021022	0.268329239	129.4895352
1.463348222	0.929082632	0.567739888	0.57739763	82.85032266
5.086899836	0.908111427	0.320954231	0.395375789	154.0245841
6.324290484	0.870459286	0.297785553	0.252463902	53.39716684
2.435228209	0.946051683	0.590221471	0.636623687	123.3774096
1.464960024	0.930054212	0.523635803	0.632989814	107.7526354
3.091561781	0.923114507	0.492340507	0.507927422	90.46314987
12.40562877	0.837260238	0.215406669	0.133931516	75.18103232
3.681029505	0.922951644	0.394521368	0.554841518	162.2533154
1.646432015	0.952067226	0.640989858	0.645505922	107.5102382
20.47894543	0.894870281	0.275169094	0.426830862	193.427241
5.530701956	0.897075129	0.335016501	0.323027548	84.13881005
9.766097488	0.866244741	0.244635226	0.301024486	108.454598
1.705403843	0.937862058	0.542632117	0.577709428	111.9518698
4.039681074	0.927770933	0.457477252	0.409204825	118.6356068
3.012985101	0.941615683	0.543734111	0.514248849	106.7661153
3.309342716	0.925899219	0.426132981	0.384537052	95.56352993
9.130932515	0.899602786	0.286843092	0.299909397	148.6820831
6.36334178	0.933448102	0.373171505	0.363432696	112.6935257
11.89453335	0.861427946	0.230972742	0.164929906	110.6113206
4.53865556	0.839290025	0.30504579	0.078947967	37.17668275
4.437921207	0.945930507	0.51927671	0.528888449	183.4698624

log.sigma.2.5.mm.3D_glcm_SumEntropy	log.sigma.2.5.mm.3D_glcm_AverageIntensity	log.sigma.2.5.mm.3D_glcm_Energy	log.sigma.2.5.mm.3D_glcm_SumSquares	log.sigma.2.5.mm.3D_glcm_ClusterProminence
4.36349868	12.24964152	0.011382011	9.923890911	2440.062911
3.842404202	5.86108388	0.019085397	7.605281843	918.5671821
4.444318145	9.797062037	0.011975667	12.84521976	3030.247364
3.794265344	6.703075611	0.023338402	8.487344997	1336.452085
4.107090258	8.581185627	0.016736697	7.349239699	985.3587566
4.319704124	14.7075258	0.016039443	22.97043673	8683.754453
3.007370296	12.92137235	0.139291554	3.239104674	821.763164
3.994910877	14.48326942	0.04465139	6.873910197	1874.093482
3.511365708	13.00379859	0.076552153	4.406884352	795.7996844
4.304043738	7.931692512	0.010805424	10.4414525	1966.697684
3.159702995	21.49300043	0.094027935	2.521990931	533.0644409
3.521136265	12.33743389	0.076912656	4.498242329	979.0895879
4.568959941	9.361010477	0.007779224	17.13658986	5969.007356
3.988070251	7.670916859	0.01583237	7.377902679	929.7106108
4.086569137	12.58172424	0.027412949	7.44376757	1530.817766
3.340702427	12.3328724	0.11248135	5.413304848	1471.280347
3.82954095	6.050158597	0.021966579	4.612271907	440.442566
3.766203519	6.852903439	0.02229347	6.559637142	650.2013358
3.902689848	7.331135276	0.025540811	4.97293387	571.2114011
3.691304833	14.95195708	0.067574992	5.047210007	1452.697097
4.008832447	7.567945115	0.028730986	6.260503519	871.2711731
3.566720837	5.719195528	0.025163654	4.521938614	373.1435964
4.128895661	8.529062128	0.018601011	7.904057807	1266.447341
3.050522965	10.71524885	0.125479836	2.420740898	464.9785651
4.067251609	12.55674737	0.052371261	10.62621309	2731.207923
4.209947868	11.89702523	0.02818909	10.30449571	6748.536661
4.303061363	10.01965198	0.013423905	8.296837831	1733.376244
3.914009852	12.68668443	0.034195274	5.351996054	996.2221342
2.997015009	6.873058046	0.058380935	12.94577795	1804.488187
4.004683882	8.094242823	0.025997699	5.881784263	930.2840091
3.891425203	10.50163409	0.047271302	6.474892831	1227.417325
4.04709512	8.251814373	0.017089346	7.633521857	1300.847118
4.124404694	8.953398421	0.023803338	7.551492293	1450.618926
3.504672313	12.48483472	0.087505308	4.911134946	1179.127541
3.916482774	12.54026649	0.036165158	5.754226294	935.9671302
4.531952599	14.57764347	0.01757663	14.20309553	6028.150433
4.665818634	12.83673569	0.006491277	28.66173656	9617.436644
3.322609203	12.79762669	0.11507605	4.943559241	1638.732199
3.892456261	8.186838415	0.023030806	5.380617084	761.5742709
3.2927029	28.83996826	0.104529415	3.614681803	1056.875487
4.126056624	9.679129558	0.015398354	6.852615572	1022.284124
3.090202198	11.0315421	0.104167643	2.415901831	390.5833886
4.424940367	11.71214623	0.012527363	10.63364243	3056.422731
4.243710052	10.86914826	0.013840272	7.955601542	1367.720997
3.616729633	8.895356726	0.064125412	4.212195063	674.968338
4.028859728	14.03954363	0.032138017	6.979025735	1360.818817
3.494223413	11.64269004	0.08892077	3.897616005	908.7797017
4.195048071	6.847041734	0.018225376	7.955332698	1613.271194
3.763117236	6.55044959	0.021987331	7.05021309	901.718472
5.057871521	11.6632878	0.006864252	66.17739723	122970.1878
3.48775246	12.0048586	0.09412711	5.075906787	1309.569525
4.376913996	12.42090198	0.015586956	11.3595115	4231.003074
2.850352654	10.85078894	0.149871838	2.063352222	308.3130414
4.368779942	8.705929478	0.01556008	8.959016427	1928.695389
4.349460192	8.872382895	0.011284685	12.34864302	3875.334277
4.1108403	8.721474589	0.014971396	6.911819092	1046.180702
3.566926594	12.41857158	0.078075631	4.433241811	1066.296973
4.130425013	10.64517352	0.028385959	8.307976247	1509.209717
4.183958936	9.297317184	0.015048999	8.183158547	1496.098819
2.865181104	12.99408821	0.135478529	2.272634319	497.9772559
4.259978416	11.17450074	0.019989064	10.19612953	2333.611885
4.571793666	12.84509018	0.011897347	14.61876809	5005.00189
4.75575496	15.00226464	0.009926171	18.79861613	8123.942916
3.847443718	19.14282974	0.057392385	7.033253395	2050.785198
4.194476742	8.689214534	0.016278599	7.978095908	1298.331737
4.986871713	13.87893833	0.005473411	25.10896754	16795.19193
3.945089698	7.708502882	0.020692317	9.48855355	1772.380716
4.181484064	12.57763215	0.03012991	9.327641249	3378.963976
3.231591472	9.784110882	0.096452659	2.783441962	394.9700598
4.352194885	10.72902754	0.012759189	10.2753198	2776.86808
4.006839226	7.879820601	0.017239148	7.685226915	877.6704624
3.485780475	15.37261822	0.096315132	5.932355316	1915.418272
4.262687414	11.52072564	0.016266267	8.73539609	1557.196529
3.888625702	9.601235583	0.032367982	5.695193539	1067.419122
3.778811481	10.37715914	0.060520719	6.438085664	2594.838573
4.254695554	8.390858417	0.015687507	8.70956403	2701.850105
5.081708602	11.37595482	0.005815864	34.97599499	35743.82605
4.351872287	7.928657624	0.014572751	16.88849976	4867.293901
4.410187195	11.24354655	0.009445807	11.3673122	2510.801203
3.485106066	8.993695113	0.061933583	3.373892426	421.2304532
4.36349868	12.24964152	0.011382011	9.923890911	2440.062911
4.249293459	7.121535369	0.01215884	10.26011194	2279.867232
3.499613183	10.94319498	0.096201021	5.634128075	1448.791819
3.747425193	10.24668208	0.039968384	4.321700694	663.245811
3.924515969	9.349200879	0.036252161	5.966496305	945.9050333
4.444087341	8.518744863	0.009646432	18.49145567	5656.876255
4.346055215	12.53505318	0.015155864	9.098362727	2918.340107
3.238388951	10.25054317	0.121397554	3.748085426	667.0092839
5.139386068	13.5723005	0.006611735	36.16420641	37895.55058
4.273274019	9.007482541	0.011878239	9.237927267	1779.908156
4.631986675	10.15559921	0.008078112	17.91445002	5535.229438
3.665545213	10.46620115	0.048251479	4.094404413	550.8383803
4.033511553	10.76967014	0.027153403	6.42113535	1184.529644
3.794283969	10.20089915	0.054597424	5.23055173	955.1593909
3.991194402	9.660969483	0.02169639	5.68172	918.36131
4.608405168	12.02684251	0.007639056	14.64883658	4356.102661
4.315519537	10.46276301	0.013971218	10.05533416	3713.862056
4.178721134	10.37404619	0.017731621	17.4806855	5119.90069
3.635466583	6.05800068	0.024664355	6.701173677	607.944011
4.016189761	13.39343042	0.053826758	7.857470374	2714.238369

log.sigma.2.5.mm.3D_glcm_SumAverage	log.sigma.2.5.mm.3D_glcm_Imc2	log.sigma.2.5.mm.3D_glcm_Imc1	log.sigma.2.5.mm.3D_glcm_DifferenceAverage	log.sigma.2.5.mm.3D_glcm_Id
24.49928304	0.754136936	-0.118130456	2.602859985	0.403617112
11.72216776	0.882398456	-0.228000988	2.779150148	0.381601251
19.59412407	0.82874716	-0.159416339	2.949630149	0.399781105
13.40615122	0.92287126	-0.289142511	2.927188675	0.381634435
17.16237125	0.716760057	-0.109539849	2.24542413	0.448283547
29.34871045	0.986472353	-0.459955186	4.627479925	0.274462619
25.84146225	0.758024196	-0.195407052	0.896157255	0.704105573
28.9647275	0.773277478	-0.154014541	1.522086742	0.561444306
26.00759718	0.777502939	-0.186407829	1.146548395	0.622518368
15.86338502	0.793460336	-0.136377057	2.976264581	0.374344232
42.8573933	0.658125763	-0.124157434	1.037226343	0.646346913
24.67486778	0.785706816	-0.184920429	1.148101284	0.626073825
18.72202095	0.844662359	-0.158316923	4.028703296	0.31732518
15.34183372	0.8223949	-0.167470317	2.61736993	0.386918219
25.16344847	0.774539403	-0.149019869	1.822292659	0.501963739
24.66574479	0.780077088	-0.199836964	1.157579097	0.64925138
12.10031719	0.706309886	-0.117834234	1.664675684	0.502019061
13.70580688	0.882840189	-0.234966908	2.633466428	0.390249202
14.66227055	0.727793239	-0.127663174	1.706012645	0.501530655
29.85413242	0.741349038	-0.143444219	1.34115278	0.60541922
15.13589023	0.723297598	-0.12206375	1.779418669	0.513887476
11.43839106	0.752461173	-0.139889653	2.12077907	0.436238923
17.05812426	0.80633679	-0.162394813	2.091489178	0.462307273
21.43049771	0.73553754	-0.180290357	0.822820209	0.689870271
25.11349474	0.853317067	-0.214860546	2.016291351	0.530358592
23.7227553	0.792788642	-0.153549289	1.965114043	0.517623418
20.03930396	0.728253943	-0.120800109	2.139955594	0.44811041
25.37336886	0.757262976	-0.147445142	1.442907123	0.553478737
13.74611609	0.987640855	-0.623706406	4.141407451	0.30240855
16.18848565	0.728643711	-0.126318175	1.70976209	0.509177017
21.00326818	0.777513127	-0.16743188	1.456671495	0.575758746
16.50362875	0.817476546	-0.16373636	2.433441203	0.424298706
17.90679684	0.754914242	-0.134432031	1.913890446	0.501393855
24.96966944	0.770663077	-0.179966161	1.173214481	0.631977208
25.08053298	0.73092637	-0.129556465	1.588914609	0.543978946
29.15142574	0.833574724	-0.1629393	2.441656632	0.459714506
25.27574741	0.916378655	-0.227831138	5.767756157	0.255993146
25.58420298	0.76977692	-0.187708487	1.072712455	0.672926508
16.37367683	0.810094955	-0.171322556	1.867723461	0.471220278
57.59443849	0.730527934	-0.156942119	1.129102615	0.648943634
19.35825912	0.72625041	-0.116057167	2.097575818	0.450753815
22.0630842	0.630297544	-0.112650238	1.044874975	0.653731946
23.42429245	0.777176309	-0.14653538	2.363950589	0.439505814
21.73829653	0.723384262	-0.113265885	2.232217986	0.436579373
17.79071345	0.795078212	-0.189629482	1.132213387	0.614976288
28.07510821	0.778970946	-0.154375386	1.712897528	0.528163042
23.28538008	0.790857521	-0.193707581	1.023377671	0.650449513
13.69408347	0.863917989	-0.208617878	2.004266942	0.449666795
13.10089918	0.89787962	-0.25472331	2.665393678	0.405166571
22.60938759	0.968549298	-0.315809761	6.618646004	0.26485396
24.00971721	0.777305106	-0.186407058	1.178279458	0.62413974
24.76528552	0.732257287	-0.109933992	2.504589147	0.456417038
21.70157788	0.719893893	-0.178984209	0.769114432	0.71120631
17.41185896	0.83396355	-0.186500246	1.984577582	0.458408168
17.74476579	0.839042985	-0.164806539	3.177448664	0.362746729
17.44294918	0.708625339	-0.106308426	2.125215617	0.451288617
24.83714316	0.753866209	-0.166100514	1.180983178	0.623582666
21.29034704	0.772347859	-0.144909186	1.89532689	0.511094193
18.59463437	0.841021933	-0.17739512	2.280013584	0.427166271
25.98199205	0.671345929	-0.14728918	0.846633075	0.689803695
22.34900147	0.790445744	-0.140145081	2.628338761	0.445235641
25.63242102	0.755317006	-0.113827139	2.98294095	0.41430245
29.73184369	0.741975727	-0.101285236	3.368494007	0.408096286
38.10792025	0.765133452	-0.147157952	1.705780348	0.576011318
17.37842907	0.811201728	-0.165869437	2.116187976	0.445128796
27.48105465	0.707511683	-0.083824067	4.011584499	0.341048793
15.41700576	0.904572253	-0.252099037	3.047608611	0.373232752
25.06450539	0.754566105	-0.130286243	2.046435839	0.517853267
19.56822176	0.73629185	-0.16652815	0.963837037	0.660760749
21.44491401	0.701467921	-0.096105527	2.606068772	0.428130623
15.7596412	0.780117842	-0.146533305	2.471243695	0.41663149
30.74263499	0.799155082	-0.197657126	1.129697629	0.652102282
23.04145128	0.75164323	-0.130080456	2.179231086	0.453371601
19.20247117	0.760363808	-0.143127827	1.631947711	0.536047209
20.73227109	0.790721663	-0.167704878	1.514263806	0.594158964
16.78171683	0.7804752	-0.140526831	2.242850579	0.449097805
22.54111672	0.785289756	-0.114440168	4.132836047	0.333694887
15.85731525	0.96642145	-0.358087791	3.799441488	0.324166317
22.48709309	0.717191078	-0.09799698	3.089649812	0.374477089
17.98739023	0.714620901	-0.142735523	1.157477045	0.605818007
24.49928304	0.754136936	-0.118130456	2.602859985	0.403617112
14.24307074	0.756796143	-0.120789543	2.952535844	0.383208701
21.88638997	0.775779862	-0.184820675	1.243413073	0.626100162
20.49336415	0.758293164	-0.155618575	1.283423749	0.569878196
18.69840176	0.741631669	-0.135697934	1.640370039	0.545626431
17.03748973	0.934533138	-0.261728799	4.354876158	0.306957717
25.07010635	0.817726795	-0.161853241	2.053916624	0.464030411
20.50108633	0.78215004	-0.199801163	0.983340925	0.669214834
26.75296146	0.860295976	-0.155686763	4.357110256	0.353377832
18.01496508	0.698982748	-0.096265207	2.621254103	0.414282282
20.31119842	0.899845481	-0.207102394	3.893941724	0.333507019
20.9324023	0.740955243	-0.154829299	1.276300093	0.586659775
21.53934027	0.683313092	-0.100552179	1.862583566	0.514375738
20.4017983	0.768176887	-0.15465539	1.418404346	0.585521239
19.32193897	0.617024556	-0.079192182	1.886748849	0.489676827
24.022175	0.722676415	-0.096754973	3.283762847	0.371962256
20.89262106	0.717967471	-0.107212907	2.447226186	0.44503699
20.74809239	0.977881169	-0.415205751	4.099337812	0.321157677
12.11600136	0.93989467	-0.329647296	2.769409037	0.387217842
26.78039792	0.779762574	-0.151138547	1.679012574	0.56548349

log.sigma.2.5.mm.3D_glcm_ClusterTendency	log.sigma.2.5.mm.3D_firstorder_InterquartileRange	log.sigma.2.5.mm.3D_firstorder_Skewness	log.sigma.2.5.mm.3D_firstorder_Uniformity
27.75830622	112.6471634	-0.186943513	0.086717348
17.48688134	108.1358337	0.524152945	0.100634349
34.8116377	143.8847485	0.163410116	0.077847648
19.42654024	97.41667938	0.663774205	0.104442344
20.32101883	107.0764399	0.251457195	0.101010573
57.94482534	167.0464325	-0.309009946	0.068715242
10.04129314	18.80056363	-1.217535358	0.284920273
22.39925458	99.47838855	-0.817378868	0.137475848
14.53861646	76.41537666	-1.059807025	0.186767985
26.59854403	113.8442851	0.258928001	0.087368675
7.006601337	30.6741097	-1.400921539	0.243393802
14.65105468	63.03202438	-0.96676813	0.190772873
41.56900626	162.8154144	0.473675306	0.066036272
18.6590828	97.95243454	0.003376481	0.099889197
23.51004285	114.9468899	-0.60593958	0.111323251
17.68975133	38.3288222	-1.309918489	0.242717849
13.46447122	85.7898221	0.246058977	0.124810622
14.87542511	76.26787567	0.404762257	0.12263615
14.57364857	75.09379005	-0.028494778	0.130477248
15.02679092	42.01025057	-0.214631008	0.185690171
18.90335282	106.2236123	0.055080677	0.117946937
10.62031323	74.39945793	0.653122907	0.1334722
23.76193491	113.9539986	-0.342501914	0.105051861
7.896072608	32.60913555	-1.422144982	0.274802829
32.98248122	145.8533207	-0.510497703	0.137528554
29.22642544	92.86067295	1.208528837	0.122204542
25.12407735	98.69799042	-0.178357949	0.096249267
17.2322129	86.78188539	-0.742566035	0.130792633
25.57922973	119.2629296	0.253064145	0.119897959
18.17903495	94.45272923	-0.283914535	0.119108455
21.17750444	102.0117084	-0.661664238	0.140237692
19.40226637	83.46967316	0.572266116	0.107481738
22.51569163	100.1752462	-0.299204806	0.115035019
15.99043699	52.44255924	-0.975324101	0.208543877
17.65514332	93.93681574	-0.389574898	0.131612489
43.92426214	142.905118	-0.620780689	0.085279287
53.98536495	175.1423378	0.576358969	0.057343853
15.64169778	29.41357934	-1.194227464	0.249405207
15.59972612	84.96658325	-0.180189454	0.113968409
10.69422609	33.2965526	-0.240346217	0.243942834
19.77900315	100.2773132	0.141804258	0.103910227
6.537297481	29.51360416	-0.246464616	0.252235811
31.88301317	122.6557446	-0.194871411	0.084395314
23.36275374	116.8632584	0.058103454	0.094394507
14.0494995	71.14313775	-0.995774237	0.176945353
21.589538	106.6325865	-0.362477925	0.119681321
12.93157147	48.85172367	-1.041094453	0.203815326
25.31576075	119.2896514	0.372935373	0.102062092
15.59992484	92.49638176	0.314199817	0.105878732
151.8614163	179.8151064	1.650592822	0.057107438
16.95960687	74.17556858	-1.312677798	0.206932545
29.26691709	98.20049882	0.673551751	0.095264585
6.4780027	23.08808687	-1.441022353	0.303849027
29.15938586	127.1634693	-0.236022701	0.091129328
31.90344678	131.9836197	0.744216486	0.083854363
19.57549819	102.4604092	0.210507308	0.103803953
14.16944479	56.29165399	-0.935635152	0.192652401
25.98725321	124.5975952	-0.290266807	0.110937673
23.83679062	107.0562782	-0.11820968	0.094912413
6.742523595	23.05742788	-1.060157742	0.298723109
26.76221033	118.769001	0.104148839	0.09510728
38.43147519	129.830917	0.2224706	0.078104101
47.4345306	128.1632841	0.17622139	0.073523153
20.65683146	79.7908898	-0.694882238	0.159617195
24.32933349	115.6987057	-0.240770705	0.098339496
65.44133143	140.6893578	0.653402607	0.064696783
22.2405971	99.00313568	0.59317892	0.11220959
25.05504071	98.44107866	0.236833499	0.113273973
8.641867336	43.72769117	-0.352419042	0.227053981
27.28875852	115.0865698	0.497595075	0.088684834
20.34315291	128.9821558	-0.017470706	0.102285481
19.99796136	61.54889917	-1.429497484	0.213835335
26.53486218	125.0757332	-0.046717834	0.097402678
17.10265557	83.97936058	-0.0642261	0.127248597
18.20873706	67.09474689	0.197036099	0.166677496
24.9498832	95.89643157	0.545114642	0.101229113
101.6405226	164.7096128	1.03375234	0.064685356
44.22872934	155.6539841	0.269185772	0.072060491
28.86451322	121.7894034	0.16611727	0.079921944
10.66771312	62.03496611	-1.040275168	0.181648514
27.75830622	112.6471634	-0.186943513	0.086717348
25.85452867	118.2569084	0.688026117	0.085773459
18.50458656	79.52783361	-1.209216515	0.213843963
14.14439758	76.94641972	-0.763522539	0.143947125
18.03294147	87.7657671	-0.524393703	0.134781961
42.14803009	186.3046455	0.222659205	0.065276343
28.40978826	104.4585953	-0.38521627	0.095020214
12.3651791	54.9842574	-1.231805363	0.246045391
97.9807598	131.1557391	0.953129363	0.066340244
24.47614392	121.501471	0.420012276	0.088659375
46.45001582	149.366771	-0.043679323	0.070074564
12.98841699	81.08509231	-0.688611187	0.151954619
18.13862362	84.94944996	-0.063190531	0.119455334
15.87124194	70.16422299	-0.359684353	0.158493767
15.81131641	77.99463606	0.399891125	0.118772219
37.66300727	135.6391697	0.16982234	0.072372626
26.73373698	104.0496826	0.581108672	0.093996203
40.89154172	142.8788071	0.421644447	0.078874356
14.31739104	87.56455231	0.126985446	0.107319976
23.96629437	64.12456405	-0.479533082	0.148431967

log.sigma.2.5.mm.3D_firstorder_MeanAbsoluteDeviation	log.sigma.2.5.mm.3D_firstorder_Energy	log.sigma.2.5.mm.3D_firstorder_RobustMeanAbsoluteDeviation	log.sigma.2.5.mm.3D_firstorder_Median
67.24383471	9669274.769	46.7247892	-77.03711319
61.19851604	4346968.999	43.97911283	-193.8740387
79.21404304	10671692.92	59.00764833	-116.2365417
63.34608897	3726048.437	43.16429993	-194.6630707
58.69713555	11211667.57	43.49676812	-105.1871986
97.1190063	3083984.673	69.01379999	-104.3495789
30.59129518	41513227.34	12.67224455	-2.282468557
58.47739134	43298055.59	41.82173015	-34.44626808
46.16318947	21254217.11	33.15138866	-18.55500031
65.57990757	3653396.108	46.72812036	-58.12807274
26.61595165	9711628.509	13.72516768	1.798409462
44.83893283	13714127.05	28.83207369	-10.43452835
93.81637236	10669055.72	67.92487576	-153.8285828
57.80310861	8294545.331	41.62228816	-193.2641296
61.24416854	11388979.43	46.98164701	-74.41712189
45.56375046	40220124.95	25.37447561	-6.712983608
46.44177582	2993260.374	34.8056828	-57.970047
51.20515125	4694082.685	35.16237838	-205.655365
45.48661263	3482690.417	31.53930357	-47.25165939
39.64766512	43810015.33	20.79641115	-5.122968197
55.11129609	14095371.11	42.06816271	-70.03937531
45.18996829	3698973.41	31.39028394	-152.5375061
60.11145179	4101310.246	46.45418366	-90.87712097
29.24875619	10618143.17	16.00682261	-7.906006336
73.74269774	9527973.684	58.33410593	-44.78744316
54.97395673	16038511.77	36.79070047	-40.71478271
58.73914954	13202174.81	42.09275781	-92.65541077
50.16669362	11365581.87	35.86387639	-36.0992012
71.50704866	302611.4502	47.47184957	-64.41941833
52.78683313	9245601.771	38.89307872	-54.48775482
56.54074864	26703498.23	43.11790725	-32.02071762
56.09628714	9600107.605	36.31013588	-184.1212921
57.42085764	11681905.57	41.33393848	-50.73849106
45.98447254	14378502.87	27.6238031	-7.599108696
52.09812269	23327331.54	38.63842902	-38.52718353
80.73757894	22796294.43	58.38323754	-82.50449753
104.9187931	23367867.7	75.003636	-222.7722778
39.8878446	44082201.64	19.24531793	-1.915474236
50.85349221	3095086.781	34.94722206	-103.0823593
33.86291865	37560709.23	17.06819489	-5.97479105
55.42301412	9350997.447	40.39351307	-103.3119659
27.17741688	14190973.24	13.78369447	-6.761959553
69.44748682	25624200.67	49.78629403	-97.49604034
61.88843013	10772764.46	46.38060817	-97.27079773
43.55317316	5079712.537	30.18364981	-22.86924076
57.49997025	18009731.62	42.97927351	-53.34638977
40.34211725	24953419.45	23.58114121	-5.66448307
62.6144897	2182187.463	47.75379538	-73.16255188
56.06067225	2156936.046	38.3887908	-120.4937096
118.4373214	8995066.19	75.26825197	-145.1266785
50.41506312	11329969.98	33.65971656	-15.85434532
64.14785161	22077828.95	41.07413784	-49.59812546
26.32969754	18112280.8	13.03688003	-4.962779045
65.94213331	5561947.526	50.25391184	-10.08447647
74.76184507	7770863.445	53.84593959	-147.5198975
56.01737879	9265775.03	41.39954996	-101.1348038
43.40924838	22769903.05	26.37064423	-11.30319118
63.6822397	17690142.08	50.23662087	-67.07022095
60.49251598	6592602.097	43.51087717	-134.5266724
25.71257267	43541592.88	12.02921584	-4.339234352
67.6724011	10852055.64	47.72560375	-73.87634277
79.33728691	22439097.08	55.06408779	-51.562397
82.90391871	69947690.52	54.79924531	-53.02481079
54.25079545	55550654.45	34.89901187	-12.36294508
61.52016979	3167669.454	45.89600607	-55.92037201
90.89457904	43632767.22	59.60837754	-83.53551483
59.50741287	5081944.738	41.39177966	-204.8520813
60.74363281	38891760.02	40.72032209	-41.93142891
33.045048	24440509.23	19.94760303	-5.571438313
66.81593065	17147186.44	46.94526946	-64.79214478
60.78169269	3694428.989	47.92181158	-121.3690987
50.8396563	38198075.93	31.24960913	-5.425123692
63.43985358	13226929.4	48.99351248	-114.3635406
50.10472998	7196767.857	35.24957843	-42.26776886
46.43017013	15678368.44	29.81289765	-13.36943722
58.07887358	4840830.021	40.23514894	-47.99865341
105.4821267	29141919.22	69.69687941	-70.3213501
89.10377898	5609030.486	66.77635245	-204.6776123
71.06809829	8604008.693	49.27129274	-70.63030243
39.15004773	6757103.579	26.97984739	-27.28825855
67.24383471	9669274.769	46.7247892	-77.03711319
71.88805321	6404298.998	50.37096652	-111.8216019
52.69102648	21229059.16	36.13812414	-10.59978962
44.96503217	8286462.399	32.27559494	-52.22691345
51.42368358	12544765.35	36.43569987	-36.37989807
95.64596341	6067078.32	71.79964354	-161.4609909
62.72756898	8739998.55	43.41387378	-68.16807556
40.41317449	15394815.59	26.06149564	-9.159034252
98.06540743	23443968.51	58.02242794	-52.11218643
66.94109743	10727712.47	48.82918584	-90.41733551
83.16909567	5115672.767	59.30773612	-86.25258255
45.20153472	8733567.535	33.40046215	-41.15037727
52.85990355	22283891.46	35.47134622	-34.33792114
46.94875447	18316604.7	30.99790125	-13.95030212
48.98620042	12841859.82	32.43340715	-45.60821533
79.30664918	26441812.37	55.20144818	-114.9533615
62.26897663	17730080.2	42.24805936	-73.20456696
86.09407753	3412607.115	58.17393847	-172.7757721
54.13173244	1736532.134	38.4911725	-145.0094147
54.07698053	60348219.76	31.88202838	-9.492529392

log.sigma.2.5.mm.3D_firstorder_TotalEnergy	log.sigma.2.5.mm.3D_firstorder_Maximum	log.sigma.2.5.mm.3D_firstorder_RootMeanSquared	log.sigma.2.5.mm.3D_firstorder_90Percentile	log.sigma.2.5.mm.3D_firstorder_Minimum
261070418.8	214.0972748	120.4924503	12.3479228	-350.532074
117368163	32.43988419	196.1345258	-81.03796082	-324.6633911
288135708.9	169.7371368	148.642532	-6.145518684	-334.662384
100603307.8	24.63498688	201.2474261	-58.08746185	-335.6242981
302715024.3	152.514267	122.5930055	-6.739838839	-299.6273804
83267586.18	165.3348389	173.0364088	12.06148119	-486.8923035
1120857138	310.0786438	50.45677979	10.45676851	-320.8938904
1169047501	237.0723572	92.63190035	6.086212397	-379.3617859
573863861.9	171.2478333	70.89305857	6.218340302	-334.9649353
98641694.93	320.0751648	100.1838435	41.70418396	-247.2906952
262213969.7	213.9498444	42.15903731	39.19039192	-502.8084106
370281430.2	224.4825439	66.80405179	11.73521996	-311.5694885
288064504.4	185.7264557	179.5349457	21.33695221	-369.1043091
223952723.9	11.15033913	208.9389905	-103.630397	-357.5968933
307502444.6	129.6646423	111.2624171	-1.48995806	-350.2928467
1085943374	232.3213196	68.67893884	10.2699379	-305.2825623
80818030.11	178.2748718	82.85696161	10.61985588	-183.4466553
126740232.5	-12.37079716	205.6429751	-105.9375992	-336.6908569
94032641.27	148.0392456	78.37289801	12.99792347	-215.2350769
1182870414	508.930481	60.60676355	43.02275314	-360.3797302
380575019.9	189.4183502	98.52692961	-0.98501628	-225.3891907
99872282.06	66.24812317	153.0072682	-60.60455666	-261.7284241
110735376.7	65.68584442	120.8114652	-14.67135048	-284.3114014
286689865.5	253.0398712	47.01352217	7.608098793	-255.2268829
257255289.5	171.0755463	114.5597254	2.48595047	-363.043396
433039817.7	447.2994995	88.99580172	12.50399532	-302.2038574
356458719.9	204.9315186	118.574129	3.676001072	-309.0629883
306870710.5	125.4020691	80.38276068	12.23755169	-334.368042
8170509.156	146.1347198	103.9593757	38.3663065	-222.6645355
249631247.8	131.8331146	89.6251571	4.725330353	-246.9171448
720994452.3	226.9183044	87.21047757	8.015465736	-280.3352661
259202905.3	59.74137878	194.0295874	-90.71217194	-369.9657593
315411450.4	256.4916382	95.56999724	7.082418346	-260.3189392
388219577.5	213.2462463	67.57258044	17.87183304	-304.6530151
629837951.6	233.4440918	81.90872875	10.11723309	-337.7560425
615499949.5	180.236145	142.1600537	-1.832378113	-441.0004883
630932427.8	264.5414429	244.7807376	-26.16561661	-507.3154602
1190219444	450.1956482	61.28223357	19.51365376	-308.1155396
83567343.1	53.29374695	125.3439285	-29.7972332	-294.7682495
1014139149	464.3850098	55.68306179	11.99844532	-722.2014771
252476931.1	135.7115326	121.7346719	-17.34131813	-303.2712402
383156277.5	324.0736389	44.28195683	21.01448555	-273.6343079
691853418.1	186.3636017	129.5405226	8.654702759	-353.3371582
290864640.5	138.3513947	123.4394208	-3.839652205	-330.8015747
137152238.5	82.37120819	68.32900873	6.6632339	-235.6704865
486262753.9	242.2197723	95.9798882	3.486956263	-383.9064331
673742325.2	293.7386169	59.83837639	13.1936573	-279.0209351
58919061.5	195.93396	99.82147221	24.65089951	-212.9792023
58237273.25	102.1238327	137.551778	-26.97849255	-259.0057068
242866787.1	555.8158569	202.2045116	48.99880676	-364.3293762
305909189.6	129.3014679	77.0997432	5.393243313	-300.7065125
596101381.7	413.3118896	101.1234461	35.16405563	-336.7580566
489031581.7	258.7658081	42.34312331	8.061117744	-257.1936646
150172583.2	191.1223297	81.03483244	73.06781616	-223.673584
209813313	209.8708649	164.5484101	-21.68803177	-334.8153076
250175925.8	205.563324	118.2181371	-8.174627686	-295.4945374
614787382.5	286.6471252	65.62600021	14.4234293	-314.9432983
477633836.3	226.4256134	107.0043464	3.698415613	-317.42276
178000256.6	110.2442551	155.6839995	-47.01360016	-334.9731445
1175623008	430.9597778	43.03265428	13.37044773	-301.2066956
293005502.4	209.3014984	116.6881744	0.033785141	-343.6842041
605855621.1	360.1505737	118.3139428	47.53967361	-363.7200623
1888587644	383.9415588	123.6897122	65.44569473	-413.6876526
1499867670	396.4838867	80.74670188	14.8774168	-477.8769226
85527075.25	110.4620667	99.33845751	22.43982697	-264.5082397
1178084715	421.6031799	141.3125254	75.72179108	-394.7762756
137212507.9	27.79561615	206.6528925	-87.03762054	-361.7510376
1050077521	589.1763306	97.06417204	12.63342981	-337.8806458
659893749.1	306.3329163	49.38058834	13.21317749	-229.7285461
462974033.9	338.9191589	105.9685316	29.4096035	-310.3556519
99749582.69	33.68404007	136.5969702	-21.74006596	-285.7774353
1031348050	259.5406799	73.35372212	17.64693108	-397.9009094
357127093.8	113.1962051	132.8888701	-7.770691109	-365.822937
194312732.1	299.6881409	81.89716836	9.29107399	-257.5930481
423315948	526.9066162	69.82202064	19.89420605	-261.3740234
130702410.6	284.9924927	89.52440583	38.40870552	-242.223938
786831818.9	427.9346924	140.2280753	170.7358688	-289.0261841
151443823.1	76.43320465	220.8487676	-52.98245087	-388.3330994
232308234.7	219.588913	114.9634864	41.69347763	-327.3566895
182441796.6	99.05999756	65.06745863	4.536275148	-247.9829407
261070418.8	214.0972748	120.4924503	12.3479228	-350.532074
172916072.9	169.6624908	131.7415039	41.70712509	-250.4692993
573184597.3	189.2740326	78.80964015	4.22472744	-291.7015991
223734484.8	79.41816711	83.97802346	-2.978915358	-289.9747009
338708664.5	155.8652802	83.36671101	7.99374752	-254.1630402
163811114.6	117.6601105	185.6664642	-4.287485719	-371.5653992
235979960.8	193.8212585	109.7960401	12.75785465	-371.0279846
415660020.9	173.1795654	61.53235802	3.528044105	-268.3743591
632987149.9	457.6512146	141.2526588	151.755896	-335.8670349
289648236.7	224.7998047	115.8000025	11.90162477	-299.2164001
138123164.7	212.9718475	136.1434767	43.57822609	-343.0882874
235806323.5	126.3138885	78.14979133	0.670814478	-289.2839966
601665069.4	226.1039276	80.6966516	21.71310768	-285.0397949
494548327	241.8171692	68.93926221	19.26591053	-266.2372437
346730215.2	261.2394409	78.82132373	29.19546432	-270.1577148
713928933.9	253.9784546	149.4409977	9.241728497	-389.5056458
478712165.5	472.2739258	110.0111077	11.23556919	-304.9460754
92140392.09	129.320755	198.0539551	1.28948648	-408.098053
46886367.62	20.92710495	156.3911732	-51.06797028	-293.2138367
1629401934	324.553833	79.26950676	46.23273239	-349.1509399

log.sigma.2.5.mm.3D_firstorder_Entropy	log.sigma.2.5.mm.3D_firstorder_StandardDeviation	log.sigma.2.5.mm.3D_firstorder_Range	log.sigma.2.5.mm.3D_firstorder_Variance	log.sigma.2.5.mm.3D_firstorder_10Percentile
3.766737292	84.92673936	564.6293488	7212.551059	-195.1754684
3.509184141	74.84392164	357.1032753	5601.612606	-270.5762878
3.879519544	95.08314335	504.3995209	9040.804148	-237.1845795
3.531615365	79.51138512	360.259285	6322.060364	-268.2798706
3.504765923	71.38683414	452.1416473	5096.080089	-185.8924103
4.051920956	118.4602218	652.2271423	14032.82414	-285.6364136
2.419941871	47.94896176	630.9725342	2299.102934	-80.54570007
3.32171557	71.88687416	616.4341431	5167.722676	-163.6115875
2.858232511	56.28401281	506.2127686	3167.890098	-132.2691833
3.696625143	81.79649651	567.36586	6690.666842	-167.4825089
2.542466131	42.15577301	716.758255	1777.109198	-43.33484497
2.910135441	58.21388073	536.0520325	3388.855909	-121.750705
4.10496336	113.9888157	554.8307648	12993.4501	-274.5351563
3.516214594	71.73756295	368.7472324	5146.277938	-295.6899536
3.414080068	73.06305669	479.957489	5338.210253	-189.3236923
2.716095848	61.51373069	537.6038818	3783.939063	-139.6205017
3.153143905	56.02974353	361.7215271	3139.33216	-133.1018219
3.288893901	65.00165405	324.3200598	4225.21503	-268.8825684
3.189321032	56.97669938	363.2743225	3246.344272	-132.5253448
3.020219177	58.81717054	869.3102112	3459.459551	-91.74178925
3.318153102	66.02122827	414.8075409	4358.802582	-166.6206329
3.13374909	56.37232373	327.9765472	3177.838883	-211.8451981
3.400701176	70.17630302	349.9972458	4924.713505	-189.8795166
2.396339679	41.55838059	508.2667542	1727.098997	-80.03615112
3.395483386	84.96919736	534.1189423	7219.764499	-197.1851044
3.407784032	75.9176661	749.5033569	5763.492026	-134.9565155
3.549365247	72.85285048	513.9945068	5307.537823	-189.7490814
3.232260809	61.69334159	459.7701111	3806.068397	-138.5933502
3.227396674	89.35546708	368.7992554	7984.399497	-167.6085449
3.320722958	63.87991733	378.7502594	4080.643839	-151.4607697
3.184749924	66.82522134	507.2535706	4465.610207	-158.7050171
3.501368428	73.91501164	429.7071381	5463.428945	-269.8187683
3.404997692	70.989664	516.8105774	5039.532395	-166.8114105
2.867401797	61.09306395	517.8992615	3732.362463	-130.4466858
3.2465037	63.02945347	571.2001343	3972.712005	-139.7195526
3.871088949	98.61709506	621.2366333	9725.331438	-244.5742432
4.313952426	129.4045227	771.8569031	16745.5305	-362.1108582
2.649740663	57.93588179	758.3111877	3356.566398	-113.9480835
3.364237566	63.74869003	348.0619965	4063.89548	-194.7988403
2.68110168	52.73675869	1186.586487	2781.165717	-87.86377792
3.471458125	68.28890535	438.9827728	4663.374594	-190.5297852
2.541747006	42.56595239	597.7079468	1811.860303	-60.76085587
3.785462338	86.09125137	539.7007599	7411.703563	-202.4268677
3.573048739	74.6701377	469.1529694	5575.629464	-194.6050049
2.913347127	53.65966683	318.0416946	2879.359844	-124.8913727
3.357306298	69.90970909	626.1262054	4887.367425	-162.2915039
2.86353156	54.54745661	572.759552	2975.425023	-103.80952
3.490782195	73.88991132	408.9131622	5459.718994	-154.9921082
3.437475854	70.87592281	361.1295395	5023.396434	-208.5829742
4.32271125	163.261942	920.1452332	26654.46169	-289.0446045
2.874781832	63.08716012	430.0079803	3979.989772	-147.1146622
3.736865302	87.02148295	750.0699463	7572.738496	-154.3989471
2.258167138	38.61995722	515.9594727	1491.501096	-73.4869751
3.615829861	78.42742109	414.7959137	6150.860378	-131.0975372
3.795140674	92.73918622	544.6861725	8600.556661	-243.3510773
3.446750277	68.24767852	501.0578613	4657.745624	-181.7957214
2.94329937	58.0410964	601.5904236	3368.768872	-118.5314819
3.418569119	74.41417887	543.8483734	5537.470017	-183.26539
3.576144543	74.18487341	445.2173996	5503.395442	-230.7309967
2.278507635	40.6556456	732.1664734	1652.881519	-64.17051392
3.699994697	84.25090714	552.9857025	7098.215355	-188.9991058
3.971146678	100.6491182	723.870636	10130.245	-196.2893707
4.097334138	107.5597106	797.6292114	11569.09134	-200.526738
3.226463447	71.61958091	874.3608093	5129.36437	-138.4589752
3.517606815	73.33582013	374.9703064	5378.142514	-166.4378052
4.242035798	119.4652961	816.3794556	14271.95696	-217.4627686
3.447779719	74.91921243	389.5466537	5612.888391	-265.1509827
3.557802132	80.35616616	927.0569763	6457.113439	-157.7195358
2.638289882	45.51040166	536.0614624	2071.196659	-84.0226593
3.742127119	84.17511838	649.2748108	7085.450554	-170.3000519
3.419507457	71.17302196	319.4614754	5065.599056	-210.5516373
2.835775121	65.68485347	657.4415894	4314.499975	-142.3134674
3.522107532	75.30697773	479.0191422	5671.140895	-204.5732605
3.272037692	63.63551789	557.281189	4049.479137	-138.6495148
3.108866249	62.85587597	788.2806396	3950.861143	-115.3477135
3.547885924	74.90585205	527.2164307	5610.886672	-142.0120178
4.268779204	135.286819	716.9608765	18302.52339	-175.3574966
3.969052123	105.1770423	464.766304	11062.21023	-334.2856384
3.872322876	89.79088889	546.9456024	8062.403728	-187.730545
2.803949397	49.06025938	347.0429382	2406.90905	-118.0491295
3.766737292	84.92673936	564.6293488	7212.551059	-195.1754684
3.745129693	89.56001228	420.1317902	8020.995799	-197.5125793
2.845481949	65.09386374	480.9756317	4237.211096	-151.4806442
3.053386495	55.45145092	369.392868	3074.863409	-145.4576019
3.238530327	63.47267998	410.0283203	4028.781104	-147.1455475
4.084440495	113.4150072	489.2255096	12862.96387	-287.7622223
3.65521701	79.5125882	564.8492432	6322.251682	-180.5068787
2.611375837	51.61013554	441.5539246	2663.60609	-118.2856293
4.288813198	135.6971073	793.5182495	18413.70493	-189.8913452
3.701882417	81.79817424	524.0162048	6690.941309	-180.183931
4.032511349	103.6615458	556.0601349	10745.71608	-231.4983444
3.014163422	54.33742289	415.5978851	2952.555527	-129.3099731
3.415040828	68.04150792	511.1437225	4629.6468	-132.9904526
3.133048299	60.62912191	508.0544128	3675.890424	-119.0804802
3.367110791	64.02968118	531.3971558	4099.800072	-124.2914169
4.01804418	99.60362256	643.4841003	9920.881627	-237.7627853
3.679466645	81.07943191	777.2200012	6573.874279	-176.1745972
3.899905701	109.5040193	537.418808	11991.13025	-304.5972839
3.403987043	67.03858675	314.1409416	4494.172114	-223.5764313
3.368512379	74.21161429	673.7047729	5507.363696	-136.5366882

log.sigma.2.5.mm.3D_firstorder_Kurtosis	log.sigma.2.5.mm.3D_firstorder_Mean	log.sigma.2.5.mm.3D_glrlm_ShortRunLowGrayLevelEmphasis	log.sigma.2.5.mm.3D_glrlm_GrayLevelVariance
3.370683438	-85.47443784	0.011920782	11.90056649
2.75117133	-181.2929662	0.064853918	9.106401502
2.628184589	-114.2532195	0.021695148	14.69736829
2.871235838	-184.87419	0.055952829	10.78357927
2.85412566	-99.66426093	0.023550978	8.326229905
2.88102907	-126.129991	0.015763636	22.79203985
8.723851236	-15.7093505	0.006031277	5.747467142
3.669005388	-58.42042696	0.006213678	9.255749826
3.757022858	-43.10377773	0.006968931	5.849400138
3.642532063	-57.84579201	0.042138887	11.02616778
15.17159733	-0.524622687	0.002019984	3.905851724
4.928910249	-32.77080143	0.008946423	6.699334568
2.627123895	-138.7059719	0.027216675	21.12084236
2.602596241	-196.2376717	0.03776447	8.667668743
2.858771962	-83.91135331	0.010272654	8.65800861
4.636491305	-30.54271726	0.007983238	7.992652686
2.925519404	-61.04051055	0.050107269	5.186031428
3.097414207	-195.0995084	0.050899577	6.814866819
3.195484943	-53.81418837	0.030563008	5.535228316
7.178560222	-14.61917362	0.004346201	7.341513839
2.649978596	-73.13517127	0.02910134	7.190239363
3.533444869	-142.2441044	0.05321391	5.340220092
2.283175329	-98.33970014	0.026716999	7.966242433
7.75893678	-21.9811799	0.009335169	3.887953413
2.47741267	-76.83857231	0.011187923	12.26085269
8.769813721	-46.44093774	0.009155885	10.64704167
2.987793261	-93.55365442	0.018417857	8.646822411
3.35815043	-51.52979544	0.008416801	6.753383158
2.74076932	-53.13334449	0.072705392	12.32342256
2.780755459	-62.86513299	0.032977589	6.999109576
2.717449508	-56.03621321	0.013833912	7.686743734
3.967155221	-179.3991412	0.028755435	9.303059635
3.29442563	-63.98509184	0.025498987	8.564582594
4.310537177	-28.8737106	0.007775659	7.555960515
3.234976902	-52.30992105	0.007593765	6.960688278
3.03913174	-102.3921356	0.008594862	16.12835339
2.966694953	-207.7789185	0.011875125	27.14432917
6.809753071	-19.97362644	0.00703246	7.545403774
2.926267279	-107.922217	0.028557989	6.943471824
12.2426839	-17.87281886	0.001082075	6.408874088
2.954521029	-100.776762	0.016697566	7.768686647
9.440549319	-12.20784167	0.008114351	4.089250439
3.029507603	-96.79381922	0.013274098	12.2235655
2.653073311	-98.29374929	0.013012735	9.058770005
3.424589499	-42.30240644	0.01999232	5.390100204
3.571710609	-65.76299502	0.006439079	8.296472207
5.271726644	-24.60094035	0.008990466	6.329843813
2.729220865	-67.11637148	0.051853826	9.081608231
2.896880721	-117.8859415	0.051500142	8.529944167
6.956901158	-119.2987963	0.029642035	43.12542025
4.032414974	-44.32133379	0.010493195	7.663994512
5.737293867	-51.50934719	0.009063163	13.45558643
7.923561594	-17.3619986	0.008224959	3.63356614
2.263588449	-20.39077464	0.030126745	10.12261394
3.596974026	-135.9250624	0.023239682	14.30531677
3.217146365	-96.52866064	0.020801647	7.739407301
4.96883571	-30.62683516	0.007810895	6.768497289
2.473698809	-76.89252327	0.013941047	9.003196034
2.824792254	-136.8726133	0.023994606	9.132572717
12.53881048	-14.10417722	0.005261388	3.940596022
3.307609229	-80.73360322	0.012828454	12.21978057
3.642085201	-62.19279754	0.009473033	17.40674373
3.596362849	-61.07416449	0.006512336	19.95953687
5.430500063	-37.29162769	0.002937882	10.24302635
2.366407249	-67.00736248	0.026729196	9.014861679
3.99536449	-75.48028144	0.010309556	23.79383172
3.242655759	-192.5942096	0.039994036	9.347878646
6.503351271	-54.44575332	0.010885152	11.92201528
6.331651095	-19.16365951	0.011110023	4.528190343
3.990034764	-64.372969	0.012706068	12.16284229
1.990800191	-116.5895931	0.031912458	8.35036927
4.728030115	-32.65376813	0.004930589	8.778383974
2.353436766	-109.4911453	0.011766243	8.981838594
4.561862787	-51.55256588	0.016722791	7.206230897
8.364161217	-30.40153652	0.013770687	7.846259811
4.625542031	-49.02787541	0.025612778	9.613707908
3.653406113	-36.89701492	0.018089185	29.67022234
2.277678248	-194.1956949	0.04805695	18.2002309
2.992489552	-71.79275372	0.011912903	13.42212521
3.757318311	-42.74184275	0.01805972	4.357639695
3.370683438	-85.47443784	0.011920782	11.90056649
2.949093565	-96.61691386	0.036998184	13.39492649
3.820304441	-44.42688694	0.015154204	8.060205482
3.085618705	-63.06698832	0.013042799	5.256773341
3.113193936	-54.04838018	0.017396898	7.172748178
2.25844169	-147.000245	0.045080748	20.85397637
3.665231343	-75.71604012	0.012125221	10.54689001
4.350802386	-33.50559646	0.01233884	5.487468838
4.360321045	-39.22510286	0.015194599	31.32481938
3.079413963	-81.96767216	0.021391999	11.21656748
2.633714217	-88.25718203	0.020044656	17.17432028
3.159317673	-56.16791218	0.011942347	5.179386511
3.848368842	-43.38551347	0.011790078	8.426148244
4.247203932	-32.81358637	0.012664409	7.109861992
4.108807988	-45.96739064	0.013333249	7.208851365
3.136525884	-111.4079448	0.012407618	16.39041071
5.361972784	-74.35435122	0.013374066	11.38676971
2.836239402	-165.0279942	0.02588889	19.85685944
2.680908733	-141.2941151	0.060635278	7.355462511
4.583933596	-27.86199933	0.007476479	11.04814963

log.sigma.2.5.mm.3D_glrlm_LowGrayLevelRunEmphasis	log.sigma.2.5.mm.3D_glrlm_GrayLevelNonUniformityNormalized	log.sigma.2.5.mm.3D_glrlm_RunVariance	log.sigma.2.5.mm.3D_glrlm_GrayLevelNonUniformity
0.012435389	0.084758396	0.12181794	51.28771004
0.068018106	0.098590938	0.081421491	10.34680115
0.022909202	0.075092562	0.161902792	32.3575957
0.057738913	0.10092658	0.09333376	8.604675065
0.025295501	0.099256259	0.235231559	63.55732747
0.015909255	0.06842611	0.035228668	6.822005622
0.007871118	0.199409311	2.354976522	1830.218593
0.007033641	0.106750831	0.729116601	403.5268389
0.00825024	0.139910276	1.008701151	407.846102
0.043825141	0.086318069	0.099413369	29.02507777
0.00253233	0.196990715	1.118960905	718.7617034
0.010408495	0.145144367	0.943697955	307.6974951
0.028498507	0.064750201	0.069149862	20.14742389
0.039368614	0.098346894	0.090198873	17.32395772
0.011064881	0.100612612	0.354478447	75.94723518
0.009682937	0.164086997	1.460461998	896.6340988
0.054771016	0.123504778	0.233181691	45.46148412
0.053219378	0.11976682	0.08457292	12.3661538
0.033296629	0.1236433	0.273461874	58.8445371
0.005326646	0.136363438	1.079293548	1112.032922
0.032373564	0.106127511	0.510577676	122.5060491
0.056608341	0.130051231	0.105187902	18.7211244
0.028624066	0.102295194	0.196651143	24.85562779
0.011779848	0.201537303	1.534561733	595.5322664
0.01216189	0.098212443	0.57884848	55.35011039
0.010333992	0.108791774	0.459350671	174.3116
0.01965981	0.096010674	0.1907778	78.57272798
0.009476095	0.11567248	0.4704742	158.292821
0.073479527	0.118581469	0.034985026	3.20218664
0.035808057	0.108952748	0.393102861	101.6769368
0.015697536	0.111976612	0.839220162	285.6784648
0.030221291	0.104365095	0.121690738	24.12310448
0.027478164	0.106496222	0.310355228	113.2682207
0.009272562	0.150465938	1.011020751	320.829727
0.008553768	0.114208375	0.53297139	310.4145921
0.009188607	0.076409584	0.296295214	73.25471416
0.012167206	0.056960789	0.034871073	21.46730396
0.008698524	0.169197615	1.779568425	1208.111682
0.030060108	0.109479107	0.153109411	19.05614686
0.001382363	0.17397863	1.330369572	1354.050715
0.017909267	0.103042631	0.179250054	56.88122878
0.010121918	0.192542032	1.164509206	924.8125534
0.014153893	0.083072723	0.235372051	110.4205048
0.013907475	0.093390091	0.173696043	58.06956972
0.02283338	0.139389924	0.717144453	109.2359626
0.007202511	0.105507747	0.496562837	162.5755533
0.010853131	0.14511502	1.323543156	657.2052242
0.05548709	0.099039975	0.153659866	19.19359074
0.054421831	0.10268575	0.086615442	10.80678289
0.031027615	0.056763228	0.053236548	11.87686085
0.011895337	0.142337622	0.891893159	191.6755312
0.00987205	0.088993752	0.273768962	162.8257019
0.010869996	0.217135642	2.016617059	1257.902917
0.031985544	0.088913303	0.197689673	65.94729945
0.024392173	0.081932993	0.086337544	21.7816998
0.022483439	0.10252898	0.195286883	58.98171228
0.00922029	0.141313902	1.039207552	515.5485588
0.015325273	0.098146323	0.520507608	119.4974914
0.025235324	0.093832807	0.138031561	23.03727899
0.007027458	0.23051255	1.561652208	3244.858819
0.013758807	0.08682275	0.313536308	58.60828098
0.010115876	0.072350494	0.213938959	101.0357555
0.00698001	0.068396125	0.215717708	274.007889
0.003439993	0.116122727	1.073617894	698.5166194
0.028585457	0.095807252	0.172523671	27.10791432
0.01082902	0.062908775	0.115334503	125.5697114
0.041899083	0.107908881	0.084656114	11.89613497
0.011939048	0.095829541	0.529945758	312.2963058
0.013996804	0.172981333	1.33971401	1095.755736
0.013659022	0.085143348	0.227114409	112.7786903
0.03364488	0.101157559	0.120955559	18.12418082
0.005960577	0.145521304	1.521709608	653.3433725
0.01264799	0.095144967	0.243690036	60.89914017
0.01839957	0.115398432	0.425294669	98.82679576
0.015656409	0.128128726	0.785568495	302.1961786
0.027599972	0.098060971	0.210628424	51.65202043
0.019223434	0.062747909	0.103741295	85.42549113
0.050272528	0.070721569	0.049453626	7.753681629
0.012573519	0.077706645	0.108149232	46.25059857
0.020527434	0.151034251	0.737067304	175.2646357
0.012435389	0.084758396	0.12181794	51.28771004
0.039776824	0.083318199	0.109531179	27.97936676
0.017139819	0.144566711	1.02765397	338.5608416
0.014626475	0.129912417	0.477212042	118.9529733
0.019454239	0.11498688	0.549224839	160.4110458
0.046947568	0.064788511	0.06199939	10.79905544
0.013115041	0.092891392	0.198691845	58.573696
0.014870376	0.158045123	1.642889043	399.9794599
0.016067553	0.063219983	0.149646417	66.78048154
0.022776145	0.086690935	0.164803359	61.36252275
0.02109176	0.070158384	0.079975663	18.06230088
0.013563536	0.130853185	0.659216221	141.2616669
0.013041254	0.105903814	0.428061739	291.8402981
0.014655881	0.122212749	0.828204767	339.8540298
0.014698097	0.112174899	0.250714871	197.3947247
0.013087062	0.071170638	0.119633235	76.61679247
0.014529186	0.090278643	0.226159662	113.860663
0.026481544	0.077002414	0.055535636	6.338909446
0.063461957	0.105870216	0.067818448	7.021788963
0.008524732	0.104883631	0.904993669	731.3771351

log.sigma.2.5.mm.3D_glrlm_LongRunEmphasis	log.sigma.2.5.mm.3D_glrlm_ShortRunHighGrayLevelEmphasis	log.sigma.2.5.mm.3D_glrlm_RunLengthNonUniformity	log.sigma.2.5.mm.3D_glrlm_ShortRunEmphasis
1.335189297	145.7726681	507.419522	0.932242317
1.24633602	47.65075848	91.08111685	0.944396306
1.425181958	102.0698369	352.120106	0.921490431
1.261147288	59.41292131	74.07341866	0.945568393
1.595327048	70.09854956	495.4375109	0.900278632
1.104950027	255.6353572	93.68524643	0.975562063
5.632272359	109.7328274	4148.832491	0.697321916
2.533604929	160.1262548	2459.229412	0.832076444
3.165188983	118.0470242	1680.18448	0.784897153
1.274699062	73.15873631	290.998959	0.943748238
3.422366998	354.5810566	1993.247684	0.764717025
3.08741502	111.1847348	1217.280006	0.783801213
1.20170968	117.4480429	276.4195898	0.95471641
1.258214006	63.56174066	153.1164617	0.945062378
1.846089248	124.6398935	549.7254895	0.876239078
3.983302357	107.0029641	2877.125465	0.751734195
1.641976907	36.13682075	272.336772	0.88277769
1.244054233	50.16735424	90.17343642	0.947041643
1.709702265	51.78089464	353.1032566	0.881710865
3.246313522	177.1628598	4703.577586	0.787077096
2.104496836	51.94099292	822.7839043	0.867183605
1.315326948	36.42027849	120.5784669	0.930583237
1.538334182	69.85354319	187.6149371	0.899680759
4.266488492	77.63695397	1450.958921	0.724348646
2.276376455	125.2294764	386.6955295	0.851470407
2.071002089	121.3878982	1101.169374	0.85379768
1.515284925	89.83983047	641.6835322	0.904538989
2.140095151	126.7640278	909.71033	0.840344206
1.119858217	65.29740432	25.23735338	0.970035446
1.937451627	57.33628791	663.8437288	0.865293196
2.761212381	79.80217924	1599.423908	0.818046842
1.344330403	74.08022241	192.9245127	0.929564709
1.76549094	73.11748724	781.6656127	0.880038904
3.239470617	112.3259132	1183.197228	0.771710819
2.181941064	128.3455746	1860.295896	0.85216072
1.687540653	189.444215	737.5902882	0.897668802
1.10737487	198.2166366	352.2267462	0.973844456
4.554587619	111.2355899	3576.16493	0.734418932
1.439047665	65.48464874	138.2968035	0.910884557
3.813081729	612.3490888	4061.067228	0.748901711
1.490943931	86.45729064	433.8500703	0.906357755
3.48631831	94.67414014	2605.401798	0.763033926
1.575414785	131.469555	1059.289683	0.908594056
1.471539747	106.734176	494.2728042	0.910666163
2.683590132	59.74573922	471.2681403	0.799995782
2.130425746	160.4495574	1063.358981	0.854339592
3.739991736	96.98427944	2429.765691	0.759948971
1.443428809	50.76717127	155.155072	0.91132176
1.262381001	53.31548015	90.12141461	0.940184253
1.160577116	155.8628333	189.7684523	0.962367626
2.936561547	102.0139341	802.2246936	0.798524282
1.6704312	149.4726079	1386.08502	0.892330988
5.162693561	76.97044158	2595.968177	0.692856357
1.525120586	75.29194677	591.6202268	0.907365776
1.255085606	93.21894461	229.5207987	0.943312035
1.526845111	72.98027794	446.7340948	0.902208261
3.188323282	114.4814713	2114.848005	0.78748962
2.143532363	89.94854182	851.9643572	0.860498726
1.37928678	83.34424093	204.2942243	0.926796372
4.430357527	113.8156219	6515.068031	0.702997735
1.714496003	119.9112981	522.1296109	0.899334602
1.53705101	167.7618567	1105.714909	0.909087158
1.522533823	225.1092339	3211.534086	0.913961017
3.113081647	283.0769958	3708.469046	0.812336514
1.471493463	77.98597678	225.3644281	0.910294968
1.314860418	194.8628062	1690.774599	0.936286989
1.252147832	68.03269984	95.15613405	0.94340009
2.151952165	133.6513034	2273.57229	0.859108591
3.905215896	68.17447913	3190.202679	0.735380031
1.560582912	117.8654298	1040.992782	0.906493792
1.347092587	63.18481059	148.8895587	0.927682711
4.072991603	158.5483192	2371.560346	0.754402571
1.620451161	111.4670327	490.3995695	0.896007191
2.003994695	77.20337727	595.9858717	0.859175162
2.664256566	88.82735723	1489.049956	0.821590604
1.545050724	75.02335562	414.4305298	0.90491616
1.290434296	146.7649146	1162.877343	0.939419591
1.151315653	94.13482369	99.73209143	0.963732217
1.306737935	141.5466535	503.6229075	0.935584837
2.66535412	59.05412398	713.1294709	0.808490779
1.335189297	145.7726681	507.419522	0.932242317
1.319514405	69.45389708	280.5484692	0.93093759
3.218674106	81.95856526	1345.875885	0.783401233
2.141831112	81.32775933	613.8033373	0.843140818
2.244171118	73.10578542	932.5401786	0.84275697
1.178370389	112.3585324	150.4224418	0.960367062
1.529825698	146.4895821	492.5484799	0.903348356
4.285648108	70.29567834	1317.445695	0.748520971
1.391557308	186.77063	874.1665298	0.926451696
1.445518384	88.42415042	568.2315363	0.915122378
1.23049609	135.298479	225.952888	0.949735418
2.453600486	84.56017194	709.2270616	0.832078165
1.979056401	104.3244372	1950.961603	0.865729367
2.775729735	84.38836197	1710.484298	0.810966738
1.636944963	90.13562768	1335.593757	0.89258998
1.331823152	149.7600994	904.5014568	0.932745462
1.585137996	109.5840329	972.2770775	0.898354909
1.175891558	133.9311839	73.74017191	0.95710901
1.218783305	52.55172823	57.90051628	0.946619098
2.8208844	151.059364	4431.212408	0.823730899

log.sigma.2.5.mm.3D_glrlm_LongRunHighGrayLevelEmphasis	log.sigma.2.5.mm.3D_glrlm_RunPercentage	log.sigma.2.5.mm.3D_glrlm_LongRunLowGrayLevelEmphasis	log.sigma.2.5.mm.3D_glrlm_RunEntropy
212.6504396	0.908523909	0.014925003	4.231739512
57.33478108	0.928522805	0.081974751	3.830334111
166.7657701	0.891861761	0.028903447	4.375745906
74.18657699	0.926421405	0.066586938	3.847375435
136.0348631	0.858527531	0.034569074	4.111973345
288.5139184	0.967886482	0.016522152	4.183517945
997.6227471	0.55998264	0.034305861	4.471520708
571.3183109	0.748178298	0.013858174	4.464406315
577.6702807	0.687687578	0.020090699	4.231584974
98.26733523	0.923710904	0.051990825	4.067460138
1590.257335	0.665953373	0.007778996	4.077172324
506.5158081	0.688327618	0.023387058	4.330811699
139.3466028	0.940041831	0.034020698	4.400191746
83.03452623	0.927125506	0.046673994	3.859763572
314.865626	0.82048495	0.015982719	4.186907811
660.1406756	0.637756989	0.028466839	4.406479851
69.67270582	0.844389555	0.079555599	3.853532456
62.4279495	0.93000693	0.063573674	3.607880631
103.0638508	0.839234839	0.049113323	3.915844545
750.4745447	0.682491567	0.01527408	4.500889516
148.8874158	0.794871795	0.054093633	4.1736283
48.65994861	0.91090555	0.071909967	3.558743264
131.4793328	0.864768683	0.03792363	3.983425516
514.3951027	0.613319029	0.039192426	4.151188166
419.9245399	0.773786819	0.019371286	4.376060245
308.0100019	0.790731244	0.01830675	4.359486391
165.7197236	0.871631031	0.025858696	4.137955292
365.7714888	0.777452224	0.016401535	4.23001843
75.47777764	0.964285714	0.076576065	3.341008833
145.444387	0.810131658	0.053199009	4.168669543
350.2358181	0.725609623	0.030767373	4.354399997
102.8292773	0.906184012	0.037268664	3.953397257
166.1645938	0.83129849	0.038169098	4.184335
541.4234733	0.674963969	0.022993065	4.372812328
373.7029259	0.781265901	0.01555729	4.222565193
402.9295762	0.849699945	0.012670394	4.562454274
218.8818498	0.966272189	0.013361311	4.492216389
808.1625795	0.605954363	0.029105855	4.487626474
107.4448996	0.883248731	0.037215349	3.890626226
3212.06167	0.640346198	0.004711072	4.380277582
148.8382637	0.874923808	0.023971926	4.055472902
434.897619	0.661950872	0.030525072	4.114900106
237.4621627	0.870837741	0.019423143	4.385645959
184.2682863	0.879447285	0.018361339	4.130547814
237.6836651	0.719386312	0.043747141	4.152350427
456.7523293	0.787369664	0.012560566	4.287577545
540.1449064	0.647957438	0.030325858	4.47094934
79.53079739	0.884439761	0.07272346	3.996719807
66.91219949	0.923076923	0.067470272	3.789804028
177.7364776	0.951048951	0.037222087	4.541207354
457.8721953	0.704213415	0.024284379	4.228953445
281.508076	0.847329604	0.014520671	4.477439198
633.6591482	0.57106742	0.045180311	4.213985282
133.7027245	0.875034057	0.041801067	4.196423061
116.545791	0.926025194	0.029643105	4.168403811
128.0926435	0.867734076	0.031082897	4.063526497
523.0294387	0.688292037	0.022782987	4.379709294
285.2671223	0.787752054	0.024834704	4.301017801
126.7270237	0.902432127	0.031156816	4.018407898
772.0151531	0.596959456	0.02682797	4.147272295
250.6082554	0.846636425	0.019334068	4.357775781
288.7477196	0.870867124	0.013638366	4.604234083
375.9026042	0.876001077	0.009580746	4.7232541
1216.549463	0.704144095	0.008758681	4.588596666
127.4661956	0.881140666	0.037676644	4.066094923
263.9055303	0.913501144	0.013308569	4.704228748
84.94785601	0.926308985	0.050117692	3.788230634
367.725148	0.788703787	0.01947429	4.529704076
401.2306511	0.630327171	0.043298567	4.313796549
207.3184578	0.867261095	0.018869558	4.377186178
95.96209134	0.904817405	0.041876061	3.845310175
1045.452545	0.630413818	0.017912897	4.532309786
232.1540791	0.854678032	0.017481	4.147877103
205.1331422	0.797763281	0.029040335	4.173563918
311.1126001	0.732323957	0.031039626	4.346938019
125.7294141	0.871625064	0.038210981	4.158186451
187.6937543	0.918561196	0.02457459	4.698657853
104.1294981	0.953177258	0.059308298	4.17343184
190.6167114	0.914214817	0.015628616	4.312026019
234.8642511	0.726286871	0.039988696	3.996946873
212.6504396	0.908523909	0.014925003	4.231739512
89.80330793	0.909943715	0.052473611	4.185975584
427.5346836	0.682990503	0.034980917	4.303894778
241.4357492	0.778919804	0.025120527	4.018171319
227.1690449	0.772299169	0.033417545	4.244019714
127.6440864	0.947115385	0.054832706	4.310691943
260.287117	0.869708223	0.017932531	4.264271287
495.069806	0.620379129	0.044125334	4.304558477
267.8635973	0.898723404	0.020194953	4.805924832
138.7554051	0.884711538	0.029760568	4.258031626
163.490311	0.932831661	0.02585425	4.32882498
292.4078782	0.754384077	0.026108877	4.074827937
253.0562599	0.804882435	0.021307062	4.329588172
319.4253157	0.720430322	0.031290274	4.398933553
163.122463	0.85117785	0.022379822	4.095305303
214.8954858	0.909173597	0.016239433	4.481923933
193.6060194	0.860540824	0.020693163	4.352835031
160.6872122	0.946065429	0.028887926	4.119318775
62.79093011	0.933911159	0.074914774	3.663905688
543.6059645	0.724858232	0.018470914	4.659319328

log.sigma.2.5.mm.3D_glrlm_HighGrayLevelRunEmphasis	log.sigma.2.5.mm.3D_glrlm_RunLengthNonUniformityNormalized	log.sigma.2.5.mm.3D_glszm_GrayLevelVariance
156.9209495	0.837931696	18.89851812
49.45958433	0.865850097	13.30056711
111.5200821	0.815882169	22.63562426
62.04653131	0.868070679	15.27836735
78.76302919	0.772776519	15.82005549
261.8032377	0.938541989	25.62402344
162.7239064	0.451022032	18.6971784
199.0014486	0.649014696	20.46507387
157.9183773	0.573711828	12.07084322
77.44048768	0.864134826	16.56686391
463.8903482	0.543712425	14.45095573
147.0378166	0.572082107	17.75908304
121.5241877	0.887939773	25.62506193
66.97001348	0.867323133	13.21561269
147.7394586	0.727052529	14.07251926
148.7444782	0.524928823	14.47380326
41.18162212	0.738187508	10.77129804
52.4114349	0.872018357	9.608987603
58.99120918	0.738054181	10.71469136
226.2168878	0.576059581	20.18000863
61.14719976	0.711395972	11.36954012
38.69140149	0.835703545	9.830178475
79.04989154	0.770929096	10.57797934
111.0228448	0.487931424	13.21904732
154.4135403	0.684773771	17.20818115
143.0312222	0.68583459	27.14613527
101.1614002	0.781541579	14.2484253
155.0757881	0.662715782	14.20792079
67.33347898	0.930029948	14.02768166
67.83863871	0.70794241	13.96098156
103.3948116	0.625593778	13.22892761
78.95954336	0.833151353	15.86094587
85.00106764	0.733557508	16.16208399
151.2889105	0.553776396	17.11981488
154.3518656	0.683285623	14.57052144
215.5020121	0.768267554	22.82941261
202.2399377	0.933809641	29.71102041
158.2600808	0.499917889	16.70755019
72.49002572	0.793454085	13.61363636
824.0981983	0.520412092	23.81658717
96.14033216	0.784346167	14.15781573
124.0380055	0.540808736	14.70062491
145.6153442	0.791766154	20.05945893
118.7015305	0.793440951	14.74365473
77.81888314	0.597895455	10.746913
192.3785638	0.687747466	17.41274475
131.7428266	0.535750692	16.84592357
55.49016775	0.797075578	15.43820862
55.87162736	0.855458243	11.61814745
160.0756529	0.906116108	48.88002232
134.7309624	0.59456338	13.43314639
167.0846753	0.756841185	27.86387454
114.8090896	0.446473774	13.22656184
83.87892888	0.791305922	13.61
97.58087971	0.862468177	20.45767092
81.39936102	0.775642104	13.57703452
149.8947175	0.577561958	16.42054071
109.2742414	0.698769274	14.40569098
90.11056757	0.828633932	13.83760684
165.7047878	0.460429659	18.29931428
134.7102255	0.772703395	21.87859365
184.7397853	0.790789742	31.00884919
245.6220148	0.800769411	31.44715715
356.5694847	0.616552886	25.02998264
85.75677572	0.793581947	13.5975346
206.4889056	0.84664695	34.44204294
71.09202667	0.862499016	12.72069943
157.8680592	0.696176376	28.08857513
94.99420836	0.501894168	16.66810514
130.0214553	0.785064755	21.02038781
68.71622345	0.829163914	9.737024221
222.1055371	0.527782216	17.64881922
127.7691186	0.764382178	13.86511296
92.05834074	0.695148021	17.50190311
109.5188799	0.630914091	20.60217329
82.40829902	0.783209618	16.70243487
153.9394899	0.853619549	31.84582533
96.08879803	0.909176041	18.86478057
150.0107754	0.845363349	19.83213174
76.42819207	0.611160788	9.635962539
156.9209495	0.837931696	18.89851812
73.1165199	0.83470066	17.90425702
111.9203722	0.572081516	12.20598847
99.71340992	0.667867247	10.13861084
89.33965614	0.666742479	14.42804584
115.1977508	0.90161202	21.89395062
163.6177357	0.778776013	18.6216409
99.47750297	0.51972451	12.9437624
199.5168805	0.826442997	45.88813243
96.21303477	0.801943389	17.86078409
140.4960906	0.876984067	18.81416667
104.8281389	0.651665965	11.23466709
121.4510181	0.70709679	18.31215357
106.1783596	0.613860661	15.70485493
100.4786695	0.757561202	14.29823616
160.4087241	0.839362539	25.4175395
121.6125355	0.769061963	23.12196121
139.1776616	0.894253318	24.1017284
54.56169878	0.870917497	10.11222222
185.0875398	0.635121513	23.76359003

log.sigma.2.5.mm.3D_glszm_SmallAreaHighGrayLevelEmphasis	log.sigma.2.5.mm.3D_glszm_GrayLevelNonUniformityNormalized	log.sigma.2.5.mm.3D_glszm_SizeZoneNonUniformityNormalized
102.1636254	0.065094646	0.398117902
51.55377126	0.080340265	0.403591682
99.45534085	0.05902497	0.38587396
47.75982691	0.07755102	0.386122449
74.07672408	0.074118113	0.477473907
191.0392795	0.062011719	0.462890625
123.2140632	0.065498569	0.387771173
113.1458258	0.065119933	0.313188356
85.08926615	0.084276844	0.319576333
60.83689646	0.073964497	0.373254438
298.0675846	0.084903753	0.386828688
106.8827372	0.071637111	0.379406358
113.0462029	0.057970362	0.431376965
54.75844978	0.082004128	0.485456934
66.65405225	0.078059547	0.28255484
98.35330449	0.078002524	0.350760171
36.11070269	0.094329481	0.339962376
48.93369171	0.104338843	0.431818182
48.7908119	0.087901235	0.405432099
179.6639871	0.06468873	0.426442714
62.05165483	0.085559344	0.345324707
39.23031983	0.104380746	0.415900487
61.57641174	0.092970522	0.357520786
69.34713759	0.082722742	0.333968661
99.89824462	0.070032871	0.3458729
141.8105033	0.060202689	0.403355388
66.94148254	0.077517427	0.384843649
89.34353081	0.082246839	0.328007058
37.35058824	0.107266436	0.453287197
53.76779531	0.075826856	0.393156531
71.07564028	0.080754519	0.351270469
78.16499772	0.0743605	0.403033908
71.99815937	0.071055939	0.414404022
95.95230904	0.073586777	0.335854545
119.9956494	0.073563577	0.397686882
151.0451046	0.059625435	0.382111066
180.7438897	0.052794669	0.554785506
99.01217914	0.077527987	0.334699297
34.07478978	0.087809917	0.271694215
599.6083088	0.060821381	0.417832233
71.92274908	0.076562207	0.418934134
104.1226261	0.082168545	0.430936437
122.58181	0.065330417	0.431030155
79.75947848	0.07922865	0.337594123
49.69182922	0.090771986	0.333584692
159.5905847	0.070501958	0.444841581
82.98496019	0.070061799	0.382442534
36.85466771	0.078231293	0.185941043
55.6425025	0.09073724	0.448015123
129.0669939	0.053252551	0.378985969
59.67611929	0.082241493	0.292911679
162.4947702	0.055633452	0.447108132
91.21008701	0.089230978	0.451684194
45.60285075	0.078828125	0.339921875
96.37014507	0.069151852	0.440676639
72.33264106	0.081506677	0.425422021
93.90460736	0.072977054	0.356763451
78.31338615	0.077160494	0.369834971
56.10678406	0.079552926	0.366863905
126.9221708	0.077721166	0.395952894
117.3584958	0.060231879	0.399519276
170.9966041	0.051424047	0.451792206
216.9729438	0.049237075	0.439565357
275.8231434	0.057533333	0.457918056
61.74338058	0.081314879	0.321366782
175.6949237	0.048556094	0.429238227
57.86153716	0.085066163	0.352551985
151.6227798	0.05502478	0.479767715
93.1808197	0.074017553	0.414255139
123.5236997	0.062132964	0.418614958
34.12181266	0.093425606	0.23644752
134.8723556	0.068983044	0.372466734
72.08064127	0.080047562	0.302259215
78.18232053	0.075709343	0.448442907
92.4860741	0.066664463	0.378848994
80.10670732	0.075816506	0.377590708
131.0552396	0.05370522	0.407115438
101.0377814	0.068389396	0.490755179
133.4866272	0.063369946	0.472234319
42.90510126	0.097273673	0.369656608
102.1636254	0.065094646	0.398117902
78.55501126	0.071269133	0.371970663
59.08303971	0.084581901	0.300641677
51.5898386	0.090332031	0.285644531
71.62997117	0.076897455	0.353964481
113.5646588	0.069382716	0.461728395
116.4333687	0.069678954	0.341926278
73.25933231	0.081283089	0.375731449
166.5365309	0.045159774	0.378632648
103.0965396	0.069713758	0.451331179
113.9853709	0.07125	0.377222222
59.18873353	0.085591262	0.267695862
104.0688507	0.064230911	0.427439819
93.15298749	0.070943841	0.436209505
82.67505484	0.074900394	0.349817856
143.1343087	0.055134652	0.433971832
121.8792282	0.060890108	0.443015546
100.421586	0.067654321	0.415308642
51.70601193	0.106666667	0.371111111
140.7973614	0.057082045	0.404961012

log.sigma.2.5.mm.3D_glszm_SizeZoneNonUniformity	log.sigma.2.5.mm.3D_glszm_GrayLevelNonUniformity	log.sigma.2.5.mm.3D_glszm_LargeAreaEmphasis	log.sigma.2.5.mm.3D_glszm_ZoneVariance
85.59534884	13.99534884	54.75348837	45.15790157
18.56521739	3.695652174	13.63043478	7.595935728
55.95172414	8.55862069	49.53793103	38.44214031
13.51428571	2.714285714	18.28571429	11.37632653
83.08045977	12.89655172	188.9655172	170.584093
29.625	3.96875	3.609375	1.019287109
376.9135802	63.66460905	73427.07613	73145.65134
176.3250444	36.6625222	4763.609236	4683.279248
94.59459459	24.94594595	9812.834459	9608.711457
48.52307692	9.615384615	26.04615385	18.20615385
163.2417062	35.82938389	14139.30806	13971.66079
103.1985294	19.48529412	5892.172794	5764.532642
64.27516779	8.637583893	15.75167785	10.81671997
35.43835616	5.98630137	20.68493151	13.91067743
42.10067114	11.63087248	378.3221477	340.1977388
226.2403101	50.31162791	25264.64341	25089.87091
20.73770492	5.754098361	262.6885246	211.6011825
19	4.590909091	19.25	12.88584711
36.48888889	7.911111111	296.4555556	256.7655556
467.8076572	70.96353692	20782.81039	20664.60176
66.64766839	16.51295337	768.4041451	711.8038605
17.88372093	4.488372093	52.65116279	39.14981071
22.52380952	5.857142857	80.14285714	60.2484253
84.49407115	20.92885375	23563.91304	23203.3635
51.18918919	10.36486486	359.3378378	335.2748356
111.326087	16.61594203	1316.641304	1262.810373
72.73544974	14.65079365	181.1058201	156.4222726
66.25742574	16.61386139	1622.905941	1547.078056
7.705882353	1.823529412	3.647058824	0.934256055
63.69135802	12.28395062	644.3888889	593.908741
114.8654434	26.40672783	4113.379205	3998.09597
33.04878049	6.097560976	34.25609756	24.58551457
99.87136929	17.12448133	375.2987552	347.1339336
92.36	20.23636364	6898.134545	6767.011226
193.6735113	35.82546201	1449.735113	1398.760791
105.8447653	16.51624549	192.9747292	176.3918988
135.922449	12.93469388	4.816326531	2.282382341
279.8086124	64.81339713	38670.10526	38472.96485
11.95454545	3.863636364	67.75	47.70402893
416.5787362	60.63891675	33066.28084	32918.64737
61.16438356	11.17808219	99.65068493	80.97171139
289.5892857	55.2172619	17713.74256	17597.76376
143.9640719	21.82035928	215.5538922	194.6520223
55.7030303	13.07272727	112.030303	93.67037649
34.3592233	9.349514563	1750.466019	1638.886794
117.8830189	18.68301887	1141.233962	1086.808544
182.8075314	33.48953975	18990.96025	18778.39849
7.80952381	3.285714286	77.69047619	50.50170068
20.60869565	4.173913043	15.60869565	9.466918715
42.44642857	5.964285714	6.428571429	2.570153061
60.33980583	16.94174757	2809.825243	2724.217834
229.3664717	28.53996101	405.0389864	387.326866
219.5185185	43.36625514	60164.11111	59732.05297
54.3875	12.6125	194.26875	166.2449609
49.79646018	7.814159292	21.01769912	14.5669982
53.6031746	10.26984127	188.3412698	160.6536281
175.8843813	35.97768763	9601.373225	9486.366091
93.1984127	19.44444444	588.3055556	550.7169785
28.61538462	6.205128205	42.1025641	29.94214333
449.010582	88.13580247	140124.1949	139694.2726
82.30097087	12.40776699	143.5776699	128.6090348
199.2403628	22.67800454	205.8888889	192.6762409
613.6332378	68.73495702	314.1375358	303.4114416
549.5016667	69.04	7264.695	7214.285
21.85294118	5.529411765	80.48529412	58.20134083
326.2210526	36.90263158	93.94342105	85.67779605
16.2173913	3.913043478	20.41304348	13.72069943
382.8546366	43.90977444	1584.250627	1557.491398
232.8113879	41.59786477	37224.51779	36906.44771
159.0736842	23.61052632	199.3973684	183.2496607
12.05882353	4.764705882	37.52941176	22.4567474
206.3465704	38.2166065	17636.27256	17472.07193
43.82758621	11.60689655	165.1655172	138.4829489
76.23529412	12.87058824	622.6647059	582.8263322
161.3896714	28.39906103	3010.140845	2953.148978
51.72992701	10.38686131	121.270073	101.832916
200.7079108	26.47667343	86.15821501	77.12166682
32.88059701	4.582089552	5.776119403	2.83002896
117.5863454	15.77911647	41.73092369	34.89553394
57.29677419	15.07741935	2472.735484	2366.711925
85.59534884	13.99534884	54.75348837	45.15790157
41.66071429	7.982142857	50.65178571	39.79711416
107.9303621	30.36490251	5623.922006	5533.274633
36.5625	11.5625	1143.882813	1059.61615
90.9688716	19.76264591	1012.587549	963.2601553
41.55555556	6.244444444	8.511111111	4.68691358
49.57931034	10.10344828	189.7310345	164.7310345
92.80566802	20.07692308	15249.02834	14978.04609
139.7154472	16.66395664	89.73170732	79.59205646
102.9035088	15.89473684	102.0526316	89.74115112
45.26666667	8.55	11.91666667	6.626666667
36.94202899	11.8115942	1616.405797	1509.028145
270.9968454	40.72239748	1291.864353	1262.731602
184.9528302	30.08018868	4520.731132	4438.109892
151.4711316	32.43187067	495.7159353	472.9279904
176.6265356	22.43980344	46.89434889	38.43153898
143.537037	19.72839506	271.212963	250.7680517
18.68888889	3.044444444	7.4	3.662222222
11.13333333	3.2	11.36666667	5.765555556
530.0939649	74.72039725	8165.436211	8111.606133

log.sigma.2.5.mm.3D_glszm_ZonePercentage	log.sigma.2.5.mm.3D_glszm_LargeAreaLowGrayLevelEmphasis	log.sigma.2.5.mm.3D_glszm_LargeAreaHighGrayLevelEmphasis	log.sigma.2.5.mm.3D_glszm_HighGrayLevelZoneEmphasis
0.322822823	0.36346515	9441.888372	153.6744186
0.407079646	0.671692108	501.3695652	65.39130435
0.300207039	0.619511095	6445.896552	132.3793103
0.380434783	0.74429434	760.1714286	77.91428571
0.233243968	3.184076208	14494.3046	94.35632184
0.621359223	0.036066168	959.5625	264.90625
0.05960996	409.4421837	13260712.79	172.2582305
0.111573524	19.12978837	1201427.81	178.5186501
0.069992906	50.47484062	1943482.118	133.2939189
0.357142857	0.573669138	2009.446154	85.58461538
0.077232796	30.23759471	6633608.671	465.4597156
0.088512854	34.06175563	1037071.941	145.3161765
0.450151057	0.319690527	1287.805369	150.1946309
0.384210526	0.484104441	1307.684932	71.64383562
0.161956522	2.000146899	77279.04698	117.4563758
0.075642078	145.8528471	4437323.902	145
0.139908257	9.728023292	10599.21311	53.7704918
0.396396396	0.744468949	728.75	64.84090909
0.158730159	5.71928594	18748.83333	67.63333333
0.091976188	89.49697839	4869597.459	251.1257976
0.13292011	16.08303205	54139.6114	90.69948187
0.272151899	2.531459112	1432.813953	52.53488372
0.224199288	1.334211142	7215.47619	86.79365079
0.052664446	190.9848498	2939382.802	102.3833992
0.203856749	1.794032364	78062.22973	146.6756757
0.136296296	9.172662257	202028.1232	191.8405797
0.201277955	1.885563112	21448.62963	95.53439153
0.114837976	9.014766391	302917.2426	135.2079208
0.607142857	0.167393933	236.3529412	65.52941176
0.140747176	9.665329621	52748.00617	74.83950617
0.093135859	30.73439444	591488.6024	98.01529052
0.321568627	0.624898309	2478.939024	101.0853659
0.18842846	3.970830304	41931.41494	92.74273859
0.087329311	39.15916427	1232946.411	144.8509091
0.140063273	8.256235264	268760.9569	164.2936345
0.245567376	0.755196659	56042.00722	212.3140794
0.628205128	0.057719301	726.5836735	223.3183673
0.071221673	210.4590865	7161537.067	147.4760766
0.223350254	1.086055661	5232.477273	69.86363636
0.082301469	38.82574774	28202737.24	859.3570712
0.231378764	1.135594242	10759.37671	100.1643836
0.092856156	142.2425037	2227130.81	143.516369
0.218729535	1.64309195	35358.47305	159.7095808
0.233380481	0.95134823	15667.87273	119.2787879
0.094669118	19.2804058	168981.6214	70.9223301
0.135549872	5.03826098	271670.9736	209.7320755
0.068589468	129.4710975	2838565.711	123.9121339
0.191780822	2.773412468	3975.928571	70.97619048
0.403508772	0.634450971	707.8695652	70.17391304
0.509090909	0.233058007	726.5625	191.3839286
0.108079748	16.64355876	482722.7621	99.49029126
0.237610005	2.528770691	68450.45419	209.7719298
0.048109285	483.6926615	7567137.356	122.3559671
0.188902007	2.824285365	19769.0875	72.9
0.393728223	0.464113747	1388.424779	124.3893805
0.190045249	2.699534879	16205.43651	97.02380952
0.093247588	56.05938029	1664510.945	139.5720081
0.163106796	4.172245405	94073.05556	110.7103175
0.286764706	0.606423657	4095.538462	88.94871795
0.048228639	790.7227021	25005255.14	173.0537919
0.25846926	1.073942637	22209.0534	161.4854369
0.27510917	1.135306214	41975.60544	222.2131519
0.305336833	1.277695502	82118.78295	290.9663324
0.14084507	17.80761282	2988921.849	369.9058333
0.211838006	1.194733937	7821.308824	93.27941176
0.347826087	0.5863933	16985.04474	244.9210526
0.386554622	0.599233271	1007.782609	86.45652174
0.193313953	8.903258159	293165.698	186.2869674
0.056071037	363.2398847	3925989.758	126.0231317
0.248853962	1.744370929	26384	168.5789474
0.257575758	0.883667174	2771.196078	69.11764706
0.07803916	64.97109706	4829075.097	194.6732852
0.193591455	1.196472269	27634.67586	115.1103448
0.158434296	6.145389816	68870.92941	101.1705882
0.132462687	24.75441712	376177.7535	129.42723
0.226821192	1.908967645	9737.321168	106.7445255
0.33265857	1.574745466	6369.026369	191.8965517
0.582608696	0.288234132	355.2089552	119.4626866
0.382488479	0.371694655	5459.060241	175.7309237
0.097117794	27.09957273	234964.0258	64.58709677
0.322822823	0.36346515	9441.888372	153.6744186
0.303523035	1.745151356	2366.071429	106.6339286
0.105032183	38.6976864	831050.8496	90.37325905
0.10893617	9.568022822	143934.8438	84.6640625
0.142382271	9.928072739	111005.1712	100.2607004
0.511363636	0.303580751	621.6	144.6111111
0.2	1.249096873	33688.94483	171.8689655
0.060747664	127.0823058	1862295.206	100.1052632
0.314042553	0.676271017	14816.7832	239.6937669
0.285	1.624930377	8269.570175	130.4254386
0.434782609	0.231938305	1244.1	164.0166667
0.096503497	13.03043298	210294.913	100.9347826
0.185271771	10.15610085	172599.5647	142.1041009
0.110015568	38.53690438	550490.2311	124.8679245
0.209482342	5.101630102	50602.08314	121.8729792
0.34375	0.359879763	7891.051597	187.0884521
0.22116041	2.433214321	34110.23148	159.7376543
0.517241379	0.112973566	914.2666667	149.0444444
0.422535211	0.511692086	465.6333333	70.43333333
0.136297376	41.29473059	1637500.834	202.7998472

log.sigma.2.5.mm.3D_glszm_SmallAreaEmphasis	log.sigma.2.5.mm.3D_glszm_LowGrayLevelZoneEmphasis	log.sigma.2.5.mm.3D_glszm_ZoneEntropy	log.sigma.2.5.mm.3D_glszm_SmallAreaLowGrayLevelEmphasis
0.654354998	0.020222295	5.642517711	0.016660612
0.658617816	0.089209887	4.74095326	0.067414748
0.643947929	0.030972683	5.642908315	0.020364929
0.63737039	0.092552178	4.536286231	0.083307416
0.717272323	0.040362406	5.240438705	0.032243788
0.705186632	0.021534461	5.103373781	0.019757289
0.646401743	0.012258199	6.020405464	0.007515196
0.5802514	0.013089194	6.341784039	0.009046268
0.586297238	0.016217607	5.820984709	0.011009855
0.633682438	0.05705942	5.451842841	0.031798987
0.644641585	0.004983945	5.60513092	0.004166539
0.640338006	0.023816637	5.784423585	0.017954716
0.682934648	0.028086452	5.455545066	0.01410362
0.717546589	0.05664576	4.85614995	0.045469888
0.548916956	0.023407242	5.780673303	0.017935199
0.614794098	0.014259304	6.024066495	0.009276648
0.602232107	0.10054524	4.877641748	0.075693492
0.681445293	0.059057167	4.464558039	0.030127452
0.658576762	0.059536634	5.04274598	0.044281421
0.679178709	0.006945176	6.034062991	0.0049275
0.608260561	0.032725729	5.441651231	0.018045048
0.659200516	0.074327219	4.487554802	0.055317453
0.616320202	0.041088337	4.760943567	0.027025565
0.5935273	0.025096519	5.613543467	0.016074811
0.608287587	0.020342575	5.679619807	0.014740159
0.659791939	0.013870498	5.886900487	0.009428148
0.64458893	0.032774189	5.476094618	0.020266058
0.592119928	0.016752754	5.566158033	0.008077268
0.634705882	0.100521406	3.969815782	0.089853695
0.648809548	0.068756689	5.453849767	0.045622689
0.615680292	0.027130924	5.790022921	0.017498001
0.655941486	0.044546803	4.960657646	0.033989282
0.667953949	0.043987391	5.642218278	0.027869084
0.601584726	0.015952187	5.902662861	0.010836532
0.655680503	0.011997406	5.779328639	0.008316936
0.642578892	0.01350791	5.876001956	0.009769771
0.77306618	0.013080696	5.482323588	0.01081657
0.600309515	0.013334696	6.173527368	0.007762644
0.533013821	0.06121951	4.777613437	0.050008007
0.672068779	0.002302586	6.071984858	0.001850033
0.670414465	0.029146324	5.410137034	0.023092354
0.682603266	0.015115117	5.596086181	0.010368136
0.681872172	0.022490341	5.752349371	0.015419813
0.601251145	0.020999948	5.617743198	0.014679494
0.594978591	0.051017992	5.142194909	0.033857847
0.693128317	0.011775209	5.533710256	0.009128125
0.6427085	0.021300649	5.82986531	0.01402513
0.423353905	0.091130777	5.154222185	0.044898727
0.68966236	0.06234564	4.430851902	0.044035345
0.623085867	0.028278674	5.555971191	0.017938631
0.558080235	0.026167477	5.796080006	0.017848609
0.695192375	0.012829301	5.964982541	0.008829968
0.698404367	0.019524752	5.381539513	0.014361344
0.604795515	0.067376128	5.545077919	0.052228461
0.688859224	0.029181867	5.376898654	0.022721659
0.675519716	0.032232249	5.139169558	0.022240269
0.619030999	0.016285035	5.979903998	0.010875827
0.629664036	0.023220814	5.699207625	0.015996237
0.627731997	0.040782036	5.221380187	0.03100334
0.654266395	0.011316067	5.884312219	0.007382358
0.648384637	0.018987979	5.635754168	0.013539102
0.698818367	0.013840821	5.933930642	0.01041615
0.689452333	0.008167906	6.250919843	0.005819636
0.703642709	0.004817324	6.018794864	0.003632562
0.585810618	0.041359741	5.101881223	0.017858622
0.681142538	0.014464084	6.235618815	0.011353867
0.607961093	0.047902584	4.778677989	0.023190059
0.719629835	0.017337712	5.932967956	0.010976328
0.668951784	0.022292176	5.709475602	0.015385627
0.672257386	0.0179278	5.784168451	0.013514105
0.48145654	0.049732207	4.99095696	0.032037707
0.632938046	0.010591737	5.973930634	0.007280353
0.567273352	0.021936396	5.613766505	0.015860422
0.69457519	0.031524659	5.378865547	0.023852482
0.638583833	0.025255676	5.960705893	0.015332735
0.63746332	0.036656981	5.450747255	0.019766331
0.663477488	0.020792158	6.11798496	0.013382956
0.727468084	0.039326307	4.836184295	0.017452896
0.71421518	0.015644737	5.496106504	0.012495985
0.630807116	0.047175532	5.24749384	0.036599192
0.654354998	0.020222295	5.642517711	0.016660612
0.632909794	0.037938392	5.295602803	0.022350154
0.565175062	0.033705729	5.958629714	0.018407289
0.551591973	0.02896979	5.550782884	0.020280219
0.616808872	0.028696353	5.679518447	0.017752242
0.704232853	0.043219889	4.940504438	0.017828681
0.604679132	0.017353058	5.632606504	0.006549473
0.636958015	0.025655968	5.5377588	0.013853584
0.638216645	0.019587714	6.27487678	0.010757547
0.697952276	0.031552606	5.448182424	0.025046063
0.634659535	0.024073738	5.392476558	0.016276862
0.520499492	0.025651302	5.580632428	0.016665661
0.67938056	0.019274523	5.796917232	0.014068557
0.686718257	0.022256154	5.576615382	0.01573804
0.613220835	0.017630169	5.859461488	0.011192133
0.684851149	0.015934775	5.878173729	0.01087562
0.691695177	0.019492231	5.73571566	0.01401839
0.665731293	0.037211377	4.74719143	0.032662076
0.628508461	0.069764097	4.056564762	0.03388115
0.661624762	0.013734733	6.197742694	0.009614726

log.sigma.2.5.mm.3D_ngtdm_Coarseness	log.sigma.2.5.mm.3D_ngtdm_Complexity	log.sigma.2.5.mm.3D_ngtdm_Strength	log.sigma.2.5.mm.3D_ngtdm_Busyness	log.sigma.2.5.mm.3D_ngtdm_Contrast
0.012215584	0.594052649	2.144245546	0.253909776	0.000125752
0.057369427	1.492022945	3.972298118	0.253695289	0.001517147
0.014553929	0.782866878	1.974745116	0.332800988	0.000326593
0.058703386	1.978850447	4.293005947	0.231014286	0.002449209
0.010010719	0.330287403	1.230784795	0.569899981	0.000108229
0.048439974	5.71596656	8.462523277	0.072809992	0.003475808
0.000462073	0.013100331	0.240214287	4.021358074	5.79E-07
0.001960501	0.061834862	0.643972552	0.931414564	5.26E-06
0.002112931	0.039161681	0.548606747	1.143290473	4.74E-06
0.019934744	0.831671511	2.569826284	0.322926884	0.000347363
0.001520187	0.04365559	0.939813271	0.778762216	1.47E-06
0.002842941	0.059760005	0.772083608	0.852831474	6.70E-06
0.019044822	1.646516017	2.891801223	0.326975432	0.000816948
0.034921617	0.968178111	2.717715903	0.242522782	0.000700206
0.010082897	0.209449008	1.546030998	0.346422833	6.60E-05
0.000874587	0.02478732	0.305696461	2.545925788	2.54E-06
0.0193517	0.223037857	1.486070225	0.585020783	0.000142341
0.051665335	1.201720254	3.213593232	0.224779499	0.001204191
0.014809076	0.18351968	1.112426537	0.540567747	0.000100782
0.00071662	0.042796739	0.602483967	1.798940907	9.49E-07
0.005381756	0.11305194	0.611325649	1.240621429	4.61E-05
0.040565101	0.676533263	2.434768816	0.371515995	0.00064744
0.02653145	0.401559725	1.803080774	0.321081796	0.000451554
0.001700477	0.029851857	0.643545814	1.5038668	1.61E-06
0.0104282	0.412954674	2.141613809	0.257637907	0.000102668
0.004472246	0.255506149	2.294800125	0.458765734	1.46E-05
0.008893249	0.224183935	1.124496077	0.609328781	8.82E-05
0.005675294	0.078793134	0.752709807	0.609323819	2.29E-05
0.137628449	5.580914783	9.049835852	0.105085903	0.02060254
0.007410192	0.107774191	0.649518211	0.909736575	5.78E-05
0.002595446	0.059090018	0.533745213	1.23953055	1.01E-05
0.025932684	0.823487395	2.56567158	0.283441457	0.00048396
0.006528522	0.188859108	1.046965288	0.653978051	4.63E-05
0.00256349	0.054641704	0.663086295	1.002389026	8.07E-06
0.002458858	0.079479415	0.58293866	0.997882344	8.61E-06
0.00755731	0.446204609	1.822666558	0.278319609	7.84E-05
0.015055155	3.028060163	3.83445851	0.223312938	0.000700111
0.000696795	0.028471347	0.516112289	2.304682792	9.34E-07
0.039926523	0.588132123	2.846642616	0.19796435	0.00042978
0.000671358	0.039933985	0.813668292	0.890524019	5.69E-07
0.01254298	0.344525775	1.545653457	0.396905856	0.000103785
0.001115232	0.029999309	0.426416463	2.066141767	1.23E-06
0.005380321	0.238034396	0.990971294	0.615633662	5.26E-05
0.011788191	0.316529102	1.424966469	0.40283224	0.000111713
0.008890169	0.060246871	0.726302159	0.693235335	3.87E-05
0.004445603	0.155744093	1.077759904	0.512584283	2.00E-05
0.001314954	0.024960166	0.407030071	1.948809993	2.34E-06
0.04104441	0.511947044	3.420861913	0.288353003	0.000480354
0.054353827	1.338517829	3.260407229	0.23221528	0.001635009
0.025480876	6.667995932	12.0868894	0.189806678	0.002141327
0.004775953	0.061590001	0.908875556	0.669065216	1.79E-05
0.004170201	0.349303956	1.667137628	0.457177743	2.19E-05
0.000742279	0.014214389	0.297097632	3.355103512	6.80E-07
0.012313038	0.158274102	1.158145071	0.542927178	0.000101584
0.022562835	1.469386227	3.892164287	0.263311102	0.000528973
0.011456221	0.293181378	1.276121807	0.581656439	0.000129583
0.001650182	0.040132083	0.515492133	1.39223698	3.60E-06
0.005063092	0.175482746	0.94694269	0.687378914	3.84E-05
0.030808832	0.633670079	2.878250037	0.212779261	0.000365593
0.000291544	0.012416327	0.315737304	5.225353869	1.97E-07
0.009402115	0.526229737	1.611856041	0.344127174	0.000114153
0.005214612	0.521530097	1.645701452	0.394909489	5.15E-05
0.001978744	0.227017318	0.701558275	0.816232879	1.79E-05
0.001022363	0.068064307	0.605814222	1.015859201	2.46E-06
0.026954426	0.430940646	2.123605505	0.267101566	0.000320615
0.003608986	0.548337398	1.435380604	0.563281675	5.26E-05
0.045113518	1.807593755	4.256564931	0.19418362	0.001348807
0.002324847	0.180655188	1.374311474	0.734041448	7.60E-06
0.000814167	0.019075952	0.315886525	3.405299179	1.03E-06
0.005247383	0.400289309	1.508802761	0.519261059	4.08E-05
0.029791948	0.703601188	1.937509361	0.340301643	0.000839343
0.001169577	0.031975643	0.484604151	1.476211835	3.02E-06
0.010662655	0.26774835	1.254275388	0.44674989	0.00011805
0.008266075	0.217308768	1.583431897	0.44488814	3.49E-05
0.002870421	0.120173561	1.434718294	0.85018562	6.78E-06
0.013658621	0.489059364	2.45031331	0.352505852	0.000109776
0.005107737	0.661743601	1.92907883	0.715722286	0.000118249
0.050126154	3.134860331	6.043194256	0.164766699	0.002542749
0.011708766	0.641044581	1.644615863	0.332436672	0.000192852
0.005525411	0.04365036	0.463508946	1.086643638	2.21E-05
0.012215584	0.594052649	2.144245546	0.253909776	0.000125752
0.018277884	0.733094065	1.904427505	0.548715276	0.00050853
0.002282568	0.048255882	0.509973692	1.321509117	9.64E-06
0.008429431	0.071407919	0.763655608	0.627363116	3.47E-05
0.004833598	0.087177445	0.563946336	0.941824633	2.81E-05
0.029366244	2.700858117	3.689094311	0.243787658	0.002076288
0.012221784	0.39052233	2.166503064	0.250087735	8.75E-05
0.002130033	0.026379799	0.374706652	1.654163122	5.17E-06
0.007300732	0.979783675	2.939097377	0.308771389	0.000125343
0.008970783	0.442937663	1.330769947	0.559842231	0.00012818
0.022930487	1.975470588	3.818262069	0.176579687	0.000667412
0.006501748	0.082011938	0.812694292	0.652062984	2.25E-05
0.002541855	0.082124848	0.440999076	1.260727912	1.33E-05
0.002295234	0.052642827	0.437247216	1.431406556	7.95E-06
0.004149302	0.127365419	0.741570019	0.941046388	2.08E-05
0.006391398	0.551702168	1.348624078	0.468671547	0.000101292
0.005599422	0.348705615	1.550565423	0.547892811	4.36E-05
0.062941827	5.082805982	8.136597692	0.094855114	0.003962771
0.081212001	1.706988699	3.700465539	0.175553218	0.002704269
0.000951516	0.040829723	0.311530887	1.995860816	3.32E-06

log.sigma.2.0.mm.3D_gldm_GrayLevelVariance	log.sigma.2.0.mm.3D_gldm_HighGrayLevelEmphasis	log.sigma.2.0.mm.3D_gldm_GrayLevelNonUniformityNormalized	log.sigma.2.0.mm.3D_gldm_DependenceEntropy
11.70862755	172.3813814	0.087695804	6.239862845
10.79630355	83.87610619	0.093742658	5.470590525
15.33554518	176.6728778	0.08017952	6.388988923
10.07124291	76.70652174	0.098534972	5.106411995
9.172214276	183.0160858	0.101510109	6.188157837
22.95070223	185.2038835	0.06456782	5.13040411
3.573106612	200.1504967	0.30888742	6.125237862
8.135799122	250.6272295	0.161488464	6.4493843
4.869956852	204.4615748	0.21374417	6.082179139
15.3291873	107.3626374	0.075806062	6.09070804
3.256085888	605.2291362	0.248918785	6.26649796
5.562161183	274.1679141	0.214140479	6.242760969
22.38597676	147.4743202	0.062732177	6.013138421
10.88612188	112.0473684	0.091024931	5.440163097
9.062566163	148.4565217	0.132615784	6.13674933
5.517501296	246.5916501	0.263957785	6.117329005
5.413996086	60.53440367	0.125768033	6.063848607
9.708465222	81.42342342	0.101046993	5.153902745
5.77589902	65.8042328	0.136387248	6.091907587
5.769159427	261.9113775	0.195625784	6.500659467
6.028126874	73.35399449	0.134385743	6.448618634
5.957058164	64.02531646	0.124419164	5.449408537
7.955319715	76.35587189	0.118172262	5.998892161
2.676511378	142.1317652	0.288649706	5.955850366
11.83704248	238.8663912	0.168286926	6.231899656
9.233215912	129.2898765	0.13383594	6.475946267
8.638238173	166.7976571	0.10032085	6.329706292
5.710040558	227.5997726	0.152298857	6.425373287
14.61607143	74.67857143	0.096938776	3.994680368
6.322957184	96.4821894	0.13136388	6.24770455
6.396220496	144.9125605	0.169254037	6.461837771
10.09802384	102.3333333	0.095301807	5.681403642
8.451034055	118.151681	0.123814601	6.44281567
5.648838905	139.7862814	0.223953407	6.253201152
6.288536468	122.8564855	0.148836319	6.426402007
16.41513804	243.3608156	0.093628967	6.652183329
31.26922419	209.1153846	0.057278107	5.89444704
5.08360641	171.3415403	0.278020634	6.273007991
7.17529439	105.4568528	0.115462908	5.567976599
4.510604188	1297.018161	0.265218182	6.116253213
8.316037985	116.1664025	0.102991001	6.198907976
3.598776909	126.1908249	0.245693044	6.164373477
10.63476159	189.2167649	0.091330082	6.578944113
9.374150994	161.3224894	0.098523754	6.215336092
4.177517267	106.8979779	0.208911724	6.105463337
7.419968734	213.8235294	0.136804181	6.403617287
4.485622479	141.2839719	0.241324076	6.245232754
7.597839912	47.94063927	0.112612331	5.759721303
8.419821484	77.28070175	0.100338566	5.204618883
45.80948347	174.0136364	0.063636364	5.825652674
5.24001634	153.4763903	0.240658813	6.048664604
13.58115291	205.2477999	0.100352457	6.679644325
2.374122076	145.0775094	0.320502837	5.917155736
8.845040974	107.433294	0.102606742	6.251370254
16.31706103	116.2473868	0.076205854	5.929247941
6.922517284	96.10105581	0.113031174	6.063872585
4.858974279	138.4675619	0.214343872	6.299610677
8.792118222	178.7695793	0.128004943	6.448640465
10.28242593	131.8492647	0.093966263	5.888099971
2.781821174	173.1091311	0.296137266	5.841231032
13.29625682	153.8243413	0.096571365	6.439280599
18.49079841	228.731753	0.082609066	6.710262111
21.36103069	401.2613736	0.074804202	6.938411058
9.000555432	600.3179577	0.176765881	6.661388497
8.487902874	137.2149533	0.106724508	6.048708062
27.7889425	281.6114416	0.060487933	6.769646015
14.09180143	99.8487395	0.088199986	5.173590769
11.80329457	174.3657946	0.126688225	6.650998307
3.279794893	101.7432904	0.251195761	6.121192893
12.28667311	144.5442043	0.094475387	6.53683114
9.092643608	68.95959596	0.102183451	5.722036186
6.409993087	244.7098183	0.23080667	6.406073324
9.435494767	111.8531375	0.105042594	6.179065888
6.539830057	101.0624418	0.149838144	6.263835977
7.391113463	163.8277363	0.184428255	6.514786743
10.14980428	89.99172185	0.102851849	6.33777789
26.77740306	229.9264507	0.063523415	6.533813794
14.29444234	63.7826087	0.079017013	5.190030951
14.03686636	183.0368664	0.079091366	6.249801085
3.746670483	87.02944862	0.201924768	6.015493919
11.70862755	172.3813814	0.087695804	6.239862845
13.01171407	84.41192412	0.085509066	6.053018745
6.391220832	177.6579871	0.246891564	6.069410597
4.561344319	77.54382979	0.153896243	6.23501033
6.37079826	101.500831	0.159210258	6.488935481
20.8986312	162.4545455	0.065728306	5.650153727
9.831693698	178.9751724	0.102672057	6.424722554
3.741770925	134.1428923	0.271287529	5.989025909
29.16551852	228.972766	0.070144319	6.813798698
12.33911094	132.32375	0.08675	6.341312263
19.53796471	174.7028986	0.066057551	5.84062911
4.358779891	98.55734266	0.172023082	6.247178842
8.378587549	155.8924605	0.122533536	6.560950108
6.105579993	115.8113648	0.177460844	6.464281249
7.630358791	88.71746493	0.117234942	6.403543357
18.76768441	300.5481419	0.070393821	6.491098629
11.38518841	111.8225256	0.099472795	6.478893346
23.97648302	123.5172414	0.0651341	5.145313002
5.891688157	53.52112676	0.123983337	4.742841269
8.807351579	283.2229279	0.164664162	6.635778544

log.sigma.2.0.mm.3D_gldm_DependenceNonUniformity	log.sigma.2.0.mm.3D_gldm_GrayLevelNonUniformity	log.sigma.2.0.mm.3D_gldm_SmallDependenceEmphasis
102.5915916	58.40540541	0.282506714
20.87610619	10.59292035	0.35485616
69.86956522	38.72670807	0.284485187
19.84782609	9.065217391	0.37696558
91.62466488	75.72654155	0.23205624
42.2038835	6.650485437	0.611987594
820.3268735	5036.718263	0.071586828
352.2160127	814.8707887	0.14705438
257.0307401	903.9240955	0.102919494
73.65384615	27.59340659	0.335710073
308.4418009	1360.09224	0.101752068
182.0868858	658.0536935	0.108711388
75.87613293	20.76435045	0.397531997
39.72631579	17.29473684	0.323042398
88.5	122.0065217	0.182638102
487.0389351	2250.768031	0.09708757
45.22477064	54.83486239	0.171434916
26.72972973	11.21621622	0.348601101
58.45679012	77.33156966	0.200903862
733.739834	2333.228725	0.122591734
119.6239669	195.1280992	0.150903096
26	19.65822785	0.250869159
32.20996441	33.20640569	0.22718984
262.6240633	1386.673189	0.066499865
62.61983471	122.1763085	0.187052743
165.3091358	271.0177778	0.153123028
118.8253461	94.20127796	0.209301619
130.5758954	267.8936896	0.150336646
14	2.714285714	0.722222222
106.1155517	151.1998262	0.155911917
224.7242951	594.2509257	0.10810881
48.97647059	24.30196078	0.299436659
123.5754496	158.3588741	0.204726777
185.1994284	705.2292791	0.110934271
269.1334484	517.5038827	0.16522958
131.322695	105.6134752	0.263127389
123.2102564	22.33846154	0.549342393
577.2414381	3263.406202	0.085199481
32.25888325	22.74619289	0.235947096
680.4391613	3212.853063	0.099066403
78.29001585	64.98732171	0.242910448
424.8922205	1778.080558	0.12347097
183.8696791	139.4610347	0.200394615
91.6562942	69.6562942	0.23744387
66.33455882	227.2959559	0.118748314
156.1508951	267.4521739	0.167418281
378.7623762	1681.787487	0.083574319
27.92237443	24.66210046	0.227434882
25.66666667	11.43859649	0.345873705
55.72727273	14	0.424612689
108.9632739	458.6956978	0.118867205
234.0356647	216.6609541	0.246374799
531.4353593	3237.719659	0.063963971
109.0448642	86.90791027	0.193790606
58.01045296	21.87108014	0.318552789
77.56410256	74.93966817	0.187216789
312.4465671	1133.236051	0.118234334
130.7190939	197.7676375	0.162468608
46.27205882	25.55882353	0.278300056
1425.948497	6963.075533	0.081698466
97.77791719	76.96737767	0.276631983
198.9700561	132.4223331	0.293543351
649.555993	342.0048119	0.317374991
526.7514085	1506.045305	0.155091301
41.43613707	34.25856698	0.24015659
406.6457666	132.1661327	0.364752374
29.92436975	10.49579832	0.404516807
336.4893411	522.9689922	0.207676592
554.9493166	2517.735109	0.079401475
179.7308448	144.2639162	0.254788233
30.74747475	20.23232323	0.261048117
369.977039	1638.496549	0.096680057
87.72096128	78.67690254	0.197728029
88.57315937	160.7763281	0.16160623
218.3215174	593.1212687	0.156662692
69.16556291	62.12251656	0.22919879
289.582996	94.1417004	0.331790654
31.55652174	9.086956522	0.417333333
111.5130568	51.48847926	0.327832437
103.0426065	322.2719298	0.108394354
102.5915916	58.40540541	0.282506714
61.36856369	31.55284553	0.308910167
207.6939731	843.8753657	0.110893784
93.95319149	180.8280851	0.127416534
133.1396122	287.3745152	0.154969154
46.625	11.56818182	0.462221779
81.51310345	74.43724138	0.223277804
212.8794884	1103.055091	0.076551897
180.1302128	82.41957447	0.308613281
104.36	69.4	0.275582279
71.19565217	18.23188406	0.449156571
104.2699301	245.993007	0.124736644
318.4476914	419.3097604	0.213650299
244.0487805	683.9340944	0.13223708
236.6139332	242.3246251	0.222106401
199.5168919	83.34628378	0.35120206
169.0300341	145.7276451	0.237216484
30.31034483	5.666666667	0.538754789
14.94366197	8.802816901	0.349936728
650.6366097	1581.434611	0.15206581

log.sigma.2.0.mm.3D_gldm_DependenceNonUniformityNormalized	log.sigma.2.0.mm.3D_gldm_DependenceVariance	log.sigma.2.0.mm.3D_gldm_LargeDependenceEmphasis
0.154041429	5.147877607	17.36636637
0.184744303	4.431200564	14.07964602
0.144657485	15.39537655	32.62732919
0.21573724	2.037334594	8.956521739
0.122821267	15.05026271	36.91152815
0.409746442	0.507116599	3.13592233
0.050308284	32.84893847	157.3850117
0.069801033	26.56503599	78.51010702
0.060778137	26.59231698	98.29865216
0.202345731	2.493841324	10.34615385
0.056449817	26.00201398	100.5347731
0.059253786	25.07343904	96.93947283
0.22923303	2.176979035	8.540785498
0.209085873	1.746703601	9.294736842
0.096195652	21.52237713	55.33913043
0.057117267	29.79810828	120.2811071
0.103726538	11.87294841	38.36238532
0.240808376	1.349078809	7.576576577
0.103098395	13.40496875	38.90123457
0.061519228	28.65104532	96.56870965
0.082385652	29.71149322	74.9862259
0.164556962	2.995032847	14.41772152
0.114626208	12.64794012	34.81494662
0.05466779	26.31254086	127.4900083
0.086253216	30.91985217	78.27548209
0.081634141	18.63147106	56.99802469
0.126544565	10.47657252	29.94355698
0.074233028	21.40795926	65.71859011
0.5	0.387755102	2.428571429
0.092194224	16.91597304	49.84622068
0.06400578	31.96547109	97.12361151
0.192064591	2.365613226	11.03921569
0.096618803	15.16832259	43.21110242
0.05881214	26.50117036	97.30358844
0.077403925	23.64363343	67.30543572
0.116420829	19.47234797	43.94326241
0.315923734	1.420092045	5.338461538
0.049177154	35.84615076	146.2160504
0.163750676	3.295678837	15.52791878
0.056169652	27.71142952	117.5306257
0.124072925	9.328789108	27.62440571
0.058711099	26.2480199	96.80848418
0.120412364	8.745065143	28.53503602
0.129641152	9.890346885	28.10042433
0.060969264	27.86547091	92.15992647
0.079872581	21.85272885	62.91099744
0.054349602	29.80605037	123.9453293
0.127499427	7.360897396	25.20091324
0.225146199	3.536472761	11.61403509
0.253305785	2.347190083	8.063636364
0.057168559	29.7064233	105.613851
0.10840003	15.09748629	39.17971283
0.052606945	29.73753099	149.8245892
0.128742461	8.27273703	26.95513577
0.202127014	3.002245991	11.46689895
0.116989597	9.157215363	30.55656109
0.059097138	25.72692411	94.16209571
0.084607828	24.54466794	65.16569579
0.170117863	4.217939014	15.11764706
0.060645111	23.77909892	118.7995152
0.122682456	23.33377203	47.98368883
0.124123553	14.10527049	34.36369308
0.142072614	12.03133226	28.57480315
0.061825283	34.29932685	102.0948357
0.129084539	8.401490669	25.85358255
0.186107902	5.461995612	14.94050343
0.251465292	1.452722265	6.949579832
0.081513891	24.8198266	65.68313953
0.055367586	27.14569679	124.6216702
0.117701928	14.38556882	36.27963327
0.155290277	9.262830323	24.15151515
0.052116783	34.49510561	126.7216509
0.117117438	16.84623022	42.7623498
0.082547213	20.77964856	60.22460391
0.067886044	30.43408734	85.69962687
0.114512521	12.35799965	34.31125828
0.195400132	2.665666814	10.81241565
0.274404537	1.327485822	6.32173913
0.171295018	5.332828379	15.92780338
0.064563037	22.33056168	81.35839599
0.154041429	5.147877607	17.36636637
0.166310471	5.540352965	16.92411924
0.060764767	29.28691836	107.46811
0.079960163	17.22394133	58.08595745
0.073761558	30.2452762	79.61828255
0.264914773	1.886234504	7
0.112431867	10.06969608	31.05241379
0.052355998	33.20149651	137.8283325
0.153302309	8.884074966	22.86212766
0.13045	10.69524375	28.105
0.257955261	1.858013022	7.101449275
0.072916035	27.55971246	79.35944056
0.09305894	17.27258865	47.68030392
0.063323503	25.49346799	86.11831863
0.11447215	11.11906479	32.25592646
0.168510888	8.031775018	19.8597973
0.115378863	15.11406306	37.9774744
0.348394768	1.196195006	4.793103448
0.210474112	3.351715929	11.36619718
0.067746419	30.0094266	88.96189088

log.sigma.2.0.mm.3D_gldm_LargeDependenceLowGrayLevelEmphasis	log.sigma.2.0.mm.3D_gldm_SmallDependenceHighGrayLevelEmphasis	log.sigma.2.0.mm.3D_gldm_LargeDependenceHighGrayLevelEmphasis
0.104145356	44.68703993	3453.309309
0.236081861	36.65539614	1107.39823
0.169179907	52.42488355	7486.432712
0.184710808	32.017968	637.5543478
0.17388183	41.02754105	8602.380697
0.047454356	116.5423409	532.1747573
0.762094635	14.65442294	32888.57365
0.27828683	31.07492325	22722.52893
0.433992799	16.8994932	22701.84725
0.179956189	39.59288514	1033.269231
0.167518167	63.20138375	60641.78551
0.329657974	26.69217028	28979.79889
0.110056147	70.44059483	1124.247734
0.116074793	34.65682456	1080.384211
0.315726043	19.38666607	10705.21957
0.461034706	21.71566218	31863.78128
0.614622016	10.54909368	2898.323394
0.137924718	28.49329329	612.8648649
0.63325343	15.24547176	2826.569665
0.367013344	34.47307621	25800.80682
0.920017912	11.65569445	7013.705234
0.27485156	16.33741452	1006.683544
0.561339499	16.78428796	3455.430605
0.863355006	8.749163361	19120.42527
0.282196759	37.72118028	22526.50275
0.428833615	24.01387021	8195.814321
0.165665259	31.83033216	6259.161874
0.26095523	29.47047148	16984.30131
0.12886529	59.73015873	155.1071429
0.475140523	14.1560654	5801.100782
0.580090535	13.0698439	17073.35118
0.14640761	32.2469304	1152.160784
0.34646566	23.98635584	6115.487099
0.663334363	14.02796051	14913.64909
0.487302099	19.19620406	9890.644809
0.170963669	63.34281489	13444.08422
0.060549901	136.4020108	788.7358974
0.810072646	13.36525349	26915.18581
0.168349025	24.32094663	1775.80203
0.089558958	130.3117635	154625.8508
0.229152354	28.66852786	3955.849445
0.777501998	18.3498557	12301.17493
0.149395693	37.54076141	6469.688278
0.154400866	33.80321002	5803.831683
0.785516767	10.94741499	11198.97335
0.26205346	33.00483865	15935.56266
0.837174321	10.90100126	18864.70971
0.892960418	13.96993412	1384.6621
0.198275429	26.59894016	940.8421053
0.173315254	92.27643687	845.3954545
0.625939616	13.55544595	18239.20567
0.194825111	61.80862773	8464.32793
1.0057389	9.056022891	22595.83003
0.275528354	19.01299406	3163.064935
0.149354044	42.81091129	1471.209059
0.306267863	18.10740141	3698.490196
0.650625567	15.39312161	14117.02553
0.307682418	24.84657864	14716.70097
0.126990878	32.22095504	2432.518382
0.670524107	13.94803639	21306.34122
0.289148275	48.86248151	8750.670013
0.154341379	79.96590912	8533.762321
0.073585524	140.7023191	11974.9556
0.159699869	91.06302127	65784.99777
0.1857684	31.6522707	4096.249221
0.069006065	119.178667	4085.883295
0.137028347	54.81497432	568.9243697
0.353845273	41.06131153	12973.15068
1.186358219	9.319782773	13499.36536
0.255967894	45.3129074	5861.640472
0.384242597	18.50125093	2381.757576
0.475183888	19.53548034	34404.4664
0.318629686	18.58944194	6805.209613
0.535705766	15.57395236	7431.998136
0.506937673	27.31605463	14895.37811
0.433218505	25.88396738	3259.031457
0.076200389	88.33747343	2158.553981
0.407773835	34.97886232	302.0086957
0.103898963	70.47039227	2878.3149
0.837368106	7.252401203	8320.291353
0.104145356	44.68703993	3453.309309
0.335019388	33.58559755	1465.01355
0.544230212	14.02006023	21773.67496
0.69051798	8.081966209	5480.645106
0.716164458	15.55899762	9530.741828
0.081106883	87.82435581	928.6704545
0.175791265	43.72673768	6427.223448
0.952992972	8.889702752	20378.591
0.130766722	83.74929209	4875.231489
0.22948151	45.49197233	4324.68125
0.09251407	93.49223784	907.2028986
0.718755938	10.26522819	9317.013287
0.297793618	36.25875808	8105.484512
0.723299393	16.46740132	10811.40192
0.412059233	24.48176779	2906.758104
0.063561514	113.2226785	7051.460304
0.341492347	31.45641614	4998.475085
0.105811268	73.66148787	474.5057471
0.406822098	25.38492487	439.4366197
0.307498968	45.63755612	26287.59569

log.sigma.2.0.mm.3D_gldm_SmallDependenceLowGrayLevelEmphasis	log.sigma.2.0.mm.3D_gldm_LowGrayLevelEmphasis	log.sigma.2.0.mm.3D_gldzm_DistanceZoneVariabilityNormalized
0.006299211	0.011404884	0.981133787
0.018515375	0.03163107	1
0.005760591	0.011677865	0.95125
0.023692817	0.036258937	0.905
0.003492611	0.008404512	0.945987654
0.015895199	0.0205757	1
0.000696639	0.005678698	0.898225793
0.001423024	0.005249294	0.884267222
0.001174069	0.005904811	0.84738474
0.014991084	0.027717912	0.946349584
0.000426414	0.00193349	0.875363002
0.001020162	0.004502365	0.917649219
0.016367927	0.024565667	0.925577523
0.014573535	0.023281295	1
0.005633335	0.014050992	0.95791421
0.0007657	0.004773419	0.837492382
0.010931742	0.030682498	0.95125
0.021722277	0.03298248	0.955578512
0.011402708	0.029076609	0.901096408
0.000736911	0.004416396	0.726361218
0.005887926	0.023993428	0.958352196
0.015136616	0.032533917	1
0.011570163	0.040184336	0.947406866
0.001108231	0.008184703	0.908784832
0.003195349	0.007220296	0.962039542
0.00328543	0.011804585	0.587861756
0.003425346	0.009195587	0.962099466
0.001654181	0.005607268	0.912709149
0.094812942	0.100583607	1
0.0048571	0.016459013	0.929199219
0.001771269	0.009268306	0.968521554
0.009983279	0.019950947	0.936909263
0.00566009	0.014917226	0.944681552
0.002331403	0.010641334	0.863076557
0.003556236	0.011882201	0.951910851
0.004149939	0.009065481	0.975834432
0.009115216	0.014228896	1
0.001242041	0.007473307	0.795867893
0.00980251	0.019675209	0.96001666
0.000163135	0.000864337	0.854260208
0.005910143	0.014226617	0.976193932
0.001686948	0.009685586	0.736519868
0.002875278	0.008569476	0.969426334
0.004278053	0.009932399	0.91933518
0.003559977	0.013269846	0.925645359
0.001543518	0.006623526	0.903766462
0.001869385	0.009727846	0.971802535
0.021890041	0.066858209	1
0.018164744	0.033755502	1
0.012422426	0.023081554	0.799470699
0.003137441	0.00962456	0.991769688
0.002963253	0.007768139	0.900606427
0.000913057	0.007768978	0.940159287
0.006480513	0.017512314	1
0.010602064	0.022108288	0.945987654
0.006273711	0.017651069	0.938991878
0.002391381	0.009785132	0.83843024
0.002508834	0.008106723	0.91595218
0.008662898	0.016959166	0.936239585
0.000814287	0.006448439	0.533241458
0.005439805	0.012284143	0.95125
0.004075352	0.008566597	0.937492944
0.001327571	0.003463869	0.907417821
0.000455921	0.00191761	0.932309157
0.007018858	0.0136543	1
0.003004621	0.006660933	0.73255564
0.014350145	0.026195042	0.92737629
0.003459243	0.009794953	0.934736271
0.001588453	0.012065238	0.804734674
0.004824527	0.012352186	0.935569731
0.020266221	0.040298906	0.967750605
0.001036192	0.005127897	0.780942069
0.007158356	0.017668732	0.932286857
0.004853366	0.016297582	0.968594387
0.002227328	0.008336125	0.910644531
0.011026658	0.024977748	0.935555556
0.003562754	0.008232117	0.578360331
0.041921923	0.082658525	1
0.004455853	0.009694093	0.959034331
0.004611002	0.01729676	0.89007795
0.006299211	0.011404884	0.981133787
0.016563427	0.036842825	0.984128
0.00236001	0.008090837	0.961638873
0.006287139	0.023097795	0.973338203
0.005542253	0.016847419	0.941421558
0.012387265	0.018583799	0.95791421
0.003310551	0.009431598	0.965919342
0.001368949	0.009137926	0.953047319
0.005190146	0.010338574	0.53736954
0.006036696	0.01618936	0.951051277
0.008884064	0.015423203	0.9720336
0.003879024	0.015666511	0.96532872
0.003121726	0.009365983	0.924951509
0.003383117	0.013074901	0.89489429
0.007158952	0.020086848	0.939817977
0.002719346	0.005416714	0.941230487
0.008262749	0.019404594	0.973479593
0.02717559	0.03771754	1
0.021482783	0.044746847	1
0.000904122	0.004335717	0.774584344

log.sigma.2.0.mm.3D_gldzm_LowIntensityEmphasis	log.sigma.2.0.mm.3D_gldzm_LargeDistanceEmphasis	log.sigma.2.0.mm.3D_gldzm_HighIntensitySmallDistanceEmphasis
0.021301649	1.028571429	153.097619
0.04773251	1	97.60416667
0.019175286	1.075	173.9921875
0.058090873	1.15	77.8125
0.015149525	1.083333333	168.8194444
0.023986156	1	189.0540541
0.010364491	1.330630631	195.4188188
0.010187489	1.309677419	180.5190771
0.012048528	1.642857143	137.6399117
0.042337953	1.082758621	113.8913793
0.004307335	1.313320826	598.9771341
0.009832612	1.290909091	228.651452
0.037487941	1.116129032	168.5887097
0.041015433	1	105.9863014
0.033034795	1.064516129	94.88978495
0.008282803	1.492840095	199.1153623
0.064989313	1.075	58.515625
0.057038093	1.068181818	81.00568182
0.059610922	1.156521739	68.59565217
0.00623312	2.412084182	243.1990259
0.041466304	1.063829787	74.85319149
0.056247752	1	62.53061224
0.065112074	1.081081081	66.43581081
0.018521561	1.247457627	118.3702448
0.016211811	1.058064516	190.016129
0.022433155	2.557755776	92.03169692
0.016546821	1.057971014	141.9347826
0.011384207	1.218867925	176.9579665
0.122512625	1	79.95454545
0.031839668	1.161458333	84.27546296
0.017801316	1.124668435	111.0578581
0.030873368	1.097826087	101.8423913
0.02796986	1.085409253	111.1895018
0.02353505	1.526315789	112.3394498
0.022611796	1.146853147	109.7479604
0.015755954	1.036697248	229.0496942
0.016229602	1	242.9669421
0.016106997	1.685714286	126.6935167
0.041788088	1.06122449	98.26530612
0.001671547	1.473191489	1223.328203
0.024046995	1.036144578	112.6159639
0.015003175	1.886133033	128.7221784
0.015121167	1.046583851	178.0535714
0.017461697	1.126315789	130.9539474
0.031401636	1.374045802	84.97084394
0.011239503	1.373831776	180.0460202
0.025928573	1.151245552	127.3276122
0.103554944	1	59.26315789
0.049030286	1	76.02
0.02702818	1.339130435	152.95
0.026697575	1.012396694	104.9917355
0.012141334	1.182608696	231.6608213
0.01576409	1.252971138	134.1856485
0.034434863	1	97.15028902
0.032591039	1.083333333	126.1157407
0.035019554	1.094488189	90.70275591
0.021042537	1.566718995	115.7674302
0.015478592	1.131868132	139.8424908
0.029766439	1.098901099	107.4423077
0.010556547	5.280533333	123.5965863
0.020018885	1.075	165.7729167
0.014113469	1.126506024	261.5245984
0.004174188	1.145976294	422.1174361
0.002965029	1.135272727	562.1909293
0.028423198	1	131.1463415
0.008271208	1.662895928	263.2203997
0.034461623	1.113207547	122.1415094
0.017809736	1.123318386	183.804995
0.021985609	2.643347051	111.3505919
0.01888421	1.139810427	174.0196979
0.071335968	1.049180328	68.1147541
0.011343046	2.328509407	166.6868021
0.03608653	1.105263158	82.78947368
0.031847572	1.04787234	89.93218085
0.014825171	1.140625	164.2260742
0.048134942	1.1	105.7433333
0.010305522	2.143636364	190.693548
0.095498474	1	79.2
0.013333578	1.062761506	208.8922594
0.047339301	1.287356322	54.61781609
0.021301649	1.028571429	153.097619
0.055997926	1.024	106.722
0.021436506	1.107055961	114.5062855
0.060740515	1.040540541	55.83108108
0.036594013	1.090604027	94.19714765
0.02518398	1.064516129	184.9247312
0.015795015	1.052023121	185.0491329
0.019572398	1.072164948	109.1005155
0.016088108	2.321167883	157.5590024
0.024210771	1.075313808	157.7761506
0.018703917	1.042553191	205.2730496
0.037055767	1.052941176	75.49558824
0.014880047	1.185806452	162.9449194
0.027759921	1.25984252	118.726501
0.033408057	1.103305785	106.7851814
0.007558205	1.090909091	306.1212121
0.035651674	1.040322581	129.2473118
0.047155118	1	135.6727273
0.055184794	1	68.64516129
0.006158653	1.843397478	266.0678046

log.sigma.2.0.mm.3D_gldzm_LowIntensityLargeDistanceEmphasis	log.sigma.2.0.mm.3D_gldzm_HighIntensityEmphasis	log.sigma.2.0.mm.3D_gldzm_DistanceZoneVariability	log.sigma.2.0.mm.3D_gldzm_ZonePercentage
0.021541508	154.1904762	206.0380952	0.315315315
0.04773251	97.60416667	48	0.424778761
0.019511654	178.4125	152.2	0.33126294
0.059154495	83.25	36.2	0.434782609
0.015547148	173.4444444	170.2777778	0.241286863
0.023986156	189.0540541	74	0.718446602
0.012092269	203.9495495	997.0306306	0.068073102
0.011065433	198.8645161	685.3070968	0.153587
0.014917913	153.9193548	367.764977	0.102624734
0.043875948	116.3586207	137.2206897	0.398351648
0.010847681	623.9774859	466.5684803	0.097547584
0.010803715	239.5969697	302.8242424	0.107386918
0.038517549	172.3677419	143.4645161	0.468277946
0.041015433	105.9863014	73	0.384210526
0.033365786	98.34946237	178.172043	0.202173913
0.01030817	216.0381862	701.8186158	0.098276064
0.065674231	60.5875	76.1	0.183486239
0.057341123	84.84090909	42.04545455	0.396396396
0.06130777	74.13913043	103.626087	0.202821869
0.011481104	281.6951799	1069.930075	0.1235013
0.043898935	75.70212766	225.212766	0.16184573
0.056247752	62.53061224	49	0.310126582
0.066572878	67.58108108	70.10810811	0.263345196
0.020423522	125.1118644	268.0915254	0.061407161
0.016443592	193.7612903	149.116129	0.213498623
0.031732859	161.4917492	178.1221122	0.14962963
0.016839982	145.0434783	199.1545894	0.220447284
0.012085184	188.5433962	241.8679245	0.150653781
0.122512625	79.95454545	22	0.785714286
0.032981213	88.72395833	178.40625	0.166811468
0.018538316	113.4509284	365.132626	0.107376816
0.031425641	106.1956522	86.19565217	0.360784314
0.028689809	114.2241993	265.455516	0.219702893
0.026862392	122.1911357	311.5706371	0.114639568
0.023494645	113.1765734	544.493007	0.164509635
0.015888879	231.6391437	319.0978593	0.289893617
0.016229602	242.9669421	242	0.620512821
0.020285463	148.4098522	807.8059113	0.08647129
0.042100458	101.2653061	47.04081633	0.248730964
0.002011773	1314.65617	1003.755745	0.096995212
0.024219522	114.5180723	162.0481928	0.263074485
0.021496142	149.048478	653.2931229	0.122564599
0.015296376	181.2981366	312.1552795	0.210870989
0.018055167	138.0315789	174.6736842	0.26874116
0.037449095	88.41984733	121.259542	0.120404412
0.012654972	190.5358255	290.1090343	0.164194373
0.027047838	129.1672598	546.1530249	0.080642847
0.103554944	59.26315789	57	0.260273973
0.049030286	76.02	50	0.438596491
0.02901443	213.6869565	91.93913043	0.522727273
0.026760824	105.5991736	240.0082645	0.126967471
0.013018463	244.3078261	517.8486957	0.266327003
0.017576896	138.286927	553.75382	0.058305286
0.034434863	97.15028902	173	0.204250295
0.033199155	129.6018519	102.1666667	0.37630662
0.035885165	93.82677165	119.2519685	0.191553544
0.024882314	127.0361068	534.0800628	0.120484207
0.016140325	146.6886447	250.0549451	0.176699029
0.030177488	113.5494505	85.1978022	0.334558824
0.034937413	169.7024	999.8277333	0.079743121
0.020281896	172.0541667	228.3	0.301129235
0.014711654	267.1807229	466.8714859	0.310667498
0.004652669	439.1709295	1454.590767	0.350612423
0.00322436	578.4370909	1281.925091	0.161384977
0.028423198	131.1463415	82	0.255451713
0.010327898	324.4468326	647.5791855	0.404576659
0.034869993	130.4339623	49.1509434	0.445378151
0.018389687	192.2085202	833.7847534	0.216085271
0.038151184	121.6927298	586.6515775	0.072732715
0.020263501	177.4549763	394.8104265	0.276358874
0.071677498	69.8852459	59.03278689	0.308080808
0.016564961	193.617945	539.6309696	0.097337653
0.036882417	86.68421053	159.4210526	0.228304406
0.032261143	91.40425532	182.0957447	0.175209692
0.01539209	175.375	466.25	0.15920398
0.049104619	111.4333333	140.3333333	0.248344371
0.018882276	272.1745455	318.0981818	0.371120108
0.095498474	79.2	60	0.52173913
0.013934086	212.0962343	229.209205	0.367127496
0.049699047	60.59195402	154.8735632	0.109022556
0.021541508	154.1904762	206.0380952	0.315315315
0.079997926	106.728	123.016	0.338753388
0.022075291	117.3065693	395.2335766	0.120245758
0.061198003	56.88513514	144.0540541	0.125957447
0.037352376	97.09395973	280.5436242	0.165096953
0.025852594	187.0215054	89.08602151	0.528409091
0.016078052	187.6589595	167.1040462	0.23862069
0.020162839	111.6082474	277.3367698	0.07156911
0.024084504	270.2360097	220.8588808	0.349787234
0.024696064	161.0460251	227.3012552	0.29875
0.019313972	206.177305	137.0567376	0.510869565
0.037405285	77.51176471	164.1058824	0.118881119
0.015962596	169.1406452	716.8374194	0.226475745
0.030436061	123.980315	454.6062992	0.131811105
0.034568275	109.6632231	454.8719008	0.234155781
0.007851428	313.9718615	434.8484848	0.390202703
0.036104425	130.4892473	362.1344086	0.253924915
0.047155118	135.6727273	55	0.632183908
0.055184794	68.64516129	31	0.436619718
0.008994302	300.9641672	1167.298607	0.156913786

log.sigma.2.0.mm.3D_gldzm_IntensityVariabilityNormalized	log.sigma.2.0.mm.3D_gldzm_LowIntensitySmallDistanceEmphasis	log.sigma.2.0.mm.3D_gldzm_IntensityVariability
0.064535147	0.021241684	13.55238095
0.081597222	0.04773251	3.916666667
0.05578125	0.019091194	8.925
0.08125	0.057824968	3.25
0.073950617	0.015050119	13.31111111
0.062819576	0.023986156	4.648648649
0.064132781	0.010124597	71.18738739
0.070458273	0.010043168	54.60516129
0.091528807	0.011754103	39.7235023
0.061212842	0.041953454	8.875862069
0.08335768	0.002736138	44.42964353
0.069972452	0.009714175	23.09090909
0.05157128	0.03723054	7.993548387
0.079001689	0.041015433	5.767123288
0.087235519	0.032952048	16.22580645
0.070083902	0.007979232	58.73031026
0.0975	0.064818084	7.8
0.080578512	0.056962335	3.545454545
0.077353497	0.05918671	8.895652174
0.0649671	0.005755663	95.69653768
0.079185152	0.040858146	18.60851064
0.099541858	0.056247752	4.87755102
0.081811541	0.064746873	6.054054054
0.083251939	0.018267263	24.55932203
0.065223725	0.016153866	10.10967742
0.067389907	0.021185183	20.41914191
0.08394595	0.016473531	17.37681159
0.078390886	0.011271969	20.77358491
0.082644628	0.122512625	1.818181818
0.079752604	0.031646395	15.3125
0.084127799	0.017722496	31.71618037
0.081758034	0.0307353	7.52173913
0.067134408	0.027789873	18.86476868
0.076603157	0.023139736	27.65373961
0.072699154	0.022481778	41.58391608
0.055204856	0.015722723	18.05198777
0.045283792	0.016229602	10.95867769
0.069822612	0.01555958	70.86995074
0.08121616	0.041709995	3.979591837
0.063550204	0.001627211	74.67148936
0.071418203	0.024003863	11.85542169
0.082630353	0.01414921	73.29312289
0.063577794	0.015077365	20.47204969
0.078116343	0.01731333	14.84210526
0.092710215	0.03084442	12.14503817
0.069302511	0.011063872	22.24610592
0.068103241	0.025846364	38.27402135
0.088334872	0.103554944	5.035087719
0.0976	0.049030286	4.88
0.049376181	0.026531617	5.67826087
0.086981764	0.026681763	21.04958678
0.052334216	0.011941389	30.09217391
0.077905344	0.015593916	45.88624788
0.072872465	0.034434863	12.60693642
0.062757202	0.03243901	6.777777778
0.082274165	0.034803151	10.4488189
0.078189808	0.020578405	49.80690738
0.077620792	0.015313159	21.19047619
0.076923077	0.029663676	7
0.121001244	0.009145665	226.8773333
0.054340278	0.019953133	13.04166667
0.045668618	0.013987612	22.74297189
0.045011128	0.004054568	72.15283843
0.052993587	0.002911111	72.86618182
0.06989887	0.028423198	5.731707317
0.04376702	0.007881931	38.69004525
0.080811677	0.034359531	4.283018868
0.052786101	0.017686419	47.08520179
0.07499045	0.021102341	54.66803841
0.057433571	0.018580711	24.23696682
0.078204784	0.071250586	4.770491803
0.067824688	0.010945635	46.86685962
0.085940973	0.035887558	14.69590643
0.080296514	0.031744179	15.09574468
0.061737061	0.014683442	31.609375
0.064	0.047892523	9.6
0.047133884	0.008810202	25.92363636
0.068888889	0.095498474	4.133333333
0.059680328	0.013183451	14.26359833
0.100938037	0.046961236	17.56321839
0.064535147	0.021241684	13.55238095
0.06112	0.049997926	7.64
0.079025106	0.021343574	32.47931873
0.103542732	0.060626142	15.32432432
0.075154272	0.036404422	22.39597315
0.060469418	0.025016826	5.623655914
0.066590932	0.015724255	11.52023121
0.081541314	0.019424788	23.72852234
0.042155801	0.014458732	17.32603406
0.058454859	0.024089448	13.9707113
0.060610633	0.018551403	8.546099291
0.100622837	0.036968387	17.10588235
0.063472216	0.014699724	49.19096774
0.064177878	0.027279546	32.6023622
0.068719691	0.033129716	33.26033058
0.051695058	0.007484899	23.88311688
0.060353798	0.035538487	22.4516129
0.058512397	0.047155118	3.218181818
0.117585848	0.055184794	3.64516129
0.056251979	0.005816038	84.77173192

log.sigma.2.0.mm.3D_gldzm_HighIntensityLargeDistanceEmphasis	log.sigma.2.0.mm.3D_gldzm_SmallDistanceEmphasis	log.sigma.2.0.mm.3D_glcm_SumVariance	log.sigma.2.0.mm.3D_glcm_Homogeneity1
158.5619048	0.992857143	494.2307804	0.40204228
97.60416667	1	198.4870744	0.380286934
196.09375	0.98125	485.1217632	0.403426417
105	0.9625	181.8994304	0.355185865
191.9444444	0.979166667	543.7994877	0.450372075
189.0540541	1	459.7246075	0.263549621
273.9315315	0.957986236	650.6148741	0.67552756
319.5432258	0.951344086	810.9935045	0.551339961
303.3041475	0.932115975	662.9366644	0.605701095
126.2275862	0.979310345	245.5047351	0.348134046
771.5234522	0.947792891	2105.10211	0.630272985
329.3424242	0.964520202	904.545721	0.608609037
187.483871	0.970967742	335.9356348	0.311338791
105.9863014	1	310.3234871	0.362808889
112.188172	0.983870968	455.1102404	0.506864186
342.7708831	0.930619862	805.9748388	0.630178222
68.875	0.98125	143.0580815	0.499003023
100.1818182	0.982954545	216.7992895	0.355060549
96.31304348	0.960869565	153.44155	0.487659633
694.2138493	0.873447978	829.2300781	0.570572494
79.09787234	0.984042553	179.420093	0.528547618
62.53061224	1	166.7611845	0.431195067
72.16216216	0.97972973	187.8055819	0.472284117
163.6881356	0.962753296	446.9877375	0.666339364
208.7419355	0.985483871	746.9372308	0.531762606
724.679868	0.792881371	366.8195284	0.506758616
157.4782609	0.985507246	492.5812202	0.447821855
258.4679245	0.964281971	723.9431384	0.546371127
79.95454545	1	156.9657826	0.25942904
112.7447917	0.971209491	258.1171126	0.50487765
135.7320955	0.98670056	435.9373368	0.578142695
123.6086957	0.975543478	269.2482093	0.374103728
126.3629893	0.978647687	315.6828333	0.491028555
210.4570637	0.939611419	421.3016167	0.604719015
139.3006993	0.979858683	349.1355936	0.542568379
241.9969419	0.990825688	738.4119901	0.45234306
242.9669421	1	532.3613567	0.256761265
299.5852217	0.910522989	535.8872512	0.657444943
113.2653061	0.984693878	287.9319639	0.434731904
2000.038298	0.93811004	4756.063511	0.627893316
122.126506	0.990963855	319.3382375	0.445640171
290.9763247	0.880492296	374.640973	0.609769337
194.2763975	0.988354037	552.1617473	0.443218135
166.3421053	0.968421053	481.5031184	0.438598063
127.519084	0.966391009	307.4662329	0.605667803
295.5264798	0.958912253	666.9283314	0.525656856
150.5266904	0.988014037	431.3031161	0.640789088
59.26315789	1	100.9131895	0.456352559
76.02	1	187.2377231	0.394325238
456.6347826	0.915217391	510.4819911	0.269156653
108.0289256	0.996900826	484.8027739	0.629255381
301.8678261	0.960144928	581.7944591	0.443649845
175.8438031	0.974612809	461.8962618	0.683560366
97.15028902	1	270.8804214	0.464054852
143.5462963	0.979166667	286.1417363	0.345749695
106.3228346	0.976377953	254.2838489	0.460002781
216.4913658	0.930414268	415.8436276	0.602371346
174.0732601	0.967032967	543.1060374	0.511409294
137.978022	0.975274725	383.7212356	0.407470277
964.7968	0.753684855	556.2934622	0.643436497
197.1791667	0.98125	414.1244779	0.446556737
297.5963855	0.975066934	633.4773276	0.406352682
507.3849033	0.963505926	1189.592865	0.385321165
661.1316364	0.973075758	2059.337687	0.572725858
131.1463415	1	371.7677416	0.450810621
679.2884615	0.880271179	828.2462453	0.312400742
163.6037736	0.971698113	256.5253147	0.318468389
230.8699552	0.974152965	508.4248148	0.512010598
303.7544582	0.909206241	293.2914013	0.643065226
199.563981	0.97434505	371.0351201	0.429326312
76.96721311	0.987704918	165.6448154	0.424975471
545.7771346	0.899761166	798.4045176	0.627508313
102.2631579	0.973684211	317.8276465	0.465318632
97.29255319	0.988031915	279.7808689	0.536887127
219.9707031	0.96484375	485.6716372	0.569371586
134.1933333	0.975	209.6564722	0.450694949
696.6927273	0.787891414	649.3573005	0.309509377
79.2	1	116.9539796	0.333272211
224.9121339	0.984309623	479.3812091	0.369538224
97.59195402	0.953703704	248.8763257	0.594263027
158.5619048	0.992857143	494.2307804	0.40204228
106.752	0.994	171.1711426	0.379228921
135.5474453	0.984049743	569.2788549	0.618521909
61.10135135	0.989864865	210.6941667	0.555435838
108.6812081	0.977348993	271.2628502	0.548578558
195.4086022	0.983870968	359.6995938	0.297903433
198.0982659	0.98699422	517.4282278	0.465787239
121.6391753	0.981958763	414.9072091	0.657710057
881.8199513	0.757654096	662.9920804	0.348594744
174.125523	0.981171548	330.4008436	0.41544329
209.7943262	0.989361702	417.5527544	0.297990094
85.57647059	0.986764706	282.59515	0.579131178
205.6283871	0.969408602	436.9761318	0.488631136
155.9350394	0.957058727	318.903583	0.571463496
122.9752066	0.976469238	216.8829722	0.470554929
345.3744589	0.977272727	902.8963605	0.354327988
135.4569892	0.989919355	280.5938034	0.442989133
135.6727273	1	281.8438501	0.272058687
68.64516129	1	100.7297801	0.405940562
583.1074983	0.899878248	888.506762	0.545693347

log.sigma.2.0.mm.3D_glcm_Homogeneity2	log.sigma.2.0.mm.3D_glcm_ClusterShade	log.sigma.2.0.mm.3D_glcm_MaximumProbability	log.sigma.2.0.mm.3D_glcm_Idmn	log.sigma.2.0.mm.3D_glcm_SumVariance2
0.321402968	-65.63686382	0.031413962	0.977947272	25.55044248
0.295456292	38.5101752	0.048449493	0.968313058	21.0463729
0.325297353	-77.35508576	0.074582723	0.972768362	33.29347776
0.264077884	2.062609953	0.043446588	0.952024653	17.31520366
0.378614195	-28.37874439	0.083451272	0.984772565	19.92242068
0.165075879	-61.17005507	0.03345809	0.94889192	54.80888883
0.655802838	-35.3874374	0.210846363	0.996647197	7.959308817
0.503593701	-100.1722629	0.16047569	0.992768373	18.91366884
0.57007566	-61.23104719	0.18749744	0.993462221	11.54953277
0.260571087	47.05357573	0.023871966	0.96767303	32.93563513
0.601463339	-27.62559584	0.169424065	0.996750861	7.211794546
0.57492842	-69.49693382	0.164614951	0.996172722	12.29718499
0.221397694	99.41017306	0.020013147	0.956445389	41.91884845
0.272183893	-42.43236848	0.033577666	0.967427832	24.10106017
0.448848002	-104.2063701	0.131717336	0.986355824	20.9598617
0.601409284	-93.91597642	0.216008547	0.993904915	13.5529085
0.436191739	-11.1942066	0.055927379	0.983234012	12.45217551
0.264931939	18.8265746	0.040294075	0.960581856	23.21872226
0.423747724	-4.575388425	0.095408317	0.982964789	13.48720188
0.528564074	-29.21928282	0.149543939	0.994797219	12.95668606
0.471060097	-27.37392405	0.148961796	0.982589017	14.75229904
0.351942038	3.949308946	0.047893485	0.973001238	13.07557625
0.405722355	-53.96816578	0.081219059	0.970935082	20.61797667
0.645803028	-23.59586322	0.236026756	0.996607743	6.157495401
0.480957612	-155.2553363	0.206061731	0.985867748	28.57490291
0.450539484	168.4696781	0.097788831	0.989091502	25.49856037
0.373301255	-54.33360775	0.041165615	0.985309323	21.62579133
0.496445414	-54.77942752	0.115346548	0.990301895	13.3633625
0.157129378	73.08854612	0.08334322	0.902948273	27.69841341
0.444708111	-39.24014045	0.104722279	0.980875887	15.02486052
0.533870859	-72.01128405	0.145935181	0.991866079	16.2289925
0.284875396	-4.347058843	0.034929081	0.963521998	20.54064487
0.42922563	-63.69623327	0.088713618	0.984644478	19.74631287
0.569668841	-73.12640217	0.159857787	0.992346326	12.54187153
0.491838302	-51.76686085	0.124895238	0.990980479	14.89152712
0.384241525	-246.4052331	0.106542599	0.982940031	39.7799784
0.166374385	218.1251741	0.019912456	0.957772996	56.15687605
0.631903057	-62.50462434	0.219844258	0.996715818	12.43054444
0.35734932	-31.27294453	0.047006878	0.974058309	15.08235281
0.599630461	-2.596176383	0.208511166	0.998406358	9.14510165
0.372014817	-23.68743341	0.052517093	0.980855367	18.91182907
0.577390751	1.031318985	0.187983078	0.99449889	7.077100185
0.369132057	-69.80546802	0.036166498	0.983714353	25.43178443
0.363578221	-47.31505459	0.056718861	0.982700959	21.65630495
0.568608994	-44.38611643	0.213442622	0.987868981	10.35302881
0.472212361	-60.15763927	0.12373721	0.991240296	17.6552808
0.612835822	-43.83045319	0.195906225	0.995524513	10.02680415
0.384107634	17.7375078	0.058808222	0.978217252	19.18204434
0.309802395	-21.51383396	0.053197643	0.953129269	15.48821525
0.184274086	2681.337393	0.027458021	0.948305539	148.1484135
0.597584809	-73.01261554	0.24044506	0.992349259	11.58091411
0.377393768	49.75494513	0.0636172	0.985026075	27.96460635
0.665676816	-21.53526691	0.257449355	0.996869148	5.078015284
0.394275239	-43.65764448	0.062264145	0.979856819	22.54871442
0.254299305	67.65376348	0.022495352	0.969097297	34.33653407
0.389460269	-23.29699205	0.060176883	0.978124644	16.87100613
0.567228981	-48.19877468	0.173642348	0.993762738	10.78167086
0.454480492	-79.55200885	0.112524365	0.98892156	21.74197645
0.32429343	-70.83028854	0.042095717	0.980235035	25.01236369
0.618690541	-20.64071092	0.190475547	0.997343338	5.770036708
0.378400229	-6.433139297	0.110462023	0.978887038	27.10854576
0.334056415	-12.9937927	0.059652034	0.979049812	36.67318008
0.312400568	42.45602356	0.053251959	0.982540392	47.56568994
0.532059776	-100.1035193	0.140267062	0.994625052	19.53733516
0.379257932	-52.84572338	0.059648106	0.976667867	20.75315165
0.228261209	290.482214	0.021635946	0.969790706	63.8383689
0.224589673	174.0654157	0.036135252	0.954563846	33.64468432
0.459262503	-11.43670286	0.118422381	0.989903459	24.37835287
0.616889575	-15.64618196	0.169910727	0.995341544	6.844882446
0.357078644	25.86472917	0.061738312	0.980644532	25.60766822
0.345896683	-23.62386123	0.071485477	0.951775624	20.32687174
0.596269992	-109.209736	0.18195867	0.99579684	15.54464179
0.395865468	-55.07116623	0.095541222	0.980856966	23.50297819
0.484343931	-51.00400164	0.119926492	0.989994536	14.82407884
0.526783747	27.73860685	0.176764911	0.993043435	17.4515713
0.380501956	82.36148456	0.071225711	0.981697182	23.91127254
0.221552107	561.3027879	0.015590911	0.96688258	75.81052102
0.242402046	63.4041135	0.03662546	0.941109598	29.83110606
0.286476472	11.50968246	0.028851673	0.970348083	27.78880771
0.556354184	-36.29325337	0.167862907	0.986879449	8.788104295
0.321402968	-65.63686382	0.031413962	0.977947272	25.55044248
0.296169001	41.26701315	0.031553348	0.962208682	23.91030949
0.585405234	-102.1441201	0.221081483	0.990289139	14.59259149
0.507428955	-34.79960458	0.110812506	0.98891599	10.86380449
0.498284126	-51.99935534	0.164757341	0.983093946	15.05539754
0.204911253	95.30284589	0.026150949	0.949441507	38.15509688
0.39700771	-78.62733652	0.056388349	0.987996171	23.61303211
0.63170763	-47.44041065	0.263784065	0.995034051	9.362240566
0.272719517	565.4032139	0.04018771	0.963158344	82.21789845
0.339372607	-2.264172958	0.042363569	0.971289486	24.13115925
0.207181669	58.37156047	0.023224682	0.947065064	44.18355473
0.534241357	-36.68461	0.179989097	0.987703156	10.26654659
0.428365392	-27.73652693	0.079381899	0.985098776	16.94600101
0.529746324	-36.24766557	0.152373952	0.989716	13.59131555
0.404441425	8.506915082	0.069022702	0.98495999	15.77658867
0.270680997	8.371514997	0.02925849	0.981850504	37.10663465
0.371902569	39.36978575	0.076530608	0.987056568	25.1421003
0.174996294	128.5907944	0.036187054	0.941058952	41.15809135
0.324488009	0.867032069	0.069918841	0.949818082	10.88126966
0.499035144	-66.27393057	0.158290041	0.989791335	20.49194416

log.sigma.2.0.mm.3D_glcm_Contrast	log.sigma.2.0.mm.3D_glcm_DifferenceEntropy	log.sigma.2.0.mm.3D_glcm_InverseVariance	log.sigma.2.0.mm.3D_glcm_Entropy	log.sigma.2.0.mm.3D_glcm_Dissimilarity
13.90706919	3.011136422	0.318424157	6.879209481	2.768934993
17.84372844	3.008262242	0.287586175	6.090317506	3.104442645
19.30895338	3.194880909	0.284697015	6.878391715	3.132748401
16.03355341	2.978046821	0.271591072	5.87003282	3.168889323
10.26201641	2.811260304	0.349285312	6.330961562	2.309510639
33.82989018	3.242728153	0.191981452	6.176258419	4.751995798
3.677212843	1.851778864	0.442717021	3.950717967	1.001320828
6.392670954	2.455005349	0.408678273	5.578495625	1.64548982
3.637121475	2.107736365	0.44440847	4.707308968	1.229668932
25.47952929	3.329952541	0.268767218	7.163839613	3.668880177
4.37469274	2.005013959	0.458289954	4.584291943	1.150764315
4.483875661	2.115367978	0.451135173	4.78661156	1.255132243
31.53806137	3.527744101	0.233686227	7.421210508	4.30198092
14.30814566	2.986468786	0.280676402	6.459208969	3.014886035
7.751723864	2.612093124	0.385581869	5.746314732	1.921131034
4.405258393	2.075526995	0.441280812	4.437813211	1.217539727
5.900886313	2.449163488	0.400013127	5.789013359	1.760301359
16.35576824	2.985928556	0.291467192	6.095155518	3.138730805
7.524037517	2.572571709	0.389958183	5.877010674	1.929332014
6.688532137	2.407629633	0.420481413	5.418785936	1.584088914
6.087135848	2.515345298	0.370987851	5.789030278	1.730753796
7.520787661	2.567080906	0.360608639	5.827654173	2.15680037
8.447079152	2.670512922	0.37173185	5.957785617	2.096373717
2.210964874	1.756239537	0.473050043	3.986329806	0.904666178
12.27703342	2.740391257	0.349306112	5.546693064	2.166262532
12.68985462	2.718028773	0.388577439	6.057078235	2.126207592
9.062421823	2.723711585	0.348776764	6.46026707	2.222923694
4.955614424	2.347102226	0.423570627	5.54765933	1.532714871
32.00518981	2.389726178	0.183949623	4.230740871	4.661456588
5.98589006	2.490090201	0.400354996	5.839298365	1.770709614
4.92383241	2.315460369	0.403456837	5.245707121	1.454510078
15.16388071	2.994626507	0.284854566	6.517691528	2.97147026
9.751573907	2.735358486	0.370304721	6.160257027	2.100334606
4.371241751	2.15457944	0.442772836	4.771520002	1.2854203
5.987431165	2.443310111	0.409349642	5.616429704	1.634550564
15.95535293	3.032050983	0.32880681	6.773431316	2.662271977
59.10693676	3.878142631	0.165363809	7.485822907	5.973382417
4.834658308	2.018326867	0.419248327	4.333916261	1.142493657
8.272063598	2.630388411	0.36402242	5.935775982	2.1907195
5.354388466	2.089951806	0.451153347	4.494508326	1.24910497
9.150258825	2.744859547	0.356801962	6.358703081	2.240348111
5.00592377	2.14089627	0.453932724	4.672794944	1.290676963
10.13525146	2.755884981	0.349323378	6.636638381	2.314902915
9.798827945	2.795224137	0.345237613	6.404400562	2.332085259
3.358111681	2.092933813	0.438480619	4.806269008	1.206693149
6.741555848	2.507501703	0.40733249	5.717397746	1.753737382
3.184665952	1.973602685	0.446559677	4.473725289	1.079654856
7.674061178	2.609610409	0.367831236	5.972501843	2.084319858
12.39260171	2.813113422	0.316067238	5.777527784	2.696833308
102.623565	4.03615186	0.178872834	7.282543742	6.94063567
3.561124894	2.067125585	0.427388593	4.48958071	1.171757145
20.56680242	3.119457066	0.343796507	6.876345071	2.885839473
2.232296262	1.710968797	0.460533865	3.741920894	0.859909159
7.056226907	2.510089859	0.385409947	6.274085976	1.992924245
21.96693373	3.261720759	0.249738716	6.99046146	3.537506515
7.726263977	2.647239942	0.373770466	6.155805466	2.074041509
4.13301085	2.137874151	0.450716884	4.852862343	1.26877167
7.934947049	2.62916995	0.381610278	5.849627856	1.927605509
10.2420592	2.78648415	0.331376655	6.471327154	2.476215561
3.083221473	1.869419332	0.478206434	4.100937591	1.01944661
18.82105148	3.109804887	0.319521538	6.649093154	2.851874846
27.45637881	3.362556494	0.3014895	7.276804242	3.467294379
35.5136755	3.53312643	0.287950657	7.806113989	3.965788608
10.58047699	2.552345367	0.395037962	5.55406687	1.824739461
8.271026561	2.652332111	0.375213551	6.187916311	2.147910098
46.95357172	3.813821084	0.231107504	8.336821293	4.947277623
25.73399349	3.301364222	0.233561649	6.345269041	3.925948789
15.46118751	2.82180409	0.379810193	6.20743958	2.290064357
3.119334932	1.92334195	0.461934666	4.35952679	1.047395819
16.9224584	3.101848906	0.323781124	6.847557024	2.820941602
11.22025162	2.84881024	0.314652787	6.127755982	2.51567471
4.547179905	2.124628005	0.427189011	4.721880515	1.235097066
9.208384139	2.745397725	0.349614119	6.20869545	2.191804078
6.231818701	2.447357273	0.404527422	5.488839554	1.665096121
11.13774618	2.550217673	0.392295302	5.518437455	1.828466223
14.46854387	2.902432644	0.342280636	6.504434409	2.509740444
41.49762267	3.717120144	0.23056559	8.226616919	4.698147243
20.35939094	3.130682659	0.265258218	6.215120298	3.556280765
20.84289848	3.272206386	0.287159255	7.20406146	3.369957669
3.161808983	2.077101379	0.457148443	4.77890865	1.214735298
13.90706919	3.011136422	0.318424157	6.879209481	2.768934993
17.37134751	3.143499737	0.29450872	6.739120383	3.127526067
4.632208688	2.17250199	0.419894609	4.522619983	1.297606116
3.817641225	2.196964257	0.444277154	5.334837544	1.385690372
6.76733039	2.506642576	0.385841645	5.609870088	1.702312191
34.90193316	3.504719822	0.215286147	6.879676647	4.522219146
9.524030086	2.710466925	0.365985232	6.375990076	2.156657491
2.760106779	1.910420514	0.433381706	4.182044322	1.006193687
51.24413301	3.767938671	0.256071991	8.040492574	4.835335348
15.99805937	3.083052726	0.320094577	6.838465479	2.833204119
36.06345163	3.618734853	0.214512787	7.334202587	4.638830324
3.801896351	2.193157584	0.420456896	5.092715532	1.332734791
10.23623262	2.754602351	0.383861714	6.265451893	2.131739649
6.633080313	2.368608251	0.429257839	5.377529928	1.550364145
9.444631613	2.739981814	0.376713341	6.345743658	2.144631849
28.74391524	3.45706897	0.268542972	7.489076967	3.819607408
15.50337202	3.001727025	0.331499347	6.682126621	2.638838747
41.39796445	3.442892684	0.184111403	6.208439521	4.996831697
10.05097041	2.594621529	0.351061376	5.30060329	2.455671362
10.00034148	2.632192584	0.392234285	5.884459235	1.9139919

log.sigma.2.0.mm.3D_glcm_DifferenceVariance	log.sigma.2.0.mm.3D_glcm_Idn	log.sigma.2.0.mm.3D_glcm_Idm	log.sigma.2.0.mm.3D_glcm_Correlation	log.sigma.2.0.mm.3D_glcm_Autocorrelation
6.162410631	0.903603501	0.321402968	0.29031845	171.011905
7.921711258	0.886179987	0.295456292	0.0800475	74.94651216
9.256657509	0.898014908	0.325297353	0.259622973	167.6116781
5.843931987	0.854365946	0.264077884	0.03926691	69.33235211
4.884240752	0.920859882	0.378614195	0.316920779	183.8939023
10.34962877	0.845355576	0.165075879	0.234857226	154.5500937
2.664706834	0.971670788	0.655802838	0.36152971	199.2890741
3.64159394	0.94963443	0.503593701	0.489057998	257.8173817
2.094417597	0.952322861	0.57007566	0.512876645	209.2826142
11.8065636	0.886624608	0.260571087	0.128018189	92.29822162
3.038883635	0.97002481	0.601463339	0.242142908	599.1535084
2.8881562	0.965394343	0.57492842	0.458463222	277.5490906
12.82617839	0.86492031	0.221397694	0.135492098	120.7168784
5.098393609	0.877068931	0.272183893	0.250276544	113.327092
3.984070548	0.928325465	0.448848002	0.455295314	155.8311681
2.880172109	0.958516381	0.601409284	0.497784112	246.5476081
2.76196095	0.916762309	0.436191739	0.352804463	59.28593552
6.330585571	0.868464569	0.264931939	0.177385954	81.8266077
3.751691655	0.91830668	0.423747724	0.282420487	62.56989146
4.159811438	0.959344932	0.528564074	0.315900926	259.5379371
3.06041354	0.918868019	0.471060097	0.412967137	71.43235844
2.809364008	0.888383273	0.351942038	0.260469333	66.26836486
3.983375725	0.89385776	0.405722355	0.415468051	74.50502811
1.385248069	0.966857814	0.645803028	0.465396824	143.1474922
7.363926738	0.934823863	0.480957612	0.386326018	238.4201787
8.095537862	0.943032489	0.450539484	0.337307099	129.6769212
3.970122836	0.920198864	0.373301255	0.402955388	170.2279165
2.57110739	0.938846428	0.496445414	0.45542898	232.2918952
9.524072328	0.791824276	0.157129378	-0.074353385	50.18029585
2.806569155	0.91233015	0.444708111	0.425333549	96.33366078
2.754926286	0.946551145	0.533870859	0.526358019	148.0550024
6.089951924	0.874834568	0.284875396	0.154534886	99.84760509
5.282660836	0.925776169	0.42922563	0.334349267	114.565564
2.695770768	0.950828169	0.569668841	0.477114122	140.0880722
3.291153763	0.942537296	0.491838302	0.422617019	123.2683578
8.795592291	0.9228965	0.384241525	0.416132123	243.3512728
21.86407979	0.865169513	0.166374385	-0.002863511	175.112023
3.509777659	0.972028049	0.631903057	0.434356216	170.4931361
3.40651923	0.893411775	0.35734932	0.286882059	105.3016917
3.779281615	0.979526617	0.599630461	0.25877068	1296.341439
4.075188922	0.909864666	0.372014817	0.343710588	116.7391479
3.325642636	0.960330066	0.577390751	0.169074984	124.2671116
4.438229066	0.917671641	0.369132057	0.411793373	188.6573961
4.266734012	0.91360091	0.363578221	0.373501457	166.2044986
1.880404139	0.935137615	0.568608994	0.506796748	107.6460839
3.617789974	0.942746496	0.472212361	0.43921592	216.9774934
2.008411538	0.962460651	0.612835822	0.512614612	142.2885258
3.233933168	0.902989782	0.384107634	0.427831514	45.36875982
5.052236035	0.860101092	0.309802395	0.093807398	71.36319519
53.32214594	0.86689671	0.184274086	0.196680125	155.8463365
2.158532523	0.950904739	0.597584809	0.523898049	157.4123281
12.13569806	0.930462953	0.377393768	0.154961619	195.5037856
1.488327608	0.969727926	0.665676816	0.384493576	145.2300725
2.738457469	0.906676082	0.394275239	0.517217278	103.3532081
9.352601264	0.885018658	0.254299305	0.218145674	106.0525657
3.391111723	0.903624533	0.389460269	0.369551127	96.06601971
2.500078331	0.954750376	0.567228981	0.435781755	138.9703761
4.165163608	0.935538409	0.454480492	0.461362278	182.0027642
4.021413431	0.904549019	0.32429343	0.415817535	137.3920554
2.037094063	0.97149648	0.618690541	0.299762392	172.9567533
10.60426854	0.916240144	0.378400229	0.176889926	144.8435324
15.31330221	0.915975723	0.334056415	0.141836174	211.4625595
19.5404262	0.922747075	0.312400568	0.14714311	373.8302802
7.229926023	0.962277493	0.532059776	0.292906655	599.0089415
3.570960939	0.900717493	0.379257932	0.425312728	133.216819
22.22895976	0.892040099	0.228261209	0.157508631	269.9113162
10.00087582	0.860271312	0.224589673	0.141843275	92.95145471
10.13435717	0.946860098	0.459262503	0.224081463	172.0306523
2.013820245	0.962239813	0.616889575	0.369051048	100.4582131
8.899778784	0.915909956	0.357078644	0.201320129	132.6408569
4.754835734	0.861373806	0.345896683	0.284487917	66.48724949
3.006082748	0.96513506	0.596269992	0.542503901	247.5244663
4.307179583	0.91216651	0.395865468	0.432969067	116.4481197
3.42730683	0.939408575	0.484343931	0.405666325	101.7141755
7.744791629	0.957950602	0.526783747	0.219993908	161.7585008
8.06310722	0.919443725	0.380501956	0.24459159	81.57624858
19.05229495	0.885878113	0.221552107	0.298475157	218.0501573
7.581066149	0.8408644	0.242402046	0.186716654	47.29409679
9.376125136	0.890512803	0.286476472	0.141403464	165.9341909
1.669694512	0.930418814	0.556354184	0.467093665	89.47736681
6.162410631	0.903603501	0.321402968	0.29031845	171.011905
7.461988392	0.875805838	0.296169001	0.149809541	68.41943824
2.908387659	0.946641074	0.585405234	0.50981275	181.4731825
1.877011383	0.932761344	0.507428955	0.475986129	80.21115685
3.828959038	0.924965879	0.498284126	0.375406683	99.33136433
13.93832351	0.854725181	0.204911253	0.032989454	125.2306995
4.807609146	0.930649975	0.39700771	0.417018469	177.6668592
1.738659514	0.960665994	0.63170763	0.539747175	135.7120299
27.30960508	0.889234029	0.272719517	0.24013395	219.7734937
7.918047161	0.89582251	0.339372607	0.200201687	120.2011554
14.38139385	0.851608041	0.207181669	0.10201803	145.0601425
1.977150474	0.932326287	0.534241357	0.447551311	101.1434057
5.659141503	0.927603051	0.428365392	0.243289302	150.8113723
4.21203144	0.944361414	0.529746324	0.340577057	112.7248258
4.812313802	0.923690716	0.404441425	0.245033987	83.93957167
14.01868254	0.915144104	0.270680997	0.128287565	290.7819092
8.419454798	0.93129999	0.371902569	0.235996223	104.4509005
15.99939248	0.842110099	0.174996294	-0.00922919	96.87409095
3.860108622	0.853884498	0.324488009	0.049403191	41.89590508
6.31267899	0.945042622	0.499035144	0.339960766	280.0055386

log.sigma.2.0.mm.3D_glcm_SumEntropy	log.sigma.2.0.mm.3D_glcm_AverageIntensity	log.sigma.2.0.mm.3D_glcm_Energy	log.sigma.2.0.mm.3D_glcm_SumSquares	log.sigma.2.0.mm.3D_glcm_ClusterProminence
4.283204445	12.96480582	0.01191748	9.864377918	2079.354023
3.906524044	8.609452208	0.017850326	9.722525337	1589.140878
4.367798154	12.80964191	0.014709494	13.15060778	3150.063364
3.791360878	8.305715818	0.019936714	8.337189268	812.8174993
4.055366743	13.47100417	0.01998056	7.546109273	1069.679274
4.319957145	12.21642457	0.015420464	22.15969475	6998.414239
2.807632725	14.08746671	0.144286252	2.965986541	690.6771093
3.774468272	15.96771589	0.054547204	6.410074739	1898.17841
3.274545758	14.3978755	0.086626885	3.796663561	720.2433954
4.440781835	9.512872636	0.008869664	14.67446094	3622.263871
3.123556619	24.47267828	0.09073296	2.781218588	854.9251314
3.332023634	16.61625656	0.085137764	4.261293303	1113.25948
4.591209464	10.8677964	0.007249434	18.36422746	5963.792579
4.144624566	10.52907834	0.013881206	9.602301459	1677.307739
3.865227632	12.3498493	0.040137853	7.17789639	1694.268684
3.108103977	15.62999231	0.115756846	4.498166985	1244.972904
3.758458606	7.592402099	0.024643873	4.588265457	423.8333309
3.990369521	8.949656457	0.01764523	9.893622624	1664.757971
3.801399381	7.814958848	0.029006823	5.252809849	696.3164593
3.552708997	16.11417187	0.066966442	5.094636214	1164.98252
3.813750986	8.322416966	0.038040924	5.209858723	597.8845826
3.716683529	8.053717378	0.021447298	5.149090978	510.4307329
3.978170817	8.45295066	0.025055247	7.266263957	1130.641917
2.850690134	11.92304982	0.126753659	2.092115069	407.6350898
3.815413351	15.32043932	0.067031915	10.29366454	2989.366343
4.017811214	11.28163716	0.032647178	10.08536152	6464.061457
4.153323627	12.92535587	0.015889385	7.672053289	1451.889018
3.687825991	15.17189141	0.042721865	4.579744231	818.5708642
2.975777724	7.12698045	0.057617122	14.9259008	2362.21791
3.808196613	9.698854974	0.032020431	5.252687645	744.8537567
3.616338455	12.05070758	0.06232458	5.288206228	947.4651694
4.083274146	9.92323927	0.013704319	8.926131395	1344.707667
3.970680393	10.58577433	0.028478848	7.374471694	1346.968378
3.281152234	11.7491492	0.085954299	4.228278321	1036.387235
3.722314245	11.0016961	0.045757033	5.219739571	821.9367613
4.386185119	15.43580477	0.022813967	14.09747385	6158.96922
4.716841324	13.56675443	0.00699835	30.78305226	10939.84492
3.089813938	12.99796552	0.12314849	4.418662529	1647.956679
3.840129042	10.17779387	0.02126065	5.838604102	786.8634683
3.085392581	36.04334097	0.110470594	3.593732226	1077.606138
4.050593976	10.69063677	0.017157397	7.015521973	1012.41678
3.077008065	11.12881846	0.091645638	3.081889669	598.3582954
4.244848613	13.59359827	0.014550012	8.891758972	2241.50459
4.110136568	12.77615766	0.017893028	7.863783224	1348.361133
3.344833955	10.29051068	0.080051232	3.427785123	492.3857702
3.794647295	14.64012849	0.042601179	6.132135253	1115.638773
3.188451752	11.85743943	0.108013281	3.314079315	720.1959797
3.998987689	6.517815215	0.021167361	6.71402638	1170.48973
3.706411732	8.400121664	0.022698006	6.970204241	820.9416296
5.006595131	12.45268194	0.007946766	68.72314807	124889.9353
3.179275753	12.46614407	0.10545706	3.785509752	892.4674831
4.298890117	14.02449982	0.018138531	12.89118903	4575.360689
2.670164778	12.02189105	0.148896349	1.832299653	259.2112552
4.19191386	9.973102826	0.019823492	7.401235332	1407.288862
4.427500019	10.14605015	0.009524043	14.07586695	4100.553952
3.961087637	9.683450409	0.019848071	6.149317527	754.164768
3.310389274	11.71770525	0.082888093	3.728670427	786.5451772
3.891963985	13.36251384	0.039905138	7.425461491	1382.15394
4.187923907	11.56207212	0.014782654	8.813605721	1936.200938
2.788472233	13.13196936	0.118719514	2.268380537	463.3750471
4.225807237	11.9620854	0.023111939	11.68039715	3242.306602
4.495743869	14.59372391	0.014515828	16.83485548	5696.353666
4.720318315	19.50603369	0.010676662	20.66095773	9225.51931
3.693909548	24.48075668	0.068931109	7.571921382	2583.242361
4.077244492	11.40559521	0.020764089	7.256044554	1274.293732
4.953182021	16.62743362	0.005318929	28.75574599	17547.87368
4.14969965	9.535761078	0.014486486	14.84466945	4908.56524
4.061213331	13.10245767	0.037499633	10.5254671	4005.041745
3.028293501	9.976278461	0.105250058	2.491054344	370.7139541
4.259799269	11.43866743	0.016647798	10.85313459	2735.085821
3.974329997	8.01212214	0.019841045	7.886780839	872.0147363
3.311281813	15.65555941	0.097911985	5.069674716	1615.062515
4.094572795	10.62325564	0.024034311	8.177840583	1350.83677
3.679755284	9.97803754	0.045585028	5.263974385	909.5480786
3.674680593	12.71154283	0.065399573	7.586296869	3396.172885
4.172302316	8.908459874	0.018560339	9.729373094	3384.371573
4.99806975	14.69474671	0.005185212	30.78007547	19684.44664
4.076207058	6.700746058	0.01550487	12.54762425	2420.10904
4.378104371	12.81359468	0.009723162	12.15792655	2526.320177
3.275173692	9.384436133	0.07053547	2.987478319	388.243109
4.283204445	12.96480582	0.01191748	9.864377918	2079.354023
4.21011987	8.171501944	0.01232879	10.32041425	1881.81094
3.205597068	13.37827907	0.107979346	4.806200045	1311.213463
3.579515835	8.856805888	0.041213099	3.670361429	469.1615608
3.718257718	9.86173813	0.050101579	5.455681983	828.2683898
4.379370722	11.1532134	0.010223932	18.26425751	5571.804238
4.17279233	13.20361596	0.018953623	8.364270614	2118.082939
3.019081549	11.57842159	0.125887441	3.030586836	504.7890481
5.024097263	14.80501178	0.008240249	34.85826448	27284.29376
4.2414941	10.87015256	0.013627219	10.03230465	1968.344138
4.596595282	11.95893282	0.00769089	20.06175159	5574.398828
3.45243073	9.975835182	0.058624709	3.517110736	444.051197
3.930100446	12.2119546	0.028632554	6.795558407	1293.512788
3.597229364	10.53482614	0.063539334	5.056098966	977.6182637
3.970715046	9.074692727	0.021629768	6.305305071	1064.851477
4.55937395	17.2731069	0.008503712	16.8929692	5710.50869
4.221909208	10.13049441	0.018341155	10.53545327	3857.945759
4.18000525	9.843984947	0.015203389	20.63901395	5807.899652
3.435630878	6.456103518	0.030325707	5.233060017	372.0730037
3.847898141	16.70096346	0.05827321	7.87621375	2340.576984

log.sigma.2.0.mm.3D_glcm_SumAverage	log.sigma.2.0.mm.3D_glcm_Imc2	log.sigma.2.0.mm.3D_glcm_Imc1	log.sigma.2.0.mm.3D_glcm_DifferenceAverage	log.sigma.2.0.mm.3D_glcm_Id
25.92961163	0.723949215	-0.105164285	2.768934993	0.40204228
17.21890442	0.912118383	-0.26197173	3.104442645	0.380286934
25.61928381	0.833835277	-0.164162561	3.132748401	0.403426417
16.61143164	0.929738112	-0.297421281	3.168889323	0.355185865
26.94200833	0.737027891	-0.119710378	2.309510639	0.450372075
24.43284914	0.987018395	-0.46378153	4.751995798	0.263549621
28.15795455	0.672776518	-0.145139776	1.001320828	0.67552756
31.91741543	0.687818252	-0.115812075	1.64548982	0.551339961
28.795751	0.671122424	-0.129624239	1.229668932	0.605701095
19.0174891	0.837045345	-0.15654036	3.668880177	0.348134046
48.92622725	0.59132526	-0.093986833	1.150764315	0.630272985
33.20196122	0.714805465	-0.144394323	1.255132243	0.608609037
21.73559281	0.864917453	-0.170431205	4.30198092	0.311338791
21.05815667	0.859074431	-0.190287582	3.014886035	0.362808889
24.6996986	0.725504612	-0.129640641	1.921131034	0.506864186
31.25738241	0.683570449	-0.144232154	1.217539727	0.630178222
15.1848042	0.683131126	-0.10826574	1.760301359	0.499003023
17.89931291	0.927118102	-0.279963427	3.138730805	0.355060549
15.6299177	0.67158817	-0.100455587	1.929332014	0.487659633
32.12280731	0.647218723	-0.097879698	1.584088914	0.570572494
16.64483393	0.678564524	-0.106346212	1.730753796	0.528547618
16.10743476	0.791793762	-0.159685863	2.15680037	0.431195067
16.90590132	0.804546201	-0.163952977	2.096373717	0.472284117
23.84609965	0.636715417	-0.127870158	0.904666178	0.666339364
30.61585126	0.840803797	-0.210474327	2.166262532	0.531762606
22.49180081	0.737698863	-0.126318332	2.126207592	0.506758616
25.85071174	0.699346502	-0.105542099	2.222923694	0.447821855
30.34378282	0.656691093	-0.103567123	1.532714871	0.546371127
14.2539609	0.99655724	-0.75186573	4.661456588	0.25942904
19.39770995	0.666511706	-0.101860183	1.770709614	0.50487765
24.10141515	0.700005787	-0.131873645	1.454510078	0.578142695
19.84647854	0.82782498	-0.163742	2.97147026	0.374103728
21.17154867	0.714590984	-0.113521146	2.100334606	0.491028555
23.4982984	0.663562889	-0.121606852	1.2854203	0.604719015
22.0033922	0.67228668	-0.106426424	1.634550564	0.542568379
30.81241808	0.818104505	-0.15483964	2.662271977	0.45234306
26.48980495	0.928124375	-0.243645921	5.973382417	0.256761265
25.96864104	0.686593456	-0.142737122	1.142493657	0.657444943
20.35558774	0.805341506	-0.16403247	2.1907195	0.434731904
71.98313854	0.651564304	-0.118640322	1.24910497	0.627893316
21.38127354	0.710725534	-0.108958313	2.240348111	0.445640171
22.24846365	0.573053098	-0.084185769	1.290676963	0.609769337
27.18719654	0.743183363	-0.128161279	2.314902915	0.443218135
25.55231532	0.720381645	-0.112557243	2.332085259	0.438598063
20.58102136	0.699695996	-0.136474729	1.206693149	0.605667803
29.27398649	0.726717978	-0.13114015	1.753737382	0.525656856
23.7129438	0.702930405	-0.147559407	1.079654856	0.640789088
13.03563043	0.808488879	-0.16611472	2.084319858	0.456352559
16.80024333	0.914385184	-0.276503653	2.696833308	0.394325238
24.02964794	0.96805099	-0.317756768	6.94063567	0.269156653
24.93228815	0.666212654	-0.130691956	1.171757145	0.629255381
27.83160422	0.721765119	-0.102739495	2.885839473	0.443649845
24.04312969	0.618323888	-0.124082421	0.859909159	0.683560366
19.94620565	0.779703532	-0.152491089	1.992924245	0.464054852
20.29210029	0.853692396	-0.171734568	3.537506515	0.345749695
19.36690082	0.690146116	-0.102347788	2.074041509	0.460002781
23.4354105	0.64122705	-0.111842963	1.26877167	0.602371346
26.72395678	0.728669846	-0.127916256	1.927605509	0.511409294
23.12414424	0.809935268	-0.156388708	2.476215561	0.407470277
26.25145352	0.528494509	-0.080082526	1.01944661	0.643436497
23.8964205	0.819511818	-0.156972334	2.851874846	0.446556737
28.92418257	0.768797899	-0.116975162	3.467294379	0.406352682
38.51239499	0.721561144	-0.091840921	3.965788608	0.385321165
48.85759752	0.744424834	-0.138342635	1.824739461	0.572725858
22.81119042	0.794921769	-0.157005379	2.147910098	0.450810621
32.59888354	0.690912682	-0.075005752	4.947277623	0.312400742
19.07152216	0.940627107	-0.294978616	3.925948789	0.318468389
26.06107895	0.740125379	-0.124065354	2.290064357	0.512010598
19.95255692	0.63256057	-0.115321	1.047395819	0.643065226
22.8442834	0.700214342	-0.095671814	2.820941602	0.429326312
16.02424428	0.820782052	-0.170976826	2.51567471	0.424975471
31.2904314	0.725054035	-0.150860485	1.235097066	0.627508313
21.24651128	0.743586083	-0.12873623	2.191804078	0.465318632
19.95607508	0.689353502	-0.115761567	1.665096121	0.536887127
25.31210744	0.741264177	-0.137091802	1.828466223	0.569371586
17.79988129	0.775302704	-0.136966735	2.509740444	0.450694949
28.9455189	0.738771915	-0.091976883	4.698147243	0.309509377
13.40149212	0.954078338	-0.32968718	3.556280765	0.333272211
25.62718937	0.724323103	-0.099405225	3.369957669	0.369538224
18.76887227	0.613963554	-0.099996913	1.214735298	0.594263027
25.92961163	0.723949215	-0.105164285	2.768934993	0.40204228
16.34300389	0.774105663	-0.128629343	3.127526067	0.379228921
26.75655814	0.683955625	-0.138942085	1.297606116	0.618521909
17.71361178	0.663436185	-0.108516401	1.385690372	0.555435838
19.72347626	0.685215225	-0.113811793	1.702312191	0.548578558
22.3064268	0.943018655	-0.278838336	4.522219146	0.297903433
26.39112412	0.777384442	-0.139362315	2.156657491	0.465787239
23.15684318	0.705731261	-0.157801988	1.006193687	0.657710057
29.12167102	0.848441316	-0.147105935	4.835335348	0.348594744
21.74030513	0.738261919	-0.110655972	2.833204119	0.41544329
23.91786563	0.907257361	-0.212077779	4.638830324	0.297990094
19.95167036	0.64847341	-0.114107099	1.332734791	0.579131178
24.42390919	0.63317548	-0.080762724	2.131739649	0.488631136
21.06965227	0.710483391	-0.126095048	1.550364145	0.571463496
18.14938545	0.587492538	-0.066700155	2.144631849	0.470554929
33.98002606	0.764903536	-0.111782974	3.819607408	0.354327988
20.20195725	0.709685449	-0.103938568	2.638838747	0.442989133
19.68796989	0.983150921	-0.43222711	4.996831697	0.272058687
12.91220704	0.91005652	-0.287237821	2.455671362	0.405940562
33.30943669	0.714816544	-0.116949219	1.9139919	0.545693347

log.sigma.2.0.mm.3D_glcm_ClusterTendency	log.sigma.2.0.mm.3D_firstorder_InterquartileRange	log.sigma.2.0.mm.3D_firstorder_Skewness	log.sigma.2.0.mm.3D_firstorder_Uniformity
25.55044248	112.7019696	-0.448380878	0.087695804
21.0463729	90.73999023	0.734948457	0.093742658
33.29347776	147.1771212	-0.14026739	0.08017952
17.31520366	111.1176014	0.371562292	0.098534972
19.92242068	112.80616	-0.10324396	0.101510109
54.80888883	173.1612873	-0.081619833	0.06456782
7.959308817	16.99630022	-0.886162789	0.30888742
18.91366884	79.21210414	-1.049090938	0.161488464
11.54953277	57.21742415	-1.356008837	0.21374417
32.93563513	121.6017327	0.572761325	0.075806062
7.211794546	30.36621761	-1.690040908	0.248918785
12.29718499	46.64080191	-1.225914284	0.214140479
41.91884845	164.4506998	0.419956612	0.062732177
24.10106017	107.0707932	-0.209567914	0.091024931
20.9598617	108.3228893	-0.927257217	0.132615784
13.5529085	28.59195161	-1.547242118	0.263957785
12.45217551	82.94430518	0.026746168	0.125768033
23.21872226	95.34886932	0.393698938	0.101046993
13.48720188	70.44949102	0.225463124	0.136387248
12.95668606	38.85344982	-0.230542401	0.195625784
14.75229904	94.46974182	-0.285446295	0.134385743
13.07557625	76.92690659	0.237440306	0.124419164
20.61797667	104.3556557	-0.510588536	0.118172262
6.157495401	26.62993228	-1.378315737	0.288649706
28.57490291	112.9318854	-0.761877164	0.168286926
25.49856037	82.3648746	1.455383211	0.13383594
21.62579133	107.6406136	-0.356999662	0.10032085
13.3633625	69.89273384	-1.014876861	0.152298857
27.69841341	107.7869582	0.176609025	0.096938776
15.02486052	84.94870496	-0.412787486	0.13136388
16.2289925	80.90599424	-0.878405222	0.169254037
20.54064487	98.59355545	0.345580633	0.095301807
19.74631287	92.17416656	-0.340696985	0.123814601
12.54187153	38.57938194	-1.168273579	0.223953407
14.89152712	82.83489749	-0.530136594	0.148836319
39.7799784	132.0060611	-0.651901646	0.093628967
56.15687605	186.01544	0.63989642	0.057278107
12.43054444	22.96478164	-1.064885891	0.278020634
15.08235281	76.71244049	-0.550707548	0.115462908
9.14510165	27.05016601	-0.043026112	0.265218182
18.91182907	106.0540457	-0.005962272	0.102991001
7.077100185	31.53904819	0.5935033	0.245693044
25.43178443	115.2869663	-0.422242642	0.091330082
21.65630495	117.2107801	-0.260173796	0.098523754
10.35302881	53.99648592	-1.259464769	0.208911724
17.6552808	92.75734806	-0.608908381	0.136804181
10.02680415	35.74837136	-1.035395587	0.241324076
19.18204434	102.4610856	0.524003777	0.112612331
15.48821525	89.10842609	-0.110175217	0.100338566
148.1484135	177.8427725	1.793529275	0.063636364
11.58091411	41.2140233	-1.706422085	0.240658813
27.96460635	92.89026503	0.851821177	0.100352457
5.078015284	20.34965676	-1.281688898	0.320502837
22.54871442	101.4695969	-0.417783257	0.102606742
34.33653407	140.7837811	0.61718529	0.076205854
16.87100613	102.7958145	-0.192951756	0.113031174
10.78167086	42.76216817	-0.873441992	0.214343872
21.74197645	111.3918889	-0.535032471	0.128004943
25.01236369	110.9015322	-0.366803125	0.093966263
5.770036708	25.82642937	-0.696623862	0.296137266
27.10854576	114.007225	0.266671149	0.096571365
36.67318008	121.8724576	0.340503728	0.082609066
47.56568994	124.3886161	0.267136201	0.074804202
19.53733516	62.82354331	-0.649883702	0.176765881
20.75315165	101.1518533	-0.527583044	0.106724508
63.8383689	144.3671145	0.668410512	0.060487933
33.64468432	116.8097305	0.805266695	0.088199986
24.37835287	88.60755756	0.532152306	0.126688225
6.844882446	32.40276241	0.103728997	0.251195761
25.60766822	111.5527123	0.501937648	0.094475387
20.32687174	130.2465711	-0.090459218	0.102183451
15.54464179	40.50986123	-1.547569545	0.23080667
23.50297819	127.5638409	-0.210489755	0.105042594
14.82407884	81.13332701	-0.169851721	0.149838144
17.4515713	55.30036944	0.634048522	0.184428255
23.91127254	89.49598578	0.932994046	0.102851849
75.81052102	158.2354078	0.824902525	0.063523415
29.83110606	133.3595886	0.509582454	0.079017013
27.78880771	118.4280469	0.251589315	0.079091366
8.788104295	53.49885112	-1.331392205	0.201924768
25.55044248	112.7019696	-0.448380878	0.087695804
23.91030949	118.3457489	0.523405353	0.085509066
14.59259149	49.24080053	-1.688541597	0.246891564
10.86380449	70.74456787	-0.788481912	0.153896243
15.05539754	73.98435712	-0.622031555	0.159210258
38.15509688	166.618608	0.218736385	0.065728306
23.61303211	100.9322348	-0.277047152	0.102672057
9.362240566	36.75921092	-1.406569697	0.271287529
82.21789845	125.1298113	0.831958468	0.070144319
24.13115925	115.0435545	0.330697039	0.08675
44.18355473	159.1487535	0.145712706	0.066057551
10.26654659	70.96471965	-1.042995245	0.172023082
16.94600101	78.13916659	-0.002713525	0.122533536
13.59131555	57.37500858	-0.160084066	0.177460844
15.77658867	77.56826472	0.609387157	0.117234942
37.10663465	139.1077881	0.290017082	0.070393821
25.1421003	99.50177479	0.640133983	0.099472795
41.15809135	148.0342751	0.656983393	0.0651341
10.88126966	69.89234543	0.08076284	0.123983337
20.49194416	54.84781051	-0.438594558	0.164664162

log.sigma.2.0.mm.3D_firstorder_MeanAbsoluteDeviation	log.sigma.2.0.mm.3D_firstorder_Energy	log.sigma.2.0.mm.3D_firstorder_RobustMeanAbsoluteDeviation	log.sigma.2.0.mm.3D_firstorder_Median
67.70026042	8104495.736	47.24422945	-59.97175789
62.21113986	3234521.65	40.58525357	-153.0220947
81.04199315	8976215.964	59.95546279	-86.92237091
65.09538629	2840339.644	45.57297246	-171.8078995
61.02359932	9341917.429	44.92356059	-81.75995636
99.1130016	2944032.061	72.26650129	-116.9280396
26.97528051	37557082.6	9.90067472	-2.133719444
54.46477273	37114468.73	35.19430232	-22.72303295
42.14308867	17648596.74	26.56388717	-13.06981659
76.11517345	4256316.758	52.29149859	-51.47993851
26.55886405	10787056.23	13.35356428	2.023118734
41.4008439	12867849.81	23.36164246	-8.473614693
96.23300159	8486203.498	69.39312454	-114.4171829
65.320866	6223986.71	45.87991039	-156.5953217
61.25809319	9333689.319	44.43194748	-44.37615776
39.51147222	33973705	18.15989011	-6.152886391
46.25075062	2624889.089	33.51810408	-47.75829887
60.32971612	3183167.17	41.11552478	-158.7640381
45.53063738	3071596.934	30.62270971	-36.07823181
38.7349536	44250356.65	19.2165918	-4.938410282
51.09745354	10122407.47	38.16407034	-46.15293503
47.41834945	2942406.39	32.08858055	-124.6123352
59.19422036	3230795.511	43.85445227	-65.96234131
26.30338978	9164073.328	13.1615865	-6.394837379
70.14175544	8016303.904	49.85671513	-20.61694908
52.88929014	14590691.93	34.55685992	-28.51312256
58.99839146	9994884.743	42.28602217	-64.92003632
45.74331772	9029564.209	30.20676623	-24.1175518
78.51422309	405559.9635	51.9253977	-77.39951324
50.53858448	7363999.748	35.86623047	-35.86063766
51.22256791	20463660.39	36.31894578	-16.79819107
61.43752531	7121079.348	41.79138213	-149.6718445
56.63149161	9981036.28	38.4656562	-33.3377533
40.51558017	12522250.43	20.52465713	-6.064640522
49.78616749	19730111.57	34.95901023	-23.33998299
80.86905982	19868030.27	56.66978768	-63.0886898
110.7774955	20122596.12	77.44307174	-193.3314743
34.51456094	39366790.71	13.68059457	-1.32333082
50.13790281	2447290.211	33.02338069	-85.13243103
30.66708139	35656712.47	13.84743322	-5.498190641
58.6120669	7057299.027	43.25430187	-71.37584686
28.72913838	16805894.51	14.27186901	-6.844456196
65.45691683	19397654.07	46.95121617	-70.66608429
63.01594118	8543697.957	47.01822936	-69.49153137
38.92712664	4022508.154	24.64909105	-16.14570045
54.36513544	14464591.78	39.00748048	-33.73829269
35.83723639	21766517.74	18.59547648	-5.281633377
55.82075479	1685060.945	40.76537953	-49.36138535
56.94121934	1856320.216	38.94598244	-107.1166534
121.4086455	8710258.272	75.81422916	-140.5225754
41.28281967	8191789.806	22.80894024	-11.16923857
64.97044687	21904403.77	39.62963714	-37.58353424
23.56782663	16264374.54	10.55069232	-4.399780512
59.48846286	4811014.857	41.69896181	-1.343168378
80.80258029	6545791.124	58.08476578	-117.408783
53.74551762	6669472.597	40.08263846	-76.01068878
38.20374006	18781412.94	20.92237829	-9.362419128
61.38619581	14128770.92	46.1559998	-39.76797485
63.92163929	5184972.435	44.50961155	-109.513607
24.77488551	42635212.86	11.83042179	-4.075191975
70.80852382	10168543.93	47.08018885	-62.76271057
81.20247524	22478723.67	52.87768324	-35.85133743
86.5301215	72313014.09	55.12298093	-41.40584373
52.33181491	55003312.98	29.88978889	-8.023273945
58.59297702	2621355.45	41.44615429	-36.90501022
99.12496949	46833246.45	63.95239856	-72.95465851
72.68685608	3883694.665	49.8588702	-172.7044067
60.9960782	38174953.24	38.00205417	-26.57383347
29.33282884	22061925.51	15.80576699	-5.350821018
67.40518615	15507417.63	45.21101664	-44.18428802
65.27246725	2860553.722	51.83074165	-95.30618668
44.86643335	33154467.05	23.75183908	-5.101898193
64.5521977	9800542.582	50.05293418	-77.11723328
49.86362599	6474034.366	34.57778378	-27.25801849
45.25229088	16622180.61	25.63250024	-10.44378519
58.80480988	4731181.825	39.00867893	-36.42774582
100.8665449	26137456.21	67.33514102	-57.47501373
77.91431037	4011923.454	57.36895508	-171.2621155
72.57688477	8180287.406	49.16833232	-64.56490326
36.41775091	5519616.16	23.23675899	-19.90600681
67.70026042	8104495.736	47.24422945	-59.97175789
71.62221948	5124513.775	49.83893042	-77.6490097
46.88079028	17675238.52	26.53982395	-7.357695103
41.51866408	6038075.303	28.65387779	-36.82027435
48.87324184	10501781	32.54185961	-25.08570671
92.78563002	4641052.635	66.03182607	-123.9029388
61.03426993	7168201.539	42.26870421	-52.99877167
35.18942181	12106380.72	20.09414098	-7.250768661
96.90585568	22927251.41	57.7896232	-38.71940231
68.83968269	9355950.951	47.22004052	-67.13964081
90.21602111	5008738.299	64.71774811	-84.34682465
41.66766272	6733180.611	29.08981602	-28.54084778
53.196997	21681021.79	33.36828458	-24.37677383
43.84281448	17000205.78	26.05768805	-10.86454439
50.85483536	12592813.7	32.58707459	-37.1354332
84.1457336	24025625.94	57.62095161	-90.81429672
63.25142048	15957196.65	41.8808474	-56.91623688
95.64394936	2957655.248	63.15013026	-152.0545807
46.2598901	1189231.555	32.45858812	-111.556694
51.18571307	57627583.23	27.73473892	-8.535187721

log.sigma.2.0.mm.3D_firstorder_TotalEnergy	log.sigma.2.0.mm.3D_firstorder_Maximum	log.sigma.2.0.mm.3D_firstorder_RootMeanSquared	log.sigma.2.0.mm.3D_firstorder_90Percentile	log.sigma.2.0.mm.3D_firstorder_Minimum
218821384.9	224.9016418	110.312794	24.63360882	-351.384552
87332084.56	199.6887512	169.1865403	-62.64892883	-325.2640991
242357831	210.3256989	136.3242387	4.89990654	-394.0130005
76689170.39	64.36178589	175.707874	-50.39762573	-331.80896
252231770.6	221.3421173	111.9047699	-0.246396072	-388.2380981
79488865.66	173.3357391	169.0645899	42.82517471	-409.7703247
1014041230	436.7935791	47.99237094	11.78187418	-336.1596985
1002090656	292.9093628	85.76261231	9.603888035	-409.6653748
476512112	188.3071594	64.60055473	10.28874531	-356.2031555
114920552.5	358.2944641	108.1349985	71.04890137	-262.304657
291250518.1	255.2328339	44.43202696	38.31902771	-589.2398071
347431944.9	398.2163391	64.71004645	14.79188995	-405.5307617
229127494.4	236.2256165	160.1189465	64.92417145	-366.2923279
168047641.2	93.69722748	180.9912285	-54.61078033	-393.5780945
252009611.6	217.5437164	100.7239519	3.770240831	-348.8126221
917290035	248.4433746	63.12092059	13.38556938	-385.2404785
70872005.42	219.545578	77.59115813	14.68796253	-207.0114899
85945513.59	109.8712997	169.343384	-54.85349274	-342.1182556
82933117.22	260.552887	73.60216398	16.18336601	-214.7230377
1194759630	471.5912476	60.91058605	41.15569	-377.1197205
273305001.6	182.3882294	83.49464057	1.724111533	-225.2716064
79444972.54	87.06038666	136.4654728	-39.03288078	-298.3540039
87231478.78	107.1650925	107.226363	-4.450591564	-260.903717
247429979.9	322.2188721	43.67599207	11.10668831	-289.0997925
216440205.4	264.2490845	105.0796884	6.412921429	-401.8006897
393948682.1	494.5610046	84.88392053	15.9606144	-278.9862976
269861888.1	220.3279572	103.1706339	14.1240263	-351.5697327
243798233.6	141.3240356	71.6474067	16.37935791	-377.2817383
10950119.02	145.1312256	120.3506727	66.38719978	-248.4384766
198827993.2	145.2053986	79.98696542	12.35786533	-250.7228241
552518830.5	261.5690613	76.34423456	10.88927746	-313.3773499
192269142.4	97.23712921	167.1101474	-51.54602127	-360.8210144
269487979.6	286.3241882	88.33901345	15.57414989	-287.0171814
338100761.7	255.7723694	63.06013007	20.14945259	-298.6655884
532713012.5	302.3612366	75.32903869	14.12887897	-277.3739319
536436817.2	253.3271179	132.7158695	8.213805676	-432.9807739
543310095.3	370.3135681	227.1484103	18.70011406	-485.6882935
1062903349	579.013855	57.91191859	18.79944553	-314.1552429
66076835.71	75.78623199	111.4575836	-20.60397224	-316.4803772
962731236.7	516.8137817	54.25338809	15.46186142	-891.4644775
190547073.7	184.9039764	105.7558938	1.998977184	-302.3399658
453759151.8	439.8125305	48.18940125	22.59566574	-266.3725891
523736659.9	181.2756653	112.7080891	14.16357727	-375.8929749
230679844.8	195.6938782	109.9292427	3.021943665	-360.5151978
108607720.2	114.7838898	60.80426161	10.57587004	-274.2702942
390543978.1	274.0437012	86.01609253	8.365039825	-398.4596863
587695978.9	327.8432617	55.88680026	13.08715801	-296.8148804
45496645.52	247.1103058	87.71739973	21.2722332	-194.5305634
50120645.82	61.17569733	127.6068598	-10.68306379	-282.9778748
235176973.3	582.024231	198.9775944	96.99289093	-366.2515259
221178324.8	198.3548431	65.55834055	9.373361588	-322.4935913
591418901.7	487.3557739	100.7254919	40.37541046	-364.5913391
439138112.5	349.4740295	40.12499592	11.01556263	-275.3921204
129897401.1	189.8904419	75.3662044	71.62020874	-228.2111206
176736360.3	274.5084839	151.0219672	3.160852957	-346.4480896
180075760.1	140.1573639	100.29724	-1.178949881	-282.29245
507098149.4	304.1210938	59.60181369	18.71160469	-299.2096863
381476814.9	253.0945282	95.62863352	8.625639343	-362.0746765
139994255.7	168.8664398	138.0666457	-17.80135345	-365.2263184
1151150747	492.3274536	42.58240595	18.53087273	-320.8468933
274550686.1	326.0854797	112.9536382	21.87166367	-336.3391724
606925539	449.0489502	118.4183657	62.38416672	-395.2404175
1952451380	541.5258789	125.7636465	87.03940201	-515.0167236
1485089450	447.8901978	80.34791788	19.5037344	-622.439209
70776597.14	119.6875229	90.36711987	25.97868538	-308.9971924
1264497654	451.7304077	146.4034838	110.6560471	-427.6584473
104859755.9	150.1029968	180.6546142	-32.2386055	-374.8371277
1030723738	562.8271484	96.16552626	15.51040583	-337.582428
595671988.8	361.6911316	46.91620138	16.01564026	-234.3071289
418700276	368.7166443	100.7744004	46.29448166	-322.5536194
77234950.5	73.11638641	120.1966764	-5.28283658	-266.981842
895170610.2	392.9759521	68.33960078	18.81545372	-399.3070984
264614649.7	187.4499054	114.3889704	0.284991491	-318.5130005
174798927.9	320.4204712	77.67614061	12.92773266	-267.5922852
448798876.3	618.7843628	71.89289601	21.36733913	-305.5128174
127741909.3	397.4152527	88.50470322	45.56073532	-245.5627289
705711317.7	434.9870605	132.8028996	159.0430359	-360.7328491
108321933.2	88.5728302	186.7787218	-34.25069427	-307.5420837
220867760	233.2209015	112.0969476	57.41344452	-357.1037292
149029636.3	104.6470261	58.80821469	9.302210808	-230.5916595
218821384.9	224.9016418	110.312794	24.63360882	-351.384552
138361871.9	205.6888123	117.8455396	53.66351776	-272.1145935
477231440.1	157.8376007	71.91121265	6.953574228	-333.6102295
163028033.2	185.8392944	71.68533666	4.938007355	-244.8131409
283548087.1	175.5376129	76.27687212	11.38343487	-272.9294128
125308421.2	191.6143494	162.3872447	26.79489422	-376.4640198
193541441.5	277.4573669	99.43427244	17.30817719	-364.4756775
326872279.4	254.0306854	54.5661706	6.139519453	-298.2689819
619035788.2	468.5253296	139.6873452	152.3528259	-363.8665771
252610675.7	216.7066803	108.1431398	30.4071907	-309.1310425
135235934.1	224.3995819	134.7130377	69.94024277	-363.0718689
181795876.5	130.438858	68.61864112	5.272089386	-274.0012817
585387588.2	282.1498718	79.59758114	33.94212341	-315.1013184
459005555.9	309.7884216	66.41577335	22.05262203	-257.3930969
340005970	348.7565613	78.05327874	38.72850037	-236.6593781
648691900.3	432.5785828	142.4496887	24.58796539	-488.8381958
430844309.6	522.621582	104.3661075	15.33351841	-296.3055115
79856691.68	200.926712	184.3801435	16.85411072	-363.835907
32109251.98	31.28841019	129.4207874	-42.83817291	-256.9656677
1555944747	322.8114014	77.46207522	45.8271244	-418.3130798

log.sigma.2.0.mm.3D_firstorder_Entropy	log.sigma.2.0.mm.3D_firstorder_StandardDeviation	log.sigma.2.0.mm.3D_firstorder_Range	log.sigma.2.0.mm.3D_firstorder_Variance	log.sigma.2.0.mm.3D_firstorder_10Percentile
3.764582371	85.04267885	576.2861938	7232.257225	-187.1786041
3.597499773	81.78251763	524.9528503	6688.38019	-243.5820129
3.906398608	97.81805238	604.3386993	9568.371372	-222.81987
3.593692465	79.05244297	396.1707458	6249.28874	-246.0493698
3.546349227	75.26341273	609.5802155	5664.581295	-178.2813263
4.123950198	120.3805659	583.1060638	14491.48064	-276.3768616
2.30144571	46.43336372	772.9532776	2156.057266	-64.18931961
3.187435014	71.06329931	702.5747375	5049.992509	-153.1424942
2.744475157	54.77597158	544.5103149	3000.407062	-122.0684372
3.929063954	97.1436949	620.5991211	9436.897458	-167.3616882
2.535025491	44.42509521	844.472641	1973.589085	-36.60281181
2.805996855	58.48625956	803.7471008	3420.642558	-112.8900742
4.199451761	118.0526814	602.5179443	13936.43558	-244.518219
3.664754345	82.24606434	487.275322	6764.415099	-277.3082214
3.31103303	75.02343199	566.3563385	5628.515348	-173.452211
2.593473211	58.25134546	633.6838531	3393.219248	-116.8843094
3.186965489	57.48693612	426.5570679	3304.747824	-130.6785507
3.583239839	77.51033115	451.9895554	6007.851435	-246.8010559
3.199594061	59.45483661	475.2759247	3534.877596	-121.8457565
2.985950404	59.63785682	848.710968	3556.673966	-87.40739136
3.211735563	61.42768018	407.6598358	3773.359893	-144.0994064
3.264220038	60.74100032	385.4143906	3689.46912	-192.9590195
3.354834617	70.98721171	368.0688095	5039.184227	-181.2502594
2.313184675	40.11797266	611.3186646	1609.451731	-66.16861343
3.275737812	85.89580512	666.0497742	7378.089338	-196.1333313
3.31335342	75.59523165	773.5473022	5714.639048	-134.0832245
3.523770216	73.10395706	571.8976898	5344.188538	-166.7027283
3.109804035	59.32024231	518.6057739	3518.891147	-123.3818954
3.56610894	99.28872412	393.5697021	9858.250738	-205.5431885
3.250168536	62.49575152	395.9282227	3905.718958	-140.1845703
3.014803143	62.83539202	574.9464111	3948.286491	-145.0719604
3.629221231	78.37717308	458.0581436	6142.98126	-245.8749908
3.397069765	72.52188509	573.3413696	5259.423817	-152.9887329
2.77478464	58.90377667	554.4379578	3469.654906	-111.5894363
3.160205103	62.30161741	579.7351685	3881.491532	-129.8500458
3.858571811	101.4514198	686.3078918	10292.39057	-226.8149536
4.401703603	139.3322446	856.0018616	19413.47437	-344.0913544
2.504514317	55.85374448	893.1690979	3119.640772	-89.6843689
3.380245519	65.43277991	392.2666092	4281.448686	-176.7335266
2.56200938	52.53458343	1408.278259	2759.882457	-72.93003159
3.504903099	71.67538661	487.2439423	5137.361046	-167.2540131
2.59795414	46.98724839	706.1851196	2207.801511	-60.43468933
3.691955332	81.01884044	557.1686401	6564.052506	-177.3983704
3.566729716	76.16399132	556.2090759	5800.953574	-180.9344727
2.762624589	50.60249761	389.054184	2560.612764	-109.1333374
3.258718057	67.50775968	672.5033875	4557.297617	-149.7103363
2.673068619	52.16162241	624.6581421	2720.834852	-91.95720367
3.373679571	69.19299368	441.6408691	4787.670375	-142.349588
3.523196423	71.5928107	344.1535721	5125.530543	-199.3025848
4.2974766	169.276079	948.2757568	28654.39092	-261.463208
2.661326204	56.68118452	520.8484344	3212.756679	-125.4898376
3.744420072	91.80998131	851.947113	8429.072668	-151.0474792
2.170110689	37.73852416	624.8661499	1424.196206	-59.85275154
3.541254498	74.04120895	418.1015625	5482.100623	-122.937146
3.93183693	100.560535	620.9565735	10112.42119	-228.8242432
3.362259772	64.90641474	422.4498138	4212.842674	-157.6657013
2.804006396	54.81291277	603.33078	3004.455406	-102.5032684
3.328457959	73.66012002	615.1692047	5425.813281	-168.7485901
3.62982587	80.59967734	534.0927582	6496.307987	-214.8228714
2.285086535	41.0941321	813.1743469	1688.727693	-53.42213669
3.772007758	91.65030089	662.4246521	8399.777654	-182.406073
4.014588948	107.3695553	844.2893677	11528.22141	-185.4276825
4.144821659	115.306015	1056.542603	13295.4771	-199.0692749
3.183616066	74.70554929	1070.329407	5580.919095	-131.6681137
3.500613582	72.24557466	428.6847153	5219.423058	-155.1451111
4.363284164	131.4195585	879.388855	17271.10037	-219.3892334
3.730578157	92.88134515	524.9401245	8626.944276	-257.2483154
3.519571032	85.54669977	900.4095764	7318.237842	-152.4863174
2.524522842	44.5963303	595.9982605	1988.832676	-72.47359467
3.740802343	87.39435835	691.2702637	7637.773871	-158.6951752
3.467218564	74.97440087	340.0982285	5621.160786	-189.4717911
2.758726911	62.96765294	792.2830505	3964.925317	-128.1137207
3.480501926	76.56927042	505.9629059	5862.853173	-191.2934723
3.125091891	63.94855782	588.0127563	4089.418047	-135.9950989
3.072337062	67.52518578	924.2971802	4559.650715	-114.1232643
3.581203788	79.23603967	642.9779816	6278.349982	-136.1326279
4.279817159	129.1058185	795.7199097	16668.31236	-168.5518875
3.814833232	93.98288318	396.1149139	8832.782331	-277.003418
3.903027572	93.2715467	590.3246307	8699.581425	-175.654007
2.70948366	47.61921568	335.2386856	2267.589702	-103.9156265
3.764582371	85.04267885	576.2861938	7232.257225	-187.1786041
3.769844171	90.10165295	477.8034058	8118.307864	-180.9419312
2.666688569	62.69536558	491.4478302	3930.708866	-136.3069061
2.997404536	52.64677973	430.6524353	2771.683416	-121.977626
3.123835645	62.73165067	448.4670258	3935.259996	-138.2177856
4.128364812	114.3646402	568.0783691	13079.27094	-265.6423645
3.58838846	77.73318675	641.9330444	6042.448322	-163.7194824
2.46225912	47.628198	552.2996674	2268.445244	-102.98806
4.271149008	135.4098309	832.3919067	18335.8223	-194.3499939
3.788274635	87.22428977	525.8377228	7608.076726	-170.8902405
4.118816281	110.7583492	587.4714508	12267.41192	-199.8543091
2.894977593	51.96113281	404.4401398	2699.959323	-118.5636551
3.451128716	72.05595339	597.2511902	5192.060419	-126.6113014
3.061163726	61.32530046	567.1815186	3760.792476	-110.2916443
3.433650069	68.51471899	585.4159393	4694.266718	-118.3798096
4.101835198	108.0676577	921.4167786	11678.61865	-229.8430786
3.669840009	84.04620193	818.9270935	7063.764058	-169.2007904
4.136124643	122.731979	564.762619	15063.13868	-277.6683655
3.225990067	58.77463324	288.2540779	3454.457512	-199.8044739
3.286058722	73.8928523	741.1244812	5460.153621	-127.707235

log.sigma.2.0.mm.3D_firstorder_Kurtosis	log.sigma.2.0.mm.3D_firstorder_Mean	log.sigma.2.0.mm.3D_glrlm_ShortRunLowGrayLevelEmphasis	log.sigma.2.0.mm.3D_glrlm_GrayLevelVariance
3.401264027	-70.26133568	0.01151196	12.21728336
5.054922542	-148.1070735	0.031855723	11.43435673
2.850943111	-94.95223373	0.011886317	16.06560582
2.838578317	-156.9202608	0.036464166	10.434385
3.41527928	-82.81362342	0.008368585	9.520621622
2.375656341	-118.7070129	0.020545495	22.97107936
12.09296298	-12.1330294	0.004762793	5.236585592
5.085393541	-48.01284371	0.005104814	9.443679905
4.977925519	-34.24652697	0.005482659	5.80949234
4.102462654	-47.50032051	0.027527134	15.80081119
26.18037976	0.784815365	0.001693047	4.285212419
8.396653469	-27.69020682	0.004176905	6.919677011
2.750631497	-108.1741256	0.024795026	23.03799671
2.965675538	-161.224718	0.023525624	11.18840441
3.679140554	-67.207136	0.01486502	9.620985684
6.464938049	-24.3111367	0.004258245	7.254289061
3.701387986	-52.11180284	0.030693842	5.778643951
3.548556297	-150.5633763	0.033275247	9.979400483
5.027234246	-43.3866448	0.028988985	6.349434347
7.542186283	-12.38650584	0.003870384	7.405621982
2.79806323	-56.55081884	0.024147037	6.578109879
3.624936179	-122.2021118	0.032474408	6.170975228
2.759138298	-80.36360304	0.040749456	8.20837333
10.89593596	-17.26674701	0.007072868	3.608431037
3.679410444	-60.52810568	0.007612121	13.48285547
11.41306807	-38.60881915	0.011475344	10.68831525
3.365395594	-72.80103823	0.009041727	8.872644541
4.668958022	-40.18034021	0.005362904	6.469861766
2.64724098	-68.01495184	0.101725	14.84022017
3.136309266	-49.9218958	0.016268499	6.891741615
3.488596084	-43.36076174	0.00900387	7.19714581
3.361349855	-147.5900407	0.019738022	10.4212881
4.059692782	-50.44162449	0.014980241	9.216651806
6.060601954	-22.51499719	0.010458547	7.061936665
4.020441981	-42.34350645	0.011729202	7.047093153
3.609829815	-85.5634936	0.009332556	17.43551714
3.296939324	-179.3960031	0.014050122	31.85776347
11.27848841	-15.3019457	0.006939652	7.141940269
3.667478244	-90.22939801	0.019777028	7.539768187
19.51245915	-13.54797635	0.000735626	6.245109568
3.19462561	-77.76212465	0.014078948	8.688793833
12.99355274	-10.69658274	0.008573743	4.721983333
3.434690247	-78.35215908	0.008399635	11.04327665
3.093030534	-79.26843531	0.009909871	9.661353329
4.625634024	-33.71268998	0.013053605	4.993969489
4.255334835	-53.30544585	0.006460512	8.163876737
7.083583202	-20.06239246	0.009355369	5.953225248
3.994340355	-53.91355897	0.065730963	7.988547261
2.672448219	-105.6313406	0.033951823	8.759683724
7.106857809	-104.5834219	0.022463667	46.47710647
5.976501611	-32.94145317	0.00975838	6.546899876
6.692615175	-41.43129312	0.00760348	15.28934971
11.52063667	-13.63154767	0.006450117	3.388576729
2.740089047	-14.06997311	0.017365137	9.435996342
3.726764158	-112.6730375	0.021941287	16.72069908
2.844182659	-76.4636755	0.017485877	7.154896562
6.358208195	-23.40770789	0.009318998	6.058669274
3.151801777	-60.98378692	0.008101027	9.362255867
3.297431011	-112.0985757	0.017105299	10.54381005
16.48953601	-11.15946251	0.005383785	3.786749573
4.191414798	-66.02080525	0.012480113	14.75217402
4.640075209	-49.94685105	0.008639746	20.44457965
4.043855931	-50.20973684	0.003326923	23.14540569
7.504493805	-29.57818137	0.00174107	11.38506566
3.149750471	-54.28437433	0.013805571	9.034834814
3.948042869	-64.52038205	0.006503057	29.09969922
3.937525066	-154.9488475	0.025842748	14.48526245
7.778256328	-43.92687787	0.00992456	13.89823832
10.2311585	-14.57042471	0.010620145	4.398631892
4.526193767	-50.17674673	0.012231275	13.47150627
2.015273054	-93.94722047	0.041117734	9.194679826
6.612794088	-26.55891033	0.00482775	8.25784705
2.776348387	-84.98225336	0.0182248	9.542224528
5.675183283	-44.09268389	0.016330275	7.26279538
12.32060543	-24.67666472	0.007976854	9.183116654
7.017961818	-39.43009651	0.025263719	11.18403424
3.459524712	-31.11748334	0.007950474	27.36913474
2.544163722	-161.4109927	0.081091177	14.56702987
3.382016196	-62.17832611	0.009503733	14.79594148
4.737828467	-34.50820791	0.017309027	4.329972107
3.401264027	-70.26133568	0.01151196	12.21728336
3.013187906	-75.95566695	0.036629876	13.60740226
5.534693403	-35.22092616	0.008141808	7.921129184
3.890472205	-48.6528938	0.023420472	4.99517437
3.84330813	-43.39240975	0.017222284	7.345309057
2.65608131	-115.2837643	0.01857164	21.38449308
4.196480575	-62.00585629	0.009272877	10.3893299
5.831378518	-26.62746196	0.008345582	4.885532605
4.326234578	-34.30352897	0.010341685	31.36847992
3.509013077	-63.92856922	0.016176653	13.22344536
2.775448979	-76.68240095	0.015135714	19.8262078
4.088577994	-44.81694531	0.015661577	4.897570727
4.6336159	-33.81884835	0.009084878	9.514760026
6.041093482	-25.50024457	0.012815151	7.486104627
5.285708522	-37.39047478	0.019618872	8.398081705
4.268078344	-92.80783992	0.005372418	19.6837671
6.37050803	-61.87503815	0.019799582	12.47478014
3.277307897	-137.5968701	0.037816937	24.45950586
2.755963314	-115.305172	0.043378906	6.086430139
5.435948663	-23.24262197	0.003972027	10.98522178

log.sigma.2.0.mm.3D_glrlm_LowGrayLevelRunEmphasis	log.sigma.2.0.mm.3D_glrlm_GrayLevelNonUniformityNormalized	log.sigma.2.0.mm.3D_glrlm_RunVariance	log.sigma.2.0.mm.3D_glrlm_GrayLevelNonUniformity
0.011962947	0.084886358	0.122625023	51.11039013
0.03294375	0.091173081	0.100485926	9.475365219
0.012437847	0.073648699	0.205935596	31.27329109
0.037418047	0.096718899	0.067718467	8.340413988
0.008918818	0.095362211	0.244301427	61.08676065
0.02077735	0.064507634	0.02385434	6.485373014
0.006155708	0.237840863	1.551145474	2369.934575
0.005725819	0.124105805	0.602402288	477.2383861
0.006436532	0.163641674	0.762255255	493.7234101
0.02848051	0.074524497	0.079923101	25.25009729
0.002045185	0.206143431	0.781039527	796.5541186
0.004908468	0.167254679	0.756713125	366.6080416
0.025337552	0.06151751	0.067331631	19.17346753
0.024128297	0.089015795	0.084365188	15.77851199
0.015753978	0.110434656	0.389525371	82.86595654
0.005181965	0.196647066	1.038512688	1130.173804
0.033030867	0.120355307	0.242102716	44.11737091
0.034021571	0.099307062	0.061657458	10.39000606
0.031107549	0.123938943	0.259140505	59.3662244
0.004621927	0.148837456	0.780861635	1281.968992
0.02655914	0.112588502	0.549166765	127.5244196
0.03386097	0.122371439	0.113085885	17.57002183
0.043274184	0.109570615	0.216198886	26.40989337
0.008868184	0.228159166	1.101175543	715.5569461
0.008219005	0.116345734	0.566607947	65.5706854
0.012687406	0.116284206	0.406058869	188.4971995
0.009648533	0.098121416	0.207799336	80.04474839
0.006017608	0.130240106	0.476790933	179.4393142
0.101991602	0.09578318	0.016410956	2.639147178
0.017738577	0.115866716	0.339039816	109.1163004
0.01028467	0.130850679	0.778141283	335.0094966
0.020501242	0.093626004	0.087313436	22.09082203
0.016093674	0.110001309	0.290122998	117.544718
0.011987833	0.174267002	0.743631617	392.6555077
0.012966199	0.124139746	0.486340221	338.7745031
0.00985227	0.078710097	0.294501846	75.35815717
0.014359545	0.055923856	0.041715296	20.99270536
0.008470921	0.205158303	1.305792307	1531.39818
0.020591891	0.111820309	0.125669658	19.9185262
0.00091307	0.203225006	0.991174157	1662.20396
0.014951823	0.099400871	0.193759219	54.82088629
0.010266684	0.197349873	0.763559568	1023.180523
0.008993845	0.088883161	0.226779111	117.7639883
0.010488924	0.093954633	0.186060245	58.08635022
0.014836945	0.159631128	0.665767404	126.938199
0.007161864	0.114751775	0.472938187	178.1055129
0.011073014	0.180148059	1.061162803	836.6770554
0.070128823	0.107987509	0.171752239	20.72198106
0.034968745	0.098194112	0.079406191	10.40502774
0.023271576	0.061325985	0.060658681	12.77408533
0.011103682	0.177446578	0.769735489	238.4615384
0.008190795	0.090197992	0.268138059	165.5179355
0.008366781	0.250311162	1.393998234	1563.097659
0.018450037	0.095708885	0.204793236	70.79003778
0.022746001	0.075106007	0.082818961	19.97222108
0.018676579	0.10849479	0.218663626	61.9016958
0.010782281	0.167969029	0.747867182	640.4056304
0.008863236	0.107009988	0.525523911	131.4741444
0.017730169	0.091779452	0.116356269	22.75525989
0.006813175	0.240069452	0.97241851	3747.838598
0.01323743	0.082741203	0.308937768	55.91148346
0.009124838	0.072653097	0.23457204	100.822847
0.003557643	0.067534019	0.20241623	272.4237706
0.002040809	0.129320876	0.844025745	796.6783637
0.014512471	0.099726068	0.18646675	28.12409553
0.006802867	0.058343722	0.110250995	117.3000439
0.026584256	0.08703734	0.057065879	9.823606963
0.010804053	0.102278735	0.478678571	334.9463924
0.013159141	0.197586532	1.0461998	1305.868062
0.013064075	0.086021459	0.249263446	112.7963668
0.042864908	0.09729868	0.155443624	17.15900351
0.005740903	0.168583837	1.087560936	802.2679714
0.019292441	0.095530031	0.283727504	60.30750792
0.017873087	0.127930037	0.416880681	109.4490585
0.009042929	0.141593949	0.664087961	343.1886514
0.026783036	0.09529472	0.247133321	49.44039127
0.008326627	0.061904989	0.086719499	85.20092085
0.083560028	0.077811757	0.048667835	8.52460687
0.009959295	0.07646868	0.112154443	45.46603001
0.019445639	0.167654428	0.602929961	198.9386885
0.011962947	0.084886358	0.122625023	51.11039013
0.038243276	0.083456711	0.115964618	27.98210044
0.009310062	0.182383058	0.826148772	436.1398571
0.025592295	0.138745512	0.404374793	129.2790805
0.018904591	0.126701774	0.588871358	175.998712
0.018978439	0.064470095	0.054960478	10.79624094
0.00993303	0.097022914	0.210237079	60.66335241
0.010208945	0.193270762	1.19762715	507.7694161
0.010825136	0.064003887	0.166242772	67.323281
0.017080193	0.082687438	0.192732446	58.09063824
0.015579896	0.065258243	0.058323171	17.11808352
0.017426999	0.142903886	0.608364949	155.8478178
0.009922957	0.107500373	0.342865767	304.1342689
0.014518343	0.138220349	0.678659827	394.0910471
0.021065038	0.108908617	0.220950968	193.9848075
0.005614673	0.06813154	0.136718911	73.10545034
0.0209148	0.090968252	0.265794181	113.9278122
0.038357577	0.063960942	0.036240488	5.374338056
0.045407766	0.120918085	0.079345039	7.982626622
0.004595465	0.119421204	0.716486165	853.0824228

log.sigma.2.0.mm.3D_glrlm_LongRunEmphasis	log.sigma.2.0.mm.3D_glrlm_ShortRunHighGrayLevelEmphasis	log.sigma.2.0.mm.3D_glrlm_RunLengthNonUniformity	log.sigma.2.0.mm.3D_glrlm_ShortRunEmphasis
1.348115661	156.4757011	498.8197665	0.927588563
1.293350698	79.7602037	88.78758007	0.937464259
1.508568437	155.8718738	343.2151097	0.91642337
1.208573996	73.6464743	75.96844041	0.950887571
1.604746899	157.2733562	496.1120249	0.900567634
1.0744748	182.5153394	95.80624397	0.9813813
4.293358235	139.0618902	4840.959358	0.723241577
2.338578868	195.877185	2539.931276	0.8383897
2.747427709	150.3723145	1789.531618	0.797071029
1.236799567	102.62824	295.2156394	0.94687234
2.805516416	479.0618288	2246.556989	0.789139673
2.745800002	206.5726175	1294.012496	0.79507781
1.197489454	143.1169805	277.7701425	0.955687882
1.239145978	105.9035321	155.7524967	0.949420323
1.9046405	117.4800813	543.8952123	0.874388149
3.278480401	179.086228	3173.120385	0.77007841
1.666962624	50.68468375	268.5789161	0.878481811
1.191893198	77.74409327	93.21558021	0.954663553
1.670286899	57.67110444	358.6559459	0.88705564
2.711405633	210.408758	5263.999457	0.809384996
2.203370424	57.71375131	779.9289751	0.854376214
1.329722764	58.75733179	120.0099109	0.929771491
1.584804273	64.81971162	184.1331055	0.894449753
3.497363252	101.0216001	1622.459354	0.745020786
2.261086671	185.4790416	384.0267376	0.849235132
1.974720126	108.5189872	1126.576683	0.858688141
1.540390372	145.401441	640.5648842	0.905052821
2.12317506	182.8488806	926.5597252	0.84565734
1.052596976	74.29999061	26.6741179	0.986850756
1.844968695	79.43791957	674.9201445	0.869423243
2.68305723	105.7247803	1590.255789	0.815176615
1.258902817	96.37924638	203.6406366	0.942637316
1.733070789	99.43669742	788.2354406	0.88133004
2.713169385	105.0409976	1334.311123	0.796975483
2.119382527	97.80084197	1864.785996	0.851947088
1.690702332	207.163679	734.6735797	0.896747578
1.12250067	207.5913991	348.6451349	0.971674617
3.825268292	118.8891498	3818.068166	0.741908977
1.353780363	96.80186816	147.85646	0.92765979
3.216546318	987.0734833	4491.655233	0.768585949
1.508832284	101.9054839	435.2580811	0.9078638
2.736059146	101.0166652	3081.701811	0.798304088
1.578270235	166.1012965	1041.534065	0.902920213
1.497655755	139.5158951	487.2545127	0.907709582
2.55736457	80.47176237	488.5254752	0.810613511
2.089925445	172.1186747	1074.458049	0.856137259
3.339863774	102.1253149	2526.691852	0.765457966
1.480961869	43.19561096	151.1989804	0.906812462
1.241336719	72.58738839	91.76341427	0.94440592
1.179694087	172.4467246	187.5691523	0.959043168
2.813175583	110.1710682	775.087109	0.786748486
1.656309893	183.8946951	1398.219495	0.894611302
4.059226047	100.6018939	3035.209961	0.722648947
1.534432772	95.75022243	586.0804001	0.906639821
1.249641633	109.3861804	229.2874435	0.942990095
1.572998578	82.7198402	438.9431084	0.898336746
2.696590482	106.2281431	2302.33951	0.803493237
2.135955546	141.5059911	867.5569682	0.863775176
1.32514623	119.8207837	209.219628	0.933685263
3.258484934	126.7596834	8233.798377	0.753209371
1.705579958	134.9768347	522.7450192	0.899696222
1.572751634	206.6363674	1091.820228	0.906955021
1.490963411	368.9301732	3274.056018	0.918810111
2.777412793	471.7060581	3830.310074	0.815805308
1.49488833	121.6658615	223.7604056	0.90907098
1.292666159	268.4289147	1729.723274	0.942213604
1.170312478	98.03719036	101.7985151	0.96051639
2.080037744	144.6948871	2286.541153	0.85995873
3.371998903	73.81354989	3484.922023	0.753026568
1.609044112	129.2645571	1016.108862	0.901146091
1.429782086	59.94684045	143.7445974	0.918077902
3.342542302	173.8470502	2640.005693	0.772299402
1.700319889	90.18815319	476.0367429	0.889484197
1.998728082	80.61219768	592.2908037	0.857123715
2.441598366	133.3869054	1577.908551	0.833302634
1.618740556	81.18947687	400.8542895	0.89873103
1.246964617	221.3899096	1196.237774	0.946440559
1.153995046	62.94264093	99.50054813	0.962315239
1.312808338	172.8034364	502.1783926	0.935151228
2.428710351	65.02986894	738.6684191	0.815791306
1.348115661	156.4757011	498.8197665	0.927588563
1.329563422	79.70640321	280.3173043	0.930961676
2.902258896	125.6944129	1375.588095	0.785096061
2.006565185	61.36127997	634.3851882	0.849909522
2.302259613	80.12332895	931.5998643	0.84394243
1.161520746	159.2857996	152.1964758	0.963081271
1.562707838	157.0939353	481.8771487	0.898054332
3.621845006	91.65072334	1368.556288	0.749147081
1.418986582	215.5583366	866.2003152	0.924823443
1.493429736	119.7789317	561.6225103	0.913203738
1.166154232	172.1301448	238.2852901	0.963242815
2.364842598	76.16146642	721.0534443	0.835670303
1.812129407	135.0658974	2074.496366	0.879527327
2.521105172	91.47300678	1786.611579	0.818889206
1.570551945	80.61598751	1375.780515	0.900096622
1.35637794	276.1814212	901.6336746	0.932760065
1.640252541	98.30387024	967.5399138	0.899603438
1.110869944	121.8119236	78.45297081	0.973324832
1.242385492	52.51789387	57.43420136	0.944833036
2.533321792	231.1430674	4610.656815	0.830169303

log.sigma.2.0.mm.3D_glrlm_LongRunHighGrayLevelEmphasis	log.sigma.2.0.mm.3D_glrlm_RunPercentage	log.sigma.2.0.mm.3D_glrlm_LongRunLowGrayLevelEmphasis	log.sigma.2.0.mm.3D_glrlm_RunEntropy
237.2915131	0.904019404	0.014100516	4.251839465
106.8894215	0.918992512	0.03793674	3.938764524
279.7950551	0.878802357	0.015490345	4.449362157
92.07904853	0.93729097	0.041364461	3.867625779
310.5365961	0.858630645	0.012015203	4.176071605
198.128664	0.976101568	0.021704771	4.215649012
881.4554893	0.609247186	0.022252108	4.153457282
621.2334308	0.761257965	0.010645555	4.322383509
593.9063531	0.712770795	0.014349101	4.074833679
131.7226889	0.9306847	0.032575846	4.278637804
1695.188615	0.706414011	0.005052983	3.919306806
782.8647733	0.712408321	0.010952747	4.169415306
173.6379126	0.941436207	0.027765737	4.496122798
139.6338582	0.932793522	0.026929195	3.987029044
309.0919528	0.814799331	0.021491792	4.153034396
838.2768846	0.67260557	0.014067266	4.166347317
105.9528656	0.840508116	0.044958151	3.911401515
97.08279777	0.942480942	0.037134136	3.842481923
111.9182536	0.844254511	0.042996896	3.937306252
715.8664227	0.721491638	0.011210754	4.330058438
176.885391	0.779667302	0.043270014	4.164542944
85.97891569	0.908471276	0.040262556	3.677290306
127.6158988	0.857377498	0.05524083	3.992429325
511.250934	0.65158842	0.025986249	3.923006443
582.3201273	0.773786819	0.012977679	4.344658267
262.533028	0.80022792	0.020551944	4.256229665
267.8380519	0.868763824	0.012964613	4.126699301
505.82475	0.782437574	0.010599739	4.120607447
77.91685451	0.983516484	0.103058011	3.625020559
187.7549598	0.817750451	0.026580424	4.095846941
424.6372176	0.727997721	0.020934083	4.241467458
128.8657203	0.925188537	0.023912622	3.994255511
214.445268	0.834786793	0.022523107	4.192157215
394.8151501	0.714830105	0.024198691	4.137820819
277.4595022	0.784318931	0.021581902	4.168769558
436.7486539	0.848199673	0.012939289	4.576936753
229.1409909	0.962327416	0.015696342	4.608385459
682.2614242	0.634395848	0.024445027	4.251909301
144.1729044	0.903943772	0.024504387	3.835062123
4199.968695	0.673950039	0.002605212	4.133934925
181.9159149	0.873948555	0.019559567	4.098109861
345.8992519	0.715383552	0.024400396	3.960966917
310.0150448	0.867361846	0.01230056	4.321239991
253.9983731	0.874333587	0.013446945	4.155379293
288.8334957	0.730062217	0.028628192	3.995074355
475.3146397	0.792012591	0.011999266	4.211944462
490.0446304	0.66528693	0.027274468	4.256644964
72.16542621	0.876009835	0.090222609	3.937998633
96.3934579	0.929149798	0.039282071	3.823557419
197.8366125	0.946503497	0.026835558	4.548597213
456.601943	0.703365889	0.022103901	4.053980837
342.1341741	0.849716749	0.011636461	4.4937594
601.5597169	0.616983689	0.029074718	3.946206899
167.6889943	0.87221869	0.024346743	4.156744483
145.4631547	0.926561244	0.026274815	4.2940206
158.2758742	0.860540666	0.025023287	4.011297063
386.4240833	0.720286334	0.022857464	4.137143323
418.236099	0.793328354	0.014568032	4.252644201
180.1368185	0.9114819	0.020626327	4.04122613
573.8528194	0.663544553	0.019680112	3.865294745
273.1699824	0.847505067	0.017938726	4.467824272
364.0507657	0.865348625	0.011893734	4.687780627
600.9974548	0.882024362	0.004886976	4.757325271
1722.371386	0.721776815	0.004854852	4.53005836
210.6039632	0.877785766	0.018217353	4.072640294
360.7232943	0.920049287	0.008235987	4.794339812
114.2241091	0.948287007	0.029880758	3.962687364
377.6938374	0.79259839	0.017022878	4.517293456
353.5581785	0.658731072	0.036313001	4.110985548
236.5947127	0.858495794	0.017799518	4.427491357
104.5811527	0.89005439	0.051118831	3.94089089
864.0082081	0.669097489	0.014706535	4.33018133
209.2511791	0.842662011	0.025365383	4.167757867
215.9618461	0.796903004	0.027571488	4.06860713
409.4018011	0.7525354	0.017805321	4.27354729
146.5755836	0.858252674	0.035278531	4.239080772
281.4212684	0.928682653	0.010053376	4.670389683
71.81819896	0.952508361	0.093486306	4.027838403
238.4314922	0.913269526	0.012096667	4.347254422
225.7696392	0.742963177	0.034918521	3.877411024
237.2915131	0.904019404	0.014100516	4.251839465
111.293927	0.908692933	0.04574212	4.205892263
549.1124114	0.69838412	0.019243927	4.10679954
165.6076685	0.792602291	0.038800559	3.93824456
249.3041312	0.768207969	0.031508017	4.193902703
185.4274645	0.951486014	0.020739105	4.356195053
286.5280854	0.862281167	0.013430927	4.242670269
511.6430906	0.645048999	0.028761647	4.098983442
319.3653719	0.894664484	0.013364812	4.816319825
201.5815395	0.877980769	0.021712299	4.370913036
198.560981	0.95039019	0.017687929	4.358479648
249.841673	0.761646046	0.030778337	3.949403742
288.7399962	0.826372342	0.015248338	4.301300653
301.8540319	0.738992455	0.027824946	4.300175151
138.8596083	0.86163522	0.028862745	4.13020643
416.0659674	0.906185031	0.006840643	4.56949678
189.4187079	0.854555001	0.027442806	4.363604724
135.2873648	0.965517241	0.040571607	4.274497033
64.07651109	0.929577465	0.054087713	3.500869869
729.4759905	0.743151892	0.010013213	4.538240703

log.sigma.2.0.mm.3D_glrlm_HighGrayLevelRunEmphasis	log.sigma.2.0.mm.3D_glrlm_RunLengthNonUniformityNormalized	log.sigma.2.0.mm.3D_glszm_GrayLevelVariance
170.3077565	0.827770615	19.85133787
84.55031907	0.851766438	18.46831597
172.548457	0.806734022	25.89
77.08711126	0.879676406	16.8275
177.8816876	0.773495473	18.30358025
185.6380043	0.95229132	23.54930606
195.8002703	0.484828187	22.1128285
240.7988194	0.659530808	20.02997544
195.6912731	0.591598859	12.17771136
107.9115885	0.870335383	23.13626635
606.1818938	0.579546214	18.24504293
266.1691529	0.588773262	20.82597796
148.7797731	0.890532968	31.60099896
111.732861	0.876933645	16.38581347
140.2065516	0.724034783	13.13472656
238.9508855	0.550788222	18.63233292
58.87723031	0.730555631	13.49359375
81.42405594	0.889095525	16.02634298
65.28585861	0.74721266	15.04982987
260.6833385	0.610644377	21.30996083
69.68330646	0.687654745	13.05658669
63.58759807	0.833994037	10.33735943
74.0646201	0.761947711	11.73575603
138.632251	0.515359455	14.82626831
226.8898401	0.680207905	22.06676379
127.4622882	0.694019081	33.04730473
163.1163212	0.782952485	14.62045789
221.0361634	0.671370256	14.90061944
75.02336339	0.967178089	18.1177686
93.4588766	0.714706508	14.24346246
136.0551797	0.620388618	13.61001625
102.2928511	0.861594093	16.76984877
115.0332234	0.736390489	18.93603171
135.4090209	0.591260329	17.8797124
118.0723751	0.6826368	15.78175889
235.8225492	0.76654248	26.80790057
211.7021754	0.928432723	39.25619835
165.3081227	0.510367361	22.88156082
104.8393937	0.82878682	15.41940858
1289.978839	0.548181778	26.30442734
113.8760852	0.787746111	16.43649296
126.2654532	0.59330117	16.86329533
185.9475545	0.780744801	19.80297828
156.8741729	0.787150261	15.17185596
102.5724851	0.613213209	11.22149059
206.1281727	0.690723851	18.03501519
136.9091763	0.543438596	19.62608123
47.68238321	0.786177849	14.81871345
77.05342724	0.864822156	11.6996
177.3205557	0.899312474	54.26888469
146.2428645	0.575734758	12.50059764
205.1706725	0.761393122	33.8812673
141.9987616	0.484592523	16.08872913
106.3917513	0.788372201	15.94206288
116.1196445	0.861293304	24.53935185
93.72735413	0.768391338	14.65881332
134.950104	0.601999388	16.67069195
169.5529022	0.705121398	16.24379772
129.7889789	0.842079375	15.08199493
170.5404059	0.526331071	15.41287822
151.2058786	0.773036479	28.95276042
227.9330588	0.786394931	40.369865
401.1152975	0.810948456	38.24712787
587.2136287	0.621571838	30.67285369
135.2560065	0.791180812	18.06127305
283.3718908	0.860033489	43.58108326
101.0915807	0.901515525	21.56639373
170.5603212	0.697337573	34.55403286
99.56971607	0.526032096	18.70781893
143.6926206	0.774362624	25.85002583
66.78781905	0.811582069	13.26578877
233.7658699	0.553884377	19.95818472
105.6635002	0.752342396	13.88639239
96.94576309	0.691477844	16.23166591
161.5404999	0.650514477	25.79063416
90.0543529	0.770519571	22.94795556
232.1917591	0.868839422	36.69157025
64.67607569	0.906683167	18.36
183.9628533	0.844047661	24.09656694
82.92011733	0.621112626	9.395461752
170.3077565	0.827770615	19.85133787
85.01600495	0.835084952	22.235136
168.178012	0.573538008	14.01965416
74.45849731	0.679069778	10.4059076
97.54080903	0.669426042	16.09395973
164.0647842	0.907910314	27.39322465
176.6207664	0.768603104	19.55949079
127.7891287	0.52025151	13.7954677
231.2786323	0.822880251	50.31649114
131.4356867	0.798713373	25.3576443
176.9958056	0.908086099	23.63995775
94.17884877	0.656937751	10.7532526
154.3666526	0.732604023	21.52598377
113.3727582	0.625990846	20.80590861
89.11822642	0.77174824	18.98088843
297.7916076	0.839695275	32.50224415
110.1826823	0.771755714	26.79991907
124.4394696	0.93287298	30.51702479
54.65957511	0.867570778	7.704474506
280.3045682	0.645252999	26.17909609

log.sigma.2.0.mm.3D_glszm_SmallAreaHighGrayLevelEmphasis	log.sigma.2.0.mm.3D_glszm_GrayLevelNonUniformityNormalized	log.sigma.2.0.mm.3D_glszm_SizeZoneNonUniformityNormalized
104.1693702	0.064535147	0.406031746
75.12754454	0.081597222	0.418402778
127.3443492	0.05578125	0.391640625
60.79268538	0.08125	0.44875
126.5627363	0.073950617	0.442716049
146.394985	0.062819576	0.549671293
154.6671469	0.064132781	0.447965263
135.6963782	0.070458273	0.380085744
99.88626578	0.091528807	0.349646414
78.11158606	0.061212842	0.380927467
432.8042336	0.08335768	0.44047816
167.7703059	0.069972452	0.413296602
128.1081172	0.05157128	0.432174818
66.24909853	0.079001689	0.368737099
58.03018645	0.087235519	0.329460053
150.1885442	0.070083902	0.38161095
41.76130337	0.0975	0.360625
53.10732263	0.080578512	0.382231405
57.17021797	0.077353497	0.451493384
196.3272432	0.0649671	0.400540805
52.02972799	0.079185152	0.336894522
38.72726285	0.099541858	0.299458559
48.97086347	0.081811541	0.381300219
88.30764208	0.083251939	0.369077851
125.5434166	0.065223725	0.351425598
122.4533852	0.067389907	0.418226971
97.80654218	0.08394595	0.389810731
129.9698135	0.078390886	0.426272695
72.24747475	0.082644628	0.685950413
56.47924993	0.079752604	0.328613281
73.17590564	0.084127799	0.304293986
67.67890099	0.081758034	0.337901701
83.61291731	0.067134408	0.415635567
84.84652213	0.076603157	0.381695966
85.38870595	0.072699154	0.447344613
175.7086387	0.055204856	0.450308148
201.9680767	0.045283792	0.570179633
107.568552	0.069822612	0.383721032
66.99595645	0.08121616	0.381091212
960.0861036	0.063550204	0.468646446
83.73855622	0.071418203	0.421686747
116.6373587	0.082630353	0.484674663
124.1912785	0.063577794	0.376547973
88.29033102	0.078116343	0.362880886
60.63766519	0.092710215	0.383019638
144.0792925	0.069302511	0.462951641
86.6808463	0.068103241	0.371759476
44.30170092	0.088334872	0.380116959
46.08870768	0.0976	0.3424
154.9674112	0.049376181	0.414139887
71.33666554	0.086981764	0.38730278
193.5266845	0.052334216	0.462182231
105.6666306	0.077905344	0.44918872
55.5611986	0.072872465	0.32353236
91.15243281	0.062757202	0.369513032
67.05688146	0.082274165	0.383718767
91.88119538	0.078189808	0.428127826
95.10615968	0.077620792	0.340793517
66.36805912	0.076923077	0.343074508
123.3556715	0.121001244	0.438204587
136.6437222	0.054340278	0.477465278
222.3676988	0.045668618	0.535547814
335.0486233	0.045011128	0.48666587
428.4946117	0.052993587	0.465952264
90.142478	0.06989887	0.422070196
248.860679	0.04376702	0.481414181
107.7585799	0.080811677	0.458882164
158.8525262	0.052786101	0.498167568
94.28024772	0.07499045	0.462969549
136.9591548	0.057433571	0.4461827
46.56753128	0.078204784	0.336737436
129.8688555	0.067824688	0.377813986
56.53297438	0.085940973	0.317943983
64.67271516	0.080296514	0.362267995
128.8354032	0.061737061	0.441627502
84.10096884	0.064	0.412977778
187.6084441	0.047133884	0.413031405
58.69527797	0.068888889	0.385555556
159.5658546	0.059680328	0.447698745
39.49273858	0.100938037	0.34892324
104.1693702	0.064535147	0.406031746
83.33906999	0.06112	0.459712
71.06764647	0.079025106	0.326880613
37.45667175	0.103542732	0.349068663
68.72939875	0.075154272	0.371334625
145.2962846	0.060469418	0.496820442
144.4969219	0.066590932	0.431721741
78.87889823	0.081541314	0.371948843
196.8260575	0.042155801	0.432563151
129.6807373	0.058454859	0.465975736
158.1253473	0.060610633	0.483023993
53.2131206	0.100622837	0.396193772
126.1690483	0.063472216	0.457458481
94.29996764	0.064177878	0.441324633
82.28966641	0.068719691	0.423007308
244.7290794	0.051695058	0.488474729
101.0292196	0.060353798	0.432448838
104.0406111	0.058512397	0.511404959
49.23931778	0.117585848	0.363163371
213.8562998	0.056251979	0.420854416

log.sigma.2.0.mm.3D_glszm_SizeZoneNonUniformity	log.sigma.2.0.mm.3D_glszm_GrayLevelNonUniformity	log.sigma.2.0.mm.3D_glszm_LargeAreaEmphasis	log.sigma.2.0.mm.3D_glszm_ZoneVariance
85.26666667	13.55238095	64.66666667	54.60870748
20.08333333	3.916666667	12.27083333	6.728732639
62.6625	8.925	50.44375	41.33089844
17.95	3.25	10.85	5.56
79.68888889	13.31111111	158.2222222	141.0458025
40.67567568	4.648648649	2.445945946	0.508582907
497.2414414	71.18738739	71141.69369	70925.89495
294.5664516	54.60516129	4437.659355	4395.266654
151.7465438	39.7235023	7861.702765	7766.752559
55.23448276	8.875862069	16.95172414	10.64989298
234.7748593	44.42964353	11624.46154	11519.37019
136.3878788	23.09090909	5586.984848	5500.269247
66.98709677	7.993548387	11.8516129	7.29132154
26.91780822	5.767123288	17.36986301	10.59560893
61.27956989	16.22580645	447.5483871	423.083131
319.7899761	58.73031026	21396.45465	21292.91553
28.85	7.8	180.15	150.4475
16.81818182	3.545454545	14.25	7.885847107
51.92173913	8.895652174	256.2869565	231.9777694
589.9966056	95.69653768	16376.6334	16311.07068
79.17021277	18.60851064	753.4212766	715.2446537
14.67346939	4.87755102	25.14285714	14.7455227
28.21621622	6.054054054	76.82432432	62.40485756
108.8779661	24.55932203	21700.66441	21435.47146
54.47096774	10.10967742	475.3806452	453.4419979
126.7227723	20.41914191	1378.848185	1334.183446
80.69082126	17.37681159	221.4347826	200.8573829
112.9622642	20.77358491	1432.464151	1388.404614
15.09090909	1.818181818	2	0.380165289
63.09375	15.3125	624.4322917	588.4947645
114.7188329	31.71618037	4515.541114	4428.809194
31.08695652	7.52173913	24.57608696	16.89354915
116.7935943	18.86476868	420.8078292	400.0907537
137.7922438	27.65373961	5688.916898	5612.826313
255.8811189	41.58391608	1456.928322	1419.978077
147.2507645	18.05198777	204.3180428	192.4187077
137.9834711	10.95867769	5.330578512	2.733419848
389.4768473	70.86995074	36173.91133	36040.17293
18.67346939	3.979591837	63.65306122	47.48937943
550.6595745	74.67148936	31320.80851	31214.5168
70	11.85542169	101.5240964	87.07493831
429.9064262	73.29312289	13032.95039	12966.38172
121.2484472	20.47204969	224.6863354	202.1975329
68.94736842	14.84210526	125.8157895	111.9695568
50.17557252	12.14503817	1624.931298	1555.952567
148.6074766	22.24610592	1195.454829	1158.362555
208.9288256	38.27402135	19577.60676	19423.83794
21.66666667	5.035087719	55.77192982	41.01015697
17.12	4.88	11.12	5.9216
47.62608696	5.67826087	7.217391304	3.557655955
93.72727273	21.04958678	3302.181818	3240.149921
265.7547826	30.09217391	410.8608696	396.7624771
264.5721562	45.88624788	53478.52971	53184.36936
55.97109827	12.60693642	231.9017341	207.9313709
39.90740741	6.777777778	19.10185185	12.04003772
48.73228346	10.4488189	227.2047244	199.9513919
272.7174254	49.80690738	8432.858713	8363.971319
93.03663004	21.19047619	771.7838828	739.7557461
31.21978022	7	31.84615385	22.91196715
821.6336	226.8773333	83470.94027	83313.68198
114.5916667	13.04166667	138.3458333	127.3178993
266.7028112	22.74297189	232.9497992	222.5886397
780.1253899	72.15283843	323.7567062	315.6219339
640.6843636	72.86618182	7345.712	7307.317078
34.6097561	5.731707317	86.76829268	71.44393218
425.5701357	38.69004525	70.72285068	64.6134536
24.32075472	4.283018868	12.20754717	7.166251335
444.3654709	47.08520179	1794.991031	1773.574474
337.5048011	54.66803841	32581.82853	32392.79432
188.2890995	24.23696682	250.7606635	237.6672413
20.54098361	4.770491803	33.67213115	23.1362537
261.0694645	46.86685962	15704.52243	15598.97729
54.36842105	14.69590643	183.6900585	164.5046339
68.10638298	15.09574468	645.4521277	612.8771786
226.1132813	31.609375	3062.488281	3023.03418
61.94666667	9.6	140.5733333	124.3592889
227.1672727	25.92363636	42.84	35.57942479
23.13333333	4.133333333	6.15	2.476388889
107	14.26359833	43.76150628	36.34215087
60.71264368	17.56321839	2581.482759	2497.349584
85.26666667	13.55238095	64.66666667	54.60870748
57.464	7.64	35.736	27.021696
134.3479319	32.47931873	6385.318735	6316.157861
51.66216216	15.32432432	1104.290541	1041.259816
110.6577181	22.39597315	1295.587248	1258.89943
46.20430108	5.623655914	9.225806452	5.644351948
74.68786127	11.52023121	176.8843931	159.3219954
108.2371134	23.72852234	14324.90034	14129.66935
177.783455	17.32603406	100.1386861	91.96548683
111.3682008	13.9707113	89.70711297	78.50282733
68.10638298	8.546099291	9.035460993	5.203862985
67.35294118	17.10588235	1568.105882	1497.348097
354.5303226	49.19096774	1189.187097	1169.690599
224.1929134	32.6023622	4335.15748	4277.600766
204.7355372	33.26033058	511.6219008	493.3833541
225.6753247	23.88311688	53.44588745	46.87809449
160.8709677	22.4516129	279.5994624	264.0902633
28.12727273	3.218181818	4.345454545	1.843305785
11.25806452	3.64516129	11.12903226	5.883454735
634.2276045	84.77173192	8403.069675	8362.455485

log.sigma.2.0.mm.3D_glszm_ZonePercentage	log.sigma.2.0.mm.3D_glszm_LargeAreaLowGrayLevelEmphasis	log.sigma.2.0.mm.3D_glszm_LargeAreaHighGrayLevelEmphasis	log.sigma.2.0.mm.3D_glszm_HighGrayLevelZoneEmphasis
0.315315315	0.37442956	12757.70476	154.1904762
0.424778761	0.232809382	983.25	97.60416667
0.33126294	0.278943571	11093.88125	178.4125
0.434782609	0.235520478	800.125	83.25
0.241286863	0.803779979	33917.56667	173.4444444
0.718446602	0.040514062	438.4324324	189.0540541
0.068073102	342.6427882	14848592.86	203.9495495
0.153587	15.35880782	1289900.228	198.8645161
0.102624734	33.88294306	1834377.189	153.9193548
0.398351648	0.2819546	1609.462069	116.3586207
0.097547584	19.15856028	7068892.199	623.9774859
0.107386918	18.71376779	1677903.382	239.5969697
0.468277946	0.171972962	1404.993548	172.3677419
0.384210526	0.213364514	2003.794521	105.9863014
0.202173913	2.42309696	86172.13978	98.34946237
0.098276064	80.48840956	5711451.113	216.0381862
0.183486239	3.029236498	12515.9375	60.5875
0.396396396	0.260923103	1121.909091	84.84090909
0.202821869	4.088280898	18115.88696	74.13913043
0.1235013	61.59386302	4377295.191	281.6951799
0.16184573	9.256035181	67416.36596	75.70212766
0.310126582	0.509107943	1641.265306	62.53061224
0.263345196	1.121345053	7519.540541	67.58108108
0.061407161	145.3321021	3264729.441	125.1118644
0.213498623	1.673369507	137882.7806	193.7612903
0.14962963	9.923182564	198157.9208	161.4917492
0.220447284	1.222934967	43394.343	145.0434783
0.150653781	5.647112383	368072.9208	188.5433962
0.785714286	0.141998871	137.0454545	79.95454545
0.166811468	5.665020088	72430.15104	88.72395833
0.107376816	25.90988355	799019.5703	113.4509284
0.360784314	0.289145747	2624.554348	106.1956522
0.219702893	3.150911939	59462.09964	114.2241993
0.114639568	37.32742572	878826.8781	122.1911357
0.164509635	10.32906927	212403.7448	113.1765734
0.289893617	0.708985368	63799.62997	231.6391437
0.620512821	0.061767233	802.8305785	242.9669421
0.08647129	197.3152613	6671649.979	148.4098522
0.248730964	0.653758853	7161.530612	101.2653061
0.096995212	23.78857764	41272559.63	1314.65617
0.263074485	0.799792474	14393.46386	114.5180723
0.122564599	103.2849138	1660458.479	149.048478
0.210870989	1.091474273	51531.20497	181.2981366
0.26874116	0.657584022	25967.28947	138.0315789
0.120404412	13.67536142	196733.4733	88.41984733
0.164194373	4.813636623	303592.8879	190.5358255
0.080642847	129.7202982	2982477.502	129.1672598
0.260273973	2.010491896	2888.368421	59.26315789
0.438596491	0.216183865	880.28	76.02
0.522727273	0.159103797	784.6869565	213.6869565
0.126967471	19.12369137	574606.7686	105.5991736
0.266327003	1.950041824	88921.90957	244.3078261
0.058305286	355.304478	8103228.772	138.286927
0.204250295	2.32744273	26117.00578	97.15028902
0.37630662	0.264382376	2119.712963	129.6018519
0.191553544	2.297572742	25620.30709	93.82677165
0.120484207	56.20263965	1277451.133	127.0361068
0.176699029	3.491041586	175749.7839	146.6886447
0.334558824	0.260774526	5027.43956	113.5494505
0.079743121	465.6340921	15050713.7	169.7024
0.301129235	0.865089172	24112.44167	172.0541667
0.310667498	0.972085158	58495.51606	267.1807229
0.350612423	0.756852392	140913.9008	439.1709295
0.161384977	11.40165923	4743907.518	578.4370909
0.255451713	0.622415547	13386.58537	131.1463415
0.404576659	0.284696163	19171.0871	324.4468326
0.445378151	0.235603633	956.3773585	130.4339623
0.216085271	9.366251176	350399.8767	192.2085202
0.072732715	306.544945	3514821.425	121.6927298
0.276358874	1.736714138	39035.6327	177.4549763
0.308080808	0.665136977	2878.557377	69.8852459
0.097337653	57.89137235	4283122.378	193.617945
0.228304406	1.308558231	29051.36842	86.68421053
0.175209692	5.434330374	80128.79787	91.40425532
0.15920398	17.57429891	538192.3789	175.375
0.248344371	1.754781004	12800.94	111.4333333
0.371120108	0.29119841	7400.816364	272.1745455
0.52173913	0.461033065	278.1166667	79.2
0.367127496	0.281613441	7622.548117	212.0962343
0.109022556	26.16368343	260657.7414	60.59195402
0.315315315	0.37442956	12757.70476	154.1904762
0.338753388	0.678262156	2860.848	106.728
0.120245758	31.57693344	1298818.105	117.3065693
0.125957447	12.50736547	101917.7297	56.88513514
0.165096953	11.31093796	152201.6275	97.09395973
0.528409091	0.106760475	1213.268817	187.0215054
0.23862069	0.960543188	35965.11561	187.6589595
0.07156911	97.18935646	2127730.931	111.6082474
0.349787234	0.522680257	21030.3674	270.2360097
0.29875	0.746979788	12711.60251	161.0460251
0.510869565	0.114696131	1159.985816	206.177305
0.118881119	14.12338997	180009.1	77.51176471
0.226475745	7.233801885	200114.0981	169.1406452
0.131811105	34.97982006	546829.6043	123.980315
0.234155781	6.063543495	45579.4814	109.6632231
0.390202703	0.166103859	18768.0671	313.9718615
0.253924915	2.381733817	35718.19355	130.4892473
0.632183908	0.105733792	440.7272727	135.6727273
0.436619718	0.419354503	454.9677419	68.64516129
0.156913786	28.41484695	2498780.109	300.9641672

log.sigma.2.0.mm.3D_glszm_SmallAreaEmphasis	log.sigma.2.0.mm.3D_glszm_LowGrayLevelZoneEmphasis	log.sigma.2.0.mm.3D_glszm_ZoneEntropy	log.sigma.2.0.mm.3D_glszm_SmallAreaLowGrayLevelEmphasis
0.662406589	0.021301649	5.589019838	0.018049424
0.668800771	0.04773251	4.704448699	0.039399894
0.649651934	0.019175286	5.748678936	0.015234743
0.687731867	0.058090873	4.593942708	0.050797952
0.690710478	0.015149525	5.451661807	0.01247325
0.766681682	0.023986156	4.996634366	0.021169205
0.695486467	0.010364491	5.960340432	0.007630639
0.640730344	0.010187489	6.012031355	0.007342342
0.613530245	0.012048528	5.750662508	0.008672911
0.640933439	0.042337953	5.667960473	0.033892621
0.690196727	0.004307335	5.577878742	0.003773521
0.668517736	0.009832612	5.803151586	0.007751687
0.681398276	0.037487941	5.673480397	0.032568112
0.628448109	0.041015433	5.041215361	0.03498437
0.591444087	0.033034795	5.531292475	0.022993169
0.642432765	0.008282803	6.063694063	0.005675539
0.622371943	0.064989313	4.973499308	0.049440375
0.632733787	0.057038093	4.697845823	0.050084019
0.694097434	0.059610922	5.024644996	0.048411983
0.657924618	0.00623312	6.084735053	0.00432335
0.599528397	0.041466304	5.602225738	0.026495621
0.561170473	0.056247752	4.874614691	0.041751216
0.639909014	0.065112074	5.064815198	0.03157044
0.631283518	0.018521561	5.592795836	0.013330787
0.612500051	0.016211811	5.756955274	0.013168923
0.672050992	0.022433155	5.8451963	0.017373238
0.648567928	0.016546821	5.502243208	0.0129776
0.677570107	0.011384207	5.453441455	0.009209691
0.851010101	0.122512625	3.879664005	0.119295298
0.593290075	0.031839668	5.562121006	0.022905253
0.571015452	0.017801316	5.962602808	0.011341566
0.598732556	0.030873368	5.310269468	0.024099659
0.67040514	0.02796986	5.704974517	0.021720239
0.640585098	0.02353505	5.790047481	0.015204134
0.694905546	0.022611796	5.660169775	0.017492299
0.697131485	0.015755954	5.872306504	0.012386098
0.782426835	0.016229602	5.581167967	0.013530526
0.644112097	0.016106997	6.098476255	0.010513694
0.638112341	0.041788088	4.849204232	0.034428277
0.711683174	0.001671547	5.906950921	0.00142568
0.673367147	0.024046995	5.48596262	0.019266886
0.723610273	0.015003175	5.449025546	0.010388349
0.63759556	0.015121167	5.960967193	0.010925795
0.622324311	0.017461697	5.575902874	0.013615278
0.642094031	0.031401636	5.21786203	0.024117255
0.706132744	0.011239503	5.51493769	0.006635785
0.633767914	0.025928573	6.010668098	0.017576393
0.637904547	0.103554944	4.825417699	0.068418797
0.595955077	0.049030286	4.66146788	0.036057003
0.666114751	0.02702818	5.533818834	0.021108003
0.645311176	0.026697575	5.444564157	0.021403589
0.706919686	0.012141334	5.990380901	0.009479011
0.696817917	0.01576409	5.561193434	0.011755711
0.588779358	0.034434863	5.613964661	0.025699709
0.627537545	0.032591039	5.466983947	0.024133205
0.642269907	0.035019554	5.315451739	0.026978339
0.680396131	0.021042537	5.703372611	0.016396502
0.60476653	0.015478592	5.844425292	0.011463729
0.596552783	0.029766439	5.267202849	0.022801279
0.688378237	0.010556547	5.394795561	0.007531456
0.717992488	0.020018885	5.682486648	0.015682353
0.757938721	0.014113469	5.894062415	0.011616143
0.725166337	0.004174188	6.210344625	0.003160236
0.709653573	0.002965029	6.109835153	0.002320151
0.672680163	0.028423198	5.093542974	0.024817523
0.721393145	0.008271208	6.176108521	0.006300513
0.701778203	0.034461623	4.722557954	0.027944844
0.733380301	0.017809736	5.917093779	0.013314656
0.707106716	0.021985609	5.644359098	0.015703589
0.69467987	0.01888421	5.79912724	0.014687739
0.598228764	0.071335968	5.035176838	0.057864737
0.637769425	0.011343046	6.014291318	0.008319725
0.581752662	0.03608653	5.644690416	0.026271371
0.623428056	0.031847572	5.559719393	0.022274679
0.690842143	0.014825171	5.878246355	0.011217841
0.667267507	0.048134942	5.449233479	0.038610101
0.667908406	0.010305522	6.260017928	0.008041452
0.636590136	0.095498474	5.017155963	0.06854238
0.695180973	0.013333578	5.613898278	0.010452612
0.607841974	0.047339301	5.187768984	0.033580567
0.662406589	0.021301649	5.589019838	0.018049424
0.697374802	0.055997926	5.375902291	0.041433468
0.591365351	0.021436506	5.872906474	0.016355869
0.610226158	0.060740515	5.3732256	0.037712033
0.632683859	0.036594013	5.692655029	0.027887122
0.731543865	0.02518398	5.055145172	0.021799547
0.682857279	0.015795015	5.408670047	0.011315023
0.633954391	0.019572398	5.724339132	0.013739841
0.683310893	0.016088108	6.238368212	0.013150506
0.70791627	0.024210771	5.491429224	0.016174742
0.722042425	0.018703917	5.408044864	0.015605722
0.653314406	0.037055767	5.178737765	0.024539106
0.703079036	0.014880047	5.770636333	0.011447721
0.690555533	0.027759921	5.76891835	0.020622054
0.676180984	0.033408057	5.737091569	0.025256102
0.725397265	0.007558205	5.932305688	0.006146506
0.682367005	0.035651674	5.780764661	0.027532488
0.738313131	0.047155118	4.883467387	0.040275569
0.617296611	0.055184794	4.180002762	0.043024661
0.674742464	0.006158653	6.18569913	0.004313488

log.sigma.2.0.mm.3D_ngtdm_Coarseness	log.sigma.2.0.mm.3D_ngtdm_Complexity	log.sigma.2.0.mm.3D_ngtdm_Strength	log.sigma.2.0.mm.3D_ngtdm_Busyness	log.sigma.2.0.mm.3D_ngtdm_Contrast
0.010928408	0.66140142	1.890821415	0.263983111	0.000144954
0.057835144	2.135677797	5.029567239	0.147882934	0.001827043
0.013528444	1.081281831	2.280335637	0.231264108	0.000321947
0.057175151	2.867137442	5.203625648	0.138333102	0.001987778
0.009705208	0.509886318	1.729638499	0.289278586	9.76E-05
0.048821628	5.56569687	7.389816625	0.093632199	0.003674308
0.00037002	0.024010301	0.39164405	3.566842909	4.05E-07
0.001594449	0.090641122	0.743163779	0.877196608	4.82E-06
0.001715564	0.051039625	0.580165448	1.177502539	4.72E-06
0.01818993	1.474628341	3.068934754	0.273285702	0.000495217
0.001303298	0.065496524	1.187850598	0.72385125	1.68E-06
0.002356022	0.113862676	1.387879297	0.586834636	5.13E-06
0.017650007	2.417904757	3.426217063	0.242531859	0.000772109
0.033683595	1.287162901	3.254872852	0.184506348	0.000902958
0.008205631	0.271227521	1.410731814	0.372267798	7.50E-05
0.000720791	0.030211115	0.32333371	2.293139479	2.32E-06
0.018531109	0.290886021	1.548513336	0.424356179	0.000152317
0.053683525	2.153892807	5.05741139	0.136859508	0.001411332
0.013017128	0.324672878	1.617744505	0.470531013	0.000106136
0.000615457	0.058546636	0.537799611	1.749156367	9.91E-07
0.005129509	0.114314014	0.552434436	1.112618872	4.11E-05
0.043333863	0.859000647	3.377577471	0.193983257	0.000539683
0.024906721	0.550240004	2.174040542	0.256878306	0.000361569
0.001405566	0.03796665	0.669913788	1.630317027	1.79E-06
0.008911594	0.635783216	2.854659905	0.203086449	9.14E-05
0.003814152	0.347374644	2.515739199	0.507847049	1.44E-05
0.008320598	0.300954657	1.299951177	0.405189151	7.76E-05
0.00469072	0.117170609	0.937444652	0.506086231	1.96E-05
0.143755723	9.193556665	11.22252385	0.076986109	0.01812725
0.006878666	0.133187769	0.685879125	0.722541086	5.32E-05
0.002165739	0.071111389	0.562577022	1.176513294	8.64E-06
0.022747118	1.142936239	2.337740045	0.265833804	0.00060518
0.006068472	0.258367798	1.13911937	0.52396938	4.58E-05
0.002178724	0.076962732	0.702127351	1.088520349	7.35E-06
0.002184606	0.090170412	0.574803549	1.244523604	9.21E-06
0.006916902	0.578874048	1.965680536	0.258457286	8.06E-05
0.014335651	3.97180465	4.724639694	0.1887604	0.000716174
0.000568722	0.04354532	0.755831494	2.370840234	7.42E-07
0.036349273	0.803082158	3.023876989	0.160050816	0.000483403
0.000547973	0.054649751	0.908139228	0.811201631	5.91E-07
0.011678565	0.44105051	1.661578086	0.351108039	0.000115638
0.000968853	0.054670901	0.612205581	1.994530007	1.38E-06
0.005173527	0.268789063	1.087725783	0.486648961	4.32E-05
0.010691215	0.446839988	1.665966651	0.298869486	0.0001084
0.007239917	0.094762962	0.887685251	0.587303159	2.98E-05
0.003966662	0.165645259	0.975313289	0.531268477	2.05E-05
0.001023603	0.036910988	0.501176031	2.049384447	1.88E-06
0.033025216	0.758915769	3.600974387	0.285118953	0.000423804
0.053996929	1.549374517	3.275499629	0.166238675	0.001535763
0.021864504	8.058852876	12.32492634	0.187499459	0.002166867
0.003870789	0.078575722	0.915141063	0.71274771	1.46E-05
0.003689396	0.437913576	1.484657895	0.446428193	2.77E-05
0.000608247	0.021876373	0.374585023	3.176891571	5.86E-07
0.010875526	0.198822207	1.159374861	0.458881041	8.99E-05
0.022825346	1.926954724	4.075975203	0.195220185	0.000589986
0.01041013	0.314810595	1.170171098	0.475262042	0.000107167
0.001335033	0.050666322	0.483958919	1.694952004	3.42E-06
0.004653223	0.229099392	1.137508339	0.504028706	3.34E-05
0.029737027	0.820932485	3.382700483	0.161337103	0.000420678
0.000253521	0.018413111	0.330682865	5.337415536	2.01E-07
0.009837948	0.798129195	2.241388184	0.26562281	0.000117719
0.005002094	0.737578923	1.796975972	0.323934448	5.83E-05
0.001806323	0.341316771	0.780793469	0.620373241	2.12E-05
0.000885319	0.119418746	0.876913849	0.721832835	2.11E-06
0.024627465	0.5446144	2.411698749	0.192791211	0.000302771
0.003194636	0.886619652	1.485003769	0.469639867	6.59E-05
0.041705061	3.788977698	6.643414663	0.173774101	0.001947741
0.002027165	0.249910325	1.389581218	0.722682877	8.59E-06
0.00067559	0.026617045	0.364869474	3.604242775	9.66E-07
0.004821546	0.448119726	1.30440687	0.526347641	5.06E-05
0.030125435	0.702315878	1.853254572	0.340969038	0.000978043
0.000989701	0.050569075	0.64486267	1.409062933	2.31E-06
0.010026731	0.372335275	1.557997922	0.395549511	0.000105411
0.0067954	0.27272861	1.73904062	0.465043667	3.40E-05
0.002423165	0.174910678	1.434860349	0.72361746	7.76E-06
0.012552902	0.708394583	2.92409542	0.340926796	0.000135794
0.004745458	0.925617361	1.80331433	0.443628661	0.00010563
0.048013179	2.525188004	4.592322658	0.274173045	0.002480527
0.010621275	0.827879528	1.616896269	0.310239085	0.000223605
0.004713475	0.056509143	0.483978265	1.070758073	2.06E-05
0.010928408	0.66140142	1.890821415	0.263983111	0.000144954
0.016805626	0.911900897	1.941200026	0.417527436	0.00050954
0.001790738	0.060833861	0.552608786	1.269504427	9.39E-06
0.007224308	0.098398603	0.740405171	0.75068005	3.32E-05
0.004302469	0.107000267	0.585995965	0.899846815	2.70E-05
0.030971118	3.606910008	4.494428953	0.152204678	0.001843535
0.01077768	0.519675209	2.395814274	0.243222923	8.80E-05
0.001705459	0.041571925	0.545596583	1.488062534	3.55E-06
0.006759875	1.181313202	2.601250094	0.282631055	0.000134599
0.009332162	0.517447348	1.271052787	0.417759315	0.000152804
0.020396798	2.652078184	3.471555214	0.186107934	0.000866749
0.005682839	0.079053659	0.619602018	0.813493859	2.56E-05
0.0023148	0.129076684	0.520721604	1.03117546	1.40E-05
0.001958561	0.081660837	0.577075739	1.34893978	7.20E-06
0.003823321	0.186379201	0.875872503	0.970553578	2.38E-05
0.0059521	1.118215635	2.345491675	0.246289833	8.62E-05
0.005104288	0.490774085	1.864534681	0.548338557	4.57E-05
0.060999274	8.087726316	9.250141937	0.097795288	0.004625893
0.083195679	1.410000122	3.35111667	0.182978068	0.002418137
0.000817699	0.055489374	0.326432535	1.696498733	3.35E-06

log.sigma.1.5.mm.3D_gldm_GrayLevelVariance	log.sigma.1.5.mm.3D_gldm_HighGrayLevelEmphasis	log.sigma.1.5.mm.3D_gldm_GrayLevelNonUniformityNormalized	log.sigma.1.5.mm.3D_gldm_DependenceEntropy
12.24320717	166.957958	0.094941789	6.100505388
12.65596366	82.59292035	0.099381314	5.426169728
13.66452769	174.3188406	0.10328391	6.249274385
12.68336484	148.4565217	0.08979206	5.044702038
9.674174687	178.9182306	0.120514055	6.091888175
24.54067301	180.184466	0.061362994	5.273655445
3.182441311	233.2666503	0.333364372	5.700340752
7.469115018	266.2479191	0.195432618	6.162353109
4.382065618	187.1399858	0.23493063	5.808176962
20.58425915	191.1593407	0.074643763	6.118028035
3.828854887	1129.834187	0.243723057	5.971223806
5.184121405	282.6293524	0.243428456	5.875904886
23.59742974	179.8096677	0.063060761	6.110348325
13.72601108	149.1421053	0.078116343	5.403166418
8.733005671	221.2586957	0.168152174	6.015992763
4.686869126	223.8497713	0.282437733	5.793665405
5.677889067	84.87155963	0.139455854	6.013346025
11.22149176	118.5585586	0.094716338	5.262010853
6.658031846	74.32098765	0.141519617	5.929544619
6.03366937	334.0210447	0.202176267	6.225672161
5.097387758	84.80096419	0.160559388	6.279894499
7.32226406	68.22151899	0.104550553	5.279794731
8.962120541	132.480427	0.125821608	5.762197976
2.52560154	171.5634888	0.303673354	5.687210087
11.06800537	173.6831956	0.209017295	6.102965698
8.998895046	161.5244444	0.161444902	6.177419797
8.61768065	190.1064963	0.11492184	6.203168135
5.172589211	211.704946	0.188122741	6.167420968
17.55102041	96	0.089285714	4.06610894
5.8709693	88.33188532	0.151401607	6.052890543
5.390331773	132.711763	0.215450226	6.094577676
10.72316801	94.85882353	0.087274125	5.629112697
8.590144152	154.3971853	0.144975581	6.247969063
5.141771733	170.9615751	0.240153966	5.916868362
6.182968088	156.6879494	0.173976588	6.16804615
14.85666513	209.4937943	0.109033122	6.407707723
33.13549638	255.9102564	0.051545036	5.922877444
4.548633123	331.0653433	0.312275344	5.856953782
8.011698317	197.248731	0.111288619	5.513961184
4.390351236	1615.31864	0.283497871	5.831204996
8.643372907	185.6893819	0.113022119	6.098783259
4.25503576	128.942794	0.23208332	5.997110301
8.866028599	241.513425	0.108474441	6.377304471
9.377007856	210.3988685	0.121134583	6.028680997
3.712524836	114.5229779	0.228366944	5.869908371
6.709901165	201.4230179	0.16792067	6.143147119
4.008904119	170.3593055	0.281306607	5.792822205
6.364337691	53.96803653	0.127582828	5.731800465
9.963450292	93.99122807	0.111265005	5.249183054
39.03355372	272.8454545	0.064173554	5.766441491
3.978860868	139.2628541	0.275736642	5.719482218
13.79148994	218.7095878	0.112714724	6.496738822
2.317866885	230.5844387	0.332966306	5.572115871
7.391566038	131.7390791	0.128187687	6.166355722
19.48422343	166.3414634	0.078051209	5.994060909
6.332684971	93.21870287	0.124756295	6.077285559
4.12038815	169.6079062	0.229728972	5.944684915
8.307158911	256.3339806	0.163298248	6.165614531
11.15176254	212.75	0.094425822	5.849291206
2.915545816	264.984179	0.268019592	5.646003489
14.48239871	199.2308657	0.111568004	6.392986685
20.59653823	346.7560823	0.090671771	6.542681737
23.48032013	554.8372703	0.082094288	6.771618253
9.486618341	950.0030516	0.199649237	6.335516394
7.284129618	126.6448598	0.139585214	5.786484412
32.19410061	338.7244851	0.059454466	6.682819643
18.53753266	118.8739496	0.072240661	5.322139642
12.53444406	215.1967054	0.148095841	6.41432139
3.281700896	126.6549935	0.272809324	5.805887196
12.22678965	184.3398821	0.10653425	6.368117437
10.02318641	104.3484848	0.102642587	5.721955315
5.677346571	319.3000423	0.251863662	6.002935027
8.822315825	111.4833111	0.137819719	6.033281543
7.166083717	206.889096	0.166747298	6.034745382
8.118100322	226.5345149	0.204122616	6.272621834
10.38060557	162.968543	0.119018903	6.253864041
25.08150027	300.0101215	0.065230813	6.488968711
13.46162571	72.0173913	0.083100189	5.268990216
15.20208305	200.0952381	0.083244259	6.247005473
3.570471841	135.4028822	0.218789455	5.825657324
12.24320717	166.957958	0.094941789	6.100505388
12.62239555	119.3441734	0.08762421	6.071504377
5.548971028	245.9803979	0.288042412	5.764413216
4.000362155	151.7004255	0.177042282	5.956289436
5.961555237	132.2842105	0.189349376	6.24468106
18.10792226	147.3693182	0.067342459	5.57918654
9.053306064	170.3958621	0.115517717	6.211053626
3.221500735	140.6460895	0.305750251	5.644268586
26.85240815	405.4280851	0.077388864	6.659251469
13.52743594	201.86875	0.095746875	6.26574364
23.76479469	194.9384058	0.060465238	5.993939189
3.934539586	153.3552448	0.199643992	5.964028307
8.944005952	220.5181181	0.13628459	6.396729464
6.411549313	196.442138	0.197404135	6.171734541
8.300165548	116.9356555	0.121799494	6.250824447
21.17797834	410.6402027	0.07914793	6.494442083
11.46799334	171.6416382	0.116223136	6.307324468
30.35301889	160.1034483	0.062755978	5.074902691
5.022812934	56.61971831	0.138266217	4.760603592
8.719487054	290.9867763	0.176942339	6.328806778

log.sigma.1.5.mm.3D_gldm_DependenceNonUniformity	log.sigma.1.5.mm.3D_gldm_GrayLevelNonUniformity	log.sigma.1.5.mm.3D_gldm_SmallDependenceEmphasis
105.5435435	63.23123123	0.321459589
21.76106195	11.2300885	0.335534285
62.2173913	49.88612836	0.303968226
22.26086957	8.260869565	0.400006039
86.54155496	89.90348525	0.246387291
35.27184466	6.32038835	0.529800431
1046.245554	5435.839446	0.079647481
354.9936583	986.1529925	0.166109753
284.6041617	993.5216363	0.129724487
73.8021978	27.17032967	0.379117606
362.8945827	1331.702782	0.130664787
206.5307517	748.0556459	0.133767745
75.66465257	20.87311178	0.439217734
47.89473684	14.84210526	0.419336257
79.35217391	154.7	0.211788862
572.6864079	2408.346546	0.112122067
42.24311927	60.80275229	0.201216033
25.55855856	10.51351351	0.36988989
62.88359788	80.24162257	0.225495877
831.8692882	2411.356334	0.156339158
108.9903581	233.1322314	0.161776198
29.78481013	16.51898734	0.305272889
34.80782918	35.35587189	0.275586126
302.7639467	1458.846794	0.091706632
56.55371901	151.7465565	0.197771207
169.7565432	326.9259259	0.192136581
114.1970181	107.9116081	0.250105851
128.2632177	330.9079022	0.161666636
11.14285714	2.5	0.60515873
101.1337967	174.2632493	0.164213335
218.8234121	756.445742	0.129342633
52.46666667	22.25490196	0.350094504
125.3831118	185.4237686	0.226841777
214.5154017	756.2448396	0.128054639
274.8912856	604.9165948	0.188548926
135.0957447	122.9893617	0.296373608
130.9333333	20.1025641	0.571613048
696.0405521	3665.487988	0.09622033
34.00507614	21.92385787	0.310771573
784.4981014	3434.293214	0.118122465
77.85895404	71.31695721	0.269131869
490.1568329	1679.586984	0.138618075
173.048461	165.6404715	0.217778276
81.95615276	85.64214993	0.240222012
71.36948529	248.4632353	0.128261766
153.7017903	328.2849105	0.174961652
448.2107906	1960.425743	0.10506175
26.39726027	27.94063927	0.22868494
21.1754386	12.68421053	0.394121498
60.75454545	14.11818182	0.46247096
120.0041973	525.5540399	0.131032991
246.279296	243.3510885	0.269429205
669.8523065	3363.625619	0.079002293
93.49586777	108.5749705	0.211711187
57.17421603	22.40069686	0.372993198
69.33484163	82.71342383	0.20791098
357.6332514	1214.577076	0.130738919
123.6174757	252.2957929	0.182957051
44.68382353	25.68382353	0.303352164
1621.025858	6301.944669	0.101225588
92.09912171	88.91969887	0.279347777
220.1353712	145.3468497	0.332244455
699.3902012	375.3350831	0.347469595
594.1159624	1701.011502	0.178954178
35.31775701	44.80685358	0.232344872
446.6036613	129.9080092	0.409654701
37.11764706	8.596638655	0.560251446
350.9898256	611.3396318	0.227708794
655.3116831	2734.367854	0.107655982
182.0438769	162.6777996	0.268897693
29.1010101	20.32323232	0.2894652
442.4968305	1787.980138	0.123687175
70.74899866	103.2269693	0.216098596
93.8555452	178.9198509	0.199605475
229.1225124	656.4583333	0.175000265
65.08609272	71.88741722	0.260081879
284.6990553	96.67206478	0.349616668
28.79130435	9.556521739	0.4595
115.2734255	54.19201229	0.376740436
109.1766917	349.1879699	0.149159639
105.5435435	63.23123123	0.321459589
60.01897019	32.33333333	0.325623077
220.7156232	984.5289643	0.119958337
94.60510638	208.0246809	0.158168626
128.5412742	341.7756233	0.170976231
43.68181818	11.85227273	0.443412054
82.55034483	83.75034483	0.238687259
251.9695032	1243.180521	0.082438038
186.3004255	90.93191489	0.343662746
105.225	76.5975	0.30942467
71.92753623	16.6884058	0.472027666
104.2923077	285.4909091	0.150963482
335.244886	466.3658679	0.239605804
272.0015568	760.7955371	0.161501057
243.9946783	251.7595549	0.258472245
199.9662162	93.71114865	0.37077818
164.7488055	170.2668942	0.256210569
27.94252874	5.459770115	0.514287995
13.95774648	9.816901408	0.301968418
743.3594336	1699.354227	0.183799328

log.sigma.1.5.mm.3D_gldm_DependenceNonUniformityNormalized	log.sigma.1.5.mm.3D_gldm_DependenceVariance	log.sigma.1.5.mm.3D_gldm_LargeDependenceEmphasis
0.158473789	6.142728314	18.06906907
0.192575769	2.604119352	10.87610619
0.128814475	18.718808	41.25672878
0.241965974	1.546786389	7.369565217
0.116007446	21.92160513	50.44235925
0.342445094	0.681496842	4.048543689
0.064163225	24.6240032	136.838955
0.070351498	22.78716663	74.07728894
0.067298217	21.80727505	83.59825018
0.202753291	3.44988528	11.3021978
0.066415553	19.65408656	75.32906296
0.067208185	22.84585764	89.48096323
0.228594117	3.769023649	10.66163142
0.252077562	1.373961219	6.736842105
0.086252363	20.80046786	59.75434783
0.067161535	24.01092206	106.4612408
0.096887888	13.86865584	41.79357798
0.230257284	1.547277007	7.774774775
0.110905816	10.56796345	32.13227513
0.069746733	24.2919238	81.09222772
0.07506223	27.31796363	75.76033058
0.188511456	2.670245153	11.51898734
0.123871278	11.48729119	31.4341637
0.063023303	22.85713751	110.2897585
0.077897685	28.73216007	81.78512397
0.083830392	18.85941631	56.3017284
0.121615568	11.50163374	31.27050053
0.072918259	21.79858123	67.20579875
0.397959184	0.515306122	3.214285714
0.087866027	14.69527725	48.12597741
0.062325096	27.3984	95.95642267
0.205751634	2.450657439	9.964705882
0.098032144	15.15992324	43.07036747
0.068121753	20.8930418	80.85836774
0.079059904	21.20891955	63.02818522
0.119765731	16.43673294	39.7464539
0.335726496	1.313583169	4.933333333
0.059298054	27.0835601	126.3658204
0.1726146	4.285140045	14.6751269
0.064759625	24.1360824	105.8598316
0.123389785	11.98369001	31.51980983
0.067729285	20.56193021	74.97319331
0.113325777	9.731515969	30.53634578
0.115921008	14.24114483	38.09476662
0.065596953	23.17479117	82.81066176
0.078619842	20.60032941	62.54168798
0.064314936	24.73633761	114.7942316
0.120535435	9.699756052	29.52054795
0.185749461	4.939673746	14.47368421
0.276157025	1.260661157	5.863636364
0.062961279	24.5887004	95.26862539
0.114071003	15.66994824	39.31773969
0.06630888	23.34694099	127.1963968
0.110384732	10.12717153	32.10743802
0.199213296	5.799002052	15.26480836
0.104577438	12.83225159	37.65158371
0.067643891	20.62303514	78.07811613
0.080011311	24.10591594	69.33915858
0.164278763	5.602508651	17.24264706
0.068941686	18.50444631	85.00187131
0.115557242	20.0327042	45.74027604
0.137327119	13.6093429	32.37991266
0.152972485	12.23189596	28.07480315
0.06973192	28.05889815	89.34647887
0.110024165	15.22353238	40.85046729
0.204395268	8.067769324	17.73318078
0.311913	1.979662453	6.428571429
0.085026605	23.19882275	64.0125969
0.065380792	23.73398372	108.3802255
0.119216684	15.00403091	37.29207597
0.146974798	8.83124171	23.7979798
0.062332276	26.36306334	103.5251444
0.094457942	19.93870599	53.46728972
0.087470219	19.80166225	56.69058714
0.071244562	25.20975308	75.46268657
0.107758432	17.81256305	43.91721854
0.192104626	2.903755548	11.08906883
0.250359168	1.742155009	7.052173913
0.177071314	9.853119743	21.93701997
0.068406448	20.98955566	74.78571429
0.158473789	6.142728314	18.06906907
0.162653036	10.16154405	23.36856369
0.064574495	24.14136806	102.3639555
0.080514984	16.79284672	54.35148936
0.071214002	28.60390697	78.64875346
0.248192149	2.000903926	7.534090909
0.113862545	11.80163805	33.36965517
0.061969873	26.4286605	127.3753074
0.158553554	9.701906021	23.66723404
0.13153125	12.1146	30.3475
0.260607015	2.218966604	7.528985507
0.072931684	22.93209644	69.95524476
0.09796753	15.80100706	43.95733489
0.070576429	23.44373795	78.88323819
0.118042902	9.889527159	29.19932269
0.168890385	12.02022462	26.13344595
0.112456522	16.09946022	40.44846416
0.321178491	0.878583697	4.563218391
0.196587979	2.816107915	11.64788732
0.077401024	24.52829903	75.01166181

log.sigma.1.5.mm.3D_gldm_LargeDependenceLowGrayLevelEmphasis	log.sigma.1.5.mm.3D_gldm_SmallDependenceHighGrayLevelEmphasis	log.sigma.1.5.mm.3D_gldm_LargeDependenceHighGrayLevelEmphasis
0.113440098	48.02317989	3548.807808
0.262211369	33.87736595	862.2920354
0.20373312	48.65274143	9105.590062
0.063469099	54.98260568	1286.543478
0.239558678	41.46950325	11182.63405
0.058117491	106.7791262	682.7864078
0.575844796	18.95564183	32768.45284
0.254564014	37.00204009	21912.37317
0.410840618	19.58282107	17271.13336
0.083203631	79.68583202	1959.923077
0.067318833	152.5147017	84684.17057
0.301596006	33.31273339	27031.63293
0.086744575	92.70634709	1950.595166
0.054908745	49.21350146	1243.615789
0.234931882	36.17529593	15674.75217
0.455859955	22.58404475	25178.84965
0.457742669	15.24920418	4259.582569
0.09595697	45.40663914	921.2792793
0.483377563	20.11422814	2523.121693
0.241591086	54.9471394	27467.20944
0.82457152	14.37802032	7553.991047
0.196491476	17.3256075	1000.791139
0.220498833	32.49451534	5055.071174
0.624842294	14.84004861	19664.75416
0.434450376	28.51327146	16432.06336
0.32972215	33.03572229	9928.816296
0.148738535	42.14339709	7028.468584
0.293210127	29.02061403	15672.49289
0.177842603	72.05753968	206.2142857
0.534322124	14.79490061	4933.108601
0.64336423	13.54677675	14767.6961
0.130364315	31.00767291	1154.521569
0.265084976	35.67312552	7471.118061
0.456225393	19.89338663	14650.90695
0.366817244	26.79697239	11175.88841
0.197392726	59.91459648	10062.16223
0.035018392	161.5353874	1095.807692
0.370008877	28.92066549	43422.82834
0.075071232	58.11593356	3294.385787
0.065052093	191.3737505	172662.8121
0.153217935	46.08388441	6890.954041
0.594572517	21.51835875	9650.554788
0.118161684	48.54120165	8447.15979
0.155777656	43.16781378	9846.560113
0.682259795	13.16468483	10322.44577
0.278455758	31.54685972	14445.12634
0.644537594	16.70339233	20708.53379
0.620451697	14.85314056	1850.296804
0.180640598	36.78809811	1479.184211
0.040966495	148.3291717	1323.4
0.643711415	13.97790856	14362.71721
0.184314951	67.38562448	8971.965725
0.542046022	17.91835813	30041.36567
0.25831733	27.10230431	4307.491145
0.099826548	59.40231435	3118.202091
0.362225806	17.37086588	4419.73454
0.445642219	21.6721446	13901.40212
0.236769282	38.97151358	20777.35663
0.077133714	55.48356631	4435.742647
0.316445805	26.46461924	23002.37524
0.219012178	56.71304843	10118.57842
0.09215665	126.3176556	11912.85714
0.05113264	205.5706397	16042.15573
0.090895546	168.1427312	88229.88732
0.303312978	27.29995807	5888.688474
0.059572096	160.3932827	5991.232494
0.089434484	73.08158635	752.5042017
0.283623045	53.1148636	14922.38905
0.844968047	15.2685923	14125.47571
0.201343197	57.39148715	7381.170269
0.262370273	28.6877073	3064.409091
0.305196932	33.63715796	35433.9452
0.399595689	18.46674205	7773.307076
0.246471492	35.98598814	13368.72507
0.328344814	40.44721474	17616.76959
0.278169846	50.11436206	7294.018212
0.050340025	121.7194831	2912.181511
0.232774982	39.95171498	448.5913043
0.118128908	85.96568557	4549.250384
0.50226677	16.18263989	11358.79762
0.113440098	48.02317989	3548.807808
0.218857228	48.02067131	3020.905149
0.385582088	21.19378227	27486.01083
0.328820764	21.50396206	9219.770213
0.550381292	21.81787353	11586.39723
0.085969223	74.28972828	1012.630682
0.184438884	42.29405391	6528.58069
0.858654177	9.835487065	19171.06222
0.063671112	150.8573457	9465.16766
0.146794989	69.47198185	6755.63625
0.063594808	99.77133139	1274.018116
0.412842193	18.37899064	12081.03497
0.197130374	56.18550721	10110.01607
0.391221244	34.14336775	16205.00934
0.272081627	36.28571947	3409.980648
0.058952675	155.3229618	12368.74831
0.223097658	47.92798992	7874.433447
0.149054038	95.4054917	627.6436782
0.270706767	18.97878815	631.4366197
0.253448073	56.23888521	22535.38953

log.sigma.1.5.mm.3D_gldm_SmallDependenceLowGrayLevelEmphasis	log.sigma.1.5.mm.3D_gldm_LowGrayLevelEmphasis	log.sigma.1.5.mm.3D_gldzm_DistanceZoneVariabilityNormalized
0.011808638	0.017270477	0.991489517
0.02541604	0.051096635	1
0.006963547	0.011879466	0.9881661
0.016537989	0.021269632	0.916824197
0.004434566	0.009252029	0.979850035
0.015757701	0.022194839	1
0.00065583	0.004762523	0.841137567
0.00143876	0.004747844	0.829973656
0.001679429	0.006559864	0.772485444
0.009846737	0.014556045	0.950648934
0.000315107	0.001092686	0.790428899
0.001016129	0.004655578	0.847683042
0.012435121	0.017961818	0.964717272
0.013180784	0.0176418	0.936909263
0.003097345	0.006980253	0.963648834
0.001155471	0.005417958	0.747207499
0.01019694	0.022023494	0.96001666
0.016569815	0.023653373	0.959201389
0.011346092	0.030802639	0.912592506
0.000703822	0.003378606	0.650578136
0.004629761	0.018109811	0.937074493
0.019079204	0.032442006	0.965527855
0.008805416	0.016306475	0.929506246
0.001137697	0.006634318	0.738913905
0.004847454	0.013016672	0.975907198
0.002969571	0.008764343	0.597439715
0.00343099	0.007941652	0.961846508
0.002058183	0.006173998	0.897301923
0.045431272	0.060835496	1
0.007421258	0.021954406	0.8346
0.002912362	0.010485926	0.900027548
0.01498118	0.025410402	0.962976148
0.004850878	0.011192996	0.943444245
0.001992267	0.007753113	0.790189292
0.003122005	0.008877613	0.898579775
0.004840545	0.011990095	0.973123269
0.008104428	0.011099807	1
0.000533307	0.003353997	0.758800762
0.007446038	0.011200467	0.942906574
0.000159477	0.000708167	0.748550037
0.003993164	0.008407621	0.96757638
0.001955879	0.0095263	0.69394177
0.002516254	0.006048777	0.922563928
0.003764614	0.007667493	0.969395152
0.004076361	0.01253067	0.851042907
0.002301089	0.006770323	0.838502184
0.001617544	0.00722741	0.866371835
0.016081144	0.039588081	0.964297521
0.020207179	0.029567588	1
0.00727424	0.010678902	0.803935651
0.004546335	0.011229936	0.899367364
0.003203739	0.007990261	0.869205294
0.000580111	0.004680087	0.770819056
0.004869489	0.012219362	0.989130759
0.012251034	0.017618741	0.969238281
0.010487832	0.0216473	0.895061728
0.001614647	0.007133692	0.750190725
0.001898173	0.005308938	0.945058594
0.006023957	0.00981528	0.959201389
0.00060329	0.004089779	0.529287747
0.005079858	0.009456553	0.950850929
0.002396032	0.004718182	0.874193263
0.001109312	0.002466562	0.891449731
0.000341036	0.001221126	0.8700566
0.007533029	0.014998547	1
0.00284637	0.005386768	0.704840226
0.024911906	0.030706605	0.947406866
0.002759016	0.007067445	0.912609342
0.001515721	0.009252983	0.631231477
0.003906279	0.009020495	0.921541328
0.011342699	0.027993245	0.970156107
0.000847332	0.003680133	0.686085306
0.011055511	0.019886257	0.946277586
0.002575055	0.006743415	0.918317933
0.001692878	0.00570334	0.922953882
0.004943604	0.010570356	0.931515003
0.002548902	0.005583823	0.542621987
0.034488026	0.055301391	1
0.006029371	0.010230447	0.950210453
0.002855834	0.009465059	0.839387627
0.011808638	0.017270477	0.991489517
0.00848893	0.017685113	0.92866941
0.001654522	0.005341144	0.903553719
0.002605336	0.0084655	0.936436673
0.004404714	0.011631781	0.951101109
0.018762065	0.024826817	0.956066153
0.00372677	0.009232292	0.936909263
0.001457886	0.008530406	0.942992834
0.002139208	0.004186566	0.537248072
0.004164208	0.00838127	0.927119972
0.010648193	0.014878569	0.9608
0.00251306	0.008286606	0.956917464
0.002143478	0.006055715	0.881865072
0.001729863	0.006490299	0.746921128
0.004594686	0.013008837	0.94341157
0.002256598	0.004015179	0.939817977
0.004873116	0.010494339	0.970738163
0.019116865	0.042147661	1
0.02269341	0.040707858	1
0.001133567	0.004232943	0.702334054

log.sigma.1.5.mm.3D_gldzm_LowIntensityEmphasis	log.sigma.1.5.mm.3D_gldzm_LargeDistanceEmphasis	log.sigma.1.5.mm.3D_gldzm_HighIntensitySmallDistanceEmphasis
0.035485975	1.012820513	144.6111111
0.080679035	1	94.54545455
0.021536463	1.017857143	154.0178571
0.034963568	1.130434783	128.6141304
0.017660252	1.055837563	160.8685843
0.02715015	1	190.4925373
0.008563991	1.608934169	218.083844
0.00883001	1.4503386	184.8517212
0.013283769	1.766956522	120.1576652
0.02394268	1.075949367	203.4984177
0.002479737	1.527007299	1074.016636
0.008805457	1.4375	222.8981481
0.026389538	1.053892216	199.7919162
0.028588126	1.097826087	113.5380435
0.014323605	1.055555556	159.2928241
0.010716061	2.29233871	164.3088938
0.048937313	1.06122449	72.25765306
0.040269037	1.0625	116
0.058533356	1.13740458	80.63549618
0.004553382	3.186886939	286.5559186
0.030114316	1.097560976	84.34552846
0.05565853	1.052631579	56.75877193
0.032832992	1.109756098	106.5213415
0.01321115	2.016786571	133.6204017
0.027435941	1.036585366	134.0396341
0.015962705	2.531328321	100.9731795
0.013709195	1.058365759	159.4406615
0.012820466	1.228187919	161.8840418
0.069239564	1	110.65
0.048692566	1.32	78.46625
0.023137519	1.360995851	93.06783022
0.040141907	1.056603774	84.01886792
0.02175137	1.087378641	146.5242718
0.016240228	1.751162791	128.5727229
0.016550201	1.303851641	130.5821247
0.018219707	1.040871935	191.3310627
0.013624395	1	276.7670683
0.005720694	2.064978903	248.5665874
0.022261422	1.088235294	178.4485294
0.001349507	2.015950069	1417.293872
0.014695115	1.049450549	162.7884615
0.014828847	2.181188119	130.1893201
0.011529018	1.121037464	206.4884726
0.014758333	1.046632124	171.2759067
0.03290533	1.431654676	91.00579536
0.013381936	1.520891365	155.6821348
0.016003554	1.818548387	142.5788634
0.07008544	1.054545455	60.76363636
0.049158896	1	91.14
0.014480657	1.330508475	248.9597458
0.034362472	1.25093633	92.9599459
0.012492201	1.271721959	229.5728015
0.007733002	2.403141361	195.7419303
0.023777194	1.016393443	123.8306011
0.029282448	1.046875	155.4414063
0.051696914	1.166666667	73.15625
0.012736669	2.04	138.557269
0.010056205	1.115625	197.8608507
0.018368921	1.0625	172.8854167
0.006282222	4.774395703	188.2390397
0.017742196	1.075630252	196.8056723
0.007181124	1.315881326	351.0393761
0.003129588	1.180990324	557.5837128
0.001889887	1.544437539	873.4585919
0.031483792	1	112.626506
0.006870405	1.719033233	306.5452053
0.041763563	1.081081081	120.0472973
0.012357419	1.19858871	215.7214452
0.014749847	4.229128015	116.3584737
0.014509436	1.14479638	204.0319884
0.044539892	1.045454545	91.81818182
0.006900911	2.938511327	211.0490651
0.049126081	1.082872928	75.05524862
0.012674302	1.210970464	165.8162213
0.009817013	1.120401338	215.6475753
0.018474215	1.106508876	183.2352071
0.007200575	2.271604938	243.9164217
0.07178109	1	83.3442623
0.015365799	1.076642336	216.4653285
0.019806643	1.362139918	91.79103795
0.035485975	1.012820513	144.6111111
0.025791785	1.111111111	138.5814815
0.013681387	1.318181818	156.7303819
0.017167649	1.152173913	124.2595109
0.026295326	1.07523511	118.9326019
0.038823946	1.06741573	161.8455056
0.015922447	1.097826087	164.4565217
0.018632723	1.279069767	109.2077277
0.006041805	2.331096197	284.5712777
0.01335346	1.132075472	210.8916143
0.020742127	1.06	206.6233333
0.016879313	1.066079295	112.2301762
0.009056236	1.313432836	217.9642891
0.01102016	2.076323988	181.4508264
0.018605438	1.096363636	135.0265657
0.005894168	1.103305785	397.9178719
0.019163764	1.044554455	178.2295792
0.042923563	1	178.2075472
0.071527773	1	62.125
0.006234578	2.415653094	255.0748181

log.sigma.1.5.mm.3D_gldzm_LowIntensityLargeDistanceEmphasis	log.sigma.1.5.mm.3D_gldzm_HighIntensityEmphasis	log.sigma.1.5.mm.3D_gldzm_DistanceZoneVariability	log.sigma.1.5.mm.3D_gldzm_ZonePercentage
0.035551386	145.2393162	232.008547	0.351351351
0.080679035	94.54545455	44	0.389380531
0.021627571	154.8928571	166.0119048	0.347826087
0.035521975	136.5217391	42.17391304	0.5
0.017878464	162.9187817	193.0304569	0.264075067
0.02715015	190.4925373	67	0.650485437
0.011241566	235.4231975	1073.291536	0.078253404
0.010121536	212.5485327	735.3566591	0.175584621
0.016731541	143.5530435	444.1791304	0.135965949
0.024211521	209.2468354	150.2025316	0.434065934
0.003334688	1168.29635	541.4437956	0.125366032
0.010356699	242.5208333	366.1990741	0.140579239
0.02665766	202.5808383	161.1077844	0.504531722
0.028891931	121.4782609	86.19565217	0.484210526
0.014513935	163.4490741	208.1481481	0.234782609
0.016199221	193.5816532	741.2298387	0.116336343
0.049527827	73.97959184	94.08163265	0.224770642
0.040513178	120	46.04166667	0.432432432
0.059393859	87.30534351	119.5496183	0.231040564
0.010951055	350.5203924	1260.16985	0.162404628
0.03291476	86.13821138	230.5203252	0.169421488
0.055969959	58.98245614	55.03508772	0.360759494
0.033320797	112.695122	76.2195122	0.291814947
0.01913245	157.9688249	308.1270983	0.086802664
0.027695361	135.3658537	160.0487805	0.225895317
0.027499538	166.9924812	238.3784461	0.197037037
0.013969437	162.9688716	247.1945525	0.273695421
0.01381442	172.5536913	267.3959732	0.16941444
0.069239564	110.65	20	0.714285714
0.052018364	86.615	166.92	0.173761946
0.025560947	99.83609959	433.813278	0.137282825
0.040430702	86.79245283	102.0754717	0.415686275
0.02225053	151	291.5242718	0.241594996
0.020471054	146.4813953	339.7813953	0.136551286
0.018148848	138.9201141	629.9044223	0.201610584
0.018386797	193.9836512	357.1362398	0.32535461
0.013624395	276.7670683	249	0.638461538
0.009023554	291.1392405	899.178903	0.100954166
0.022536288	185.6176471	64.11764706	0.345177665
0.001961251	1614.371706	1079.409154	0.119035826
0.014873577	166.2252747	176.0989011	0.288431062
0.024051339	154.6623762	700.8811881	0.139560591
0.011914081	216.7896254	320.129683	0.22724296
0.014897342	175.1968912	187.0932642	0.272984441
0.044137244	98.73381295	118.294964	0.127757353
0.01559574	172.7855153	301.0222841	0.183631714
0.020698741	153.641129	644.5806452	0.106758502
0.070630895	62.12727273	53.03636364	0.251141553
0.049158896	91.14	50	0.438596491
0.015151677	316.0084746	94.86440678	0.536363636
0.036128375	99.54681648	240.1310861	0.140083945
0.013890629	245.6635071	550.206951	0.293191292
0.01381215	222.2133508	588.9057592	0.075628588
0.023860834	124.6338798	181.010929	0.216056671
0.02950223	158.125	124.0625	0.445993031
0.052751185	79.97916667	128.8888889	0.21719457
0.018530331	160.9310345	543.8882759	0.137128807
0.010397799	205.296875	302.41875	0.207119741
0.018525171	179.1354167	92.08333333	0.352941176
0.020697212	257.3871979	1182.428827	0.09501127
0.018299061	202.9159664	226.302521	0.298619824
0.008174589	369.6544503	500.91274	0.357454772
0.003521717	586.2128628	1566.277177	0.384295713
0.002455501	928.7395898	1399.921069	0.188849765
0.031483792	112.626506	83	0.258566978
0.009244619	381.9446123	699.9063444	0.454462243
0.042056044	125.9864865	70.10810811	0.621848739
0.013152481	227.5856855	905.3084677	0.240310078
0.040248659	141.8756957	680.4675325	0.107552629
0.015347053	210.3574661	407.321267	0.289456451
0.044771803	94.04545455	64.03030303	0.333333333
0.012680793	264.6256742	636.0010787	0.130581772
0.049548902	79.1160221	171.2762431	0.241655541
0.013445869	175.0506329	217.6413502	0.220876048
0.010248226	227.2525084	551.9264214	0.185945274
0.019066354	191.8579882	157.4260355	0.279801325
0.014422006	349.3915344	307.6666667	0.382591093
0.07178109	83.3442623	61	0.530434783
0.015758249	221.8467153	260.3576642	0.420890937
0.02201869	103.5802469	203.9711934	0.152255639
0.035551386	145.2393162	232.008547	0.351351351
0.032197626	141.1037037	125.3703704	0.365853659
0.01502249	167.2045455	397.5636364	0.128730252
0.018052594	129.4456522	172.3043478	0.156595745
0.026777041	122.1724138	303.4012539	0.176731302
0.039683841	163.9101124	85.08988764	0.505681818
0.01630458	170.7173913	172.3913043	0.253793103
0.020751862	113.1627907	324.3895349	0.084604033
0.016817955	435.3780761	240.1498881	0.380425532
0.013863518	219.0603774	245.6867925	0.33125
0.021317305	209.2733333	144.12	0.543478261
0.017172998	115.9471366	217.2202643	0.158741259
0.010468573	230.9506315	768.1044776	0.254529515
0.01646225	208.0202492	479.5233645	0.166580176
0.019343821	138.9763636	518.8763636	0.266086115
0.006108129	410.1590909	454.8719008	0.408783784
0.019577296	180.0990099	392.1782178	0.275767918
0.042923563	178.2075472	53	0.609195402
0.071527773	62.125	24	0.338028169
0.011033144	303.1671074	1328.113696	0.196897126

log.sigma.1.5.mm.3D_gldzm_IntensityVariabilityNormalized	log.sigma.1.5.mm.3D_gldzm_LowIntensitySmallDistanceEmphasis	log.sigma.1.5.mm.3D_gldzm_IntensityVariability
0.061984075	0.035469622	14.5042735
0.07231405	0.080679035	3.181818182
0.060019841	0.021513686	10.08333333
0.080340265	0.034823966	3.695652174
0.066505192	0.017625215	13.10152284
0.058587659	0.02715015	3.925373134
0.06877512	0.008265517	87.75705329
0.075737966	0.008618586	67.10383747
0.09845293	0.012827875	56.61043478
0.05151418	0.023875469	8.139240506
0.091544568	0.002309133	62.7080292
0.083236883	0.008577383	35.95833333
0.048944028	0.026322508	8.173652695
0.073960302	0.028512174	6.804347826
0.083633402	0.014276023	18.06481481
0.086988245	0.010228128	86.29233871
0.092461474	0.048789684	9.06122449
0.079861111	0.040208002	3.833333333
0.081172426	0.058318231	10.63358779
0.065905928	0.004056961	127.6597832
0.084043889	0.029414206	20.67479675
0.098184057	0.055580672	5.596491228
0.072575848	0.032711041	5.951219512
0.097378213	0.012559166	40.60671463
0.065809042	0.027371086	10.79268293
0.072958084	0.014424997	29.11027569
0.076352405	0.013644134	19.62256809
0.086685284	0.012644989	25.83221477
0.085	0.069239564	1.7
0.08175	0.047998291	16.35
0.085811195	0.02285189	41.36099585
0.075827697	0.040069708	8.037735849
0.065070538	0.02162658	20.10679612
0.085278529	0.015715175	36.66976744
0.075752797	0.016316492	53.10271041
0.057205859	0.018177935	20.99455041
0.045208948	0.013624395	11.25702811
0.079748616	0.005382294	94.5021097
0.085640138	0.022192705	5.823529412
0.081570134	0.001281332	117.6241331
0.077949523	0.0146505	14.18681319
0.082856583	0.01374885	83.68514851
0.066598012	0.011432753	23.10951009
0.075572499	0.01472358	14.58549223
0.095906009	0.031642179	13.33093525
0.075798605	0.013081316	27.21169916
0.072761302	0.015658663	54.1344086
0.091570248	0.069949076	5.036363636
0.076	0.049158896	3.8
0.048118357	0.014312902	5.677966102
0.103115488	0.034079797	27.53183521
0.054543549	0.012199525	34.52606635
0.095960911	0.007285852	73.31413613
0.07097853	0.023756284	12.98907104
0.0625	0.029227503	8
0.08285108	0.051433346	11.93055556
0.091538644	0.012119009	66.36551724
0.077402344	0.009990043	24.76875
0.076822917	0.018329858	7.375
0.118465419	0.005350717	264.6517457
0.052715204	0.017602979	12.54621849
0.044933588	0.007012956	25.7469459
0.043008388	0.003034363	75.56573705
0.052702693	0.001831158	84.79863269
0.087240528	0.031483792	7.240963855
0.040134922	0.006400503	39.85397784
0.064280497	0.041690443	4.756756757
0.053350189	0.012218275	52.9233871
0.092757494	0.013315023	99.99257885
0.053633218	0.014319671	23.70588235
0.074839302	0.044481914	4.939393939
0.08377583	0.006458522	77.66019417
0.086413724	0.049020375	15.64088398
0.086257544	0.012546299	20.44303797
0.059848324	0.00970921	35.78929766
0.062707888	0.01832618	10.59763314
0.044639163	0.005916142	25.31040564
0.072292395	0.07178109	4.409836066
0.057008898	0.015267687	15.62043796
0.103795153	0.019399196	25.22222222
0.061984075	0.035469622	14.5042735
0.060411523	0.024190325	8.155555556
0.0825	0.013507599	36.3
0.107455104	0.017030283	19.77173913
0.072984739	0.026174897	23.28213166
0.058957202	0.038608973	5.247191011
0.072896975	0.015826914	13.41304348
0.088713494	0.018468108	30.51744186
0.044347352	0.003656054	19.82326622
0.055179779	0.013247337	14.62264151
0.051022222	0.020598333	7.653333333
0.099419744	0.016805891	22.56828194
0.059196642	0.008828147	51.56027555
0.06952087	0.010406798	44.63239875
0.065824793	0.018427832	36.20363636
0.049091592	0.005844664	23.76033058
0.057531124	0.01906038	23.24257426
0.055891776	0.042923563	2.962264151
0.128472222	0.071527773	3.083333333
0.057634748	0.005754256	108.9873083

log.sigma.1.5.mm.3D_gldzm_HighIntensityLargeDistanceEmphasis	log.sigma.1.5.mm.3D_gldzm_SmallDistanceEmphasis	log.sigma.1.5.mm.3D_glcm_SumVariance	log.sigma.1.5.mm.3D_glcm_Homogeneity1
147.7521368	0.996794872	480.8437502	0.395313767
94.54545455	1	203.2281948	0.365871848
158.3928571	0.995535714	499.3123628	0.428030827
168.1521739	0.967391304	440.1277952	0.329225349
177.6395939	0.991680767	543.2932967	0.470295432
190.4925373	1	464.0743028	0.271753683
382.8675549	0.931337833	781.2850202	0.662316174
384.9638826	0.927036305	880.2860689	0.547861882
320.5895652	0.898686957	607.6498164	0.588777916
232.2405063	0.981012658	520.7588246	0.328645888
1710.516788	0.908901054	4095.565645	0.588344846
370.2708333	0.934510031	961.3113259	0.584798091
213.7365269	0.986526946	455.7243739	0.311040593
153.2391304	0.975543478	448.2525403	0.327351192
180.0740741	0.986111111	724.9993541	0.521857107
510.2701613	0.882910918	735.4465053	0.621601565
80.86734694	0.984693878	227.8591539	0.497994677
136	0.984375	344.8751579	0.348336772
113.9847328	0.965648855	181.942717	0.468079636
1142.338668	0.830845103	1085.386287	0.544789899
93.30894309	0.975609756	225.3342917	0.541000185
67.87719298	0.986842105	186.1341688	0.402802271
137.3902439	0.972560976	379.446432	0.460257541
350.9688249	0.881508127	558.8624541	0.644794206
140.6707317	0.990853659	530.1446618	0.552421585
742.2305764	0.799046227	484.432317	0.500034042
177.0817121	0.98540856	580.5706015	0.447842955
227.909396	0.957867263	678.9036472	0.548572342
110.65	1	225.177624	0.261934665
122.81	0.931111111	238.0815553	0.507346954
156.9232365	0.957378976	405.0210914	0.590213647
97.88679245	0.985849057	255.1728676	0.353427333
168.9029126	0.97815534	442.91619	0.489476843
287.7604651	0.905878553	537.4021146	0.584795912
200.2838802	0.957528531	473.5089387	0.538738696
204.5940054	0.989782016	633.3110445	0.450431731
276.7670683	1	711.7118218	0.2521377
676.649789	0.889830052	1120.729041	0.640409671
214.2941176	0.977941176	615.223012	0.407800818
3334.275312	0.885762342	6008.887094	0.61797759
179.9725275	0.987637363	565.2580605	0.449617794
339.4386139	0.857731702	382.4844778	0.572013716
257.9942363	0.969740634	754.3653472	0.450318774
190.880829	0.988341969	666.9767901	0.460705965
143.6546763	0.936300959	341.5640196	0.594291409
302.5654596	0.930628288	636.4381229	0.529848136
304.0846774	0.940092394	547.883186	0.637780126
67.58181818	0.986363636	125.0158279	0.463716872
91.14	1	248.6977656	0.398768643
584.2033898	0.917372881	840.6811977	0.261015414
137.2134831	0.958073242	443.9020049	0.626069608
324.8341232	0.946111989	637.228764	0.436220945
558.6662304	0.893338958	778.5610187	0.662995895
127.8469945	0.995901639	357.3664556	0.480963038
168.859375	0.98828125	462.1467246	0.347044403
107.2708333	0.958333333	253.5930223	0.471245302
358.24	0.885781226	532.114808	0.582042593
245.06875	0.978038194	834.7766239	0.5264688
204.1354167	0.984375	680.5083258	0.40633852
1295.691137	0.753543562	885.7232964	0.584023011
227.3571429	0.981092437	579.5640374	0.441602056
482.5811518	0.947268276	1032.705925	0.388912939
705.8594195	0.956649592	1775.917508	0.376747145
1466.617154	0.944406982	3384.225671	0.558196141
112.626506	1	355.2377449	0.484278875
799.5347432	0.865663813	1006.155767	0.29906786
149.7432432	0.97972973	316.7527862	0.291424475
289.6955645	0.964388721	664.1468108	0.501321769
579.7903525	0.815191586	383.5908637	0.622853663
240.8076923	0.968828557	514.0113406	0.43035594
102.9545455	0.988636364	287.1021259	0.428625529
935.5641855	0.849239005	1094.298378	0.592591646
95.35911602	0.979281768	323.2441557	0.495245819
233.6666667	0.966010314	656.2011303	0.527365344
273.6722408	0.969899666	711.7139872	0.548445472
226.3491124	0.973372781	454.2976522	0.463676285
899.3156966	0.760606506	875.587873	0.299235035
83.3442623	1	153.344213	0.32829101
243.3722628	0.980839416	552.5120031	0.36953526
165.9176955	0.932327389	423.6094625	0.581850927
147.7521368	0.996794872	480.8437502	0.395313767
151.1925926	0.972222222	288.6704477	0.385972378
246.7340909	0.958491162	831.161951	0.62538214
157.1467391	0.974033816	472.4210111	0.545168659
135.1316614	0.981191223	385.4678691	0.553050644
172.1685393	0.983146067	340.2492732	0.311484185
195.7608696	0.975543478	500.0790484	0.467157243
151.9360465	0.97565407	448.9414317	0.655732956
1229.946309	0.755856948	1257.255674	0.339216791
255.0226415	0.971174004	568.4095863	0.416385153
219.8733333	0.985	511.9097197	0.275324166
130.814978	0.983480176	481.7323656	0.570513733
310.4902411	0.950464026	667.635235	0.474352941
446.0451713	0.884476462	604.1479857	0.556238855
157.1109091	0.977929293	309.6168873	0.45601782
464.4132231	0.976469238	1292.508521	0.365289971
187.5767327	0.988861386	489.9583445	0.445065026
178.2075472	1	404.0356492	0.258545189
62.125	1	123.2689865	0.417571748
768.6377578	0.860819085	927.008369	0.523994884

log.sigma.1.5.mm.3D_glcm_Homogeneity2	log.sigma.1.5.mm.3D_glcm_ClusterShade	log.sigma.1.5.mm.3D_glcm_MaximumProbability	log.sigma.1.5.mm.3D_glcm_Idmn	log.sigma.1.5.mm.3D_glcm_SumVariance2
0.315195523	-93.93766773	0.043964987	0.976238595	23.12900374
0.280736891	54.8920265	0.050355255	0.969162457	23.50044078
0.356664509	-101.1543791	0.101098279	0.973327648	26.8987703
0.236651885	-24.93601151	0.041847804	0.957649914	21.42311848
0.404266423	-50.95681945	0.127108797	0.984198987	17.99658728
0.177998379	-45.72823088	0.030508256	0.945054069	50.81356347
0.643147308	-18.74125806	0.205030369	0.996918308	6.00066732
0.501882946	-72.48497035	0.157021267	0.993010572	14.92252329
0.55159461	-49.0790447	0.154699011	0.992424239	8.60635556
0.242634228	134.0211144	0.032644275	0.964357157	41.32863744
0.551984503	-26.72943824	0.14039941	0.997547971	7.628602608
0.55077243	-52.41418324	0.205341626	0.996498161	8.77128619
0.224401683	69.32519661	0.026997774	0.95868453	42.77695072
0.233062064	-59.35788073	0.025302562	0.960201665	29.13386973
0.469960412	-97.92324658	0.141209608	0.988844909	16.91554977
0.593197355	-69.90955444	0.1910811	0.993953245	9.680491731
0.436893324	-25.3180517	0.084340857	0.982220876	10.93860996
0.256664138	13.28138221	0.036791832	0.968413441	24.84819043
0.40274309	0.582714455	0.090693607	0.983881292	12.9820184
0.499033496	-22.00813152	0.132216873	0.995275312	11.64744227
0.488161382	-28.92352175	0.158968777	0.982913362	10.72321463
0.319229458	-13.40719505	0.038148841	0.966160799	15.66658801
0.393500838	-80.10860851	0.101631586	0.976111981	18.6011197
0.620838681	-15.75050536	0.20640581	0.996351528	4.826217978
0.509084694	-149.8978988	0.206765763	0.982867588	22.49518017
0.444971108	124.2679672	0.110512157	0.98893522	21.25830244
0.376301518	-61.44234783	0.068529296	0.985409004	17.85104319
0.500323506	-45.98872226	0.136170263	0.989966763	9.947885698
0.163915533	66.70440243	0.091750518	0.899131213	30.36718341
0.449586049	-33.62928367	0.101405246	0.982921393	11.7762287
0.551781887	-58.15817223	0.151331254	0.990954532	11.43766052
0.263338404	-25.67332327	0.02865999	0.957301519	21.35115516
0.430249605	-58.20284268	0.099847215	0.983260436	16.76393461
0.546925908	-57.45200572	0.144676459	0.99240059	9.306079112
0.489912118	-49.31643024	0.121884733	0.990984612	12.31492084
0.385220037	-186.6979922	0.101398919	0.980823687	31.20157653
0.160281256	87.77270775	0.018286188	0.960506562	55.56043586
0.615200287	-38.3586773	0.197806884	0.997019795	9.917355277
0.325998954	-35.19445632	0.052543423	0.976921575	15.35957729
0.589610691	3.297834514	0.186569052	0.998689656	7.744010609
0.380261369	-34.83394992	0.079797827	0.984981466	16.76395664
0.532431576	17.17366732	0.150254163	0.993919485	7.837290387
0.37793953	-61.19826934	0.057791765	0.986475864	18.40090229
0.39383634	-66.42772258	0.084007894	0.984498848	18.32808085
0.556254511	-31.52952391	0.198630087	0.987159879	7.574247707
0.479235781	-57.8004532	0.132596646	0.990714219	13.38934523
0.612460023	-29.49032642	0.186352442	0.995959585	7.425265973
0.392775008	6.756711606	0.075787177	0.977331636	14.08401553
0.315617167	-30.03384987	0.063695609	0.957381971	17.75924413
0.173962727	1574.090764	0.018709948	0.951600124	110.3943026
0.596849782	-43.31665121	0.222126668	0.991370209	7.202034615
0.369915041	41.08156482	0.074781488	0.983729091	24.98803607
0.643465654	-13.82419999	0.222567707	0.997596129	4.130994943
0.416700739	-37.29154932	0.077045435	0.982528523	15.51083523
0.264464058	22.58237328	0.043696655	0.9650445	37.9845846
0.402744504	-34.83605716	0.07212892	0.972466963	13.43630971
0.543422475	-27.27303054	0.151158602	0.993194112	7.665462452
0.475746869	-84.45550047	0.132129287	0.990067201	17.04115187
0.325406888	-92.82425685	0.049524803	0.979725801	23.51696346
0.545496977	-12.86377753	0.140528709	0.997542851	5.085711814
0.377640602	6.698061667	0.113530899	0.981916272	26.34312752
0.316179743	20.05380664	0.054922697	0.980039455	34.89345061
0.306349261	0.423585849	0.065560995	0.98565079	44.74571295
0.518037967	-78.1270649	0.140385201	0.995901487	17.65094925
0.420479825	-52.62123542	0.119797196	0.976256788	15.57261938
0.216952092	319.444111	0.035262636	0.964324796	65.30339952
0.198643936	125.0210378	0.030470225	0.946935861	39.25592447
0.450813977	-0.003197746	0.140597313	0.990970188	21.06147052
0.593971367	-5.863749088	0.173074532	0.995571117	5.705832516
0.361123671	11.4991136	0.071683978	0.981899594	22.69766568
0.353868322	-59.73816089	0.068642486	0.962738433	19.05780376
0.559959568	-73.04537612	0.204649788	0.995998787	10.51099971
0.435291368	-67.95680523	0.125693548	0.983233724	18.18349669
0.475918935	-53.26647222	0.114430303	0.992832766	14.02239514
0.504886545	25.09916696	0.167230321	0.991476501	16.04134023
0.398065726	80.73305563	0.111599856	0.984167555	21.13249389
0.211849162	287.3759571	0.016602408	0.96596593	56.6280853
0.231596758	24.2531193	0.033978577	0.949447393	24.61181997
0.289040848	28.63831034	0.053958895	0.971084744	28.23538236
0.54190605	-31.4264845	0.148316597	0.988130987	6.888235848
0.315195523	-93.93766773	0.043964987	0.976238595	23.12900374
0.304182242	15.61930783	0.059701598	0.969014523	21.91219652
0.597339604	-80.52351572	0.199330062	0.992474231	10.51245652
0.495814812	-23.15834727	0.124686711	0.992058899	7.794098939
0.506124814	-44.89132933	0.17493873	0.986086865	11.7602851
0.21969475	42.60309847	0.024079939	0.950677409	31.43563328
0.400085502	-53.57130851	0.071774647	0.988964261	18.17191888
0.632069237	-32.2070374	0.246804964	0.995923951	6.666916832
0.263541429	414.9156007	0.05110156	0.971176053	68.01505363
0.344180116	-3.847459995	0.060342846	0.975815369	23.61144225
0.187808039	90.74451506	0.019598974	0.957430338	50.51558011
0.526522738	-30.84811757	0.167882668	0.989078005	7.742889039
0.413352337	-18.29070128	0.090517579	0.987169331	16.0153065
0.513190242	-15.13545225	0.141278161	0.992158246	12.28771939
0.388274056	16.08578474	0.07207841	0.984923601	15.21985968
0.287497735	43.46988707	0.054537523	0.985095223	42.08748094
0.377209409	6.622561645	0.092605935	0.985309611	22.07513876
0.168359145	154.2642773	0.031615118	0.92746262	50.62078422
0.33512328	-8.391052502	0.076292118	0.954868691	9.213915029
0.475361016	-47.29782253	0.133211712	0.98883214	17.0874335

log.sigma.1.5.mm.3D_glcm_Contrast	log.sigma.1.5.mm.3D_glcm_DifferenceEntropy	log.sigma.1.5.mm.3D_glcm_InverseVariance	log.sigma.1.5.mm.3D_glcm_Entropy	log.sigma.1.5.mm.3D_glcm_Dissimilarity
16.50164836	3.114875218	0.312809042	6.7578053	2.959015308
22.75667896	3.10373746	0.279364154	6.021332432	3.423651549
18.7690024	3.177697956	0.291883913	6.396028353	3.036571566
21.30944995	3.154469445	0.248868892	6.041634129	3.642409238
11.6703861	2.874437595	0.340853471	6.012217736	2.351406094
40.53051747	3.430415269	0.186364528	6.2318562	5.050691733
4.267819594	1.871617954	0.468097525	3.792219033	1.064994491
8.077883196	2.527035317	0.413967699	5.335495309	1.763455515
4.279712349	2.171669516	0.458342161	4.625101882	1.318518453
41.34732358	3.606355758	0.238935983	7.224708684	4.495380933
6.452106802	2.182382391	0.464830945	4.746440976	1.383388427
5.187693333	2.137916057	0.490521524	4.487667948	1.34472723
37.70216579	3.641297878	0.232365523	7.305100781	4.599567869
19.80125409	3.173663675	0.252279015	6.696447756	3.547700179
9.422620808	2.658480921	0.39039074	5.399635051	1.982174685
4.739456365	2.085783086	0.460436646	4.290133309	1.249313665
6.99343561	2.56132539	0.392562499	5.609645166	1.875123941
18.98429006	3.041273606	0.271469924	6.161893785	3.326050475
10.39731552	2.729340788	0.386642291	5.898714109	2.184942146
8.735476654	2.544453257	0.419914095	5.453256819	1.798867649
6.017714064	2.48536833	0.386263303	5.437718448	1.67701332
9.582371176	2.731599726	0.341908033	6.063435172	2.444551392
10.81946226	2.786157634	0.370404932	5.918200086	2.293841871
2.805280598	1.857801437	0.481441841	3.95015109	1.00663675
13.14508008	2.699028252	0.366484935	5.124713536	2.139206567
15.47514297	2.818282591	0.388181365	5.853999716	2.303866897
10.64425391	2.823770416	0.352918987	6.278827896	2.338195535
5.697559049	2.389453571	0.424793891	5.228974239	1.577731686
37.76962327	2.621713712	0.158792326	4.178332172	5.046845788
6.715587761	2.540358805	0.406261074	5.62562139	1.814568229
5.108391266	2.277887178	0.425084961	4.788650905	1.420082066
17.70050333	3.127642147	0.27086803	6.636382105	3.277839941
11.70962093	2.815438795	0.374521601	5.983976456	2.233837735
5.193091139	2.22411865	0.453463099	4.686924378	1.38804776
7.047864911	2.494810081	0.420016827	5.42076619	1.716339763
16.87779889	3.059535751	0.344338844	6.515643644	2.7289154
57.88325086	3.887490339	0.164502542	7.636484876	5.946123022
5.91739577	2.068067387	0.445575272	4.150819032	1.246380761
11.26711695	2.790910422	0.336040734	6.040158315	2.528009963
6.43845649	2.135145532	0.461988651	4.370619225	1.333526327
10.9109704	2.845065893	0.362131828	6.185610511	2.355724884
7.303155972	2.320384586	0.451877306	4.921557129	1.540155859
9.750767221	2.761082351	0.353209254	6.348890655	2.264553134
11.24998543	2.868788578	0.349770935	6.027976042	2.371371731
4.061658131	2.175272569	0.440665771	4.672952634	1.302082721
7.200195526	2.52123592	0.416432861	5.41657126	1.770206625
3.870484532	1.978528319	0.466609695	4.150032803	1.124957963
8.121873077	2.634176086	0.360930173	5.717629093	2.092117231
14.43681781	2.934630808	0.295025431	5.682372663	2.841559173
86.79884291	4.00120639	0.184512267	7.272350011	6.621227413
3.723421235	2.042825761	0.454851768	4.180209534	1.171187094
23.72528256	3.20801955	0.335097266	6.732108309	3.091424775
2.927912817	1.78694952	0.481271939	3.706836435	0.956230632
7.599401993	2.59547237	0.391698072	5.960907835	1.975321359
29.67606799	3.438715791	0.262365915	6.899480186	3.972045111
7.856191734	2.67415021	0.365595029	5.976642718	2.057876473
4.54992735	2.191562659	0.460288052	4.762157653	1.348289095
8.91974194	2.630090429	0.396552021	5.469384546	1.929774383
12.65957364	2.915928147	0.322128473	6.423344409	2.663984521
4.35580229	2.105444864	0.471707172	4.470762278	1.283627531
24.96591804	3.241115588	0.322742891	6.515297092	3.191891312
34.41444324	3.496868308	0.295714598	7.23184782	3.849852633
43.82348474	3.639381351	0.295009135	7.68933641	4.331279714
13.5380438	2.634051362	0.408322471	5.439291735	2.019216294
8.507733733	2.690664741	0.367205503	5.721037578	2.074663506
63.07176113	4.012146016	0.212455936	8.417445741	5.67730688
33.05465661	3.452573307	0.216657909	6.566187492	4.525565437
18.4954439	2.869461937	0.403352869	5.900500741	2.455363334
4.024722087	2.019674693	0.470537562	4.313557911	1.163992843
19.43983569	3.158621663	0.332518634	6.709538781	2.942073548
12.6371614	2.895892903	0.332735613	5.986174417	2.57839911
5.200971526	2.146382685	0.483206156	4.505636387	1.341225716
9.768138746	2.743617829	0.366757787	5.725262665	2.117263948
8.446581586	2.551914616	0.410599042	5.398527439	1.831943435
14.53397482	2.681158876	0.398066633	5.492579172	2.085631667
17.91852197	2.973809121	0.338348981	6.245265336	2.625646151
47.96331908	3.833307565	0.217619918	8.249399327	5.064421882
21.45810064	3.165668674	0.223672193	6.21456984	3.646735081
25.7105165	3.404226168	0.275564716	7.154921188	3.644975425
3.692407713	2.14844401	0.457812429	4.675937443	1.293650924
16.50164836	3.114875218	0.312809042	6.7578053	2.959015308
18.52025994	3.197966449	0.283335057	6.717372145	3.178981886
5.096183871	2.118105557	0.445888086	4.166116944	1.287130044
4.445916018	2.263455221	0.444995969	5.133013459	1.464941457
7.436369324	2.514446151	0.398898643	5.305121715	1.723806993
31.22259985	3.447612618	0.228814723	6.858587501	4.253525932
10.87329304	2.772890344	0.365199361	6.162151977	2.235238356
3.146496665	1.904756468	0.451519416	3.893444228	1.030615343
55.97962637	3.853861335	0.248065598	7.938643431	5.090907165
20.29955937	3.182159395	0.324757454	6.729899171	3.052290521
53.61575864	3.854116934	0.185729491	7.468719298	5.608299025
4.218307053	2.234258282	0.437518231	4.883363654	1.386477243
13.51272427	2.886514465	0.378024461	6.235672912	2.379613735
9.540812043	2.490836839	0.428112762	5.304836271	1.756600417
12.1009661	2.869362456	0.366143877	6.364325247	2.369201106
36.08566821	3.576608102	0.264789838	7.378778082	4.09899068
17.84255763	3.083421289	0.332673293	6.461710185	2.775070136
57.21217582	3.733837462	0.179806246	6.324078437	5.845483106
8.826828772	2.579638123	0.339833215	5.208332885	2.321706562
12.57036399	2.746668466	0.396884493	5.85343867	2.135099807

log.sigma.1.5.mm.3D_glcm_DifferenceVariance	log.sigma.1.5.mm.3D_glcm_Idn	log.sigma.1.5.mm.3D_glcm_Idm	log.sigma.1.5.mm.3D_glcm_Correlation	log.sigma.1.5.mm.3D_glcm_Autocorrelation
7.710912548	0.90203582	0.315195523	0.163252804	164.7701346
10.82734229	0.889686052	0.280736891	0.015118708	75.43148407
9.396657547	0.901320032	0.356664509	0.171665313	169.2152964
7.899678128	0.862593455	0.236651885	0.005128339	148.5175866
6.117206715	0.923248228	0.404266423	0.211662885	181.2268326
14.26418008	0.844653852	0.177998379	0.116908959	153.9054114
3.130374247	0.973137473	0.643147308	0.16505164	232.3689015
4.949011421	0.95269821	0.501882946	0.294345082	273.0520771
2.530681643	0.949332355	0.55159461	0.329492026	190.8590328
21.01083886	0.885622467	0.242634228	0.001947879	174.7712722
4.534797914	0.973968236	0.551984503	0.083498885	1123.358532
3.37211006	0.966796584	0.55077243	0.254102423	287.1995627
16.41483107	0.870877131	0.224401683	0.060519322	156.0614839
7.106122004	0.864961302	0.233062064	0.189829768	155.4731765
5.443571066	0.93890347	0.469960412	0.280092283	229.7714416
3.160363915	0.959020984	0.593197355	0.331405407	223.7600015
3.462398832	0.91666615	0.436893324	0.217556897	84.94223912
7.807567512	0.882496564	0.256664138	0.138428636	121.5298402
5.607139548	0.922935783	0.40274309	0.11002104	70.40938222
5.491889432	0.961359386	0.499033496	0.141818356	328.465221
3.19483078	0.921558104	0.488161382	0.27875662	83.82956939
3.56748672	0.876059126	0.319229458	0.236320629	73.03195155
5.534209003	0.905812356	0.393500838	0.262870076	132.28273
1.790576669	0.9659756	0.620838681	0.261345179	172.4652782
8.427969818	0.932387522	0.509084694	0.248172855	171.9135814
10.13047045	0.943783024	0.444971108	0.159487289	161.8988745
5.107117494	0.922773658	0.376301518	0.248309758	193.2573074
3.190155363	0.940173279	0.500323506	0.269990843	215.0946776
11.36634947	0.789605898	0.163915533	-0.100918766	68.78156498
3.410644005	0.91924308	0.449586049	0.269207724	88.16520006
3.066598354	0.94615085	0.551781887	0.37400905	135.0006193
6.681034068	0.863804665	0.263338404	0.094650927	94.96442756
6.700236456	0.925075849	0.430249605	0.174201235	150.6540151
3.256976385	0.951146953	0.546925908	0.279206591	170.8493782
4.093833668	0.944322244	0.489912118	0.269214319	157.3824808
9.402456367	0.919019845	0.385220037	0.288343064	209.3864842
21.72812647	0.868376746	0.160281256	-0.007968726	229.8542845
4.354162508	0.973654289	0.615200287	0.250490953	328.753864
4.822253873	0.899545926	0.325998954	0.154264198	201.5330913
4.654689678	0.981861998	0.589610691	0.092543314	1615.374741
5.345392282	0.922421953	0.380261369	0.208060344	187.9869664
4.923094248	0.958658762	0.532431576	0.036397365	126.4354778
4.426555711	0.924696536	0.37793953	0.286525504	244.107402
5.588578089	0.922218174	0.39383634	0.237049814	217.0999279
2.358432427	0.934544666	0.556254511	0.300351753	115.2992314
4.045775588	0.942562922	0.479235781	0.294387755	204.4547991
2.601959202	0.966084658	0.612460023	0.312445441	171.5678719
3.702494554	0.903309051	0.392775008	0.269597815	52.54966761
6.25634241	0.869154637	0.315617167	0.104082662	91.11343412
42.35658669	0.86722801	0.173962727	0.132050077	260.047998
2.34209835	0.948954061	0.596849782	0.315224227	142.0299437
14.11883432	0.928122391	0.369915041	0.028247494	209.0010775
2.012802838	0.974028227	0.643465654	0.168982111	230.9277745
3.544683855	0.916343463	0.416700739	0.339716182	127.1239897
13.83133021	0.882809315	0.264464058	0.12346204	158.7257984
3.61226133	0.894934361	0.402744504	0.260262458	94.07672019
2.724031267	0.952061332	0.543422475	0.248403823	169.8616038
5.166738836	0.942116643	0.475746869	0.30966985	261.1252334
5.519193083	0.906491741	0.325406888	0.297677398	223.5853341
2.706852528	0.97091316	0.545496977	0.077791713	264.1447997
14.73255934	0.924331352	0.377640602	0.027834209	191.3800252
19.54914381	0.918410766	0.316179743	0.008205937	324.033874
24.94341695	0.930641038	0.306349261	0.0122874	533.2896698
9.454966881	0.967560051	0.518037967	0.130419205	947.9478091
4.16622327	0.905059854	0.420479825	0.288612615	124.8274253
30.72738139	0.884781547	0.216952092	0.023828766	317.7889264
12.33222552	0.848302754	0.198643936	0.089460118	111.6128172
12.41785159	0.950719235	0.450813977	0.06904223	212.7850567
2.668290631	0.963789306	0.593971367	0.169948018	124.9283245
10.75988153	0.920669491	0.361123671	0.075693257	173.6890186
5.930068947	0.880190855	0.353868322	0.200412744	103.7949452
3.397299787	0.965230023	0.559959568	0.336620059	323.9652249
5.239990696	0.922432942	0.435291368	0.298069538	115.0833473
5.075229343	0.950809884	0.475918935	0.24820505	209.8443346
10.15038769	0.953918547	0.504886545	0.051252653	224.1602018
10.98718677	0.929111355	0.398065726	0.082571686	154.6366604
22.16707199	0.884305205	0.211849162	0.089187009	282.3500803
8.104034645	0.851109347	0.231596758	0.065109426	60.04095497
12.37570962	0.894513594	0.289040848	0.046717374	186.2381065
2.014537447	0.934241662	0.54190605	0.299647804	138.7162675
7.710912548	0.90203582	0.315195523	0.163252804	164.7701346
8.347763547	0.888241462	0.304182242	0.078428087	105.6422869
3.420981743	0.955063298	0.597339604	0.340263337	250.2569577
2.293705006	0.943349663	0.495814812	0.271630302	155.6671169
4.447862927	0.933657578	0.506124814	0.220915367	130.8787353
12.74547467	0.8571602	0.21969475	-0.008497298	119.2877727
5.851158313	0.935177888	0.400085502	0.245657844	169.2237893
2.081701292	0.965458218	0.632069237	0.355260533	142.1599906
29.79104613	0.900411177	0.263541429	0.105187042	390.3205075
10.95522743	0.907717052	0.344180116	0.075461352	190.0181353
22.00415068	0.866273142	0.187808039	-0.026666802	170.1925698
2.273126413	0.93657316	0.526522738	0.283352002	156.9478246
7.836848674	0.934337617	0.413352337	0.084569704	216.0734507
6.451547722	0.953609384	0.513190242	0.125293506	193.5267618
6.472624233	0.925309874	0.388274056	0.11119531	111.6553832
19.23572385	0.925700689	0.287497735	0.076791437	399.8493111
10.07903605	0.92864781	0.377209409	0.104441321	166.0926459
22.63269718	0.828216443	0.168359145	-0.06436848	131.6735074
3.331394756	0.85984061	0.33512328	0.026593771	49.31134577
8.00526838	0.942926136	0.475361016	0.151114352	287.6307133

log.sigma.1.5.mm.3D_glcm_SumEntropy	log.sigma.1.5.mm.3D_glcm_AverageIntensity	log.sigma.1.5.mm.3D_glcm_Energy	log.sigma.1.5.mm.3D_glcm_SumSquares	log.sigma.1.5.mm.3D_glcm_ClusterProminence
4.149920846	12.77118112	0.014492793	9.907663025	2061.987213
3.946850701	8.673131025	0.019046226	11.56427993	2590.559664
4.126510067	12.92901247	0.02594002	11.41694317	2328.864446
3.911707327	12.1846647	0.017493713	10.68314211	1254.863458
3.887514596	13.40726965	0.034346036	7.461709834	1109.61414
4.277489134	12.30018306	0.014576009	22.83602023	6309.197656
2.615090111	15.25143711	0.153726494	2.654174559	535.0737271
3.527982262	16.50070759	0.065864396	5.933219546	1588.558004
3.076890243	13.77586946	0.086604451	3.221516977	580.8199299
4.545089184	13.38117819	0.009535176	22.30399975	7330.073061
3.087338375	33.54212067	0.080107861	3.520376321	1577.705188
2.977952449	17.00756583	0.102314416	3.560958199	975.0762106
4.562638449	12.50147732	0.00847858	20.81105791	6504.184918
4.278759562	12.37447443	0.011462826	12.23378095	2396.26931
3.583085536	15.10198595	0.058062531	6.646182382	1519.890844
2.894749122	14.91873258	0.117334095	3.615694353	940.3998144
3.598043321	9.16262643	0.034339066	4.483011392	417.8292044
4.026758207	10.95684741	0.016150549	10.95812012	2147.808439
3.706824963	8.352162206	0.030757536	5.844833481	964.1607556
3.439235204	18.16943271	0.06511613	4.935212974	1110.833357
3.533645559	9.091249227	0.051131077	4.185232172	410.8427361
3.859115138	8.455347174	0.017544739	6.312239795	606.8860745
3.837842308	11.4161577	0.029831451	7.35514549	1303.750465
2.688929946	13.1145901	0.127656515	1.924541201	330.6080601
3.513974625	13.02495017	0.083937377	8.937758846	2597.33967
3.812743022	12.72557657	0.043726996	9.822767902	5181.851261
3.953017575	13.83952671	0.022184121	7.157052479	1224.634062
3.395813558	14.62977388	0.057040667	3.911361187	646.0456109
2.889812059	8.36264348	0.060051777	17.03420167	2829.067599
3.601345484	9.3218084	0.039568305	4.622954114	590.798956
3.263089206	11.5504626	0.086638522	4.136512947	712.7440005
4.108989138	9.696590542	0.012277296	9.762914623	1306.484974
3.802223371	12.22229779	0.037645744	7.118388885	1284.722509
3.082880483	13.03147246	0.085156844	3.624792563	862.3637423
3.510144572	12.49378121	0.057079683	4.856603704	789.0247251
4.155864077	14.3678851	0.029490351	12.19955371	4330.52839
4.754720933	15.52136052	0.006050223	29.90553757	10315.29866
2.879236006	18.15829571	0.130721218	3.801642394	1546.061512
3.82883292	14.15984823	0.019579583	6.656673559	907.6577178
2.908340596	40.23768792	0.115781815	3.572721972	1013.34304
3.895369477	13.66289197	0.024179945	6.992493238	1059.598464
3.121178384	11.25655324	0.076211019	3.976918722	884.2401392
3.983945208	15.55444051	0.019924064	7.047558174	1432.254618
3.880188999	14.67626117	0.030784871	7.423229318	1303.369175
3.11835577	10.69668867	0.086093075	2.90897646	337.5863123
3.52898711	14.24677788	0.056269093	5.172046903	902.5299075
2.88124647	13.07042442	0.126520345	2.881635984	574.7453704
3.760298919	7.145013945	0.02839614	5.551472152	740.897017
3.811345745	9.500473356	0.025256487	8.049015483	1122.304148
4.861229428	16.39615392	0.007621583	53.56332305	65669.2077
2.865025233	11.88105088	0.116946458	2.731363963	518.4721541
4.148688693	14.56789556	0.023579276	12.93525358	4158.70529
2.544330474	15.19545093	0.148379605	1.810306362	246.4784181
3.885690274	11.18644845	0.029586911	5.777559307	943.8710213
4.43756065	12.5783902	0.011839746	17.69066275	5277.682918
3.758265156	9.626706866	0.02536004	5.323125362	579.5642867
3.105889117	13.00311042	0.08140727	3.053847451	522.5410823
3.597302578	16.10432108	0.059273111	6.54748782	1275.172934
4.092738727	14.86120935	0.01622143	9.044134276	1970.815179
2.81833156	16.30038851	0.084941588	2.367232106	465.7856618
4.123079158	13.9727158	0.028161772	13.60541893	3977.328212
4.407317646	18.25382886	0.016374464	17.53458246	6801.060812
4.56943349	23.3994482	0.015377312	22.62031496	10448.55014
3.522072119	30.85930132	0.077354375	7.821123861	2981.986593
3.757235008	11.09304171	0.037933827	6.020088278	964.2832246
4.949034706	18.23495737	0.005939249	32.85587813	18734.02825
4.325327084	10.48947273	0.011969754	18.07764527	5593.775337
3.771381927	14.72333453	0.053307949	10.46565597	3810.183908
2.877517777	11.16232973	0.10798239	2.479723929	410.097457
4.131214692	13.20350663	0.021969124	11.0299525	2504.14211
3.847876603	10.1082917	0.023414434	7.923741291	1089.859049
3.003440301	18.04132694	0.105791264	3.996814277	1178.957783
3.793545573	10.62860116	0.042258672	6.987908859	1112.62387
3.534676503	14.45567832	0.053688609	5.801061035	1160.623696
3.543387082	15.06706717	0.069158293	8.118969566	3213.151272
3.99347369	12.44206273	0.028273358	10.19888216	3409.219162
4.860231129	17.064096	0.005203899	27.14816798	12321.72362
4.052968914	7.6955883	0.01522758	11.51748015	1718.553428
4.350882015	13.67352321	0.012077997	13.99014324	2997.777692
3.074159492	11.74375132	0.075436849	2.64516089	331.6459672
4.149920846	12.77118112	0.014492793	9.907663025	2061.987213
4.141575845	10.23650089	0.014428372	10.10811411	1605.865015
2.906434555	15.77658053	0.12667862	3.902160097	1081.456753
3.331464649	12.44293285	0.050480468	3.060003739	333.5545647
3.454686708	11.39272431	0.064948607	4.799163607	710.1784727
4.268534671	10.91815022	0.010128786	15.66455828	3820.070937
3.925762912	12.94717321	0.025685183	7.379404762	1426.206162
2.74201968	11.88720604	0.142018116	2.467921249	395.3406904
4.875319731	20.03687143	0.010219897	31.37354123	20000.29901
4.168840744	13.79650575	0.018286732	11.36435039	2528.341547
4.671744847	13.34001812	0.006778783	27.77460762	7863.88734
3.215189706	12.49241827	0.066942356	2.990299023	341.7029377
3.829736512	14.7253285	0.032876468	7.716328802	1517.595145
3.445360267	13.9223149	0.067807819	5.71610131	1331.082241
3.902413059	10.53461928	0.023001725	6.895115985	1211.568711
4.556933535	20.16905044	0.012612486	19.91983699	9169.484104
4.063416024	12.90820469	0.026207209	10.48355919	3152.249727
4.298438785	11.54512146	0.013684909	26.95824001	8218.709318
3.35403342	7.015063087	0.033591407	4.51018595	249.5451737
3.687993332	17.00218252	0.057985355	7.727602927	1984.118353

log.sigma.1.5.mm.3D_glcm_SumAverage	log.sigma.1.5.mm.3D_glcm_Imc2	log.sigma.1.5.mm.3D_glcm_Imc1	log.sigma.1.5.mm.3D_glcm_DifferenceAverage	log.sigma.1.5.mm.3D_glcm_Id
25.54236225	0.687219787	-0.091473279	2.959015308	0.395313767
17.34626205	0.92826892	-0.283346958	3.423651549	0.365871848
25.85802493	0.782709881	-0.141428793	3.036571566	0.428030827
24.3693294	0.936157985	-0.299682671	3.642409238	0.329225349
26.80581973	0.710793111	-0.113017873	2.351406094	0.470295432
24.60036611	0.989043422	-0.475672126	5.050691733	0.271753683
30.45882755	0.581273983	-0.104490394	1.064994491	0.662316174
32.94465638	0.541059025	-0.066152672	1.763455515	0.547861882
27.55173891	0.504330046	-0.066489024	1.318518453	0.588777916
26.43883066	0.891641537	-0.196833954	4.495380933	0.328645888
67.02430165	0.490843066	-0.057576729	1.383388427	0.588344846
33.84091543	0.564868772	-0.084358723	1.34472723	0.584798091
24.88158864	0.898340817	-0.202387311	4.599567869	0.311040593
24.74894886	0.887719931	-0.210061108	3.547700179	0.327351192
30.19223293	0.644890664	-0.099744615	1.982174685	0.521857107
29.83431379	0.551285285	-0.088020409	1.249313665	0.621601565
18.32525286	0.655947441	-0.096648092	1.875123941	0.497994677
21.91369482	0.92643854	-0.276295569	3.326050475	0.348336772
16.70432441	0.668519939	-0.096302755	2.184942146	0.468079636
36.20693469	0.557688233	-0.066875041	1.798867649	0.544789899
18.18249845	0.608748777	-0.084410122	1.67701332	0.541000185
16.91069435	0.773821404	-0.142255818	2.444551392	0.402802271
22.8323154	0.774781586	-0.147102383	2.293841871	0.460257541
26.22663576	0.493369434	-0.070143349	1.00663675	0.644794206
26.04336994	0.75833244	-0.163167184	2.139206567	0.552421585
25.33383458	0.657490982	-0.093583979	2.303866897	0.500034042
27.67266508	0.636540218	-0.081521019	2.338195535	0.447842955
29.25954777	0.537911304	-0.066135577	1.577731686	0.548572342
16.72528696	0.996201466	-0.765086445	5.046845788	0.261934665
18.6436168	0.592327152	-0.076957908	1.814568229	0.507346954
23.1009252	0.575261256	-0.087537481	1.420082066	0.590213647
19.39318108	0.815195978	-0.153274218	3.277839941	0.353427333
24.44459559	0.67010507	-0.095889583	2.233837735	0.489476843
26.06294492	0.525409409	-0.070214045	1.38804776	0.584795912
24.98503151	0.577786329	-0.074518602	1.716339763	0.538738696
28.68941999	0.761038466	-0.127382167	2.7289154	0.450431731
30.33407226	0.932490072	-0.239175514	5.946123022	0.2521377
36.20786659	0.541073924	-0.082924193	1.246380761	0.640409671
28.31969646	0.807632232	-0.162062648	2.528009963	0.407800818
80.37531926	0.542211288	-0.077953087	1.333526327	0.61797759
27.31434036	0.677800406	-0.096406946	2.355724884	0.449617794
22.47678509	0.523145218	-0.063619337	1.540155859	0.572013716
31.10683879	0.646016166	-0.091359113	2.264553134	0.450318774
29.34791555	0.659211128	-0.09199826	2.371371731	0.460705965
21.39337734	0.572971452	-0.084538024	1.302082721	0.594291409
28.48877485	0.63123846	-0.093655713	1.770206625	0.529848136
26.12877721	0.56733222	-0.092102835	1.124957963	0.637780126
14.29002789	0.768551208	-0.146029242	2.092117231	0.463716872
19.00094671	0.905141786	-0.263535733	2.841559173	0.398768643
31.87956214	0.96517969	-0.30761861	6.621227413	0.261015414
23.76210176	0.502946014	-0.070104995	1.171187094	0.626069608
28.8916539	0.691648028	-0.091966597	3.091424775	0.436220945
30.37279277	0.496469438	-0.074457699	0.956230632	0.662995895
22.37289689	0.680156049	-0.105414982	1.975321359	0.480963038
25.03058733	0.868794942	-0.185148286	3.972045111	0.347044403
19.25341373	0.622588982	-0.07962208	2.057876473	0.471245302
26.00622085	0.499336878	-0.062333711	1.348289095	0.582042593
32.19252343	0.657090239	-0.101844486	1.929774383	0.5264688
29.72241871	0.791567859	-0.143565275	2.663984521	0.40633852
32.49382376	0.3901778	-0.036938941	1.283627531	0.584023011
27.64217237	0.822776843	-0.159699403	3.191891312	0.441602056
35.99504725	0.7519952	-0.109683883	3.849852633	0.388912939
46.17557004	0.72291563	-0.091932247	4.331279714	0.376747145
61.5440071	0.6778687	-0.108129299	2.019216294	0.558196141
22.18608343	0.742024513	-0.134401934	2.074663506	0.484278875
35.62142321	0.717430452	-0.08228215	5.67730688	0.29906786
20.97894546	0.965233459	-0.341484159	4.525565437	0.291424475
29.13006436	0.676309931	-0.100404031	2.455363334	0.501321769
22.31659601	0.486816895	-0.062355493	1.163992843	0.622853663
26.29602592	0.675193507	-0.087253497	2.942073548	0.43035594
20.2165834	0.856648592	-0.200298338	2.57839911	0.428625529
35.92417255	0.574758577	-0.087229012	1.341225716	0.592591646
21.25720232	0.661941538	-0.099819182	2.117263948	0.495245819
28.87537764	0.610068923	-0.084500092	1.831943435	0.527365344
29.91858287	0.682864491	-0.108166492	2.085631667	0.548445472
24.80556173	0.78221369	-0.142341132	2.625646151	0.463676285
33.47693029	0.700403472	-0.077915693	5.064421882	0.299235035
15.3911766	0.936005137	-0.29341997	3.646735081	0.32829101
27.24686943	0.764280017	-0.115505233	3.644975425	0.36953526
23.48750264	0.491755158	-0.059263124	1.293650924	0.581850927
25.54236225	0.687219787	-0.091473279	2.959015308	0.395313767
20.47300178	0.775097963	-0.129345214	3.178981886	0.385972378
31.55316105	0.551876642	-0.089700695	1.287130044	0.62538214
24.88586571	0.540774817	-0.066846821	1.464941457	0.545168659
22.78544862	0.617267038	-0.089586957	1.723806993	0.553050644
21.83630045	0.933793708	-0.262967876	4.253525932	0.311484185
25.87538717	0.702452102	-0.106703441	2.235238356	0.467157243
23.77217029	0.58382304	-0.104917601	1.030615343	0.655732956
39.36018553	0.83714256	-0.141793894	5.090907165	0.339216791
27.50868363	0.746446575	-0.11448755	3.052290521	0.416385153
26.14835289	0.934385566	-0.243150615	5.608299025	0.275324166
24.98483654	0.528487353	-0.070935344	1.386477243	0.570513733
29.35617846	0.584778025	-0.06506231	2.379613735	0.474352941
27.77324471	0.622155856	-0.089093023	1.756600417	0.556238855
21.05905197	0.556429107	-0.057394765	2.369201106	0.45601782
39.91551611	0.781297432	-0.120684338	4.09899068	0.365289971
25.69234571	0.679708914	-0.09368978	2.775070136	0.445065026
23.09024293	0.986352077	-0.447930426	5.845483106	0.258545189
14.03012617	0.886200822	-0.272491574	2.321706562	0.417571748
33.85259865	0.621763747	-0.080534164	2.135099807	0.523994884

log.sigma.1.5.mm.3D_glcm_ClusterTendency	log.sigma.1.5.mm.3D_firstorder_InterquartileRange	log.sigma.1.5.mm.3D_firstorder_Skewness	log.sigma.1.5.mm.3D_firstorder_Uniformity
23.12900374	101.321367	-0.736687972	0.094941789
23.50044078	93.94117355	0.998655113	0.099381314
26.8987703	132.7914226	-0.37561497	0.10328391
21.42311848	139.2932596	-0.101615166	0.08979206
17.99658728	105.3329775	-0.3398981	0.120514055
50.81356347	171.9305234	0.071986637	0.061362994
6.00066732	16.85826194	-0.465430045	0.333364372
14.92252329	57.49326685	-1.055670434	0.195432618
8.60635556	38.20716619	-1.664269178	0.23493063
41.32863744	116.2492924	0.662607772	0.074643763
7.628602608	31.93553615	-1.778620202	0.243723057
8.77128619	33.94404507	-1.307966771	0.243428456
42.77695072	156.5065222	0.331515793	0.063060761
29.13386973	128.417408	-0.273870527	0.078116343
16.91554977	84.78280458	-1.158975888	0.168152174
9.680491731	26.33067846	-1.825890477	0.282437733
10.93860996	78.13943335	-0.384461755	0.139455854
24.84819043	120.7083569	0.19138754	0.094716338
12.9820184	68.62768424	0.538620383	0.141519617
11.64744227	37.36110497	-0.148662617	0.202176267
10.72321463	72.67898634	-0.575914682	0.160559388
15.66658801	104.3361015	0.067231609	0.104550553
18.6011197	93.62744236	-0.694809412	0.125821608
4.826217978	24.00842381	-1.269734053	0.303673354
22.49518017	65.58550197	-1.053375692	0.209017295
21.25830244	66.82063437	1.33576092	0.161444902
17.85104319	92.02519768	-0.591041917	0.11492184
9.947885698	51.14430356	-1.280177904	0.188122741
30.36718341	148.0775434	0.142145648	0.089285714
11.7762287	71.71978313	-0.411063495	0.151401607
11.43766052	54.35323775	-1.179976781	0.215450226
21.35115516	117.0506821	0.036006853	0.087274125
16.76393461	77.0895946	-0.339017203	0.144975581
9.306079112	33.02517605	-1.287765387	0.240153966
12.31492084	63.96297312	-0.663178507	0.173976588
31.20157653	120.4057274	-0.591293897	0.109033122
55.56043586	184.1277037	0.390858164	0.051545036
9.917355277	19.85696173	-0.847630493	0.312275344
15.35957729	84.47502136	-0.761514166	0.111288619
7.744010609	25.65463555	0.278145475	0.283497871
16.76395664	94.67569065	-0.263623529	0.113022119
7.837290387	34.57113457	1.276041912	0.23208332
18.40090229	98.21502256	-0.680211308	0.108474441
18.32808085	102.405625	-0.712031117	0.121134583
7.574247707	39.32934374	-1.333194226	0.228366944
13.38934523	73.15325272	-0.889501759	0.16792067
7.425265973	24.08554554	-0.917777913	0.281306607
14.08401553	83.28712869	0.563576905	0.127582828
17.75924413	94.13184166	-0.203342416	0.111265005
110.3943026	154.034956	1.466739684	0.064173554
7.202034615	28.37074953	-2.093822376	0.275736642
24.98803607	82.1365335	0.744991729	0.112714724
4.130994943	20.63220763	-0.915438557	0.332966306
15.51083523	68.07602406	-0.588801471	0.128187687
37.9845846	153.3524904	0.366746557	0.078051209
13.43630971	87.77198362	-0.608820442	0.124756295
7.665462452	36.06371021	-0.776260378	0.229728972
17.04115187	86.76791954	-0.830244025	0.163298248
23.51696346	116.2798681	-0.658701122	0.094425822
5.085711814	30.93839359	-0.311374571	0.268019592
26.34312752	102.2399025	0.374858795	0.111568004
34.89345061	106.5438056	0.413886893	0.090671771
44.74571295	114.6088729	0.270770795	0.082094288
17.65094925	43.82580519	-0.490929601	0.199649237
15.57261938	81.599962	-0.808785475	0.139585214
65.30339952	147.5462689	0.654901983	0.059454466
39.25592447	136.0536041	0.496630981	0.072240661
21.06147052	72.26231956	0.655075864	0.148095841
5.705832516	28.29745555	0.559901877	0.272809324
22.69766568	91.17420411	0.446000163	0.10653425
19.05780376	118.32795	-0.415193908	0.102642587
10.51099971	31.58099508	-1.673570861	0.251863662
18.18349669	105.9990919	-0.446221668	0.137819719
14.02239514	70.20820141	-0.100898743	0.166747298
16.04134023	39.38072431	0.624174606	0.204122616
21.13249389	78.81667536	0.928932902	0.119018903
56.6280853	140.3270264	0.699737118	0.065230813
24.61181997	111.4944992	0.477018487	0.083100189
28.23538236	108.8551867	0.340668022	0.083244259
6.888235848	42.65405625	-1.636248104	0.218789455
23.12900374	101.321367	-0.736687972	0.094941789
21.91219652	115.2007275	0.419746719	0.08762421
10.51245652	24.50620437	-2.199185269	0.288042412
7.794098939	57.88740921	-0.864319346	0.177042282
11.7602851	56.26734126	-0.728002787	0.189349376
31.43563328	151.1512513	0.110646989	0.067342459
18.17191888	92.14981914	-0.072224871	0.115517717
6.666916832	22.3289572	-1.502370218	0.305750251
68.01505363	114.5720348	0.721092781	0.077388864
23.61144225	101.6457475	0.305515896	0.095746875
50.51558011	154.8045611	0.309913609	0.060465238
7.742889039	52.27462265	-1.342649494	0.199643992
16.0153065	66.85271168	0.0627916	0.13628459
12.28771939	43.2372601	0.189577698	0.197404135
15.21985968	72.9893899	0.753720481	0.121799494
42.08748094	137.1685529	0.37356253	0.07914793
22.07513876	94.96283436	0.357953472	0.116223136
50.62078422	170.7623816	0.5790409	0.062755978
9.213915029	75.13012314	-0.195951025	0.138266217
17.0874335	45.41014147	-0.280503395	0.176942339

log.sigma.1.5.mm.3D_firstorder_MeanAbsoluteDeviation	log.sigma.1.5.mm.3D_firstorder_Energy	log.sigma.1.5.mm.3D_firstorder_RobustMeanAbsoluteDeviation	log.sigma.1.5.mm.3D_firstorder_Median
67.45214858	6803067.527	45.00227486	-34.92164803
66.44109087	2135350.947	43.0661439	-99.57028198
75.20883793	6529928.157	54.33077558	-54.25565338
71.98595179	2080710.969	50.50388535	-121.1985703
62.25061935	7384103.55	44.69357092	-45.79697227
102.8894995	2602907.018	74.66163183	-102.4599304
23.26581687	32399913.37	8.196539542	-1.730873525
48.58999478	29654995.25	27.59529918	-13.19054985
36.15332438	13920014.23	19.21052472	-9.006277084
82.4963304	5104553.052	51.61899769	-40.24850845
27.40096292	12689975.52	13.86179182	1.24342066
36.51506344	11201879.22	17.87142282	-5.921519279
97.49500778	6780715.635	67.90964898	-74.35955811
73.90559175	4396461.737	53.27656047	-122.3312988
57.03062729	7043657.846	37.75289966	-16.85733891
32.74179988	27064166.08	13.05000965	-4.851878166
46.4261373	2262748.907	31.36553486	-29.40703392
66.2074565	1946690.977	47.06571345	-103.147789
46.59838523	2892692.965	29.66792812	-22.75483894
37.93507108	45474449.29	17.93891226	-4.366164684
44.77890321	6754428.131	31.12694884	-21.8371315
55.28213081	2081745.452	41.3032961	-90.61342621
58.42675732	2568757.564	39.42094219	-40.31932449
23.64600265	7950163.702	11.32348518	-4.748538971
61.97594535	6366201.074	36.43762463	-10.80855131
49.43270029	12911994.57	29.67400627	-15.94121265
57.08104786	7433598.283	38.88014948	-35.013237
40.20897727	6985907.8	23.17044355	-14.71697712
90.17091649	435766.4904	67.56322374	-88.27492523
46.25280923	5606361.344	30.83045142	-20.69840813
43.25528567	14781165.04	26.21825963	-8.859713554
65.56502399	4680907.322	46.75047807	-106.0680008
53.46872388	8374113.531	33.23335902	-18.16511345
35.38248728	10835732.93	16.10744944	-4.138548851
45.70281589	16469084.98	28.54192191	-13.68460846
75.58846893	15065203.73	51.22423047	-34.26021767
113.6014331	15686069.71	78.01086218	-147.736557
28.58878097	33868521.26	9.781015888	-1.00674504
53.90413557	1877450.854	36.78759702	-57.50579453
28.08160193	33657959.8	12.02803861	-4.190812349
57.36709846	5197683.032	40.0226929	-40.18634796
30.49408517	19342457.34	15.23404401	-6.039315701
58.39211479	13202415.78	40.73053905	-43.12800598
60.21844878	6419249.92	42.34954275	-38.66246796
34.39966278	3115388.553	19.47016301	-10.14775372
48.9962417	10981749.01	32.00592191	-18.09861374
30.31655413	18599399.13	13.35061379	-4.364914417
48.89697298	1193057.415	34.61490281	-30.41308022
60.79781594	1506873.992	40.07468576	-78.73296738
113.2984182	6733121.185	70.03781018	-112.0767059
32.20366798	5522983.172	14.65174492	-7.45580554
62.82867563	20360968.43	36.00032356	-22.48634911
21.42218112	15025416.26	9.458590233	-3.396903872
49.73527246	3959419.729	30.18855415	-1.110107303
87.82312757	5494839.296	62.65112328	-83.26818085
50.28189479	4527600.831	35.77180865	-42.19664764
32.87369481	14729285.68	16.89698273	-6.586256981
56.09475819	10864611.36	37.87582721	-15.43506908
67.48691018	3730018.36	47.81268137	-63.73999214
25.30864216	43170355.56	13.23156559	-3.020016432
69.93303094	9009096.539	44.0634776	-32.66322708
80.07809278	22551029.45	47.48140895	-23.15680313
86.84769411	73065374.31	52.12902034	-25.00362301
48.43498222	53815130.52	23.45547021	-5.612375498
52.26529362	1944308.365	34.33609585	-21.02841187
105.4438292	49231895.95	66.71254195	-52.99651337
85.34953948	2848236.945	58.87101892	-122.8392029
58.71907036	36252133.38	33.17633223	-15.48845863
26.81653423	20966731.54	13.13925407	-4.341143131
63.84909892	13474968.52	39.7758405	-24.93951607
64.9530358	2183165.143	46.70608917	-54.88307381
37.82552988	27517094.56	16.99900973	-4.264638901
60.65657992	6723323.349	44.69622191	-33.57039261
48.4378882	5992110.61	30.4440534	-14.42337036
43.42747182	17176360.46	20.98806183	-6.919412613
56.95660735	4411774.197	35.24951211	-22.1046381
94.87539475	23918558.72	60.8905096	-39.7595005
73.51911393	2648672.08	52.2906341	-131.6550598
74.22188786	7656818.515	48.85904303	-46.78409195
33.59461199	4524784.077	19.54806538	-12.26069403
67.45214858	6803067.527	45.00227486	-34.92164803
69.94495763	3967896.037	47.92183894	-53.62445831
38.98238944	13830178.61	16.58725774	-4.728638887
37.14700865	4213815.835	24.03746377	-22.26339912
44.18432405	8451852.284	26.30643287	-13.62129879
85.05743996	3065808.839	59.64202602	-82.02160645
57.32834611	5527260.522	38.54265769	-30.19769287
29.4097899	9412814.32	13.59848159	-5.623152256
91.07641104	20495274.78	53.55856534	-25.06916046
68.08575602	8257846.117	43.21850593	-37.15597534
95.95558023	5112801.818	65.95279259	-64.23311996
36.72494017	4854422.263	23.07938193	-17.22767544
51.60182733	20950274.9	29.57706958	-14.66601229
40.35446796	16480355.52	21.02532603	-7.956493616
51.09842089	12060594.72	31.17131393	-24.79687309
86.32628911	21304550.89	56.96388353	-58.69619942
62.12383336	13571751.61	39.88446478	-32.60715103
106.9235384	2587079.946	68.0477782	-118.3704605
43.54762144	687449.8599	30.0597085	-83.67868805
47.87599663	54884000.02	23.78725482	-6.906374216

log.sigma.1.5.mm.3D_firstorder_TotalEnergy	log.sigma.1.5.mm.3D_firstorder_Maximum	log.sigma.1.5.mm.3D_firstorder_RootMeanSquared	log.sigma.1.5.mm.3D_firstorder_90Percentile	log.sigma.1.5.mm.3D_firstorder_Minimum
183682823.2	256.6290894	101.0683734	36.90057564	-348.2312622
57654475.56	315.4522095	137.4660359	-16.43114319	-282.3383179
176308060.2	229.3517761	116.2734707	13.61323032	-369.4929199
56179196.16	104.9855042	150.3875779	-9.2194664	-386.8587646
199370795.8	273.2818298	99.49001021	6.484195471	-374.9009705
70278489.49	219.8996735	158.9683677	70.10276489	-379.8834229
874797661.1	518.1782227	44.57570345	13.50970078	-372.6604309
800684871.7	392.6808167	76.66114584	14.93674231	-414.2654114
375840384.2	211.6885834	57.37213307	16.59661331	-326.3161926
137822932.4	404.5882568	118.4208487	98.58199234	-332.180542
342629339	375.5552368	48.19201072	40.05758438	-803.0335693
302450738.9	488.4112244	60.37597577	18.63004532	-420.6207275
183079322.1	322.7776794	143.1277252	100.1971817	-356.0169067
118704466.9	120.0308914	152.1159831	-5.406634927	-377.7955017
190178761.8	282.8050232	87.49942761	12.47172327	-397.8082581
730732484.1	289.3730774	56.33771045	17.56359787	-370.6448669
61094220.48	222.2473907	72.04020454	17.56572723	-242.8778839
52560656.39	203.1044922	132.430195	-3.008300543	-340.2675781
78102710.07	358.2812805	71.42654452	26.99738083	-222.2511749
1227810131	585.6517334	61.74732025	39.30250092	-446.2838135
182369559.5	192.2449646	68.20417881	5.135476732	-248.1228485
56207127.21	113.7004242	114.7850344	-3.47792592	-252.5279999
69356454.23	168.1501923	95.61111857	8.345436096	-319.1108093
214654420	328.844574	40.68052381	14.78869925	-315.3022461
171887429	296.1596985	93.64225543	11.16347885	-349.2171631
348623853.3	525.1757202	79.85169762	21.06225853	-308.3669128
200707153.7	261.6179504	88.97474414	22.35263557	-352.138916
188619510.6	177.358551	63.02001541	20.80125008	-350.0316467
11765695.24	137.4393463	124.75211	74.50522232	-265.6412964
151371756.3	205.6748352	69.791556	18.97273254	-240.6537781
399091456.1	265.3294373	64.88419463	13.35890579	-292.8351135
126384497.7	149.5337524	135.4861591	-6.37759366	-321.6738892
226101065.3	289.0991516	80.91595236	22.91131096	-315.9365234
292564789	296.0802002	58.66010227	24.98634987	-323.724823
444665294.5	348.2331848	68.8228083	18.95116196	-323.0198364
406760500.8	298.3175354	115.5667645	17.46985435	-395.2680054
423523882.1	387.7662964	200.55097	62.55850372	-485.5354614
914450073.9	623.307373	53.7156784	18.83824043	-430.0439148
50691173.06	114.6466904	97.62278105	6.712318707	-389.7402649
908764914.7	710.866333	52.71086086	19.41754608	-975.3607788
140337441.9	267.9650269	90.75910743	16.18330193	-351.6542358
522246348.1	543.2921143	51.69833275	27.51646385	-269.1756592
356465226	217.7455444	92.98377722	20.57868729	-413.1477356
173319747.8	237.1899719	95.28673263	11.91346207	-376.7177124
84115490.92	149.1152802	53.51082691	15.27205238	-255.5894623
296507223.2	292.1734619	74.94840171	14.70911827	-364.2058411
502183776.5	412.7303162	51.66116847	14.55706081	-317.1862488
32212550.21	234.1988983	73.80887992	22.62966537	-186.7809601
40685597.78	107.8693771	114.9704001	10.78500547	-292.7199402
181794272	475.8741455	174.943123	115.264447	-425.0924988
149120545.6	190.5171967	53.83012792	12.30471754	-299.3616943
549746147.7	510.454834	97.11199904	46.74178238	-372.6546631
405686239	453.1119385	38.56644199	14.56099892	-367.9159851
106904332.7	201.1852875	68.37133599	67.83191833	-265.5680542
148360661	298.3458557	138.3682822	19.88797798	-350.0589905
122245222.4	122.2688065	82.63752495	11.60278397	-274.9363708
397690713.3	294.6748657	52.78204195	22.72135162	-310.0401611
293344506.7	276.3637695	83.85768249	13.58795013	-415.0302429
100710495.7	165.6583557	117.103812	8.099351692	-412.0177002
1165599600	615.2626953	42.84881253	25.32169762	-390.9495544
243245606.6	484.4637756	106.3191411	42.01172791	-365.5053101
608877795.3	485.2153015	118.608667	73.75137177	-473.405365
1972765106	704.4937744	126.4161896	107.1264053	-598.8540649
1453008524	601.1046143	79.47534129	24.64334641	-761.7989502
52496325.86	130.3666534	77.82695809	22.42921829	-293.8862305
1329261191	481.8505554	150.1058299	137.3768707	-457.1860962
76902397.52	204.3511658	154.7086431	14.70379829	-333.8878174
978807601.2	690.7301636	93.71237408	22.87037334	-353.5891113
566101751.7	440.4610291	45.7368765	19.85377731	-271.5462036
363824150	409.7781677	93.93866005	57.5685997	-337.069397
58945458.86	107.6691742	105.0051741	13.70310965	-278.9804688
742961553.2	410.138855	62.25908017	20.77912636	-430.6322632
181529730.4	262.7895813	94.74386347	7.333130646	-286.6268005
161786986.5	445.3209229	74.72915126	18.20773926	-352.2657166
463761732.5	597.6796265	73.08151684	26.55813885	-371.0043945
119117903.3	429.6766052	85.46497479	57.79031906	-304.5057373
645801085.4	430.3948975	127.0408531	156.5429398	-420.2045898
71514146.15	152.74823	151.7627462	-1.796358836	-292.5716248
206734099.9	315.1191406	108.4510303	77.19319153	-371.0372925
122169170.1	122.1043625	53.24544809	15.40653753	-282.7812805
183682823.2	256.6290894	101.0683734	36.90057564	-348.2312622
107133193	258.4813232	103.6971831	55.04367142	-279.0812683
373414822.4	222.0037842	63.61036172	10.80733156	-389.8020325
113773027.5	240.1190491	59.88510869	12.77604866	-301.8798218
228200011.7	247.4324341	68.42855135	15.23700104	-299.9291687
82776838.65	203.2319794	131.9824549	55.0853405	-342.8245544
149236034.1	377.8978271	87.31441815	24.3723877	-340.2556763
254145986.6	355.0558777	48.11450905	9.879348278	-287.5092163
553372419.1	510.8686829	132.0711439	136.1267426	-478.7886963
222961845.2	288.2260742	101.5987581	48.40753479	-364.4778442
138045649.1	433.0534668	136.1052691	98.82545471	-357.7634277
131069401.1	132.0500641	58.26406168	11.56275072	-307.4570007
565657422.3	397.3864441	78.2446875	40.35856056	-363.3389587
444969598.9	459.5774231	65.39242348	25.56211109	-338.6704102
325636057.4	397.403595	76.38606244	43.45986252	-270.465332
575222874.1	616.0355225	134.1406297	34.43684883	-527.2602539
366437293.5	455.340271	96.24964557	19.45879211	-349.9351196
69851158.53	247.6470184	172.4428923	100.3725342	-363.7454224
18561146.22	50.20315933	98.39914833	-11.02209663	-225.3208313
1481868000	362.6088867	75.59564941	42.22402725	-405.0617981

log.sigma.1.5.mm.3D_firstorder_Entropy	log.sigma.1.5.mm.3D_firstorder_StandardDeviation	log.sigma.1.5.mm.3D_firstorder_Range	log.sigma.1.5.mm.3D_firstorder_Variance	log.sigma.1.5.mm.3D_firstorder_10Percentile
3.716778985	87.05414409	604.8603516	7578.424004	-177.3730316
3.621581075	88.73283356	597.7905273	7873.515752	-212.1766785
3.690333879	92.26582081	598.844696	8512.98169	-200.4103333
3.708343214	89.66783809	491.8442688	8040.321187	-220.1678818
3.469299823	77.63848747	648.1828003	6027.734736	-167.1319046
4.170615738	124.0186327	599.7830963	15380.62125	-261.3959839
2.166768145	43.76501104	890.8386536	1915.376192	-46.4483223
2.990908123	68.01988808	806.946228	4626.705174	-136.71492
2.626619708	51.89866525	538.004776	2693.471455	-99.45011292
4.097325894	112.8902579	736.7687988	12744.21032	-170.217067
2.567038917	48.17190787	1178.588806	2320.532708	-35.14490776
2.650888284	56.58185066	909.0319519	3201.505824	-96.51573486
4.223025964	121.7075143	678.7945862	14812.71905	-226.8483734
3.855696519	91.66859425	497.8263931	8403.131172	-242.6115631
3.131161498	73.3168671	680.6132813	5375.363001	-156.5277039
2.459195759	53.56653085	660.0179443	2869.373227	-79.06932831
3.156191323	59.6974043	465.1252747	3563.780081	-126.1349792
3.607536305	83.59986645	543.3720703	6988.93767	-195.3515167
3.229284572	63.85978498	580.5324554	4078.072137	-114.5884598
2.963603853	60.97713587	1031.935547	3718.211099	-78.22384338
3.047907341	55.90684445	440.3678131	3125.575256	-120.3032211
3.402478056	67.24117205	366.2284241	4521.375218	-179.9045349
3.393646162	74.74453307	487.2610016	5586.745225	-167.1312866
2.239510245	38.75165019	644.1468201	1501.690393	-53.25451469
3.031058753	83.02193785	645.3768616	6892.642164	-174.881134
3.185823621	74.45689548	833.5426331	5543.829284	-123.6511597
3.455902658	73.03126724	613.7568665	5333.565995	-149.5318939
2.919057424	56.38774481	527.3901978	3179.577765	-106.5233307
3.628085279	105.5936679	403.0806427	11150.02271	-206.6353439
3.140923901	59.95985085	446.3286133	3595.183713	-123.5384293
2.773450779	57.33629956	558.1645508	3287.451248	-122.6871719
3.701622651	81.20326474	471.2076416	6593.970205	-213.1874756
3.323715134	72.6926278	605.035675	5284.218136	-135.5821533
2.672854458	56.53305263	619.8050232	3195.986039	-85.88011932
3.052799223	61.46764439	671.2530212	3778.271307	-118.6539841
3.726129868	96.50420732	693.5855408	9313.06203	-202.5942642
4.486394992	144.2011335	873.3017578	20793.96691	-320.5809692
2.310403472	52.64330168	1053.351288	2771.317212	-54.32644844
3.420538627	69.65678477	504.3869553	4852.067664	-153.6123413
2.450183433	51.91879963	1686.227112	2695.561755	-56.6877552
3.464839368	72.52723649	619.6192627	5260.200033	-154.8157196
2.674733042	51.01853831	812.4677734	2602.891251	-59.04056396
3.512499771	73.91950754	630.89328	5464.093596	-151.0082001
3.409821624	75.92930657	613.9076843	5765.259596	-160.8506989
2.657460804	47.75077294	404.7047424	2280.136316	-95.46004181
3.083618741	64.2442994	656.379303	4127.330005	-128.4277832
2.449348323	49.39086074	729.9165649	2439.457125	-72.69022369
3.241974801	62.14375512	420.9798584	3861.846301	-119.5392181
3.500365181	78.76025491	400.5893173	6203.177754	-190.075061
4.298891749	155.7855197	900.9666443	24269.12816	-228.5976456
2.438029268	49.22389923	489.878891	2422.992255	-86.91181946
3.676809287	92.51285442	883.1094971	8558.628233	-139.8388641
2.100811564	37.33902691	821.0279236	1394.202931	-44.41304436
3.366501873	67.82385642	466.7533417	4600.0755	-108.0148376
3.956065606	109.6960367	648.4048462	12033.22046	-211.2582886
3.268088355	62.20426021	397.2051773	3869.369988	-135.2364471
2.686662704	50.35371756	604.7150269	2535.496872	-79.2572998
3.160828557	71.46095723	691.3940125	5106.668408	-150.2988403
3.620108996	82.91575707	577.6760559	6875.02277	-183.8170059
2.384897688	42.12458643	1006.21225	1774.480782	-42.71703033
3.703961809	95.05689116	849.9690857	9035.812556	-166.9058899
4.008193179	112.986104	958.6206665	12765.85969	-177.1292206
4.143281377	121.1390862	1303.347839	14674.67821	-187.3970871
3.08716477	76.69755583	1362.903564	5882.51507	-116.6676414
3.273070287	66.93600232	424.2528839	4480.428406	-133.6634369
4.436984415	141.5876658	939.0366516	20047.06712	-217.0816223
4.031519001	107.0215948	538.2389832	11453.62175	-237.8717621
3.423741953	88.17543113	1044.319275	7774.906654	-140.4706055
2.437979957	44.66745181	712.0072327	1995.181251	-58.11382675
3.683637551	87.29659537	746.8475647	7620.695563	-144.0215271
3.552184731	78.78589175	386.6496429	6207.216739	-167.9566757
2.636447824	59.01042829	840.7711182	3482.230646	-99.86712341
3.26859247	74.02667619	549.4163818	5479.948788	-167.7971252
3.069889511	66.34619755	797.5866394	4401.81793	-128.259494
3.012642563	70.83308237	968.684021	5017.325558	-104.490078
3.508192794	80.5873273	734.1823425	6494.317322	-119.943158
4.273289995	124.9894766	850.5994873	15622.36927	-162.3394516
3.760329454	90.74474793	445.3198547	8234.609277	-233.4441406
3.927843396	97.36311028	686.1564331	9479.575244	-172.3946533
2.635528418	46.74939699	404.885643	2185.506119	-88.18615341
3.716778985	87.05414409	604.8603516	7578.424004	-177.3730316
3.792343898	88.57984142	537.5625916	7846.388305	-158.9866821
2.437024604	58.55373122	611.8058167	3428.53944	-111.5776031
2.870724356	49.54630683	541.9988708	2454.83652	-98.77891998
2.98081447	60.8018422	547.3616028	3696.864015	-116.619043
4.065474812	105.8635069	546.0565338	11207.0821	-208.5206985
3.466927833	74.91050484	718.1535034	5611.583735	-144.3008942
2.275993487	44.14705538	642.565094	1948.962499	-82.40277481
4.201069508	129.5072921	989.6573792	16772.13871	-177.316983
3.779007973	91.51534585	652.7039185	8375.058527	-158.4518158
4.255624218	121.932169	790.8168945	14867.45385	-208.4399109
2.758082145	48.80957367	439.5070648	2382.374482	-101.6888039
3.411970063	74.52647973	760.7254028	5554.196181	-116.7555267
2.973042211	62.87177105	798.2478333	3952.859595	-95.46496124
3.439633778	71.44224225	667.868927	5103.993978	-112.1846619
4.072619481	114.8744044	1143.295776	13196.12878	-215.0894928
3.577299048	84.4460866	805.2753906	7131.141542	-150.1556885
4.19651602	138.0499529	611.3924408	19057.78951	-254.4835785
3.091942346	55.3663996	275.5239906	3065.438205	-159.5055695
3.212926912	73.50396278	767.6706848	5402.832544	-114.8598343

log.sigma.1.5.mm.3D_firstorder_Kurtosis	log.sigma.1.5.mm.3D_firstorder_Mean	log.sigma.1.5.mm.3D_glrlm_ShortRunLowGrayLevelEmphasis	log.sigma.1.5.mm.3D_glrlm_GrayLevelVariance
3.929254317	-51.34580901	0.017956087	12.92574038
6.602941768	-104.9923582	0.051621075	13.29753812
3.197709217	-70.7568958	0.012413105	14.73976338
3.229806956	-120.7315302	0.021797301	12.95091377
4.048840362	-62.21356279	0.009475095	10.52138867
2.439429138	-99.45009137	0.022206346	24.74879148
16.59037635	-8.462691451	0.003913913	4.652723915
6.921461636	-35.35853656	0.004529498	8.976980291
7.030547807	-24.45588269	0.006140284	5.366542856
4.734034866	-35.76712282	0.014746878	21.56565901
43.08125895	1.391829426	0.000999149	4.874810508
12.54845147	-21.06543676	0.004397357	6.603146963
3.025443327	-75.31816963	0.018223331	24.54637112
2.732716469	-121.3925085	0.017943921	13.89751575
5.027778272	-47.75758402	0.007115001	9.829020677
9.240872213	-17.45177333	0.004922146	6.236019604
4.039334648	-40.32382658	0.022535311	6.204726649
3.936253405	-102.7074432	0.023981926	11.52327657
7.056703133	-31.99498592	0.031224048	7.408629679
8.725806093	-9.722163254	0.002975786	7.67531114
3.526654047	-39.06705455	0.017798359	5.81338967
2.580815052	-93.02810817	0.032675808	7.394821981
3.935841979	-59.62164681	0.016871812	9.66585793
14.1060058	-12.37798951	0.005700091	3.399959275
4.873066768	-43.31546882	0.014177136	13.32934964
13.09073041	-28.85245794	0.008543773	10.6652452
4.160717027	-50.8226239	0.007889703	9.231205945
6.390246598	-28.14150986	0.005953117	6.119494842
2.132540773	-66.43091323	0.060268239	17.6294076
4.050645072	-35.71662884	0.022262897	6.579414131
5.073726956	-30.37280799	0.010380669	6.508735296
2.882253618	-108.455194	0.025597496	10.92591548
5.153679934	-35.54114812	0.011315023	9.674451505
8.734696248	-15.65316449	0.007338537	6.474855401
5.534852165	-30.9565443	0.008710258	7.199347159
3.978715569	-63.58156209	0.012272322	16.17021004
3.133317195	-139.3797857	0.011098748	33.8885601
18.44735359	-10.67974225	0.002817151	6.444982855
5.190835093	-68.39692769	0.011471798	8.382146603
28.52468106	-9.10346624	0.000613457	6.054876393
4.101109151	-54.56203395	0.008408856	9.301478291
16.74262079	-8.356216703	0.008558891	5.383718969
4.37506267	-56.40823726	0.005952803	9.500637727
4.13313108	-57.56997324	0.007776546	10.03821687
5.720723306	-24.15103062	0.012315623	4.542120331
5.431479808	-38.59964914	0.006643129	7.700001861
10.79494898	-15.14659047	0.006610591	5.475819448
4.722783932	-39.8234159	0.039577422	6.842249775
2.947561649	-83.75568731	0.030280243	10.53664515
5.642745257	-79.59879483	0.010677471	39.60875051
8.931811391	-21.78739123	0.011532025	5.111216973
6.745933563	-29.53154457	0.007956842	15.65813474
17.32825697	-9.652332199	0.00377992	3.277902108
3.787358177	-8.635049834	0.011990111	8.182707047
3.946436644	-84.33600098	0.01814866	20.21445145
3.364792723	-54.40211891	0.022382693	6.747923835
8.549423701	-15.82551991	0.006516117	5.150864045
4.608764842	-43.87986444	0.00526375	9.486779862
3.445608692	-82.69389352	0.010033909	11.55083544
21.48795045	-7.844740489	0.003462853	3.763709621
5.606394321	-47.62296943	0.009713644	16.38885267
5.430508489	-36.08540126	0.004702594	23.02646365
4.874922988	-36.143807	0.002389755	25.74312265
10.38955291	-20.82822131	0.001107693	12.22549535
3.859068879	-39.70651079	0.015416784	8.094089463
4.019029228	-49.84669543	0.005327094	34.20731064
3.324474197	-111.7190337	0.031179951	19.12042494
8.914498425	-31.73487673	0.007022072	15.09686661
14.63250784	-9.832630399	0.008018071	4.460232677
5.221182564	-34.69547937	0.008960656	13.71154945
2.860083458	-69.41808008	0.028562364	10.52307525
9.616252761	-19.84848653	0.003330778	7.401149982
3.832939175	-59.13079466	0.021441549	9.578398004
9.39386342	-34.38936055	0.006651034	8.228511539
12.43491577	-17.98840033	0.005371793	10.16473676
7.966709115	-28.45952553	0.010544481	11.88156847
3.741450515	-22.73783365	0.005421316	26.0681028
3.00737872	-121.644243	0.055268962	13.79009198
3.783837447	-47.77081472	0.010305171	16.33091822
6.492043722	-25.48669503	0.009055608	4.251694477
3.929254317	-51.34580901	0.017956087	12.92574038
3.513280739	-53.91398227	0.017638607	13.46002954
9.067609241	-24.85434929	0.005157763	7.197077618
5.964177949	-33.63613714	0.008097365	4.554380875
5.522925373	-31.39430879	0.011755199	7.192924605
2.81983746	-78.81805824	0.025246366	18.73202228
5.390494884	-44.85781851	0.009140462	9.865643503
9.293787266	-19.13226287	0.007644219	4.370385715
4.745824729	-25.89687888	0.004145584	29.11262486
4.403073569	-44.12764575	0.008370987	14.88123586
3.362972682	-60.47470893	0.014968467	24.46657116
5.56496117	-31.8170772	0.007955752	4.569014866
6.023491736	-23.83348361	0.005794977	10.30776994
9.840001143	-17.98080795	0.006055922	8.058565513
6.165882709	-27.03398894	0.01252452	9.201836917
5.785754898	-69.26456339	0.004030763	22.73150762
6.41452625	-46.18281856	0.010649911	12.82438528
3.171291923	-103.3380936	0.041329042	30.92762566
2.822327371	-81.34466293	0.040806686	5.254107095
6.509676904	-17.65983196	0.003910424	10.8569508

log.sigma.1.5.mm.3D_glrlm_LowGrayLevelRunEmphasis	log.sigma.1.5.mm.3D_glrlm_GrayLevelNonUniformityNormalized	log.sigma.1.5.mm.3D_glrlm_RunVariance	log.sigma.1.5.mm.3D_glrlm_GrayLevelNonUniformity
0.018413465	0.089352134	0.122396562	53.90350495
0.053092401	0.096531985	0.081924595	10.12522636
0.012979159	0.086861874	0.25591056	35.93441234
0.022097431	0.088775452	0.059530837	7.724719444
0.010080885	0.100002602	0.322285392	62.17950062
0.022523917	0.061224037	0.031970803	6.103768077
0.005061495	0.269865386	1.22516985	2778.728664
0.005126912	0.15257321	0.529379541	587.5733244
0.007106997	0.18935993	0.617338787	589.5446024
0.01511776	0.071290071	0.082126488	24.15299409
0.001159321	0.206537473	0.543148051	848.3278358
0.005100238	0.19295146	0.670206888	429.7335982
0.018613834	0.061074055	0.076292641	18.9540975
0.018217494	0.077490251	0.053075799	13.97938195
0.007712441	0.133558752	0.395638608	98.1581317
0.005896972	0.222893353	0.863966004	1310.350622
0.024034921	0.126643295	0.274955878	46.15753527
0.024440824	0.093799517	0.067734408	9.815499348
0.033031277	0.126857731	0.224578723	61.87205954
0.003500611	0.157909653	0.626301928	1410.196412
0.019846507	0.129219038	0.528906584	144.6565828
0.033709437	0.104155588	0.083869802	15.20757398
0.017643403	0.110866662	0.200865185	27.01890252
0.007064287	0.24868142	0.861029123	811.3167962
0.015269328	0.151754227	0.549629593	83.65450529
0.00941273	0.133221019	0.389163847	216.7269521
0.008383759	0.105212093	0.212072273	85.72653605
0.006661974	0.155668905	0.474947953	213.5911765
0.061203412	0.089127059	0.024559007	2.433876835
0.024050531	0.131015871	0.321782689	123.0887888
0.011813136	0.165711144	0.742005565	419.2710848
0.026298164	0.086356102	0.073666123	20.54912974
0.012105435	0.123691723	0.294495694	132.1837878
0.008439578	0.194475878	0.581220327	453.5938845
0.009620718	0.141956772	0.446350319	389.8767418
0.01297847	0.090206006	0.265790003	86.83437957
0.011269397	0.050765229	0.037133159	19.1118872
0.003563075	0.247371175	1.02603933	1903.115469
0.011789549	0.107378625	0.108890057	19.34807623
0.000749923	0.225868306	0.841288253	1890.865511
0.008903855	0.102905266	0.207688569	56.40526849
0.009991111	0.192915063	0.558933919	1054.2602
0.006370545	0.101331613	0.223032516	133.6232251
0.008257117	0.106666387	0.234556411	64.17036568
0.013936683	0.18007555	0.578310781	145.3069784
0.007342816	0.136902832	0.467971629	211.7386739
0.007960042	0.220421044	0.935629001	1034.712031
0.042031783	0.119767955	0.196527027	22.76859668
0.03105924	0.104502606	0.095827387	10.96052354
0.010884979	0.062853223	0.04978655	13.22056672
0.012981453	0.217223728	0.665696527	296.2953765
0.008497994	0.097600029	0.273221251	179.499962
0.00489522	0.272598434	1.065741765	1780.833628
0.012870861	0.115250169	0.221499423	83.82920665
0.018564901	0.073892573	0.100591989	19.5163559
0.023630679	0.115991655	0.240370287	65.13587976
0.007579196	0.187695027	0.580969897	741.3313984
0.00580921	0.127698333	0.53565494	154.2278244
0.010359042	0.090146092	0.119113761	22.2475148
0.004218207	0.225918677	0.641861353	3850.369141
0.010268342	0.091046414	0.302061496	61.23167747
0.004974907	0.077528438	0.211638205	108.3618237
0.002541103	0.071466406	0.19484472	289.3668869
0.001290386	0.148863658	0.703295707	935.5462934
0.01633624	0.119496588	0.259362241	32.42489414
0.005540018	0.055462328	0.118952737	111.3658691
0.031560833	0.071221439	0.046675464	8.115029137
0.007700179	0.115685733	0.454825899	378.719779
0.009850224	0.218060296	0.863472124	1496.233663
0.009556061	0.092865831	0.249298093	121.5300493
0.029771312	0.096354427	0.157791345	16.99204501
0.003972173	0.194931005	0.81268823	969.7260741
0.022551191	0.11343641	0.352854279	69.36768176
0.007286485	0.137744177	0.370352015	118.9950666
0.006093271	0.158716425	0.563919848	391.2230038
0.011307566	0.101787942	0.27958994	51.79551512
0.005665787	0.062947081	0.085376475	86.61118082
0.056519886	0.082006157	0.058510599	8.956973311
0.010703603	0.077035609	0.138893011	45.38444349
0.010287808	0.18264513	0.527715649	220.5270474
0.018413465	0.089352134	0.122396562	53.90350495
0.018459153	0.082319125	0.146931181	27.30323481
0.006000252	0.228384217	0.780006457	545.2960015
0.009005897	0.155938872	0.371991863	147.1113267
0.012955201	0.147207515	0.579171756	203.974947
0.025643253	0.066275032	0.059196167	11.05841453
0.009766258	0.104733066	0.222680125	65.31563417
0.009410954	0.234676166	1.037018492	622.2710568
0.004353175	0.068606022	0.164586187	72.16382736
0.008843201	0.086596014	0.206558015	60.57862457
0.015260968	0.059217203	0.057087571	15.52474112
0.008934235	0.165370613	0.499766859	183.3457156
0.006337487	0.11636812	0.315504171	332.3608365
0.006949259	0.155227664	0.589433737	449.9557594
0.01347983	0.111563594	0.20180508	200.521867
0.004208662	0.071435898	0.174856879	75.63706574
0.011301427	0.100183165	0.279129823	124.6037997
0.042345044	0.062144965	0.037138531	5.21514265
0.042194489	0.133420405	0.082706596	8.759679451
0.00447139	0.134072768	0.557488469	985.3233752

log.sigma.1.5.mm.3D_glrlm_LongRunEmphasis	log.sigma.1.5.mm.3D_glrlm_ShortRunHighGrayLevelEmphasis	log.sigma.1.5.mm.3D_glrlm_RunLengthNonUniformity	log.sigma.1.5.mm.3D_glrlm_ShortRunEmphasis
1.343568319	151.3676806	502.4759223	0.929673575
1.247955868	78.6469545	90.87698918	0.943886682
1.627053535	147.6091932	319.6980648	0.899572539
1.180467039	140.0942889	78.0702515	0.957948291
1.767103111	148.8948659	464.3982542	0.886366116
1.10171366	176.6939109	93.44587644	0.974571585
3.749784917	167.1102434	5122.940212	0.732523673
2.250020928	210.0088812	2502.501948	0.833225841
2.466006019	142.9158679	1908.214046	0.810809871
1.237300207	183.5178286	295.6071846	0.947912645
2.318019418	929.9718142	2581.52698	0.820483538
2.579870294	217.7161897	1337.259133	0.80295415
1.216178824	172.7066376	275.290949	0.953721807
1.163804089	140.0988895	162.6724893	0.960539735
1.96998012	175.6967148	508.1942336	0.856890894
2.976148758	167.015885	3312.111487	0.778764536
1.717817767	70.92450061	267.0101549	0.87873185
1.195948161	113.5900598	93.62072405	0.956889444
1.579871673	66.55728634	376.2736775	0.899453166
2.412782553	275.0806373	5713.59426	0.827311488
2.216355488	67.03949309	745.9936194	0.842531246
1.256746637	62.25650575	125.0381486	0.940570732
1.535500399	114.3391289	189.5568189	0.902745538
3.039832645	127.179193	1766.748288	0.763839741
2.300241045	132.7930876	357.0765413	0.830833612
1.942753436	135.4023435	1138.277652	0.861424393
1.545141393	165.883216	637.4713492	0.904724878
2.130449168	170.8515876	916.282882	0.843400622
1.079631821	96.00363467	26.0249222	0.980092045
1.826451778	73.07518628	664.3230472	0.865811369
2.68147407	97.05197058	1526.195705	0.804502292
1.226640596	87.97716144	208.089327	0.947424623
1.730980848	132.3046832	790.81045	0.883306049
2.405666664	134.1918265	1434.107823	0.812410856
2.051958549	127.4040439	1883.274733	0.853931458
1.644245443	179.6419219	741.3222839	0.898638761
1.110987181	251.6102041	351.1859517	0.973525954
3.361295054	240.0938555	4008.474532	0.749657607
1.30815893	181.6372672	152.8718452	0.936079765
2.942779561	1249.276788	4709.256725	0.778484529
1.536180152	162.4032978	429.7355321	0.905728924
2.320367704	107.1920801	3481.772168	0.825525322
1.579839089	211.4108276	1022.920372	0.899303851
1.62002502	177.2284878	454.3586306	0.891257273
2.401840414	88.69088711	497.6671738	0.813051401
2.088469718	162.8372424	1063.237968	0.853939085
3.143192472	124.9684497	2561.183069	0.767125192
1.534386357	47.77046664	147.7294773	0.901510442
1.279675248	87.54502357	89.50856687	0.939125099
1.145355787	267.6945199	193.0497209	0.966934274
2.629306176	103.5728517	793.7212433	0.790722448
1.65609501	196.1464219	1413.586495	0.89776722
3.464819498	167.0453609	3327.673806	0.74111197
1.587831873	117.5380151	557.6068573	0.895918075
1.283896936	152.7462116	226.077886	0.940158644
1.636745495	78.07065893	419.6158297	0.88758926
2.378483249	135.5683272	2477.14538	0.819403674
2.196292739	203.2024205	823.3996154	0.851080015
1.337994652	191.6491576	205.9250723	0.929663753
2.546178747	209.3812992	10129.72877	0.799565518
1.710126868	174.448256	513.1246853	0.894800814
1.528087195	313.7556764	1108.098705	0.910282812
1.472009043	511.0535242	3305.26121	0.921383397
2.54588088	767.3198651	3983.78681	0.823417453
1.672334475	108.2349523	202.5770637	0.886499293
1.303828087	322.309726	1732.600899	0.943255588
1.139110853	115.5714923	104.6954742	0.967532789
2.049418353	180.2255908	2270.93895	0.858177158
2.999349957	96.41981204	3801.045467	0.772761561
1.612860025	165.0101967	1012.69678	0.900650584
1.430721144	92.64547677	143.7163097	0.919326565
2.853182205	239.6382085	2873.200278	0.788372067
1.862834438	87.55631139	438.9125959	0.870761437
1.916390715	169.5643849	604.4458912	0.861739053
2.274978615	187.688397	1628.103896	0.839269295
1.696255662	145.6026192	381.8073715	0.888199055
1.245937476	288.579108	1194.397939	0.946085108
1.16796786	70.26228779	99.02760707	0.962382099
1.361868633	187.423176	494.4459821	0.932400739
2.280246024	105.1351859	767.5661443	0.824721719
1.343568319	151.3676806	502.4759223	0.929673575
1.387355415	110.9744893	274.5545283	0.926982017
2.838024537	179.0298174	1355.731443	0.781542793
1.927669867	125.0974992	653.2232081	0.857589194
2.294786085	105.9185909	920.1208078	0.840717349
1.17246796	143.2160727	150.6613556	0.960977681
1.580095633	149.0505349	479.6165929	0.897918712
3.392483383	98.25687666	1370.587601	0.746950392
1.415696396	377.4233338	868.908586	0.926182845
1.516790901	182.1583121	556.0657996	0.911292087
1.16583572	189.7223861	237.3054376	0.962162919
2.179899298	122.0533188	740.2746046	0.842005994
1.754549382	193.8853231	2123.958476	0.885192387
2.35866522	159.7958815	1856.433931	0.827747056
1.526484805	106.7655894	1406.288752	0.905288488
1.427670588	373.8582083	878.0609694	0.927459731
1.670531591	149.9554993	948.4630828	0.894953575
1.112699307	157.568788	78.1816015	0.972854933
1.256992306	53.49246051	56.06173838	0.939357693
2.266274106	241.5789798	4860.082459	0.839916455

log.sigma.1.5.mm.3D_glrlm_LongRunHighGrayLevelEmphasis	log.sigma.1.5.mm.3D_glrlm_RunPercentage	log.sigma.1.5.mm.3D_glrlm_LongRunLowGrayLevelEmphasis	log.sigma.1.5.mm.3D_glrlm_RunEntropy
230.1366	0.905636406	0.020562349	4.211347307
102.0432982	0.927842069	0.059444441	3.944854165
301.1792515	0.855868769	0.016279607	4.368683196
178.0635061	0.945652174	0.02336559	3.948500847
336.7260069	0.833058363	0.013921912	4.232760899
197.2645444	0.967886482	0.0237942	4.292828945
887.3088237	0.631032466	0.01668916	3.924542001
624.6734118	0.763011067	0.009497489	4.158957307
481.5985305	0.736126016	0.014417005	3.88324476
233.6459776	0.9306847	0.01677221	4.454428003
2613.338736	0.751478207	0.002334347	3.77136407
750.9672059	0.724498736	0.010534743	3.981526254
217.5172789	0.937485475	0.020377637	4.53114337
177.2279185	0.949392713	0.019350119	4.105256706
462.9332513	0.798411371	0.011633117	4.100311041
684.3356165	0.68922247	0.014451014	3.960197783
152.8212748	0.835215243	0.032411136	3.916063868
141.4000696	0.942480942	0.026615859	3.863153728
118.2819811	0.859856193	0.042684611	3.922588558
810.0368029	0.748592399	0.007739076	4.205912057
199.3693248	0.770767112	0.034191179	4.084667049
88.38105315	0.924050633	0.038052602	3.748156735
212.3192795	0.866684917	0.021649795	4.029078188
531.391611	0.678825338	0.018690944	3.746721684
424.5232084	0.75831744	0.022738641	4.211558814
322.595891	0.80311491	0.015093606	4.154406854
304.9837351	0.867453101	0.011098484	4.10246241
467.5382392	0.779288932	0.011662736	3.979080351
101.4563138	0.975274725	0.064944106	3.681258561
168.1780638	0.816079663	0.034165138	4.023801376
380.9863505	0.720000876	0.023471123	4.091085697
118.6601543	0.933031674	0.029293998	4.029100701
275.6106398	0.835267938	0.016817371	4.153802366
421.2491725	0.74062584	0.016512115	3.937798204
335.7352231	0.789739165	0.015925946	4.078268596
362.1711768	0.852768685	0.016741057	4.462632895
281.4167186	0.965285996	0.011977176	4.669446099
1134.867557	0.655228908	0.010512263	3.974678218
262.2164829	0.914486529	0.013320394	3.820402848
4774.614656	0.690764659	0.001944028	3.952227649
295.5570066	0.868462758	0.011622929	4.093512834
298.4584529	0.754753882	0.020595075	3.873128435
393.2647243	0.86302957	0.008692905	4.18450559
358.9274587	0.850614732	0.010900067	4.11779429
284.7194493	0.741445136	0.02572654	3.869181207
442.6905041	0.789376353	0.012309512	4.093240955
550.8543561	0.673466009	0.020113129	4.016676718
84.85114752	0.867228662	0.054357299	3.842277657
121.3383968	0.919703104	0.034533496	3.886907649
307.4491062	0.955944056	0.011789667	4.517710756
379.672055	0.715110178	0.024080556	3.792473093
364.5871233	0.851426943	0.011748982	4.433192458
808.714421	0.646513257	0.015439275	3.754452688
210.2356098	0.858141858	0.017713872	4.068872146
219.106004	0.920128652	0.020458562	4.340466993
161.2289331	0.846849983	0.030268756	3.985179682
411.123377	0.746926423	0.015550595	3.912530191
599.3810592	0.77978591	0.009989972	4.207442925
293.6515493	0.907239819	0.011880676	4.064156421
680.5316612	0.724823257	0.010001558	3.688772174
349.8540715	0.843451404	0.013803129	4.470139005
535.1484053	0.871826863	0.006521749	4.674566836
819.4698532	0.885372502	0.003422482	4.761231613
2458.930704	0.737360058	0.002870756	4.405683547
219.3091569	0.843757489	0.021513131	3.998233891
438.6612223	0.91888752	0.006626362	4.873754144
134.7404613	0.957336781	0.033189277	4.229337793
452.6359363	0.792747466	0.012493235	4.46013538
384.6177903	0.684602338	0.025623461	3.935903781
300.6945861	0.856883784	0.013048667	4.3995323
155.1514535	0.88966589	0.035508209	4.037659934
941.8335349	0.700607886	0.009485548	4.08487322
226.8026433	0.815754339	0.029385303	4.121618102
413.9285229	0.804860563	0.011366239	4.009412977
521.1625383	0.765810371	0.011703484	4.187956958
276.221931	0.841951095	0.015883722	4.256187738
367.4349838	0.928423129	0.006775957	4.672545998
82.9412963	0.949832776	0.061825126	3.982936725
272.2422478	0.904761905	0.012746117	4.409499809
323.1371952	0.756362059	0.019044923	3.769137063
230.1366	0.905636406	0.020562349	4.211347307
166.0650691	0.898686679	0.022546515	4.273631013
728.1074488	0.698294099	0.01298477	3.898386806
302.9016865	0.802749591	0.014757592	3.801095823
317.0981834	0.766375453	0.022262785	4.100434942
171.000374	0.94798951	0.027343514	4.309020463
276.7772184	0.859840849	0.013174709	4.147418138
494.2746083	0.652029967	0.025741862	3.904108116
570.968436	0.894729951	0.005495964	4.752572192
311.0569461	0.874230769	0.011463491	4.400600844
224.6321137	0.949832776	0.016518732	4.511798518
349.8601382	0.77471759	0.016034328	3.806448329
390.8865825	0.83437486	0.009792293	4.256763477
471.3020581	0.752085745	0.013795652	4.185901822
177.6210483	0.869450337	0.018598544	4.114179128
600.9440803	0.893970894	0.005210365	4.613465588
295.6491658	0.848674193	0.015205271	4.327335827
176.0365997	0.964633068	0.046437655	4.328181561
70.74723512	0.924160347	0.048040051	3.41990074
667.0672618	0.76518598	0.008808172	4.392675556

log.sigma.1.5.mm.3D_glrlm_HighGrayLevelRunEmphasis	log.sigma.1.5.mm.3D_glrlm_RunLengthNonUniformityNormalized	log.sigma.1.5.mm.3D_glszm_GrayLevelVariance
164.5902584	0.832402916	21.40236686
83.01772887	0.864747014	24.59504132
168.3130162	0.771930252	24.00836168
147.1945453	0.896034371	17.42722117
172.4055796	0.746469051	20.74039527
180.8080376	0.936058394	27.64446425
230.472387	0.497055551	22.80291811
258.3108203	0.649482346	20.3851026
181.1664892	0.612432701	13.27563251
192.5110275	0.872283957	33.76818619
1131.751541	0.628053668	20.91671586
276.3155307	0.600003988	19.53021584
180.4755928	0.886327331	36.06267704
147.3314932	0.901196449	16.19281664
212.1808325	0.690872104	15.08314043
218.808847	0.562819802	18.47299261
82.48014169	0.731060622	12.56518117
118.6694125	0.89379041	17.484375
74.09482377	0.770976166	18.45335353
333.0668615	0.63965927	22.94216237
81.67973854	0.665926098	12.290039
67.07014871	0.85561748	9.736534318
129.1460103	0.777399144	17.75148721
168.8583978	0.540990447	14.83084036
165.929367	0.64687522	23.3158834
158.9463312	0.699176761	31.40717709
186.1709714	0.78144426	16.05938016
206.5545107	0.667143614	14.95852664
97.0941705	0.950881985	19.4475
86.07593258	0.706488959	15.970975
125.9378392	0.602677561	13.94704378
93.75851263	0.872545704	15.34211463
151.6958251	0.739496784	21.45287544
167.831003	0.614694027	18.68625744
152.3656235	0.685470339	16.47215614
203.7503299	0.769899219	26.32702002
257.3318659	0.932500721	43.99016145
325.3551591	0.52066841	23.28527408
195.4464304	0.847146754	15.08477509
1609.556926	0.562231495	25.33759553
181.9813595	0.783387884	16.76793262
129.3572237	0.636596241	19.80359671
237.7030427	0.772325152	19.82012972
203.8038423	0.754632947	17.58382775
111.5374357	0.616281602	12.33745665
195.1085563	0.686590512	17.89050364
166.3141596	0.545414846	20.43105959
53.3500629	0.775679527	14.64264463
93.54718622	0.853000061	16.8356
275.1392016	0.917258599	49.32296754
134.9238276	0.581255501	11.94562976
218.38552	0.768155598	33.82996788
227.8891086	0.508928348	16.38099799
131.406312	0.76466921	17.51703544
164.1048102	0.855309137	27.66577148
90.18762931	0.746894628	13.17124807
167.2909489	0.626494328	16.56666635
246.0490999	0.681488011	17.52124023
209.0244491	0.833007272	15.7750651
263.2777709	0.59424875	17.27594236
196.5646815	0.762788606	33.25986865
345.3550305	0.792633971	45.90571043
554.4927757	0.816085807	44.43888516
939.4162833	0.633703426	34.32443515
124.020884	0.74608042	15.02162868
340.3106808	0.862778342	53.54305719
119.1966005	0.918333222	26.25657414
212.046035	0.693415634	37.6072603
125.5572119	0.553695463	17.71243043
183.5349259	0.773515885	29.75305583
102.2056054	0.813015116	16.55211203
310.9083384	0.577283709	20.29538861
105.1874853	0.716816554	15.46912487
201.1189139	0.699405818	17.47928573
224.8520957	0.660225731	28.95147985
163.3766676	0.74945337	25.1578026
302.8333065	0.86788532	41.37974861
72.57461493	0.90606928	18.29078205
200.5082275	0.839043975	26.66338111
131.0072791	0.635182665	10.05622449
164.5902584	0.832402916	21.40236686
119.4489452	0.827232411	22.37443073
237.0403211	0.567162665	15.57890496
148.3607157	0.691751093	12.24149338
128.6148918	0.663372723	18.94936174
148.2769396	0.902689408	27.88637798
167.8126932	0.767989652	20.9210775
135.9222724	0.516751334	13.743341
406.6232971	0.825902443	48.16020299
200.5584373	0.794585702	29.86813813
196.1907872	0.905045329	32.73782222
148.5826799	0.665816478	9.502920685
219.5298213	0.743273029	24.85840075
194.412033	0.64027996	24.16861492
117.4413224	0.781950463	21.27359008
405.948744	0.829084629	38.32277081
169.093379	0.762192978	28.1506715
161.143722	0.931082653	39.14560342
56.80206928	0.853048346	8.956597222
288.9674455	0.661182914	27.88092613

log.sigma.1.5.mm.3D_glszm_SmallAreaHighGrayLevelEmphasis	log.sigma.1.5.mm.3D_glszm_GrayLevelNonUniformityNormalized	log.sigma.1.5.mm.3D_glszm_SizeZoneNonUniformityNormalized
103.7304394	0.061984075	0.469135802
73.41260569	0.07231405	0.411157025
115.4945558	0.060019841	0.464427438
88.79729598	0.080340265	0.395085066
119.1258215	0.066505192	0.430441392
147.2815091	0.058587659	0.470706171
181.8834717	0.06877512	0.491498462
154.531052	0.075737966	0.458302972
98.17354051	0.09845293	0.410691871
150.0751925	0.05151418	0.447925012
870.4681	0.091544568	0.499574831
173.1876187	0.083236883	0.455386231
164.2825047	0.048944028	0.501667324
73.6458915	0.073960302	0.449432892
115.6476159	0.083633402	0.450145748
142.3190584	0.086988245	0.444296306
49.1716018	0.092461474	0.385880883
80.15684822	0.079861111	0.395833333
70.36452387	0.081172426	0.462385642
255.7613788	0.065905928	0.454733768
64.37519239	0.084043889	0.397646903
33.13650859	0.098184057	0.35118498
90.32340606	0.072575848	0.545508626
120.7392688	0.097378213	0.491077641
99.28148059	0.065809042	0.428762641
132.8440815	0.072958084	0.471561108
111.5400535	0.076352405	0.421580947
117.3370363	0.086685284	0.418945093
95.06805556	0.085	0.54
62.37337523	0.08175	0.36335
68.72586321	0.085811195	0.392727398
56.96882324	0.075827697	0.387860449
117.6008537	0.065070538	0.463495355
102.3667773	0.085278529	0.39592212
100.0297921	0.075752797	0.447091072
155.0422923	0.057205859	0.508705239
229.8826667	0.045208948	0.58465186
206.1281397	0.079748616	0.432226317
128.1166287	0.085640138	0.435553633
1221.612865	0.081570134	0.513766709
121.8220627	0.077949523	0.463772491
119.3324004	0.082856583	0.45006764
150.7458661	0.066598012	0.429195492
113.9296989	0.075572499	0.382802223
71.11056802	0.095906009	0.428601004
124.7413371	0.075798605	0.423344015
118.2020251	0.072761302	0.474718898
47.44657923	0.091570248	0.386446281
70.32708966	0.076	0.5336
243.4853644	0.048118357	0.474288997
70.71405256	0.103115488	0.445384281
191.8876821	0.054543549	0.482401563
169.8014308	0.095960911	0.480325375
86.33922049	0.07097853	0.429484308
108.2572563	0.0625	0.423339844
57.78341558	0.08285108	0.429783951
113.253167	0.091538644	0.406185018
134.7779223	0.077402344	0.376972656
115.6198217	0.076822917	0.399305556
183.7236483	0.118465419	0.439482115
155.1890892	0.052715204	0.517089189
307.5290928	0.044933588	0.55838808
453.0926008	0.043008388	0.514386717
711.7457844	0.052702693	0.515150368
78.22181189	0.087240528	0.404267673
308.6625972	0.040134922	0.520912247
108.8377293	0.064280497	0.626734843
188.003403	0.053350189	0.535499724
111.6748101	0.092757494	0.488558486
164.4944816	0.053633218	0.475174546
68.01336847	0.074839302	0.416896235
183.5737581	0.08377583	0.435149983
56.79710989	0.086413724	0.413265773
119.1039022	0.086257544	0.409425128
166.9094052	0.059848324	0.453596716
150.1541744	0.062707888	0.497846714
258.7386075	0.044639163	0.462961408
68.28170056	0.072292395	0.506046762
176.2530827	0.057008898	0.507432468
75.63635323	0.103795153	0.477789632
103.7304394	0.061984075	0.469135802
111.8081401	0.060411523	0.452620027
113.2924961	0.0825	0.433254132
92.73257792	0.107455104	0.432301512
93.60646799	0.072984739	0.453700337
126.4770813	0.058957202	0.487185961
130.3490782	0.072896975	0.44860586
77.42117287	0.088713494	0.36430165
330.4632459	0.044347352	0.492935754
178.3522676	0.055179779	0.529711641
159.1219217	0.051022222	0.506311111
73.67232454	0.099419744	0.390013391
178.7367484	0.059196642	0.500989269
161.5011472	0.06952087	0.478377539
112.9268674	0.065824793	0.515702479
322.9135086	0.049091592	0.525015368
142.5635425	0.057531124	0.467883051
139.8355608	0.055891776	0.480242079
42.01279914	0.128472222	0.378472222
224.1536489	0.057634748	0.456859442

log.sigma.1.5.mm.3D_glszm_SizeZoneNonUniformity	log.sigma.1.5.mm.3D_glszm_GrayLevelNonUniformity	log.sigma.1.5.mm.3D_glszm_LargeAreaEmphasis	log.sigma.1.5.mm.3D_glszm_ZoneVariance
109.7777778	14.5042735	46.00854701	37.90795529
18.09090909	3.181818182	18.43181818	11.83626033
78.02380952	10.08333333	84.19642857	75.93080357
18.17391304	3.695652174	7.347826087	3.347826087
84.79695431	13.10152284	209.6345178	195.2946481
31.53731343	3.925373134	3.029850746	0.666518155
627.1520376	87.75705329	67681.0094	67517.70664
406.0564334	67.10383747	5019.255079	4986.819097
236.1478261	56.61043478	6696.537391	6642.444564
70.7721519	8.139240506	18.4556962	13.14821343
342.2087591	62.7080292	9549.20292	9485.576097
196.7268519	35.95833333	4904.094907	4853.494079
83.77844311	8.173652695	11.91017964	7.98171322
41.34782609	6.804347826	8.173913043	3.90879017
97.23148148	18.06481481	555.1388889	536.9975995
440.7419355	86.29233871	19796.24899	19722.3618
37.81632653	9.06122449	170.7959184	151.002499
19	3.833333333	9.854166667	4.506510417
60.57251908	10.63358779	262.5267176	243.7930191
880.8193082	127.6597832	12602.5684	12564.65409
97.82113821	20.67479675	989.3577236	954.5189371
20.01754386	5.596491228	17.50877193	9.825176978
44.73170732	5.951219512	87.23170732	75.48854848
204.7793765	40.60671463	16118.15348	15985.43424
70.31707317	10.79268293	586.1707317	566.5739143
188.1528822	29.11027569	1368.24812	1342.490587
108.3463035	19.62256809	199.7081712	186.3586731
124.8456376	25.83221477	1608.077181	1573.235496
10.8	1.7	2.4	0.44
72.67	16.35	753.245	720.124975
189.2946058	41.36099585	5003.23029	4950.170249
41.11320755	8.037735849	13.80188679	8.014684941
143.2200647	20.10679612	563.0517799	545.9191462
170.2465116	36.66976744	5180.072093	5126.442017
313.4108417	53.10271041	1451.487874	1426.885708
186.6948229	20.99455041	242.1035422	232.6567129
145.5783133	11.25702811	4.835341365	2.382155127
512.1881857	94.5021097	35436.08608	35337.96744
29.61764706	5.823529412	32.27941176	23.88646194
740.851595	117.6241331	27577.22885	27506.65487
84.40659341	14.18681319	146.3681319	134.3478143
454.5683168	83.68514851	10544.68416	10493.34197
148.9308357	23.10951009	337.3170029	317.9519305
73.88082902	14.58549223	198.4818653	185.0627399
59.57553957	13.33093525	1749.05036	1687.783138
151.9805014	27.21169916	1446.4039	1416.748442
353.1908602	54.1344086	17535.78898	17448.0495
21.25454545	5.036363636	77.18181818	61.32694215
26.68	3.8	15.76	10.5616
55.96610169	5.677966102	7.050847458	3.574834818
118.917603	27.53183521	3548.044944	3497.085665
305.3601896	34.52606635	470.4249605	458.7917961
366.9685864	73.31413613	43155.08639	42980.25154
78.59562842	12.98907104	339.1202186	317.6979904
54.1875	8	19.4296875	14.40228271
61.88888889	11.93055556	242.6458333	221.4474826
294.4841379	66.36551724	7962.616552	7909.437253
120.63125	24.76875	1009.871875	986.561084
38.33333333	7.375	38.14583333	30.11805556
981.8030439	264.6517457	61188.32453	61077.54749
123.0672269	12.54621849	190.0378151	178.8237589
319.95637	25.7469459	263.08726	255.2609364
903.7774616	75.56573705	408.464428	401.6931769
828.8769422	84.79863269	7452.400249	7424.360953
33.55421687	7.240963855	124.1084337	109.1511105
517.265861	39.85397784	79.21349446	74.37172189
46.37837838	4.756756757	5.148648649	2.562636961
531.2157258	52.9233871	2003.504032	1986.187695
526.6660482	99.99257885	23924.31262	23837.86402
210.0271493	23.70588235	321.9615385	310.0262331
27.51515152	4.939393939	34.84848485	25.84848485
403.3840345	77.66019417	12870.41532	12811.76979
74.80110497	15.64088398	314.9889503	297.8649003
97.03375527	20.44303797	627.1687764	606.6711887
271.2508361	35.78929766	3054.842809	3025.920717
84.13609467	10.59763314	157.1242604	144.3510381
262.4991182	25.31040564	53.9047619	47.07303827
30.86885246	4.409836066	7.62295082	4.06879871
139.0364964	15.62043796	41.75547445	36.11051468
116.1028807	25.22222222	1955.053498	1911.916171
109.7777778	14.5042735	46.00854701	37.90795529
61.1037037	8.155555556	40.18518519	32.71407407
190.6318182	36.3	7375.427273	7315.082624
79.54347826	19.77173913	1129.146739	1088.367409
144.7304075	23.28213166	1567.043887	1535.027447
43.35955056	5.247191011	8.831460674	4.920843328
82.54347826	13.41304348	199.9728261	184.447513
125.3197674	30.51744186	14063.09302	13923.38612
220.3422819	19.82326622	121.0178971	114.1081733
140.3735849	14.62264151	129.6226415	120.509078
75.94666667	7.653333333	8.386666667	5.001066667
88.53303965	22.56828194	1363.110132	1323.425683
436.3616533	51.56027555	1212.776119	1197.340513
307.1183801	44.63239875	3965.766355	3929.728962
283.6363636	36.20363636	554.42	540.2960694
254.107438	23.76033058	84.31818182	78.33389113
189.0247525	23.24257426	368.4084158	355.2588165
25.45283019	2.962264151	4.056603774	1.362050552
9.083333333	3.083333333	22.04166667	13.28993056
863.9212057	108.9873083	7417.860391	7392.06624

log.sigma.1.5.mm.3D_glszm_ZonePercentage	log.sigma.1.5.mm.3D_glszm_LargeAreaLowGrayLevelEmphasis	log.sigma.1.5.mm.3D_glszm_LargeAreaHighGrayLevelEmphasis	log.sigma.1.5.mm.3D_glszm_HighGrayLevelZoneEmphasis
0.351351351	0.28092936	9046.641026	145.2393162
0.389380531	0.410433834	1477.795455	94.54545455
0.347826087	0.407668982	18865.59524	154.8928571
0.5	0.076005513	1285.173913	136.5217391
0.264075067	0.977543498	46729.69036	162.9187817
0.650485437	0.051066483	532.4328358	190.4925373
0.078253404	283.8365243	16208093.12	235.4231975
0.175584621	16.88768322	1498075.653	212.5485327
0.135965949	32.4771818	1388643.908	143.5530435
0.434065934	0.135643074	3223.088608	209.2468354
0.125366032	8.489399238	10755338.93	1168.29635
0.140579239	16.25497845	1485980.157	242.5208333
0.504531722	0.101827692	2180.580838	202.5808383
0.484210526	0.074136054	1510.25	121.4782609
0.234782609	2.118735776	147173.0278	163.4490741
0.116336343	83.72558261	4700774.477	193.5816532
0.224770642	1.798860859	17529.08163	73.97959184
0.432432432	0.129202523	1205.916667	120
0.231040564	3.591133114	20732.79389	87.30534351
0.162404628	37.22087871	4281200.009	350.5203924
0.169421488	10.21830883	98927.62195	86.13821138
0.360759494	0.347482278	1407.736842	58.98245614
0.291814947	0.598214521	14023.64634	112.695122
0.086802664	90.57957011	2884788.463	157.9688249
0.225895317	2.982019772	118029.8293	135.3658537
0.197037037	7.82422594	242024.2932	166.9924812
0.273695421	0.914989074	44939.12062	162.9688716
0.16941444	6.892431876	377642.4799	172.5536913
0.714285714	0.129703566	189.2	110.65
0.173761946	7.505869073	78608.29	86.615
0.137282825	32.73253961	772027.3091	99.83609959
0.415686275	0.201673149	1426.198113	86.79245283
0.241594996	3.228273904	100119.2751	151
0.136551286	28.64750802	943020.2093	146.4813953
0.201610584	8.1967217	259655.2553	138.9201141
0.32535461	0.985574165	63245.26158	193.9836512
0.638461538	0.037056836	1056.839357	276.7670683
0.100954166	103.400155	12180609.01	291.1392405
0.345177665	0.169444293	7171.970588	185.6176471
0.119035826	16.90078833	45028099.8	1614.371706
0.288431062	0.690806556	32192.87912	166.2252747
0.139560591	82.25078393	1364150.025	154.6623762
0.22724296	1.227813673	95699.08069	216.7896254
0.272984441	0.777548272	52260.09326	175.1968912
0.127757353	14.03342354	220640.1942	98.73381295
0.183631714	6.254487778	337501.9443	172.7855153
0.106758502	97.75226299	3164731.801	153.641129
0.251141553	1.717824486	4575.472727	62.12727273
0.438596491	0.215058985	1600.34	91.14
0.536363636	0.05159446	1543.559322	316.0084746
0.140083945	23.58336359	537658.4082	99.54681648
0.293191292	2.081209266	108260.0221	245.6635071
0.075628588	182.8460281	10227713.81	222.2133508
0.216056671	2.650273959	44770.34426	124.6338798
0.445993031	0.136028781	3910.21875	158.125
0.21719457	2.323921057	27402.31944	79.97916667
0.137128807	44.5168551	1433132.859	160.9310345
0.207119741	3.363822961	305465.8313	205.296875
0.352941176	0.170942772	9721.916667	179.1354167
0.09501127	226.3270024	16606665.57	257.3871979
0.298619824	0.883681553	42344.66807	202.9159664
0.357454772	0.722925845	97252.40489	369.6544503
0.384295713	0.704773178	238297.6892	586.2128628
0.188849765	7.537439388	7377360.031	928.7395898
0.258566978	0.916068615	17855.21687	112.626506
0.454462243	0.242911136	27065.87009	381.9446123
0.621848739	0.085850604	613	125.9864865
0.240310078	8.614959752	469446.7036	227.5856855
0.107552629	184.7607826	3123743.762	141.8756957
0.289456451	1.658873012	64145.35973	210.3574661
0.333333333	0.41043828	4390.318182	94.04545455
0.130581772	37.70676147	4407266.031	264.6256742
0.241655541	2.239667054	46352.42541	79.1160221
0.220876048	2.667801863	148967.3165	175.0506329
0.185945274	13.0454757	718820.6773	227.2525084
0.279801325	0.99136343	25927.31953	191.8579882
0.382591093	0.237940909	13198.40741	349.3915344
0.530434783	0.25861041	503.1803279	83.3442623
0.420890937	0.222199815	8623.927007	221.8467153
0.152255639	12.9943969	297099.2387	103.5802469
0.351351351	0.28092936	9046.641026	145.2393162
0.365853659	0.38771098	5025.422222	141.1037037
0.128730252	27.5339029	1983779.62	167.2045455
0.156595745	6.841314682	189044.0543	129.4456522
0.176731302	10.75438849	231015.3009	122.1724138
0.505681818	0.113192563	1177.292135	163.9101124
0.253793103	1.066722274	39473.94022	170.7173913
0.084604033	93.44121012	2129801.491	113.1627907
0.380425532	0.312581114	48276.08949	435.3780761
0.33125	0.616872263	28373.63396	219.0603774
0.543478261	0.075719247	1419.153333	209.2733333
0.158741259	7.856529287	238566.5903	115.9471366
0.254529515	5.226947113	283680.0677	230.9506315
0.166580176	19.34640574	818452.2181	208.0202492
0.266086115	4.893398852	65144.92727	138.9763636
0.408783784	0.182005586	40569.33058	410.1590909
0.275767918	1.893634938	73509.18564	180.0990099
0.609195402	0.133142107	565.5660377	178.2075472
0.338028169	0.532601537	1177.666667	62.125
0.196897126	24.65351591	2240781.155	303.1671074

log.sigma.1.5.mm.3D_glszm_SmallAreaEmphasis	log.sigma.1.5.mm.3D_glszm_LowGrayLevelZoneEmphasis	log.sigma.1.5.mm.3D_glszm_ZoneEntropy	log.sigma.1.5.mm.3D_glszm_SmallAreaLowGrayLevelEmphasis
0.710935359	0.035485975	5.537477092	0.031641414
0.663163945	0.080679035	4.879664005	0.055440026
0.707907706	0.021536463	5.464636524	0.018526433
0.65077145	0.034963568	4.762267391	0.031794684
0.68012415	0.017660252	5.633867208	0.01481274
0.709162521	0.02715015	5.101474407	0.022770769
0.728517033	0.008563991	5.729667933	0.006764516
0.70410841	0.00883001	5.64423017	0.006953308
0.665722482	0.013283769	5.328309549	0.010228366
0.695378682	0.02394268	5.698471119	0.021280593
0.734361532	0.002479737	5.393008279	0.00223548
0.701458092	0.008805457	5.514455289	0.005564849
0.734987998	0.026389538	5.59855862	0.023164337
0.693694719	0.028588126	5.007432136	0.025796671
0.697508288	0.014323605	5.261362798	0.011815661
0.693249577	0.010716061	5.581401021	0.008120382
0.641263381	0.048937313	5.08329964	0.040510236
0.645017955	0.040269037	4.855388542	0.035912417
0.706555073	0.058533356	5.099687549	0.039070943
0.701280007	0.004553382	5.921042809	0.003399787
0.655613164	0.030114316	5.452113955	0.020836359
0.607877964	0.05565853	4.784215672	0.047634314
0.758198296	0.032832992	4.823837146	0.027388421
0.727584434	0.01321115	5.262769956	0.010646293
0.679689578	0.027435941	5.414227605	0.017445226
0.713766956	0.015962705	5.667508988	0.012614479
0.674757775	0.013709195	5.515471754	0.010824584
0.672298275	0.012820466	5.449802027	0.010472352
0.761111111	0.069239564	4.021928095	0.061164459
0.62345709	0.048692566	5.551190554	0.03505418
0.65127625	0.023137519	5.552070507	0.017343727
0.645153283	0.040141907	5.217050323	0.03252296
0.706776011	0.02175137	5.633343974	0.017513984
0.65033625	0.016240228	5.612766377	0.011910609
0.695250142	0.016550201	5.602570444	0.013301953
0.739954302	0.018219707	5.678771187	0.012588882
0.791789697	0.013624395	5.568943236	0.011959504
0.682806794	0.005720694	5.710015272	0.004214354
0.683998615	0.022261422	4.970575534	0.020270881
0.744583939	0.001349507	5.563035003	0.001178162
0.707510056	0.014695115	5.358370127	0.012231356
0.697506267	0.014828847	5.629680827	0.010867068
0.680871609	0.011529018	5.735482412	0.009666587
0.641418364	0.014758333	5.482509777	0.012324779
0.677514732	0.03290533	5.134139588	0.027691894
0.676461761	0.013381936	5.572539872	0.01061797
0.716333167	0.016003554	5.594777071	0.012791501
0.642068594	0.07008544	4.876369941	0.053828364
0.753437016	0.049158896	4.538171939	0.043481337
0.714495393	0.014480657	5.597999193	0.012610576
0.693649616	0.034362472	5.035663549	0.02978689
0.722087246	0.012492201	5.906372661	0.009229964
0.720134511	0.007733002	5.389490999	0.005943736
0.67891476	0.023777194	5.469640053	0.019221618
0.673773464	0.029282448	5.435835942	0.025810467
0.680012096	0.051696914	5.184863464	0.043092967
0.661253027	0.012736669	5.604428776	0.009469771
0.637132346	0.010056205	5.650068783	0.007839984
0.6504715	0.018368921	5.223063288	0.015743441
0.689576362	0.006282222	5.508555493	0.004724229
0.744732413	0.017742196	5.678669886	0.015624858
0.774866794	0.007181124	5.874076607	0.00605882
0.745108847	0.003129588	6.19667931	0.002522989
0.745568604	0.001889887	5.967318236	0.001565004
0.65807593	0.031483792	4.906203251	0.026146723
0.749816508	0.006870405	6.164645486	0.005545432
0.814133714	0.041763563	4.864859367	0.03885132
0.759410783	0.012357419	5.842666251	0.010058656
0.726611594	0.014749847	5.398518289	0.010890264
0.716077495	0.014509436	5.756703179	0.011720503
0.670423345	0.044539892	4.877822628	0.027203248
0.68607906	0.006900911	5.686841575	0.00542837
0.667041813	0.049126081	5.278183432	0.042030844
0.663588708	0.012674302	5.382522734	0.010089543
0.699987736	0.009817013	5.881793736	0.007681191
0.730167867	0.018474215	5.404103088	0.015920979
0.707480472	0.007200575	6.122329967	0.00569008
0.736460707	0.07178109	4.76214369	0.059006357
0.739520968	0.015365799	5.561362309	0.01317719
0.718396836	0.019806643	4.891664263	0.016072245
0.710935359	0.035485975	5.537477092	0.031641414
0.698491842	0.025791785	5.319418843	0.020264936
0.68445566	0.013681387	5.437080451	0.011417685
0.68321552	0.017167649	4.98704603	0.013930506
0.699883898	0.026295326	5.456220216	0.021804695
0.724477793	0.038823946	5.145355588	0.035407147
0.696021793	0.015922447	5.385134436	0.012504479
0.626578898	0.018632723	5.578946001	0.013264575
0.729703532	0.006041805	6.061631356	0.00501668
0.754467875	0.01335346	5.485754064	0.011289974
0.738825828	0.020742127	5.45063048	0.018507233
0.647766939	0.016879313	5.202857595	0.013319936
0.735437558	0.009056236	5.744444226	0.007198096
0.718898449	0.01102016	5.656438451	0.008599082
0.744652603	0.018605438	5.543335478	0.014255867
0.752157315	0.005894168	5.847401764	0.005031814
0.71058078	0.019163764	5.745256208	0.015556755
0.719339623	0.042923563	4.817266445	0.025144093
0.625062159	0.071527773	3.918295834	0.059397452
0.702768629	0.006234578	6.006113438	0.004701187

log.sigma.1.5.mm.3D_ngtdm_Coarseness	log.sigma.1.5.mm.3D_ngtdm_Complexity	log.sigma.1.5.mm.3D_ngtdm_Strength	log.sigma.1.5.mm.3D_ngtdm_Busyness	log.sigma.1.5.mm.3D_ngtdm_Contrast
0.010149684	0.737153768	1.737734802	0.2735215	0.000168794
0.048893833	3.246388394	6.484697492	0.137061434	0.001934681
0.011405923	1.132562804	2.31800804	0.23874354	0.000283523
0.053900087	4.081032804	6.047538411	0.093557433	0.002642508
0.008522327	0.651417455	1.995807954	0.274178507	9.34E-05
0.050310945	6.14453259	7.068814016	0.106362647	0.004354083
0.000322617	0.029859269	0.451120841	3.500612399	3.44E-07
0.001239004	0.138062281	0.939430323	0.912221541	3.86E-06
0.001439101	0.056992747	0.459012009	1.445966252	4.86E-06
0.019528104	2.684958752	4.566362309	0.130468702	0.000484547
0.00120472	0.127015778	1.922943309	0.420482746	1.36E-06
0.001977786	0.148503744	1.550824925	0.620613206	4.64E-06
0.017857589	3.0903389	3.941517277	0.171956519	0.000745408
0.03323769	2.025081911	4.111304876	0.134712781	0.000957038
0.006502945	0.390141789	1.708150589	0.323541532	6.69E-05
0.000638233	0.038600949	0.353060829	2.420040628	1.81E-06
0.015931587	0.419246197	1.654872334	0.320171929	0.000143621
0.047145645	2.730130288	5.485475902	0.13707535	0.001830085
0.0119611	0.556842126	2.225444768	0.379995413	0.00010574
0.000568373	0.063733634	0.449690778	1.735283563	1.28E-06
0.004605132	0.116924204	0.508087064	1.059391724	3.58E-05
0.039333921	0.873112859	2.567741997	0.273518256	0.00090402
0.023628546	0.924261001	3.134590674	0.155765639	0.00033685
0.00120559	0.052953618	0.708685238	1.485905834	1.51E-06
0.007063261	0.641310836	2.364986613	0.300890442	0.000100702
0.003221266	0.475822769	2.876361456	0.462615844	1.31E-05
0.007301349	0.375683151	1.32039488	0.373686254	7.91E-05
0.003792682	0.155737074	1.002512966	0.544363467	1.63E-05
0.131202115	10.94185154	11.54102873	0.115984345	0.025243925
0.006082951	0.18846566	0.846381469	0.69396825	4.27E-05
0.001729448	0.074507864	0.510530817	1.473111418	7.77E-06
0.02314111	1.388644076	2.467867088	0.248856147	0.000614881
0.005328806	0.358348112	1.341200477	0.429554685	4.01E-05
0.001866021	0.097725476	0.693401393	1.073350744	7.07E-06
0.001893575	0.10389668	0.559662579	1.177087715	9.13E-06
0.006045534	0.626005534	1.778423109	0.306910865	7.82E-05
0.015835231	3.783086085	4.325489615	0.166402896	0.000837325
0.000454031	0.045753544	0.680613657	2.180695338	7.79E-07
0.031693818	1.17836456	3.444035007	0.122366392	0.000578685
0.000485319	0.070988702	1.054870991	0.744732473	5.42E-07
0.010917448	0.567537944	1.858050251	0.258883927	0.000121688
0.000926647	0.076242199	0.706981963	1.93266921	1.67E-06
0.00462986	0.287799437	1.133297069	0.438644017	3.67E-05
0.008674394	0.493915165	1.589274356	0.314838985	0.00013424
0.006092932	0.108923164	0.738777725	0.66396305	3.07E-05
0.003254195	0.193901356	1.031091399	0.601918741	1.82E-05
0.000843154	0.04771357	0.570551338	2.016558837	1.59E-06
0.029112537	0.822428561	3.104279314	0.273089824	0.000385018
0.053002093	1.972349668	3.995179097	0.125239226	0.001707417
0.022348124	8.305244811	10.77848464	0.107521374	0.001625857
0.003192857	0.085316217	0.750523555	0.876970706	1.22E-05
0.003439837	0.549916294	1.603097885	0.416834483	2.74E-05
0.000540697	0.027859087	0.43092655	2.706063143	5.95E-07
0.009207592	0.285075578	1.309001969	0.376218999	6.80E-05
0.01887219	2.339674079	3.813781297	0.184003989	0.000830634
0.00989688	0.24733918	0.83463126	0.564960969	0.000130066
0.001194112	0.059456177	0.469457409	1.600418685	2.94E-06
0.003891113	0.277761701	1.262497078	0.456391374	3.11E-05
0.025267038	0.900670205	2.759900899	0.164945653	0.000611525
0.000249183	0.026982328	0.383127503	3.99760306	2.10E-07
0.008816595	1.121241437	2.890448527	0.213421601	0.000112195
0.004613413	1.115347634	2.325703095	0.230669058	5.53E-05
0.001710557	0.597368493	1.204920852	0.411859205	1.76E-05
0.000778601	0.154892522	1.0336165	0.612785469	2.26E-06
0.020550235	0.657168789	2.500605266	0.191185766	0.000241005
0.003105717	1.071015389	1.392592005	0.433942361	8.53E-05
0.045054422	5.722429721	7.621285413	0.106074305	0.002075853
0.001778378	0.312574839	1.421839401	0.684343587	9.26E-06
0.000597212	0.038536844	0.446726061	3.173406675	8.81E-07
0.004733466	0.499629207	1.359076875	0.433736282	4.94E-05
0.033977119	1.071333283	2.906716198	0.162632595	0.0007593
0.000825835	0.074147523	0.769577726	1.247107731	1.78E-06
0.008255823	0.475749308	1.765860598	0.399854748	9.54E-05
0.006116588	0.343080151	2.072790713	0.356018566	4.22E-05
0.002179562	0.267424093	1.728181565	0.560504061	7.11E-06
0.011994742	1.032679789	3.659131891	0.193515323	0.000107415
0.004743518	0.996461084	1.605211634	0.361208243	0.000109707
0.04560208	2.821587797	4.485280624	0.226050091	0.002478035
0.010320686	1.182615195	2.124662871	0.245280072	0.000207609
0.00412011	0.075810846	0.572433795	0.891399374	1.96E-05
0.010149684	0.737153768	1.737734802	0.2735215	0.000168794
0.016832923	1.455263443	2.888674649	0.230595462	0.000346642
0.001472069	0.09368319	0.829804371	1.038782063	6.09E-06
0.006209942	0.150649601	1.014438896	0.511142583	2.58E-05
0.003718171	0.158627296	0.797579823	0.719777222	2.02E-05
0.031807769	3.537898575	4.430277011	0.148879065	0.001541686
0.010005305	0.550045891	2.247779062	0.274178575	9.64E-05
0.00140901	0.051799192	0.605946109	1.633031313	3.05E-06
0.006453522	1.483119141	2.826704107	0.185015091	0.00011876
0.009156863	0.817708713	1.854450312	0.248545314	0.000130063
0.021389471	3.709775156	4.27360308	0.153976574	0.001039846
0.00465556	0.102024513	0.671252682	0.70348129	2.28E-05
0.002175583	0.200792443	0.734283142	0.742607941	1.23E-05
0.001753561	0.152443736	1.025961456	0.857711427	5.45E-06
0.003544629	0.270608745	1.011124911	0.758229157	2.31E-05
0.005317712	1.548964054	2.99082226	0.199480488	9.06E-05
0.004530037	0.56970346	1.842024202	0.42453687	4.24E-05
0.053279544	10.08494684	9.21645769	0.109693461	0.006914393
0.083728436	1.137508459	2.988368444	0.170825852	0.002274793
0.000730074	0.076499198	0.373730051	1.636035031	3.07E-06

log.sigma.1.0.mm.3D_gldm_GrayLevelVariance	log.sigma.1.0.mm.3D_gldm_HighGrayLevelEmphasis	log.sigma.1.0.mm.3D_gldm_GrayLevelNonUniformityNormalized	log.sigma.1.0.mm.3D_gldm_DependenceEntropy
4.937363039	94.84534535	0.161291923	5.821050406
4.862401128	34.19469027	0.152008771	5.142842185
4.663631804	69.16149068	0.158777311	5.95670985
6.11944707	87.31521739	0.120274102	5.040913826
3.923741635	71.37935657	0.178629186	5.738511593
10.59873692	97.10679612	0.087755679	4.983207416
1.310230738	108.0242242	0.383161661	5.28432553
2.874669646	98.53904082	0.275281834	5.697288401
1.706023353	101.8526838	0.322837991	5.381353304
9.94985509	105.0659341	0.116637483	5.810508679
1.805015733	603.8588946	0.330049047	5.54130181
2.018848014	147.3966808	0.324392913	5.489167911
10.02113891	110.081571	0.098985953	5.899509334
6.17232687	86.51052632	0.115290859	5.29180202
3.353799622	94.59782609	0.246013705	5.603100452
1.733134723	104.6317579	0.36156387	5.33245369
2.474260374	46.92889908	0.216427489	5.71548537
4.481779076	59.99099099	0.156074994	4.91812657
3.004675121	37.18694885	0.207394343	5.687175041
2.555029007	153.5603253	0.277460171	5.816753627
1.881261905	47.06336088	0.243357694	5.831056129
3.268546707	34.43037975	0.161432463	5.222050908
3.914717392	72.13879004	0.203885462	5.518500151
1.087283417	68.99188177	0.379043605	5.252336684
4.136283951	78.01101928	0.278419052	5.732729065
3.522203567	66.08444444	0.242379393	5.824367883
3.559631675	113.4632588	0.185719746	5.910450771
1.97586036	100.593519	0.288057423	5.739600677
7.952806122	45.25	0.114795918	3.682005815
2.25748169	48.12250217	0.234947739	5.709607323
2.06591531	64.39988607	0.289878959	5.667112212
4.146005383	43.56470588	0.145251826	5.356195205
3.453932259	80.68491009	0.225764607	5.929487163
2.117701124	86.4436329	0.324637328	5.504651953
2.534237852	81.13977567	0.251608396	5.845961201
5.389365946	109.6356383	0.175542918	6.042920706
13.43419461	104.074359	0.077935569	5.699273446
1.784666183	178.363435	0.378761562	5.375863335
3.414723389	105.0456853	0.171274704	5.320738181
1.802922154	642.8197127	0.356886939	5.387284128
3.401448158	73.8177496	0.189860383	5.791999009
1.887240292	71.19510847	0.319178391	5.650823646
3.159252212	90.78454486	0.186573568	6.045910945
3.735816216	73.18811881	0.187402596	5.798370202
1.475981293	37.60845588	0.312136746	5.491718805
2.645680235	97.0286445	0.245690439	5.799394386
1.640090802	68.54211508	0.353171923	5.368807533
2.238944142	24.05936073	0.208148287	5.342566396
5.000923361	50.38596491	0.149122807	5.190132177
13.18039256	110.7409091	0.099297521	5.399208612
1.49098528	83.5996852	0.366132685	5.309692814
5.318298554	103.5108847	0.184282175	6.167244741
1.041372575	128.6897644	0.394589344	5.160699696
2.750960749	56.41912633	0.220854492	5.859894363
8.420230912	85.57839721	0.120810013	5.812075626
2.475015299	43.62443439	0.201881843	5.756205038
1.580026723	68.75676187	0.32234538	5.508179369
3.171506792	115.9572816	0.243731423	5.804501314
4.395423335	101.1139706	0.15643923	5.644826246
1.277369614	153.7635776	0.358919997	5.231270675
5.674151342	117.2057716	0.169095526	6.084617109
8.534945978	171.6188397	0.147783962	6.210961487
9.90591419	228.385608	0.130779885	6.457550079
3.928281371	448.771831	0.26415267	5.930550603
2.550324628	47.07788162	0.225783911	5.610461666
14.02428331	169.3203661	0.092824071	6.435412348
7.873314031	76.1092437	0.104300544	5.286085071
5.155794553	102.4275678	0.217608594	6.018304043
1.409953874	70.45754764	0.352025471	5.328706574
4.797177545	82.51866405	0.170612022	6.079779852
4.256325885	53.75252525	0.169829609	5.520506497
2.205470055	125.0470489	0.334097299	5.524493961
3.419084814	72.51535381	0.208675207	5.678426958
3.101551338	98.35041938	0.254195803	5.674161732
3.392160776	105.8196517	0.272583367	5.907282727
4.153622648	83.1986755	0.182990878	5.859226352
10.18913967	132.2901484	0.105892391	6.251583197
5.650964083	41.23478261	0.125141777	5.255861738
6.520220575	96.98924731	0.126807157	6.026641367
1.486304656	65.11591479	0.317016696	5.430851697
4.937363039	94.84534535	0.161291923	5.821050406
4.730047517	45.84281843	0.141868817	5.828722391
2.06924558	123.2817437	0.347282363	5.390493372
1.578405432	79.66382979	0.269807515	5.582742334
2.328925039	80.62936288	0.274260326	5.851455208
6.016238378	55.41477273	0.114927686	5.420573006
3.527845898	77.27448276	0.184201665	5.892630073
1.338863983	66.89867191	0.363118602	5.305662165
10.12724237	178.5965957	0.124539973	6.42538273
5.556485938	118.26125	0.159565625	6.019780541
10.52188353	77.75	0.092470069	5.63914056
1.470084601	80.07132867	0.298197467	5.610790289
3.741430804	104.2036821	0.211786184	6.002436954
2.742463703	105.5433316	0.280057867	5.702665378
3.489720395	66.64876633	0.188812891	5.920612557
8.769300128	154.6402027	0.127846227	6.128872639
4.512967653	95.5883959	0.178224091	6.064203897
13.42475888	83.25287356	0.090764962	5.112499749
1.998016267	25.74647887	0.204919659	4.801175205
3.619052809	127.4808413	0.242678282	5.958654607

log.sigma.1.0.mm.3D_gldm_DependenceNonUniformity	log.sigma.1.0.mm.3D_gldm_GrayLevelNonUniformity	log.sigma.1.0.mm.3D_gldm_SmallDependenceEmphasis
67.67567568	107.4204204	0.23311892
15.83185841	17.17699115	0.227708011
44.39544513	76.68944099	0.211143739
16.73913043	11.06521739	0.309117652
60.08310992	133.2573727	0.169419426
30.53398058	9.038834951	0.520347896
1255.81884	6247.834049	0.050110188
316.9825604	1389.072136	0.11051759
296.2229842	1365.281863	0.085416038
57.30769231	42.45604396	0.308391563
356.8235725	1803.387994	0.09145094
206.8268793	996.8594208	0.084996284
53.22960725	32.76435045	0.340643078
35.98947368	21.90526316	0.290718376
64.34565217	226.3326087	0.145899485
632.3639029	3083.055119	0.068854231
30.2293578	94.36238532	0.137030626
19.34234234	17.32432432	0.221466609
45.31040564	117.5925926	0.163450544
767.9813029	3309.26746	0.102937331
88.0523416	353.3553719	0.109088679
18.64556962	25.50632911	0.205847721
22.90035587	57.29181495	0.172520321
344.6765196	1820.925479	0.055552454
46.30578512	202.1322314	0.124932325
133.0424691	490.8182716	0.128921884
79.07667732	174.3908413	0.169915224
104.2427516	506.6930074	0.098545235
11.64285714	3.214285714	0.529761905
75.64639444	270.424848	0.119323393
215.9193962	1017.765024	0.088496306
35.76078431	37.03921569	0.26284977
91.86317435	288.752932	0.162716194
211.560813	1022.282947	0.093430883
224.677308	874.8423929	0.131688088
96.57446809	198.0124113	0.21372014
96.76410256	30.39487179	0.460011091
828.3383881	4445.90322	0.058388055
23.56852792	33.74111675	0.204378528
874.1338947	4323.32838	0.074307228
52.340729	119.8019017	0.167097134
457.1113721	2309.894017	0.098933942
120.2364113	284.8978389	0.132900826
55.37057992	132.4936351	0.176167775
68.66727941	339.6047794	0.091239167
126.5907928	480.3248082	0.117215359
512.9896685	2461.25513	0.06797903
20.47945205	45.58447489	0.132421361
16.66666667	17	0.334838062
49.15454545	21.84545455	0.34514765
121.8677859	697.8488982	0.089444351
172.4849467	397.8652154	0.203184802
799.2611364	3986.141556	0.054201078
63.45336482	187.0637544	0.138717158
38.79790941	34.67247387	0.263911897
50.51432881	133.8476621	0.150017488
349.6597314	1704.240023	0.092815522
96.78899676	376.5650485	0.123879384
27.68382353	42.55147059	0.213480182
1825.71739	8439.285884	0.076175342
65.23337516	134.7691343	0.200852547
150.8116032	236.8976918	0.239406457
500.1084864	597.9256343	0.26625923
547.7666667	2250.580751	0.12830731
25.82866044	72.47663551	0.131367106
324.205492	202.820595	0.317441299
25.01680672	12.41176471	0.349750233
288.815407	898.2882752	0.176634634
750.3640627	3528.351292	0.066857475
126.2167649	260.524558	0.189546126
19.98989899	33.62626263	0.230657369
480.0967742	2371.756726	0.075730116
52.78638184	156.2977303	0.150070842
73.63932898	272.7520969	0.138010582
200.971393	876.6281095	0.138399662
49.47682119	110.5264901	0.177924967
201.1066127	156.9325236	0.271925469
22.28695652	14.39130435	0.388552918
83.74961598	82.55145929	0.301502011
103.1378446	505.9586466	0.096299788
67.67567568	107.4204204	0.23311892
42.76693767	52.3495935	0.213513046
235.6898771	1187.011118	0.082881635
74.77191489	317.0238298	0.109930406
102.9058172	495.0398892	0.124639116
29.81818182	20.22727273	0.322318589
60.10896552	133.5462069	0.174756801
289.0231185	1476.440236	0.061257969
130.373617	146.3344681	0.240882568
73.675	127.6525	0.222670832
57.43478261	25.52173913	0.373631337
82.66013986	426.4223776	0.091269321
249.3056692	724.7323203	0.183448803
254.3674105	1079.34302	0.108760168
174.5907112	390.2762458	0.17975927
138.3902027	151.3699324	0.274521304
115.6238908	261.0982935	0.188142802
22.51724138	7.896551724	0.441733716
9.76056338	14.54929577	0.217248793
639.9506456	2330.682216	0.12167745

log.sigma.1.0.mm.3D_gldm_DependenceNonUniformityNormalized	log.sigma.1.0.mm.3D_gldm_DependenceVariance	log.sigma.1.0.mm.3D_gldm_LargeDependenceEmphasis
0.101615129	13.79018658	39.3033033
0.140104942	5.23016681	20.18584071
0.091916035	20.52482543	53.81987578
0.18194707	3.81758034	13.89130435
0.080540362	21.70571915	64.04289544
0.296446413	1.605429352	6.126213592
0.077015751	18.97280783	144.7988471
0.062818581	22.73768826	96.4130004
0.070045634	20.82237639	111.4209033
0.157438715	5.919846637	18.32417582
0.065304461	20.1337143	100.6863104
0.067304549	21.74991595	116.7188415
0.160814523	10.13466471	23.87613293
0.189418283	3.504709141	13.14736842
0.069940926	20.69028355	77.79130435
0.074160186	20.6698374	130.1319339
0.069333389	21.42277586	71.71559633
0.174255336	5.848551254	19.55855856
0.079912532	16.39204452	54.60493827
0.064390149	24.62389176	107.9647019
0.060642109	24.13074395	92.9338843
0.118009934	9.762217593	30.75949367
0.081495928	18.02869771	57.83985765
0.071747818	19.21509622	131.2822648
0.063782073	23.93970509	90.16253444
0.065699985	21.84565377	82.1782716
0.084213714	17.19912308	52.80404686
0.059262508	24.36939111	99.89823764
0.415816327	0.418367347	3.357142857
0.065722324	19.67331094	78.67332754
0.061497977	24.06077169	112.8689832
0.14023837	6.665497885	21.76862745
0.071824218	20.98276055	67.96794371
0.067183491	21.0701804	109.2654811
0.06461815	23.41624839	88.53005464
0.085615663	20.1860539	57.88652482
0.248113083	2.211019066	7.8
0.070568955	20.45297596	138.1213154
0.119637198	8.33734443	29.16243655
0.072158981	20.82268889	125.9351164
0.082948857	19.0067435	57.659271
0.063163102	22.27429098	105.8058588
0.078740282	14.93745028	54.81663392
0.078317652	18.91941766	58.58981612
0.063113308	22.65984199	103.8253676
0.064752324	21.89095414	85.73554987
0.073610227	20.44983173	132.2010331
0.09351348	13.24275974	46.18721461
0.14619883	7.183748846	21.54385965
0.223429752	1.856280992	8.663636364
0.063939027	22.13199935	116.4847849
0.079891129	23.66073918	66.04029643
0.079119099	18.59872206	142.4155613
0.074915425	17.57824616	61.99645809
0.135184353	9.812647962	26.86062718
0.076190541	18.670343	61.50075415
0.066135754	21.15547995	107.9268016
0.0626466	24.8979353	94.2776699
0.101778763	11.83801903	36.69117647
0.077647148	19.07468501	121.8728788
0.081848651	22.25790881	62.3902133
0.09408085	19.13482012	51.98066126
0.109385058	18.05371086	44.52712161
0.064291862	26.8104108	106.9129108
0.080463117	20.32691841	63.41121495
0.1483778	11.77423058	27.42105263
0.210225267	2.110161712	9.386554622
0.069964973	25.39236299	83.93265504
0.074864219	20.86411048	134.8186172
0.08265669	20.02438448	58.81925344
0.100959086	15.19365371	42.88888889
0.067628789	23.16298277	130.7594027
0.07047581	21.15604429	71.62483311
0.068629384	21.82365597	80.75396086
0.062491105	24.143876	90.22014925
0.081915267	17.99862945	55.0794702
0.135699469	6.192833116	20.84075574
0.193799622	3.54026465	12.02608696
0.128647644	12.73311295	31.80491551
0.06462271	21.55720913	104.9887218
0.101615129	13.79018658	39.3033033
0.11589956	12.00613979	33.91056911
0.068955494	20.03338331	113.1462844
0.063635672	20.8933273	81.99914894
0.057011533	29.31633244	103.333518
0.169421488	4.568052686	15.05681818
0.082908918	17.28358811	53.56551724
0.071082912	21.18477149	134.0688637
0.11095627	14.60115093	38.3906383
0.09209375	21.633275	56.8575
0.208097038	2.468126444	9.695652174
0.057804294	24.55941317	99.43636364
0.07285379	22.77078354	71.62361192
0.066000885	23.98894792	105.4566684
0.084465753	15.12920689	48.79390421
0.116883617	14.92291077	37.63006757
0.078924158	19.65208494	59.14880546
0.258818866	1.527810807	6.655172414
0.137472724	6.323745289	22.43661972
0.066633762	24.82916431	97.3059142

log.sigma.1.0.mm.3D_gldm_LargeDependenceLowGrayLevelEmphasis	log.sigma.1.0.mm.3D_gldm_SmallDependenceHighGrayLevelEmphasis	log.sigma.1.0.mm.3D_gldm_LargeDependenceHighGrayLevelEmphasis
0.382378281	19.9909062	4293.145646
0.809607192	10.41742481	666.6725664
0.700873014	14.45793861	4431.906832
0.158550201	23.10647203	1459.402174
0.806256434	11.64258083	5335.510724
0.093389704	54.3643096	595.5533981
1.336637141	5.824558681	15875.97222
0.918985898	9.007620673	10320.87475
1.038780618	7.082135516	12123.12864
0.225483158	42.68072811	1694.302198
0.168195683	57.06225011	60469.04484
0.766925744	11.21283982	17942.47966
0.236534052	44.55112107	2741.344411
0.150447458	19.69322562	1431.636842
0.744868615	10.32199448	8347.084783
1.218190504	6.483306051	14100.93667
1.424846235	6.190966219	3824.158257
0.330368664	12.65621273	1367.072072
1.566999114	7.284962577	2077.21164
0.706692536	17.19007724	16676.57131
1.900169029	5.240496265	4750.287879
0.824355845	5.663382732	1412.848101
0.741141895	11.50385147	4737.725979
1.901742853	3.682717183	9220.618027
1.082648252	8.68308697	7827.710744
1.208907212	8.935209817	5812.45284
0.425091184	16.62420854	6772.042599
0.954861301	8.95671143	10614.09551
0.181497871	21.86904762	150.6785714
1.552381657	6.074219605	4212.009557
1.628933166	4.501398976	8059.142694
0.490670901	9.584993968	1199.509804
0.817366152	14.02100441	5874.148554
1.236698754	7.590584175	9862.451889
1.029813245	10.17174263	7807.878919
0.484059052	22.07482022	7252.378546
0.126627688	56.29240123	687.0948718
0.760366768	9.907016579	25283.79758
0.269052557	20.18505807	3363.634518
0.195478101	48.85227818	81318.38567
0.725799618	11.56794014	4781.706815
1.534993241	8.577771254	7441.457095
0.576396959	11.45875225	5527.489194
0.706611365	10.07110354	5094.336634
2.733026261	3.126214248	4107.204044
0.822937177	9.599080004	9147.790793
1.896107189	4.392266145	9421.007892
1.980422499	3.556367704	1234.013699
0.457357073	14.66096164	1176.754386
0.132167209	45.90366922	766.4863636
1.363480891	6.008801433	10089.62172
0.651586643	24.84982664	6917.579435
1.104143706	6.95681377	18530.04237
1.161123124	7.42923764	3473.689492
0.318433756	25.50386813	2750.379791
1.292931341	5.477953128	3125.479638
1.548302215	6.455899019	7681.248534
0.740383512	12.07045823	12247.99741
0.319526094	18.16967853	4490.459559
0.788531382	11.66969421	18985.57398
0.526316274	26.02431453	7644.611041
0.300604072	45.71563481	9265.966937
0.198925554	65.81188679	10269.00153
0.233703998	58.32085148	49129.76714
1.331753473	5.604966713	3192.623053
0.177547289	61.96347082	4575.017391
0.179337396	29.55854809	641.1008403
0.803833162	20.44367612	8996.356831
1.924956278	5.572970251	9616.493665
0.728505981	18.92709727	4976.556647
0.739123064	10.40980998	2754.348485
1.001356669	8.255208381	17294.81462
0.871099586	8.506933411	6116.739653
0.763083364	11.57228776	8737.931034
0.862422723	16.043896	9606.049129
0.696576498	18.43734164	4532.711921
0.195383345	42.49065629	2467.053981
0.47137126	16.21384428	463.3217391
0.346339821	33.73850071	3118.156682
1.535117032	4.765611721	7327.956767
0.382378281	19.9909062	4293.145646
0.821258501	11.87190188	1622.439024
0.874395666	7.687477697	14825.70948
0.979632537	7.627074772	6999.857872
1.237229292	9.674138126	8791.843767
0.409186544	22.12494076	705.9034091
0.647831172	13.86691356	4641.02069
1.959891818	3.470476664	9347.266109
0.230366637	47.15952123	6713.837447
0.474253914	30.01299258	7026.75375
0.231306026	33.26138165	668.4094203
1.191384047	6.087392074	8434.332867
0.687807661	20.88424327	7628.495909
0.994899654	13.0465197	11362.45407
0.771351611	15.01770529	3240.01016
0.230244323	44.78085025	6453.114865
0.600368121	18.90963233	6103.210239
0.16597948	47.12066411	431.6896552
1.031287826	6.327486542	555.2676056
0.764231892	17.00436889	12611.74781

log.sigma.1.0.mm.3D_gldm_SmallDependenceLowGrayLevelEmphasis	log.sigma.1.0.mm.3D_gldm_LowGrayLevelEmphasis	log.sigma.1.0.mm.3D_gldzm_DistanceZoneVariabilityNormalized
0.008339537	0.017722482	0.974526535
0.029860103	0.078260737	1
0.009605564	0.024425061	1
0.017507868	0.026317925	0.941230487
0.006895481	0.020991559	1
0.021848555	0.028833052	1
0.000842606	0.010006155	0.952346854
0.0031589	0.012948691	0.873159522
0.001802128	0.011136329	0.797279384
0.011236277	0.020816417	0.919497211
0.000344982	0.001862531	0.816154514
0.001302643	0.00768965	0.939086914
0.009475437	0.017983103	0.98374093
0.012637928	0.022495632	1
0.004616561	0.014491171	0.985816327
0.001332795	0.010779848	0.867834889
0.008685422	0.031254179	1
0.018721417	0.034320375	1
0.014709737	0.049914908	0.938836565
0.000987921	0.007154257	0.73529627
0.005163307	0.027297592	0.974030471
0.022311722	0.054163586	1
0.009126867	0.02338678	1
0.002083542	0.016537385	0.92174925
0.00632991	0.021487123	1
0.004971577	0.021278193	0.597750089
0.00373945	0.011480179	0.974687705
0.002182247	0.011671223	0.943280151
0.066492967	0.085684424	1
0.006757277	0.028872374	0.89986634
0.004178331	0.019957069	0.993464123
0.023943416	0.046922224	1
0.005534651	0.017806061	0.963482964
0.002937293	0.01456981	0.800333272
0.003523428	0.015306253	0.953394397
0.004871797	0.013281226	1
0.016855672	0.02626086	1
0.0005543	0.005987647	0.853762763
0.008026306	0.016002262	0.953514739
0.000204528	0.001655183	0.878213218
0.005920145	0.019027212	0.981310075
0.002509647	0.016343008	0.693471947
0.003689387	0.015297032	0.940941534
0.011413928	0.023776495	0.98425296
0.009073278	0.037477109	0.879224377
0.002952731	0.01291085	0.921637854
0.003237323	0.018058303	0.974479176
0.015042827	0.070821149	1
0.02559539	0.04415034	1
0.009264243	0.01862743	0.752839506
0.002976063	0.014245128	0.945987654
0.004447993	0.013747719	0.875002469
0.00067637	0.008183074	0.940002687
0.007549555	0.025686394	0.98333451
0.012699513	0.025891554	1
0.015738055	0.038765158	0.922456892
0.003322759	0.017488011	0.811503519
0.002704854	0.010711284	1
0.00734627	0.01593466	1
0.000692028	0.006833353	0.460695377
0.004351009	0.012284642	0.964508637
0.003592274	0.008716941	0.909934723
0.002367421	0.00608105	0.887748974
0.00046074	0.002441004	0.938826277
0.013070881	0.036227085	1
0.004229036	0.009611524	0.734298192
0.018668374	0.030565267	0.958352196
0.004404542	0.013796842	0.943748293
0.001872861	0.016070752	0.842210716
0.005027749	0.017415253	0.9345645
0.018118317	0.038066323	1
0.001537621	0.009329067	0.813713308
0.006909982	0.020061465	0.966676242
0.003684792	0.01300696	0.987421888
0.00263699	0.01158661	0.911768131
0.004861178	0.017115603	0.963648834
0.004544888	0.011841218	0.592317026
0.046154044	0.069162177	1
0.00697245	0.01607984	0.935257892
0.003909326	0.018440444	0.885794483
0.008339537	0.017722482	0.974526535
0.015638803	0.042106417	0.953514739
0.001870494	0.009535053	0.924173834
0.003026401	0.014711434	0.966396377
0.003725579	0.015406405	0.990740941
0.020940992	0.041545639	0.911952663
0.005665559	0.017980698	0.969238281
0.002324215	0.017312724	0.991379472
0.00301038	0.008108947	0.573991517
0.004198019	0.011863758	0.959201389
0.022594062	0.041857539	1
0.002547503	0.014465765	1
0.003360574	0.012185563	0.883995288
0.0020781	0.011299502	0.86236655
0.004688128	0.019653041	0.932930625
0.004272878	0.009753699	0.977211006
0.005610344	0.016229175	0.98566383
0.026925218	0.042366107	1
0.026609446	0.069565748	1
0.001799875	0.009419992	0.777252276

log.sigma.1.0.mm.3D_gldzm_LowIntensityEmphasis	log.sigma.1.0.mm.3D_gldzm_LargeDistanceEmphasis	log.sigma.1.0.mm.3D_gldzm_HighIntensitySmallDistanceEmphasis
0.03710075	1.038709677	83.0516129
0.146386034	1	43.27586207
0.047647901	1	65.77142857
0.051736413	1.090909091	68.15909091
0.043347619	1	65.21538462
0.039378451	1	102.4166667
0.017948346	1.101626016	116.6961518
0.029969449	1.351493849	68.2197935
0.022374256	1.823033708	67.15200843
0.036132268	1.12605042	126.4180672
0.003849443	1.439583333	587.9114005
0.016091263	1.1328125	123.3084852
0.027159894	1.024590164	127.0348361
0.03942179	1	69.67164179
0.032311045	1.021428571	66.4
0.020730463	1.39011925	83.6971891
0.064775512	1	43.46153846
0.077242551	1	58.32142857
0.101438017	1.094736842	42.43421053
0.009876639	2.263422819	145.7804766
0.05015138	1.039473684	47.99671053
0.105380375	1	25.81081081
0.054434683	1	61.71698113
0.041704306	1.226720648	61.68981556
0.054594844	1	61.25490196
0.042709586	2.630188679	40.13139413
0.022947728	1.038461538	92.15064103
0.023560487	1.11627907	83.44186047
0.106949182	1	43.42105263
0.060371055	1.231343284	47.49067164
0.050154826	1.009836066	48.03360656
0.09109775	1	36
0.035195315	1.055813953	82.24418605
0.033378114	1.712837838	69.01918262
0.027926987	1.140086207	72.7236141
0.023277436	1	99.7734375
0.03480101	1	119.5147059
0.010040416	1.48733234	148.8885681
0.038328918	1.071428571	95.05357143
0.002806348	1.336384439	618.2264572
0.03706859	1.028301887	66.41745283
0.026739378	2.241531664	74.85404885
0.031753676	1.091370558	81.35532995
0.066001016	1.023809524	53.62698413
0.107463832	1.421052632	30.90299708
0.027095963	1.22972973	75.75957207
0.050916771	1.100858369	62.11428529
0.12200561	1	27.09090909
0.073554435	1	44.34090909
0.024415701	1.433333333	109.4833333
0.033947668	1.083333333	60.2
0.022365288	1.222222222	113.7914198
0.013443121	1.180327869	122.0118966
0.057836994	1.025210084	51.26260504
0.045987152	1	92.15116279
0.109161411	1.121212121	33.37121212
0.037958883	1.648305085	61.87398835
0.022453222	1	92.98453608
0.032752473	1	81.25757576
0.009376566	5.81875	104.082467
0.021741717	1.054216867	129.1129518
0.014916622	1.201970443	182.254584
0.008770179	1.186401833	235.0421229
0.003612011	1.118705036	440.0359275
0.101295059	1	41.09090909
0.013290063	1.666666667	160.184144
0.049438802	1.063829787	83.2393617
0.025452198	1.112712975	109.1957187
0.029813792	2.191522763	79.27927917
0.027697102	1.134680135	97.38935653
0.075252441	1	45.17647059
0.021491744	2.206378987	92.58364161
0.046891386	1.050847458	51.41949153
0.026825665	1.018987342	79.21518987
0.019678885	1.13876652	108.2296256
0.028238601	1.055555556	104.0763889
0.016914438	2.062200957	119.421252
0.108778977	1	42.62745098
0.02298683	1.100478469	108.138756
0.043042386	1.390728477	41.50666851
0.03710075	1.038709677	83.0516129
0.07336565	1.071428571	56.9672619
0.023790338	1.230496454	81.7857565
0.029574305	1.051282051	66.42094017
0.031714564	1.013953488	75.09418605
0.06113912	1.138461538	65.68846154
0.033396387	1.046875	76.32421875
0.04237146	1.012987013	52.66341991
0.012268834	2.148026316	138.6826572
0.018789602	1.0625	129.5299479
0.060832326	1	88.93162393
0.029692972	1	62.20769231
0.018959606	1.237795276	108.2124016
0.01988087	1.518159806	112.359891
0.02699256	1.130319149	80.81213061
0.015464753	1.034582133	157.9329971
0.032258478	1.02166065	99.17870036
0.057908689	1	101.8888889
0.121484395	1	27.27777778
0.015296981	1.91838488	124.4214261

log.sigma.1.0.mm.3D_gldzm_LowIntensityLargeDistanceEmphasis	log.sigma.1.0.mm.3D_gldzm_HighIntensityEmphasis	log.sigma.1.0.mm.3D_gldzm_DistanceZoneVariability	log.sigma.1.0.mm.3D_gldzm_ZonePercentage
0.037369567	84.44516129	151.0516129	0.232732733
0.146386034	43.27586207	29	0.256637168
0.047647901	65.77142857	105	0.217391304
0.052274337	72	31.06060606	0.358695652
0.043347619	65.21538462	130	0.174262735
0.039378451	102.4166667	60	0.582524272
0.018711436	119.4525745	702.8319783	0.045259414
0.032732629	76.88400703	496.827768	0.112762584
0.030144504	78.07022472	283.8314607	0.084180657
0.036896645	135.1344538	109.4201681	0.326923077
0.00493342	631.33125	391.7541667	0.087847731
0.016978883	127.1953125	240.40625	0.083306215
0.027305398	128.0737705	120.0163934	0.36858006
0.03942179	69.67164179	67	0.352631579
0.032459855	67.17142857	138.0142857	0.152173913
0.02431376	90.17717206	509.4190801	0.068840155
0.064775512	43.46153846	65	0.149082569
0.077242551	58.32142857	28	0.252252252
0.102482419	45.12631579	89.18947368	0.167548501
0.018485485	168.3708054	876.4731544	0.09994131
0.052588027	48.44078947	148.0526316	0.104683196
0.105380375	25.81081081	37	0.234177215
0.054434683	61.71698113	53	0.1886121
0.04651371	64.24696356	227.6720648	0.051415487
0.054594844	61.25490196	102	0.140495868
0.076835013	68.43018868	158.4037736	0.130864198
0.023175312	93.77564103	152.0512821	0.166134185
0.0246192	86.37209302	162.244186	0.097782831
0.106949182	43.42105263	19	0.678571429
0.064839454	50.02985075	120.5820896	0.116420504
0.050355562	48.15409836	303.0065574	0.086869838
0.09109775	36	72	0.282352941
0.035978142	83.64651163	207.1488372	0.168100078
0.041668025	77.83445946	236.8986486	0.093998095
0.029354399	74.68318966	442.375	0.133448375
0.023277436	99.7734375	256	0.226950355
0.03480101	119.5147059	204	0.523076923
0.01301803	164.2041729	572.8748137	0.057164764
0.038751572	98.07142857	40.04761905	0.21319797
0.00331704	655.3832952	767.5583524	0.072147928
0.03730249	67.27358491	104.0188679	0.167987322
0.045498278	88.64064801	470.8674521	0.093823407
0.032557157	84.17258883	185.3654822	0.129011133
0.06637304	54.00793651	124.015873	0.178217822
0.21201427	33.11578947	83.52631579	0.087316176
0.029684489	79.15315315	204.6036036	0.113554987
0.052687522	62.72317597	454.1072961	0.066867556
0.12200561	27.09090909	33	0.150684932
0.073554435	44.34090909	44	0.385964912
0.027564725	136.7333333	67.75555556	0.409090909
0.034759049	62.48333333	170.2777778	0.094438615
0.024620414	122.6	393.7511111	0.208429829
0.014872577	125.7889344	458.7213115	0.048307266
0.05814823	51.77310924	117.0168067	0.140495868
0.045987152	92.15116279	86	0.299651568
0.111125496	35.48484848	91.32323232	0.149321267
0.047005848	68.62076271	383.029661	0.089275582
0.022453222	92.98453608	194	0.125566343
0.032752473	81.25757576	66	0.242647059
0.041745483	152.0198864	810.8238636	0.074852209
0.02279466	130.3554217	160.1084337	0.208281054
0.016401325	188.5418719	369.4334975	0.253275109
0.010167753	247.7754011	1162.063407	0.286307962
0.003889237	452.0341727	1043.97482	0.130516432
0.101295059	41.09090909	44	0.137071651
0.018015726	193.4149933	548.5207497	0.34187643
0.049816493	85.93617021	45.04255319	0.394957983
0.026599942	113.2581913	720.0799476	0.184835271
0.047760268	84.76609105	536.4882261	0.063553826
0.029221442	100.3232323	277.5656566	0.194499018
0.075252441	45.17647059	51	0.257575758
0.031520898	104.1444653	433.7091932	0.075080997
0.047311613	52.95762712	114.0677966	0.157543391
0.026957522	79.89873418	156.0126582	0.147250699
0.020900649	114.7334802	413.9427313	0.141169154
0.028537614	107.6944444	104.0740741	0.178807947
0.03755023	158.4736842	247.5885167	0.282051282
0.108778977	42.62745098	51	0.443478261
0.023982126	111.6124402	195.4688995	0.321044547
0.047983595	46.15231788	133.7549669	0.094611529
0.037369567	84.44516129	151.0516129	0.232732733
0.110071999	57.29761905	80.0952381	0.227642276
0.026064232	85.57446809	260.6170213	0.082504389
0.030186856	67.60683761	113.0683761	0.099574468
0.031829882	75.51627907	213.0093023	0.119113573
0.064124875	67.84615385	59.27692308	0.369318182
0.033783784	77.7421875	124.0625	0.176551724
0.042448306	53.21212121	229.008658	0.056812592
0.034591554	196.6315789	174.4934211	0.258723404
0.019200208	132.1197917	184.1666667	0.24
0.060832326	88.93162393	117	0.423913043
0.029692972	62.20769231	130	0.090909091
0.021359215	114.3070866	561.3370079	0.185563998
0.024854483	120.4140436	356.157385	0.107161391
0.028431248	83.44148936	350.7819149	0.181906144
0.015617984	160.3602305	339.092219	0.293074324
0.032755776	99.70397112	273.0288809	0.189078498
0.057908689	101.8888889	45	0.517241379
0.121484395	27.27777778	18	0.253521127
0.022510309	141.0721649	904.7216495	0.1211995

log.sigma.1.0.mm.3D_gldzm_IntensityVariabilityNormalized	log.sigma.1.0.mm.3D_gldzm_LowIntensitySmallDistanceEmphasis	log.sigma.1.0.mm.3D_gldzm_IntensityVariability
0.096524454	0.037033546	14.96129032
0.117717004	0.146386034	3.413793103
0.090068027	0.047647901	9.457142857
0.112947658	0.051601932	3.727272727
0.10591716	0.043347619	13.76923077
0.08	0.039378451	4.8
0.094171606	0.017804999	69.49864499
0.113278004	0.029530398	64.45518453
0.141412069	0.021550421	50.34269663
0.067721206	0.035941174	8.058823529
0.140347222	0.003644922	67.36666667
0.107055664	0.015927373	27.40625
0.069067455	0.027123518	8.426229508
0.10670528	0.03942179	7.149253731
0.128061224	0.032273843	17.92857143
0.123084201	0.020212865	72.25042589
0.135621302	0.064775512	8.815384615
0.109693878	0.077242551	3.071428571
0.119113573	0.101176916	11.31578947
0.088083026	0.009086246	104.9949664
0.137378809	0.049542218	20.88157895
0.161431702	0.105380375	5.972972973
0.102171591	0.054434683	5.41509434
0.129013752	0.041160562	31.86639676
0.091887735	0.054594844	9.37254902
0.109633321	0.038177495	29.05283019
0.112754767	0.022890833	17.58974359
0.119321255	0.023375561	20.52325581
0.119113573	0.106949182	2.263157895
0.117509468	0.059586654	15.74626866
0.128750336	0.050104642	39.26885246
0.122299383	0.09109775	8.805555556
0.092309356	0.034999608	19.84651163
0.132121987	0.032371141	39.10810811
0.107043326	0.027717016	49.66810345
0.082550049	0.023277436	21.1328125
0.063244906	0.03480101	12.90196078
0.106749496	0.009656425	71.62891207
0.120181406	0.038223254	5.047619048
0.099419277	0.002731208	86.89244851
0.106087576	0.037010115	11.24528302
0.108946927	0.02448131	73.97496318
0.10386766	0.031552806	20.46192893
0.108465608	0.06590801	13.66666667
0.143711911	0.096494351	13.65263158
0.114722831	0.026761645	25.46846847
0.107903995	0.050706269	50.2832618
0.144168962	0.12200561	4.757575758
0.115702479	0.073554435	5.090909091
0.074814815	0.023628445	6.733333333
0.152037037	0.033744823	27.36666667
0.076444444	0.021854697	34.4
0.115174012	0.013273614	56.20491803
0.112774522	0.057759185	13.42016807
0.072471606	0.045987152	6.23255814
0.121518212	0.108670389	12.03030303
0.133411735	0.036786785	62.97033898
0.10277394	0.022453222	19.93814433
0.112947658	0.032752473	7.454545455
0.197824122	0.007422762	348.1704545
0.077151981	0.021478482	12.80722892
0.064864471	0.014657386	26.33497537
0.059810993	0.008428053	78.29258976
0.072539271	0.003555566	80.66366906
0.135330579	0.101295059	5.954545455
0.054330665	0.012429279	40.58500669
0.07831598	0.049344379	3.680851064
0.074790997	0.025236464	57.0655308
0.114752482	0.028741316	73.09733124
0.08222517	0.027377854	24.42087542
0.109573241	0.075252441	5.588235294
0.102372848	0.020801868	54.56472795
0.124102269	0.046786329	14.6440678
0.132350585	0.026792701	20.91139241
0.079004056	0.019373444	35.86784141
0.102880658	0.028163848	11.11111111
0.063803484	0.012989165	26.66985646
0.109573241	0.108778977	5.588235294
0.077631007	0.022738005	16.22488038
0.163194597	0.042481537	24.64238411
0.096524454	0.037033546	14.96129032
0.092403628	0.064189062	7.761904762
0.119963785	0.023488445	33.82978723
0.147344583	0.029421168	17.23931624
0.101871282	0.031685734	21.90232558
0.098698225	0.060392681	6.415384615
0.103149414	0.033299538	13.203125
0.125016398	0.042352249	28.87878788
0.0611366	0.007839937	18.58552632
0.078179253	0.018686951	15.01041667
0.072101687	0.060832326	8.435897436
0.167928994	0.029692972	21.83076923
0.092913386	0.018502819	59
0.086516307	0.019310063	35.73123487
0.097088615	0.026682961	36.50531915
0.068408508	0.015426445	23.73775216
0.086368909	0.032134153	23.92418773
0.066666667	0.057908689	3
0.216049383	0.121484395	3.888888889
0.076460481	0.014524297	89

log.sigma.1.0.mm.3D_gldzm_HighIntensityLargeDistanceEmphasis	log.sigma.1.0.mm.3D_gldzm_SmallDistanceEmphasis	log.sigma.1.0.mm.3D_glcm_SumVariance	log.sigma.1.0.mm.3D_glcm_Homogeneity1
90.01935484	0.990322581	261.902078	0.502467319
43.27586207	1	70.32315269	0.462719232
65.77142857	1	177.304543	0.515403633
87.36363636	0.977272727	246.236453	0.413577567
65.21538462	1	194.1428294	0.548734892
102.4166667	1	235.8821128	0.340097863
134.4119241	0.981422764	344.552005	0.693516768
134.0474517	0.946482621	298.2914657	0.618448307
172.7303371	0.908888889	320.7223867	0.65662559
170	0.968487395	262.1319324	0.402548559
870.0708333	0.921426505	2165.948695	0.65377734
148.2460938	0.975477431	481.7461574	0.665588536
132.2295082	0.993852459	271.6759638	0.400032776
69.67164179	1	244.0821428	0.408651655
70.25714286	0.994642857	285.8433653	0.596936875
136.1584327	0.944367783	329.5385234	0.677110125
43.46153846	1	119.5133789	0.588238885
58.32142857	1	157.5640939	0.458848446
55.89473684	0.976315789	82.28698748	0.550977422
379.8355705	0.878790876	481.0011558	0.619946398
50.21710526	0.990131579	120.613602	0.617381602
25.81081081	1	82.82495518	0.512855686
61.71698113	1	197.2803502	0.561438399
78.29554656	0.968223122	209.0199529	0.691824827
61.25490196	1	221.4951067	0.610362568
337.7018868	0.799213836	177.505958	0.583978442
100.275641	0.990384615	335.9379968	0.540469619
100.1860465	0.977390181	308.1969858	0.635640093
43.42105263	1	93.09870911	0.319828075
64.06716418	0.958747927	124.4386645	0.599797343
48.63606557	0.997540984	184.8631635	0.647522582
36	1	103.8900478	0.455644435
89.25581395	0.986046512	220.3601321	0.577992538
145.6418919	0.910373498	261.1096863	0.652597771
89.54525862	0.980618415	233.5081262	0.615045908
99.7734375	1	314.408315	0.535502885
119.5147059	1	249.9405068	0.334620003
272.4321908	0.937218082	596.2455409	0.687855323
110.1428571	0.982142857	310.8433069	0.503532595
891.4313501	0.949036995	2339.336984	0.67007055
70.69811321	0.992924528	202.8380393	0.551520799
199.2164948	0.857252688	203.5068377	0.644167769
95.44162437	0.97715736	258.5842763	0.556307102
55.53174603	0.994047619	204.3432891	0.549709391
46.07368421	0.947733918	96.80816899	0.654081906
100.9369369	0.967905405	290.0160811	0.605243782
69.22317597	0.989240582	204.141241	0.682227093
27.09090909	1	48.21139673	0.55868165
44.34090909	1	121.1966936	0.476448321
245.7333333	0.891666667	287.7820463	0.343644756
71.61666667	0.979166667	261.4849626	0.683700832
160.1355556	0.949382716	281.238135	0.534922841
150.4118852	0.975562955	424.0845464	0.69995275
53.81512605	0.993697479	139.8923786	0.58616335
92.15116279	1	217.7612347	0.434073343
43.93939394	0.96969697	107.7113183	0.564766473
121.5720339	0.917321681	200.4164124	0.654492941
92.98453608	1	356.3631047	0.606465021
81.25757576	1	302.0245661	0.514956593
915.7193182	0.704367296	507.0770834	0.664179832
135.3253012	0.986445783	328.9762149	0.531474526
221.5837438	0.963191024	490.9982478	0.487054214
300.355233	0.95509719	677.8325955	0.459325692
509.8714029	0.97585057	1550.315542	0.614501932
41.09090909	1	116.4163932	0.577290941
379.9825971	0.881024096	468.5774704	0.382853153
96.72340426	0.984042553	193.0276787	0.366340705
131.9895151	0.977646716	290.6177808	0.580522017
174.4740973	0.928089011	207.6375179	0.677916534
115.6801347	0.973812196	210.6548933	0.521564477
45.17647059	1	137.5061851	0.527104651
260.945591	0.91533364	400.2234656	0.669992101
59.11016949	0.987288136	204.9985788	0.577066204
82.63291139	0.995253165	294.695134	0.607344486
140.7488987	0.96530837	309.972586	0.615506115
122.1666667	0.986111111	218.9741473	0.539558714
362	0.796999601	343.8364896	0.391138417
42.62745098	1	85.19606877	0.411947933
125.507177	0.974880383	247.2022372	0.448260495
80.1589404	0.950377116	192.9071507	0.656470881
90.01935484	0.990322581	261.902078	0.502467319
58.61904762	0.982142857	95.15108002	0.477053065
110.7234043	0.968109732	399.3093468	0.668275699
72.35042735	0.987179487	237.9212615	0.626715724
77.20465116	0.996511628	232.5448528	0.628854572
76.47692308	0.965384615	111.662864	0.41582433
83.4140625	0.98828125	209.1515051	0.554156864
55.40692641	0.996753247	200.6426821	0.687141142
481.8355263	0.784379569	513.3567669	0.432995485
142.4791667	0.984375	324.8577943	0.512790313
88.93162393	1	170.2095393	0.345207564
62.20769231	1	242.7963212	0.654229078
143.1307087	0.952909011	296.3302905	0.564635859
184.6924939	0.940951708	313.5309788	0.62706546
96.84308511	0.973330378	169.486453	0.54178714
170.0691643	0.991354467	444.8563422	0.45603239
101.8050542	0.994584838	261.6584394	0.533687634
101.8888889	1	194.7750531	0.324955268
27.27777778	1	50.32650794	0.511429891
281.3582474	0.900469406	379.6995395	0.596695078

log.sigma.1.0.mm.3D_glcm_Homogeneity2	log.sigma.1.0.mm.3D_glcm_ClusterShade	log.sigma.1.0.mm.3D_glcm_MaximumProbability	log.sigma.1.0.mm.3D_glcm_Idmn	log.sigma.1.0.mm.3D_glcm_SumVariance2
0.442817425	-25.41750175	0.106385388	0.978466745	8.751195839
0.393032739	13.88978216	0.075449744	0.971054057	8.726143625
0.457381146	-19.35702382	0.125200964	0.97942408	8.676619665
0.334588387	-12.98496656	0.057565413	0.959872808	10.10058895
0.498802933	-13.54003036	0.153441515	0.984172298	6.768746489
0.247171479	-20.81487404	0.04232818	0.94625504	20.04999523
0.680976442	-3.379921754	0.224545025	0.997004541	2.270582694
0.5870006	-14.06488058	0.192529799	0.993252983	5.269647555
0.634741712	-10.3231525	0.204971589	0.992770237	3.096119599
0.323753717	48.40899602	0.058636586	0.968258487	18.31101414
0.632320746	-2.652391948	0.192134085	0.998103461	3.330703639
0.645688214	-11.23253018	0.213316559	0.99635829	3.327624256
0.320365593	5.131353134	0.076101712	0.964743879	17.13800482
0.32479825	-21.91226503	0.04622291	0.957979865	12.66280679
0.560722673	-21.3279136	0.173618033	0.988411239	5.90368432
0.660120213	-13.49925493	0.229292239	0.994090197	3.297008738
0.546120071	-7.7476384	0.162244397	0.982144168	4.463614796
0.387538299	-2.603436516	0.083171097	0.970674591	9.121412234
0.503211948	1.240848433	0.152197663	0.984530782	5.593177321
0.589506066	-4.161993374	0.178598225	0.99578593	4.491566064
0.583728759	-5.925664495	0.197218643	0.986485296	3.660691882
0.453807615	-7.506410433	0.088491733	0.967049393	6.493149629
0.515998187	-22.09906528	0.179881876	0.981939944	7.209197242
0.678451546	-3.246273542	0.246034199	0.994773138	1.917804983
0.57687878	-33.83314143	0.209485054	0.981842669	7.823306394
0.54511468	21.55673145	0.15516111	0.989359458	7.422086022
0.488918295	-14.9648765	0.112346474	0.987780071	6.617920586
0.607539092	-9.222328534	0.212002565	0.990958846	3.484358776
0.221655055	18.04800472	0.086548348	0.893512408	13.43451657
0.563244999	-7.025224098	0.159587394	0.984358118	4.121951203
0.622347726	-12.8296519	0.184429848	0.99158396	4.002697848
0.381539476	-9.115339358	0.078321539	0.963816766	7.936566834
0.536763753	-12.64307126	0.161452725	0.984428256	6.235200424
0.630077864	-13.39610745	0.19412788	0.992971295	3.496415459
0.582515217	-11.59887173	0.170662386	0.991692273	4.614204496
0.484924258	-35.01194656	0.138969594	0.98336472	10.3997858
0.243058851	15.51829078	0.027948616	0.956250144	23.01928644
0.673089866	-5.834907287	0.223613027	0.997479471	3.440394428
0.441104027	-9.224488449	0.095300837	0.97892619	6.284420577
0.652620164	1.383941588	0.222568128	0.998711245	2.956242326
0.502653685	-9.268515472	0.155890029	0.985314051	6.106769954
0.619969226	4.672327945	0.209313758	0.994395085	3.291037512
0.509210393	-12.21420452	0.112056089	0.988865579	6.025113578
0.50036071	-17.57765007	0.125266974	0.982661732	6.653130797
0.630044865	-6.308544053	0.238133508	0.982462745	2.75528784
0.571107984	-13.96691049	0.173797318	0.990753599	4.782818784
0.666527113	-6.336108865	0.222776231	0.995612334	2.769589804
0.512183319	-0.250438732	0.14343154	0.971757039	4.513048963
0.410723795	-5.559036551	0.104793813	0.962285654	8.545304809
0.256676049	242.1748319	0.035119342	0.955426912	33.85412417
0.666998725	-8.358575324	0.270696173	0.993654043	2.494356763
0.48525304	6.534575879	0.145148238	0.984464919	8.985314535
0.688766709	-2.784488264	0.246359885	0.997607036	1.7359945
0.54453594	-8.078231125	0.150297534	0.982290136	5.263883473
0.360481859	-6.339796029	0.073487088	0.96992711	14.85537779
0.518985532	-8.149297127	0.142076135	0.978842566	4.774367926
0.633095528	-4.79030596	0.197310295	0.993429577	2.734424964
0.571509079	-19.65066439	0.169052651	0.990425405	5.88111014
0.456352483	-24.7820653	0.106928389	0.978369666	8.512471196
0.64526906	-2.830521673	0.201121055	0.997809285	2.158468227
0.480335589	3.462071321	0.159080268	0.987102015	9.568482554
0.428171704	1.621279914	0.102600387	0.982340251	14.03819267
0.397760138	3.654430866	0.103213499	0.984706495	18.86860668
0.583613677	-15.63808204	0.17645296	0.995994465	6.78482326
0.53323145	-10.23270883	0.193802647	0.974859386	4.98784951
0.30663695	73.06260814	0.063759884	0.966180534	28.26564159
0.275986294	15.03190578	0.039725552	0.952541883	15.5063744
0.541878397	2.338849931	0.166837741	0.991223565	8.527644539
0.66141105	-0.645411688	0.218459552	0.996301084	2.262614786
0.467489547	-0.594319731	0.125793686	0.983194062	8.172808644
0.472554646	-18.85224346	0.15236701	0.966630939	7.387530075
0.650837929	-15.99602857	0.203634627	0.99547903	3.981200499
0.535798225	-16.7091943	0.161799655	0.986699002	6.343359305
0.572685998	-14.26959074	0.171850184	0.993898071	5.477094216
0.583526806	2.665087129	0.206278306	0.991332182	6.188585722
0.489532569	12.97470218	0.148077636	0.98475856	7.649510456
0.312256764	64.34852993	0.041090041	0.971174058	21.87482906
0.327725902	1.364572476	0.057304782	0.958391449	10.11038145
0.377873749	5.13965494	0.08117418	0.975545818	11.33295077
0.634026222	-7.140342697	0.218129967	0.990070287	2.642086723
0.442817425	-25.41750175	0.106385388	0.978466745	8.751195839
0.410211334	1.929328286	0.085840163	0.971610234	8.059562745
0.649266347	-16.37237466	0.214737138	0.992763294	3.644278083
0.598281467	-4.79828222	0.1960344	0.991450702	2.856993541
0.597482717	-9.47283262	0.232237252	0.989911681	4.216404346
0.338056339	5.647454037	0.05562688	0.949044316	10.06144364
0.506462749	-12.55354035	0.120353041	0.989405708	6.524085141
0.671871611	-6.759138446	0.251240261	0.995343206	2.507049722
0.365530216	75.62039094	0.086328783	0.975331672	24.09110622
0.455991915	-3.951819365	0.137395649	0.981647334	8.959015857
0.256289528	33.1723437	0.030947235	0.963829176	21.10182371
0.630036849	-5.997098428	0.242177959	0.99131106	2.632058584
0.521336839	-4.774669965	0.148314228	0.989475413	6.123368352
0.59887976	-1.717066916	0.192549359	0.993613164	4.731299969
0.491881886	3.819513267	0.128968106	0.986330243	6.042874134
0.38987917	16.54265461	0.091404288	0.985376415	16.08774491
0.481657643	-5.438037555	0.131301915	0.98625879	7.888884989
0.236419385	52.45081753	0.05242877	0.939466019	23.80317506
0.449709705	-2.205033934	0.124237478	0.949568067	3.743184724
0.561088384	-10.40820218	0.171495571	0.989433632	6.481401134

log.sigma.1.0.mm.3D_glcm_Contrast	log.sigma.1.0.mm.3D_glcm_DifferenceEntropy	log.sigma.1.0.mm.3D_glcm_InverseVariance	log.sigma.1.0.mm.3D_glcm_Entropy	log.sigma.1.0.mm.3D_glcm_Dissimilarity
6.921575773	2.559956468	0.397599319	5.468076868	1.850492622
8.724395119	2.563676845	0.378074386	5.235077572	2.09806015
6.528300959	2.530667154	0.385712219	5.326640727	1.799324756
10.30009972	2.726440659	0.344651314	5.536785222	2.475531611
4.984884522	2.357105249	0.417145346	4.997023371	1.534924677
18.40176755	3.044436625	0.267821293	5.930593163	3.368705073
1.815810057	1.562882113	0.48947807	3.165304768	0.784141109
3.460377669	2.052514642	0.456241066	4.201671387	1.169903152
1.962419831	1.751006581	0.485180465	3.621627355	0.906709512
18.19059813	3.115438831	0.311121641	6.349023275	2.990971651
3.095289323	1.793862477	0.487656199	3.755282981	0.972634553
2.233323025	1.739133277	0.480932412	3.585237462	0.899508652
16.1469485	3.109559889	0.298654837	6.43358285	2.964915644
9.478574556	2.70734289	0.334820064	5.907503581	2.407791592
4.063465423	2.158800289	0.447593948	4.313981488	1.297517255
2.038076122	1.683926595	0.480148063	3.369881563	0.862338559
3.28035908	2.121830489	0.432817842	4.512222807	1.256065773
7.437794877	2.532552714	0.380194394	5.229164806	2.036427129
4.973636663	2.300630484	0.437723692	4.910929845	1.49765967
3.696371267	2.058914148	0.458701594	4.322376435	1.18125849
2.443229496	1.950717894	0.460400822	4.29881884	1.075698564
4.431956588	2.28819222	0.422635841	4.965560586	1.59262001
5.161153288	2.307681995	0.425512937	4.733348274	1.507063422
1.416404403	1.534380274	0.49354872	3.168394243	0.75063806
5.268746094	2.216747914	0.418156681	4.191540843	1.389820596
5.837249557	2.279868614	0.4397342	4.667799662	1.443143844
4.729803315	2.320023213	0.427622905	5.049196691	1.523612338
2.469726703	1.899623258	0.467273523	4.013573002	1.023784611
16.88444997	2.37705344	0.259444798	4.187937546	3.387287808
2.868065075	2.029258242	0.463784873	4.380229482	1.165347371
2.282546671	1.845281802	0.468972555	3.835161504	0.975702681
6.910912097	2.548043639	0.367890741	5.442872203	2.018295752
5.005206593	2.292947006	0.430841996	4.77449922	1.438880426
2.482112282	1.811143624	0.480637312	3.683972822	0.962089311
3.189131982	2.038477948	0.459029931	4.329621072	1.154695852
6.606235304	2.498550017	0.410643598	5.285316004	1.719212695
22.11068717	3.295565098	0.245112295	6.963433465	3.669196629
2.41849902	1.654689571	0.47561887	3.273803284	0.850011904
5.230107723	2.342453329	0.407327	5.121848615	1.680273126
2.806951892	1.745660506	0.48343684	3.506064144	0.931302492
4.567611045	2.308462152	0.426886782	4.946079494	1.486351237
3.225542118	1.901952861	0.47419821	3.915896404	1.046617351
3.821625452	2.18634999	0.449052407	4.950745945	1.383371703
4.823440323	2.346336787	0.420138018	4.911512872	1.522851757
1.952907556	1.779891637	0.472781169	3.733988408	0.920297128
3.184286004	2.045210587	0.465734421	4.333051898	1.179647953
1.854642921	1.637511523	0.485009046	3.343590305	0.82486799
3.156739977	2.081502846	0.462846678	4.584809323	1.305316969
7.441662494	2.541268994	0.387893122	5.16417565	1.987119048
25.83954082	3.315825874	0.275698042	6.57177353	3.732956539
1.719081252	1.644545446	0.475475589	3.270421324	0.815653518
8.398002206	2.585701795	0.402976303	5.39944952	1.841603301
1.432641304	1.487364611	0.494670305	3.035201137	0.727746351
3.270982266	2.103880795	0.44224146	4.631697566	1.2533975
12.39025872	2.913148604	0.33268985	5.931563449	2.547228076
3.334931225	2.137147485	0.451458548	4.754474547	1.313999627
2.016256525	1.745385446	0.493523151	3.678992063	0.910903618
3.687985782	2.116715477	0.445373544	4.295733585	1.237415168
5.350845602	2.399064464	0.407185358	5.203629613	1.673938241
1.926364434	1.66801379	0.499799173	3.463140498	0.859985678
8.870193522	2.616511493	0.390558069	5.390620708	1.895429024
13.48165473	2.884253699	0.367194575	5.96965095	2.354651605
19.40405846	3.12476313	0.339747464	6.564328992	2.849007136
5.778082977	2.198664046	0.440141163	4.469714238	1.35944069
3.389765288	2.14793673	0.436735106	4.548095547	1.296377435
26.61060155	3.425486555	0.288058374	7.323431706	3.622284774
14.03551776	2.943494967	0.288205989	6.0725537	2.958269584
7.587449263	2.396035589	0.424362244	4.875507572	1.596238074
1.892001889	1.649603749	0.488577128	3.413421322	0.83328905
7.446984617	2.572659702	0.401527182	5.487782017	1.822666586
5.613217638	2.398834782	0.40956369	4.919591637	1.664249522
2.323553314	1.751206071	0.474235946	3.590162956	0.905069261
4.175850441	2.212413194	0.445678153	4.610782518	1.363343793
3.761414462	2.091883751	0.45055003	4.237961394	1.22130437
5.557622795	2.194333137	0.434047334	4.474081472	1.338539397
6.987576376	2.4419907	0.41909037	5.18646556	1.683204011
18.86274439	3.212305829	0.310106989	7.048245648	3.125339621
9.258788295	2.693662332	0.325023148	5.574156267	2.394279881
10.89123951	2.854485546	0.354196299	6.110952336	2.371109212
1.783546073	1.733408074	0.48460635	3.623778301	0.890187775
6.921575773	2.559956468	0.397599319	5.468076868	1.850492622
7.190389189	2.597009322	0.381953655	5.578891121	1.965681716
2.249625439	1.743607539	0.477555632	3.433342644	0.908834684
2.030347676	1.812366787	0.494767724	3.98962565	0.985160247
3.146357735	2.023815448	0.441072262	4.183211537	1.121012428
10.27358762	2.760088271	0.358574108	5.849839634	2.440458974
4.578142707	2.26880262	0.437664478	4.986823678	1.455284475
1.58819268	1.594760508	0.483792985	3.261527719	0.786423953
20.54472369	3.200408389	0.324542008	6.77462756	3.036001791
8.155951816	2.633852149	0.384329043	5.514020299	1.920948811
21.91553198	3.281141611	0.261264466	6.783441292	3.566406874
1.797933275	1.750495505	0.476094785	3.732054472	0.898817055
5.512620164	2.345088681	0.435694241	4.97996695	1.509877177
4.032687541	2.027381115	0.464162608	4.203144965	1.166418748
5.36297225	2.381227475	0.428451896	5.19708632	1.573020167
14.60640905	2.963444726	0.352475631	6.172064629	2.538504075
6.674295425	2.493254493	0.408411747	5.28896596	1.711136677
26.55678695	3.268195332	0.259467036	5.92242173	3.952627143
3.751059124	2.09328427	0.425435121	4.432513874	1.510043097
5.098243144	2.240003373	0.440009805	4.72941923	1.380217084

log.sigma.1.0.mm.3D_glcm_DifferenceVariance	log.sigma.1.0.mm.3D_glcm_Idn	log.sigma.1.0.mm.3D_glcm_Idm	log.sigma.1.0.mm.3D_glcm_Correlation	log.sigma.1.0.mm.3D_glcm_Autocorrelation
3.489651393	0.909662441	0.442817425	0.114116568	93.99330524
4.290876497	0.893903404	0.393032739	0.001972197	31.28830248
3.265286531	0.911848543	0.457381146	0.138939846	67.71843477
4.145208476	0.869116827	0.334588387	-0.006501655	88.69722777
2.62515236	0.923416346	0.498802933	0.151592981	71.96850651
6.84103666	0.847534572	0.247171479	0.046522249	86.74775455
1.200350656	0.97001092	0.680976442	0.109796469	107.4314238
2.089059939	0.95277098	0.5870006	0.206352593	100.1116162
1.138269965	0.949723961	0.634741712	0.220927766	103.3718173
9.198861326	0.891329952	0.323753717	0.002513431	95.93801933
2.147227441	0.976771401	0.632320746	0.037859307	600.635016
1.422795991	0.965790576	0.645688214	0.195198025	148.5977907
7.307967955	0.881786077	0.320365593	0.028431783	99.35258794
3.6413655	0.863879488	0.32479825	0.144977044	90.24670132
2.370113626	0.937980039	0.560722673	0.182493648	97.06120265
1.290411388	0.957305831	0.660120213	0.227340557	104.5240855
1.700288882	0.91878498	0.546120071	0.152440338	47.04766729
3.255591924	0.889384015	0.387538299	0.104316	61.38797699
2.724522034	0.925327182	0.503211948	0.058184209	35.37591413
2.298934569	0.963025829	0.589506066	0.096589628	151.8765529
1.285146853	0.929015525	0.583728759	0.198699798	46.88208817
1.887023976	0.883260662	0.453807615	0.185411236	36.26039461
2.883848539	0.921373712	0.515998187	0.166605658	72.03564577
0.852586346	0.957750141	0.678451546	0.149408716	69.09058511
3.309659781	0.928668962	0.57687878	0.185171928	77.40063387
3.749643982	0.943681718	0.54511468	0.119590812	65.54888221
2.394169876	0.930501307	0.488918295	0.16390921	114.7963247
1.417885086	0.943949263	0.607539092	0.170720763	101.6848034
4.820612423	0.78355362	0.221655055	-0.102553565	33.38724672
1.508477914	0.923908418	0.563244999	0.177054161	48.27550736
1.325576928	0.946408935	0.622347726	0.266854133	65.19252555
2.776285251	0.875605177	0.381539476	0.07022933	43.86803369
2.932042147	0.928793992	0.536763753	0.107593399	79.11272569
1.553663724	0.952751644	0.630077864	0.167638117	86.16649382
1.854654161	0.946522952	0.582515217	0.18178517	81.35709692
3.6468492	0.923852123	0.484924258	0.218663513	109.7691215
8.409196524	0.862412653	0.243058851	0.024866459	92.91393798
1.694730086	0.973922509	0.673089866	0.171918616	178.0688884
2.386152149	0.906206679	0.441104027	0.093208861	107.3118536
1.93749848	0.980930044	0.652620164	0.028873726	643.3977535
2.35549093	0.925305329	0.502653685	0.142865956	74.44643088
2.127344467	0.959437037	0.619969226	0.011398984	69.88845348
1.865658585	0.932912437	0.509210393	0.207381566	91.57448831
2.499575327	0.919773959	0.50036071	0.158275659	74.71969519
1.1039277	0.923135375	0.630044865	0.170140254	37.56432768
1.788602018	0.942568686	0.571107984	0.197892892	98.02173627
1.17325809	0.962668287	0.666527113	0.197725221	68.72415308
1.447633169	0.893672063	0.512183319	0.176490653	23.51831568
3.423326777	0.879334702	0.410723795	0.074294087	49.04196789
11.79064437	0.868332812	0.256676049	0.138273389	103.5140913
1.052605795	0.954630253	0.666998725	0.183409826	84.75182989
5.000587207	0.929672831	0.48525304	0.034691391	99.22577789
0.901991072	0.971847723	0.688766709	0.096198921	128.6785228
1.665819881	0.918889948	0.54453594	0.23313807	54.46689444
5.894569331	0.891686645	0.360481859	0.090995851	81.93566041
1.6062761	0.908720819	0.518985532	0.176810619	43.87749735
1.185451301	0.952233614	0.633095528	0.1486789	68.5754355
2.152728233	0.943234881	0.571509079	0.227651215	117.6460527
2.543687619	0.907032514	0.456352483	0.226959216	105.7075074
1.185631705	0.972340593	0.64526906	0.057254888	153.4759793
5.271185537	0.935353625	0.480335589	0.037483007	113.5100252
7.928301279	0.924620578	0.428171704	0.018916002	163.4184117
11.25696179	0.929364936	0.397760138	-0.013733267	219.531821
3.92747651	0.96680622	0.583613677	0.081306144	446.0811071
1.701796992	0.903665247	0.53323145	0.188829969	46.30046225
13.46391389	0.889492238	0.30663695	0.030844924	160.8402497
5.199283682	0.855414282	0.275986294	0.050367245	73.54378839
5.027660307	0.95061823	0.541878397	0.060488097	100.1666379
1.196914859	0.96543793	0.66141105	0.089130742	69.55924055
4.121568246	0.9233605	0.467489547	0.04578756	77.94420039
2.832931218	0.889876845	0.472554646	0.135241946	53.78769578
1.503570227	0.962875962	0.650837929	0.261665396	125.9479362
2.30997726	0.931351994	0.535798225	0.204603175	74.40666301
2.267868758	0.954530422	0.572685998	0.186440882	99.15287713
3.758707788	0.951766001	0.583526806	0.054591325	104.0300133
4.14907188	0.929088424	0.489532569	0.045113996	79.66355638
9.069671489	0.894406689	0.312256764	0.074068657	123.1747996
3.506952613	0.864368467	0.327725902	0.042523995	37.20415127
5.257751028	0.902392863	0.377873749	0.020812303	91.02265898
0.990590328	0.94023677	0.634026222	0.192993005	66.30134827
3.489651393	0.909662441	0.442817425	0.114116568	93.99330524
3.314846188	0.893273977	0.410211334	0.055077068	41.0712631
1.421154153	0.952890466	0.649266347	0.234213379	124.8242252
1.058436966	0.941894796	0.598281467	0.168787129	81.10429313
1.887016716	0.942853931	0.597482717	0.142258869	80.39752555
4.205025259	0.855654791	0.338056339	-0.019694354	46.33573015
2.454722956	0.936540483	0.506462749	0.172176125	76.56674296
0.96852651	0.960474761	0.671871611	0.223053217	67.3642485
11.28794201	0.908638414	0.365530216	0.08053462	173.5271148
4.457430324	0.919748931	0.455991915	0.047039642	112.9773143
9.152909826	0.875860316	0.256289528	-0.019114672	66.32662827
0.987312837	0.943466488	0.630036849	0.181951466	81.41873068
3.229851004	0.940531643	0.521336839	0.053685282	101.8609854
2.670597876	0.95701231	0.59887976	0.080005681	103.9620013
2.884419171	0.929078356	0.491881886	0.058346729	64.33771437
8.153884806	0.928065166	0.38987917	0.046656665	151.0118985
3.737144552	0.930452658	0.481657643	0.082090624	93.27131831
10.79058518	0.843392288	0.236419385	-0.055180645	71.29425885
1.43163331	0.854071817	0.449709705	0.001378701	23.15377147
3.191156348	0.943440095	0.561088384	0.118798315	125.407322

log.sigma.1.0.mm.3D_glcm_SumEntropy	log.sigma.1.0.mm.3D_glcm_AverageIntensity	log.sigma.1.0.mm.3D_glcm_Energy	log.sigma.1.0.mm.3D_glcm_SumSquares	log.sigma.1.0.mm.3D_glcm_ClusterProminence
3.432532735	9.671254749	0.041186486	3.918192903	340.7420634
3.341393483	5.59303734	0.033969525	4.362634686	385.3286024
3.408226308	8.196070663	0.044689376	3.801230156	262.5944316
3.475837271	9.420122667	0.025972507	5.100172168	284.7843589
3.226035556	8.45699701	0.059919085	2.938407753	178.2995836
3.89411293	9.290833682	0.018790858	9.612940696	1094.484435
2.218075134	10.35941886	0.182930394	1.021598188	75.08044848
2.848048378	9.98292343	0.112104885	2.182506306	219.3442407
2.484517517	10.1532145	0.141452821	1.264634857	83.13871726
3.971966667	9.79297211	0.019783353	9.125403065	1723.08487
2.509366814	24.51415916	0.135445682	1.59717868	332.2227022
2.484945295	12.17882237	0.148716462	1.39023682	140.1214449
3.956359668	9.9549096	0.019253601	8.32123833	1098.542255
3.703751957	9.457692218	0.020497039	5.535345336	478.5223615
2.925952103	9.828535969	0.100257337	2.491787436	209.5505788
2.354545244	10.20825427	0.168773879	1.333771215	117.8099677
2.949300546	6.837485432	0.076994156	1.935993469	79.33011365
3.433756038	7.807850341	0.034651425	4.139801778	304.3773959
3.112186306	5.934602923	0.061892342	2.641703496	194.334923
2.802451189	12.31637554	0.11237804	2.055947628	188.7439396
2.835315294	6.824741123	0.089191716	1.525980344	55.90795942
3.223181451	5.978372233	0.039966753	2.731276554	111.600317
3.128028564	8.456982438	0.072058073	3.092587632	240.8627958
2.218095642	8.304517884	0.175982354	0.833552346	46.05401047
2.905660494	8.761190006	0.119340808	3.273013122	357.7646458
3.101882289	8.071695197	0.086074791	3.314833895	671.2919211
3.237949701	10.69211397	0.053361213	2.836930975	194.929661
2.686685711	10.07128463	0.116897634	1.48852137	85.48550029
2.832339385	5.82577397	0.060980369	7.579741634	503.65551
2.882343404	6.92539544	0.085951791	1.747504069	81.36029751
2.646934947	8.047434533	0.128137577	1.57131113	105.919875
3.414533352	6.603424883	0.031263358	3.711869733	186.6332848
3.121713432	8.877135145	0.079320134	2.810101754	205.3832659
2.487596569	9.2688904	0.141236319	1.494631935	148.4727092
2.870980702	9.000012062	0.10091634	1.95083412	126.6278828
3.428583931	10.43124472	0.059045638	4.251505276	490.0684916
4.197740269	9.620725194	0.010120099	11.2824934	1680.154305
2.321774789	13.33812696	0.177228678	1.504933174	231.5997542
3.241626604	10.34625628	0.039140142	2.878632075	177.0525704
2.393001763	25.38540273	0.162476768	1.516479027	150.5311378
3.18606619	8.605832136	0.060439717	2.66859525	161.5203573
2.568195002	8.35894482	0.131681752	1.629144907	158.9202454
3.19200814	9.540129529	0.052625789	2.461684758	170.0490531
3.175133866	8.617484115	0.061530223	2.86914278	199.7846433
2.527170031	6.112539885	0.134349576	1.177048849	46.19229382
2.87235794	9.880337179	0.096645705	1.991776197	143.4993748
2.361906177	8.276184694	0.16797865	1.156058181	85.89012857
3.019332076	4.814272449	0.062575045	1.917447235	73.1194326
3.355323029	6.982788087	0.040421804	3.996741826	293.2343401
4.215686573	10.07444273	0.013700934	14.92341625	5689.497132
2.297940964	9.195535867	0.172952038	1.053359504	72.5016735
3.407677763	9.953797718	0.057672523	4.345829185	555.4831396
2.129531917	11.34030871	0.187667436	0.792158951	41.40777395
3.090521489	7.346182285	0.076413064	2.133716435	131.6076864
3.791332629	9.017472457	0.026490558	6.811409128	855.23306
3.04823145	6.596622073	0.059886082	2.027324788	84.33667845
2.480597404	8.270156122	0.138884918	1.187670372	72.07909594
2.921350701	10.82111381	0.102433602	2.39227398	189.7595412
3.354327531	10.24267309	0.043467575	3.4658292	292.1112114
2.302039242	12.38724776	0.15012258	1.032526814	84.00625633
3.420008625	10.64573701	0.055634002	4.609669019	619.3865082
3.716862364	12.78401906	0.041116235	6.948950641	1190.507377
3.972343987	14.92522981	0.032040618	10.01310176	1931.65122
2.941620605	21.15529725	0.112888113	2.982023507	489.4440272
2.994517559	6.774924543	0.080344473	2.0944037	114.5730119
4.348447441	12.68740985	0.014105522	13.96118315	3447.681283
3.785462055	8.553665204	0.017635769	7.385473041	755.1587449
3.197660341	9.999636462	0.086406257	4.076848843	656.4342143
2.338438037	8.334655589	0.15998526	1.038654169	73.38092557
3.407275819	8.818249788	0.048360806	3.904948315	353.0616433
3.20191549	7.303408118	0.056506307	3.250186928	207.3275903
2.502552985	11.20416104	0.153790164	1.576188453	181.2339062
3.094941934	8.594295676	0.077174925	2.629802437	174.7969342
2.865711814	9.935947618	0.104374801	2.309627169	211.6835437
2.954280934	10.19174511	0.109772523	2.936552129	431.2370004
3.295448584	8.916117648	0.054567141	3.659271708	455.3024089
4.185663558	11.0643521	0.013810122	10.18439336	1997.991809
3.500664302	6.081215871	0.025579003	4.842292435	301.3654717
3.711666818	9.534643162	0.025209327	5.556047569	514.0275472
2.465204019	8.129353436	0.135612212	1.106408199	51.03712556
3.432532735	9.671254749	0.041186486	3.918192903	340.7420634
3.45151543	6.39153085	0.031501239	3.812487984	230.7508041
2.422397379	11.15683989	0.161382711	1.473475881	150.5185245
2.657013331	8.994264898	0.101590091	1.221835304	49.75573114
2.792777173	8.951482711	0.113764073	1.84069052	110.0811303
3.545103371	6.810171527	0.023143647	5.083757813	371.6284201
3.211179642	8.722158206	0.057134512	2.775556962	199.6269353
2.311096994	8.193554291	0.171506138	1.0238106	60.60695116
4.159272741	13.16299107	0.02389845	11.42207204	2717.131948
3.465911943	10.61951272	0.047336993	4.278741918	408.9852861
4.102760025	8.155997508	0.011633824	10.75433892	1569.178436
2.526772938	9.011595268	0.126706247	1.107497965	45.50544418
3.134762388	10.08503023	0.071900113	2.908997129	260.2017181
2.802556257	10.18758354	0.116723001	2.190996878	251.9864434
3.236850117	8.010401623	0.052225048	2.851461596	199.4896347
3.840905793	12.30067746	0.029973949	7.986200722	1639.380417
3.354177751	9.641821383	0.052158544	3.640795103	404.4585204
3.895278475	8.483505706	0.019894466	12.5899905	1769.05557
2.801615316	4.811893624	0.058766092	1.873560962	42.50764476
3.047416418	11.1830686	0.095619724	2.894911069	319.3334561

log.sigma.1.0.mm.3D_glcm_SumAverage	log.sigma.1.0.mm.3D_glcm_Imc2	log.sigma.1.0.mm.3D_glcm_Imc1	log.sigma.1.0.mm.3D_glcm_DifferenceAverage	log.sigma.1.0.mm.3D_glcm_Id
19.3425095	0.559449509	-0.066853824	1.850492622	0.502467319
11.18607468	0.790971597	-0.174951091	2.09806015	0.462719232
16.39214133	0.60981796	-0.086368741	1.799324756	0.515403633
18.84024533	0.847057076	-0.210154692	2.475531611	0.413577567
16.91399402	0.576760989	-0.078952819	1.534924677	0.548734892
18.58166736	0.95494033	-0.34573207	3.368705073	0.340097863
20.71883772	0.453536073	-0.071856328	0.784141109	0.693516768
19.96584686	0.403651872	-0.043762066	1.169903152	0.618448307
20.30642899	0.38206551	-0.04653059	0.906709512	0.65662559
19.58594422	0.779038787	-0.137708463	2.990971651	0.402548559
49.01330904	0.393473137	-0.044872948	0.972634553	0.65377734
24.35764475	0.453597634	-0.064495925	0.899508652	0.665588536
19.9098192	0.780932161	-0.137427499	2.964915644	0.400032776
18.91538444	0.767008342	-0.140807773	2.407791592	0.408651655
19.65707194	0.482360461	-0.062761342	1.297517255	0.596936875
20.41650854	0.42952583	-0.06598977	0.862338559	0.677110125
13.67497086	0.523181204	-0.069442856	1.256065773	0.588238885
15.61570068	0.785660277	-0.173082775	2.036427129	0.458848446
11.86920585	0.569243451	-0.077788421	1.49765967	0.550977422
24.63146738	0.473838459	-0.058054199	1.18125849	0.619946398
13.64948225	0.444953192	-0.051605453	1.075698564	0.617381602
11.95674447	0.605462663	-0.091036636	1.59262001	0.512855686
16.91396488	0.664656747	-0.11870727	1.507063422	0.561438399
16.60903577	0.377957214	-0.048545851	0.75063806	0.691824827
17.52238001	0.599414859	-0.108567489	1.389820596	0.610362568
16.14339039	0.517348914	-0.065962848	1.443143844	0.583978442
21.38422795	0.507321388	-0.059053838	1.523612338	0.540469619
20.14256925	0.418372986	-0.048046236	1.023784611	0.635640093
11.65154794	0.989359797	-0.653270196	3.387287808	0.319828075
13.85079088	0.452568934	-0.053353885	1.165347371	0.599797343
16.09486907	0.448523291	-0.062595016	0.975702681	0.647522582
13.20684977	0.659430116	-0.101254062	2.018295752	0.455644435
17.75427029	0.541278158	-0.071226235	1.438880426	0.577992538
18.5377808	0.421388497	-0.054520438	0.962089311	0.652597771
18.00002412	0.465494301	-0.055851966	1.154695852	0.615045908
20.86248944	0.597115833	-0.081308654	1.719212695	0.535502885
19.24145039	0.809550937	-0.146453231	3.669196629	0.334620003
26.66931747	0.41713814	-0.0593839	0.850011904	0.687855323
20.69251257	0.643606396	-0.099887233	1.680273126	0.503532595
50.72909827	0.438148005	-0.059672433	0.931302492	0.67007055
17.21166427	0.521001395	-0.063707748	1.486351237	0.551520799
16.71788964	0.44419608	-0.05523105	1.046617351	0.644167769
19.08025906	0.510112897	-0.06733379	1.383371703	0.556307102
17.23496823	0.525878902	-0.064973727	1.522851757	0.549709391
12.22507977	0.424377975	-0.054142113	0.920297128	0.654081906
19.76067436	0.497841911	-0.065761859	1.179647953	0.605243782
16.55236939	0.41774788	-0.057064294	0.82486799	0.682227093
9.628544897	0.572534479	-0.084368058	1.305316969	0.55868165
13.96557617	0.801824703	-0.184457928	1.987119048	0.476448321
20.14888547	0.859718877	-0.188881589	3.732956539	0.343644756
18.39107173	0.363070509	-0.043888934	0.815653518	0.683700832
19.90759544	0.549783703	-0.065120442	1.841603301	0.534922841
22.68061743	0.382766236	-0.051681242	0.727746351	0.69995275
14.69236457	0.52004766	-0.070392396	1.2533975	0.58616335
18.03494491	0.769062613	-0.141411052	2.547228076	0.434073343
13.19324415	0.474473468	-0.052861718	1.313999627	0.564766473
16.54031224	0.374475516	-0.042767949	0.910903618	0.654492941
21.64222762	0.502256082	-0.068029329	1.237415168	0.606465021
20.48534617	0.662243504	-0.106934004	1.673938241	0.514956593
24.77239641	0.303886253	-0.028583515	0.859985678	0.664179832
21.29147401	0.690971098	-0.114472703	1.895429024	0.531474526
25.55604728	0.618569567	-0.078564108	2.354651605	0.487054214
29.64197005	0.628137861	-0.073817694	2.849007136	0.459325692
42.2292986	0.571221886	-0.084940098	1.35944069	0.614501932
13.54984909	0.554430749	-0.080084764	1.296377435	0.577290941
25.33168707	0.587682019	-0.056321778	3.622284774	0.382853153
17.10733041	0.883203255	-0.225819194	2.958269584	0.366340705
19.9930742	0.548841192	-0.07250308	1.596238074	0.580522017
16.66931118	0.385008408	-0.047011989	0.83328905	0.677916534
17.63649958	0.528101149	-0.058494494	1.822666586	0.521564477
14.60681624	0.721952346	-0.140824823	1.664249522	0.527104651
22.40832208	0.471017453	-0.069057671	0.905069261	0.669992101
17.18859135	0.535599376	-0.072988889	1.363343793	0.577066204
19.87189524	0.484089668	-0.061779798	1.22130437	0.607344486
20.38349022	0.555497742	-0.080443022	1.338539397	0.615506115
17.8322353	0.652588897	-0.102497506	1.683204011	0.539558714
22.1287042	0.544372215	-0.048850693	3.125339621	0.391138417
12.16243174	0.830206153	-0.19395609	2.394279881	0.411947933
19.06928632	0.621107789	-0.077124325	2.371109212	0.448260495
16.25870687	0.361780688	-0.038846275	0.890187775	0.656470881
19.3425095	0.559449509	-0.066853824	1.850492622	0.502467319
12.7830617	0.620285733	-0.084389092	1.965681716	0.477053065
22.31367977	0.425741027	-0.061724498	0.908834684	0.668275699
17.9885298	0.388276955	-0.040847474	0.985160247	0.626715724
17.90296542	0.494032957	-0.067085809	1.121012428	0.628854572
13.62034305	0.790852067	-0.157281474	2.440458974	0.41582433
17.44431641	0.562092968	-0.074692186	1.455284475	0.554156864
16.38710858	0.437544603	-0.065258189	0.786423953	0.687141142
26.27843128	0.697466412	-0.094333097	3.036001791	0.432995485
21.23902543	0.592890571	-0.075958638	1.920948811	0.512790313
16.31199502	0.8050757	-0.143401264	3.566406874	0.345207564
18.02319054	0.39234264	-0.046904392	0.898817055	0.654229078
20.17006045	0.469233098	-0.048937242	1.509877177	0.564635859
20.37516707	0.500940473	-0.067183655	1.166418748	0.62706546
16.02080325	0.428332875	-0.039173319	1.573020167	0.54178714
24.54651566	0.638491137	-0.081807302	2.538504075	0.45603239
19.28364277	0.534692558	-0.063713589	1.711136677	0.533687634
16.96701141	0.963315812	-0.370551346	3.952627143	0.324955268
9.623787249	0.743262738	-0.178250438	1.510043097	0.511429891
22.36613721	0.528044559	-0.067644058	1.380217084	0.596695078

log.sigma.1.0.mm.3D_glcm_ClusterTendency	log.sigma.1.0.mm.3D_firstorder_InterquartileRange	log.sigma.1.0.mm.3D_firstorder_Skewness	log.sigma.1.0.mm.3D_firstorder_Uniformity
8.751195839	60.17130637	-0.860680123	0.161291923
8.726143625	59.72813225	1.005694646	0.152008771
8.676619665	73.37323962	-0.423805909	0.158777311
10.10058895	79.06711674	-0.323958719	0.120274102
6.768746489	59.56332517	-0.393522645	0.178629186
20.04999523	107.1444288	0.072106531	0.087755679
2.270582694	10.63818479	-0.335058435	0.383161661
5.269647555	29.58566964	-0.987715411	0.275281834
3.096119599	22.77637339	-1.770069127	0.322837991
18.31101414	74.81707239	0.68284627	0.116637483
3.330703639	21.29277658	-0.932389539	0.330049047
3.327624256	20.63514566	-1.348031905	0.324392913
17.13800482	96.80207717	0.235775424	0.098985953
12.66280679	83.64982128	-0.392902876	0.115290859
5.90368432	42.58058488	-1.242856831	0.246013705
3.297008738	16.9811883	-1.896717712	0.36156387
4.463614796	44.91492742	-0.588914517	0.216427489
9.121412234	76.94778347	0.005082933	0.156074994
5.593177321	41.16684687	0.587257309	0.207394343
4.491566064	23.48632431	-0.061075627	0.277460171
3.660691882	38.66504475	-0.662214625	0.243357694
6.493149629	65.6717124	-0.183065012	0.161432463
7.209197242	55.85057414	-0.734267601	0.203885462
1.917804983	15.22525275	-1.210252857	0.379043605
7.823306394	25.79476917	-1.213691635	0.278419052
7.422086022	35.81508255	1.136665117	0.242379393
6.617920586	53.19992459	-0.740405197	0.185719746
3.484358776	27.08108783	-1.364525835	0.288057423
13.43451657	104.5800194	0.193677461	0.114795918
4.121951203	38.12148964	-0.393147019	0.234947739
4.002697848	25.357674	-1.377731403	0.289878959
7.936566834	74.76023388	-0.173658218	0.145251826
6.235200424	43.12219143	-0.341248983	0.225764607
3.496415459	21.68743896	-1.31262183	0.324637328
4.614204496	34.08258343	-0.731546367	0.251608396
10.3997858	68.94419643	-0.514687742	0.175542918
23.01928644	120.3681455	0.236321029	0.077935569
3.440394428	12.59461486	-0.670319534	0.378761562
6.284420577	55.59001255	-0.776149918	0.171274704
2.956242326	16.70381463	0.421666122	0.356886939
6.106769954	53.58470923	-0.423711469	0.189860383
3.291037512	22.57700586	1.347846487	0.319178391
6.025113578	53.31906301	-0.732035898	0.186573568
6.653130797	55.98330438	-0.976158971	0.187402596
2.75528784	22.4487204	-1.248843979	0.312136746
4.782818784	37.54732692	-1.069906589	0.245690439
2.769589804	14.72428274	-0.909629519	0.353171923
4.513048963	47.1972802	0.480560789	0.208148287
8.545304809	60.08861279	-0.07802861	0.149122807
33.85412417	96.52460808	1.15419046	0.099297521
2.494356763	16.19789237	-2.268488998	0.366132685
8.985314535	46.02798748	0.56695749	0.184282175
1.7359945	13.73830044	-0.721040278	0.394589344
5.263883473	33.57882214	-0.577243937	0.220854492
14.85537779	93.90098763	0.253048908	0.120810013
4.774367926	47.90784007	-0.719760225	0.201881843
2.734424964	21.8140707	-0.683380372	0.32234538
5.88111014	41.7353394	-1.026453658	0.243731423
8.512471196	70.57603288	-0.735218227	0.15643923
2.158468227	21.72837543	-0.271356073	0.358919997
9.568482554	56.32603693	0.356391398	0.169095526
14.03819267	62.76082945	0.38296278	0.147783962
18.86860668	67.32117581	0.21536218	0.130779885
6.78482326	23.15170074	-0.414045109	0.26415267
4.98784951	41.78905725	-0.950447621	0.225783911
28.26564159	93.28812408	0.581856219	0.092824071
15.5063744	92.24897861	0.220232958	0.104300544
8.527644539	39.50264609	0.62484412	0.217608594
2.262614786	18.02984571	0.693059971	0.352025471
8.172808644	52.41769433	0.411285761	0.170612022
7.387530075	66.00165915	-0.597802271	0.169829609
3.981200499	19.4368372	-1.709372176	0.334097299
6.343359305	55.12753993	-0.720369527	0.208675207
5.477094216	36.5626111	-0.077121199	0.254195803
6.188585722	21.40882409	0.478532385	0.272583367
7.649510456	43.48489422	0.761245448	0.182990878
21.87482906	80.86411667	0.686885745	0.105892391
10.11038145	76.70635509	0.348902497	0.125141777
11.33295077	66.1961602	0.300007036	0.126807157
2.642086723	24.99483454	-1.697441514	0.317016696
8.751195839	60.17130637	-0.860680123	0.161291923
8.059562745	66.28267288	0.432074273	0.141868817
3.644278083	13.53328884	-2.431506329	0.347282363
2.856993541	33.29351401	-0.962386131	0.269807515
4.216404346	29.6426971	-0.803472151	0.274260326
10.06144364	82.30675602	0.07507088	0.114927686
6.524085141	54.4247241	-0.01715868	0.184201665
2.507049722	13.31452739	-1.5591891	0.363118602
24.09110622	69.06323195	0.638687664	0.124539973
8.959015857	59.07631826	0.27330529	0.159565625
21.10182371	92.91795516	0.406311725	0.092470069
2.632058584	29.45493945	-1.438071896	0.298197467
6.123368352	37.79580164	0.068507376	0.211786184
4.731299969	24.97096825	0.360308762	0.280057867
6.042874134	45.35013747	0.736717292	0.188812891
16.08774491	82.3869825	0.389784464	0.127846227
7.888884989	53.58893728	0.09927504	0.178224091
23.80317506	105.150679	0.524418538	0.090764962
3.743184724	43.02563953	-0.426788207	0.204919659
6.481401134	27.97787142	-0.222447979	0.242678282

log.sigma.1.0.mm.3D_firstorder_MeanAbsoluteDeviation	log.sigma.1.0.mm.3D_firstorder_Energy	log.sigma.1.0.mm.3D_firstorder_RobustMeanAbsoluteDeviation	log.sigma.1.0.mm.3D_firstorder_Median
41.47880478	2481162.307	26.39653905	-15.45021963
41.15358621	646596.519	25.79237462	-54.52239609
42.87634975	2024607.683	29.97355074	-22.7676239
48.04908174	700320.161	33.26840364	-63.46938705
38.39411923	2554417.522	26.6264484	-17.81154346
65.85858633	983861.1673	46.32067938	-58.13329315
13.66366547	11878234.14	4.907887232	-0.891165912
28.59598133	10439550.81	15.19138521	-5.746036768
21.15069502	4859057.365	10.9212747	-4.603210449
55.36653082	2339693.148	33.15927821	-22.23768616
18.15930826	5848641.637	9.27753795	0.355971411
21.59173321	4073581.859	10.184116	-3.213861704
61.54117361	2486881.098	42.10537794	-32.49389648
49.54479784	1490960.694	35.58743563	-57.49826241
33.59510248	2405734.247	19.93466183	-6.981692314
18.82662818	9277866.18	7.626681023	-2.412990332
29.30345998	837673.191	18.94716519	-12.8176775
41.80335985	581093.5793	30.41193091	-54.72853851
30.07980244	1186542.991	18.29307326	-11.87301159
23.85785714	18567398.73	11.04538601	-2.246622086
25.79814983	2139099.138	16.8823218	-7.393392324
37.57929304	689867.047	28.24537688	-41.80814934
36.92616173	943169.8014	23.36144392	-16.73468208
14.45123301	3051863.833	6.866467466	-2.517841697
36.01313137	2179415.876	18.85295367	-4.72264576
29.76013986	4766715.335	16.77666937	-7.007400513
35.31464115	2637911.521	22.94789476	-13.13631725
23.56177756	2434677.911	12.69571099	-6.170268536
60.14756929	178287.1192	46.04607588	-42.006464
27.52760311	1941488.104	17.47147043	-9.430249214
24.68509912	5042314.376	13.27240285	-3.740053654
40.87444416	1415976.624	29.87503252	-54.17359543
32.35000353	3077855.491	19.1270323	-8.088446617
21.36563832	4161830.817	9.849602148	-1.649622202
27.45123795	6116659.794	15.85787488	-6.440989971
44.63385238	4885114.041	29.66789371	-13.77207661
72.88991325	5360426.1	50.89011272	-77.28874969
16.44196294	12292521.74	5.706349638	-0.470015034
34.76412527	657955.4182	23.6003957	-33.80688095
17.21873737	12973831.14	7.445946038	-1.964999974
35.29682447	1808671.079	23.41241656	-16.7048912
19.81753478	8266842.654	9.900735988	-3.056877375
33.617289	4156322.355	22.45062884	-19.27033615
36.40162204	2203988.589	24.01265967	-16.05436134
20.50304793	1102286.483	11.0515592	-4.880075693
28.88529268	3810183.52	17.29870168	-7.674808502
17.77151242	6995880.475	7.397342185	-2.204720497
28.63582042	396224.1044	19.69060886	-16.04476547
41.90286669	564786.0044	27.18702483	-39.26001167
68.17478486	2190135.738	43.63180924	-56.56941605
17.95421893	1832582.036	7.752316487	-3.51121521
38.16081192	7539109.26	20.86010155	-9.769227028
13.32490305	5969395.48	6.052156784	-1.759373486
28.47011231	1423102.465	16.06097664	-0.868063092
56.95264846	2053977.019	39.42755547	-39.50979996
30.63817793	1504758.501	20.77251749	-17.72919273
19.30090545	5181262.112	9.917565285	-3.150848866
32.48122496	3716404.685	19.69824514	-6.713664055
41.97311157	1215748.493	29.89468353	-28.18526363
16.64708951	17872098.38	9.152854023	-1.431347847
42.42852922	3249571.138	25.74081856	-12.89189529
49.76135942	9004929.855	27.96565789	-10.36227798
54.32980996	29448617.35	31.12690619	-11.25631094
29.06120701	21249960.98	12.83266751	-2.715770841
29.61094078	629514.0924	18.1208124	-10.33896923
68.24386416	20414545.12	42.2528771	-25.27824783
55.96857463	961689.3151	38.84264712	-60.23696518
36.05151092	14104478.14	19.03363067	-6.3829
16.63311886	8382771.874	8.091240953	-2.280618429
38.16644954	4914938.509	22.60439719	-11.66411304
40.48711017	780427.8171	27.02696848	-24.06370831
22.02552449	10008948.65	9.484851692	-2.237184763
35.51177054	2209993.239	24.10731798	-13.30410576
29.86961984	2346852.027	17.31017374	-6.325921059
26.66303943	6802723.346	11.81533916	-3.364722133
34.32757688	1640497.504	20.02041711	-9.937788963
58.62265707	9563825.501	36.17865588	-19.82893372
47.43709387	874267.7253	33.14578516	-63.97673798
47.40733962	2984391.024	30.43220273	-23.54990959
20.62355071	1682785.834	11.4444779	-6.097310066
41.47880478	2481162.307	26.39653905	-15.45021963
42.53642585	1387357.503	28.57733676	-26.0062294
21.54522242	4629628.032	8.175563745	-2.101521492
22.4451588	1420226.941	14.21212878	-9.417000771
25.88821579	2982968.105	14.2173694	-6.046909332
48.20121518	891960.5331	33.63956658	-38.45861816
34.58024665	1917369.087	22.42180105	-14.88866425
16.94160724	3416028.794	7.217520553	-2.672791362
55.07093516	7595537.358	31.7395046	-11.29482841
42.20677758	3137346.228	25.56271421	-13.44688177
62.05655079	2045941.417	41.81881624	-33.82642555
21.56690124	1616282.816	12.84949414	-7.900542498
31.87135883	8268388.724	17.31907796	-6.758286953
24.73526572	6685388.488	12.05373896	-4.064971447
32.54602692	4801959.575	19.42529687	-11.67903233
54.35303646	7938831.118	34.90976102	-23.77261543
37.76930189	4870735.321	23.25397309	-12.71801758
70.77255676	960552.8913	45.53389175	-64.90356445
27.41654193	201748.8767	18.7631713	-37.95711517
29.61497314	21865794.97	14.21594989	-3.44808495

log.sigma.1.0.mm.3D_firstorder_TotalEnergy	log.sigma.1.0.mm.3D_firstorder_Maximum	log.sigma.1.0.mm.3D_firstorder_RootMeanSquared	log.sigma.1.0.mm.3D_firstorder_90Percentile	log.sigma.1.0.mm.3D_firstorder_Minimum
66991382.3	165.8331146	61.03661958	26.52083778	-236.7216949
17458106.01	213.5525513	75.6445179	8.456394291	-169.118515
54664407.45	181.7204437	64.74360458	10.34264526	-215.6401367
18908644.35	77.51288605	87.24778314	10.32648106	-252.4059448
68969273.1	182.3033752	58.51625564	6.181394577	-202.7276154
26564251.52	145.6149445	97.73459044	62.62998657	-253.283371
320712321.9	329.481781	26.98995882	9.11330986	-245.4981079
281867872	277.9862366	45.48490408	11.73488998	-241.5673828
131194548.9	136.5443115	33.89668056	13.42726078	-227.1186981
63171715	288.6207275	80.17311537	68.80079117	-248.6339264
157913324.2	358.8805847	32.71690105	26.24942417	-594.8515015
109986710.2	296.2541809	36.40884851	13.12494946	-284.5336304
67145789.64	212.9916229	86.67892446	59.88735962	-255.3138275
40255938.74	60.94202042	88.58420597	10.70126057	-266.3151245
64954824.66	190.2404022	51.13637188	11.45965109	-239.0245819
250502386.9	193.5858154	32.98571639	12.68085384	-228.1895905
22617176.16	127.4564819	43.83228019	13.7004838	-150.040802
15689526.64	136.8273773	72.35383398	7.890477657	-200.9378662
32036660.75	250.917984	45.74569284	21.79625664	-149.3589478
501319765.6	404.5939636	39.4557154	24.50622253	-292.8419189
57755676.72	132.0731964	38.38240186	4.94985404	-153.629837
18626410.27	84.94498444	66.07758419	7.928041124	-158.0379486
25465584.64	151.7107849	57.93510302	11.07224464	-215.1373749
82400323.48	192.5963287	25.20467355	10.94309959	-195.6595154
58844228.64	166.1864014	54.79005605	10.15730143	-216.3421326
128701314	347.2878723	48.51735255	15.82068748	-188.7918701
71223611.06	181.8000031	53.00261734	17.66139221	-256.776062
65736303.59	143.1607819	37.203846	14.57292919	-237.6545105
4813752.218	90.03623962	79.79597179	62.75592613	-150.8139038
52420178.8	129.884491	41.07047305	14.50476265	-161.5246124
136142488.1	184.2967224	37.89653867	9.564950943	-180.5663757
38231368.86	108.4782333	74.51744432	1.066190577	-191.8942719
83102098.27	189.3829956	49.05562807	17.7378212	-214.8208618
112369432	210.9174194	36.35430785	18.25039177	-204.154953
165149814.4	230.3644257	41.94254871	14.69190655	-223.844162
131898079.1	191.848465	65.80862299	13.49231606	-252.0651703
144731504.7	218.0080566	117.2377171	50.78333321	-292.0558472
331898086.9	416.3758545	32.36111225	12.00663948	-307.6677551
17764796.29	84.4068985	57.79165354	15.13546581	-257.550354
350293440.7	519.5818481	32.72580481	13.89854584	-603.9848633
48834119.14	183.0210571	53.53836664	14.92860222	-223.6814117
223204751.7	368.4029541	33.79796481	19.13163757	-197.0248871
112220703.6	188.6570587	52.17171257	15.27828255	-246.5545197
59507691.89	158.2389069	55.83351425	12.49536476	-221.9902344
29761735.05	98.94779968	31.82971817	12.92769318	-149.1961212
102874955	185.5898285	44.14683428	11.71917439	-239.9321136
188888772.8	279.1418152	31.6837048	10.42314377	-194.6904297
10698050.82	123.5800171	42.53519115	14.87426682	-113.8053741
15249222.12	106.9583588	70.3865271	15.370226	-175.1415558
59133664.93	273.7329407	99.77556037	92.58086166	-271.7284851
49479714.96	150.0531464	31.00775075	9.920307636	-205.6700287
203555950	294.7459106	59.09268146	32.70734482	-235.4606628
161173678	309.8388977	24.30868658	10.7794013	-251.9780579
38423766.56	148.8845673	40.98985484	41.197155	-173.8148804
55457379.52	220.4954681	84.59736611	16.49733772	-238.4517975
40628479.53	104.4974899	47.64053595	11.65605507	-169.4637451
139894077	200.899765	31.30495807	16.04890137	-191.9747162
100342926.5	188.4352112	49.04528444	9.656485939	-272.8842468
32825209.31	82.01813507	66.85554261	13.66532021	-259.5715942
482546656.3	421.9528503	27.56980861	18.72440109	-278.0358887
87738420.73	344.671936	63.85337598	32.43868027	-250.6876831
243133106.1	306.2861938	74.95030524	46.4804985	-315.6516418
795112668.6	464.559906	80.25633876	74.93947525	-366.517395
573748946.5	392.4402771	49.94123423	17.07213898	-520.0335083
16996880.5	78.58924866	44.28434364	17.36398315	-159.9341888
551192718.3	324.7600708	96.65940847	92.53867493	-306.5968323
25965611.51	130.6424561	89.89673418	30.19552002	-233.4694519
380820909.8	445.9364929	58.45325051	17.4555685	-231.842392
226334840.6	329.1983948	28.91977825	14.68929558	-199.0265961
132703339.7	268.7092285	56.73349333	34.27019653	-214.2112427
21071551.06	96.18847656	62.78180176	9.655354977	-184.9650726
270241613.6	257.6984253	37.54876372	13.9969017	-258.1174316
59669817.46	175.1643677	54.31934991	8.631342316	-205.416153
63365004.72	328.570343	46.76737485	14.04614944	-228.4897156
183673530.3	342.3821411	45.99211533	19.42958736	-238.8244324
44293432.61	258.5074768	52.11578907	39.39453087	-206.4531097
258223288.5	323.7431946	80.33258088	92.1064949	-261.8051758
23605228.58	131.7654266	87.19133011	2.109396029	-188.3439178
80578557.66	228.9184723	67.70759197	55.70562363	-228.9960785
45435217.52	124.9418182	32.4711732	12.53011847	-177.2306976
66991382.3	165.8331146	61.03661958	26.52083778	-236.7216949
37458652.58	185.1196442	61.31701583	29.7985569	-165.5045929
124999956.9	142.4837189	36.8033219	8.409375954	-262.3569031
38346127.41	144.574646	34.76641741	10.86956654	-210.5744629
80540138.83	189.5827484	40.65235428	10.97896957	-206.3968811
24082934.39	112.2001495	71.18958895	40.39931488	-199.3431854
51768965.35	266.7123718	51.42613165	21.14791985	-201.6394806
92232777.43	247.2848969	28.98525158	8.108894348	-190.7307587
205079508.7	348.7411804	80.4007905	79.7509201	-324.1347351
84708348.15	221.2343292	62.62334057	34.28373871	-254.5943298
55240418.26	327.9466553	86.09780033	69.01059723	-218.7297211
43639636.03	104.4618454	33.61945424	10.05294085	-206.8340454
223246495.6	277.3872375	49.15531012	27.49486847	-234.4259796
180505489.2	332.9764404	41.64927725	17.69244251	-231.0481262
129652908.5	252.2247925	48.19910917	31.50092278	-175.7239075
214348440.2	415.8943176	81.88463745	27.03397427	-310.5179749
131509853.7	257.9797974	57.66050648	16.3561245	-239.1553955
25934928.07	208.966217	105.0753912	91.11301117	-237.2679596
5447219.672	35.49354935	53.30603601	5.69916296	-147.3970337
590376464.1	247.5133972	47.71517929	26.28275013	-252.4639893

log.sigma.1.0.mm.3D_firstorder_Entropy	log.sigma.1.0.mm.3D_firstorder_StandardDeviation	log.sigma.1.0.mm.3D_firstorder_Range	log.sigma.1.0.mm.3D_firstorder_Variance	log.sigma.1.0.mm.3D_firstorder_10Percentile
3.0275563	55.31669438	402.5548096	3059.936677	-104.7509575
2.967849928	55.89130559	382.6710663	3123.838041	-119.159848
3.003730542	53.74780466	397.3605804	2888.826505	-110.5890518
3.229581467	60.24943797	329.9188309	3629.994775	-137.2416306
2.871997567	48.81513262	385.0309906	2382.917172	-99.0987854
3.702675361	81.22218676	398.8983154	6597.043621	-153.9222229
1.795645909	26.65857596	574.9798889	710.6796723	-23.66773224
2.370753463	41.78982002	519.5536194	1746.389057	-76.95334244
2.054447925	31.58241793	363.6630096	997.4491222	-53.27124329
3.532022629	77.95514124	537.2546539	6077.004046	-109.5585648
2.044732277	32.7051842	953.7320862	1069.629074	-23.6792696
2.094396381	34.73913797	580.7878113	1206.807707	-52.4831192
3.643242523	78.16123796	468.3054504	6109.179119	-137.5830994
3.285056354	61.42651236	327.2571449	3773.216421	-151.1783295
2.497507437	45.35513327	429.2649841	2057.088114	-91.1630928
1.927390126	31.78729104	421.7754059	1010.431872	-37.34398193
2.561360479	38.32099126	277.4972839	1468.498371	-78.88936996
2.937422708	52.02429772	337.7652435	2706.527554	-103.2986145
2.682603461	42.65983556	400.2769318	1819.86157	-69.83208313
2.399471897	39.11023195	697.4358826	1529.610243	-46.53634644
2.395604527	33.18511319	285.7030334	1101.251737	-65.68905029
2.76576506	45.190897	242.982933	2042.217172	-112.9159409
2.759121775	49.06671265	366.8481598	2407.54229	-104.5253372
1.781457436	24.40269567	388.2558441	595.4915559	-28.71169147
2.431708665	50.16243913	382.5285339	2516.270299	-102.1485329
2.544727311	46.1699396	536.0797424	2131.663323	-73.22221985
2.798987296	46.55675093	438.5760651	2167.531057	-89.41609497
2.257074888	34.44750513	380.8152924	1186.63061	-57.31122894
3.281317209	70.84566673	240.8501434	5019.108495	-126.5277924
2.468235874	36.91261816	291.4091034	1362.54138	-69.10362244
2.218932235	34.66054469	364.8630981	1201.353358	-66.83937073
2.999909191	50.24684299	300.3725052	2524.74523	-127.6620117
2.656067677	45.7153349	404.2038574	2089.891845	-79.28105011
2.113246275	35.51237135	415.0723724	1261.128519	-39.90929184
2.45415495	38.83661912	454.2085876	1508.282985	-70.88018799
2.996859211	57.33401009	443.9136353	3287.188713	-113.375058
3.888671814	91.18187477	510.0639038	8314.134286	-191.4992172
1.846949506	31.91778147	724.0436096	1018.744774	-24.72129402
2.808131528	45.4360469	341.9572525	2064.434358	-90.02171478
1.959594815	32.43049828	1123.566711	1051.737219	-30.13375187
2.777269555	45.89726467	406.7024689	2106.558905	-89.82774353
2.132644977	33.51546384	565.4278412	1123.286316	-35.38136749
2.747363202	43.8692245	435.2115784	1924.508858	-88.18949127
2.792066584	47.62800195	380.2291412	2268.42657	-95.13276367
2.1016042	29.42964198	248.1439209	866.103827	-54.05504494
2.471609488	39.63446073	425.5219421	1570.890477	-74.19922791
1.948806888	30.65339073	473.8322449	939.6303633	-40.35043182
2.538998305	37.09359788	237.3853912	1375.935004	-69.98446503
3.0768766	54.80693089	282.0999146	3003.799673	-125.7231537
3.646708756	91.18627474	545.4614258	8314.936701	-131.1695648
1.890568343	29.16941814	355.723175	850.8549549	-39.47592163
2.985420003	57.23728824	530.2065735	3276.107165	-82.87688599
1.707206977	23.82194673	561.8169556	567.4851459	-23.9891222
2.617803512	40.78758046	322.6994476	1663.62672	-59.1663269
3.391188815	72.33964025	458.9472656	5233.023551	-132.4470032
2.614429708	38.94957509	273.961235	1517.0694	-79.61236115
2.076617417	30.35279484	392.8744812	921.2921546	-41.74834442
2.504606679	43.74785075	461.319458	1913.874445	-84.49261017
2.963104812	51.64529896	341.5897293	2667.236905	-112.4633682
1.87533478	27.29506719	699.988739	745.0206926	-26.45933075
3.082448516	59.3397503	595.3596191	3521.205966	-97.40970306
3.327380028	72.67909224	621.9378357	5282.25045	-111.9686386
3.49376719	78.22902805	831.077301	6119.780829	-113.326725
2.546200431	48.86580086	912.4737854	2387.866494	-67.77094803
2.56378983	39.35689224	238.5234375	1548.964967	-72.23375702
3.832035243	93.0383861	631.3569031	8656.141288	-136.426825
3.480215183	70.02012248	364.111908	4902.817552	-140.4594727
2.809798909	56.22748531	677.7788849	3161.530105	-84.58829117
1.936415619	28.52757755	528.2249908	813.8226811	-32.73811798
3.019347552	54.10149887	482.9204712	2926.97218	-84.15299835
2.911255522	51.31736375	281.1535492	2633.471822	-104.9239052
2.073929279	36.08746647	515.8158569	1302.305237	-51.37717133
2.644619933	45.66419723	380.5805206	2085.218909	-93.57602692
2.444379059	43.06811162	557.0600586	1854.862239	-79.71692352
2.480233487	45.06822615	581.2065735	2031.145008	-62.58168411
2.890539989	50.0992054	464.9605865	2509.930381	-70.77430573
3.626195806	79.53831077	585.5483704	6326.342881	-101.0437134
3.17626402	59.40750305	320.1093445	3529.251418	-138.8512543
3.328887104	63.02719782	457.9145508	3972.427665	-104.0367966
2.041963987	29.66717086	302.1725159	880.1410265	-50.46941948
3.0275563	55.31669438	402.5548096	3059.936677	-104.7509575
3.101281503	54.96801814	350.6242371	3021.483018	-97.95405121
1.983577072	34.69726142	404.8406219	1203.89995	-57.71970863
2.223735093	30.51440869	355.1491089	931.1291377	-55.99034042
2.360001988	37.38308408	395.9796295	1397.494975	-66.89675293
3.302116644	60.2339624	311.543335	3628.130227	-111.9392052
2.806272286	46.26738781	468.3518524	2140.671175	-84.46142578
1.86466683	27.30112706	438.0156555	745.3515388	-43.77424431
3.517911454	79.33490827	672.8759155	6294.027671	-106.0698608
3.108324308	58.77815562	475.8286591	3454.871578	-96.80776443
3.660738138	79.75842672	546.6763763	6361.406633	-126.6062622
2.117475323	29.67285153	311.2958908	880.4781177	-56.01593094
2.765988353	47.66783651	511.8132172	2272.222638	-69.20634232
2.381711721	40.65832057	564.0245667	1653.099032	-55.82510071
2.811405026	46.23383382	427.9487	2137.56739	-68.89828949
3.415845413	73.79959006	726.4122925	5446.379493	-125.4506996
2.935479492	52.59311214	497.1351929	2766.035444	-87.43180389
3.748921873	90.65758585	446.2341766	8218.797872	-154.3825439
2.513049968	35.28968592	182.890583	1245.361933	-83.3447113
2.639527377	46.8079826	499.9773865	2190.987235	-67.20427704

log.sigma.1.0.mm.3D_firstorder_Kurtosis	log.sigma.1.0.mm.3D_firstorder_Mean	log.sigma.1.0.mm.3D_glrlm_ShortRunLowGrayLevelEmphasis	log.sigma.1.0.mm.3D_glrlm_GrayLevelVariance
4.563612249	-25.7979118	0.017916049	5.499090143
6.625699427	-50.9730816	0.078595618	5.283998503
3.789820555	-36.09581456	0.024783511	5.217631464
3.229996018	-63.10452352	0.027033193	6.372834616
4.297924159	-32.26817321	0.0209228	4.5169975
2.63413673	-54.35997192	0.029090895	10.8891512
18.92185249	-4.216420849	0.007679973	1.89910025
8.291220028	-17.95793534	0.012169741	3.660252611
8.491516559	-12.30998905	0.009622364	2.221181985
5.044717403	-18.72763686	0.020800548	10.7733041
51.17407425	0.875523181	0.001568344	2.431045008
13.82563471	-10.89938269	0.006558254	2.747060102
3.215543998	-37.47074628	0.018009649	10.81213874
2.907414068	-63.82746374	0.022722358	6.293468549
5.935950822	-23.61864549	0.014179931	4.007987536
10.70584433	-8.810539943	0.008941166	2.412372303
4.004342974	-21.27840256	0.030509746	2.888224121
3.94246513	-50.28468691	0.034808469	4.713758774
7.515092135	-16.51686542	0.049933224	3.547981876
9.516652054	-5.209916904	0.005921499	3.452365605
4.244482081	-19.28618768	0.025112557	2.299735926
2.459384719	-48.20819392	0.054473881	3.377040398
4.837921829	-30.80477027	0.02381613	4.547185436
15.29633311	-6.307456932	0.013530589	1.50724234
5.392557614	-22.03814744	0.022149658	5.176089005
13.49799273	-14.90872818	0.020351338	4.415422933
5.123989951	-25.33271379	0.01103356	4.034395836
7.53610274	-14.05331092	0.010395037	2.526896089
1.926409015	-36.71904982	0.086097685	7.972060368
4.693134863	-18.00673143	0.027533242	2.700293005
6.363743306	-15.32299857	0.018756977	2.626688109
3.004453175	-55.02821347	0.047665285	4.322120609
5.828502894	-17.79221178	0.017293831	4.142179085
11.20626539	-7.778636141	0.013189164	2.842722218
6.45676318	-15.83964671	0.014160501	3.146396702
4.152828186	-32.30458398	0.013105047	6.16729049
2.848019716	-73.69225212	0.02620856	13.89545506
22.44735867	-5.33824055	0.00464841	2.574780719
5.734036506	-35.7133149	0.016094087	3.713323667
31.66341718	-4.386465803	0.0012994	2.586030312
4.5541596	-27.56442993	0.018486421	3.903029967
17.93220494	-4.360746413	0.013773726	2.555549431
5.174861421	-28.23789536	0.014629634	3.570112018
4.950505338	-29.13682797	0.024709178	4.238803537
5.959233037	-12.12547451	0.035809487	1.897917217
6.464590581	-19.4435722	0.012079395	3.232041305
13.4547982	-8.014161604	0.016023438	2.310719293
4.575434105	-20.81603906	0.069157119	2.510561672
3.234491287	-44.16405239	0.044959962	5.393902163
4.553050706	-40.49970057	0.01819806	13.4501784
11.74812192	-10.51787295	0.012497811	2.027905997
6.49093205	-14.69142053	0.01316489	6.475055018
19.93670519	-4.84015467	0.006123158	1.491677636
4.713144438	-4.067121806	0.024387735	3.291834558
4.245417936	-43.8598997	0.026337411	9.04546992
3.893880728	-27.43266785	0.039590502	2.816418611
10.23029843	-7.662130571	0.015410166	2.10058147
5.889756541	-22.17127602	0.010100846	3.884661481
3.59572628	-42.45499585	0.016054811	4.698580058
22.94885436	-3.882480439	0.005296555	1.77096506
6.507376128	-23.58066281	0.011908839	6.780735568
5.678331815	-18.31113887	0.008558338	10.07370074
5.433515405	-17.92481749	0.0058523	11.37581136
12.3440482	-10.30826766	0.002073279	5.327227657
4.29853626	-20.30118531	0.036640522	3.002228246
4.152566848	-26.20877637	0.009406689	15.3598762
3.128976123	-56.37912082	0.03054631	8.179162178
9.46533669	-15.97662013	0.01334074	6.587477798
17.48752907	-4.746671799	0.012959837	2.014770819
5.626016932	-17.07972734	0.016609784	5.71283931
3.531153252	-36.16742747	0.039528548	4.709131392
11.48233866	-10.37325504	0.00809936	3.061984367
4.901416051	-29.41722056	0.020065512	3.955834067
11.54958832	-18.22978638	0.012319749	3.798405454
11.32933248	-9.172222429	0.010402273	4.436138103
7.581767728	-14.3570571	0.016228223	4.928192652
4.314497567	-11.26856998	0.011361694	10.98874799
3.244143433	-63.82065988	0.069658023	5.863158139
3.862562282	-24.73641738	0.015693222	7.241157732
7.467609931	-13.19985085	0.016611738	1.907998226
4.563612249	-25.7979118	0.017916049	5.499090143
3.96424025	-27.17155522	0.041762977	5.215965631
11.59383365	-12.27128978	0.008435381	2.764077159
7.062855088	-16.66057148	0.013225242	1.906209686
6.95382887	-15.97244294	0.014193714	3.024801656
2.771230268	-37.94505696	0.04104202	6.332907229
6.36035045	-22.44940627	0.017399404	4.044273758
11.97265968	-9.736183558	0.014655944	1.847360422
5.07411132	-13.04835018	0.007779196	11.39685876
4.959184103	-21.60581419	0.011451309	6.557801237
3.931976973	-32.42567792	0.041856824	10.92894929
6.767626267	-15.80473301	0.012821546	1.839887163
6.966604345	-12.00091145	0.011303848	4.63209355
12.16451381	-9.031238243	0.009916724	3.704212476
6.407403477	-13.62302224	0.018139071	4.063608459
6.39055991	-35.47836463	0.009648692	9.808041116
6.43919138	-23.63680526	0.016052207	5.326738725
3.110325334	-53.12287604	0.042450294	13.87434857
3.218468225	-39.95211562	0.067989048	2.150293865
7.266092455	-9.260188943	0.008344371	4.759060939

log.sigma.1.0.mm.3D_glrlm_LowGrayLevelRunEmphasis	log.sigma.1.0.mm.3D_glrlm_GrayLevelNonUniformityNormalized	log.sigma.1.0.mm.3D_glrlm_RunVariance	log.sigma.1.0.mm.3D_glrlm_GrayLevelNonUniformity
0.019099731	0.142610626	0.263459555	80.21461951
0.082501281	0.14539218	0.133062261	14.62125931
0.026711405	0.137879614	0.342282863	54.40186804
0.02774242	0.117833124	0.103743945	9.94022057
0.023005609	0.150092831	0.423482741	88.27651861
0.029544484	0.086548923	0.046329165	8.530578351
0.010459178	0.331448189	1.288170259	3281.421244
0.014264938	0.222872713	0.70838618	796.677566
0.011950746	0.269655225	0.86434271	766.9107235
0.02171564	0.107260002	0.128009579	35.2708716
0.001948184	0.284233613	0.763972296	1077.188626
0.008227511	0.26943397	0.914640893	549.5240649
0.018823396	0.090980852	0.159445555	26.98798969
0.02345607	0.113453755	0.094513786	19.81495243
0.016046403	0.202088498	0.53208642	139.1190895
0.011531862	0.303929685	1.090808505	1648.867837
0.034324009	0.184143094	0.455285638	61.51782048
0.036263894	0.151524211	0.124247532	15.07682209
0.054603332	0.178335514	0.362618551	81.03423459
0.00740296	0.221947883	0.869503922	1819.692394
0.029561647	0.202822277	0.666036478	211.8740595
0.057913191	0.157681135	0.177493698	21.4824369
0.025935424	0.168768421	0.364815032	37.77268102
0.017681968	0.327422263	1.055198959	993.1606181
0.024811213	0.224188745	0.612966205	118.1619382
0.023326259	0.199943549	0.591284756	299.5715834
0.012211026	0.16424254	0.362214947	124.8255714
0.01250237	0.236237175	0.761488565	292.8703667
0.086999938	0.115496035	0.027302492	3.14426599
0.031606609	0.200041462	0.547940152	171.0884654
0.022343991	0.237175983	0.898044121	562.9503259
0.049781173	0.139319973	0.151117216	31.60092146
0.019342584	0.188915934	0.474353322	187.2878739
0.016010959	0.271149074	0.817355411	578.3707133
0.016535292	0.207720984	0.653301801	525.8896127
0.014371995	0.145050472	0.395205561	131.3113384
0.026850459	0.076095125	0.056045725	28.12513318
0.006255439	0.324788846	1.144311528	2368.673969
0.017066684	0.16299356	0.199617225	27.71656164
0.001711585	0.301133761	1.041854191	2350.519865
0.020465185	0.16235828	0.372543918	81.91060807
0.017119252	0.267871921	0.846167679	1332.384144
0.016367524	0.169061857	0.409304549	205.6742272
0.026637675	0.162832973	0.365039918	91.6747303
0.042115704	0.256264961	0.745617034	192.9983406
0.014023086	0.203775169	0.65894064	291.7404975
0.020083426	0.29721259	1.112934243	1309.008132
0.07582174	0.189554599	0.29951669	33.95767109
0.046840242	0.13630302	0.129911215	13.87838716
0.018892913	0.096870932	0.067628274	19.99391806
0.015510729	0.305347977	0.842289969	387.1058907
0.014689547	0.15051619	0.467473527	256.2576614
0.008447517	0.34410807	1.199886336	2123.057732
0.027552297	0.189331056	0.406169835	125.6115326
0.027590087	0.111720699	0.168173259	28.21114602
0.043200601	0.179199875	0.397555522	93.48505768
0.018869154	0.27185755	0.835271935	977.6541427
0.011740707	0.196485306	0.72918339	218.178092
0.017162609	0.142459448	0.213712015	32.82031733
0.007015876	0.307316128	0.986438134	4687.002894
0.013189116	0.136038137	0.422743872	86.20822229
0.009324359	0.121078735	0.356351535	158.7940296
0.00636142	0.109102954	0.307830485	419.4315418
0.002546375	0.206331902	0.869455904	1220.657422
0.04009384	0.18847523	0.439240931	47.60329197
0.009961246	0.083340451	0.18526524	161.4133764
0.031453728	0.102072102	0.071970388	11.35481136
0.01513663	0.170726946	0.617962347	524.559345
0.017037127	0.295560944	1.128494301	1860.445426
0.018501304	0.143329175	0.407138649	174.8601341
0.041858481	0.146310622	0.278916006	24.24349545
0.01020278	0.274072496	1.072092832	1244.500653
0.022307025	0.174370916	0.475254831	99.99779529
0.014151272	0.210505722	0.55763086	167.8741619
0.012306019	0.220574443	0.679605464	515.147215
0.018076156	0.156411443	0.397672799	76.03040407
0.012126079	0.098592849	0.150487784	130.2362547
0.071730568	0.122102222	0.087520944	13.00909707
0.016765043	0.114997472	0.215955709	65.19160072
0.02008316	0.266385812	0.779095014	292.176325
0.019099731	0.142610626	0.263459555	80.21461951
0.044606011	0.131693916	0.218004761	41.72462415
0.01039176	0.295232718	0.872620805	673.4169588
0.015605714	0.233938592	0.576535669	202.8840393
0.016740859	0.217663888	0.779446305	278.3148665
0.04284905	0.110984094	0.111238223	17.85759223
0.019229959	0.162243087	0.361925284	94.97078874
0.018797372	0.304513941	1.115689056	780.0806706
0.008446446	0.107436745	0.247765047	107.4368028
0.012607943	0.132596668	0.390069402	85.97182955
0.043062255	0.090022386	0.080275894	23.23456732
0.015438961	0.249062307	0.754550655	251.510829
0.012889709	0.173966622	0.535391552	458.2890163
0.012064156	0.22477578	0.826010883	598.9425192
0.020379031	0.166788241	0.332433678	281.1437825
0.010310399	0.111811419	0.257069349	113.2255028
0.017630474	0.152371341	0.414978664	177.9092723
0.043329426	0.088204082	0.059911652	7.305015232
0.072482367	0.193083558	0.136072879	12.12572524
0.009995247	0.190041011	0.75819163	1297.890631

log.sigma.1.0.mm.3D_glrlm_LongRunEmphasis	log.sigma.1.0.mm.3D_glrlm_ShortRunHighGrayLevelEmphasis	log.sigma.1.0.mm.3D_glrlm_RunLengthNonUniformity	log.sigma.1.0.mm.3D_glrlm_ShortRunEmphasis
1.669937231	81.29892393	421.304775	0.888186517
1.400019514	31.66383436	80.50547942	0.913831765
1.849037142	56.88081907	283.1119637	0.870290467
1.301522631	79.4641254	71.65984503	0.936064097
2.035081081	57.07262593	399.9883065	0.850839887
1.141536752	94.11415632	90.43475889	0.966405811
4.006474842	74.99567211	4552.689078	0.703275666
2.703580583	73.0563556	2061.383589	0.787845059
3.080664005	72.82642298	1510.615337	0.756618274
1.357009764	99.99988929	272.3172055	0.927326797
2.850566154	468.6993401	2100.130236	0.772526631
3.188011288	106.433288	1063.785644	0.749827522
1.408338207	102.3679959	245.6648113	0.926792728
1.280541518	78.52294647	148.4626267	0.937588386
2.328835292	71.38019533	426.8486722	0.814863827
3.569723167	73.35285254	2671.398879	0.728204464
2.171631797	36.55613234	213.2053318	0.825802927
1.3733729	53.98568911	80.62917395	0.918514946
1.928810829	31.68442588	312.4392203	0.854542218
2.987428119	119.2536692	4595.883489	0.777381364
2.600596112	35.8732483	618.6087339	0.797647366
1.525561499	28.55434561	102.7850291	0.891364246
1.950288151	57.76885302	150.8605589	0.846639725
3.570088694	48.46290984	1450.465105	0.716878744
2.524014015	57.97858224	314.0714802	0.798936805
2.421988171	52.062422	923.5343503	0.813134654
1.896777475	94.03295948	533.9094785	0.862454125
2.79014691	75.80096306	710.0654095	0.784695851
1.08817883	44.17242494	25.79985098	0.977955293
2.362594194	37.06264955	524.7092787	0.810990317
3.092468885	45.38873241	1291.549746	0.765747768
1.425588106	38.11743023	182.8636963	0.915325307
2.144230335	65.97988934	656.3983901	0.840234332
2.999425974	63.25114525	1136.913673	0.758028576
2.540919435	62.40859871	1531.135902	0.806001722
1.951558143	89.9301658	633.1785935	0.861358373
1.171412529	101.4826764	332.3465059	0.959272241
3.738546621	123.6646625	3436.340943	0.711957425
1.547599849	92.31725885	130.3512152	0.896742115
3.45740899	470.3657429	3908.676342	0.734834947
1.940145552	60.43543485	346.511918	0.854764157
2.971755407	55.77081248	2759.771238	0.77329795
2.018262987	74.44380463	832.4331499	0.849328877
1.944509922	57.84908928	382.3930264	0.850435676
2.83930444	27.82143969	416.2943336	0.77143236
2.548419988	74.18432382	873.9105639	0.808231336
3.620870412	47.828253	2147.862103	0.72507436
1.800526704	20.30500262	126.8607398	0.865735324
1.388499863	45.31178631	81.90467447	0.915758387
1.205233877	107.6383528	182.0635906	0.952080245
3.111330732	59.65482089	651.5223716	0.743758634
2.082098782	88.79306487	1169.417073	0.85439135
3.893499689	88.9678944	2829.491495	0.701353607
2.058077927	47.12137268	436.2121569	0.836001199
1.46394828	75.94265684	200.4020769	0.91029114
2.01463359	34.6151214	348.2630267	0.843886141
3.000130573	51.26225837	1950.391644	0.764571685
2.679521591	86.40253668	664.6101899	0.801497282
1.612856662	84.28832309	169.5329319	0.881529432
3.367772906	111.9022246	7665.705171	0.736151182
2.008053188	99.56325834	438.776523	0.857555309
1.851889166	149.3304036	948.5850665	0.874497314
1.725261525	204.002308	2904.183035	0.891174116
2.946099952	347.9299677	3397.162191	0.786383513
2.067657952	38.82044327	171.431765	0.848701165
1.458712024	157.5015594	1579.585428	0.921309916
1.217614432	73.131018	97.44801757	0.949524299
2.425387953	82.83720533	1940.939503	0.822484735
3.667620534	50.40858906	3029.527554	0.720136922
1.974997625	71.07991459	848.6855218	0.859559846
1.718829526	44.72115859	122.6169295	0.882300139
3.520013278	86.78356704	2260.042541	0.732706344
2.19203878	54.98941263	368.5479826	0.828106249
2.369651913	75.71056393	492.3262909	0.813640894
2.583722203	85.05429525	1410.957618	0.805231113
1.950135489	72.71925742	341.3106063	0.862788407
1.4099746	124.3113984	1072.220496	0.919941732
1.254811387	39.23726916	92.29486878	0.944397954
1.537942289	88.70454788	447.4212442	0.908603058
2.899645989	47.19329526	600.8323083	0.768296546
1.669937231	81.29892393	421.304775	0.888186517
1.578616479	41.14043599	242.8860528	0.896839239
3.121341759	87.51463864	1194.144262	0.75119936
2.418725678	61.80752636	526.42417	0.806800273
2.791684671	61.69259484	750.6868043	0.793804269
1.312635657	53.22866985	136.7204729	0.936203917
1.909500559	64.21074408	409.5073468	0.860287013
3.642155915	46.07118009	1240.75442	0.722419605
1.63046542	160.8614892	758.7898794	0.893457782
1.916639479	102.1800889	464.1431413	0.869978497
1.225069448	74.99143366	227.72107	0.951988967
2.784190907	60.25978736	584.4714623	0.786682012
2.225982027	87.27693303	1748.272145	0.841330421
2.919555159	81.3220282	1498.108052	0.778511622
1.839972136	58.22891613	1192.044166	0.865988476
1.625595525	136.5362358	782.1505701	0.899953275
1.99508001	80.25777004	809.1091042	0.857647205
1.167773201	82.05595374	75.53105362	0.963974007
1.421593299	23.54350371	49.44007461	0.9066483
2.737269029	100.796433	4023.844077	0.796167536

log.sigma.1.0.mm.3D_glrlm_LongRunHighGrayLevelEmphasis	log.sigma.1.0.mm.3D_glrlm_RunPercentage	log.sigma.1.0.mm.3D_glrlm_LongRunLowGrayLevelEmphasis	log.sigma.1.0.mm.3D_glrlm_RunEntropy
164.2577042	0.844190344	0.025856535	3.788763208
47.35741546	0.889720899	0.099964619	3.465584474
134.6455526	0.816531295	0.038571146	3.843399474
116.5429201	0.91638796	0.031074986	3.584244553
153.0130087	0.788203753	0.036690523	3.829892583
110.5554574	0.956684093	0.031430442	3.886518005
436.2388005	0.607029975	0.038324867	3.584830235
276.5870093	0.708329522	0.030758304	3.742722958
324.2355666	0.672372083	0.031515686	3.549964043
139.026028	0.903000845	0.026149419	4.039599425
1716.482726	0.693265007	0.005037599	3.472764055
479.9771486	0.663646149	0.022674618	3.648277734
155.0382732	0.895886591	0.023148762	4.137811733
114.3755255	0.91902834	0.026709839	3.656648161
231.9338319	0.747826087	0.029060681	3.671444031
380.3255617	0.636061019	0.035779545	3.601946598
106.5924284	0.765702188	0.058823735	3.606043442
83.99887672	0.896049896	0.042837069	3.381340076
71.97855105	0.800705467	0.083005278	3.618015595
459.8526591	0.687341584	0.02047418	3.881585638
126.3146502	0.719432083	0.063460828	3.650649441
55.78988606	0.862220058	0.073763022	3.352647166
146.6886239	0.795784287	0.038587401	3.701351015
248.6203236	0.631300839	0.055060249	3.443771736
206.243665	0.725471498	0.043834963	3.737258257
164.4404197	0.7397151	0.044891253	3.771689219
223.6129083	0.808962071	0.019636499	3.693655366
287.8864574	0.704202563	0.029776973	3.619161256
49.3315801	0.972527473	0.09060895	3.32909619
118.1586817	0.743032814	0.059686588	3.63530045
209.304499	0.676007274	0.053275071	3.690318752
64.63012656	0.888989442	0.060049283	3.518986
177.250171	0.774824081	0.033469365	3.741132268
264.5815697	0.67726018	0.03894552	3.617008767
213.5477387	0.728103361	0.034819559	3.724062972
223.4340879	0.802304965	0.022642468	3.950145743
119.8733741	0.947534517	0.029535473	4.174348083
676.2491623	0.621249853	0.021393553	3.591713054
165.6077364	0.862944162	0.022171591	3.436363598
2227.300605	0.644137108	0.005527647	3.614581776
148.5146047	0.799341704	0.032781499	3.701464878
210.1800543	0.686939977	0.045861017	3.59953877
189.3809617	0.795577049	0.027588742	3.701584667
150.5903511	0.796213687	0.03838932	3.731315924
109.4142612	0.691954186	0.091377018	3.494970456
257.937171	0.731143026	0.029156805	3.715416844
253.0580084	0.631875228	0.058056151	3.637836889
44.39696646	0.817702845	0.113982529	3.334301469
71.1638113	0.892712551	0.055263865	3.56658426
130.523727	0.938111888	0.021915045	3.961521102
264.7838672	0.664863992	0.040302771	3.421220493
215.617741	0.788078526	0.025636023	4.016370378
503.9561688	0.610488403	0.030921042	3.45985919
116.0533085	0.782126964	0.047610177	3.646102255
128.7189773	0.879656928	0.033749443	3.966309865
92.40336535	0.786750203	0.06546655	3.579445794
209.603416	0.680187979	0.047810138	3.553113496
327.1695162	0.718098083	0.025181298	3.797587862
170.6757336	0.846719457	0.022730362	3.667606677
521.2680367	0.648502138	0.022369892	3.475564903
238.2441553	0.794807451	0.021917511	4.056201464
320.6712695	0.818033495	0.014359067	4.231263039
394.194797	0.840568006	0.009664577	4.313079138
1336.707199	0.694230769	0.006816163	4.023333742
99.62465819	0.786005272	0.06329336	3.52967255
245.0972515	0.88632283	0.013064465	4.424835264
91.5767632	0.934712346	0.035338465	3.790776739
252.6532904	0.744186047	0.02905777	4.05050589
259.9509727	0.62788663	0.055340648	3.635827945
163.5173107	0.798901819	0.031036852	3.985497019
97.18458075	0.836052836	0.055127015	3.649909121
453.2600297	0.639505022	0.029675573	3.746245044
168.9778798	0.765225429	0.037362351	3.703814696
242.1909805	0.743207398	0.027320304	3.637781543
273.8859041	0.725818025	0.027570605	3.810571845
160.9569362	0.804253693	0.030448277	3.808375099
182.5069239	0.891259213	0.016015338	4.204180532
51.37379558	0.926421405	0.081147063	3.517632129
148.7104716	0.870495096	0.022850771	3.969872012
195.0884079	0.687150569	0.04812009	3.449961317
164.2577042	0.844190344	0.025856535	3.788763208
72.4246667	0.8584532	0.060027475	3.763654734
396.3420385	0.667326822	0.026923863	3.520001257
197.9692821	0.737806874	0.032813305	3.401028029
230.9200244	0.707564458	0.03842108	3.707901748
70.90357538	0.913898601	0.051466678	3.696567196
152.3745887	0.806790451	0.030726307	3.708201269
249.2092066	0.629819516	0.057690468	3.533523472
289.0960557	0.85086743	0.012315477	4.272976223
229.2294537	0.810192308	0.020455054	4.004296214
93.76630161	0.935061315	0.048510122	3.991414931
228.9861943	0.705648198	0.037023735	3.433758487
233.5520687	0.769635391	0.024822737	3.880615119
310.8405476	0.691309728	0.030339463	3.840396047
122.0452125	0.815302743	0.034040242	3.7147855
257.0257575	0.855184511	0.014298994	4.137749138
195.2866961	0.796744552	0.028110227	3.905745372
94.79406145	0.951370469	0.047256415	3.9554306
36.34937423	0.884073673	0.0923831	3.031393261
351.1391965	0.711025855	0.023798005	4.029140573

log.sigma.1.0.mm.3D_glrlm_HighGrayLevelRunEmphasis	log.sigma.1.0.mm.3D_glrlm_RunLengthNonUniformityNormalized	log.sigma.1.0.mm.3D_glszm_GrayLevelVariance
92.90226951	0.748552871	12.68786681
34.43747905	0.799350679	13.21521998
66.95578222	0.716176992	11.43346939
85.97384123	0.84756288	9.924701561
68.91432693	0.679990644	12.03763314
97.25921666	0.916349202	14.99416667
107.2830859	0.459687242	13.1782669
95.44588296	0.576409499	10.87195184
98.92192303	0.530824489	7.009113433
106.6094728	0.827546358	22.48923099
605.3315059	0.553773959	13.08374566
144.570496	0.52129026	11.96850586
110.2328486	0.827591221	18.85413867
84.9548078	0.849311408	8.037424816
90.82186669	0.619195495	8.10122449
102.6812597	0.491952537	9.391680621
45.44846738	0.637124941	7.276686391
59.31336734	0.80923958	9.820153061
37.11630785	0.68685027	10.25418283
153.4123918	0.560479723	13.75242951
45.91392914	0.591768845	7.126341759
33.11266248	0.753686605	4.948137327
70.01272828	0.673138343	11.11925952
68.30314068	0.477839821	8.693176417
75.07835472	0.594689543	12.25490196
64.90083137	0.61610003	14.94718405
110.8944349	0.70137642	10.10005753
98.47372203	0.572084287	8.481206057
45.20425597	0.945395017	8.049861496
46.77829451	0.612965943	8.648474048
61.65210574	0.543604272	7.654436979
42.50101183	0.803696961	7.258487654
79.43344659	0.661605818	11.67299081
84.8905037	0.532862884	10.71629611
79.06524353	0.604615589	9.400968899
106.6145668	0.699001252	13.05078125
105.0360078	0.898546114	19.61272107
175.9944319	0.470998684	13.94992015
103.867496	0.765390707	9.621882086
641.4287309	0.500532189	15.24697857
72.06077971	0.686549946	9.599590602
71.65376793	0.554601689	12.03303828
88.97971061	0.679819556	10.23004973
70.3224385	0.678824825	9.45760897
36.79835659	0.552307041	6.294293629
94.08398742	0.609149779	9.408083759
67.22223692	0.487496933	11.50550296
23.70878939	0.707240873	5.875114784
49.88358514	0.803433331	8.067665289
112.0555327	0.881564276	17.68098765
81.96741689	0.513486117	6.483055556
103.66577	0.686621843	17.46915556
127.8067418	0.458074751	10.44661633
56.40136754	0.655220661	9.139891251
84.32086167	0.792571316	18.31111412
42.15717195	0.667397785	6.932557902
67.84056638	0.541949884	9.180582986
111.408109	0.598055467	10.77386013
98.10084355	0.73507296	7.973599633
152.8544545	0.502427359	9.013620545
116.4803213	0.692230333	17.56078531
170.9981896	0.723144251	24.75231139
228.5933339	0.755160844	23.21063729
445.1559824	0.574163469	19.6784425
46.32294629	0.677708255	7.766528926
170.204433	0.81550073	28.97814551
76.61663614	0.875631128	13.8669081
101.3808577	0.631598663	19.55173154
70.1756582	0.481208384	11.78031836
82.53491817	0.695508613	15.70801165
52.01068543	0.738823456	8.229142637
121.6183062	0.497575118	12.26522674
69.18945525	0.641436234	8.701307096
95.46283174	0.617050119	10.31357154
105.6937335	0.603718212	15.56873314
83.74839005	0.70161101	13.71219136
133.9003757	0.811548443	22.17643827
41.40745277	0.86531391	8.485198001
97.29952983	0.789035049	14.36830659
63.19600525	0.54754115	5.480022806
92.90226951	0.748552871	12.68786681
45.84824515	0.765743553	11.41312358
119.8291031	0.523223353	8.732458126
78.00783623	0.606412906	6.044707429
79.02772979	0.586227118	10.62014062
56.22468627	0.848377327	9.617514793
75.74834845	0.697826904	11.090271
65.33922288	0.484188813	8.072337475
179.3958572	0.758646544	23.50138504
117.6905899	0.715541778	15.77210829
78.35002872	0.881932707	17.18927606
78.22777138	0.576430187	5.030828402
103.8725834	0.663462448	13.58136524
104.9038257	0.562178064	15.36395242
66.9440457	0.706659968	11.5527388
152.8556638	0.772154955	21.09302461
94.35558687	0.692306776	15.09053943
84.32609189	0.911112358	20.08691358
25.86374531	0.785483517	3.916666667
126.9549878	0.589120591	16.3777205

log.sigma.1.0.mm.3D_glszm_SmallAreaHighGrayLevelEmphasis	log.sigma.1.0.mm.3D_glszm_GrayLevelNonUniformityNormalized	log.sigma.1.0.mm.3D_glszm_SizeZoneNonUniformityNormalized
64.49779869	0.096524454	0.53057232
32.73500069	0.117717004	0.357907253
52.73379178	0.090068027	0.478820862
45.45949963	0.112947658	0.397612489
49.8961918	0.10591716	0.427337278
84.37037715	0.08	0.578888889
88.08339429	0.094171606	0.451079972
56.22919586	0.113278004	0.428442586
57.53373402	0.141412069	0.447418255
110.1803537	0.067721206	0.474472142
450.7381961	0.140347222	0.473498264
96.17012024	0.107055664	0.48059082
104.0627167	0.069067455	0.515049718
34.66022447	0.10670528	0.321452439
48.62581953	0.128061224	0.462653061
64.94833652	0.123084201	0.41361527
30.51944901	0.135621302	0.371360947
31.59074373	0.109693878	0.290816327
33.85059408	0.119113573	0.425373961
124.2012182	0.088083026	0.460227017
36.28968296	0.137378809	0.450831025
16.43561985	0.161431702	0.348429511
46.38210536	0.102171591	0.399074404
47.00377545	0.129013752	0.43303447
47.36644988	0.091887735	0.368319877
51.63601201	0.109633321	0.441708793
65.17701373	0.112754767	0.448553583
60.3507054	0.119321255	0.386425095
26.77777778	0.119113573	0.473684211
40.37476687	0.117509468	0.468478503
34.28170363	0.128750336	0.414630476
23.7792776	0.122299383	0.437885802
65.95739676	0.092309356	0.460508383
60.5346781	0.132121987	0.47694485
57.2260649	0.107043326	0.463101962
78.1315281	0.082550049	0.502838135
95.43767528	0.063244906	0.508554402
116.6568903	0.106749496	0.402506658
67.50390637	0.120181406	0.43537415
481.9910869	0.099419277	0.471168095
48.69934129	0.106087576	0.433250267
71.32468794	0.108946927	0.528354311
57.89148296	0.10386766	0.389780721
38.95333899	0.108465608	0.464978584
24.19114202	0.143711911	0.415401662
56.78221098	0.114722831	0.448746043
47.45958516	0.107903995	0.45264234
14.78491933	0.144168962	0.232323232
30.88630352	0.115702479	0.502066116
91.68008505	0.074814815	0.381481481
45.5951083	0.152037037	0.445987654
98.17204469	0.076444444	0.516730864
101.1135154	0.115174012	0.528848092
33.30986665	0.112774522	0.388743733
68.41160591	0.072471606	0.386154678
24.0328123	0.121518212	0.422507907
54.66574001	0.133411735	0.514067438
69.11332131	0.10277394	0.449888405
54.68542249	0.112947658	0.391184573
113.5266105	0.197824122	0.484686209
100.0729597	0.077151981	0.497750036
150.3519144	0.064864471	0.518442573
186.9263565	0.059810993	0.495471499
342.3312142	0.072539271	0.501337599
23.35567947	0.135330579	0.309917355
150.3043124	0.054330665	0.492854058
59.29575242	0.07831598	0.423268447
93.572648	0.074790997	0.526927748
66.98491084	0.114752482	0.483898474
77.81910865	0.08222517	0.473160335
30.69649428	0.109573241	0.420991926
76.41172386	0.102372848	0.428728321
38.26706345	0.124102269	0.42114335
56.14533133	0.132350585	0.41908348
89.63629107	0.079004056	0.500834482
82.58298391	0.102880658	0.483367627
118.9609729	0.063803484	0.483390948
30.12561547	0.109573241	0.496347559
89.100837	0.077631007	0.528536435
33.93657882	0.163194597	0.47686505
64.49779869	0.096524454	0.53057232
40.00625771	0.092403628	0.384070295
63.15947168	0.119963785	0.467230019
48.10895681	0.147344583	0.47329973
60.21001103	0.101871282	0.51601947
49.50443575	0.098698225	0.416331361
59.25546578	0.103149414	0.446289063
39.42244973	0.125016398	0.431719795
141.0721611	0.0611366	0.442239439
102.3952088	0.078179253	0.468967014
65.74149404	0.072101687	0.452114837
40.07679813	0.167928994	0.359763314
90.90447139	0.092913386	0.537687395
94.06716924	0.086516307	0.47523876
66.12506891	0.097088615	0.466854346
125.8242494	0.068408508	0.504605137
78.74533117	0.086368909	0.519946826
80.95632253	0.066666667	0.45382716
17.49661819	0.216049383	0.320987654
104.6691864	0.076460481	0.454482115

log.sigma.1.0.mm.3D_glszm_SizeZoneNonUniformity	log.sigma.1.0.mm.3D_glszm_GrayLevelNonUniformity	log.sigma.1.0.mm.3D_glszm_LargeAreaEmphasis	log.sigma.1.0.mm.3D_glszm_ZoneVariance
82.23870968	14.96129032	367.4709677	349.0086993
10.37931034	3.413793103	49.34482759	34.16171225
50.27619048	9.457142857	262.5238095	241.3638095
13.12121212	3.727272727	20.18181818	12.40955005
55.55384615	13.76923077	591.6615385	558.7315976
34.73333333	4.8	6.116666667	3.169722222
332.897019	69.49864499	136225.4932	135737.3108
243.7838313	64.45518453	11614.96661	11536.32182
159.2808989	50.34269663	15626.58146	15485.46575
56.46218487	8.058823529	72.1512605	62.79485912
227.2791667	67.36666667	19619.60417	19490.02389
123.03125	27.40625	11543.98828	11399.89452
62.83606557	8.426229508	47.56557377	40.2045821
21.53731343	7.149253731	20.3880597	12.34617955
64.77142857	17.92857143	1368.471429	1325.287755
242.7921635	72.25042589	44018.89779	43807.88133
24.13846154	8.815384615	542.3692308	497.3760947
8.142857143	3.071428571	41.89285714	26.17729592
40.41052632	11.31578947	610.0526316	574.4305817
548.590604	104.9949664	30953.73238	30853.6149
68.52631579	20.88157895	2926.460526	2835.207756
12.89189189	5.972972973	67.94594595	49.71073776
21.1509434	5.41509434	261.3396226	233.2296191
106.9595142	31.86639676	34830.91498	34452.6361
37.56862745	9.37254902	1374.647059	1323.986159
117.0528302	29.05283019	3425.135849	3366.743183
69.97435897	17.58974359	869.7115385	833.4803994
66.46511628	20.52325581	4857.947674	4753.361378
9	2.263157895	2.526315789	0.354570637
62.7761194	15.74626866	2057.858209	1984.077801
126.4622951	39.26885246	11073.8459	10941.33184
31.52777778	8.805555556	68.84722222	56.30381944
99.00930233	19.84651163	1478.897674	1443.50901
141.1756757	39.10810811	10607.48311	10494.30514
214.8793103	49.66810345	3557.519397	3501.366338
128.7265625	21.1328125	676.8203125	657.4052734
103.745098	12.90196078	9.5	5.845155709
270.0819672	71.62891207	77050.40835	76744.39303
18.28571429	5.047619048	112.7857143	90.78514739
411.8009153	86.89244851	58666.22426	58474.11324
45.9245283	11.24528302	561.6509434	526.2147561
358.7525773	73.97496318	23446.19293	23332.59312
76.78680203	20.46192893	1685.832487	1625.750316
58.58730159	13.66666667	583.3412698	551.8567019
39.46315789	13.65263158	3710.210526	3579.047756
99.62162162	25.46846847	3810.427928	3732.876897
210.9313305	50.2832618	36047.70601	35824.05591
7.666666667	4.757575758	236.8787879	192.8374656
22.09090909	5.090909091	32.45454545	25.74173554
34.33333333	6.733333333	21.31111111	15.33580247
80.27777778	27.36666667	7229.022222	7116.897654
232.5288889	34.4	1593.735556	1570.716884
258.0778689	56.20491803	81431.87705	81003.35311
46.2605042	13.42016807	1165.369748	1114.708848
33.20930233	6.23255814	61.52325581	50.38628989
41.82828283	12.03030303	674.7171717	629.8677686
242.6398305	62.97033898	18363.64619	18238.17771
87.27835052	19.93814433	2728.417526	2664.993543
25.81818182	7.454545455	126.7575758	109.7731864
853.0477273	348.1704545	110407.304	110228.8235
82.62650602	12.80722892	508.8975904	485.8460226
210.4876847	26.33497537	723.8300493	708.241167
648.5721925	78.29258976	1151.303285	1139.104034
557.4874101	80.66366906	14356.53058	14297.82632
13.63636364	5.954545455	466.9318182	413.7081612
368.1619813	40.58500669	258.0120482	249.4562274
19.89361702	3.680851064	17.21276596	10.80217293
402.0458716	57.0655308	4287.090433	4257.819922
308.2433281	73.09733124	54398.95604	54151.37545
140.5286195	24.42087542	1094.084175	1067.650036
21.47058824	5.588235294	100.8627451	85.79008074
228.5121951	54.56472795	30779.43902	30602.04461
49.69491525	14.6440678	833.7542373	793.464019
66.21518987	20.91139241	1679.322785	1633.203213
227.3788546	35.86784141	5689.973568	5639.794756
52.2037037	11.11111111	526.2592593	494.9821674
202.0574163	26.66985646	301.5215311	288.9512832
25.31372549	5.588235294	15.19607843	10.11149558
110.4641148	16.22488038	153.9760766	144.2738948
72.00662252	24.64238411	5104.066225	4992.351125
82.23870968	14.96129032	367.4709677	349.0086993
32.26190476	7.761904762	149.8452381	130.5480442
131.7588652	33.82978723	14133.87234	13986.96419
55.37606838	17.23931624	2846.042735	2745.186208
110.944186	21.90232558	3621.669767	3551.187885
27.06153846	6.415384615	26.61538462	19.28378698
57.125	13.203125	581.2421875	549.1605835
99.72727273	28.87878788	25353.84416	25044.0232
134.4407895	18.58552632	300.3585526	285.4193105
90.04166667	15.01041667	397.5833333	380.2222222
52.8974359	8.435897436	19.62393162	14.0591716
46.76923077	21.83076923	4305.492308	4184.492308
341.4314961	59	3384.897638	3355.856571
196.2736077	35.73123487	9579.307506	9492.226524
175.537234	36.50531915	1440.704787	1410.484035
175.0979827	23.73775216	276.0345821	264.3921302
144.0252708	23.92418773	1036.574007	1008.60251
20.42222222	3	7.311111111	3.573333333
5.777777778	3.888888889	46.5	30.94135802
529.0171821	89	17681.13574	17613.05906

log.sigma.1.0.mm.3D_glszm_ZonePercentage	log.sigma.1.0.mm.3D_glszm_LargeAreaLowGrayLevelEmphasis	log.sigma.1.0.mm.3D_glszm_LargeAreaHighGrayLevelEmphasis	log.sigma.1.0.mm.3D_glszm_HighGrayLevelZoneEmphasis
0.232732733	3.529746152	39590.53548	84.44516129
0.256637168	1.974012721	1603.103448	43.27586207
0.217391304	3.419139866	21323.47619	65.77142857
0.358695652	0.254777092	2048.939394	72
0.174262735	7.351181536	49096.65385	65.21538462
0.582524272	0.101860841	594.9	102.4166667
0.045259414	1254.384107	14930227.26	119.4525745
0.112762584	108.6902497	1252829.659	76.88400703
0.084180657	145.2584955	1696698.778	78.07022472
0.326923077	0.823786868	6846.87395	135.1344538
0.087847731	32.73693605	11779092.1	631.33125
0.083306215	75.34770341	1780836.117	127.1953125
0.36858006	0.47793333	5353.106557	128.0737705
0.352631579	0.249437675	2147.686567	69.67164179
0.152173913	12.84297637	147785.2143	67.17142857
0.068840155	408.7650471	4783144.775	90.17717206
0.149082569	10.69818565	28781.36923	43.46153846
0.252252252	0.838028818	2602.107143	58.32142857
0.167548501	17.20840956	22885.44211	45.12631579
0.09994131	201.4061496	4789584.919	168.3708054
0.104683196	59.39991107	148326.9211	48.44078947
0.234177215	2.012809896	2874.27027	25.81081081
0.1886121	3.290295835	21570.88679	61.71698113
0.051415487	501.4776514	2451880.579	64.24696356
0.140495868	15.98209371	120491.2745	61.25490196
0.130864198	49.37629859	241953.7245	68.43018868
0.166134185	6.986631064	110232.9359	93.77564103
0.097782831	46.02462467	517647.2209	86.37209302
0.678571429	0.169127511	115.8421053	43.42105263
0.116420504	39.73552481	109944.3657	50.02985075
0.086869838	157.4945025	790935.1279	48.15409836
0.282352941	1.597091734	3545.347222	36
0.168100078	17.17004728	129618.2791	83.64651163
0.093998095	118.2988612	962467.1453	77.83445946
0.133448375	40.79428443	314306.3728	74.68318966
0.226950355	5.423292164	86036.59766	99.7734375
0.523076923	0.161601923	812.5343137	119.5147059
0.057164764	423.1546635	14107537.47	164.2041729
0.21319797	1.11341918	12251.80952	98.07142857
0.072147928	90.90622228	37917981.99	655.3832952
0.167987322	6.973343255	46514.04717	67.27358491
0.093823407	337.0322109	1654415.277	88.64064801
0.129011133	17.13000554	171238.9848	84.17258883
0.178217822	6.912316969	50719.7619	54.00793651
0.087316176	95.58502904	147789.9158	33.11578947
0.113554987	36.1320406	406692.4279	79.15315315
0.066867556	513.275727	2567904.365	62.72317597
0.150684932	10.30121707	6141.909091	27.09090909
0.385964912	0.706705578	1738.363636	44.34090909
0.409090909	0.332148029	1648.855556	136.7333333
0.094438615	84.05042041	627966.0944	62.48333333
0.208429829	15.58219374	165620.9778	122.6
0.048307266	628.9673425	10621376.73	125.7889344
0.140495868	21.35104399	65248.46218	51.77310924
0.299651568	0.721164453	6329.848837	92.15116279
0.149321267	14.16905841	33682.15152	35.48484848
0.089275582	261.5883051	1308096.544	68.62076271
0.125566343	21.17727986	355240.3196	92.98453608
0.242647059	1.106275639	15314.40909	81.25757576
0.074852209	712.5572071	17217170.12	152.0198864
0.208281054	4.209137999	62765.65663	130.3554217
0.253275109	4.088989466	129546.9286	188.5418719
0.286307962	4.948597999	269833.4217	247.7754011
0.130516432	31.2471028	6611344.309	452.0341727
0.137071651	9.468516251	24091.5	41.09090909
0.34187643	1.55798945	43836.39759	193.4149933
0.394957983	0.359397509	1079.340426	85.93617021
0.184835271	40.38021927	460164.114	113.2581913
0.063553826	776.6199786	3865872.863	84.76609105
0.194499018	13.53803525	90980.14815	100.3232323
0.257575758	1.776229499	6428.568627	45.17647059
0.075080997	234.6733895	4069105.856	104.1444653
0.157543391	9.970059023	71423.44068	52.95762712
0.147250699	15.71097466	181577.9114	79.89873418
0.141169154	53.43911607	611384.2599	114.7334802
0.178807947	6.761829792	42207.55556	107.6944444
0.282051282	2.722683933	34581.50239	158.4736842
0.443478261	0.623808705	558.4313725	42.62745098
0.321044547	1.726849878	14303.22488	111.6124402
0.094611529	74.13117833	356448.1258	46.15231788
0.232732733	3.529746152	39590.53548	84.44516129
0.227642276	3.603278128	7035.428571	57.29761905
0.082504389	108.7350354	1851557.23	85.57446809
0.099574468	34.09380577	241379.7692	67.60683761
0.119113573	42.9735424	308731.3953	75.51627907
0.369318182	0.67948981	1331.953846	67.84615385
0.176551724	7.006556862	49924.04688	77.7421875
0.056812592	367.5480224	1772436.589	53.21212121
0.258723404	1.743216597	52892.28618	196.6315789
0.24	3.31039973	48715.70313	132.1197917
0.423913043	0.405313582	1408.452991	88.93162393
0.090909091	50.91941835	368370.0615	62.20769231
0.185563998	32.12049619	361273.9449	114.3070866
0.107161391	89.82011078	1032463.516	120.4140436
0.181906144	21.76242587	97718.82181	83.44148936
0.293074324	1.648457952	47341.91354	160.3602305
0.189078498	10.19754254	107602.1119	99.70397112
0.517241379	0.193340826	484.5111111	101.8888889
0.253521127	2.18257283	1153.777778	27.27777778
0.1211995	137.320096	2296893.519	141.0721649

log.sigma.1.0.mm.3D_glszm_SmallAreaEmphasis	log.sigma.1.0.mm.3D_glszm_LowGrayLevelZoneEmphasis	log.sigma.1.0.mm.3D_glszm_ZoneEntropy	log.sigma.1.0.mm.3D_glszm_SmallAreaLowGrayLevelEmphasis
0.753603889	0.03710075	4.796703059	0.031987817
0.608938875	0.146386034	4.064203409	0.09244035
0.717183948	0.047647901	4.896149839	0.037422348
0.641252283	0.051736413	4.150158513	0.045686214
0.678925747	0.043347619	4.757541263	0.032740646
0.785203305	0.039378451	4.558659712	0.035837014
0.697965628	0.017948346	5.321700913	0.013727512
0.680629747	0.029969449	5.19663684	0.023194758
0.695406353	0.022374256	4.775773571	0.016973923
0.715058619	0.036132268	5.273749516	0.030864925
0.715340127	0.003849443	4.968956996	0.003384679
0.719545306	0.016091263	4.995023563	0.012981559
0.740936663	0.027159894	5.054816285	0.022998094
0.576991017	0.03942179	4.790209437	0.031879282
0.705089008	0.032311045	4.680448324	0.025915267
0.668896094	0.020730463	5.162501564	0.014594741
0.629671002	0.064775512	4.577504014	0.0476992
0.552001261	0.077242551	4.494680368	0.067067409
0.677212074	0.101438017	4.748457519	0.067512262
0.705475095	0.009876639	5.450327981	0.007351993
0.696982629	0.05015138	4.494654837	0.037497283
0.609407666	0.105380375	4.195675663	0.081279572
0.654120257	0.054434683	4.543128237	0.041712893
0.68163701	0.041704306	4.808325852	0.032404125
0.624247906	0.054594844	5.042475897	0.035958679
0.690814458	0.042709586	5.081534847	0.029762448
0.693962563	0.022947728	4.899396022	0.01893635
0.645637563	0.023560487	5.039645459	0.017371465
0.676900585	0.106949182	3.681880803	0.093743781
0.706169179	0.060371055	4.724200882	0.046412253
0.669371488	0.050154826	4.981441053	0.038750977
0.679784458	0.09109775	4.493716017	0.075015907
0.705479794	0.035195315	5.183314784	0.027691223
0.716246624	0.033378114	4.848209078	0.025768104
0.706396948	0.027926987	5.142913271	0.021243068
0.736126529	0.023277436	5.120300317	0.018572027
0.740195007	0.03480101	5.279131451	0.029554616
0.659674973	0.010040416	5.421197147	0.007044644
0.680499092	0.038328918	4.183486472	0.034577559
0.713720965	0.002806348	5.280298965	0.002310813
0.681156644	0.03706859	4.834139372	0.028856715
0.754894664	0.026739378	4.958040582	0.021245968
0.647709023	0.031753676	5.212195036	0.021650506
0.706835529	0.066001016	4.840752589	0.058470097
0.66959249	0.107463832	4.527623564	0.085176702
0.696366403	0.027095963	4.925472229	0.020679507
0.699232229	0.050916771	5.106228699	0.040669795
0.470721191	0.12200561	4.513794877	0.063418131
0.733082211	0.073554435	4.180689289	0.061268458
0.636014967	0.024415701	5.10987758	0.019651914
0.69333202	0.033947668	4.456006273	0.027311126
0.746180948	0.022365288	5.256530977	0.017998678
0.753674604	0.013443121	4.826694037	0.010556961
0.64722278	0.057836994	4.868711147	0.045047266
0.641466671	0.045987152	5.145392918	0.036771502
0.675009253	0.109161411	4.672222922	0.093423855
0.743899293	0.037958883	4.764155272	0.031064448
0.695559568	0.022453222	5.024360089	0.017961995
0.648389881	0.032752473	4.663554303	0.027089166
0.723825428	0.009376566	4.6110319	0.007009815
0.732005526	0.021741717	5.161346916	0.018165999
0.747352179	0.014916622	5.439403475	0.01259829
0.731659693	0.008770179	5.801366724	0.007136585
0.735452249	0.003612011	5.597812098	0.002912512
0.562636308	0.101295059	4.328194402	0.080726838
0.7296786	0.013290063	5.813770533	0.010615079
0.672592295	0.049438802	4.894598426	0.043300471
0.753905412	0.025452198	5.346021933	0.020648838
0.723199808	0.029813792	5.022357736	0.022999952
0.714956068	0.027697102	5.199195498	0.020966925
0.671287911	0.075252441	4.461161861	0.06107436
0.680435087	0.021491744	5.309944272	0.016527399
0.673291651	0.046891386	4.84772132	0.037667681
0.670409235	0.026825665	4.769895403	0.021305636
0.734841187	0.019678885	5.325371521	0.015542639
0.72150819	0.028238601	4.802203384	0.022128242
0.722482248	0.016914438	5.629413377	0.0135704
0.728535106	0.108778977	4.375582881	0.098585826
0.754660275	0.02298683	5.035856152	0.01897734
0.716951634	0.043042386	4.203790912	0.03464302
0.753603889	0.03710075	4.796703059	0.031987817
0.636005693	0.07336565	4.886323453	0.05573522
0.710413779	0.023790338	4.812451427	0.019255053
0.714259884	0.029574305	4.360143357	0.024283196
0.744324544	0.031714564	4.813264854	0.026113766
0.667926943	0.06113912	4.652589929	0.049814945
0.692019243	0.033396387	4.964871283	0.026394031
0.682767261	0.04237146	4.867560895	0.031763844
0.691140215	0.012268834	5.781916617	0.009859784
0.7112093	0.018789602	5.136748792	0.015000678
0.698956796	0.060832326	5.181859862	0.046573854
0.620221348	0.029692972	4.617765806	0.021629304
0.761389276	0.018959606	4.996432636	0.015099918
0.715725804	0.01988087	5.260125538	0.015380821
0.710270575	0.02699256	5.122548683	0.019785757
0.73758386	0.015464753	5.411302614	0.012786982
0.748168602	0.032258478	5.089515614	0.025266165
0.699760802	0.057908689	4.889199419	0.047488241
0.561729103	0.121484395	3.413833864	0.086571962
0.701042928	0.015296981	5.631864943	0.011574325

log.sigma.1.0.mm.3D_ngtdm_Coarseness	log.sigma.1.0.mm.3D_ngtdm_Complexity	log.sigma.1.0.mm.3D_ngtdm_Strength	log.sigma.1.0.mm.3D_ngtdm_Busyness	log.sigma.1.0.mm.3D_ngtdm_Contrast
0.009849267	0.264494605	0.991374631	0.461023535	8.19E-05
0.054553468	0.777130338	2.789470826	0.332375219	0.001281964
0.012506627	0.337742479	1.1887764	0.468672194	0.000121939
0.061529052	1.214842138	2.78913879	0.175619993	0.00203123
0.009045539	0.194654833	0.883781986	0.610240701	5.79E-05
0.059218884	2.578027073	4.445830255	0.123953111	0.001948779
0.000303003	0.010657989	0.174179619	8.367430477	2.54E-07
0.001154371	0.045033359	0.412966916	2.414175571	2.40E-06
0.001302052	0.022500841	0.240800196	2.959466993	2.67E-06
0.018521699	1.194487158	2.802044292	0.211591569	0.000271845
0.001044313	0.064057138	1.245013379	0.791037074	7.16E-07
0.001799097	0.056351762	0.728667871	1.297297382	2.55E-06
0.019636819	1.106785895	2.183611855	0.237804954	0.000383762
0.033363739	0.608632149	1.818827829	0.280642878	0.000728303
0.00628535	0.143854108	0.860852886	0.683024333	3.37E-05
0.000580951	0.013709074	0.15700225	5.65045393	1.04E-06
0.015398412	0.147303247	0.80179803	0.612578596	9.09E-05
0.044405846	0.896241208	2.743652736	0.234622762	0.000979957
0.01249826	0.222609649	1.28435851	0.660084909	6.03E-05
0.000509744	0.021576416	0.205292512	4.232164714	8.62E-07
0.004722278	0.046170287	0.316485863	1.685110555	1.50E-05
0.037734686	0.330275588	1.286823832	0.467522222	0.000565662
0.023275779	0.423967912	2.067348333	0.246823594	0.000167501
0.001076997	0.016728116	0.219403691	4.234248563	1.28E-06
0.006574829	0.187371921	0.912304312	0.725709979	5.83E-05
0.003027106	0.123517956	1.008414532	1.257095187	9.26E-06
0.007159523	0.149175437	0.778331821	0.595937769	3.86E-05
0.003529024	0.050506662	0.439610937	1.230273933	9.39E-06
0.143315639	3.960890296	5.681545778	0.138759202	0.012829442
0.005810987	0.059795905	0.380917094	1.393075554	2.43E-05
0.001628982	0.028883121	0.255022645	3.052552356	4.15E-06
0.022204942	0.429697208	1.209643881	0.484308787	0.000327973
0.005004308	0.112150085	0.607941491	0.910093636	2.39E-05
0.001668811	0.040597654	0.380244894	2.207031892	3.85E-06
0.001805385	0.038632634	0.300986074	2.384916006	5.33E-06
0.005494837	0.194839405	0.82010925	0.658382308	4.07E-05
0.015504447	1.221062383	1.973138562	0.349187545	0.000468135
0.000408555	0.02210957	0.416224886	4.182777279	3.87E-07
0.033277721	0.411411226	1.934410582	0.217738584	0.000337256
0.000437273	0.024290798	0.465285978	1.983990408	3.74E-07
0.010767559	0.171507926	0.8535406	0.595512031	7.30E-05
0.000802288	0.029751039	0.362451068	4.055839259	1.01E-06
0.004374882	0.094382256	0.585611823	0.9876234	1.68E-05
0.008956509	0.169909935	0.784963903	0.641889513	6.38E-05
0.005616745	0.030271712	0.231071856	2.118492815	2.40E-05
0.003112982	0.067124379	0.481478856	1.25587969	1.01E-05
0.00075824	0.019520548	0.276702611	5.046994441	1.04E-06
0.030670614	0.191366254	1.005824154	0.61248801	0.000230531
0.058086476	0.77426115	2.235696389	0.211577304	0.001122476
0.023339662	1.967707897	3.792939831	0.237826487	0.00082959
0.002870357	0.040708012	0.474038417	1.565116354	5.48E-06
0.003411731	0.145208389	0.637224651	0.926378662	1.64E-05
0.00049374	0.013177222	0.226409928	5.173582218	3.67E-07
0.00811223	0.081322495	0.514419896	0.970535878	4.19E-05
0.018890819	1.064139791	2.517298661	0.258940639	0.00036006
0.010065997	0.097170733	0.482297005	0.984151292	6.11E-05
0.001049004	0.020224991	0.208967319	4.218465833	1.77E-06
0.003687929	0.092392001	0.621914724	0.979560069	1.62E-05
0.02422967	0.349581363	1.511436349	0.271682142	0.000281736
0.000218068	0.011857099	0.215503569	7.71740254	1.17E-07
0.009402318	0.39014248	1.604889369	0.340399696	5.48E-05
0.004320653	0.317916813	0.983122354	0.521094323	3.54E-05
0.001669326	0.171472453	0.515884955	0.984836489	1.15E-05
0.000726277	0.066915363	0.630008538	1.194262206	1.09E-06
0.020061234	0.157359806	0.841084213	0.529904953	0.000153057
0.003197285	0.331721898	0.619499569	0.831990845	5.41E-05
0.046674917	1.805009399	3.479685819	0.17556461	0.001243334
0.001657921	0.098296183	0.629060119	1.559944832	5.78E-06
0.000543133	0.015053663	0.218839632	6.560127801	5.67E-07
0.004824162	0.148179928	0.616318207	0.902375482	2.77E-05
0.033815127	0.381240859	1.415460046	0.297922438	0.000459085
0.000739958	0.025889795	0.325507053	3.243081267	1.09E-06
0.008386893	0.176934956	0.999468131	0.603990397	4.33E-05
0.00540087	0.172547937	1.494013402	0.671188156	1.98E-05
0.002016525	0.091146496	0.765790077	1.250649235	4.24E-06
0.012264346	0.356018781	1.818597441	0.340989602	5.46E-05
0.004771272	0.355786877	0.856401991	0.658443821	5.67E-05
0.04654331	0.941647717	2.2150319	0.354798533	0.001416884
0.01087129	0.431364934	1.15064059	0.419744979	0.000112346
0.003766893	0.031924071	0.290649201	1.82923622	1.07E-05
0.009849267	0.264494605	0.991374631	0.461023535	8.19E-05
0.017544183	0.395303117	1.326848075	0.518197801	0.000193882
0.001452851	0.032503313	0.364865231	2.226784402	3.65E-06
0.005702737	0.047394113	0.421639415	1.146266471	1.66E-05
0.00358357	0.066645198	0.521800774	1.180946191	9.02E-06
0.033255885	0.706012265	1.459959666	0.391905834	0.000887641
0.009354555	0.222478275	1.274000069	0.514856328	4.34E-05
0.001298152	0.021975089	0.287672356	3.580149887	1.94E-06
0.006369344	0.421246756	1.229839662	0.399389899	6.57E-05
0.008982269	0.270041042	0.963238377	0.449352449	7.46E-05
0.021455012	1.085462864	2.038543933	0.377867681	0.00073674
0.004446802	0.042543951	0.406829429	1.274651918	1.02E-05
0.002027243	0.080485021	0.46065028	1.449915769	6.10E-06
0.001611299	0.065291694	0.642212024	1.614480544	2.84E-06
0.003450396	0.093272023	0.513705924	1.435683053	1.41E-05
0.005366964	0.566559027	1.73970364	0.398763826	4.37E-05
0.004661591	0.184954796	0.874013761	0.723258661	2.08E-05
0.060500041	4.125557517	5.825997978	0.133421373	0.003064178
0.084964655	0.387498318	1.422029541	0.307221177	0.001176584
0.000675455	0.02307035	0.149735221	4.097604541	2.11E-06

log.sigma.0.5.mm.3D_gldm_GrayLevelVariance	log.sigma.0.5.mm.3D_gldm_HighGrayLevelEmphasis	log.sigma.0.5.mm.3D_gldm_GrayLevelNonUniformityNormalized	log.sigma.0.5.mm.3D_gldm_DependenceEntropy
0.471645068	11.05855856	0.411470029	5.277013108
0.353669042	4.283185841	0.552509985	4.671733394
0.382658419	10.01449275	0.468221819	5.112404556
0.422967864	8.782608696	0.437854442	4.53236043
0.343460026	10.2077748	0.503291909	4.969657674
0.759355264	9.242718447	0.318126119	4.582077295
0.284627895	12.16822029	0.487571679	4.79141425
0.344192957	11.19916766	0.476225699	4.915177601
0.295681705	11.5306219	0.490586259	4.733720487
0.673733547	11.20054945	0.39379302	5.055070082
0.312753757	56.77708638	0.4833385	4.983142437
0.310906037	11.64367068	0.485162277	4.839666079
0.724199304	10.33232628	0.34378109	5.189279661
0.460415512	8.931578947	0.410470914	4.801999829
0.361945888	10.90326087	0.46419896	4.892818352
0.306479646	11.78702944	0.476941112	4.766085057
0.284550964	5.440366972	0.517475381	4.941206634
0.341043746	9.558558559	0.543868193	4.51002873
0.313603265	5.578483245	0.51125544	4.85585499
0.337651318	12.11025405	0.465134387	4.894398885
0.237773585	5.294077135	0.569852355	4.853686898
0.340330075	4.189873418	0.49238904	4.717074064
0.355466623	10.09608541	0.498283963	4.784047427
0.266295052	5.998751041	0.504108428	4.73863324
0.419933748	10.99586777	0.426412889	5.202439091
0.361481481	5.805925926	0.4826057	4.99889972
0.33488371	10.79552716	0.486000674	5.100981048
0.290720249	11.23820352	0.506186813	4.956777194
0.452806122	4.892857143	0.400510204	3.869331261
0.270712356	5.559513467	0.53046684	4.792037791
0.306686695	5.753346625	0.48408927	4.933225348
0.296409073	4.003921569	0.548204537	4.719966175
0.361337074	11.3252541	0.470611141	5.198242263
0.336308229	12.05970149	0.461206162	4.842754461
0.316232336	11.38711533	0.487211785	5.091496725
0.459141266	10.43085106	0.42726724	5.293046135
0.915062459	8.484615385	0.295016437	4.976250353
0.324612157	20.16800136	0.468771172	4.875835554
0.252931021	10.03045685	0.597155299	4.547736364
0.309015872	55.51634473	0.485779323	4.793874455
0.30865906	10.41521395	0.520882256	4.979868778
0.303671491	6.19856294	0.488441182	4.890903678
0.308706544	10.50098232	0.523223325	5.243042396
0.357680019	10.59405941	0.480383076	4.824496615
0.257250723	5.703125	0.52381765	4.829965796
0.315749897	11.05268542	0.499162355	4.992869229
0.294854315	6.055818625	0.485684352	4.794885332
0.233356269	5.383561644	0.601467859	4.515678439
0.394044321	4.5	0.485841797	4.802378694
0.929669421	10.42727273	0.335785124	4.830880861
0.274629793	11.38457503	0.510376466	4.811712056
0.467144547	11.46225104	0.447402978	5.32521384
0.272789562	11.98148881	0.497047655	4.702290639
0.333249234	6.375442739	0.459666662	5.094897844
0.691498015	10.26480836	0.352669087	5.066428207
0.258571464	5.04826546	0.563385953	4.824360971
0.284930841	6.080574995	0.491811159	4.843787564
0.348607995	11.02394822	0.47062138	4.982482477
0.362240484	9.808823529	0.479509083	4.912429902
0.286174589	12.23042572	0.486670087	4.59367607
0.446690774	10.804266	0.465983637	5.215234937
0.6903346	19.2345602	0.368877522	5.385435791
0.737678002	19.21150481	0.358841026	5.607085083
0.416958441	41.25985915	0.444437226	5.147110801
0.27899574	5.323987539	0.524111761	4.86895235
0.969952191	18.79496568	0.320118763	5.577620837
0.609985171	9.260504202	0.36572276	4.765159181
0.46782461	11.46778101	0.441037127	5.261713759
0.28449456	12.05357677	0.493381565	4.730805694
0.417697768	11.32023576	0.459765693	5.11435224
0.331012142	4.681818182	0.498673605	4.781426625
0.338515663	11.84054092	0.463482801	4.910031562
0.350690997	10.46461949	0.497302144	4.907427935
0.360261923	11.2693383	0.473375551	4.89702539
0.378762283	11.72325871	0.462047466	5.149523007
0.365433534	11.30794702	0.509495198	5.037887102
0.725714876	12.04925776	0.371407861	5.545424997
0.419962193	3.947826087	0.439395085	4.734585154
0.491777981	10.86789555	0.432511957	5.175023838
0.267713457	5.682957393	0.51570813	4.671491578
0.471645068	11.05855856	0.411470029	5.277013108
0.396559955	5.346883469	0.46911377	4.942405669
0.323321171	11.59625512	0.475664066	4.857826555
0.244345858	10.98042553	0.557740878	4.850228822
0.289861496	11.07590028	0.517027033	5.036267036
0.439533833	4.835227273	0.426717459	5.015901127
0.353225208	11.03034483	0.477209037	5.208045478
0.266117001	5.832513527	0.512039058	4.707536527
0.748084382	19.76510638	0.362230874	5.516548434
0.442275	10.9075	0.45445625	5.119245464
0.745155955	10.75	0.34089477	5.182778067
0.244679446	10.97832168	0.554669666	4.870430003
0.383030643	11.67475161	0.458798417	5.193216081
0.346284156	11.80487805	0.476018015	4.982330059
0.347196213	5.780358007	0.493029941	5.099759786
0.67649459	17.65033784	0.37567482	5.367761514
0.391918834	10.84709898	0.487217789	5.088269243
1.033954287	9.896551724	0.292641036	4.680871945
0.178139258	4.464788732	0.686570125	4.327799987
0.387640647	11.87369846	0.448013839	5.041953005

log.sigma.0.5.mm.3D_gldm_DependenceNonUniformity	log.sigma.0.5.mm.3D_gldm_GrayLevelNonUniformity	log.sigma.0.5.mm.3D_gldm_SmallDependenceEmphasis
39.65165165	274.039039	0.061412743
6.061946903	62.43362832	0.091648183
23.77846791	226.1511387	0.049975755
6.826086957	40.2826087	0.06981495
39.01072386	375.4557641	0.059069433
10.84466019	32.76699029	0.175759569
1453.791733	7950.3438	0.017256225
349.5747126	2403.034879	0.030257206
369.2804445	2074.689288	0.021735793
21.58791209	143.3406593	0.091012346
410.3781113	2640.961567	0.022710123
248.8945656	1490.903677	0.024597372
20.27492447	113.7915408	0.096022263
13.89473684	77.98947368	0.055806168
64.34130435	427.0630435	0.035964029
744.0531254	4066.876862	0.017574448
26.11926606	225.6192661	0.030917999
6.891891892	60.36936937	0.082971536
33.25044092	289.8818342	0.043202064
959.8711327	5547.657835	0.025949999
78.91184573	827.4256198	0.036436155
9.215189873	77.79746835	0.055161065
16.43772242	140.0177936	0.058550621
399.9550375	2421.736886	0.017213781
42.79063361	309.5757576	0.033333631
133.4681481	977.2765432	0.030787433
54.26304579	456.3546326	0.046432286
113.6185333	890.3826038	0.027496325
4.785714286	11.21428571	0.086065613
80.3032146	610.5673328	0.030268728
262.4212475	1699.637425	0.024784823
14.68627451	139.7921569	0.072210257
68.51524629	601.9116497	0.048931037
270.340108	1452.338203	0.022238398
224.3465631	1694.035375	0.037559995
61.0177305	481.9574468	0.060771425
34.0974359	115.0564103	0.142190745
1000.064406	5502.43602	0.016586071
11.49746193	117.6395939	0.070880192
1019.652468	5884.730725	0.022868895
32.8637084	328.6767036	0.047481924
542.1333425	3534.848832	0.02584089
71.97969876	798.962017	0.038306221
47.79773692	339.6308345	0.051085753
75.31617647	569.9136029	0.024574686
123.9974425	975.8624041	0.034330358
608.5376668	3384.734252	0.020932139
14.52511416	131.7214612	0.026237547
7.263157895	55.38596491	0.099039129
17.80909091	73.87272727	0.083196845
138.6463799	972.7775446	0.024017551
106.5006948	965.9430292	0.054587822
910.8204316	5021.175411	0.018135569
56.23730815	389.3376623	0.033233478
18.99303136	101.2160279	0.08286822
38.00452489	373.5248869	0.043386926
428.6973709	2600.205599	0.021505512
105.0841424	727.1100324	0.035582677
18.34558824	130.4264706	0.060001391
2439.314379	11443.07375	0.014970881
38.33249686	371.3889586	0.06385845
90.48845914	591.3106675	0.072676223
250.6684164	1640.621172	0.071282935
600.8302817	3786.605164	0.036139055
17.85358255	168.2398754	0.03487816
124.6421053	699.4594966	0.084740806
9.386554622	43.5210084	0.085008415
239.1947674	1820.60126	0.05732348
902.5955303	4945.163424	0.018672617
82.01768173	702.0622135	0.053319902
11.3030303	98.73737374	0.069369588
594.319059	3290.264403	0.021003851
45.93190921	372.4793057	0.045755423
81.30754893	507.9319664	0.03274599
199.8905473	1485.944652	0.037576404
30.87086093	307.7350993	0.052078711
74.84615385	550.4264507	0.067961105
7.817391304	50.53043478	0.093983557
32.79109063	281.5652842	0.083402434
132.1165414	823.0701754	0.023491493
39.65165165	274.039039	0.061412743
18.61517615	173.102981	0.059368964
278.1047396	1625.819778	0.025548342
72.72595745	655.3455319	0.02910136
96.32520776	933.233795	0.033855189
9.931818182	75.10227273	0.07994471
40.74068966	345.9765517	0.051095911
328.4977865	2081.950812	0.019736666
66.01787234	425.6212766	0.060500498
40.81	363.565	0.062825319
21.42753623	94.08695652	0.107923674
85.00699301	793.1776224	0.028573434
192.0263004	1570.008182	0.046035756
278.0389206	1834.57343	0.030179007
111.2380261	1019.092888	0.051713488
65.14189189	444.7989865	0.08590959
76.81843003	713.7740614	0.051801336
8.517241379	25.45977011	0.11813637
4.774647887	48.74647887	0.071244313
686.4454394	4302.724906	0.031922628

log.sigma.0.5.mm.3D_gldm_DependenceNonUniformityNormalized	log.sigma.0.5.mm.3D_gldm_DependenceVariance	log.sigma.0.5.mm.3D_gldm_LargeDependenceEmphasis
0.059537014	22.66642318	113.5165165
0.053645548	46.0731459	169.8141593
0.049230782	34.14923121	161.0496894
0.074196597	24.19281664	92.43478261
0.052293196	33.68721115	180.077748
0.105287963	10.42039778	37.60194175
0.089156858	12.77743884	177.3045505
0.069277589	18.49496907	161.3083631
0.087320985	13.59152215	164.9763537
0.059307451	27.75483033	103.4065934
0.075105804	15.00514803	148.9612006
0.080994001	15.53073794	164.755288
0.061253548	29.37349969	100.2688822
0.073130194	15.94060942	86.14736842
0.0699362	17.69809074	136.223913
0.087258488	13.29037155	163.8453149
0.059906574	23.27579749	161.2844037
0.062089116	32.15648081	136.7117117
0.058642753	23.00768611	146.7389771
0.080478841	15.50932461	166.7228138
0.054347001	31.02095144	225.2892562
0.058323987	32.59477648	132.8481013
0.058497233	25.77019035	141.8113879
0.083254587	13.5303494	170.7497918
0.058940267	21.30204373	128.2506887
0.065910197	20.21360988	166.2474074
0.057788121	24.4107762	148.6656017
0.064592685	20.578204	171.9982945
0.170918367	5.418367347	27.64285714
0.069768214	19.1066447	175.3892268
0.074742594	17.16266402	175.6385645
0.057593233	34.67767782	146.2078431
0.053569387	26.11021487	143.0172009
0.08584951	12.78107473	147.4191807
0.064523026	21.12065855	161.7241875
0.054093733	26.08502779	136.5212766
0.087429323	12.38332676	46.44102564
0.085198876	12.91882944	164.2100869
0.058362751	26.0761679	148.6446701
0.084171411	14.08108384	163.2862803
0.052081947	30.87835323	167.4088748
0.074911337	15.54285824	154.8167749
0.047137982	34.67697833	190.8860511
0.067606417	20.20881506	137.5035361
0.069224427	18.34574705	167.7334559
0.063425802	22.71280538	174.3616368
0.087320658	13.7451185	172.7930837
0.066324722	18.88663706	157.1369863
0.063711911	36.11511234	121.5964912
0.080950413	14.64247934	64.40909091
0.072742067	15.62160252	152.043022
0.049328715	32.05854228	158.0199166
0.090162387	12.77295921	175.581667
0.066395877	18.10059255	132.2396694
0.06617781	19.17583071	84.35191638
0.057322059	29.44769536	196.9607843
0.081085185	14.40983918	160.8944581
0.068015626	19.88778668	166.2116505
0.067447016	22.03498054	125.1470588
0.103743222	9.89823116	167.14341
0.048095981	38.09605028	166.4930991
0.056449444	26.59697409	126.4847162
0.054826863	27.47120783	111.7156605
0.070519986	20.00666446	157.5786385
0.055618637	25.66450248	154.3208723
0.057044442	31.01362127	109.3340961
0.07887861	17.99887014	71.57142857
0.057944469	26.11862244	152.2781008
0.090052432	13.14942584	182.6218697
0.053711645	31.35774097	165.5199738
0.057086012	28.52025304	129.9393939
0.083718701	14.8487983	173.7494013
0.061324311	23.8933157	164.105474
0.075775908	16.0751323	135.5219012
0.062155021	20.69931901	142.988806
0.051110697	32.38720889	169.5562914
0.050503478	31.66769202	133.511471
0.067977316	29.71236295	93.29565217
0.050370339	36.77557155	137.9155146
0.082779788	14.39918719	162.8847118
0.059537014	22.66642318	113.5165165
0.050447632	33.85979833	147.9268293
0.081364757	14.46419448	148.2867174
0.061894432	21.78257963	179.3438298
0.053365766	28.22348816	181.6127424
0.056430785	44.02259814	139.3068182
0.056194055	26.48044899	144.7048276
0.080791389	15.21956314	182.9813084
0.056185423	23.73615862	115.0646809
0.0510125	37.359775	169.265
0.077636001	22.46676118	72.38405797
0.05944545	22.62501638	177.6013986
0.056115225	24.71323963	149.993571
0.072142948	19.07561355	173.1987545
0.053816171	25.70459702	141.8998549
0.05501849	29.54042355	118.6706081
0.052435788	30.8728097	169.887372
0.097899326	16.15008588	53.96551724
0.067248562	35.42511406	163.3380282
0.071474952	19.02528153	163.9162849

log.sigma.0.5.mm.3D_gldm_LargeDependenceLowGrayLevelEmphasis	log.sigma.0.5.mm.3D_gldm_SmallDependenceHighGrayLevelEmphasis	log.sigma.0.5.mm.3D_gldm_LargeDependenceHighGrayLevelEmphasis
10.77500459	0.553202719	1304.86036
43.60581121	0.764547501	678.2477876
17.58510582	0.566119467	1531.910973
10.60190217	0.585362778	834.7391304
19.33087169	0.637225155	1732.258713
5.038247033	2.132672044	320.2912621
16.24302711	0.231105432	2095.437201
16.0536393	0.328806828	1730.670036
15.76650292	0.210176504	1857.990778
11.43691468	1.398574742	974.2774725
2.686758163	1.334876722	8408.210835
15.8482913	0.273573493	1841.863651
11.60887966	1.248299315	919.776435
9.786915205	0.304836889	823.7105263
13.61059058	0.334594064	1459.311957
15.45706019	0.216673321	1877.90618
36.26587984	0.153416081	795.4311927
15.20375125	0.827532115	1242.693694
33.98963551	0.335337605	691.0141093
15.56696967	0.385318071	1931.256561
53.15915787	0.21297848	1018.294077
34.0221519	0.229349719	552.9620253
15.47478055	0.714047551	1343.434164
35.40319121	0.113015715	947.4796003
12.99363445	0.404626027	1369.488981
36.20391193	0.221223037	866.2
15.09786593	0.442544314	1552.571885
17.27191333	0.292150768	1816.428653
7.979166667	0.473677136	124.3928571
39.20190776	0.194923107	872.3536056
36.68009866	0.137287886	974.7974936
37.89033224	0.301897983	589.2823529
14.61625228	0.639347	1480.163409
13.25589358	0.258824483	1785.58336
15.89826015	0.432445802	1759.114466
14.6913389	0.772390889	1358.01773
7.420369658	1.597523763	354.5564103
8.518341452	0.353285126	3331.70506
16.17033277	0.437800449	1389.492386
3.063126353	1.372875785	8846.894585
17.87786714	0.488916467	1619.812995
31.72201776	0.246847952	872.6371425
20.34611348	0.419730607	1858.899149
14.07683915	0.412313181	1418.659123
36.62684462	0.131020155	863.2527574
17.67420574	0.379166441	1820.743734
34.65469827	0.142389175	1003.53494
37.0323757	0.22278267	709.9406393
31.66008772	0.499604983	489.1491228
8.10622096	1.194840791	586.1454545
15.08657427	0.237734858	1631.707765
16.4668769	0.780034031	1595.590088
16.54225133	0.251835017	2008.371115
24.62745966	0.245682949	837.3175915
9.151875726	1.166826151	851.0034843
46.74003896	0.211616559	884.1553544
32.55198222	0.15537037	922.4978249
16.55006778	0.373130219	1786.810356
13.47832414	0.498982544	1224.911765
15.04030486	0.198505709	2014.543104
17.98908841	0.845311859	1591.757842
7.285159962	1.817988752	2302.166563
6.663505334	1.636979398	1963.698819
3.973422617	1.612768827	6393.333099
36.42281066	0.175710369	705.1931464
6.809613523	2.098327038	1833.406407
8.581339869	0.95340205	644.8907563
15.28700263	0.891259572	1620.0797
17.12606213	0.276699436	2100.311384
17.28926963	0.788680487	1659.870334
32.24116162	0.362174793	555.0757576
15.90231351	0.237273476	2063.670658
17.12899199	0.466820898	1648.94526
13.19656091	0.395750967	1496.296365
14.07372312	0.548963414	1556.886816
17.97039597	0.791629331	1655.549669
13.93940185	1.110225041	1372.697031
27.09233092	0.643695603	362.573913
14.97255718	1.196306688	1310.963134
34.84132641	0.099310932	865.7280702
10.77500459	0.553202719	1304.86036
35.95831451	0.432412278	645.7425474
14.0323516	0.234315619	1692.318315
18.56163168	0.290943881	1811.640851
18.80560265	0.397416613	1835.338504
37.30034722	0.544698936	562.6420455
14.69559195	0.647555358	1511.902069
39.08179066	0.119748908	973.8885883
6.709859842	1.478188541	2077.802553
18.16311389	0.930518738	1633.9725
8.279697534	1.604760989	682.7717391
18.47879157	0.296408182	1780.162937
14.96867256	0.643155697	1601.04062
16.84914813	0.461015262	1909.246497
32.3624703	0.44707079	684.6250605
7.287896486	1.894912909	2004.548986
17.96825256	0.681576411	1671.787713
6.672844828	1.699917255	487.6206897
40.88967136	0.425235468	657.4507042
15.80227738	0.45975537	1835.068617

log.sigma.0.5.mm.3D_gldm_SmallDependenceLowGrayLevelEmphasis	log.sigma.0.5.mm.3D_gldm_LowGrayLevelEmphasis	log.sigma.0.5.mm.3D_gldzm_DistanceZoneVariabilityNormalized
0.016682003	0.117388639	1
0.02973713	0.351632252	1
0.008694063	0.11984242	1
0.019879744	0.146588164	1
0.010134661	0.114495457	1
0.051335617	0.172281553	1
0.002229991	0.090769323	0.976051423
0.005791891	0.10330691	0.89716199
0.00367118	0.097322916	1
0.016721767	0.119117063	0.939453125
0.000628428	0.018187244	0.750385802
0.004096641	0.097500362	1
0.023441794	0.138651393	1
0.019654158	0.146125731	1
0.007709707	0.107918478	1
0.002189227	0.0952137	1
0.016921751	0.237847222	1
0.020064593	0.1272998	1
0.01735079	0.233046247	0.900277008
0.003401571	0.093467349	0.851811601
0.01648003	0.230324074	0.957466919
0.03222871	0.371308017	1
0.01018655	0.1178707	1
0.006343363	0.20212034	1
0.005213073	0.109401974	1
0.015752575	0.231103704	0.570665717
0.008494104	0.107021654	1
0.00456821	0.099672162	1
0.03098372	0.344246032	1
0.013828944	0.223784873	1
0.012083706	0.228359679	1
0.046965085	0.37333878	1
0.007346529	0.102329945	0.9608
0.00346014	0.094465968	1
0.005533797	0.099160994	1
0.011320171	0.120374557	1
0.048286258	0.217087607	1
0.001149271	0.053185872	0.835061728
0.018518692	0.114001692	1
0.000506218	0.018488182	0.968594387
0.0083895	0.110324441	1
0.007335836	0.198722575	0.821270408
0.006557527	0.109781161	1
0.012464969	0.112292551	1
0.011632398	0.213854422	0.895061728
0.005896609	0.103913896	1
0.008751967	0.208073532	1
0.007329261	0.216609589	1
0.053249112	0.349780702	1
0.012388691	0.138309343	0.745
0.00398911	0.09749009	1
0.007806802	0.105607792	0.92866941
0.002034522	0.091688952	1
0.011674058	0.207431457	1
0.022481815	0.141552458	1
0.024196699	0.255289509	1
0.007425471	0.203333543	1
0.006581997	0.10535365	1
0.012927895	0.122727737	1
0.00207898	0.090485419	0.967455621
0.011362361	0.112336703	1
0.006500009	0.061941761	0.9802
0.006449417	0.0629994	0.942496571
0.001165763	0.025418721	0.974899454
0.017370215	0.244375216	1
0.007145072	0.06719632	0.851800554
0.024480894	0.151451914	1
0.009086173	0.105620468	0.943971893
0.002081355	0.091278184	1
0.007048315	0.103628575	0.972978045
0.033871125	0.31523569	1
0.00403611	0.097455236	0.978725864
0.010512881	0.113081887	1
0.005611414	0.102493391	1
0.00483036	0.098192539	0.97727573
0.00663119	0.101357156	1
0.011709728	0.110139815	0.6228125
0.035209598	0.432427536	1
0.012678276	0.113596604	0.9608
0.013758086	0.218797863	1
0.016682003	0.117388639	1
0.024718417	0.273957392	1
0.0056427	0.099331318	1
0.0049303	0.099997163	1
0.005011757	0.101129578	1
0.026755451	0.338304924	0.875555556
0.007601654	0.105189655	1
0.008310162	0.209769293	1
0.005238097	0.060094049	0.545123967
0.008711868	0.110572917	1
0.019624426	0.133135537	1
0.004593515	0.100023116	1
0.006715507	0.099627817	0.941230487
0.004660806	0.096633652	0.957010023
0.019176112	0.227673494	1
0.008038115	0.067883817	0.97727573
0.007983344	0.110200986	0.971020761
0.032029558	0.183074713	1
0.029042821	0.274647887	1
0.005251494	0.098557343	0.891420835

log.sigma.0.5.mm.3D_gldzm_LowIntensityEmphasis	log.sigma.0.5.mm.3D_gldzm_LargeDistanceEmphasis	log.sigma.0.5.mm.3D_gldzm_HighIntensitySmallDistanceEmphasis
0.333973064	1	7.606060606
0.38982906	1	7.384615385
0.180959596	1	11.31818182
0.283730159	1	7.714285714
0.194371693	1	9.619047619
0.293317901	1	12.05555556
0.1692651	1.036363636	13.70454545
0.237876984	1.3125	8.918216766
0.231363248	1	7.215384615
0.193654514	1.09375	15.3828125
0.034621679	1.791666667	58.17399691
0.235597484	1	9.20754717
0.239833333	1	14.5
0.367361111	1	5.1
0.261914683	1	7.392857143
0.154747863	1	12.93846154
0.752480159	1	3.428571429
0.233861111	1	9.9
0.476154971	1.157894737	7.421052632
0.157106278	1.325991189	14.10841899
0.537137681	1.065217391	5.35326087
0.494444444	1	4.8
0.191631944	1	11.25
0.616975309	1	5.822222222
0.177100694	1	13.125
0.702573375	2.754716981	2.91889413
0.225269097	1	7.8125
0.212192192	1	8.756756757
0.368055556	1	5.75
0.599279835	1	5.925925926
0.65338694	1	4.859649123
0.670428241	1	4.083333333
0.176272222	1.06	12.485
0.189292929	1	12.13636364
0.172097612	1	10.71962617
0.213079502	1	12.77586207
0.358115079	1	11.85714286
0.083317257	1.377777778	20.92469136
0.28258547	1	5.076923077
0.024736103	1.04787234	59.86968085
0.209950397	1	8.571428571
0.380171635	1.37398374	10.04719964
0.206168981	1	10.16666667
0.2653003	1	6.972972973
0.708719136	1.444444444	3.728395062
0.222106061	1	9.127272727
0.591672167	1	6.653465347
0.297222222	1	9.2
0.531944444	1	5.4
0.135	1.45	13.5625
0.230727969	1	7.172413793
0.153847737	1.111111111	14.98842593
0.152471038	1	13.92079208
0.531415344	1	7.047619048
0.330614035	1	14.73684211
0.707047325	1	3.740740741
0.51727037	1	7.266666667
0.217184343	1	9.431818182
0.224151235	1	7.722222222
0.205656576	1.076923077	12.89575702
0.190786848	1	13.53333333
0.092036749	1.08	27.55
0.101379731	1.088888889	23.19259259
0.035311887	1.038135593	44.81991525
0.684027778	1	3.375
0.090012774	1.368421053	24.84046053
0.269111111	1	12.9
0.174350505	1.086538462	15.59014423
0.13514119	1	16.04273504
0.145547945	1.04109589	15.01369863
0.56547619	1	4.642857143
0.262368578	1.032258065	10.46505376
0.253261649	1	9.483870968
0.20553045	1	11.57142857
0.151655394	1.034482759	15.6091954
0.143305556	1	15.4
0.192571074	1.9	14.14444444
0.352430556	1	6.75
0.162977778	1.06	14.145
0.838734568	1	2.555555556
0.333973064	1	7.606060606
0.517939815	1	6.666666667
0.312095104	1	6.830508475
0.219837963	1	7.958333333
0.198622222	1	9.32
0.422685185	1.2	5.683333333
0.177962963	1	11.83333333
0.618153292	1	5.259259259
0.093866986	2.2	18.25151515
0.143743961	1	15.43478261
0.198205215	1	16.64
0.21210728	1	8.103448276
0.172436869	1.090909091	13.64204545
0.178725487	1.065934066	15.81593407
0.468082437	1	8.774193548
0.098486527	1.034482759	23.04885057
0.188590686	1.044117647	13
0.292884615	1	14.15384615
0.430555556	1	5.5
0.188628093	1.288209607	14.83324236

log.sigma.0.5.mm.3D_gldzm_LowIntensityLargeDistanceEmphasis	log.sigma.0.5.mm.3D_gldzm_HighIntensityEmphasis	log.sigma.0.5.mm.3D_gldzm_DistanceZoneVariability	log.sigma.0.5.mm.3D_gldzm_ZonePercentage
0.333973064	7.606060606	33	0.04954955
0.38982906	7.384615385	13	0.115044248
0.180959596	11.31818182	22	0.045548654
0.283730159	7.714285714	7	0.076086957
0.194371693	9.619047619	42	0.056300268
0.293317901	12.05555556	18	0.174757282
0.171128736	13.89090909	161.0484848	0.010118975
0.249722222	10.14285714	100.4821429	0.022195799
0.231363248	7.215384615	65	0.015370064
0.197404514	15.96875	30.0625	0.087912088
0.062246422	62.06944444	54.02777778	0.01317716
0.235597484	9.20754717	53	0.01724699
0.239833333	14.5	30	0.090634441
0.367361111	5.1	10	0.052631579
0.261914683	7.392857143	28	0.030434783
0.154747863	12.93846154	65	0.007622845
0.752480159	3.428571429	14	0.032110092
0.233861111	9.9	10	0.09009009
0.486023392	8.052631579	17.10526316	0.0335097
0.17814978	15.57709251	193.3612335	0.019032447
0.602355072	5.369565217	44.04347826	0.031680441
0.494444444	4.8	10	0.063291139
0.191631944	11.25	16	0.056939502
0.616975309	5.822222222	45	0.009367194
0.177100694	13.125	16	0.022038567
1.086844864	6.622641509	30.24528302	0.02617284
0.225269097	7.8125	32	0.034078807
0.212192192	8.756756757	37	0.021034679
0.368055556	5.75	4	0.142857143
0.599279835	5.925925926	27	0.023457863
0.65338694	4.859649123	57	0.016234691
0.670428241	4.083333333	24	0.094117647
0.178672222	12.86	48.04	0.039093041
0.189292929	12.13636364	44	0.01397269
0.172097612	10.71962617	107	0.030773655
0.213079502	12.77586207	58	0.05141844
0.358115079	11.85714286	56	0.143589744
0.096040997	24.25555556	75.15555556	0.007667405
0.28258547	5.076923077	13	0.065989848
0.025224562	61.07446809	182.0957447	0.015519234
0.209950397	8.571428571	28	0.04437401
0.470198735	11.21138211	101.0162602	0.016995993
0.206168981	10.16666667	48	0.031434185
0.2653003	6.972972973	37	0.052333805
1.15316358	3.777777778	16.11111111	0.016544118
0.222106061	9.127272727	55	0.028132992
0.591672167	6.653465347	101	0.014492754
0.297222222	9.2	5	0.02283105
0.531944444	5.4	15	0.131578947
0.182666667	16	14.9	0.090909091
0.230727969	7.172413793	29	0.01521511
0.157952675	15.75925926	100.2962963	0.050023159
0.152471038	13.92079208	101	0.00999802
0.531415344	7.047619048	21	0.024793388
0.330614035	14.73684211	19	0.066202091
0.707047325	3.740740741	27	0.040723982
0.51727037	7.266666667	75	0.014185739
0.217184343	9.431818182	44	0.028478964
0.224151235	7.722222222	18	0.066176471
0.208331714	13.38461538	176.0769231	0.007740399
0.190786848	13.53333333	45	0.056461731
0.100925638	27.63	98.02	0.062383032
0.107781471	23.99259259	254.4740741	0.059055118
0.036175922	45.36016949	230.0762712	0.027699531
0.684027778	3.375	8	0.024922118
0.101018711	27.35526316	129.4736842	0.069565217
0.269111111	12.9	10	0.084033613
0.180205751	16.22115385	196.3461538	0.050387597
0.13514119	16.04273504	117	0.011673152
0.155821918	15.05479452	71.02739726	0.047806156
0.56547619	4.642857143	14	0.070707071
0.263658901	10.66666667	91.02150538	0.013100437
0.253261649	9.483870968	31	0.041388518
0.20553045	11.57142857	28	0.026095061
0.160276084	15.64367816	85.02298851	0.027052239
0.143305556	15.4	30	0.049668874
0.471848852	18.0125	49.825	0.053981107
0.352430556	6.75	12	0.104347826
0.165377778	14.52	48.04	0.076804916
0.838734568	2.555555556	27	0.016917293
0.333973064	7.606060606	33	0.04954955
0.517939815	6.666666667	24	0.06504065
0.312095104	6.830508475	59	0.017261556
0.219837963	7.958333333	24	0.020425532
0.198622222	9.32	50	0.027700831
0.444907407	6.133333333	13.13333333	0.085227273
0.177962963	11.83333333	30	0.04137931
0.618153292	5.259259259	54	0.013280866
0.263103999	27.05454545	29.98181818	0.046808511
0.143743961	15.43478261	46	0.0575
0.198205215	16.64	25	0.09057971
0.21210728	8.103448276	29	0.02027972
0.18084596	14.09090909	124.2424242	0.038573933
0.180959919	16.31868132	87.08791209	0.023611832
0.468082437	8.774193548	93	0.044992743
0.099190256	23.47126437	85.02298851	0.07347973
0.199620098	13.04411765	66.02941176	0.046416382
0.292884615	14.15384615	13	0.149425287
0.430555556	5.5	6	0.084507042
0.212674672	15.81659389	204.1353712	0.023844232

log.sigma.0.5.mm.3D_gldzm_IntensityVariabilityNormalized	log.sigma.0.5.mm.3D_gldzm_LowIntensitySmallDistanceEmphasis	log.sigma.0.5.mm.3D_gldzm_IntensityVariability
0.360881543	0.333973064	11.90909091
0.396449704	0.38982906	5.153846154
0.32231405	0.180959596	7.090909091
0.306122449	0.283730159	2.142857143
0.421768707	0.194371693	17.71428571
0.25308642	0.293317901	4.555555556
0.323195592	0.168799191	53.32727273
0.422353316	0.236316964	47.30357143
0.589585799	0.231363248	38.32307692
0.240234375	0.192717014	7.6875
0.242283951	0.030275186	17.44444444
0.469562122	0.235597484	24.88679245
0.233333333	0.239833333	7
0.42	0.367361111	4.2
0.538265306	0.261914683	15.07142857
0.338224852	0.154747863	21.98461538
0.540816327	0.752480159	7.571428571
0.28	0.233861111	2.8
0.296398892	0.473687865	5.631578947
0.365405888	0.152628564	82.94713656
0.439508507	0.520833333	20.2173913
0.36	0.494444444	3.6
0.3046875	0.191631944	4.875
0.415308642	0.616975309	18.68888889
0.265625	0.177100694	4.25
0.489498042	0.635804107	25.94339623
0.54296875	0.225269097	17.375
0.495982469	0.212192192	18.35135135
0.375	0.368055556	1.5
0.393689986	0.599279835	10.62962963
0.452754694	0.65338694	25.80701754
0.479166667	0.670428241	11.5
0.3304	0.175672222	16.52
0.326446281	0.189292929	14.36363636
0.363088479	0.172097612	38.85046729
0.272889417	0.213079502	15.82758621
0.260204082	0.358115079	14.57142857
0.286666667	0.080522125	25.8
0.609467456	0.28258547	7.923076923
0.301889995	0.024613988	56.75531915
0.43877551	0.209950397	12.28571429
0.314032653	0.358794038	38.62601626
0.361979167	0.206168981	17.375
0.493060628	0.2653003	18.24324324
0.5	0.65933642	9
0.392396694	0.222106061	21.58181818
0.393196745	0.591672167	39.71287129
0.28	0.297222222	1.4
0.386666667	0.531944444	5.8
0.26	0.123083333	5.2
0.550535077	0.230727969	15.96551724
0.28377915	0.152821502	30.64814815
0.363199686	0.152471038	36.68316832
0.35600907	0.531415344	7.476190476
0.218836565	0.330614035	4.157894737
0.514403292	0.707047325	13.88888889
0.329777778	0.51727037	24.73333333
0.430785124	0.217184343	18.95454545
0.401234568	0.224151235	7.222222222
0.369037556	0.205231992	67.16483516
0.259259259	0.190786848	11.66666667
0.2192	0.091049095	21.92
0.200246914	0.099779295	54.06666667
0.263537777	0.035095879	62.19491525
0.46875	0.684027778	3.75
0.199013158	0.088073505	30.25
0.26	0.269111111	2.6
0.238350592	0.172886694	49.57692308
0.315070495	0.13514119	36.86324786
0.321448677	0.142979452	23.46575342
0.43877551	0.56547619	6.142857143
0.263729911	0.262045998	24.52688172
0.365244537	0.253261649	11.32258065
0.405612245	0.20553045	11.35714286
0.287620558	0.149500222	25.02298851
0.257777778	0.143305556	7.733333333
0.2159375	0.140498543	17.275
0.347222222	0.352430556	4.166666667
0.3168	0.162377778	15.84
0.679012346	0.838734568	18.33333333
0.360881543	0.333973064	11.90909091
0.322916667	0.517939815	7.75
0.473139902	0.312095104	27.91525424
0.458333333	0.219837963	11
0.4616	0.198622222	23.08
0.351111111	0.41712963	5.266666667
0.32	0.177962963	9.6
0.426611797	0.618153292	23.03703704
0.176859504	0.062071029	9.727272727
0.256143667	0.143743961	11.7826087
0.248	0.198205215	6.2
0.464922711	0.21210728	13.48275862
0.316460055	0.170334596	41.77272727
0.279314093	0.178166878	25.41758242
0.311134235	0.468082437	28.93548387
0.197516184	0.098310594	17.18390805
0.292820069	0.185833333	19.91176471
0.195266272	0.292884615	2.538461538
0.388888889	0.430555556	2.333333333
0.293796838	0.184708879	67.27947598

log.sigma.0.5.mm.3D_gldzm_HighIntensityLargeDistanceEmphasis	log.sigma.0.5.mm.3D_gldzm_SmallDistanceEmphasis	log.sigma.0.5.mm.3D_glcm_SumVariance	log.sigma.0.5.mm.3D_glcm_Homogeneity1
7.606060606	1	22.70421456	0.724793513
7.384615385	1	7.001265103	0.80680012
11.31818182	1	20.7296577	0.756725583
7.714285714	1	18.08722535	0.717650573
9.619047619	1	22.46036136	0.769197544
12.05555556	1	14.29850743	0.635226857
14.63636364	0.990909091	28.77309489	0.748017725
18.54464286	0.956907242	25.06953727	0.746586392
7.215384615	1	26.98960276	0.752587409
18.3125	0.9765625	20.43284947	0.694047113
89.26388889	0.88744213	179.4478751	0.748794349
9.20754717	1	27.02295579	0.752703466
14.5	1	17.10460617	0.675084912
5.1	1	17.81828878	0.71522894
7.392857143	1	24.32211337	0.738885631
12.93846154	1	27.07050851	0.744359322
3.428571429	1	9.515544408	0.774296571
9.9	1	21.63526612	0.783830522
10.57894737	0.960526316	9.381038134	0.754222558
23.40969163	0.938081253	27.22660359	0.738116312
5.434782609	0.983695652	9.653589395	0.799857078
4.8	1	6.431372326	0.770475675
11.25	1	21.69769866	0.757170891
5.822222222	1	11.21832735	0.755351857
13.125	1	23.03581237	0.726169443
42.98113208	0.78341195	9.367078632	0.747657027
7.8125	1	23.94566419	0.757252798
8.756756757	1	26.12922272	0.764319337
5.75	1	7.053600057	0.650307063
5.925925926	1	10.05020289	0.770476597
4.859649123	1	9.906291933	0.758068116
4.083333333	1	6.200522085	0.78225984
14.36	0.985	24.56187216	0.750282101
12.13636364	1	27.66156894	0.737740531
10.71962617	1	25.8093111	0.758184072
12.77586207	1	20.89068979	0.733123831
11.85714286	1	11.76769169	0.628004972
43.9	0.930246914	52.85705252	0.744156965
5.076923077	1	24.31729573	0.797475827
65.89361702	0.988031915	176.2556754	0.743917546
8.571428571	1	23.52750087	0.773463062
17.02439024	0.924570912	11.10256023	0.744062282
10.16666667	1	23.71195936	0.786971735
6.972972973	1	23.46600932	0.747598536
4.222222222	0.950617284	10.52933382	0.767570065
9.127272727	1	25.16548596	0.760324972
6.653465347	1	10.89487725	0.751925325
9.2	1	10.52697904	0.793174548
5.4	1	6.577008092	0.771249237
25.75	0.8875	16.68784515	0.634621463
7.172413793	1	27.02489587	0.757838261
18.84259259	0.972222222	23.42605223	0.734991855
13.92079208	1	28.64881647	0.752423581
7.047619048	1	10.7718481	0.759664179
14.73684211	1	18.12802828	0.671918382
3.740740741	1	8.853454092	0.79290782
7.266666667	1	11.10599473	0.750186761
9.431818182	1	24.29633143	0.750263594
7.722222222	1	21.47816485	0.760504854
15.95054945	0.986874237	29.12248735	0.742718423
13.53333333	1	22.16431736	0.747272921
28.35	0.991111111	43.00205145	0.693130112
27.19259259	0.977777778	41.49308	0.683864162
47.52118644	0.990466102	121.5755251	0.734727777
3.375	1	9.175141643	0.774070841
41.625	0.937134503	38.61351977	0.645368647
12.9	1	16.42496923	0.670981123
18.74519231	0.978365385	23.88028443	0.732754486
16.04273504	1	28.52854949	0.753073855
15.21917808	0.989726027	23.47584269	0.74047324
4.642857143	1	7.4747152	0.760480732
11.47311828	0.991935484	26.83553285	0.749089474
9.483870968	1	23.07873897	0.75937725
11.57142857	1	24.83702372	0.744559791
15.7816092	0.99137931	25.47235045	0.743231814
15.4	1	24.71698596	0.766595982
35.7625	0.816666667	21.19496143	0.683331545
6.75	1	4.739117796	0.738206246
16.02	0.985	21.31546587	0.724023462
2.555555556	1	10.4877817	0.761801298
7.606060606	1	22.70421456	0.724793513
6.666666667	1	7.667016991	0.748832012
6.830508475	1	26.68619111	0.744217132
7.958333333	1	26.64169469	0.785404131
9.32	1	25.38571433	0.770632535
7.933333333	0.95	6.096140165	0.749300107
11.83333333	1	23.97849865	0.760842983
5.259259259	1	10.71366006	0.762198993
66.87272727	0.760606061	42.72710385	0.670930207
15.43478261	1	22.14104593	0.748500437
16.64	1	17.51169044	0.644084003
8.103448276	1	26.67319584	0.78444757
15.88636364	0.977272727	25.45455647	0.737533717
18.32967033	0.983516484	26.40926916	0.748012338
8.774193548	1	9.515770946	0.756630186
25.16091954	0.99137931	39.10288644	0.695280669
13.22058824	0.988970588	22.95618304	0.756442052
14.15384615	1	15.33977382	0.616523692
5.5	1	8.839243485	0.849925033
22.02620087	0.954785298	25.58975602	0.734684754

log.sigma.0.5.mm.3D_glcm_Homogeneity2	log.sigma.0.5.mm.3D_glcm_ClusterShade	log.sigma.0.5.mm.3D_glcm_MaximumProbability	log.sigma.0.5.mm.3D_glcm_Idmn	log.sigma.0.5.mm.3D_glcm_SumVariance2
0.717115162	-0.383351851	0.286520057	0.973225256	0.919997659
0.800409697	0.326673435	0.601690439	0.978886723	0.58714928
0.751001426	-0.042359978	0.440798183	0.977405708	0.736468148
0.707490957	-0.079585062	0.394865529	0.957311548	0.758140506
0.765270104	0.035972923	0.46531976	0.979541894	0.647948709
0.611807508	-0.184647834	0.221219475	0.953001417	1.374715193
0.746689668	0.013923513	0.288096821	0.989336918	0.557990402
0.743369759	-0.034143639	0.351254864	0.984572853	0.678245482
0.751380134	-0.042507214	0.328966778	0.980124731	0.595898499
0.674971104	0.806507347	0.363512372	0.969386192	1.229905327
0.746753077	-0.030483635	0.261728174	0.995282302	0.614029463
0.75118551	-0.038899905	0.330742935	0.985702677	0.600622024
0.65416058	0.315124287	0.317952875	0.969524358	1.248219769
0.705680009	-0.146568073	0.339188212	0.957144056	0.972833365
0.734596555	-0.124527659	0.342069399	0.976987155	0.701897084
0.74281881	-0.089670477	0.301769739	0.979263329	0.612792222
0.772355272	0.081498676	0.423872361	0.971676648	0.584797424
0.776305875	0.03729224	0.549312198	0.977719772	0.628560771
0.750014194	0.156341357	0.411796215	0.97793258	0.617831457
0.734789009	0.013551082	0.28964124	0.990802934	0.647836185
0.799020974	0.160755107	0.508219798	0.975691444	0.500645758
0.765288034	-0.018203772	0.487823031	0.947311296	0.658391154
0.752457758	-0.085336644	0.447028543	0.977812881	0.688816504
0.754549323	0.051229479	0.334604209	0.970462008	0.531187074
0.719935035	-0.211978715	0.316357221	0.974470331	0.811498457
0.742600025	0.412394685	0.361160264	0.982825043	0.77169444
0.754188943	-0.021311644	0.390424354	0.979166716	0.682795536
0.762592754	0.042327676	0.387391669	0.980678296	0.587541594
0.633831868	0.104693722	0.286985894	0.908516927	0.85468014
0.769065158	0.107171994	0.414002187	0.971699663	0.553671495
0.756535798	-0.048439866	0.341344674	0.970120051	0.642329776
0.777362804	0.016476994	0.536141061	0.969806681	0.570551059
0.744984307	0.021380255	0.378435544	0.976965644	0.697057091
0.73565047	-0.155686059	0.263876852	0.984620468	0.636458306
0.755720787	0.012888099	0.360920418	0.985606566	0.63667982
0.725832354	-0.284886199	0.362758697	0.973990833	0.904901574
0.598511685	0.303690715	0.194987476	0.961109346	1.608666093
0.742248808	-0.079295012	0.25626772	0.99309941	0.653788494
0.795067707	0.066646025	0.546030846	0.973833757	0.488537926
0.741752649	0.098782305	0.306500178	0.996643826	0.578570251
0.770513474	0.038542119	0.467314432	0.980601826	0.603061692
0.741339767	0.145679782	0.315136981	0.984452821	0.588429911
0.784259948	0.058961282	0.48227436	0.981786354	0.619938372
0.743648322	-0.066179834	0.383638642	0.977938639	0.699391581
0.766665433	0.086536713	0.390399279	0.97183049	0.532409173
0.75801924	-0.021964463	0.39166042	0.979962123	0.627773749
0.750700347	-0.001495403	0.310280071	0.980041875	0.586420246
0.791097111	0.271489299	0.503003145	0.97377346	0.493041664
0.762469751	0.211595988	0.520681677	0.964405306	0.695544935
0.604958808	3.743982561	0.254020016	0.957752282	2.347722134
0.756938292	0.019513791	0.365148708	0.980765103	0.552775894
0.725368734	0.268873841	0.382674076	0.979674687	0.853195484
0.751530076	0.045211183	0.314361455	0.989695468	0.536197973
0.757384551	-0.10044204	0.278119522	0.96955529	0.694608153
0.654714838	-0.054240577	0.272094567	0.972079657	1.261059414
0.791313956	0.119721698	0.504309667	0.97416483	0.542361236
0.748893863	0.031182836	0.313898054	0.979893036	0.560868803
0.746972781	-0.100019616	0.355974237	0.978511101	0.708157876
0.756727747	-0.14183936	0.423809476	0.968268778	0.749882599
0.741578306	-0.006686312	0.274760063	0.99162843	0.549693044
0.737877192	0.336443879	0.437618606	0.985276557	0.806906861
0.677685315	0.115105551	0.288890654	0.983401199	1.195388821
0.664139647	0.269579102	0.298871304	0.984627815	1.396938767
0.729431944	-0.102901025	0.296080453	0.993286352	0.764859615
0.772172693	0.053554909	0.453956443	0.952331973	0.555151096
0.615455111	1.226016582	0.280341487	0.974240502	1.928338552
0.652292499	0.215666821	0.295141576	0.960835133	1.199541564
0.724967196	0.206143691	0.344604106	0.988355613	0.848475357
0.751745026	0.075842026	0.313404786	0.989529492	0.554278687
0.732638377	0.197779412	0.393626252	0.981435192	0.762264954
0.756913619	-0.029478355	0.464974497	0.94743146	0.617793405
0.747183257	-0.162619815	0.28601639	0.985356391	0.675206665
0.755153771	-0.038040363	0.418624044	0.978445192	0.697071261
0.74158744	-0.057768419	0.332789742	0.98830403	0.71181996
0.73776942	0.19370241	0.339680946	0.986885856	0.739918534
0.760384909	0.468454616	0.457949422	0.983083596	0.711122101
0.660712319	1.20466279	0.315868445	0.975060341	1.571580042
0.730369012	0.038211817	0.423792016	0.961579514	0.729658294
0.711876367	0.230154158	0.403126556	0.978046063	0.866018907
0.761157769	0.044476183	0.368424054	0.971382459	0.550718351
0.717115162	-0.383351851	0.286520057	0.973225256	0.919997659
0.741882605	0.137520194	0.437703431	0.963768827	0.717793233
0.742364514	-0.124814087	0.307857242	0.979011812	0.631338067
0.784628316	0.134175546	0.45528208	0.982933825	0.514639708
0.768336256	0.09046476	0.431161114	0.980769894	0.578205383
0.738074796	0.119267013	0.458576367	0.95957576	0.748738474
0.757501268	-0.016625159	0.392170758	0.985126705	0.705173284
0.761498713	0.063831151	0.364009456	0.9811888	0.545500554
0.648172058	1.180485726	0.275702735	0.978723693	1.710809792
0.740190443	0.167565629	0.430303298	0.981604294	0.773343979
0.616228167	0.601762183	0.267653355	0.971168172	1.504566787
0.783716733	0.125713091	0.456777084	0.982905696	0.504217681
0.732028119	0.084082928	0.342346341	0.98255951	0.698382543
0.744169791	0.116718463	0.336507157	0.987963853	0.665167526
0.751356439	0.233155709	0.404896922	0.977407221	0.659388569
0.679576719	0.376405498	0.331541607	0.986860064	1.233695846
0.750185767	0.128541373	0.432270279	0.983056669	0.735606367
0.581318179	0.86170943	0.243926801	0.951654354	1.820957099
0.848296706	0.049907627	0.685677974	0.967855656	0.340927131
0.729563283	-0.035946341	0.306054395	0.982684086	0.744469579

log.sigma.0.5.mm.3D_glcm_Contrast	log.sigma.0.5.mm.3D_glcm_DifferenceEntropy	log.sigma.0.5.mm.3D_glcm_InverseVariance	log.sigma.0.5.mm.3D_glcm_Entropy	log.sigma.0.5.mm.3D_glcm_Dissimilarity
0.729847052	1.278246791	0.487063684	2.723662802	0.593039683
0.609823923	1.172053539	0.331103389	2.045351688	0.433088068
0.607643622	1.208871992	0.439790077	2.494693442	0.516271561
0.774814258	1.300146665	0.481890219	2.641205538	0.616650782
0.54638836	1.154530108	0.429458734	2.335651876	0.482281221
1.355678612	1.545478081	0.533426446	3.385554159	0.872303943
0.537018688	1.07441947	0.492938982	2.17058107	0.511642338
0.580801889	1.14743629	0.480329988	2.385476519	0.524505081
0.521543574	1.064330248	0.484922655	2.202589229	0.501271139
1.264014121	1.587179835	0.447263944	3.142015251	0.749629651
0.585789123	1.112098438	0.484626766	2.219059661	0.518225295
0.536530166	1.083136308	0.48186469	2.217282793	0.503926045
1.220008121	1.575650878	0.474209483	3.306066384	0.779112532
0.782090346	1.303653078	0.49114154	2.819554802	0.62088171
0.613753466	1.176393975	0.487162853	2.455018847	0.544631319
0.545003358	1.07932639	0.4986546	2.253965424	0.51946921
0.49023283	1.078752416	0.435633807	2.164824964	0.461113351
0.614128535	1.224173814	0.370017915	2.258484363	0.475178298
0.593536754	1.176483995	0.456695798	2.313905531	0.515528865
0.605421752	1.15485588	0.496198241	2.381476203	0.542764959
0.417007919	1.015874868	0.393492502	1.947843802	0.404466363
0.562801477	1.167769672	0.416899063	2.350970301	0.484986856
0.598204839	1.185564816	0.446672724	2.415321969	0.51227121
0.506151496	1.046063216	0.482745282	2.092863391	0.493443044
0.689367688	1.237511662	0.496363103	2.631461257	0.581669556
0.666487298	1.225558679	0.461424981	2.474964754	0.539500498
0.553727846	1.13235041	0.460337571	2.367710413	0.501973069
0.508876441	1.080889846	0.457222403	2.191142533	0.480479685
1.028889779	1.248079907	0.565524913	2.605704202	0.78176185
0.487631714	1.065976378	0.447565397	2.118631094	0.466163355
0.515185502	1.075180639	0.471388152	2.256695152	0.49163792
0.537150535	1.154894139	0.395550891	2.227439854	0.460587083
0.621432598	1.211857123	0.455793197	2.460618078	0.528556124
0.577338442	1.105157623	0.507144959	2.308314097	0.536696622
0.540284805	1.117592507	0.463343438	2.296893885	0.497154207
0.710205939	1.275663651	0.473355103	2.73754455	0.575294437
1.579552388	1.686727115	0.4953169	3.678517655	0.931237159
0.572171492	1.104697448	0.495420953	2.29759166	0.524582941
0.458018441	1.065079812	0.385300598	1.969033055	0.417890229
0.573994026	1.112971983	0.493967263	2.228946564	0.525732272
0.514342242	1.118233779	0.429022404	2.238182078	0.46820125
0.588632978	1.134936566	0.488959194	2.251053028	0.528985861
0.482944398	1.092555585	0.403922986	2.198032858	0.440057487
0.587042477	1.16199969	0.47258512	2.423456078	0.52509321
0.482952519	1.045488044	0.45750973	2.069553238	0.469383032
0.529692857	1.104309702	0.460456125	2.267312749	0.491583409
0.523976678	1.067343898	0.486072286	2.198822856	0.502820386
0.455199655	1.066132579	0.396771724	1.909083255	0.424038092
0.655691724	1.259838885	0.385313706	2.39781808	0.505165703
1.773587833	1.772511638	0.473539886	3.668105062	0.950066839
0.503235338	1.049741382	0.476976734	2.123088396	0.488975403
0.79695584	1.349732947	0.449126658	2.716213626	0.590027564
0.51797783	1.052263726	0.487713396	2.107474147	0.500408201
0.529311637	1.09286375	0.46203444	2.321503634	0.492577689
1.103336893	1.491127924	0.51260554	3.251343537	0.758770813
0.446666811	1.053192479	0.401211331	2.067125002	0.422254542
0.52758233	1.070193276	0.489043122	2.170253914	0.50643686
0.571033098	1.143104693	0.472517781	2.409475639	0.516884215
0.558248762	1.147983369	0.448160789	2.427983741	0.498495215
0.548146919	1.065986985	0.504931093	2.15783857	0.521899721
0.772988008	1.336506399	0.426295047	2.630607559	0.564891228
1.14930328	1.52246181	0.480334568	3.188953581	0.725910558
1.339274946	1.622192849	0.461179579	3.418594252	0.778825369
0.691728596	1.23367477	0.485640838	2.556505799	0.56537108
0.489821297	1.073965281	0.436435857	2.124063091	0.461349062
1.82768183	1.812991943	0.447982529	3.826004654	0.9390865
1.102087181	1.486619134	0.503535605	3.185573451	0.763193699
0.788928036	1.316516994	0.467774633	2.680053108	0.588636387
0.527641632	1.074057621	0.482798405	2.146938574	0.501630707
0.716808431	1.292974132	0.453877266	2.604106977	0.564899747
0.550380803	1.136557797	0.45005574	2.28946766	0.496874103
0.548803385	1.099258749	0.486007841	2.319002218	0.512780125
0.578067366	1.172351492	0.446462712	2.408662736	0.504421609
0.597266493	1.14608868	0.48579455	2.400175643	0.529857767
0.679742868	1.236939354	0.46712154	2.5044079	0.549767489
0.664038719	1.246959122	0.413759828	2.369003625	0.509231543
1.353975837	1.650404735	0.439946082	3.483181889	0.788719061
0.696182425	1.258474656	0.459310705	2.535631685	0.565415384
0.857506866	1.393647569	0.45090479	2.801938218	0.623053467
0.489515223	1.035435409	0.471159389	2.101613886	0.479656255
0.729847052	1.278246791	0.487063684	2.723662802	0.593039683
0.652849923	1.247042084	0.445436272	2.505791105	0.539003979
0.553227457	1.094541902	0.496342704	2.283710924	0.52157832
0.446952145	1.027566266	0.422803395	1.986954094	0.433444831
0.508622339	1.100208082	0.439930252	2.186719634	0.470864784
0.74908243	1.312706336	0.409317862	2.578532326	0.561389078
0.563855353	1.146470047	0.45049025	2.382031454	0.497966483
0.492263547	1.039500681	0.469812798	2.092392751	0.479541228
1.498663973	1.685159903	0.459805141	3.610292319	0.831028485
0.711956747	1.296777753	0.433891818	2.607278172	0.551535942
1.570731197	1.704366209	0.474976013	3.554582796	0.897654451
0.447299213	1.025343926	0.425107164	1.973316723	0.43502198
0.667728994	1.230497769	0.479005884	2.494256801	0.557717639
0.618385228	1.180155478	0.471436699	2.372359326	0.529006416
0.610849004	1.210050094	0.443195151	2.37169991	0.516167963
1.135816171	1.521296997	0.474683683	3.191403638	0.719897917
0.654494528	1.245869102	0.434767632	2.492522054	0.525080873
2.041385658	1.801467499	0.459946724	3.685233597	1.035036001
0.332716466	0.922476265	0.28691978	1.577409554	0.308291567
0.660458626	1.217006619	0.488008416	2.543985097	0.560680069

log.sigma.0.5.mm.3D_glcm_DifferenceVariance	log.sigma.0.5.mm.3D_glcm_Idn	log.sigma.0.5.mm.3D_glcm_Idm	log.sigma.0.5.mm.3D_glcm_Correlation	log.sigma.0.5.mm.3D_glcm_Autocorrelation
0.377222322	0.904309038	0.717115162	0.113812244	10.96394858
0.419129011	0.931557719	0.800409697	-0.013487152	4.067768358
0.339735684	0.916104028	0.751001426	0.095884862	9.867962295
0.391945705	0.881898003	0.707490957	-0.00979006	8.913723164
0.313574941	0.921121416	0.765270104	0.085218278	10.22312588
0.577744341	0.865451958	0.611807508	0.011019593	8.427688142
0.274892939	0.936383378	0.746689668	0.019161852	11.94427147
0.305478666	0.926049855	0.743369759	0.077425555	11.20972054
0.269619347	0.916925842	0.751380134	0.066558088	11.52560564
0.699080321	0.901234947	0.674971104	-0.014195528	10.57137954
0.316146356	0.957197707	0.746753077	0.023921358	56.32825449
0.28226776	0.928552171	0.75118551	0.05653816	11.54387236
0.611723876	0.896137754	0.65416058	0.009921975	9.469688364
0.392522503	0.881060329	0.705680009	0.109613635	9.328062386
0.316216797	0.910851088	0.734596555	0.067136354	11.01177476
0.274619432	0.9140182	0.74281881	0.058269423	11.61703961
0.276593506	0.908747979	0.772355272	0.088054093	5.345192743
0.38592733	0.923987763	0.776305875	0.012110654	9.589407871
0.326884492	0.915862405	0.750014194	0.020035731	5.364341782
0.310535828	0.940364022	0.734789009	0.033881578	11.81911018
0.253313106	0.919524779	0.799020974	0.091176954	5.141099259
0.326305784	0.882644017	0.765288034	0.077759871	4.251927055
0.334710039	0.916594948	0.752457758	0.070901015	10.05495666
0.26235832	0.901729898	0.754549323	0.024086431	5.832341814
0.349788366	0.905551782	0.719935035	0.079891137	10.85337862
0.375050937	0.925005235	0.742600025	0.073246292	5.642131182
0.300152281	0.917542474	0.754188943	0.105226066	10.84182946
0.276753341	0.920583446	0.762592754	0.072124559	11.20377465
0.399923511	0.816915934	0.633831868	-0.102285926	4.146963872
0.270101912	0.90748068	0.769065158	0.063366309	5.470717477
0.272995263	0.902453047	0.756535798	0.109304978	5.655604547
0.322760663	0.910411019	0.777362804	0.032155983	3.989982493
0.341836834	0.91405186	0.744984307	0.056956006	11.0727921
0.288500841	0.924019853	0.73565047	0.048862356	11.87500483
0.292939221	0.929727302	0.755720787	0.081863043	11.30409919
0.378824165	0.907206809	0.725832354	0.118698403	10.33888843
0.698126614	0.877831645	0.598511685	0.012990319	7.689083228
0.296641712	0.947943614	0.742248808	0.066393292	19.94279639
0.281226214	0.917729036	0.795067707	0.032935268	10.13607547
0.297105479	0.962666122	0.741752649	0.004065341	55.33621371
0.2947545	0.923056019	0.770513474	0.078397434	10.41261293
0.307835636	0.925425083	0.741339767	1.37E-05	5.939966856
0.28638111	0.927667678	0.784259948	0.12171763	10.46066223
0.31067833	0.913946609	0.743648322	0.08727704	10.72789407
0.262266481	0.90657571	0.766665433	0.048554893	5.578171904
0.286684184	0.918960021	0.75801924	0.083980874	11.03535484
0.270729033	0.916686485	0.750700347	0.056230699	5.897734242
0.273950898	0.9162311	0.791097111	0.040465513	5.307247339
0.395482394	0.903840898	0.762469751	0.035872428	4.28574617
0.862787377	0.877588079	0.604958808	0.142399383	10.00235475
0.263817608	0.918840001	0.756938292	0.046752676	11.3593408
0.448678979	0.919181028	0.725368734	0.034140799	11.04764502
0.266779896	0.937682391	0.751530076	0.017279771	11.79195353
0.282391996	0.902689579	0.757384551	0.13655937	6.089304456
0.526654626	0.897453501	0.654714838	0.067385733	9.941782755
0.267946511	0.916358992	0.791313956	0.096877027	4.987338826
0.270864641	0.916088715	0.748893863	0.03055362	5.895635003
0.302647291	0.915119096	0.746972781	0.106913523	11.02407054
0.308575087	0.902269146	0.756727747	0.145825514	10.15860376
0.274832649	0.942283449	0.741578306	0.0014626	12.01129366
0.453467592	0.93209933	0.737877192	0.021693726	10.44380031
0.621641034	0.923658985	0.677685315	0.019479977	18.54363415
0.730974408	0.926801612	0.664139647	0.020087271	18.42747494
0.371723822	0.949500948	0.729431944	0.050317218	41.05704894
0.274323598	0.886086346	0.772172693	0.06373067	5.129895665
0.944141886	0.904643274	0.615455111	0.027304702	18.20481466
0.51453012	0.880591066	0.652292499	0.04216635	9.09941979
0.441567375	0.936652153	0.724967196	0.037287753	11.21873321
0.275595539	0.937641427	0.751745026	0.024690397	11.83158808
0.397479887	0.921895182	0.732638377	0.030653459	10.88009027
0.301644388	0.878456809	0.756913619	0.056140464	4.618028094
0.285515463	0.927363022	0.747183257	0.103279366	11.74632602
0.322544139	0.917634185	0.755153771	0.093007439	10.57059416
0.316345981	0.93463968	0.74158744	0.087645412	11.23402766
0.37663452	0.932956938	0.73776942	0.042831993	11.46448251
0.403779227	0.929781425	0.760384909	0.034452895	10.9339747
0.730461445	0.90867083	0.660712319	0.074352739	11.40773575
0.373006375	0.891175188	0.730369012	0.026461078	3.615644939
0.468752828	0.91500973	0.711876367	0.005329218	10.35877722
0.258995196	0.904395811	0.761157769	0.058677226	5.634703235
0.377222322	0.904309038	0.717115162	0.113812244	10.96394858
0.36191811	0.895920889	0.741882605	0.046354032	4.899858
0.280800314	0.913804142	0.742364514	0.066075608	11.55311473
0.258376647	0.928071892	0.784628316	0.070216427	10.99963225
0.286022318	0.92240492	0.768336256	0.063568445	10.91892941
0.428084326	0.893831478	0.738074796	-0.007836959	4.169466253
0.315016228	0.929979985	0.757501268	0.110685938	10.89709991
0.261631353	0.920368474	0.761498713	0.051302055	5.694714947
0.80586239	0.914413746	0.648172058	0.066890004	19.33722765
0.407222557	0.923953444	0.740190443	0.041464635	10.42577049
0.761026747	0.896434836	0.616228167	-0.02172154	9.903877191
0.25685684	0.927782391	0.783716733	0.059692167	10.96276351
0.356419632	0.922193972	0.732028119	0.022648009	11.37803188
0.338124363	0.935028795	0.744169791	0.036653253	11.5520171
0.344013229	0.916148928	0.751356439	0.037856586	5.497329533
0.616875201	0.931496437	0.679576719	0.040275648	17.29923345
0.378278228	0.927168467	0.750185767	0.058588648	10.58766756
0.958316551	0.868443606	0.581318179	-0.057078359	9.032066806
0.235057989	0.924148353	0.848296706	0.010338109	4.250831245
0.345553037	0.92160562	0.729563283	0.059819873	11.58194885

log.sigma.0.5.mm.3D_glcm_SumEntropy	log.sigma.0.5.mm.3D_glcm_AverageIntensity	log.sigma.0.5.mm.3D_glcm_Energy	log.sigma.0.5.mm.3D_glcm_SumSquares	log.sigma.0.5.mm.3D_glcm_ClusterProminence
1.941142253	3.303970849	0.190681577	0.412461178	2.721309454
1.505278699	2.018212607	0.391712048	0.299243301	2.341130576
1.801547865	3.136149398	0.254204233	0.336027943	1.736781485
1.810524406	2.986202084	0.217960166	0.383238691	1.640011109
1.716526564	3.193375621	0.280986962	0.298584267	1.334056931
2.212001962	2.902100822	0.121353425	0.682598451	5.304535623
1.598840874	3.455288344	0.242781055	0.273752272	0.817427511
1.750216547	3.344445804	0.237295799	0.314761843	1.502393475
1.64701716	3.392189927	0.245117184	0.279360518	0.980922455
2.123794064	3.252614747	0.188160467	0.623479862	6.925161317
1.636651104	7.504744185	0.23878882	0.299954646	1.815926189
1.650311528	3.395263751	0.245330486	0.284288048	1.110154557
2.171120288	3.07610741	0.153807429	0.617056972	5.537337144
1.989569333	3.046332099	0.184610277	0.438730928	2.548077099
1.770286815	3.315066267	0.226779575	0.328912638	1.500383298
1.66828396	3.405881482	0.236301151	0.289448895	1.071877501
1.625593454	2.306835264	0.275412761	0.268757564	0.910102078
1.609709131	3.096045282	0.34012178	0.310672327	1.822764894
1.669557229	2.314774821	0.26516282	0.302842053	1.287280095
1.717326377	3.436346186	0.22761499	0.313314484	1.363648494
1.500234619	2.262775493	0.333891929	0.229413419	0.712831484
1.711528946	2.056184235	0.286725957	0.305298158	1.420840054
1.751392018	3.167342897	0.266901328	0.321755336	1.610414037
1.558404643	2.413721554	0.255306774	0.259334643	0.66779347
1.86609654	3.28977158	0.204004783	0.375216536	2.045015861
1.8078687	2.369766568	0.235949395	0.359545435	2.944684986
1.752843775	3.287752543	0.247499763	0.309130846	1.344070807
1.634751654	3.34425899	0.262354937	0.274104509	0.939522288
1.638590312	2.042570245	0.188344318	0.47089248	1.87015824
1.589295384	2.335413176	0.277245663	0.260325802	0.819236463
1.699749027	2.371437361	0.241428408	0.28937882	1.112114796
1.619174752	1.99534175	0.327848672	0.276925398	1.144646079
1.764436986	3.324717089	0.239541609	0.329622422	1.511421918
1.689311296	3.443861694	0.228967491	0.303449187	1.302613948
1.699966584	3.358564955	0.245377577	0.294241156	1.129610957
1.946209527	3.207732817	0.209830333	0.403776878	2.855499676
2.361825385	2.770178908	0.105911289	0.79705462	8.062415899
1.701781296	4.463448186	0.230156901	0.306489996	1.598668314
1.483915435	3.182491786	0.357721563	0.236639092	0.864276078
1.623210796	7.43875418	0.242432527	0.288141069	1.12203191
1.659147941	3.223411455	0.290879742	0.279350984	1.169833341
1.632051296	2.437213297	0.242613432	0.294265722	1.119840815
1.653891034	3.228915806	0.302631625	0.275720693	1.349438285
1.77083037	3.271044181	0.240584316	0.321608515	1.508664626
1.556643856	2.359178737	0.271277131	0.253840423	0.678057532
1.68317213	3.318247863	0.258623662	0.289366652	1.242178817
1.640030194	2.425305412	0.242848316	0.277599231	0.897786289
1.436012651	2.301653966	0.3358222	0.23706033	0.746244647
1.71329268	2.067722335	0.31028365	0.337809165	2.089537811
2.493188225	3.139686756	0.119897501	1.030327492	24.31579362
1.591978695	3.36852209	0.260590865	0.264002808	0.821791016
1.892373562	3.321677087	0.226090536	0.412537831	3.057367275
1.56449116	3.433276752	0.250845543	0.263543951	0.699795543
1.745154569	2.459217219	0.229882627	0.305979947	1.233763569
2.18700172	3.146755901	0.145304011	0.591099077	5.182862521
1.572984485	2.227840962	0.322496058	0.247257012	0.881495156
1.605556154	2.426370625	0.245979476	0.272112783	0.810496294
1.77357593	3.315071848	0.233557682	0.319797744	1.479963326
1.807181736	3.179666471	0.251927726	0.32703284	1.860341558
1.586101207	3.465674979	0.242735023	0.274459991	0.87731093
1.839421233	3.23034458	0.252604154	0.394973717	3.128244768
2.144176648	4.304881631	0.165274671	0.586173025	6.137446024
2.250582098	4.291016524	0.153518287	0.684053428	8.531809108
1.820951373	6.406146884	0.216465099	0.364147053	2.585739251
1.586949544	2.261300292	0.293715419	0.261243098	0.91832953
2.470933942	4.263740722	0.122725442	0.939005096	14.82319697
2.124080247	3.012364079	0.151151189	0.575407186	4.371955473
1.895352545	3.347206614	0.216879957	0.409350848	3.183041409
1.588431816	3.438737839	0.247876942	0.27048008	0.811368295
1.8277748	3.296766422	0.235610326	0.369768346	2.073010705
1.672719408	2.144969173	0.282233027	0.292043552	1.199098911
1.730695805	3.422674405	0.229593465	0.306002512	1.468319643
1.762787646	3.246637754	0.255694961	0.318784657	1.535389923
1.783184162	3.347436652	0.228748577	0.327271613	1.621367047
1.794377283	3.383693472	0.228457939	0.35491535	2.31504613
1.710301532	3.304868601	0.284426077	0.343790205	2.42204862
2.309283446	3.369453316	0.151253131	0.73138897	9.683344308
1.799086005	1.89908343	0.241355247	0.35646018	1.577458267
1.914471447	3.218145443	0.222181282	0.430881443	2.699237475
1.588827478	2.370519729	0.261993021	0.260058393	0.749635719
1.941142253	3.303970849	0.190681577	0.412461178	2.721309454
1.783993259	2.20984935	0.256383424	0.342660789	1.679109632
1.687868247	3.39610813	0.235607001	0.296141381	1.254062239
1.516646214	3.314003506	0.303997274	0.240397963	0.711996382
1.622946937	3.301737517	0.279803259	0.27170693	0.961166135
1.776504463	2.041836513	0.255717979	0.374455226	2.117774639
1.767637013	3.295676273	0.247385839	0.317257159	1.531134034
1.578475332	2.383566287	0.262178704	0.259441025	0.735417144
2.378516407	4.391365083	0.13610525	0.802368441	12.00688096
1.830642757	3.226502501	0.249005268	0.371325181	2.229913261
2.299087888	3.149554293	0.124318492	0.768824496	7.940690204
1.502425018	3.308842374	0.305979496	0.237879223	0.677379022
1.768516696	3.371993176	0.230059981	0.341527884	1.719232136
1.720402805	3.39709939	0.237700177	0.320888189	1.685967727
1.708414133	2.342035923	0.257584286	0.317559393	1.468775211
2.159292711	4.156221309	0.171032369	0.592378004	7.491935034
1.787900035	3.250720911	0.259729261	0.347525224	2.343475982
2.357789346	3.014291882	0.113710971	0.965585689	10.87184188
1.209836668	2.061200196	0.496517355	0.168410899	0.517203543
1.815881993	3.400134732	0.217802271	0.351232051	1.952507053

log.sigma.0.5.mm.3D_glcm_SumAverage	log.sigma.0.5.mm.3D_glcm_Imc2	log.sigma.0.5.mm.3D_glcm_Imc1	log.sigma.0.5.mm.3D_glcm_DifferenceAverage	log.sigma.0.5.mm.3D_glcm_Id
6.607941698	0.240671446	-0.023764802	0.593039683	0.724793513
4.036425213	0.35881457	-0.069564708	0.433088068	0.80680012
6.272298797	0.224637597	-0.022103495	0.516271561	0.756725583
5.972404168	0.278040602	-0.036799157	0.616650782	0.717650573
6.386751243	0.181171633	-0.014802229	0.482281221	0.769197544
5.804201644	0.448148168	-0.069656962	0.872303943	0.635226857
6.910576688	0.133104296	-0.008448159	0.511642338	0.748017725
6.688891609	0.167447965	-0.013171864	0.524505081	0.746586392
6.784379854	0.16472105	-0.013649495	0.501271139	0.752587409
6.505229494	0.325936604	-0.036730484	0.749629651	0.694047113
15.00948837	0.153811427	-0.011281336	0.518225295	0.748794349
6.790527503	0.179618304	-0.015589764	0.503926045	0.752703466
6.15221482	0.299237724	-0.029463633	0.779112532	0.675084912
6.092664197	0.305881762	-0.039689846	0.62088171	0.71522894
6.630132534	0.17736252	-0.014679929	0.544631319	0.738885631
6.811762964	0.176005531	-0.016220954	0.51946921	0.744359322
4.613670529	0.163098177	-0.01464818	0.461113351	0.774296571
6.192090564	0.376251409	-0.070620265	0.475178298	0.783830522
4.629549643	0.183868178	-0.015749287	0.515528865	0.754222558
6.872692372	0.143181163	-0.009094918	0.542764959	0.738116312
4.525550986	0.129193233	-0.009051075	0.404466363	0.799857078
4.112368471	0.241843184	-0.02743555	0.484986856	0.770475675
6.334685794	0.276585544	-0.033970094	0.51227121	0.757170891
4.827443108	0.104806122	-0.006028588	0.493443044	0.755351857
6.57954316	0.246342084	-0.027113693	0.581669556	0.726169443
4.739533135	0.196423013	-0.016358728	0.539500498	0.747657027
6.575505085	0.209186126	-0.020769789	0.501973069	0.757252798
6.688517981	0.161025335	-0.013170739	0.480479685	0.764319337
4.085140489	0.601861883	-0.207774061	0.78176185	0.650307063
4.670826353	0.12610667	-0.008308052	0.466163355	0.770476597
4.742874721	0.17954448	-0.018279816	0.49163792	0.758068116
3.990683499	0.206069149	-0.022412073	0.460587083	0.78225984
6.649434179	0.191721409	-0.015881302	0.528556124	0.750282101
6.887723387	0.208707466	-0.020860002	0.536696622	0.737740531
6.717129909	0.162139424	-0.012043268	0.497154207	0.758184072
6.415465635	0.278937034	-0.030755582	0.575294437	0.733123831
5.540357816	0.319529792	-0.0307315	0.931237159	0.628004972
8.926896372	0.198338132	-0.018328457	0.524582941	0.744156965
6.364983572	0.219672708	-0.02796806	0.417890229	0.797475827
14.87750836	0.140192176	-0.009356582	0.525732272	0.743917546
6.446822911	0.170766935	-0.013966249	0.46820125	0.773463062
4.874426594	0.13485934	-0.009075883	0.528985861	0.744062282
6.457831612	0.229246201	-0.030837727	0.440057487	0.786971735
6.542088361	0.18309921	-0.015044907	0.52509321	0.747598536
4.718357475	0.103327906	-0.005771753	0.469383032	0.767570065
6.636495727	0.181200643	-0.016542173	0.491583409	0.760324972
4.850610825	0.139471252	-0.009354582	0.502820386	0.751925325
4.603307932	0.157720239	-0.015243035	0.424038092	0.793174548
4.135444669	0.28646736	-0.039786566	0.505165703	0.771249237
6.279373512	0.365026403	-0.040409949	0.950066839	0.634621463
6.73704418	0.13818168	-0.010393262	0.488975403	0.757838261
6.643354174	0.218924357	-0.018364286	0.590027564	0.734991855
6.866553505	0.118517498	-0.007121485	0.500408201	0.752423581
4.918434437	0.244373769	-0.030195314	0.492577689	0.759664179
6.293511802	0.318910991	-0.033294165	0.758770813	0.671918382
4.455681924	0.15704502	-0.012780112	0.422254542	0.79290782
4.85274125	0.119832021	-0.007101988	0.50643686	0.750186761
6.630143696	0.199055949	-0.018814721	0.516884215	0.750263594
6.359332942	0.255073444	-0.029937349	0.498495215	0.760504854
6.931349957	0.138360399	-0.009639144	0.521899721	0.742718423
6.460689161	0.260468538	-0.026680023	0.564891228	0.747272921
8.609763263	0.289714522	-0.027754095	0.725910558	0.693130112
8.582033048	0.297505735	-0.027531889	0.778825369	0.683864162
12.81229377	0.219550778	-0.019434837	0.56537108	0.734727777
4.522600585	0.186422137	-0.01944967	0.461349062	0.774070841
8.527481444	0.267786145	-0.01969101	0.9390865	0.645368647
6.024728157	0.384867221	-0.050478676	0.763193699	0.670981123
6.694413228	0.224720597	-0.019635767	0.588636387	0.732754486
6.877475678	0.116930956	-0.006701536	0.501630707	0.753073855
6.593532843	0.16723718	-0.011070821	0.564899747	0.74047324
4.289938347	0.202553648	-0.022833737	0.496874103	0.760480732
6.845348809	0.209944961	-0.019632752	0.512780125	0.749089474
6.493275507	0.221736749	-0.022494138	0.504421609	0.75937725
6.694873303	0.210850373	-0.019238972	0.529857767	0.744559791
6.767386945	0.212655224	-0.019316807	0.549767489	0.743231814
6.609737202	0.250672261	-0.028068015	0.509231543	0.766595982
6.738906632	0.263188511	-0.021272896	0.788719061	0.683331545
3.79816686	0.272198617	-0.032002329	0.565415384	0.738206246
6.436290886	0.206393987	-0.016376817	0.623053467	0.724023462
4.741039459	0.128731692	-0.008435716	0.479656255	0.761801298
6.607941698	0.240671446	-0.023764802	0.593039683	0.724793513
4.419698701	0.182312038	-0.015437284	0.539003979	0.748832012
6.792216259	0.168606511	-0.013230308	0.52157832	0.744217132
6.628007012	0.158011279	-0.014407042	0.433444831	0.785404131
6.603475034	0.151822804	-0.011947311	0.470864784	0.770632535
4.083673026	0.339736884	-0.057194509	0.561389078	0.749300107
6.591352546	0.257177085	-0.029589077	0.497966483	0.760842983
4.767132574	0.141641616	-0.010190622	0.479541228	0.762198993
8.782730165	0.314465534	-0.029125511	0.831028485	0.670930207
6.453005002	0.220969343	-0.019886421	0.551535942	0.748500437
6.299108587	0.330305159	-0.033590252	0.897654451	0.644084003
6.617684748	0.135312026	-0.011823213	0.43502198	0.78444757
6.743986352	0.158347795	-0.010291943	0.557717639	0.737533717
6.79419878	0.184407556	-0.014786781	0.529006416	0.748012338
4.684071846	0.131808828	-0.008122509	0.516167963	0.756630186
8.312442617	0.283686755	-0.026738274	0.719897917	0.695280669
6.501441823	0.215099919	-0.019296751	0.525080873	0.756442052
6.028583764	0.57679026	-0.112209614	1.035036001	0.616523692
4.122400393	0.308802004	-0.069104833	0.308291567	0.849925033
6.800269465	0.193205617	-0.015535562	0.560680069	0.734684754

log.sigma.0.5.mm.3D_glcm_ClusterTendency	log.sigma.0.5.mm.3D_firstorder_InterquartileRange	log.sigma.0.5.mm.3D_firstorder_Skewness	log.sigma.0.5.mm.3D_firstorder_Uniformity
0.919997659	14.92574286	-0.861716517	0.411470029
0.58714928	14.89179754	1.004112513	0.552509985
0.736468148	18.10297842	-0.418249225	0.468221819
0.758140506	19.42564857	-0.329071046	0.437854442
0.647948709	14.74858296	-0.388398216	0.503291909
1.374715193	26.89206387	0.069039705	0.318126119
0.557990402	2.674896806	-0.336184511	0.487571679
0.678245482	7.3563447	-0.981487506	0.476225699
0.595898499	5.74391222	-1.769041044	0.490586259
1.229905327	19.11304861	0.688925702	0.39379302
0.614029463	5.403107107	-0.857701188	0.4833385
0.600622024	5.126717806	-1.349153695	0.485162277
1.248219769	24.42114228	0.231749036	0.34378109
0.972833365	20.74302173	-0.40342329	0.410470914
0.701897084	10.52410343	-1.243623705	0.46419896
0.612792222	4.2422629	-1.894522174	0.476941112
0.584797424	11.17276447	-0.59728044	0.517475381
0.628560771	19.29637873	-0.00429715	0.543868193
0.617831457	10.18634748	0.58284927	0.51125544
0.647836185	5.879566312	-0.056627177	0.465134387
0.500645758	9.575140841	-0.660450007	0.569852355
0.658391154	16.54009312	-0.198908112	0.49238904
0.688816504	13.92510217	-0.730525369	0.498283963
0.531187074	3.837322056	-1.212351846	0.504108428
0.811498457	6.297496766	-1.225692192	0.426412889
0.77169444	8.962752938	1.137033495	0.4826057
0.682795536	13.33031696	-0.743830966	0.486000674
0.587541594	6.724206448	-1.365439788	0.506186813
0.85468014	26.23223147	0.201815193	0.400510204
0.553671495	9.388093382	-0.395157177	0.53046684
0.642329776	6.316132843	-1.389185513	0.48408927
0.570551059	18.58204865	-0.187830833	0.548204537
0.697057091	10.76511985	-0.342317142	0.470611141
0.636458306	5.450748205	-1.312198903	0.461206162
0.63667982	8.45912075	-0.736279029	0.487211785
0.904901574	17.29683242	-0.508657185	0.42726724
1.608666093	29.73437619	0.234691839	0.295016437
0.653788494	3.175256491	-0.65576276	0.468771172
0.488537926	13.93447745	-0.770368193	0.597155299
0.578570251	4.206311226	0.426706796	0.485779323
0.603061692	13.540342	-0.431152367	0.520882256
0.588429911	5.71302712	1.342385626	0.488441182
0.619938372	13.3076555	-0.725879825	0.523223325
0.699391581	13.71420148	-0.987723783	0.480383076
0.532409173	5.661563113	-1.241234964	0.52381765
0.627773749	9.30220744	-1.075005385	0.499162355
0.586420246	3.705019712	-0.921388943	0.485684352
0.493041664	11.67787777	0.474127523	0.601467859
0.695544935	15.29788131	-0.062007853	0.485841797
2.347722134	24.27993774	1.140218569	0.335785124
0.552775894	4.068362832	-2.280629945	0.510376466
0.853195484	11.5519312	0.554781007	0.447402978
0.536197973	3.480960369	-0.712257733	0.497047655
0.694608153	8.326729059	-0.574979245	0.459666662
1.261059414	23.70125011	0.254279126	0.352669087
0.542361236	11.95155151	-0.712346936	0.563385953
0.560868803	5.465233922	-0.672913116	0.491811159
0.708157876	10.30256796	-1.036306326	0.47062138
0.749882599	17.95728308	-0.735588936	0.479509083
0.549693044	5.482224703	-0.273778636	0.486670087
0.806906861	13.89929926	0.356138991	0.465983637
1.195388821	15.63118601	0.379098186	0.368877522
1.396938767	16.91891438	0.211242781	0.358841026
0.764859615	5.811298698	-0.4133858	0.444437226
0.555151096	10.51272583	-0.955327216	0.524111761
1.928338552	23.37497807	0.575745896	0.320118763
1.199541564	23.37203968	0.207090789	0.36572276
0.848475357	9.88068977	0.620923257	0.441037127
0.554278687	4.544373989	0.695856144	0.493381565
0.762264954	13.05567253	0.41196574	0.459765693
0.617793405	16.49987318	-0.60190894	0.498673605
0.675206665	4.902462184	-1.712503742	0.463482801
0.697071261	13.62265828	-0.742234195	0.497302144
0.71181996	8.949843884	-0.068535635	0.473375551
0.739918534	5.316117406	0.466683669	0.462047466
0.711122101	10.69194829	0.754425808	0.509495198
1.571580042	20.27808511	0.685434103	0.371407861
0.729658294	19.28116536	0.340235313	0.439395085
0.866018907	16.52712199	0.294503066	0.432511957
0.550718351	6.284587145	-1.69438298	0.51570813
0.919997659	14.92574286	-0.861716517	0.411470029
0.717793233	16.59626955	0.439471557	0.46911377
0.631338067	3.375595599	-2.439189445	0.475664066
0.514639708	8.33163777	-0.966766019	0.557740878
0.578205383	7.318463624	-0.809102154	0.517027033
0.748738474	20.49061449	0.079861113	0.426717459
0.705173284	13.61749554	-0.012252501	0.477209037
0.545500554	3.375806794	-1.565916277	0.512039058
1.710809792	17.25347984	0.634842231	0.362230874
0.773343979	14.96353614	0.273864399	0.45445625
1.504566787	23.20080239	0.413137519	0.34089477
0.504217681	7.176703706	-1.438142029	0.554669666
0.698382543	9.487594903	0.067563508	0.458798417
0.665167526	6.248950005	0.364990802	0.476018015
0.659388569	11.31640548	0.733735838	0.493029941
1.233695846	20.42406933	0.395470474	0.37567482
0.735606367	13.44274199	0.087969533	0.487217789
1.820957099	26.51227692	0.523384749	0.292641036
0.340927131	10.89359367	-0.436405274	0.686570125
0.744469579	6.97824806	-0.223976969	0.448013839

log.sigma.0.5.mm.3D_firstorder_MeanAbsoluteDeviation	log.sigma.0.5.mm.3D_firstorder_Energy	log.sigma.0.5.mm.3D_firstorder_RobustMeanAbsoluteDeviation	log.sigma.0.5.mm.3D_firstorder_Median
10.36612531	155270.8123	6.568935596	-3.841409802
10.25990335	40110.40505	6.405416977	-13.37861824
10.6446595	125255.7724	7.413633869	-5.579176426
12.00789635	43690.53778	8.291697143	-16.00063658
9.605968903	159843.6568	6.647403394	-4.350051165
16.52438275	61887.28195	11.57968262	-14.33216
3.419080361	744805.0571	1.230661406	-0.223006204
7.141617644	652921.4351	3.785631309	-1.450836957
5.289742575	304075.3568	2.735758093	-1.130916595
13.94507433	148622.3443	8.326924837	-5.574467897
4.573170043	371600.7681	2.340087066	0.082566619
5.395762704	254937.0715	2.545228214	-0.806277812
15.43767833	156495.4502	10.54380806	-7.955579758
12.47261882	93762.13927	8.944744879	-14.33091831
8.385247791	150446.0388	4.941095551	-1.752134919
4.702827659	578931.8808	1.912476151	-0.6113801
7.338613794	52530.22557	4.730016116	-3.162063599
10.4454413	36184.15223	7.601906654	-13.35344887
7.56508225	75066.7935	4.589767935	-3.008999348
5.984481511	1170076.237	2.768915608	-0.554311097
6.437920673	133441.8869	4.201021378	-1.842421234
9.438584173	43237.75377	7.069133362	-10.63315153
9.257597378	59329.08	5.829939625	-4.180524826
3.623608409	192174.33	1.722262694	-0.63781321
8.976906397	135964.9271	4.670493754	-1.164690316
7.439529007	298604.1618	4.184642215	-1.728384256
8.844473317	165693.4148	5.736889009	-3.217982769
5.887764129	152338.8122	3.164238976	-1.538831353
15.20395529	11301.39793	11.6130309	-10.71055984
6.87927701	121487.4198	4.356323502	-2.351895332
6.172164801	316546.4758	3.302411652	-0.949265063
10.20162545	87985.55259	7.463172126	-13.26037216
8.103140997	193298.836	4.779428353	-1.977308273
5.34978194	262215.9614	2.470026446	-0.423217833
6.874900356	384491.3933	3.959554798	-1.615988016
11.13802424	304110.117	7.395539184	-3.393746376
18.28742883	335661.2339	12.79442896	-19.42163563
4.108003923	769691.157	1.432496314	-0.138342097
8.711423431	41204.66	5.909302341	-8.459986687
4.31402809	814119.3229	1.86905575	-0.496593177
8.844029055	113563.4548	5.851213748	-4.109729767
4.976267041	521690.5523	2.484923553	-0.763932109
8.384379731	258912.4681	5.583806407	-4.823422432
9.102882031	137999.2869	5.978590038	-4.12321043
5.131474489	69081.34663	2.763327418	-1.228860199
7.220555016	238625.8324	4.30586283	-1.89403677
4.449873671	439861.2986	1.851160069	-0.555363715
7.161918853	24807.533	4.90890099	-3.961563349
10.60371044	35618.19605	6.908168688	-9.788841248
17.07698247	136586.6252	10.9511734	-14.35617447
4.484311471	114837.943	1.937319125	-0.888984501
9.54360644	471782.1217	5.206030421	-2.455511093
3.346009136	376142.8459	1.523339998	-0.440431431
7.115848199	89233.95771	4.001829346	-0.236306965
14.28671443	129301.8801	9.861507223	-9.466054916
7.681777024	94395.04405	5.19429031	-4.384081364
4.825133013	324337.4481	2.480551085	-0.786179245
8.090392833	232067.5036	4.87601537	-1.701379418
10.4982821	75893.67224	7.477522453	-6.948803186
4.18708029	1127605.675	2.308426869	-0.354361534
10.59356779	203007.7746	6.401513487	-3.145803213
12.47500771	566781.8546	6.996142166	-2.527439356
13.62135169	1856166.455	7.779488039	-2.806363463
7.273358205	1336589.607	3.202482312	-0.668037385
7.374574058	39152.0404	4.501328127	-2.648622036
17.14226053	1288455.527	10.60005313	-6.386258602
14.04239477	60181.59685	9.75411192	-15.25849056
9.022170602	886490.2218	4.743358154	-1.581838787
4.169182526	527281.4469	2.030453251	-0.583053231
9.541266533	308007.7864	5.635447214	-3.007028103
10.14675023	49077.12264	6.734643403	-5.873129606
5.510246917	628272.6366	2.372922293	-0.553757906
8.870804478	138358.9486	5.987767195	-3.368919134
7.474984331	147557.9095	4.313403463	-1.655840039
6.672319919	426979.6962	2.946570797	-0.857716471
8.576366759	102527.0438	4.985897984	-2.487272263
14.71302533	603751.5614	9.069154501	-4.966334581
11.93443463	54947.37387	8.322358932	-16.10761261
11.89225166	187896.8988	7.622203226	-5.982357025
5.17367417	105832.1383	2.865369361	-1.507379234
10.36612531	155270.8123	6.568935596	-3.841409802
10.64161198	87024.42064	7.13313923	-6.226285934
5.35286944	287490.1029	2.03280128	-0.55272159
5.624238225	88903.88055	3.561063532	-2.397055149
6.464410603	186682.4412	3.538139718	-1.530180931
11.97665732	55148.61232	8.352956706	-9.642344952
8.647463471	120032.1161	5.595460981	-3.734057188
4.239716981	214913.5015	1.802780124	-0.668987423
13.79590364	476715.2068	7.937180288	-2.647344828
10.57700501	197360.915	6.386430959	-3.261815667
15.56702373	128707.0611	10.48466055	-8.256816387
5.386755493	100932.3478	3.202968771	-1.986935437
7.98521889	520342.09	4.328282585	-1.655988872
6.205724395	421702.6694	3.018923622	-1.026605487
8.173500204	302852.1918	4.876808689	-2.912578106
13.61663382	498054.1593	8.728309793	-5.722602129
9.434420392	304631.1809	5.785723047	-3.231035948
17.7750392	60220.18733	11.47753921	-16.26940918
6.908418529	12624.53713	4.740051448	-9.456907272
7.427823941	1378564.25	3.561168802	-0.865532935

log.sigma.0.5.mm.3D_firstorder_TotalEnergy	log.sigma.0.5.mm.3D_firstorder_Maximum	log.sigma.0.5.mm.3D_firstorder_RootMeanSquared	log.sigma.0.5.mm.3D_firstorder_90Percentile	log.sigma.0.5.mm.3D_firstorder_Minimum
4192311.931	41.61726379	15.26890166	6.622424603	-59.52997589
1082980.936	53.33534622	18.84036456	2.245520949	-42.1907959
3381905.856	46.12325668	16.10368658	2.358504486	-54.64083862
1179644.52	19.59584618	21.79213505	2.416035652	-63.12727737
4315778.734	45.80529022	14.63788361	1.532909989	-50.55142975
1670956.613	36.5236702	24.51218875	16.23232021	-63.89095306
20109736.54	82.36353302	6.758457535	2.285734773	-61.76890564
17628878.75	70.12409973	11.37514235	2.935578346	-61.68493271
8210034.635	35.02639008	8.479529784	3.380273342	-57.01690674
4012803.297	73.32468414	20.20651241	17.81763191	-62.90032959
10033220.74	91.98886108	8.246752412	6.590253258	-150.6501923
6883300.929	73.59693909	9.10825574	3.291514826	-72.05291748
4225377.156	53.15353775	21.74387091	15.12959671	-64.6162262
2531577.76	15.65911865	22.21452101	2.732941508	-67.87983704
4062043.047	47.76145554	12.78781854	2.870320511	-59.33955765
15631160.78	48.86177063	8.23977972	3.203813887	-56.90325928
1418316.09	31.62173271	10.97643698	3.401551247	-37.55541992
976972.1102	34.13229752	18.05500909	2.154720306	-50.0566597
2026803.424	63.2084465	11.50621284	5.611150169	-37.47507095
31592058.4	101.7454071	9.904703288	6.188358021	-73.62926483
3602930.947	33.24323654	9.586559653	1.228413069	-38.37494659
1167419.352	21.65955353	16.54257143	1.952179015	-39.80774307
1601885.16	38.87177658	14.53050295	2.737040043	-54.03317261
5188706.911	48.03395081	6.324791138	2.732608891	-49.53081894
3671053.03	40.88431931	13.68501055	2.567718625	-54.38779831
8062312.369	87.94332886	12.14326337	3.923353434	-47.19674683
4473722.201	45.89940643	13.28372353	4.473106861	-64.89021301
4113147.929	36.32268906	9.306199593	3.630043221	-59.68536377
305137.7442	23.00544739	20.09032988	16.21826677	-37.46021271
3280160.335	32.44190598	10.27372632	3.681367159	-40.62062073
8546754.847	46.71108627	9.495182724	2.376444101	-45.64068985
2375609.92	27.09728432	18.57528957	0.09385424	-48.187603
5219068.571	47.69664383	12.29360761	4.440598297	-53.87094879
7079830.957	53.79976273	9.125217648	4.575844097	-51.94975662
10381267.62	57.98163223	10.51576727	3.718660307	-57.22505569
8210973.159	48.02550888	16.41953615	3.380526042	-63.29645157
9062853.315	53.93698883	29.33717489	13.01578236	-73.31461334
20781661.24	104.6495667	8.097690914	3.007838082	-77.65254974
1112525.82	21.27283859	14.46238952	3.749842024	-64.52837372
21981221.72	130.3555145	8.197855269	3.506426072	-150.676239
3066213.281	45.89641571	13.41543065	3.690213919	-56.04413605
14085644.91	92.64977264	8.490381396	4.846788311	-49.61993027
6990636.639	48.14719772	13.02137853	3.808253431	-61.55477905
3725980.746	39.62225723	13.9710393	3.11500783	-56.45014572
1865196.359	25.15850258	7.968305012	3.233092713	-36.99947357
6442897.475	46.57168198	11.04804278	2.942198181	-60.72428131
11876255.06	70.14081573	7.944611119	2.632057333	-49.31888962
669803.3909	30.8394165	10.64313893	3.654026461	-28.51898575
961691.2933	27.40426064	17.67598134	4.081796169	-43.66109848
3687838.88	68.41119385	24.91682757	23.72707615	-68.07666779
3100624.46	37.96724319	7.762136115	2.505013227	-52.1832428
12738117.29	73.15708923	14.78238191	8.173526382	-59.26683044
10155856.84	77.91589355	6.102007273	2.729159689	-63.17291641
2409316.858	37.48106003	10.26415904	10.27237015	-43.62602997
3491150.762	55.93783569	21.22567311	4.060180855	-60.21878815
2548666.189	26.61589622	11.93212605	2.955424643	-42.31724548
8757111.099	50.51704788	7.832382574	4.039960003	-47.94367981
6265822.597	47.14827728	12.25583551	2.420230961	-68.6184082
2049129.151	19.88500977	16.70391732	3.383127856	-64.95296478
30445353.23	106.0318222	6.925076967	4.741679573	-70.30722046
5481209.915	86.78620148	15.95979007	8.406739616	-63.65539169
15303110.07	77.08271027	18.80360882	11.75502071	-79.68824768
50116494.28	116.3924255	20.14908593	18.89438419	-91.88887024
36087919.4	98.24262238	12.52504349	4.305580235	-131.023468
1057105.091	19.99416161	11.04395641	4.402472973	-39.6231308
34788299.24	81.66644287	24.28337216	23.43008842	-77.78363037
1624903.115	32.70237732	22.48839044	7.678234386	-58.63753128
23935235.99	111.5409927	14.65436925	4.404433107	-58.6076622
14236599.07	83.22083282	7.253078536	3.707132864	-50.50751495
8316210.232	67.44889069	14.20238656	8.480858421	-53.77376556
1325082.311	24.53062248	15.74370527	2.392485309	-46.44672394
16963361.19	65.34879303	9.407527239	3.511550665	-64.37216187
3735691.613	43.61112595	13.5913538	2.165967274	-51.9285202
3984063.556	83.2461319	11.72685043	3.512243414	-56.99920273
11528451.8	85.29210663	11.52247015	4.863315821	-59.80494308
2768230.184	64.07489014	13.02868993	9.759456348	-51.60474396
16301292.16	81.66070557	20.18389748	23.05224991	-65.33760834
1483579.094	33.50617599	21.8587111	0.529444087	-47.40738297
5073216.267	57.64597702	16.9890585	14.26385689	-57.24206924
2857467.734	32.21743011	8.143148261	3.154497385	-44.20204544
4192311.931	41.61726379	15.26890166	6.622424603	-59.52997589
2349659.357	46.58047104	15.35703547	7.599859238	-41.20814133
7762232.779	35.35041046	9.17118428	2.118807817	-66.14020538
2400404.775	35.72632599	8.698441071	2.748620367	-53.11898041
5040425.913	48.04339981	10.16981687	2.7752985	-52.34024811
1489012.533	27.81188774	17.70153632	10.18642902	-49.41504288
3240867.135	67.28009033	12.86707191	5.32476387	-50.31368256
5802664.539	62.20244598	7.270230209	2.035745978	-48.29377365
12871310.58	87.76625061	20.14237001	20.16058388	-81.33761597
5328744.706	55.85527802	15.70672289	8.607542992	-64.24846649
3475090.649	82.53102112	21.59467368	17.40473652	-55.36719894
2725173.39	26.30473328	8.401313075	2.640255713	-52.10434723
14049236.43	69.66811371	12.33117542	6.992458248	-58.61087036
11385972.07	83.97635651	10.46037659	4.45511384	-58.1257019
8177009.178	63.25086212	12.10445171	7.883180523	-43.98770905
13447462.3	104.2291031	20.50984765	6.994726753	-77.1758728
8225041.884	64.41028595	14.42010307	4.156065178	-60.24147797
1625945.058	52.80643845	26.30942913	22.97216949	-59.53997421
340862.5024	8.889029503	13.33455594	1.333167195	-37.10702515
37221234.74	62.08264542	11.98084446	6.605853653	-63.36644745

log.sigma.0.5.mm.3D_firstorder_Entropy	log.sigma.0.5.mm.3D_firstorder_StandardDeviation	log.sigma.0.5.mm.3D_firstorder_Range	log.sigma.0.5.mm.3D_firstorder_Variance	log.sigma.0.5.mm.3D_firstorder_10Percentile
1.473724342	13.85517605	101.1472397	191.9659034	-25.88537025
1.185734568	13.96959738	95.52614212	195.1496509	-29.41327133
1.355284321	13.36879616	100.7640953	178.7247108	-27.55099945
1.421548383	15.09295841	82.72312355	227.7973936	-34.46921844
1.276223082	12.22709361	96.35671997	149.5018183	-24.99507809
1.843032359	20.42046269	100.4146233	416.9952964	-38.51832047
1.122001084	6.676260742	144.1324387	44.5724575	-5.87719655
1.267932132	10.46030447	131.8090324	109.4179695	-19.179389
1.155782321	7.90490078	92.04329681	62.48745634	-13.32713871
1.688490211	19.65853042	136.2250137	386.4578183	-27.57699184
1.139630038	8.243910587	242.6390533	67.96206176	-6.019572401
1.182066567	8.694797349	145.6498566	75.59950093	-12.96381283
1.798310258	19.63296015	117.7697639	385.4531242	-34.55236053
1.478400976	15.47064911	83.53895569	239.3409839	-37.61795273
1.30896903	11.35925124	107.1010132	129.0325886	-22.48884544
1.18252835	7.941736895	105.7650299	63.07118491	-9.279392624
1.137787801	9.606787012	69.17715263	92.2903567	-19.49799442
1.248548625	12.99597188	84.18895721	168.8952852	-25.7197628
1.191851724	10.7431892	100.6835175	115.4161142	-17.46441765
1.252740943	9.818946159	175.3746719	96.41170368	-11.65637226
1.008612808	8.294801591	71.61818314	68.80373344	-16.35863705
1.265226643	11.37514469	61.4672966	129.3939167	-28.65165596
1.299371416	12.33277377	92.90494919	152.0973089	-25.87534714
1.075226482	6.125858718	97.56476974	37.52614503	-7.154797363
1.416931719	12.5318218	95.27211761	157.0465575	-25.64124584
1.263777546	11.56003036	135.1400757	133.6343019	-18.26979294
1.256731512	11.68538113	110.7896194	136.5481322	-22.53705482
1.150212687	8.620940059	96.00805283	74.32060751	-14.37000656
1.442604943	17.91016317	60.4656601	320.773945	-31.73614826
1.097036012	9.242565436	73.0625267	85.42501584	-17.34763336
1.185441979	8.689839633	92.35177612	75.51331285	-16.63410759
1.16788593	12.5430726	75.28488731	157.3286702	-31.54493484
1.307624261	11.46505403	101.5675926	131.447464	-20.0395649
1.243242166	8.916533236	105.7495193	79.50456495	-9.805401611
1.210375126	9.745527033	115.2066879	94.97529714	-17.78072968
1.483991125	14.31697573	111.3219604	204.9757942	-28.27109756
1.967368807	22.85394811	127.2516022	522.3029444	-47.75048828
1.205219089	7.987631199	182.3021164	63.80225217	-6.157471085
1.044250662	11.39329683	85.80121231	129.8072128	-22.58901443
1.163318284	8.124854344	281.0317535	66.01325811	-7.522162294
1.199832335	11.51539811	101.9405518	132.6043937	-22.60231209
1.156162766	8.420731142	142.2697029	70.90871297	-8.843536949
1.196092869	10.96237314	109.7019768	120.1736249	-21.99924965
1.29911433	11.93830671	96.07240295	142.523167	-23.89449539
1.05381818	7.374051927	62.15797615	54.37664182	-13.63719797
1.206925752	9.931098017	107.2959633	98.62670783	-18.52635918
1.153958942	7.687979349	119.4597054	59.10502647	-10.10190411
0.944666375	9.285058608	59.35840225	86.21231335	-17.25072823
1.351041992	13.84040896	71.06535912	191.5569202	-31.6722044
1.855493089	22.7937722	136.4878616	519.5560511	-32.86167221
1.102411495	7.30547298	90.15048599	53.36993546	-9.791908264
1.45960018	14.32284236	132.4239197	205.1438132	-20.64701233
1.088145669	5.981632825	141.08881	35.77993126	-5.99317646
1.243503652	10.21364503	81.10709	104.3185448	-14.90105343
1.767038078	18.18610695	116.1566238	330.7344861	-33.16345749
1.076465505	9.774589329	68.93314171	95.54259655	-20.19272003
1.127437643	7.596505374	98.46072769	57.7068939	-10.39420166
1.283731356	10.94116174	115.7666855	119.7090203	-20.97088165
1.308831742	12.91896057	84.83797455	166.8995421	-27.92508793
1.116812385	6.857268861	176.3390427	47.02213623	-6.653949547
1.426513007	14.8456964	150.4415932	220.3947017	-24.38282433
1.739250506	18.2406353	156.7709579	332.7207762	-28.04321709
1.796646068	19.64991545	208.2812958	386.119177	-28.32943535
1.381669986	12.25932002	229.2660904	150.2909273	-16.88000565
1.120654262	9.814781289	59.6172924	96.32993175	-17.90890121
1.972054801	23.3877359	159.4500732	546.9861906	-34.27536392
1.68290645	17.56103667	91.3399086	308.3900088	-35.10709152
1.453579709	14.1042527	170.1486549	198.9299443	-21.13013897
1.114513431	7.156271818	133.7283478	51.21222633	-8.129977036
1.392332592	13.54984373	121.2226563	183.5982652	-20.86191788
1.245759904	12.89318832	70.97734642	166.2343051	-26.64197655
1.242047432	9.044069221	129.7209549	81.79518808	-12.8284277
1.285955505	11.44312255	95.53964615	130.9450536	-23.56276016
1.268950649	10.80836863	140.2453346	116.8208325	-19.7642086
1.318389776	11.29324142	145.0970497	127.5373018	-15.77566242
1.258099787	12.52780558	115.6796341	156.9459127	-17.87747593
1.757862841	19.9888805	146.9983139	399.5553438	-25.40120831
1.400873375	14.9572516	80.91355896	223.7193755	-34.80986023
1.513402268	15.833197	114.8880463	250.6901272	-25.9481678
1.0847077	7.446847352	76.41947556	55.45553548	-12.70985556
1.473724342	13.85517605	101.1472397	191.9659034	-25.88537025
1.366298417	13.78056399	87.78861237	189.9039439	-24.3906002
1.211682209	8.64865734	101.4906158	74.79927379	-14.17706747
1.019203675	7.643094446	88.8453064	58.41689271	-14.05193329
1.149960462	9.357094346	100.3836479	87.5552146	-16.70531616
1.447679716	14.97897166	77.22693062	224.369592	-27.78592587
1.287552609	11.59101546	117.5937729	134.3516395	-21.14729843
1.076242608	6.852244295	110.4962196	46.95325189	-11.05575371
1.786592967	19.87979467	169.1038666	395.2062362	-26.39797325
1.438789436	14.75697921	120.1037445	217.7684355	-24.12184963
1.803028195	20.01782002	137.8982201	400.7131182	-31.74413204
1.021065788	7.423990188	78.40908051	55.11563031	-13.91427259
1.338529529	11.96208836	128.2789841	143.0915579	-17.25254421
1.25209249	10.21520762	142.1020584	104.3504667	-13.9608345
1.253125618	11.61905172	107.2385712	135.0023629	-17.36571655
1.728879549	18.50959898	181.4049759	342.6052543	-31.34055729
1.341556486	13.1647287	124.6517639	173.3100818	-21.94987259
2.037753662	22.74045889	112.3464127	517.1284704	-38.85508118
0.849856659	8.884476566	45.99605465	78.93392385	-21.08860779
1.353931024	11.75580771	125.4490929	138.1990148	-16.78570442

log.sigma.0.5.mm.3D_firstorder_Kurtosis	log.sigma.0.5.mm.3D_firstorder_Mean	log.sigma.0.5.mm.3D_glrlm_ShortRunLowGrayLevelEmphasis	log.sigma.0.5.mm.3D_glrlm_GrayLevelVariance
4.611493069	-6.416654455	0.102712778	0.563388396
6.598731514	-12.64158558	0.314007956	0.55395918
3.861801211	-8.977973636	0.093251594	0.529589421
3.232571542	-15.71940699	0.130106982	0.528012386
4.313000581	-8.047721306	0.08565784	0.497175573
2.658499475	-13.55920725	0.160658459	0.846643824
19.02356289	-1.050852395	0.057704546	0.314278039
8.396197726	-4.469440003	0.0744943	0.432363039
8.557399438	-3.068382151	0.065932003	0.342694451
5.063859586	-4.673898319	0.097386109	0.861109582
51.66700423	0.216479976	0.01246674	0.353646602
13.84238246	-2.71308343	0.066452122	0.367661805
3.232467001	-9.345736887	0.121116323	0.910960899
2.945867327	-15.94189323	0.129009174	0.531193564
5.979369695	-5.873305233	0.082593346	0.443583877
10.7620924	-2.196083998	0.064142022	0.349914354
4.001369352	-5.309596222	0.175297064	0.354307491
3.941582892	-12.5334779	0.103584779	0.49597609
7.516580567	-4.120293639	0.169056835	0.395922055
9.562206735	-1.300555088	0.06272482	0.402544101
4.289677132	-4.806078708	0.142673986	0.323859509
2.486239428	-12.01094304	0.345417494	0.455082418
4.898744024	-7.683632417	0.09002267	0.483482215
15.3231868	-1.573797288	0.128611508	0.292426071
5.426336891	-5.498450358	0.084953361	0.50698279
13.58504208	-3.718137094	0.172170908	0.464075482
5.197218665	-6.317371182	0.080325973	0.422472881
7.57941619	-3.504959824	0.06669246	0.355965838
1.92717656	-9.102055254	0.314824234	0.476439605
4.727010164	-4.486026821	0.149971215	0.333179591
6.448015166	-3.826902417	0.169851372	0.363789544
3.033375942	-13.7008289	0.347540467	0.424616167
5.859909315	-4.436814638	0.075239846	0.449883181
11.44416961	-1.940369082	0.067488894	0.384147606
6.508900117	-3.950451136	0.068967822	0.383373081
4.163936801	-8.038990817	0.096741429	0.588693737
2.832175693	-18.39475159	0.202789368	1.035957314
22.63074511	-1.330543484	0.03570921	0.370411016
5.7314315	-8.908058034	0.086008605	0.360391147
31.57800456	-1.09159191	0.012041866	0.35770623
4.566899654	-6.882542099	0.080154726	0.425372386
17.96119643	-1.085294099	0.132648372	0.346997446
5.22010459	-7.027280682	0.077750424	0.433367012
4.992679522	-7.257187611	0.088472329	0.457812973
5.951342936	-3.019477264	0.141735276	0.295803997
6.515871889	-4.840717043	0.072633436	0.405625531
13.57272484	-2.002952661	0.144739842	0.336439411
4.557552395	-5.202316122	0.127533919	0.29016706
3.245718025	-10.99469854	0.315306535	0.539929624
4.502277584	-10.06440486	0.115778975	1.04895065
11.94542863	-2.623132022	0.065258261	0.31603378
6.469000865	-3.657184962	0.077845665	0.626014436
19.99386744	-1.206052033	0.057087469	0.299401481
4.760286782	-1.017062404	0.162236983	0.378180408
4.282658798	-10.9450771	0.128255501	0.840094307
3.906403541	-6.843466629	0.187891778	0.367294157
10.34604432	-1.907700941	0.138573951	0.317834711
5.986758893	-5.522362148	0.076362185	0.439743058
3.608936494	-10.58873513	0.098216841	0.456673459
22.96716825	-0.966723726	0.058976743	0.314085557
6.560220761	-5.858344257	0.084904503	0.630533098
5.682261669	-4.566719657	0.049468344	0.903842493
5.459162977	-4.45718373	0.051050061	0.927574759
12.4618003	-2.566278864	0.017646896	0.527499689
4.318371902	-5.06350091	0.17875991	0.356031826
4.158502752	-6.534215558	0.055560444	1.231674604
3.124543741	-14.04769361	0.134410831	0.72532745
9.489512926	-3.977511027	0.080123509	0.619617427
17.6700221	-1.181068125	0.055702407	0.319855327
5.642665574	-4.255528042	0.073401909	0.563882342
3.570511482	-9.034929476	0.277107466	0.446667455
11.58074437	-2.589668063	0.068819688	0.400211897
4.97048796	-7.33347424	0.085529272	0.475934241
11.69869196	-4.54952618	0.076295936	0.437582997
11.25226718	-2.286922978	0.069878062	0.46503947
7.529474486	-3.577827361	0.067521614	0.49125005
4.347941949	-2.798995132	0.08707893	0.938120448
3.259780818	-15.94000864	0.395270875	0.523717395
3.86144583	-6.15938159	0.090106478	0.663738101
7.51068347	-3.294742496	0.15254281	0.308769989
4.611493069	-6.416654455	0.102712778	0.563388396
4.003561134	-6.777506505	0.225780639	0.538465086
11.73711362	-3.051450035	0.072630105	0.377476184
7.068078608	-4.152828476	0.062796525	0.301071095
7.039092623	-3.983711911	0.066581008	0.37456285
2.759213888	-9.432645238	0.285108406	0.585324168
6.424801948	-5.586582142	0.078531669	0.44484277
12.11111766	-2.429608075	0.134465437	0.304790898
5.085289822	-3.24173309	0.046763271	0.939123653
4.987275051	-5.378913302	0.082192761	0.62221037
3.952855075	-8.100420553	0.117560149	0.881898259
6.835041105	-3.932738368	0.063228797	0.302585048
7.011246802	-2.994382963	0.072214685	0.481177457
12.23749254	-2.251446561	0.06447066	0.430833031
6.418988886	-3.393433119	0.168945613	0.435294778
6.415964227	-8.834511672	0.055830829	0.884703162
6.450865318	-5.884665735	0.081558083	0.547723484
3.106983384	-13.23093311	0.17293952	1.201022353
3.223981188	-9.943664224	0.20258389	0.278862266
7.31537893	-2.311194336	0.069696813	0.484070948

log.sigma.0.5.mm.3D_glrlm_LowGrayLevelRunEmphasis	log.sigma.0.5.mm.3D_glrlm_GrayLevelNonUniformityNormalized	log.sigma.0.5.mm.3D_glrlm_RunVariance	log.sigma.0.5.mm.3D_glrlm_GrayLevelNonUniformity
0.127477611	0.375021207	0.809682115	167.9694685
0.402905202	0.410557505	1.005016358	28.50392784
0.126128533	0.373179494	1.253192532	109.2498774
0.157095226	0.373230432	0.503308074	24.78137317
0.120271109	0.391760583	1.424004293	167.6396883
0.178611859	0.297841128	0.2642695	25.74553803
0.090730392	0.472149329	1.669287419	4197.035292
0.106787807	0.423470411	1.380163529	1236.951209
0.099253251	0.459497665	1.38780732	1098.554076
0.122614554	0.322344725	0.669352011	82.71690809
0.018309662	0.473117375	1.247254472	1533.970288
0.099360847	0.453027877	1.388125036	791.6784941
0.146122324	0.288374773	0.677003755	68.25414284
0.157256544	0.370957381	0.526152161	50.51784215
0.112986777	0.414397166	1.010133653	236.411192
0.09636171	0.451712159	1.464892254	2182.442307
0.249928971	0.456532549	1.113126872	116.8651375
0.136036122	0.42084028	0.758454947	30.23239753
0.237392301	0.445658228	1.111289882	154.482513
0.09431622	0.43511673	1.47323044	2934.857499
0.23050403	0.470963689	2.118066075	343.8552905
0.423155302	0.400593693	0.791946662	41.41476228
0.123028183	0.404050802	0.963630625	70.95008068
0.201338696	0.481661368	1.453996843	1287.175339
0.113387321	0.383102475	0.935621118	178.2024773
0.243499605	0.427926695	1.391126787	497.3203394
0.110842713	0.424418029	1.167854166	243.5890457
0.100495844	0.454692458	1.490670075	452.363072
0.349811177	0.387920724	0.20068753	9.321704178
0.22763464	0.471697532	1.430660708	302.9882161
0.2438239	0.44614594	1.553661403	868.4667069
0.431398142	0.431760527	1.013214514	69.85431107
0.103579456	0.415644761	1.13579884	331.1291557
0.097296366	0.440270389	1.172313203	821.0887737
0.100592796	0.442874573	1.356171216	897.0740006
0.126901128	0.361893626	1.103469884	259.1293921
0.227501706	0.272747032	0.330182372	86.7872441
0.054037408	0.45101959	1.422636851	2993.237792
0.118665059	0.488310155	1.05959314	59.15232316
0.018398855	0.462126409	1.455078564	3183.762719
0.113722633	0.424730792	1.294705338	158.0491616
0.197478767	0.464075882	1.343520493	1963.597173
0.113219375	0.424231936	2.055021871	363.5808723
0.11889184	0.414146816	0.966758877	182.2451135
0.214460262	0.484046119	1.333327609	299.4660573
0.10693838	0.435870733	1.504205439	481.9387497
0.215475026	0.461166058	1.555513462	1778.629792
0.207408983	0.527000899	1.005217822	67.84111259
0.38524561	0.384205951	0.700009968	30.02634389
0.140249344	0.298237528	0.396355748	50.34610816
0.097525614	0.474531074	1.160251587	533.0867952
0.107423238	0.36811579	1.354843839	482.7186485
0.090763223	0.479146909	1.571183311	2651.251479
0.219886044	0.439691227	0.947775671	234.0096607
0.152109473	0.309563972	0.55967539	64.72539272
0.273453691	0.454959679	1.553362505	163.3961705
0.206195451	0.470467707	1.387507688	1425.380311
0.109944388	0.414512571	1.393392516	367.3715401
0.130312407	0.408901864	0.771614161	72.21295499
0.091207366	0.474664223	1.4892744	6207.701844
0.115756256	0.363882976	1.354543299	174.9352924
0.064108623	0.311503253	0.991876761	326.7176421
0.064550384	0.303911548	0.885966102	952.8827447
0.025635101	0.404079121	1.38596388	2022.28295
0.252829105	0.451873862	1.08688883	87.53952992
0.069035787	0.259821233	0.846326298	396.4228673
0.159505717	0.324905573	0.401207678	29.2863879
0.109454978	0.378179871	1.299553003	946.9358977
0.090208207	0.472844769	1.7054868	2548.718612
0.104172909	0.378360038	1.398871268	342.7872916
0.348551095	0.405960853	0.865683331	52.55094896
0.101974633	0.436204501	1.539190764	1714.470735
0.119914582	0.411700741	1.294291688	180.0112597
0.10584651	0.434304293	1.006849927	288.1215819
0.098847374	0.419724913	1.163339925	827.8953063
0.099721473	0.420354234	1.391209421	149.5153847
0.113481194	0.301685821	1.036918379	290.9803243
0.46884101	0.379368734	0.502342343	31.9564983
0.116637275	0.344318232	1.094511622	146.2497903
0.225238816	0.478730971	1.316275	435.4643741
0.127477611	0.375021207	0.809682115	167.9694685
0.293000153	0.377203593	1.056086178	87.45060458
0.10292956	0.446881894	1.22008673	906.663275
0.098922232	0.493852598	1.457385649	322.7521647
0.100869387	0.445562521	1.587729401	452.5471146
0.362208131	0.349937366	0.867965751	40.82436628
0.10795396	0.419887058	1.165415974	189.3272266
0.210837202	0.477462091	1.639864929	1048.930362
0.061121313	0.309012697	0.843907618	243.72177
0.113328529	0.355638248	1.392151551	170.0015629
0.137892365	0.301614652	0.502769869	63.87886143
0.098740559	0.489035979	1.540674807	392.0909583
0.100765503	0.408885509	1.292659069	850.994741
0.097342898	0.434185921	1.524537467	938.780178
0.231400221	0.432609136	1.155046086	558.6509633
0.070596162	0.309442824	0.92738918	247.5674589
0.114148446	0.3922274	1.447508812	336.6030405
0.194855972	0.259741328	0.304961368	18.1349621
0.290535572	0.5532653	0.840838483	23.87664403
0.100920579	0.405430313	1.459467242	2240.89456

log.sigma.0.5.mm.3D_glrlm_LongRunEmphasis	log.sigma.0.5.mm.3D_glrlm_ShortRunHighGrayLevelEmphasis	log.sigma.0.5.mm.3D_glrlm_RunLengthNonUniformity	log.sigma.0.5.mm.3D_glrlm_ShortRunEmphasis
3.054205507	7.958360035	233.0037523	0.74634746
3.774535554	3.258194316	31.46267768	0.688306792
4.030626871	7.248342135	134.4373805	0.700491151
2.451673386	6.676863291	37.63826014	0.776854861
4.48393737	7.161567029	181.3547569	0.673531355
1.707829248	8.372557892	63.23047913	0.876023434
5.044336836	7.936949819	3404.794007	0.633723716
4.371157923	7.645249247	1226.324104	0.668215557
4.534244822	7.541403343	951.3827961	0.646363446
2.709763103	9.5407181	143.1773608	0.773057022
4.1095719	38.55170716	1396.15282	0.675123497
4.487277849	7.791852232	703.0595116	0.650800728
2.648961362	8.765761972	139.4139979	0.794324031
2.524297285	6.467243921	75.97174155	0.766213432
3.641446229	7.527266272	259.2277614	0.694091599
4.598891554	7.834507018	1956.266263	0.653248579
4.067945227	3.837140942	105.8884715	0.657640669
3.191300081	7.050257707	34.16819772	0.709352315
3.827017504	4.359095829	156.4123059	0.692655678
4.605576847	8.24676225	2730.604664	0.654809658
6.112413374	3.832679148	256.2749892	0.605759544
3.160712412	2.99195593	51.01796583	0.725882795
3.556243209	7.366065533	80.8837518	0.698883406
4.69930144	4.088036875	1035.380991	0.636242341
3.392359332	8.030625326	226.7394111	0.721539486
4.446076295	4.200745982	481.5492456	0.663306803
3.92308152	7.618851524	261.5990228	0.695420025
4.668363727	7.646157517	401.8759276	0.651226316
1.599377126	4.419958249	18.6380156	0.888017009
4.672345301	3.918814726	249.8880531	0.63763583
4.834973452	3.769718996	765.8928548	0.64306977
3.590399198	2.799070921	76.80594506	0.709482862
3.732556279	8.525079732	377.6047287	0.713026102
4.031285635	7.988215311	789.8709306	0.669199615
4.315199262	7.889509232	857.8798553	0.671214246
3.61475736	7.767060699	350.9435117	0.724892144
1.875572183	7.638833666	224.3741333	0.859261023
4.556922234	12.9847624	2651.197719	0.648670486
3.800968101	7.248713693	54.85165655	0.690693
4.561384389	37.19902675	2804.500056	0.655908917
4.212579479	7.437658483	161.4713463	0.678468086
4.289357649	4.512739438	1796.589544	0.671230563
5.555645141	7.372197946	359.7356684	0.660118695
3.568578994	7.359201533	200.8194258	0.697237853
4.44840091	4.050766197	249.59381	0.65191613
4.716801646	7.55499026	451.2979129	0.654813793
4.827937344	4.010187528	1506.890364	0.64126896
3.994925616	4.125851458	52.43620507	0.646975911
2.887455297	3.680772877	41.54852007	0.753844556
2.111413316	9.048392515	106.0335111	0.818133226
4.058018297	7.84700411	469.4198052	0.663384757
4.093737656	8.944215191	612.7843989	0.708414894
4.924657843	7.905722299	2119.545569	0.6324194
3.577847177	4.39050867	245.4555016	0.691704046
2.459992771	8.233838442	122.9363113	0.793143904
5.018701735	3.534830679	135.2747603	0.627605224
4.446672624	4.187169214	1238.164453	0.656890762
4.47835493	7.339059265	365.3080873	0.660305278
3.207671343	6.814586762	83.99728228	0.70763528
4.740695424	7.912226819	5103.518416	0.638961193
4.121050178	8.458879008	225.0185951	0.709981536
3.337415126	14.97285678	542.5554737	0.746882243
3.029205606	15.59662371	1743.421824	0.773035021
4.290953263	28.47329762	2170.454662	0.680980302
3.941071411	4.002940453	84.79152986	0.674156398
2.902647755	15.72369538	878.6821667	0.787225107
2.160399934	7.605151667	55.05334481	0.805534401
4.02484883	8.565027441	1154.258736	0.7041576
5.171331842	7.897996174	2010.429791	0.622916166
4.255493735	8.695756176	407.7302299	0.69564744
3.320772882	3.574196394	64.75601355	0.724733526
4.808506248	7.470098269	1531.288397	0.639683941
4.278720443	7.041302806	183.3518924	0.664330696
3.632410048	7.861835251	299.9763063	0.694005187
3.843880031	8.654294199	902.2422851	0.698884796
4.337554884	8.863768876	156.748976	0.685864538
3.413110227	9.95128386	496.1217566	0.744804829
2.382821016	3.231074422	49.49144762	0.792234971
3.467339503	8.907651376	223.1339499	0.752406751
4.41238103	3.874990657	365.2579237	0.64950598
3.054205507	7.958360035	233.0037523	0.74634746
3.62622653	4.389379558	112.0832032	0.719871494
4.063966075	7.838210385	871.45409	0.675946544
4.734468604	7.541846535	254.7376807	0.636674776
4.808800961	7.894328066	414.6473656	0.656669881
3.211455366	4.134670392	62.43983429	0.74968184
3.852561997	8.048824023	212.0506357	0.705782057
5.077275181	3.967721496	826.9366728	0.626889852
3.078200519	15.63968519	413.5213779	0.75053494
4.225324086	8.532403434	221.1984218	0.7058986
2.217532041	9.381198911	136.8462414	0.82989603
4.83390483	7.635433181	319.9419564	0.643142661
4.01314915	8.746118672	961.703308	0.704044393
4.707945983	8.201928734	868.4173795	0.652287957
3.762566818	4.620874335	611.7214211	0.710856144
3.130580824	13.91020619	434.987927	0.765064163
4.400249801	8.063888468	376.4384996	0.685672109
1.876445891	8.715848113	47.32627484	0.845261116
3.706589416	3.288758694	18.27631452	0.650852009
4.485270543	8.297945472	2317.54639	0.668070328

log.sigma.0.5.mm.3D_glrlm_LongRunHighGrayLevelEmphasis	log.sigma.0.5.mm.3D_glrlm_RunPercentage	log.sigma.0.5.mm.3D_glrlm_LongRunLowGrayLevelEmphasis	log.sigma.0.5.mm.3D_glrlm_RunEntropy
34.29925039	0.671864172	0.326370718	2.868707308
15.52965662	0.610619469	1.144245126	2.653102389
39.15181923	0.60519191	0.461660397	2.962051422
21.73191437	0.720735786	0.32995133	2.549228429
44.25339383	0.573107857	0.494445153	3.042140064
15.43341475	0.83793876	0.275369839	2.56043732
60.28283849	0.545122607	0.4605546	2.994414334
47.65992728	0.578828623	0.442934278	3.043803039
51.60423446	0.565236372	0.436411254	2.95875986
28.29572074	0.703930685	0.312065365	2.952567134
232.4442612	0.593310057	0.074402396	2.829401275
51.11355907	0.568624997	0.433455978	2.97664675
25.9982688	0.714617709	0.342240465	2.981101126
23.20944409	0.716194332	0.336222834	2.645704527
39.3445691	0.619732441	0.377614293	2.908647508
53.24944202	0.566535259	0.436514441	2.974653406
21.08927085	0.586626676	0.934739504	2.841668107
29.83321589	0.645183645	0.380487495	2.679737904
19.62984172	0.610636277	0.886114935	2.820927701
54.37520089	0.565504253	0.429950935	3.074787836
29.20911088	0.50238398	1.431304219	3.029192502
13.21883773	0.653359299	1.005019604	2.633095462
34.82065767	0.624144539	0.402688392	2.869602333
26.96872832	0.556203164	0.963338005	2.891615804
36.70258911	0.640707777	0.359091955	2.916103465
24.32025182	0.573675214	0.991158247	3.062380756
41.57217179	0.609732121	0.408477496	2.896850399
50.62975549	0.565181266	0.467008327	2.956347397
7.669304203	0.857142857	0.537448968	1.987183724
24.31973929	0.557642184	1.045366884	2.930965779
27.07215139	0.554279955	1.054256065	3.028241621
14.4183095	0.632277526	1.127250517	2.668521744
40.37167116	0.622601792	0.381181345	2.905796526
48.6714166	0.592178225	0.371781496	2.941996924
47.87872288	0.582398619	0.42568873	2.962392591
36.62152536	0.634274414	0.412232713	3.040331415
15.26492608	0.814003945	0.378591021	2.801962542
92.21960562	0.565382649	0.239011569	3.010722311
36.93640434	0.612651308	0.420077047	2.640742228
249.563633	0.568655465	0.085070288	2.961049936
42.20839942	0.589052786	0.455407141	2.907152316
25.24123182	0.584560113	0.866067815	2.888489215
55.54690579	0.557755277	0.599481784	3.045906212
37.28864064	0.621912741	0.381823689	2.89255194
24.02321097	0.568368213	0.959941252	2.8157301
50.65567168	0.564823923	0.48114443	3.027981109
28.44431177	0.553406846	0.984691256	3.003459789
19.84318171	0.58623112	0.900279752	2.626014297
12.36032997	0.682860999	0.894084112	2.621726653
21.04187846	0.767132867	0.285057562	2.859100567
44.7742739	0.589232384	0.399174802	2.802440667
43.69471752	0.606798019	0.429332911	3.143821634
57.35188065	0.547705709	0.459239831	2.943561345
22.68452389	0.627554264	0.703713863	2.794191348
24.94329845	0.727954972	0.316378191	2.94490801
23.6153637	0.540665971	1.226172302	2.963911333
26.09315731	0.572958345	0.902685465	2.893504108
48.72656641	0.573213841	0.456716516	3.061960174
31.47855164	0.64790724	0.370182346	2.765299919
57.48211093	0.556163693	0.427468683	2.945973747
41.66633913	0.602644532	0.452295015	3.092138458
62.36840428	0.654062095	0.199446081	3.236012784
55.80849715	0.685443166	0.185936063	3.184182366
175.2994293	0.587342001	0.108565997	3.145798823
19.46664127	0.602204649	0.943755072	2.71429993
51.75627813	0.698081324	0.188451941	3.289535084
19.77167322	0.756948933	0.304739828	2.703462237
44.22062572	0.606458706	0.413384501	3.150599776
60.48830525	0.537763145	0.480488753	3.01734382
44.96648875	0.592967609	0.443224881	3.097971396
14.93095585	0.651126651	0.936274957	2.652000763
56.93042698	0.553631606	0.452473593	3.101455661
43.68452275	0.583033789	0.462971471	3.042326372
40.48645764	0.618108825	0.362658476	2.881939328
43.33421331	0.613136242	0.377746761	2.965246695
45.49198874	0.588003057	0.448724974	2.964287581
38.02383757	0.65031662	0.365698616	3.267722683
9.289378442	0.731772575	0.913062479	2.490759989
35.20100062	0.651660168	0.384531835	2.971863608
24.15685047	0.569789859	0.951603802	2.859106434
34.29925039	0.671864172	0.326370718	2.868707308
17.62573661	0.627683969	0.932910741	2.887370252
46.63961571	0.59353198	0.393338342	2.912348387
49.57552775	0.555679214	0.482632404	2.855621525
50.66303578	0.562113786	0.491653452	2.95887343
14.29961191	0.663024476	0.989385666	2.692608563
41.1327911	0.620265252	0.398329562	2.888320221
28.12699811	0.540296644	1.073743001	2.960755094
58.24531333	0.670900164	0.182463774	3.232797079
43.1674292	0.596730769	0.458584279	3.097405682
22.77505087	0.766722408	0.279413564	2.82082999
50.37573938	0.559655729	0.494419756	2.838027146
44.63204071	0.607966551	0.399659227	3.002319024
53.43697459	0.56097561	0.456249083	3.09823313
19.7941727	0.623869599	0.856198687	2.859228404
53.99141504	0.675350832	0.202719503	3.133232655
45.20995291	0.584982935	0.473192254	3.096534436
18.08304449	0.801945181	0.311183499	2.877924937
15.71687937	0.603466956	0.969841665	2.365842669
51.34642499	0.575497389	0.437623654	3.149830177

log.sigma.0.5.mm.3D_glrlm_HighGrayLevelRunEmphasis	log.sigma.0.5.mm.3D_glrlm_RunLengthNonUniformityNormalized	log.sigma.0.5.mm.3D_glszm_GrayLevelVariance
10.86403429	0.518251286	1.581267218
4.488920552	0.452095465	1.325443787
10.22352892	0.457242819	1.481404959
8.696011983	0.564207807	1.102040816
10.46386505	0.423601124	1.455782313
9.40406482	0.728435207	2.027777778
12.35182941	0.382586625	2.206942149
11.36386012	0.419453303	2.081313776
11.61980851	0.397132167	1.080236686
11.80432223	0.556619861	2.600585938
56.94537024	0.429290929	3.928819444
11.82896329	0.402010234	1.825560698
10.72401525	0.587757774	2.25
8.691263366	0.552051887	0.69
10.90838769	0.452793952	1.320153061
11.91496524	0.403846883	1.793136095
5.652683294	0.411451411	0.959183673
9.751958272	0.474922943	1.49
5.9576383	0.449615116	1.933518006
12.36650433	0.404538315	2.464398688
5.825485489	0.350531983	1.104442344
4.169723357	0.492287534	0.8
10.33736731	0.459084238	1.484375
6.218442582	0.386876913	1.642469136
11.08852999	0.48555394	1.734375
6.088641368	0.413602461	2.546813813
10.91213781	0.452566988	1.24609375
11.52944763	0.402356946	1.452154858
4.944846077	0.764787935	0.6875
5.865380275	0.388433682	1.624142661
5.833849937	0.392264622	1.335795629
3.980623105	0.471604085	1.020833333
11.68845919	0.473023371	1.97
12.01364335	0.4230498	2.012396694
11.59642122	0.423020322	1.60712726
10.60866895	0.488484102	1.931331748
8.707761899	0.699044674	2.423469388
20.0974451	0.399169128	3.197654321
10.28619304	0.450663106	0.437869822
56.15717131	0.4063935	2.893277501
10.72600295	0.43227362	1.204081633
6.470336876	0.423636434	2.308678697
10.89167101	0.411892012	1.414930556
10.62075421	0.455519121	1.056245435
5.969594623	0.402538029	1
11.31596218	0.405266093	1.489586777
6.168954545	0.390353099	1.908832467
5.842229107	0.403718129	1.36
4.697116536	0.53109007	1.128888889
10.76610018	0.625683408	1.56
11.63943295	0.416866663	1.008323424
12.10355233	0.466337965	2.314814815
12.25505429	0.382118053	2.185079894
6.373369654	0.454395255	1.823129252
10.33496041	0.58658803	3.390581717
5.36082309	0.375840013	0.962962963
6.239456449	0.408027937	1.822222222
11.09535006	0.410933826	1.617252066
9.747651287	0.472594264	0.904320988
12.31242581	0.389246294	2.875618887
11.41018177	0.467854544	2.273580247
19.67817831	0.516910565	3.3251
19.76124677	0.554842756	3.306941015
41.56018573	0.433365766	3.275262137
5.651001888	0.432886547	0.734375
19.46945906	0.575602391	3.653739612
9.350607811	0.608491665	2.01
11.84660705	0.460727005	2.729659763
12.36516599	0.372592773	2.346409526
11.96409378	0.449789173	2.271720773
4.821626155	0.494383456	0.923469388
11.77827401	0.389215667	2.112614175
10.58678327	0.4177159	1.787721124
11.32296505	0.451575005	2.571428571
12.07800683	0.456157562	2.534284582
12.1251153	0.439272184	1.955555556
12.77008942	0.514067171	3.09359375
4.007175298	0.586713188	0.909722222
11.4199057	0.525135811	2.1296
5.840492705	0.40074709	0.677640604
10.86403429	0.518251286	1.581267218
5.759846784	0.481863084	1.604166667
11.62416024	0.429292898	1.280091928
11.43038257	0.388046733	1.067708333
11.57194779	0.406341955	1.48
5.190843557	0.529610521	0.995555556
11.25290423	0.466459402	1.805555556
6.090884095	0.375874227	1.406035665
20.36910533	0.523219066	3.664132231
11.54295321	0.462748765	1.776937618
11.0624755	0.64472798	2.5024
11.45423427	0.395085227	1.053507729
12.09162746	0.461268565	2.365472911
12.2030424	0.401380386	2.766574085
6.17856942	0.471647458	2.1144641
17.94273892	0.543133861	3.1695072
11.34078354	0.438038294	2.095804498
10.1067628	0.674257273	2.698224852
4.726384867	0.419691858	0.805555556
12.18344808	0.418908171	2.553002422

log.sigma.0.5.mm.3D_glszm_SmallAreaHighGrayLevelEmphasis	log.sigma.0.5.mm.3D_glszm_GrayLevelNonUniformityNormalized	log.sigma.0.5.mm.3D_glszm_SizeZoneNonUniformityNormalized
5.464545946	0.360881543	0.390266299
5.610505112	0.396449704	0.266272189
6.078456099	0.32231405	0.256198347
2.729999349	0.306122449	0.224489796
7.342071758	0.421768707	0.386621315
8.872900485	0.25308642	0.462962963
9.660031045	0.323195592	0.450651974
7.790290412	0.422353316	0.413265306
5.081945519	0.589585799	0.361893491
10.45917158	0.240234375	0.36328125
41.17868077	0.242283951	0.452546296
6.977784371	0.469562122	0.416162335
8.366004281	0.233333333	0.357777778
1.107930338	0.42	0.22
5.044756324	0.538265306	0.318877551
9.592054803	0.338224852	0.432426036
1.63297697	0.540816327	0.346938776
5.513245745	0.28	0.32
6.28980327	0.296398892	0.501385042
11.70953652	0.365405888	0.439965844
3.532726593	0.439508507	0.4536862
1.56080853	0.36	0.22
8.727294001	0.3046875	0.421875
4.613403163	0.415308642	0.500246914
7.131563993	0.265625	0.2265625
4.469955134	0.489498042	0.311498754
6.247201882	0.54296875	0.52734375
6.303078614	0.495982469	0.370343316
0.639444444	0.375	0.25
4.529645438	0.393689986	0.46776406
4.132443934	0.452754694	0.492767005
1.927014547	0.479166667	0.215277778
9.853758986	0.3304	0.4664
8.061557587	0.326446281	0.41322314
8.076750835	0.363088479	0.458467988
10.67960258	0.272889417	0.53686088
7.474306168	0.260204082	0.361607143
17.42628617	0.286666667	0.42962963
2.923961294	0.609467456	0.609467456
43.78444497	0.301889995	0.447204617
5.794609986	0.43877551	0.303571429
8.935930427	0.314032653	0.507171657
6.461894658	0.361979167	0.327256944
4.171017181	0.493060628	0.329437546
2.503476245	0.5	0.432098765
6.92585885	0.392396694	0.369256198
5.225398733	0.393196745	0.418292324
4.200648147	0.28	0.28
2.079329815	0.386666667	0.244444444
7.410632535	0.26	0.24
5.475758139	0.550535077	0.493460166
8.842981314	0.28377915	0.343106996
12.42638514	0.363199686	0.605136751
5.523733984	0.35600907	0.283446712
11.63395047	0.218836565	0.423822715
2.670483886	0.514403292	0.368998628
5.218307326	0.329777778	0.376355556
8.016628941	0.430785124	0.537190083
4.349948462	0.401234568	0.283950617
10.64317523	0.369037556	0.446322908
9.223693995	0.259259259	0.418271605
20.00048176	0.2192	0.4672
15.71106266	0.200246914	0.372784636
32.44756747	0.263537777	0.426709279
1.515573297	0.46875	0.3125
19.4358107	0.199013158	0.441135734
6.930596375	0.26	0.32
12.60907601	0.238350592	0.464080991
12.42157916	0.315070495	0.537584922
10.04673363	0.321448677	0.344342278
2.919262681	0.43877551	0.255102041
8.337400118	0.263729911	0.411261418
7.290407751	0.365244537	0.411030177
8.228790003	0.405612245	0.308673469
11.11294008	0.287620558	0.422644999
9.455809793	0.257777778	0.328888889
12.95840597	0.2159375	0.4696875
4.390254356	0.347222222	0.236111111
10.51144105	0.3168	0.4104
1.532820176	0.679012346	0.423868313
5.464545946	0.360881543	0.390266299
4.129661361	0.322916667	0.253472222
4.628354396	0.473139902	0.376615915
5.604173044	0.458333333	0.534722222
6.239445638	0.4616	0.32
3.570135779	0.351111111	0.253333333
9.6660518	0.32	0.426666667
3.683226683	0.426611797	0.399862826
18.85955098	0.176859504	0.479008264
10.60650861	0.256143667	0.374291115
12.06009961	0.248	0.4368
5.644639559	0.464922711	0.448275862
10.15362646	0.316460055	0.41402663
12.29382275	0.279314093	0.464074387
6.89666485	0.311134235	0.444328824
18.12456606	0.197516184	0.509050073
9.336760563	0.292820069	0.384948097
8.001162799	0.195266272	0.230769231
3.215995607	0.388888889	0.333333333
11.44556352	0.293796838	0.458744112

log.sigma.0.5.mm.3D_glszm_SizeZoneNonUniformity	log.sigma.0.5.mm.3D_glszm_GrayLevelNonUniformity	log.sigma.0.5.mm.3D_glszm_LargeAreaEmphasis	log.sigma.0.5.mm.3D_glszm_ZoneVariance
12.87878788	11.90909091	5354.545455	4947.239669
3.461538462	5.153846154	516.6923077	441.1360947
5.636363636	7.090909091	4623.5	4141.497934
1.571428571	2.142857143	504	331.2653061
16.23809524	17.71428571	6470.095238	6154.609977
8.333333333	4.555555556	106.3888889	73.64506173
74.35757576	53.32727273	782203.3333	772437.1024
46.28571429	47.30357143	107380.0536	105350.2293
23.52307692	38.32307692	134398.7846	130165.7808
11.625	7.6875	1549.125	1419.734375
32.58333333	17.44444444	199336.3889	193577.2654
22.05660377	24.88679245	85952.4717	82590.66002
10.73333333	7	1104.3	982.5655556
2.2	4.2	1324.8	963.8
8.928571429	15.07142857	13868	12788.40816
28.10769231	21.98461538	530373.0308	513163.6275
4.857142857	7.571428571	6968.142857	5998.265306
3.2	2.8	653.1	529.89
9.526315789	5.631578947	8563.736842	7673.185596
99.8722467	82.94713656	289983.5463	287222.9002
20.86956522	20.2173913	25464.30435	24467.9414
2.2	3.6	1119	869.36
6.75	4.875	2382.4375	2073.996094
22.51111111	18.68888889	257795.3333	246398.5847
3.625	4.25	13697.25	11638.35938
16.50943396	25.94339623	37155.03774	35695.22108
16.875	17.375	13244.21875	12383.16309
13.7027027	18.35135135	42081.43243	39821.32944
1	1.5	72.5	23.5
12.62962963	10.62962963	25817.81481	24000.52949
28.0877193	25.80701754	103645.3509	99851.22315
5.166666667	11.5	1384.708333	1271.817708
23.32	16.52	15098.62	14444.2836
18.18181818	14.36363636	103241.5227	98119.51808
49.05607477	38.85046729	32592.58879	31536.6425
31.13793103	15.82758621	8887.310345	8509.074911
20.25	14.57142857	628.1071429	579.6058673
38.66666667	25.8	715510.7111	698500.7551
7.923076923	7.923076923	1774.384615	1544.745562
84.07446809	56.75531915	377490.2447	373338.2246
8.5	12.28571429	7240.75	6732.891582
62.38211382	38.62601626	206869.813	203407.9736
15.70833333	17.375	24523.47917	23511.44401
12.18918919	18.24324324	6341.054054	5975.934259
7.777777778	9	34264.88889	30611.35802
20.30909091	21.58181818	34257.90909	32994.42975
42.24752475	39.71287129	231855.1386	227094.1386
1.4	1.4	5768.6	3850.16
3.666666667	5.8	388	330.24
4.8	5.2	617.2	496.2
14.31034483	15.96551724	63732.62069	59412.95838
37.05555556	30.64814815	18695.52778	18295.89806
61.11881188	36.68316832	501028.7327	491024.7719
5.952380952	7.476190476	15590.80952	13964.03175
8.052631579	4.157894737	1436.578947	1208.409972
9.962962963	13.88888889	9021.518519	8418.54321
28.22666667	24.73333333	181524.6	176555.29
23.63636364	18.95454545	25198.88636	23965.9189
5.111111111	7.222222222	1853.888889	1625.54321
81.23076923	67.16483516	1477230.467	1460539.815
18.82222222	11.66666667	6292.511111	5978.827654
46.72	21.92	9040.49	8783.5291
100.6518519	54.06666667	25660.56296	25373.82519
100.7033898	62.19491525	105829.161	104525.8286
2.5	3.75	6710.875	5100.859375
67.05263158	30.25	9307.072368	9100.431743
3.2	2.6	501.9	360.29
96.52884615	49.57692308	34734.43269	34340.56287
62.8974359	36.86324786	422311.3419	414972.5641
25.1369863	23.46575342	14193.13699	13755.58229
3.571428571	6.142857143	1329.714286	1129.693878
38.24731183	24.52688172	248341.5376	242514.7599
12.74193548	11.32258065	8849.580645	8265.812695
8.642857143	11.35714286	19123.46429	17654.9324
36.77011494	25.02298851	54330.78161	52964.33214
9.866666667	7.733333333	6057.866667	5652.515556
37.575	17.275	9816.8	9473.624375
2.833333333	4.166666667	298.9166667	207.0763889
20.52	15.84	3464.18	3294.6596
11.44444444	18.33333333	48360.07407	44865.95062
12.87878788	11.90909091	5354.545455	4947.239669
6.083333333	7.75	2548.625	2312.234375
22.22033898	27.91525424	93765.49153	90409.35134
12.83333333	11	31925.79167	29528.87326
16	23.08	33363.22	32060.01
3.8	5.266666667	778.8	641.1288889
12.8	9.6	8182.233333	7598.205556
21.59259259	23.03703704	155310.3704	149640.8381
26.34545455	9.727272727	8800.454545	8344.049587
17.2173913	11.7826087	5997.565217	5695.10775
10.92	6.2	910.64	788.7584
13	13.48275862	38827.44828	36395.93817
54.65151515	41.77272727	40078.74242	39406.67608
42.23076923	25.41758242	77112.57143	75318.90931
41.32258065	28.93548387	21485.17204	20991.18557
44.28735632	17.18390805	5446.183908	5260.973709
26.17647059	19.91176471	15007.54412	14543.39511
3	2.538461538	153.4615385	108.6745562
2	2.333333333	570.8333333	430.8055556
105.0524017	67.27947598	178168.4716	176409.6032

log.sigma.0.5.mm.3D_glszm_ZonePercentage	log.sigma.0.5.mm.3D_glszm_LargeAreaLowGrayLevelEmphasis	log.sigma.0.5.mm.3D_glszm_LargeAreaHighGrayLevelEmphasis	log.sigma.0.5.mm.3D_glszm_HighGrayLevelZoneEmphasis
0.04954955	513.3968939	60227	7.606060606
0.115044248	135.0223077	2060.307692	7.384615385
0.045548654	508.3457323	43495.13636	11.31818182
0.076086957	61.53373016	4467.714286	7.714285714
0.056300268	691.042586	62398.07143	9.619047619
0.174757282	14.81762346	904.4444444	12.05555556
0.010118975	71558.98423	9250717.703	13.89090909
0.022195799	10681.80682	1146873.527	10.14285714
0.015370064	13020.15821	1485232.169	7.215384615
0.087912088	163.072526	15508.96875	15.96875
0.01317716	3576.959295	11307739.94	62.06944444
0.01724699	8250.732846	960880.0566	9.20754717
0.090634441	137.6056944	9815.8	14.5
0.052631579	150.6611111	12410.3	5.1
0.030434783	1393.166429	146340.3929	7.392857143
0.007622845	49793.81468	6093971.969	12.93846154
0.032110092	1571.054067	34090	3.428571429
0.09009009	72.87969444	5975.9	9.9
0.0335097	1939.086901	41750.47368	8.052631579
0.019032447	26767.52856	3395785.96	15.57709251
0.031680441	5990.894626	115410.413	5.369565217
0.063291139	305.5111111	4537.4	4.8
0.056939502	257.1846875	22783.9375	11.25
0.009367194	53193.3179	1436517.556	5.822222222
0.022038567	1367.127344	147764.0625	13.125
0.02617284	8059.473344	193034.2075	6.622641509
0.034078807	1357.579219	135984.1563	7.8125
0.021034679	4223.867973	443895.027	8.756756757
0.142857143	21.42361111	321.25	5.75
0.023457863	5819.70499	126212.8519	5.925925926
0.016234691	21960.88487	558536.8772	4.859649123
0.094117647	354.8139468	5535.125	4.083333333
0.039093041	1515.2986	159483.94	12.86
0.01397269	9309.102159	1242229.773	12.13636364
0.030773655	3235.564273	349036.7757	10.71962617
0.05141844	934.6381466	88998.27586	12.77586207
0.143589744	107.2123958	4394.446429	11.85714286
0.007667405	37153.12547	14479388.29	24.25555556
0.065989848	191.9615385	16741.38462	5.076923077
0.015519234	7055.815715	20529419.38	61.07446809
0.04437401	767.422748	70615.28571	8.571428571
0.016995993	41932.37285	1179821.195	11.21138211
0.031434185	2611.451406	237558.4792	10.16666667
0.052333805	656.4935811	64090.16216	6.972972973
0.016544118	7482.596065	176118.3889	3.777777778
0.028132992	3475.471636	356113.2364	9.127272727
0.014492754	46879.14338	1326690.307	6.653465347
0.02283105	1361.1125	26001.8	9.2
0.131578947	100.5740741	1570.333333	5.4
0.090909091	83.73509722	5225.6	16
0.01521511	6318.713487	683536.5172	7.172413793
0.050023159	1911.684558	192408.1759	15.75925926
0.00999802	46952.25518	5764645.95	13.92079208
0.024793388	2848.046958	100260.7619	7.047619048
0.066202091	157.9468713	14405.15789	14.73684211
0.040723982	2146.838735	40082.62963	3.740740741
0.014185739	36839.89088	1033691.867	7.266666667
0.028478964	2527.675991	266422.8636	9.431818182
0.066176471	200.5096451	17706.33333	7.722222222
0.007740399	132961.1426	17784519.67	13.38461538
0.056461731	671.7112128	61271.84444	13.53333333
0.062383032	519.4449358	165144.41	27.63
0.059055118	1498.126975	457807.5741	23.99259259
0.027699531	2655.001287	4312389.966	45.36016949
0.024922118	1536.263889	32214.875	3.375
0.069565217	585.7263745	156243.8487	27.35526316
0.084033613	67.65194444	4274	12.9
0.050387597	3478.643527	367760.6971	16.22115385
0.011673152	39573.71491	4859223.41	16.04273504
0.047806156	1455.366564	145446.137	15.05479452
0.070707071	327.5813492	5752.214286	4.642857143
0.013100437	22872.69866	2916837.892	10.66666667
0.041388518	931.8101613	87364.32258	9.483870968
0.026095061	1901.375491	204606.9286	11.57142857
0.027052239	5270.624664	599619.1264	15.64367816
0.049668874	631.7739167	60510.86667	15.4
0.053981107	1010.527079	102281.1625	18.0125
0.104347826	99.52372685	1121.5	6.75
0.076804916	365.3509278	34200.82	14.52
0.016917293	10530.72762	249657.6296	2.555555556
0.04954955	513.3968939	60227	7.606060606
0.06504065	594.2491319	11876.875	6.666666667
0.017261556	8932.423861	1058126.203	6.830508475
0.020425532	3314.782708	320846.4167	7.958333333
0.027700831	3414.417522	342456.92	9.32
0.085227273	195.1740741	3481.933333	6.133333333
0.04137931	831.6619907	84981.96667	11.83333333
0.013280866	33181.35504	824602.0185	5.259259259
0.046808511	508.1570993	159902.9455	27.05454545
0.0575	634.1624034	59324.6087	15.43478261
0.09057971	106.3659719	8870.52	16.64
0.02027972	4027.133803	390812.2414	8.103448276
0.038573933	3904.954729	439757.2652	14.09090909
0.023611832	7436.021303	857259.4286	16.31868132
0.044992743	4776.074713	107422.172	8.774193548
0.07347973	339.0893486	90706.34483	23.47126437
0.046416382	1571.308791	149177.8971	13.04411765
0.149425287	22.31019231	1274.538462	14.15384615
0.084507042	142.7777778	2320.833333	5.5
0.023844232	16851.28545	2029076.397	15.81659389

log.sigma.0.5.mm.3D_glszm_SmallAreaEmphasis	log.sigma.0.5.mm.3D_glszm_LowGrayLevelZoneEmphasis	log.sigma.0.5.mm.3D_glszm_ZoneEntropy	log.sigma.0.5.mm.3D_glszm_SmallAreaLowGrayLevelEmphasis
0.641387102	0.333973064	3.403819408	0.23826962
0.516452845	0.38982906	2.807390451	0.081724204
0.495739375	0.180959596	3.697845823	0.10801083
0.358476308	0.283730159	2.521640636	0.169811165
0.639086422	0.194371693	3.117344208	0.118610701
0.669800281	0.293317901	2.816340114	0.246574504
0.697797804	0.1692651	3.487190286	0.126489747
0.665578545	0.237876984	3.541112313	0.160792577
0.618563282	0.231363248	3.003881451	0.136958127
0.616732407	0.193654514	3.593139062	0.138915278
0.694035498	0.034621679	3.726379636	0.02950148
0.667795102	0.235597484	3.150501703	0.142096833
0.613159886	0.239833333	3.68591758	0.200627369
0.426712151	0.367361111	2.921928095	0.256660605
0.564759122	0.261914683	3.145593314	0.164761115
0.676295747	0.154747863	3.43603544	0.099493007
0.418655785	0.752480159	2.359879029	0.335814171
0.551760795	0.233861111	3.121928095	0.16840481
0.723997876	0.476154971	2.905307776	0.358502522
0.68843631	0.157106278	3.424061768	0.104727575
0.69788214	0.537137681	2.759425943	0.414602313
0.283563628	0.494444444	2.921928095	0.131226857
0.634888532	0.191631944	2.89939747	0.120749525
0.731458662	0.616975309	2.803038801	0.434824781
0.445647669	0.177100694	3.452819531	0.089211138
0.572561344	0.702573375	3.369493222	0.383490772
0.741486644	0.225269097	2.625	0.170840323
0.623066688	0.212192192	2.939723114	0.134381519
0.080555556	0.368055556	2	0.017993827
0.705568779	0.599279835	2.770301986	0.422453753
0.71528984	0.65338694	2.811687196	0.425152834
0.422316802	0.670428241	3.450825795	0.285509578
0.706560182	0.176272222	3.160959863	0.126673352
0.665168391	0.189292929	3.375554699	0.135669374
0.703132707	0.172097612	3.434395707	0.12059589
0.748922924	0.213079502	3.153309507	0.155706798
0.618839082	0.358115079	3.800147279	0.248161532
0.673715266	0.083317257	3.874651338	0.059189712
0.788518369	0.28258547	1.505876256	0.254811417
0.692196323	0.024736103	3.631396945	0.018321681
0.558022358	0.209950397	3.209888638	0.117390301
0.738846617	0.380171635	3.197596108	0.259431994
0.588528658	0.206168981	3.610320756	0.123563335
0.581603298	0.2653003	3.103289831	0.177579724
0.670139401	0.708719136	2.46132014	0.506944518
0.627297886	0.222106061	3.509169426	0.128164107
0.670525234	0.591672167	3.339803355	0.353895542
0.450076303	0.297222222	2.321928095	0.21563455
0.430117305	0.531944444	3.189898095	0.25901931
0.480557545	0.135	3.541446071	0.083721979
0.722387553	0.230727969	2.623058791	0.172579629
0.593970984	0.153847737	3.823181414	0.097026503
0.802140837	0.152471038	2.694222479	0.106739058
0.535635503	0.531415344	3.141619208	0.17857484
0.663935578	0.330614035	3.110578015	0.24399328
0.621580784	0.707047325	2.619748965	0.420930987
0.623214706	0.51727037	3.466627139	0.289014522
0.742793293	0.217184343	2.934510965	0.160300575
0.541129192	0.224151235	3.350209029	0.136821545
0.694431301	0.205656576	3.501343197	0.14113951
0.669921235	0.190786848	3.695299011	0.147010548
0.70993369	0.092036749	3.86319864	0.073692164
0.633144459	0.101379731	4.481290602	0.070969102
0.678527524	0.035311887	4.089146839	0.025273706
0.515449333	0.684027778	2.405639062	0.404323289
0.689948335	0.090012774	4.070342858	0.064187066
0.550439575	0.269111111	2.921928095	0.219110791
0.706115286	0.174350505	3.920410091	0.1256868
0.759802283	0.13514119	3.182250061	0.102643892
0.607140135	0.145547945	3.734192711	0.089543948
0.490237848	0.56547619	2.950212065	0.220344466
0.664961584	0.262368578	3.899538931	0.178088514
0.661308649	0.253261649	3.139207197	0.183653582
0.561706408	0.20553045	3.378211621	0.119798501
0.669319761	0.151655394	3.61646552	0.100440977
0.577316331	0.143305556	3.581076012	0.086625105
0.711049931	0.192571074	3.782788667	0.150422622
0.42704126	0.352430556	2.918295834	0.047259076
0.664211524	0.162977778	3.440518555	0.114362414
0.669115385	0.838734568	2.334211708	0.600154344
0.641387102	0.333973064	3.403819408	0.23826962
0.491886364	0.517939815	3.688721876	0.202826739
0.635902124	0.312095104	3.365637484	0.212873834
0.752604593	0.219837963	2.317534343	0.178151071
0.551527859	0.198622222	3.024067294	0.105723617
0.469680799	0.422685185	3.132914564	0.130391411
0.674844024	0.177962963	3.327421329	0.112240947
0.653391215	0.618153292	3.124288086	0.395608569
0.716218648	0.093866986	3.823057952	0.076570928
0.629202244	0.143743961	3.698036555	0.093229256
0.675846207	0.198205215	3.317405811	0.140668521
0.68702131	0.21210728	2.676152417	0.15481563
0.667233933	0.172436869	3.643509107	0.113450948
0.70583891	0.178725487	3.468372186	0.135933718
0.690523706	0.468082437	3.313998913	0.297358621
0.736180068	0.098486527	3.839241372	0.077519385
0.642196335	0.188590686	3.597216723	0.101705051
0.456646745	0.292884615	3.39274741	0.134244694
0.521969423	0.430555556	2.251629167	0.222612537
0.702653659	0.188628093	3.776454243	0.143758718

log.sigma.0.5.mm.3D_ngtdm_Coarseness	log.sigma.0.5.mm.3D_ngtdm_Complexity	log.sigma.0.5.mm.3D_ngtdm_Strength	log.sigma.0.5.mm.3D_ngtdm_Busyness	log.sigma.0.5.mm.3D_ngtdm_Contrast
0.008775098	0.008116524	0.072644073	6.312091697	3.71E-05
0.102824563	0.026179336	0.591767231	1.116828455	0.000195365
0.015778627	0.009592751	0.110799448	3.396696448	3.54E-05
0.092863074	0.030560834	0.331842445	0.982843208	0.000366657
0.009894605	0.005941108	0.074815269	5.206810632	1.95E-05
0.060455	0.061042031	0.37868492	1.271452679	0.000522998
0.000261583	0.000727548	0.007858963	111.210107	4.17E-07
0.001036663	0.001531534	0.016461537	36.82008997	2.17E-06
0.001126218	0.001278072	0.012149259	44.07952277	3.29E-06
0.017999538	0.023974142	0.191231904	2.508402494	7.78E-05
0.000783899	0.004831477	0.078227389	10.61138901	6.42E-07
0.001533785	0.002578719	0.026311861	24.43282533	3.25E-06
0.022912412	0.027448103	0.216861383	2.152312053	9.27E-05
0.040784771	0.014823164	0.168502136	2.331632426	0.000189623
0.006001557	0.005734198	0.053897029	8.619745214	1.92E-05
0.000534558	0.000667083	0.005832672	94.44111175	1.78E-06
0.012897982	0.006409483	0.068340765	8.165153839	4.64E-05
0.089065204	0.034654318	0.532125595	0.582372873	0.000121843
0.009171034	0.0094108	0.104668786	7.918171984	2.62E-05
0.000379824	0.00109731	0.013187215	78.70343061	6.99E-07
0.004155251	0.001734874	0.022611116	24.08251877	1.03E-05
0.057443059	0.006550156	0.096183131	3.863131874	0.000285869
0.026742994	0.015839733	0.194843157	1.942940953	5.48E-05
0.000914347	0.000678218	0.005307695	115.7784246	4.43E-06
0.007692422	0.007647489	0.065526296	7.070616873	3.02E-05
0.002573335	0.004285524	0.05754208	21.56054047	5.69E-06
0.006265009	0.005215475	0.056537275	8.099874745	1.61E-05
0.002924308	0.002902075	0.030146383	16.81698328	7.44E-06
0.249089417	0.050562031	0.462967888	1.124094325	0.002821757
0.004509137	0.002469185	0.025002137	23.1759102	1.70E-05
0.001458593	0.000855118	0.007954618	74.95996528	6.61E-06
0.039227687	0.009776495	0.161540756	2.941407564	7.06E-05
0.004222281	0.004140816	0.039173735	12.26980125	1.39E-05
0.001424106	0.002695865	0.024012558	25.86696791	3.68E-06
0.001488304	0.002182529	0.024276172	25.29302992	2.81E-06
0.005610052	0.00455319	0.043208721	9.969638034	2.07E-05
0.016077914	0.027330294	0.183760266	3.896061459	0.000115041
0.000382125	0.001686873	0.022300049	42.08184817	3.85E-07
0.037693924	0.013413693	0.211751913	1.802174667	7.78E-05
0.000355939	0.002002578	0.033942981	27.13196687	3.63E-07
0.010721053	0.007050963	0.090074077	4.647517139	2.01E-05
0.000593906	0.001302098	0.014117261	88.23751115	1.44E-06
0.004319518	0.002829588	0.038561029	11.48958816	8.05E-06
0.008771155	0.006882453	0.074001919	5.85963337	2.32E-05
0.004438931	0.002786808	0.025328109	23.35659069	1.76E-05
0.002796814	0.002546555	0.027226908	17.80825706	7.26E-06
0.000654668	0.000809407	0.007912566	106.9901966	2.04E-06
0.024012034	0.012214402	0.140980455	4.097226734	7.07E-05
0.076314563	0.023637166	0.287342691	1.620192089	0.000262875
0.02999503	0.044633143	0.321233955	1.894253218	0.000192357
0.002480143	0.002808262	0.027080939	19.64478697	6.66E-06
0.002662484	0.003735822	0.035216021	15.09993974	7.78E-06
0.000425808	0.001152504	0.013021657	69.28018974	6.30E-07
0.006087426	0.003459249	0.032905315	16.15644017	2.98E-05
0.023791666	0.031178286	0.252639293	1.991916754	0.000100299
0.01024902	0.003667061	0.051594785	9.949110206	2.44E-05
0.000832765	0.001087163	0.010167348	83.78730378	2.64E-06
0.003700777	0.003261763	0.033909365	13.85687375	1.04E-05
0.026643537	0.010635803	0.133468587	2.943729798	9.49E-05
0.000176351	0.000713435	0.008792539	136.2262874	2.23E-07
0.008443438	0.01409479	0.175114259	3.798198507	1.35E-05
0.003804999	0.011115654	0.092210793	5.691386886	9.95E-06
0.001390106	0.005274336	0.046919219	13.31885821	2.97E-06
0.000575515	0.003098505	0.041307164	17.45200801	5.85E-07
0.018244032	0.004375593	0.044776481	10.75476452	0.000121693
0.003123019	0.009097634	0.061782165	7.716373305	1.18E-05
0.063900728	0.044825332	0.382902168	1.070266433	0.000294002
0.001319768	0.004094942	0.05072961	19.93742238	2.20E-06
0.000434175	0.001159507	0.013070472	68.1025701	6.66E-07
0.003842347	0.00518331	0.051662302	10.31492327	9.36E-06
0.040420967	0.005649743	0.073262389	5.167139103	0.00023166
0.000672732	0.001125173	0.011363808	55.73896487	1.54E-06
0.009017154	0.006007931	0.073578391	5.676865902	2.02E-05
0.005012473	0.010265655	0.133758514	6.097356276	7.56E-06
0.001556981	0.003645805	0.040505095	19.75606252	2.88E-06
0.009521421	0.011967559	0.141063892	3.990683662	1.89E-05
0.004524525	0.008601298	0.077992941	7.676670647	1.45E-05
0.065551218	0.028179612	0.29586536	2.139455314	0.000294301
0.010439027	0.012557732	0.117778135	4.004246384	2.75E-05
0.003026484	0.001910877	0.017245374	34.56637902	1.26E-05
0.008775098	0.008116524	0.072644073	6.312091697	3.71E-05
0.018679141	0.007110161	0.080105513	6.393092319	8.35E-05
0.001371943	0.001629752	0.014556932	36.82825567	4.66E-06
0.004547037	0.003995382	0.04862122	10.40968482	8.45E-06
0.00303555	0.002739496	0.029865526	16.15465266	7.06E-06
0.046206716	0.016430894	0.172433566	2.756128872	0.00020323
0.007949852	0.010182522	0.119364356	4.831867244	1.50E-05
0.001142498	0.001338506	0.014326349	61.63611329	3.01E-06
0.005442942	0.014837994	0.128520204	4.021533287	1.45E-05
0.008679774	0.009454392	0.103077092	4.66722181	1.84E-05
0.02470924	0.039515943	0.313656948	1.958946075	0.000121946
0.003748806	0.003304045	0.0398112	12.63597273	6.98E-06
0.001449736	0.002431063	0.022015543	26.83587515	3.95E-06
0.001247562	0.002988696	0.034184365	24.27414705	2.15E-06
0.0025174	0.002568963	0.027880753	29.29936164	8.15E-06
0.006047299	0.019024943	0.2035063	3.108565508	9.62E-06
0.004338432	0.004928368	0.058550895	9.003348759	8.43E-06
0.081968693	0.121600219	0.724755082	0.736047734	0.000636942
0.134933542	0.011639673	0.251918082	1.174519995	0.000238403
0.000509399	0.000865909	0.00788793	76.76226426	1.41E-06

original_gldm_GrayLevelVariance	original_gldm_HighGrayLevelEmphasis	original_gldm_GrayLevelNonUniformityNormalized	original_gldm_DependenceEntropy	original_gldm_DependenceNonUniformity
26.36405099	603.7342342	0.089968347	6.23229929	110.2882883
34.98222257	396.3185841	0.060850497	5.377410657	35.26548673
48.57712108	831.3022774	0.146895053	6.429299487	49.83643892
32.11472117	459.076087	0.113185255	5.164479756	16.69565217
27.30021958	730.9718499	0.183053138	6.167870525	72.38337802
66.56141012	545.8737864	0.061740032	5.409577775	35.48543689
12.65439982	1584.057709	0.268328756	6.505091492	754.7936956
6.148355659	1015.828577	0.235371326	6.234213277	302.8406659
4.738968246	1026.560889	0.258266892	5.980674494	261.782218
57.21068561	836.7554945	0.067579399	6.300080417	78.01648352
11.29891434	717.9011713	0.224090292	6.335670183	335.3967789
5.926119274	1056.525545	0.254699482	6.139065966	170.5053693
101.2522157	895.265861	0.068299851	6.377076024	76.26283988
17.04930748	228.0631579	0.106260388	5.461156564	35.26315789
5.812049858	495.6163043	0.230132325	6.019522117	59.1826087
12.72528343	1507.177436	0.299059332	5.990868415	469.1240765
14.25852201	695.9954128	0.143538002	6.019402597	43.85321101
19.53786219	279.009009	0.150231312	5.298215866	15.7027027
16.3196999	451.5608466	0.14928349	6.131234572	56.40388007
21.58904273	1212.976105	0.179699239	6.715943076	764.9280624
35.85278926	657.4483471	0.198292087	6.462303078	120.5385675
11.02038936	277.8670886	0.147732735	5.508040618	20.3164557
19.73389395	535.7188612	0.172325578	5.749899329	27.81850534
4.3691707	423.8940466	0.327780815	5.897130303	222.1253122
27.97734672	1118.614325	0.28304457	5.912189593	44.54820937
18.0916192	1418.403457	0.190082975	6.357243729	143.0088889
14.58679333	743.5292865	0.120660617	6.399459676	105.85836
9.649926424	568.2103468	0.149114067	6.565778466	143.233087
28.24489796	140	0.086734694	4.235926351	12.28571429
13.49917157	472.5977411	0.183352066	6.102035832	93.07645526
9.223395633	1116.761322	0.23278923	6.413496059	193.7126175
25.25188774	598.6196078	0.129719339	5.883840503	35.0627451
27.6766275	772.1258796	0.134613939	6.527377303	126.0977326
8.366264863	1112.276913	0.248369007	6.050313	196.501429
14.15990168	827.5004314	0.187208534	6.493450212	242.2418752
39.67108986	923.4680851	0.15145585	6.463480804	117.0248227
90.23971729	846.7717949	0.037080868	6.216328259	148.2461538
9.610965449	1402.487732	0.267493698	6.451747814	569.680184
12.91525162	215.7969543	0.117524286	5.734149854	28.93908629
12.29260433	1245.534918	0.305630425	6.031008123	598.1013703
22.72674119	650.1061807	0.120536667	6.235409463	69.95087163
10.30394174	910.7351112	0.191656597	6.345009684	503.3775045
29.68766267	941.5140799	0.099136306	6.662866153	175.97315
14.3168797	616.1272984	0.144225556	6.435991118	65.71004243
7.059256055	373.6691176	0.278541644	5.746062236	63.02941176
19.17089344	714.484399	0.198178453	6.252274879	139.857289
6.321754454	1050.495336	0.339985222	5.93318084	325.3969006
6.980713496	166.6164384	0.184128771	5.466650554	23.52054795
21.92797784	325.4210526	0.082333026	5.364492238	27.64912281
82.58039256	1210.013636	0.051859504	5.961562376	67.72727273
2.366838745	113.9207765	0.247157317	6.168250076	102.9233998
49.31030376	1631.463177	0.084220411	6.926689891	258.7582214
5.532522703	945.5176203	0.340046552	5.900148257	476.7646011
8.486829689	516.5714286	0.17180855	6.186895023	73.91381346
45.49823356	832.8188153	0.101579478	6.194686842	44.49825784
23.63338634	447.3846154	0.13532574	6.15739688	71.42232278
7.443855224	693.9790051	0.224642677	6.236258522	336.4991489
17.78989747	903.4783172	0.18780239	6.49817639	104.4757282
14.13965182	320.1323529	0.105157872	5.910983209	40.29411765
5.479417503	1399.20427	0.236426272	6.078899294	1535.556628
50.94481974	1044.262233	0.164719014	6.40487654	83.3161857
67.07150621	1282.613849	0.12029729	6.589069509	188.3300062
92.74800318	1473.36133	0.071215658	7.094466362	671.4488189
30.50407128	1568.690376	0.177744716	6.985263835	521.0112676
13.4252385	317.9875389	0.14686387	5.94380392	33.90342679
96.03603433	1167.796796	0.064330232	6.848694527	429.4411899
32.92422851	417.9411765	0.077607514	5.476950983	28.39495798
32.11028108	1532.621609	0.188221111	6.536758705	302.7533915
8.111011978	1024.742891	0.25218598	6.261371558	553.9464232
55.50542366	1071.975115	0.112107796	6.687562159	185.1100196
25.74484746	402.0656566	0.12962963	5.770619316	23.87878788
8.908965094	1427.520496	0.226212358	6.391147943	416.9056205
14.59233406	676.9158879	0.228578915	6.07530927	51.83845127
8.270205997	879.5377446	0.203065327	6.165608531	75.24231128
20.91448531	1389.75342	0.20247565	6.555153711	209.6094527
30.74769473	735.544702	0.170672339	6.351352301	60.35430464
118.7328523	1216.970985	0.038942342	6.980331035	351.388664
80.23969754	619.2869565	0.044688091	5.578979264	35.22608696
59.63281351	685.0168971	0.080139028	6.554606109	115.0614439
3.388560295	308.8276942	0.256505141	5.853127886	97.01503759
26.36405099	603.7342342	0.089968347	6.23229929	110.2882883
67.75302767	648.1842818	0.076563774	6.309454873	67.80758808
4.743539948	668.2053833	0.360985845	5.966340959	153.9666472
5.786654595	252.8782979	0.179333997	6.092704989	91.3387234
19.4032133	748.9717452	0.137137376	6.683117629	137.9052632
92.88003616	584.4090909	0.040741219	5.695131835	68.625
21.71368561	1026.997241	0.130037574	6.284483359	81.52137931
6.473178535	591.4800787	0.392820842	5.675991992	183.0034432
136.7946401	1469.510638	0.067354278	7.055404403	180.7770213
50.62996094	839.88875	0.1058375	6.581835292	106.27
100.4715921	567.0942029	0.04652384	6.260478636	78.34782609
5.950417624	370.1195804	0.182611375	6.321995548	99.43076923
33.35827557	906.3690824	0.111911751	6.700903418	372.9853887
23.13078029	1188.377011	0.22966481	6.227929293	240.4353918
19.75021164	817.8195452	0.115675664	6.298552403	272.0827286
59.88358291	1076.976351	0.102542629	6.541406806	176.6993243
36.96546075	1276.005461	0.148678261	6.481060192	149.5365188
71.53335976	685.091954	0.055621614	5.329221048	27.66666667
25.62626463	370.2253521	0.075580242	4.774412746	25.11267606
49.44737399	1101.924198	0.175968867	6.659903983	665.137651

original_gldm_GrayLevelNonUniformity	original_gldm_SmallDependenceEmphasis	original_gldm_DependenceNonUniformityNormalized	original_gldm_DependenceVariance	original_gldm_LargeDependenceEmphasis
59.91891892	0.34067884	0.16559803	4.990684378	15.78378378
6.876106195	0.562513445	0.312083953	1.981674368	6.380530973
70.95031056	0.289671232	0.103181033	33.52851613	74.53623188
10.41304348	0.410092868	0.18147448	7.576086957	19.82608696
136.5576408	0.287247052	0.097028657	35.37575919	88.74798928
6.359223301	0.604080202	0.344518805	2.590442077	7.194174757
4375.368699	0.090999297	0.046289323	39.62314968	177.6460199
1187.68371	0.125924973	0.060015986	23.63671707	92.58660325
1092.210688	0.108023145	0.061901683	20.68224654	89.68337668
24.5989011	0.438653635	0.214330999	4.353127642	12.23626374
1224.429356	0.136324047	0.061383012	26.14770967	86.69912152
782.6915067	0.119356295	0.055484988	28.43061933	105.5193622
22.60725076	0.485423152	0.230401329	11.34892891	22.65558912
20.18947368	0.408093542	0.185595568	6.135623269	16.85263158
211.7217391	0.136021813	0.064328922	23.83300567	81.26304348
2550.078926	0.10723852	0.05501631	28.60376789	122.4734373
62.58256881	0.211859643	0.100580759	10.00707011	35.46788991
16.67567568	0.329954487	0.14146579	10.50693937	27.16216216
84.64373898	0.25408965	0.099477743	15.5989474	44.32627866
2143.272826	0.158538034	0.064134155	25.95199682	84.77177832
287.9201102	0.252004624	0.083015542	80.84067952	186.9931129
23.34177215	0.255629548	0.128585163	7.698605993	24.51898734
48.42348754	0.253711352	0.09899824	15.08640975	44.69395018
1574.659034	0.101947699	0.046237575	41.94446742	150.244796
205.4903581	0.188474571	0.06136117	43.50389697	122.1900826
384.9180247	0.181102013	0.070621674	22.8722085	72.28790123
113.3003195	0.253309314	0.112735208	9.614544737	30.10969116
262.291643	0.178372997	0.081428702	18.55387367	55.66060262
2.428571429	0.615079365	0.43877551	0.387755102	2.857142857
211.0382276	0.195182403	0.08086573	16.06526112	55.33709818
817.3229849	0.133722072	0.055173061	31.0904835	118.6576474
33.07843137	0.341733709	0.137500961	12.92524414	35.47843137
172.1712275	0.250479772	0.098590878	16.33717947	44.91243159
782.1140044	0.102699562	0.062401216	20.66519366	84.50778025
650.9240725	0.178177679	0.069669794	22.59833415	72.70779408
170.8421986	0.295074023	0.10374541	27.06435479	63.72695035
14.46153846	0.629608974	0.380118343	1.424194609	4.738461538
3139.841029	0.082262806	0.048532986	33.09060692	147.7655478
23.15228426	0.294186875	0.146898915	5.763508465	19.01015228
3702.406967	0.112742259	0.04937274	36.76930042	133.7480601
76.05863708	0.272119413	0.110857166	14.7628271	38.48019017
1387.018792	0.150135625	0.069556101	19.49798134	68.20823546
151.3811395	0.286824993	0.115241094	12.1070579	33.98886706
101.9674682	0.245035527	0.092942069	22.73786684	56.41725601
303.0533088	0.15158823	0.057931445	32.8263949	105.9540441
387.4388747	0.196882795	0.071538255	25.87760258	76.34936061
2369.35701	0.091746952	0.046692051	38.73982669	164.6051083
40.32420091	0.273467681	0.107399762	13.48825087	38.67123288
9.385964912	0.473040786	0.242536165	2.811326562	9.105263158
11.40909091	0.553240453	0.30785124	2.478842975	7.081818182
471.0818468	0.106231946	0.053999685	29.20849606	110.5299056
181.8318666	0.302045758	0.119850959	15.85474422	38.6720704
3435.150267	0.091880774	0.04719507	37.07029722	156.2219363
145.5218418	0.201626588	0.087265423	18.13062005	51.96812279
29.1533101	0.382548403	0.155046195	18.2279984	38.77700348
89.72096531	0.281754046	0.107725977	18.38873533	47.30165913
1187.685833	0.132881284	0.06364652	21.60419748	80.6587857
290.1546926	0.197381859	0.067621831	30.98478483	90.0407767
28.60294118	0.34288518	0.148140138	7.682093426	22.30147059
5559.090928	0.099351382	0.065306708	17.97062575	81.6464509
131.281054	0.306334421	0.104537247	53.10402403	107.923463
192.8365565	0.317143192	0.117485968	19.44259457	48.59451029
325.5979878	0.356706544	0.146861071	11.5561834	28.54024497
1514.384977	0.185406924	0.061151557	38.19139583	113.6079812
47.14330218	0.276064893	0.105618152	15.33096534	42.36448598
140.5615561	0.434984399	0.19654059	15.35345234	30.49748284
9.235294118	0.475224329	0.238613092	3.249064332	9.991596639
776.9767442	0.2162312	0.073341422	29.07698888	85.2252907
2527.660082	0.111155657	0.055267527	27.77560524	113.0435
171.1886051	0.325468019	0.121224636	22.97340814	52.89652914
25.66666667	0.285151191	0.120599939	9.885725946	29.90909091
1605.881533	0.102889756	0.058727373	23.11834488	103.1929849
171.2056075	0.20073926	0.069210215	31.56746601	89.82777036
217.889096	0.154611604	0.07012331	19.32483591	68.03821062
651.1616915	0.187788695	0.065177069	34.64683114	97.22201493
103.0860927	0.290294396	0.099924345	47.16179115	98.88741722
57.71255061	0.436081173	0.237104362	2.439206602	8.44534413
5.139130435	0.556462265	0.3063138	2.753572779	7.747826087
52.17050691	0.414764303	0.17674569	25.65553172	46.84946237
409.3822055	0.131885533	0.060786364	25.05121199	87.77443609
59.91891892	0.34067884	0.16559803	4.990684378	15.78378378
28.25203252	0.41510256	0.183760401	14.49044881	30.42547425
1233.84962	0.088380011	0.04504583	40.37604381	164.863663
210.7174468	0.182482971	0.077735084	19.69315636	59.6893617
247.532964	0.189050396	0.076401808	20.13216166	62.12686981
7.170454545	0.631524052	0.389914773	5.16322314	9.727272727
94.27724138	0.275690458	0.112443282	11.9935201	34.52
1597.209543	0.107122851	0.045008225	56.27193414	213.1805214
79.1412766	0.354835345	0.153852784	12.54419267	28.83829787
84.67	0.346556879	0.1328375	18.90109375	43.5275
12.84057971	0.493002735	0.283868935	5.365469439	11.72463768
261.1342657	0.153647068	0.069532006	22.16710059	70.52307692
382.9620105	0.277018818	0.108996315	14.41606397	39.33430742
885.1281785	0.181296606	0.062385935	32.71618809	102.2096523
239.1015965	0.304687695	0.131631702	10.41211341	27.86405418
121.410473	0.372678936	0.149239294	14.66712701	35.00844595
217.8136519	0.291164975	0.102072709	25.56107584	64.52764505
4.83908046	0.534744572	0.318007663	1.228695997	5.091954023
5.366197183	0.601173469	0.353699663	2.26740726	6.323943662
1690.004998	0.202486186	0.069256315	31.60227933	91.35047897

original_gldm_LargeDependenceLowGrayLevelEmphasis	original_gldm_SmallDependenceHighGrayLevelEmphasis	original_gldm_LargeDependenceHighGrayLevelEmphasis	original_gldm_SmallDependenceLowGrayLevelEmphasis
0.026880074	171.1245172	10520.3018	0.003172339
0.03170816	175.0014683	3493.433628	0.020729278
0.080405074	161.1766667	74370.15942	0.005561558
0.05035357	138.5239782	11255.40217	0.015803207
0.104011743	137.9757633	77559.90751	0.002561161
0.035329741	246.7276659	5425.601942	0.026575178
0.109114861	107.9857729	290093.6869	0.000194869
0.088286326	106.8667143	97589.93242	0.000381062
0.085676994	94.22161086	94367.2996	0.000408743
0.01820868	297.897093	12736.32143	0.005654943
0.120351851	94.95169557	63361.82723	0.000777602
0.097585794	105.7283516	114734.8529	0.000538208
0.030659549	300.0436017	28922.89728	0.006333989
0.076105046	74.77740636	4490.789474	0.012243881
0.160789186	56.28842785	41556.00761	0.001632808
0.079004365	128.5233701	190757.4671	0.000293632
0.048992711	113.4024142	27250.52752	0.002839524
0.162224897	66.27686462	9350.522523	0.016428072
0.089053905	79.30590731	23063.71252	0.003865025
0.064781987	146.9004815	111882.7382	0.000341745
0.231405631	101.7159511	154671.0861	0.002548534
0.084768737	48.70855411	7916.791139	0.008794017
0.078812036	92.21292276	27492.03915	0.006434566
0.342000256	33.67522697	66315.40654	0.001193555
0.102148998	148.5124824	149037.259	0.002076146
0.048998469	206.0277981	108392.8321	0.000785558
0.039890186	159.0876208	23791.23962	0.001629321
0.09476558	84.88616511	33285.43036	0.001185459
0.100515246	80.83928571	450.9285714	0.057311698
0.109250881	62.80545954	28791.25282	0.002234459
0.103070606	117.1698764	137346.3204	0.000481282
0.08281817	145.7436283	25020.47059	0.003598218
0.054993757	142.4913021	39274.07428	0.003169678
0.075683209	100.9157185	95658.29216	0.000933961
0.083940216	116.2604567	63643.53724	0.000755382
0.061003557	187.8005527	69357.27305	0.002317251
0.010260312	438.9853996	5413.171795	0.005029912
0.104240025	95.01897547	210497.3453	0.000353082
0.090070416	56.07570346	4653.878173	0.00914326
0.104094525	114.7755581	172602.8389	0.000350412
0.053036944	121.3483657	29595.76704	0.002968625
0.071555367	110.2549778	65605.62526	0.000572543
0.033486451	203.1774717	36223.28225	0.001576643
0.086877944	123.7439606	37613.19943	0.002167971
0.265941426	40.79940369	42597.8989	0.002173753
0.098186369	98.94330142	60378.12737	0.001383663
0.152109056	76.90631272	178551.8097	0.000295033
0.216886739	33.03769081	7204.872146	0.008642349
0.034403695	119.9016417	3663.929825	0.012622671
0.04386406	545.3574692	11049.48636	0.003554028
0.95514753	9.701845016	13181.33945	0.003154121
0.021044947	375.7346657	74596.11811	0.000910222
0.161250521	69.92836265	151664.966	0.000275519
0.101607389	107.1145336	27122.27863	0.001830305
0.043943492	235.1405717	39161.03484	0.005397472
0.095204859	84.15472292	25542.44344	0.004637722
0.113645113	76.64520482	57896.75449	0.000941859
0.093079579	130.3054473	88189.69579	0.001148101
0.071260775	92.42148552	7774.683824	0.00710234
0.057525164	125.2031911	116236.1268	0.000147688
0.086510151	218.3668172	138508.0916	0.002252185
0.034145548	273.1127624	74933.00811	0.002647533
0.016945169	378.1104538	53123.65792	0.000934367
0.068705544	224.3714276	189720.6512	0.000536875
0.120926903	62.44648094	15619.55763	0.005503427
0.023104629	366.8372636	50302.18764	0.001873717
0.030737222	150.1990437	5313.067227	0.01089012
0.051986866	240.9954606	143233.1725	0.000870779
0.108163124	92.61007625	119514.2889	0.000230626
0.041902714	242.3883475	69842.64833	0.001518058
0.073922179	71.43363221	14348.77273	0.011546365
0.070559276	119.8395373	151632.8477	0.000255269
0.124684962	99.68121807	65779.01202	0.002077632
0.076168978	117.530398	61874.37465	0.00128187
0.065600159	207.4736861	145413.9804	0.000568709
0.115118107	147.5255151	88311.14735	0.004241087
0.205232101	464.8675769	12003.16667	0.003406756
0.084830238	280.9130591	7116.26087	0.007599733
0.053298805	194.3879986	46725.79724	0.004476352
0.275339285	32.34589565	28173.73872	0.001322861
0.026880074	171.1245172	10520.3018	0.003172339
0.04674572	169.6120586	27819.95122	0.012841246
0.242956072	48.79492224	112528.017	0.000716756
0.214221162	34.07523748	16944.26723	0.0023264
0.075696145	98.43002554	52106.2482	0.001246173
0.030548865	283.7903045	10118.01136	0.011390467
0.032474462	232.6981535	38917.64828	0.001936831
0.342687716	44.44704839	132912.0915	0.000704375
0.077057113	390.1353395	55485.11404	0.002596668
0.045224847	196.4213771	45701.39125	0.002904405
0.038839772	206.5477649	11568.05797	0.008011926
0.179077358	43.95086524	28170.07203	0.001475731
0.038899347	184.5639662	41329.42694	0.001238267
0.079511763	156.1694857	132468.0646	0.00066006
0.031000048	203.5508772	25928.28302	0.001143237
0.028959934	288.8059206	45550.88514	0.002019099
0.045048661	272.9528791	94816.03959	0.001180451
0.021209236	292.3671552	4458.609195	0.014212331
0.027681894	173.9179371	3247.774648	0.016799412
0.073332328	143.0204376	117773.4858	0.001002692

original_gldm_LowGrayLevelEmphasis	original_gldzm_DistanceZoneVariabilityNormalized	original_gldzm_LowIntensityEmphasis	original_gldzm_LargeDistanceEmphasis	original_gldzm_HighIntensitySmallDistanceEmphasis
0.004268117	0.958696078	0.009071506	1.063291139	487.4757384
0.021704607	0.942906574	0.034652216	1.088235294	298.7242647
0.006404298	0.974443288	0.017549038	1.070967742	557.7096774
0.016997486	1	0.034136628	1	353.744186
0.003462629	1	0.008465094	1	481.5869565
0.027165724	1	0.040403299	1	435.2647059
0.000764953	0.836313527	0.002205556	1.785665529	1030.518509
0.001229331	0.830153678	0.00315613	1.525619835	756.008517
0.001266896	0.80372241	0.004022197	1.700471698	753.7704789
0.006418346	0.923618285	0.011874516	1.119318182	670.2528409
0.002137305	0.862626042	0.006141709	1.34478022	626.575435
0.001358129	0.865228367	0.004684862	1.407407407	801.6440131
0.008198988	0.977145912	0.013107972	1.034682081	610.2947977
0.014952219	0.976473923	0.028330196	1.035714286	182.6458333
0.003356812	0.968262227	0.012140748	1.048387097	404.3346774
0.000888525	0.775959552	0.002825788	2.132960894	1004.704495
0.003935954	0.9608	0.012539915	1.06	523.9
0.032067927	0.95125	0.058414681	1.075	196.13125
0.005416138	0.921699292	0.01503064	1.12244898	295.127551
0.001037954	0.688885643	0.002171858	2.37057919	734.6623423
0.004056846	0.985366649	0.009796787	1.022113022	397.5042998
0.011343161	1	0.029696566	1	196
0.007716199	0.973338203	0.024539739	1.040540541	354.652027
0.003314456	0.798797739	0.012350976	1.788008565	273.1856715
0.002769092	0.958639159	0.010676127	1.063380282	769.1338028
0.001357227	0.54711995	0.004385091	2.713114754	897.8467858
0.002626146	0.991902968	0.006319351	1.012195122	619.0081301
0.002643649	0.905019096	0.006622555	1.223602484	445.90625
0.065633967	1	0.08391577	1	131.25
0.003953234	0.908241966	0.011481909	1.186956522	291.4573671
0.001239258	0.912501858	0.003656105	1.252155172	813.5506466
0.010432381	1	0.015340247	1	434.688172
0.004193091	0.953106259	0.012399561	1.072072072	540.1328829
0.001744612	0.832524415	0.010052473	1.597938144	873.8107341
0.001736509	0.947400602	0.004241181	1.127962085	620.7528085
0.003081123	0.988668923	0.007611479	1.017094017	626.1631054
0.005709141	1	0.007541641	1	716.3159851
0.001014821	0.719609457	0.004580376	2.24039517	955.7175034
0.012908972	0.937565036	0.02993814	1.096774194	179.3145161
0.001072114	0.785265683	0.003302001	1.817270625	869.6644375
0.004030865	1	0.010363044	1	443.0978261
0.001493443	0.771984115	0.003868406	1.680184332	627.4009834
0.002298727	0.944135004	0.005391814	1.097130243	672.6208609
0.003365869	0.967922968	0.008474494	1.048913043	495.9538043
0.004403854	0.883774539	0.014584359	1.359756098	231.5434451
0.002510349	0.898000464	0.007039056	1.241116751	452.5788388
0.00115201	0.868471501	0.003389465	1.54950495	739.430231
0.012842122	0.968757874	0.030578365	1.047619048	119.984127
0.014073094	1	0.024330941	1	261.15
0.011097551	0.788343038	0.009934849	1.360902256	958.443609
0.011258687	0.891970254	0.03130166	1.196891192	82.28641336
0.001336331	0.880895759	0.002906473	1.218390805	1151.466156
0.0012271	0.75931549	0.003124615	2.50896861	623.6972554
0.00341065	1	0.009201472	1	532.8928571
0.006103541	0.983194484	0.013256575	1.025423729	615.0105932
0.006709948	0.933412604	0.016061978	1.103448276	279.6083744
0.002213306	0.798811734	0.0074811	1.904903418	493.9590969
0.002003419	0.974785995	0.005727035	1.054140127	640.5525478
0.009131367	0.943444245	0.019107322	1.087378641	271.1383495
0.0007891	0.581006566	0.001597668	4.546807489	916.6574456
0.002914596	0.9559048	0.006914324	1.067669173	703.7227444
0.00315109	0.900222496	0.007976013	1.230483271	798.1579926
0.001447688	0.895460306	0.002610858	1.165994236	986.7845821
0.00105914	0.873214771	0.002850739	1.406079027	1104.874096
0.00773432	0.937565036	0.019323837	1.096774194	214.1774194
0.002694187	0.675962136	0.004185857	1.837452471	722.7926766
0.01222231	0.936576189	0.021489372	1.098360656	313.6762295
0.00145193	0.885680572	0.004064717	1.218985976	1039.903931
0.001256155	0.774185848	0.002618515	2.755877034	694.7426837
0.002174022	0.934868568	0.004496009	1.119402985	703.5226472
0.013168235	1	0.038898447	1	252.8983051
0.000876191	0.81056987	0.002642621	1.994100295	999.0266756
0.003210333	0.987097318	0.010184026	1.019480519	488.2077922
0.002240244	0.98870093	0.007929391	1.017045455	750.3196023
0.001149107	0.891043879	0.002938836	1.180967239	1045.475862
0.005175468	0.940649729	0.013302516	1.091836735	499.7359694
0.012371772	0.553056	0.007996768	2.236	891.0653056
0.021705444	1	0.01939996	1	519.2916667
0.005519395	0.954422222	0.010057804	1.07	463.6608333
0.004095015	0.863276463	0.010292004	1.291262136	220.9761327
0.004268117	0.958696078	0.009071506	1.063291139	487.4757384
0.013794359	0.943230396	0.028036767	1.087719298	403.6140351
0.002179676	0.821519047	0.008813117	1.558219178	482.3202293
0.005537516	0.938925412	0.012729074	1.094594595	171.3096847
0.002305696	0.9616585	0.006443947	1.058659218	494.9713687
0.013915396	0.952783559	0.017267	1.072580645	450.3447581
0.002622942	1	0.006744491	1	834.8151659
0.002185484	0.879481596	0.006769526	1.428571429	363.3022014
0.008588781	0.517817089	0.008293233	2.430107527	920.4841249
0.003815565	0.941488889	0.008063567	1.123333333	546.8940741
0.012170899	0.963203125	0.015767149	1.05625	419.1046875
0.003767106	1	0.009940307	1	273.4814815
0.002027383	0.891231361	0.004385212	1.24749499	604.3917975
0.001329403	0.849881633	0.003684985	1.462857143	749.0865607
0.001969179	0.918680107	0.003599177	1.134502924	637.8596491
0.002581529	0.953807377	0.005240382	1.070967742	755.1467742
0.001710414	0.978310634	0.003879298	1.032894737	928.2889254
0.015153308	1	0.023705535	1	565.7735849
0.017641875	0.959201389	0.025105699	1.0625	297.5885417
0.001876006	0.722873986	0.005024054	2.060068932	550.1839933

original_gldzm_LowIntensityLargeDistanceEmphasis	original_gldzm_HighIntensityEmphasis	original_gldzm_DistanceZoneVariability	original_gldzm_ZonePercentage	original_gldzm_IntensityVariabilityNormalized
0.009157844	499.9029536	227.2109705	0.355855856	0.047125639
0.034769006	315.4117647	64.11764706	0.601769912	0.053200692
0.017613176	569.5483871	151.0387097	0.320910973	0.046742976
0.034136628	353.744186	43	0.467391304	0.065440779
0.008465094	481.5869565	230	0.308310992	0.058223062
0.040403299	435.2647059	68	0.660194175	0.047577855
0.002703086	1143.277133	1225.199317	0.089844229	0.048696199
0.00378928	824.5818182	502.2429752	0.119896948	0.077970084
0.004695256	845.2169811	340.7783019	0.100260109	0.093761125
0.012397753	687.0170455	162.5568182	0.483516484	0.04222624
0.006535433	684.5041209	627.9917582	0.133235725	0.0584395
0.005059884	865.6210826	303.6951567	0.114220631	0.074634134
0.01313199	622.8150289	169.0462428	0.52265861	0.032443449
0.028488926	184.6547619	82.02380952	0.442105263	0.06547619
0.012250469	409.6693548	120.0645161	0.134782609	0.086108221
0.0035454	1167.509497	694.4837989	0.104960713	0.077377111
0.012625332	534.97	96.08	0.229357798	0.069
0.058674197	201.55	38.05	0.36036036	0.07
0.015511901	305.3061224	135.4897959	0.259259259	0.064926651
0.00338799	897.6986161	1344.015889	0.163578435	0.044552058
0.009832281	400.963145	401.044226	0.28030303	0.051258987
0.029696566	196	48	0.303797468	0.088541667
0.02459535	362.0405405	72.02702703	0.263345196	0.061723886
0.014145416	314.012848	373.0385439	0.097210658	0.085666861
0.010725032	789.6690141	136.1267606	0.195592287	0.055742908
0.050417878	1116.234973	200.2459016	0.180740741	0.06013915
0.006332901	621.7520325	244.0081301	0.261980831	0.058496926
0.007011973	469.5248447	291.4161491	0.183058556	0.062594036
0.08391577	131.25	20	0.714285714	0.08
0.011852302	310.626087	208.8956522	0.199826238	0.065557656
0.003875065	855.9633621	423.4008621	0.132156081	0.066587396
0.015340247	434.688172	93	0.364705882	0.061625621
0.012499762	554.3963964	317.3843844	0.260359656	0.048922797
0.010580827	960.1443299	242.2646048	0.092410289	0.083926737
0.004392041	639.6461295	599.7045814	0.182053494	0.051294146
0.007626802	630.968661	347.022792	0.311170213	0.042637641
0.007541641	716.3159851	269	0.68974359	0.032130568
0.009361577	1129.501647	655.5642151	0.077611177	0.054199135
0.030358491	185.7258065	58.12903226	0.314720812	0.074401665
0.00471384	976.7740941	1018.489591	0.107066204	0.060088253
0.010363044	443.0978261	184	0.291600634	0.062086484
0.004762887	712.397235	837.602765	0.149924002	0.062962475
0.005479444	697.602649	427.6931567	0.296660118	0.047098324
0.008543743	504.6684783	178.0978261	0.260254597	0.060491493
0.015315074	256.6280488	144.9390244	0.150735294	0.089976205
0.007343008	485.8604061	353.8121827	0.201534527	0.055322219
0.003893125	799.2739274	526.2937294	0.086956522	0.072258711
0.030909052	121.6984127	61.03174603	0.287671233	0.091458806
0.024330941	261.15	60	0.526315789	0.063888889
0.036676378	1008.428571	104.8496241	0.604545455	0.037367856
0.032960342	87.90673575	172.1502591	0.101259182	0.123305324
0.003079874	1228.954023	613.1034483	0.322371468	0.038933479
0.004679185	733.338565	677.309417	0.088299347	0.077281365
0.009201472	532.8928571	168	0.198347107	0.058460884
0.013279921	621.9322034	116.0169492	0.411149826	0.049554726
0.01625529	294.3497537	189.4827586	0.306184012	0.058991968
0.00875459	559.7161961	537.6002972	0.127293361	0.070697615
0.005776242	651.5987261	306.0828025	0.203236246	0.052659337
0.019415553	277.4951456	97.17475728	0.378676471	0.060231879
0.004113565	1227.591455	1210.236678	0.088589291	0.086531137
0.007022134	717.3721805	254.2706767	0.333751568	0.042879756
0.008124111	863.2156134	484.3197026	0.335620711	0.034155139
0.002901826	1036.500288	1553.623631	0.379483815	0.029038693
0.003108275	1195.350152	1436.438298	0.193075117	0.035092433
0.019536781	225.1935484	87.19354839	0.289719626	0.074343855
0.014891506	848.5228137	711.1121673	0.481464531	0.031023652
0.021782497	322.147541	57.13114754	0.512605042	0.052405267
0.004236289	1106.609493	821.02589	0.224563953	0.043558404
0.004301188	807.7106691	856.2495479	0.110346204	0.06571422
0.004591002	737.0503731	501.0895522	0.351015062	0.0382045
0.038898447	252.8983051	59	0.297979798	0.070956622
0.00335387	1118.90413	549.5663717	0.095506409	0.056099407
0.010212844	491.5	152.012987	0.205607477	0.065103727
0.00794966	753.9034091	174.0113636	0.164026095	0.069214876
0.003179702	1091.455538	571.1591264	0.19931592	0.052913131
0.014048035	513.3163265	184.3673469	0.324503311	0.051853394
0.036586643	1076.906667	414.792	0.506072874	0.031975111
0.01939996	519.2916667	72	0.626086957	0.043209877
0.010192738	473.9433333	286.3266667	0.460829493	0.040488889
0.011341882	237.6990291	177.8349515	0.129072682	0.106560468
0.009157844	499.9029536	227.2109705	0.355855856	0.047125639
0.028131297	424.5789474	161.2923977	0.463414634	0.038678568
0.009590858	538.9726027	239.8835616	0.085430076	0.083458435
0.013056263	178.7387387	208.4414414	0.18893617	0.095446798
0.006510956	508.1173184	344.273743	0.19833795	0.05012328
0.017334141	470.1532258	118.1451613	0.704545455	0.036810614
0.006744491	834.8151659	211	0.291034483	0.057433571
0.007468275	394.7704918	375.5386417	0.105017216	0.075429581
0.037982422	1083.683871	240.7849462	0.395744681	0.028465719
0.008186928	570.9866667	282.4466667	0.375	0.038777778
0.015850608	428.94375	154.1125	0.579710145	0.03453125
0.009940307	273.4814815	216	0.151048951	0.096707819
0.004638536	649.1272545	889.4488978	0.291642314	0.04242754
0.004106221	828.7057143	594.9171429	0.181629476	0.04655102
0.003775556	664.4327485	628.377193	0.330914369	0.05735098
0.00530558	777.1548387	443.5204301	0.392736486	0.03458897
0.003905028	939.4517544	446.1096491	0.311262799	0.045119652
0.023705535	565.7735849	53	0.609195402	0.045211819
0.025205699	307.3541667	46.04166667	0.676056338	0.066840278
0.006325872	670.3791236	1468.157065	0.211474386	0.035231606

original_gldzm_LowIntensitySmallDistanceEmphasis	original_gldzm_IntensityVariability	original_gldzm_HighIntensityLargeDistanceEmphasis	original_gldzm_SmallDistanceEmphasis	original_glcm_SumVariance
0.009049921	11.16877637	549.6118143	0.984177215	2012.99527
0.034623018	3.617647059	382.1617647	0.977941176	1389.472691
0.017539586	7.24516129	648.1290323	0.989426523	3014.161532
0.034136628	2.813953488	353.744186	1	1606.13414
0.008465094	13.39130435	481.5869565	1	2655.776774
0.040403299	3.235294118	435.2647059	1	1949.761261
0.002158966	71.33993174	2407.049147	0.928964327	5882.852091
0.003064919	47.17190083	1310.624793	0.925757576	3710.587112
0.00393848	39.75471698	1585.497642	0.913268475	3765.433878
0.011743706	7.431818182	754.0738636	0.970170455	2847.969645
0.006074332	42.54395604	1029.35989	0.942088294	2537.063025
0.004633195	26.1965812	1313.917379	0.942861665	3882.783931
0.013101968	5.612716763	672.8959538	0.99132948	3251.164903
0.028290514	5.5	192.6904762	0.991071429	728.2759219
0.012113318	10.67741935	431.0080645	0.987903226	1729.688096
0.002760098	69.25251397	2962.305028	0.896935754	5664.904613
0.012518561	6.9	579.25	0.985	2458.859753
0.058349802	2.8	223.225	0.98125	926.3930459
0.014910325	9.544217687	346.0204082	0.969387755	1556.754219
0.002024778	86.92106612	2580.796515	0.854719962	4361.834817
0.009787913	20.86240786	414.7985258	0.994471744	2380.555348
0.029696566	4.25	196	1	946.0765852
0.024525836	4.567567568	391.5945946	0.989864865	1899.487179
0.012138594	40.00642398	680.0107066	0.911001665	1501.820136
0.010663901	7.915492958	871.8098592	0.98415493	4126.434715
0.001107643	22.01092896	2581.346995	0.766962659	5104.693509
0.006315963	14.3902439	632.7276423	0.99695122	2565.658297
0.006557222	20.1552795	602.7453416	0.961568323	1949.49925
0.08391577	1.6	131.25	1	427.4531358
0.011407995	15.07826087	407.9	0.962922705	1650.447108
0.003624252	30.89655172	1149.795259	0.963451868	4077.684808
0.015340247	5.731182796	434.688172	1	2095.150491
0.012374511	16.29129129	611.4504505	0.981981982	2683.247372
0.009987004	24.42268041	1645.635739	0.925329324	4080.111877
0.0042163	32.46919431	750.4028436	0.978541338	2965.632328
0.007607648	14.96581197	650.1908832	0.995726496	3379.327117
0.007541641	8.643122677	716.3159851	1	2982.721868
0.00352543	49.37541164	2872.282108	0.869416697	5185.642071
0.029833053	4.612903226	211.3709677	0.975806452	678.2508434
0.003043574	77.93446415	1938.126446	0.904511152	4586.695893
0.010363044	11.42391304	443.0978261	1	2291.547715
0.003724177	68.31428571	1310.919816	0.899380952	3266.999949
0.005371798	21.33554084	810.2450331	0.978170223	3370.175842
0.008457182	11.13043478	539.5271739	0.987771739	2145.290093
0.014480238	14.75609756	456.1280488	0.951134824	1314.137992
0.006985235	21.79695431	681.248731	0.958139453	2564.226475
0.003334008	43.78877888	1413.689769	0.943783003	3888.987478
0.030495693	5.761904762	128.5555556	0.988095238	520.4677408
0.024330941	3.833333333	261.15	1	1095.249543
0.003249467	4.969924812	1208.368421	0.909774436	4075.496193
0.030921055	23.79792746	114.2797927	0.956534254	346.0496891
0.002866568	27.09770115	1587.551724	0.951787995	5917.450369
0.003007646	68.93497758	2212.745516	0.887452647	3496.040671
0.009201472	9.821428571	532.8928571	1	1741.127331
0.013250738	5.847457627	649.6186441	0.993644068	3003.221177
0.01601365	11.97536946	353.3152709	0.974137931	1579.845001
0.007352589	47.5794948	1217.2526	0.909372214	2464.710333
0.005717983	16.53503185	711.1974522	0.990003539	3289.331011
0.019030264	6.203883495	302.9223301	0.97815534	1032.597658
0.001442411	180.2443591	6338.200672	0.785666627	5186.514118
0.006887371	11.40601504	771.9699248	0.983082707	3791.015904
0.007948915	18.37546468	1236.754647	0.95890128	4733.571564
0.002538116	50.38213256	1235.363112	0.958501441	5169.695205
0.002815789	57.72705167	1876.41155	0.946745356	5748.167135
0.019270601	6.913978495	269.2580645	0.975806452	1075.063727
0.002802703	32.63688213	1569.760456	0.849420416	3971.121353
0.021416091	3.196721311	356.0327869	0.975409836	1404.904166
0.004027365	40.37864078	1425.058252	0.953643773	5667.736822
0.002517785	72.67992767	2659.384268	0.894055422	3743.65775
0.004475847	20.47761194	890.3320896	0.974295191	3890.48375
0.038898447	4.186440678	252.8983051	1	1399.386512
0.002580473	38.03539823	2541.862832	0.915615539	5269.728523
0.010176822	10.02597403	504.6688312	0.99512987	2426.496375
0.007924324	12.18181818	768.2386364	0.995738636	3165.030665
0.002879703	33.91731669	1286.468019	0.956491593	5032.059123
0.013116136	10.16326531	567.6377551	0.977040816	2595.898242
0.00238372	23.98133333	2046.382667	0.768916667	4018.393952
0.01939996	3.111111111	519.2916667	1	2240.924053
0.01002407	12.14666667	515.0733333	0.9825	2414.05674
0.010092742	21.95145631	321.1601942	0.943365696	1056.851396
0.009049921	11.16877637	549.6118143	0.984177215	2012.99527
0.028013134	6.614035088	508.4385965	0.978070175	2313.285805
0.008702263	24.36986301	947.5650685	0.92156583	2424.870878
0.012647277	21.18918919	208.454955	0.976351351	840.282938
0.006427194	17.94413408	560.7011173	0.985335196	2619.148859
0.017250214	4.564516129	549.3870968	0.981854839	2145.563356
0.006744491	12.11848341	834.8151659	1	3699.187176
0.006684706	32.20843091	662.1358314	0.949059329	2145.450501
0.002490047	13.23655914	1919.350538	0.741860812	4776.470496
0.008039785	11.63333333	698.72	0.976574074	3048.844163
0.015746284	5.525	468.3	0.9859375	1889.489569
0.009940307	20.88888889	273.4814815	1	1256.034801
0.004337594	42.34268537	906.3136273	0.955487364	3181.14122
0.0036164	32.58571429	1441.91	0.936242857	4362.380668
0.003557903	39.22807018	774.4678363	0.9679987	2863.756127
0.005224082	16.08387097	865.1870968	0.982258065	3830.55893
0.003872865	20.5745614	984.1030702	0.991776316	4646.525198
0.023705535	2.396226415	565.7735849	1	2360.102715
0.025080699	3.208333333	346.4166667	0.984375	1332.445924
0.004821411	71.55539143	1820.007878	0.873466401	3941.029889

original_glcm_Homogeneity1	original_glcm_Homogeneity2	original_glcm_ClusterShade	original_glcm_MaximumProbability	original_glcm_Idmn	original_glcm_SumVariance2	original_glcm_Contrast	original_glcm_DifferenceEntropy
0.378854829	0.301672707	-560.1887525	0.0360931	0.977114639	52.4654815	30.73936327	3.359173187
0.267276513	0.17720383	-480.2574291	0.034390804	0.944018898	66.65566254	54.52394357	3.670614682
0.494249459	0.448680922	-1674.175879	0.144866751	0.966743579	85.37411675	55.86822463	3.264737056
0.391088224	0.323007839	-663.0439025	0.093680487	0.960012664	54.64762552	39.31820758	3.220441937
0.524620287	0.482768932	-644.1654831	0.16393799	0.97394437	50.00769743	30.06757664	3.042050244
0.289495875	0.215773907	-1599.034195	0.046663618	0.927677726	125.9639159	92.7715258	3.75784556
0.683577871	0.666911942	-629.1680213	0.209728768	0.997389054	26.12650855	10.14914513	1.93819608
0.591086915	0.558059281	-123.6735076	0.150692397	0.995510269	10.76339236	7.555887107	2.181212098
0.612961404	0.582955856	-99.18826016	0.147469465	0.99650716	8.236574466	5.038836971	1.999427529
0.312678083	0.23332035	-2168.170531	0.030665135	0.970155881	139.376934	67.49104329	3.89159703
0.57715633	0.540215405	-209.0645811	0.18552695	0.998059438	24.7559742	10.43001212	2.314474398
0.607271807	0.577181017	-132.043846	0.201977159	0.996212659	9.666703615	6.477927026	2.073850326
0.313830128	0.246866889	-3346.327648	0.054402095	0.94005064	200.799685	116.281783	4.162472692
0.38719376	0.309927114	-162.6602572	0.063632514	0.958640344	26.95159432	23.93073428	3.253046223
0.586837628	0.549682164	-85.555598	0.155936565	0.991740937	9.111856315	6.987462227	2.218146497
0.63629046	0.611989415	-577.8746357	0.179383524	0.995930544	21.4952386	10.35215024	2.025450768
0.48572213	0.426445209	-443.7742968	0.08751657	0.988815062	30.36571698	11.83543019	2.703948669
0.438008119	0.374998777	-303.8901367	0.113432025	0.9568679	32.20509505	23.61537125	3.092785077
0.483383205	0.42677438	-376.4206809	0.101978048	0.978356079	31.58422212	17.33784459	2.903614252
0.545095461	0.502577499	-891.8228225	0.132911148	0.993675276	54.79029476	15.13518831	2.668618278
0.584283151	0.542067492	-1371.447732	0.301859281	0.975823538	80.33670366	23.77237399	2.931764325
0.459365844	0.393692178	-136.7745275	0.104315963	0.97536241	18.21090335	12.46408346	2.807623852
0.489601892	0.437277552	-428.122403	0.133025801	0.975666326	31.18835104	22.46309777	2.878034732
0.667683216	0.64508582	-89.32562734	0.324441625	0.994320863	7.793128978	4.02012254	1.897903328
0.611042039	0.582801002	-1360.564949	0.306569071	0.983594771	53.60732233	31.66465903	2.521814602
0.532075755	0.488481249	-1466.07924	0.130978911	0.987764268	54.71970102	27.15357149	2.81036755
0.449557241	0.384915211	-277.7817608	0.055488278	0.985146766	27.8731324	19.6133241	2.999678845
0.511877196	0.457024765	-184.4527099	0.087259527	0.991110197	19.56458378	9.889258342	2.627634418
0.224293419	0.136399141	-121.268555	0.070703509	0.875829905	59.79590124	60.33414045	2.78844372
0.526407477	0.481094267	-294.9136956	0.111173089	0.983999044	23.92318815	12.66617212	2.59532489
0.621124964	0.59280229	-232.3559198	0.179799465	0.994640035	15.94735764	9.491527154	2.235384201
0.456099454	0.400404139	-709.9372181	0.082783739	0.967589179	45.78796854	35.0789992	3.183251392
0.479968791	0.427676425	-1013.461003	0.094821534	0.979021724	59.88430187	29.23492286	3.072156594
0.601481617	0.568191049	-255.5390099	0.158605028	0.996514625	12.46153383	7.580831546	2.05529843
0.548812478	0.507443297	-391.1243155	0.119544398	0.992816552	26.08192478	13.97268451	2.560178952
0.481773262	0.434261686	-1449.213256	0.144101442	0.976318027	77.09774716	41.55251084	3.204675014
0.206418841	0.129471768	-1568.772686	0.018717004	0.93037551	186.8580039	152.598979	4.473395203
0.659299568	0.638608977	-458.1743289	0.199257902	0.996568336	18.57364529	9.996342601	1.919757197
0.433865116	0.364704419	-69.23048953	0.071432033	0.985159073	27.49892992	14.53578012	2.89380866
0.636734553	0.612206558	-590.9383588	0.276898822	0.998815891	26.75253273	12.02950789	2.151658385
0.450702152	0.390493864	-511.5620936	0.09448704	0.977336656	43.34946131	25.48084368	3.131101614
0.545289421	0.501061487	-365.0433299	0.130435065	0.993388353	24.29608879	11.15112328	2.502775362
0.420122725	0.355142838	-894.7086466	0.04756892	0.978714757	55.67577062	35.89694276	3.333210742
0.503787659	0.448326869	-251.6185444	0.124044815	0.987414752	25.14165086	15.37751325	2.824691742
0.619196059	0.589060633	-115.3989208	0.269707199	0.993206793	11.61224276	6.736242895	2.1777538
0.54233941	0.501766649	-608.9364893	0.169537278	0.98728238	36.7465856	18.90939785	2.70114844
0.685678412	0.668562435	-200.2696986	0.321820891	0.99730963	12.02990721	4.900757372	1.822352277
0.499147623	0.443579698	-68.59433425	0.124895002	0.972083072	11.77412081	9.540601278	2.62816667
0.336123441	0.248371789	-313.0257311	0.049292853	0.958535721	39.86395816	28.17625337	3.275355385
0.233784919	0.158014858	-4819.647404	0.028229495	0.934018789	243.1857818	177.9699089	4.421309666
0.655767158	0.631358061	-13.73499657	0.220709483	0.994786718	5.20195098	2.20953518	1.800150601
0.406447699	0.343309314	-2124.098089	0.062590078	0.981199129	113.4100996	51.72942686	3.603906962
0.675698666	0.656808861	-154.2440471	0.302226217	0.996392799	10.11456656	5.536142102	1.859622053
0.53832591	0.491349132	-40.47240333	0.13780261	0.993052057	15.54610441	9.783484847	2.502576279
0.421228449	0.362838998	-1509.346497	0.101626292	0.966530019	89.63936341	56.49592144	3.491847859
0.469449764	0.412726876	-585.5379134	0.106442533	0.973899066	42.64712147	21.19009513	3.041292063
0.584790539	0.548749386	-180.2277126	0.154095762	0.994952041	13.41381283	8.144270962	2.224239042
0.556810732	0.515282783	-472.6568547	0.16118728	0.989848021	29.80401491	16.7867383	2.632162936
0.426832323	0.352912353	-152.5386511	0.052786551	0.979951082	23.30226011	15.14388791	2.959546662
0.573191034	0.533977131	-149.9516546	0.134511441	0.997670773	10.22845269	6.087912356	2.123066264
0.494265465	0.445153891	-2126.239552	0.235415534	0.970058884	106.7752803	52.9628071	3.428244851
0.425914464	0.37207667	-2936.701265	0.098012166	0.974745574	131.0874114	69.36471355	3.588989836
0.3693993	0.308049263	-5112.095664	0.040627569	0.971672824	245.7212562	92.81808784	3.961946061
0.568893409	0.533830043	-1291.286982	0.142298062	0.995381555	61.63443752	27.59215111	2.764336357
0.488689796	0.432143143	-223.4307969	0.11434788	0.975694984	24.14277956	15.18851742	2.835472148
0.307136847	0.240005087	-4225.369525	0.064818712	0.959254786	274.1547155	110.5456476	4.3258996
0.296840567	0.213888574	-384.4630058	0.040975161	0.950985253	59.20423906	51.48423316	3.675635441
0.548226711	0.510800811	-1520.61165	0.17345796	0.986120651	65.36102993	33.68492771	2.828974975
0.615109342	0.585985614	-217.3890608	0.155404778	0.996890794	14.58065048	7.674247967	2.16073391
0.426932947	0.371385924	-2133.614573	0.089205095	0.97201388	118.7190555	55.38948438	3.592404901
0.470273443	0.413495185	-606.7718659	0.080030234	0.969454313	43.3554006	24.65353044	2.892128529
0.612457426	0.583445725	-290.3111366	0.134878423	0.996834189	16.78729076	7.950083278	2.08237683
0.569963112	0.53331205	-367.0608246	0.214218323	0.983975501	24.84864715	17.71132918	2.582042068
0.577948128	0.540468499	-168.7656083	0.139852093	0.993659526	13.51823339	9.000526269	2.286857523
0.563015529	0.523735826	-1024.064078	0.179362017	0.991840292	49.73912656	23.7761484	2.769817108
0.508310131	0.460931742	-1022.628951	0.245252769	0.969262355	71.42724611	38.80349697	3.257389943
0.247860711	0.16577188	-11795.05701	0.009284801	0.95994226	415.316116	120.7126415	4.404763917
0.276127975	0.196599688	-2370.01093	0.033546345	0.95004729	220.2923148	72.53939291	3.815005511
0.380442653	0.318167056	-1596.564586	0.126657809	0.956012524	141.1948624	61.03634177	3.838379209
0.60473628	0.570469689	-29.43751706	0.209982541	0.993181231	5.353877044	3.919237137	2.069817344
0.378854829	0.301672707	-560.1887525	0.0360931	0.977114639	52.4654815	30.73936327	3.359173187
0.362909112	0.296813407	-2020.108686	0.067631426	0.955077584	140.9874147	67.69088881	3.819900588
0.703666532	0.687711192	-124.7707571	0.347752147	0.995861929	8.515646026	4.911454412	1.75788895
0.544688784	0.497200392	-58.00663336	0.140461734	0.985960992	10.76145925	6.266240275	2.410487537
0.516619469	0.468561602	-628.1431769	0.09489443	0.986190407	43.49052609	20.44855161	2.840546763
0.226468446	0.148669165	-1637.462375	0.039324786	0.921461427	204.6566991	122.5618405	4.306051772
0.457640288	0.396967888	-644.6589231	0.075107888	0.987558317	39.26943007	22.57914437	3.028457503
0.705688292	0.687611434	-155.4364787	0.423826668	0.993016065	11.81547805	6.298510098	1.915940418
0.328560829	0.264885629	-14159.73489	0.043629555	0.957881448	499.7480907	130.2940508	4.265260123
0.420322737	0.361463678	-1628.630275	0.078583834	0.968495515	98.25137157	52.61816104	3.550021823
0.251928068	0.180040369	-1461.870576	0.035107964	0.920843982	256.9918422	122.8929639	4.348614399
0.573155286	0.532167644	-74.18694172	0.142673054	0.99129574	11.20632795	5.995780615	2.290322042
0.437384627	0.376469408	-1212.130674	0.064843173	0.986810619	76.12544343	30.65084526	3.27743938
0.575150331	0.541404722	-1081.755381	0.220345955	0.992149788	50.31643824	20.22757083	2.58956163
0.42968879	0.362929552	-475.4212565	0.074255058	0.98429774	44.07709627	22.69083704	3.178488896
0.39279084	0.335833123	-2042.764739	0.075427783	0.963729453	115.987488	77.69186363	3.806054231
0.478625343	0.430039312	-1422.065815	0.108421988	0.979972433	75.61531535	43.3982215	3.306036441
0.24004138	0.158827798	-1361.957035	0.029944886	0.928936646	136.7436852	112.5239336	3.989968497
0.291618888	0.203722396	-309.3949513	0.046536277	0.952166231	46.21189931	37.36572395	3.336874696
0.530594788	0.48976848	-3569.763066	0.178857504	0.988591444	141.0105473	28.12831571	2.936004397

original_glcm_InverseVariance	original_glcm_Entropy	original_glcm_Dissimilarity	original_glcm_DifferenceVariance	original_glcm_Idn	original_glcm_Idm	original_glcm_Correlation	original_glcm_Autocorrelation
0.311931228	7.011374673	3.652429222	17.33435604	0.912211601	0.301672707	0.260421173	601.2548269
0.193397253	6.476265629	5.577629072	22.64755484	0.848925173	0.17720383	0.113765385	420.1351064
0.331520263	5.782365943	4.014601817	39.32396924	0.917065359	0.448680922	0.218369115	853.9357645
0.314280262	5.506217713	4.057673333	21.89200744	0.88943816	0.323007839	0.188795042	472.8646441
0.342335428	5.381064058	3.094321418	20.43492331	0.92234944	0.482768932	0.247193972	758.2503647
0.227829932	6.047665999	6.786066347	45.68391637	0.843689177	0.215773907	0.152309979	563.1132087
0.423758875	4.30409911	1.279933917	8.500981447	0.980934547	0.666911942	0.432390393	1592.906693
0.478703404	4.676906647	1.439945774	5.474144617	0.967004245	0.558059281	0.178925193	1024.116416
0.48461985	4.39966965	1.208617736	3.575305533	0.969523339	0.582955856	0.238377491	1033.696557
0.235799066	7.34515244	5.593601572	35.7128784	0.898354229	0.23332035	0.35260839	832.5135197
0.45906811	5.004311302	1.617558692	7.800878326	0.978944347	0.540215405	0.40584442	720.393183
0.482158721	4.454025065	1.31239831	4.750962527	0.969693556	0.577181017	0.199108661	1065.003212
0.225653219	7.18324532	7.337454768	61.55122464	0.863039915	0.246866889	0.268509774	930.3970394
0.290784889	6.087159367	3.458848187	11.84250123	0.878017117	0.309927114	0.055118681	232.8473646
0.452964931	4.685160895	1.45176983	4.863146619	0.953444208	0.549682164	0.132769499	498.1484073
0.4671967	4.148596129	1.358640471	8.48408298	0.974603134	0.611989415	0.345030621	1523.077357
0.400192925	5.724461055	2.181639562	6.968851154	0.939324742	0.426445209	0.420132964	709.2803551
0.325264208	5.298749067	3.213784085	13.17752343	0.883870771	0.374998777	0.145068398	284.6670395
0.379840988	5.854365151	2.53496317	10.85825985	0.921078507	0.42677438	0.292901162	465.9331459
0.414962167	5.896246433	2.109214059	10.6655906	0.960618352	0.502577499	0.567891184	1221.451349
0.230608511	5.326010691	2.742233561	16.1660722	0.925963602	0.542067492	0.541110712	685.5586232
0.366982971	5.413601041	2.40297699	6.612993523	0.906697166	0.393692178	0.168211224	291.5116419
0.393041836	5.294545646	2.691049772	15.16239983	0.92144339	0.437277552	0.167719607	551.0440762
0.437304986	3.908929459	1.034206183	2.947481491	0.963602055	0.64508582	0.309616185	428.7636912
0.38326724	4.457631334	2.437395449	25.46151474	0.951572616	0.582801002	0.256926732	1129.601494
0.403340967	5.626831942	2.572939607	20.46387734	0.950694628	0.488481249	0.342596364	1408.150329
0.369190372	6.407894904	2.743739827	11.93173414	0.932128436	0.384915211	0.182156136	744.0563461
0.404039386	5.89058934	1.971236014	5.958041428	0.946151978	0.457024765	0.331191436	573.0238066
0.160276508	4.329606005	6.446795243	16.76857197	0.767838584	0.136399141	0.001534037	123.8737105
0.440981953	5.351423984	2.04734154	8.422812223	0.93499054	0.481094267	0.286173607	485.667631
0.434664477	4.736936509	1.50843301	7.199642333	0.966937926	0.59280229	0.253867658	1126.802994
0.341132355	5.754493853	3.493634077	22.54830643	0.907656462	0.400404139	0.154025718	610.1283779
0.379694512	6.227215345	3.046070133	19.88026577	0.928378541	0.427676425	0.347508715	775.9262915
0.477643424	4.544378308	1.321514032	5.824065597	0.972339292	0.568191049	0.243104002	1117.312092
0.436130304	5.399790584	1.990876709	9.986239126	0.958802756	0.507443297	0.297793889	839.3782957
0.369510942	5.947093987	3.550624337	28.84060941	0.92676969	0.434261686	0.299660111	955.3287396
0.134575883	7.91180522	9.511327585	57.30962553	0.832855146	0.129471768	0.133135123	858.4522377
0.459967988	4.336011312	1.242098201	8.442395456	0.978118907	0.638608977	0.304569681	1408.916548
0.370440122	5.935199164	2.609777067	7.686375848	0.925821341	0.364704419	0.309425095	220.6780391
0.442264162	4.30365027	1.497036685	9.774125509	0.985937195	0.612206558	0.37971803	1247.432978
0.358074661	6.231643982	3.101273877	15.80143852	0.919313457	0.390493864	0.259284445	670.2872768
0.439145923	5.464689845	1.842274718	7.736375889	0.958518108	0.501061487	0.372925302	919.3901936
0.333886669	6.780008224	3.658123066	21.45387671	0.921933675	0.355142838	0.221423179	964.7622936
0.373249118	5.835965589	2.331425583	9.916659611	0.940183734	0.448326869	0.235772322	625.3380781
0.437953524	4.284973552	1.382545136	4.819340299	0.959801064	0.589060633	0.264107679	383.128648
0.417757703	5.306105101	2.306168725	13.49900408	0.946806499	0.501766649	0.304189804	729.8765466
0.440598698	3.770492297	1.023813404	3.849271647	0.977389664	0.668562435	0.396642298	1058.26727
0.409289207	5.093297692	2.039464159	5.352885053	0.903966504	0.443579698	0.104107579	168.7177085
0.256178908	6.064978431	3.92825155	12.52723442	0.87168509	0.248371789	0.176328435	336.7448104
0.165103572	7.425422878	9.412049321	87.30680841	0.848209959	0.158014858	0.170990324	1144.224959
0.465437309	4.256433292	0.932609655	1.336829957	0.958011029	0.631358061	0.401382898	115.9657887
0.308819042	7.41007391	4.37089372	32.43913647	0.926893076	0.343309314	0.379404141	1657.430707
0.446735698	3.784288284	1.069082353	4.390708975	0.974245807	0.656808861	0.288164301	953.1533312
0.430050598	5.421591628	1.830345473	6.394123898	0.95521107	0.491349132	0.226500434	512.3137127
0.315682506	6.288449273	4.50442106	36.05972933	0.906809041	0.362838998	0.223848472	860.7077908
0.372431288	5.985183867	2.821922707	13.18719076	0.913812521	0.412726876	0.332716168	472.2174707
0.46510378	4.87396501	1.491443708	5.911618261	0.965153051	0.548749386	0.233746931	697.7374459
0.402374585	5.376926172	2.123359778	12.23571725	0.952844689	0.515282783	0.275566332	926.2214605
0.335999621	6.134719237	2.698631755	7.789542443	0.91345163	0.352912353	0.20315598	321.3599339
0.484135872	4.758822838	1.372647285	4.199289605	0.974067091	0.533977131	0.253080156	1409.069805
0.261369393	5.958311479	4.140839471	35.60999522	0.916550931	0.445153891	0.340107676	1067.442021
0.337214081	6.700969997	4.791272236	46.15380153	0.921652431	0.37207667	0.31185121	1323.900955
0.283677535	8.202311644	5.996791117	56.25727949	0.910860302	0.308049263	0.459126013	1465.773248
0.387229829	5.869824946	2.550433474	21.06637415	0.970015855	0.533830043	0.380970883	1587.525573
0.378744341	5.582779477	2.426502973	9.236942027	0.915282875	0.432143143	0.229302076	329.0529455
0.205632026	8.515156277	7.331990533	56.2990958	0.88180231	0.240005087	0.433831005	1141.263187
0.213167964	6.33264483	5.395120281	22.19443295	0.861538238	0.213888574	0.077600923	423.7590519
0.401090981	5.638321281	2.788245825	25.82784465	0.949916689	0.510800811	0.320182648	1559.180766
0.458162121	4.645950052	1.397721104	5.711158584	0.973614605	0.585985614	0.306761992	1033.417705
0.311103165	6.888994481	4.529685407	34.67089111	0.913273028	0.371385924	0.366893389	1106.053654
0.385917207	5.519998222	2.871365989	16.28084877	0.911577034	0.413495185	0.267121063	420.1715633
0.47674349	4.748422652	1.364406616	6.082408067	0.974336249	0.583445725	0.349038425	1438.484397
0.410908699	4.832882325	2.125391341	13.14292691	0.942949575	0.53331205	0.172863393	686.8787283
0.455908487	4.911871548	1.571423392	6.524985959	0.960664923	0.540468499	0.20084776	885.552655
0.376596335	5.557511567	2.417359422	17.86322518	0.959618809	0.523735826	0.356698209	1392.269408
0.282197612	5.774954929	3.585472868	25.74218559	0.914249211	0.460931742	0.296971735	746.0663205
0.173336926	9.192214308	7.747978523	59.49702878	0.879298549	0.16577188	0.557766095	1159.505196
0.197105149	6.501268318	6.36279667	31.02488807	0.860570763	0.196599688	0.503755293	656.2290544
0.241140838	7.229295318	5.291792262	32.75106245	0.881656114	0.318167056	0.397804893	712.3588366
0.463313413	4.396138695	1.218557786	2.430808856	0.953022959	0.570469689	0.148969318	312.4293078
0.311931228	7.011374673	3.652429222	17.33435604	0.912211601	0.301672707	0.260421173	601.2548269
0.258441901	6.957519331	5.455464355	36.92247003	0.882374146	0.296813407	0.343084393	680.812058
0.420906396	3.630739134	0.958632219	3.987445549	0.973643934	0.687711192	0.271990401	672.4016186
0.432997574	5.18223385	1.622969433	3.615873438	0.931032536	0.497200392	0.259046105	260.4924028
0.390501247	6.085374418	2.505861902	14.07259286	0.94219536	0.468561602	0.361201659	759.1887627
0.143928321	7.204437089	8.511938275	48.87691214	0.823950885	0.148669165	0.253029312	627.8481929
0.373677073	6.073488941	2.841101226	14.45221651	0.939743385	0.396967888	0.267942115	1044.599584
0.372598312	3.564722909	1.113014541	5.05313924	0.96626049	0.687611434	0.304738025	597.7616233
0.237915117	8.305079144	7.397205356	74.14248561	0.888025074	0.264885629	0.595344728	1355.499958
0.315537227	6.738084574	4.389187251	33.22536264	0.908256258	0.361463678	0.303995997	878.4008369
0.163615856	7.59923946	8.394526747	51.12488656	0.827530046	0.180040369	0.35523991	559.7700108
0.443320659	5.158466376	1.482946568	3.760305296	0.948274543	0.532167644	0.292882732	377.3431578
0.351271029	6.901485018	3.389633287	19.07338277	0.937644024	0.376469408	0.427646224	918.6867601
0.419894184	5.106965867	2.189785841	15.40239075	0.961120446	0.541404722	0.427354985	1205.645216
0.348690591	6.665404572	3.061665224	13.24731198	0.928465034	0.362929552	0.317398322	828.5496619
0.302286521	7.086907243	5.466793961	47.4811731	0.900648368	0.335833123	0.203539301	1088.339356
0.33872731	6.197945698	3.723353785	29.1877116	0.930506796	0.430039312	0.279899011	1298.0977
0.159876879	6.299878369	7.862107683	49.44146644	0.836489595	0.158827798	0.106556391	676.5509524
0.237380493	5.84659272	4.742718306	13.95043551	0.858061125	0.203722396	0.099915102	400.6677265
0.385882211	6.110266069	2.810599697	20.15873903	0.949951007	0.48976848	0.669307109	1112.174112

original_glcm_SumEntropy	original_glcm_AverageIntensity	original_glcm_Energy	original_glcm_SumSquares	original_glcm_ClusterProminence	original_glcm_SumAverage	original_glcm_Imc2	original_glcm_Imc1
4.541686503	24.51682504	0.013109444	19.40980502	16058.52595	48.81832368	0.811989193	-0.142637897
4.478424504	20.75367002	0.013131466	29.33329288	15916.46775	40.84301135	0.977374944	-0.385767791
4.073864725	29.33567889	0.048890619	31.0374521	56321.33165	58.19026744	0.850976692	-0.199671678
3.9279992	21.94810008	0.031826792	22.05023365	17295.2439	43.3099854	0.955162218	-0.357493088
3.845191328	27.61762247	0.070911998	17.92598758	15526.61024	54.89040942	0.733312764	-0.134252274
4.410159294	23.94048084	0.017698941	50.66174697	62426.72304	47.08874836	0.989792085	-0.486784534
3.1932318	39.90666211	0.104042352	8.306139812	22281.33991	79.72216531	0.691584029	-0.142884029
3.152413729	32.0791555	0.076311569	4.29179085	2649.560501	63.9785677	0.446751252	-0.047816135
2.981510871	32.14873602	0.083484814	3.120699511	2479.133864	64.27741602	0.436905534	-0.04876186
5.038084517	28.83400039	0.009229864	48.23425158	89438.44007	57.0752265	0.949771265	-0.270356301
3.4239439	26.88014842	0.07662784	8.729706594	16305.46224	53.54657265	0.616175832	-0.092526528
3.010046295	32.71348141	0.090082808	3.685029331	3576.921821	65.24431863	0.478235432	-0.057092588
5.080352618	30.70527609	0.012582466	73.73267231	150924.1474	60.30279442	0.955777229	-0.286380369
3.987593578	15.23393105	0.022482686	12.72058215	3260.309428	30.46786209	0.880930616	-0.220973845
3.134907415	22.36625865	0.073463566	4.060431581	1693.9596	44.61464735	0.547953682	-0.074675119
2.859397104	39.03393054	0.112152175	7.525917856	21553.45243	77.98170464	0.465671838	-0.061597049
3.811242535	26.58324577	0.033677603	10.20367778	11698.49599	53.08984189	0.78485289	-0.159245146
3.714862489	16.80692256	0.0418444	13.95511658	6221.323291	33.61384513	0.87940959	-0.250069152
3.952428612	21.52471733	0.035033574	12.320177	8762.793854	43.00528265	0.722135753	-0.118353499
3.986401244	34.88352094	0.056049841	16.38591048	25192.82559	69.61410238	0.749567755	-0.131865169
3.863006667	26.07912259	0.115050072	24.51490906	41747.12396	51.82293505	0.804974354	-0.179351524
3.603662444	17.03080318	0.036915237	7.668746702	2439.793433	34.06160637	0.825132951	-0.194385766
3.632300541	23.57594442	0.048539096	12.49399214	10238.85304	46.85499338	0.767937799	-0.153260833
2.715209769	20.68377888	0.146707505	2.95331288	1677.112406	41.36755776	0.446161119	-0.058485656
3.237533008	33.68680335	0.127460144	18.90058063	48670.39057	67.05481004	0.727060984	-0.158600602
3.80367984	37.5585628	0.051187286	17.96381495	58418.37793	74.86674317	0.681563634	-0.106010992
4.102680662	27.32346265	0.02085978	10.70706667	6911.180602	54.47887436	0.696724633	-0.100651766
3.84379033	23.94927842	0.033924965	6.880991259	4465.508765	47.77458383	0.629312201	-0.085579653
3.087876052	11.10045738	0.052669574	30.03251042	8329.536682	22.20091476	0.993513611	-0.6850982
3.618359974	21.99158265	0.050460725	9.201336394	6257.491227	43.94696325	0.604591565	-0.087406205
3.356017636	33.62182131	0.077906501	5.653961837	6242.718838	67.08761174	0.595118262	-0.090542452
4.023852457	24.87078515	0.031865805	18.08339995	21590.62687	49.29217648	0.854654268	-0.203066646
4.216636218	27.87687627	0.032985087	19.73764846	30602.55817	55.43482402	0.761725847	-0.130687282
3.037961388	33.43535448	0.075925768	4.525381653	9036.549841	66.81580512	0.479376728	-0.057039143
3.621987684	29.02051699	0.053665726	9.391921551	10042.17444	57.83936691	0.587985982	-0.077697171
4.063934971	31.0040906	0.04986602	26.62746283	44566.55394	61.52805918	0.762089464	-0.137905572
5.430191855	29.97385716	0.005179565	79.00353059	98103.32124	58.29201111	0.980735836	-0.350729226
3.13164916	37.5814648	0.094573887	6.373007177	17250.89079	75.01389479	0.595654121	-0.098774745
3.98296941	14.76797747	0.025031323	10.86933557	4178.1359	29.4894289	0.854000082	-0.19954478
3.006580733	35.34783816	0.121429843	8.594225019	24273.28032	70.53364096	0.503127419	-0.066792007
4.19245323	25.97570883	0.027824989	15.44445615	13124.13336	51.60621673	0.778910925	-0.139184379
3.589780623	30.33301237	0.051302225	8.327983772	8990.902449	60.53434331	0.569744921	-0.071238624
4.3909561	31.23004658	0.017610924	21.10001127	28480.86128	61.96073822	0.729160522	-0.115674875
3.87130544	25.02161488	0.037714583	9.464044681	5694.849879	49.91517947	0.71948272	-0.11867825
2.994636227	19.55322942	0.114912421	4.639751933	2221.753231	39.08492318	0.538514488	-0.077838464
3.593914162	27.01762646	0.067969701	12.64094317	15866.88214	53.86642008	0.637850601	-0.098979476
2.742216311	32.52861683	0.153137555	3.971203436	5064.015071	65.00706244	0.511725176	-0.081170894
3.381484087	12.96739955	0.051916129	5.328680522	939.0971562	25.9347991	0.711032463	-0.130731583
4.057436916	18.26947913	0.018614244	17.01005288	7130.750962	36.53895825	0.947077762	-0.31897695
5.263778533	34.35518911	0.007307222	95.76877034	290181.062	67.16507421	0.983941236	-0.373809037
3.006168292	10.73389104	0.093804118	1.85287154	175.1047561	21.46778209	0.620697959	-0.110223782
4.859938406	40.85956369	0.015577792	36.53262765	86987.98067	81.04190707	0.802222312	-0.130089084
2.667655589	30.87257443	0.15078125	3.577593038	3543.236745	61.70922784	0.434763685	-0.056066519
3.665101345	22.62950226	0.047390372	6.078930852	2302.216611	45.20491845	0.699045519	-0.119187204
4.416294927	29.34917045	0.027334256	34.08088389	50314.94826	58.3897488	0.906517668	-0.243375224
4.007018674	21.64703125	0.036634712	16.03816244	14806.38062	43.21280265	0.735638083	-0.122959624
3.269188619	26.4180527	0.0676549	4.958555899	4672.239909	52.77944526	0.483696249	-0.055936924
3.668817862	30.44278199	0.060869261	10.93078577	12412.22583	60.76057686	0.670126151	-0.1135029
3.970497112	17.87921229	0.021776465	9.69510293	3913.131603	35.73807182	0.845853653	-0.188976313
3.101202338	37.59959007	0.065743562	3.892328658	4540.5988	75.04755888	0.401651823	-0.037433778
4.232940083	32.73046144	0.071350567	37.01018535	74654.73394	64.92934326	0.835271499	-0.179736316
4.504313302	36.54400391	0.032322786	44.95260068	115208.7937	72.34479147	0.774618309	-0.127843869
5.39518198	38.29128269	0.010673676	76.29087135	242566.7231	75.5643925	0.812021884	-0.123550036
4.06481487	39.91910727	0.054915506	20.23785672	53092.44806	79.47326921	0.739006274	-0.128098069
3.738422467	18.07794906	0.039654827	9.832824246	4108.13217	36.15589811	0.778311091	-0.157035852
5.541022312	33.82661745	0.010799986	88.4607882	229958.2547	66.34210121	0.841404238	-0.132994051
4.394042087	20.95127149	0.015213093	26.05942203	12017.9426	41.07476148	0.967216734	-0.352477551
3.923767016	39.54842572	0.060791445	21.85514347	55833.79571	78.77095528	0.669438908	-0.10266274
3.173845953	32.16007215	0.087078582	5.106442523	5878.755628	64.23983932	0.507838963	-0.065173128
4.622620758	33.31844486	0.028683466	39.82993667	74155.63191	66.03630443	0.779162481	-0.126442123
3.944577863	20.38280015	0.03457174	17.00223276	15927.23047	40.76560031	0.863121492	-0.228525811
3.319306833	37.94099913	0.067511562	5.905038653	8554.704759	75.79640683	0.598007161	-0.090726522
3.342078683	26.2997538	0.083561231	9.141885491	8366.015828	52.34854117	0.647751632	-0.106694997
3.340194791	29.75593616	0.062308695	5.305891793	4041.644935	59.47848643	0.59484499	-0.085795997
3.866769803	37.39517955	0.064383327	16.4702211	33933.98207	74.45175136	0.703364585	-0.115476624
4.08529137	27.33382038	0.075627965	25.10443862	29161.97212	54.32826361	0.827206085	-0.183365018
5.879101035	33.57963934	0.002582153	128.5335945	738678.3837	65.90363795	0.904944359	-0.168805197
4.825445852	25.25655883	0.012594427	69.23537417	130508.2394	49.75244479	0.995236196	-0.528631435
4.950267754	26.51013182	0.025127708	48.17519297	62816.90253	52.6218295	0.898370704	-0.204250854
2.904391948	17.66551005	0.089594571	2.318278545	422.8065987	35.3310201	0.414653439	-0.043361183
4.541686503	24.51682504	0.013109444	19.40980502	16058.52595	48.81832368	0.811989193	-0.142637897
4.871579452	26.0614771	0.017659683	47.30572241	73872.03013	51.47205144	0.940355791	-0.269874601
2.67026489	25.93515414	0.170195416	3.063077708	3130.593572	51.82662989	0.523533675	-0.085879092
3.408844741	16.10484274	0.053470838	4.256924881	774.1332052	32.20968547	0.526789304	-0.063905236
4.146579173	27.56929283	0.032925735	14.04801316	16415.58411	54.89710472	0.743864495	-0.132885681
5.235011687	25.15546649	0.008597767	76.56757449	112597.2206	49.27999706	0.991359851	-0.437677046
4.014646519	32.3960157	0.028953931	14.30562915	19838.77151	64.51071004	0.735531354	-0.120072496
2.650629983	24.48858343	0.211729842	3.960826619	3027.534966	48.8418511	0.499010383	-0.077422837
5.684929604	36.22995059	0.009712574	147.5917674	911858.7732	71.07967809	0.950287739	-0.244316831
4.570775091	29.6535807	0.026055412	34.64726811	54545.8005	58.88810144	0.834619518	-0.163245168
5.476585183	23.33322072	0.00744867	92.64200531	137603.0128	45.8766784	0.982887797	-0.363161006
3.501801009	19.39170282	0.05296142	4.300527143	1372.975729	38.78340563	0.597033089	-0.09213403
4.521280932	30.34557293	0.023308134	24.01741139	37331.05925	60.24274512	0.708300346	-0.097137182
3.561718271	34.70484383	0.085248395	15.97339546	35070.33633	69.2275792	0.664114202	-0.109559326
4.282969093	28.7639754	0.023051171	15.58015158	11129.86878	57.38224116	0.647820852	-0.080160827
4.741990877	33.23717173	0.022259128	43.65435485	74636.62203	65.68874721	0.818600423	-0.142581982
4.226102681	36.13742389	0.043171644	26.53249463	48414.15406	71.83383609	0.741668226	-0.122097245
4.637545844	26.30161979	0.013946923	57.04111856	60852.681	51.78117611	0.993203513	-0.50922887
4.06170164	20.10012449	0.019683334	21.06154491	9043.476848	39.91810462	0.981156976	-0.442656846
4.202762343	33.09307661	0.060536782	39.12780967	139994.0931	65.84647488	0.791891619	-0.149874826

original_glcm_DifferenceAverage	original_glcm_Id	original_glcm_ClusterTendency	original_firstorder_InterquartileRange	original_firstorder_Skewness	original_firstorder_Uniformity	original_firstorder_MeanAbsoluteDeviation
3.652429222	0.378854829	52.4654815	99	-1.724359531	0.089977365	88.43562481
5.577629072	0.267276513	66.65566254	202	-0.875980876	0.060850497	119.030621
4.014601817	0.494249459	85.37411675	98.5	-2.064341276	0.146895053	124.8177154
4.057673333	0.391088224	54.64762552	149.5	-1.644454448	0.113185255	109.4603025
3.094321418	0.524620287	50.00769743	103.75	-1.869054797	0.183053138	98.45708659
6.786066347	0.289495875	125.9639159	270	-1.038638205	0.061740032	167.8401357
1.279933917	0.683577871	26.12650855	34	-4.688220105	0.268328756	41.43738876
1.439945774	0.591086915	10.76339236	38	-4.29500486	0.235371326	34.17380221
1.208617736	0.612961404	8.236574466	33	-4.990094451	0.258266892	29.02535673
5.593601572	0.312678083	139.376934	210	-1.450739955	0.067579399	144.7645212
1.617558692	0.57715633	24.7559742	35	-1.196834086	0.224090292	41.0190527
1.31239831	0.607271807	9.666703615	32	-4.777366549	0.254699482	31.74588813
7.337454768	0.313830128	200.799685	388.5	-0.999752156	0.068299851	214.396674
3.458848187	0.38719376	26.95159432	114.75	-1.150787911	0.106260388	80.57473684
1.45176983	0.586837628	9.111856315	37	-3.8267854	0.230132325	33.44066163
1.358640471	0.63629046	21.4952386	27	-5.498120016	0.299059332	38.71895091
2.181639562	0.48572213	30.36571698	70	-2.600140639	0.143538002	62.15466922
3.213784085	0.438008119	32.20509505	110	-1.768484684	0.150231312	83.68785001
2.53496317	0.483383205	31.58422212	69	-2.250189465	0.14928349	69.07858123
2.109214059	0.545095461	54.79029476	51	-2.443693985	0.179699239	76.84366532
2.742233561	0.584283151	80.33670366	148.5	-1.68038349	0.198292087	116.6450607
2.40297699	0.459365844	18.21090335	86.5	-1.870567429	0.147732735	60.59445602
2.691049772	0.489601892	31.18835104	62	-2.521811617	0.172325578	74.06124542
1.034206183	0.667683216	7.793128978	26	-4.251672358	0.327780815	27.78519566
2.437395449	0.611042039	53.60732233	30	-3.359847102	0.28304457	74.57782938
2.572939607	0.532075755	54.71970102	45	-3.922585245	0.190082975	58.60089328
2.743739827	0.449557241	27.8731324	77	-2.309509896	0.120660617	64.05791401
1.971236014	0.511877196	19.56458378	58	-2.358401065	0.149114067	49.41640183
6.446795243	0.224293419	59.79590124	170.5	-0.207866721	0.086734694	108.1147959
2.04734154	0.526407477	23.92318815	48	-2.457349219	0.183352066	59.93193544
1.50843301	0.621124964	15.94735764	35	-3.936928904	0.23278923	40.60108731
3.493634077	0.456099454	45.78796854	104	-2.290324338	0.129719339	88.20650519
3.046070133	0.479968791	59.88430187	74	-2.391482044	0.134613939	88.07398152
1.321514032	0.601481617	12.46153383	34	-6.320742904	0.248369007	31.58547734
1.990876709	0.548812478	26.08192478	45	-2.977499697	0.187208534	54.98003007
3.550624337	0.481773262	77.09774716	102	-2.057312869	0.15145585	113.4963564
9.511327585	0.206418841	186.8580039	374.25	-0.611972599	0.037080868	200.5910585
1.242098201	0.659299568	18.57364529	31	-6.169668762	0.267493698	32.27882964
2.609777067	0.433865116	27.49892992	90	-0.387221139	0.117524286	63.86106316
1.497036685	0.636734553	26.75253273	28	-3.467019723	0.305630425	39.4594879
3.101273877	0.450702152	43.34946131	94	-1.962681794	0.120536667	85.54070841
1.842274718	0.545289421	24.29608879	46	-3.24468185	0.191656597	47.42456588
3.658123066	0.420122725	55.67577062	102	-2.119170443	0.099136306	94.86836764
2.331425583	0.503787659	25.14165086	65.5	-2.29962907	0.144225556	61.85213134
1.382545136	0.619196059	11.61224276	31	-3.136631324	0.278541644	38.99007218
2.306168725	0.54233941	36.7465856	47	-2.643196345	0.198178453	70.41508206
1.023813404	0.685678412	12.02990721	24	-4.966257051	0.339985222	30.0600995
2.039464159	0.499147623	11.77412081	54.5	-1.747557995	0.184128771	47.57490461
3.92825155	0.336123441	39.86395816	148.75	-1.198983462	0.082333026	93.28947368
9.412049321	0.233784919	243.1857818	298	-1.339179356	0.051859504	178.3912397
0.932609655	0.655767158	5.20195098	37	-1.362841435	0.247157317	26.50924512
4.37089372	0.406447699	113.4100996	160.5	-1.992556016	0.084220411	128.2144882
1.069082353	0.675698666	10.11456656	23	-5.147521972	0.340046552	28.16908421
1.830345473	0.53832591	15.54610441	51	-1.023606376	0.17180855	42.94055135
4.50442106	0.421228449	89.63936341	141	-1.791931151	0.101579478	125.065765
2.821922707	0.469449764	42.64712147	104	-1.864976519	0.13532574	90.39694337
1.491443708	0.584790539	13.41381283	36	-4.179129925	0.224642677	35.75072918
2.123359778	0.556810732	29.80401491	47	-2.847711933	0.18780239	64.95046093
2.698631755	0.426832323	23.30226011	84.25	-1.432152771	0.105157872	66.44842128
1.372647285	0.573191034	10.22845269	36	-5.113187655	0.236426272	30.40755741
4.140839471	0.494265465	106.7752803	142	-1.871921279	0.164719014	132.9369137
4.791272236	0.425914464	131.0874114	151.5	-1.886368776	0.12029729	152.4247385
5.996791117	0.3693993	245.7212562	247	-1.438379794	0.071215658	190.1445678
2.550433474	0.568893409	61.63443752	47	-2.590503967	0.177744716	80.99279599
2.426502973	0.488689796	24.14277956	71	-1.895015193	0.14686387	64.15599616
7.331990533	0.307136847	274.1547155	356	-1.028824829	0.064330232	204.0402382
5.395120281	0.296840567	59.20423906	204	-0.928365547	0.077607514	119.1288751
2.788245825	0.548226711	65.36102993	49	-2.924336778	0.188221111	88.32562646
1.397721104	0.615109342	14.58065048	32	-4.002959073	0.25218598	37.00616191
4.529685407	0.426932947	118.7190555	194	-1.502124293	0.112107796	147.0930421
2.871365989	0.470273443	43.3554006	83.75	-1.996990351	0.12962963	88.37251301
1.364406616	0.612457426	16.78729076	40	-4.480782685	0.226212358	38.31932101
2.125391341	0.569963112	24.84864715	38	-3.224590078	0.228578915	56.77474015
1.571423392	0.577948128	13.51823339	42	-4.180647046	0.203065327	39.93431591
2.417359422	0.563015529	49.73912656	46	-3.153668174	0.20247565	68.12313413
3.585472868	0.508310131	71.42724611	126.25	-1.746194326	0.170672339	106.3237194
7.747978523	0.247860711	415.316116	295.25	-1.331264968	0.038942342	209.1855446
6.36279667	0.276127975	220.2923148	351.5	-0.57758273	0.044688091	190.6061248
5.291792262	0.380442653	141.1948624	287	-0.958411821	0.080139028	161.8294483
1.218557786	0.60473628	5.353877044	32	-2.733229668	0.256505141	28.34755828
3.652429222	0.378854829	52.4654815	99	-1.724359531	0.089977365	88.43562481
5.455464355	0.362909112	140.9874147	269	-1.113977597	0.076563774	169.5854613
0.958632219	0.703666532	8.515646026	22	-5.776825913	0.360985845	24.41458978
1.622969433	0.544688784	10.76145925	57	-1.879857931	0.179333997	43.51276994
2.505861902	0.516619469	43.49052609	66	-2.462498317	0.137137376	70.63607554
8.511938275	0.226468446	204.6566991	413	-0.386033188	0.040741219	208.084517
2.841101226	0.457640288	39.26943007	73	-2.639488908	0.130037574	76.00368704
1.113014541	0.705688292	11.81547805	22	-4.109688825	0.392820842	32.97895122
7.397205356	0.328560829	499.7480907	308	-1.504276055	0.067354278	228.5684788
4.389187251	0.420322737	98.25137157	182.5	-1.57061924	0.1058375	138.268475
8.394526747	0.251928068	256.9918422	484	-0.259260408	0.04652384	224.7066793
1.482946568	0.573155286	11.20632795	49	-2.397085	0.182611375	40.03605457
3.389633287	0.437384627	76.12544343	112	-1.872816789	0.111911751	105.322536
2.189785841	0.575150331	50.31643824	42	-2.992358196	0.22966481	74.08750975
3.061665224	0.42968879	44.07709627	107	-1.92673303	0.115675664	80.93633009
5.466793961	0.39279084	115.987488	181.5	-1.610532461	0.102542629	149.2854387
3.723353785	0.478625343	75.61531535	105	-2.180677457	0.148678261	108.7606351
7.862107683	0.24004138	136.7436852	303	-1.005967471	0.055621614	172.3791782
4.742718306	0.291618888	46.21189931	204.5	-0.873158893	0.076770482	102.6415394
2.810599697	0.530594788	141.0105473	90	-2.111583762	0.175968867	125.6229149

original_firstorder_Energy	original_firstorder_RobustMeanAbsoluteDeviation	original_firstorder_Median	original_firstorder_TotalEnergy	original_firstorder_Maximum	original_firstorder_RootMeanSquared
10975300	47.24601271	45	296333100	250	128.37223
4626317	84.54008332	-121	124910559	85	202.3384749
16592645	61.32383876	11	448001415	129	185.3464385
1913951	64.54552469	41	51676677	165	144.2352773
14568657	57.76112562	12	393353739	68	139.7463088
6350941	116.2505716	-56	171475407	97	248.3135545
141049198	14.09037022	47	3808328346	526	93.00613383
35147117	16.54083616	69	948972159	241	83.45862598
17778646	14.29196485	46	480023442	149	64.83813267
14488722	92.87429293	9.5	391195494	340	199.5098527
42248412	16.17128491	37	1140707124	1144	87.93258682
17985300	14.08985392	58	485603100	209	76.50284095
31991328	162.4855821	-47	863765856	99	310.886696
2043048	51.18433081	35	55162296	177	103.6961147
3503866	16.3346314	25	94604382	130	61.71345072
74333883	11.87875246	46	2007014841	238	93.36740284
3999565	31.53082487	43	107988255	142	95.7774202
1510421	48.07271809	11	40781367	84	116.6507454
5888349	35.32708141	18	158985423	102	101.9072875
160301082	32.92184914	39	4328129214	324	115.9318102
38488668	74.53304904	17	1039194036	49	162.8107709
1212580	36.47530864	0	32739660	79	87.60454875
3550220	35.32567155	19	95855940	91	112.4021124
15775519	11.5964313	36	425939013	142	57.30471127
12854776	17.33577727	30	347078952	134	133.0650331
23892211	21.9169302	49	645089697	171	108.6214653
8678241	35.02911276	35	234312507	198	96.13533791
11812983	25.50818251	41	318950541	246	81.94960597
1335809	81.2231405	-154.5	36066843	26	218.4203843
9717823	26.21924429	26	262381221	119	91.88546775
21368341	15.82575259	35	576945207	183	78.01354051
5058697	47.40607032	-18	136584819	53	140.8475326
22593376	40.79584628	64	610021152	188	132.9092761
22037008	14.66040589	51	594999216	294	83.65463032
32827034	21.39017612	48	886329918	352	97.16581039
30208787	59.02295957	24	815637249	173	163.6485244
37744960	153.5897436	-149.5	1019113920	163	311.0979729
93895381	13.36194082	55	2535175287	350	89.43861425
1603917	39.38311168	40	43305759	408	90.23142834
95230640	12.33876272	32	2571227280	1624	88.66343788
11395335	48.31292474	-20	307674045	89	134.3844038
47234516	21.16311506	32	1275331932	238	80.78866813
28753317	51.21496036	61	776339559	212	137.2222252
6305369	30.7179875	27	170244963	219	94.43773159
5044009	15.7317124	34	136188243	275	68.08845485
23311744	27.95020468	33	629417088	247	109.1978284
30070559	10.59085489	33	811905093	240	65.68792126
954880	26.8330449	23	25781760	101	66.03167913
1889253	62.54824297	-13.5	51009831	109	128.7338135
17895939	126.1778151	-114.5	483190353	112	285.2107594
7677281	16.72105496	59	207286587	285	63.46616701
67218257	75.63711844	47	1814892939	202	176.4482284
42716367	10.63069835	41	1153341909	194	65.02696317
5026161	21.82838235	29	135706347	357	77.03294347
9187050	74.55384146	11	248050350	156	178.9151396
10216662	51.88504155	27	275849874	114	124.1360004
33601231	15.99138726	53	907233237	343	79.72103693
17674962	25.18870968	12	477223974	179	106.9584258
2559534	37.76123114	41.5	69107418	247	97.0054198
127565760	15.45923143	53	3444275520	345	73.65683245
29904558	75.38046875	11	807423066	84	193.7044211
74126575	83.71502171	27	2001417525	257	215.0404726
297338685	121.1918934	27	8028144495	322	255.0190346
162481419	26.62593097	29	4386998313	895	138.0963037
2888858	33.14524368	2	77999166	116	94.86599829
213672070	152.6034931	-99	5769145890	193	312.7146
3069845	87.03180055	-15	82885815	177	160.6144975
84118392	31.15705306	29	2271196584	145	142.7498422
62035668	14.08973872	48	1674963036	439	78.6723035
65498107	94.22367613	1	1768448889	149	207.1070371
3303266	44.05150113	16.5	89188182	111	129.1633137
59449610	17.18438214	68	1605139470	324	91.51155116
6827939	21.236104	35	184354353	104	95.47813064
8192337	18.51668898	63	221193099	191	87.37839106
42298403	25.47348136	22	1142056881	335	114.6842993
11947739	65.56782324	45	322588953	124	140.6450313
160600451	132.9209037	-96.5	4336212177	243	329.1920045
11891250	148.1301775	-190	321063750	65	321.5620841
41445700	123.0782138	-92	1119033900	63	252.3185878
7188025	14.36798989	57	194076675	177	67.11017108
10975300	47.24601271	45	296333100	250	128.37223
22369299	121.8981442	-40	603971073	131	246.2141477
13236881	9.546577647	40	357395787	192	62.23100334
4270515	25.65243096	23	115303905	105	60.28665566
22003130	32.32610421	18	594084510	207	110.4087846
24382175	164.7521429	-234.5	658318725	44	372.2030161
9780714	37.42639108	41	264079278	205	116.1492144
17335815	10.14390914	34	468067005	105	65.29628075
118938602	145.2910249	13	3211342254	217	318.1577315
31107670	88.26272066	0.5	839907090	139	197.1917531
41846516	189.6694171	-259	1129855932	38	389.381319
7523712	22.31840756	53	203140224	186	72.53507471
73062472	58.02588364	32	1972686744	379	146.1191639
55998481	25.80263686	31	1511958987	340	120.5403
25541854	47.7830894	33	689630058	176	111.1619024
49263468	90.00082515	23	1330113636	198	203.9795548
38761459	57.27881276	4	1046559393	146	162.6601789
6354171	120.005041	-95	171562617	80	270.2525639
2192279	77.85410896	-94	59191533	50	175.7190058
322668354	60.64294424	25	8712045558	317	183.2956301

original_firstorder_90Percentile	original_firstorder_Minimum	original_firstorder_Entropy	original_firstorder_StandardDeviation	original_firstorder_Range	original_firstorder_Variance	original_firstorder_10Percentile
122	-574	3.960803036	127.7591098	824	16322.39015	-174
21.8	-596	4.252780062	147.8850286	681	21869.98167	-346.8
47.8	-746	3.608638245	173.9835552	875	30270.27749	-333.2
85.9	-511	3.704210225	142.575985	676	20327.91151	-227.6
38.5	-690	3.280038011	130.4371896	758	17013.86043	-246
49.6	-661	4.35574115	204.1028487	758	41657.97285	-498.8
74	-938	2.463866855	88.64528963	1464	7857.987374	10
99	-704	2.581851359	61.58908196	945	3793.215017	14
74.2	-733	2.413722409	53.92130094	882	2907.506695	2
96	-747	4.397153985	188.5903348	1087	35566.31439	-337.4
72	-608	2.864835286	83.78823892	1752	7020.468981	-23
88	-727	2.518838148	60.19637403	936	3623.603446	11
33	-860	4.519859443	251.0221036	959	63012.09649	-594
97	-329	3.690205085	103.6959219	506	10752.84421	-161.2
61	-523	2.629810748	59.92324312	653	3590.795066	-24
70	-909	2.338376508	88.91651968	1147	7906.147473	2
88	-625	3.309975541	94.54813871	767	8939.350533	-82.5
49	-421	3.310317496	110.9830062	505	12317.22766	-200
60	-511	3.328774376	100.6018475	613	10120.73173	-146
74	-831	3.303846908	115.9318084	1155	13440.18419	-156.4
36.9	-655	3.295473966	149.2475229	704	22274.8231	-304
44.3	-404	3.235612829	82.50857214	483	6807.664477	-147
55	-564	3.194502987	110.5159083	655	12213.76599	-154
57	-461	2.209713013	51.99630524	603	2703.615759	-9
50	-824	2.704360898	132.5115559	958	17559.31246	-103.5
88	-898	3.063042895	106.236153	1069	11286.1202	-60
93	-648	3.528744533	95.37633552	846	9096.645376	-95.6
95.2	-534	3.255163929	77.18221326	780	5957.094044	-48.2
7.8	-423	3.69951385	131.8869481	449	17394.16709	-355.3
63	-505	3.072880125	91.86307026	624	8438.823678	-121
68	-777	2.689117743	75.67590189	960	5726.842127	-31
29	-639	3.493410522	125.2724733	692	15693.19256	-216
109	-645	3.566758641	131.5343538	833	17301.28623	-152.2
84	-765	2.48967228	72.02645008	1059	5187.809512	12
86	-655	3.078090657	93.91943111	1007	8820.85954	-58
58.3	-763	3.587840196	157.3426741	936	24756.7171	-284.4
63.2	-851	4.994168243	237.7501368	1014	56525.12755	-540.4
86	-868	2.427211694	77.21868435	1218	5962.725213	21
92	-305	3.48636927	88.39605044	713	7813.861733	-94.2
55	-838	2.382626283	87.42348892	2462	7642.866415	-14
32	-670	3.619985511	119.1968689	759	14207.89355	-250
68	-725	2.958728848	79.86862208	963	6378.996793	-56
126	-707	3.855035549	136.1737657	919	18543.29446	-167
74	-587	3.366042609	94.43661279	806	8918.273835	-102
57	-449	2.496759977	66.20290158	724	4382.824177	-45
64	-628	3.096464193	109.1269226	875	11908.68523	-138.2
54	-768	2.159279929	62.58187691	1008	3916.491317	-1
56.2	-293	2.964627234	66.00487436	394	4356.643439	-99.2
59	-458	3.857266896	117.5420977	567	13816.14474	-215.7
43.4	-994	4.644099706	227.0248748	1106	51540.29378	-460.3
86	-196	2.408770054	37.65931573	481	1418.224061	4
120	-980	4.149425138	175.4632986	1182	30787.36915	-267
61	-716	2.136759351	58.33776412	910	3403.294723	0
76	-525	3.104831553	72.68639813	882	5283.312473	-34
67.4	-729	3.960386004	168.1128943	885	28261.94522	-312.8
67	-503	3.542420729	121.8570295	617	14849.13564	-208
89	-592	2.693743671	67.64742575	935	4576.17421	4
49	-747	3.150769529	105.2440983	926	11076.32022	-142
113.9	-384	3.687251237	94.00650808	631	8837.223562	-92.2
86	-855	2.551761147	58.06103758	1200	3371.084084	11
42	-846	3.595500762	178.5328653	930	31873.98398	-362.8
72	-914	3.947649896	204.5727616	1171	41850.01477	-391.2
105	-982	4.485549245	240.6871519	1304	57930.30507	-508.9
69	-958	3.332576415	137.8816707	1853	19011.3551	-156
48	-445	3.320976082	90.98410891	561	8278.108073	-159
27	-976	4.661548011	244.9100017	1169	59980.90894	-579.6
58.8	-538	4.0492332	143.0921927	715	20475.37561	-322
62	-961	3.254903026	141.4186339	1106	19999.23002	-194.3
78	-745	2.604448522	70.88415128	1184	5024.562903	-5
48	-851	3.986657798	186.0413161	1000	34611.37129	-415.8
66.9	-496	3.49884545	126.3549412	607	15965.57117	-200
100	-854	2.65176439	74.19245203	1178	5504.519938	19
62	-607	2.851236425	95.32466724	711	9086.792184	-93
101	-668	2.823065157	71.83694465	859	5160.546617	3.2
52	-908	3.095916652	113.9367512	1243	12981.58328	-127
73	-664	3.473855911	138.5496651	788	19196.0097	-245.4
67	-998	5.026799078	272.5377253	1241	74276.81172	-616.4
26.8	-798	4.679909566	222.8788127	863	49674.96514	-576.2
16	-775	4.316986593	192.7283633	838	37144.22204	-476
87	-362	2.46349663	45.77756523	539	2095.585478	9
122	-574	3.960803036	127.7591098	824	16322.39015	-174
43.2	-703	4.338242503	205.5653594	834	42257.11699	-499.6
59	-581	2.059981109	53.89359343	773	2904.519413	7
57	-361	2.919789689	60.06469303	466	3607.767349	-79.6
71	-651	3.431816143	109.6358411	858	12020.01766	-141
10.5	-802	4.825667057	241.1229614	846	58140.28251	-610.5
88	-751	3.527334812	116.0563863	956	13469.08479	-145
50	-559	2.056591456	63.05499435	664	3975.932312	-17.5
99	-1023	4.647004019	292.3940363	1240	85494.27247	-598.6
50	-751	4.017347018	177.5808396	890	31534.9546	-380.1
-4	-820	4.75858472	250.3249645	858	62662.58785	-637
92	-413	2.923836092	60.80884239	599	3697.715313	-22
83	-743	3.84570954	144.0597312	1122	20753.20617	-244.9
58	-845	2.965209812	120.1599257	1185	14438.40774	-154
84	-676	3.665411476	110.9515704	852	12310.25096	-159
75	-844	4.102234918	193.1622465	1042	37311.65348	-394.4
42	-909	3.584544764	151.8681638	1055	23063.93916	-273
31.2	-772	4.415349025	210.57826	852	44343.20359	-519.2
17	-571	3.9058951	125.3535053	621	15713.50129	-288
56	-839	3.462428812	175.7612302	1156	30892.01005	-331

original_firstorder_Kurtosis	original_firstorder_Mean	original_glrlm_ShortRunLowGrayLevelEmphasis	original_glrlm_GrayLevelVariance	original_glrlm_LowGrayLevelRunEmphasis	original_glrlm_GrayLevelNonUniformityNormalized
6.41550194	12.53153153	0.004428851	28.16969081	0.004529728	0.084411752
3.232124037	-138.0973451	0.022524053	35.27155876	0.022586178	0.059420959
6.54409798	-63.89855072	0.007677228	56.79782433	0.007823339	0.107861908
5.183571896	-21.81521739	0.018485059	34.08193102	0.018621752	0.098834705
6.010904527	-50.15147453	0.004010112	32.04249172	0.00420238	0.128908932
2.959274988	-141.4271845	0.028326544	67.64346699	0.028373833	0.059305127
29.79875307	28.14522262	0.000691848	20.25766465	0.000871748	0.21507631
30.38290919	56.3216409	0.001141809	8.037005436	0.001336807	0.201089358
43.99765589	36.00662095	0.001191508	6.24070531	0.001389528	0.222772877
4.806542239	-65.0989011	0.00675505	59.17726449	0.006814743	0.063518751
30.8293756	26.67715959	0.002133496	14.81015368	0.002395459	0.177086397
40.30104849	47.21314676	0.001348501	8.043141707	0.001544839	0.204808559
2.684848865	-183.407855	0.008744846	104.3034295	0.008858067	0.05804411
3.840723917	-0.2	0.015742043	18.10298773	0.01601089	0.096658369
25.22091796	14.75652174	0.003471014	7.481973623	0.003830349	0.192291059
38.43002648	28.48375748	0.000855338	18.35171214	0.001010687	0.241838506
11.95900027	15.29587156	0.004266996	16.07198656	0.004424361	0.128116453
5.608331286	-35.91891892	0.034325389	20.99507638	0.035193251	0.131322752
8.396010365	-16.25925926	0.00587954	18.51024469	0.006117261	0.127841416
9.744288258	0.020793158	0.000990776	26.75912415	0.001129422	0.136652922
5.042407854	-65.05785124	0.005323935	43.05764044	0.005595386	0.096266267
7.197722652	-29.44303797	0.012166949	11.92893446	0.012462214	0.132444035
9.854213059	-20.50533808	0.008769974	22.62722212	0.008980006	0.143507475
27.19921897	24.0876353	0.003353278	6.406915565	0.003902479	0.232082571
14.63374843	-12.12396694	0.0034509	37.84831267	0.003618667	0.19375373
22.57830364	22.63851852	0.001452296	22.57518411	0.001564829	0.156085975
10.90063786	12.05644302	0.00270568	16.21470026	0.002837214	0.111460941
12.21610524	27.54349062	0.002635114	11.38897416	0.002875718	0.130435798
2.043628323	-174.1071429	0.066355851	28.36312724	0.06673382	0.086408665
9.508302648	-2.028670721	0.00416358	15.98190455	0.004460789	0.155738433
23.70757483	18.95442894	0.001216535	12.84418702	0.001418153	0.189335012
8.695383412	-64.38039216	0.011165708	27.80086477	0.011598462	0.112860005
9.066617042	19.06802189	0.004654544	31.50876883	0.004798078	0.114047751
56.31472505	42.54747539	0.001884113	11.05911971	0.002059817	0.214646746
14.17323575	24.90652862	0.001721758	17.64654889	0.001915302	0.156138783
6.747503011	-44.99024823	0.003474526	45.71743464	0.003598756	0.113398923
2.451266199	-200.6410256	0.00581543	90.3952493	0.005849673	0.036019547
51.70517076	45.12804566	0.001007204	14.77878865	0.001200939	0.220719102
6.859842708	18.10659898	0.01350923	13.85509749	0.013859419	0.108930344
40.73548655	14.77629189	0.00104237	17.86329229	0.001222373	0.22790459
7.008136477	-62.05863708	0.004359776	24.98763185	0.004504663	0.103499704
17.05718337	12.15780019	0.001451013	12.66972438	0.001626305	0.162236336
8.011997592	16.93058284	0.002426639	32.9456628	0.002525078	0.088808606
10.00425624	0.459688826	0.003585634	16.79579623	0.003784876	0.120891717
15.81966448	15.91268382	0.004681541	9.308060463	0.005182418	0.205523699
10.37643015	-3.934526854	0.002680932	23.51071589	0.002891114	0.153246958
35.59156734	19.96025255	0.001045106	9.736615389	0.001298709	0.248509214
6.1870623	1.881278539	0.013585751	7.905852221	0.014199352	0.158072282
4.139527071	-52.5	0.014686882	22.59948597	0.014798265	0.07935967
4.815238845	-172.6409091	0.010886783	83.27389749	0.011196517	0.04914168
9.96832453	51.08551941	0.010446447	2.974708014	0.012411704	0.20795156
7.392279532	-18.61741547	0.001415623	53.71110733	0.001471537	0.072297708
37.83907886	28.7264898	0.001075713	8.429891585	0.001348097	0.253279082
14.80228156	25.51003542	0.003486317	10.17183118	0.003746836	0.149471621
5.827909908	-61.22648084	0.006805344	49.64665452	0.006905495	0.082589076
6.082771706	-23.67722474	0.007397252	26.27523088	0.007652407	0.109186143
28.69933848	42.18138831	0.002229477	9.597912166	0.002492319	0.19115799
12.38205492	-19.07313916	0.002165991	22.60294513	0.002347545	0.142484073
6.512904444	23.93382353	0.009650887	15.42649225	0.009892333	0.09680946
48.17793211	45.32377834	0.000679904	7.103401033	0.0008198	0.206145799
5.695292441	-75.14930991	0.003487235	59.53025601	0.003606249	0.098531792
5.860626379	-66.27510917	0.00357974	75.5137985	0.003658608	0.093213186
3.955080929	-84.2876203	0.00150717	98.41643247	0.001561303	0.062327922
13.29016058	-7.696361502	0.00113104	40.93887926	0.001250524	0.134414479
6.502393798	-26.85981308	0.008357775	15.10781248	0.008694791	0.125434344
3.075574981	-194.4466819	0.002851546	98.06196649	0.002922063	0.050300309
2.995501911	-72.94957983	0.012780009	33.65999132	0.012885115	0.073282007
12.40647724	-19.4496124	0.001621361	40.19843069	0.001730012	0.141934185
26.27360399	34.12870398	0.001160644	11.19438403	0.001385376	0.202872604
4.261093394	-91.00523903	0.002365501	61.2912873	0.002459788	0.084032923
6.411227874	-26.78787879	0.014685035	28.3571525	0.01490253	0.116212959
30.25268259	53.57092548	0.00080543	12.15152523	0.000961128	0.195642982
14.67956856	5.411214953	0.003603177	18.5859459	0.003844031	0.172200026
30.20336922	49.74370923	0.002390463	10.29957633	0.00258019	0.174615168
15.10295558	-13.07307214	0.001200084	26.73072676	0.001321453	0.152115537
5.605533293	-24.18708609	0.006269219	35.57213016	0.006445323	0.103347859
4.0877824	-184.6363023	0.010879612	117.764433	0.011785966	0.038008634
2.328390653	-231.7913043	0.02133458	79.39930646	0.02196109	0.043370948
2.88645814	-162.8509985	0.006103385	60.80407782	0.00621679	0.057391828
15.64386753	49.07330827	0.003842209	4.355866199	0.004442114	0.209399582
6.41550194	12.53153153	0.004428851	28.16969081	0.004529728	0.084411752
3.059684407	-135.5149051	0.015341714	70.66023456	0.015442512	0.062212518
48.93668383	31.11556466	0.002214174	7.411921114	0.002628596	0.26624319
7.604432897	5.168510638	0.005486673	6.659732602	0.006026819	0.152770876
9.668341393	-13.04155125	0.002411831	22.91377096	0.00260162	0.118958809
1.951164429	-283.5397727	0.014277217	90.98468576	0.014395871	0.038625051
12.21437999	4.642758621	0.002820566	24.31636503	0.002915871	0.115078747
22.941737	16.96089523	0.00218731	10.62257355	0.002643235	0.235139202
4.251428539	-125.4195745	0.008690773	142.4386923	0.009105899	0.056452162
4.708869527	-85.73	0.00420447	55.34007223	0.004317581	0.082921827
1.656142665	-298.2536232	0.012451683	98.68505736	0.012656377	0.043088532
12.43282802	39.54265734	0.003703937	7.144191037	0.004130677	0.157241097
6.321863022	-24.44593805	0.00210824	37.07855903	0.00221616	0.094655963
13.06404482	-9.568500259	0.001398225	29.99995199	0.001545934	0.164561869
7.372537199	-6.835026609	0.001992246	21.37804491	0.00208965	0.102157724
4.756554236	-65.54391892	0.002783916	65.46012383	0.002860114	0.083720554
7.779956782	-58.26143345	0.001872064	42.88220267	0.001969214	0.113513817
3.038655386	-169.3908046	0.015628892	72.37173526	0.015676443	0.054251632
3.613648619	-123.1408451	0.018229811	25.76328299	0.018290816	0.073162915
6.683032443	-52.01228655	0.002087733	59.89795331	0.002237787	0.121952203

original_glrlm_RunVariance	original_glrlm_GrayLevelNonUniformity	original_glrlm_LongRunEmphasis	original_glrlm_ShortRunHighGrayLevelEmphasis	original_glrlm_RunLengthNonUniformity	original_glrlm_ShortRunEmphasis
0.120580692	51.29229155	1.324481646	555.2952945	514.5979432	0.936029615
0.05078223	6.433584266	1.142904261	372.5747108	100.020586	0.969135919
0.462231921	41.33656764	2.063440438	653.370078	268.5409989	0.861158019
0.115518129	8.224652262	1.341873684	405.2901674	68.56369051	0.925514665
0.585817848	72.94842505	2.335838316	546.4838487	371.5909425	0.836778207
0.045461246	5.842619127	1.142079991	508.0464717	90.07880895	0.965370316
1.712115128	2059.239332	4.644512581	1071.728353	4448.971256	0.707708208
0.681902107	729.8872073	2.622105709	788.9860492	2143.540438	0.796503426
0.656266023	677.4038186	2.594897098	799.2725903	1776.990777	0.792662831
0.092233084	21.52165006	1.249086504	771.1704218	296.9343387	0.949279489
0.644171534	715.5690669	2.48225505	580.0145498	2516.373567	0.81642992
0.829824192	441.232052	2.874789697	813.6694629	1244.344298	0.787692952
0.13360776	17.47273415	1.344465982	779.0474425	255.3166786	0.936412056
0.113088611	16.77156984	1.317314026	206.5312153	146.0737208	0.933709384
0.563130484	132.245295	2.367627346	397.9285612	431.00572	0.818128838
1.0021809	1373.719391	3.269589789	1109.383271	3044.977844	0.759969263
0.237488422	47.21590244	1.650511832	593.7613848	272.4015542	0.881993367
0.168871645	12.85658064	1.458040303	241.6087373	77.86158006	0.911811336
0.298420168	60.39584812	1.748345175	376.6379917	346.7812932	0.879808642
0.649486381	1212.164387	2.461621413	947.7368312	5604.245494	0.82247477
1.860133403	90.03603778	4.299174024	418.5392862	579.7864658	0.811382806
0.154781564	18.45006995	1.450683885	242.4213409	110.0392024	0.907861725
0.273828904	33.48888974	1.736644833	442.4439916	168.4219248	0.872931889
1.481235735	712.0012642	3.950013546	309.0503889	1647.502291	0.760545193
0.909502433	98.24735282	2.979750248	832.2402636	301.3531922	0.798095497
0.484886314	242.9507744	2.186561423	1145.99427	1012.50678	0.833526968
0.20048841	90.50108276	1.54346147	656.0916771	624.4186102	0.898468995
0.385594394	184.6506263	1.943566044	477.0925137	986.786184	0.859231604
0.021632723	2.366449078	1.073307035	136.5543639	26.29147491	0.981673241
0.36125291	143.0516881	1.941114155	382.733046	624.6359868	0.850115321
0.898861284	451.6769249	3.091433256	826.0226753	1304.657991	0.766963076
0.208937178	24.66885292	1.581392246	503.8392146	164.9796651	0.890958864
0.298080923	121.5594567	1.744404097	649.7638641	785.0700189	0.881355879
0.618653844	494.234886	2.493134743	883.4149092	1383.095491	0.803021596
0.500973509	416.1294582	2.209553636	666.4973098	1729.723934	0.832614336
0.438104992	103.169257	1.984569537	745.7499834	652.7001747	0.870960024
0.034790772	13.6075514	1.101929959	808.0773831	355.1876304	0.976347795
1.229382239	1624.521317	3.797773816	997.0015346	3603.490125	0.725545912
0.141909787	19.29839674	1.384541983	194.9734034	145.7170773	0.924596755
1.233926686	1821.870397	3.539306846	929.271407	4409.963258	0.770757847
0.252799115	55.63564889	1.638658376	550.369751	408.6398374	0.893432669
0.485080099	904.5140034	2.177770383	737.7171933	3630.347702	0.833863581
0.238340345	116.6227224	1.610677602	812.3906027	1014.150629	0.897699497
0.351215655	69.71445797	1.859818701	519.0712347	412.5150132	0.8699574
0.816709835	158.7826555	2.810573209	282.7458136	462.479618	0.801361142
0.574915179	229.8603197	2.30397083	564.6499718	992.4328248	0.838564762
1.6031707	1051.570556	4.330275445	735.4120849	2074.677381	0.72728505
0.256942899	29.29899921	1.660964839	142.0173857	139.0769641	0.888293132
0.074015088	8.528737927	1.205929126	303.1803289	96.47291919	0.957296538
0.055141286	10.33808126	1.150269164	1143.531698	193.6199852	0.968273928
0.807652236	274.2634516	2.912839266	84.99775475	739.105379	0.775582959
0.255997417	133.466761	1.627239219	1395.011013	1421.149315	0.898596476
1.494046068	1583.641967	4.118142415	672.6772984	3138.330453	0.736037114
0.346803973	103.24598	1.862644947	447.730765	491.1380887	0.867028162
0.22618503	20.5048711	1.569280464	710.9650898	194.34509	0.905077332
0.297111192	60.26166743	1.746374592	365.5388848	405.5011828	0.880744268
0.59334946	750.9569078	2.411095843	553.3106462	2432.945591	0.814489712
0.68563581	164.0297198	2.527576925	705.5562968	733.8344439	0.824196568
0.148114604	23.49009933	1.41180332	288.1849703	196.8825807	0.918667572
0.605796063	3545.979759	2.476572757	1110.051232	10297.03851	0.80206147
0.809189413	59.29231558	2.576171267	792.5379268	418.4688466	0.85720811
0.320065428	124.2024378	1.771150086	1051.991641	984.7237469	0.882862015
0.188690103	250.8711058	1.482343396	1270.135997	3229.048978	0.914840336
0.931555631	807.446587	2.955645688	1196.982841	3615.707208	0.80355615
0.258895037	33.81209805	1.6894743	265.0614099	198.3643	0.880890049
0.194890117	97.74119514	1.462214975	996.4738638	1606.664549	0.926156633
0.069606579	8.187293987	1.20728711	384.9600608	98.88526988	0.952972745
0.606873048	439.8620649	2.388342018	1196.47292	1986.373963	0.827784837
0.903055238	1389.573268	3.051389904	770.3260966	3810.978206	0.774024981
0.349767074	106.3047112	1.809767749	872.5115118	940.0304923	0.884695549
0.195775264	19.9601017	1.53870271	341.5087601	132.4843689	0.898359389
0.763930439	964.605556	2.846620364	1077.758813	2737.755825	0.772908488
0.62811693	96.19427315	2.441408658	529.8951248	355.9301282	0.824954802
0.443109019	144.3373548	2.135819773	715.8121784	531.9388708	0.829176541
0.752120858	359.7351332	2.619026925	1089.004593	1495.471687	0.821978556
0.689206854	47.70671495	2.42541635	558.580664	314.8376162	0.850844166
0.064082274	53.18647999	1.186450277	1147.2804	1253.311413	0.957943703
0.05443966	4.752092936	1.158228651	574.6726057	99.79617623	0.964040283
0.290492557	32.21049896	1.640505063	556.3491493	447.5168338	0.911677644
0.627429331	245.2781658	2.488924328	243.6196157	715.8902048	0.809581716
0.120580692	51.29229155	1.324481646	555.2952945	514.5979432	0.936029615
0.192768649	20.34330581	1.473585881	544.3880809	266.2184249	0.920410652
1.466111401	554.999976	4.179085987	469.731739	1011.748447	0.723105176
0.400155501	142.7951164	1.987316906	205.076377	641.4377624	0.853466201
0.431007137	169.8256864	2.061838715	599.4779968	964.9125979	0.846199693
0.05763549	6.503468981	1.152743682	534.9008777	155.498112	0.96914679
0.229322404	71.46946245	1.601359138	892.871869	474.6540838	0.894878693
2.590054851	532.7356846	5.832096518	393.3045454	1083.820122	0.719010552
0.189171754	58.63332364	1.474562405	1265.214746	839.8798846	0.917784748
0.286099132	56.31465611	1.683370417	694.5439488	517.6630935	0.894809208
0.073806057	11.20147385	1.203744338	507.4004564	232.5860443	0.957104802
0.509192875	173.6860892	2.214271062	297.3962927	724.4329975	0.835096378
0.27557852	274.2753327	1.674342819	770.4297825	2195.922805	0.892445467
0.838141522	455.3672687	2.781715736	905.5850727	1700.279053	0.811643044
0.192404143	185.4507106	1.493320624	723.5025519	1449.133358	0.912790149
0.227940446	85.76913073	1.565508612	920.7873165	803.0991414	0.905852856
0.437077801	133.0278804	2.013809157	1026.721666	818.6319749	0.861058055
0.040782111	4.544936925	1.120189345	652.0900897	77.869307	0.972024742
0.03882588	4.994069352	1.124184016	347.6475583	63.0318896	0.968953996
0.725531332	868.3791701	2.548157079	825.2090601	4579.904955	0.82896754

original_glrlm_LongRunHighGrayLevelEmphasis	original_glrlm_RunPercentage	original_glrlm_LongRunLowGrayLevelEmphasis	original_glrlm_RunEntropy	original_glrlm_HighGrayLevelRunEmphasis
813.8021648	0.912104412	0.005036833	4.439507015	597.7948072
468.3621178	0.957794418	0.022868346	4.419024799	389.4490049
1845.401918	0.792164357	0.008914779	4.639506647	789.764674
641.7319756	0.903846154	0.019244222	4.143846878	447.4761598
1850.895266	0.757475768	0.005747785	4.471046341	687.0753895
645.4547716	0.955937267	0.028567874	4.550510238	535.0089009
7498.333871	0.586603327	0.003110389	4.394557035	1549.174431
2709.864285	0.719092045	0.002882306	3.912271874	1002.240119
2694.258952	0.718973389	0.002909512	3.736273129	1017.00758
1079.098576	0.930473373	0.00709697	4.75733578	822.7219712
1795.482746	0.739173893	0.004468638	4.160729763	713.8114212
3081.617856	0.70076848	0.003272799	3.924809206	1043.903721
1294.87759	0.909133163	0.009379985	4.992792739	858.6503801
309.2796863	0.912550607	0.017278165	4.12599413	224.226712
1189.071058	0.746989967	0.006521753	3.822027649	490.62031
5015.02912	0.665821689	0.002475925	3.940464886	1482.590219
1181.223821	0.844389555	0.00528399	4.077053841	683.4676175
426.5852335	0.881496881	0.038961997	3.872764698	271.1345322
825.4020038	0.832315832	0.007583526	4.155440899	438.4493202
3100.413445	0.743484402	0.002252768	4.613465008	1179.263254
3289.371798	0.642191142	0.00974068	4.908425609	568.8329028
416.7843424	0.880720545	0.013899811	3.804690014	271.9488663
970.8015493	0.829181495	0.010192472	4.044506405	519.9916572
1715.042837	0.638202139	0.010617545	3.875386449	414.3848204
3486.961513	0.697287561	0.005248652	4.177597631	1076.573151
3172.090902	0.768091168	0.002361868	4.223125406	1394.739727
1165.718253	0.864340133	0.003537872	4.226484096	736.2150553
1124.928943	0.804171951	0.004477324	4.212643318	561.072727
151.2685733	0.978021978	0.068245695	3.749946198	139.4972058
950.0862887	0.79743367	0.006306621	4.087919876	460.5400175
3519.57728	0.678548737	0.003228141	4.216301678	1096.402392
990.7640193	0.855806938	0.013534207	4.201815818	580.551103
1400.851082	0.832862212	0.00568276	4.430612387	752.5780677
2795.064352	0.731147861	0.003385086	3.76149511	1105.74042
1871.738346	0.766199863	0.003307458	4.251751223	812.9052225
1952.971493	0.805578287	0.004525114	4.596734018	884.9003191
955.7940651	0.968441815	0.005994932	5.155639788	835.926977
5374.355326	0.626590823	0.003171727	4.172426783	1388.559679
305.5283073	0.898477157	0.015602633	3.980252549	213.0252932
4498.998164	0.659700791	0.003200643	4.013957505	1223.743621
1118.128245	0.851152018	0.005357397	4.362807838	630.7904981
2026.84485	0.770027955	0.00286052	4.096686759	896.5124974
1569.632542	0.858546169	0.003110771	4.57826729	921.0946257
1177.044934	0.815254053	0.005092245	4.272524346	605.2435313
1089.208581	0.709558824	0.009716277	3.867569309	362.325062
1720.662039	0.765217391	0.004564367	4.276689013	691.5256465
4640.070365	0.606962703	0.004375978	3.994486196	1029.84311
286.404017	0.845451352	0.017807357	3.715472581	162.8934568
404.0343806	0.94197031	0.01532816	4.107684327	320.3920395
1430.680233	0.955944056	0.012449481	4.865321135	1193.154293
339.809657	0.6916216	0.028945871	3.781308284	111.6281756
2783.596055	0.854740443	0.001809158	4.894630691	1586.021875
3955.36064	0.618628451	0.004566323	3.915452522	929.357859
964.5101512	0.814730724	0.005420442	4.025200549	516.2870753
1379.002024	0.864111498	0.007491444	4.627933529	805.749623
830.4498866	0.831651004	0.009170731	4.383203514	429.2858847
1698.524112	0.742896219	0.004475903	3.938850977	686.0923274
2371.538011	0.742892706	0.003916888	4.415409196	877.2666821
460.1517596	0.891402715	0.011110324	4.226081336	316.5859289
3491.807838	0.731549487	0.001860187	3.82986212	1391.070336
2983.532851	0.753691729	0.004860243	4.780632733	971.0172426
2417.160141	0.830798023	0.004169449	4.857171788	1231.155596
2311.847851	0.879954909	0.001853739	5.137378293	1424.670105
4791.092122	0.704451065	0.002431174	4.826397508	1525.444502
561.7227543	0.838485502	0.010618421	4.097543029	308.5502652
1861.553307	0.888787185	0.003304564	5.252992617	1113.134882
521.3922823	0.938590821	0.013340457	4.336813854	410.1206614
3814.72275	0.750260883	0.002580451	4.534365423	1484.620419
3177.250072	0.683305321	0.003373127	4.128379167	1009.479923
2086.641825	0.828069115	0.003099235	4.886528124	1022.605343
648.0864135	0.866355866	0.016053657	4.123313455	390.0988876
4122.172544	0.694290637	0.002222149	4.094253556	1410.268273
1712.887695	0.744890623	0.005823375	4.095125511	657.9833253
1903.513816	0.770019356	0.003836512	3.933112827	870.8499425
3771.667331	0.734213548	0.002411648	4.43011547	1353.265687
1955.04224	0.761844116	0.008072712	4.608516854	688.9631001
1468.249785	0.944202222	0.01611361	5.331536	1205.997647
747.7604571	0.952508361	0.024479578	4.857175986	605.9632583
1266.126941	0.861396668	0.006953015	4.981168688	639.8251933
782.3889595	0.733853865	0.009107607	3.710471139	304.5717022
813.8021648	0.912104412	0.005036833	4.439507015	597.7948072
1044.191082	0.88492808	0.016011566	4.903049073	615.2489748
2827.737096	0.609330693	0.007326619	3.879845262	658.8236129
523.5651673	0.795220949	0.009602983	3.890549537	245.8436607
1612.414228	0.789217984	0.00389261	4.448497779	726.6356378
720.1121198	0.956293706	0.01489597	4.979515497	565.1891046
1686.838342	0.855915119	0.003457379	4.265868606	1010.671405
3578.165192	0.556680162	0.010410067	4.106817033	567.2986647
2310.544522	0.883207856	0.011177165	5.272043302	1416.693353
1516.719773	0.847596154	0.005018999	4.808982098	803.494266
734.4578875	0.941471572	0.01350369	5.032325142	545.5554869
844.5213822	0.771005917	0.007228849	3.999240211	362.3423742
1587.009977	0.846468552	0.002878806	4.661304479	881.7876585
3452.802342	0.717835615	0.002929503	4.383953301	1147.970151
1258.657729	0.877786461	0.002634231	4.319624033	803.4670957
1777.602342	0.864994802	0.003308976	4.838229639	1042.534555
2713.289571	0.798372276	0.002669285	4.626466808	1228.967387
784.34348	0.962864721	0.01587599	4.569017015	677.0434464
428.8685959	0.96099675	0.018534834	4.109624361	363.8917658
3026.350327	0.74116554	0.003506632	4.808993477	1041.356369

original_glrlm_RunLengthNonUniformityNormalized	original_glszm_GrayLevelVariance	original_glszm_SmallAreaHighGrayLevelEmphasis	original_glszm_GrayLevelNonUniformityNormalized
0.846494003	49.63962328	352.2035114	0.047125639
0.923143481	37.30709343	242.5706449	0.053200692
0.700540443	82.13594173	389.6784055	0.046742976
0.8236112	43.82044348	248.2335937	0.065440779
0.656945444	35.64274102	378.7111307	0.058223062
0.91358472	72.02595156	328.0726138	0.047577855
0.463893875	63.89675081	860.1874238	0.048696199
0.589919748	26.63604945	641.2072075	0.077970084
0.583893037	24.88091848	622.4149846	0.093761125
0.876031	75.85998838	502.0379445	0.04222624
0.62192139	61.08279307	517.1412131	0.0584395
0.576719473	29.24149966	643.0192737	0.074634134
0.848106886	102.8168666	481.0144977	0.032443449
0.841266222	25.71471088	139.683431	0.06547619
0.625292974	27.21455515	275.3608885	0.086108221
0.535356628	70.82672076	857.2296242	0.077377111
0.737308702	32.7619	343.5693391	0.069
0.79514681	28.6275	138.9690653	0.07
0.733333582	29.79129067	223.7673514	0.064926651
0.631554004	53.69337335	629.9643578	0.044552058
0.62190346	40.80295082	296.4855562	0.051258987
0.78848038	17.109375	104.2525633	0.088541667
0.720637177	38.52684441	260.6848865	0.061723886
0.536339233	21.14129553	229.0008637	0.085666861
0.593920968	82.18394168	570.442138	0.055742908
0.649995664	56.60796082	843.0566249	0.06013915
0.768160989	32.99928944	437.6711467	0.058496926
0.695533395	29.43741561	317.5037941	0.062594036
0.956734554	30.2475	96.9875	0.08
0.679000641	27.21618147	223.4942021	0.065557656
0.545826654	34.47387318	674.6508598	0.066587396
0.754270857	34.68817204	334.3262224	0.061625621
0.735966321	52.79782485	403.0784158	0.048922797
0.600120239	61.38101817	675.2847568	0.083926737
0.648435504	42.83589517	462.9721748	0.051294146
0.717474544	60.69793265	487.4752943	0.042637641
0.939899631	86.8470585	556.9727271	0.032130568
0.488406951	80.90786472	826.9164764	0.054199135
0.822023878	26.23335068	132.7750241	0.074401665
0.551089683	68.52087226	749.1637311	0.060088253
0.759422495	32.59499055	297.3403286	0.062086484
0.6504745	34.23883285	492.5345217	0.062962475
0.769561706	51.56765054	520.5086249	0.047098324
0.714905292	34.6743561	361.6115262	0.060491493
0.59817573	21.08042088	192.7524298	0.089976205
0.660286838	42.40522173	356.7508862	0.055322219
0.489655185	32.91611933	620.5288938	0.072258711
0.749921252	13.60443437	84.97107767	0.091458806
0.896251722	27.56972222	185.1825037	0.063888889
0.920349574	81.61524111	755.74124	0.037367856
0.55898333	9.221562995	63.99635553	0.123305324
0.76942997	72.75861243	893.9577278	0.038933479
0.501475989	30.49426894	541.6202847	0.077281365
0.709693321	35.70904195	414.9711077	0.058460884
0.782762697	63.69146797	476.8866651	0.049554726
0.734714477	32.16506103	206.8231909	0.058991968
0.618535485	35.08159557	408.0727095	0.070697615
0.636033914	43.6778774	489.1709684	0.052659337
0.81027332	27.05683853	198.6218202	0.060231879
0.598306803	35.90006797	911.3939014	0.086531137
0.696114541	73.24304653	540.5121881	0.042879756
0.738933524	100.306035	654.0266154	0.034155139
0.801960884	111.0783015	795.0260506	0.029038693
0.60157339	88.86777986	912.811735	0.035092433
0.735151515	23.43137935	163.6328114	0.074343855
0.82693986	91.22682488	636.6565487	0.031023652
0.884245321	35.37274926	238.25077	0.052405267
0.640724187	71.74154253	852.1874363	0.043558404
0.555763009	35.61450448	575.320654	0.06571422
0.74307529	68.30691343	565.5668554	0.0382045
0.769401696	42.89399598	156.6452263	0.070956622
0.554531938	48.61070866	861.520128	0.056099407
0.636620796	38.14188733	365.9236614	0.065103727
0.642706467	31.33235408	500.5429322	0.069214876
0.631830991	56.1420898	795.7209481	0.052913131
0.683228156	42.24916701	367.0267834	0.051853394
0.895324181	107.1240249	736.2806751	0.031975111
0.91025591	69.49826389	392.2643364	0.043209877
0.797586081	55.19532222	356.4598251	0.040488889
0.610585232	12.40767273	166.049127	0.106560468
0.846494003	49.63962328	352.2035114	0.047125639
0.814283787	72.41311857	292.0233301	0.038678568
0.484522228	32.41433665	383.4691788	0.083458435
0.685183129	11.37440143	120.2106121	0.095446798
0.673283418	42.13635654	339.398265	0.05012328
0.923222499	76.25877992	351.314902	0.036810614
0.762825961	43.36627659	629.0233696	0.057433571
0.478286696	24.27561167	304.7696977	0.075429581
0.808355403	138.8817019	780.6726466	0.028465719
0.762307107	64.13648889	425.0811736	0.038777778
0.894247543	81.35433594	289.2304865	0.03453125
0.653749827	15.99785665	192.7275171	0.096707819
0.757305551	53.77703403	476.8437306	0.04242754
0.614267811	58.40533878	668.8472744	0.04655102
0.797779104	32.6895113	468.1399034	0.05735098
0.783846578	82.80400509	608.0470126	0.03458897
0.698768566	59.08039012	727.6946783	0.045119652
0.929149449	78.44713421	384.5126449	0.045211819
0.92234824	26.09331597	222.3340336	0.066840278
0.643075868	78.5315643	509.459378	0.035231606

original_glszm_SizeZoneNonUniformityNormalized	original_glszm_SizeZoneNonUniformity	original_glszm_GrayLevelNonUniformity	original_glszm_LargeAreaEmphasis	original_glszm_ZoneVariance	original_glszm_ZonePercentage
0.53607862	127.0506329	11.16877637	85.79746835	77.90065695	0.355855856
0.662197232	45.02941176	3.617647059	5.867647059	3.106185121	0.601769912
0.551550468	85.49032258	7.24516129	190.2903226	180.5800624	0.320910973
0.570578691	24.53488372	2.813953488	16.79069767	12.21308816	0.467391304
0.657429112	151.2086957	13.39130435	414.5565217	404.0363705	0.308310992
0.689878893	46.91176471	3.235294118	4.985294118	2.690960208	0.660194175
0.582810749	853.8177474	71.33993174	45949.00478	45825.11952	0.089844229
0.602043576	364.2363636	47.17190083	8673.434711	8603.870839	0.119896948
0.54752581	232.1509434	39.75471698	9912.266509	9812.784704	0.100260109
0.569860537	100.2954545	7.431818182	18.43181818	14.15444215	0.483516484
0.509622932	371.0054945	42.54395604	8420.203297	8363.870849	0.133235725
0.563250298	197.7008547	26.1965812	6203.279202	6126.629427	0.114220631
0.663871162	114.849711	5.612716763	26.17919075	22.51849377	0.52265861
0.600907029	50.47619048	5.5	30.61904762	25.50283447	0.442105263
0.49817898	61.77419355	10.67741935	1415.919355	1360.872529	0.134782609
0.558810274	500.1351955	69.25251397	23235.80223	23145.03137	0.104960713
0.457	45.7	6.9	222.76	203.7504	0.229357798
0.545	21.8	2.8	42.025	34.324375	0.36036036
0.604979407	88.93197279	9.544217687	273.7210884	258.8435374	0.259259259
0.496731427	969.1230138	86.92106612	11755.40595	11718.03381	0.163578435
0.55790859	227.0687961	20.86240786	961.4496314	948.7220931	0.28030303
0.386284722	18.54166667	4.25	56.125	45.28993056	0.303797468
0.607377648	44.94594595	4.567567568	163.7972973	149.3778305	0.263345196
0.534960498	249.8265525	40.00642398	15521.67452	15415.85343	0.097210658
0.596508629	84.70422535	7.915492958	978.8873239	952.7478675	0.195592287
0.587879602	215.1639344	22.01092896	1945.576503	1914.964772	0.180740741
0.518177011	127.4715447	14.3902439	215.1097561	200.5397085	0.261980831
0.474846649	152.9006211	20.1552795	1090.468944	1060.627493	0.183058556
0.54	10.8	1.6	2.4	0.44	0.714285714
0.572854442	131.7565217	15.07826087	935.8217391	910.778242	0.199826238
0.632450208	293.4568966	30.89655172	5663.575431	5606.318812	0.132156081
0.673025783	62.59139785	5.731182796	52.35483871	44.83662851	0.364705882
0.561561562	187	16.29129129	458.3993994	443.647341	0.260359656
0.509807395	148.3539519	24.42268041	7827.584192	7710.483532	0.092410289
0.547035232	346.2733017	32.46919431	1983.176935	1953.005084	0.182053494
0.640847071	224.9373219	14.96581197	422.2393162	411.9116079	0.311170213
0.657412142	176.8438662	8.643122677	5.784386617	3.682425616	0.68974359
0.565305613	514.9934138	49.37541164	36963.77168	36797.7551	0.077611177
0.48074922	29.80645161	4.612903226	54.11290323	44.01690947	0.314720812
0.57735216	748.8257517	77.93446415	32860.86045	32773.62456	0.107066204
0.53172259	97.83695652	11.42391304	190.5815217	178.8210952	0.291600634
0.493952303	535.9382488	68.31428571	7998.098618	7953.609103	0.149924002
0.59550507	269.7637969	21.33554084	324.1169978	312.7542944	0.296660118
0.53774811	98.94565217	11.13043478	263.6358696	248.8718986	0.260254597
0.576293873	94.51219512	14.75609756	1919.121951	1875.110054	0.150735294
0.571310778	225.0964467	21.79695431	1703.814721	1679.193982	0.201534527
0.589980285	357.5280528	43.78877888	26527.55281	26395.30281	0.086956522
0.572688335	36.07936508	5.761904762	118.1111111	106.0272109	0.287671233
0.579444444	34.76666667	3.833333333	9.5	5.89	0.526315789
0.61818079	82.21804511	4.969924812	8.180451128	5.44428741	0.604545455
0.526483932	101.611399	23.79792746	4176.569948	4079.041531	0.101259182
0.554560543	385.9741379	27.09770115	312.5186782	302.8962029	0.322371468
0.542862314	484.2331839	68.93497758	36983.23318	36854.97502	0.088299347
0.559098639	93.92857143	9.821428571	520.7321429	495.3137401	0.198347107
0.646078713	76.23728814	5.847457627	54.1440678	48.22845447	0.411149826
0.544225776	110.4778325	11.97536946	234.7438424	224.077022	0.306184012
0.544259696	366.2867756	47.5794948	8235.127786	8173.413104	0.127293361
0.60872652	191.1401274	16.53503185	1261.117834	1236.907674	0.203236246
0.561504383	57.83495146	6.203883495	36.38834951	29.41464794	0.378676471
0.5373069	1119.210274	180.2443591	58383.1469	58255.72692	0.088589291
0.608146306	161.7669173	11.40601504	353.8759398	344.8984821	0.333751568
0.639446663	344.0223048	18.37546468	475.6078067	466.7300652	0.335620711
0.606848657	1052.882421	50.38213256	422.6697406	415.7256803	0.379483815
0.594844098	978.518541	57.72705167	6263.725228	6236.899755	0.193075117
0.585616834	54.46236559	6.913978495	140.1827957	128.2691641	0.289719626
0.593851653	624.7319392	32.63688213	179.4001901	175.0862769	0.481464531
0.627519484	38.27868852	3.196721311	12.27868852	8.472991131	0.512605042
0.630448873	584.4261057	40.37864078	3088.142395	3068.312523	0.224563953
0.511196531	565.3833635	72.67992767	21245.10398	21162.97712	0.110346204
0.607909612	325.8395522	20.47761194	405.7033582	397.5872376	0.351015062
0.546681988	32.25423729	4.186440678	76.84745763	65.58517667	0.297979798
0.598150034	405.5457227	38.03539823	15770.10177	15660.47037	0.095506409
0.60364311	92.96103896	10.02597403	768.1103896	744.4554309	0.205607477
0.452027376	79.55681818	12.18181818	994.8352273	957.6668066	0.164026095
0.534561102	342.6536661	33.91731669	2792.870515	2767.698613	0.19931592
0.553206997	108.4285714	10.16326531	282.9897959	273.4933361	0.324503311
0.479399111	359.5493333	23.98133333	11.36533333	7.460757333	0.506072874
0.61882716	44.55555556	3.111111111	4.569444444	2.018325617	0.626086957
0.598777778	179.6333333	12.14666667	83.49	78.7811	0.460829493
0.525073051	108.1650485	21.95145631	2922.203883	2862.178999	0.129072682
0.53607862	127.0506329	11.16877637	85.79746835	77.90065695	0.355855856
0.580657296	99.29239766	6.614035088	45.93567251	41.27916282	0.463414634
0.522870144	152.6780822	24.36986301	13470.42466	13333.40641	0.085430076
0.477680383	106.045045	21.18918919	870.9954955	842.9818399	0.18893617
0.509581474	182.4301676	17.94413408	721.0027933	695.5820433	0.19833795
0.6355359	78.80645161	4.564516129	4.193548387	2.178980229	0.704545455
0.573280025	120.9620853	12.11848341	199.7298578	187.9236315	0.291034483
0.61308404	261.7868852	32.20843091	14755.12412	14664.45091	0.105017216
0.524569314	243.9247312	13.23655914	127.0946237	120.7094924	0.395744681
0.615622222	184.6866667	11.63333333	169.8	162.6888889	0.375
0.50375	80.6	5.525	8.8875	5.911875	0.579710145
0.514660494	111.1666667	20.88888889	1443.768519	1399.939215	0.151048951
0.565439496	564.3086172	42.34268537	920.0841683	908.3271031	0.291642314
0.648885714	454.22	32.58571429	4640.071429	4609.758539	0.181629476
0.512307206	350.4181287	39.22807018	286.3084795	277.1764197	0.330914369
0.665746329	309.572043	16.08387097	227.9784946	221.495174	0.392736486
0.630780625	287.6359649	20.5745614	587.2083333	576.886768	0.311262799
0.540761837	28.66037736	2.396226415	4.509433962	1.81488074	0.609195402
0.623263889	29.91666667	3.208333333	3.979166667	1.791232639	0.676056338
0.576177811	1170.217134	71.55539143	7498.424421	7476.06377	0.211474386

original_glszm_LargeAreaLowGrayLevelEmphasis	original_glszm_LargeAreaHighGrayLevelEmphasis	original_glszm_HighGrayLevelZoneEmphasis	original_glszm_SmallAreaEmphasis	original_glszm_LowGrayLevelZoneEmphasis
0.136821452	57343.96203	499.9029536	0.759450931	0.009071506
0.04401059	3024.264706	315.4117647	0.832207573	0.034652216
0.208695697	188814.7806	569.5483871	0.770250956	0.017549038
0.06222007	9414.767442	353.744186	0.779150116	0.034136628
0.484393081	360507.4043	481.5869565	0.836525837	0.008465094
0.045755351	3529.044118	435.2647059	0.85224674	0.040403299
27.98123553	75524408.94	1143.277133	0.790593345	0.002205556
8.241341536	9142562.041	824.5818182	0.80149748	0.00315613
9.448861752	10414649.75	845.2169811	0.767188703	0.004022197
0.029472807	19334.91477	687.0170455	0.778980329	0.011874516
11.50829388	6174620.775	684.5041209	0.741140202	0.006141709
5.71841405	6740974.39	865.6210826	0.777432327	0.004684862
0.037972011	33334.46821	622.8150289	0.839350909	0.013107972
0.139236394	8297.02381	184.6547619	0.800524549	0.028330196
2.794662682	721665.7339	409.6693548	0.729132397	0.012140748
14.93249693	36185598.45	1167.509497	0.774297682	0.002825788
0.301432386	171489.22	534.97	0.701990273	0.012539915
0.255124155	14124.95	201.55	0.760959609	0.058414681
0.545591526	141148.2653	305.3061224	0.801727177	0.01503064
8.89739025	15550234.14	897.6986161	0.732443361	0.002171858
1.178107343	793890.7002	400.963145	0.775083506	0.009796787
0.199086262	18329.22917	196	0.643309295	0.029696566
0.291090387	100131.1216	362.0405405	0.801470597	0.024539739
35.22021231	6852696.949	314.012848	0.758711982	0.012350976
0.816873876	1187733.268	789.6690141	0.798125382	0.010676127
1.302642573	2917342.904	1116.234973	0.793966802	0.004385091
0.277614806	170622.2033	621.7520325	0.74686588	0.006319351
1.846575259	648277.8075	469.5248447	0.716226312	0.006622555
0.108834209	372.3	131.25	0.761111111	0.08391577
1.810472426	488109.7652	310.626087	0.783554894	0.011481909
4.888833115	6573379.825	855.9633621	0.819702948	0.003656105
0.12010462	36933.82796	434.688172	0.834949553	0.015340247
0.534112953	403159.1381	554.3963964	0.775912786	0.012399561
6.93881235	8853614.832	960.1443299	0.740708065	0.010052473
2.259319257	1746616.589	639.6461295	0.767114106	0.004241181
0.392995336	462114.5014	630.968661	0.825832804	0.007611479
0.012345347	6891.200743	716.3159851	0.837177601	0.007541641
25.91698726	52776430.52	1129.501647	0.778638556	0.004580376
0.252427568	13339.22581	185.7258065	0.712893282	0.02993814
25.53580679	42319142.34	976.7740941	0.787692888	0.003302001
0.255560902	147754.6793	443.0978261	0.755727281	0.010363044
8.333070448	7693117.159	712.397235	0.730266468	0.003868406
0.306140291	349872.1589	697.602649	0.798911638	0.005391814
0.401710054	176645.1739	504.6684783	0.76008137	0.008474494
4.796615008	771355.8049	256.6280488	0.786238569	0.014584359
2.149172704	1356536.708	485.8604061	0.783803602	0.007039056
24.5153265	28728220.58	799.2739274	0.795806725	0.003389465
0.65753122	22185.98413	121.6984127	0.778129741	0.030578365
0.04545986	3779.166667	261.15	0.785499322	0.024330941
0.038370537	12656.42857	1008.428571	0.812477547	0.009934849
34.58397828	511215.6632	87.90673575	0.752172804	0.03130166
0.162495288	611748.5129	1228.954023	0.772075799	0.002906473
38.02697709	35995607.45	733.338565	0.764667843	0.003124615
1.004309819	272957.7738	532.8928571	0.771559913	0.009201472
0.06583674	54937.78814	621.9322034	0.826569457	0.013256575
0.449864463	127847.3793	294.3497537	0.76530969	0.016061978
11.52130227	5901471.473	559.7161961	0.76489935	0.0074811
1.296315276	1232681.592	651.5987261	0.804996851	0.005727035
0.124773115	12338.4466	277.4951456	0.773336058	0.019107322
41.02860166	83175777.71	1227.591455	0.760719397	0.001597668
0.283469161	452136.8308	717.3721805	0.807347924	0.006914324
0.313880668	738526.3903	863.2156134	0.825852097	0.007976013
0.227976832	794983.3533	1036.500288	0.806469893	0.002610858
3.741456926	10504434.21	1195.350152	0.798685343	0.002850739
0.406144926	50770.64516	225.1935484	0.790834947	0.019323837
0.112276014	302839.1331	848.5228137	0.798625659	0.004185857
0.044437755	6556.377049	322.147541	0.810714946	0.021489372
1.849151367	5172806.36	1106.609493	0.820417901	0.004064717
20.10874377	22474164.06	807.7106691	0.742677229	0.002618515
0.309651931	539361.1828	737.0503731	0.80722728	0.004496009
0.202179042	35882.55932	252.8983051	0.762869964	0.038898447
10.7132001	23249721.83	1118.90413	0.799999215	0.002642621
1.058076889	562969.1818	491.5	0.802521751	0.010184026
1.097155076	909063.2614	753.9034091	0.697568018	0.007929391
1.867514027	4184872.301	1091.455538	0.758951542	0.002938836
0.330420016	251910.1378	513.3163265	0.771739452	0.013302516
0.167877292	16652.5	1076.906667	0.719718282	0.007996768
0.067647444	3738.138889	519.2916667	0.80285108	0.01939996
0.094608985	83173.20667	473.9433333	0.800974914	0.010057804
9.140481355	938416.5485	237.6990291	0.751682272	0.010292004
0.136821452	57343.96203	499.9029536	0.759450931	0.009071506
0.077977281	41872.2924	424.5789474	0.789769331	0.028036767
19.77028561	9189421.63	538.9726027	0.750103491	0.008813117
3.085749229	248216.8468	178.7387387	0.717711726	0.012729074
0.872155136	601612.2179	508.1173184	0.741340457	0.006443947
0.026561826	3746.935484	470.1532258	0.824782846	0.017267
0.182192077	226608.545	834.8151659	0.783150574	0.006744491
23.71534407	9187546.749	394.7704918	0.80980103	0.006769526
0.141781451	248988.6516	1083.683871	0.752399679	0.008293233
0.170138151	178111.97	570.9866667	0.810711602	0.008063567
0.038327171	8299.2625	428.94375	0.736094184	0.015767149
3.686681629	569954.5324	273.4814815	0.74399754	0.009940307
0.876689705	971617.9148	649.1272545	0.77997127	0.004385212
3.587123357	6011651.819	828.7057143	0.831134889	0.003684985
0.307165951	269542.0365	664.4327485	0.743397312	0.003599177
0.179695052	297260.9634	777.1548387	0.841181571	0.005240382
0.401707026	865932.5439	939.4517544	0.820313873	0.003879298
0.028836008	3813.603774	565.7735849	0.762507701	0.023705535
0.03085271	1887.291667	307.3541667	0.814892618	0.025105699
5.809337974	9700093.485	670.3791236	0.786814802	0.005024054

original_glszm_ZoneEntropy	original_glszm_SmallAreaLowGrayLevelEmphasis	original_ngtdm_Coarseness	original_ngtdm_Complexity	original_ngtdm_Strength	original_ngtdm_Busyness	original_ngtdm_Contrast
5.686359748	0.008569657	0.009759907	1.912742308	5.536462527	0.092202035	0.000207655
4.846233018	0.034201183	0.044423777	8.053181062	10.10132988	0.049330596	0.004458733
5.5584142	0.017130288	0.008501239	3.909957258	10.4861405	0.075838607	0.00058533
4.58290051	0.033574813	0.05358273	7.739416673	18.1873566	0.031893806	0.004732208
5.079739316	0.008155677	0.005027192	1.243567597	4.778053494	0.18258987	0.00027534
4.98608265	0.040151146	0.039907675	17.65980277	18.97776056	0.034174905	0.00884513
5.971409643	0.002007006	0.000391096	0.089969103	2.352937777	0.689971106	6.47E-07
5.222288277	0.002923035	0.001235098	0.119902388	1.952239271	0.477486985	2.95E-06
5.083003045	0.003768199	0.001490475	0.121139631	2.667116577	0.435985223	2.92E-06
5.754644406	0.011482438	0.016069047	6.6803266	13.82467061	0.041857757	0.000802864
6.04751143	0.005217058	0.00122265	0.275814404	4.722185792	0.354667993	2.12E-06
5.271588034	0.004454888	0.002224923	0.17199767	3.514046242	0.290911748	5.41E-06
5.692112604	0.011581922	0.011107985	11.21194112	12.0933405	0.060045909	0.002371428
4.713132008	0.027026647	0.02841335	2.839189408	5.353654188	0.086337312	0.0011167
5.072169296	0.011540125	0.007282563	0.325699086	3.763363097	0.175764264	3.20E-05
5.690854353	0.002600909	0.000610926	0.160899484	3.594238376	0.502637538	1.69E-06
5.323305065	0.012037129	0.015815908	1.131379098	8.231154134	0.069117011	0.000194338
4.565311532	0.038809024	0.034751139	3.275532059	7.143641866	0.08102841	0.003532397
5.099066531	0.014490484	0.009553175	1.067592223	5.186060454	0.133366873	0.000195472
6.298009678	0.001852933	0.00055904	0.12658346	1.313372759	0.702628807	3.08E-06
5.547100996	0.008587414	0.003368225	0.588999255	3.883013771	0.261586104	0.000127939
4.714661719	0.02818603	0.03836082	1.562744974	6.861365683	0.072457705	0.000949481
4.947506366	0.0241274	0.016495551	2.256117848	9.718478816	0.072692461	0.000579475
5.232755505	0.011481851	0.001416172	0.062457199	1.355472366	0.793800113	2.62E-06
5.505064677	0.010458324	0.005682192	1.854229809	14.70018972	0.08243772	0.000117579
5.48740449	0.004184252	0.002834037	0.75783864	7.401048774	0.141858219	2.12E-05
5.549182965	0.005910087	0.00699555	0.890597051	4.206387744	0.124149249	7.96E-05
5.654455616	0.005975105	0.004322675	0.354526333	2.971076157	0.217722844	1.98E-05
4.121928095	0.078243431	0.101967628	18.03753356	15.37150038	0.065819021	0.041217771
5.226535397	0.010664282	0.004748131	0.501559588	3.449631928	0.239213382	6.27E-05
5.292290064	0.003460526	0.001845526	0.212465161	3.329550367	0.277690228	5.76E-06
4.794483076	0.007023268	0.017441994	3.238905817	9.613879765	0.069408307	0.001012679
5.747817407	0.011850319	0.004584481	0.958477361	5.170698024	0.149846285	8.71E-05
5.540657132	0.009756164	0.00200168	0.290325132	6.059038366	0.221977724	4.09E-06
5.83790422	0.003868002	0.001748542	0.325505784	2.584299798	0.302957421	8.91E-06
5.6370306	0.007272345	0.003886087	1.645289157	5.616091394	0.146759539	0.000155899
5.836817561	0.007137803	0.012735068	10.00547528	7.91557995	0.070001461	0.001930249
6.014929843	0.004314838	0.000552178	0.105054556	3.038545347	0.539658798	6.92E-07
4.903208255	0.027620461	0.031854541	2.117610407	7.915327923	0.080137377	0.00062028
5.824752499	0.003011473	0.000466112	0.176830029	3.654440378	0.556223591	6.88E-07
5.278980333	0.009883886	0.009406996	1.370893285	5.955093023	0.104071682	0.000226962
5.846011144	0.003499465	0.000914706	0.136277996	1.584446718	0.576454804	2.91E-06
5.669181444	0.005126398	0.003988953	1.11867206	4.080710132	0.154162799	8.52E-05
5.357229818	0.008019989	0.00936751	0.931144663	4.813715921	0.102873325	0.000101287
4.892368148	0.01373706	0.005370368	0.345655928	3.937537467	0.229598561	2.57E-05
5.548425295	0.006526619	0.002745325	0.496270788	3.261588838	0.252692129	3.51E-05
5.325699685	0.003123044	0.000944654	0.085833971	2.655468075	0.517977646	1.30E-06
4.369706315	0.028911275	0.023211673	0.864501346	3.313797278	0.153308441	0.000462051
4.804574504	0.023656038	0.042612797	4.493664285	7.67172172	0.069020967	0.003642311
5.519746587	0.003810146	0.017025759	17.46421803	15.25915296	0.042142331	0.003696807
4.666812729	0.028053057	0.004185825	0.055684617	0.775624463	0.84921613	6.91E-06
6.128029298	0.002705208	0.00292728	1.415308757	5.417098487	0.126271085	7.14E-05
5.434962173	0.002800802	0.000574467	0.063457293	1.729021487	0.894233076	8.34E-07
5.449289832	0.008735805	0.008402879	0.717841605	5.311300395	0.107389036	3.10E-05
5.236084207	0.012996713	0.015570111	5.680095073	11.50027835	0.052826806	0.001308617
5.385022266	0.014457482	0.008093474	1.071864475	4.238638056	0.156629492	0.000264007
5.554182483	0.006933047	0.001202754	0.141937232	1.84831323	0.521161794	2.85E-06
5.497548505	0.005451327	0.004025481	0.616230554	4.642178205	0.154556206	4.00E-05
5.134011907	0.018320256	0.026208054	1.852316956	6.439450307	0.070049196	0.000403939
5.555664212	0.001401196	0.00027775	0.051326073	1.163871915	1.186770884	2.45E-07
5.6357955	0.006605954	0.004786669	2.388046132	7.13868699	0.121397597	0.000359077
5.950484363	0.007780766	0.002450917	2.342785069	5.451668	0.160512635	0.00015897
6.350094813	0.002317193	0.001157703	0.938976085	1.902445779	0.334707009	7.62E-05
6.271184912	0.002650037	0.000725003	0.481796527	3.489706684	0.301436129	3.35E-06
4.828054683	0.018385806	0.018106081	1.311839249	5.366514509	0.100131316	0.000368314
6.23066188	0.003659528	0.002139353	2.11521251	2.992912629	0.228919044	0.000214394
4.868540821	0.020886248	0.033924681	7.477220564	8.887686635	0.059292945	0.004396677
5.813016786	0.003708316	0.001124639	0.509204215	3.560866608	0.324872146	2.07E-05
5.790010858	0.001571522	0.000626073	0.097505965	1.591005117	0.687573604	1.13E-06
5.866678333	0.004160471	0.002999585	1.474143676	3.950822902	0.186712877	0.000178595
4.814952906	0.038253848	0.027678845	2.908120905	10.62493009	0.056934495	0.00125877
5.671824934	0.002470065	0.000893661	0.132647208	2.54377075	0.432377724	1.92E-06
5.116534091	0.009829685	0.007150483	0.828478644	6.560868541	0.122407677	9.56E-05
5.466081466	0.00751876	0.006201308	0.486748161	5.866708695	0.129868149	3.17E-05
5.898016065	0.00269881	0.001730444	0.469660162	4.221278296	0.217771043	1.32E-05
5.456812363	0.012867936	0.00673745	1.643637544	5.711841028	0.134757793	0.000369441
6.566708396	0.005170354	0.004536621	3.376122026	5.456966517	0.105381237	0.00031163
5.050075134	0.008432307	0.045566073	15.67439265	21.88498761	0.03055901	0.006836046
5.692619182	0.0094493	0.00705655	3.046508767	5.470509596	0.127034811	0.000661737
4.79169596	0.00934368	0.004426544	0.125869082	1.410858406	0.401277876	1.13E-05
5.686359748	0.008569657	0.009759907	1.912742308	5.536462527	0.092202035	0.000207655
5.600136364	0.027477755	0.013993638	4.988974091	9.103202982	0.065319911	0.001311043
5.366305544	0.007727652	0.001812722	0.118089637	3.405745555	0.398287623	2.62E-06
5.020918961	0.011193702	0.005589732	0.177066592	1.278968023	0.43656305	4.23E-05
5.799571234	0.005965415	0.003945858	0.460147908	3.217650953	0.194827497	4.25E-05
5.377605892	0.015575021	0.024355209	16.31256684	12.82041452	0.053916944	0.005925422
5.473170764	0.006484428	0.008192196	1.529128177	7.989276545	0.078987919	0.000131713
5.052557837	0.006299753	0.00152212	0.089859955	2.282877837	0.566101039	4.09E-06
6.465563001	0.004255337	0.005057284	4.024219606	9.171962685	0.074484021	0.000411106
5.750582253	0.007507612	0.00579059	2.365386474	5.749590652	0.121120338	0.000384165
5.823722124	0.012593188	0.014117642	10.96803438	9.295543996	0.08555053	0.003596349
5.00071409	0.008926015	0.005028957	0.194709627	1.91308611	0.309653754	2.15E-05
5.951968874	0.004029364	0.001822248	0.480057907	2.194656209	0.321206444	3.41E-05
5.696942479	0.003472011	0.00130885	0.41846675	3.691998834	0.291765833	1.11E-05
5.729445247	0.003224089	0.002986045	0.572503432	2.632258795	0.238694884	4.05E-05
5.805020366	0.005025415	0.003399093	2.493134303	4.420779252	0.157938168	0.000281789
5.651720938	0.003689654	0.002907011	1.372966941	5.063387191	0.163105034	0.000105665
5.119153237	0.023084054	0.040773457	23.99537502	18.40666384	0.035562351	0.013899015
4.433208073	0.024684309	0.058808575	7.652937058	9.849772458	0.053505891	0.007848649
6.252732648	0.004414361	0.000650196	0.193169826	1.40163845	0.6446749	1.11E-05

wavelet.LHL_gldm_GrayLevelVariance	wavelet.LHL_gldm_HighGrayLevelEmphasis	wavelet.LHL_gldm_GrayLevelNonUniformityNormalized	wavelet.LHL_gldm_DependenceEntropy	wavelet.LHL_gldm_DependenceNonUniformity
82.27605083	753.2882883	0.039115692	6.500942478	216.3393393
99.55783538	616.4159292	0.034850027	5.768904737	41
62.63930147	632.3954451	0.041189255	6.52025888	138.3167702
83.33400284	409.2934783	0.040170132	5.302868885	40.89130435
43.92808832	381.8766756	0.049209726	6.731963595	161.8150134
115.1007635	894.7572816	0.038175134	5.501905688	53.09708738
17.34300466	864.0794799	0.241445078	6.583663021	889.4295351
44.88874314	940.5539041	0.047077391	7.069329382	1049.332937
10.62013266	447.1820761	0.200490303	6.26876636	296.5138331
61.82139838	621.0659341	0.038416254	6.212092248	126.956044
10.39354569	2067.889275	0.127536635	6.536922913	566.5552709
27.51154238	876.0022779	0.07217423	6.849751642	470.1897169
70.57036719	1090.706949	0.03617163	6.476890137	101.3081571
40.73639889	330.4526316	0.048476454	5.814181103	62.82105263
54.51512169	811.3032609	0.076238185	6.782233187	150.9978261
22.51956794	1320.200422	0.266863641	6.131938354	542.0552363
19.95111417	355.5298165	0.077266223	6.126281265	83.16972477
56.65254444	596.9009009	0.049590131	5.76965365	31.84684685
23.05675155	304.8465608	0.077268585	6.128675835	112.4250441
24.65850127	1067.227719	0.143906432	6.774579005	1016.460552
18.09221203	245.7238292	0.099783523	6.7192114	162.9242424
31.61676815	341.8670886	0.064572985	5.692018271	39.63291139
30.52843809	377.0711744	0.065817302	6.135122908	59.48398577
11.98310434	454.9173605	0.209189227	6.468942387	308.3338884
24.43690094	403.8815427	0.144214497	6.365205981	76.56749311
27.20995604	493.3007407	0.076910959	6.948566492	278.3017284
21.45135253	333.0734824	0.077898791	6.451987013	159.6645367
19.32975704	247.6162592	0.087195196	6.723419634	225.7617965
37.73852041	290.3214286	0.084183673	4.164497779	11.92857143
14.9586904	614.4213727	0.129907058	6.379537621	114.8644657
12.94682903	329.1503845	0.137892944	6.748882754	302.0401595
59.23961553	398.5215686	0.041937716	6.047533428	86.42745098
27.77974632	618.4331509	0.090279557	6.615839888	199.5441751
45.50227794	940.688155	0.054313643	7.083891662	568.1051127
21.9677597	573.2922059	0.116845826	6.806511526	356.2090883
93.71423407	1951.721631	0.075131407	6.973423509	183
136.8073702	976.7666667	0.02504931	6.34120295	197.1948718
19.04329215	653.4668598	0.131654922	6.975325547	964.9904583
29.55314489	349.9086294	0.063980005	5.817678412	48.52284264
30.18138016	5704.098564	0.071958353	7.145832997	1696.517418
28.03928562	293.7654517	0.066704172	6.513099139	116.0396197
13.22788999	566.0333011	0.112077559	6.68178705	792.4213072
18.76874371	595.2089064	0.099189486	6.564355822	195.4963982
48.49087224	550.7072136	0.043203047	6.477885086	198.0777935
10.01114346	197.3336397	0.143086681	6.208169872	105.3694853
28.27852356	412.6992327	0.060958	6.844713047	339.6792839
16.87360751	1739.403501	0.226771591	6.277832275	464.5468503
19.78424136	270.4063927	0.084923167	6.05252572	37.68493151
32.45283164	182.622807	0.064943059	5.548304681	33.05263158
126.2128926	1416.981818	0.030867769	6.118850371	101.6363636
5.481492971	407.1411333	0.223960564	6.158115515	126.7460651
59.71076191	1435.746179	0.049389213	7.04590689	458.5229273
4.220275081	303.7171847	0.289270016	5.916299709	628.2829143
12.5307377	519.498229	0.106743852	6.135207428	124.5419126
25.53154706	366.815331	0.066323495	6.112635537	65.83623693
45.56445518	424.7269985	0.047821844	6.688672912	146.7616893
19.54022874	699.4505391	0.098662013	6.864396401	620.9122376
44.3987365	458.3961165	0.051478724	6.958404942	310.9055016
43.49718858	257.6029412	0.050956964	6.049864282	80.90441176
8.423351534	752.4648067	0.180241555	6.418829355	1690.511589
52.31120466	518.0476788	0.047907067	6.627728247	195.7779172
44.68751238	853.8646288	0.065959062	6.771048356	293.1709295
66.52945905	782.2909011	0.036398857	7.014483992	1268.120297
51.42159073	1371.703286	0.04578509	7.29357387	1648.228169
32.51057346	435.7570093	0.068137926	6.185145252	76.22741433
63.30748844	1465.835698	0.040158769	6.912054744	588.6118993
77.02492762	483.4369748	0.038627216	5.722138141	49.16806723
28.94060392	680.0557171	0.088902222	6.898773707	555.3100775
11.99469666	657.5592138	0.156800558	6.667361449	740.5236955
31.919796	612.2658808	0.077301007	6.78905679	231.5579568
39.95949393	261.9393939	0.052800735	5.917426146	53.61616162
11.47117519	969.1727004	0.197576957	6.480697281	462.7523595
17.92789674	307.0186916	0.104347408	6.391689266	99.376502
42.37225849	481.7688723	0.059485169	6.803699455	196.8863001
28.97786217	814.0578358	0.127421235	6.738601762	363.1467662
55.19756535	849.3360927	0.042119863	6.6935478	152.307947
83.23371643	1232.041161	0.032627244	7.017289777	420.9136302
61.25640832	369.1826087	0.045444234	5.465765786	47.4
55.9503399	474.5330261	0.043985267	6.633375134	171.749616
13.00115381	224.033208	0.098869511	6.503685276	205.952381
82.27605083	753.2882883	0.039115692	6.500942478	216.3393393
27.57059657	274.4607046	0.060406431	6.182729095	80.20867209
25.05856682	576.8548859	0.101417957	6.890897359	386.4956115
6.210170032	191.7029787	0.144909009	6.262863408	109.3114894
27.29307479	384.3041551	0.061070434	6.768566091	327.9229917
101.9327221	934.3863636	0.03441374	5.978286397	73.59090909
26.73154055	748.08	0.081151011	6.311998758	137.1958621
10.8811836	569.2978357	0.149502225	6.709409464	296.03394
80.91911272	1132.610213	0.044361793	7.00651639	293.5923404
38.30746094	581.48875	0.0632125	6.699290808	149.0925
70.6390858	668.5833333	0.037570888	6.045807545	111.442029
6.8659421	148.4426573	0.166766101	6.253454256	122.1300699
27.18115028	585.104325	0.0849777	6.748437302	506.7194623
13.53869856	268.0960042	0.115091337	6.714574043	403.0871821
17.22325745	561.2162554	0.082640587	6.440007455	354.1698113
77.67679134	1469.39527	0.037894049	6.881219314	335.9746622
57.55564305	758.268942	0.053459912	6.937573541	304.5167235
119.2463998	614.4367816	0.038182058	5.332263145	45.57471264
25.92422138	276.0985915	0.072009522	5.042293732	19.98591549
25.11458249	1251.003957	0.133714698	6.764115569	916.6697209

wavelet.LHL_gldm_GrayLevelNonUniformity	wavelet.LHL_gldm_SmallDependenceEmphasis	wavelet.LHL_gldm_DependenceNonUniformityNormalized	wavelet.LHL_gldm_DependenceVariance
26.05105105	0.542246362	0.324833843	1.309056805
3.938053097	0.596238938	0.362831858	0.819014801
19.89440994	0.496184995	0.286370125	1.479786874
3.695652174	0.667421498	0.444470699	0.530718336
36.71045576	0.417390632	0.216910206	6.264905232
3.932038835	0.743932039	0.515505703	0.538410783
3937.003434	0.136454428	0.054546151	39.235746
237.5525168	0.371376037	0.207953416	3.239866003
847.8734926	0.17507575	0.070114408	23.87290204
13.98351648	0.573671398	0.348780341	0.950640019
696.8601757	0.199246135	0.103688739	10.01357039
221.791409	0.307015493	0.153006742	5.063900665
11.97280967	0.527473539	0.30606694	1.320232565
9.210526316	0.56203655	0.330637119	1.39601108
70.13913043	0.374861477	0.164128072	12.83090265
2275.546265	0.136550321	0.063569278	25.82593127
33.68807339	0.377476542	0.190756249	3.377788065
5.504504505	0.508385886	0.28690853	1.317750183
43.81128748	0.36776417	0.198280501	2.538587634
1716.372013	0.222919971	0.085223489	23.09104197
144.8856749	0.252013724	0.112206779	21.04567463
10.20253165	0.458650902	0.250841211	2.292100625
18.49466192	0.376677191	0.211686782	3.853522625
1004.945046	0.155348062	0.064182741	28.06783914
104.6997245	0.281000195	0.105464867	21.27697713
155.7446914	0.301392653	0.137432952	11.08373333
73.14696486	0.360489228	0.17003678	9.412312965
153.3763502	0.274334702	0.128346672	9.782929406
2.357142857	0.636904762	0.426020408	0.459183673
149.5230235	0.236297001	0.099795365	14.55977162
484.1421248	0.218369933	0.086026818	25.29306024
10.69411765	0.56224183	0.33893118	0.994632834
115.4675528	0.341454579	0.156015774	10.72193813
171.0336615	0.349209504	0.180408102	4.307145448
406.2729364	0.25706583	0.10244725	18.10950021
84.74822695	0.37977281	0.162234043	12.79016398
9.769230769	0.731980057	0.505627876	0.456147272
1545.36548	0.198283648	0.082210807	22.83174743
12.60406091	0.437652284	0.246308846	1.915792728
871.7034836	0.282405233	0.140046014	9.123817433
42.09033281	0.352222719	0.183897971	7.939853476
811.1052922	0.232128037	0.109495828	12.64214617
151.4623445	0.260621521	0.128026456	8.558654972
30.54455446	0.482224948	0.28016661	1.35612555
155.6783088	0.223635138	0.096846953	16.49987835
119.17289	0.322893879	0.173748994	3.916087938
1580.371215	0.166578171	0.06665904	28.2583301
18.59817352	0.356259745	0.172077313	6.296282396
7.403508772	0.527090494	0.289935365	2.190827947
6.790909091	0.696338384	0.461983471	0.558760331
426.8688353	0.148657954	0.06649846	23.92674814
106.6313108	0.421361184	0.212377456	5.540825365
2922.205702	0.102206957	0.062193914	26.18895094
90.4120425	0.306152641	0.147038858	6.870156354
19.03484321	0.4527781	0.229394554	3.438502349
31.70588235	0.410739174	0.221360014	3.594211603
521.6260639	0.259487581	0.117441316	14.09526012
79.53462783	0.380349637	0.201233334	4.172603974
13.86029412	0.522995495	0.29744269	1.699340398
4238.019691	0.137058482	0.071896891	17.60270099
38.18193225	0.452178223	0.24564356	2.473919608
105.7323768	0.398894058	0.182888914	9.125003065
166.4155731	0.487657753	0.277366644	1.787558297
390.0889671	0.372956175	0.19345401	4.405402312
21.87227414	0.457753326	0.23746858	3.314350598
87.74691076	0.492319894	0.269387597	2.94354246
4.596638655	0.647350607	0.413177036	0.621707507
366.9883721	0.322622949	0.13452279	17.40476811
1571.611992	0.16140395	0.07388244	21.43541906
118.0386379	0.348211068	0.151642408	12.91159393
10.45454545	0.480384314	0.270788695	1.905621875
1402.598817	0.15772836	0.06518557	27.85366658
78.15620828	0.298431288	0.132678908	17.79940143
63.82758621	0.384016981	0.183491426	6.753134855
409.7866915	0.289804407	0.112918771	22.27280227
25.44039735	0.459096857	0.252165475	2.239024604
48.35357625	0.480545893	0.284017294	1.513927818
5.226086957	0.62397343	0.412173913	0.516446125
28.6344086	0.488244989	0.263824295	3.385192579
157.7957393	0.250658295	0.129042845	7.480420977
26.05105105	0.542246362	0.324833843	1.309056805
22.2899729	0.400724242	0.217367675	2.617357393
346.6465769	0.257559184	0.113076539	16.07639691
170.2680851	0.190872551	0.093031055	14.73441376
110.232133	0.344020232	0.181674788	3.596670989
6.056818182	0.664655934	0.418130165	0.948217975
58.83448276	0.363206554	0.189235672	4.576483234
607.8760453	0.166510137	0.072807167	25.83585788
52.12510638	0.488484472	0.249865822	6.382141421
50.57	0.388855033	0.186365625	10.67699375
10.36956522	0.642889493	0.403775467	0.7268431
238.4755245	0.198915726	0.085405643	19.48585065
290.7936879	0.332155384	0.148076991	11.85582956
443.5620135	0.242109913	0.104589305	15.1589851
170.8180939	0.357198924	0.171344853	5.928450606
44.86655405	0.51801672	0.283762384	2.060354273
78.31877133	0.431971387	0.207861245	10.09184498
3.32183908	0.74201788	0.523847272	0.353018893
5.112676056	0.408967136	0.281491768	1.644911724
1284.19596	0.247656173	0.09544666	21.88580373

wavelet.LHL_gldm_LargeDependenceEmphasis	wavelet.LHL_gldm_LargeDependenceLowGrayLevelEmphasis	wavelet.LHL_gldm_SmallDependenceHighGrayLevelEmphasis	wavelet.LHL_gldm_LargeDependenceHighGrayLevelEmphasis
5.084084084	0.011602989	443.8569869	3525.357357
3.920353982	0.027636026	444.2707104	1788.823009
5.948240166	0.016394886	360.5628098	3182.614907
2.913043478	0.040020626	288.5129831	1120.054348
14.57104558	0.047880274	185.4084808	5669.10992
2.631067961	0.015292634	637.3446602	2775.378641
135.6694468	0.156839835	126.1902368	118376.9359
10.810543	0.013214713	361.3243591	10053.27408
77.23646252	0.170194208	80.26807203	35449.92079
4.258241758	0.013592094	368.1658425	2489.505495
33.66691069	0.016591801	415.4073733	69370.93906
17.03644647	0.021495865	292.0032998	14257.10674
5.332326284	0.008710011	580.0342783	6024.779456
5.147368421	0.074295693	202.2221447	1518.484211
28.50217391	0.036900983	310.2340135	24211.87065
103.3431453	0.078300208	184.8065558	137133.5538
11.7293578	0.041018607	148.2851292	3870.502294
5.648648649	0.021242365	318.3267392	3274.720721
10.15696649	0.053288844	112.0544514	3073.873016
63.22268802	0.06053762	254.3610654	66574.06297
48.7892562	0.195108238	68.38368081	13036.60537
7.835443038	0.036311841	164.3880162	2627.246835
11.6975089	0.040596167	135.1641634	4398.672598
91.99458784	0.203214264	77.68868394	42142.43568
54.11019284	0.137147597	122.9110589	22598.03306
27.76641975	0.061824897	169.4417318	13410.28444
21.48775293	0.06403779	115.7875386	8083.256656
27.11483798	0.123662577	74.11445921	7125.575327
2.928571429	0.047020915	144.7857143	1106.75
41.07645526	0.070708302	161.8588163	24378.84014
64.36713187	0.191192623	75.41051128	22166.13244
4.435294118	0.025833627	239.4181797	1597.862745
25.15480844	0.041578918	215.9770493	16002.62236
13.75071451	0.019225865	347.1847106	12468.1826
46.37935001	0.0804089	149.7085615	27524.47023
28.59219858	0.015895188	756.0780843	56067.39362
2.517948718	0.010412918	763.793287	2163.048718
61.30175498	0.095170766	137.5926382	40065.00852
7.487309645	0.0342683	169.7904766	2269.685279
24.39788674	0.004392978	1615.0444	138700.4966
18.98415214	0.078099737	108.7956182	6179.759113
35.32195661	0.066170362	141.070493	19207.07821
25.61820563	0.047887672	162.3196132	15939.57826
5.851485149	0.026025298	283.4502238	3114.181047
46.87132353	0.238991952	48.11952985	9579.953125
13.72531969	0.040216531	147.4180584	5566.614322
96.58329746	0.056246051	299.6522061	166702.4226
17.25570776	0.113575038	109.4385836	4099.611872
7	0.089998579	111.1823351	1126.587719
2.863636364	0.008421435	985.0328914	4013.218182
80.29485834	0.19420567	57.27404143	33481.31847
13.76887448	0.010681849	642.8985491	19512.24734
118.1453178	0.390229134	33.78612787	35996.53801
20.23966942	0.040786551	156.1994966	10530.51358
10.35888502	0.036629517	178.7363372	3672.425087
10.66515837	0.033417512	190.2949396	4501.535445
35.70096463	0.052478133	193.6801809	24834.93683
12.33851133	0.038889081	200.0949894	4987.897735
6.029411765	0.056091031	153.9977055	1255.485294
63.48628418	0.084927217	104.643768	47759.03003
8.247176913	0.022661264	269.919556	3802.328733
21.05614473	0.0261592	356.4768037	18140.72926
6.516185477	0.012604017	406.7194872	4787.130796
13.03051643	0.01058366	529.6227029	17369.86432
9.728971963	0.029078161	209.5560206	4036.049844
8.351029748	0.006855231	736.1266121	11893.69382
3.25210084	0.022344384	349.028303	1320.848739
38.74612403	0.057455665	234.3541938	26858.31638
66.20103761	0.10266475	112.0234082	43115.74199
29.11656843	0.048350597	215.6935307	18539.33726
6.888888889	0.061786942	143.4963219	1578.242424
87.93308917	0.088947588	147.010212	87385.93788
40.27636849	0.125115295	97.73091627	13654.6235
16.97763281	0.043347592	201.9189511	8380.78658
51.60634328	0.065239286	250.5022833	41553.39739
7.735099338	0.012482774	402.6786239	6308.19702
6.043184885	0.007580169	609.9513701	7060.278677
3.104347826	0.031206245	257.1761473	951.4608696
9.061443932	0.030314484	257.2596375	3826.858679
23.8433584	0.121939244	65.32709624	5037.088346
5.084084084	0.011602989	443.8569869	3525.357357
9.428184282	0.046260586	113.9967178	2590.617886
40.33879462	0.070399004	161.1979014	23899.63897
44.44851064	0.230421278	38.34416329	8845.914043
12.6099723	0.042434613	145.9272226	4521.476454
3.738636364	0.012255611	650.8947112	3308.920455
13.6262069	0.020376785	281.6982102	10117.76552
73.92424988	0.126935928	101.9330539	43593.69429
13.84085106	0.016534595	580.3097411	14557.7166
22.77	0.042333515	225.9392371	13545.15375
3.456521739	0.012640353	438.9261524	2179.271739
55.83916084	0.386020066	32.35030623	8531.407692
28.01519579	0.049621458	204.4237779	16546.50584
40.34094447	0.159510051	71.58079185	10641.24494
16.64005806	0.032130822	212.1856296	9097.424286
6.793918919	0.006230596	799.8062085	9558.760135
20.67098976	0.029981663	340.3161612	16330.99113
2.287356322	0.02477202	483.0810983	1265.172414
6.915492958	0.046377353	126.497723	1871
57.05351937	0.045880697	323.1921235	71615.76885

wavelet.LHL_gldm_SmallDependenceLowGrayLevelEmphasis	wavelet.LHL_gldm_LowGrayLevelEmphasis	wavelet.LHL_gldzm_DistanceZoneVariabilityNormalized	wavelet.LHL_gldzm_LowIntensityEmphasis
0.003019374	0.003942422	0.880225062	0.005077619
0.010562053	0.012824943	0.950032873	0.016166656
0.003276124	0.004576701	0.806238185	0.006114989
0.019065582	0.022899157	0.916824197	0.026998483
0.003832993	0.006529092	0.818950617	0.008837
0.012411624	0.012894075	1	0.015403284
0.00039953	0.001512364	0.658553686	0.002963742
0.001012766	0.001798326	0.589362901	0.002553782
0.000990698	0.002968475	0.836240442	0.005646305
0.005248332	0.006260145	0.854201389	0.008301732
0.000287123	0.000679063	0.747013903	0.001429632
0.000779417	0.001667906	0.641595571	0.002477099
0.003758605	0.004294349	0.8872	0.006383009
0.017147482	0.026742381	0.983472222	0.03173869
0.0019835	0.002982768	0.841398892	0.005066443
0.000278028	0.000961659	0.65616128	0.001997357
0.003746785	0.005924505	0.862465306	0.009620217
0.010608486	0.011724124	0.970156107	0.018165068
0.0040063	0.009242211	0.828707085	0.013001326
0.000355738	0.001133982	0.627625386	0.00157463
0.003276928	0.007562882	0.857359565	0.012937197
0.008790456	0.011148287	0.975613474	0.017741077
0.006758349	0.008949144	0.984128	0.015988403
0.000845796	0.002875107	0.79484375	0.005373127
0.004627571	0.006789623	0.898596939	0.015441064
0.001412478	0.003235729	0.579277476	0.004652681
0.005111241	0.007283171	0.963554236	0.01319867
0.004635608	0.009781439	0.820149726	0.017821577
0.040581839	0.041661443	1	0.057184788
0.001335431	0.002663918	0.912343781	0.00549292
0.001485729	0.004172646	0.808286306	0.006736165
0.00757716	0.009914674	0.897445202	0.012494256
0.001781352	0.002954994	0.876956938	0.004904843
0.001113794	0.002412051	0.569173293	0.003443194
0.001093317	0.002576603	0.888960833	0.004211026
0.001274933	0.001630082	0.833443201	0.003129479
0.00446147	0.005431949	0.993650858	0.005900597
0.000612919	0.001952396	0.630315927	0.003080896
0.007167437	0.00931947	1	0.014821244
0.000134865	0.000262184	0.472290003	0.000453704
0.004792949	0.008921458	0.880636815	0.013710809
0.000668165	0.002128452	0.766896001	0.002847996
0.001033086	0.003414875	0.907504937	0.005086525
0.002214293	0.005721637	0.794727153	0.005659753
0.003545307	0.008194397	0.833478106	0.015346226
0.001798004	0.004038807	0.705738739	0.005531578
0.00031453	0.000801595	0.833287139	0.001798871
0.004727078	0.014476821	0.978024691	0.017716469
0.016138848	0.022384081	1	0.028940838
0.006581129	0.006837294	0.757508651	0.008598654
0.001031602	0.003170427	0.949246447	0.006691468
0.000846367	0.001320038	0.72514474	0.00189157
0.000541985	0.003623141	0.789022861	0.0053368
0.001948362	0.003330119	0.964800041	0.006058897
0.005684804	0.007541326	0.920138889	0.011773468
0.003545415	0.005663416	0.767164717	0.008091497
0.00068629	0.001846814	0.68119643	0.002609676
0.003130782	0.005198128	0.623255538	0.007801918
0.014737367	0.018770529	0.906111873	0.025674258
0.000276844	0.001455167	0.616927069	0.0020598
0.003270245	0.004761269	0.734697904	0.006737898
0.001596746	0.002401463	0.815900526	0.003820562
0.002270449	0.003230144	0.809418351	0.004337722
0.000513574	0.001034953	0.547324569	0.001326138
0.004828527	0.006331534	0.827664399	0.009603699
0.000967763	0.001364213	0.671322148	0.001841811
0.012833758	0.014141272	0.910311419	0.018319338
0.000937171	0.002076879	0.815727615	0.002842332
0.000531224	0.001874909	0.598634272	0.003242841
0.00204054	0.003256795	0.755570238	0.005549093
0.010997267	0.016973234	0.8828125	0.021858177
0.000354485	0.001240682	0.809198797	0.002209349
0.003168521	0.005979663	0.938286694	0.010252412
0.00283965	0.004764398	0.857988166	0.007173342
0.000993058	0.001944983	0.838629275	0.003268538
0.002632839	0.003395459	0.808496452	0.005217003
0.001321237	0.001929684	0.548823696	0.002526125
0.016703739	0.018857382	0.976193932	0.023909514
0.004099885	0.005860973	0.824459877	0.00784299
0.002677396	0.006791353	0.795924165	0.010504341
0.003019374	0.003942422	0.880225062	0.005077619
0.006619671	0.009823442	0.837372449	0.015542446
0.000996325	0.002481093	0.676731275	0.003766215
0.002442743	0.007023619	0.916477565	0.012800358
0.002060452	0.004482671	0.699276876	0.005953496
0.007520276	0.008087132	0.883660838	0.010422867
0.002054569	0.002980453	0.872799916	0.005256884
0.000602225	0.002160474	0.738133897	0.003574558
0.002888903	0.00351093	0.559634055	0.005470253
0.00298342	0.004273017	0.801355102	0.007148371
0.005985848	0.006899285	0.931459198	0.008682546
0.003398513	0.011267045	0.919686884	0.019323643
0.001572383	0.002872315	0.806246804	0.004535844
0.002296749	0.005676051	0.651289265	0.009397579
0.001512598	0.002759991	0.903664855	0.00403872
0.00133092	0.001727354	0.810800173	0.002403078
0.002880147	0.003881656	0.864600429	0.006219981
0.015130235	0.016942223	0.945248959	0.019253633
0.017493598	0.020470461	0.944489796	0.036418518
0.000358555	0.000996107	0.689584113	0.001415227

wavelet.LHL_gldzm_LargeDistanceEmphasis	wavelet.LHL_gldzm_HighIntensitySmallDistanceEmphasis	wavelet.LHL_gldzm_LowIntensityLargeDistanceEmphasis	wavelet.LHL_gldzm_HighIntensityEmphasis
1.191943128	764.389218	0.005470843	799.5094787
1.076923077	694.474359	0.016373792	703.4358974
1.326086957	651.0036232	0.006814036	706.9927536
1.130434783	423.8586957	0.039333046	426.1304348
1.327777778	409.6804012	0.011286635	428.825
1	863.3095238	0.015403284	863.3095238
2.208818636	809.6154322	0.007173432	916.218802
2.227461859	773.8960131	0.00440289	968.2274619
1.468193384	424.8223335	0.006919361	452.9020356
1.2375	621.6583333	0.026145527	636.5083333
1.675749319	1868.500032	0.001786032	2096.986376
2.038461538	803.7337396	0.003872581	947.800789
1.18	1059.34	0.006682662	1092.07
1.025	351.3666667	0.03180119	353.8666667
1.260526316	771.9381579	0.005680369	812.9394737
2.1408	1180.21498	0.003315405	1340.8632
1.222857143	375.7471429	0.010514671	394.96
1.045454545	617.5340909	0.018250994	623.5454545
1.283783784	287.6722973	0.015689508	306.972973
2.360416667	963.3516628	0.003147461	1136.399653
1.231884058	252.1394928	0.014719012	266.8695652
1.037037037	355.8549383	0.018496935	356.308642
1.024	359.29	0.016071448	361.024
1.4925	462.7754806	0.007037183	498.225
1.160714286	413.1618304	0.01620267	425.8214286
2.384384384	439.5802678	0.014246784	555.8243243
1.055702918	314.1187003	0.013358331	319.5994695
1.353965184	249.6309908	0.021495812	264.8181818
1	240.55	0.057184788	240.55
1.154929577	659.2897105	0.005705775	688.528169
1.441636582	313.8197841	0.011831834	340.7376655
1.162650602	398.9533133	0.013360478	418.9277108
1.207392197	607.9404517	0.0056529	630.3080082
2.353229763	777.0097631	0.01036809	990.5143091
1.211716341	558.8739294	0.004932973	578.7677287
1.275053305	1913.23081	0.003428153	1995.345416
1.00955414	1017.249204	0.005913703	1018.990446
2.220606061	582.9330139	0.006988956	694.220202
1	382.969697	0.014821244	382.969697
4.071926999	4091.888002	0.001022221	5714.609501
1.19123506	290.1673307	0.01494349	301.3665339
1.66065389	549.8345176	0.004099361	611.1775648
1.168202765	588.1902202	0.005466319	606.8018433
1.371794872	533.9084046	0.006867396	580.4153846
1.328301887	199.1263103	0.017684029	211.6377358
1.789550073	392.8718396	0.00884539	452.8476052
1.748997594	1671.636775	0.002227541	1808.128308
1.033333333	301.3555556	0.017947951	302.5555556
1	206.6911765	0.028940838	206.6911765
1.423529412	1257.047059	0.031742778	1420.335294
1.126213592	371.7095469	0.007005432	380.3656958
1.535679374	1365.774166	0.002450386	1514.793744
1.998151571	305.0153091	0.009373424	330.935305
1.053763441	509.9426523	0.006154596	517.7383513
1.125	375.7552083	0.012335581	386.2291667
1.446202532	428.1552391	0.011097317	456.4335443
1.939824206	628.7898062	0.004039994	745.1724138
2.075528701	431.5093991	0.011274111	518.2915408
1.148148148	285.8132716	0.031907767	290.9197531
3.348508634	618.2785064	0.005240942	764.3117739
1.547677262	515.4826134	0.008517252	583.1320293
1.340974212	832.4886581	0.004457787	884.3567335
1.330847724	772.7619811	0.005190416	825.7323391
2.944646352	1122.618931	0.003082025	1418.901314
1.285714286	417.1205357	0.010507824	455.6071429
1.814415907	1267.646023	0.002596121	1497.280862
1.141176471	504.8794118	0.018575777	524.9529412
1.392491468	669.8213026	0.003592811	720.6450512
3.215167549	556.2090902	0.006635087	701.1087596
1.472881356	567.085452	0.007067199	615.1101695
1.1875	286.0066964	0.032836202	291.2232143
1.820683903	836.3996756	0.003047017	921.3269391
1.09561753	309.440239	0.010824819	315.8286853
1.230769231	499.9510989	0.008776854	518.5164835
1.265564202	793.5756323	0.004364258	858.7636187
1.321766562	772.5930599	0.005561974	868.1388013
2.291666667	903.7254464	0.003676704	1265.746429
1.036144578	395.8222892	0.024009638	399.0843373
1.291666667	492.5722222	0.009213703	521.2138889
1.383027523	240.1443202	0.01324339	258.9426606
1.191943128	764.389218	0.005470843	799.5094787
1.267857143	266.3630952	0.017531111	281.6845238
1.784473953	531.4560989	0.005761933	616.4586313
1.131004367	196.3591703	0.013779995	202.1528384
1.670014347	358.721465	0.008057467	421.0703013
1.186046512	934.751938	0.010779498	969.4496124
1.204778157	733.8020478	0.00561224	770.334471
1.765957447	549.0630323	0.005129039	615.3319149
2.244929797	885.4428844	0.008634045	1182.812793
1.348571429	540.9460317	0.007981739	581.3885714
1.106598985	669.8147208	0.00938605	679.7664975
1.157051282	155.4112358	0.03031142	158.7852564
1.390749601	569.5055932	0.005524312	609.34689
1.959163347	250.5784515	0.019550555	296.7679283
1.176108374	568.1875684	0.004376715	590.9544335
1.372058824	1436.592157	0.002746828	1534.425
1.219101124	751.7721208	0.006807392	779.9087079
1.084507042	620.4964789	0.019368187	636.6056338
1.085714286	297.7714286	0.036855835	301.9714286
2.015714833	1112.229624	0.002268463	1297.390954

wavelet.LHL_gldzm_DistanceZoneVariability	wavelet.LHL_gldzm_ZonePercentage	wavelet.LHL_gldzm_IntensityVariabilityNormalized	wavelet.LHL_gldzm_LowIntensitySmallDistanceEmphasis	wavelet.LHL_gldzm_IntensityVariability
371.4549763	0.633633634	0.032771052	0.004979313	13.82938389
74.1025641	0.690265487	0.033201841	0.016114872	2.58974359
222.5217391	0.571428571	0.032976265	0.005940227	9.101449275
63.26086957	0.75	0.034656585	0.023914842	2.391304348
294.8222222	0.482573727	0.037145062	0.008259625	13.37222222
84	0.815533981	0.037414966	0.015403284	3.142857143
1583.163062	0.147430394	0.037026476	0.002102478	89.01164725
1274.791956	0.428656361	0.033996934	0.002229591	73.53536755
657.2849873	0.185859541	0.056413444	0.005438374	44.34096692
205.0083333	0.659340659	0.033854167	0.003840783	8.125
822.462307	0.201500732	0.057220708	0.001370013	63
650.5779093	0.329970713	0.039004237	0.002232536	39.55029586
177.44	0.604229607	0.03175	0.006308096	6.35
118.0166667	0.631578947	0.040833333	0.031723065	4.9
319.7315789	0.413043478	0.033185596	0.004912962	12.61052632
820.2016	0.146593175	0.04494848	0.00175922	56.1856
150.9314286	0.401376147	0.050187755	0.009396603	8.782857143
64.03030303	0.594594595	0.041322314	0.018143587	2.727272727
183.972973	0.391534392	0.049793036	0.01232928	11.05405405
1807.561111	0.241468936	0.036648341	0.001335268	105.5472222
354.9468599	0.285123967	0.046675535	0.012491743	19.3236715
79.02469136	0.512658228	0.050754458	0.017552113	4.111111111
123.016	0.444839858	0.045632	0.015967641	5.704
635.875	0.166527893	0.0503125	0.005033267	40.25
201.2857143	0.308539945	0.040457589	0.015250663	9.0625
385.7987988	0.328888889	0.041280019	0.002613388	27.49249249
363.2599469	0.401490948	0.048582626	0.013158755	18.31564987
424.0174081	0.293916998	0.046575055	0.017102927	24.07930368
20	0.714285714	0.075	0.057184788	1.5
259.1056338	0.246741964	0.050733981	0.00544779	14.4084507
671.6859206	0.236684705	0.04964804	0.005551441	41.25752106
148.9759036	0.650980392	0.03592684	0.0122777	5.963855422
427.0780287	0.380766224	0.0407178	0.004720958	19.82956879
696.098937	0.388377263	0.033597664	0.001848771	41.08994276
864.95889	0.279838942	0.04420378	0.004042711	43.01027749
390.8848614	0.415780142	0.026009156	0.00305481	12.19829424
312.0063694	0.805128205	0.024159195	0.00589732	7.585987261
1560.031919	0.210853638	0.0402834	0.002315564	99.70141414
99	0.502538071	0.046423834	0.014821244	4.595959596
1759.75255	0.307578009	0.038437159	0.000401465	143.2168545
221.0398406	0.3977813	0.042396152	0.013402639	10.64143426
1360.473506	0.245129197	0.050379463	0.002649927	89.37316798
393.8571429	0.28421742	0.052740555	0.004997886	22.88940092
309.9435897	0.551626591	0.036607495	0.005365927	14.27692308
220.8716981	0.243566176	0.064179423	0.01484733	17.00754717
486.2539913	0.352429668	0.042224802	0.004899364	29.09288824
1039.109062	0.178935285	0.046005522	0.001758866	57.36888532
88.02222222	0.410958904	0.052098765	0.017658599	4.688888889
68	0.596491228	0.053633218	0.028940838	3.647058824
128.7764706	0.772727273	0.027404844	0.002812623	4.658823529
293.3171521	0.162119622	0.079251369	0.006640328	24.48867314
741.8230694	0.473830477	0.030511338	0.001760587	31.21309873
853.7227357	0.107107503	0.071024426	0.004861746	76.84842884
269.1792115	0.329397875	0.064451896	0.006034972	17.98207885
132.5	0.50174216	0.049382716	0.01163294	7.111111111
242.4240506	0.476621418	0.036172088	0.007372049	11.43037975
1007.48952	0.279742765	0.044824523	0.002367043	66.29546991
412.5951662	0.428478964	0.035925192	0.007162758	23.78247734
146.7901235	0.595588235	0.04199055	0.02411588	6.802469136
1964.912716	0.135456981	0.058781326	0.001791935	187.2185243
300.4914425	0.513174404	0.033901041	0.006348561	13.86552567
569.4985673	0.435433562	0.032397107	0.003669666	22.61318052
2062.397959	0.557305337	0.030518904	0.004128112	77.76216641
1957.779983	0.419835681	0.032142343	0.001103854	114.9731619
139.047619	0.523364486	0.050524376	0.009377668	8.488095238
810.2858326	0.552402746	0.030849457	0.001687556	37.23529412
77.37647059	0.714285714	0.032802768	0.018255228	2.788235294
1195.040956	0.354893411	0.038084078	0.002682714	55.79317406
1018.276896	0.169709668	0.048442991	0.002932264	82.40152851
445.7864407	0.38637852	0.037150244	0.005193758	21.91864407
98.875	0.565656566	0.044802296	0.019113671	5.017857143
970.2293578	0.168897028	0.050938334	0.002123175	61.07506255
235.5099602	0.335113485	0.049411914	0.010109311	12.40239044
390.3846154	0.424044734	0.038763434	0.006772464	17.63736264
862.1108949	0.319651741	0.038531999	0.002994608	39.61089494
256.2933754	0.524834437	0.031555693	0.00513076	10.00315457
461.0119048	0.566801619	0.029484127	0.00229914	24.76666667
81.02409639	0.72173913	0.039628393	0.023884483	3.289156627
296.8055556	0.552995392	0.033055556	0.007500312	11.9
347.0229358	0.273182957	0.057634038	0.009848959	25.12844037
371.4549763	0.633633634	0.032771052	0.004979313	13.82938389
140.6785714	0.455284553	0.045209751	0.01504528	7.595238095
662.5199183	0.28642481	0.04104478	0.00336864	40.18283963
209.8733624	0.194893617	0.073015389	0.012555449	16.72052402
487.3959828	0.386149584	0.042261465	0.005489416	29.45624103
113.9922481	0.732954545	0.030587104	0.010333709	3.945736434
255.7303754	0.404137931	0.049866626	0.005168045	14.6109215
520.3843972	0.17338908	0.047677682	0.003310033	33.61276596
358.725429	0.545531915	0.027791502	0.004897579	17.81435257
280.4742857	0.4375	0.038432653	0.006944725	13.45142857
183.4974619	0.713768116	0.033213945	0.00850667	6.543147208
286.9423077	0.218181818	0.075423241	0.016613038	23.53205128
1011.033493	0.366452367	0.039929641	0.004317027	50.07177033
653.8944223	0.260508563	0.050640069	0.00715636	50.84262948
733.7758621	0.392839865	0.051193307	0.003966407	41.56896552
551.3441176	0.574324324	0.02825692	0.002328048	19.21470588
615.5955056	0.486006826	0.030859424	0.006073128	21.97191011
67.11267606	0.816091954	0.035905574	0.019224995	2.549295775
33.05714286	0.492957746	0.056326531	0.036309188	1.971428571
1799.124952	0.271657643	0.037855076	0.00129126	98.76389421

wavelet.LHL_gldzm_HighIntensityLargeDistanceEmphasis	wavelet.LHL_gldzm_SmallDistanceEmphasis	wavelet.LHL_glcm_SumVariance	wavelet.LHL_glcm_Homogeneity1	wavelet.LHL_glcm_Homogeneity2
939.9905213	0.952014218	2374.66866	0.214954489	0.128569506
739.2820513	0.980769231	1788.331778	0.209833996	0.124936632
930.9492754	0.918478261	1925.170357	0.226589404	0.13886119
435.2173913	0.967391304	1074.710198	0.205474992	0.120592715
510.8972222	0.924228395	1061.268184	0.294857578	0.208897208
863.3095238	1	3082.025258	0.172636151	0.096293297
1805.475874	0.838858782	3007.033543	0.597410246	0.564454682
2137.781322	0.796289875	3065.937164	0.279971593	0.192801538
647.6386768	0.929954764	1478.827315	0.54270243	0.498457974
695.9083333	0.940625	1817.236248	0.204555081	0.119908795
3455.654859	0.887356191	7525.671177	0.446869981	0.378619295
1863.705128	0.829118179	2874.489082	0.34162957	0.259686706
1222.99	0.955	3560.760077	0.224755088	0.136517862
363.8666667	0.99375	901.6588711	0.249516876	0.159048739
976.9447368	0.934868421	2537.610323	0.344063282	0.272588247
2646.0568	0.837333778	4704.365315	0.587577894	0.557975373
471.8114286	0.944285714	1071.246975	0.329227355	0.24015072
647.5909091	0.988636364	1749.452671	0.244577337	0.158269404
384.1756757	0.929054054	909.5253426	0.314625818	0.223658836
2510.388889	0.820115946	3655.850111	0.466747252	0.411828685
325.7898551	0.942028986	707.7029608	0.438945858	0.372074028
358.1234568	0.990740741	943.4176137	0.286131968	0.197761136
367.96	0.994	1147.144956	0.32185483	0.234793636
706.12375	0.911864236	1487.265407	0.561811842	0.521866383
476.4598214	0.959821429	1233.995463	0.455336199	0.39983657
1489.765766	0.789873207	1506.859087	0.364938417	0.288993651
341.5225464	0.986074271	1003.878355	0.353051423	0.268694069
332.3926499	0.924403611	702.5160858	0.392020661	0.314268032
240.55	1	956.5201088	0.219521761	0.126242633
813.056338	0.965179969	2064.30812	0.456979484	0.394252195
487.1034898	0.918559634	1014.648607	0.476095672	0.416600754
498.8253012	0.959337349	1082.786836	0.217318224	0.129064444
726.4312115	0.950433493	1956.038793	0.373677591	0.300007111
2375.520033	0.782675343	3064.156402	0.297849575	0.213923795
679.5282631	0.95506452	1866.166137	0.44636364	0.384189328
2323.803838	0.931236674	6526.586398	0.348046157	0.279694955
1025.955414	0.997611465	2808.198531	0.160701317	0.083307163
1471.800404	0.822069024	2143.360867	0.4640801	0.404997235
382.969697	1	999.3080902	0.299909477	0.20910422
22989.14144	0.714423004	21300.62357	0.348618195	0.267382768
346.1633466	0.952191235	841.4385582	0.342068504	0.259750132
1001.492108	0.897627458	1871.200761	0.423446904	0.351291436
697.8894009	0.963069636	1933.710859	0.400303587	0.323954977
786.7282051	0.912749288	1651.853099	0.24309535	0.152958192
270.6226415	0.930503145	568.3775028	0.480765083	0.421765161
802.8548621	0.865916384	1220.109169	0.329574741	0.243785411
3138.678428	0.9272261	6389.08264	0.562801472	0.525141833
307.3555556	0.991666667	800.1630008	0.356952231	0.276832208
206.6911765	1	455.0701772	0.279189596	0.190723813
2073.488235	0.894117647	4585.578484	0.157768023	0.080434706
432.3365696	0.979234088	1375.00246	0.574420115	0.534337798
2167.967742	0.876941458	4755.695294	0.286602649	0.202859506
620.4787431	0.905459797	1005.342594	0.618915979	0.589264299
548.921147	0.98655914	1711.773394	0.409365088	0.332962532
428.125	0.96875	1062.53341	0.308611966	0.219621106
585.3132911	0.89899789	1188.799531	0.284277871	0.196176316
1499.46856	0.852070468	2303.167192	0.403367928	0.331354045
1040.555891	0.817931353	1326.783568	0.286667734	0.199997783
311.345679	0.962962963	614.0870692	0.264056282	0.172320879
2588.928414	0.81180658	2615.045138	0.514448487	0.461153072
891.9584352	0.882097256	1568.74556	0.253280009	0.166709774
1122.56447	0.922715696	2761.802418	0.321826154	0.245315788
1045.742151	0.9199045	2395.632583	0.237821543	0.151539184
3947.381884	0.768562653	4601.058589	0.287215815	0.201108587
609.5535714	0.928571429	1354.094555	0.287988086	0.19983593
2753.848384	0.846767698	4906.42253	0.236330593	0.151744745
605.2470588	0.964705882	1371.471816	0.17563227	0.093140195
991.8860068	0.922046834	2183.819004	0.389901551	0.319759607
2260.37331	0.801481432	2249.72364	0.497547029	0.442554571
831.5932203	0.893079096	1931.107524	0.363990596	0.290801208
312.0892857	0.953125	657.6656548	0.28101189	0.190983043
1749.28774	0.916697241	3442.589347	0.549047606	0.50596101
341.3824701	0.976095618	933.2433581	0.418470946	0.349638385
592.778022	0.942307692	1416.030618	0.326103012	0.241605507
1119.515564	0.933608949	2707.242781	0.433688644	0.375652776
1250.321767	0.91955836	2815.028475	0.256259486	0.169324371
3397	0.766575728	4188.055887	0.219765597	0.13254283
412.1325301	0.990963855	983.8142359	0.213022087	0.124317432
635.7805556	0.927083333	1345.270859	0.251910111	0.166840854
345.2729358	0.913242227	641.6480821	0.39526736	0.315563372
939.9905213	0.952014218	2374.66866	0.214954489	0.128569506
342.9702381	0.933035714	765.930675	0.293775409	0.206239429
1052.390194	0.849953184	1830.675567	0.408118362	0.340917976
225.3275109	0.967248908	573.5595007	0.498213333	0.438487602
752.4806313	0.862784951	1109.461742	0.312130584	0.224870401
1108.24031	0.953488372	2917.573813	0.196111363	0.112768261
916.4641638	0.948805461	2464.230725	0.328589584	0.243683586
1070.526241	0.882910165	1889.858355	0.514552649	0.462938601
2816.302652	0.774733489	3713.891542	0.249058408	0.170823859
751.7428571	0.916031746	1819.053918	0.339253321	0.259591126
719.5736041	0.973350254	1982.27022	0.196413594	0.112718171
177.8653846	0.967859687	420.3269858	0.522913195	0.471007291
804.1132376	0.917956982	1847.567586	0.37261448	0.298297124
564.1942231	0.834718625	785.3822481	0.427510266	0.358116663
692.2463054	0.96144636	1818.318905	0.365315708	0.282848243
1990.266176	0.92005719	4847.447088	0.239672325	0.153491489
892.4550562	0.945224719	2345.091351	0.300297745	0.221904542
701.0422535	0.978873239	1785.259338	0.157632656	0.080270334
318.7714286	0.978571429	810.601797	0.306055804	0.214762056
2597.499042	0.856514359	4363.590854	0.449925537	0.391719429

wavelet.LHL_glcm_ClusterShade	wavelet.LHL_glcm_MaximumProbability	wavelet.LHL_glcm_Idmn	wavelet.LHL_glcm_SumVariance2	wavelet.LHL_glcm_Contrast	wavelet.LHL_glcm_DifferenceEntropy
3360.09054	0.01028459	0.979028146	196.7456761	129.9410891	4.403371566
1489.055439	0.023482315	0.954483181	259.3222265	111.7274513	4.077363296
937.6478171	0.014059771	0.969024713	136.0112157	98.761358	4.258972784
1121.775322	0.025434053	0.924029875	170.2480639	137.421782	4.005658269
607.9694097	0.026442954	0.969491333	100.7863015	56.22828275	3.861821902
-153.2722908	0.027053254	0.944809243	236.2011705	207.910751	4.211769729
-28.53644617	0.186332097	0.996052038	42.79287771	20.61546679	2.571360982
51.234393	0.011406788	0.985652089	108.0627825	65.65860528	4.008482319
-72.59101444	0.134922586	0.992138203	21.11520396	15.79755695	2.658615922
105.9780604	0.011954485	0.949020181	127.4059459	128.5625307	4.366535912
-105.7284546	0.057081663	0.996504018	24.12194175	16.57843612	2.949222218
204.5221205	0.023005709	0.986913185	58.66108753	44.76636715	3.66875711
132.3403838	0.012617076	0.972163436	157.08122	89.77458673	4.150255359
-11.48828425	0.022171482	0.954599973	68.43401739	66.53422484	3.845130839
-187.2566681	0.059698848	0.983553058	104.7416245	88.24604451	3.97349903
-357.7651735	0.182717519	0.994261594	51.13726602	36.61775908	2.534395824
117.0064646	0.027416212	0.977850369	37.14536939	32.2147748	3.473302483
113.8258612	0.027647583	0.954236553	103.9512606	98.90911178	3.93348332
221.3188352	0.023820462	0.980119959	47.38566759	42.16627924	3.59148631
149.3046273	0.104688517	0.992142065	51.4229474	38.90679476	3.243946488
-1.806644683	0.087637961	0.981486025	38.95510844	24.56444777	3.204802705
-5.320636934	0.034132347	0.957143679	56.3034799	56.88627279	3.69916933
-204.1636473	0.033448609	0.974158685	62.25734829	35.63489344	3.534709138
-32.36470481	0.152444863	0.993179495	27.22353106	15.47901341	2.619746441
-128.3804086	0.114305643	0.98227004	46.49016435	39.68211611	3.348249256
202.3620704	0.042939696	0.986046446	67.81243311	42.79315435	3.647185325
-69.6355719	0.050741025	0.983356409	38.97407299	30.77176106	3.481876168
13.51057278	0.044570168	0.988699507	38.0712461	25.04090462	3.285512702
-382.2391009	0.077381941	0.908702431	86.62436202	73.29093858	2.885778776
195.6799472	0.076622739	0.991601186	29.69485123	19.16812988	3.007228569
-47.5856454	0.105529029	0.988949422	24.59465733	17.45343054	3.002129199
776.23917	0.016001632	0.952475872	131.717949	92.44352269	4.188935282
-86.66462169	0.054819747	0.98243026	58.4850675	46.70703068	3.597161911
43.51172389	0.017100308	0.985198917	106.0579003	73.52055617	4.002907657
-129.2870689	0.104002503	0.990383206	42.94376037	30.46009456	3.190619278
-122.7792633	0.057644493	0.977159854	199.5182279	157.4694822	4.095530837
1084.48684	0.011915818	0.941611125	291.0187582	250.4509416	4.669757399
61.43088597	0.092503394	0.991014154	41.07339755	30.33489762	3.190708183
275.6162942	0.025609893	0.974291023	53.9525205	36.94438596	3.519656324
-35.12615455	0.027151704	0.996752178	72.00413953	43.1412063	3.638019065
-100.1796742	0.038211409	0.974122213	51.84640381	41.51576731	3.604370372
72.54863734	0.062300263	0.993432496	26.08985827	19.5065448	3.118413632
-164.737147	0.041947406	0.991668914	36.67170085	28.5170641	3.222935641
20.97300803	0.012550034	0.974864524	110.9711636	71.97720364	4.082254649
-36.04933032	0.102580726	0.986571298	20.12474589	14.36579223	2.874023303
269.1112142	0.014789475	0.982864749	71.35007143	40.45085909	3.629612419
103.3849159	0.168892194	0.995020078	31.22088513	26.13507728	2.661476897
91.79711747	0.052768283	0.968503293	36.85910261	32.82277541	3.402712988
346.1876698	0.031576741	0.951958105	74.94658142	51.10691452	3.63670816
137.7886426	0.013380801	0.945669566	330.981457	274.2453061	4.724205656
-48.14710015	0.151570774	0.994284109	9.393154258	7.011169217	2.369622941
769.6757415	0.018326048	0.986778703	139.8094029	76.85222348	4.06713542
3.902443615	0.189394713	0.996378509	7.887491982	6.185530243	2.15133521
26.93753941	0.052350249	0.989653354	24.42974507	19.16988533	3.068104457
91.14186993	0.02720895	0.970687814	47.32954313	41.35238408	3.612214695
640.8959208	0.014477034	0.975394104	100.360459	58.59093963	3.906370513
86.23852249	0.056058581	0.990574557	40.76634845	33.3638989	3.410405405
713.8684951	0.014349827	0.975922614	97.60256906	68.53811097	4.000105407
528.8468924	0.02025141	0.960872214	89.01809114	59.48347293	3.868099596
39.80189567	0.096579743	0.998063571	18.20827982	10.45599175	2.5618139
952.1988687	0.012120619	0.967688494	118.5250409	90.0451185	4.203156506
100.7877795	0.038803959	0.983409459	89.56375421	70.21105092	3.976716085
432.5146793	0.006911137	0.977785441	164.4557932	97.35705266	4.31520003
203.6553801	0.01394933	0.988637384	125.954542	64.78662833	3.977616385
558.5039063	0.025360193	0.968714191	71.30794536	65.54843414	3.853876751
-45.27803304	0.012027426	0.97681111	142.7814848	109.0456206	4.363770248
771.9197314	0.022304704	0.925577812	154.1173815	154.5739922	4.318700388
18.23965961	0.065625603	0.984677998	57.32725039	42.63251713	3.594971089
107.2503912	0.100366611	0.993884666	25.04220972	16.05278874	2.823533917
-187.3669939	0.051400456	0.977089105	69.34375322	48.50992511	3.691989517
305.0527652	0.023478989	0.95996031	86.11980678	54.96163873	3.750723891
-178.9380916	0.138236816	0.995284244	23.68216221	14.83928507	2.641859222
-85.51704587	0.098242117	0.982326474	32.44249207	24.19508143	3.248571414
277.1957733	0.026918388	0.987185609	97.18511676	48.67108671	3.728642121
297.1630177	0.099274543	0.991402796	65.68566545	58.31027883	3.542551394
82.93225494	0.013241332	0.971119353	120.2218136	93.8276564	4.164689257
457.5569573	0.006541584	0.976055863	240.5491546	100.9555821	4.344462299
712.3176462	0.02615527	0.962698474	122.5124636	89.12076486	3.916843197
626.8313042	0.017119759	0.959926373	125.4591792	86.22225008	4.173564155
74.50326107	0.038116229	0.988067163	25.62116822	19.87328847	3.161231455
3360.09054	0.01028459	0.979028146	196.7456761	129.9410891	4.403371566
63.73688275	0.019078273	0.959353879	54.48005788	48.89815956	3.744208663
134.3373465	0.071243533	0.989529628	49.5824956	41.41784723	3.498707751
-4.76006294	0.083280561	0.989097087	11.91444738	8.695617668	2.622454396
187.37991	0.015097373	0.979257054	63.03814093	42.3155045	3.726925493
-448.6422031	0.016984716	0.950709834	218.29212	153.5926619	4.243830701
77.19392846	0.026690072	0.990619925	48.04217812	48.86150925	3.672631266
-16.30326557	0.110288625	0.992439866	24.31632784	15.952081	2.837653106
123.7885018	0.022497354	0.975298188	199.5694026	141.8150306	4.473906448
-54.15339033	0.041838023	0.97930482	80.28815118	44.2795511	3.707986046
515.7277266	0.012060659	0.948740496	164.2396894	129.7102147	4.341951514
-21.49590553	0.117000288	0.989585504	13.57290597	9.745231726	2.611625352
-35.22476388	0.052210885	0.98799206	48.55352892	41.05185457	3.568559178
68.86908958	0.063758432	0.989364118	26.94227362	22.41856891	3.18684035
-36.10844801	0.035955098	0.985267381	32.67874112	27.08039167	3.406296444
678.1545645	0.01216908	0.979036195	166.9995918	101.1554581	4.294160661
105.822264	0.035537789	0.977188852	115.4242474	81.78738852	4.078528874
676.2383243	0.029652891	0.925902913	202.850747	200.4089287	4.224874716
12.09302746	0.041362743	0.961275424	53.56005407	32.49862725	3.288720961
-39.50646423	0.098008859	0.992477475	52.67906831	37.77838531	3.32051631

wavelet.LHL_glcm_InverseVariance	wavelet.LHL_glcm_Entropy	wavelet.LHL_glcm_Dissimilarity	wavelet.LHL_glcm_DifferenceVariance	wavelet.LHL_glcm_Idn	wavelet.LHL_glcm_Idm	wavelet.LHL_glcm_Correlation
0.134845609	8.743748345	8.222735367	61.1463287	0.907048304	0.128569506	0.200513086
0.130482878	6.870731323	8.197080182	41.09691978	0.860465983	0.124936632	0.394077768
0.141573399	8.425868626	7.465249673	41.53511671	0.885661368	0.13886119	0.163540547
0.143020157	6.563882925	8.755134489	53.68292443	0.829131953	0.120592715	0.106211212
0.206448818	8.243430366	5.403308195	25.00630962	0.891031177	0.208897208	0.290256169
0.106081841	6.396516074	11.19884064	74.44272644	0.847084319	0.096293297	0.088965021
0.386333408	5.304884628	2.090462132	16.07173426	0.973152963	0.564454682	0.347776429
0.201401024	9.072853421	5.762294669	31.62060474	0.923813846	0.192801538	0.248208225
0.410874636	5.488210837	2.103345279	11.21690205	0.956755273	0.498457974	0.138748824
0.125973594	8.228607781	8.665133058	48.9579882	0.854986697	0.119908795	0.012004004
0.364754492	6.66974958	2.568074879	9.872165354	0.964812428	0.378619295	0.185462761
0.262639859	8.074991541	4.471741414	24.25245019	0.931480633	0.259686706	0.133623184
0.141269482	8.157323919	7.285151177	34.13054762	0.888938587	0.136517862	0.275208227
0.175014191	7.233416397	6.168267963	26.56072289	0.862653604	0.159048739	0.043756446
0.245188462	7.643047749	5.870093104	51.48996952	0.929054961	0.272588247	0.080744198
0.434390311	4.939590172	2.422093928	30.36721783	0.972202883	0.557975373	0.156530202
0.252302755	7.195238762	4.079825238	15.2852417	0.905740102	0.24015072	0.070248853
0.15808395	6.638908594	7.207977914	43.10987216	0.867639958	0.158269404	0.030294064
0.234560507	7.413840075	4.491248498	21.67248122	0.912767658	0.223658836	0.063674057
0.353656083	6.787428736	3.405479489	26.78130485	0.955982689	0.411828685	0.131978314
0.317029344	6.917712375	3.135859718	14.16351306	0.92369894	0.372074028	0.228725679
0.200300664	6.76640644	5.457158569	25.27494875	0.871462458	0.197761136	-0.000579486
0.24705695	7.256339017	4.342786267	15.98908417	0.898077915	0.234793636	0.2800018
0.407009695	5.519438747	2.041444509	11.20153516	0.960566473	0.521866383	0.275198999
0.317970353	6.318588482	3.717685006	25.39402234	0.930734405	0.39983657	0.081267302
0.270066468	7.91123679	4.337645313	23.50085326	0.930217872	0.288993651	0.231358991
0.25396027	7.356394324	3.920346292	14.93991694	0.918807857	0.268694069	0.120955968
0.30482321	7.24991927	3.339873634	13.55547278	0.935642054	0.314268032	0.212311848
0.114332147	4.361494654	6.911813867	20.42021287	0.801704361	0.126242633	0.08833348
0.366617787	6.345602039	2.719799831	11.56865958	0.948201891	0.394252195	0.218714751
0.357220753	6.437743963	2.605475367	10.55216418	0.941016592	0.416600754	0.171672721
0.13014262	7.762963016	7.537878311	33.63322516	0.856446716	0.129064444	0.18283505
0.286798466	7.458318772	4.311044709	27.31838922	0.925358791	0.300007111	0.111852052
0.218686224	8.77033962	5.75746552	39.14095096	0.926878624	0.213923795	0.182122752
0.354386677	6.727084928	3.173219487	20.0082794	0.948475971	0.384189328	0.166564309
0.251282262	7.763329761	6.94555195	105.3935885	0.925902888	0.279694955	0.073297397
0.092492001	8.274146345	12.06949494	85.43394913	0.845371784	0.083307163	0.110065675
0.346624073	6.934259104	3.12160197	20.37283396	0.951040367	0.404997235	0.150299234
0.223688877	6.965951042	4.586041602	15.06603557	0.89468279	0.20910422	0.198203002
0.262016859	8.297053869	4.319065283	23.96020938	0.964716535	0.267382768	0.255026295
0.255464478	7.455193008	4.392117167	21.40602759	0.903585116	0.259750132	0.113268399
0.33791784	6.913120959	2.899802217	10.91572497	0.95108705	0.351291436	0.145133147
0.316079516	6.925619627	3.301316181	17.06371406	0.947327637	0.323954977	0.120587963
0.165118728	8.568894654	6.443857739	29.31837238	0.895216837	0.152958192	0.219342735
0.376250061	6.10537228	2.402110564	8.442148538	0.934134076	0.421765161	0.167599661
0.253892672	8.218641337	4.386307025	20.81356128	0.919459672	0.243785411	0.277746924
0.411634429	5.291478593	2.295908091	20.73016916	0.970098573	0.525141833	0.078109195
0.267311364	6.660892807	4.039850223	15.79403235	0.8922331	0.276832208	0.068182476
0.191348609	6.468063993	5.441823779	20.66390912	0.859478909	0.190723813	0.182580481
0.08423799	7.847437211	12.50759339	106.5641904	0.851164006	0.080434706	0.115133028
0.429248674	4.933222291	1.578581209	4.460933112	0.958599362	0.534337798	0.149547837
0.204700672	8.799329276	6.029111608	39.02907845	0.928909034	0.202859506	0.28995987
0.447258447	4.430653636	1.338464338	4.347024389	0.969736555	0.589264299	0.1248404
0.33394988	6.640794042	2.911787522	10.48105874	0.938051417	0.332962532	0.133747091
0.233376681	7.236060352	4.639376583	19.10913996	0.892567274	0.219621106	0.081219445
0.200488111	8.264285731	5.531349664	26.90785448	0.900876897	0.196176316	0.266116013
0.307309268	7.416207741	3.616550034	20.02468296	0.944760078	0.331354045	0.101811895
0.200270952	8.63732949	5.792097353	33.75211741	0.904822646	0.199997783	0.181082717
0.180835275	7.599695601	5.760326133	24.93519353	0.87343534	0.172320879	0.188844232
0.426733466	5.745562848	1.907023631	6.743693336	0.975263724	0.461153072	0.267261522
0.166716671	8.550977233	6.859480912	41.91305658	0.88766097	0.166709774	0.140308906
0.236279346	8.176675547	5.636985172	37.46471431	0.923377616	0.245315788	0.126661835
0.159570351	9.631184787	7.260908387	43.21119411	0.903856277	0.151539184	0.260114935
0.20549292	9.137734617	5.673684114	30.89956542	0.932214054	0.201108587	0.319593752
0.205183464	7.462747096	5.627140578	32.67947475	0.89288442	0.19983593	0.054920286
0.156180312	9.303272744	7.617221968	48.56381821	0.902746398	0.151744745	0.141631377
0.091222723	6.983789544	9.932911674	53.86498742	0.82104578	0.093140195	0.005389184
0.28739144	7.617020406	4.168294143	24.71453154	0.929828856	0.319759607	0.151127039
0.394176572	6.232157934	2.317491831	10.57815341	0.958068486	0.442554571	0.216781156
0.270941462	7.749873647	4.591323347	26.32504226	0.912845405	0.290801208	0.174748887
0.197690917	7.24736626	5.440091518	22.95772619	0.874246704	0.190983043	0.230743592
0.408883539	5.597098692	2.056869252	10.46820274	0.966041587	0.50596101	0.229008661
0.328013736	6.593868325	3.236689733	13.47210898	0.922717864	0.349638385	0.149011007
0.237817121	7.993187816	4.744282029	25.47910903	0.930725755	0.241605507	0.331638091
0.311318753	7.146688691	4.274323332	39.16843554	0.952688738	0.375652776	0.066292986
0.174961396	8.483954879	6.846694374	44.97884186	0.895547422	0.169324371	0.126086678
0.136444827	9.544388572	7.659510093	40.63391793	0.897052431	0.13254283	0.414744718
0.140532554	6.620734607	7.402209103	31.01120985	0.870850347	0.124317432	0.181194197
0.164017081	8.496501809	6.907440948	35.79565455	0.873461524	0.166840854	0.19013667
0.314319514	7.007770665	3.059907393	10.35705857	0.931969307	0.315563372	0.128090232
0.134845609	8.743748345	8.222735367	61.1463287	0.907048304	0.128569506	0.200513086
0.207631855	7.624815631	5.18128417	20.48692711	0.872759695	0.206239429	0.064888698
0.305673989	7.395023547	3.93176133	25.43997267	0.943553456	0.340917976	0.086469907
0.394488074	5.875129574	1.960941221	4.767969279	0.936914755	0.438487602	0.160324757
0.228190914	8.27563798	4.705614105	19.64743689	0.908418172	0.224870401	0.202943342
0.122778462	7.462626176	9.344843282	55.78580418	0.858757861	0.112768261	0.1558218
0.245074678	7.461891334	4.638206147	26.88175471	0.941027287	0.243683586	-0.002753135
0.378117442	6.205949969	2.316907081	10.46859298	0.953935187	0.462938601	0.207345725
0.164163397	9.038361852	8.30715186	69.46605575	0.904197006	0.170823859	0.176521344
0.243666506	7.785530829	4.661014368	21.74180445	0.911828405	0.259591126	0.286197053
0.12517198	7.966386129	8.809342588	48.33148301	0.852253696	0.112718171	0.124497228
0.40516863	5.61553516	1.929330533	5.92903941	0.942552929	0.471007291	0.160747093
0.291683628	7.581834666	4.156357427	23.01206754	0.936186822	0.298297124	0.085445598
0.334416901	6.935952321	3.044418454	12.95003778	0.940060525	0.358116663	0.090153465
0.278818756	7.455418871	3.643812231	13.56545495	0.923926193	0.282848243	0.093164624
0.16350524	9.017399595	7.286085701	46.94954394	0.907660936	0.153491489	0.247219617
0.208860923	8.430307243	6.18360691	41.17314265	0.909216662	0.221904542	0.169615389
0.088592558	6.448896049	11.3989334	63.82029781	0.820372353	0.080270334	0.013850796
0.214255444	5.855661124	4.397141366	12.4114195	0.870258247	0.214762056	0.231608056
0.341898522	6.984174934	3.514386609	25.03222009	0.954863784	0.391719429	0.163197545

wavelet.LHL_glcm_Autocorrelation	wavelet.LHL_glcm_SumEntropy	wavelet.LHL_glcm_AverageIntensity	wavelet.LHL_glcm_Energy	wavelet.LHL_glcm_SumSquares	wavelet.LHL_glcm_ClusterProminence	wavelet.LHL_glcm_SumAverage
697.7705758	5.526121559	26.8115395	0.002877149	83.52850336	295877.7188	52.19239892
526.6376105	5.157566252	22.65610521	0.00935926	94.01926599	150103.1137	44.25540309
577.1849641	5.361502974	24.40873595	0.003646044	61.00432232	73118.1842	47.65864798
311.834834	4.774623689	17.94096578	0.011469137	77.48828397	88209.75955	34.84550076
338.4761273	5.192719932	18.46125571	0.005216389	39.60115619	41403.46347	36.1841114
852.8101109	4.819477446	29.98133294	0.013071035	110.864049	182918.5026	58.13998511
846.7062569	3.560715015	29.12361387	0.098654167	15.66011175	36206.67798	58.00547814
903.1373378	5.364441526	30.32644142	0.003065634	43.25665567	49099.66383	59.75053491
436.5853185	3.545864435	20.9472981	0.066340736	8.889470661	6005.025482	41.72543694
537.729607	5.282506478	23.88662405	0.003862512	63.72757545	54177.1724	46.38930287
2059.647849	4.113678223	45.50918404	0.021149071	10.22572768	8456.374073	90.7251076
841.2588294	4.824608223	29.19702331	0.007308612	26.28696652	21259.55974	57.88865269
1032.968301	5.413465825	32.3702902	0.004105627	62.32335803	79862.37191	63.75312853
282.8005971	4.739752293	17.23423206	0.007748819	34.49027323	18699.87068	33.60356371
739.5156492	4.911485835	27.58647394	0.013782341	46.85586846	69534.01861	54.23465495
1282.301987	3.30246785	35.92321926	0.104691628	21.04572812	47188.35537	71.51678707
336.1831762	4.446141001	18.57158453	0.009757516	17.51764825	7748.431035	36.60312473
513.1569299	4.686943709	23.11212599	0.011175768	50.00898494	43503.67215	45.24547267
289.5287101	4.592518673	17.28923352	0.008335709	23.24098701	15316.26264	33.9540318
1034.620952	4.196813741	32.34245253	0.039614743	22.57366764	30043.96114	64.2334528
232.7496771	4.416149563	15.24687683	0.023604345	16.50460377	8028.669003	30.27536386
294.4346221	4.540611705	17.44540866	0.011271745	28.57085007	14371.08858	34.32352531
361.4983934	4.736736308	19.08026659	0.009214948	24.29098791	16807.69897	37.67374453
440.8110275	3.640521413	21.0054851	0.076298846	10.65667117	9974.344034	41.85077848
375.2717996	4.195575478	19.55934489	0.043974114	21.08731912	14633.76894	38.65555897
463.825771	4.847073271	21.65521936	0.010566983	28.34989468	27051.2779	42.7815293
318.6262469	4.523241515	18.02240443	0.010923437	16.88014668	7482.066968	35.5844373
231.8345181	4.460951974	15.31680237	0.01288209	15.85677806	10117.81267	30.23730341
271.7233893	3.272038875	16.37224264	0.051727716	39.97882515	19035.00424	32.74448529
606.9508974	4.059211979	24.70738375	0.028914688	12.73272346	8628.350837	49.16567519
316.7649826	4.030376156	17.90848406	0.036182582	10.35296138	4177.50192	35.49525428
334.8041328	5.21530126	18.45830104	0.005304043	56.52821793	51307.15324	36.05374397
583.8915822	4.645029539	24.36810058	0.014924215	25.97808472	22681.86684	48.20533263
897.2713649	5.248556039	30.29130259	0.004391279	44.76407209	62958.98918	59.63653228
553.6197438	4.226691302	23.68310149	0.031293612	18.28302504	17942.86429	46.9250033
1801.094958	5.08738839	42.91167061	0.014800817	86.56485388	299740.7726	84.62812977
790.1513793	5.688050629	29.1724581	0.003729752	135.844249	253511.5373	55.84378427
631.1235537	4.286682832	25.28114896	0.028656401	17.76327417	18271.69373	50.13721126
315.9163247	4.561913678	17.92314843	0.009779897	22.75071826	15703.70504	35.30691471
5683.206679	4.977944603	75.70013387	0.007022638	28.39765288	45417.2092	150.6783299
270.487298	4.636860686	16.64125389	0.009881221	23.69331811	13918.47512	32.73515947
556.4801629	4.15524684	23.74183513	0.019704276	11.61514203	5657.020094	47.10980511
574.1048089	4.282259518	24.18332994	0.015173905	15.98304078	15778.14124	47.83527738
505.7161518	5.286845169	22.72835683	0.003396639	46.11817544	42798.20954	44.54043968
187.7537953	3.884959756	13.67519401	0.0384856	8.864149154	2294.508899	27.29926684
386.3904296	5.025430738	19.69584162	0.005162852	28.00152646	25953.4834	38.91858023
1732.937535	3.490373533	41.75782733	0.088256031	14.17715396	21883.66009	83.22649725
256.623859	4.347838622	16.1753031	0.014902717	18.45215622	6436.752046	31.97514011
150.5265991	4.550605221	12.0917597	0.012867164	32.2905439	16835.93547	24.03788591
1268.146418	5.595818045	36.47903165	0.004816526	148.9180693	407165.7171	70.81943128
403.9030237	3.21108543	20.15502512	0.0784467	3.843810239	1074.375511	40.16501899
1360.235769	5.394372596	37.14307578	0.004376107	53.90365426	117364.4551	73.33437908
297.9436827	2.915055928	17.32086334	0.114646642	3.468730442	1222.066532	34.49741697
512.2883531	4.132364312	22.67093002	0.017982722	11.00261294	4404.166146	45.2091495
332.9401242	4.549852285	18.55297631	0.008802063	22.03580006	11327.41896	36.41094674
374.8166712	5.185043695	19.45879673	0.004389891	39.93365549	42152.96086	38.17563582
679.153896	4.485608836	26.24551425	0.015355861	18.83034722	13067.14754	52.05006194
412.6999347	5.210981172	20.50341686	0.003926153	42.40988251	46845.97599	40.26997889
201.8457078	4.974223082	14.15985998	0.006251722	38.50373106	31049.86599	27.88481171
746.6620204	3.620248516	27.40566258	0.044744552	7.099573568	6716.145257	54.579252
477.7069262	5.304452058	22.16371781	0.003603885	54.5523368	62070.35663	43.38565472
808.7702427	5.013692341	28.76807519	0.008908585	40.02519902	47269.15252	56.70717628
716.6404641	5.674307708	27.04807311	0.001842107	65.97688593	97947.58864	52.90951281
1323.365106	5.438255116	36.61895546	0.003254892	47.49375716	79473.82046	72.33440399
413.1431717	4.765215908	20.6685728	0.007386623	35.32924369	27926.12192	40.57995594
1396.689318	5.499922134	37.90959374	0.002528473	62.12710299	91097.7785	74.51837647
397.8379148	5.008109451	20.67612133	0.008648081	78.2004557	69544.65575	39.89428177
647.5274762	4.63488267	25.6701742	0.018126962	25.20849922	21226.56017	50.74848251
654.2463958	3.902211927	25.67539636	0.03922034	10.59059328	8117.250047	51.06853104
580.3797584	4.817473306	24.23304904	0.013233162	29.13378373	22913.70786	47.96540415
214.1872155	4.825624273	14.54996499	0.007912492	35.94994811	32633.88659	28.73012859
966.6367922	3.639229589	31.13946298	0.065073974	9.579748495	7332.018479	62.11033845
293.9309687	4.155317904	17.27346171	0.028316135	14.42858084	5876.675341	34.16824737
439.5517009	5.032771706	21.02036533	0.006954712	36.80539193	60221.39781	41.34810678
782.868909	4.497736077	28.28511354	0.032429085	31.23278429	51392.58154	55.8933597
825.9163353	5.337428562	29.12860181	0.003647512	53.00853942	54667.8933	57.24536421
1216.263722	5.913187546	35.00040382	0.001722206	85.2154246	175322.963	68.74164257
298.8103274	4.739551767	17.52196553	0.011393928	53.54640464	53472.35811	34.07965027
415.5606566	5.360584536	20.61923294	0.003832352	53.73658021	51620.11163	40.28599655
212.7445417	4.253047111	14.70453153	0.01328198	12.01987096	4561.216511	29.07270582
697.7705758	5.526121559	26.8115395	0.002877149	83.52850336	295877.7188	52.19239892
247.9492614	4.732505418	15.92883448	0.00652199	26.84437755	11803.25908	31.40323763
548.2538925	4.539483419	23.65451495	0.019607122	22.6758128	19666.13882	46.74212183
188.45767	3.698663089	13.71093666	0.032482421	5.276914282	800.7400354	27.39721683
353.1792097	4.961069802	18.93770804	0.004993118	26.04075857	16246.03705	37.30910047
836.5872091	5.330574429	29.27349519	0.006293535	94.60917531	164879.3176	57.2841436
721.9201799	4.590111788	27.21615328	0.009007785	24.58432657	20888.65557	53.74451919
557.8965577	3.959242155	23.63924918	0.043469141	10.24239685	4190.289292	47.15096805
1068.549112	5.652646833	33.18445922	0.003590208	84.70941987	181289.258	64.93405057
553.034062	4.95050842	23.51065855	0.009622726	31.1831772	32758.33823	46.64878451
584.3308792	5.347806593	24.58331598	0.004474114	73.08645755	92329.90219	47.98013888
142.1292388	3.595743536	11.88909454	0.049762204	5.910746474	1507.498018	23.76303377
553.1422817	4.543515737	23.77198015	0.015017771	22.25057532	16630.08303	46.95805139
252.4440213	4.166148602	16.01472497	0.020395285	12.32423687	6509.446719	31.70564261
546.7265434	4.447952249	23.45131958	0.010320882	14.972377	5908.439338	46.70436927
1385.89386	5.597532855	37.56260489	0.002805615	67.23338173	109313.8281	74.01080853
696.6976468	5.251147738	26.68021911	0.006887503	48.85039737	69135.86679	52.47000811
498.6948927	4.859641821	23.23792092	0.01253867	102.2325236	124194.2234	44.62770403
255.9205368	4.149814566	15.93733549	0.019201889	22.52758181	9386.268632	31.65863733
1227.903112	4.319290022	35.22954602	0.035346177	22.56294377	26037.10507	69.97638827

wavelet.LHL_glcm_Imc2	wavelet.LHL_glcm_Imc1	wavelet.LHL_glcm_DifferenceAverage	wavelet.LHL_glcm_Id	wavelet.LHL_glcm_ClusterTendency	wavelet.LHL_firstorder_InterquartileRange	wavelet.LHL_firstorder_Skewness
0.957482721	-0.249059063	8.222735367	0.214954489	196.7456761	245.5040035	1.223347482
0.998358059	-0.593026557	8.197080182	0.209833996	259.3222265	438.8580565	0.163578507
0.952644001	-0.246532487	7.465249673	0.226589404	136.0112157	243.7921777	0.637921073
0.998577915	-0.619403784	8.755134489	0.205474992	170.2480639	356.0187205	0.253090726
0.903059756	-0.187783908	5.403308195	0.294857578	100.7863015	183.5334588	0.53597213
0.999108802	-0.662706211	11.19884064	0.172636151	236.2011705	330.3053423	-0.027797921
0.768834446	-0.157426033	2.090462132	0.597410246	42.79287771	27.89011139	0.459262595
0.709653975	-0.077034489	5.762294669	0.279971593	108.0627825	188.6483866	0.143859497
0.691717059	-0.115977304	2.103345279	0.54270243	21.11520396	40.31916486	-0.085523264
0.978137454	-0.321704806	8.665133058	0.204555081	127.4059459	268.5161882	0.085582888
0.59346071	-0.065371804	2.568074879	0.446869981	24.12194175	65.86972933	-1.131608903
0.723559919	-0.089305077	4.471741414	0.34162957	58.66108753	116.2156184	0.544751862
0.980636853	-0.334643768	7.285151177	0.224755088	157.08122	255.3103989	-0.081480176
0.978241264	-0.36273097	6.168267963	0.249516876	68.43401739	197.9597228	0.072503514
0.920547767	-0.221230683	5.870093104	0.344063282	104.7416245	131.9329292	0.243098422
0.79454992	-0.186490452	2.422093928	0.587577894	51.13726602	27.46166934	-0.046898589
0.855410988	-0.167564644	4.079825238	0.329227355	37.14536939	112.8585819	0.494503991
0.994773401	-0.513288486	7.207977914	0.244577337	103.9512606	202.229892	0.148581566
0.839132804	-0.152465842	4.491248498	0.314625818	47.38566759	117.1001654	0.698089202
0.748772044	-0.119399509	3.405479489	0.466747252	51.4229474	51.97545855	0.664617862
0.803416731	-0.145519097	3.135859718	0.438945858	38.95510844	99.99507155	0.388975286
0.97408417	-0.362455768	5.457158569	0.286131968	56.3034799	141.1219198	0.202482077
0.928147532	-0.239453723	4.342786267	0.32185483	62.25734829	136.9092707	-0.297890106
0.756304283	-0.144885739	2.041444509	0.561811842	27.22353106	34.32628381	0.446089532
0.887078475	-0.219321077	3.717685006	0.455336199	46.49016435	76.0339125	0.22253007
0.802307896	-0.123019557	4.337645313	0.364938417	67.81243311	113.2745592	0.528085767
0.778023094	-0.119973631	3.920346292	0.353051423	38.97407299	124.2749372	-0.040089358
0.72647869	-0.101099129	3.339873634	0.392020661	38.0712461	102.7886674	0.439580399
0.997443193	-0.763758863	6.911813867	0.219521761	86.62436202	198.2076622	-0.441184847
0.745186931	-0.122206553	2.719799831	0.456979484	29.69485123	59.22980196	1.031783641
0.660641867	-0.087128012	2.605475367	0.476095672	24.59465733	66.17902356	-0.030439015
0.983661745	-0.362767867	7.537878311	0.217318224	131.717949	271.621303	0.361670308
0.859352608	-0.166514554	4.311044709	0.373677591	58.4850675	101.0470176	0.032877785
0.793311262	-0.109884636	5.75746552	0.297849575	106.0579003	155.4394291	0.239134576
0.752897479	-0.121157705	3.173219487	0.44636364	42.94376037	80.13473596	0.11455723
0.963807348	-0.293256748	6.94555195	0.348046157	199.5182279	130.4742742	0.090745166
0.996006858	-0.461537592	12.06949494	0.160701317	291.0187582	463.6196253	0.153949578
0.692011043	-0.092554163	3.12160197	0.4640801	41.07339755	61.47602148	0.505931129
0.953563213	-0.294291011	4.586041602	0.299909477	53.9525205	140.2765524	0.507228925
0.647563218	-0.068195733	4.319065283	0.348618195	72.00413953	117.5516517	-0.467193227
0.876085153	-0.180223376	4.392117167	0.342068504	51.84640381	139.3837024	0.209703455
0.602492788	-0.065128512	2.899802217	0.423446904	26.08985827	73.20710883	0.461882346
0.784301713	-0.132837613	3.301316181	0.400303587	36.67170085	91.19205318	0.328103299
0.907591737	-0.184253623	6.443857739	0.24309535	110.9711636	215.9207978	0.186401504
0.733311354	-0.122184087	2.402110564	0.480765083	20.12474589	64.59051298	-0.003474444
0.771735776	-0.106805202	4.386307025	0.329574741	71.35007143	147.8071976	0.57214964
0.710899581	-0.128694941	2.295908091	0.562801472	31.22088513	30.0468121	-0.816676671
0.922741276	-0.251525306	4.039850223	0.356952231	36.85910472	102.8836831	0.143752804
0.984567411	-0.429387085	5.441823779	0.279189596	74.94658142	226.8934144	0.58462484
0.997839178	-0.518180637	12.50759339	0.157768023	330.981457	353.6749322	-0.024070013
0.587537967	-0.08506999	1.578581209	0.574420115	9.393154258	33.96300009	-1.447828084
0.855442998	-0.141100937	6.029111608	0.286602649	139.8094029	172.5246523	0.476717435
0.574526307	-0.087782655	1.338464338	0.618915979	7.887491982	23.74732488	0.758536544
0.738868114	-0.113919409	2.911787522	0.409365088	24.42974507	78.08801957	0.057603379
0.932633113	-0.248036318	4.639376583	0.308611966	47.32954313	141.3683402	0.136895005
0.913813706	-0.197310449	5.531349664	0.284277871	100.360459	189.1483677	0.61892925
0.665268341	-0.077353512	3.616550034	0.403367928	40.76634845	85.82869128	0.607831707
0.826691947	-0.125876244	5.792097353	0.286667734	97.60256906	172.0881544	0.774828352
0.962022158	-0.292085455	5.760326133	0.264056282	89.01809114	186.9973339	0.686353557
0.572505589	-0.070324756	1.907023631	0.514448487	18.20827982	44.63507106	0.531752033
0.906642677	-0.183271593	6.859480912	0.253280009	118.5250409	183.9819287	0.766174578
0.847770792	-0.145592421	5.636985172	0.321826154	89.56375421	133.8980736	0.121441378
0.750292004	-0.083579191	7.260908387	0.237821543	164.4557932	252.9651016	0.174706287
0.713389296	-0.081755815	5.673684114	0.287215815	125.954542	195.2307366	0.18803903
0.941245786	-0.254838623	5.627140578	0.287988086	71.30794536	126.936034	0.882501346
0.836000679	-0.123321405	7.617221968	0.236330593	142.7814848	225.3052701	-0.100570882
0.99759195	-0.555428454	9.932911674	0.17563227	154.1173815	333.9280128	0.351650284
0.737737209	-0.100338908	4.168294143	0.389901551	57.32725039	100.9794191	0.372569861
0.647974216	-0.084967495	2.317491831	0.497547029	25.04220972	50.23504481	0.639396211
0.833240887	-0.147077842	4.591323347	0.363990596	69.34375322	118.6721202	-0.233082588
0.979985974	-0.365684044	5.440091518	0.28101189	86.11980678	168.9385211	0.443542031
0.669423757	-0.106600666	2.056869252	0.549047606	23.68216221	38.51207184	-0.915895942
0.81789955	-0.156033697	3.236689733	0.418470946	32.44249207	99.30515184	0.099013027
0.880224642	-0.172351379	4.744282029	0.326103012	97.18511676	153.3841814	0.77073005
0.832827121	-0.155907833	4.274323332	0.433688644	65.68566545	60.97752405	0.669401991
0.950083244	-0.24239203	6.846694374	0.256259486	120.2218136	219.0946356	-0.016943949
0.899466985	-0.160846655	7.659510093	0.219765597	240.5491546	303.0349044	0.072910464
0.995657328	-0.530622748	7.402209103	0.213022087	122.5124636	272.251609	0.448778869
0.927210179	-0.20921277	6.907440948	0.251910111	125.4591792	208.4548245	0.356867896
0.649493657	-0.075622145	3.059907393	0.39526736	25.62116822	88.95908897	0.880300502
0.957482721	-0.249059063	8.222735367	0.214954489	196.7456761	245.5040035	1.223347482
0.910850217	-0.208602799	5.18128417	0.293775409	54.48005788	142.4667421	0.106158054
0.7838607	-0.123751665	3.93176133	0.408118362	49.5824956	100.6538269	0.748613788
0.610719911	-0.080927511	1.960941221	0.498213333	11.91444738	60.23272803	0.000403809
0.731419786	-0.090156568	4.705614105	0.312130584	63.03814093	147.9906176	0.383848849
0.995789838	-0.486600153	9.344843282	0.196111363	218.29212	349.975377	-0.113815359
0.849007396	-0.157863425	4.638206147	0.328589584	48.04217812	104.7055136	1.052582937
0.746639697	-0.125682227	2.316907081	0.514552649	24.31632784	62.87088198	0.259441381
0.936523962	-0.209187551	8.30715186	0.249058408	199.5694026	199.454826	0.001182104
0.881309901	-0.179210427	4.661014368	0.339253321	80.28815118	136.9736216	-0.070878353
0.989396075	-0.390886203	8.809342588	0.196413594	164.2396894	251.4771748	0.064165107
0.663093043	-0.101763454	1.929330533	0.522913195	13.57290597	54.02952837	0.203333604
0.737733748	-0.102202698	4.156357427	0.37261448	48.55352892	103.559213	0.134646273
0.65403119	-0.078452226	3.044418454	0.427510266	26.94227362	72.37030142	0.516217879
0.65732332	-0.074732553	3.643812231	0.365315708	32.67874112	103.3703421	-0.150177529
0.909007981	-0.177322091	7.286085701	0.239672325	166.9995918	240.421447	0.283779253
0.884744488	-0.170221927	6.18360691	0.300297745	115.4242474	166.6067604	0.18236548
0.998614594	-0.631552586	11.3989334	0.157632656	202.850747	425.3139314	0.249341966
0.986605106	-0.477357421	4.397141366	0.306055804	53.56005407	171.1026782	-0.123193384
0.738192992	-0.110217428	3.514386609	0.449925537	52.67906831	59.24480066	0.054992368

wavelet.LHL_firstorder_Uniformity	wavelet.LHL_firstorder_MeanAbsoluteDeviation	wavelet.LHL_firstorder_Energy	wavelet.LHL_firstorder_RobustMeanAbsoluteDeviation	wavelet.LHL_firstorder_Median
0.039115692	162.123239	35285188.67	100.7249296	-55.77992114
0.034850027	215.3905329	7531432.304	169.5507293	-100.1220949
0.041189255	153.9071968	19017927.37	102.0511146	-38.94008722
0.040170132	184.9501964	5484406.608	131.8887408	-120.6954139
0.049209726	126.2793196	22545501.04	82.07566378	-59.91875352
0.038175134	212.3659365	7844666.664	144.8730715	-39.98945724
0.241445078	49.74038017	177594059	15.04998417	-1.609416074
0.047077391	125.3739454	142530532.3	80.39150639	-16.63738213
0.200490303	45.90945373	28804967.08	19.66574031	-5.118044835
0.038416254	156.3179915	14275838.17	108.2073337	-18.21271105
0.127536635	51.89367506	35372679.01	28.66466094	-1.636506775
0.07217423	90.60352769	53059891.46	51.11975217	-15.35365029
0.03617163	165.728334	15927446.27	114.4061412	-58.75991978
0.048476454	123.9061018	5594640.158	83.02046512	-61.99317632
0.076238185	123.2685244	33527401.71	64.49185542	-21.91715311
0.266863641	50.82980783	120834605.8	12.55833978	-3.024511905
0.077266223	81.28985488	5414616.653	50.6421976	-8.840864947
0.049590131	141.9543318	4726412.9	89.3233805	-94.67852606
0.077268585	82.78040275	8334596.465	49.62821244	-19.90443911
0.143906432	69.25325687	183623839.5	27.20498513	-3.542615999
0.099783523	75.9880407	18179890.05	45.42148616	-21.53826321
0.064572985	105.0954464	3474902.944	64.239045	-55.98680794
0.065817302	99.04857689	5439343.934	59.01517252	-13.61225516
0.209189227	46.57546922	36312040.11	18.13385369	-4.023160857
0.144214497	77.9216818	11544705.44	37.69550902	-7.363295623
0.076910959	90.72832332	35248914.6	53.00171233	-16.92878423
0.077898791	85.05395217	14417752.71	54.22424207	-32.19502353
0.087195196	77.2132219	23305712.04	46.18263513	-22.87881529
0.084183673	128.5740962	681324.8963	93.20153037	41.01166123
0.129907058	58.74411342	10668459.83	27.72627466	-6.552278796
0.137892944	59.4249831	29352856.07	31.15038246	-5.0900675
0.041937716	155.4873598	10136530.31	111.1617843	-83.72213965
0.090279557	88.49632723	23398686.91	47.69095693	-15.27231905
0.054313643	117.6742708	89902081.76	68.96023716	-18.01121868
0.116845826	73.62730376	49722375.22	36.51141656	-10.35315075
0.075131407	149.2726013	67622166.84	65.83652921	-18.52523382
0.02504931	243.6897248	36284467.82	181.4837286	-102.7856127
0.131654922	64.1117694	139567751	29.42112431	-1.966328557
0.063980005	101.5577391	3729016.386	63.47143717	-19.02692337
0.071958353	91.20980069	228142786.3	51.48391314	-3.202023374
0.066704172	97.13998933	12937175.61	59.63041249	-47.09005425
0.112077559	60.30778944	59503599.43	32.85629401	-3.475996135
0.099189486	70.42629265	20057935.81	39.8798677	-27.0446827
0.043203047	135.7260063	21843346.36	92.5496627	-23.12009772
0.143086681	53.61439403	7191232.308	29.51373923	-9.442893524
0.060958	97.95863491	35425827.24	62.68190367	-24.93343626
0.226771591	46.92113693	73207370.5	14.69653168	-0.207956311
0.084923167	80.25127556	2728032.395	48.39784904	-2.914673672
0.064943059	118.6790346	2371364.533	86.39635964	-14.39725065
0.030867769	219.9004248	17826844.63	148.5173883	-50.74823838
0.223960564	34.82514425	6665756.724	15.52581822	-3.338340757
0.049389213	134.6465375	83235077.85	77.61707306	-39.76853981
0.289270016	26.79479471	26317222.87	10.84385185	-1.840324571
0.106743852	59.63657675	6561885.89	34.24315327	2.028257695
0.066323495	94.52284497	5335316.306	59.58314306	-47.62805798
0.047821844	127.0216759	22587369.46	81.13697927	-84.74765373
0.098662013	71.31152456	64931097.05	38.09271642	-7.232063519
0.051478724	121.7886818	44082818.92	74.63400972	-43.68459465
0.050956964	124.4244862	7946941.118	78.25421725	-62.53828486
0.180241555	41.24097746	123339430	19.67569641	-3.207462526
0.047907067	131.9548907	26349788.73	81.0318738	-33.42927667
0.065959062	114.8787	46059917.36	63.76760152	-12.04931273
0.036398857	158.3299277	191646191.2	106.5709145	-24.85523625
0.04578509	131.4628678	273833931.2	82.70710007	-6.049774488
0.068137926	97.86856161	6899777.738	55.75913516	-44.93977404
0.040158769	148.8765637	87809795.89	96.17715483	-25.24004032
0.038627216	181.272807	5807746.776	130.3005448	-52.36971248
0.088902222	90.54622541	76959266.62	49.41254227	-11.99083707
0.156800558	50.4239241	74792298.74	23.30604931	-1.788787674
0.077301007	99.20078011	32322975.82	55.66010668	-18.52673556
0.052800735	118.770848	5765400.911	75.41766698	-67.46454822
0.197576957	48.17843444	51994898.4	19.42516083	-1.664015516
0.104347408	75.72452502	9479048.989	44.80309173	-18.49670562
0.059485169	112.6653419	29938807.2	65.67969985	-29.95521337
0.127421235	77.66858034	58661030.18	33.7225251	-5.352840286
0.042119863	141.2845136	20837714.37	91.45366888	-12.14591505
0.032627244	180.9350273	77160921.66	125.1917894	-5.974189608
0.045444234	159.2179231	5560450.593	114.0687657	-113.7492596
0.043985267	145.0408805	23182871.05	97.15372447	-37.1883442
0.098869511	62.64111349	12859654.54	37.27281901	-2.459615132
0.039115692	162.123239	35285188.67	100.7249296	-55.77992114
0.060406431	100.2567732	7097499.78	65.66360295	-38.32545835
0.101417957	82.73159728	55737278.6	45.90468921	-10.58647116
0.144909009	43.58636478	5030455.158	26.0965621	-17.0590937
0.061070434	97.76129185	31225329.39	61.99617158	-18.68287315
0.03441374	204.2992049	11950057.7	143.9066914	-61.48706577
0.081151011	83.36873313	12330588.9	45.83224181	-17.68770583
0.149502225	54.20198485	29240422.71	29.3677047	-9.265990704
0.044361793	156.4103521	59361059.84	88.7404379	-6.419999245
0.0632125	109.8445925	19940557.21	64.21824125	-20.2075981
0.037570888	163.4331384	12320632.55	109.471772	-33.62519627
0.166766101	42.81632108	6374263.717	23.18598145	-7.47374904
0.0849777	87.60099193	59609800.88	47.68251233	-11.9946397
0.115091337	60.80916567	33226449.87	32.67682859	-10.58425517
0.082640587	74.2818757	22036027.92	45.9131667	-6.442775546
0.037894049	164.7357905	60938670.72	105.1182296	-62.74285291
0.053459912	135.0609724	56257427.89	79.57838108	-41.29382394
0.038182058	233.6442168	7518606.846	176.7620178	-95.93674118
0.072009522	102.9424069	1266220.119	73.2005451	-35.13667449
0.133714698	73.41235924	151898658.1	31.3657929	-4.647441135

wavelet.LHL_firstorder_TotalEnergy	wavelet.LHL_firstorder_Maximum	wavelet.LHL_firstorder_RootMeanSquared	wavelet.LHL_firstorder_90Percentile	wavelet.LHL_firstorder_Minimum	wavelet.LHL_firstorder_Entropy
952700094.2	1152.018834	230.1755065	206.2083389	-653.4380486	5.013231868
203348672.2	501.1962239	258.1663096	266.269387	-608.0757246	4.968217463
513484039	710.4919792	198.4303173	243.5058167	-598.4596316	4.889958085
148078978.4	393.5832606	244.157972	264.9223522	-503.9580622	4.865460462
608728528	478.5147575	173.8443305	177.0697099	-484.2695866	4.662104293
211805999.9	635.1855943	275.9742964	282.3206262	-727.7559761	4.975159635
4795039594	988.7610066	104.3615445	29.13550241	-708.2268611	2.984782286
3848324373	865.1185088	168.0661806	186.4145149	-733.3088642	4.746836743
777734111.2	502.4808378	82.53057085	30.20494729	-509.1688795	3.076280562
385447630.5	527.1651485	198.0387225	219.2480267	-581.5504804	4.905572328
955062333.3	541.9037834	80.45974118	72.87111483	-1114.484212	3.474839382
1432617069	669.1635148	131.4019767	135.6351968	-705.7048105	4.270180643
430041049.2	481.5549342	219.3608283	195.2422553	-840.5135314	5.019837996
151055284.3	392.914618	171.5968371	140.042849	-474.4580399	4.589816373
905239846	976.1508763	190.900047	106.7090555	-711.2677939	4.466892679
3262534357	943.4772643	119.041265	24.66235178	-891.7092436	2.792514209
146194649.6	436.4030541	111.4398741	131.2349429	-438.8425872	4.0559193
127613148.3	378.8382029	206.3499367	168.7669198	-649.6822605	4.616407169
225034104.5	628.3768096	121.2413488	94.01856399	-420.5355551	4.085765002
4957843666	868.7705403	124.0791822	88.86959054	-782.4607132	3.697409887
490857031.4	427.2021873	111.8954219	65.90842796	-391.4235054	3.884812343
93822379.49	348.7806249	148.3005617	144.9954801	-470.4217772	4.306206014
146862286.2	384.574939	139.1297806	127.9461434	-452.9508412	4.289697619
980425083	575.1406462	86.94083869	34.55783355	-515.7447751	3.090040511
311707047	577.5483995	126.102332	59.18163025	-495.3431575	3.683986735
951720694.1	753.2395219	131.935103	128.9102539	-541.3979377	4.230889226
389279323.2	536.5606304	123.9127485	64.34348934	-457.6843648	4.093277916
629254225	707.7126693	115.1060811	59.96869416	-397.8259219	3.982438461
18395772.2	241.8939143	155.9904869	213.8061793	-365.687303	3.726474118
288048415.4	528.600803	96.27492774	87.41406731	-580.8183633	3.571398087
792527113.9	478.7314127	91.43443125	57.53916096	-446.3617628	3.551235851
273686318.4	512.6863744	199.3767771	238.5783693	-476.2118942	4.800761664
631764546.6	572.3365809	135.2572244	94.53209028	-601.6752468	4.135001454
2427356208	909.938937	168.9656969	176.2404957	-747.4706342	4.657603122
1342504131	711.6168265	119.5841409	60.40592065	-575.724595	3.826332547
1825798505	734.5438174	244.8442964	211.2256875	-1097.485784	4.669838861
979680631.1	663.6640443	305.0198305	312.3014577	-792.8901817	5.474143215
3768329278	730.3682072	109.0424216	74.33091631	-617.7553805	3.679153739
100683442.4	446.5965343	137.5827649	158.4850833	-436.6581884	4.30132624
6159855231	925.2366671	137.233325	139.2833502	-1850.440805	4.299891091
349303741.5	450.8098924	143.1874837	75.25195387	-448.2382378	4.305169591
1606597185	728.0354021	90.67599553	101.7027488	-559.1546362	3.695651058
541564266.7	736.1738169	114.610287	44.70047439	-615.397048	3.833159758
589770351.6	682.5902677	175.7720748	181.5771784	-566.5889119	4.784738212
194163272.3	405.7071677	81.29937588	49.20537577	-327.0354603	3.399010104
956497335.5	644.1446261	134.612881	121.9220611	-481.3672643	4.372203617
1976599003	629.979568	102.4925201	54.72054377	-1016.344843	3.022392269
73656874.67	360.4799744	111.6098962	160.0694048	-367.082926	3.961873615
64026842.38	459.5976337	144.2270546	205.6835805	-253.0598983	4.21720773
481324804.9	671.4147493	284.6596423	333.999308	-921.8331567	5.285152319
179975431.6	335.252325	59.13754365	32.45267482	-478.1796893	2.793320017
2247347102	873.2084615	196.3481772	189.4740404	-944.4407469	4.827478322
710565017.6	571.7503155	51.04066771	22.81322188	-419.6396069	2.432134197
177170919	452.6419651	88.01823018	91.83164708	-546.9994969	3.667678617
144053540.3	352.2846404	136.3449753	100.7888315	-486.8192007	4.274227641
609858975.5	587.1482265	184.5763543	158.7463375	-537.2983721	4.691430426
1753139620	755.9438175	110.8209108	91.96979622	-639.173712	3.915171439
1190236111	716.6355399	168.9158633	200.2367253	-520.9928235	4.661090696
214567410.2	489.1021586	170.9289188	192.9052473	-389.3233055	4.576797941
3330164610	1101.497983	72.42640797	46.11885255	-654.6924266	3.09353087
711444295.8	656.3656	181.8274332	215.1621063	-526.0783429	4.742122588
1243617769	813.9427703	169.5098018	169.6284318	-722.8007358	4.528095029
5174447163	876.3968738	204.7373065	249.9596261	-665.9494985	5.051361967
7393516143	911.5816295	179.2766933	209.248929	-895.9948258	4.823461361
186293998.9	513.4137242	146.6104863	104.5767998	-509.7789448	4.281960745
2370864489	653.0724844	200.4683257	227.6532079	-927.3462192	4.977781346
156809163	524.3598593	220.9176198	296.0975482	-494.7126904	4.922874777
2077900199	593.182052	136.5402255	114.946697	-628.4655622	4.176998649
2019392066	589.3067388	86.38325717	69.84533196	-605.3364335	3.37290086
872720347	436.2745004	145.4910071	129.3428124	-616.8838974	4.302713802
155665824.6	401.788725	170.6405181	119.9189795	-422.82877	4.52725678
1403862257	555.8783355	85.5818714	38.92143163	-769.7771713	3.122118563
255934322.7	411.1795727	112.4971383	52.82760807	-425.0246854	3.843397944
808347794.5	903.7248742	167.0388094	103.6805455	-532.3907937	4.517864006
1583847815	1245.051911	135.0569142	104.0098049	-677.9964837	3.871543624
562618287.9	605.6499224	185.7404832	225.9911888	-692.4673812	4.875889415
2083344885	709.4548236	228.1784373	314.8737161	-808.9333052	5.181350555
150132166	600.4077285	219.8903006	155.9917246	-515.4792024	4.673382489
625937518.2	527.7195883	188.7092182	265.3367176	-506.8107091	4.817098217
347210672.6	603.3326546	89.7631754	98.89699769	-328.2796069	3.7457076
952700094.2	1152.018834	230.1755065	206.2083389	-653.4380486	5.013231868
191632494.1	374.7540982	138.6881997	103.9881656	-407.764704	4.335141479
1504906522	878.6982694	127.6988102	76.97192538	-580.6200801	3.997864955
135822289.3	304.3392746	65.43117324	40.97189511	-326.9037137	3.215176744
843083893.5	585.4517678	131.526999	150.4404161	-455.4583038	4.365593223
322651557.9	483.3725046	260.5725525	271.3420401	-750.4105082	5.059946979
332925900.4	1032.873299	130.4136068	99.83752788	-668.0618342	4.095843454
789491413.1	486.6467427	84.80239858	39.62071422	-598.0593114	3.398812681
1602748616	959.9687933	224.7666589	275.8251207	-796.558289	5.013237916
538395044.6	488.6210079	157.8787399	151.6404155	-579.684559	4.479414975
332657079	504.1081584	211.2817385	265.3474151	-620.5843321	4.984869413
172105120.4	414.7273457	66.76471429	38.31173599	-288.8889572	3.15001644
1609464624	785.7191243	131.9832544	117.7939547	-576.7272536	4.176040417
897114146.4	694.6994018	92.85089948	77.93777014	-388.4100492	3.687367864
594972753.8	430.7522449	103.2515117	121.9902507	-564.2194832	3.993192422
1645344110	664.0648561	226.866639	251.5150489	-951.537295	5.082024578
1518950553	690.4827694	195.9616663	182.8075777	-691.9622886	4.770218311
203002384.8	508.900346	293.9740947	279.7559706	-627.0097038	4.9025611
34187943.22	245.8464813	133.5443229	118.7967032	-407.2114517	4.081449937
4101263768	818.4354028	125.7624221	96.15577982	-859.5887414	3.798449372

wavelet.LHL_firstorder_StandardDeviation	wavelet.LHL_firstorder_Range	wavelet.LHL_firstorder_Variance	wavelet.LHL_firstorder_10Percentile	wavelet.LHL_firstorder_Kurtosis	wavelet.LHL_firstorder_Mean
226.69194	1805.456882	51389.23566	-277.3354824	7.03477176	-39.89396082
248.6506442	1109.271949	61827.14284	-357.9227094	1.966469459	-69.44566619
197.8145341	1308.951611	39130.5899	-245.1101224	3.651283822	-15.62052911
228.7047055	897.5413228	52305.84233	-396.4680384	2.197728665	-85.48258869
165.6315901	962.7843442	27433.82365	-248.2478717	3.500385845	-52.80177655
268.3642799	1362.94157	72019.38671	-457.0195691	2.936192942	-64.36167777
103.8654783	1696.987868	10788.03757	-89.55197717	17.80816007	-10.1633849
167.4538622	1598.427373	28040.79597	-216.1739533	4.385946636	-14.33335529
81.16535505	1011.649717	6587.81486	-95.70034947	12.31358274	-14.94925632
196.8834797	1108.715629	38763.10457	-262.8201061	2.965596016	-21.3595658
80.38522543	1656.387995	6461.784467	-82.99127891	19.11650057	-3.46200566
131.0852859	1374.868325	17183.35219	-149.083711	5.930463011	-9.117417254
209.8417033	1322.068466	44033.54045	-333.3922796	3.248409535	-63.91895305
159.6702282	867.3726579	25494.58176	-261.4313096	3.319154272	-62.85612744
184.3608162	1687.41867	33988.91054	-291.3629506	5.876324344	-49.53703057
118.4630223	1835.186508	14033.48765	-56.9087908	16.6105289	-11.71900693
111.345498	875.2456412	12397.81992	-120.3191811	5.099511684	-4.585370126
189.435447	1028.520463	35885.78859	-331.0584297	3.523822605	-81.81997193
119.9318139	1048.912365	14383.63999	-151.3398353	7.925435567	-17.77145672
123.9630582	1651.231253	15366.8398	-121.8318438	10.03260523	-5.366903232
106.1596523	818.6256927	11269.87178	-165.3349581	5.243361989	-35.36543016
140.6910585	819.2024021	19793.97393	-217.1078307	3.699455638	-46.89437789
137.3535121	837.5257802	18865.9873	-220.2087192	4.114349089	-22.16096916
86.18393324	1090.885421	7427.670349	-92.20986083	12.84180434	-11.44723039
123.5179679	1072.891557	15256.6884	-175.0043905	7.200662182	-25.39901046
129.9775234	1294.63746	16894.15658	-183.0230915	5.567237206	-22.64320709
115.0081003	994.2449952	13226.86313	-194.3264158	5.235891973	-46.1248968
109.7797991	1105.538591	12051.60428	-173.2052071	6.889188542	-34.60932865
155.6645927	607.5812173	24231.46541	-189.5265194	2.545118304	10.07802564
96.27471334	1109.419166	9268.820429	-86.06275003	9.327574394	-0.203179326
89.63996293	925.0931756	8035.322954	-129.3929338	6.572251578	-18.02587762
192.4625719	988.8982687	37041.84158	-290.776796	2.520491523	-52.05053014
131.8947334	1174.011828	17396.22069	-180.041673	6.171555518	-29.97158733
168.3455683	1657.409571	28340.23037	-197.6507704	5.871987244	-14.46293013
116.8214006	1287.341422	13647.23964	-151.2041929	8.346922251	-25.55635177
242.3598816	1832.029601	58738.31223	-287.2294436	5.824848076	-34.79105113
292.0893352	1456.554226	85316.17971	-444.1288199	2.334941881	-87.8687502
108.7572762	1348.123588	11828.14513	-117.2699092	10.30716576	-7.880645921
136.7584864	883.2547227	18702.8836	-159.302993	4.309298023	-15.03773884
137.1657583	2775.677472	18814.44525	-148.4336561	12.49229242	-4.30583836
132.1651911	899.0481302	17467.63775	-225.9886903	4.27570333	-55.09099505
90.67508517	1287.190038	8221.971071	-92.80028945	8.160716838	0.406317342
108.3527958	1351.570865	11740.32835	-151.8776243	11.45210657	-37.35223593
173.6420385	1249.17918	30151.55753	-248.6799815	3.487945898	-27.28121622
78.64829836	732.742628	6185.554835	-126.0170878	6.367559286	-20.59207816
132.7656698	1125.51189	17626.72307	-180.4310199	5.419541678	-22.22396614
102.492509	1646.324411	10504.7144	-60.61109591	24.40893539	-0.047681251
111.3253846	727.5629004	12393.34125	-112.1154427	4.502538753	7.964149208
143.1017554	712.657532	20478.11239	-142.3926868	2.809018383	17.98140375
281.7939853	1593.247906	79407.85012	-347.3643081	3.409285967	-40.28972348
58.06184273	813.4320142	3371.177581	-65.30581265	11.66209683	-11.228156
193.1641607	1817.649208	37312.393	-251.8540707	5.403307071	-35.21666789
50.81564085	991.3899224	2582.229355	-44.57026619	18.26593771	-4.787525917
88.01822449	999.641462	7747.207843	-98.73433478	8.381879877	0.031633781
126.9803971	839.1038411	16124.02124	-198.0974288	4.153604149	-49.65814175
168.3329477	1124.446599	28335.98128	-269.4455367	3.845047481	-75.71294003
110.325629	1395.11753	12171.74441	-128.2761322	9.018239506	-10.46565111
166.5017479	1237.628363	27722.83205	-208.4288908	4.58085826	-28.45587512
164.5891235	878.4254641	27089.57956	-242.8630826	3.540132205	-46.12066485
72.21519386	1756.190409	5215.034224	-64.08479093	19.91899718	-5.527236824
180.415747	1182.443943	32549.84175	-220.5351624	4.387640995	-22.61357385
167.428258	1536.743506	28032.22157	-237.7586723	5.502324542	-26.48303848
203.8901341	1542.346372	41571.18679	-265.999376	3.398218699	-18.60585583
179.2286322	1807.576455	32122.9026	-213.6590893	4.799644328	-4.150923536
142.220456	1023.192669	20226.6581	-185.3209847	5.789057696	-35.60865909
198.7166161	1580.418704	39488.29351	-265.028613	4.004576078	-26.44345097
220.7794501	1019.07255	48743.5656	-250.3660411	2.553174546	-7.812116188
134.336944	1221.647614	18046.41452	-190.8389595	5.558436291	-24.42987257
86.35562357	1194.643172	7457.293723	-79.55740523	12.03299485	-2.184810584
141.2324817	1053.158398	19946.61388	-223.3940929	4.54126435	-34.94308618
157.7012782	824.617495	24869.69315	-269.7013665	3.518494269	-65.1804669
84.47806009	1325.655507	7136.542636	-97.59146642	12.28609966	-13.70087865
105.9020285	836.2042581	11215.23964	-169.853226	5.394313533	-37.95216052
162.5764622	1436.115668	26431.10605	-231.3350527	6.806748926	-38.35176393
134.5524927	1923.048395	18104.3733	-151.4536251	11.82400074	-11.66176567
185.5672908	1298.117304	34435.21942	-229.0449513	3.63454241	-8.019206905
227.9611097	1518.388129	51966.26752	-262.9228748	3.133698112	9.956490711
196.476455	1115.886931	38602.99737	-320.9558465	3.324526759	-98.73574286
186.958283	1034.530297	34953.39959	-258.0382881	2.88829125	-25.64701638
89.75888801	931.6122615	8056.657977	-94.4221088	7.669991323	0.877314388
226.69194	1805.456882	51389.23566	-277.3354824	7.03477176	-39.89396082
131.4814433	782.5188022	17287.36993	-217.8847969	3.576596964	-44.12535343
125.144432	1459.318349	15661.12885	-172.7083066	8.192361028	-25.41372228
61.67593515	631.2429883	3803.920977	-96.17125886	6.454285817	-21.8475961
130.6339866	1040.910072	17065.23845	-171.238272	4.081806084	-15.30075211
251.9235017	1233.783013	63465.45071	-397.4206537	2.674519697	-66.57780725
129.43388	1700.935133	16753.1293	-140.8700356	13.34571306	-15.95554863
81.96826755	1084.706054	6718.796885	-124.8755155	7.893497277	-21.74051332
224.7603226	1756.527082	50517.20263	-256.7892434	4.708873537	-1.687690636
154.9171983	1068.305567	23999.33832	-217.2978303	4.586319959	-30.43613305
209.7122403	1124.692491	43979.22375	-267.8812787	3.091024762	-25.70504377
65.12141579	703.616303	4240.798795	-87.96114006	8.265063516	-14.72169419
130.157462	1362.446378	16940.96491	-181.1877843	6.330325892	-21.87726068
91.85310262	1083.109451	8436.992461	-117.748301	7.341582169	-13.57560584
103.169652	994.9717281	10643.9771	-120.9496167	5.781186539	-4.110663785
220.1553226	1615.602151	48468.36606	-314.9882365	3.680699217	-54.77322187
189.3071724	1382.445058	35837.20551	-276.0305239	4.422018389	-50.63367611
274.5298443	1135.91005	75366.63542	-430.6469733	2.204747791	-105.1386367
126.8140005	653.057933	16081.79071	-170.3424587	3.159896555	-41.86042849
125.1547232	1678.024144	15663.70475	-143.1467874	9.022930551	-12.34836246

wavelet.LHL_glrlm_ShortRunLowGrayLevelEmphasis	wavelet.LHL_glrlm_GrayLevelVariance	wavelet.LHL_glrlm_LowGrayLevelRunEmphasis	wavelet.LHL_glrlm_GrayLevelNonUniformityNormalized	wavelet.LHL_glrlm_RunVariance
0.003964141	84.10132727	0.004017998	0.038480917	0.038856682
0.01293609	100.0460887	0.013056034	0.034675112	0.029214425
0.004593623	64.13713688	0.004672661	0.040232295	0.050230395
0.023016877	84.10115489	0.023177177	0.039671641	0.020885142
0.006557014	46.04276291	0.006755104	0.046936897	0.099826359
0.013073087	115.3639026	0.013094548	0.038046133	0.018746132
0.001415814	25.49653243	0.00168822	0.167097203	1.284633654
0.001781888	46.70389334	0.001842028	0.045803391	0.082449967
0.002819908	13.57626694	0.003206475	0.156337509	0.565433965
0.006334463	62.73766156	0.006389973	0.038009839	0.033659368
0.000659652	11.73032141	0.000712677	0.115994787	0.243846134
0.001622916	29.47706127	0.001711624	0.068012122	0.123431242
0.004393998	71.76813453	0.004425184	0.035729516	0.042933279
0.026753321	41.8138308	0.027178416	0.047609677	0.041198069
0.003086969	59.94806341	0.003196593	0.063297159	0.188488632
0.000883532	31.53795831	0.001050023	0.205929428	0.845161962
0.005948954	21.02501427	0.006127601	0.074003543	0.09063597
0.012074279	58.12171931	0.012143761	0.048577491	0.042939869
0.009192437	24.19597116	0.009494814	0.074754283	0.081701124
0.001043655	30.35549261	0.001178433	0.109314424	0.491374646
0.007673271	20.66354642	0.0081853	0.081737699	0.332562155
0.011385223	32.58157357	0.011545777	0.062843265	0.057107727
0.009222129	31.99841176	0.009384705	0.062462687	0.085391242
0.002685463	15.78359703	0.003109726	0.157391836	0.712171854
0.007400373	29.13889498	0.007736451	0.109954738	0.347342979
0.003156703	29.78496964	0.003356757	0.068027647	0.191224824
0.00753249	22.94777034	0.007738372	0.071602022	0.14725606
0.009952351	21.08917576	0.010426805	0.07963125	0.188801219
0.04251072	37.85752798	0.042554418	0.083651654	0.027840059
0.002651096	17.44561376	0.002845604	0.11146611	0.286495908
0.004029793	15.5402936	0.004451878	0.107770952	0.483762008
0.009965364	59.96285462	0.010086791	0.041298181	0.036003054
0.002984838	30.50874912	0.003112887	0.078747976	0.170204198
0.002397619	48.142144	0.002489719	0.05177121	0.101554906
0.002526793	25.48141635	0.0027354	0.095530574	0.323872047
0.001731537	103.9497436	0.00177331	0.061525436	0.199532633
0.005421762	137.1899841	0.005466197	0.024966522	0.017559432
0.001829047	23.01359554	0.002046639	0.105278774	0.450177887
0.009474261	30.53666899	0.009625895	0.062279856	0.059625374
0.000258861	32.95223711	0.000272999	0.066310161	0.175171725
0.009000106	29.8477159	0.009324888	0.06188122	0.137121947
0.001978989	14.94103889	0.002171339	0.099093684	0.255258116
0.003380861	20.68487996	0.003584196	0.091543602	0.184922124
0.005581561	49.46464969	0.005749549	0.042601328	0.048937954
0.008147144	11.65342494	0.008807899	0.117602833	0.320298157
0.00394459	29.68804958	0.004130045	0.059068725	0.102928836
0.000775983	23.15341608	0.000889402	0.168193104	0.804717919
0.014410654	21.21697693	0.01503613	0.078226455	0.129888093
0.022478694	33.13724743	0.022897846	0.063396281	0.05156144
0.006946888	127.5197051	0.006959635	0.030580159	0.020632502
0.002982896	6.993906745	0.003424286	0.18035532	0.580655925
0.001323116	62.98617795	0.001363035	0.046391881	0.096608742
0.002974089	6.017618786	0.003773603	0.22773703	1.043658594
0.003340202	13.63811075	0.003486757	0.098684074	0.14369398
0.007683416	26.6972392	0.007834236	0.06403334	0.078052802
0.005679648	47.33262658	0.005832985	0.046564767	0.078207637
0.001757305	22.0596177	0.001904543	0.086418107	0.252123539
0.005190621	46.29404945	0.005357895	0.049551499	0.090776898
0.019001268	44.51675059	0.019243994	0.04979473	0.04923385
0.00127258	10.37210466	0.001487511	0.152028024	0.467232961
0.004781073	54.38563967	0.004886154	0.046386234	0.062599359
0.002442166	48.55886067	0.002523879	0.057911556	0.145274051
0.003239058	67.9647081	0.003299178	0.035807805	0.051645022
0.001009132	53.79966425	0.001053263	0.043994708	0.098769881
0.006443927	33.85408672	0.006563216	0.065727139	0.068967241
0.001368011	65.32646686	0.001396543	0.038757414	0.063182002
0.014333069	78.01380193	0.014400861	0.038137213	0.023882663
[truncated: 1,193,420 more chars]
